# Evolution and Unprecedented Variants of the Mitochondrial Genetic Code in a Lineage of Green Algae

**DOI:** 10.1093/gbe/evz210

**Published:** 2019-10-16

**Authors:** David Žihala, Marek Eliáš

**Affiliations:** 1 Department of Biology and Ecology, Faculty of Science, University of Ostrava, Czech Republic; 2 Institute of Environmental Technologies, Faculty of Science, University of Ostrava, Czech Republic

**Keywords:** codon reassignments, genetic code, green algae, mitogenomes, release factor, Sphaeropleales

## Abstract

Mitochondria of diverse eukaryotes have evolved various departures from the standard genetic code, but the breadth of possible modifications and their phylogenetic distribution are known only incompletely. Furthermore, it is possible that some codon reassignments in previously sequenced mitogenomes have been missed, resulting in inaccurate protein sequences in databases. Here we show, considering the distribution of codons at conserved amino acid positions in mitogenome-encoded proteins, that mitochondria of the green algal order Sphaeropleales exhibit a diversity of codon reassignments, including previously missed ones and some that are unprecedented in any translation system examined so far, necessitating redefinition of existing translation tables and creating at least seven new ones. We resolve a previous controversy concerning the meaning the UAG codon in Hydrodictyaceae, which beyond any doubt encodes alanine. We further demonstrate that AGG, sometimes together with AGA, encodes alanine instead of arginine in diverse sphaeroplealeans. Further newly detected changes include Arg-to-Met reassignment of the AGG codon and Arg-to-Leu reassignment of the CGG codon in particular species. Analysis of tRNAs specified by sphaeroplealean mitogenomes provides direct support for and molecular underpinning of the proposed reassignments. Furthermore, we point to unique mutations in the mitochondrial release factor mtRF1a that correlate with changes in the use of termination codons in Sphaeropleales, including the two independent stop-to-sense UAG reassignments, the reintroduction of UGA in some Scenedesmaceae, and the sense-to-stop reassignment of UCA widespread in the group. Codon disappearance seems to be the main drive of the dynamic evolution of the mitochondrial genetic code in Sphaeropleales.

## Introduction

Changes in the genetic code, that is, the meaning of particular codons, are surprisingly common. Many deviations from the standard genetic code were discovered even before the genomics era (reviewed by Knight et al. 2001), but the full breadth of the diversity of the genetic code across the tree of life is becoming apparent only thanks to large-scale sequencing of (meta)genomes and (meta)transcriptomes (Ivanova et al. 2014; Kollmar and Mühlhausen 2017). The unanticipated degree of evolutionary malleability of the genetic code employed by nuclear genomes was recently documented by studies of various microbial eukaryotes, unveiling codes with no dedicated termination codons (Heaphy et al. 2016; Swart et al. 2016; Záhonová et al. 2016) or a hotspot of code evolution in a lineage of yeasts entailing parallel sense-to-sense codon reassignments and stochastic decoding (Krassowski et al. 2018; Mühlhausen et al. 2018). Changes in the genetic code employed by plastids were considered very rare, but this perspective is changing with a series of recent discoveries afforded by sequencing of plastid genomes of exotic algae and plants (Del Cortona et al. 2017; Turmel and Lemieux 2017; Su et al. 2019; Turmel et al. 2019).

However, the most important playground for code evolution are mitochondria and their translation apparatus. Human mitochondria provided the first known deviation from the standard code (Barrell et al. 1979), and a plethora of code modifications have been described from mitochondria of diverse eukaryotes over the following 40 decades (Knight et al. 2001; Sengupta et al. 2007), amounting for 13 different mitochondrial code variants included in the present list of alternative translation tables maintained by NCBI (https://www.ncbi.nlm.nih.gov/Taxonomy/Utils/wprintgc.cgi). This list is, however, incomplete, as it ignores a growing number of additional variants that have appeared in the literature (Jacob et al. 2009; Turmel et al. 2010; Lavrov et al. 2013; Ling et al. 2014; Fučíková, Lewis, Gonzalez-Halphen, et al. 2014; Noutahi et al. 2017). In contrast to changes of the nuclear genetic code, which are virtually restricted to stop-to-sense reassignments of termination codons, mitochondria sometimes feature changes in the meaning of particular sense codons, including sense-to-sense reassignments (switches to different amino acids), but rarely even sense-to-stop reassignments (when a codon switches to signal translation termination).

Continuing exploration of mitochondrial genome (mitogenome) sequences from remote corners of the eukaryote phylogeny will certainly lead to discoveries of hitherto unknown code variants. Nevertheless, newly reported mitogenome sequences are not always scrutinized with a rigor necessary to detect departures from the standard code, and especially sense-to-sense and sense-to-stop reassignments can be quite inconspicuous. Indeed, a recent careful reanalysis of mitogenome sequences available in public databases led the authors to recognize previously missed genetic code changes in some species (Noutahi et al. 2017). We hypothesized that exploration of previously sequenced mitogenomes of other eukaryotes may unveil further cases, possibly including unprecedented code variants. Such reanalysis may not only improve our understanding of genetic code evolution, but may also detect inaccurate conceptual translations resulting in wrong amino acid identities in protein sequences stored in database. With this in mind we developed a simple bioinformatic pipeline enabling rapid screening of mitogenome sequences and flagging cases potentially representing missed codon reassignments. Application of the pipeline to available mitogenome sequences drew our attention to green algae of the order Sphaeropleales (predominantly unicellular coccoid algae including some important components of freshwater phytoplankton; Fučíková, Lewis, and Lewis 2014), which manifested a surprisingly high number of discrepancies between current conceptual translations of their mitochondrial genes and the likely meaning of particular codons suggested by our comparative method. The monophyly of Sphaeropleales as traditionally circumscribed has been recently challenged by phylogenomic analyses of plastid genome data (Fučíková et al. 2019). For the sake of simplicity, we stick to the traditional concept of the order, which is in our analyses represented by two clearly defined subgroups: Scenedesminia (most of the species analyzed) and the family Sphaeropleaceae represented by *Atractomorpha echinata*.

Nonstandard reading of certain codons in this group had been noticed before, namely translation of UAG (one of the standard termination codons) as leucine or possibly alanine and interpretation of UCA and UCG, normally encoding serine, as termination codons ( Hayashi-Ishimaru et al. 1996; Kück et al. 2000; Nedelcu et al. 2000; Fučíková, Lewis, Gonzalez-Halphen, et al. 2014). However, our dedicated analyses revealed a much more complex picture reflecting a highly dynamic evolution of the genetic code in this algal lineage. We not only solved a previous controversy concerning the meaning of the UAG codon in certain representatives of the group, but also found out that some sphaeroplealean lineages exhibit changed amino acid specificities of certain codons, that is sense-to-sense codon reassignments, which were previously unknown in this group and that are even unprecedented among all code variants characterized to date. As revealed by our analyses of tRNA specified by sphaeroplealean mitogenomes, these reassignments are linked to the emergence of novel tRNA species plausibly providing a means for the changed codon reading. Finally, we show that unique sense-to-stop codon reassignments previous reported from sphaeroplealean mitogenomes correlate with unusual modifications of the mitochondrial release factor 1a (mtRF1a), pointing to a specific molecular mechanism behind the changed meaning of the respective codons. We provide a scenario of the origin of these changes, fitting the codon capture model of genetic code evolution.

When this paper was in the final stage of preparation, a paper significantly overlapping in its core findings with our own work was published by another team (Noutahi et al. 2019). We first describe our results, obtained in complete independence on that other study, and then discuss how our conclusions differ from those reached by Noutahi et al. (2019).

## Materials and Methods

Annotated mitochondrial protein-coding genes available for 29 representatives of Sphaeropleales were downloaded directly from GenBank (supplementary table S1, Supplementary Material online). Seven additional sphaeroplealen mitogenomes that lacked annotation completely or the annotation was incomplete (*Stauridium tetras, Coelastrum* sp. F187, *Coelastrella* sp. M60, *Coelastrella* sp. UTEX B 3026, *Graesiella* sp. 549, *Kirchneriella aperta*, and *Monoraphidium neglectum*; supplementary table S1, Supplementary Material online) were annotated by manual curation of the output of the MFannot tool (http://megasun.bch.umontreal.ca/cgi-bin/mfannot/mfannotInterface.pl) employing the “*Scenedesmus obliquus* Mitochondrial Code” (NCBI translation table 22, https://www.ncbi.nlm.nih.gov/Taxonomy/Utils/wprintgc.cgi#SG22). Genes associated with mobile genetic elements or being limited to particular taxa (“ORFans”) were excluded, the remaining genes were sorted (based on existing annotation confirmed by similarity searches) into 13 groups of orthologs representing standard mitochondrial genes encoding components of the respiratory chain complexes or the ATP synthase (supplementary table S1, Supplementary Material online). Mitogenome-encoded protein sequences of other 995 diverse eukaryotic taxa were gathered from GenBank, deliberately omitting from the sample Metazoa, *Saccharomyces* spp. and *Plasmodium* spp. represented by overwhelming numbers of sequenced mitogenomes that could bias the pattern of amino acid conservation at homologous protein positions. These sequences were searched with BlastP (Altschul et al. 1997) against the standard mitochondrial protein sequences from Sphaeropleales to identify putative orthologs. Those sequences having all 10 best hits to sphaeroplealean sequences representing the same gene with e-value lower than 1e-30 were considered putative orthologs. No nonsphaeroplealean sequences met these criteria for the *atp6* gene, owing to its high divergence in Sphaeropleales. Orthologous protein sequences (for paneukaryotic or Sphaeropleales-only alignments) were aligned using MAFFT v7.407 with –auto option (Katoh and Standley 2013). Analyses of codon occurrence at conserved positions in the alignments were performed by using custom Python scripts (available upon request from the corresponding author). Sequence alignments analyzed are available (as a zipped file) at the following URL: http://www1.osu.cz/∼elias/Mitochondrial_genetic_codes_Sphaeropleales.zip. FACIL and CoreTracker analyses were performed using default settings and nucleotide sequences of the 13 conserved mitochondrial genes.

For consistency, tRNA genes in sphaeroplealean mitogenomes were (re)annotated by using tRNAscan-SE v2.0 with the -O (organellar) option (Lowe and Eddy 1997). The amino acid specificity of the tRNAs was assessed with tRNAscan-SE employing the bacterial models (an organelle-specific option is not available for such an analysis). A multiple alignment of tRNA sequences (excluding six highly divergent ones with uncertain functionality) was computed using LocARNA (Will et al. 2012). After manual alignment trimming (to remove poorly conserved regions), a phylogenetic tree was inferred with the maximum likelihood (ML) method implemented in MEGA X v10.0.5 (Kumar et al. 2018), using the GTR+G model and 100 bootstrap replications. tRNA secondary structures were modeled with tRNAscan-SE and visualized with VARNA v3-93 (Ponty and Leclerc 2015). For comparison of the genomic location of tRNA genes, mitogenome sequences from GenBank were reannotated for consistency with MFannot (with genetic code table 22), the genomic maps were visualized with OrganellarGenomeDRAW (Greiner et al. 2019) and further modified for graphical presentation by using Inkscape 0.92.4 (https://inkscape.org/).

The phylogenetic relationships of the Sphaeropleales representatives analyzed were reconstructed from nucleotide sequences of the 13 conserved mitochondrial genes (supplementary table S1, Supplementary Material online). The alignments were built at the level of amino acid sequences using MUSCLE v3.8.425 (Edgar 2004) and converted to corresponding nucleotide sequence alignments using AliView software v1.25 (Larsson 2014) and the translation table 22. The alignments were manually trimmed and concatenated with a custom Python script, yielding 11,124 aligned nucleotide positions. The ML phylogenetic tree was inferred using IQ-TREE v1.6.9 (Nguyen et al. 2015) with 100 non-parametric bootstraps and the “auto” model selection option (which chose GTR+F + I+G4 as the best model).

Homologs of mitochondrial release factors were searched with TBlastN in genome and transcriptome assemblies available in NCBI databases and in the transcriptome assemblies provided by the OneKP project (https://db.cngb.org/onekp/). A partial mRF1a sequence from *Hariotina reticulata* was assembled from RNAseq Illumina reads available in the SRA database (https://www.ncbi.nlm.nih.gov/sra). In case of the unannotated mRF1a gene from *Coelastrella* sp. UTEX B 3026, the gene mode (exon-intron structure) was deduced manually by considering sequence conservation and canonical intron splice sites GT and AG. All sequences were identified by BlastP searches as orthologs of the sole chlorophyte mitochondrial release factor mRF1a (details on the sequences are provided in supplementary table S5, Supplementary Material online). The sequences were aligned with MAFFT v7.407 (–auto option), the alignment was converted into the svg format with Jalview v2.10.15 (Waterhouse et al. 2009) and further modified with vector graphics software Inkscape 0.92.4.

## Results and Discussion

### Signatures of Codon Reassignments Detected by a Comprehensive In Silico Analysis of Codon Meaning in Sphaeroplealean Mitochondria

We gathered from public sequence repositories complete mitogenome sequences of 24 representatives of Sphaeropleales (different species or different strains of the same species) and partial mitogenome data, that is separate gene sequences (sometimes truncated), from additional 12 representatives (supplementary table S1, Supplementary Material online). For inferences on the likely meaning of individual codons, we employed sequences of 12 sufficiently conserved mitochondrial genes and aligned their existing conceptual translations with orthologs encoded by mitogenomes of nearly 1,000 other eukaryotes. We then identified alignment positions with a particular amino acid conserved in at least 70% of aligned sequences, collected information about the underlying codons in sequences from Sphaeropleales, and plotted for each sphaeroplealean genome (all twelve genes collectively) the occurrence of each codon in the conserved positions sorted by the amino acid (supplementary data set S1, Supplementary Material online). Most Sphaeropleales representatives exhibited at least one codon with a signature of a possible reassignment, that is 1) codon normally signaling translation termination yet here present in-frame and even at conserved positions; or 2) codon not at all or very rarely occurring at conserved positions dominated by the amino acid corresponding to the codon in the standard genetic code (supplementary figs. S1–S5, Supplementary Material online). The most common putative reassignment type (stop-to-alanine UAG reassignment) was seen in 16 out of 36 taxa analyzed, others were less common or limited to a single organism.

For some codons the signal of their meaning derived from the analysis of conserved positions was weak or ambiguous due to too few positions being occupied by the given codon, reflecting the inherently limited number of sufficiently conserved positions in paneukaryotic alignments of mitochondrial proteins. We reasoned that restricting the analysis to comparison of sequences from closely related taxa, that is, Sphaeropleales only, will provide a higher number of conserved alignment positions and hence a clearer signal of the likely codon meaning. For this second step, we made conceptual translations of the sphaeroplealean mitochondrial genes (now including the *atp6* gene not considered in the initial step due to its little similarity to nonsphaeroplealean homologs) that reflected the signal for codon reassignments from the initial analysis. To avoid introducing a mistake in the alternative codon assignment that would propagate in further steps, we applied the following stringent criteria. First, the codon (in a given genome) was considered potentially reassigned if it occurred in at least five conserved alignment positions and no more than 10% of these positions were conserved for the standard amino acid (the one normally encoded by the codon). Second, a specific alternative translation of such a codon was employed if the most frequent conserved amino acid position with this codon was present at least five times and if it was at least twice as frequent as the second most frequent conserved amino acid position with the codon. In other cases, the likely reassigned codon (meeting the first criterion) was translated as an unknown amino acid (X).

**Fig. 1.**
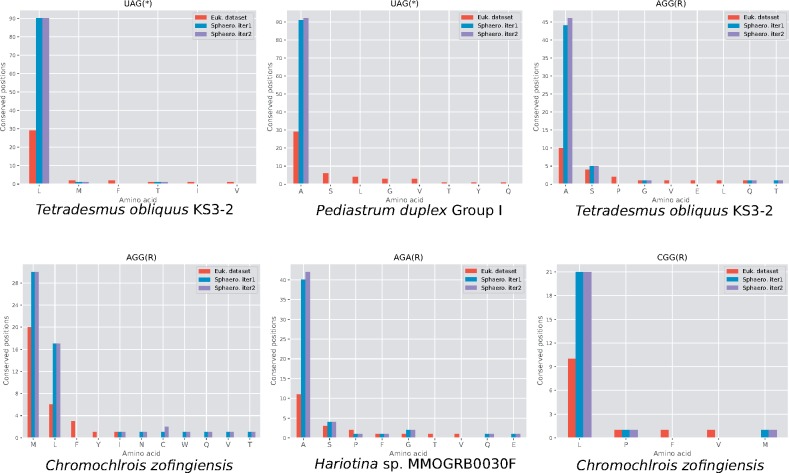
—The variety of codon reassignment in the mitochondria of Sphaeropleales supported by an analysis of conserved amino acid positions. Each type of reassignment is documented by a plot showing the distribution of the codon in a representative example (specific organism) at conserved amino acid positions in alignments of conserved mitogenome-encoded proteins. Euk. data set—multiple alignments of sequences of 12 proteins from Sphaeropleales (sequences as available in GenBank or obtained by conceptual translation with the translation table 22) and nearly 1,000 other eukaryotes; Sphaero. iter1—multiple alignments of sequences of thirteen proteins from Sphaeropleales obtained by conceptual translation considering codon reassignments suggested by the Euk. data set; Sphaero. iter2—multiple alignments of sequences of 13 proteins from Sphaeropleales obtained by conceptual translation considering codon reassignments suggested by the Sphaero. iter1. See text for further explanation. Plots for all proposed reassignment cases are provided in supplementary figures S1–S5, Supplementary Material online.

The deduced protein sequences were aligned and the codon occupancy of conserved amino acid positions (with ≥70% conservation, as in the first step) was investigated. The Sphaeropleales-only analysis, in its first iteration, supported all reassignments assumed in the initial step, except for potential reassignments of UGU and AUA to undefined weakly suggested by the paneukaryotic alignment for a few taxa (supplementary figs. S1–S5, table S2, and data set S1, Supplementary Material online). The Sphaeropleales-only analysis strongly supported UGU as the standard cysteine codon. The signal for possible AUA reassignment in the paneukaryotic alignment dropped below the threshold in the respective species in the Sphaeropleales-only analysis, but some signal for AUA reassignment (to an undefined amino acid) became newly apparent for *Ourococcus multisporus* (supplementary table S2 and data set S1, Supplementary Material online). In case of the AGG codon in *Chromochloris zofingiensis*, the initial strong signal for methionine dropped relatively to the second strongest signal (for leucine), thus no more formally fulfilling our stringent criteria for assigning a specific amino acid meaning (although it remained clear the codon does not encode the standard arginine; fig. 1). On the other hand, evidence for Arg-to-Ala AGG reassignment became apparent for *Chlorotetraedron incus* and both *Bracteacoccus* spp. (supplementary fig. S3, Supplementary Material online). We then performed a second recoding iteration applying the same rules as in the first iteration, but no further changes in the assigned codon meaning were suggested (supplementary figs. S1–S5, table S2, and data set S1, Supplementary Material online).

**Fig. 2.**
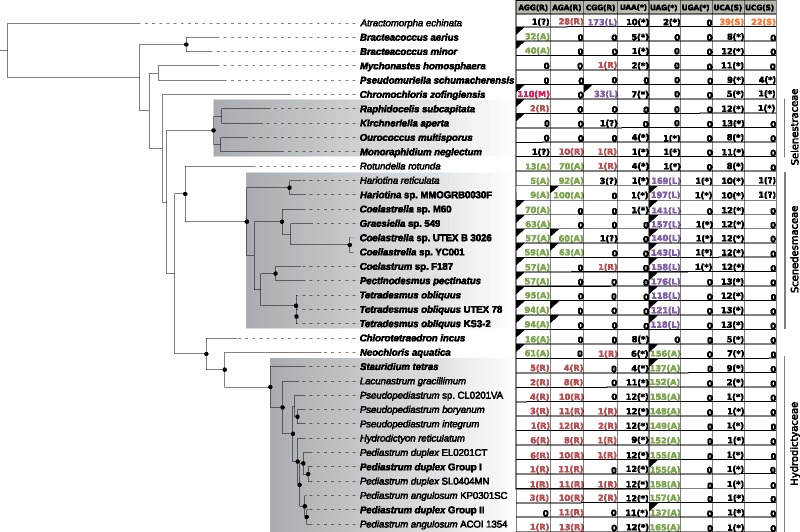
—Proposed codon reassignments in the mitochondria of Sphaeropleales representatives in relation to the phylogeny of the group. The tree shown was inferred from a nucleotide sequence alignment of 13 conserved mitochondrial genes using the maximum likelihood method implemented in IQ-TREE v1.6.9 (GTR+F + I+G4 substitution model, 100 nonparametric bootstraps). Root is placed arbitrarily between *Atractomorpha echinata* (Sphaeropleaceae) and the remaining Sphaeropleales (i.e. Scenedeseminia) following recent broader phylogenomic analyses of Chlorophyceae (Fučíková et al. 2019). Nodes marked with a black dot received support ≥98%. The table on the right shows the occurrence and the proposed meaning of the eight codons with evidence for reassignment in at least one sphaeroplealean representative. For each taxon, the number of the codon occurrences in the 13 conserved mitochondrial genes is indicated together with the most likely meaning of the codon (encoding an amino acid indicated by its one-letter abbreviation or translation termination shown as an asterisk) as inferred from the analyses described in the article; the standard meaning of the codon is indicated on the top. The small black triangles in the left upper corners of some of the cells of the table indicate the presence in the mitogenome of the respective organism of a tRNA with the anticodon cognate to the codon given codon. The absence for such a triangle in organisms with a complete mitogenome sequence available (species names highlighted in bold) means true absence of the respective cognate tRNA, in other cases (with only partial mitogenome data available) the status of the tRNA is unknown.

Plots for representative cases of each type of codon reassignments detected by our procedure are shown in figure 1 and all proposed reassignments in each species are displayed in figure 2. In the following sections, we discuss all the reassignments in more detail.

### Two Independent and Different Reassignments of the Mitochondrial UAG Codon in Sphaeropleales

UAG is one of the three standard termination codons, but an early study of the *cox1* gene from representatives of two sphaeroplealean families, Scenedesmaceae and Hydrodictyaceae, led the authors to propose that in the mitochondria of these taxa this codon has been reassigned to encode leucine and alanine, respectively (Hayashi-Ishimaru et al. 1996). The reassignment proposed for Scenedesmaceae was subsequently corroborated by obtaining a complete mitogenome sequence of *Tetradesmus* (=*Scenedesmus*) *obliquus* (Kück et al. 2000; Nedelcu et al. 2000), and our analysis of mitogenome sequences from multiple Scenedesmaceae genera demonstrate that this trait is common to the whole family (fig. 2; supplementary fig. S1, Supplementary Material online). On the other hand, having analyzed multiple genes from the mitogenome of *Pediastrum duplex*, a member of Hydrodictyaceae, Fučíková, Lewis, Gonzalez-Halphen, et al. (2014) challenged the previous hypothesis on the UAG encoding alanine in this family, and instead proposed that the codon means leucine like in Scenedesmaceae. Our analysis of the more recently published mitogenome sequences from multiple representatives of Hydrodictyaceae unambiguously demonstrates that UAG is decoded as alanine in all members of the family (fig. 1; supplementary fig. S2, Supplementary Material online), in agreement with the initial insight of Hayashi-Ishimaru et al. (1996). In addition, we show that the same reassignment is shared by *Neochloris aquatica*, a species closely related to Hydrodictyaceae (fig. 2; supplementary fig. S2, Supplementary Material online). Other sphaeroplealean lineages do not exhibit UAG within coding sequences (supplementary table S3, Supplementary Material online), and, disregarding nonconserved ORFs and intron-encoded proteins, most species apparently lack this codon completely in their mitogenomes (i.e. do not use it even as a termination codon). The only exceptions are the *nad1* gene from *Rotundella rotunda* and *A. echinata*, as observed before (Fučíková, Lewis, Gonzalez-Halphen, et al. 2014), and possibly also the *nad5* gene from *Monoraphidium neglectum*, *O. multisporus* (previously proposed to employ UAA as translation stop) and *A. echinata* (table 1; supplementary fig. S6*A*, Supplementary Material online).

**Table 1:** Termination Codons Employed by the 13 Standard Mitochondrial Genes in Sphaeropleales

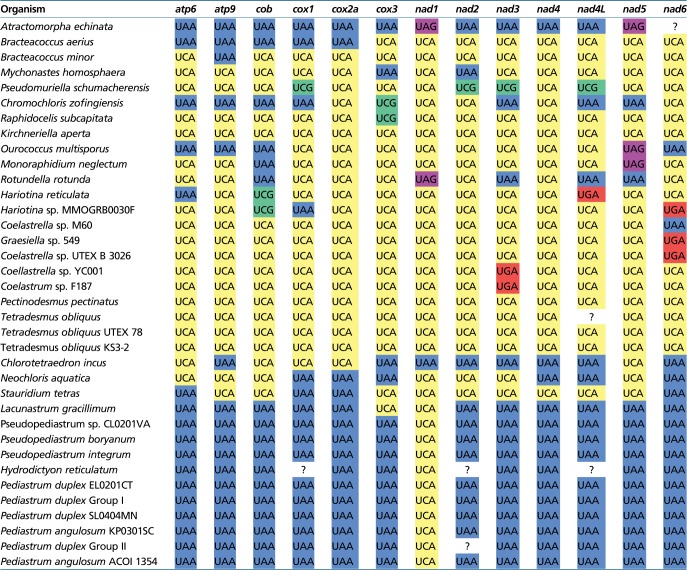

Note.—Question marks indicate unknown identity of the termination codon because of incompleteness of the available gene sequence.

### Unprecedented Sense-to-Sense Codon Reassignments in Mitochondria of Sphaeropleales

The codons AGA and AGG (collectively AGR) normally encode arginine, but rarely their meaning has changed: of both to glycine in mitochondria of ascidians (Kondow et al. 1999) and of AGG to serine or lysine in mitochondria of various invertebrates (Abascal et al. 2006). Here we show the existence of an unprecedented AGR reassignment in mitochondria of some Sphaeropleales, specifically of both AGA and AGG or AGG only to encode alanine (fig. 2; supplementary figs. S3 and S4, Supplementary Material online). The AGG-only reassignment is more widespread, encountered in *Bracteacoccus* spp., *Chl. incus*, *N. aquatica* and a subset of Scenedesmaceae (fig. 2; supplementary fig. S3, Supplementary Material online). Rather than keeping its original meaning (encoding arginine), the AGA codon is completely absent from all these species (supplementary table S3, Supplementary Material online). Both AGR codons have apparently been reassigned in *Ro. rotunda* and some members of Scenedesmaceae, namely *Coelastrella* sp. YC001, *Coelastrella* sp. UTEX B 3026 and *Hariotina* spp. The evidence for the proposed AGG reassignment in *Hariotina reticulata* is weak, because the codon is present only five times in the genome and occurs at only one conserved position (supplementary fig. S4, Supplementary Material online), but consistent with the phylogenetic position of the taxon (nested among relatives with a clear AGG=Ala signal) and with the fact that the tRNA cognate to the AGA codon (which certainly encodes alanine in this species) would be able to recognize AGG via wobble pairing of the third codon (G) position with the first anticodon position (U).

Analysis of conserved amino acid positions in mitochondrial genes additionally provided clear signal for the CGG codon having been reassigned from arginine to leucine in two sphaeroplealean representatives, *A. echinata* and *Chr. zofingiensis* (fig. 1; supplementary fig. S5, Supplementary Material online). The latter species furthermore exhibits a strong signal for a reassignment of the AGG codon from arginine to another amino acid. The difference in the strength of the signal for the two most likely candidates, methionine and leucine, does not exceed our arbitrary threshold (fig. 1), but decoding of AGG as methionine in *Chr. zofingiensis* is clearly supported by the identity of the cognate tRNA (see below). To the best of our knowledge, CGG as a leucine codon and AGG as a methionine codon have not been described from any other translation system.

### Unusual tRNAs Specified by Sphaeroplealean Mitogenomes Provide Molecular Underpinnings for the Observed Codon Reassignments

In order to provide independent evidence for the various codon reassignments proposed above, and to possibly explain the molecular mechanism behind the changed translation of the codons, we analyzed tRNAs specified by the sequenced sphaeroplealean mitogenomes. All species hypothesized to exhibit one or more sense-to-sense reassignments proved to lack mitochondrial genes that would specify tRNAs with the anticodon cognate to the respective codon and predicted to be charged by an amino acid corresponding to the codon in the standard genetic code. However, in each species there is a tRNA cognate to each putatively reassigned codon (including the UAG codon) that has its predicted amino acid specificity agreeing with the proposed reassignment or at least that it is plausibly charged by the expected amino acid (supplementary fig. S7 and table S4, Supplementary Material online). Note that we could not analyze mitochondrial tRNAs in species for which only individual protein-coding mitochondrial genes have been determined (fig. 2; supplementary table S1, Supplementary Material online). The following discussion thus concerns only Sphaeropleales with complete mitogenome sequences available, unless stated otherwise.

All genomes with the stop-to-sense UAG reassignment exhibit a tRNA with the expected anticodon CUA (supplementary fig. S7, Supplementary Material online). In addition, in agreement with the proposed meaning of the reassigned codon, the tRNA (CUA) species from Scenedesmaceae all have leucine as the most likely cognate amino acid (supplementary table S4, Supplementary Material online) and cluster together with canonical tRNA^Leu^—the one with the anticodon CAA cognate to the UUG codon (supplementary fig. S8, Supplementary Material online). Members of Hydrodictyaceae and their relative *N. aquatica* instead specify a tRNA (CUA) species that is (together with another unusual tRNA also presumably specific for alanine, see below) phylogenetically closest to canonical tRNA^Ala^ (UGC) (supplementary figs. S7 and S8, Supplementary Material online) and predicted to be charged by alanine (in one case only as the second best candidate for the cognate amino acid, but the difference from the highest-scoring amino acid—valine—is not high; supplementary table S4, Supplementary Material online). Fučíková, Lewis, Gonzalez-Halphen, et al. (2014) stated “the presence of a Leucine-type tRNA with the anticodon CUA” in the mitogenome of *Pediastrum duplex* (Hydrodictyaceae), but did not provide any evidence for their classification. Our findings build a convincing case for a separate origin of the UAG-cognate tRNAs in Scenedesmaceae and the Hydrodictyaceae/*Neochloris* clade, and for their amino acid specificity being consistent with the UAG meaning predicted for these two lineages by the analysis of conserved amino acid positions, that is, that the tRNA with the anticodon CUA is in fact of an “Alanine type” in the Hydrodictyaceae/*Neochloris* clade.

**Fig. 3.—:**
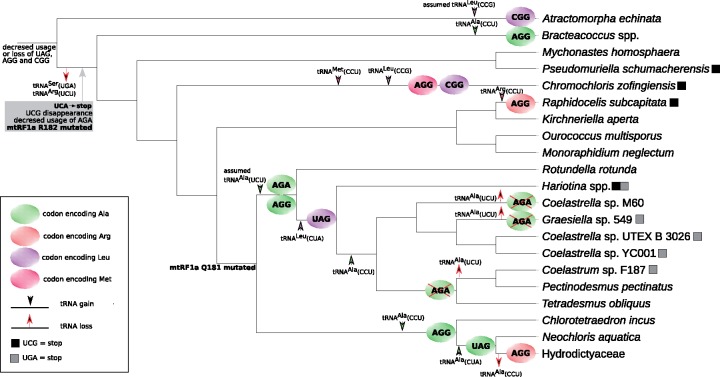
Evolutionary changes in the mitochondrial genetic code in Sphaeropleales. Various evolutionary events affecting the usage and meaning of different codons, gains and losses of tRNAs, and modifications of the mitochondrial release factor mtRF1a are mapped onto the phylogenetic tree of Sphaeropleales (the topology of the tree reflects phylogenetic relationships inferred from nucleotide sequences of mitochondrial genes, see fig.2). The events mapped on the deepest branch did not necessarily occur in the sphaeroplealean stem lineage (i.e., may be evolutionarily older). Mapping of some of the events is inferred indirectly by parsimony reasoning in the absence of relevant data (e.g., the gain of tRNA^Ala^ (UCU) most likely preceding the divergence of Scenedesmaceae and *Rotundella rotunda*, although data on tRNA genes in the *R. rotunda* mitogenome are lacking).

Interestingly, the phylogenetic analysis of tRNA sequences (supplementary fig. S8, Supplementary Material online) suggests that the “suppressor” tRNA reading UAG in the Hydrodictyaceae/*Neochloris* clade shares its origin with another non-canonical tRNA species, tRNA (CCU) present in *Chl. incus* and *N. aquatica* and presumably decoding the AGG codon (supplementary fig. S7, Supplementary Material online). The predicted amino acid specificity of this tRNA species is consistent with the evidence for AGG meaning alanine rather than arginine in the respective algae, although in the case of *N. aquatica* alanine is predicted as only the second most likely amino acid attached to the tRNA (supplementary table S4, Supplementary Material online). Considering the topology of the Sphaeropleales phylogeny as inferred from mitochondrial sequence data (fig. 2), the most parsimonious scenario for the origin of these non-canonical tRNAs assumes the following steps: 1) duplication of the gene for the canonical tRNA^Ala^ (UGC) in a common ancestor of *Chl. incus*, *N. aquatica*, and Hydrodictyaceae, followed by neofunctionalization of one of the copies including a mutation of the anticodon that makes it cognate to the AGG codon; 2) additional duplication of the newly evolved tRNA^Ala^ (CCU) after the divergence of the *Chl. incus* followed by neofunctionalization of one of the copies due to another mutation changing the specificity of the anticodon (to pair with the UAG codon) and yielding tRNA^Ala^ (CUA); and 3) loss of tRNA^Ala^ (CCU) in the stem lineage of Hydrodictyaceae (fig. 3). However, previous phylogenetic analyses based on plastid or nuclear sequence data retrieved alternative branching orders of the three lineages concerned, including *N. aquatic* being basal to *Chl. incus* and Hydrodictyaceae combined, or *Chl. incus* and *N. aquatica* belonging to a single clade (Farwagi et al., 2015, Fučíková et al. 2016, McManus et al. 2018). These alternative topologies would change the interpretation such that the origin of tRNA^Ala^ (CUA) would have preceded the radiation of all three lineages, with secondary loss of the tRNA from *Chl. incus*.

Another important insight of our analysis of tRNA sequences is that the Arg-to-Ala AGG reassignment observed in different Sphaeropleales subgroups results from multiple independent evolutionary events. This is indicated by the apparent lack of phylogenetic affinity of the tRNA^Ala^ (CCU) species from *Chl. incus* and *N. aquatica* to tRNAs cognate to the AGG codon in other taxa. A tRNA (CCU) species is found in all mitogenomes of Scenedesmaceae with the exception of *Hariotina* sp. MMOGRB0030F (supplementary figs. S7 and S8, Supplementary Material online), which may relate to a much smaller number of AGG codons in the latter organism compared with the relatives that do have the tRNA (supplementary table S4, Supplementary Material online). However, this alga, together with two strains of *Coelastrella* sp. (YC001 and UTEX B 3016) and two strains of *T. obliquus*, possesses a tRNA apparently pairing with the AGA codon (supplementary fig. S7, Supplementary Material online). The position of these two noncanonical tRNA species in phylogenetic analyses is very unstable (depending on particular alignment trimming, data not shown), but they do tend to cluster together (supplementary fig. S8, Supplementary Material online). Threonine, tryptophan, or glutamate are predicted as the best candidate for the amino acid cognate to different individual representatives of the tRNA (CCU) and tRNA (UCU) species in Scenedesmaceae (supplementary table S4, Supplementary Material online), but all these assignments are most likely wrong, as the signal for both AGG and AGA encoding alanine is strong in most Scenedesmaceae species that employ these codons (fig. 1; supplementary figs. S3 and S4, Supplementary Material online). The putative tRNA^Ala^ (UCU) from *Hariotina* sp. MMOGRB0030F is likely responsible for decoding both AGA and the less frequent AGG codon (assuming wobble pairing at the third codon position). The presence of candidates for both tRNA^Ala^ (CCU) and tRNA^Ala^ (UCU) in *Coelastrella* sp. YC001 and UTEX B 3026 is consistent with this organisms utilizing both AGG and AGA codons, but the putative tRNA^Ala^ (UCU) found in two out of the three *T. obliquus* strains may be functionally redundant with tRNA^Ala^ (CCU), given the complete absence of the AGA codon from standard mitochondrial genes in *T. obliquus* (supplementary table S3, Supplementary Material online).

Full mitogenome sequence is unavailable for *Ro. rotunda*, which branches off as a sister lineage of Scenedesmaceae and shares with them the same reassignment of the AGR codons (fig. 2; supplementary figs. S3 and S4, Supplementary Material online), so we cannot directly assess tRNAs that are responsible for decoding the AGR codons in this organism. However, based on parsimony reasoning, we predict that this organism possesses tRNAs directly related to the predicted scenedesmacean tRNA^Ala^ (UCU) and possibly also tRNA^Ala^ (CCU), and hence that the changes in the AGR meaning in *Ro. rotunda* and in Scenedesmaceae reflect the same evolutionary event (fig. 3). Whether this notion can be extended to the AGG reassignment observed in *Bracteacoccus* spp. is less clear. The mitogenome of each *Bracteacoccus* species specifies a single tRNA with the CCU anticodon, characterized by an unusually small D-arm (supplementary fig. S7, Supplementary Material online). Phylogenetic analysis is not decisive concerning the relationship of this tRNA species to the tRNA^Ala^ (CCU) from Scenedesmaceae. However, its specific clustering with *Bracteacoccus* tRNA^Trp^ (CCA) in the tree (supplementary fig. S8, Supplementary Material online) and physical neighborhood of the respective genes in the *Bracteacoccus* mitogenomes (supplementary fig. S9, Supplementary Material online) are compatible with its emergence via duplication of tRNA^Trp^ (CCA) followed by neofunctionalization of one of the copy in the *Bracteacoccus* lineage. In contrast, the tRNA^Ala^ (CCU) gene does not physically cluster with the tRNA^Trp^ (CCA) in the mitogenomes of Scenedesmaceae and the Hydrodictyaceae/*Neochloris* clade, supporting separate origins. Tryptophan is predicted as the best candidate for the cognate amino acid of the novel *Bracteacoccus* tRNA (CCU) species (supplementary table S4, Supplementary Material online), but because this amino acid specificity is discordant with AGG clearly encoding alanine rather than tryptophan in *Bracteacoccus* spp. (supplementary fig. S3, Supplementary Material online), we propose that these tRNAs are charged with alanine. Altogether, there is evidence for at least three independent evolutionary events turning AGG into an alanine codon in Sphaeropleales (fig. 3).

The putative reassignment of AGG and CGG as methionine and leucine codons, respectively, in the mitochondrion of *Chr. zofingiensis* is readily explained by the presence in this organism of tRNAs displaying the required combination of the anticodon and the predicted amino acid specificity (supplementary fig. S7 and table S4, Supplementary Material online) supported also by the phylogenetic position of these tRNAs with respect to canonical species (supplementary fig. S8, Supplementary Material online). The full sequence of the mitogenome of *A. echinata*, which would provide an access to the repertoire of mitochondrial tRNAs in this alga, is not available, rendering it impossible to test whether the Arg-to-Leu reassignment of the CGG codon observed in this organism is of the same evolutionary origin as the equivalent change in *Chr. zofingiensis*.

### Reassessment of Sense-to-Stop Codon Reassignments in Sphaeroplealean Mitogenomes

Analysis of the mitogenome of *T. obliquus* indicated that UCA, normally a serine codon, has changed its meaning in this species to serve as a termination codon (Kück et al. 2000; Nedelcu et al. 2000). The more recent exploration of sphaeroplealean mitogenomes showed that this modification is common in this group and unveiled another sense-to-stop reassignment, specifically of UCG (also normally encoding serine) in the mitogenome of *Pseudomuriella schumacherensis* and possibly also *Chr. zofingiensis* (Fučíková, Lewis, Gonzalez-Halphen, et al. 2014). The expansion of the set of fully sequenced sphaeroplealean mitogenomes because that study enabled us to reassess the usage of these two codons in this group. UCA is employed as a termination codon in at least one gene in every sphaeroplealean mitogenome investigated, except probably for *A. echinata* (table 1), that is, in all Scenedesminia. The use of UCA as a termination codon could not be formally excluded in the latter species because the termination codon of its *nad6* gene could not be determined (the available sequence is truncated at the 3′ end). However, *A. echinata* apparently employs UCA as a standard serine codon within coding sequences (fig. 2; supplementary fig. S10, Supplementary Material online), in contrast to Scenedesminia, which use UCA strictly as a termination codon. Hence, the most likely scenario is that the sense-to-stop UCA reassignment occurred only after the divergence of the *A. echinata* and Scenedesminia lineages (fig. 3). Furthermore, we refine the observation by Fučíková, Lewis, Gonzalez-Halphen, et al. (2014) that UCG is not used as a sense codon in any of the sphaeroplealean mitochondrial genes by showing that this is true for all Scenedesminia (but not *A. echinata*, where UCG occurs within coding sequences, most likely as a standard serine codon; fig. 2; supplementary fig. S10, Supplementary Material online). On the other hand, our analyses of multiple sequence alignments expand the probable use of UCG as a termination codon beyond *Pseudomur. schumacherensis* and the *cox3* gene of *Chr. zofingiensis* (table 1; supplementary fig. S6*B* and *C*, Supplementary Material online), specifically to one gene of *Raphidocelis subcapitata* (also *cox3*) and both *Hariotina* spp. (*cob* gene). The use of UCG in these species is consistent with the absence of the codon within coding sequences of their standard mitochondrial genes (supplementary table S3, Supplementary Material online) and the lack of genes for a cognate tRNA in their mitogenomes (in fact mitogenomes of all Sphaeropleales investigated).

### Unique Features of Scenedesminian mtRF1a Proteins Suggest the Molecular Mechanism behind the Changes in Termination Codon Usage

**Fig. 4.—:**
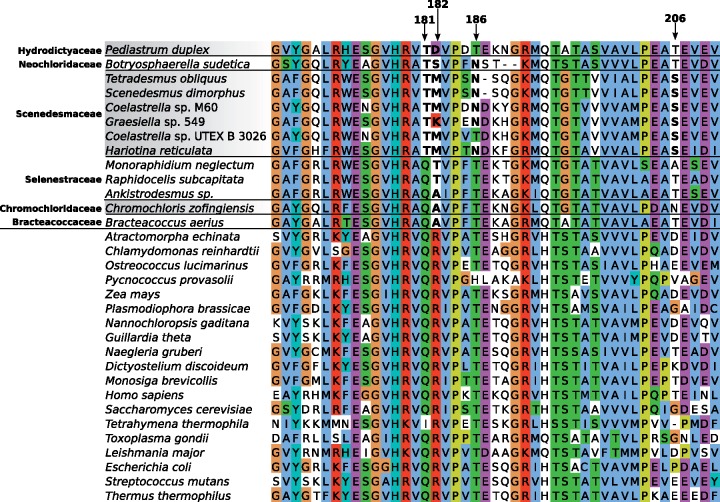
Changes in the mitochondrial release factor mtRF1a from Scenedesminia potentially related to non-standard sets of termination codons used by mitochondrial genes in this taxon. The figure shows a segment of a multiple alignment of mtRF1a protein sequences (from Scenedesminia on the top and a selection of other eukaryotes on the bottom) together with representative bacterial RF1 sequences. Amino acid positions of particular interest (those known or hypothesized to be directly implicated in recognition of a termination codon) are marked above the alignment, with the position number corresponding to the RF1 protein from *Thermus thermophilus*. Notable changes in the amino acid identity at these positions in mRF1a sequences from Scenedesminia are highlighted by bold letters. The mutation of the conserved Gln181 residue in a subset of scenedesminia mRF1a proteins apparently explains the ability of the respective species to read UAG as a sense codon. The mutation of the conserved Thr186 residue together with the appearance of serine at position 206 is probably mediates the ability of the respective mRF1a proteins to recognize UGA as a termination codon. The mutation of the conserved Arg182 residue, unique for mRF1a from Scenedesminia, is here hypothesized to enable the protein to recognize nonstandard termination codons (UCA, UCG). For additional details see main text. Note that the Glu-to-Ile mutation at the position 181 in mRF1a from the ciliate *Tetrahymena thermophila* is in agreement with the lack of the UAG codon from the mitogenome of this species. Sequence accession numbers are provided in supplementary table S5, Supplementary Material online.

The stop-to-sense and sense-to-stop codon reassignments exhibited by mitochondria of various Sphaeropleales imply a specific adaptation of the mitochondrial translation apparatus of these organisms that enable them to ignore as a termination signal the UAG codon (in Scenedesmaceae and the Hydrodictyaceae/*Neochloris* clade) and/or to recognize as efficient signals of translation termination the UCA codon (in all Scenedesminia) and the UCG codon (in up to four independent lineages within the order; fig. 2). Eubacterial translation termination is mediated by two paralogous release factors with a different stop codon specificity: RF1, recognizing UAA and UAG, and RF2, cognate to UAA and UGA. Eukaryotic organelles—mitochondria and plastids—generally inherited this system, with the prototypical versions of the release factors in mitochondria called mtRF1a and mtRF2a (Duarte et al. 2012). We searched nuclear genome or transcriptome data available for Sphaeropleales to gather genes for mitochondrial release factors (supplementary table S3, Supplementary Material online). Some transcriptome assemblies did not provide any mtRF sequence, most likely due to incomplete coverage of the actual gene repertoire of the organism, but when they did, exactly one mitochondrial release factor was found, always an ortholog of mtRF1a (fig. 4; supplementary table S5, Supplementary Material online). mtRF2a was absent, consistent with the notion that mtRF2a was lost in the early evolution of the Chlorophyta lineage (Duarte et al. 2012). It is, therefore, likely, that recognition of all termination codons in sphaeroplealean mitochondria is mediated by the mtRF1a ortholog.

We could study mtRF1a sequences (sometimes partial) from representatives of both sphaeroplealean lineages that employ UAG as a sense codon, namely six Scenedesmaceae species, one Hydrodictyaceae member and *Botryosphaerella sudetica*, a specific relative of *N. aquatica* (also classified in the family Neochloridaceae; Fučíková, Lewis, and Lewis 2014). Interestingly, all these sequences have mutated the canonical Gln181 residues (numbering based on the reference protein from *Thermus thermophilus*), which is critical for recognition of the guanine base at the third position of a termination codon (Zhou et al. 2012), whereas the residue is conserved in other sphaeroplealean mtRF1a sequences investigated (all from Selenastraceae representatives; fig. 4). This is consistent with the presumed inability of mtRF1a in Scenedesmaceae and the Hydrodictyaceae/*Neochloris* clade to recognize UAG (otherwise this codon could not serve as an efficient sense codon in these organisms), as well as with the fact that one of the species with the Gln181 residue conserved, *Mo. neglectum*, has kept UAG as the termination codon (in the *nad5* gene; table 1).

Another notable conserved position is Thr186 responsible for discrimination of adenine against guanine at the second position of a termination codon by RF1 (Zhou et al. 2012). Except one case, mtRF1a proteins in Scenedesmaceae have this position mutated to asparagine, which they, strikingly, combine with a conserved serine residue at the position 206 (occupied by various other amino acids in mtRF1a sequences from other organisms compared; fig. 4). Ser206 is a residue critical for the function of RF2, enabling the protein to accept both adenine and guanine at the second position of a termination codon (Zhou et al. 2012). In this context it is interesting to note that the codon UGA, previously not known from sphaeroplealean mitogenomes (Fučíková, Lewis, Gonzalez-Halphen, et al. 2014), is apparently employed as the termination codon in some Scenedesmaceae members (fig. 2; table 1; supplementary fig. S6*D*–*F*, Supplementary Material online). It thus seems that mtRF1 in this group has adopted features of RF2, which allowed for reintroduction of UGA as a termination codon. The mtRF1a protein from *Bo. sudetica* also lacks the conserved Thr186, which is, however, not accompanied by the presence of serine at the position 206. The mitogenome sequence is unavailable for this alga, so it is unknown whether it also employs UGA as a termination codon.

The mechanism of recognition of nonstandard termination codons by any release factor has, to our knowledge, not been studied, so we can only speculate about specific adaptations of mtRF1a in Scenedesminia enabling it to recognize UCA (and UCG in some species). Nevertheless, we noticed that uniquely among all mtRF1a sequences compared (including that from *A. echinata*), those from Scenedesminia do not have an arginine residue at the position 182 (fig. 4). We hypothesize that this mutation may contribute to the ability of the scenedesminian mtRF1a to accept cytosine at the second codon position. Another unanswered question concerns the mechanism of recognition by mtRF1a of UCG in *Hariotina* spp. (where it is postulated to serve as a termination codon of the *cob* gene, see above and table 1). *H. reticulata* mtRF1a shares the mutation of Gln181 with other Scenedesmaceae, in agreement with UAG being a sense rather than stop codon in this taxon, but it is then unclear how the same release factor can accept guanine at the third position of UCG. Additional specific modifications in the mtRF1a protein, not readily discernible from sequence analysis alone, may account for the differential recognition of UAG and UCG. Finally, it is interesting to note that the “prasinophyte” *Pycnococcus provasolii*, which exhibits a sense-to-stop reassignment independent on Scenedesminia (affecting the meaning of the UUR codons; Turmel et al. 2010), shows unusual mutations in the mRF1a amino acid sequence (fig. 4), providing a potential explanation for its changed codon specificity.

### Evaluation of the Newly Proposed Codon Reassignments by Alternative Bioinformatic Methods: Evidence for Ile-to-Met Reassignment of the AUA Codon in Some Sphaeropleales?

In order to further validate our conclusions concerning the changed meaning of various codons in Sphaeropleales, we employed two previously published tools for genetic code analysis: FACIL (Dutilh et al. 2011) and CoreTracker (Noutahi et al. 2017). The output of the former, which addresses all forms of codon reassignments (stop-to-sense, sense-to-sense, sense-to-stop) is provided in supplementary table S6, Supplementary Material online. CoreTracker is dedicated to detecting sense-to-sense reassignments only; for the output of this tool see supplementary table S7, Supplementary Material online. Both tools are congruent with our results in that they indicate a (potentially) changed codon meaning for all cases detected by us. They usually also agree with the specific change inferred by our analyses described above. However, in some cases, the programs returned ambiguous prediction concerning the amino acid specificity of the change. For example, FACIL does not assign any amino acid to the AGG codon in *Ch. zofingiensis*, because the best scoring candidate, leucine (rather than the standard arginine), receives a probability value that is too low (0.26; supplementary table S6, Supplementary Material online). CoreTracker in this case suggests two alternative meanings, methionine and leucine, both with high scores (0.979 and 0.969, respectively; supplementary table S7, Supplementary Material online). Our analyses of both multiple sequence alignments (fig. 1) and tRNAs (supplementary fig. S8, Supplementary Material online) clearly prefer Arg-to-Met reassignment of this codon (fig. 1). In fact, CoreTracker frequently provides competing high-scoring predictions for the same codon, but our analyses, taking into consideration the nature of tRNAs in the respective taxa, provide unambiguous support for only one of these alternatives.

The only case of an obvious disagreement between our conclusions and the outcome of any of these programs is the prediction for the AGG codon in *Graesiella* sp. 549 provided by FACIL, which assigns the codon to serine with high probability (0.95), whereas we inferred alanine as the most likely amino acid encoded by the codon. We prefer our prediction not only because of the vastly more frequent occurrence of the codon at conserved Ala positions as compared with Ser positions (supplementary fig. S3, Supplementary Material online), but also because the only tRNA cognate to the AGG codon specified by the *Graesiella* sp. 549 mitogenome lacks the long variable arm characteristic of tRNA^Ser^ (supplementary fig. S7, Supplementary Material online). In addition, interpretation of AGG as an alanine codon in *Graesiella* sp. 549 is consistent with the same meaning predicted for this codon in other members of Scenedesmaceae (fig. 2).

FACIL and CoreTracker additionally suggested possible codon reassignments that we disregarded because of the stringent thresholds we applied in our procedure. Thus, according to FACIL the codons UGU, and sometimes also UGC, might in some species encode leucine rather than the standard cysteine (supplementary table S6, Supplementary Material online). However, tRNAs with the respective anticodon (GCA, cognate to both UGY codons) are predicted to be charged by cysteine in all the species (supplementary table S4, Supplementary Material online) and indeed lack the long variable arm expected for a tRNA^Leu^ (an example provided in supplementary fig. S7, Supplementary Material online). Cys-to-Leu reassignment of both UGU and UGC predicted by FACIL for some species would in fact mean they completely lack a codon for cysteine, which is unrealistic given the key role of this amino acid in many mitochondrial proteins. In support of our interpretation of the FACIL prediction as an artefact, CoreTracker does not signal any departure of the UGY codons from the norm.

A more likely bona fide reassignment not directly proposed by our procedure yet pointed to by CoreTracker (and to a lesser extent also by FACIL) concerns the codon AUA. This codon is frequent in *A. echinata* (supplementary table S3, Supplementary Material online), where it most likely serves as the standard isoleucine codon (signal for isoleucine only marginally surpassed by that for leucine in our Sphaeropleales-only analysis, see supplementary data set S1, Supplementary Material online; no change proposed by FACIL and CoreTracker). In Scenedesminia the AUA codon is much rarer or completely absent (supplementary table S3, Supplementary Material online), and if present the number of conserved amino acid positions with this codon is too low to be decisive concerning its amino acid identity (supplementary data set S1, Supplementary Material online). Still, CoreTracker predicts, sometimes with a rather high probability (e.g., 0.991 in *O. multisporus*), AUA as a methionine codon (supplementary table S7, Supplementary Material online). In some species, the probability value for this reassignment is not persuasive, and sometimes leucine competes with a similar score as an alternative amino acid encoded by the codon. FACIL predicts leucine as the likely amino acid encoded by AUA in a few Scenedesminia, together with one case when it instead predicts methionine (supplementary table S6, Supplementary Material online).

So how do scenedesminian mitochondria read the AUA codon? The standard reading of AUA as isoleucine in mitochondria depends on a tRNA (CAU) species seemingly cognate to the AUG codon, whose first codon position is modified to contain the unique base lysidine pairing with A instead of G at the third codon position (Lang et al. 2012). However, all scenedesminian mitogenomes examined include genes for only two tRNA (CAU) species that presumably represent the essential initiator tRNA decoding AUG as formylmethionine and the elongator tRNA decoding in-frame AUG codons as methionine (supplementary fig. S8, Supplementary Material online). Although we cannot rule out the possibility that a tRNA^Ile^ specific for the AUA codon is imported from the cytoplasm in these organisms, it is tempting to speculate that a nonstandard decoding means is employed. Because no candidate tRNA that would translate AUA as leucine is specified by any of the scenedesminian mitogenome, the mitochondrial tRNA^Met^ (CAU) edited or modified at the first anticodon position becomes the obvious alternative. Indeed, this is the mechanism occurring in vertebrates that have reassigned AUA as a methionine codon (Jackman and Alfonzo 2013). Interestingly, mitogenomes of two *T. obliquus* strains exhibit duplication of a gene for tRNA^Met^ (CAU), with one of the copy having the anticodon mutated into UAU (supplementary fig. S8, Supplementary Material online), although *T. obliquus* and its closest relatives do not use the AUA codon in their standard mitochondrial genes (supplementary table S3, Supplementary Material online). It is possible that we witness the first step of an evolutionary path leading to the establishment of AUA as a methionine codon with its own dedicated tRNA.

### Evolution of the Mitochondrial Genetic Code in Sphaeropleales from the Perspective of General Codon Reassignment Models

Several different models have been proposed to explain how a codon may change its meaning in the course of evolution without detrimentally effecting translation efficiency and the function of proteins encoded by the organism (Sengupta et al. 2007; Kollmar and Mühlhausen 2017). One is called “codon disappearance” (or “codon capture”) mechanism and assumes elimination of a codon from the genome (e.g. due to mutational bias favoring alternative synonymous codons), followed by its reintroduction with a changed meaning. Based on the very limited data available at that time, this pathway was previously proposed to explain the reassignment of the UAG codon in sphaeroplealean taxa (Sengupta et al. 2007). The rich sample of sphaeroplealean genomes analyzed here provides obvious support for this model, because the UAG codon is scarce, if used at all, in all sphaeroplealeans except for the two groups that independently reassigned UAG as a sense codon (fig. 2; supplementary table S3, Supplementary Material online). We propose that disappearance of the UAG codon in a common ancestor of Scenedesmaceae and the Hydrodictyaceae/*Neochloris* clade allowed for mutation of the Gln181 residue of the mRF1a protein, predisposing the lineage to subsequently reintroduce UAG as a sense codon (which itself required emergence of novel tRNAs cognate to the codon).

The analysis of codon usage in Sphaeropleales that the codon disappearance model is a likely explanation for the reassignment of the AGG and AGA codons in Sphaeropleales, which seem to have been rarely used already before the radiation of the whole order (AGG) or became rare (if not disappeared completely) in the stem lineage subtending the Scenedesminia clade (after the divergence of the lineage leading to *A. echinata*, which exhibits a decent number of AGA codons apparently with the standard meaning). The disappearance of the codons was correlated with the loss of the respective tRNA(s), paving the way for subsequent reintroduction of the codons concomitant with the emergence of novel cognate tRNAs reading the codons as alanine. The apparent reading of AGG or AGA as the standard amino acid arginine in Hydrodictyaceae (both codons), *Mo. neglectum* (at least AGA) and *R. subcapitata* (AGG; fig. 2; supplementary table S3, Supplementary Material online) is interpreted by us as secondary reversion to the initial state rather than due to retention of the original meaning of the codon in these lineages. The actual molecular mechanism for AGR decoding in Hydrodictyaceae and *Mo. neglectum* remains unclear, as cognate tRNAs are not specified by any of the respective mitogenomes and the tRNA^Arg^ species employed to decode CGN box codons, that is, tRNA^Arg^ (ACG), is unlikely to decode the AGR codons efficiently. An obvious explanation is thus import of the cognate tRNAs from the cytoplasm, as known from multiple organisms (Lang et al. 2012). In contrast, *R. subcapitata* exhibits a tRNA specified by its mitogenome with the anticodon paring with the AGG codon and predicted to have arginine specificity (supplementary table S4, Supplementary Material online). This tRNA has a relative in the close species *Kirchneriella aperta* (supplementary fig. S8, Supplementary Material online), which however lacks the AGG codon in standard mitochondrial genes (supplementary table S3, Supplementary Material online). This tRNA species appears to be novel and of an unclear origin, given the lack of obvious relatives in other Sphaeropleales as well as other green algae (as documented by no significant hits in a BlastN search against the NCBI nr database).

Whether the codon disappearance model is an appropriate explanation for the two potentially independent reassignments of the CGG codon in *A. echinata* and *C. zofingiensis* is less clear. It was previously recognized that mitochondria generally rely on a single tRNA decoding all codons of the CGN box, and in protists this tRNA has the anticodon ACG, with the adenine deaminated to inosine (Jia and Higgs 2008). All sphaeroplealean mitogenomes investigated contain tRNA^Arg^ (ACG)—potentially edited to make tRNA^Arg^ (ICG)—as the only tRNA^Arg^ species cognate to the CGN box. It is, however, likely that neither ACG nor ICG provide a truly efficient decoding of the CGG codon. Except for *A. echinata* and *C. zofingiensis*, the CGG codon is indeed completely absent or occurs very rarely in standard mitochondrial genes of Sphaeropleales, although when present, it apparently encodes the standard arginine (fig. 2; supplementary table S3, Supplementary Material online). Strictly speaking, the codon disappearance model requires loss of the tRNA cognate to the codon to be reassigned (Sengupta et al. 2007), so if tRNA^Arg^ (ACG) is considered cognate to the CGG codon, then the reassignment observed in *C. zofingiensis* (and presumably also *A. echinata*, which needs to be confirmed by determining the set of tRNAs specified by its mitogenome) does not completely fit the model. The emergence of the novel tRNA^Leu^ (CCG) would thus presumably mean ambiguous decoding of the CGG codon (as arginine and leucine) reminiscent of the ambiguous intermediate model of codon reassignment (Sengupta et al. 2007). However, this model assumes that the ambiguity is transient and eventually resolved by loss of the originally present tRNA, whereas tRNA^Arg^ (ACG) has been preserved by *C. zofingiensis* (and presumably also *A. echinata*). Does this mean the CGG decoding is still ambiguous in these algae? This question can apparently be answered only by direct proteomic analysis, but it cannot be ruled out that specific molecular mechanisms, not apparent from the sequences of the tRNA genes themselves, have been employed to avoid the ambiguity. One such possibility is tRNA^Leu^ (CCG) molecules being present in high abundance, another (not mutually exclusive) is a specific modification of tRNA^Arg^ (ACG) minimizing its affinity to the CGG codon.

Present sampling does not allow us to define the actual evolutionary path to the sense-to-stop reassignments of the UCA (and in some cases also UCG) codon observed in Scenedesminia. The common occurrence of these two codons (with their original meaning) in *A. echinata* (fig. 2) means there is no direct evidence for their decrease use or disappearance before the change was initiated. It is instead possible to generalize the scenarios discussed by Sengupta et al. (2007) when dealing with the then known UCA reassignment in *Scenedesmus* (=*Tetradesmus*) *obliquus*. One possibility is that the process was initiated by mRF1a having gained the ability to read UCA (and perhaps also UCG), which led to a transient phase of ambiguous decoding resolved by the disappearance of the codons from within coding sequences loss of the cognate tRNA (ambiguous intermediate model). Alternatively, the evolutionary route to UCA (UCG) being read as termination codons might have followed the “unassigned codon” model, whereby the cognate tRNA was lost first, promoting decreased usage of the codons eventually permitting mRF1a to start recognizing the codons by a gain-of-function mutation. Data from the various hitherto unsampled lineages from the phylogenetic vicinity of Scenedesminia (i.e., Microsporaceae, Treubarinia, *Jenufa*, *Golenkinia*, etc.; Fučíková et al. 2019) are critical for discriminating between the difference possible scenarios.

### Comparison to Previously Published Analysis of Mitochondrial Genetic Codes in Sphaeropleales

Curiously, the genetic code in mitochondrial of Sphaeropleales was recently addressed by an independent study in frame of a broader analysis of the mitochondrial genetic code in green algae by Noutahi et al. (2019). Using CoreTracker combined with an analysis of tRNA genes, the authors detected novel or previously misinterpreted codon reassignments in two lineages: the prasinophyte *Pycnococcus provasolii*, shown to exhibit an Ile-to-Met reassignment of the AUA codon, and Sphaeropleales. For the latter group, Noutahi et al. recognized that UAG means alanine in *N. aquatica* and Hydrodictyaceae, that AGG is decoded as arginine in multiple representatives of the order, and that *Chr. zofingiensis* exhibits two unique reassignments, reading AGG as methionine and CGG as leucine. Their results concerning Sphaeropleales are thus highly congruent with our own findings. Nevertheless, our work, initially pursued without awareness of the effort by the Canadian colleagues, offers some further insights not provided by that parallel study. This is partly because our sampling of sphaeroplealean mitogenome data is much broader (24 complete mitogenomes plus sets of mitochondrial genes from other 12 taxa in our study versus just 14 genomes in Noutahi et al. 2019). We could thus detect codon changes in Sphaeropleales not reported by Noutahi et al., namely the reassignment of CGG as a leucine codon in *A. echinata* (potentially independent on an equivalent change in *Chr. zofingiensis*, see above) and, even more importantly, the unprecedented Arg-to-Ala AGA reassignment in some species. In addition, we also brought evidence for possible Ile-to-Met reassignment of the AUA codon in Scenedesminia, which was not noticed before. Finally, the other study did not address the termination codons in sphaeroplealean mitochondrial genes and the related modifications of the mitochondrial release factor mtRF1a. Combining the results reported herein and by Noutahi et al. (2019) thus makes our understanding of the variation in molecular mechanisms of translation in green algal mitochondria, and of the evolutionary processes generating this diversity, much more complete than ever before.

## Conclusions

Our analysis clearly shows that some codons in mitochondrial genes of most sphaeroplealean representatives have a different meaning than presently assumed, which results in inaccurate conceptual translation of the genes (i.e. corresponding protein sequences) in the respective database records. Of the 29 sphaeroplealean annotated complete mitogenomes or sets of mitochondrial genes, 28 use the translation table 22 and only one, that from *Mychonastes homosphaera*, is represented in GenBank by correctly deduced protein sequences, although the translation table 22 is in fact inappropriate in this case, because *M. homosphaera* does not exhibit Stop-to-Leu UAG reassignment (all the genes are correctly translated only because the UAG codon is simply completely absent from the genome; supplementary table S3, Supplementary Material online). The translation table 22 (“Scenedesmus obliquus Mitochondrial Code”) as presently defined is in fact not used by any sphaeroplealean species (and no evidence for its employment outside Sphaeropleales is known to us), so it will have to be modified by adding Arg-to-Ala reassignment of the AGG codon to correctly translate the mitogenome of *Scenedesmus* (=*Tetradesmus*) *obliquus* and its relatives (fig. 2). The translation table 16 used to annotate mitochondrial genes from *A. echinata*, presently called “Chlorophycean Mitochondrial Code” and differing from the current translation table 22 by the lack of Ser-to-Stop UCA reassignment is, to our knowledge, not employed by any chlorophycean or generally green algal mitochondrial genome. However, it is apparently used by the mitogenome of *Spizellomyces punctatus* and possibly other chytrids (Laforest et al. 1997) and the nuclear genome of an unidentified rhizarian (Pánek et al. 2017), so it should be kept with a changed name. Hence, at least seven new translation tables presently not defined in the NCBI list of genetic code variants will have to be added to the list of existing variants to allow for correct annotation of mitochondrial genes various sphaeroplealean algae (table 2). These new tables, however, need to be considered as provisional and may be subject to revision, given the uncertain status of the AUA codon decoding in Scenedesminia (see above). We hope that the results reported in this article will inspire direct experimental testing of the codon meaning in sphaeroplealean mitochondria, which should be able to resolve any uncertainties left, including that regarding the AUA codon.

**Table 2:** The Minimal Set of Translation Tables Required for Correct Conceptual Translation of Mitochondrial Genes in Sphaeropleales

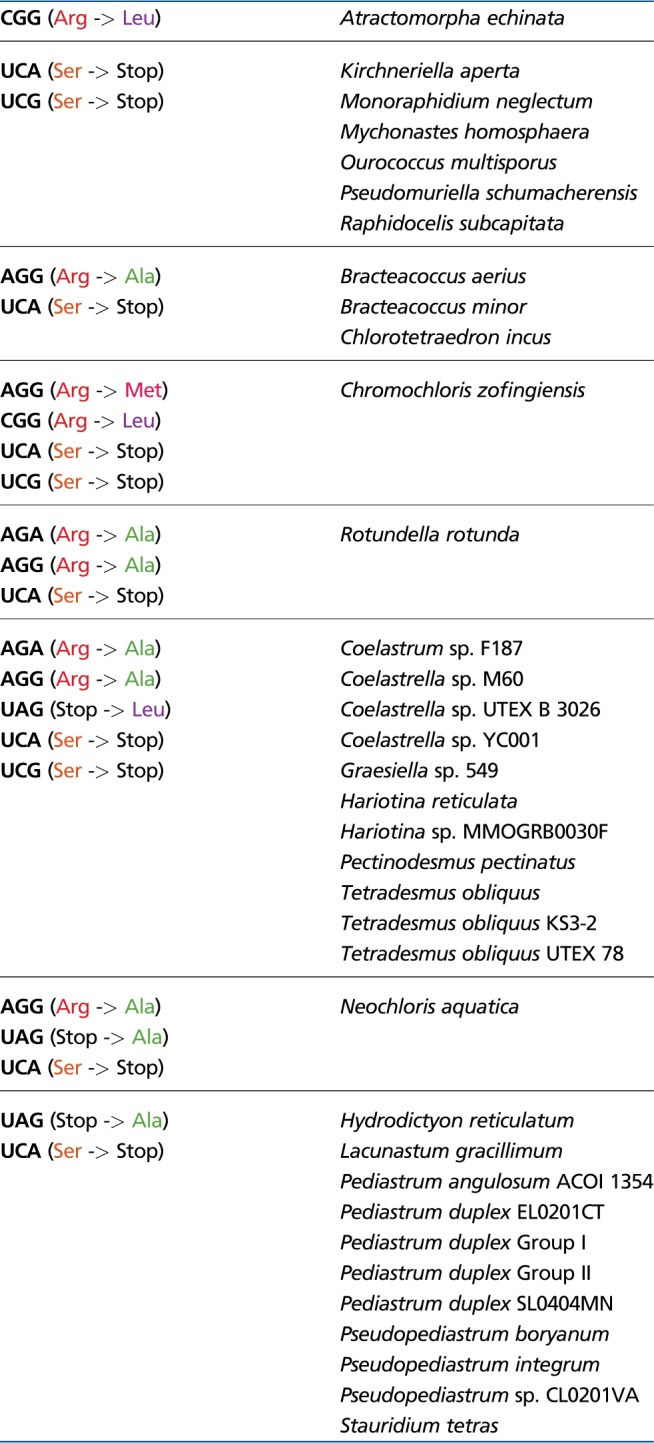

Note.—Changes in codon meaning compared with the standard genetic code (NCBI translation table 1) are indicated on the left. Note that the different species listed for each translation table do not necessarily utilize all the codons with the changed meaning. It is also possible that some of the tables will need be updated by incorporating a reassignment of the AUA codon (see main text for the controversy regarding its meaning in some Sphaeropleales).

## Supplementary Material

Supplementary data are available at *Genome Biology and Evolution* online.

## Supplementary Material

evz210_Supplementary_Data
