# Supplementary material for: Evolution and Unprecedented Variants of the Mitochondrial Genetic Code in a Lineage of Green Algae
Source: Genome Biol Evol. 2019 Oct 16;11(10):2992–3007. doi: 10.1093/gbe/evz210 (PMC6821328; doi:10.1093/gbe/evz210)
Supplement: evz210_Supplementary_Data [file evz210_supplementary_data.zip › Supplementary_figures.pdf]

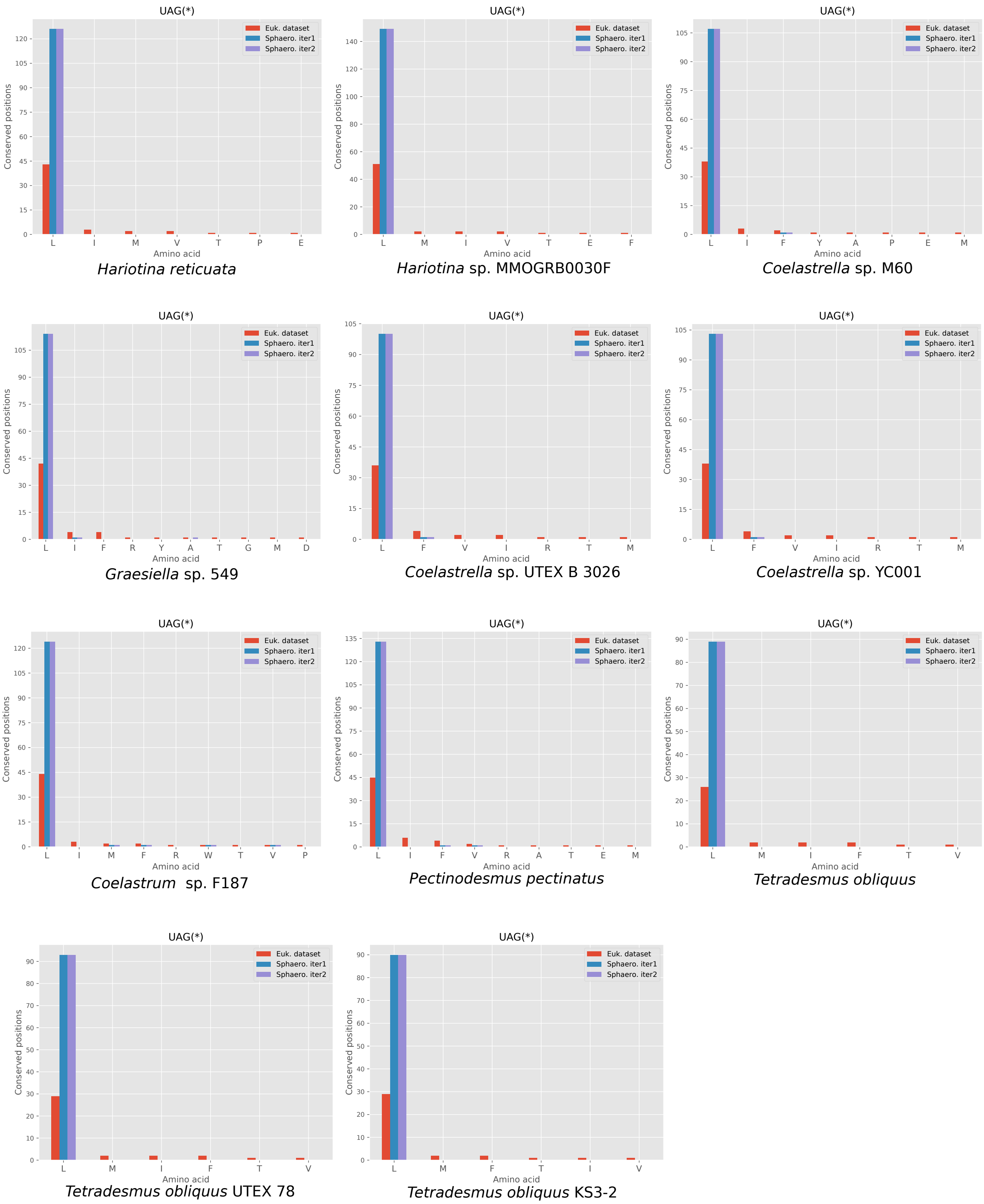


**Fig. S1.** UAG as a leucine codon in mitochondria of Scenedesmaceae. Evidence for the reassignment was obtained by analysing the occurrence of the codon at conserved amino acid positions in alignments of conserved mitogenome-encoded proteins. Euk. dataset – multiple alignments of sequences 12 proteins from Sphaeropleales (sequences as available in GenBank or obtained by conceptual translation with the translation table 22) and nearly 1,000 other eukaryotes; Sphaero. iter1 – multiple alignments of sequences of 13 proteins from Sphaeropleales obtained by conceptual translation considering codon reassignments suggested by the Euk. dataset; Sphaero. iter2 – multiple alignments of sequences of 13 proteins from Sphaeropleales obtained by conceptual translation considering codon reassignments suggested by the Sphaero. iter1.

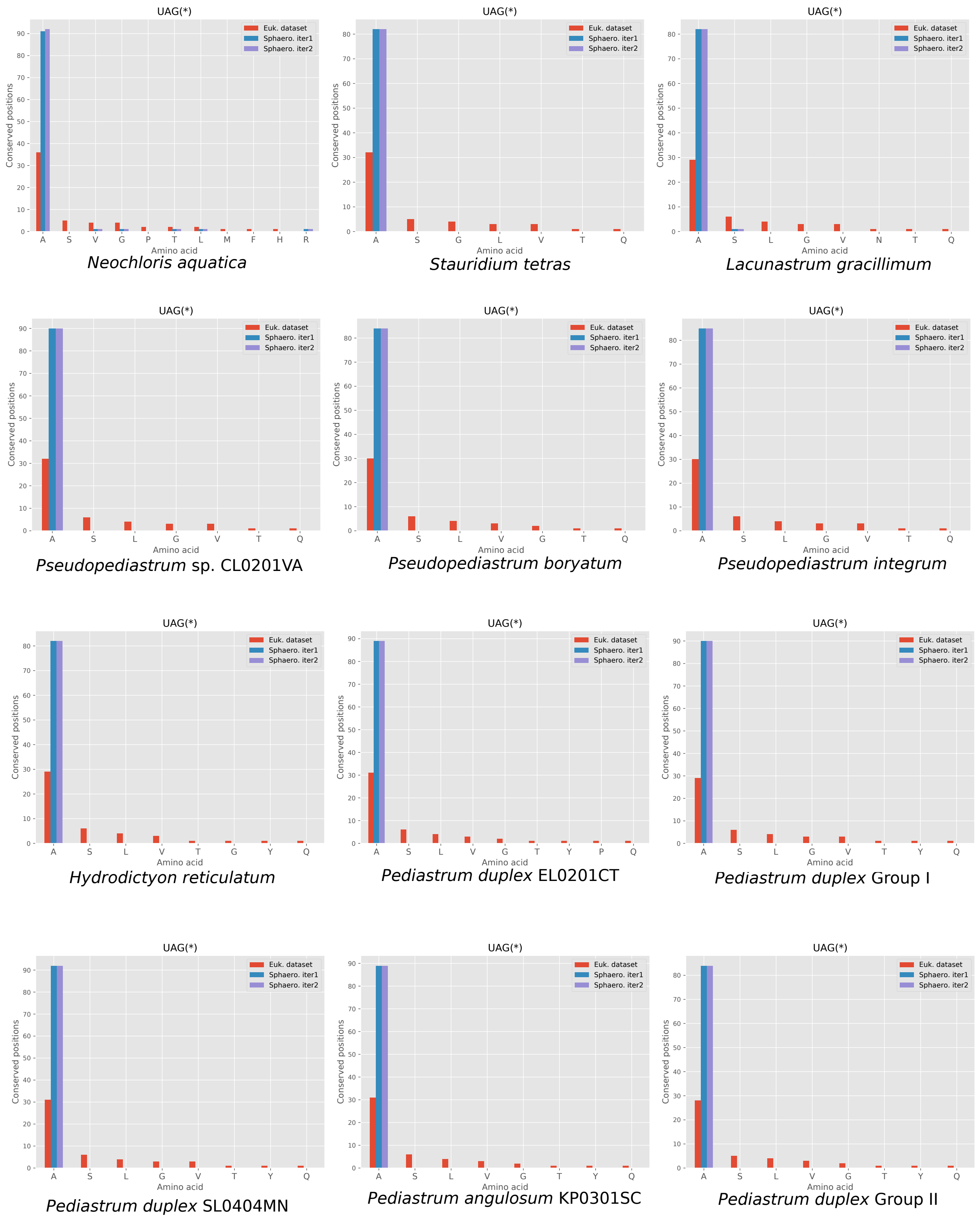


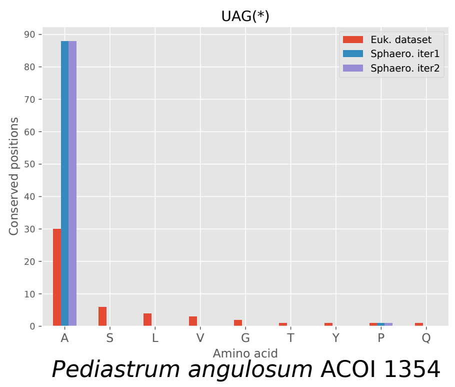


**Fig. S2.** UAG as an alanine codon in mitochondria of Hydrodictyaceae and *Neochloris aquatica*. The meaning of the plots is analogous to those shown in supplementary fig. S1.

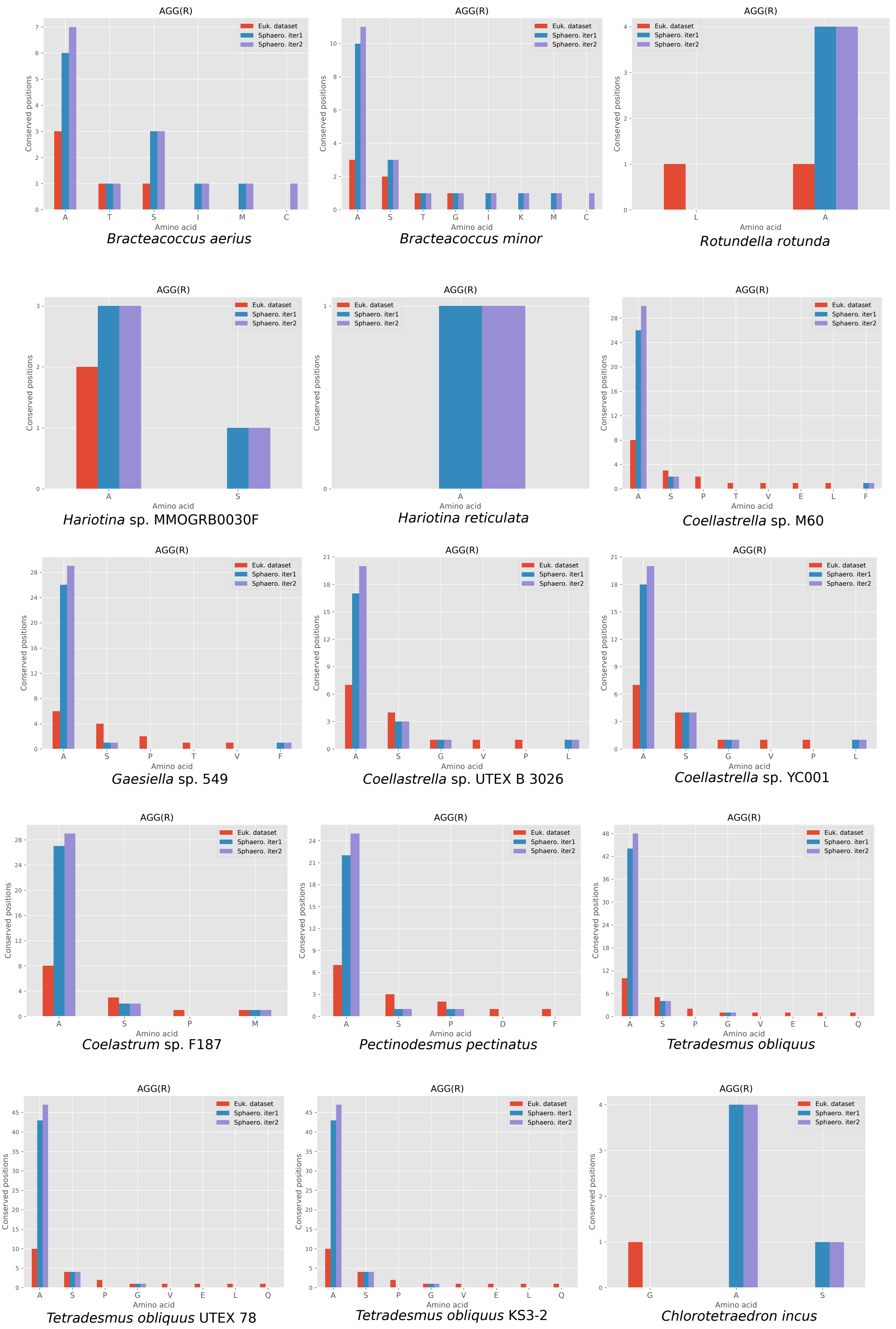


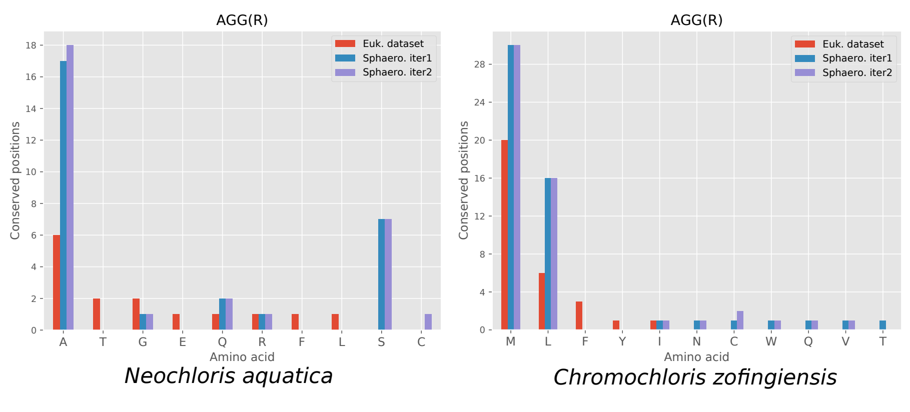


**Fig. S3.** Reassignments of the AGG codon in mitochondria of various Sphaeropleales. The meaning of the plots is analogous to those shown in supplementary fig. S1. Note the obvious signal for Arg-to-Ala reassignment of their AGG codon in all species included, except for *Chromochloris zofingiensis*, where the codon most likely means methionine.

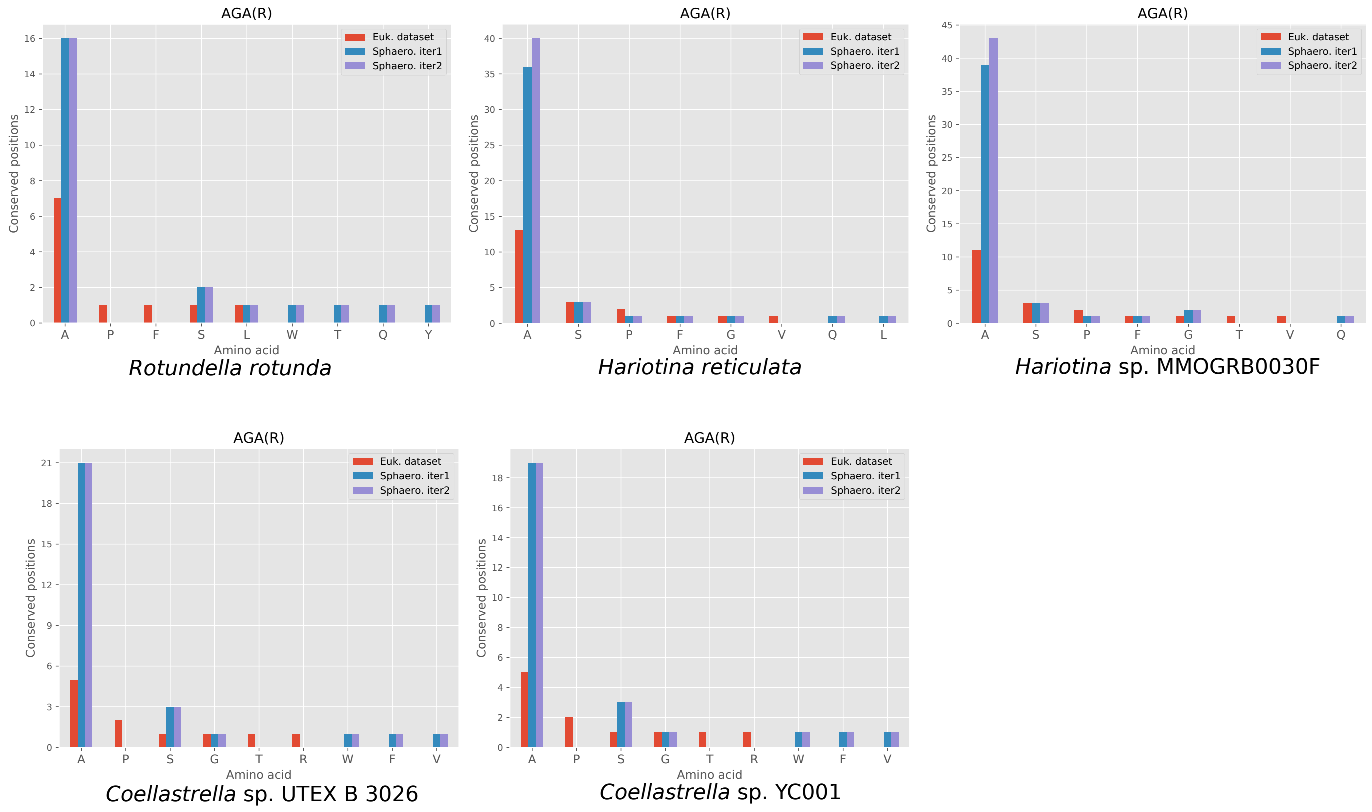


**Fig. S4.** Arg-to-Ala reassignment of the AGA codon in mitochondria of various Sphaeropleales. The meaning of the plots is analogous to those shown in supplementary fig. S1.

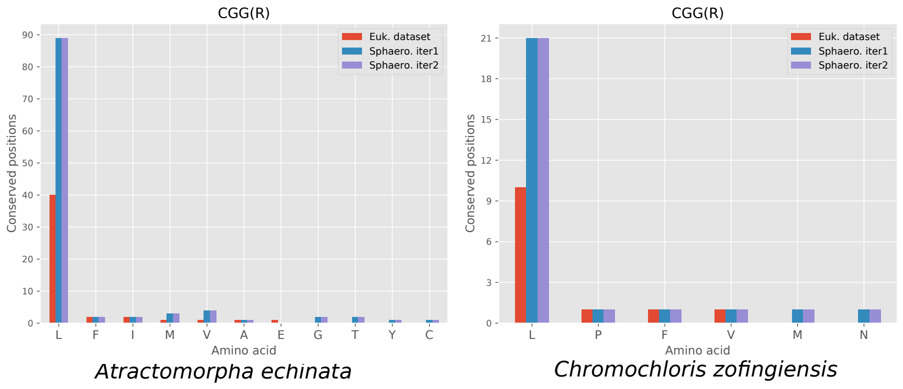


**Fig. S5.** Arg-to-Leu reassignment of the CGG codon in mitochondria of *Atractomorpha echinata* and *Chromochloris zofingiensis*. The meaning of the plots is analogous to those shown in supplementary fig. S1.

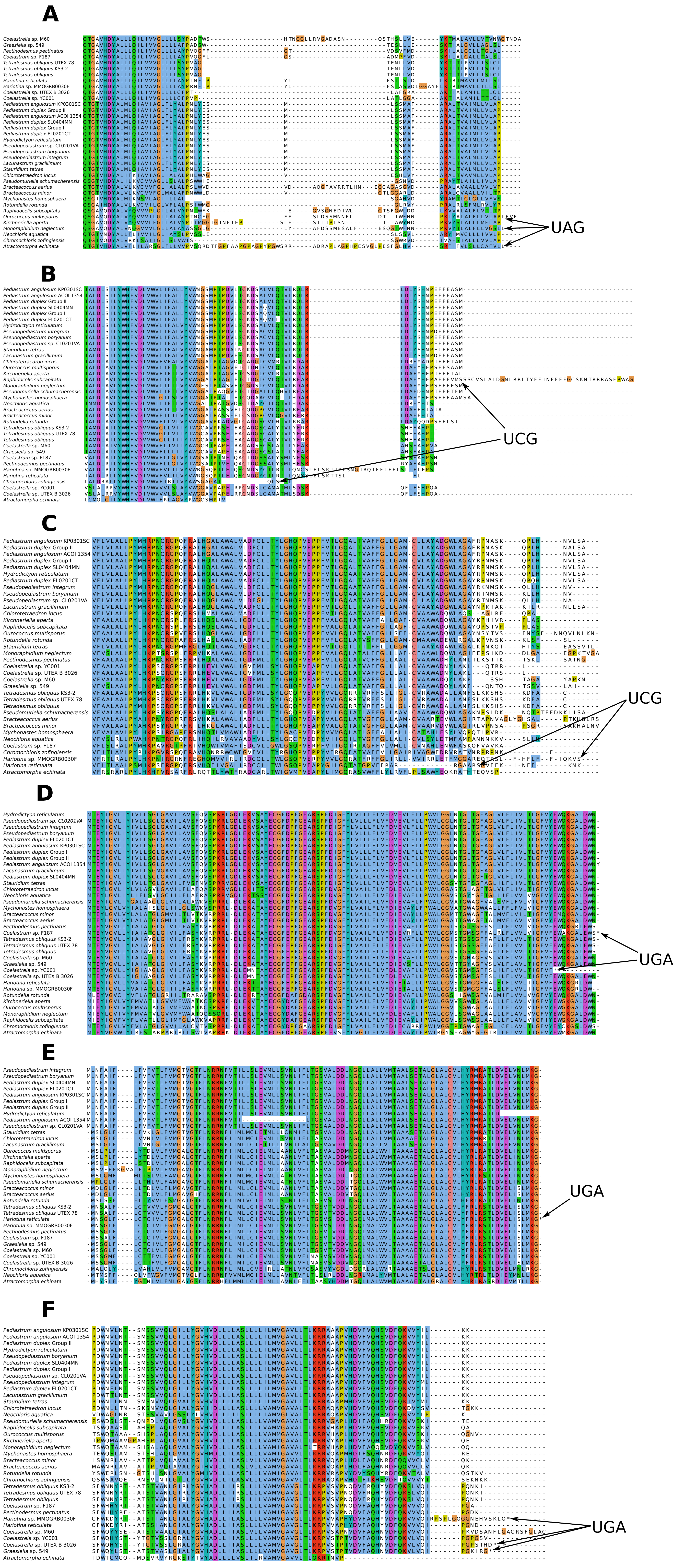


**Fig. S6.** Reassessment of termination codons in several sphaeroplealean mitochondrial genes. A) UAG is the likely termination codon of the *nad5* gene of some Sphaeropleales. B) UCG serves as a termination codon in the *cox3* gene of *Raphidocelis subcapitata*. C) UCG as a potential termination codon in the *cob* gene of *Hariotina* spp. D) UGA as a termination codon in the *nad3* gene of *Coelastrella* sp. YC001 and *Coelastrum* sp. F187. E) UGA as a termination codon in the *nad4L* gene of *Hariotina reticulata*. F) UGA as a termination codon in the *nad6* gene of *Hariotina* sp. MMOGRB0030F, *Graesiella* sp. 549 and *Coelastrella* sp. UTEX B 3026. The alignments are based on conceptual translations (protein sequences) presently featured in GenBank, i.e. obtained using NCBI translation table 22 (except for sequences from *Atractomorpha echinata*, which were inferred using the translation table 16). The presumed positions of the termination codons are indicated by arrows.

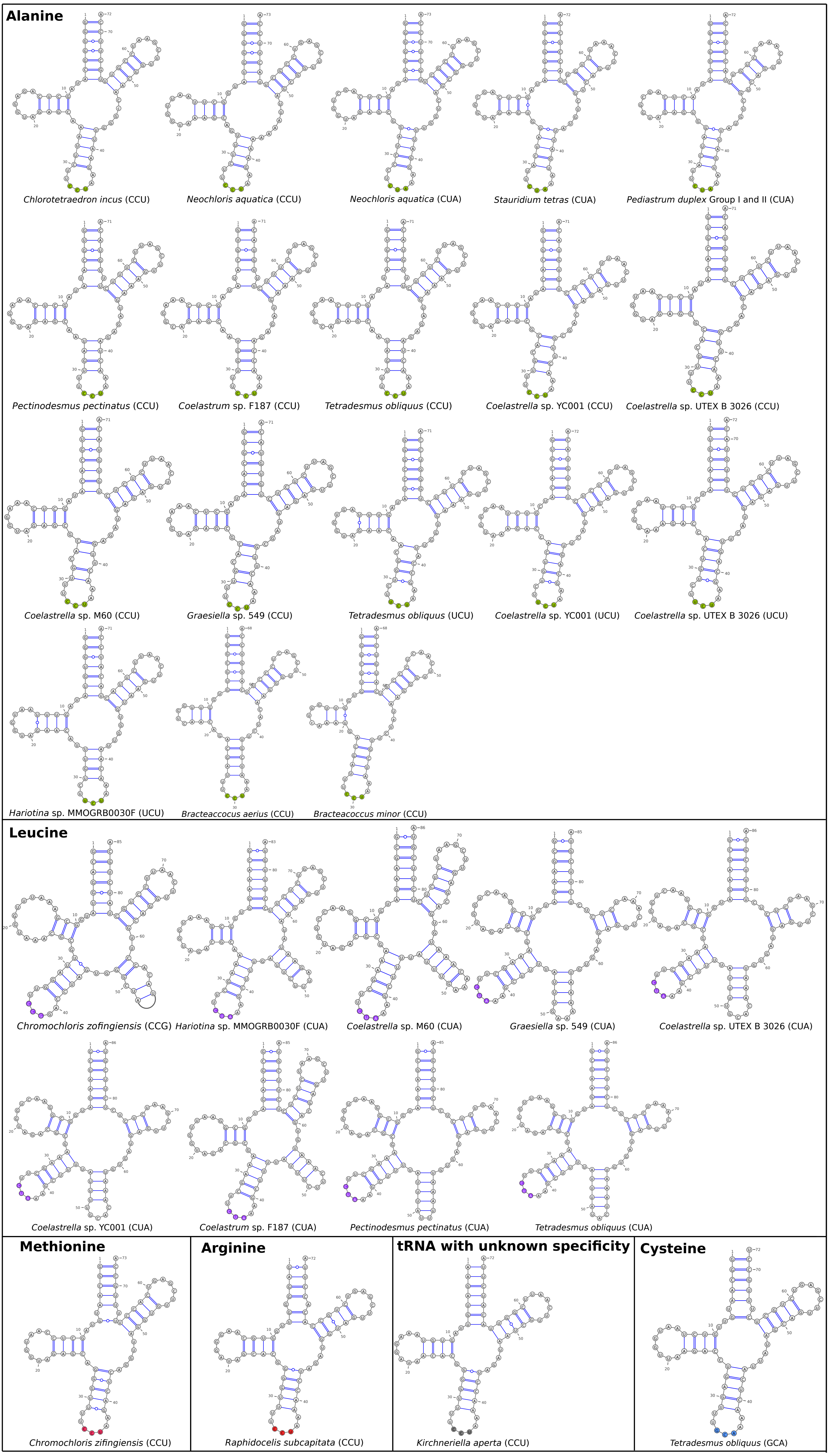


**Fig. S7.** Predicted structures of tRNAs presumably mediating predicted codon reassignments in Sphaeropleales. The models were obtained using tRNAscan-SE v2.0. Nucleotides corresponding to the anticodon are highlighted in colour. The tRNAs are grouped based on the amino acid most likely charged to them, which is inferred based on evaluation by tRNAscan-SE, phylogenetic position of the tRNA (supplementary fig. S8), and the likely meaning of the cognate codon derived from the analysis of its occurrence at conserved amino acid positions (supplementary figs. S1-S5). The tRNA from *Kirchneriella aperta* with the CCU anticodon is closely related to the putative $tRNA^{Arg}$(CCU) from *Raphidocelis subcapitata* (supplementary fig. S8), but tRNAscan-SE failed to assign its amino acid specificity with any confidence and the cognate codon (AGG) is absent from standard mitochondrial genes in this species, making the functionality of the tRNA uncertain. The tRNA from *Tetradesmus obliquus* with the GCA anticodon is shown as a representative of standard $tRNA^{Cys}$ in Scenedesminia.

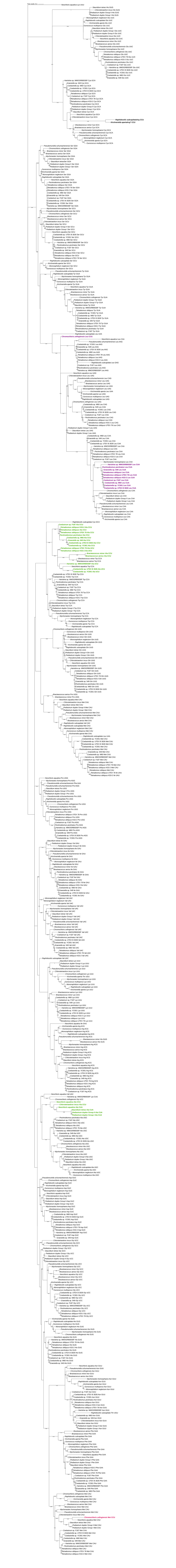

**Fig. S8.** Phylogenetic tree of tRNA genes in sphaeroplealean mitogenomes. The tree was inferred using the maximum likelihood method (GTR+G substitution model) from a manually trimmed multiple alignment generated using the LocARNA software. Numbers at branches indicate bootstrap support values (on a scale 0-1.0). The tRNAs highlighted by boldface are novel lineage-specific tRNA species, usually with a combination of the predicted amino acid specificity (distinguished by different colours) and the anticodon disagreeing with the standard genetic code (see also supplementary fig. S7) but in accord with evidence for reassignment of the respective codons in the give taxa (see supplementary figs. S1-S5).

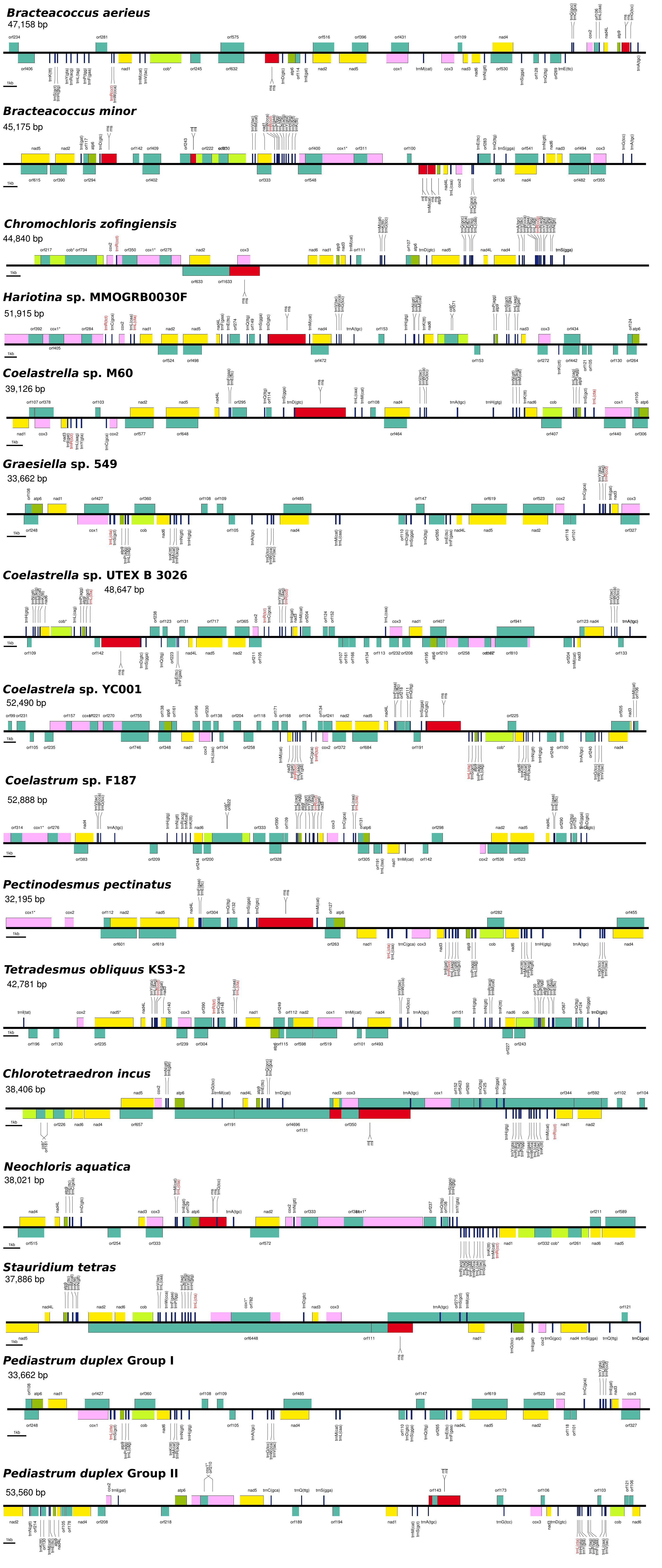


**Fig. S9.** Genomic location of genes for novel tRNAs implicated in decoding reassigned codons in sphaeroplealen mitogenomes. The figure shows linearized mitogenome maps (based on reannotation of the genome sequences using MFannot), with the salient tRNA genes highlighted in red. Accession numbers of the mitogenome sequences are provided in supplementary table S1. For complexes of closely related taxa (different strains of the same species) only one representative is included.

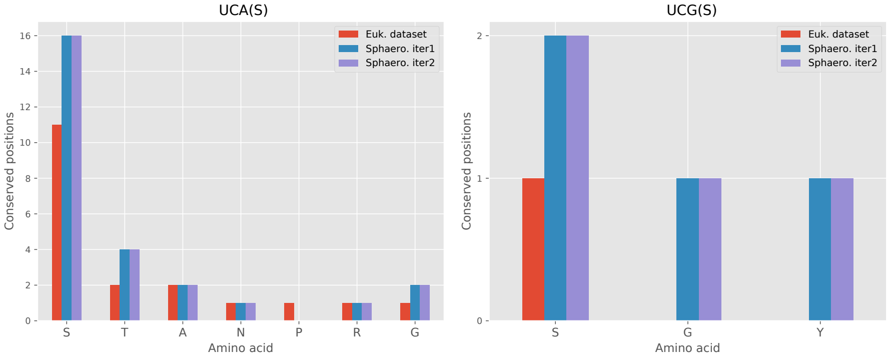


**Fig. S10.** The meaning of UCA and UCG codon in *Atractomorpha echinata*. In contrast to other Sphaeropleales, these two codons do not serve as termination ones and encode clearly (UCA) and probably (UCG) the standard amino acid serine. The meaning of the plots is analogous to those shown in supplementary fig. S1.
