# Supplementary material for: Evolution and Unprecedented Variants of the Mitochondrial Genetic Code in a Lineage of Green Algae
Source: Genome Biol Evol. 2019 Oct 16;11(10):2992–3007. doi: 10.1093/gbe/evz210 (PMC6821328; doi:10.1093/gbe/evz210)
Supplement: evz210_Supplementary_Data [file Supplemenrary_dataset_S1.pdf › Supplemenrary_dataset_S1.pdf]

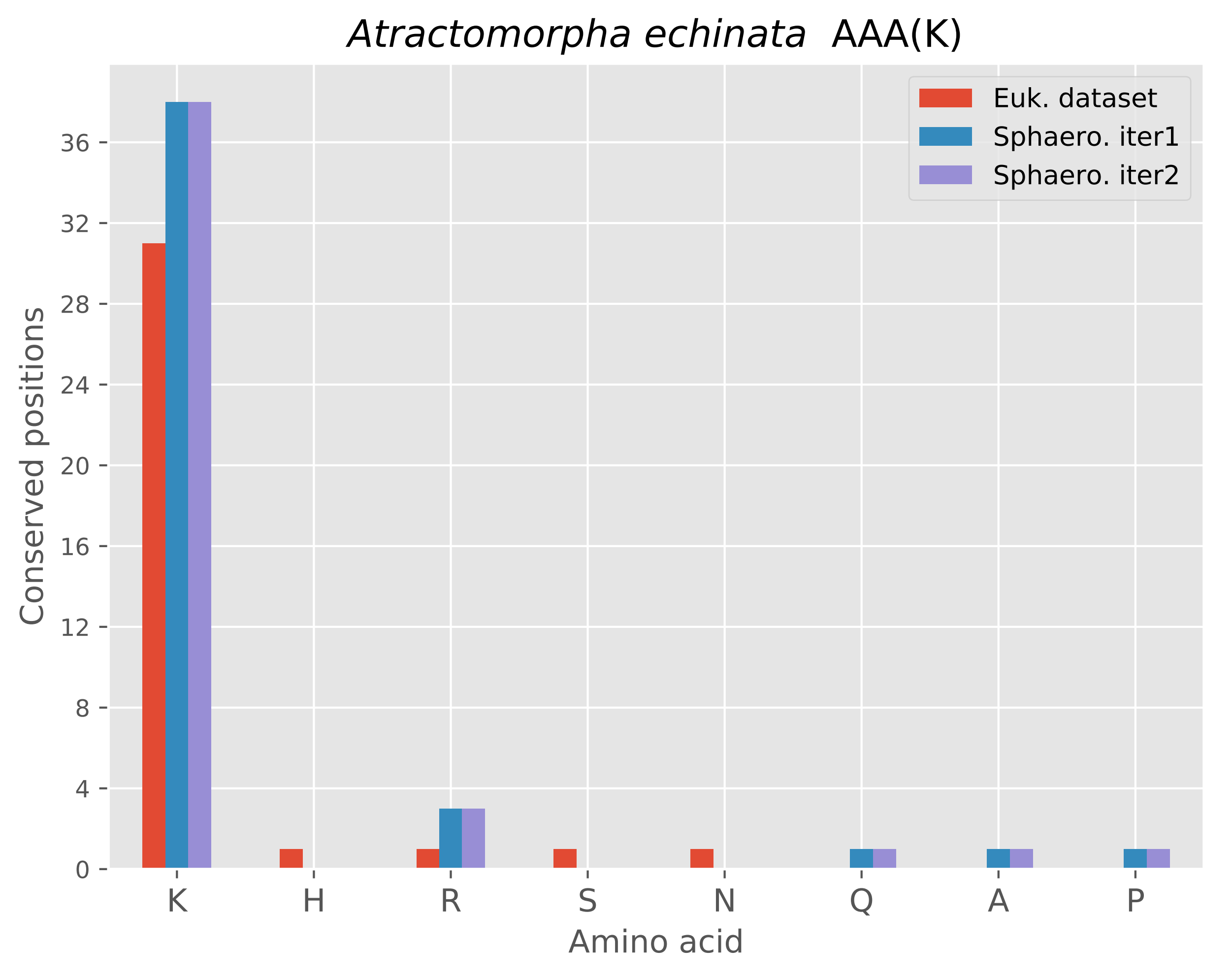

Atractomorpha echinata AAA(K)
Euk. dataset
Sphaero. iter1
Sphaero. iter2
Conserved positions
0
4
8
12
16
20
24
28
32
36
K
H
R
S
N
Q
A
P
Amino acid

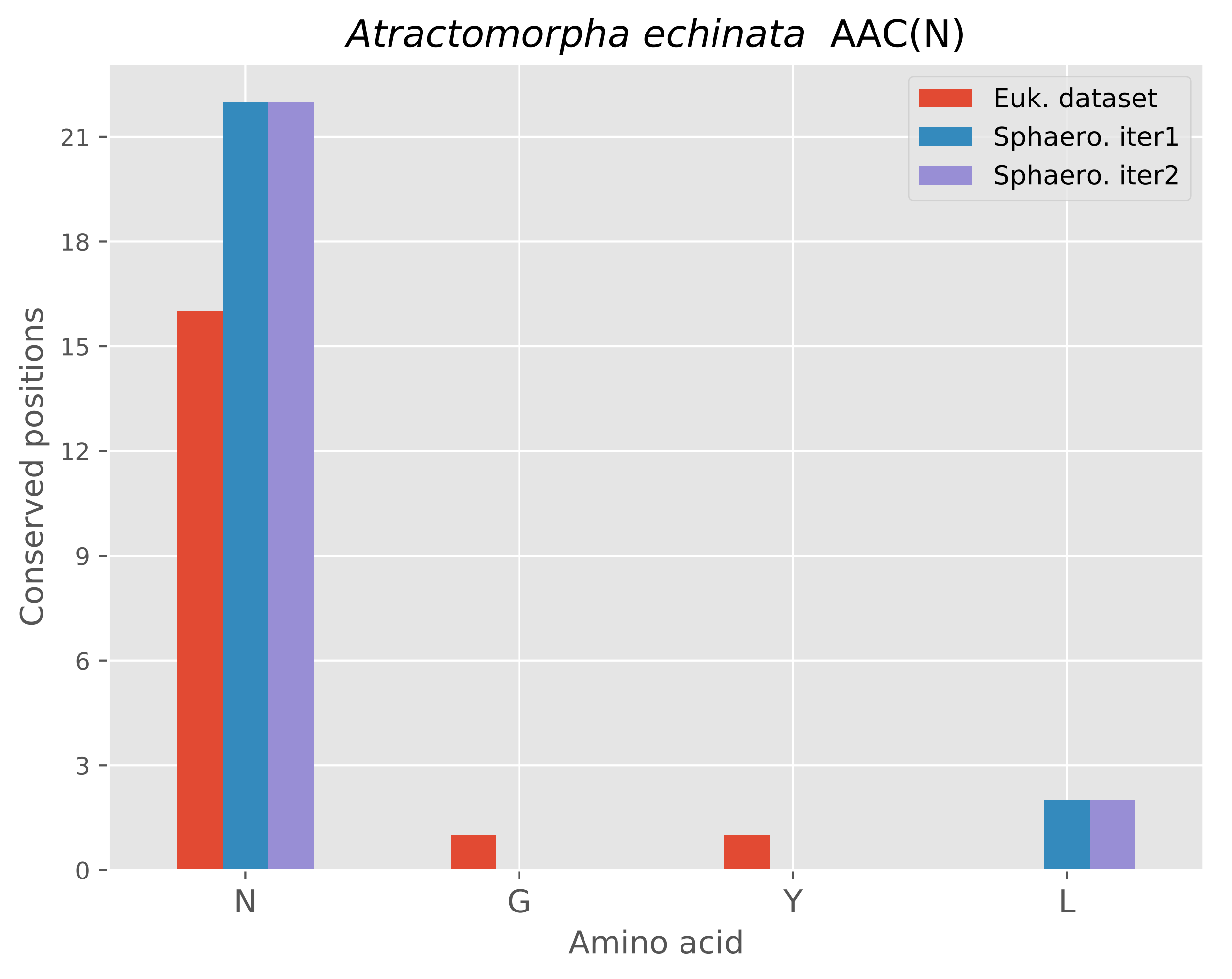

Atractomorpha echinata AAC(N)
Euk. dataset
Sphaero. iter1
Sphaero. iter2
Conserved positions
0
3
6
9
12
15
18
21
N
G
Y
L
Amino acid

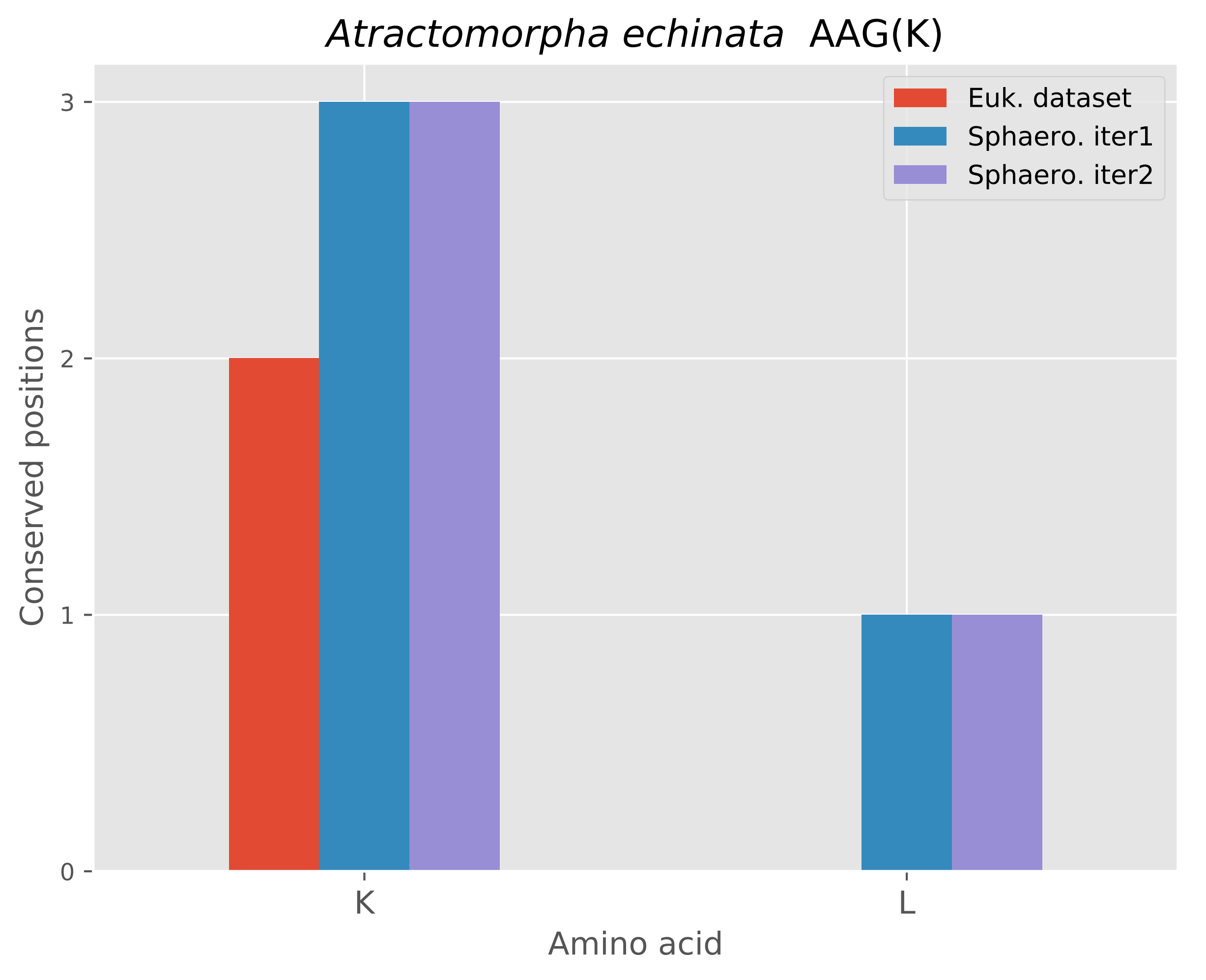

Atractomorpha echinata AAG(K)
Euk. dataset
Sphaero. iter1
Sphaero. iter2
Conserved positions
0
1
2
3
K
L
Amino acid

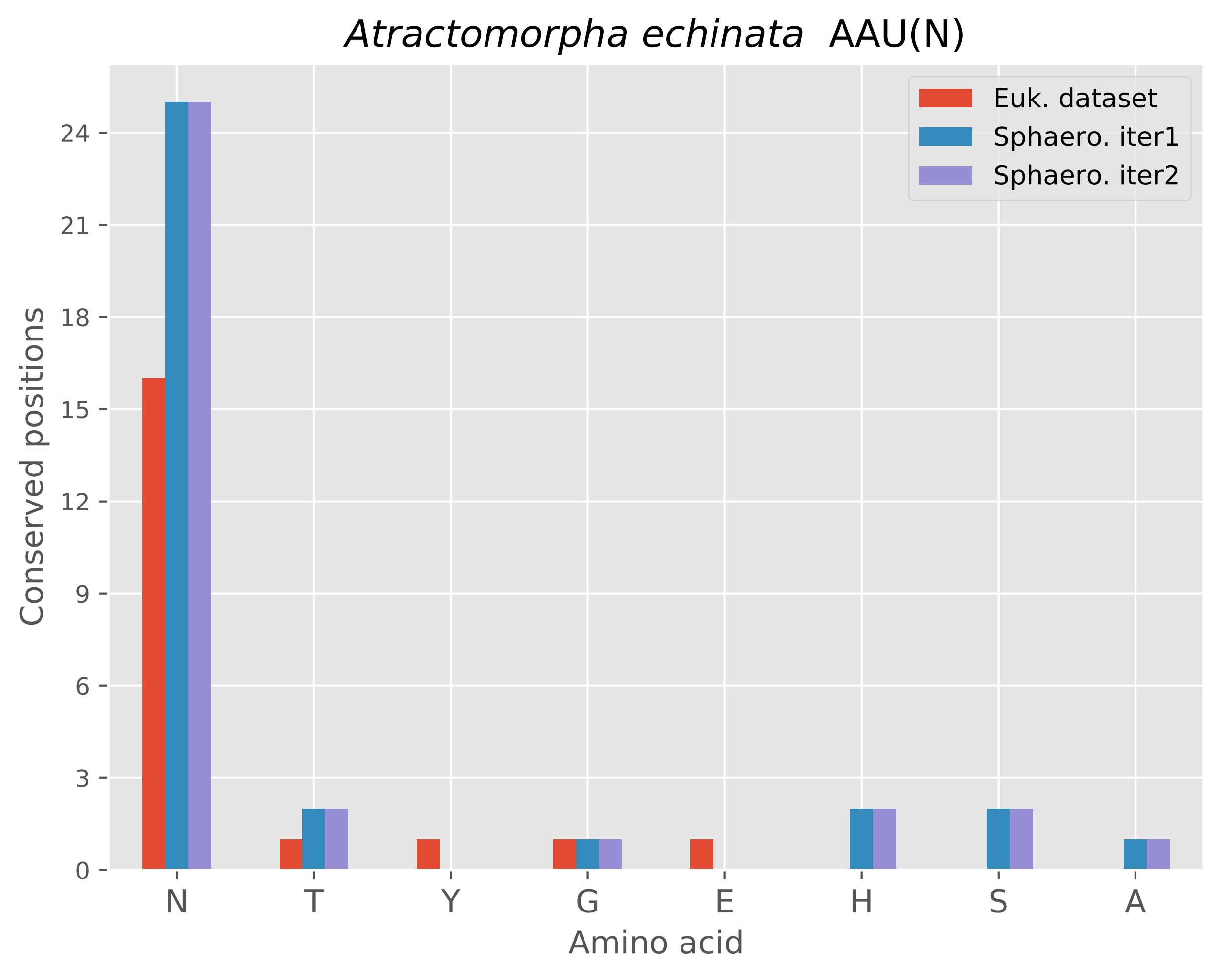

Atractomorpha echinata AAU(N)
Euk. dataset
Sphaero. iter1
Sphaero. iter2
Conserved positions
0
3
6
9
12
15
18
21
24
N
T
Y
G
E
H
S
A
Amino acid

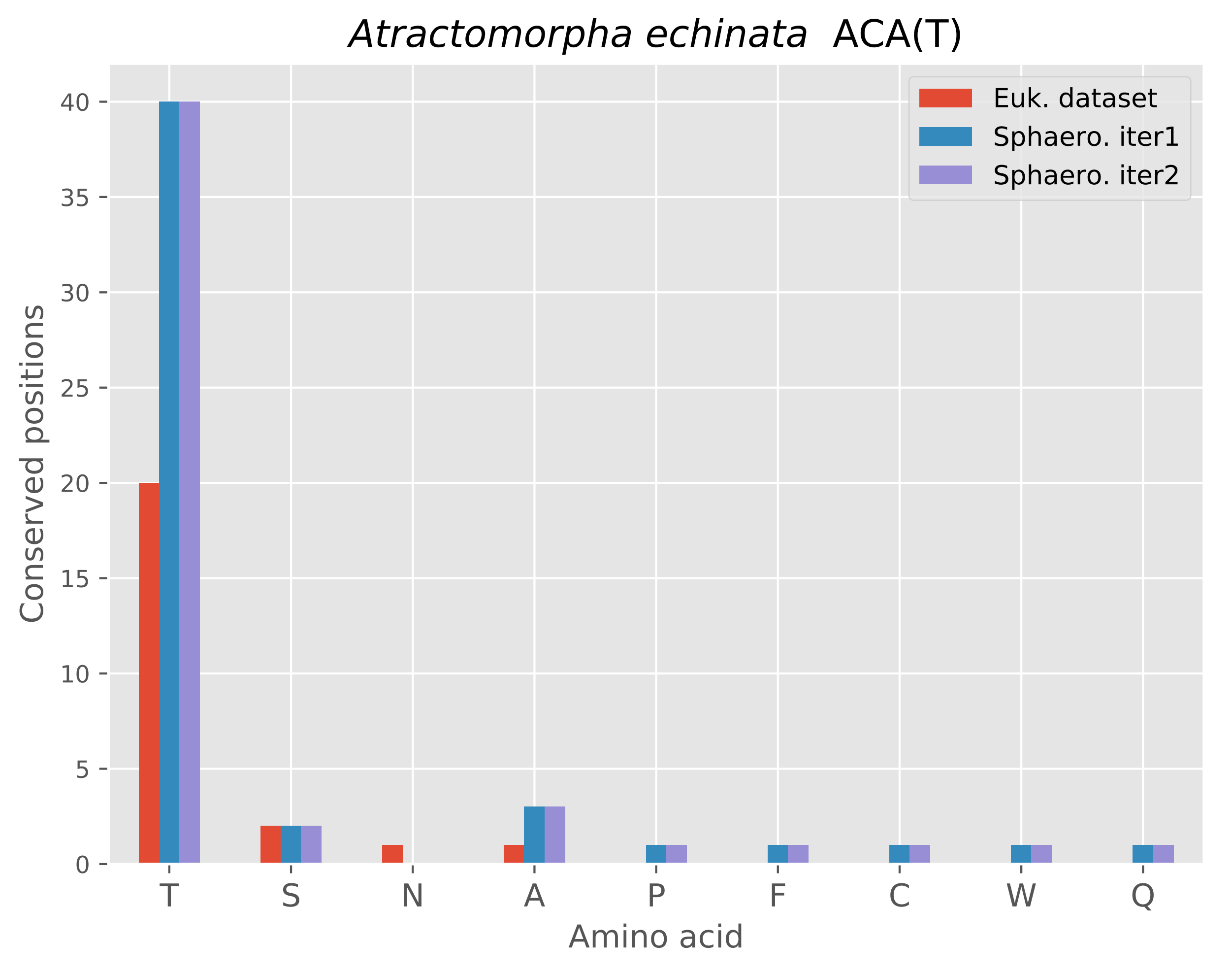

Atractomorpha echinata ACA(T)
Euk. dataset
Sphaero. iter1
Sphaero. iter2
Conserved positions
0
5
10
15
20
25
30
35
40
T
S
N
A
P
F
C
W
Q
Amino acid

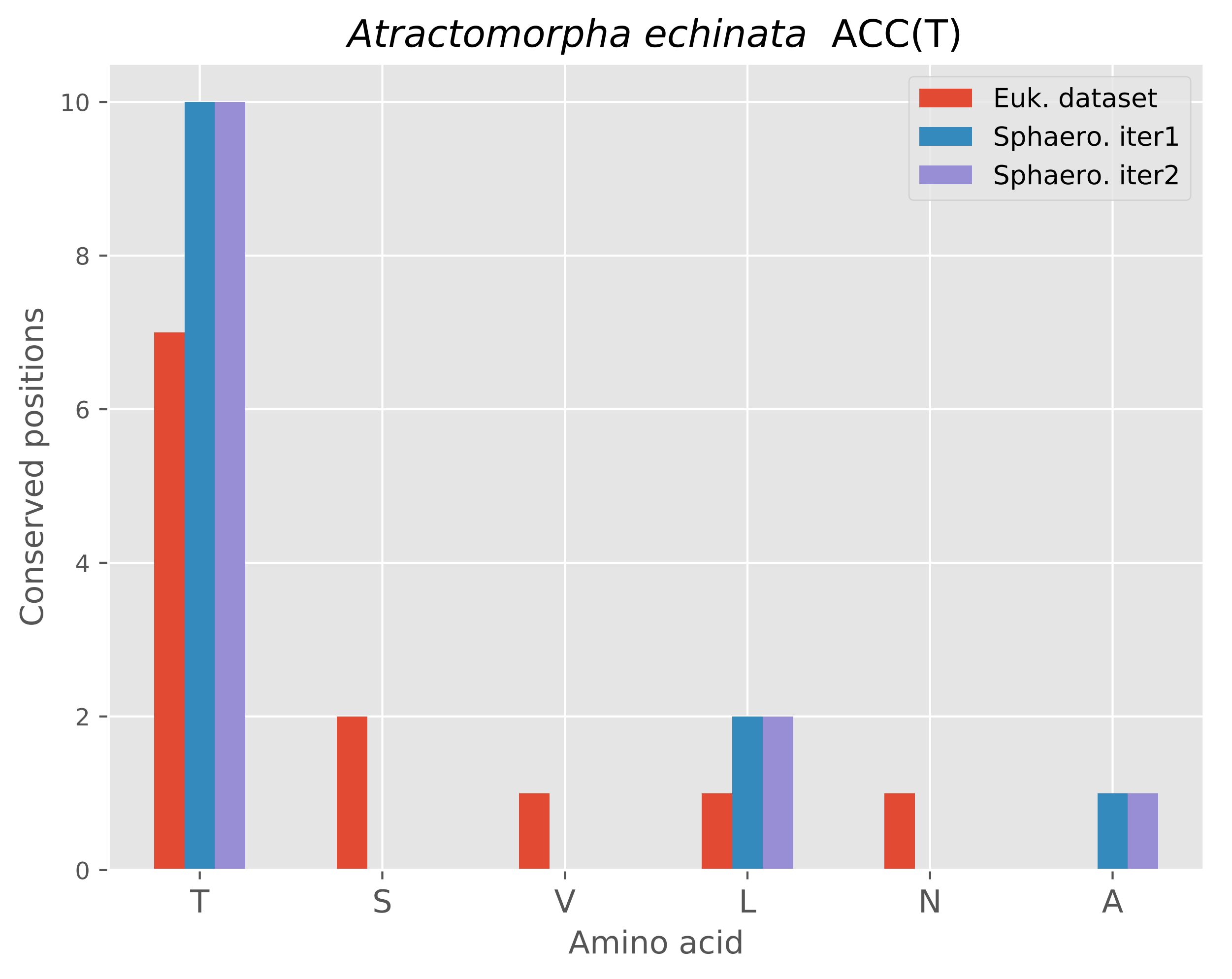

Atractomorpha echinata ACC(T)
Euk. dataset
Sphaero. iter1
Sphaero. iter2
Conserved positions
0
2
4
6
8
10
T
S
V
L
N
A
Amino acid

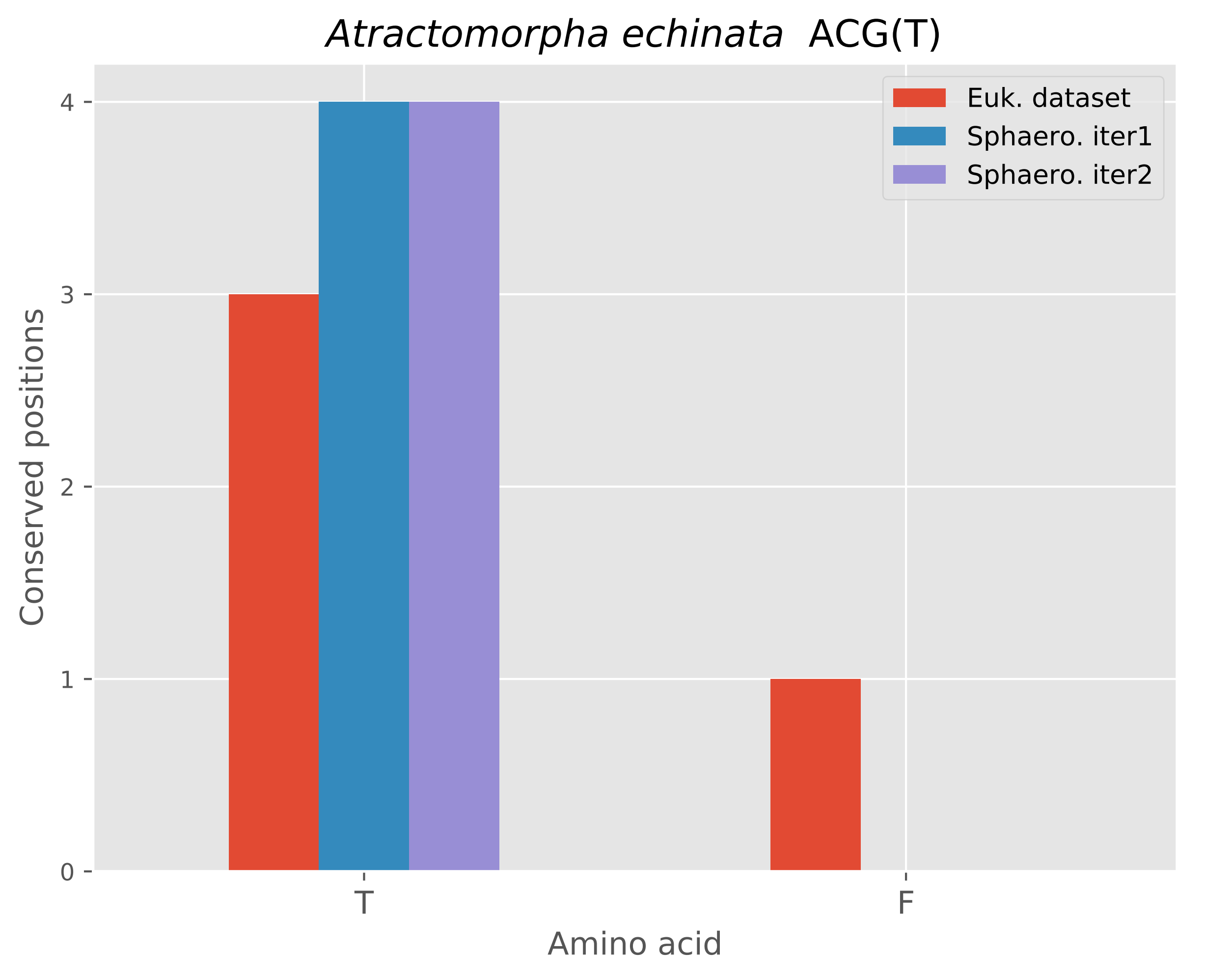

Atractomorpha echinata ACG(T)
Euk. dataset
Sphaero. iter1
Sphaero. iter2
Conserved positions
0
1
2
3
4
T
F
Amino acid

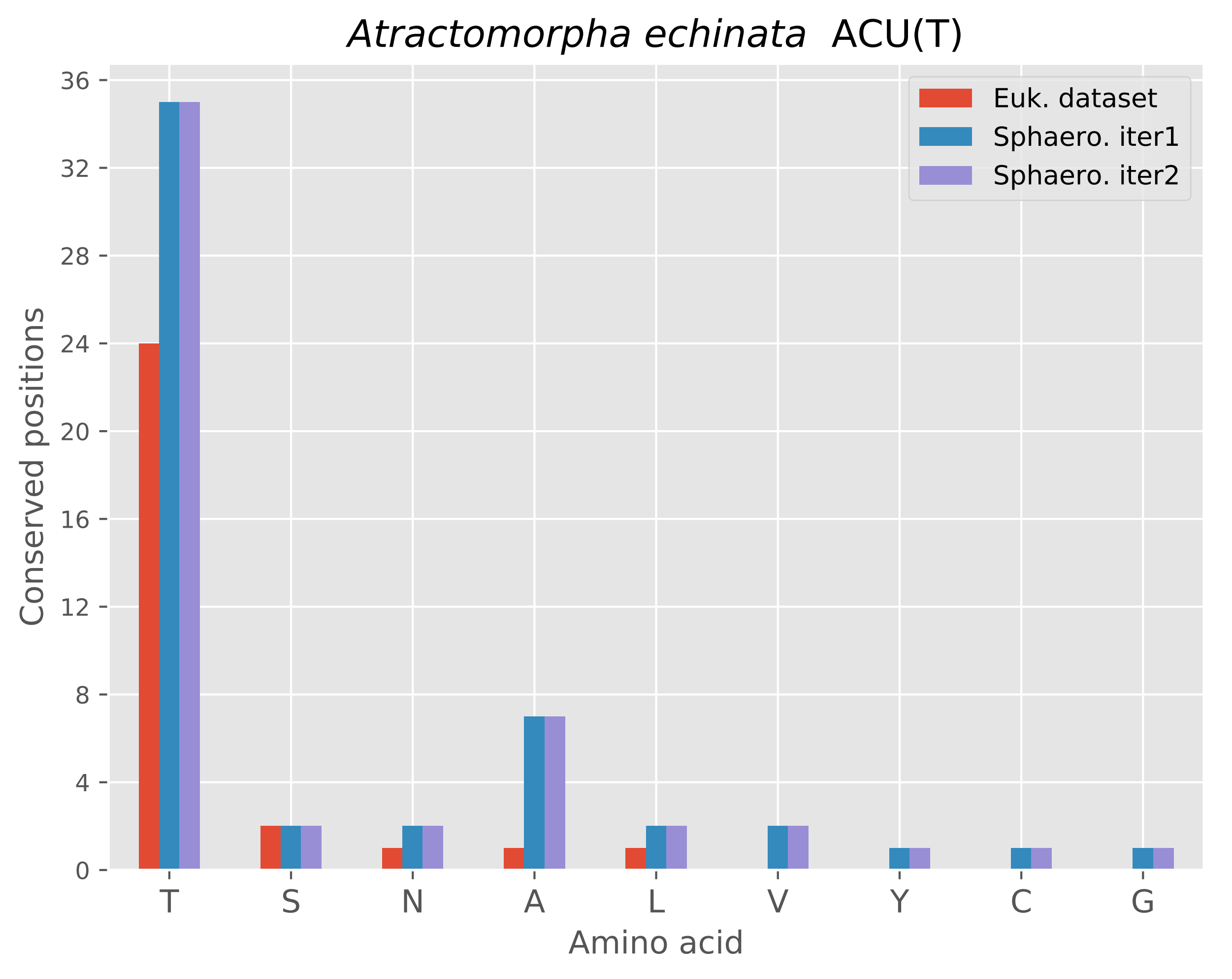

Atractomorpha echinata ACU(T)
Euk. dataset
Sphaero. iter1
Sphaero. iter2
Conserved positions
0
4
8
12
16
20
24
28
32
36
T
S
N
A
L
V
Y
C
G
Amino acid

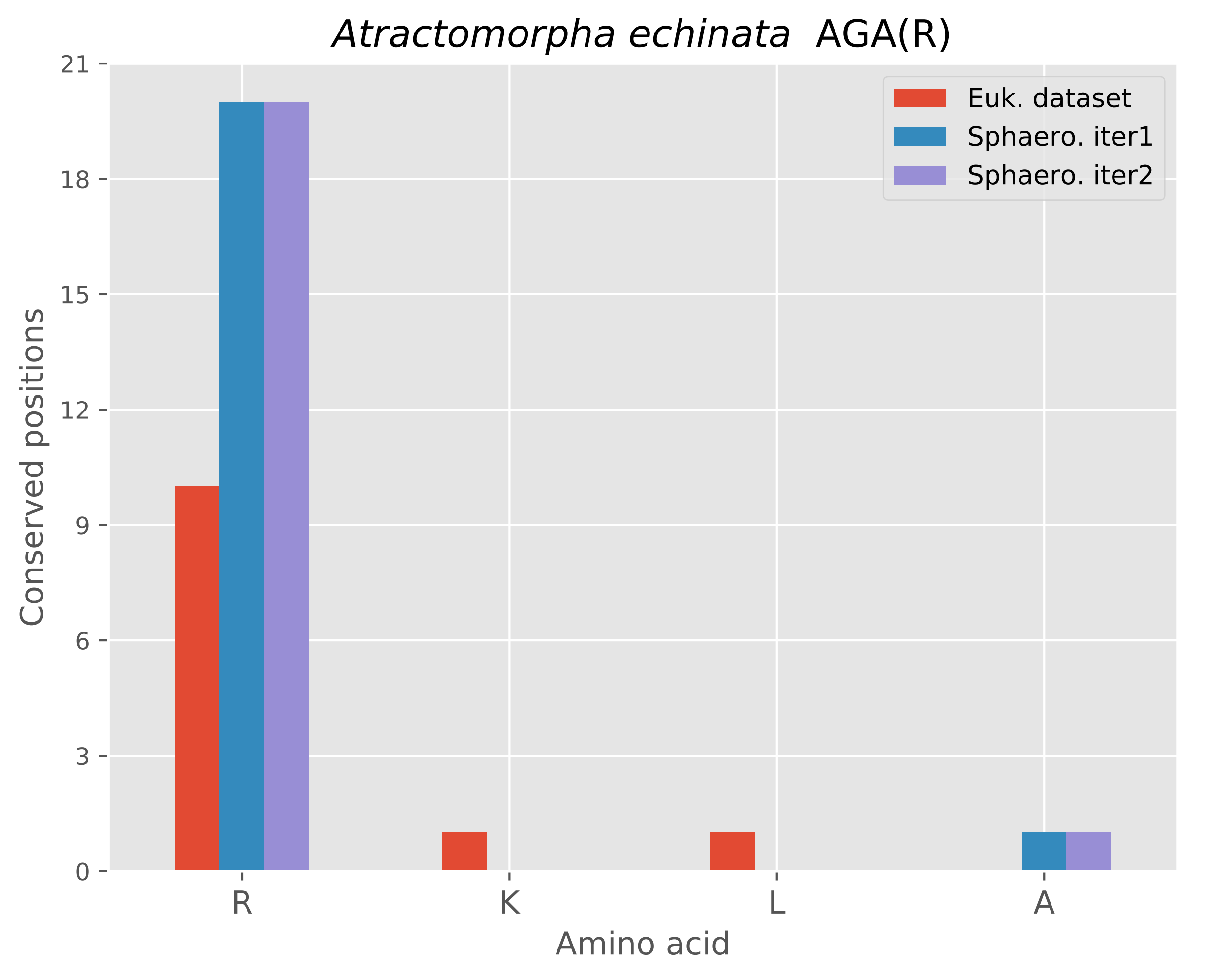

Atractomorpha echinata AGA(R)
Euk. dataset
Sphaero. iter1
Sphaero. iter2
Conserved positions
0
3
6
9
12
15
18
21
R
K
L
A
Amino acid

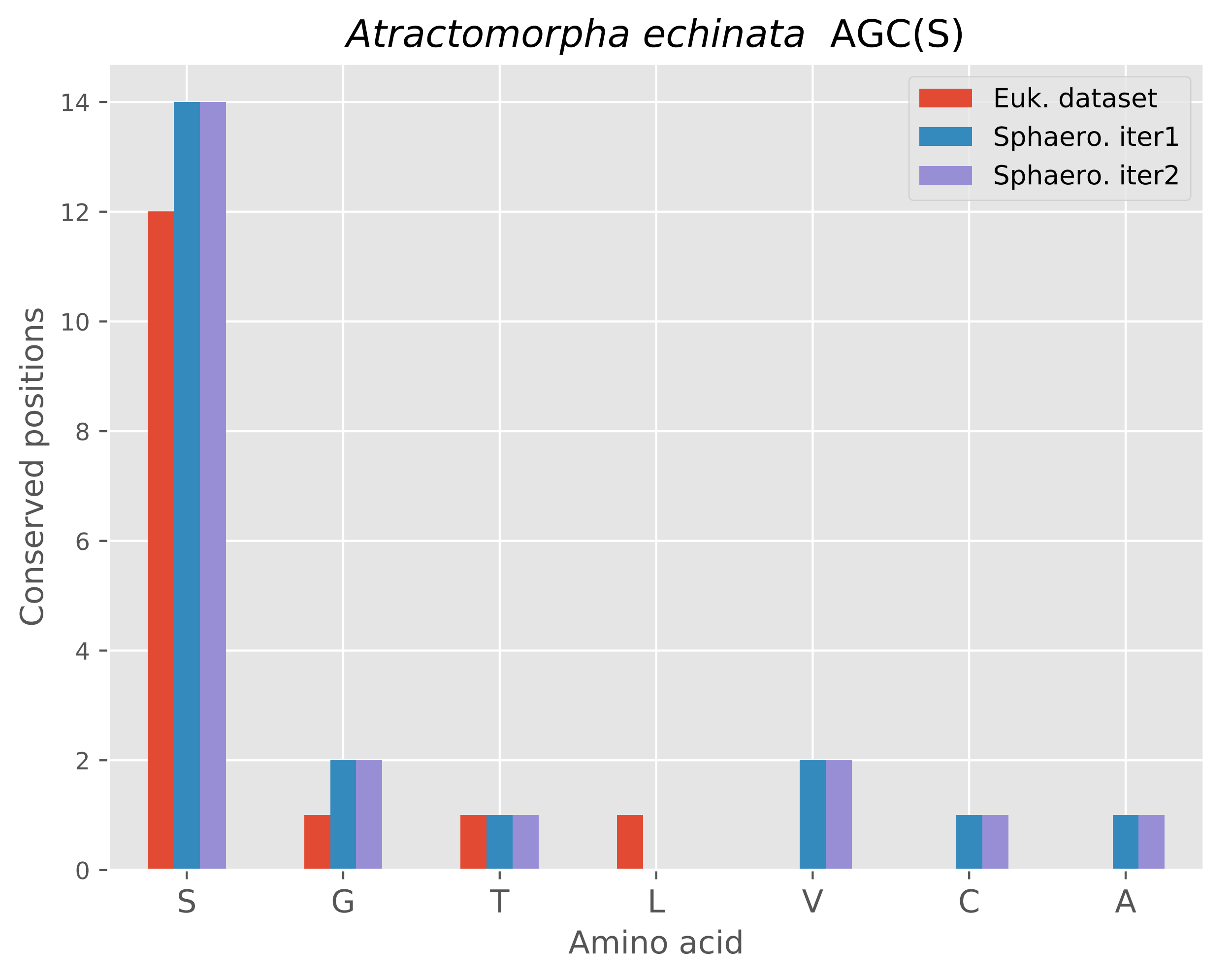

Atractomorpha echinata AGC(S)
Euk. dataset
Sphaero. iter1
Sphaero. iter2
Conserved positions
0
2
4
6
8
10
12
14
S
G
T
L
V
C
A
Amino acid

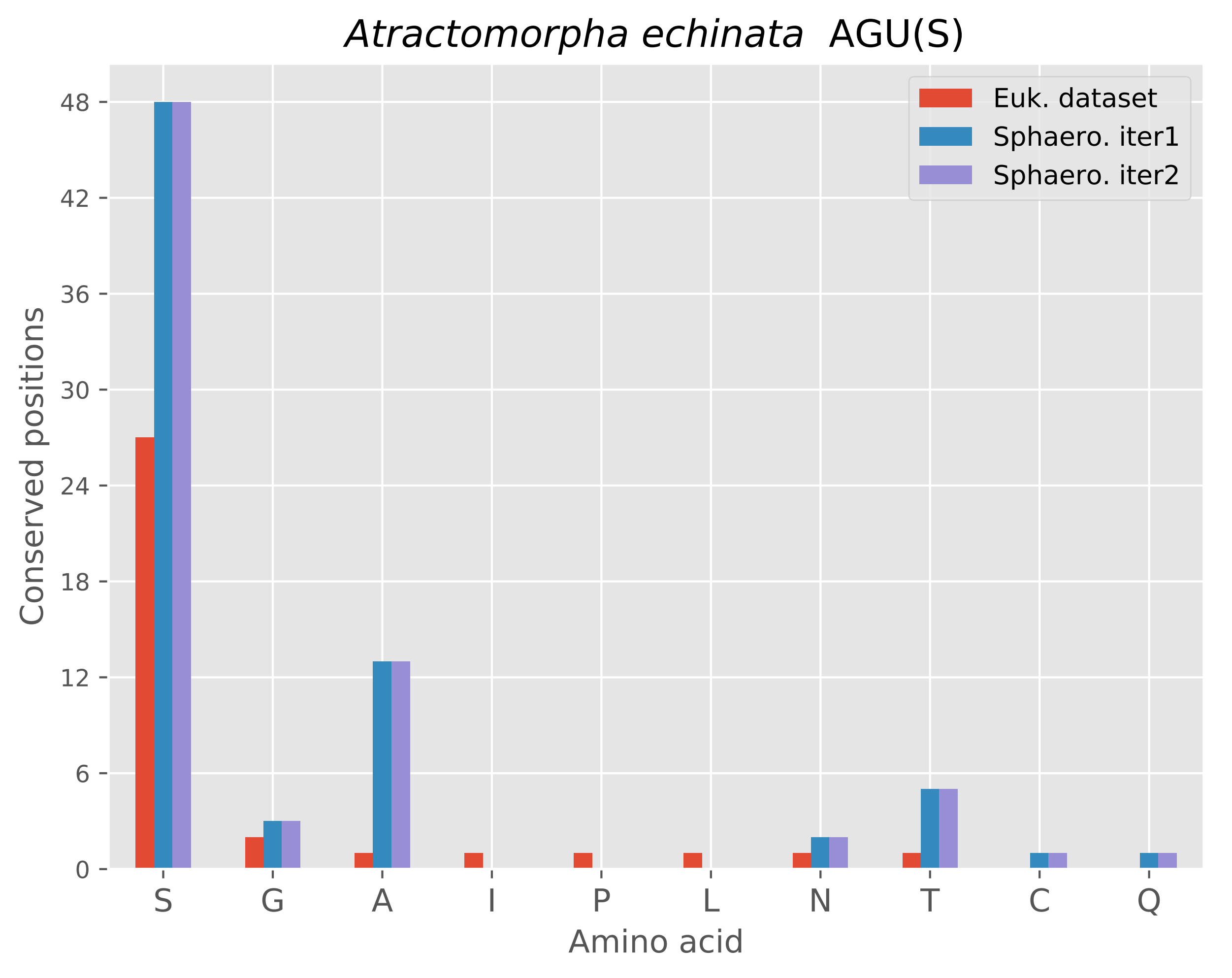

Atractomorpha echinata AGU(S)
Euk. dataset
Sphaero. iter1
Sphaero. iter2
Conserved positions
0
6
12
18
24
30
36
42
48
S
G
A
I
P
L
N
T
C
Q
Amino acid

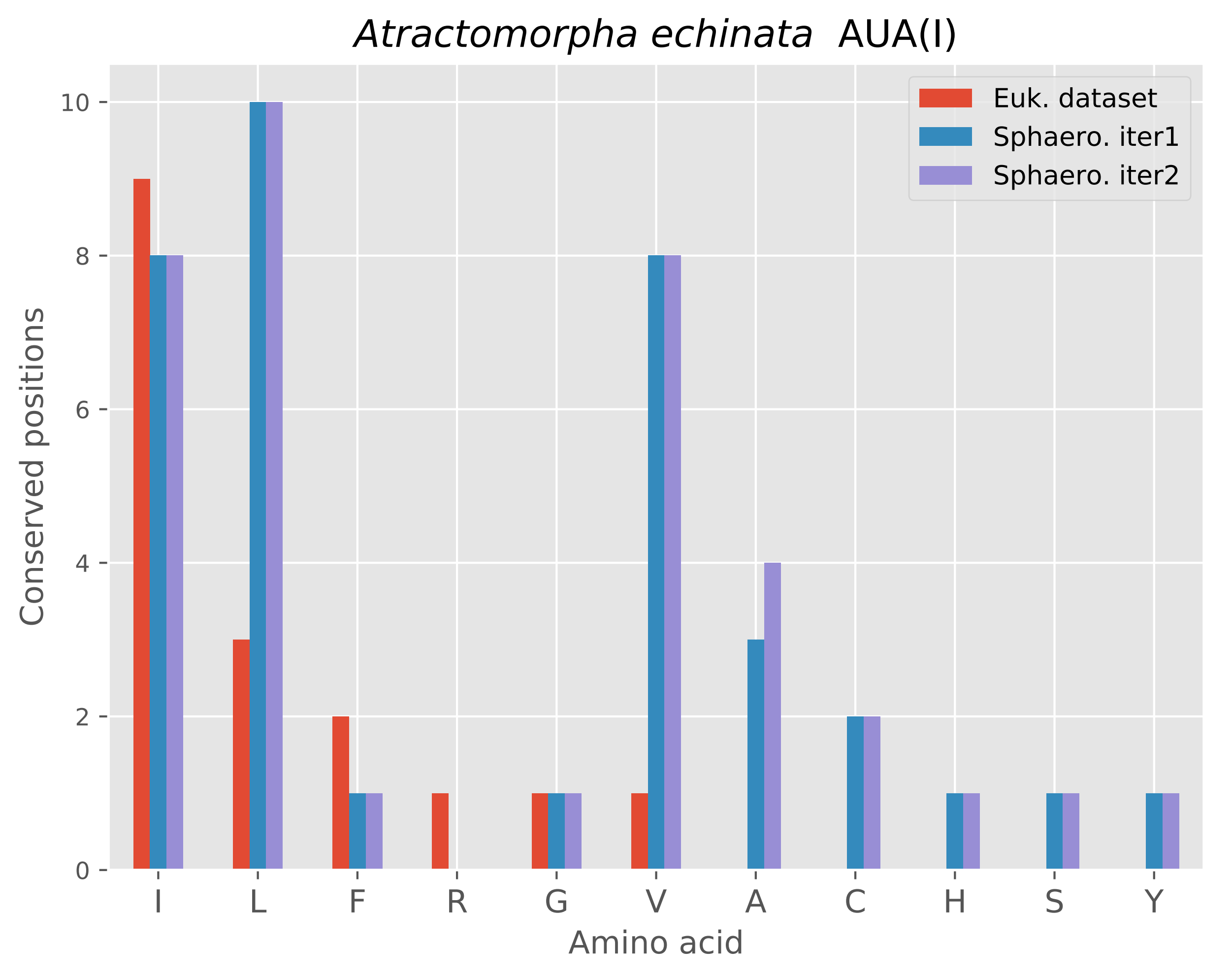

Atractomorpha echinata AUA(I)
Euk. dataset
Sphaero. iter1
Sphaero. iter2
Conserved positions
0
2
4
6
8
10
I
L
F
R
G
V
A
C
H
S
Y
Amino acid

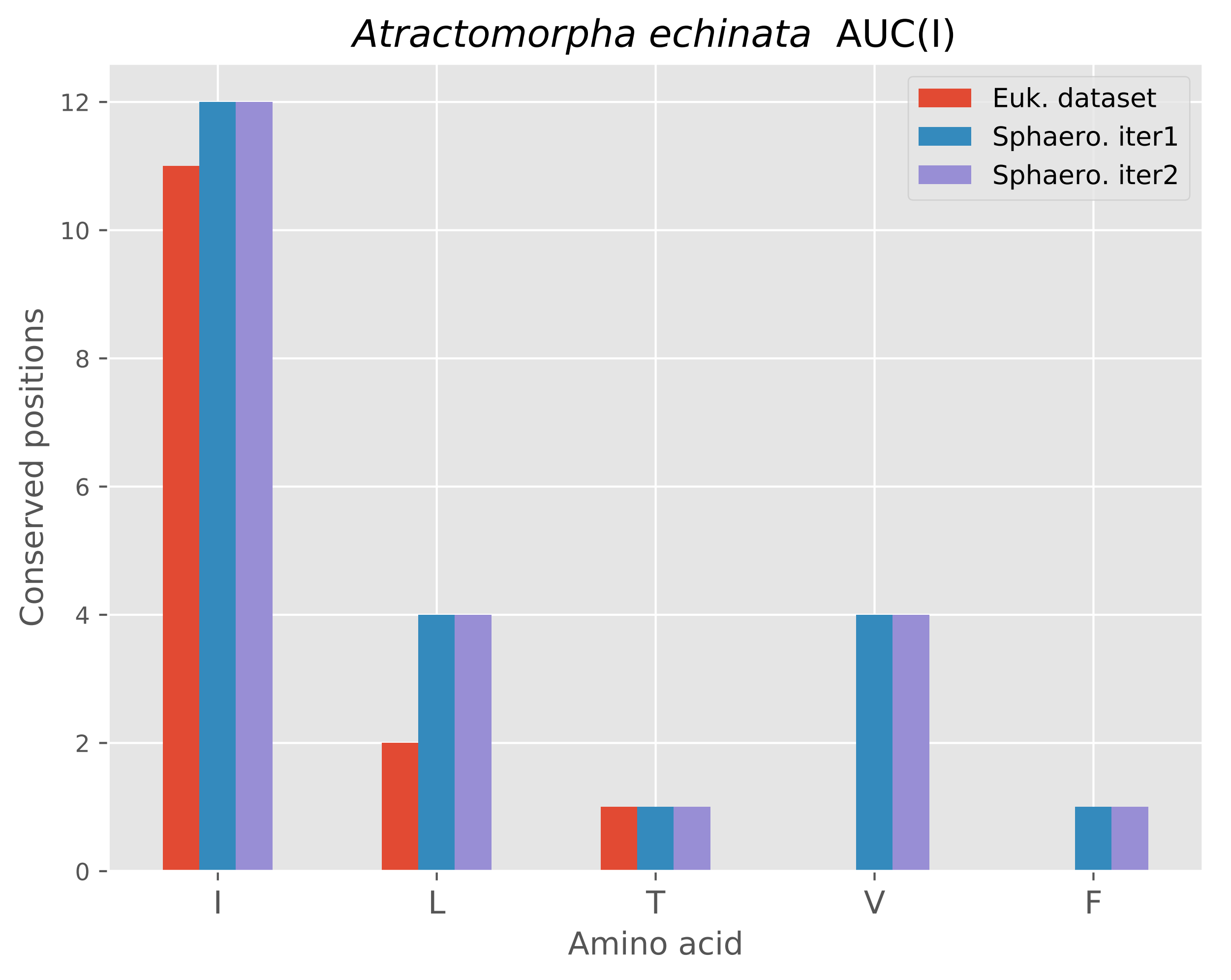

Atractomorpha echinata AUC(I)
Euk. dataset
Sphaero. iter1
Sphaero. iter2
Conserved positions
0
2
4
6
8
10
12
I
L
T
V
F
Amino acid

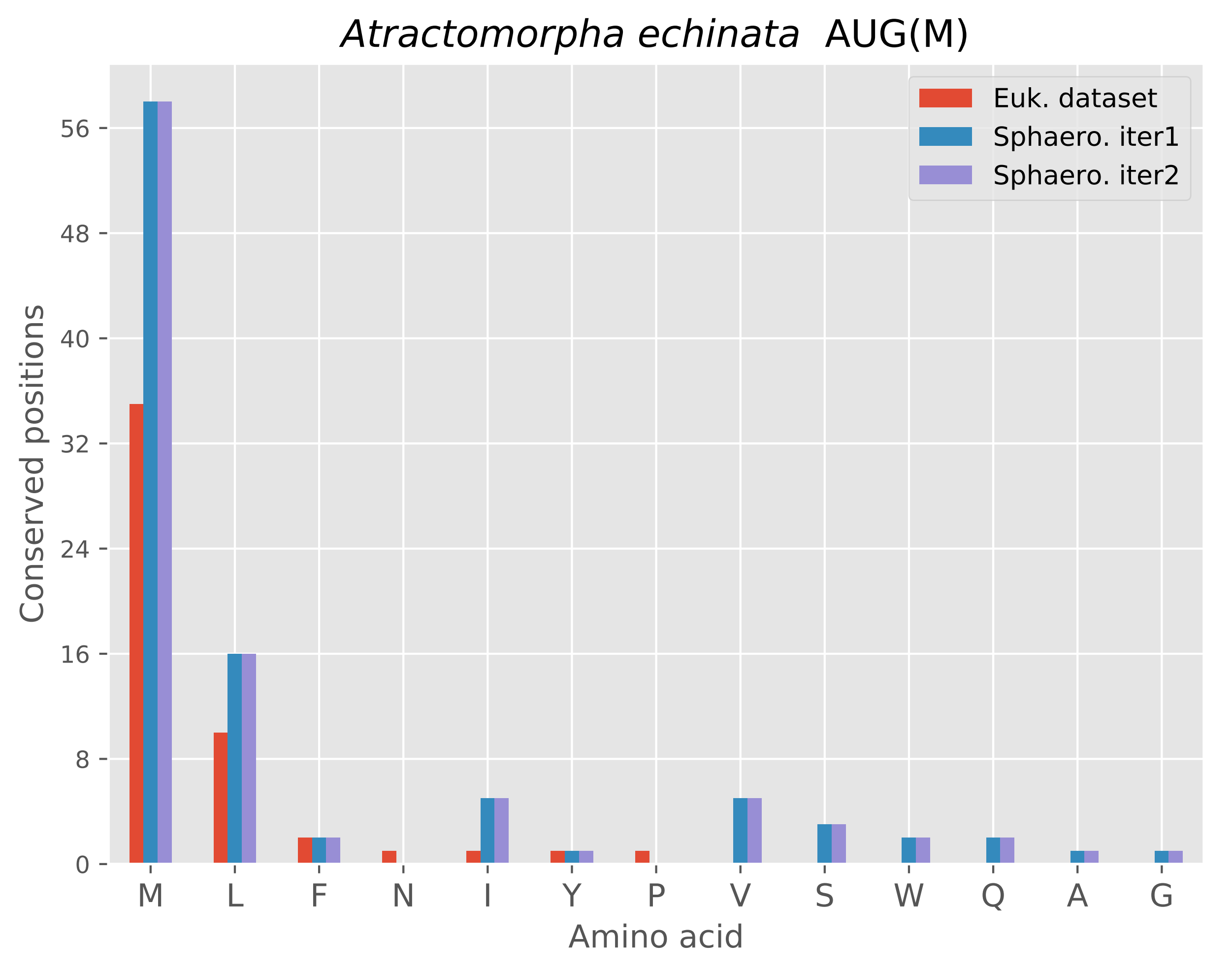

Atractomorpha echinata AUG(M)
Euk. dataset
Sphaero. iter1
Sphaero. iter2
Conserved positions
0
8
16
24
32
40
48
56
M
L
F
N
I
Y
P
V
S
W
Q
A
G
Amino acid

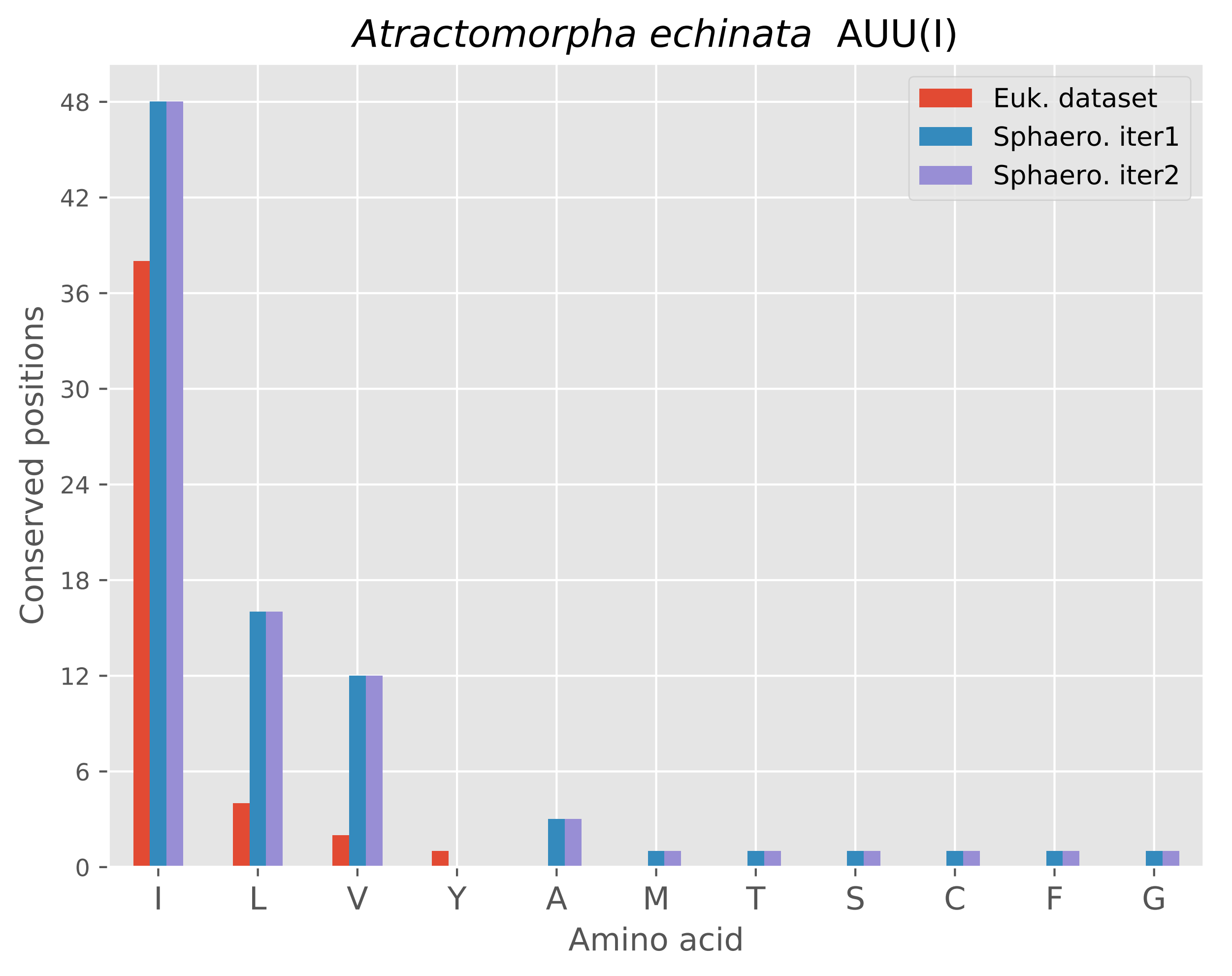

Atractomorpha echinata AUU(I)
Euk. dataset
Sphaero. iter1
Sphaero. iter2
Conserved positions
0
6
12
18
24
30
36
42
48
I
L
V
Y
A
M
T
S
C
F
G
Amino acid

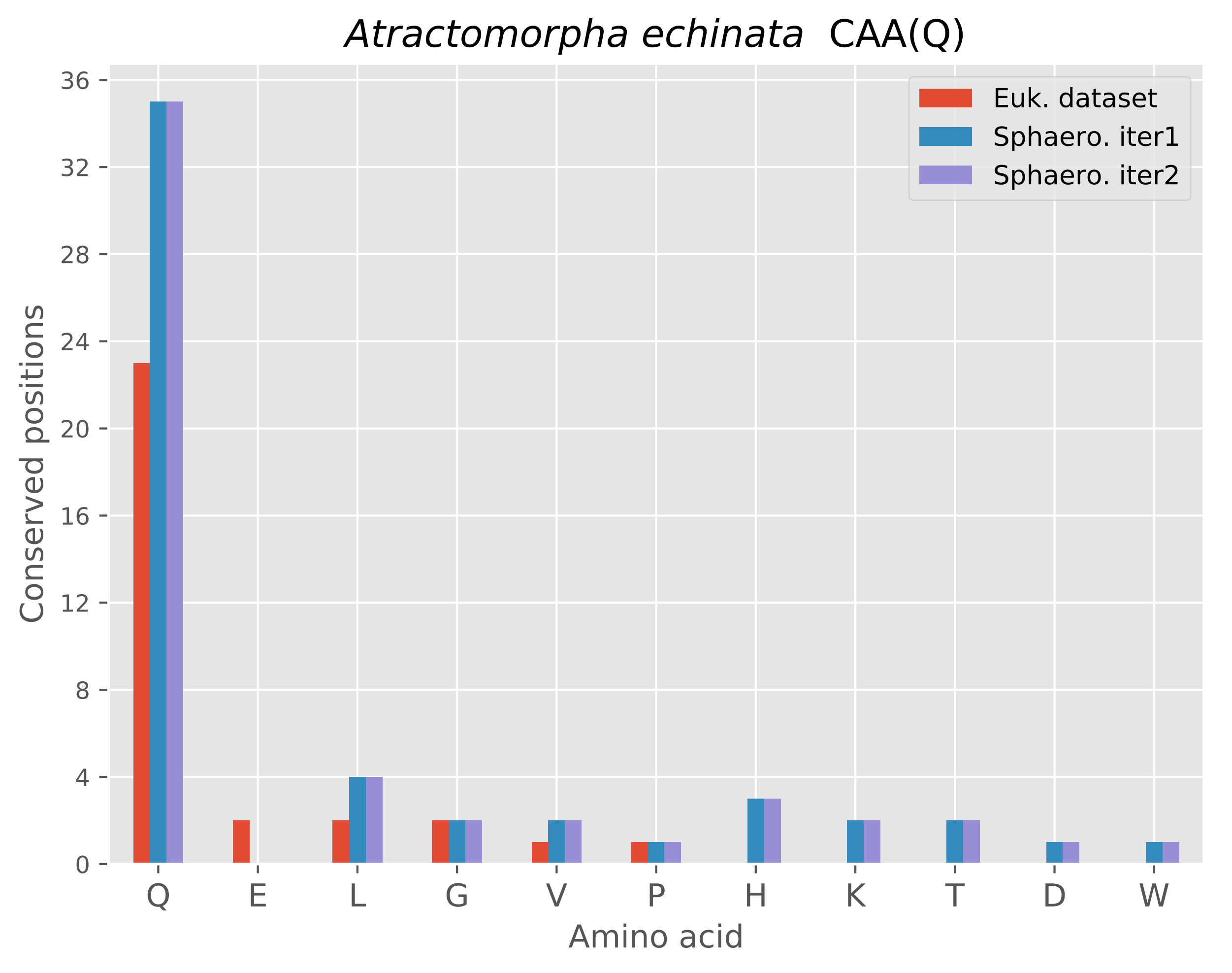

Atractomorpha echinata CAA(Q)
Euk. dataset
Sphaero. iter1
Sphaero. iter2
Conserved positions
0
4
8
12
16
20
24
28
32
36
Q
E
L
G
V
P
H
K
T
D
W
Amino acid

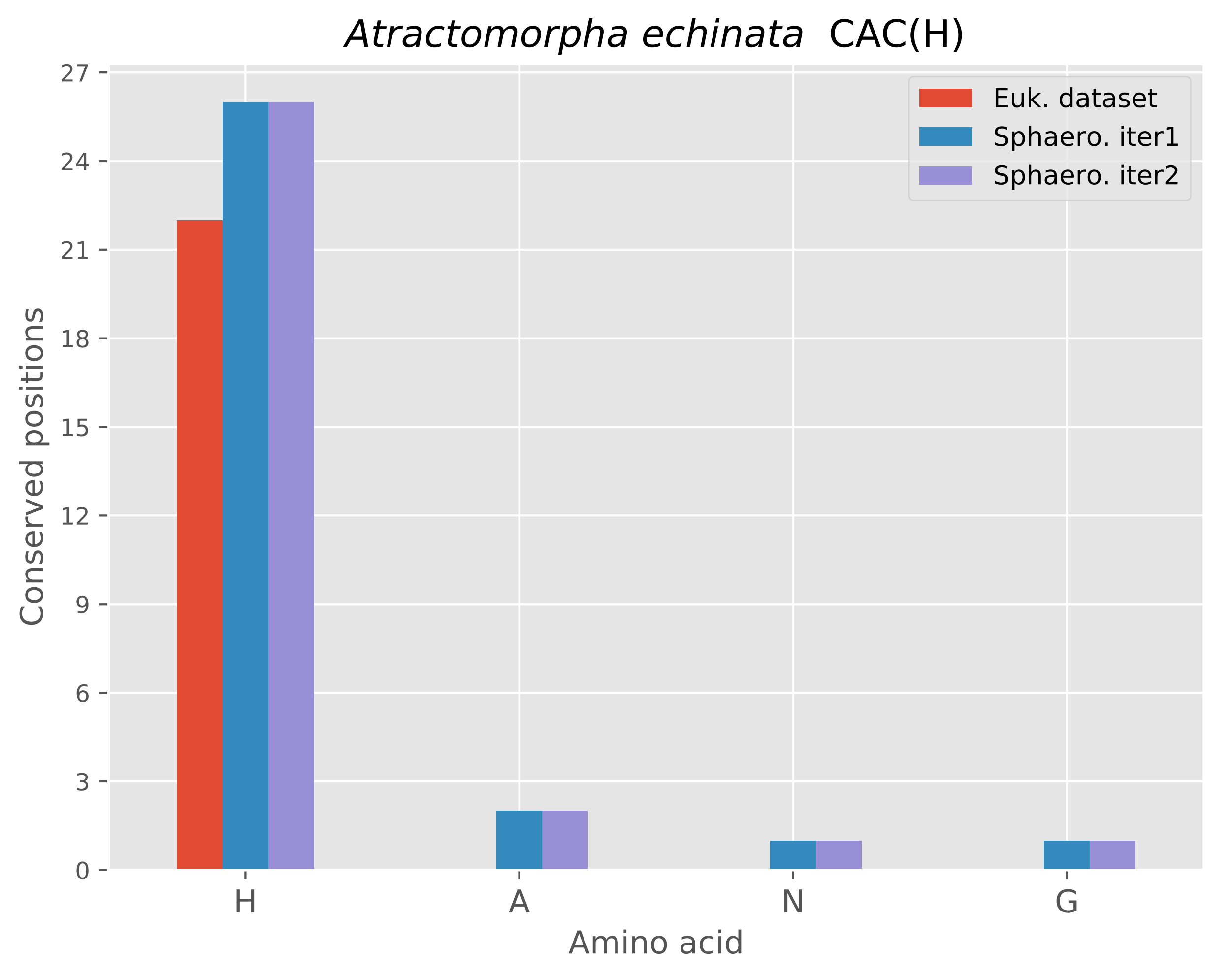
Atractomorpha echinata CAC(H)
Euk. dataset
Sphaero. iter1
Sphaero. iter2
Conserved positions
0
3
6
9
12
15
18
21
24
27
H
A
N
G
Amino acid

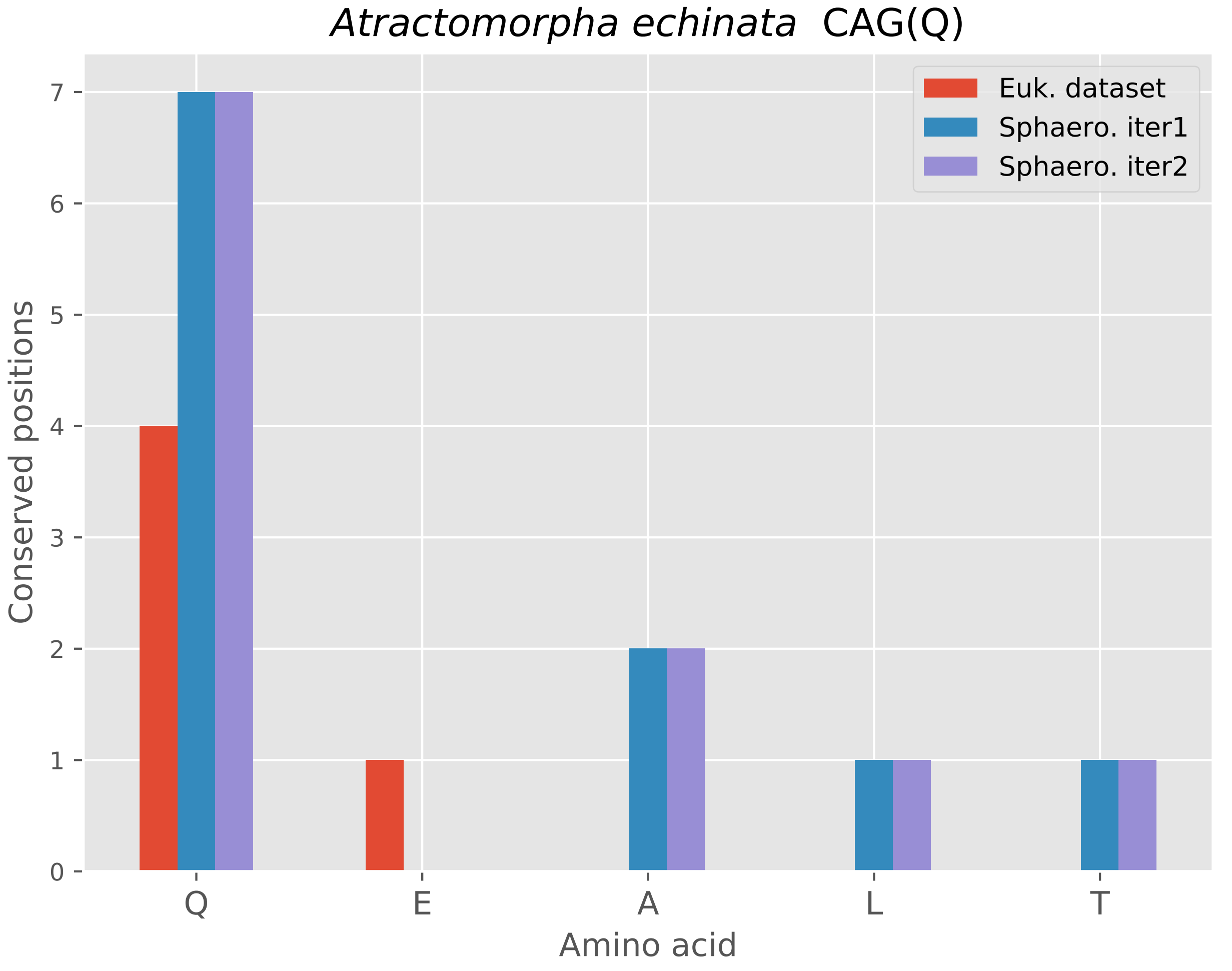
Atractomorpha echinata CAG(Q)
Euk. dataset
Sphaero. iter1
Sphaero. iter2
Conserved positions
0
1
2
3
4
5
6
7
Q
E
A
L
T
Amino acid

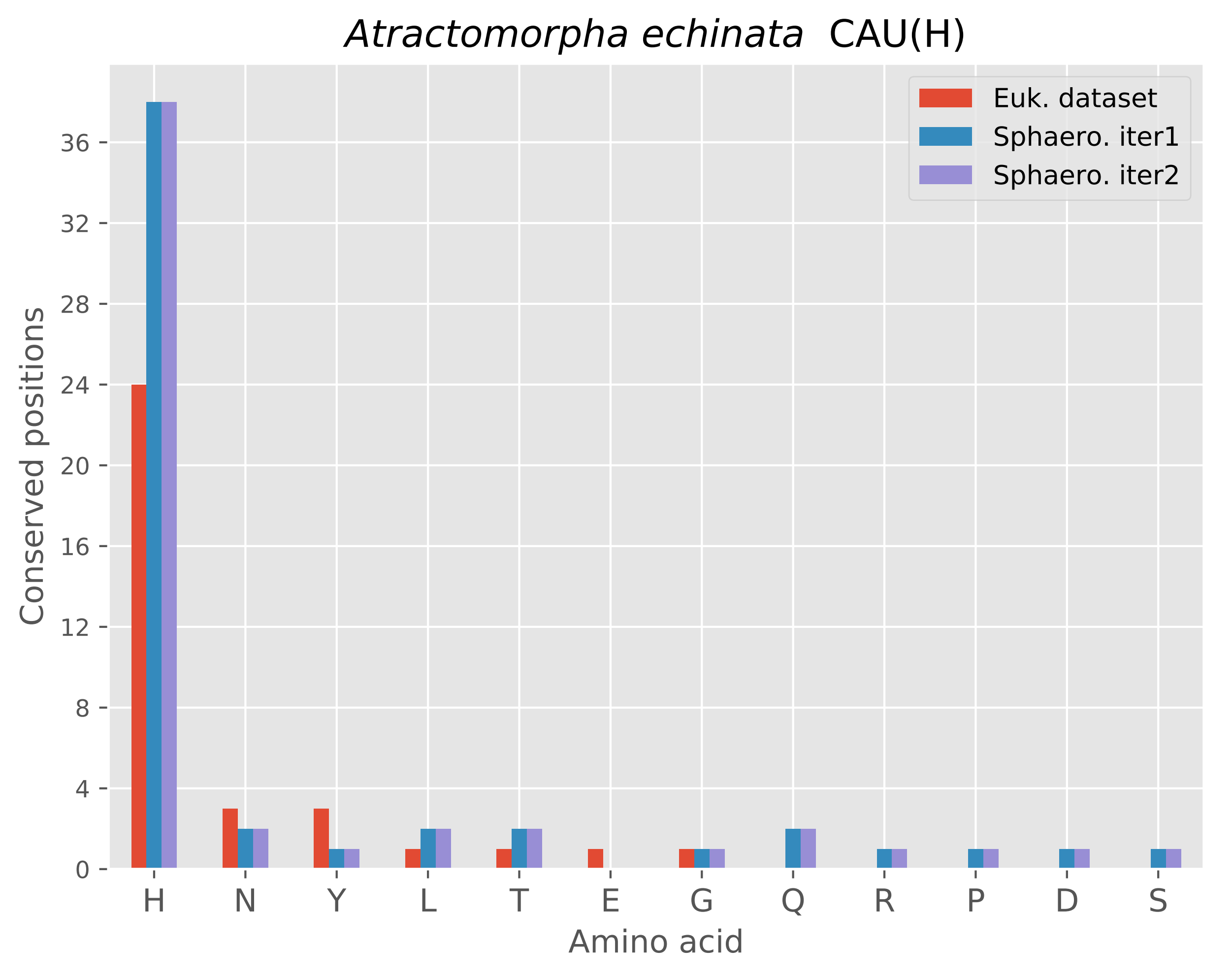

Atractomorpha echinata CAU(H)
Euk. dataset
Sphaero. iter1
Sphaero. iter2
Conserved positions
0
4
8
12
16
20
24
28
32
36
H
N
Y
L
T
E
G
Q
R
P
D
S
Amino acid

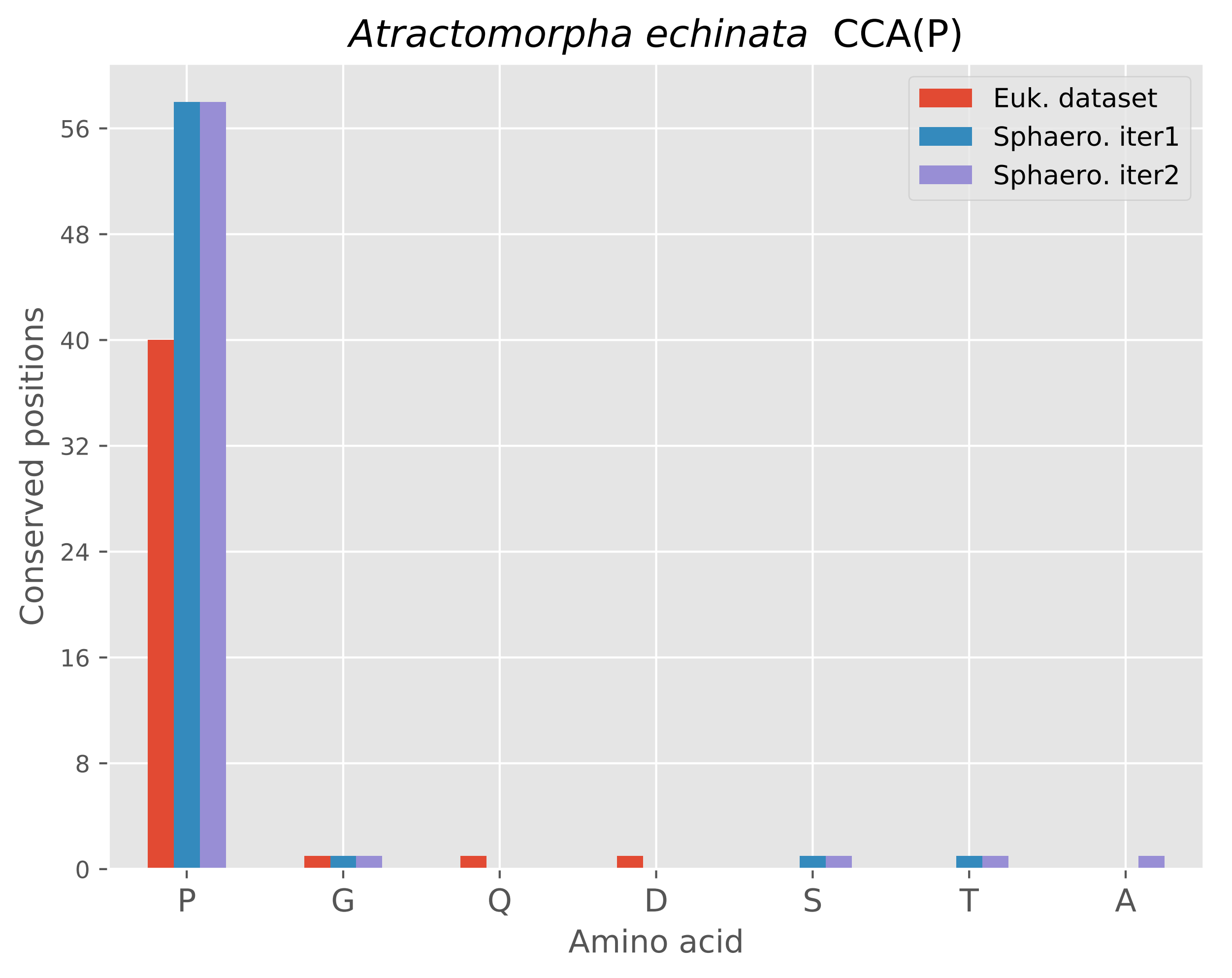

Atractomorpha echinata CCA(P)
Euk. dataset
Sphaero. iter1
Sphaero. iter2
Conserved positions
0
8
16
24
32
40
48
56
P
G
Q
D
S
T
A
Amino acid

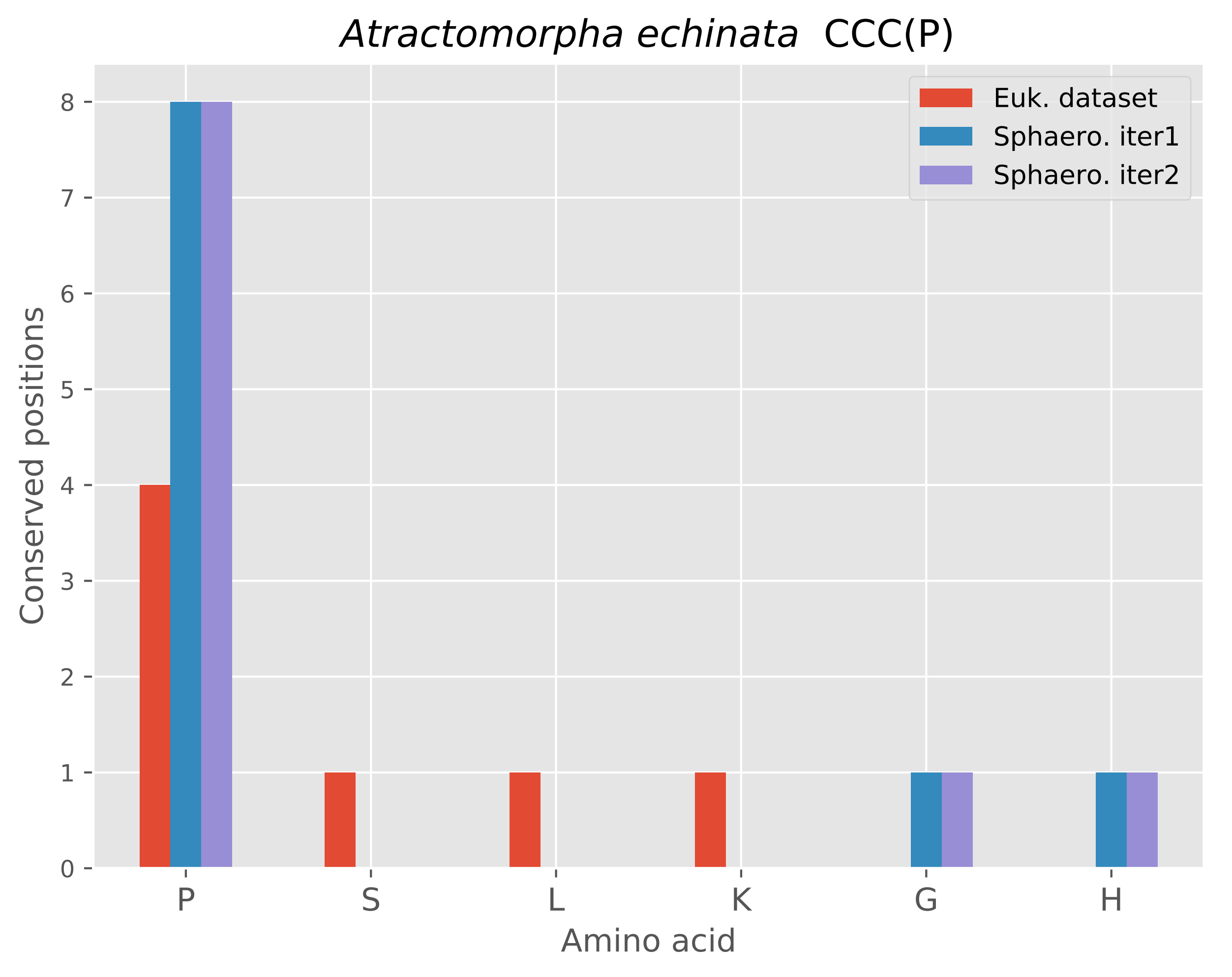

Atractomorpha echinata CCC(P)
Euk. dataset
Sphaero. iter1
Sphaero. iter2
Conserved positions
Amino acid
0
1
2
3
4
5
6
7
8
P
S
L
K
G
H

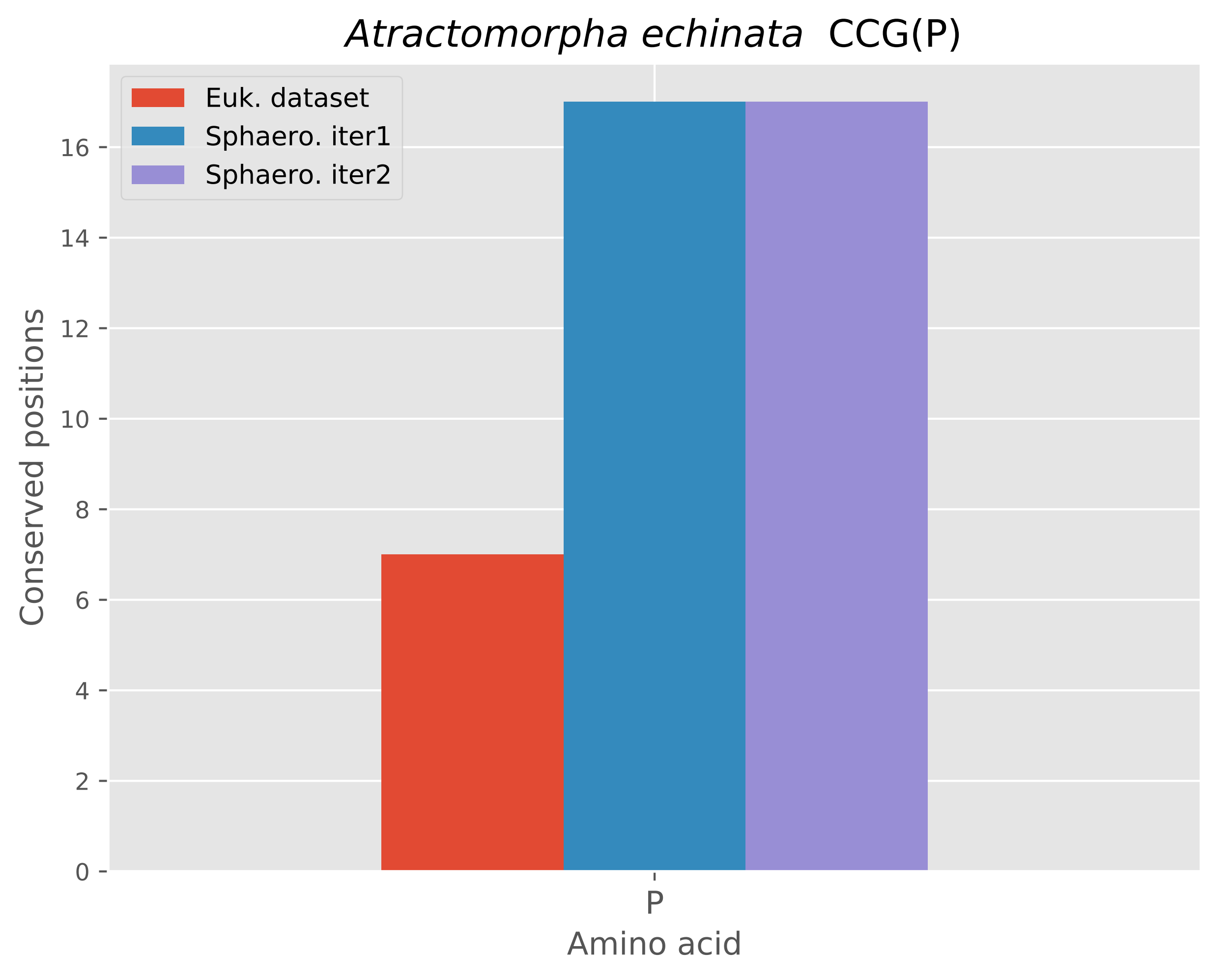

Atractomorpha echinata CCG(P)
Euk. dataset
Sphaero. iter1
Sphaero. iter2
Conserved positions
0
2
4
6
8
10
12
14
16
P
Amino acid

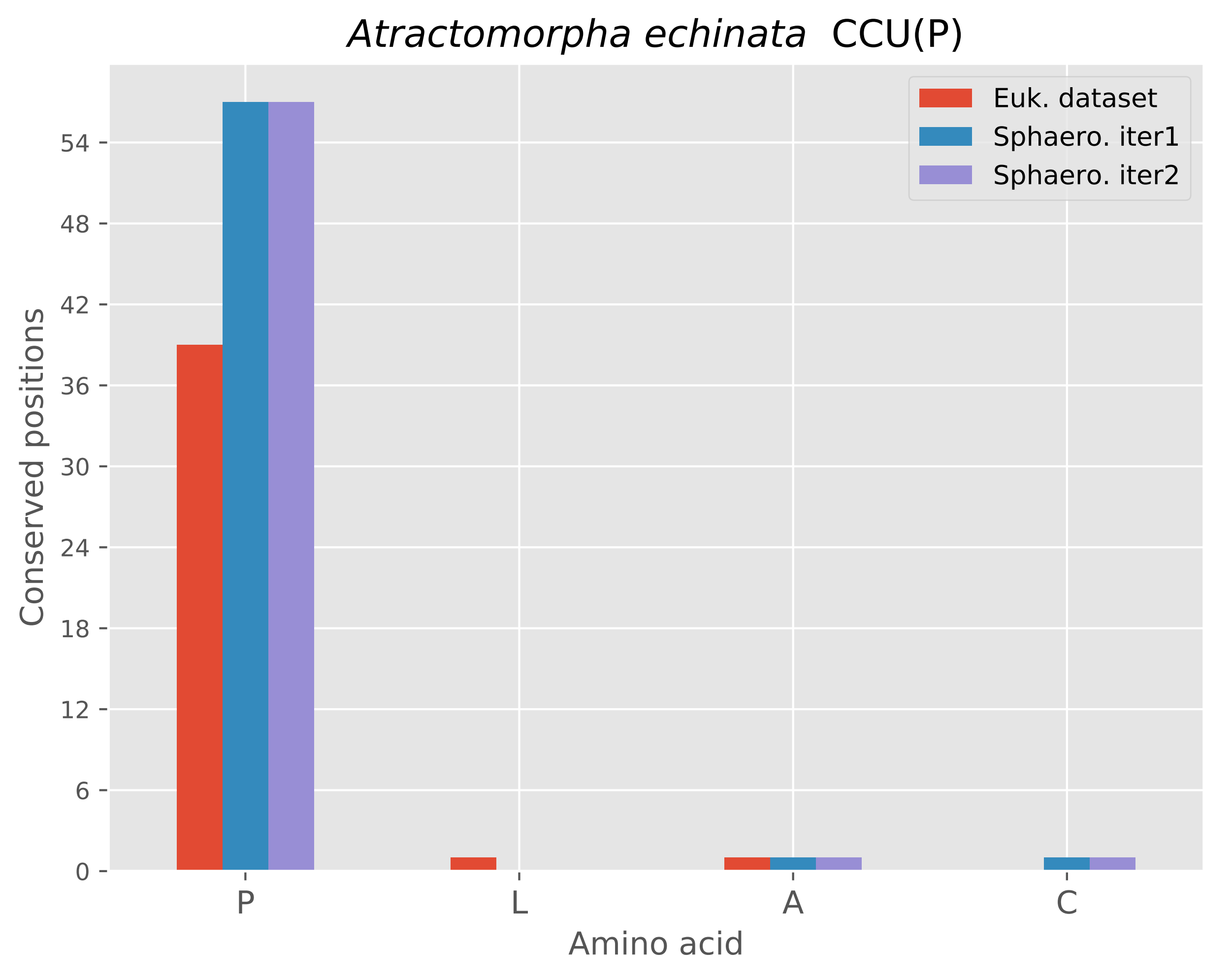
Atractomorpha echinata CCU(P)
Euk. dataset
Sphaero. iter1
Sphaero. iter2
Conserved positions
0
6
12
18
24
30
36
42
48
54
P
L
A
C
Amino acid

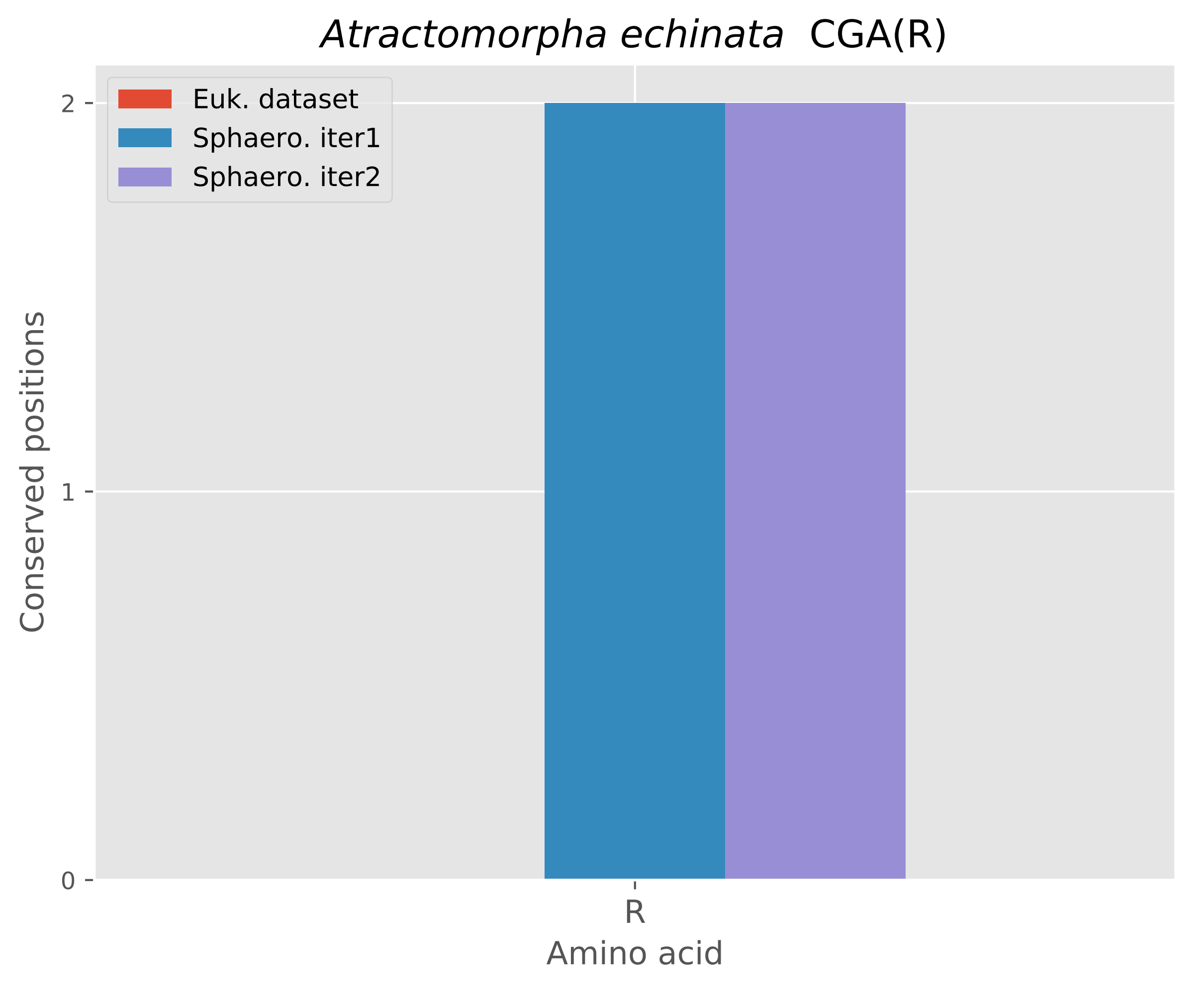
Atractomorpha echinata CGA(R)
Euk. dataset
Sphaero. iter1
Sphaero. iter2
Conserved positions
2
1
0
R
Amino acid

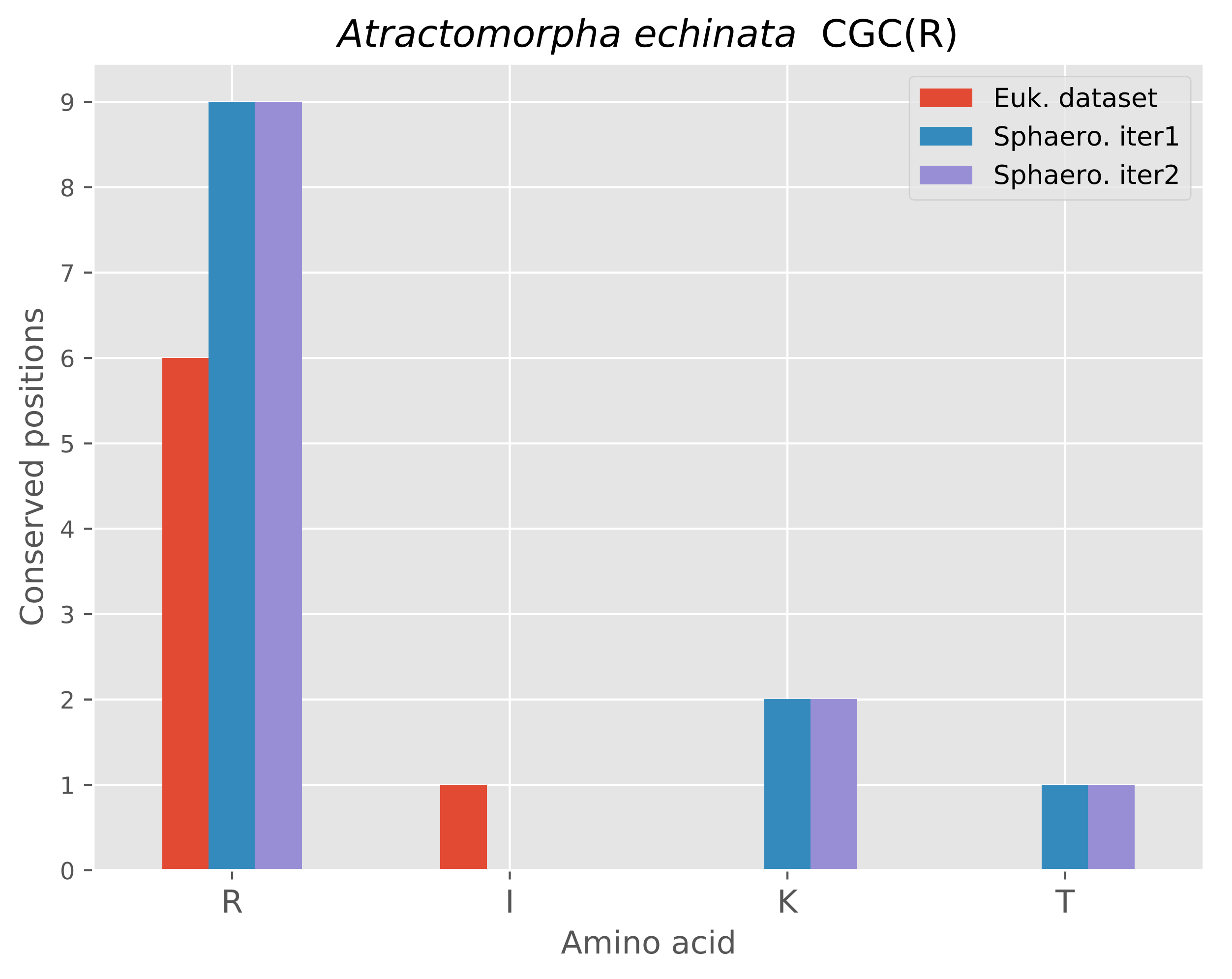

Atractomorpha echinata CGC(R)
Euk. dataset
Sphaero. iter1
Sphaero. iter2
Conserved positions
0
1
2
3
4
5
6
7
8
9
R
I
K
T
Amino acid

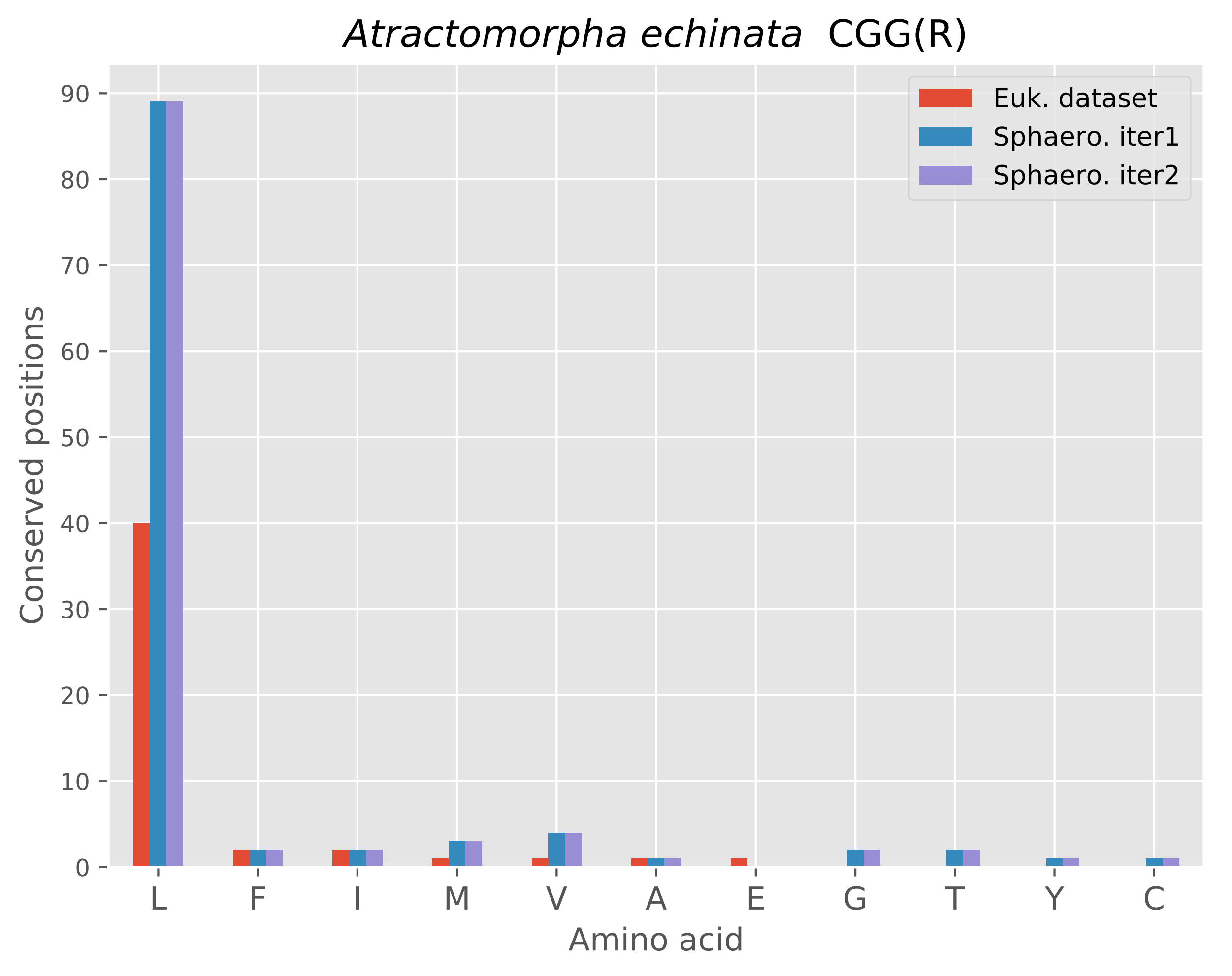

Atractomorpha echinata CGG(R)
Euk. dataset
Sphaero. iter1
Sphaero. iter2
Conserved positions
0
10
20
30
40
50
60
70
80
90
L
F
I
M
V
A
E
G
T
Y
C
Amino acid

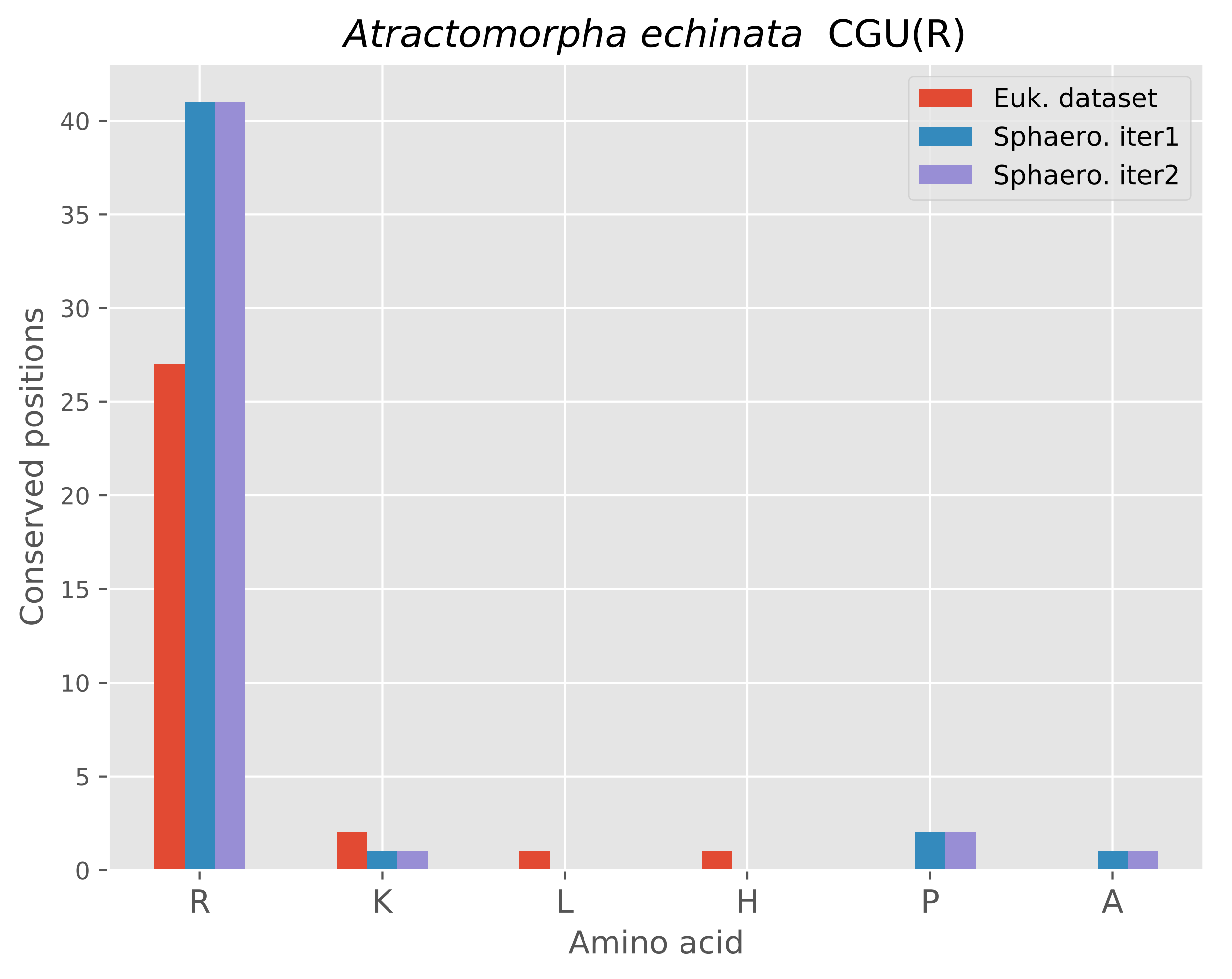

Atractomorpha echinata CGU(R)
Euk. dataset
Sphaero. iter1
Sphaero. iter2
Conserved positions
0
5
10
15
20
25
30
35
40
R
K
L
H
P
A
Amino acid

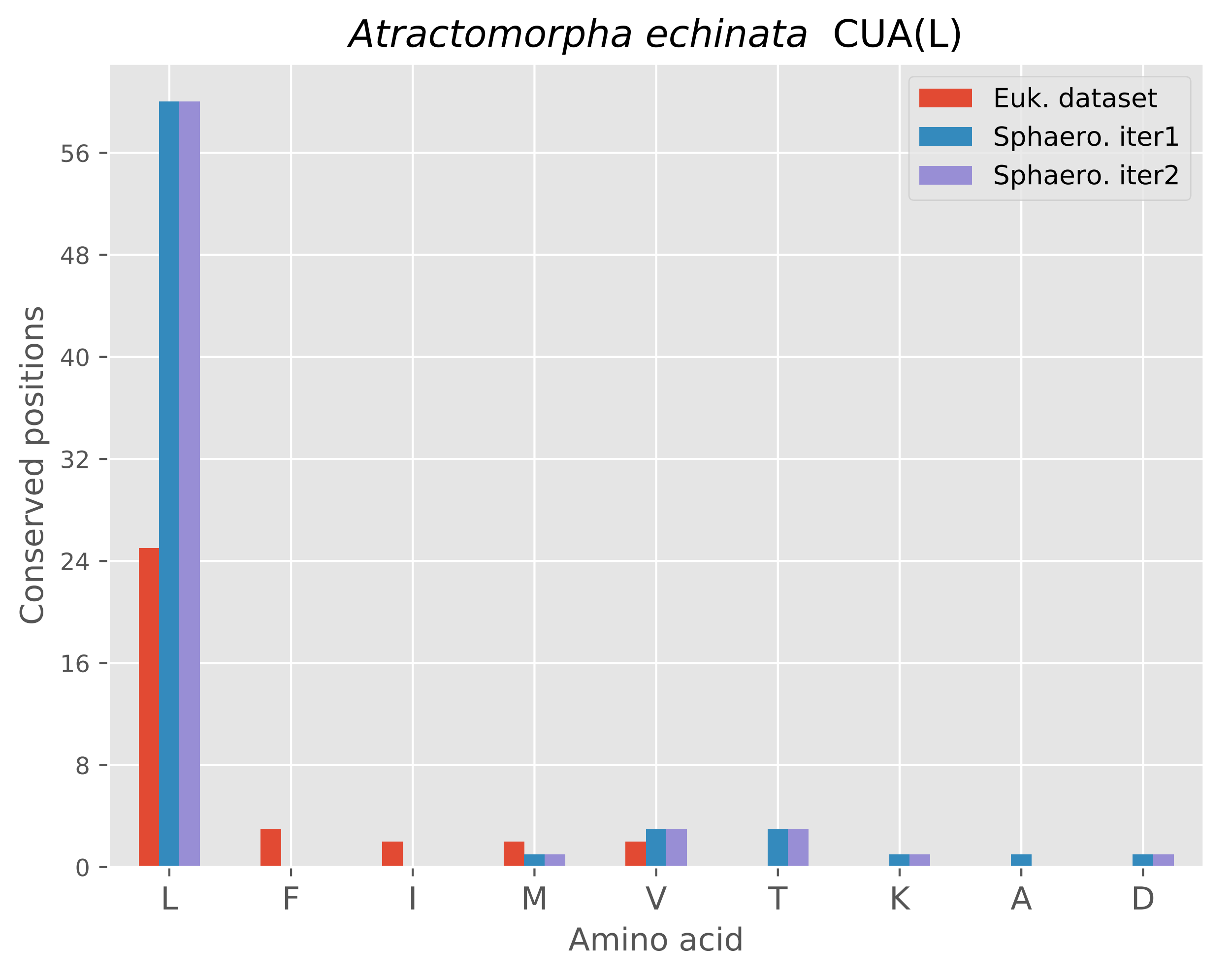

Atractomorpha echinata CUA(L)
Euk. dataset
Sphaero. iter1
Sphaero. iter2
Conserved positions
0
8
16
24
32
40
48
56
L
F
I
M
V
T
K
A
D
Amino acid

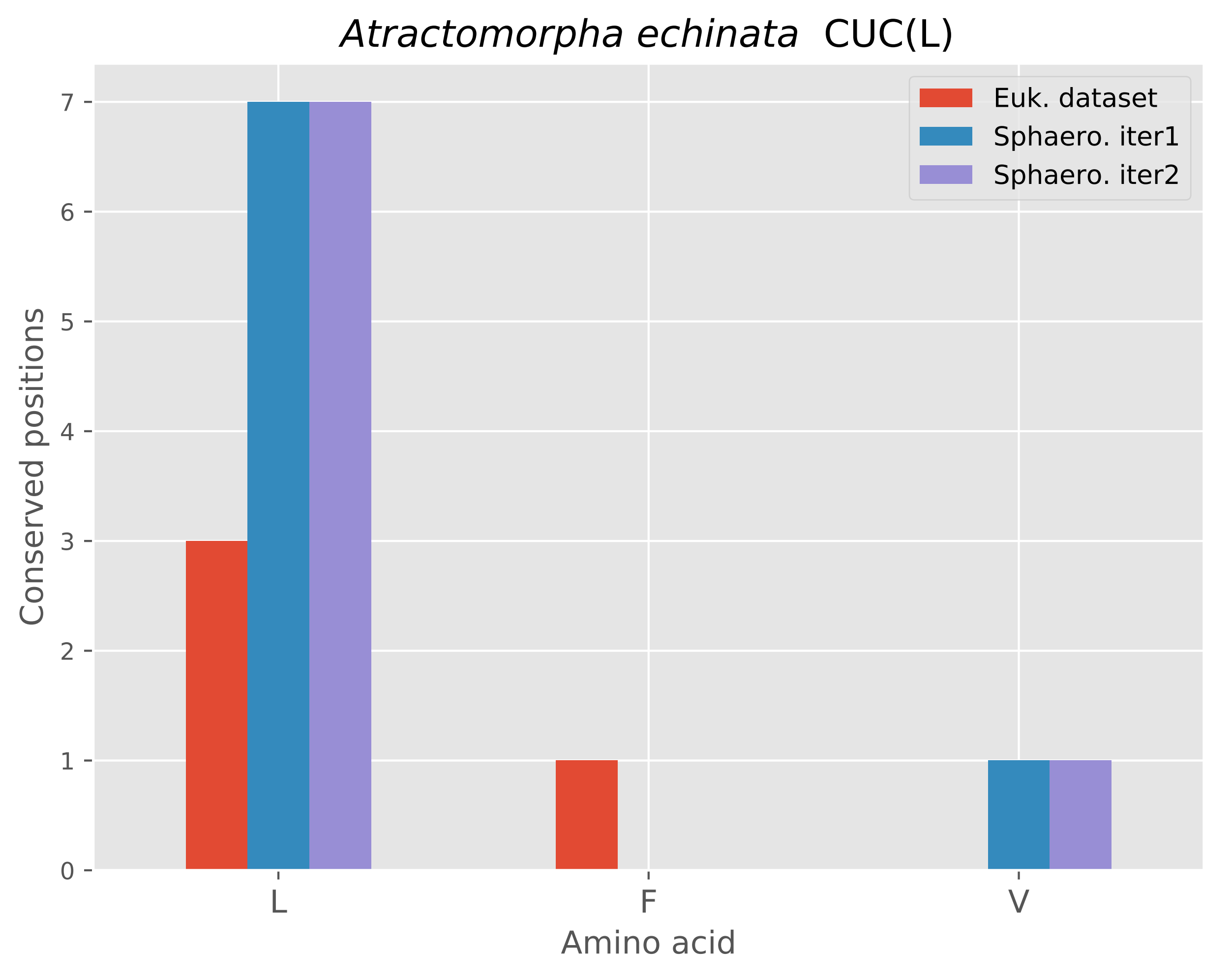

Atractomorpha echinata CUC(L)
Euk. dataset
Sphaero. iter1
Sphaero. iter2
Conserved positions
0
1
2
3
4
5
6
7
L
F
V
Amino acid

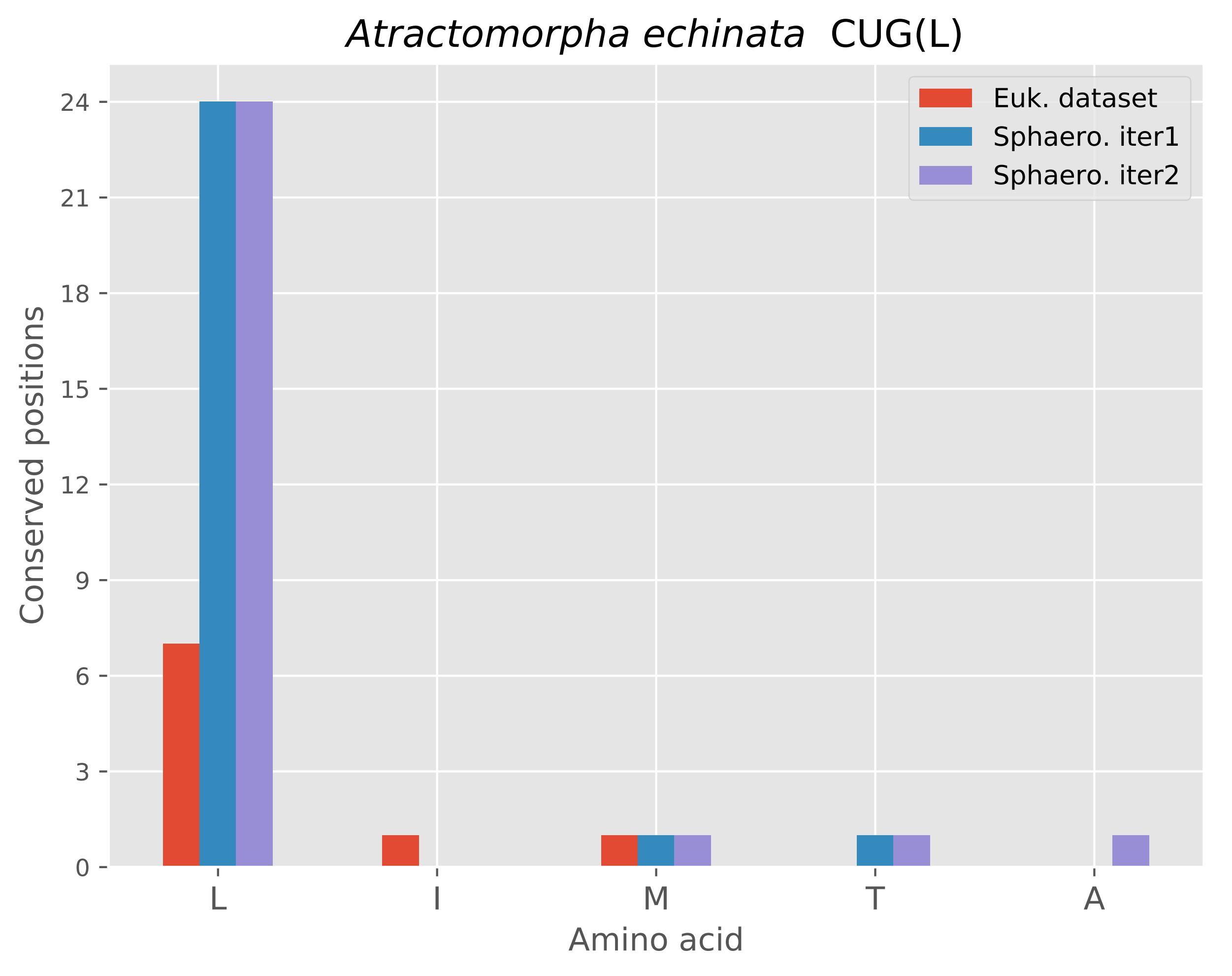

Atractomorpha echinata CUG(L)
Euk. dataset
Sphaero. iter1
Sphaero. iter2
Conserved positions
0
3
6
9
12
15
18
21
24
L
I
M
T
A
Amino acid

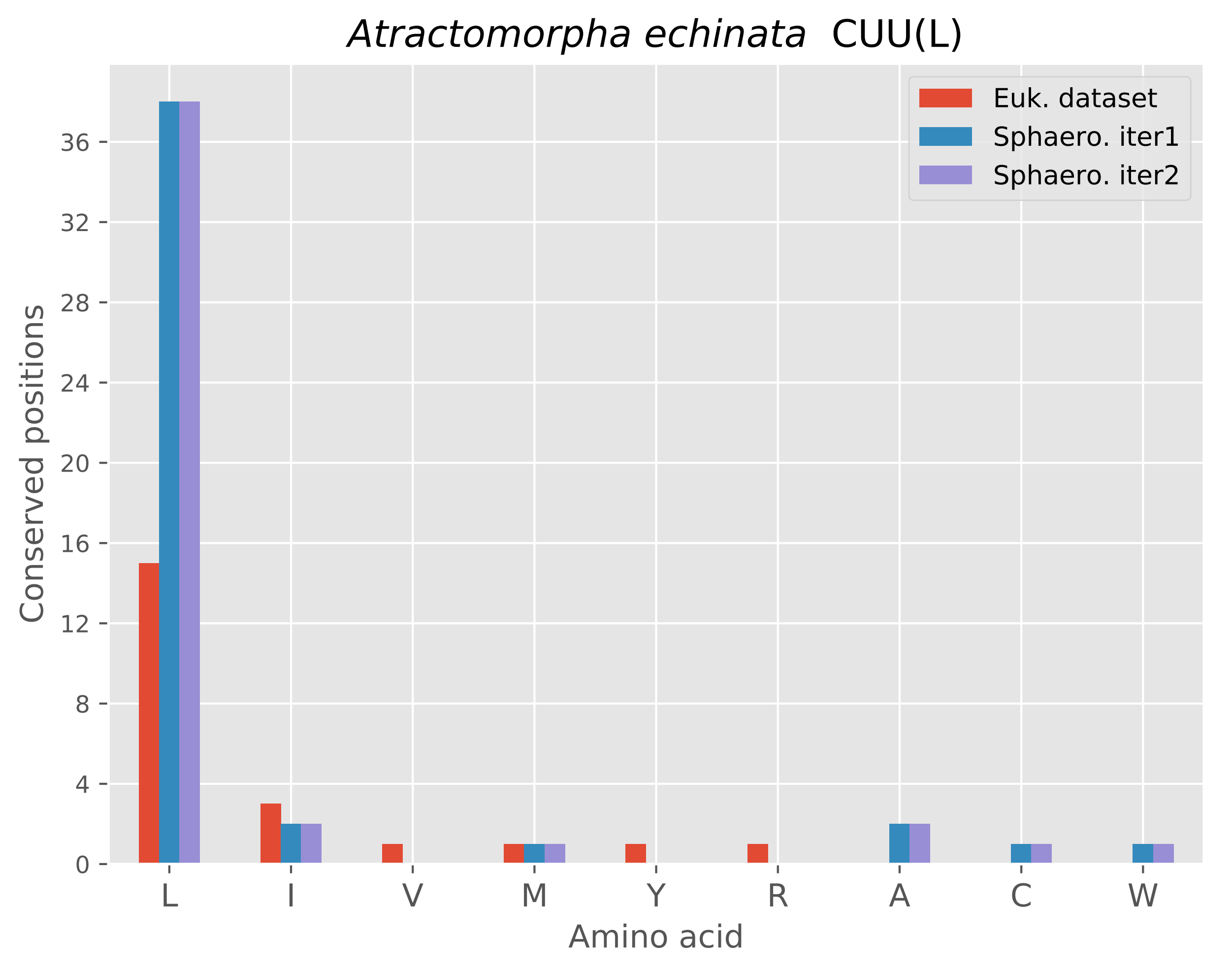

Atractomorpha echinata CUU(L)
Euk. dataset
Sphaero. iter1
Sphaero. iter2
Conserved positions
0
4
8
12
16
20
24
28
32
36
L
I
V
M
Y
R
A
C
W
Amino acid

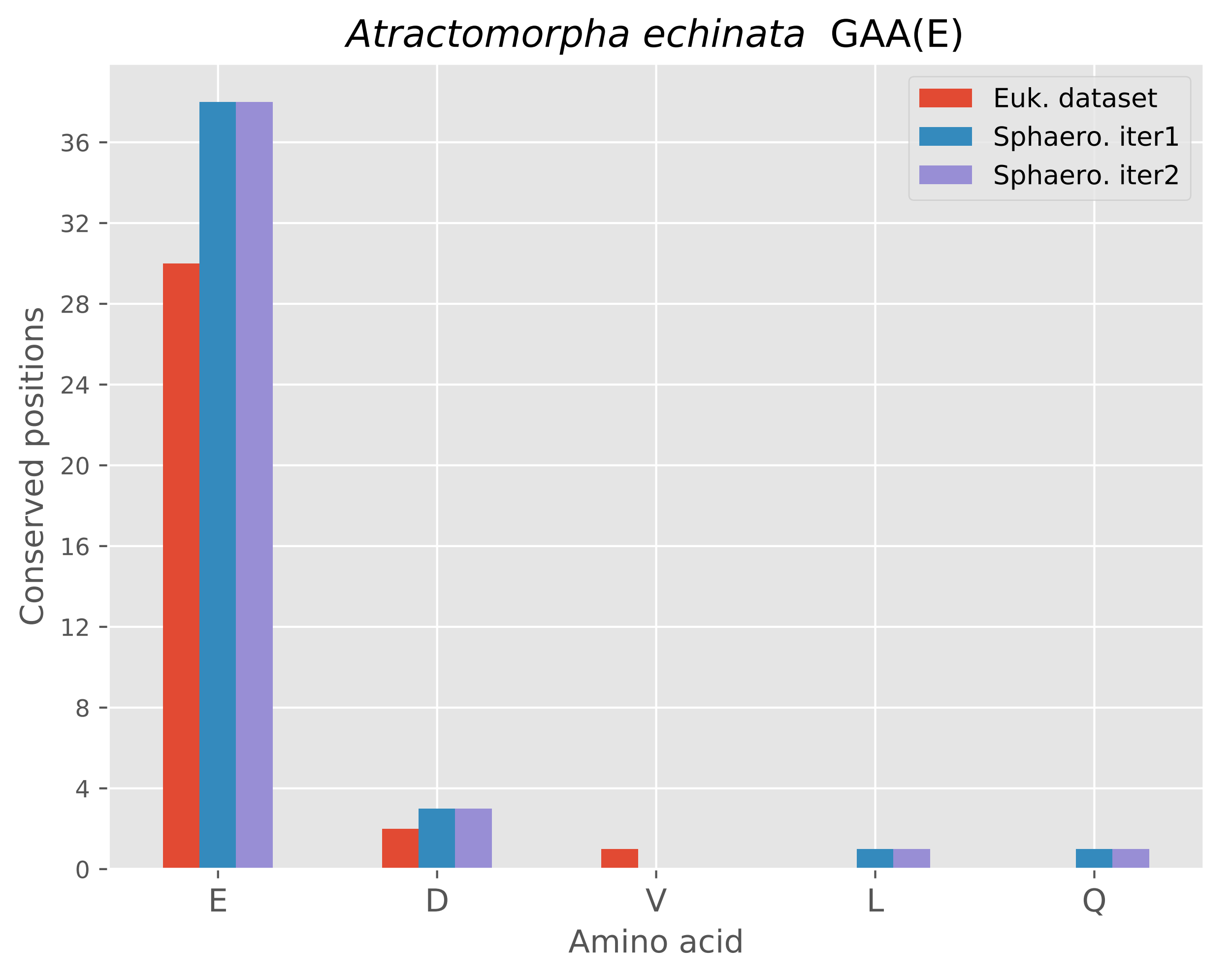
Atractomorpha echinata GAA(E)
Euk. dataset
Sphaero. iter1
Sphaero. iter2
Conserved positions
0
4
8
12
16
20
24
28
32
36
E
D
V
L
Q
Amino acid

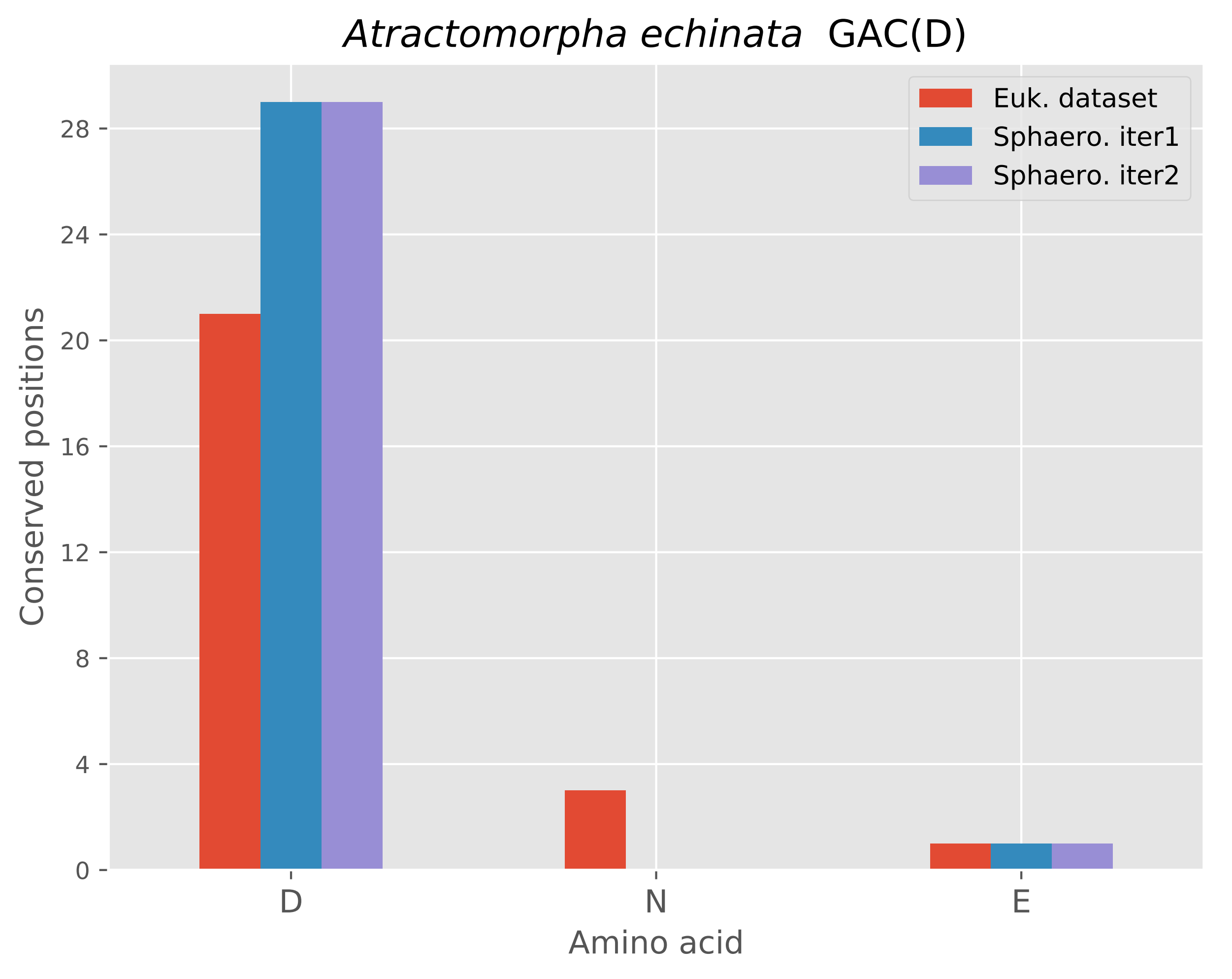

Atractomorpha echinata GAC(D)
Euk. dataset
Sphaero. iter1
Sphaero. iter2
Conserved positions
0
4
8
12
16
20
24
28
D
N
E
Amino acid

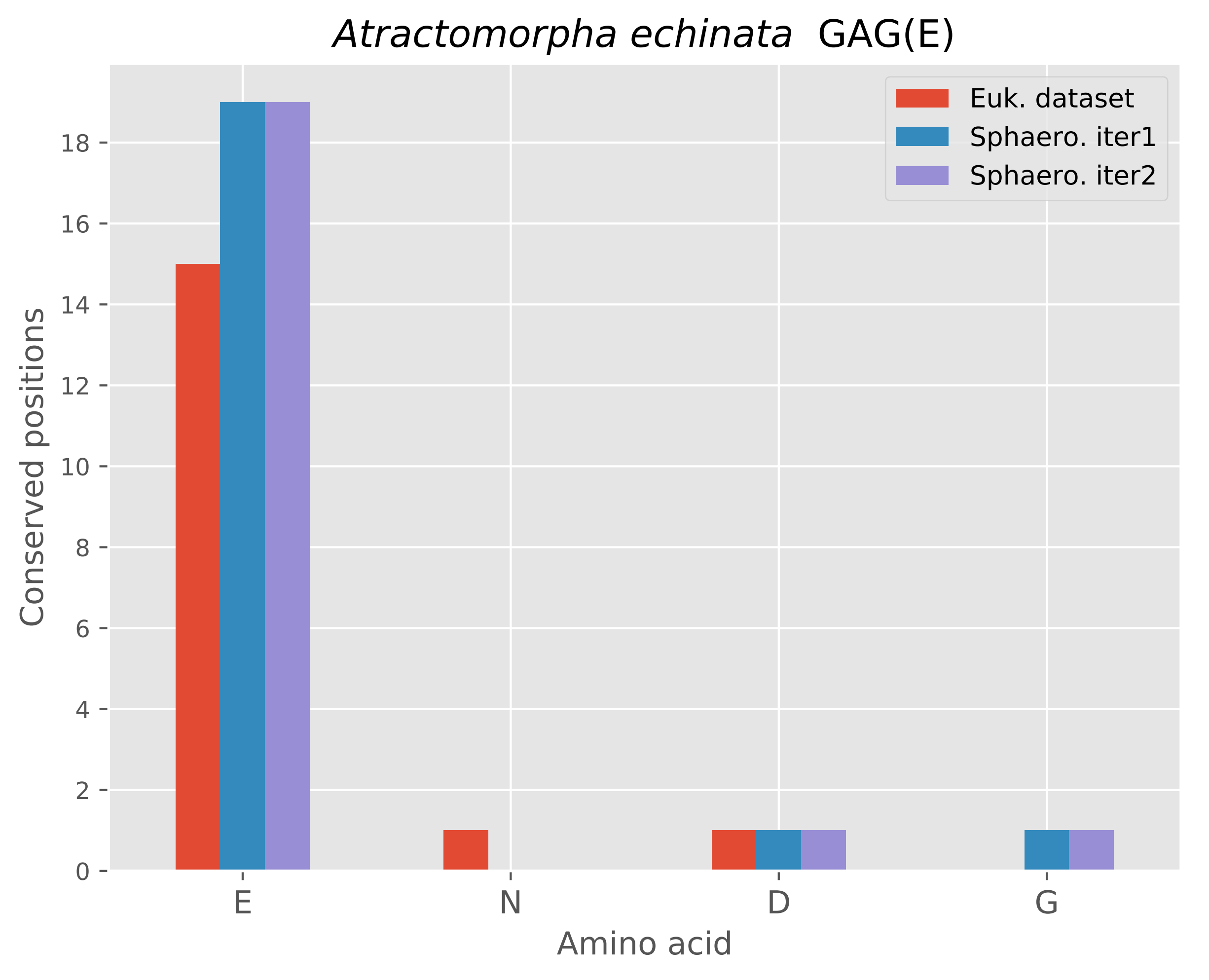

Atractomorpha echinata GAG(E)
Euk. dataset
Sphaero. iter1
Sphaero. iter2
Conserved positions
0
2
4
6
8
10
12
14
16
18
E
N
D
G
Amino acid

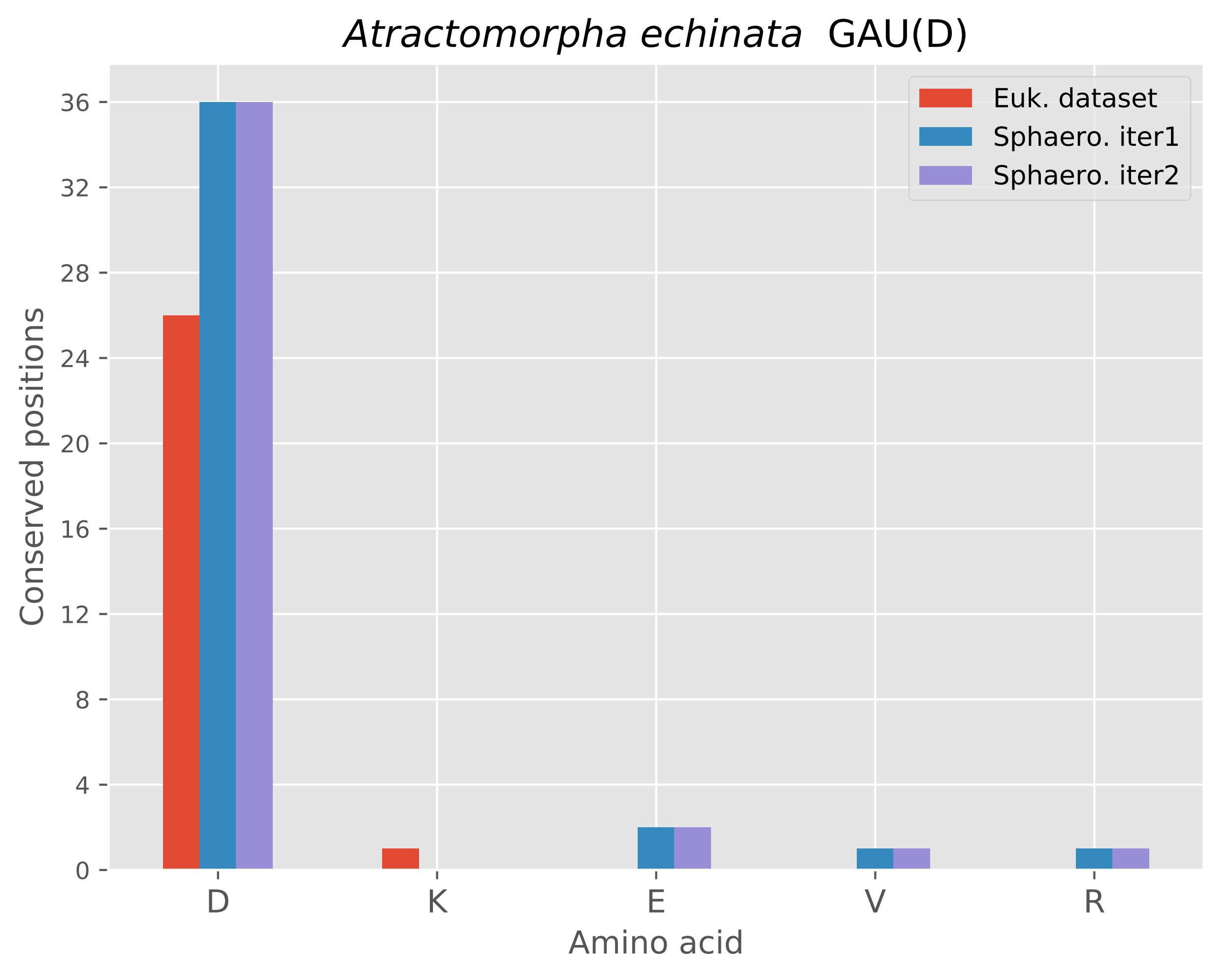

Atractomorpha echinata GAU(D)
Euk. dataset
Sphaero. iter1
Sphaero. iter2
Conserved positions
0
4
8
12
16
20
24
28
32
36
D
K
E
V
R
Amino acid

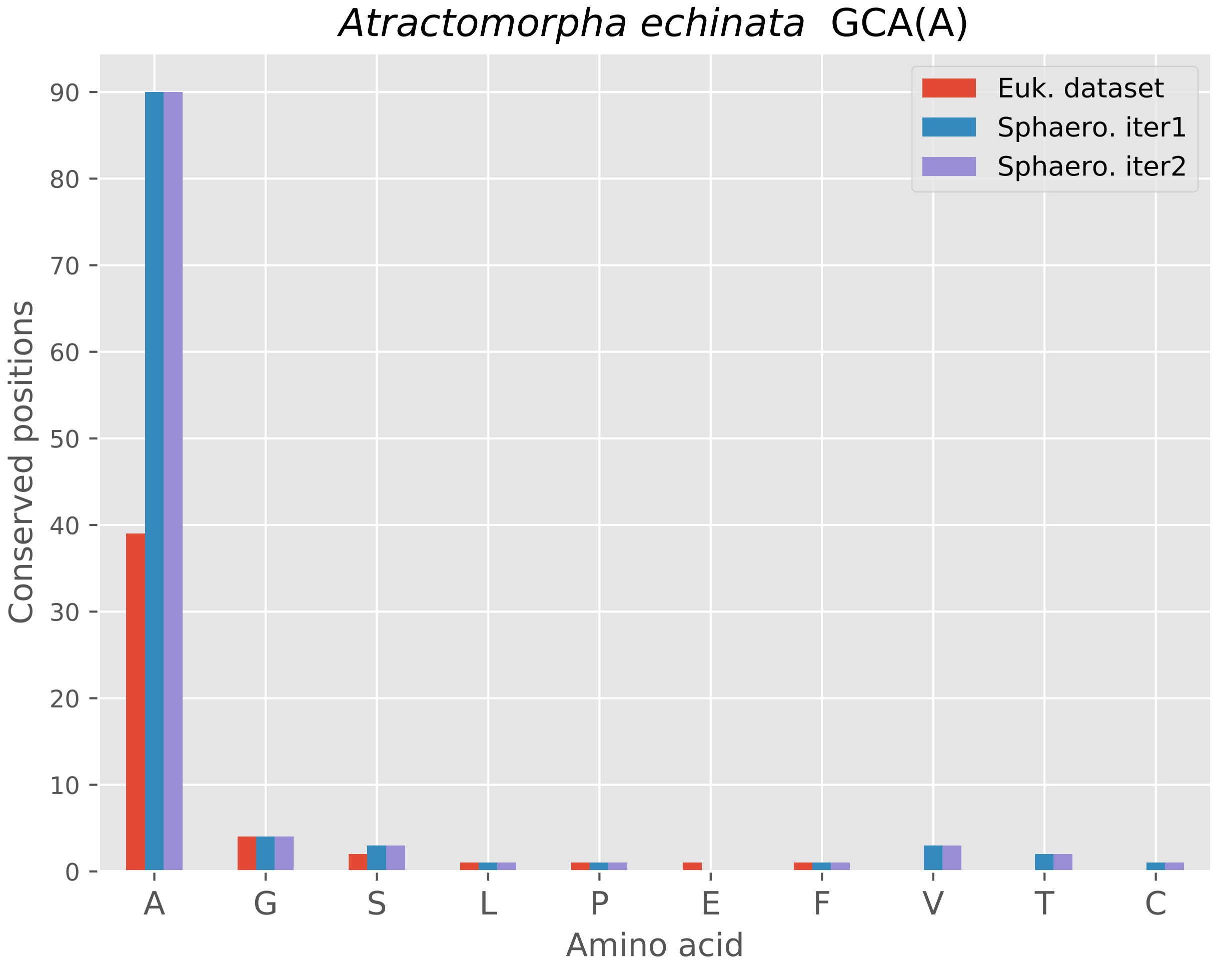
Atractomorpha echinata GCA(A)
Euk. dataset
Sphaero. iter1
Sphaero. iter2
Conserved positions
0
10
20
30
40
50
60
70
80
90
A
G
S
L
P
E
F
V
T
C
Amino acid

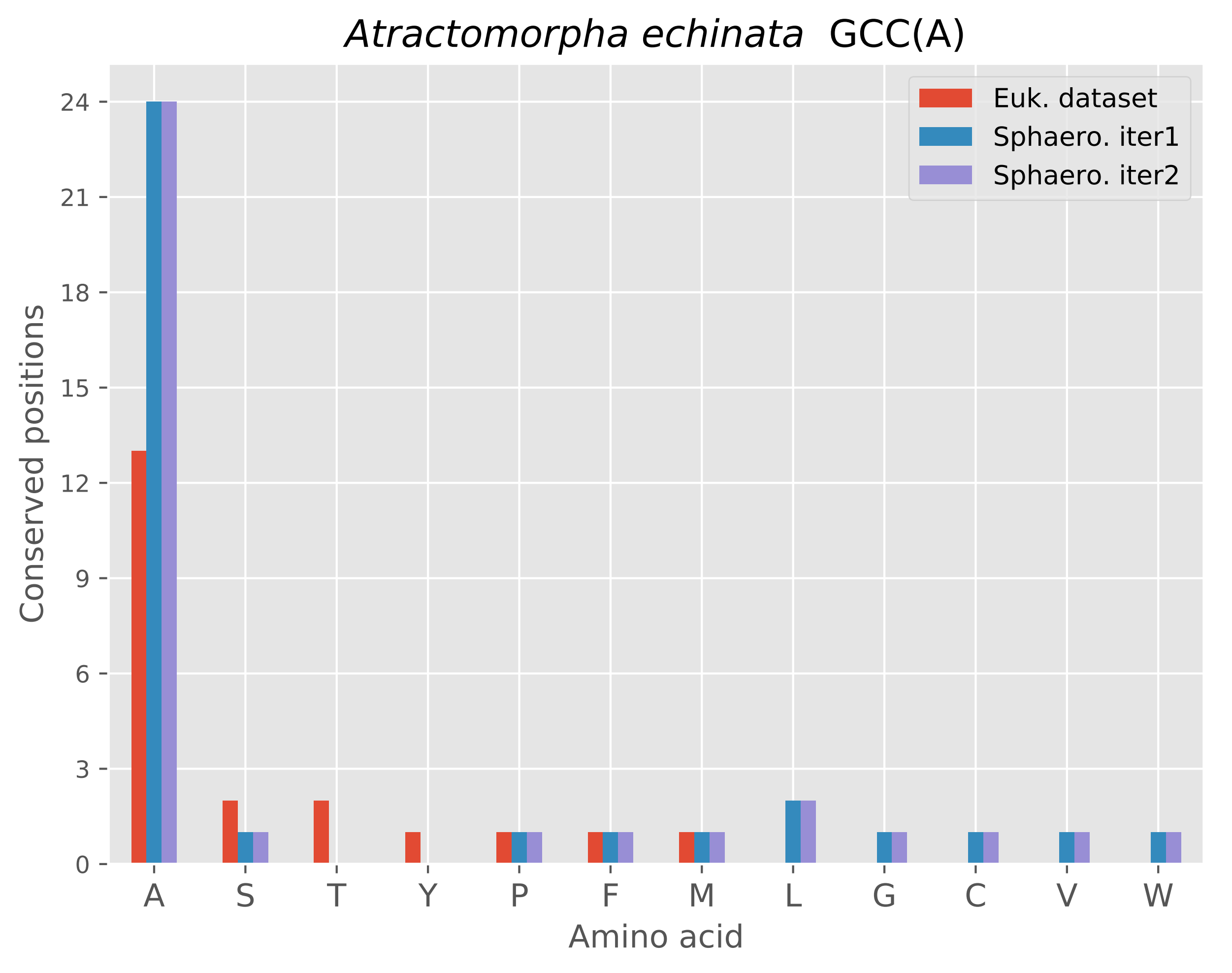

Atractomorpha echinata GCC(A)
Euk. dataset
Sphaero. iter1
Sphaero. iter2
Conserved positions
0
3
6
9
12
15
18
21
24
A
S
T
Y
P
F
M
L
G
C
V
W
Amino acid

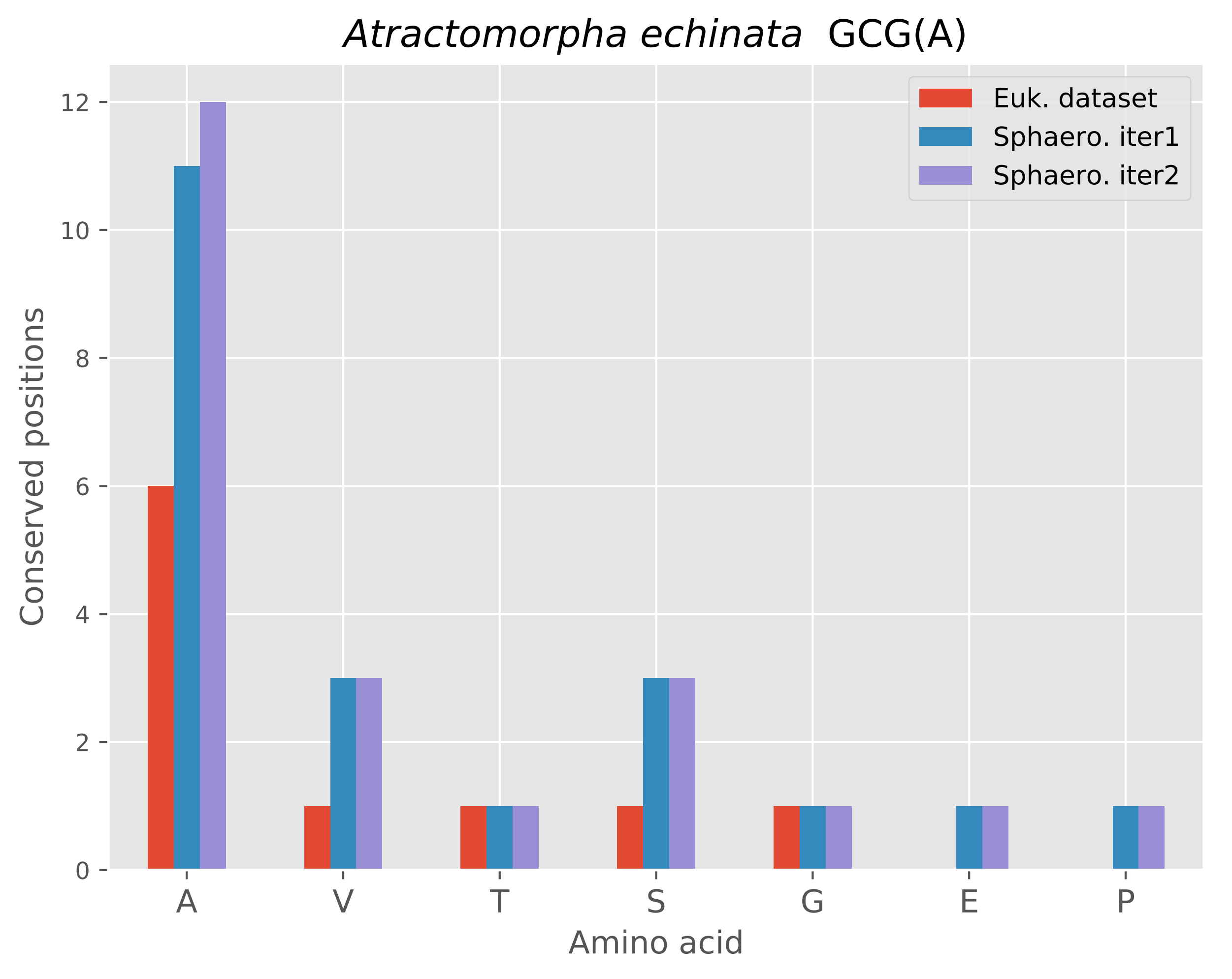

Atractomorpha echinata GCG(A)
Euk. dataset
Sphaero. iter1
Sphaero. iter2
Conserved positions
0
2
4
6
8
10
12
A
V
T
S
G
E
P
Amino acid

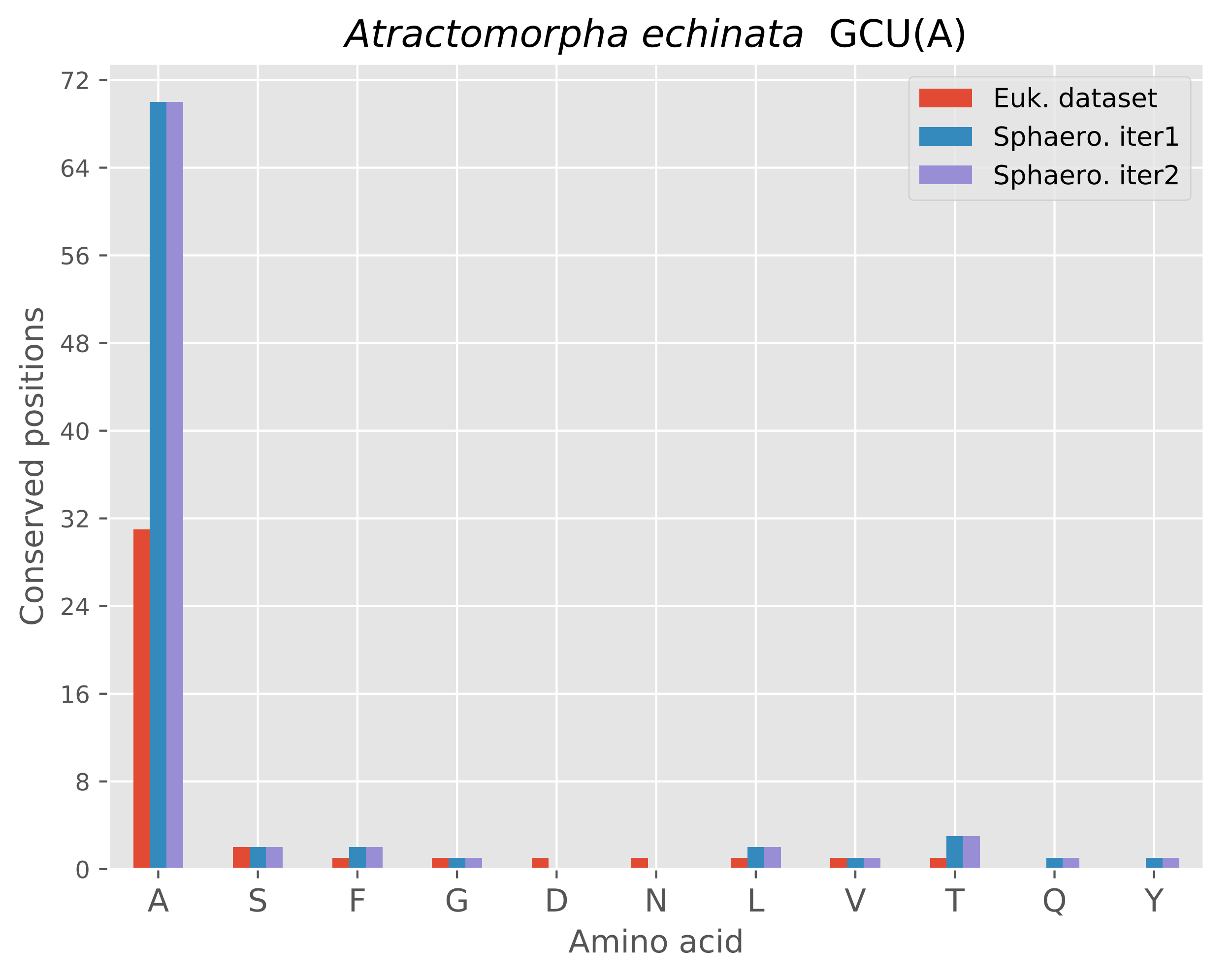

Atractomorpha echinata GCU(A)
Euk. dataset
Sphaero. iter1
Sphaero. iter2
Conserved positions
0
8
16
24
32
40
48
56
64
72
A
S
F
G
D
N
L
V
T
Q
Y
Amino acid

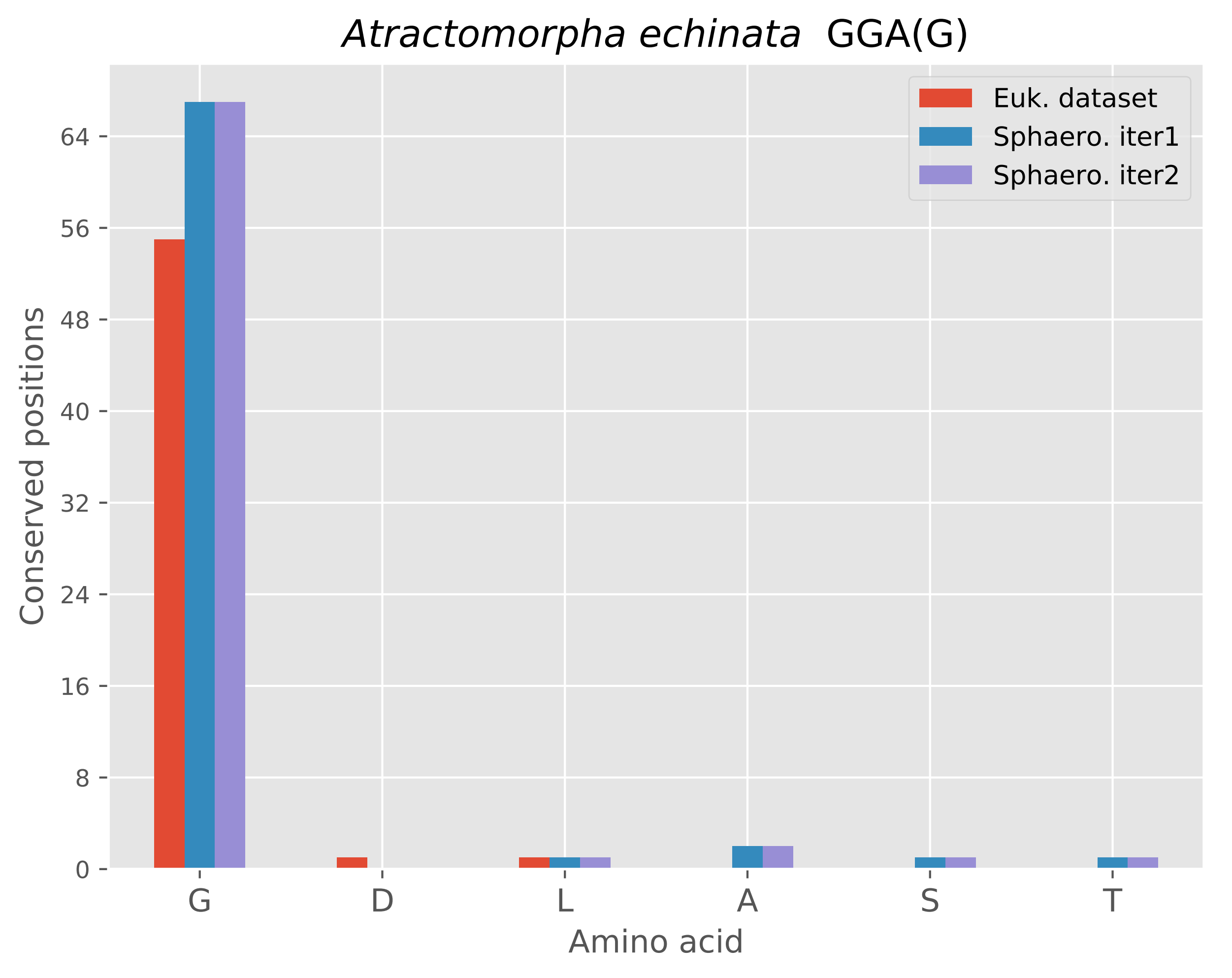

Atractomorpha echinata GGA(G)
Euk. dataset
Sphaero. iter1
Sphaero. iter2
Conserved positions
0
8
16
24
32
40
48
56
64
G
D
L
A
S
T
Amino acid

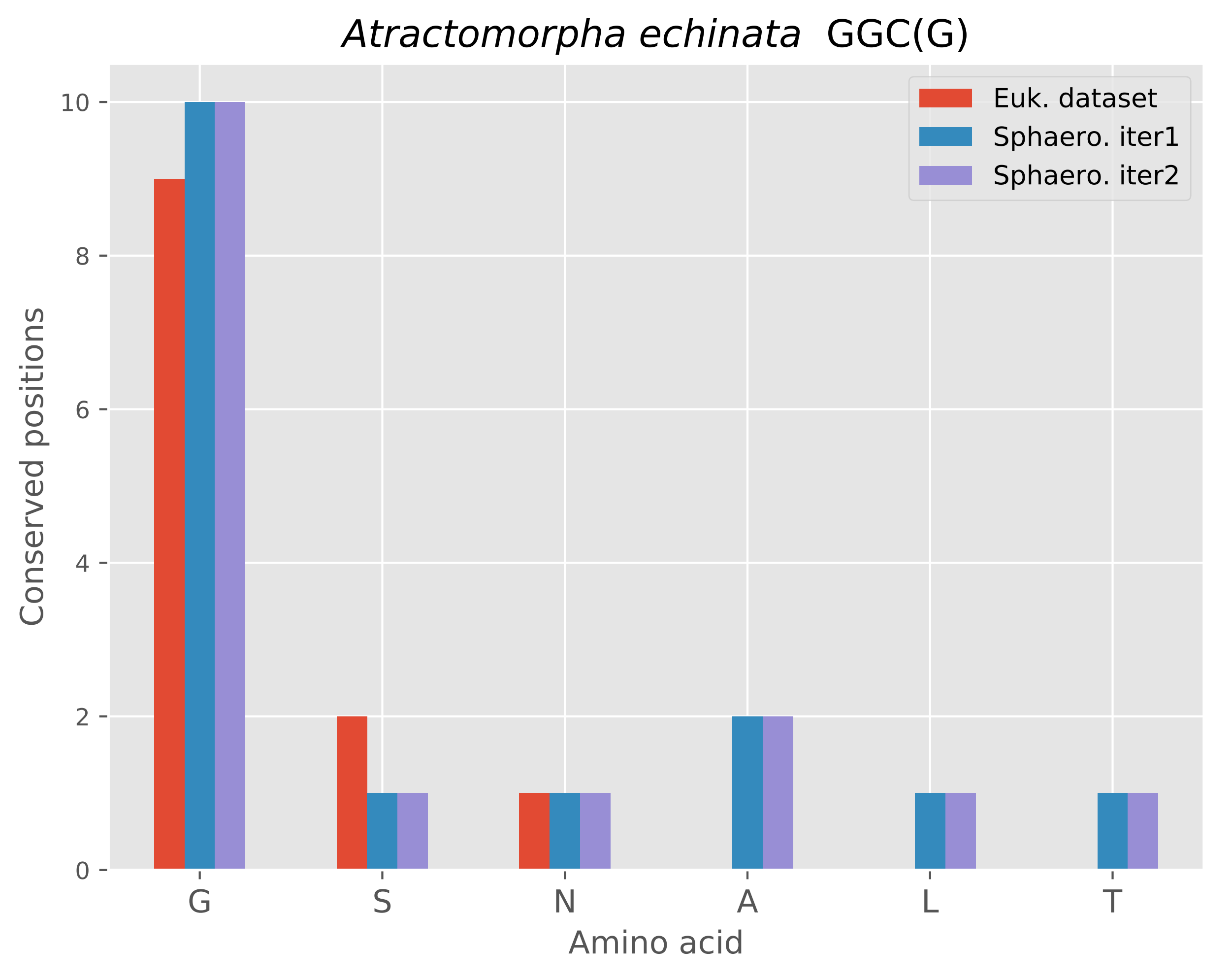

Atractomorpha echinata GGC(G)
Euk. dataset
Sphaero. iter1
Sphaero. iter2
Conserved positions
0
2
4
6
8
10
G
S
N
A
L
T
Amino acid

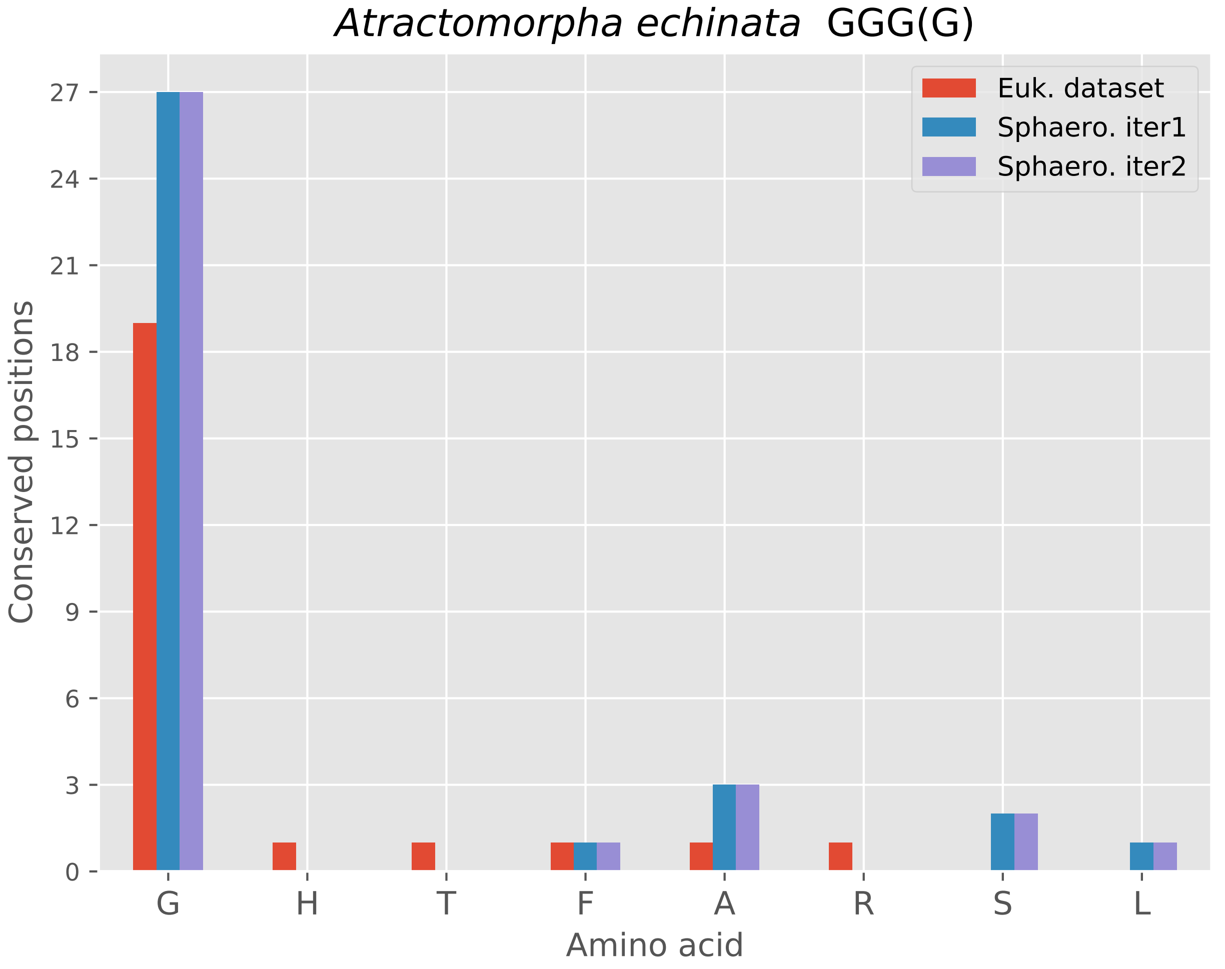

Atractomorpha echinata GGG(G)
Euk. dataset
Sphaero. iter1
Sphaero. iter2
Conserved positions
0
3
6
9
12
15
18
21
24
27
G
H
T
F
A
R
S
L
Amino acid

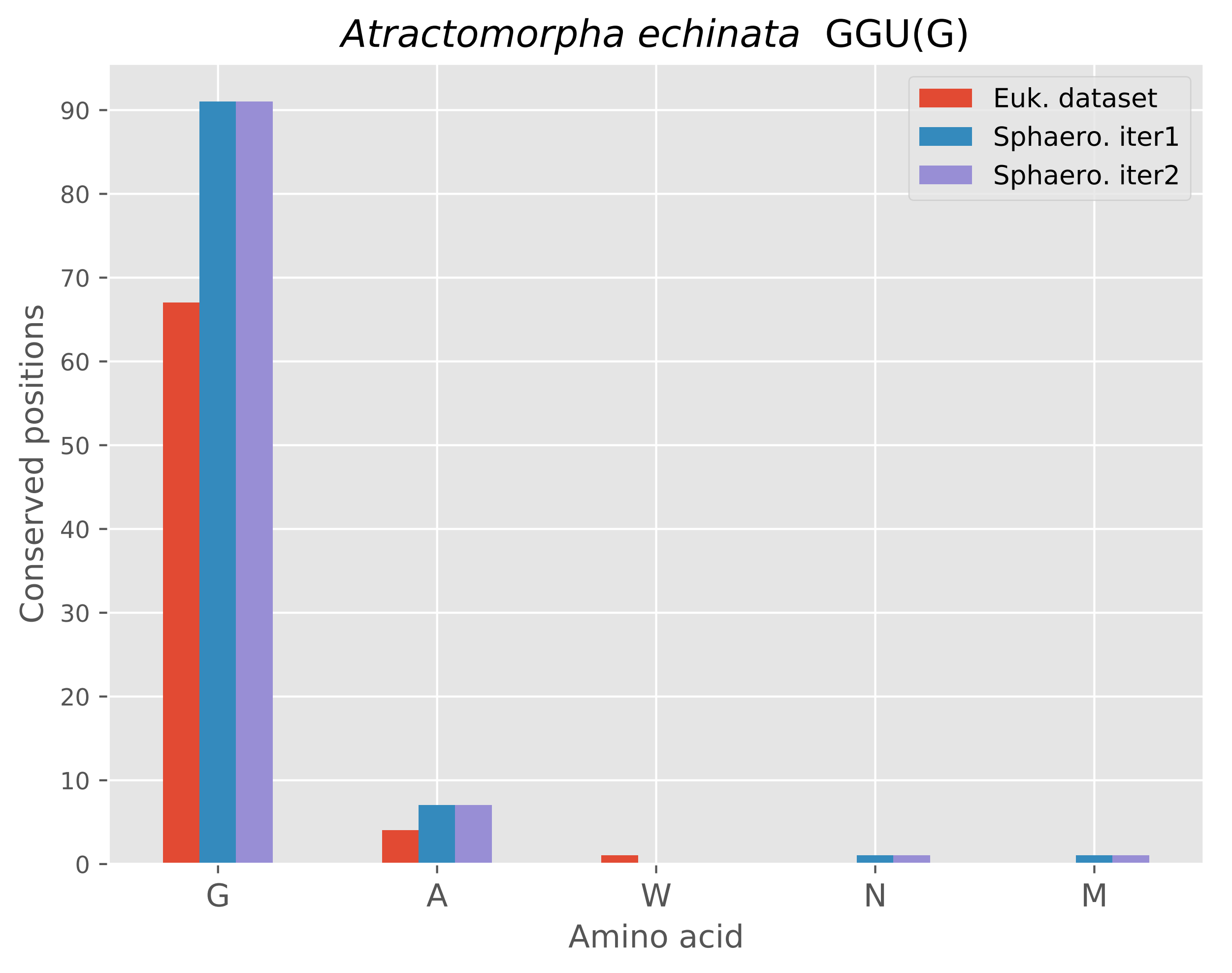
Atractomorpha echinata GGU(G)
Euk. dataset
Sphaero. iter1
Sphaero. iter2
Conserved positions
0
10
20
30
40
50
60
70
80
90
G
A
W
N
M
Amino acid

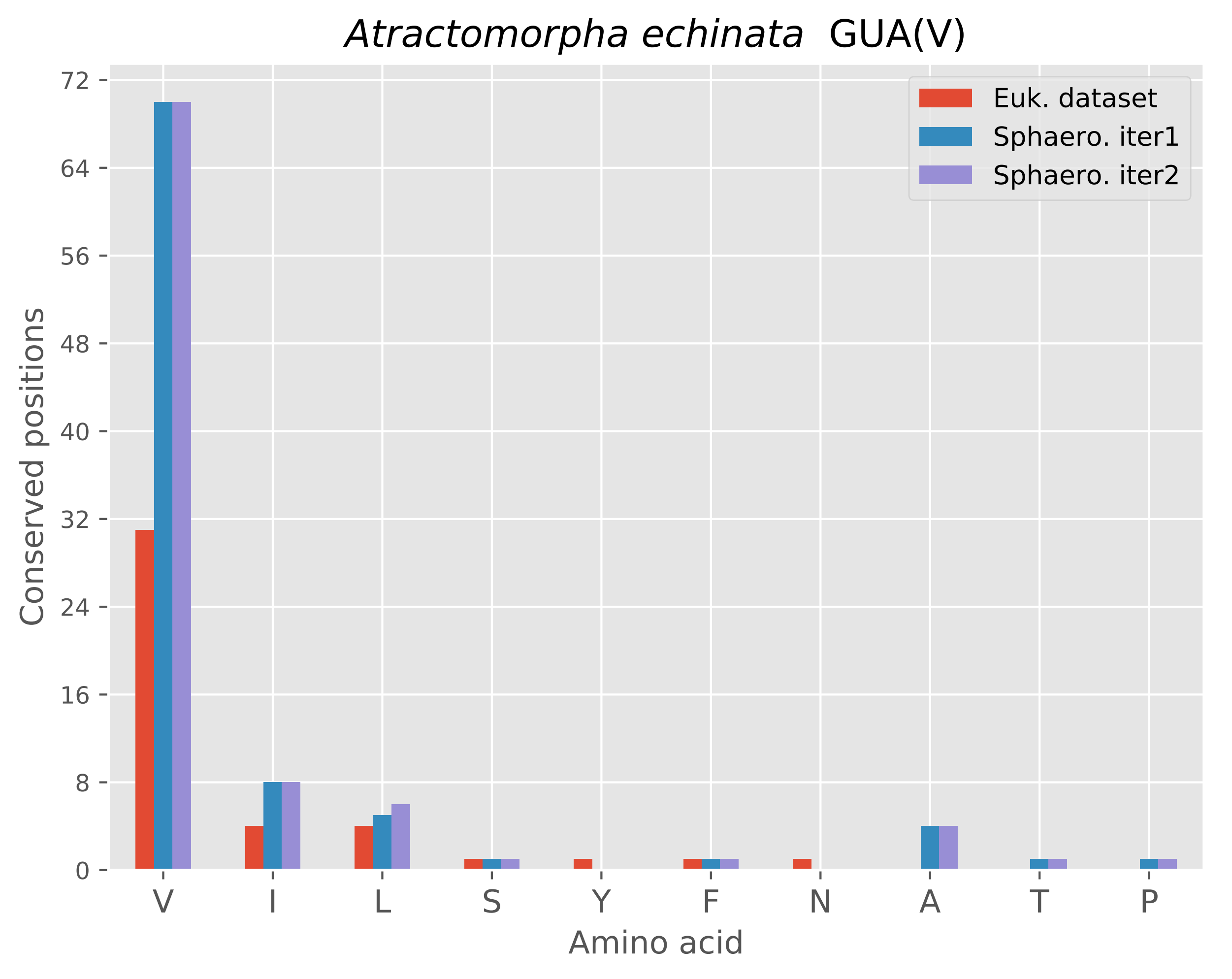

Atractomorpha echinata GUA(V)
Euk. dataset
Sphaero. iter1
Sphaero. iter2
Conserved positions
Amino acid
0
8
16
24
32
40
48
56
64
72
V
I
L
S
Y
F
N
A
T
P

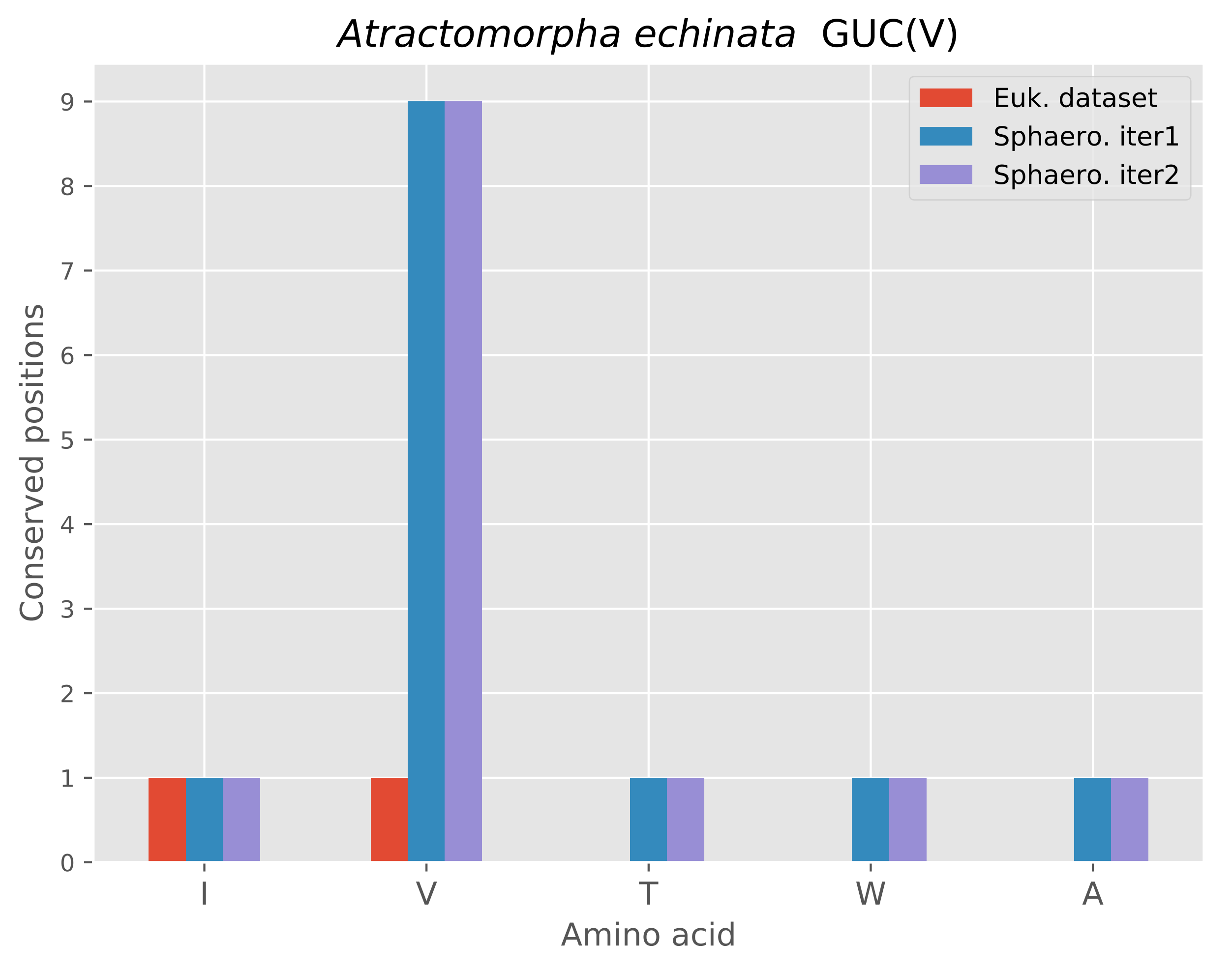

Atractomorpha echinata GUC(V)
Euk. dataset
Sphaero. iter1
Sphaero. iter2
Conserved positions
0
1
2
3
4
5
6
7
8
9
I
V
T
W
A
Amino acid

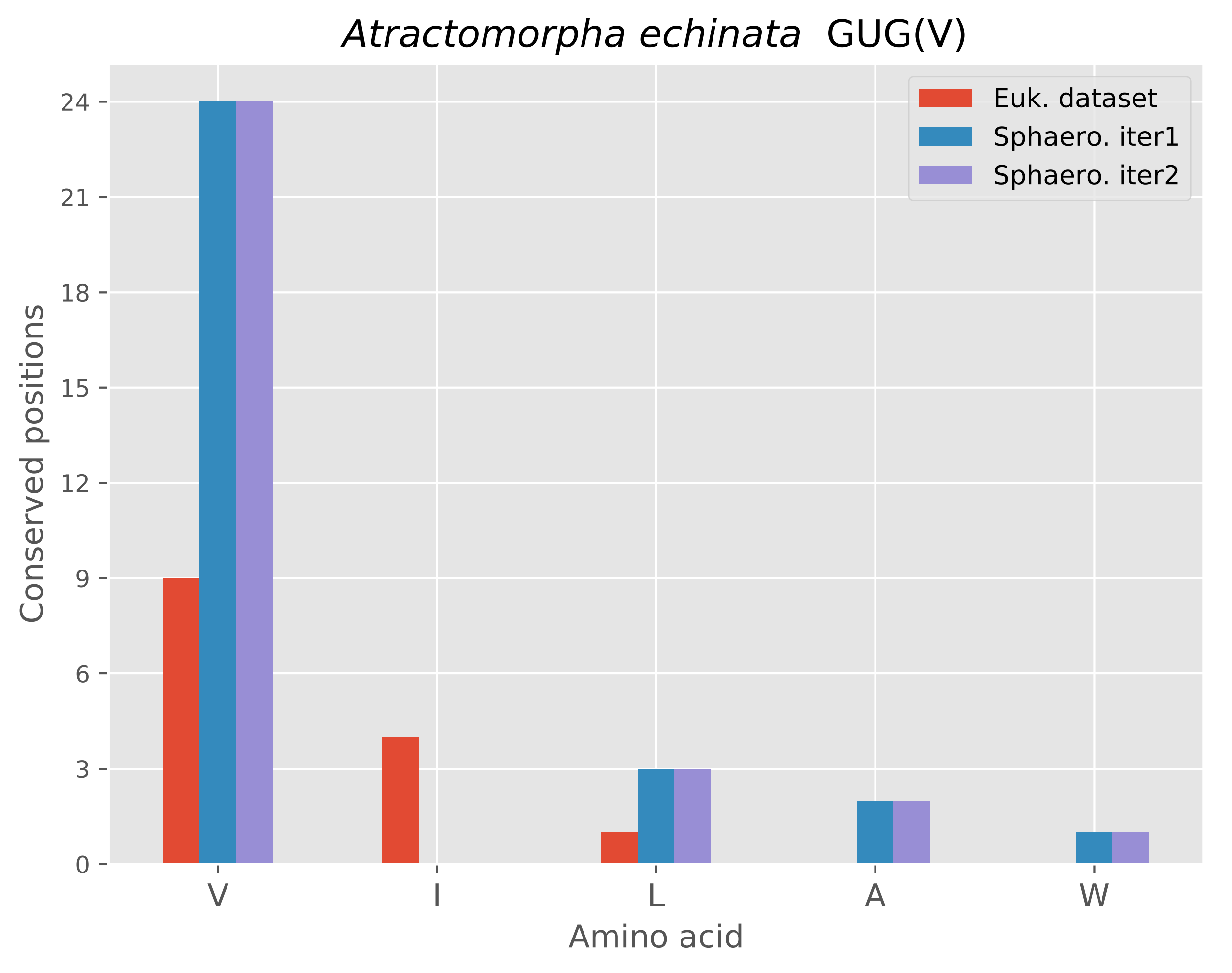

Atractomorpha echinata GUG(V)
Euk. dataset
Sphaero. iter1
Sphaero. iter2
Conserved positions
0
3
6
9
12
15
18
21
24
V
I
L
A
W
Amino acid

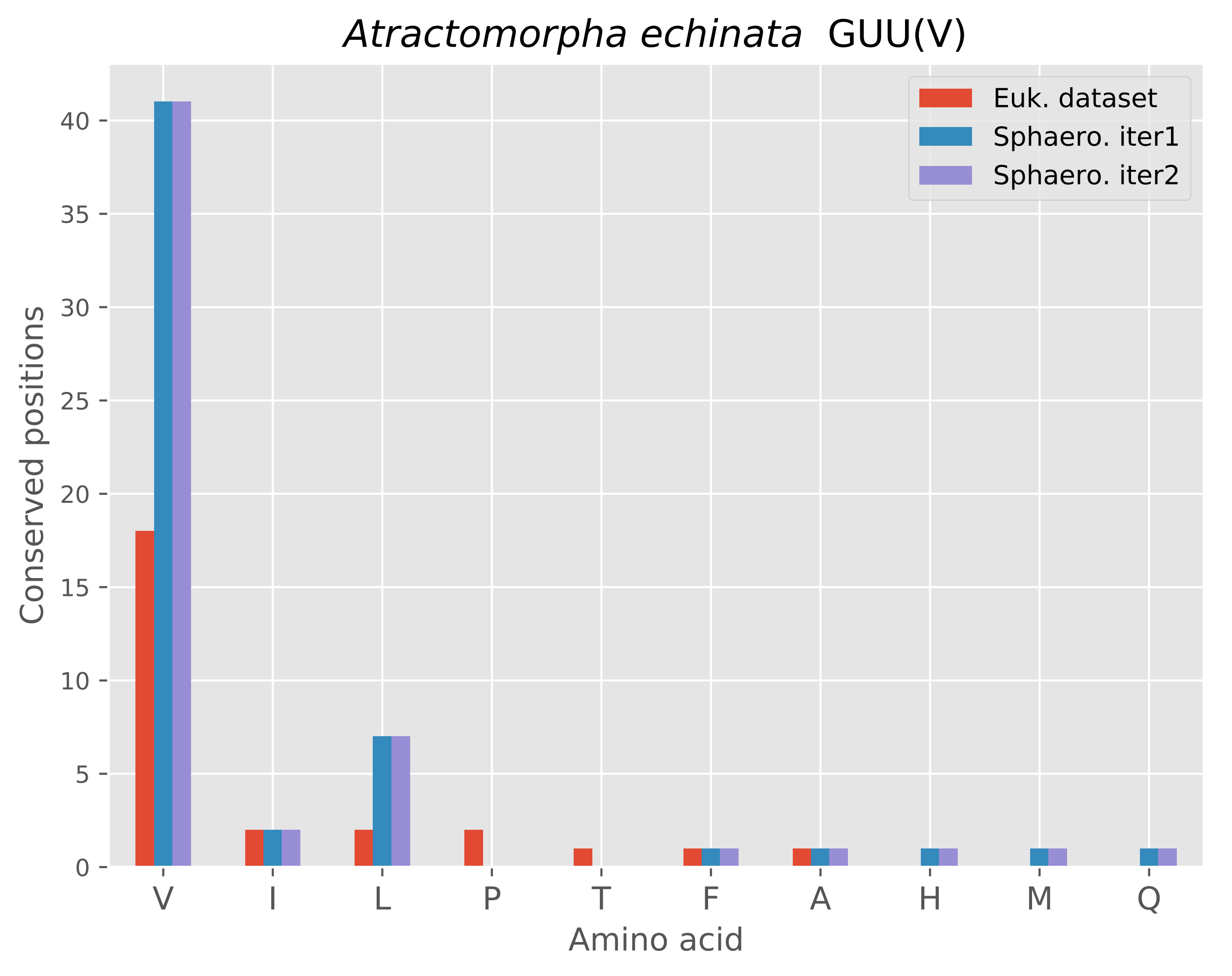

Atractomorpha echinata GUU(V)
Euk. dataset
Sphaero. iter1
Sphaero. iter2
Conserved positions
0
5
10
15
20
25
30
35
40
V
I
L
P
T
F
A
H
M
Q
Amino acid

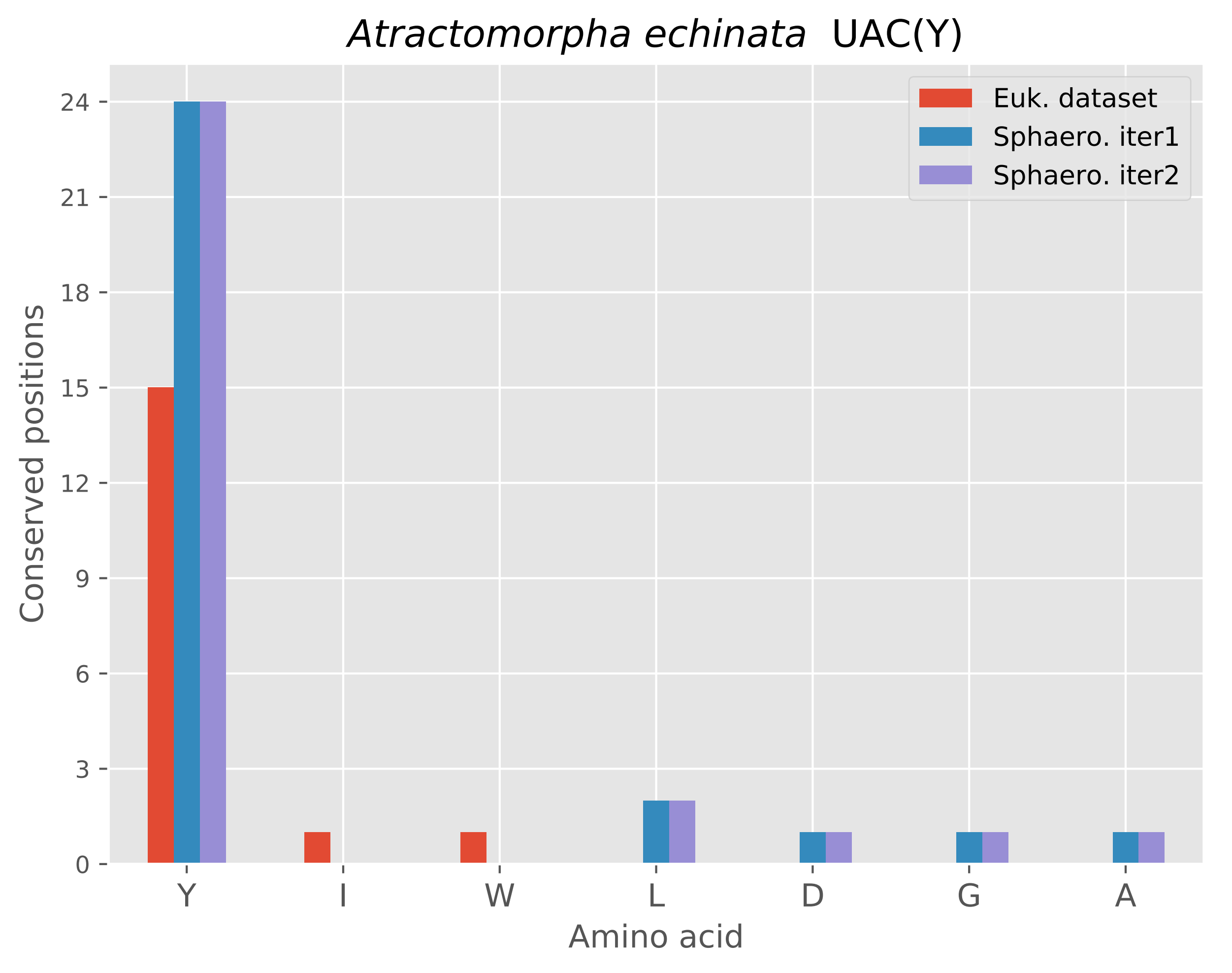

Atractomorpha echinata UAC(Y)
Euk. dataset
Sphaero. iter1
Sphaero. iter2
Conserved positions
0
3
6
9
12
15
18
21
24
Y
I
W
L
D
G
A
Amino acid

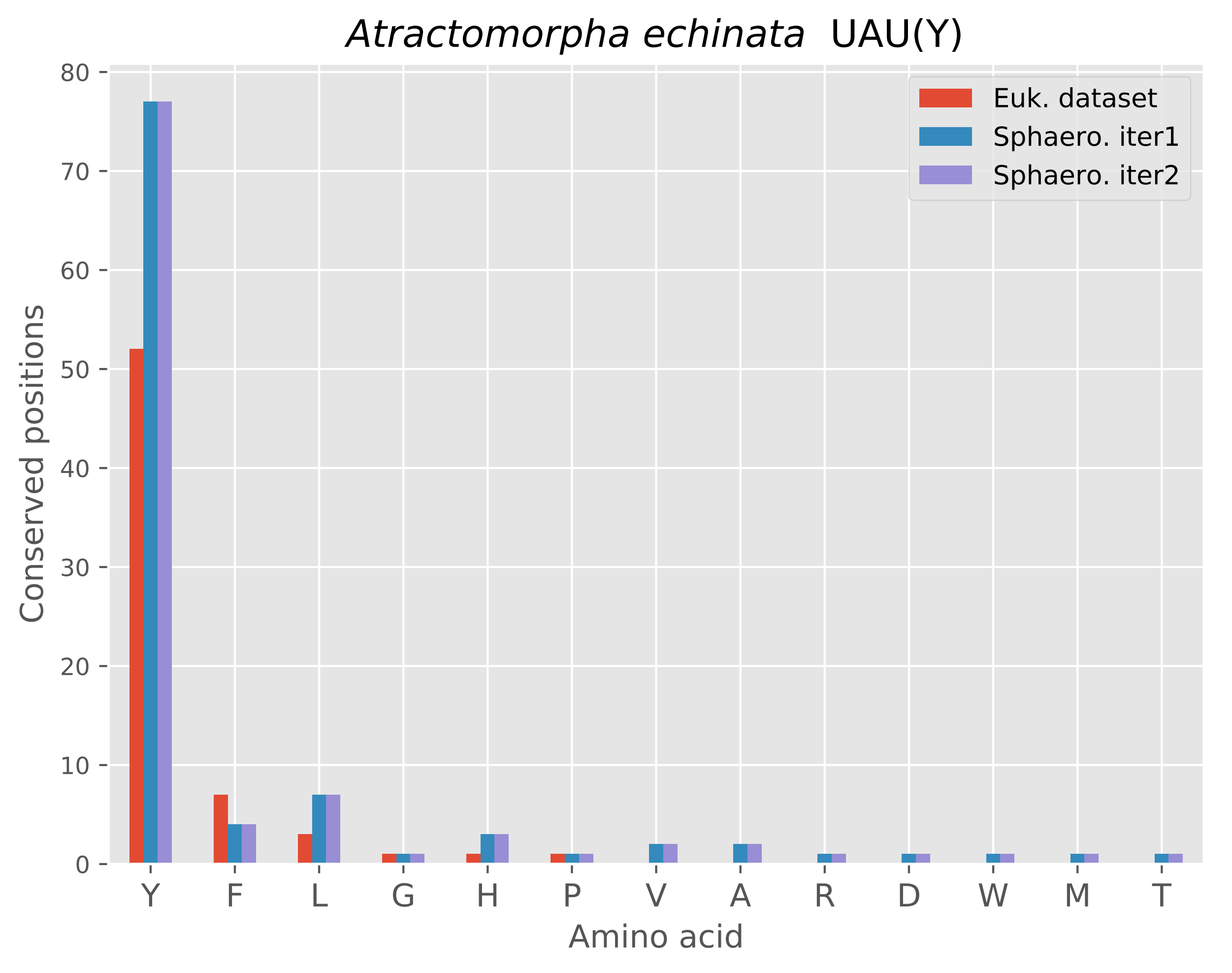

Atractomorpha echinata UAU(Y)
Euk. dataset
Sphaero. iter1
Sphaero. iter2
Conserved positions
Amino acid
0
10
20
30
40
50
60
70
80
Y
F
L
G
H
P
V
A
R
D
W
M
T

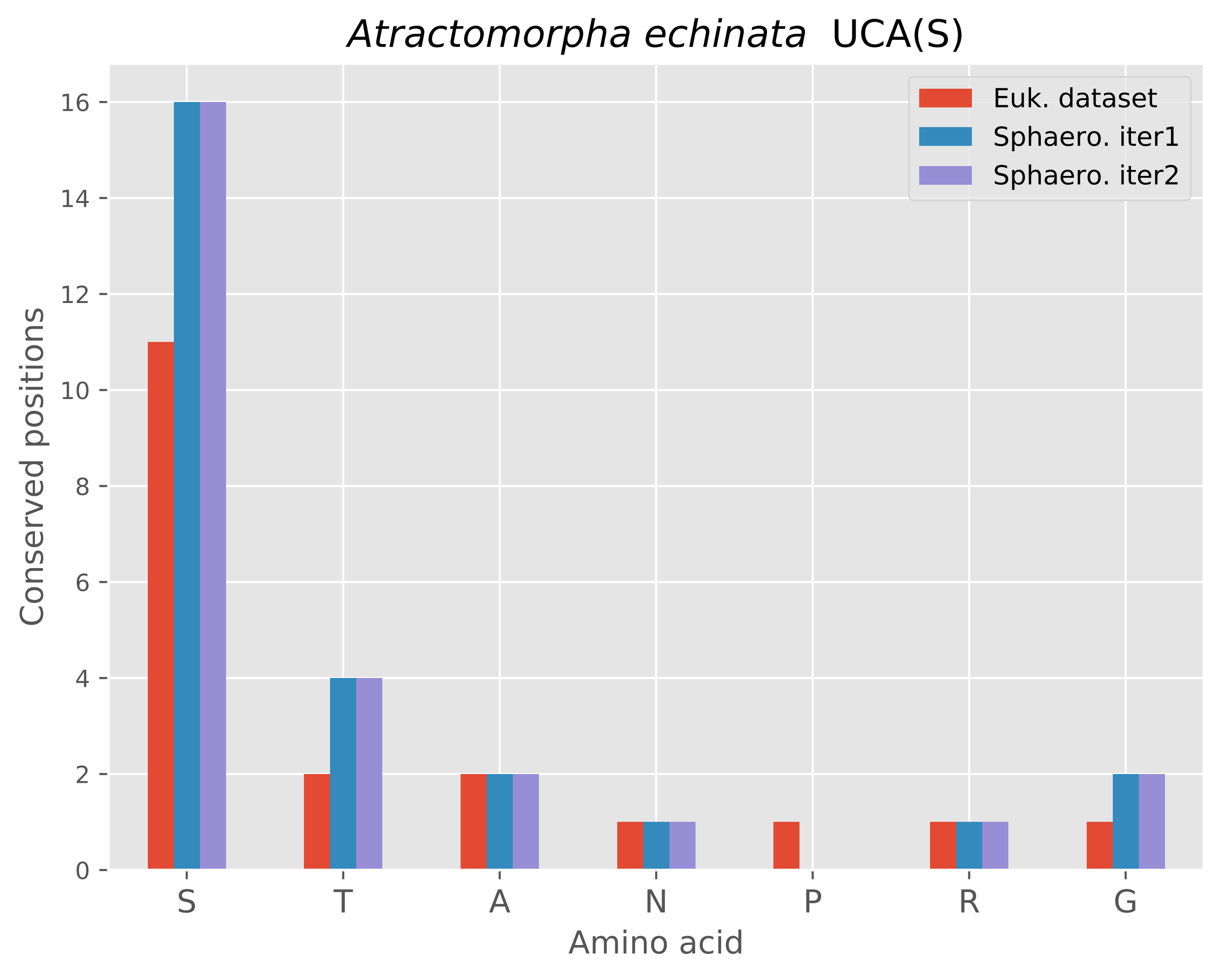

Atractomorpha echinata UCA(S)
Euk. dataset
Sphaero. iter1
Sphaero. iter2
Conserved positions
0
2
4
6
8
10
12
14
16
S
T
A
N
P
R
G
Amino acid

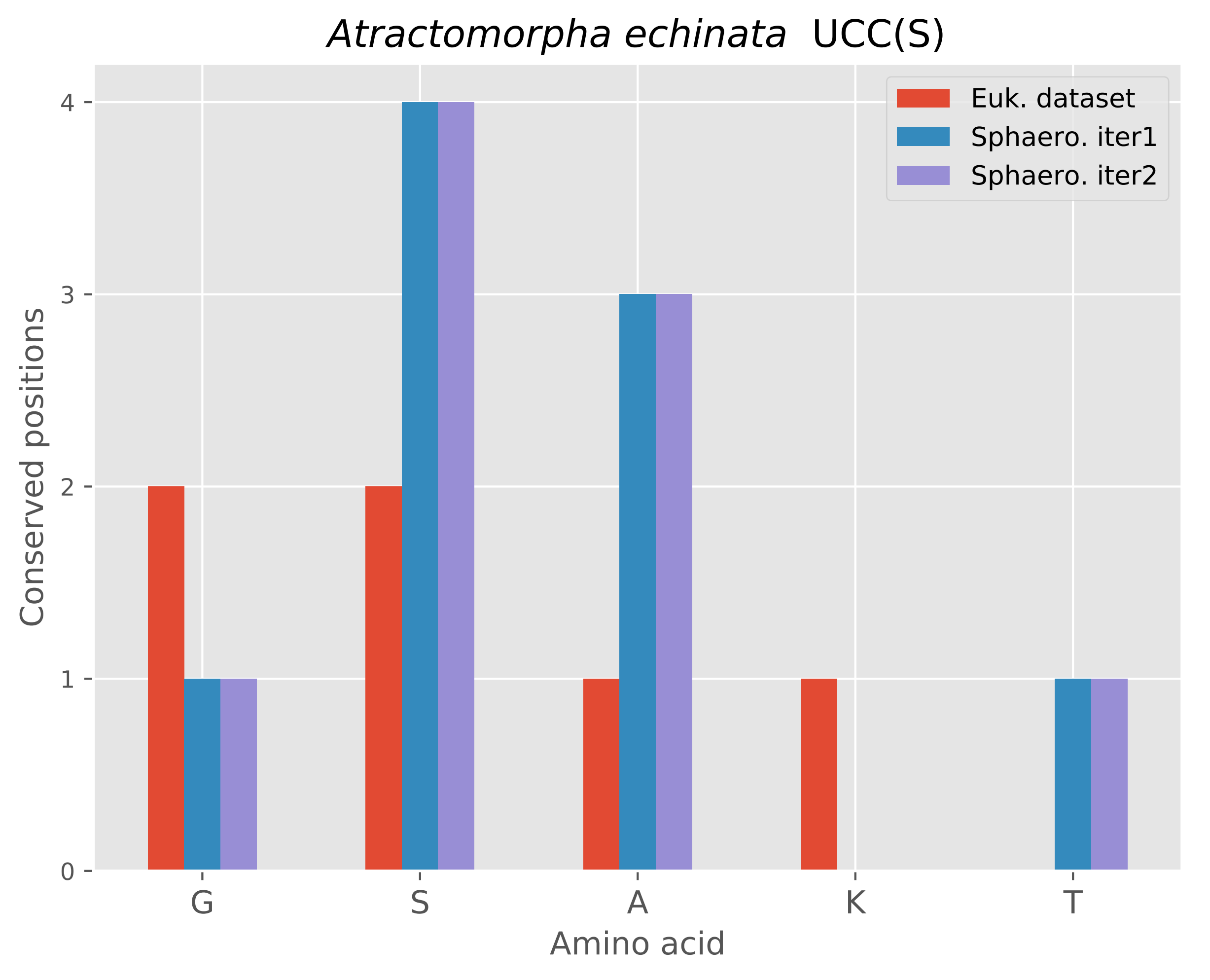
Atractomorpha echinata UCC(S)
Euk. dataset
Sphaero. iter1
Sphaero. iter2
Conserved positions
0
1
2
3
4
G
S
A
K
T
Amino acid

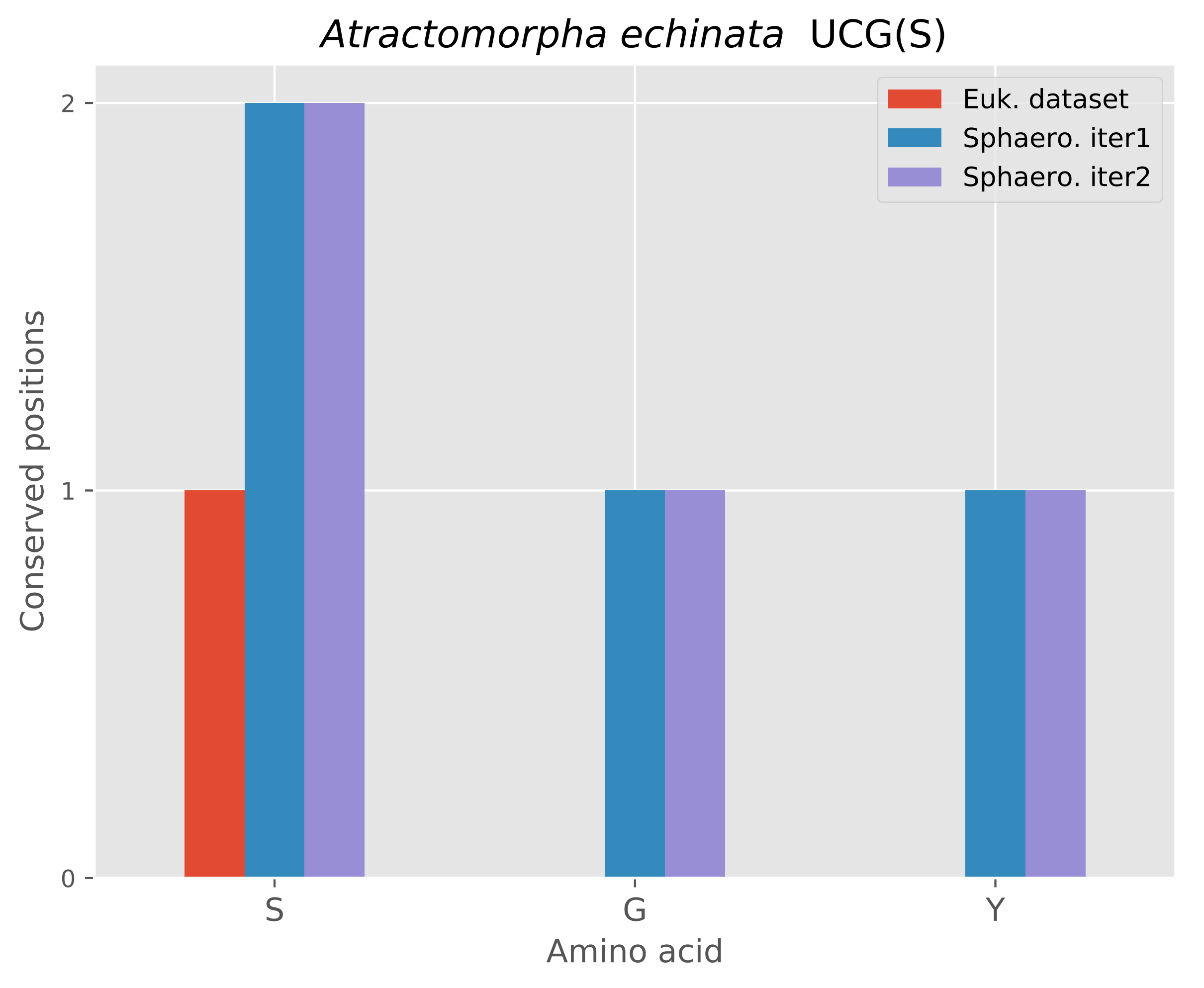
Atractomorpha echinata UCG(S)
Euk. dataset
Sphaero. iter1
Sphaero. iter2
Conserved positions
0
1
2
S
G
Y
Amino acid

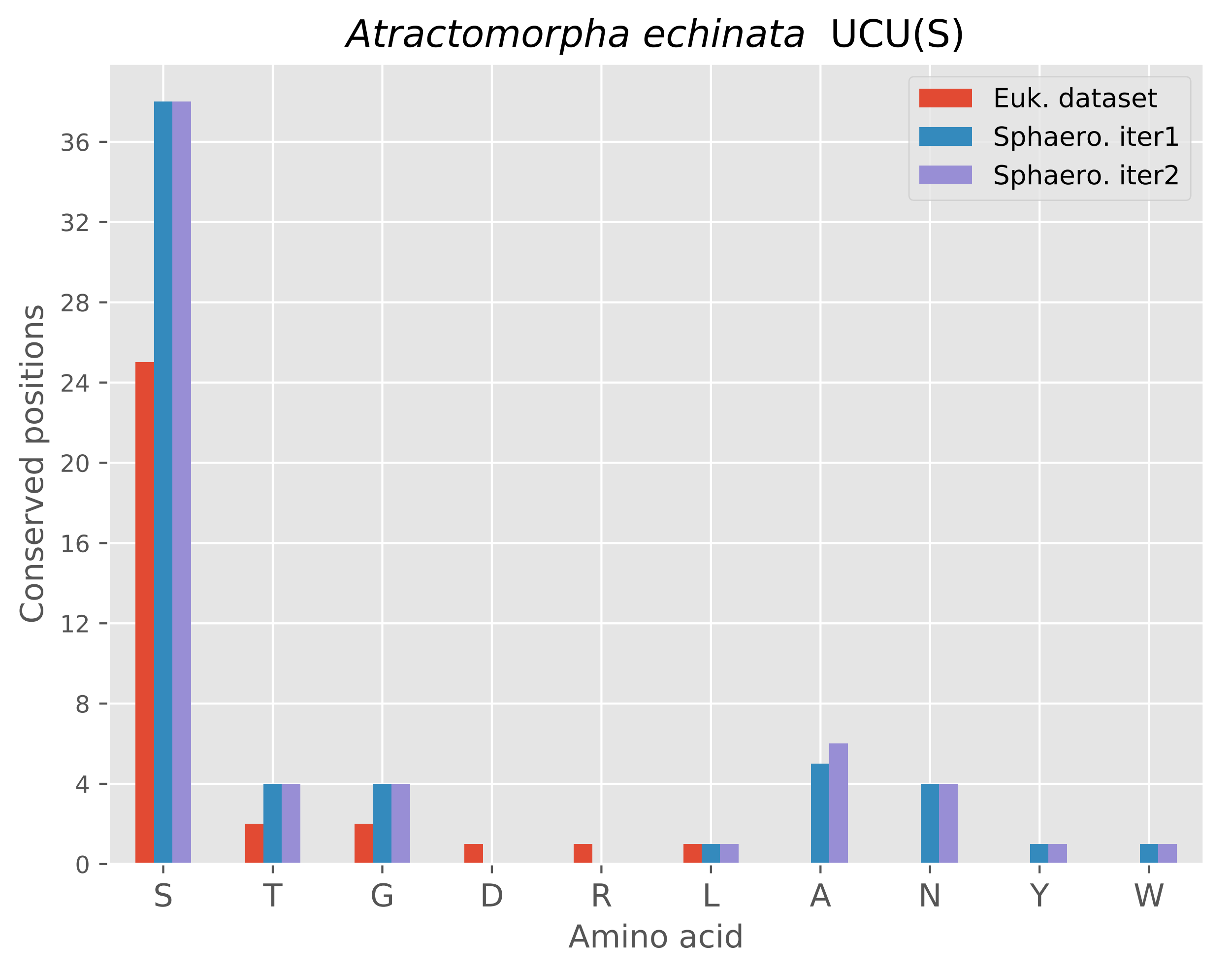

Atractomorpha echinata UCU(S)
Euk. dataset
Sphaero. iter1
Sphaero. iter2
Conserved positions
0
4
8
12
16
20
24
28
32
36
S
T
G
D
R
L
A
N
Y
W
Amino acid

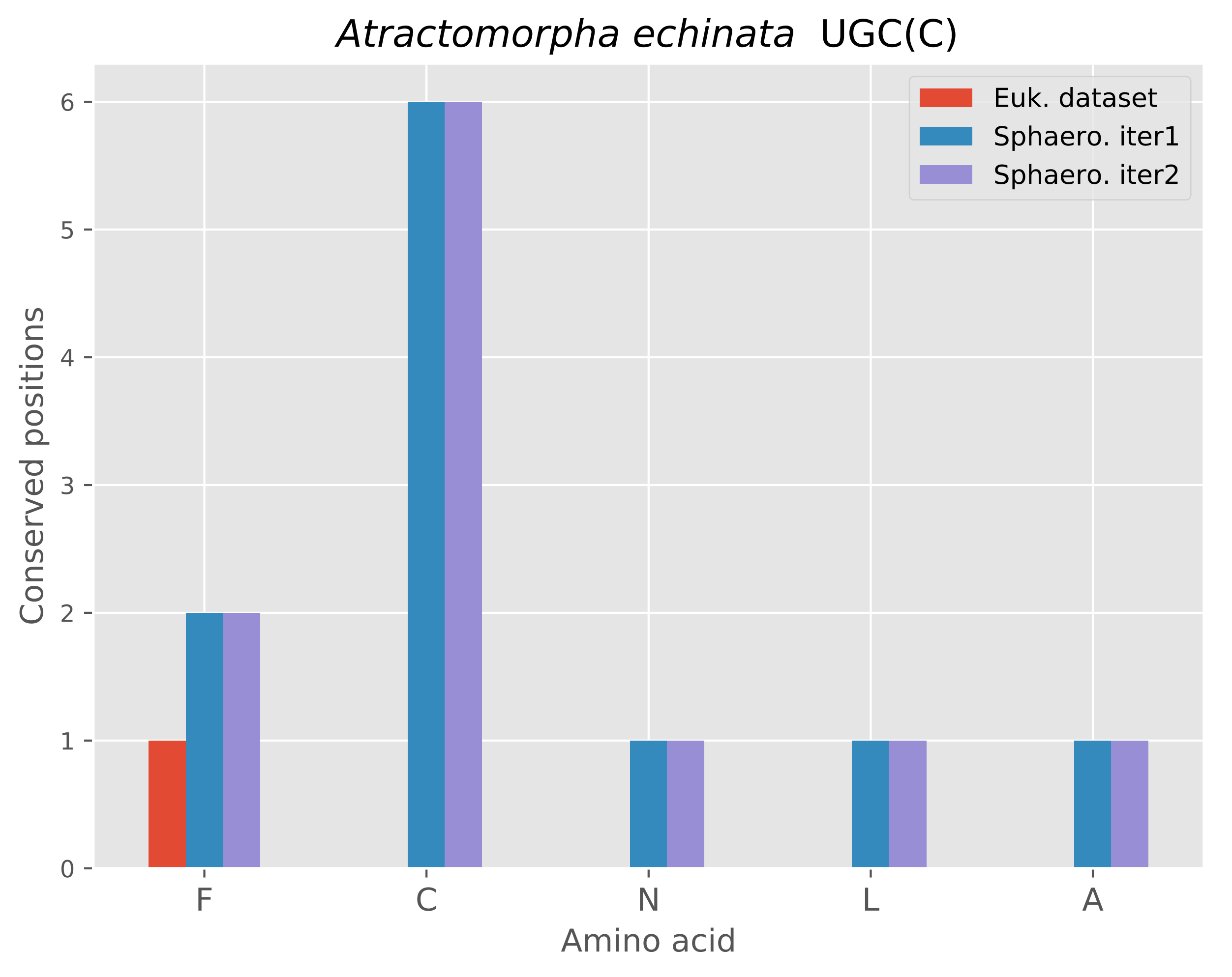

Atractomorpha echinata UGC(C)
Euk. dataset
Sphaero. iter1
Sphaero. iter2
Conserved positions
0
1
2
3
4
5
6
F
C
N
L
A
Amino acid

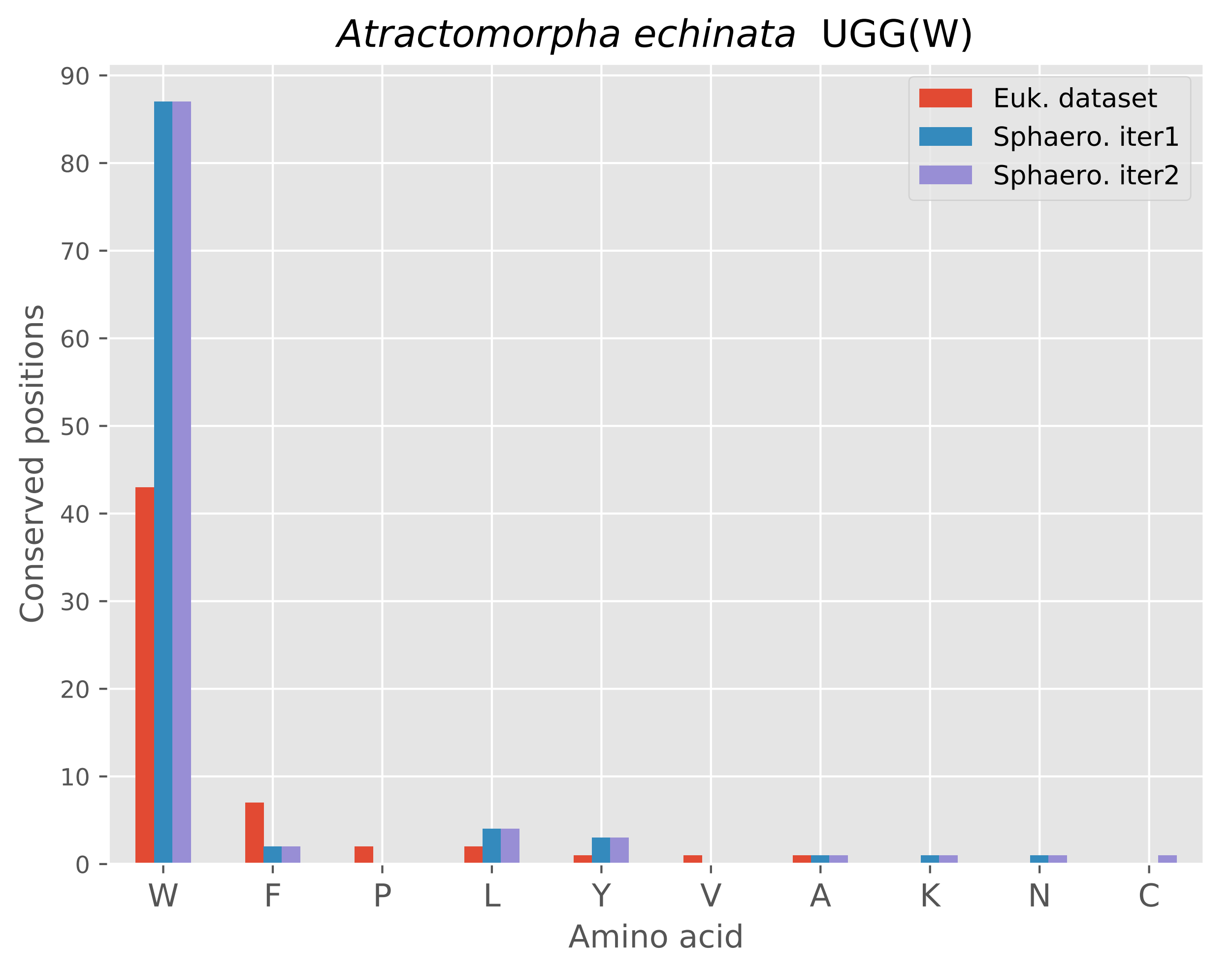

Atractomorpha echinata UGG(W)
Euk. dataset
Sphaero. iter1
Sphaero. iter2
Conserved positions
0
10
20
30
40
50
60
70
80
90
W
F
P
L
Y
V
A
K
N
C
Amino acid

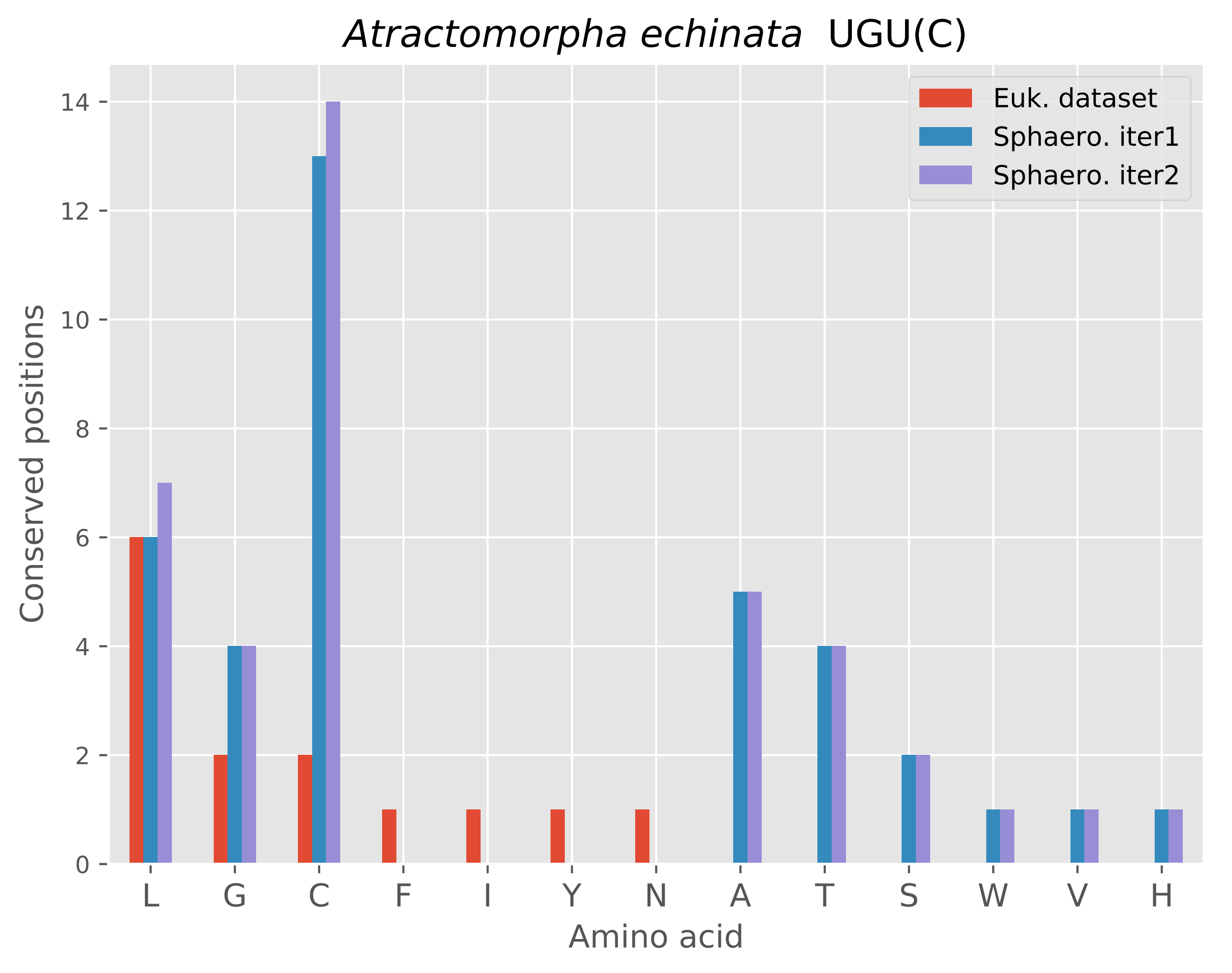
Atractomorpha echinata UGU(C)
Euk. dataset
Sphaero. iter1
Sphaero. iter2
Conserved positions
0
2
4
6
8
10
12
14
L
G
C
F
I
Y
N
A
T
S
W
V
H
Amino acid

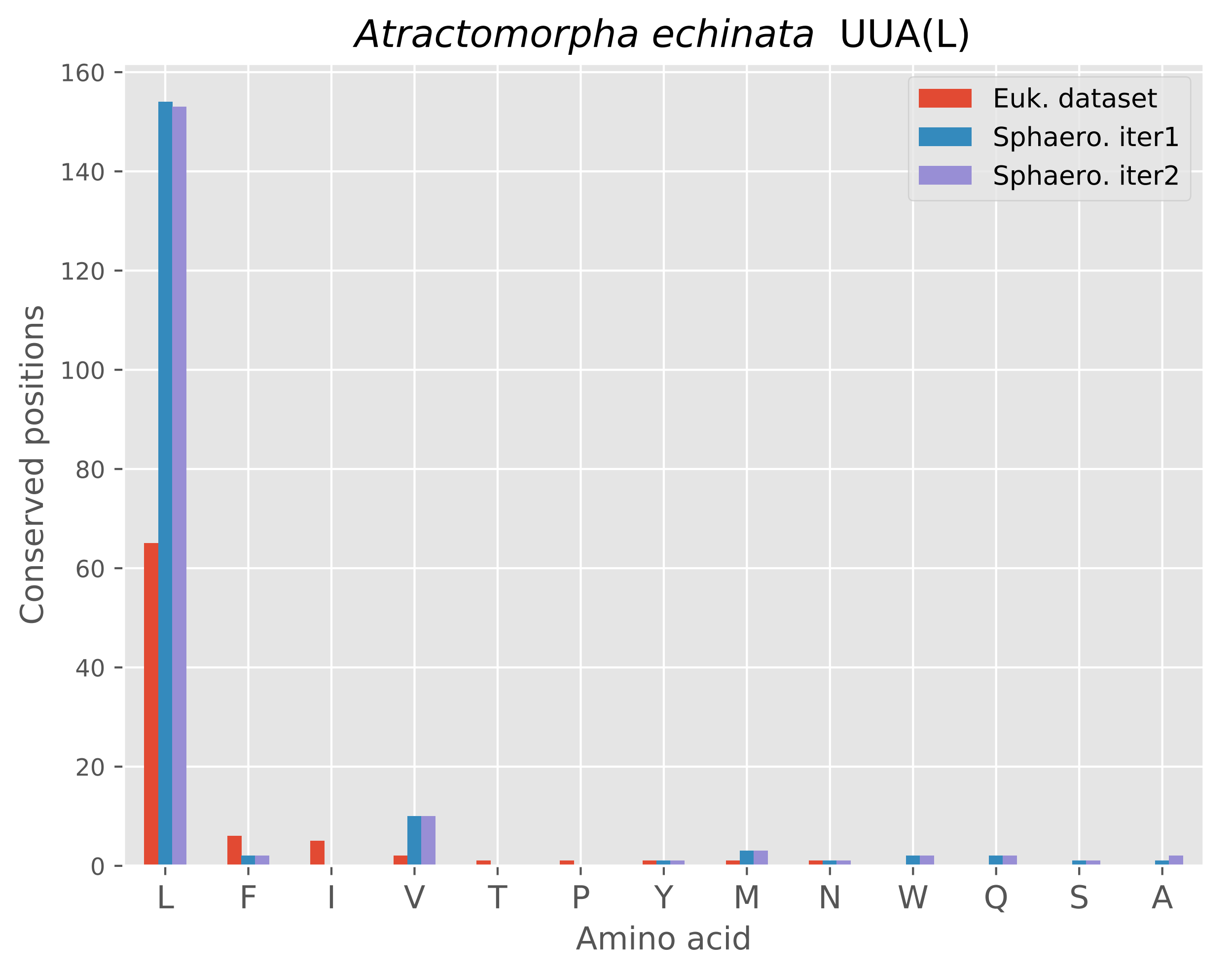

Atractomorpha echinata UUA(L)
Euk. dataset
Sphaero. iter1
Sphaero. iter2
Conserved positions
Amino acid
0
20
40
60
80
100
120
140
160
L
F
I
V
T
P
Y
M
N
W
Q
S
A

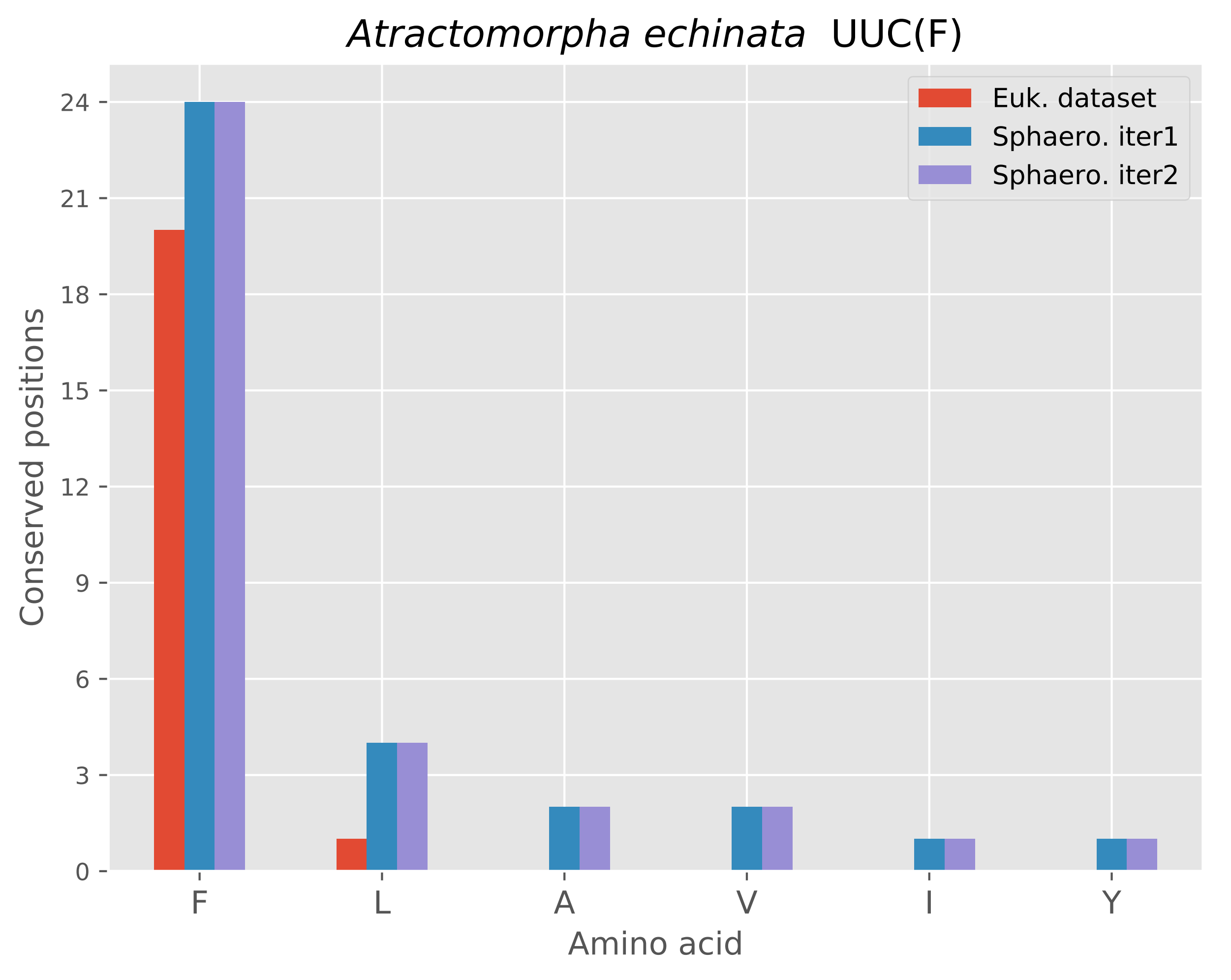

Atractomorpha echinata UUC(F)
Euk. dataset
Sphaero. iter1
Sphaero. iter2
Conserved positions
0
3
6
9
12
15
18
21
24
F
L
A
V
I
Y
Amino acid

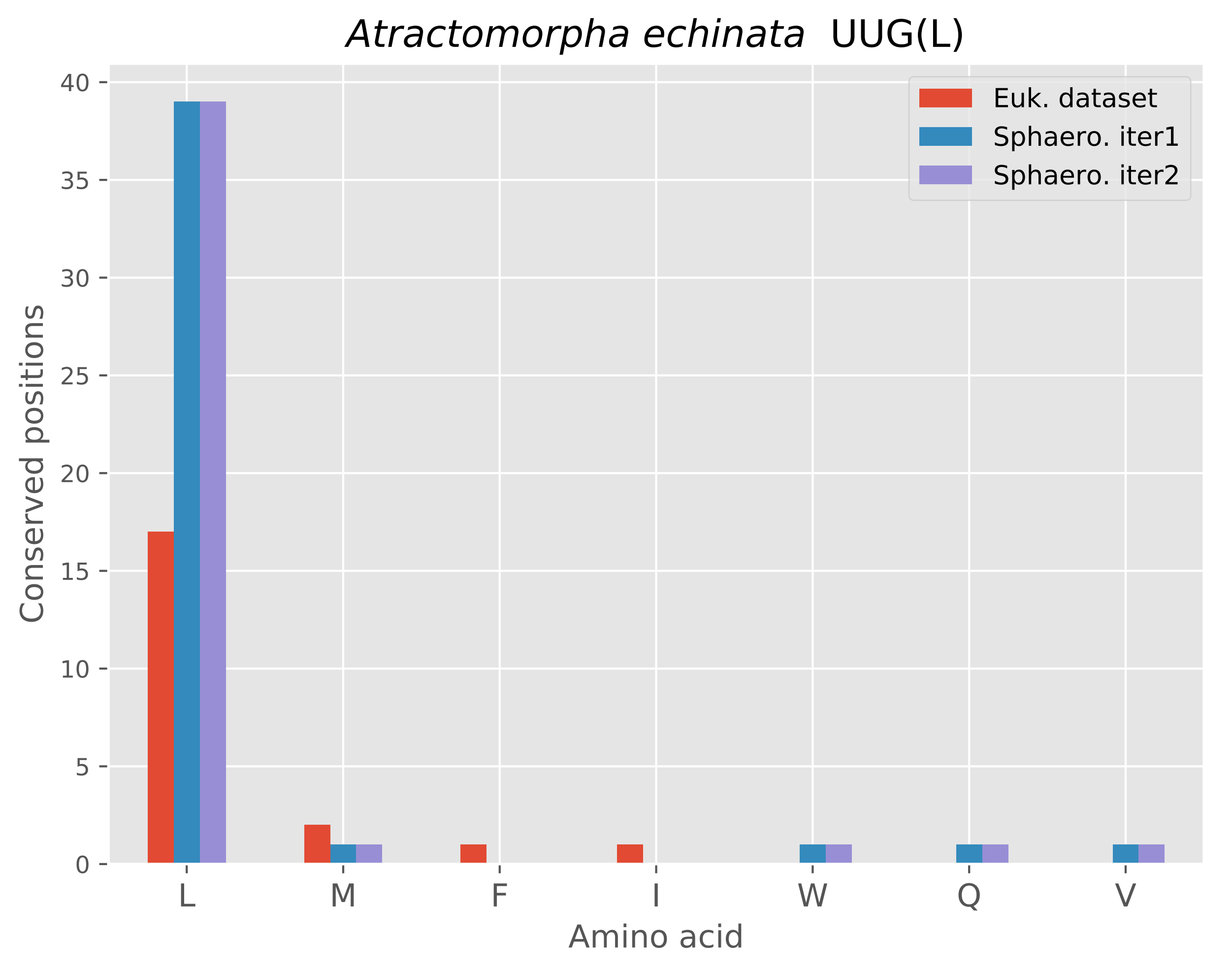

Atractomorpha echinata UUG(L)
Euk. dataset
Sphaero. iter1
Sphaero. iter2
Conserved positions
0
5
10
15
20
25
30
35
40
L
M
F
I
W
Q
V
Amino acid

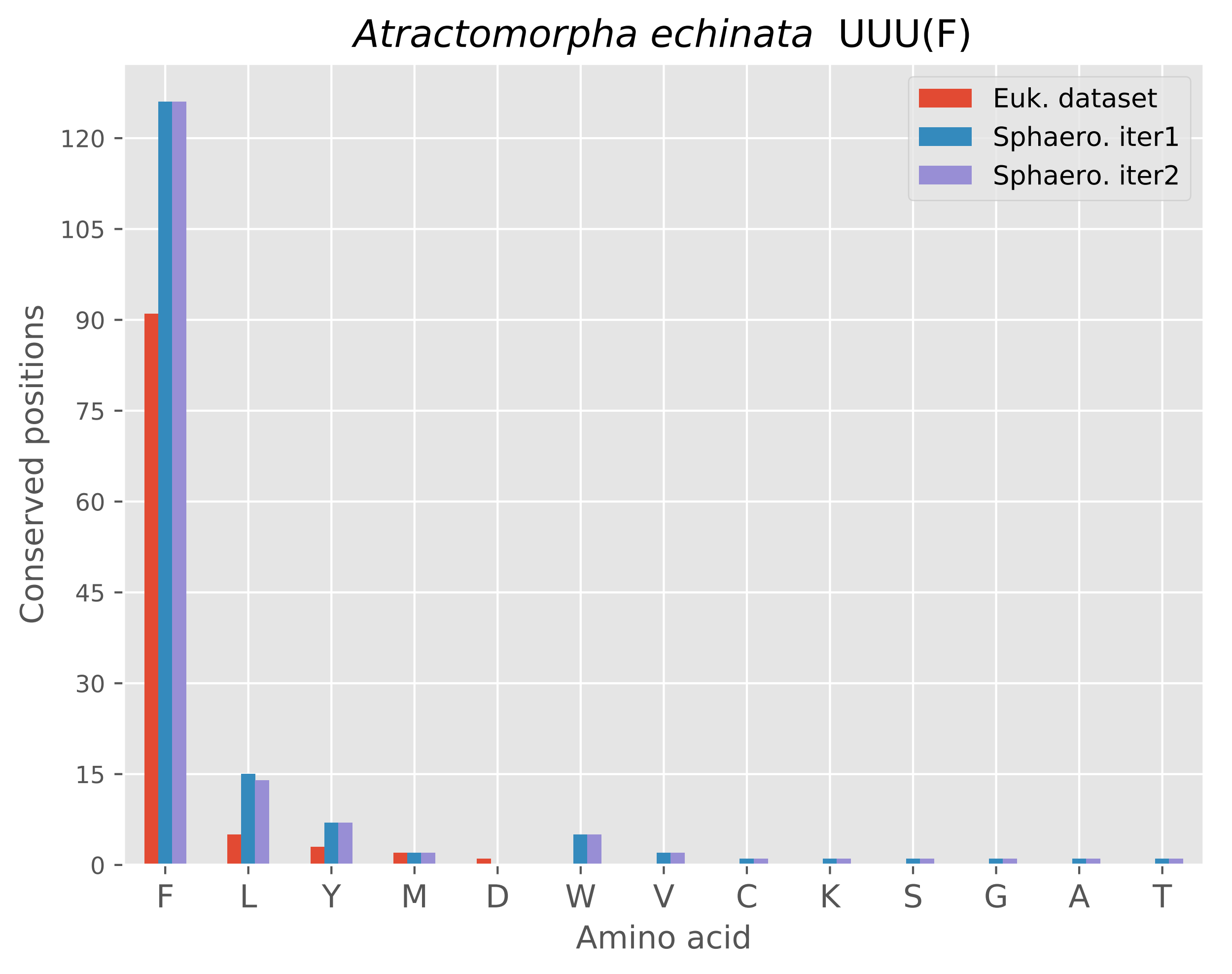
Atractomorpha echinata UUU(F)
Euk. dataset
Sphaero. iter1
Sphaero. iter2
Conserved positions
0
15
30
45
60
75
90
105
120
F
L
Y
M
D
W
V
C
K
S
G
A
T
Amino acid

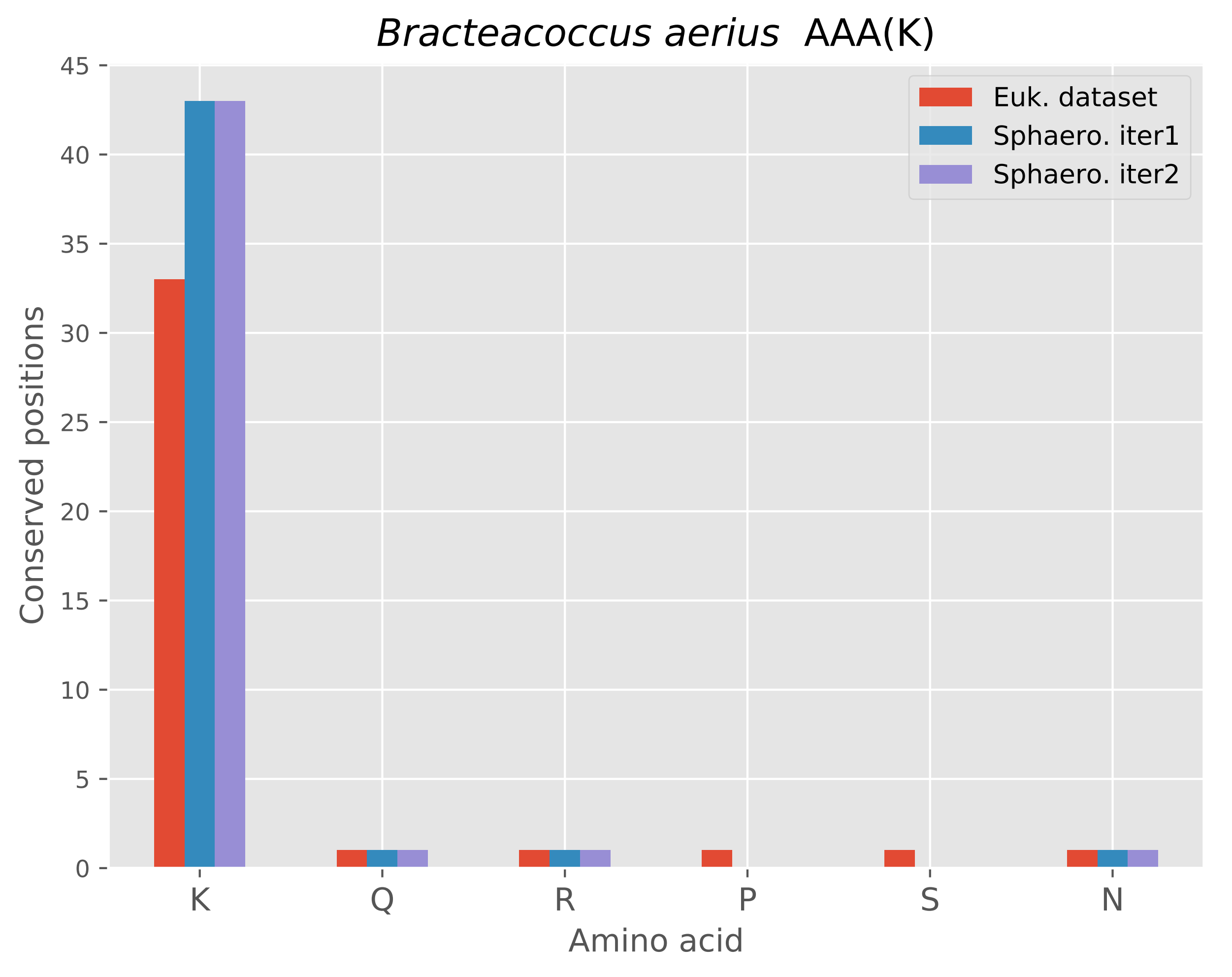

Bracteacoccus aerius AAA(K)
Euk. dataset
Sphaero. iter1
Sphaero. iter2
Conserved positions
0
5
10
15
20
25
30
35
40
45
K
Q
R
P
S
N
Amino acid

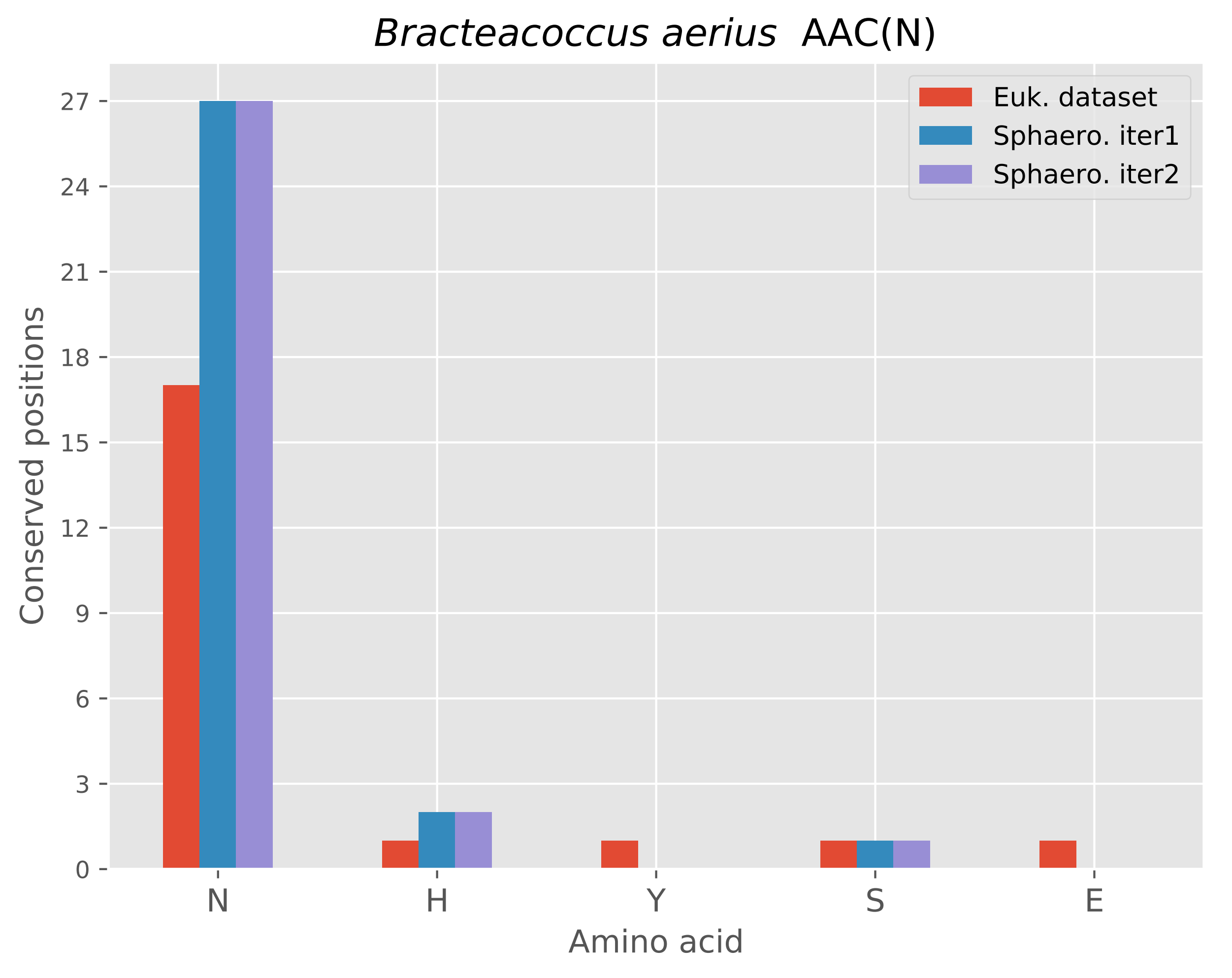

Bracteacoccus aerius AAC(N)
Euk. dataset
Sphaero. iter1
Sphaero. iter2
Conserved positions
0
3
6
9
12
15
18
21
24
27
N
H
Y
S
E
Amino acid

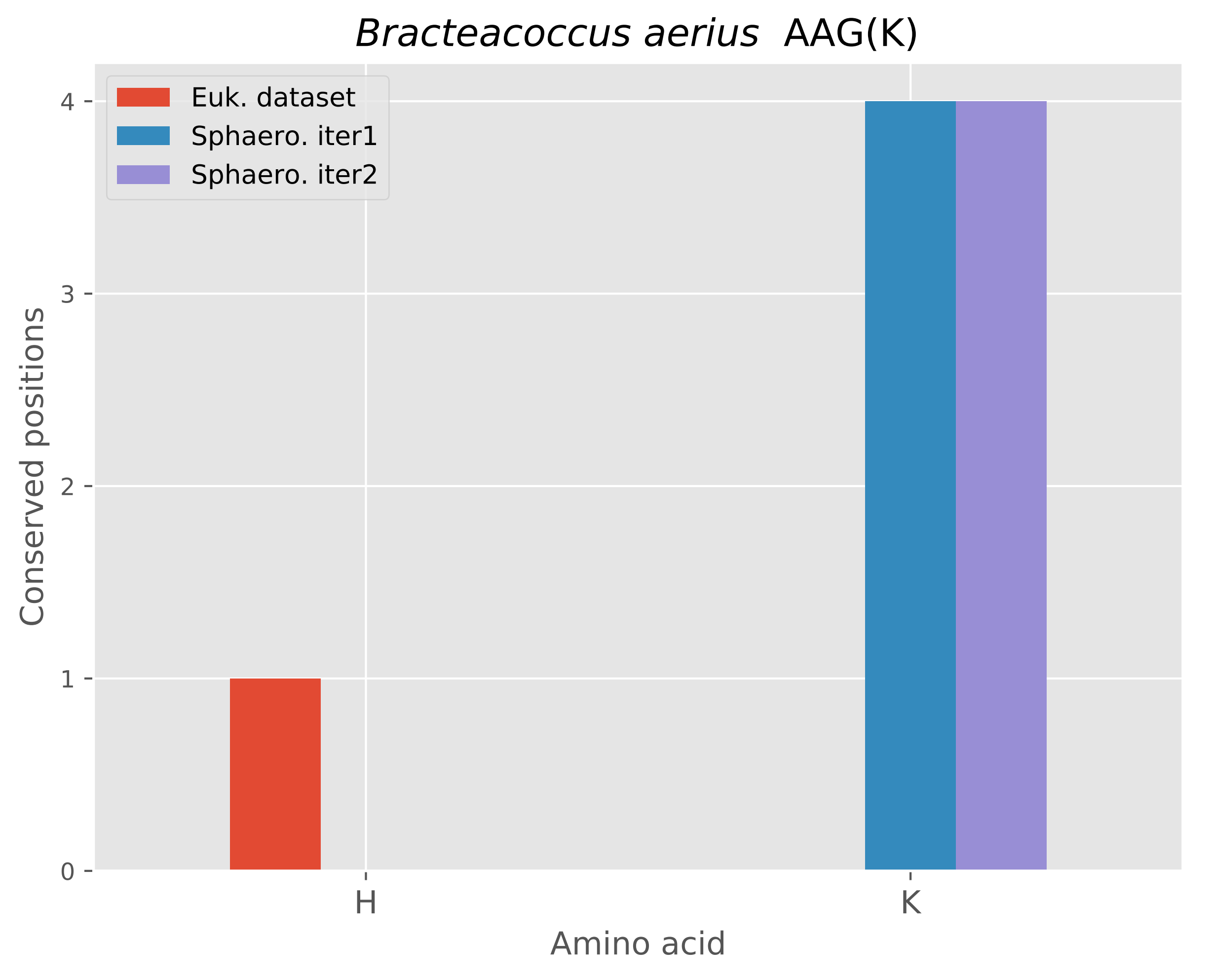

Bracteacoccus aerius AAG(K)
Euk. dataset
Sphaero. iter1
Sphaero. iter2
Conserved positions
0
1
2
3
4
H
K
Amino acid

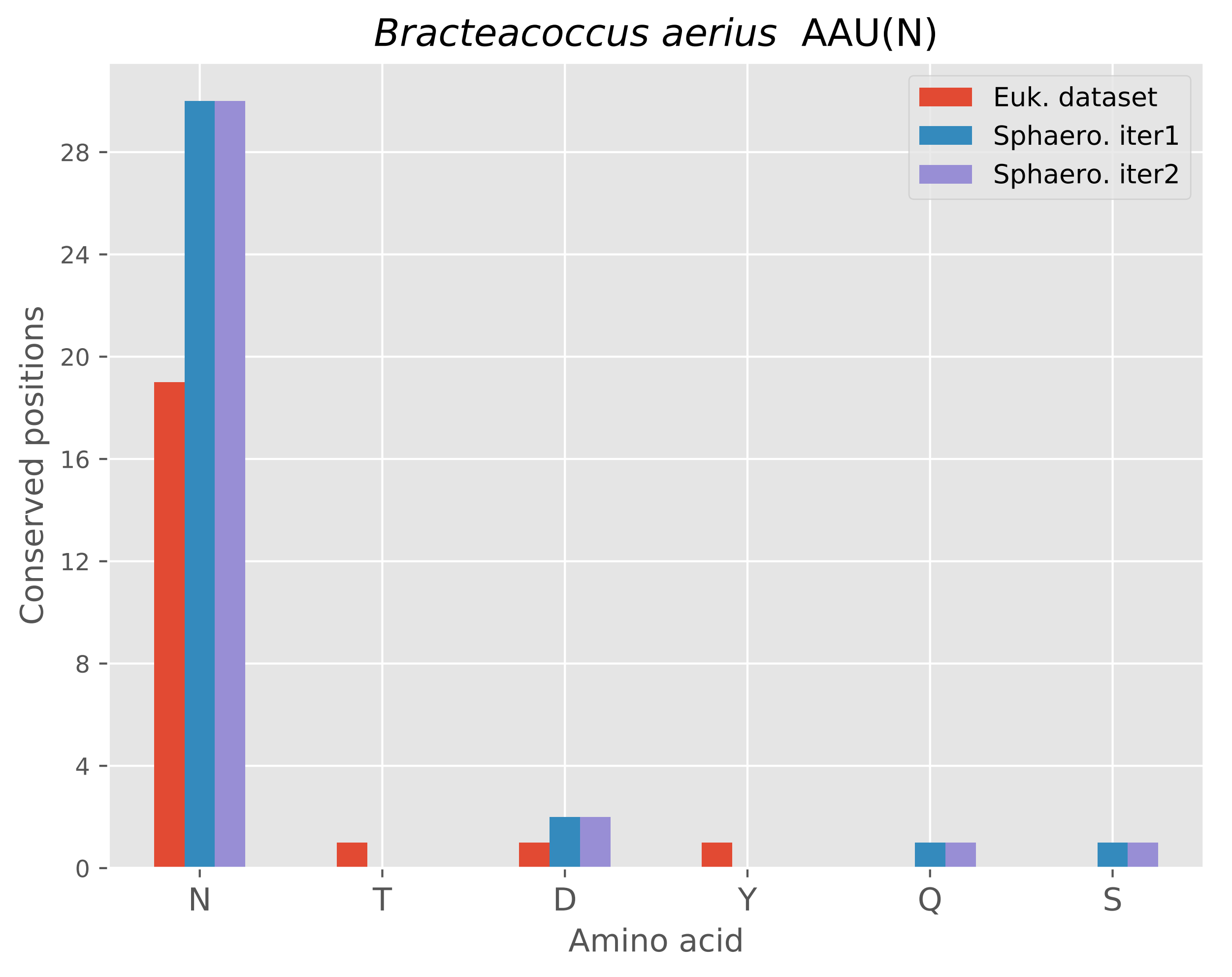

Bracteacoccus aerius AAU(N)
Euk. dataset
Sphaero. iter1
Sphaero. iter2
Conserved positions
0
4
8
12
16
20
24
28
N
T
D
Y
Q
S
Amino acid

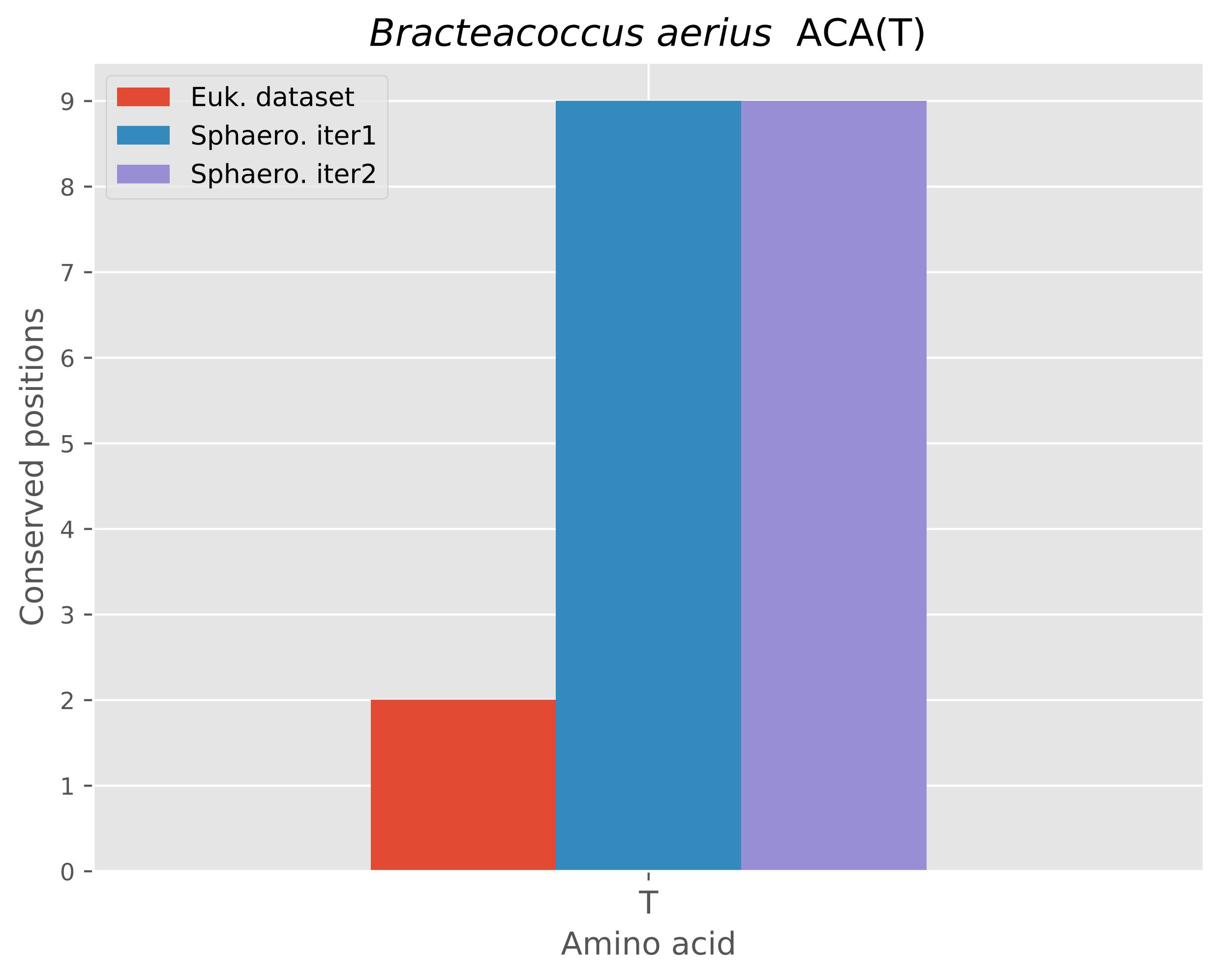

Bracteacoccus aerius ACA(T)
Euk. dataset
Sphaero. iter1
Sphaero. iter2
Conserved positions
0
1
2
3
4
5
6
7
8
9
T
Amino acid

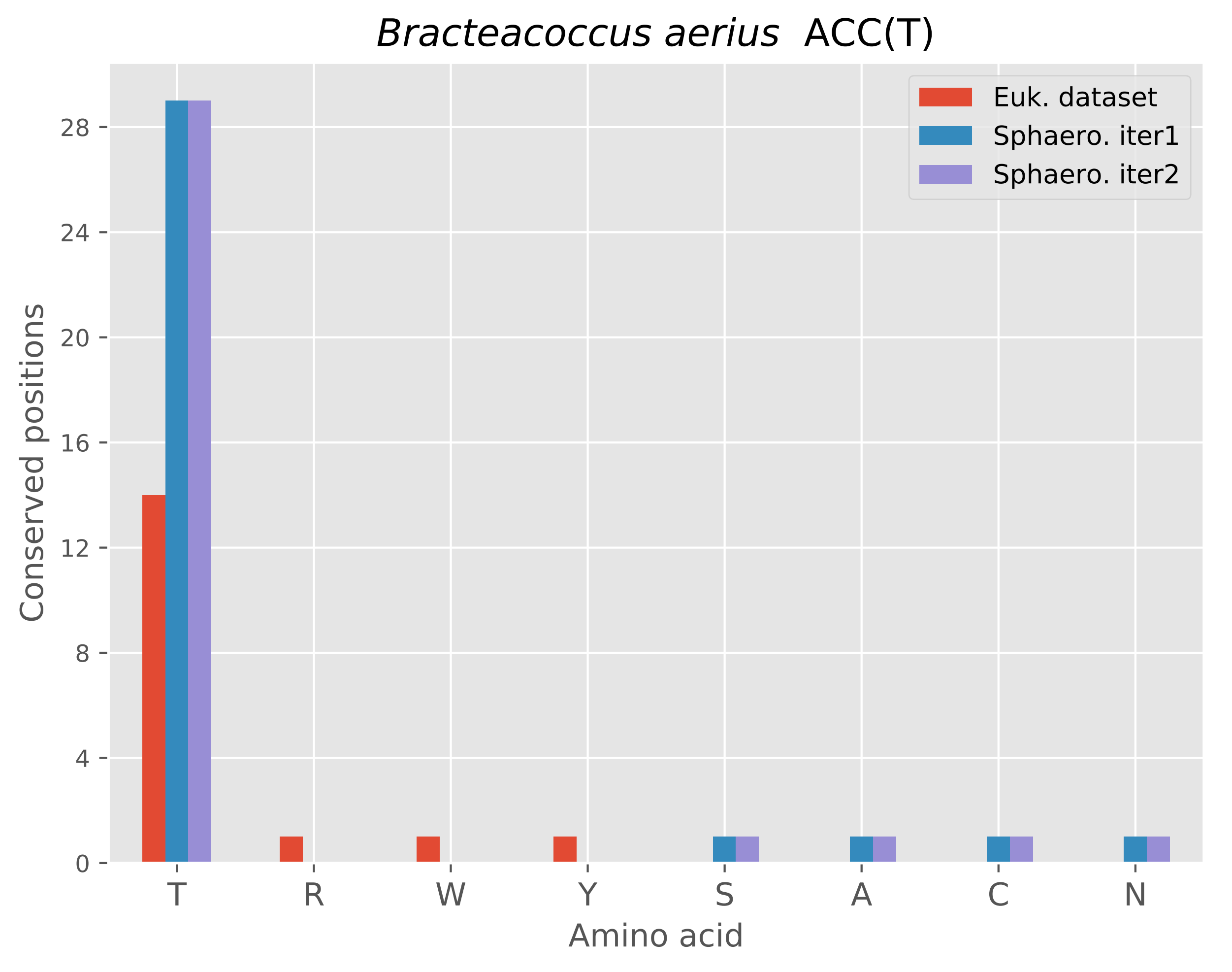

Bracteacoccus aerius ACC(T)
Euk. dataset
Sphaero. iter1
Sphaero. iter2
Conserved positions
0
4
8
12
16
20
24
28
T
R
W
Y
S
A
C
N
Amino acid

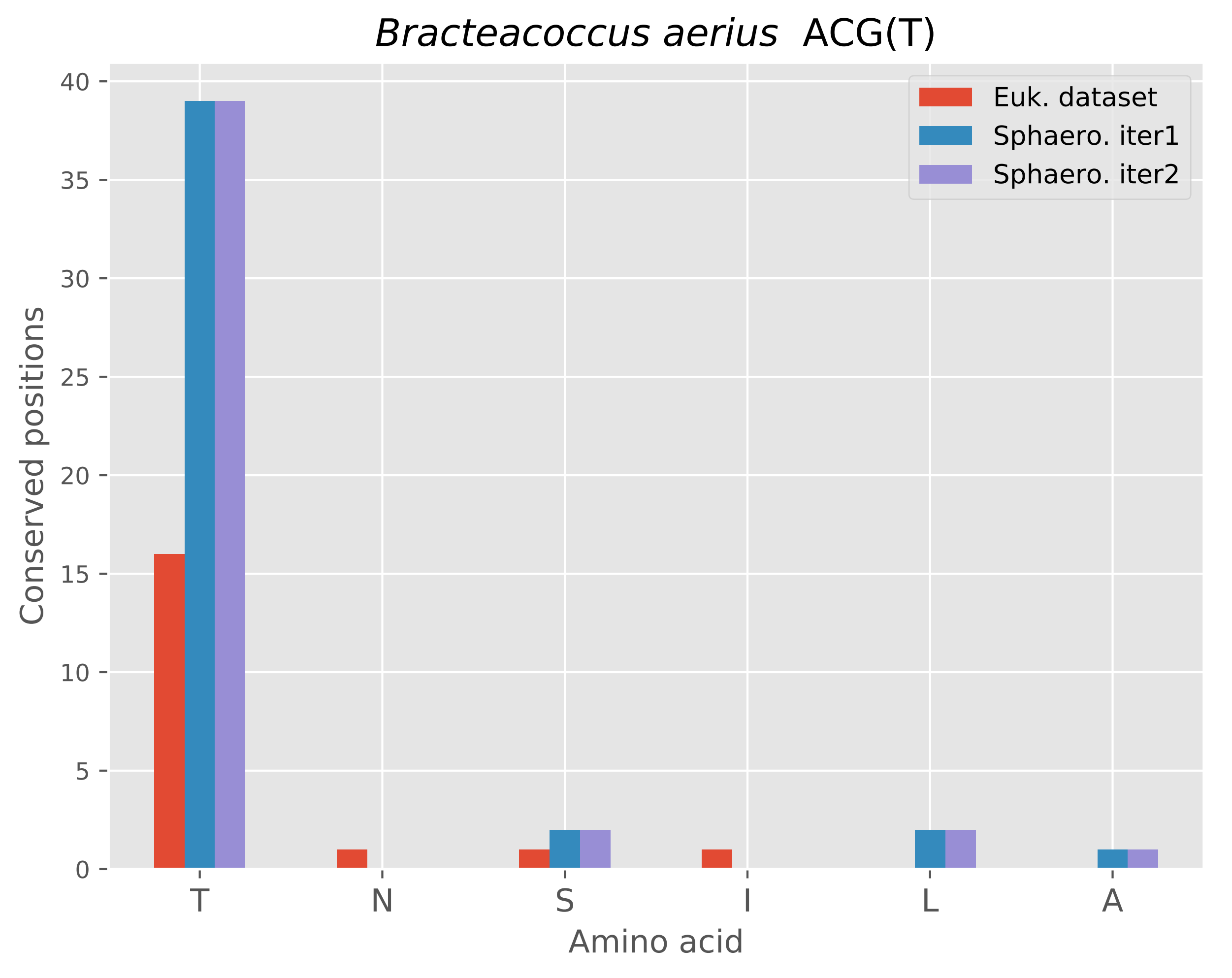

Bracteacoccus aerius ACG(T)
Conserved positions
Amino acid
T
N
S
I
L
A
0
5
10
15
20
25
30
35
40
Euk. dataset
Sphaero. iter1
Sphaero. iter2

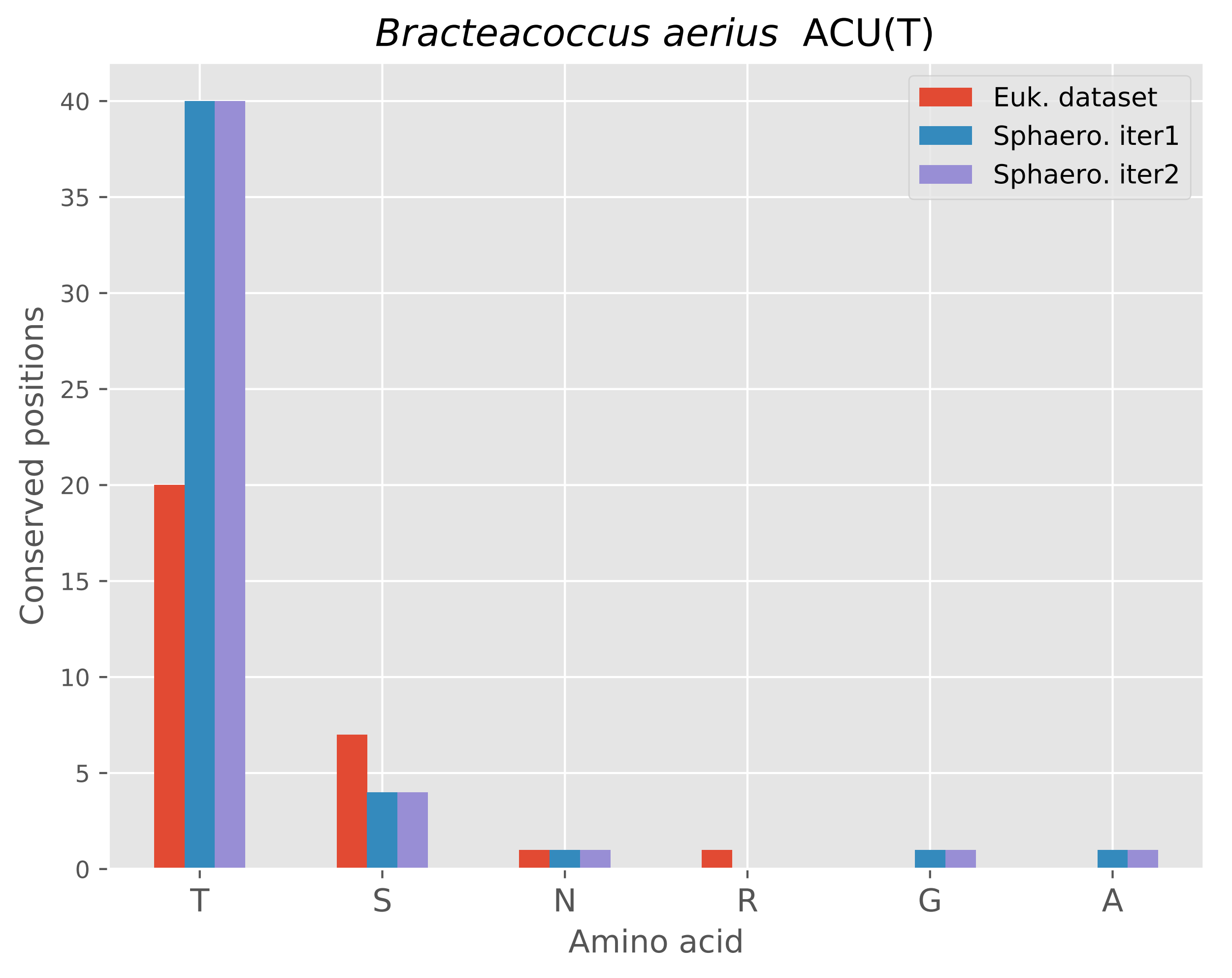
Bracteacoccus aerius ACU(T)
Euk. dataset
Sphaero. iter1
Sphaero. iter2
Conserved positions
0
5
10
15
20
25
30
35
40
T
S
N
R
G
A
Amino acid

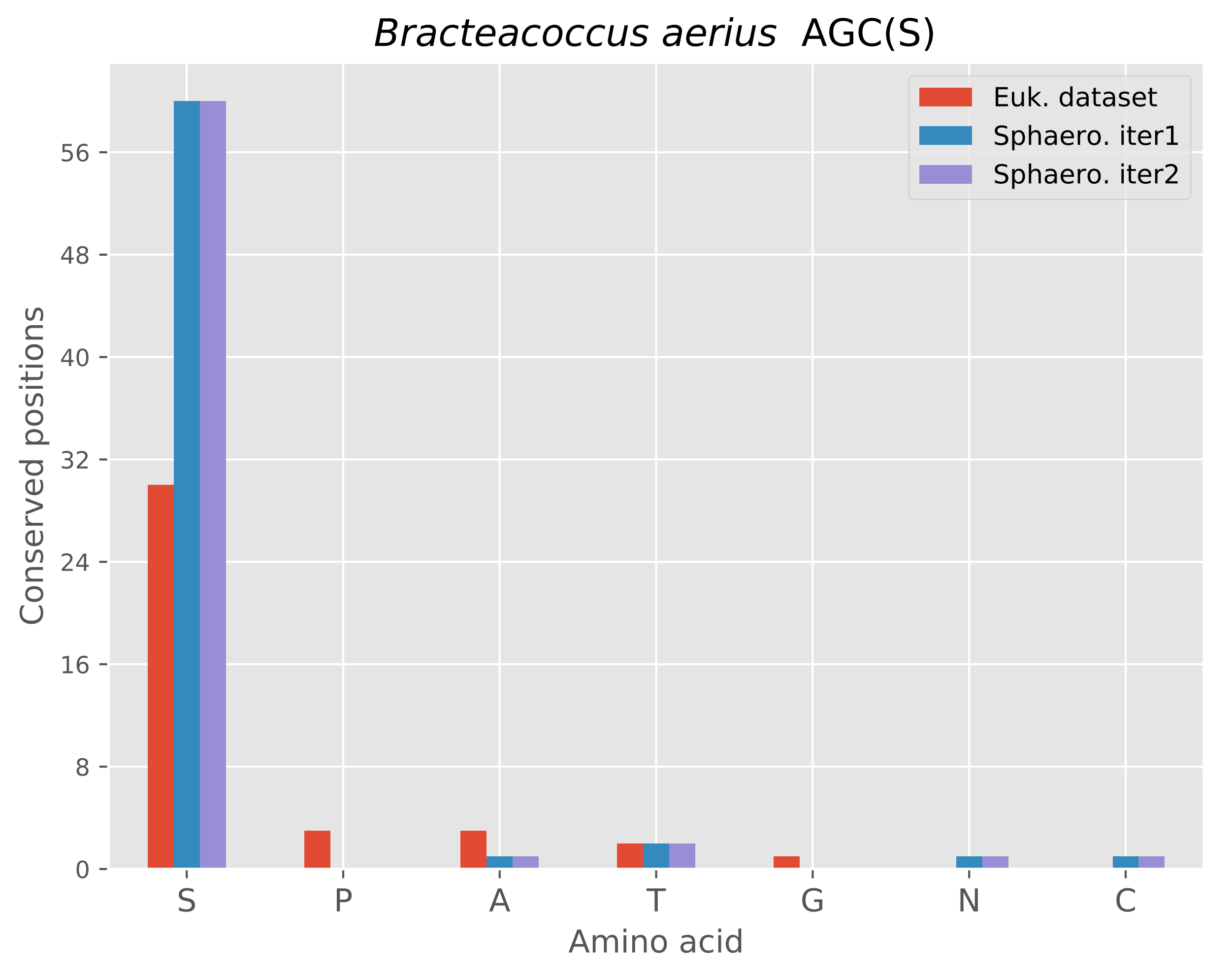

Bracteacoccus aerius AGC(S)
Euk. dataset
Sphaero. iter1
Sphaero. iter2
Conserved positions
0
8
16
24
32
40
48
56
S
P
A
T
G
N
C
Amino acid

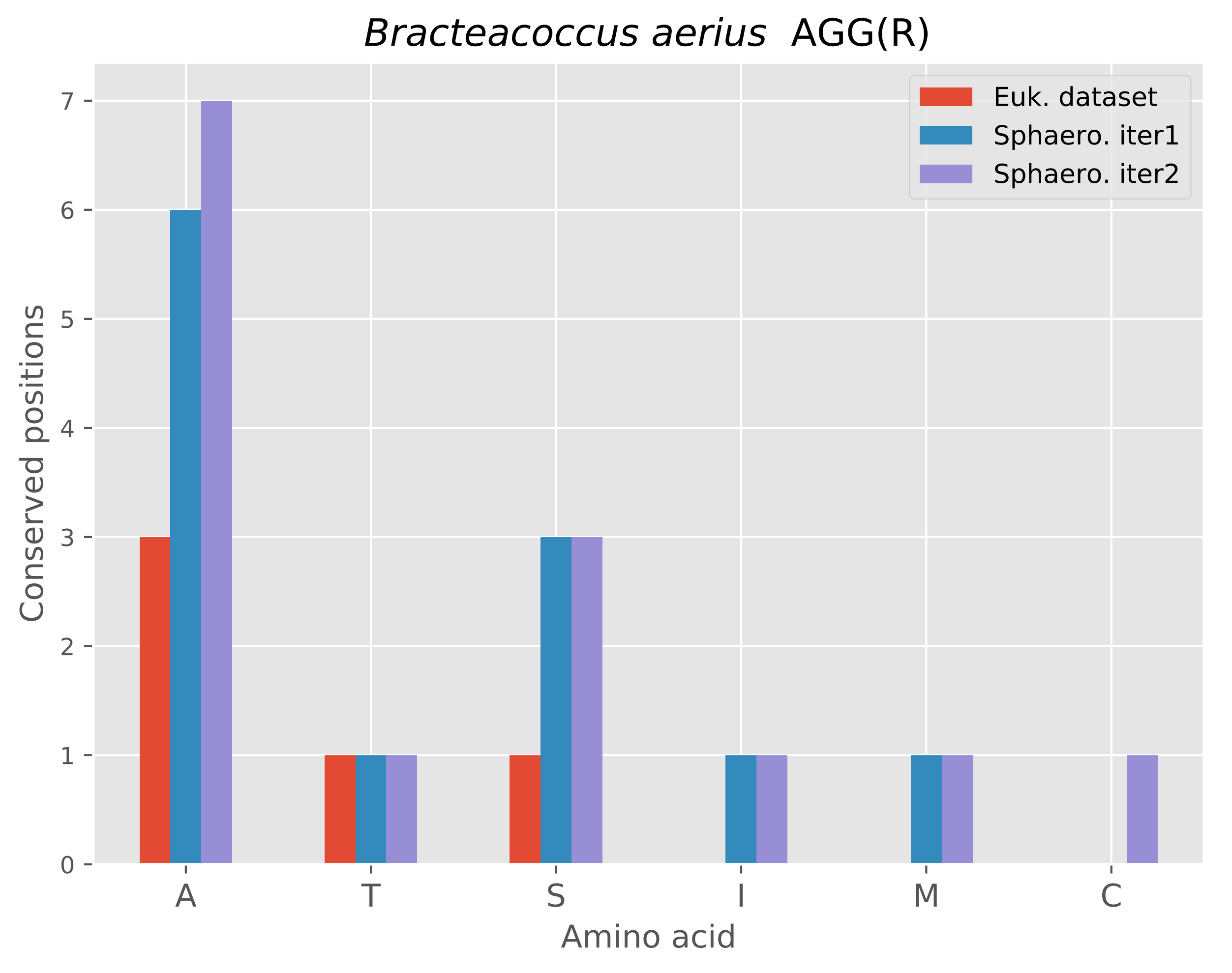

Bracteacoccus aerius AGG(R)
Euk. dataset
Sphaero. iter1
Sphaero. iter2
Conserved positions
0
1
2
3
4
5
6
7
A
T
S
I
M
C
Amino acid

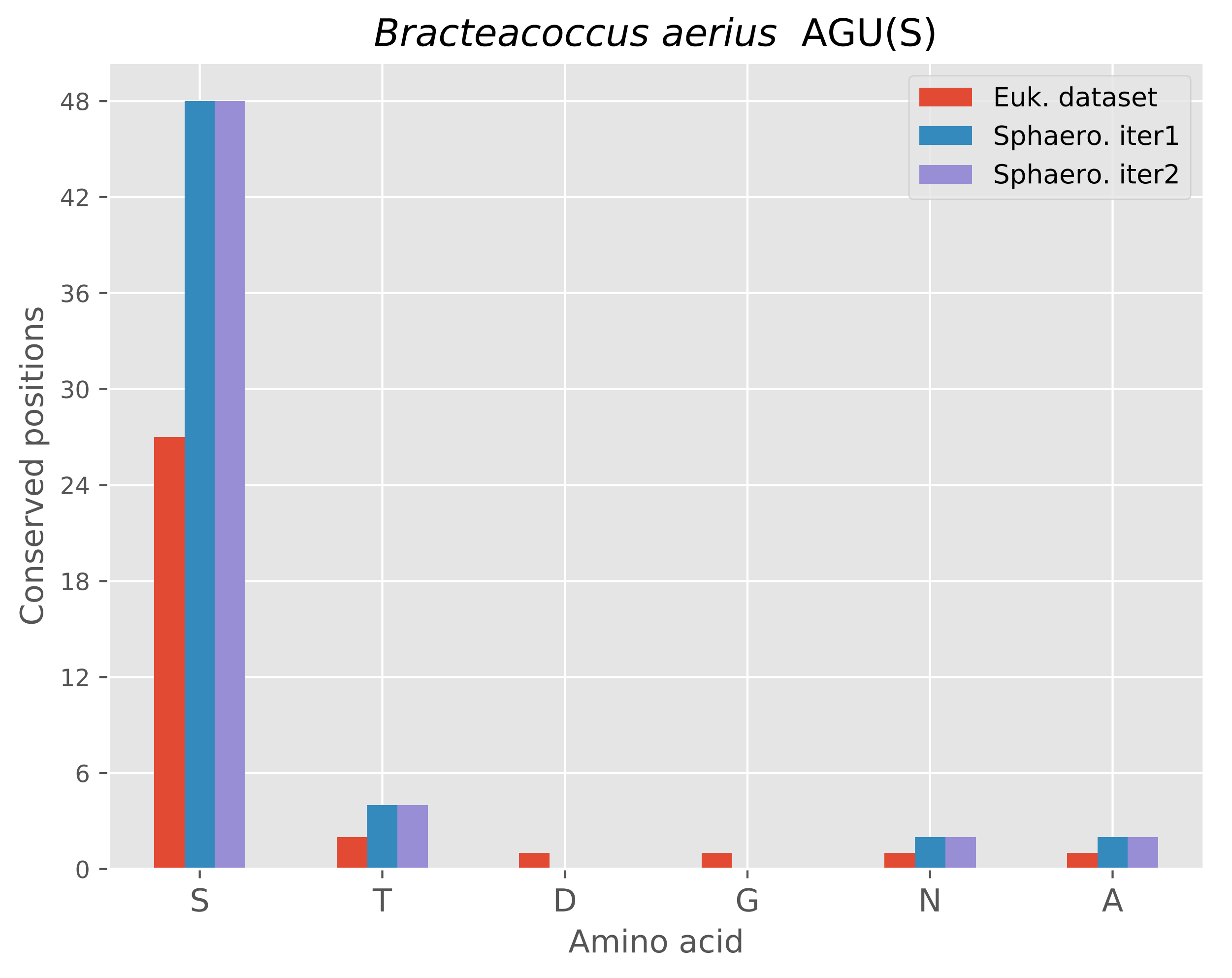

Bracteacoccus aerius AGU(S)
Euk. dataset
Sphaero. iter1
Sphaero. iter2
Conserved positions
0
6
12
18
24
30
36
42
48
S
T
D
G
N
A
Amino acid

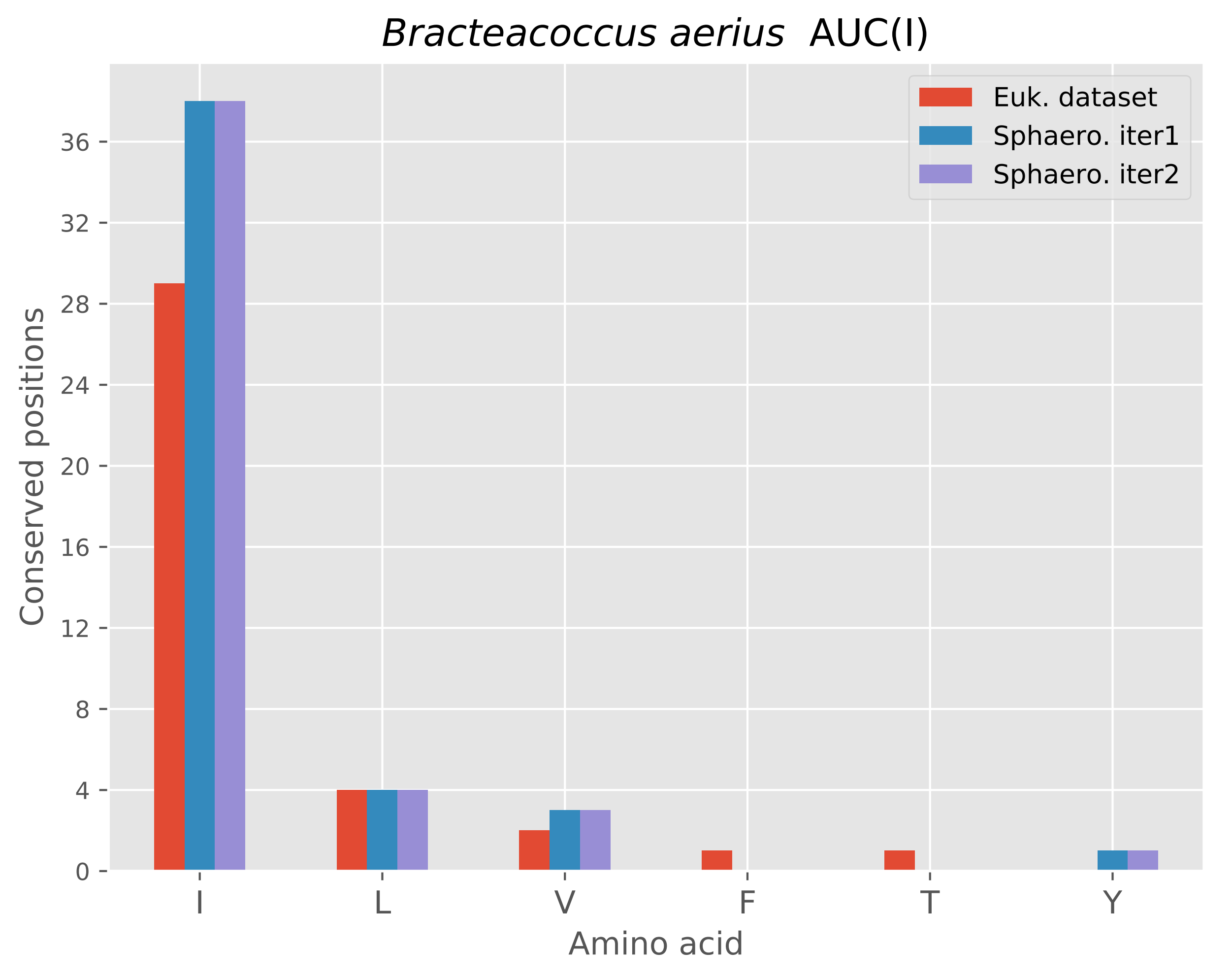

Bracteacoccus aerius AUC(I)
Euk. dataset
Sphaero. iter1
Sphaero. iter2
Conserved positions
0
4
8
12
16
20
24
28
32
36
I
L
V
F
T
Y
Amino acid

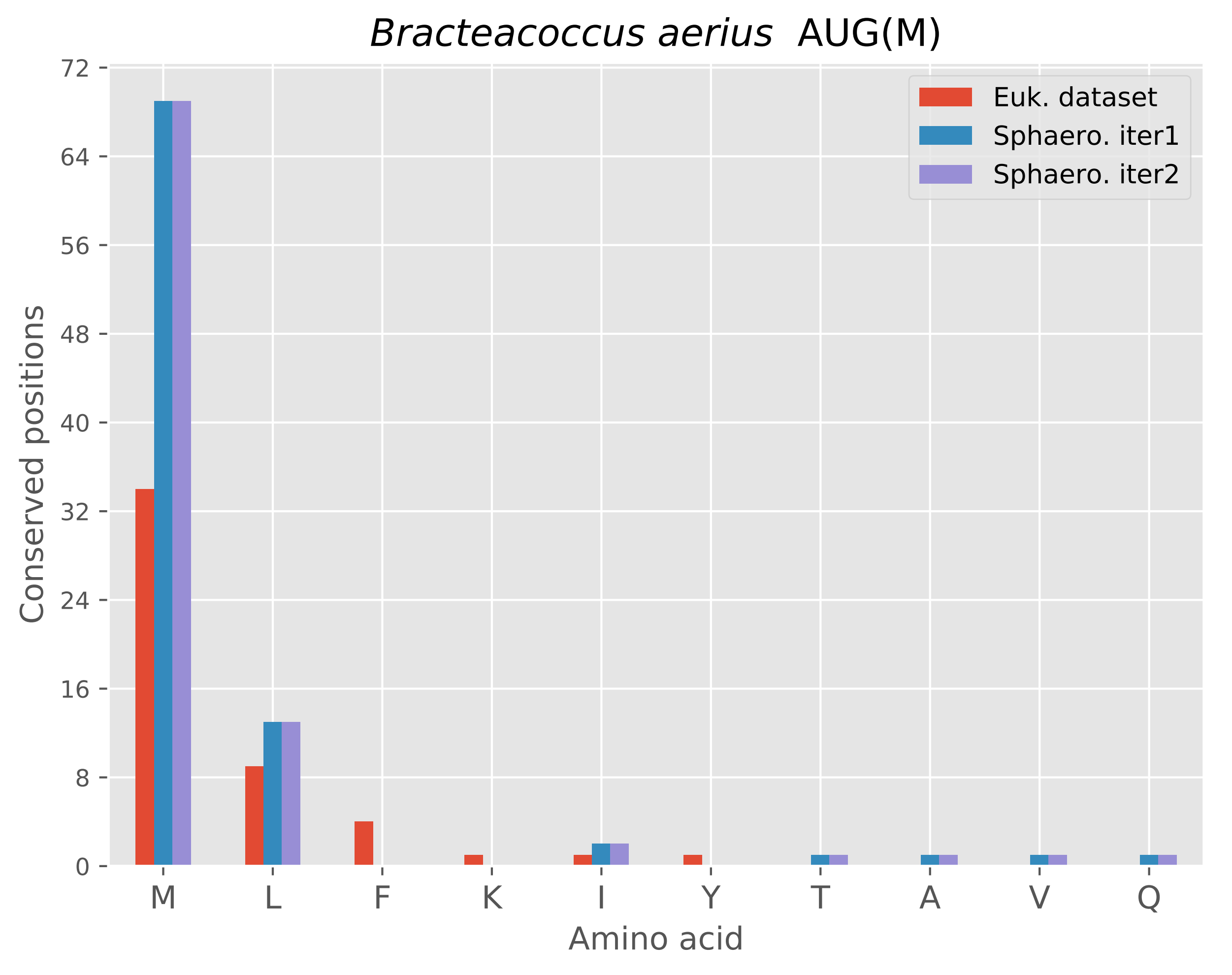

Bracteacoccus aerius AUG(M)
Euk. dataset
Sphaero. iter1
Sphaero. iter2
Conserved positions
0
8
16
24
32
40
48
56
64
72
M
L
F
K
I
Y
T
A
V
Q
Amino acid

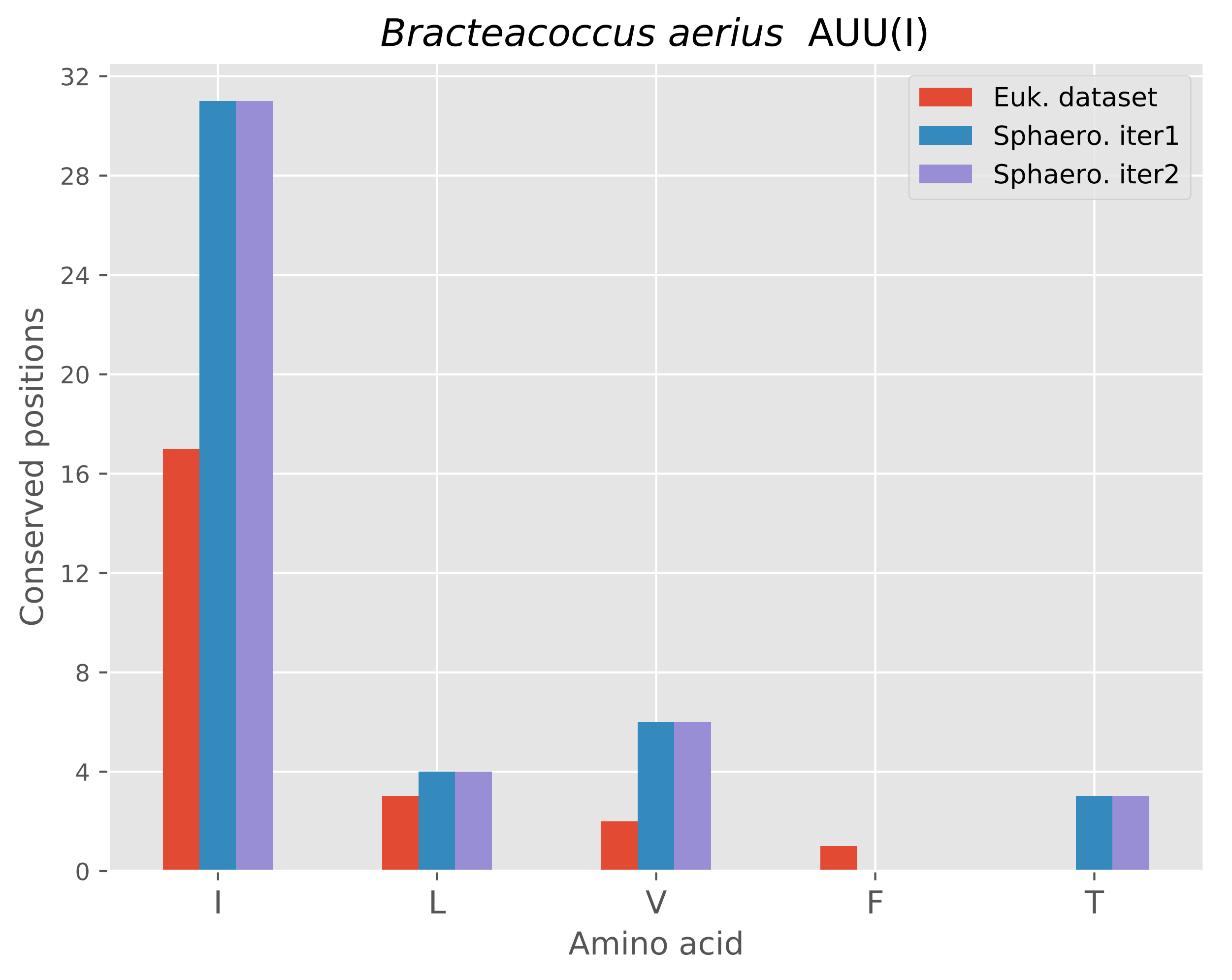
Bracteacoccus aerius AUU(I)
Euk. dataset
Sphaero. iter1
Sphaero. iter2
Conserved positions
0
4
8
12
16
20
24
28
32
I
L
V
F
T
Amino acid

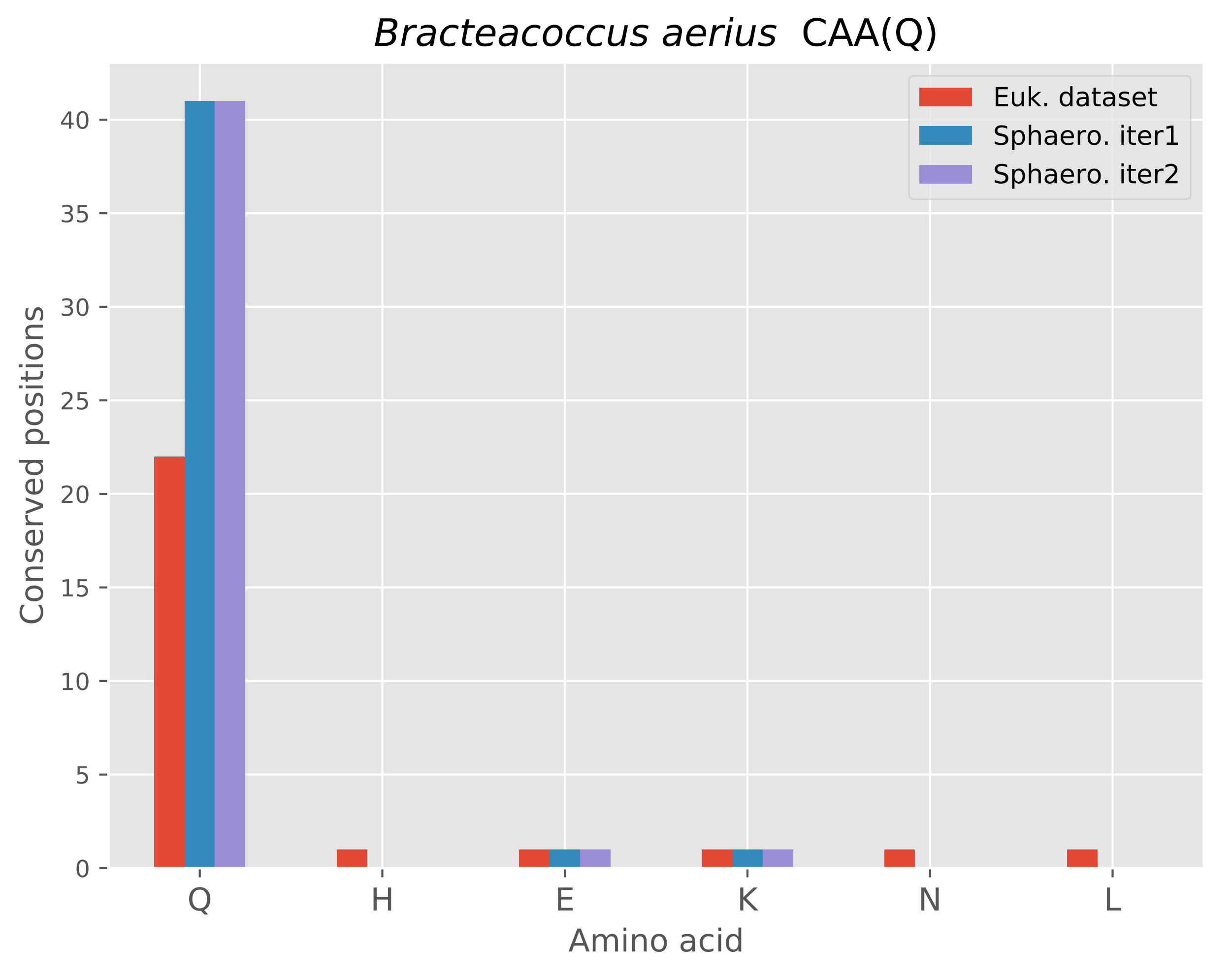

Bracteacoccus aerius CAA(Q)
Euk. dataset
Sphaero. iter1
Sphaero. iter2
Conserved positions
0
5
10
15
20
25
30
35
40
Q
H
E
K
N
L
Amino acid

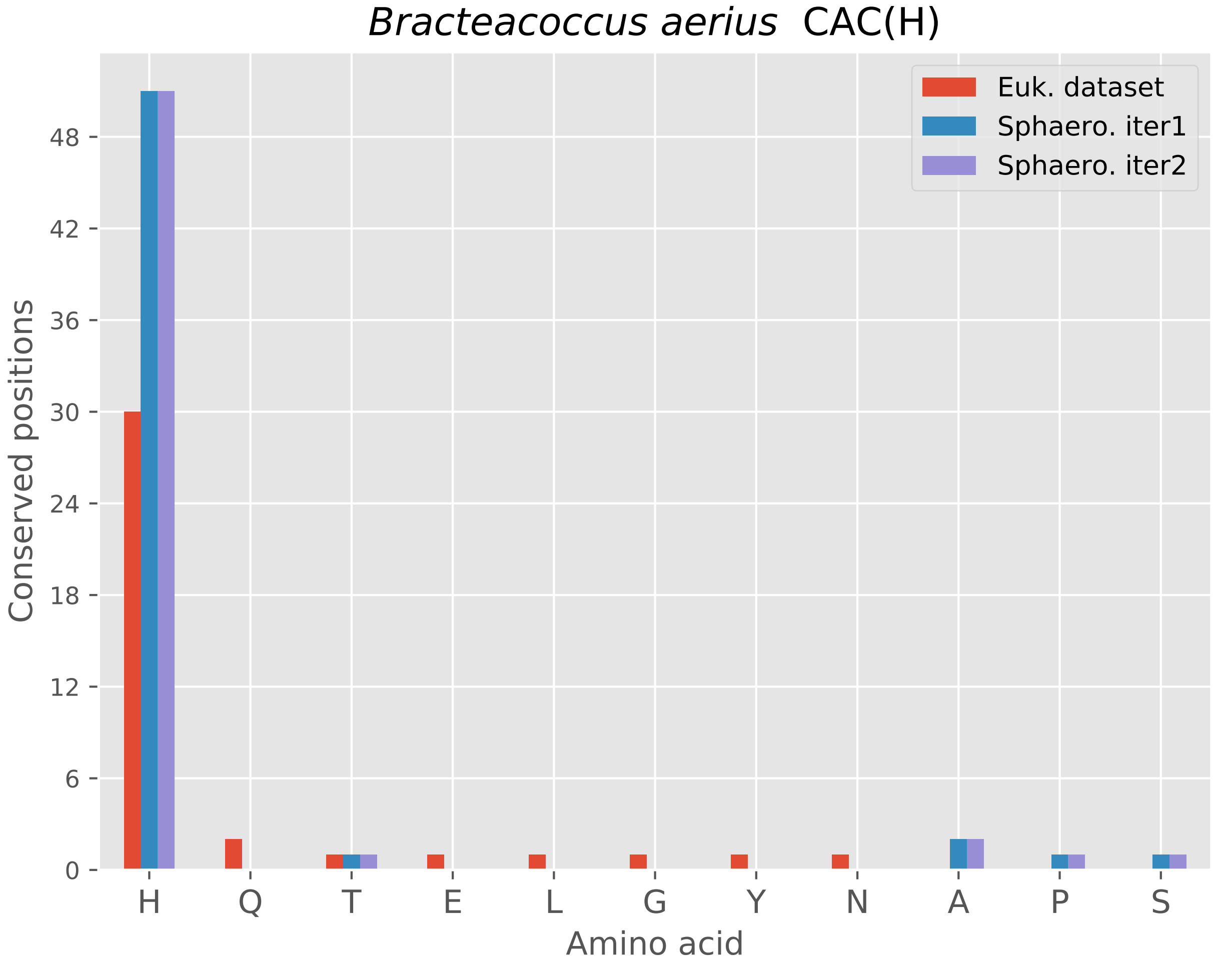
Bracteacoccus aerius CAC(H)
Euk. dataset
Sphaero. iter1
Sphaero. iter2
Conserved positions
0
6
12
18
24
30
36
42
48
H
Q
T
E
L
G
Y
N
A
P
S
Amino acid

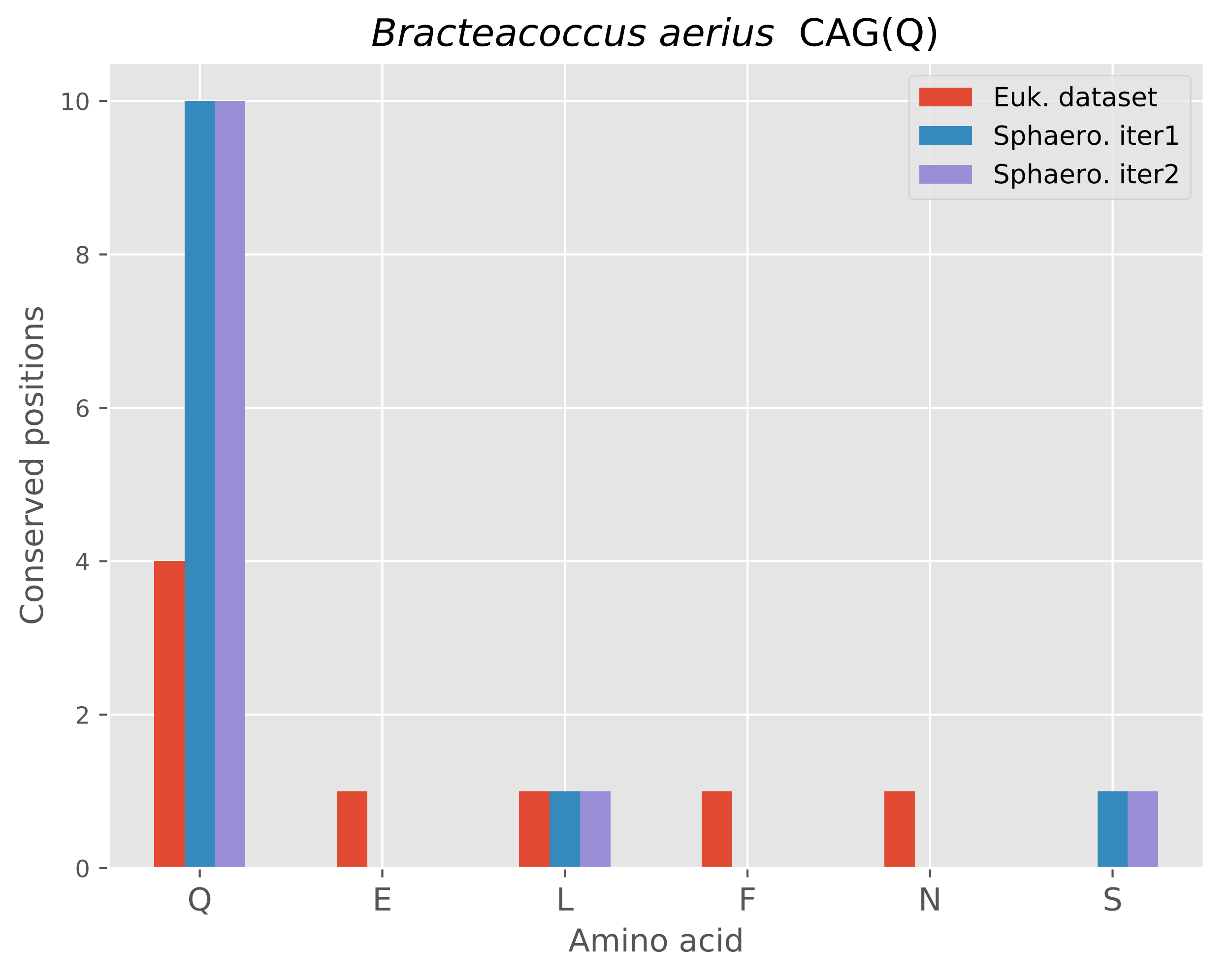

Bracteacoccus aerius CAG(Q)
Euk. dataset
Sphaero. iter1
Sphaero. iter2
Conserved positions
0
2
4
6
8
10
Q
E
L
F
N
S
Amino acid

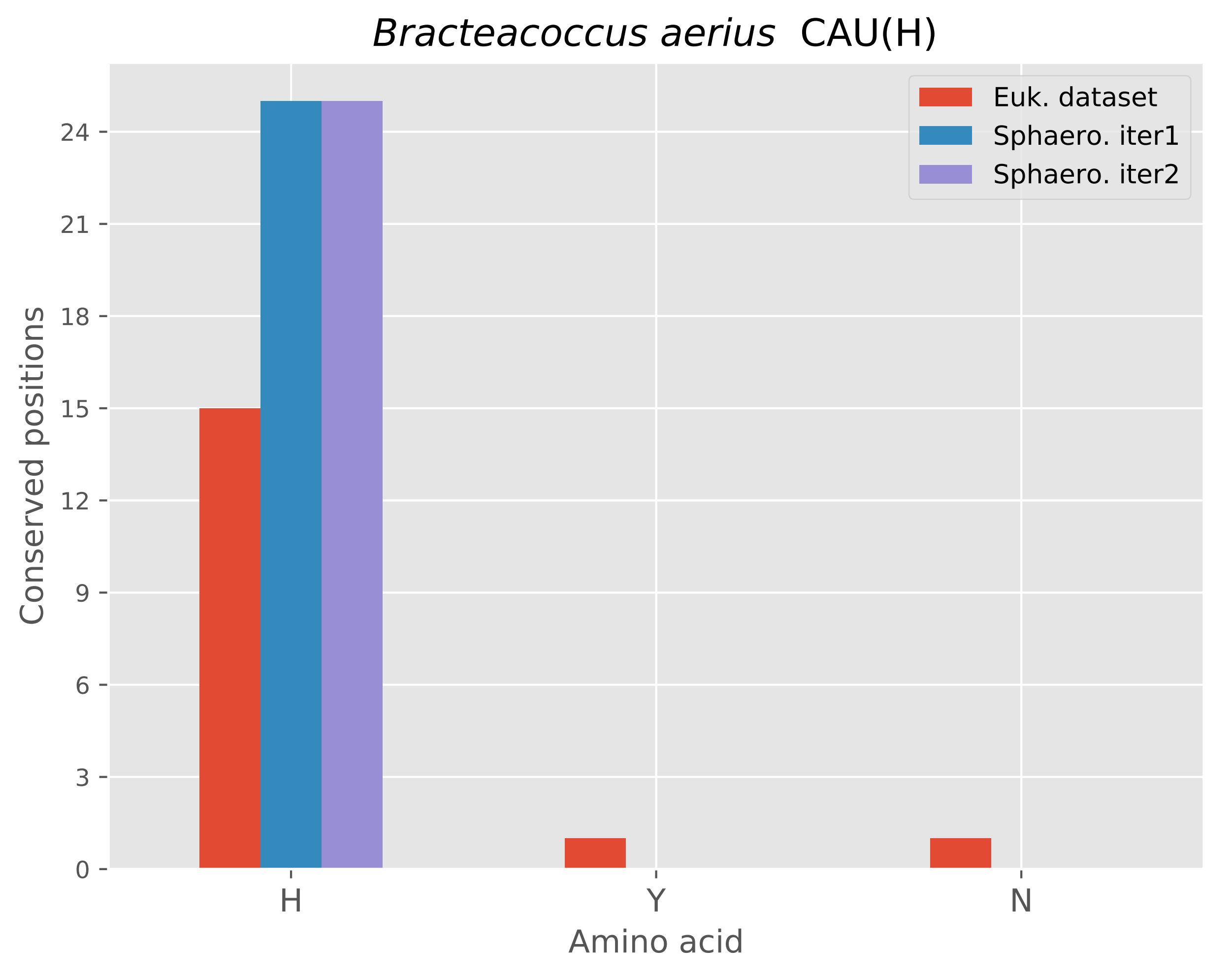

Bracteacoccus aerius CAU(H)
Euk. dataset
Sphaero. iter1
Sphaero. iter2
Conserved positions
0
3
6
9
12
15
18
21
24
H
Y
N
Amino acid

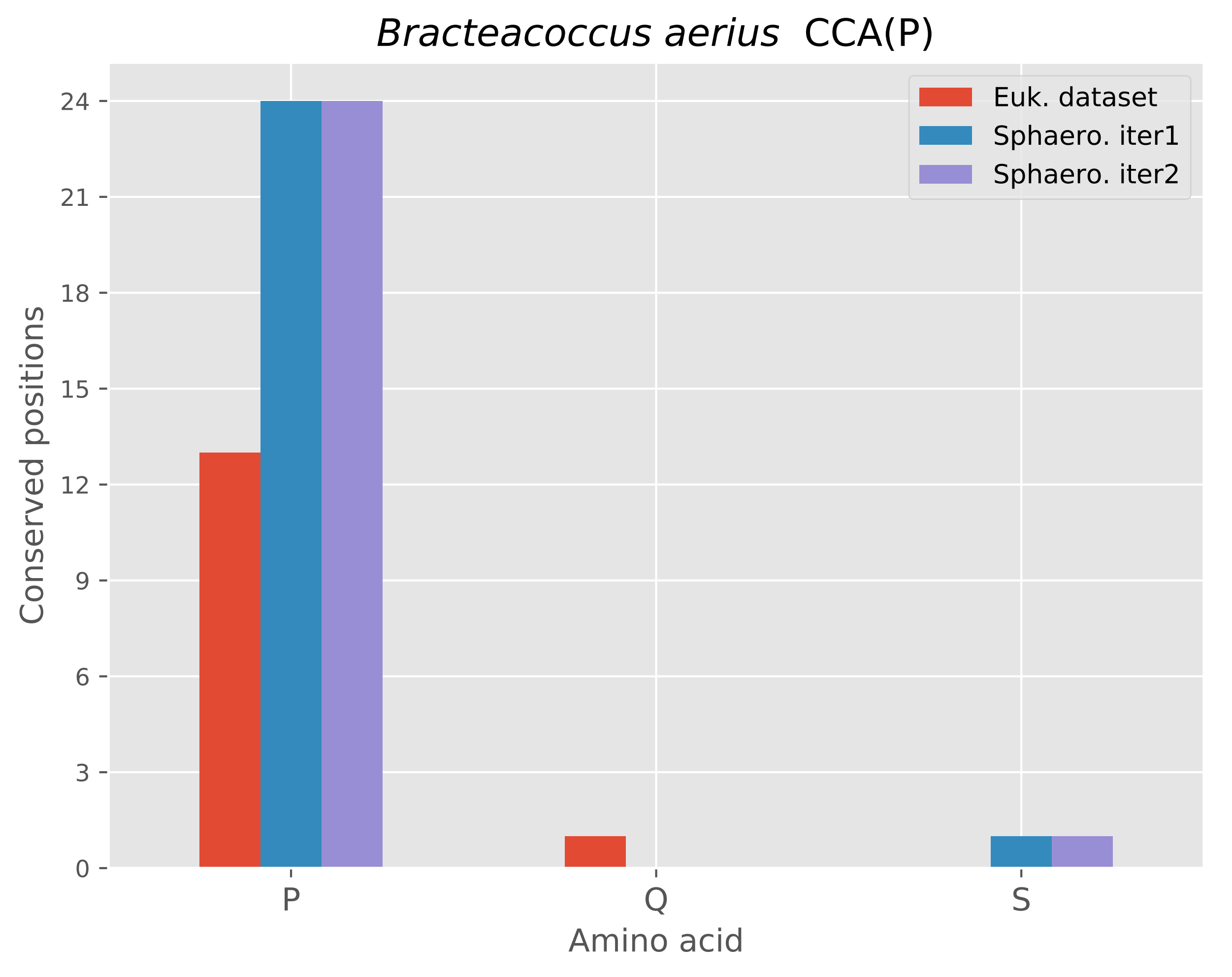

Bracteacoccus aerius CCA(P)
Euk. dataset
Sphaero. iter1
Sphaero. iter2
Conserved positions
0
3
6
9
12
15
18
21
24
P
Q
S
Amino acid

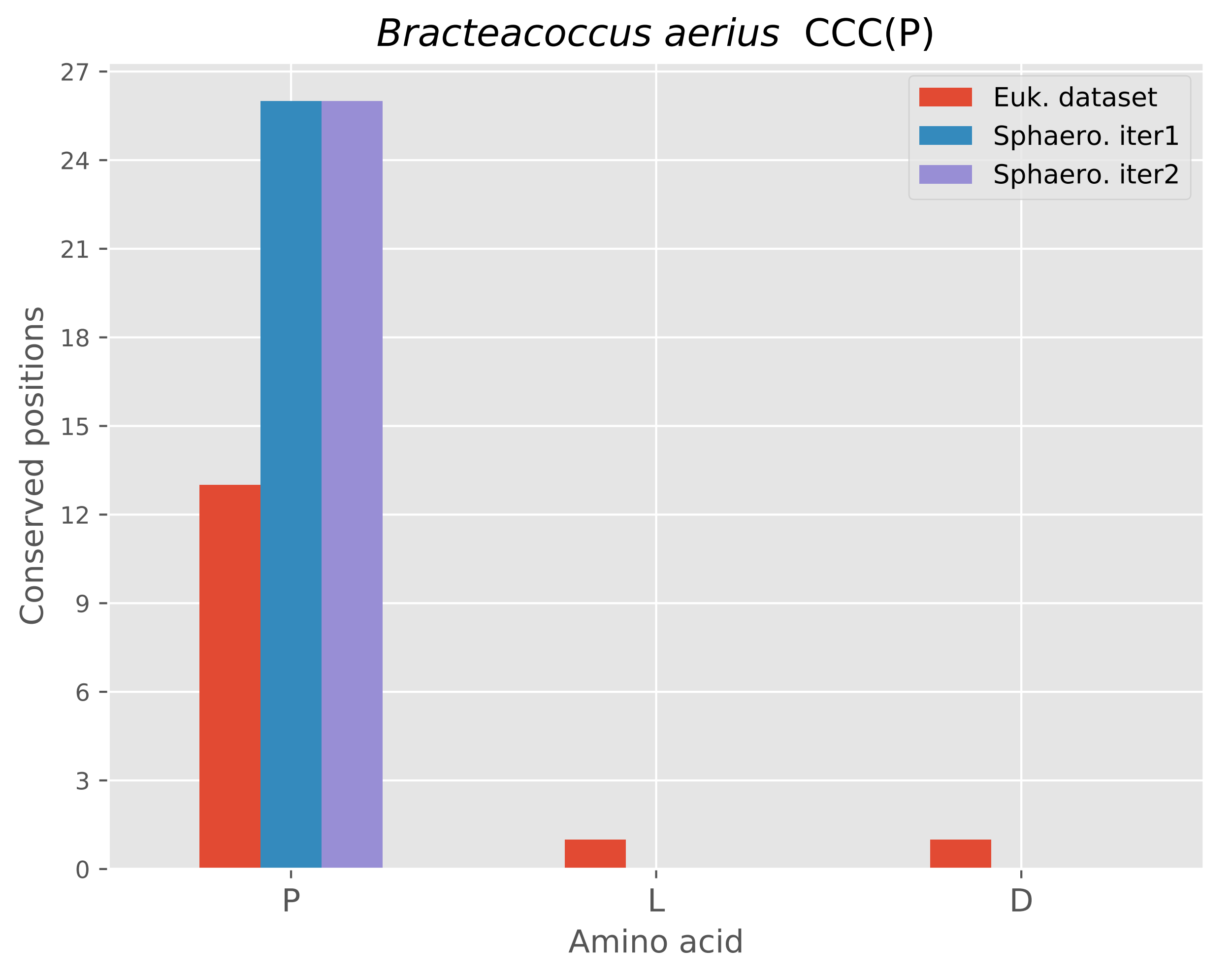

Bracteacoccus aerius CCC(P)
Euk. dataset
Sphaero. iter1
Sphaero. iter2
Conserved positions
0
3
6
9
12
15
18
21
24
27
P
L
D
Amino acid

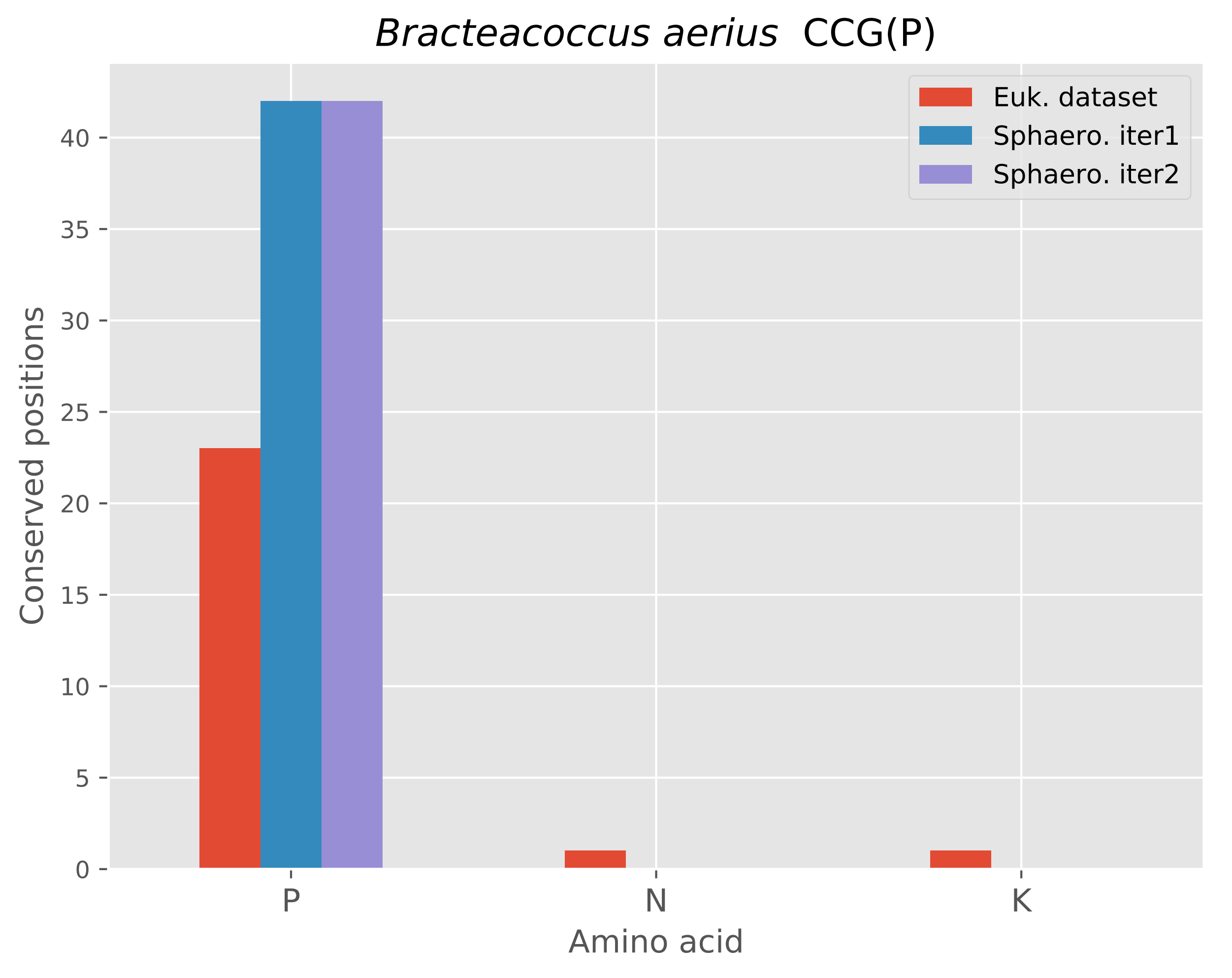

Bracteacoccus aerius CCG(P)
Euk. dataset
Sphaero. iter1
Sphaero. iter2
Conserved positions
0
5
10
15
20
25
30
35
40
P
N
K
Amino acid

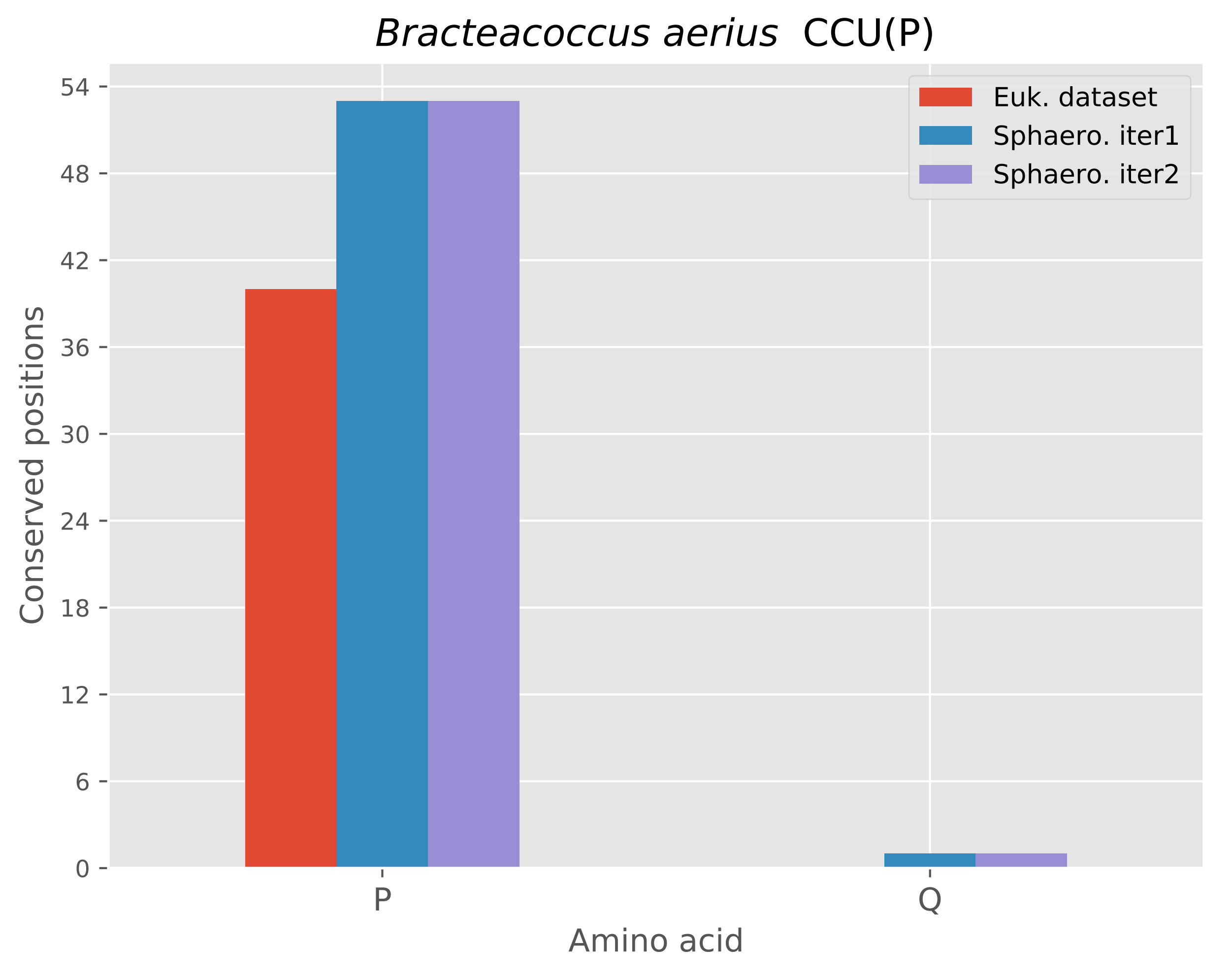

Bracteacoccus aerius CCU(P)
Euk. dataset
Sphaero. iter1
Sphaero. iter2
Conserved positions
Amino acid
P
Q
0
6
12
18
24
30
36
42
48
54

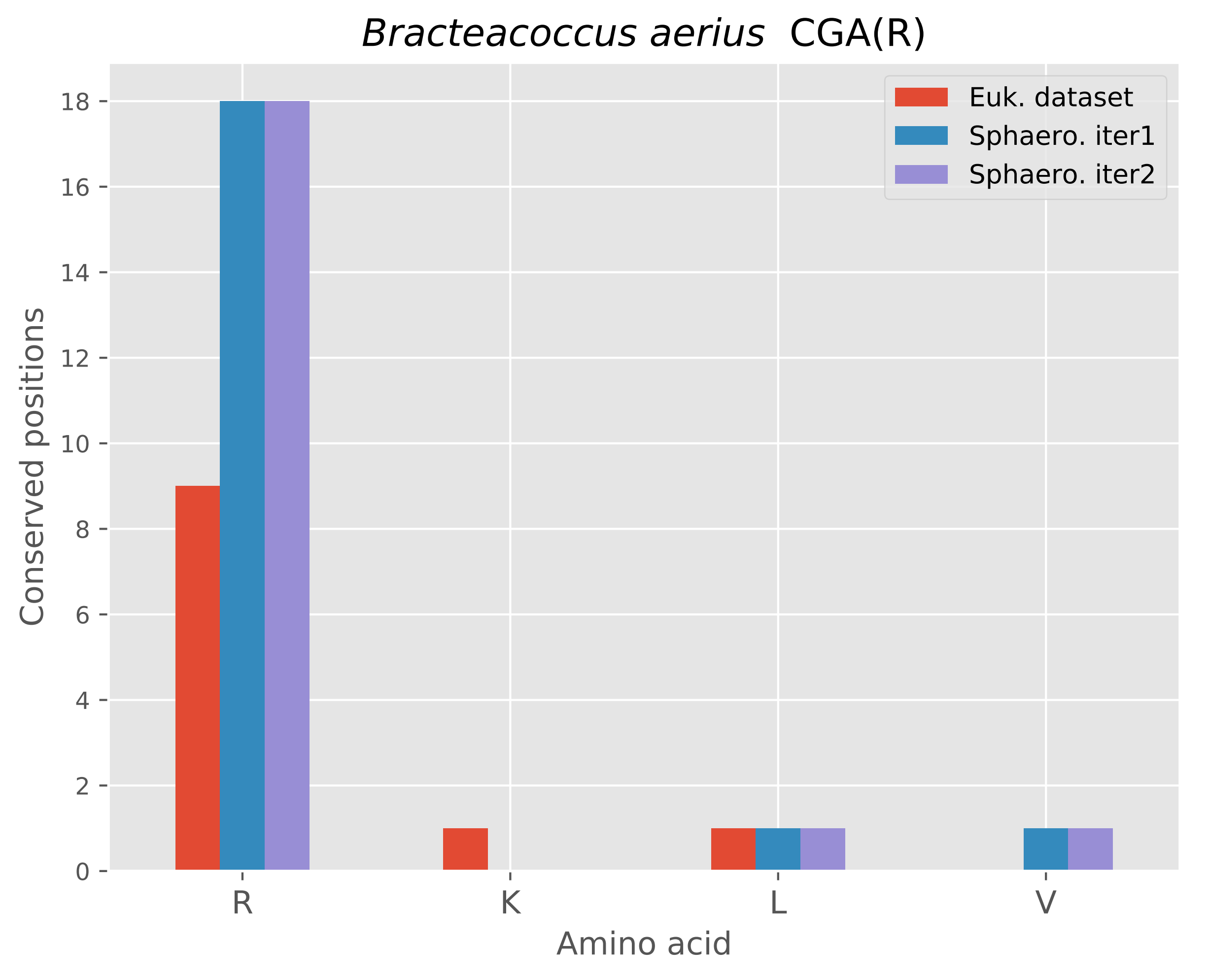
Bracteacoccus aerius CGA(R)
Euk. dataset
Sphaero. iter1
Sphaero. iter2
Conserved positions
0
2
4
6
8
10
12
14
16
18
R
K
L
V
Amino acid

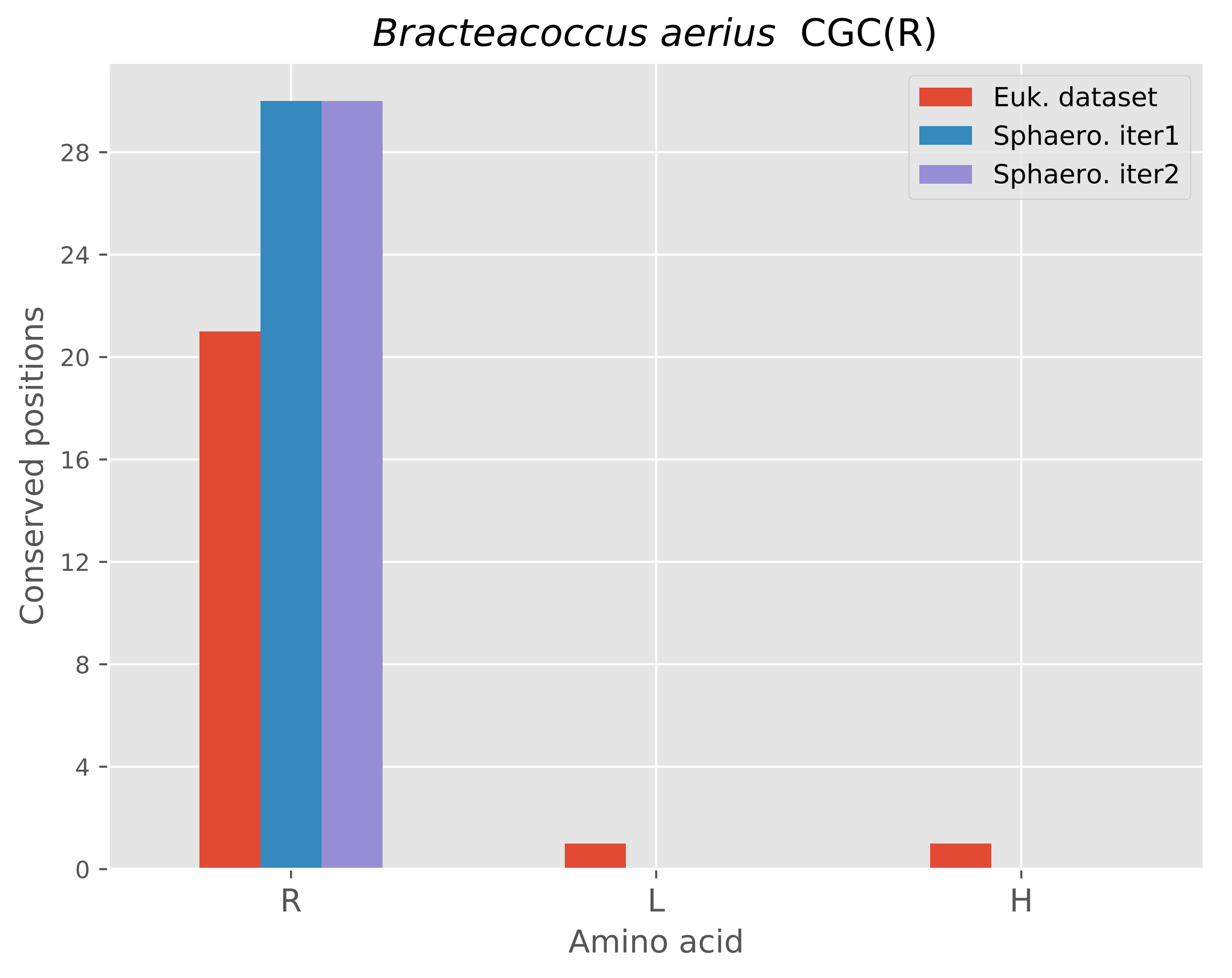

Bracteacoccus aerius CGC(R)
Euk. dataset
Sphaero. iter1
Sphaero. iter2
Conserved positions
0
4
8
12
16
20
24
28
R
L
H
Amino acid

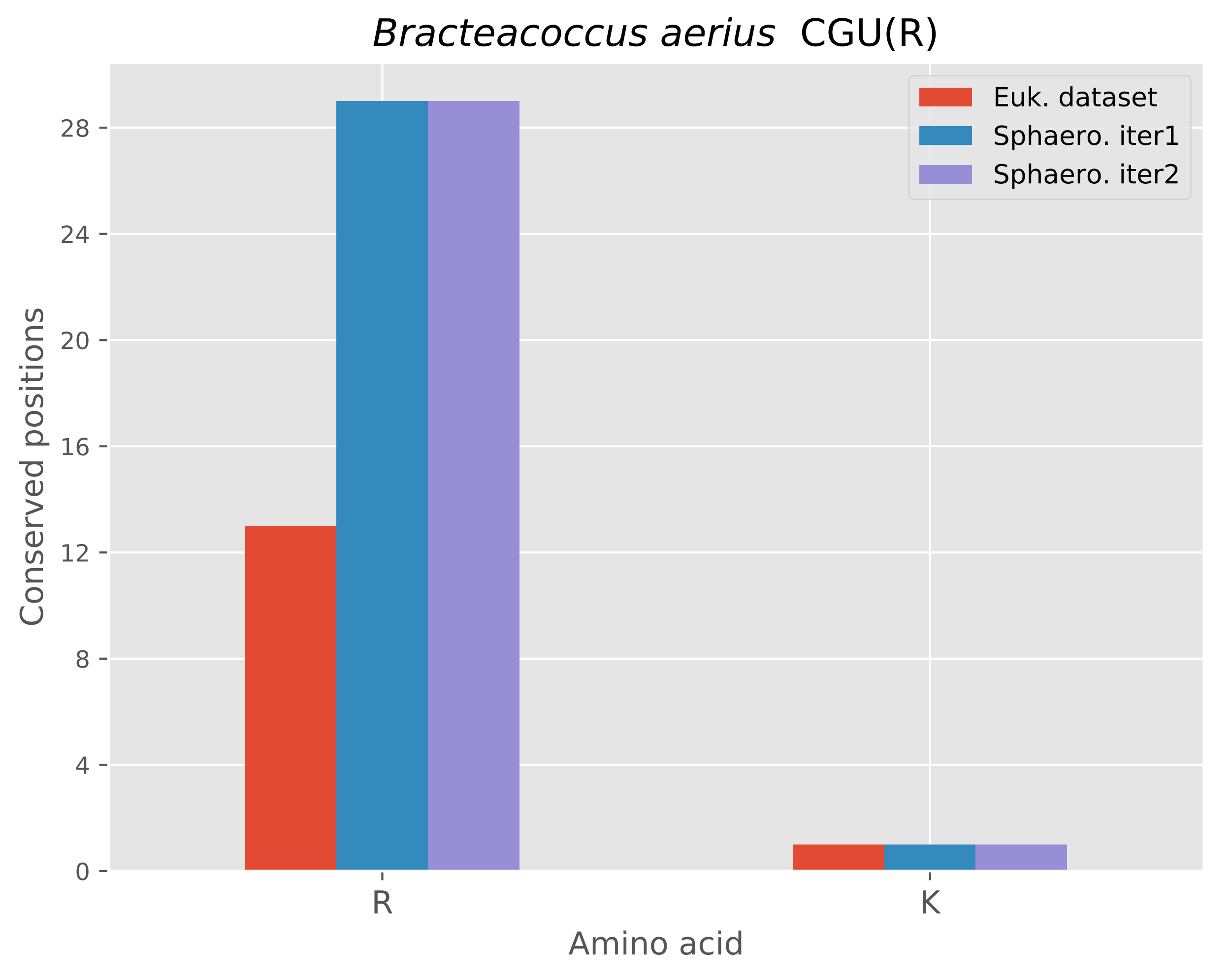

Bracteacoccus aerius CGU(R)
Euk. dataset
Sphaero. iter1
Sphaero. iter2
Conserved positions
0
4
8
12
16
20
24
28
R
K
Amino acid

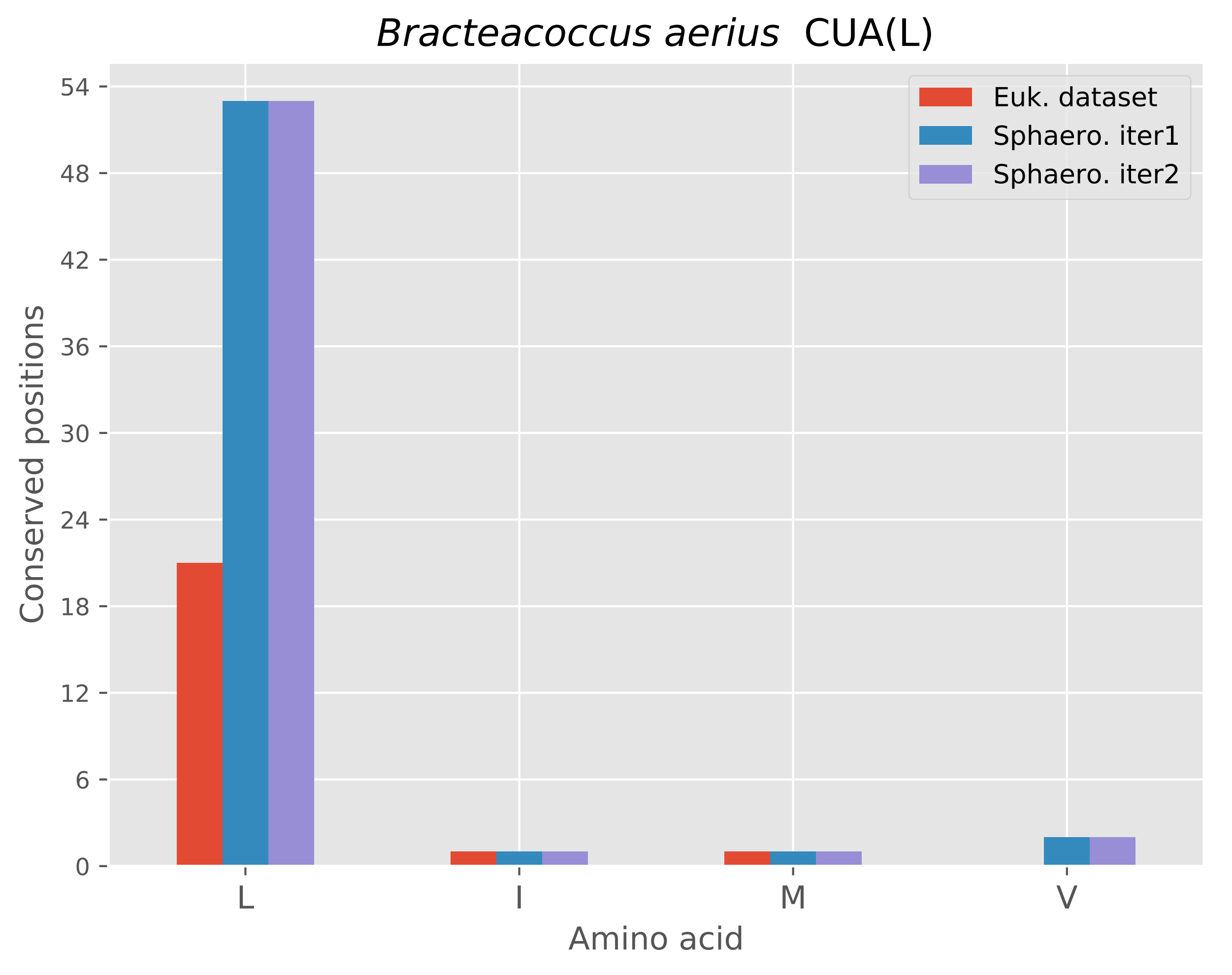
Bracteacoccus aerius CUA(L)
Euk. dataset
Sphaero. iter1
Sphaero. iter2
Conserved positions
0
6
12
18
24
30
36
42
48
54
L
I
M
V
Amino acid

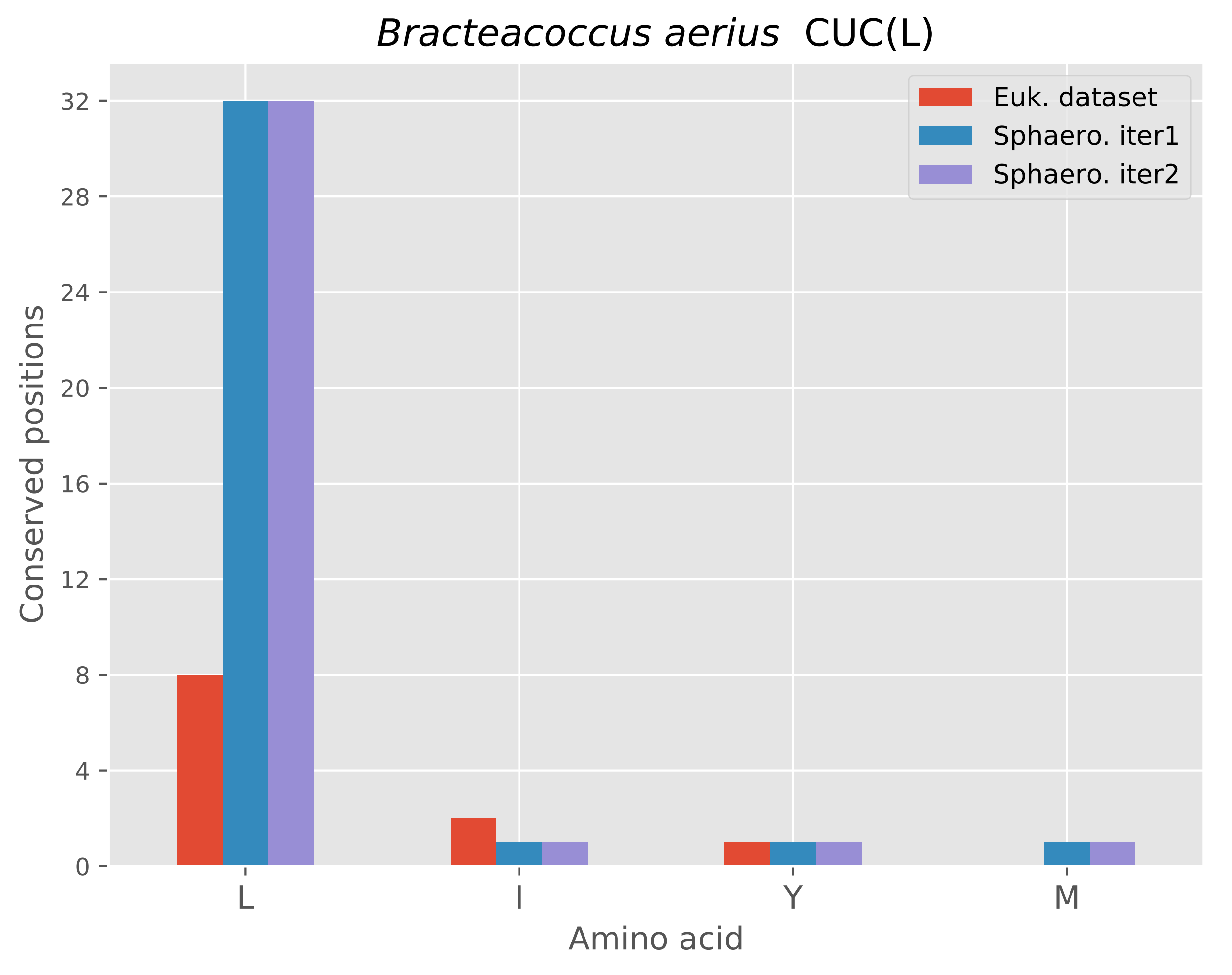

Bracteacoccus aerius CUC(L)
Euk. dataset
Sphaero. iter1
Sphaero. iter2
Conserved positions
0
4
8
12
16
20
24
28
32
L
I
Y
M
Amino acid

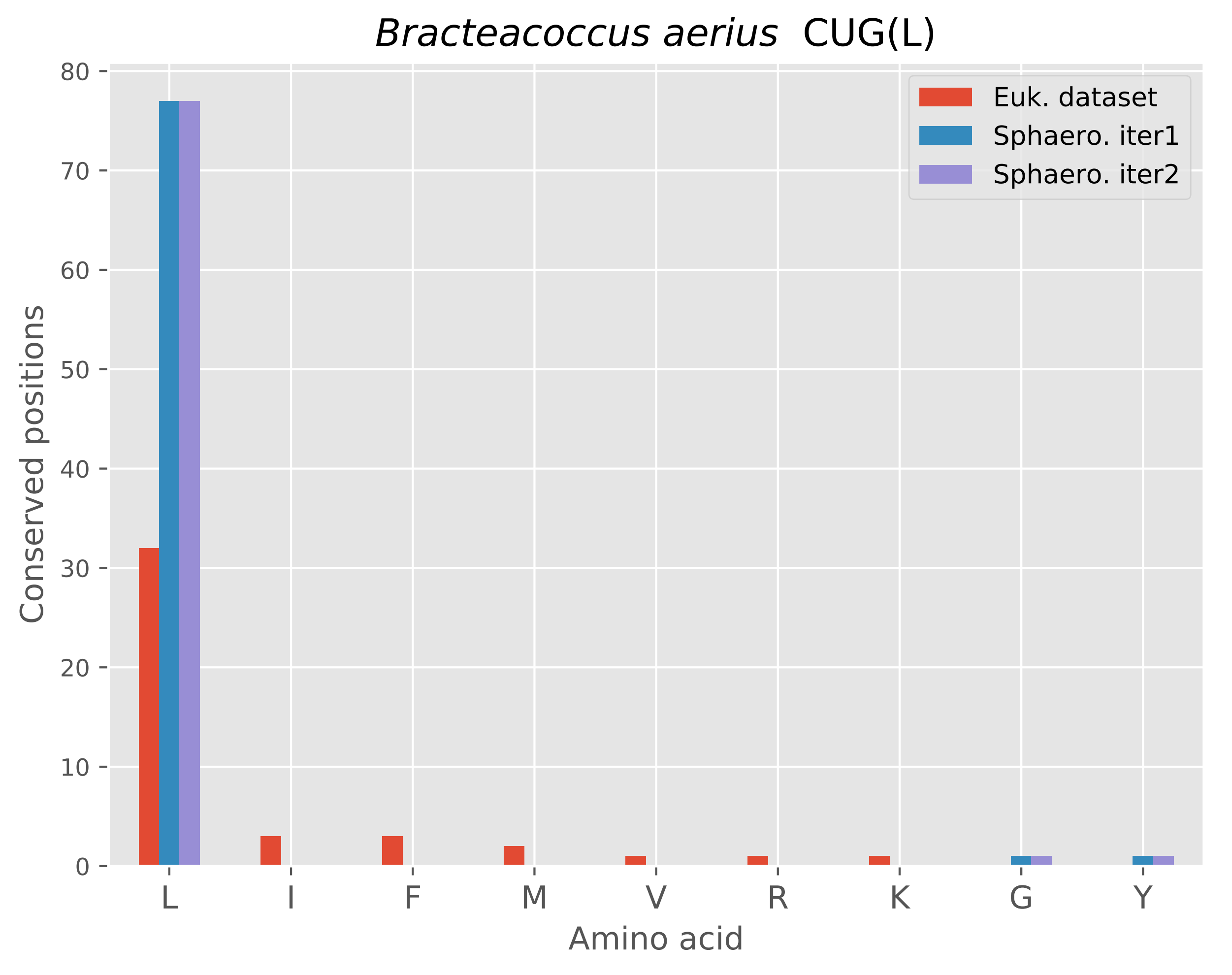
Bracteacoccus aerius CUG(L)
Euk. dataset
Sphaero. iter1
Sphaero. iter2
Conserved positions
0
10
20
30
40
50
60
70
80
L
I
F
M
V
R
K
G
Y
Amino acid

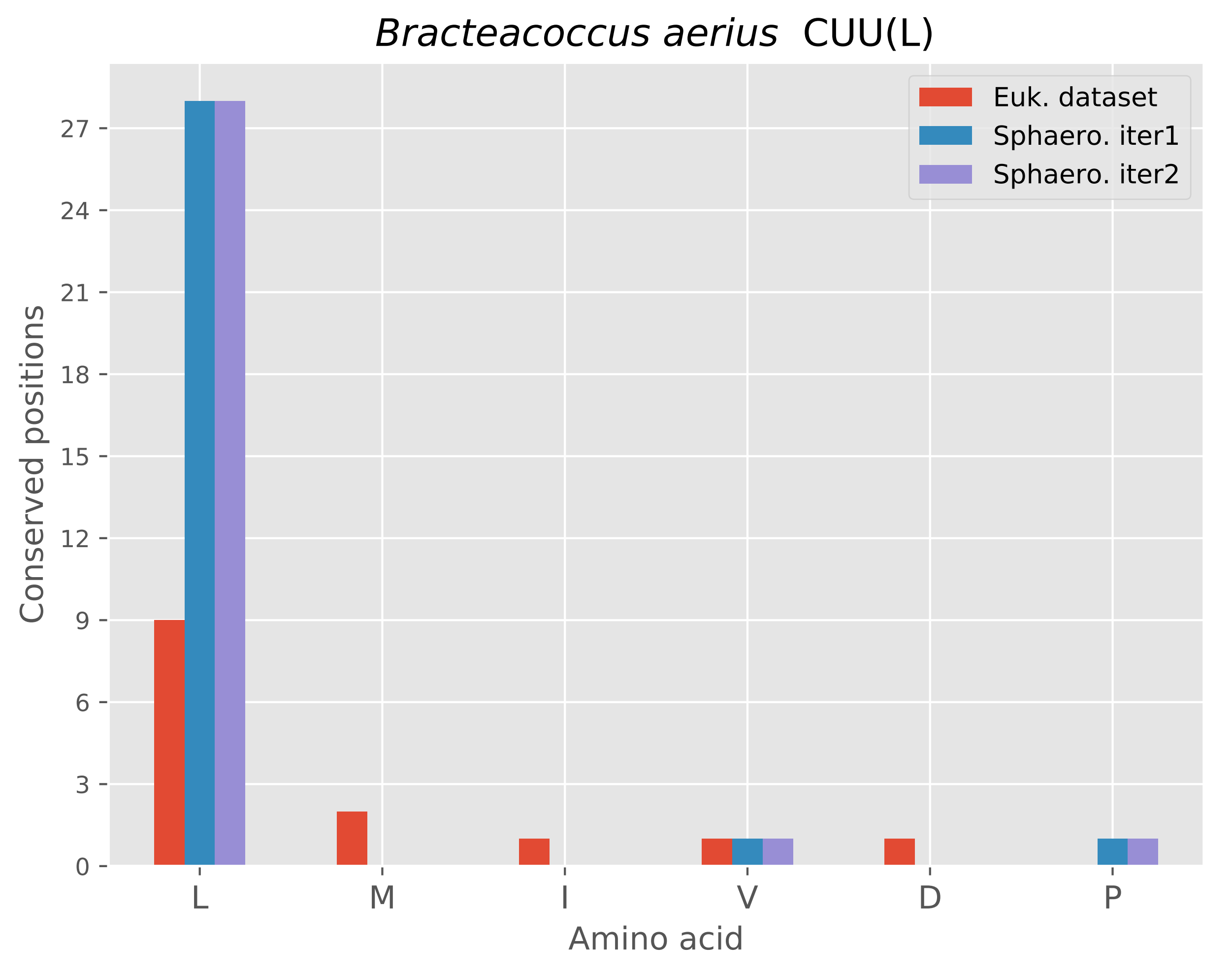

Bracteacoccus aerius CUU(L)
Euk. dataset
Sphaero. iter1
Sphaero. iter2
Conserved positions
0
3
6
9
12
15
18
21
24
27
L
M
I
V
D
P
Amino acid

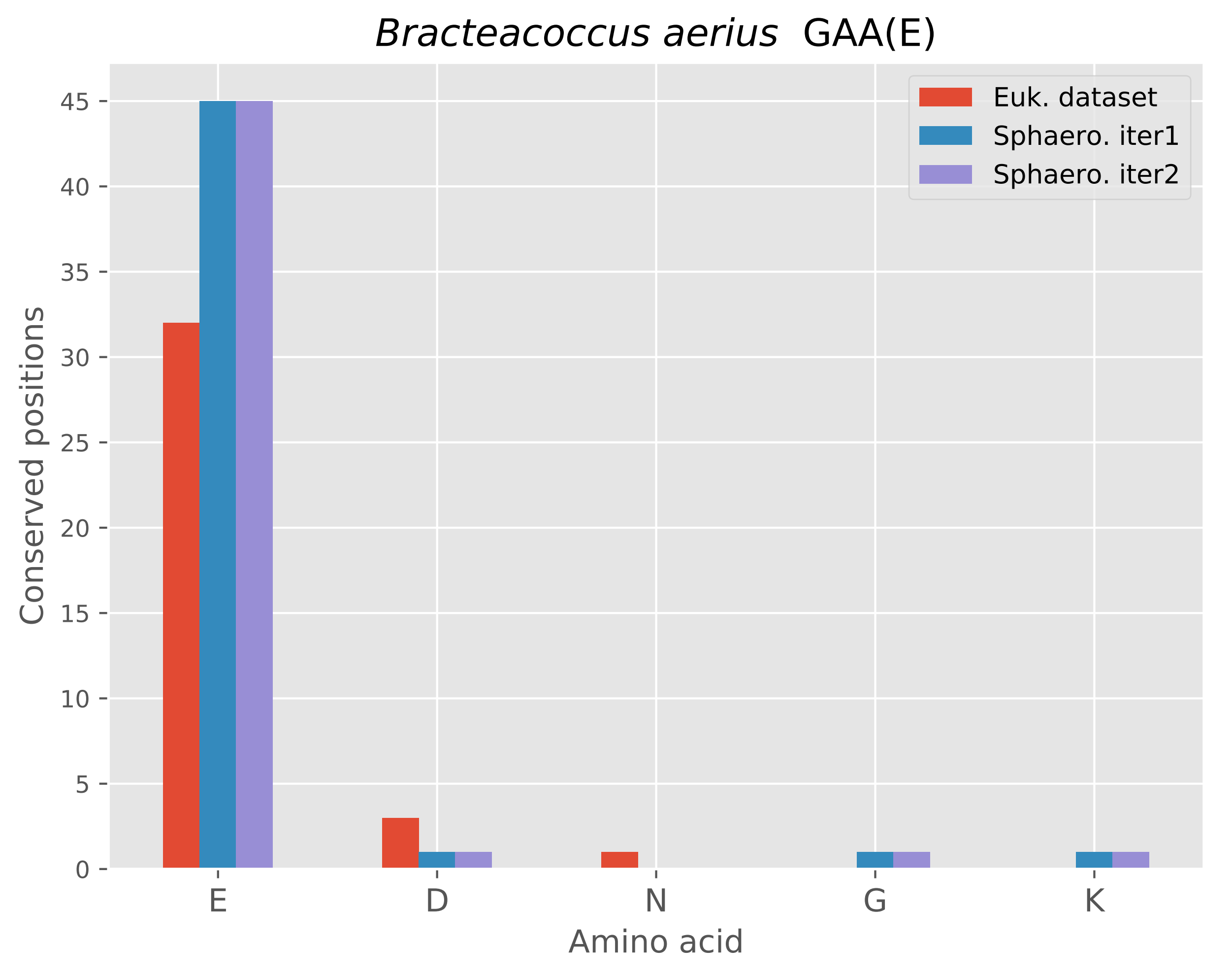

Bracteacoccus aerius GAA(E)
Euk. dataset
Sphaero. iter1
Sphaero. iter2
Conserved positions
0
5
10
15
20
25
30
35
40
45
E
D
N
G
K
Amino acid

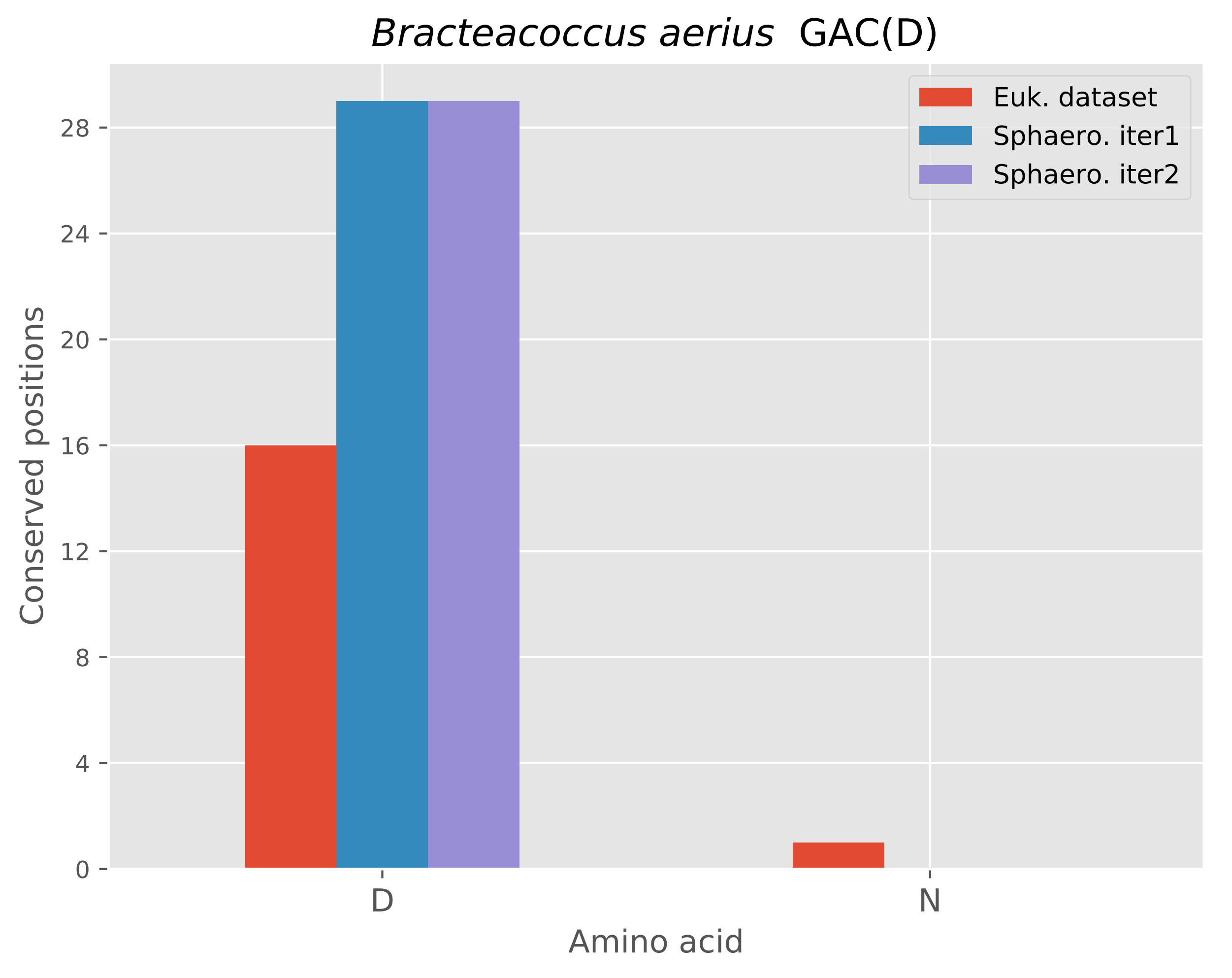
Bracteacoccus aerius GAC(D)
Euk. dataset
Sphaero. iter1
Sphaero. iter2
Conserved positions
0
4
8
12
16
20
24
28
D
N
Amino acid

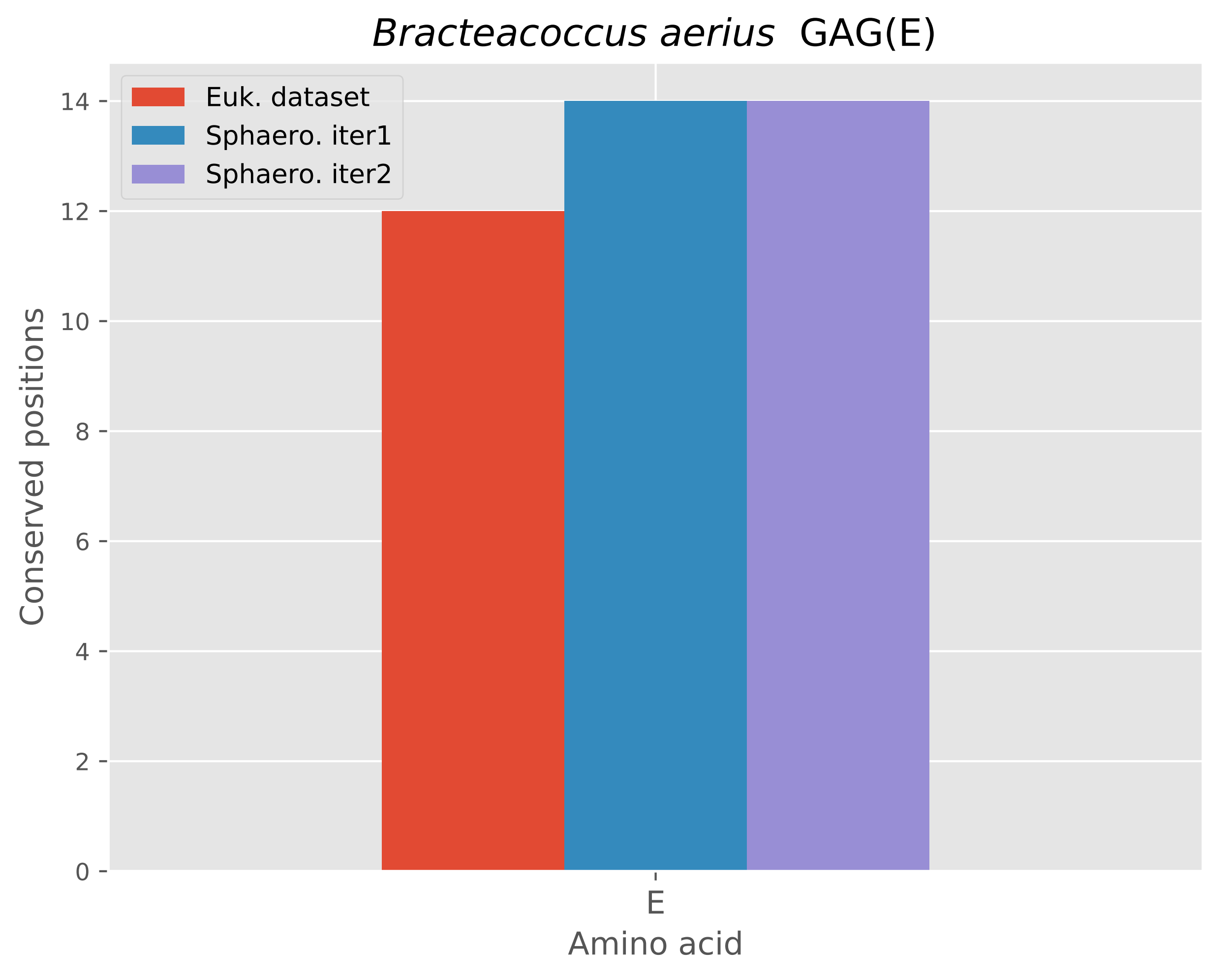

Bracteacoccus aerius GAG(E)
Euk. dataset
Sphaero. iter1
Sphaero. iter2
Conserved positions
0
2
4
6
8
10
12
14
E
Amino acid

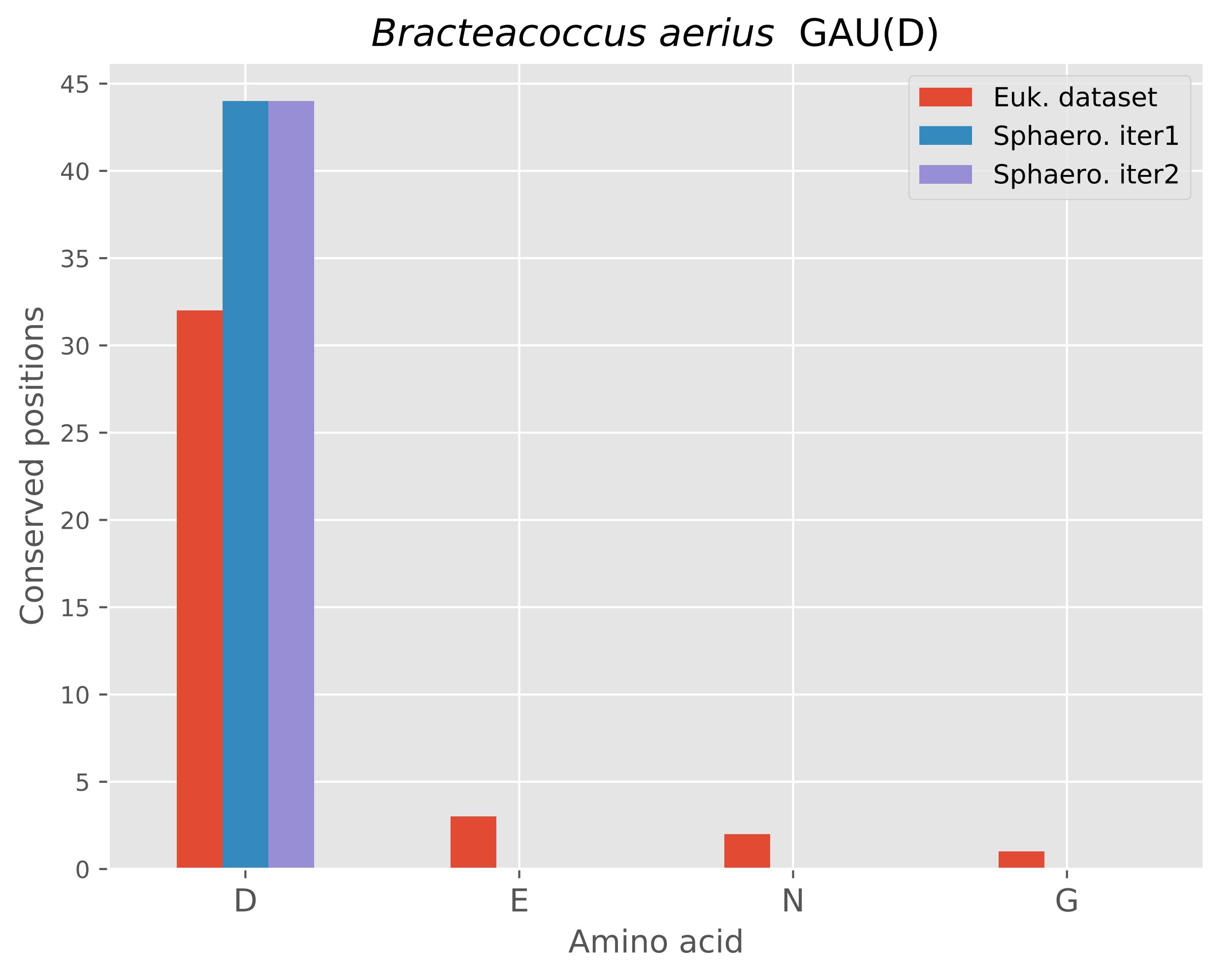

Bracteacoccus aerius GAU(D)
Euk. dataset
Sphaero. iter1
Sphaero. iter2
Conserved positions
0
5
10
15
20
25
30
35
40
45
D
E
N
G
Amino acid

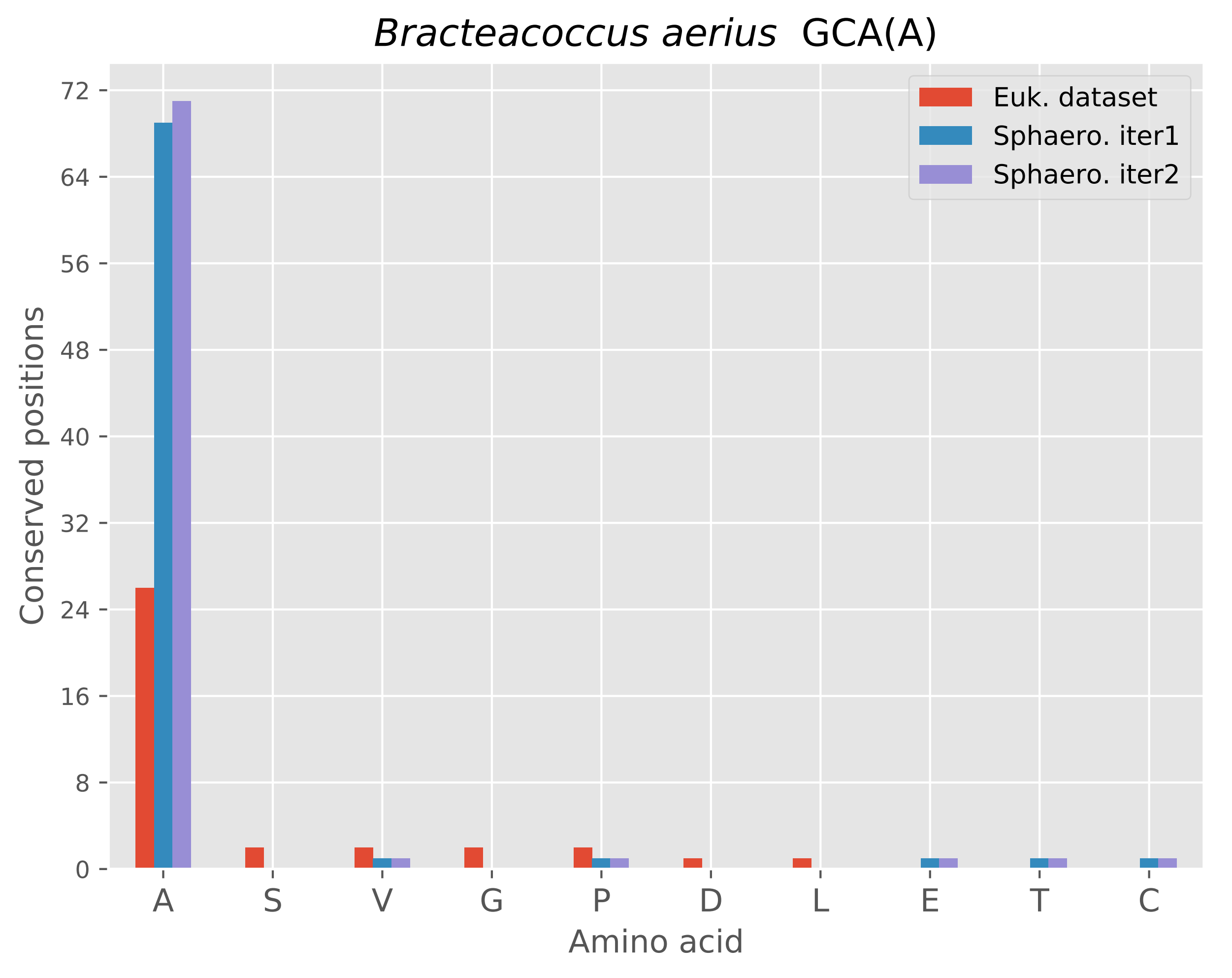
Bracteacoccus aerius GCA(A)
Euk. dataset
Sphaero. iter1
Sphaero. iter2
Conserved positions
Amino acid
0
8
16
24
32
40
48
56
64
72
A
S
V
G
P
D
L
E
T
C

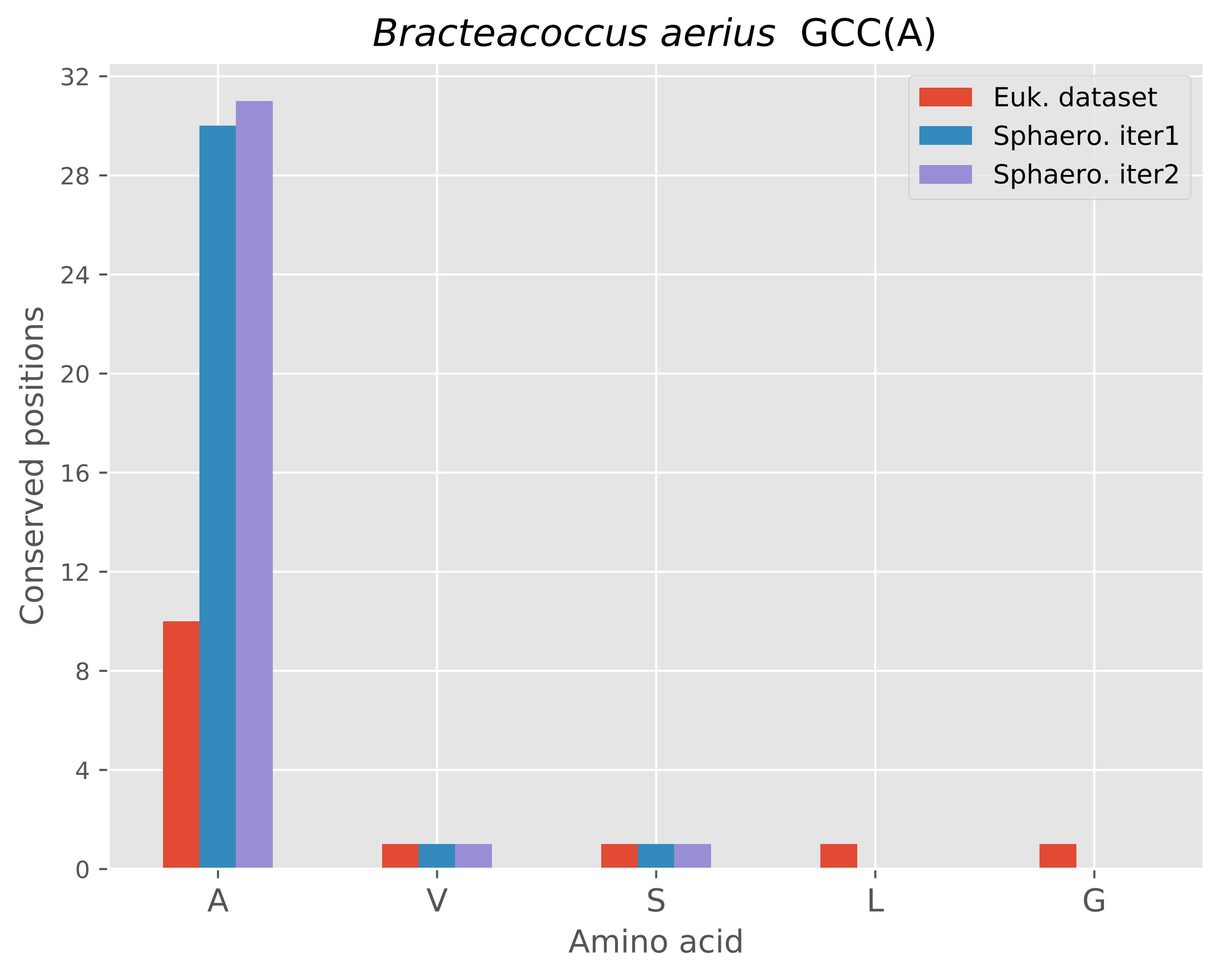

Bracteacoccus aerius GCC(A)
Euk. dataset
Sphaero. iter1
Sphaero. iter2
Conserved positions
Amino acid
0
4
8
12
16
20
24
28
32
A
V
S
L
G

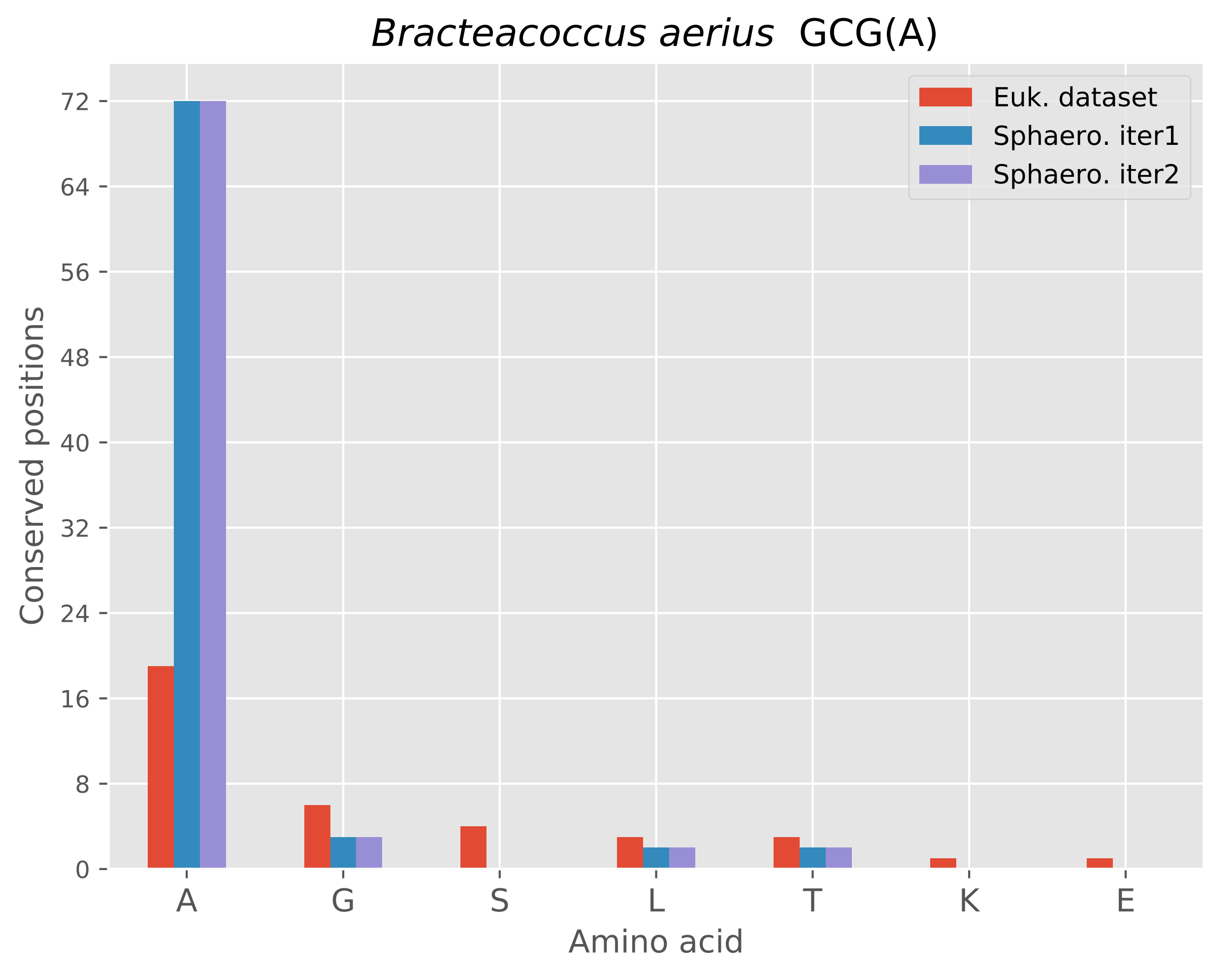
Bracteacoccus aerius GCG(A)
Euk. dataset
Sphaero. iter1
Sphaero. iter2
Conserved positions
0
8
16
24
32
40
48
56
64
72
A
G
S
L
T
K
E
Amino acid

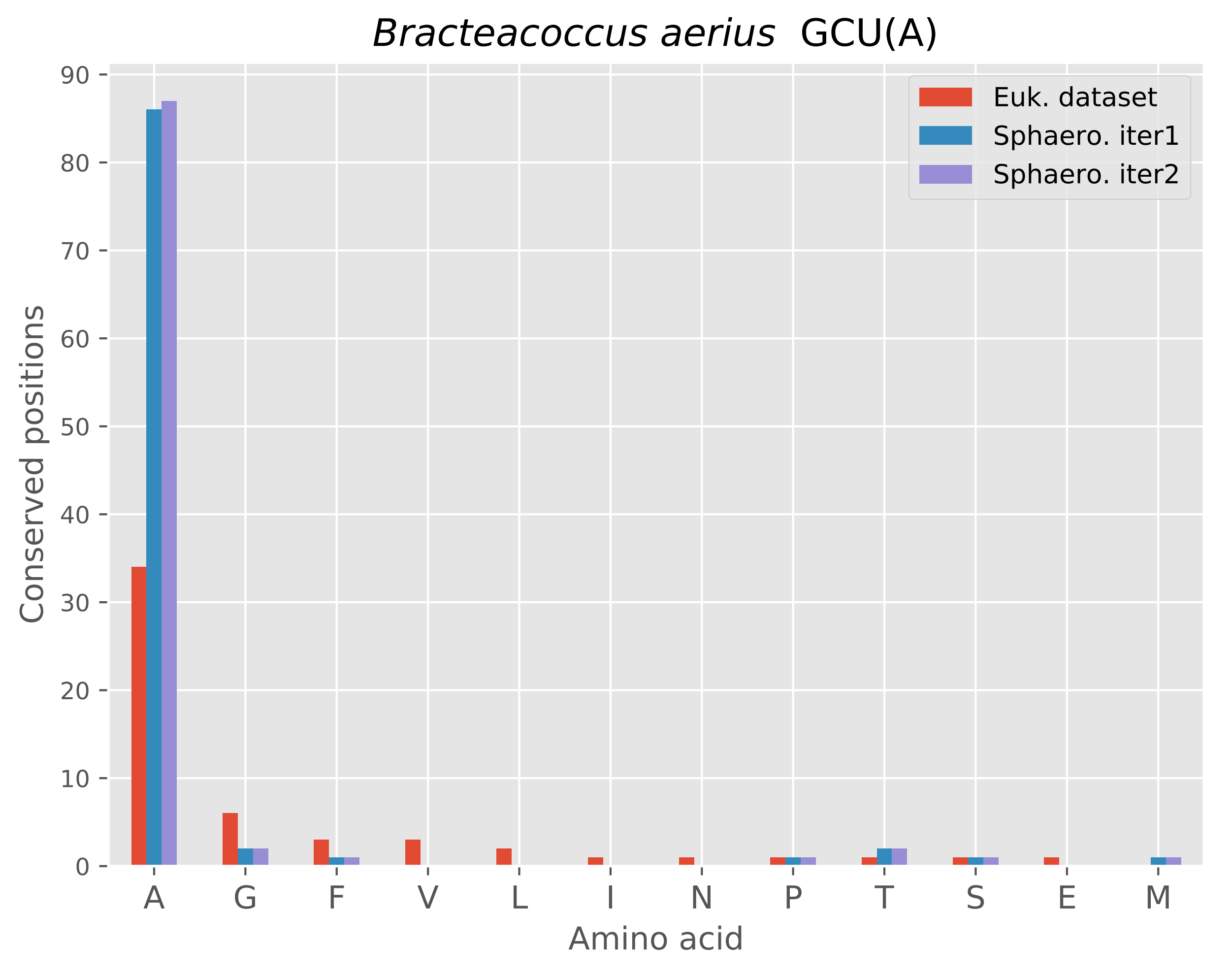
Bracteacoccus aerius GCU(A)
Euk. dataset
Sphaero. iter1
Sphaero. iter2
Conserved positions
0
10
20
30
40
50
60
70
80
90
A
G
F
V
L
I
N
P
T
S
E
M
Amino acid

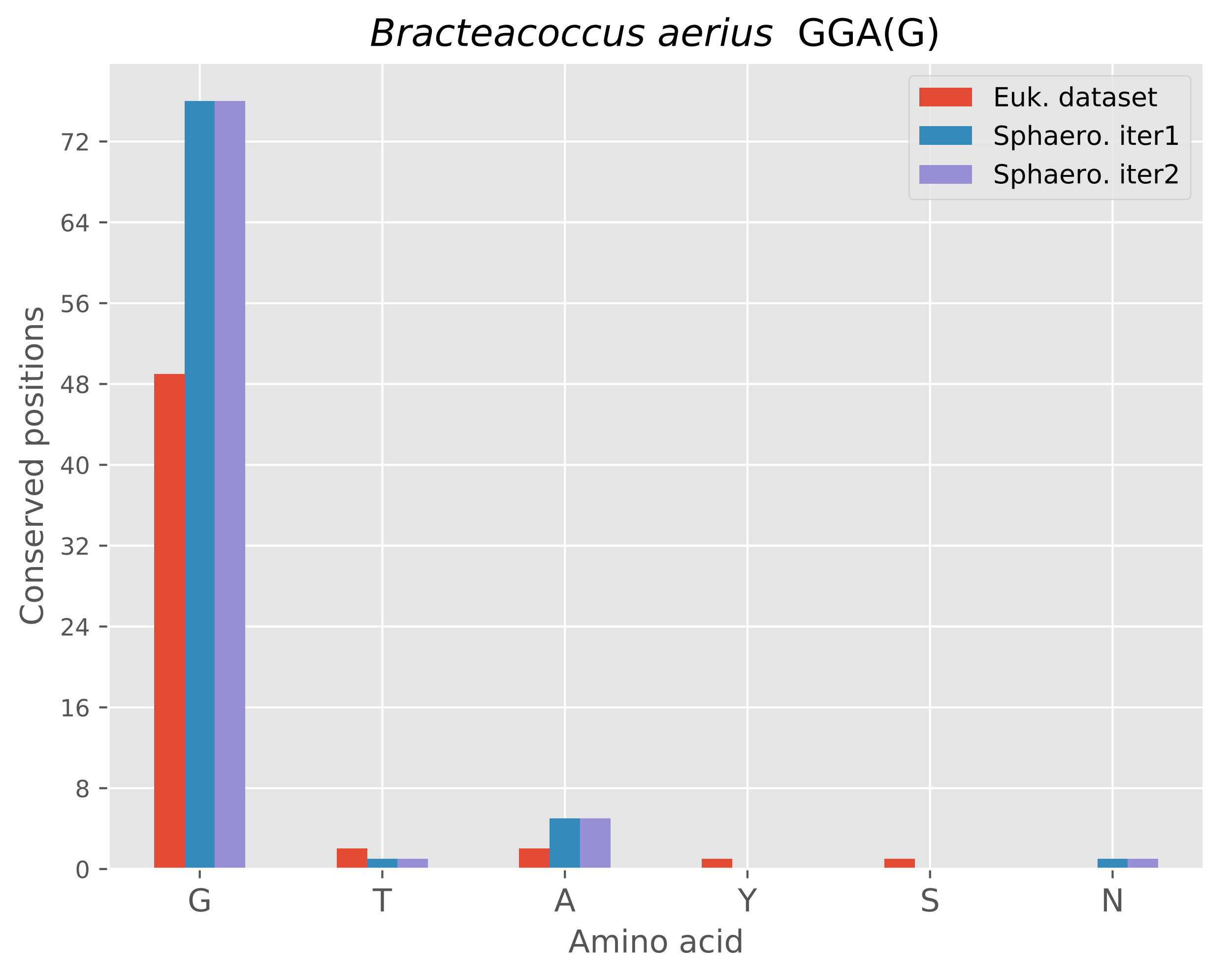

Bracteacoccus aerius GGA(G)
Euk. dataset
Sphaero. iter1
Sphaero. iter2
Conserved positions
0
8
16
24
32
40
48
56
64
72
G
T
A
Y
S
N
Amino acid

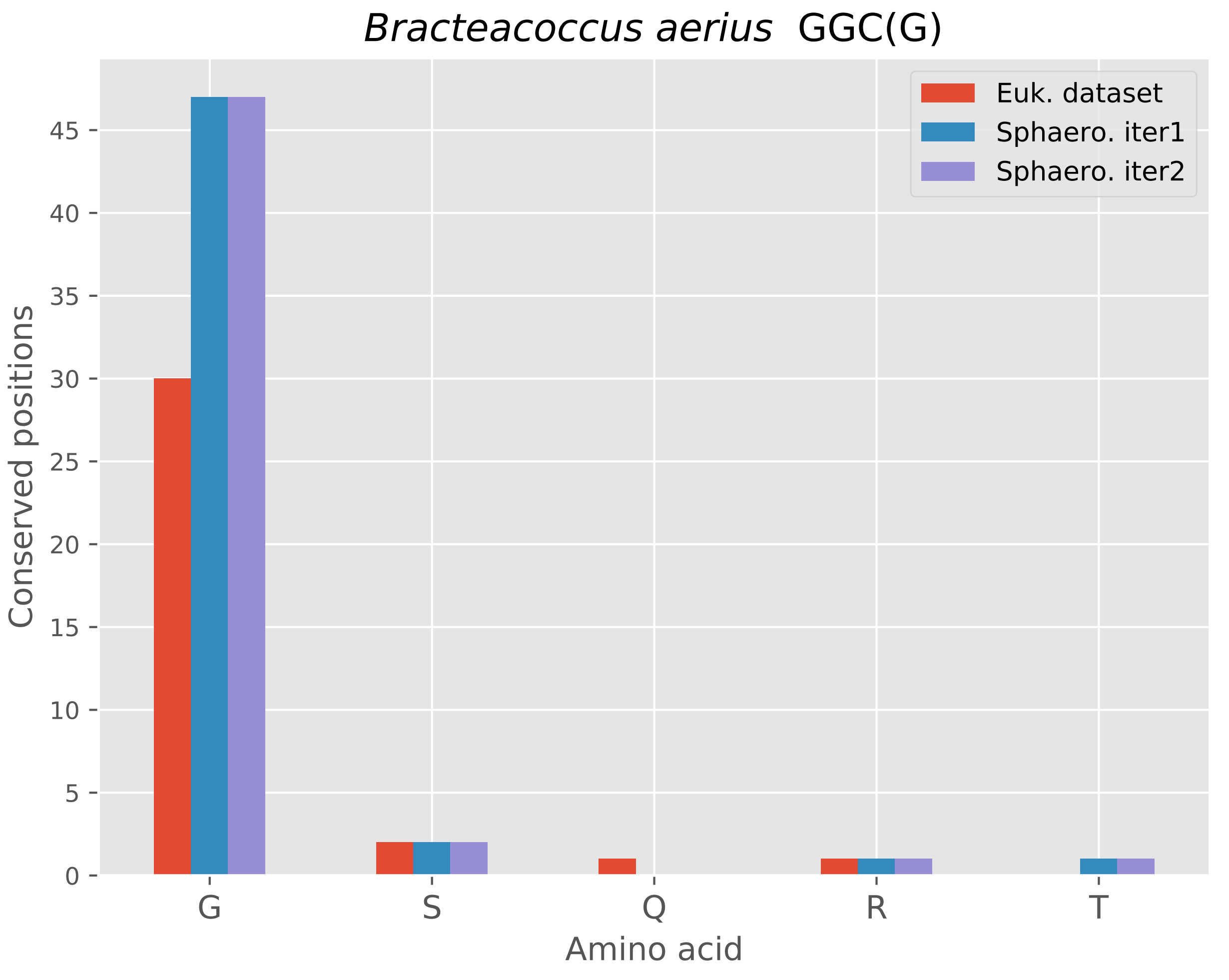

Bracteacoccus aerius GGC(G)
Euk. dataset
Sphaero. iter1
Sphaero. iter2
Conserved positions
0
5
10
15
20
25
30
35
40
45
G
S
Q
R
T
Amino acid

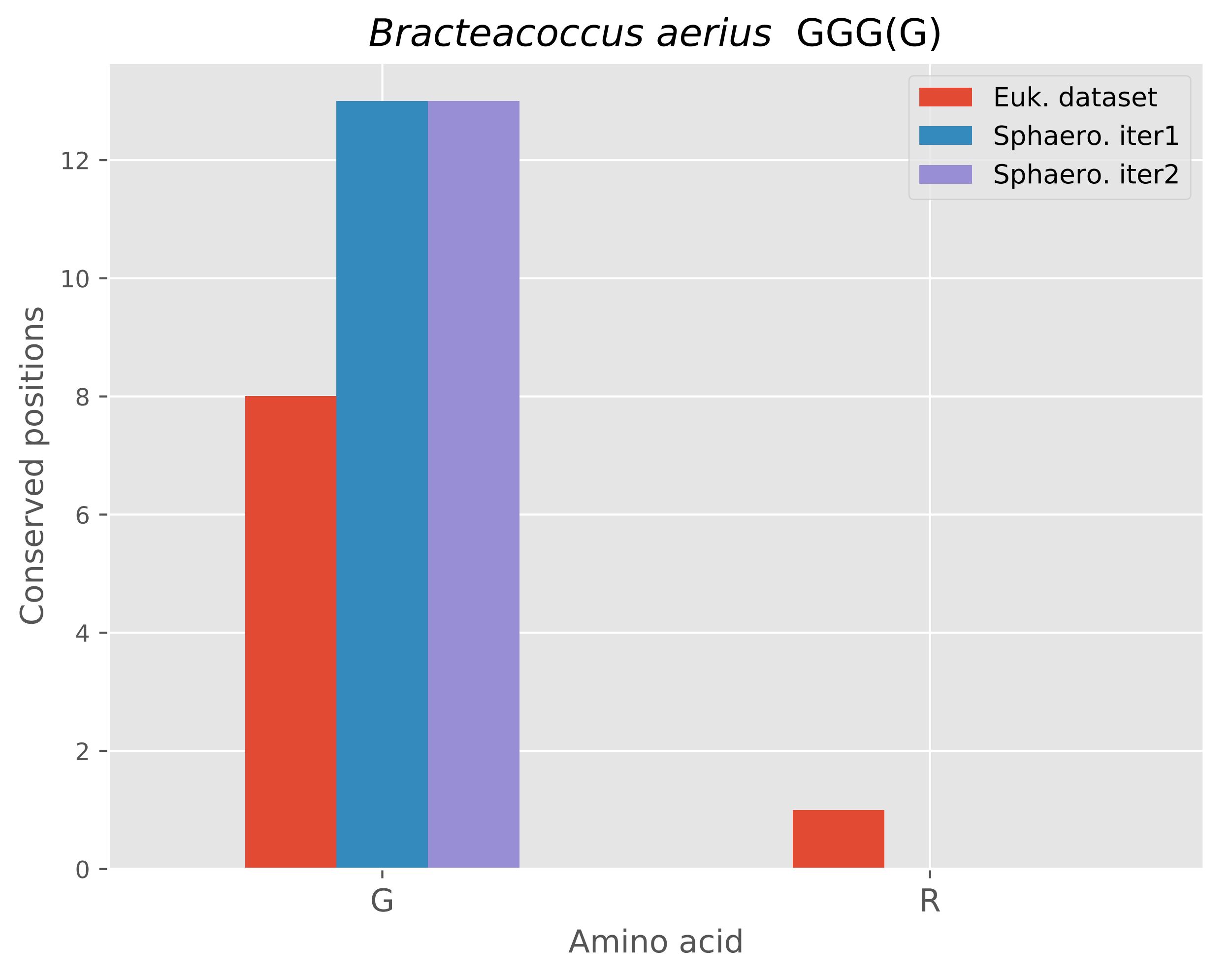

Bracteacoccus aerius GGG(G)
Euk. dataset
Sphaero. iter1
Sphaero. iter2
Conserved positions
0
2
4
6
8
10
12
G
R
Amino acid

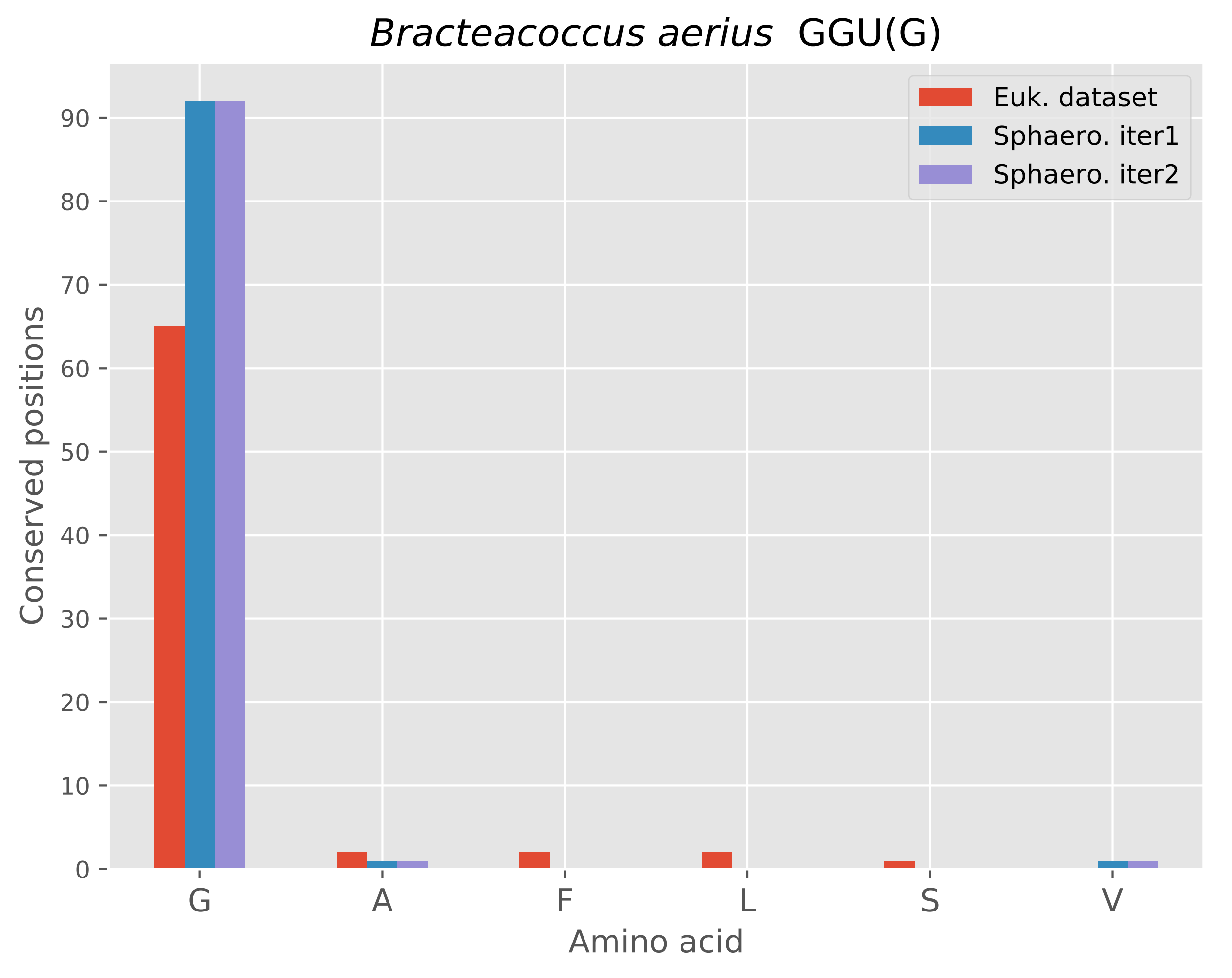

Bracteacoccus aerius GGU(G)
Euk. dataset
Sphaero. iter1
Sphaero. iter2
Conserved positions
0
10
20
30
40
50
60
70
80
90
G
A
F
L
S
V
Amino acid

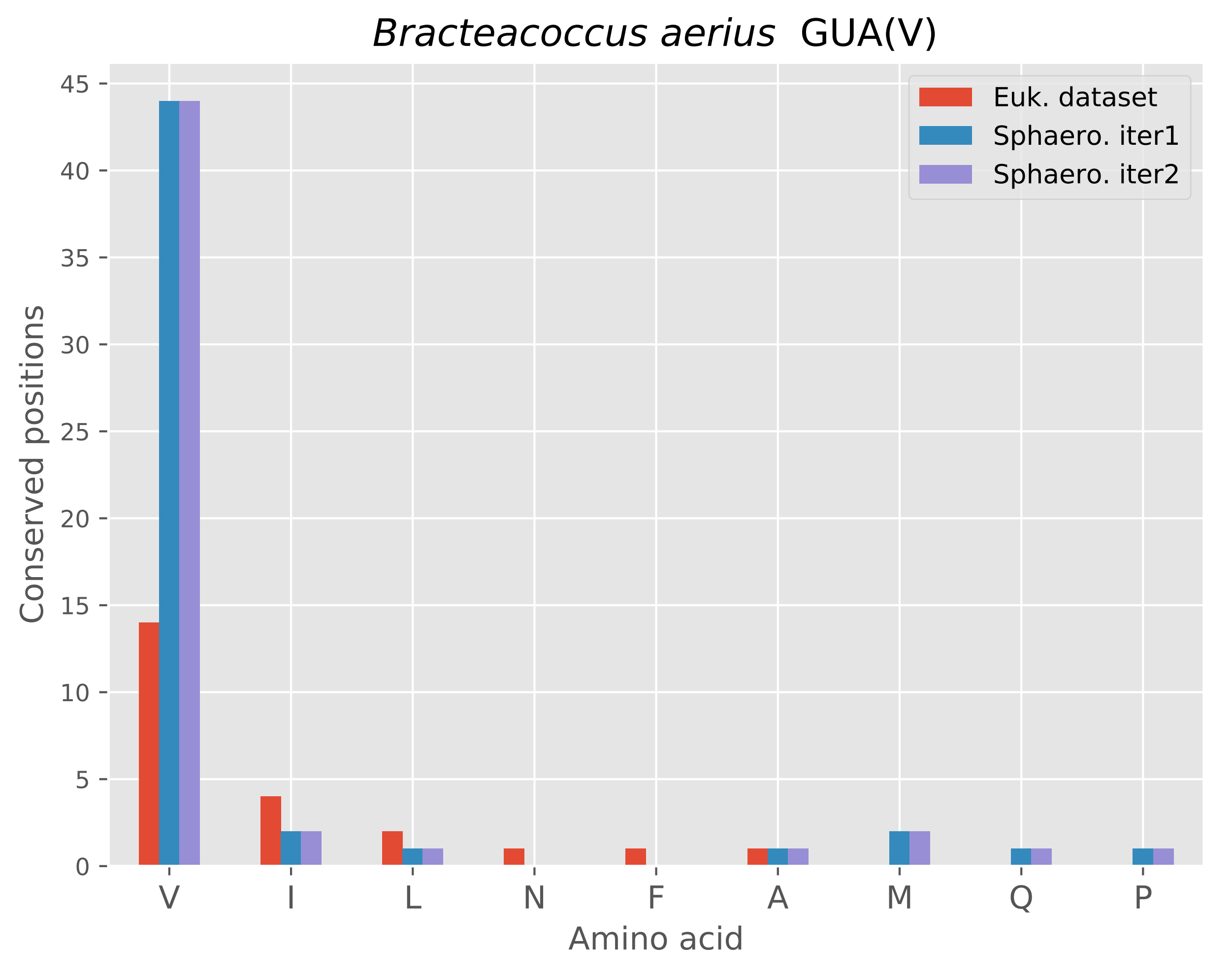
Bracteacoccus aerius GUA(V)
Euk. dataset
Sphaero. iter1
Sphaero. iter2
Conserved positions
0
5
10
15
20
25
30
35
40
45
V
I
L
N
F
A
M
Q
P
Amino acid

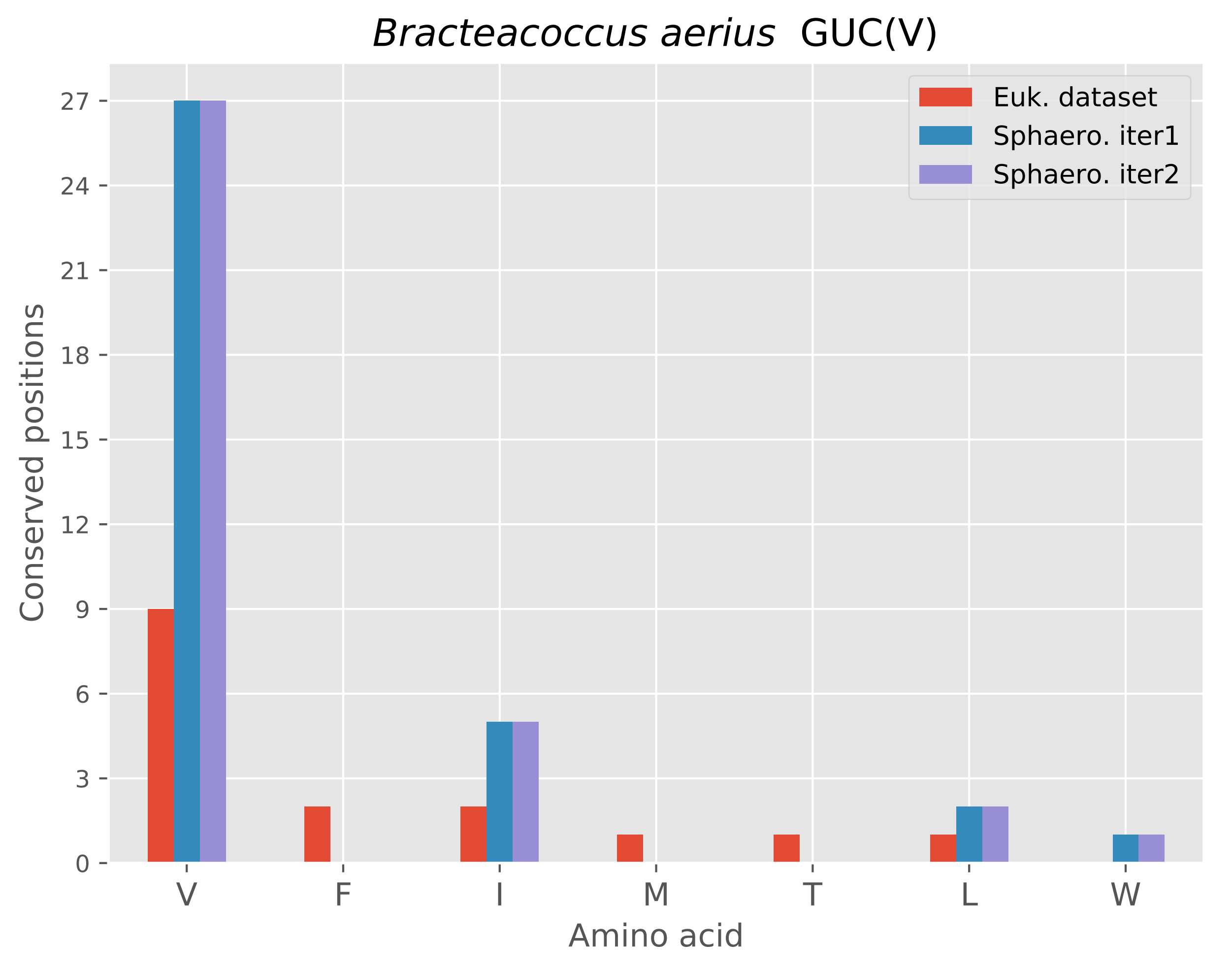
Bracteacoccus aerius GUC(V)
Euk. dataset
Sphaero. iter1
Sphaero. iter2
Conserved positions
0
3
6
9
12
15
18
21
24
27
V
F
I
M
T
L
W
Amino acid

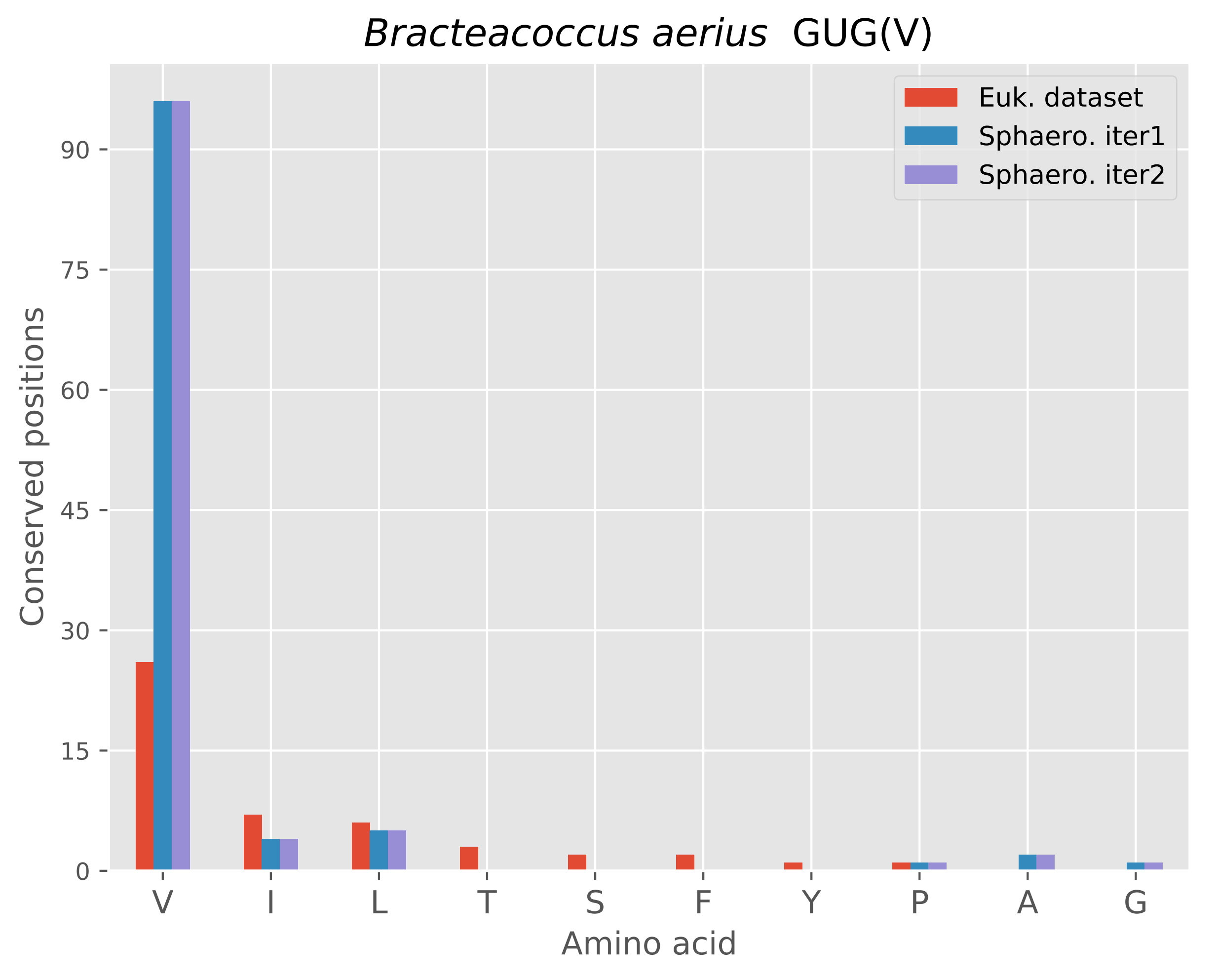

Bracteacoccus aerius GUG(V)
Euk. dataset
Sphaero. iter1
Sphaero. iter2
Conserved positions
0
15
30
45
60
75
90
V
I
L
T
S
F
Y
P
A
G
Amino acid

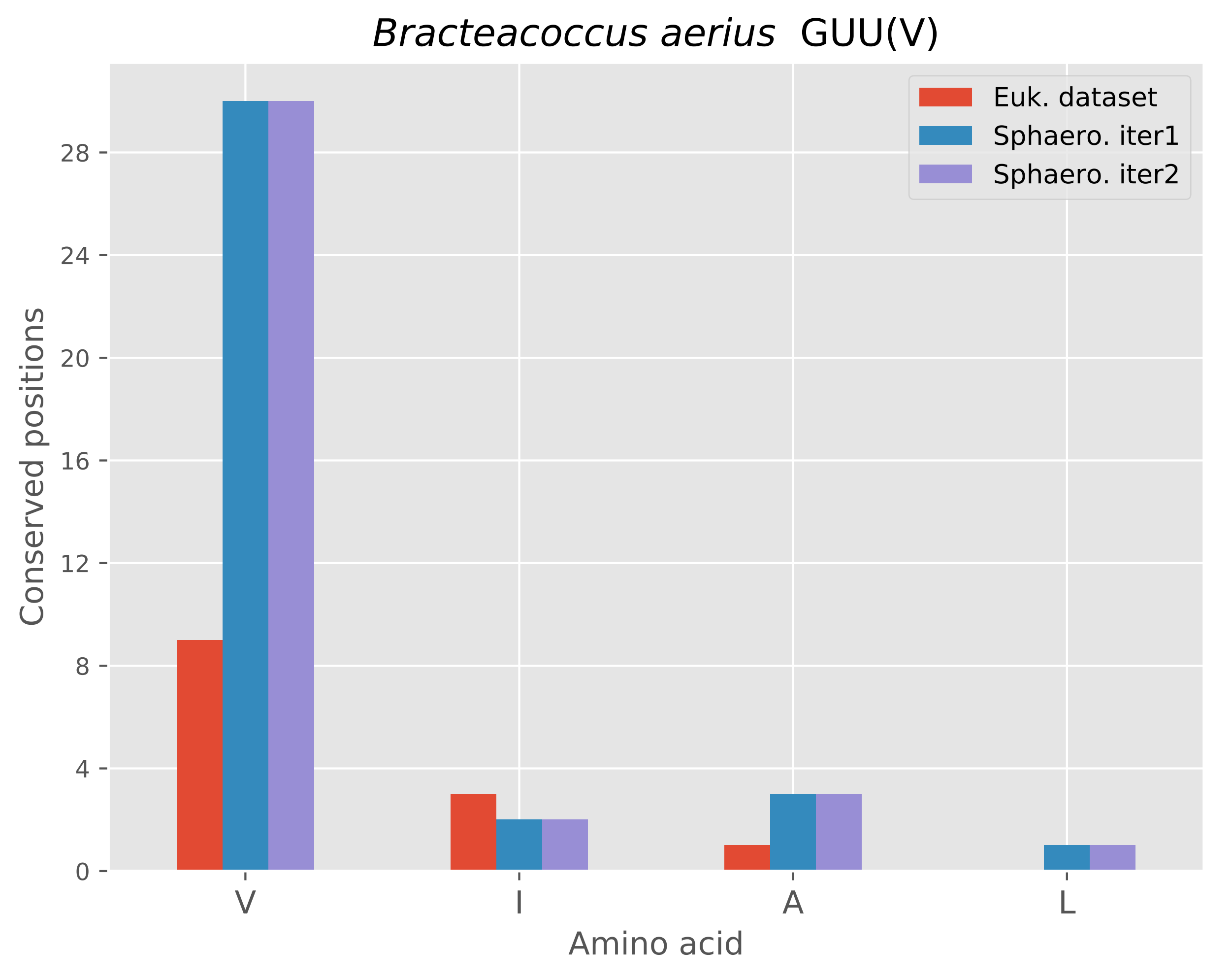

Bracteacoccus aerius GUU(V)
Euk. dataset
Sphaero. iter1
Sphaero. iter2
Conserved positions
0
4
8
12
16
20
24
28
V
I
A
L
Amino acid

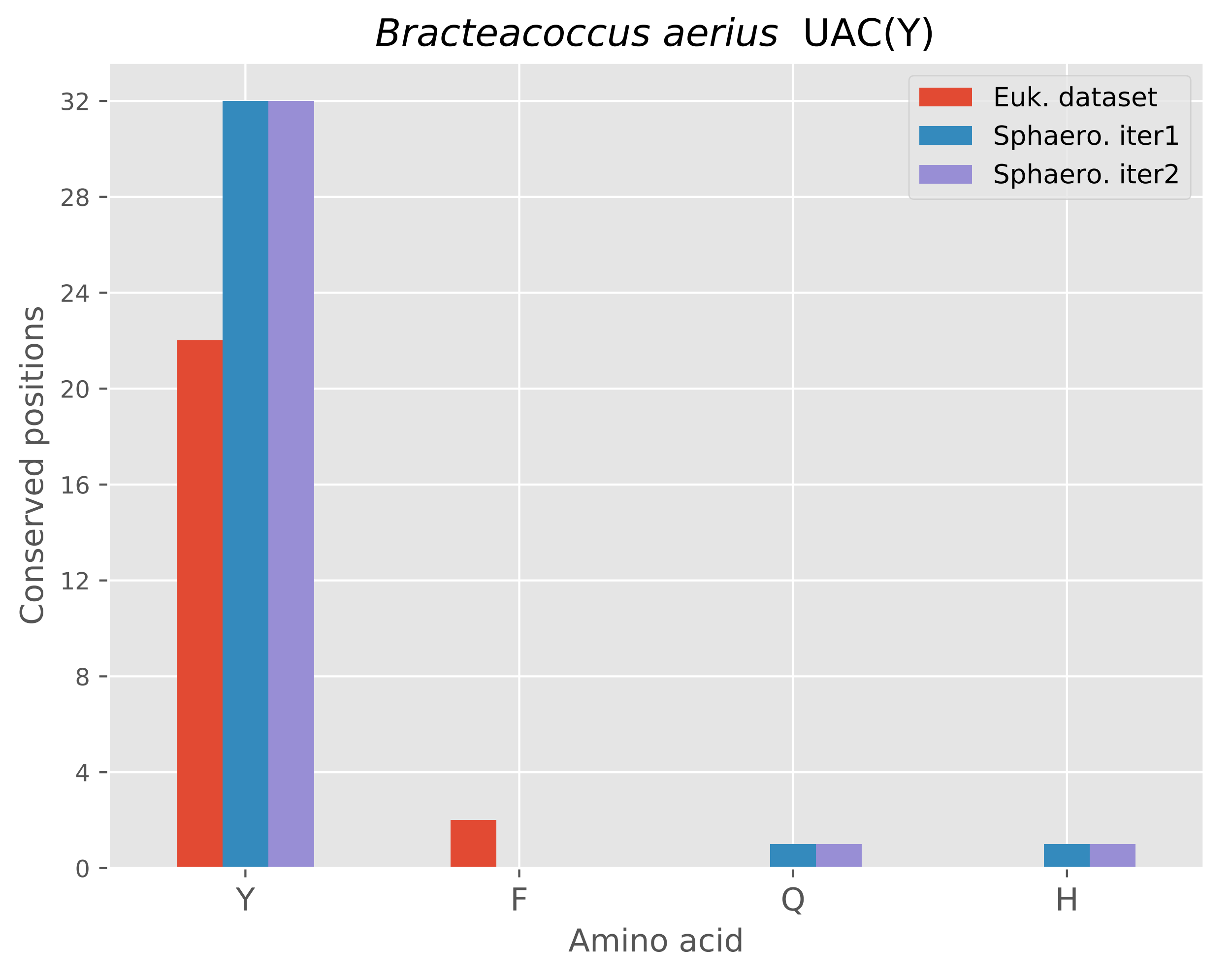

Bracteacoccus aerius UAC(Y)
Euk. dataset
Sphaero. iter1
Sphaero. iter2
Conserved positions
0
4
8
12
16
20
24
28
32
Y
F
Q
H
Amino acid

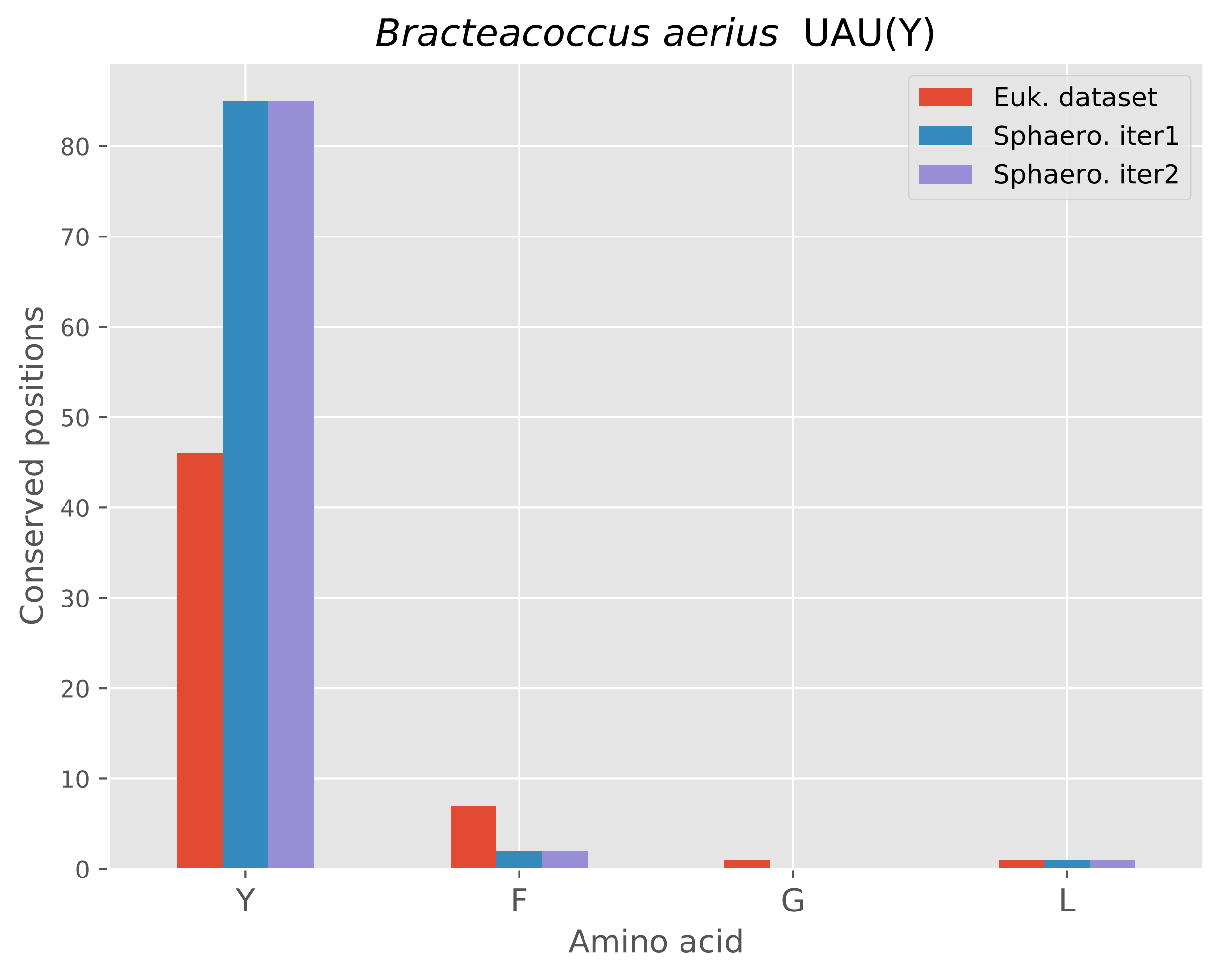
Bracteacoccus aerius UAU(Y)
Euk. dataset
Sphaero. iter1
Sphaero. iter2
Conserved positions
0
10
20
30
40
50
60
70
80
Y
F
G
L
Amino acid

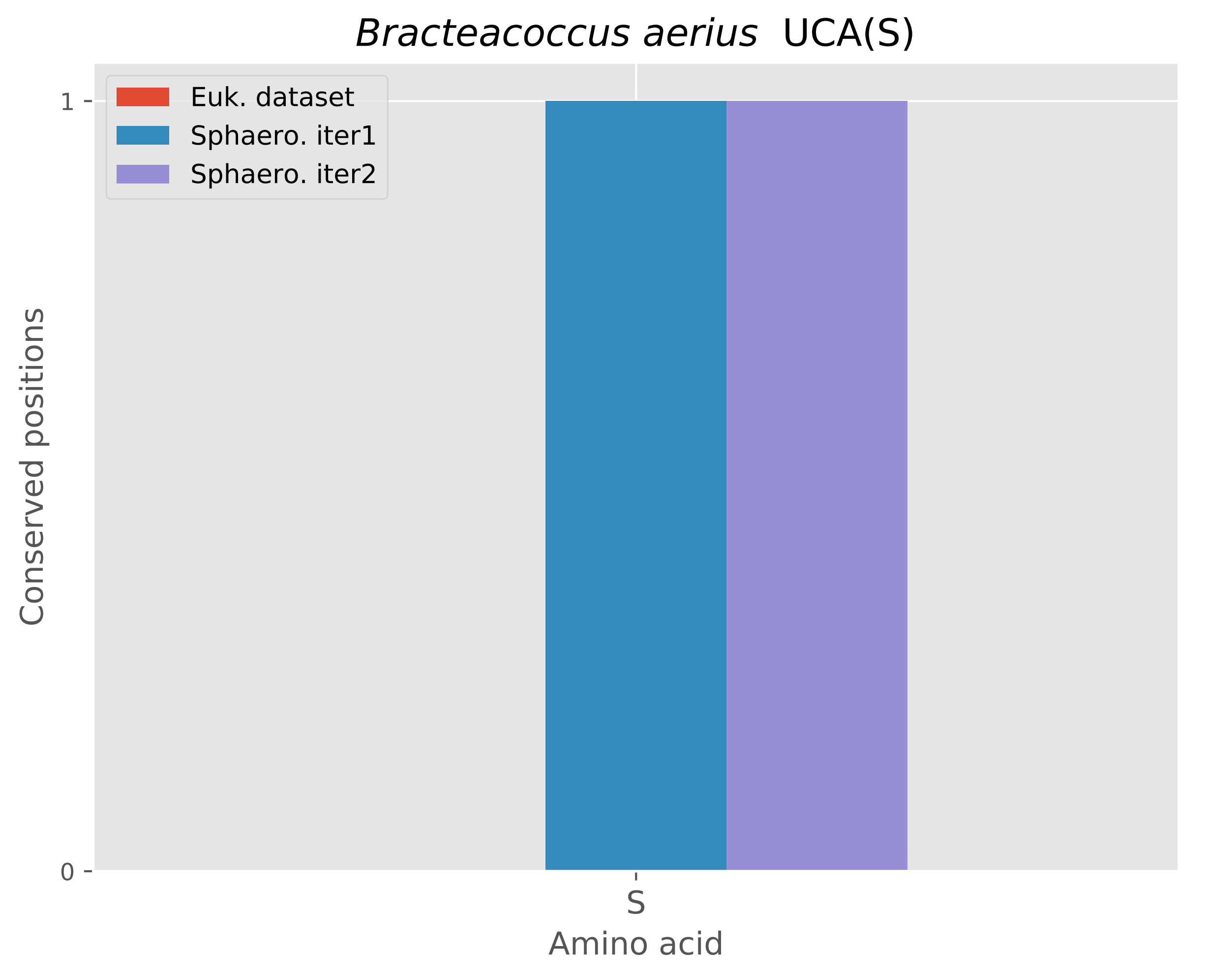

Bracteacoccus aerius UCA(S)
Euk. dataset
Sphaero. iter1
Sphaero. iter2
1
0
Conserved positions
S
Amino acid

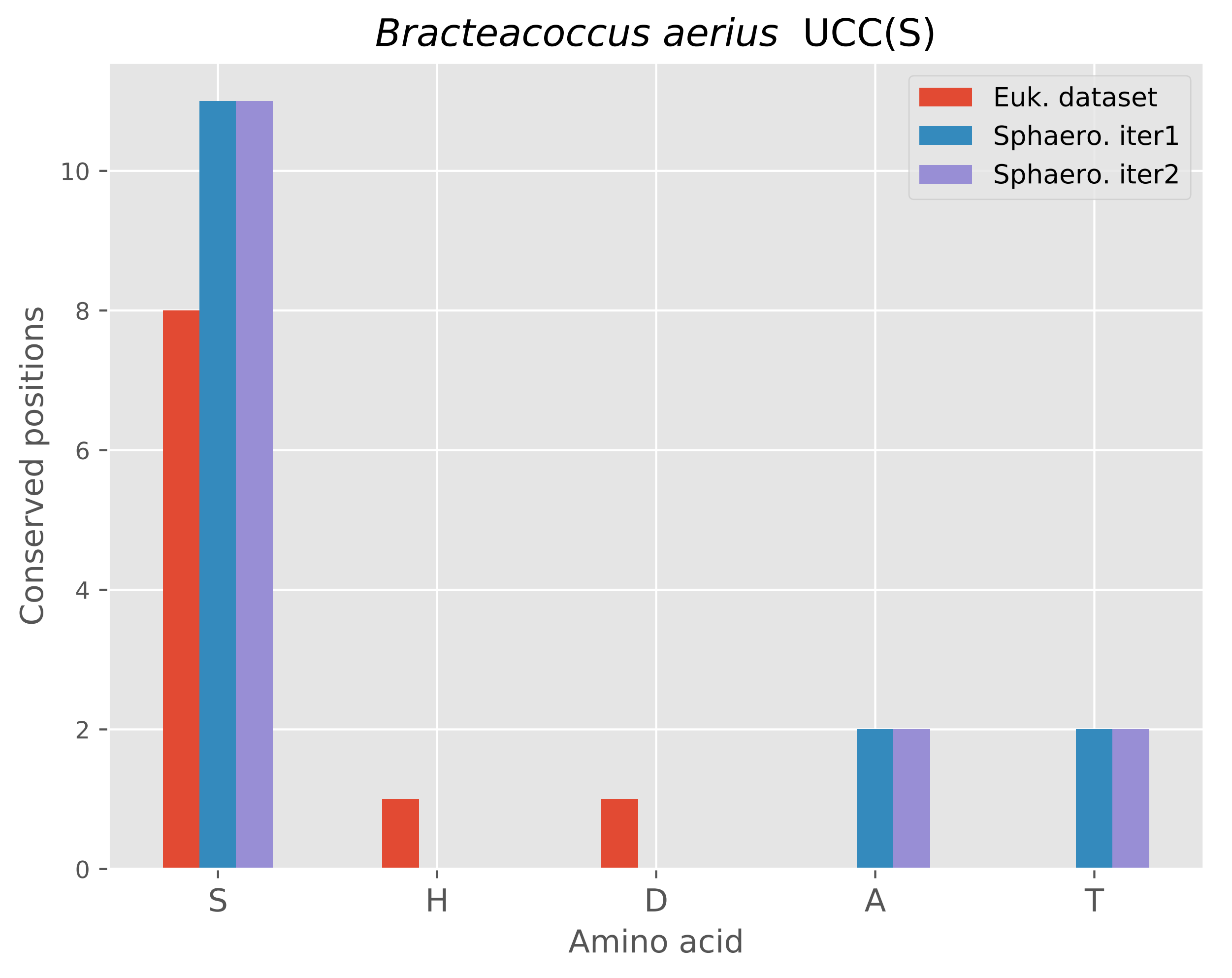

Bracteacoccus aerius UCC(S)
Euk. dataset
Sphaero. iter1
Sphaero. iter2
Conserved positions
0
2
4
6
8
10
S
H
D
A
T
Amino acid

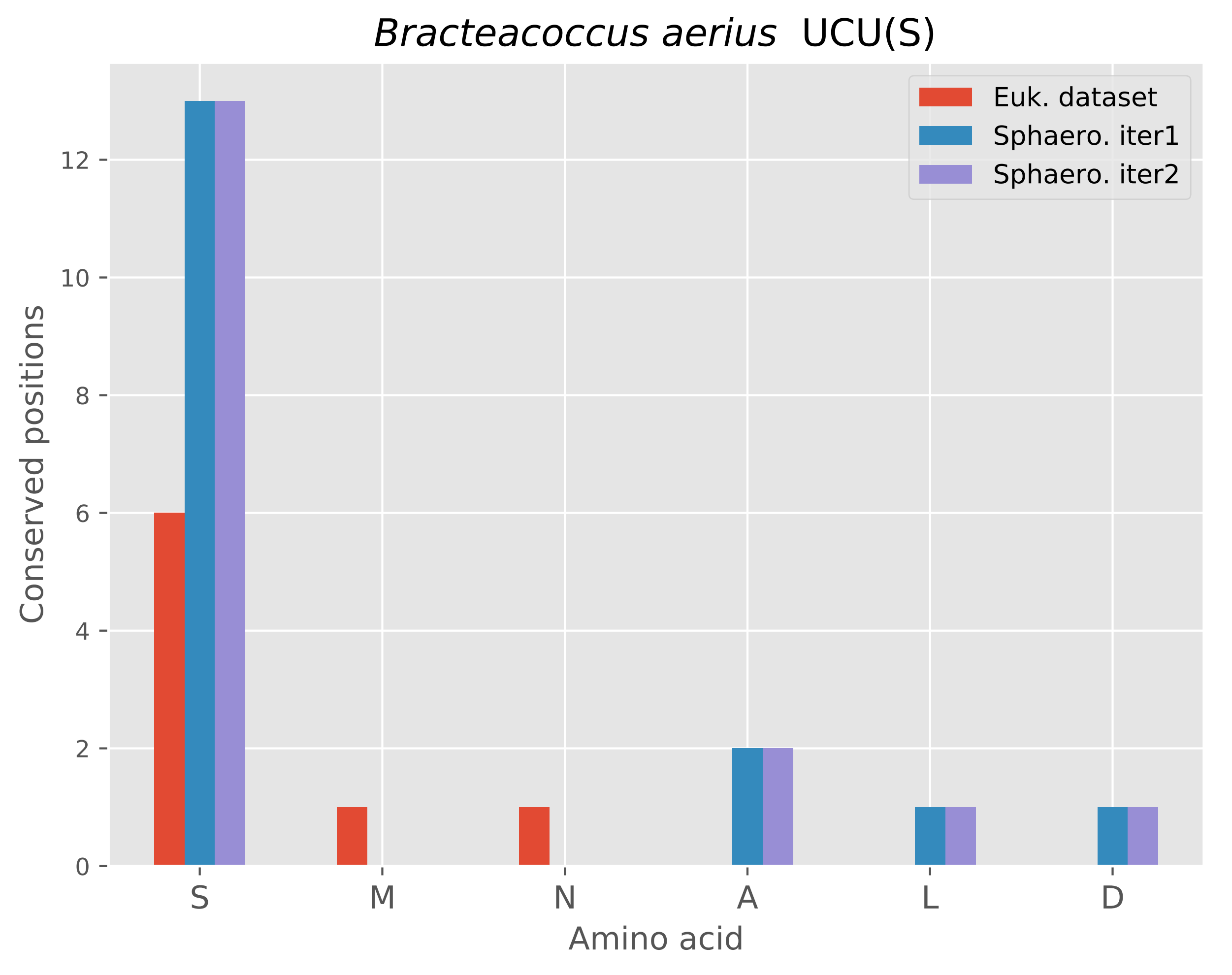

Bracteacoccus aerius UCU(S)
Euk. dataset
Sphaero. iter1
Sphaero. iter2
Conserved positions
0
2
4
6
8
10
12
S
M
N
A
L
D
Amino acid

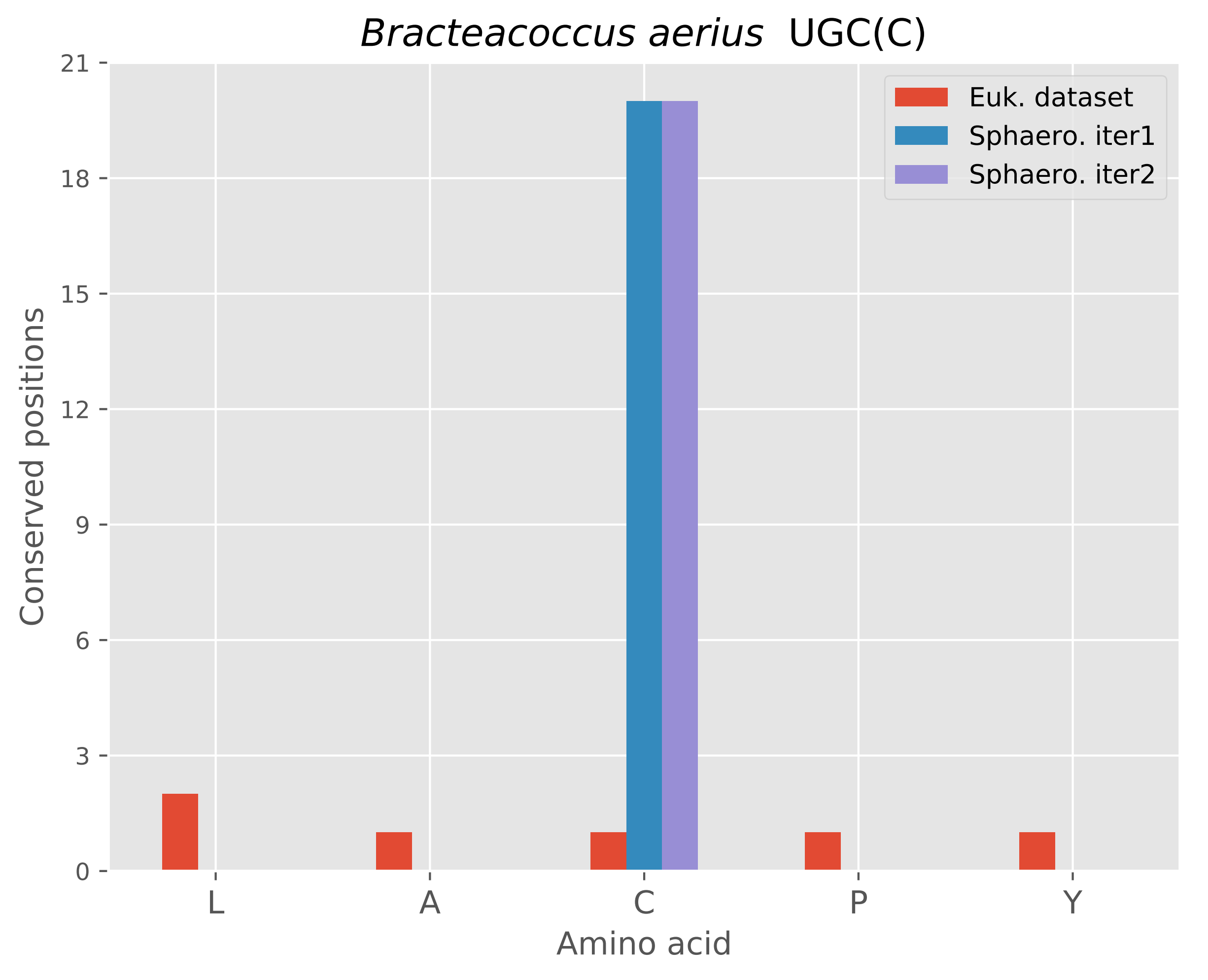
Bracteacoccus aerius UGC(C)
Euk. dataset
Sphaero. iter1
Sphaero. iter2
Conserved positions
0
3
6
9
12
15
18
21
L
A
C
P
Y
Amino acid

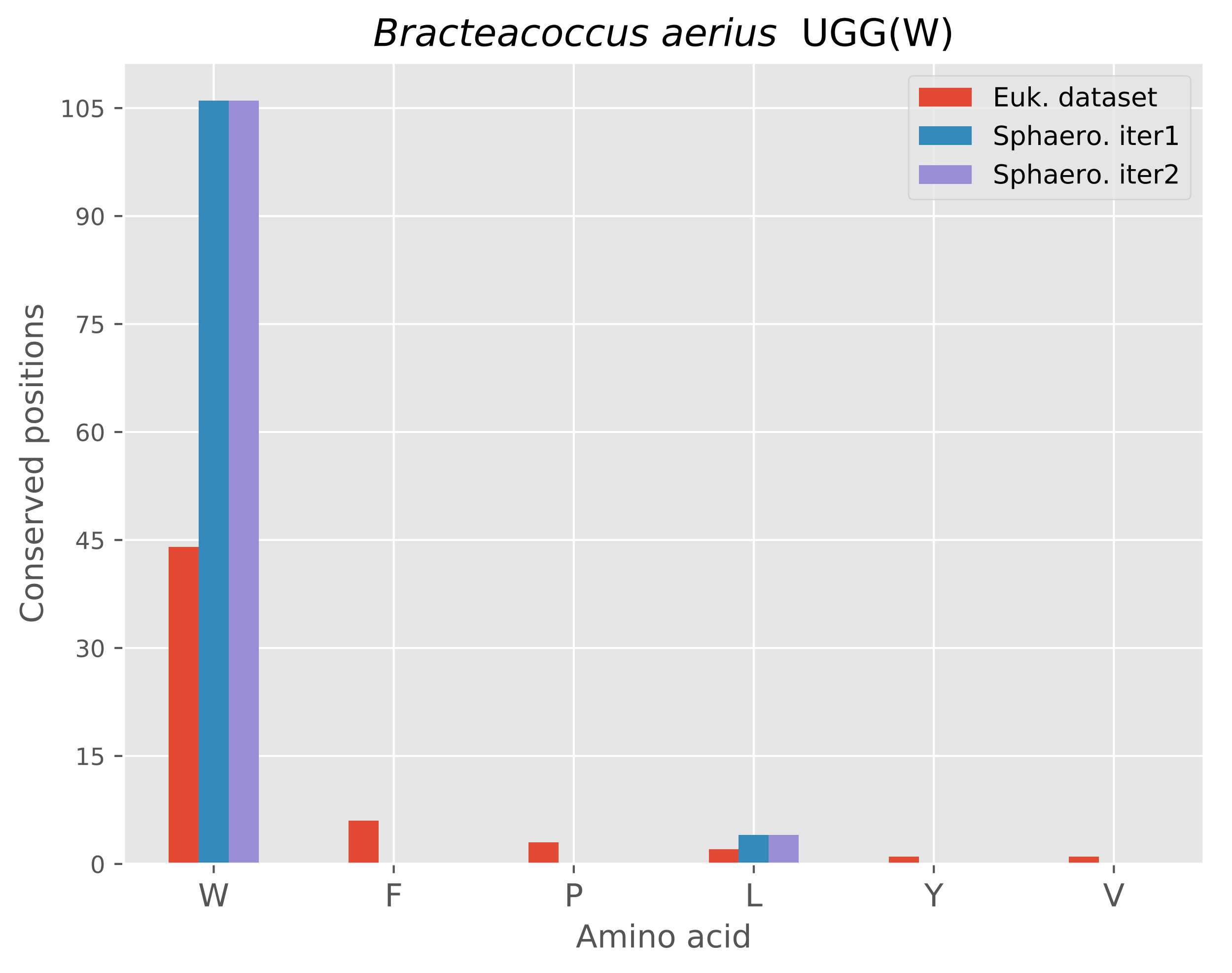

Bracteacoccus aerius UGG(W)
Euk. dataset
Sphaero. iter1
Sphaero. iter2
Conserved positions
0
15
30
45
60
75
90
105
W
F
P
L
Y
V
Amino acid

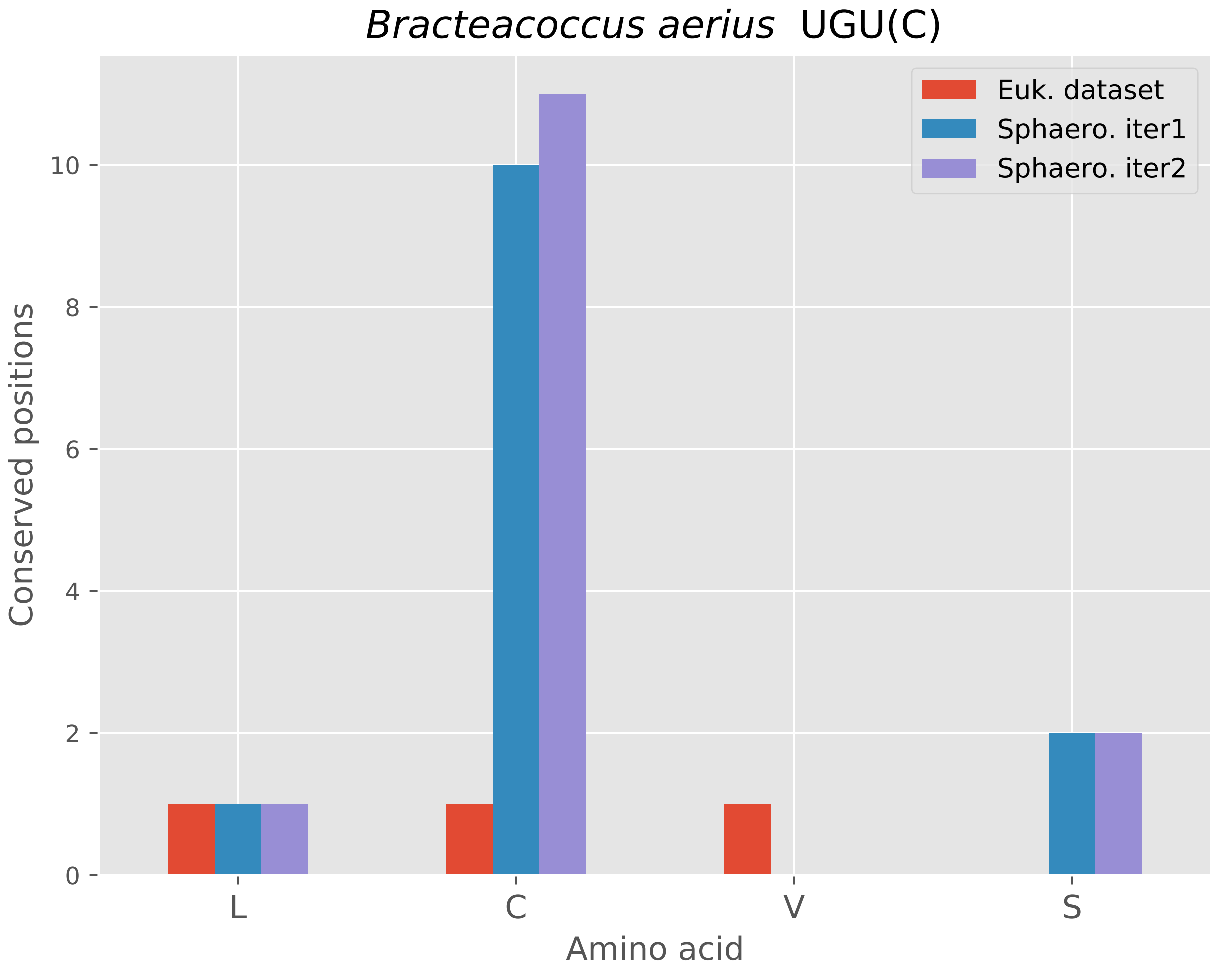
Bracteacoccus aerius UGU(C)
Euk. dataset
Sphaero. iter1
Sphaero. iter2
Conserved positions
0
2
4
6
8
10
L
C
V
S
Amino acid

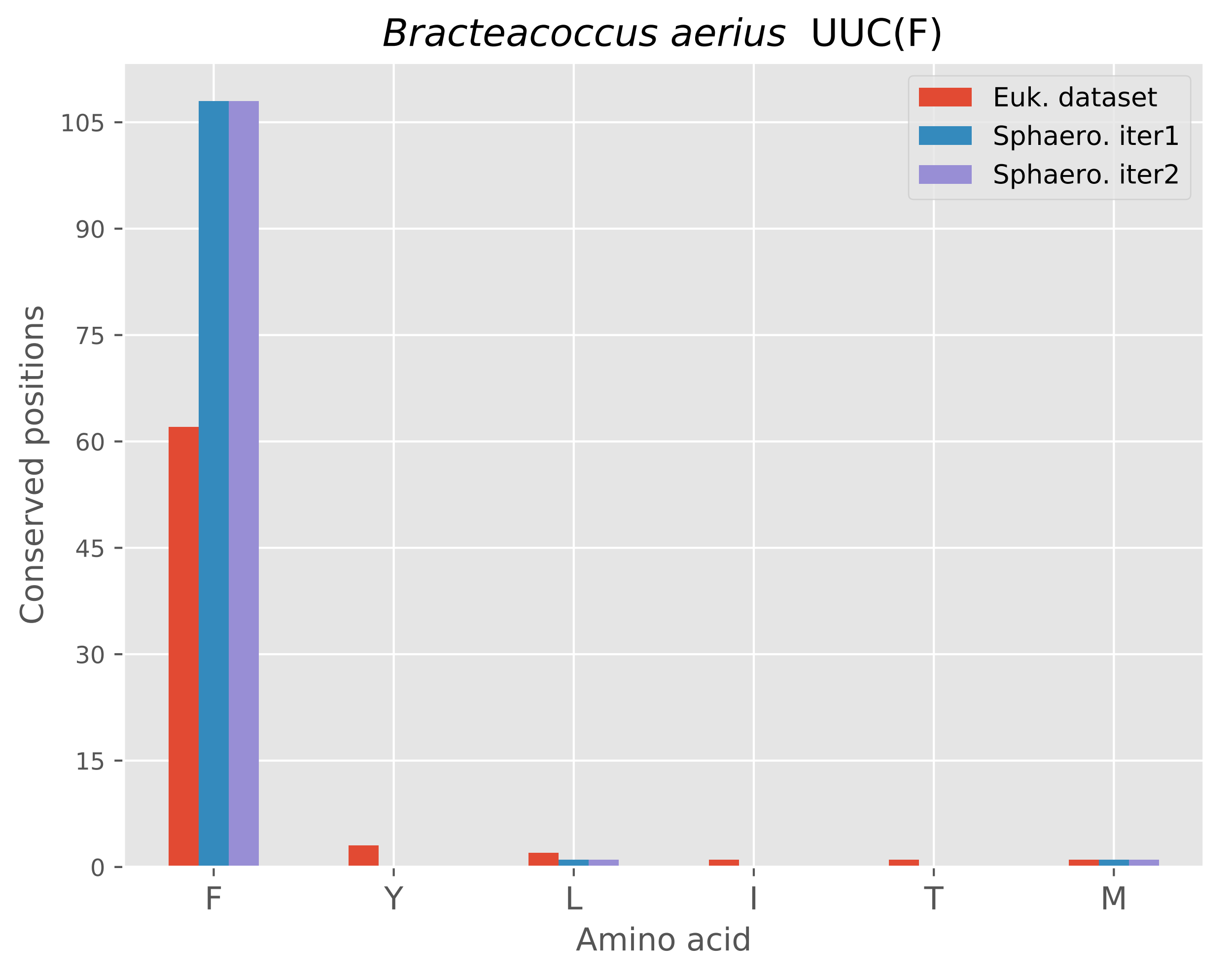

Bracteacoccus aerius UUC(F)
Euk. dataset
Sphaero. iter1
Sphaero. iter2
Conserved positions
0
15
30
45
60
75
90
105
F
Y
L
I
T
M
Amino acid

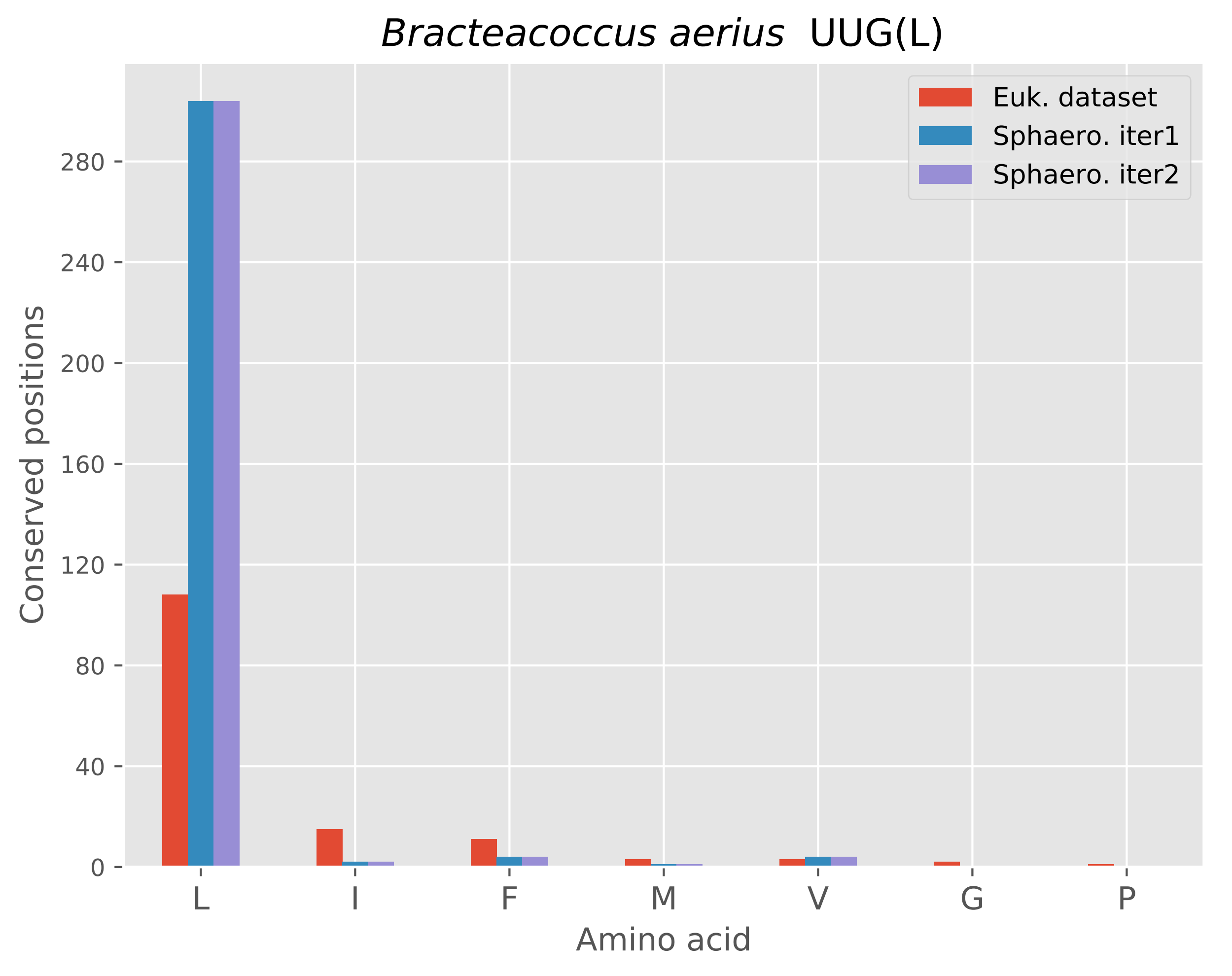

Bracteacoccus aerius UUG(L)
Euk. dataset
Sphaero. iter1
Sphaero. iter2
Conserved positions
0
40
80
120
160
200
240
280
L
I
F
M
V
G
P
Amino acid

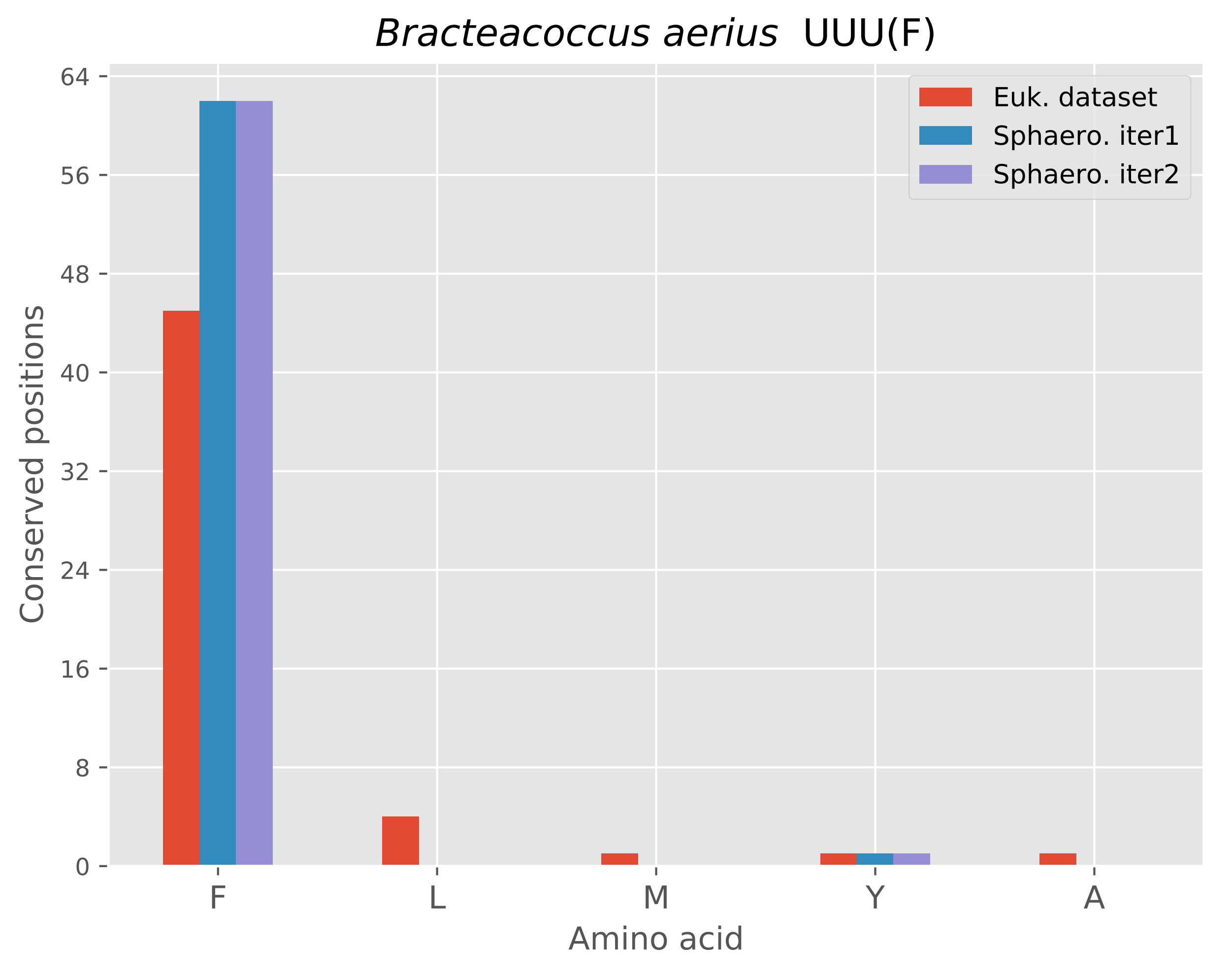

Bracteacoccus aerius UUU(F)
Euk. dataset
Sphaero. iter1
Sphaero. iter2
Conserved positions
0
8
16
24
32
40
48
56
64
F
L
M
Y
A
Amino acid

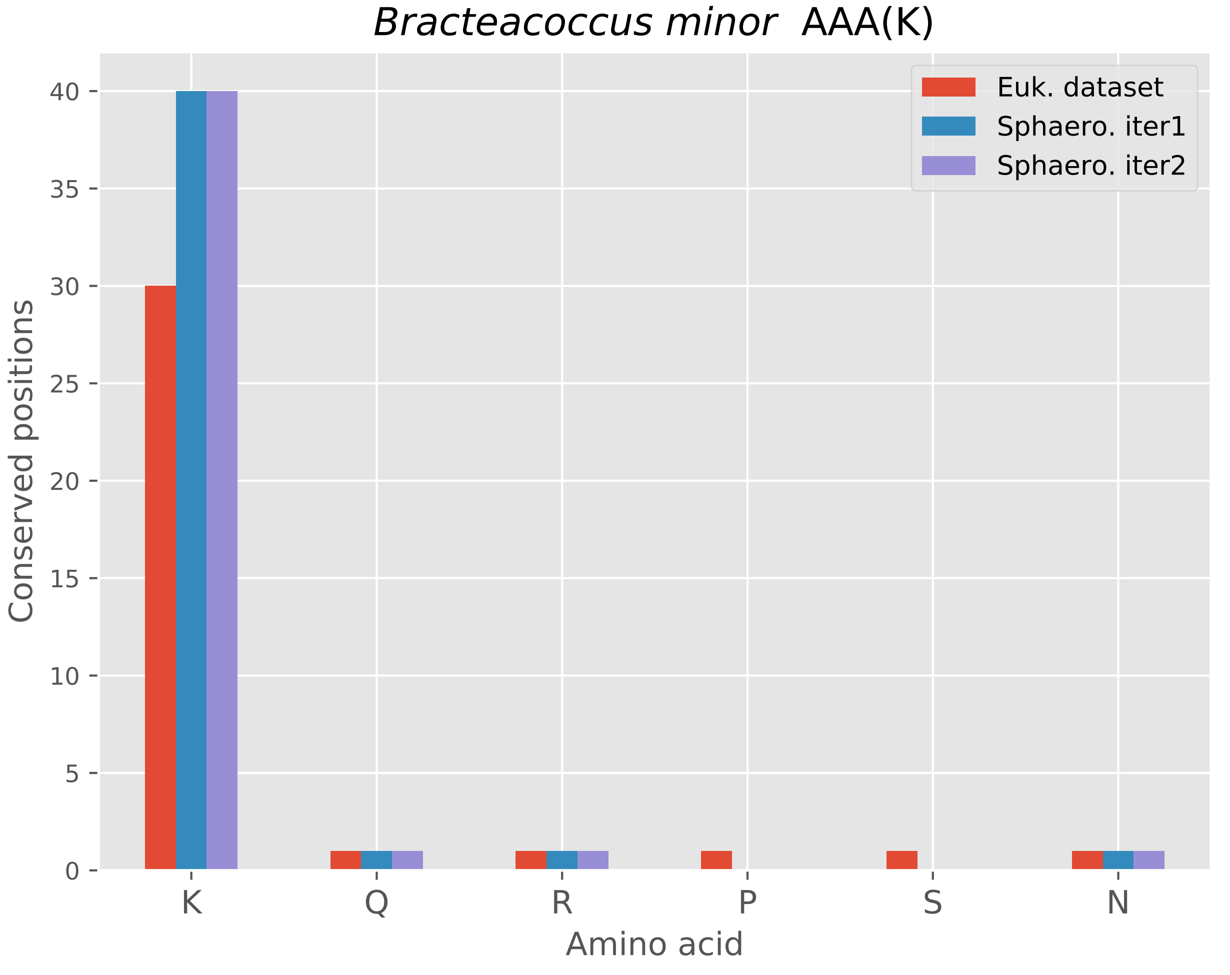

Bracteacoccus minor AAA(K)
Euk. dataset
Sphaero. iter1
Sphaero. iter2
Conserved positions
0
5
10
15
20
25
30
35
40
K
Q
R
P
S
N
Amino acid

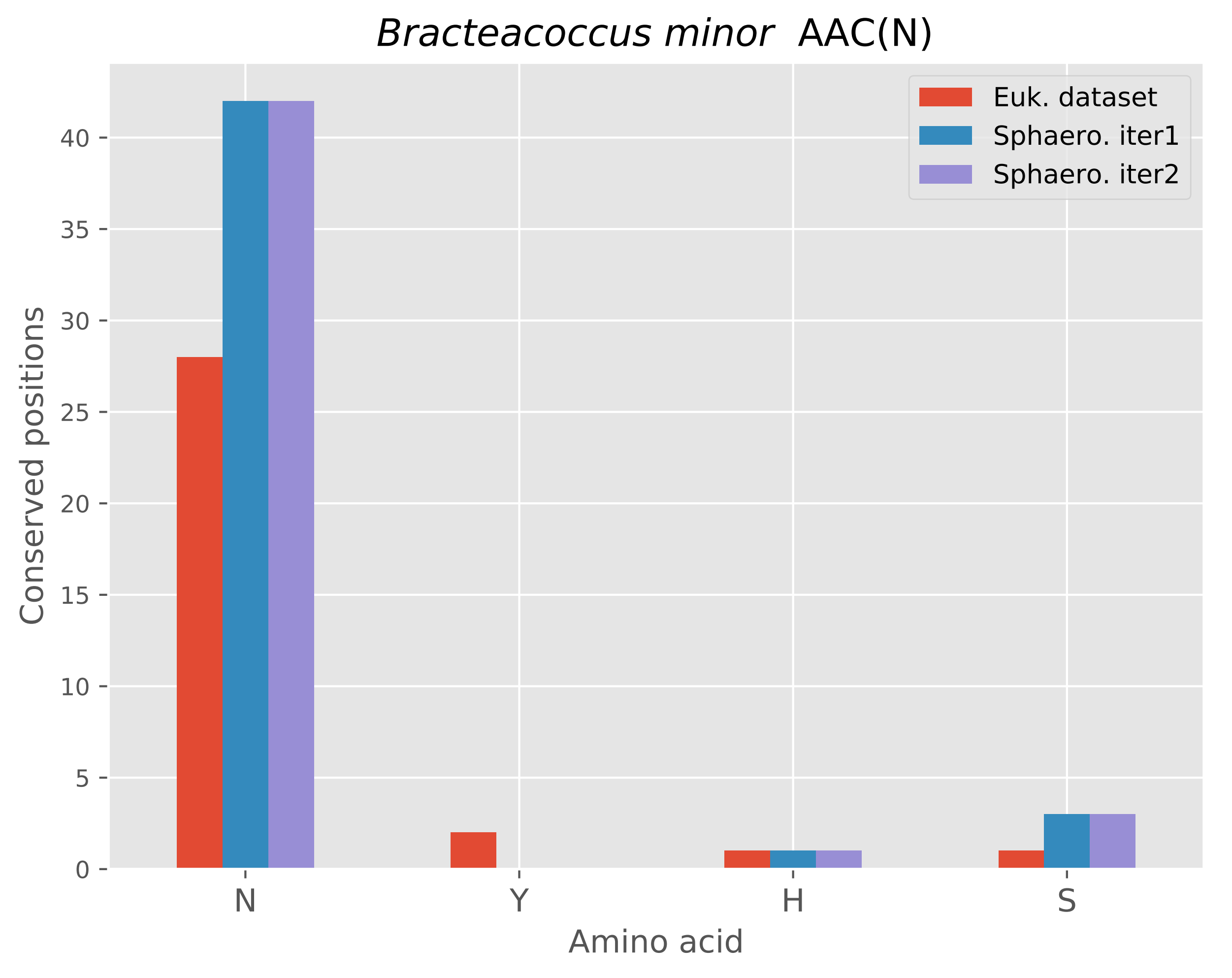

Bracteacoccus minor AAC(N)
Euk. dataset
Sphaero. iter1
Sphaero. iter2
Conserved positions
0
5
10
15
20
25
30
35
40
N
Y
H
S
Amino acid

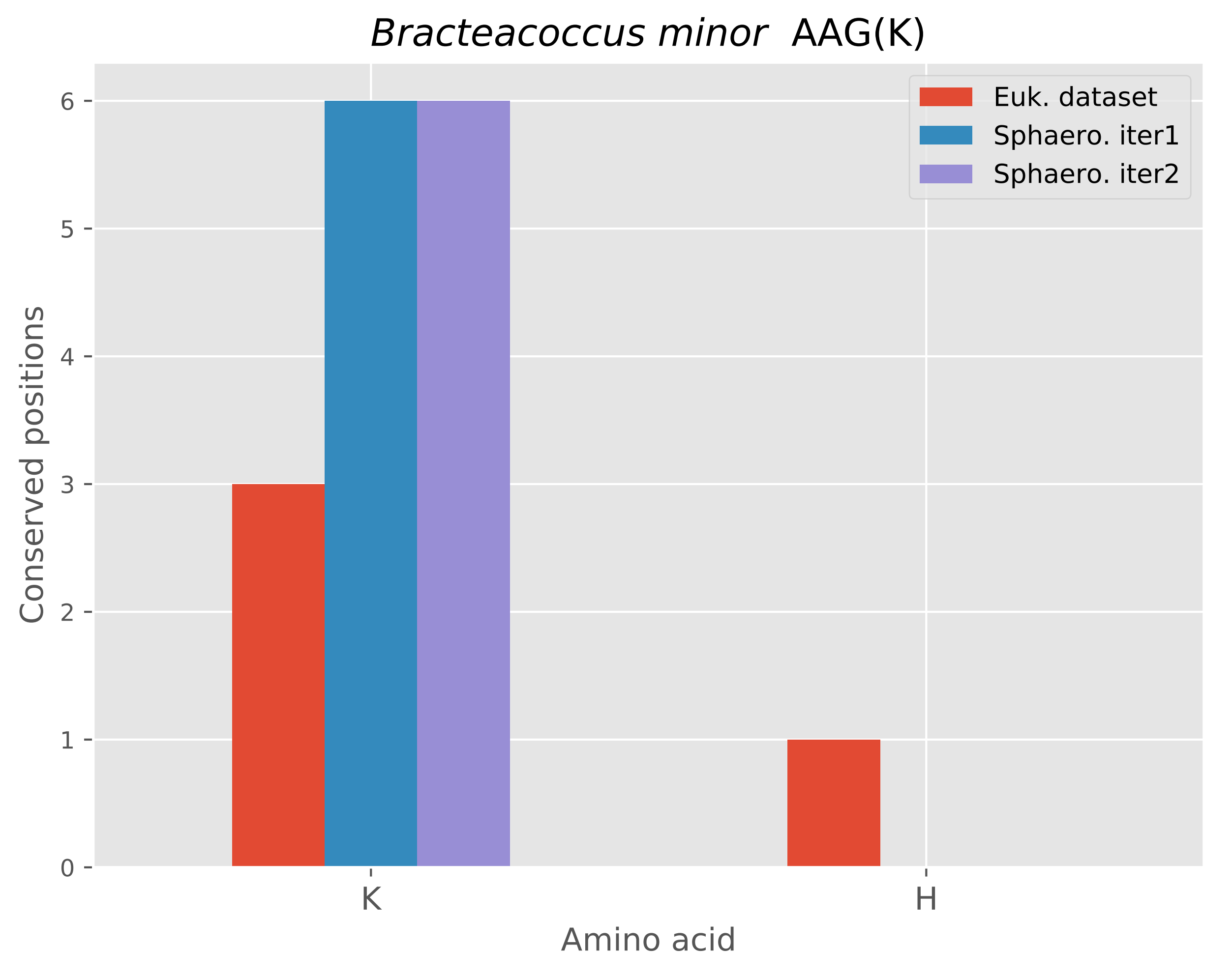

Bracteacoccus minor AAG(K)
Euk. dataset
Sphaero. iter1
Sphaero. iter2
Conserved positions
0
1
2
3
4
5
6
K
H
Amino acid

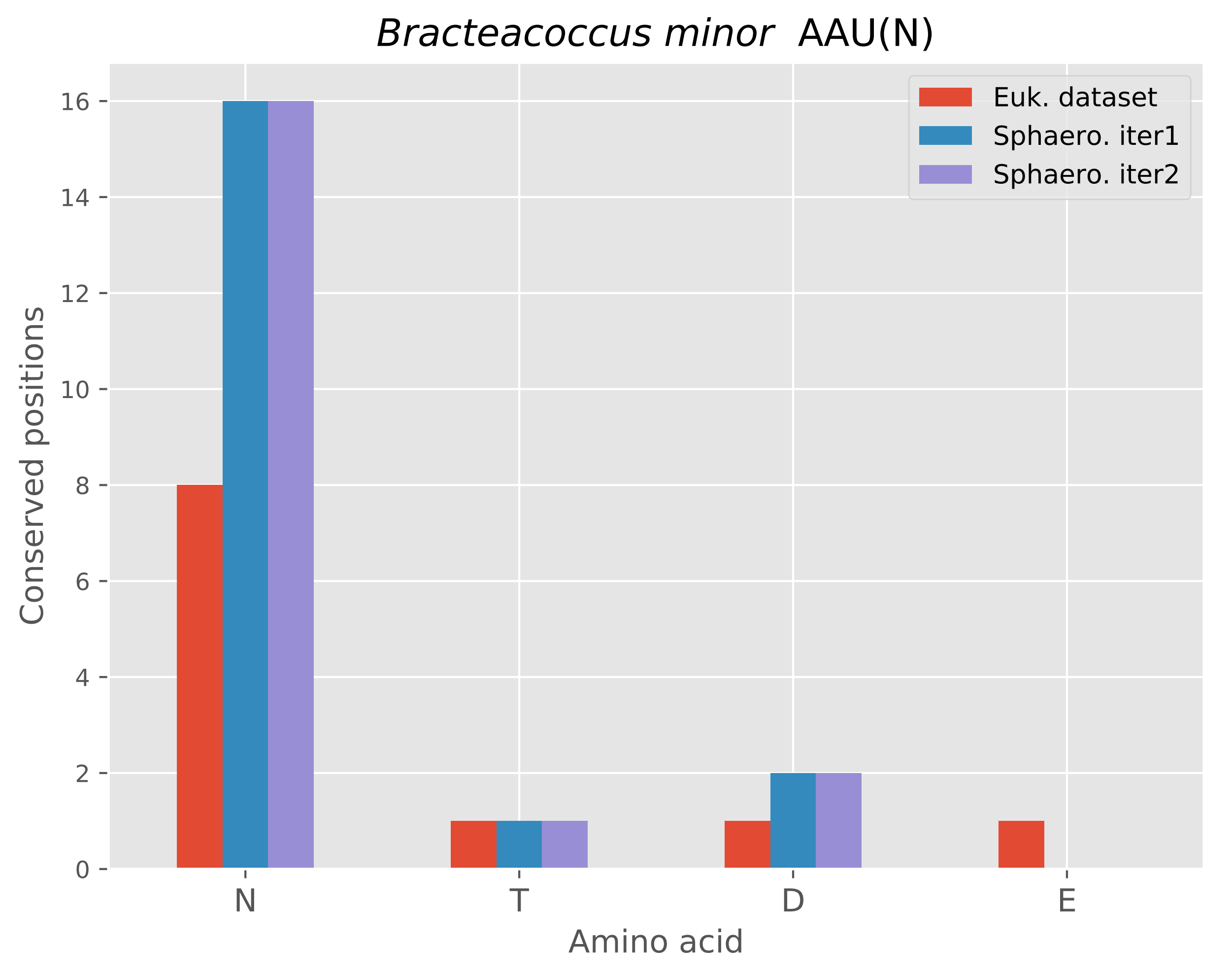

Bracteacoccus minor AAU(N)
Euk. dataset
Sphaero. iter1
Sphaero. iter2
Conserved positions
0
2
4
6
8
10
12
14
16
N
T
D
E
Amino acid

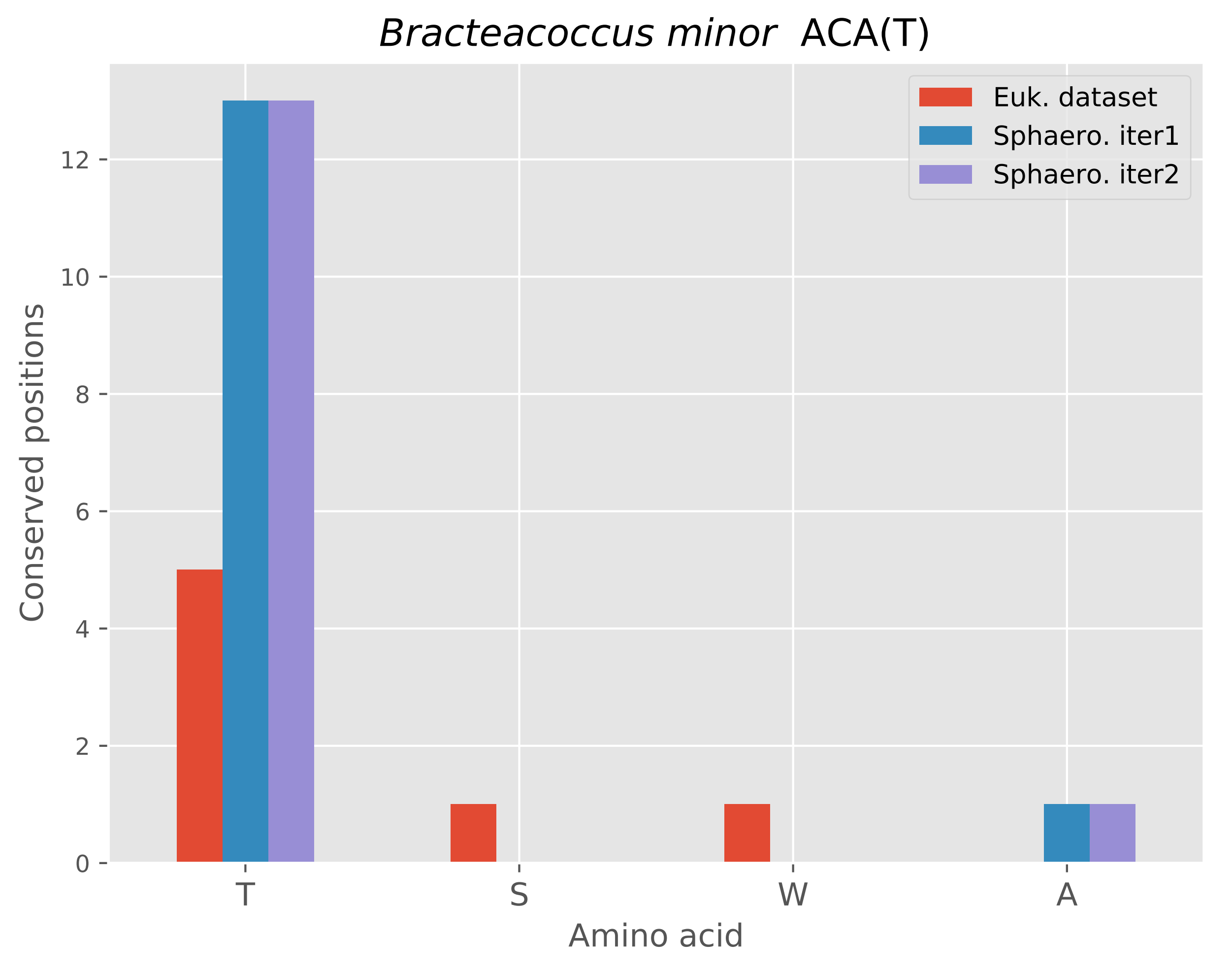

Bracteacoccus minor ACA(T)
Euk. dataset
Sphaero. iter1
Sphaero. iter2
Conserved positions
0
2
4
6
8
10
12
T
S
W
A
Amino acid

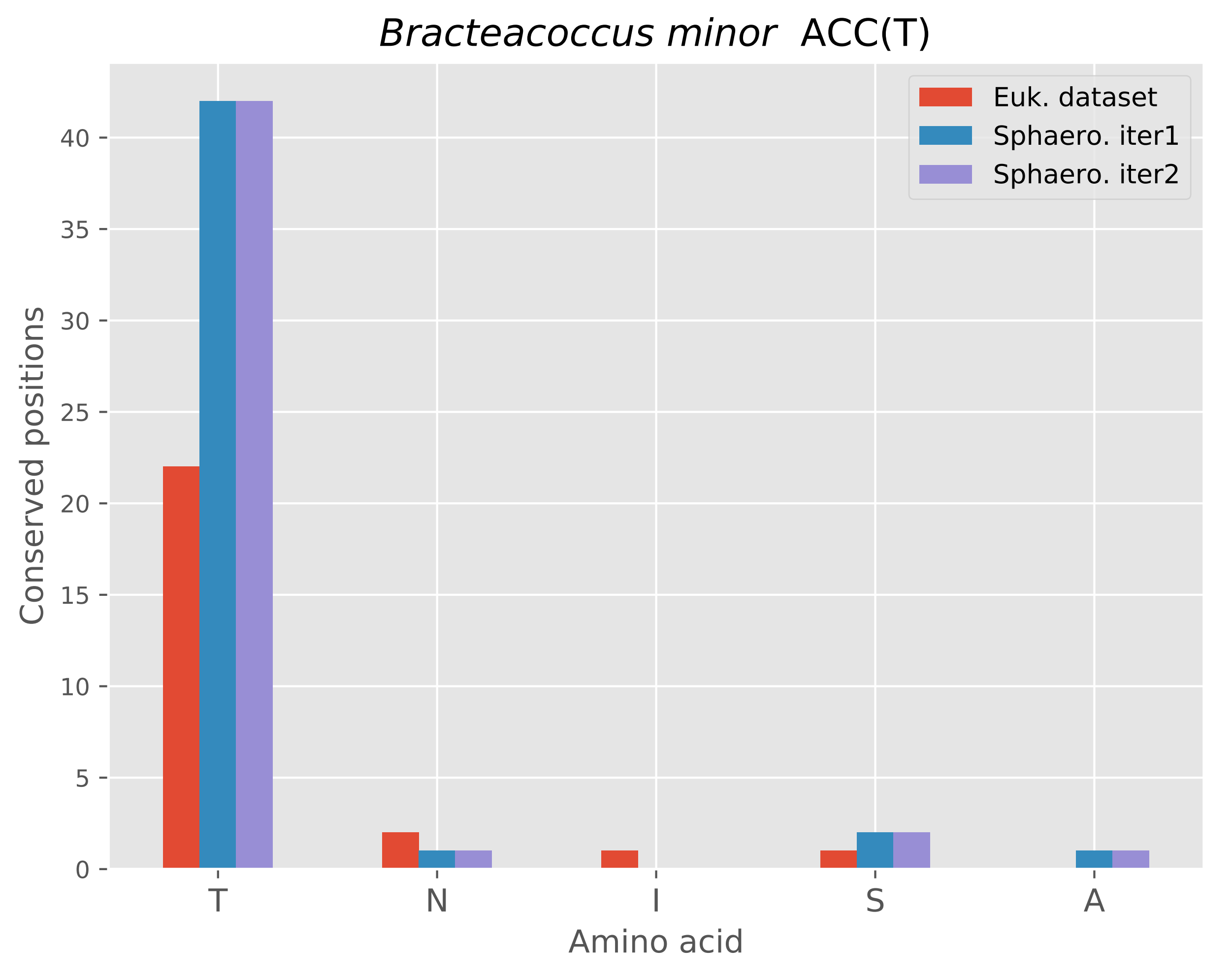

Bracteacoccus minor ACC(T)
Euk. dataset
Sphaero. iter1
Sphaero. iter2
Conserved positions
0
5
10
15
20
25
30
35
40
T
N
I
S
A
Amino acid

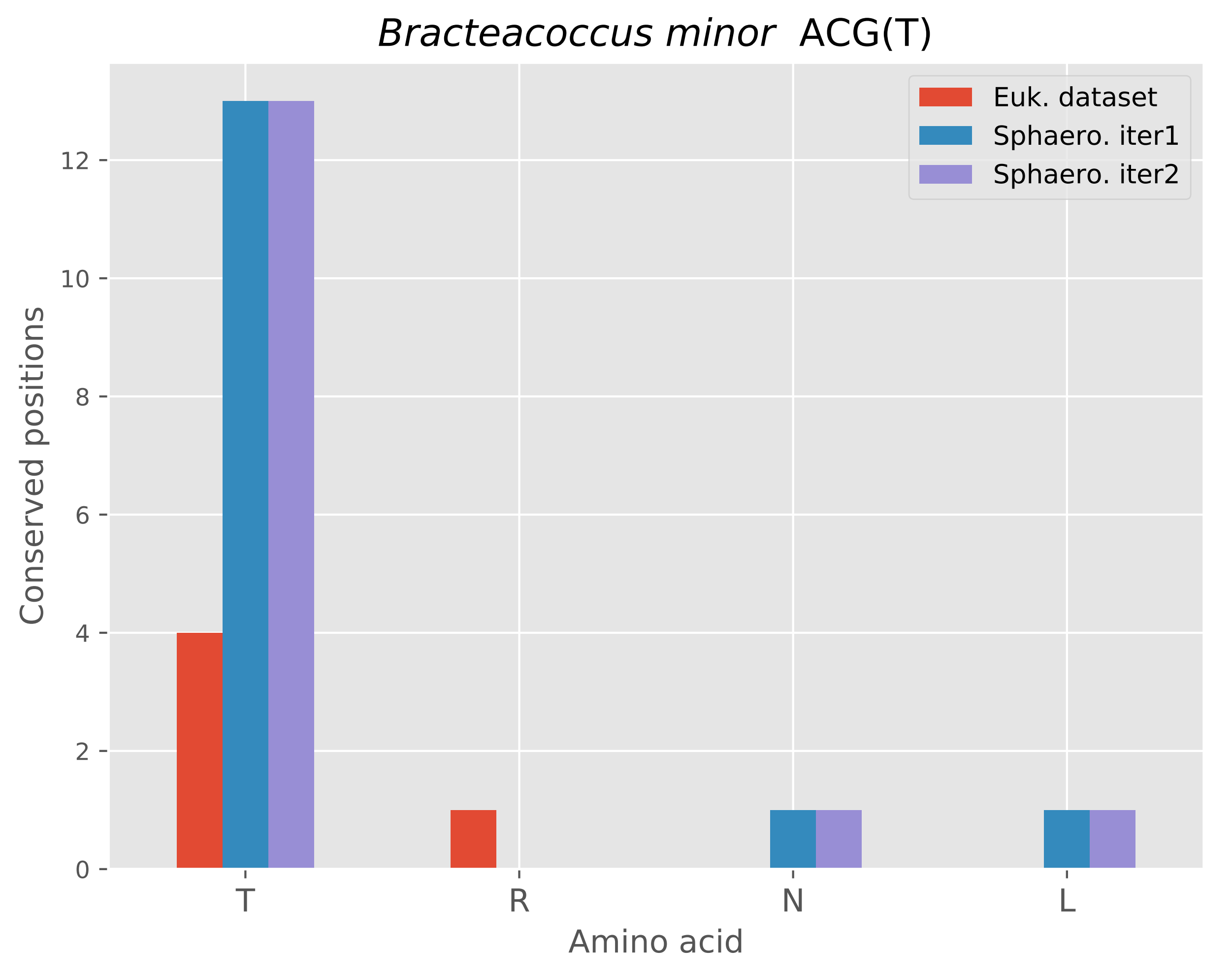

Bracteacoccus minor ACG(T)
Euk. dataset
Sphaero. iter1
Sphaero. iter2
Conserved positions
0
2
4
6
8
10
12
T
R
N
L
Amino acid

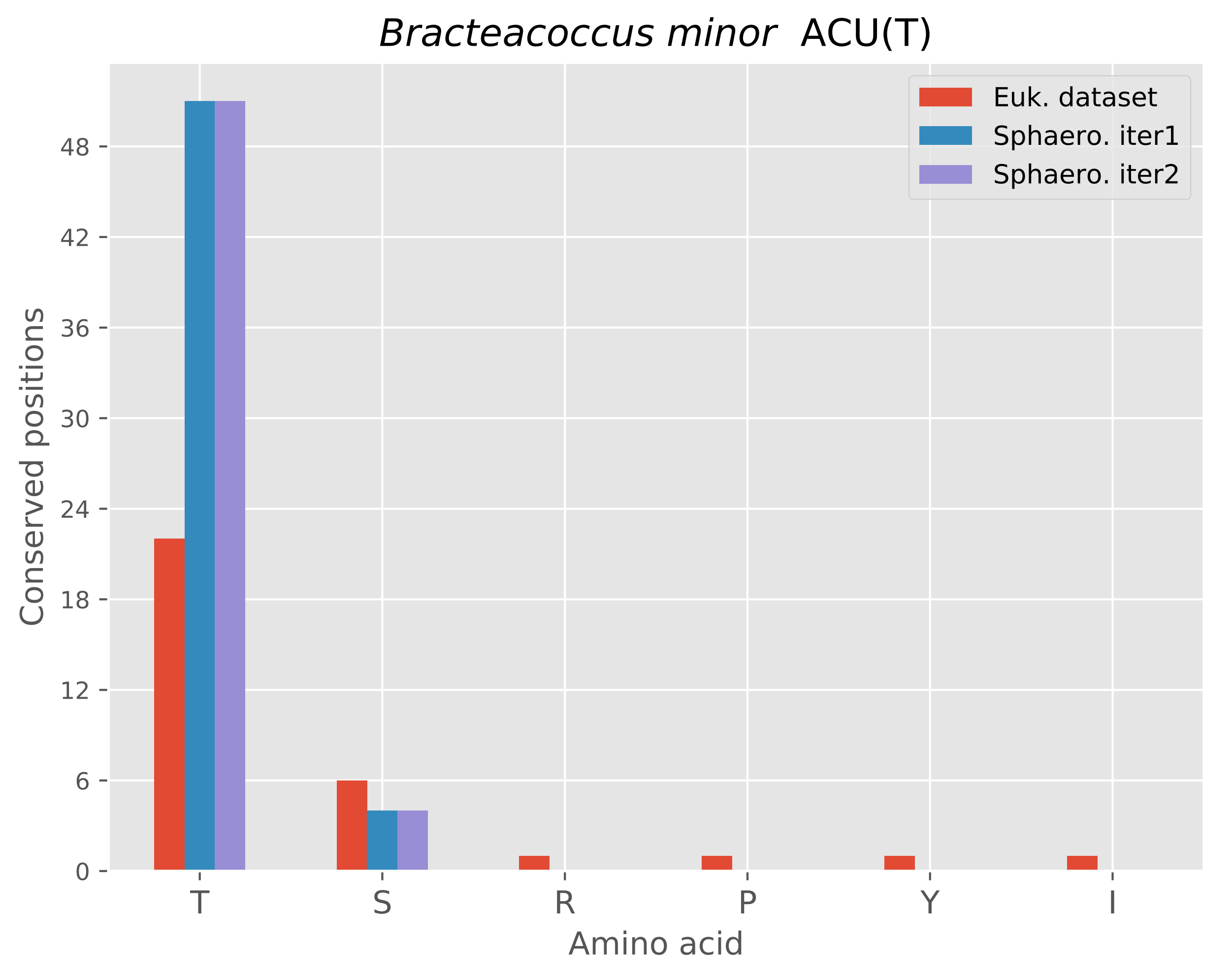
Bracteacoccus minor ACU(T)
Euk. dataset
Sphaero. iter1
Sphaero. iter2
Conserved positions
0
6
12
18
24
30
36
42
48
T
S
R
P
Y
I
Amino acid

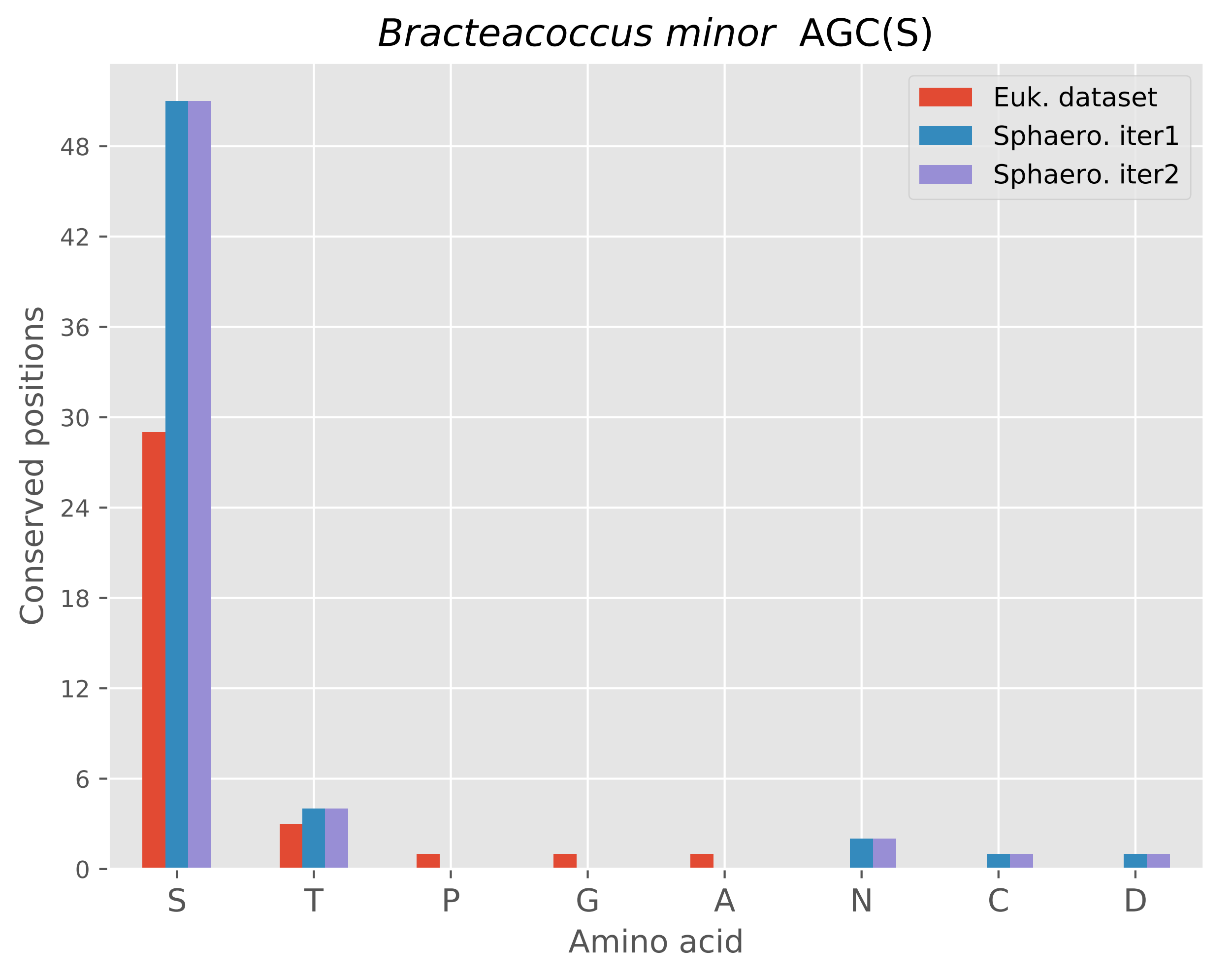

Bracteacoccus minor AGC(S)
Euk. dataset
Sphaero. iter1
Sphaero. iter2
Conserved positions
0
6
12
18
24
30
36
42
48
S
T
P
G
A
N
C
D
Amino acid

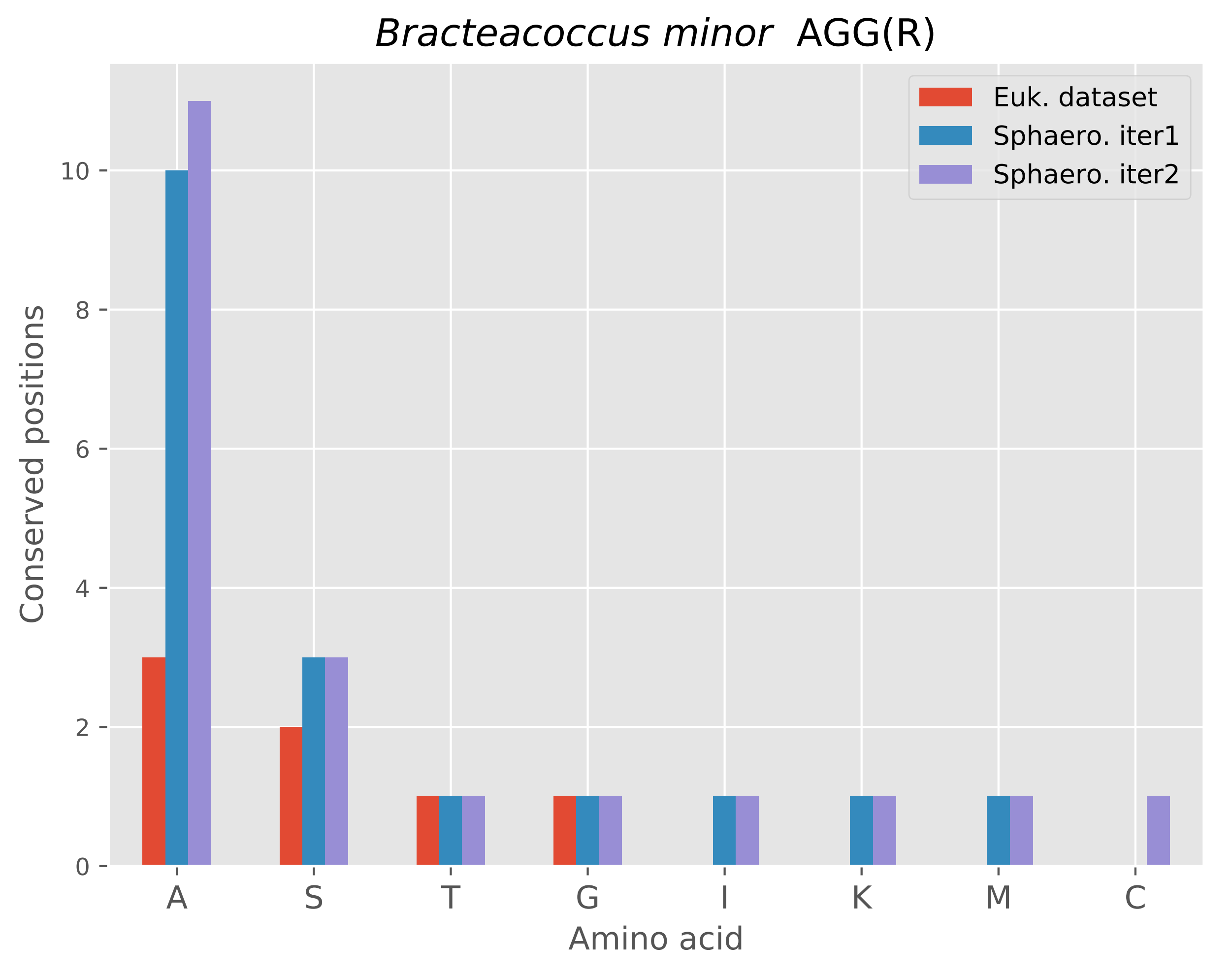

Bracteacoccus minor AGG(R)
Euk. dataset
Sphaero. iter1
Sphaero. iter2
Conserved positions
0
2
4
6
8
10
A
S
T
G
I
K
M
C
Amino acid

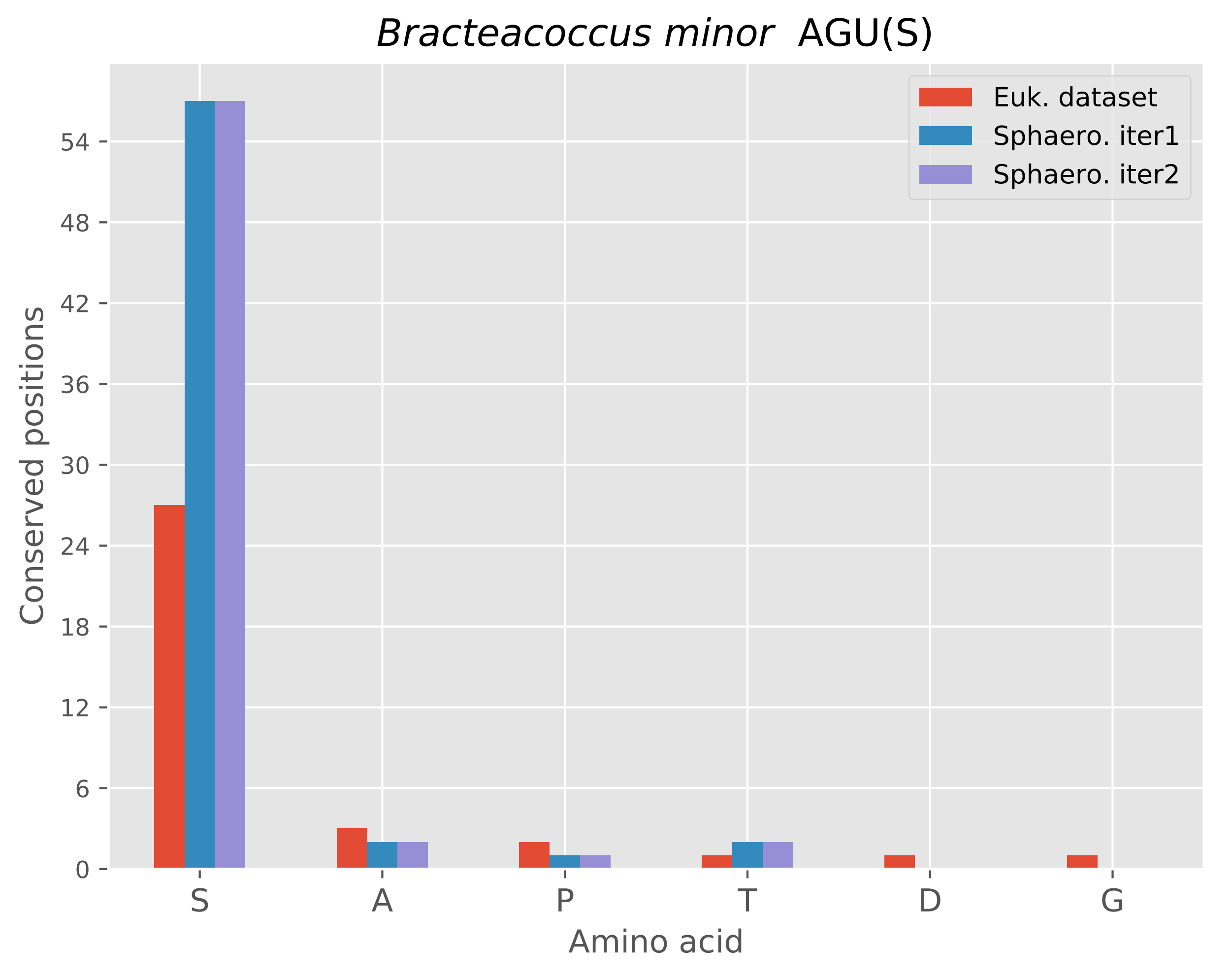

Bracteacoccus minor AGU(S)
Euk. dataset
Sphaero. iter1
Sphaero. iter2
Conserved positions
0
6
12
18
24
30
36
42
48
54
S
A
P
T
D
G
Amino acid

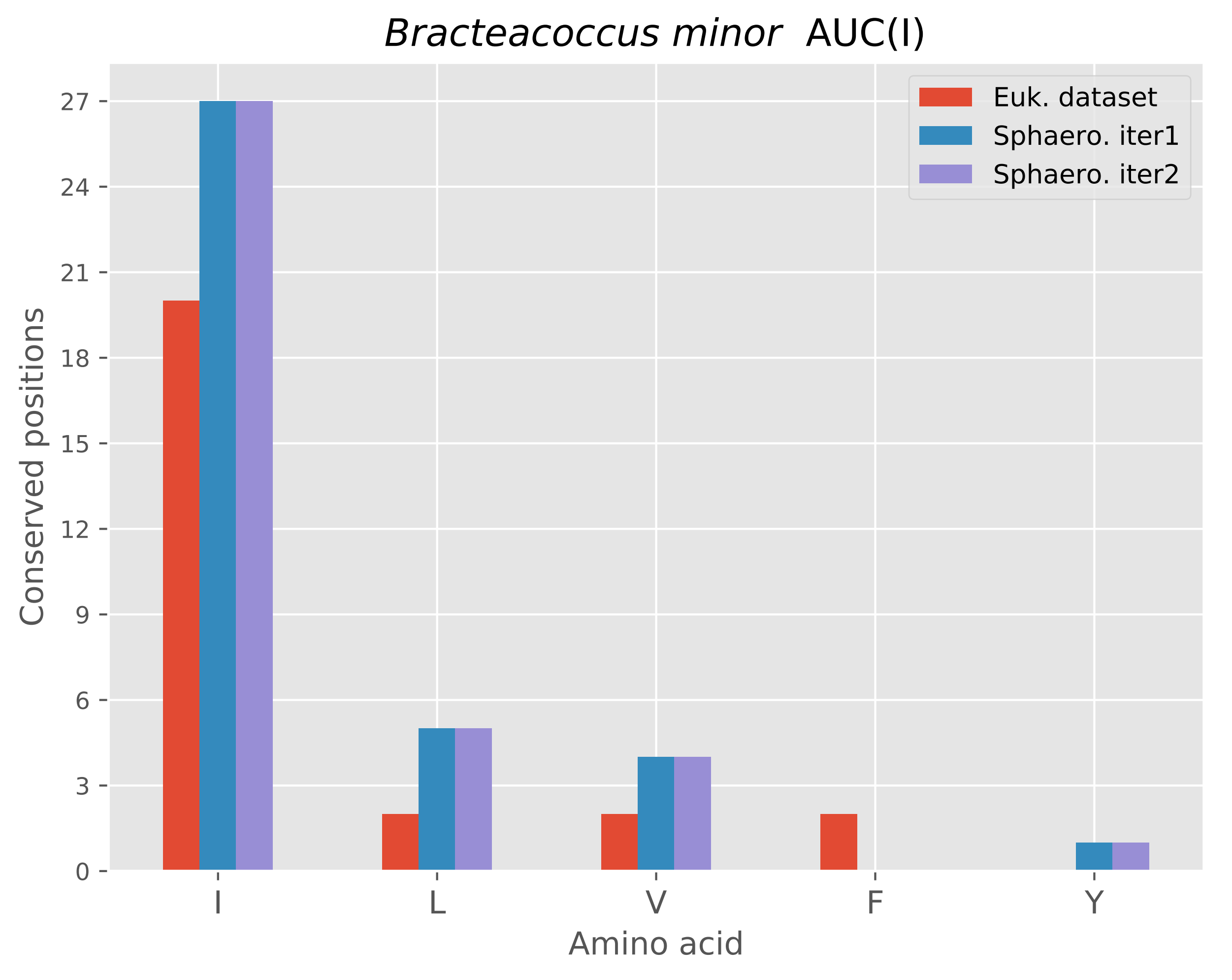

Bracteacoccus minor AUC(I)
Euk. dataset
Sphaero. iter1
Sphaero. iter2
Conserved positions
0
3
6
9
12
15
18
21
24
27
I
L
V
F
Y
Amino acid

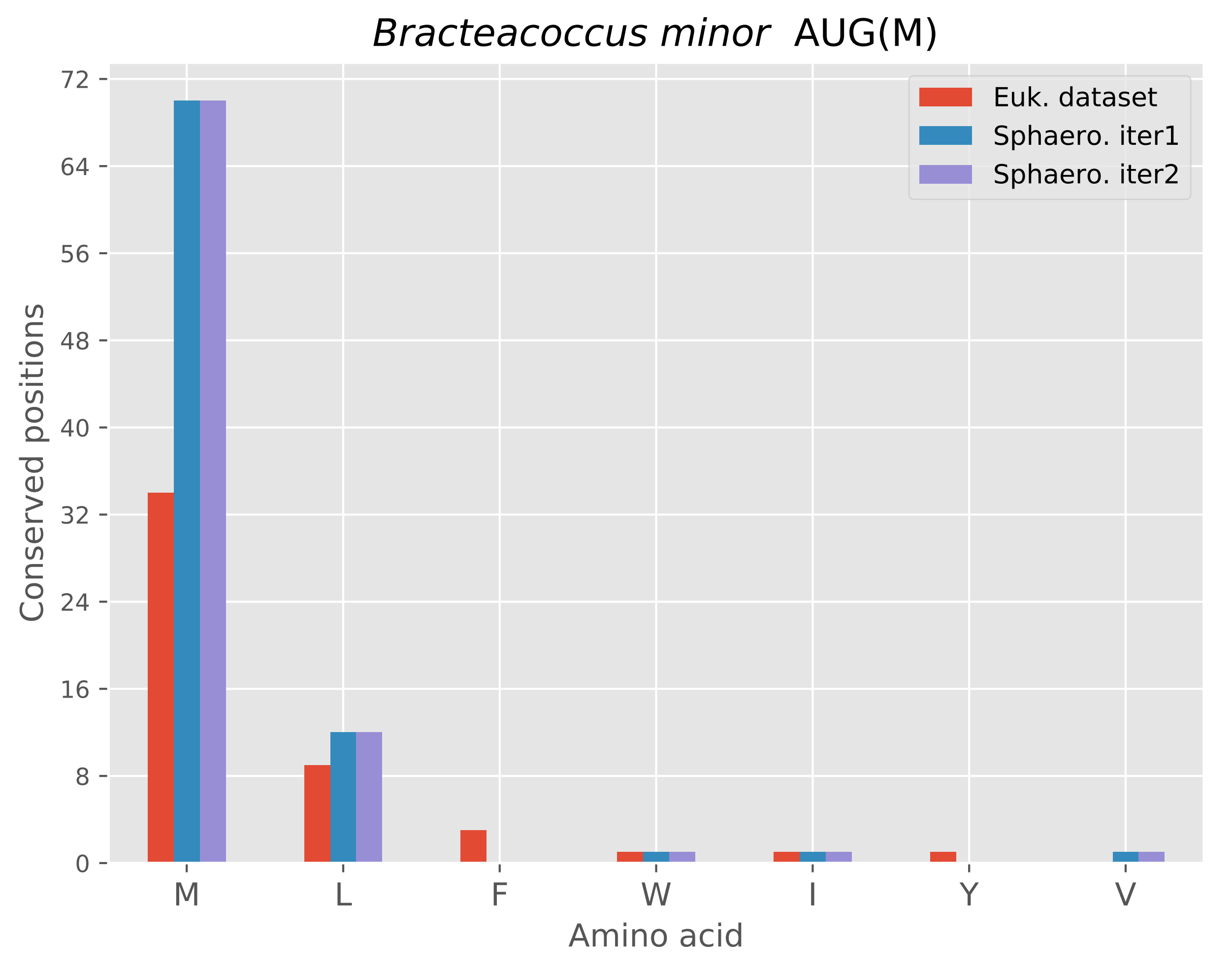

Bracteacoccus minor AUG(M)
Euk. dataset
Sphaero. iter1
Sphaero. iter2
Conserved positions
72
64
56
48
40
32
24
16
8
0
M
L
F
W
I
Y
V
Amino acid

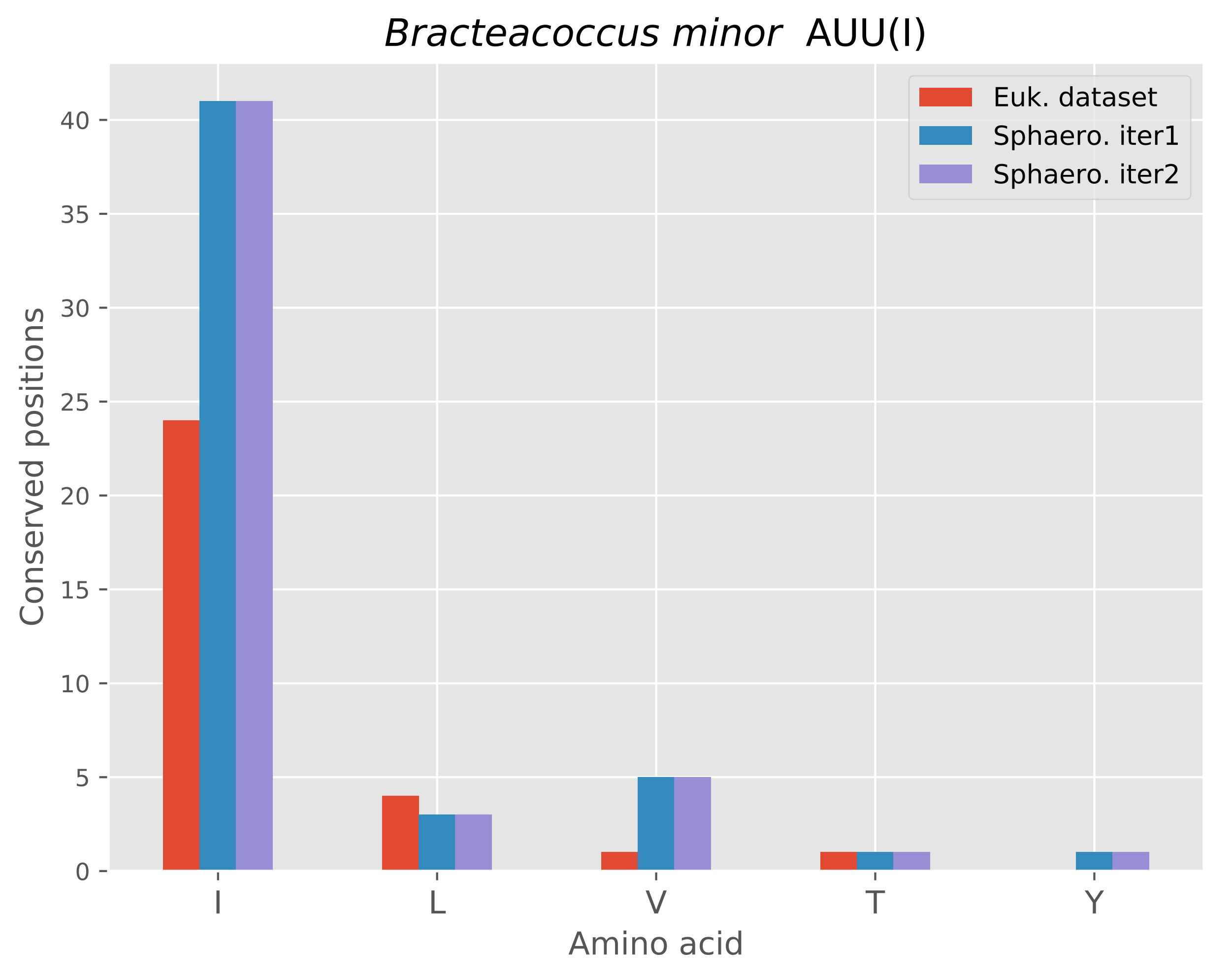
Bracteacoccus minor AUU(I)
Euk. dataset
Sphaero. iter1
Sphaero. iter2
Conserved positions
0
5
10
15
20
25
30
35
40
I
L
V
T
Y
Amino acid

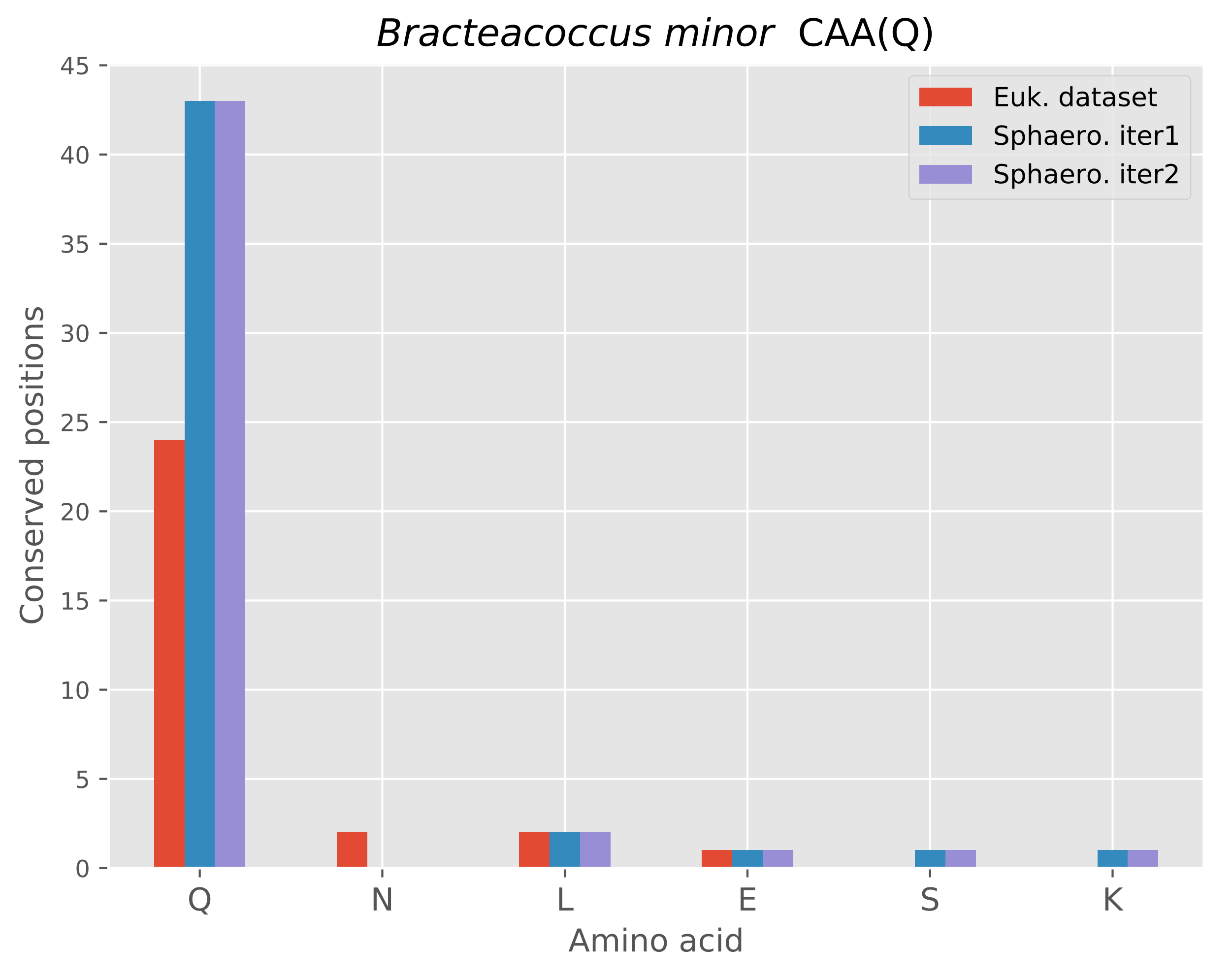
Bracteacoccus minor CAA(Q)
Conserved positions
Amino acid
Euk. dataset
Sphaero. iter1
Sphaero. iter2
0
5
10
15
20
25
30
35
40
45
Q
N
L
E
S
K

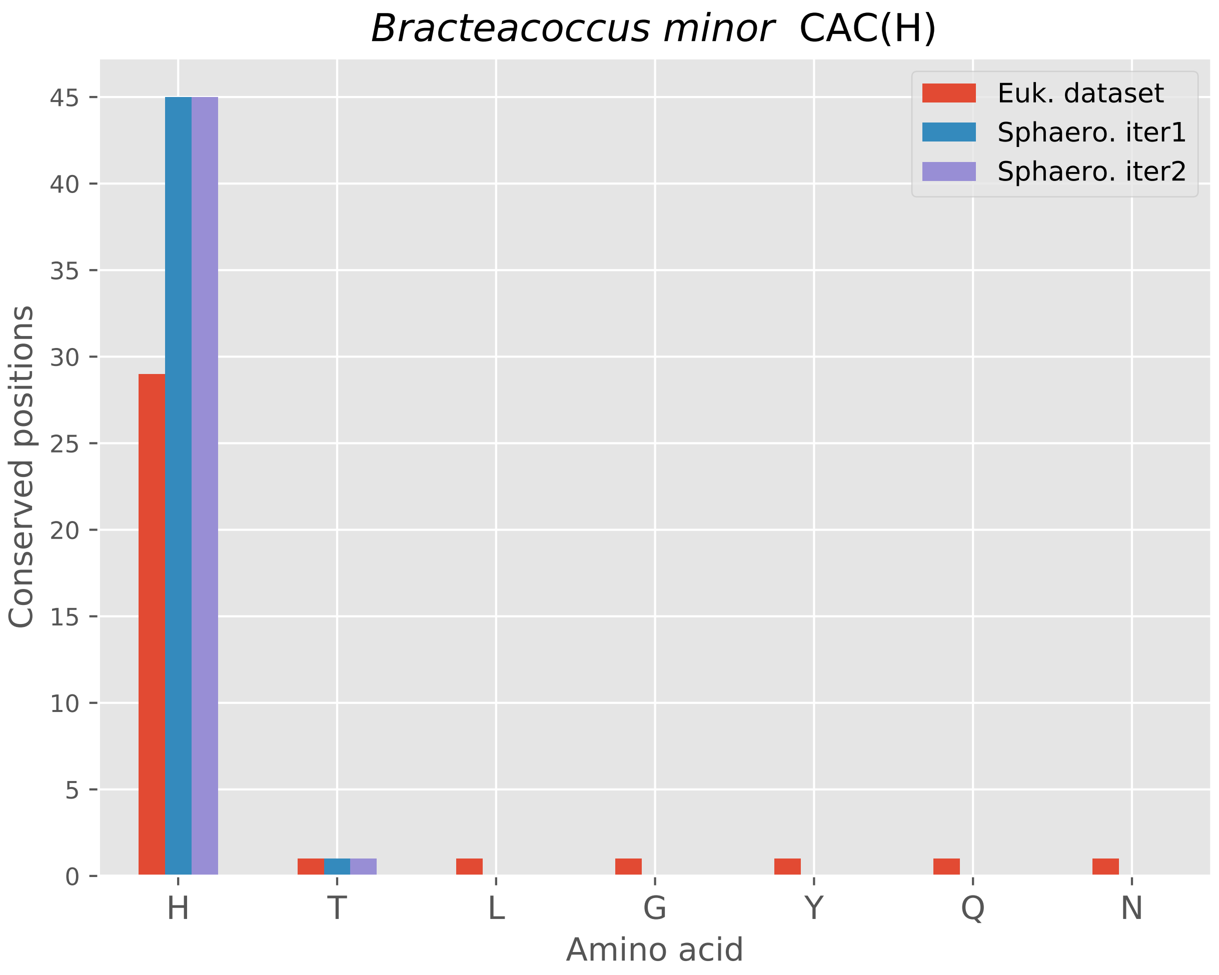
Bracteacoccus minor CAC(H)
Euk. dataset
Sphaero. iter1
Sphaero. iter2
Conserved positions
0
5
10
15
20
25
30
35
40
45
H
T
L
G
Y
Q
N
Amino acid

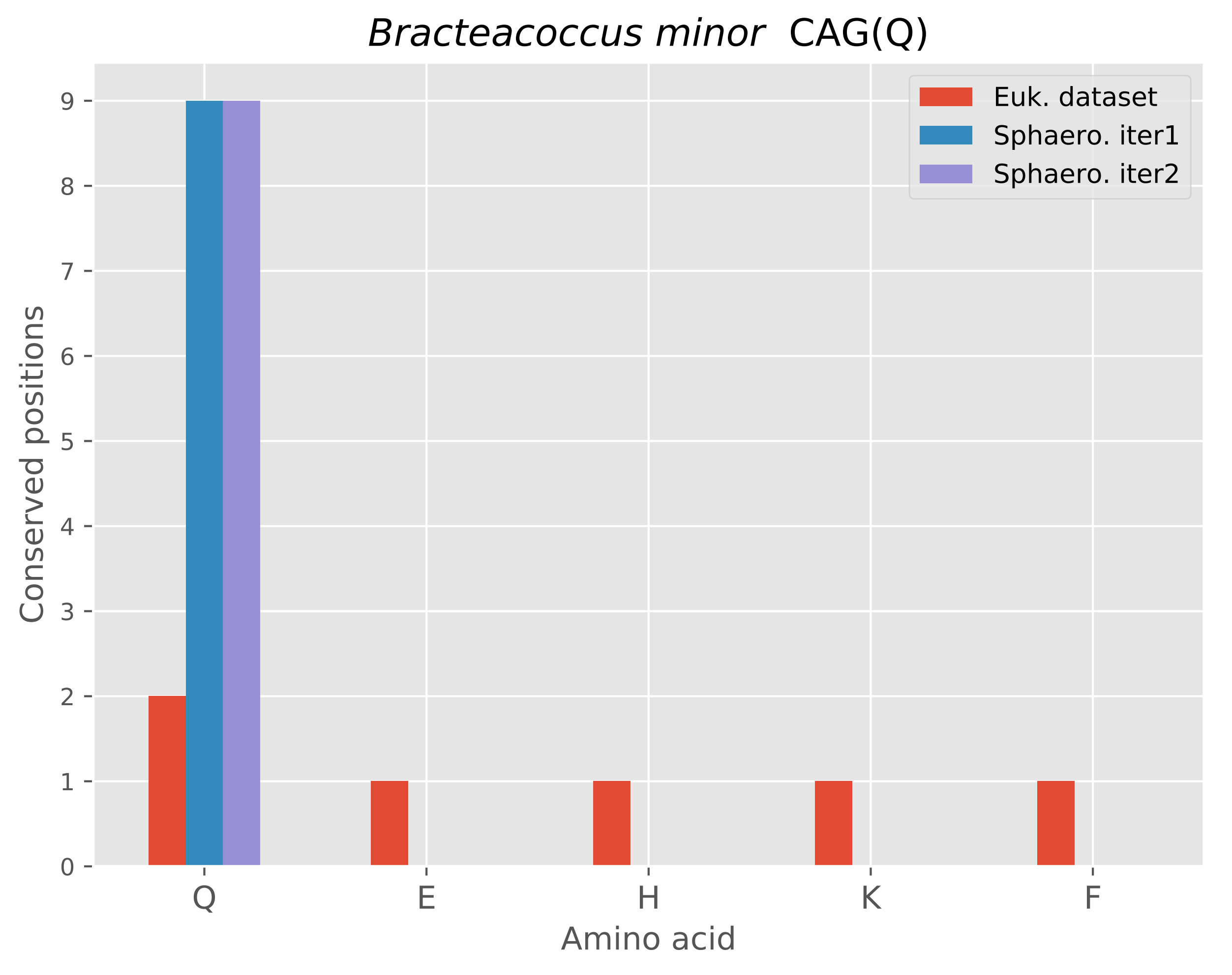

Bracteacoccus minor CAG(Q)
Euk. dataset
Sphaero. iter1
Sphaero. iter2
Conserved positions
0
1
2
3
4
5
6
7
8
9
Q
E
H
K
F
Amino acid

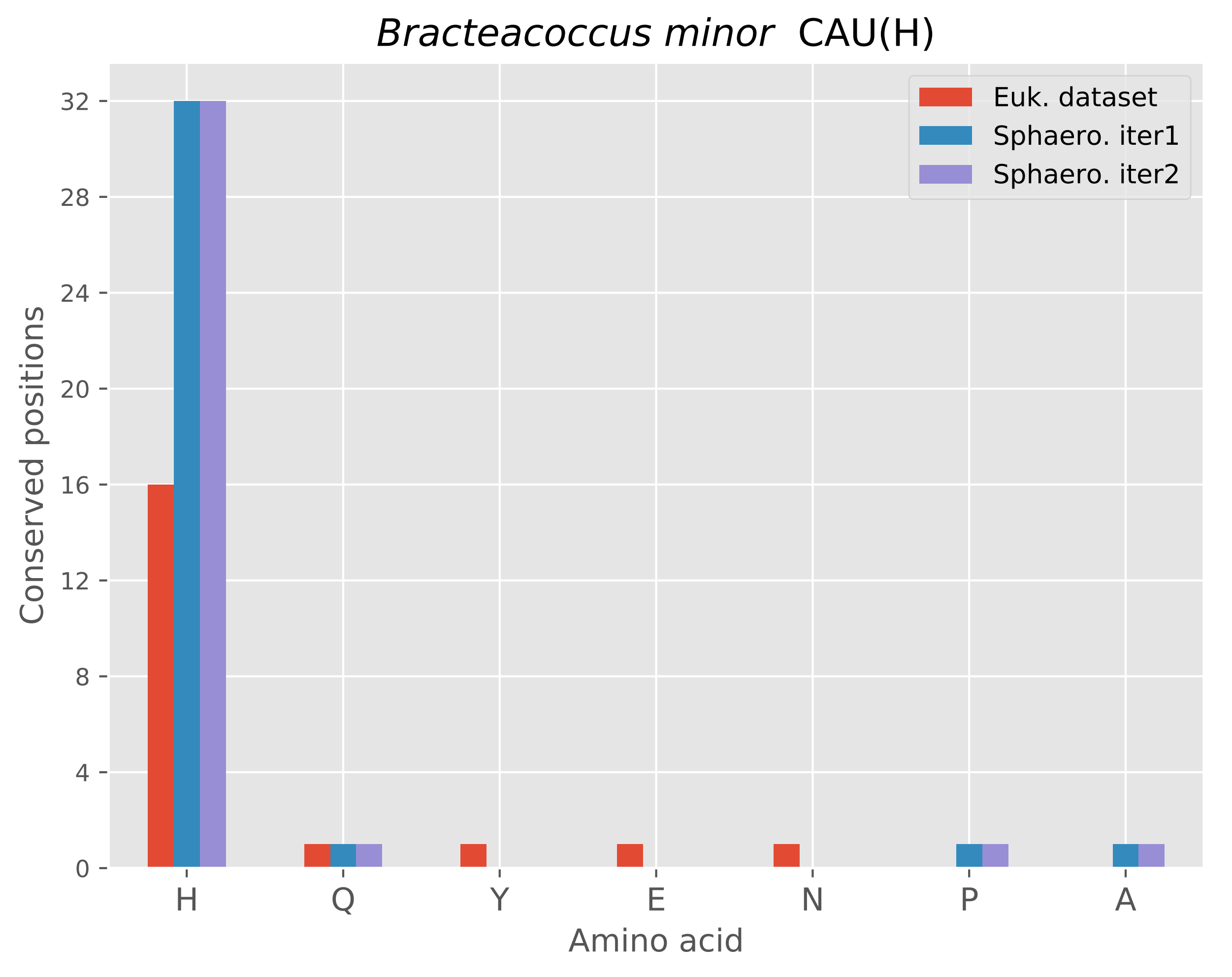

Bracteacoccus minor CAU(H)
Euk. dataset
Sphaero. iter1
Sphaero. iter2
Conserved positions
0
4
8
12
16
20
24
28
32
H
Q
Y
E
N
P
A
Amino acid

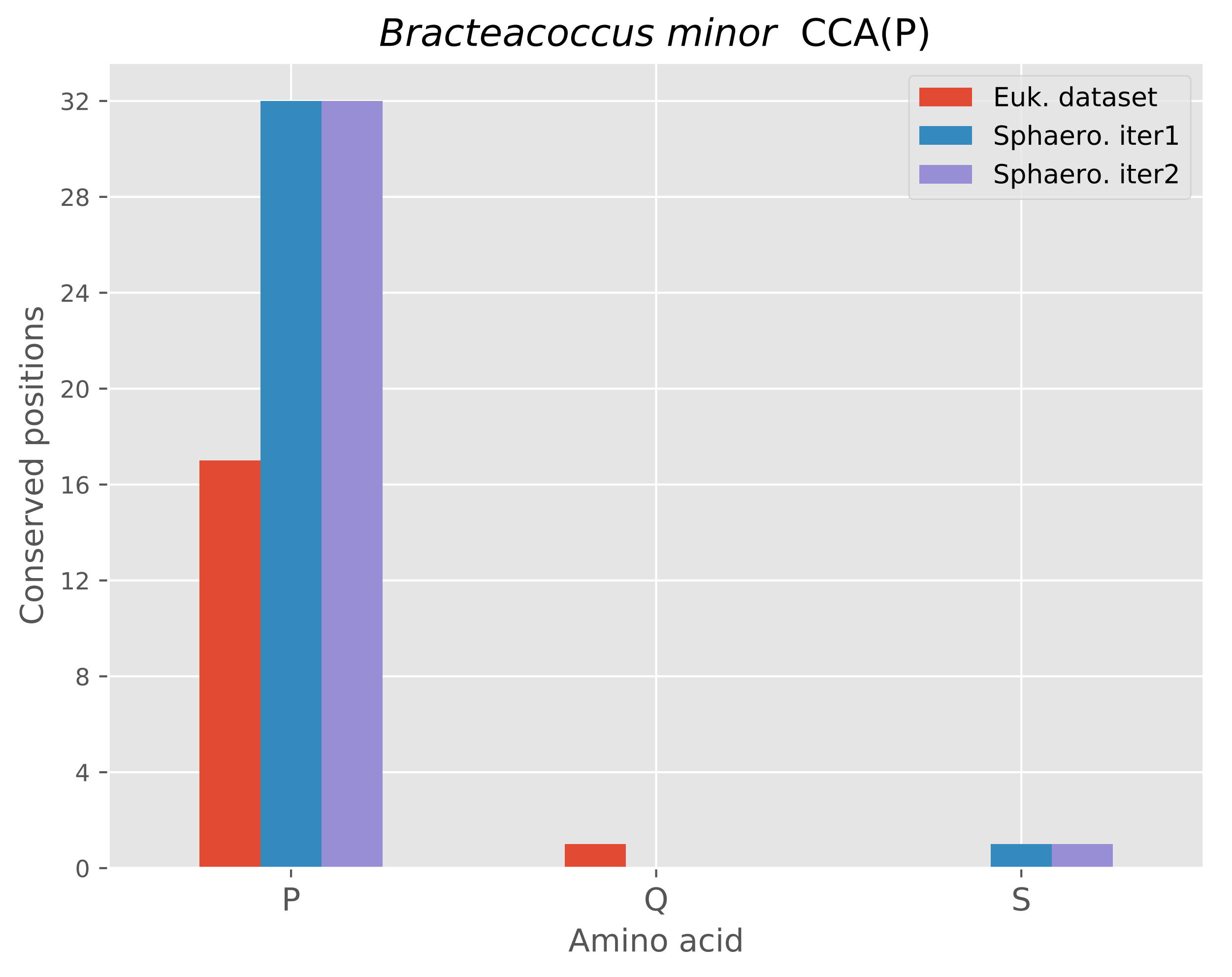

Bracteacoccus minor CCA(P)
Euk. dataset
Sphaero. iter1
Sphaero. iter2
Conserved positions
0
4
8
12
16
20
24
28
32
P
Q
S
Amino acid

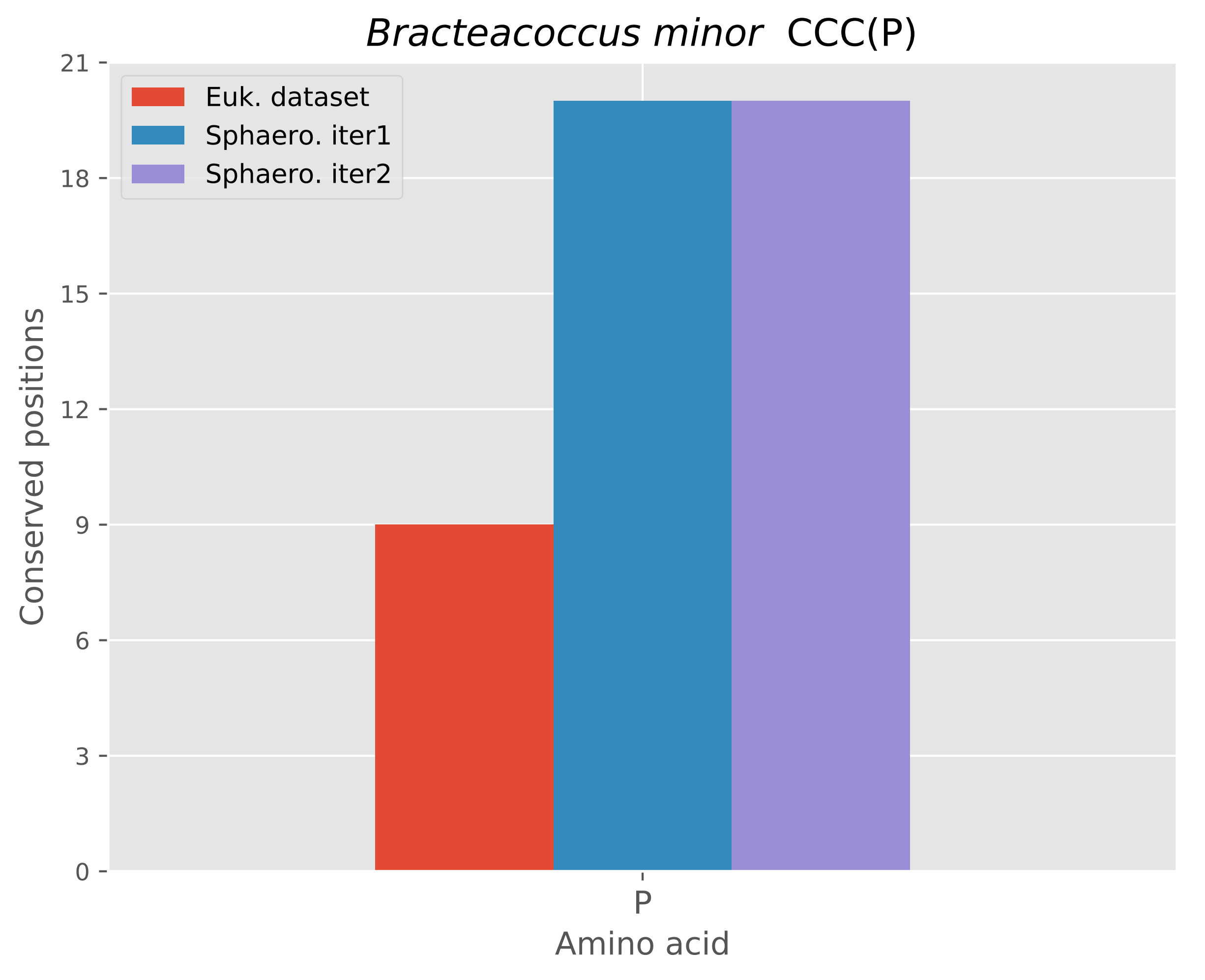

Bracteacoccus minor CCC(P)
Conserved positions
21
18
15
12
9
6
3
0
Euk. dataset
Sphaero. iter1
Sphaero. iter2
P
Amino acid

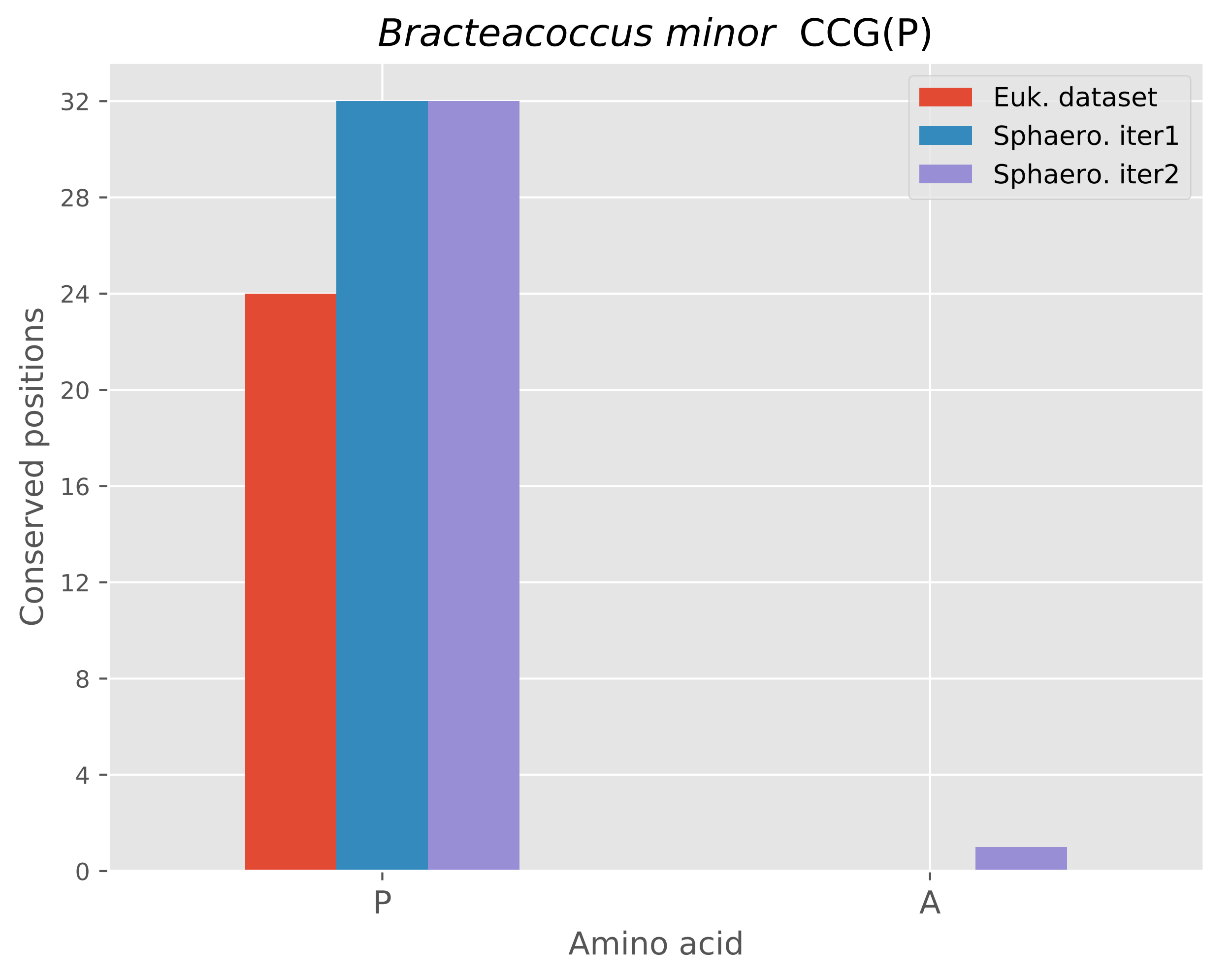

Bracteacoccus minor CCG(P)
Euk. dataset
Sphaero. iter1
Sphaero. iter2
Conserved positions
0
4
8
12
16
20
24
28
32
P
A
Amino acid

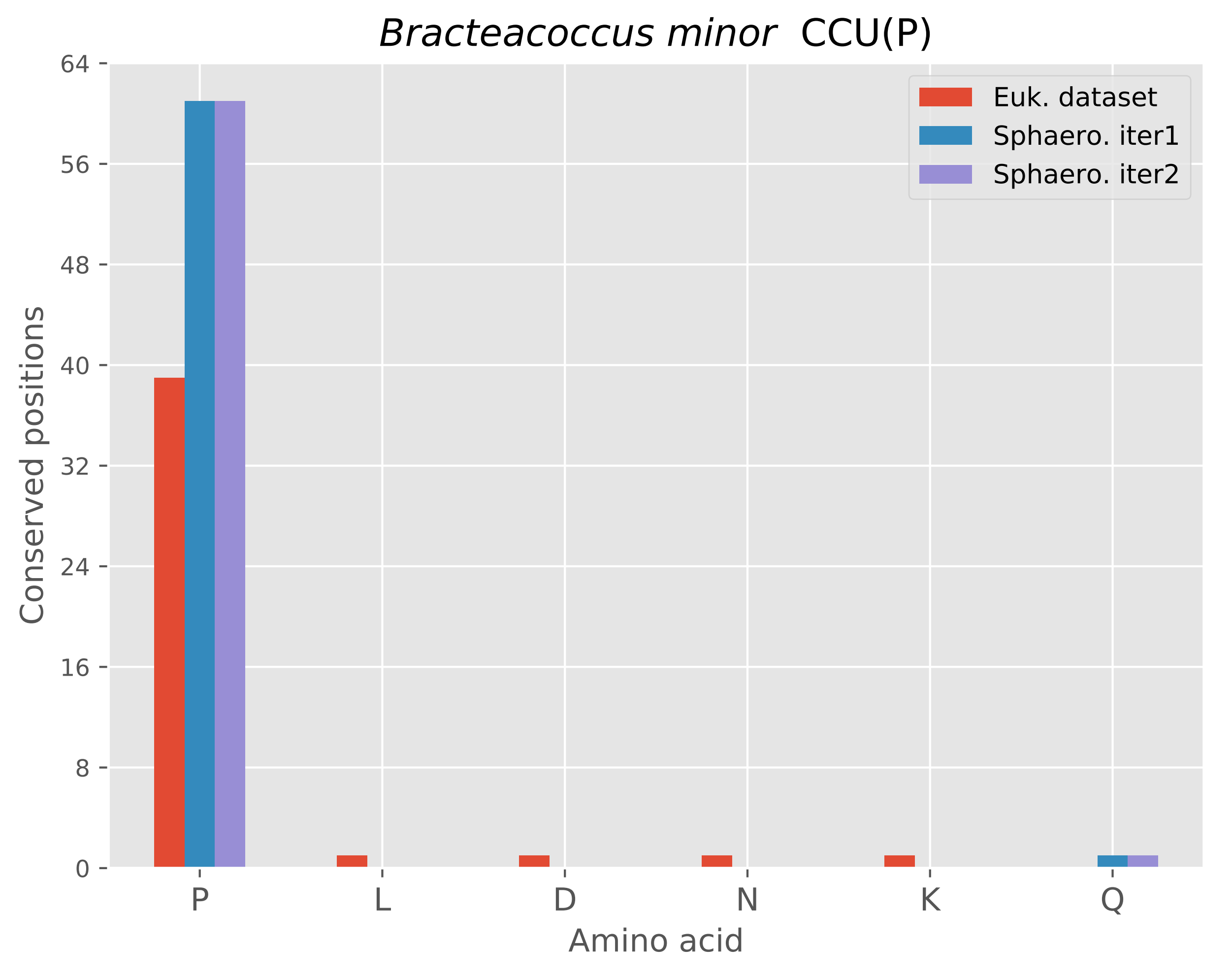

Bracteacoccus minor CCU(P)
Conserved positions
Amino acid
0
8
16
24
32
40
48
56
64
P
L
D
N
K
Q
Euk. dataset
Sphaero. iter1
Sphaero. iter2

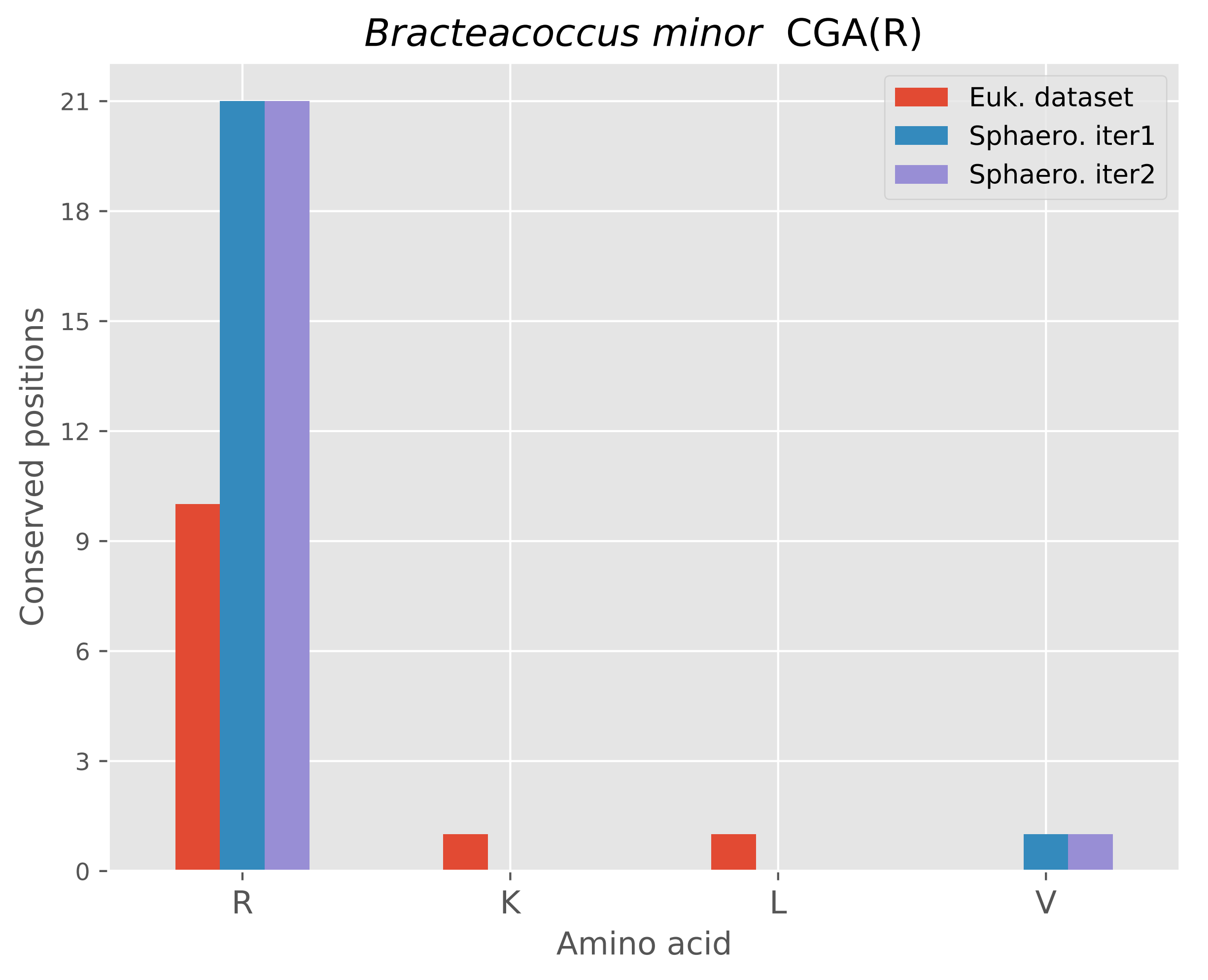

Bracteacoccus minor CGA(R)
Euk. dataset
Sphaero. iter1
Sphaero. iter2
Conserved positions
0
3
6
9
12
15
18
21
R
K
L
V
Amino acid

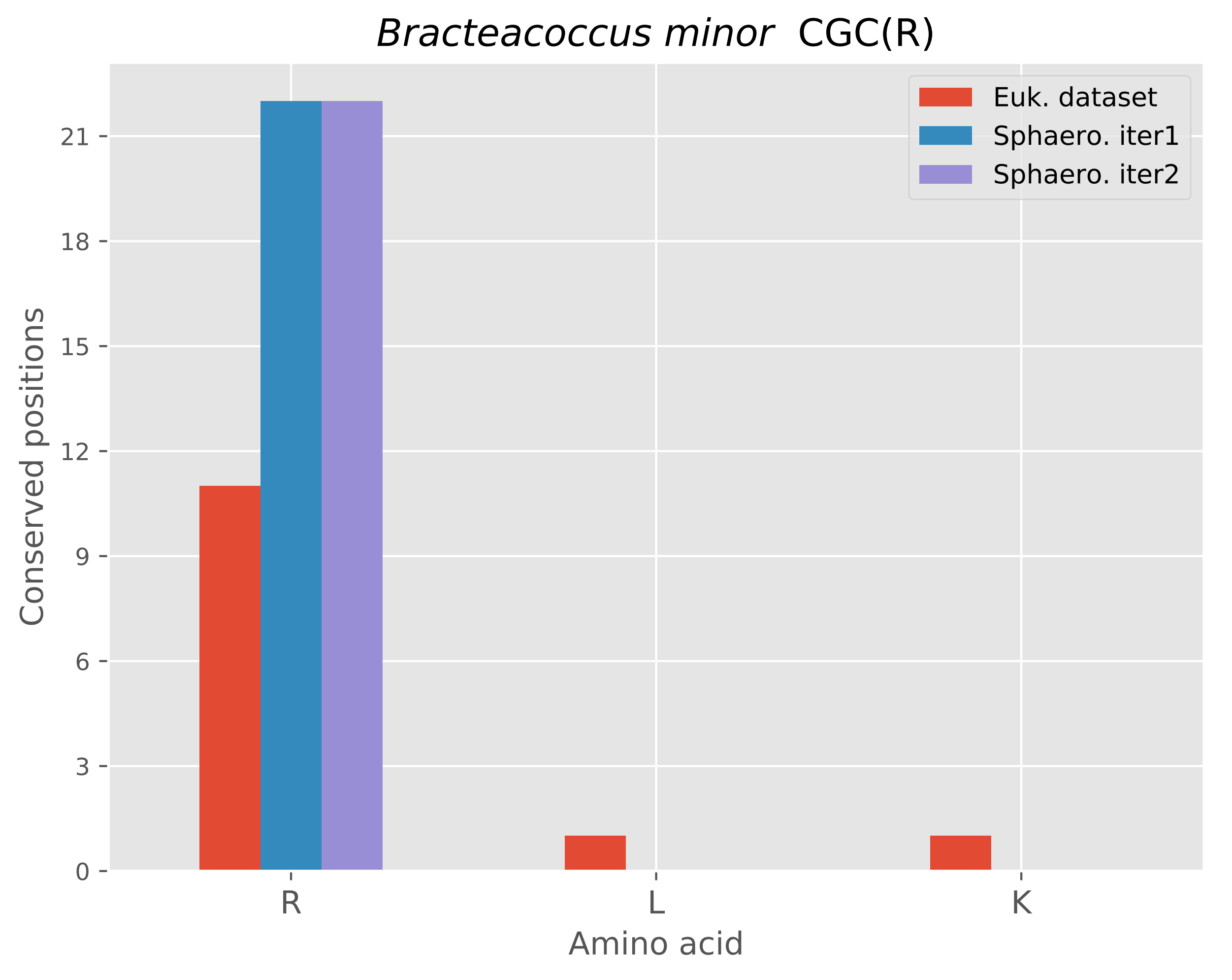

Bracteacoccus minor CGC(R)
Euk. dataset
Sphaero. iter1
Sphaero. iter2
Conserved positions
0
3
6
9
12
15
18
21
R
L
K
Amino acid

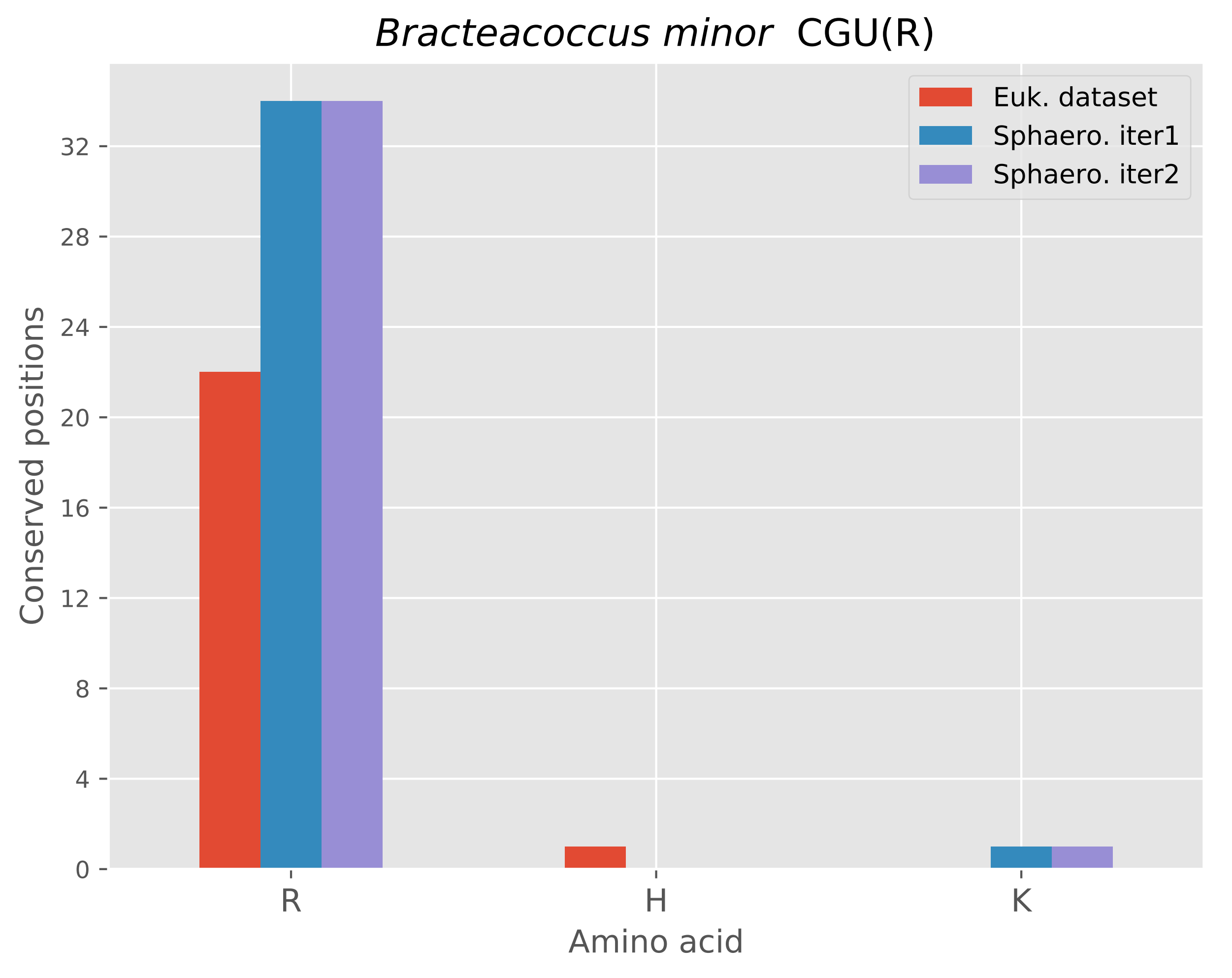

Bracteacoccus minor CGU(R)
Euk. dataset
Sphaero. iter1
Sphaero. iter2
Conserved positions
0
4
8
12
16
20
24
28
32
R
H
K
Amino acid

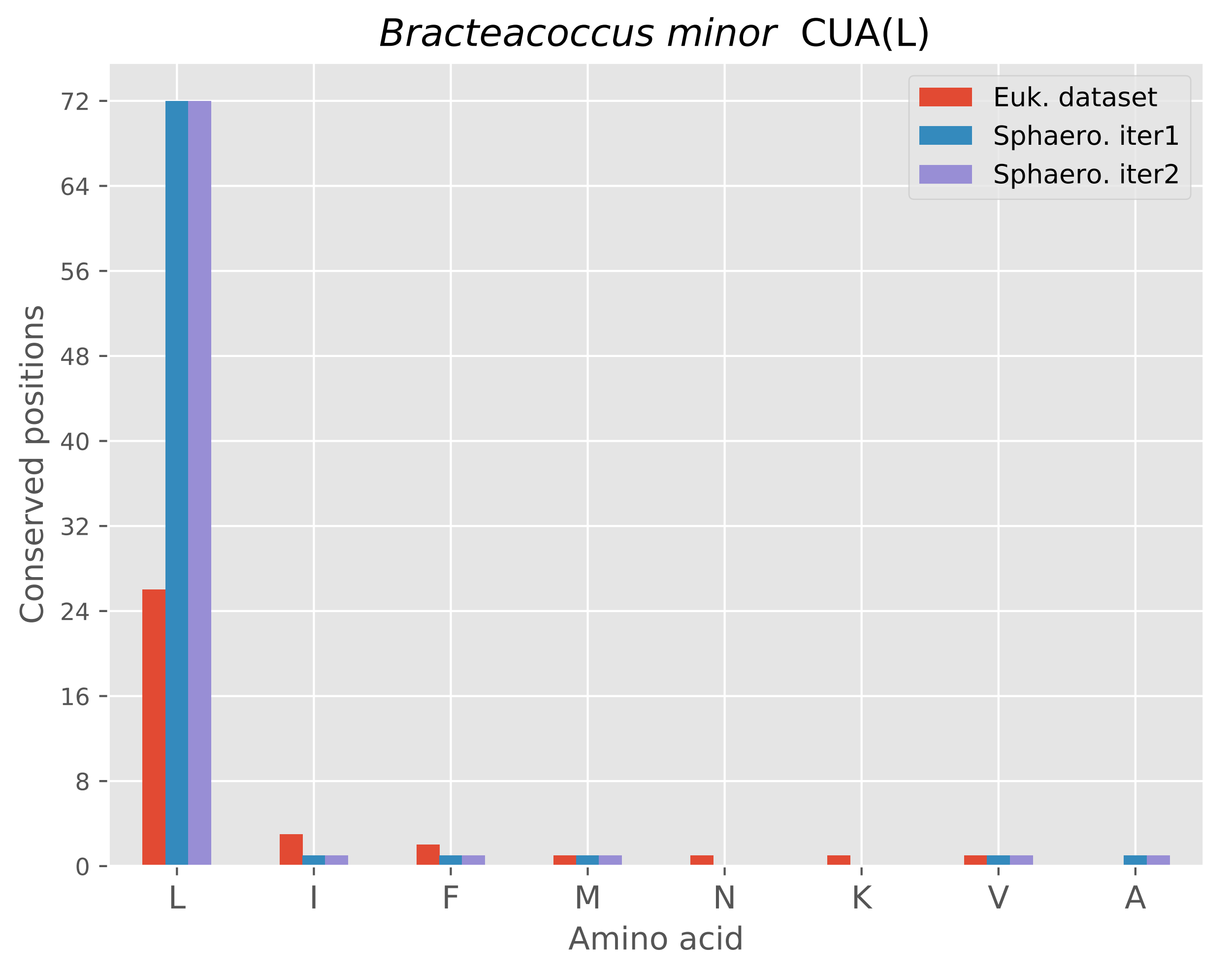

Bracteacoccus minor CUA(L)
Euk. dataset
Sphaero. iter1
Sphaero. iter2
Conserved positions
72
64
56
48
40
32
24
16
8
0
L
I
F
M
N
K
V
A
Amino acid

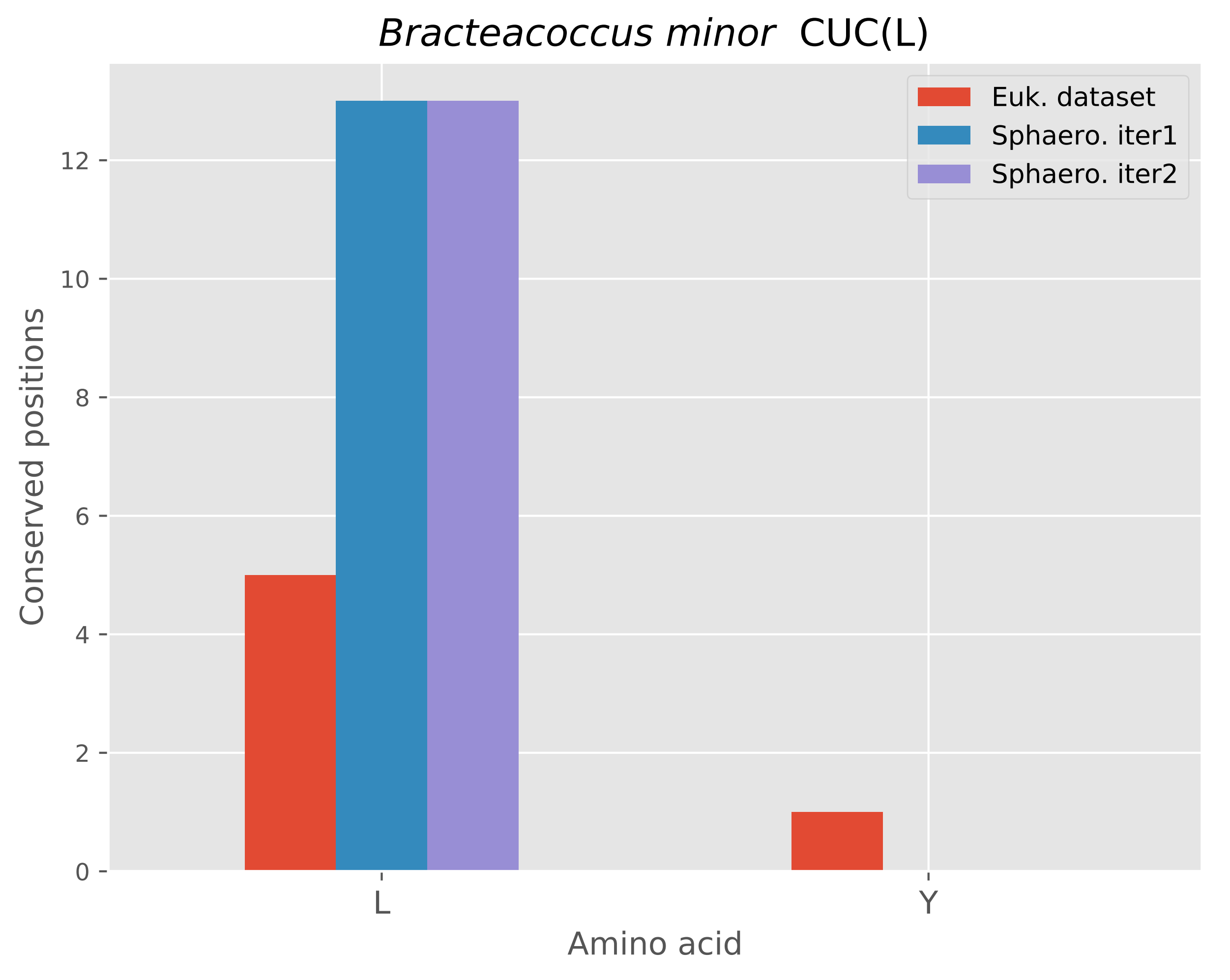

Bracteacoccus minor CUC(L)
Euk. dataset
Sphaero. iter1
Sphaero. iter2
Conserved positions
0
2
4
6
8
10
12
L
Y
Amino acid

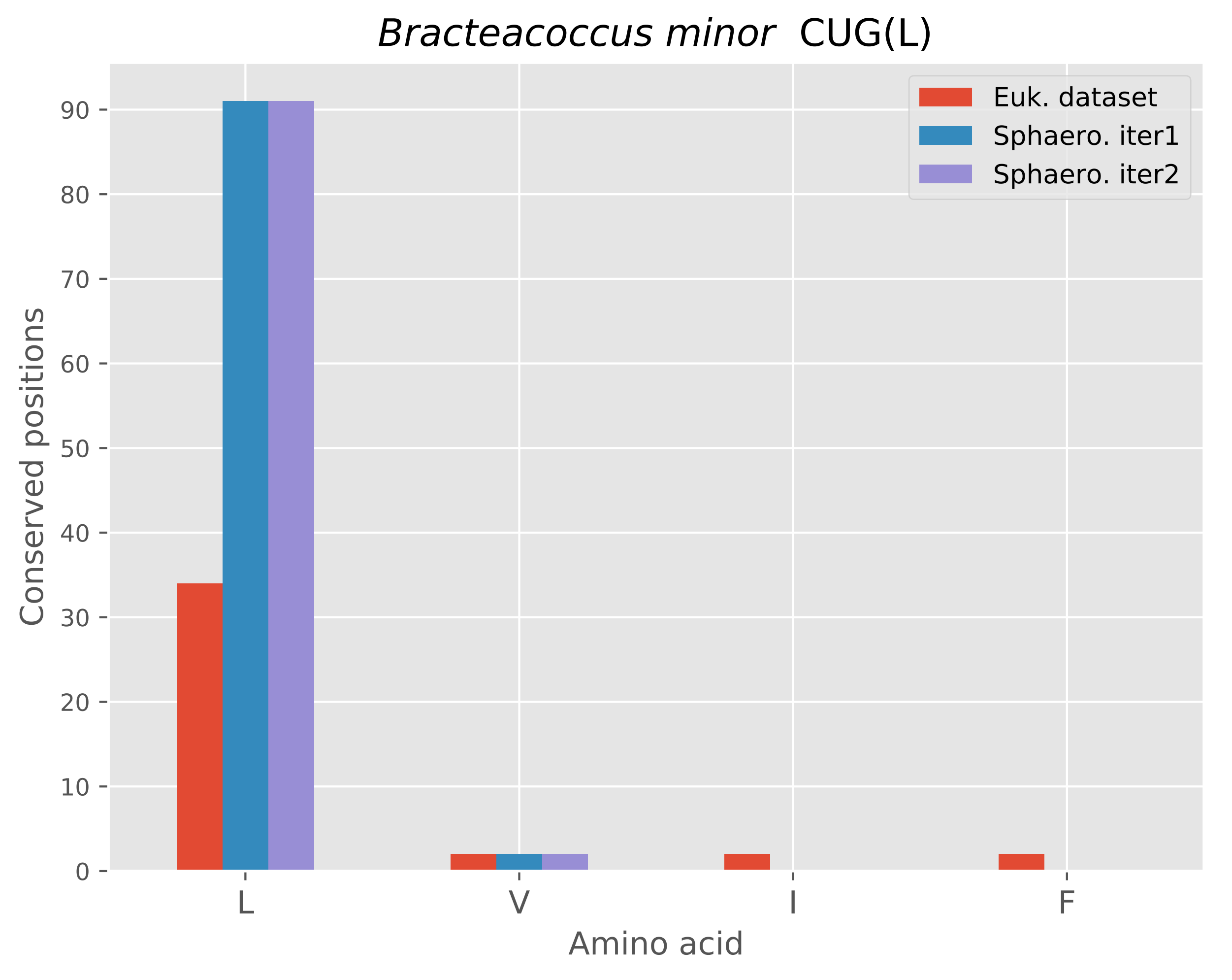

Bracteacoccus minor CUG(L)
Euk. dataset
Sphaero. iter1
Sphaero. iter2
Conserved positions
0
10
20
30
40
50
60
70
80
90
L
V
I
F
Amino acid

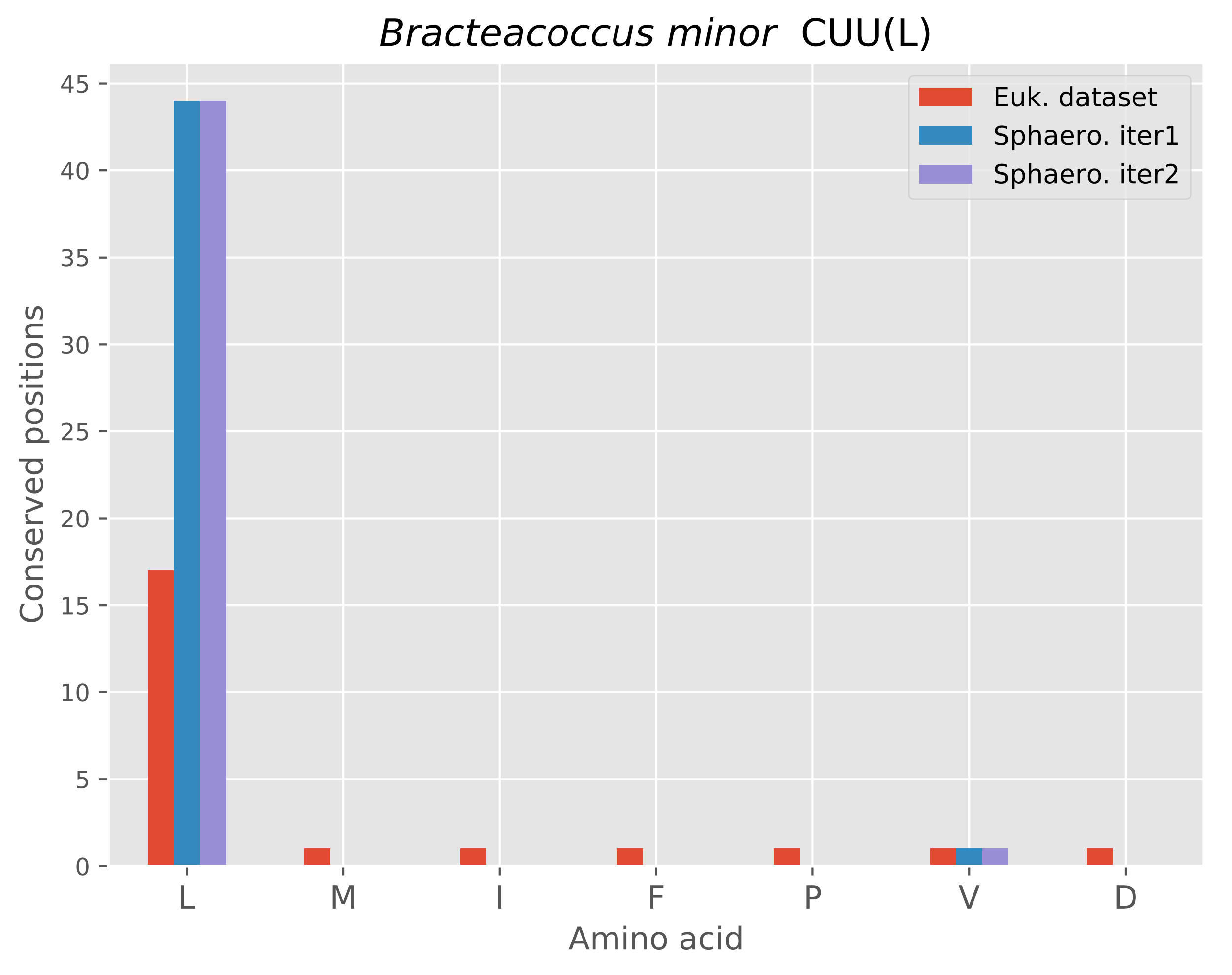

Bracteacoccus minor CUU(L)
Euk. dataset
Sphaero. iter1
Sphaero. iter2
Conserved positions
0
5
10
15
20
25
30
35
40
45
L
M
I
F
P
V
D
Amino acid

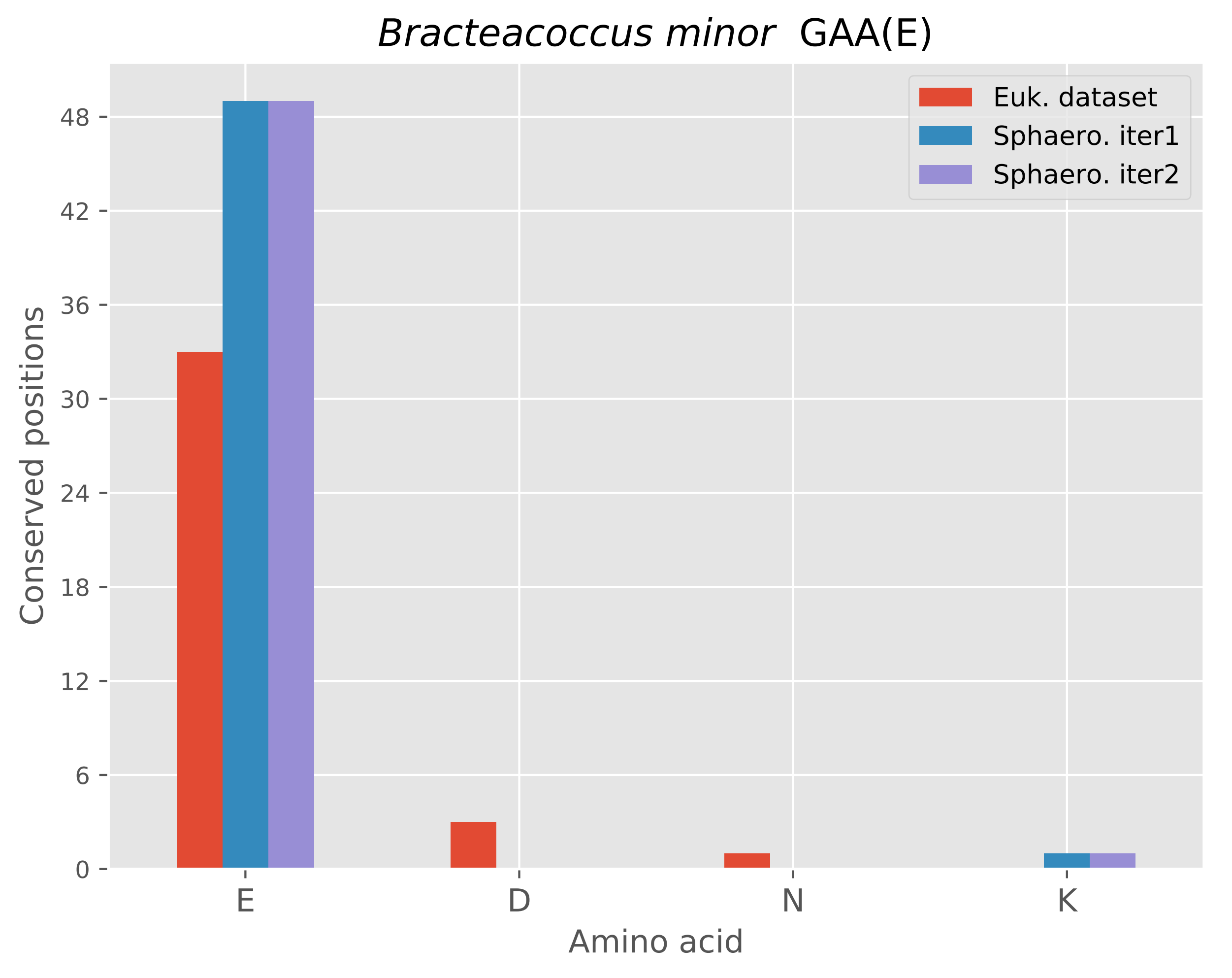
Bracteacoccus minor GAA(E)
Euk. dataset
Sphaero. iter1
Sphaero. iter2
Conserved positions
0
6
12
18
24
30
36
42
48
E
D
N
K
Amino acid

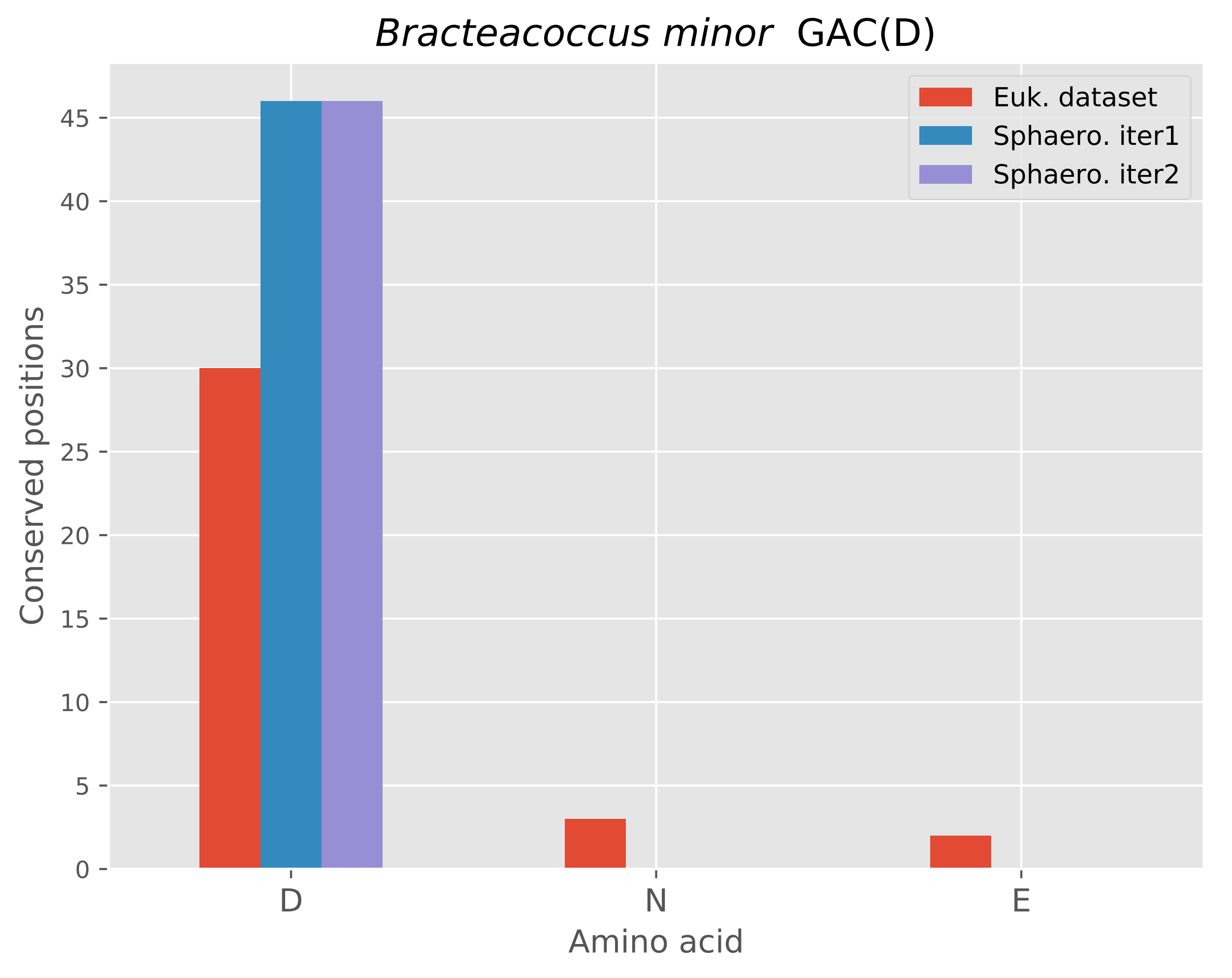

Bracteacoccus minor GAC(D)
Euk. dataset
Sphaero. iter1
Sphaero. iter2
Conserved positions
0
5
10
15
20
25
30
35
40
45
D
N
E
Amino acid

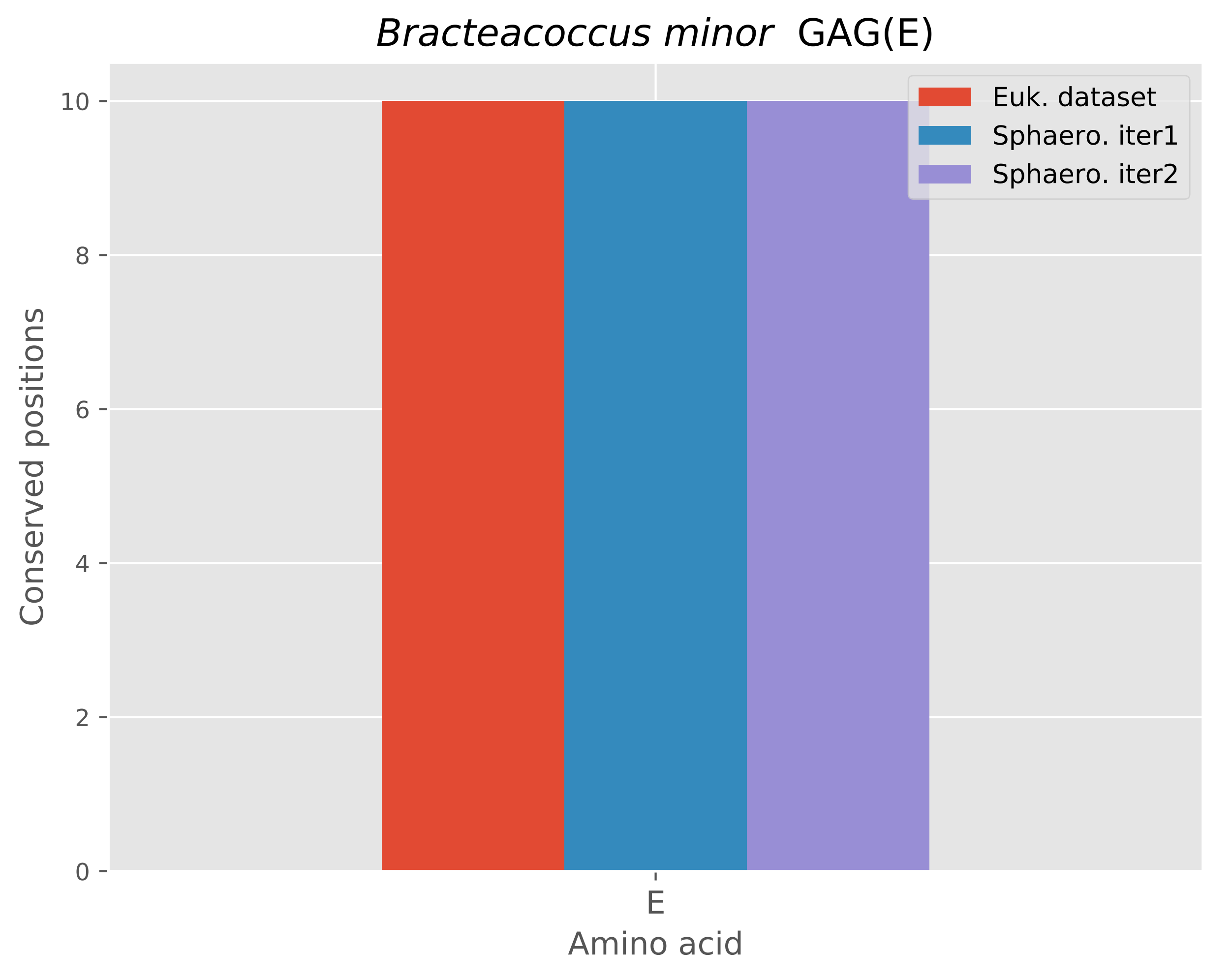

Bracteacoccus minor GAG(E)
Euk. dataset
Sphaero. iter1
Sphaero. iter2
Conserved positions
0
2
4
6
8
10
E
Amino acid

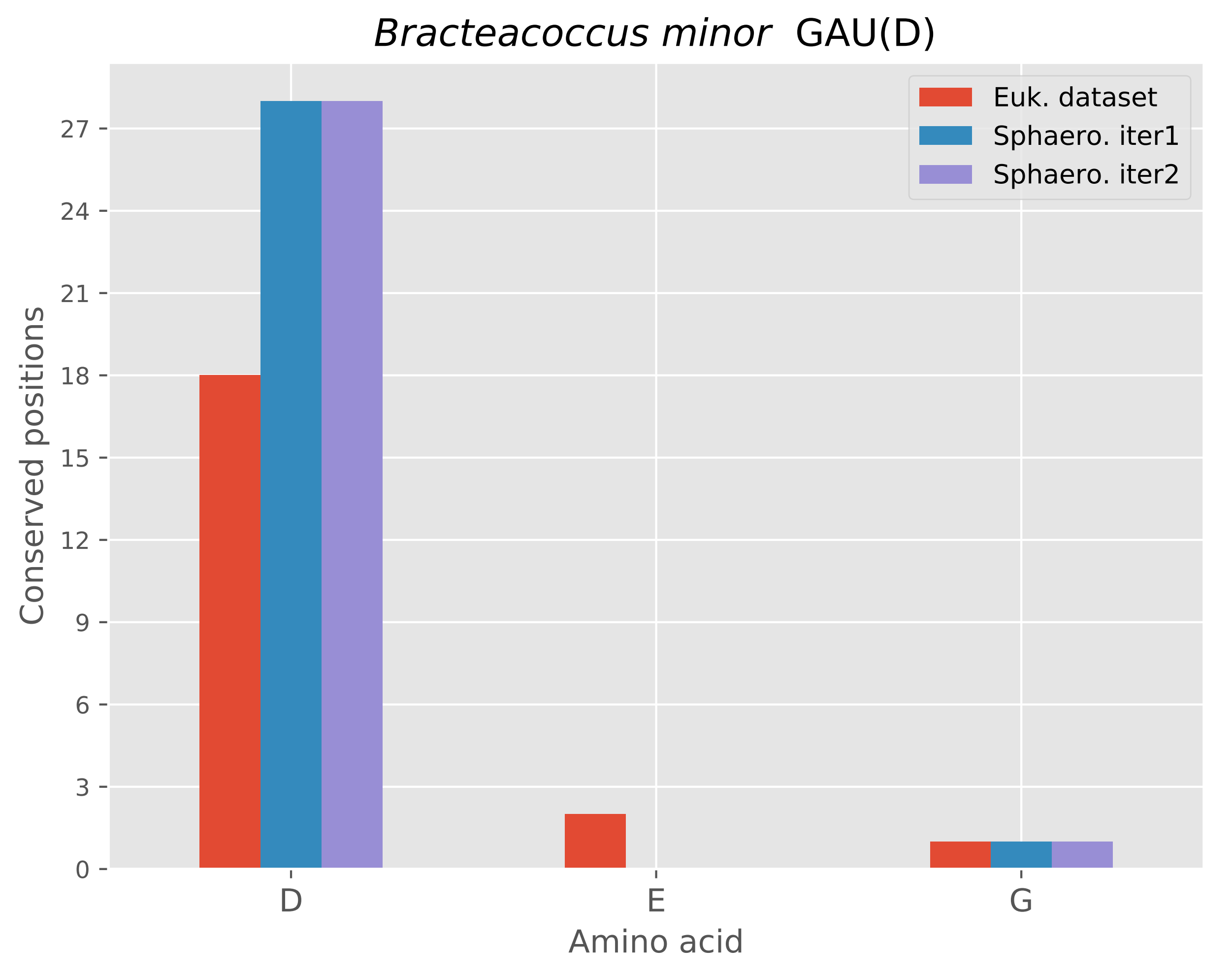

Bracteacoccus minor GAU(D)
Euk. dataset
Sphaero. iter1
Sphaero. iter2
Conserved positions
0
3
6
9
12
15
18
21
24
27
D
E
G
Amino acid

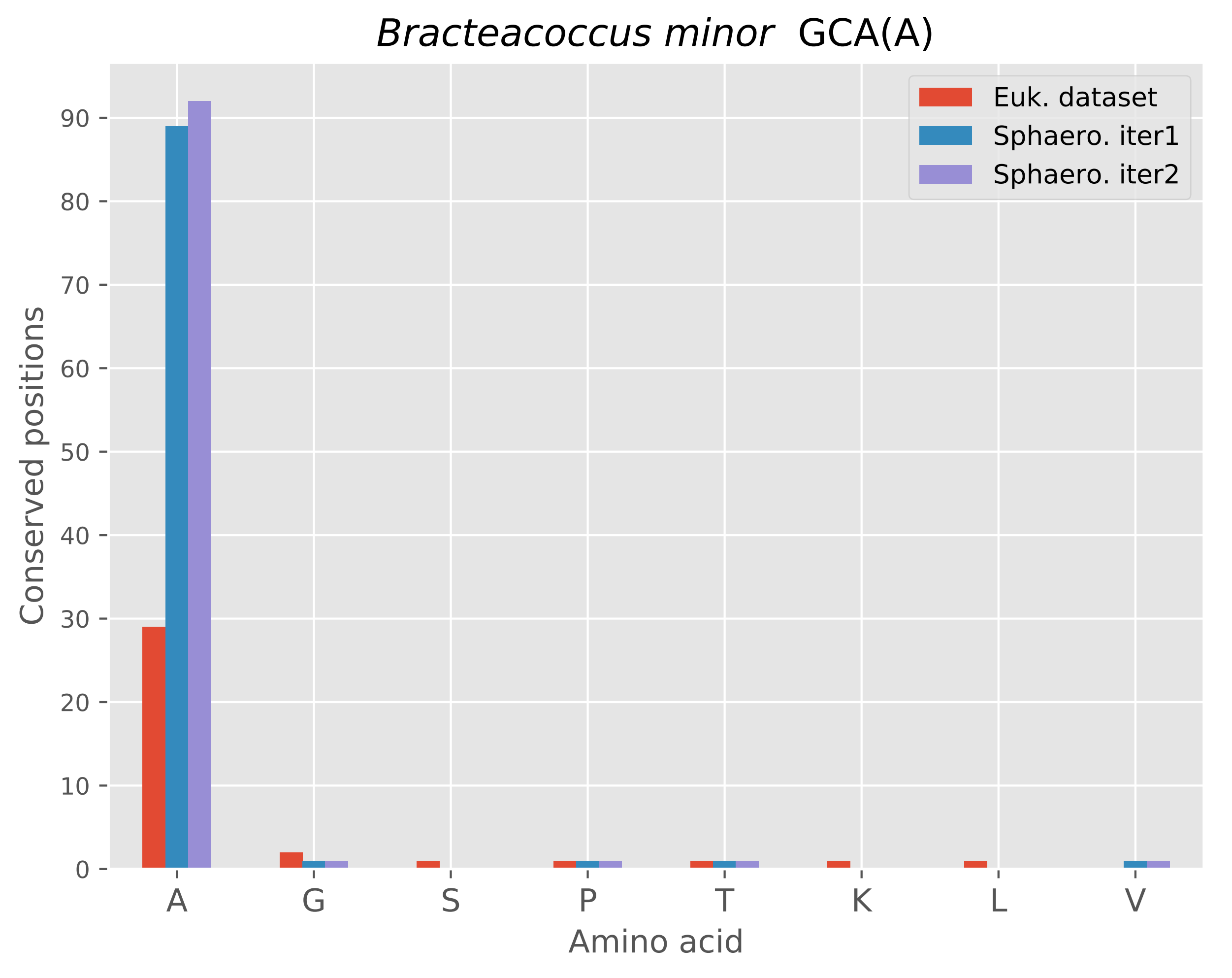

Bracteacoccus minor GCA(A)
Euk. dataset
Sphaero. iter1
Sphaero. iter2
Conserved positions
0
10
20
30
40
50
60
70
80
90
A
G
S
P
T
K
L
V
Amino acid

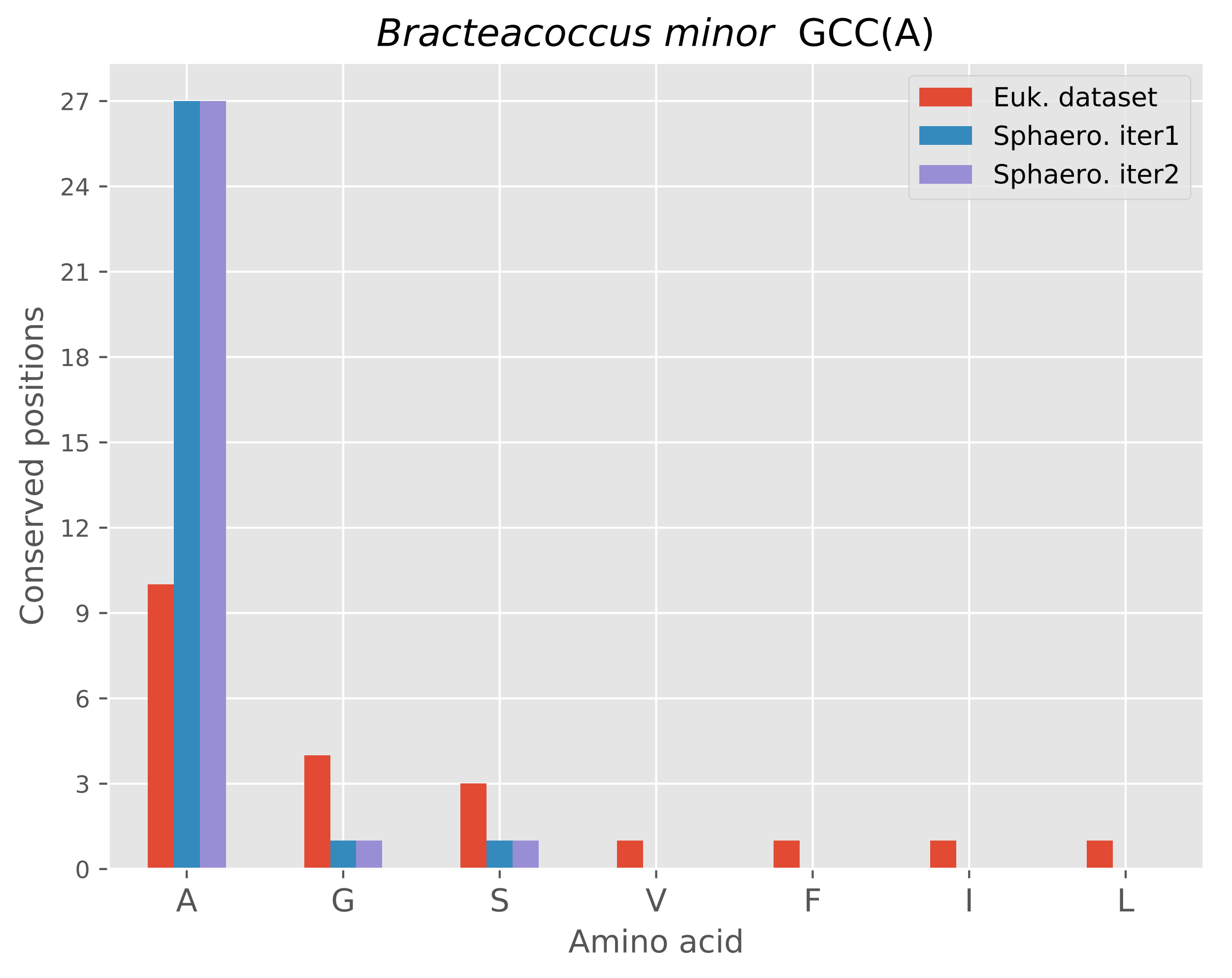
Bracteacoccus minor GCC(A)
Euk. dataset
Sphaero. iter1
Sphaero. iter2
Conserved positions
0
3
6
9
12
15
18
21
24
27
A
G
S
V
F
I
L
Amino acid

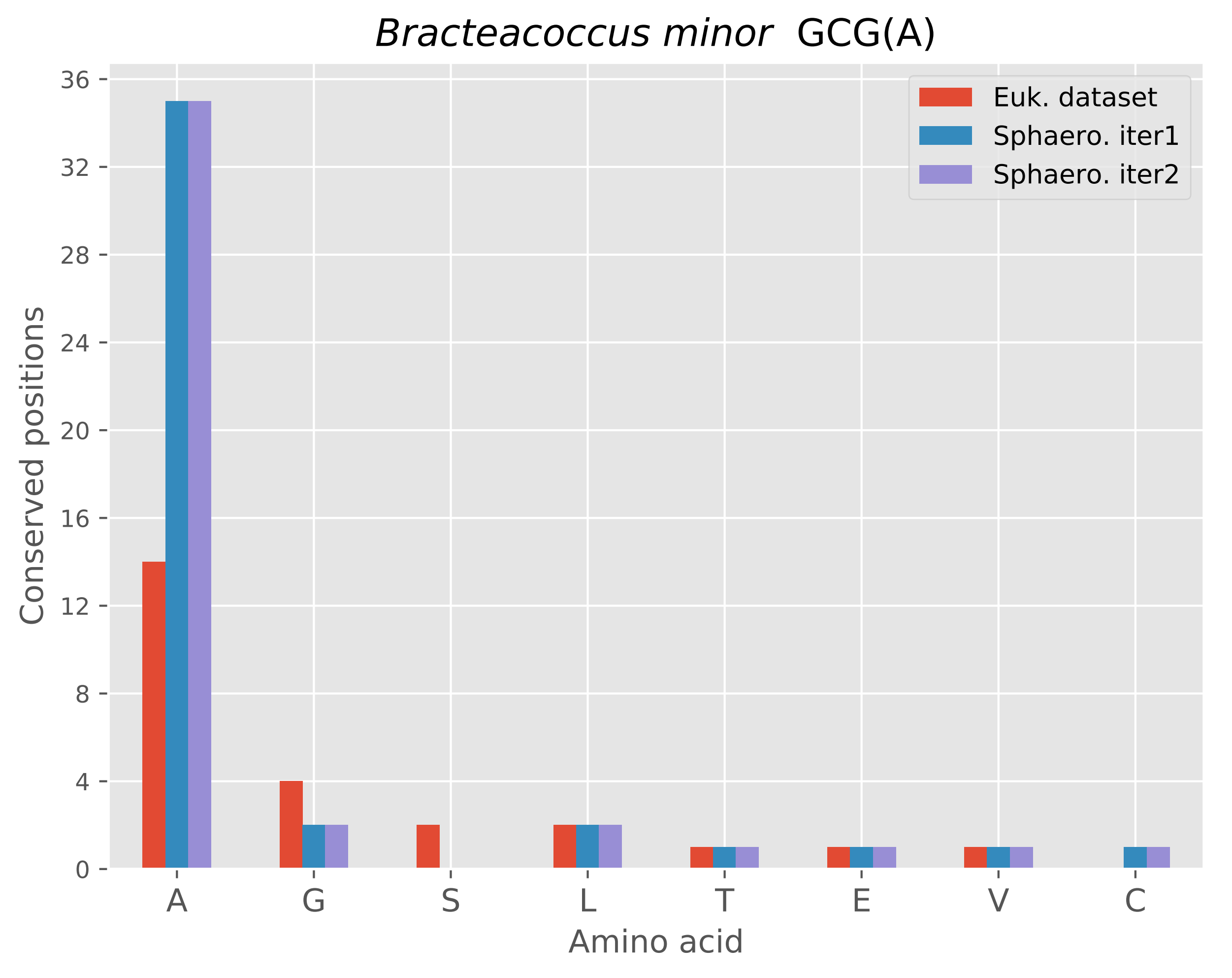

Bracteacoccus minor GCG(A)
Euk. dataset
Sphaero. iter1
Sphaero. iter2
Conserved positions
0
4
8
12
16
20
24
28
32
36
A
G
S
L
T
E
V
C
Amino acid

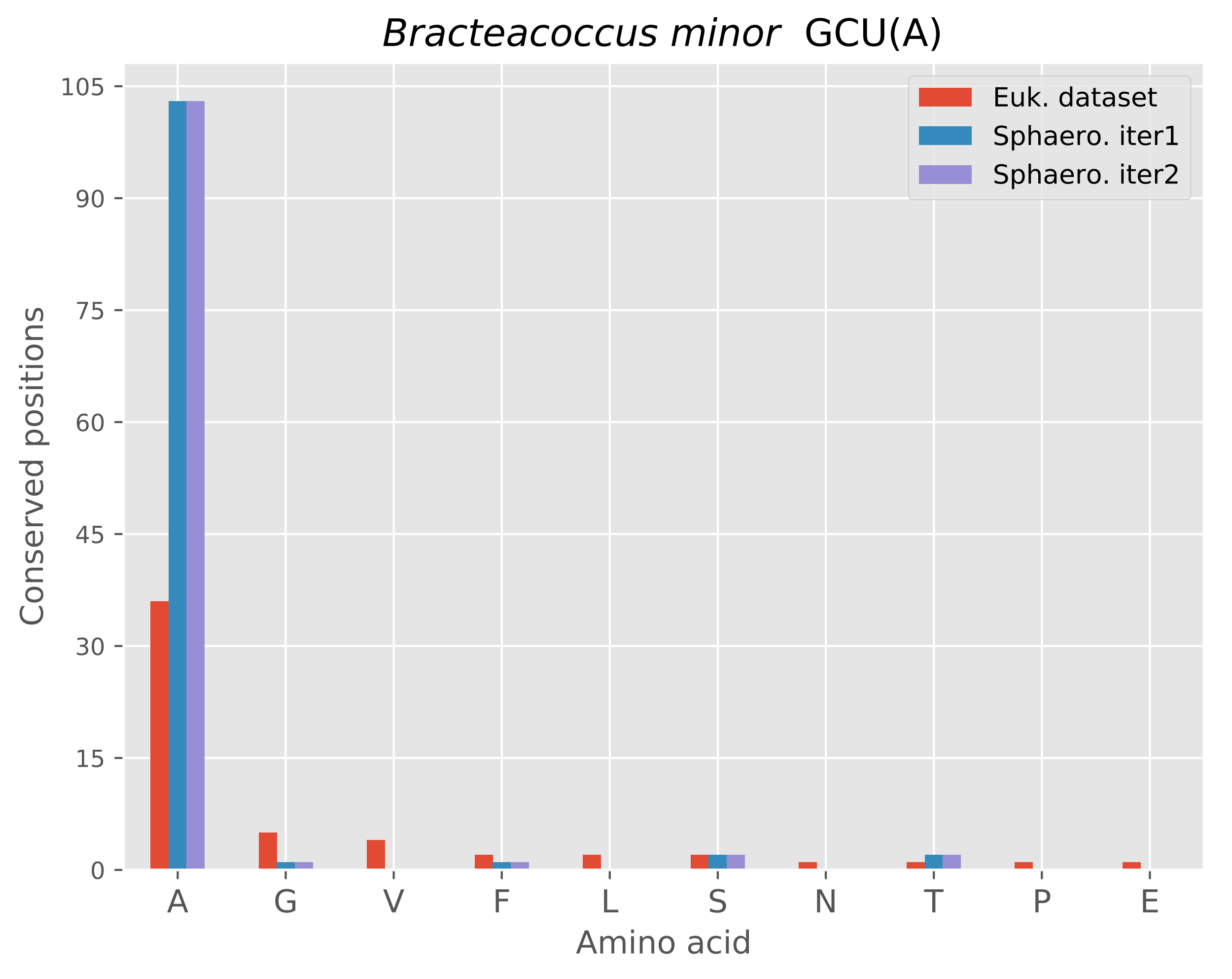

Bracteacoccus minor GCU(A)
Euk. dataset
Sphaero. iter1
Sphaero. iter2
Conserved positions
0
15
30
45
60
75
90
105
A
G
V
F
L
S
N
T
P
E
Amino acid

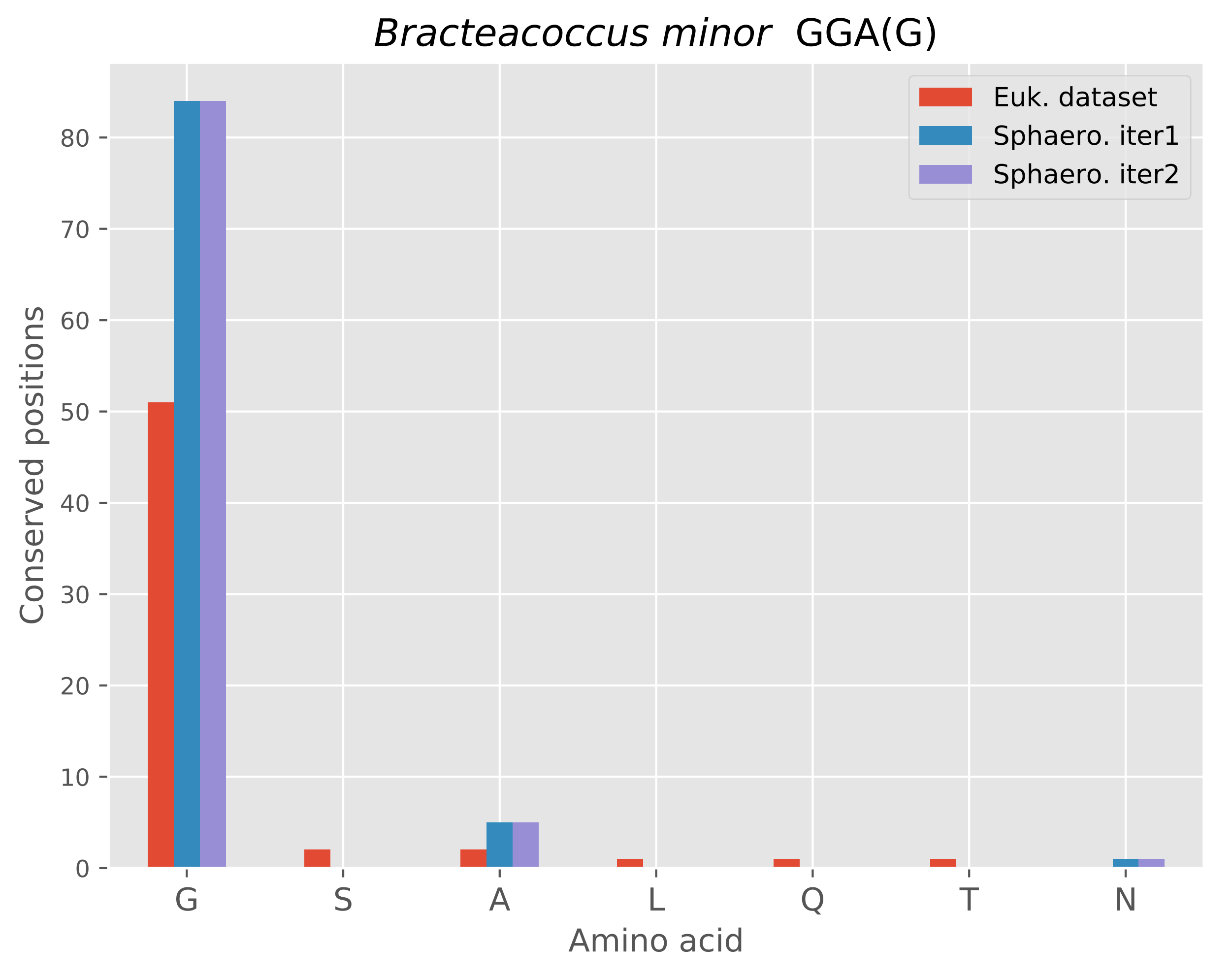

Bracteacoccus minor GGA(G)
Euk. dataset
Sphaero. iter1
Sphaero. iter2
Conserved positions
0
10
20
30
40
50
60
70
80
G
S
A
L
Q
T
N
Amino acid

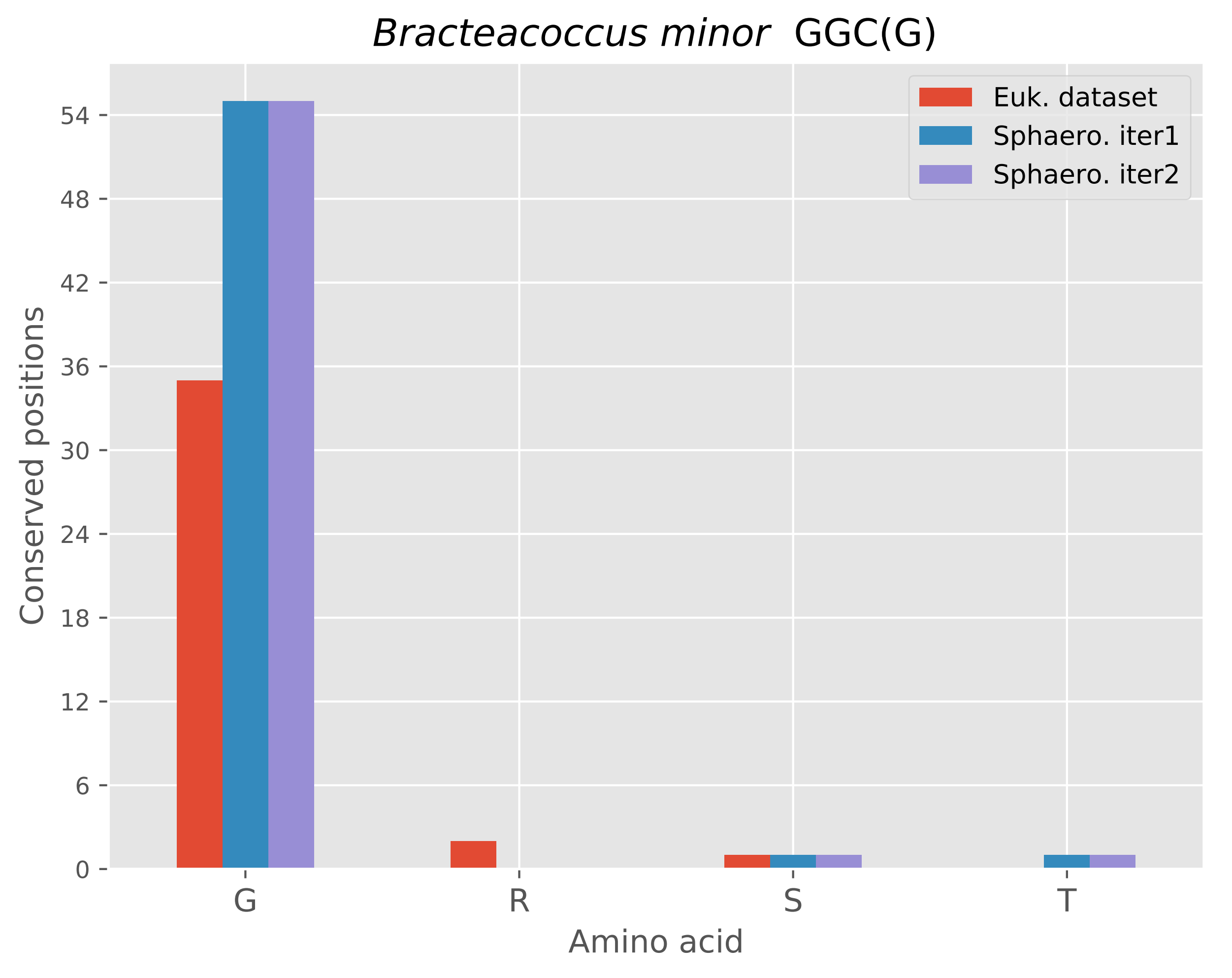

Bracteacoccus minor GGC(G)
Euk. dataset
Sphaero. iter1
Sphaero. iter2
Conserved positions
0
6
12
18
24
30
36
42
48
54
G
R
S
T
Amino acid

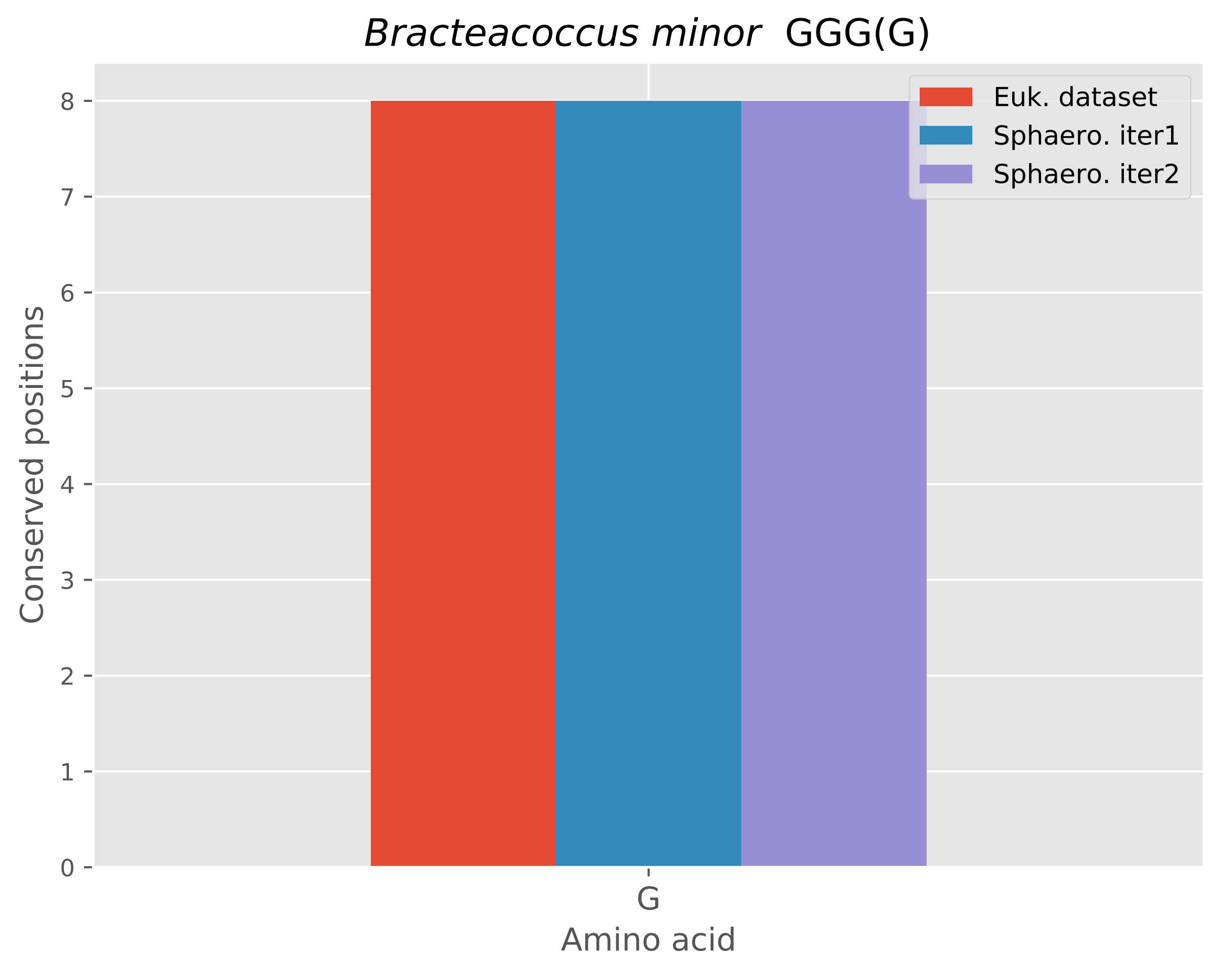

Bracteacoccus minor GGG(G)
Euk. dataset
Sphaero. iter1
Sphaero. iter2
Conserved positions
0
1
2
3
4
5
6
7
8
G
Amino acid

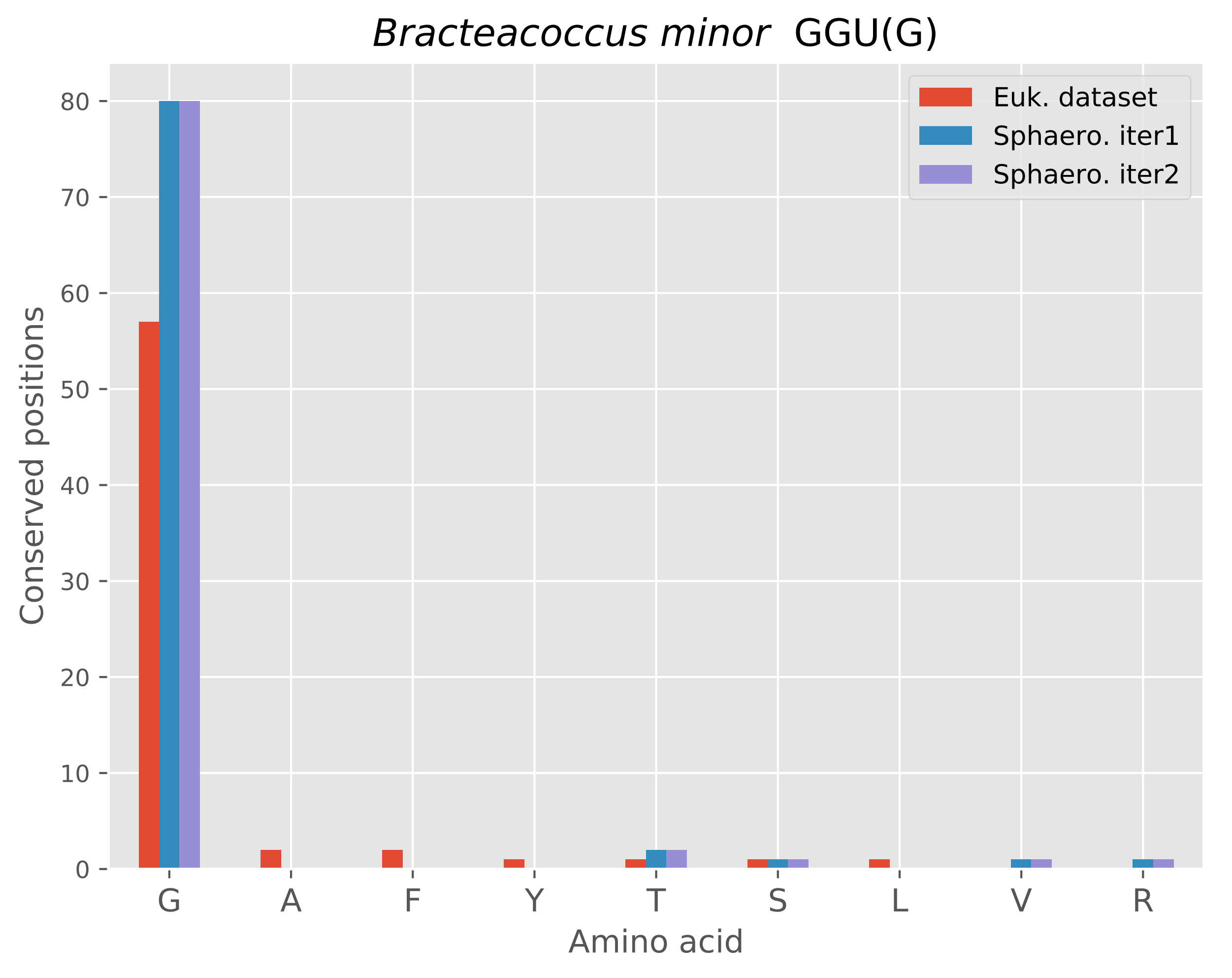
Bracteacoccus minor GGU(G)
Euk. dataset
Sphaero. iter1
Sphaero. iter2
Conserved positions
Amino acid
0
10
20
30
40
50
60
70
80
G
A
F
Y
T
S
L
V
R

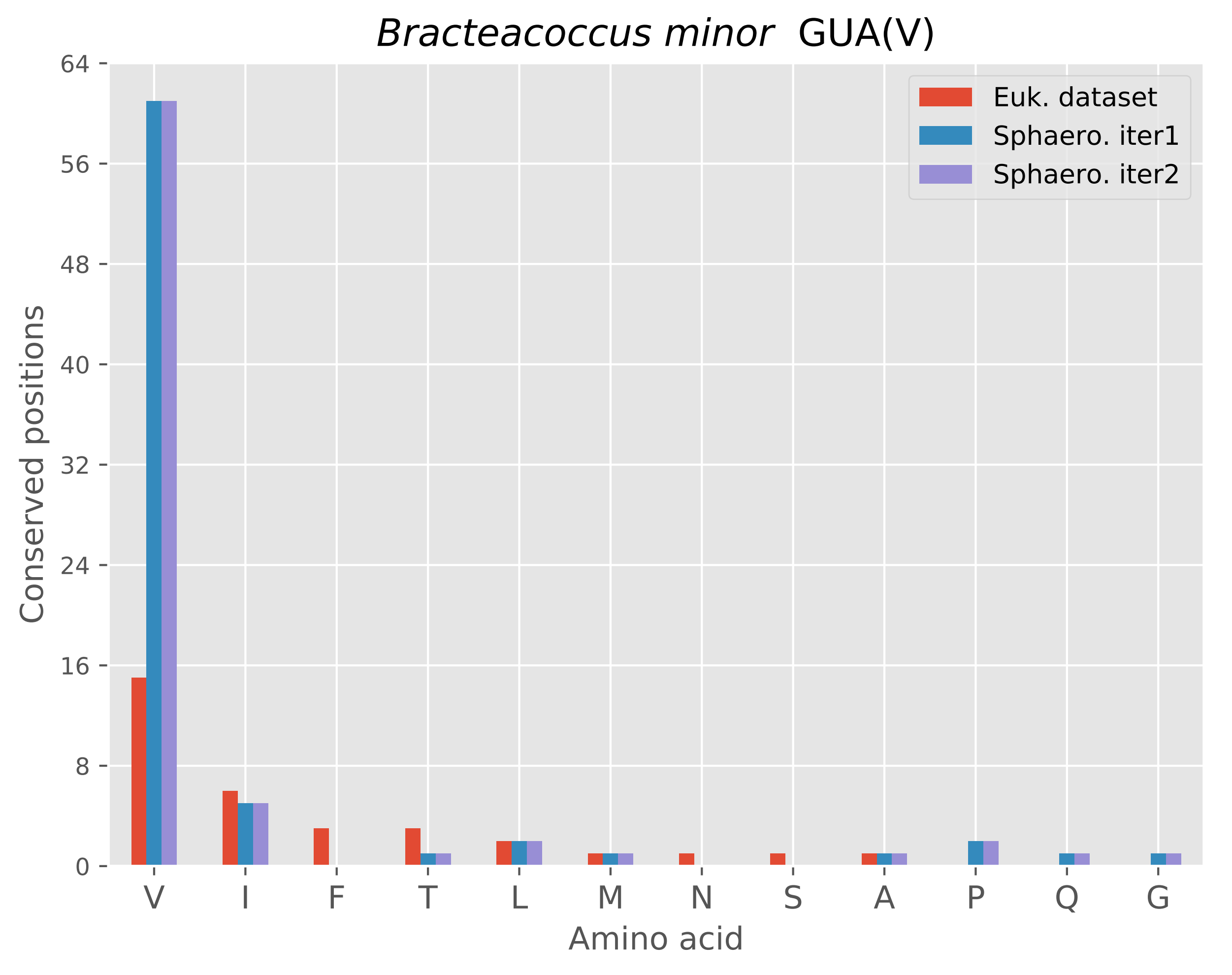

Bracteacoccus minor GUA(V)
Conserved positions
0
8
16
24
32
40
48
56
64
Euk. dataset
Sphaero. iter1
Sphaero. iter2
V
I
F
T
L
M
N
S
A
P
Q
G
Amino acid

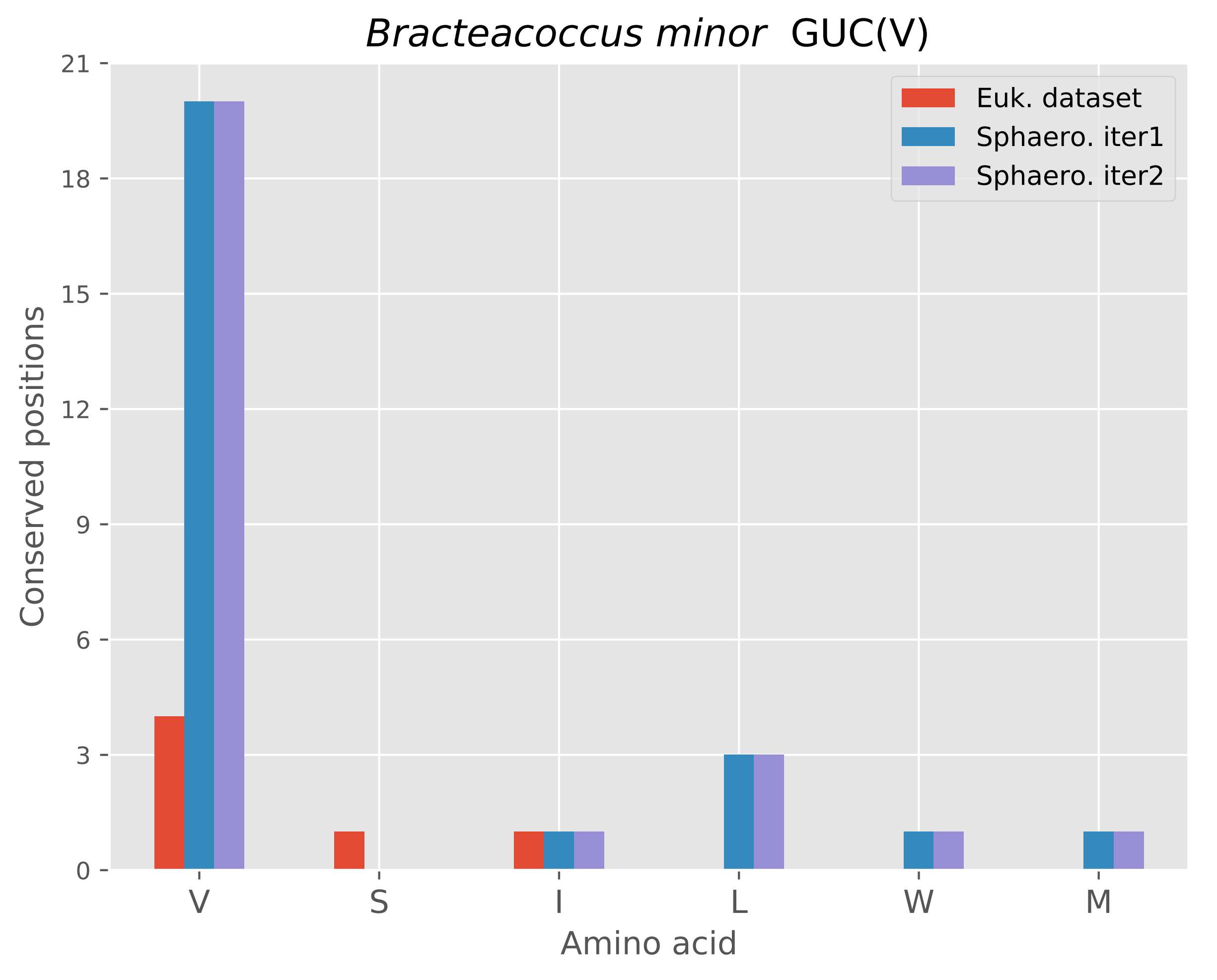
Bracteacoccus minor GUC(V)
21
18
15
12
9
6
3
0
Conserved positions
Euk. dataset
Sphaero. iter1
Sphaero. iter2
V
S
I
L
W
M
Amino acid

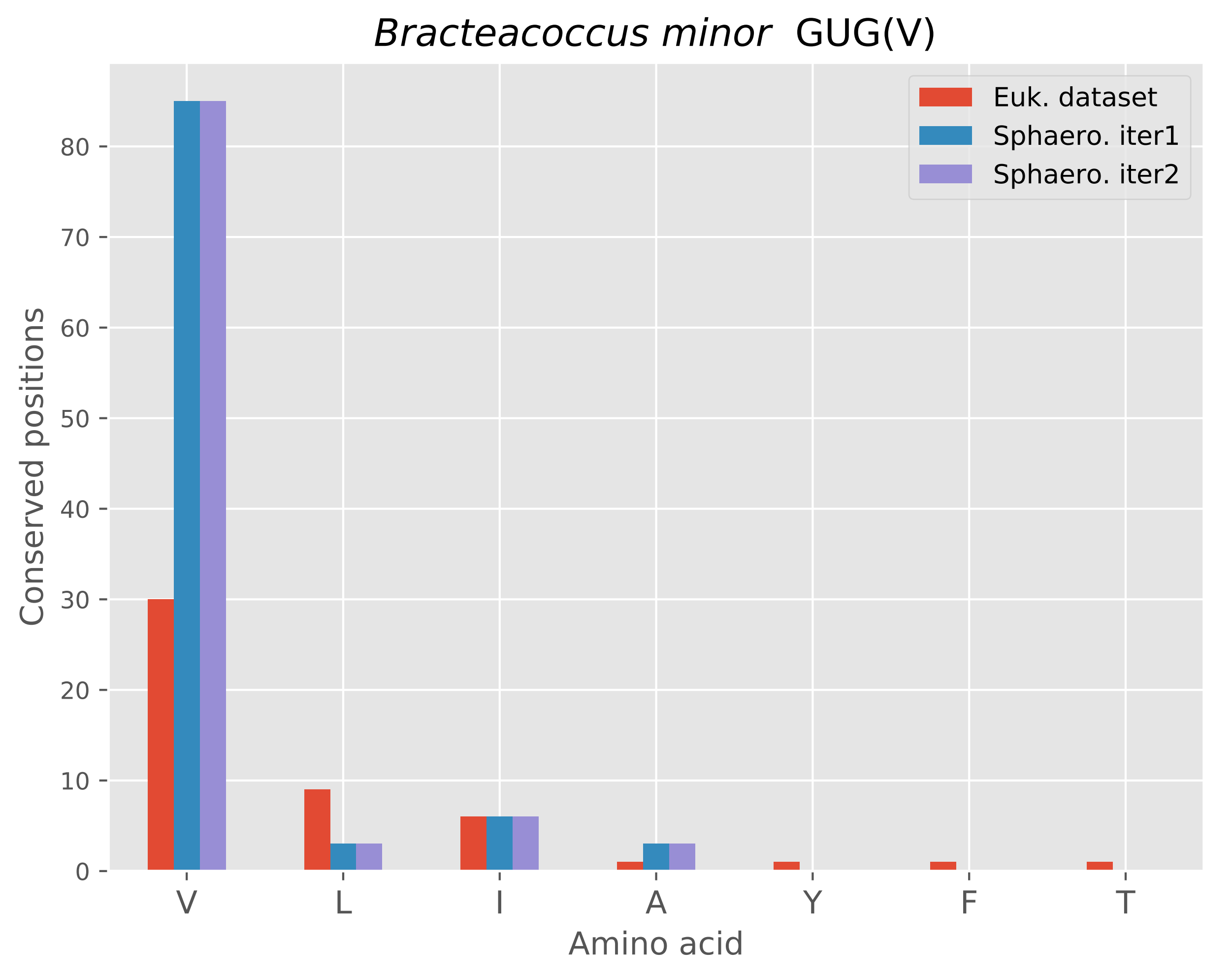

Bracteacoccus minor GUG(V)
Euk. dataset
Sphaero. iter1
Sphaero. iter2
Conserved positions
0
10
20
30
40
50
60
70
80
V
L
I
A
Y
F
T
Amino acid

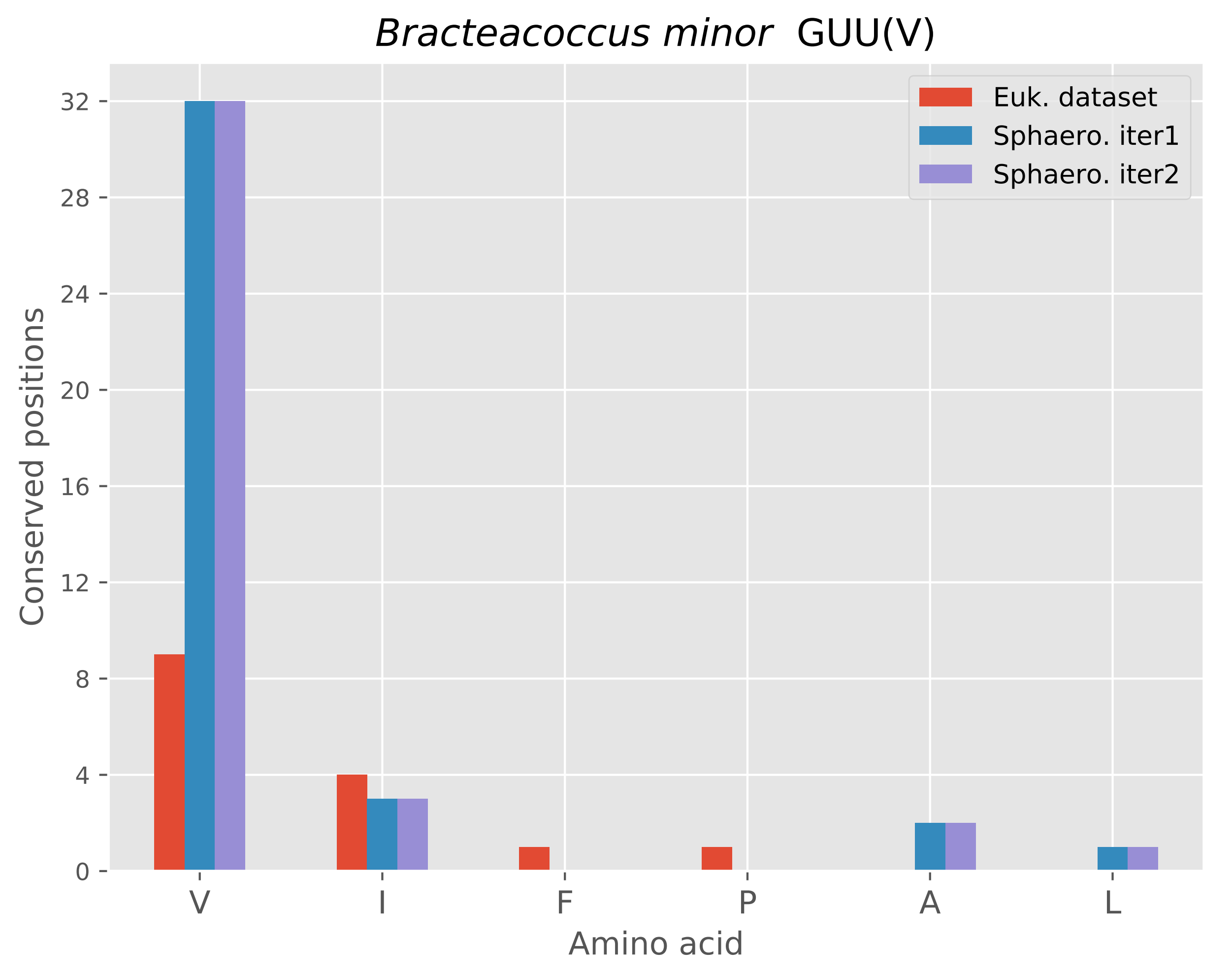

Bracteacoccus minor GUU(V)
Euk. dataset
Sphaero. iter1
Sphaero. iter2
Conserved positions
0
4
8
12
16
20
24
28
32
V
I
F
P
A
L
Amino acid

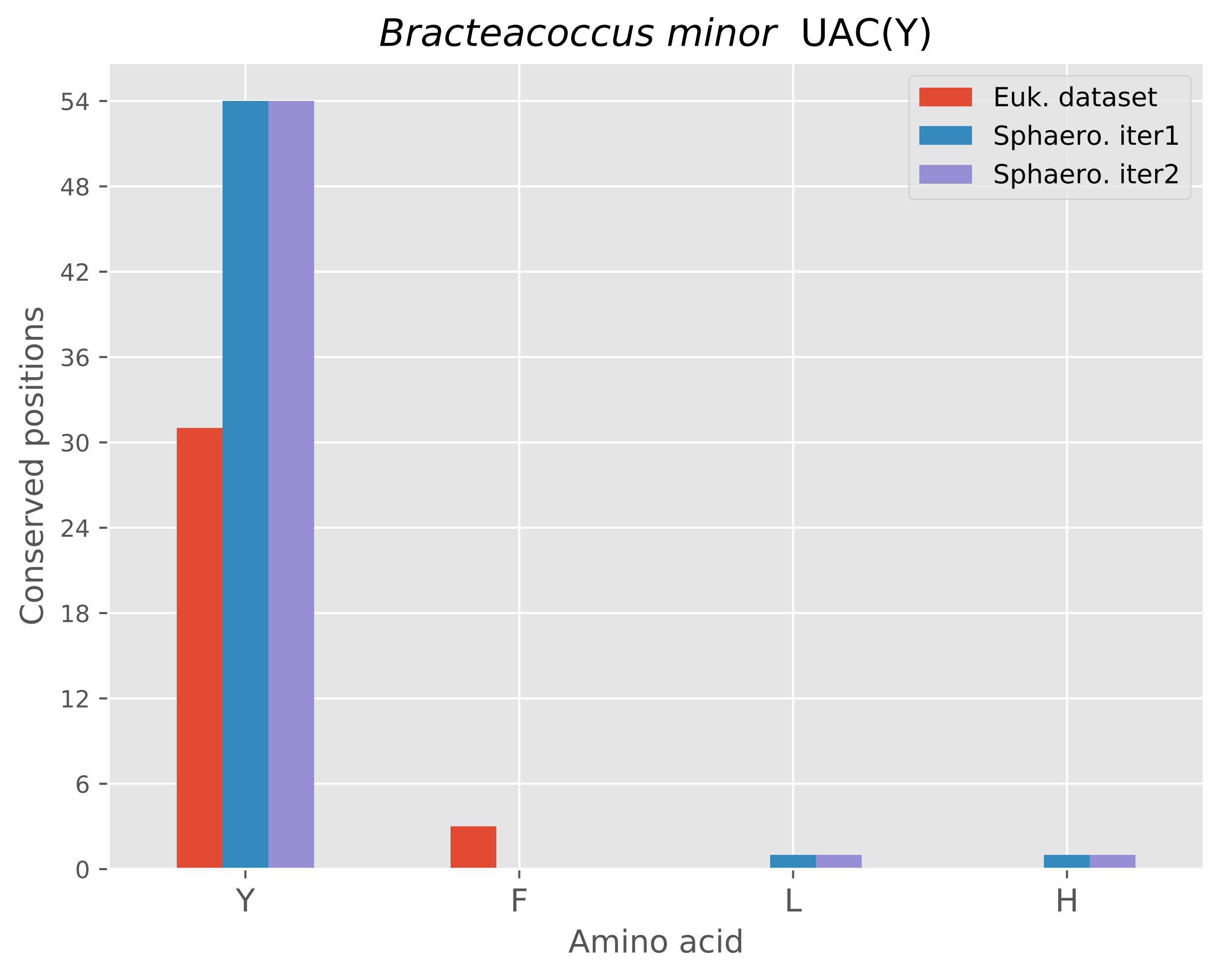
Bracteacoccus minor UAC(Y)
Euk. dataset
Sphaero. iter1
Sphaero. iter2
Conserved positions
0
6
12
18
24
30
36
42
48
54
Y
F
L
H
Amino acid

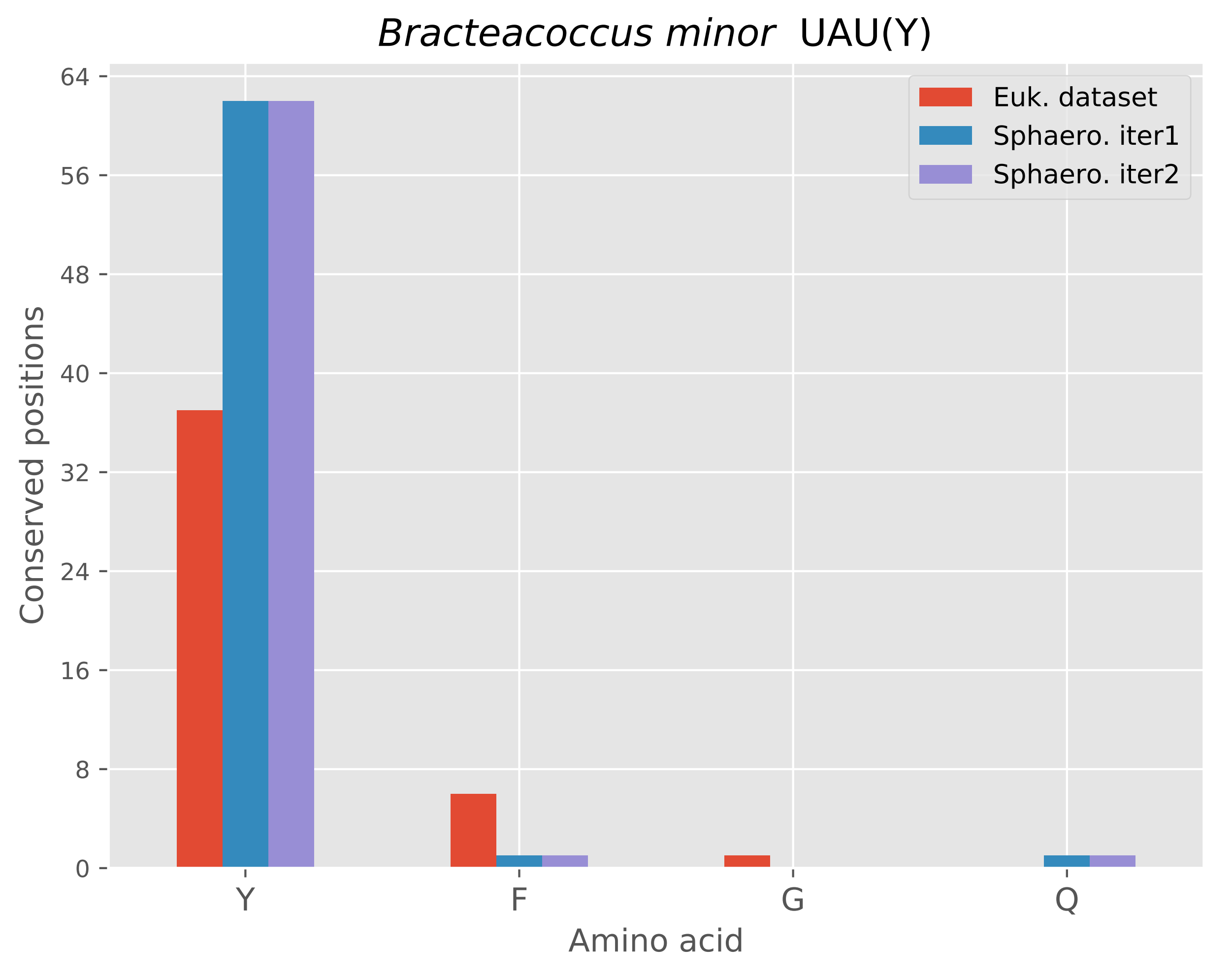

Bracteacoccus minor UAU(Y)
Euk. dataset
Sphaero. iter1
Sphaero. iter2
Conserved positions
Amino acid
0
8
16
24
32
40
48
56
64
Y
F
G
Q

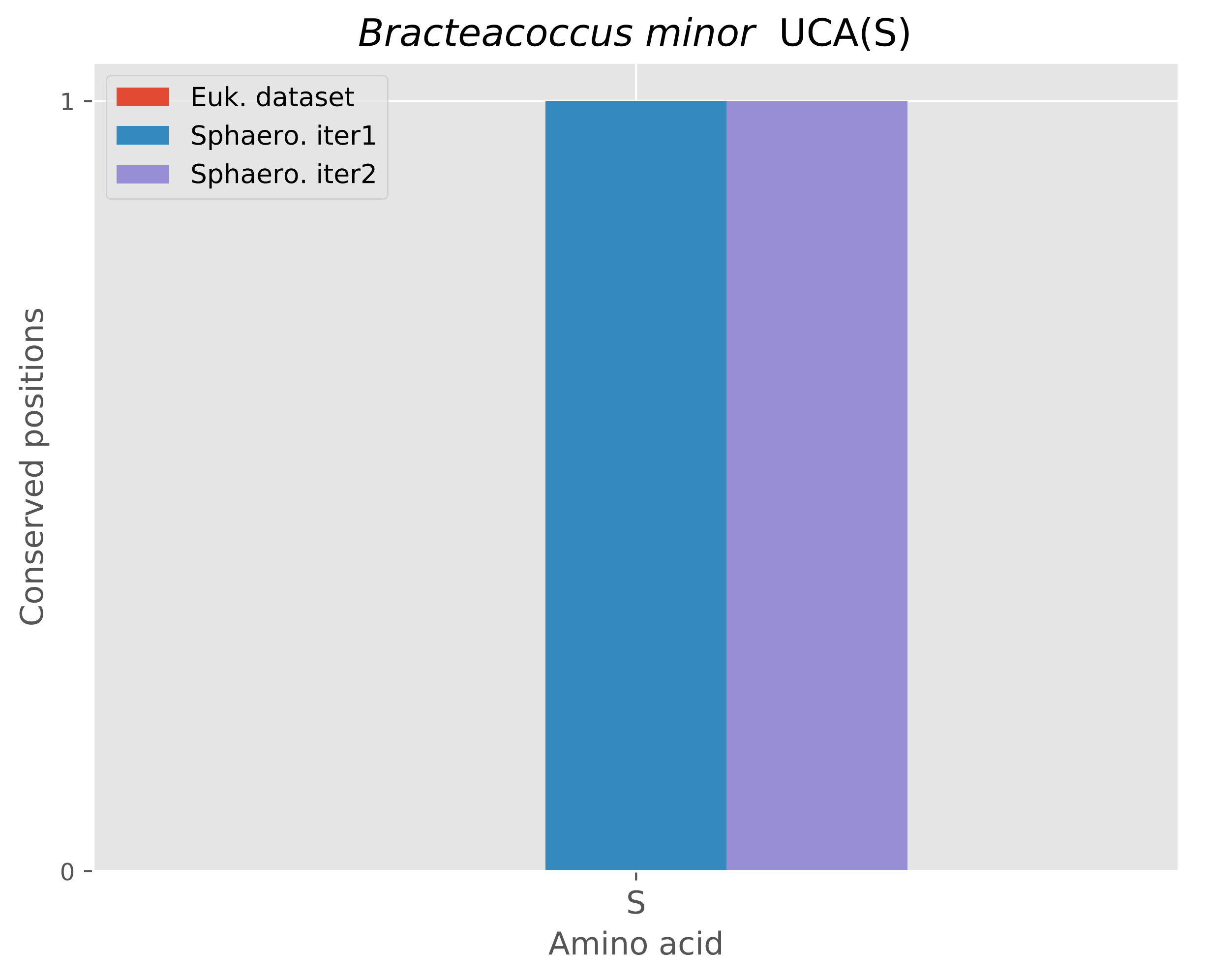
Bracteacoccus minor UCA(S)
Euk. dataset
Sphaero. iter1
Sphaero. iter2
1
0
Conserved positions
S
Amino acid

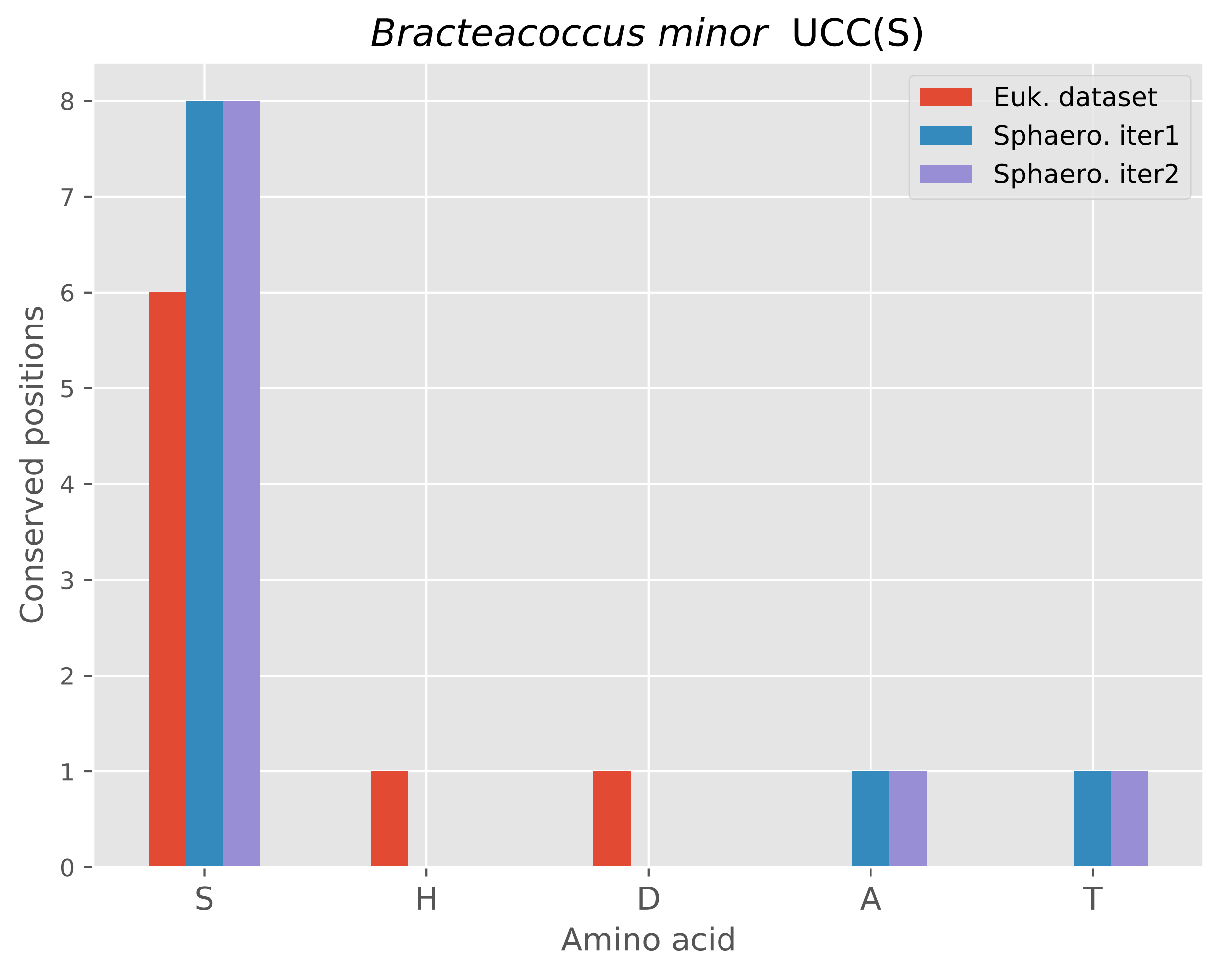

Bracteacoccus minor UCC(S)
Euk. dataset
Sphaero. iter1
Sphaero. iter2
Conserved positions
0
1
2
3
4
5
6
7
8
S
H
D
A
T
Amino acid

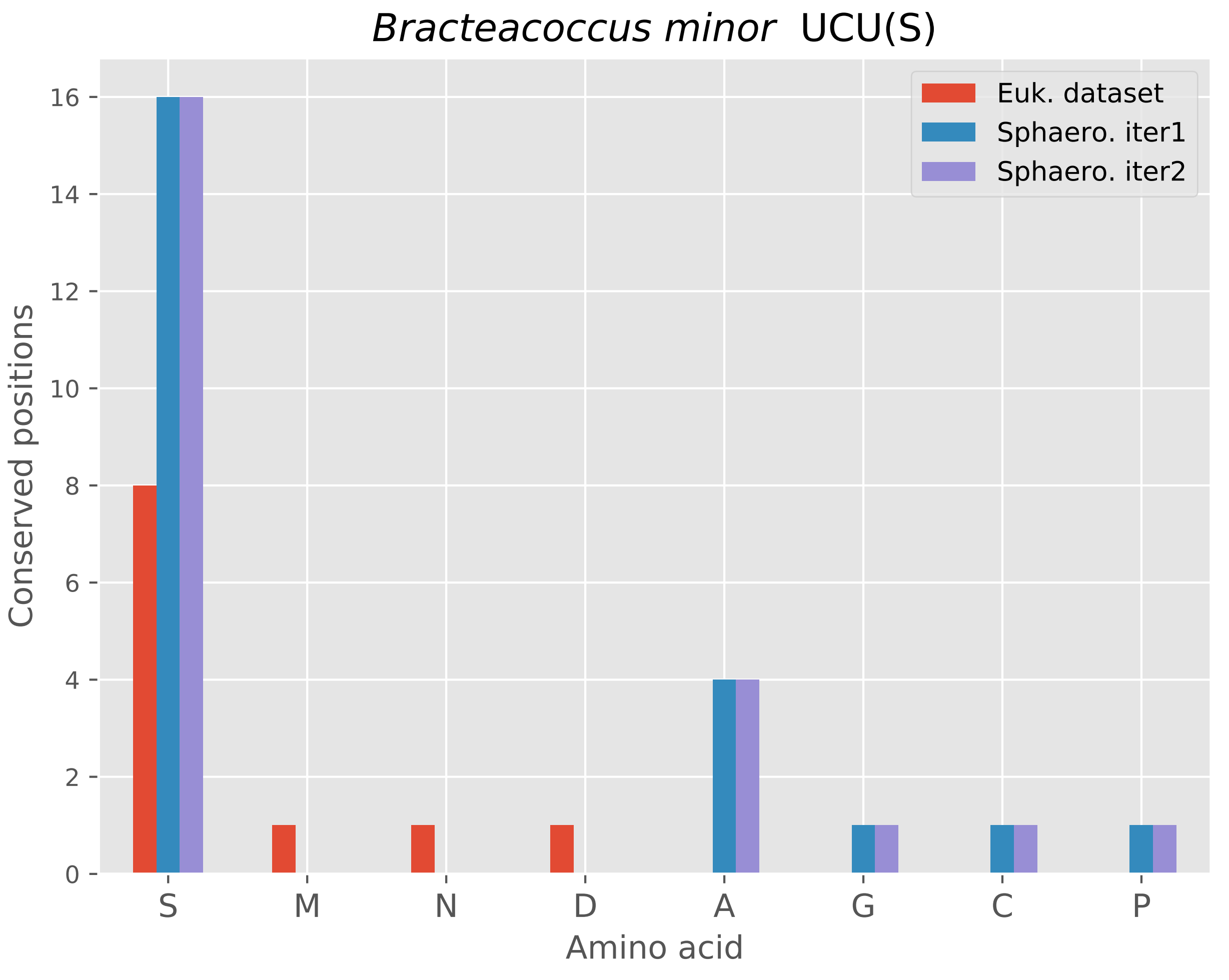
Bracteacoccus minor UCU(S)
Euk. dataset
Sphaero. iter1
Sphaero. iter2
Conserved positions
0
2
4
6
8
10
12
14
16
S
M
N
D
A
G
C
P
Amino acid

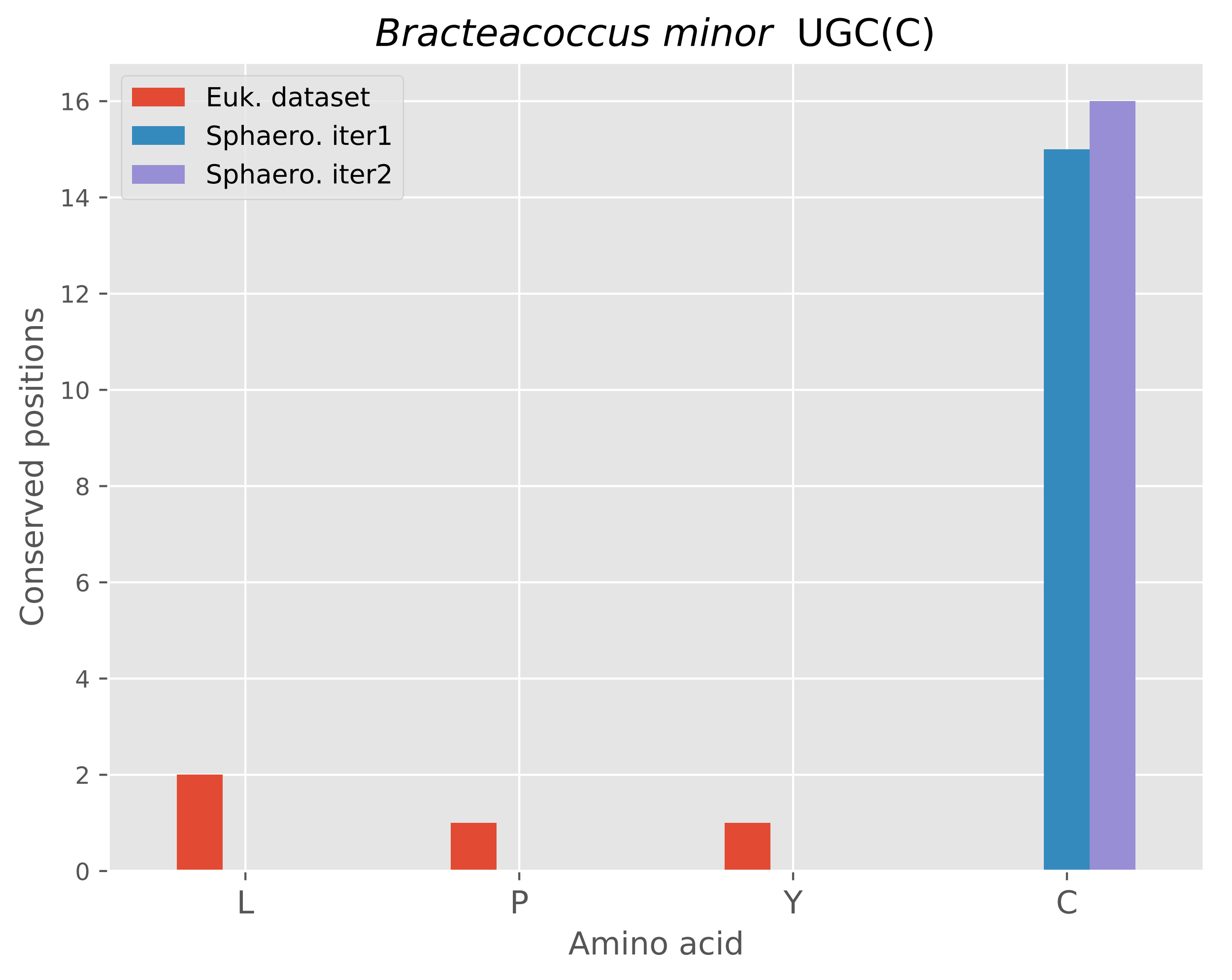

Bracteacoccus minor UGC(C)
Euk. dataset
Sphaero. iter1
Sphaero. iter2
Conserved positions
0
2
4
6
8
10
12
14
16
L
P
Y
C
Amino acid

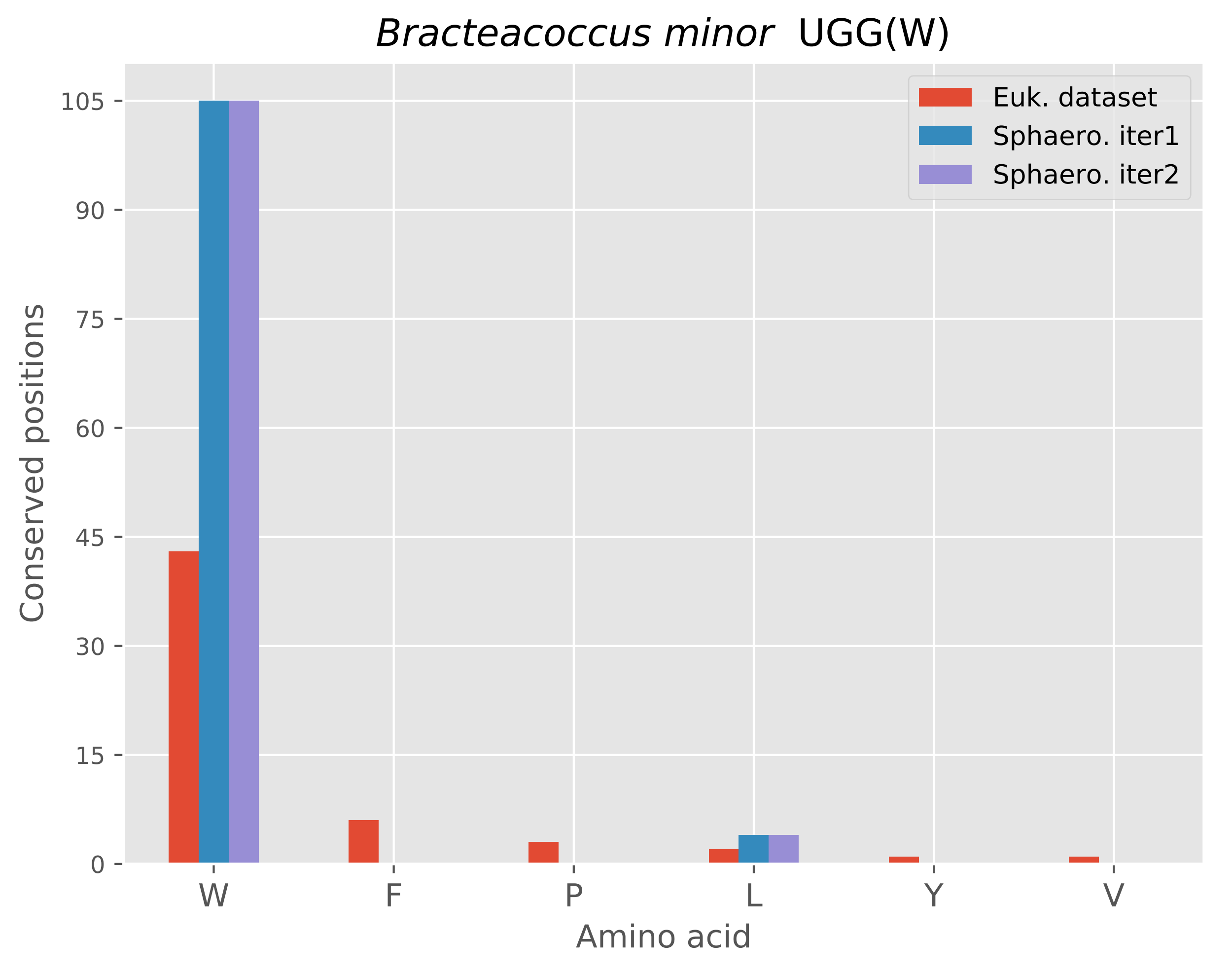
Bracteacoccus minor UGG(W)
Euk. dataset
Sphaero. iter1
Sphaero. iter2
Conserved positions
0
15
30
45
60
75
90
105
W
F
P
L
Y
V
Amino acid

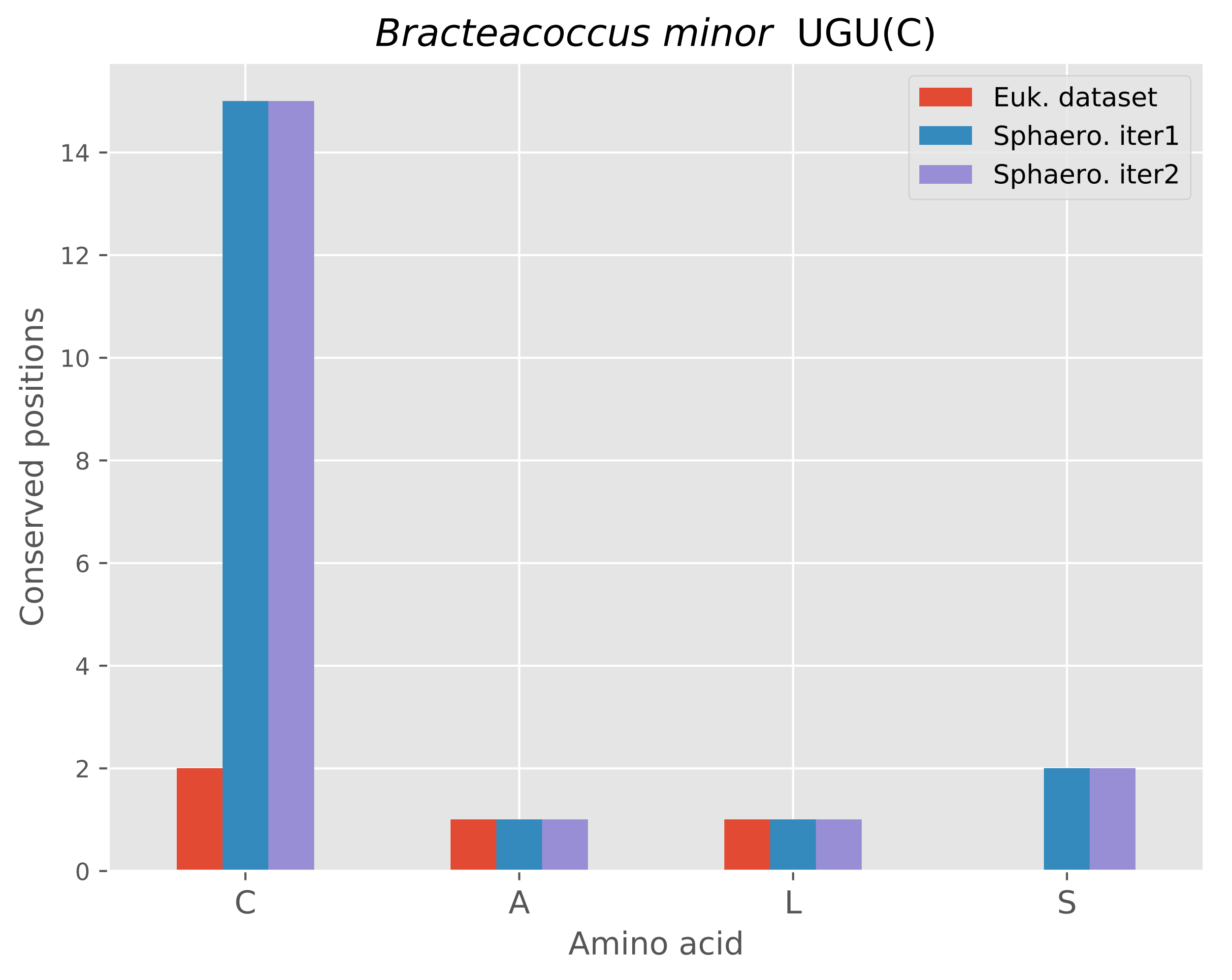

Bracteacoccus minor UGU(C)
Euk. dataset
Sphaero. iter1
Sphaero. iter2
Conserved positions
0
2
4
6
8
10
12
14
C
A
L
S
Amino acid

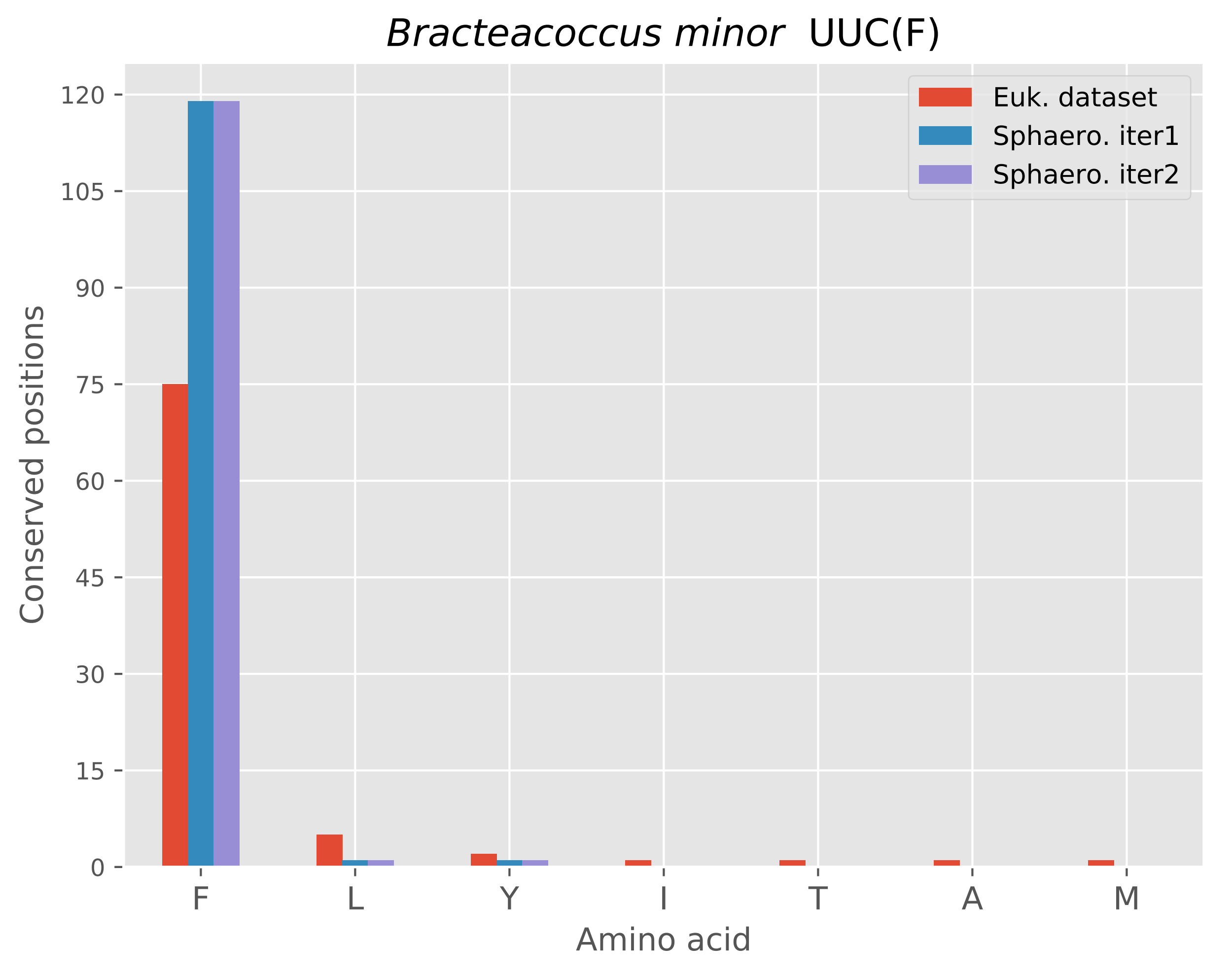

Bracteacoccus minor UUC(F)
Euk. dataset
Sphaero. iter1
Sphaero. iter2
Conserved positions
0
15
30
45
60
75
90
105
120
F
L
Y
I
T
A
M
Amino acid

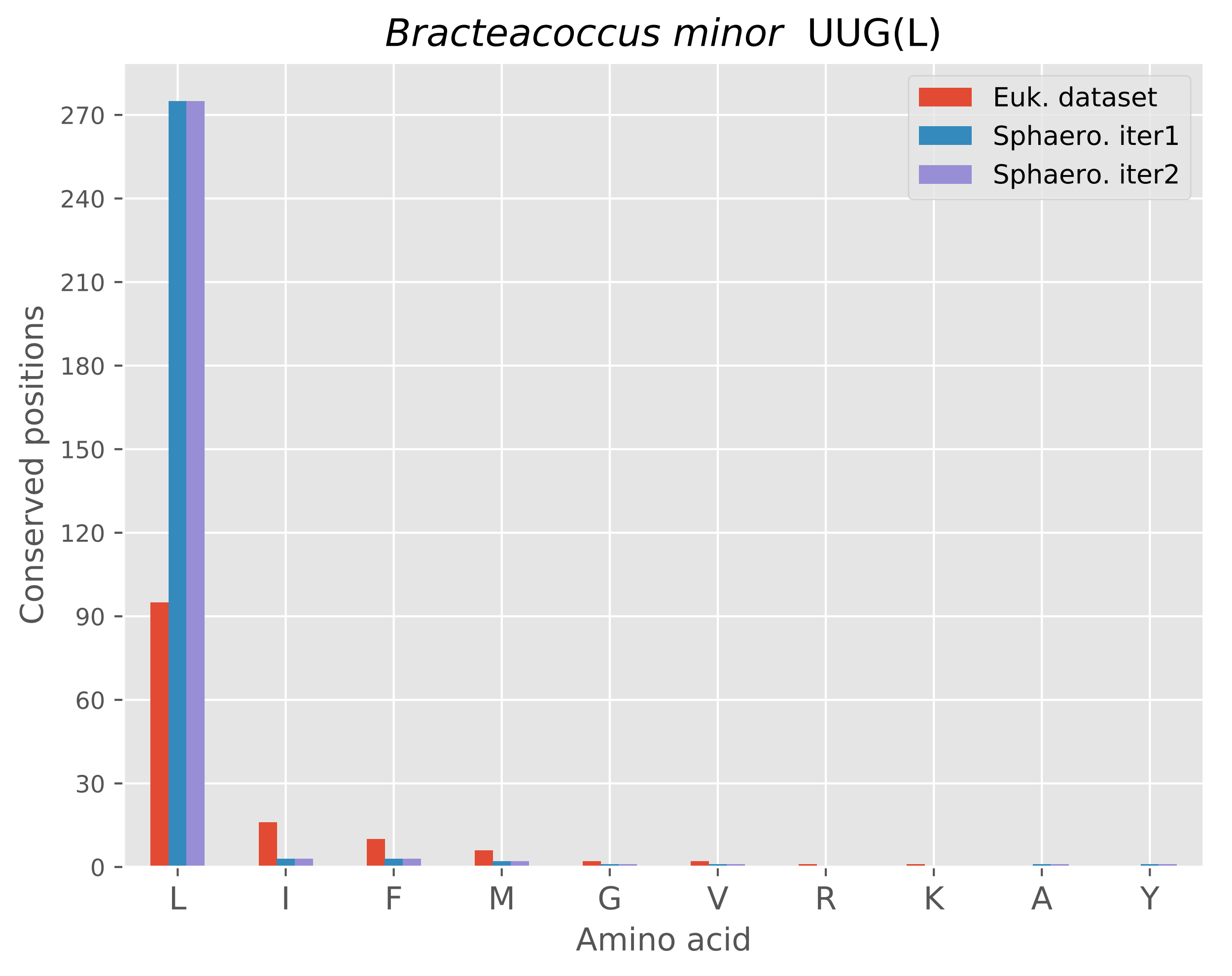
Bracteacoccus minor UUG(L)
Euk. dataset
Sphaero. iter1
Sphaero. iter2
Conserved positions
0
30
60
90
120
150
180
210
240
270
L
I
F
M
G
V
R
K
A
Y
Amino acid

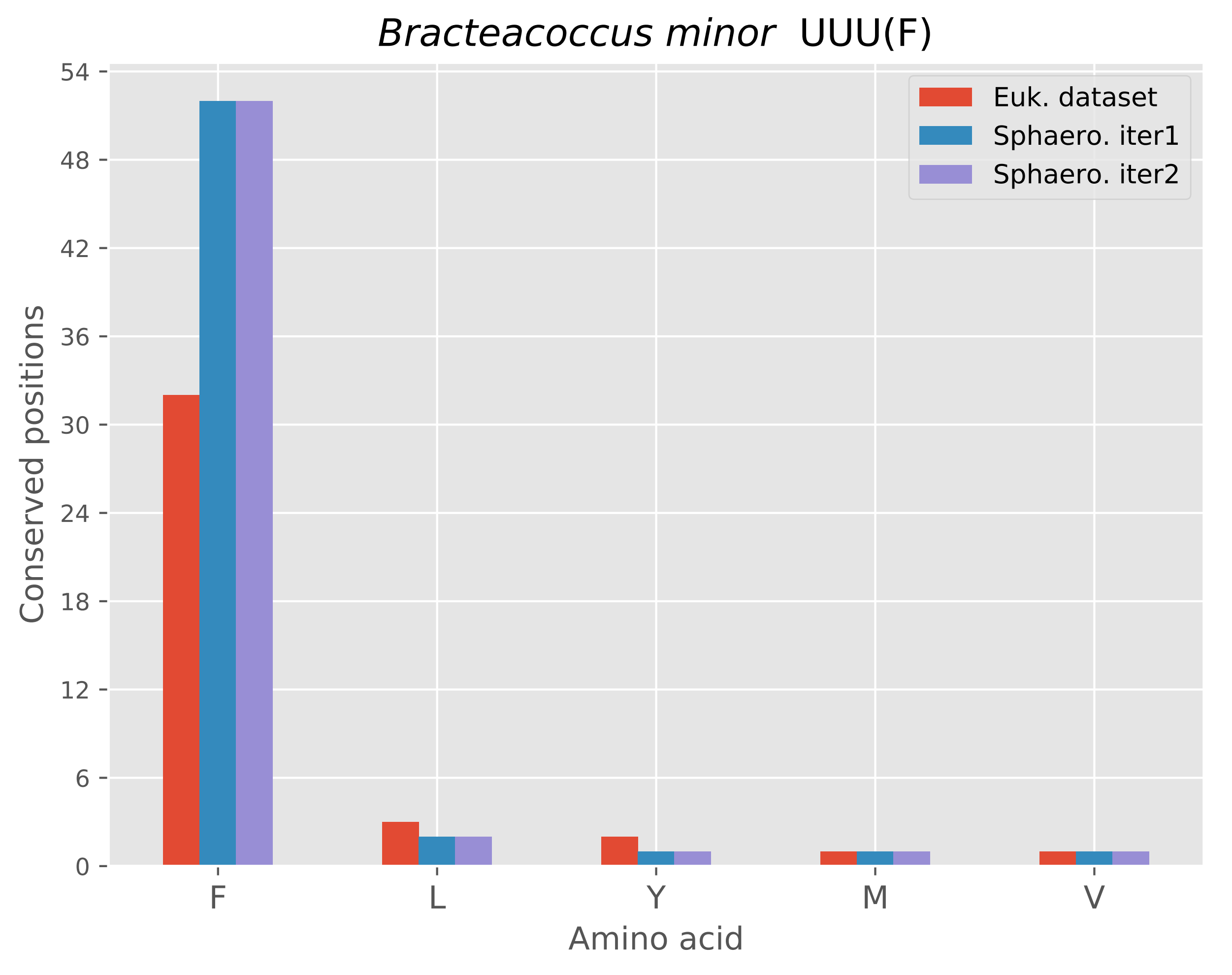

Bracteacoccus minor UUU(F)
Euk. dataset
Sphaero. iter1
Sphaero. iter2
Conserved positions
0
6
12
18
24
30
36
42
48
54
F
L
Y
M
V
Amino acid

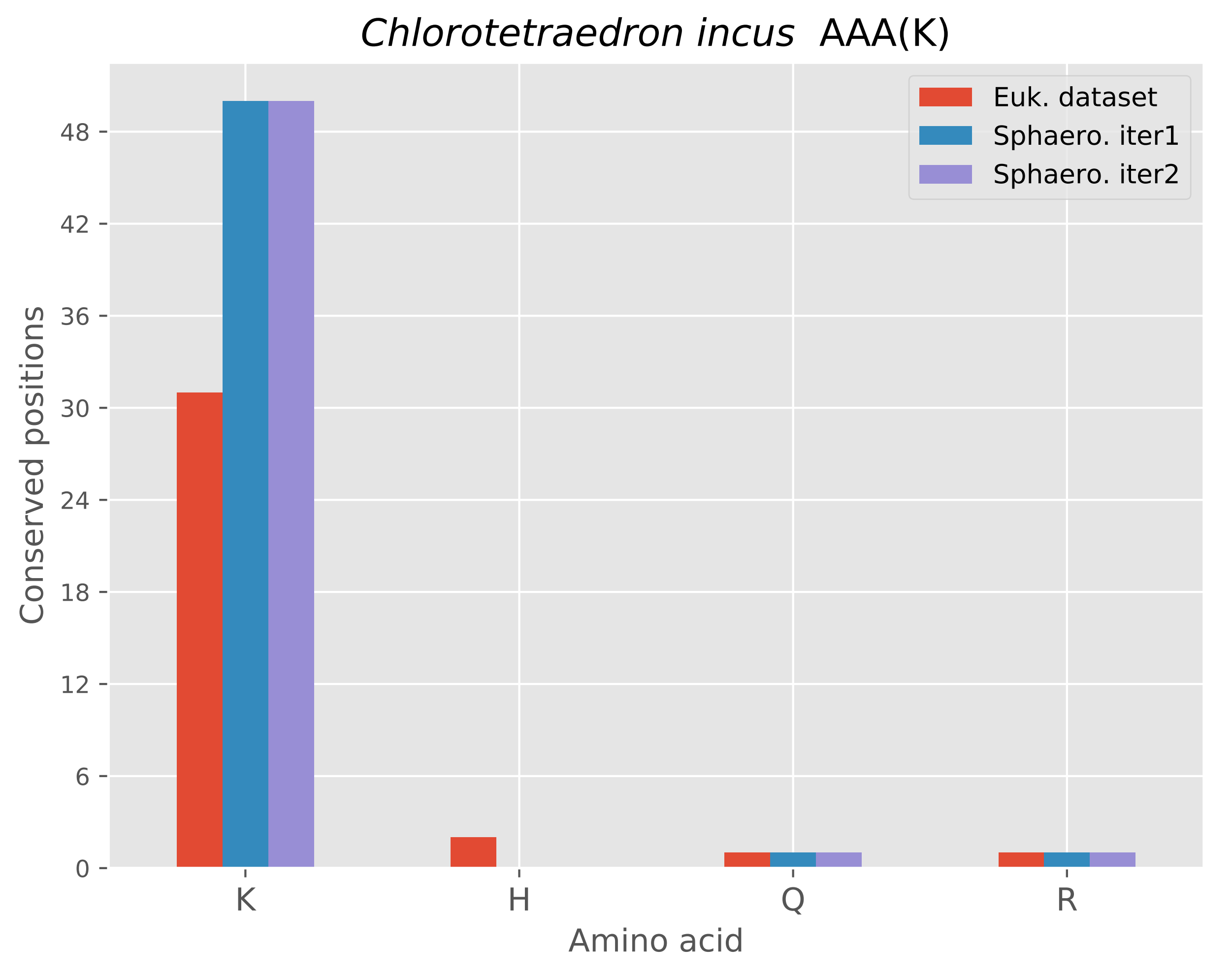

Chlorotetraedron incus AAA(K)
Euk. dataset
Sphaero. iter1
Sphaero. iter2
Conserved positions
0
6
12
18
24
30
36
42
48
K
H
Q
R
Amino acid

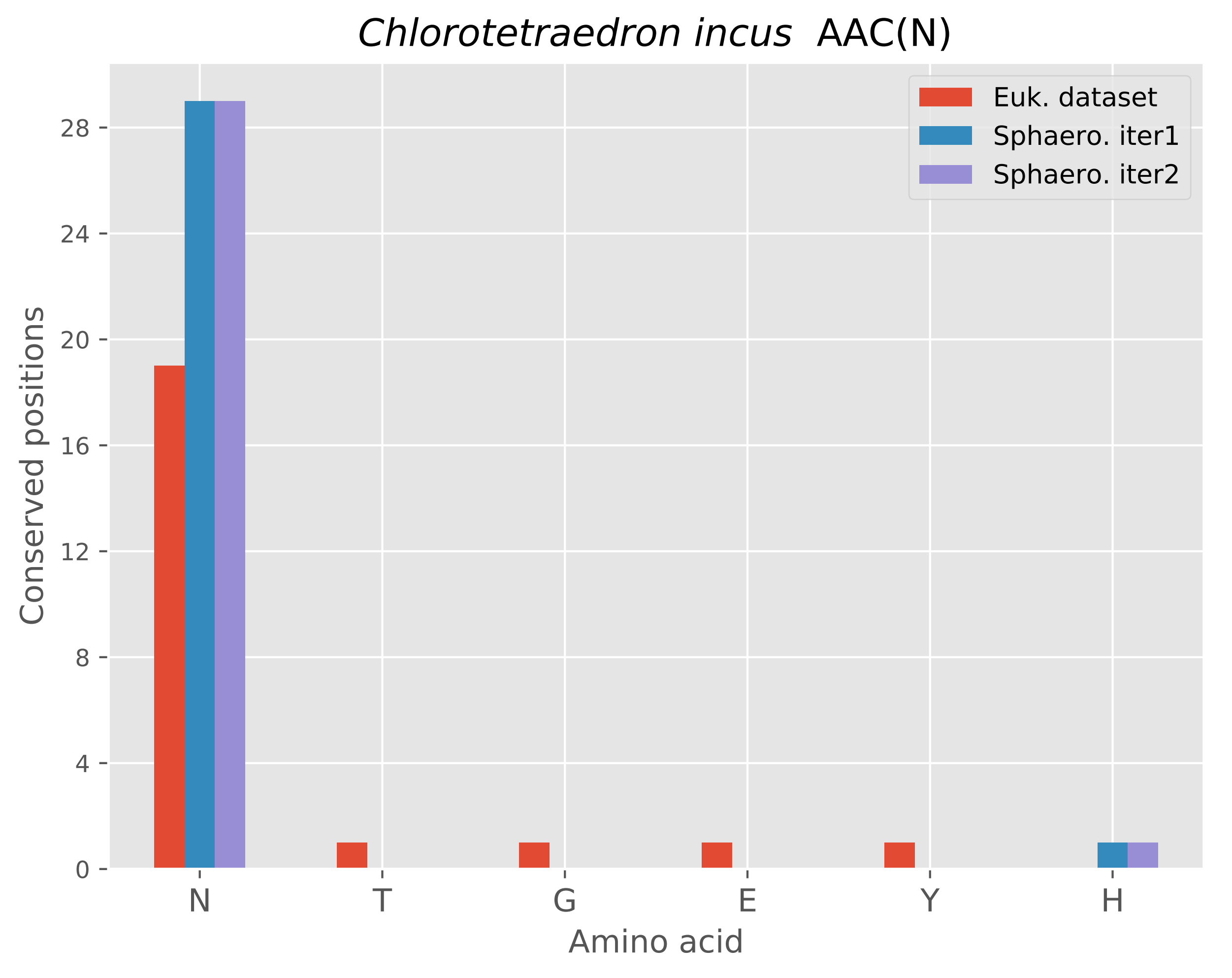

Chlorotetraedron incus AAC(N)
Euk. dataset
Sphaero. iter1
Sphaero. iter2
Conserved positions
0
4
8
12
16
20
24
28
N
T
G
E
Y
H
Amino acid

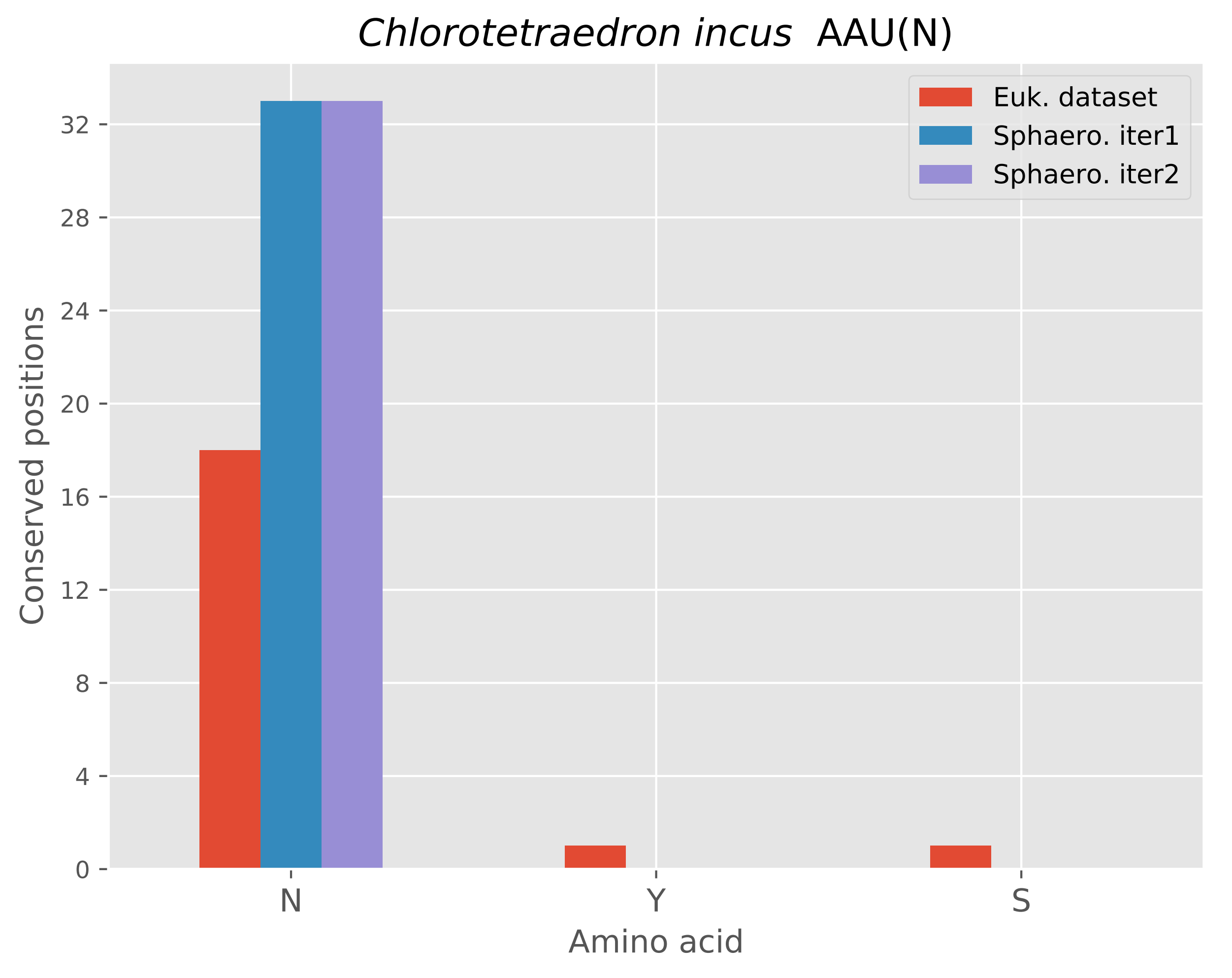

Chlorotetraedron incus AAU(N)
Euk. dataset
Sphaero. iter1
Sphaero. iter2
Conserved positions
0
4
8
12
16
20
24
28
32
N
Y
S
Amino acid

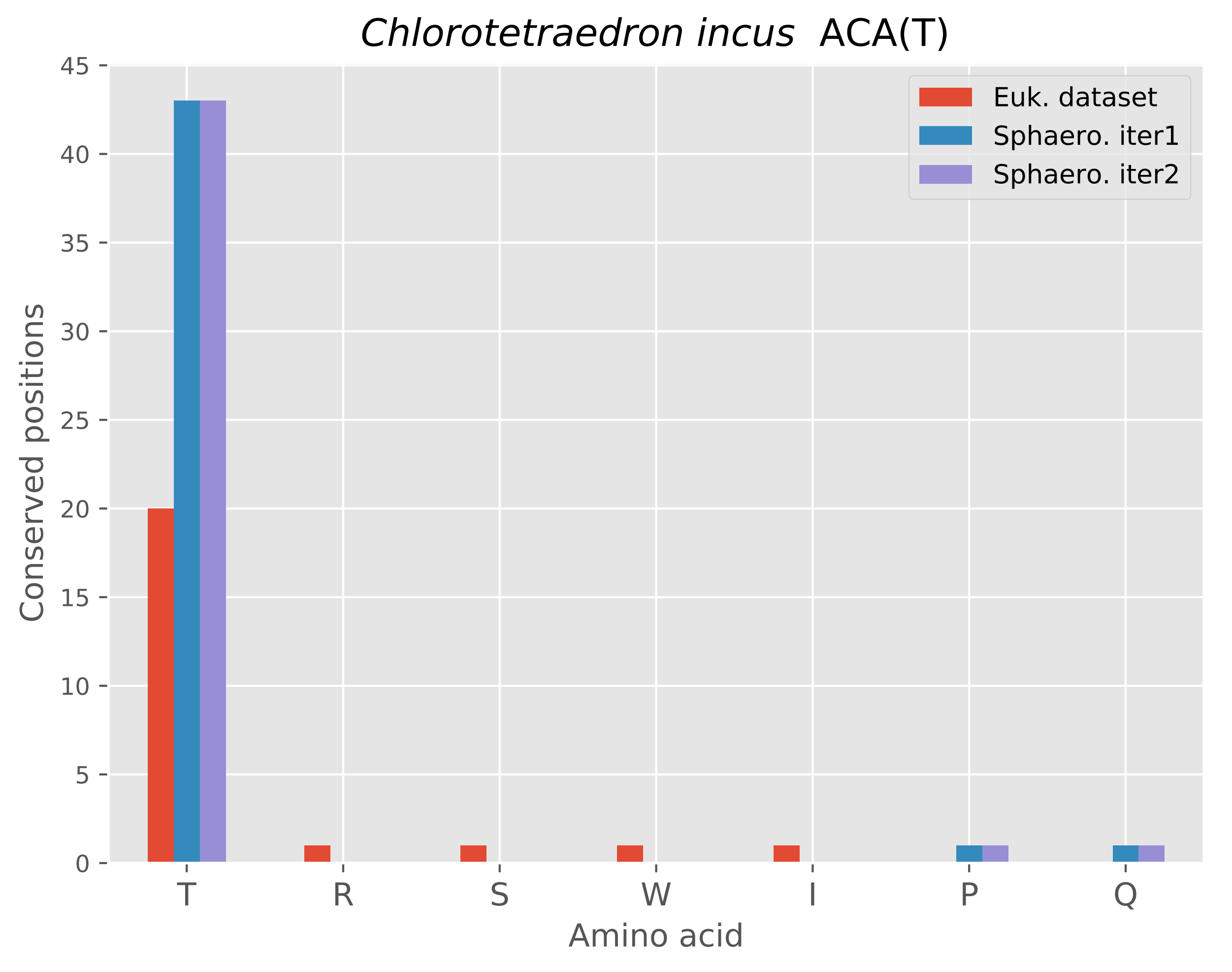
Chlorotetraedron incus ACA(T)
Euk. dataset
Sphaero. iter1
Sphaero. iter2
Conserved positions
0
5
10
15
20
25
30
35
40
45
T
R
S
W
I
P
Q
Amino acid

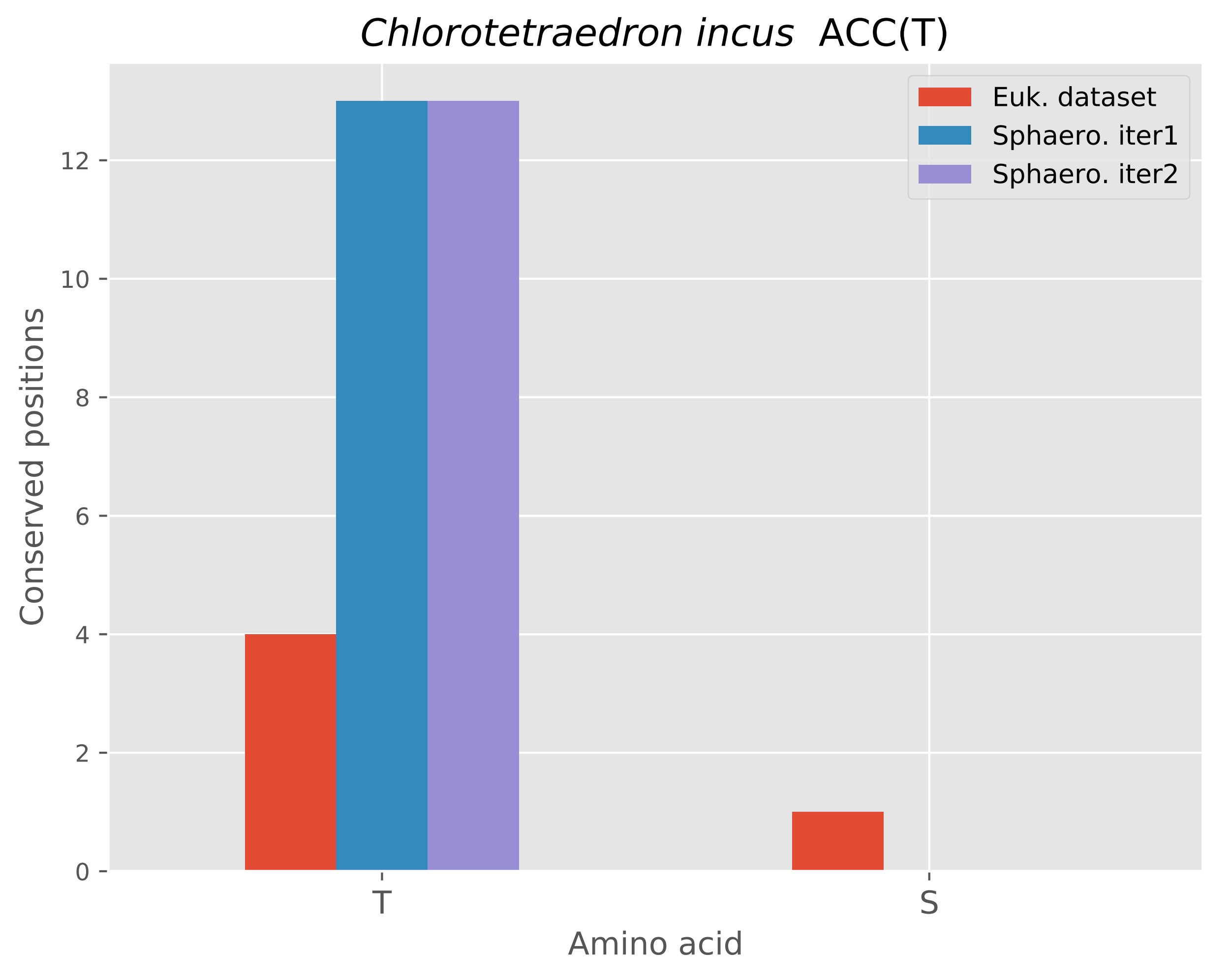
Chlorotetraedron incus ACC(T)
Euk. dataset
Sphaero. iter1
Sphaero. iter2
Conserved positions
0
2
4
6
8
10
12
T
S
Amino acid

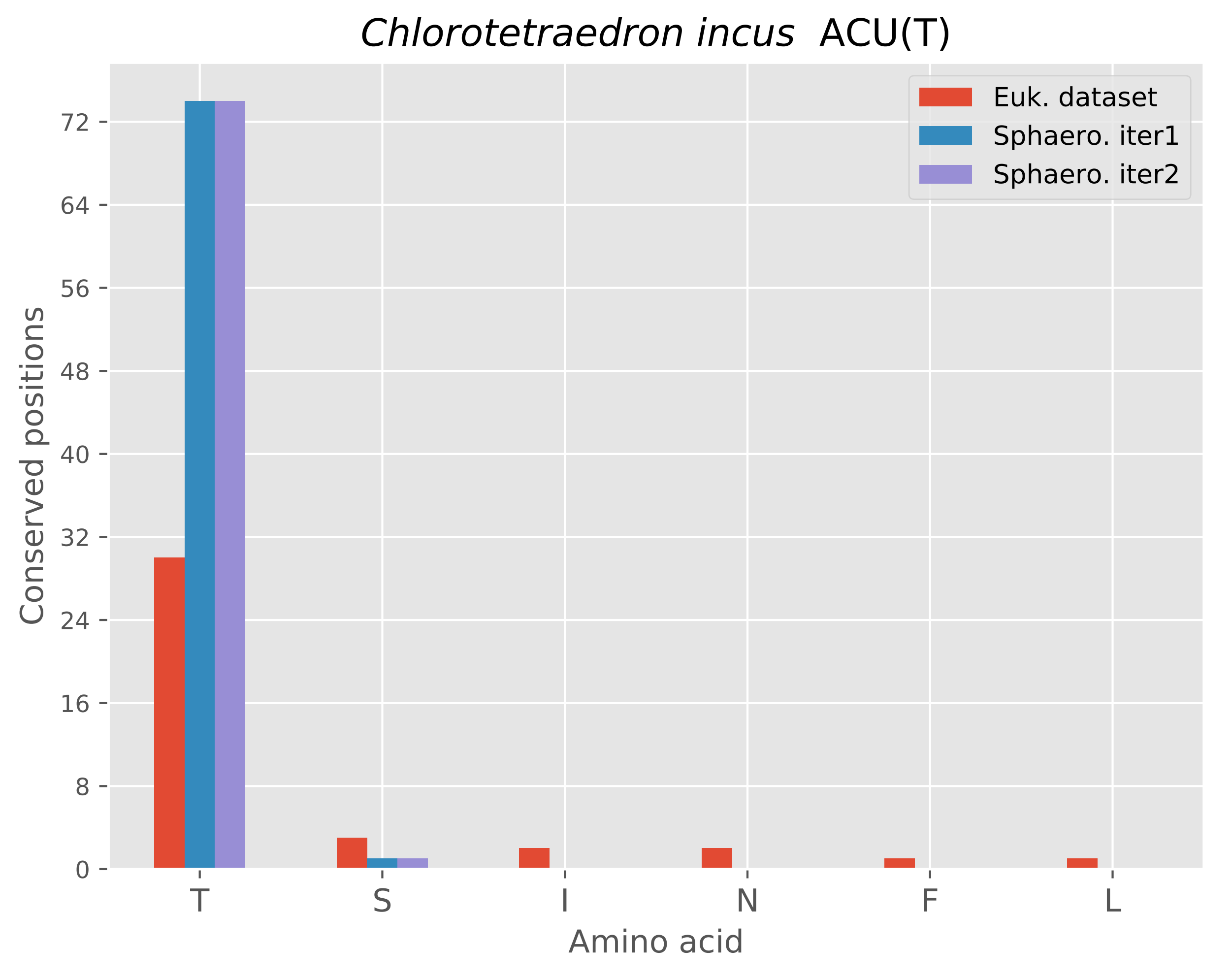

Chlorotetraedron incus ACU(T)
Euk. dataset
Sphaero. iter1
Sphaero. iter2
Conserved positions
0
8
16
24
32
40
48
56
64
72
T
S
I
N
F
L
Amino acid

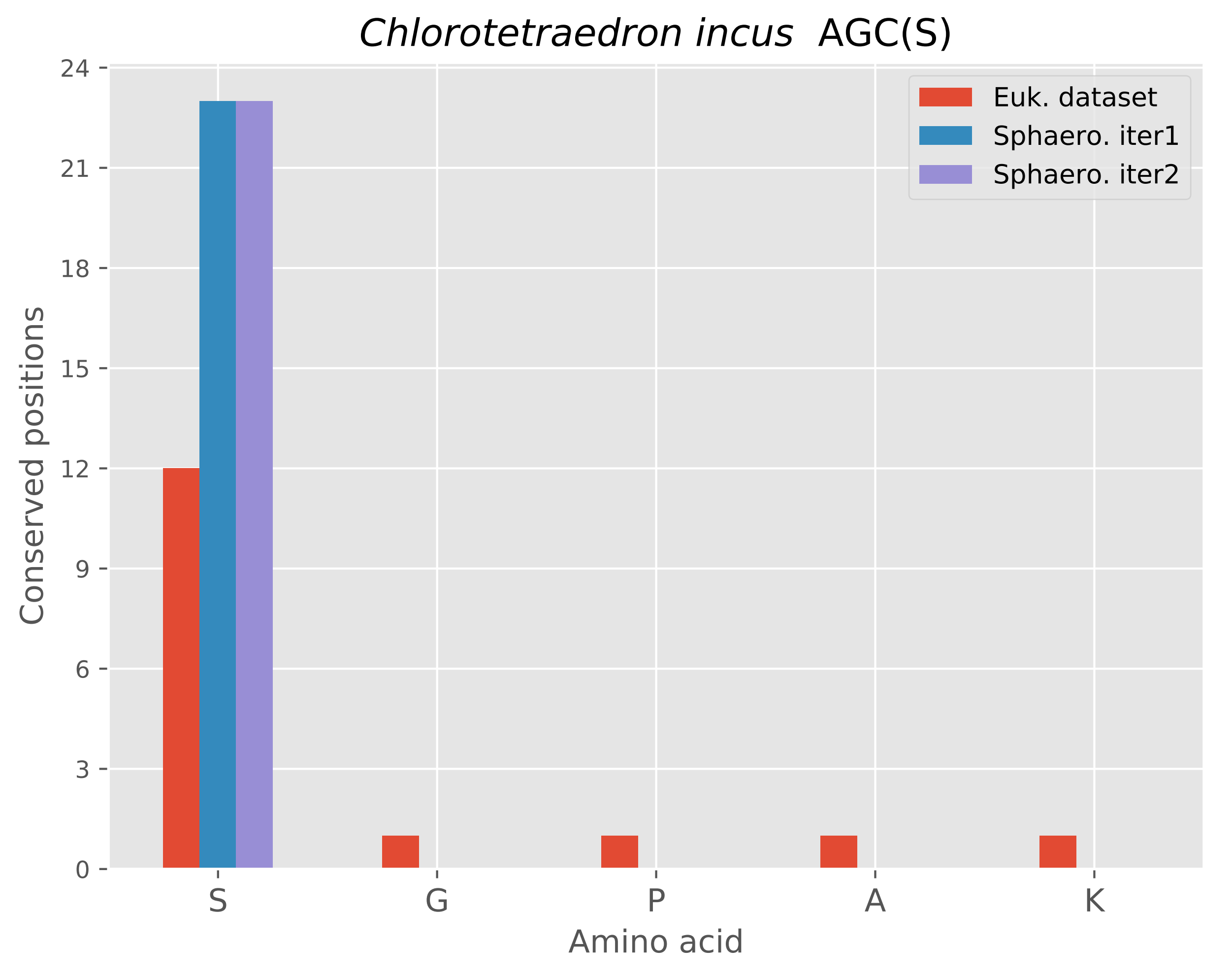

Chlorotetraedron incus AGC(S)
Euk. dataset
Sphaero. iter1
Sphaero. iter2
Conserved positions
0
3
6
9
12
15
18
21
24
S
G
P
A
K
Amino acid

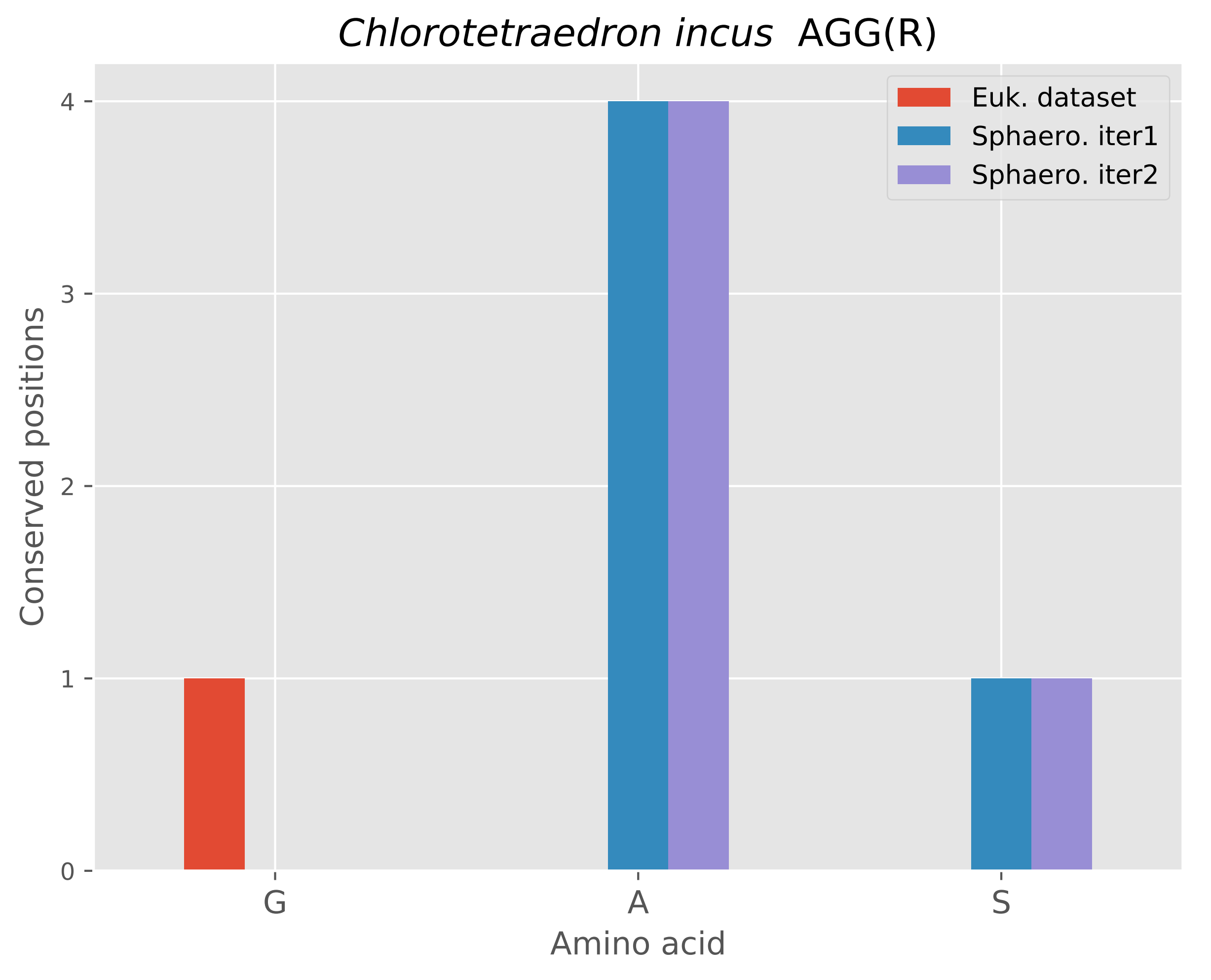
Chlorotetraedron incus AGG(R)
Euk. dataset
Sphaero. iter1
Sphaero. iter2
Conserved positions
0
1
2
3
4
G
A
S
Amino acid

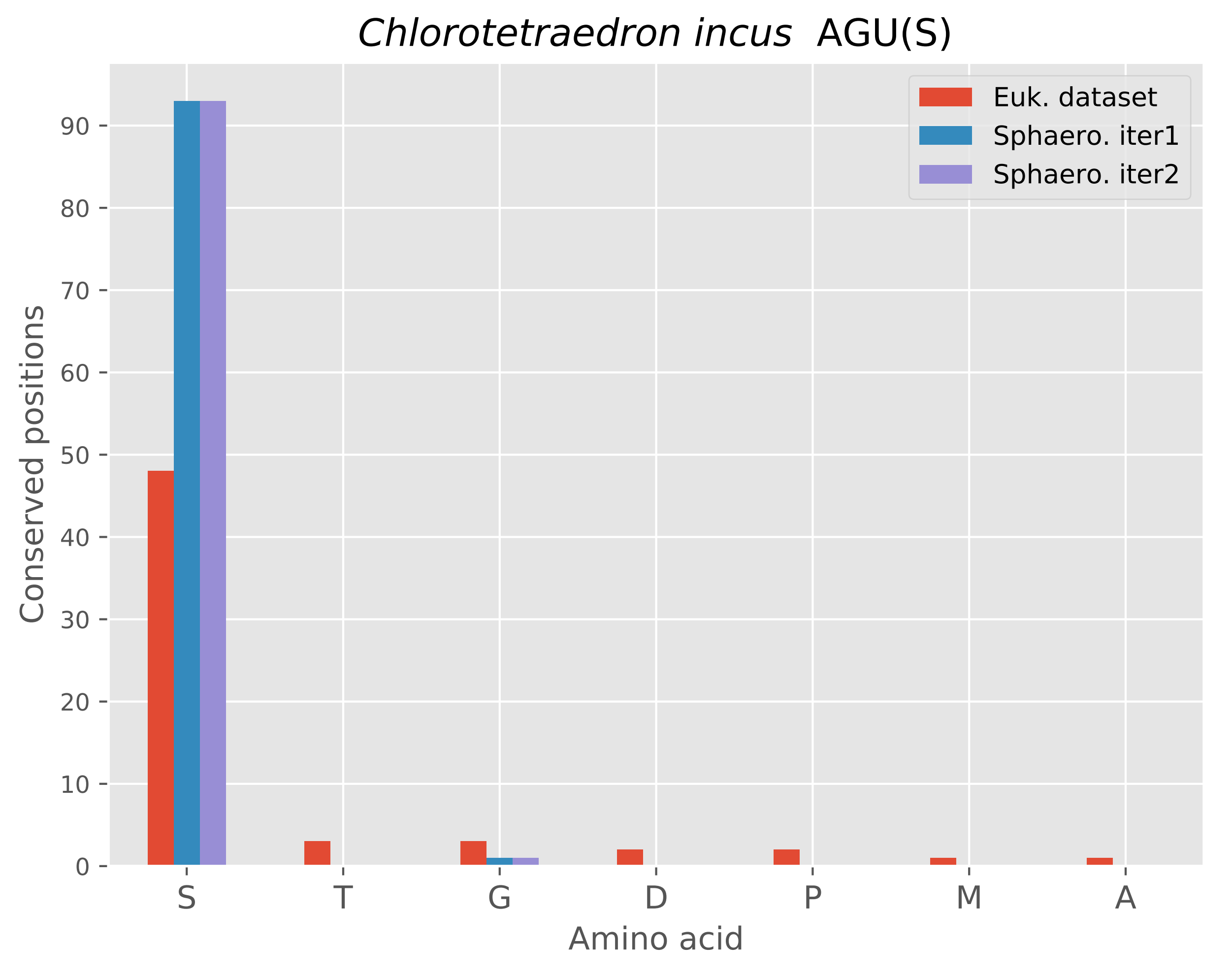

Chlorotetraedron incus AGU(S)
Euk. dataset
Sphaero. iter1
Sphaero. iter2
Conserved positions
0
10
20
30
40
50
60
70
80
90
S
T
G
D
P
M
A
Amino acid

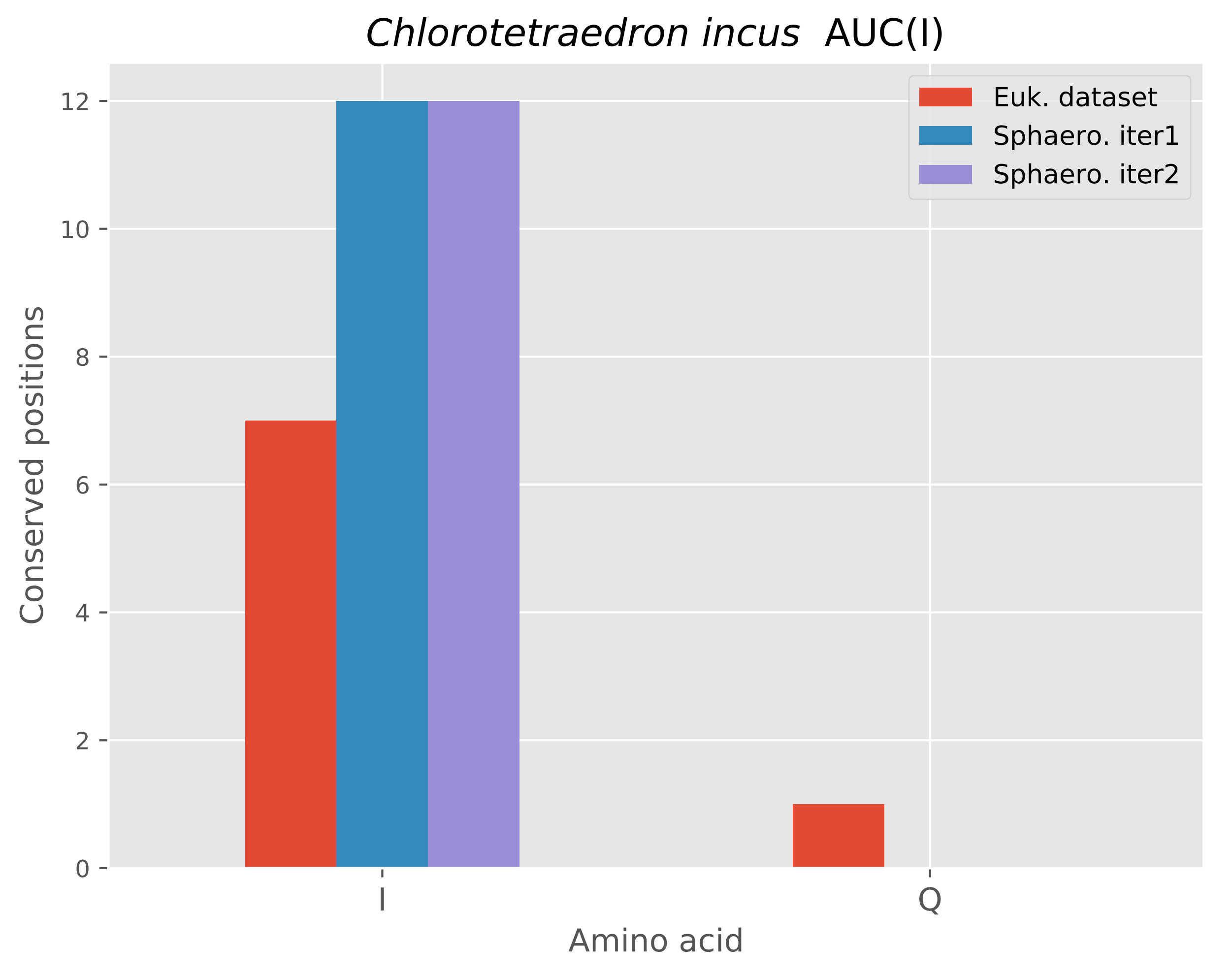

Chlorotetraedron incus AUC(I)
Euk. dataset
Sphaero. iter1
Sphaero. iter2
Conserved positions
0
2
4
6
8
10
12
I
Q
Amino acid

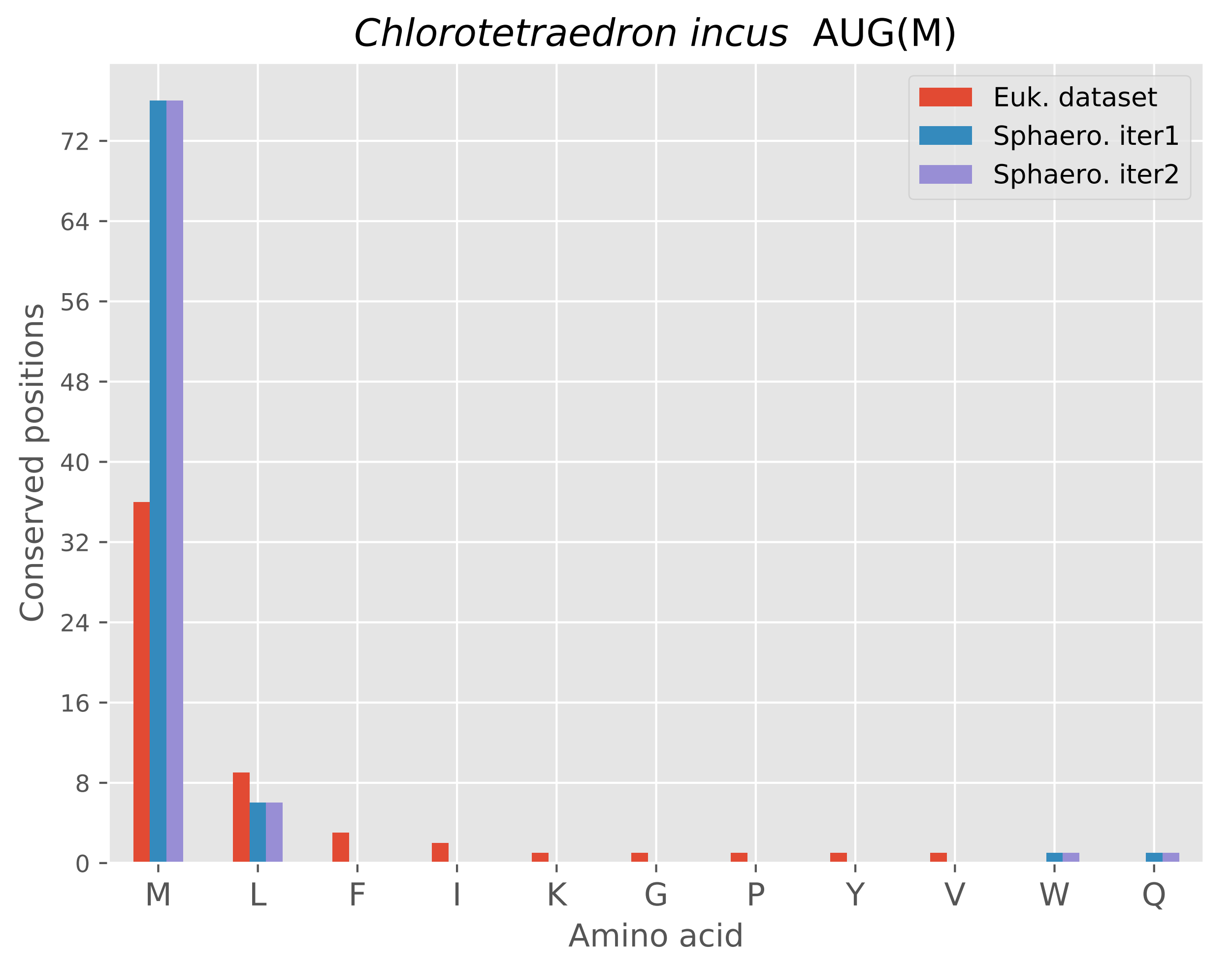
Chlorotetraedron incus AUG(M)
Euk. dataset
Sphaero. iter1
Sphaero. iter2
Conserved positions
0
8
16
24
32
40
48
56
64
72
M
L
F
I
K
G
P
Y
V
W
Q
Amino acid

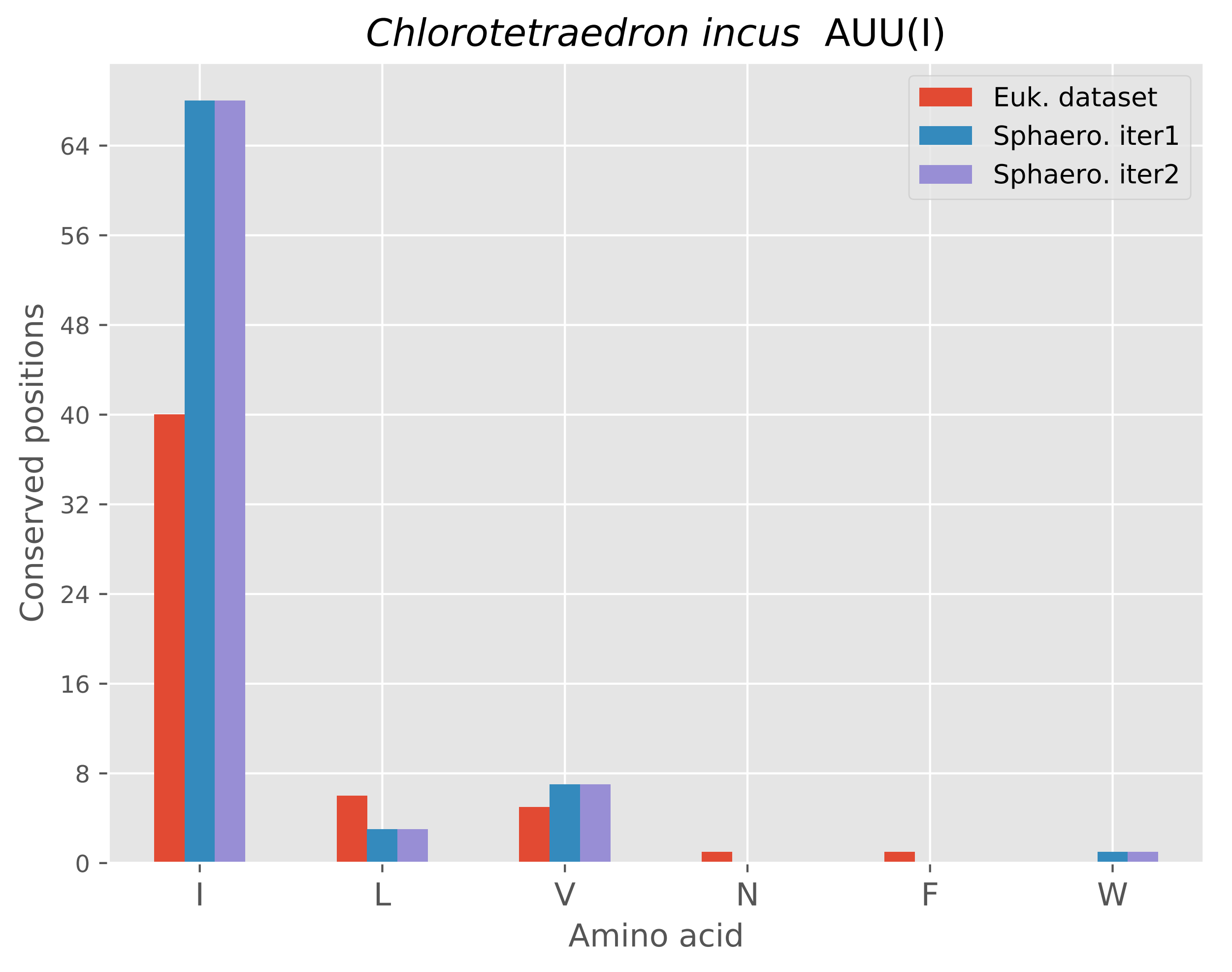

Chlorotetraedron incus AUU(I)
Euk. dataset
Sphaero. iter1
Sphaero. iter2
Conserved positions
0
8
16
24
32
40
48
56
64
I
L
V
N
F
W
Amino acid

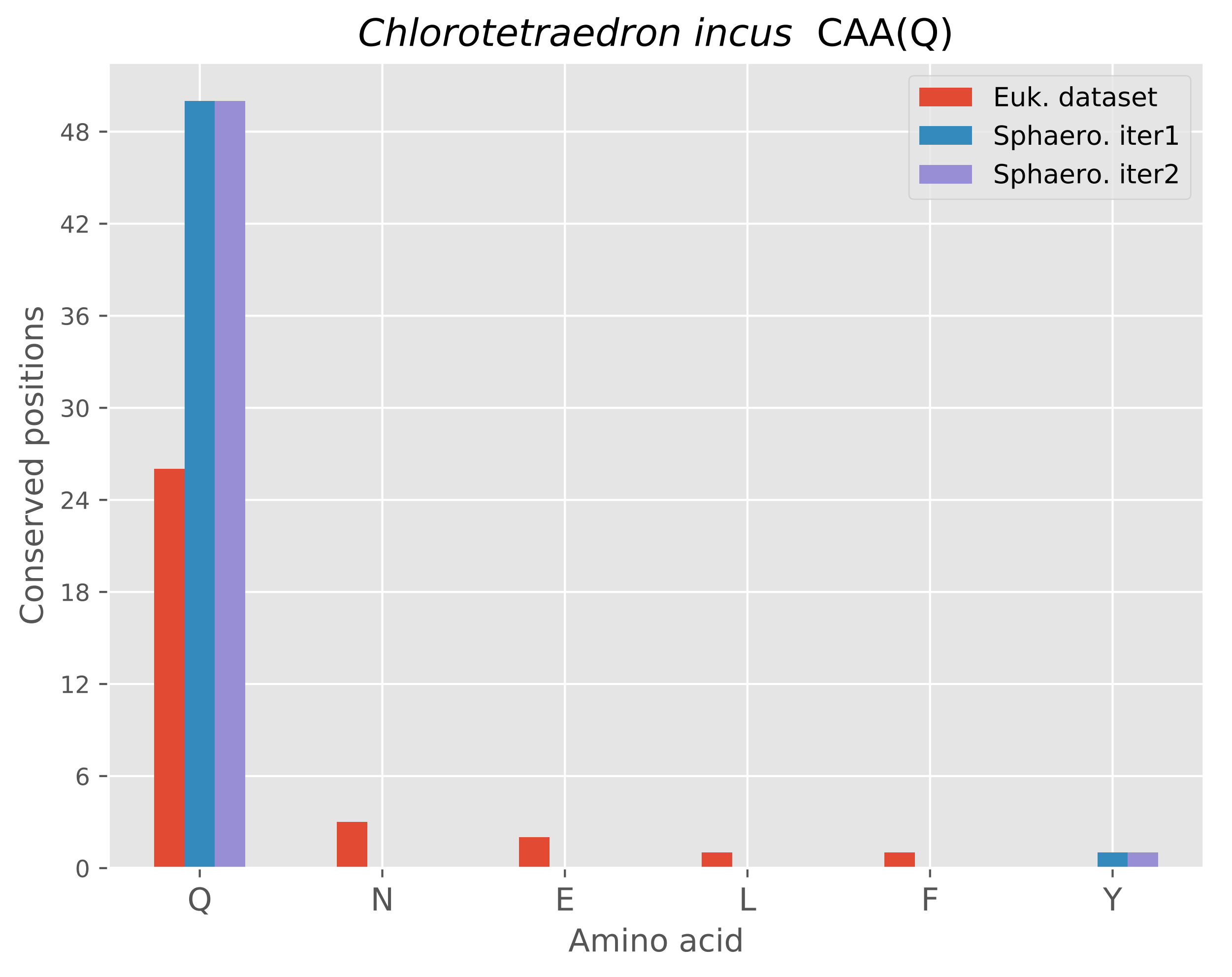

Chlorotetraedron incus CAA(Q)
Euk. dataset
Sphaero. iter1
Sphaero. iter2
Conserved positions
0
6
12
18
24
30
36
42
48
Q
N
E
L
F
Y
Amino acid

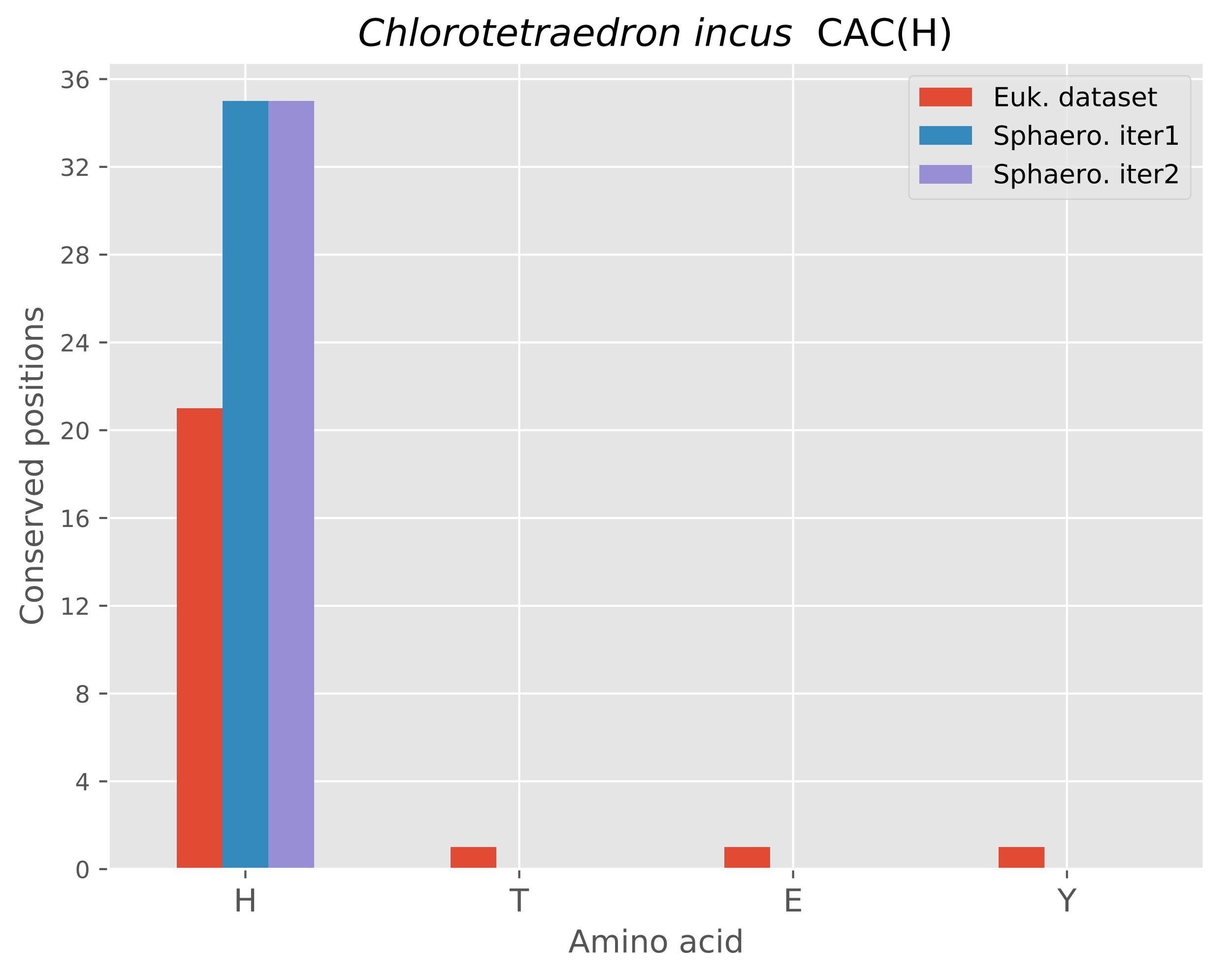
Chlorotetraedron incus CAC(H)
Euk. dataset
Sphaero. iter1
Sphaero. iter2
Conserved positions
0
4
8
12
16
20
24
28
32
36
H
T
E
Y
Amino acid

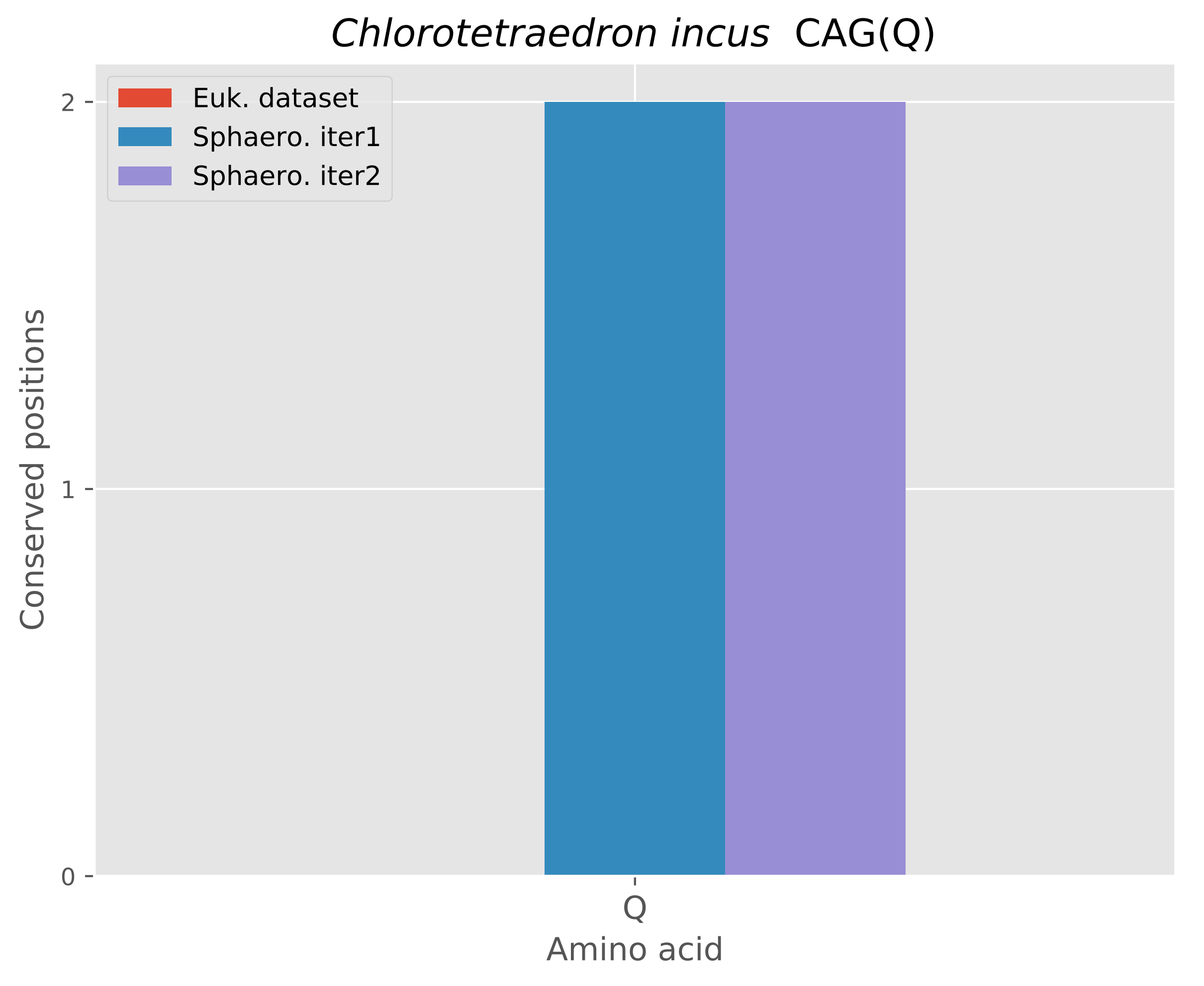
Chlorotetraedron incus CAG(Q)
Euk. dataset
Sphaero. iter1
Sphaero. iter2
Conserved positions
0
1
2
Q
Amino acid

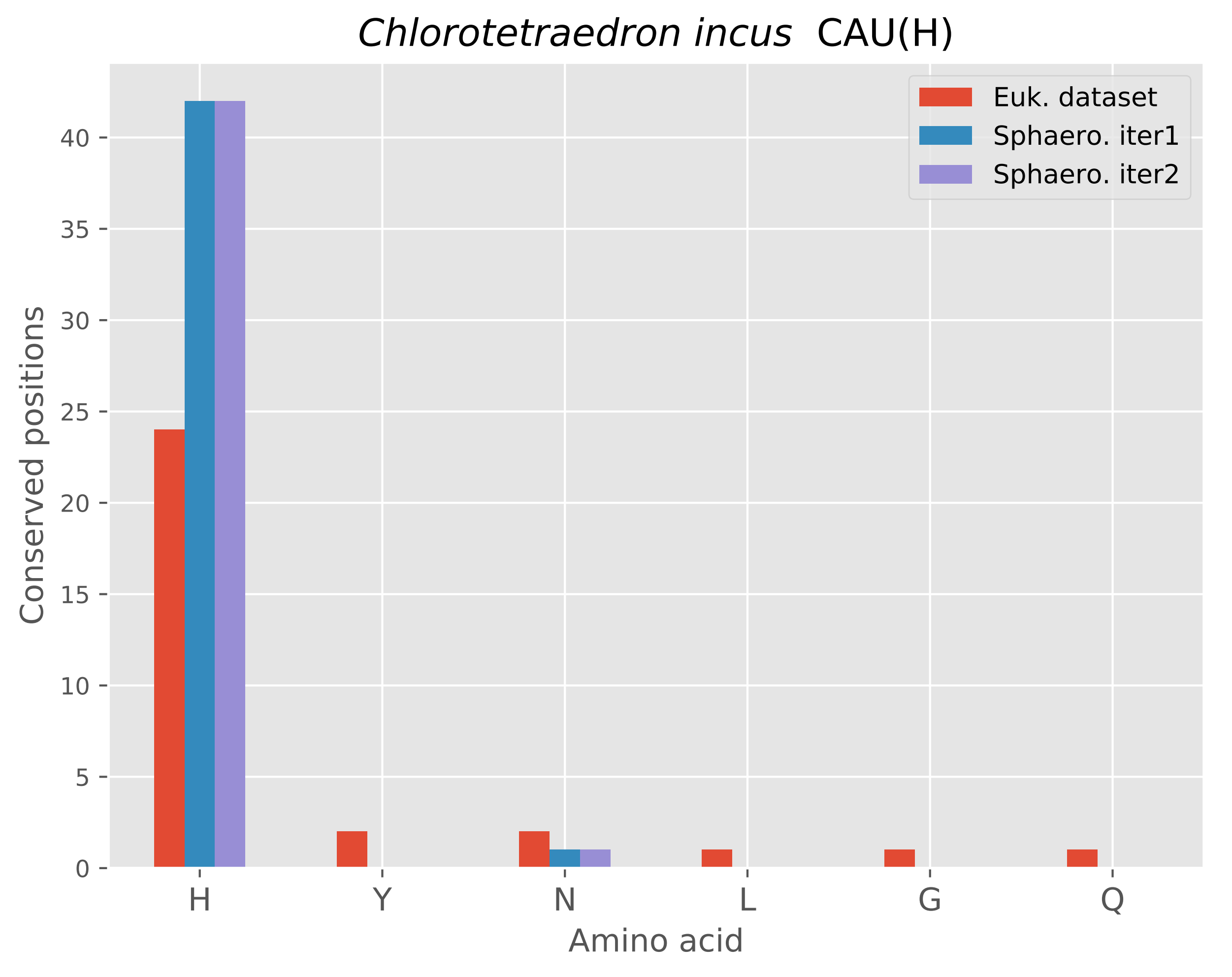

Chlorotetraedron incus CAU(H)
Euk. dataset
Sphaero. iter1
Sphaero. iter2
Conserved positions
0
5
10
15
20
25
30
35
40
H
Y
N
L
G
Q
Amino acid

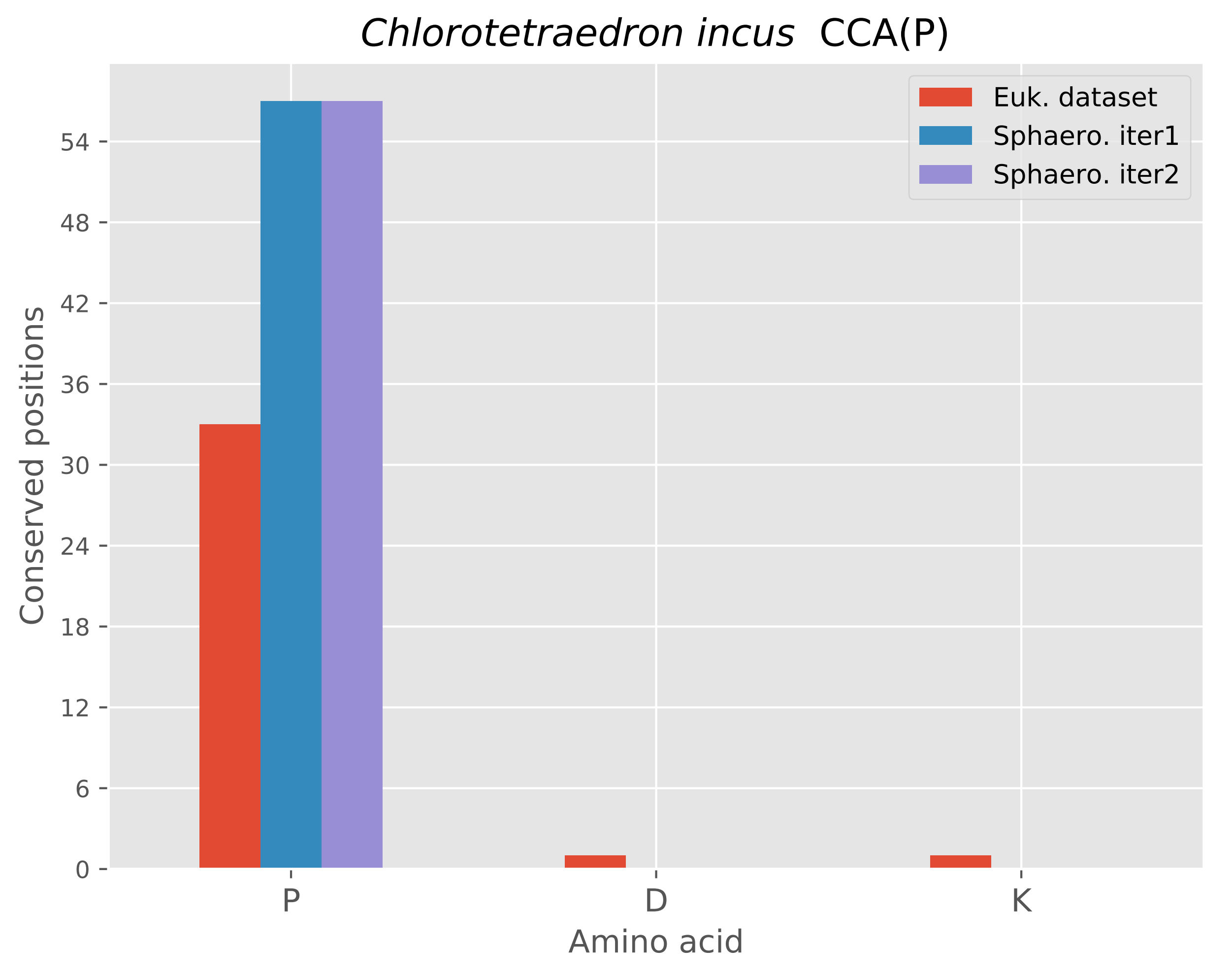

Chlorotetraedron incus CCA(P)
Euk. dataset
Sphaero. iter1
Sphaero. iter2
Conserved positions
0
6
12
18
24
30
36
42
48
54
P
D
K
Amino acid

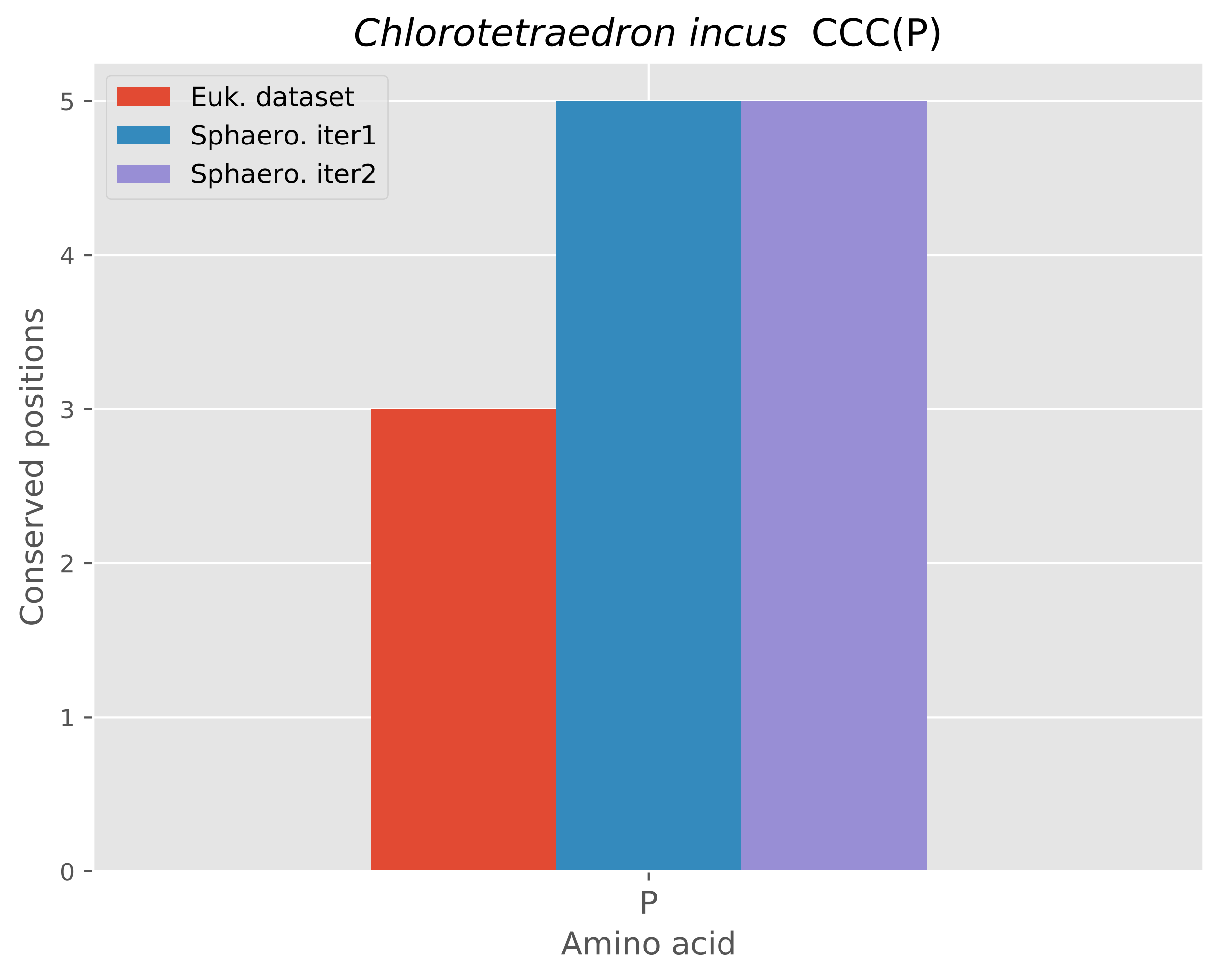

Chlorotetraedron incus CCC(P)
Euk. dataset
Sphaero. iter1
Sphaero. iter2
Conserved positions
0
1
2
3
4
5
P
Amino acid

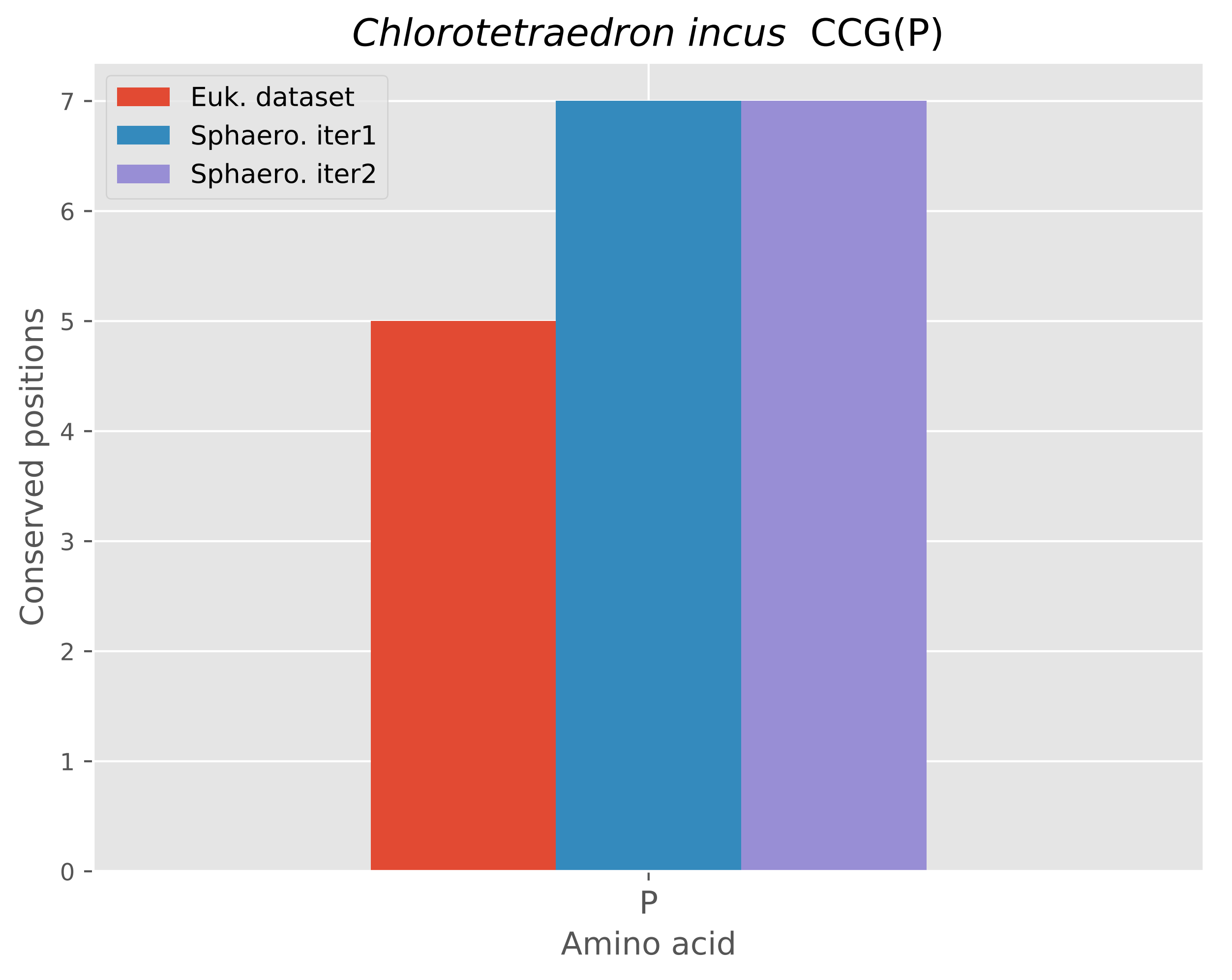

Chlorotetraedron incus CCG(P)
Euk. dataset
Sphaero. iter1
Sphaero. iter2
Conserved positions
0
1
2
3
4
5
6
7
P
Amino acid

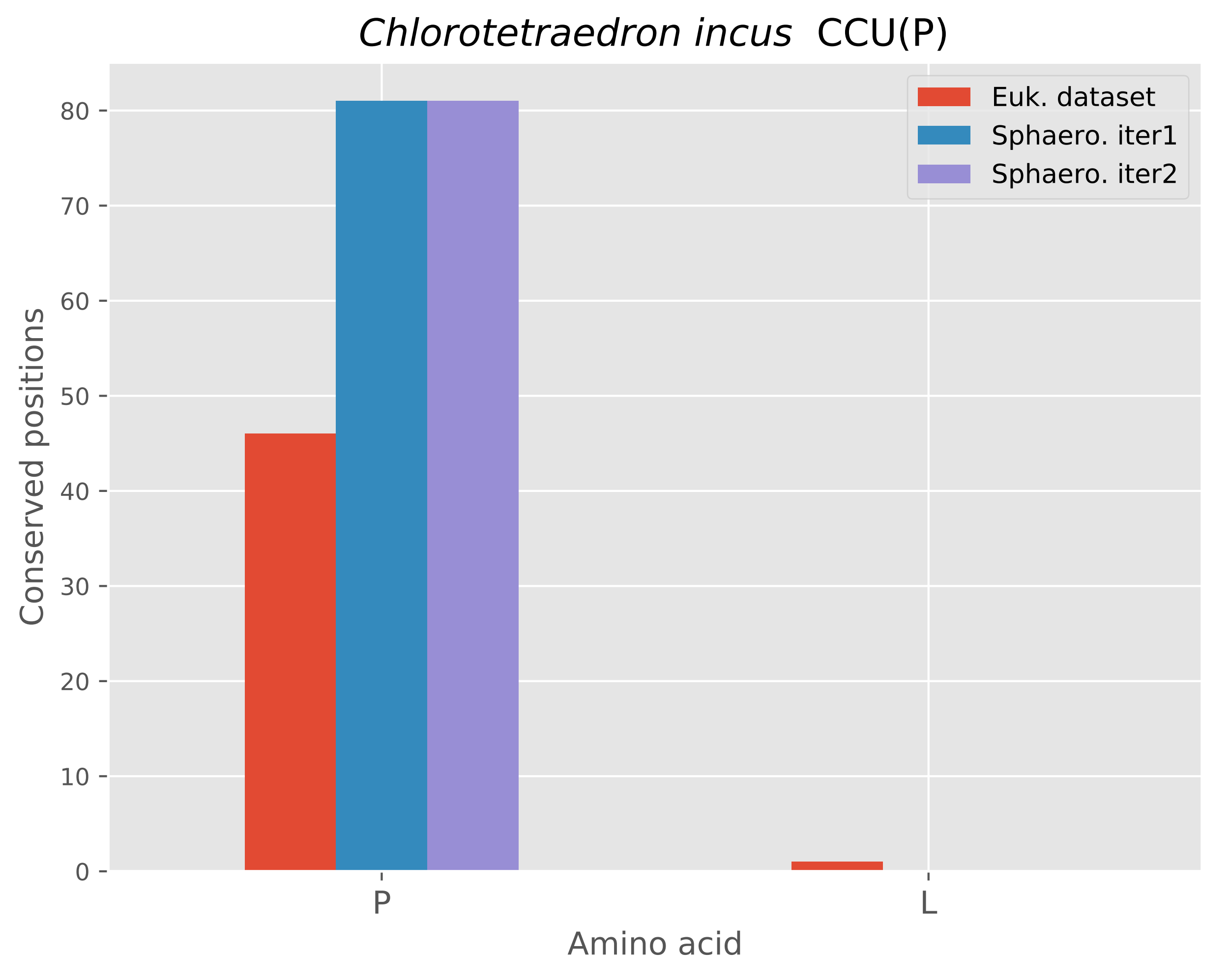
Chlorotetraedron incus CCU(P)
Euk. dataset
Sphaero. iter1
Sphaero. iter2
Conserved positions
0
10
20
30
40
50
60
70
80
P
L
Amino acid

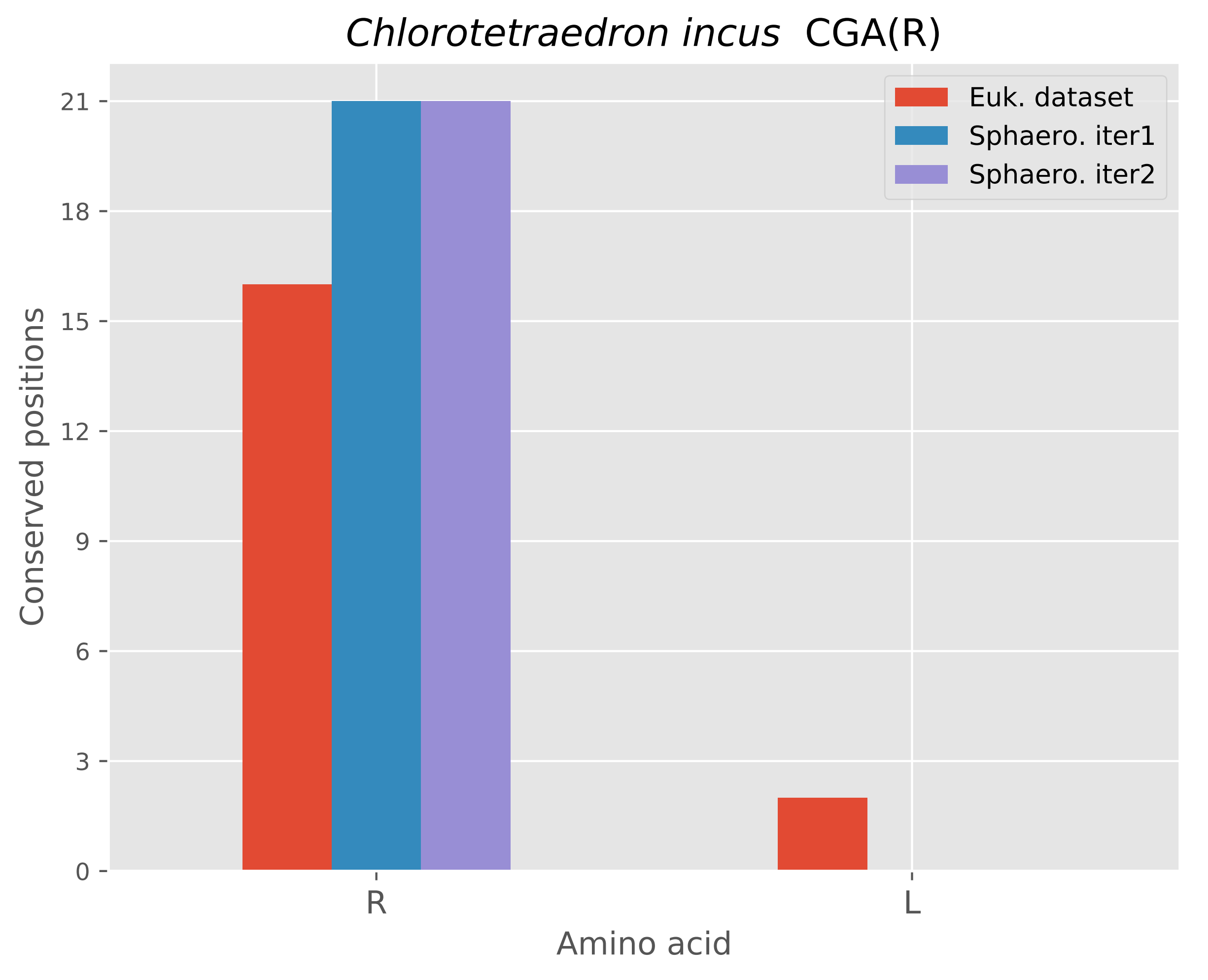

Chlorotetraedron incus CGA(R)
Euk. dataset
Sphaero. iter1
Sphaero. iter2
Conserved positions
0
3
6
9
12
15
18
21
R
L
Amino acid

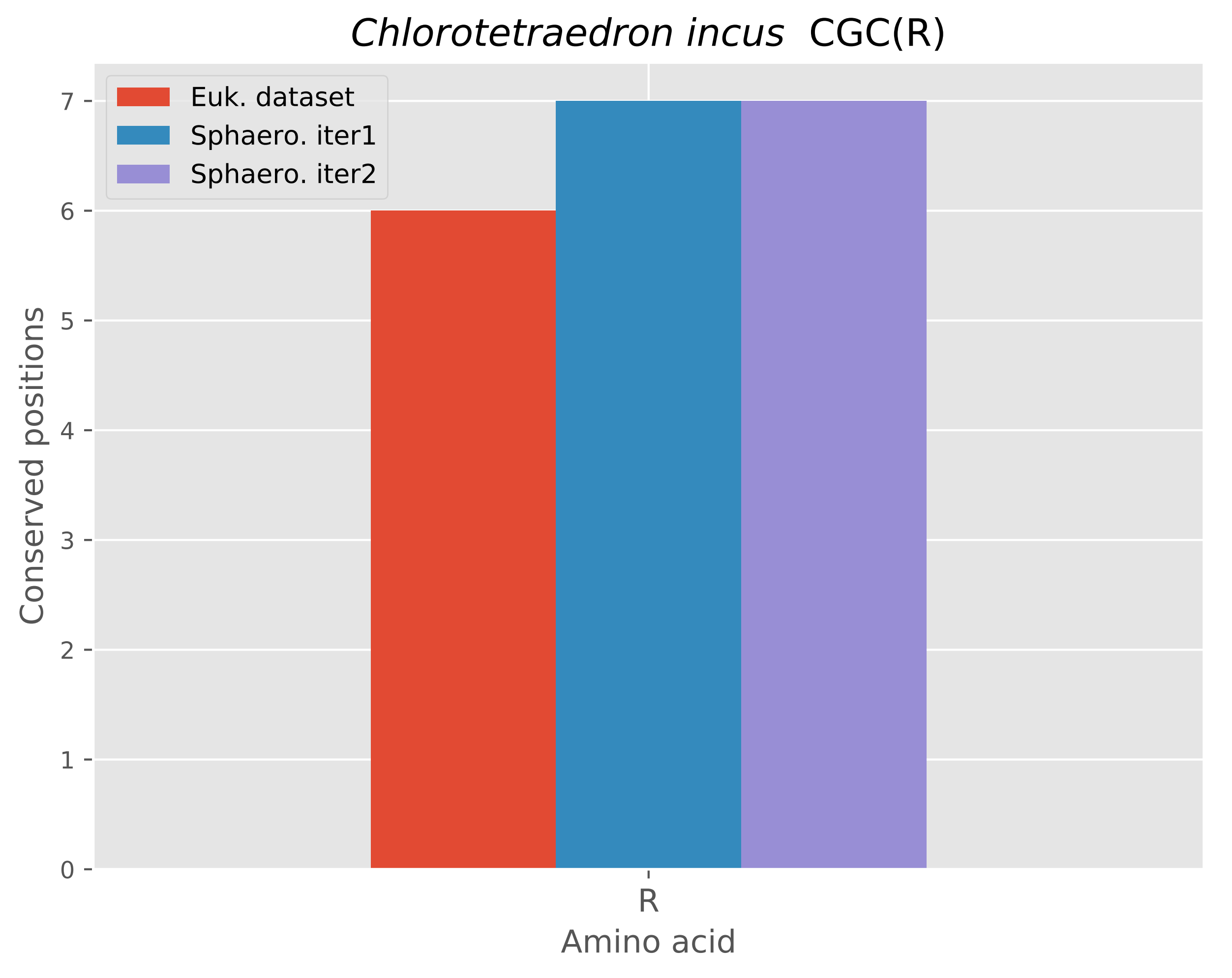

Chlorotetraedron incus CGC(R)
Euk. dataset
Sphaero. iter1
Sphaero. iter2
Conserved positions
0
1
2
3
4
5
6
7
R
Amino acid

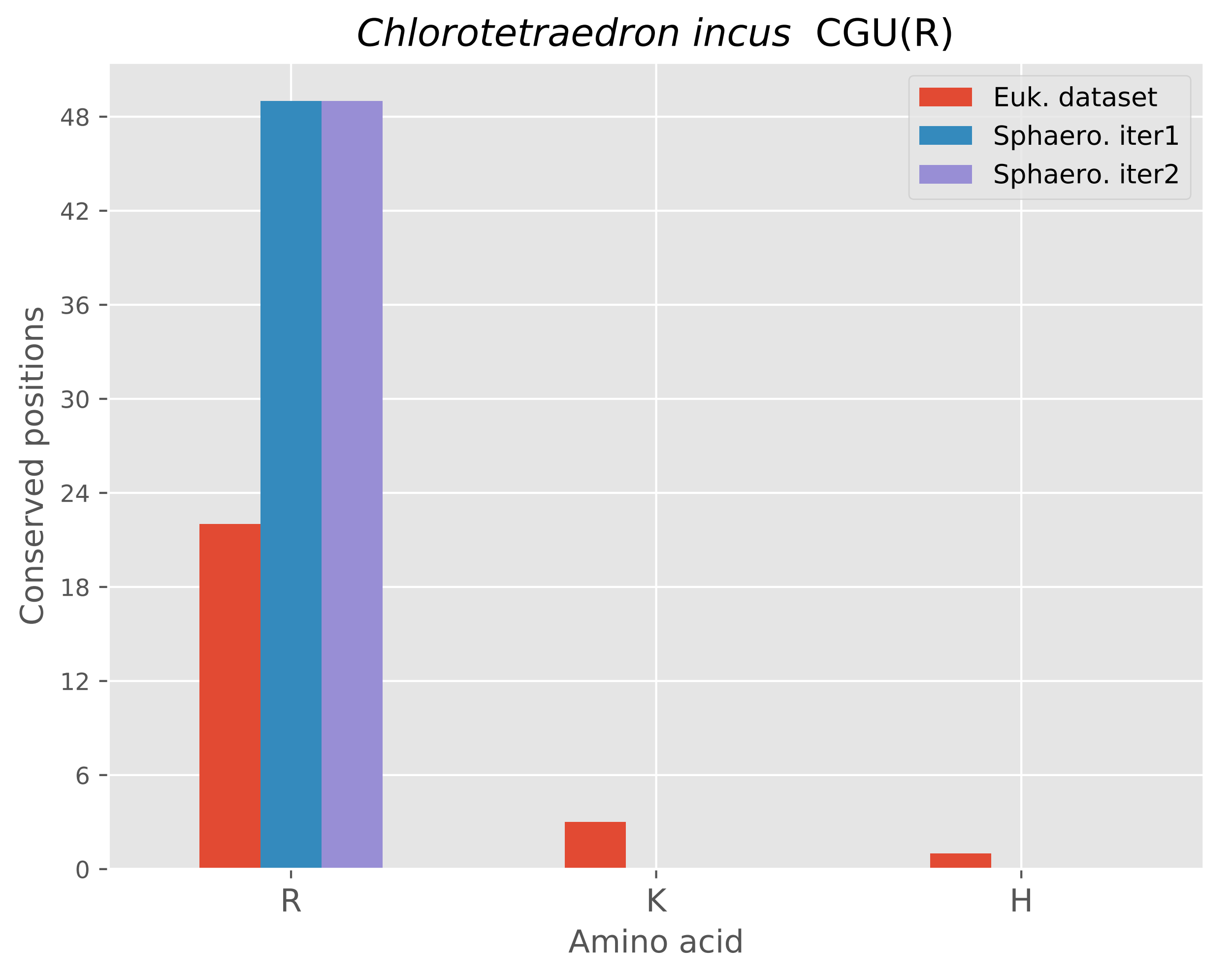

Chlorotetraedron incus CGU(R)
Euk. dataset
Sphaero. iter1
Sphaero. iter2
Conserved positions
48
42
36
30
24
18
12
6
0
R
K
H
Amino acid

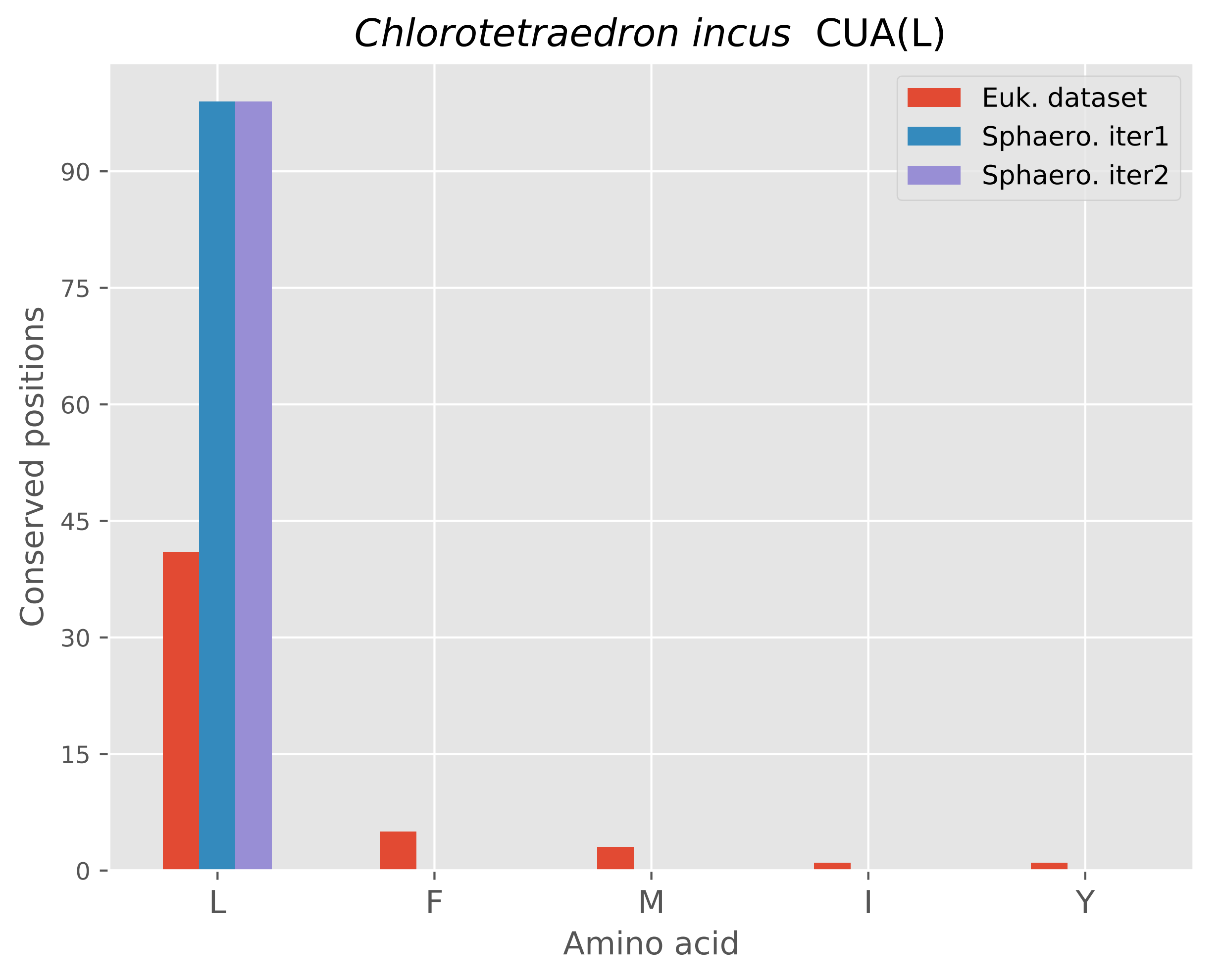

Chlorotetraedron incus CUA(L)
Euk. dataset
Sphaero. iter1
Sphaero. iter2
Conserved positions
0
15
30
45
60
75
90
L
F
M
I
Y
Amino acid

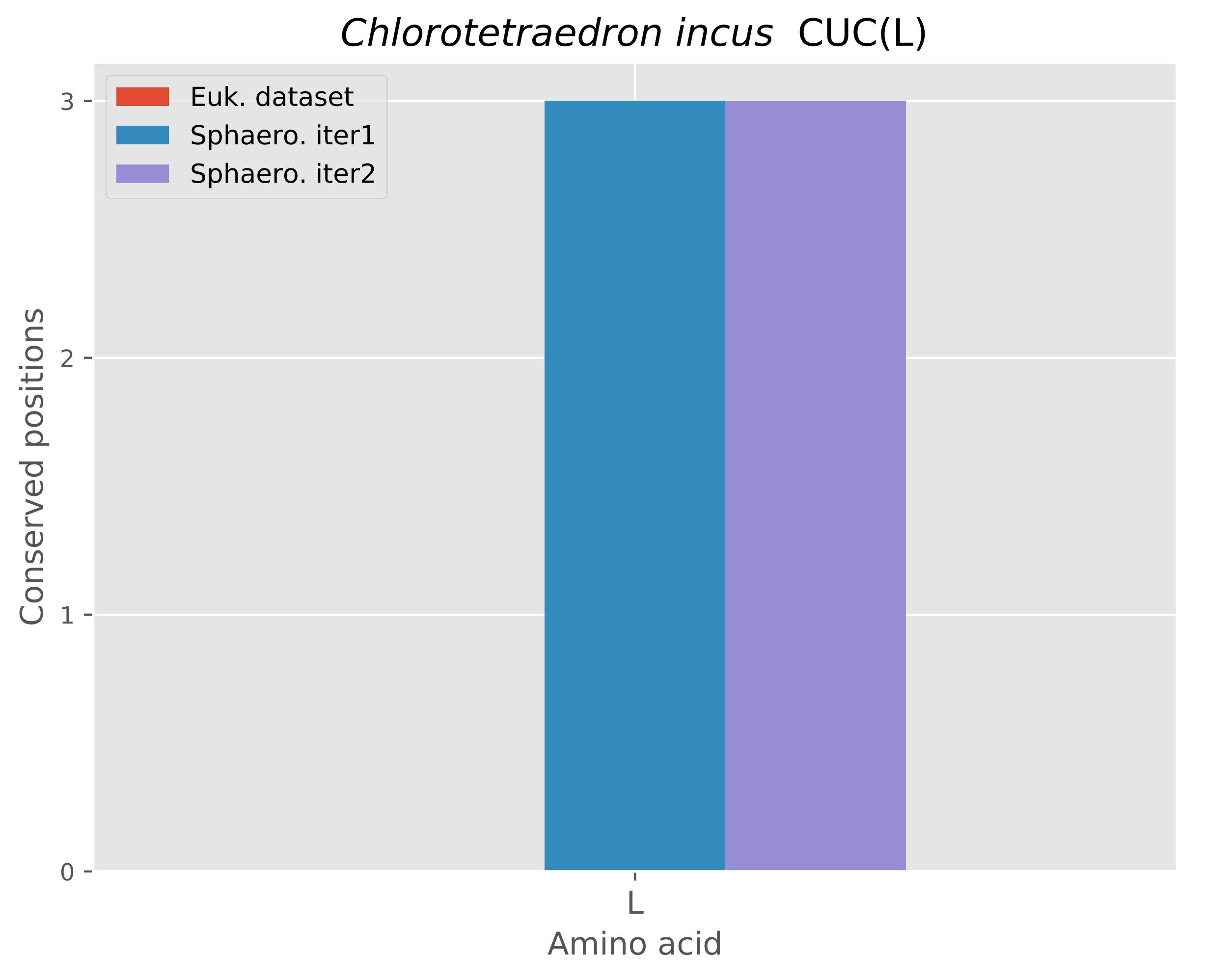

Chlorotetraedron incus CUC(L)
Euk. dataset
Sphaero. iter1
Sphaero. iter2
Conserved positions
0
1
2
3
L
Amino acid

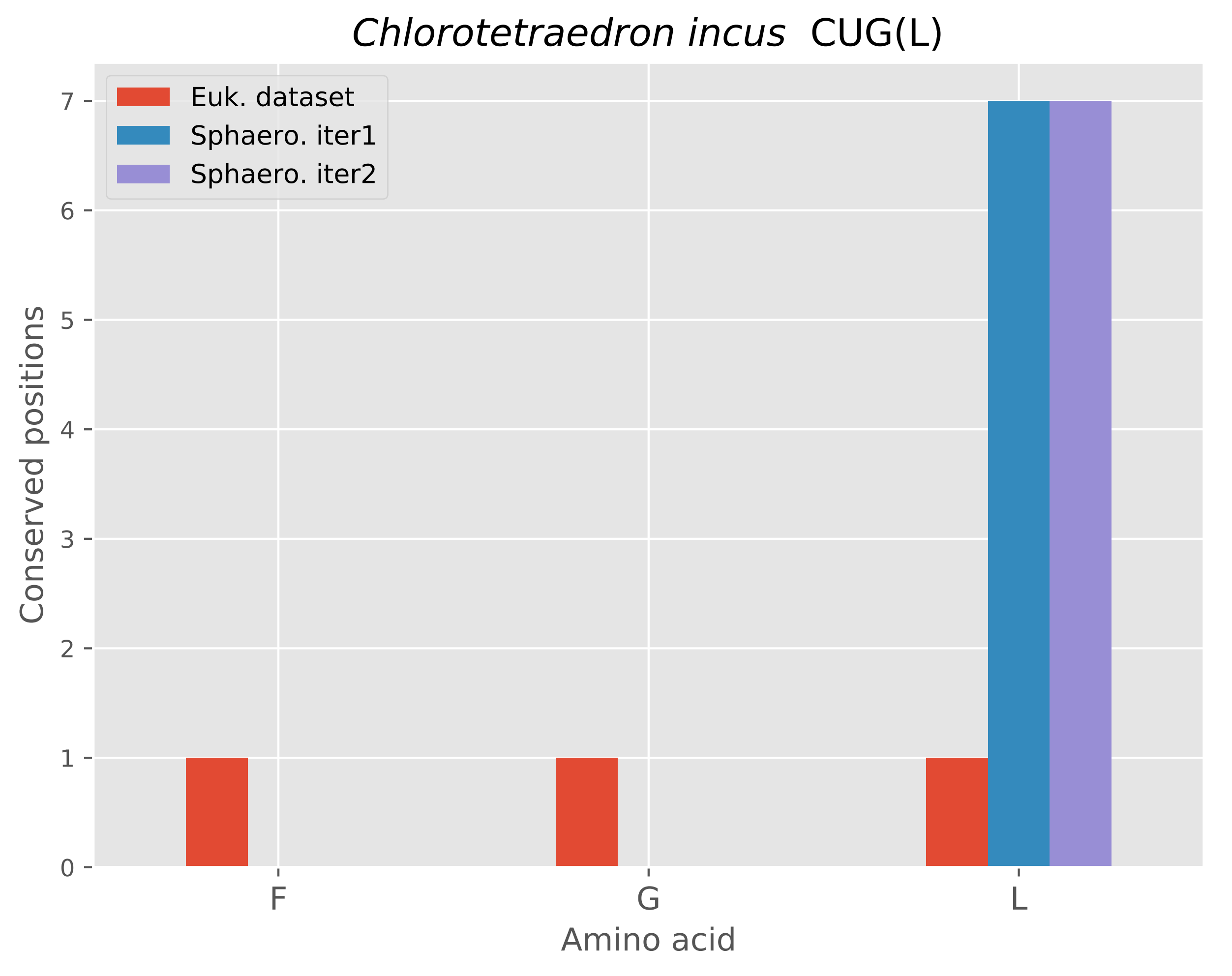

Chlorotetraedron incus CUG(L)
Euk. dataset
Sphaero. iter1
Sphaero. iter2
Conserved positions
0
1
2
3
4
5
6
7
F
G
L
Amino acid

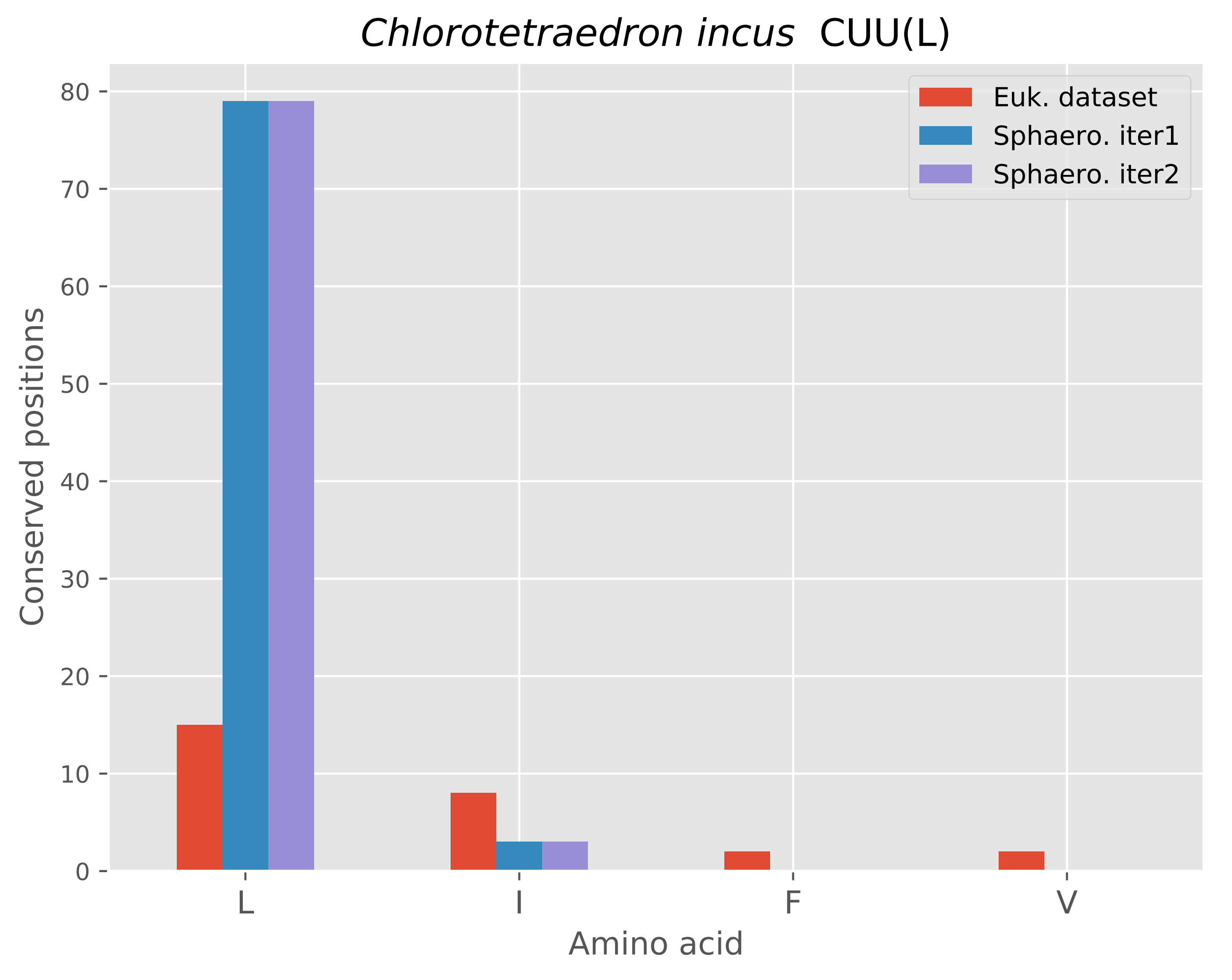

Chlorotetraedron incus CUU(L)
Euk. dataset
Sphaero. iter1
Sphaero. iter2
Conserved positions
0
10
20
30
40
50
60
70
80
L
I
F
V
Amino acid

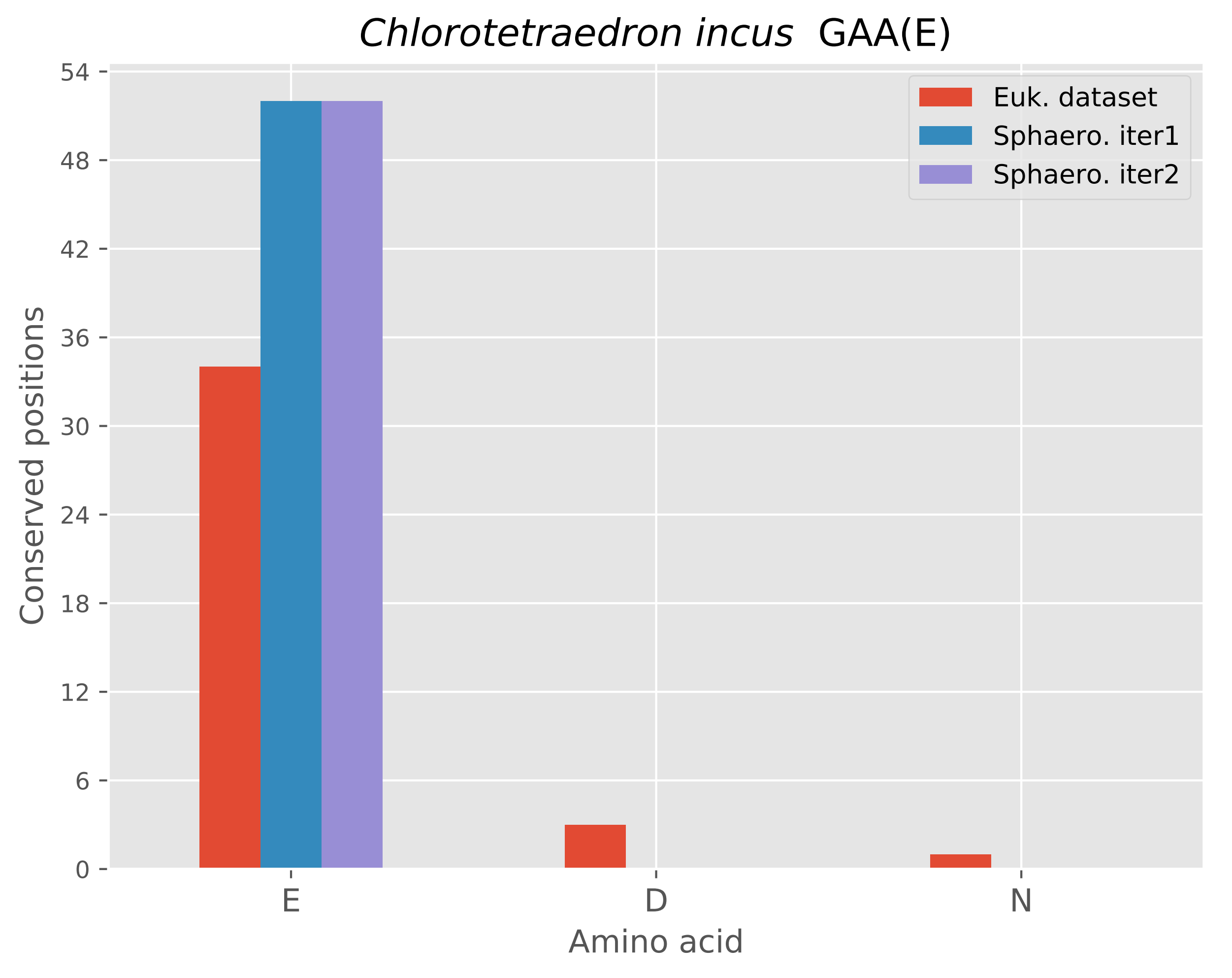
Chlorotetraedron incus GAA(E)
Conserved positions
0
6
12
18
24
30
36
42
48
54
Euk. dataset
Sphaero. iter1
Sphaero. iter2
E
D
N
Amino acid

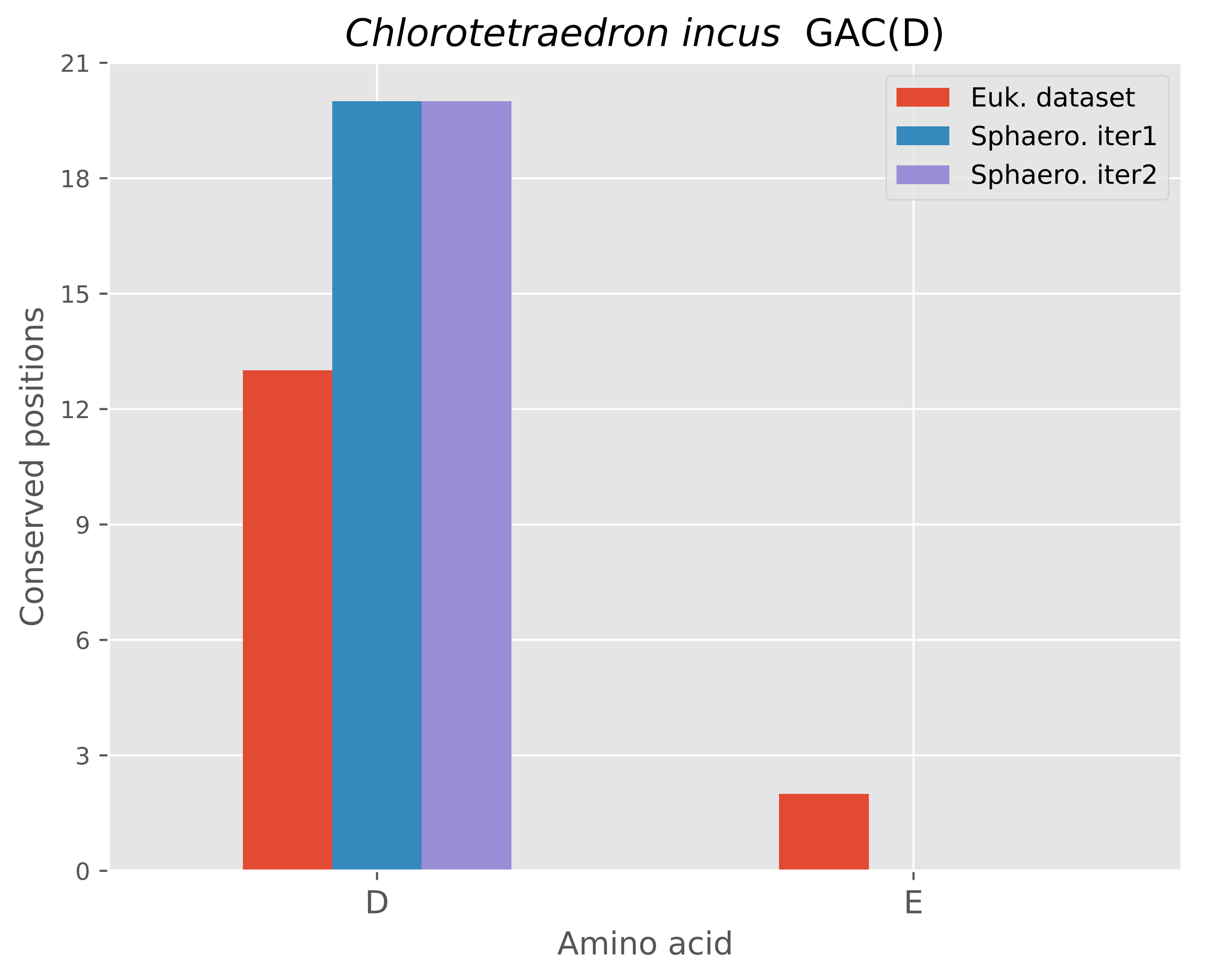
Chlorotetraedron incus GAC(D)
Euk. dataset
Sphaero. iter1
Sphaero. iter2
Conserved positions
Amino acid
D
E
0
3
6
9
12
15
18
21

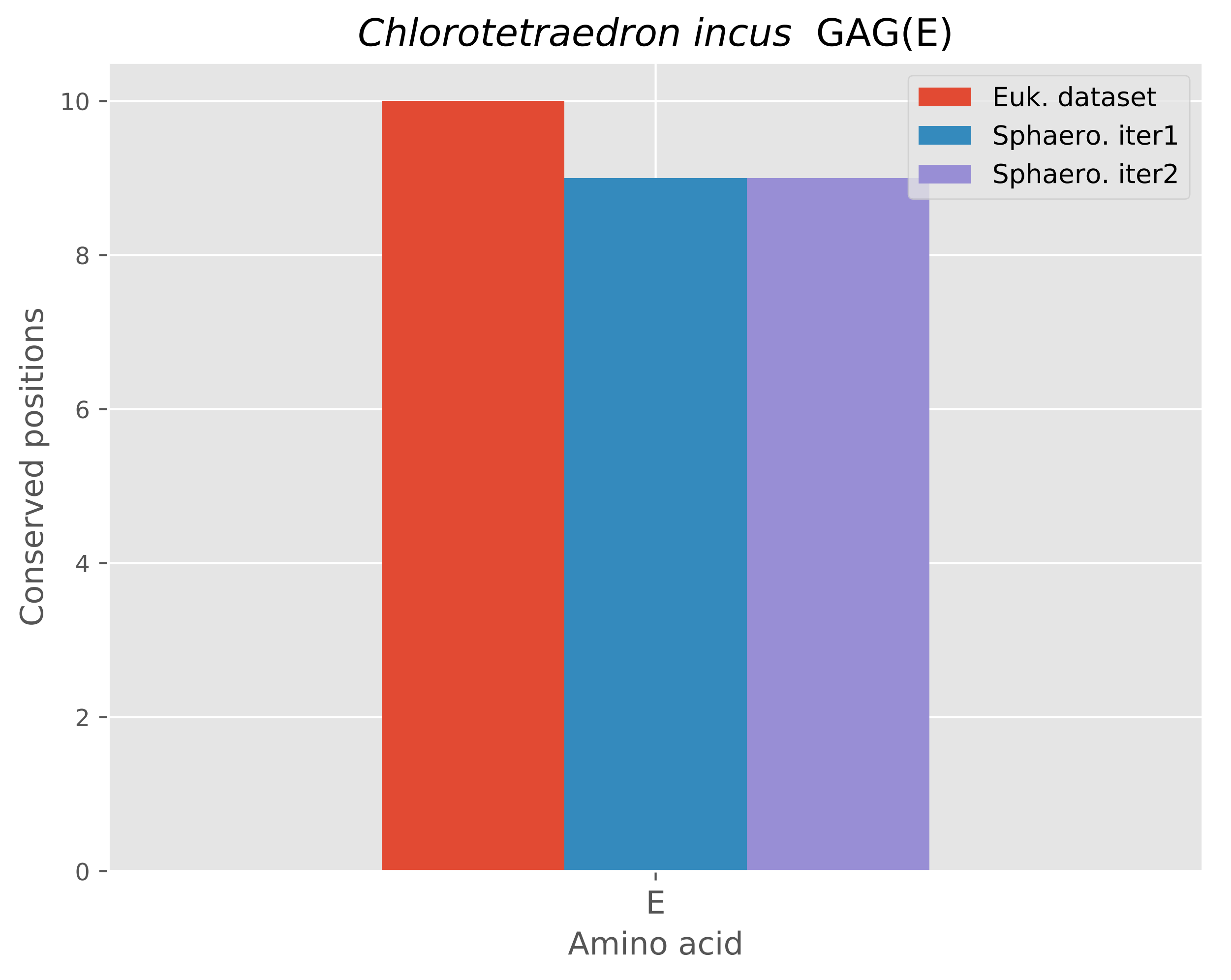

Chlorotetraedron incus GAG(E)
Euk. dataset
Sphaero. iter1
Sphaero. iter2
Conserved positions
0
2
4
6
8
10
E
Amino acid

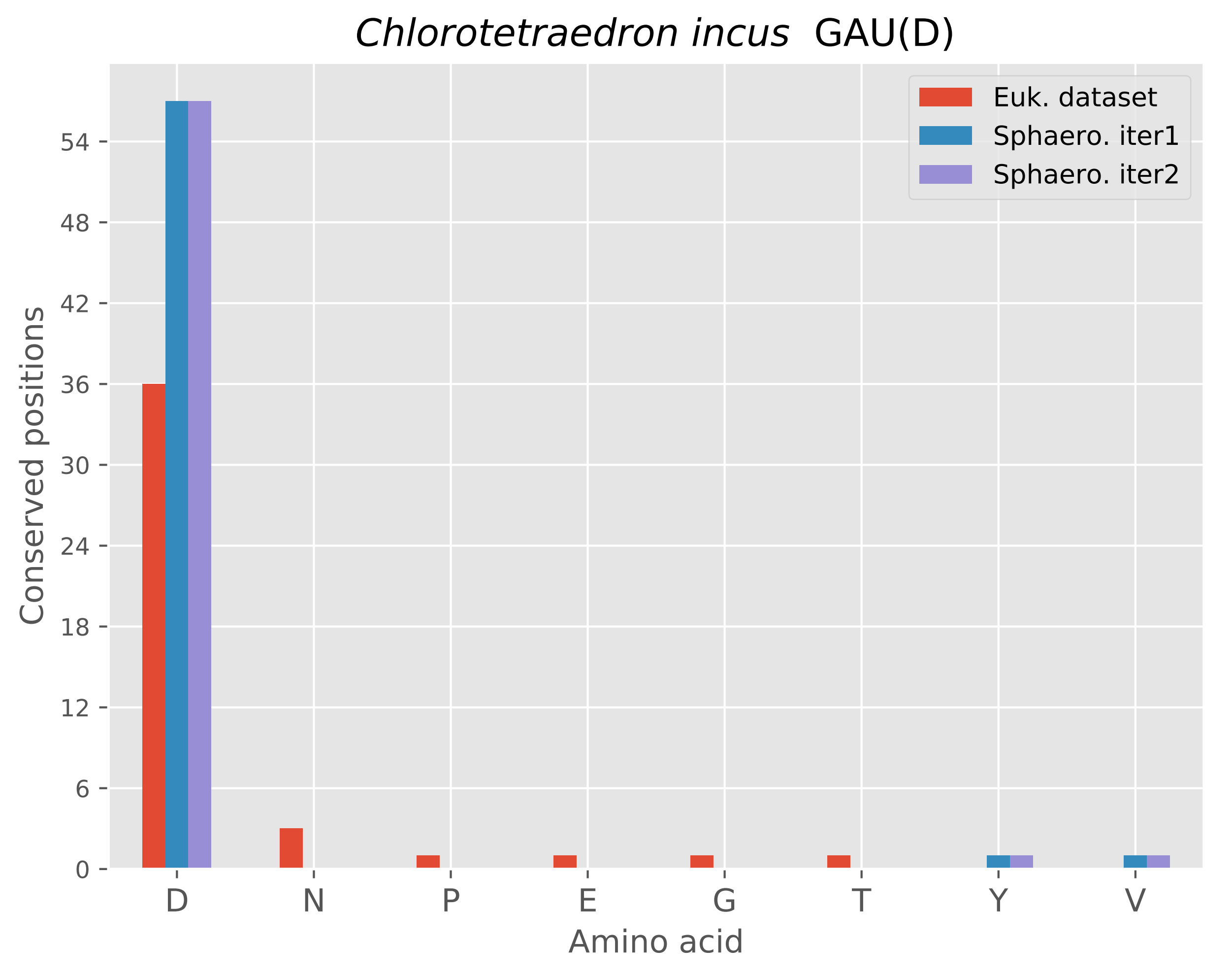
Chlorotetraedron incus GAU(D)
Euk. dataset
Sphaero. iter1
Sphaero. iter2
Conserved positions
0
6
12
18
24
30
36
42
48
54
D
N
P
E
G
T
Y
V
Amino acid

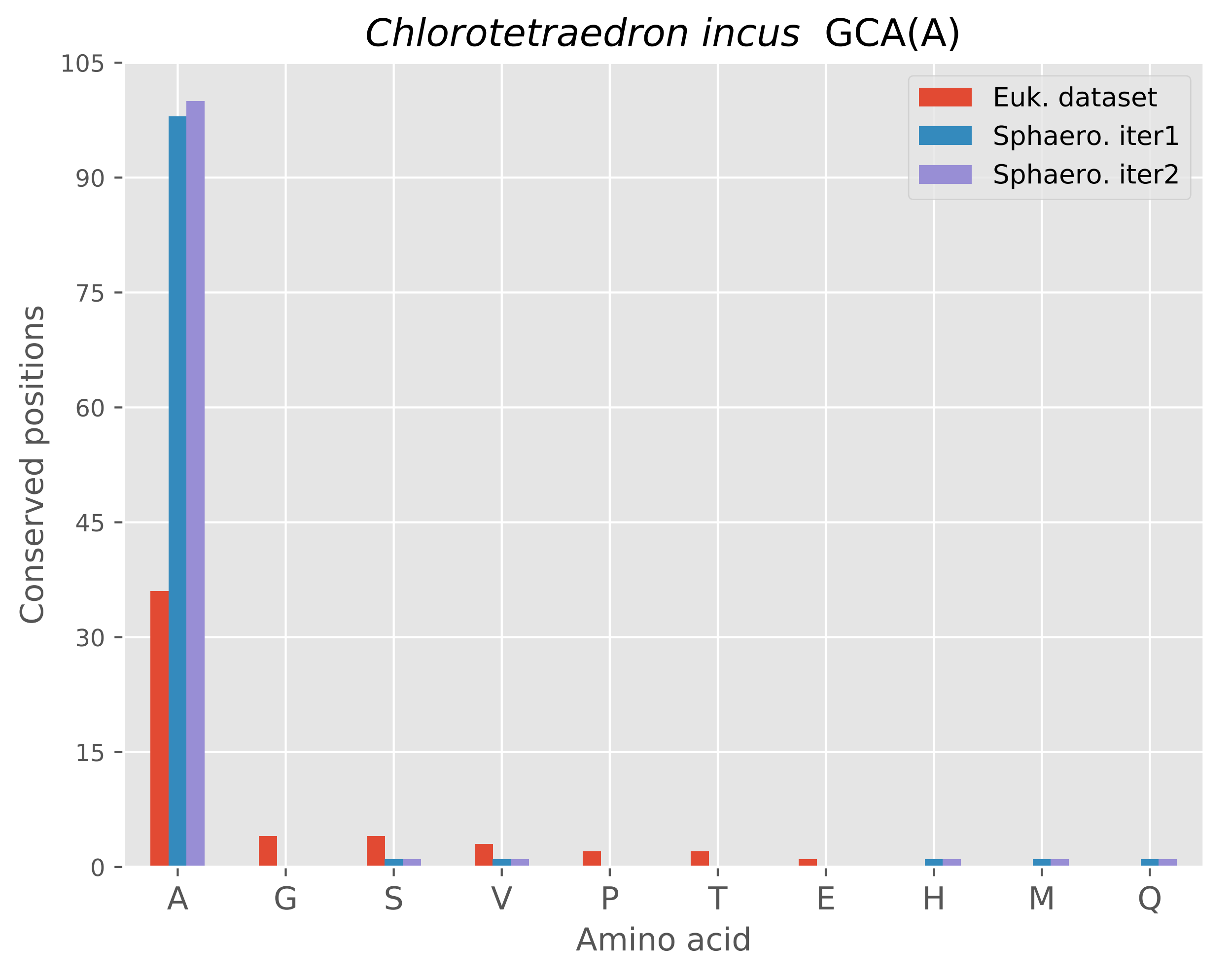

Chlorotetraedron incus GCA(A)
Conserved positions
Amino acid
0
15
30
45
60
75
90
105
A
G
S
V
P
T
E
H
M
Q
Euk. dataset
Sphaero. iter1
Sphaero. iter2

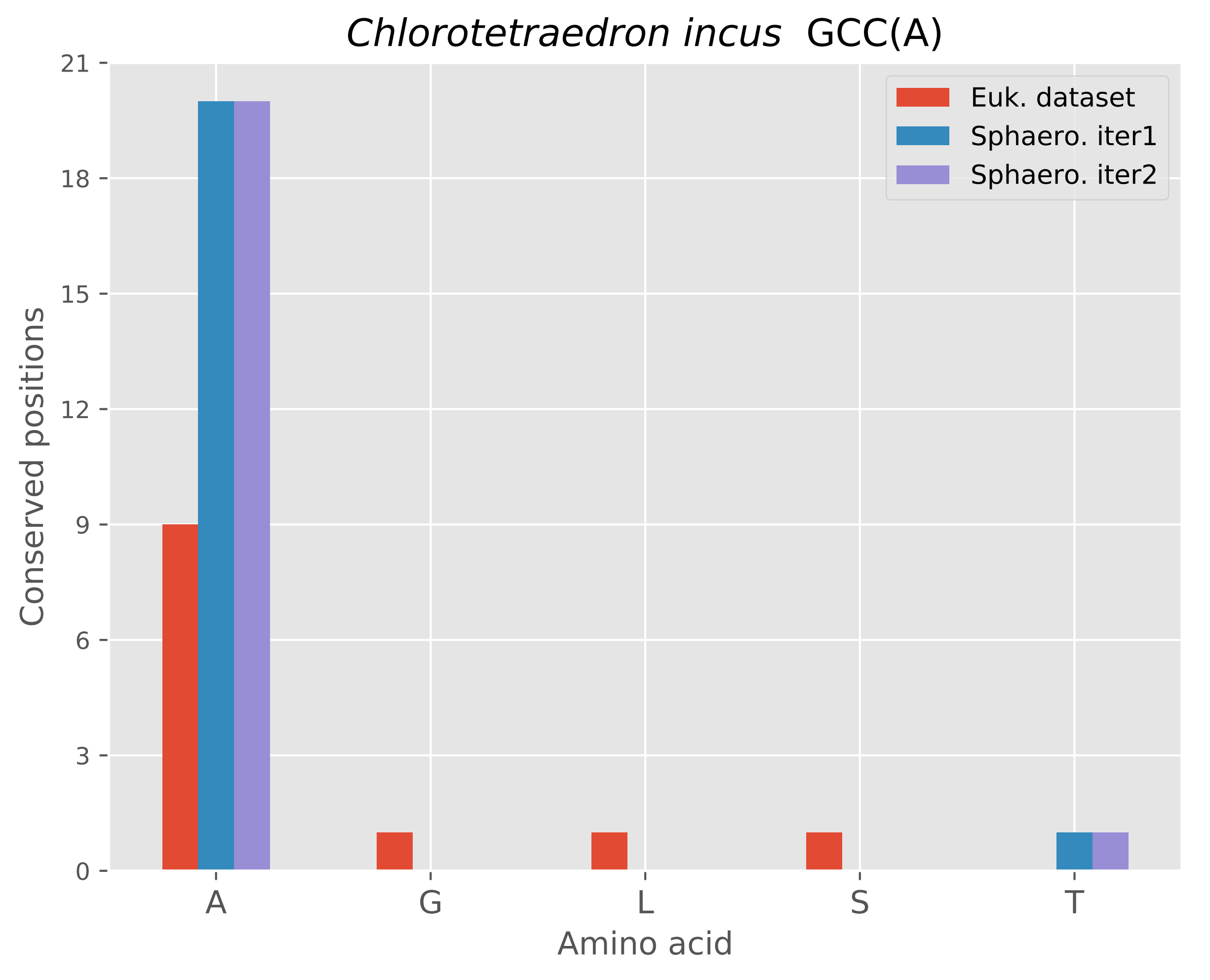

Chlorotetraedron incus GCC(A)
Conserved positions
Amino acid
0
3
6
9
12
15
18
21
A
G
L
S
T
Euk. dataset
Sphaero. iter1
Sphaero. iter2

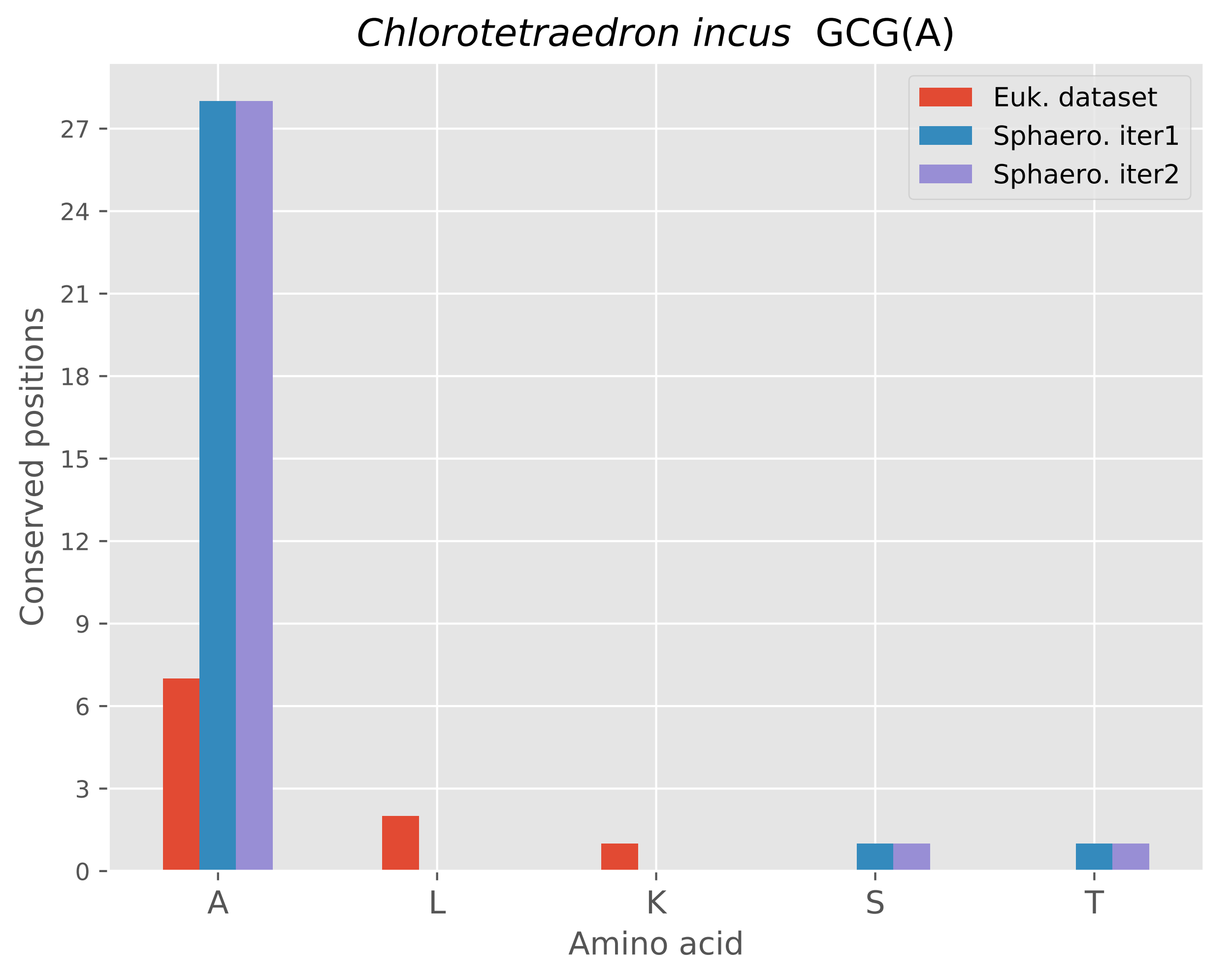

Chlorotetraedron incus GCG(A)
Euk. dataset
Sphaero. iter1
Sphaero. iter2
Conserved positions
0
3
6
9
12
15
18
21
24
27
A
L
K
S
T
Amino acid

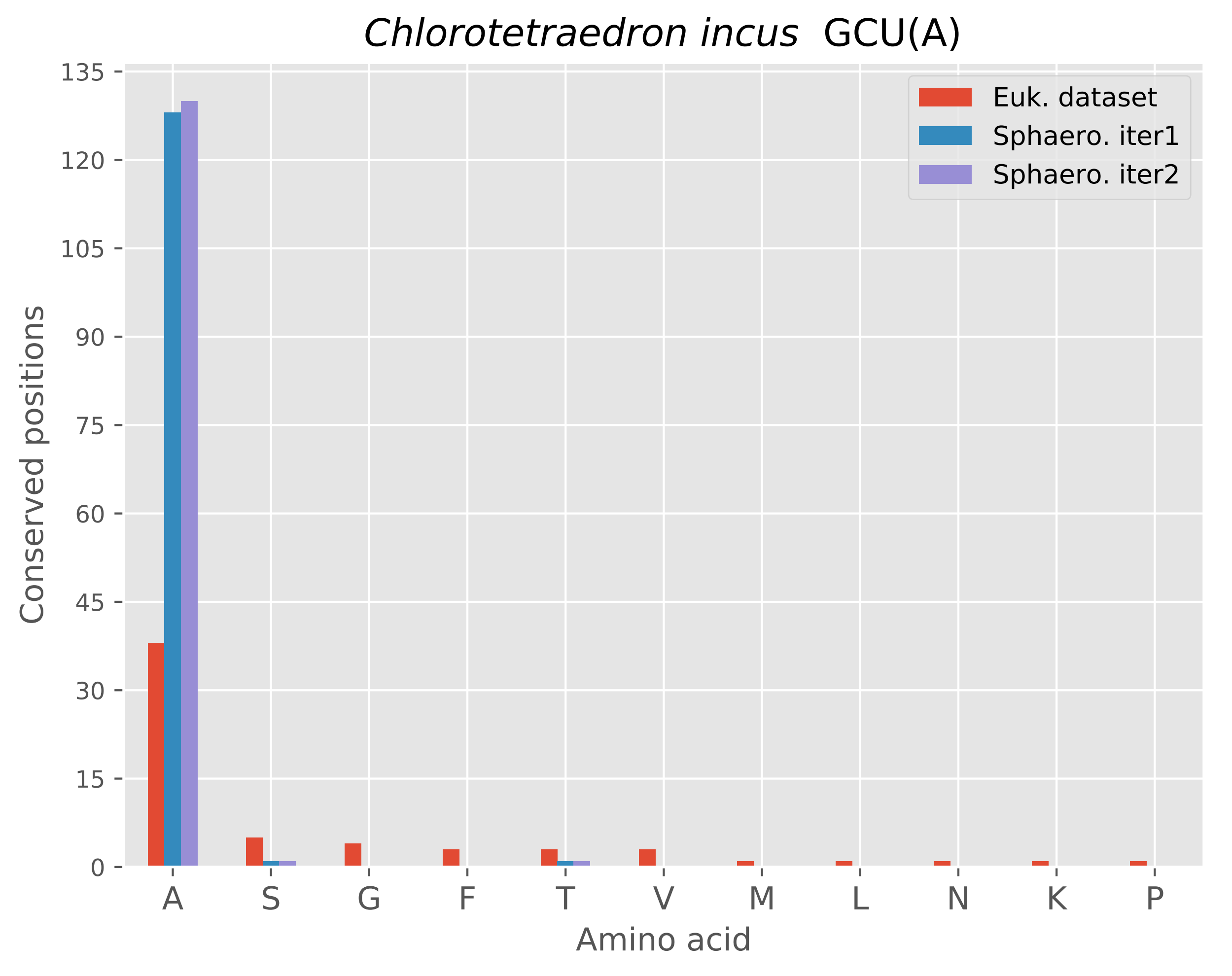

Chlorotetraedron incus GCU(A)
Euk. dataset
Sphaero. iter1
Sphaero. iter2
Conserved positions
135
120
105
90
75
60
45
30
15
0
A
S
G
F
T
V
M
L
N
K
P
Amino acid

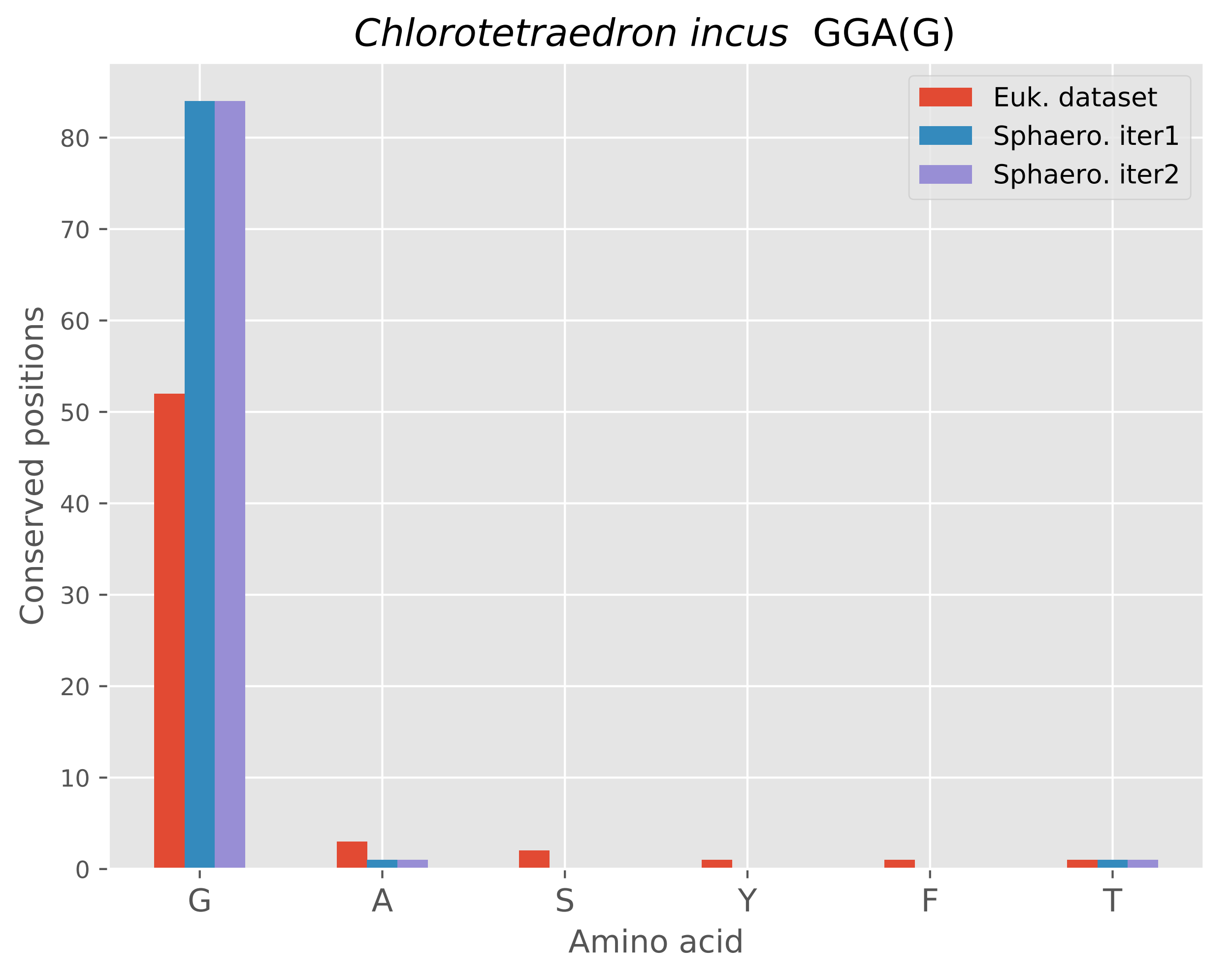

Chlorotetraedron incus GGA(G)
Euk. dataset
Sphaero. iter1
Sphaero. iter2
Conserved positions
Amino acid
0
10
20
30
40
50
60
70
80
G
A
S
Y
F
T

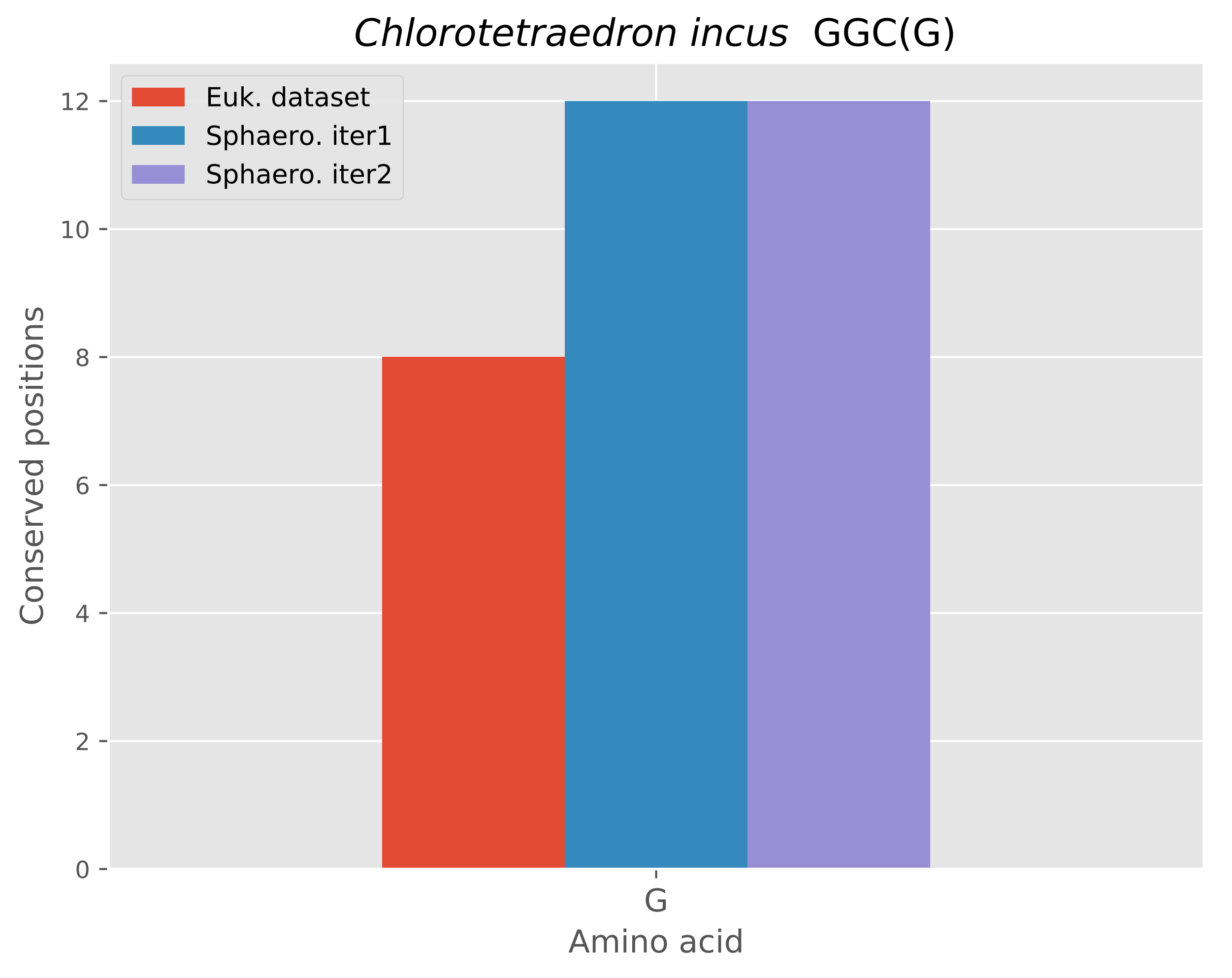

Chlorotetraedron incus GGC(G)
Euk. dataset
Sphaero. iter1
Sphaero. iter2
Conserved positions
0
2
4
6
8
10
12
G
Amino acid

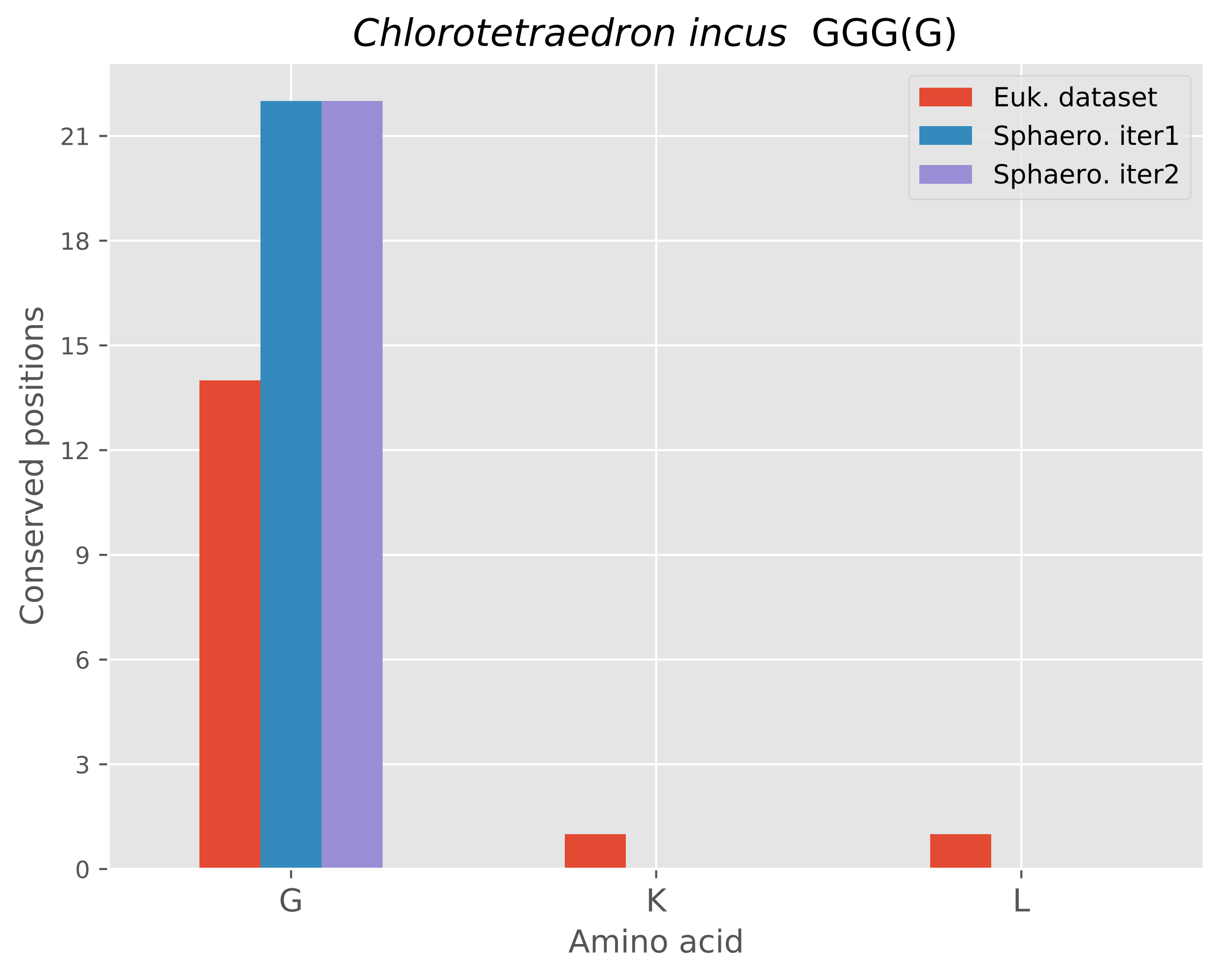

Chlorotetraedron incus GGG(G)
Euk. dataset
Sphaero. iter1
Sphaero. iter2
Conserved positions
0
3
6
9
12
15
18
21
G
K
L
Amino acid

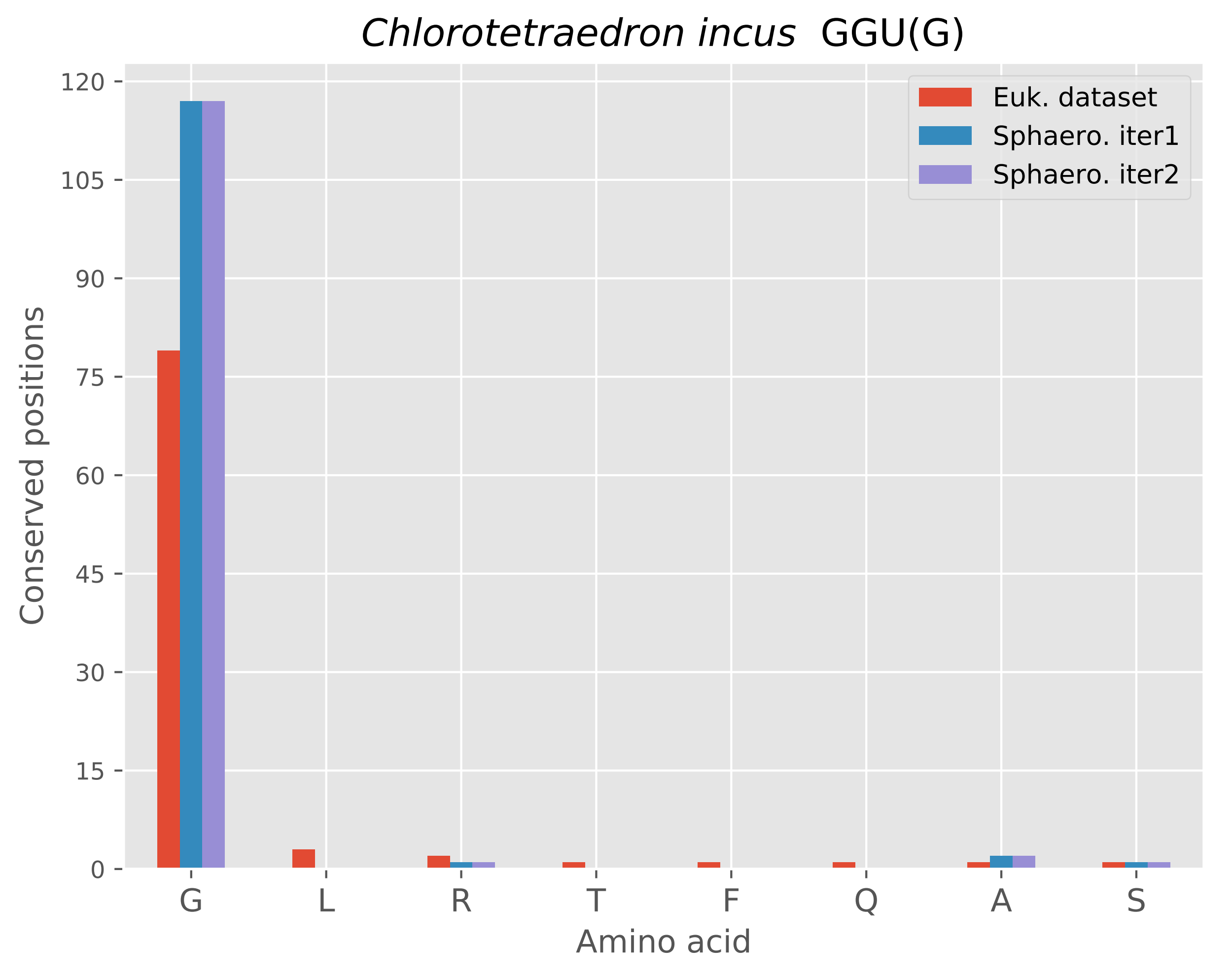

Chlorotetraedron incus GGU(G)
Euk. dataset
Sphaero. iter1
Sphaero. iter2
Conserved positions
0
15
30
45
60
75
90
105
120
G
L
R
T
F
Q
A
S
Amino acid

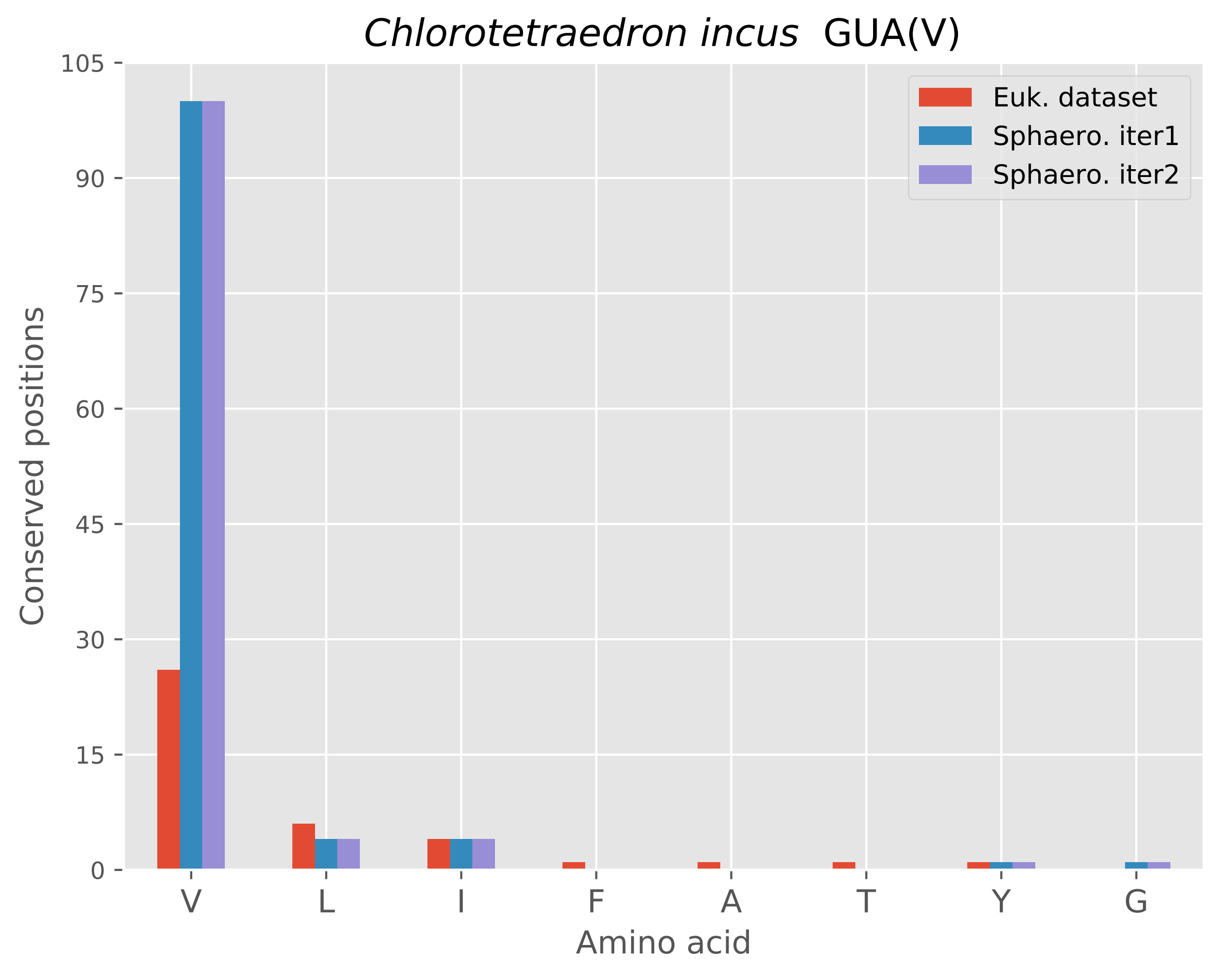
Chlorotetraedron incus GUA(V)
Euk. dataset
Sphaero. iter1
Sphaero. iter2
Conserved positions
0
15
30
45
60
75
90
105
V
L
I
F
A
T
Y
G
Amino acid

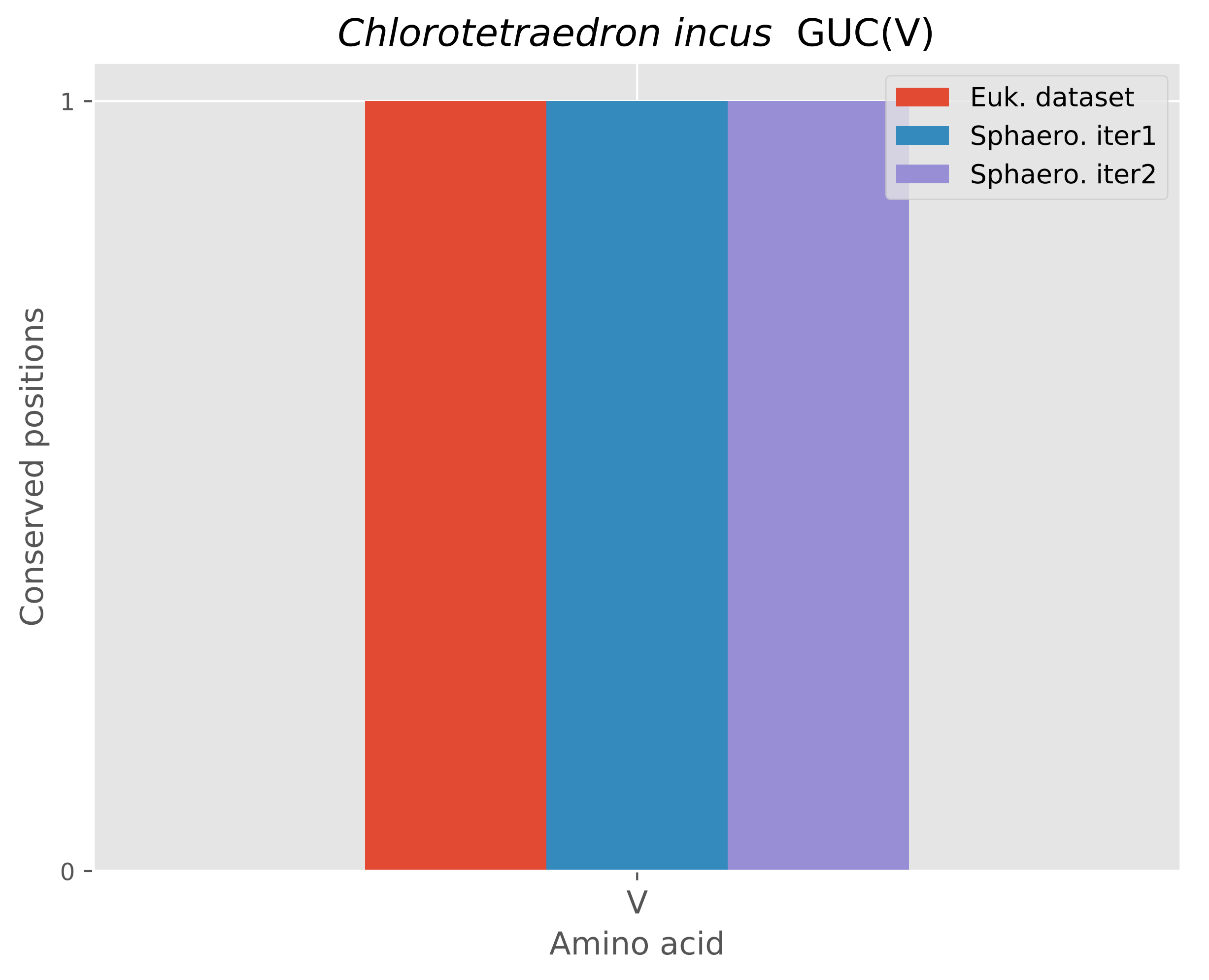

Chlorotetraedron incus GUC(V)
Euk. dataset
Sphaero. iter1
Sphaero. iter2
Conserved positions
1
0
V
Amino acid

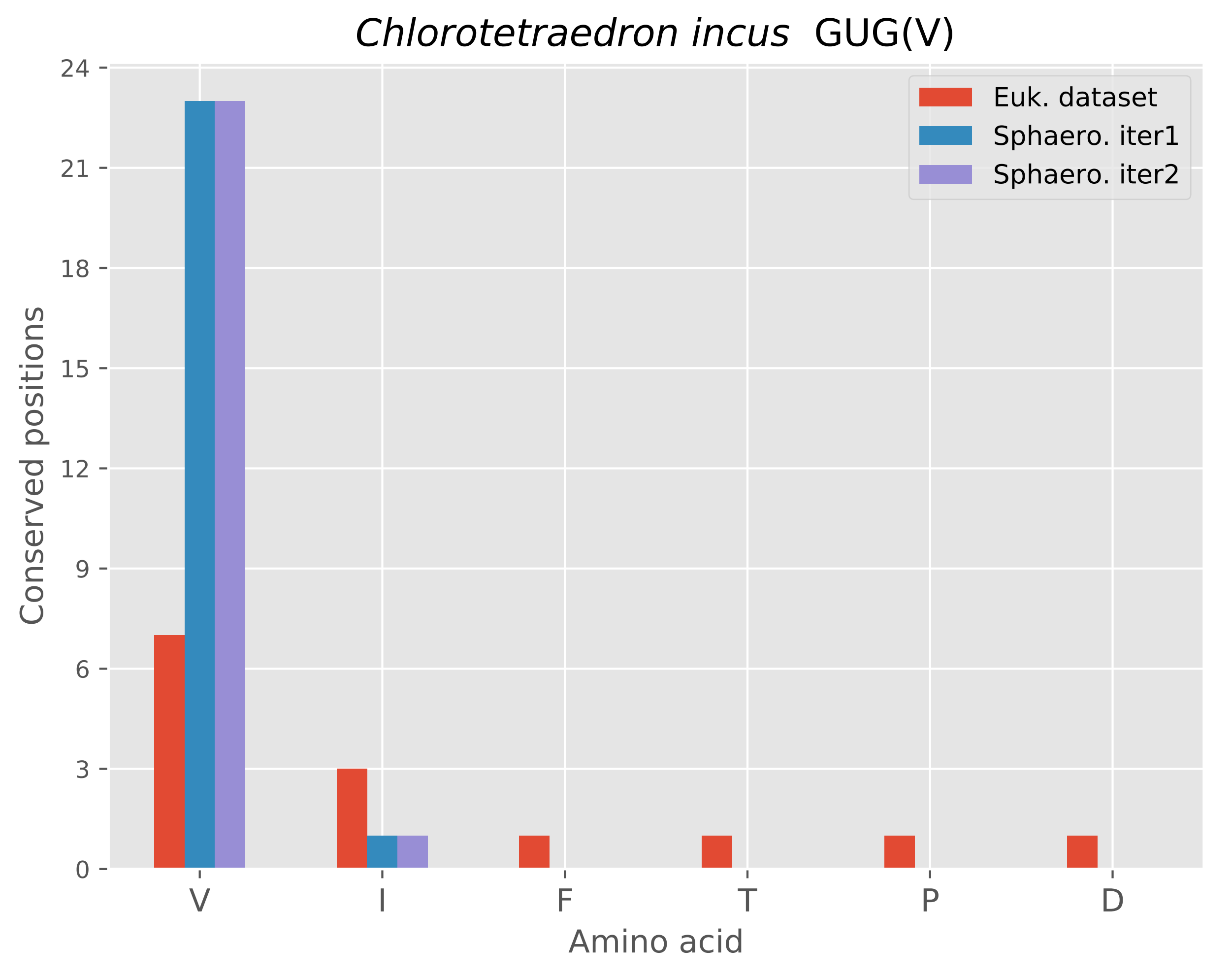

Chlorotetraedron incus GUG(V)
Euk. dataset
Sphaero. iter1
Sphaero. iter2
Conserved positions
0
3
6
9
12
15
18
21
24
V
I
F
T
P
D
Amino acid

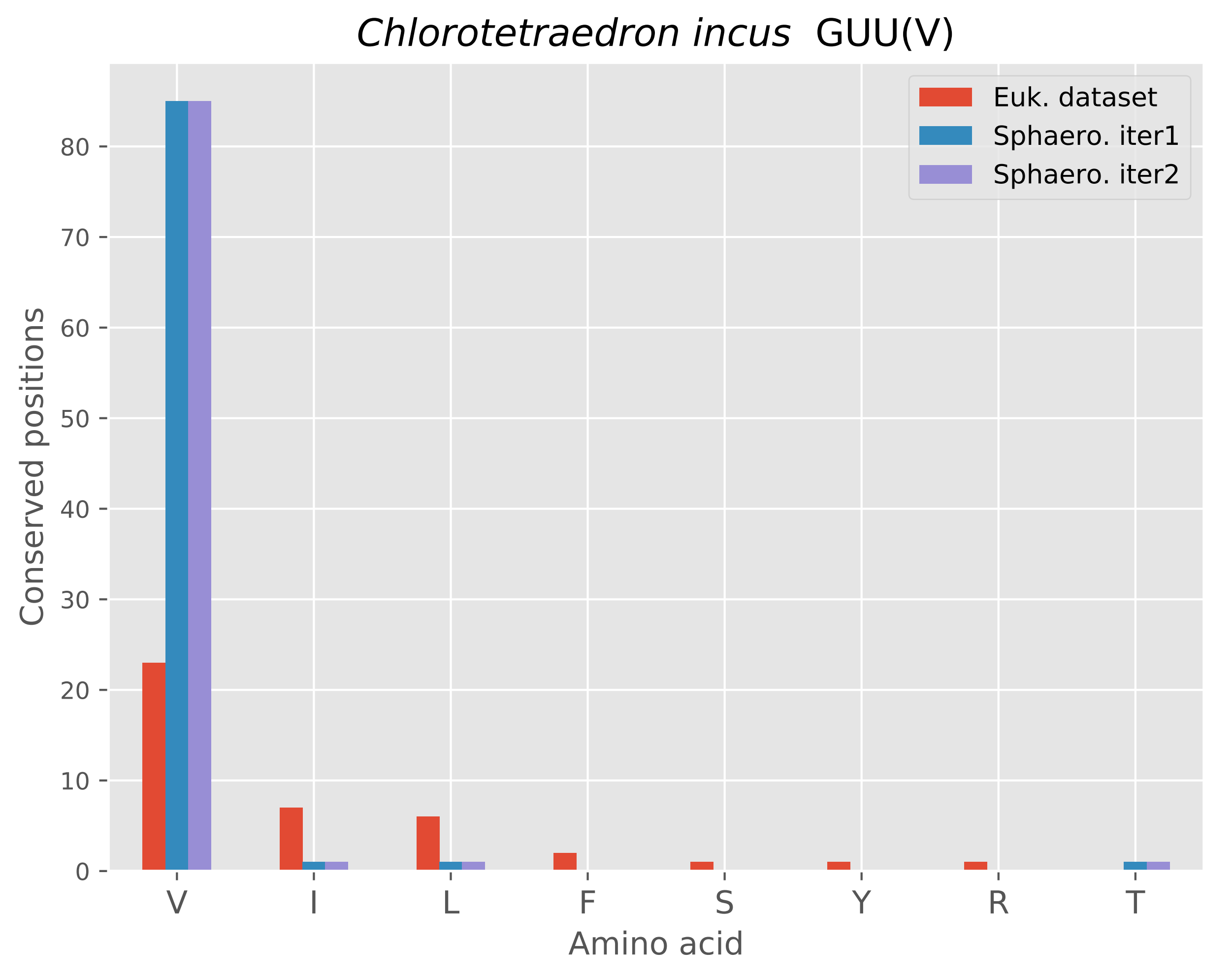

Chlorotetraedron incus GUU(V)
Euk. dataset
Sphaero. iter1
Sphaero. iter2
Conserved positions
0
10
20
30
40
50
60
70
80
V
I
L
F
S
Y
R
T
Amino acid

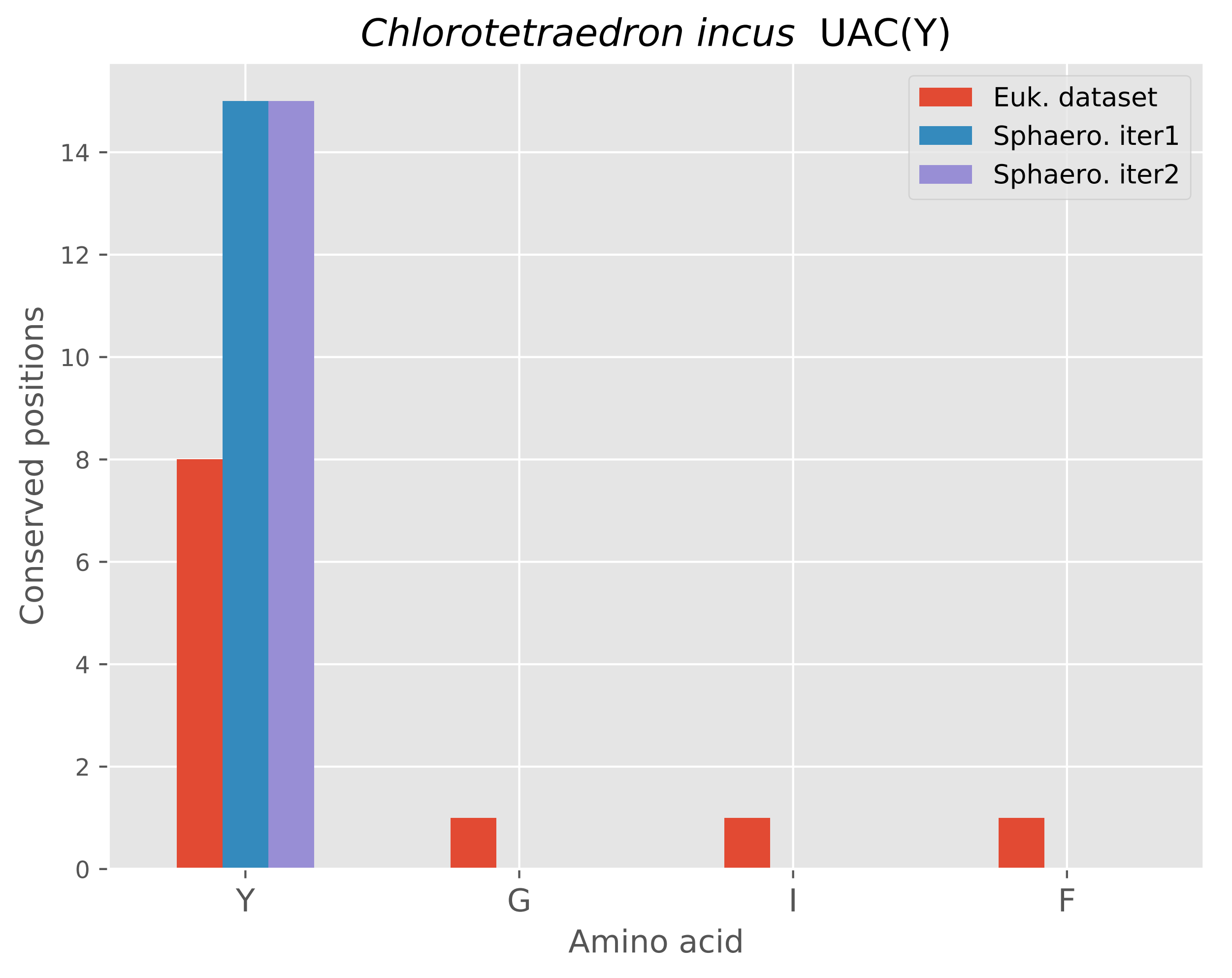

Chlorotetraedron incus UAC(Y)
Conserved positions
Amino acid
0
2
4
6
8
10
12
14
Y
G
I
F
Euk. dataset
Sphaero. iter1
Sphaero. iter2

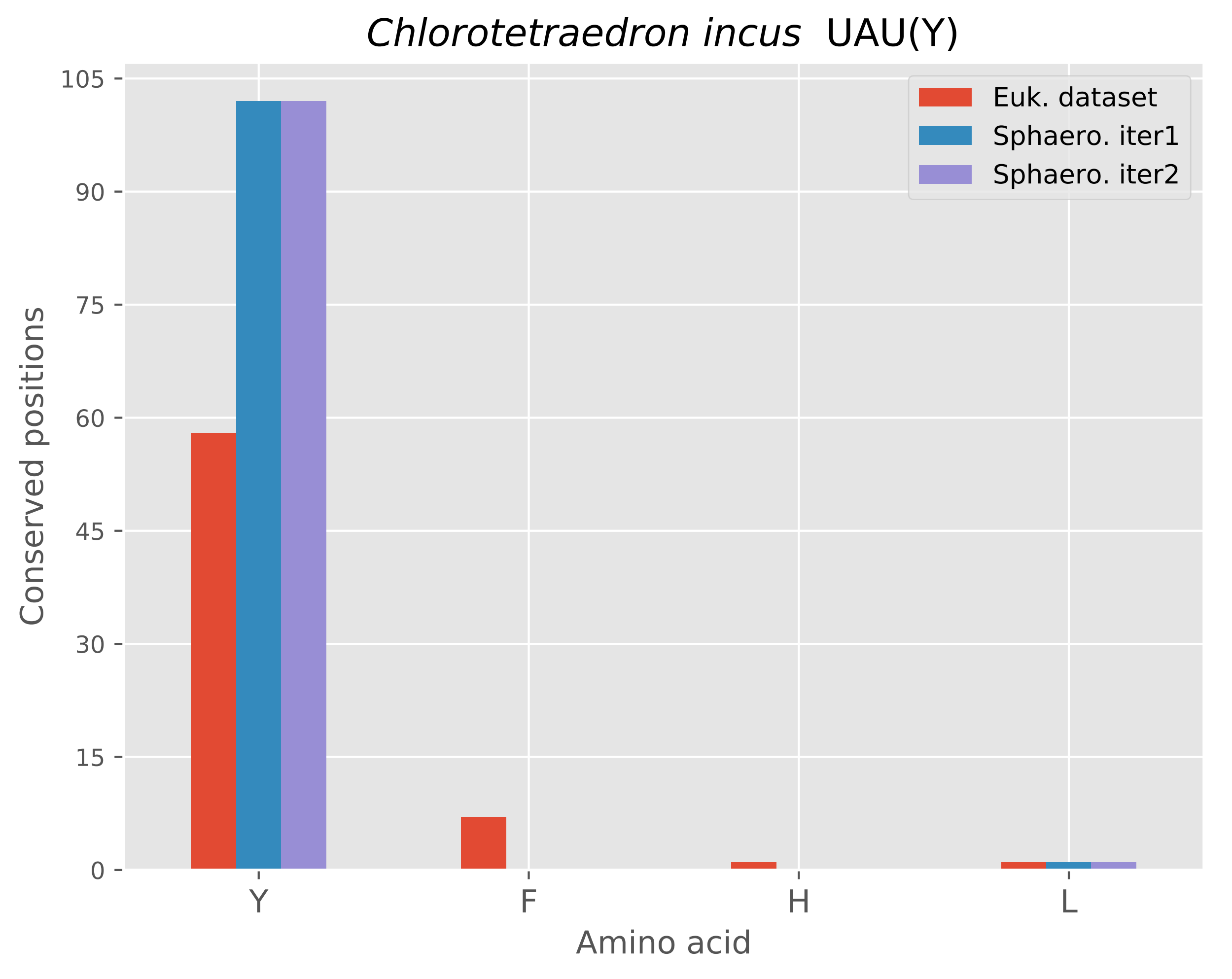

Chlorotetraedron incus UAU(Y)
Euk. dataset
Sphaero. iter1
Sphaero. iter2
Conserved positions
0
15
30
45
60
75
90
105
Y
F
H
L
Amino acid

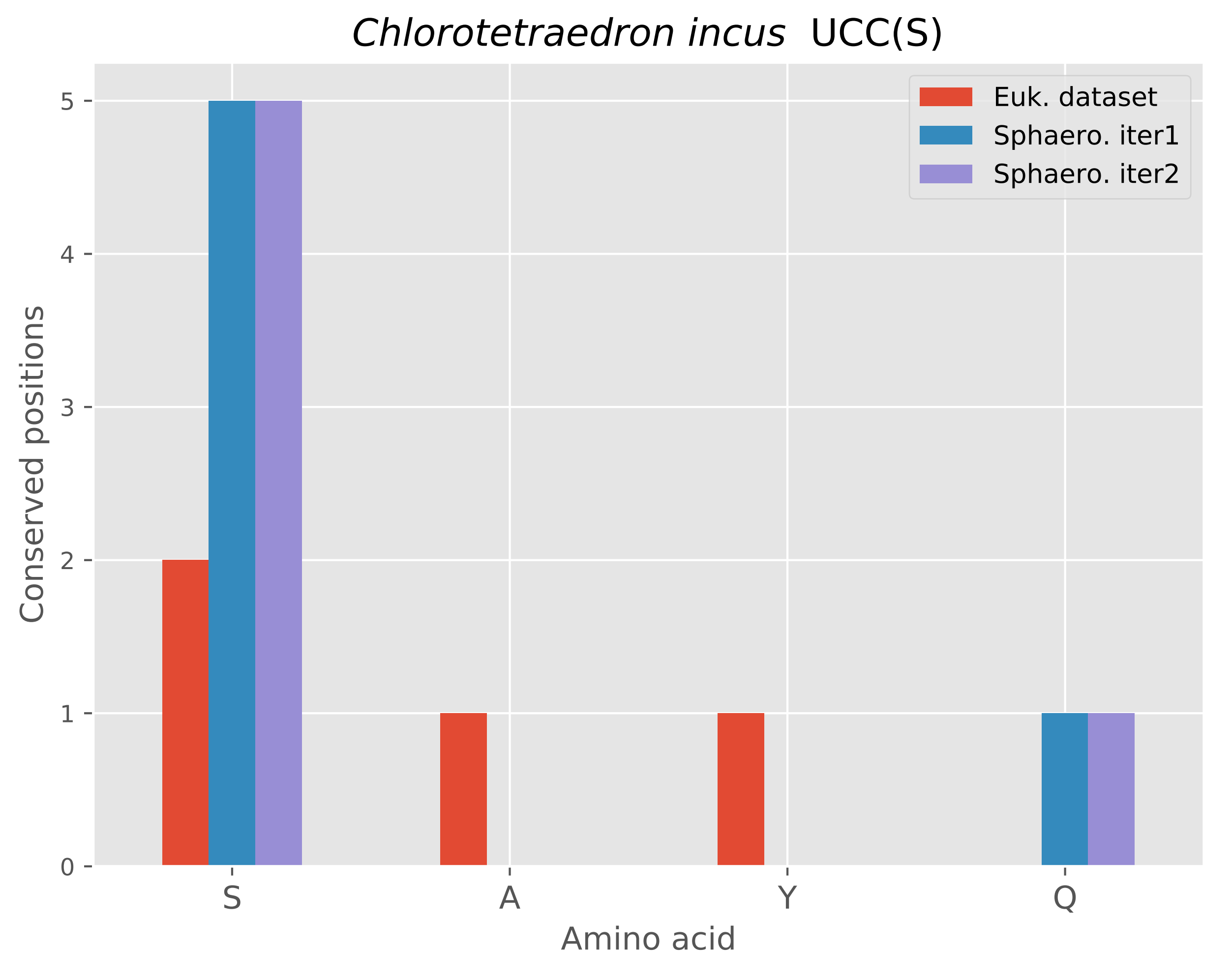
Chlorotetraedron incus UCC(S)
Euk. dataset
Sphaero. iter1
Sphaero. iter2
Conserved positions
0
1
2
3
4
5
S
A
Y
Q
Amino acid

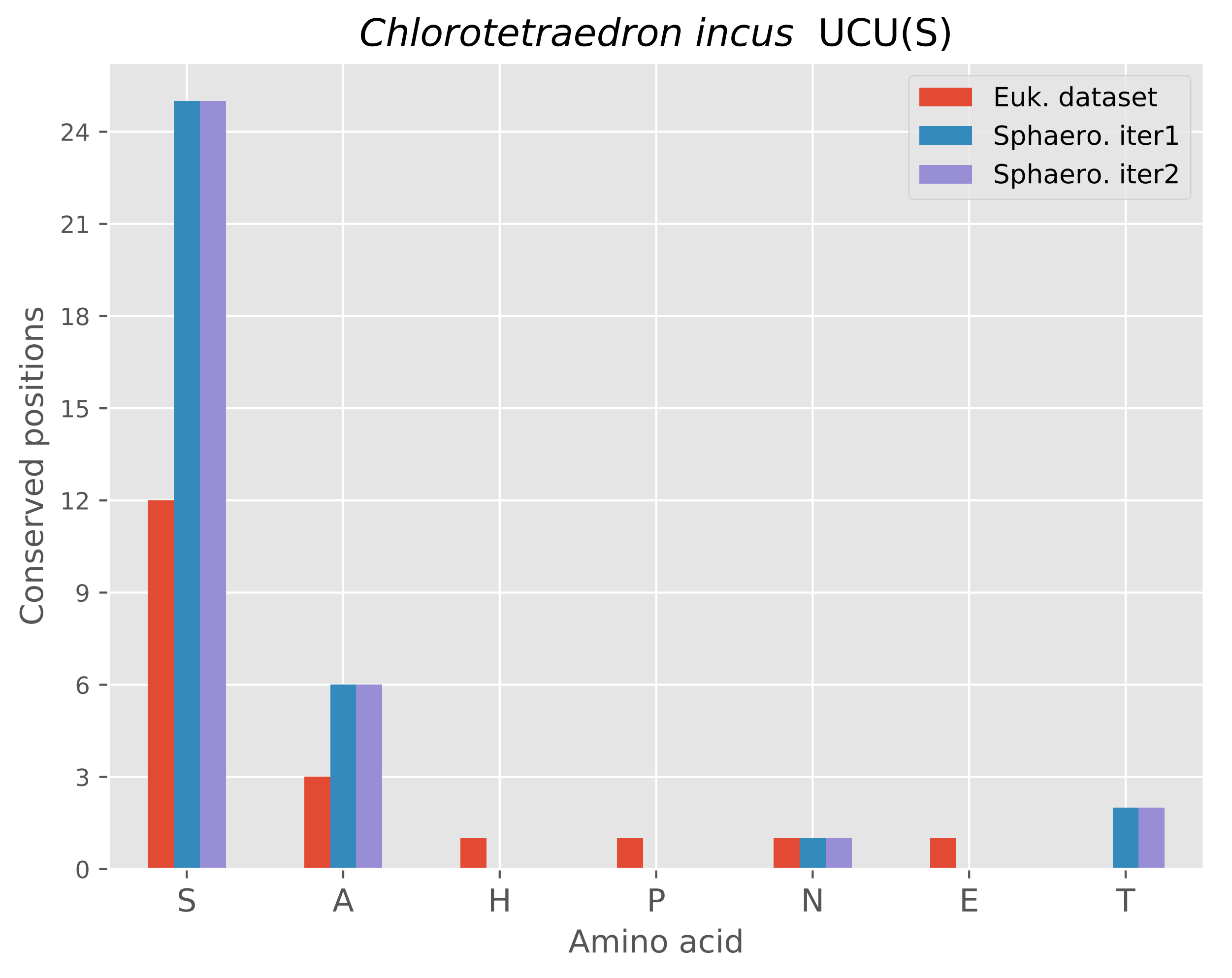

Chlorotetraedron incus UCU(S)
Euk. dataset
Sphaero. iter1
Sphaero. iter2
Conserved positions
0
3
6
9
12
15
18
21
24
S
A
H
P
N
E
T
Amino acid

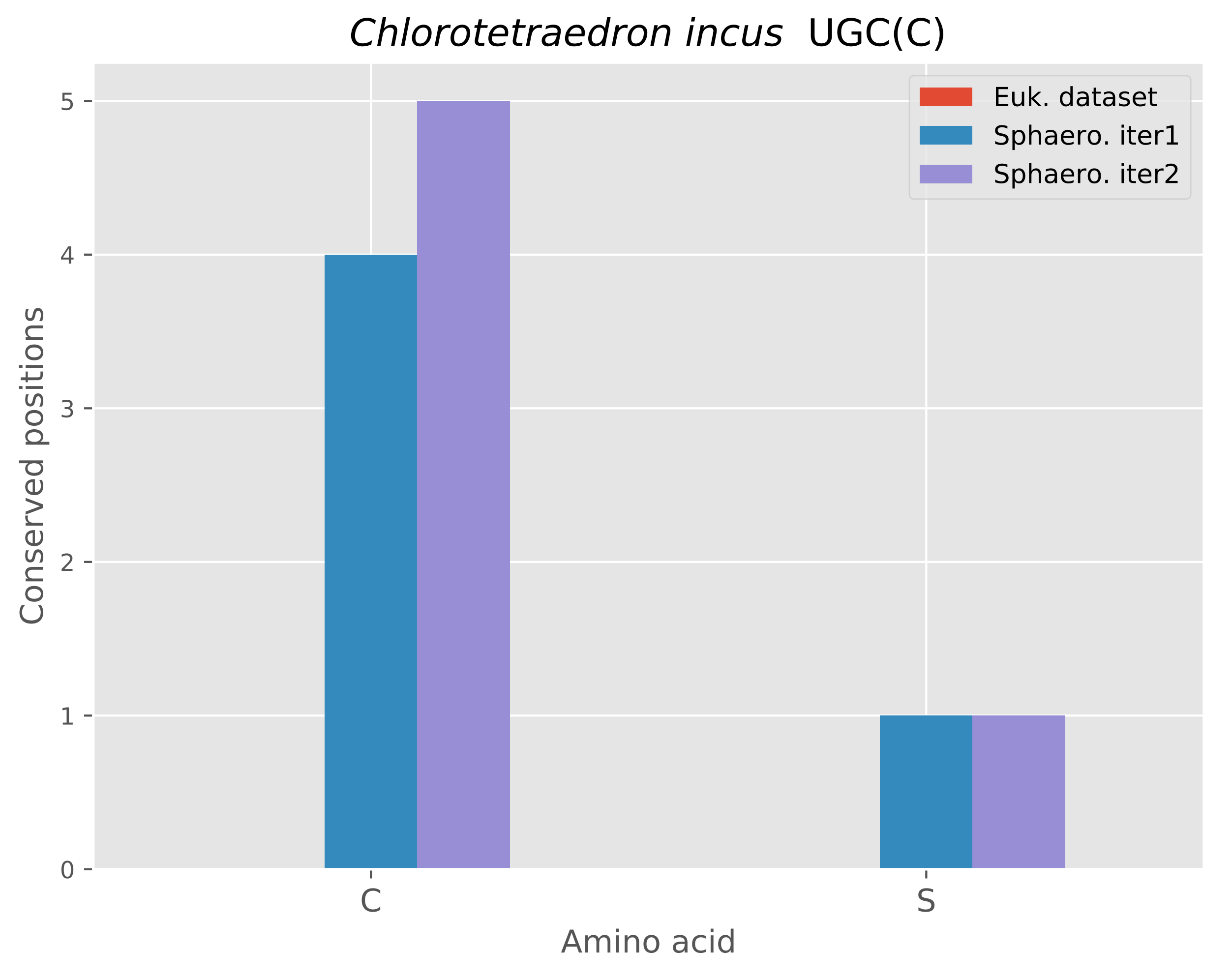

Chlorotetraedron incus UGC(C)
Euk. dataset
Sphaero. iter1
Sphaero. iter2
Conserved positions
0
1
2
3
4
5
C
S
Amino acid

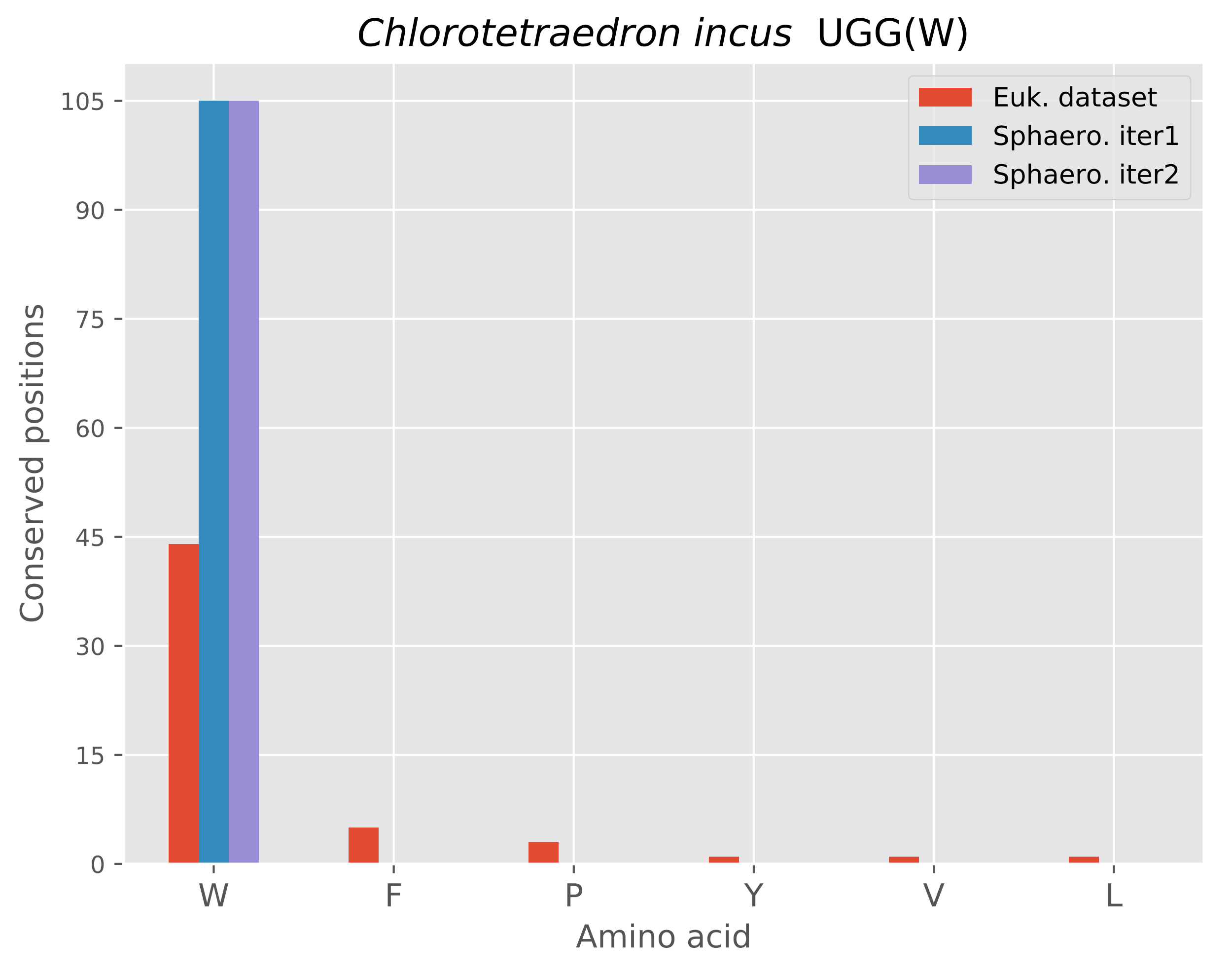

Chlorotetraedron incus UGG(W)
Conserved positions
Amino acid
0
15
30
45
60
75
90
105
W
F
P
Y
V
L
Euk. dataset
Sphaero. iter1
Sphaero. iter2

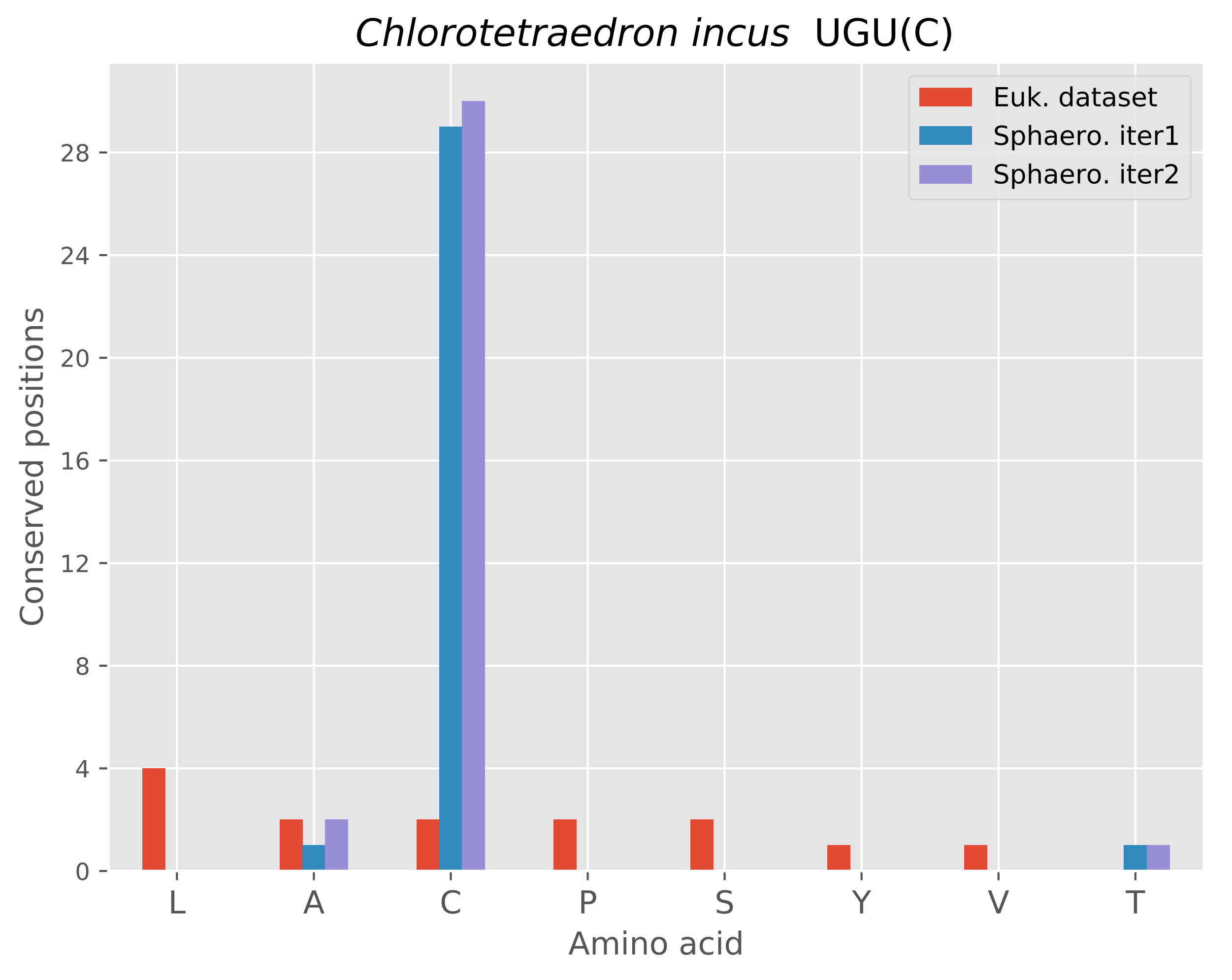

Chlorotetraedron incus UGU(C)
Euk. dataset
Sphaero. iter1
Sphaero. iter2
Conserved positions
0
4
8
12
16
20
24
28
L
A
C
P
S
Y
V
T
Amino acid

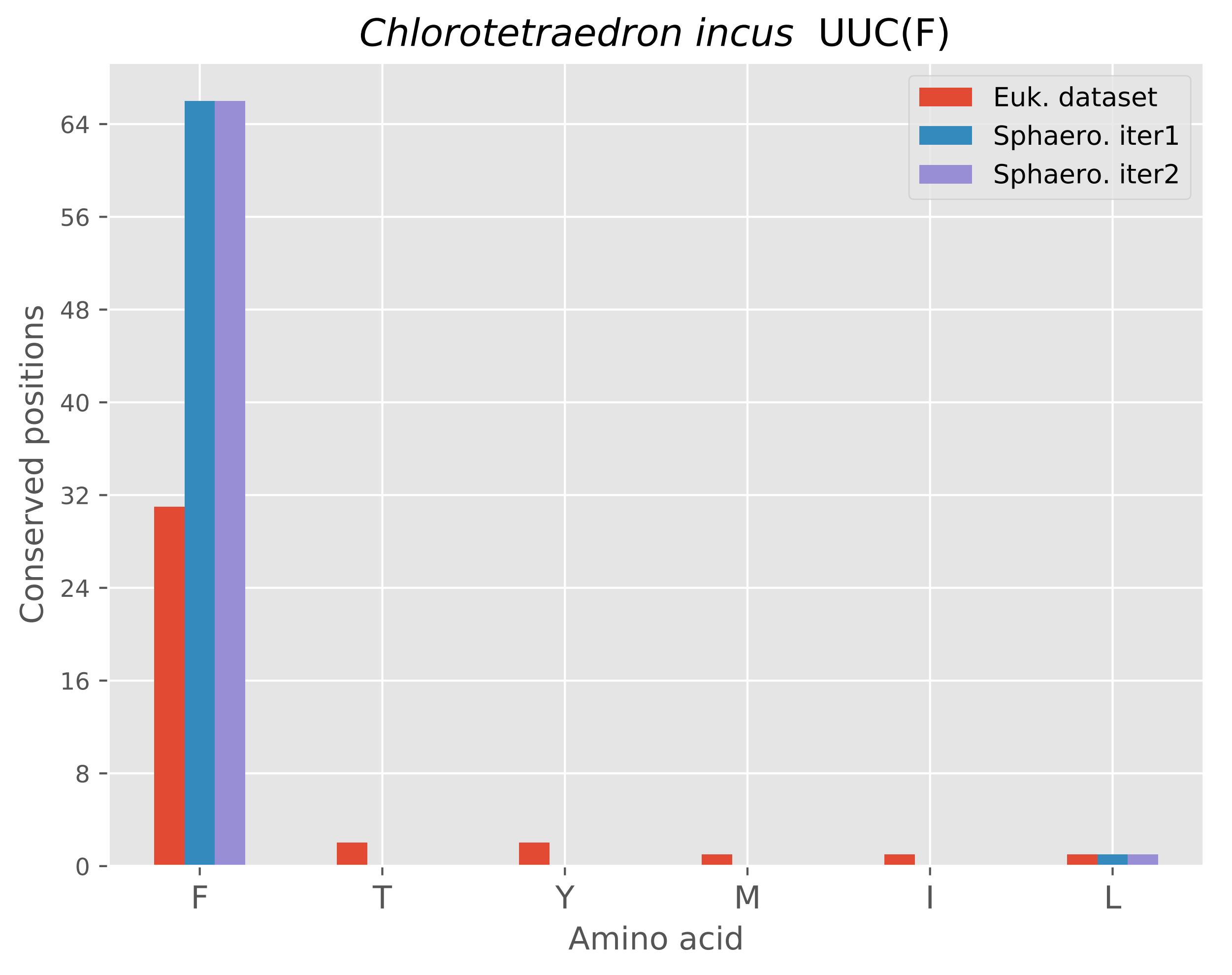

Chlorotetraedron incus UUC(F)
Euk. dataset
Sphaero. iter1
Sphaero. iter2
Conserved positions
0
8
16
24
32
40
48
56
64
F
T
Y
M
I
L
Amino acid

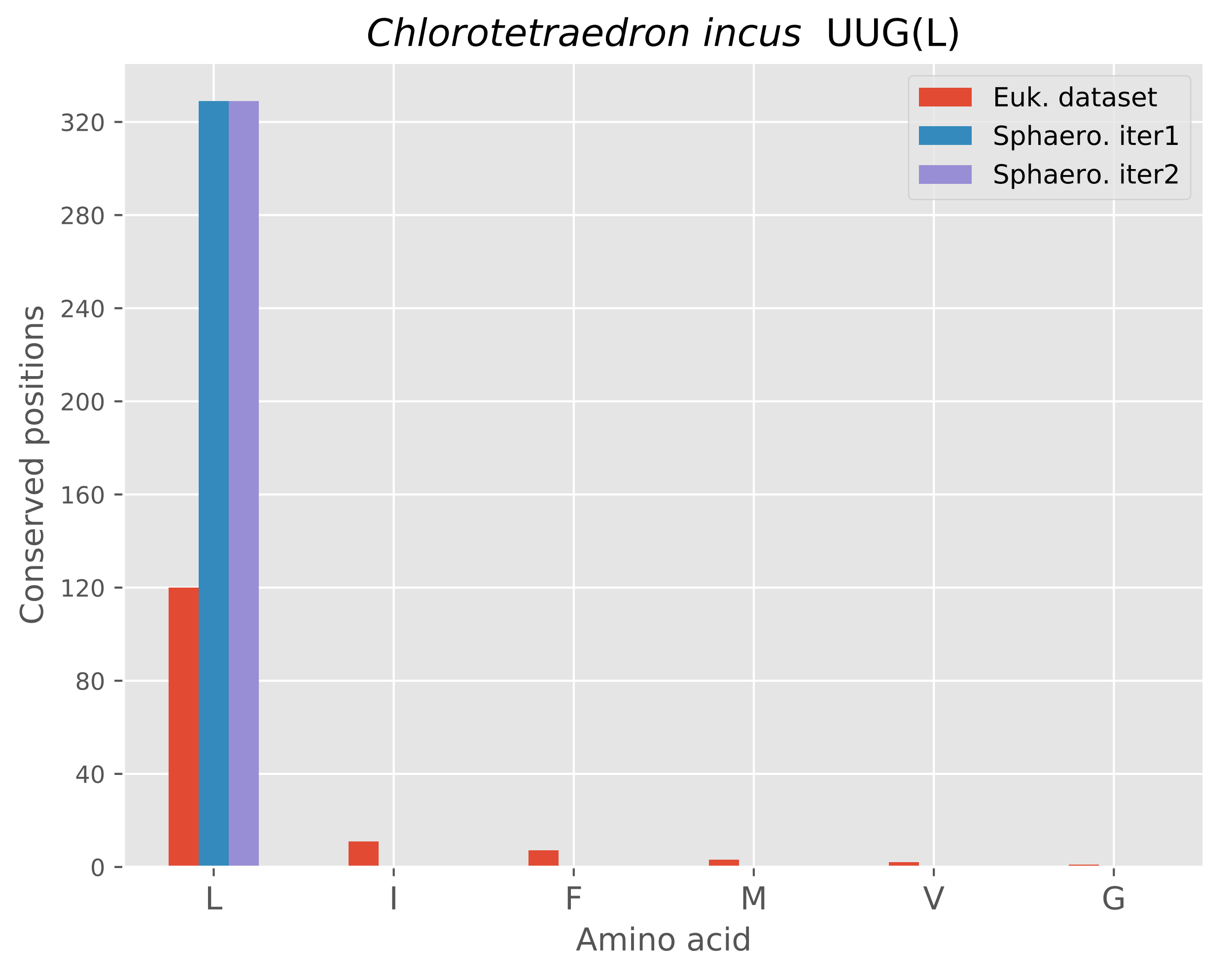

Chlorotetraedron incus UUG(L)
Euk. dataset
Sphaero. iter1
Sphaero. iter2
Conserved positions
0
40
80
120
160
200
240
280
320
L
I
F
M
V
G
Amino acid

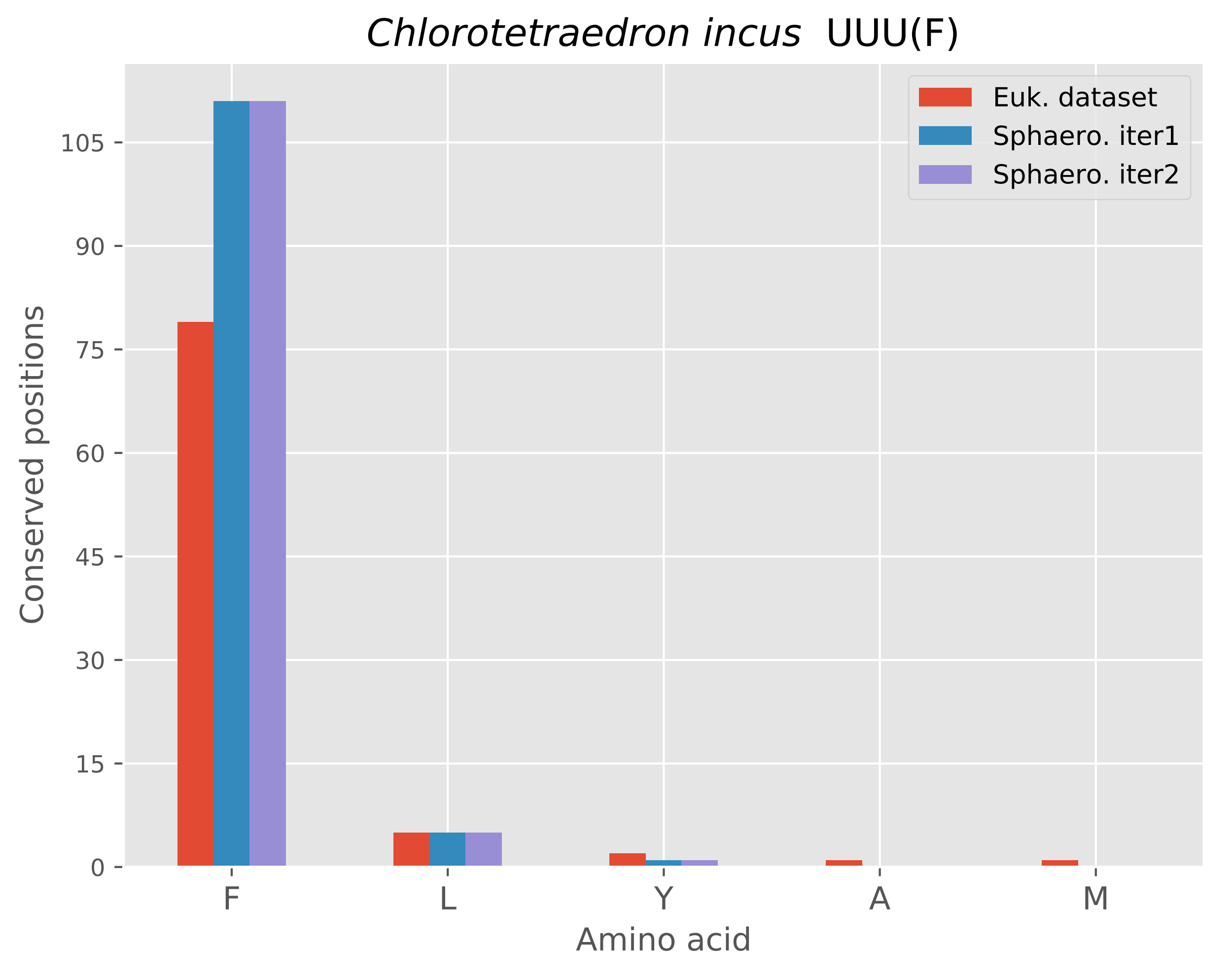

Chlorotetraedron incus UUU(F)
Euk. dataset
Sphaero. iter1
Sphaero. iter2
Conserved positions
0
15
30
45
60
75
90
105
F
L
Y
A
M
Amino acid

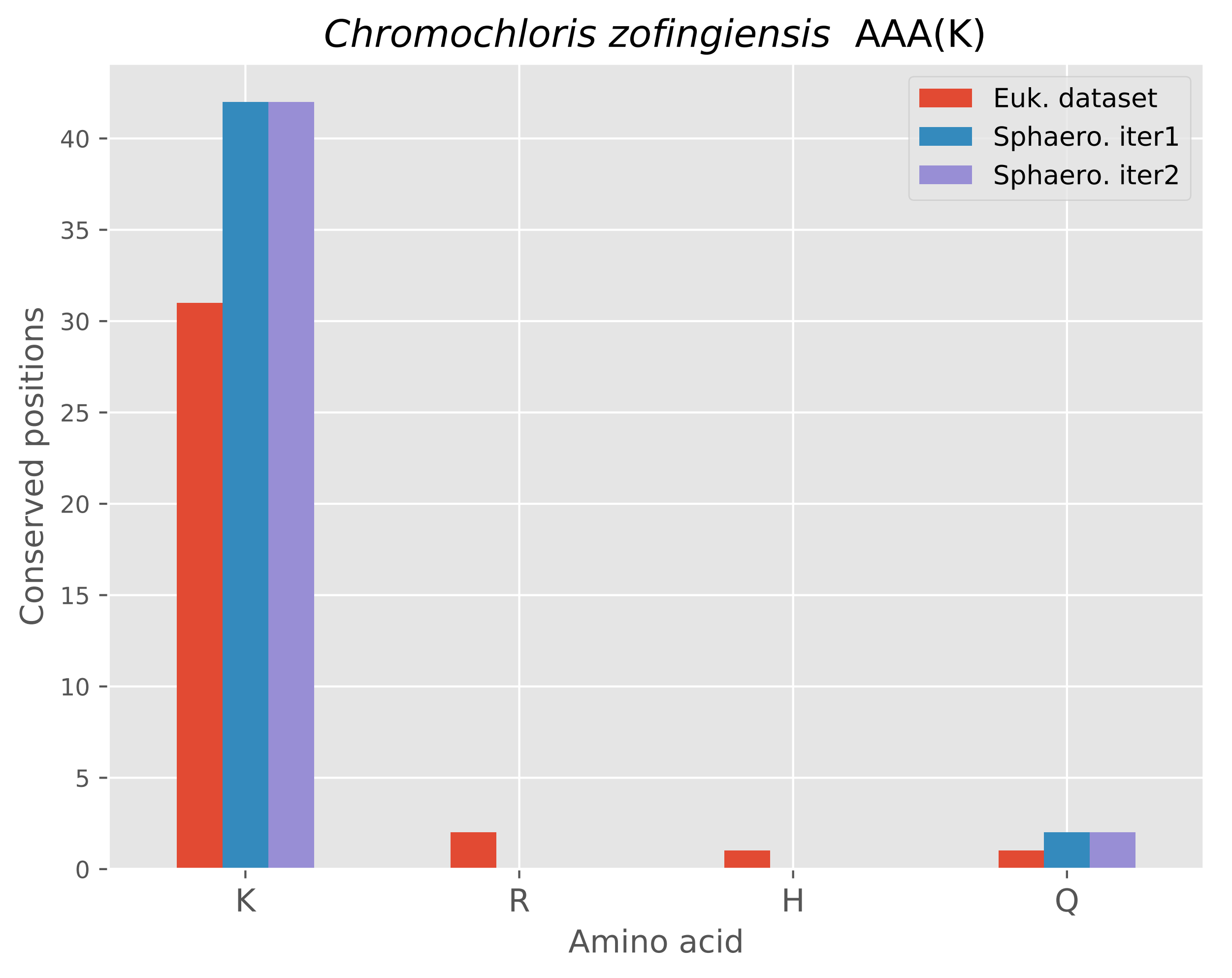

Chromochloris zofingiensis AAA(K)
Euk. dataset
Sphaero. iter1
Sphaero. iter2
Conserved positions
0
5
10
15
20
25
30
35
40
K
R
H
Q
Amino acid

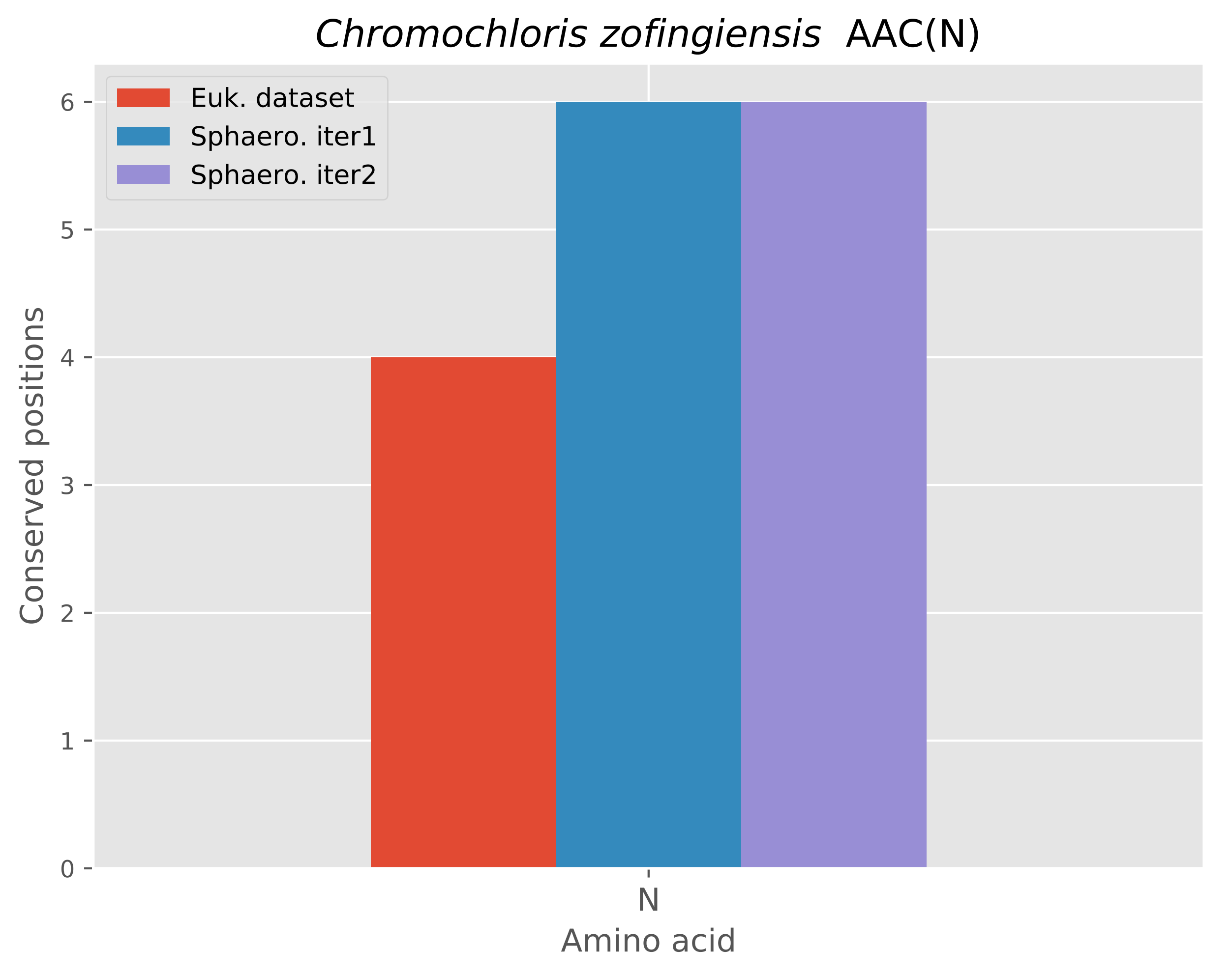

Chromochloris zofingiensis AAC(N)
Euk. dataset
Sphaero. iter1
Sphaero. iter2
Conserved positions
0
1
2
3
4
5
6
N
Amino acid

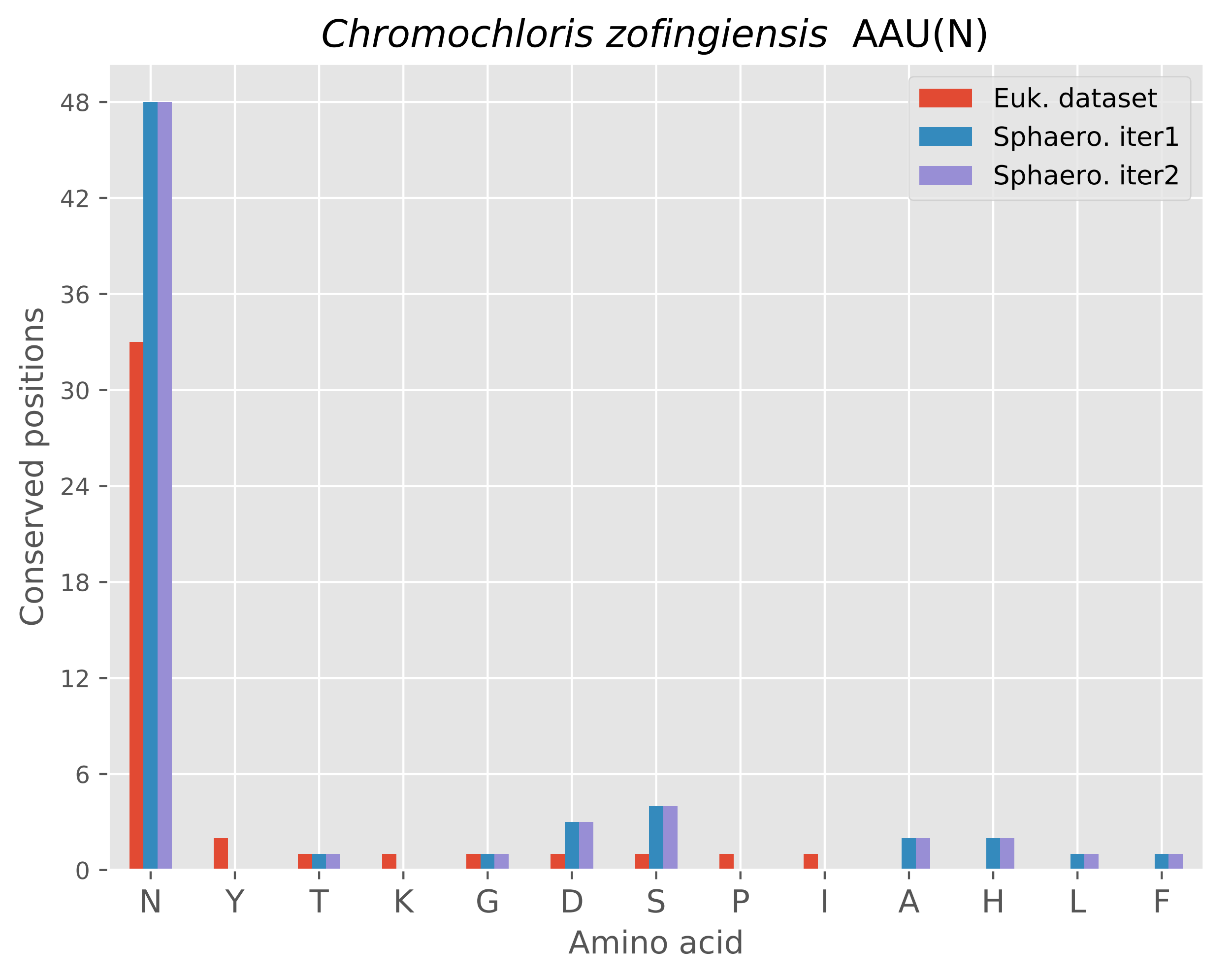

Chromochloris zofingiensis AAU(N)
Euk. dataset
Sphaero. iter1
Sphaero. iter2
Conserved positions
48
42
36
30
24
18
12
6
0
N
Y
T
K
G
D
S
P
I
A
H
L
F
Amino acid

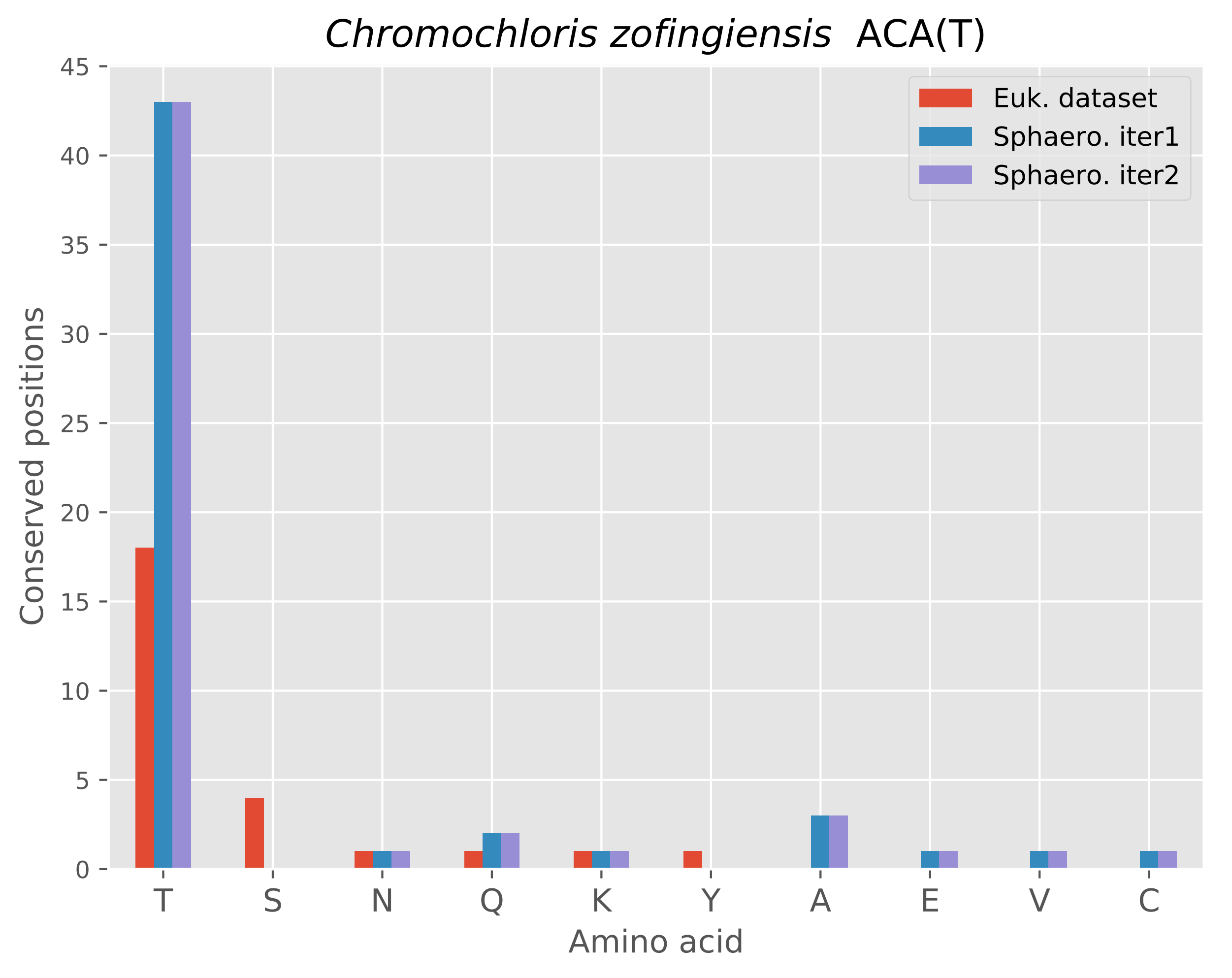

Chromochloris zofingiensis ACA(T)
Euk. dataset
Sphaero. iter1
Sphaero. iter2
Conserved positions
0
5
10
15
20
25
30
35
40
45
T
S
N
Q
K
Y
A
E
V
C
Amino acid

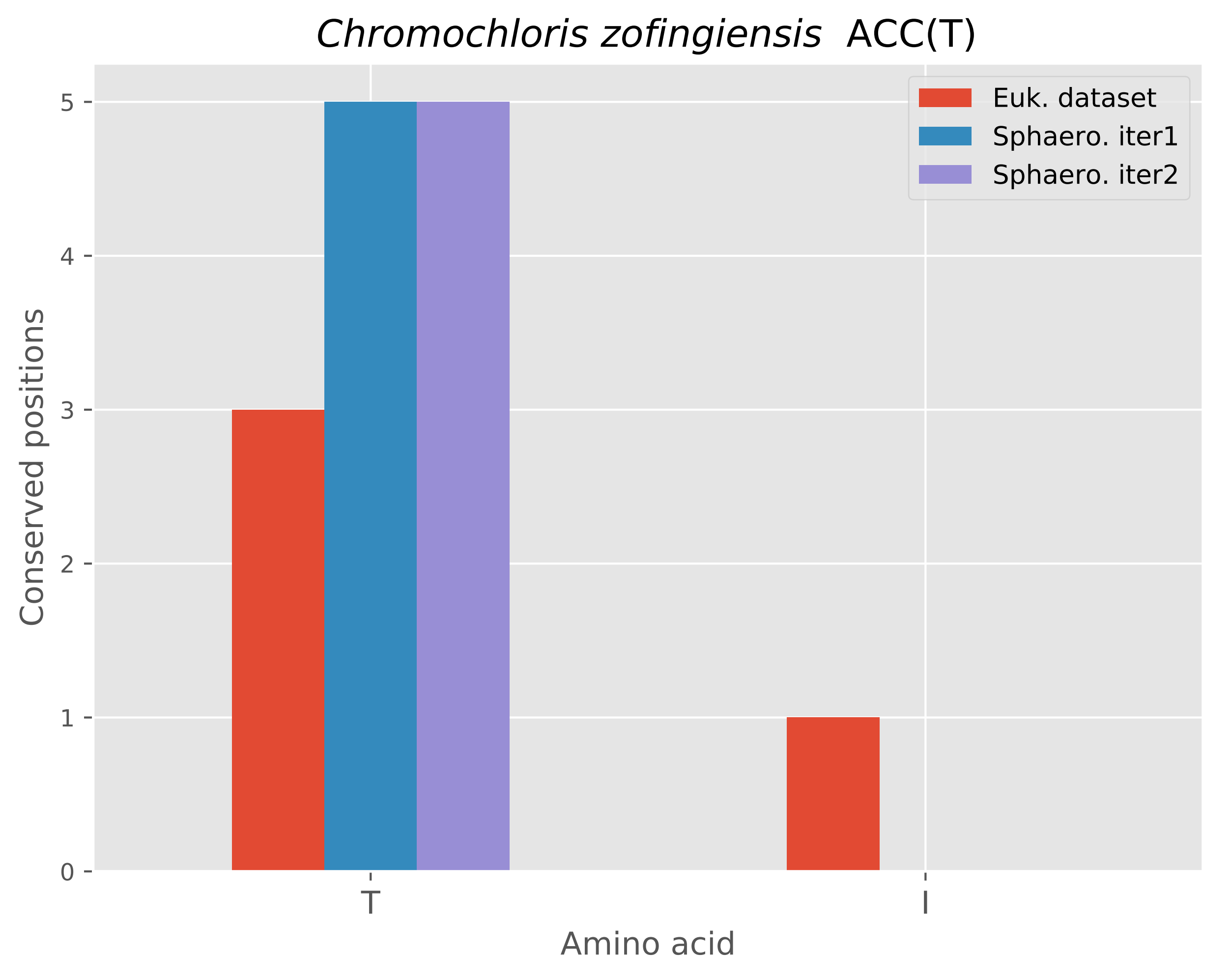

Chromochloris zofingiensis ACC(T)
Euk. dataset
Sphaero. iter1
Sphaero. iter2
Conserved positions
0
1
2
3
4
5
T
I
Amino acid

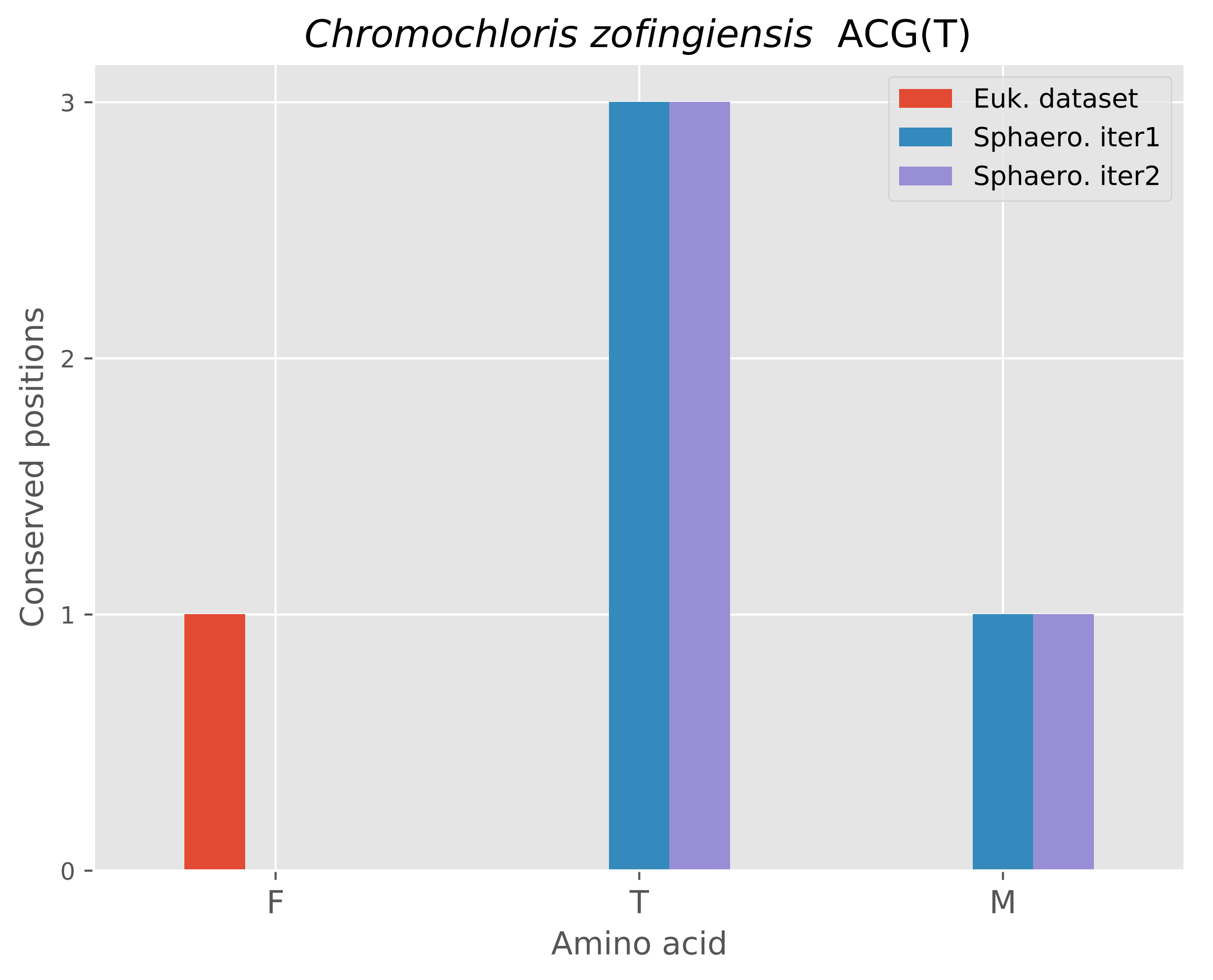

Chromochloris zofingiensis ACG(T)
Euk. dataset
Sphaero. iter1
Sphaero. iter2
Conserved positions
0
1
2
3
F
T
M
Amino acid

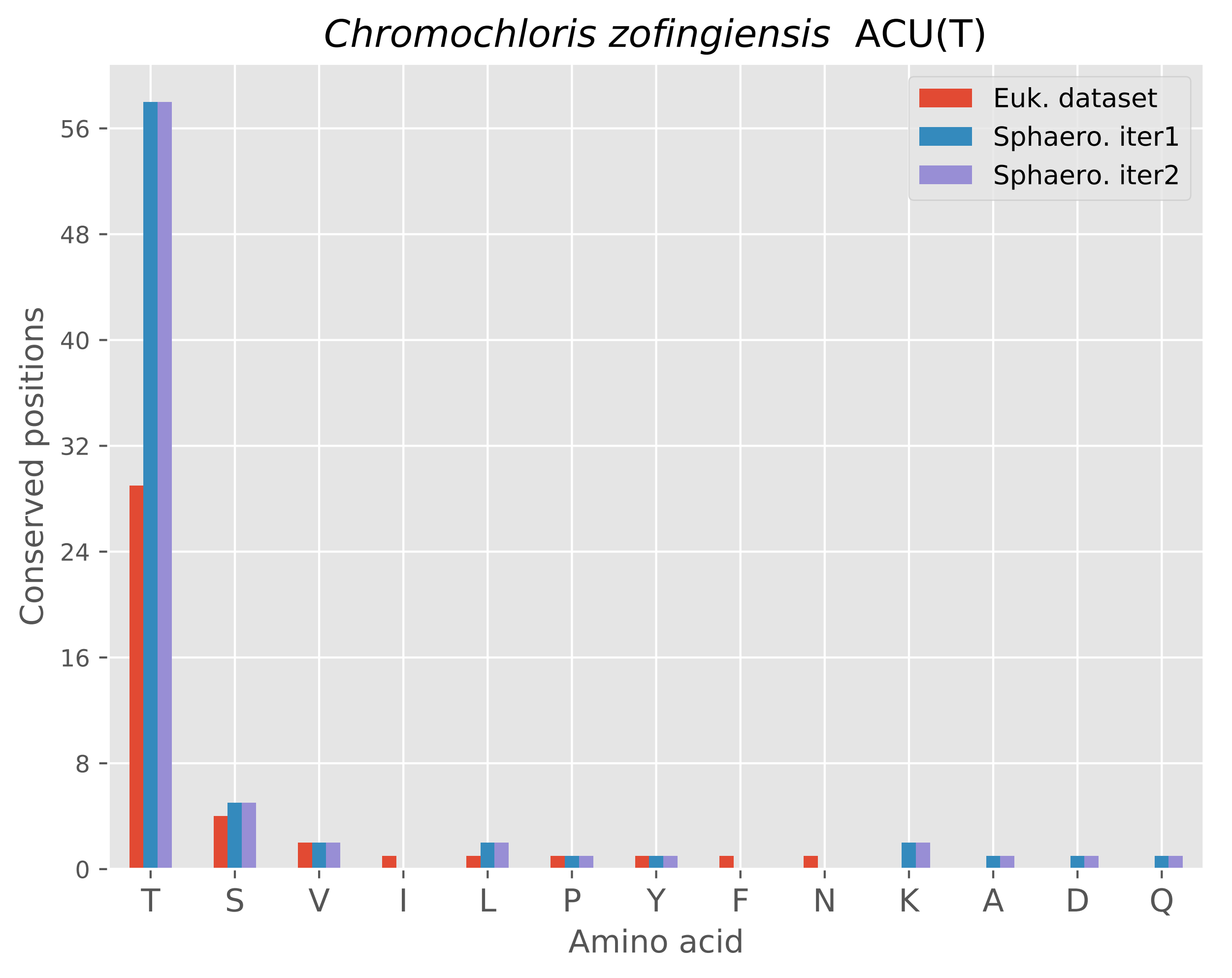

Chromochloris zofingiensis ACU(T)
Euk. dataset
Sphaero. iter1
Sphaero. iter2
Conserved positions
0
8
16
24
32
40
48
56
T
S
V
I
L
P
Y
F
N
K
A
D
Q
Amino acid

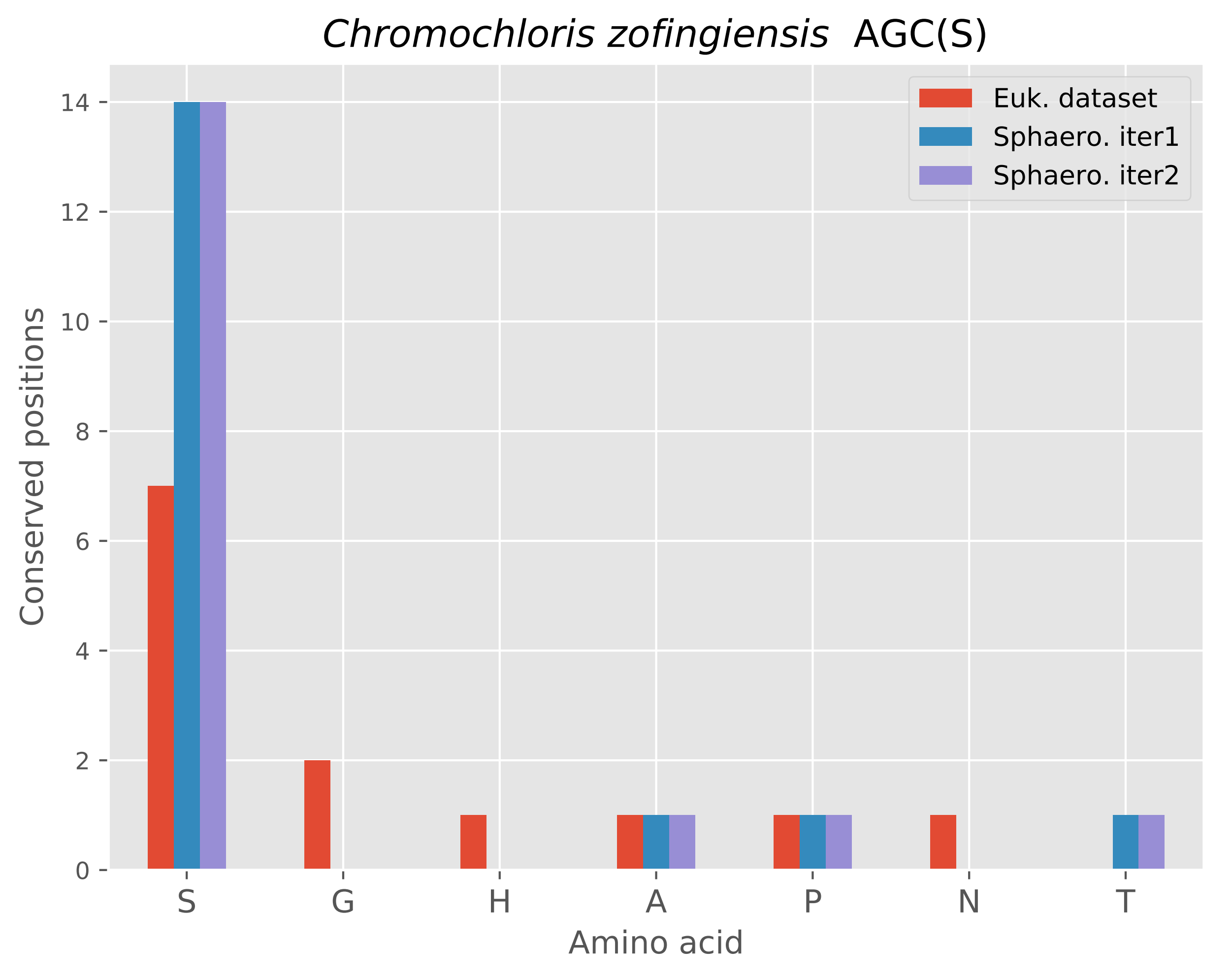
Chromochloris zofingiensis AGC(S)
Euk. dataset
Sphaero. iter1
Sphaero. iter2
Conserved positions
0
2
4
6
8
10
12
14
S
G
H
A
P
N
T
Amino acid

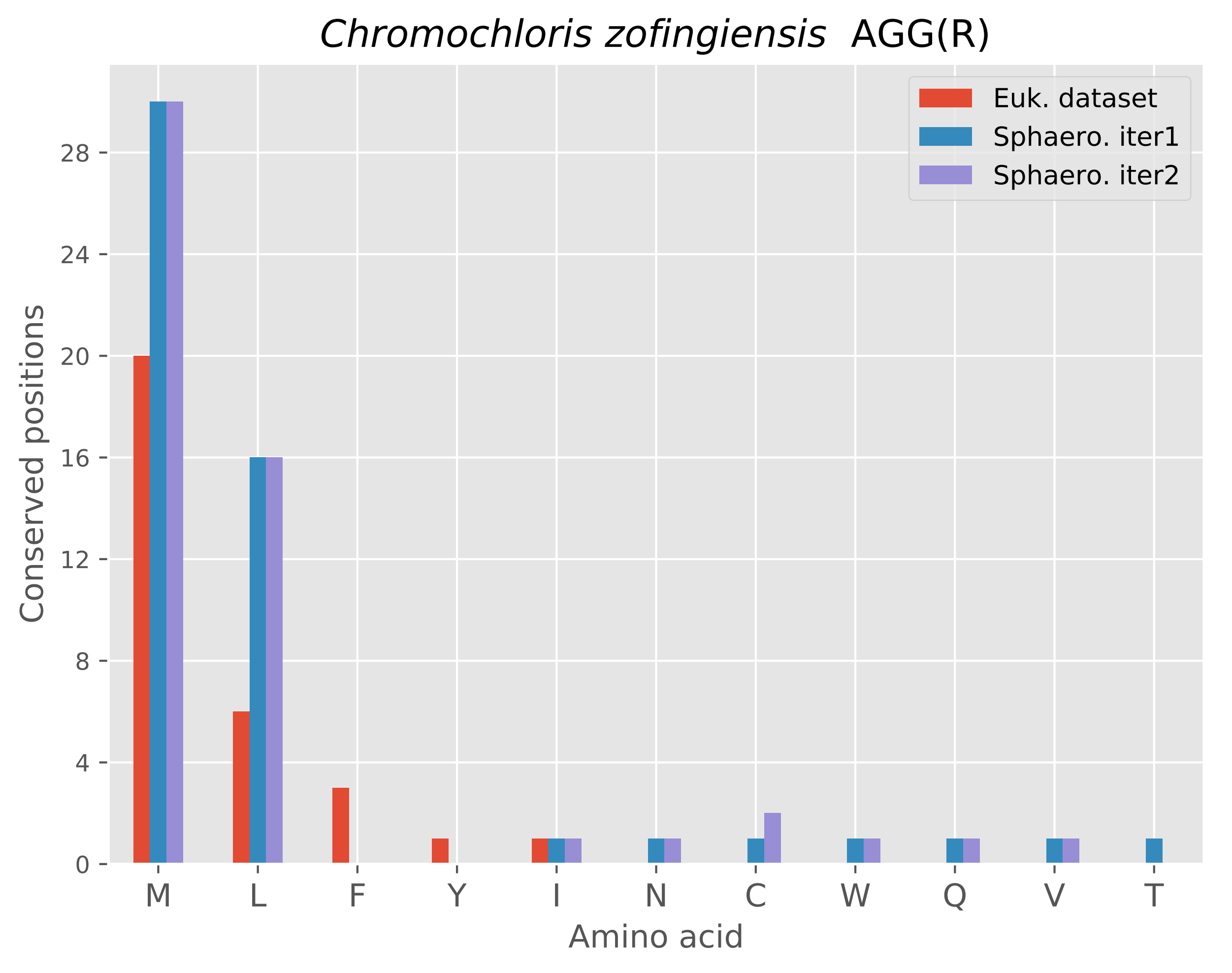

Chromochloris zofingiensis AGG(R)
Euk. dataset
Sphaero. iter1
Sphaero. iter2
Conserved positions
0
4
8
12
16
20
24
28
M
L
F
Y
I
N
C
W
Q
V
T
Amino acid

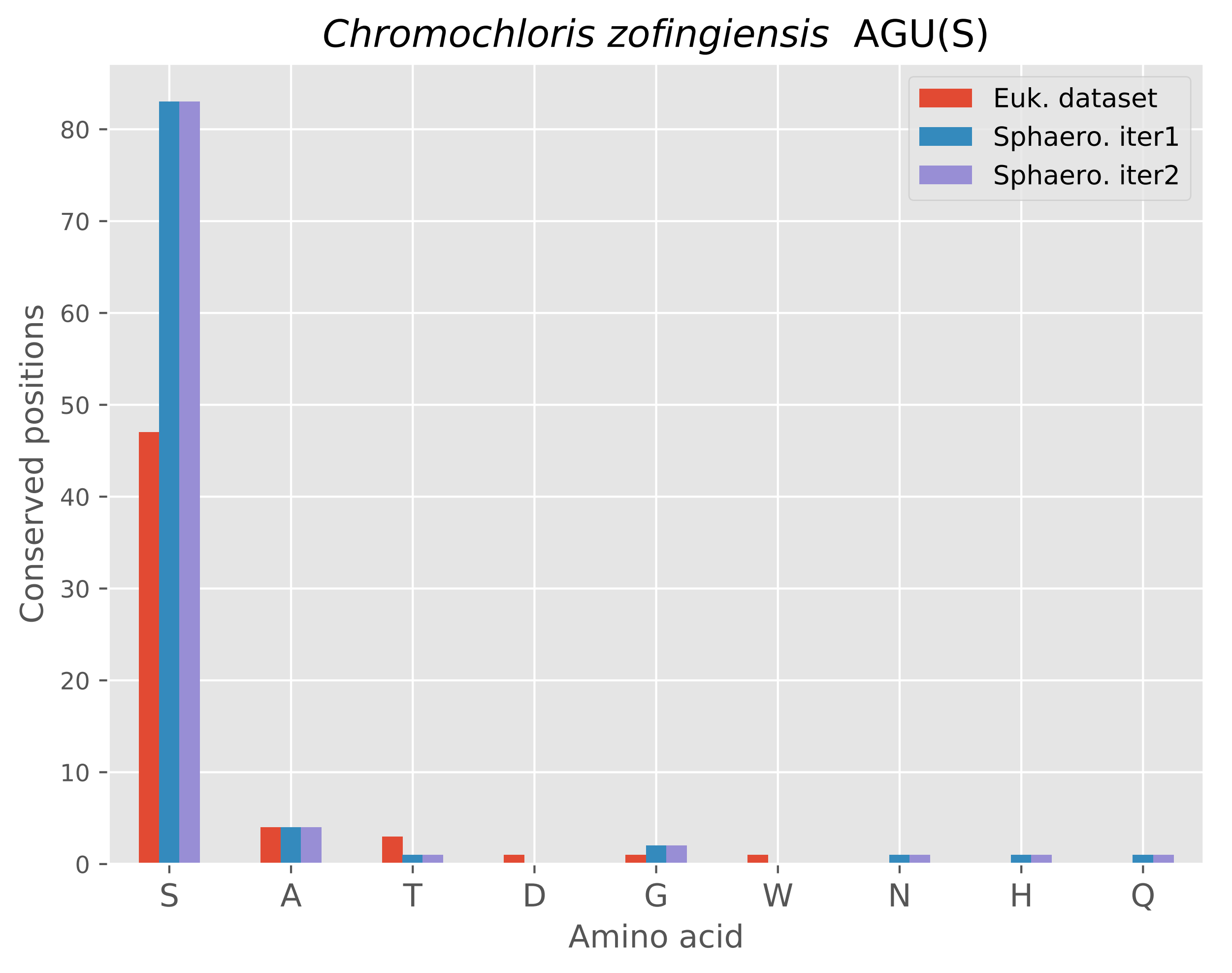

Chromochloris zofingiensis AGU(S)
Euk. dataset
Sphaero. iter1
Sphaero. iter2
Conserved positions
0
10
20
30
40
50
60
70
80
S
A
T
D
G
W
N
H
Q
Amino acid

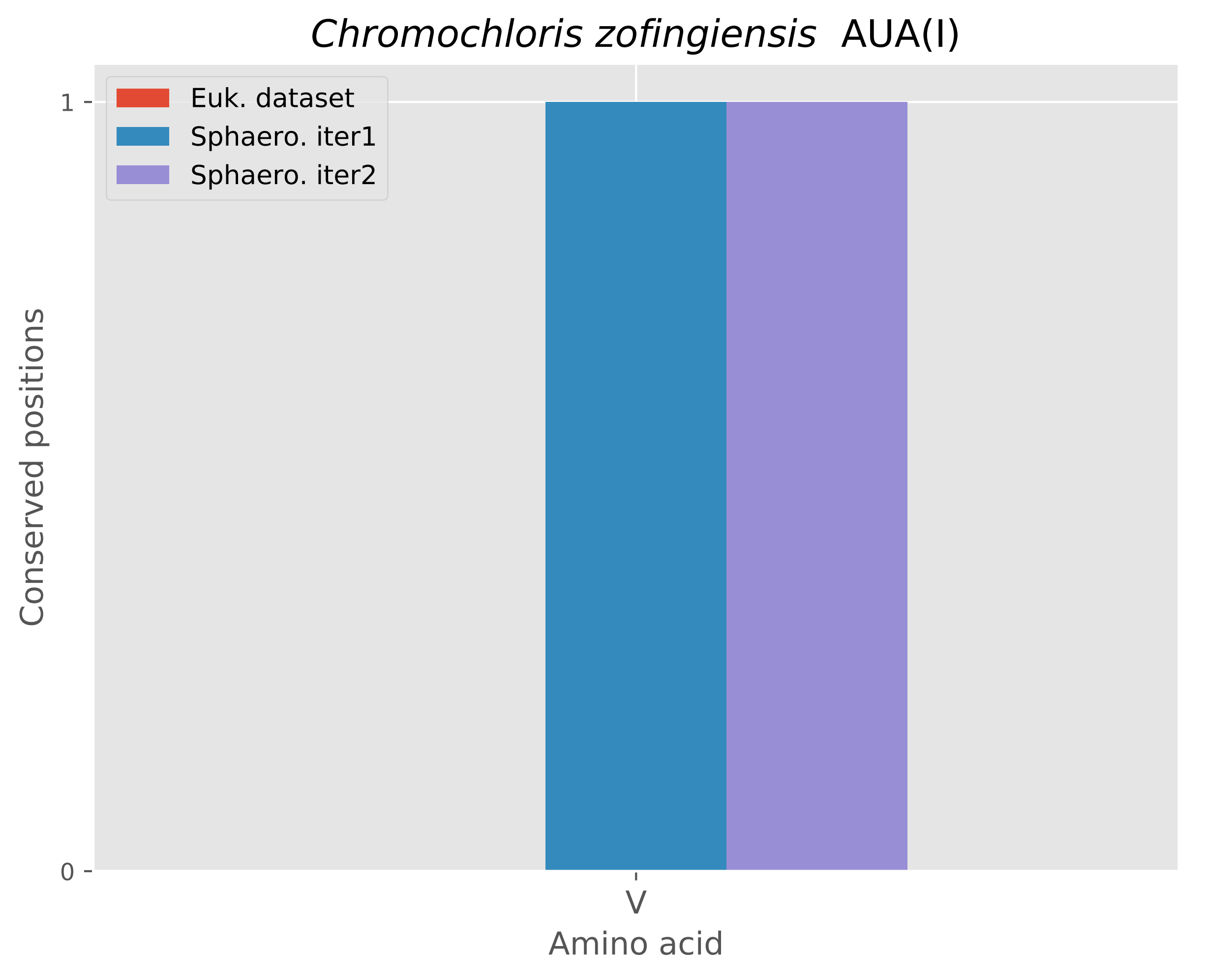

Chromochloris zofingiensis AUA(I)
Euk. dataset
Sphaero. iter1
Sphaero. iter2
Conserved positions
1
0
V
Amino acid

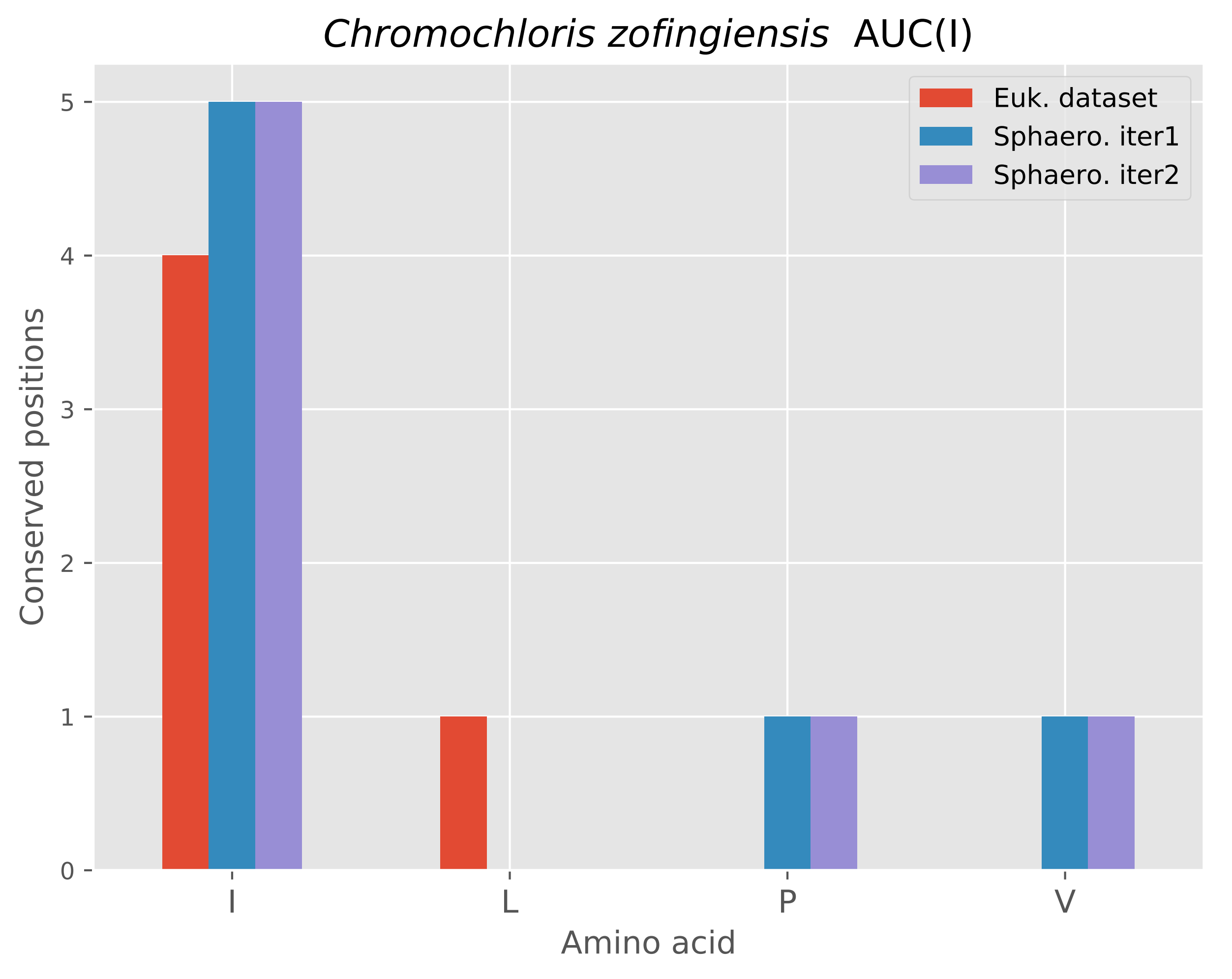

Chromochloris zofingiensis AUC(I)
Euk. dataset
Sphaero. iter1
Sphaero. iter2
Conserved positions
0
1
2
3
4
5
I
L
P
V
Amino acid

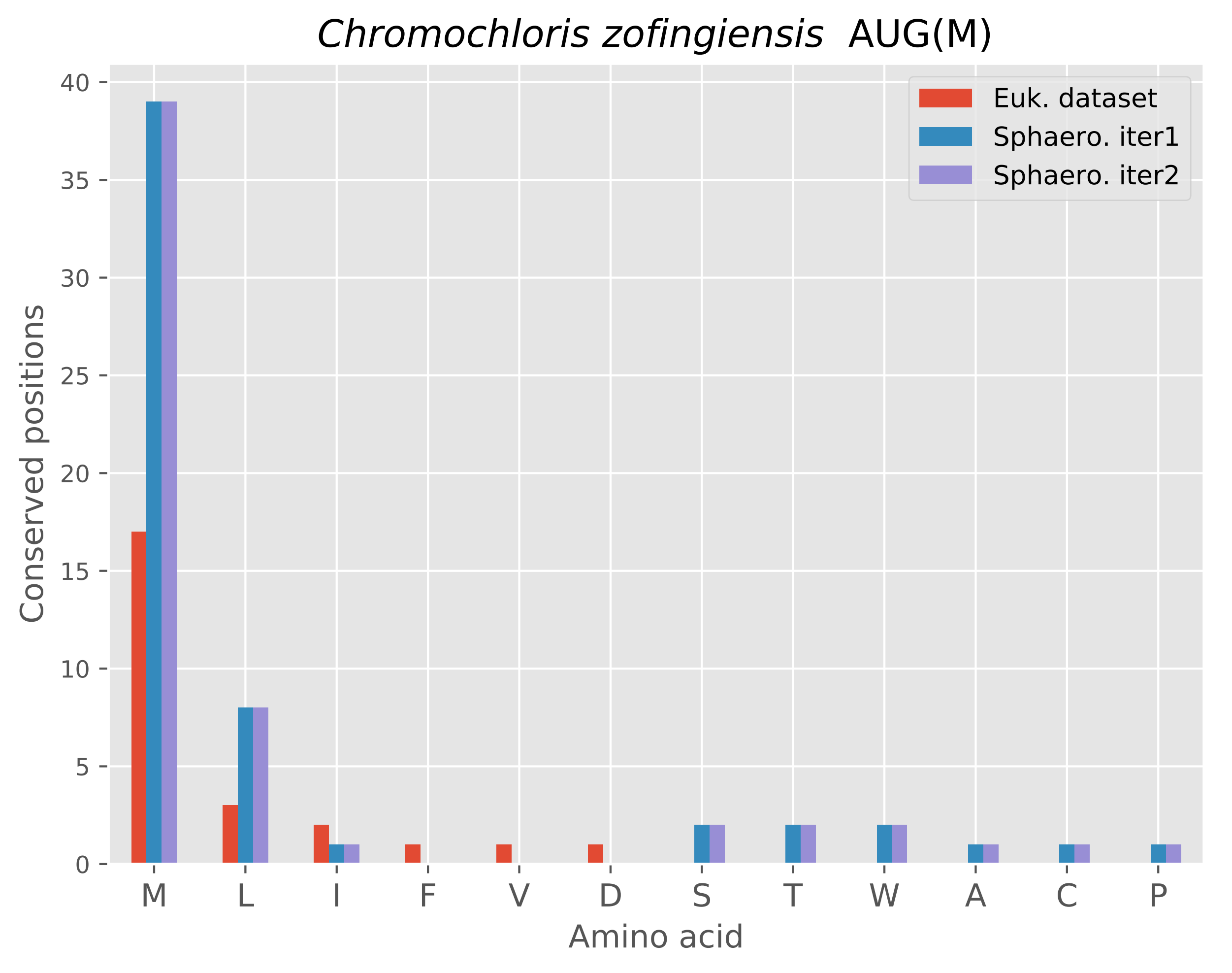
Chromochloris zofingiensis AUG(M)
Euk. dataset
Sphaero. iter1
Sphaero. iter2
Conserved positions
0
5
10
15
20
25
30
35
40
M
L
I
F
V
D
S
T
W
A
C
P
Amino acid

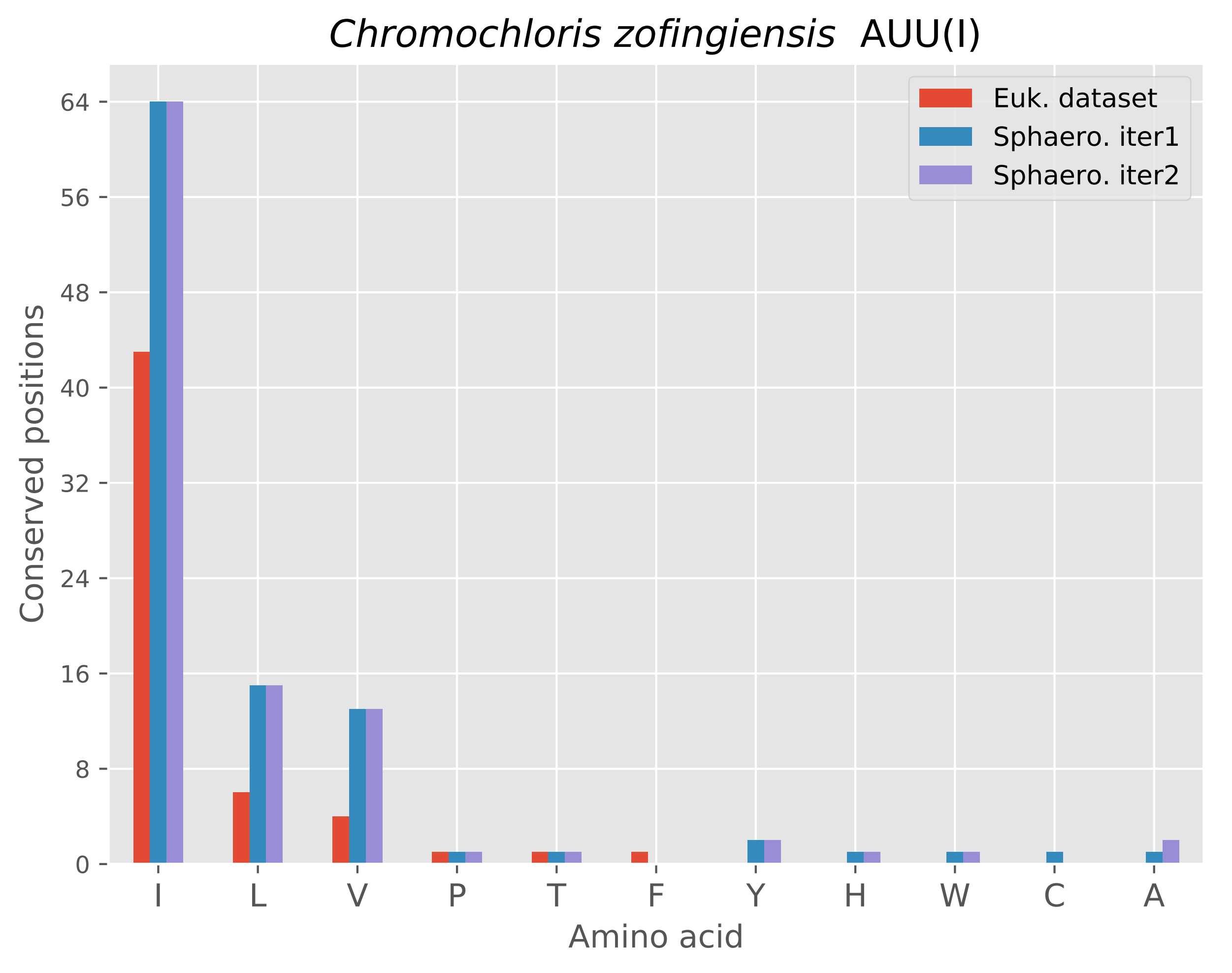
Chromochloris zofingiensis AUU(I)
Euk. dataset
Sphaero. iter1
Sphaero. iter2
Conserved positions
0
8
16
24
32
40
48
56
64
I
L
V
P
T
F
Y
H
W
C
A
Amino acid

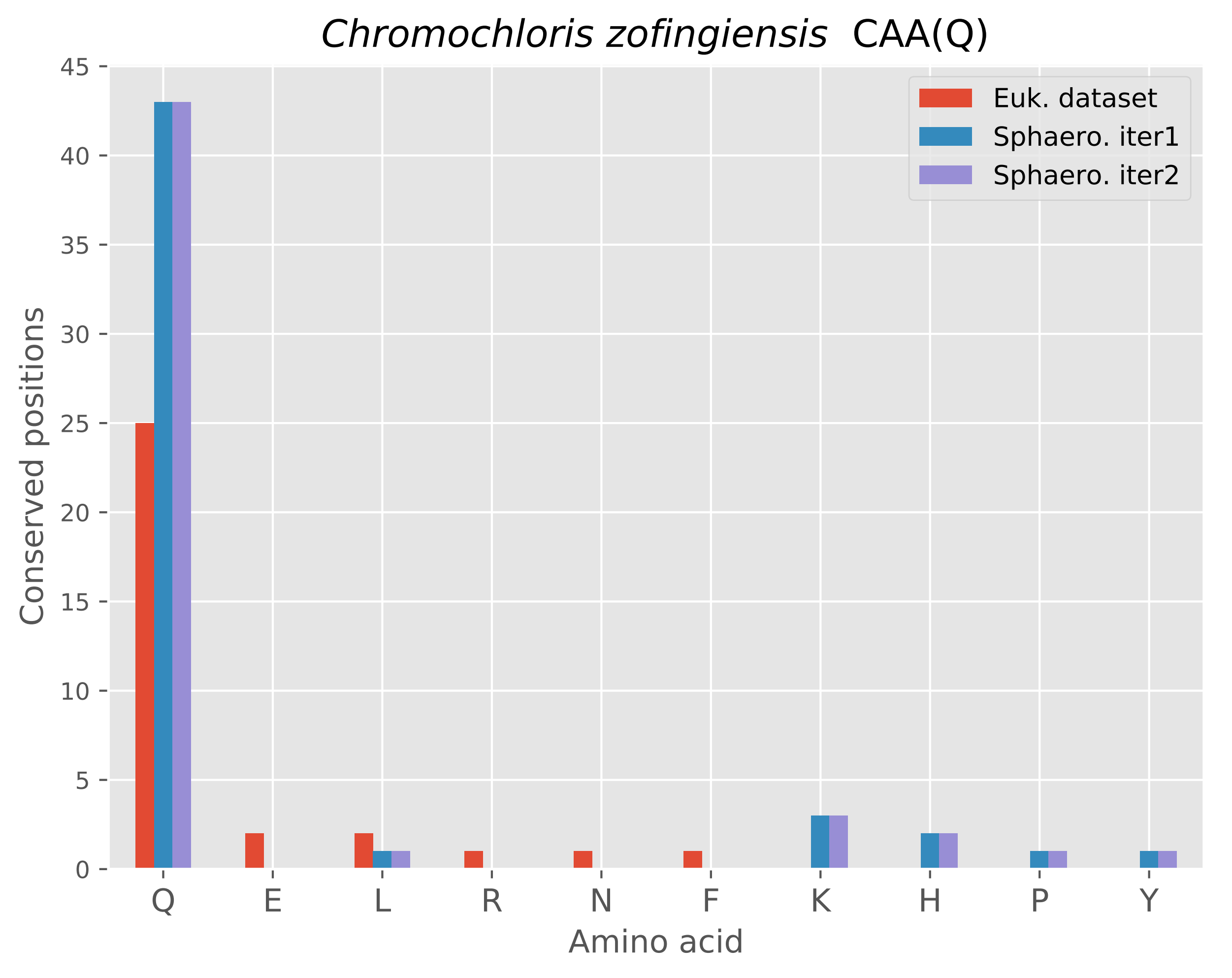

Chromochloris zofingiensis CAA(Q)
Conserved positions
Amino acid
Euk. dataset
Sphaero. iter1
Sphaero. iter2
0
5
10
15
20
25
30
35
40
45
Q
E
L
R
N
F
K
H
P
Y

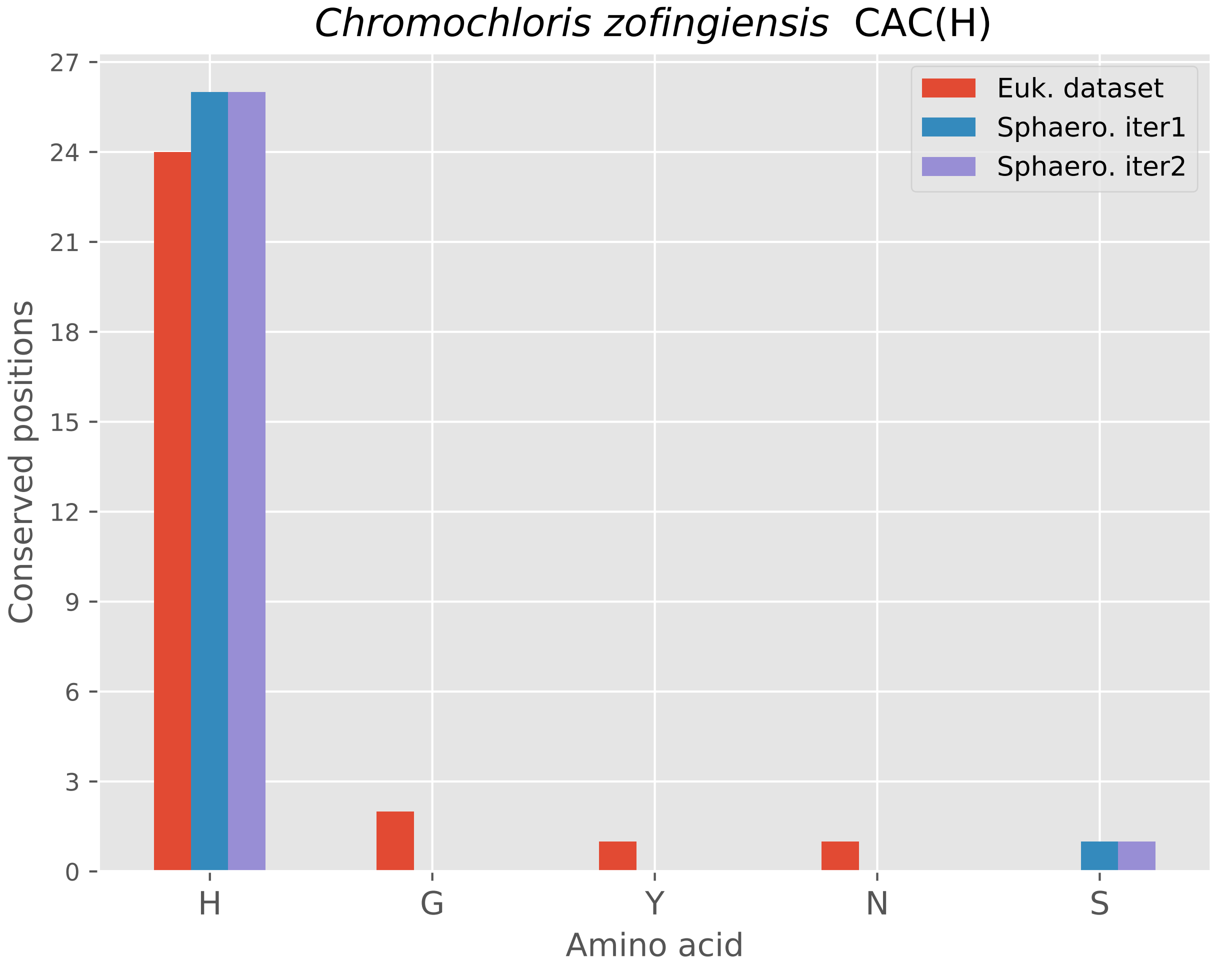

Chromochloris zofingiensis CAC(H)
Euk. dataset
Sphaero. iter1
Sphaero. iter2
Conserved positions
0
3
6
9
12
15
18
21
24
27
H
G
Y
N
S
Amino acid

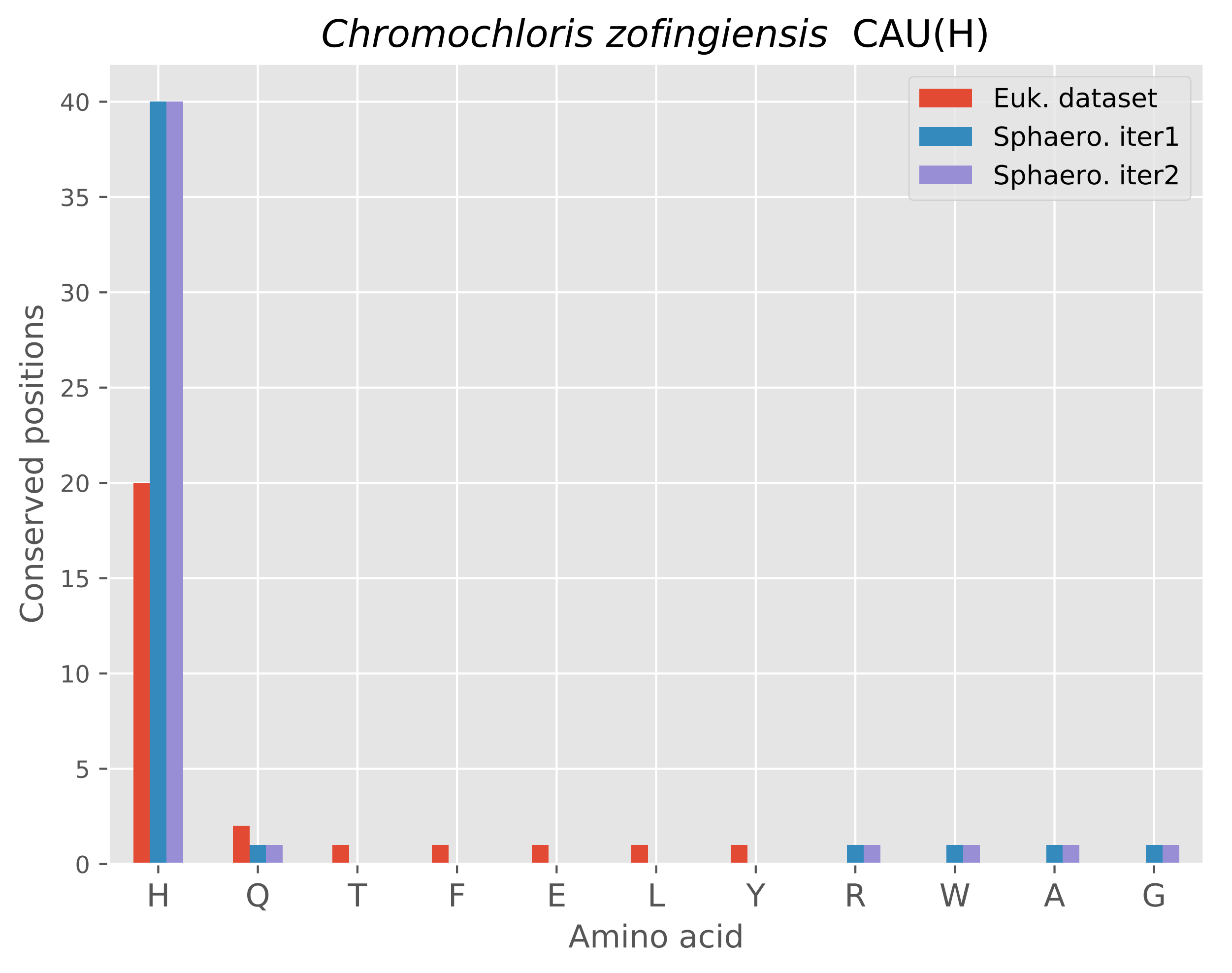

Chromochloris zofingiensis CAU(H)
Euk. dataset
Sphaero. iter1
Sphaero. iter2
Conserved positions
0
5
10
15
20
25
30
35
40
H
Q
T
F
E
L
Y
R
W
A
G
Amino acid

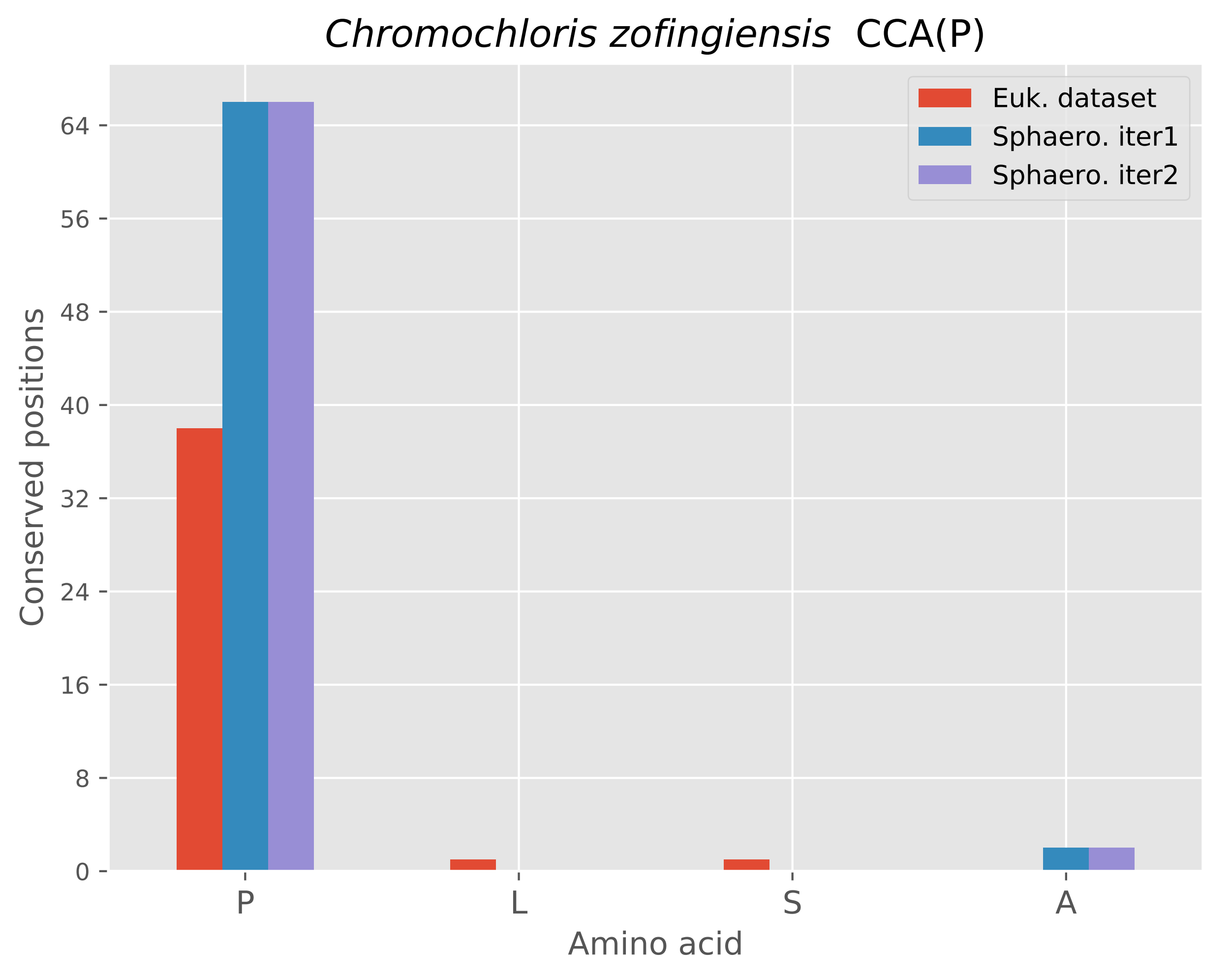
Chromochloris zofingiensis CCA(P)
Euk. dataset
Sphaero. iter1
Sphaero. iter2
Conserved positions
0
8
16
24
32
40
48
56
64
P
L
S
A
Amino acid

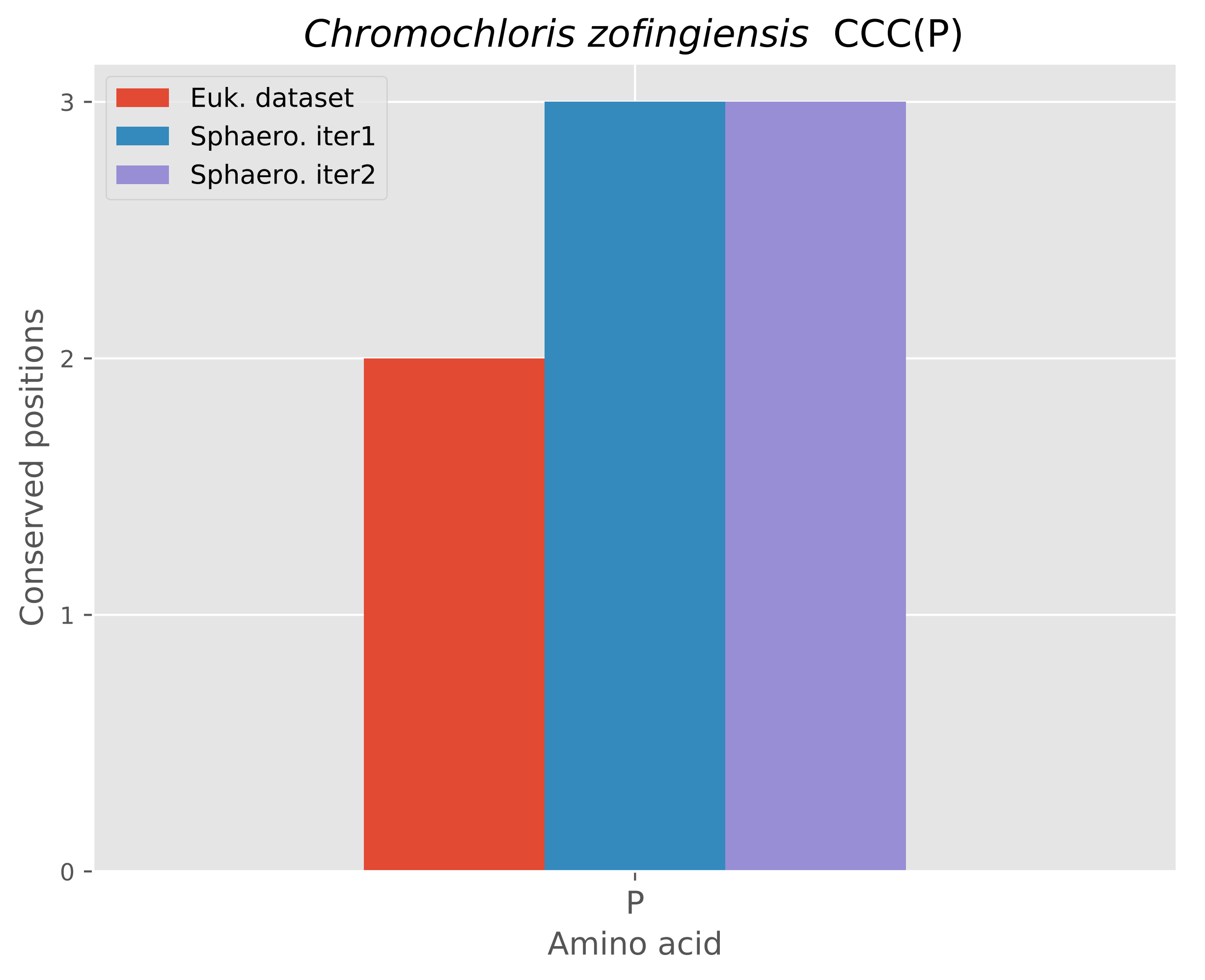

Chromochloris zofingiensis CCC(P)
Euk. dataset
Sphaero. iter1
Sphaero. iter2
Conserved positions
0
1
2
3
P
Amino acid

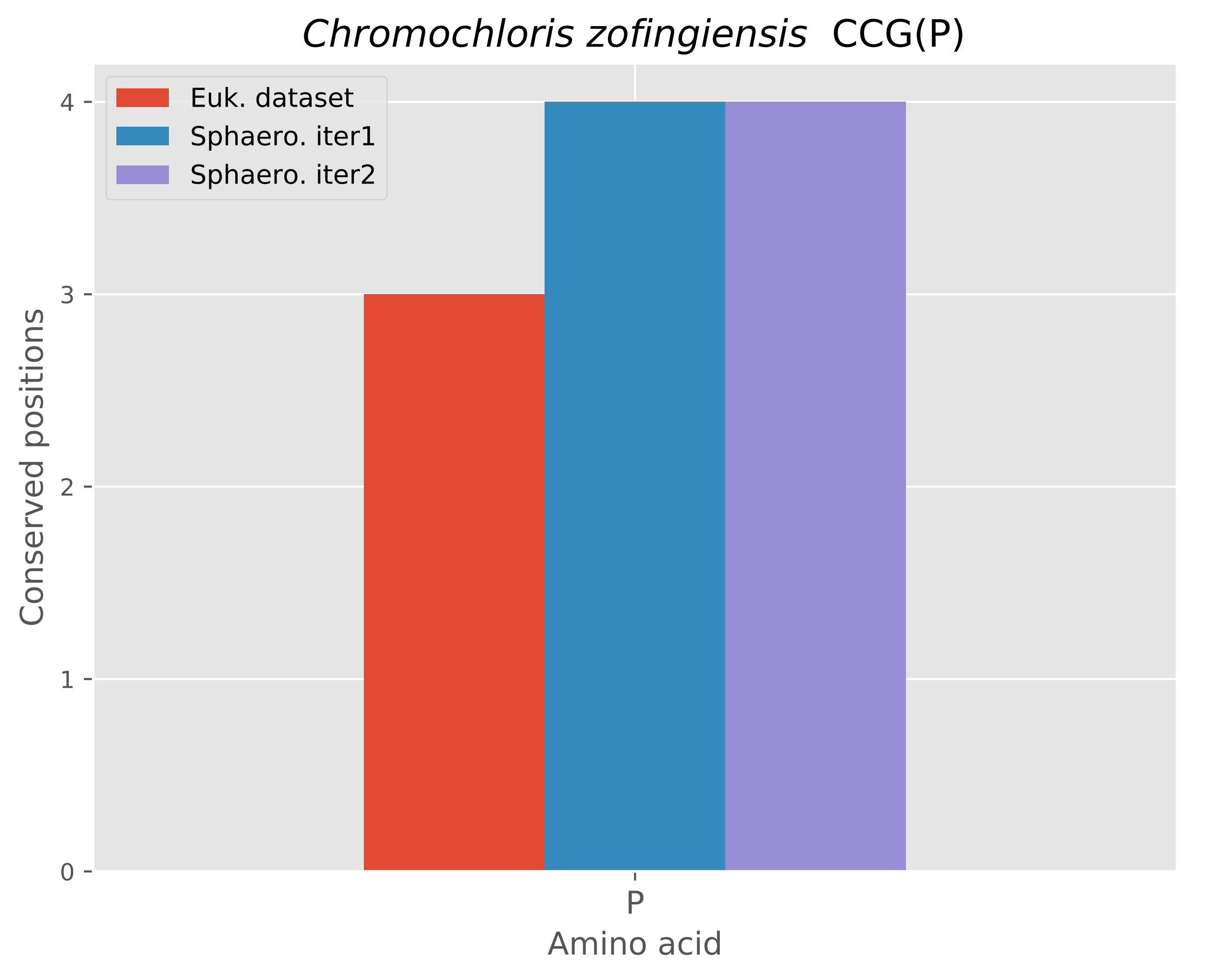

Chromochloris zofingiensis CCG(P)
Euk. dataset
Sphaero. iter1
Sphaero. iter2
Conserved positions
0
1
2
3
4
P
Amino acid

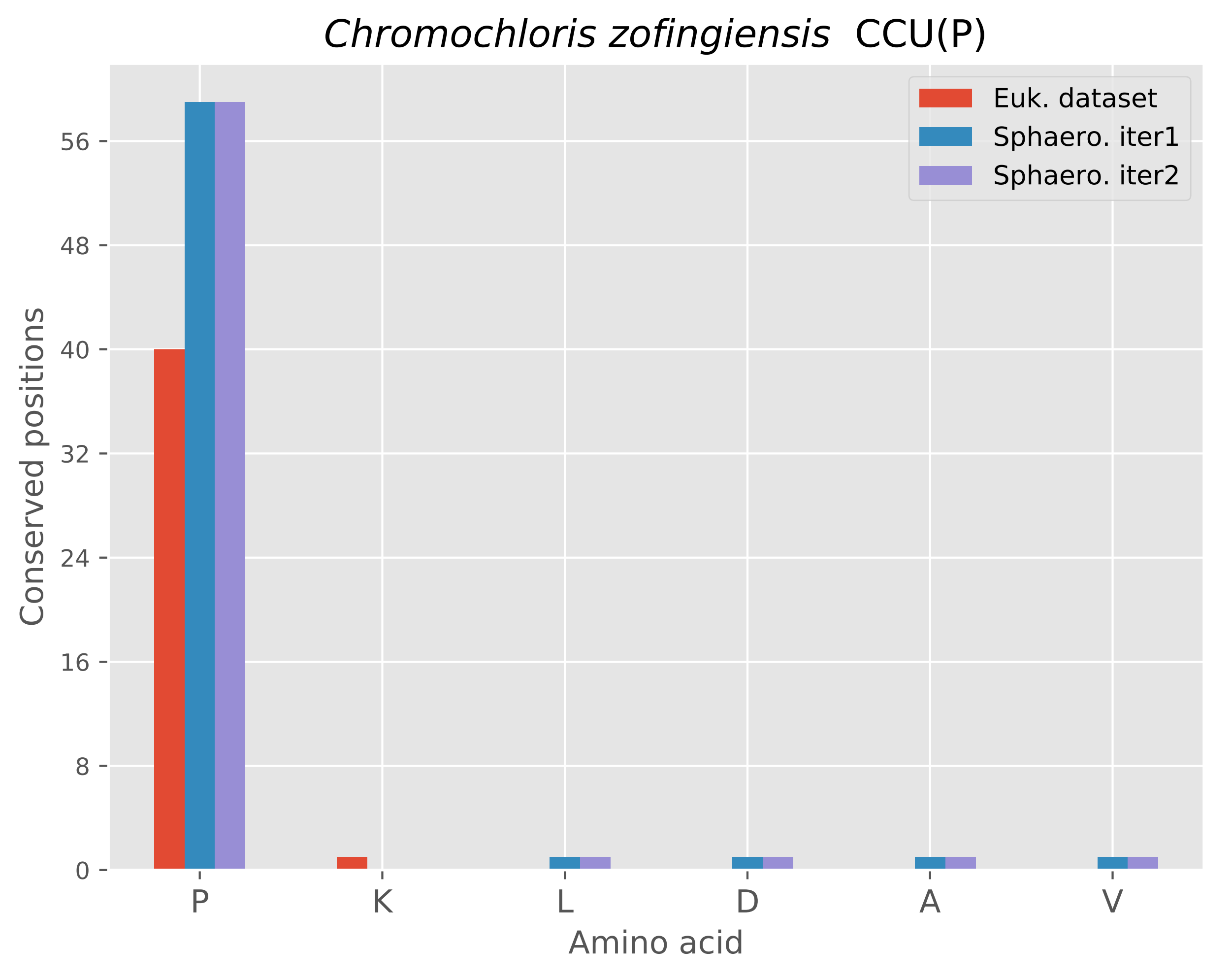

Chromochloris zofingiensis CCU(P)
Euk. dataset
Sphaero. iter1
Sphaero. iter2
Conserved positions
0
8
16
24
32
40
48
56
P
K
L
D
A
V
Amino acid

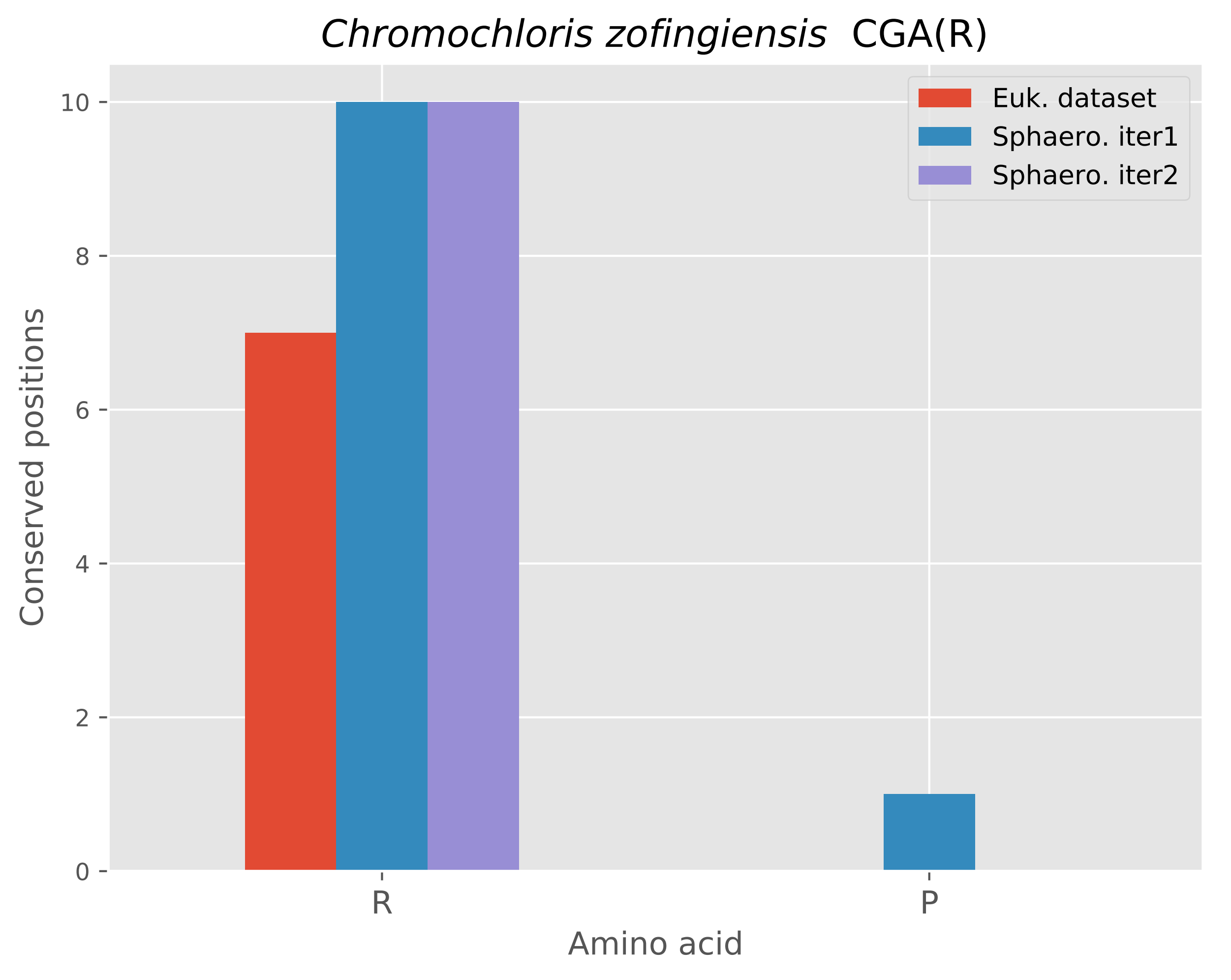

Chromochloris zofingiensis CGA(R)
Euk. dataset
Sphaero. iter1
Sphaero. iter2
Conserved positions
0
2
4
6
8
10
R
P
Amino acid

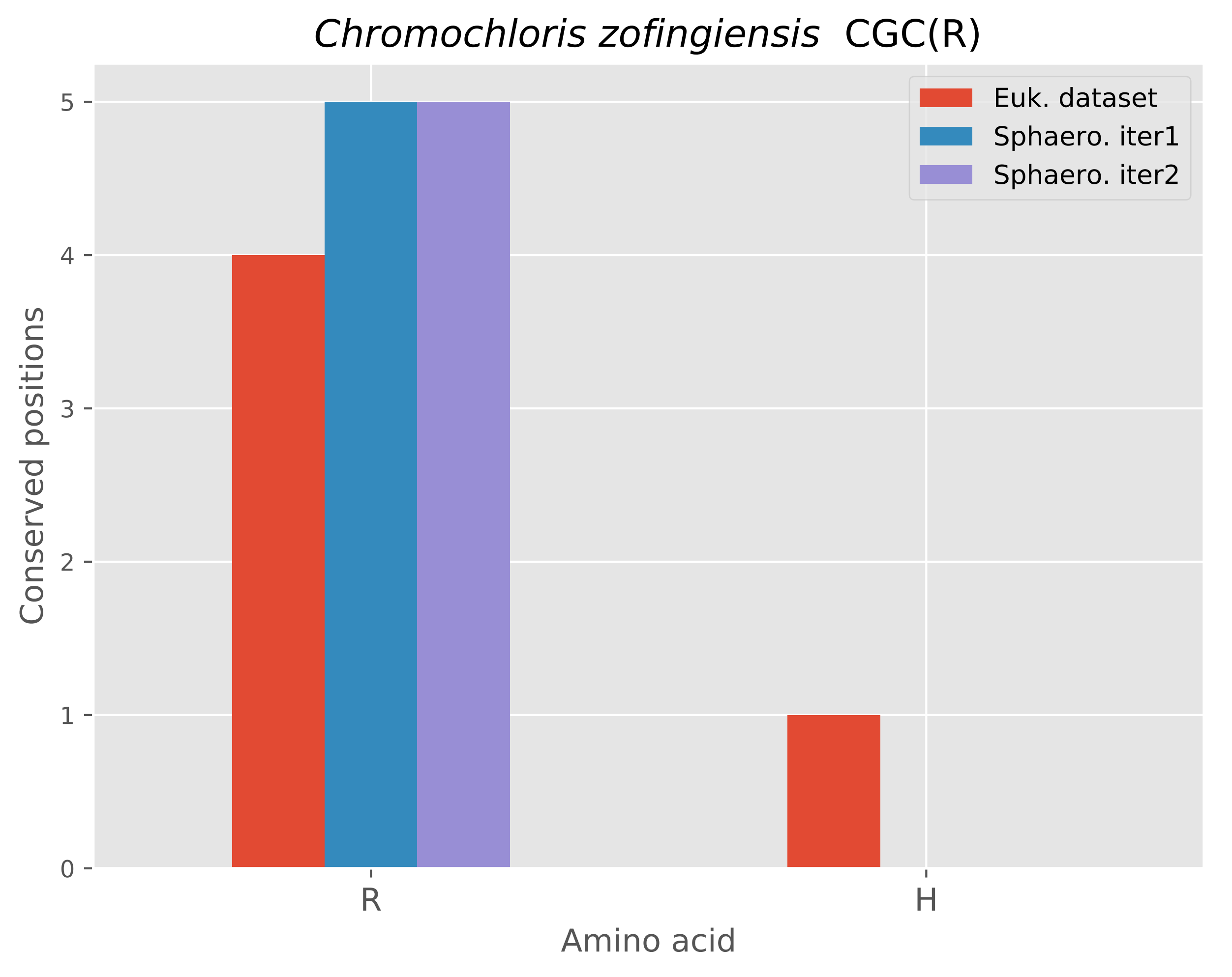

Chromochloris zofingiensis CGC(R)
Euk. dataset
Sphaero. iter1
Sphaero. iter2
Conserved positions
0
1
2
3
4
5
R
H
Amino acid

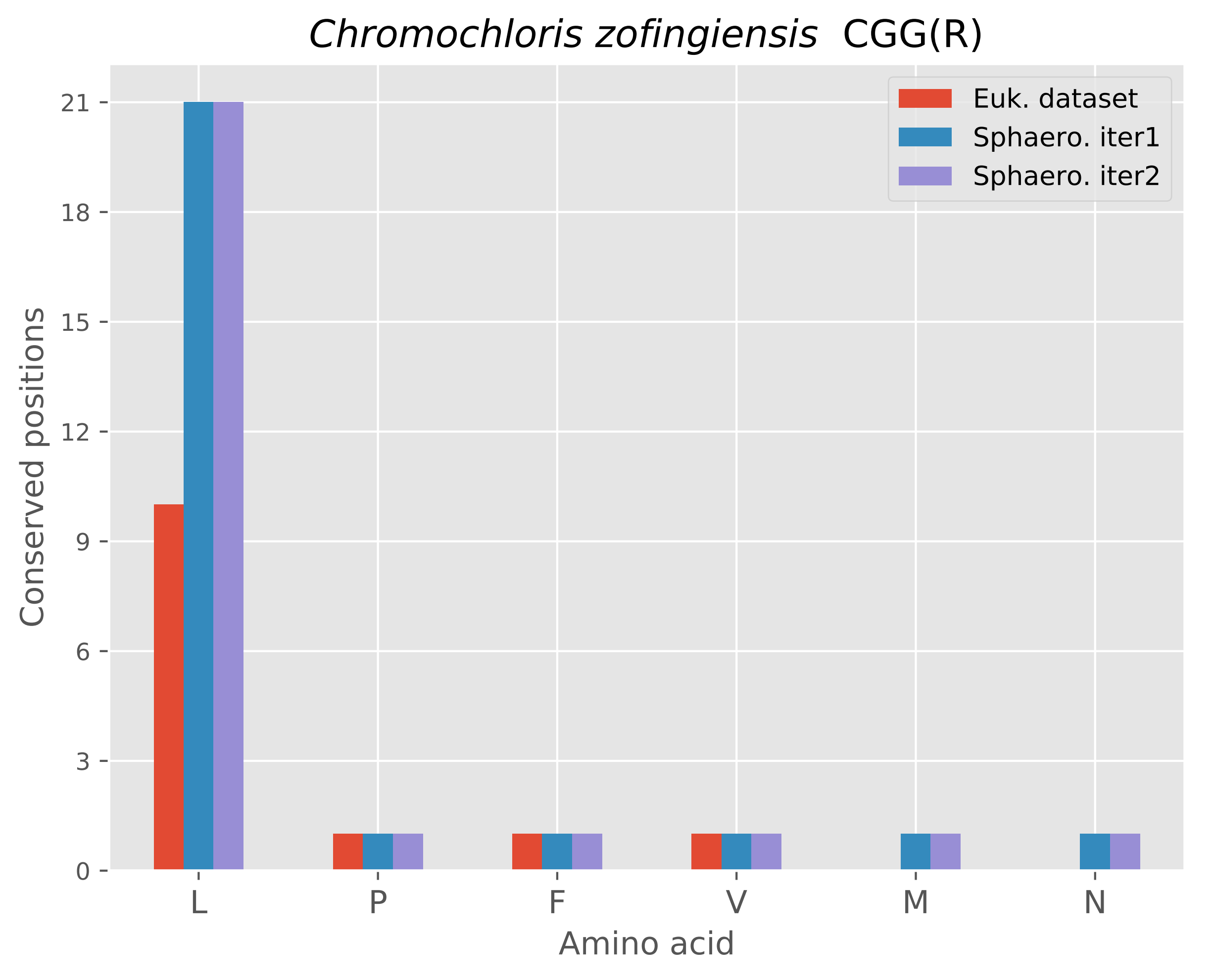

Chromochloris zofingiensis CGG(R)
Euk. dataset
Sphaero. iter1
Sphaero. iter2
Conserved positions
0
3
6
9
12
15
18
21
L
P
F
V
M
N
Amino acid

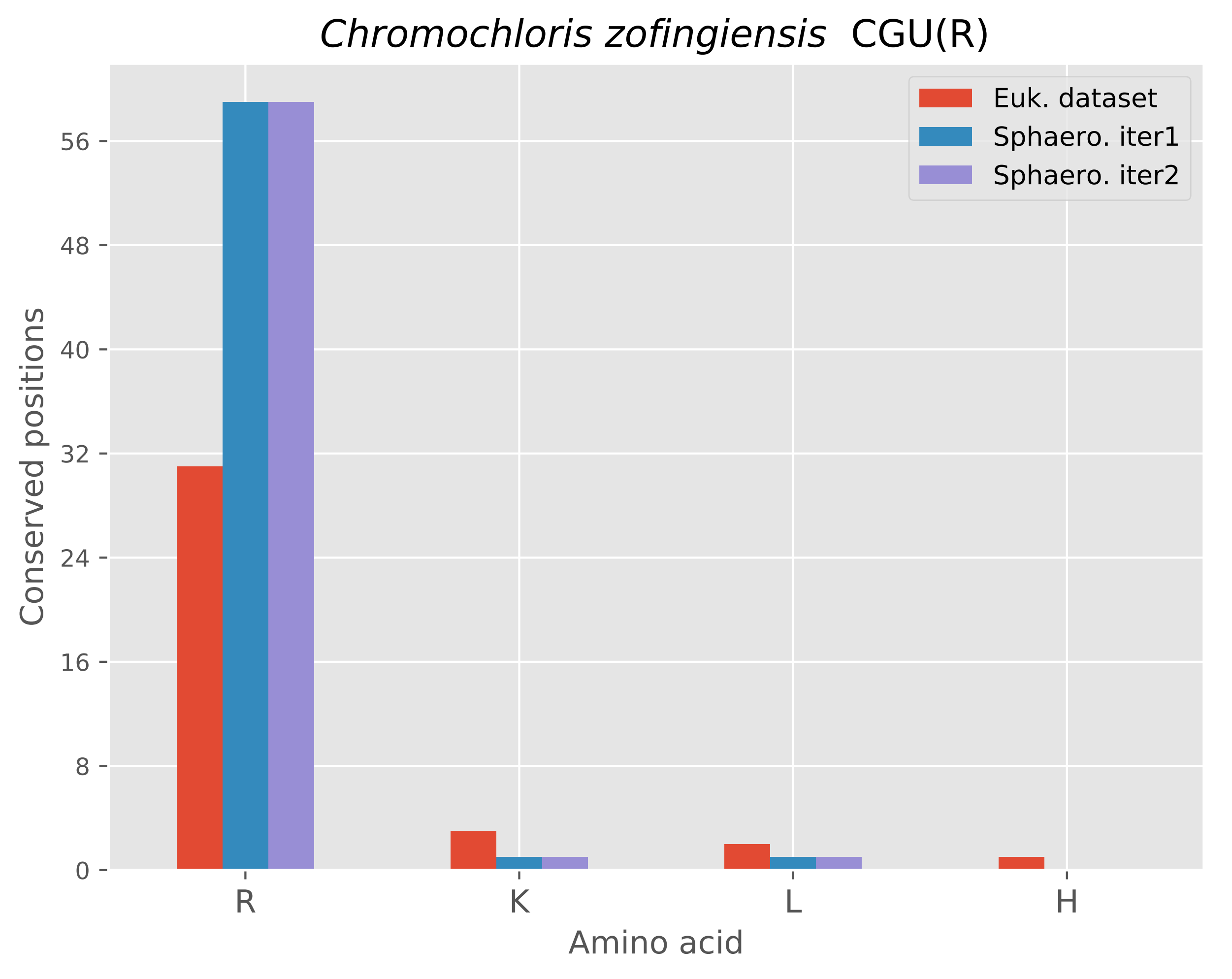

Chromochloris zofingiensis CGU(R)
Euk. dataset
Sphaero. iter1
Sphaero. iter2
Conserved positions
0
8
16
24
32
40
48
56
R
K
L
H
Amino acid

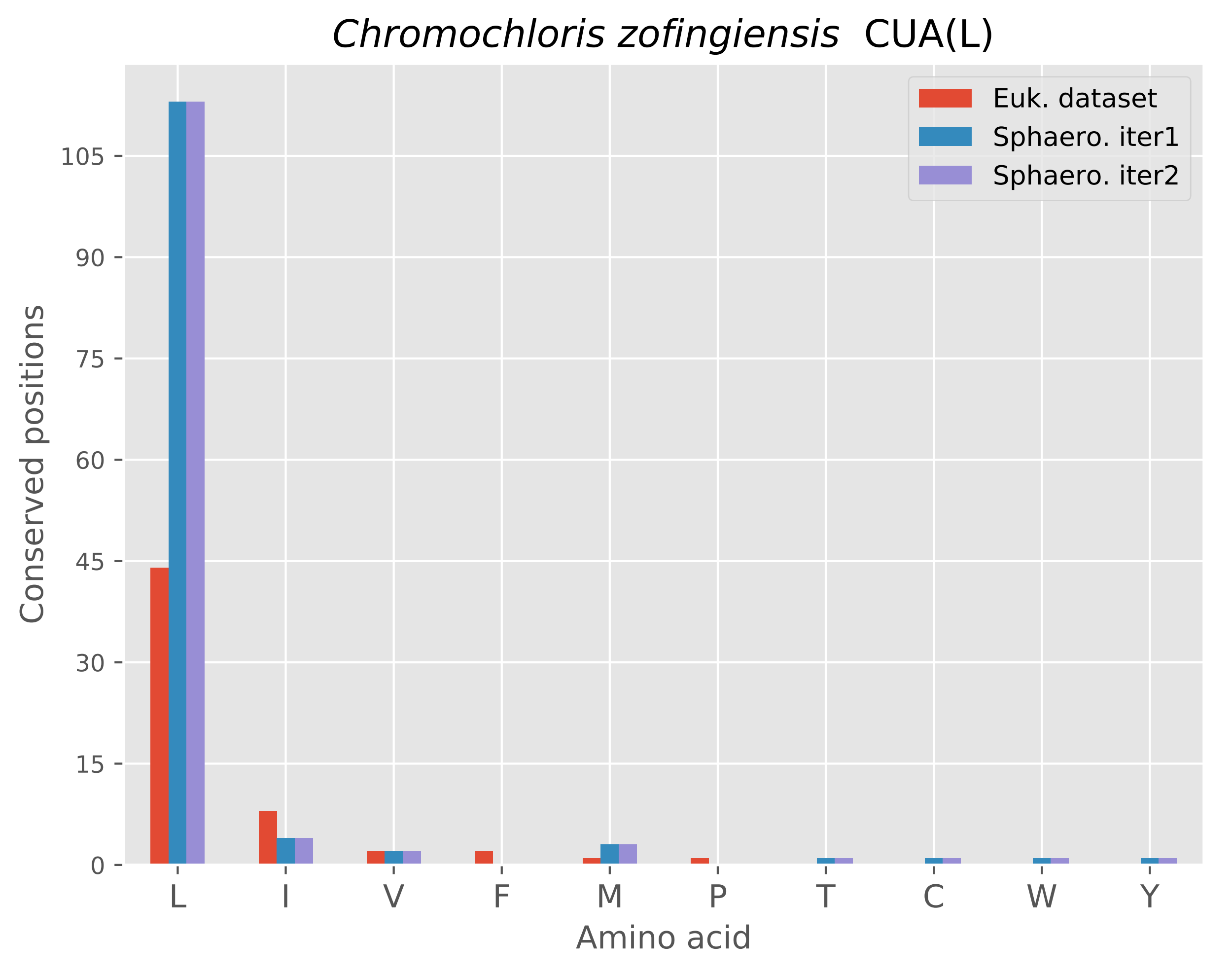

Chromochloris zofingiensis CUA(L)
Euk. dataset
Sphaero. iter1
Sphaero. iter2
Conserved positions
0
15
30
45
60
75
90
105
L
I
V
F
M
P
T
C
W
Y
Amino acid

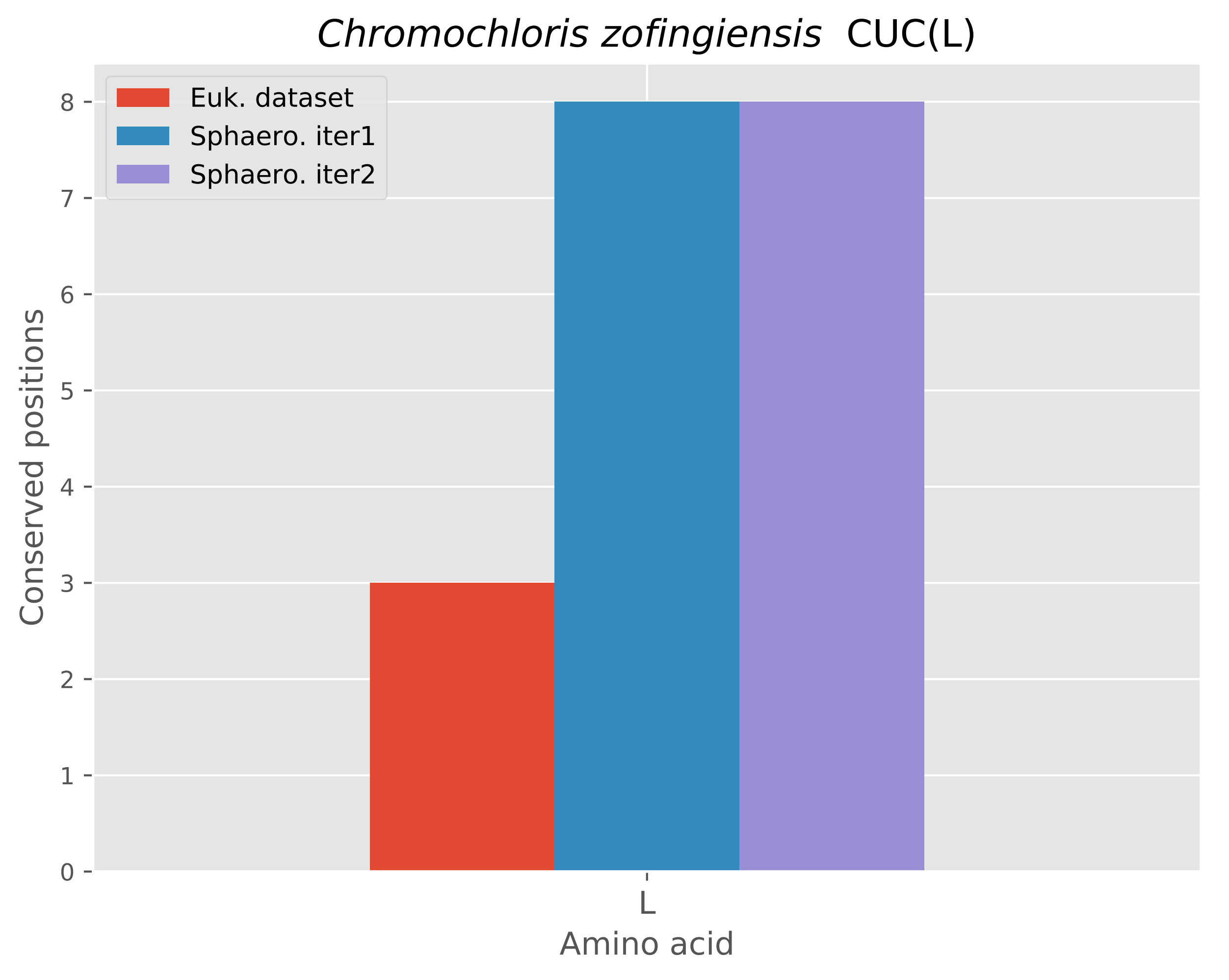
Chromochloris zofingiensis CUC(L)
Euk. dataset
Sphaero. iter1
Sphaero. iter2
Conserved positions
0
1
2
3
4
5
6
7
8
L
Amino acid

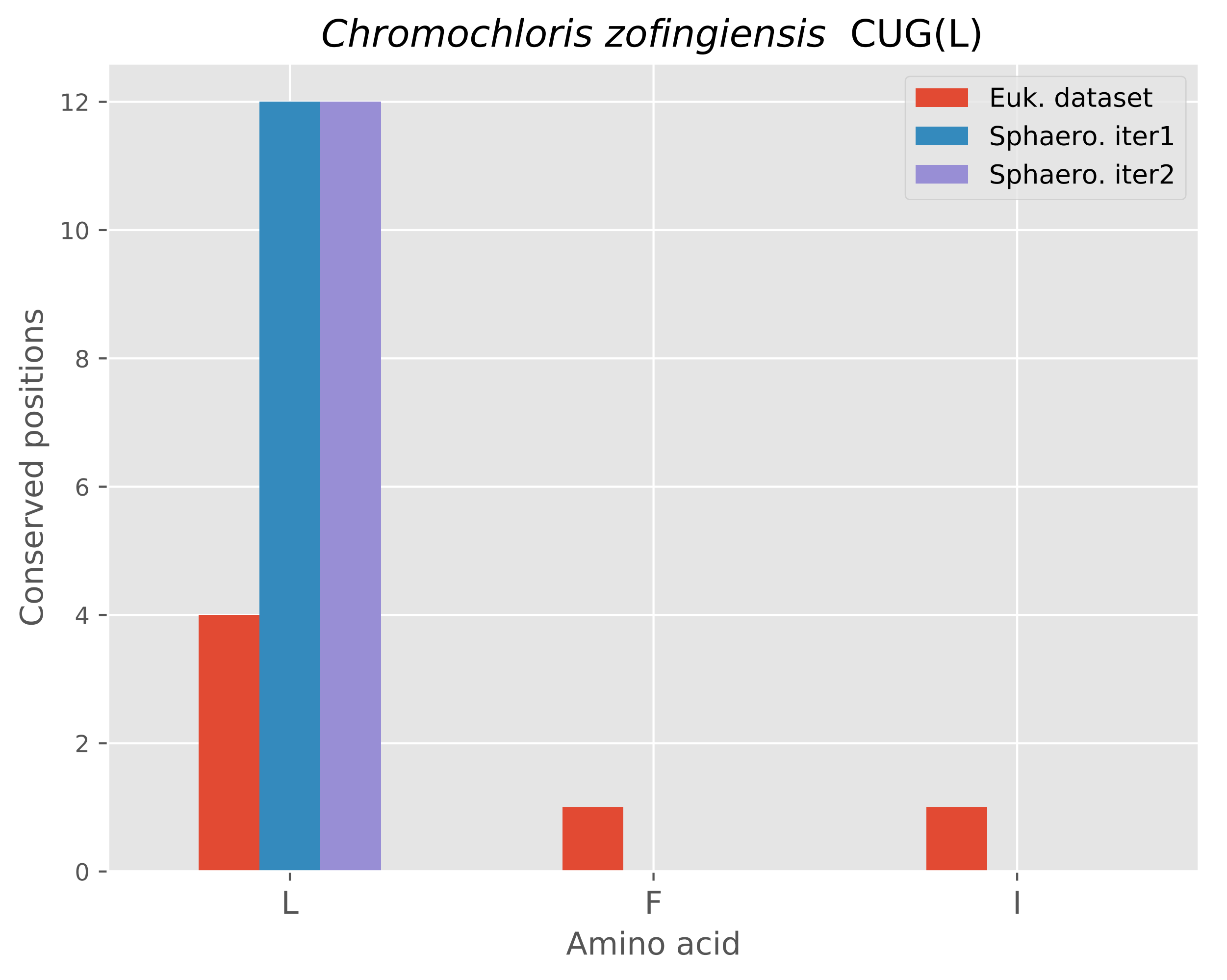

Chromochloris zofingiensis CUG(L)
Euk. dataset
Sphaero. iter1
Sphaero. iter2
Conserved positions
0
2
4
6
8
10
12
L
F
I
Amino acid

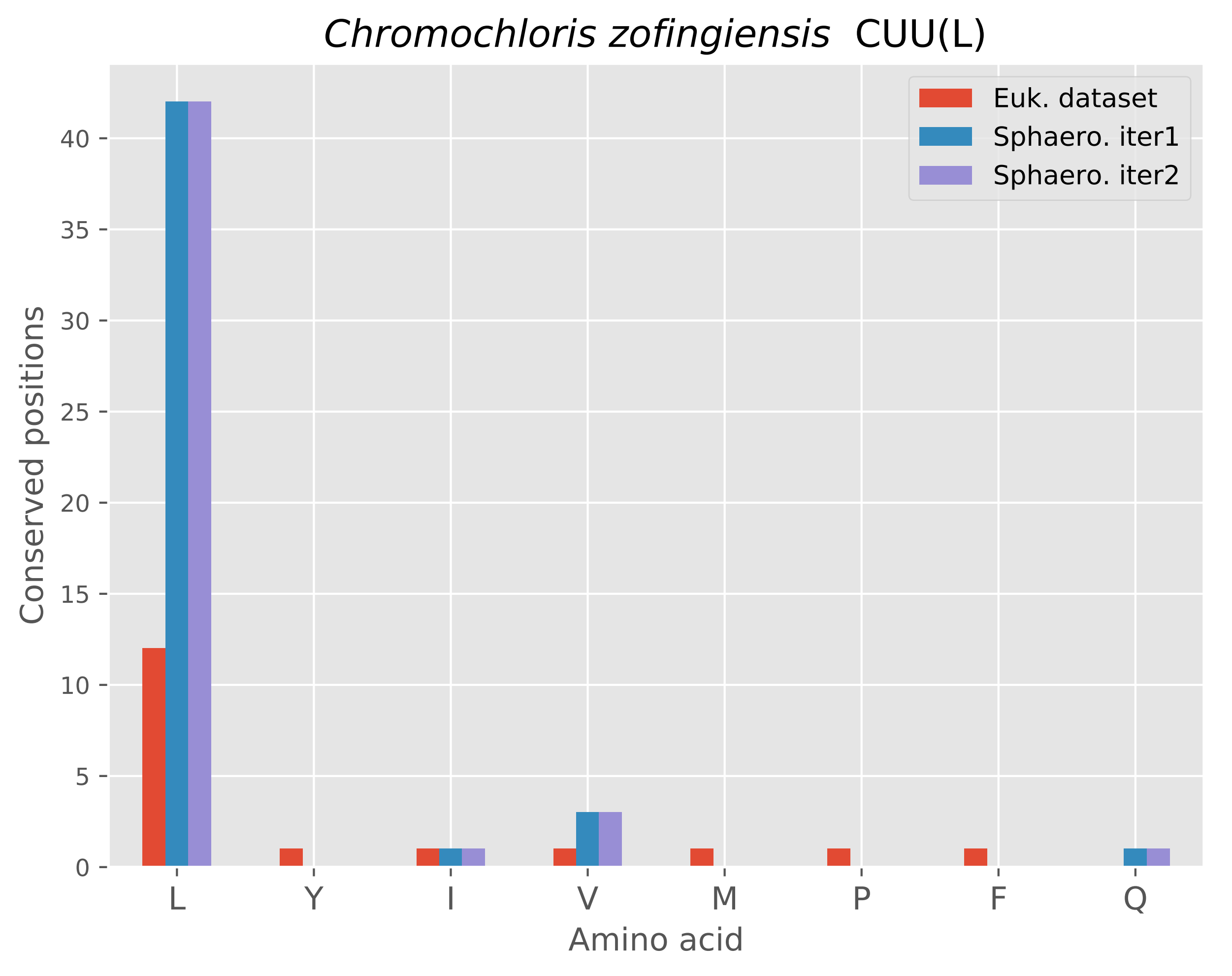

Chromochloris zofingiensis CUU(L)
Euk. dataset
Sphaero. iter1
Sphaero. iter2
Conserved positions
0
5
10
15
20
25
30
35
40
L
Y
I
V
M
P
F
Q
Amino acid

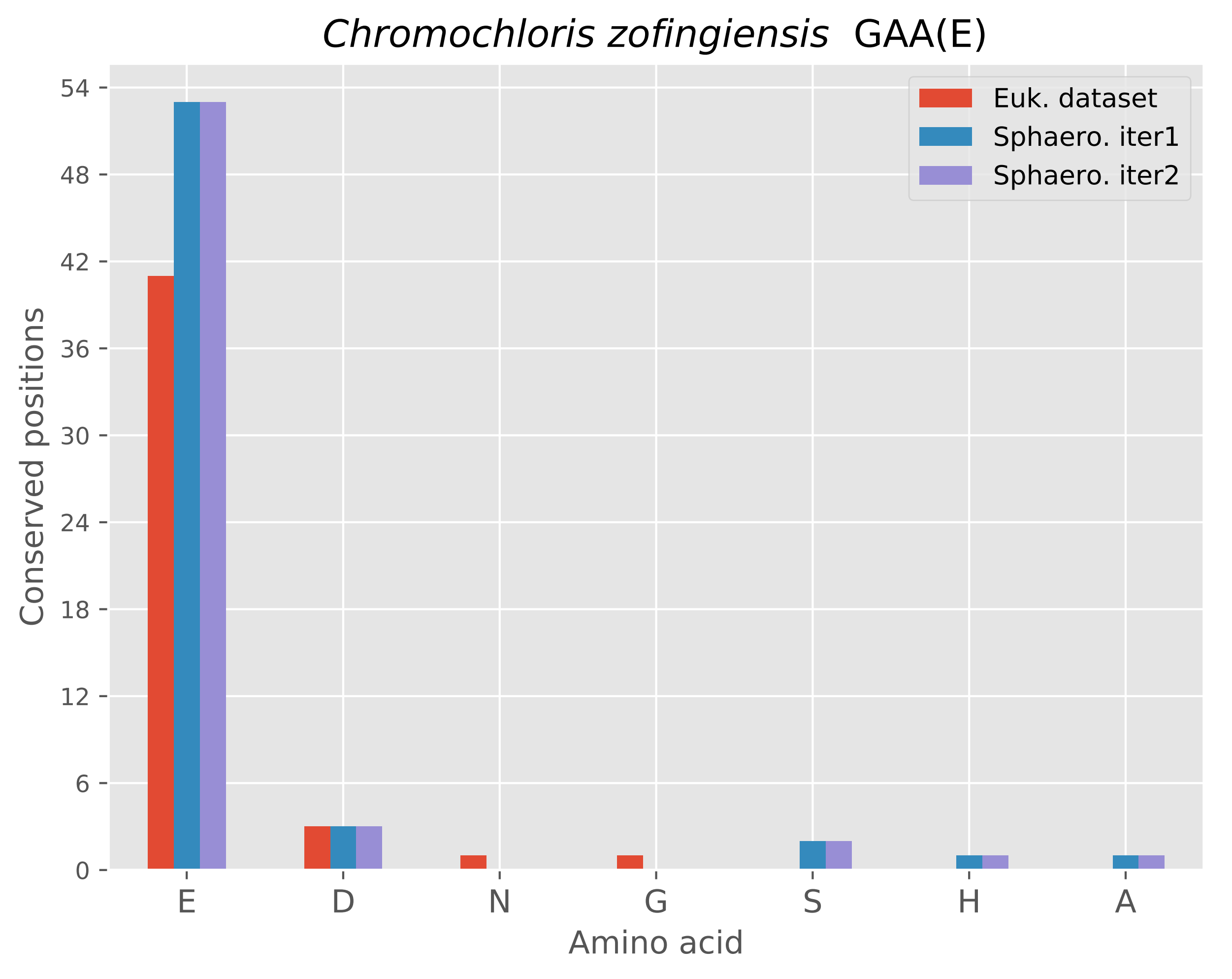

Chromochloris zofingiensis GAA(E)
Euk. dataset
Sphaero. iter1
Sphaero. iter2
Conserved positions
0
6
12
18
24
30
36
42
48
54
E
D
N
G
S
H
A
Amino acid

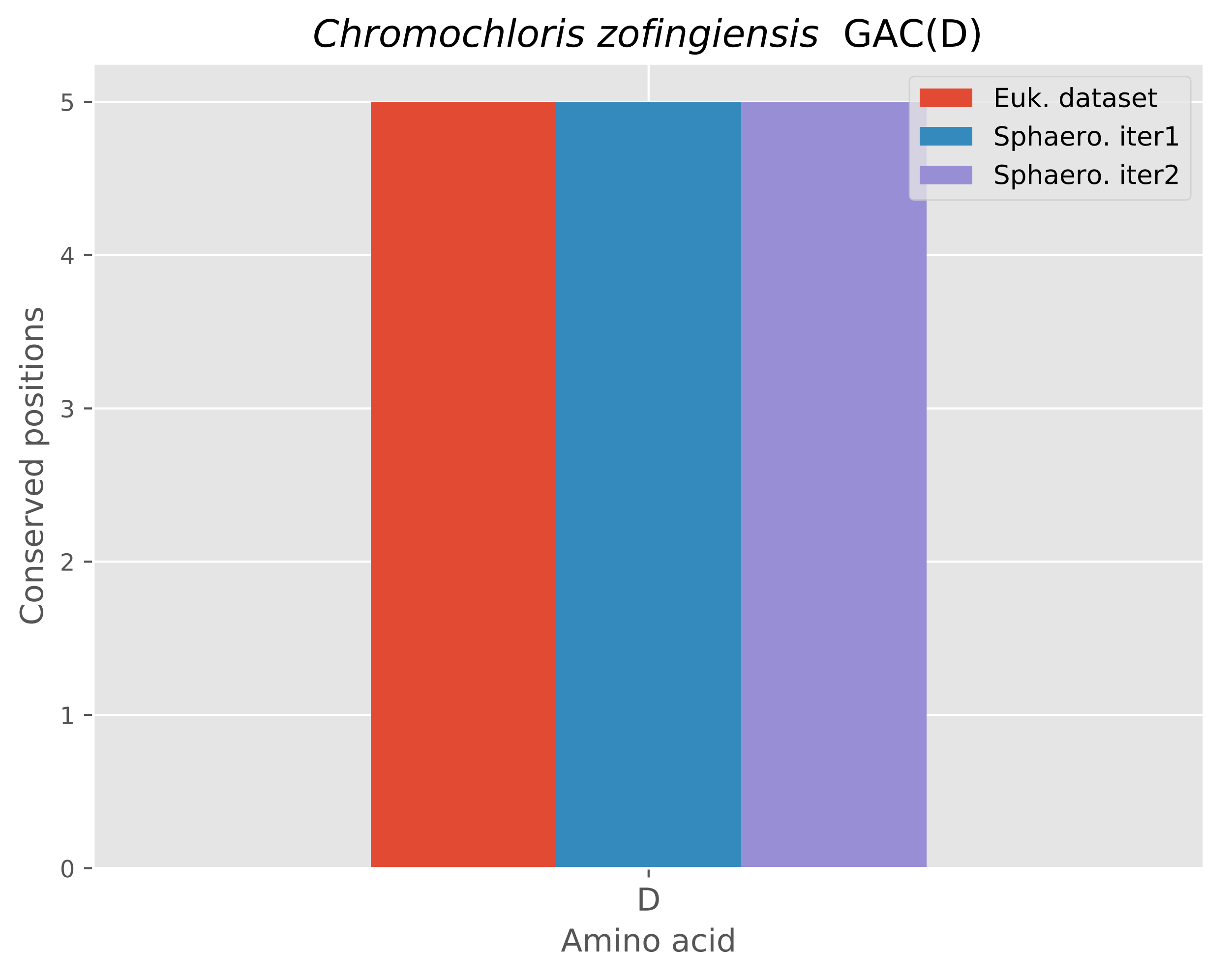

Chromochloris zofingiensis GAC(D)
Euk. dataset
Sphaero. iter1
Sphaero. iter2
Conserved positions
0
1
2
3
4
5
D
Amino acid

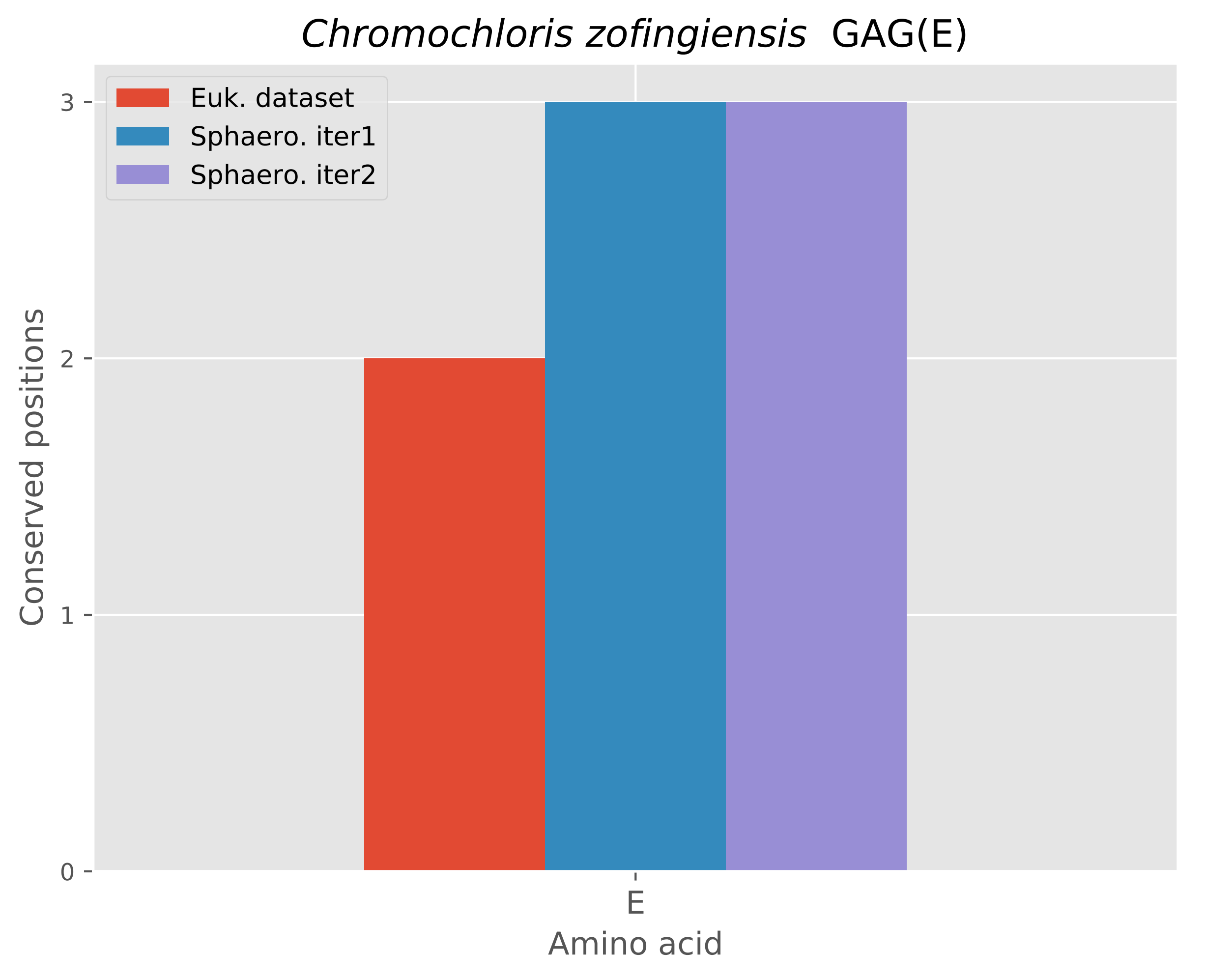

Chromochloris zofingiensis GAG(E)
Euk. dataset
Sphaero. iter1
Sphaero. iter2
Conserved positions
0
1
2
3
E
Amino acid

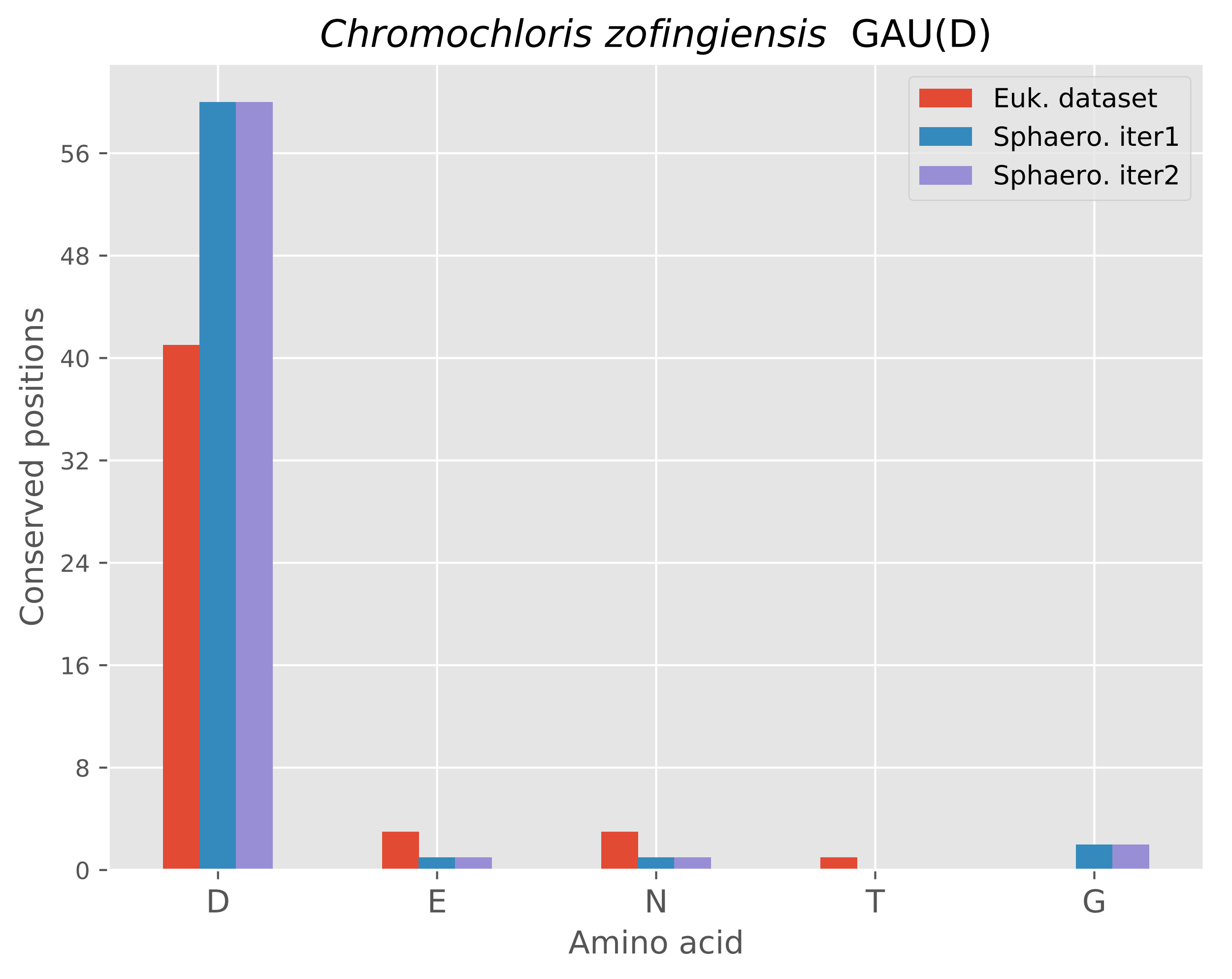

Chromochloris zofingiensis GAU(D)
Euk. dataset
Sphaero. iter1
Sphaero. iter2
Conserved positions
0
8
16
24
32
40
48
56
D
E
N
T
G
Amino acid

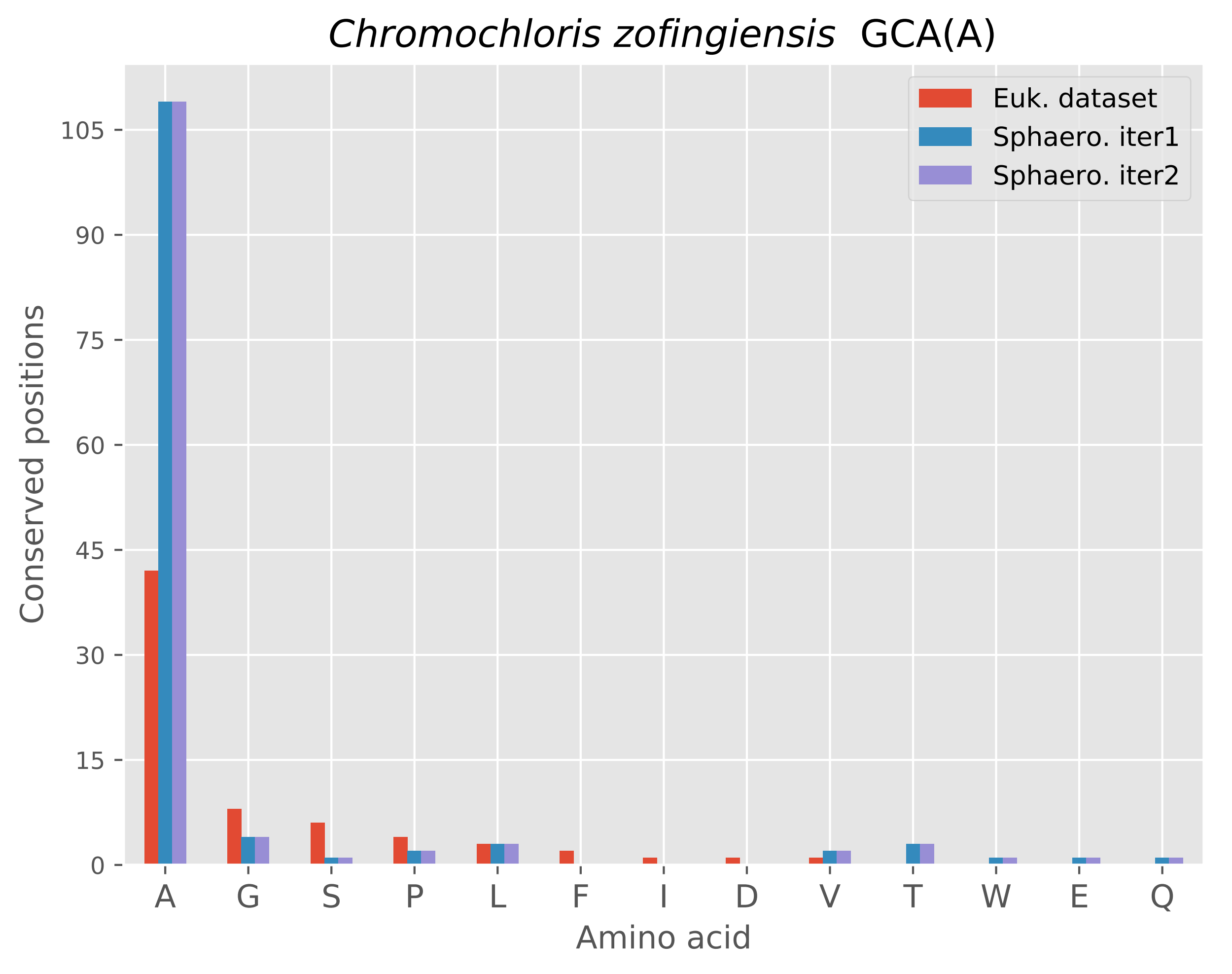

Chromochloris zofingiensis GCA(A)
Euk. dataset
Sphaero. iter1
Sphaero. iter2
Conserved positions
0
15
30
45
60
75
90
105
A
G
S
P
L
F
I
D
V
T
W
E
Q
Amino acid

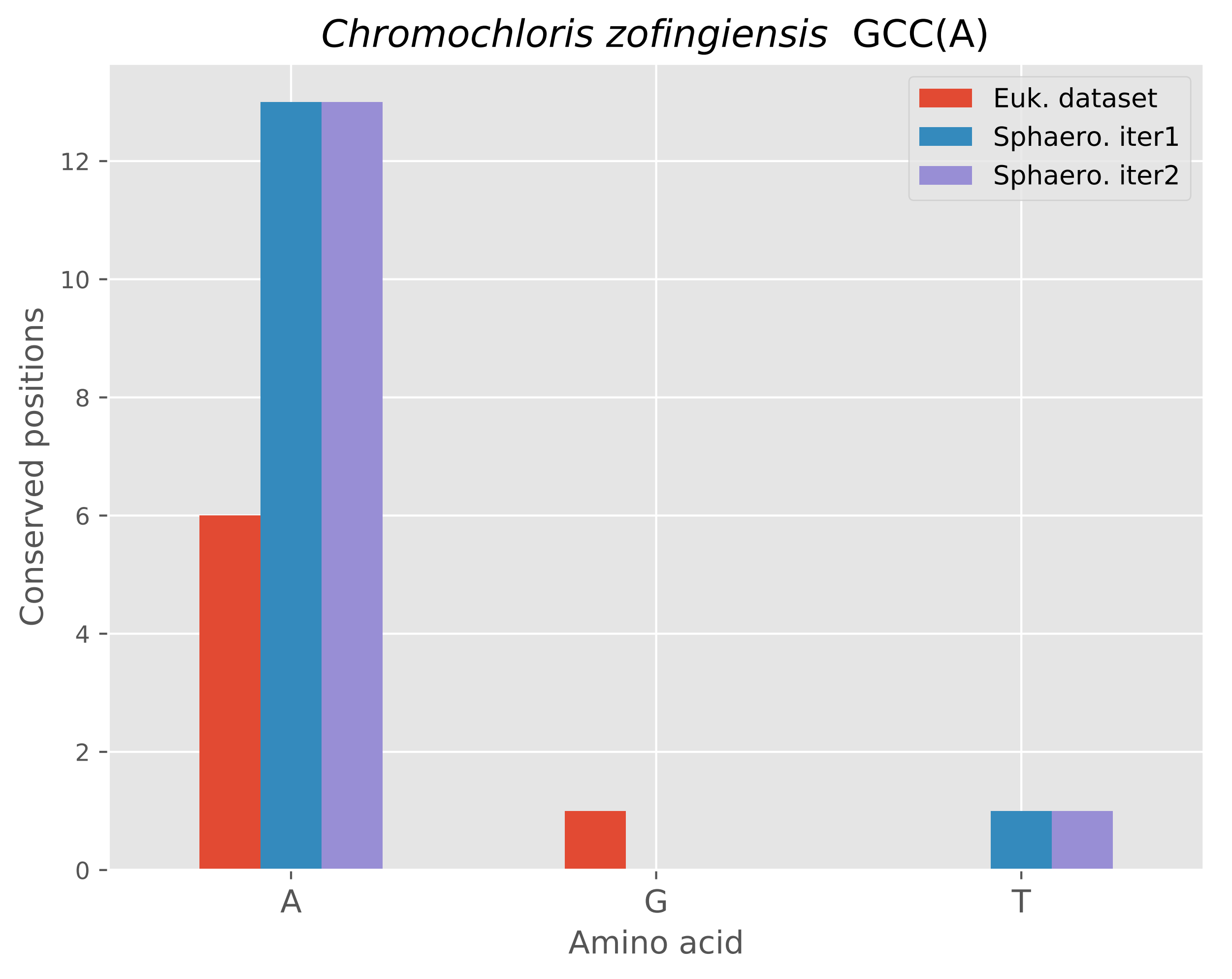

Chromochloris zofingiensis GCC(A)
Euk. dataset
Sphaero. iter1
Sphaero. iter2
Conserved positions
0
2
4
6
8
10
12
A
G
T
Amino acid

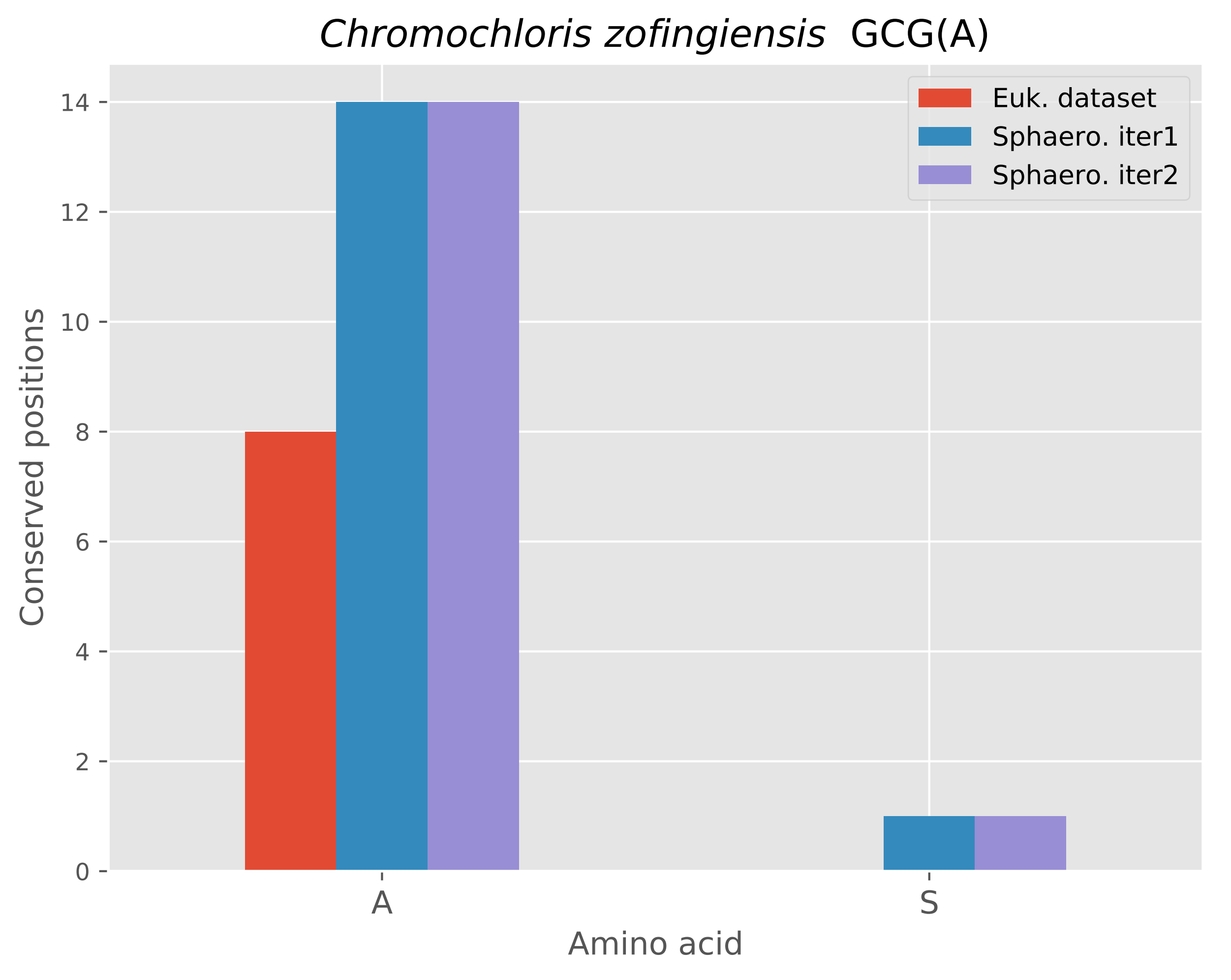

Chromochloris zofingiensis GCG(A)
Euk. dataset
Sphaero. iter1
Sphaero. iter2
Conserved positions
0
2
4
6
8
10
12
14
A
S
Amino acid

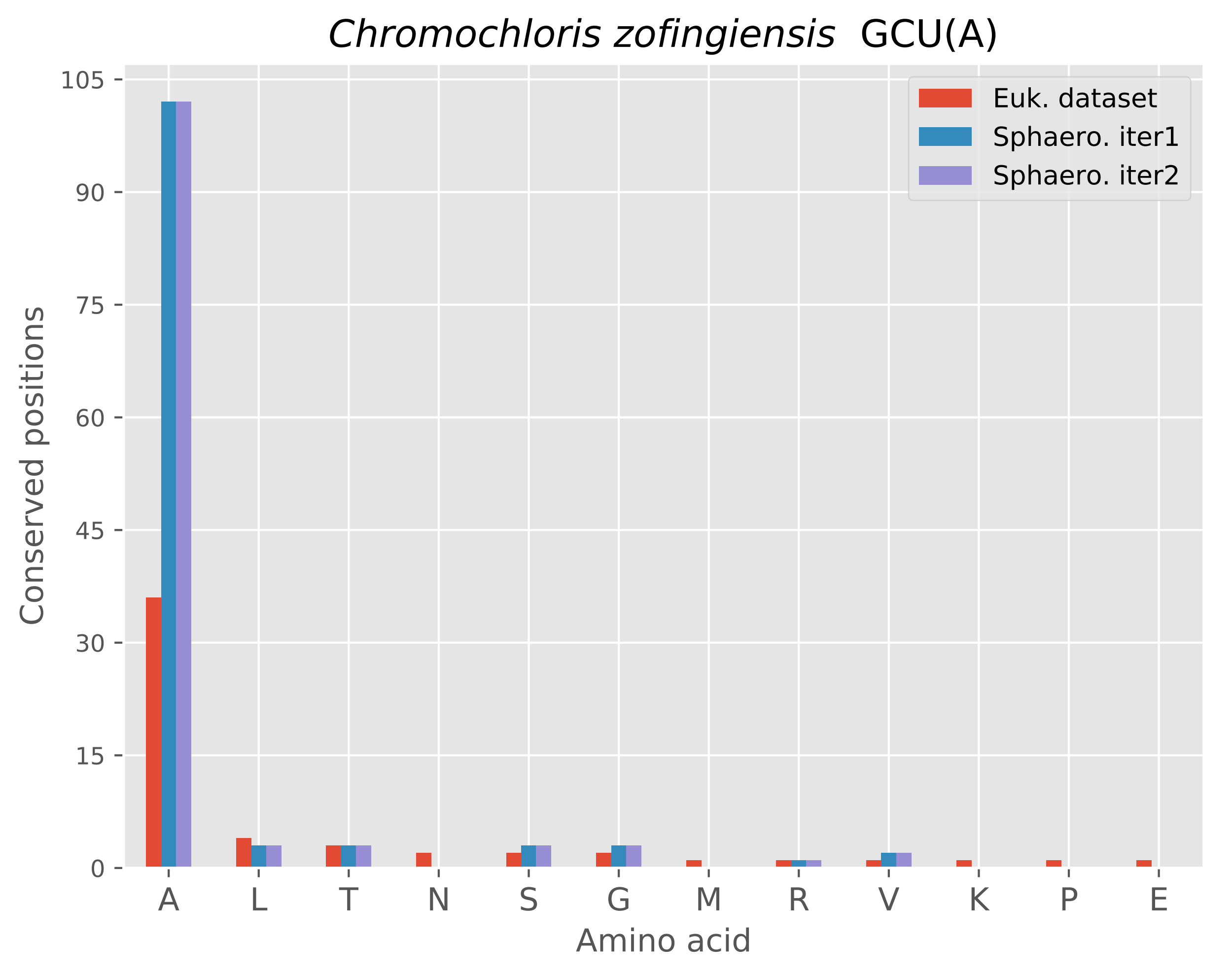

Chromochloris zofingiensis GCU(A)
Euk. dataset
Sphaero. iter1
Sphaero. iter2
Conserved positions
0
15
30
45
60
75
90
105
A
L
T
N
S
G
M
R
V
K
P
E
Amino acid

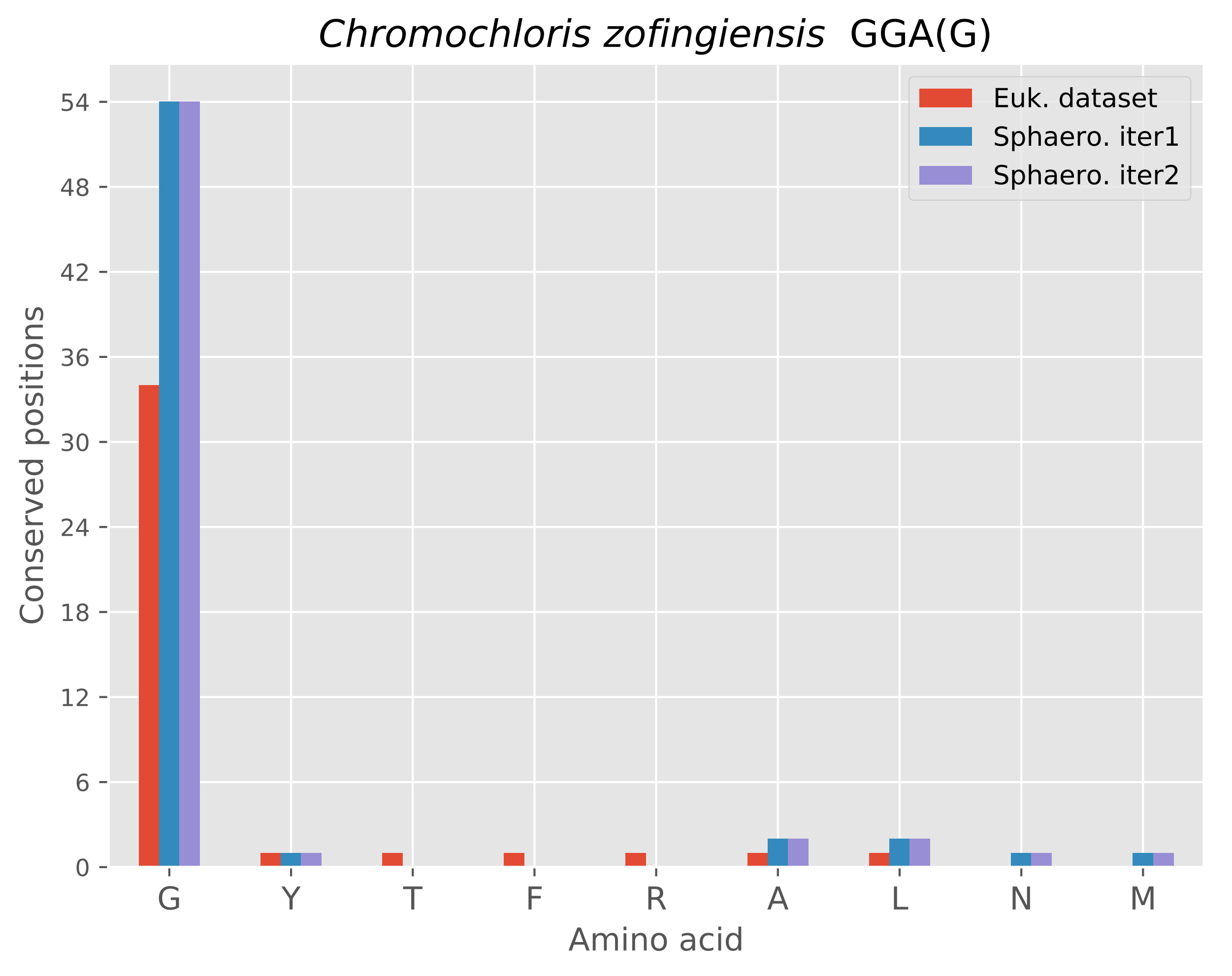

Chromochloris zofingiensis GGA(G)
Euk. dataset
Sphaero. iter1
Sphaero. iter2
Conserved positions
Amino acid
0
6
12
18
24
30
36
42
48
54
G
Y
T
F
R
A
L
N
M

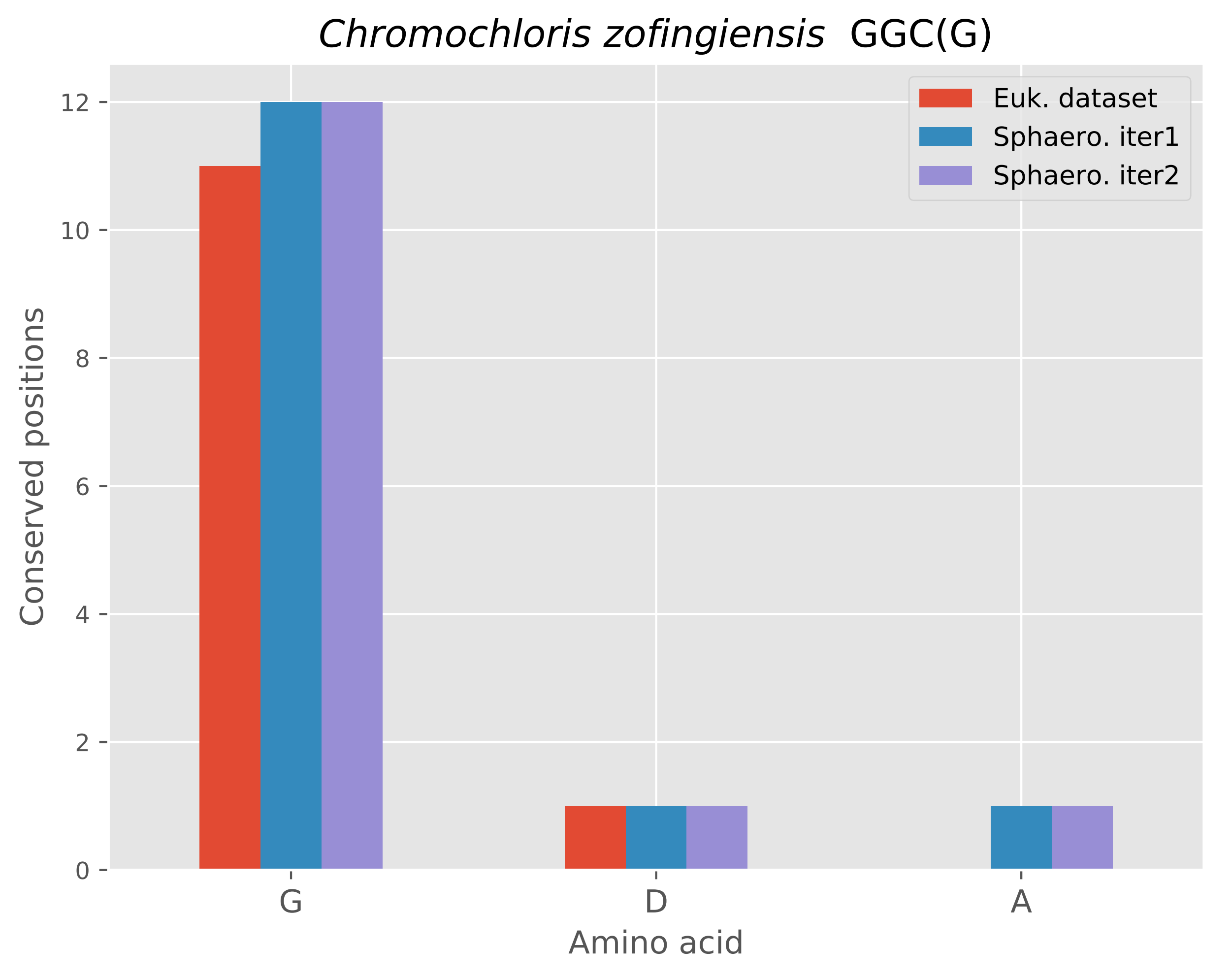

Chromochloris zofingiensis GGC(G)
Euk. dataset
Sphaero. iter1
Sphaero. iter2
Conserved positions
0
2
4
6
8
10
12
G
D
A
Amino acid

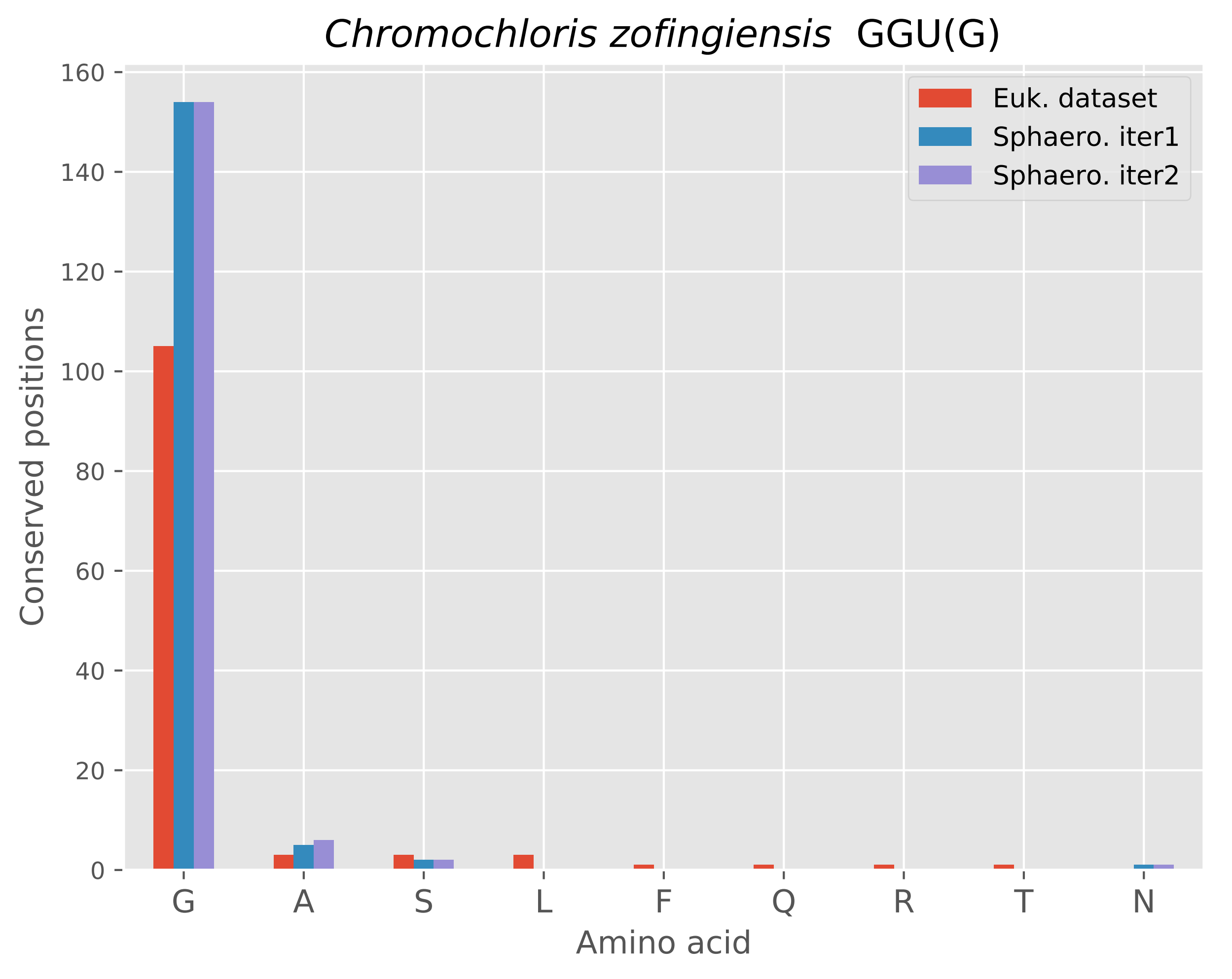

Chromochloris zofingiensis GGU(G)
Euk. dataset
Sphaero. iter1
Sphaero. iter2
Conserved positions
0
20
40
60
80
100
120
140
160
G
A
S
L
F
Q
R
T
N
Amino acid

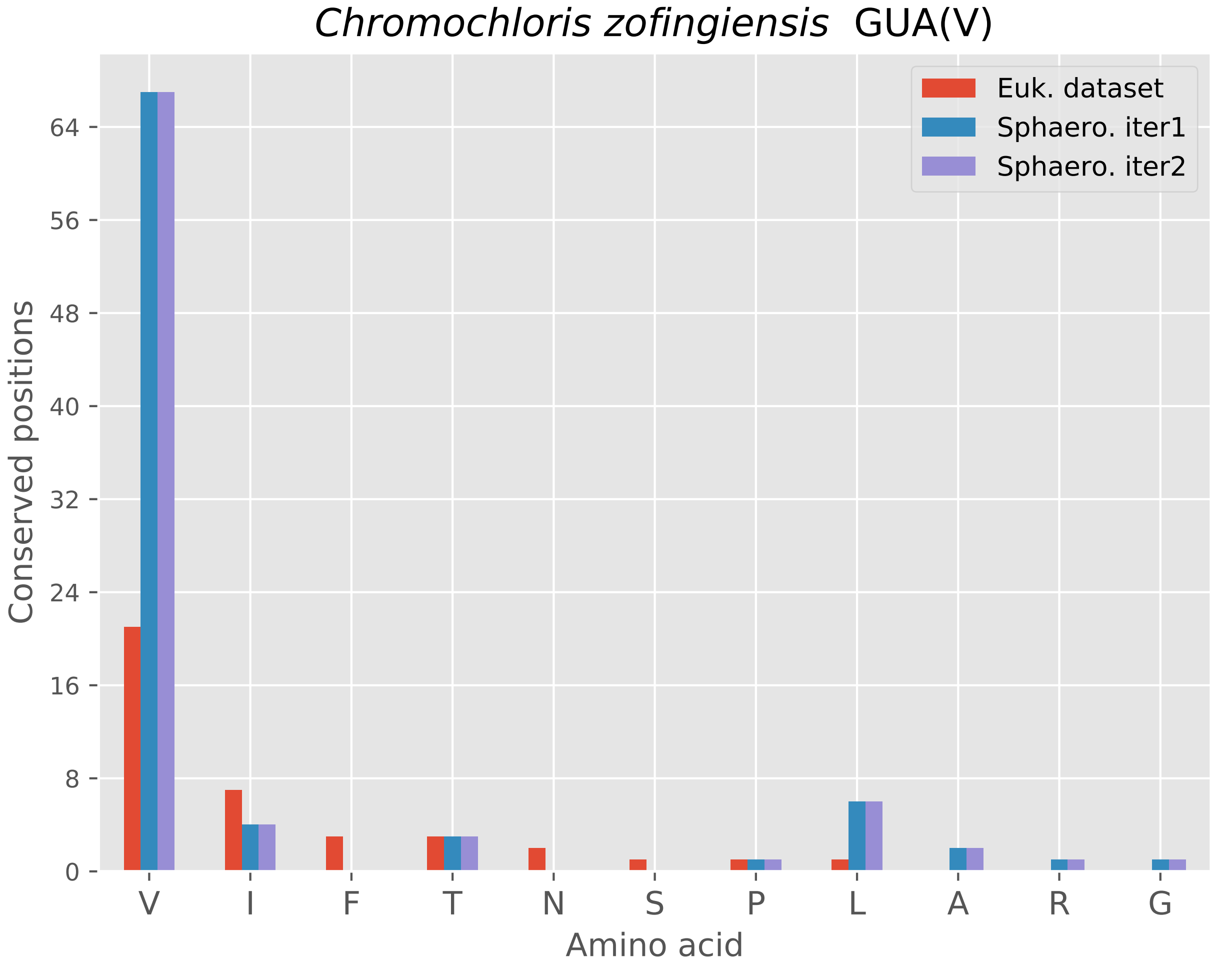

Chromochloris zofingiensis GUA(V)
Euk. dataset
Sphaero. iter1
Sphaero. iter2
Conserved positions
0
8
16
24
32
40
48
56
64
V
I
F
T
N
S
P
L
A
R
G
Amino acid

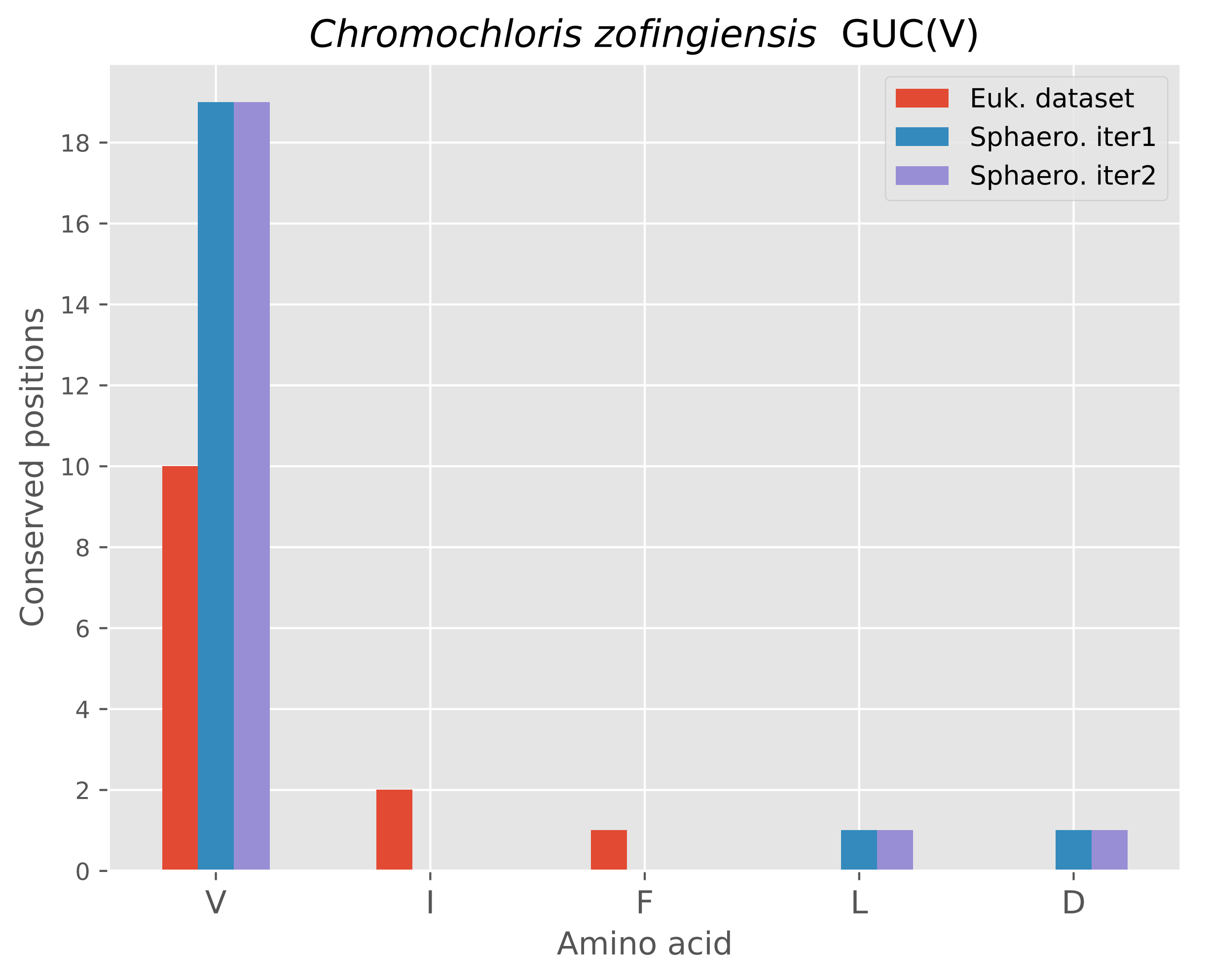

Chromochloris zofingiensis GUC(V)
Euk. dataset
Sphaero. iter1
Sphaero. iter2
Conserved positions
0
2
4
6
8
10
12
14
16
18
V
I
F
L
D
Amino acid

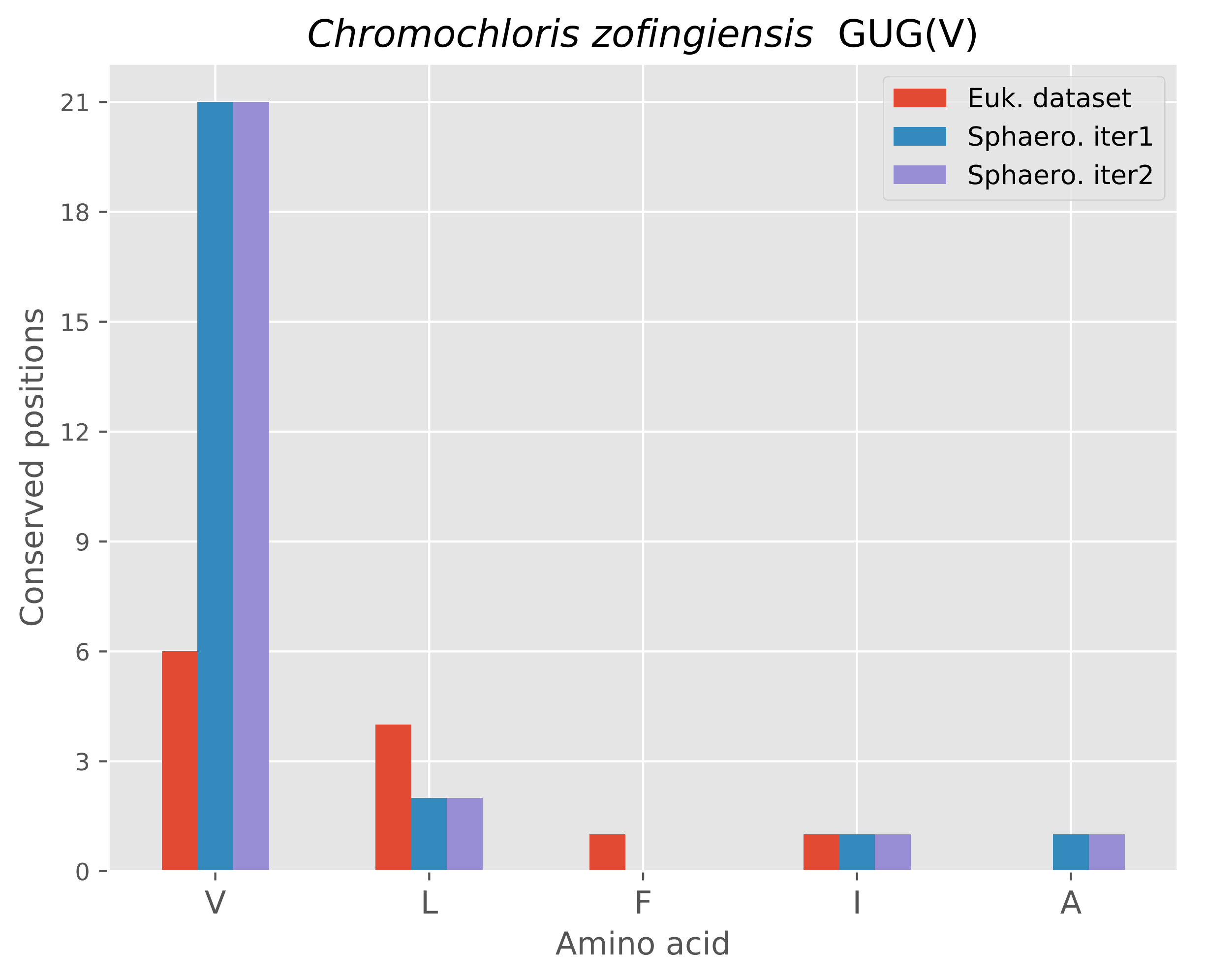

Chromochloris zofingiensis GUG(V)
Euk. dataset
Sphaero. iter1
Sphaero. iter2
Conserved positions
0
3
6
9
12
15
18
21
V
L
F
I
A
Amino acid

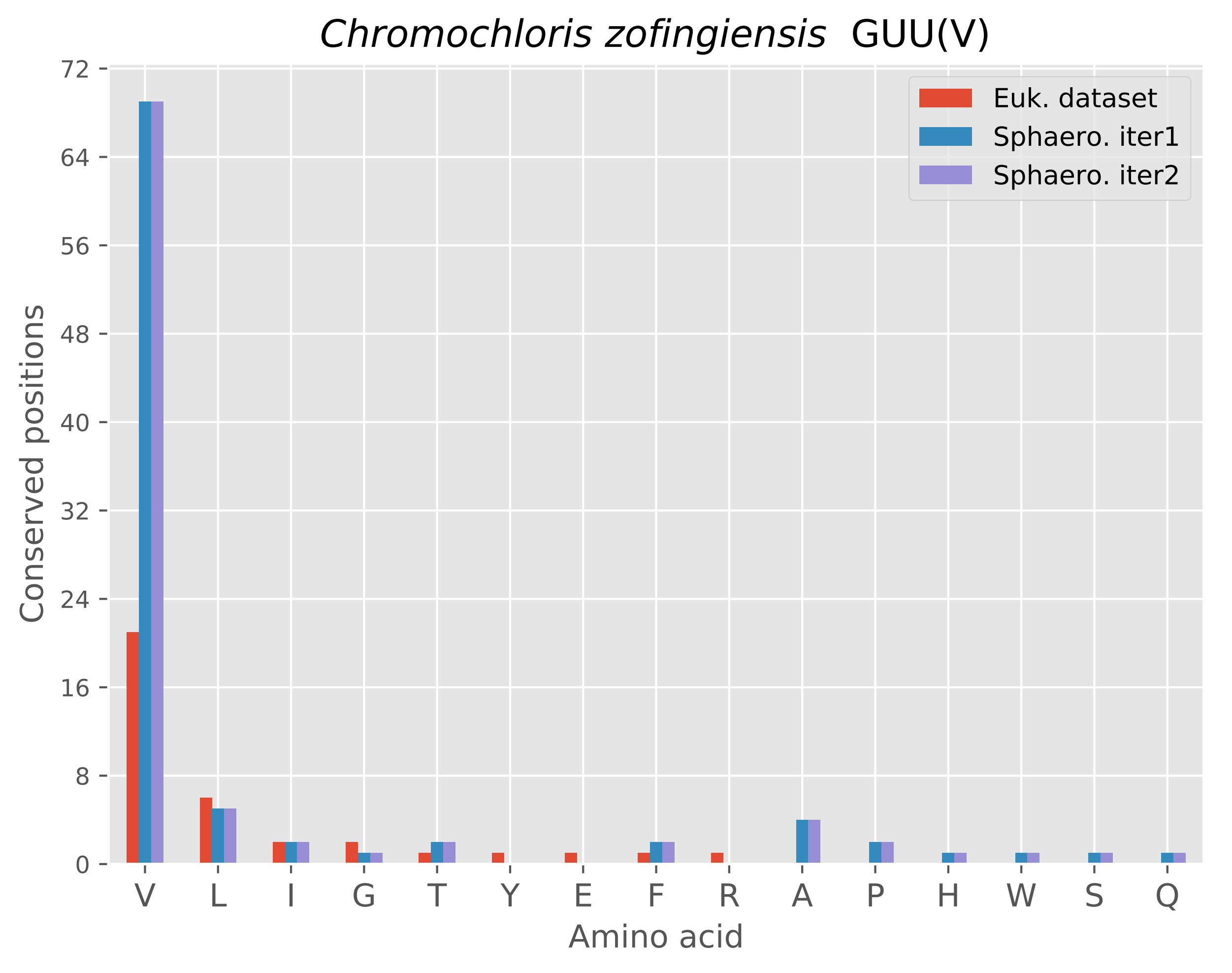

Chromochloris zofingiensis GUU(V)
Euk. dataset
Sphaero. iter1
Sphaero. iter2
Conserved positions
0
8
16
24
32
40
48
56
64
72
V
L
I
G
T
Y
E
F
R
A
P
H
W
S
Q
Amino acid

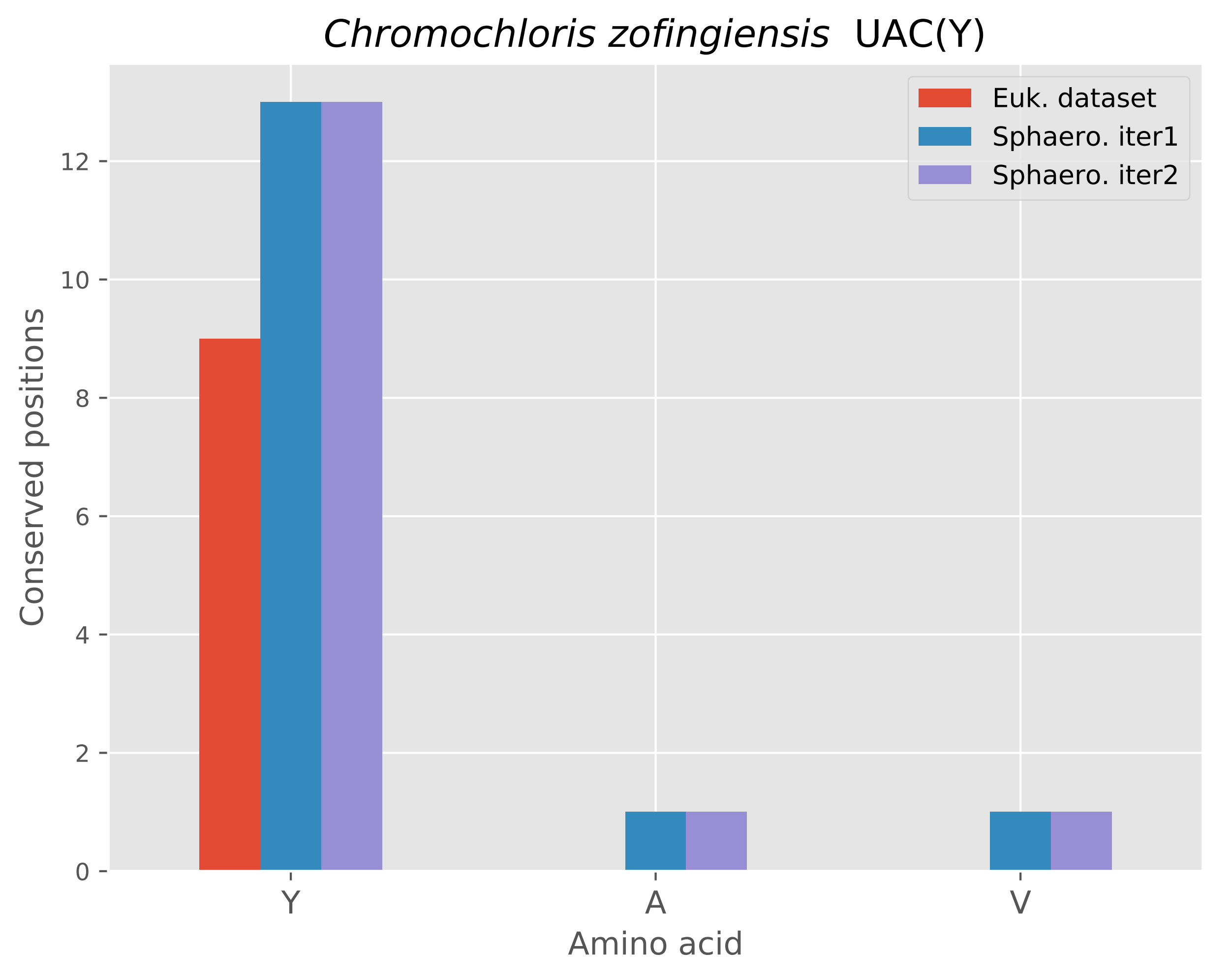

Chromochloris zofingiensis UAC(Y)
Euk. dataset
Sphaero. iter1
Sphaero. iter2
Conserved positions
0
2
4
6
8
10
12
Y
A
V
Amino acid

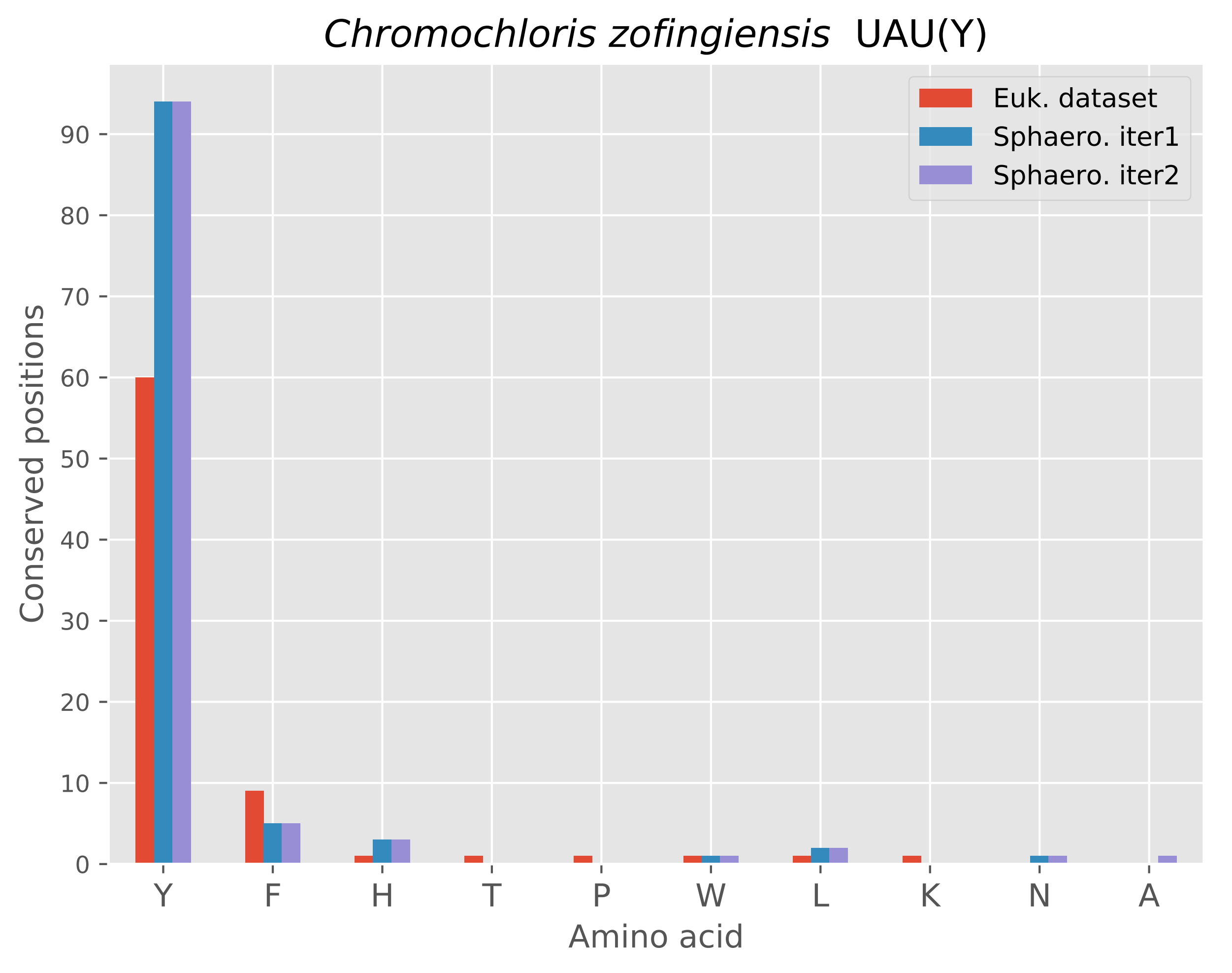

Chromochloris zofingiensis UAU(Y)
Euk. dataset
Sphaero. iter1
Sphaero. iter2
Conserved positions
0
10
20
30
40
50
60
70
80
90
Y
F
H
T
P
W
L
K
N
A
Amino acid

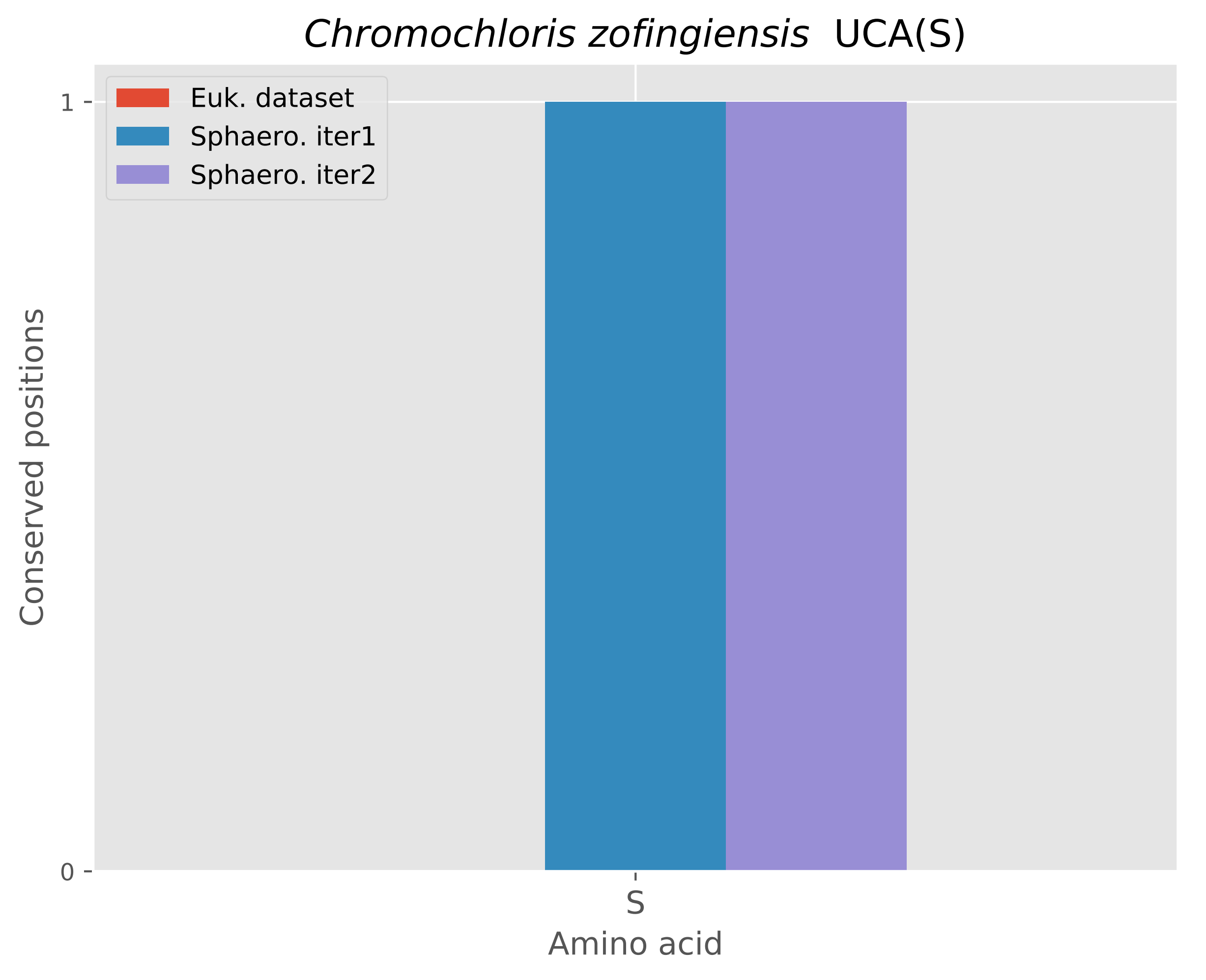
Chromochloris zofingiensis UCA(S)
Euk. dataset
Sphaero. iter1
Sphaero. iter2
1
0
Conserved positions
S
Amino acid

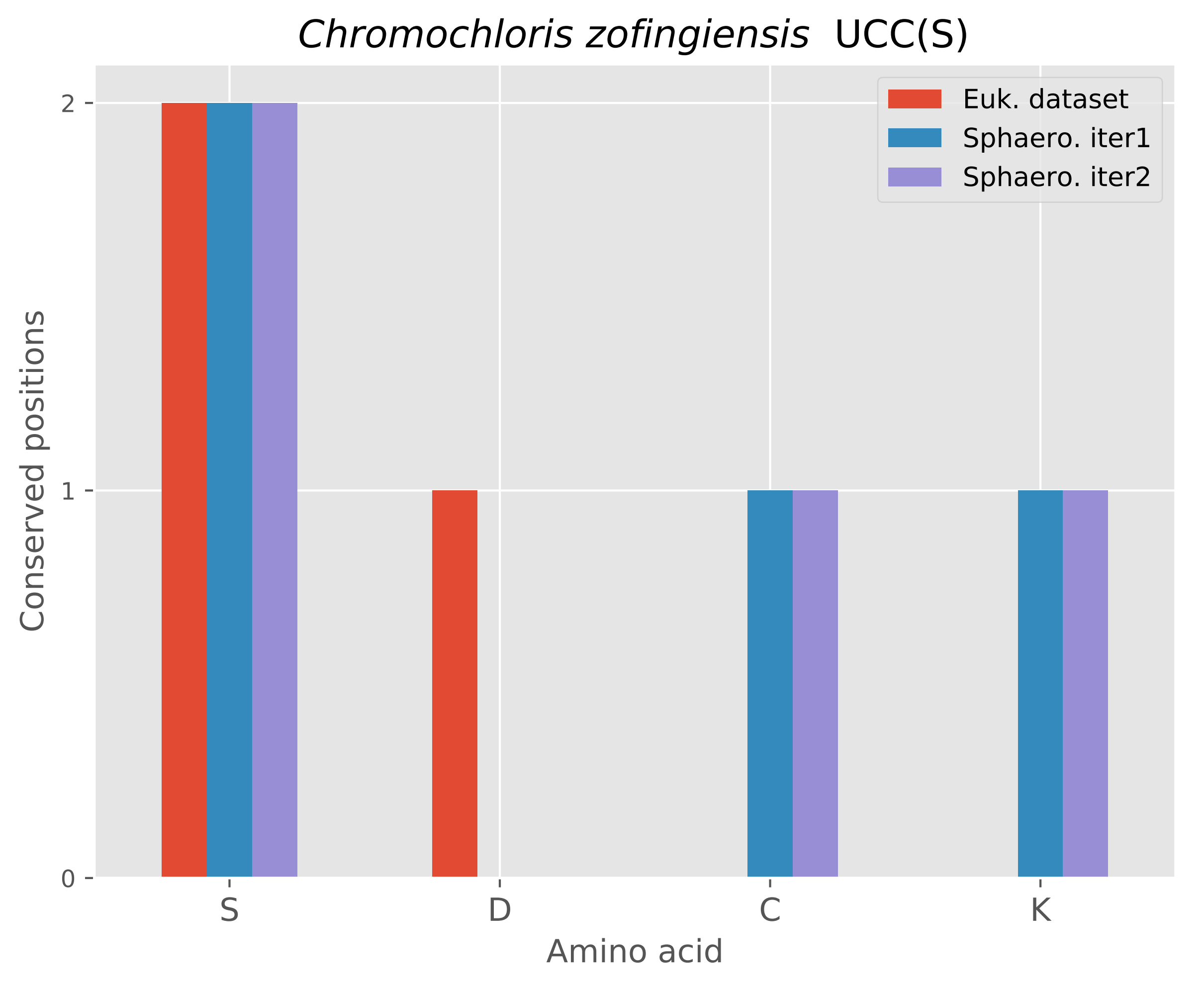
Chromochloris zofingiensis UCC(S)
Euk. dataset
Sphaero. iter1
Sphaero. iter2
Conserved positions
0
1
2
S
D
C
K
Amino acid

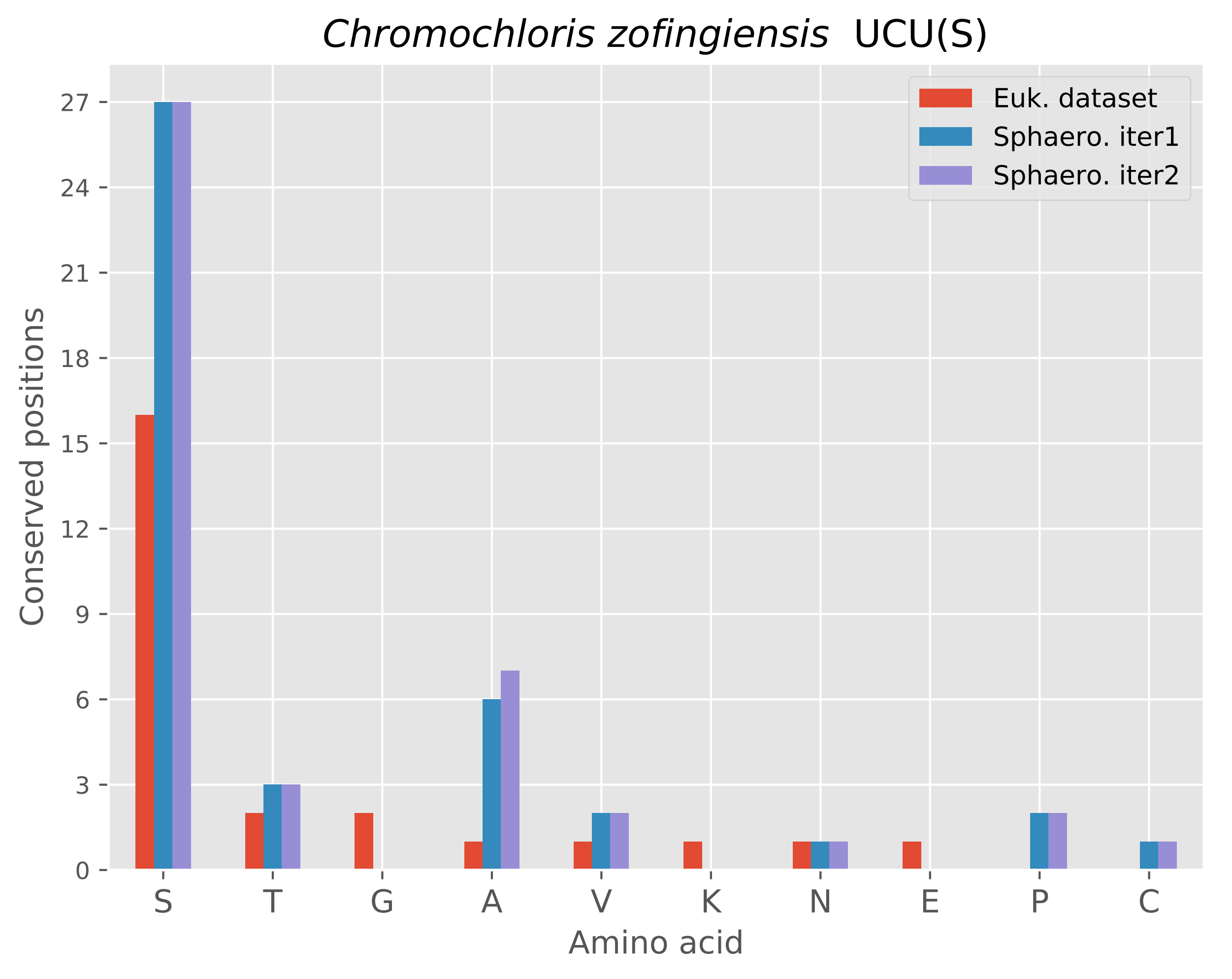

Chromochloris zofingiensis UCU(S)
Euk. dataset
Sphaero. iter1
Sphaero. iter2
Conserved positions
0
3
6
9
12
15
18
21
24
27
S
T
G
A
V
K
N
E
P
C
Amino acid

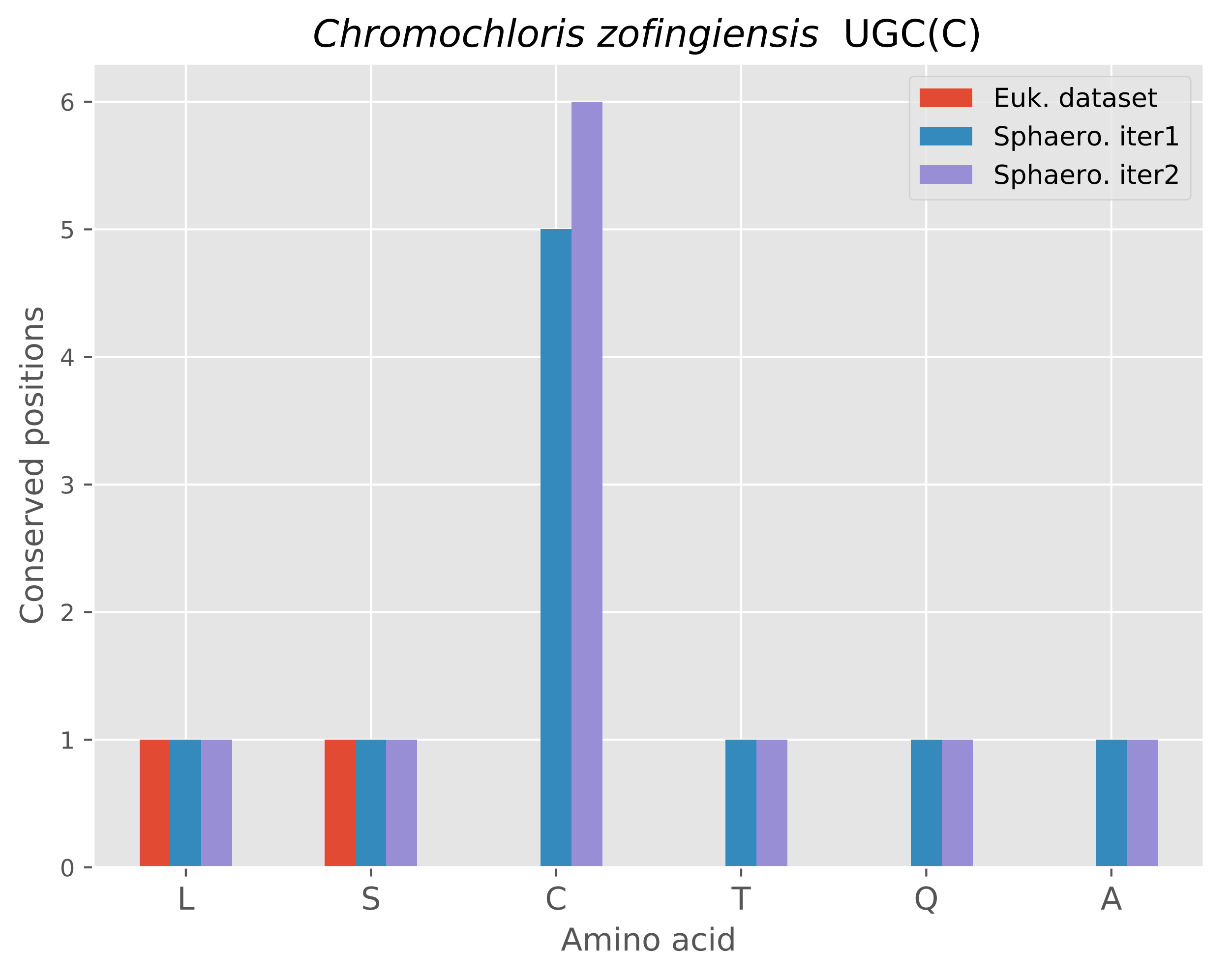

Chromocholoris zofingiensis UGC(C)
Euk. dataset
Sphaero. iter1
Sphaero. iter2
Conserved positions
0
1
2
3
4
5
6
L
S
C
T
Q
A
Amino acid

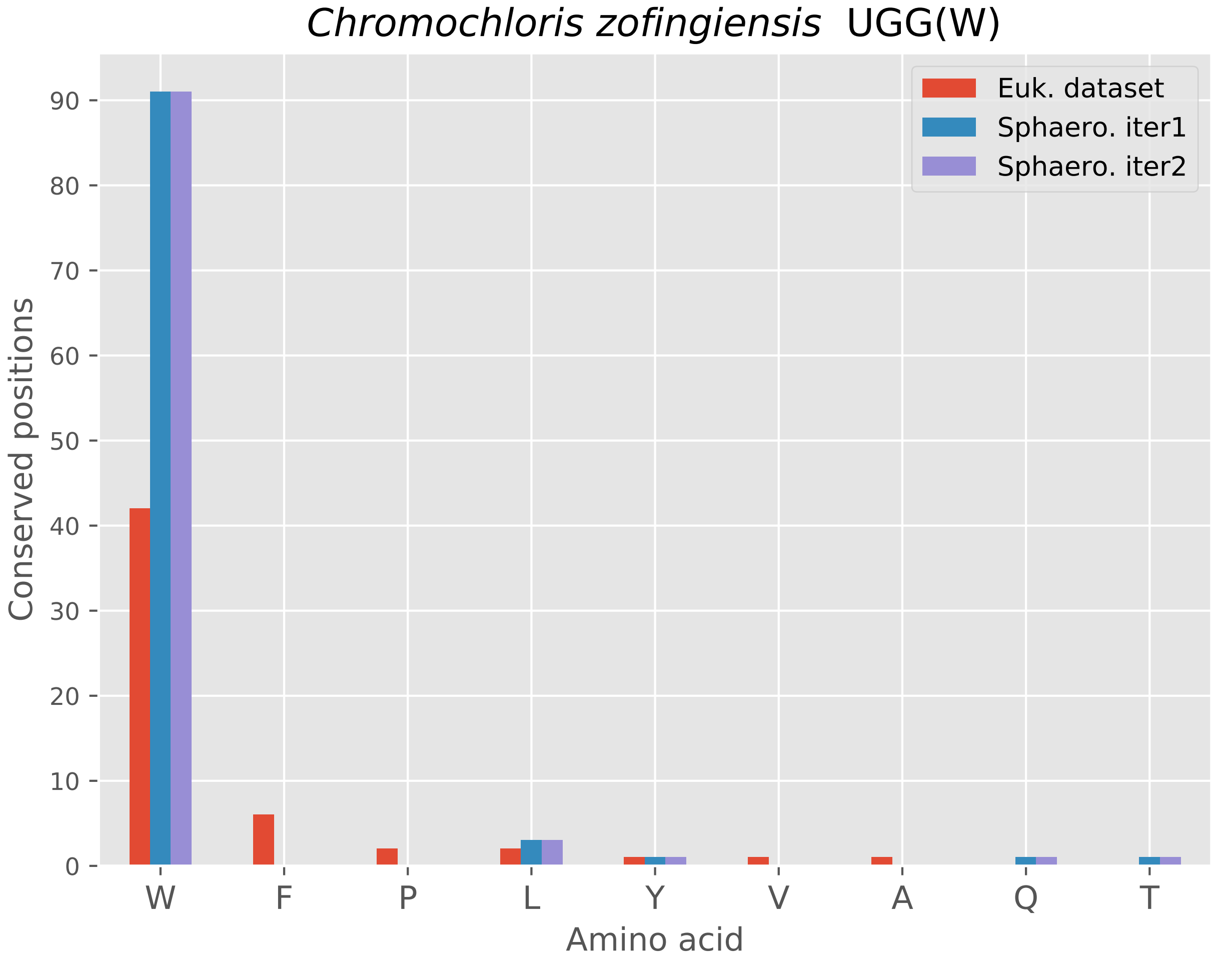

Chromochloris zofingiensis UGG(W)
Euk. dataset
Sphaero. iter1
Sphaero. iter2
Conserved positions
0
10
20
30
40
50
60
70
80
90
W
F
P
L
Y
V
A
Q
T
Amino acid

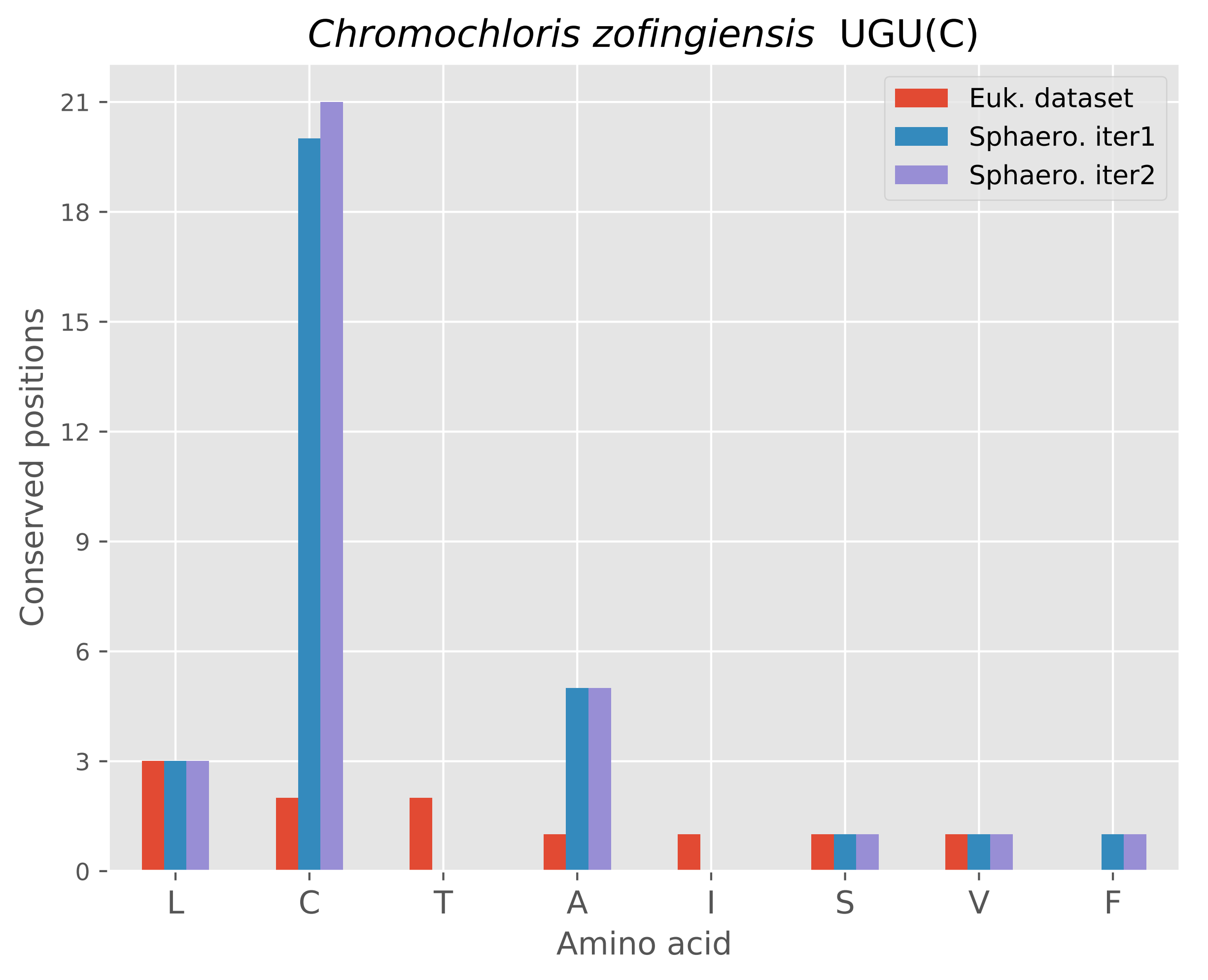

Chromochloris zofingiensis UGU(C)
Euk. dataset
Sphaero. iter1
Sphaero. iter2
Conserved positions
0
3
6
9
12
15
18
21
L
C
T
A
I
S
V
F
Amino acid

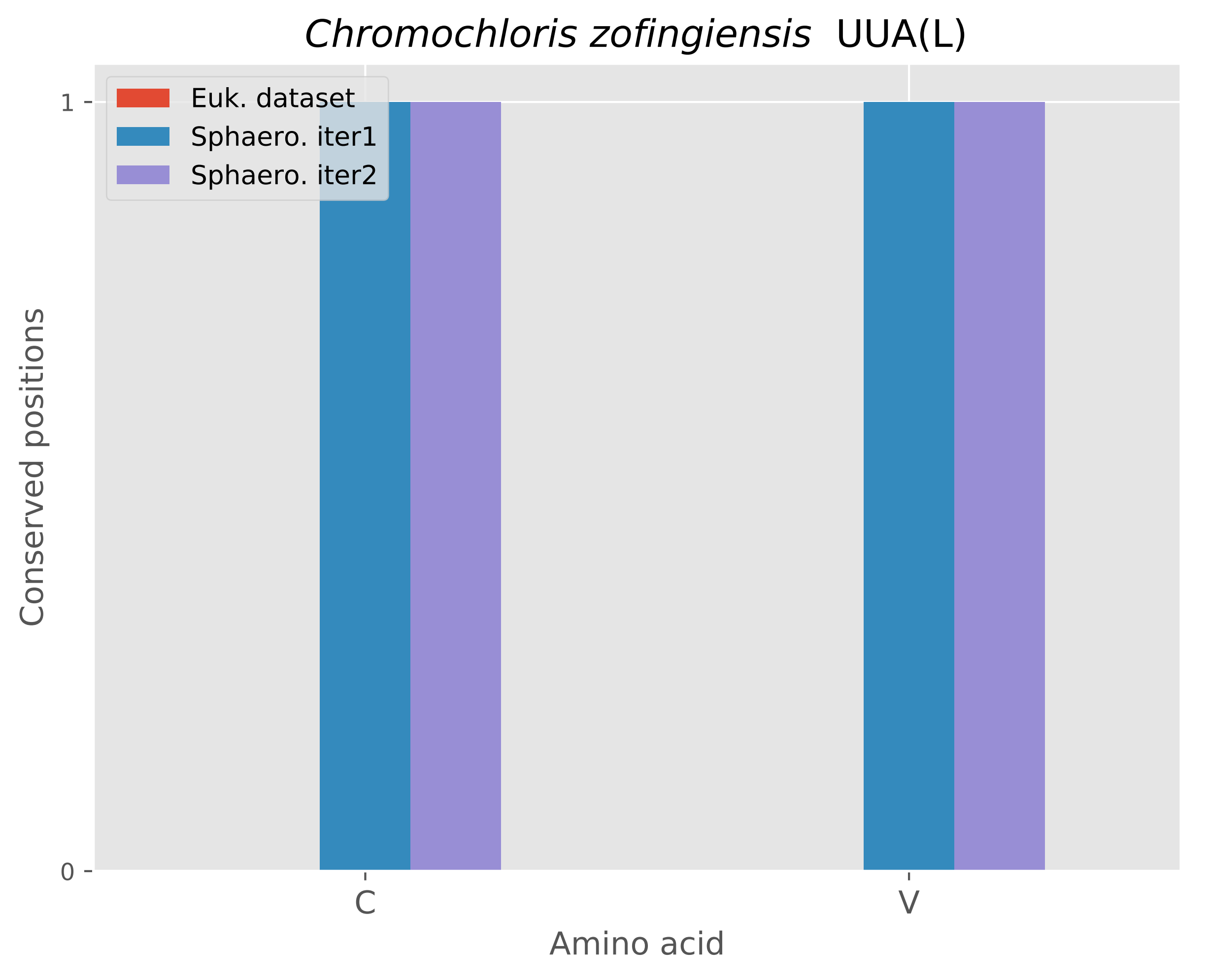

Chromochloris zofingiensis UUA(L)
Euk. dataset
Sphaero. iter1
Sphaero. iter2
1
0
Conserved positions
C
V
Amino acid

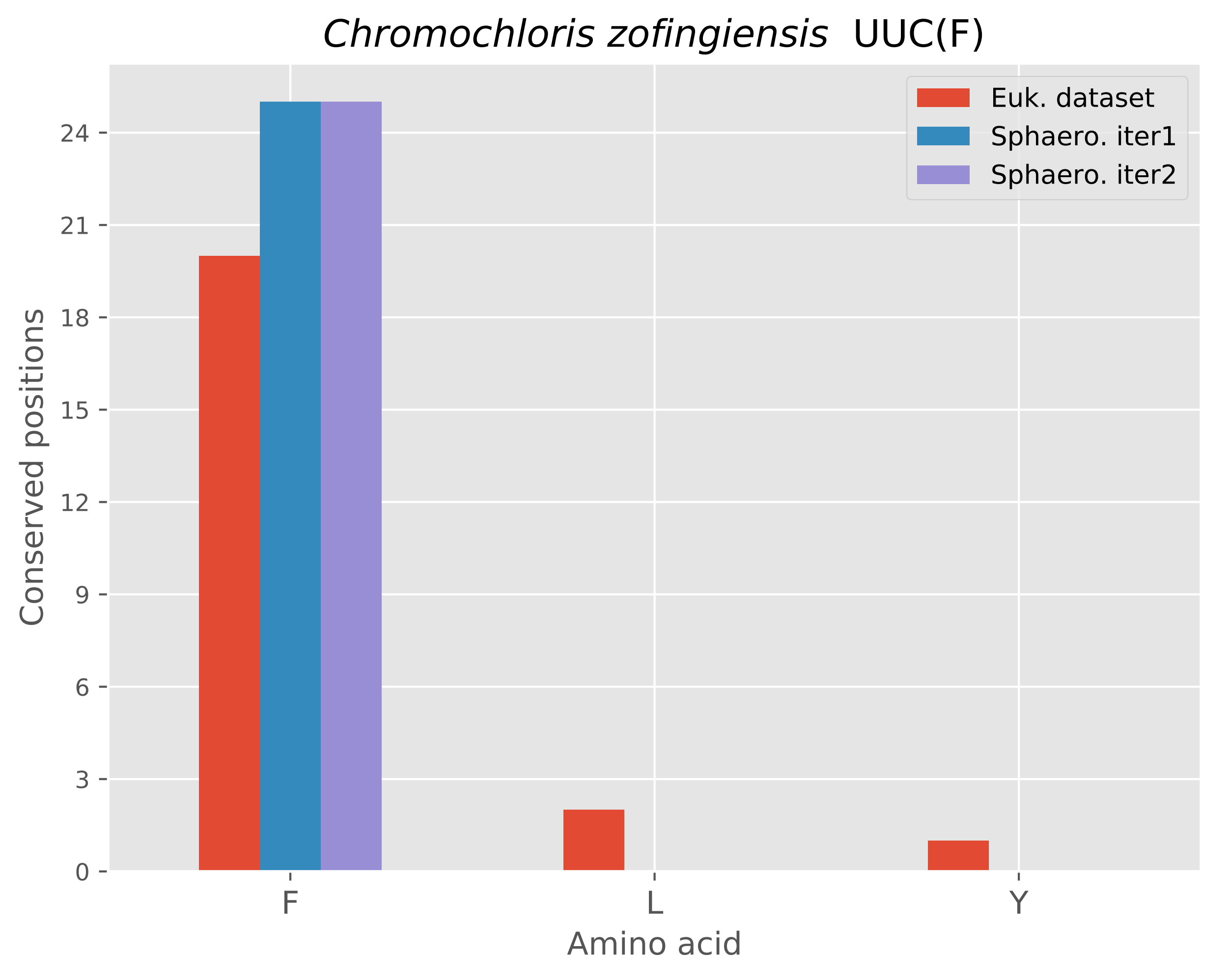

Chromochloris zofingiensis UUC(F)
Euk. dataset
Sphaero. iter1
Sphaero. iter2
Conserved positions
0
3
6
9
12
15
18
21
24
F
L
Y
Amino acid

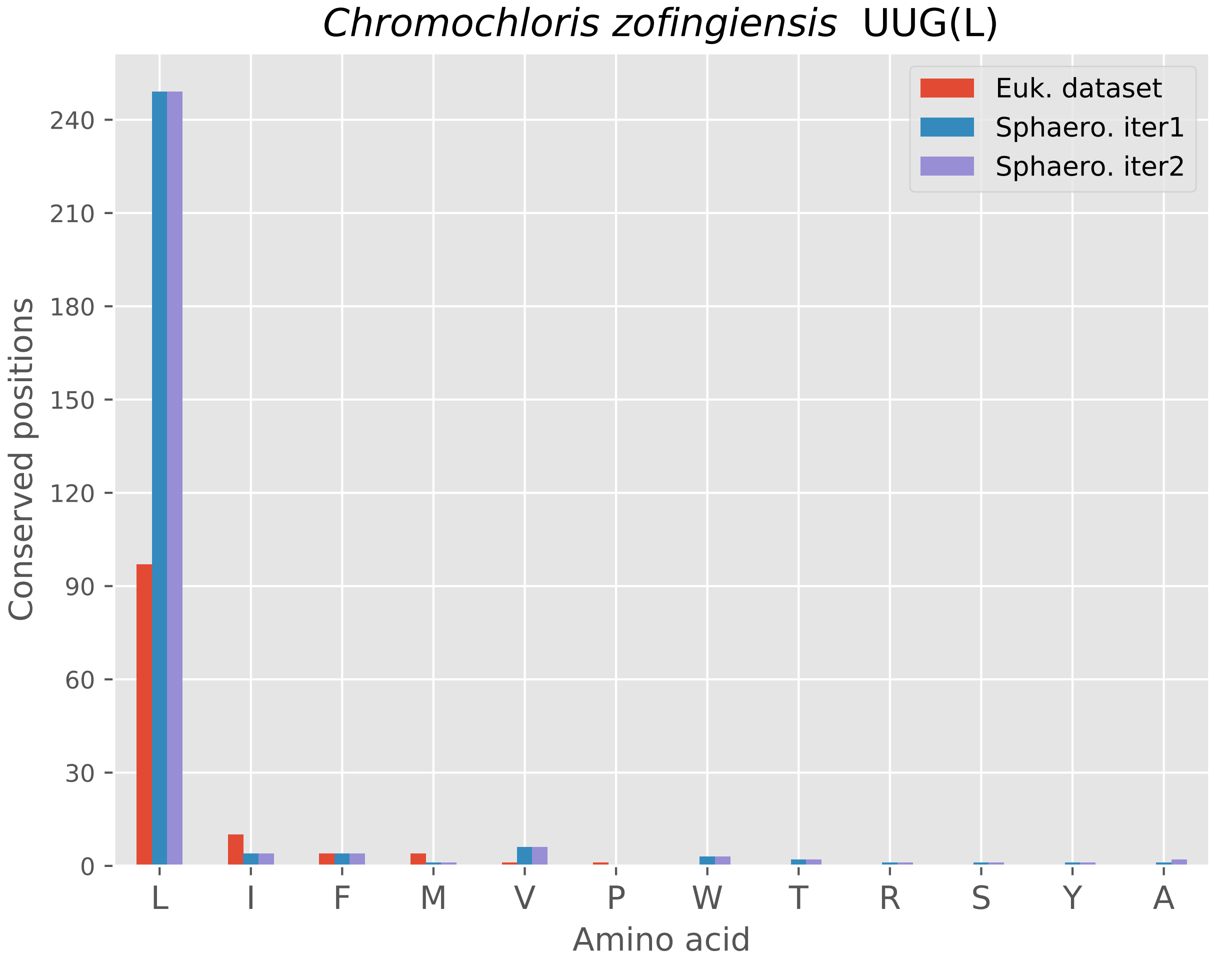

Chromochloris zofingiensis UUG(L)
Euk. dataset
Sphaero. iter1
Sphaero. iter2
Conserved positions
0
30
60
90
120
150
180
210
240
L
I
F
M
V
P
W
T
R
S
Y
A
Amino acid

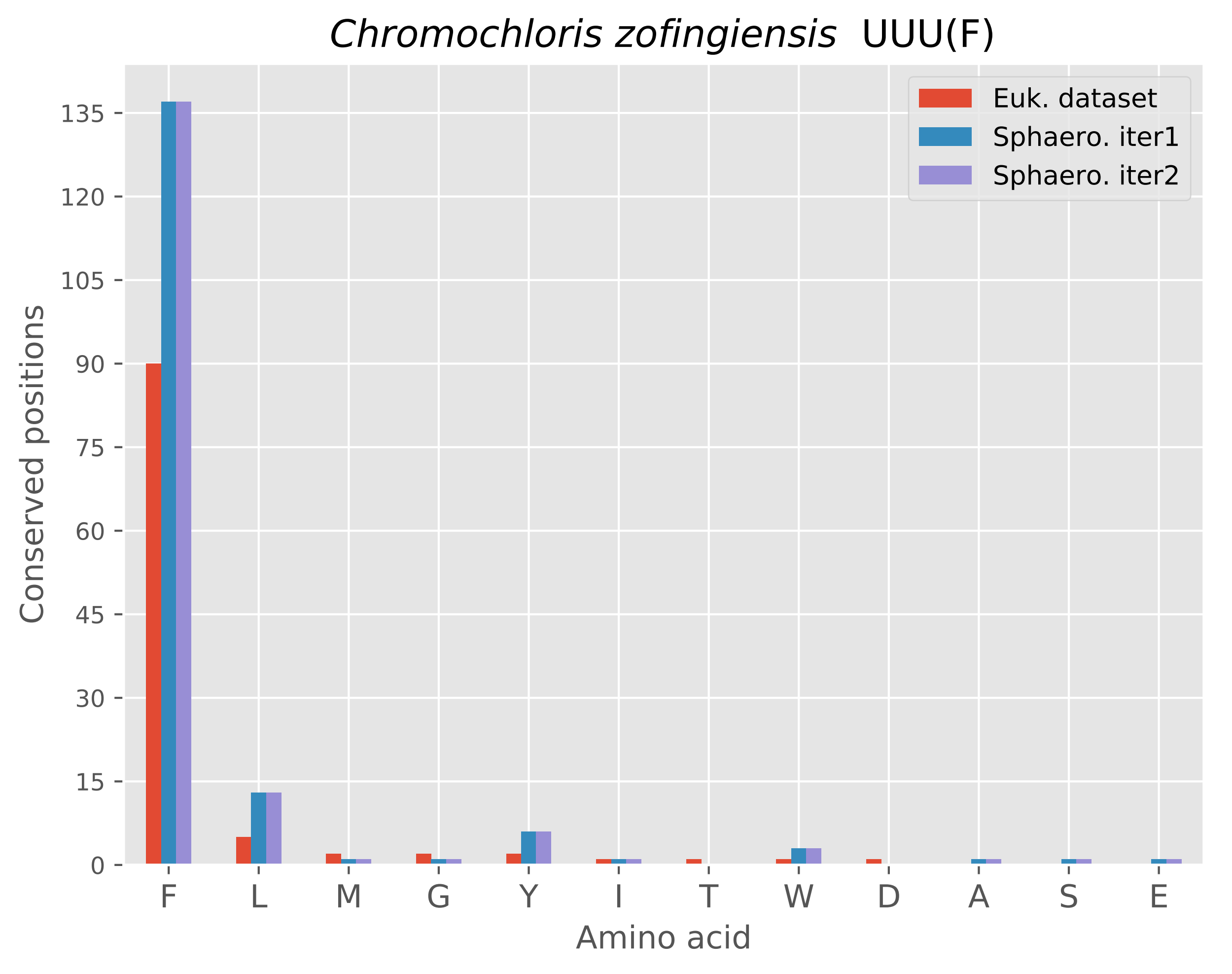
Chromochloris zofingiensis UUU(F)
Euk. dataset
Sphaero. iter1
Sphaero. iter2
Conserved positions
0
15
30
45
60
75
90
105
120
135
F
L
M
G
Y
I
T
W
D
A
S
E
Amino acid

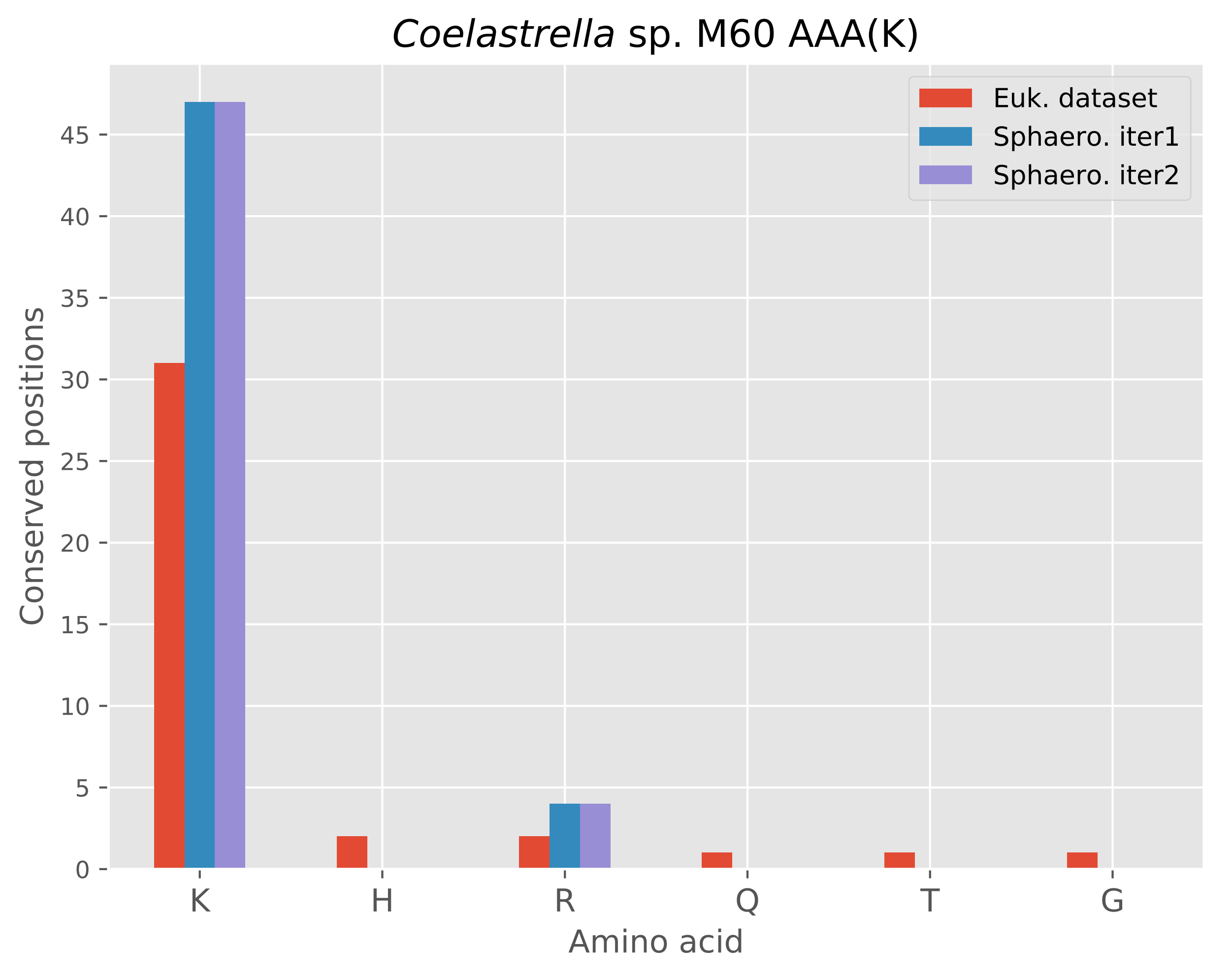

Coelastrella sp. M60 AAA(K)
Euk. dataset
Sphaero. iter1
Sphaero. iter2
Conserved positions
0
5
10
15
20
25
30
35
40
45
K
H
R
Q
T
G
Amino acid

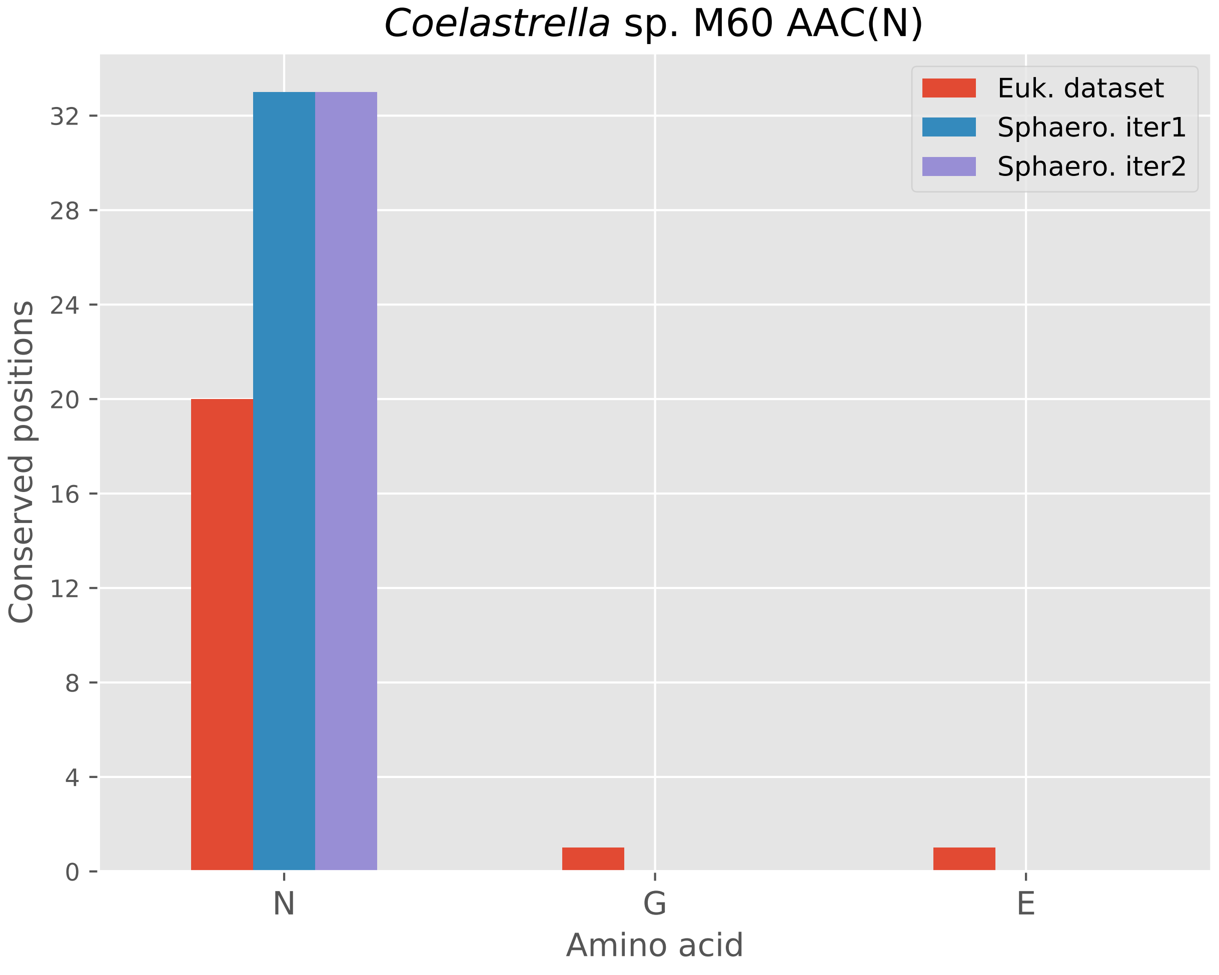

Coelastrella sp. M60 AAC(N)
Euk. dataset
Sphaero. iter1
Sphaero. iter2
Conserved positions
0
4
8
12
16
20
24
28
32
N
G
E
Amino acid

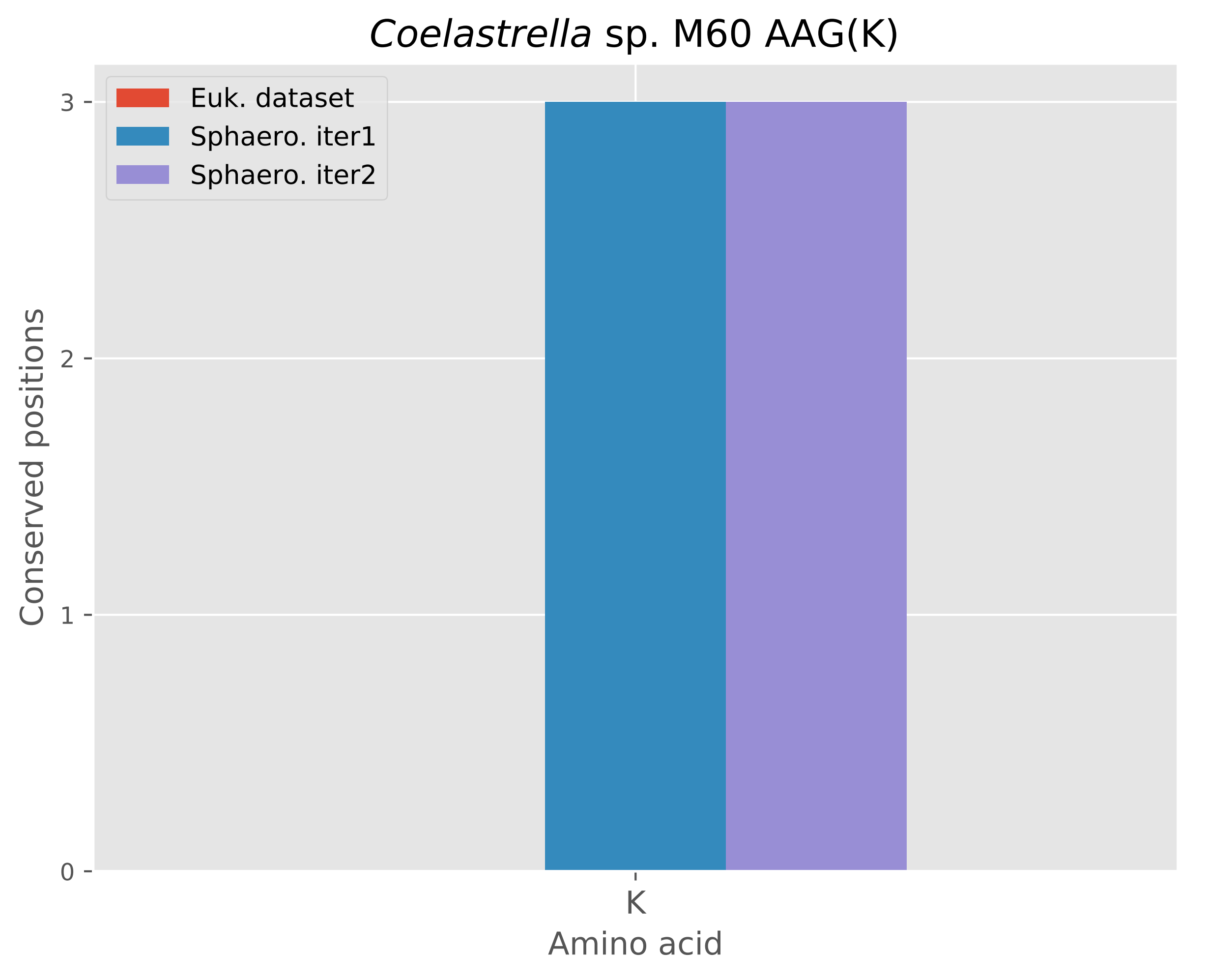

Coelastrella sp. M60 AAG(K)
Euk. dataset
Sphaero. iter1
Sphaero. iter2
Conserved positions
0
1
2
3
K
Amino acid

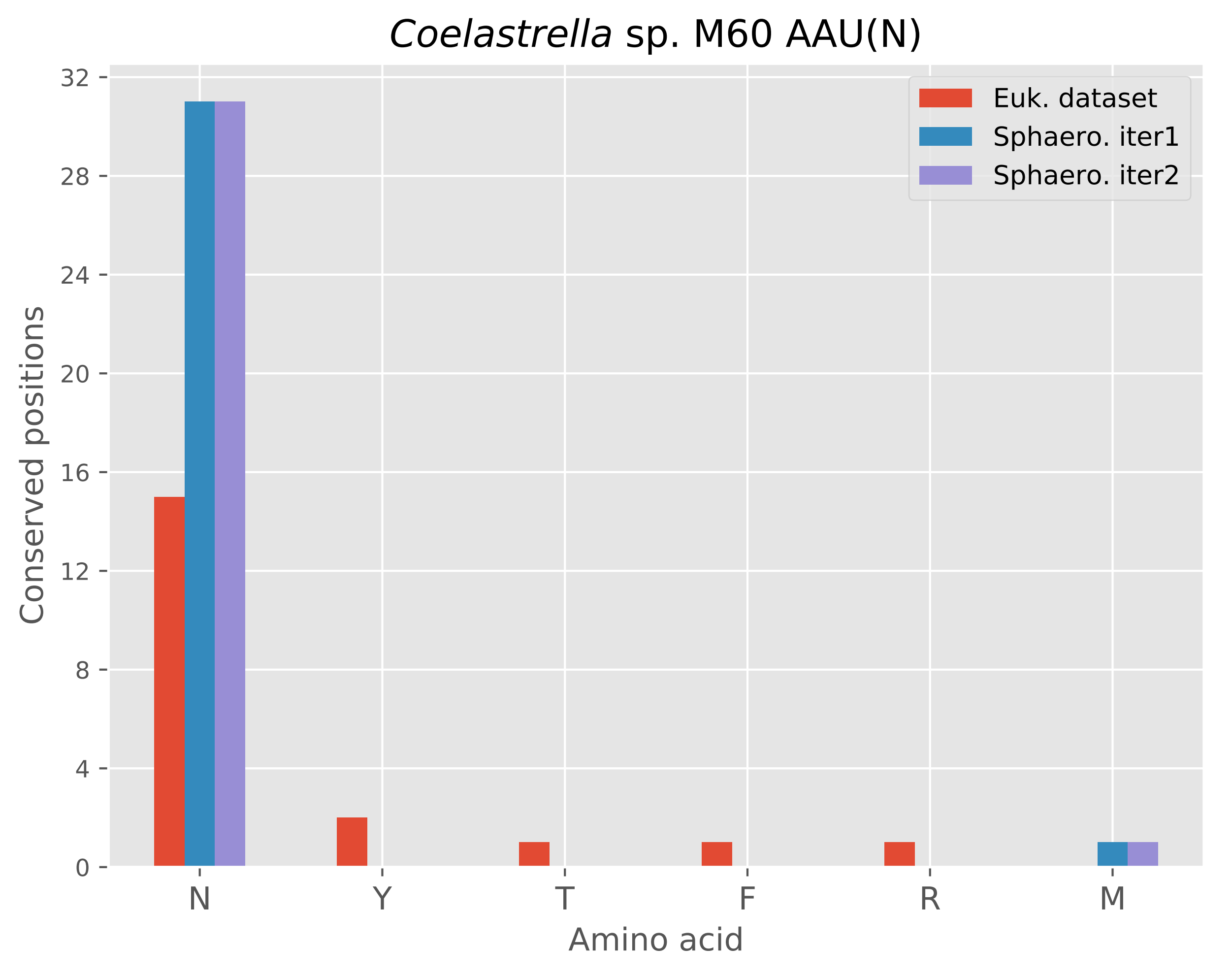
Coelastrella sp. M60 AAU(N)
Euk. dataset
Sphaero. iter1
Sphaero. iter2
Conserved positions
0
4
8
12
16
20
24
28
32
N
Y
T
F
R
M
Amino acid

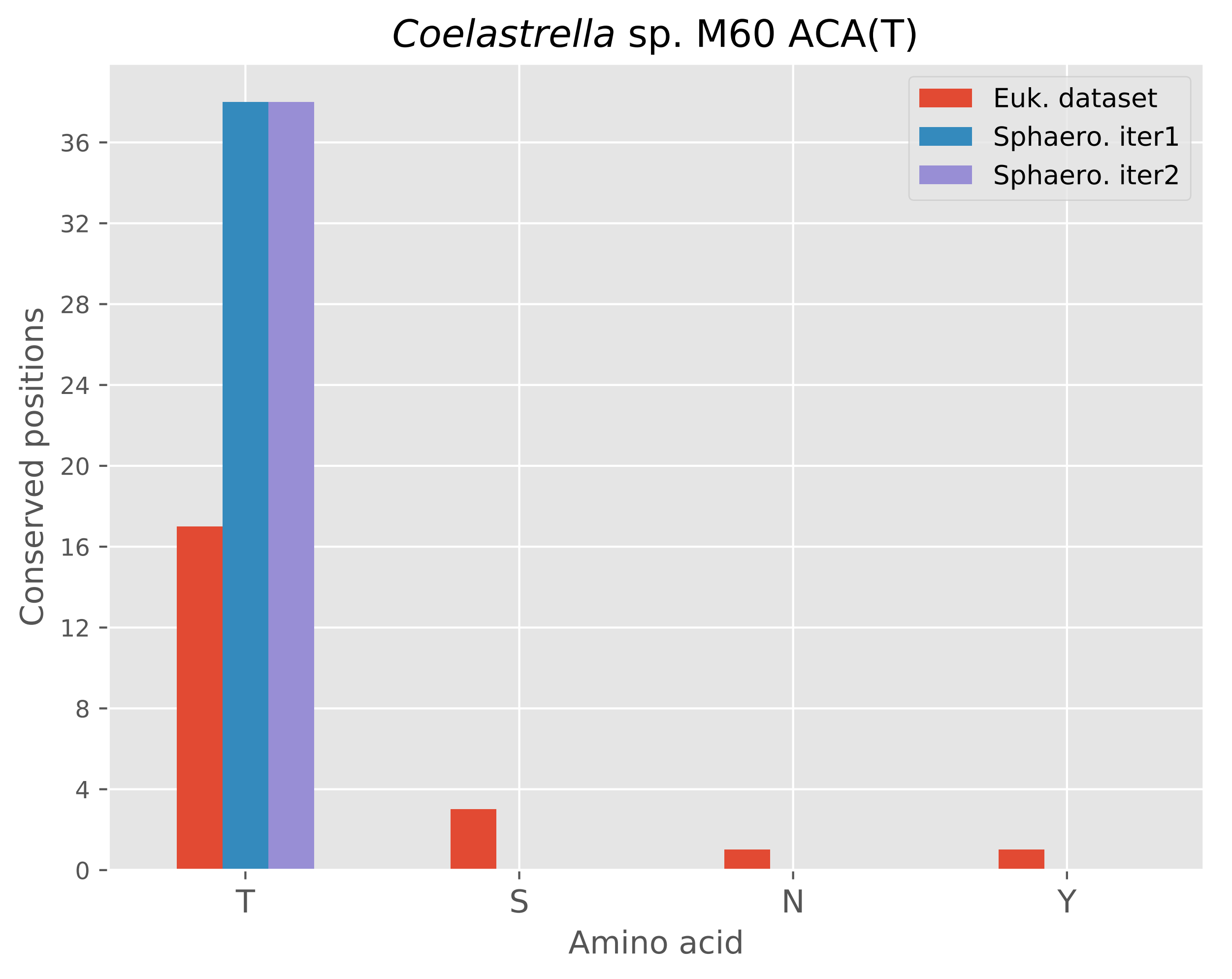
Coelastrella sp. M60 ACA(T)
Euk. dataset
Sphaero. iter1
Sphaero. iter2
Conserved positions
0
4
8
12
16
20
24
28
32
36
T
S
N
Y
Amino acid

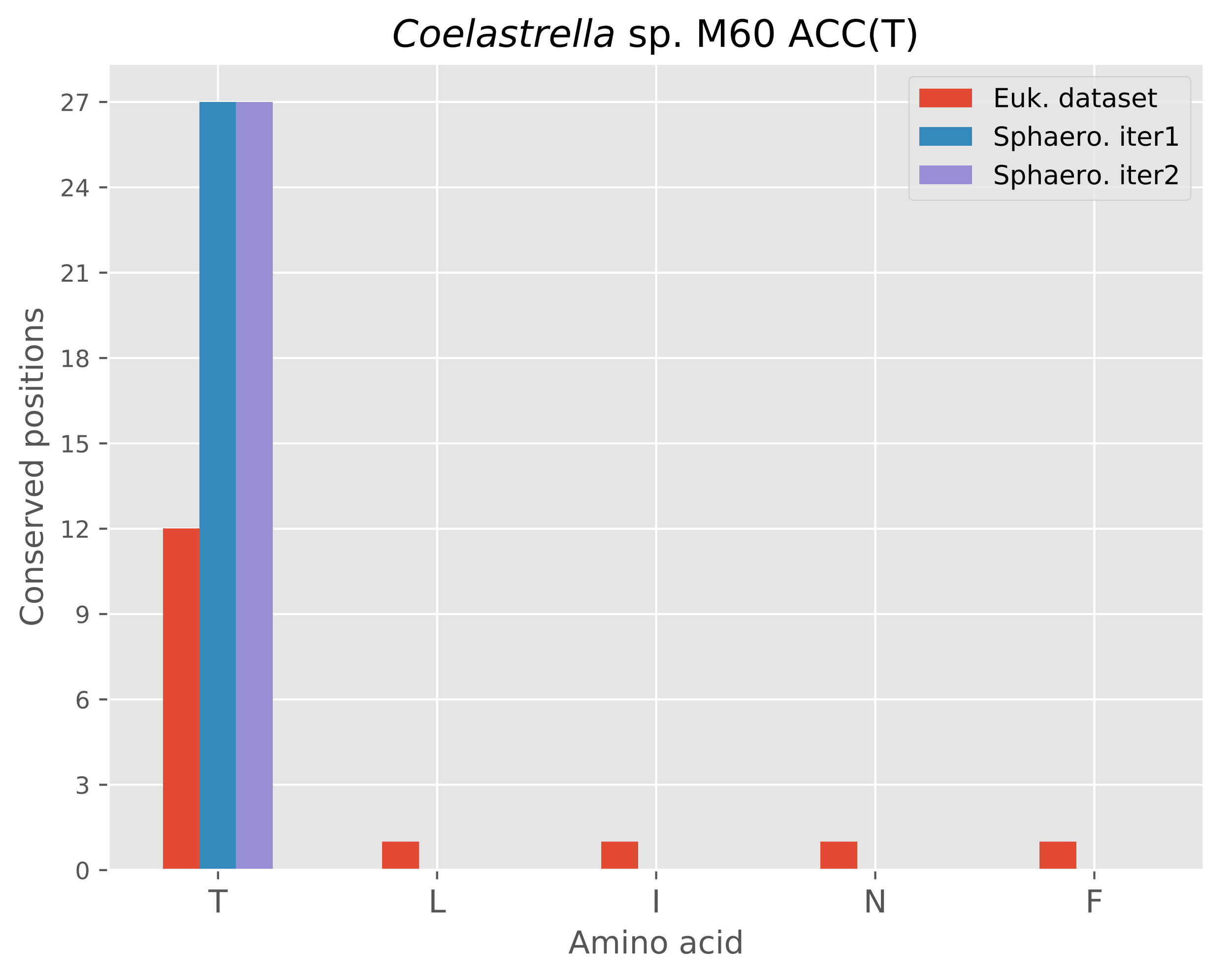

Coelastrella sp. M60 ACC(T)
Euk. dataset
Sphaero. iter1
Sphaero. iter2
Conserved positions
0
3
6
9
12
15
18
21
24
27
T
L
I
N
F
Amino acid

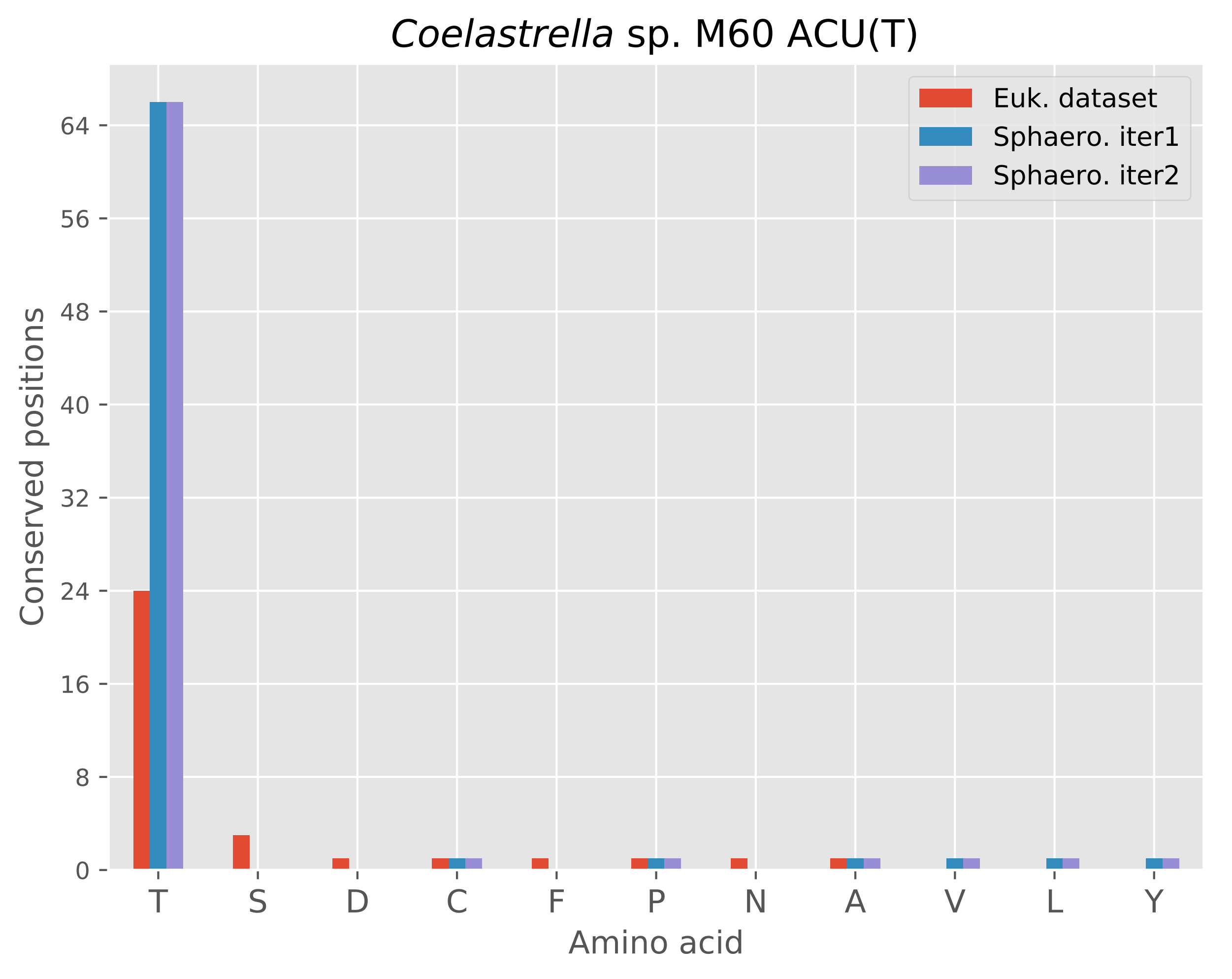

Coelastrella sp. M60 ACU(T)
Euk. dataset
Sphaero. iter1
Sphaero. iter2
Conserved positions
0
8
16
24
32
40
48
56
64
T
S
D
C
F
P
N
A
V
L
Y
Amino acid

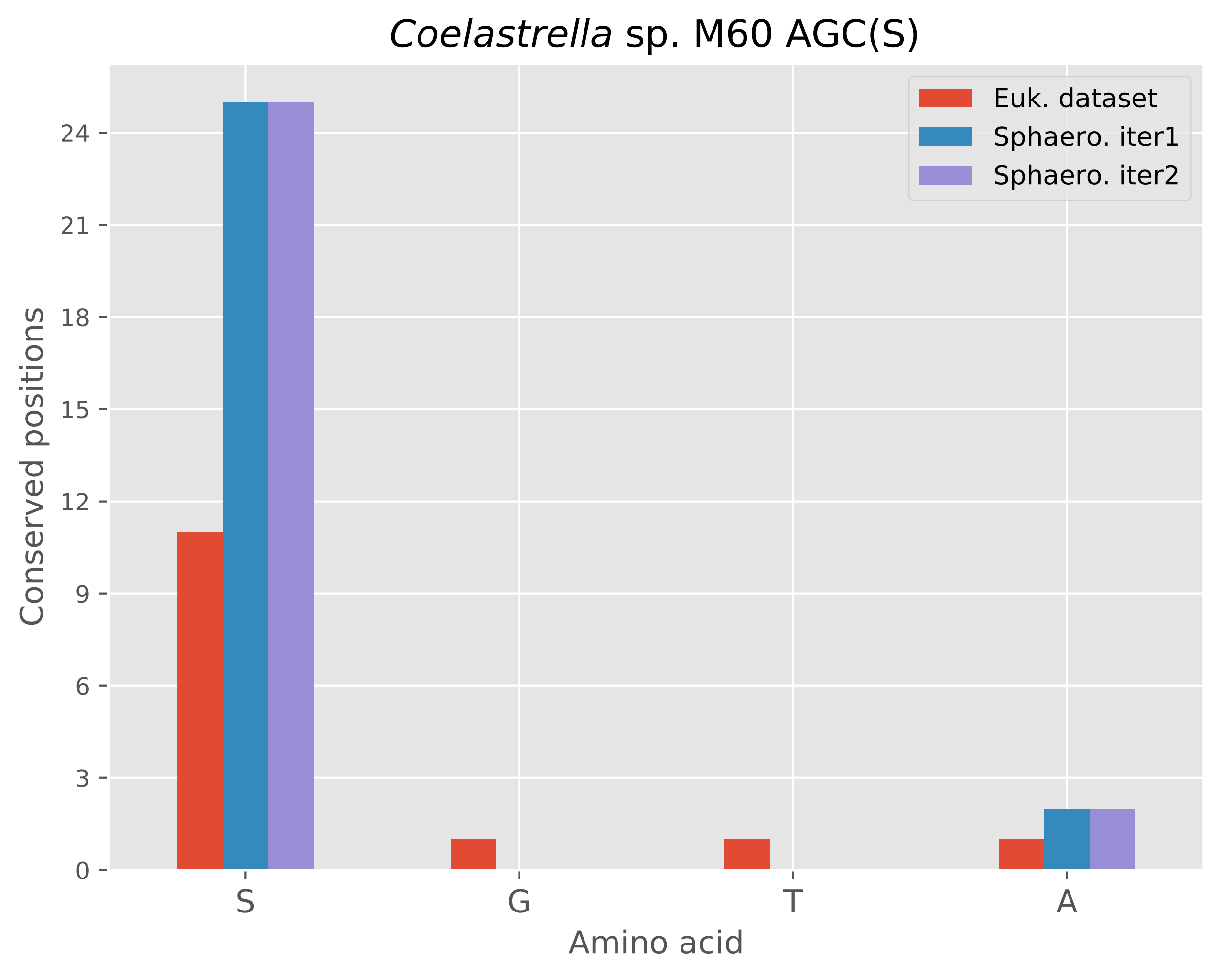

Coelastrella sp. M60 AGC(S)
Euk. dataset
Sphaero. iter1
Sphaero. iter2
Conserved positions
0
3
6
9
12
15
18
21
24
S
G
T
A
Amino acid

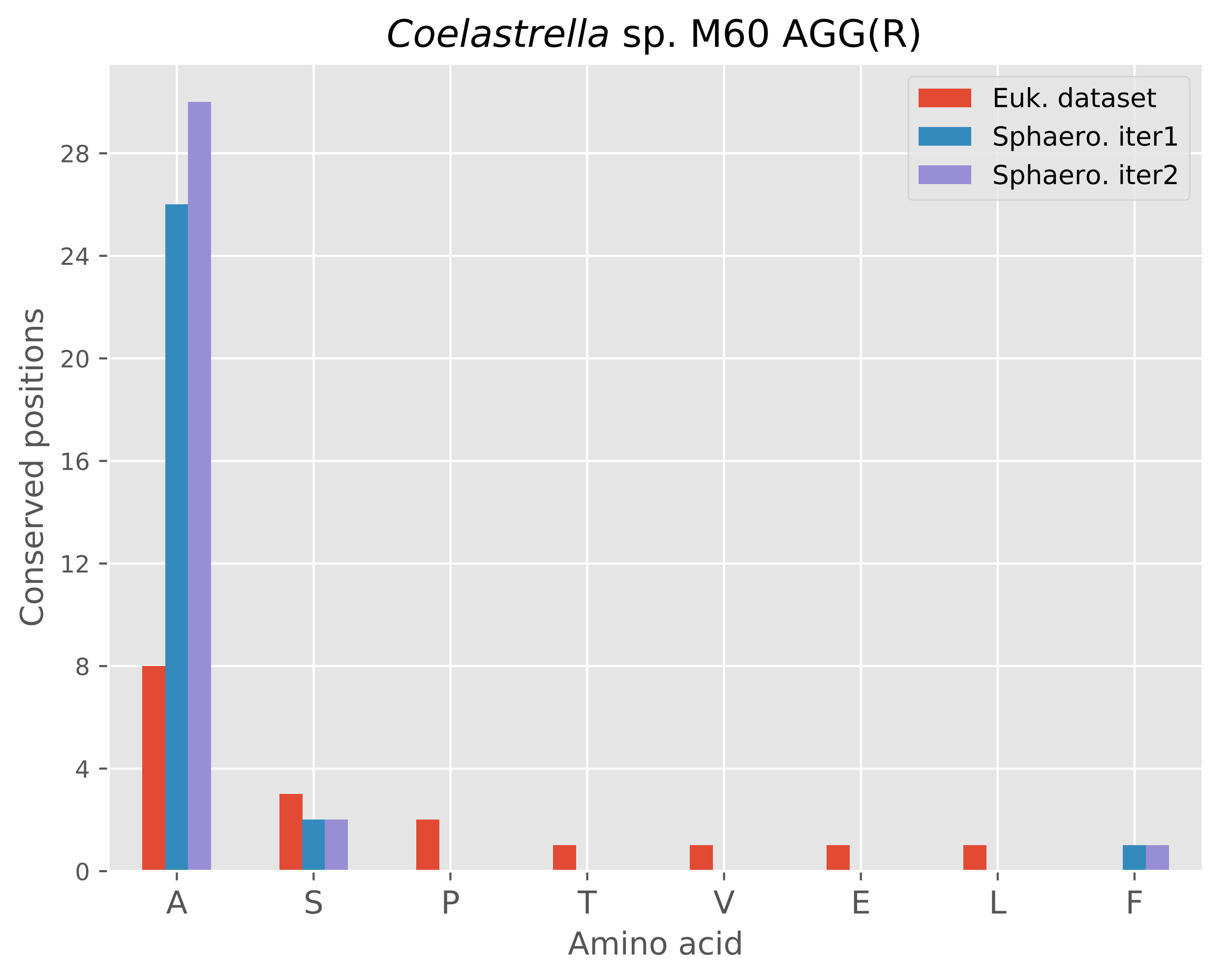

Coelastrella sp. M60 AGG(R)
Euk. dataset
Sphaero. iter1
Sphaero. iter2
Conserved positions
0
4
8
12
16
20
24
28
A
S
P
T
V
E
L
F
Amino acid

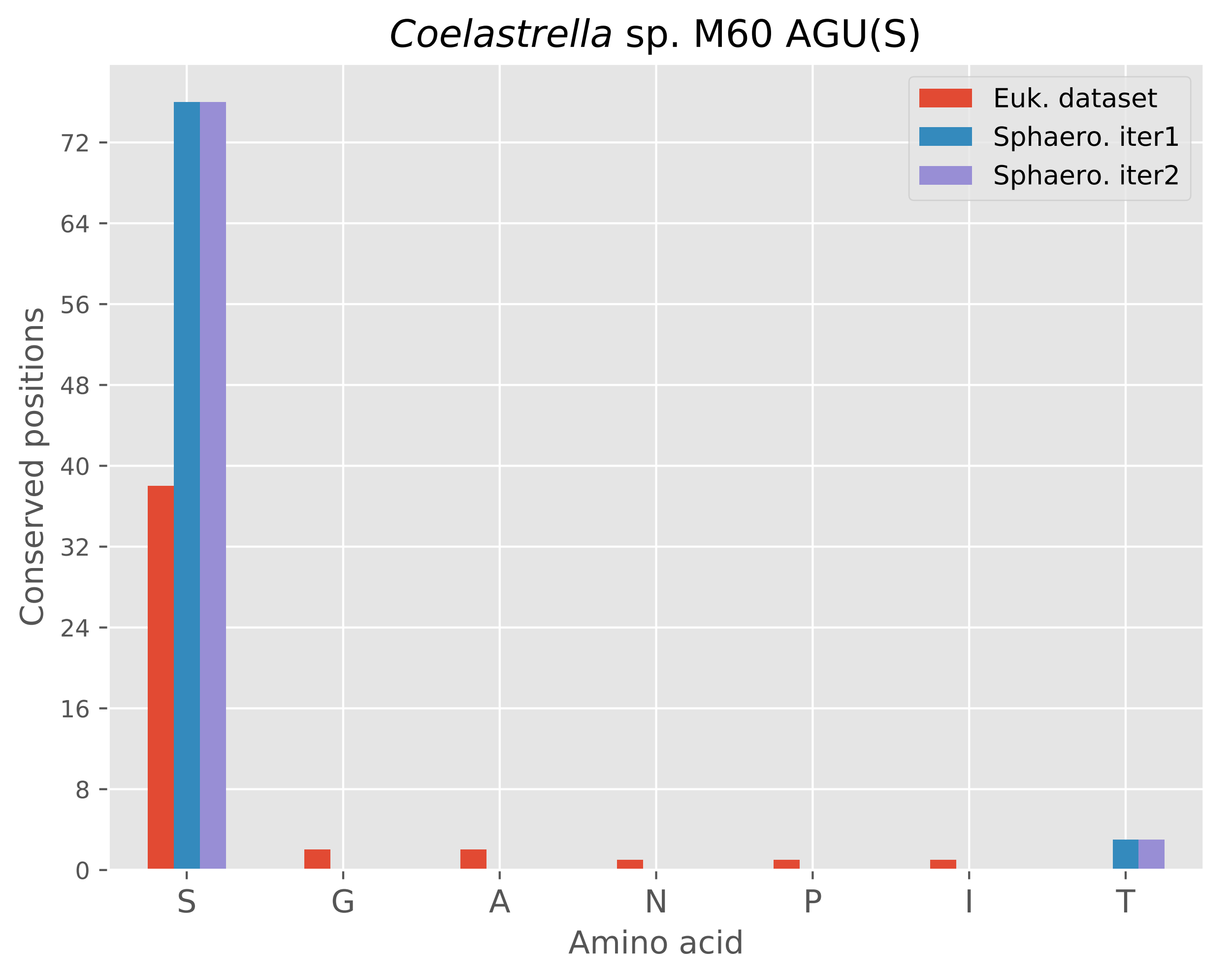

Coelastrella sp. M60 AGU(S)
Euk. dataset
Sphaero. iter1
Sphaero. iter2
Conserved positions
0
8
16
24
32
40
48
56
64
72
S
G
A
N
P
I
T
Amino acid

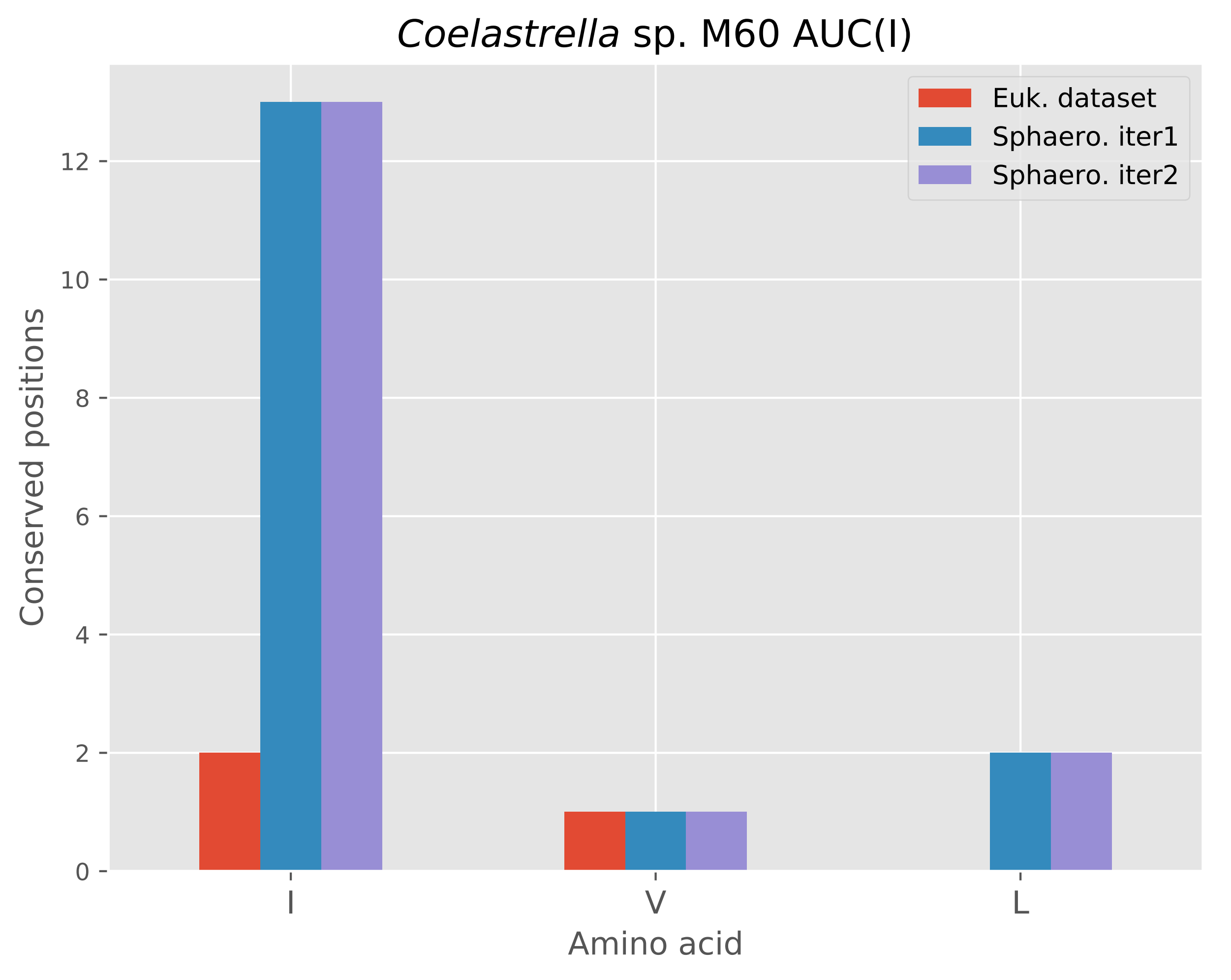

Coelastrella sp. M60 AUC(I)
Euk. dataset
Sphaero. iter1
Sphaero. iter2
Conserved positions
0
2
4
6
8
10
12
I
V
L
Amino acid

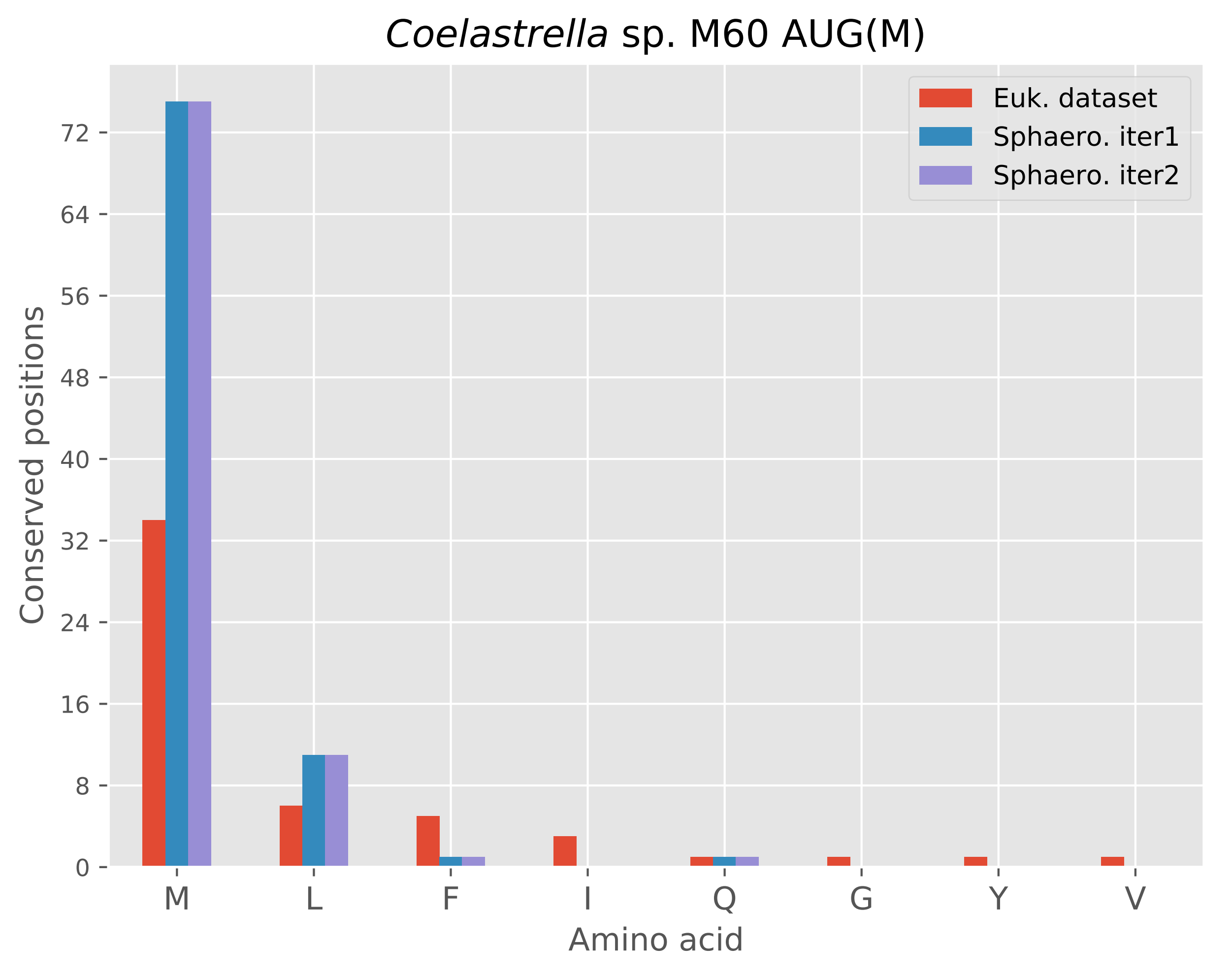

Coelastrella sp. M60 AUG(M)
Euk. dataset
Sphaero. iter1
Sphaero. iter2
Conserved positions
0
8
16
24
32
40
48
56
64
72
M
L
F
I
Q
G
Y
V
Amino acid

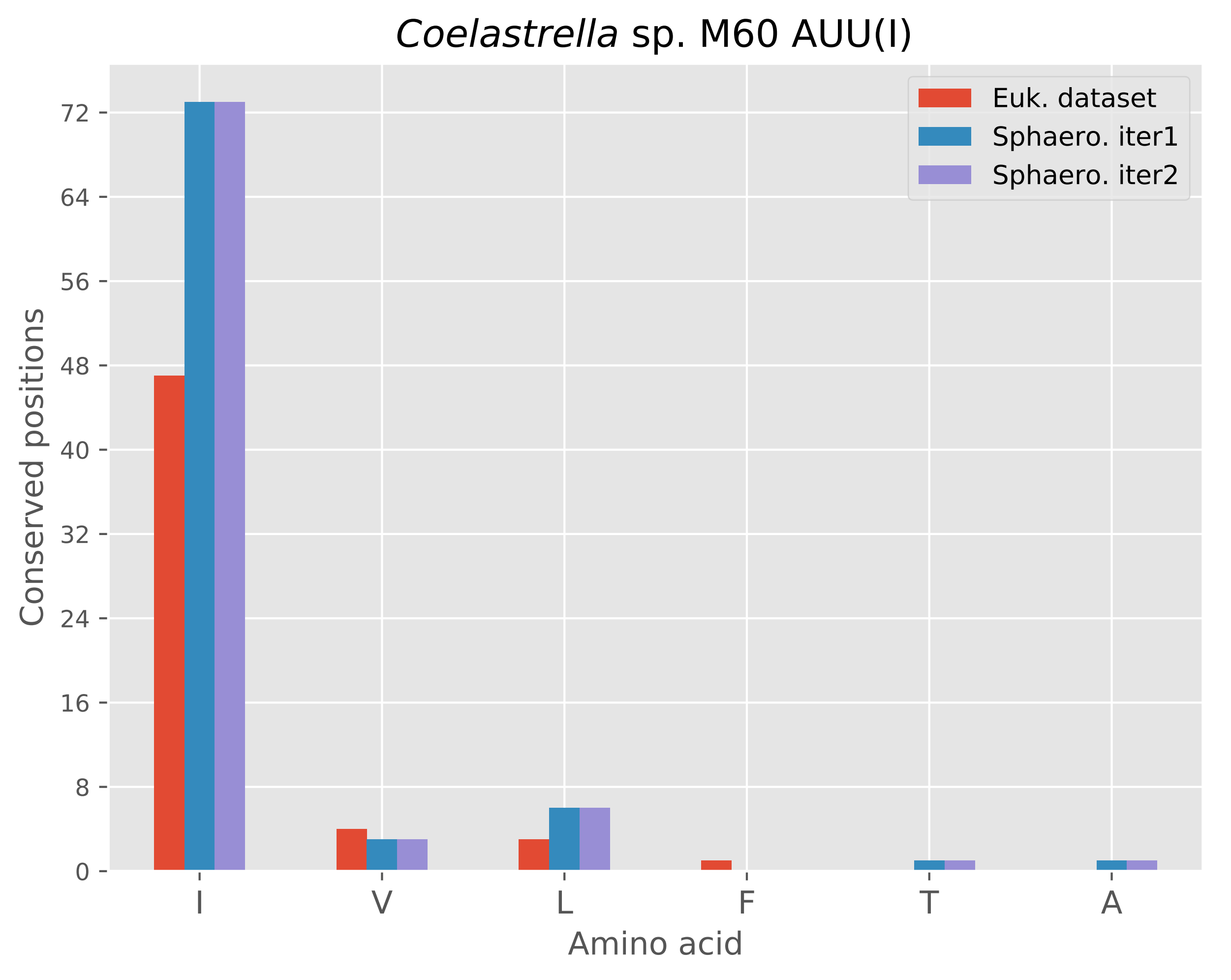

Coelastrella sp. M60 AUU(I)
Euk. dataset
Sphaero. iter1
Sphaero. iter2
Conserved positions
0
8
16
24
32
40
48
56
64
72
I
V
L
F
T
A
Amino acid

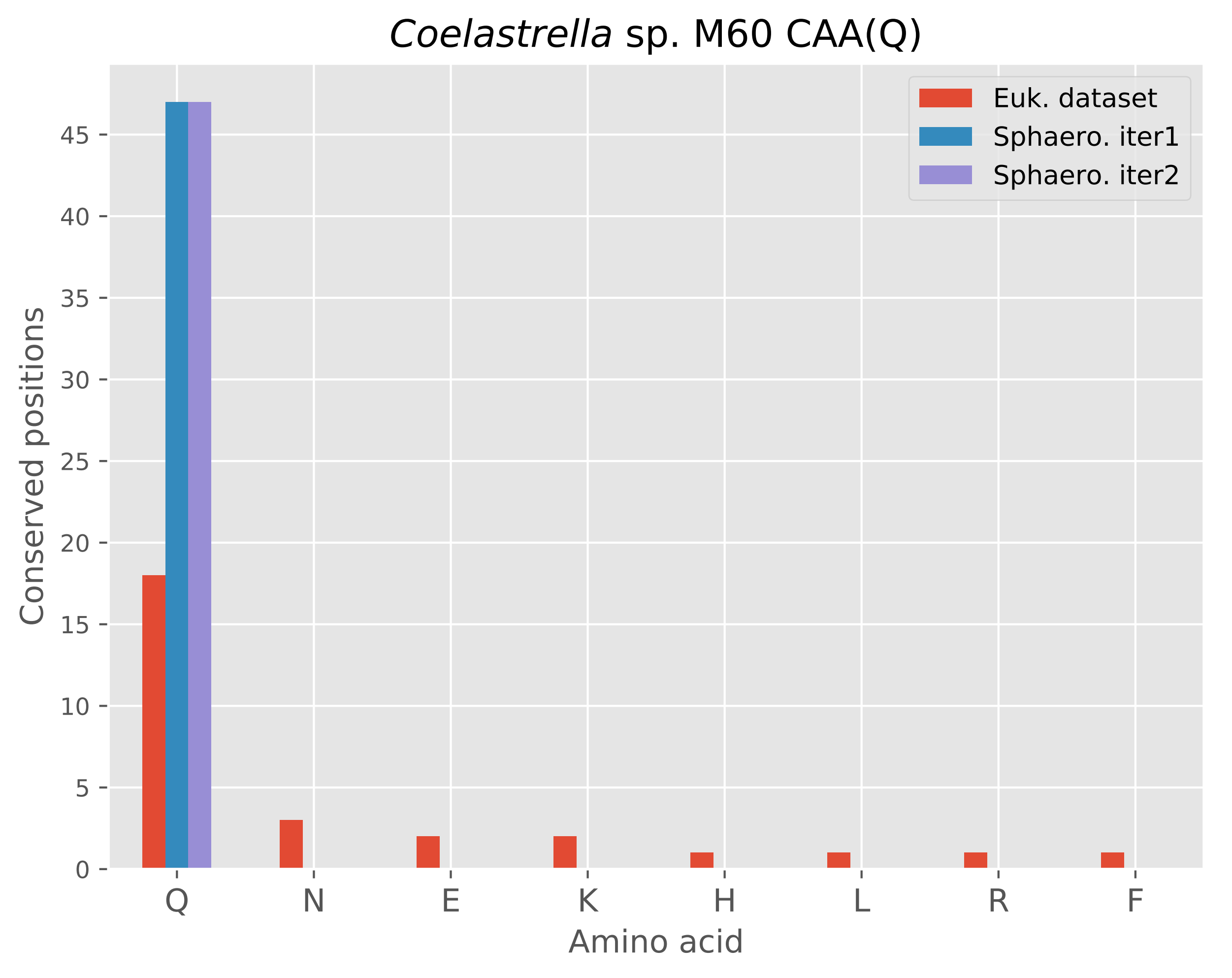

Coelastrella sp. M60 CAA(Q)
Euk. dataset
Sphaero. iter1
Sphaero. iter2
Conserved positions
0
5
10
15
20
25
30
35
40
45
Q
N
E
K
H
L
R
F
Amino acid

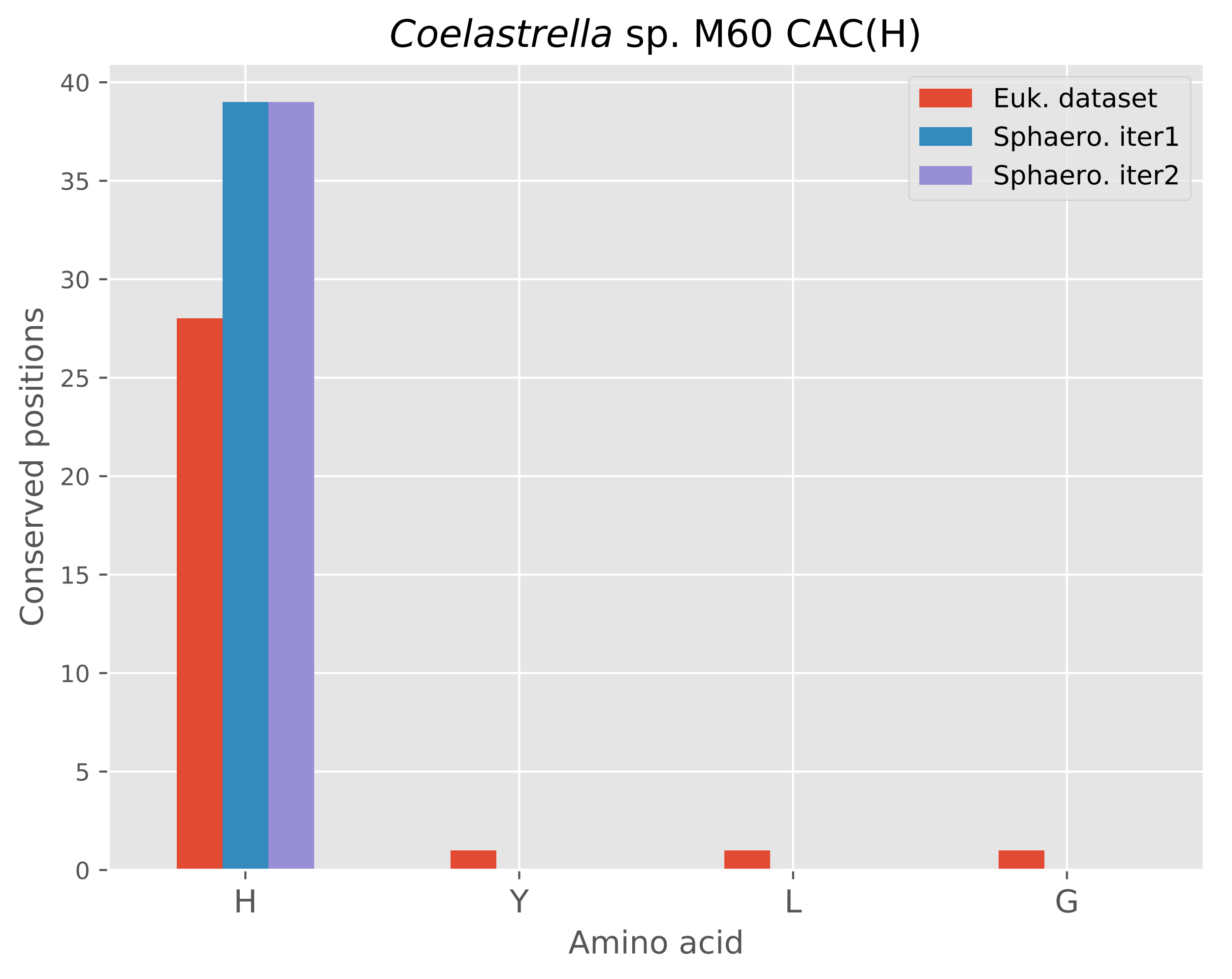

Coelastrella sp. M60 CAC(H)
Euk. dataset
Sphaero. iter1
Sphaero. iter2
Conserved positions
0
5
10
15
20
25
30
35
40
H
Y
L
G
Amino acid

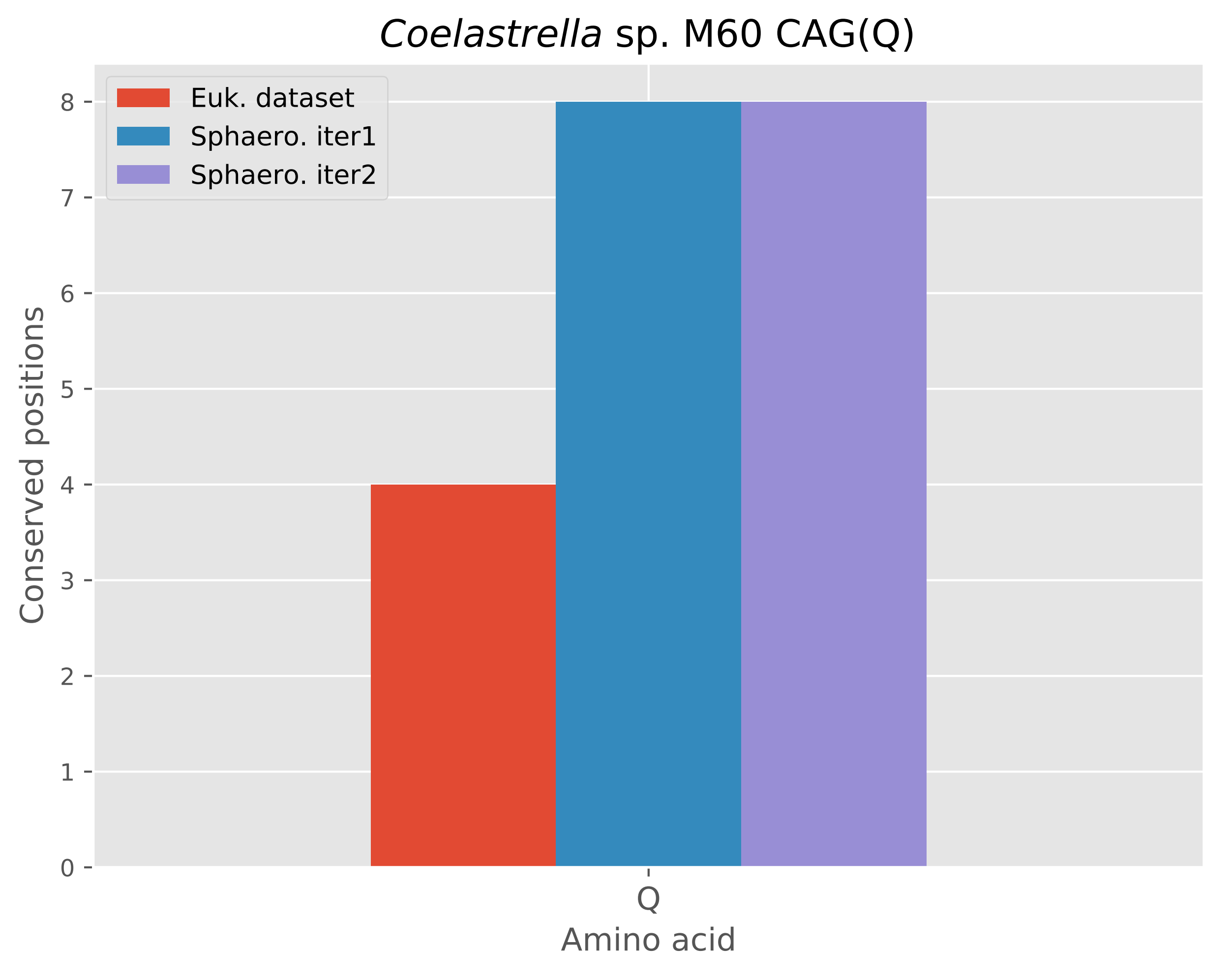

Coelastrella sp. M60 CAG(Q)
Euk. dataset
Sphaero. iter1
Sphaero. iter2
Conserved positions
0
1
2
3
4
5
6
7
8
Q
Amino acid

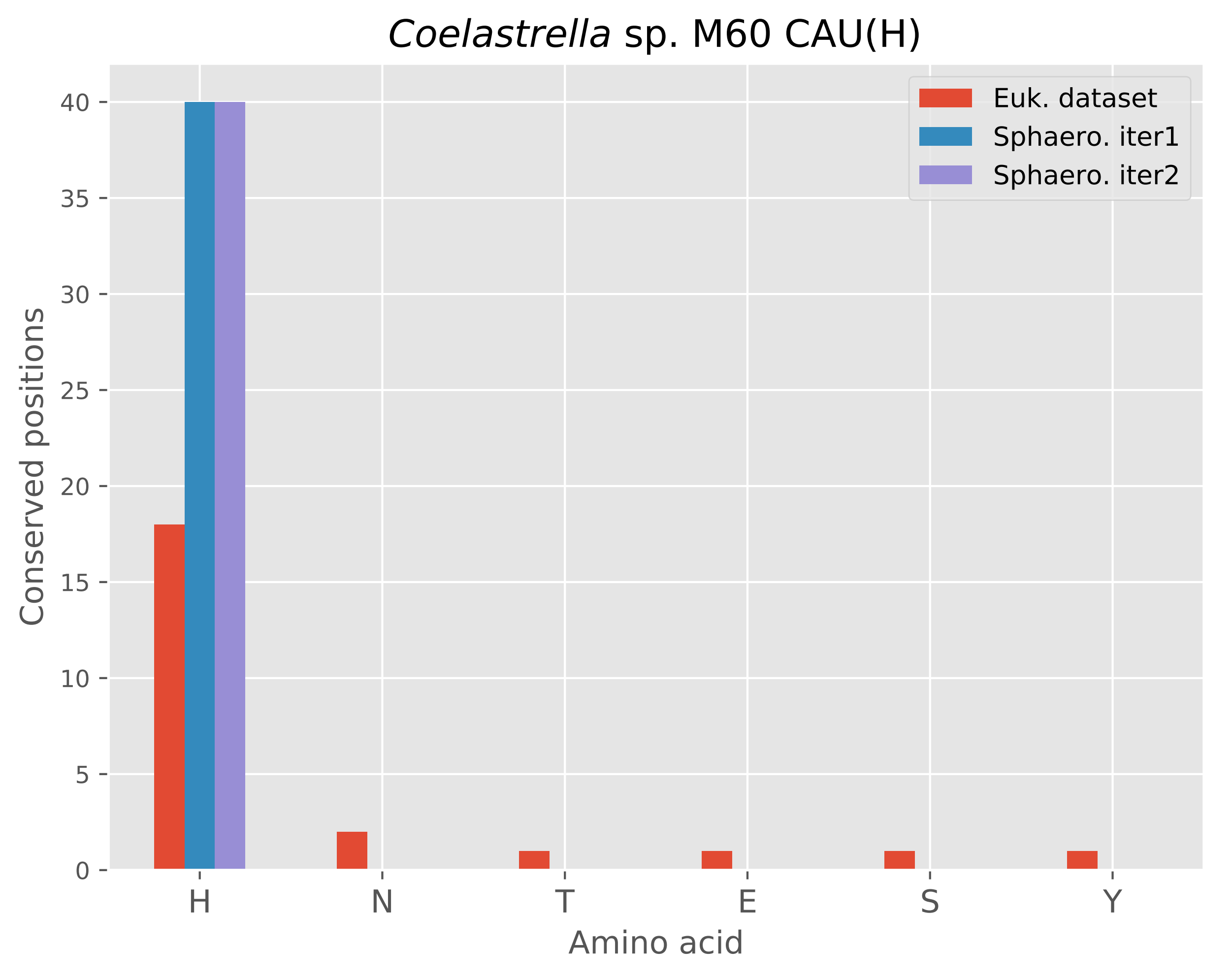

Coelastrella sp. M60 CAU(H)
Euk. dataset
Sphaero. iter1
Sphaero. iter2
Conserved positions
0
5
10
15
20
25
30
35
40
H
N
T
E
S
Y
Amino acid

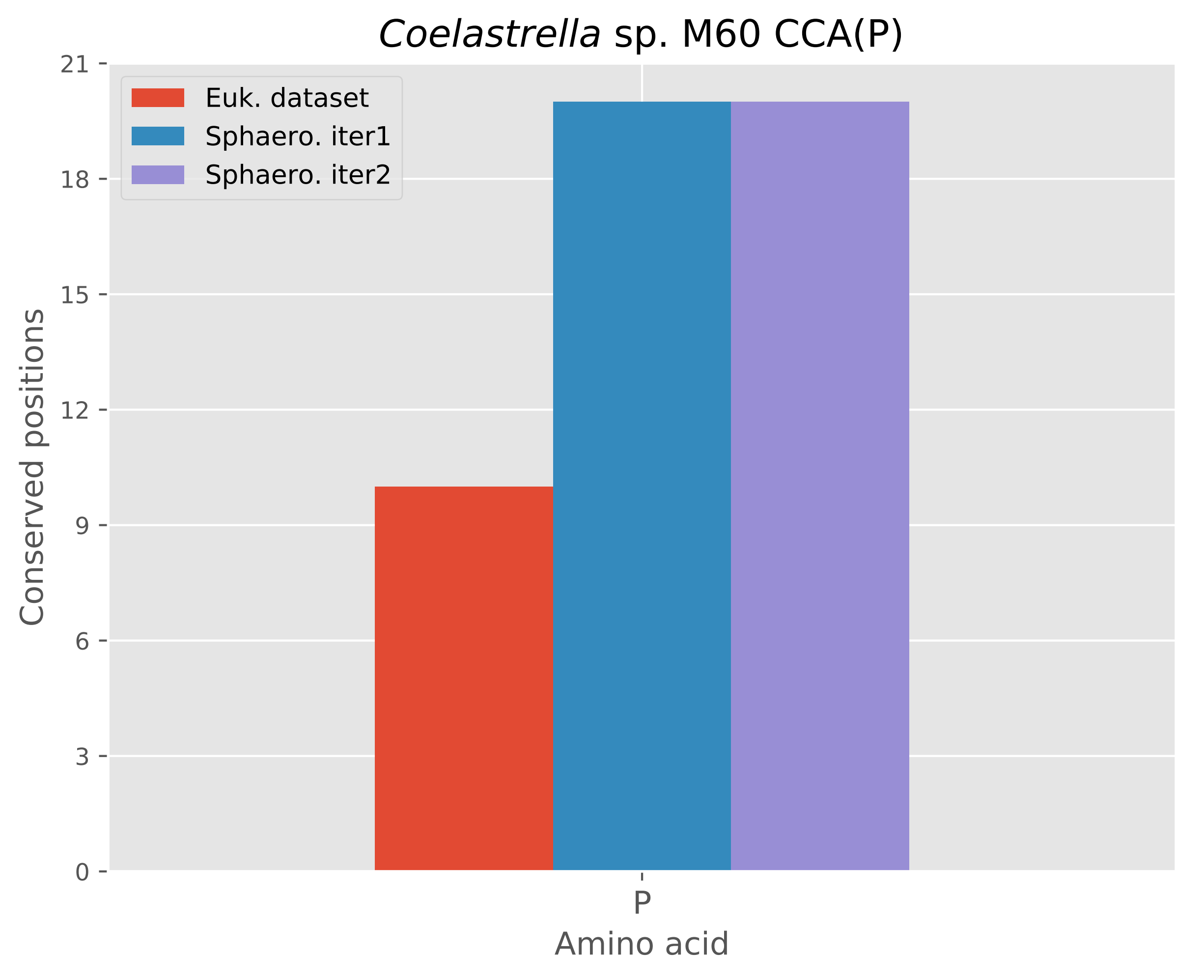
Coelastrella sp. M60 CCA(P)
Euk. dataset
Sphaero. iter1
Sphaero. iter2
Conserved positions
0
3
6
9
12
15
18
21
P
Amino acid

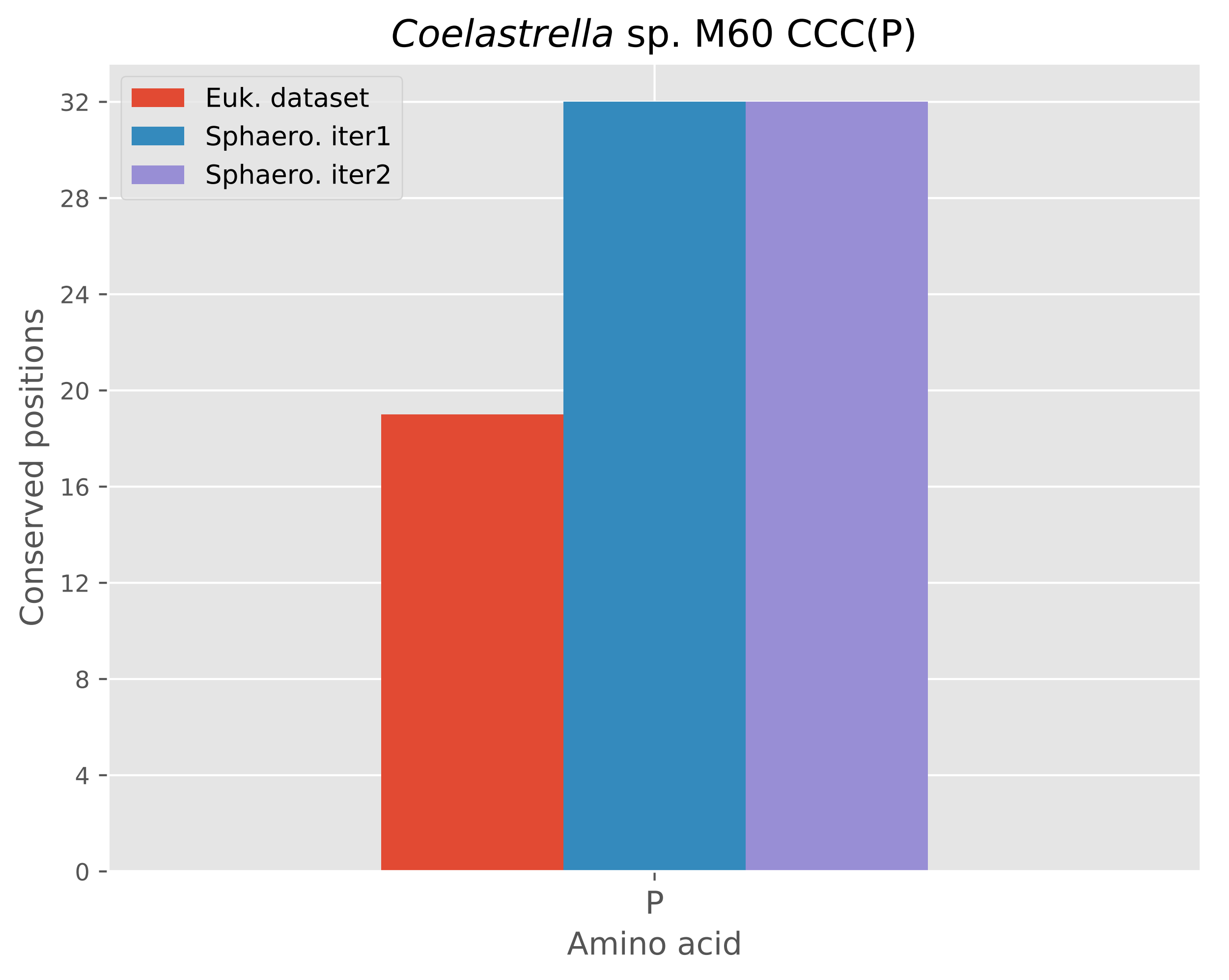

Coelastrella sp. M60 CCC(P)
Euk. dataset
Sphaero. iter1
Sphaero. iter2
Conserved positions
0
4
8
12
16
20
24
28
32
P
Amino acid

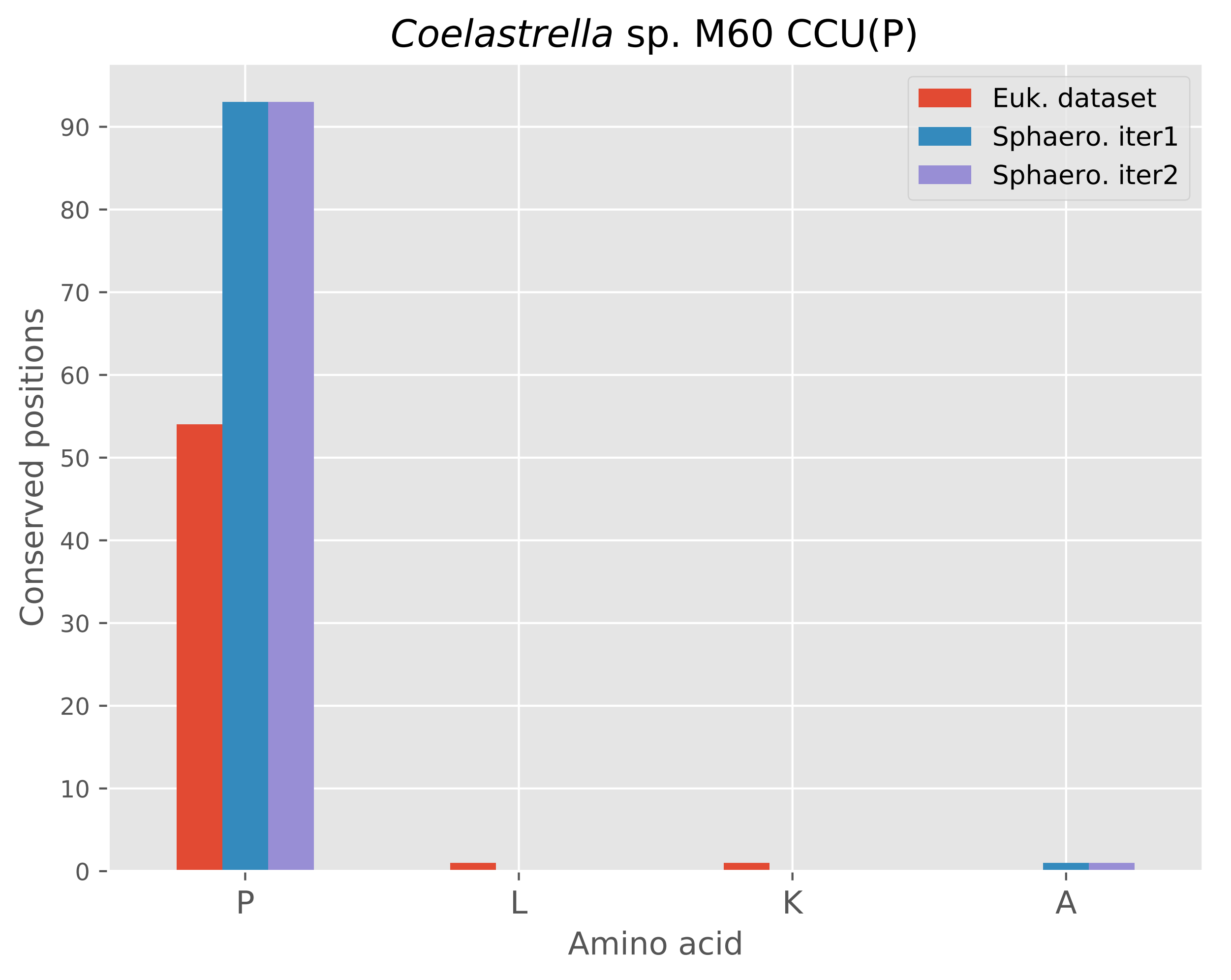

Coelastrella sp. M60 CCU(P)
Euk. dataset
Sphaero. iter1
Sphaero. iter2
Conserved positions
0
10
20
30
40
50
60
70
80
90
P
L
K
A
Amino acid

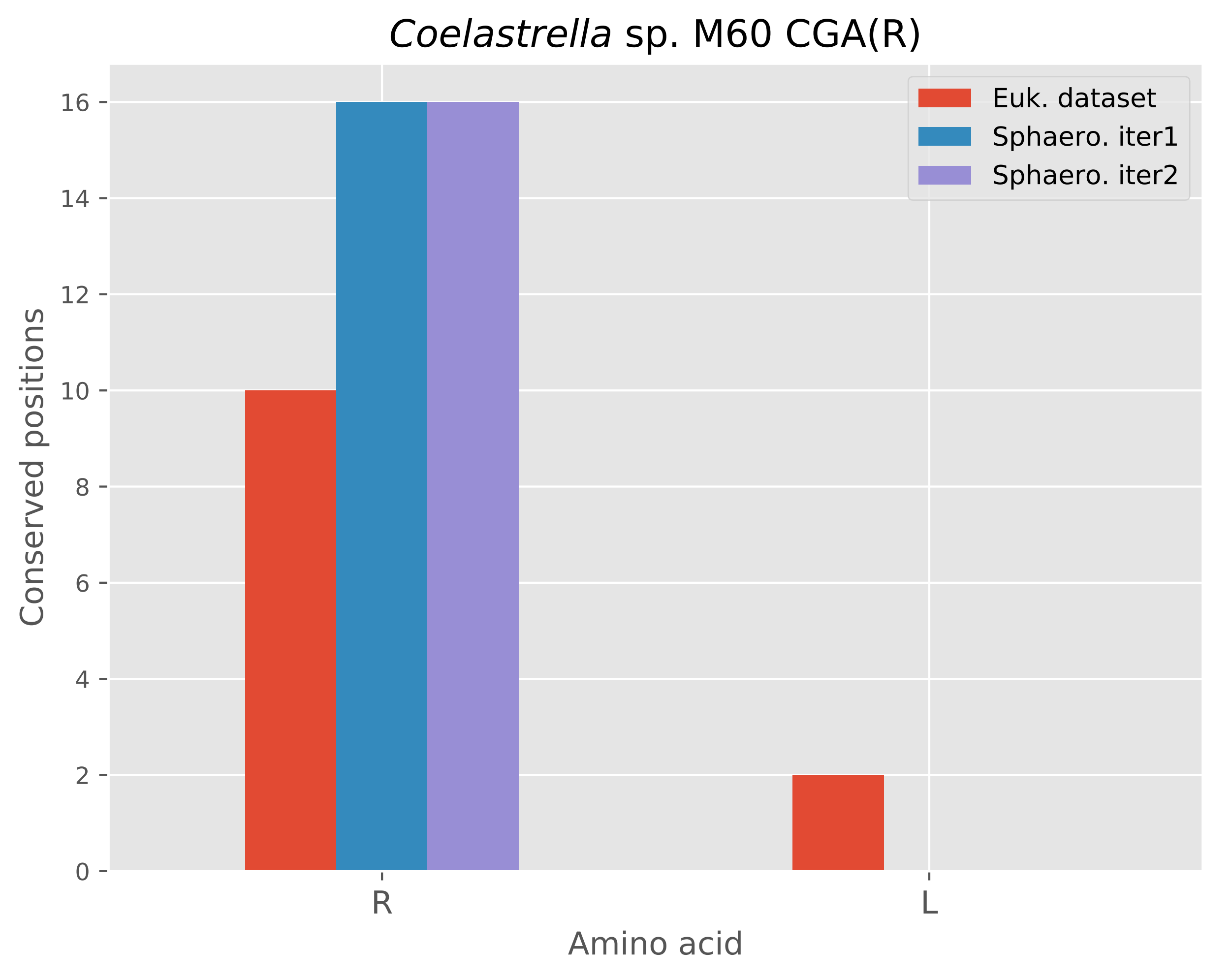
Coelastrella sp. M60 CGA(R)
Euk. dataset
Sphaero. iter1
Sphaero. iter2
Conserved positions
0
2
4
6
8
10
12
14
16
R
L
Amino acid

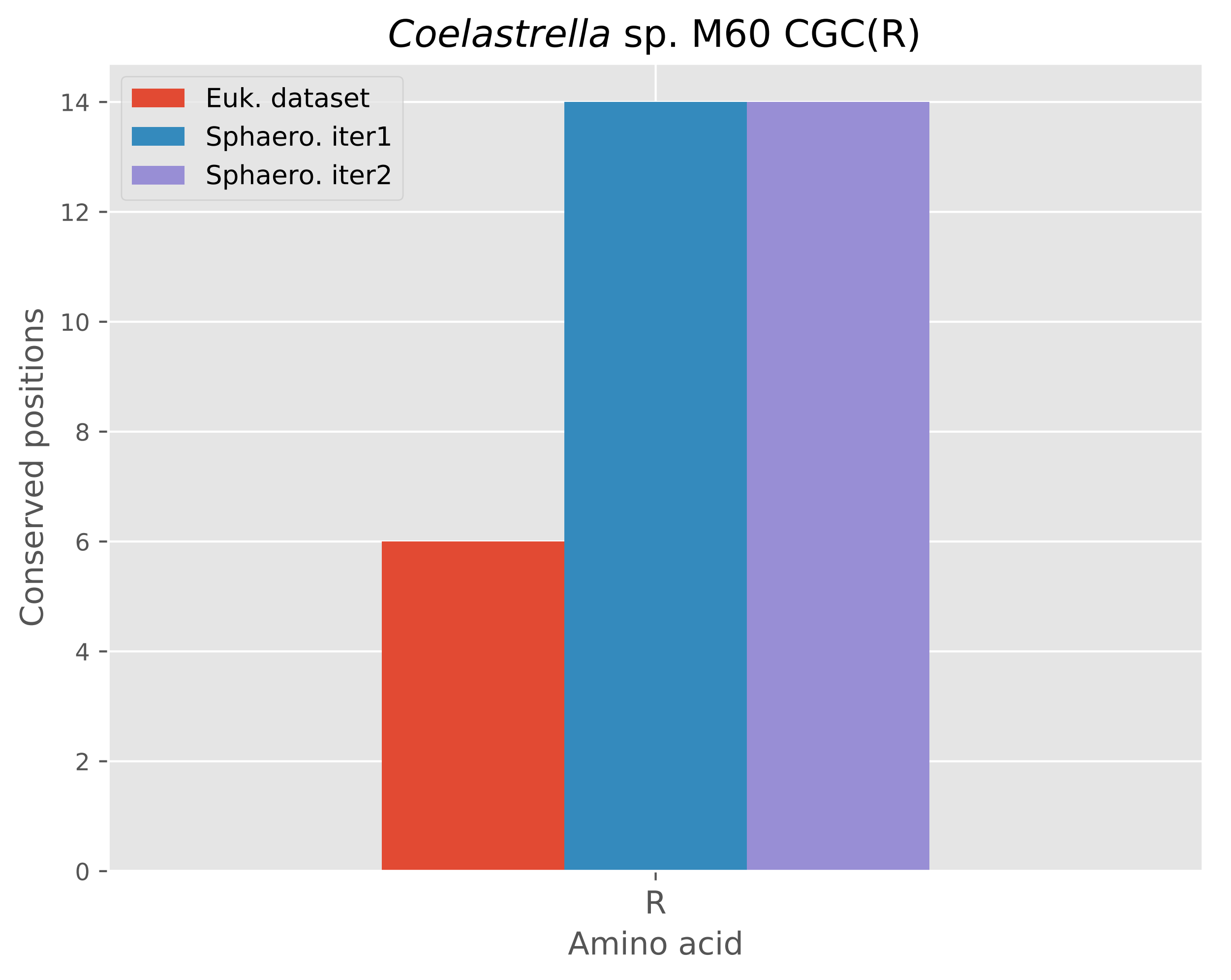

Coelastrella sp. M60 CGC(R)
Euk. dataset
Sphaero. iter1
Sphaero. iter2
Conserved positions
0
2
4
6
8
10
12
14
R
Amino acid

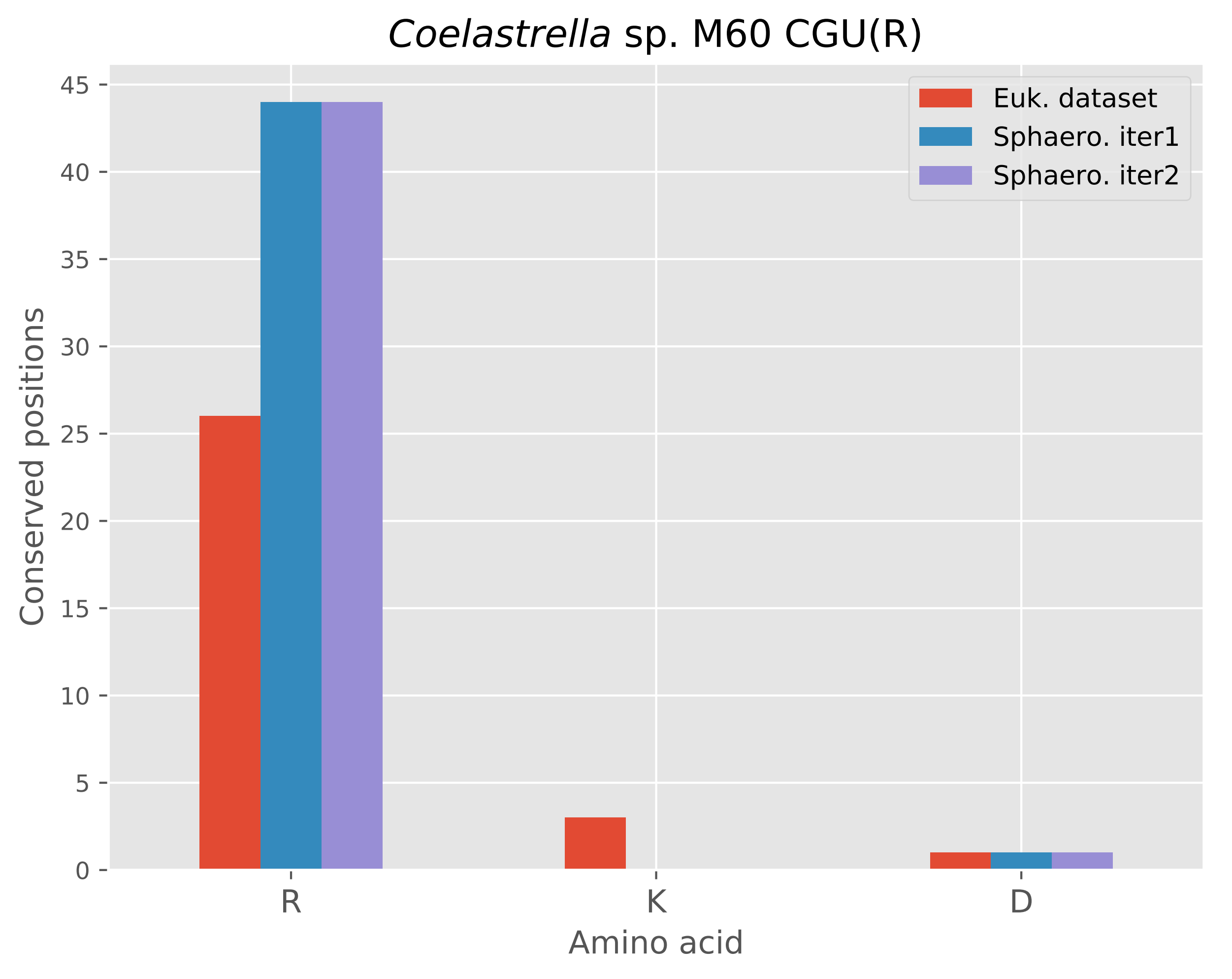

Coelastrella sp. M60 CGU(R)
Euk. dataset
Sphaero. iter1
Sphaero. iter2
Conserved positions
0
5
10
15
20
25
30
35
40
45
R
K
D
Amino acid

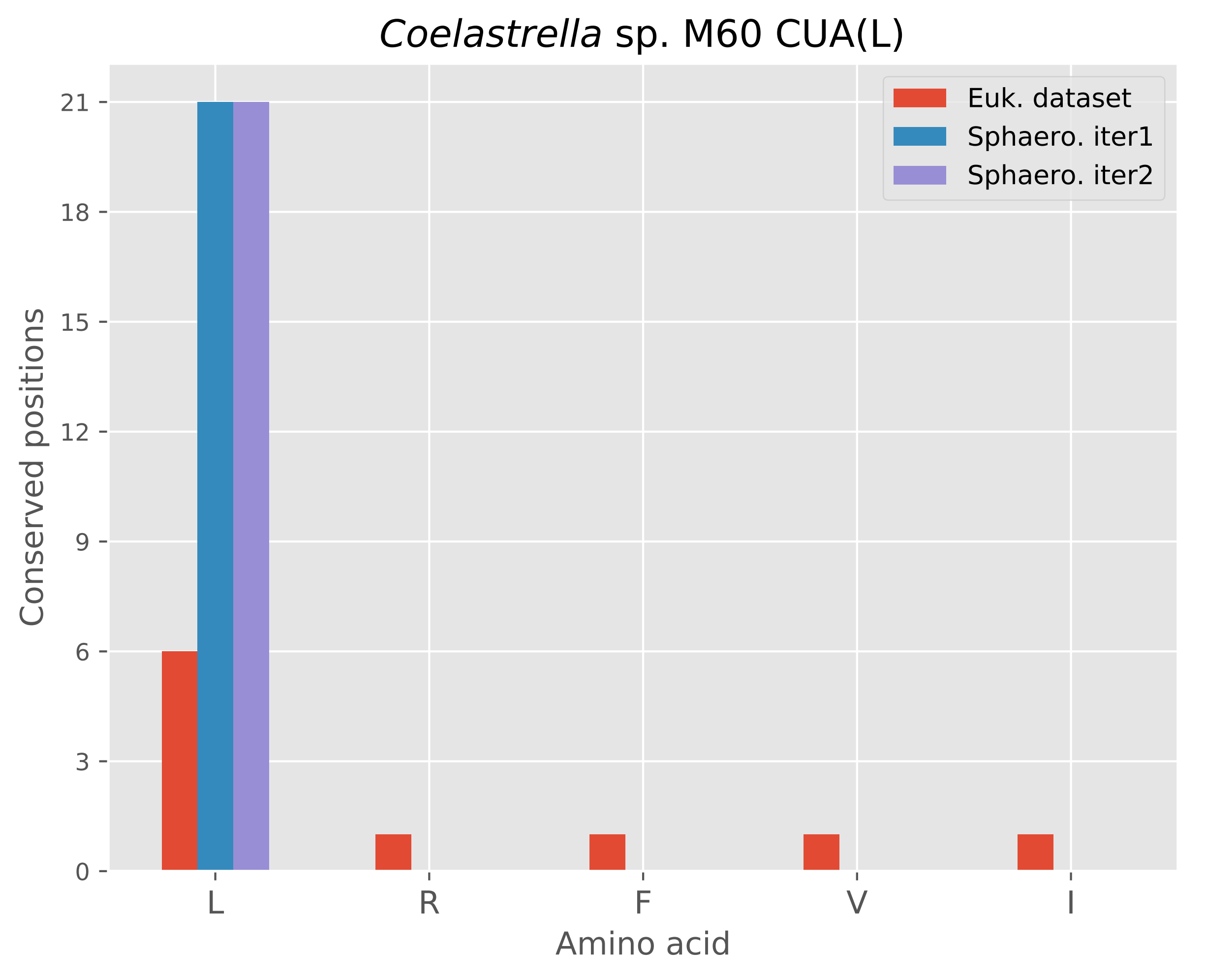

Coelastrella sp. M60 CUA(L)
Euk. dataset
Sphaero. iter1
Sphaero. iter2
Conserved positions
0
3
6
9
12
15
18
21
L
R
F
V
I
Amino acid

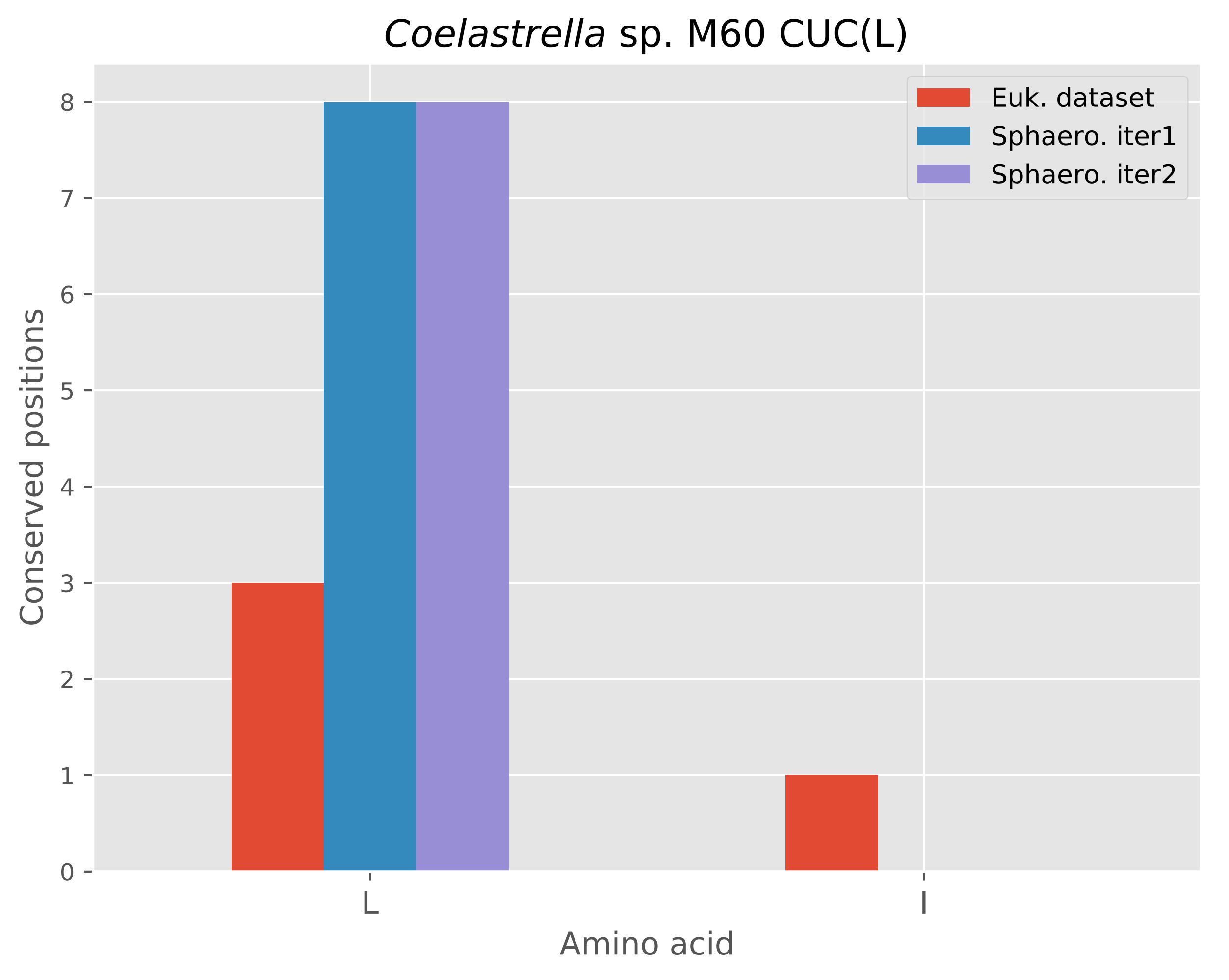
Coelastrella sp. M60 CUC(L)
Euk. dataset
Sphaero. iter1
Sphaero. iter2
Conserved positions
0
1
2
3
4
5
6
7
8
L
I
Amino acid

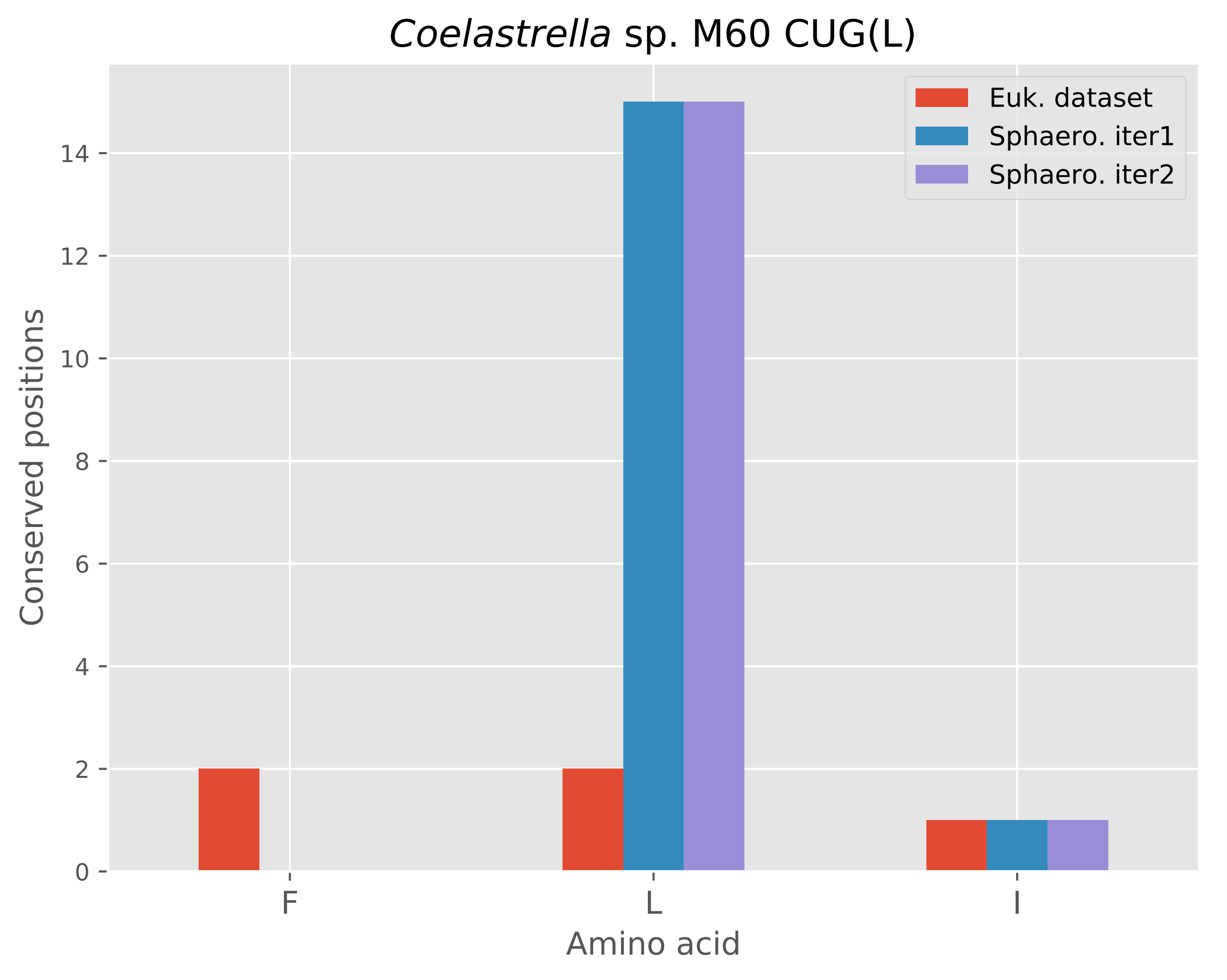
Coelastrella sp. M60 CUG(L)
Euk. dataset
Sphaero. iter1
Sphaero. iter2
Conserved positions
0
2
4
6
8
10
12
14
F
L
I
Amino acid

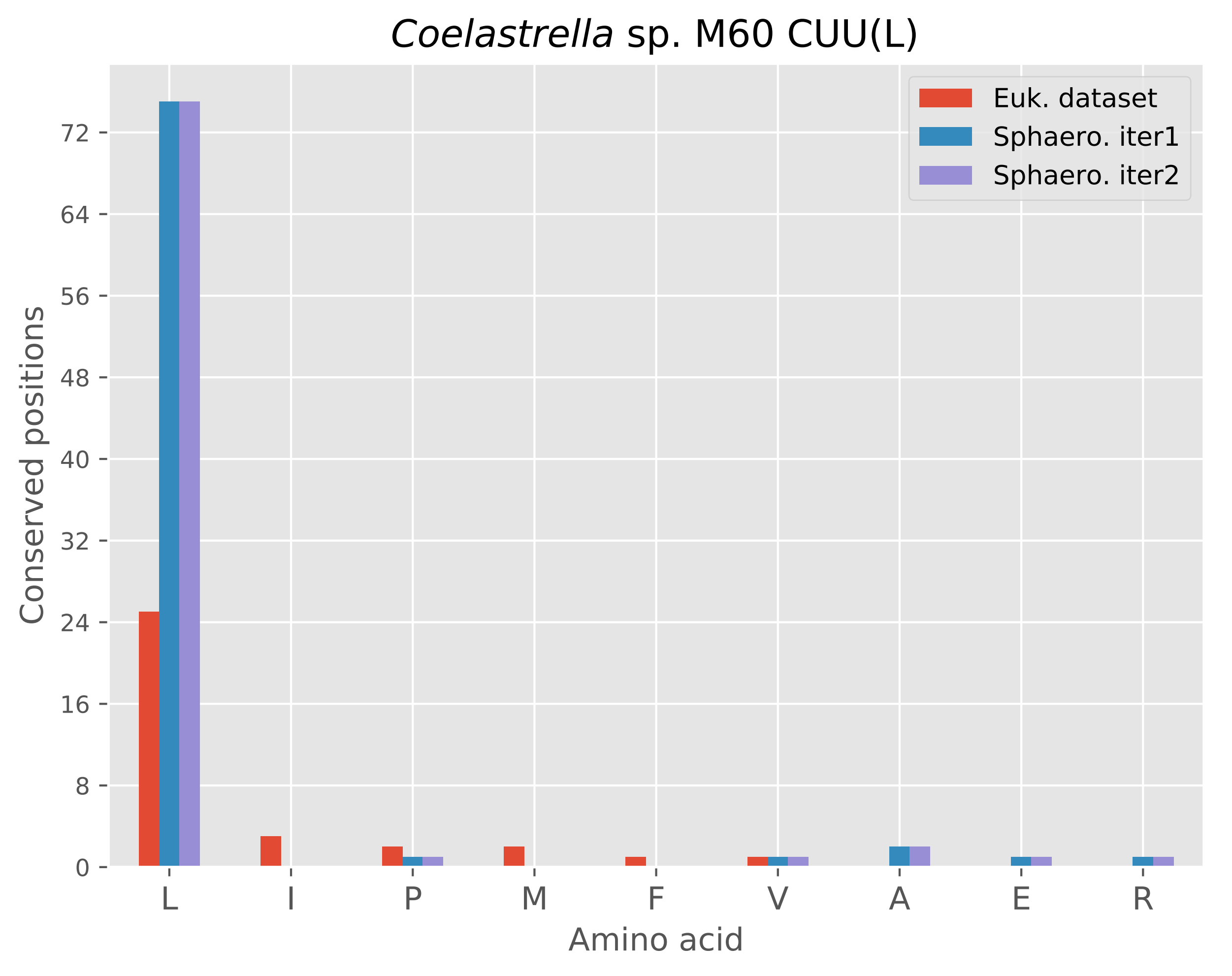

Coelastrella sp. M60 CUU(L)
Euk. dataset
Sphaero. iter1
Sphaero. iter2
Conserved positions
0
8
16
24
32
40
48
56
64
72
L
I
P
M
F
V
A
E
R
Amino acid

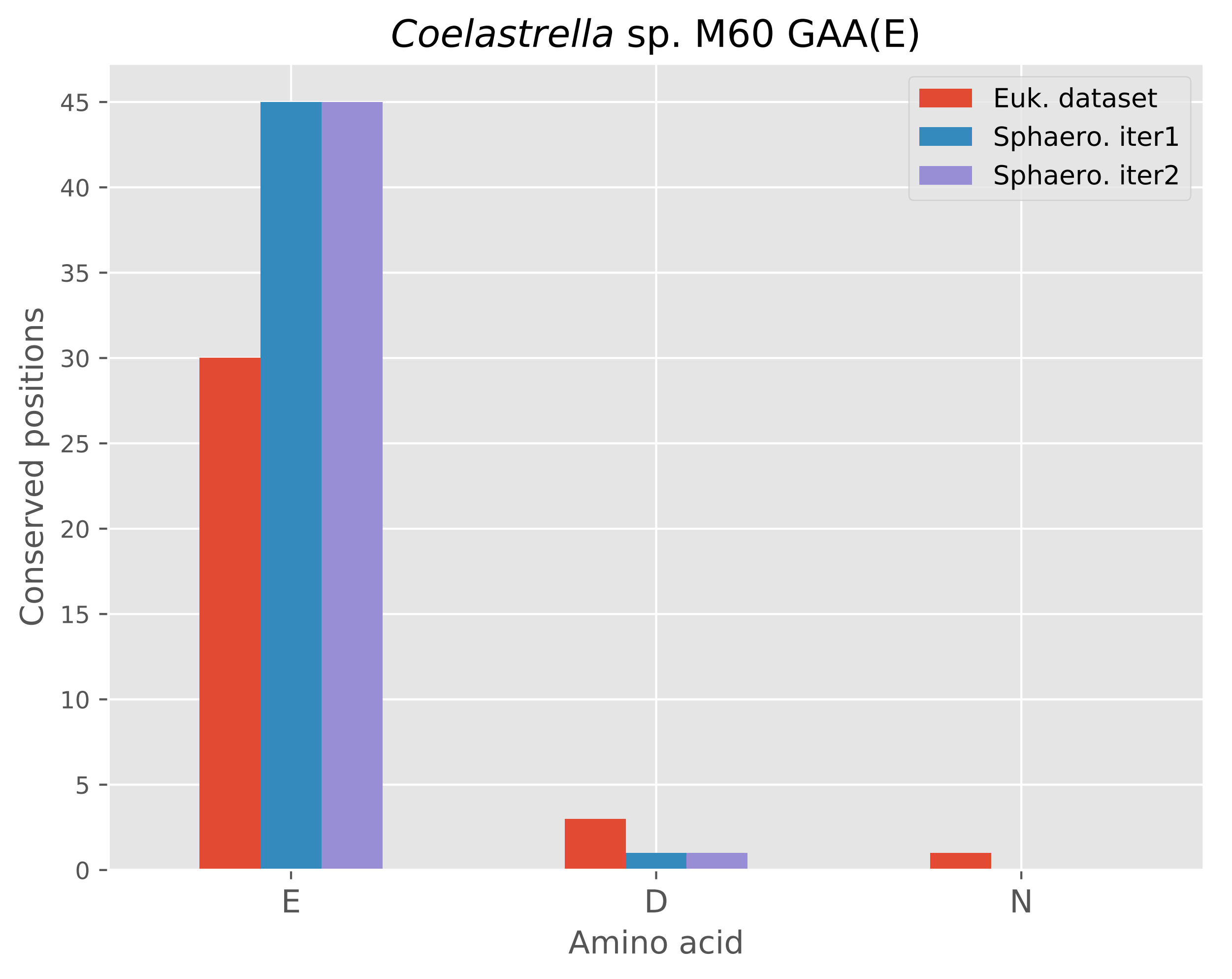

Coelastrella sp. M60 GAA(E)
Euk. dataset
Sphaero. iter1
Sphaero. iter2
Conserved positions
0
5
10
15
20
25
30
35
40
45
E
D
N
Amino acid

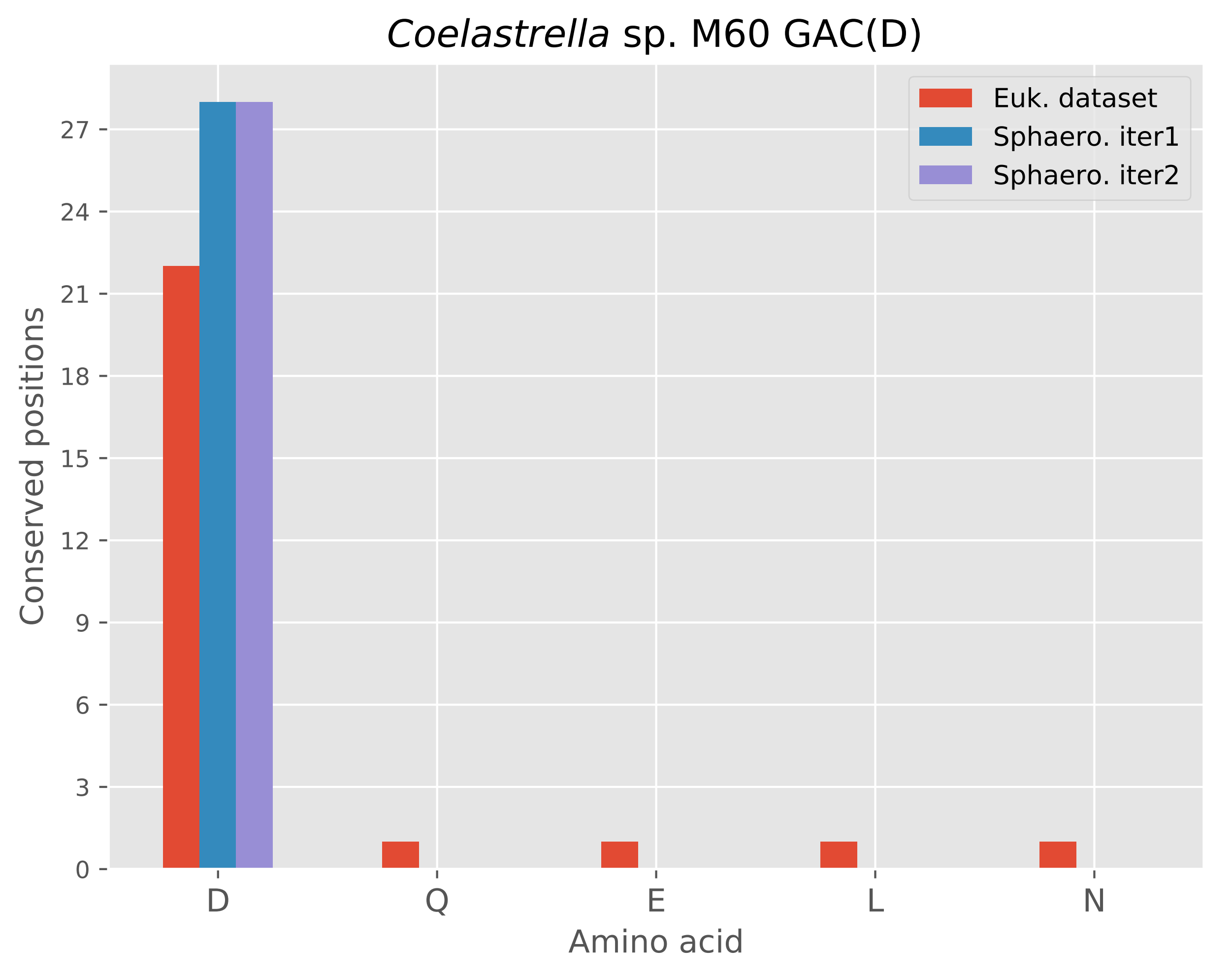

Coelastrella sp. M60 GAC(D)
Euk. dataset
Sphaero. iter1
Sphaero. iter2
Conserved positions
0
3
6
9
12
15
18
21
24
27
D
Q
E
L
N
Amino acid

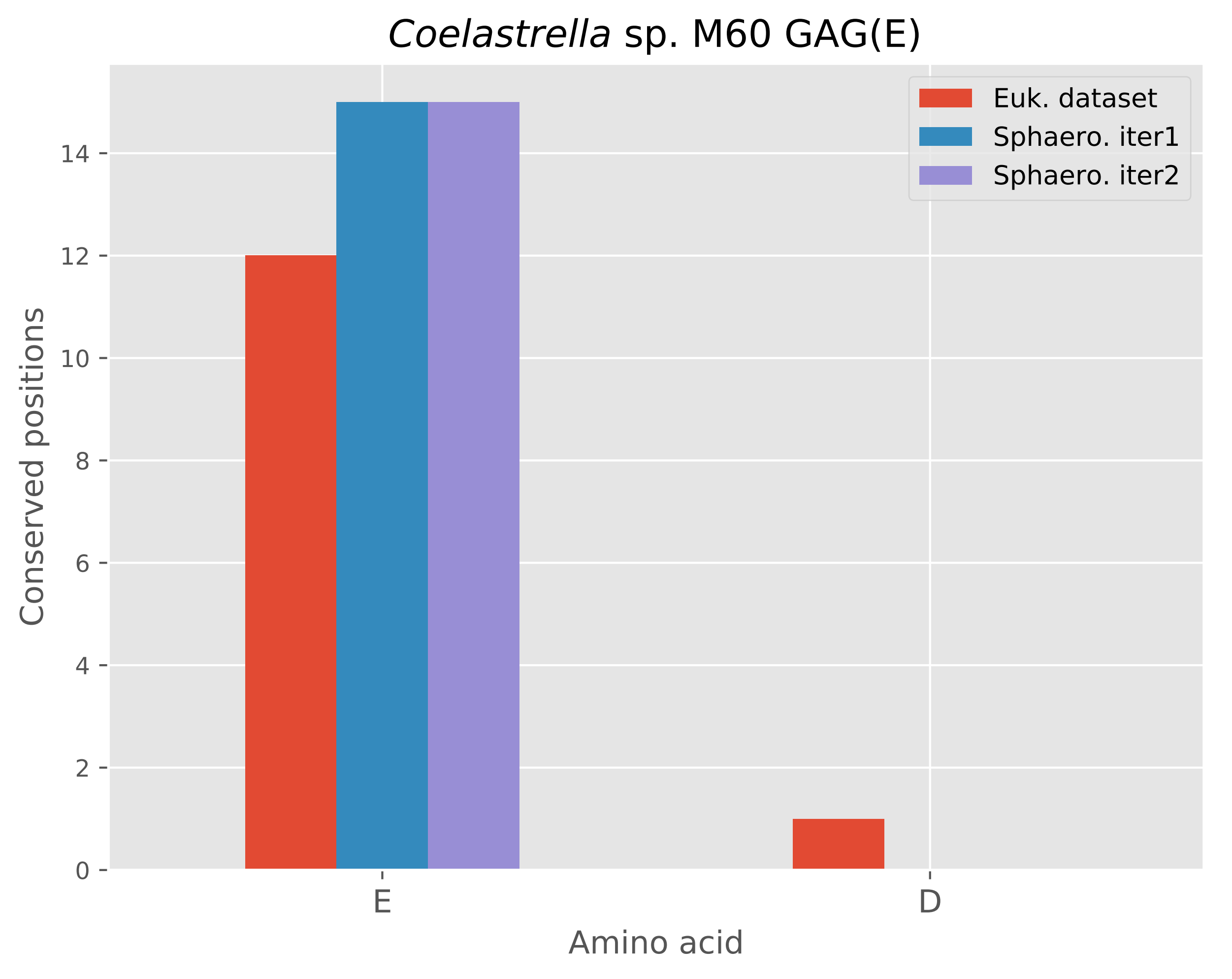
Coelastrella sp. M60 GAG(E)
Euk. dataset
Sphaero. iter1
Sphaero. iter2
Conserved positions
0
2
4
6
8
10
12
14
E
D
Amino acid

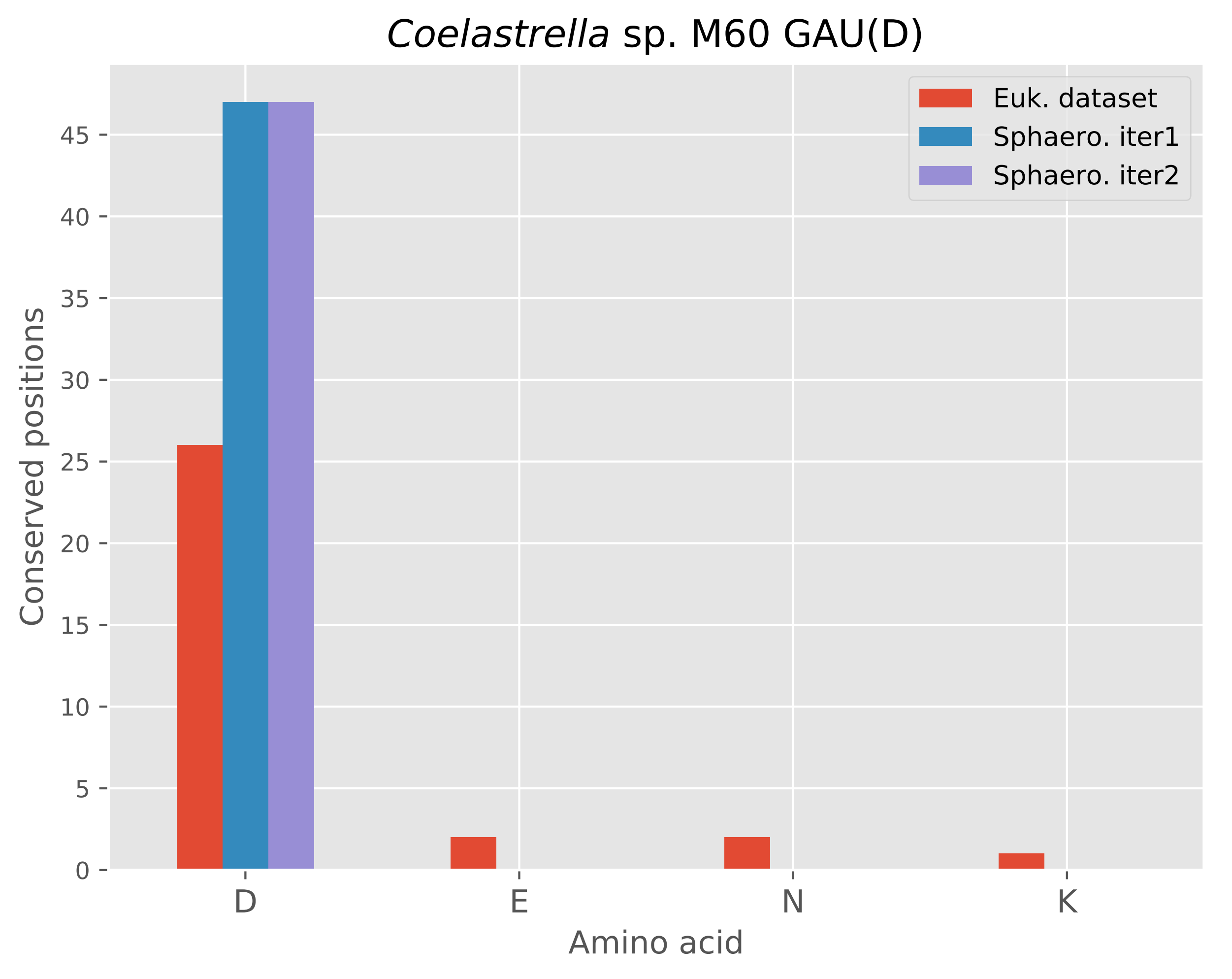
Coelastrella sp. M60 GAU(D)
Euk. dataset
Sphaero. iter1
Sphaero. iter2
Conserved positions
0
5
10
15
20
25
30
35
40
45
D
E
N
K
Amino acid

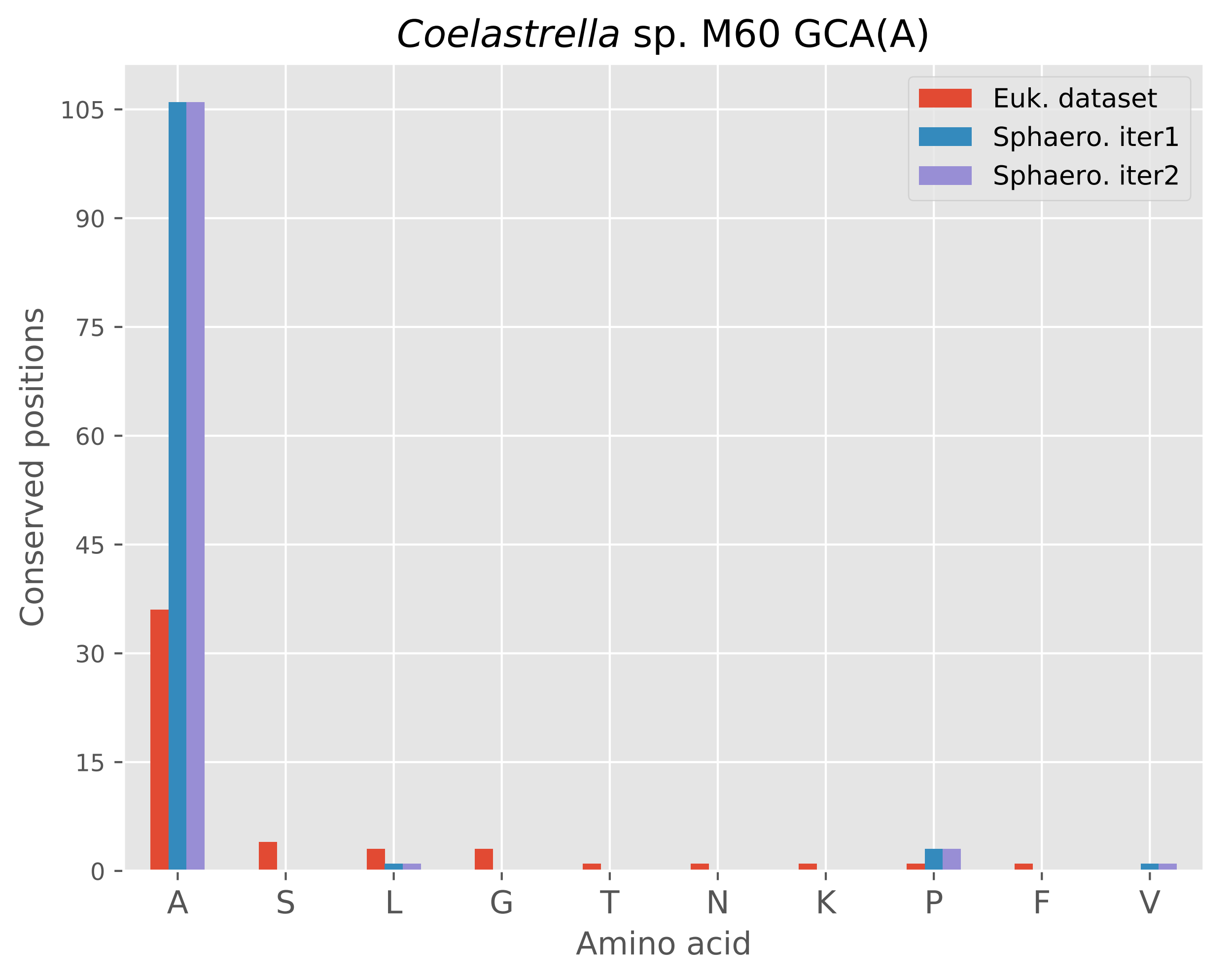

Coelastrella sp. M60 GCA(A)
Euk. dataset
Sphaero. iter1
Sphaero. iter2
Conserved positions
0
15
30
45
60
75
90
105
A
S
L
G
T
N
K
P
F
V
Amino acid

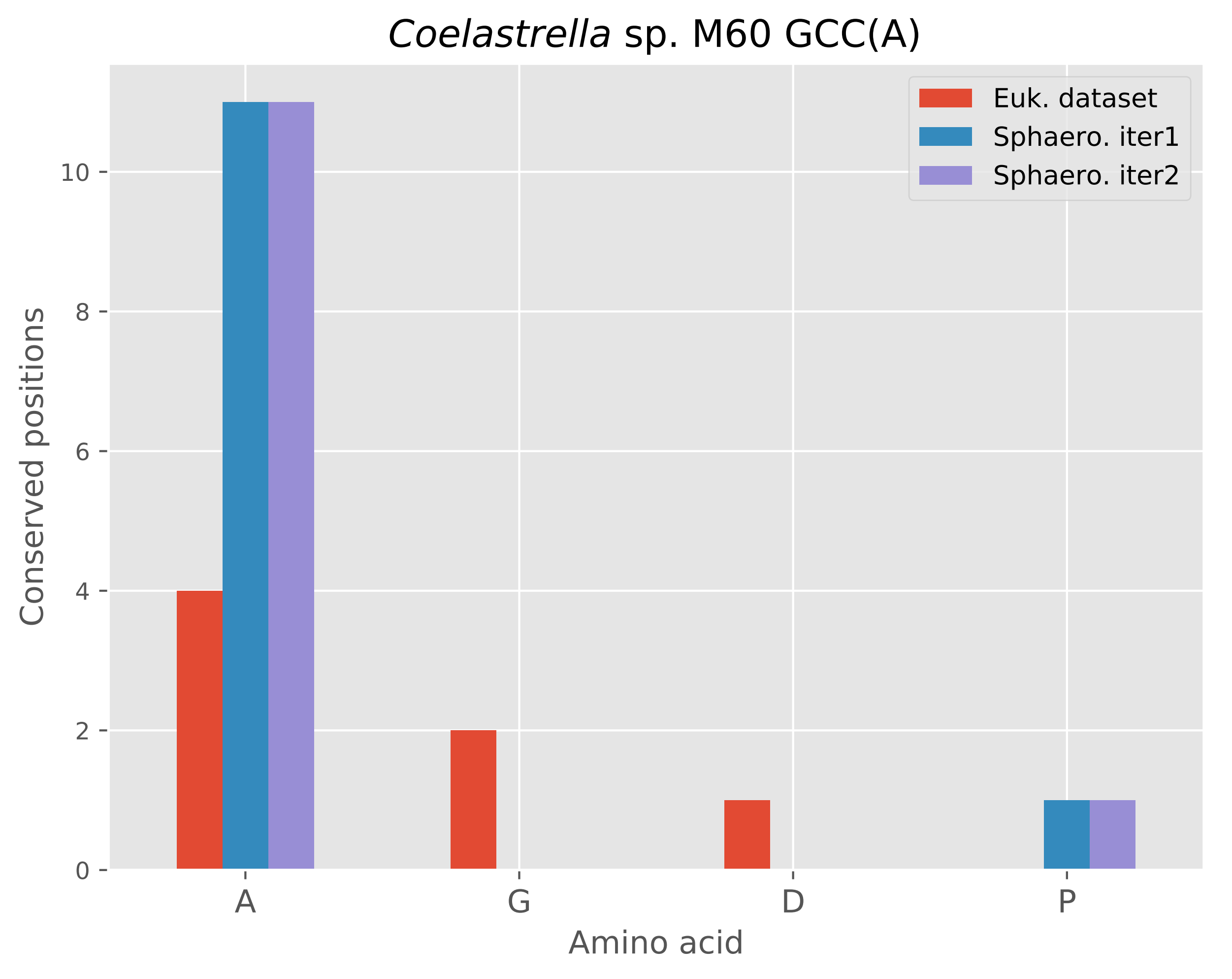

Coelastrella sp. M60 GCC(A)
Euk. dataset
Sphaero. iter1
Sphaero. iter2
Conserved positions
0
2
4
6
8
10
A
G
D
P
Amino acid

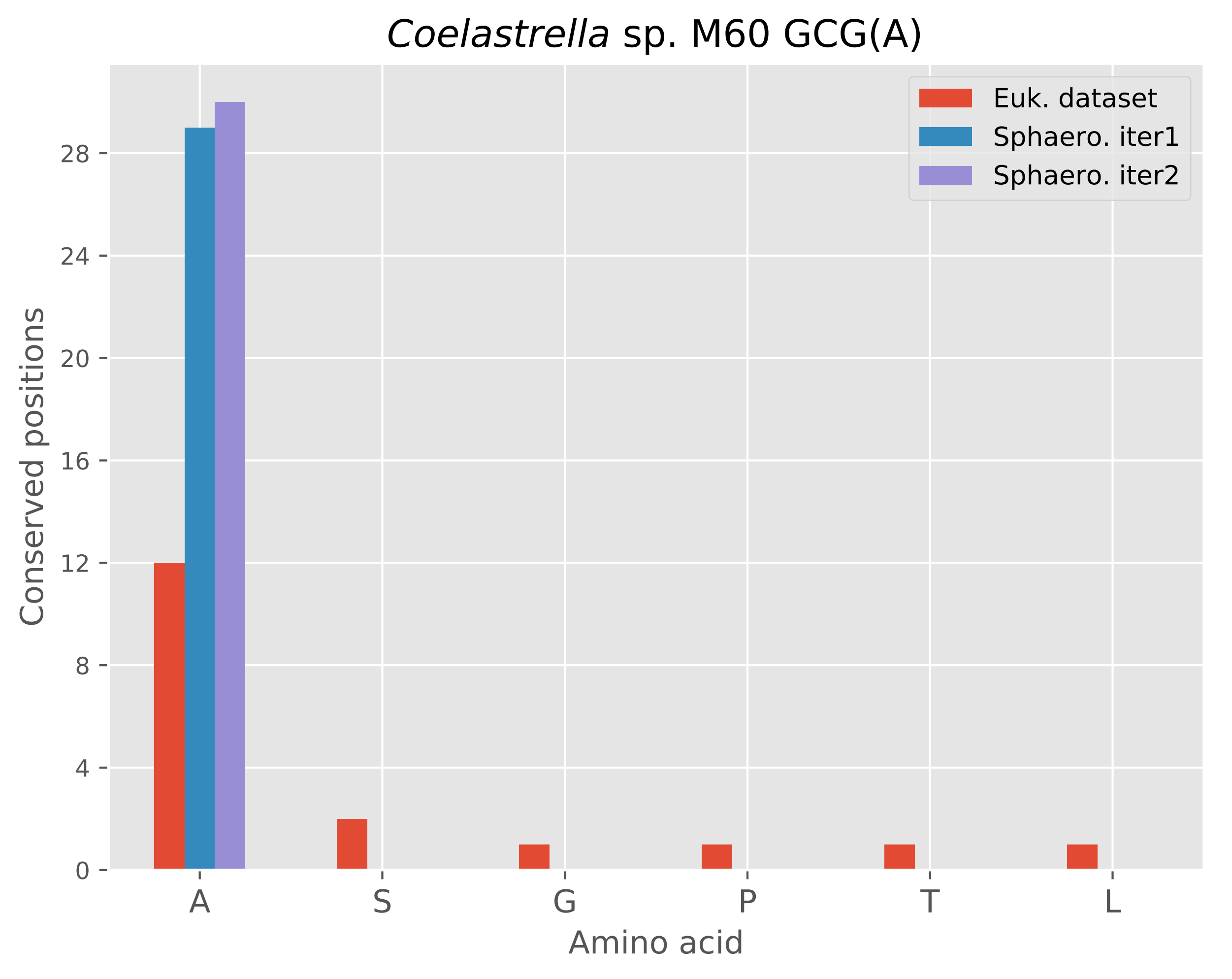

Coelastrella sp. M60 GCG(A)
Euk. dataset
Sphaero. iter1
Sphaero. iter2
Conserved positions
0
4
8
12
16
20
24
28
A
S
G
P
T
L
Amino acid

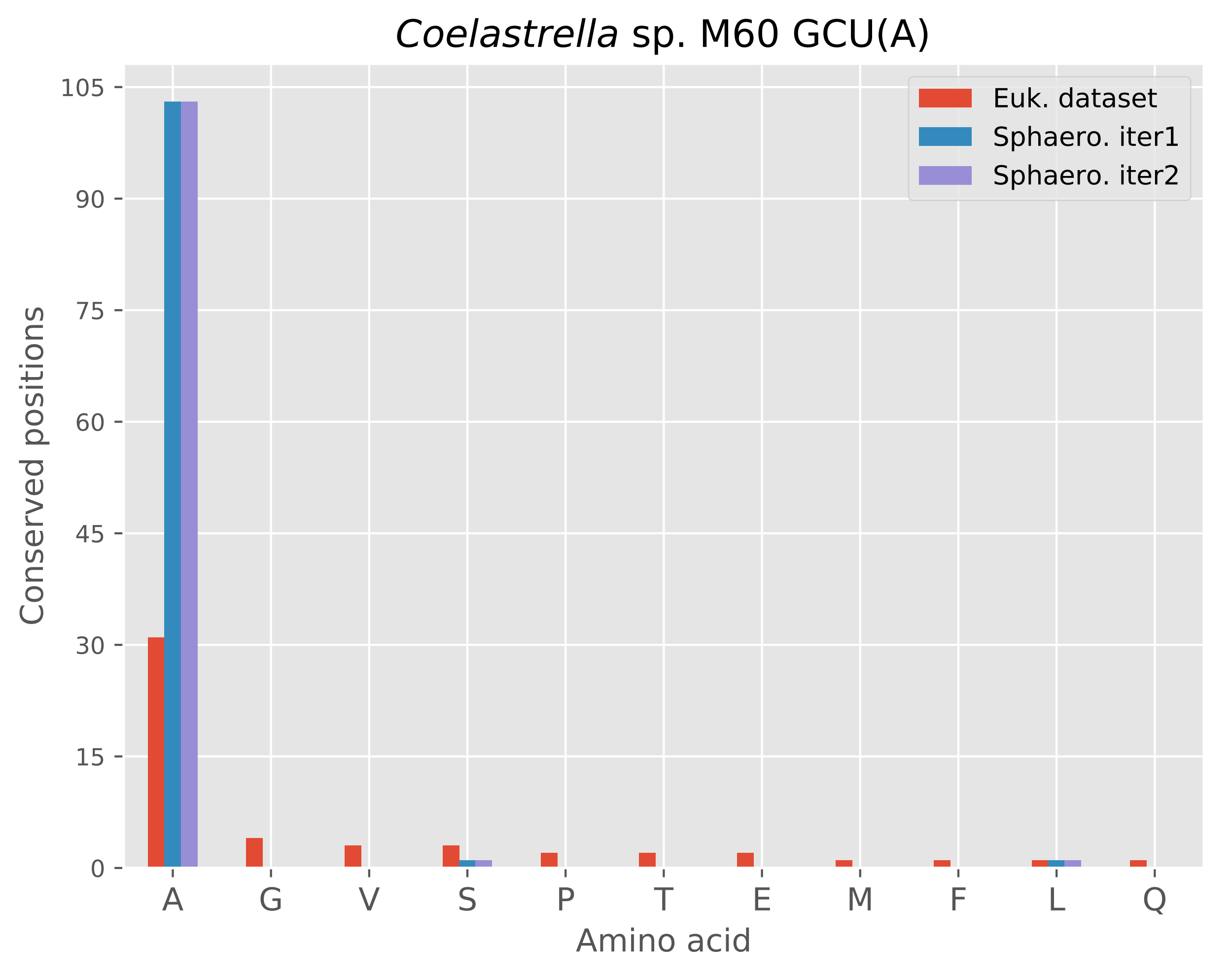

Coelastrella sp. M60 GCU(A)
Euk. dataset
Sphaero. iter1
Sphaero. iter2
Conserved positions
105
90
75
60
45
30
15
0
A
G
V
S
P
T
E
M
F
L
Q
Amino acid

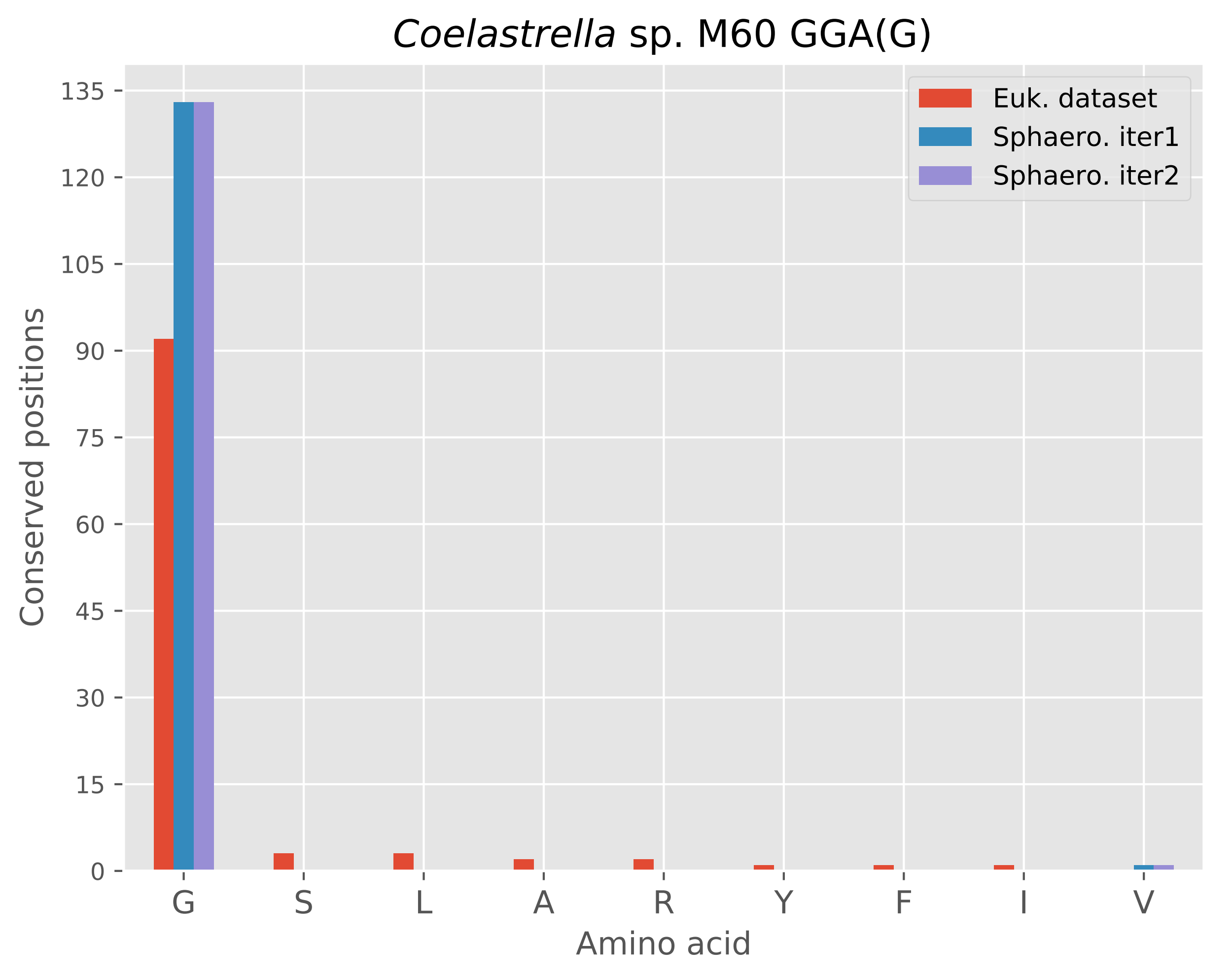
Coelastrella sp. M60 GGA(G)
Euk. dataset
Sphaero. iter1
Sphaero. iter2
Conserved positions
0
15
30
45
60
75
90
105
120
135
G
S
L
A
R
Y
F
I
V
Amino acid

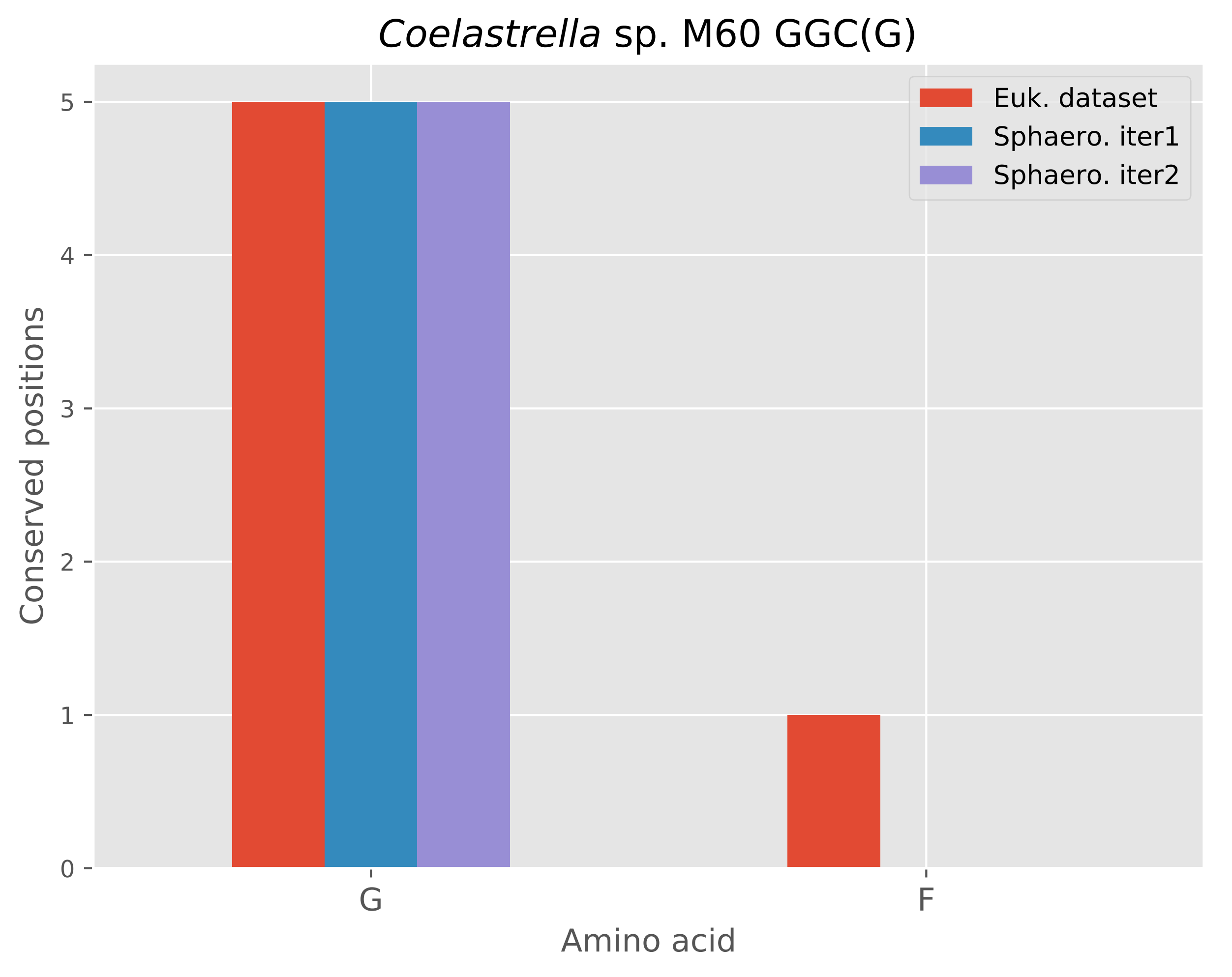

Coelastrella sp. M60 GGC(G)
Euk. dataset
Sphaero. iter1
Sphaero. iter2
Conserved positions
0
1
2
3
4
5
G
F
Amino acid

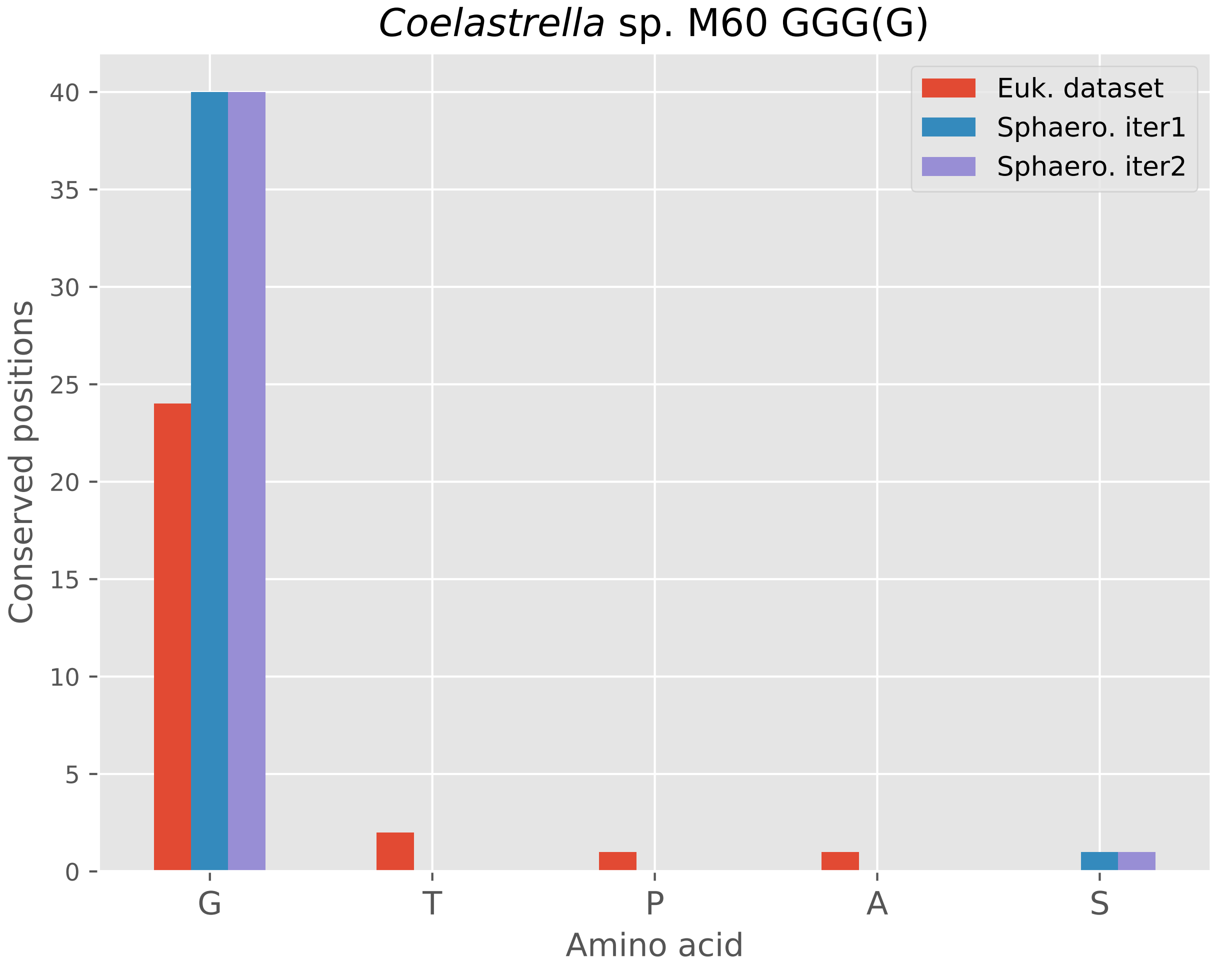

Coelastrella sp. M60 GGG(G)
Conserved positions
0
5
10
15
20
25
30
35
40
Euk. dataset
Sphaero. iter1
Sphaero. iter2
G
T
P
A
S
Amino acid

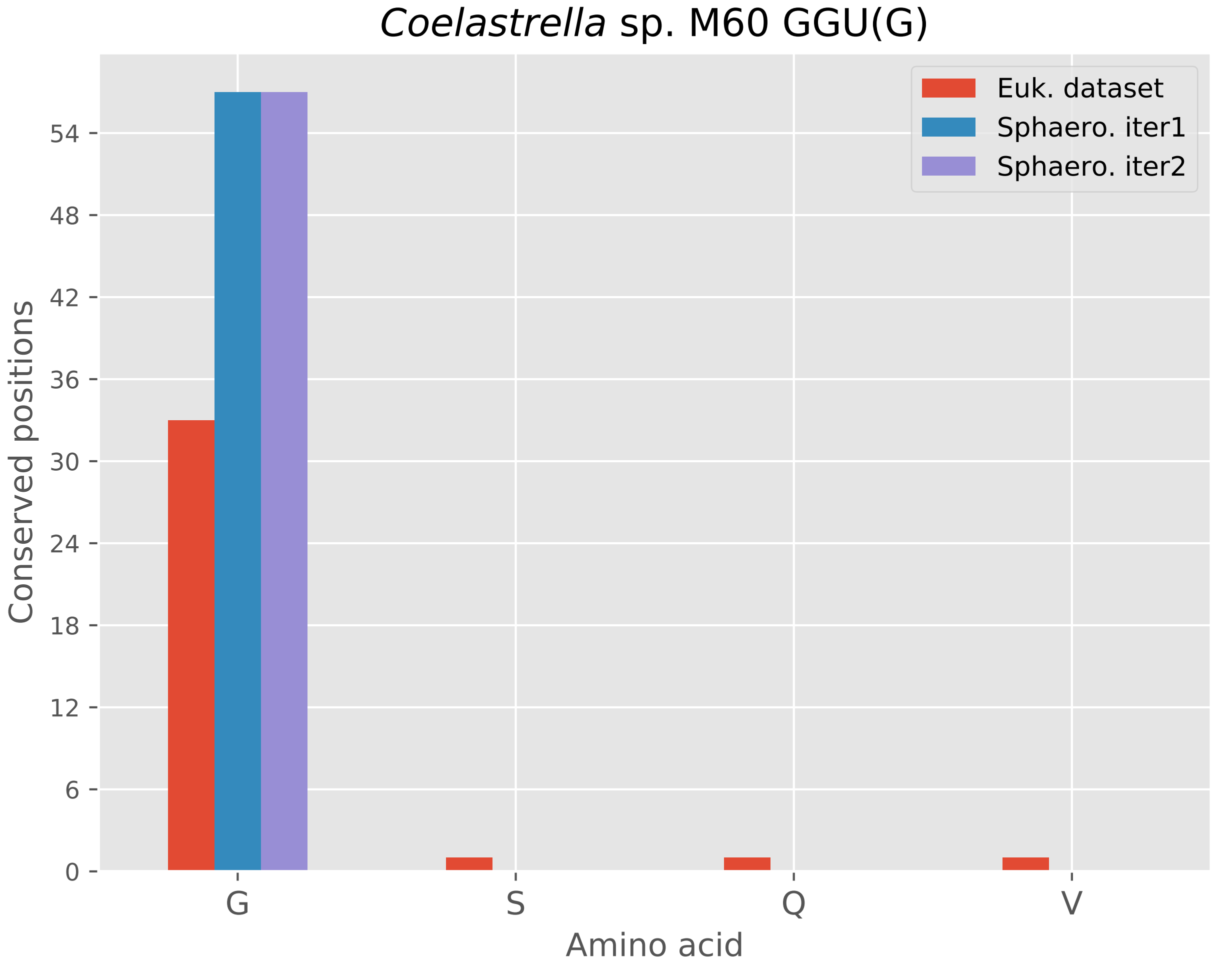
Coelastrella sp. M60 GGU(G)
Euk. dataset
Sphaero. iter1
Sphaero. iter2
Conserved positions
0
6
12
18
24
30
36
42
48
54
G
S
Q
V
Amino acid

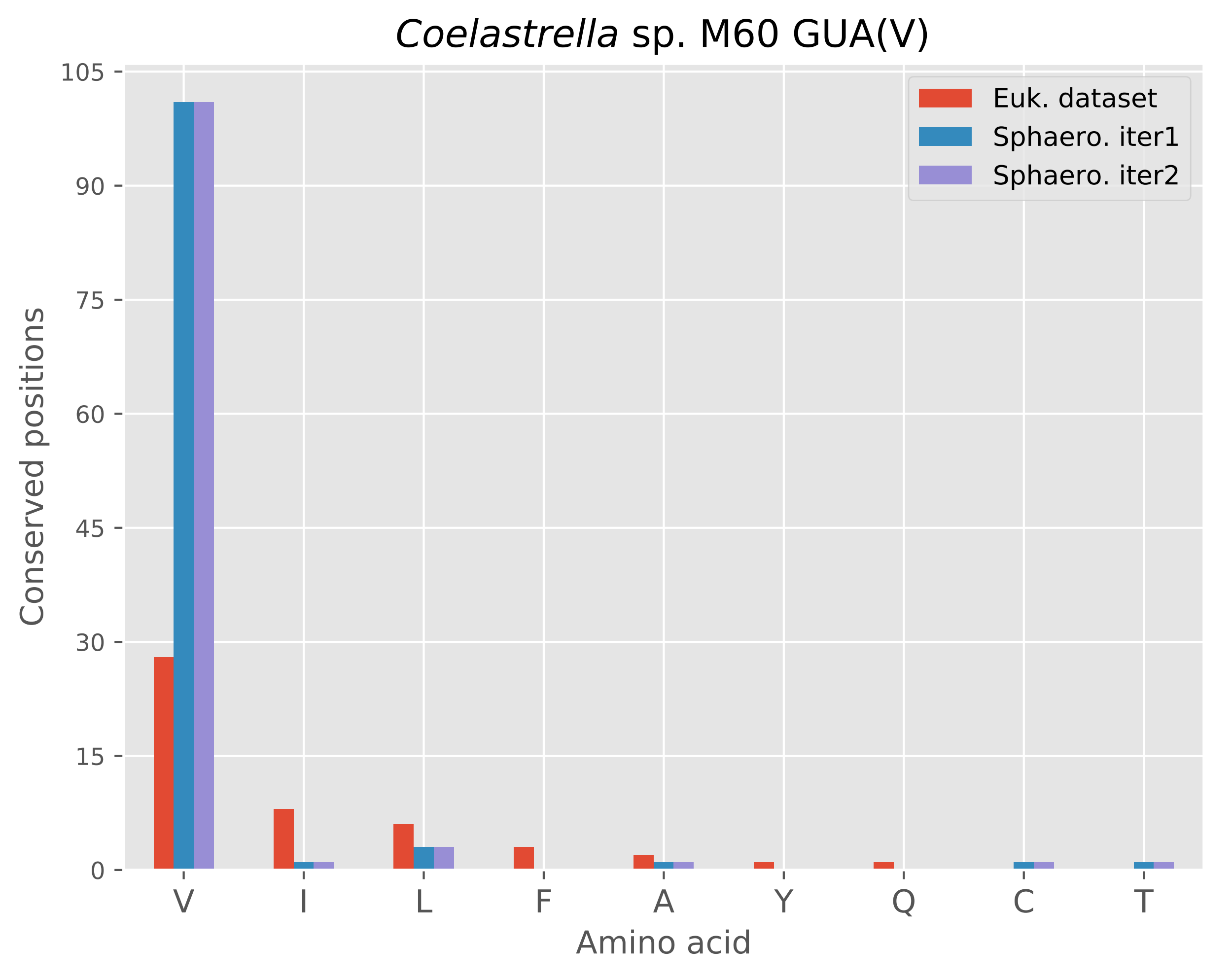

Coelastrella sp. M60 GUA(V)
Euk. dataset
Sphaero. iter1
Sphaero. iter2
Conserved positions
0
15
30
45
60
75
90
105
V
I
L
F
A
Y
Q
C
T
Amino acid

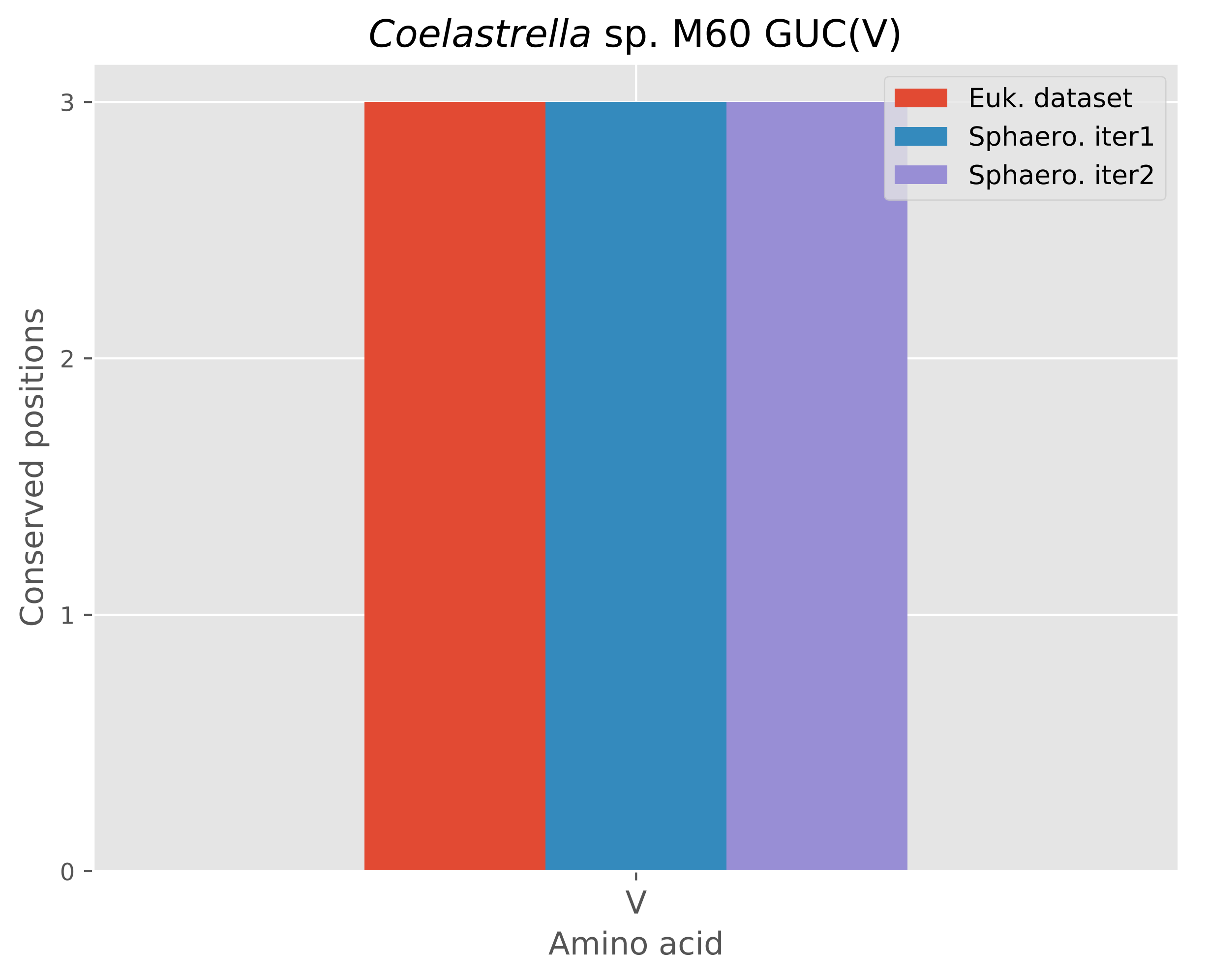

Coelastrella sp. M60 GUC(V)
Euk. dataset
Sphaero. iter1
Sphaero. iter2
Conserved positions
0
1
2
3
V
Amino acid

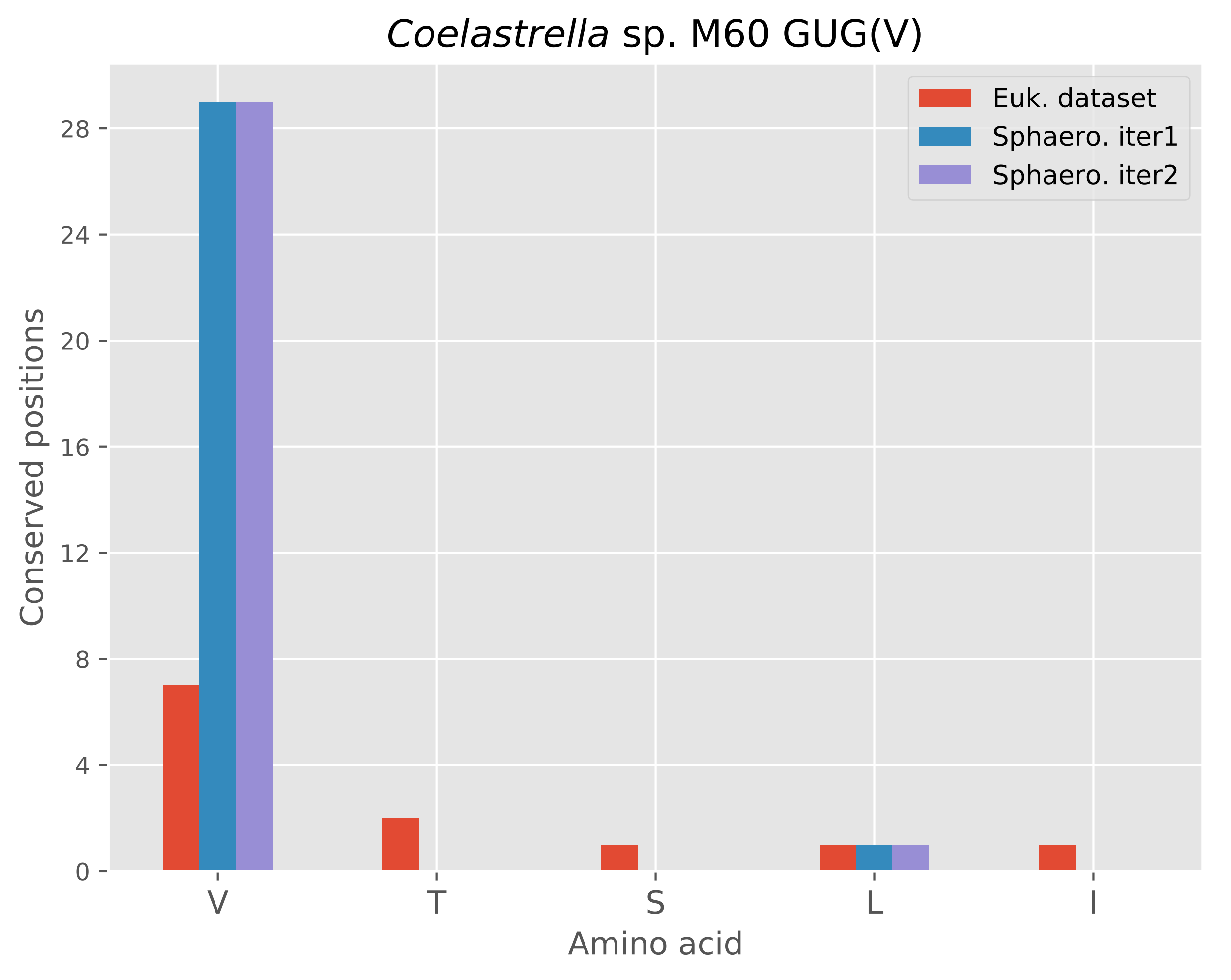

Coelastrella sp. M60 GUG(V)
Euk. dataset
Sphaero. iter1
Sphaero. iter2
Conserved positions
0
4
8
12
16
20
24
28
V
T
S
L
I
Amino acid

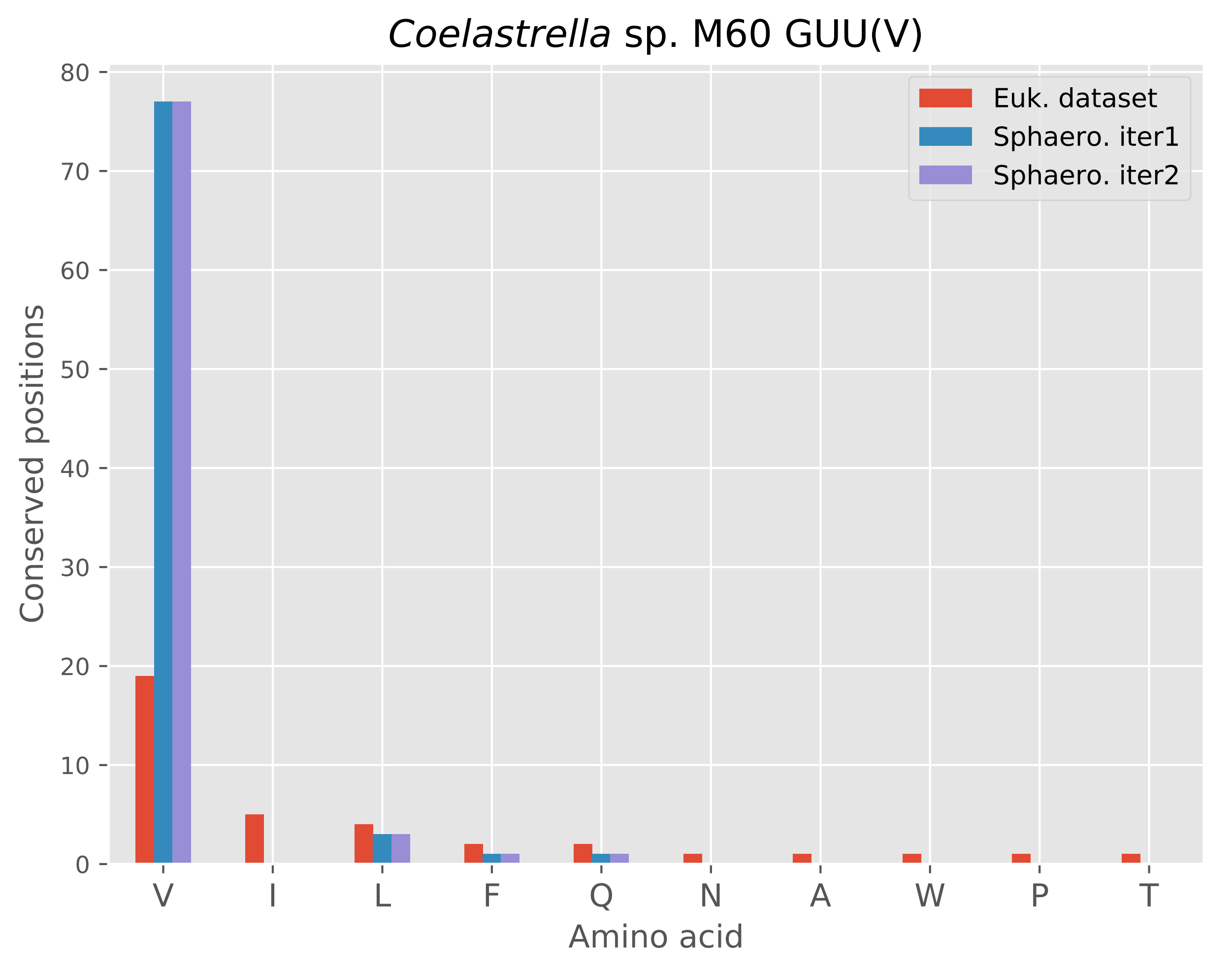

Coelastrella sp. M60 GUU(V)
Euk. dataset
Sphaero. iter1
Sphaero. iter2
Conserved positions
0
10
20
30
40
50
60
70
80
V
I
L
F
Q
N
A
W
P
T
Amino acid

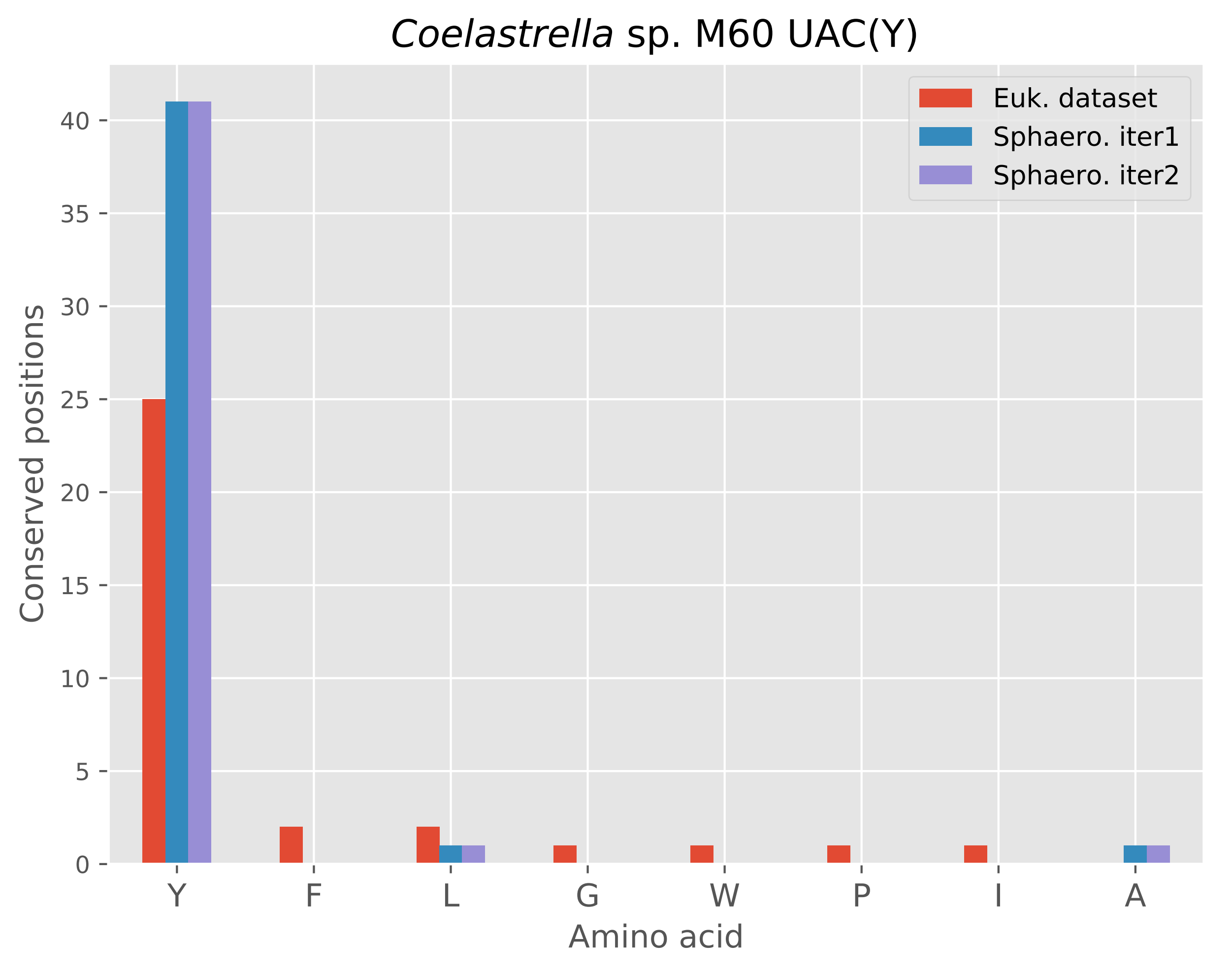

Coelastrella sp. M60 UAC(Y)
Euk. dataset
Sphaero. iter1
Sphaero. iter2
Conserved positions
0
5
10
15
20
25
30
35
40
Y
F
L
G
W
P
I
A
Amino acid

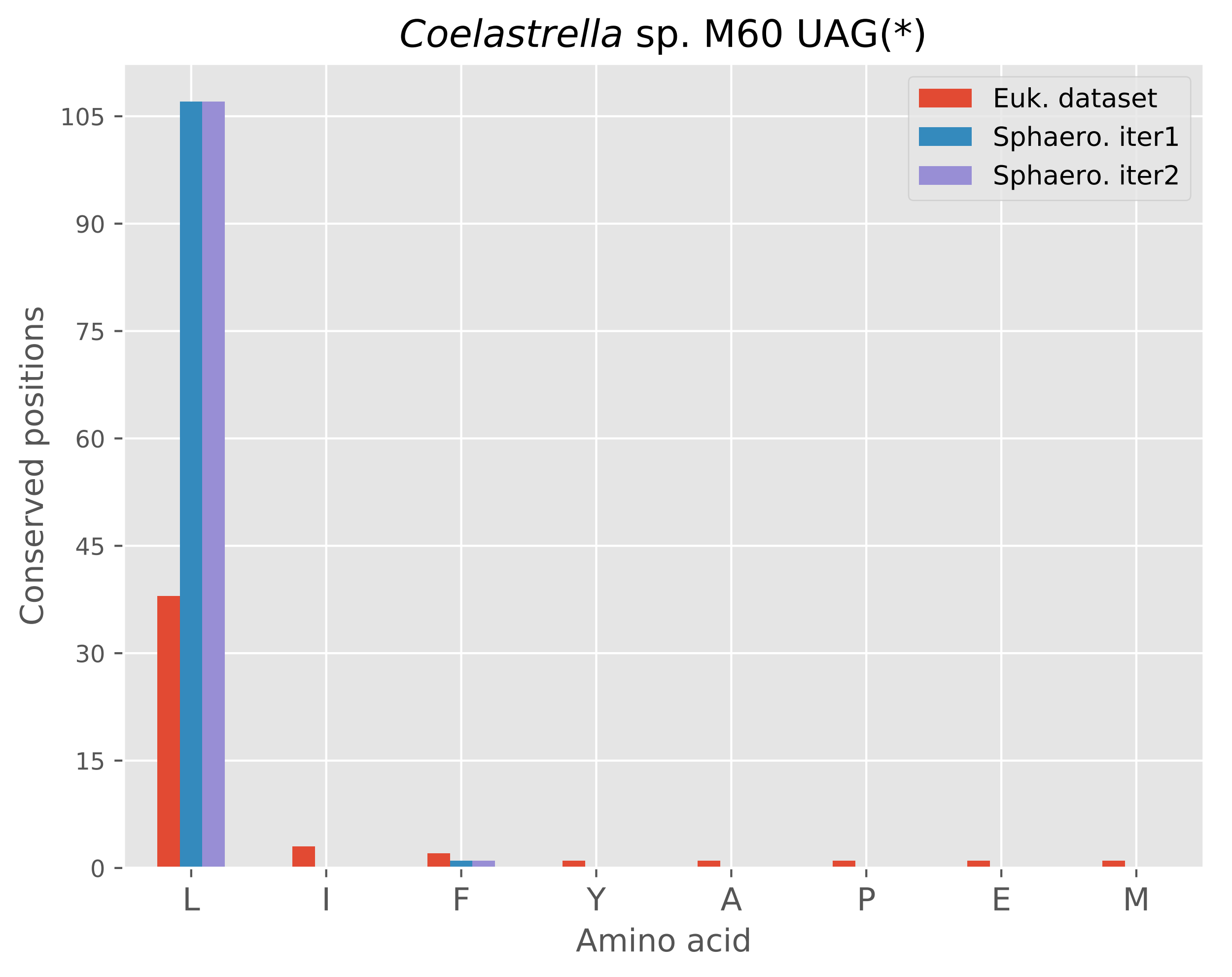

Coelastrella sp. M60 UAG(*)
Euk. dataset
Sphaero. iter1
Sphaero. iter2
Conserved positions
0
15
30
45
60
75
90
105
L
I
F
Y
A
P
E
M
Amino acid

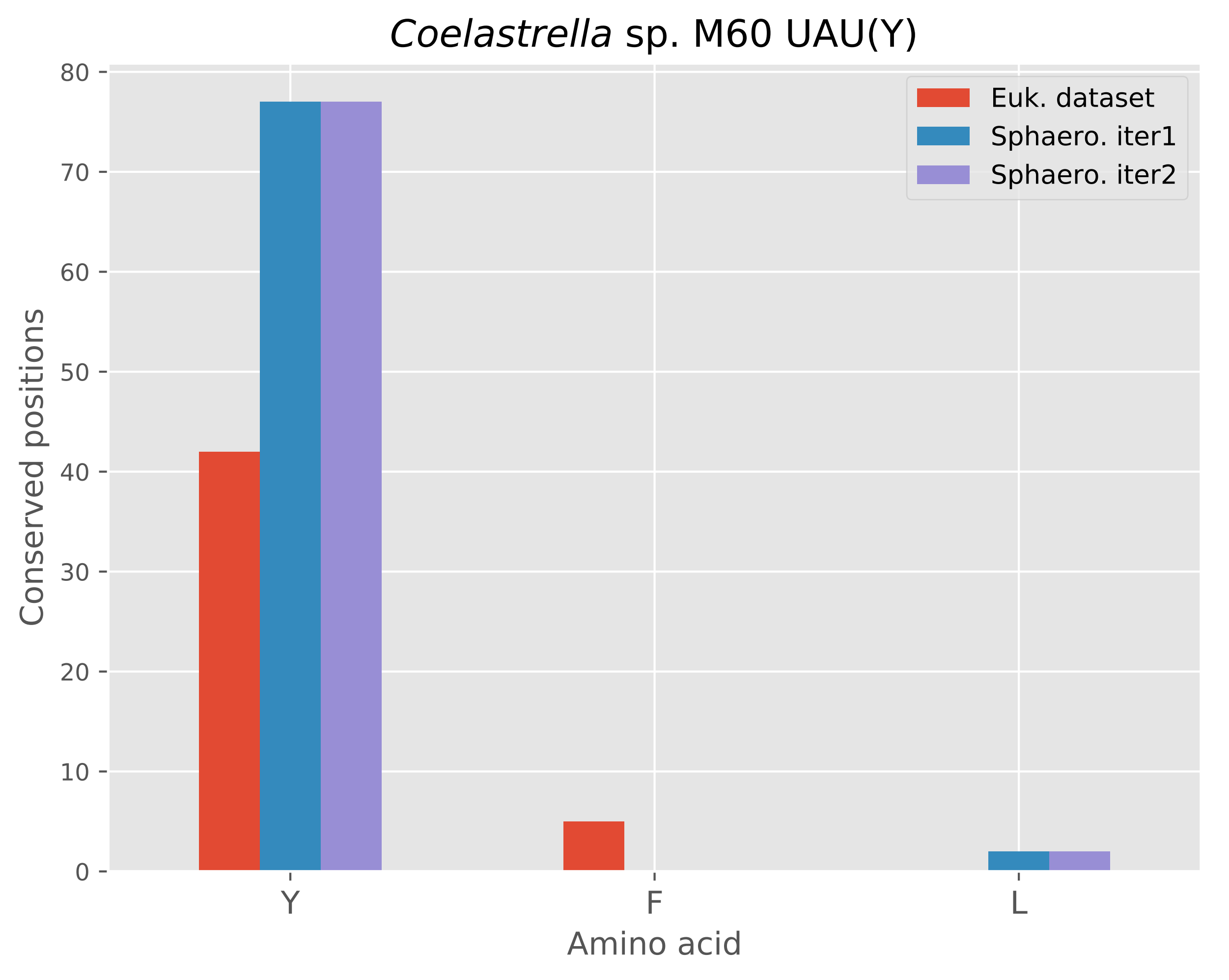

Coelastrella sp. M60 UAU(Y)
Euk. dataset
Sphaero. iter1
Sphaero. iter2
Conserved positions
0
10
20
30
40
50
60
70
80
Y
F
L
Amino acid

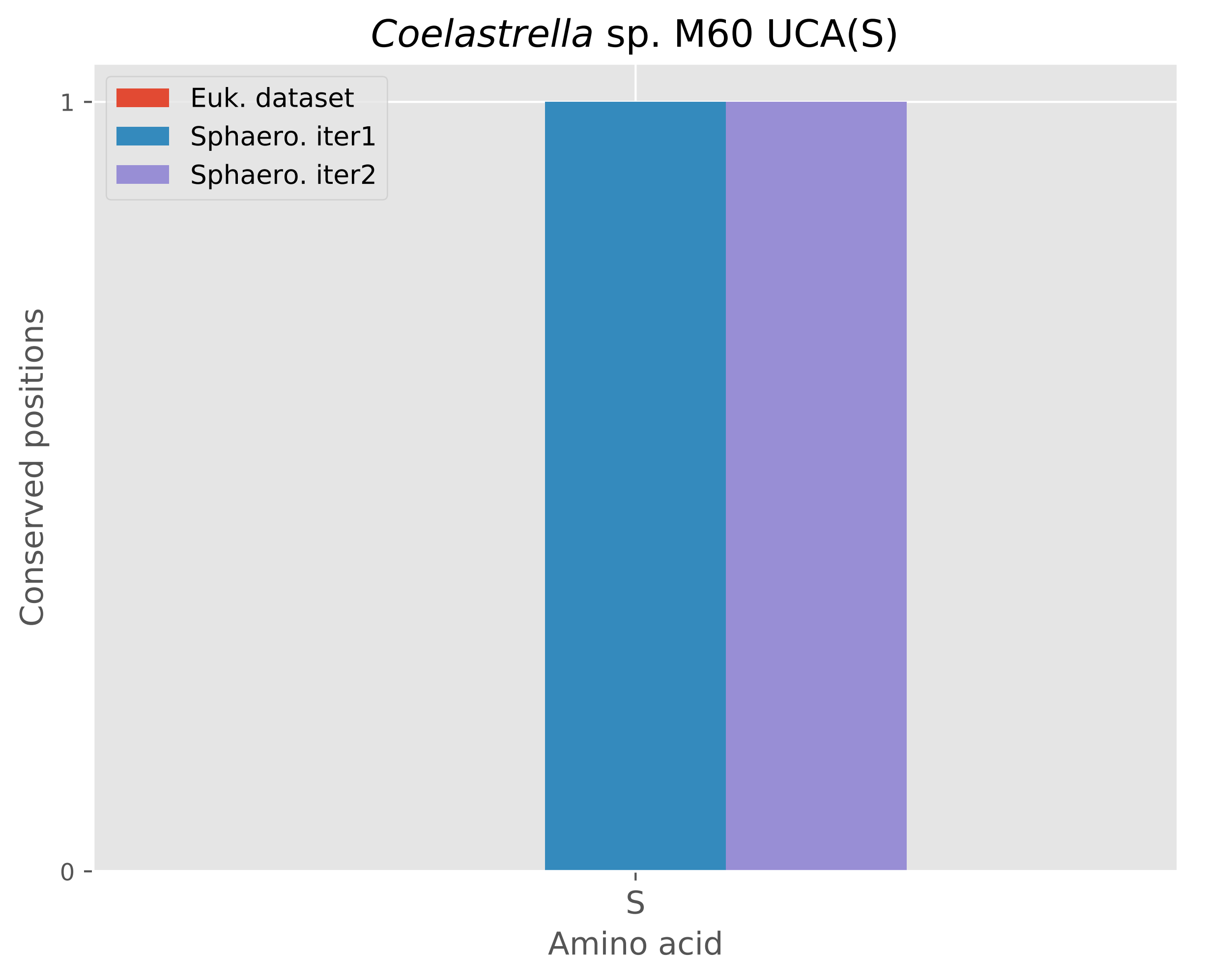

Coelastrella sp. M60 UCA(S)
Euk. dataset
Sphaero. iter1
Sphaero. iter2
1
0
Conserved positions
S
Amino acid

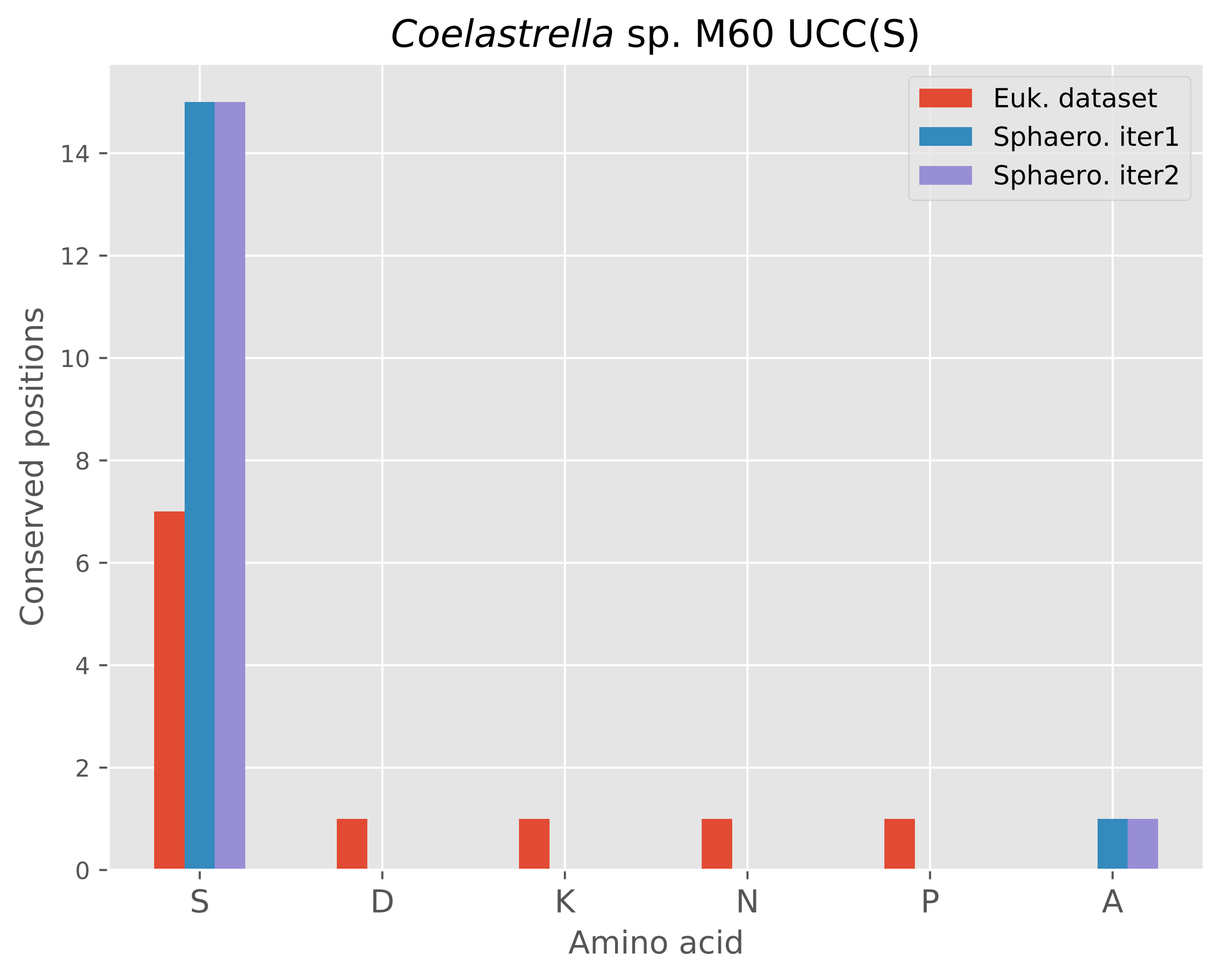

Coelastrella sp. M60 UCC(S)
Euk. dataset
Sphaero. iter1
Sphaero. iter2
Conserved positions
0
2
4
6
8
10
12
14
S
D
K
N
P
A
Amino acid

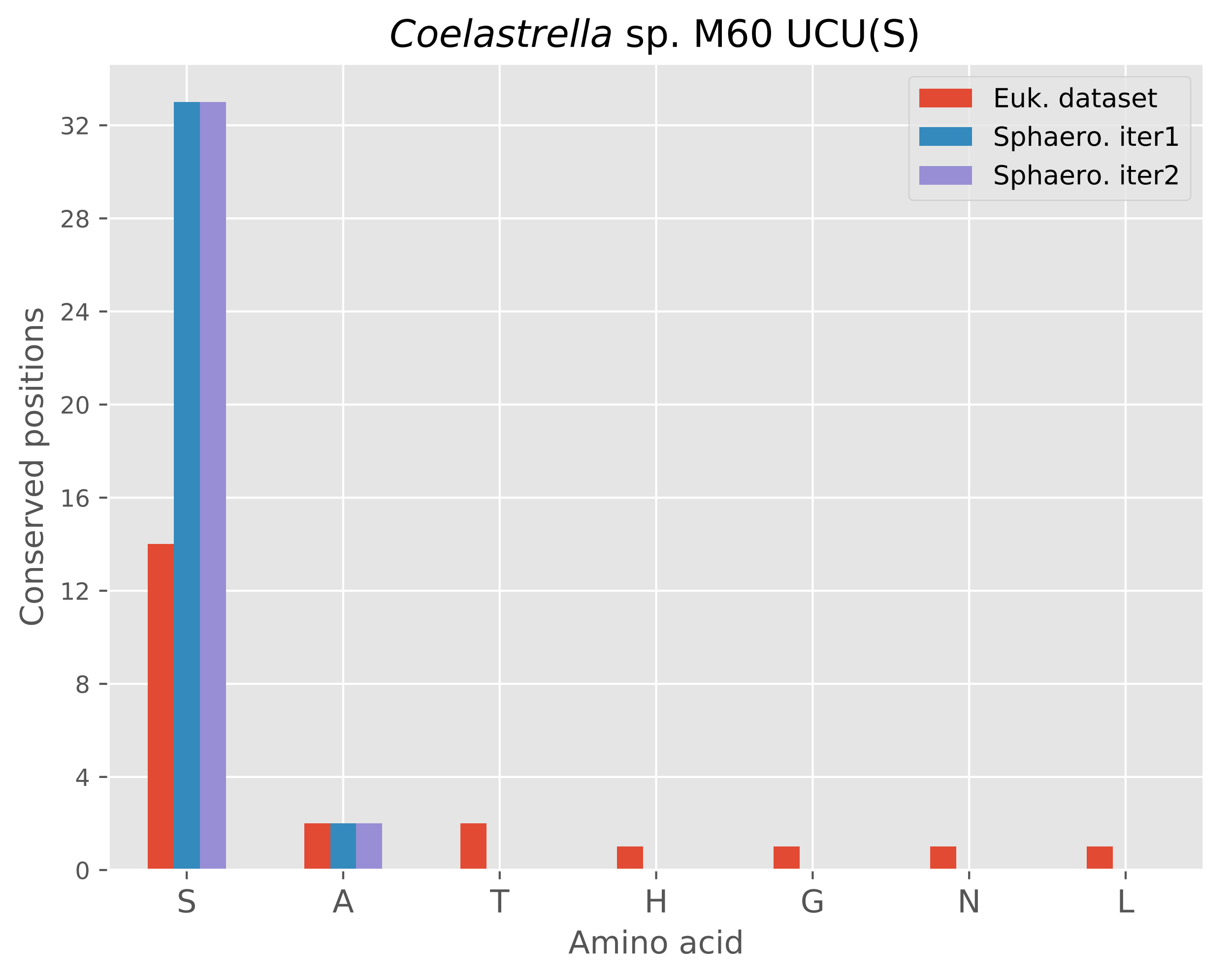

Coelastrella sp. M60 UCU(S)
Euk. dataset
Sphaero. iter1
Sphaero. iter2
Conserved positions
0
4
8
12
16
20
24
28
32
S
A
T
H
G
N
L
Amino acid

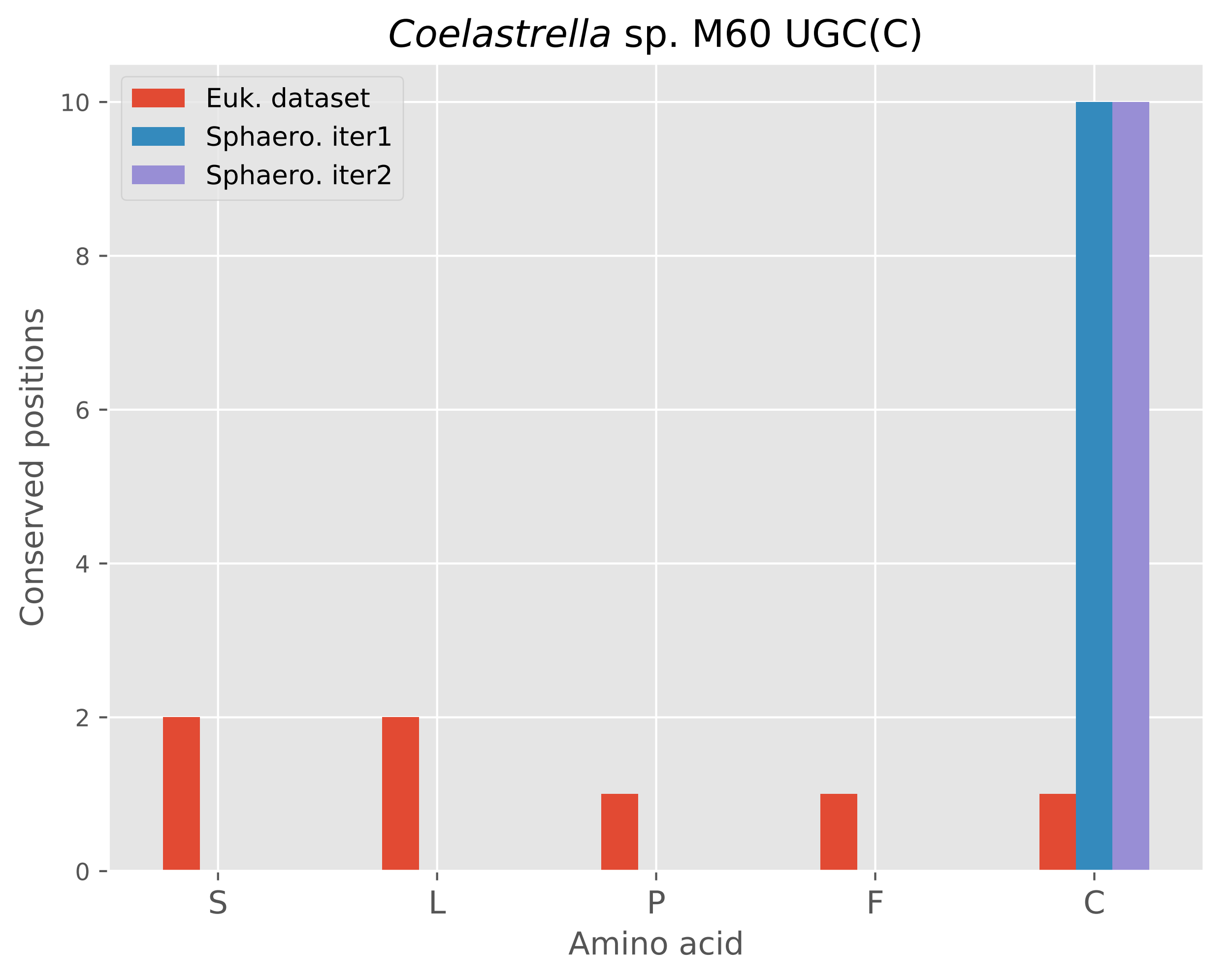

Coelastrella sp. M60 UGC(C)
Euk. dataset
Sphaero. iter1
Sphaero. iter2
Conserved positions
0
2
4
6
8
10
S
L
P
F
C
Amino acid

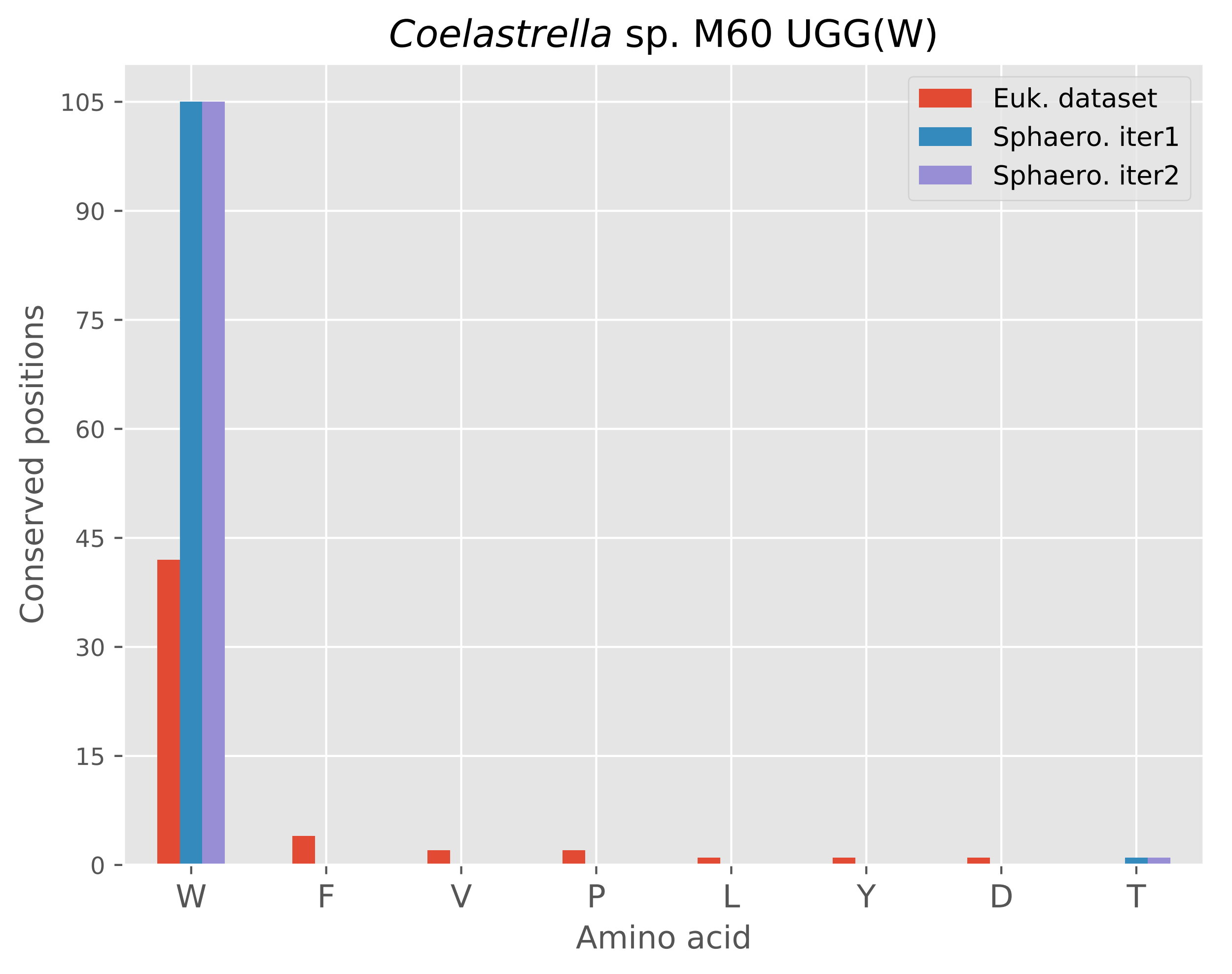
Coelastrella sp. M60 UGG(W)
Euk. dataset
Sphaero. iter1
Sphaero. iter2
Conserved positions
105
90
75
60
45
30
15
0
W
F
V
P
L
Y
D
T
Amino acid

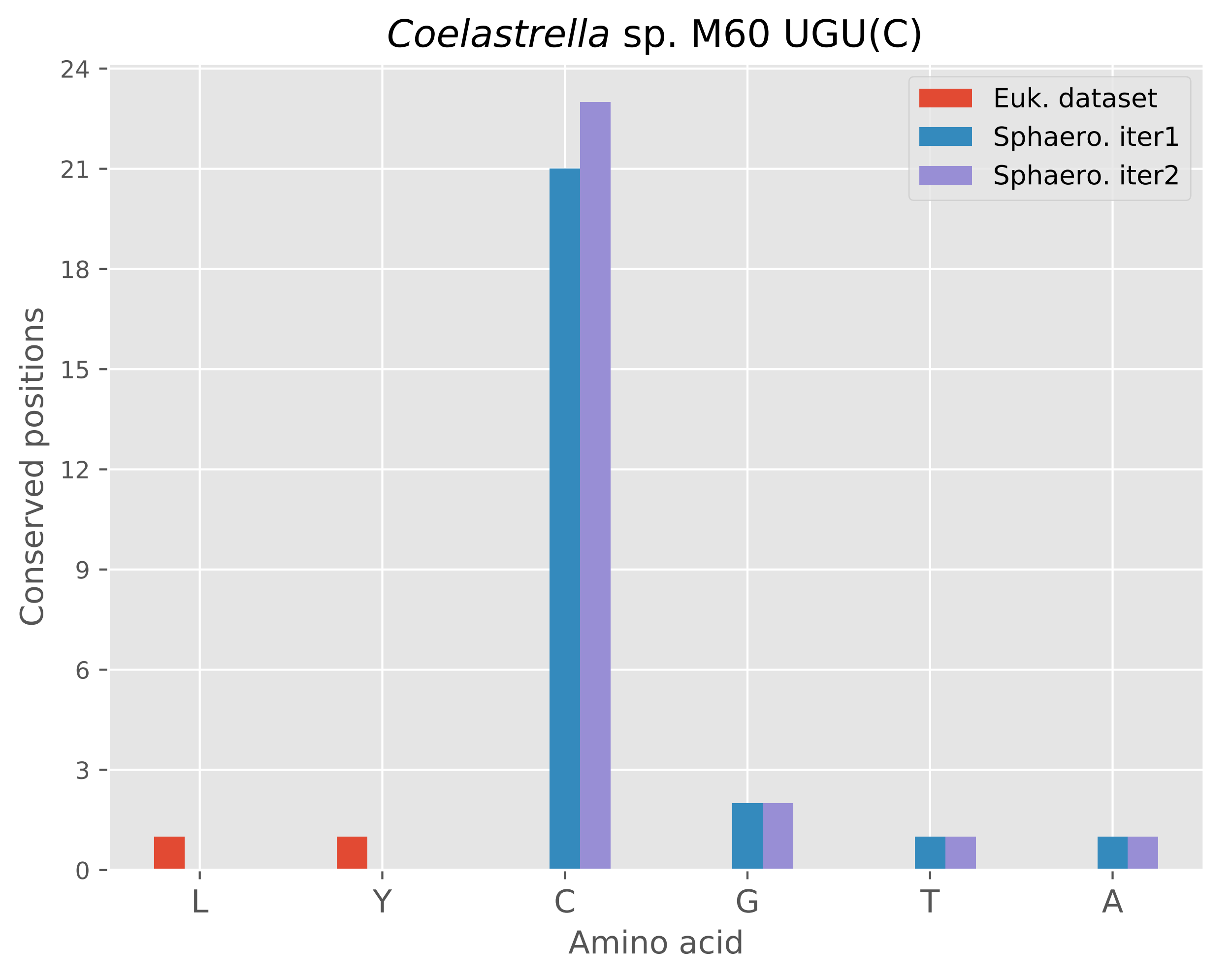
Coelastrella sp. M60 UGU(C)
Conserved positions
Amino acid
0
3
6
9
12
15
18
21
24
L
Y
C
G
T
A
Euk. dataset
Sphaero. iter1
Sphaero. iter2

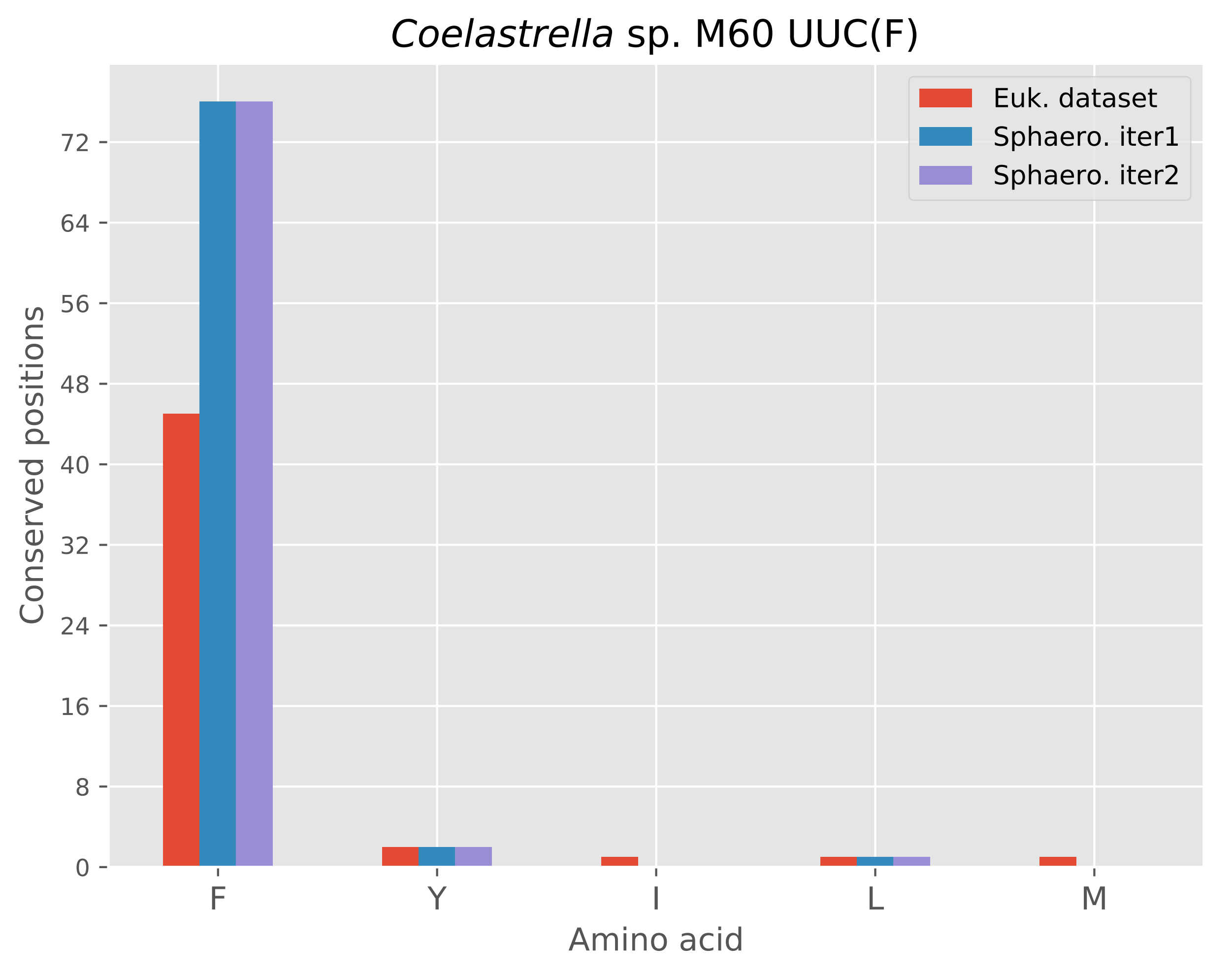

Coelastrella sp. M60 UUC(F)
Euk. dataset
Sphaero. iter1
Sphaero. iter2
Conserved positions
0
8
16
24
32
40
48
56
64
72
F
Y
I
L
M
Amino acid

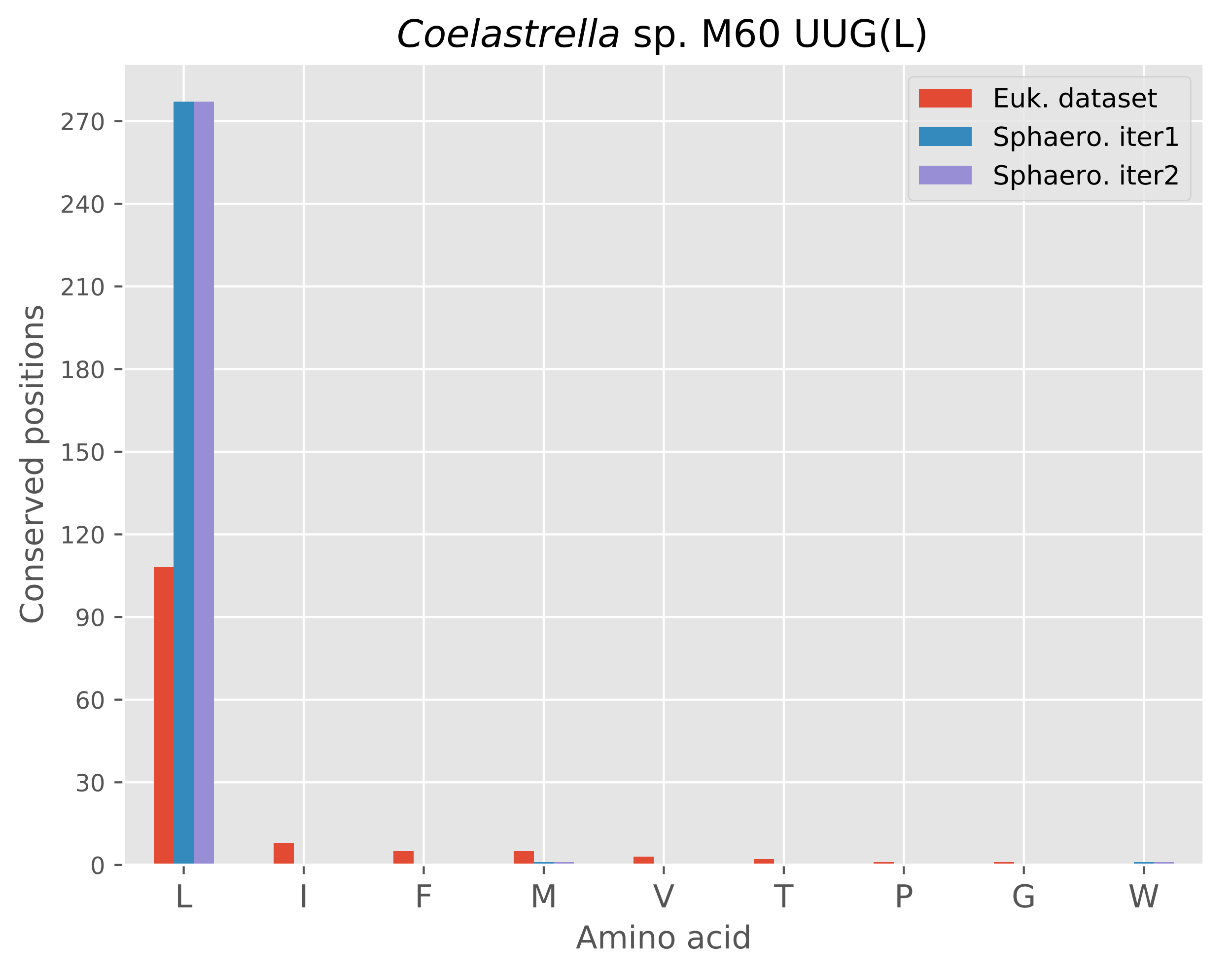
Coelastrella sp. M60 UUG(L)
Euk. dataset
Sphaero. iter1
Sphaero. iter2
Conserved positions
0
30
60
90
120
150
180
210
240
270
L
I
F
M
V
T
P
G
W
Amino acid

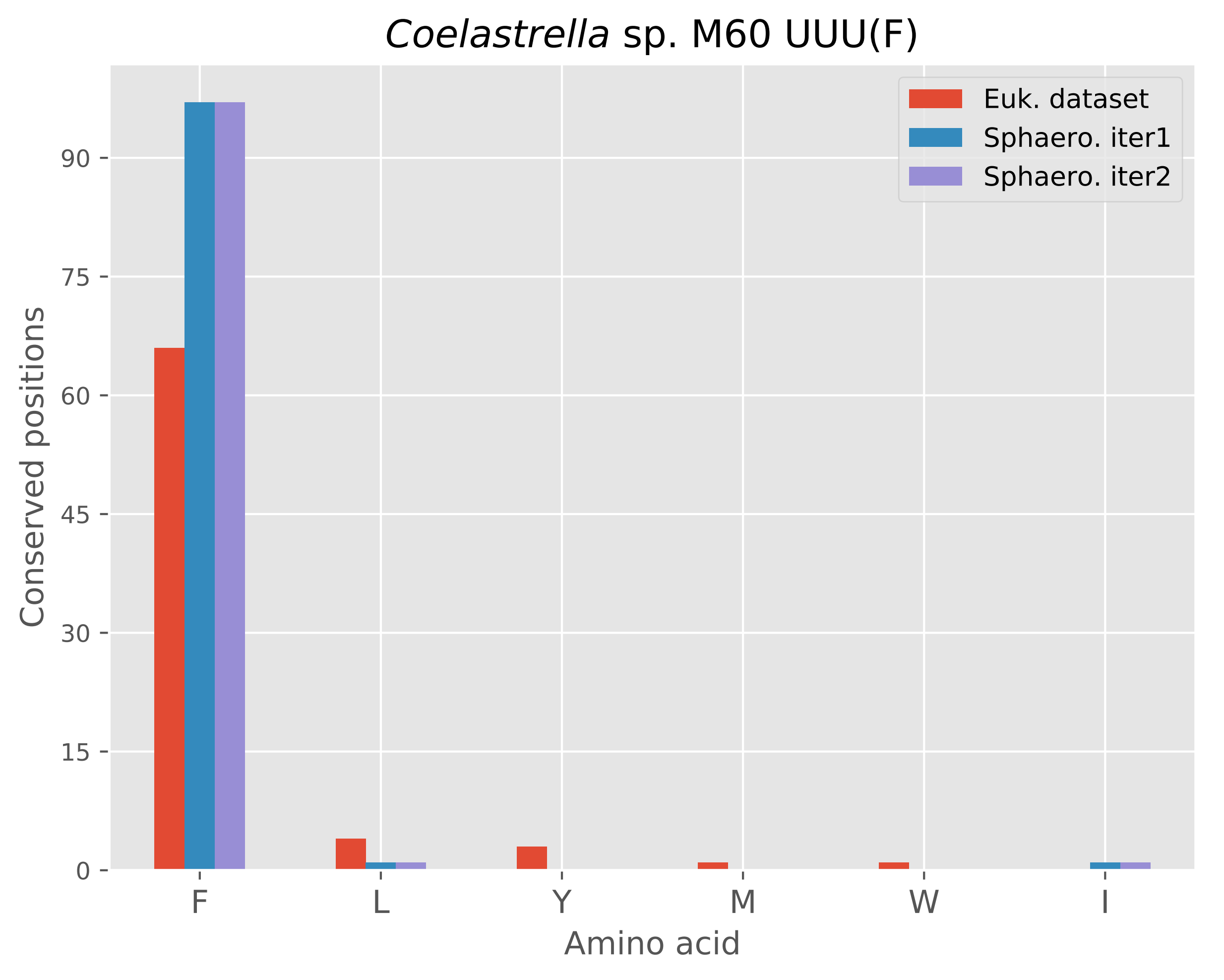

Coelastrella sp. M60 UUU(F)
Euk. dataset
Sphaero. iter1
Sphaero. iter2
Conserved positions
0
15
30
45
60
75
90
F
L
Y
M
W
I
Amino acid

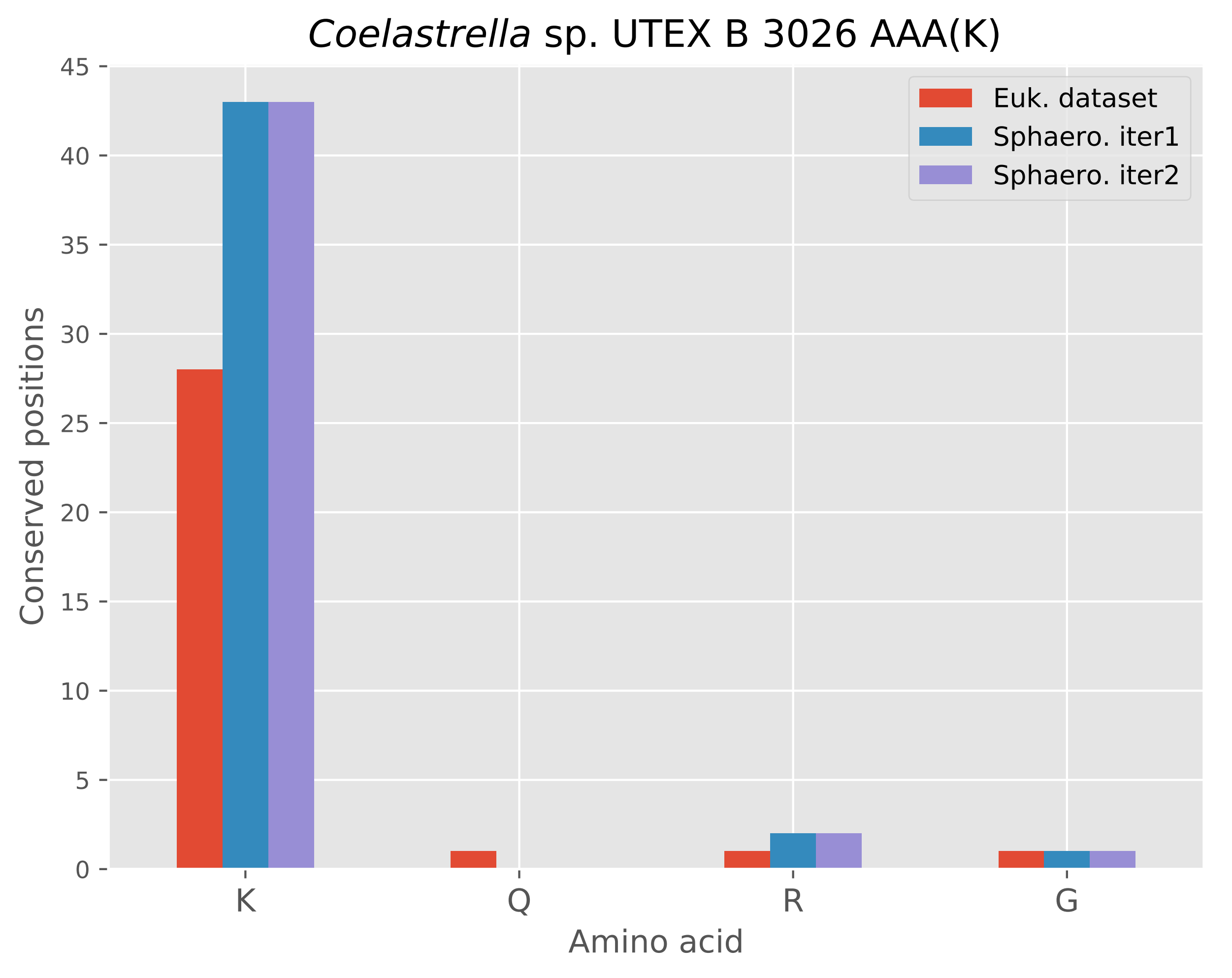

Coelastrella sp. UTEX B 3026 AAA(K)
Conserved positions
0
5
10
15
20
25
30
35
40
45
K
Q
R
G
Amino acid
Euk. dataset
Sphaero. iter1
Sphaero. iter2

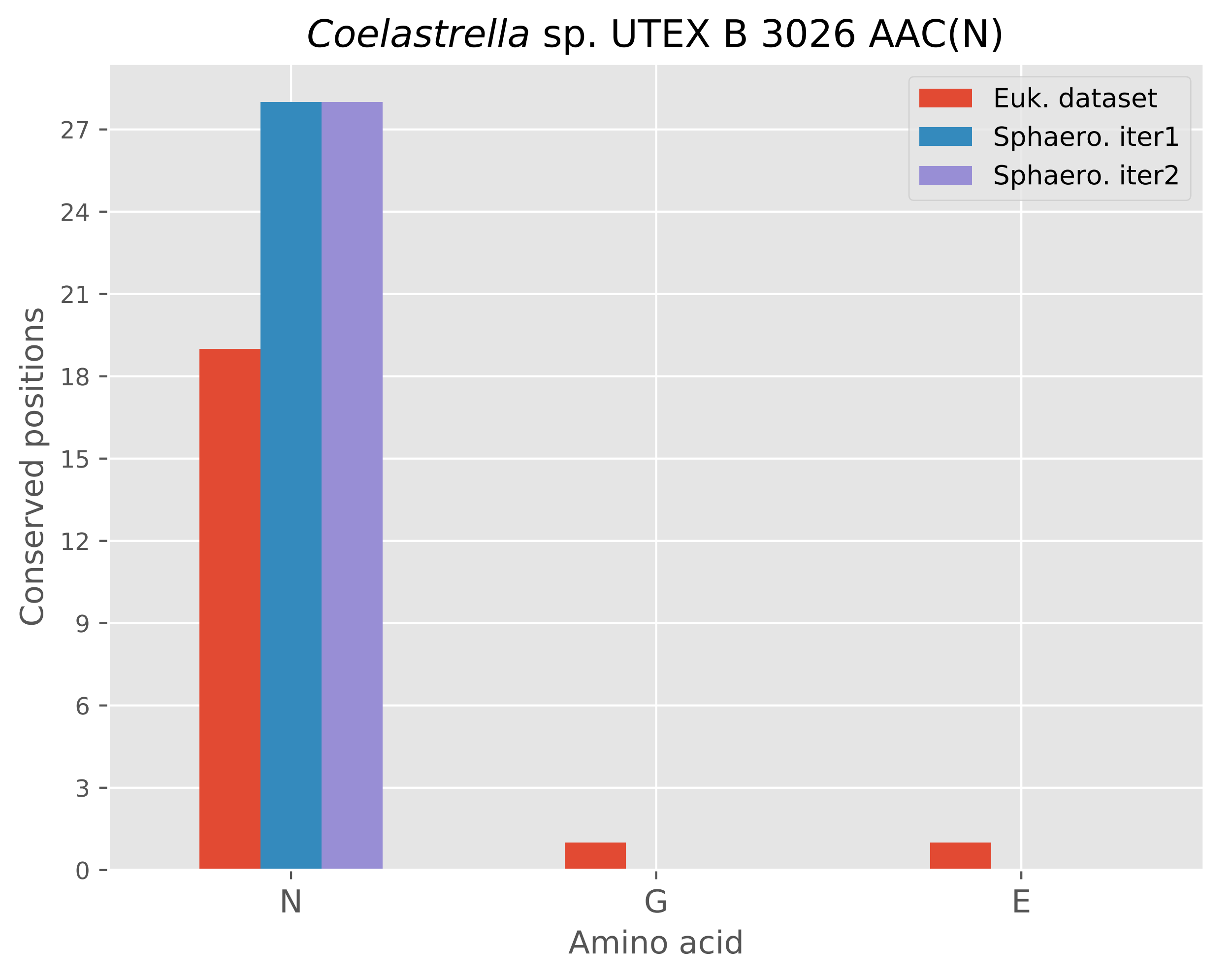

Coelastrella sp. UTEX B 3026 AAC(N)
Euk. dataset
Sphaero. iter1
Sphaero. iter2
Conserved positions
0
3
6
9
12
15
18
21
24
27
N
G
E
Amino acid

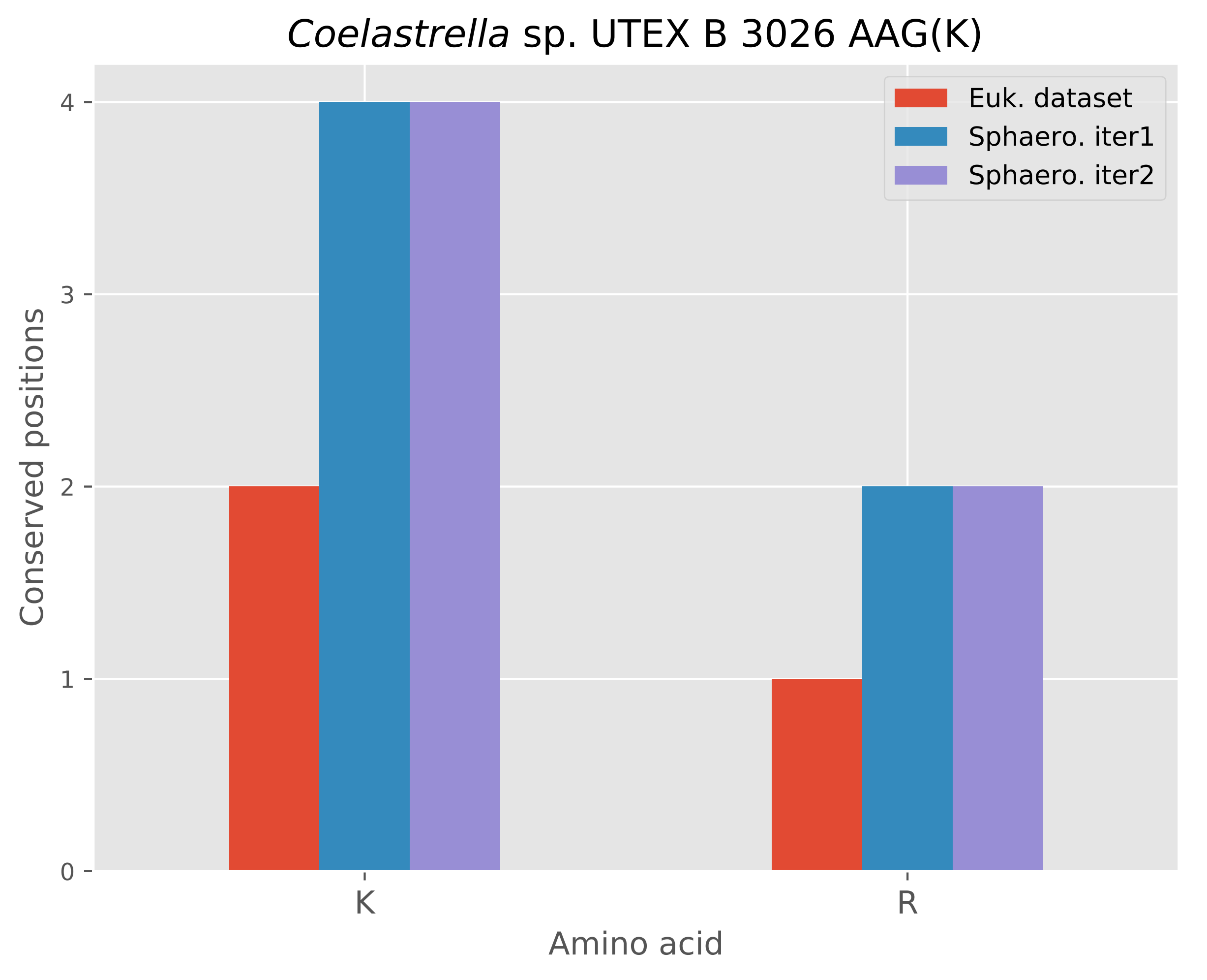
Coelastrella sp. UTEX B 3026 AAG(K)
Euk. dataset
Sphaero. iter1
Sphaero. iter2
Conserved positions
0
1
2
3
4
K
R
Amino acid

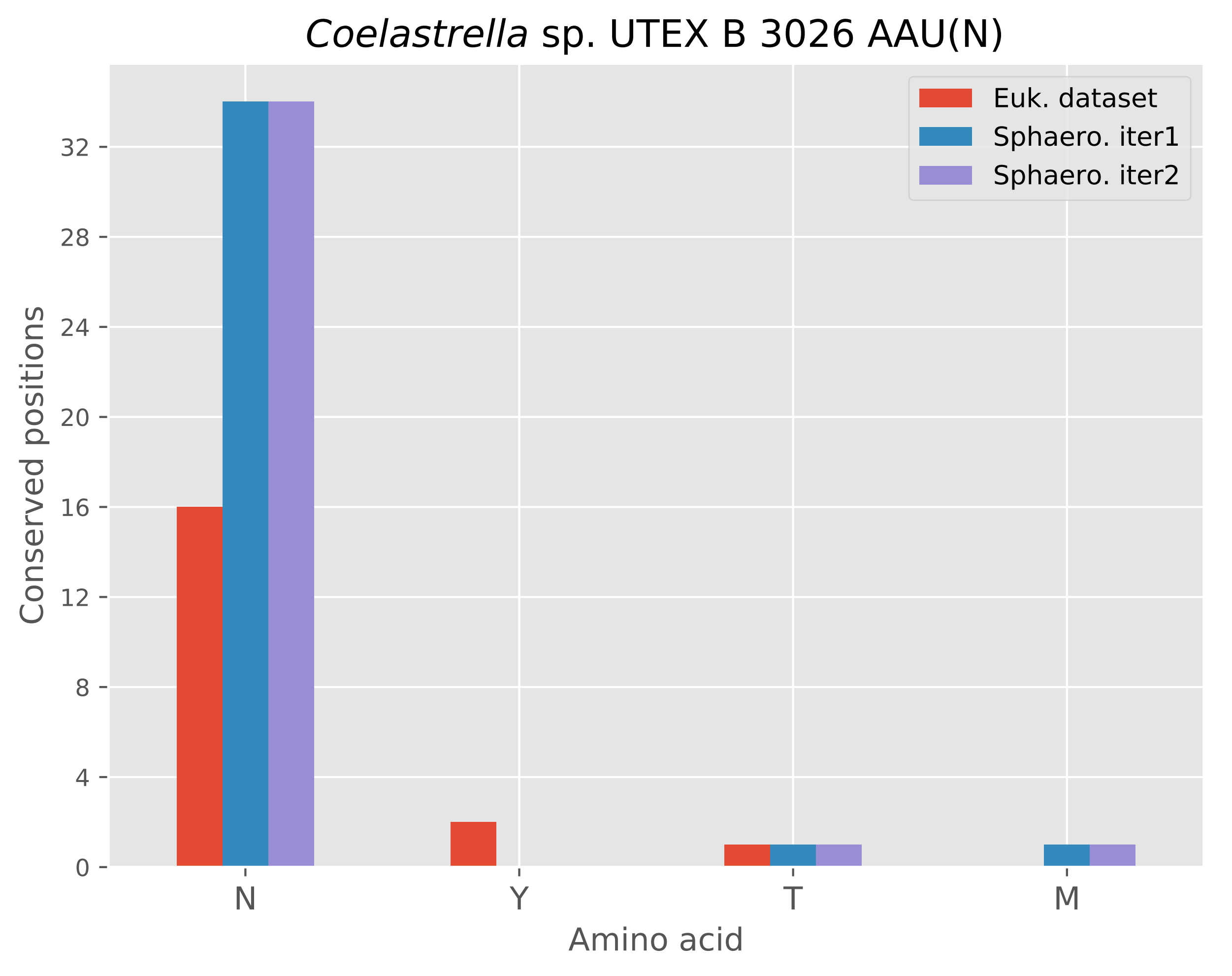

Coelastrella sp. UTEX B 3026 AAU(N)
Euk. dataset
Sphaero. iter1
Sphaero. iter2
Conserved positions
0
4
8
12
16
20
24
28
32
N
Y
T
M
Amino acid

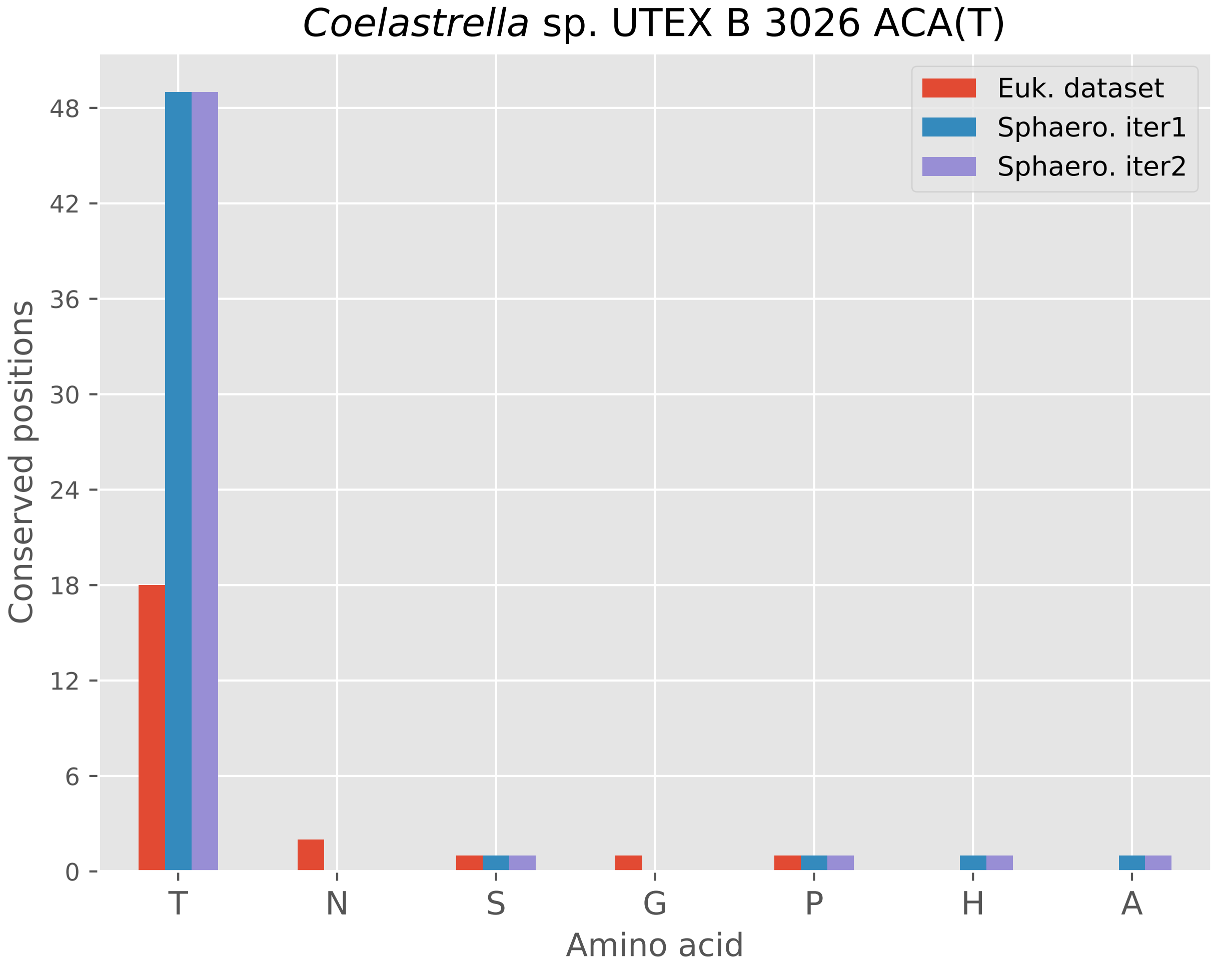

Coelastrella sp. UTEX B 3026 ACA(T)
Euk. dataset
Sphaero. iter1
Sphaero. iter2
Conserved positions
0
6
12
18
24
30
36
42
48
T
N
S
G
P
H
A
Amino acid

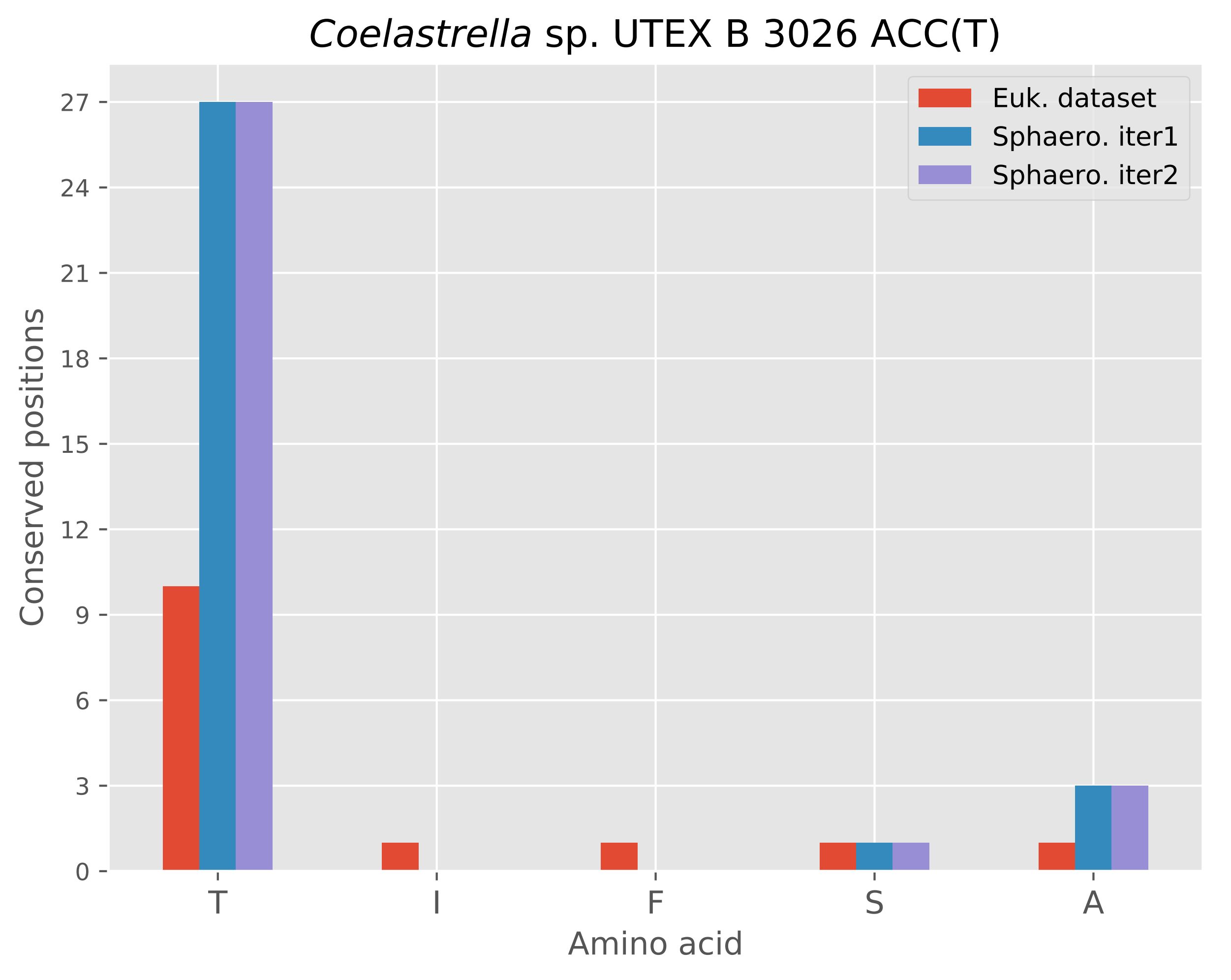

Coelastrella sp. UTEX B 3026 ACC(T)
Euk. dataset
Sphaero. iter1
Sphaero. iter2
Conserved positions
0
3
6
9
12
15
18
21
24
27
T
I
F
S
A
Amino acid

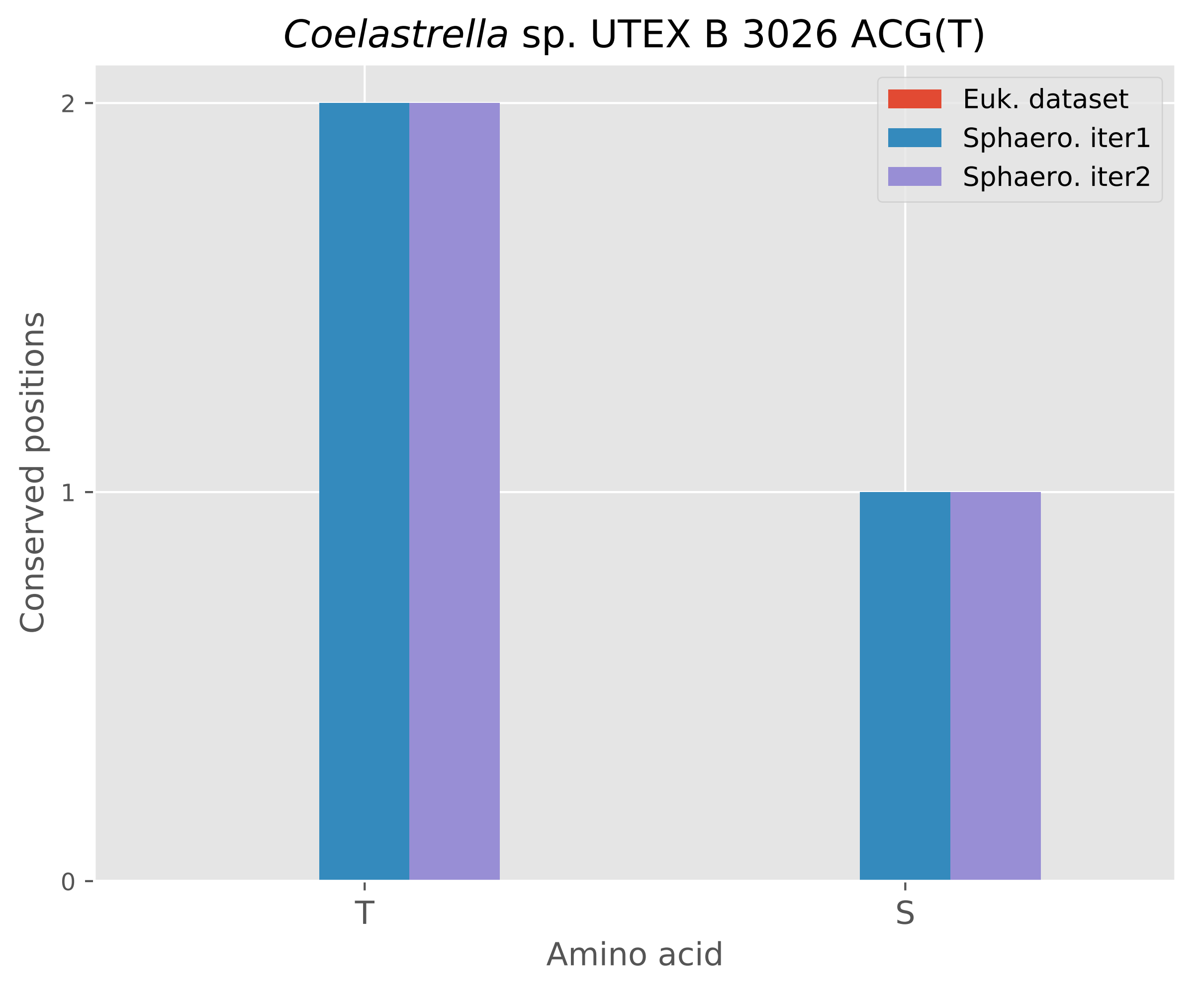

Coelastrella sp. UTEX B 3026 ACG(T)
Euk. dataset
Sphaero. iter1
Sphaero. iter2
2
1
0
Conserved positions
T
S
Amino acid

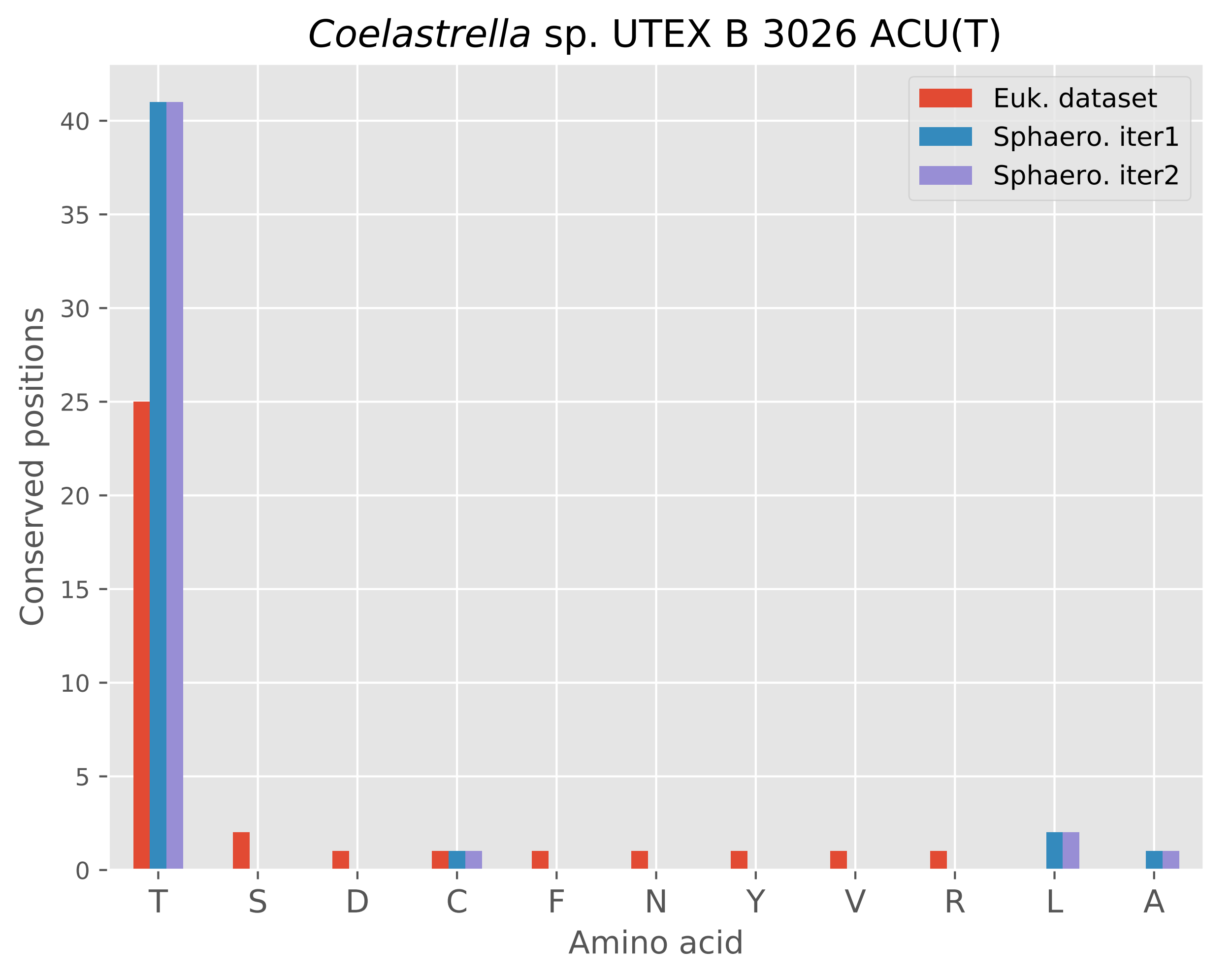

Coelastrella sp. UTEX B 3026 ACU(T)
Euk. dataset
Sphaero. iter1
Sphaero. iter2
Conserved positions
0
5
10
15
20
25
30
35
40
T
S
D
C
F
N
Y
V
R
L
A
Amino acid

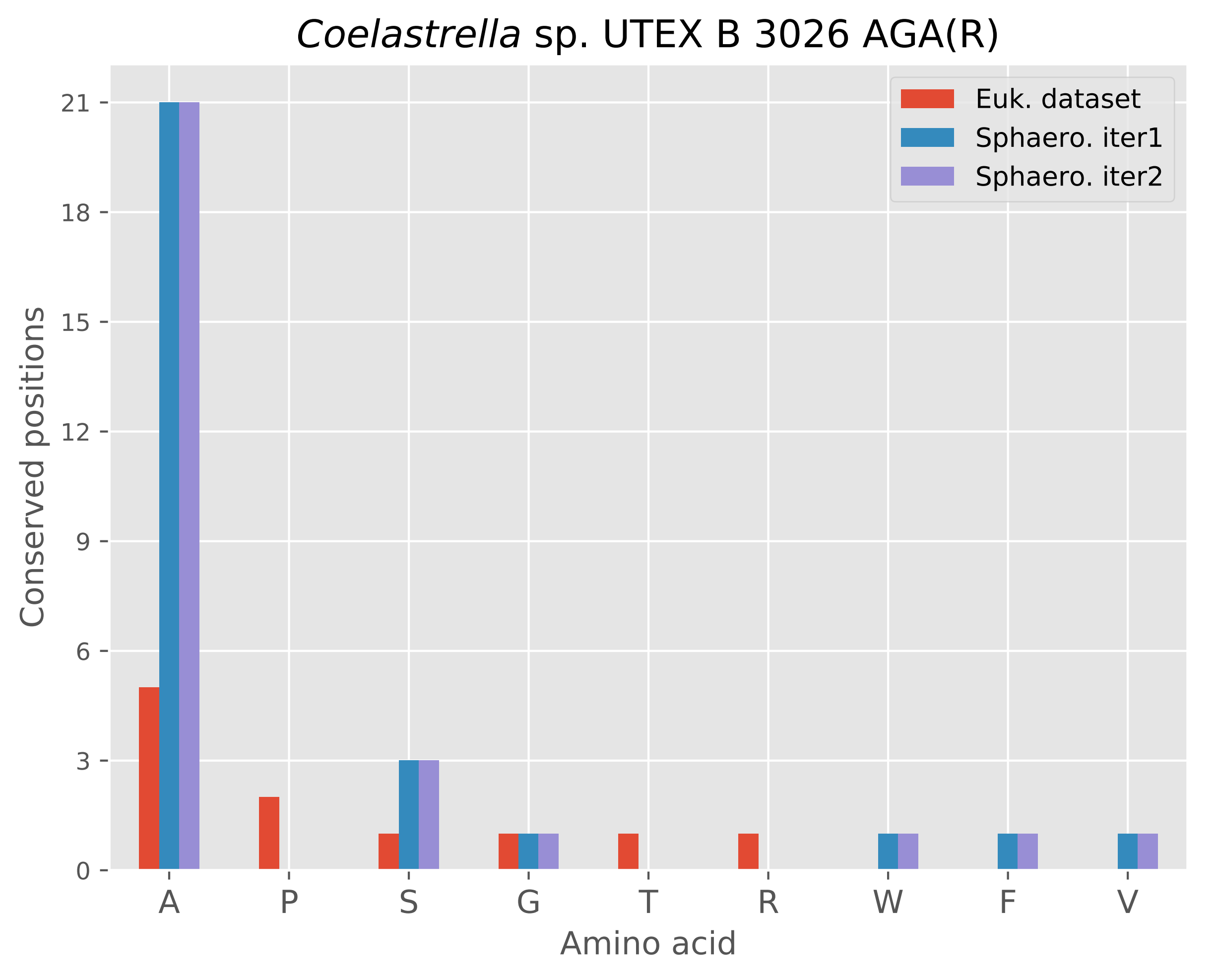

Coelastrella sp. UTEX B 3026 AGA(R)
Euk. dataset
Sphaero. iter1
Sphaero. iter2
Conserved positions
0
3
6
9
12
15
18
21
A
P
S
G
T
R
W
F
V
Amino acid

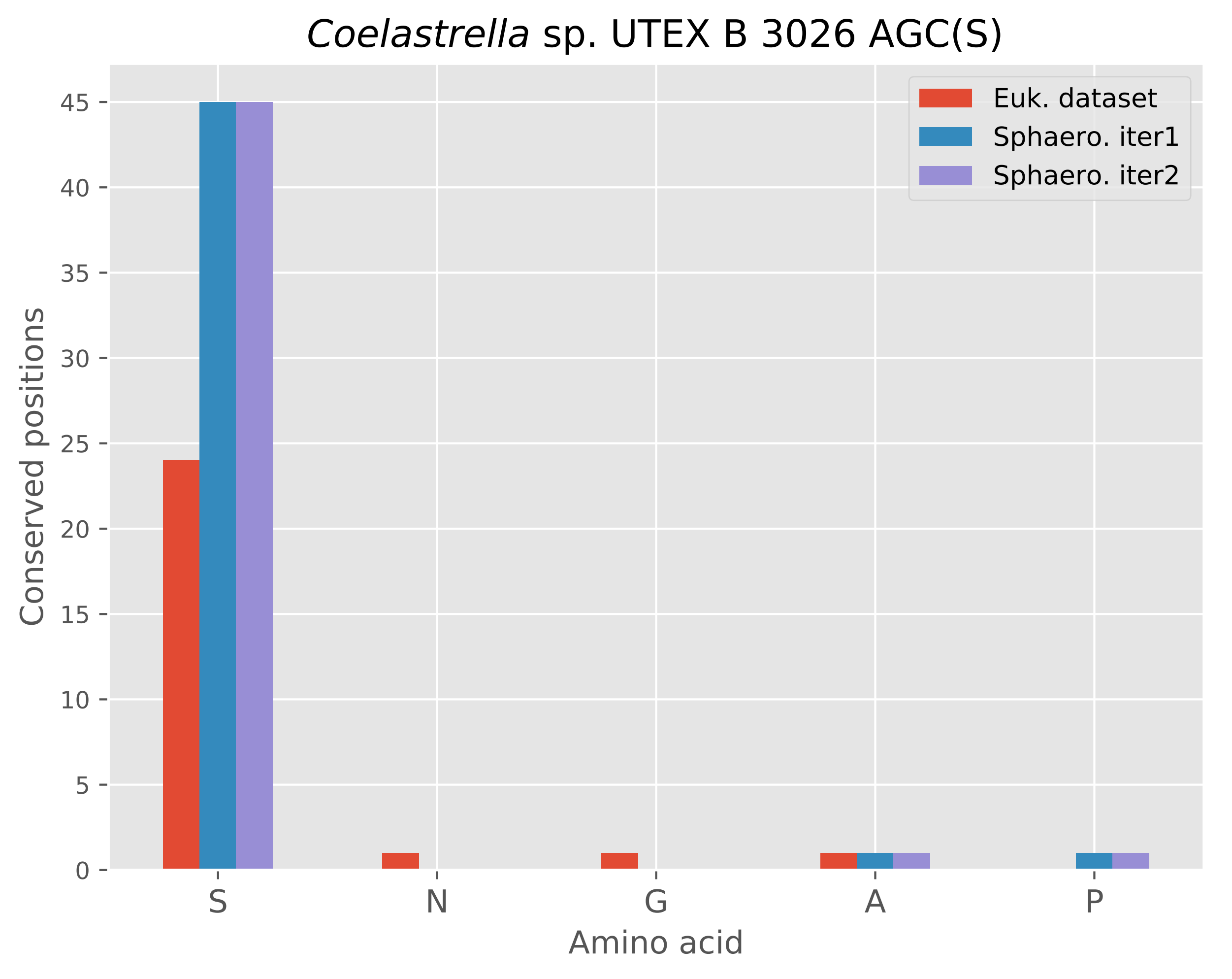

Coelastrella sp. UTEX B 3026 AGC(S)
Euk. dataset
Sphaero. iter1
Sphaero. iter2
Conserved positions
0
5
10
15
20
25
30
35
40
45
S
N
G
A
P
Amino acid

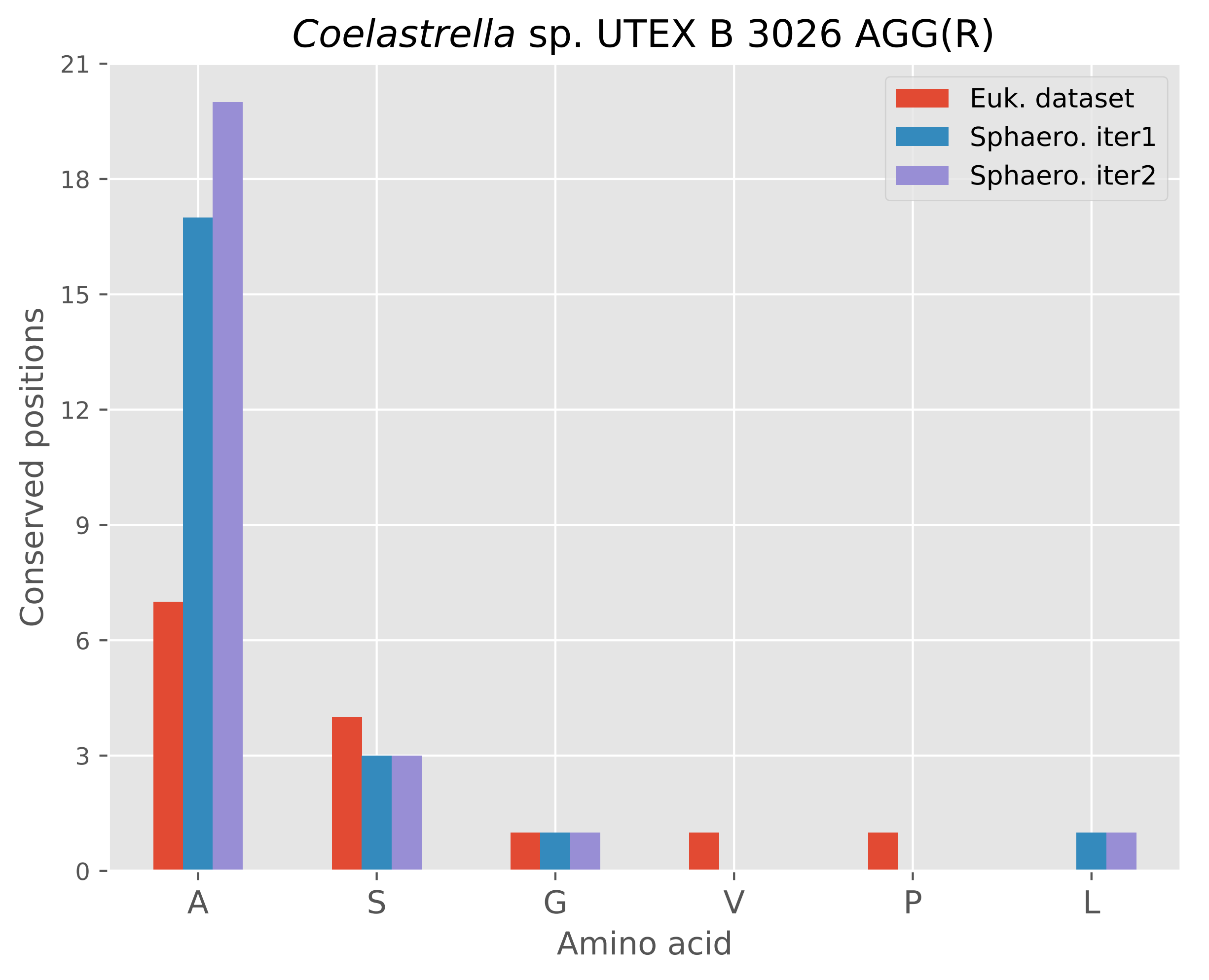

Coelastrella sp. UTEX B 3026 AGG(R)
Conserved positions
0
3
6
9
12
15
18
21
A
S
G
V
P
L
Amino acid
Euk. dataset
Sphaero. iter1
Sphaero. iter2

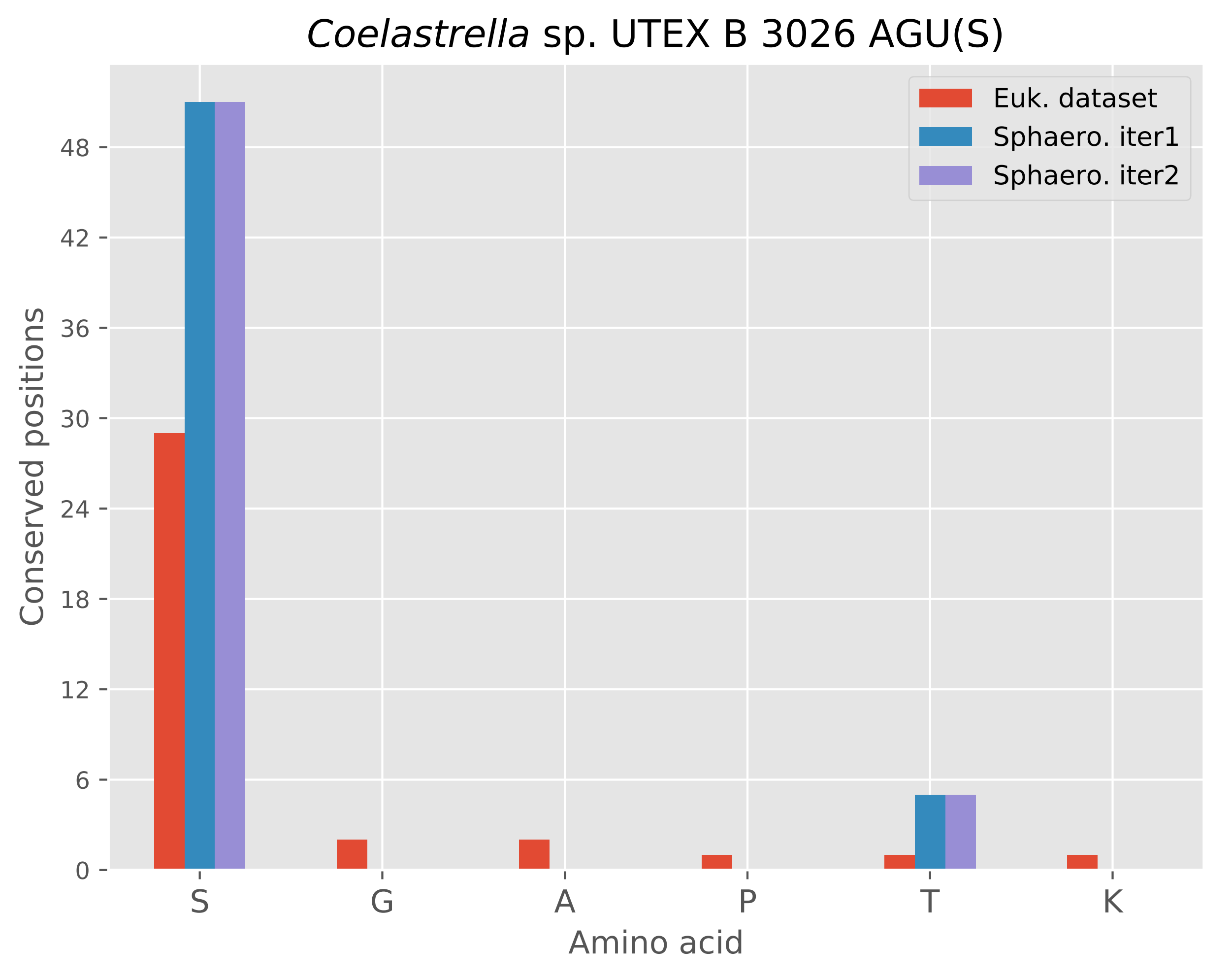

Coelastrella sp. UTEX B 3026 AGU(S)
Euk. dataset
Sphaero. iter1
Sphaero. iter2
Conserved positions
0
6
12
18
24
30
36
42
48
S
G
A
P
T
K
Amino acid

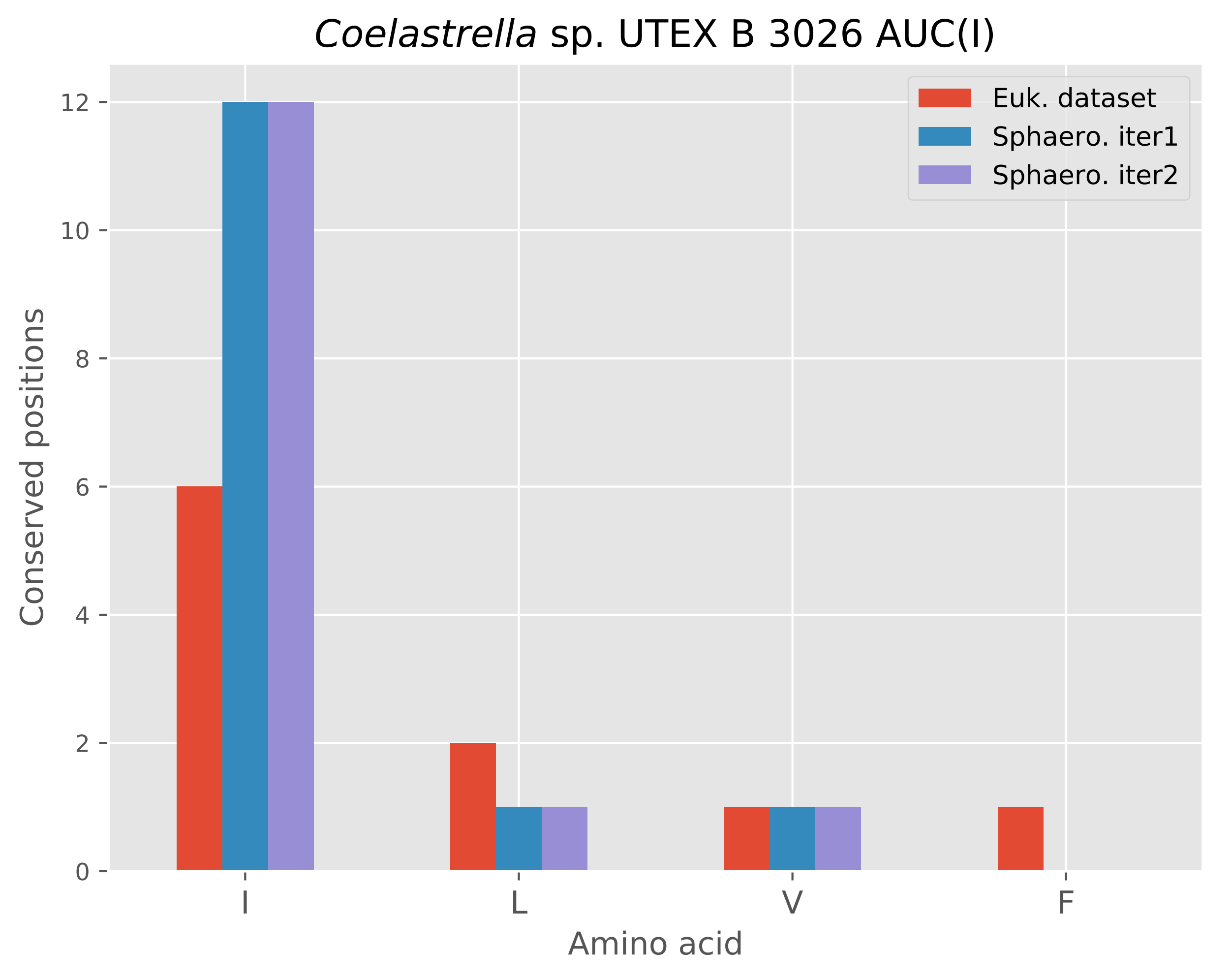

Coelastrella sp. UTEX B 3026 AUC(I)
Euk. dataset
Sphaero. iter1
Sphaero. iter2
Conserved positions
0
2
4
6
8
10
12
I
L
V
F
Amino acid

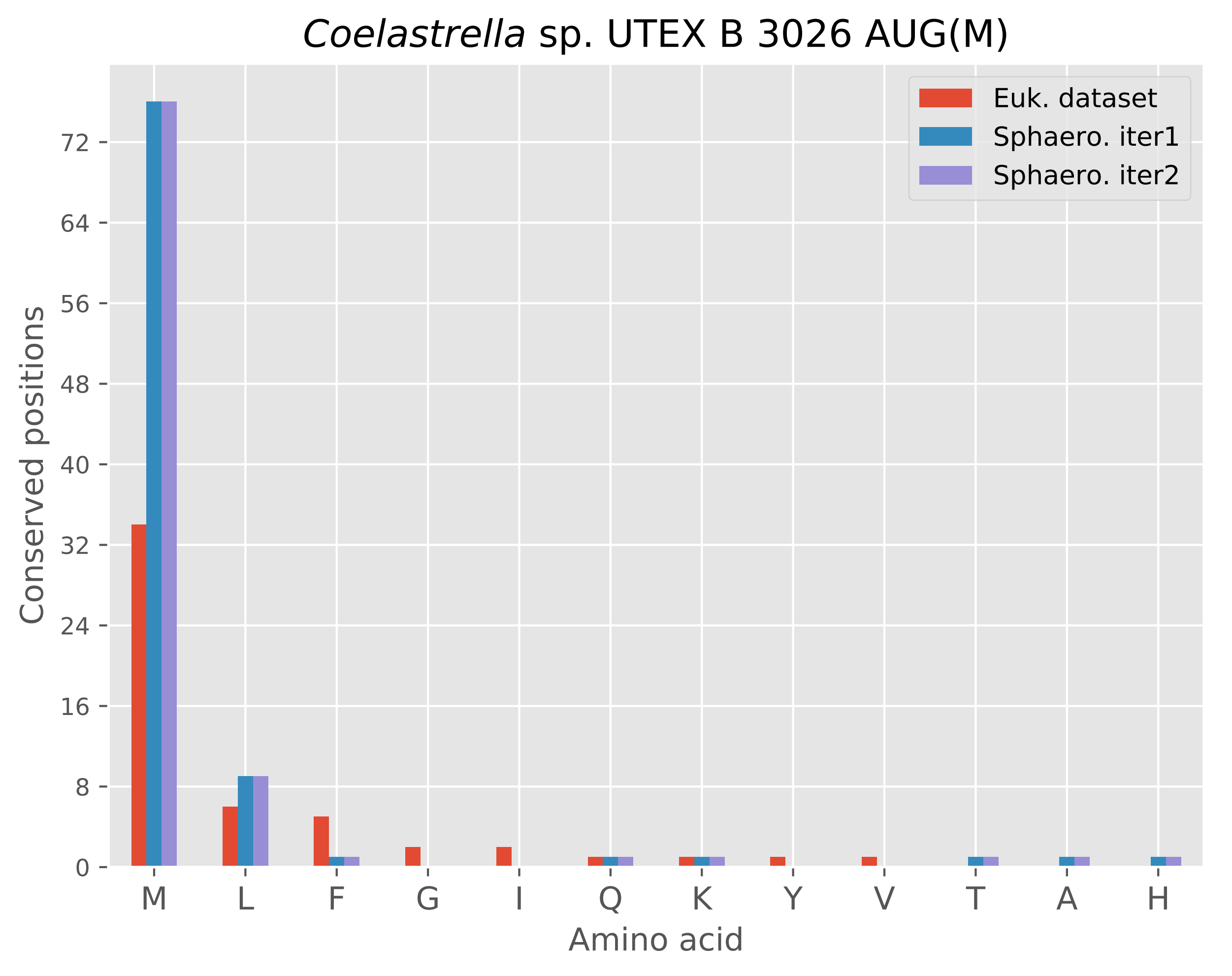

Coelastrella sp. UTEX B 3026 AUG(M)
Euk. dataset
Sphaero. iter1
Sphaero. iter2
Conserved positions
0
8
16
24
32
40
48
56
64
72
M
L
F
G
I
Q
K
Y
V
T
A
H
Amino acid

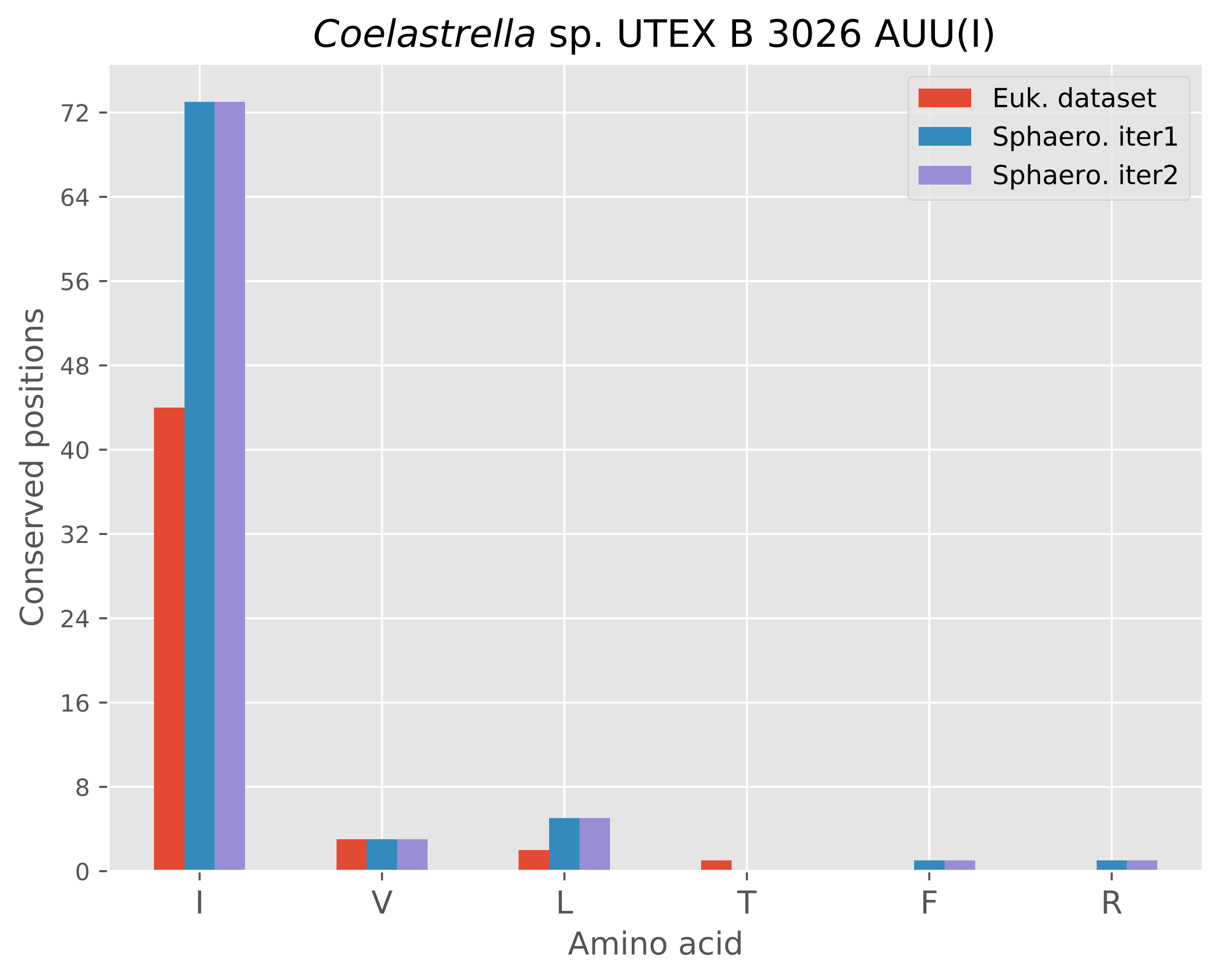

Coelastrella sp. UTEX B 3026 AUU(I)
Euk. dataset
Sphaero. iter1
Sphaero. iter2
Conserved positions
0
8
16
24
32
40
48
56
64
72
I
V
L
T
F
R
Amino acid

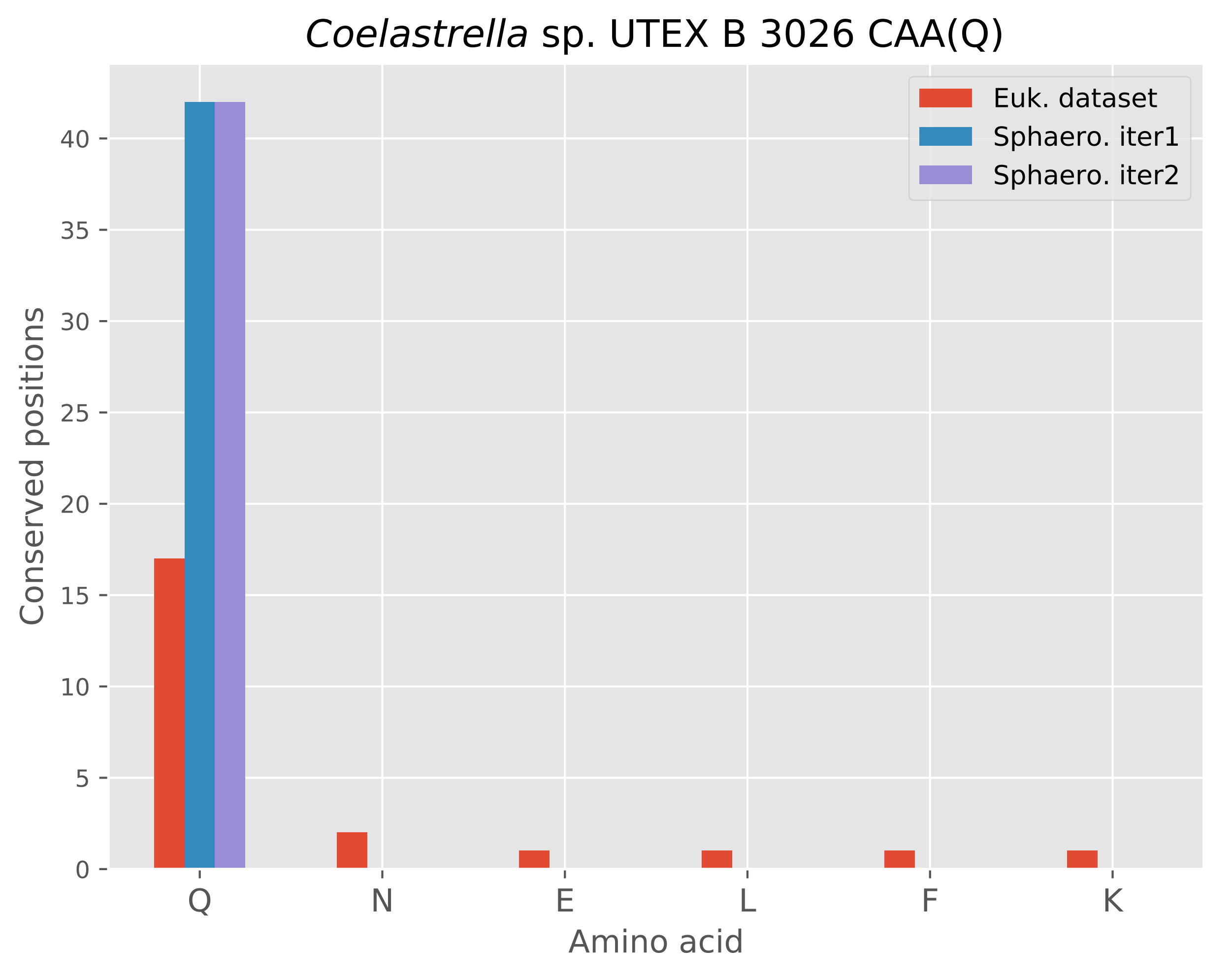

Coelastrella sp. UTEX B 3026 CAA(Q)
Euk. dataset
Sphaero. iter1
Sphaero. iter2
Conserved positions
0
5
10
15
20
25
30
35
40
Q
N
E
L
F
K
Amino acid

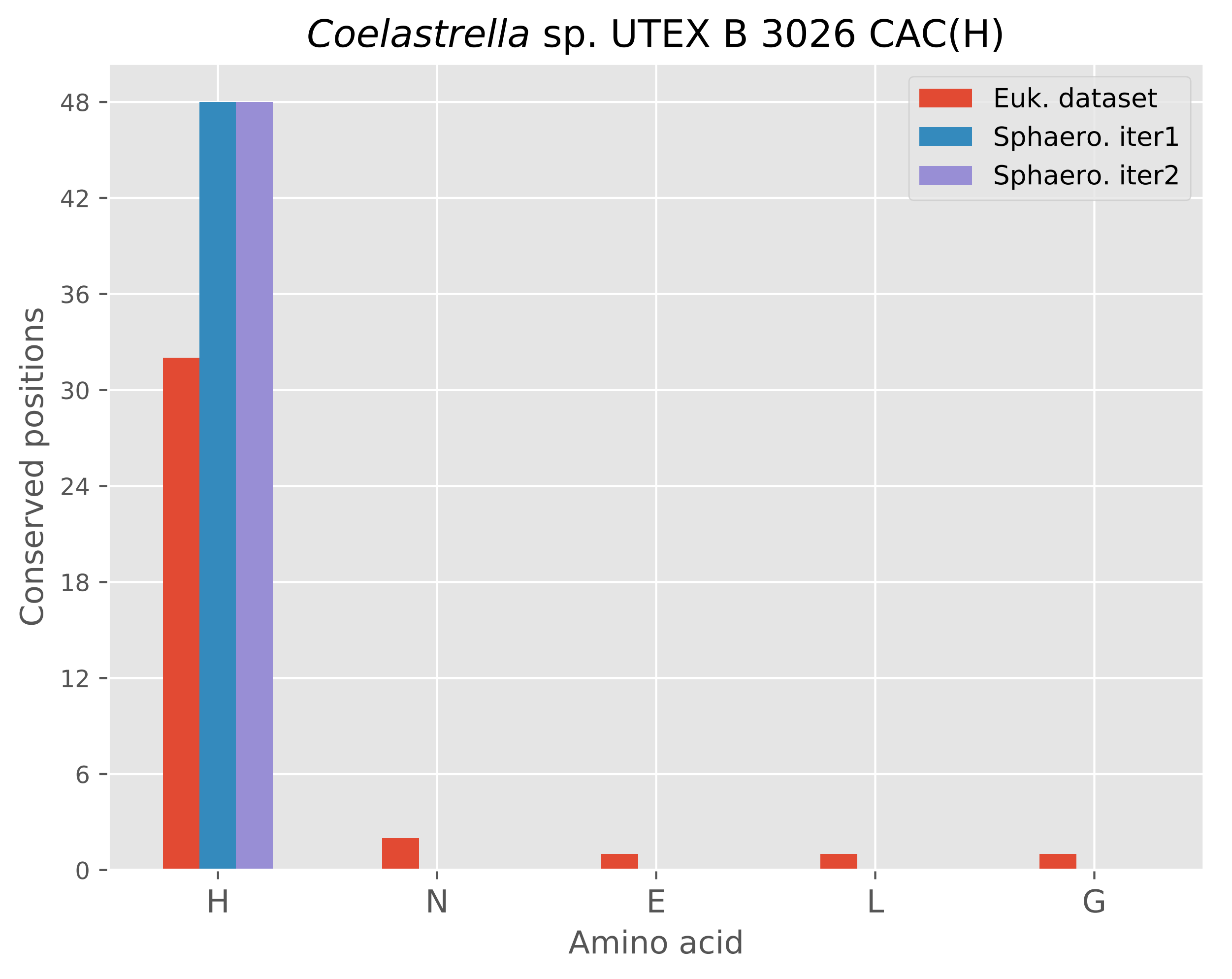

Coelastrella sp. UTEX B 3026 CAC(H)
Euk. dataset
Sphaero. iter1
Sphaero. iter2
Conserved positions
0
6
12
18
24
30
36
42
48
H
N
E
L
G
Amino acid

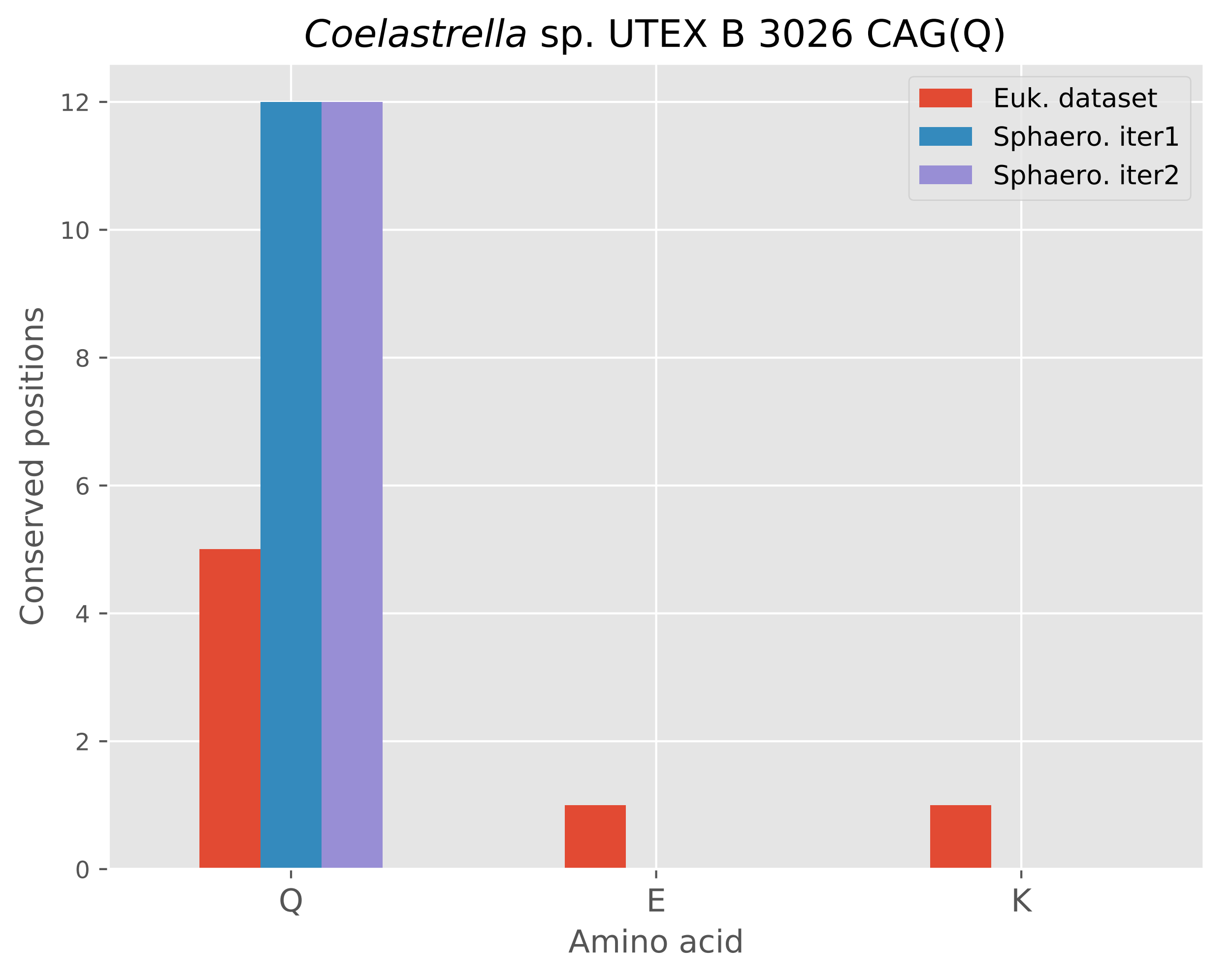

Coelastrella sp. UTEX B 3026 CAG(Q)
Euk. dataset
Sphaero. iter1
Sphaero. iter2
Conserved positions
0
2
4
6
8
10
12
Q
E
K
Amino acid

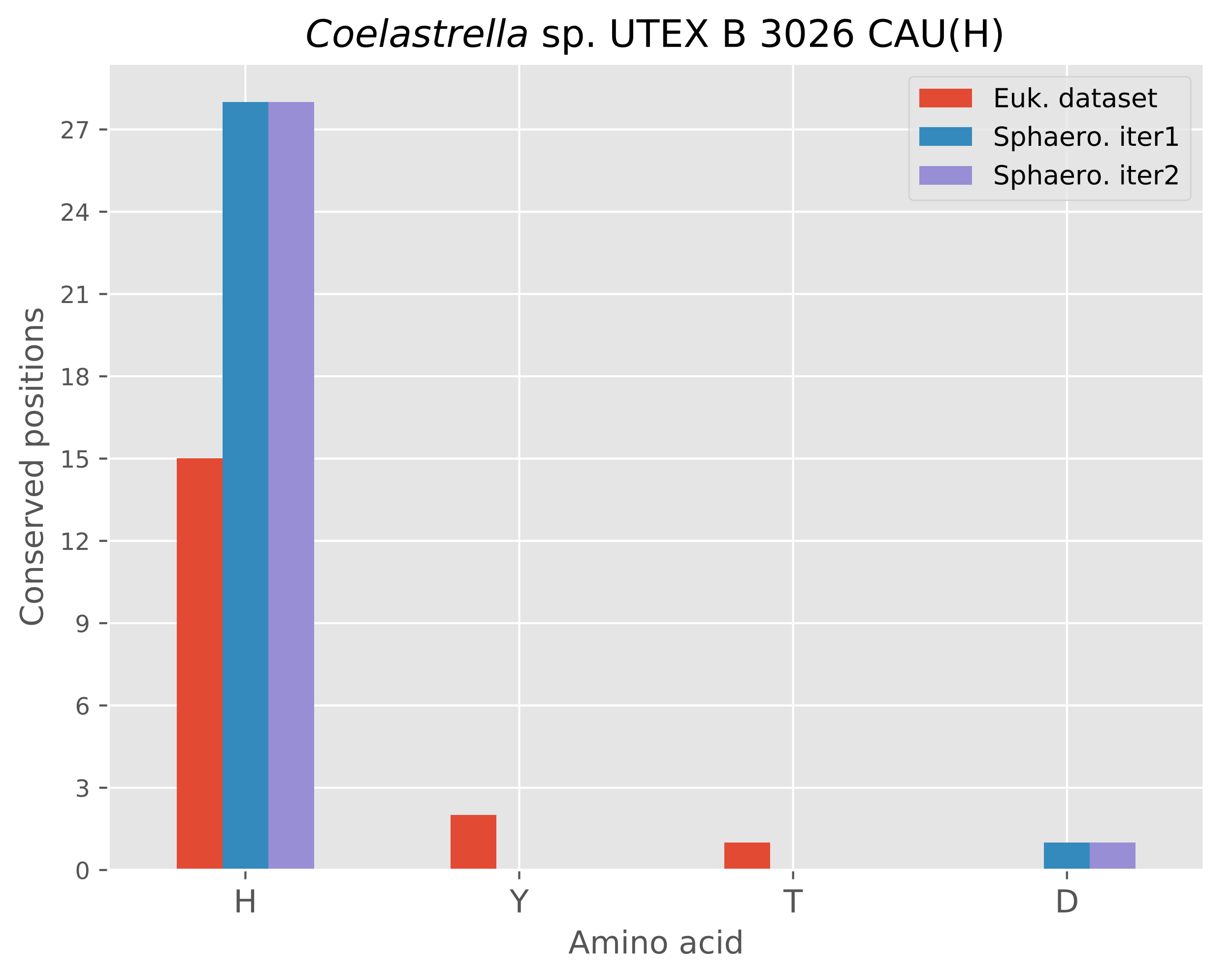
Coelastrella sp. UTEX B 3026 CAU(H)
Euk. dataset
Sphaero. iter1
Sphaero. iter2
Conserved positions
0
3
6
9
12
15
18
21
24
27
H
Y
T
D
Amino acid

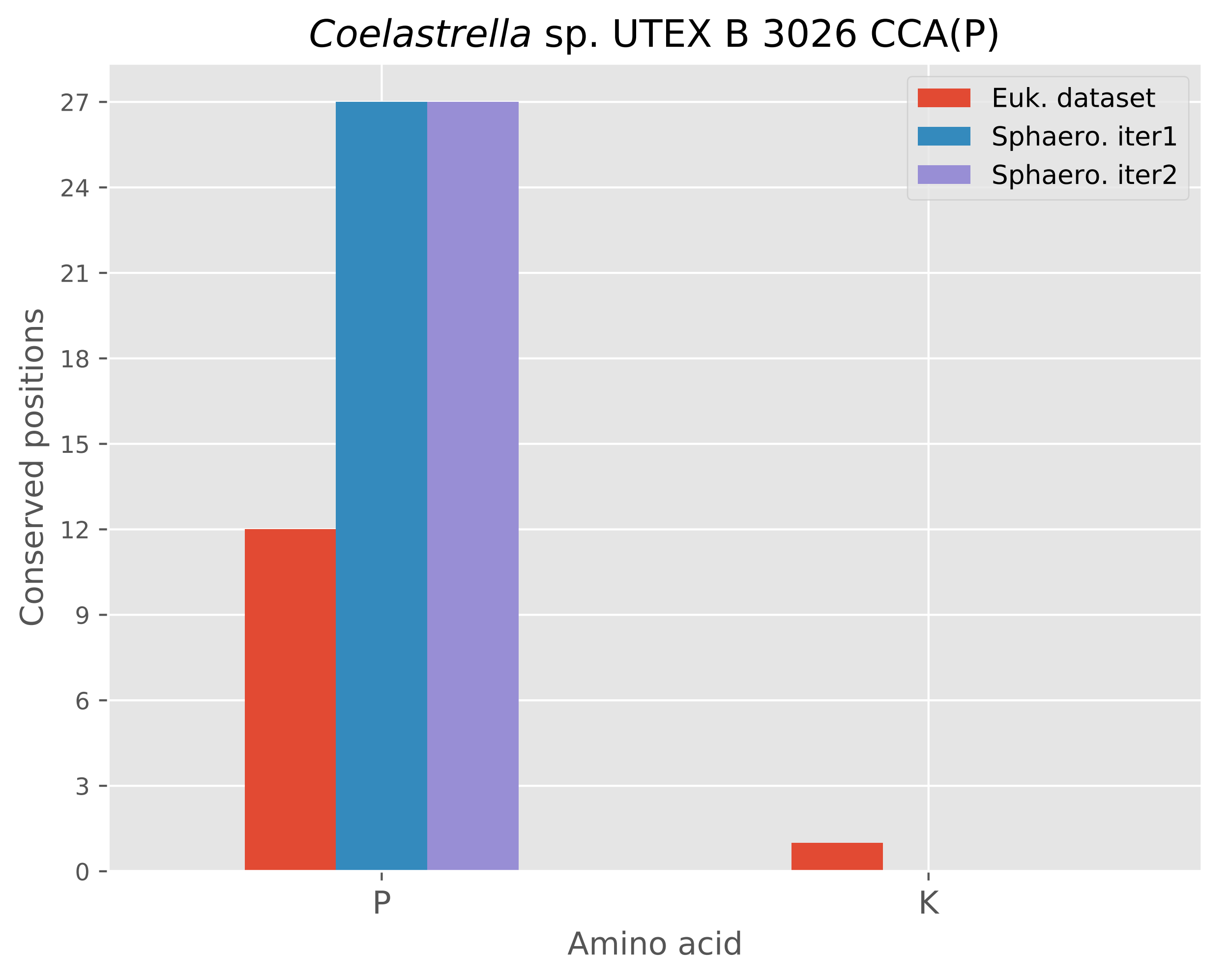

Coelastrella sp. UTEX B 3026 CCA(P)
Euk. dataset
Sphaero. iter1
Sphaero. iter2
Conserved positions
0
3
6
9
12
15
18
21
24
27
P
K
Amino acid

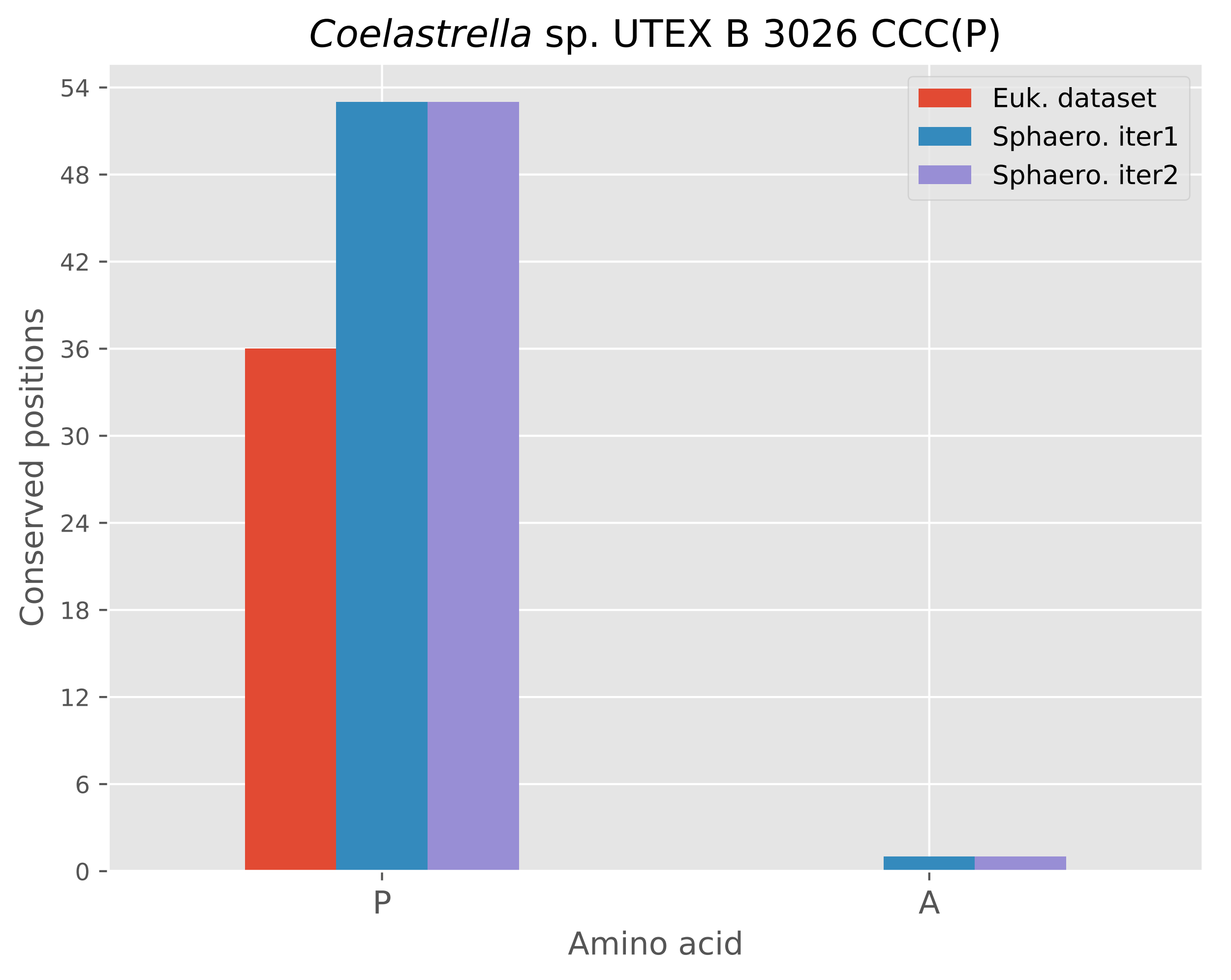
Coelastrella sp. UTEX B 3026 CCC(P)
Euk. dataset
Sphaero. iter1
Sphaero. iter2
Conserved positions
0
6
12
18
24
30
36
42
48
54
P
A
Amino acid

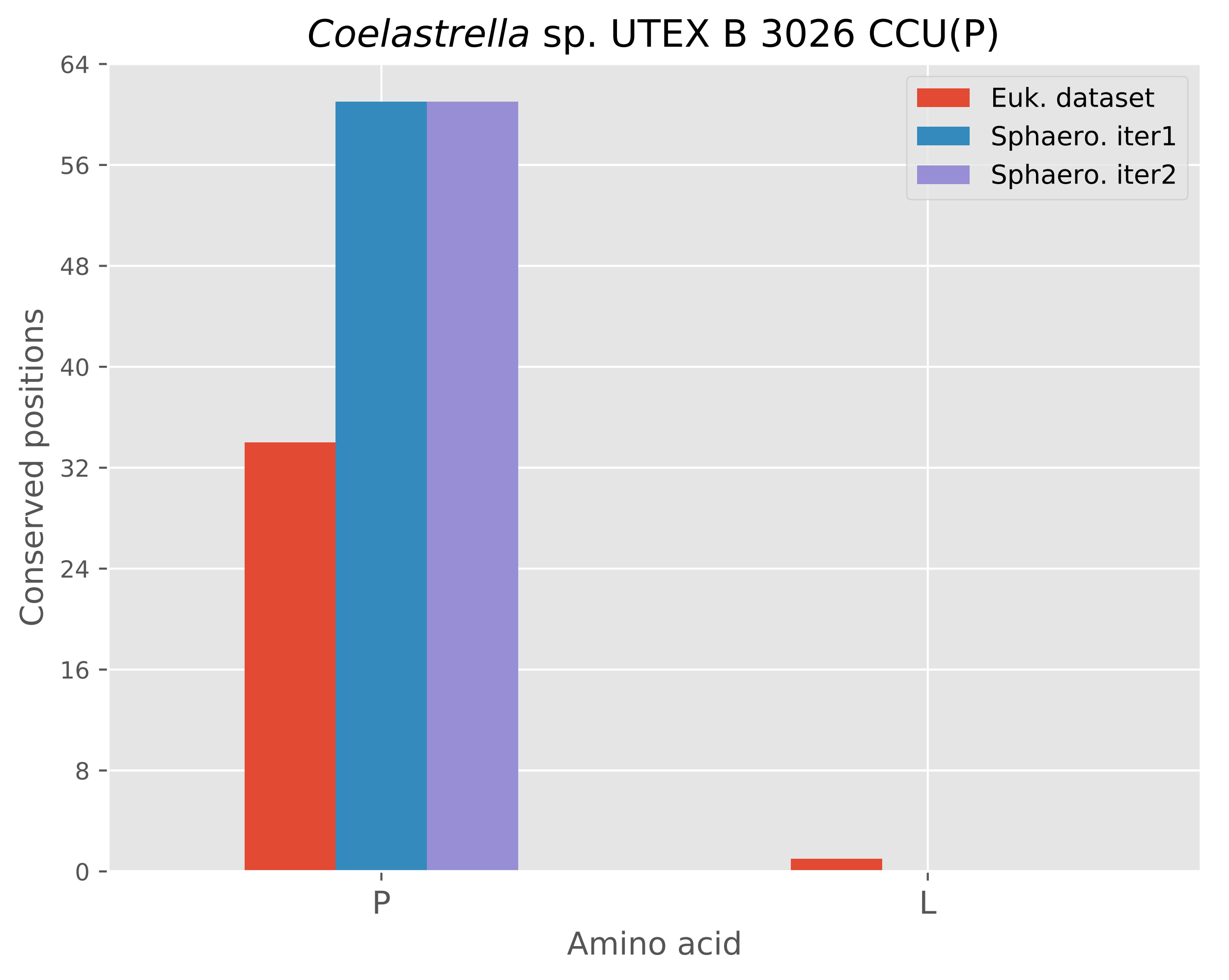

Coelastrella sp. UTEX B 3026 CCU(P)
Conserved positions
Amino acid
P
L
0
8
16
24
32
40
48
56
64
Euk. dataset
Sphaero. iter1
Sphaero. iter2

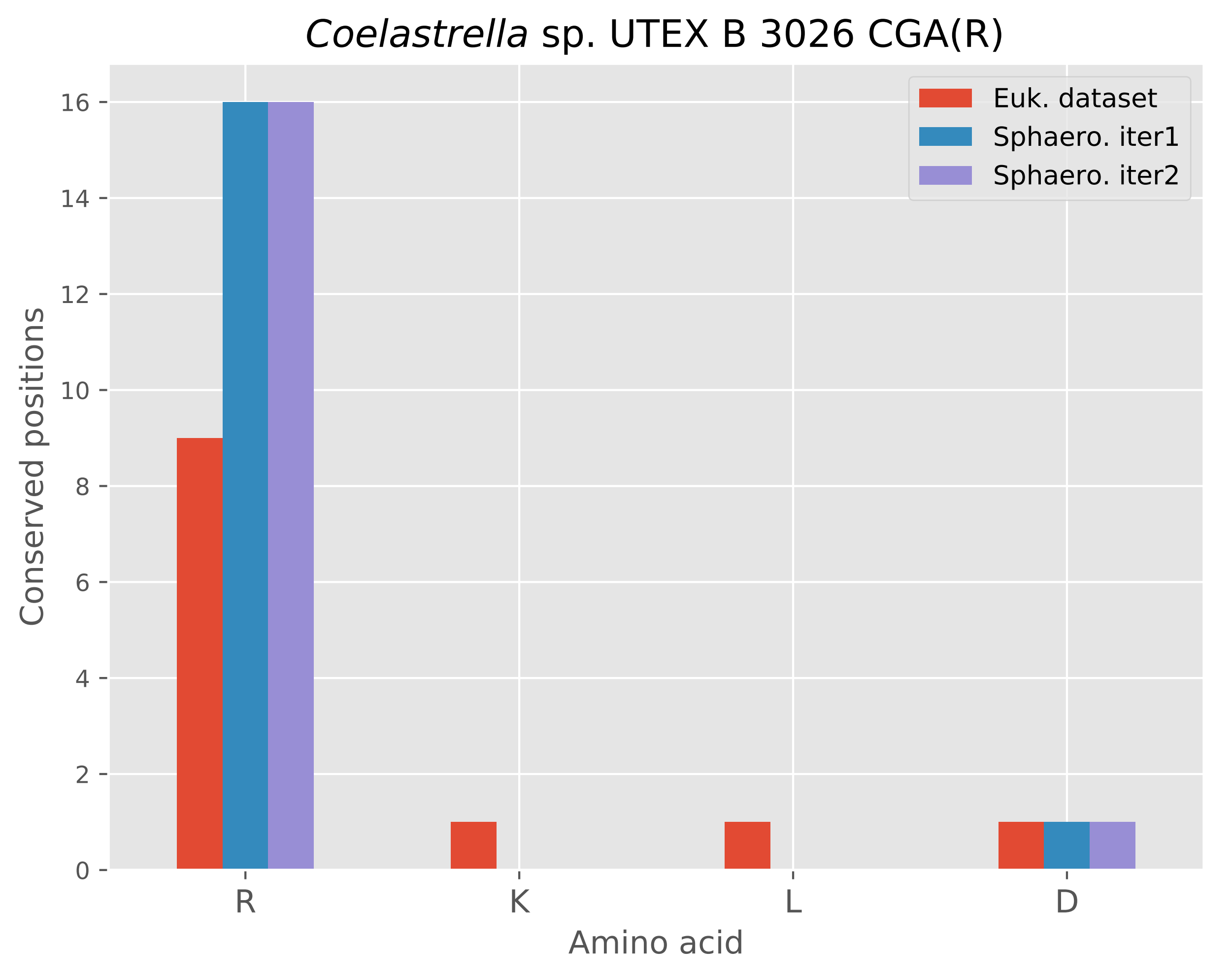
Coelastrella sp. UTEX B 3026 CGA(R)
Euk. dataset
Sphaero. iter1
Sphaero. iter2
Conserved positions
0
2
4
6
8
10
12
14
16
R
K
L
D
Amino acid

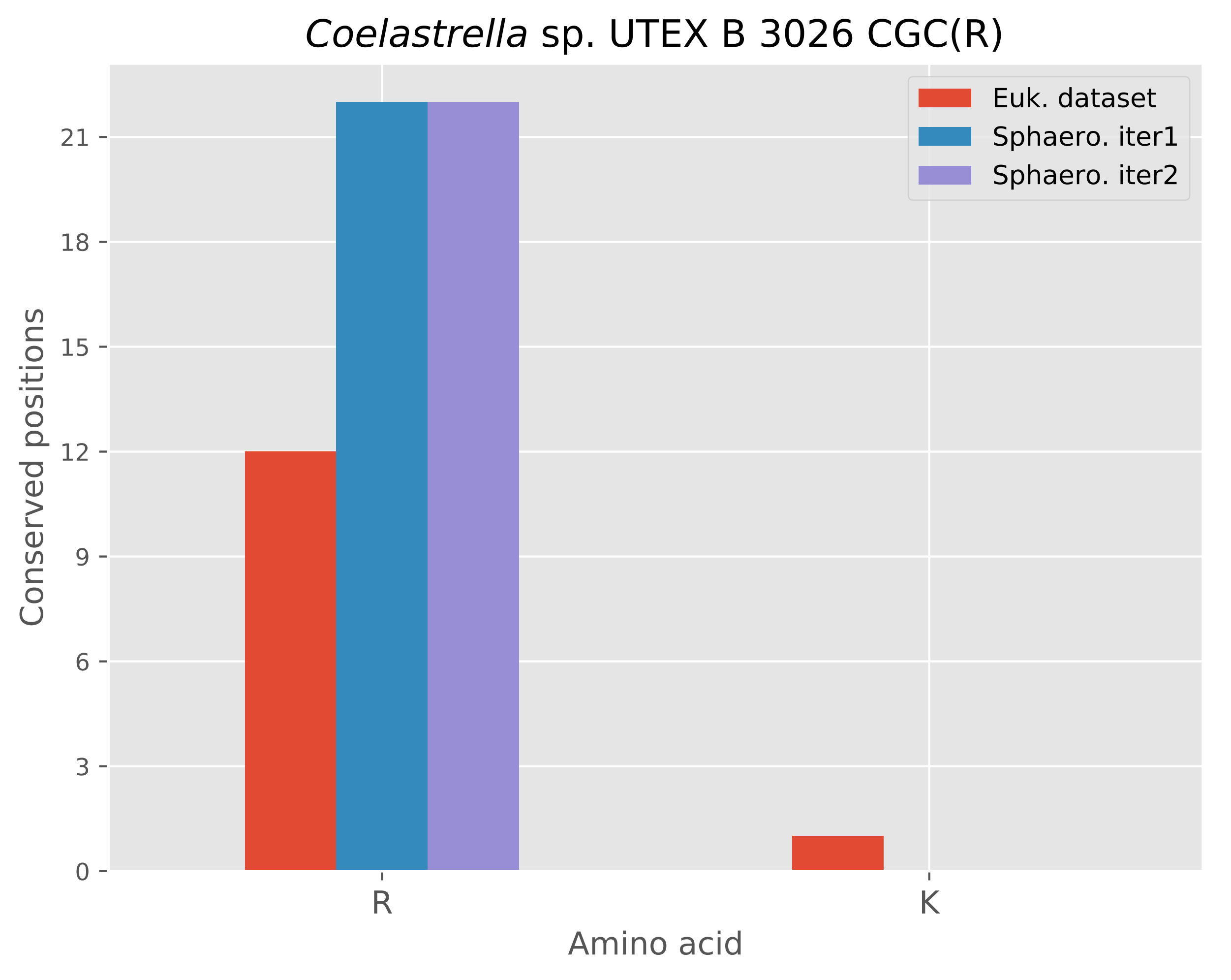

Coelastrella sp. UTEX B 3026 CGC(R)
Euk. dataset
Sphaero. iter1
Sphaero. iter2
Conserved positions
0
3
6
9
12
15
18
21
R
K
Amino acid

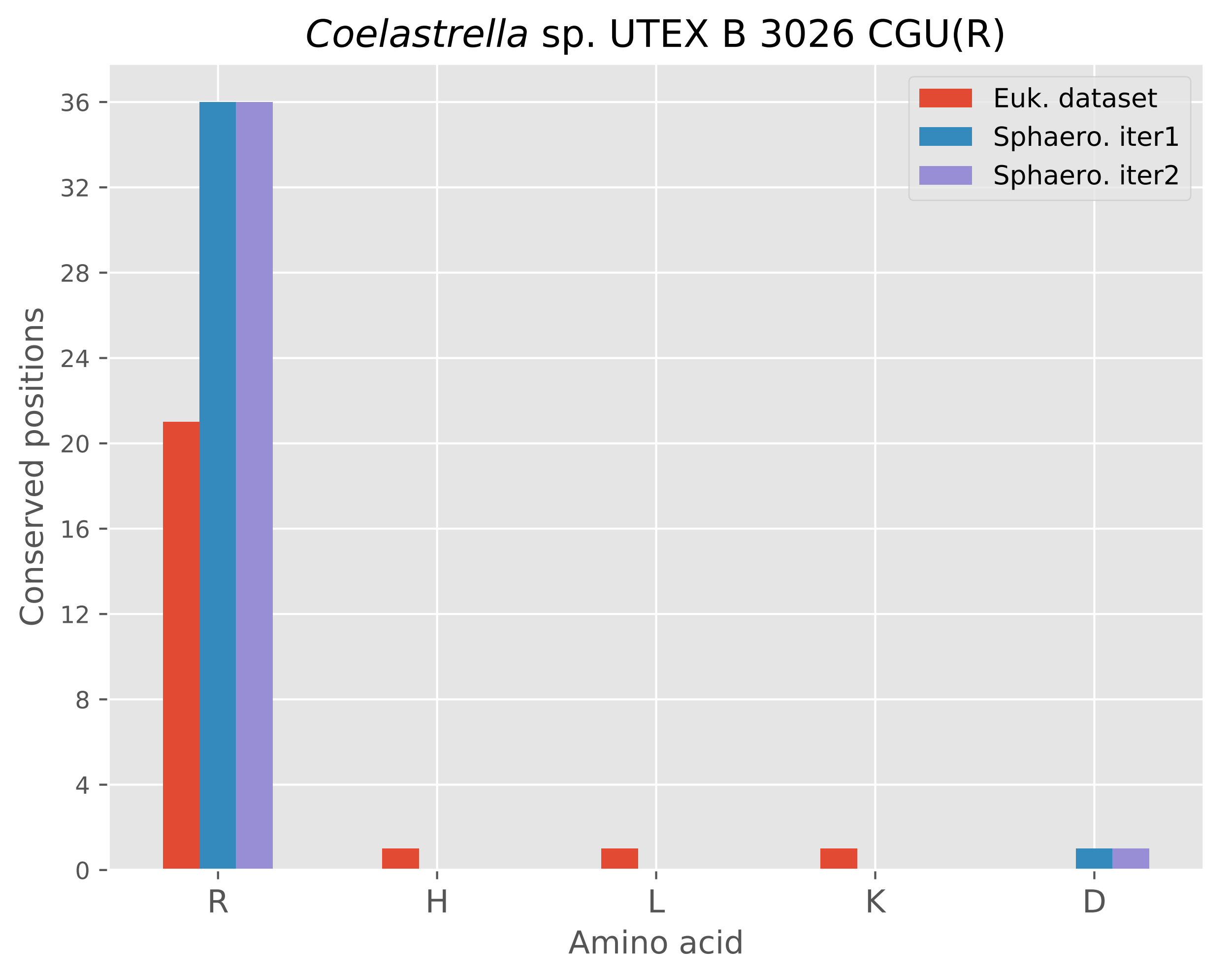

Coelastrella sp. UTEX B 3026 CGU(R)
Conserved positions
0
4
8
12
16
20
24
28
32
36
Euk. dataset
Sphaero. iter1
Sphaero. iter2
R
H
L
K
D
Amino acid

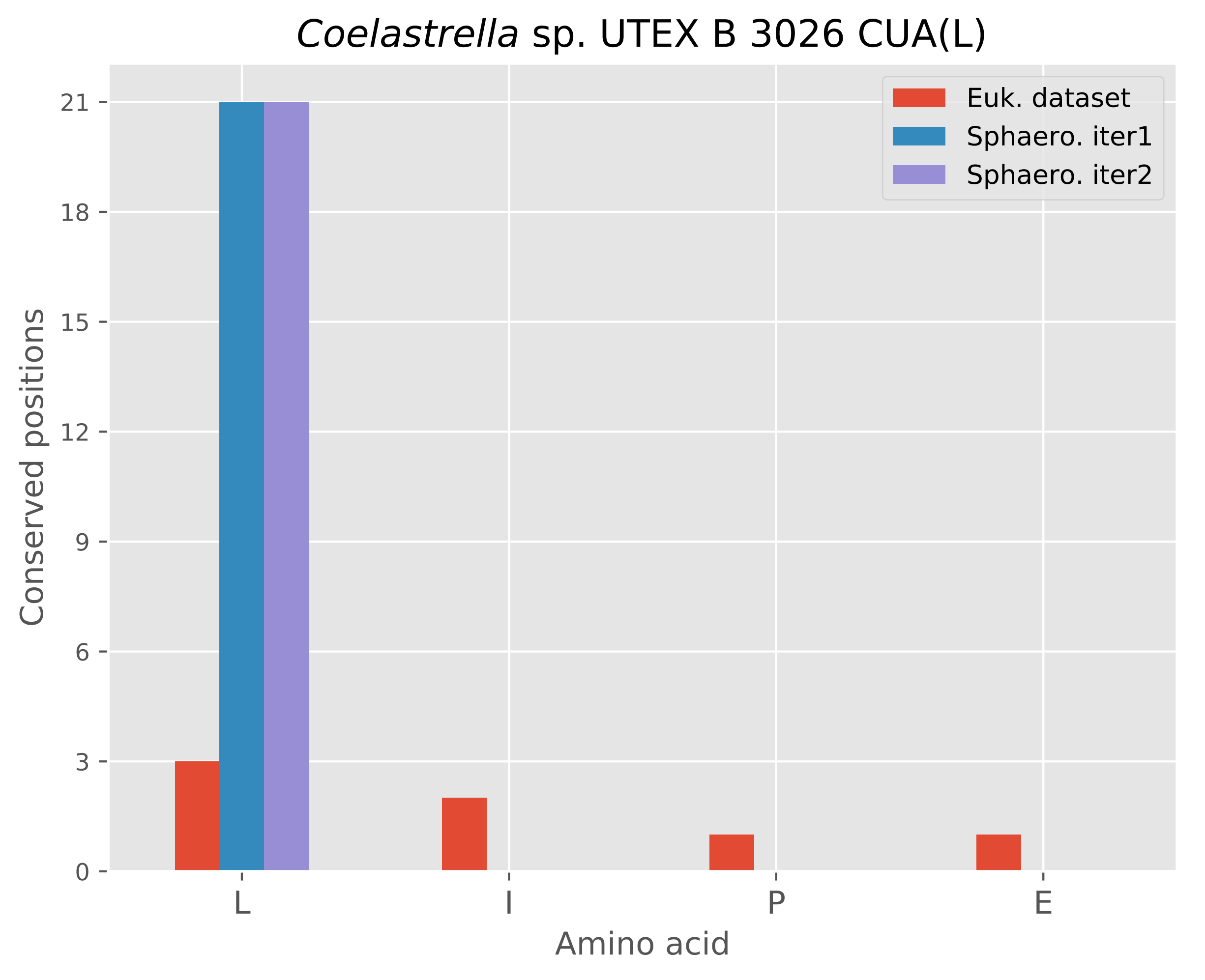

Coelastrella sp. UTEX B 3026 CUA(L)
Euk. dataset
Sphaero. iter1
Sphaero. iter2
Conserved positions
0
3
6
9
12
15
18
21
L
I
P
E
Amino acid

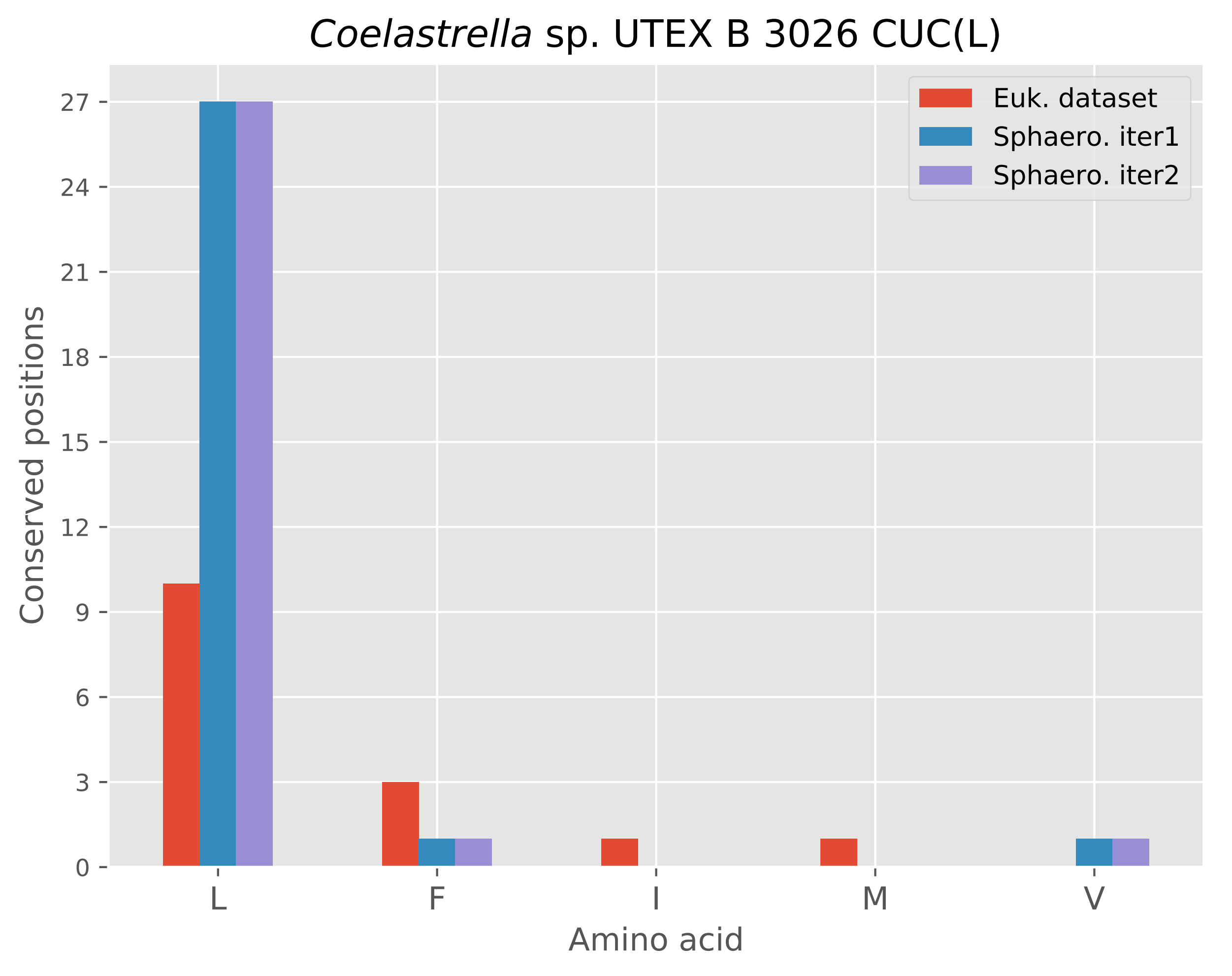

Coelastrella sp. UTEX B 3026 CUC(L)
Euk. dataset
Sphaero. iter1
Sphaero. iter2
Conserved positions
0
3
6
9
12
15
18
21
24
27
L
F
I
M
V
Amino acid

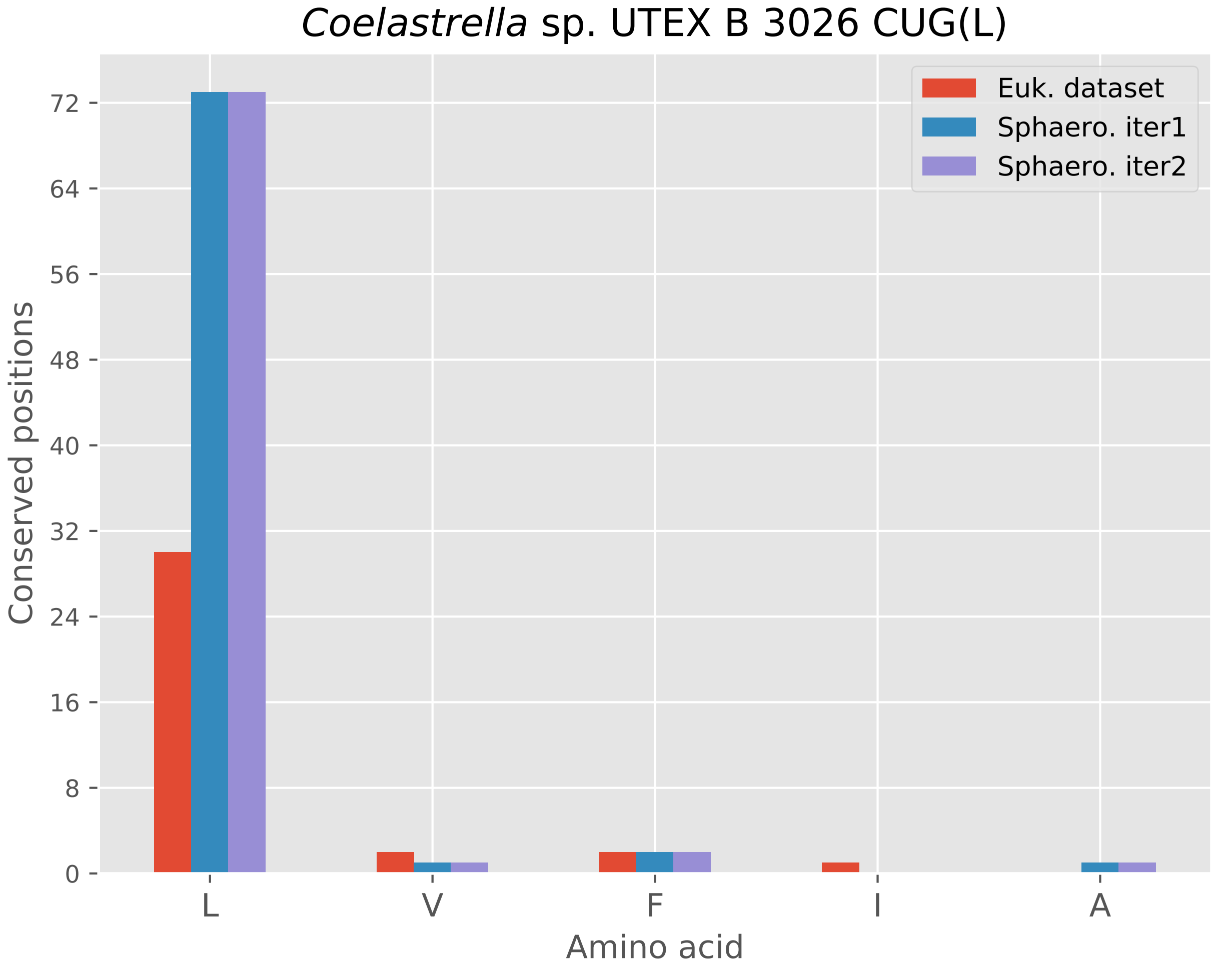

Coelastrella sp. UTEX B 3026 CUG(L)
Euk. dataset
Sphaero. iter1
Sphaero. iter2
Conserved positions
0
8
16
24
32
40
48
56
64
72
L
V
F
I
A
Amino acid

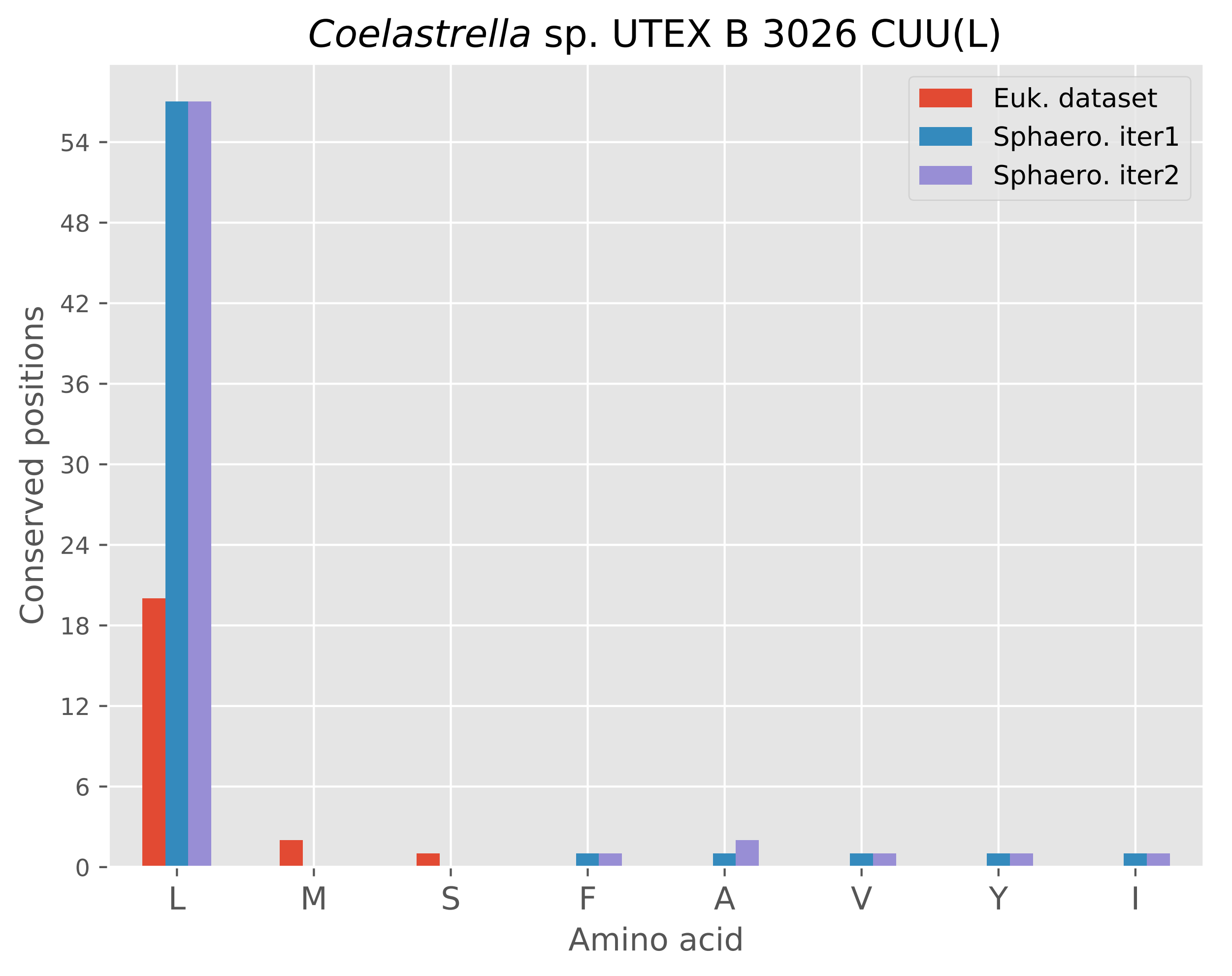
Coelastrella sp. UTEX B 3026 CUU(L)
Euk. dataset
Sphaero. iter1
Sphaero. iter2
Conserved positions
0
6
12
18
24
30
36
42
48
54
L
M
S
F
A
V
Y
I
Amino acid

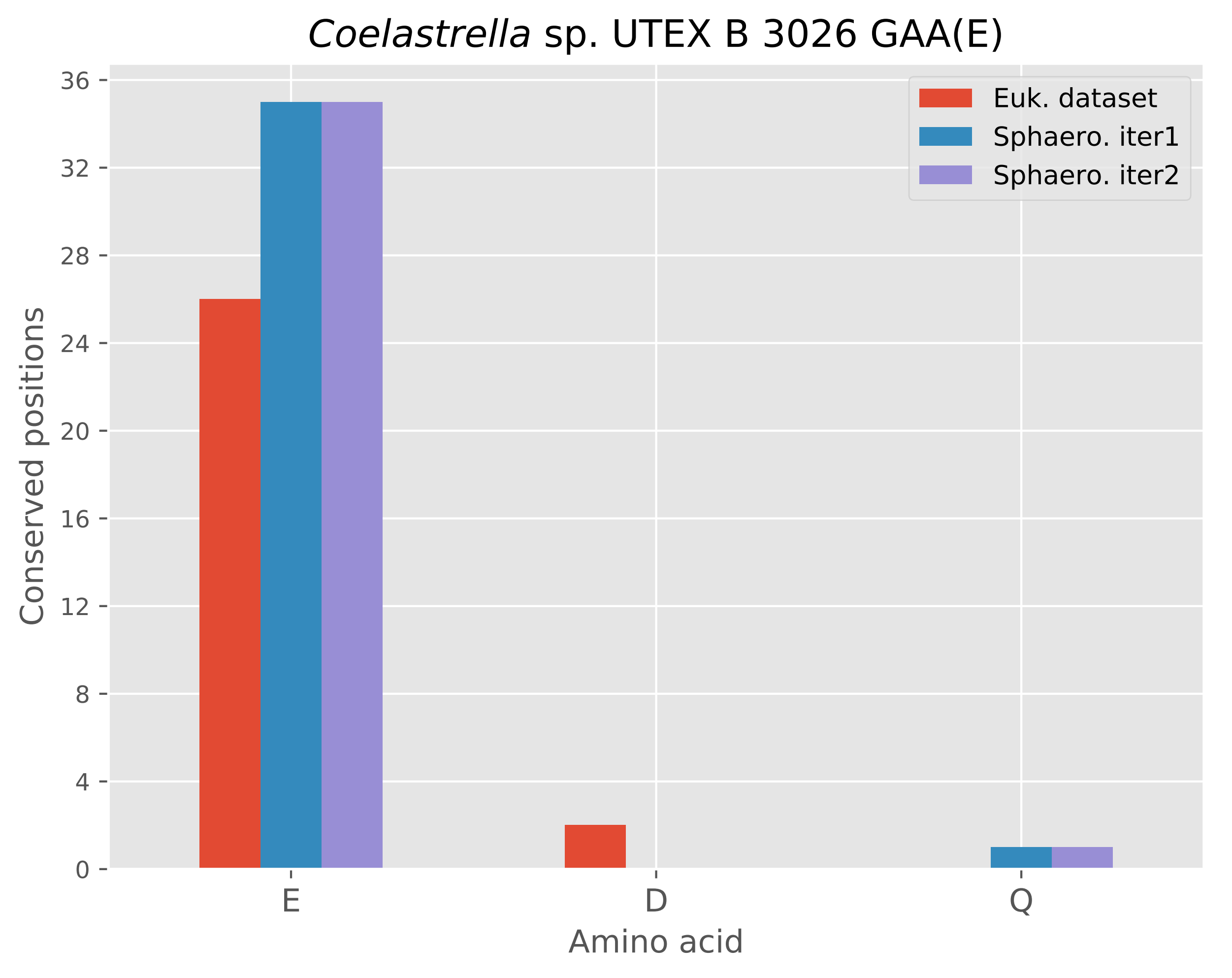
Coelastrella sp. UTEX B 3026 GAA(E)
Euk. dataset
Sphaero. iter1
Sphaero. iter2
Conserved positions
0
4
8
12
16
20
24
28
32
36
E
D
Q
Amino acid

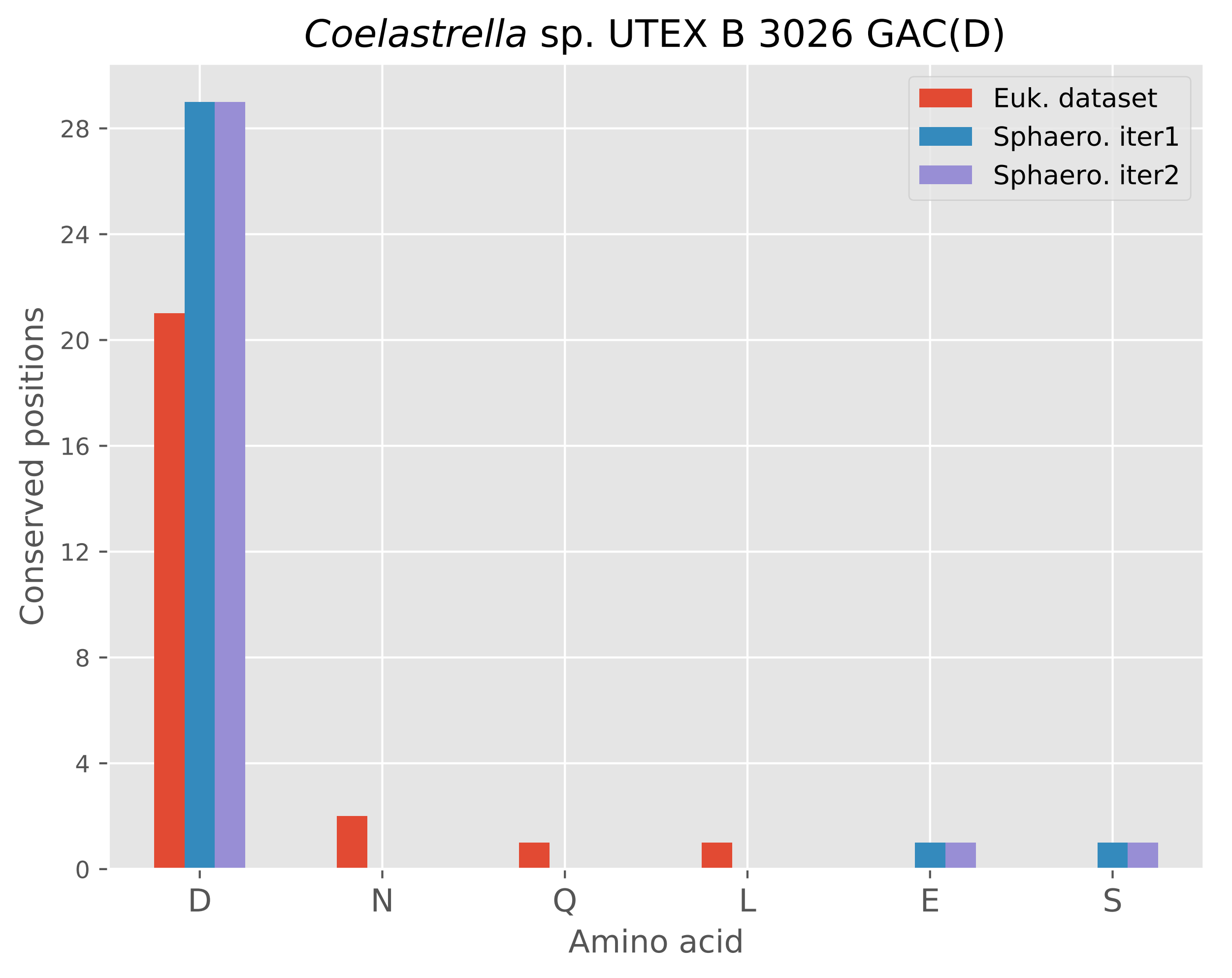

Coelastrella sp. UTEX B 3026 GAC(D)
Euk. dataset
Sphaero. iter1
Sphaero. iter2
Conserved positions
0
4
8
12
16
20
24
28
D
N
Q
L
E
S
Amino acid

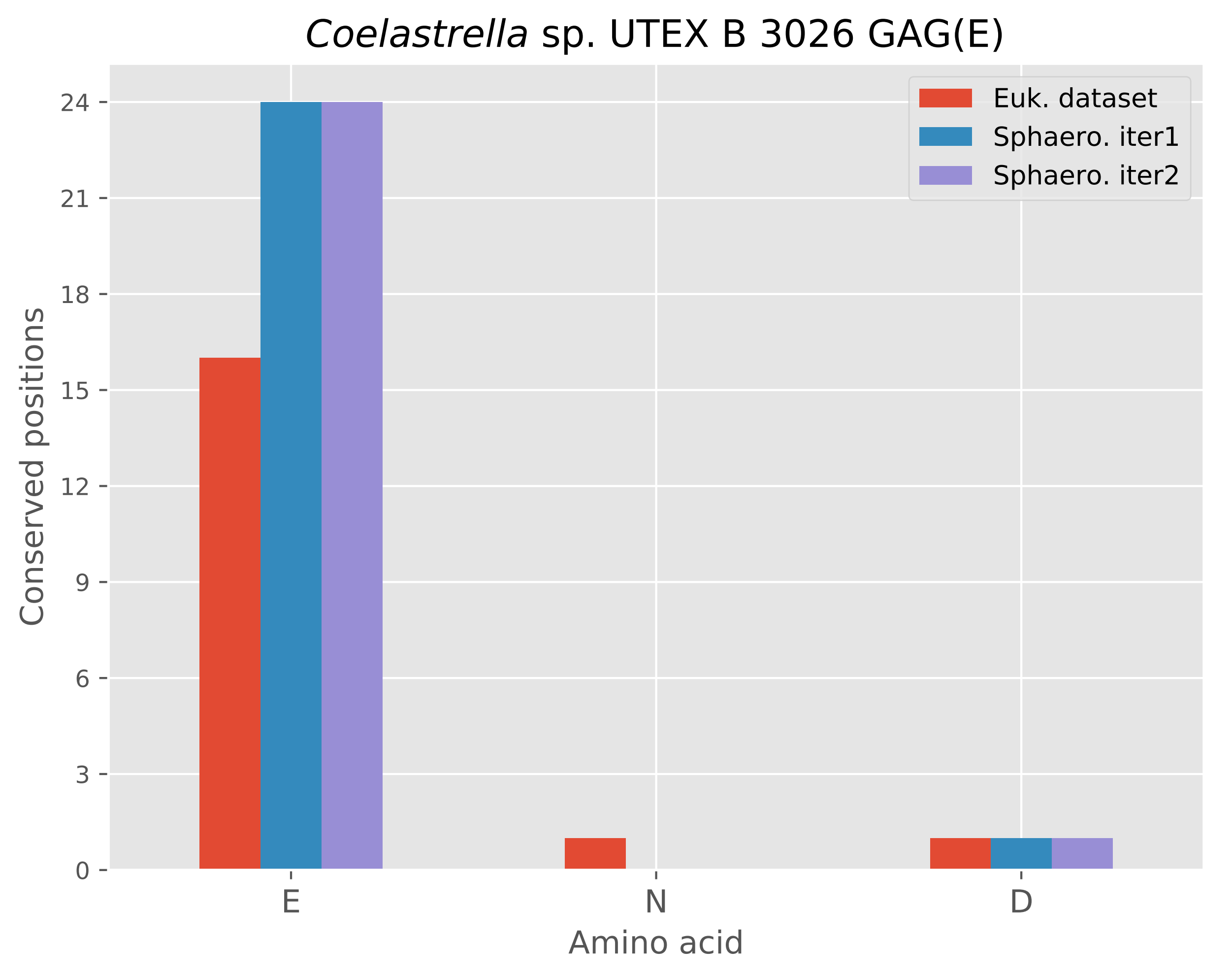

Coelastrella sp. UTEX B 3026 GAG(E)
Euk. dataset
Sphaero. iter1
Sphaero. iter2
Conserved positions
0
3
6
9
12
15
18
21
24
E
N
D
Amino acid

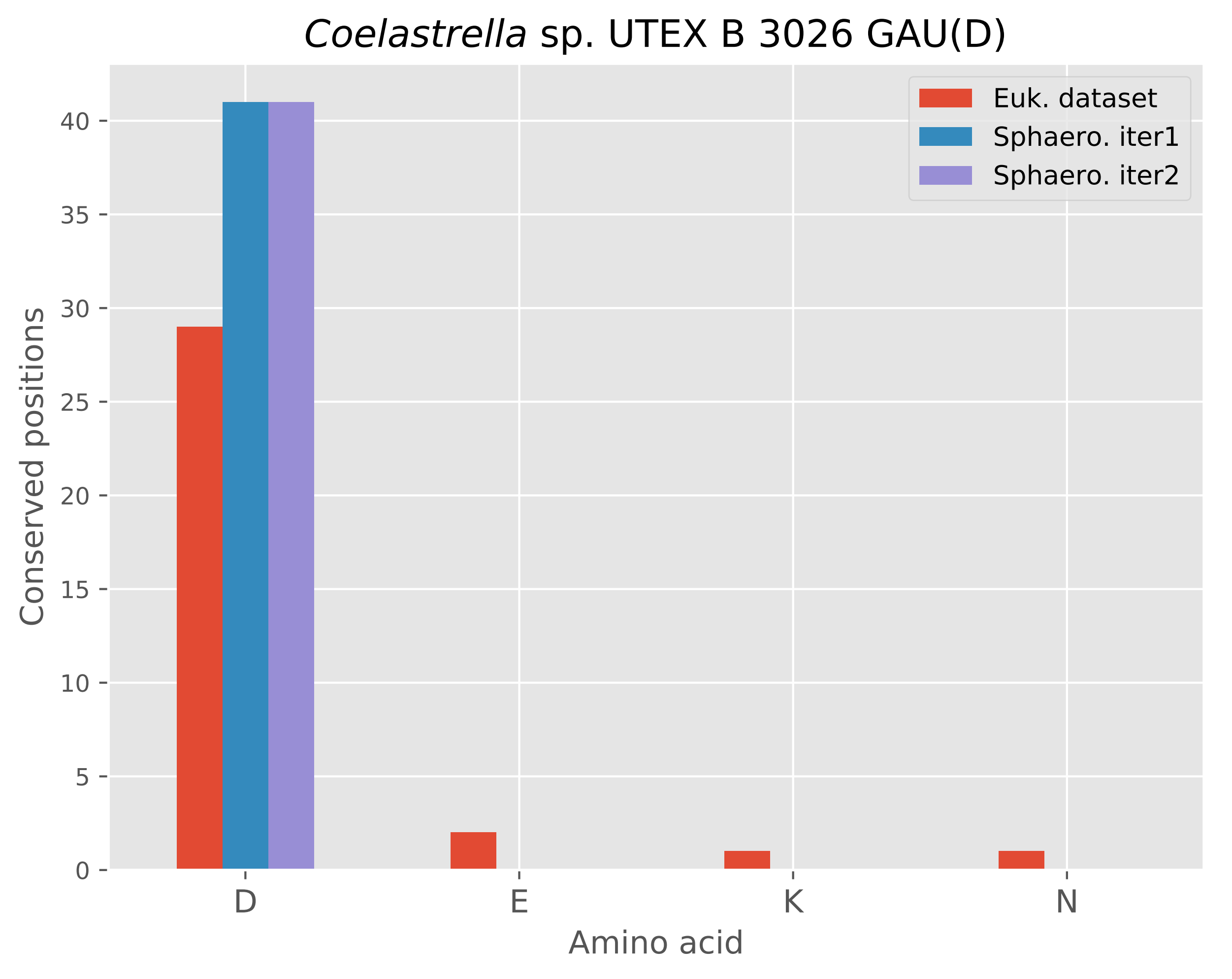

Coelastrella sp. UTEX B 3026 GAU(D)
Euk. dataset
Sphaero. iter1
Sphaero. iter2
Conserved positions
0
5
10
15
20
25
30
35
40
D
E
K
N
Amino acid

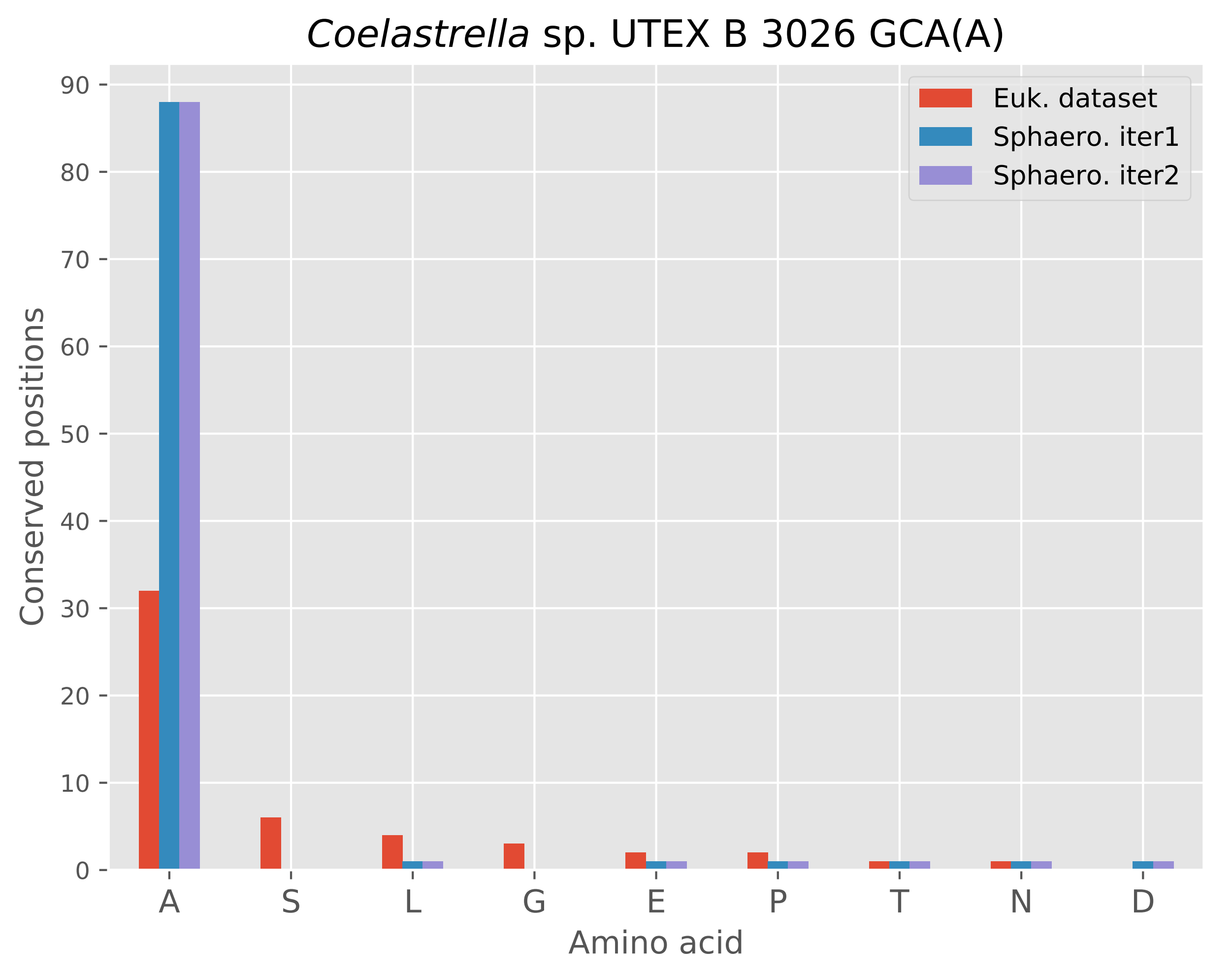

Coelastrella sp. UTEX B 3026 GCA(A)
Euk. dataset
Sphaero. iter1
Sphaero. iter2
Conserved positions
0
10
20
30
40
50
60
70
80
90
A
S
L
G
E
P
T
N
D
Amino acid

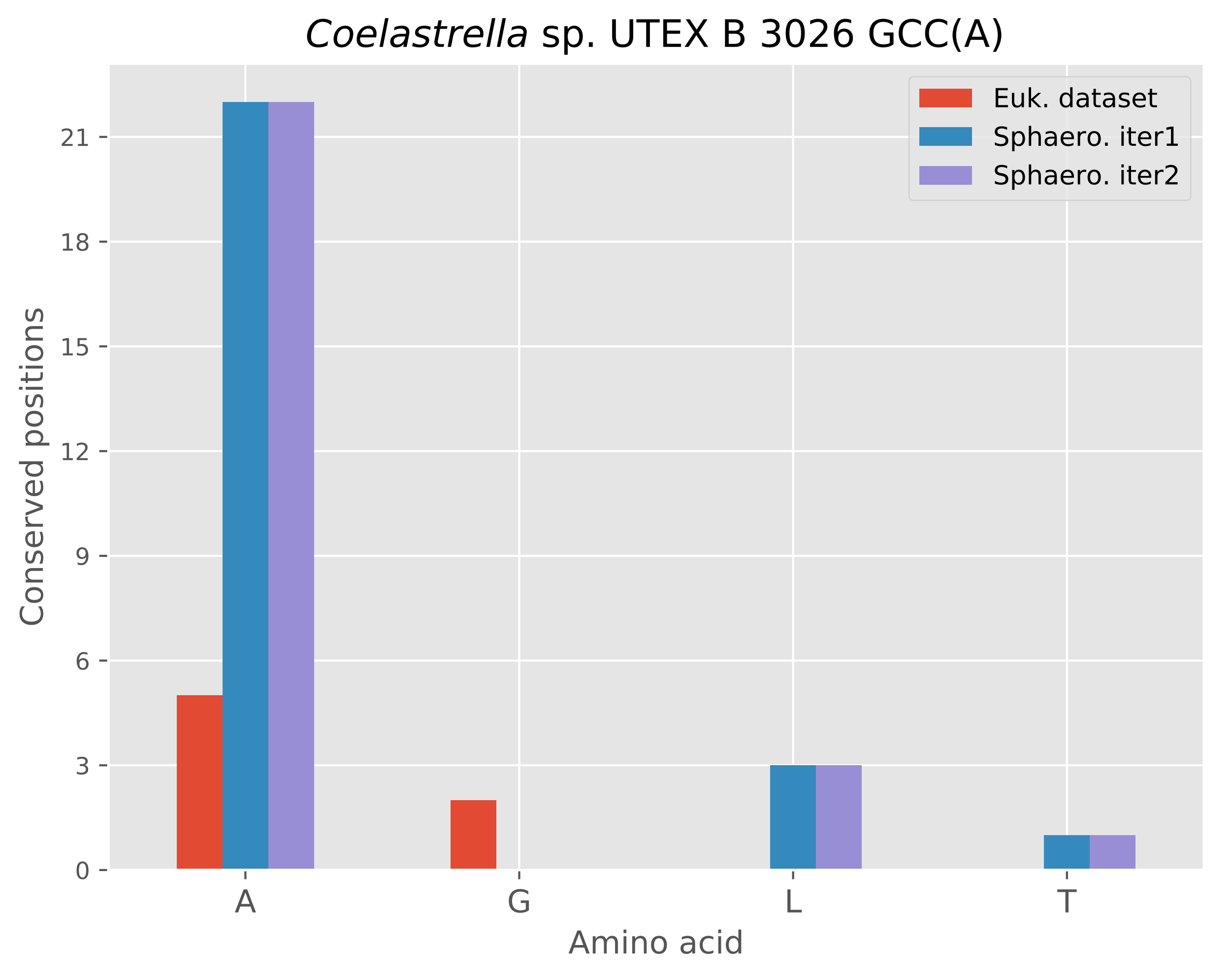

Coelastrella sp. UTEX B 3026 GCC(A)
Conserved positions
0
3
6
9
12
15
18
21
A
G
L
T
Amino acid
Euk. dataset
Sphaero. iter1
Sphaero. iter2

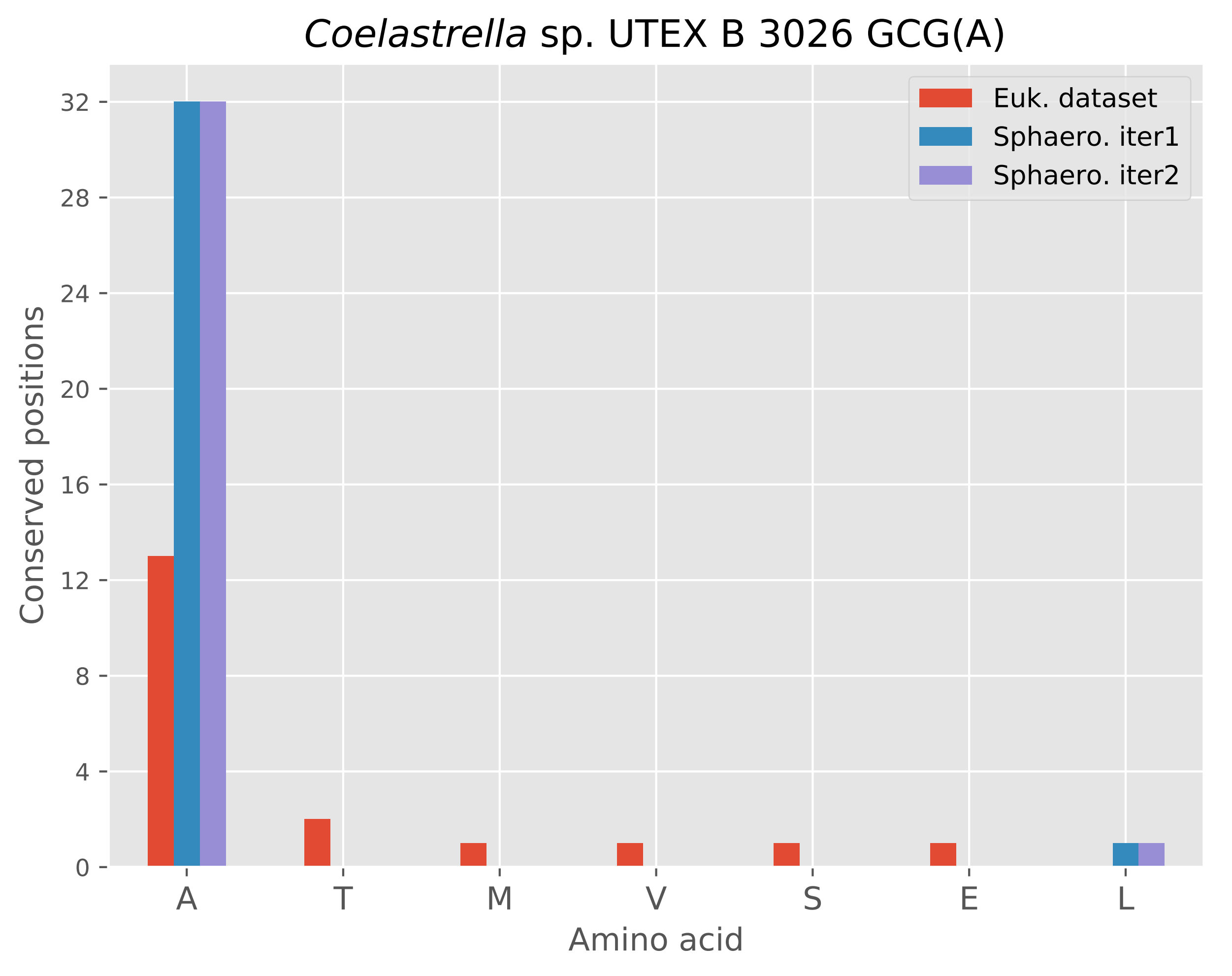

Coelastrella sp. UTEX B 3026 GCG(A)
Euk. dataset
Sphaero. iter1
Sphaero. iter2
Conserved positions
0
4
8
12
16
20
24
28
32
A
T
M
V
S
E
L
Amino acid

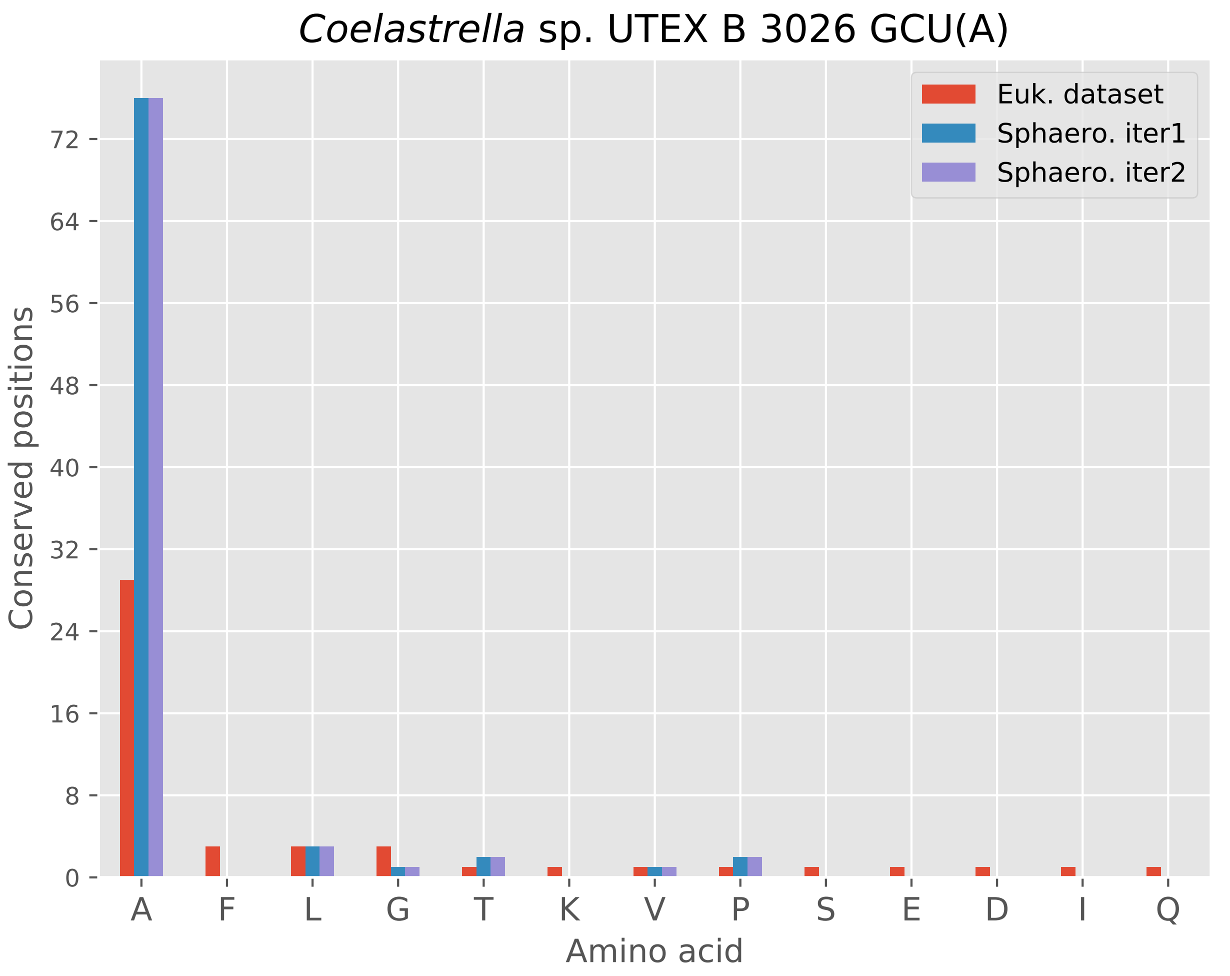

Coelastrella sp. UTEX B 3026 GCU(A)
Euk. dataset
Sphaero. iter1
Sphaero. iter2
Conserved positions
0
8
16
24
32
40
48
56
64
72
A
F
L
G
T
K
V
P
S
E
D
I
Q
Amino acid

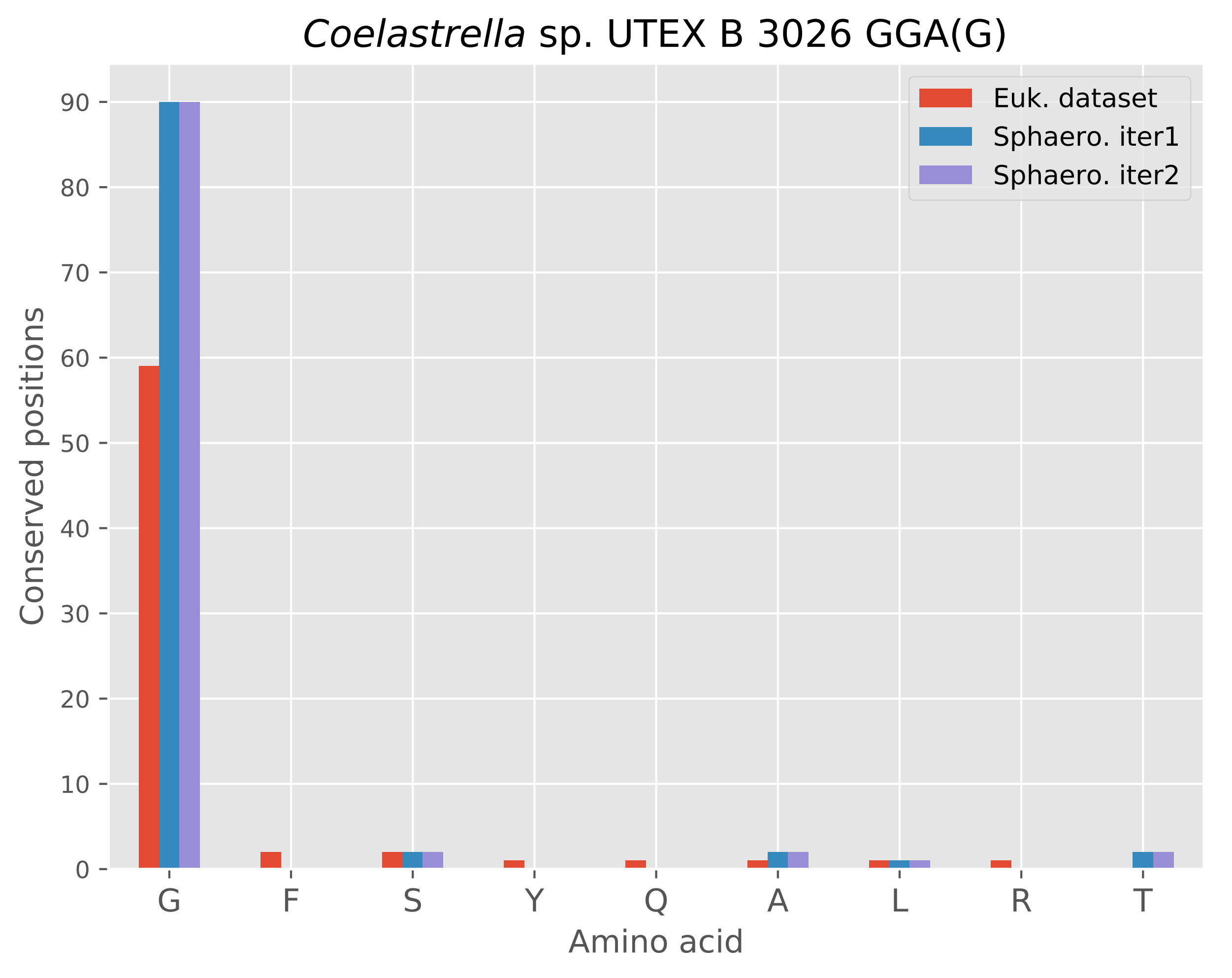

Coelastrella sp. UTEX B 3026 GGA(G)
Euk. dataset
Sphaero. iter1
Sphaero. iter2
Conserved positions
0
10
20
30
40
50
60
70
80
90
G
F
S
Y
Q
A
L
R
T
Amino acid

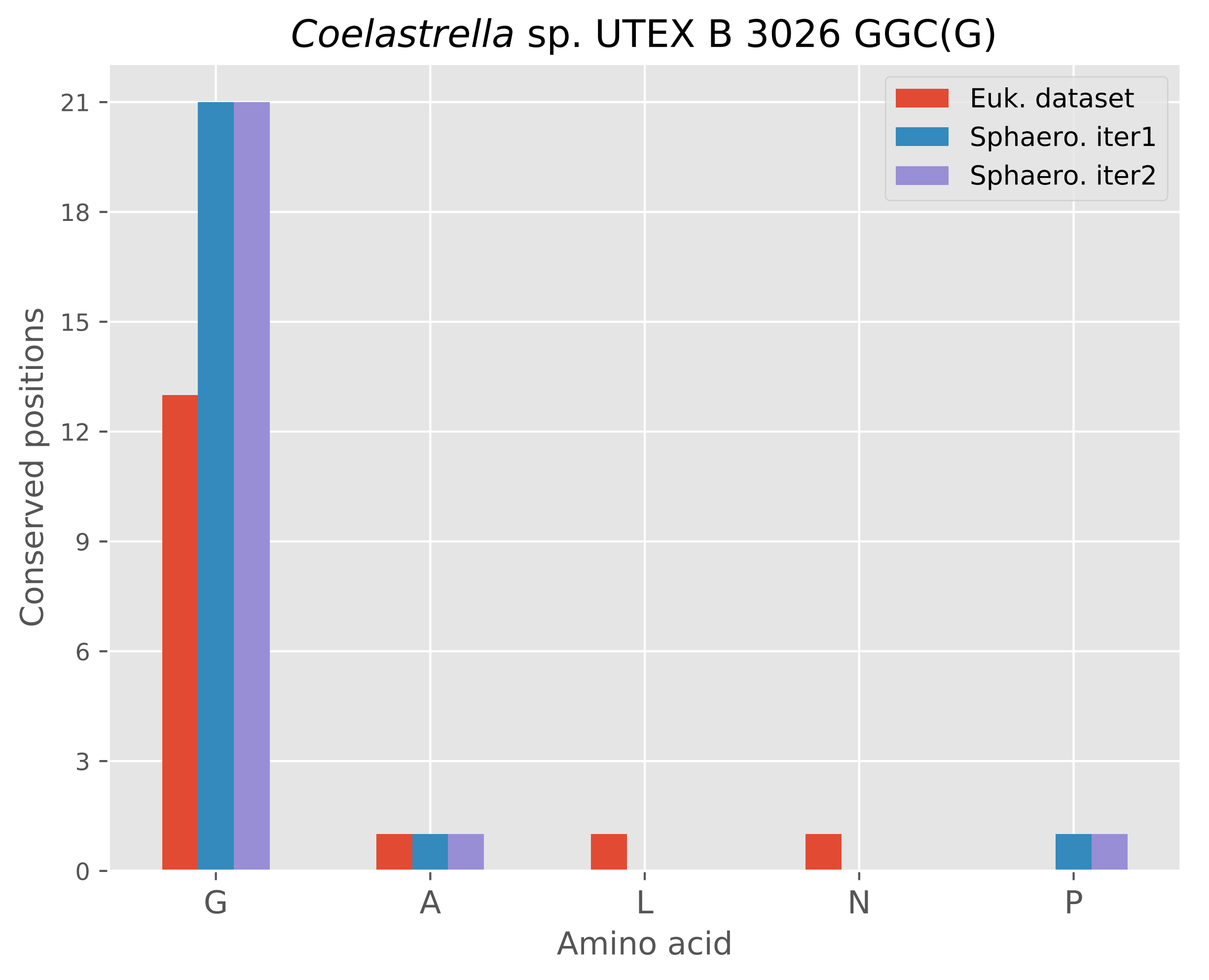

Coelastrella sp. UTEX B 3026 GGC(G)
Euk. dataset
Sphaero. iter1
Sphaero. iter2
Conserved positions
Amino acid
0
3
6
9
12
15
18
21
G
A
L
N
P

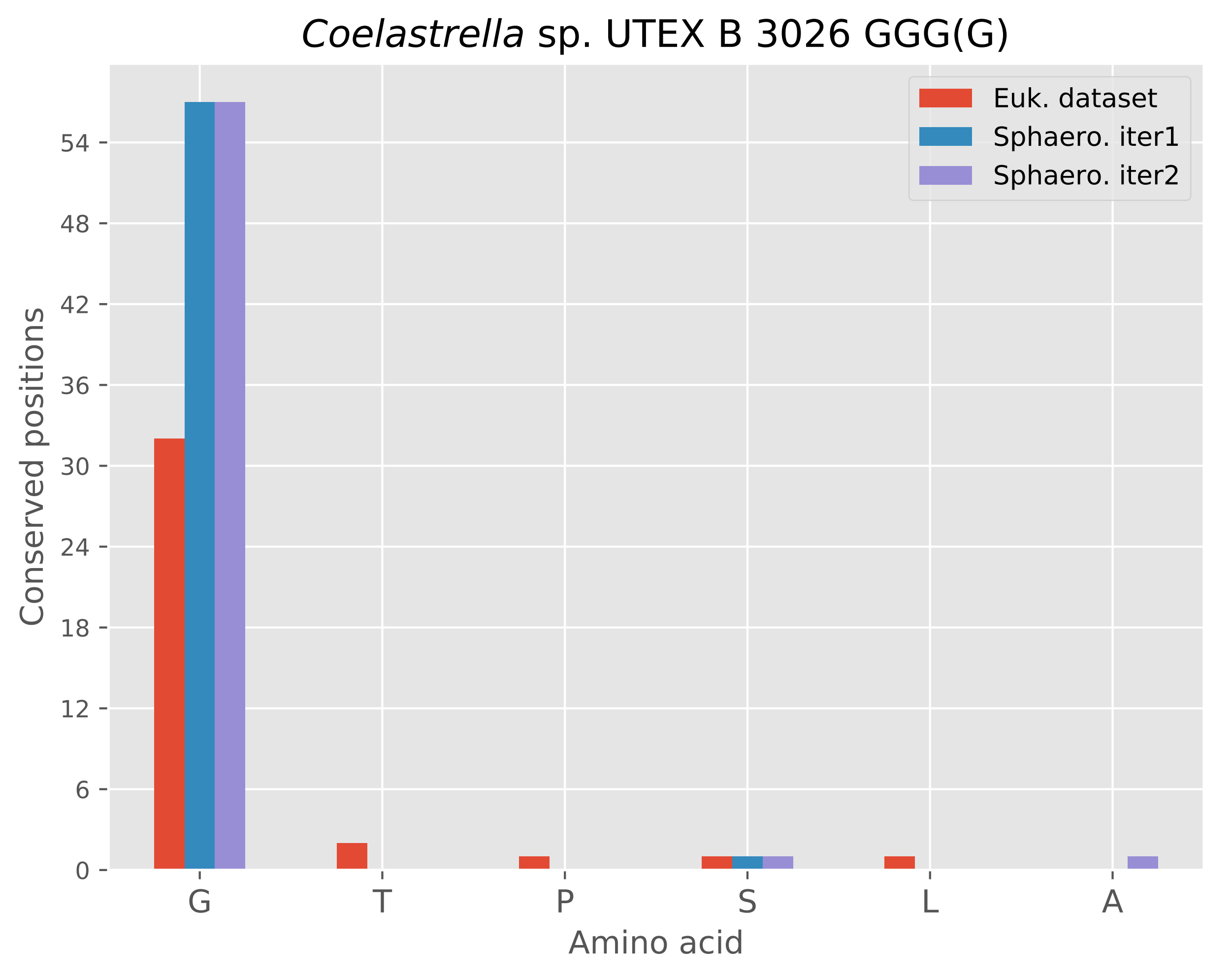

Coelastrella sp. UTEX B 3026 GGG(G)
Euk. dataset
Sphaero. iter1
Sphaero. iter2
Conserved positions
0
6
12
18
24
30
36
42
48
54
G
T
P
S
L
A
Amino acid

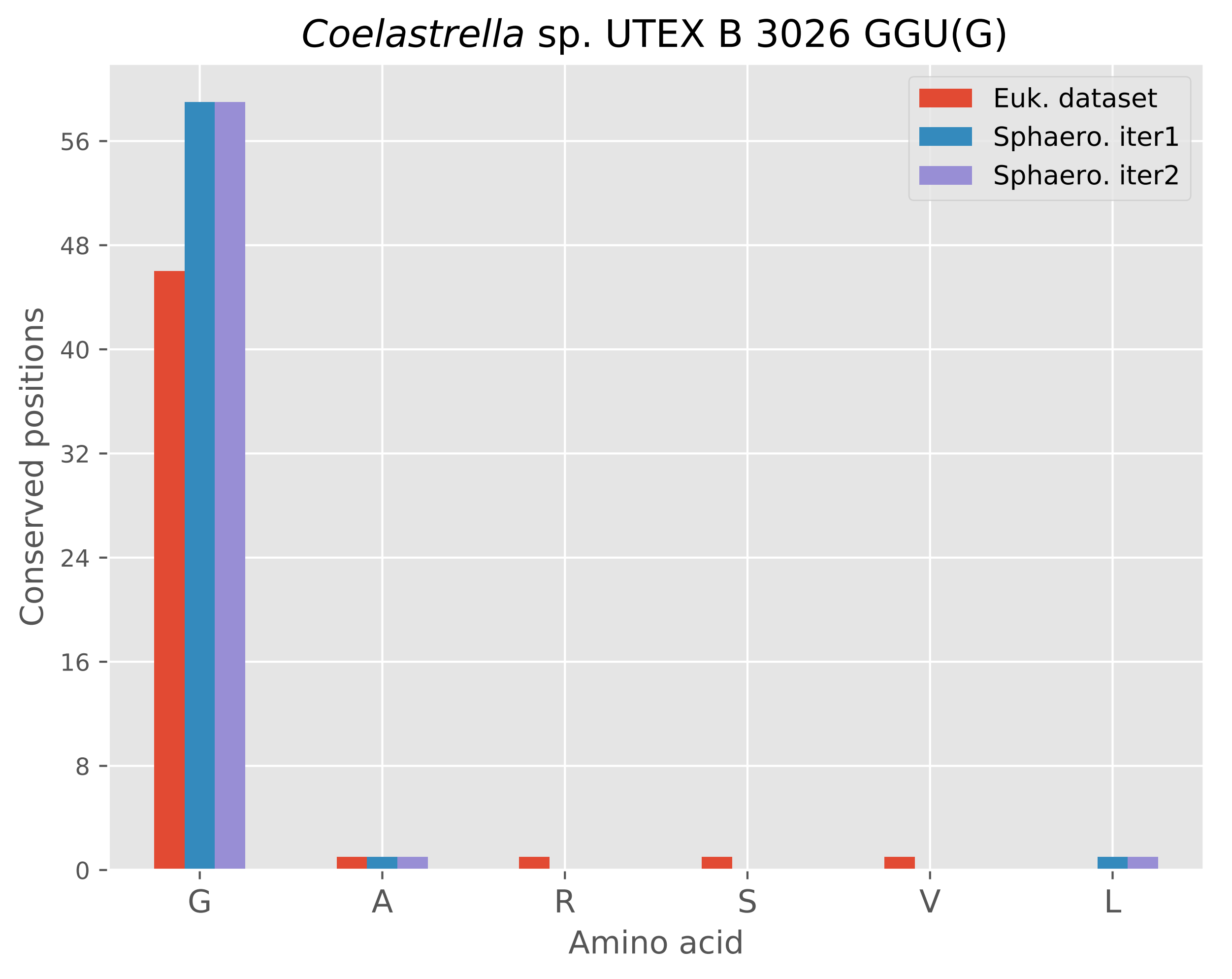

Coelastrella sp. UTEX B 3026 GGU(G)
Euk. dataset
Sphaero. iter1
Sphaero. iter2
Conserved positions
0
8
16
24
32
40
48
56
G
A
R
S
V
L
Amino acid

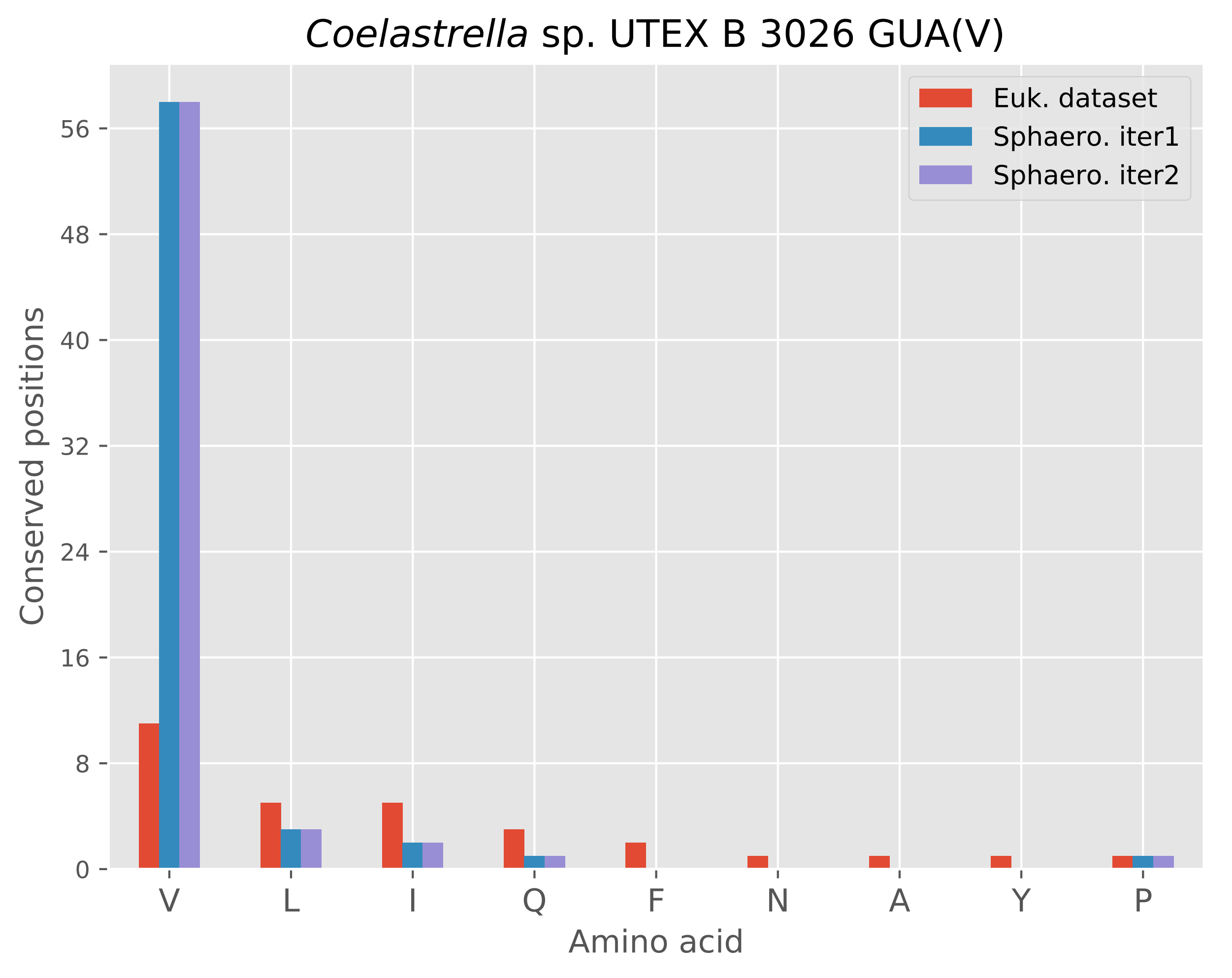

Coelastrella sp. UTEX B 3026 GUA(V)
Euk. dataset
Sphaero. iter1
Sphaero. iter2
Conserved positions
0
8
16
24
32
40
48
56
V
L
I
Q
F
N
A
Y
P
Amino acid

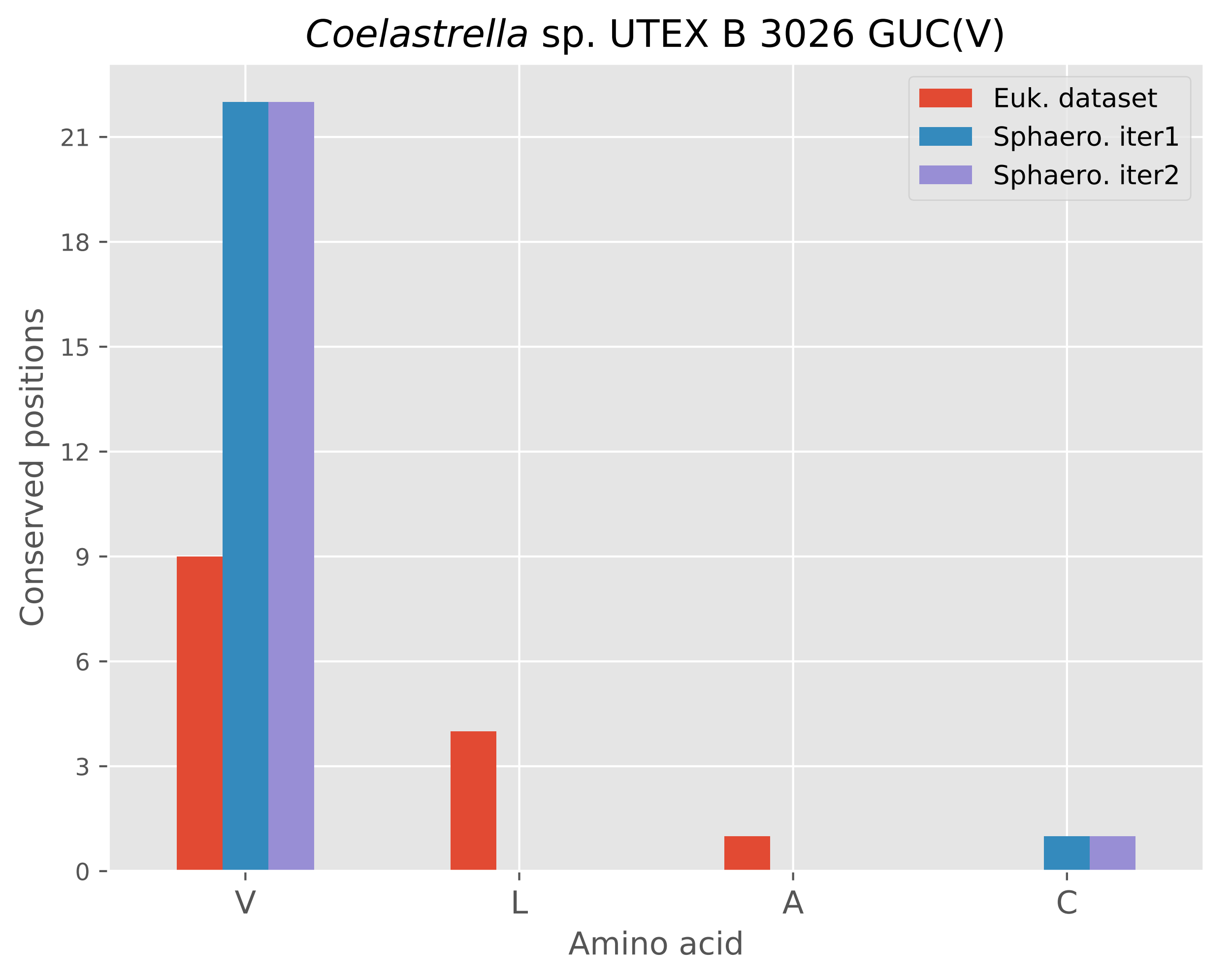

Coelastrella sp. UTEX B 3026 GUC(V)
Euk. dataset
Sphaero. iter1
Sphaero. iter2
Conserved positions
0
3
6
9
12
15
18
21
V
L
A
C
Amino acid

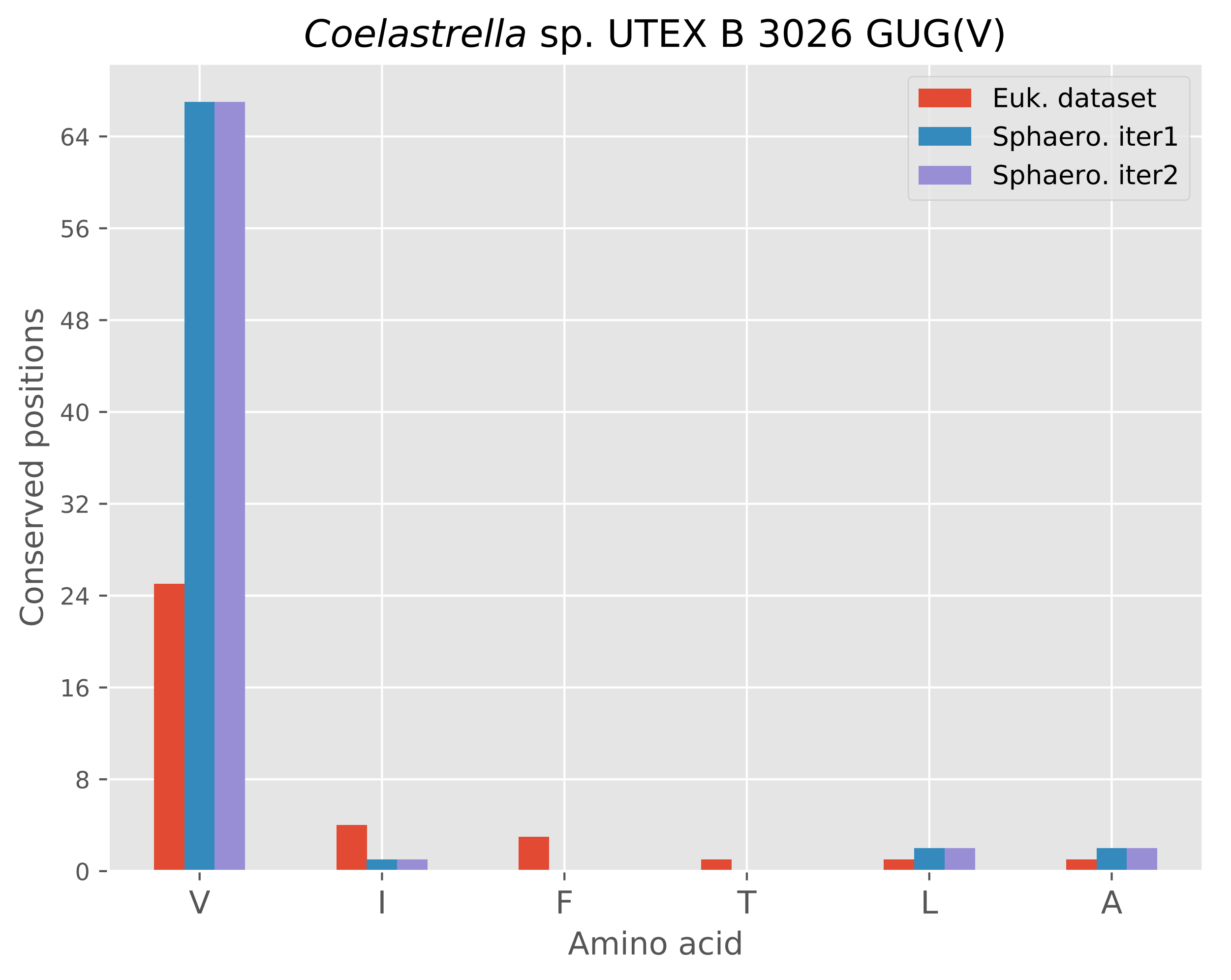
Coelastrella sp. UTEX B 3026 GUG(V)
Euk. dataset
Sphaero. iter1
Sphaero. iter2
Conserved positions
0
8
16
24
32
40
48
56
64
V
I
F
T
L
A
Amino acid

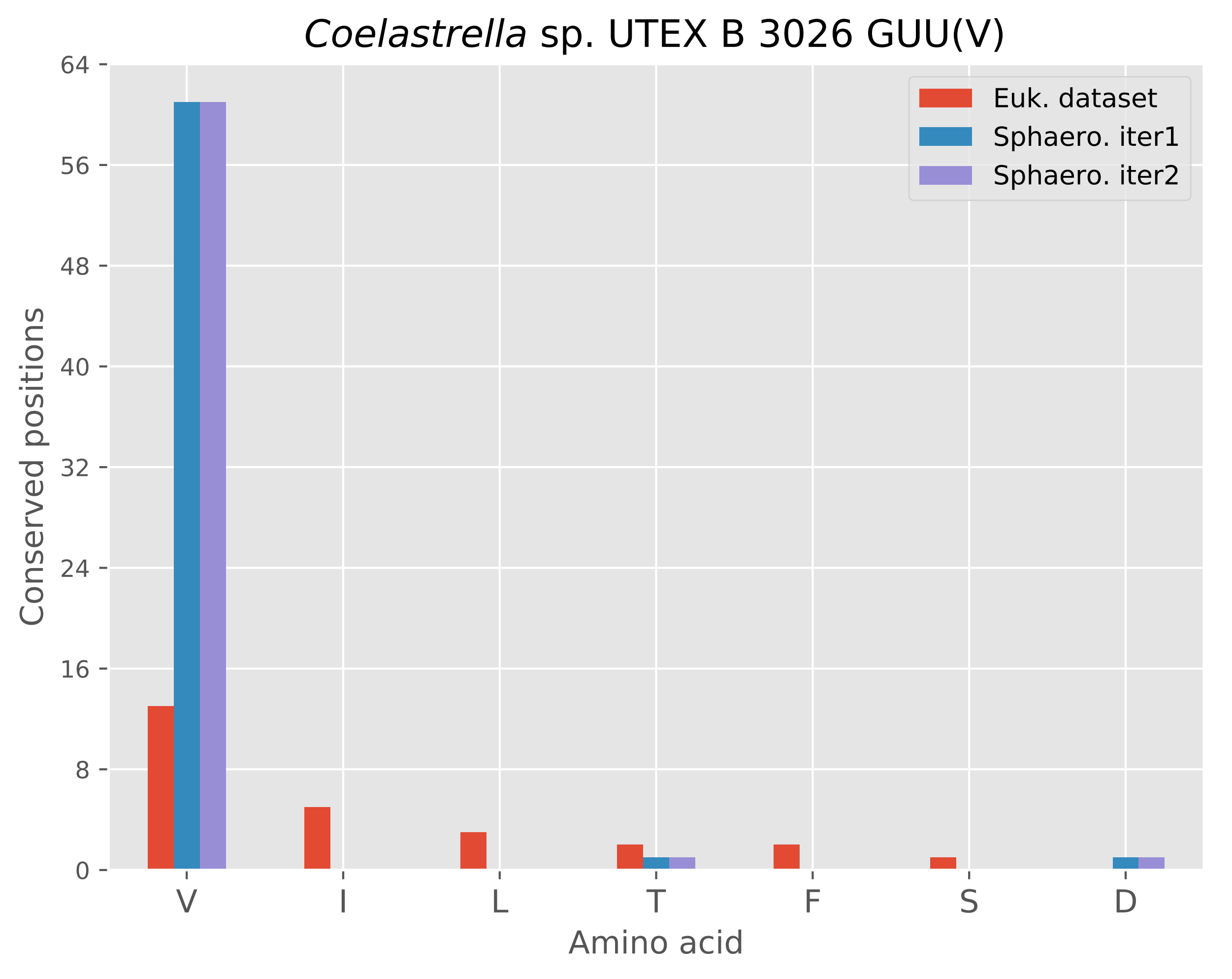

Coelastrella sp. UTEX B 3026 GUU(V)
Euk. dataset
Sphaero. iter1
Sphaero. iter2
Conserved positions
0
8
16
24
32
40
48
56
64
V
I
L
T
F
S
D
Amino acid

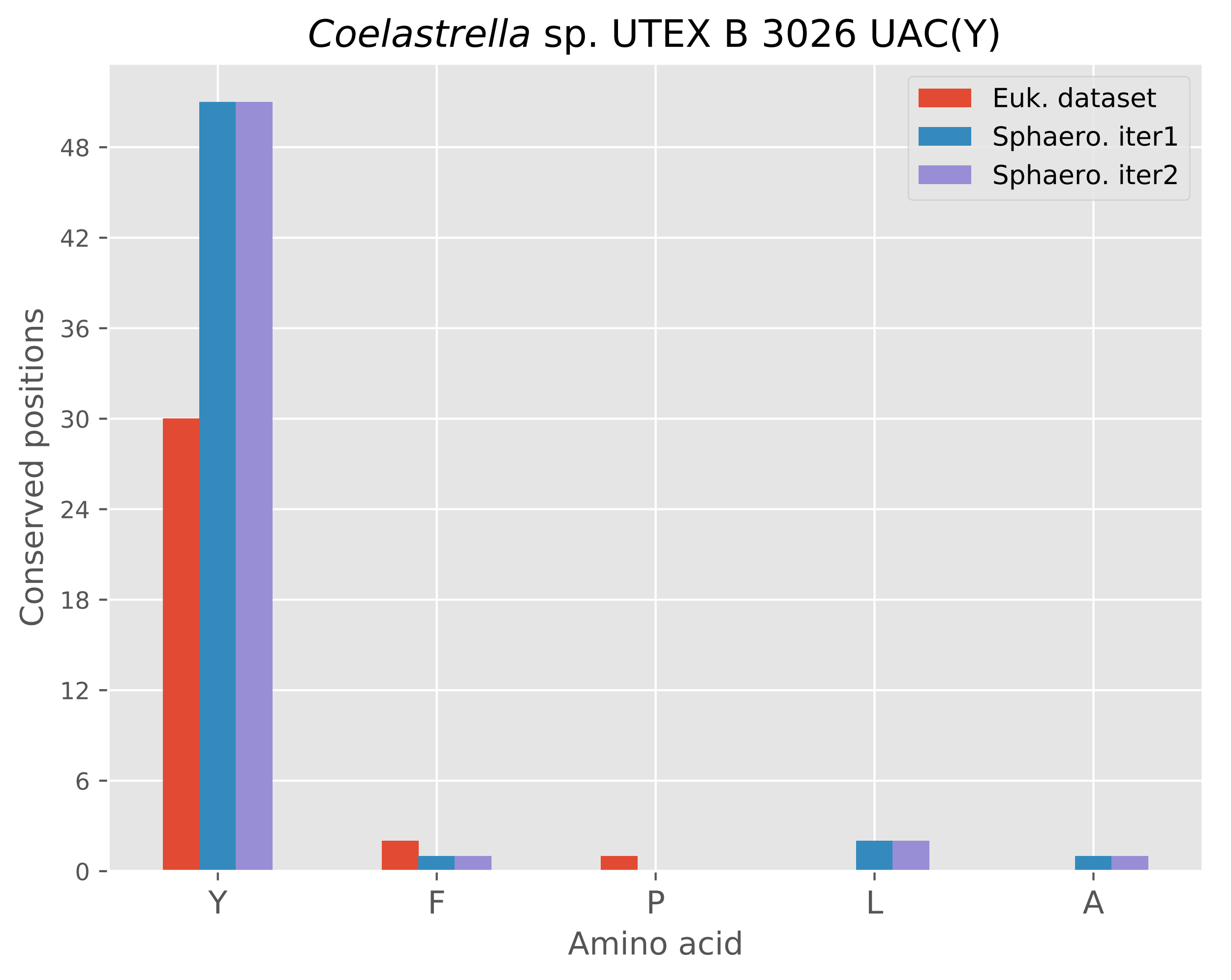

Coelastrella sp. UTEX B 3026 UAC(Y)
Euk. dataset
Sphaero. iter1
Sphaero. iter2
Conserved positions
0
6
12
18
24
30
36
42
48
Y
F
P
L
A
Amino acid

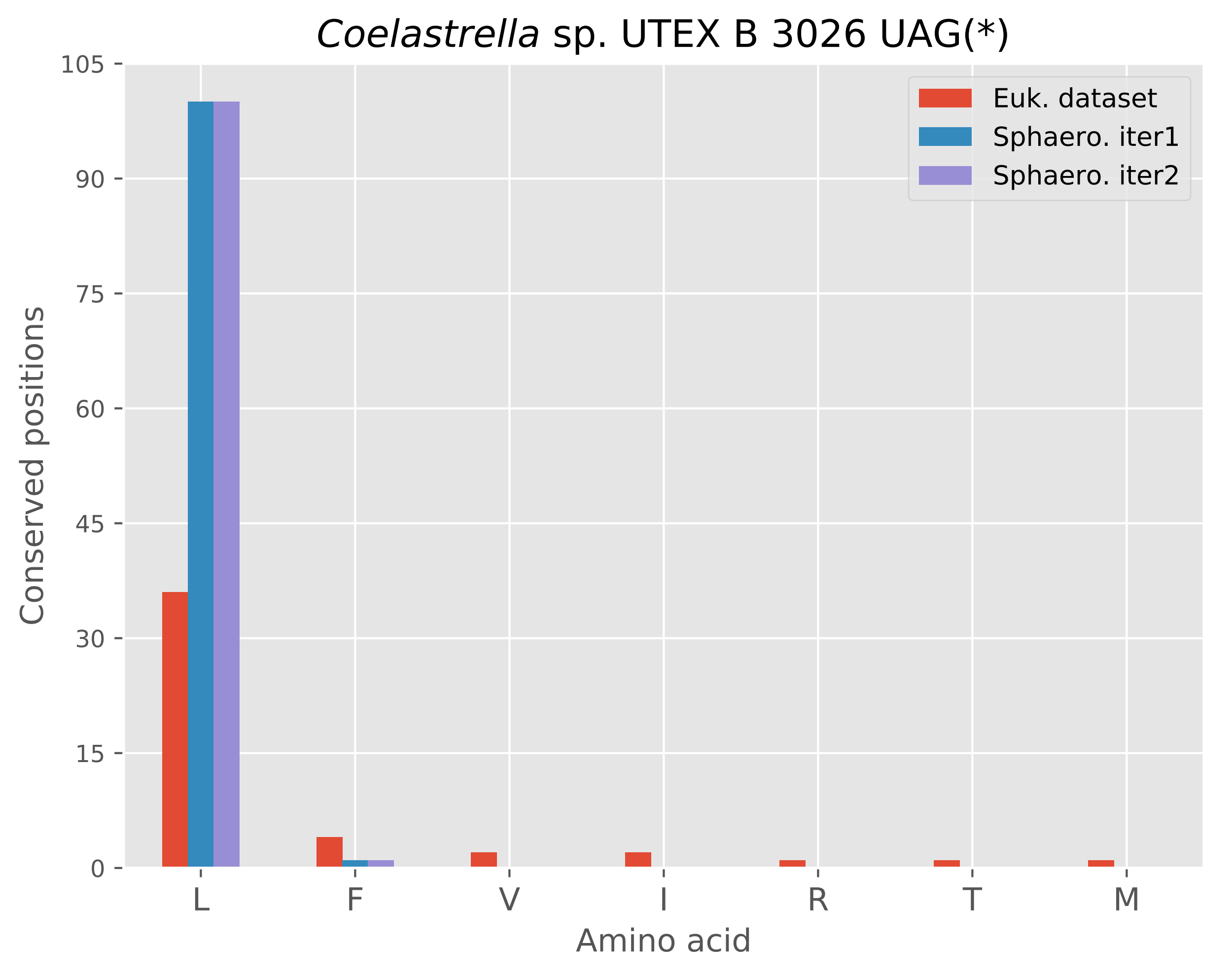
Coelastrella sp. UTEX B 3026 UAG(*)
Euk. dataset
Sphaero. iter1
Sphaero. iter2
Conserved positions
0
15
30
45
60
75
90
105
L
F
V
I
R
T
M
Amino acid

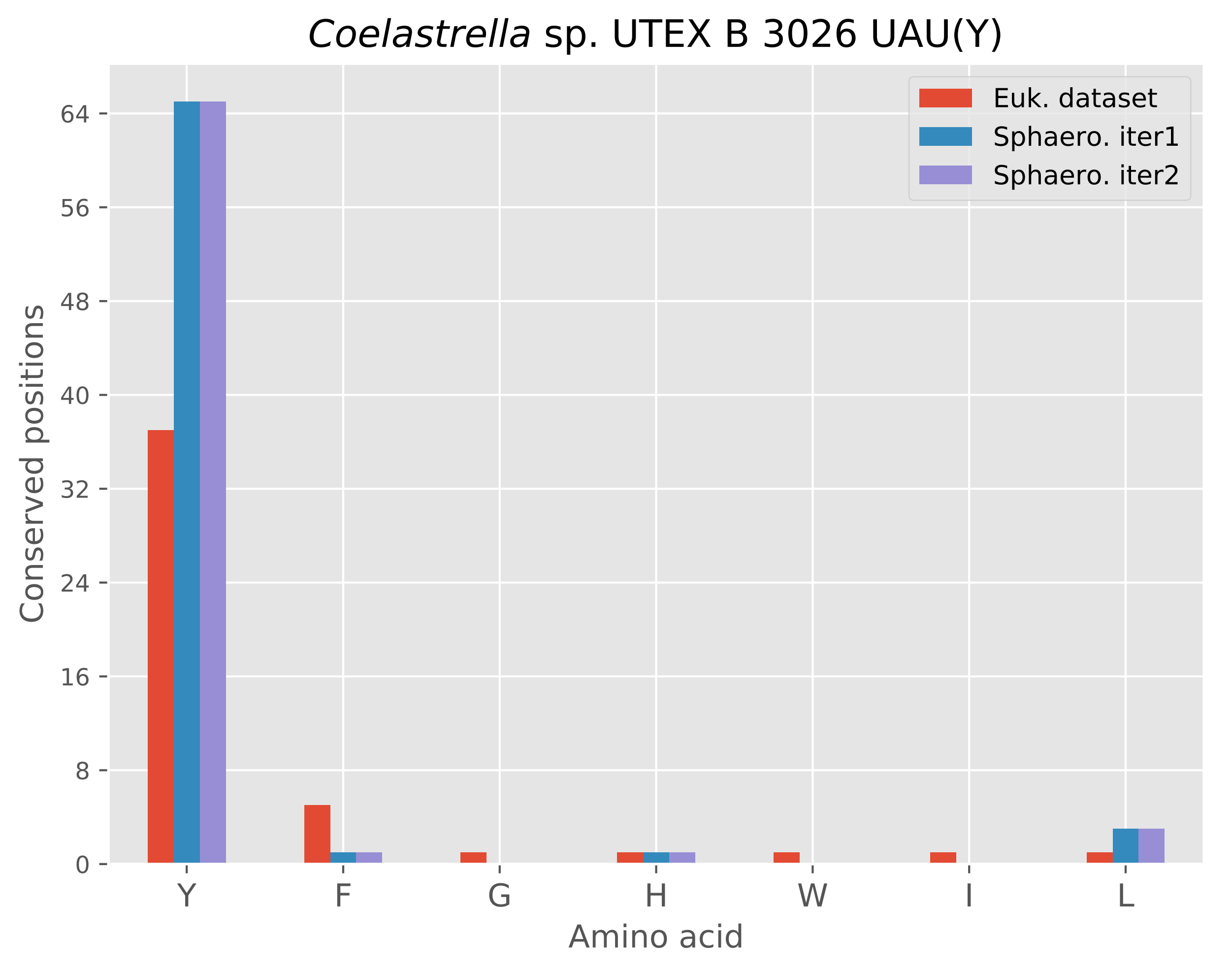
Coelastrella sp. UTEX B 3026 UAU(Y)
Euk. dataset
Sphaero. iter1
Sphaero. iter2
Conserved positions
0
8
16
24
32
40
48
56
64
Y
F
G
H
W
I
L
Amino acid

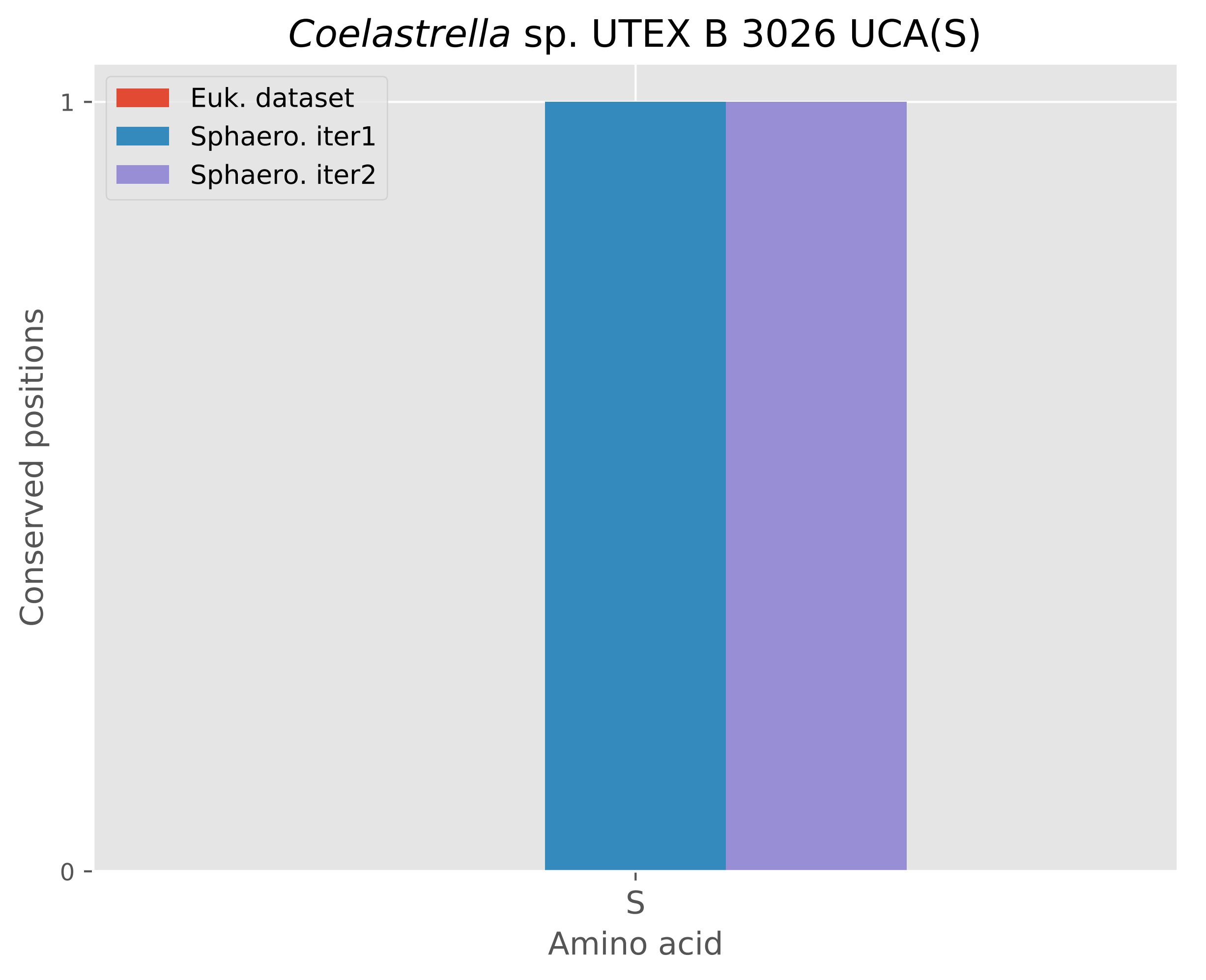

Coelastrella sp. UTEX B 3026 UCA(S)
Euk. dataset
Sphaero. iter1
Sphaero. iter2
1
0
Conserved positions
S
Amino acid

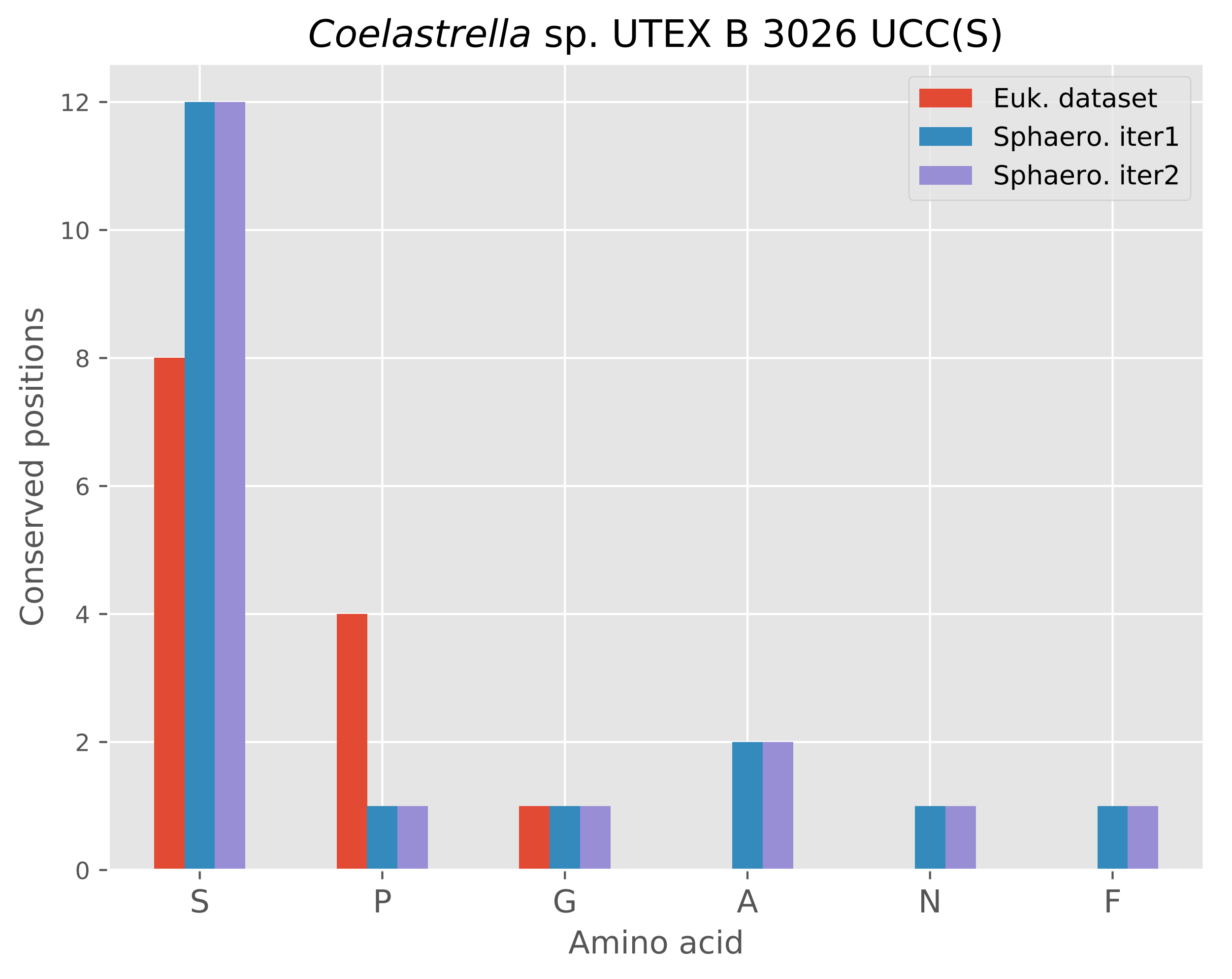

Coelastrella sp. UTEX B 3026 UCC(S)
Euk. dataset
Sphaero. iter1
Sphaero. iter2
Conserved positions
0
2
4
6
8
10
12
S
P
G
A
N
F
Amino acid

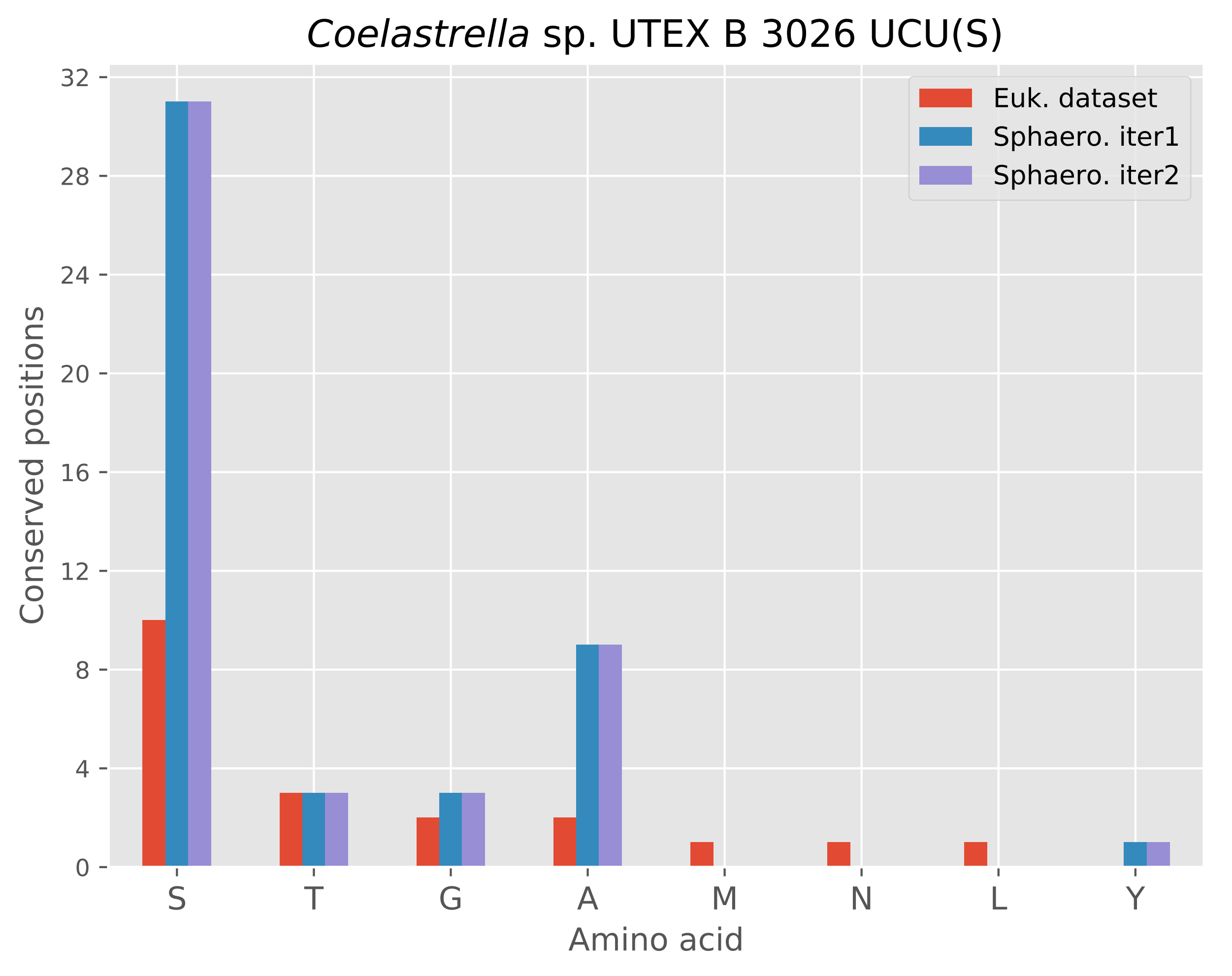
Coelastrella sp. UTEX B 3026 UCU(S)
Euk. dataset
Sphaero. iter1
Sphaero. iter2
Conserved positions
0
4
8
12
16
20
24
28
32
S
T
G
A
M
N
L
Y
Amino acid

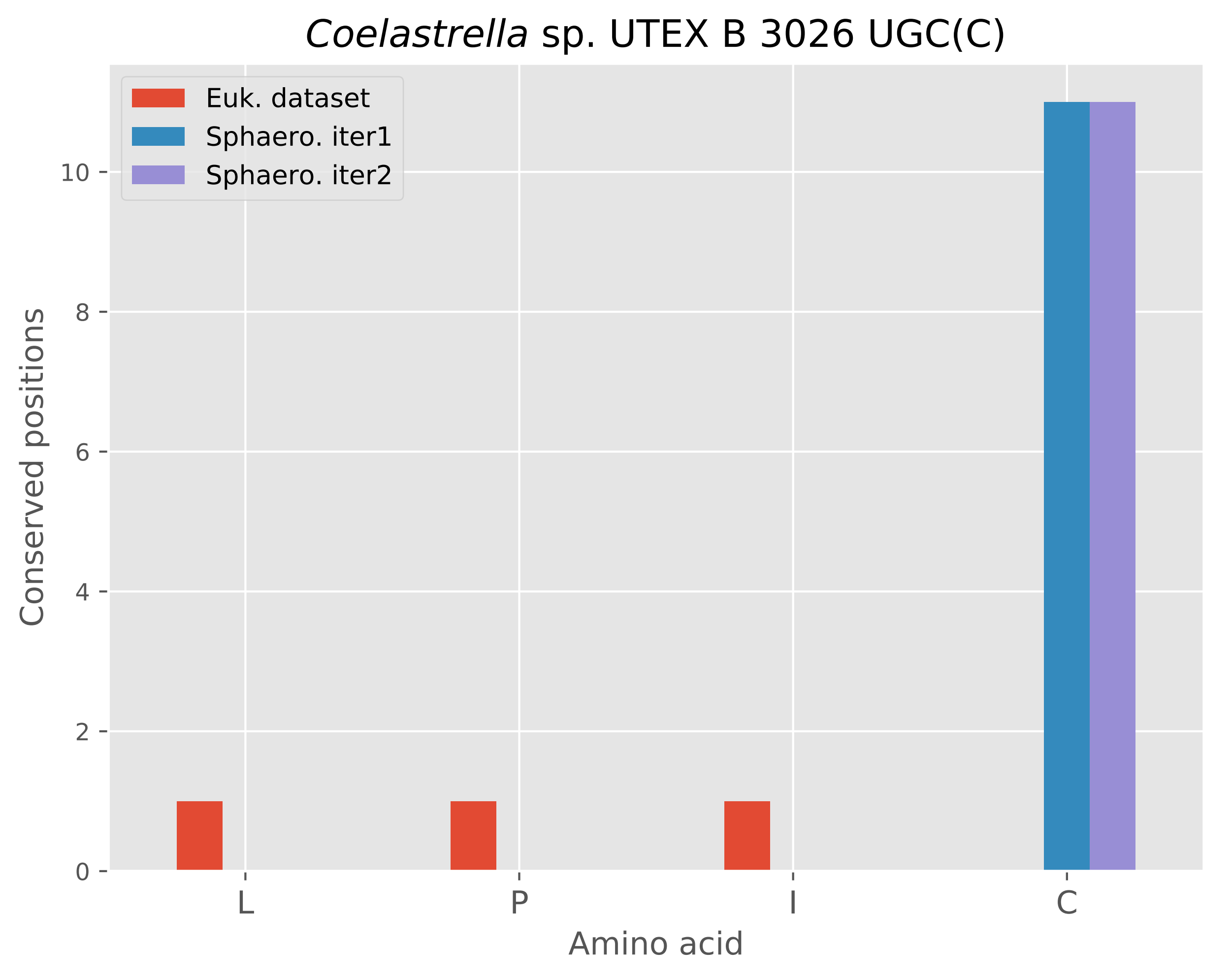

Coelastrella sp. UTEX B 3026 UGC(C)
Euk. dataset
Sphaero. iter1
Sphaero. iter2
Conserved positions
0
2
4
6
8
10
L
P
I
C
Amino acid

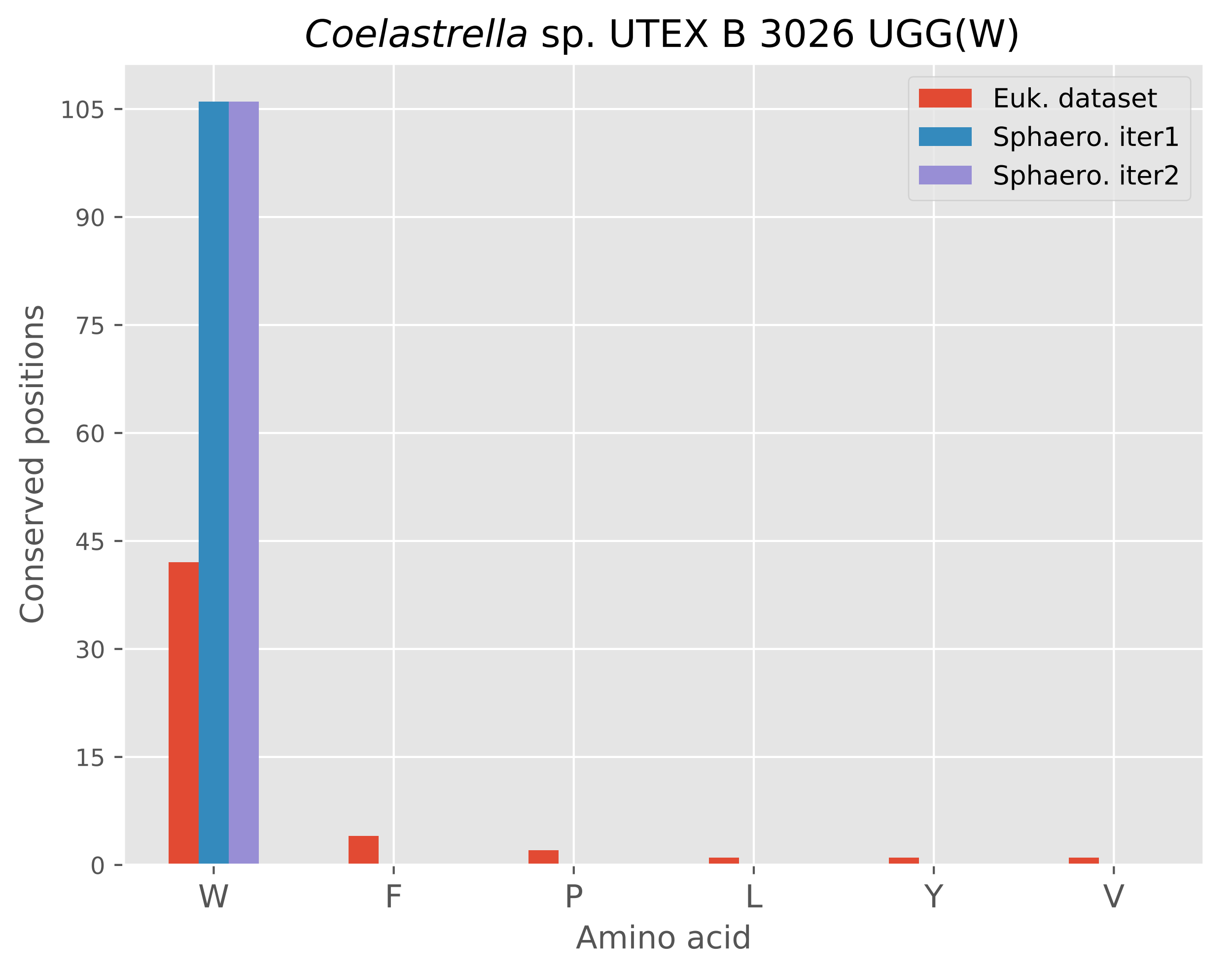
Coelastrella sp. UTEX B 3026 UGG(W)
Euk. dataset
Sphaero. iter1
Sphaero. iter2
Conserved positions
0
15
30
45
60
75
90
105
W
F
P
L
Y
V
Amino acid

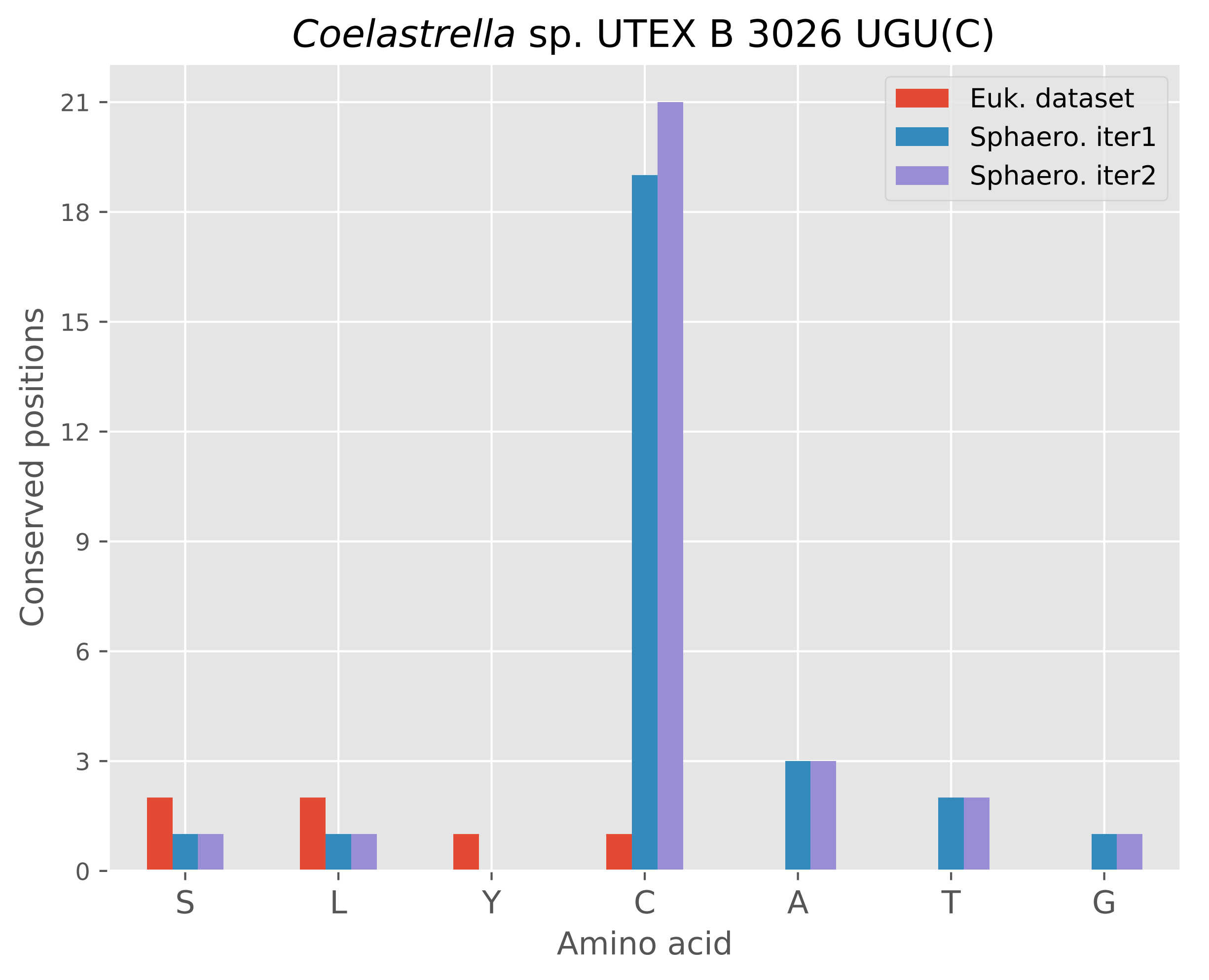

Coelastrella sp. UTEX B 3026 UGU(C)
Euk. dataset
Sphaero. iter1
Sphaero. iter2
Conserved positions
0
3
6
9
12
15
18
21
S
L
Y
C
A
T
G
Amino acid

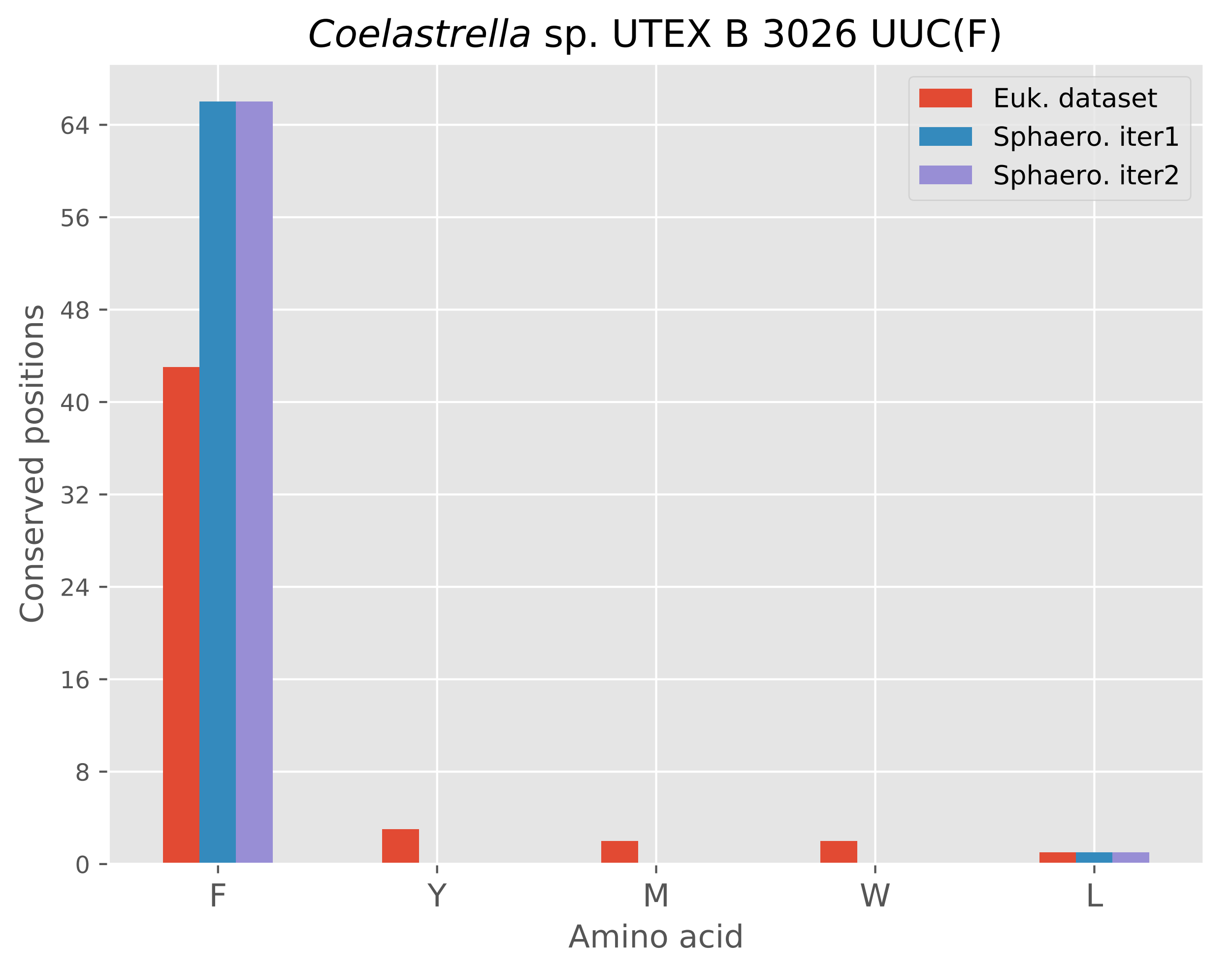

Coelastrella sp. UTEX B 3026 UUC(F)
Euk. dataset
Sphaero. iter1
Sphaero. iter2
Conserved positions
0
8
16
24
32
40
48
56
64
F
Y
M
W
L
Amino acid

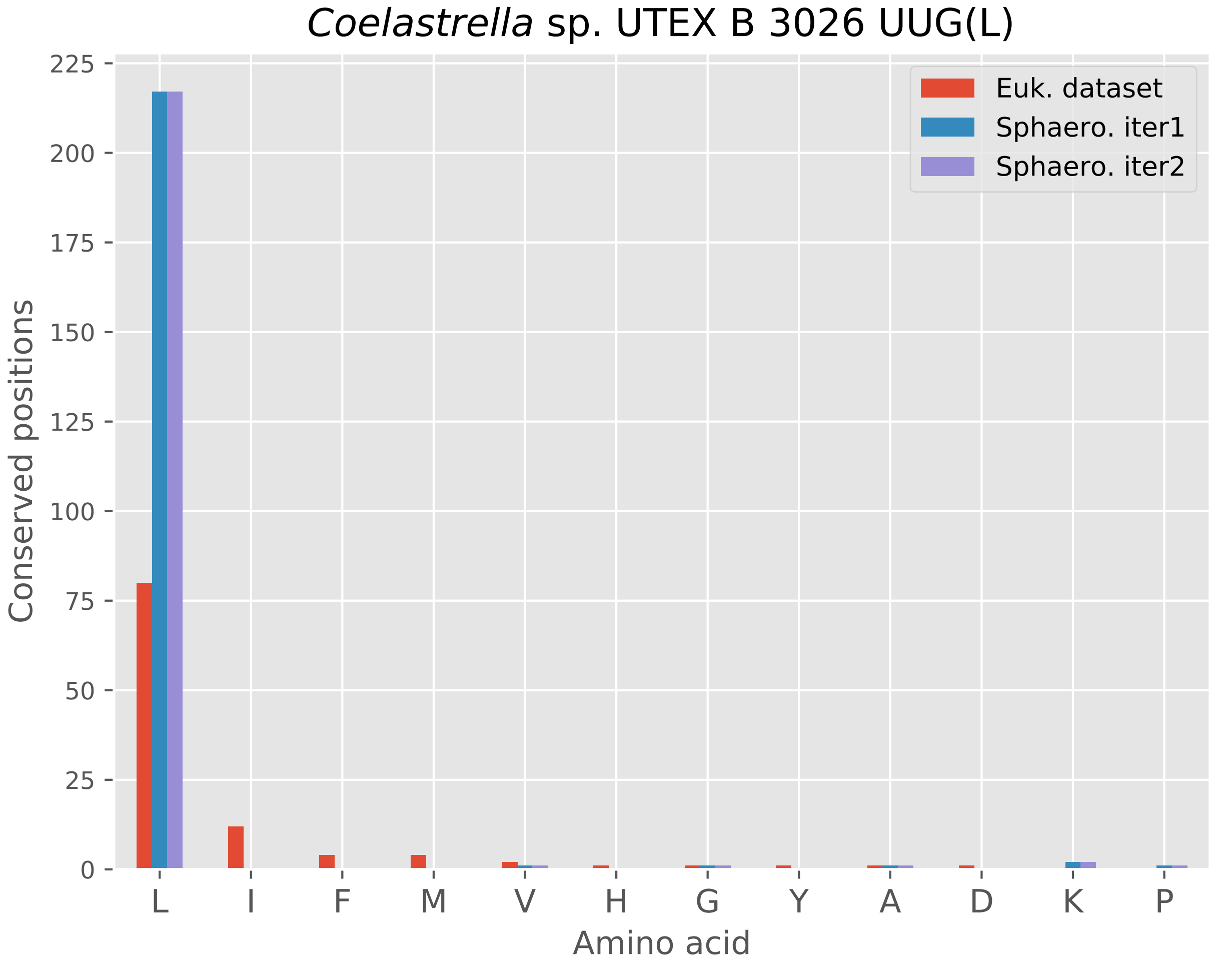

Coelastrella sp. UTEX B 3026 UUG(L)
Euk. dataset
Sphaero. iter1
Sphaero. iter2
Conserved positions
225
200
175
150
125
100
75
50
25
0
L
I
F
M
V
H
G
Y
A
D
K
P
Amino acid

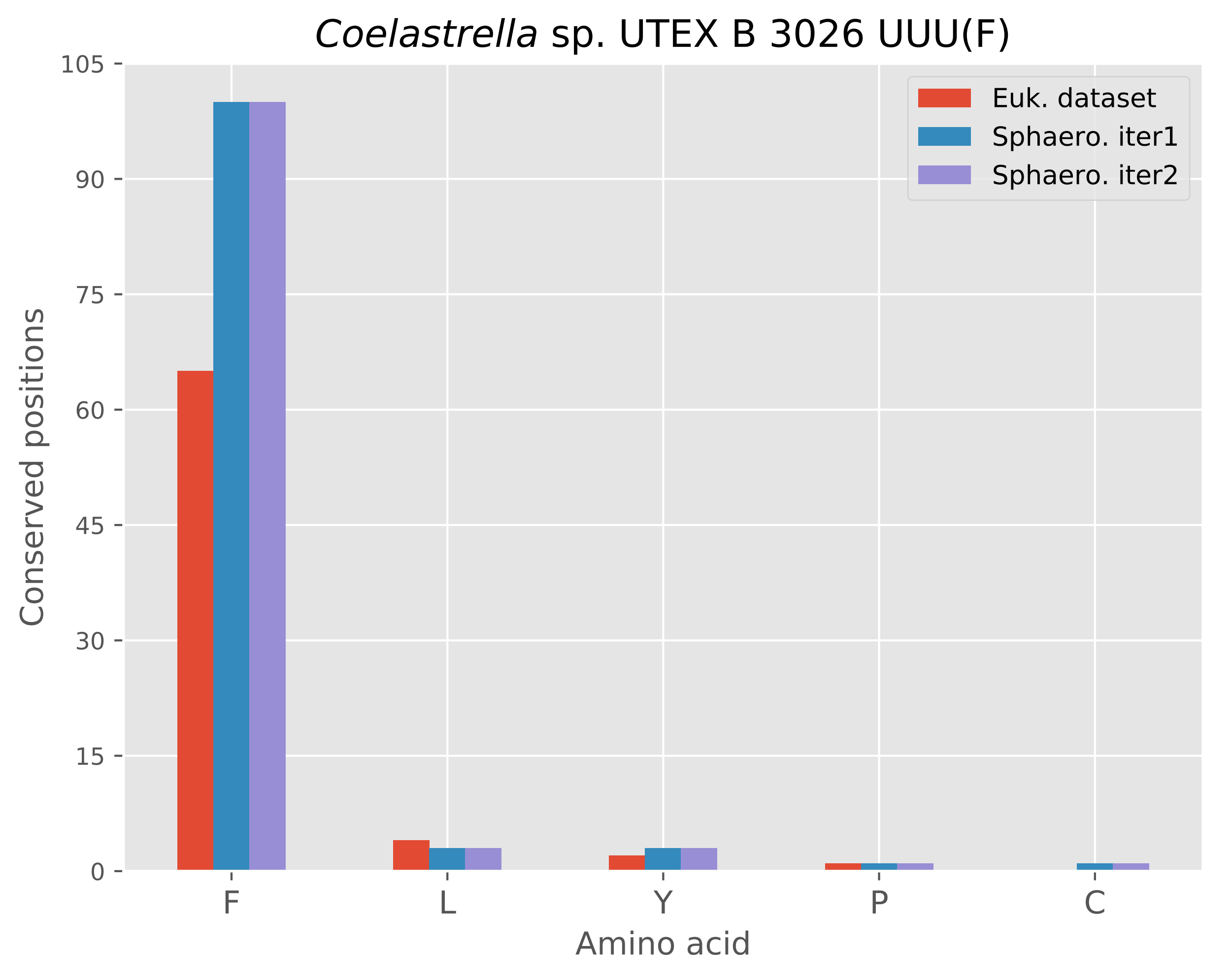

Coelastrella sp. UTEX B 3026 UUU(F)
Euk. dataset
Sphaero. iter1
Sphaero. iter2
Conserved positions
0
15
30
45
60
75
90
105
F
L
Y
P
C
Amino acid

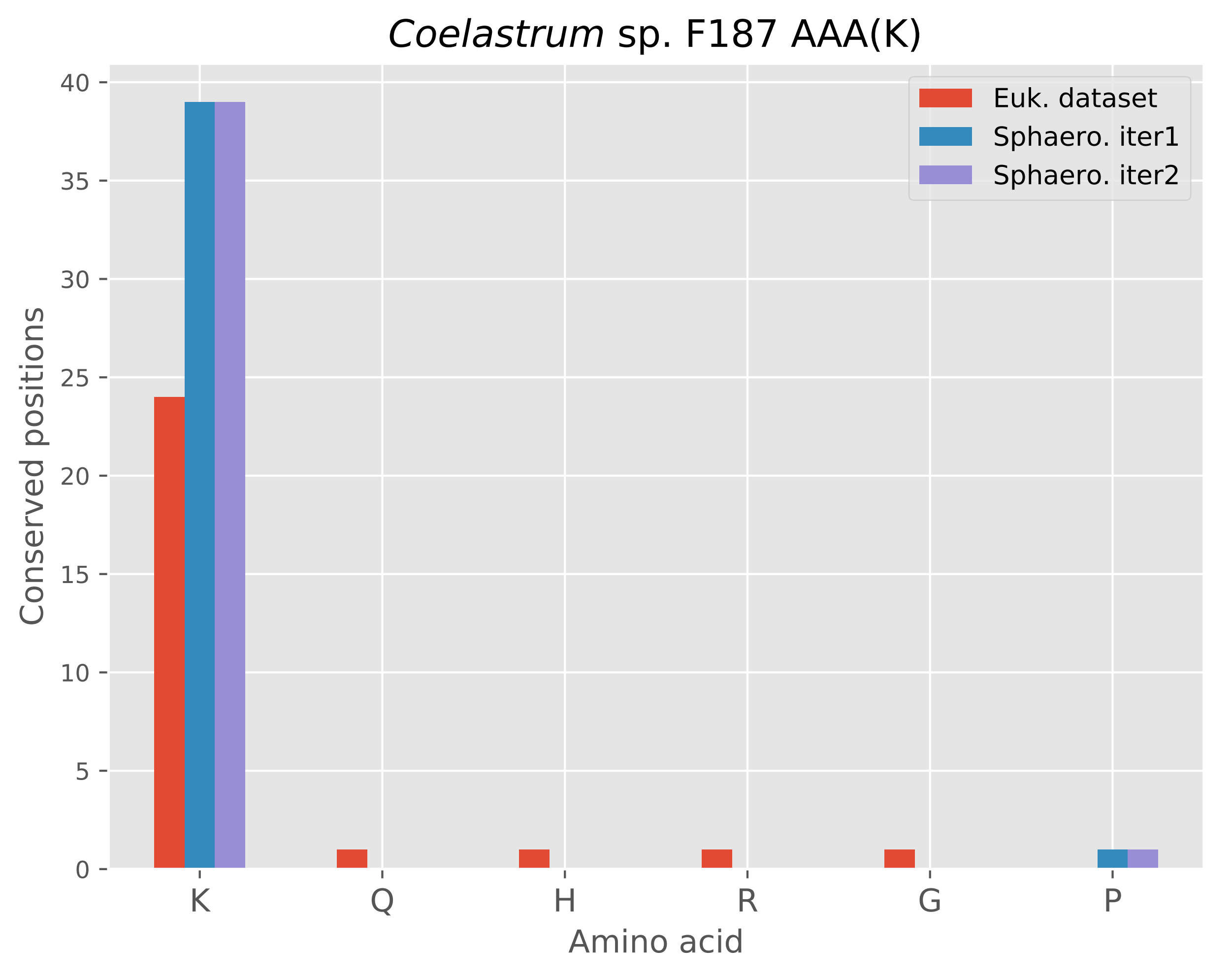

Coelastrum sp. F187 AAA(K)
Euk. dataset
Sphaero. iter1
Sphaero. iter2
Conserved positions
0
5
10
15
20
25
30
35
40
K
Q
H
R
G
P
Amino acid

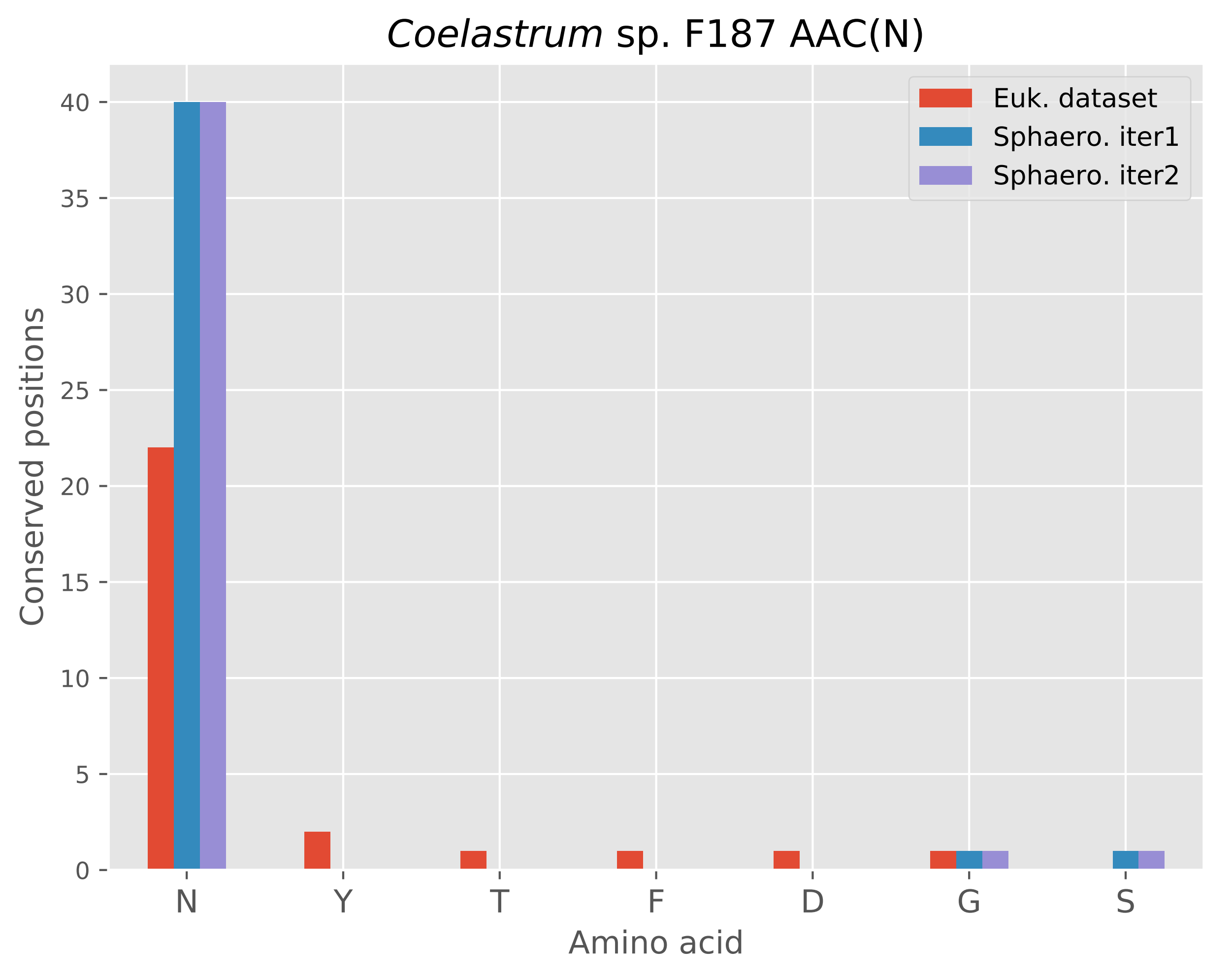

Coelastrum sp. F187 AAC(N)
Euk. dataset
Sphaero. iter1
Sphaero. iter2
Conserved positions
0
5
10
15
20
25
30
35
40
N
Y
T
F
D
G
S
Amino acid

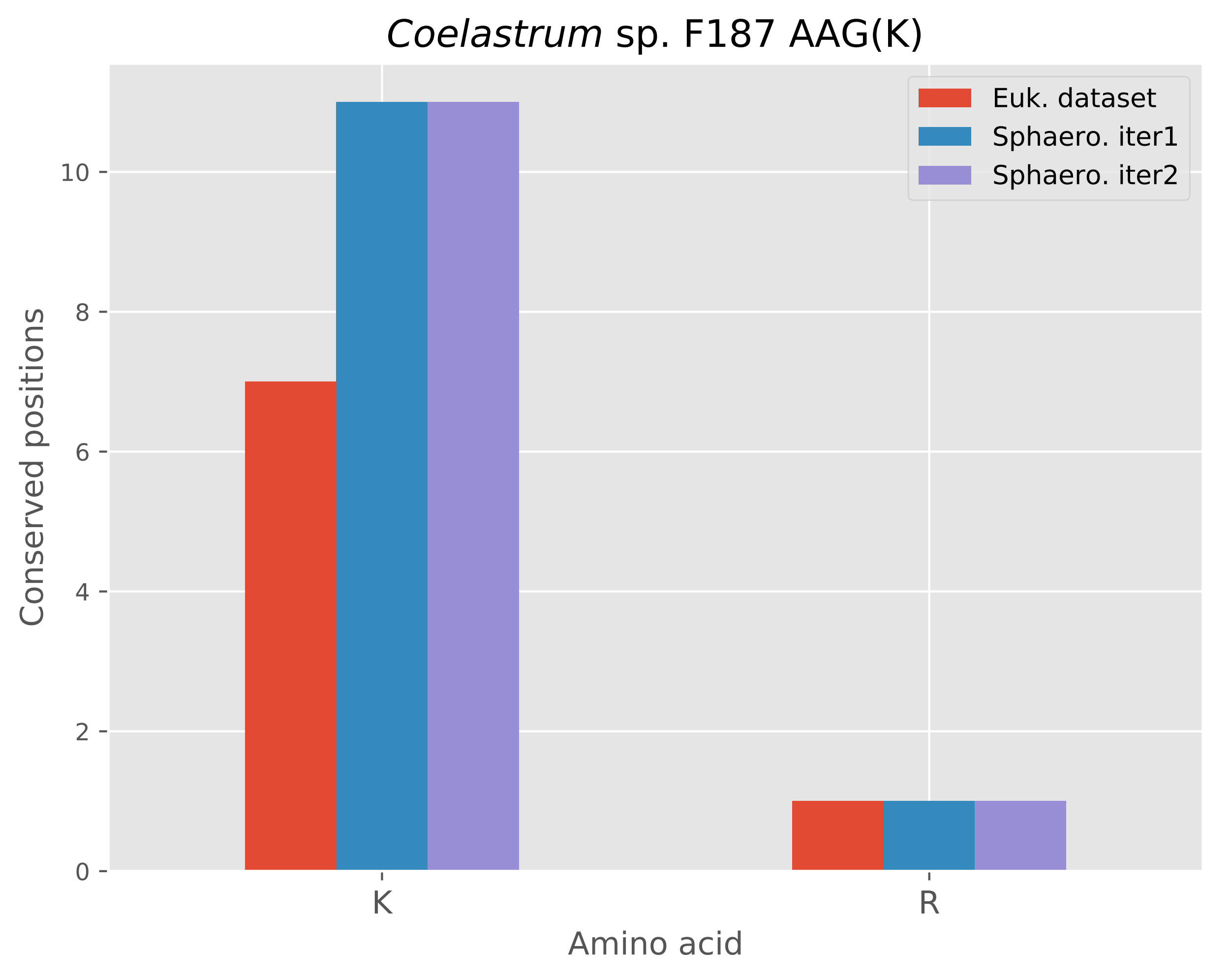
Coelastrum sp. F187 AAG(K)
Euk. dataset
Sphaero. iter1
Sphaero. iter2
Conserved positions
0
2
4
6
8
10
K
R
Amino acid

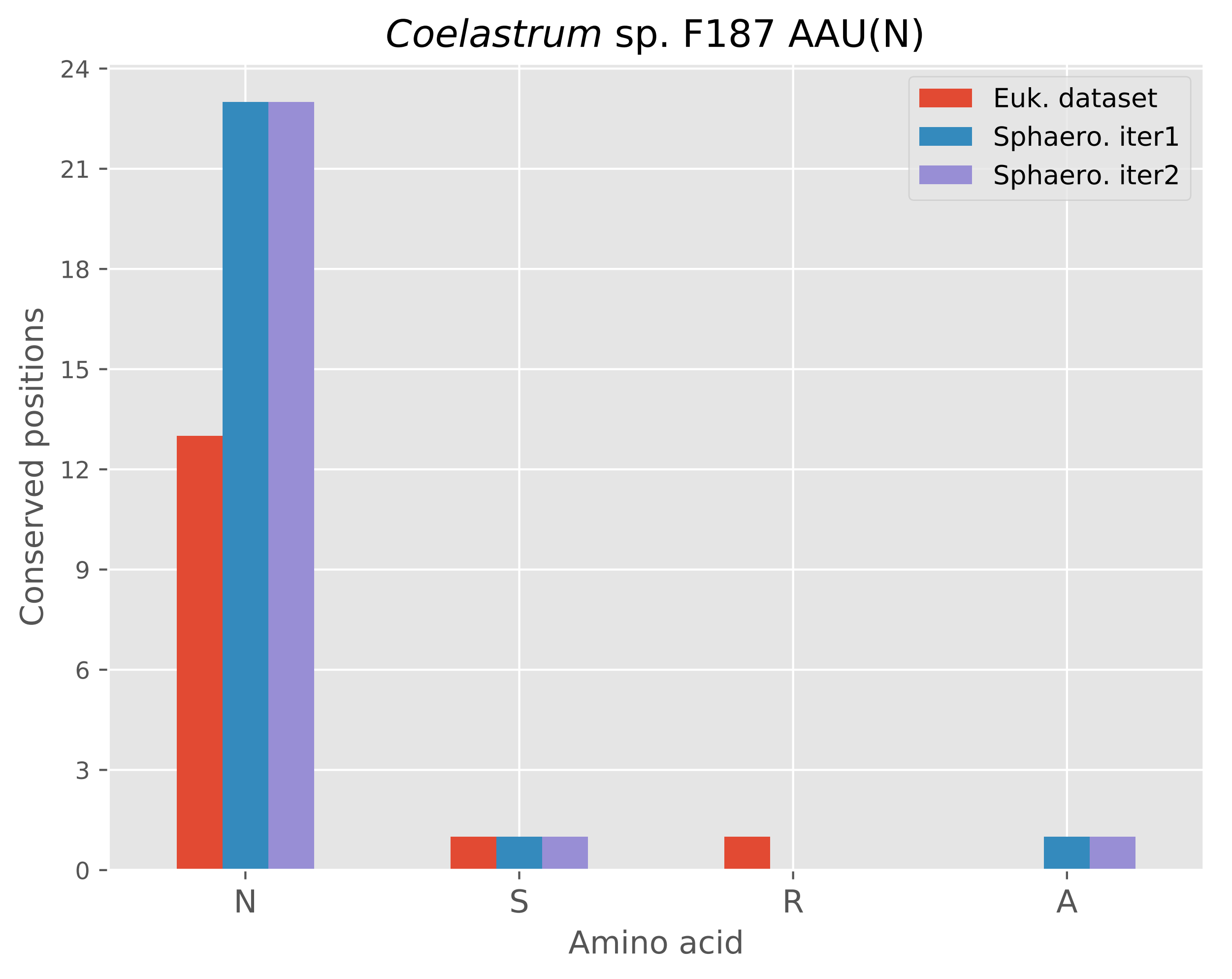

Coelastrum sp. F187 AAU(N)
Conserved positions
Amino acid
N
S
R
A
0
3
6
9
12
15
18
21
24
Euk. dataset
Sphaero. iter1
Sphaero. iter2

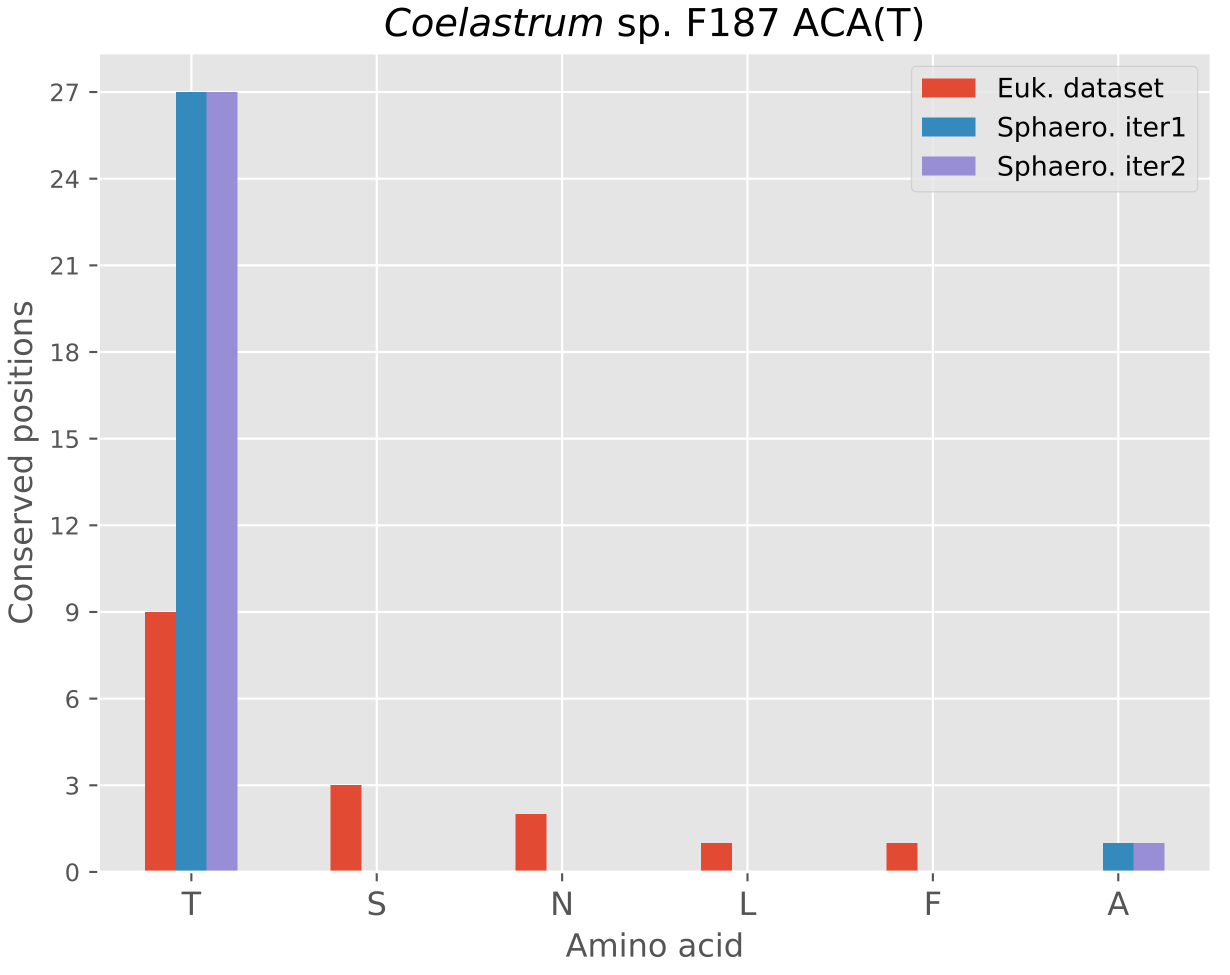

Coelastrum sp. F187 ACA(T)
Conserved positions
Amino acid
Euk. dataset
Sphaero. iter1
Sphaero. iter2
0
3
6
9
12
15
18
21
24
27
T
S
N
L
F
A

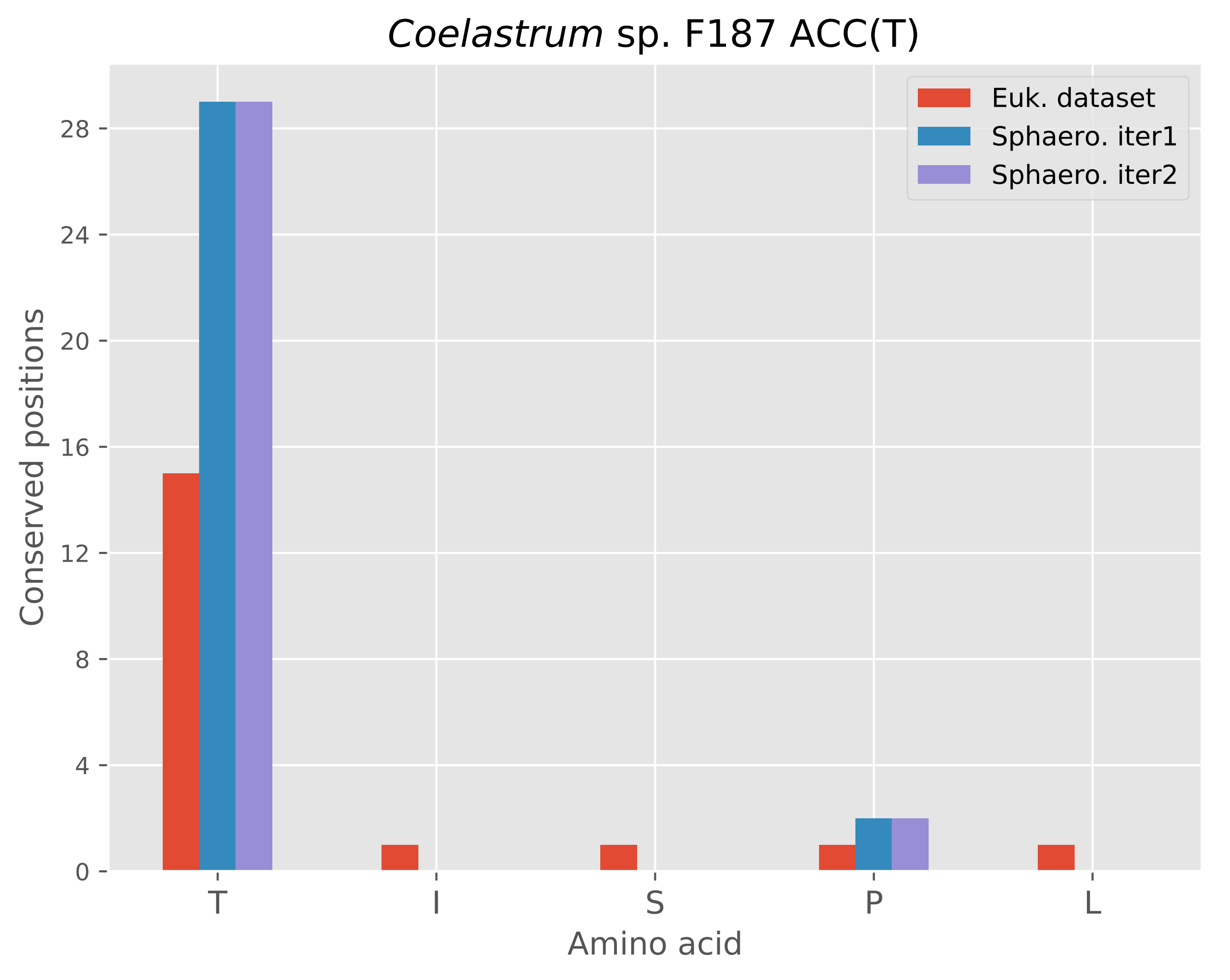

Coelastrum sp. F187 ACC(T)
Euk. dataset
Sphaero. iter1
Sphaero. iter2
Conserved positions
0
4
8
12
16
20
24
28
T
I
S
P
L
Amino acid

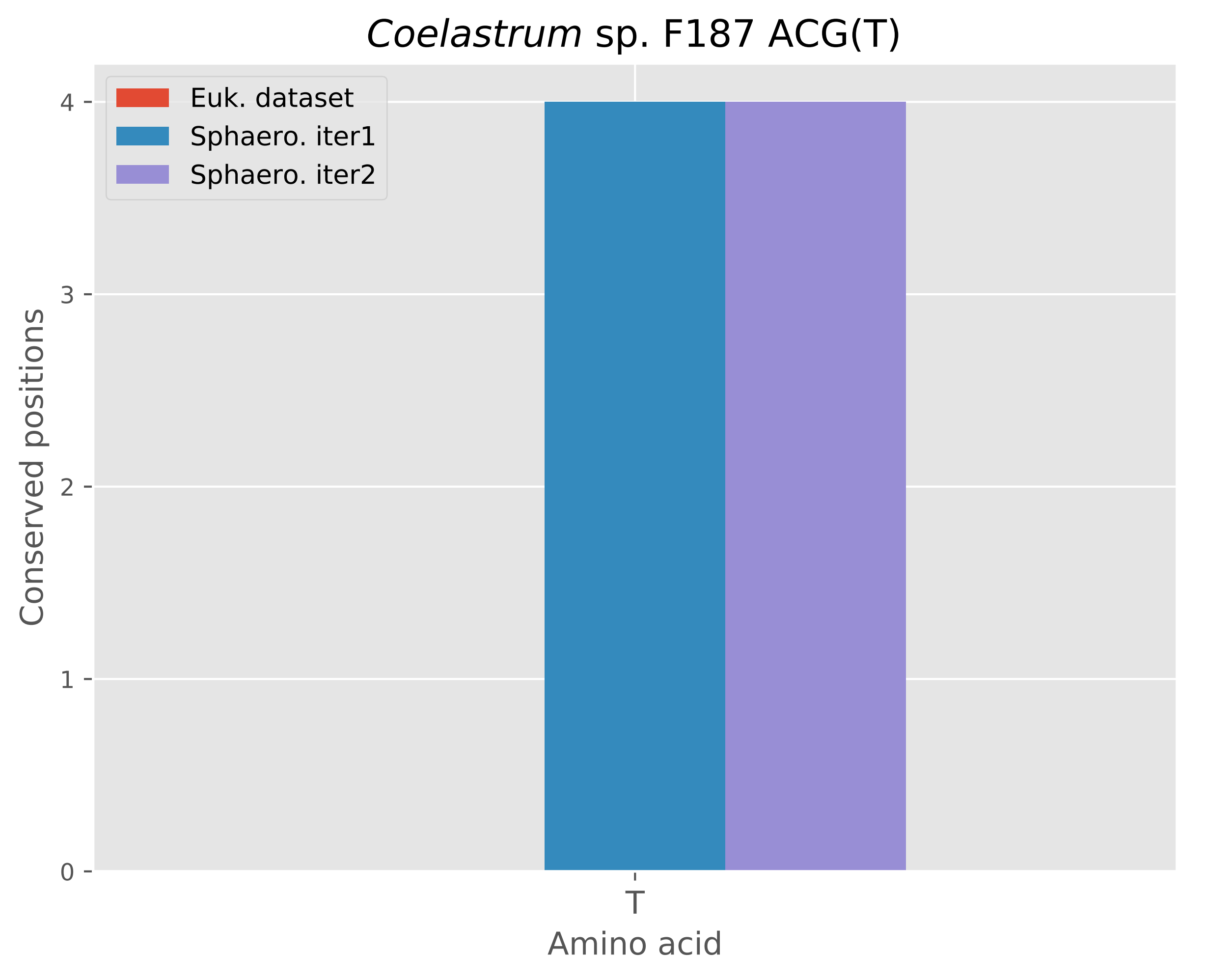

Coelastrum sp. F187 ACG(T)
Euk. dataset
Sphaero. iter1
Sphaero. iter2
Conserved positions
0
1
2
3
4
T
Amino acid

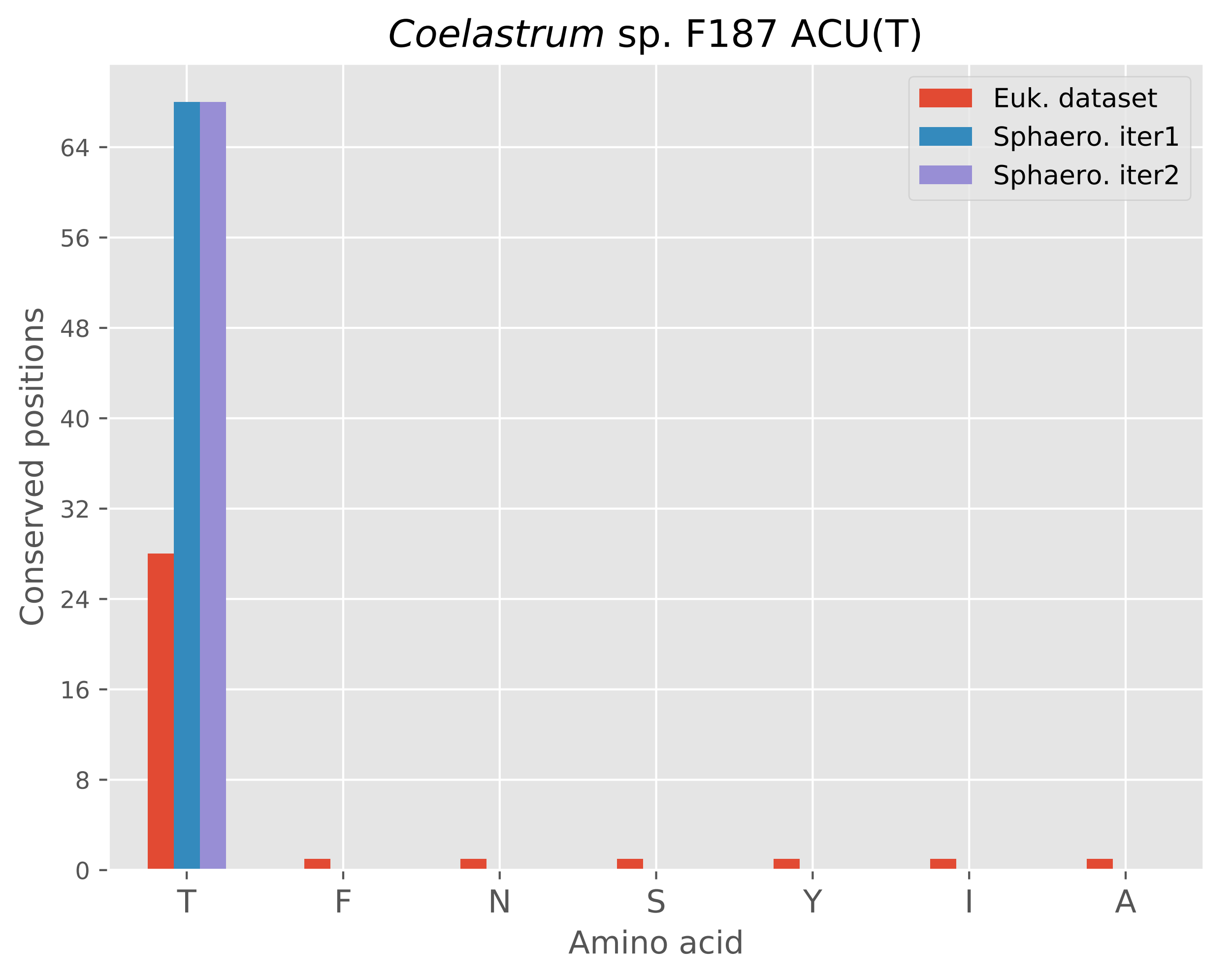
Coelastrum sp. F187 ACU(T)
Euk. dataset
Sphaero. iter1
Sphaero. iter2
Conserved positions
0
8
16
24
32
40
48
56
64
T
F
N
S
Y
I
A
Amino acid

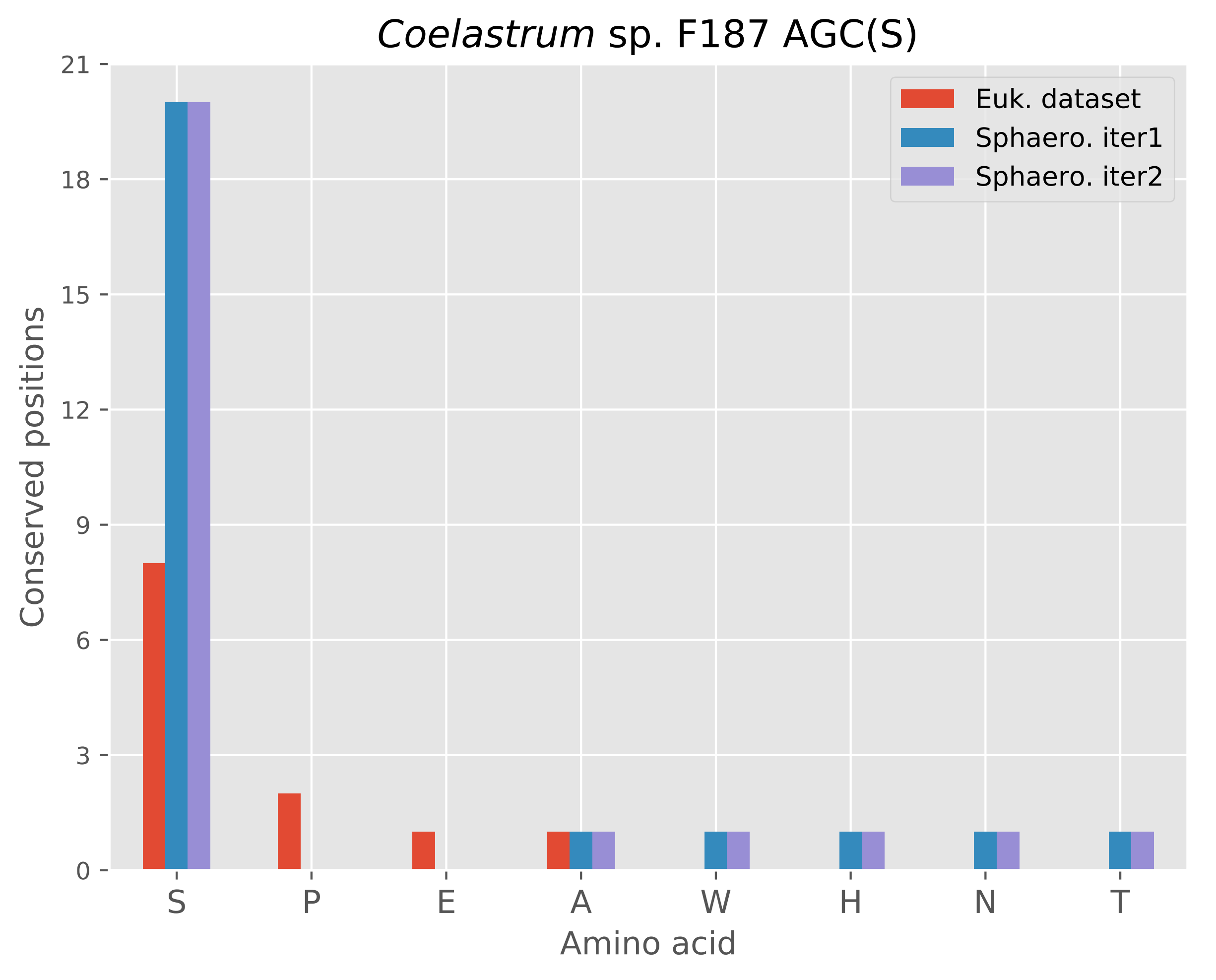

Coelastrum sp. F187 AGC(S)
Euk. dataset
Sphaero. iter1
Sphaero. iter2
Conserved positions
0
3
6
9
12
15
18
21
S
P
E
A
W
H
N
T
Amino acid

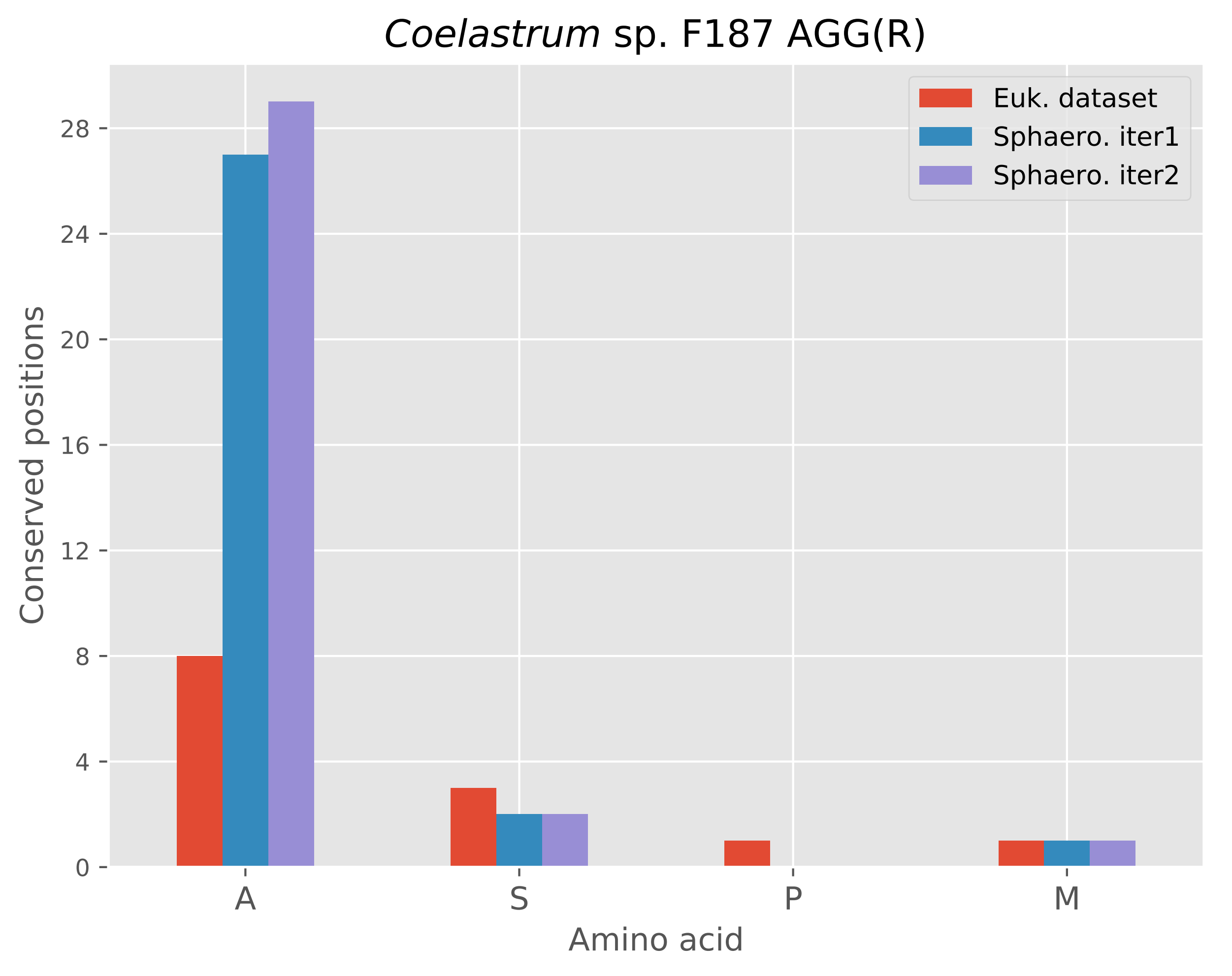
Coelastrum sp. F187 AGG(R)
Euk. dataset
Sphaero. iter1
Sphaero. iter2
Conserved positions
0
4
8
12
16
20
24
28
A
S
P
M
Amino acid

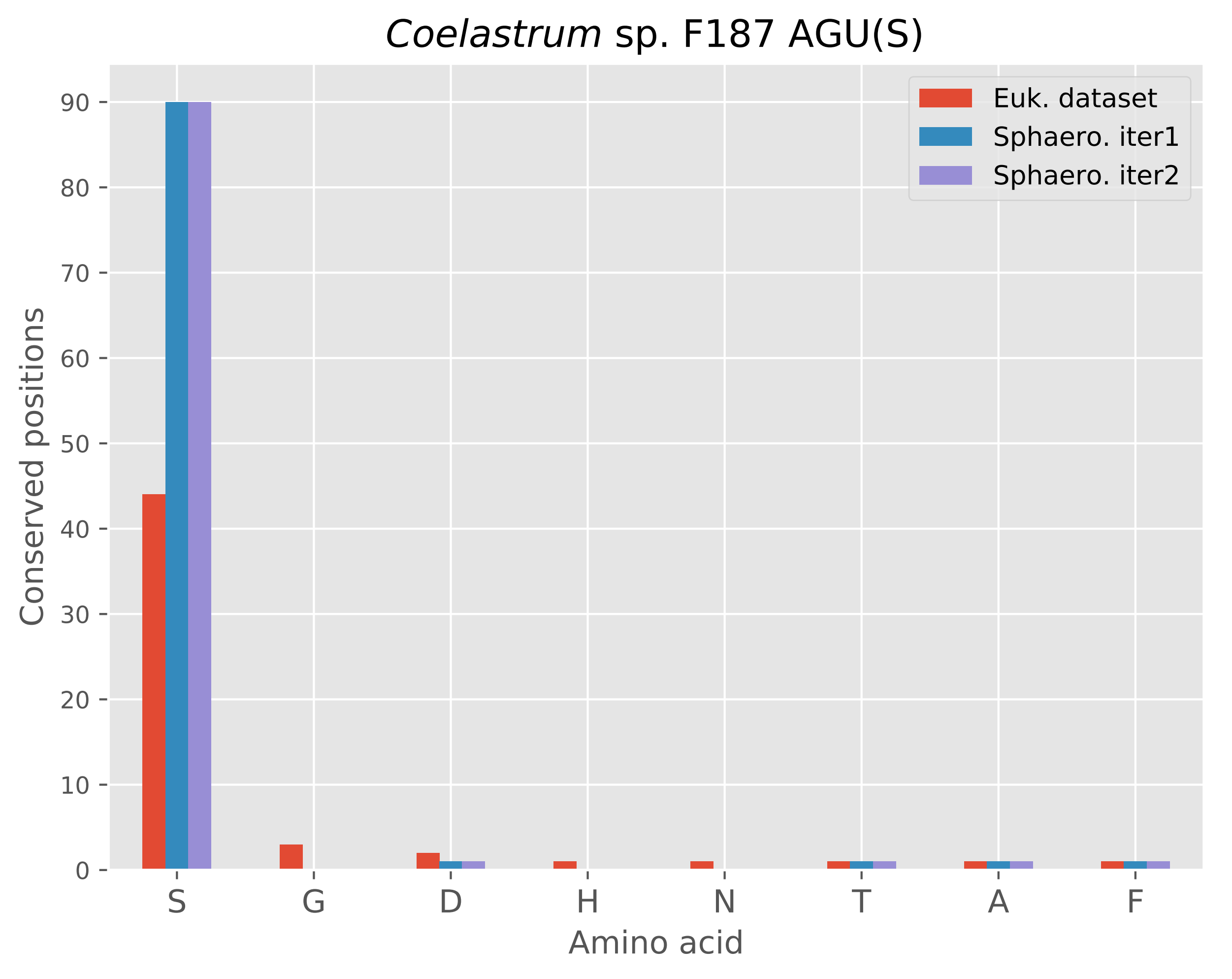

Coelastrum sp. F187 AGU(S)
Euk. dataset
Sphaero. iter1
Sphaero. iter2
Conserved positions
Amino acid
0
10
20
30
40
50
60
70
80
90
S
G
D
H
N
T
A
F

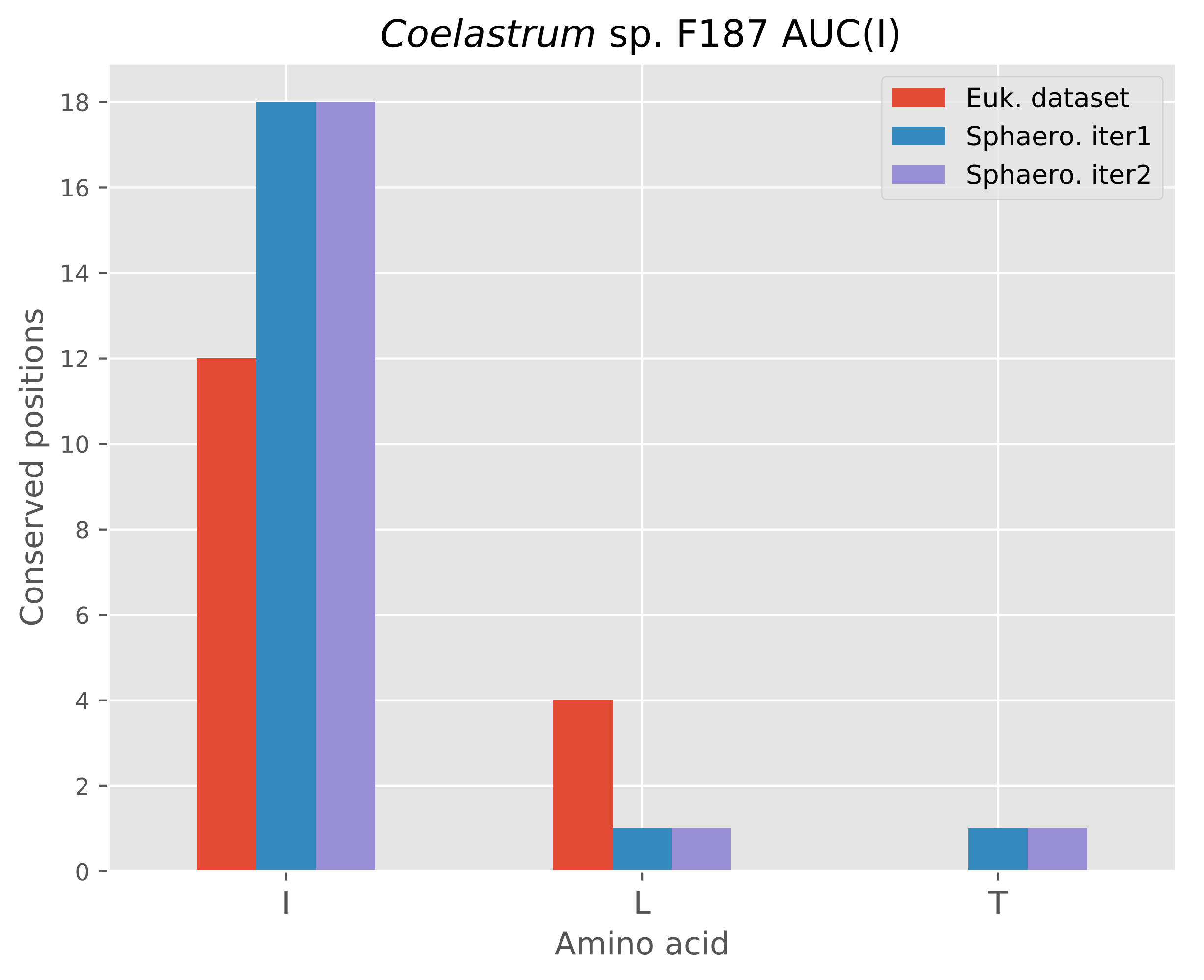

Coelastrum sp. F187 AUC(I)
Euk. dataset
Sphaero. iter1
Sphaero. iter2
Conserved positions
0
2
4
6
8
10
12
14
16
18
I
L
T
Amino acid

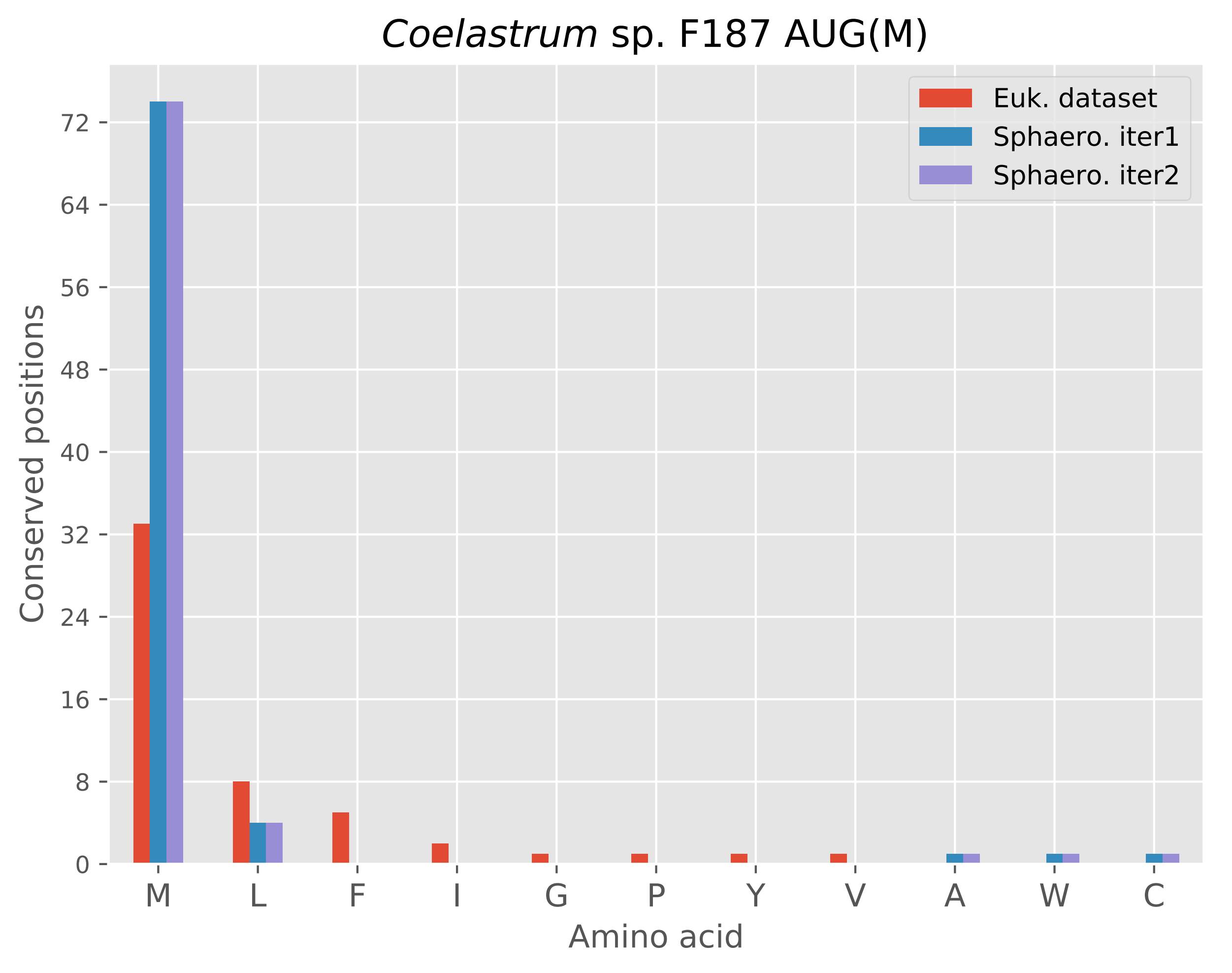

Coelastrum sp. F187 AUG(M)
Euk. dataset
Sphaero. iter1
Sphaero. iter2
Conserved positions
0
8
16
24
32
40
48
56
64
72
M
L
F
I
G
P
Y
V
A
W
C
Amino acid

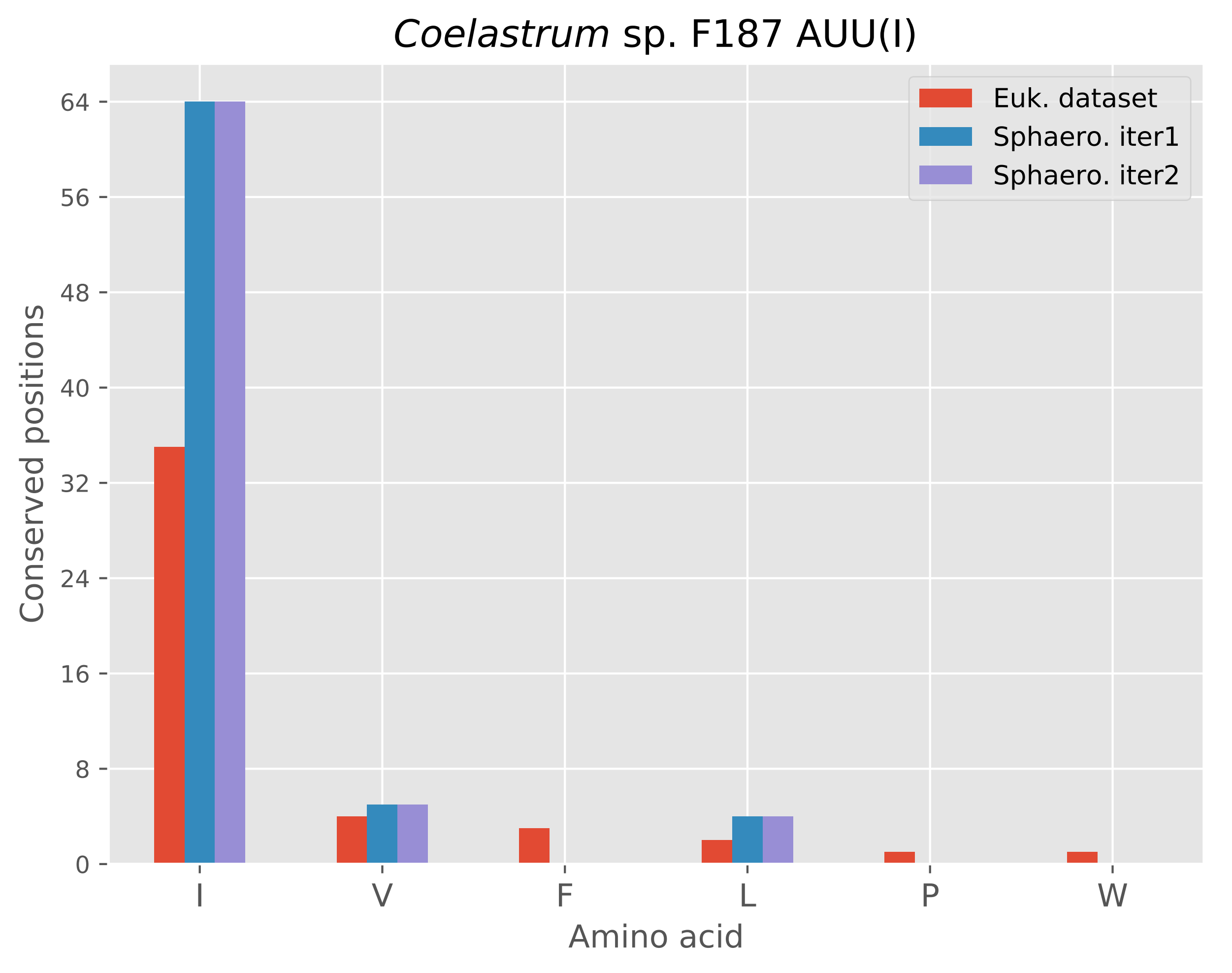

Coelastrum sp. F187 AUU(I)
Euk. dataset
Sphaero. iter1
Sphaero. iter2
Conserved positions
0
8
16
24
32
40
48
56
64
I
V
F
L
P
W
Amino acid

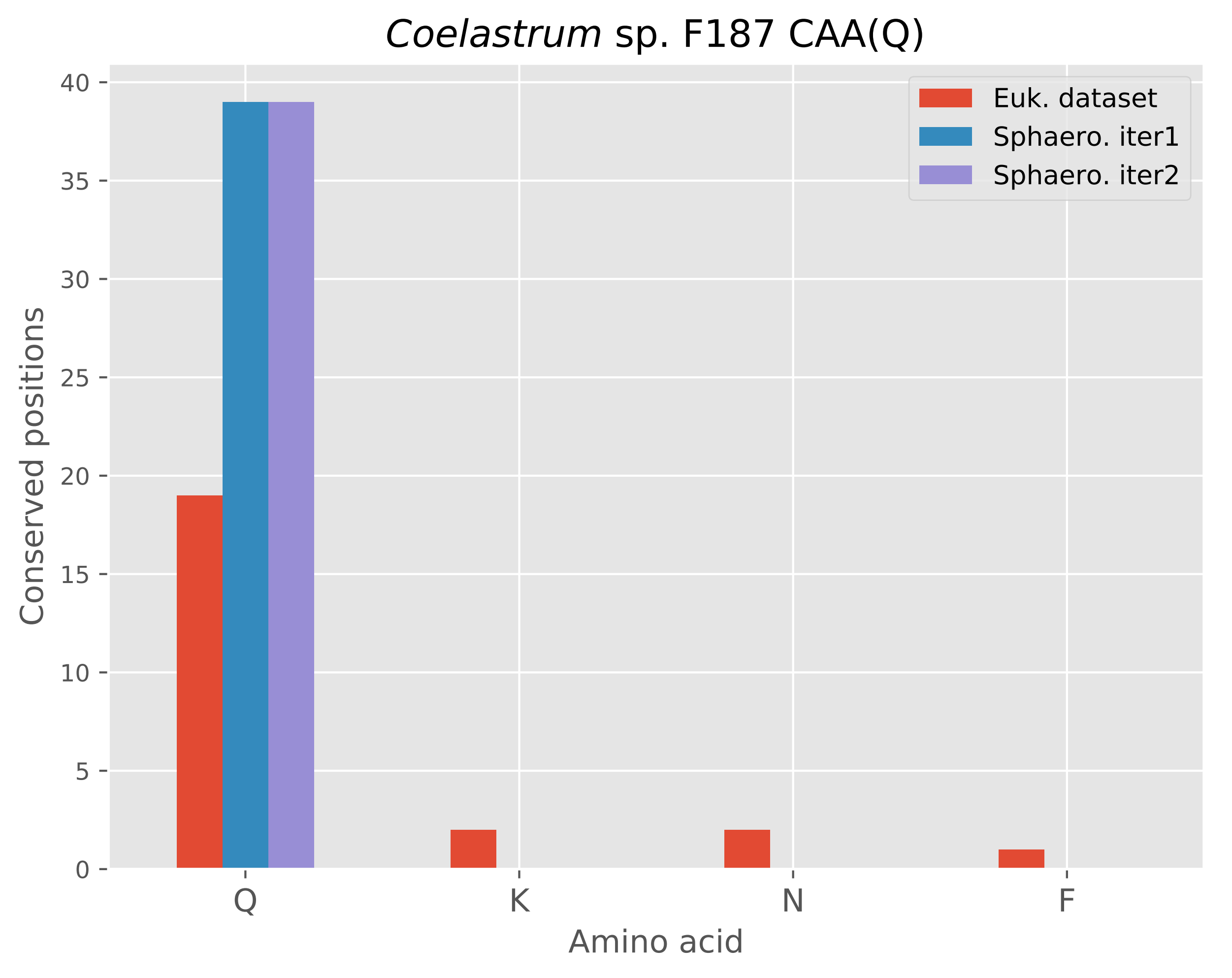

Coelastrum sp. F187 CAA(Q)
Euk. dataset
Sphaero. iter1
Sphaero. iter2
Conserved positions
0
5
10
15
20
25
30
35
40
Q
K
N
F
Amino acid

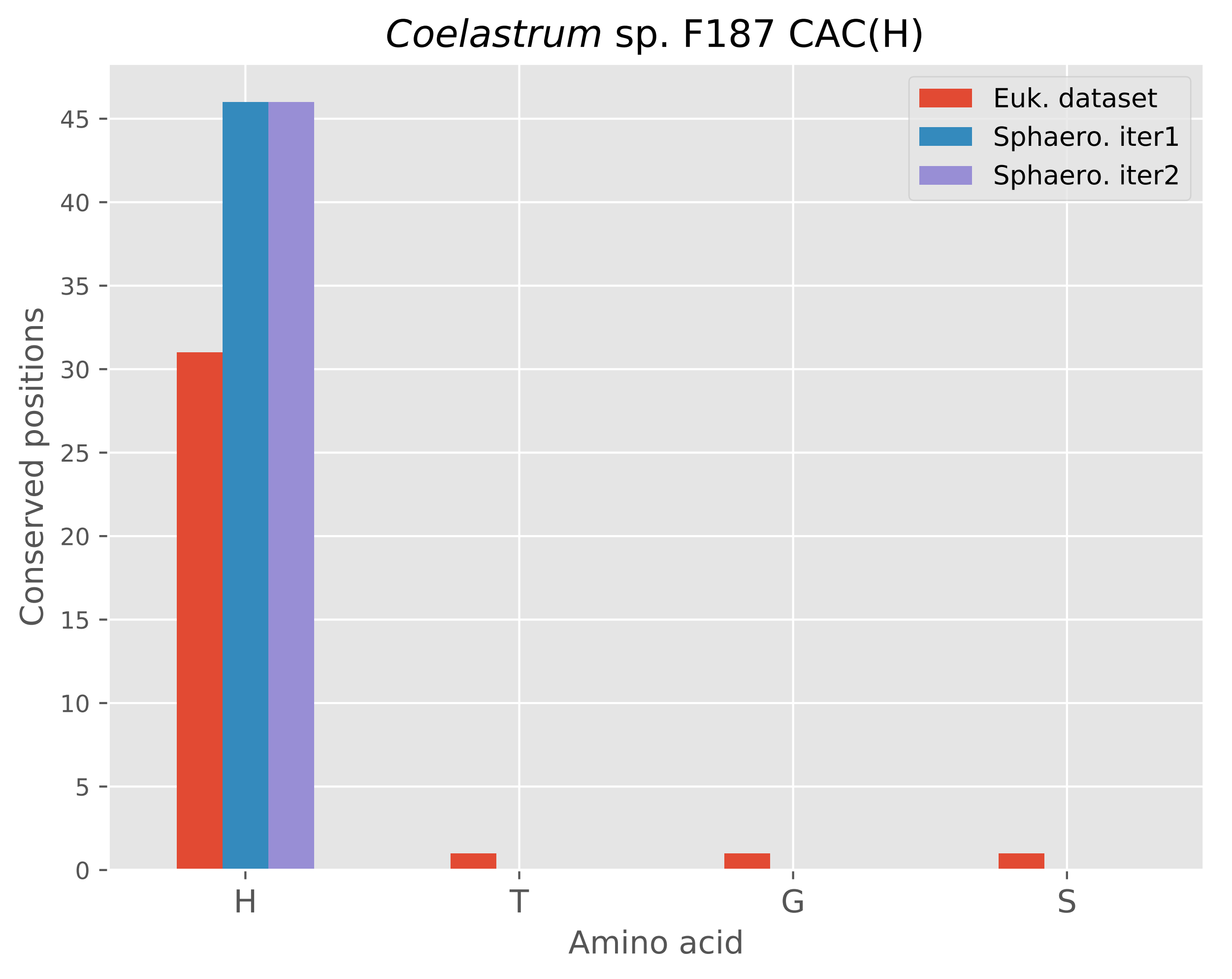
Coelastrum sp. F187 CAC(H)
Euk. dataset
Sphaero. iter1
Sphaero. iter2
Conserved positions
0
5
10
15
20
25
30
35
40
45
H
T
G
S
Amino acid

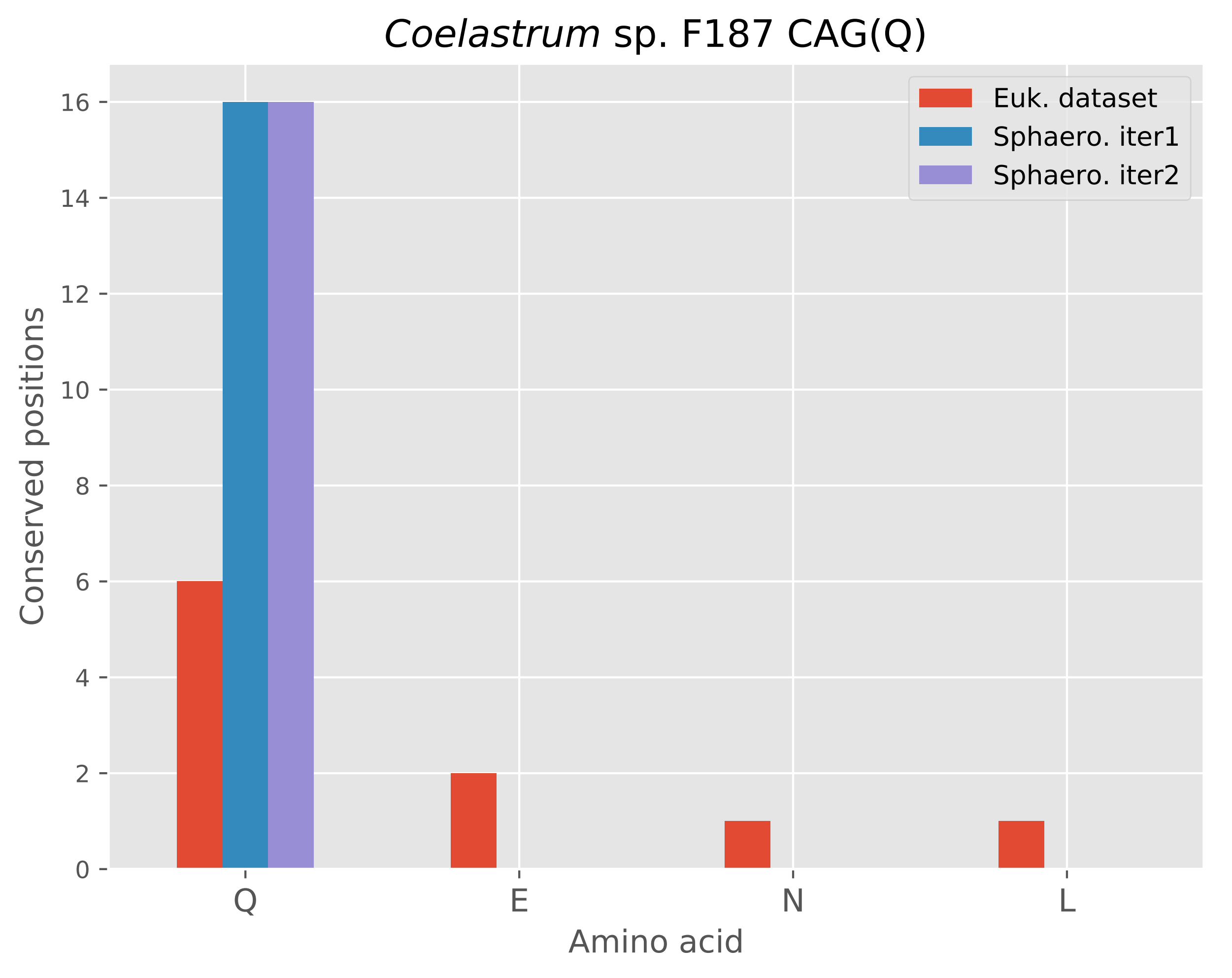

Coelastrum sp. F187 CAG(Q)
Euk. dataset
Sphaero. iter1
Sphaero. iter2
Conserved positions
0
2
4
6
8
10
12
14
16
Q
E
N
L
Amino acid

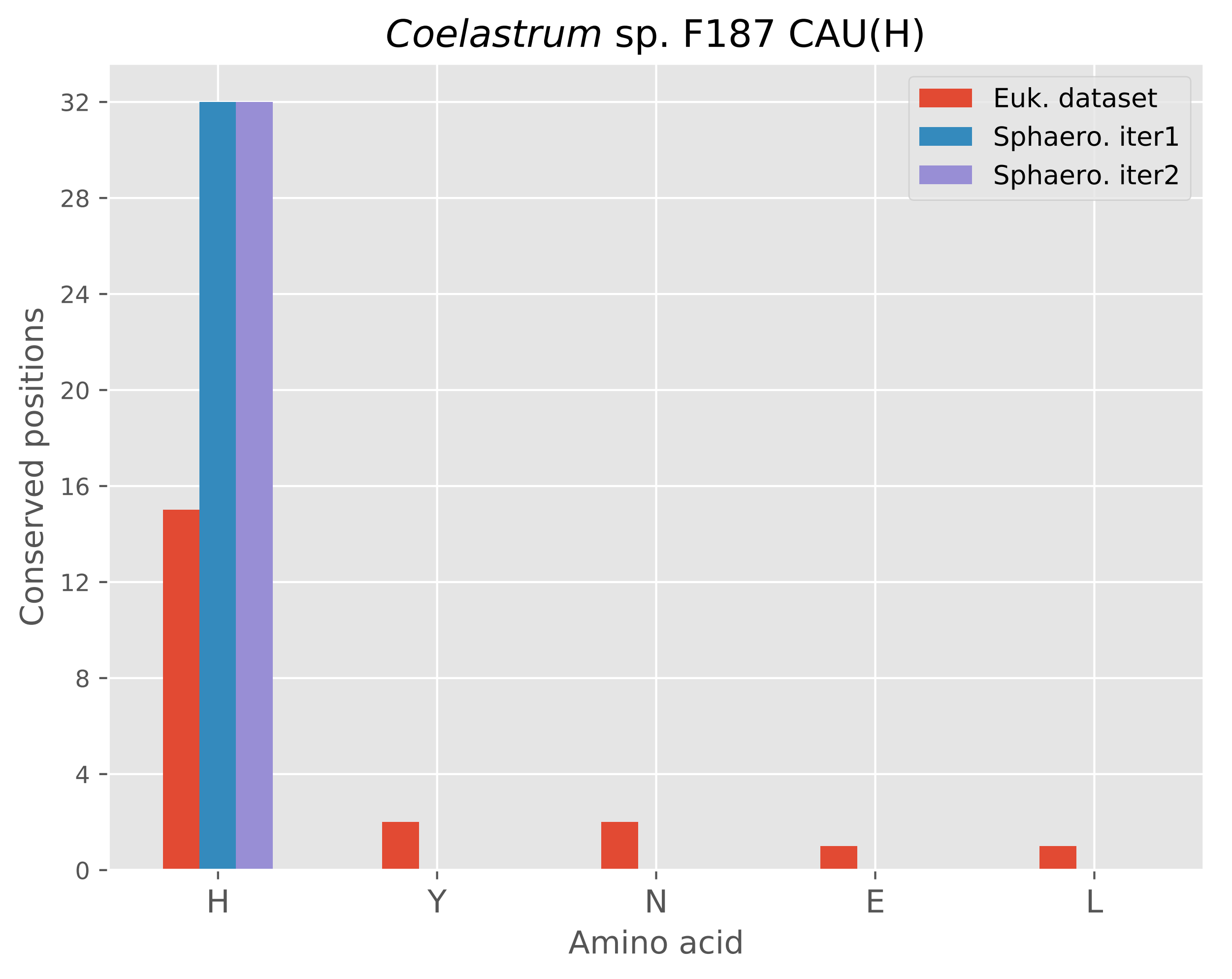

Coelastrum sp. F187 CAU(H)
Euk. dataset
Sphaero. iter1
Sphaero. iter2
Conserved positions
0
4
8
12
16
20
24
28
32
H
Y
N
E
L
Amino acid

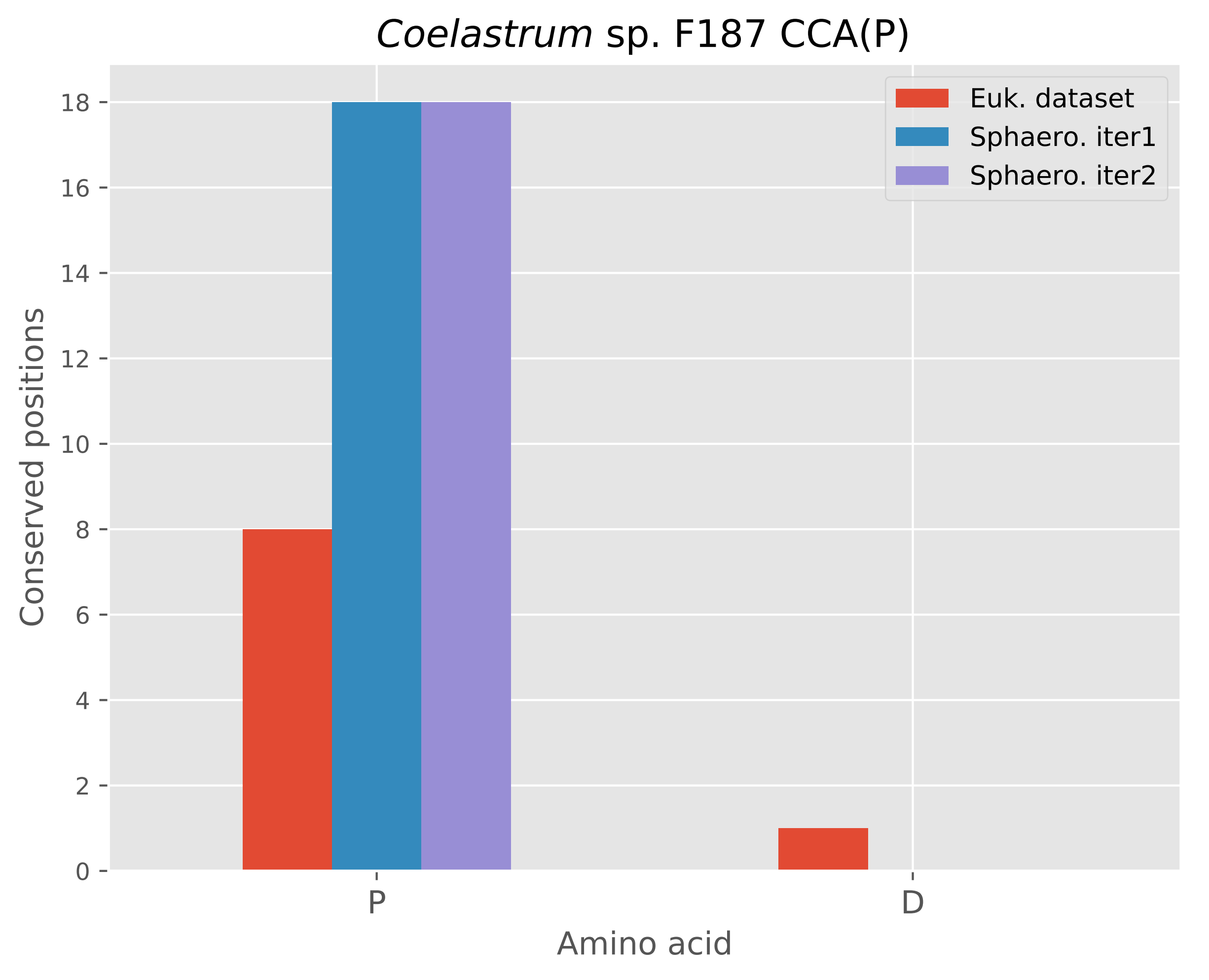

Coelastrum sp. F187 CCA(P)
Euk. dataset
Sphaero. iter1
Sphaero. iter2
Conserved positions
0
2
4
6
8
10
12
14
16
18
P
D
Amino acid

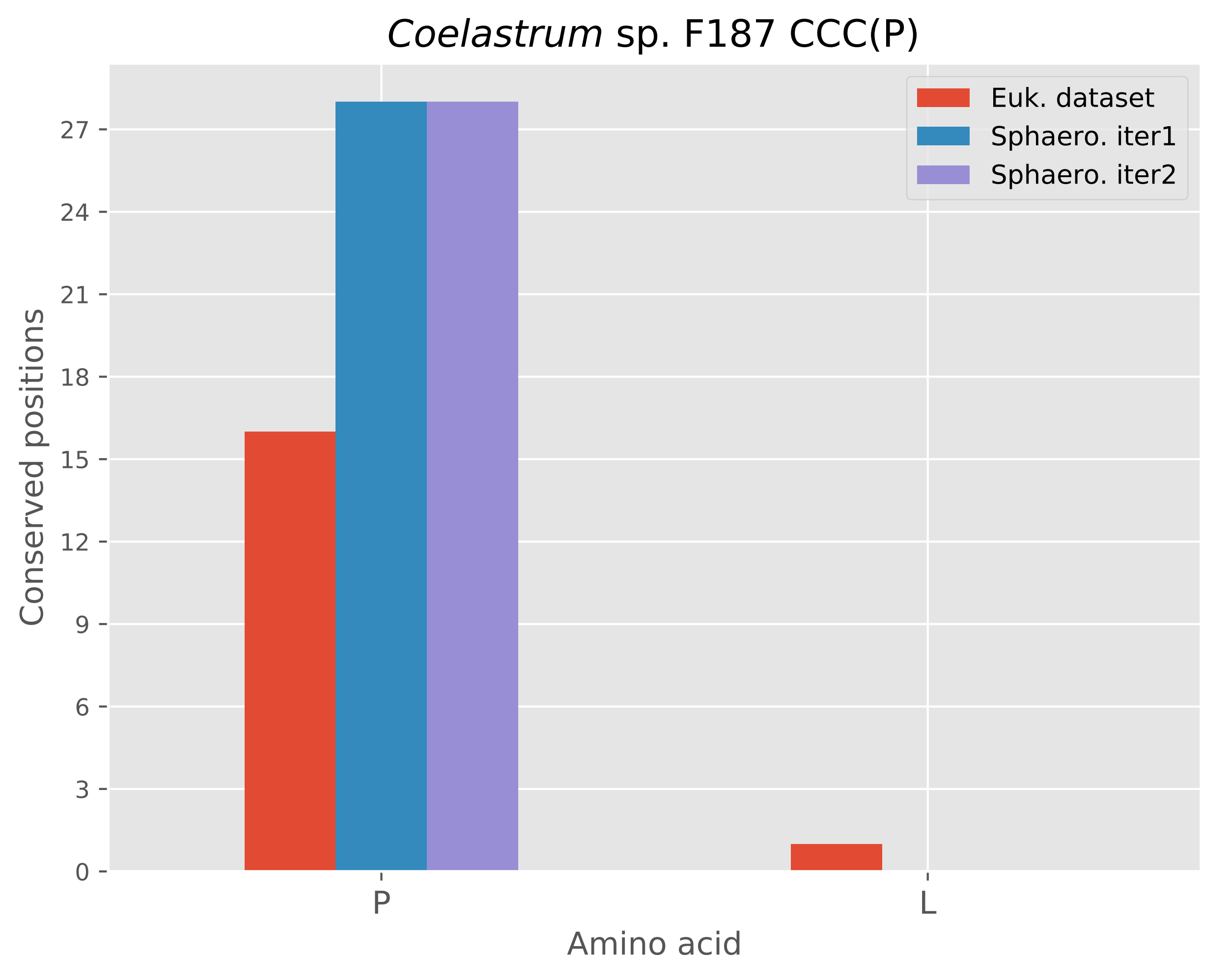

Coelastrum sp. F187 CCC(P)
Euk. dataset
Sphaero. iter1
Sphaero. iter2
Conserved positions
0
3
6
9
12
15
18
21
24
27
P
L
Amino acid

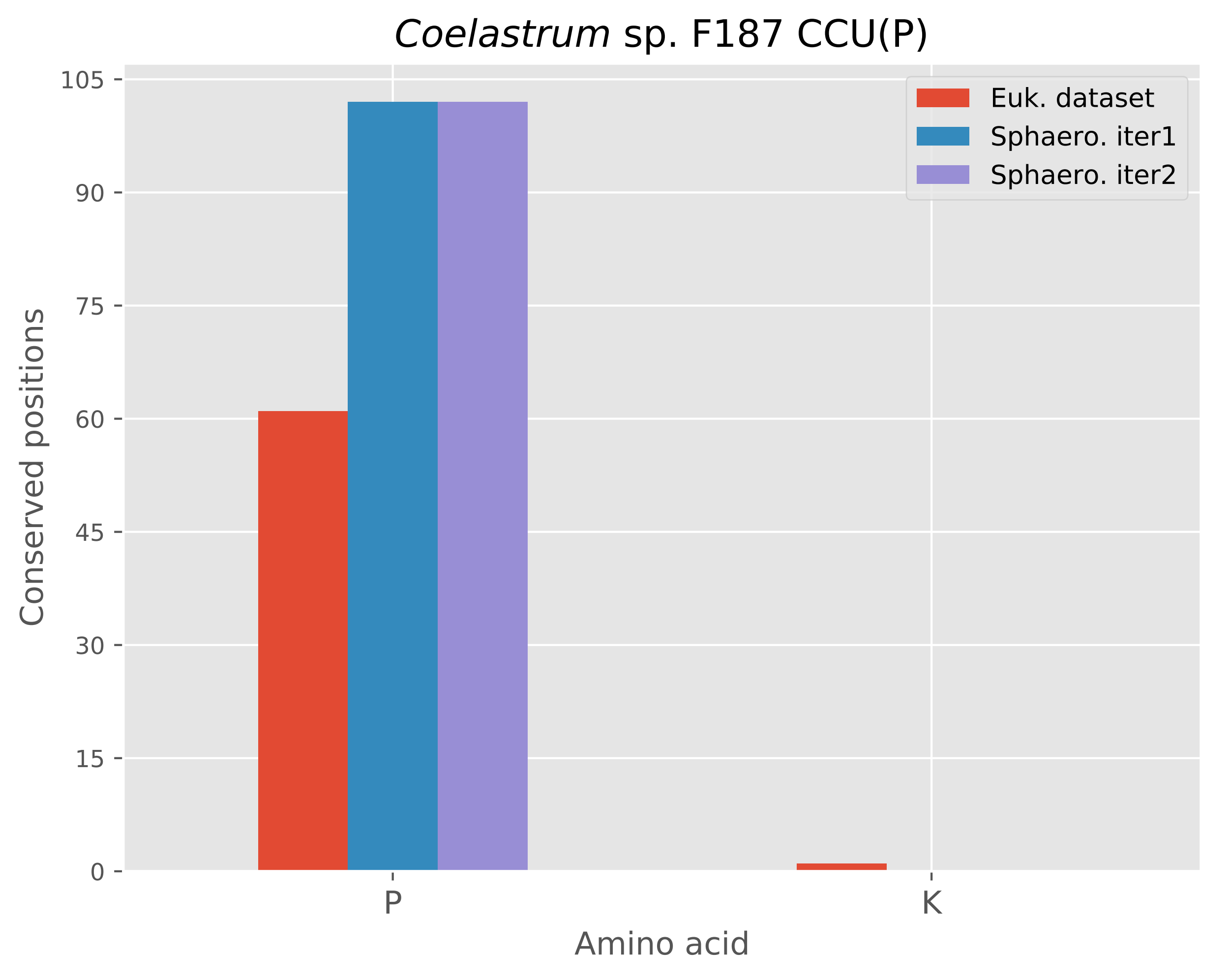

Coelastrum sp. F187 CCU(P)
Conserved positions
Amino acid
P
K
0
15
30
45
60
75
90
105
Euk. dataset
Sphaero. iter1
Sphaero. iter2

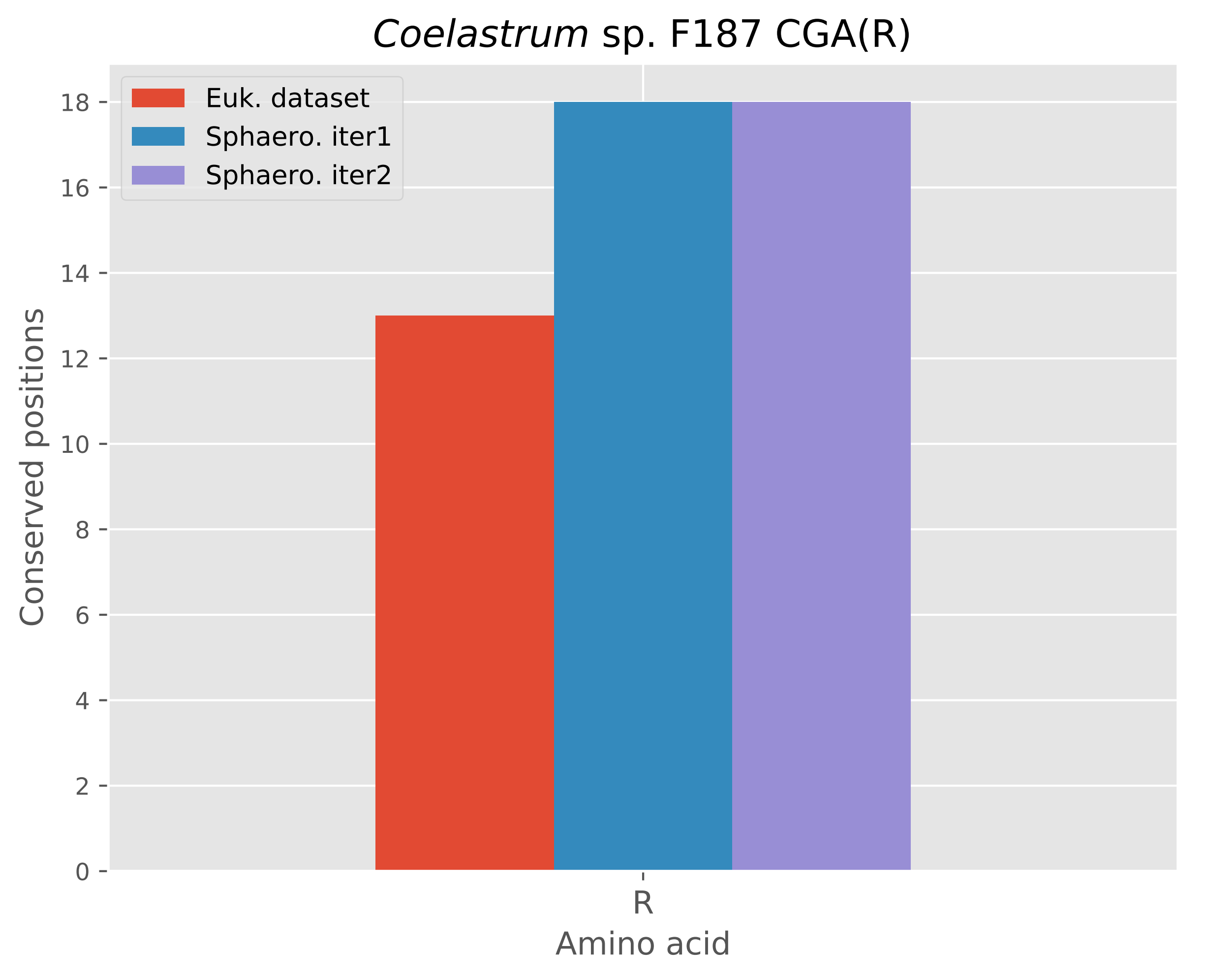
Coelastrum sp. F187 CGA(R)
Euk. dataset
Sphaero. iter1
Sphaero. iter2
Conserved positions
0
2
4
6
8
10
12
14
16
18
R
Amino acid

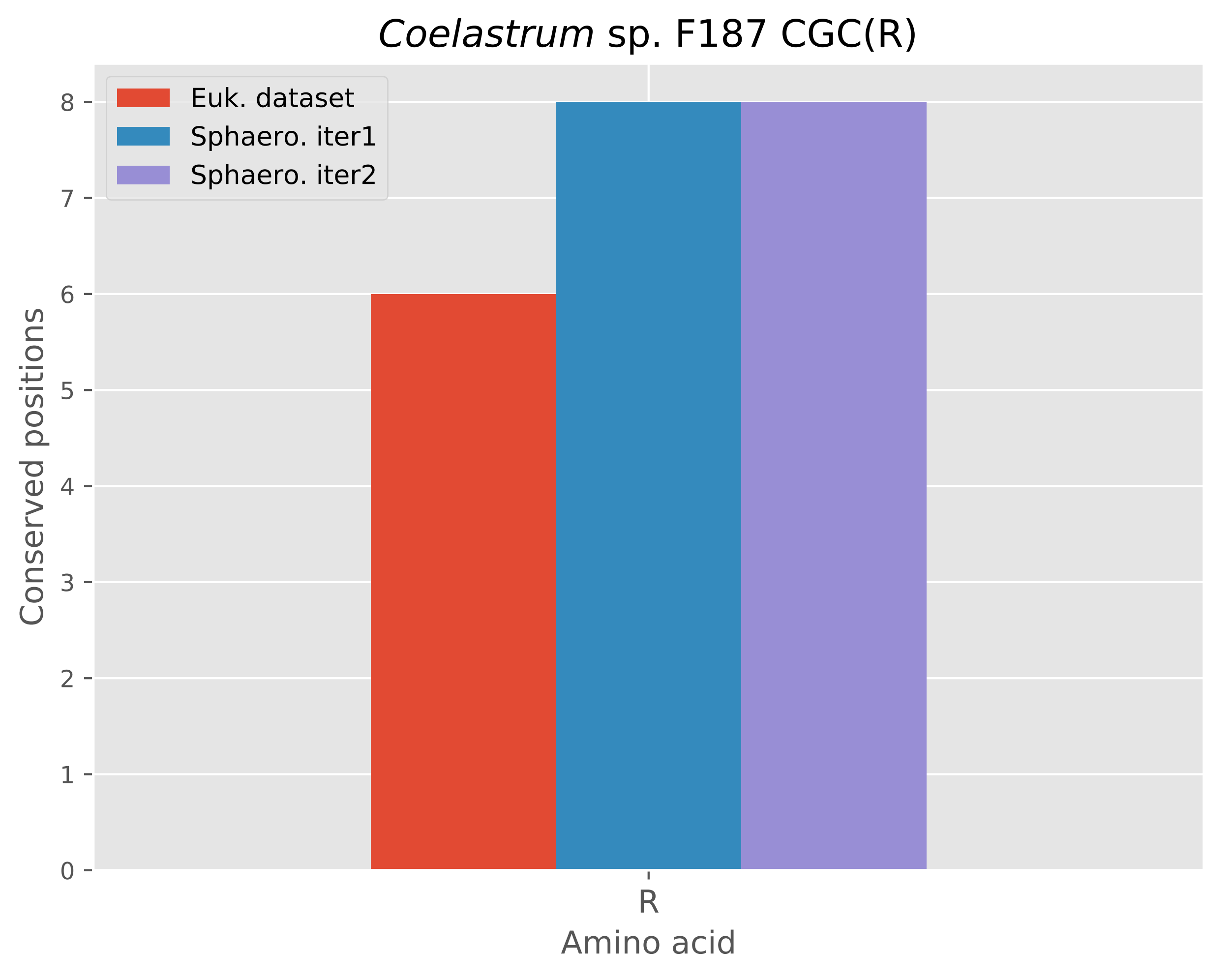

Coelastrum sp. F187 CGC(R)
Euk. dataset
Sphaero. iter1
Sphaero. iter2
Conserved positions
0
1
2
3
4
5
6
7
8
R
Amino acid

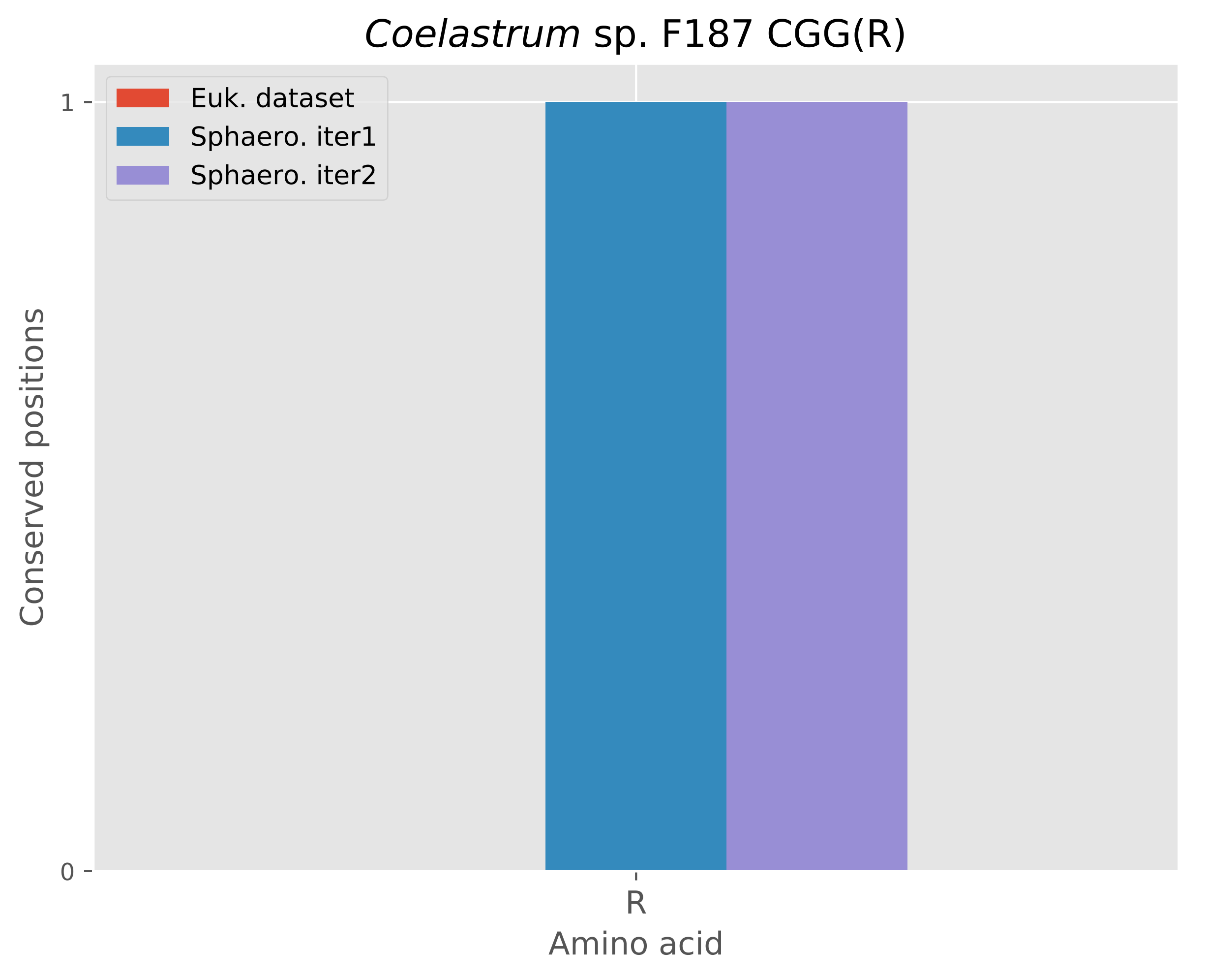

Coelastrum sp. F187 CGG(R)
Euk. dataset
Sphaero. iter1
Sphaero. iter2
1
0
Conserved positions
R
Amino acid

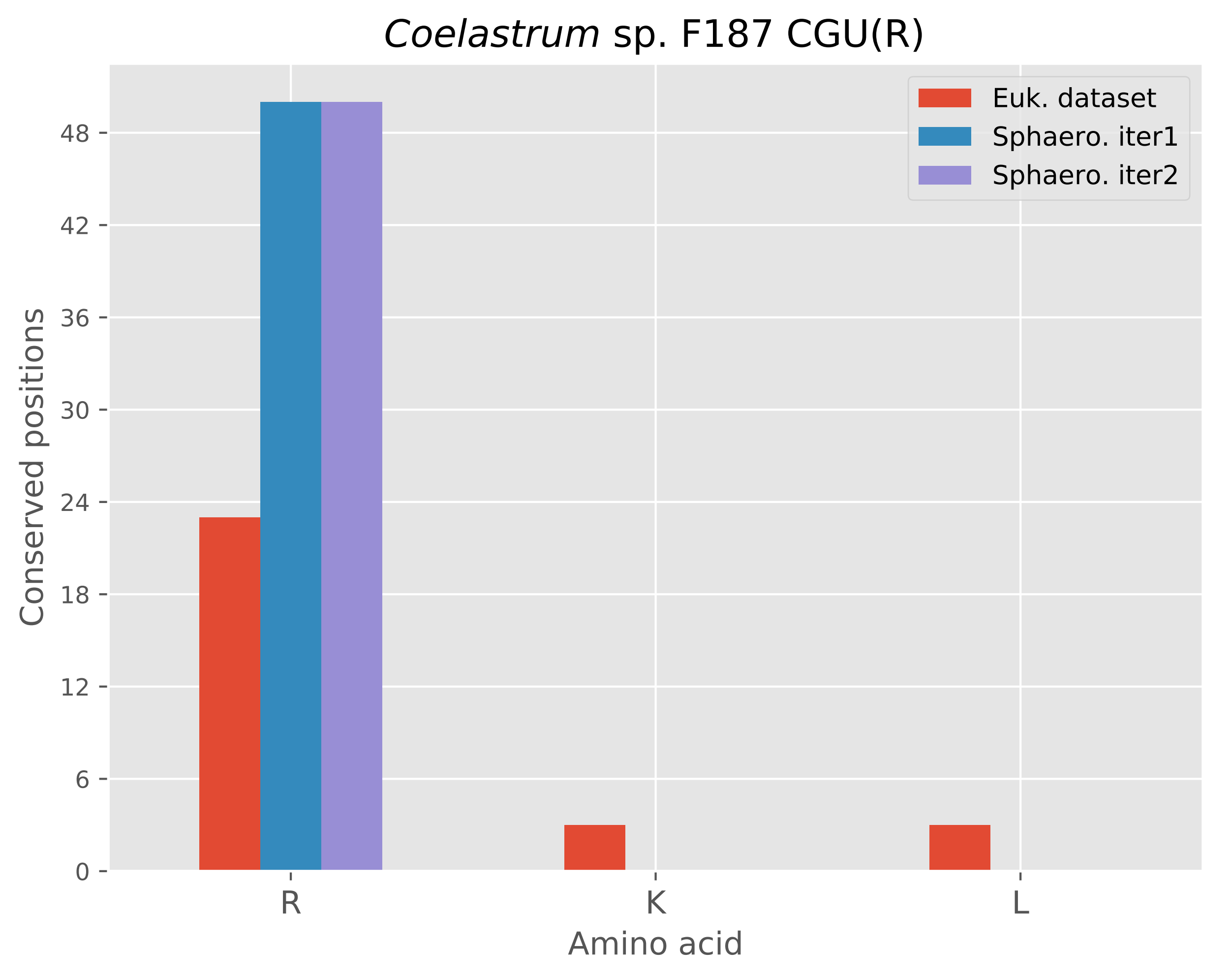
Coelastrum sp. F187 CGU(R)
Conserved positions
0
6
12
18
24
30
36
42
48
R
K
L
Amino acid
Euk. dataset
Sphaero. iter1
Sphaero. iter2

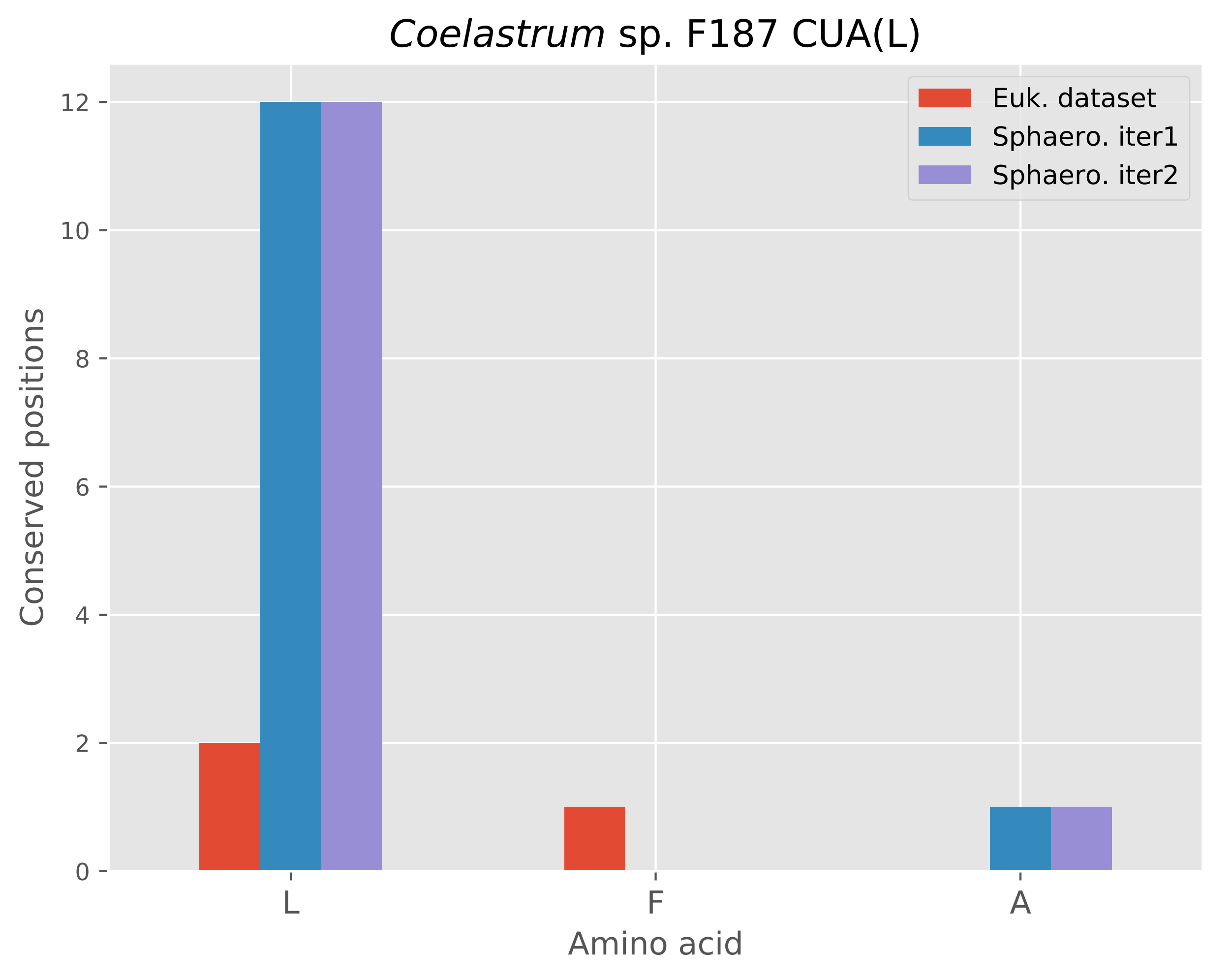

Coelastrum sp. F187 CUA(L)
Euk. dataset
Sphaero. iter1
Sphaero. iter2
Conserved positions
0
2
4
6
8
10
12
L
F
A
Amino acid

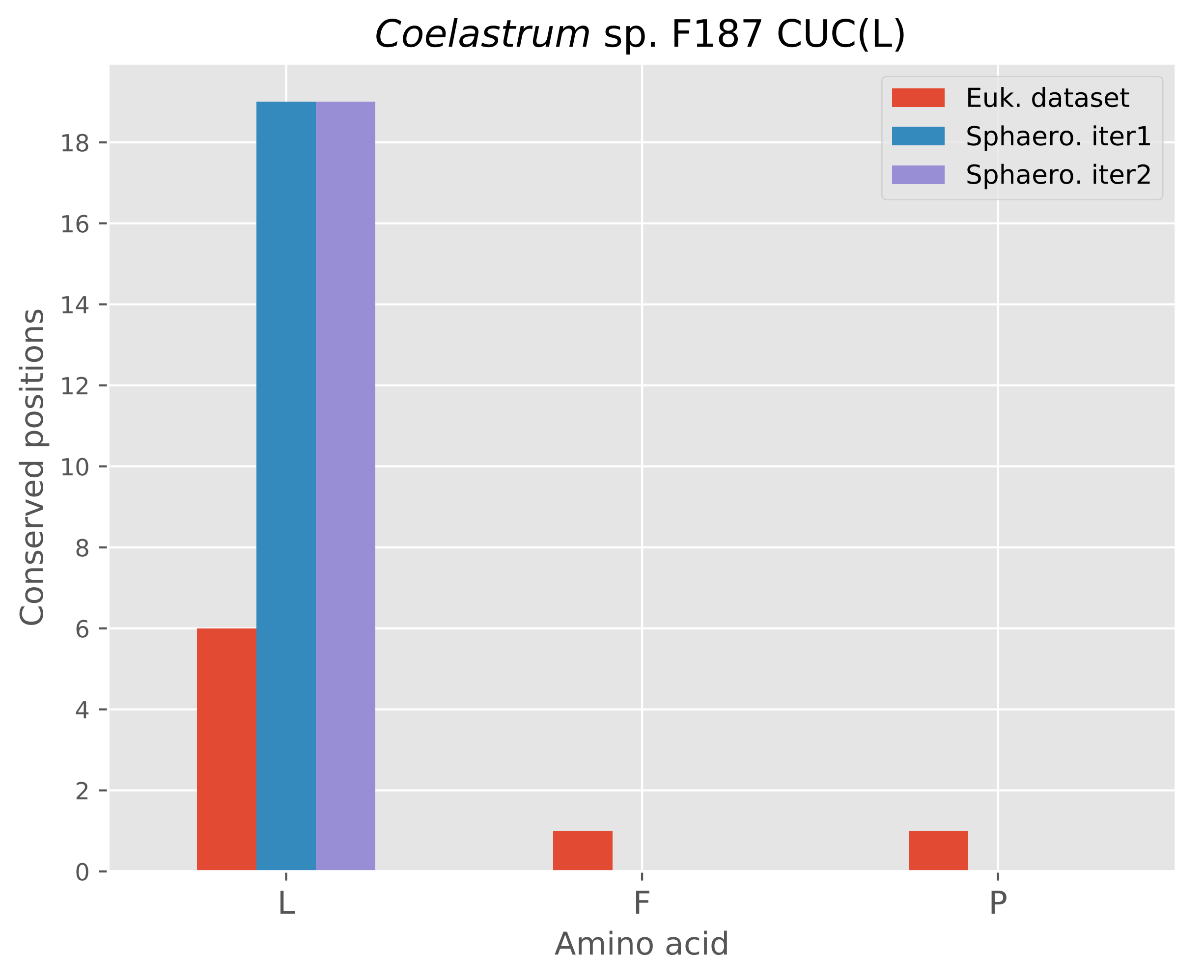

Coelastrum sp. F187 CUC(L)
Euk. dataset
Sphaero. iter1
Sphaero. iter2
Conserved positions
0
2
4
6
8
10
12
14
16
18
L
F
P
Amino acid

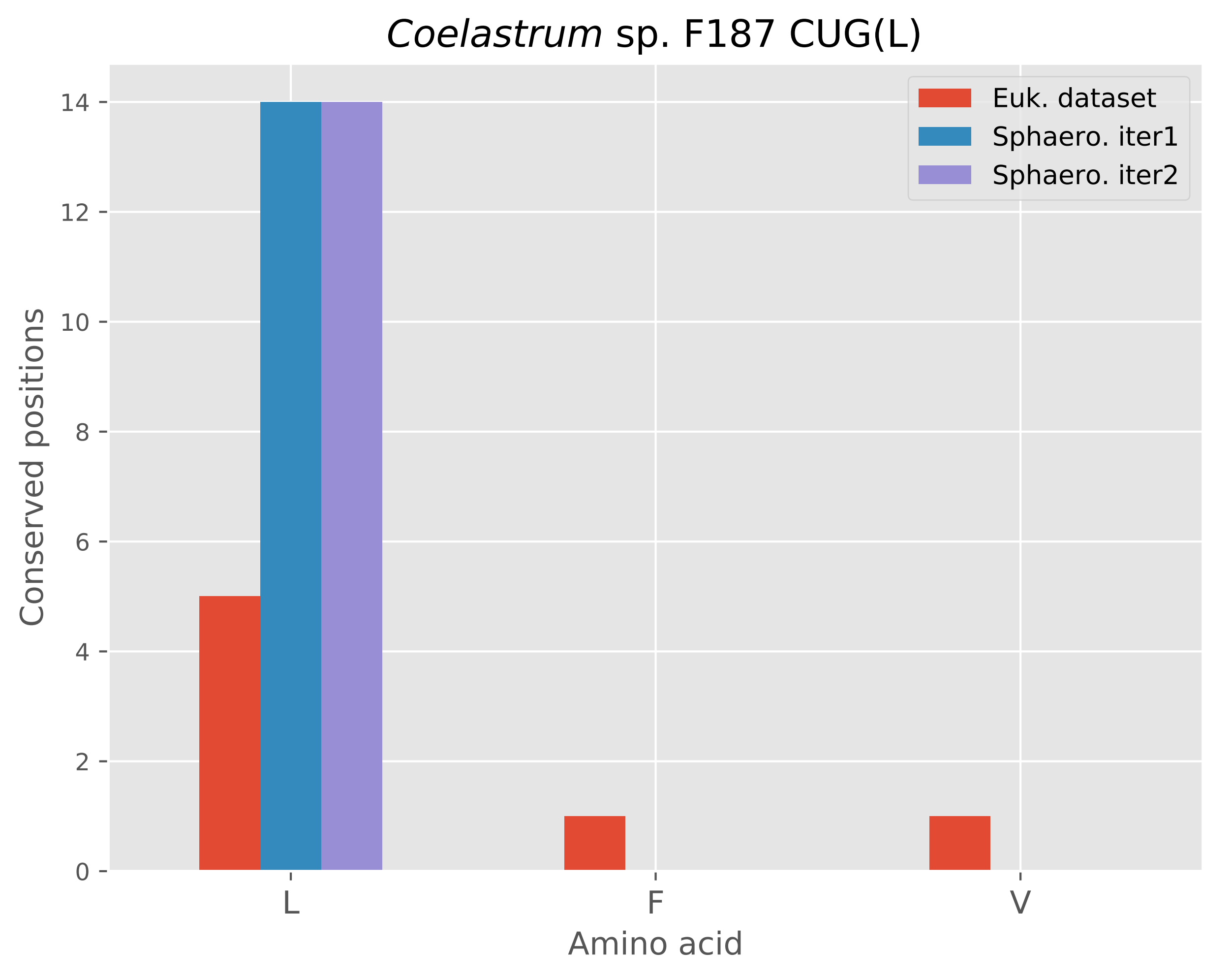

Coelastrum sp. F187 CUG(L)
Euk. dataset
Sphaero. iter1
Sphaero. iter2
Conserved positions
0
2
4
6
8
10
12
14
L
F
V
Amino acid

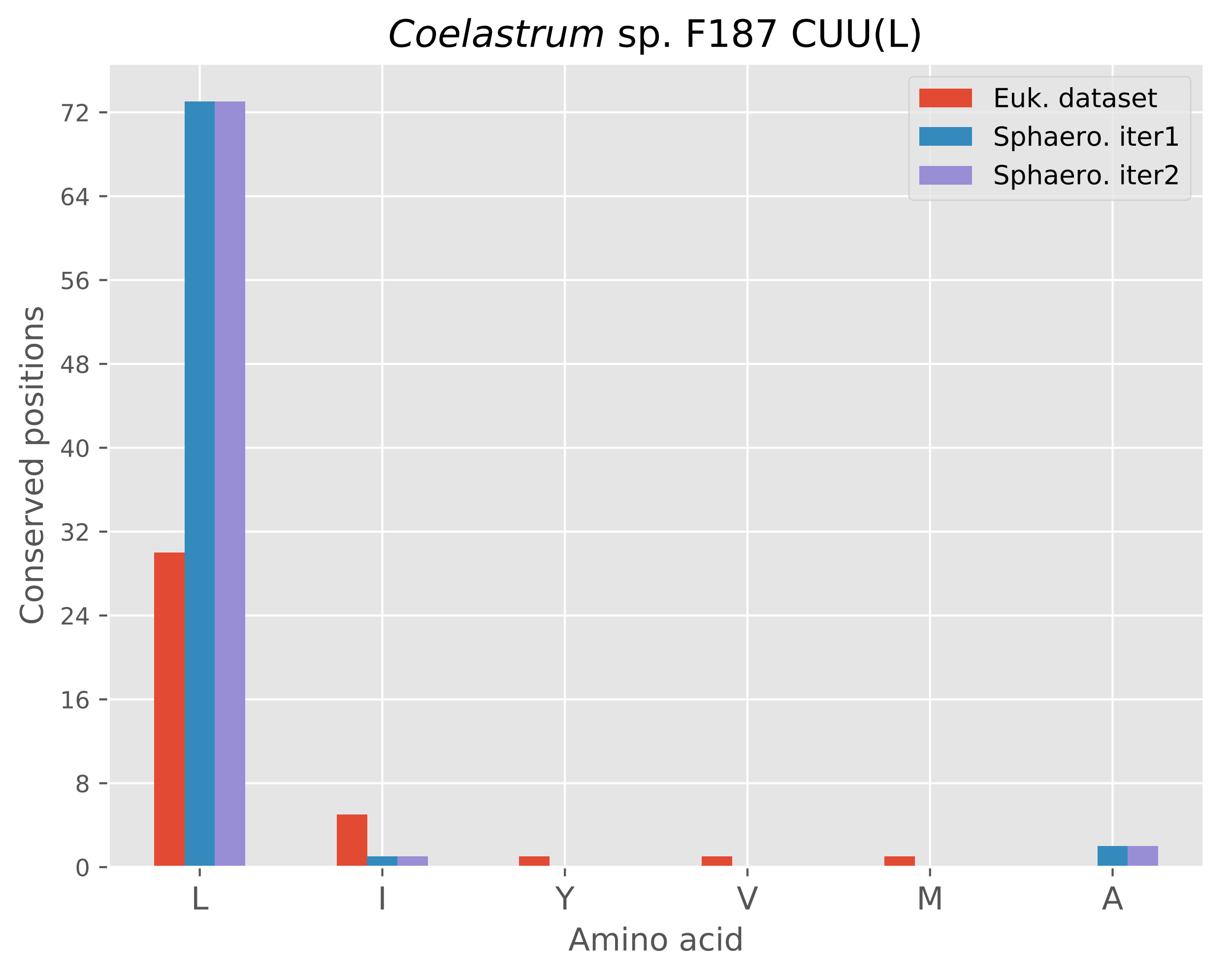

Coelastrum sp. F187 CUU(L)
Euk. dataset
Sphaero. iter1
Sphaero. iter2
Conserved positions
0
8
16
24
32
40
48
56
64
72
L
I
Y
V
M
A
Amino acid

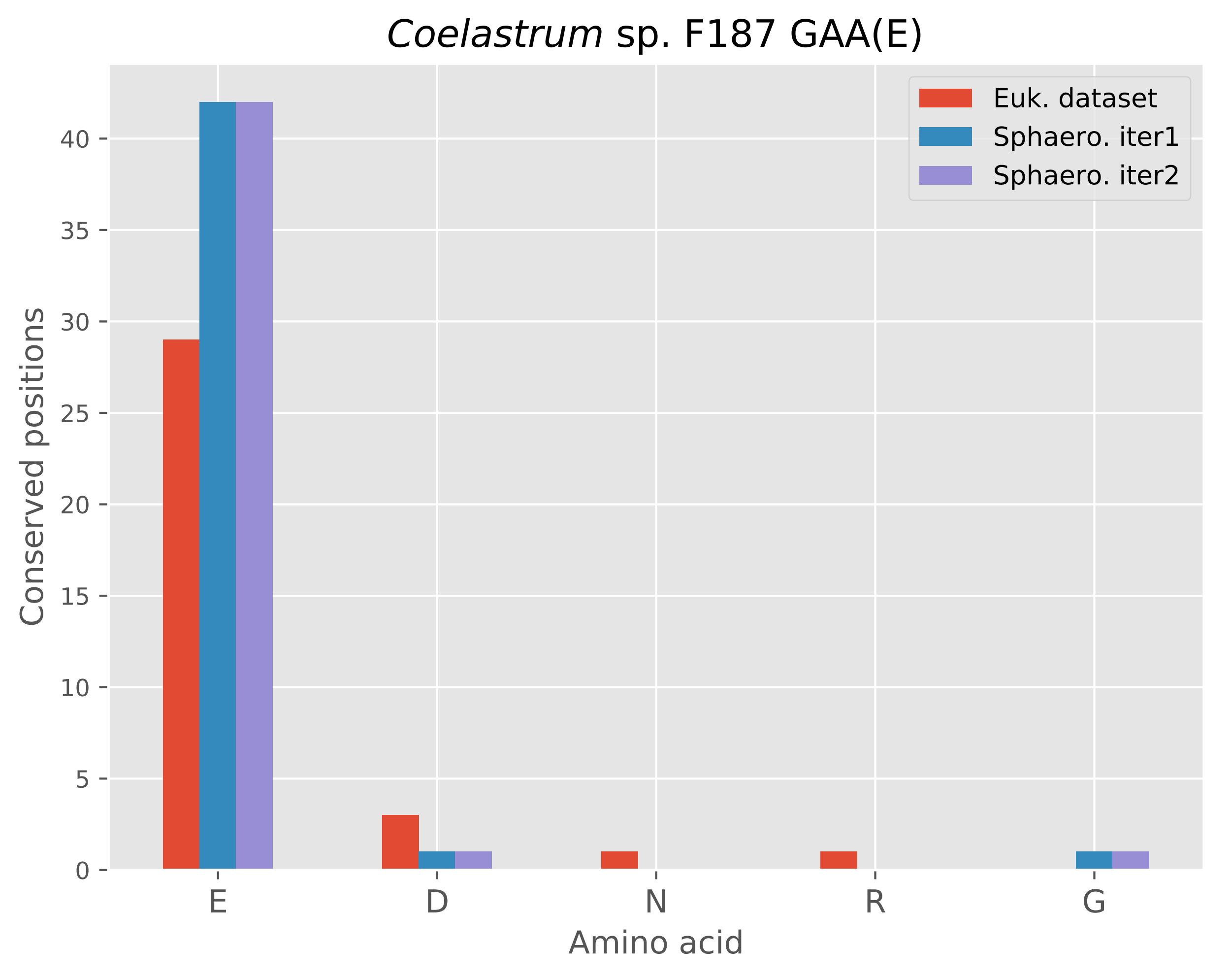

Coelastrum sp. F187 GAA(E)
Euk. dataset
Sphaero. iter1
Sphaero. iter2
Conserved positions
0
5
10
15
20
25
30
35
40
E
D
N
R
G
Amino acid

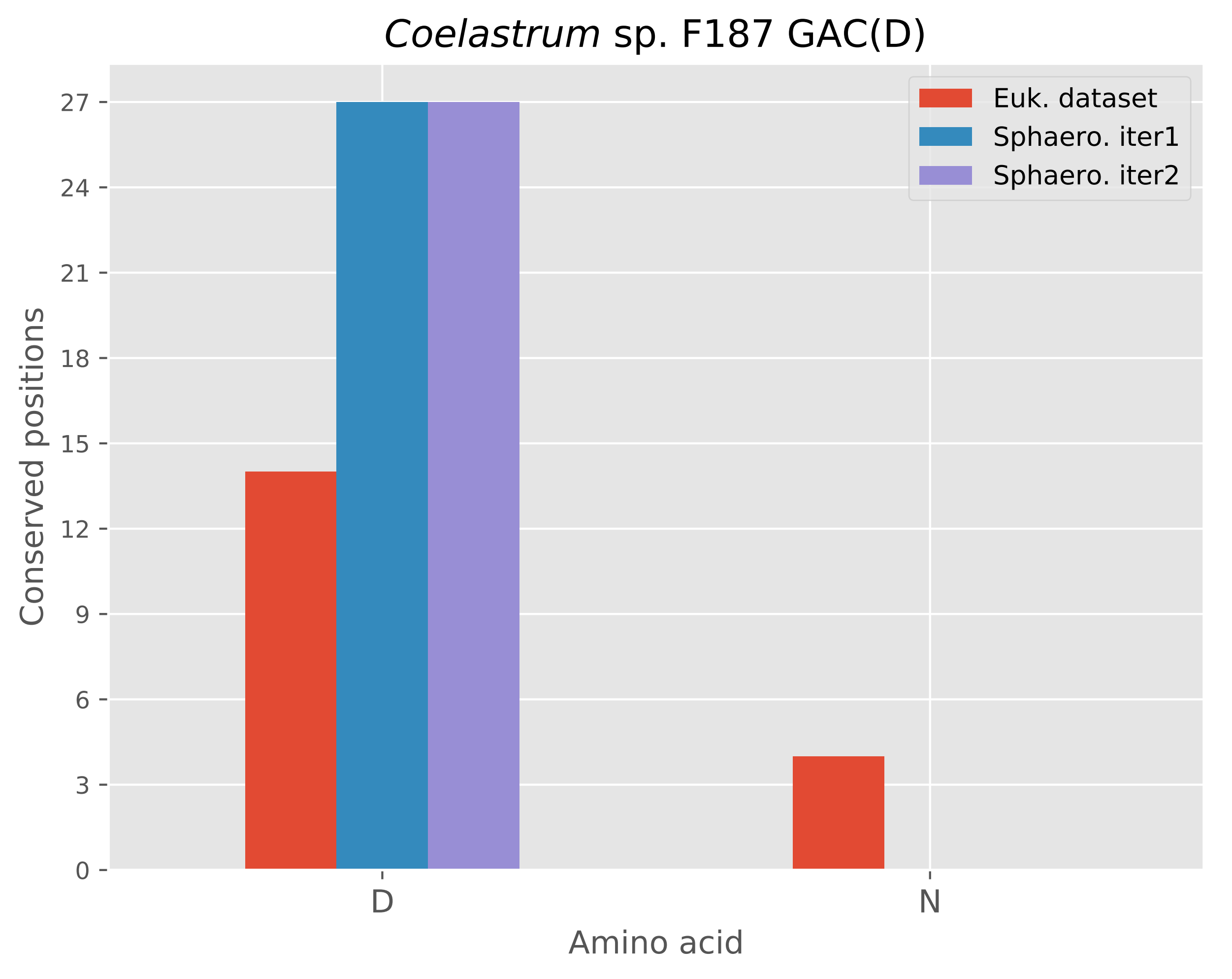

Coelastrum sp. F187 GAC(D)
Euk. dataset
Sphaero. iter1
Sphaero. iter2
Conserved positions
0
3
6
9
12
15
18
21
24
27
D
N
Amino acid

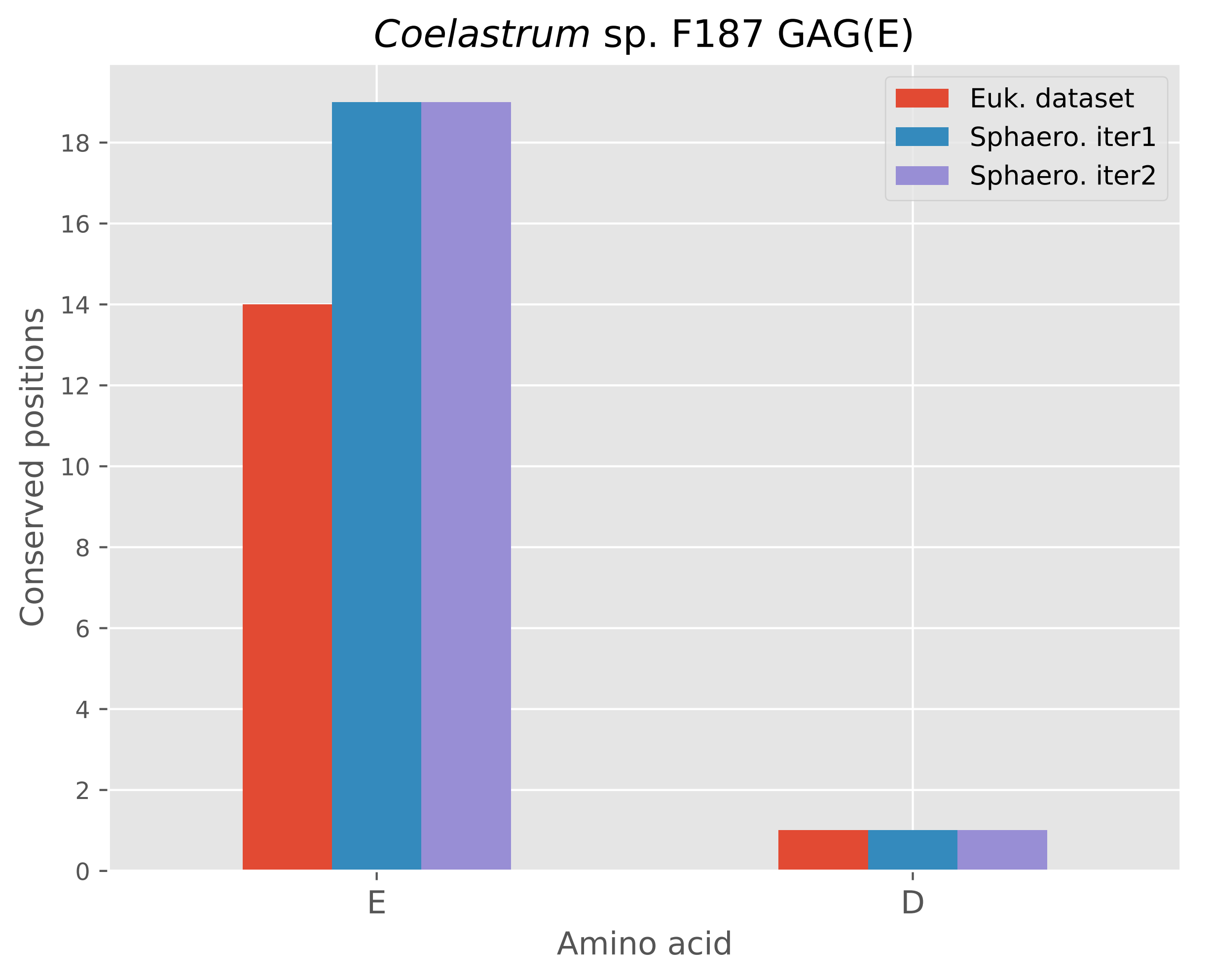

Coelastrum sp. F187 GAG(E)
Euk. dataset
Sphaero. iter1
Sphaero. iter2
Conserved positions
0
2
4
6
8
10
12
14
16
18
E
D
Amino acid

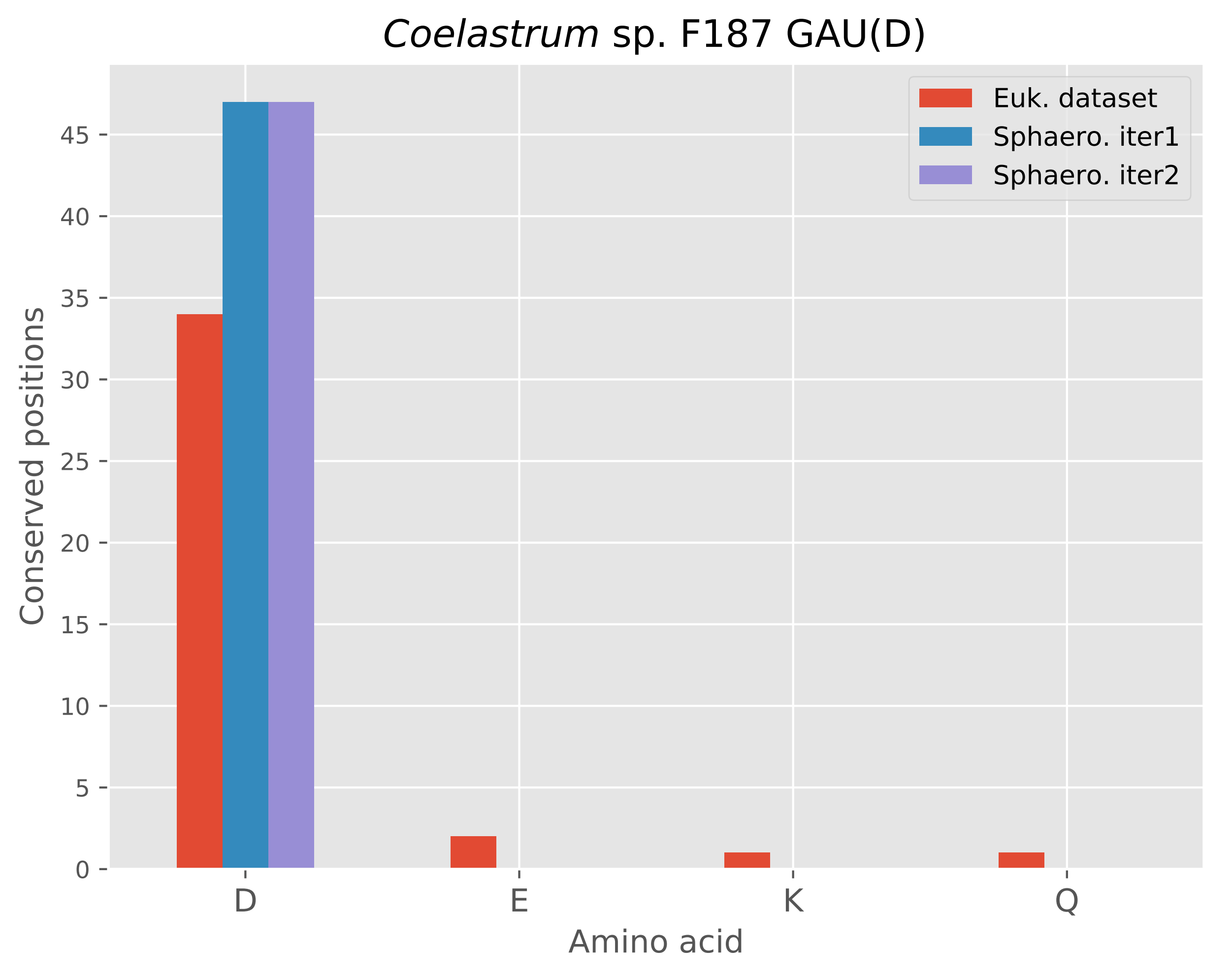

Coelastrum sp. F187 GAU(D)
Euk. dataset
Sphaero. iter1
Sphaero. iter2
Conserved positions
0
5
10
15
20
25
30
35
40
45
D
E
K
Q
Amino acid

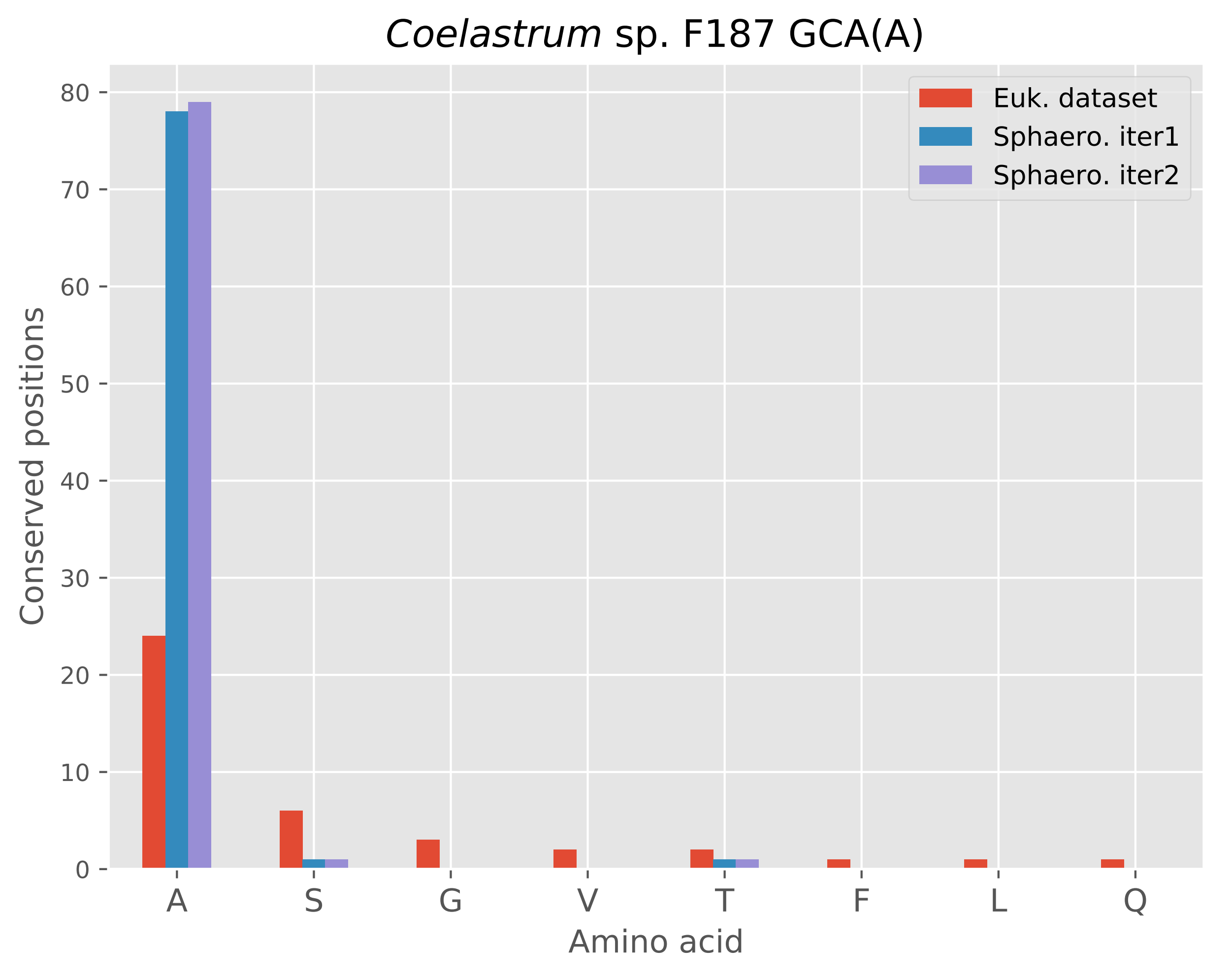

Coelastrum sp. F187 GCA(A)
Euk. dataset
Sphaero. iter1
Sphaero. iter2
Conserved positions
0
10
20
30
40
50
60
70
80
A
S
G
V
T
F
L
Q
Amino acid

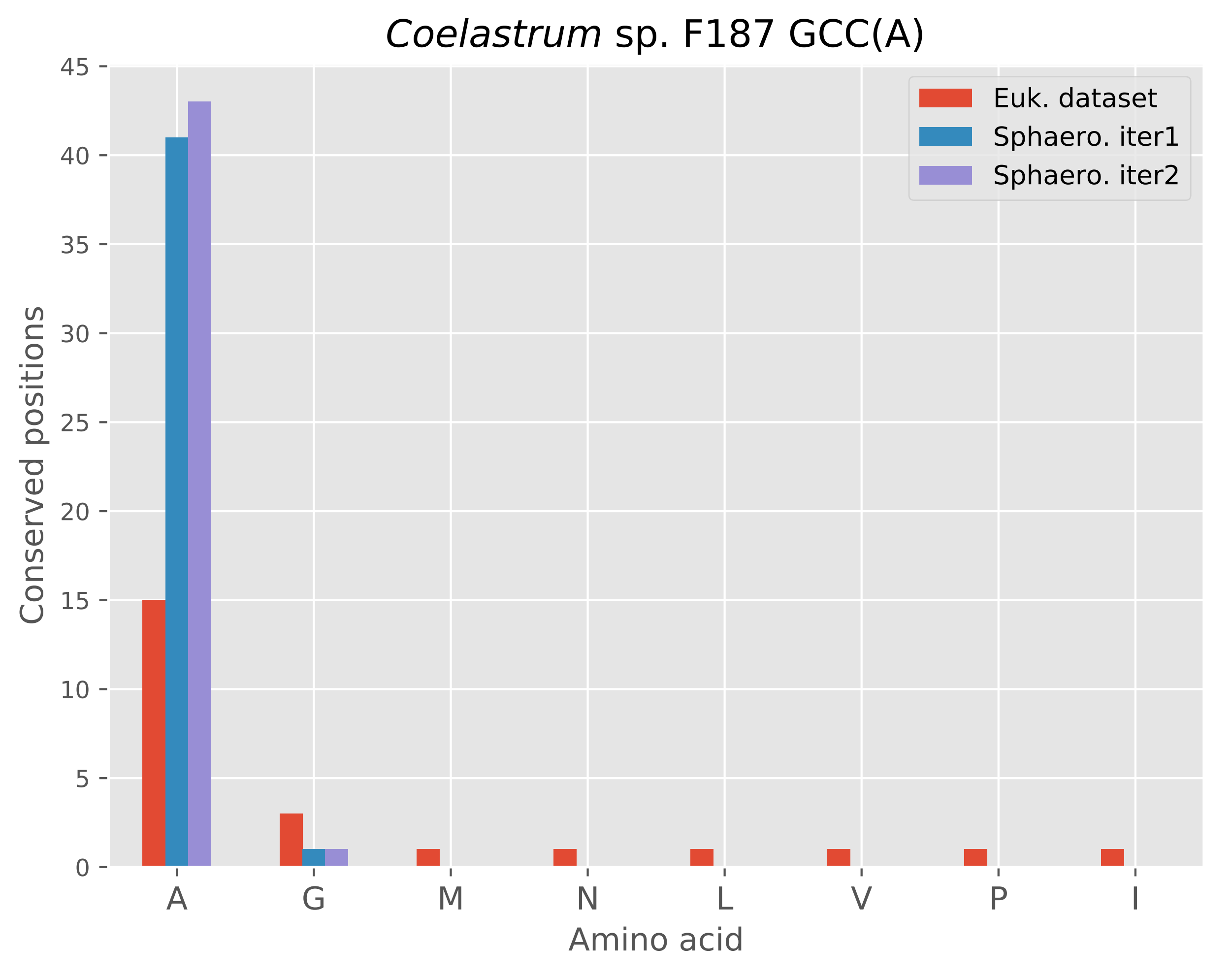

Coelastrum sp. F187 GCC(A)
Euk. dataset
Sphaero. iter1
Sphaero. iter2
Conserved positions
0
5
10
15
20
25
30
35
40
45
A
G
M
N
L
V
P
I
Amino acid

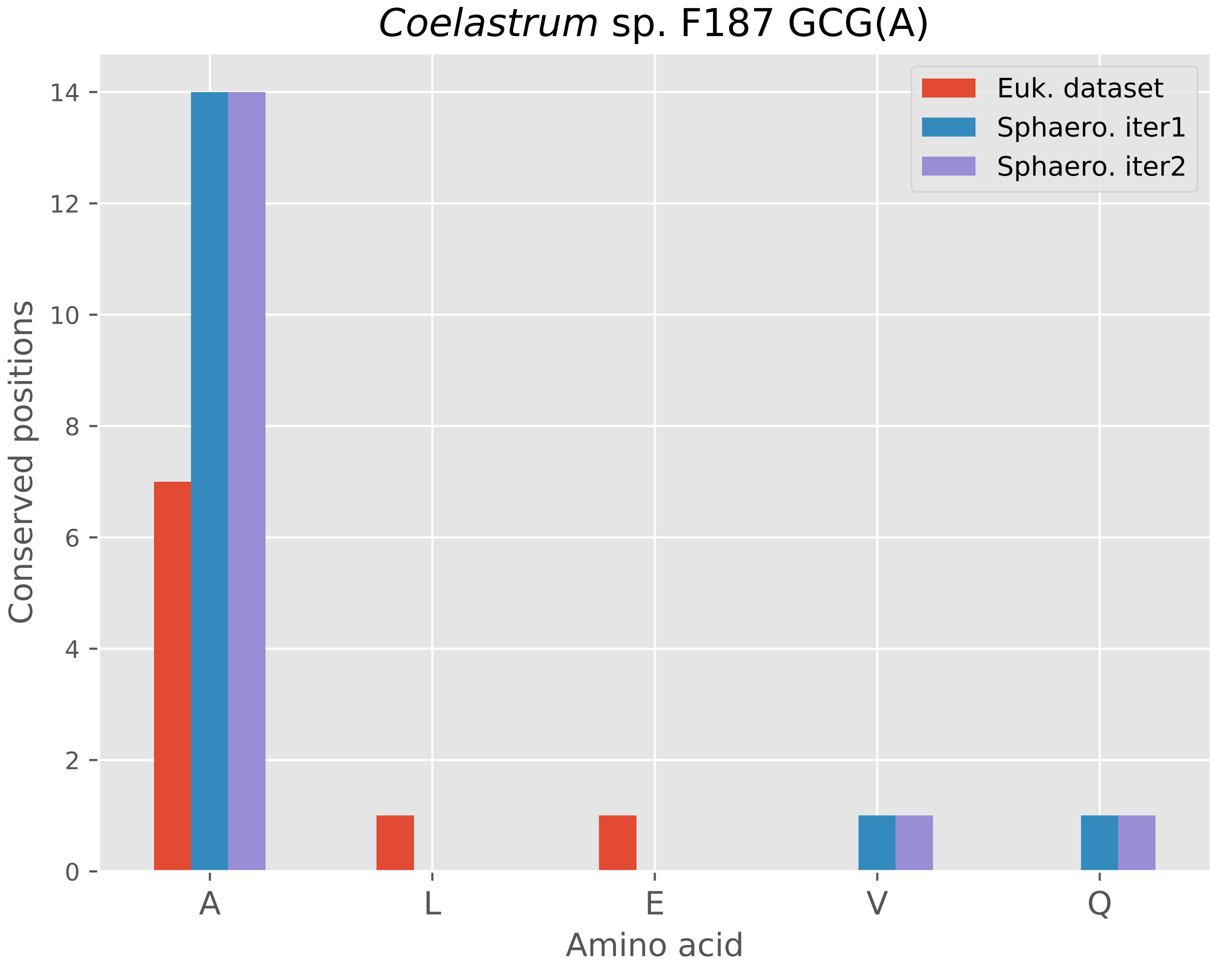
Coelastrum sp. F187 GCG(A)
Euk. dataset
Sphaero. iter1
Sphaero. iter2
Conserved positions
0
2
4
6
8
10
12
14
A
L
E
V
Q
Amino acid

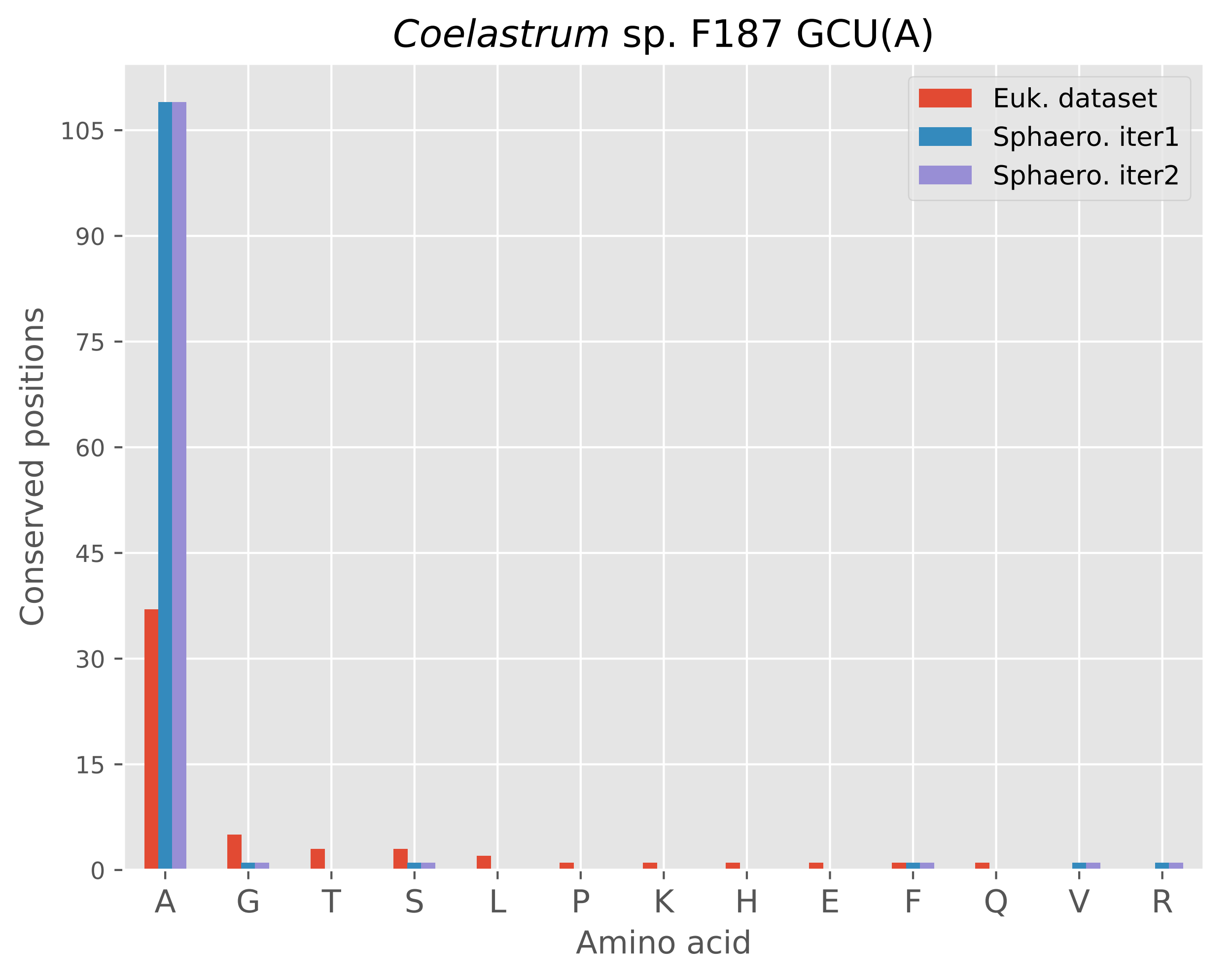

Coelastrum sp. F187 GCU(A)
Euk. dataset
Sphaero. iter1
Sphaero. iter2
Conserved positions
0
15
30
45
60
75
90
105
A
G
T
S
L
P
K
H
E
F
Q
V
R
Amino acid

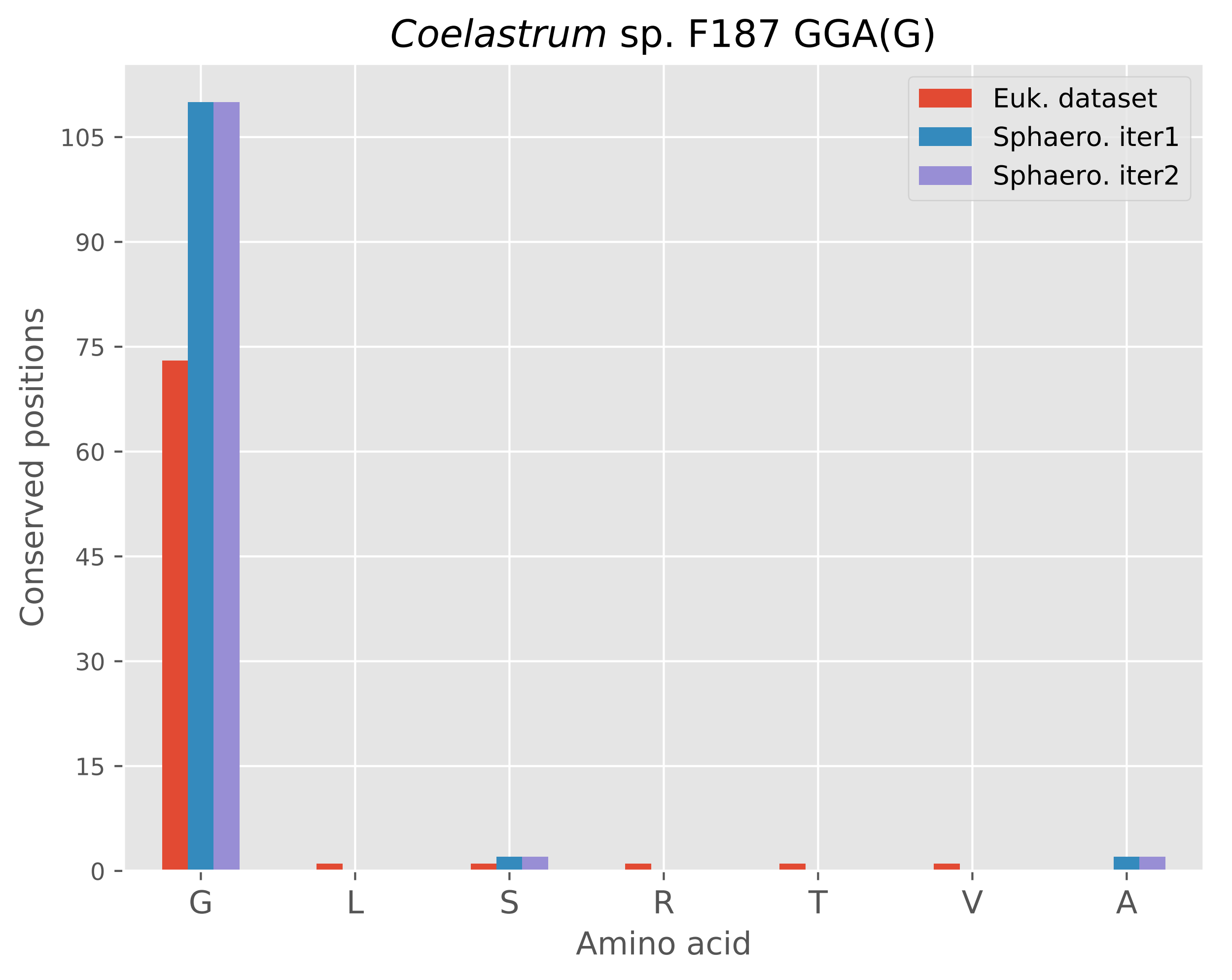

Coelastrum sp. F187 GGA(G)
Euk. dataset
Sphaero. iter1
Sphaero. iter2
Conserved positions
0
15
30
45
60
75
90
105
G
L
S
R
T
V
A
Amino acid

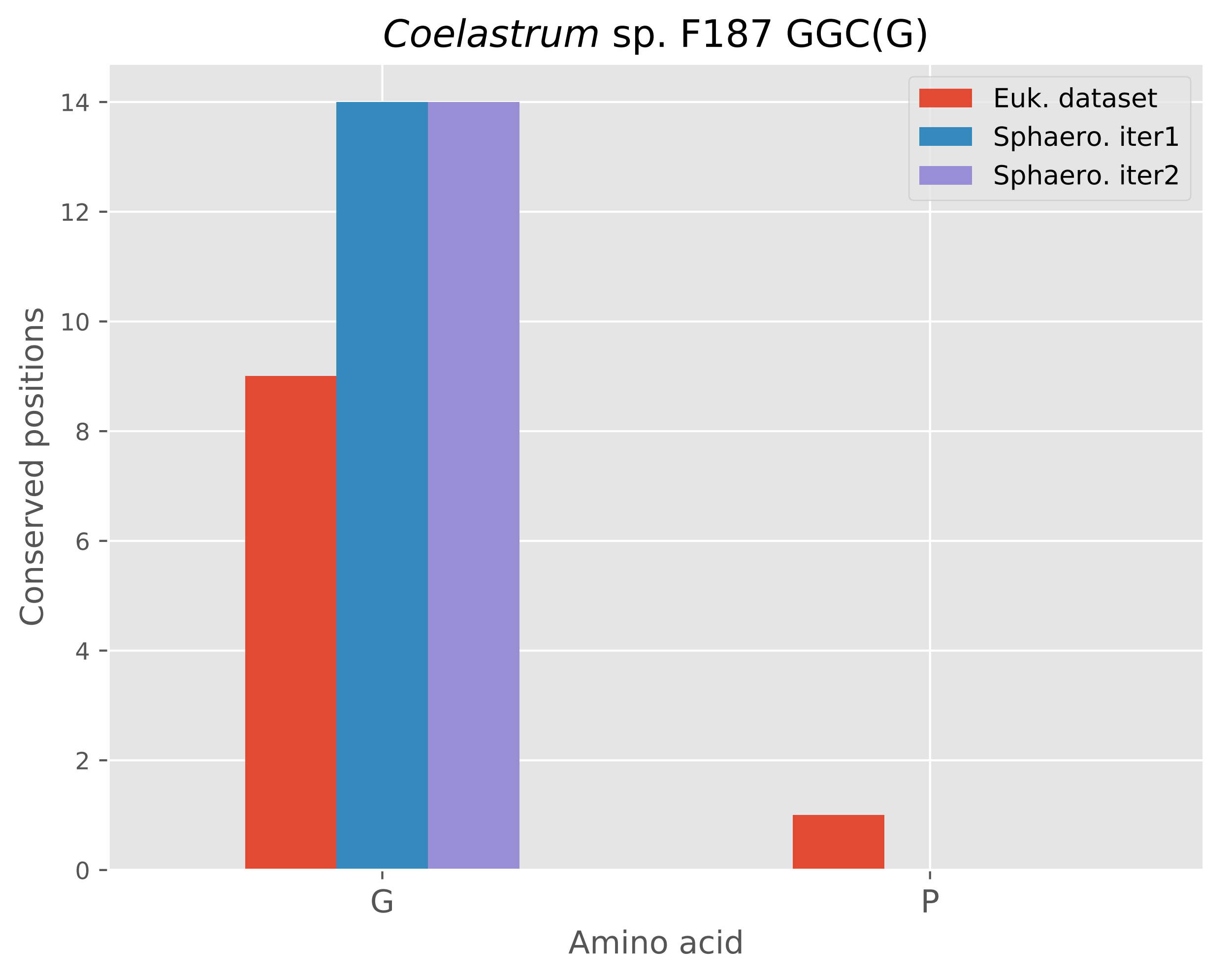

Coelastrum sp. F187 GGC(G)
Euk. dataset
Sphaero. iter1
Sphaero. iter2
Conserved positions
0
2
4
6
8
10
12
14
G
P
Amino acid

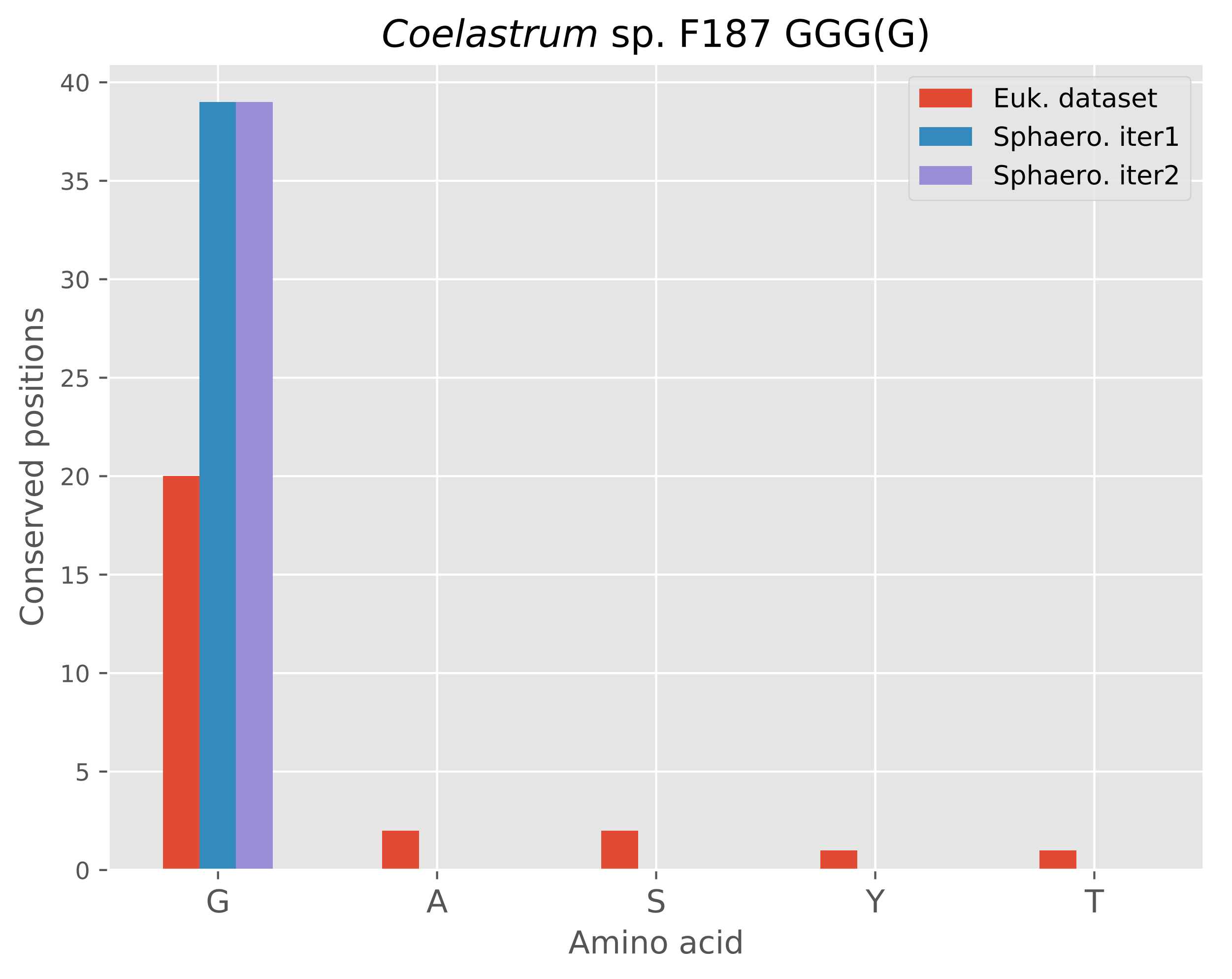

Coelastrum sp. F187 GGG(G)
Euk. dataset
Sphaero. iter1
Sphaero. iter2
Conserved positions
0
5
10
15
20
25
30
35
40
G
A
S
Y
T
Amino acid

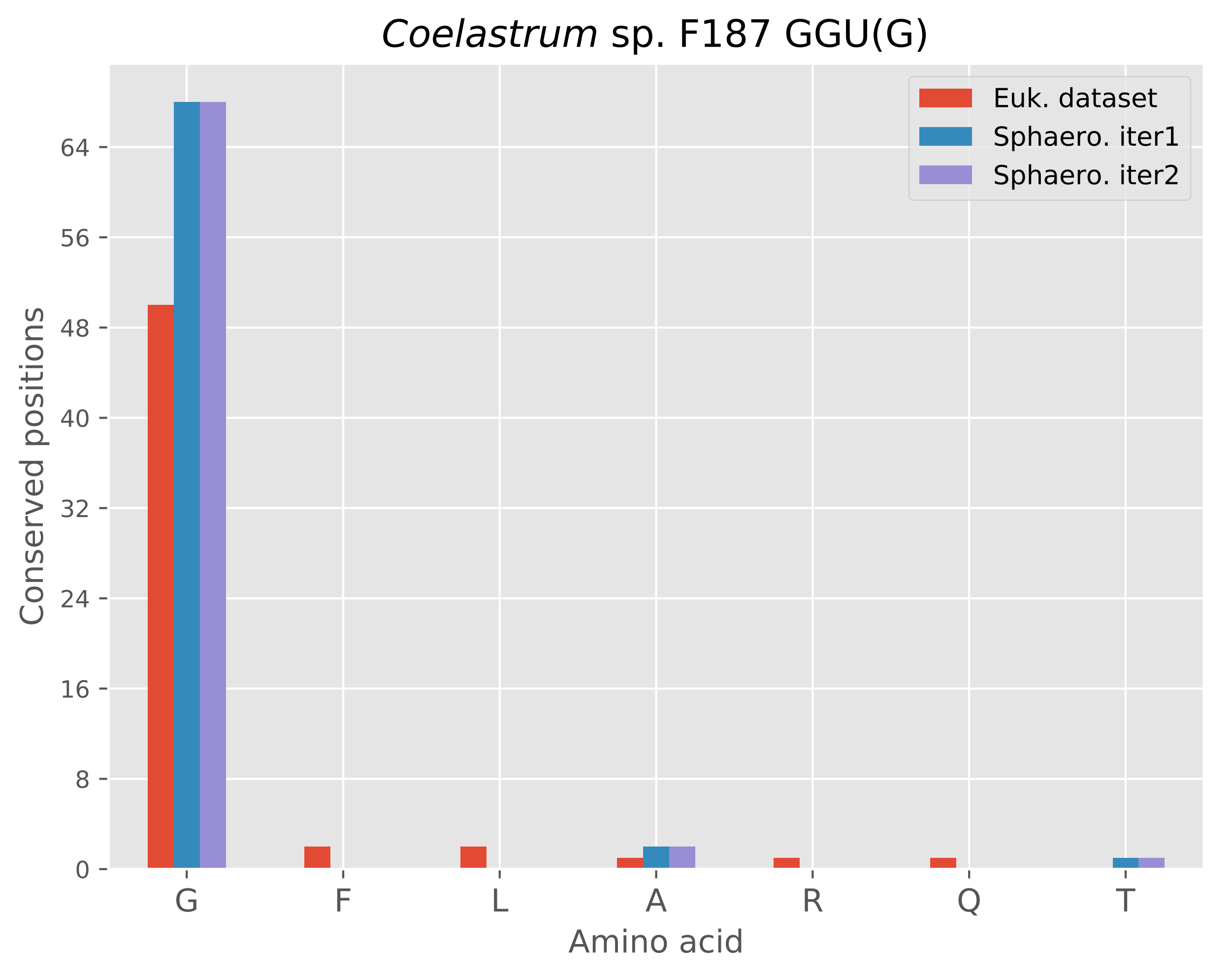

Coelastrum sp. F187 GGU(G)
Euk. dataset
Sphaero. iter1
Sphaero. iter2
Conserved positions
0
8
16
24
32
40
48
56
64
G
F
L
A
R
Q
T
Amino acid

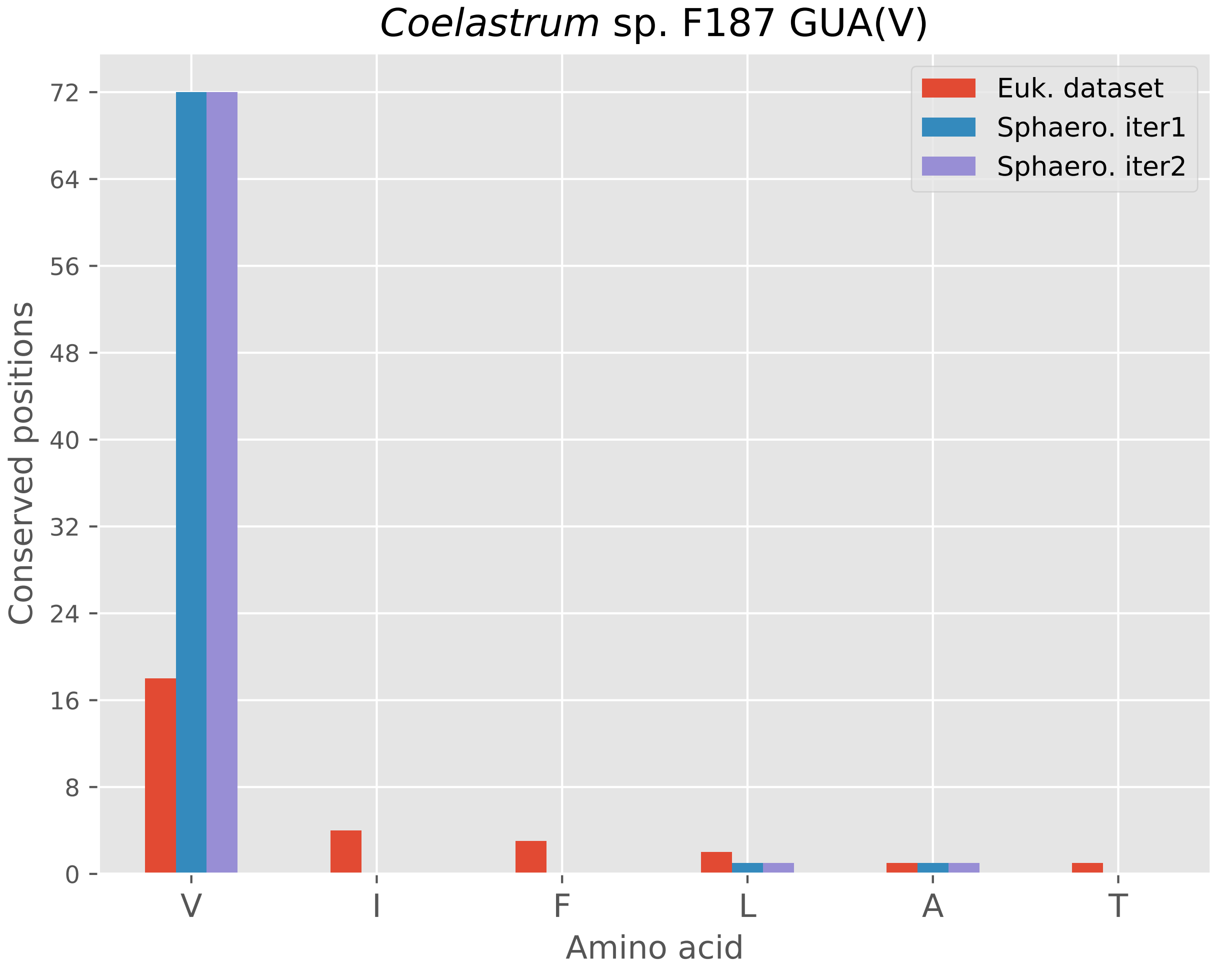
Coelastrum sp. F187 GUA(V)
Euk. dataset
Sphaero. iter1
Sphaero. iter2
Conserved positions
0
8
16
24
32
40
48
56
64
72
V
I
F
L
A
T
Amino acid

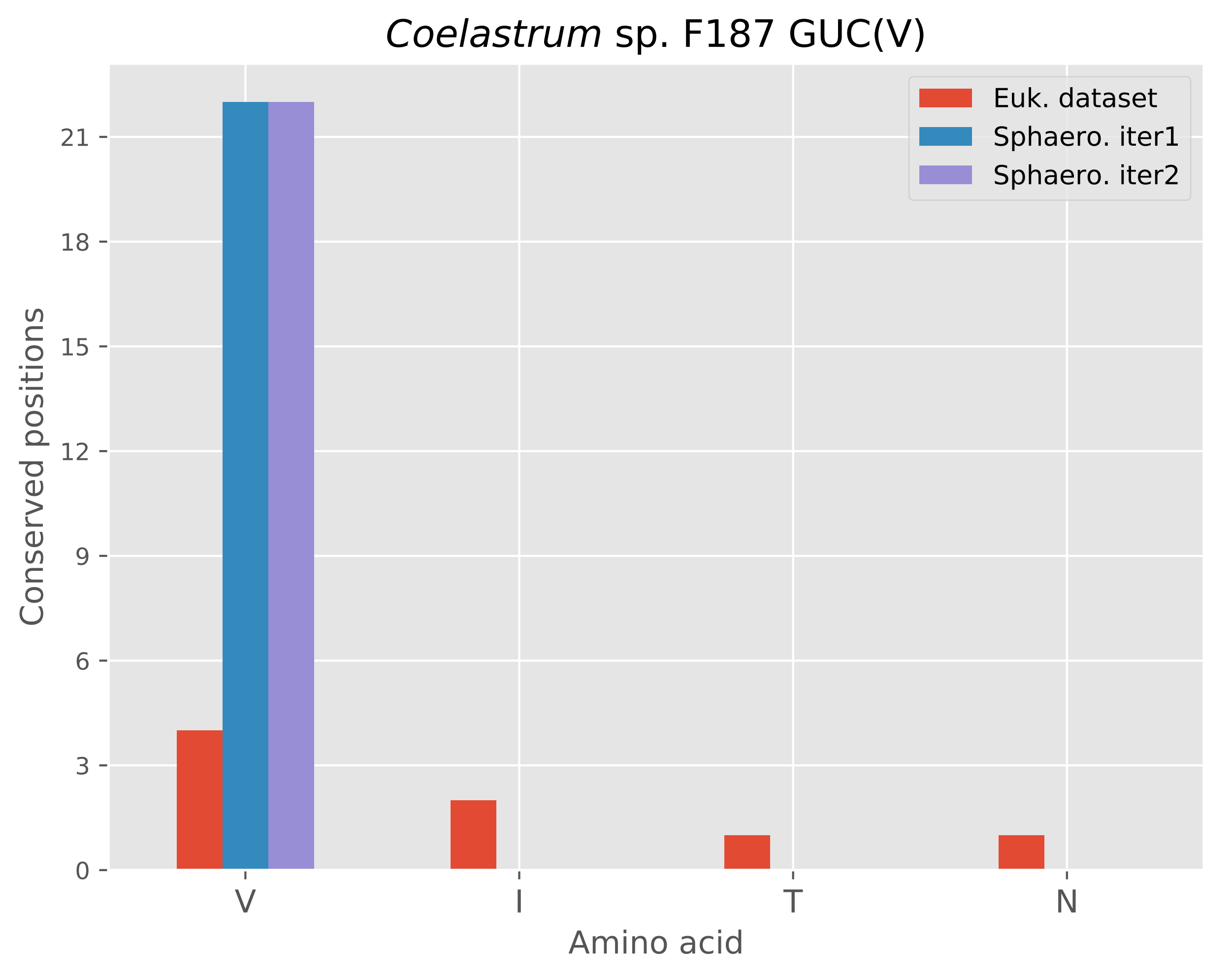

Coelastrum sp. F187 GUC(V)
Euk. dataset
Sphaero. iter1
Sphaero. iter2
Conserved positions
0
3
6
9
12
15
18
21
V
I
T
N
Amino acid

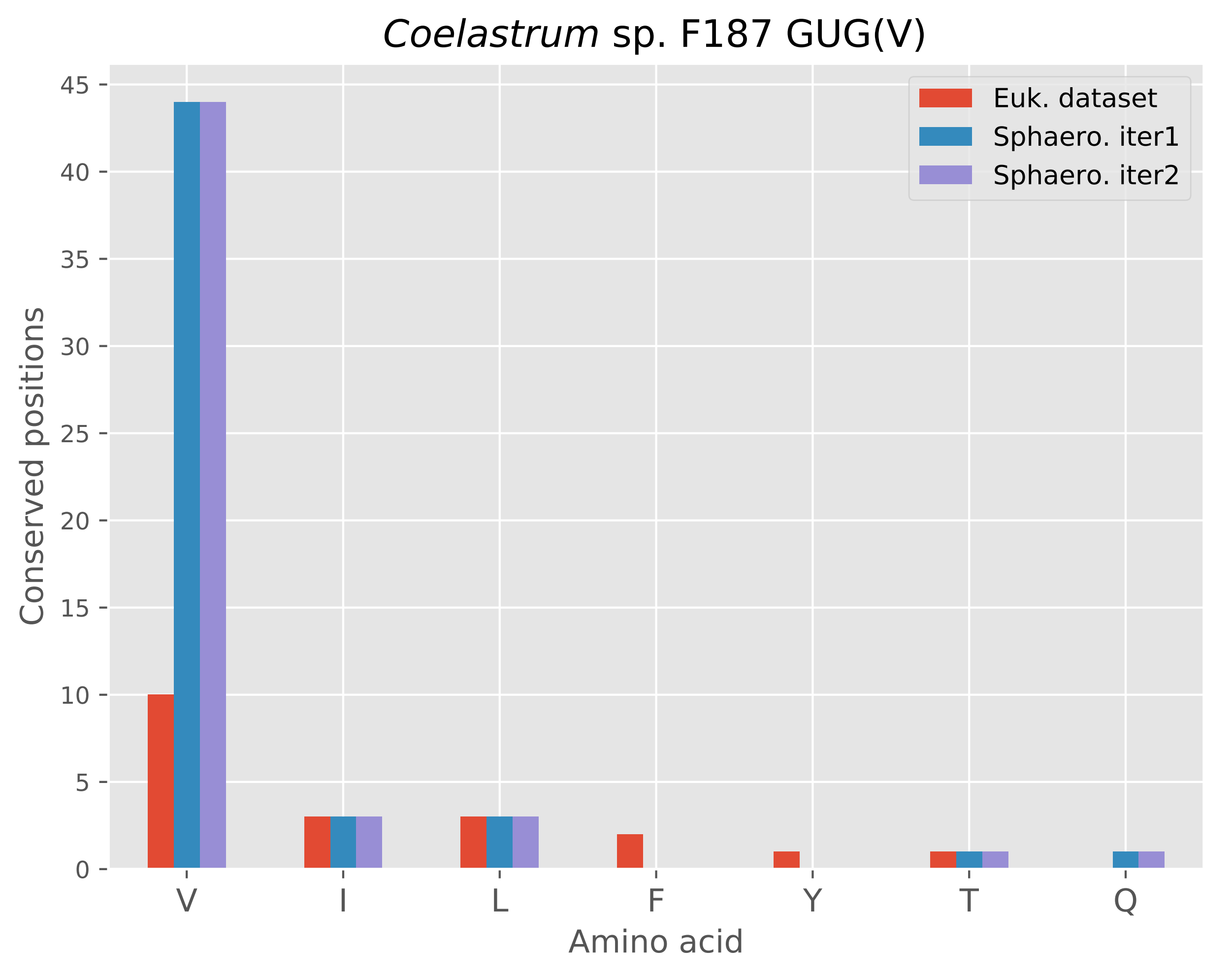

Coelastrum sp. F187 GUG(V)
Conserved positions
Amino acid
0
5
10
15
20
25
30
35
40
45
V
I
L
F
Y
T
Q
Euk. dataset
Sphaero. iter1
Sphaero. iter2

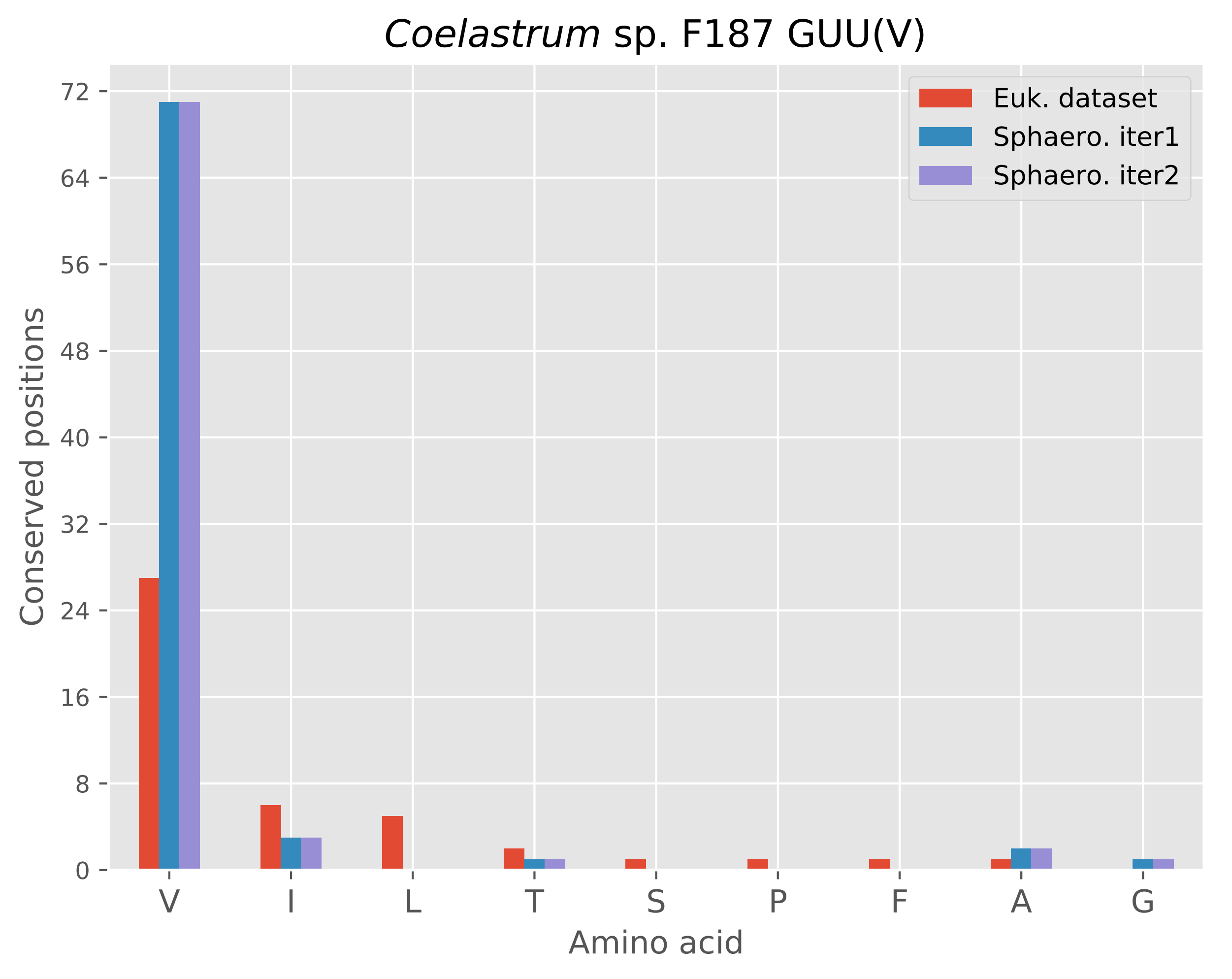

Coelastrum sp. F187 GUU(V)
Euk. dataset
Sphaero. iter1
Sphaero. iter2
Conserved positions
0
8
16
24
32
40
48
56
64
72
V
I
L
T
S
P
F
A
G
Amino acid

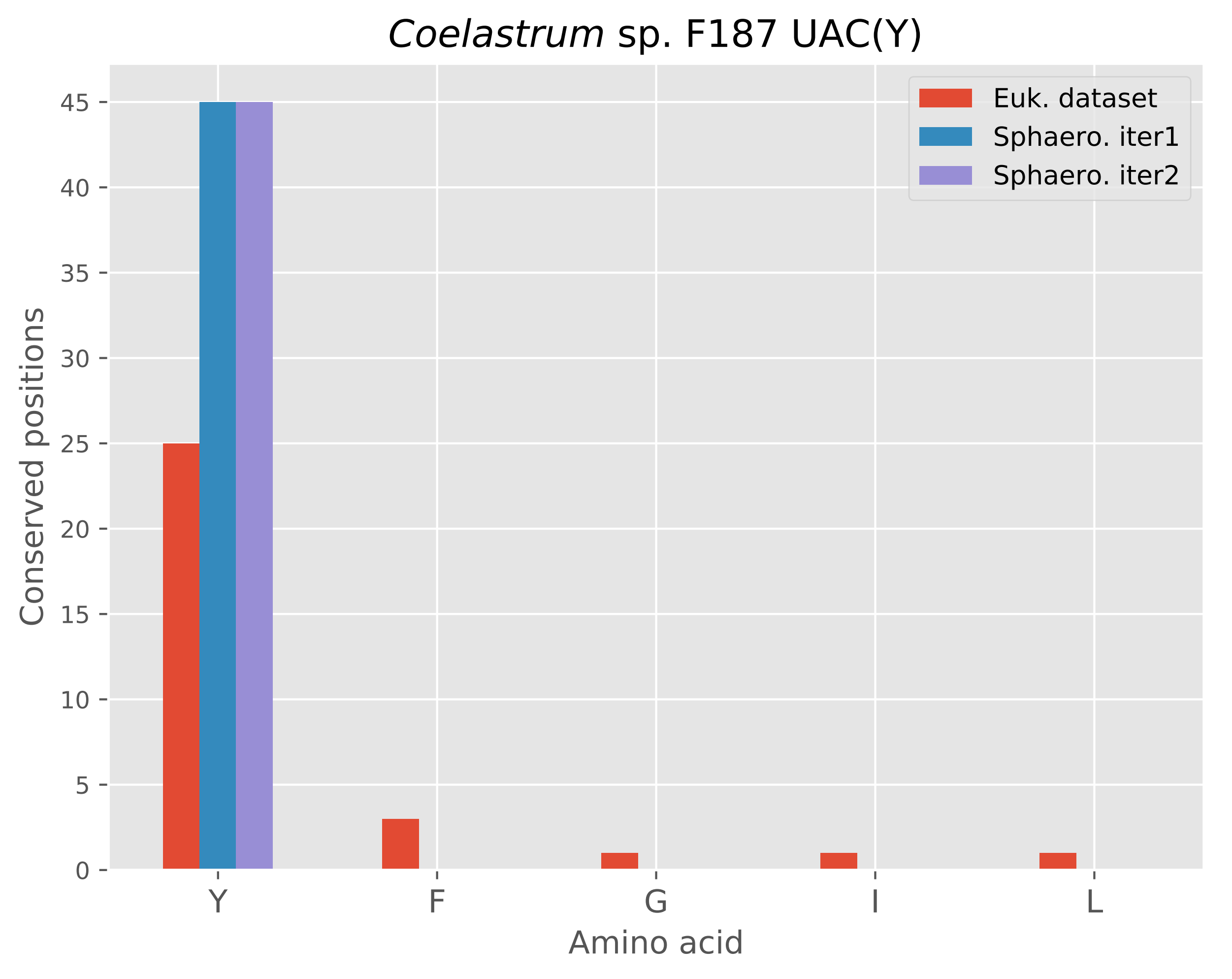

Coelastrum sp. F187 UAC(Y)
Euk. dataset
Sphaero. iter1
Sphaero. iter2
Conserved positions
0
5
10
15
20
25
30
35
40
45
Y
F
G
I
L
Amino acid

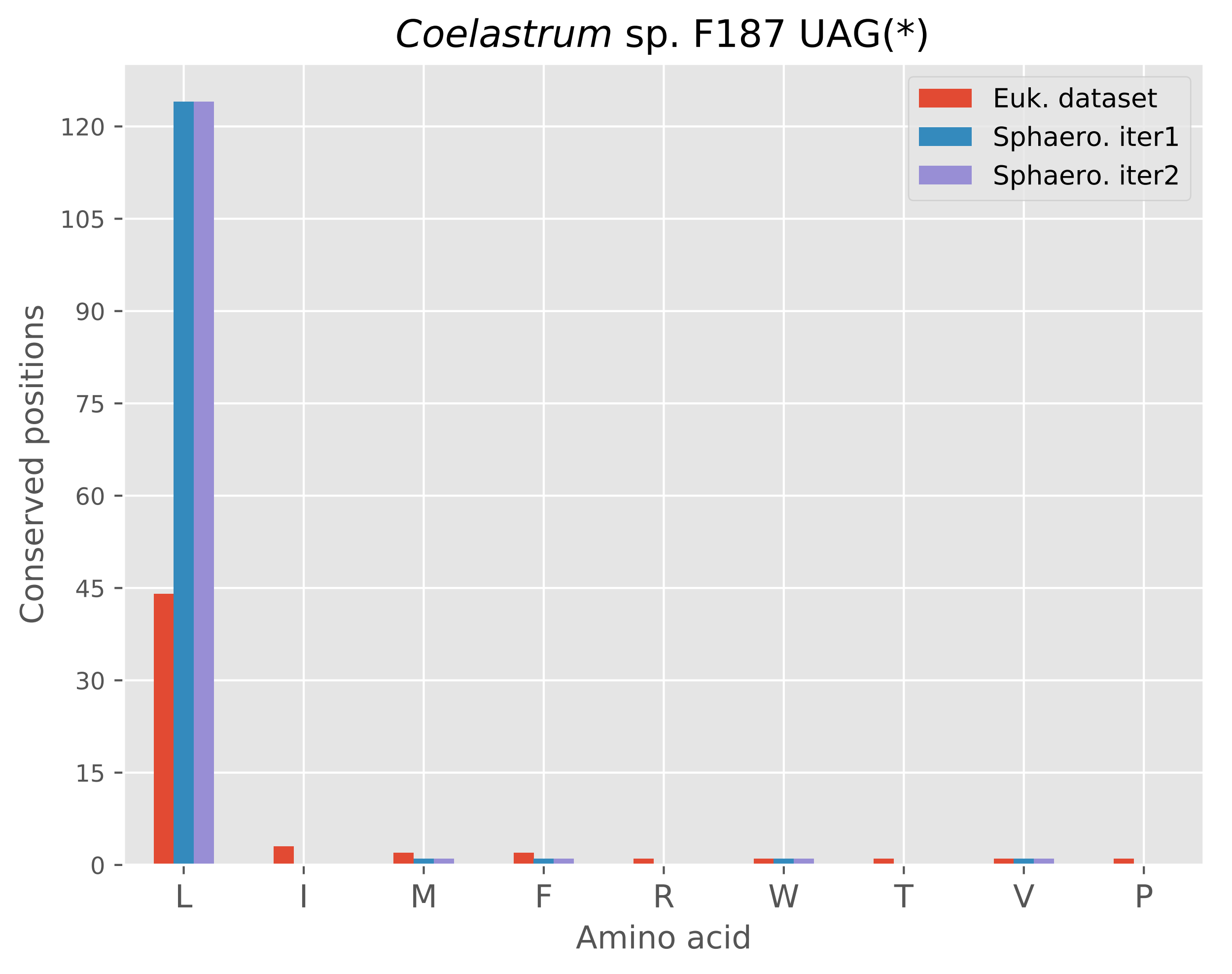
Coelastrum sp. F187 UAG(*)
Euk. dataset
Sphaero. iter1
Sphaero. iter2
Conserved positions
0
15
30
45
60
75
90
105
120
L
I
M
F
R
W
T
V
P
Amino acid

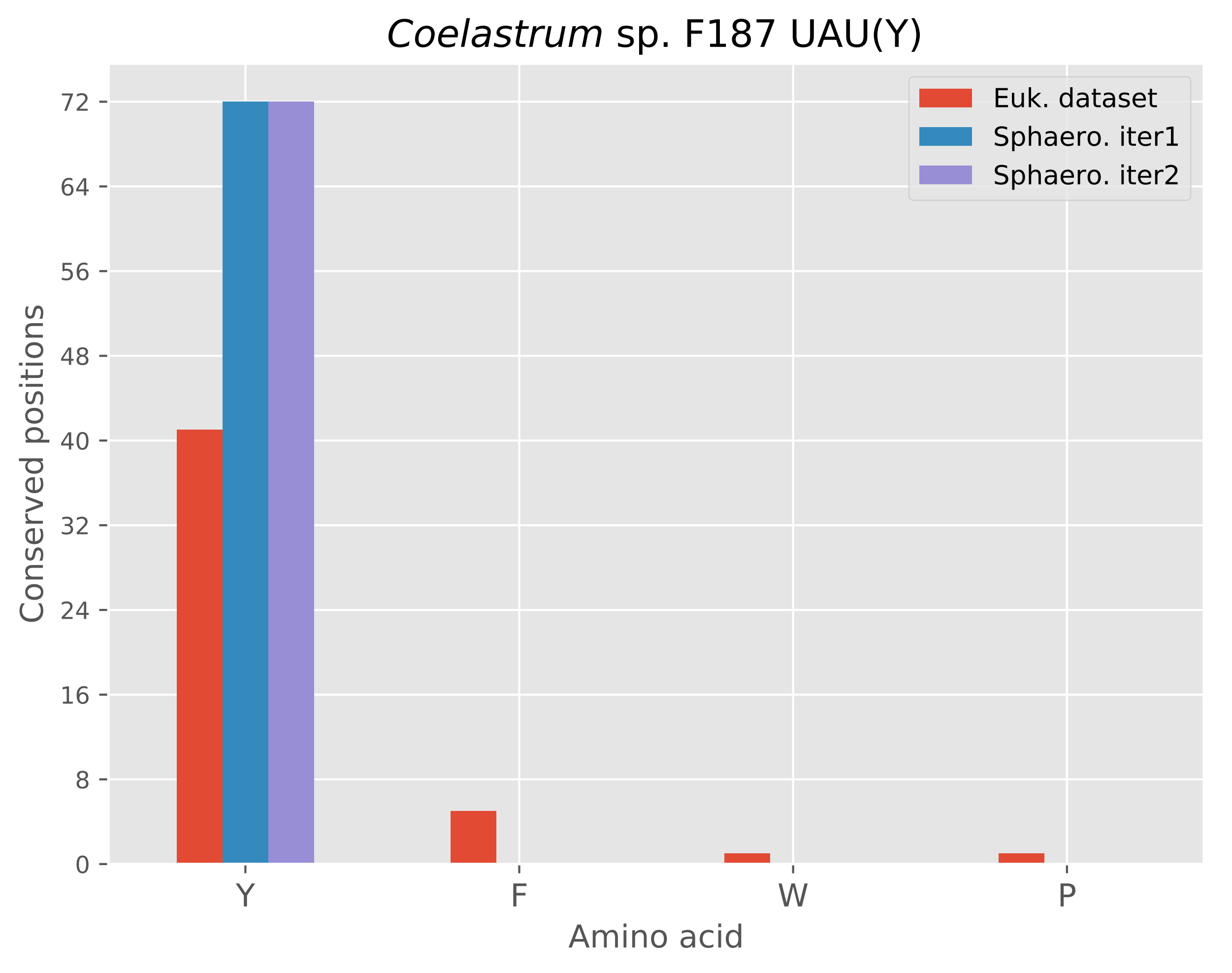

Coelastrum sp. F187 UAU(Y)
Euk. dataset
Sphaero. iter1
Sphaero. iter2
Conserved positions
0
8
16
24
32
40
48
56
64
72
Y
F
W
P
Amino acid

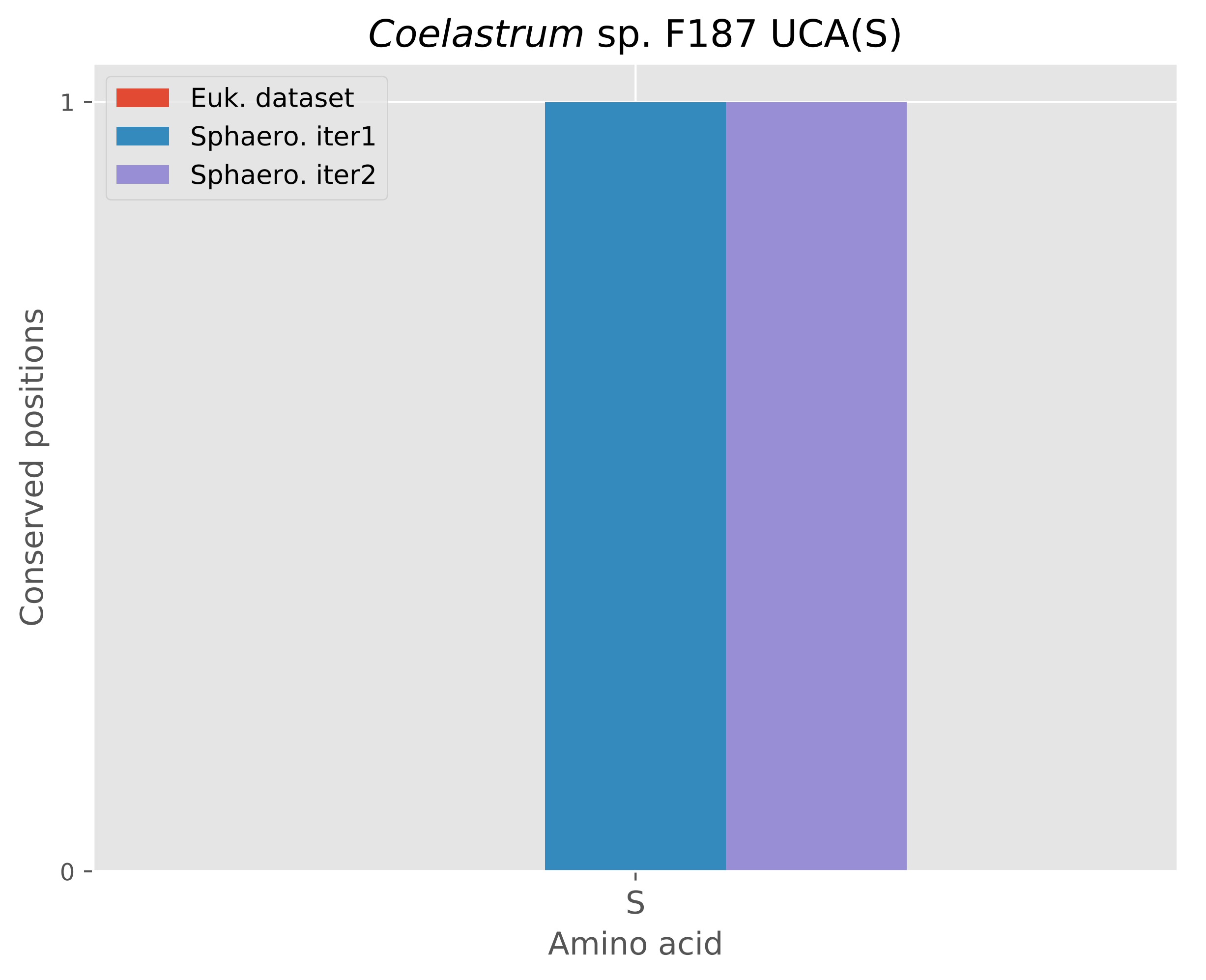

Coelastrum sp. F187 UCA(S)
Euk. dataset
Sphaero. iter1
Sphaero. iter2
1
0
Conserved positions
S
Amino acid

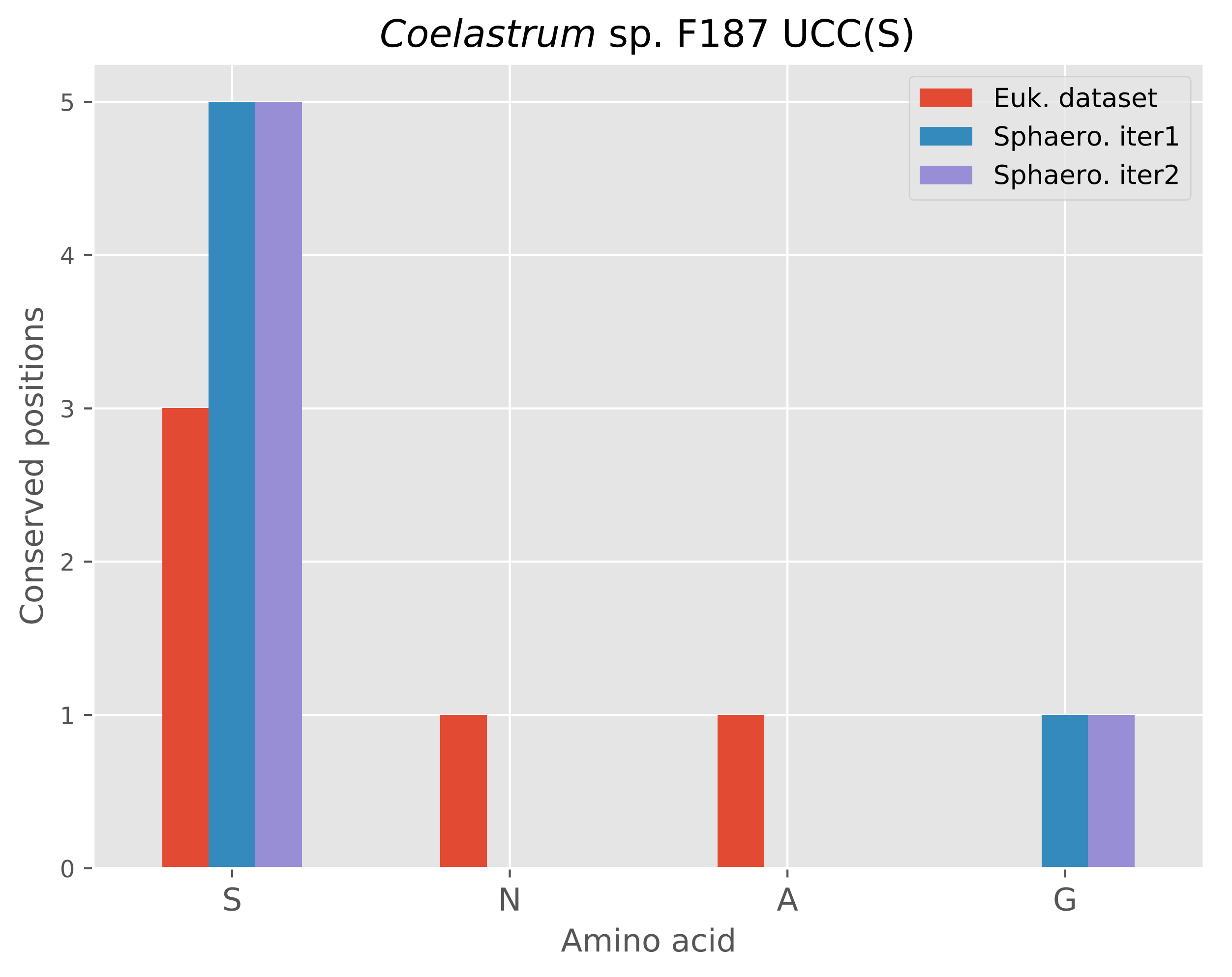

Coelastrum sp. F187 UCC(S)
Euk. dataset
Sphaero. iter1
Sphaero. iter2
Conserved positions
0
1
2
3
4
5
S
N
A
G
Amino acid

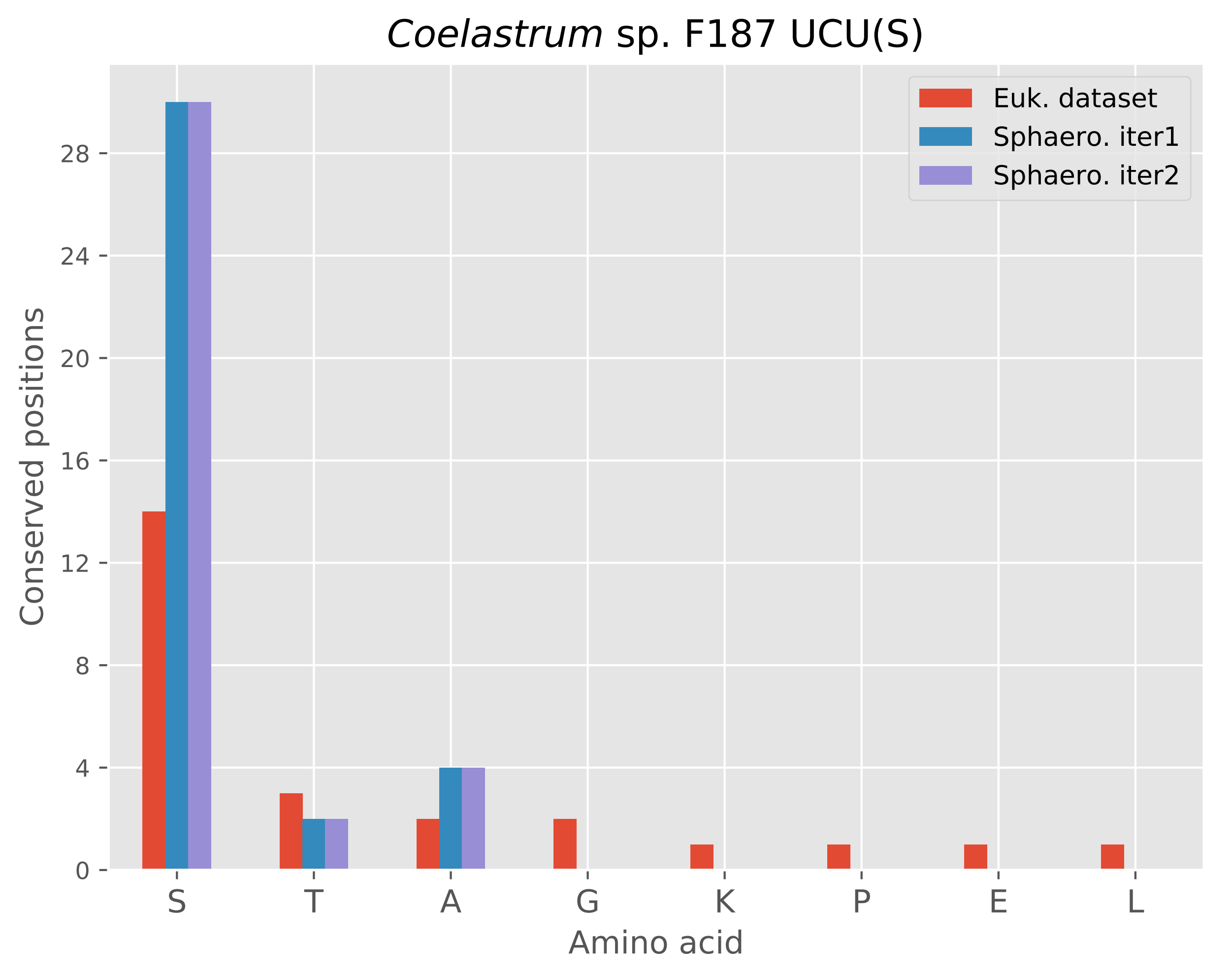
Coelastrum sp. F187 UCU(S)
Euk. dataset
Sphaero. iter1
Sphaero. iter2
Conserved positions
0
4
8
12
16
20
24
28
S
T
A
G
K
P
E
L
Amino acid

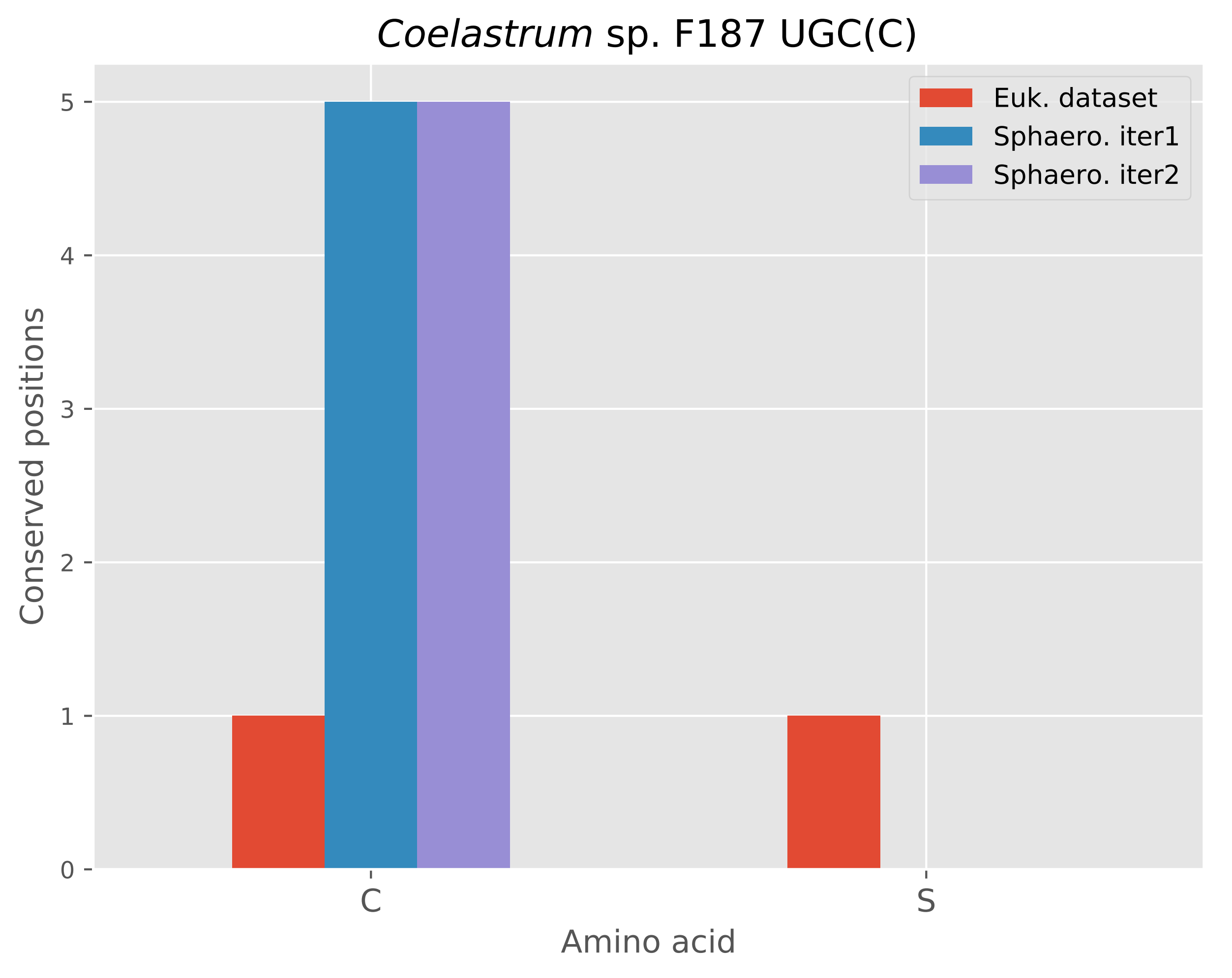

Coelastrum sp. F187 UGC(C)
Euk. dataset
Sphaero. iter1
Sphaero. iter2
Conserved positions
0
1
2
3
4
5
C
S
Amino acid

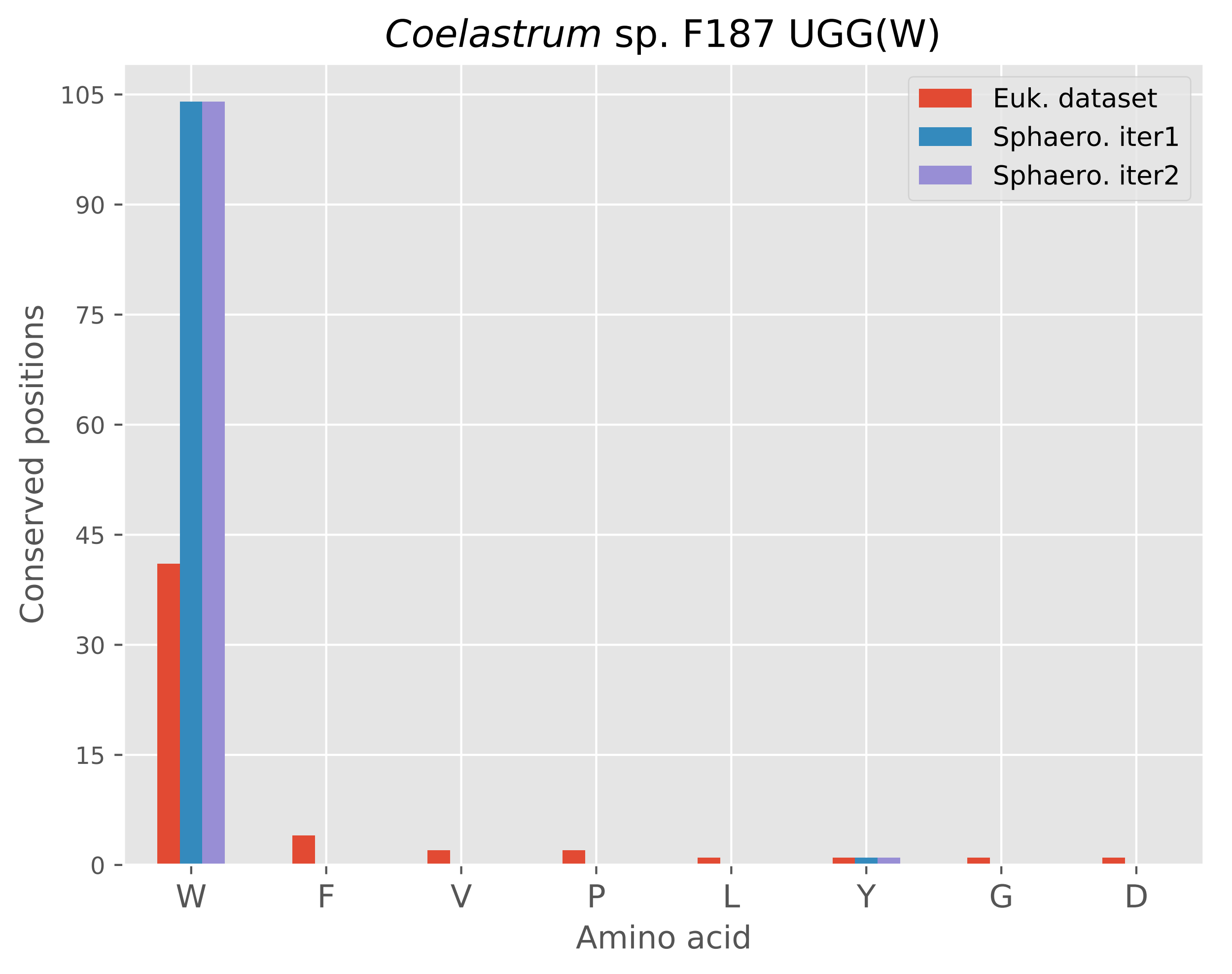

Coelastrum sp. F187 UGG(W)
Euk. dataset
Sphaero. iter1
Sphaero. iter2
Conserved positions
0
15
30
45
60
75
90
105
W
F
V
P
L
Y
G
D
Amino acid

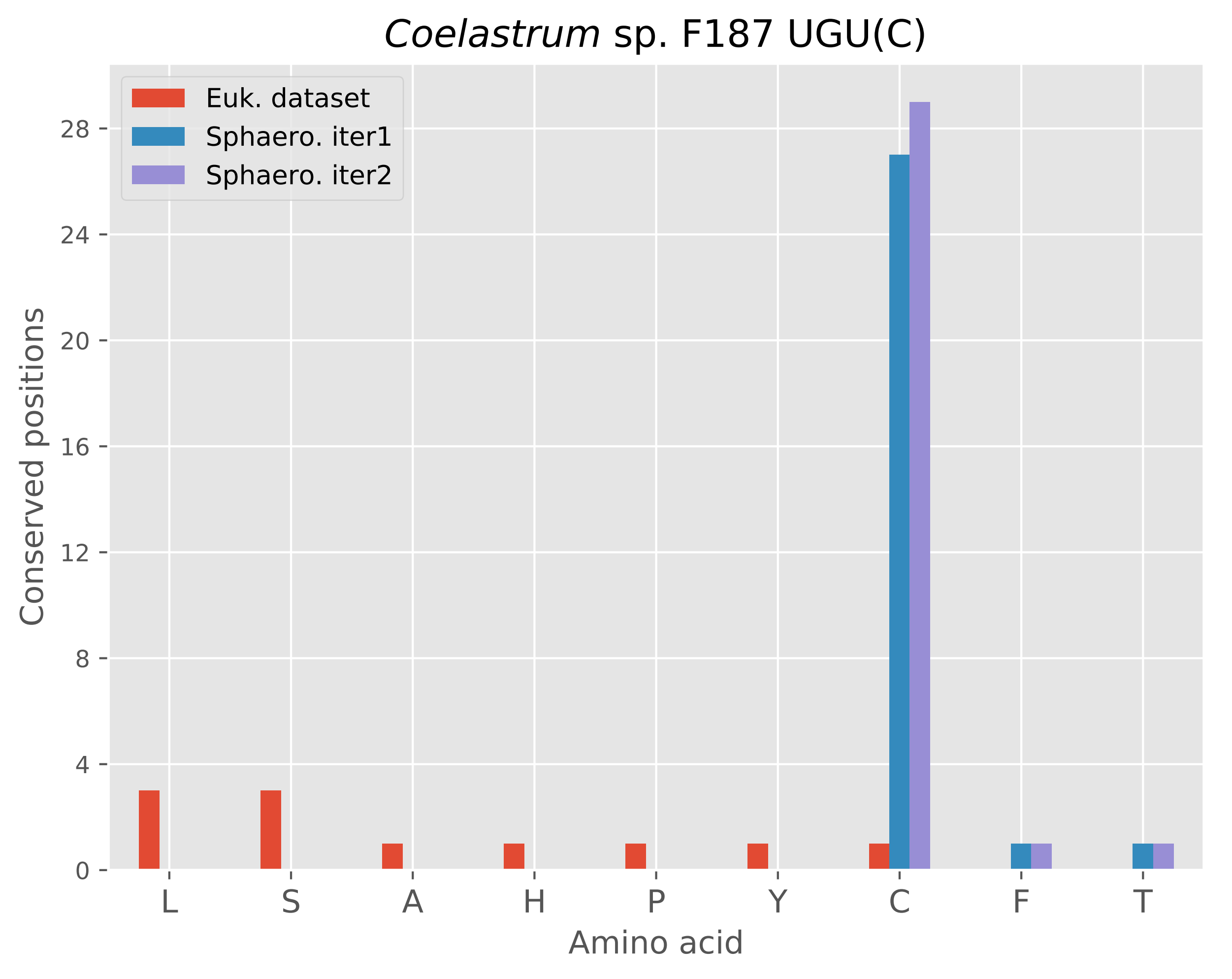
Coelastrum sp. F187 UGU(C)
Euk. dataset
Sphaero. iter1
Sphaero. iter2
Conserved positions
0
4
8
12
16
20
24
28
L
S
A
H
P
Y
C
F
T
Amino acid

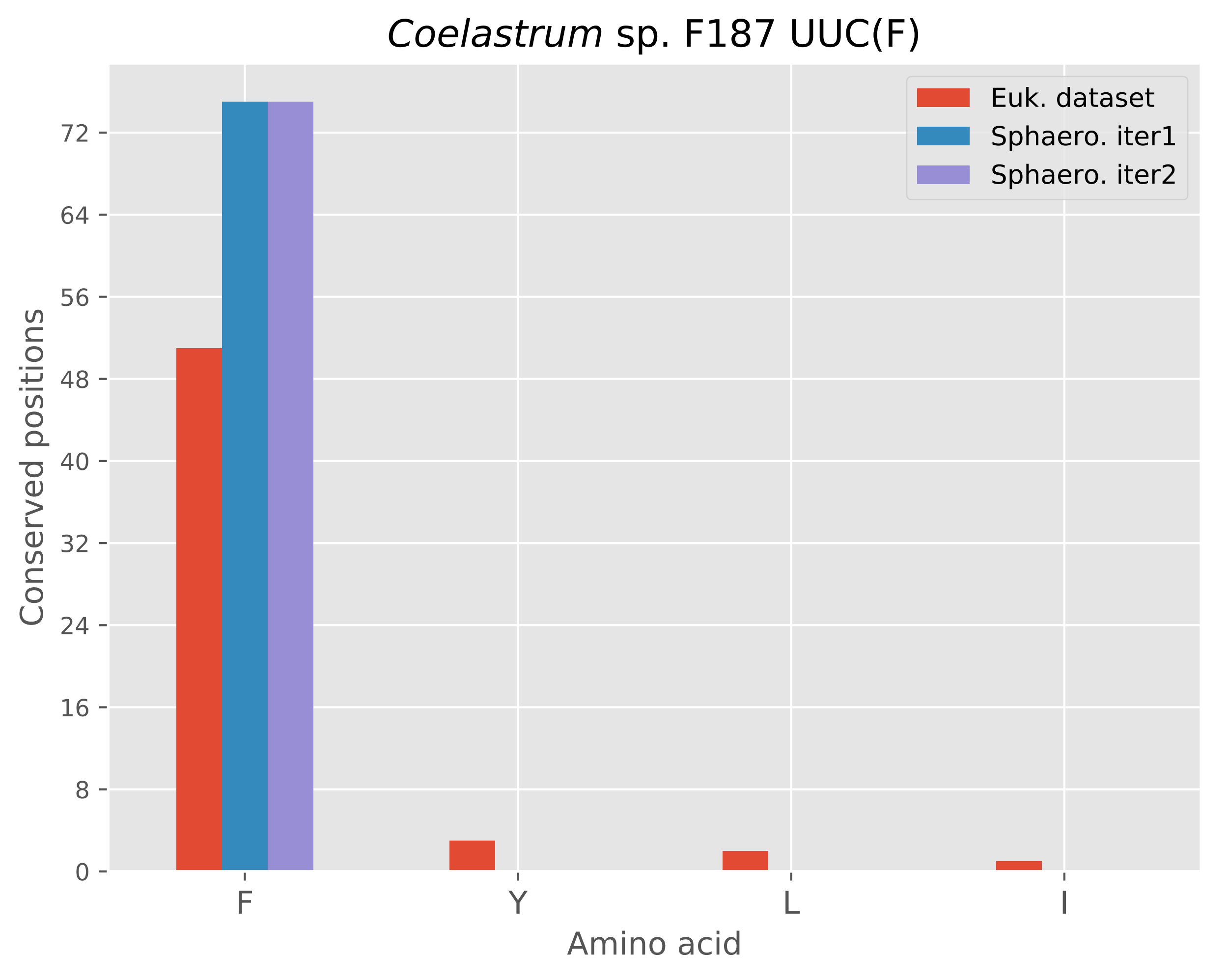

Coelastrum sp. F187 UUC(F)
Euk. dataset
Sphaero. iter1
Sphaero. iter2
Conserved positions
0
8
16
24
32
40
48
56
64
72
F
Y
L
I
Amino acid

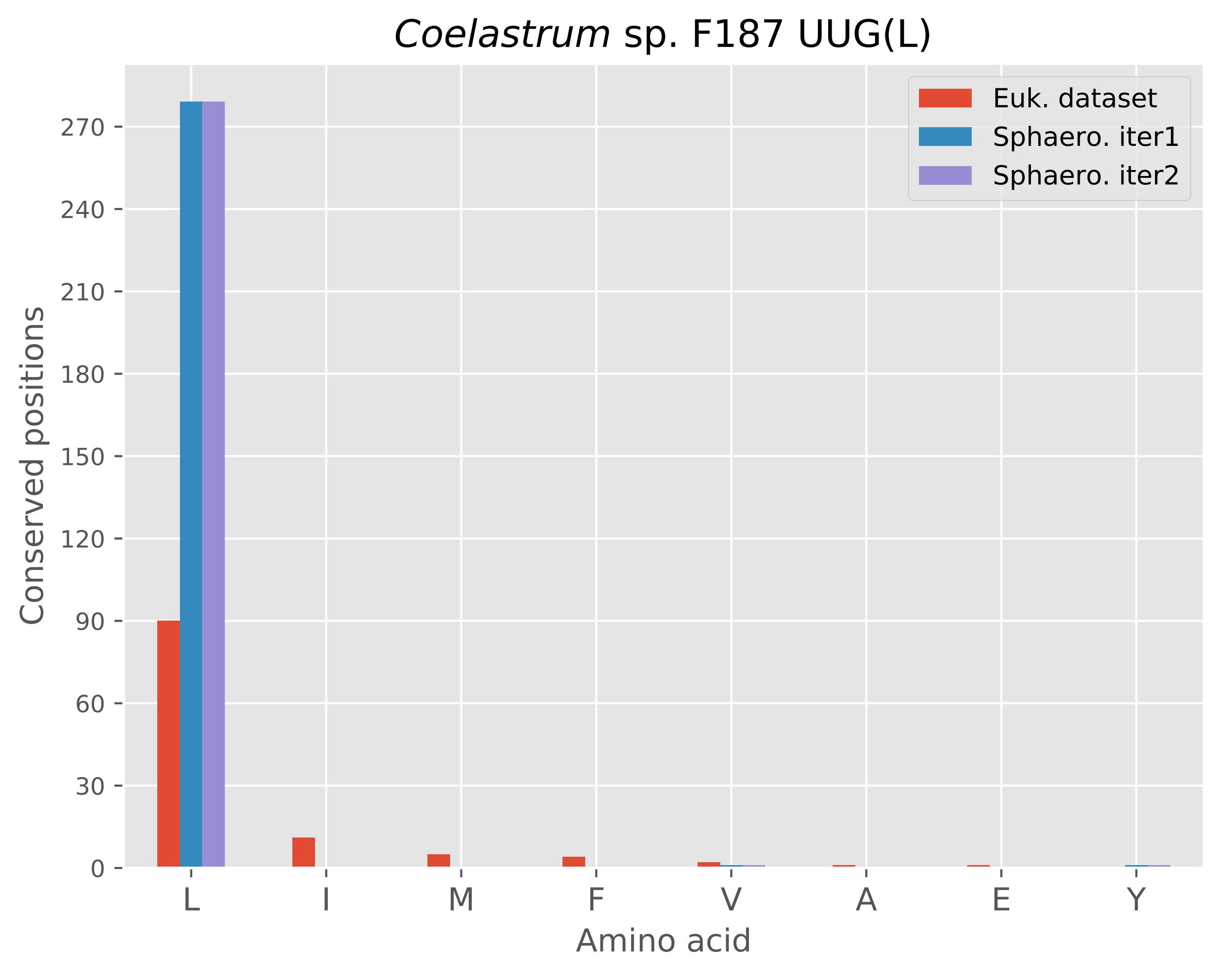

Coelastrum sp. F187 UUG(L)
Euk. dataset
Sphaero. iter1
Sphaero. iter2
Conserved positions
0
30
60
90
120
150
180
210
240
270
L
I
M
F
V
A
E
Y
Amino acid

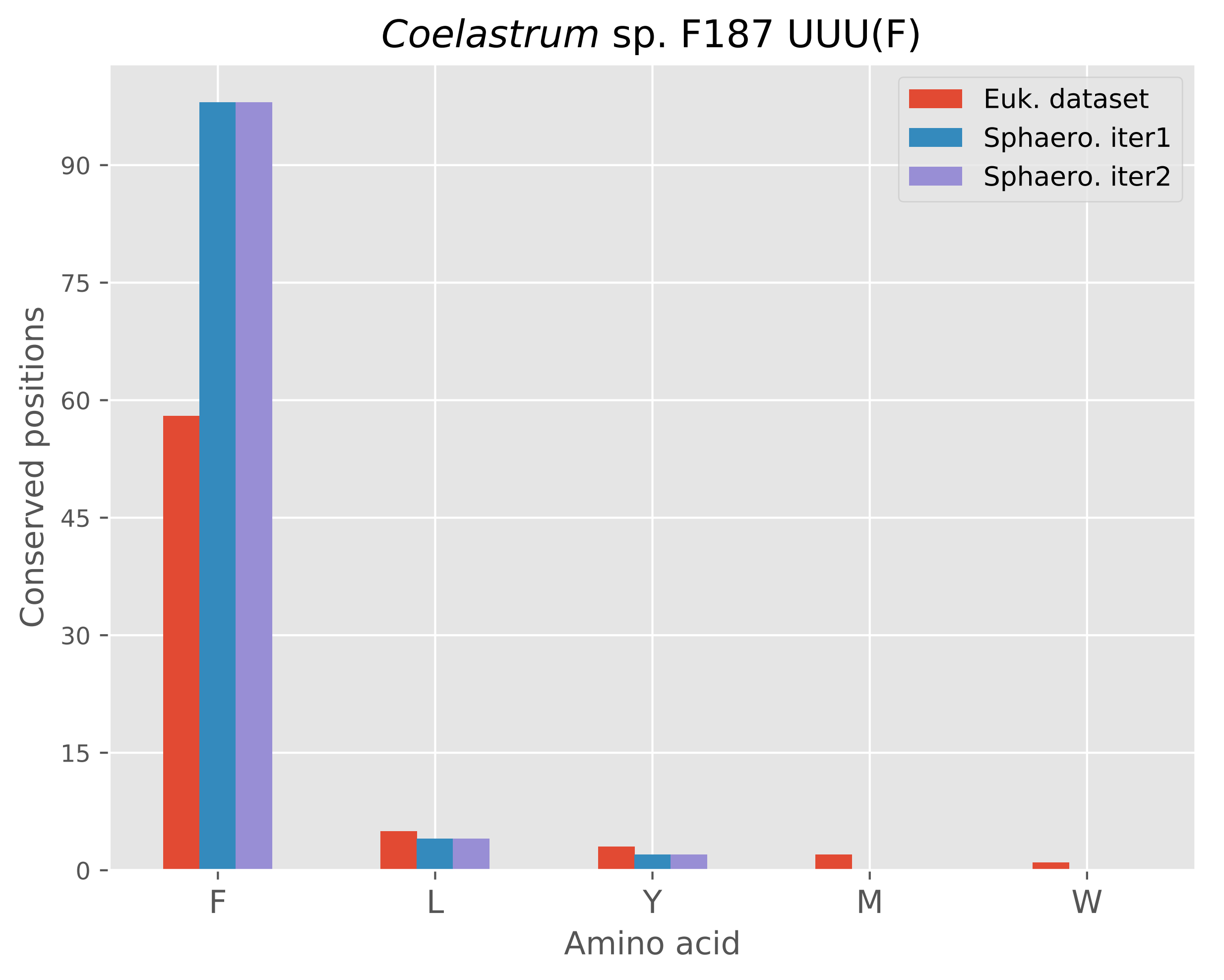

Coelastrum sp. F187 UUU(F)
Euk. dataset
Sphaero. iter1
Sphaero. iter2
Conserved positions
0
15
30
45
60
75
90
F
L
Y
M
W
Amino acid

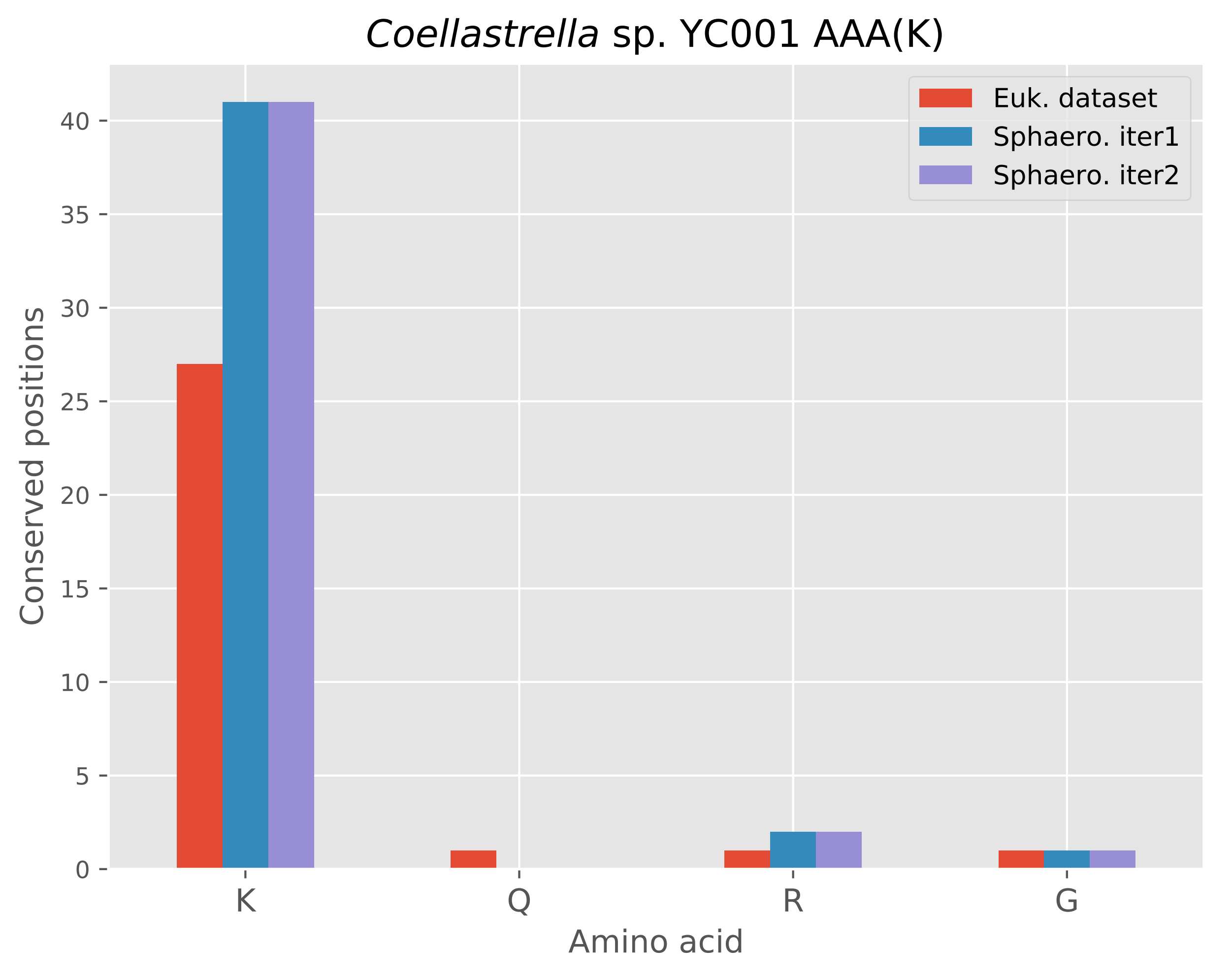

Coellastrella sp. YC001 AAA(K)
Euk. dataset
Sphaero. iter1
Sphaero. iter2
Conserved positions
0
5
10
15
20
25
30
35
40
K
Q
R
G
Amino acid

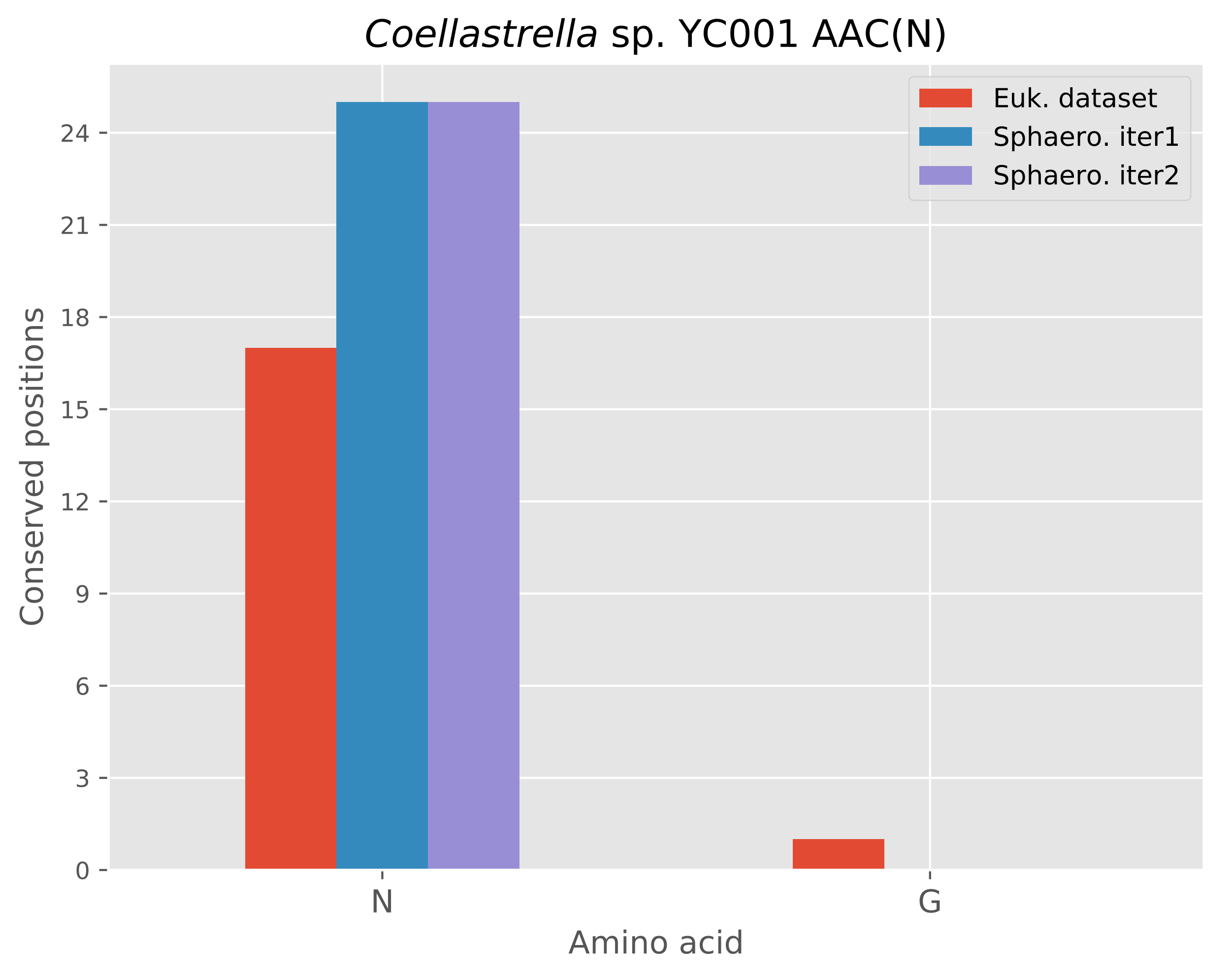
Coellastrella sp. YC001 AAC(N)
Euk. dataset
Sphaero. iter1
Sphaero. iter2
Conserved positions
0
3
6
9
12
15
18
21
24
N
G
Amino acid

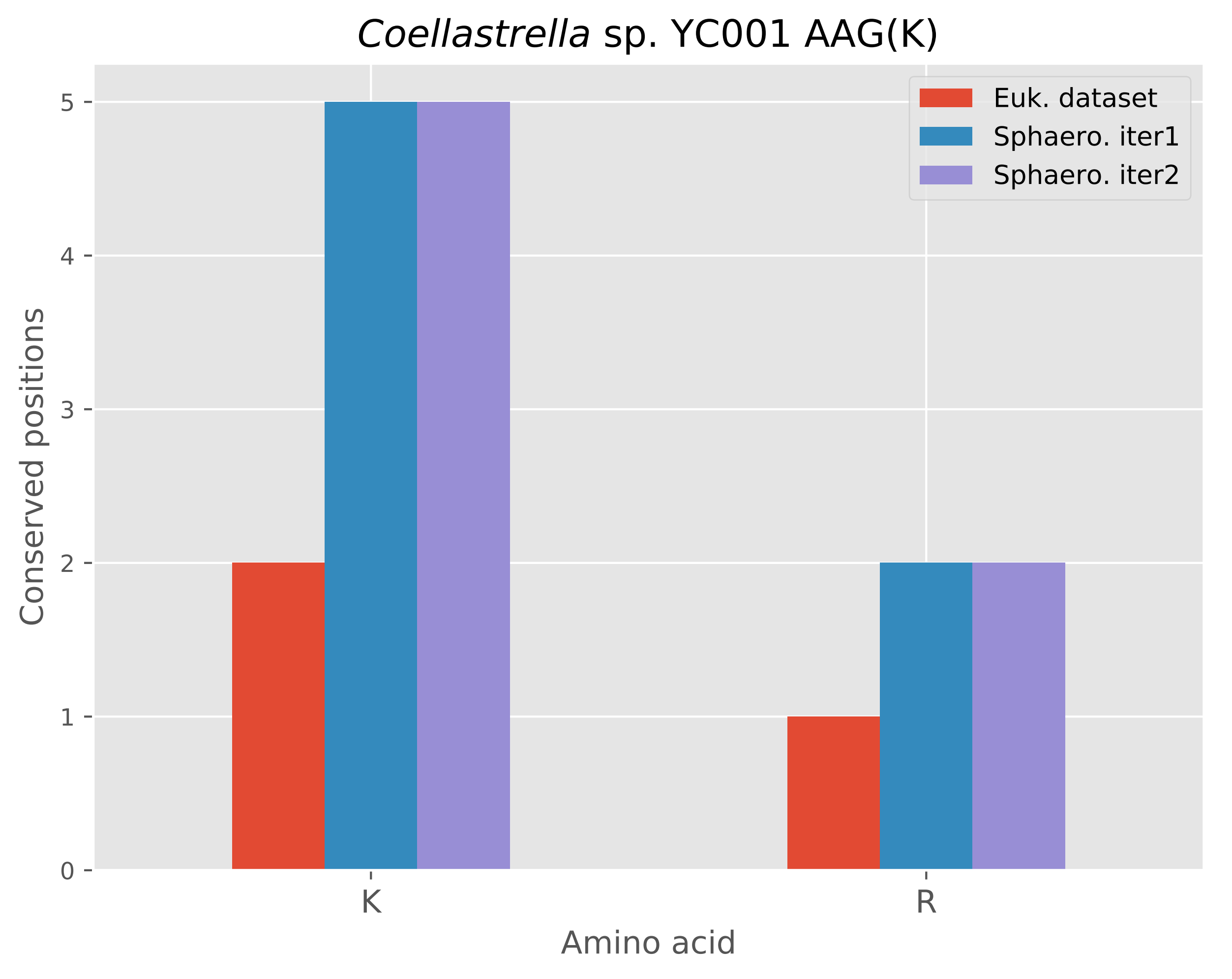

Coellastrella sp. YC001 AAG(K)
Euk. dataset
Sphaero. iter1
Sphaero. iter2
Conserved positions
0
1
2
3
4
5
K
R
Amino acid

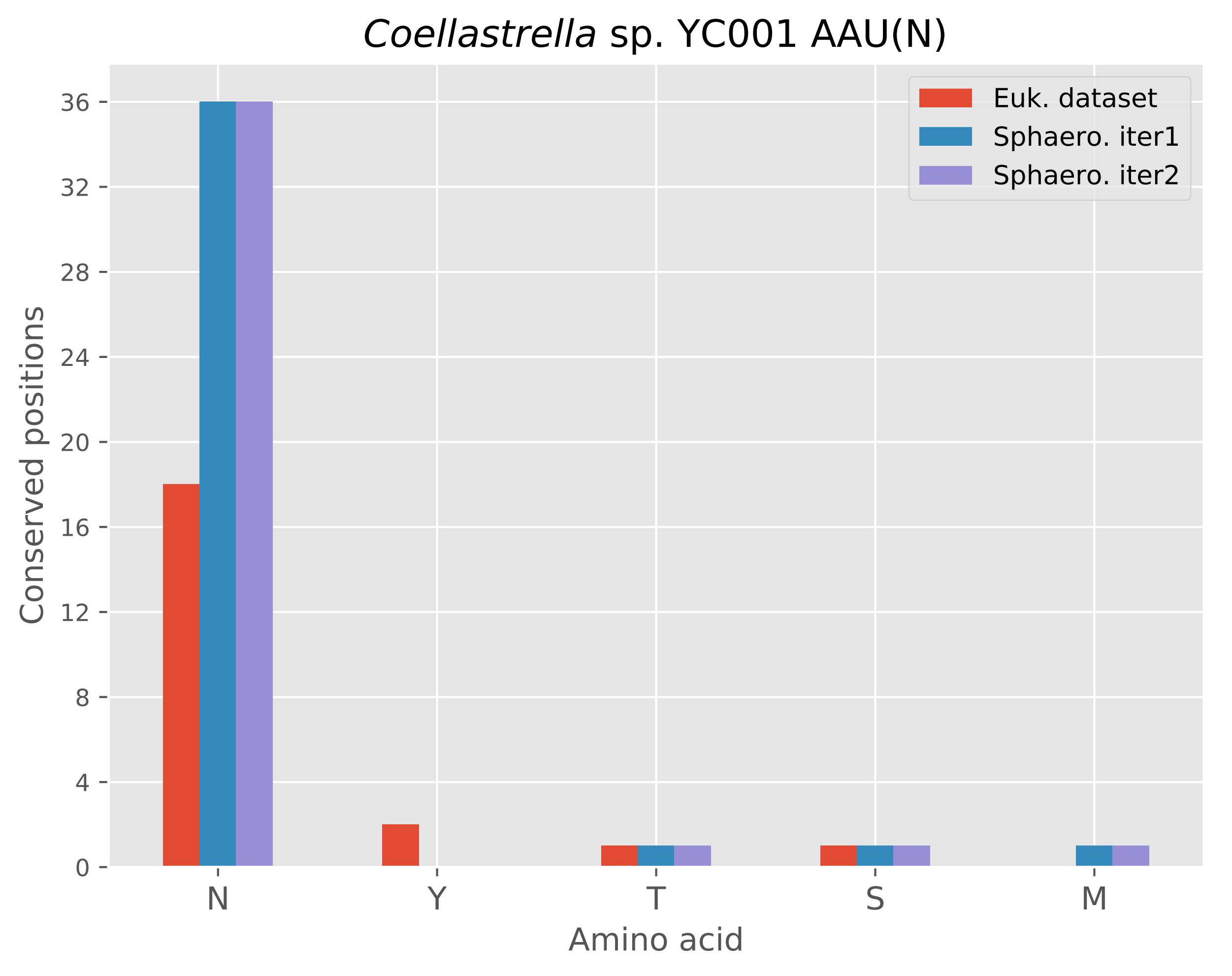
Coellastrella sp. YC001 AAU(N)
Euk. dataset
Sphaero. iter1
Sphaero. iter2
Conserved positions
0
4
8
12
16
20
24
28
32
36
N
Y
T
S
M
Amino acid

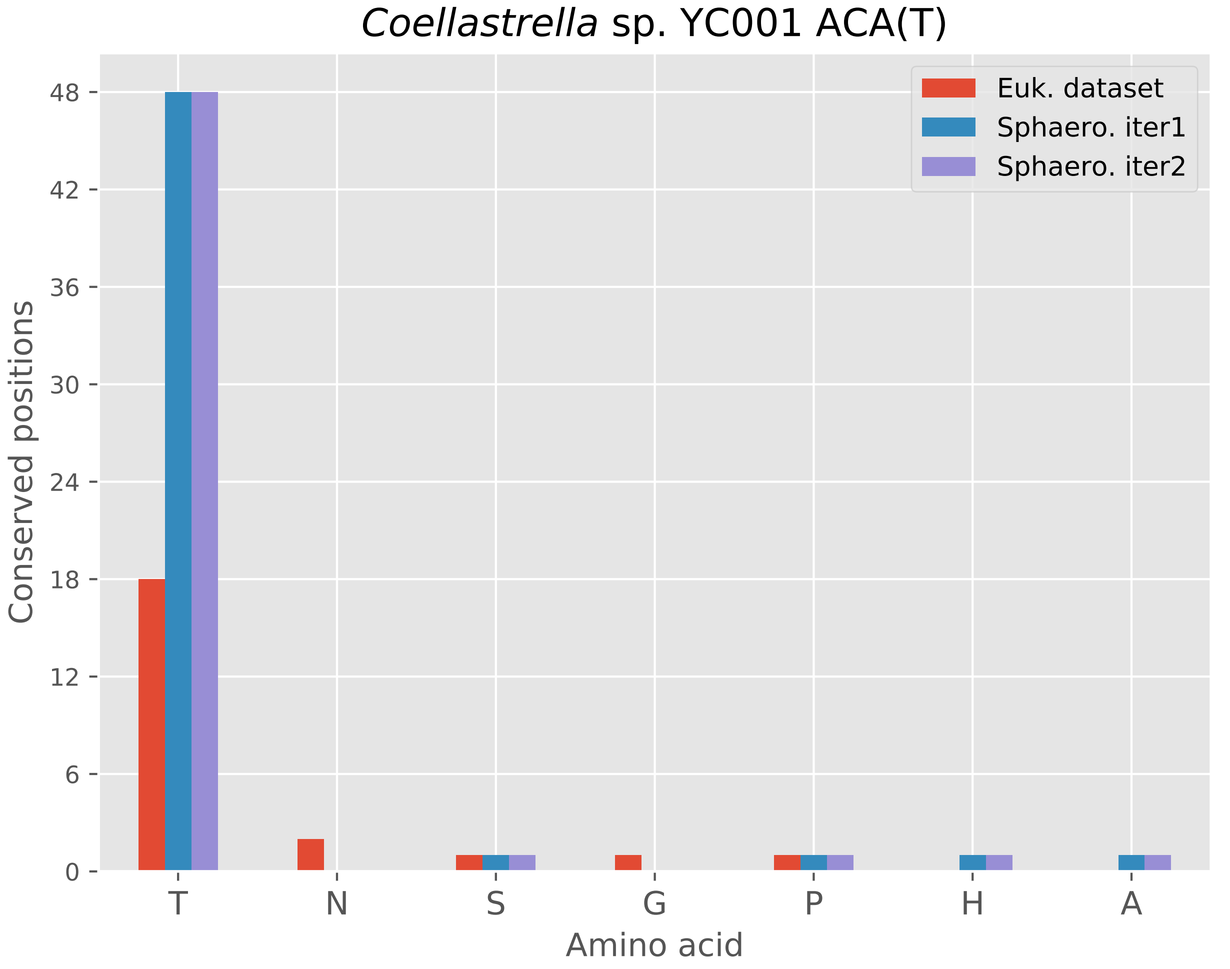
Coellastrella sp. YC001 ACA(T)
Euk. dataset
Sphaero. iter1
Sphaero. iter2
Conserved positions
0
6
12
18
24
30
36
42
48
T
N
S
G
P
H
A
Amino acid

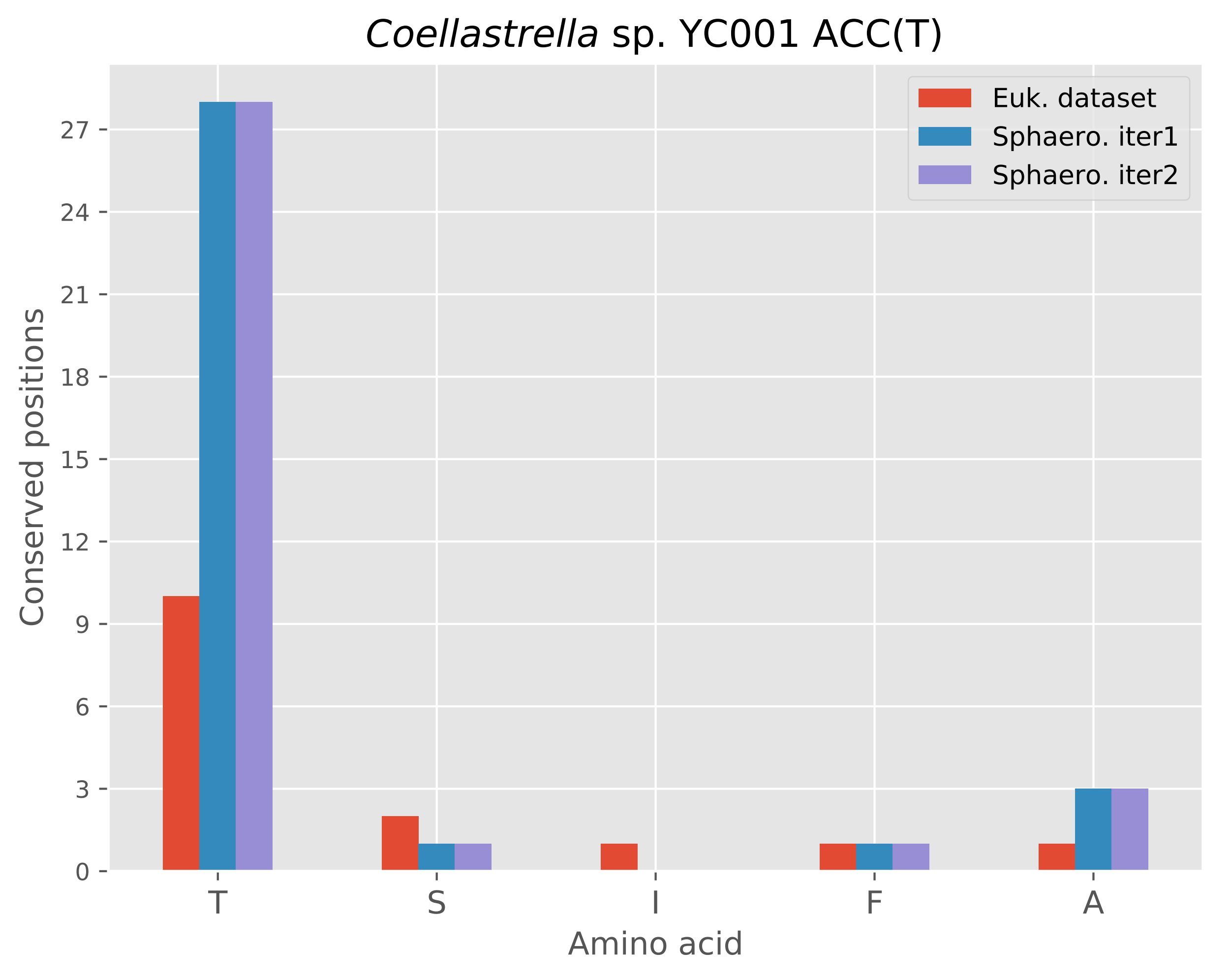

Coellastrella sp. YC001 ACC(T)
Euk. dataset
Sphaero. iter1
Sphaero. iter2
Conserved positions
0
3
6
9
12
15
18
21
24
27
T
S
I
F
A
Amino acid

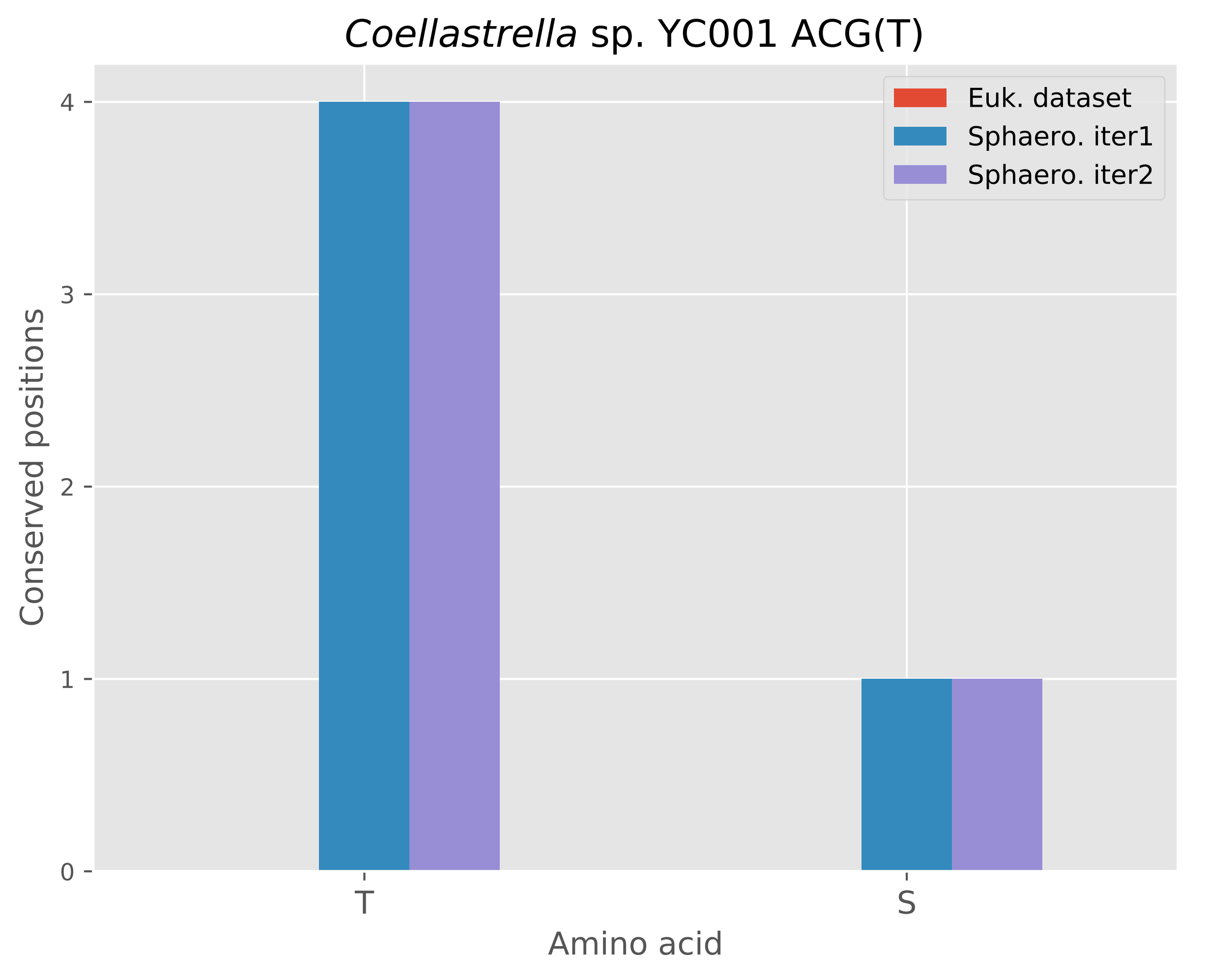

Coellastrella sp. YC001 ACG(T)
Euk. dataset
Sphaero. iter1
Sphaero. iter2
Conserved positions
0
1
2
3
4
T
S
Amino acid

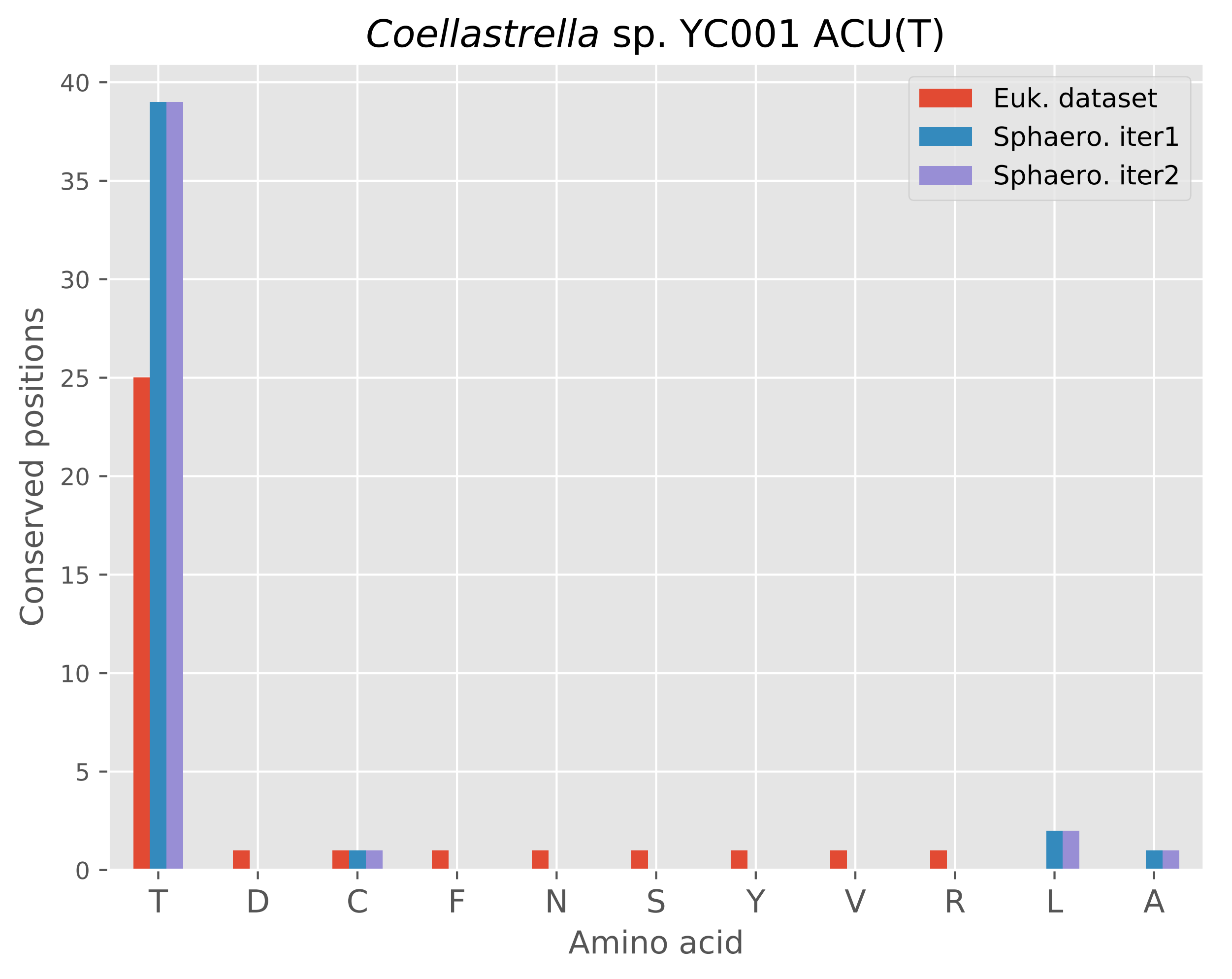

Coellastrella sp. YC001 ACU(T)
Euk. dataset
Sphaero. iter1
Sphaero. iter2
Conserved positions
0
5
10
15
20
25
30
35
40
T
D
C
F
N
S
Y
V
R
L
A
Amino acid

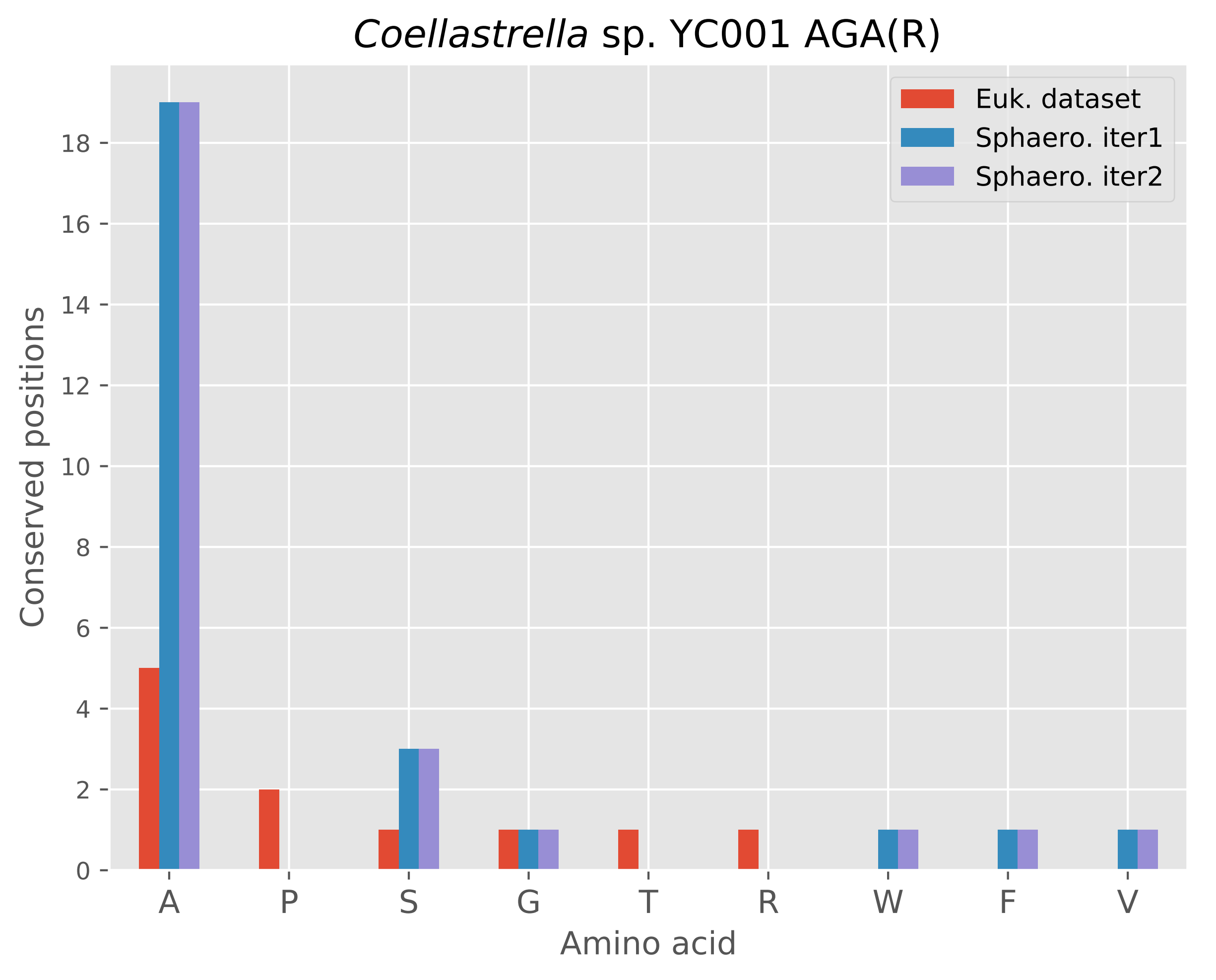

Coellastrella sp. YC001 AGA(R)
Euk. dataset
Sphaero. iter1
Sphaero. iter2
Conserved positions
0
2
4
6
8
10
12
14
16
18
A
P
S
G
T
R
W
F
V
Amino acid

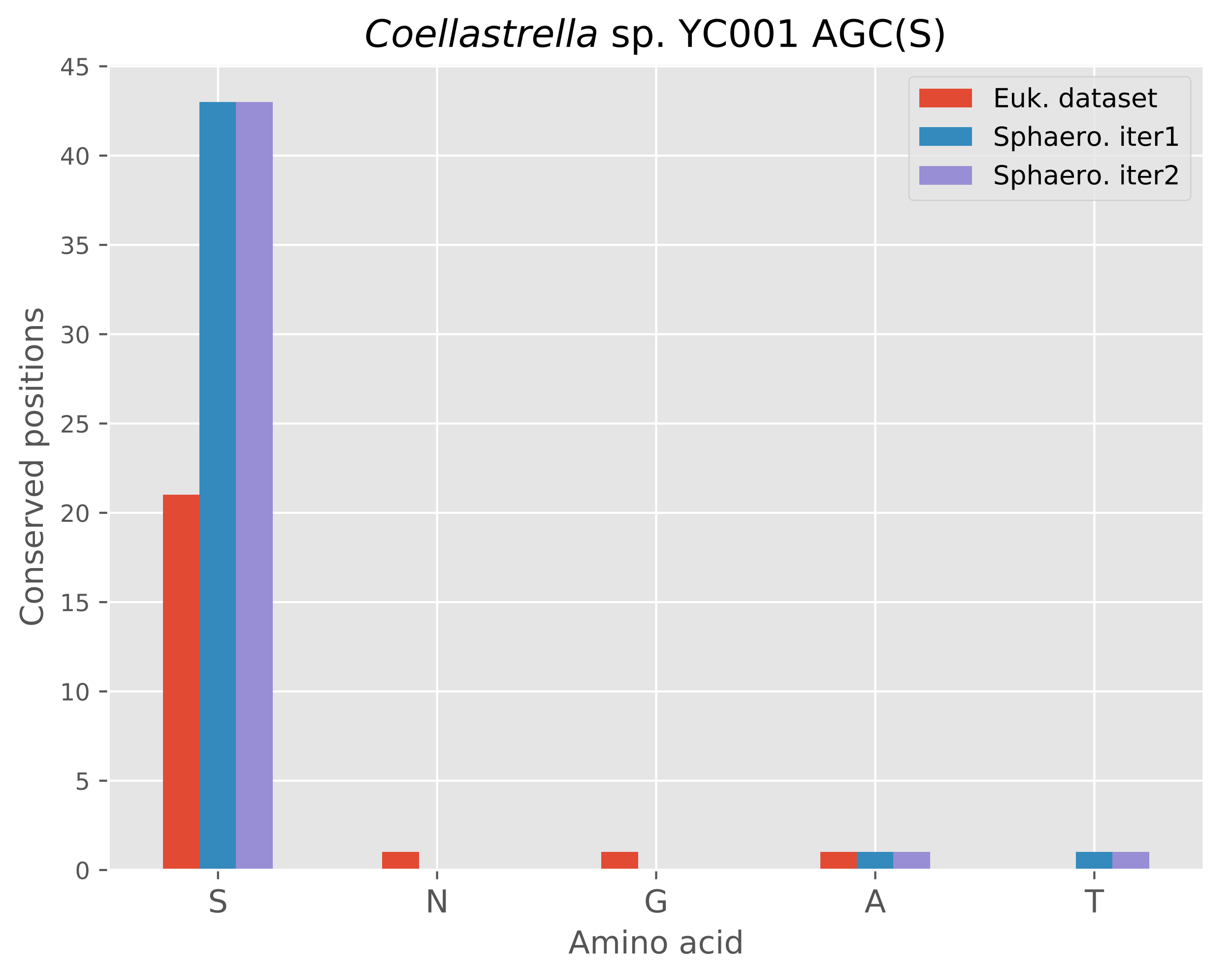

Coellastrella sp. YC001 AGC(S)
Conserved positions
Amino acid
S
N
G
A
T
Euk. dataset
Sphaero. iter1
Sphaero. iter2
0
5
10
15
20
25
30
35
40
45

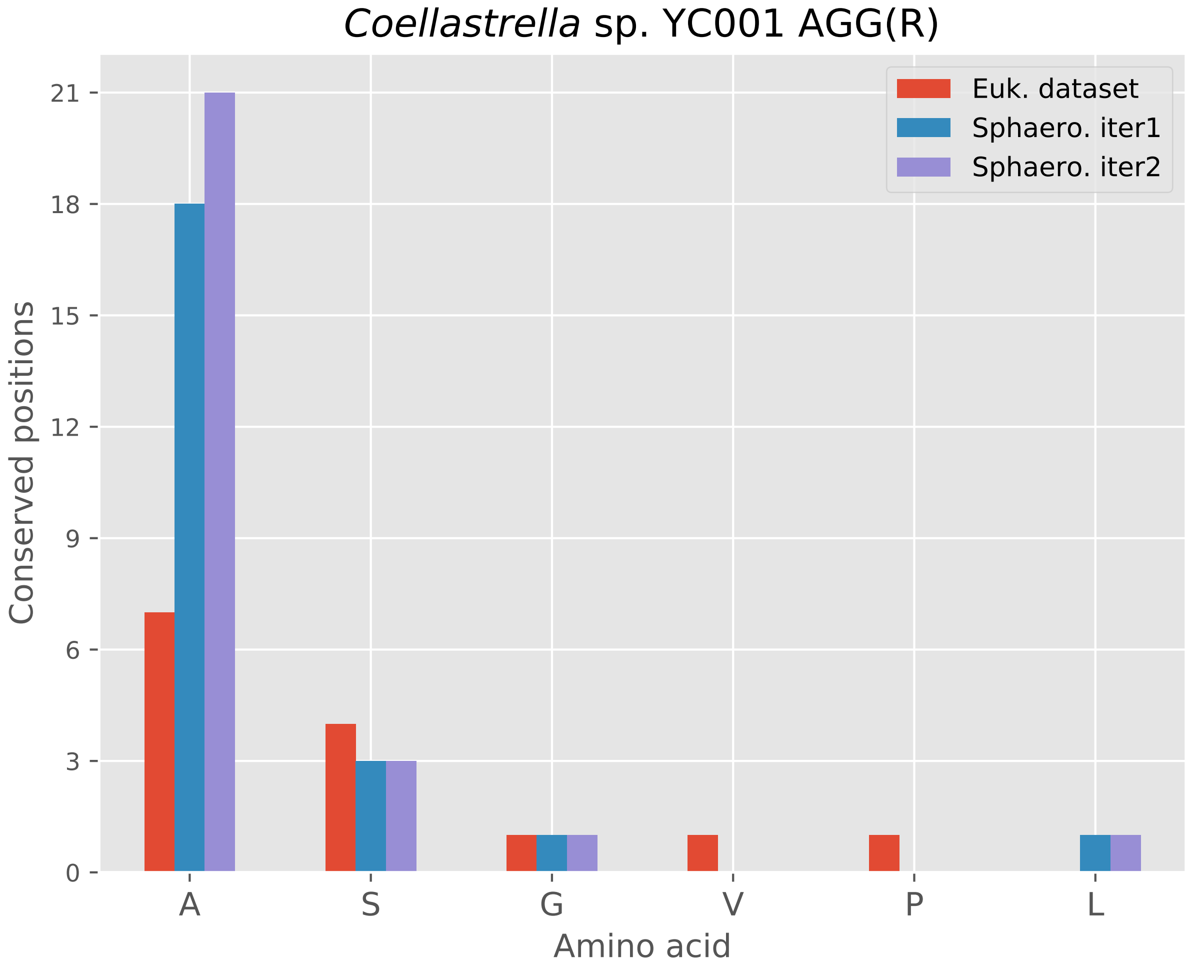

Coellastrella sp. YC001 AGG(R)
Euk. dataset
Sphaero. iter1
Sphaero. iter2
Conserved positions
0
3
6
9
12
15
18
21
A
S
G
V
P
L
Amino acid

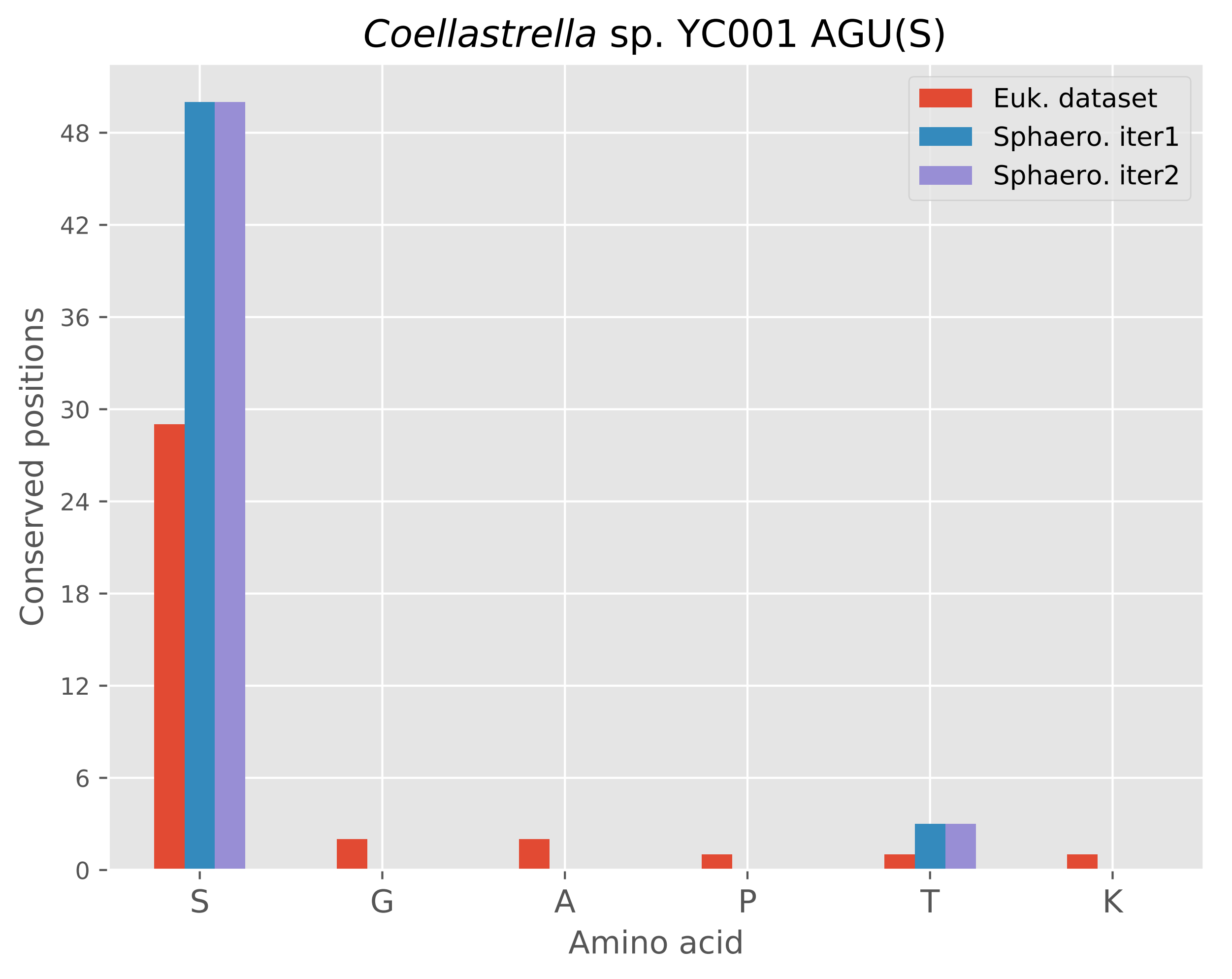

Coellastrella sp. YC001 AGU(S)
Euk. dataset
Sphaero. iter1
Sphaero. iter2
Conserved positions
0
6
12
18
24
30
36
42
48
S
G
A
P
T
K
Amino acid

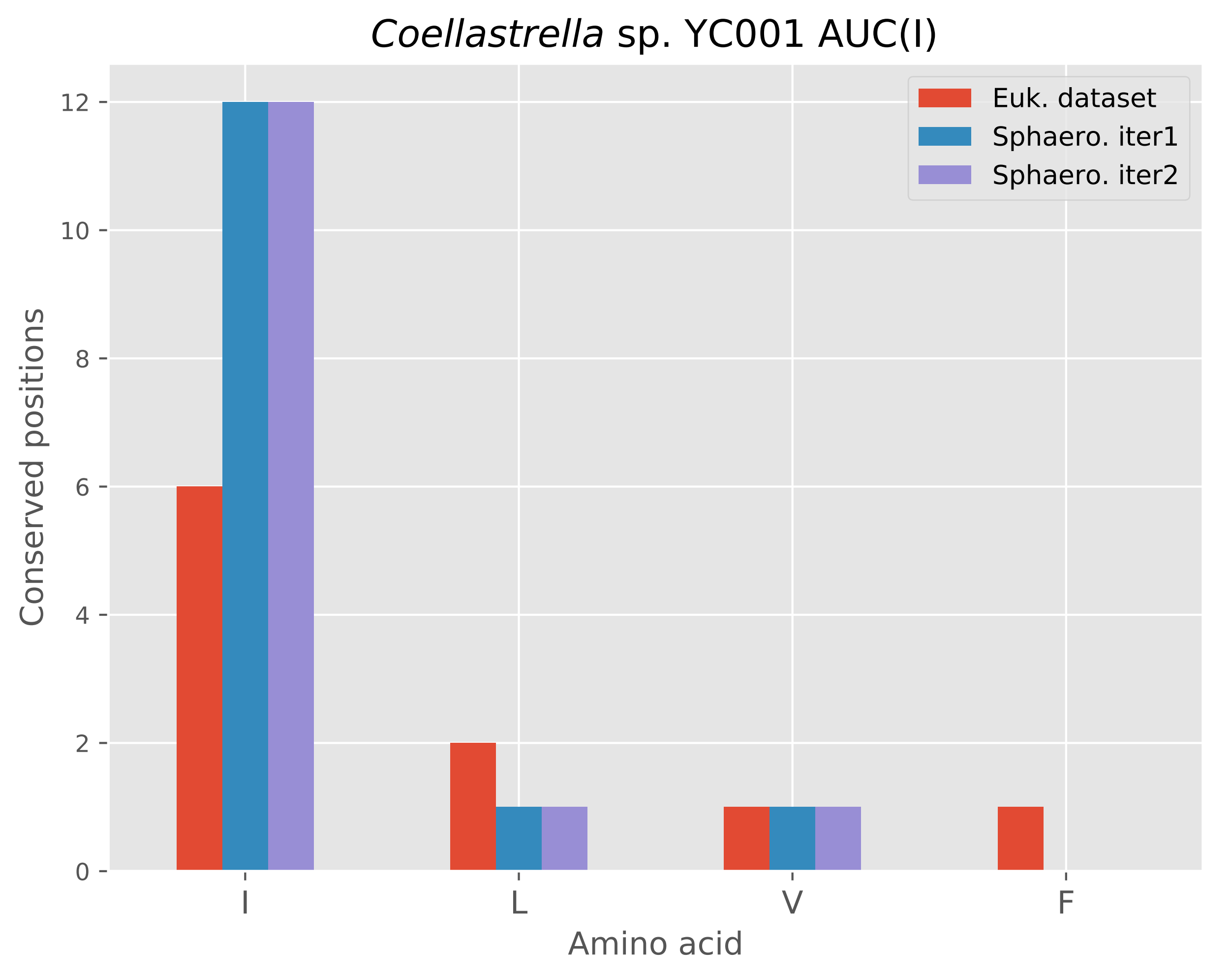
Coellastrella sp. YC001 AUC(I)
Conserved positions
Amino acid
I
L
V
F
0
2
4
6
8
10
12
Euk. dataset
Sphaero. iter1
Sphaero. iter2

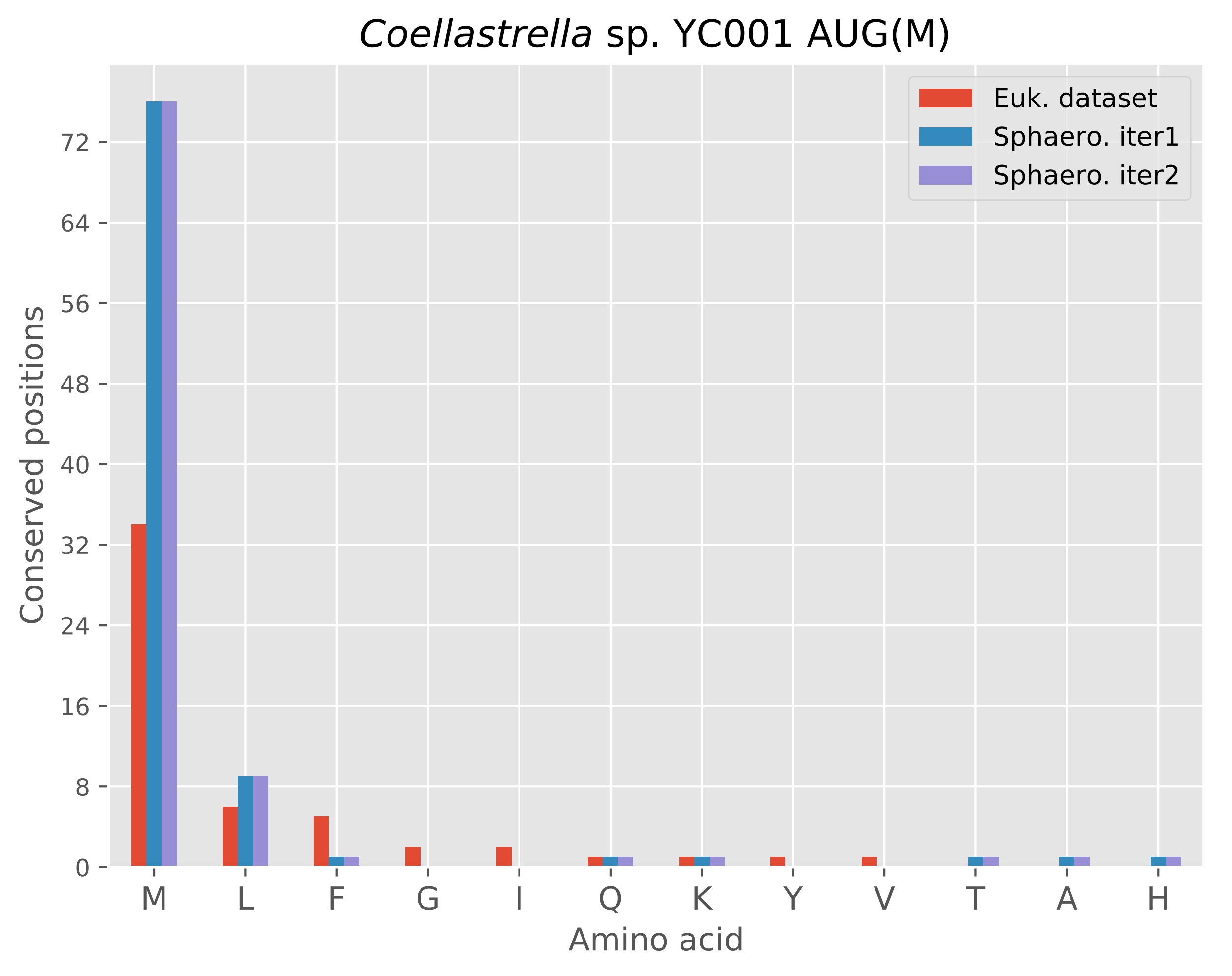

Coellastrella sp. YC001 AUG(M)
Euk. dataset
Sphaero. iter1
Sphaero. iter2
Conserved positions
0
8
16
24
32
40
48
56
64
72
M
L
F
G
I
Q
K
Y
V
T
A
H
Amino acid

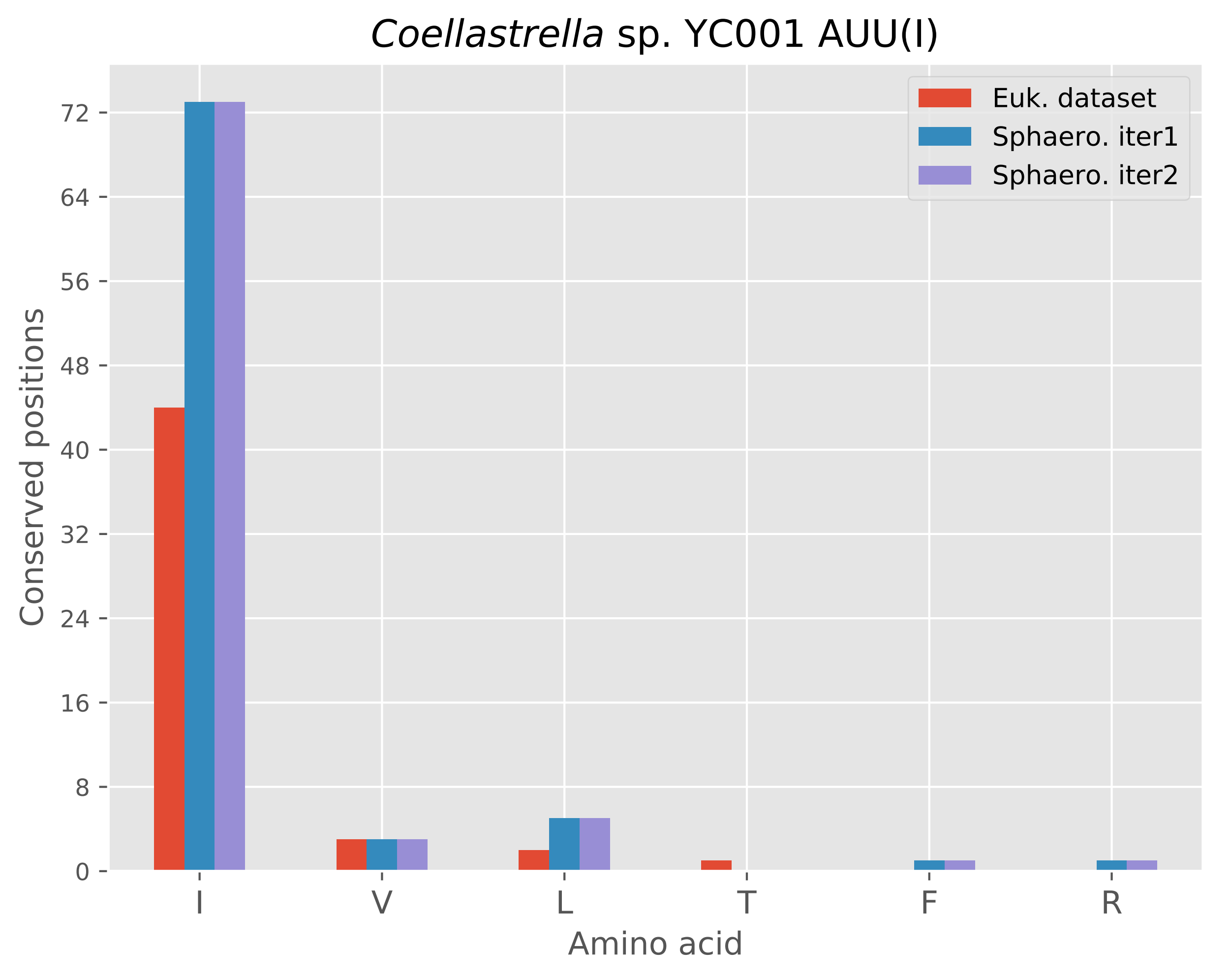

Coellastrella sp. YC001 AUU(I)
Euk. dataset
Sphaero. iter1
Sphaero. iter2
Conserved positions
0
8
16
24
32
40
48
56
64
72
I
V
L
T
F
R
Amino acid

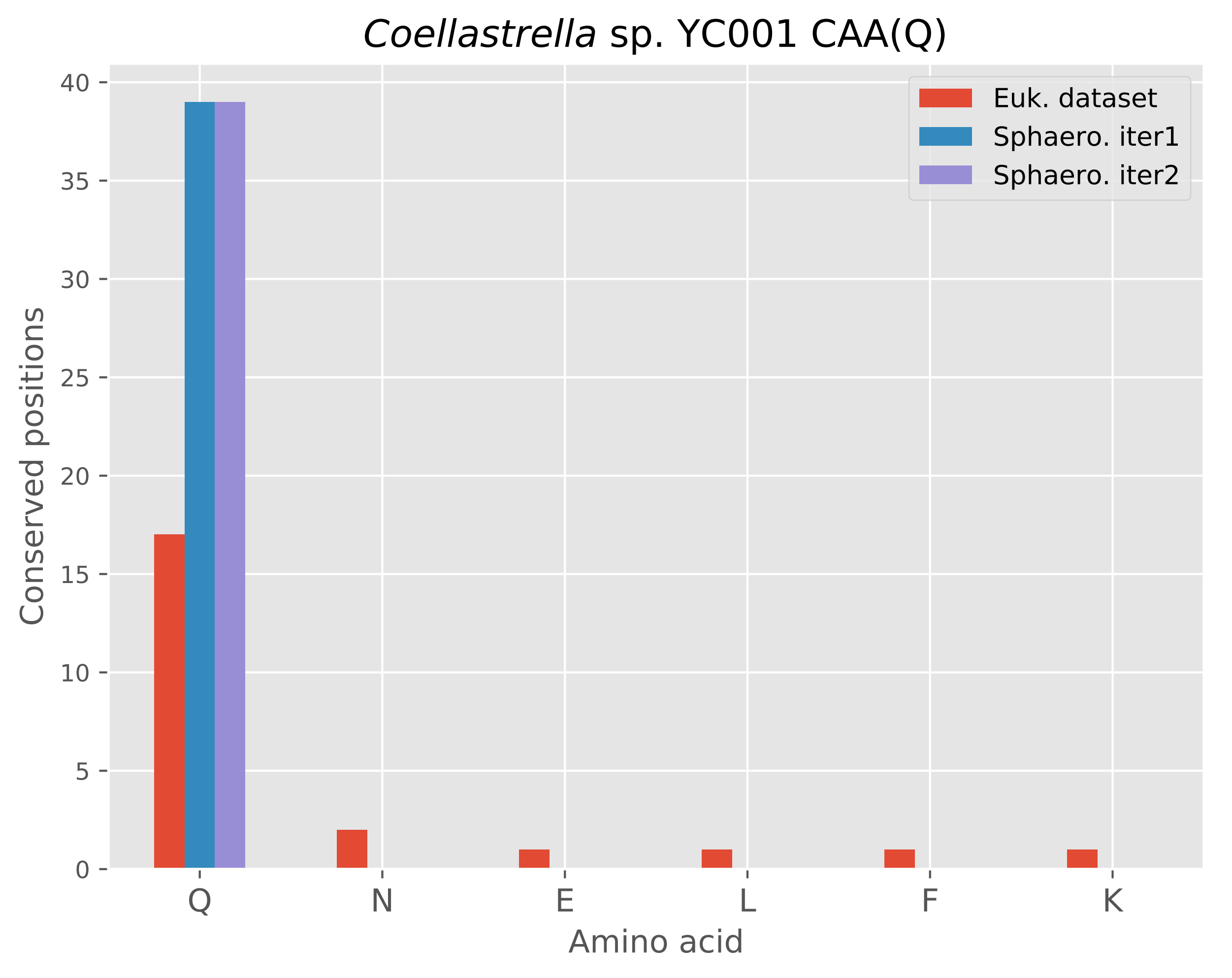

Coellastrella sp. YC001 CAA(Q)
Euk. dataset
Sphaero. iter1
Sphaero. iter2
Conserved positions
0
5
10
15
20
25
30
35
40
Q
N
E
L
F
K
Amino acid

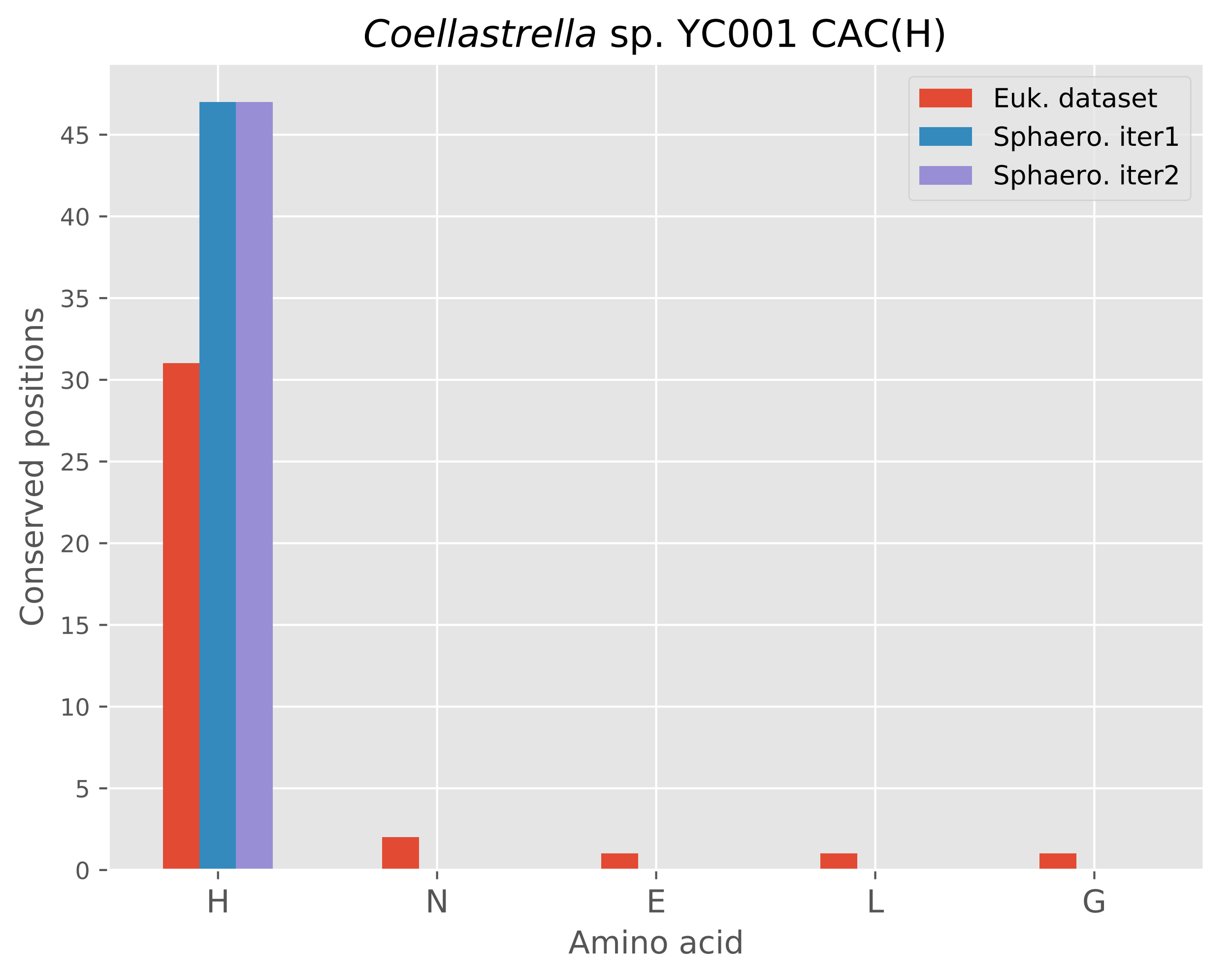

Coellastrella sp. YC001 CAC(H)
Euk. dataset
Sphaero. iter1
Sphaero. iter2
Conserved positions
0
5
10
15
20
25
30
35
40
45
H
N
E
L
G
Amino acid

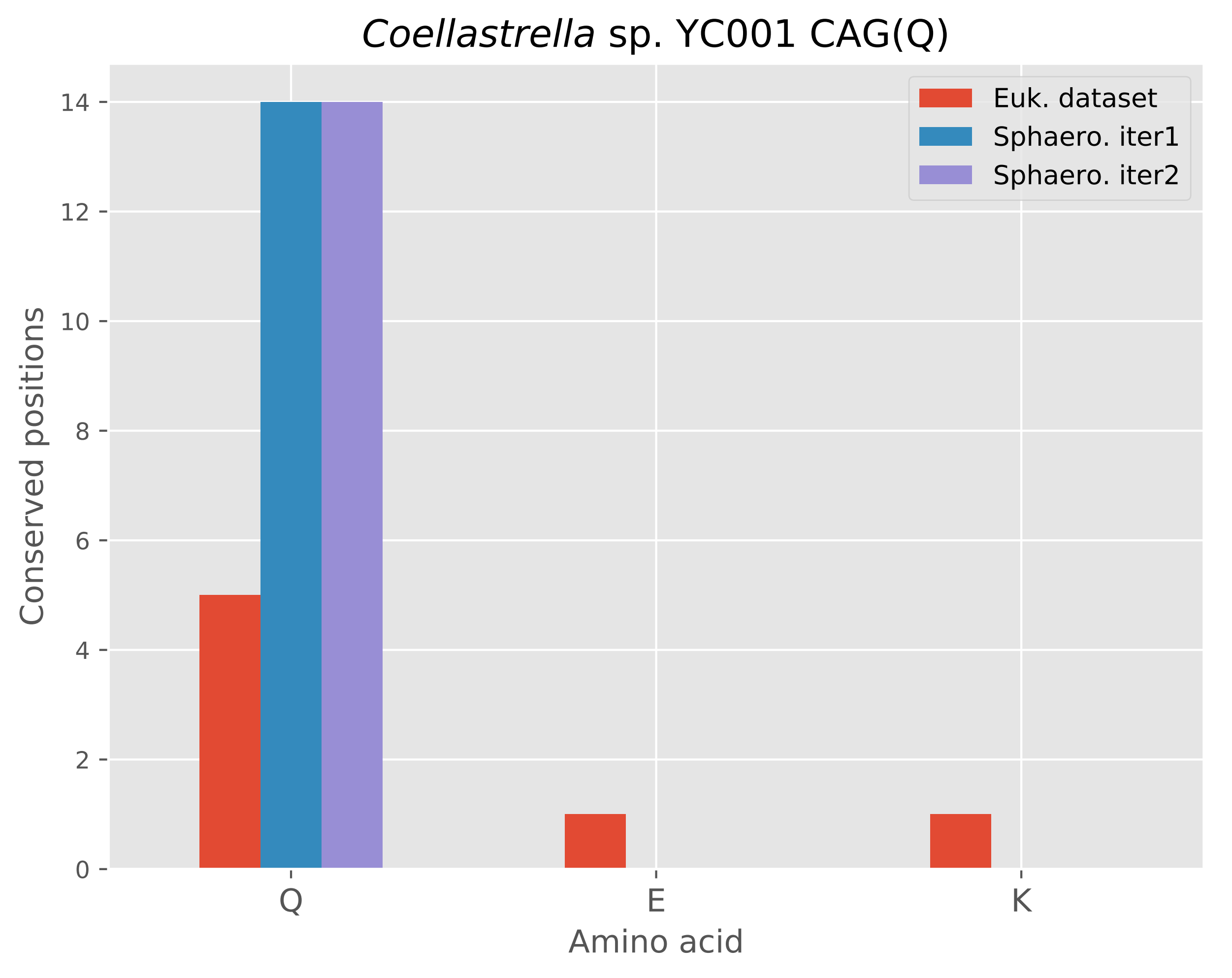
Coellastrella sp. YC001 CAG(Q)
Euk. dataset
Sphaero. iter1
Sphaero. iter2
Conserved positions
0
2
4
6
8
10
12
14
Q
E
K
Amino acid

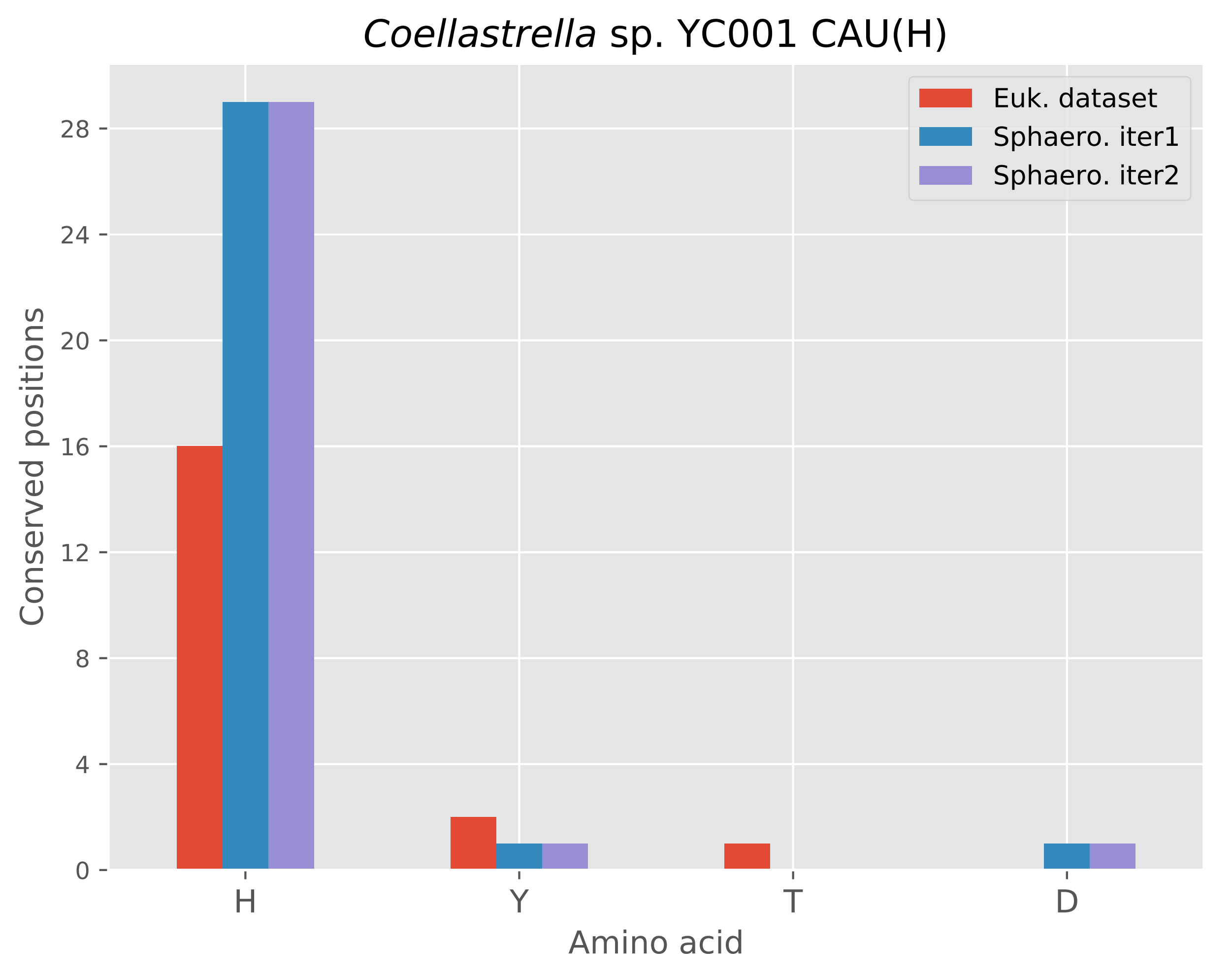

Coellastrella sp. YC001 CAU(H)
Euk. dataset
Sphaero. iter1
Sphaero. iter2
Conserved positions
0
4
8
12
16
20
24
28
H
Y
T
D
Amino acid

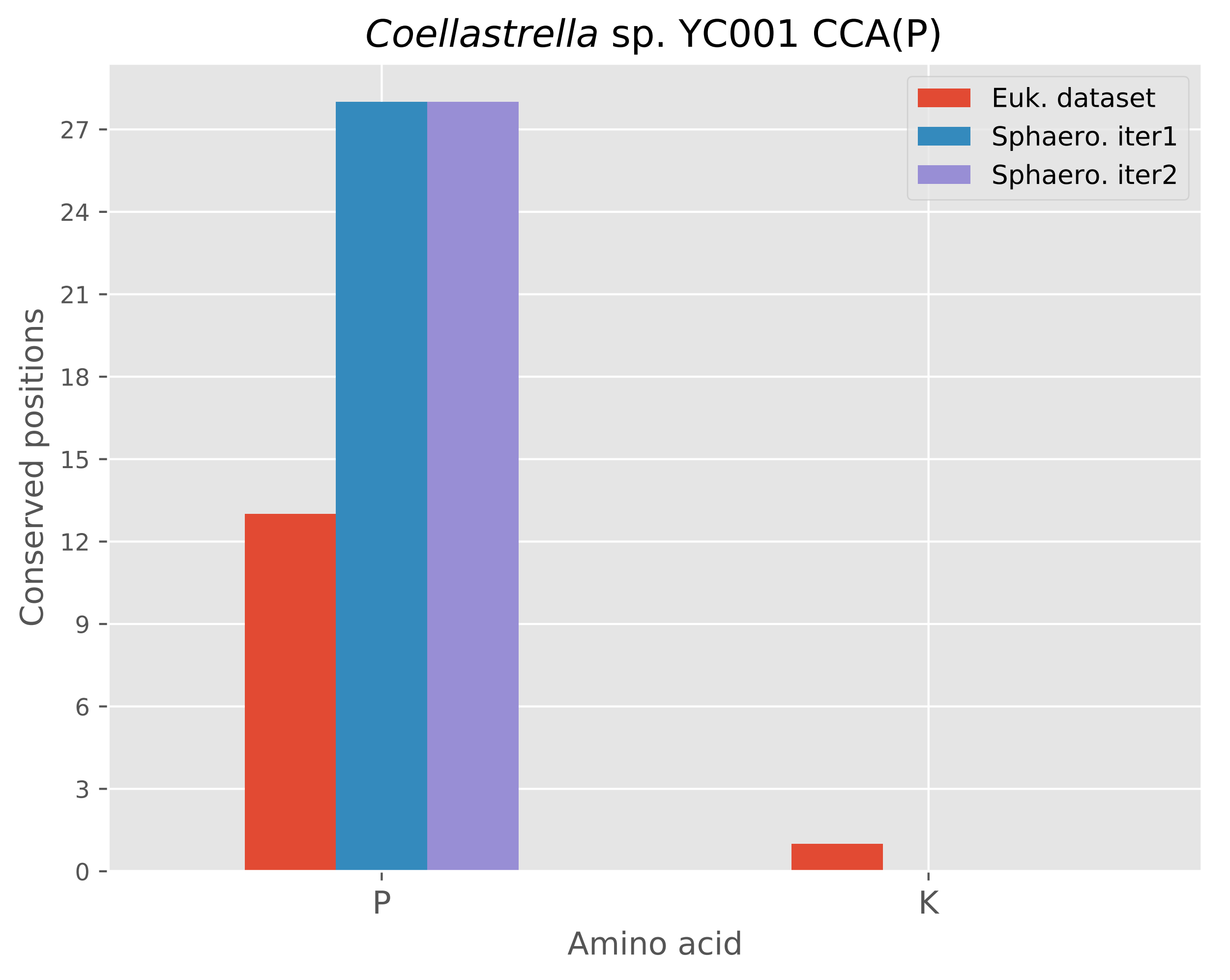

Coellastrella sp. YC001 CCA(P)
Euk. dataset
Sphaero. iter1
Sphaero. iter2
Conserved positions
0
3
6
9
12
15
18
21
24
27
P
K
Amino acid

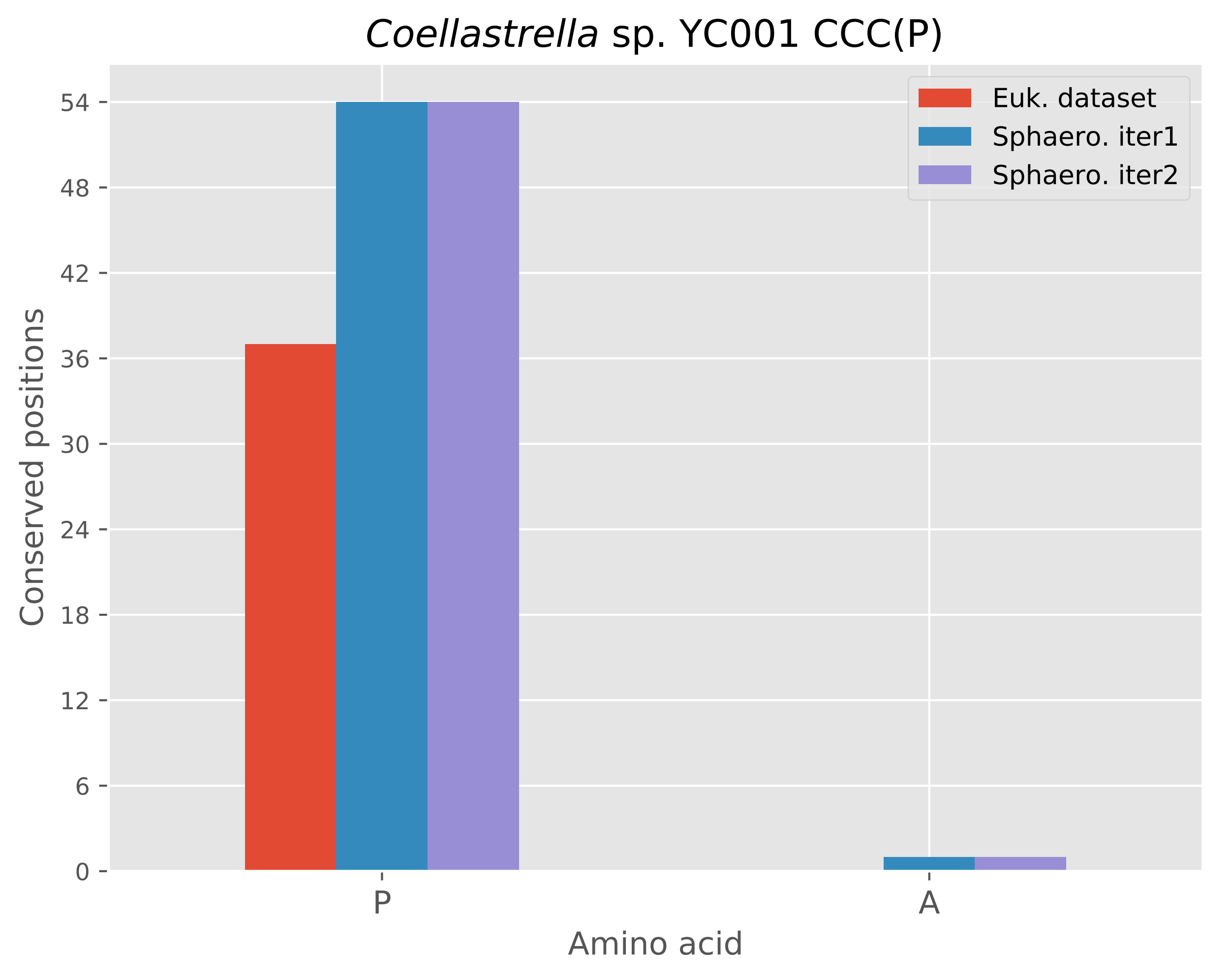

Coellastrella sp. YC001 CCC(P)
Euk. dataset
Sphaero. iter1
Sphaero. iter2
Conserved positions
0
6
12
18
24
30
36
42
48
54
P
A
Amino acid

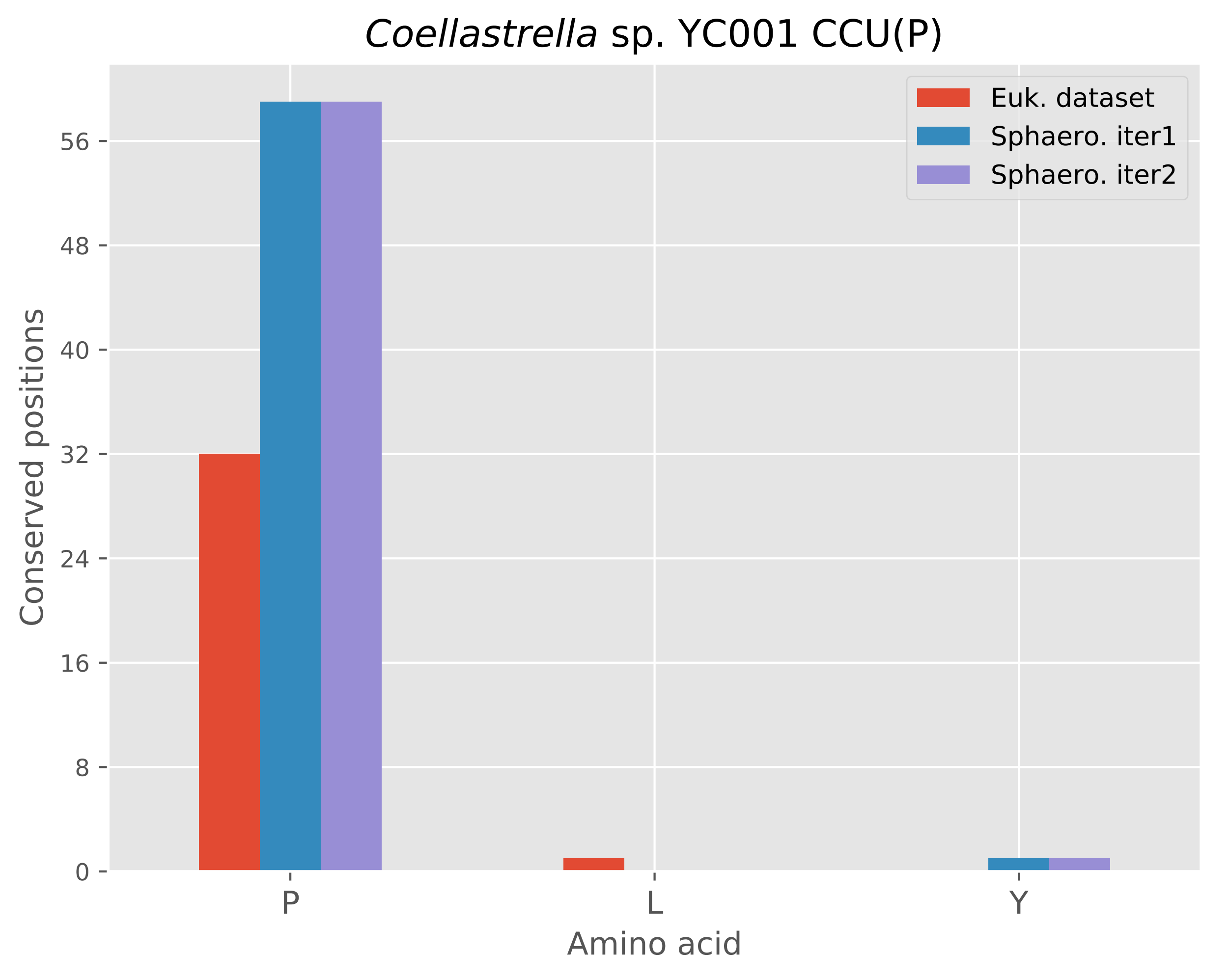

Coellastrella sp. YC001 CCU(P)
Euk. dataset
Sphaero. iter1
Sphaero. iter2
Conserved positions
0
8
16
24
32
40
48
56
P
L
Y
Amino acid

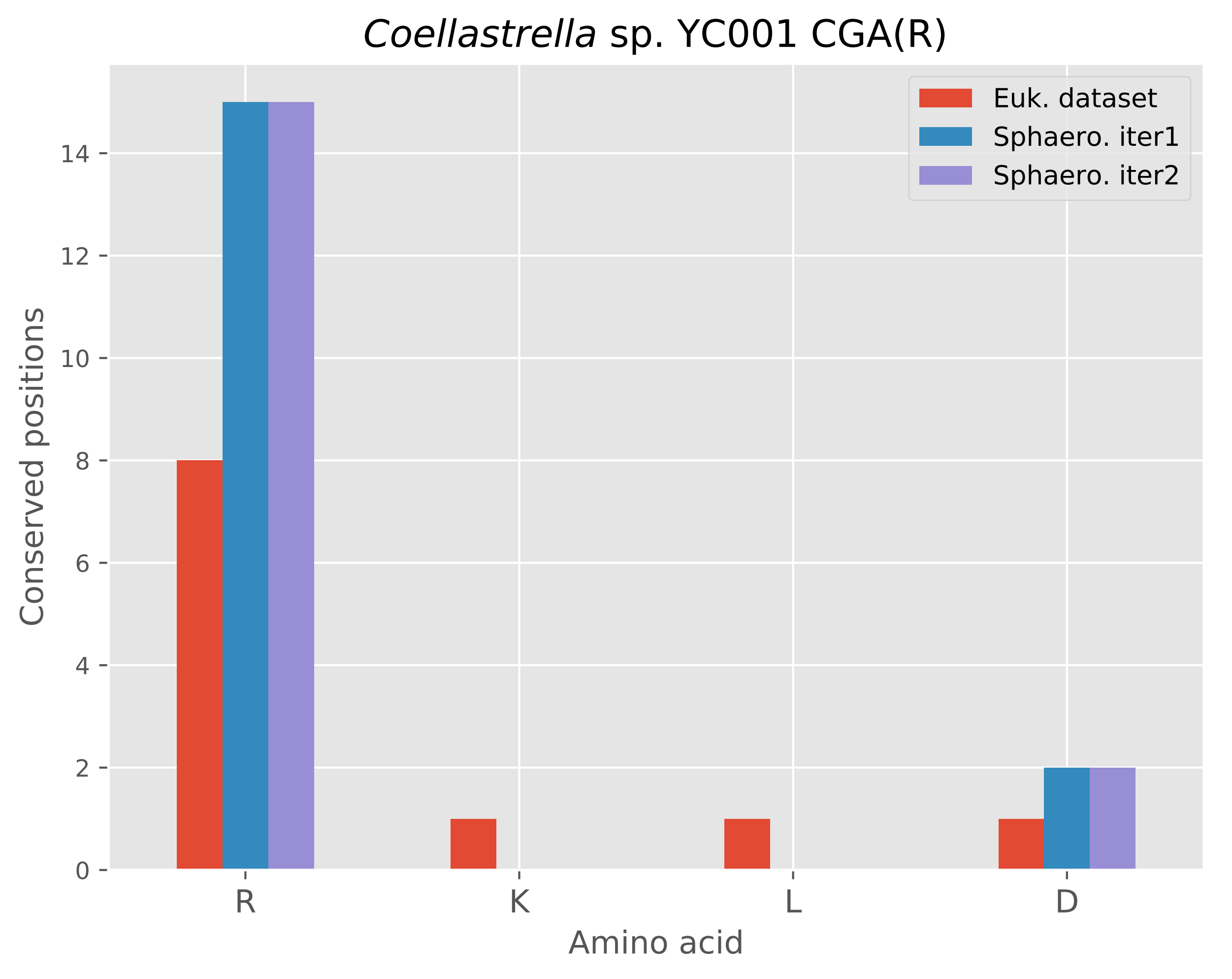

Coellastrella sp. YC001 CGA(R)
Euk. dataset
Sphaero. iter1
Sphaero. iter2
Conserved positions
0
2
4
6
8
10
12
14
R
K
L
D
Amino acid

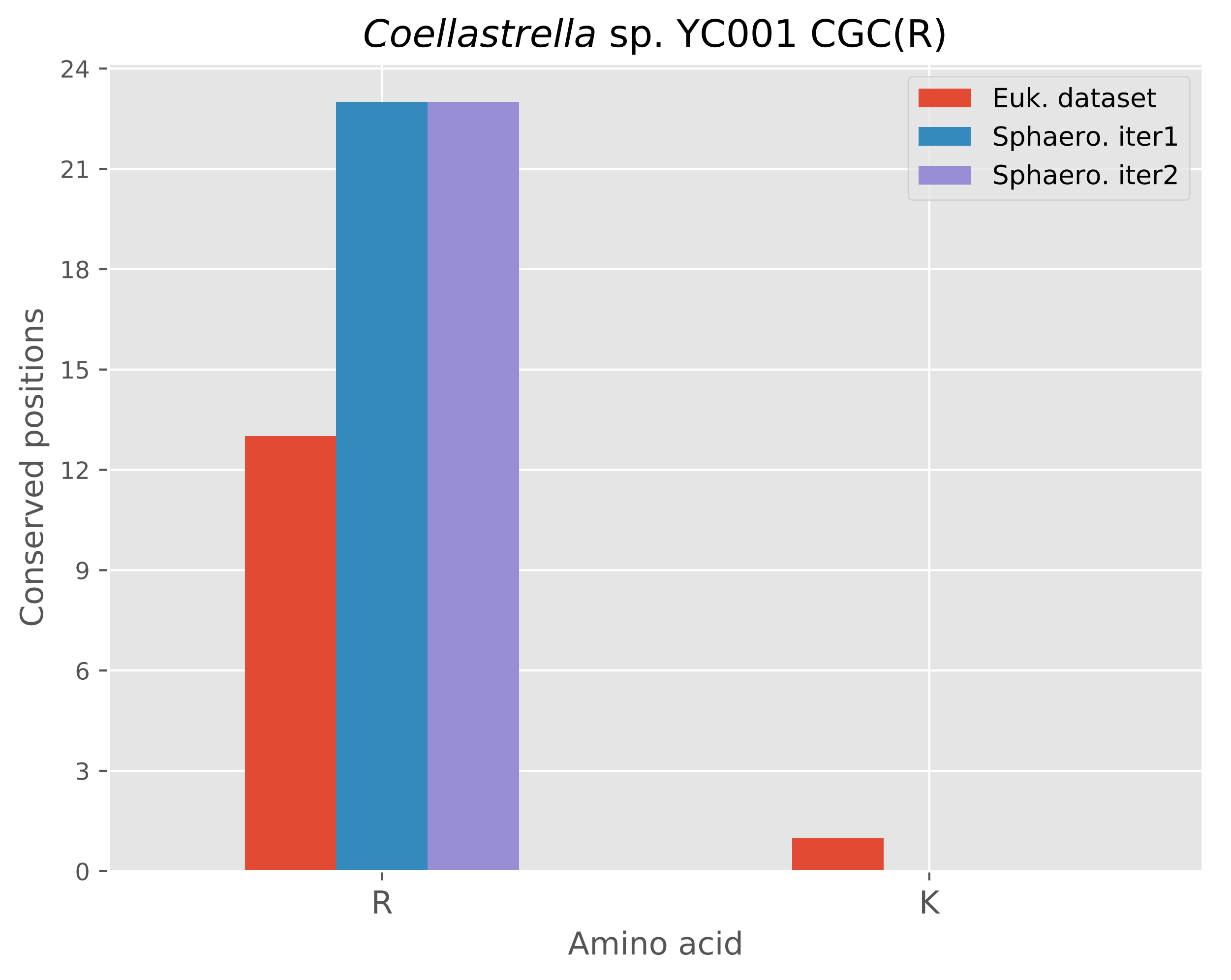

Coellastrella sp. YC001 CGC(R)
Euk. dataset
Sphaero. iter1
Sphaero. iter2
Conserved positions
0
3
6
9
12
15
18
21
24
R
K
Amino acid

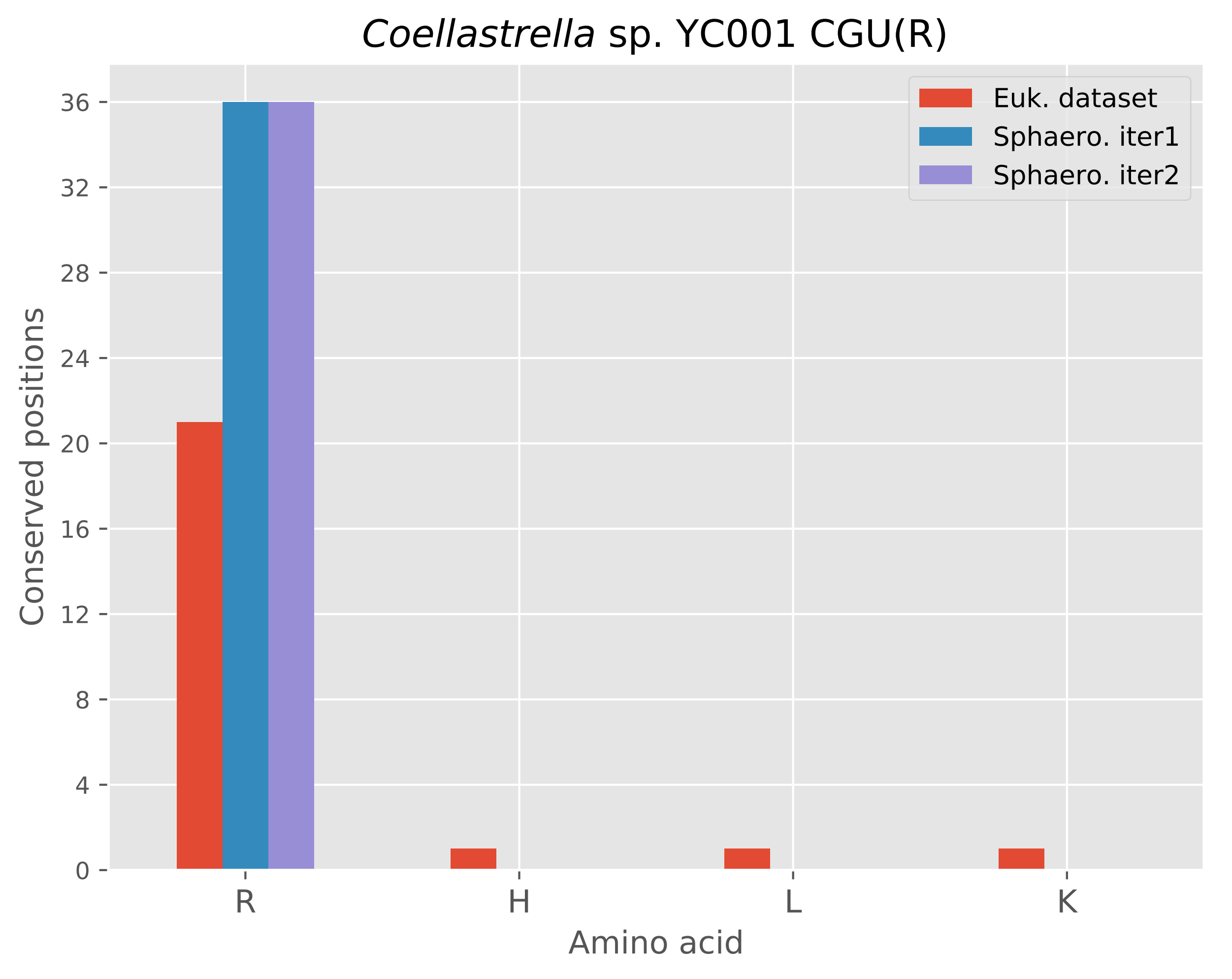

Coellastrella sp. YC001 CGU(R)
Euk. dataset
Sphaero. iter1
Sphaero. iter2
Conserved positions
0
4
8
12
16
20
24
28
32
36
R
H
L
K
Amino acid

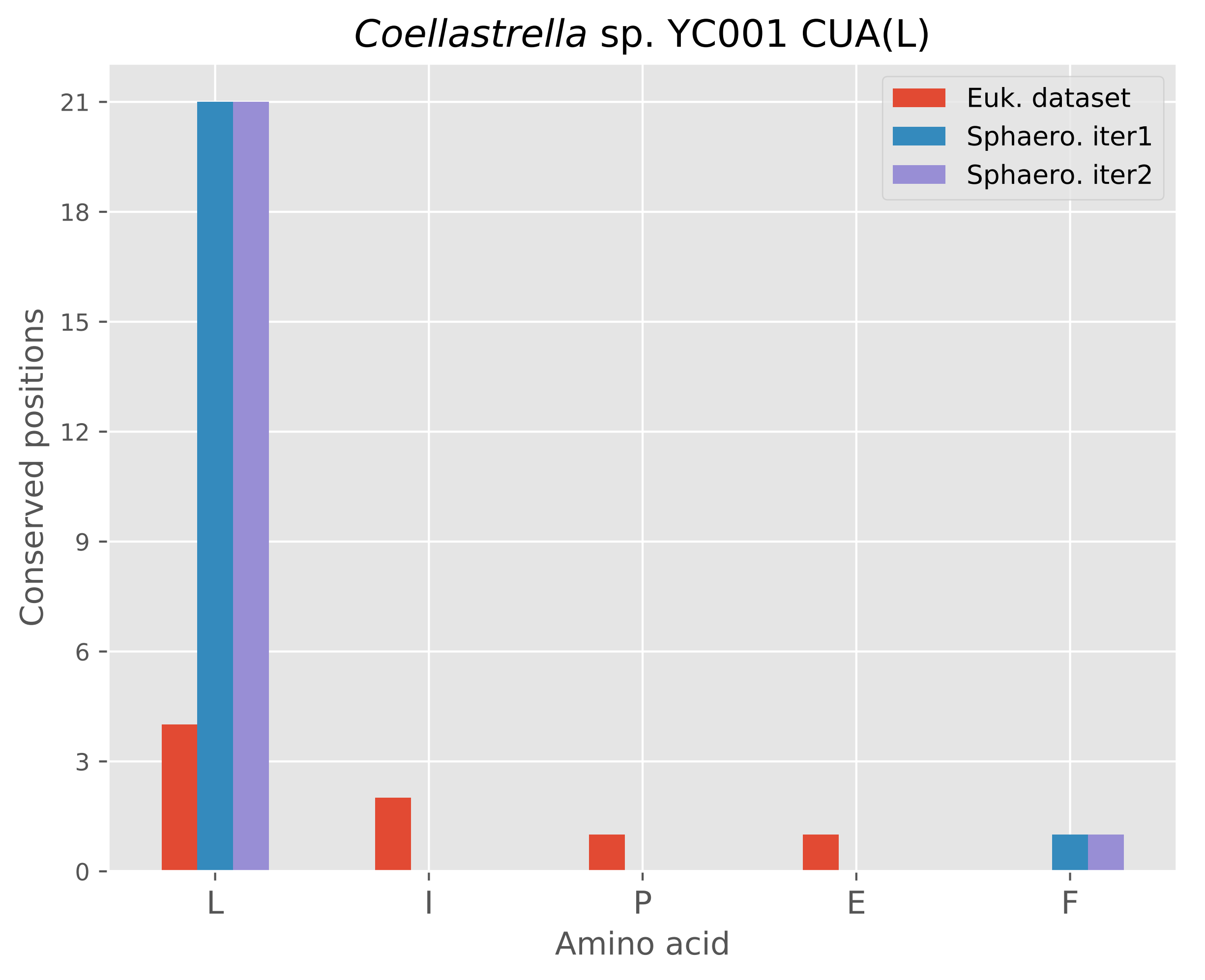

Coellastrella sp. YC001 CUA(L)
Euk. dataset
Sphaero. iter1
Sphaero. iter2
Conserved positions
0
3
6
9
12
15
18
21
L
I
P
E
F
Amino acid

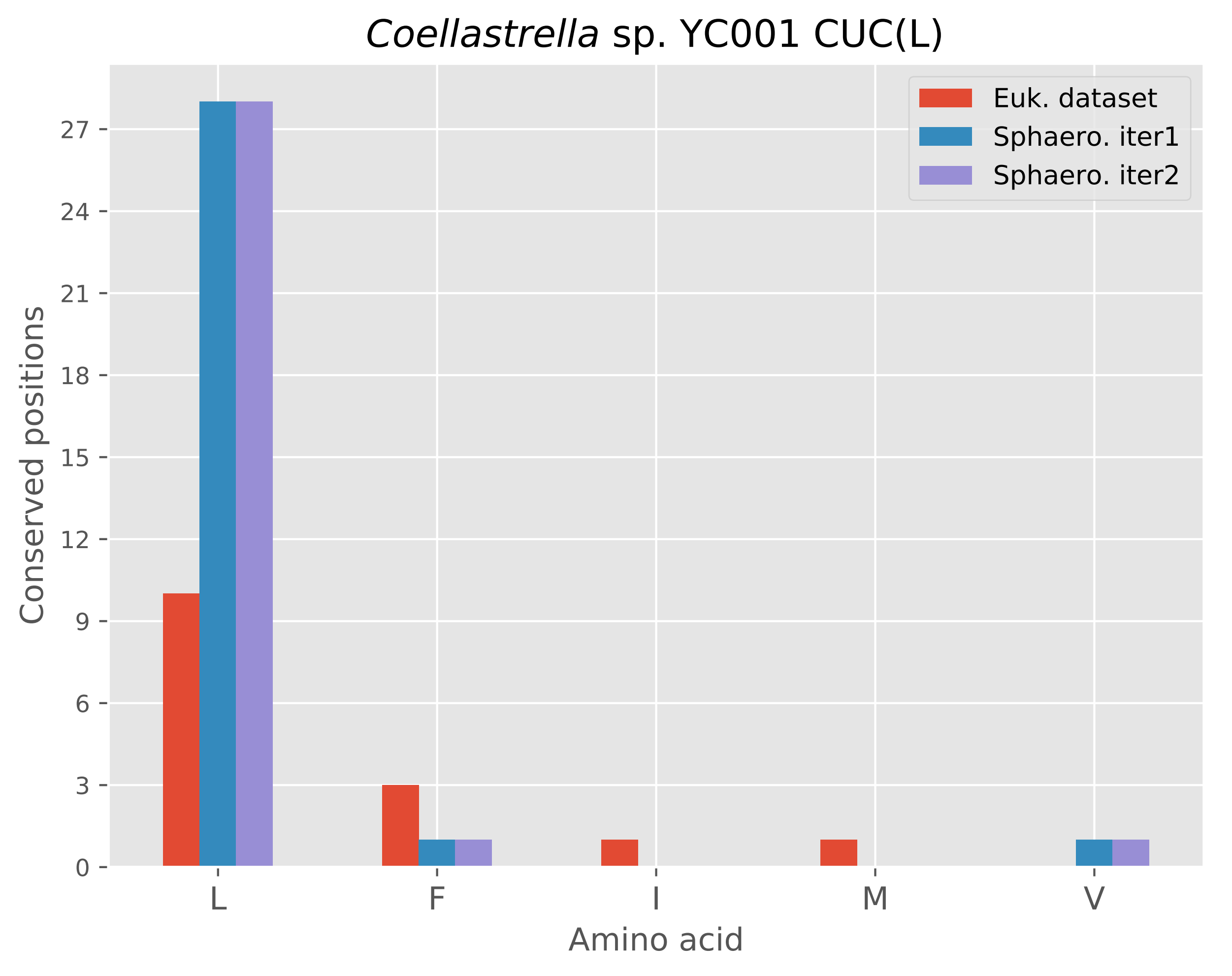

Coellastrella sp. YC001 CUC(L)
Euk. dataset
Sphaero. iter1
Sphaero. iter2
Conserved positions
0
3
6
9
12
15
18
21
24
27
L
F
I
M
V
Amino acid

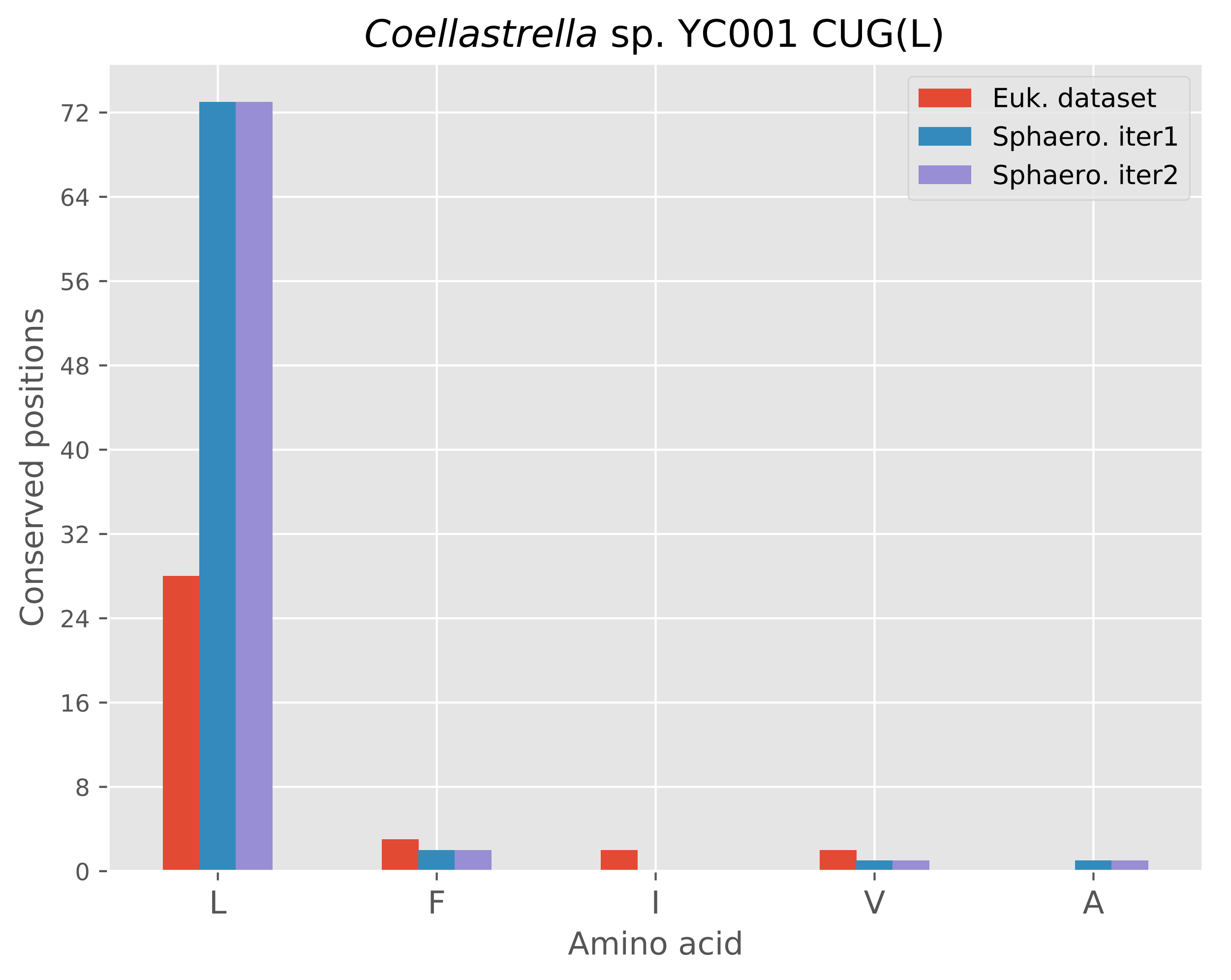

Coellastrella sp. YC001 CUG(L)
Euk. dataset
Sphaero. iter1
Sphaero. iter2
Conserved positions
0
8
16
24
32
40
48
56
64
72
L
F
I
V
A
Amino acid

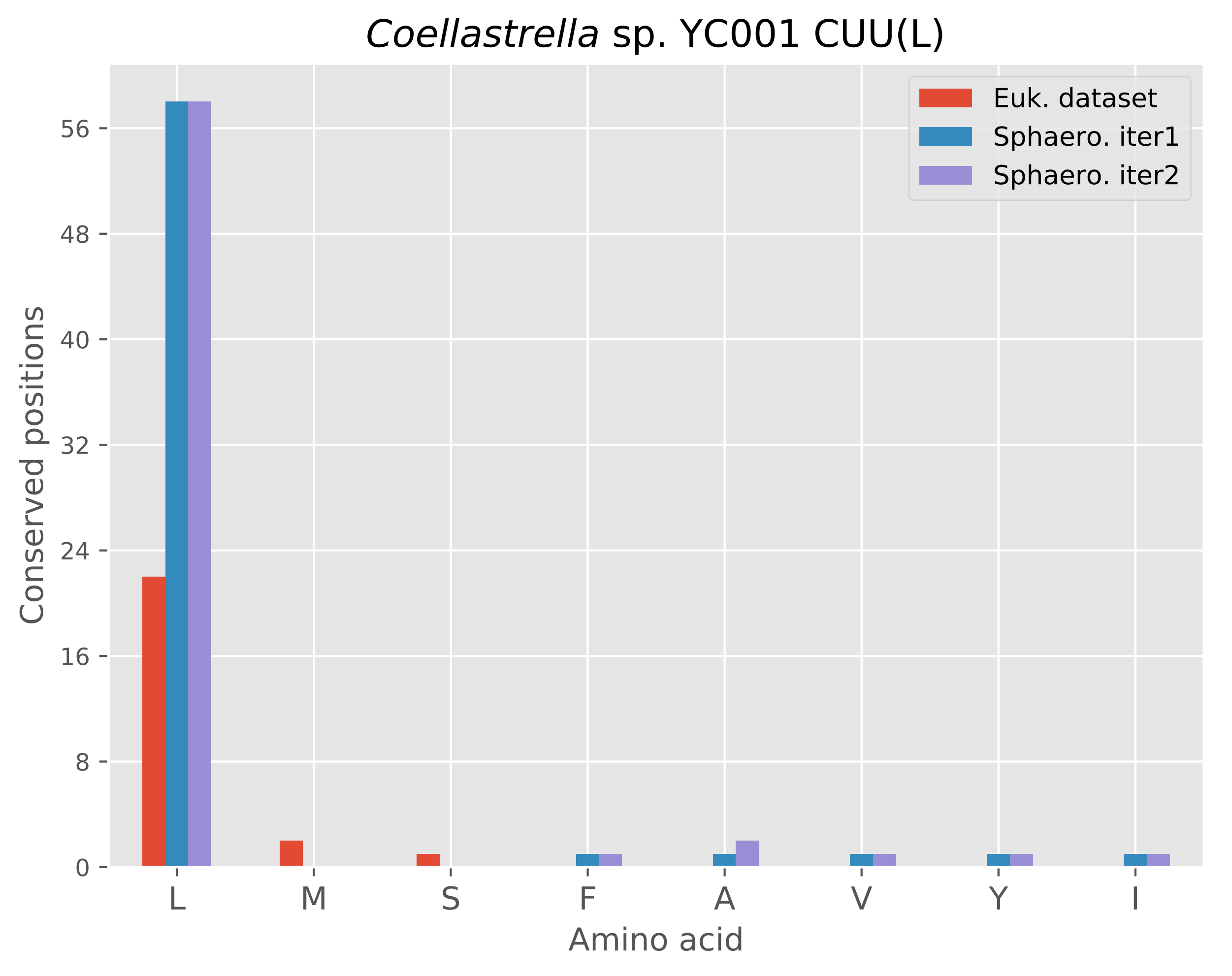

Coellastrella sp. YC001 CUU(L)
Euk. dataset
Sphaero. iter1
Sphaero. iter2
Conserved positions
0
8
16
24
32
40
48
56
L
M
S
F
A
V
Y
I
Amino acid

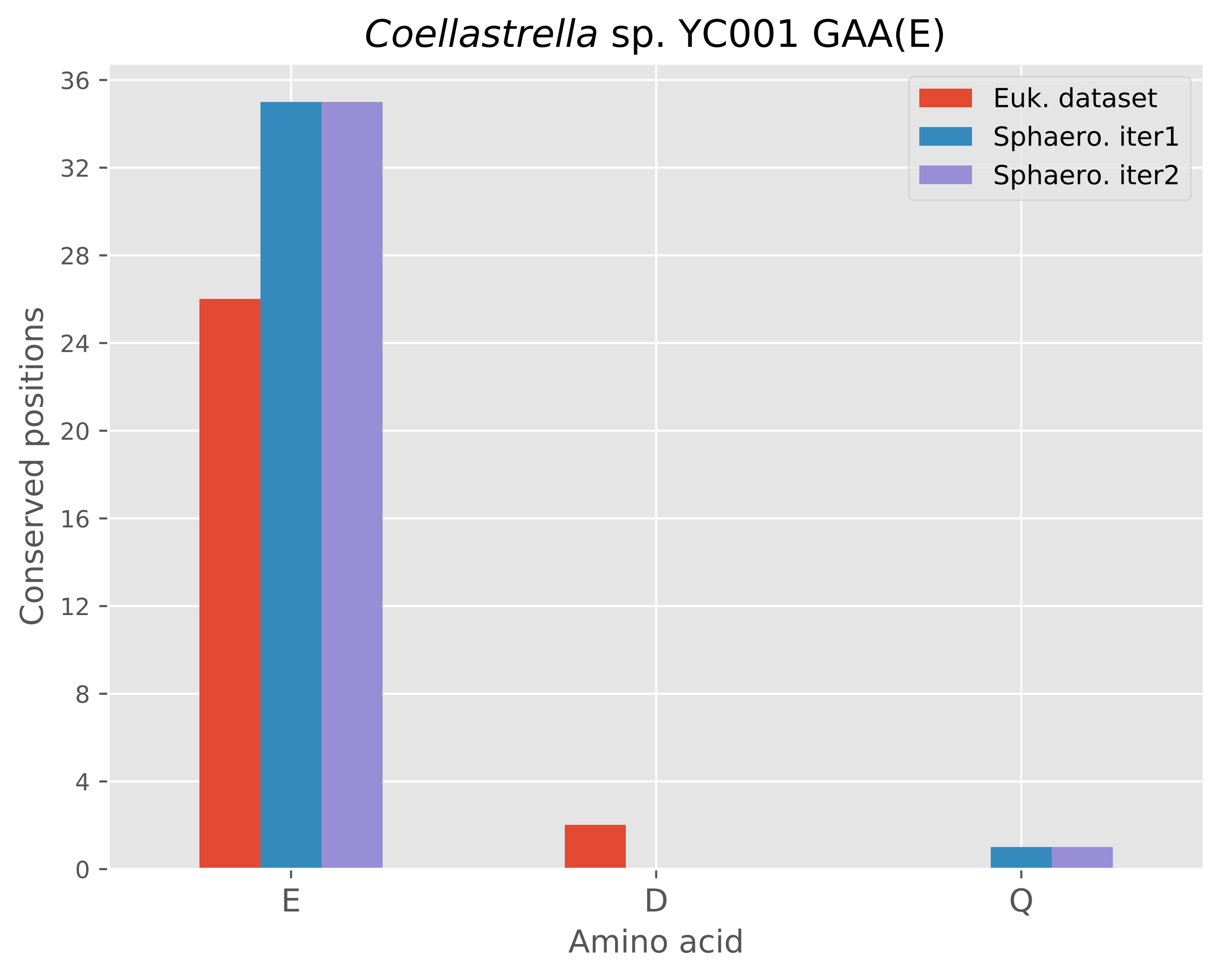
Coellastrella sp. YC001 GAA(E)
Euk. dataset
Sphaero. iter1
Sphaero. iter2
Conserved positions
0
4
8
12
16
20
24
28
32
36
E
D
Q
Amino acid

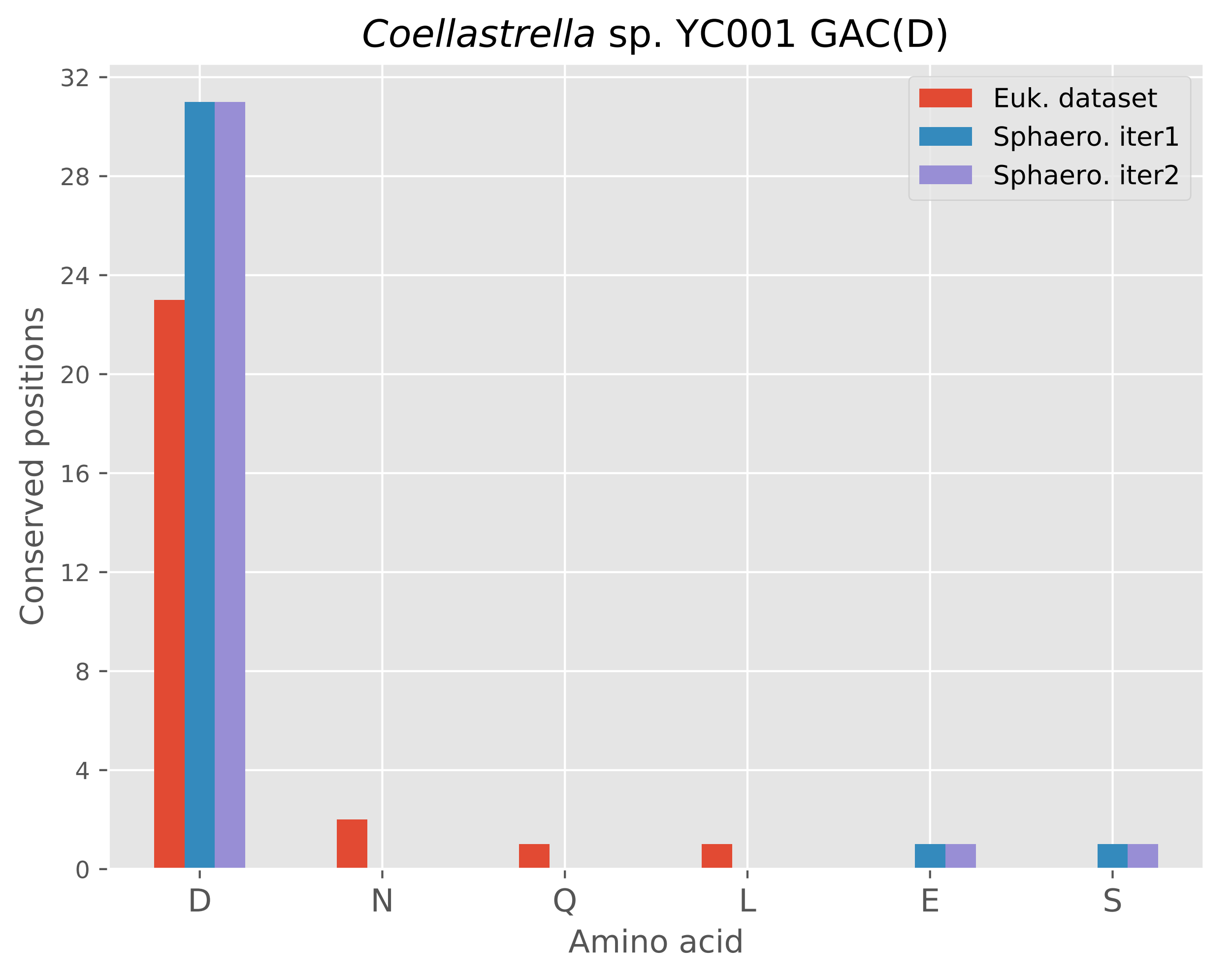

Coellastrella sp. YC001 GAC(D)
Euk. dataset
Sphaero. iter1
Sphaero. iter2
Conserved positions
0
4
8
12
16
20
24
28
32
D
N
Q
L
E
S
Amino acid

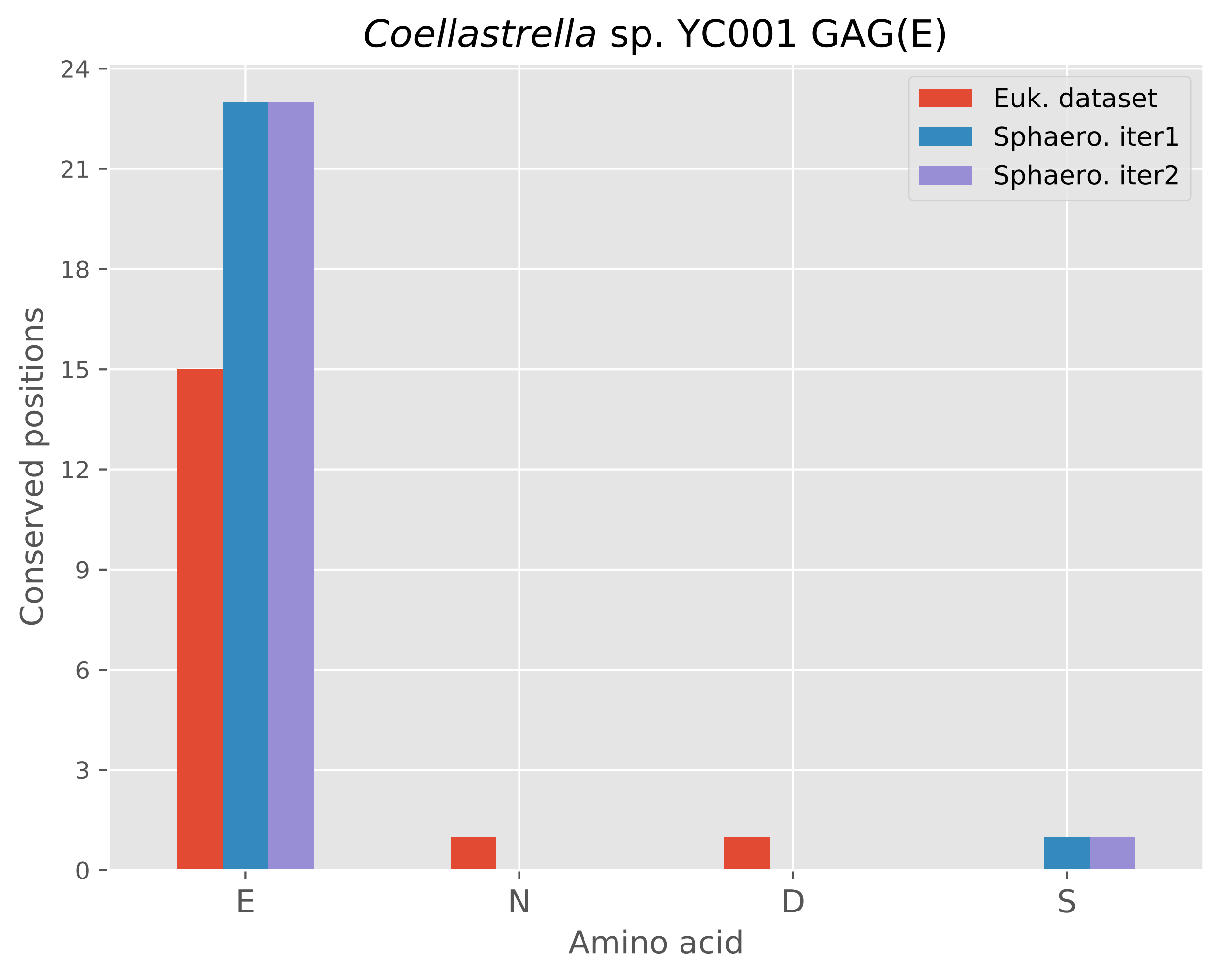

Coellastrella sp. YC001 GAG(E)
Conserved positions
0
3
6
9
12
15
18
21
24
Amino acid
E
N
D
S
Euk. dataset
Sphaero. iter1
Sphaero. iter2

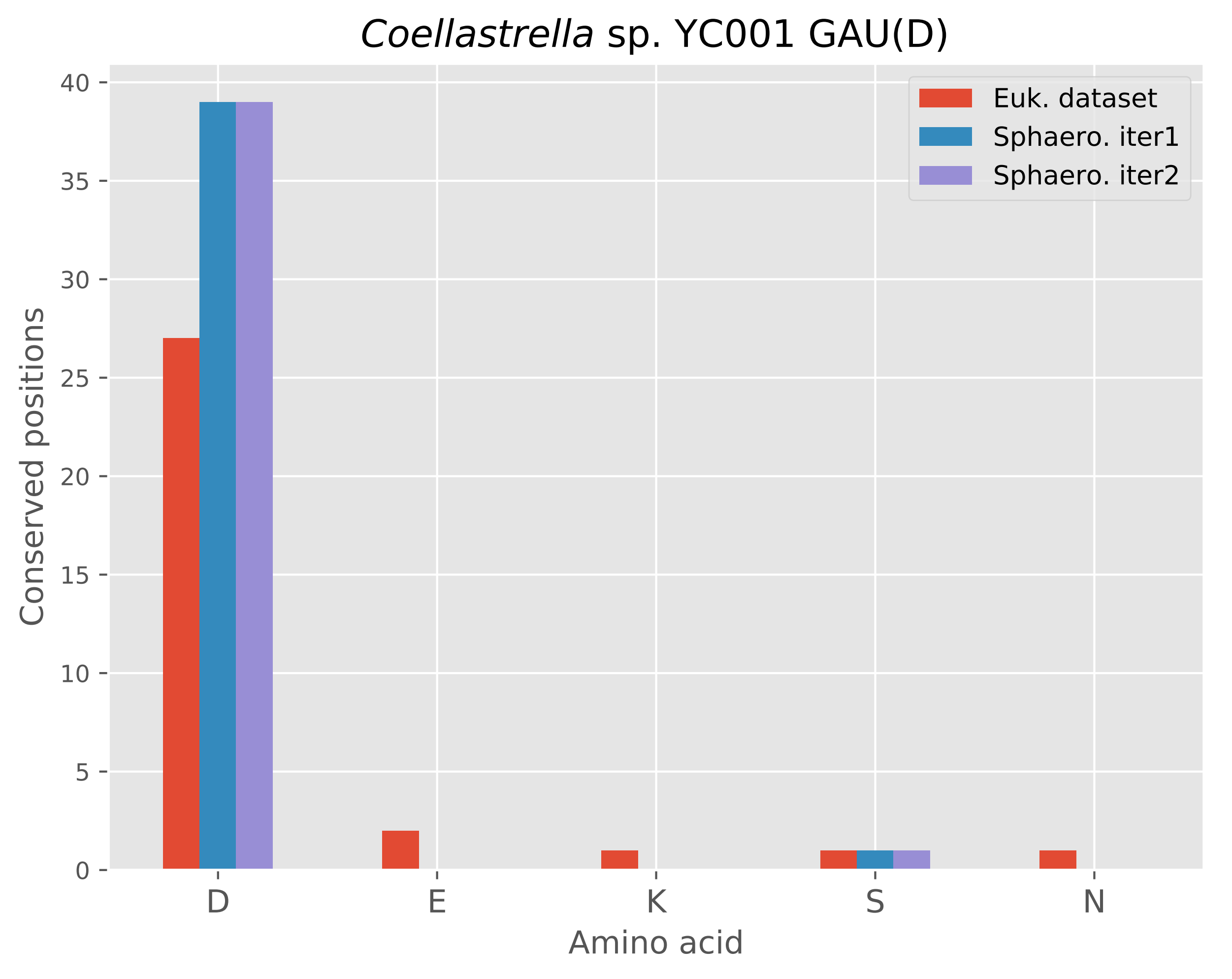

Coellastrella sp. YC001 GAU(D)
Euk. dataset
Sphaero. iter1
Sphaero. iter2
Conserved positions
0
5
10
15
20
25
30
35
40
D
E
K
S
N
Amino acid

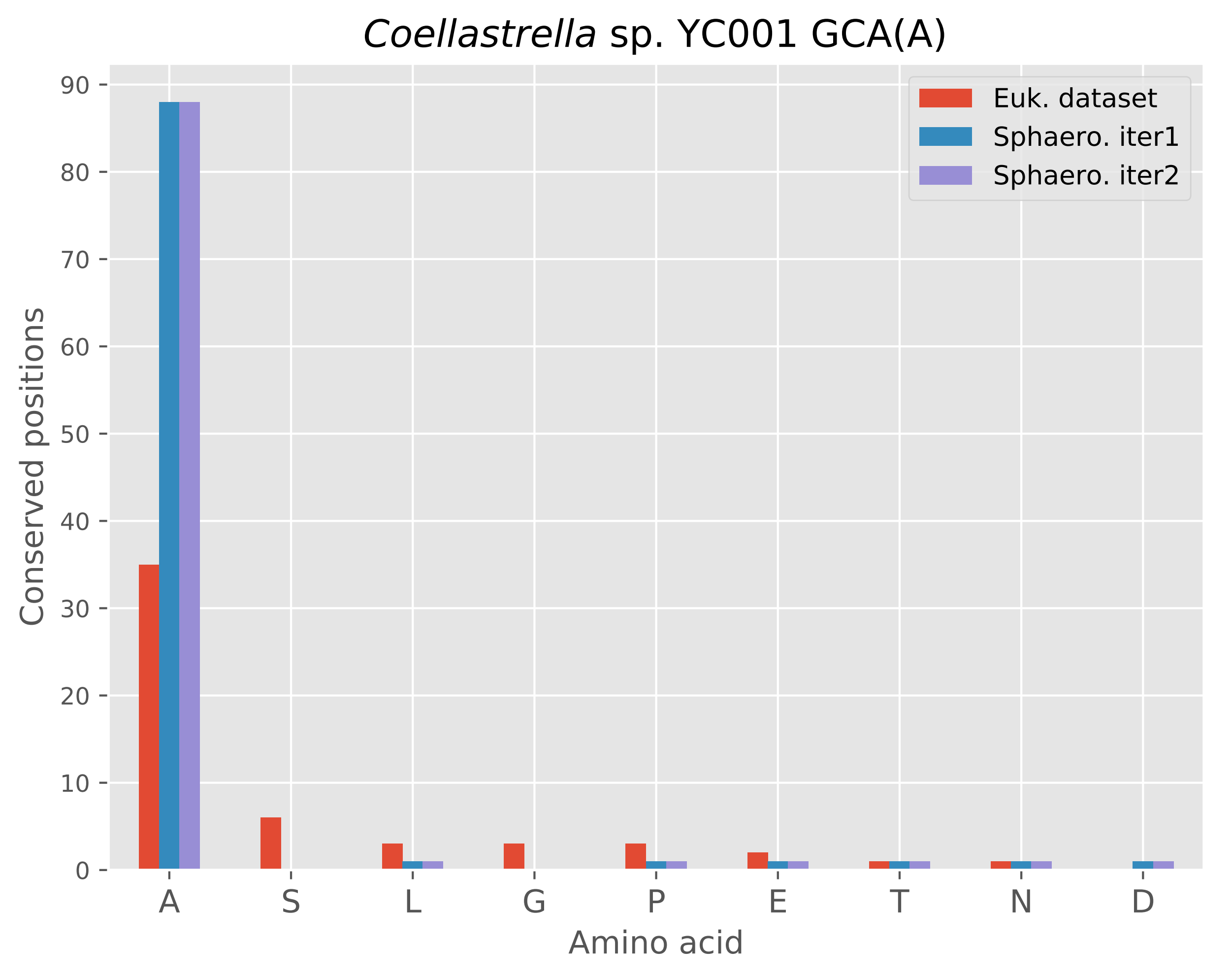

Coellastrella sp. YC001 GCA(A)
Euk. dataset
Sphaero. iter1
Sphaero. iter2
Conserved positions
0
10
20
30
40
50
60
70
80
90
A
S
L
G
P
E
T
N
D
Amino acid

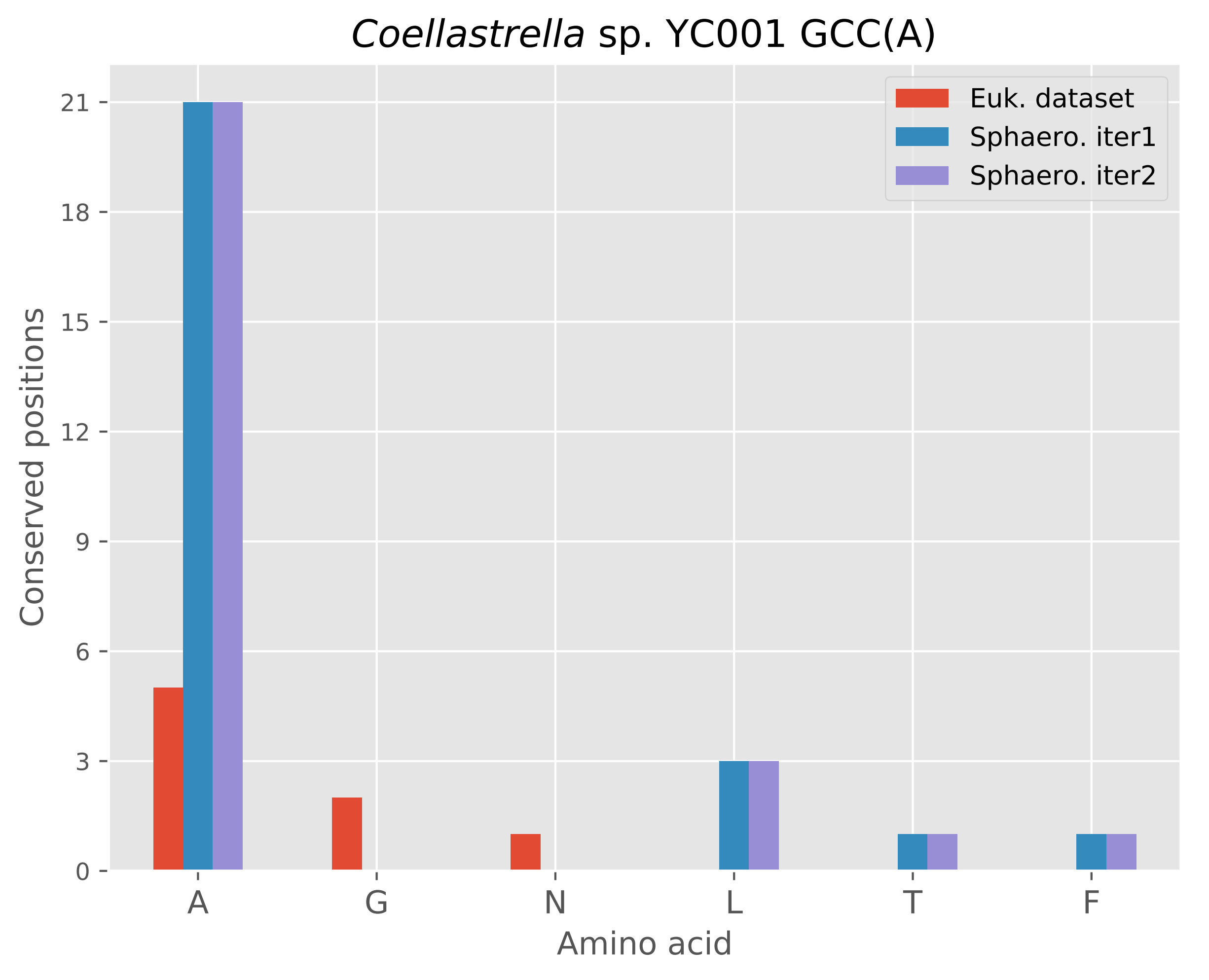

Coellastrella sp. YC001 GCC(A)
Euk. dataset
Sphaero. iter1
Sphaero. iter2
Conserved positions
21
18
15
12
9
6
3
0
A
G
N
L
T
F
Amino acid

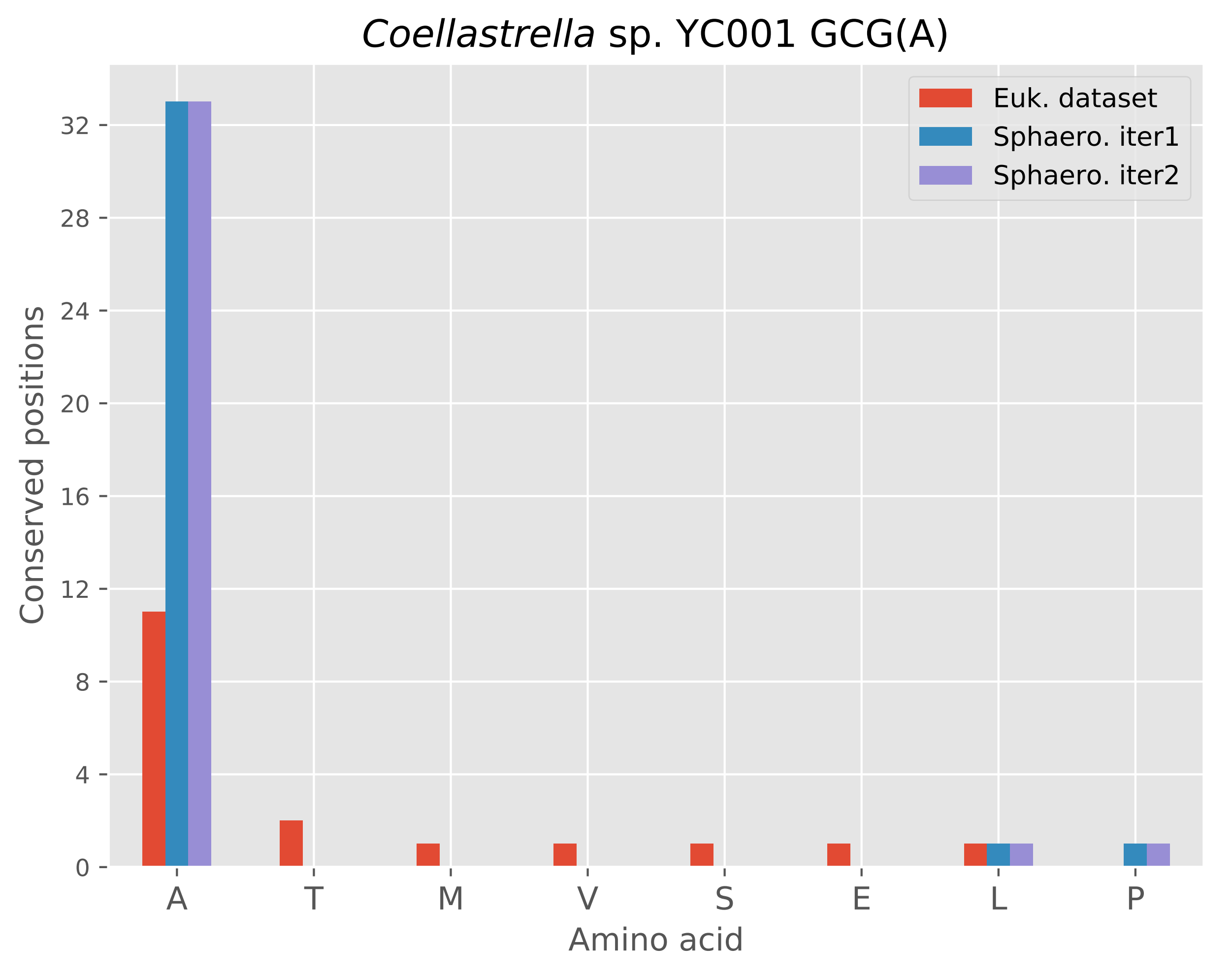

Coellastrella sp. YC001 GCG(A)
Euk. dataset
Sphaero. iter1
Sphaero. iter2
Conserved positions
0
4
8
12
16
20
24
28
32
A
T
M
V
S
E
L
P
Amino acid

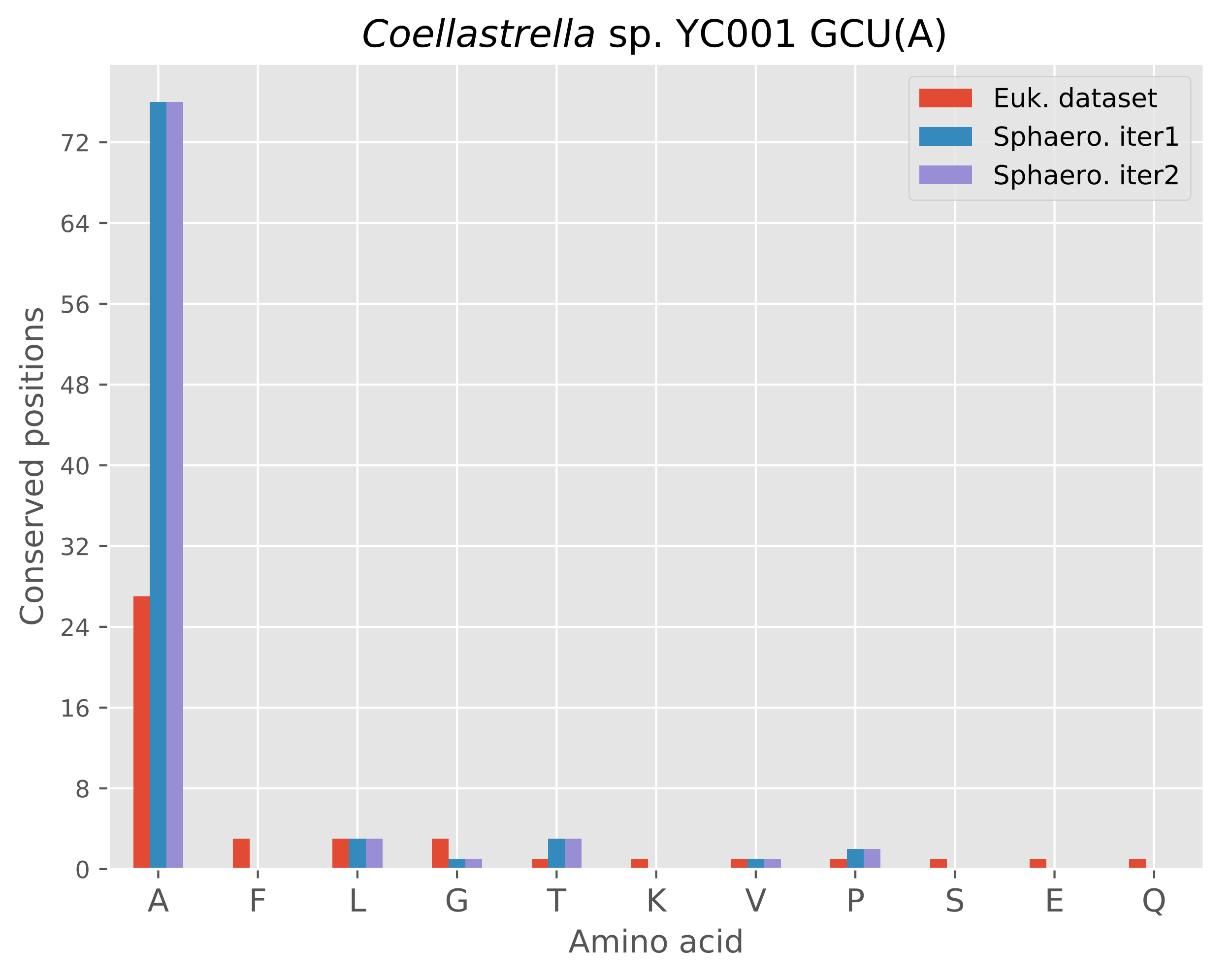

Coellastrella sp. YC001 GCU(A)
Euk. dataset
Sphaero. iter1
Sphaero. iter2
Conserved positions
0
8
16
24
32
40
48
56
64
72
A
F
L
G
T
K
V
P
S
E
Q
Amino acid

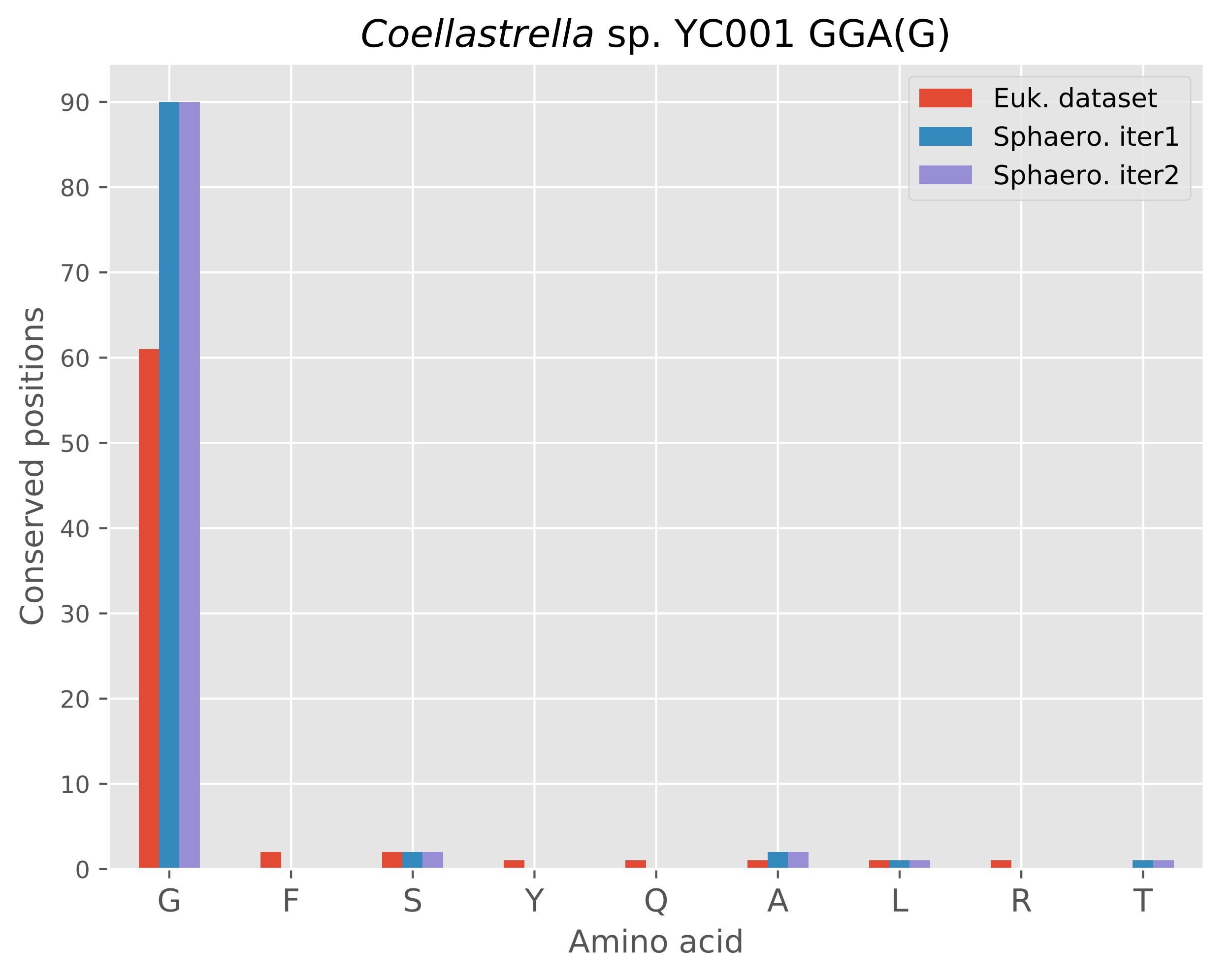

Coellastrella sp. YC001 GGA(G)
Euk. dataset
Sphaero. iter1
Sphaero. iter2
Conserved positions
0
10
20
30
40
50
60
70
80
90
G
F
S
Y
Q
A
L
R
T
Amino acid

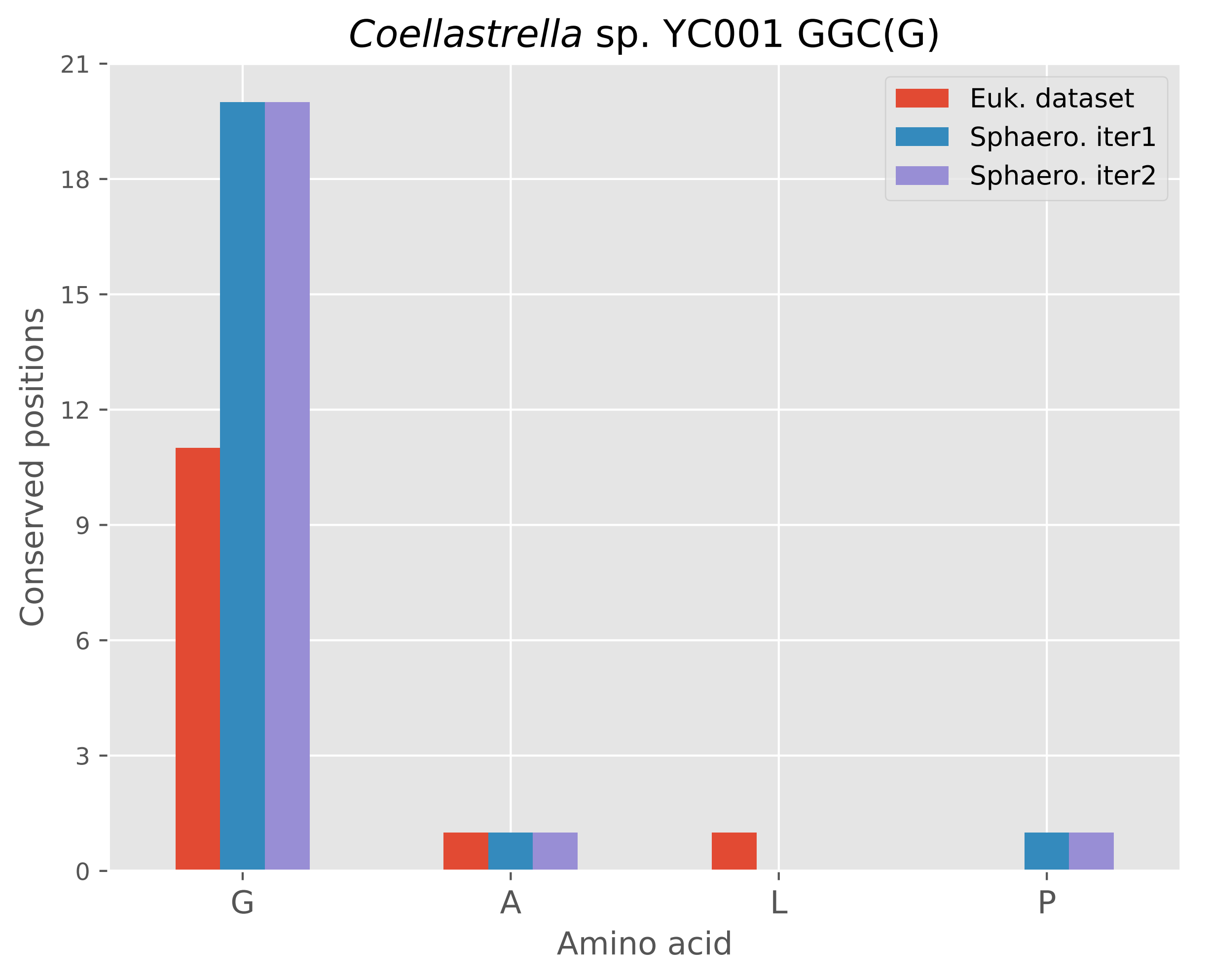
Coellastrella sp. YC001 GGC(G)
Conserved positions
Amino acid
0
3
6
9
12
15
18
21
G
A
L
P
Euk. dataset
Sphaero. iter1
Sphaero. iter2

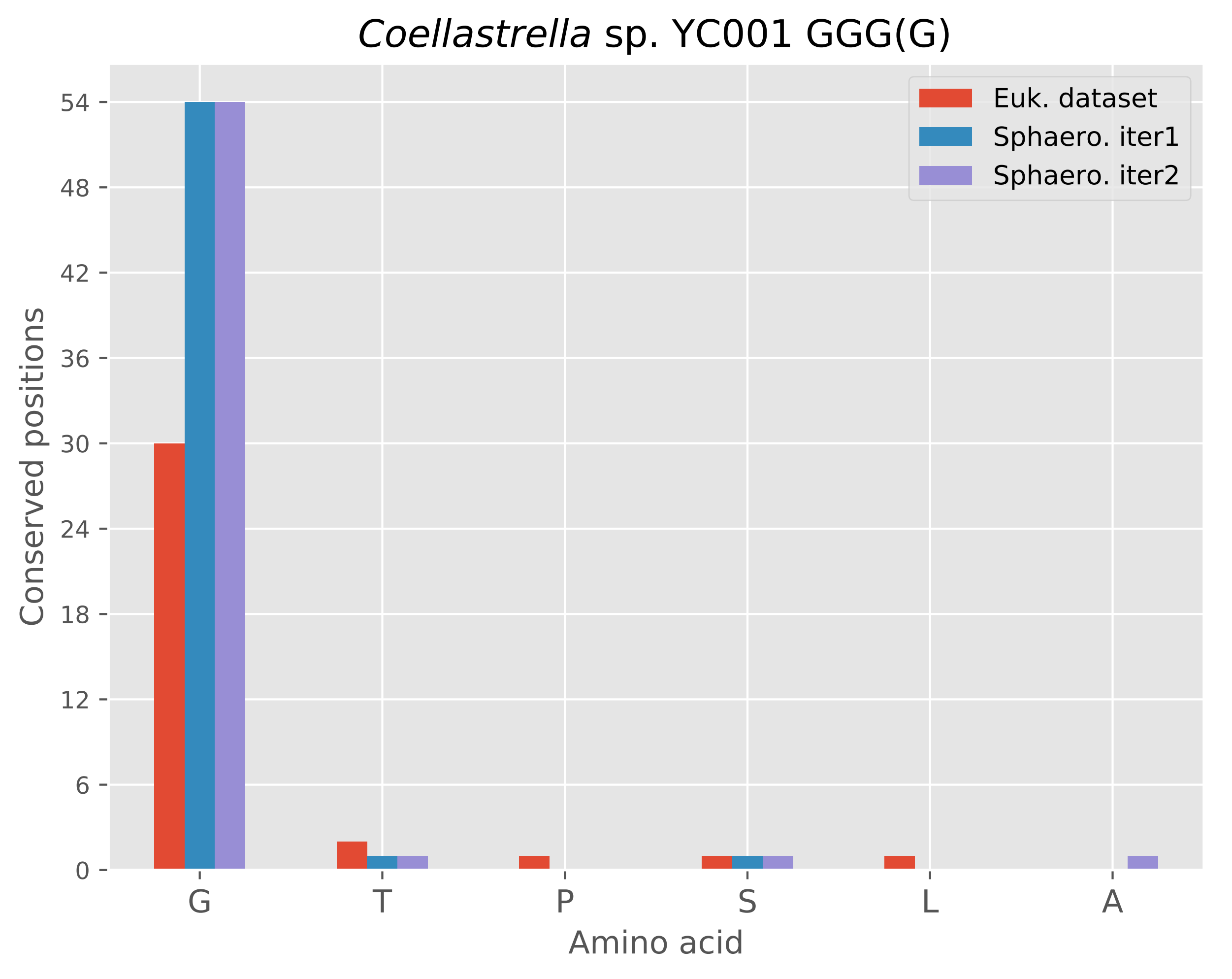

Coellastrella sp. YC001 GGG(G)
Euk. dataset
Sphaero. iter1
Sphaero. iter2
Conserved positions
0
6
12
18
24
30
36
42
48
54
G
T
P
S
L
A
Amino acid

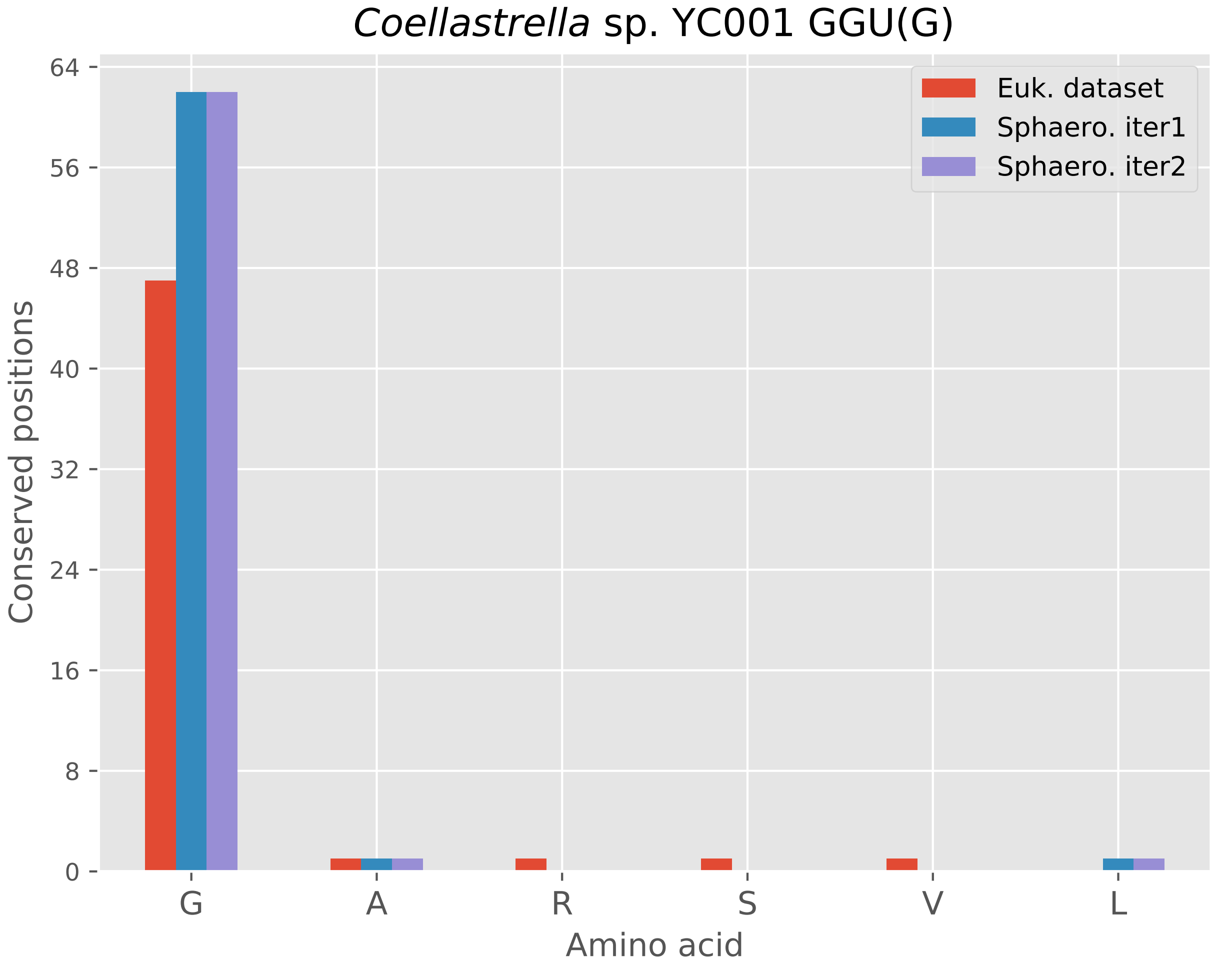

Coellastrella sp. YC001 GGU(G)
Conserved positions
Amino acid
64
56
48
40
32
24
16
8
0
G
A
R
S
V
L
Euk. dataset
Sphaero. iter1
Sphaero. iter2

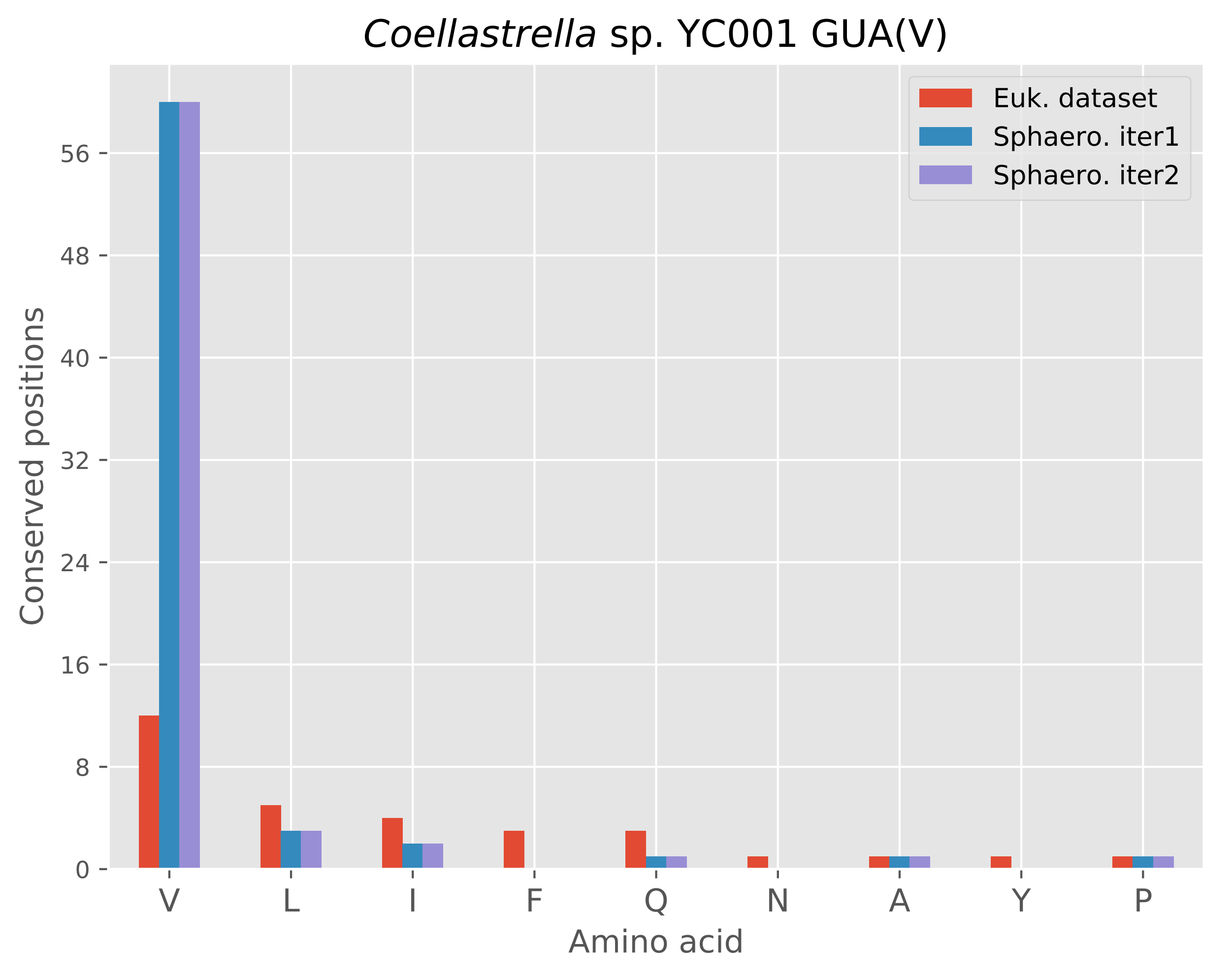

Coellastrella sp. YC001 GUA(V)
Euk. dataset
Sphaero. iter1
Sphaero. iter2
Conserved positions
0
8
16
24
32
40
48
56
V
L
I
F
Q
N
A
Y
P
Amino acid

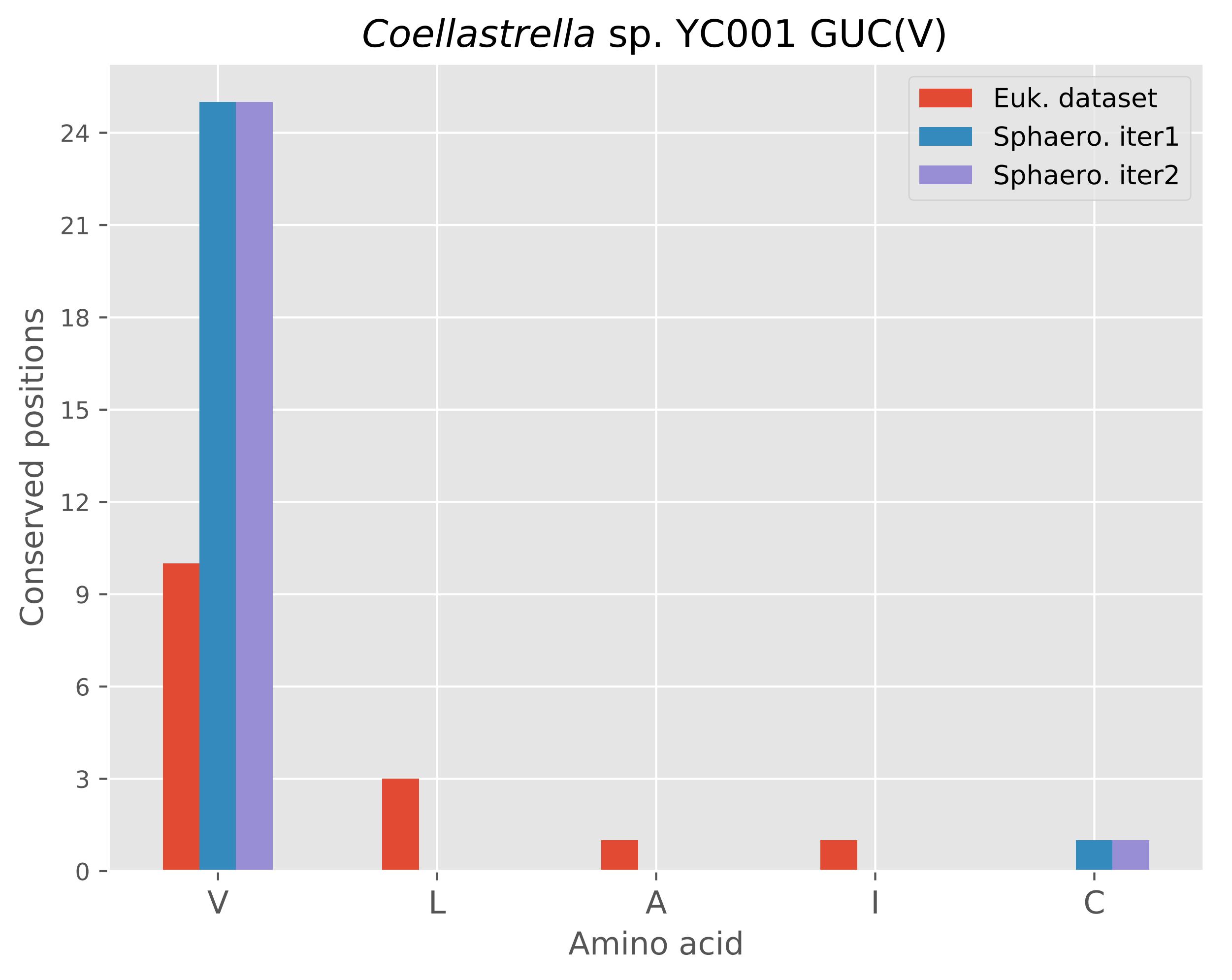
Coellastrella sp. YC001 GUC(V)
Euk. dataset
Sphaero. iter1
Sphaero. iter2
Conserved positions
0
3
6
9
12
15
18
21
24
V
L
A
I
C
Amino acid

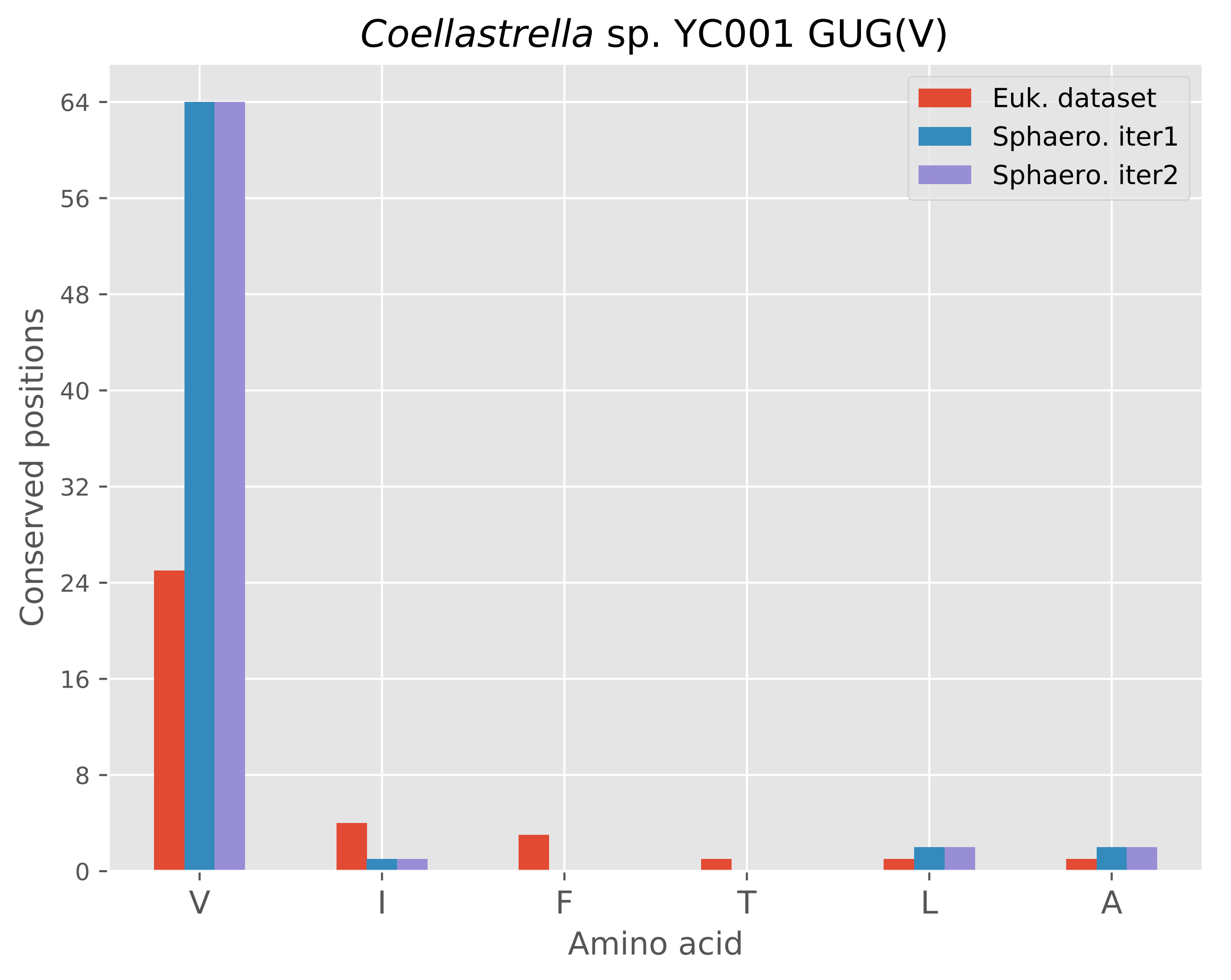
Coellastrella sp. YC001 GUG(V)
Conserved positions
Amino acid
0
8
16
24
32
40
48
56
64
V
I
F
T
L
A
Euk. dataset
Sphaero. iter1
Sphaero. iter2

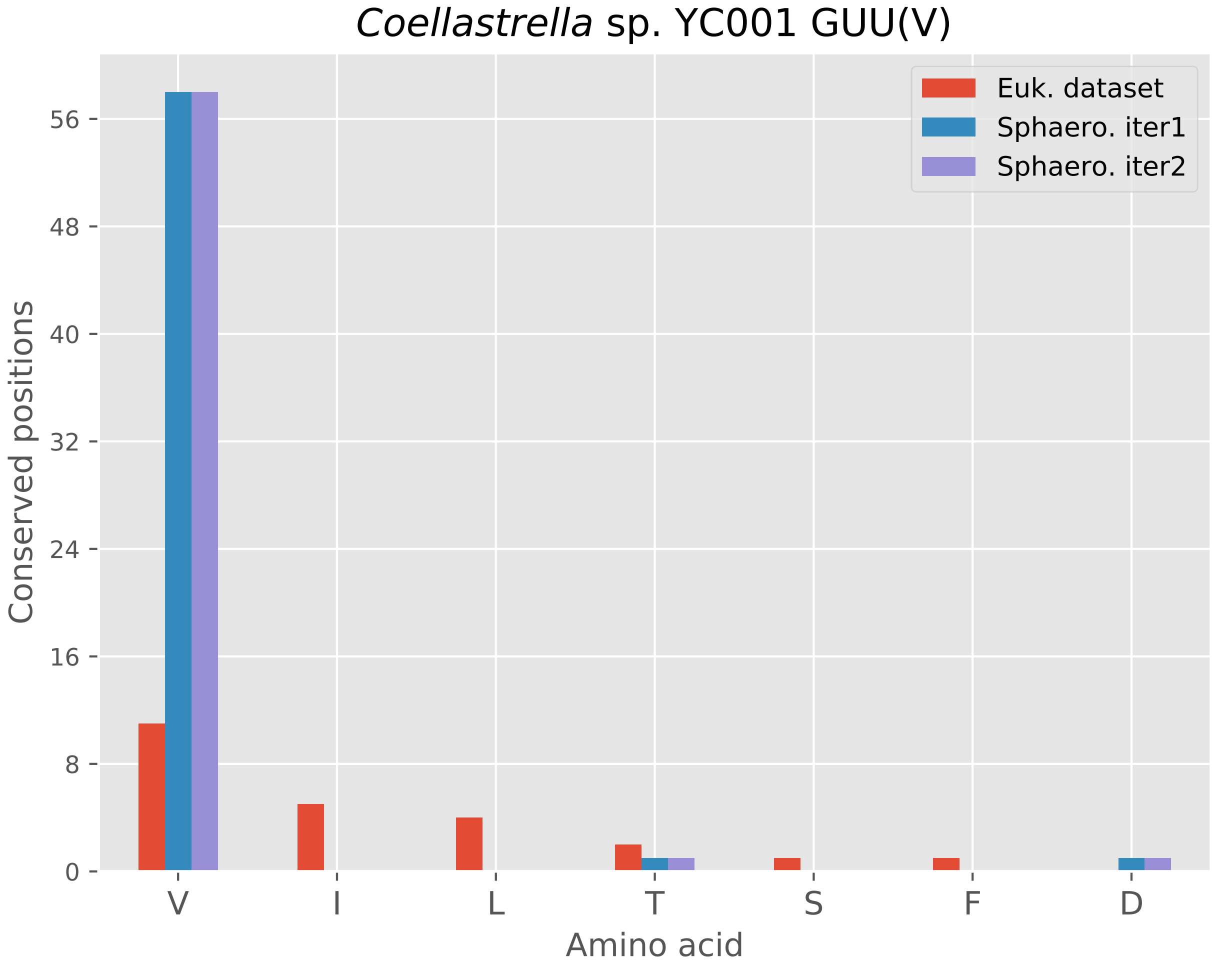

Coellastrella sp. YC001 GUU(V)
Euk. dataset
Sphaero. iter1
Sphaero. iter2
Conserved positions
0
8
16
24
32
40
48
56
V
I
L
T
S
F
D
Amino acid

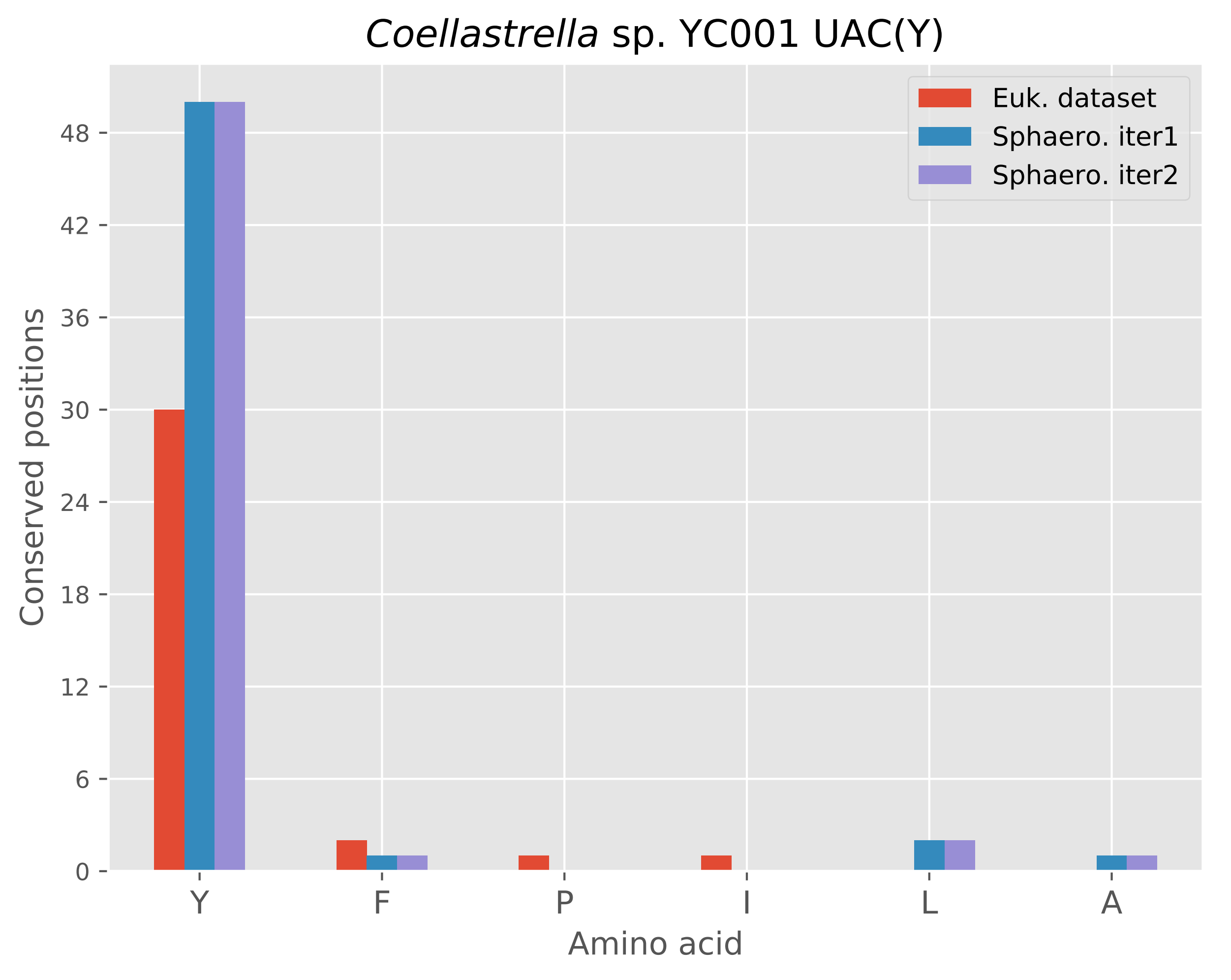

Coellastrella sp. YC001 UAC(Y)
Euk. dataset
Sphaero. iter1
Sphaero. iter2
Conserved positions
0
6
12
18
24
30
36
42
48
Y
F
P
I
L
A
Amino acid

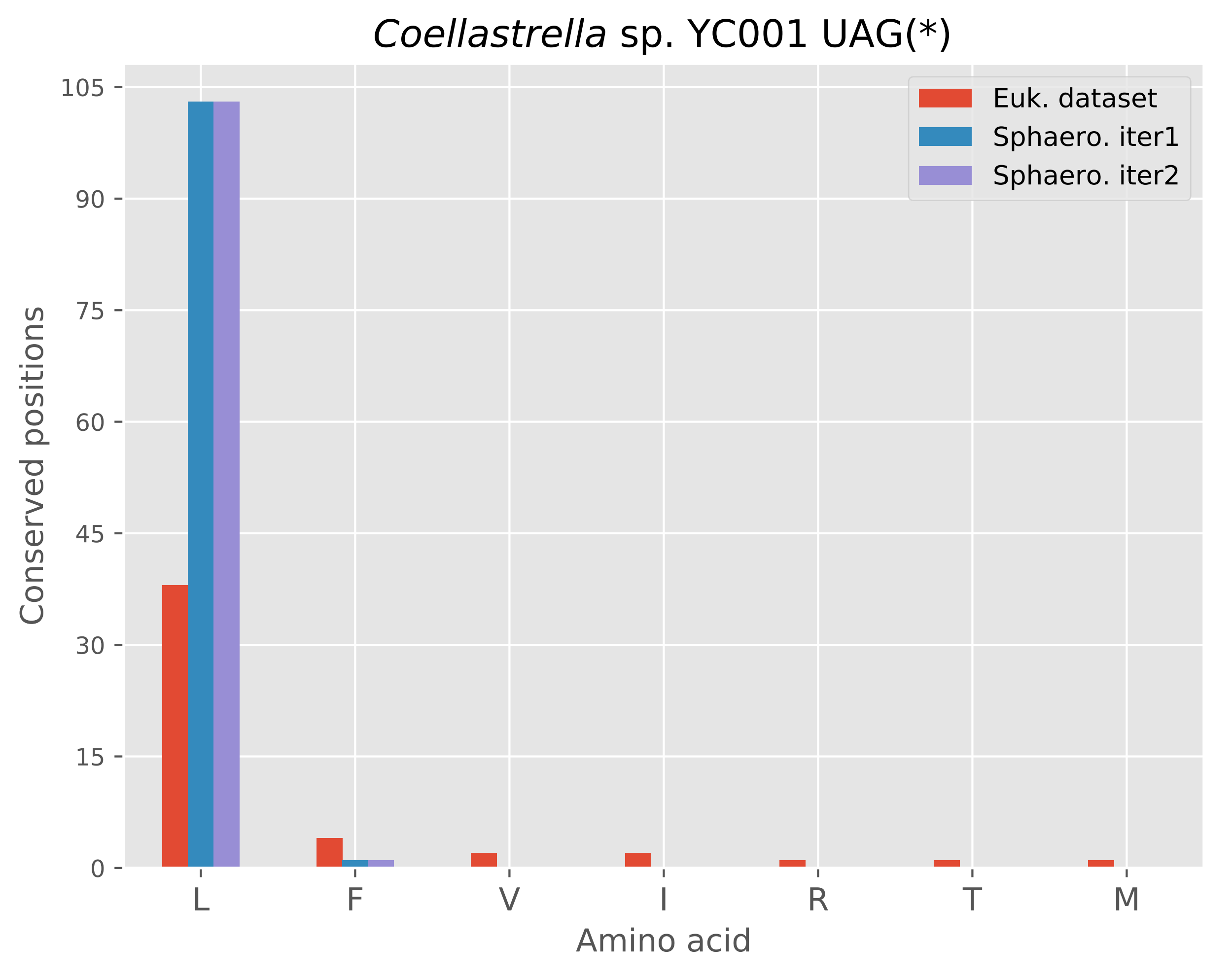

Coellastrella sp. YC001 UAG(*)
Euk. dataset
Sphaero. iter1
Sphaero. iter2
Conserved positions
0
15
30
45
60
75
90
105
L
F
V
I
R
T
M
Amino acid

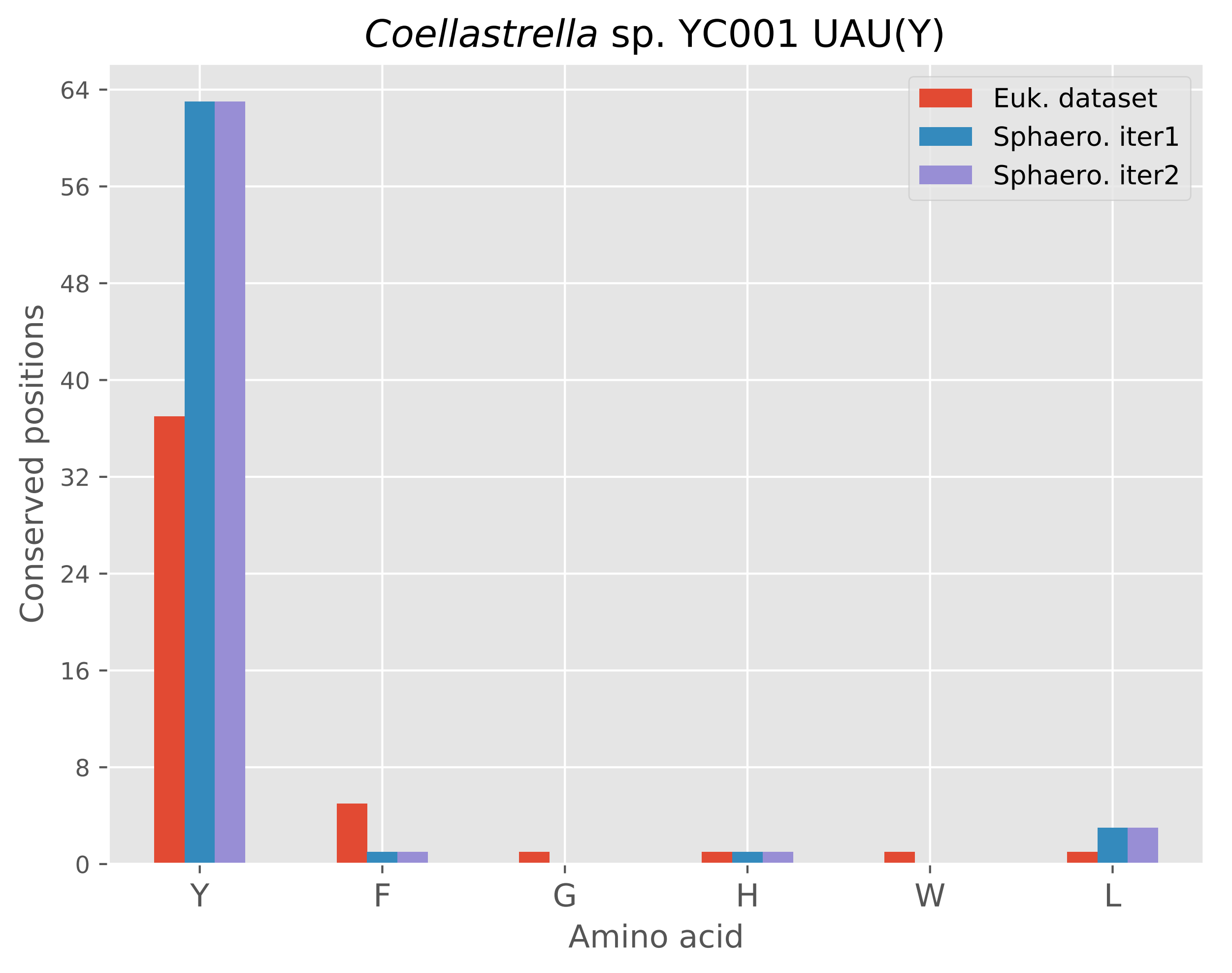

Coellastrella sp. YC001 UAU(Y)
Euk. dataset
Sphaero. iter1
Sphaero. iter2
Conserved positions
0
8
16
24
32
40
48
56
64
Y
F
G
H
W
L
Amino acid

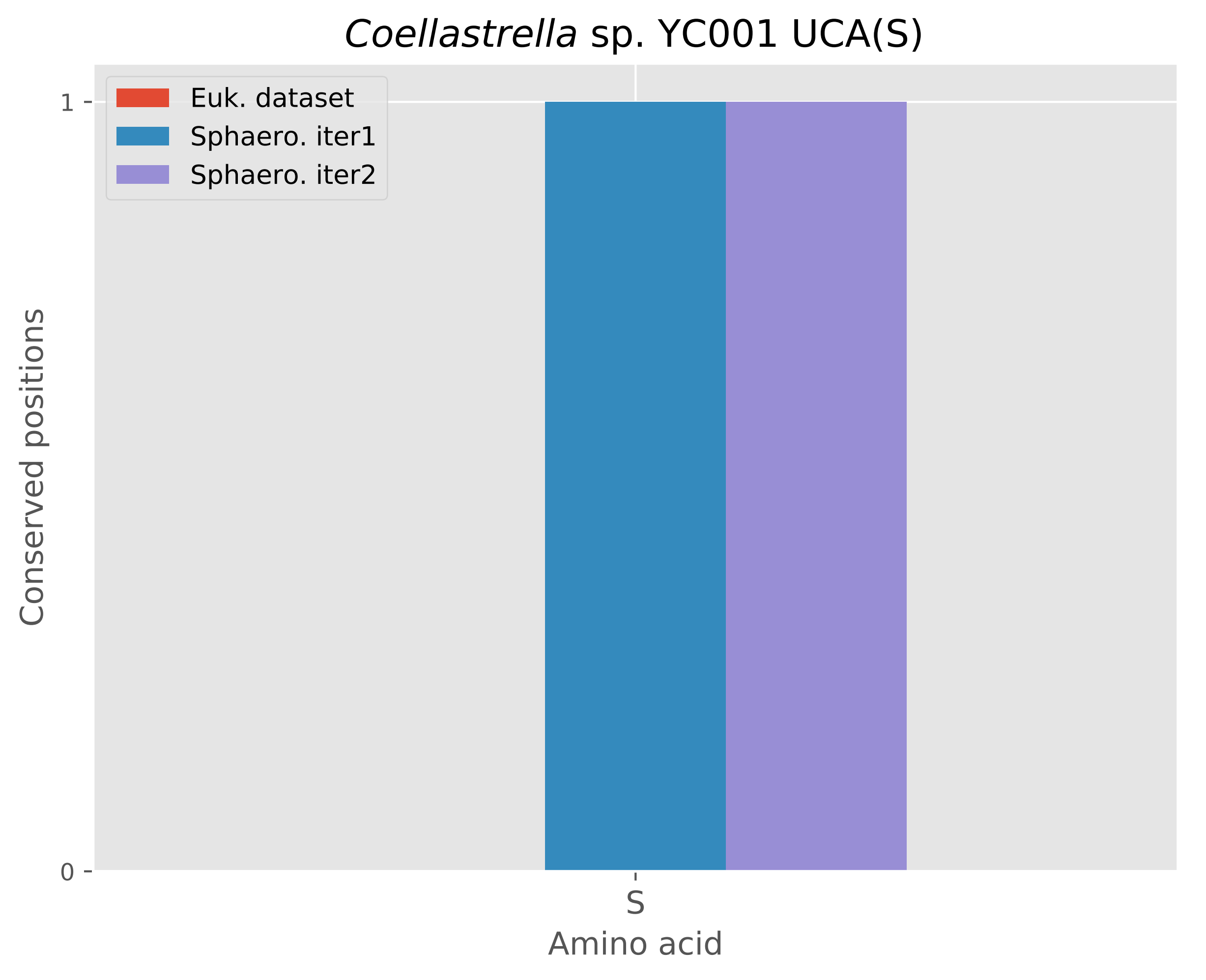

Coellastrella sp. YC001 UCA(S)
Euk. dataset
Sphaero. iter1
Sphaero. iter2
1
0
Conserved positions
S
Amino acid

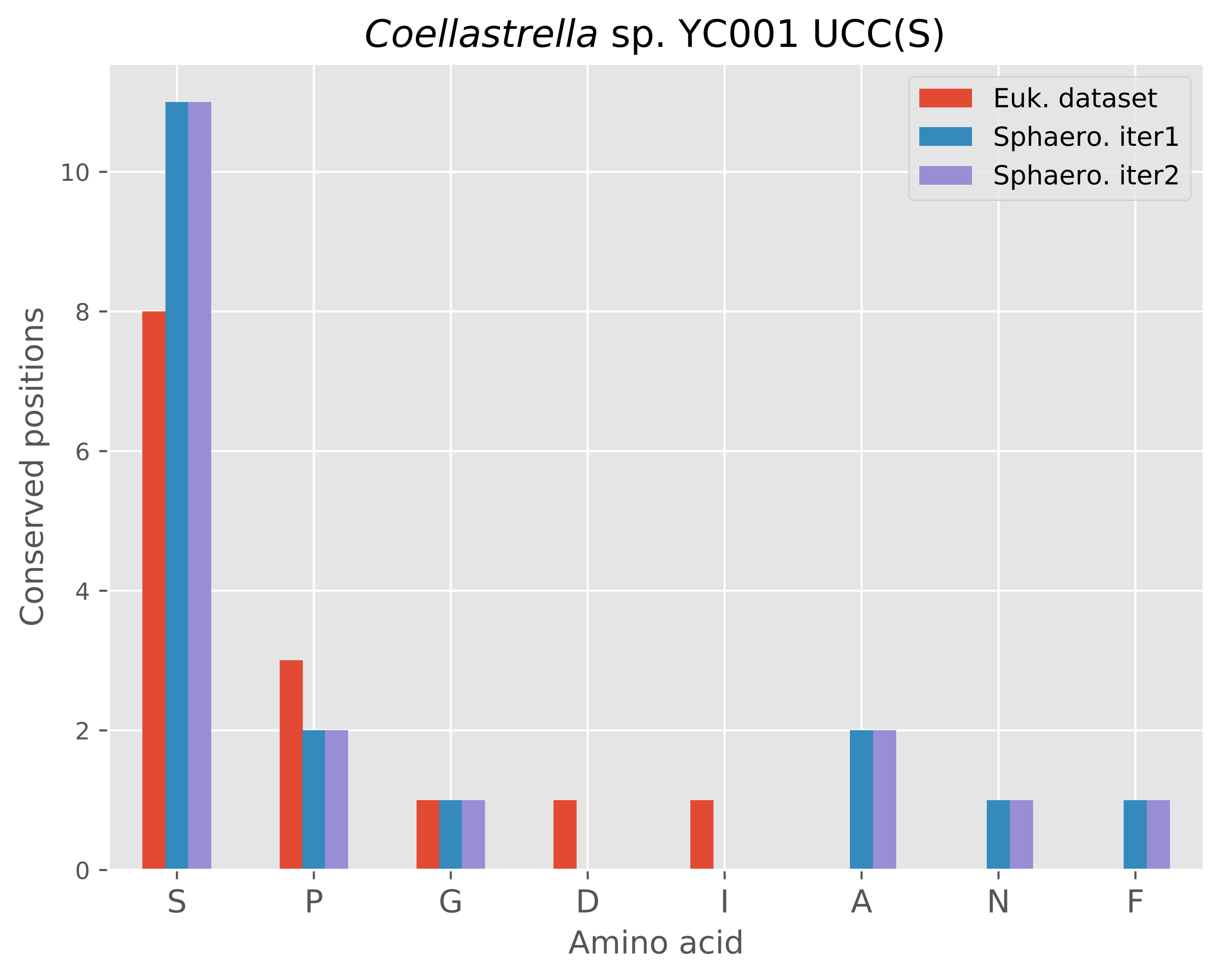
Coellastrella sp. YC001 UCC(S)
Euk. dataset
Sphaero. iter1
Sphaero. iter2
Conserved positions
0
2
4
6
8
10
S
P
G
D
I
A
N
F
Amino acid

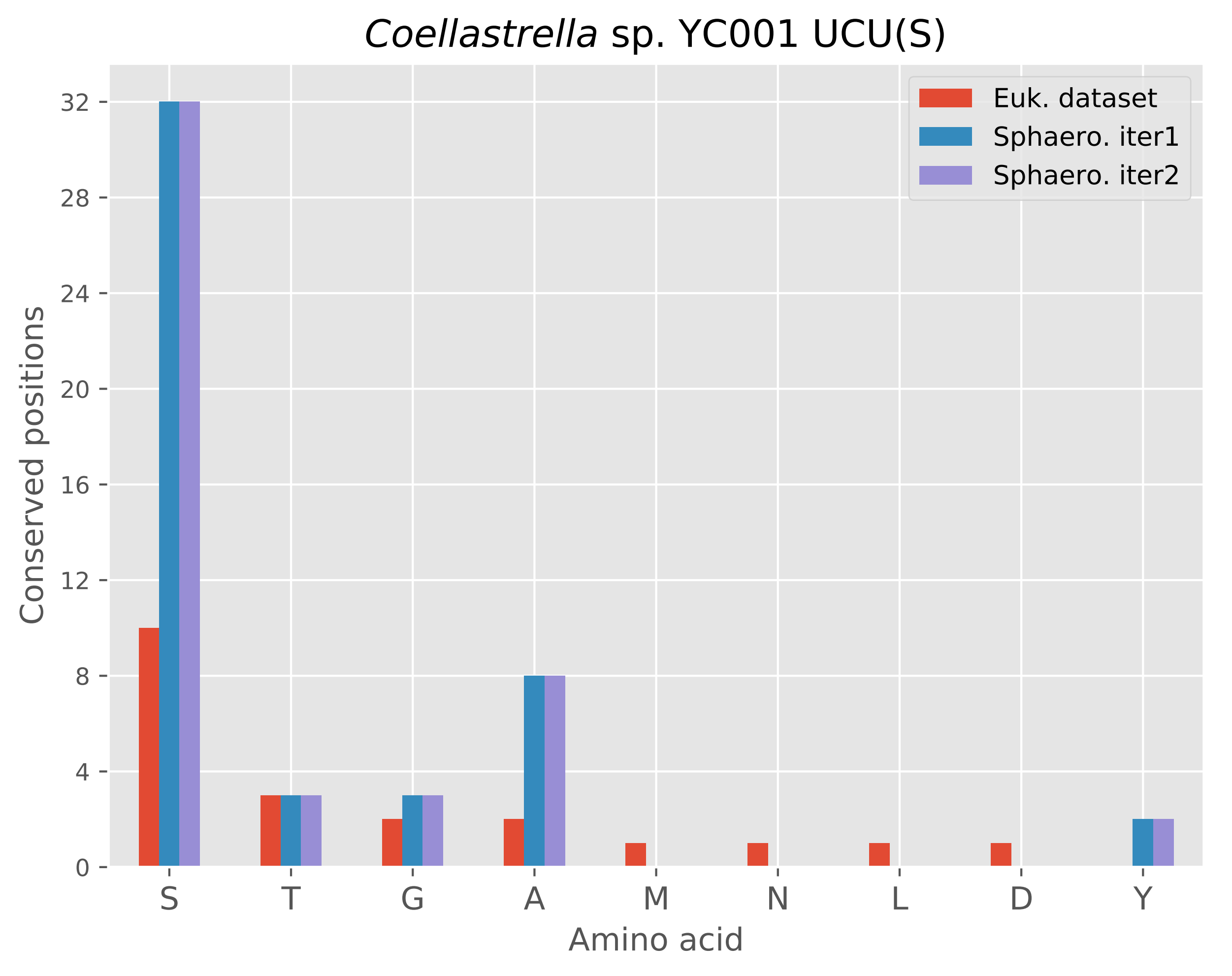

Coellastrella sp. YC001 UCU(S)
Euk. dataset
Sphaero. iter1
Sphaero. iter2
Conserved positions
0
4
8
12
16
20
24
28
32
S
T
G
A
M
N
L
D
Y
Amino acid

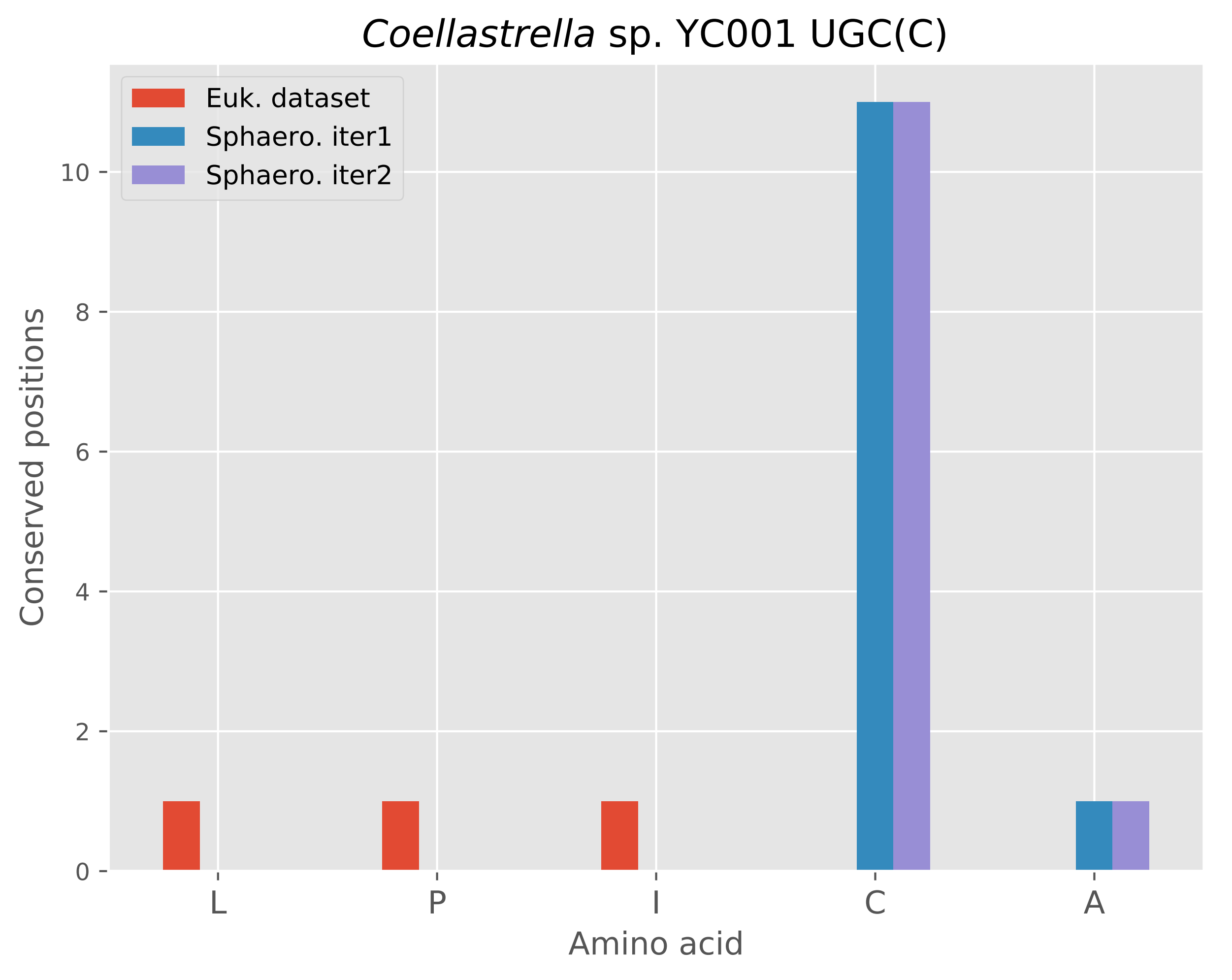

Coellastrella sp. YC001 UGC(C)
Euk. dataset
Sphaero. iter1
Sphaero. iter2
Conserved positions
0
2
4
6
8
10
L
P
I
C
A
Amino acid

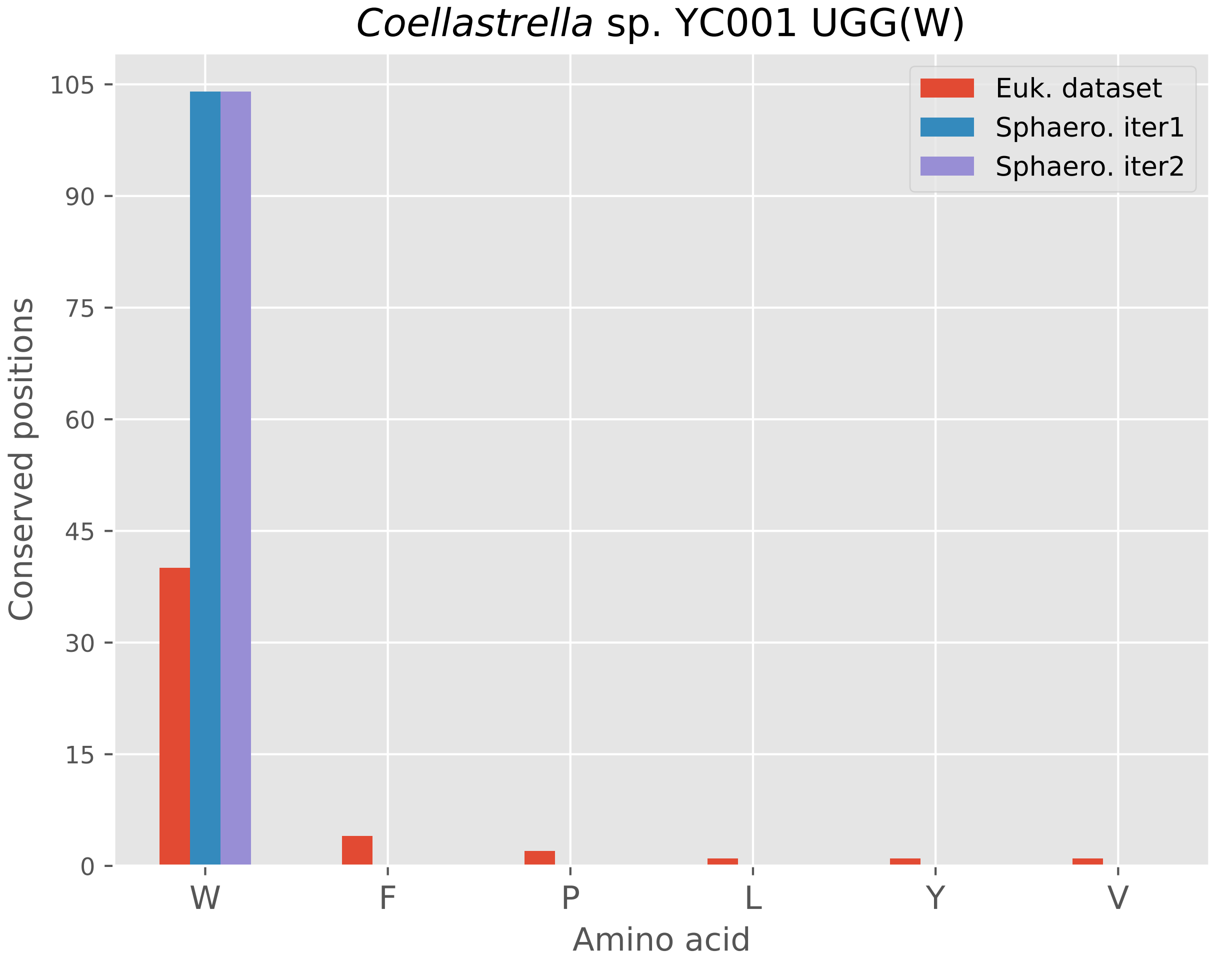

Coellastrella sp. YC001 UGG(W)
Euk. dataset
Sphaero. iter1
Sphaero. iter2
Conserved positions
0
15
30
45
60
75
90
105
W
F
P
L
Y
V
Amino acid

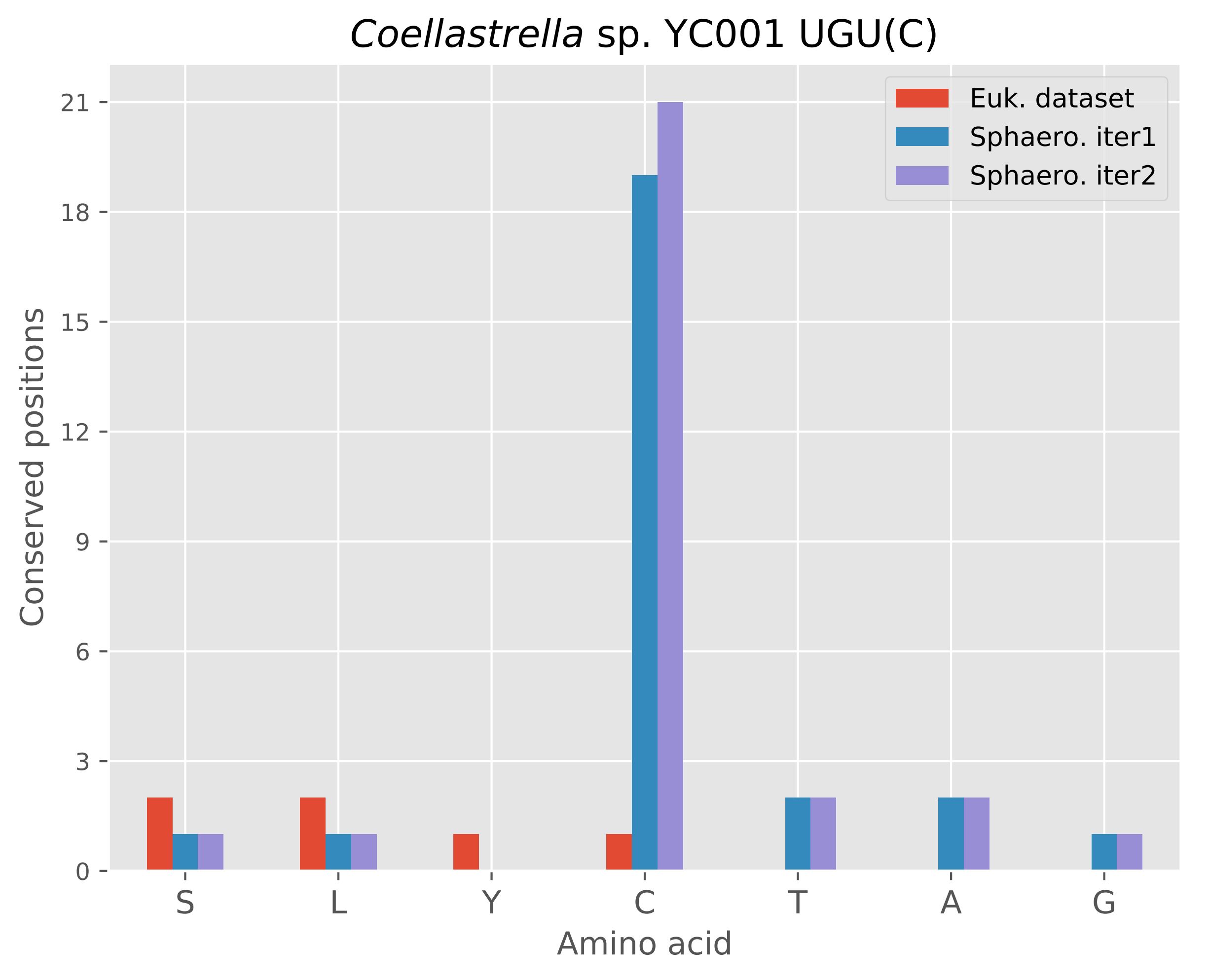
Coellastrella sp. YC001 UGU(C)
Euk. dataset
Sphaero. iter1
Sphaero. iter2
Conserved positions
0
3
6
9
12
15
18
21
S
L
Y
C
T
A
G
Amino acid

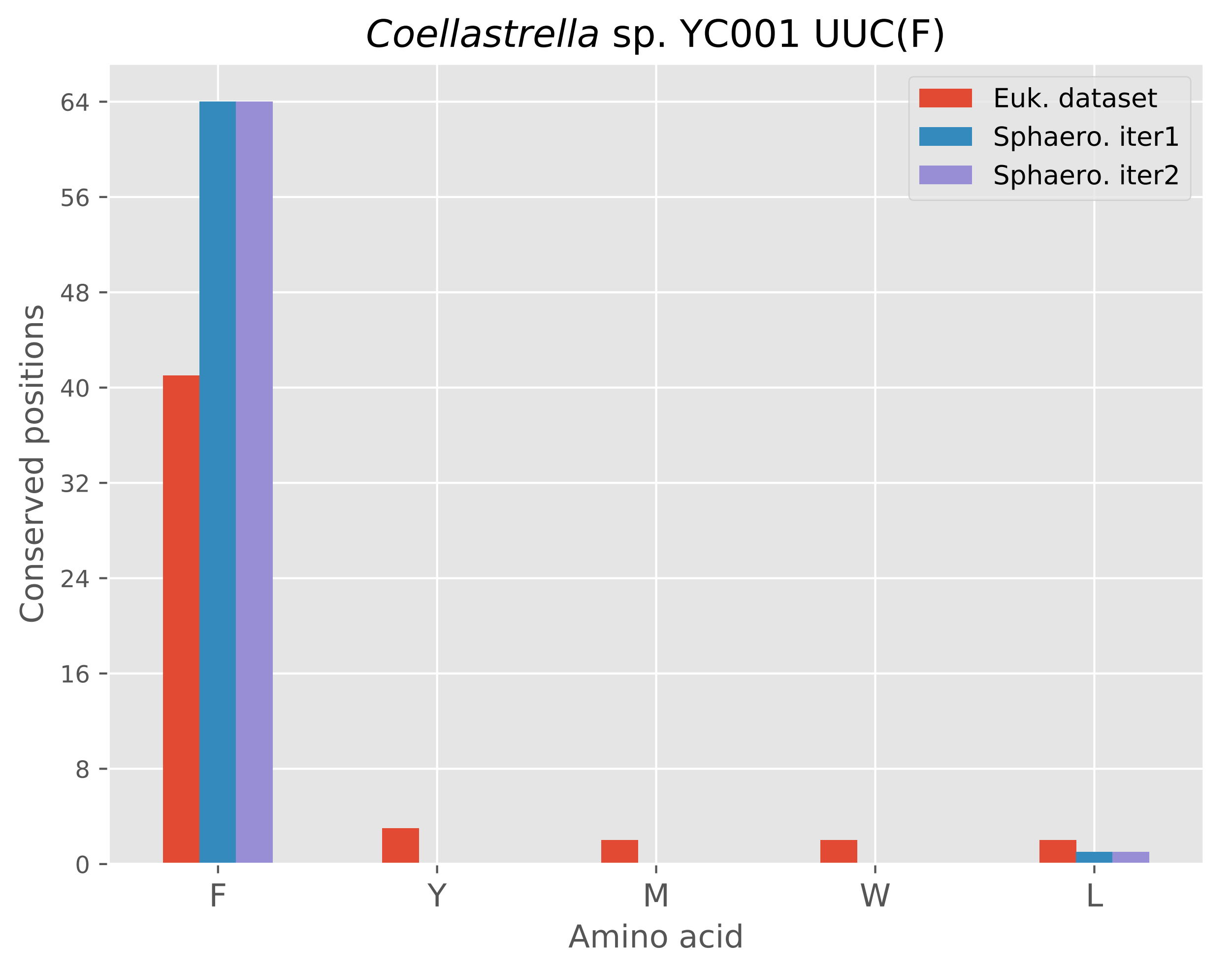

Coellastrella sp. YC001 UUC(F)
Conserved positions
0
8
16
24
32
40
48
56
64
F
Y
M
W
L
Amino acid
Euk. dataset
Sphaero. iter1
Sphaero. iter2

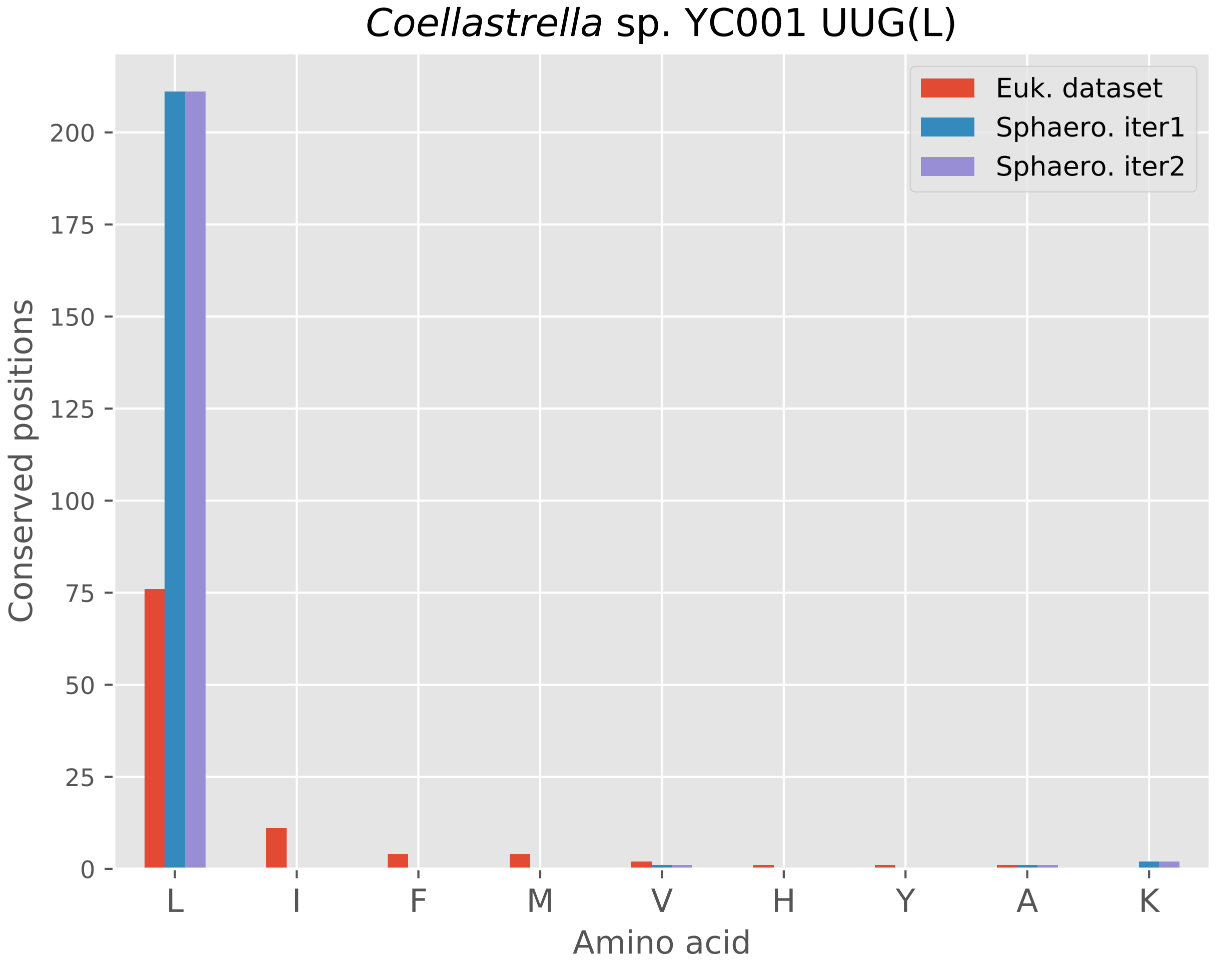

Coellastrella sp. YC001 UUG(L)
Euk. dataset
Sphaero. iter1
Sphaero. iter2
Conserved positions
0
25
50
75
100
125
150
175
200
L
I
F
M
V
H
Y
A
K
Amino acid

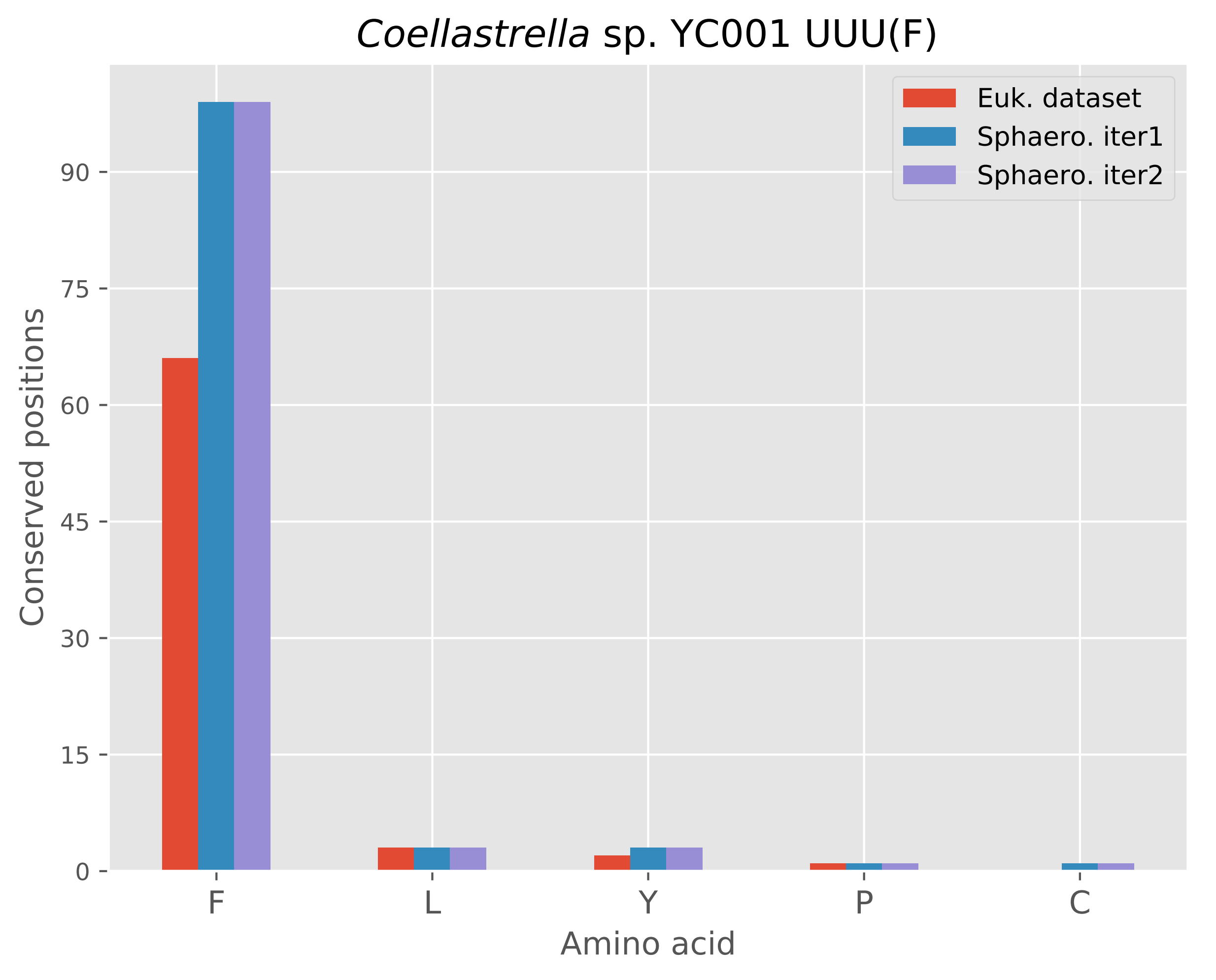

Coellastrella sp. YC001 UUU(F)
Euk. dataset
Sphaero. iter1
Sphaero. iter2
Conserved positions
0
15
30
45
60
75
90
F
L
Y
P
C
Amino acid

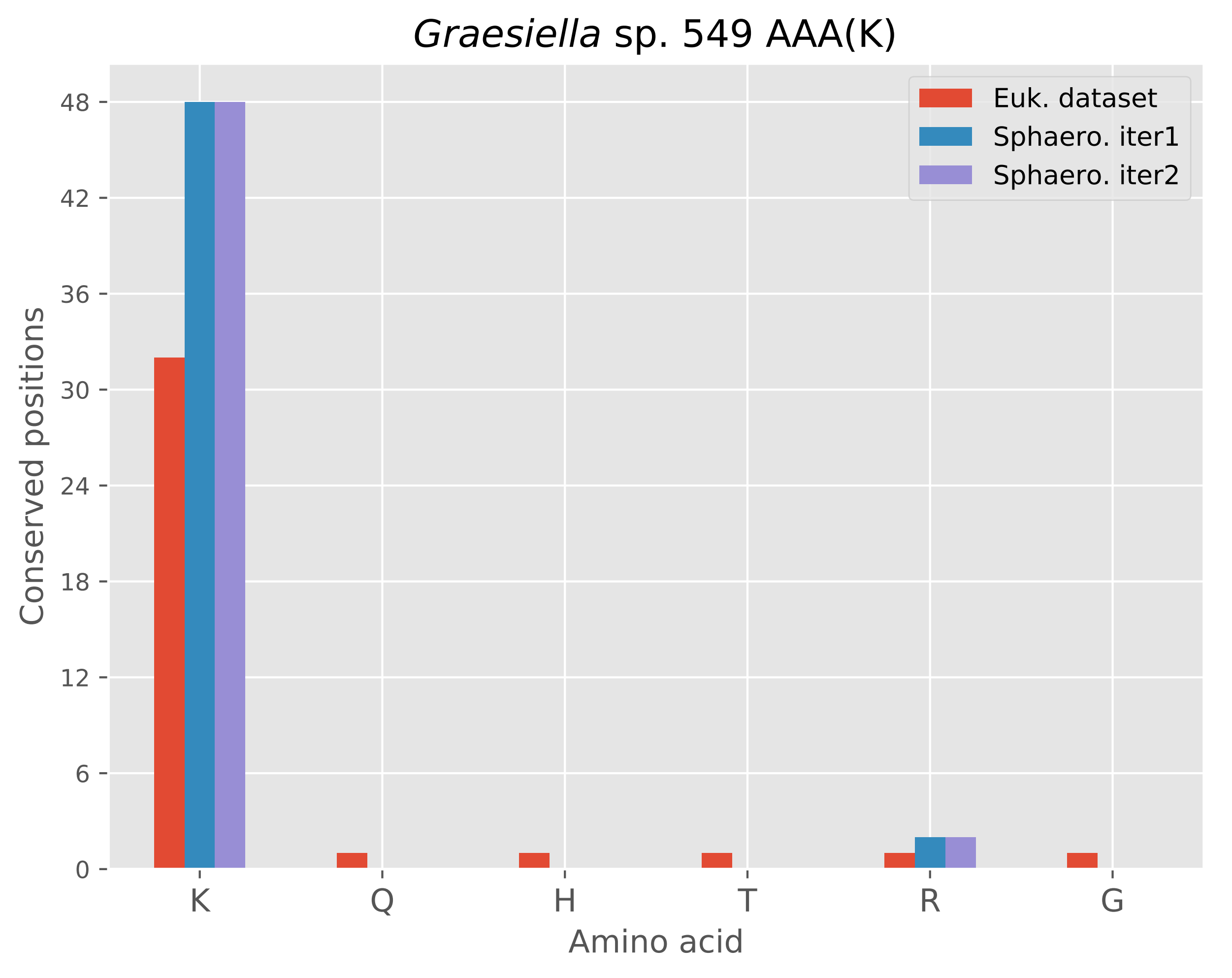

Graesiella sp. 549 AAA(K)
Euk. dataset
Sphaero. iter1
Sphaero. iter2
Conserved positions
0
6
12
18
24
30
36
42
48
K
Q
H
T
R
G
Amino acid

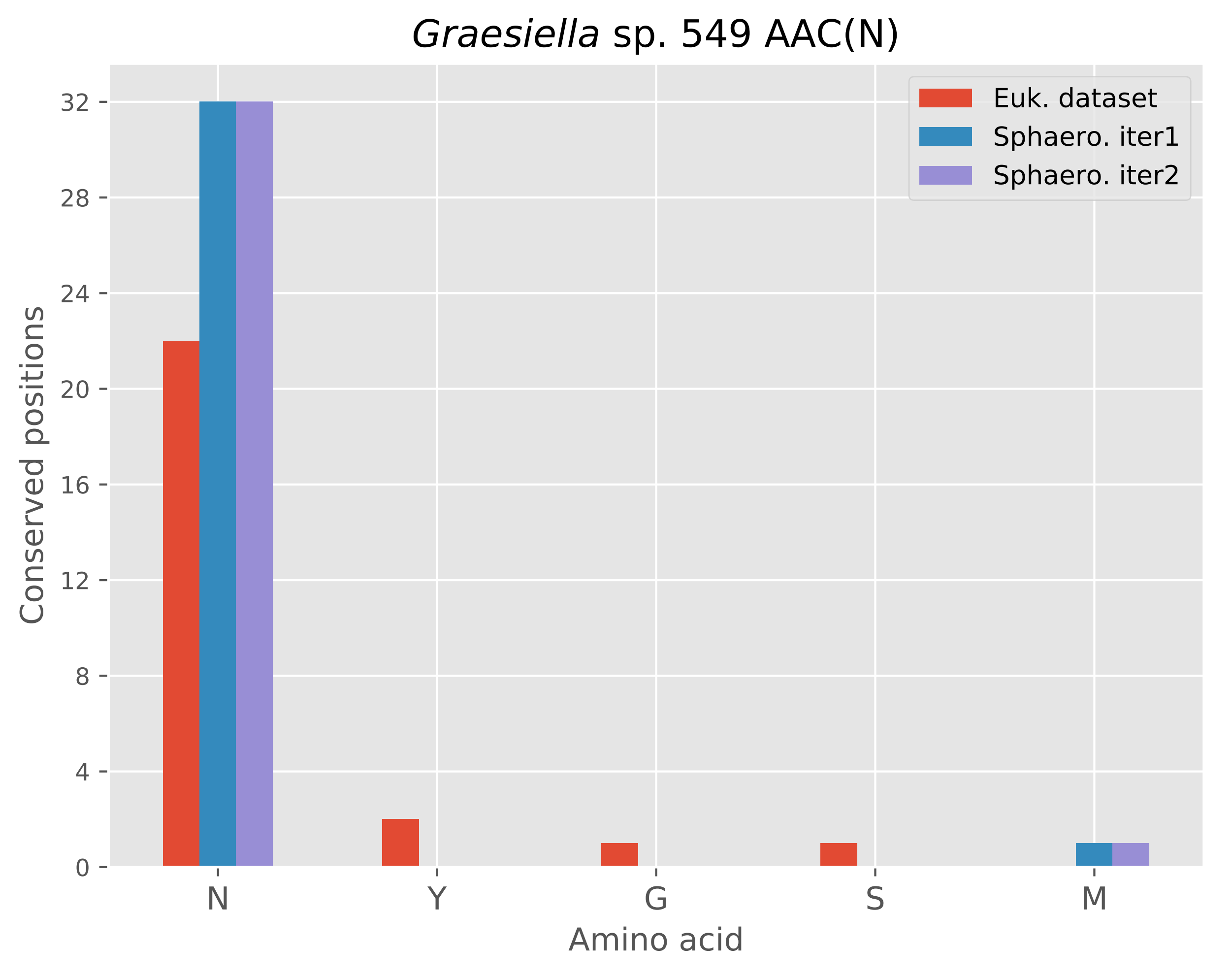

Graesiella sp. 549 AAC(N)
Euk. dataset
Sphaero. iter1
Sphaero. iter2
Conserved positions
0
4
8
12
16
20
24
28
32
N
Y
G
S
M
Amino acid

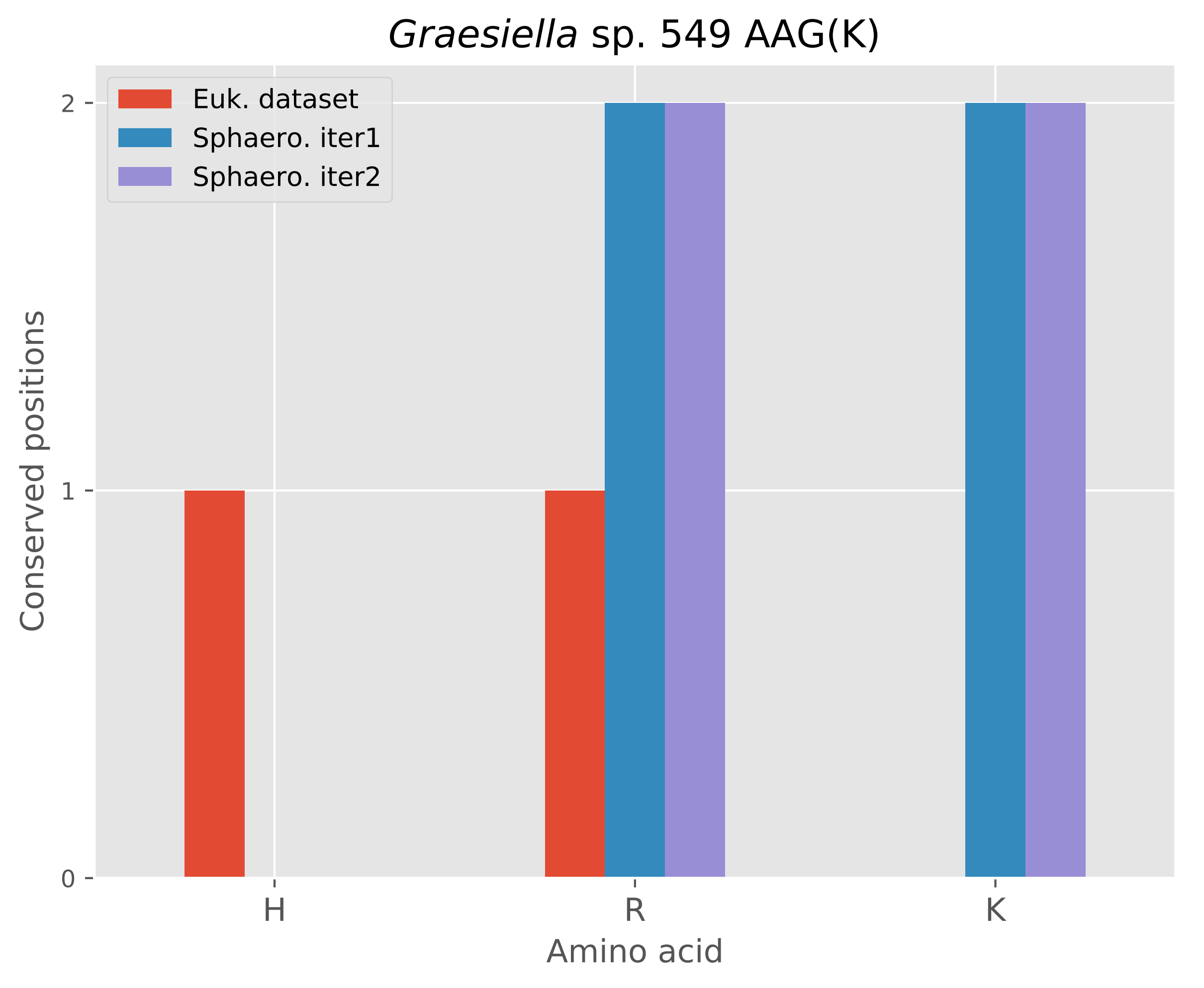

Graesiella sp. 549 AAG(K)
Euk. dataset
Sphaero. iter1
Sphaero. iter2
Conserved positions
0
1
2
H
R
K
Amino acid

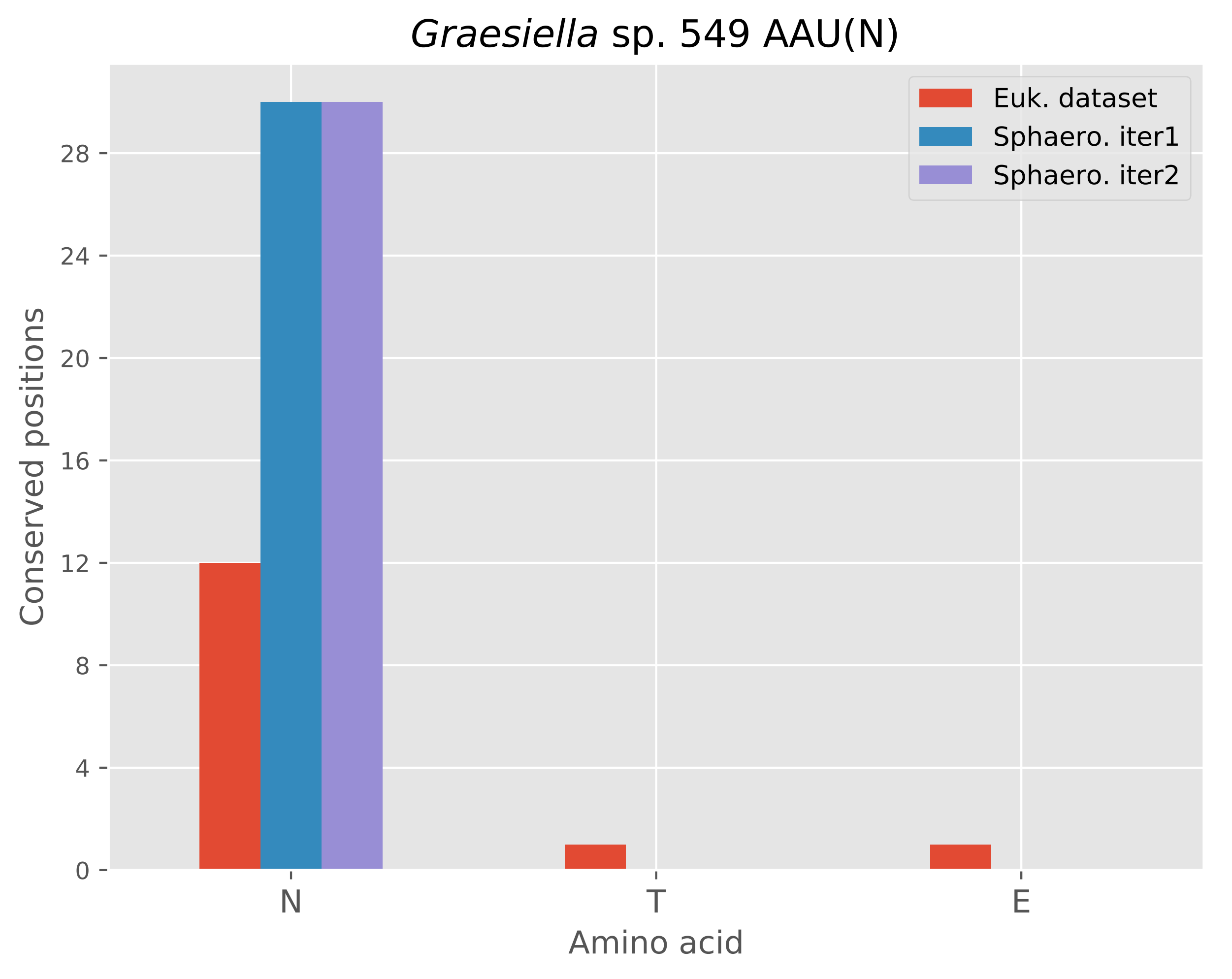

Graesiella sp. 549 AAU(N)
Euk. dataset
Sphaero. iter1
Sphaero. iter2
Conserved positions
0
4
8
12
16
20
24
28
N
T
E
Amino acid

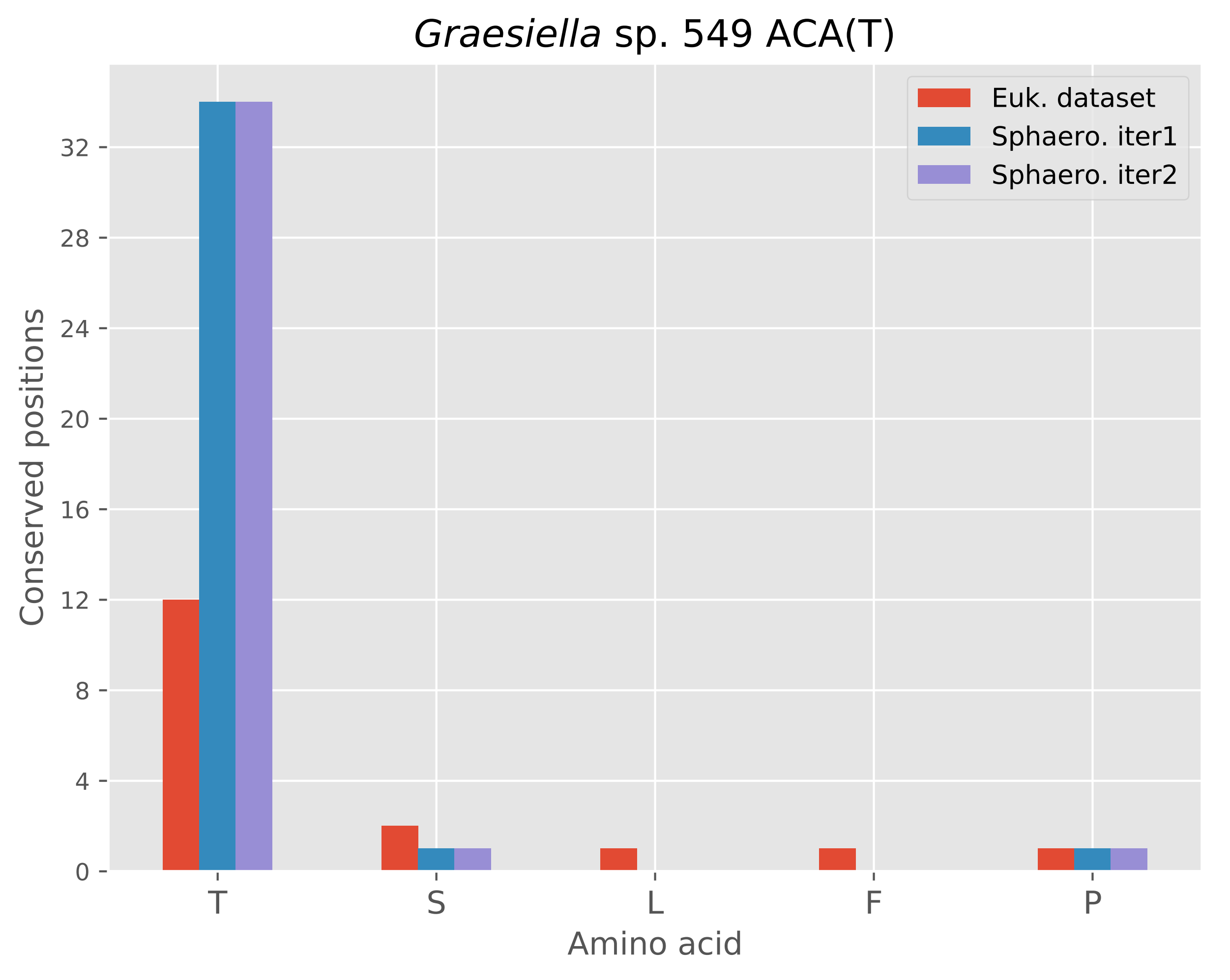

Graesiella sp. 549 ACA(T)
Euk. dataset
Sphaero. iter1
Sphaero. iter2
Conserved positions
0
4
8
12
16
20
24
28
32
T
S
L
F
P
Amino acid

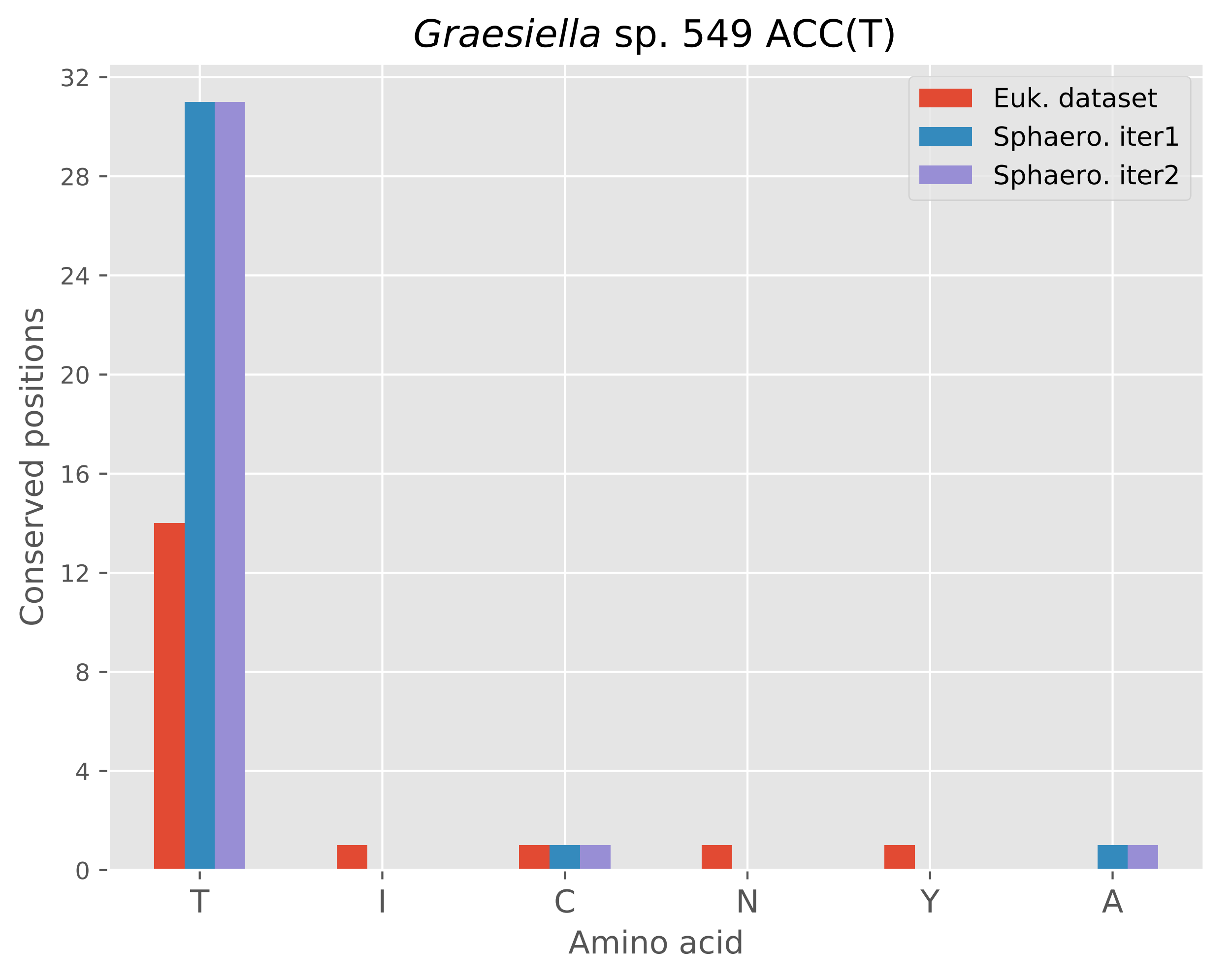

Graesiella sp. 549 ACC(T)
Euk. dataset
Sphaero. iter1
Sphaero. iter2
Conserved positions
0
4
8
12
16
20
24
28
32
T
I
C
N
Y
A
Amino acid

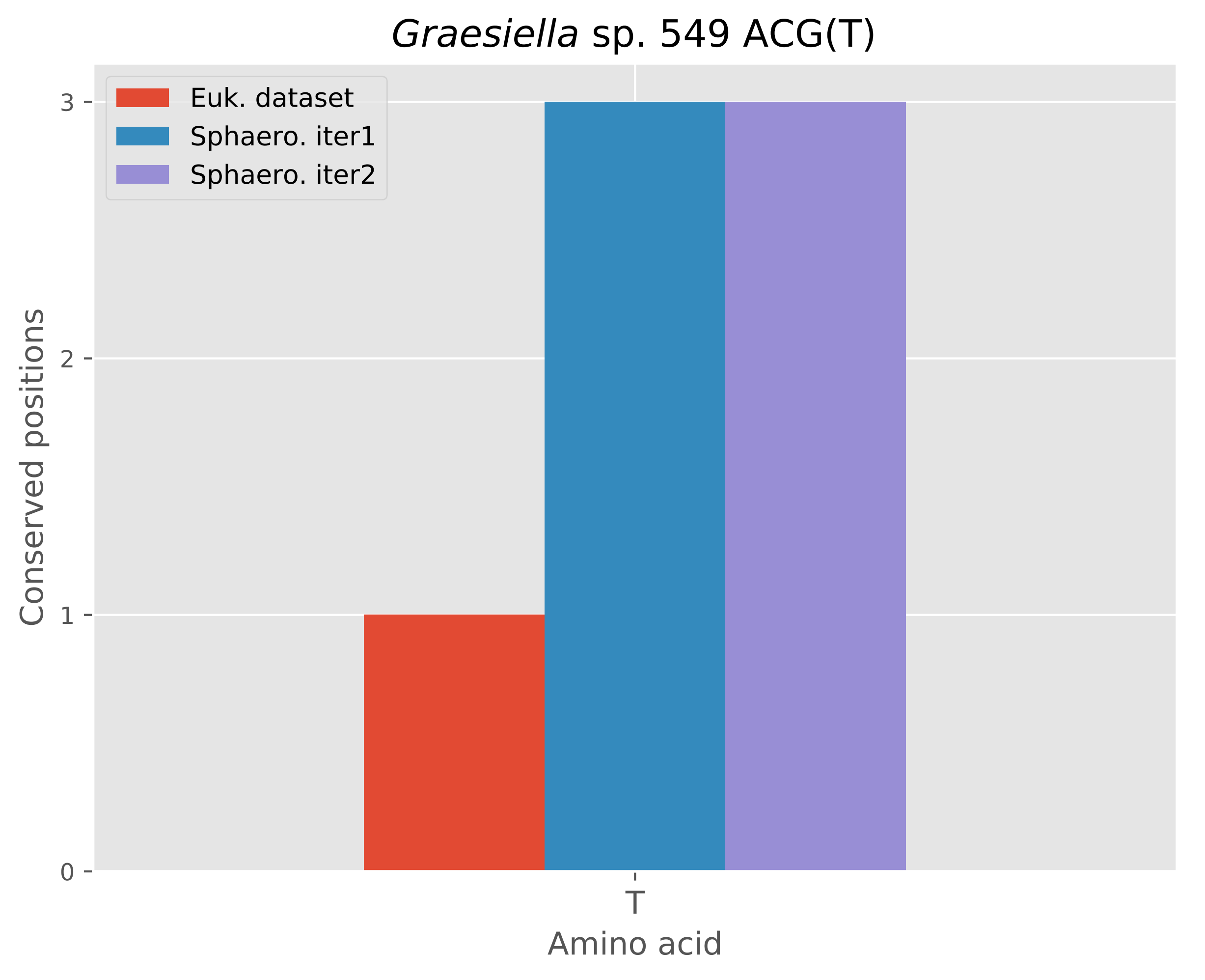

Graesiella sp. 549 ACG(T)
Euk. dataset
Sphaero. iter1
Sphaero. iter2
Conserved positions
0
1
2
3
T
Amino acid

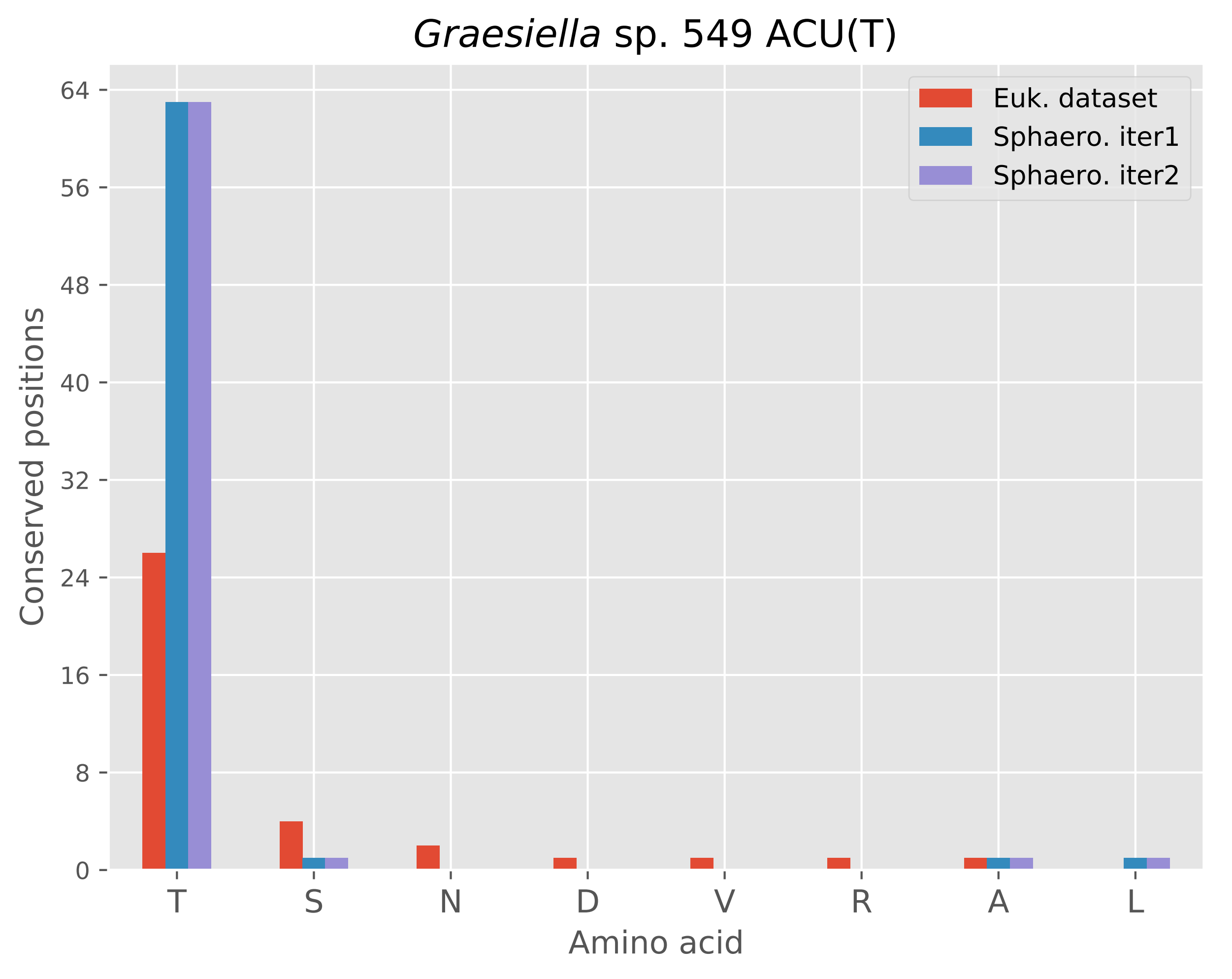
Graesiella sp. 549 ACU(T)
Conserved positions
Amino acid
64
56
48
40
32
24
16
8
0
T
S
N
D
V
R
A
L
Euk. dataset
Sphaero. iter1
Sphaero. iter2

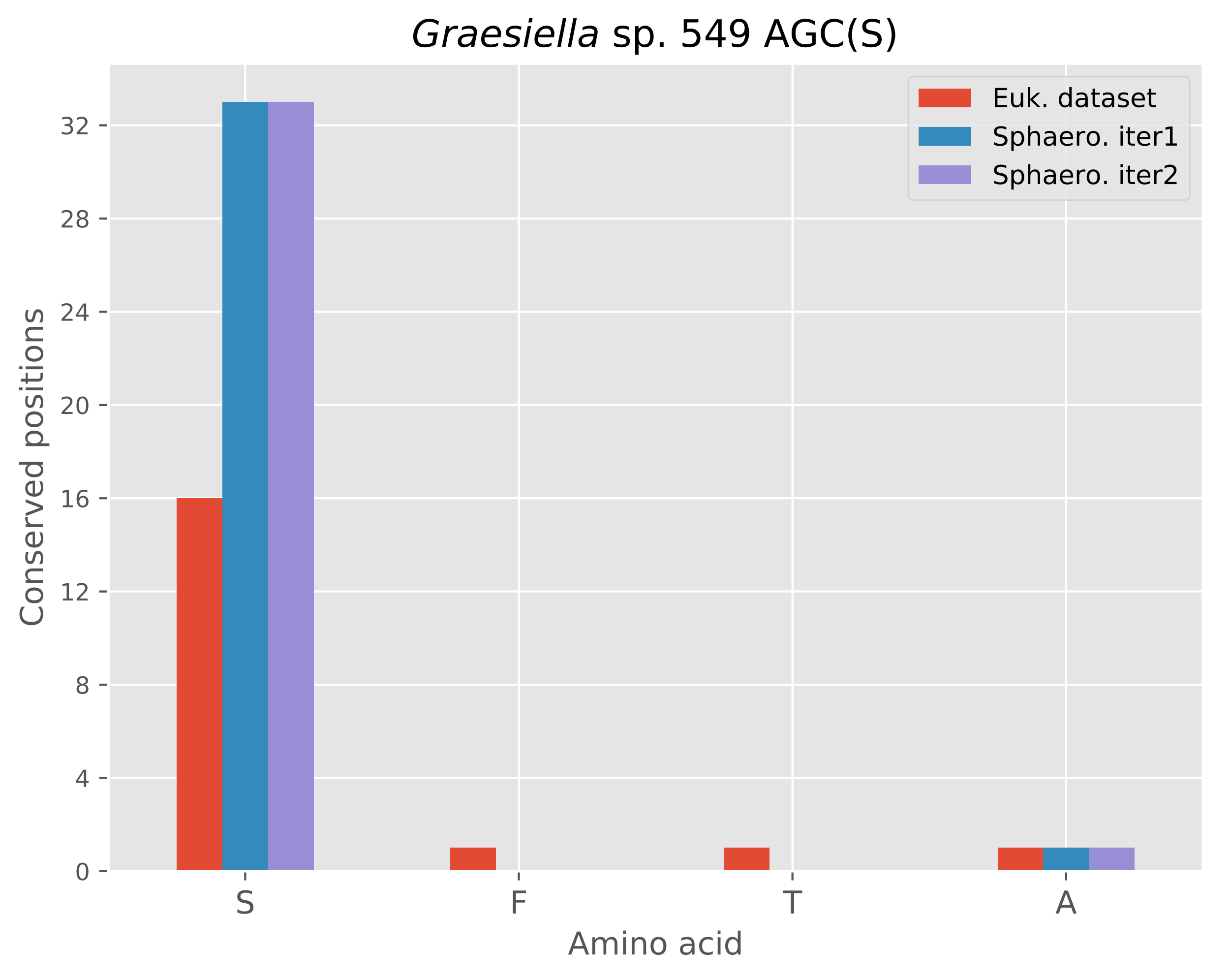
Graesiella sp. 549 AGC(S)
Conserved positions
0
4
8
12
16
20
24
28
32
S
F
T
A
Amino acid
Euk. dataset
Sphaero. iter1
Sphaero. iter2

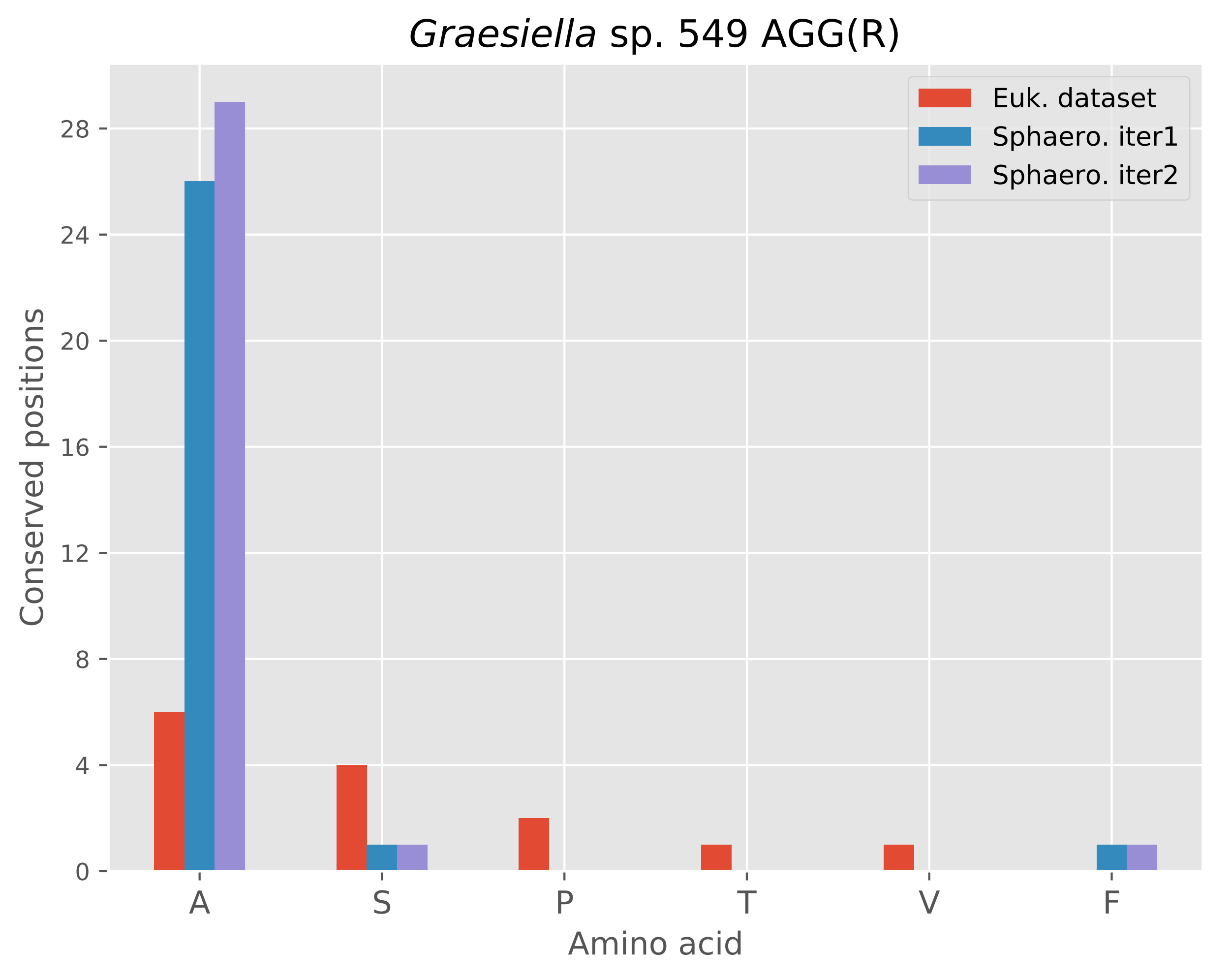

Graesiella sp. 549 AGG(R)
Euk. dataset
Sphaero. iter1
Sphaero. iter2
Conserved positions
0
4
8
12
16
20
24
28
A
S
P
T
V
F
Amino acid

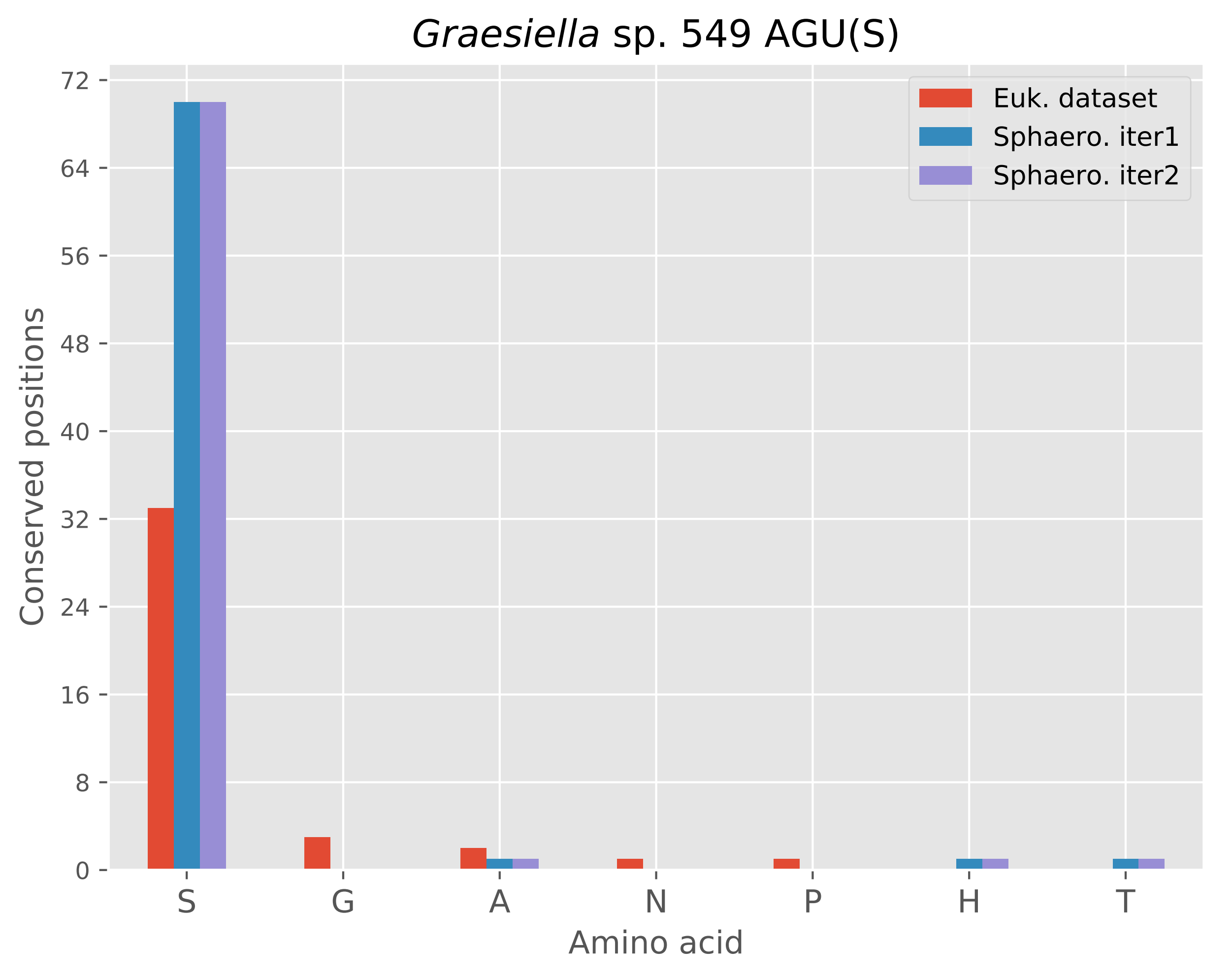

Graesiella sp. 549 AGU(S)
Euk. dataset
Sphaero. iter1
Sphaero. iter2
Conserved positions
0
8
16
24
32
40
48
56
64
72
S
G
A
N
P
H
T
Amino acid

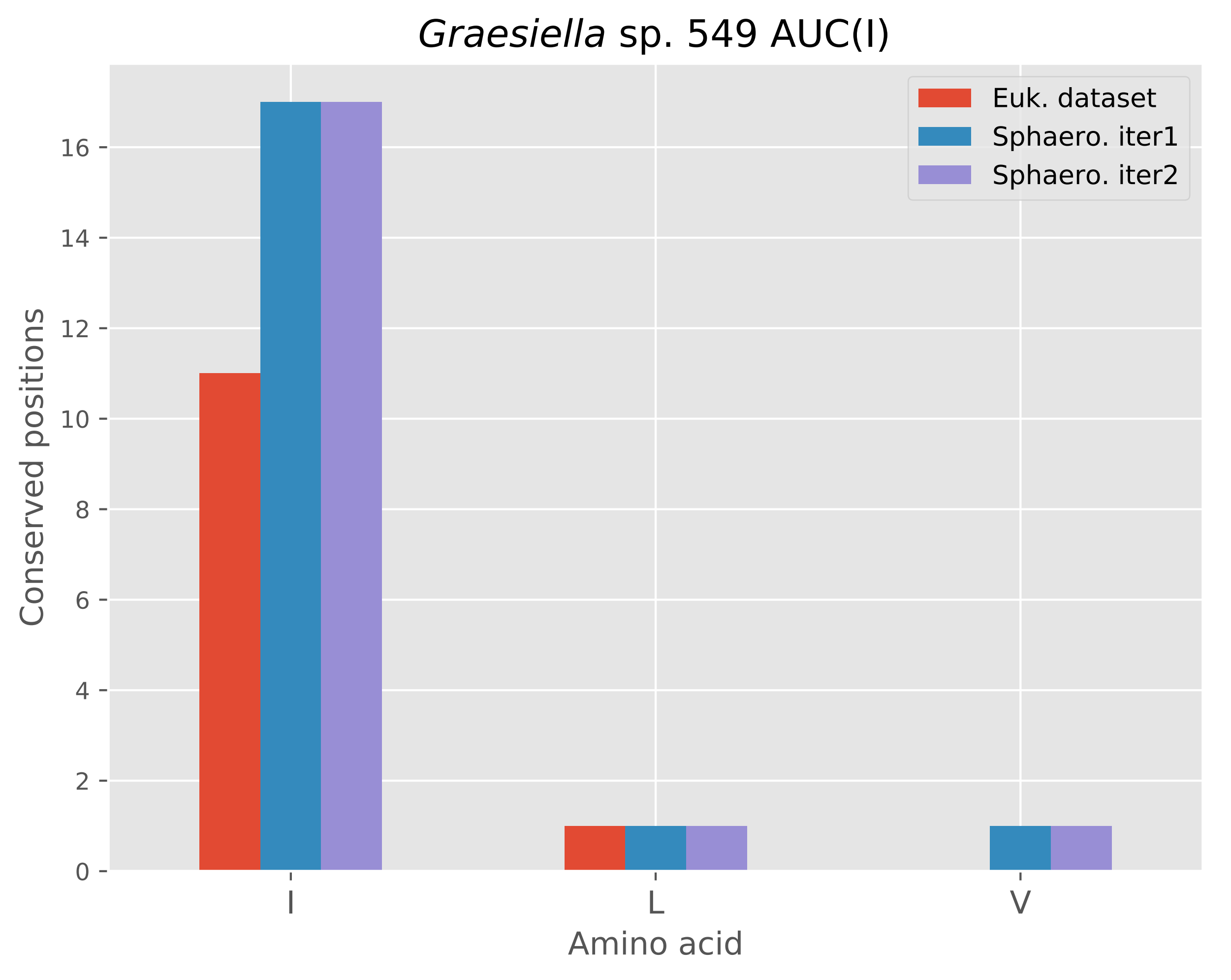

Graesiella sp. 549 AUC(I)
Euk. dataset
Sphaero. iter1
Sphaero. iter2
Conserved positions
0
2
4
6
8
10
12
14
16
I
L
V
Amino acid

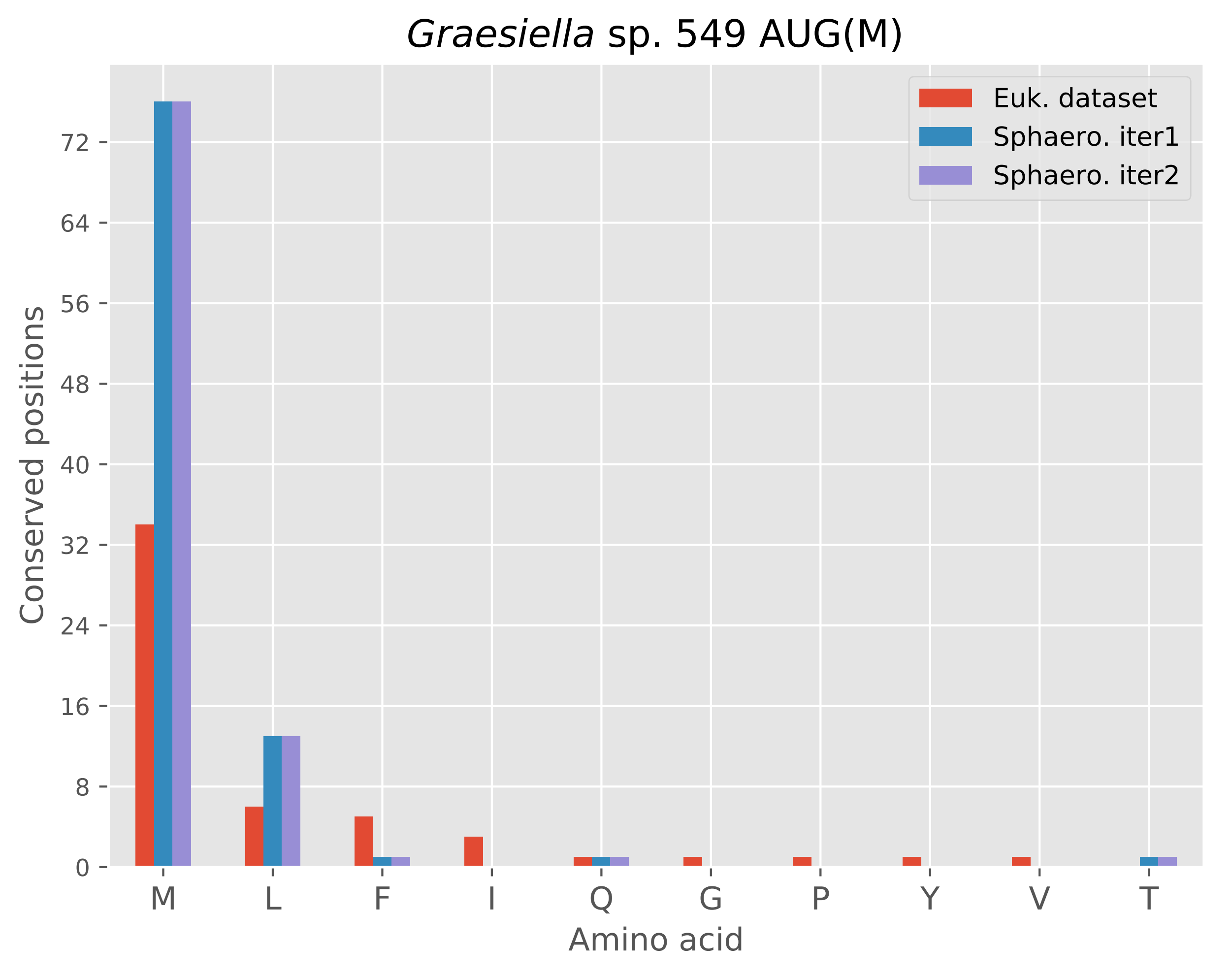

Graesiella sp. 549 AUG(M)
Euk. dataset
Sphaero. iter1
Sphaero. iter2
Conserved positions
0
8
16
24
32
40
48
56
64
72
M
L
F
I
Q
G
P
Y
V
T
Amino acid

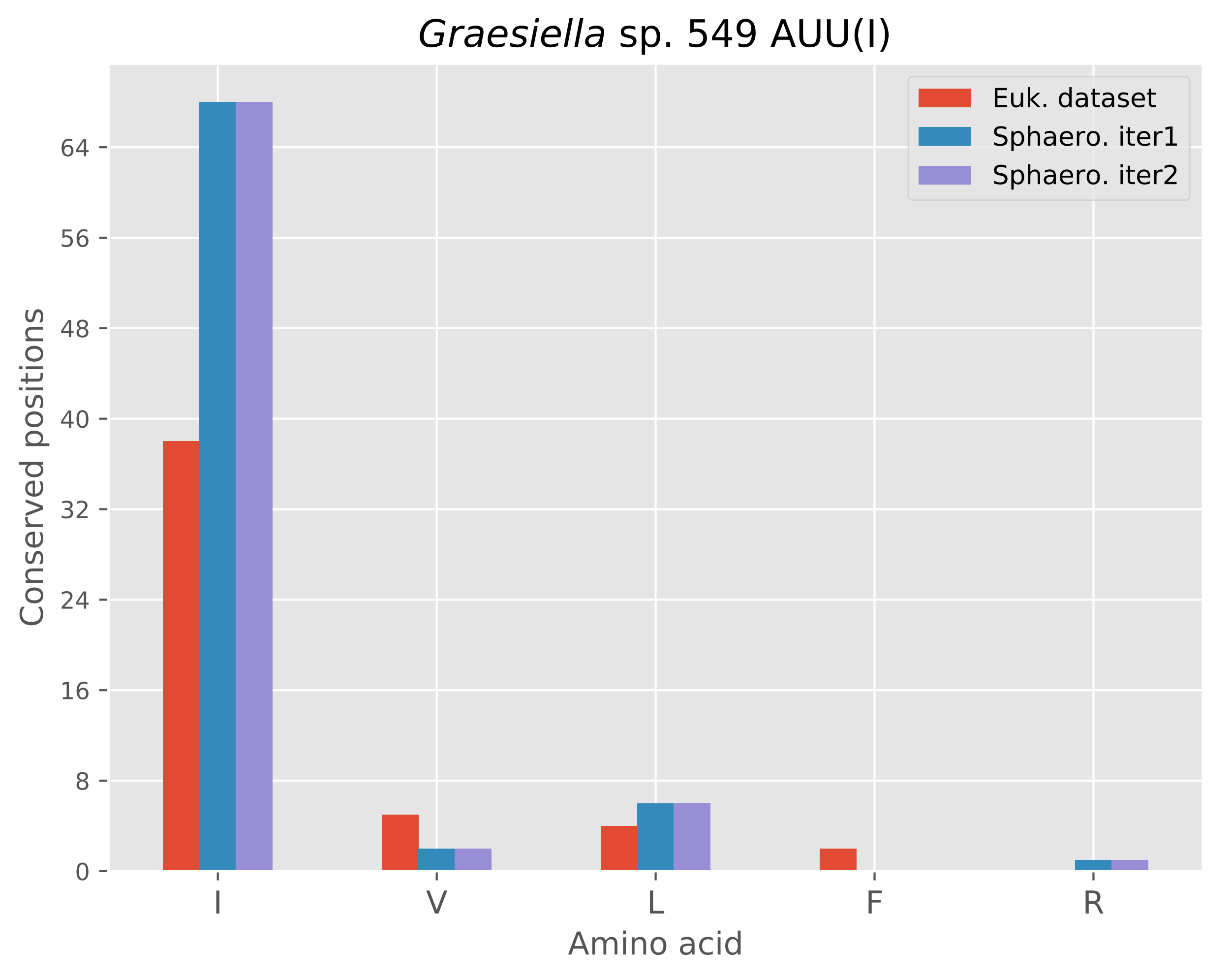

Graesiella sp. 549 AUU(I)
Euk. dataset
Sphaero. iter1
Sphaero. iter2
Conserved positions
0
8
16
24
32
40
48
56
64
I
V
L
F
R
Amino acid

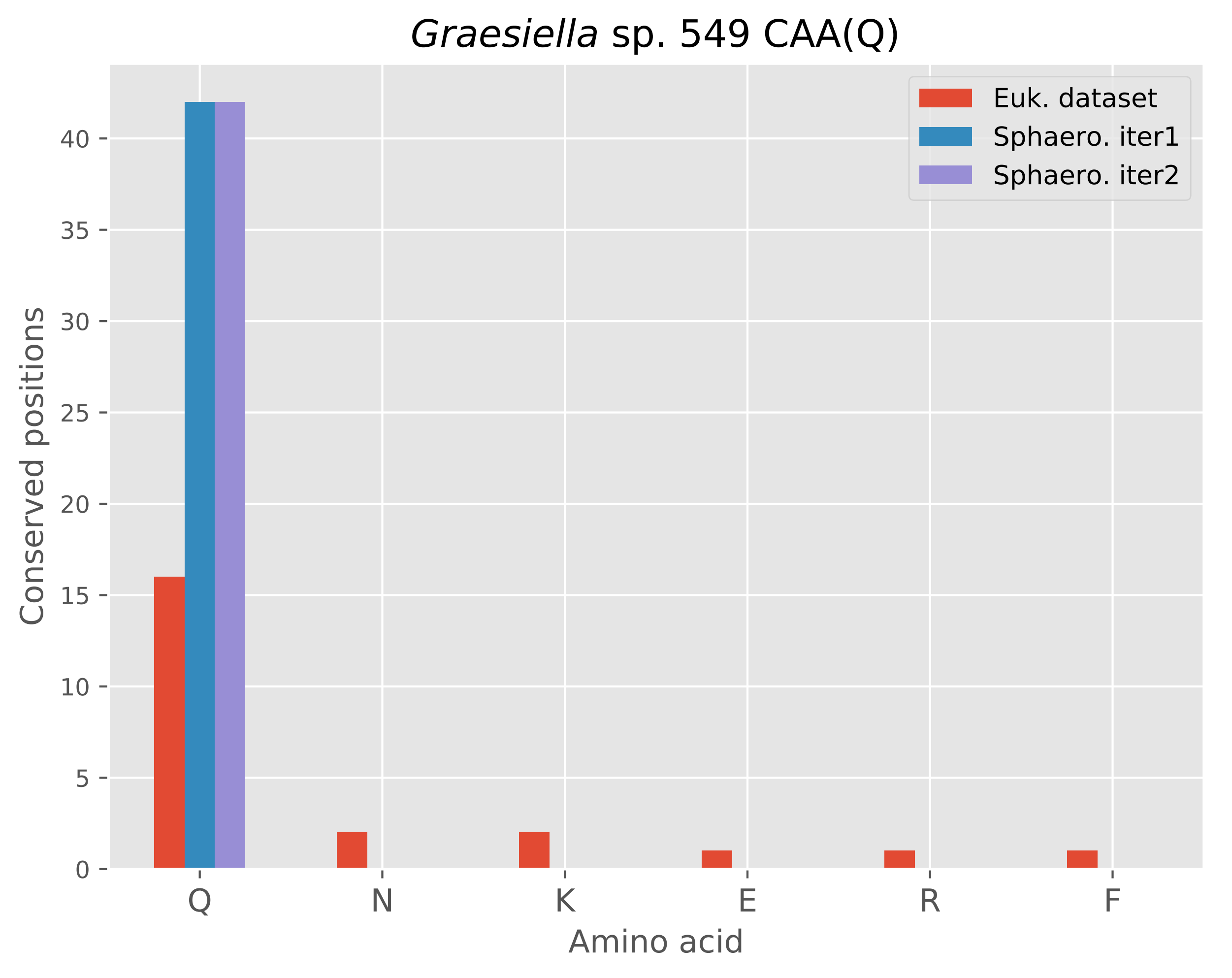

Graesiella sp. 549 CAA(Q)
Euk. dataset
Sphaero. iter1
Sphaero. iter2
Conserved positions
0
5
10
15
20
25
30
35
40
Q
N
K
E
R
F
Amino acid

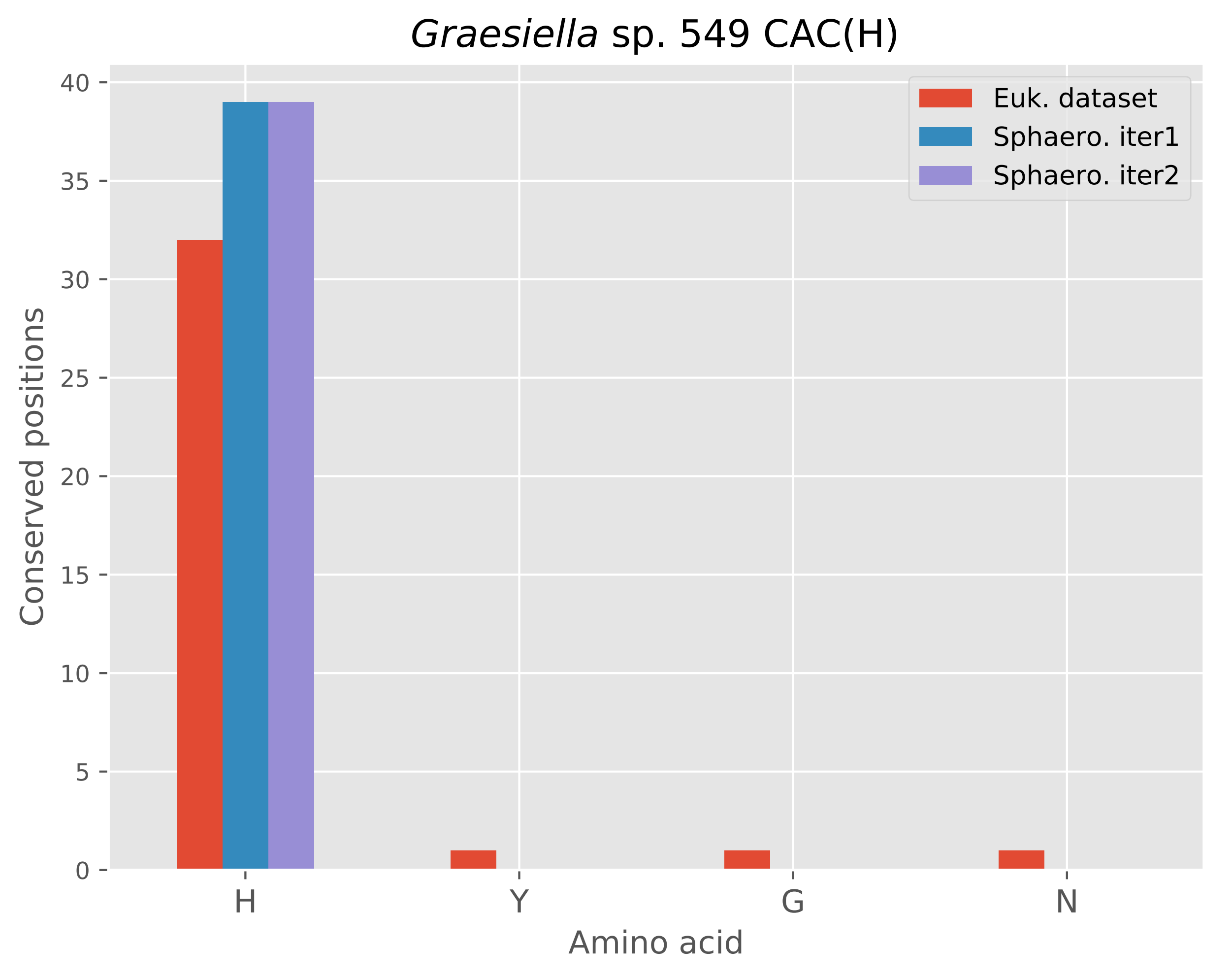

Graesiella sp. 549 CAC(H)
Euk. dataset
Sphaero. iter1
Sphaero. iter2
Conserved positions
0
5
10
15
20
25
30
35
40
H
Y
G
N
Amino acid

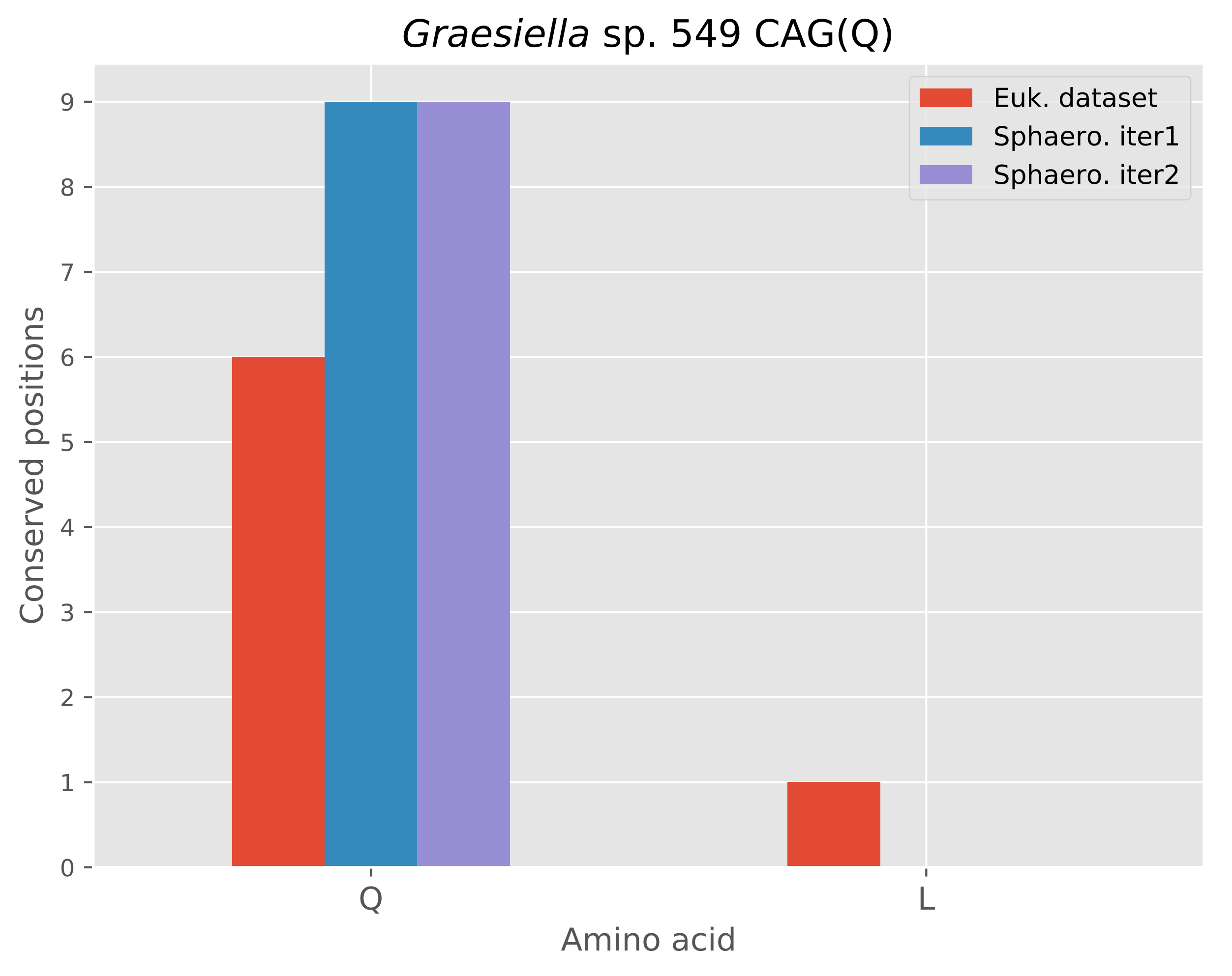

Graesiella sp. 549 CAG(Q)
Euk. dataset
Sphaero. iter1
Sphaero. iter2
Conserved positions
0
1
2
3
4
5
6
7
8
9
Q
L
Amino acid

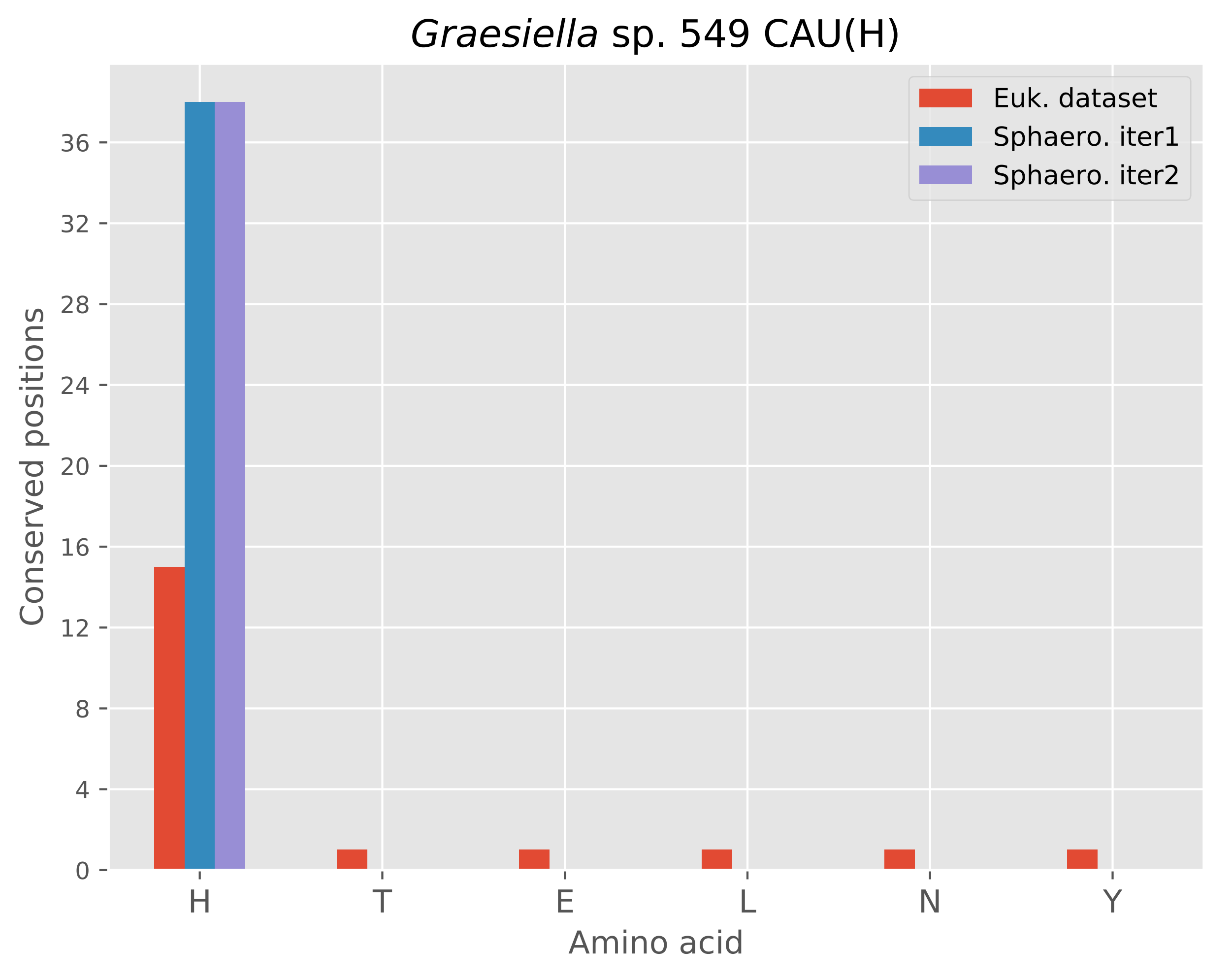

Graesiella sp. 549 CAU(H)
Euk. dataset
Sphaero. iter1
Sphaero. iter2
Conserved positions
0
4
8
12
16
20
24
28
32
36
H
T
E
L
N
Y
Amino acid

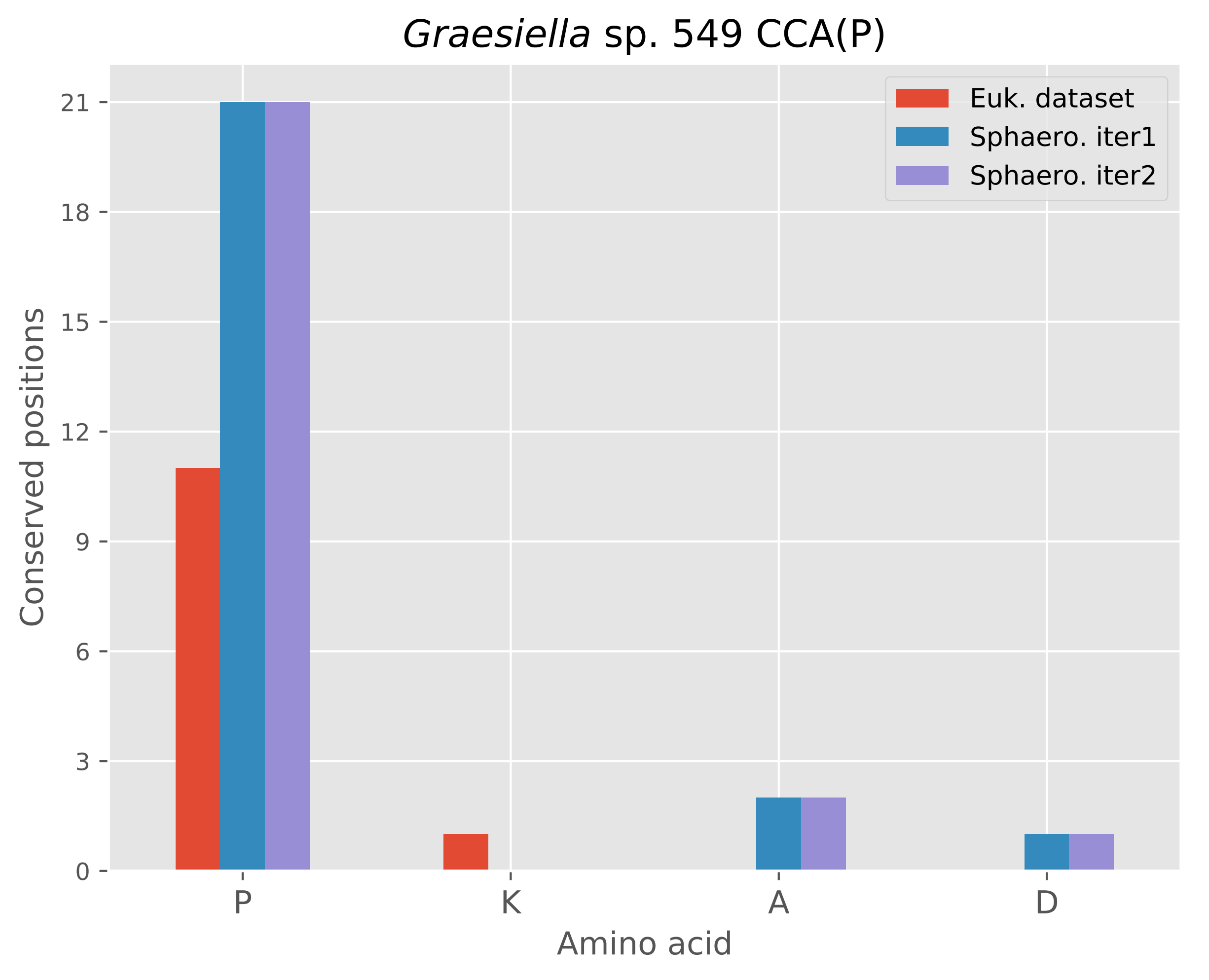
Graesiella sp. 549 CCA(P)
Euk. dataset
Sphaero. iter1
Sphaero. iter2
Conserved positions
0
3
6
9
12
15
18
21
P
K
A
D
Amino acid

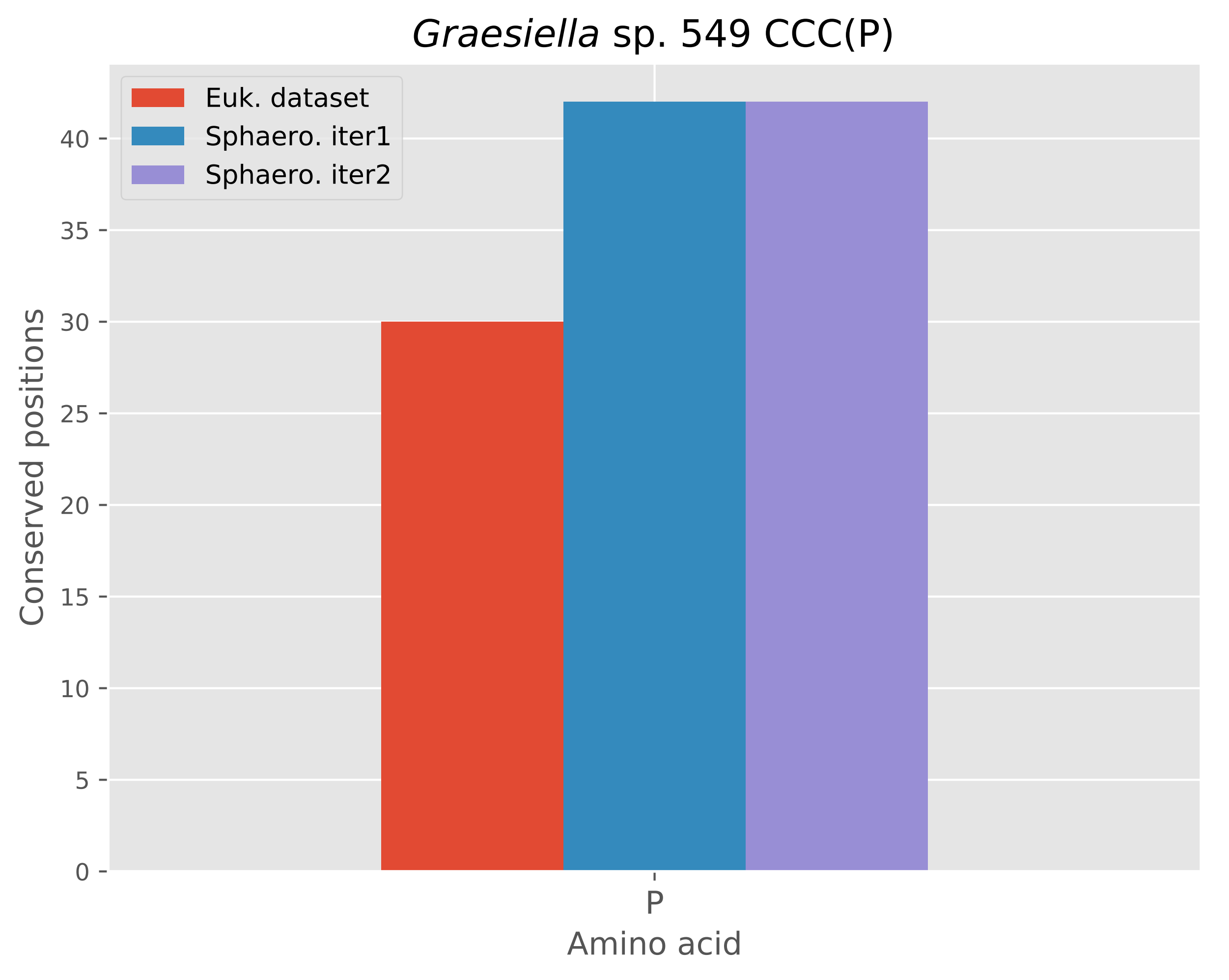
Graesiella sp. 549 CCC(P)
Euk. dataset
Sphaero. iter1
Sphaero. iter2
Conserved positions
0
5
10
15
20
25
30
35
40
P
Amino acid

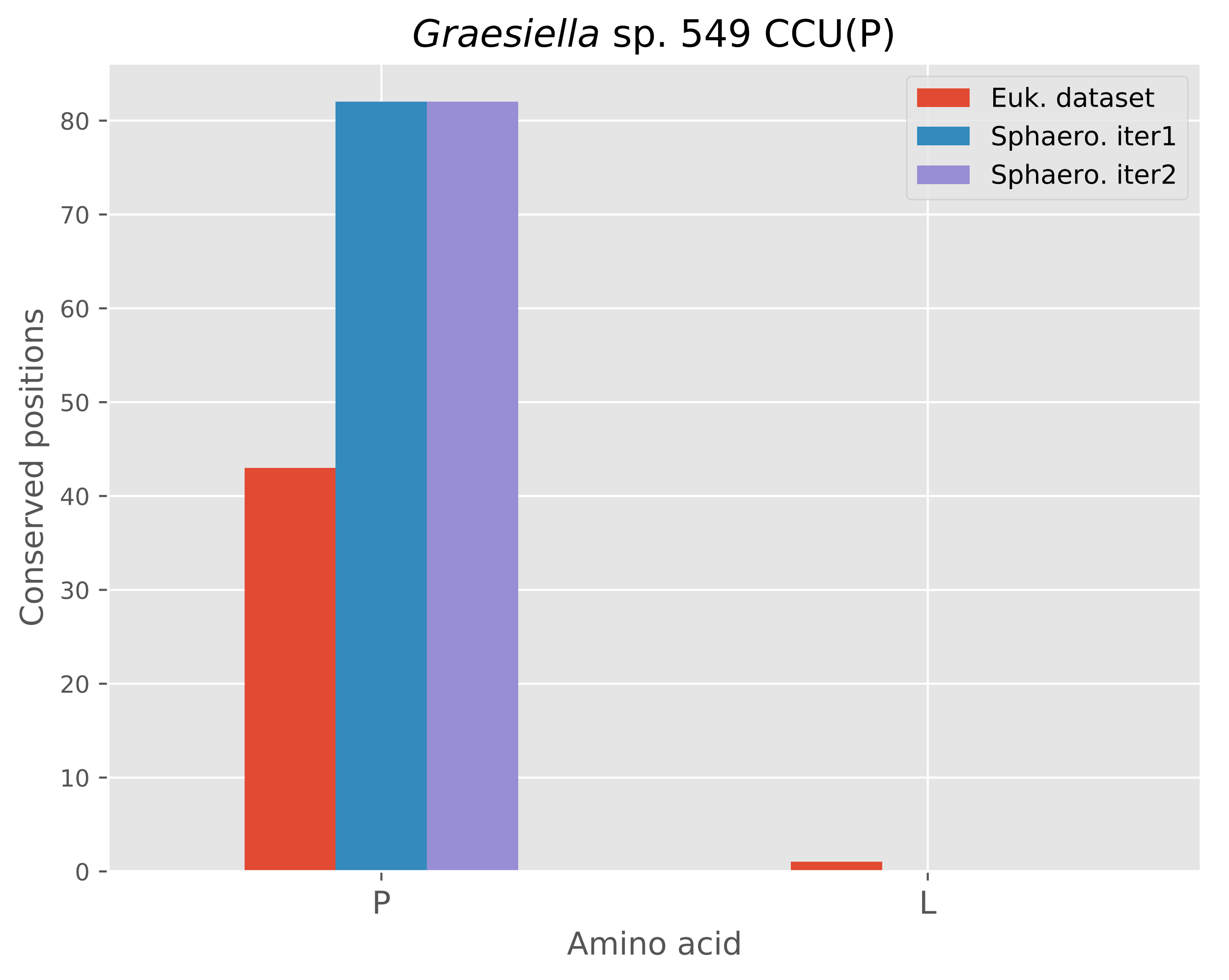

Graesiella sp. 549 CCU(P)
Euk. dataset
Sphaero. iter1
Sphaero. iter2
Conserved positions
0
10
20
30
40
50
60
70
80
P
L
Amino acid

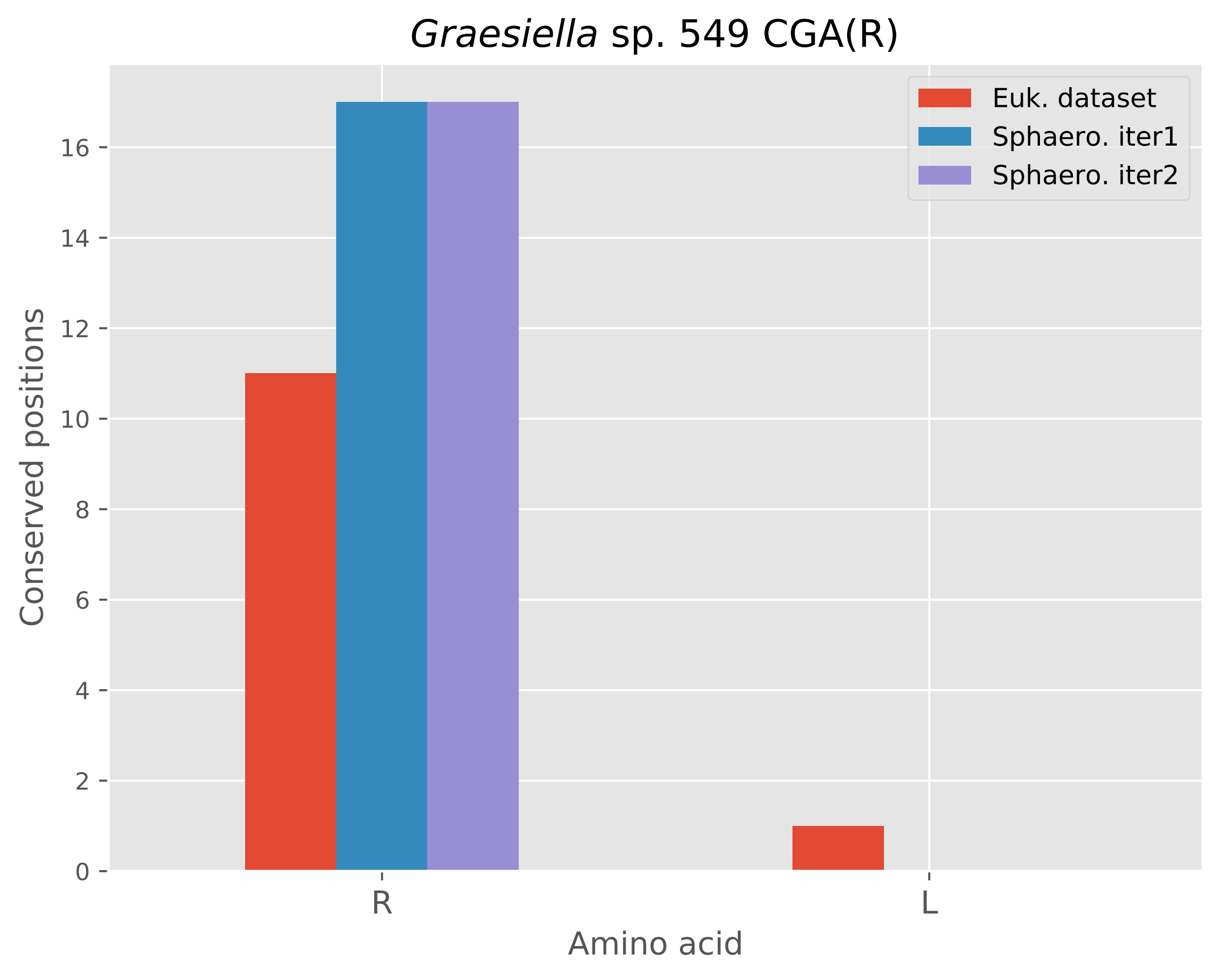

Graesiella sp. 549 CGA(R)
Euk. dataset
Sphaero. iter1
Sphaero. iter2
Conserved positions
0
2
4
6
8
10
12
14
16
R
L
Amino acid

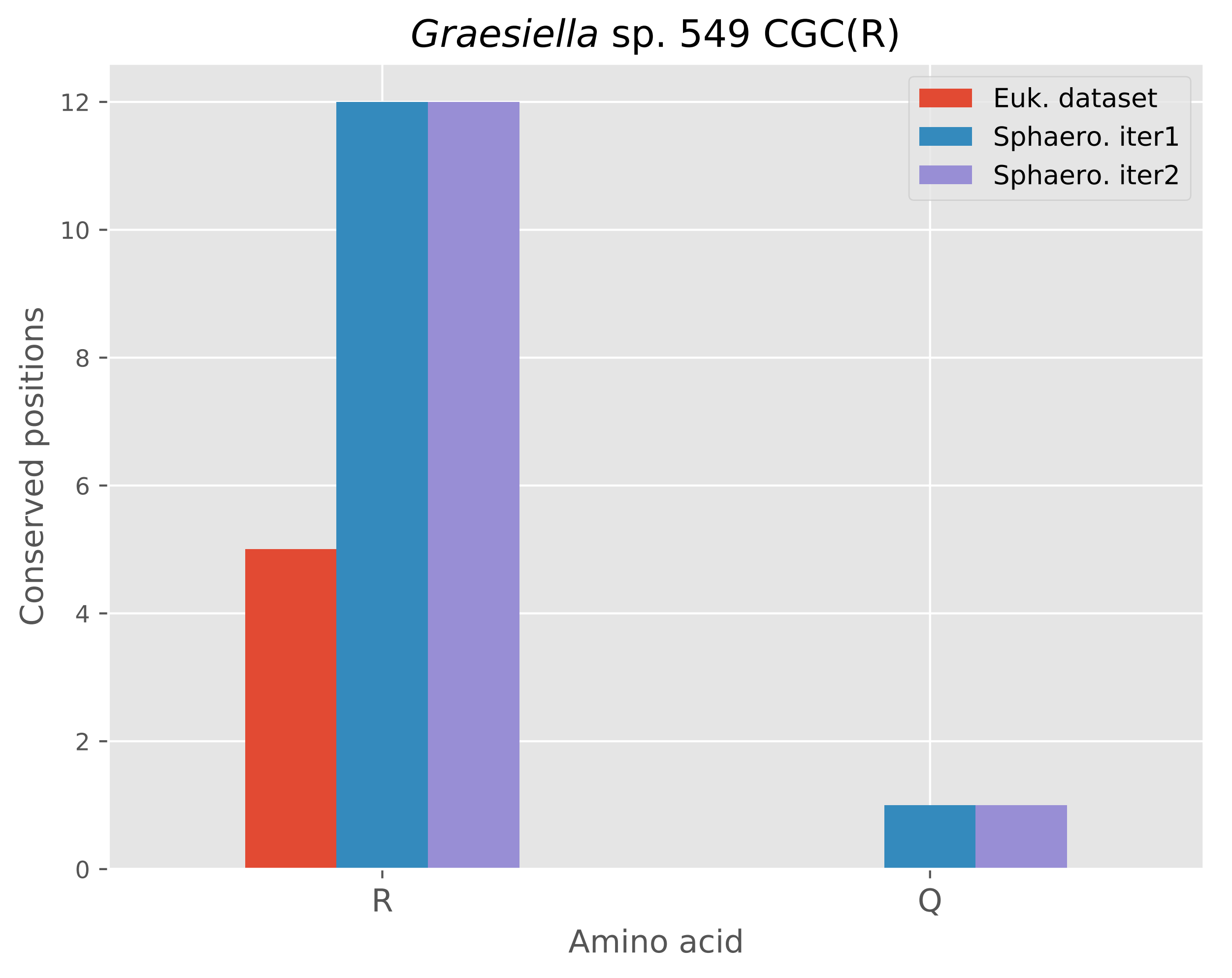
Graesiella sp. 549 CGC(R)
Euk. dataset
Sphaero. iter1
Sphaero. iter2
Conserved positions
0
2
4
6
8
10
12
R
Q
Amino acid

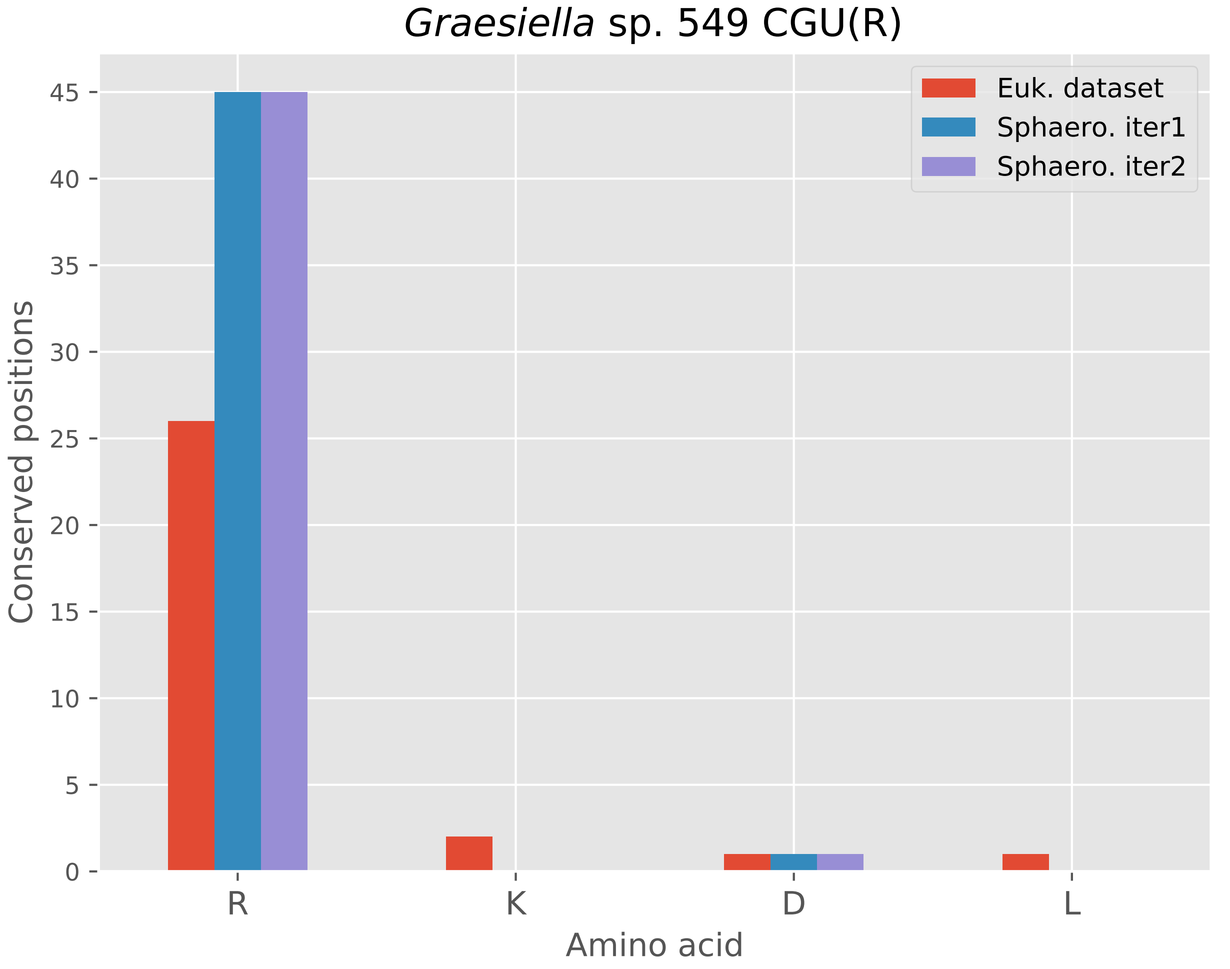

Graesiella sp. 549 CGU(R)
Euk. dataset
Sphaero. iter1
Sphaero. iter2
Conserved positions
0
5
10
15
20
25
30
35
40
45
R
K
D
L
Amino acid

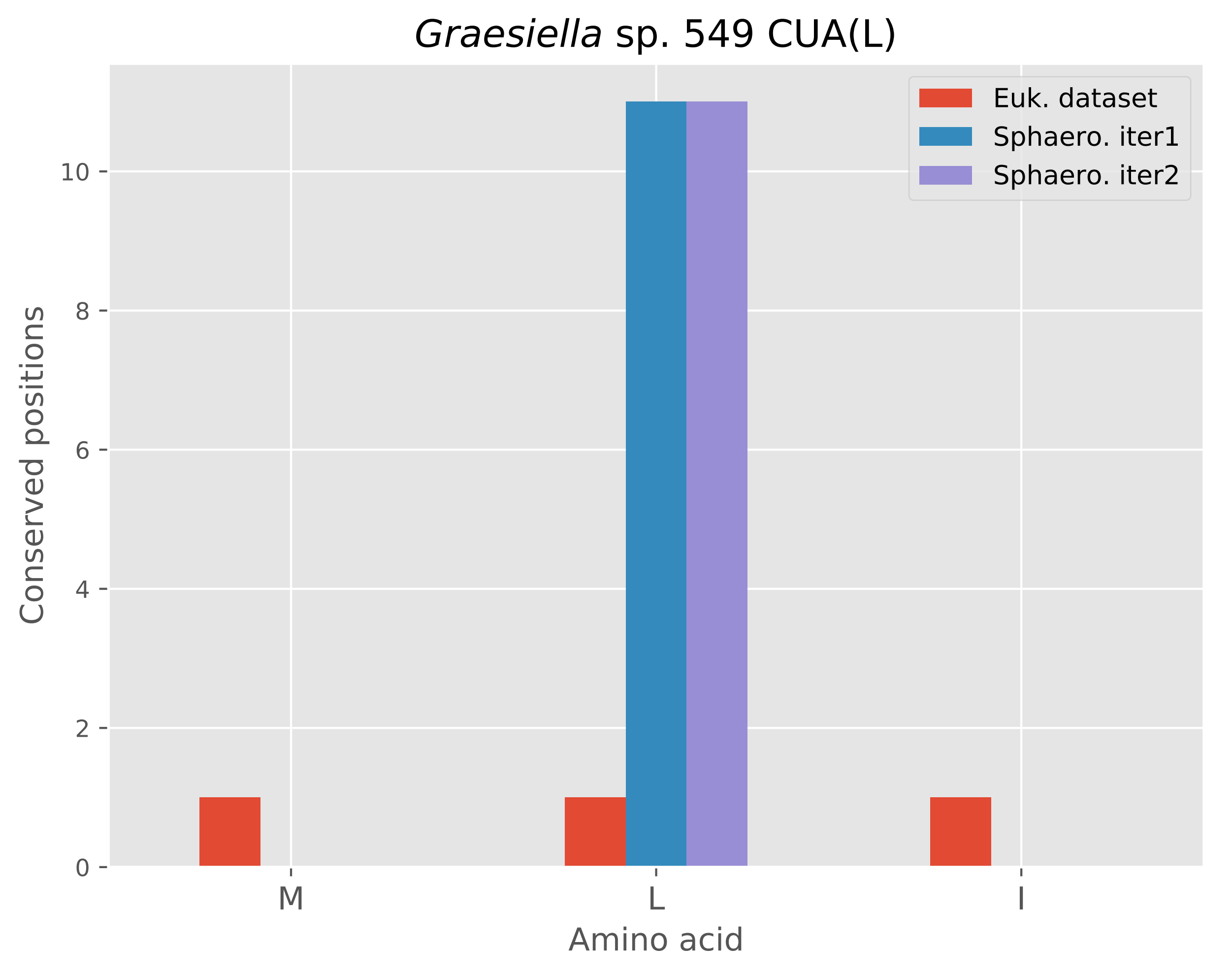

Graesiella sp. 549 CUA(L)
Euk. dataset
Sphaero. iter1
Sphaero. iter2
Conserved positions
0
2
4
6
8
10
M
L
I
Amino acid

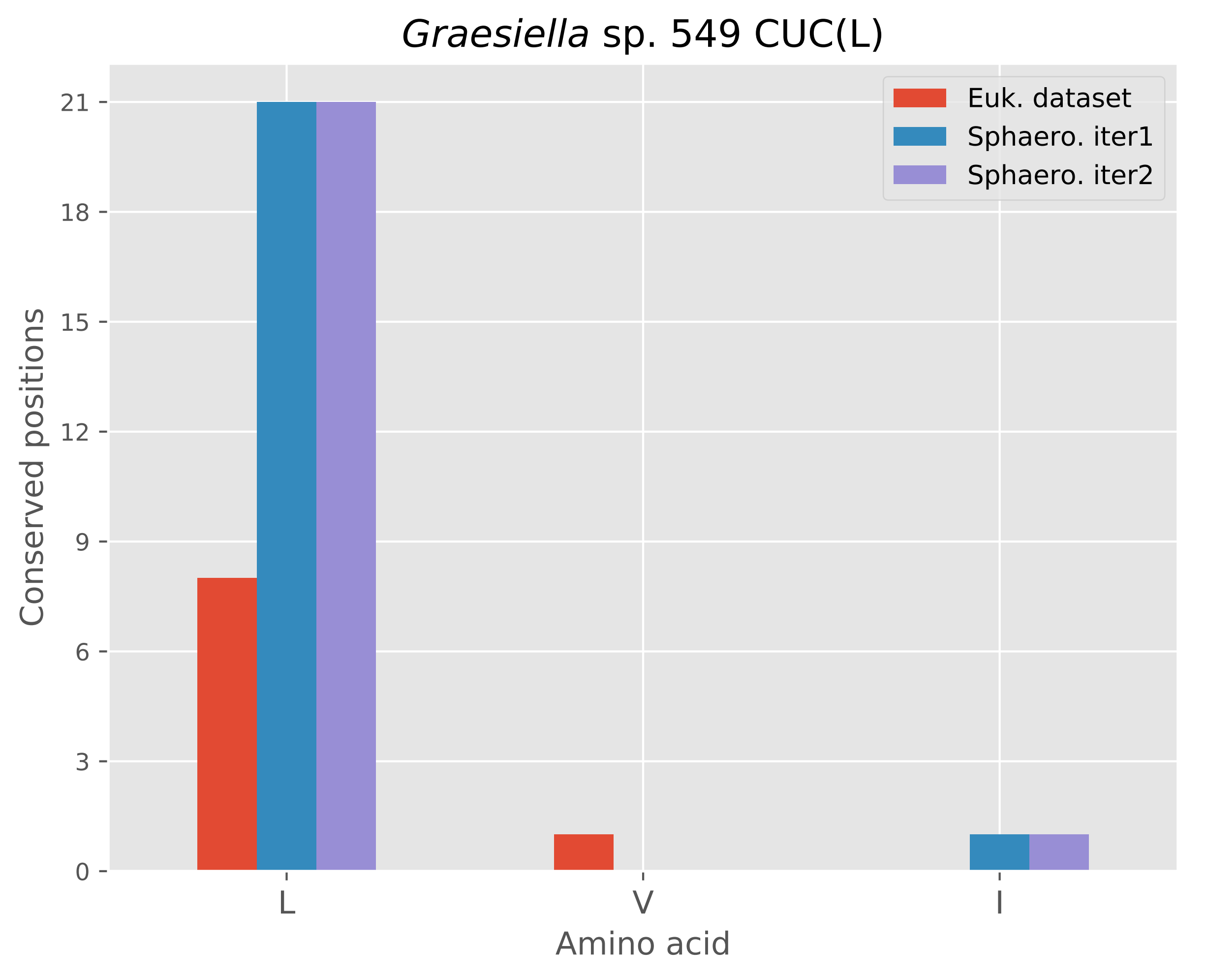

Graesiella sp. 549 CUC(L)
Euk. dataset
Sphaero. iter1
Sphaero. iter2
Conserved positions
0
3
6
9
12
15
18
21
L
V
I
Amino acid

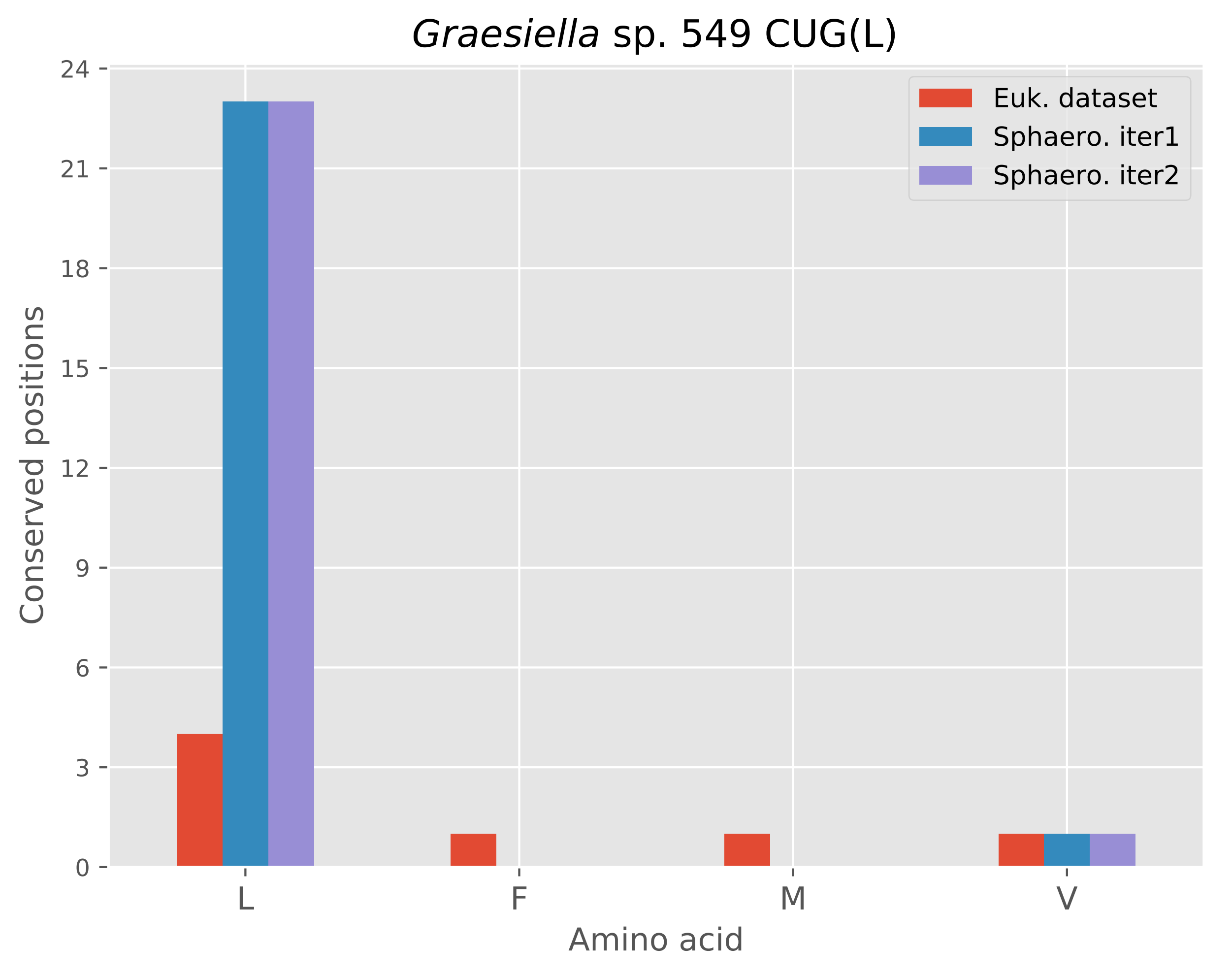
Graesiella sp. 549 CUG(L)
Conserved positions
Amino acid
Euk. dataset
Sphaero. iter1
Sphaero. iter2
0
3
6
9
12
15
18
21
24
L
F
M
V

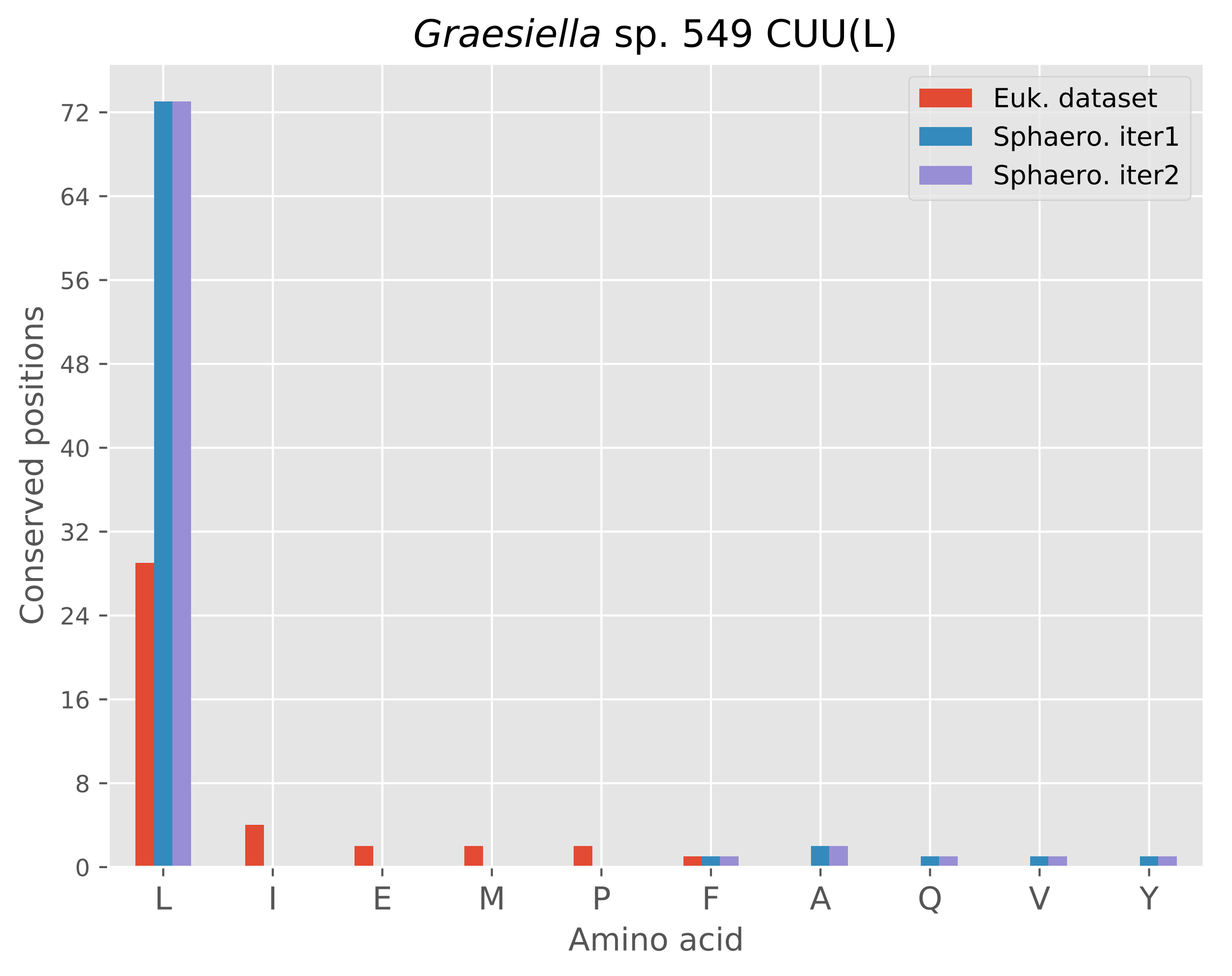
Graesiella sp. 549 CUU(L)
Euk. dataset
Sphaero. iter1
Sphaero. iter2
Conserved positions
0
8
16
24
32
40
48
56
64
72
L
I
E
M
P
F
A
Q
V
Y
Amino acid

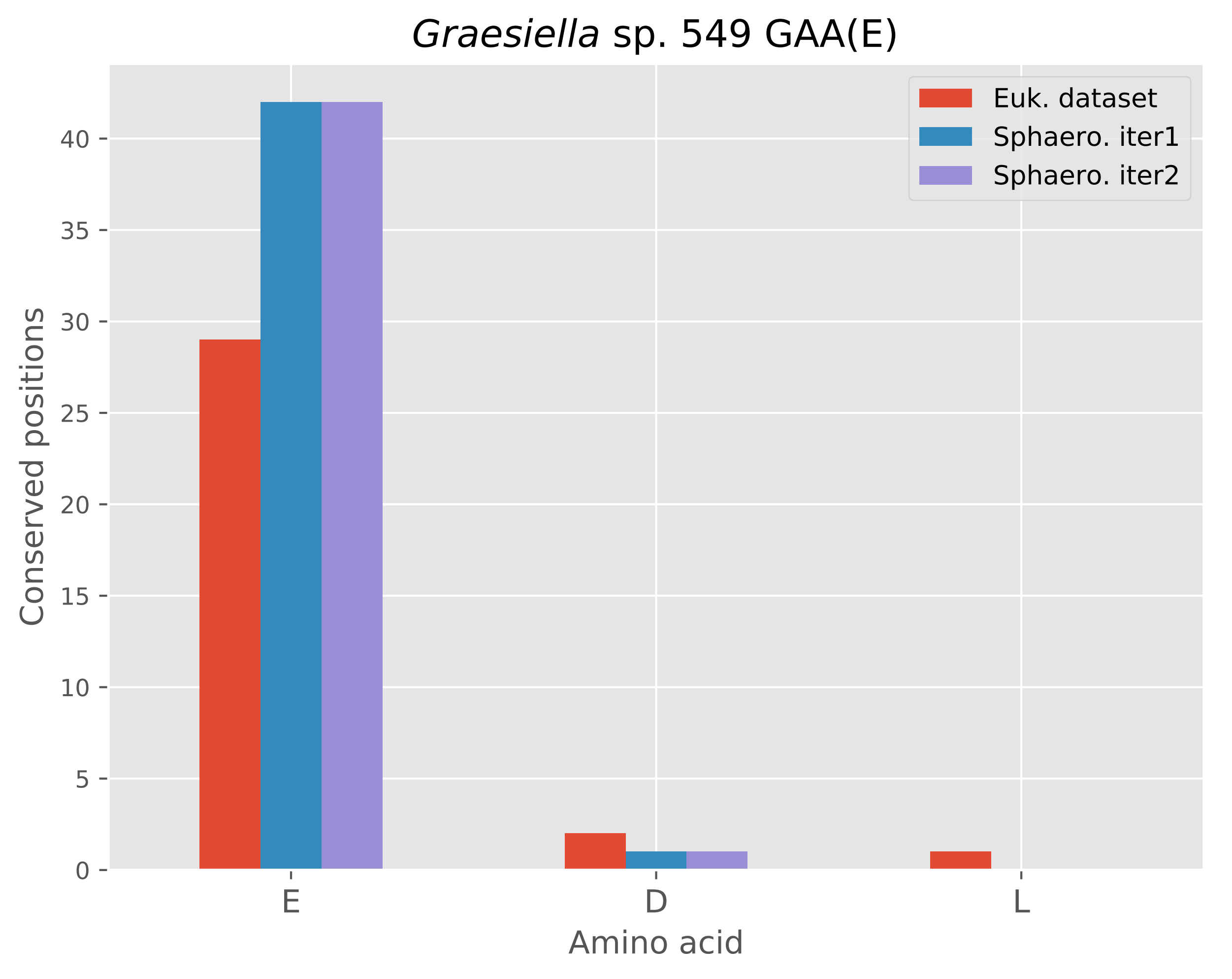
Graesiella sp. 549 GAA(E)
Euk. dataset
Sphaero. iter1
Sphaero. iter2
Conserved positions
0
5
10
15
20
25
30
35
40
E
D
L
Amino acid

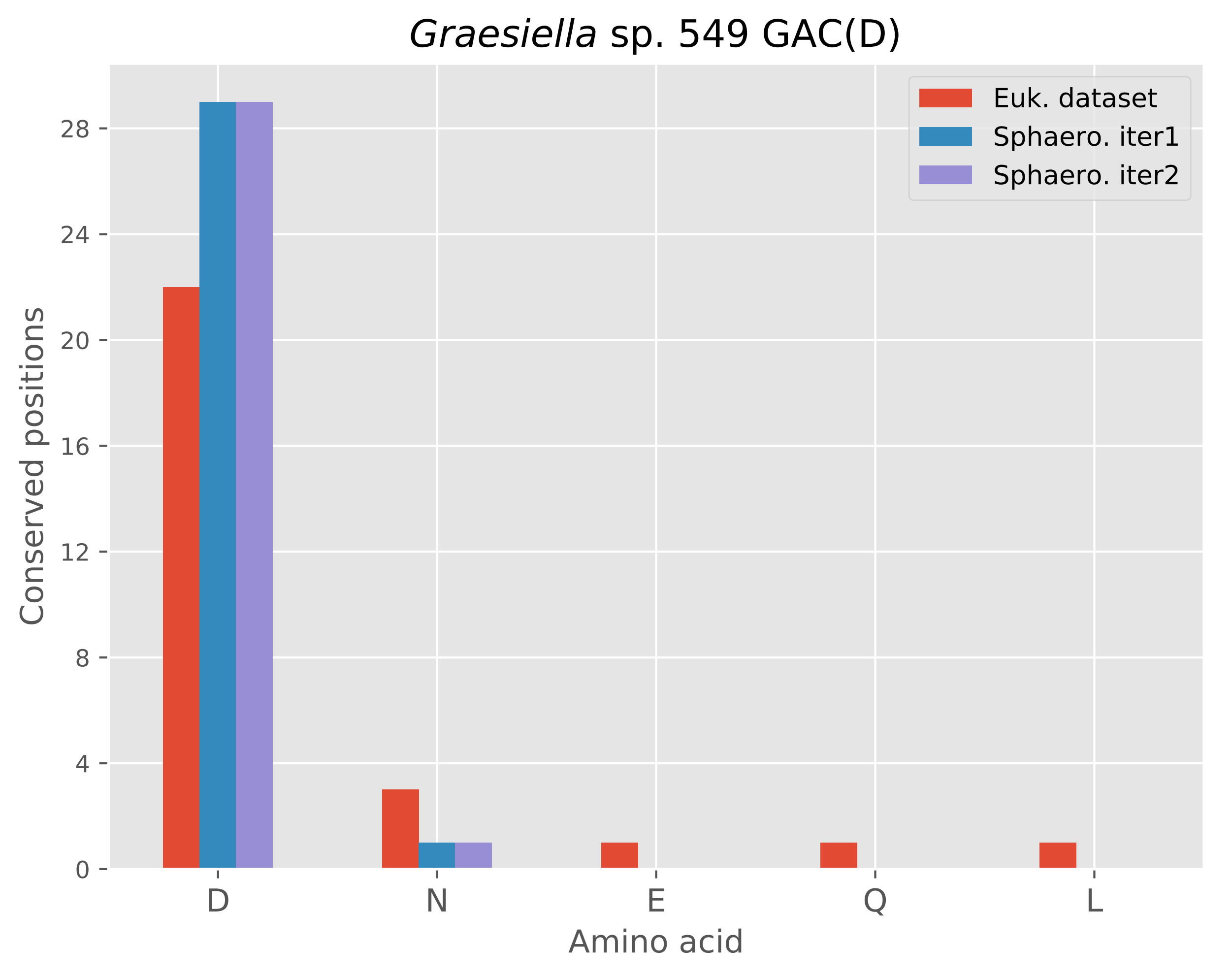

Graesiella sp. 549 GAC(D)
Euk. dataset
Sphaero. iter1
Sphaero. iter2
Conserved positions
0
4
8
12
16
20
24
28
D
N
E
Q
L
Amino acid

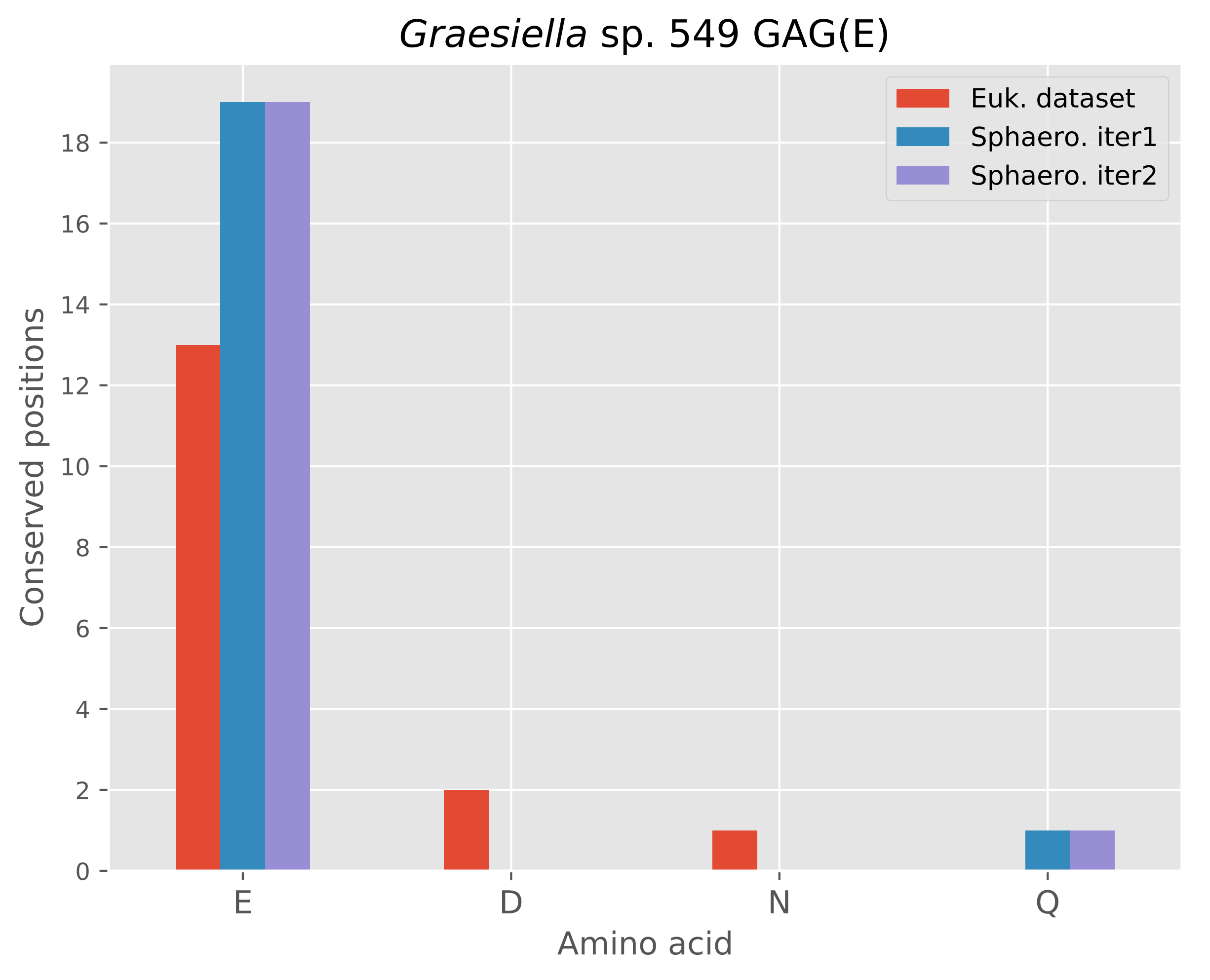

Graesiella sp. 549 GAG(E)
Euk. dataset
Sphaero. iter1
Sphaero. iter2
Conserved positions
0
2
4
6
8
10
12
14
16
18
E
D
N
Q
Amino acid

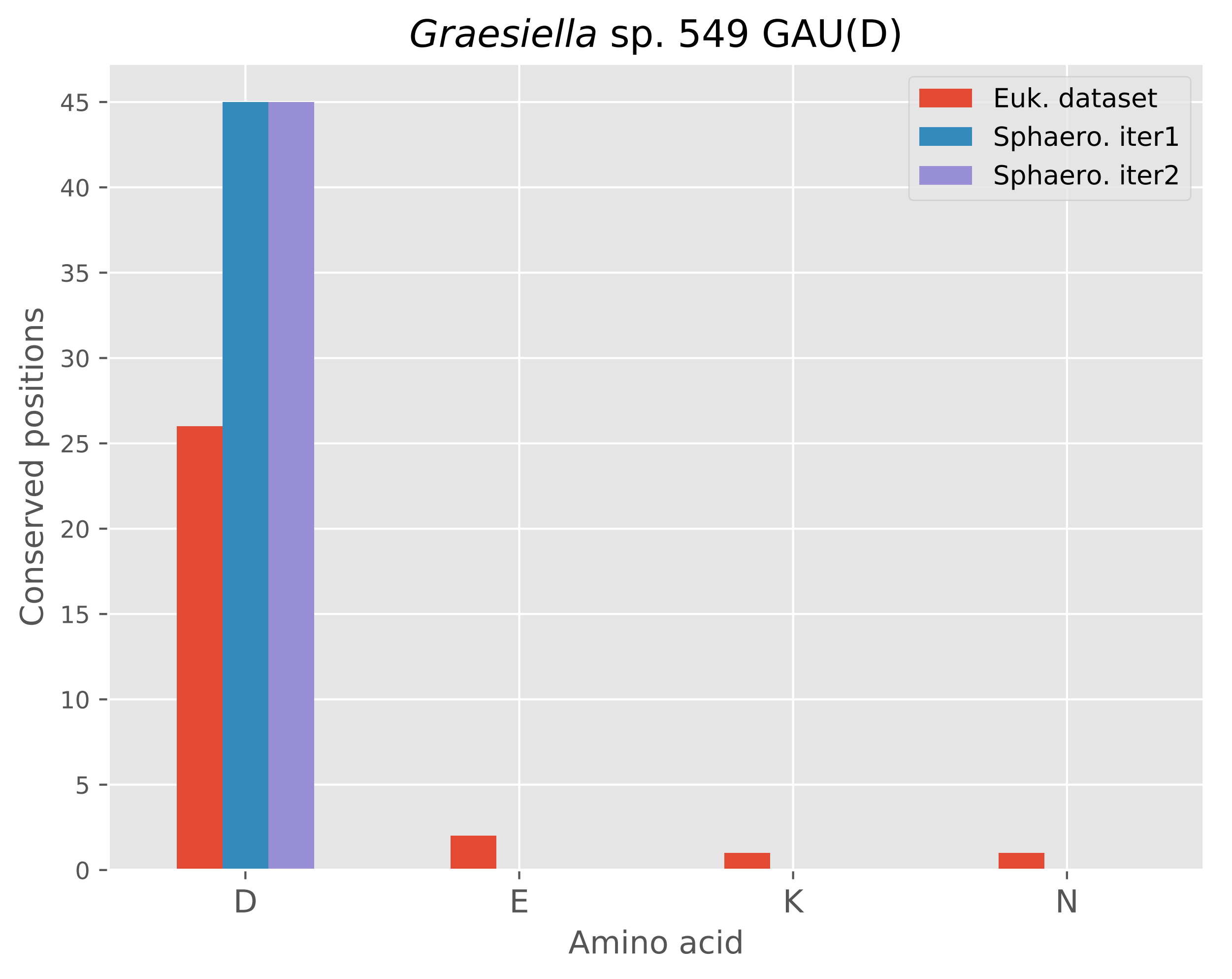

Graesiella sp. 549 GAU(D)
Euk. dataset
Sphaero. iter1
Sphaero. iter2
Conserved positions
0
5
10
15
20
25
30
35
40
45
D
E
K
N
Amino acid

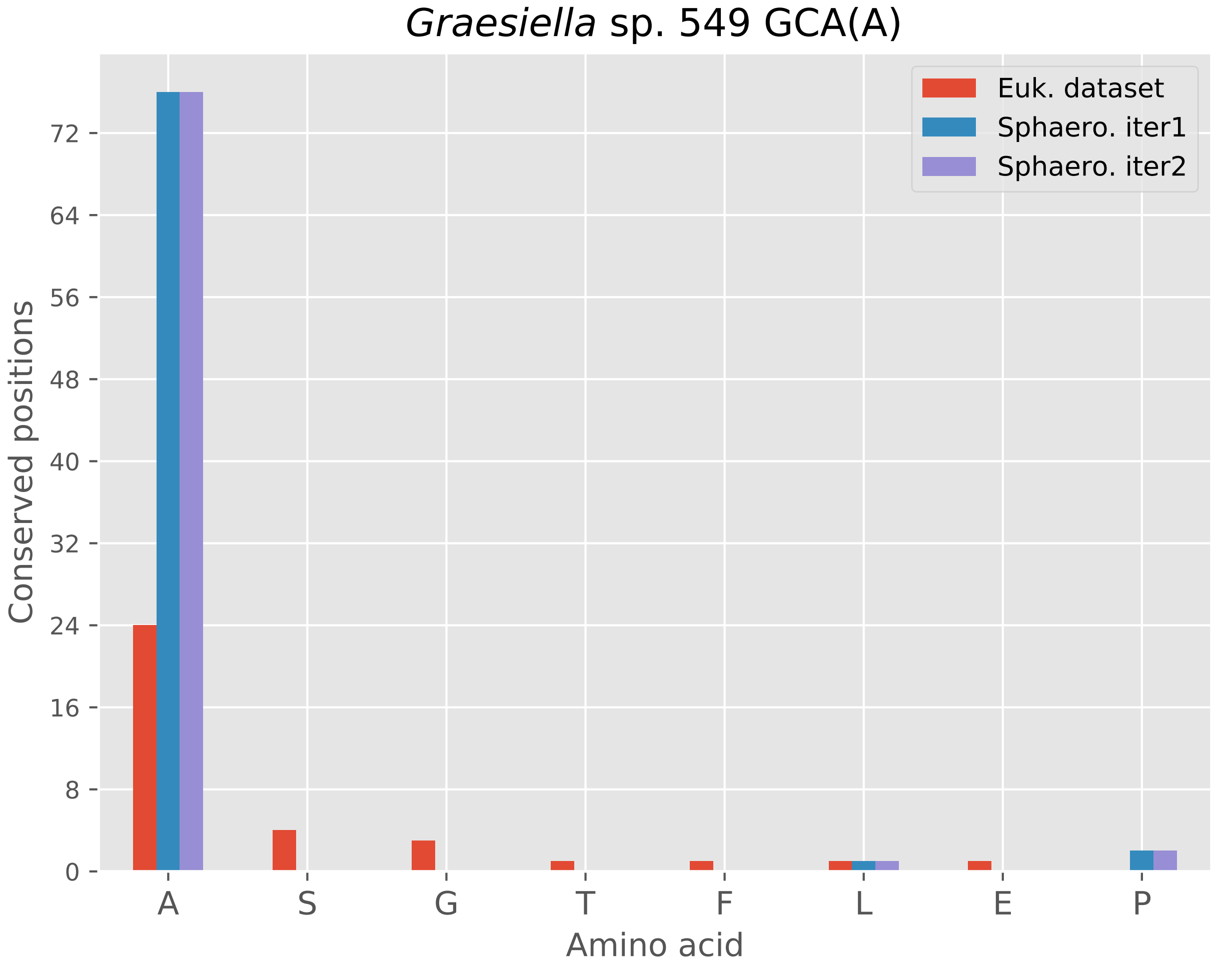

Graesiella sp. 549 GCA(A)
Euk. dataset
Sphaero. iter1
Sphaero. iter2
Conserved positions
0
8
16
24
32
40
48
56
64
72
A
S
G
T
F
L
E
P
Amino acid

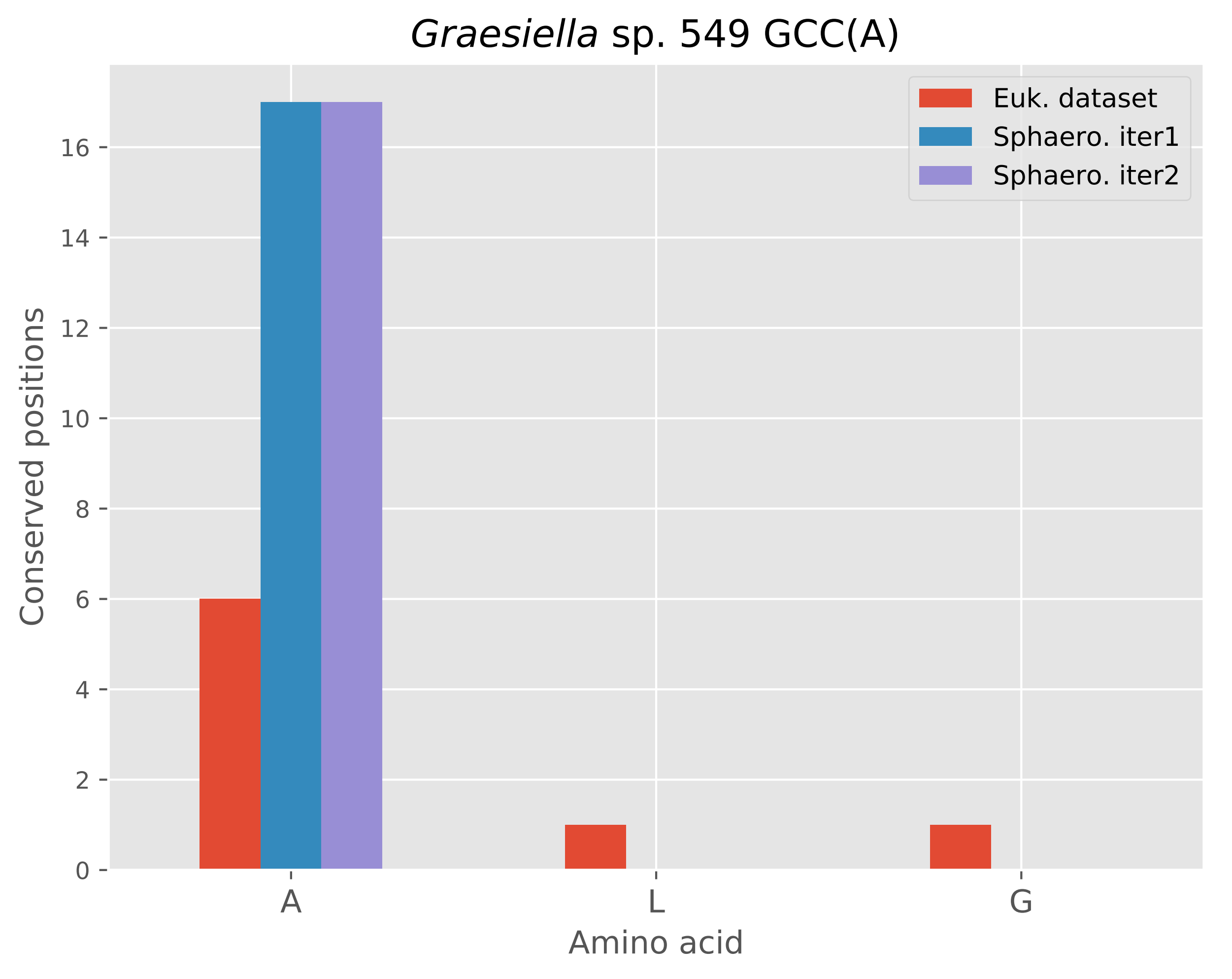

Graesiella sp. 549 GCC(A)
Euk. dataset
Sphaero. iter1
Sphaero. iter2
Conserved positions
0
2
4
6
8
10
12
14
16
A
L
G
Amino acid

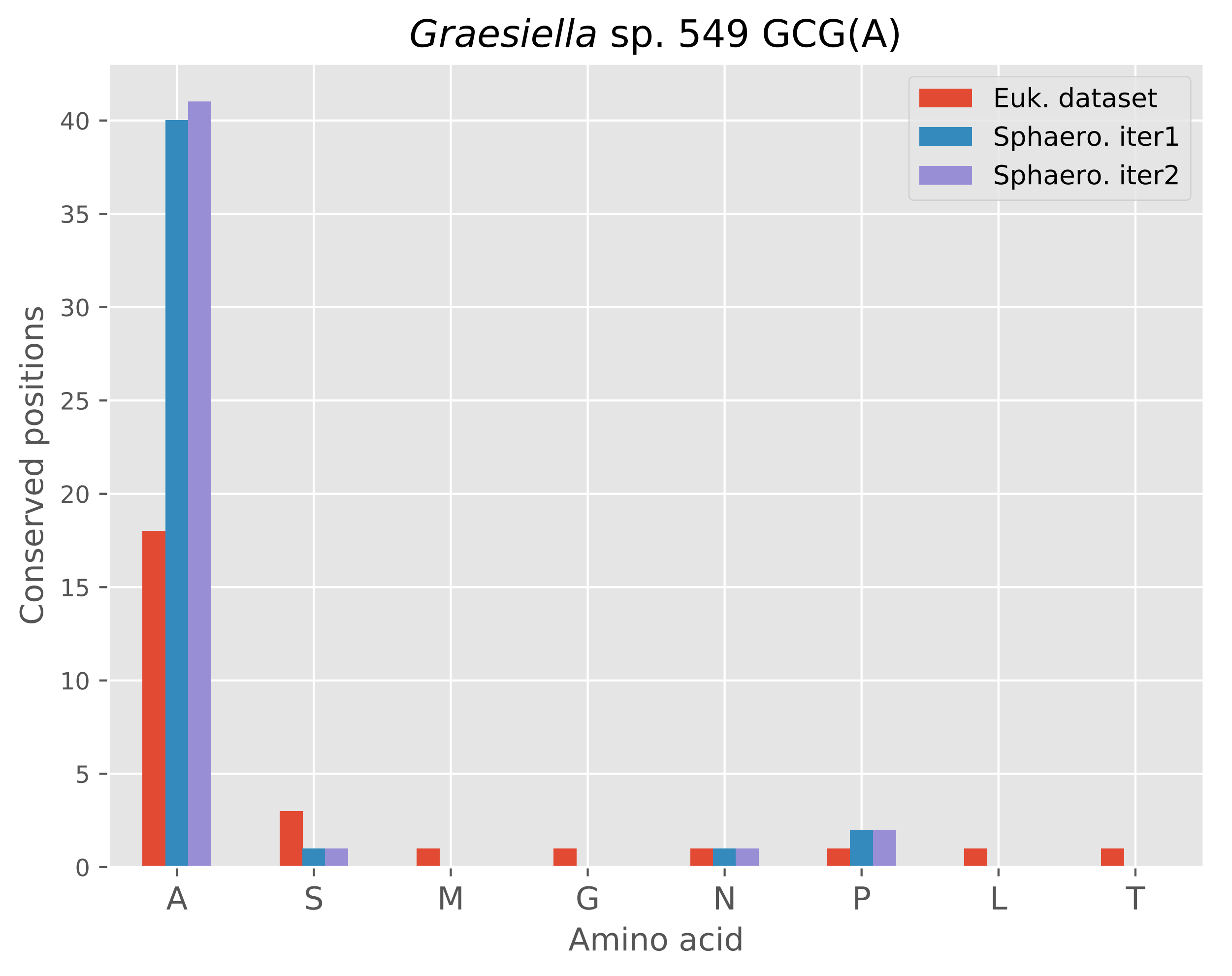

Graesiella sp. 549 GCG(A)
Euk. dataset
Sphaero. iter1
Sphaero. iter2
Conserved positions
0
5
10
15
20
25
30
35
40
A
S
M
G
N
P
L
T
Amino acid

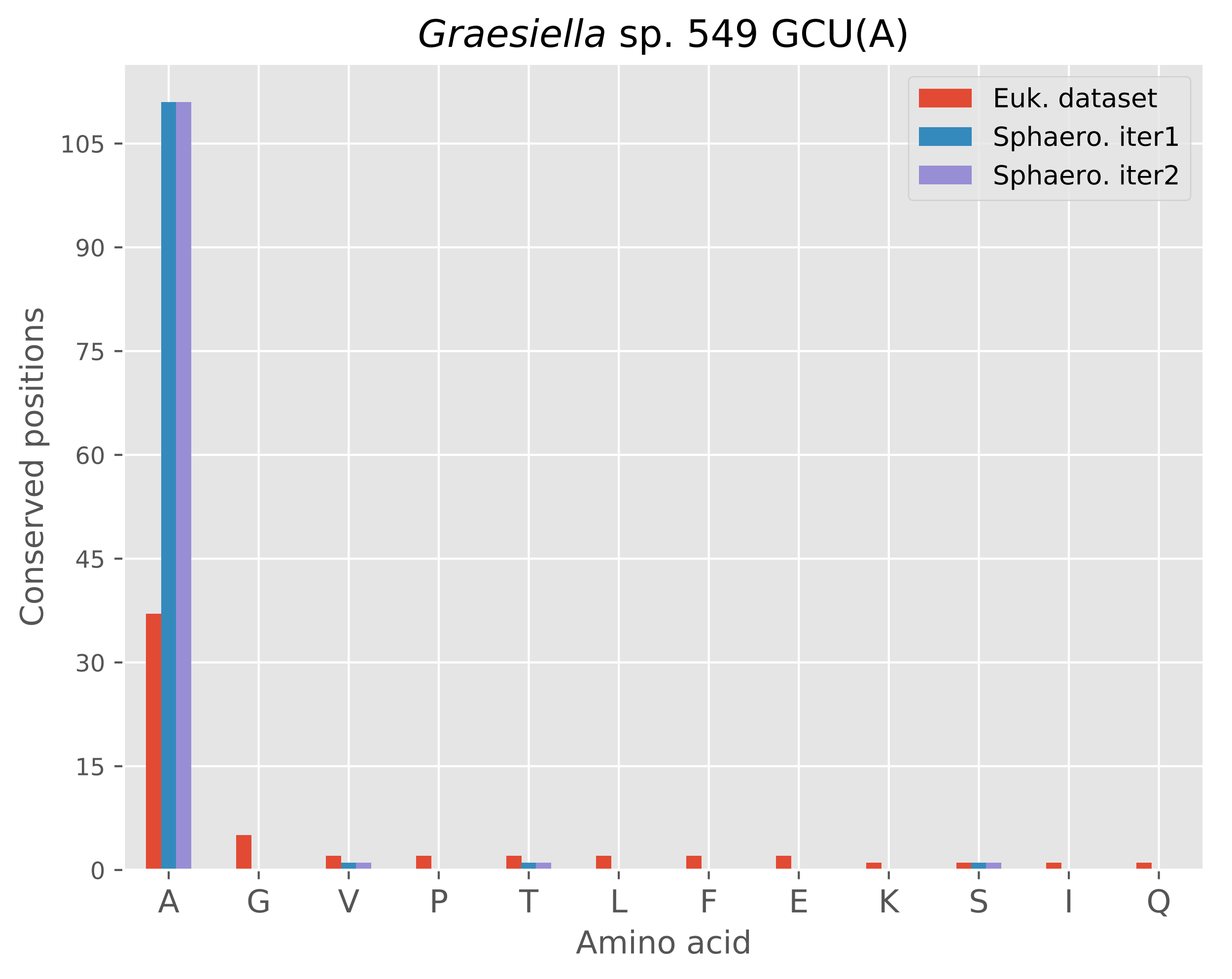

Graesiella sp. 549 GCU(A)
Euk. dataset
Sphaero. iter1
Sphaero. iter2
Conserved positions
0
15
30
45
60
75
90
105
A
G
V
P
T
L
F
E
K
S
I
Q
Amino acid

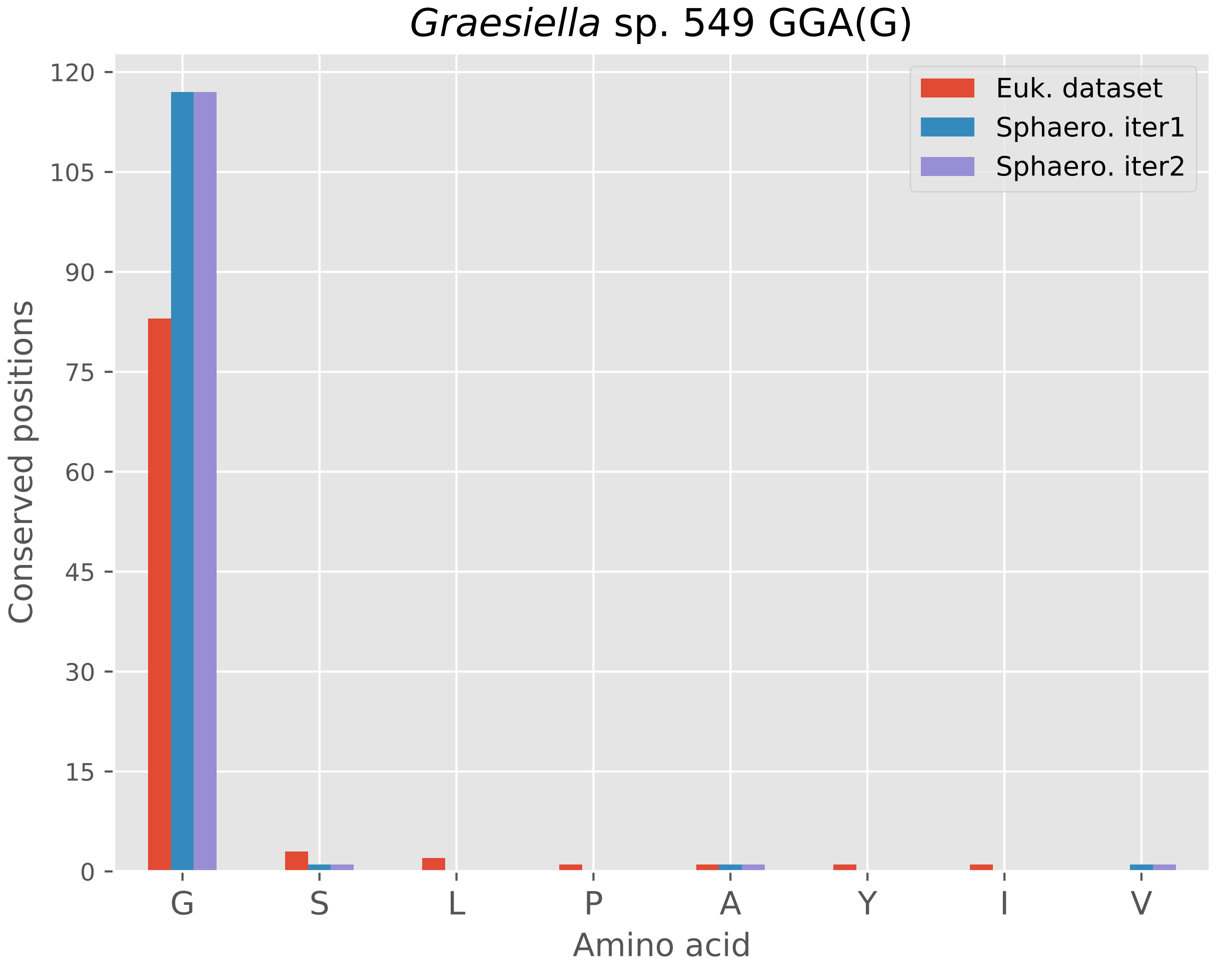

Graesiella sp. 549 GGA(G)
Euk. dataset
Sphaero. iter1
Sphaero. iter2
Conserved positions
120
105
90
75
60
45
30
15
0
G
S
L
P
A
Y
I
V
Amino acid

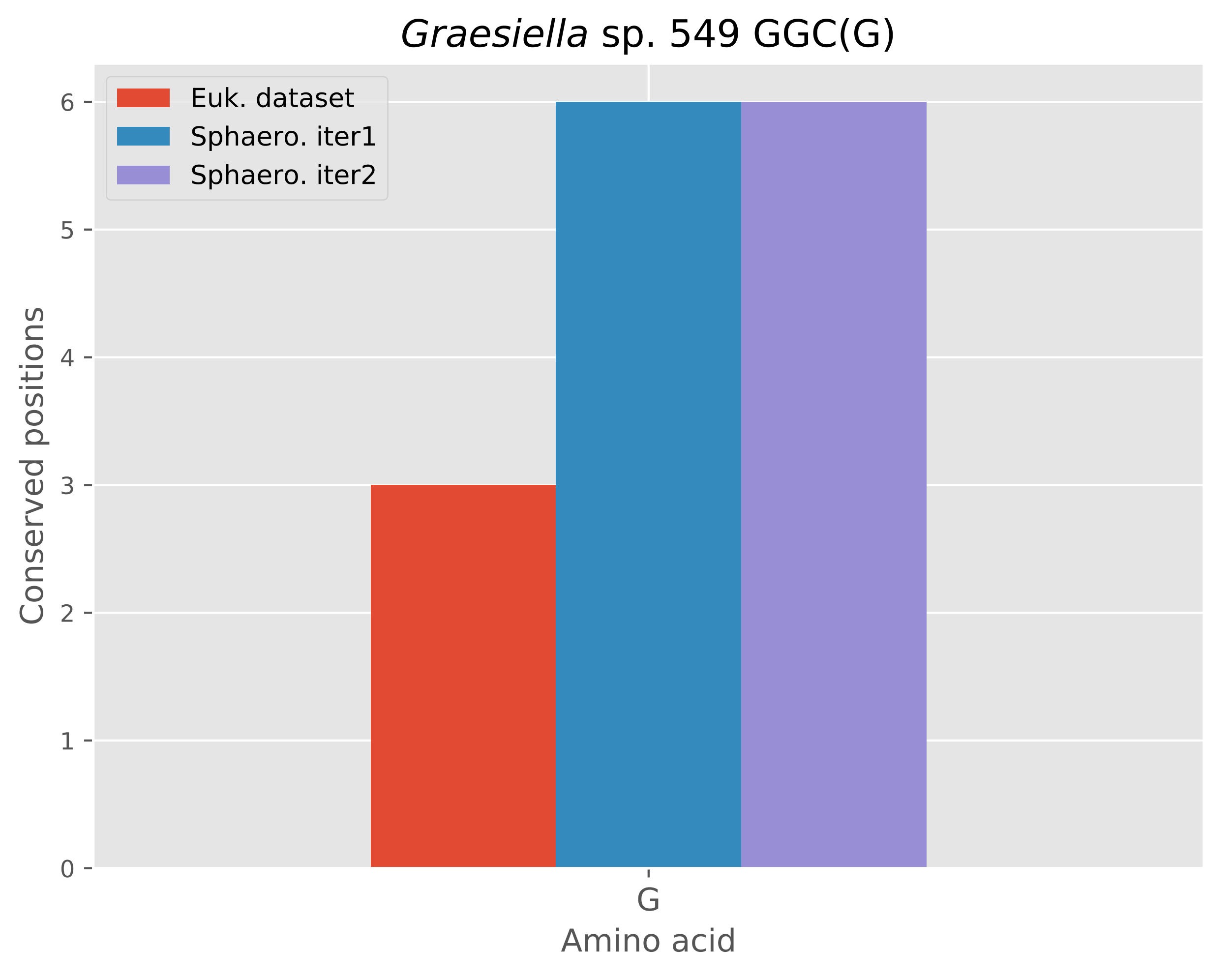

Graesiella sp. 549 GGC(G)
Euk. dataset
Sphaero. iter1
Sphaero. iter2
Conserved positions
0
1
2
3
4
5
6
G
Amino acid

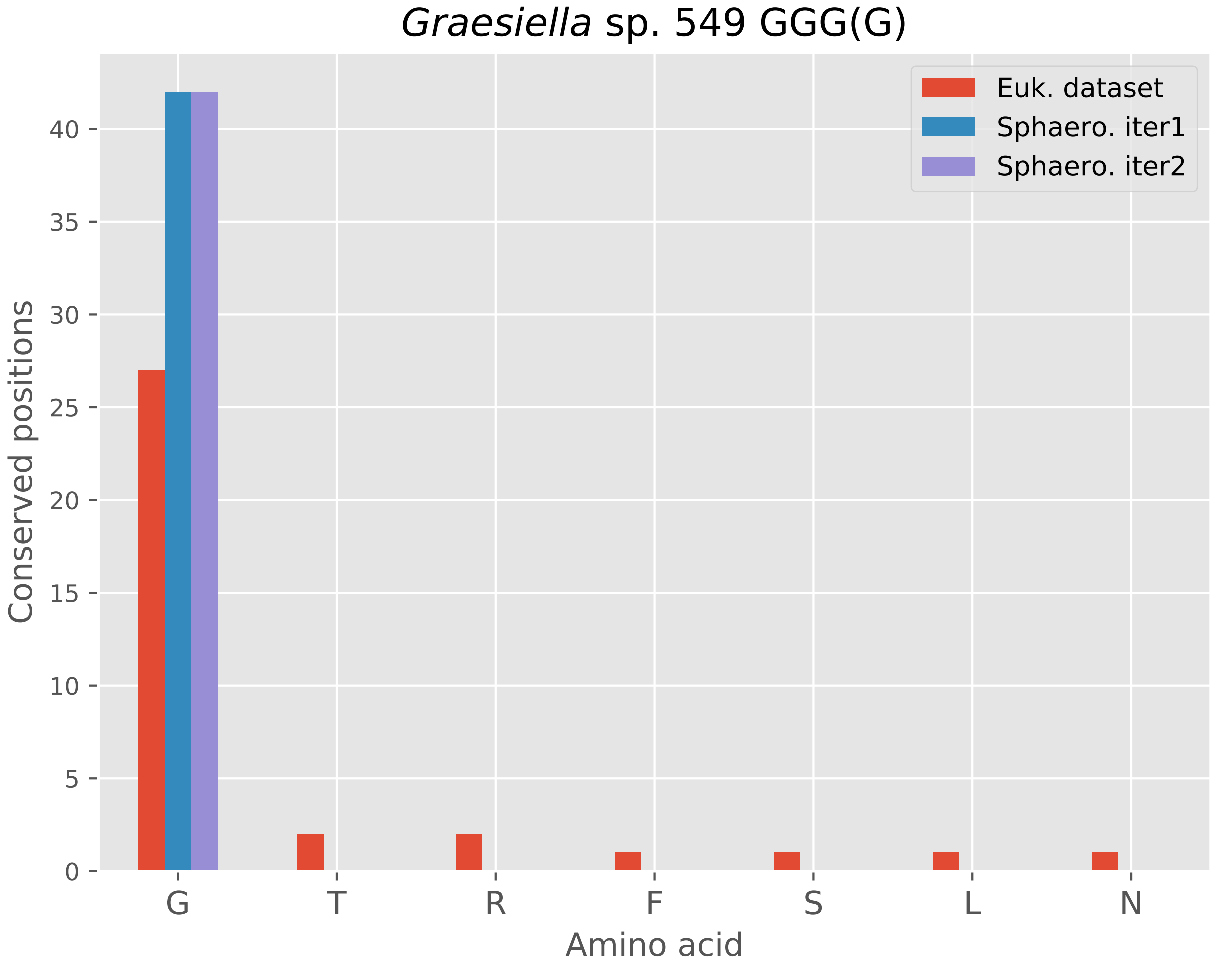
Graesiella sp. 549 GGG(G)
Euk. dataset
Sphaero. iter1
Sphaero. iter2
Conserved positions
0
5
10
15
20
25
30
35
40
G
T
R
F
S
L
N
Amino acid

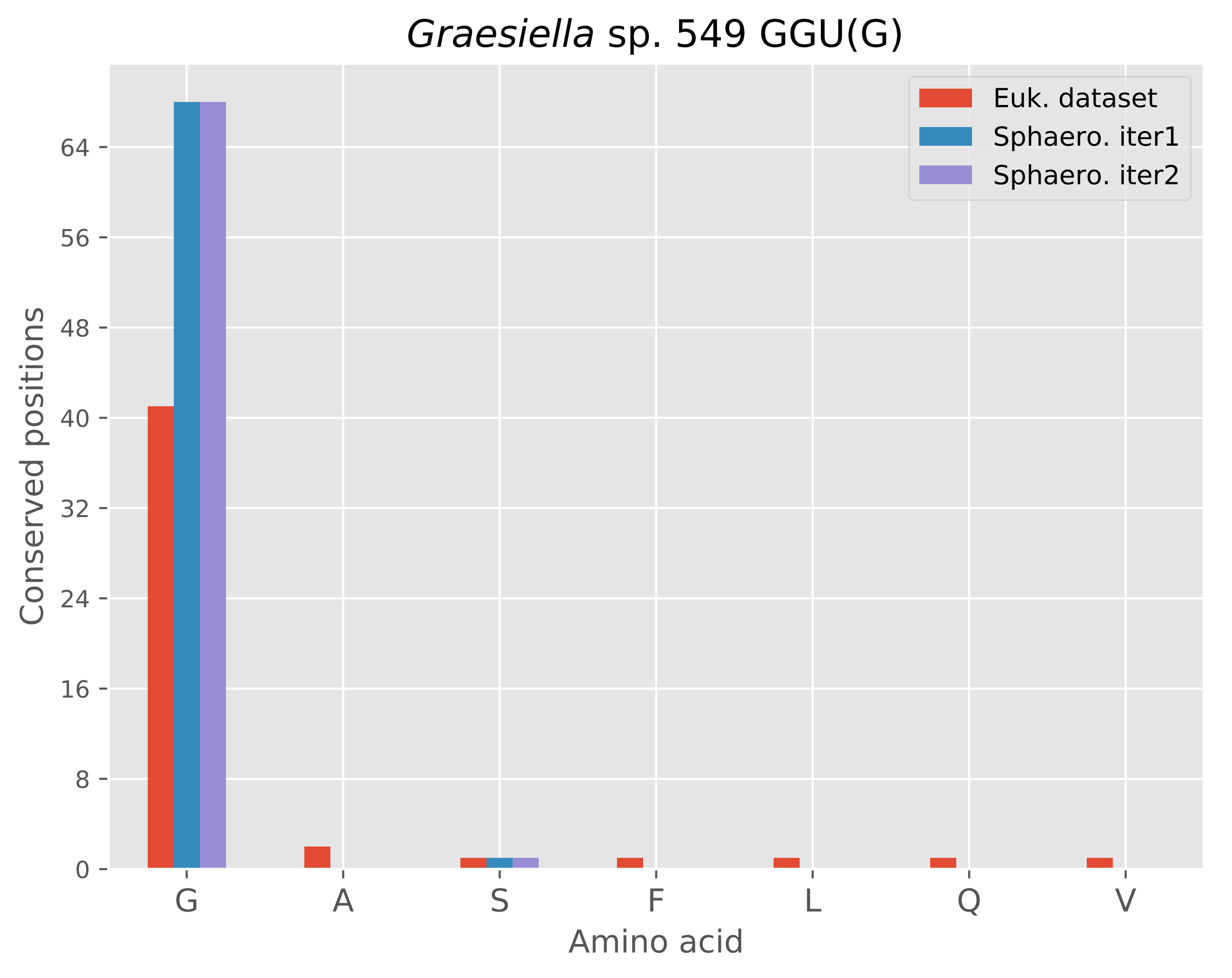

Graesiella sp. 549 GGU(G)
Euk. dataset
Sphaero. iter1
Sphaero. iter2
Conserved positions
0
8
16
24
32
40
48
56
64
G
A
S
F
L
Q
V
Amino acid

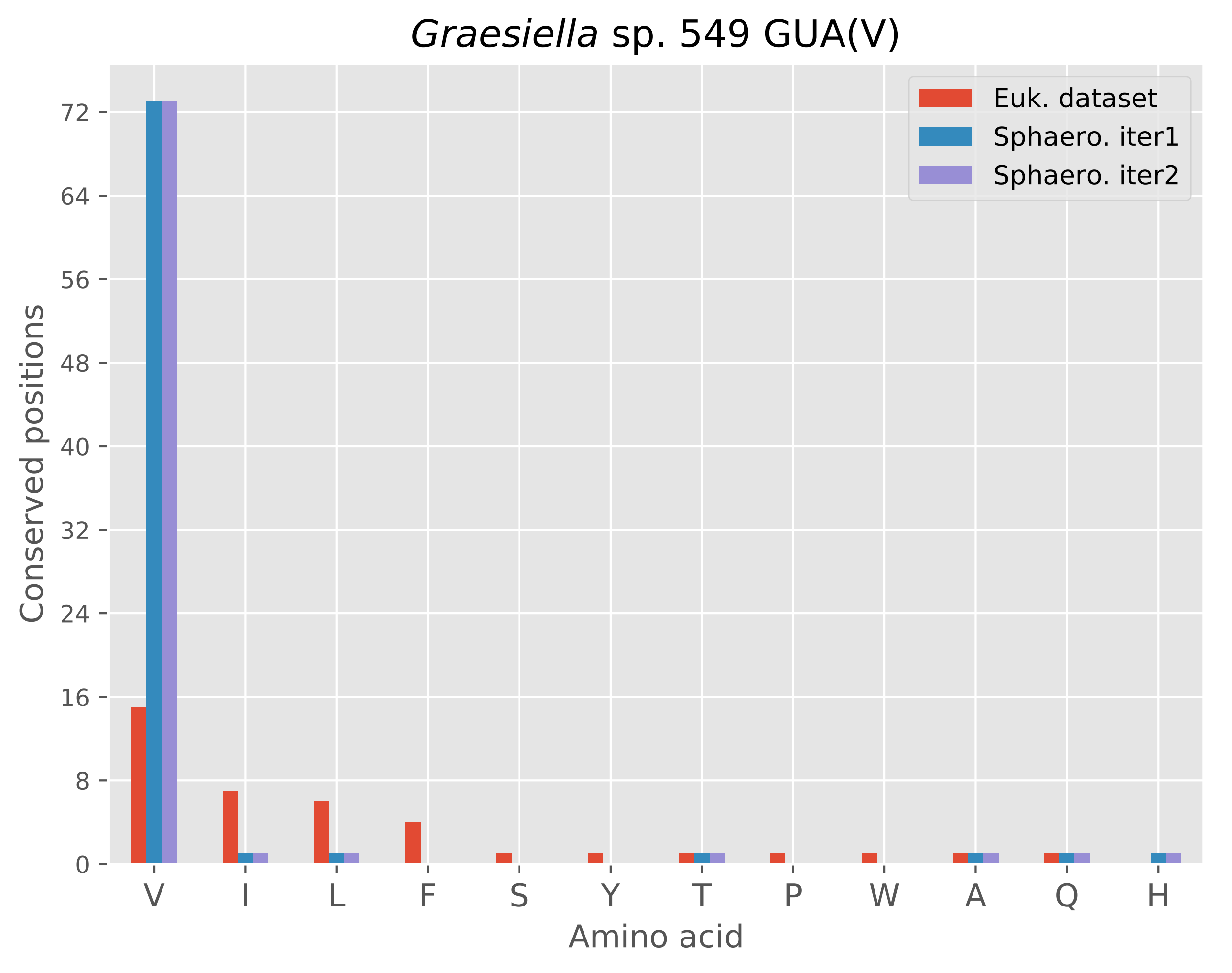

Graesiella sp. 549 GUA(V)
Euk. dataset
Sphaero. iter1
Sphaero. iter2
Conserved positions
0
8
16
24
32
40
48
56
64
72
V
I
L
F
S
Y
T
P
W
A
Q
H
Amino acid

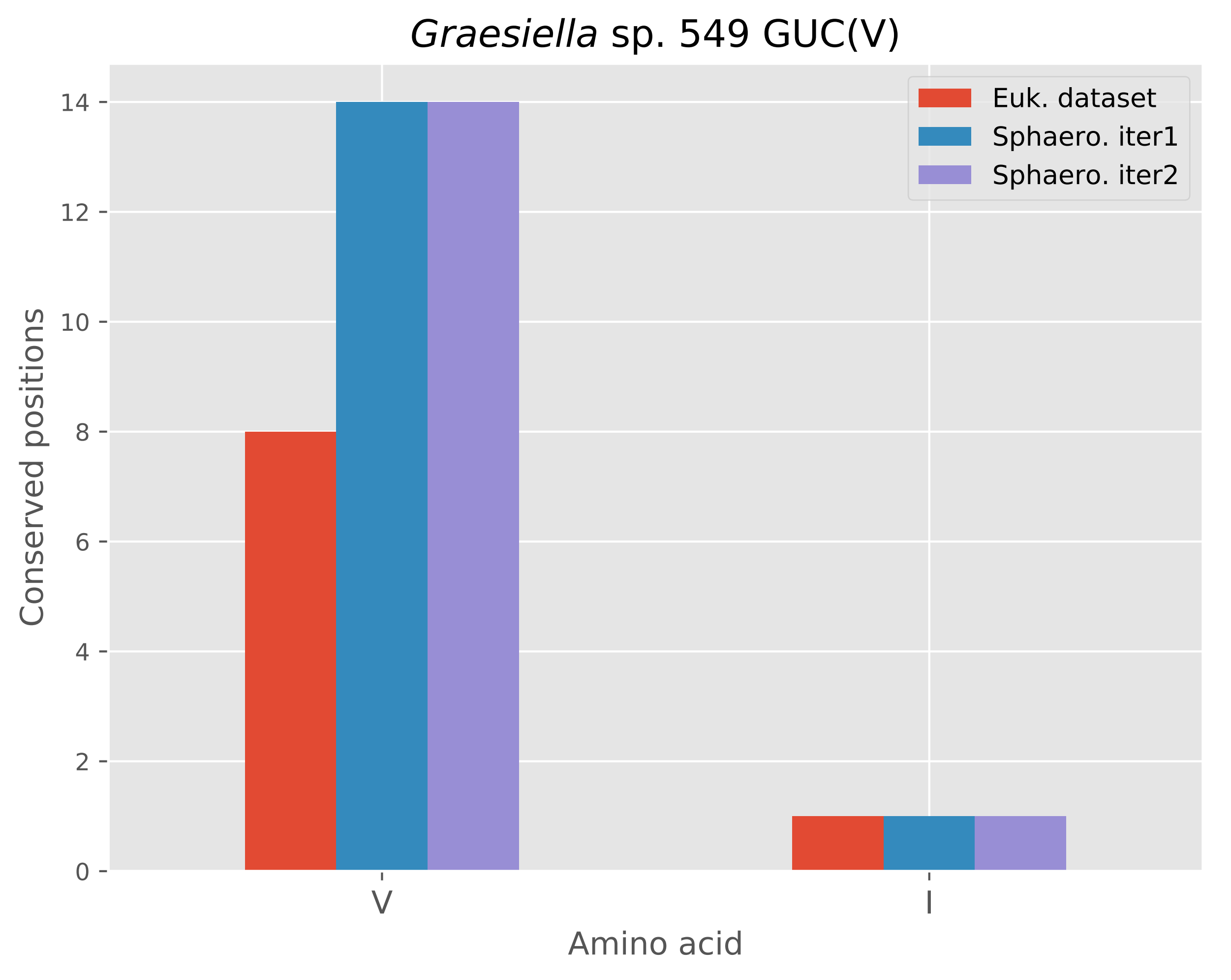

Graesiella sp. 549 GUC(V)
Euk. dataset
Sphaero. iter1
Sphaero. iter2
Conserved positions
0
2
4
6
8
10
12
14
V
I
Amino acid

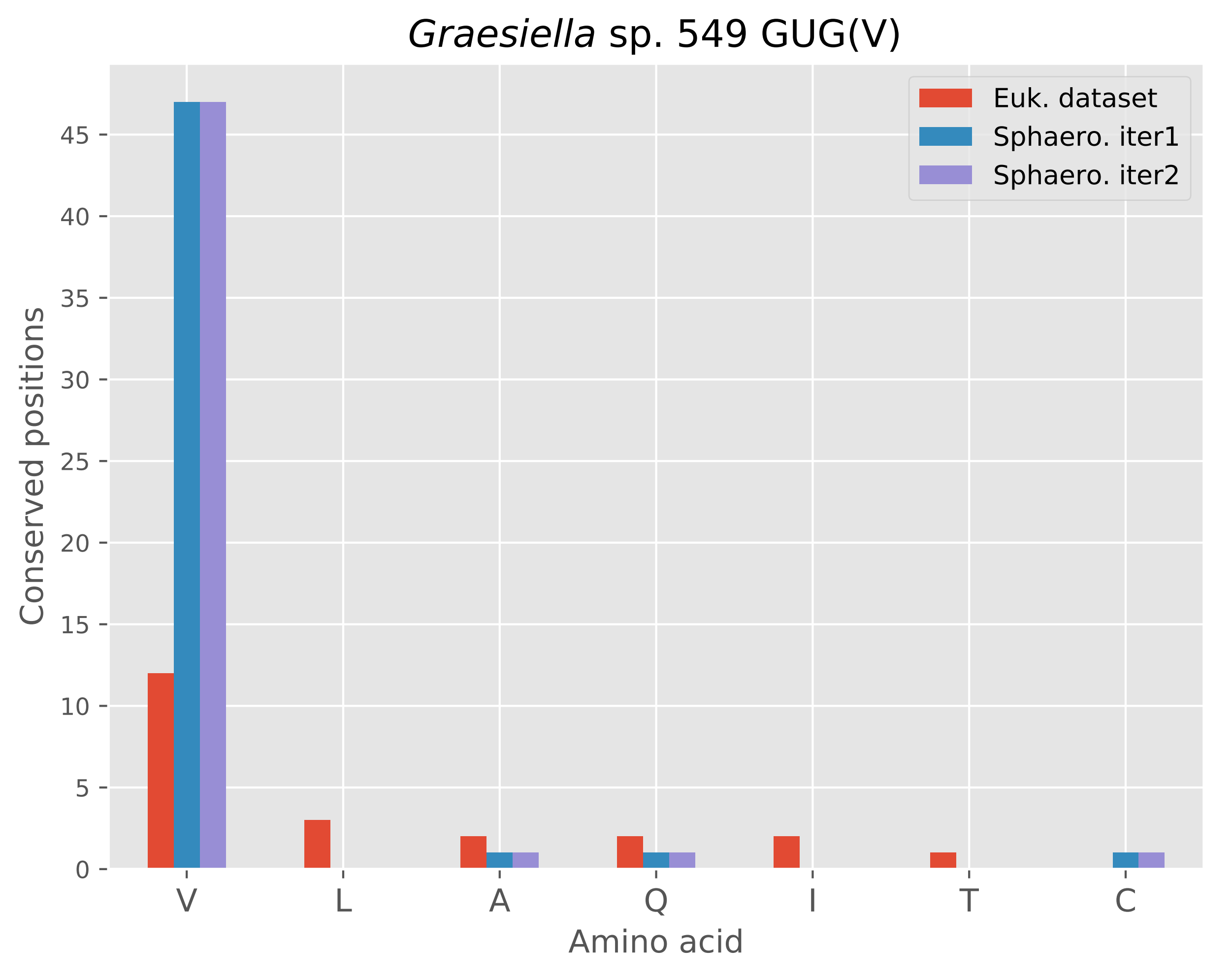

Graesiella sp. 549 GUG(V)
Euk. dataset
Sphaero. iter1
Sphaero. iter2
Conserved positions
0
5
10
15
20
25
30
35
40
45
V
L
A
Q
I
T
C
Amino acid

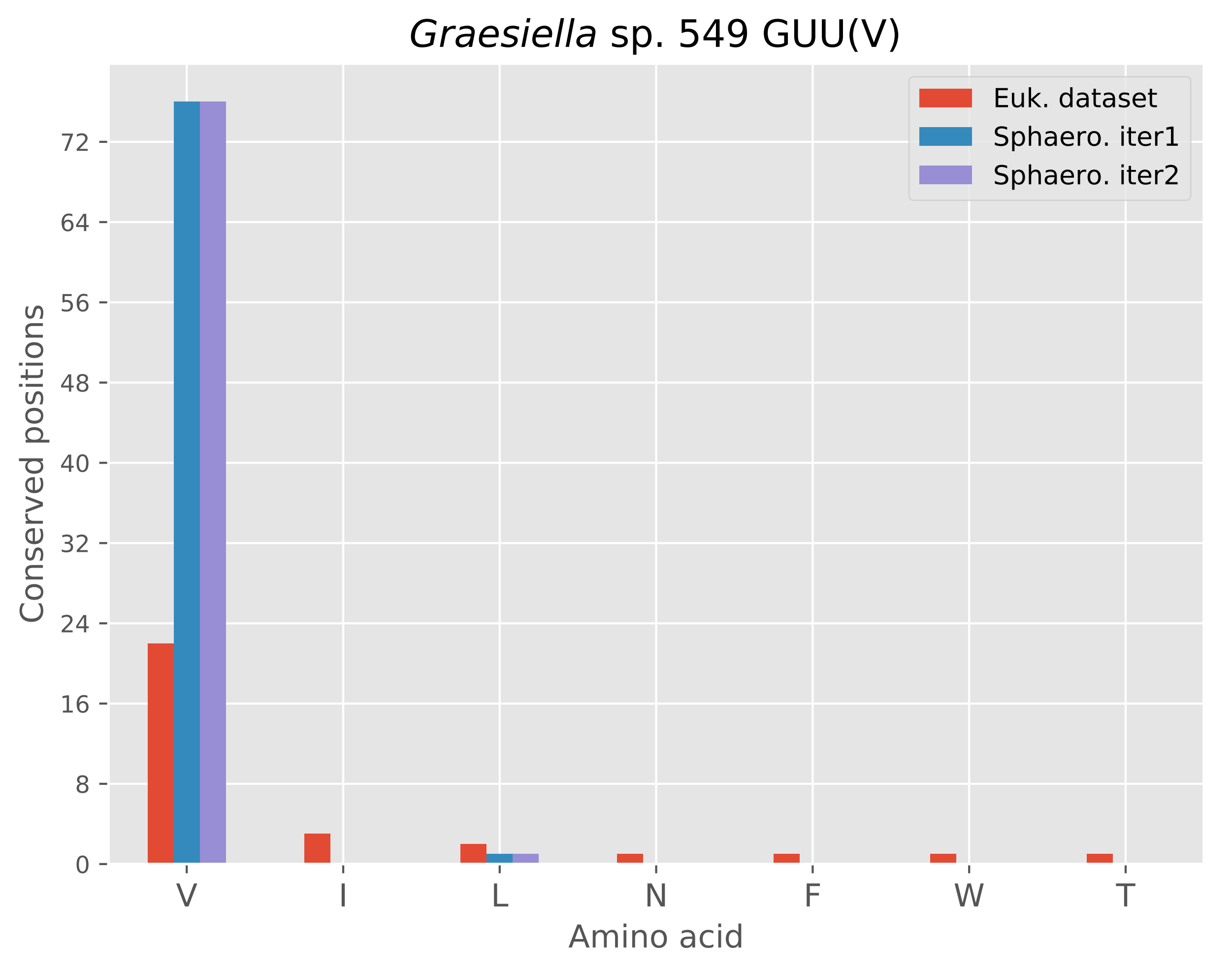
Graesiella sp. 549 GUU(V)
Euk. dataset
Sphaero. iter1
Sphaero. iter2
Conserved positions
0
8
16
24
32
40
48
56
64
72
V
I
L
N
F
W
T
Amino acid

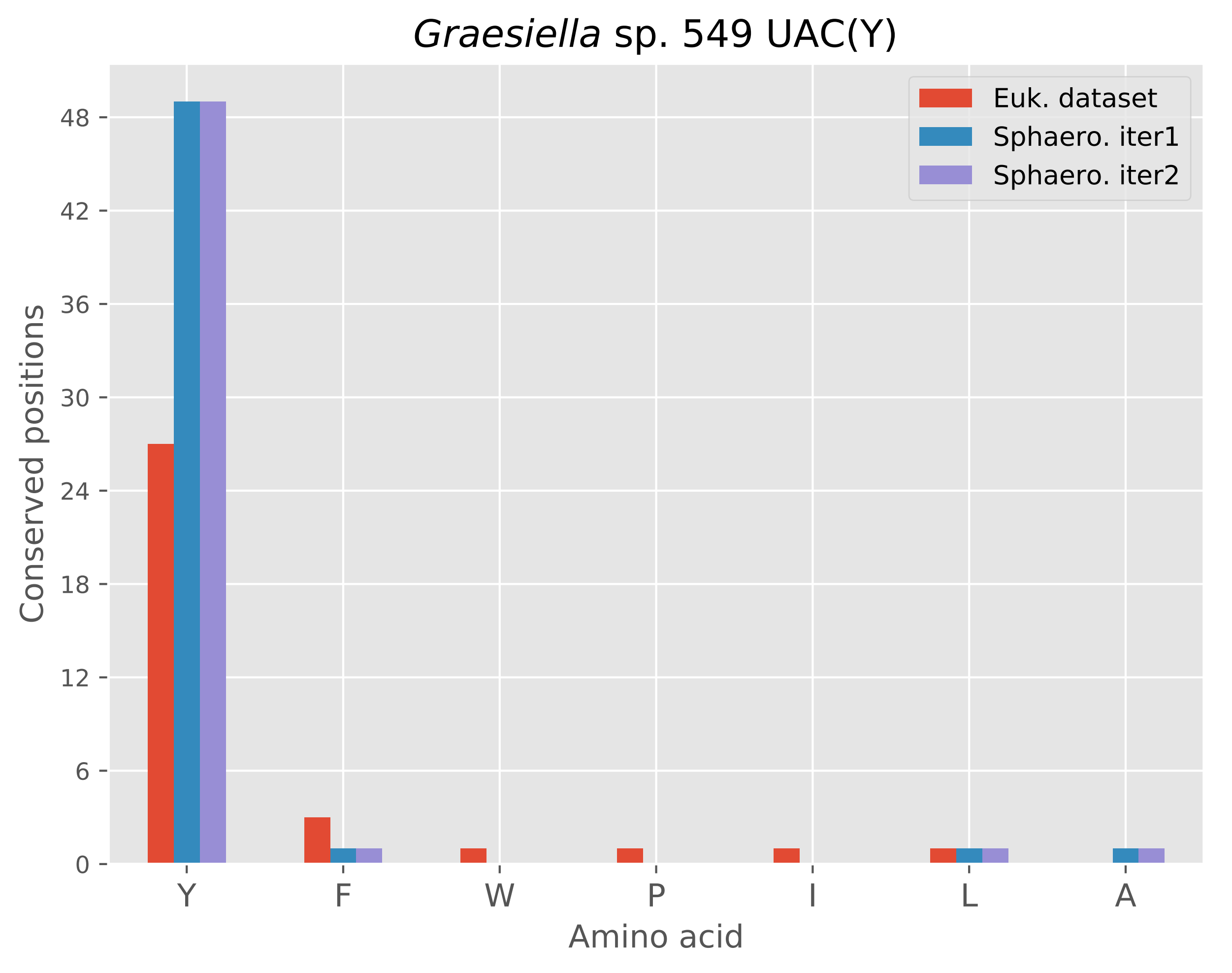

Graesiella sp. 549 UAC(Y)
Euk. dataset
Sphaero. iter1
Sphaero. iter2
Conserved positions
0
6
12
18
24
30
36
42
48
Y
F
W
P
I
L
A
Amino acid

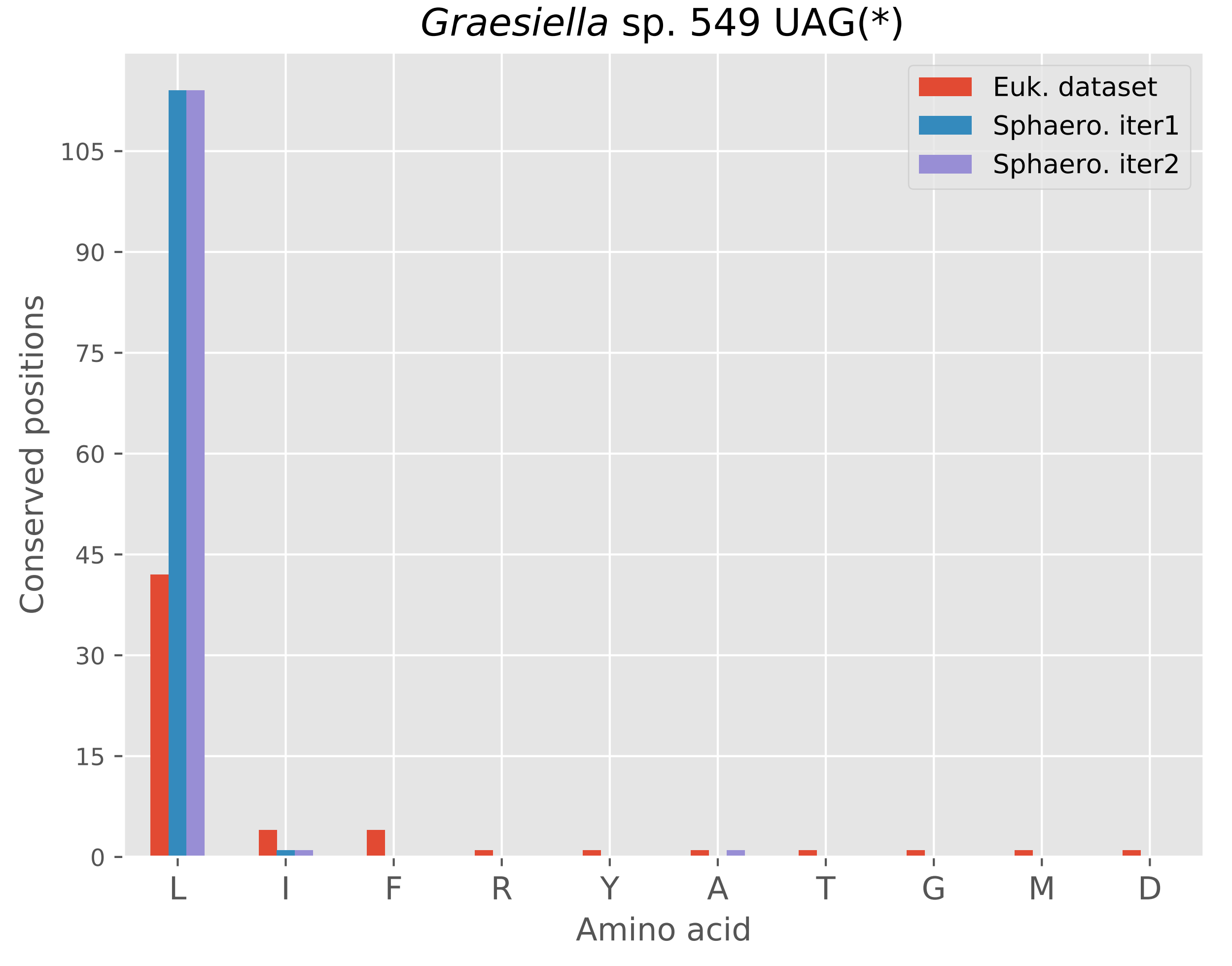

Graesiella sp. 549 UAG(*)
Euk. dataset
Sphaero. iter1
Sphaero. iter2
Conserved positions
0
15
30
45
60
75
90
105
L
I
F
R
Y
A
T
G
M
D
Amino acid

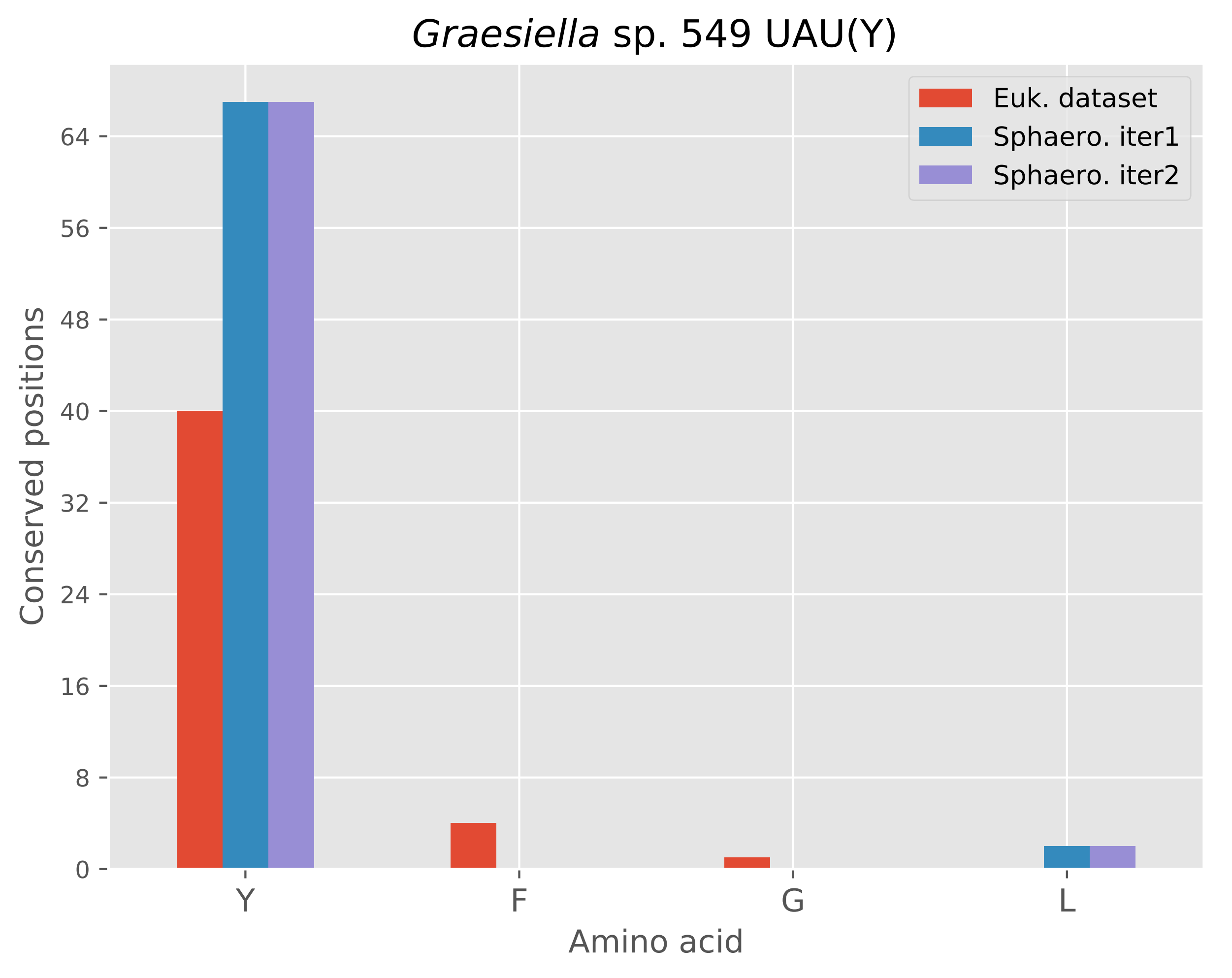

Graesiella sp. 549 UAU(Y)
Euk. dataset
Sphaero. iter1
Sphaero. iter2
Conserved positions
0
8
16
24
32
40
48
56
64
Y
F
G
L
Amino acid

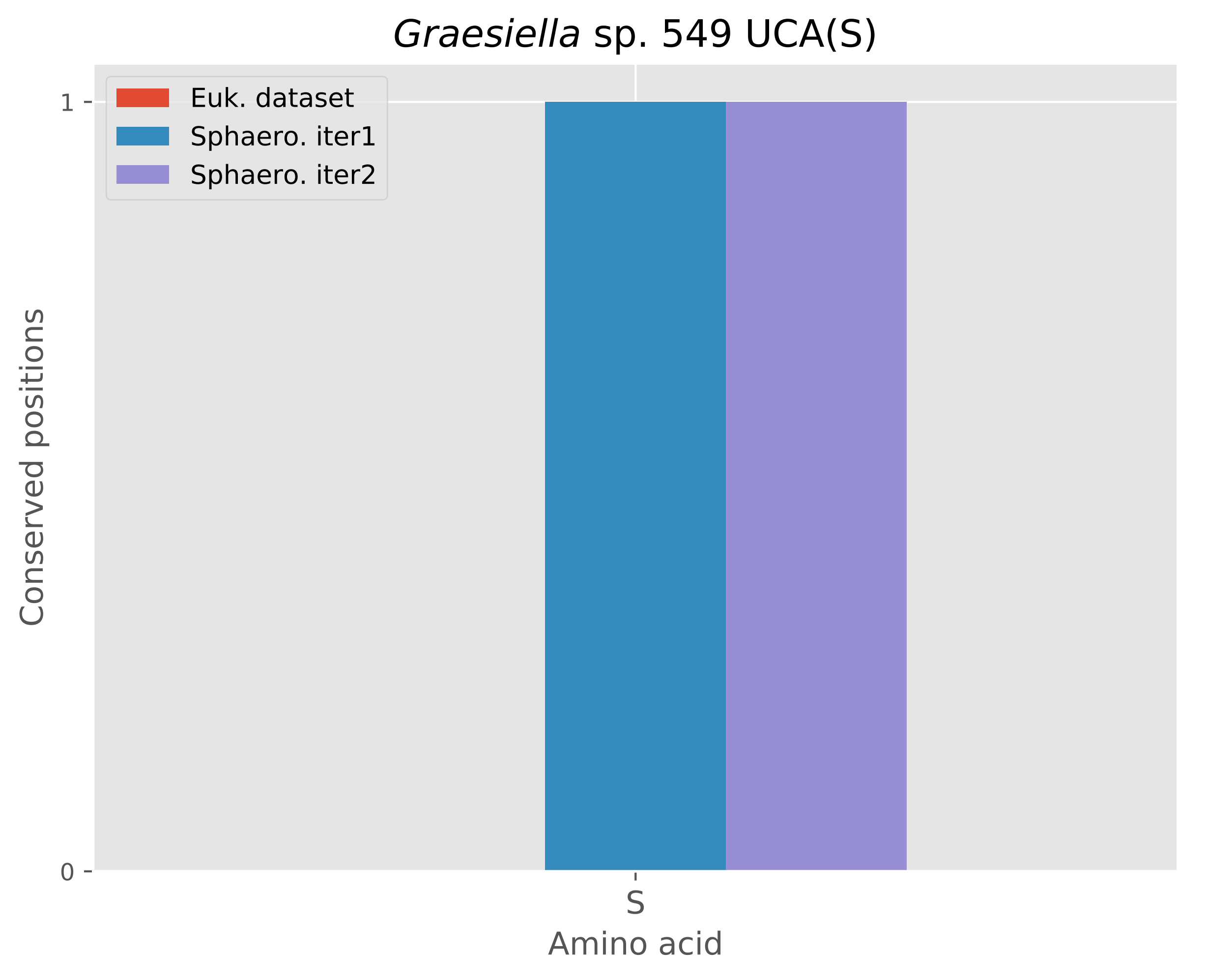
Graesiella sp. 549 UCA(S)
Euk. dataset
Sphaero. iter1
Sphaero. iter2
1
0
Conserved positions
S
Amino acid

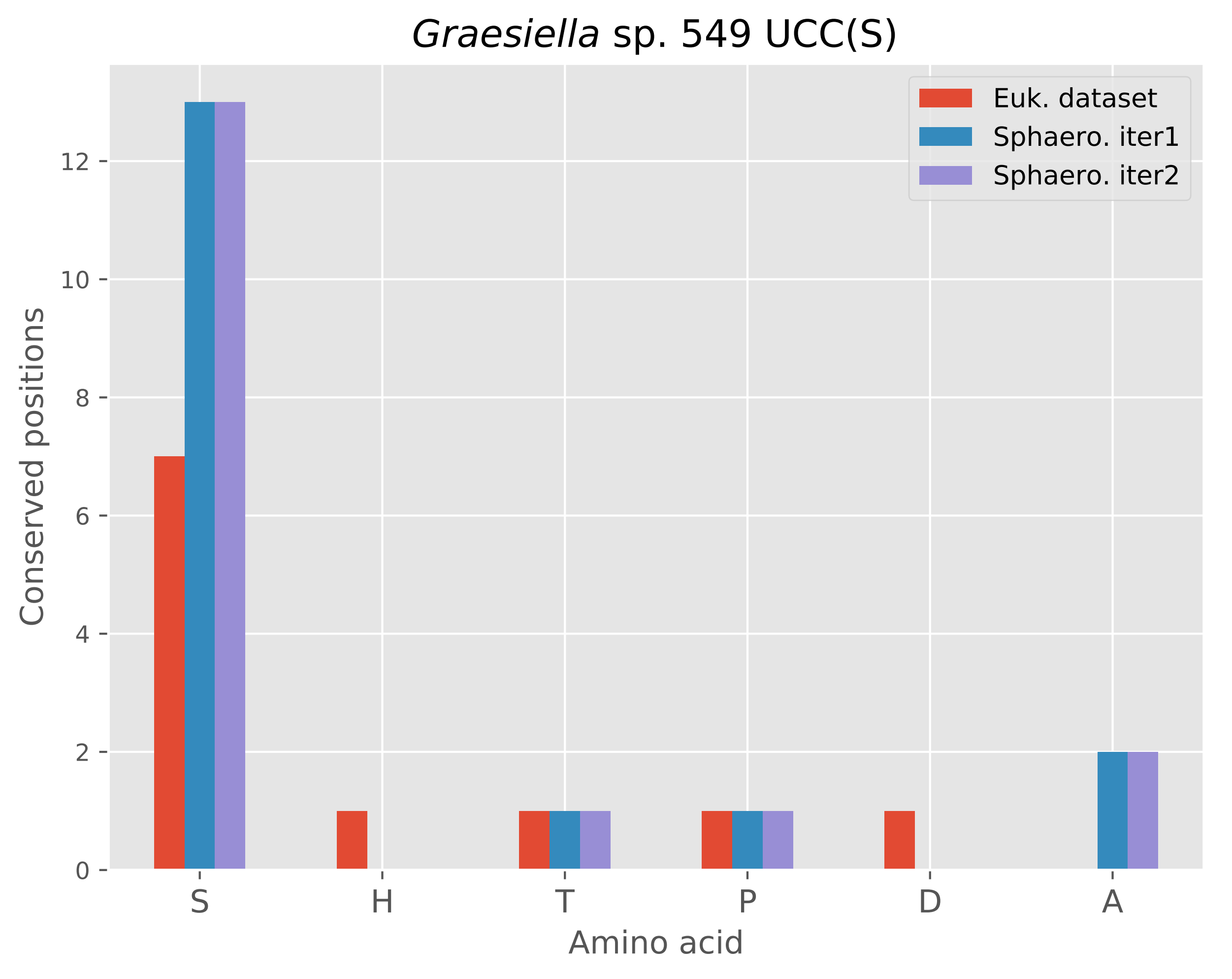

Graesiella sp. 549 UCC(S)
Euk. dataset
Sphaero. iter1
Sphaero. iter2
Conserved positions
0
2
4
6
8
10
12
S
H
T
P
D
A
Amino acid

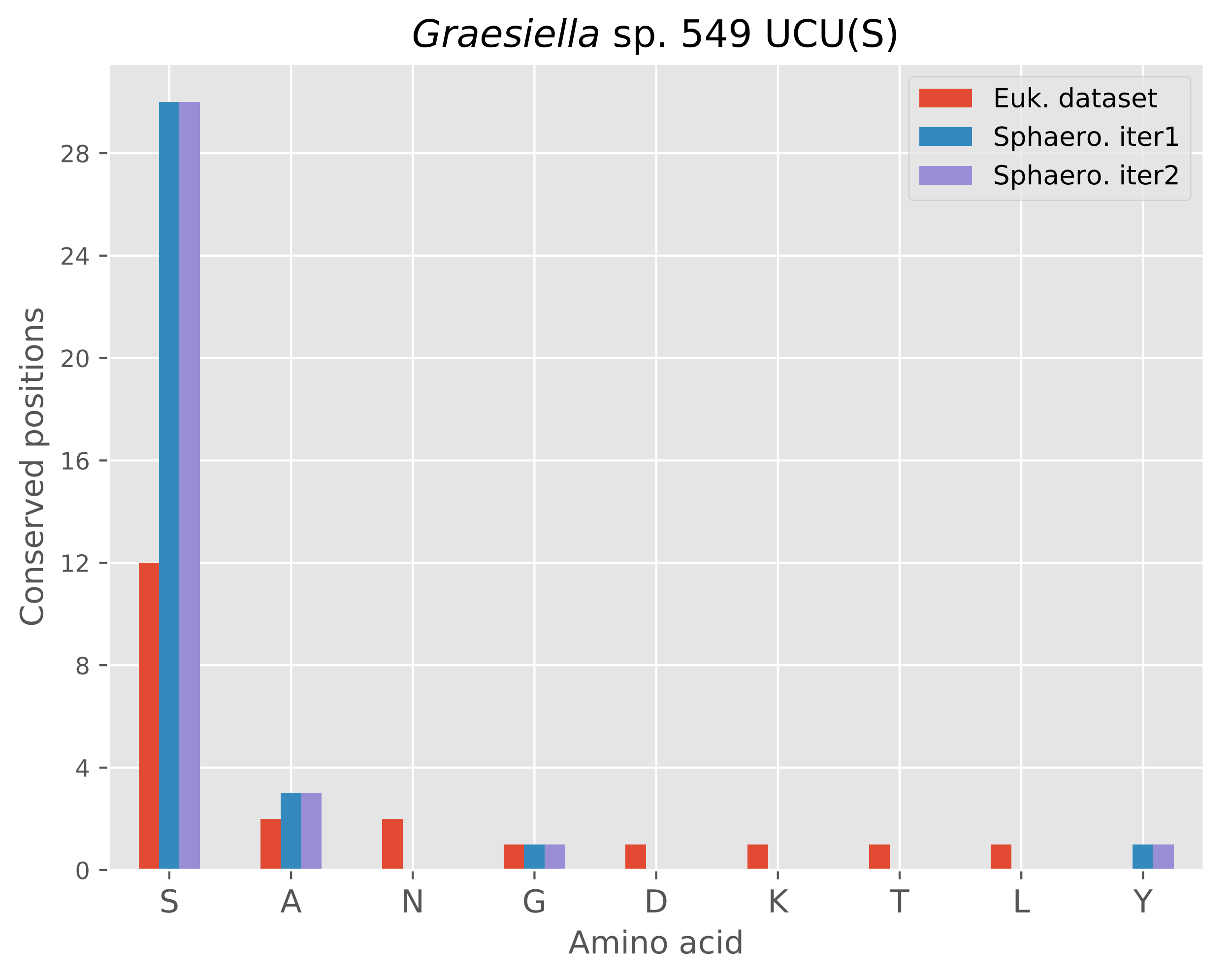

Graesiella sp. 549 UCU(S)
Euk. dataset
Sphaero. iter1
Sphaero. iter2
Conserved positions
0
4
8
12
16
20
24
28
S
A
N
G
D
K
T
L
Y
Amino acid

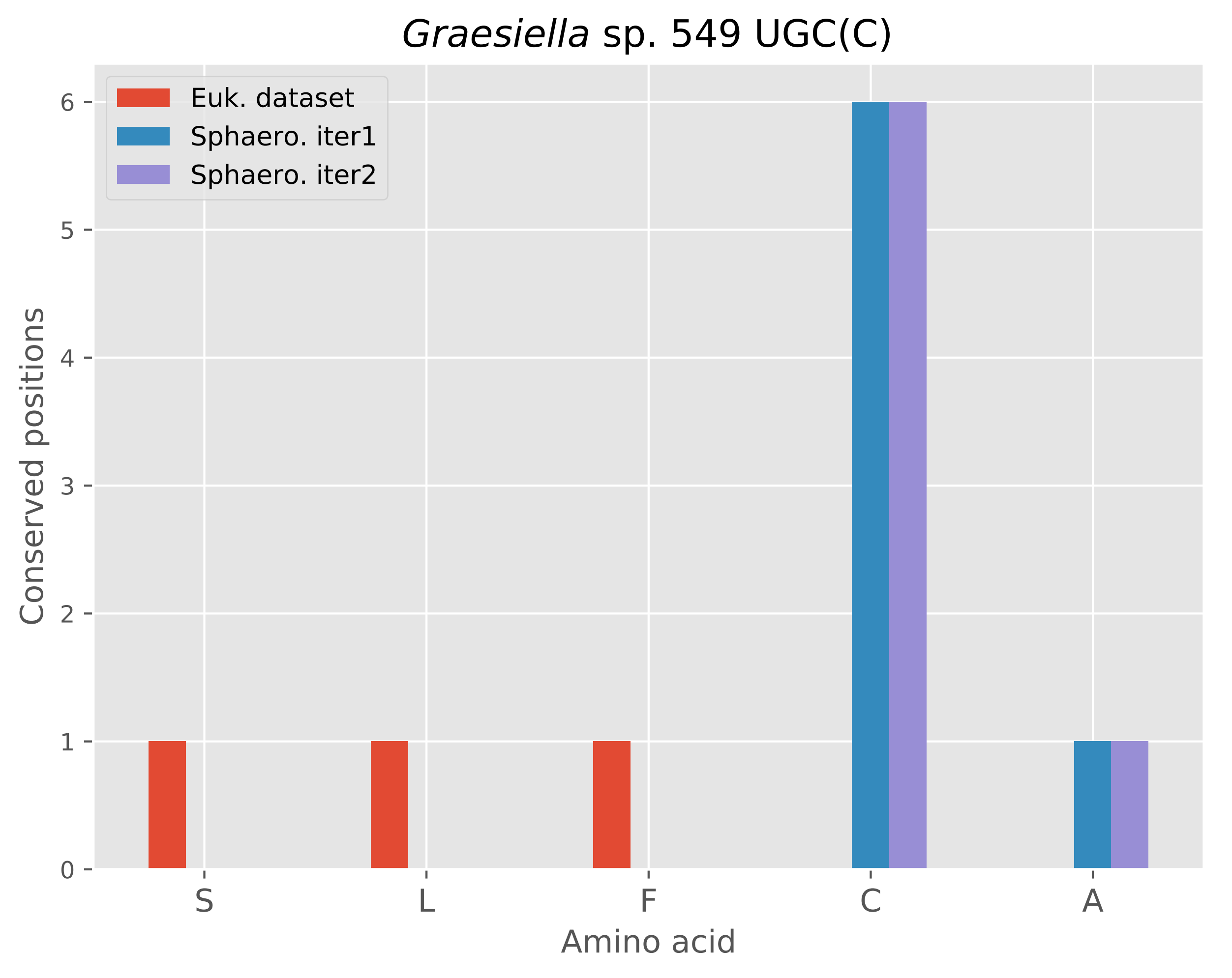

Graesiella sp. 549 UGC(C)
Euk. dataset
Sphaero. iter1
Sphaero. iter2
Conserved positions
0
1
2
3
4
5
6
S
L
F
C
A
Amino acid

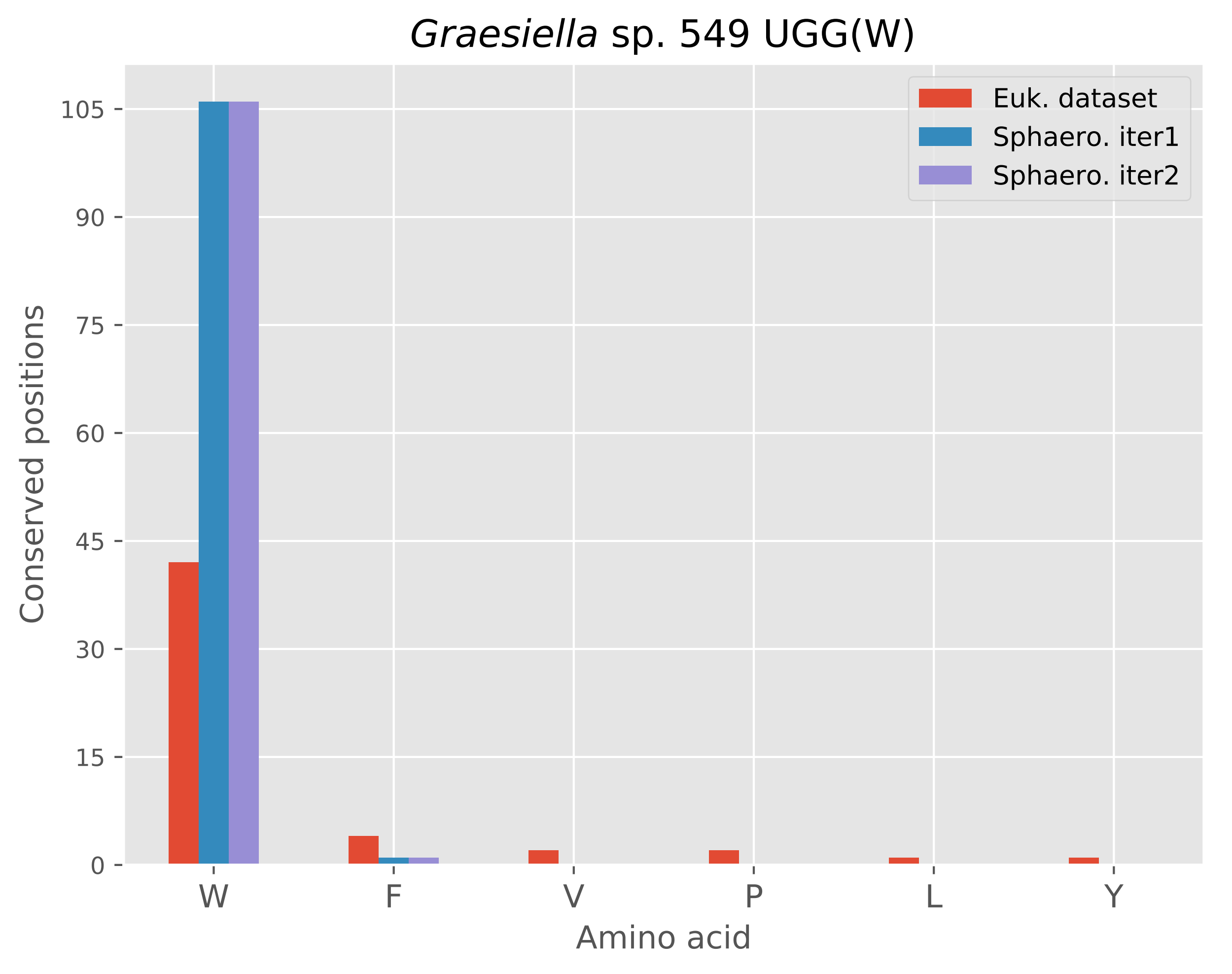

Graesiella sp. 549 UGG(W)
Euk. dataset
Sphaero. iter1
Sphaero. iter2
Conserved positions
105
90
75
60
45
30
15
0
W
F
V
P
L
Y
Amino acid

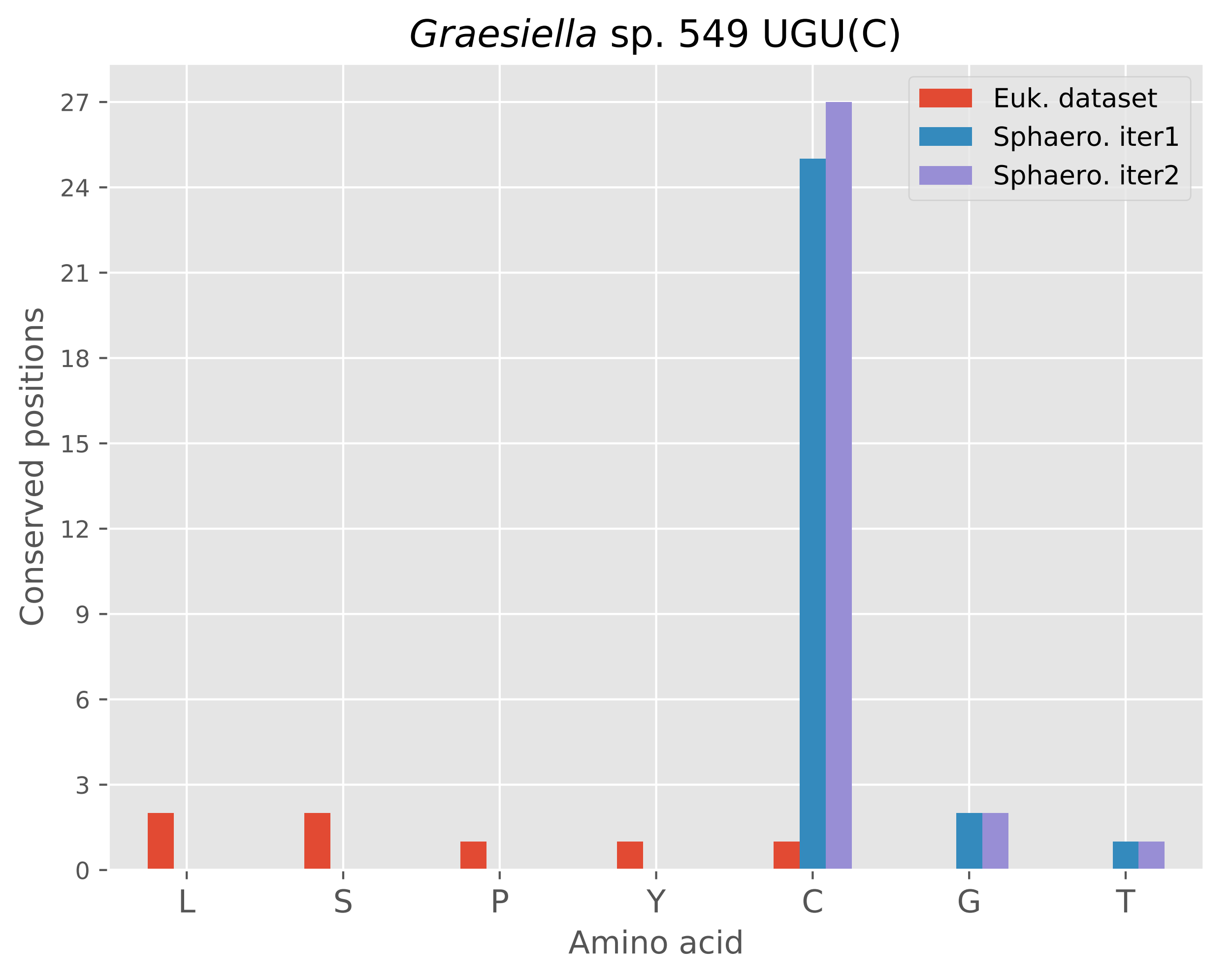

Graesiella sp. 549 UGU(C)
Euk. dataset
Sphaero. iter1
Sphaero. iter2
Conserved positions
0
3
6
9
12
15
18
21
24
27
L
S
P
Y
C
G
T
Amino acid

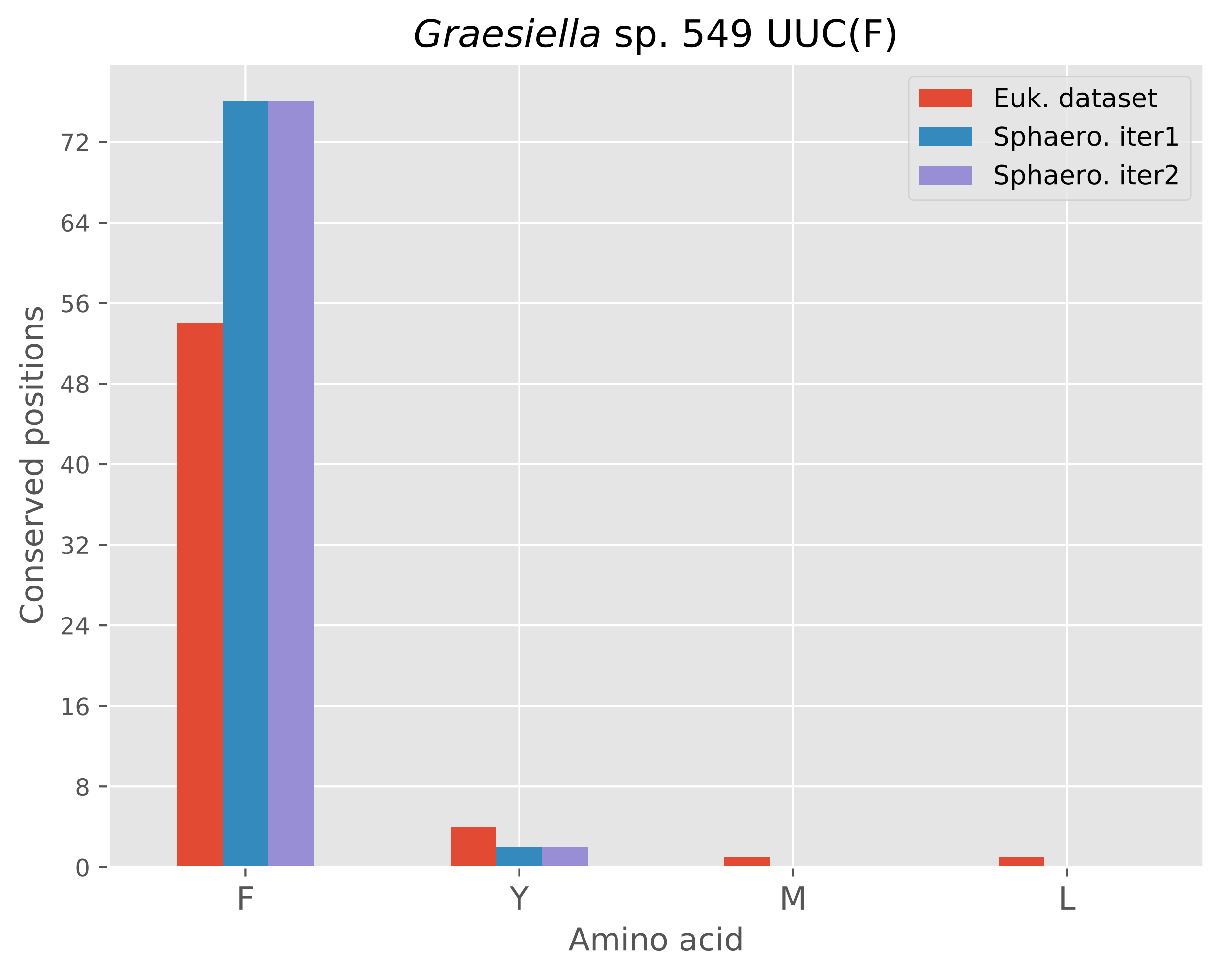

Graesiella sp. 549 UUC(F)
Euk. dataset
Sphaero. iter1
Sphaero. iter2
Conserved positions
0
8
16
24
32
40
48
56
64
72
F
Y
M
L
Amino acid

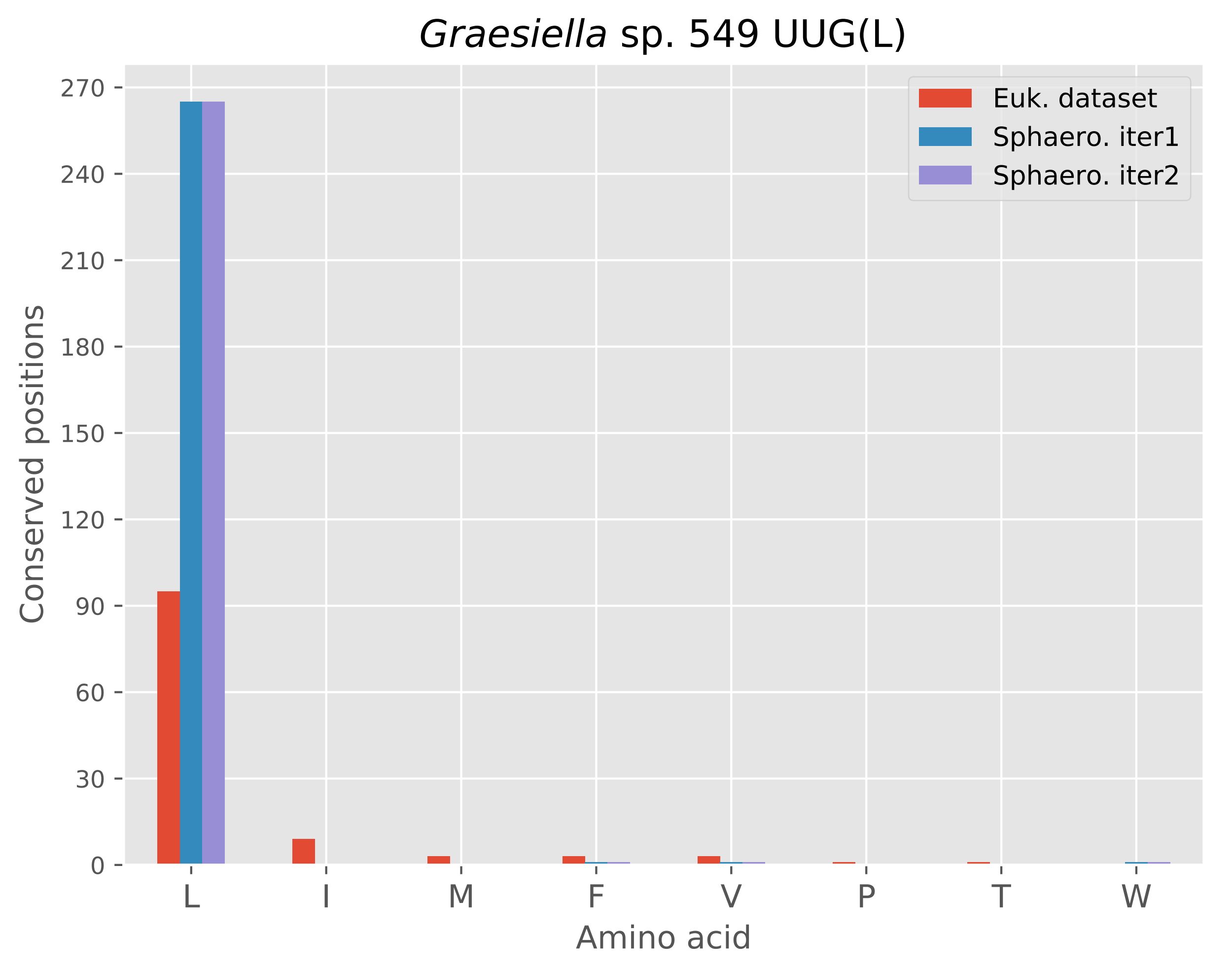

Graesiella sp. 549 UUG(L)
Euk. dataset
Sphaero. iter1
Sphaero. iter2
Conserved positions
0
30
60
90
120
150
180
210
240
270
L
I
M
F
V
P
T
W
Amino acid

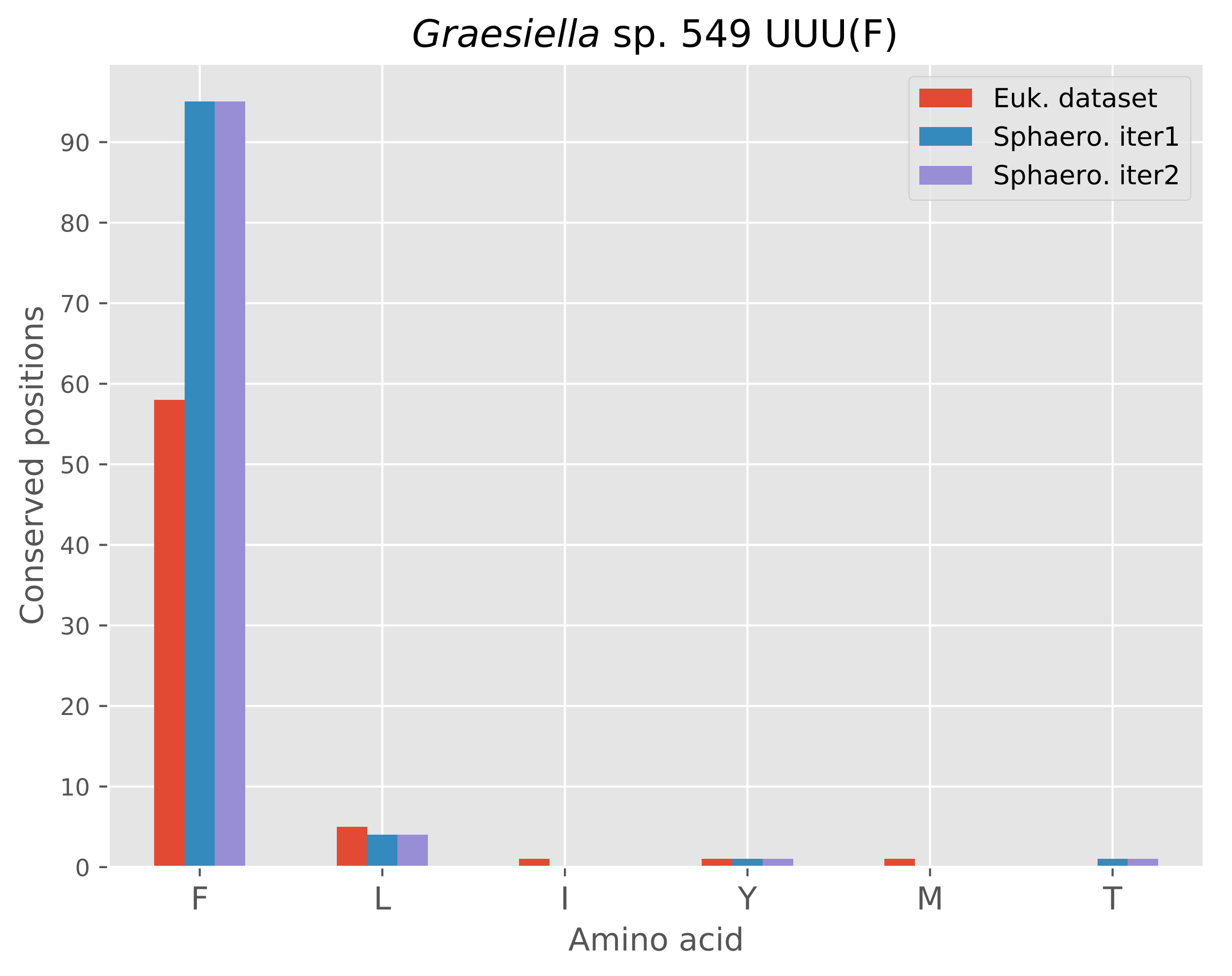

Graesiella sp. 549 UUU(F)
Euk. dataset
Sphaero. iter1
Sphaero. iter2
Conserved positions
0
10
20
30
40
50
60
70
80
90
F
L
I
Y
M
T
Amino acid

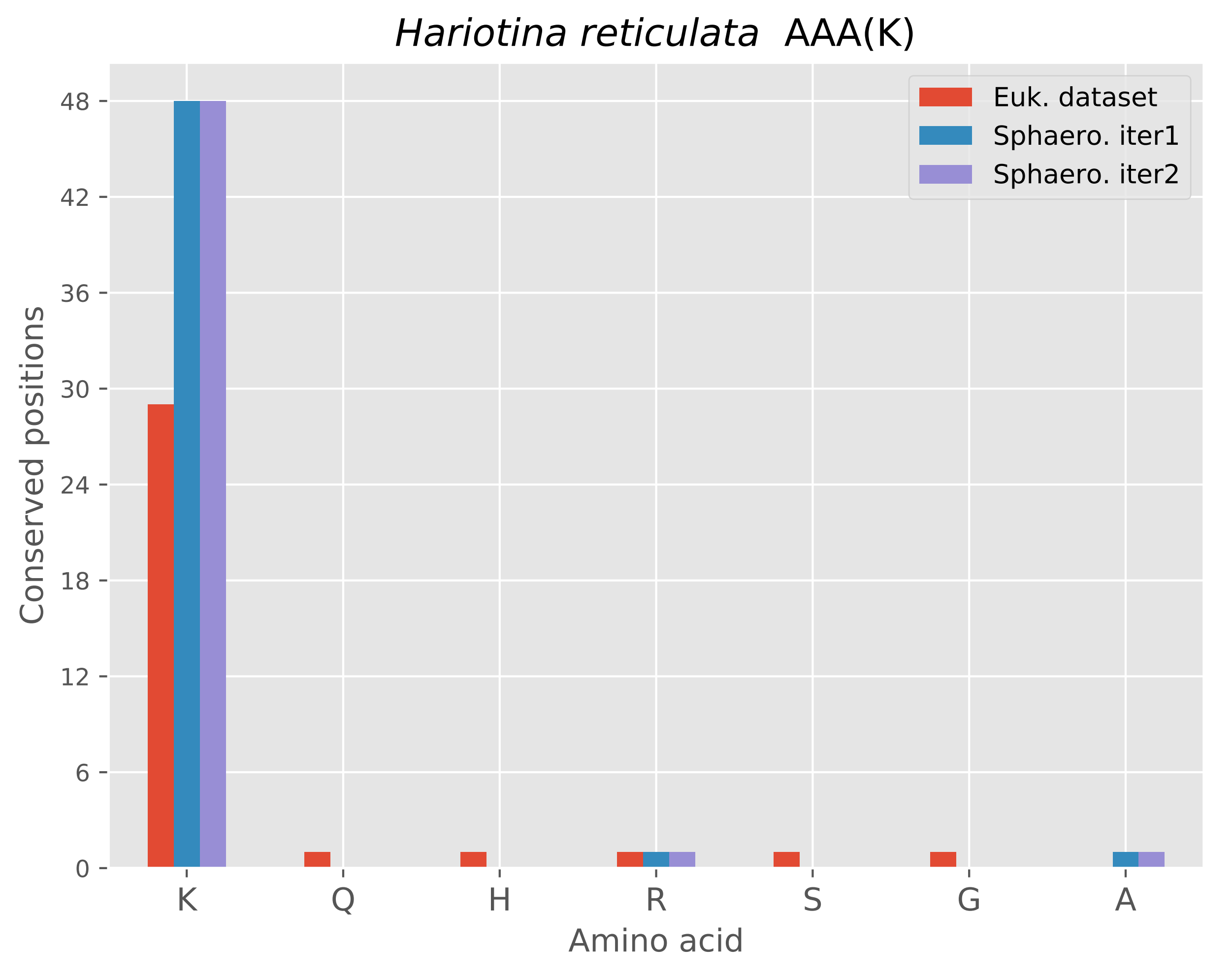

Hariotina reticulata AAA(K)
Euk. dataset
Sphaero. iter1
Sphaero. iter2
Conserved positions
0
6
12
18
24
30
36
42
48
K
Q
H
R
S
G
A
Amino acid

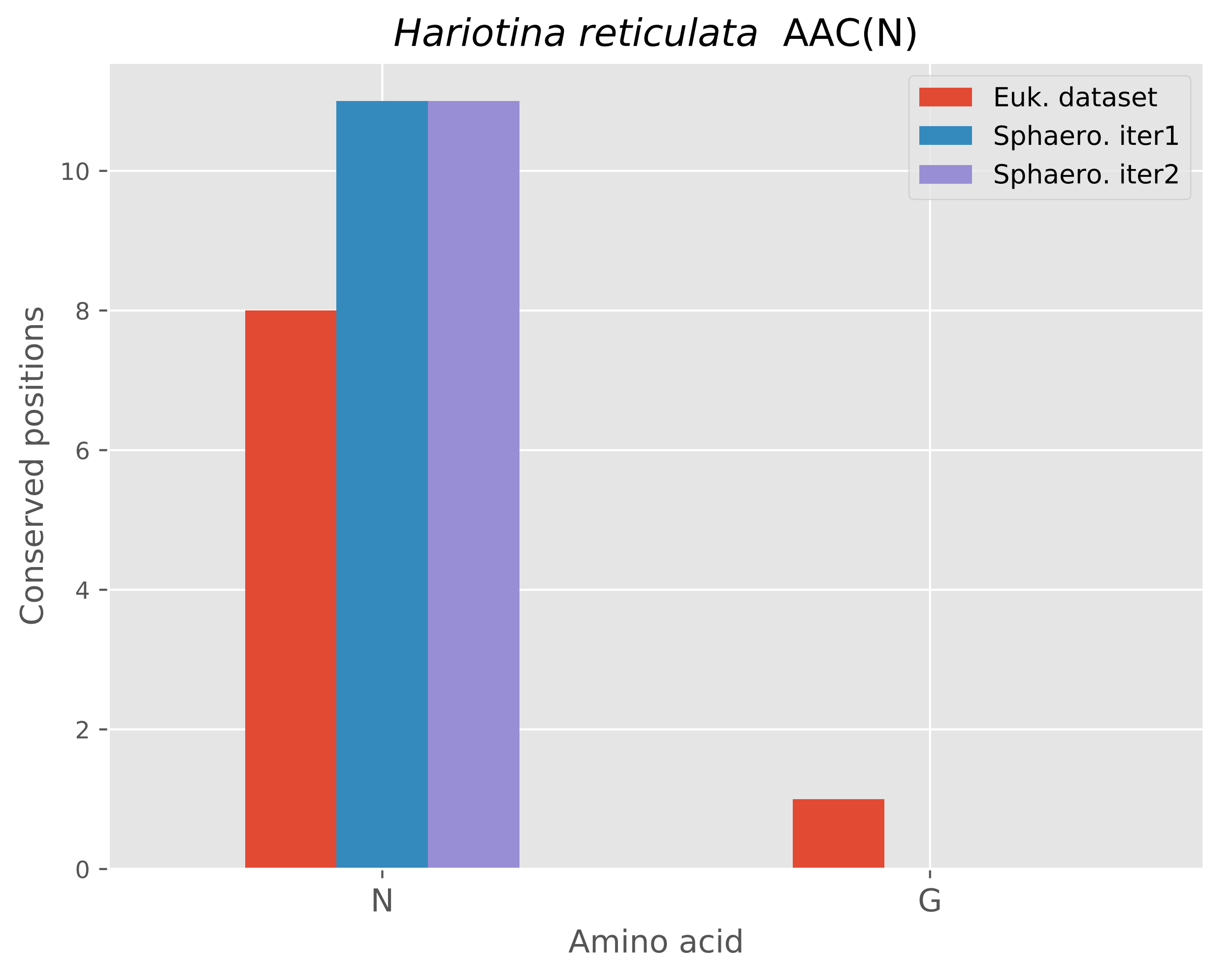

Hariotina reticulata AAC(N)
Euk. dataset
Sphaero. iter1
Sphaero. iter2
Conserved positions
0
2
4
6
8
10
N
G
Amino acid

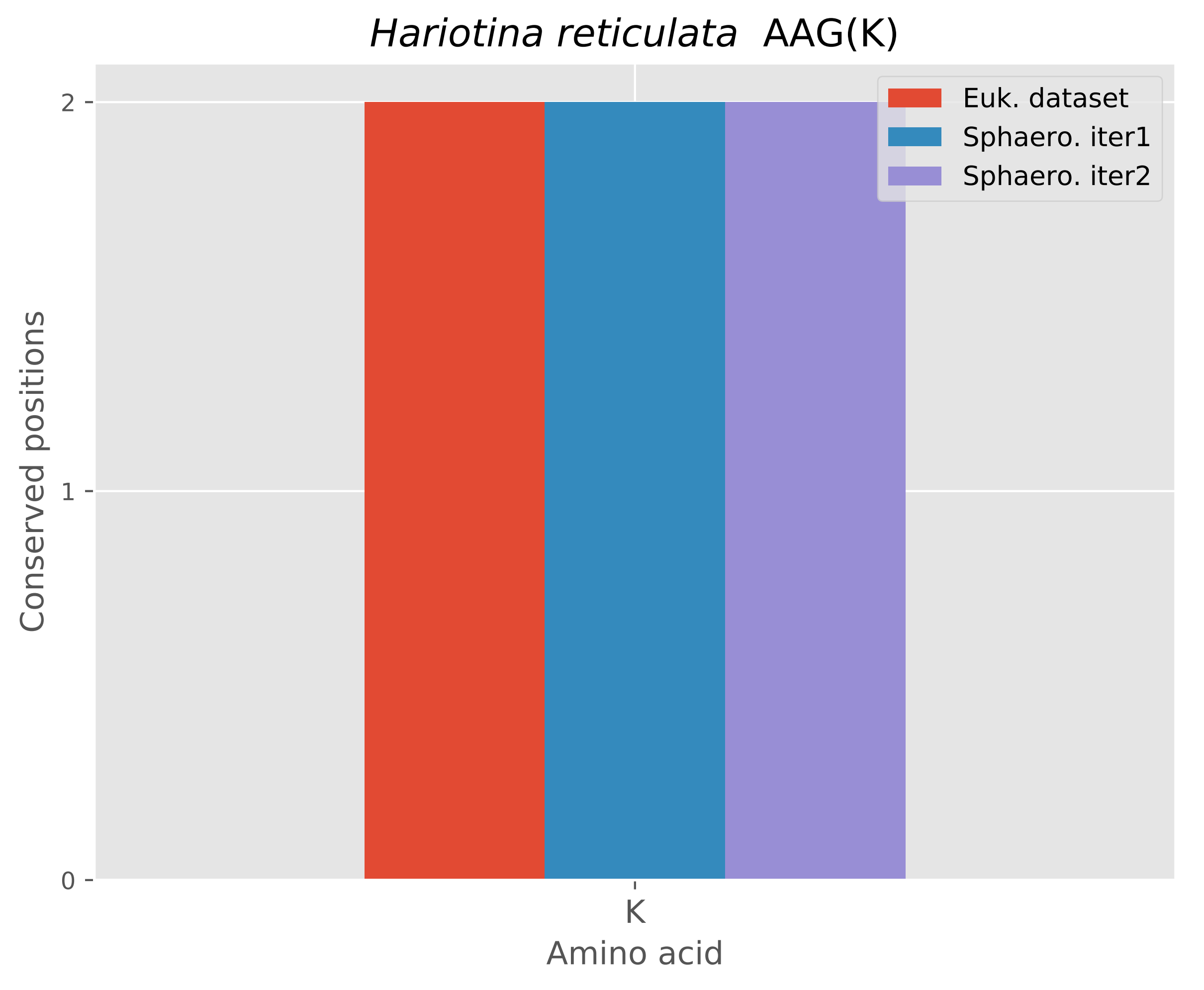
Hariotina reticulata AAG(K)
Euk. dataset
Sphaero. iter1
Sphaero. iter2
Conserved positions
0
1
2
K
Amino acid

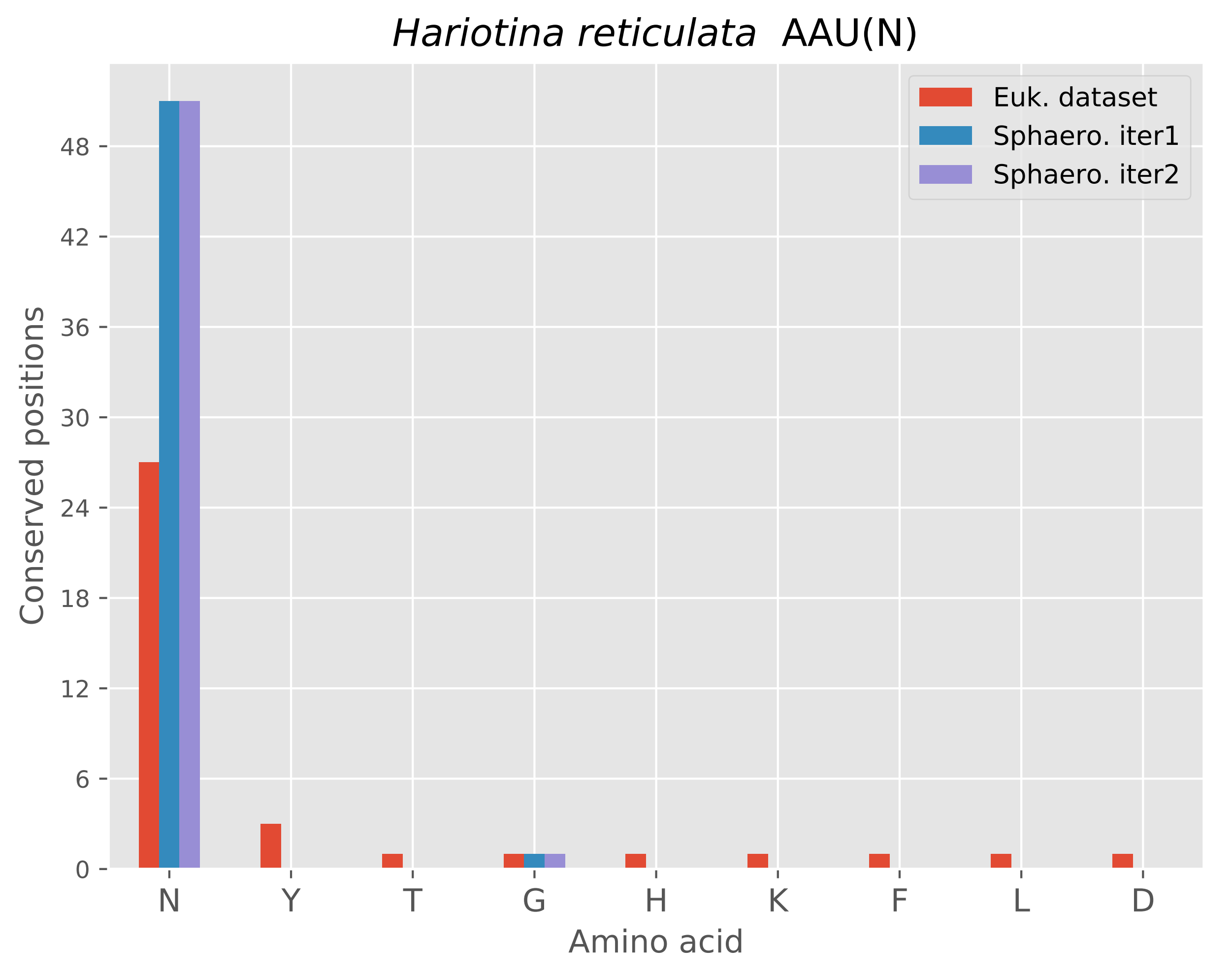

Hariotina reticulata AAU(N)
Euk. dataset
Sphaero. iter1
Sphaero. iter2
Conserved positions
0
6
12
18
24
30
36
42
48
N
Y
T
G
H
K
F
L
D
Amino acid

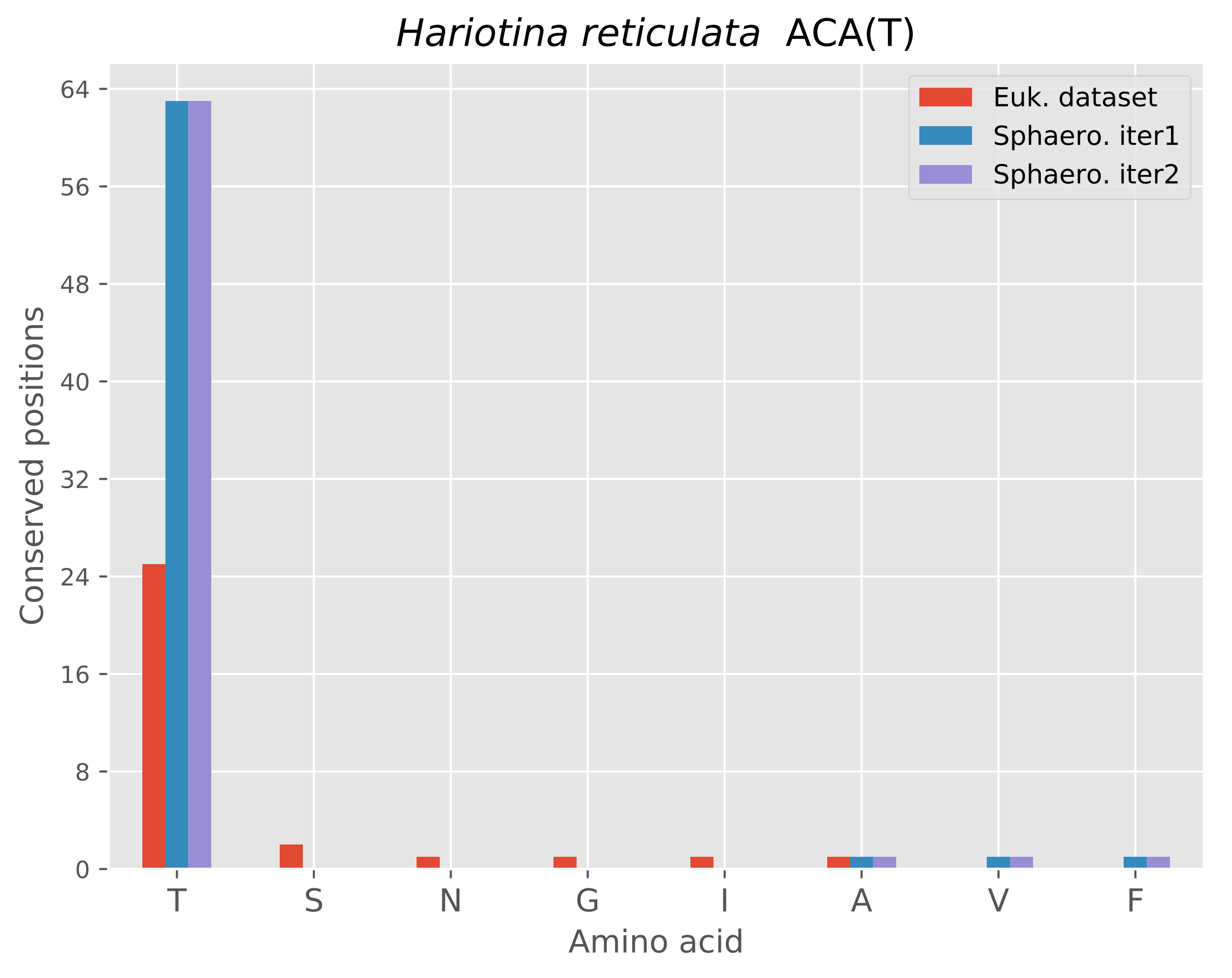

Hariotina reticulata ACA(T)
Euk. dataset
Sphaero. iter1
Sphaero. iter2
Conserved positions
0
8
16
24
32
40
48
56
64
T
S
N
G
I
A
V
F
Amino acid

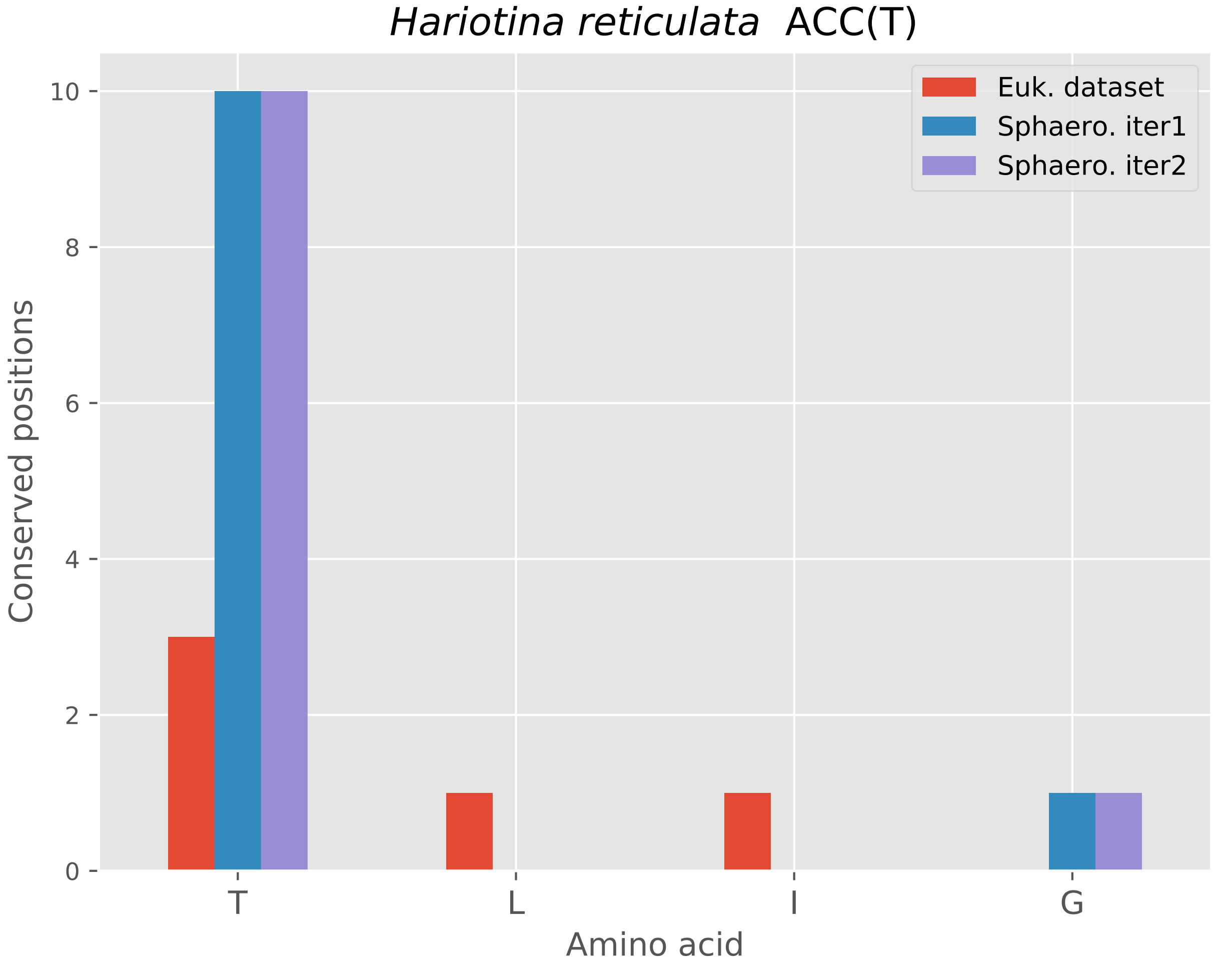

Hariotina reticulata ACC(T)
Conserved positions
Amino acid
Euk. dataset
Sphaero. iter1
Sphaero. iter2
0
2
4
6
8
10
T
L
I
G

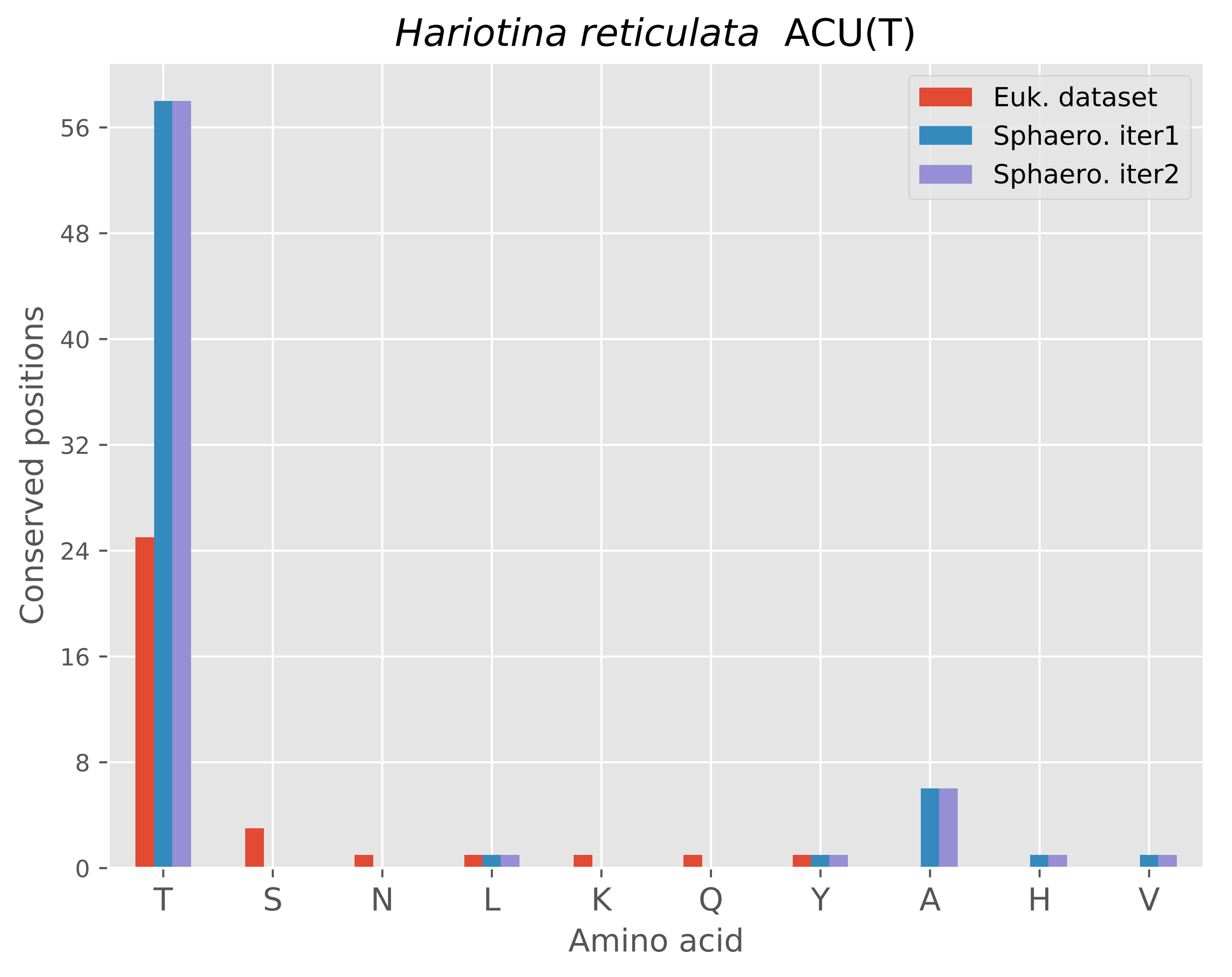

Hariotina reticulata ACU(T)
Euk. dataset
Sphaero. iter1
Sphaero. iter2
Conserved positions
0
8
16
24
32
40
48
56
T
S
N
L
K
Q
Y
A
H
V
Amino acid

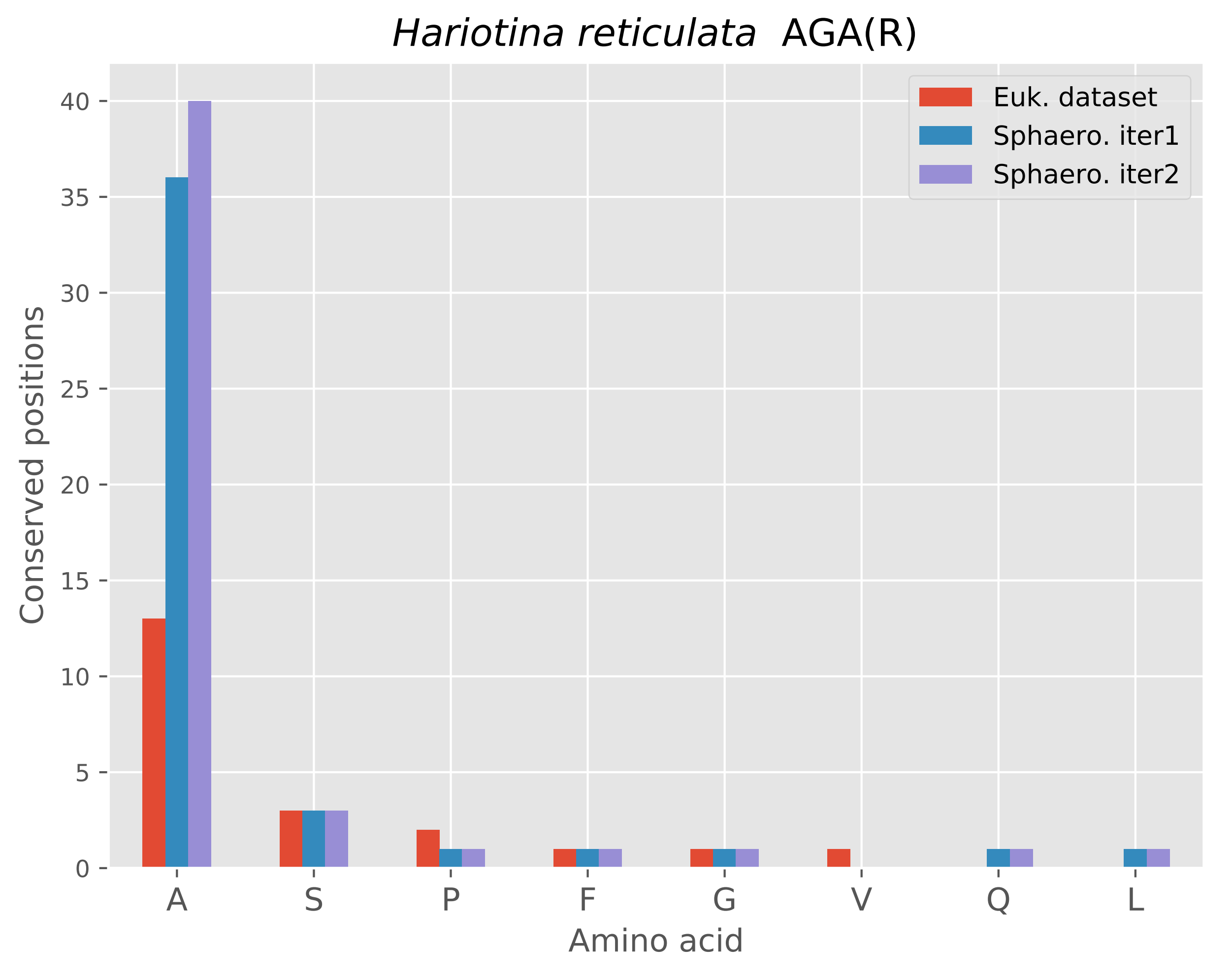

Hariotina reticulata AGA(R)
Euk. dataset
Sphaero. iter1
Sphaero. iter2
Conserved positions
0
5
10
15
20
25
30
35
40
A
S
P
F
G
V
Q
L
Amino acid

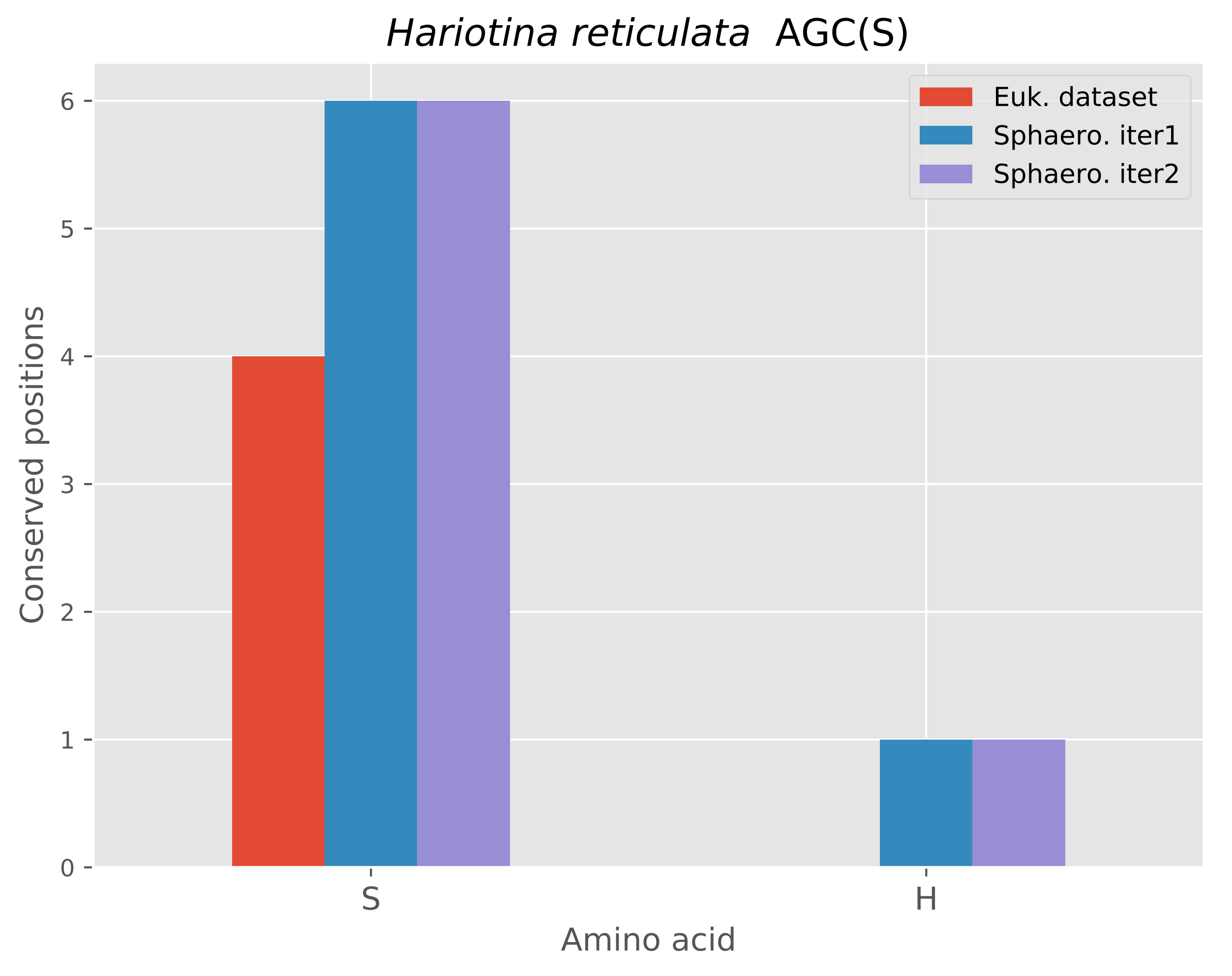

Hariotina reticulata AGC(S)
Euk. dataset
Sphaero. iter1
Sphaero. iter2
Conserved positions
0
1
2
3
4
5
6
S
H
Amino acid

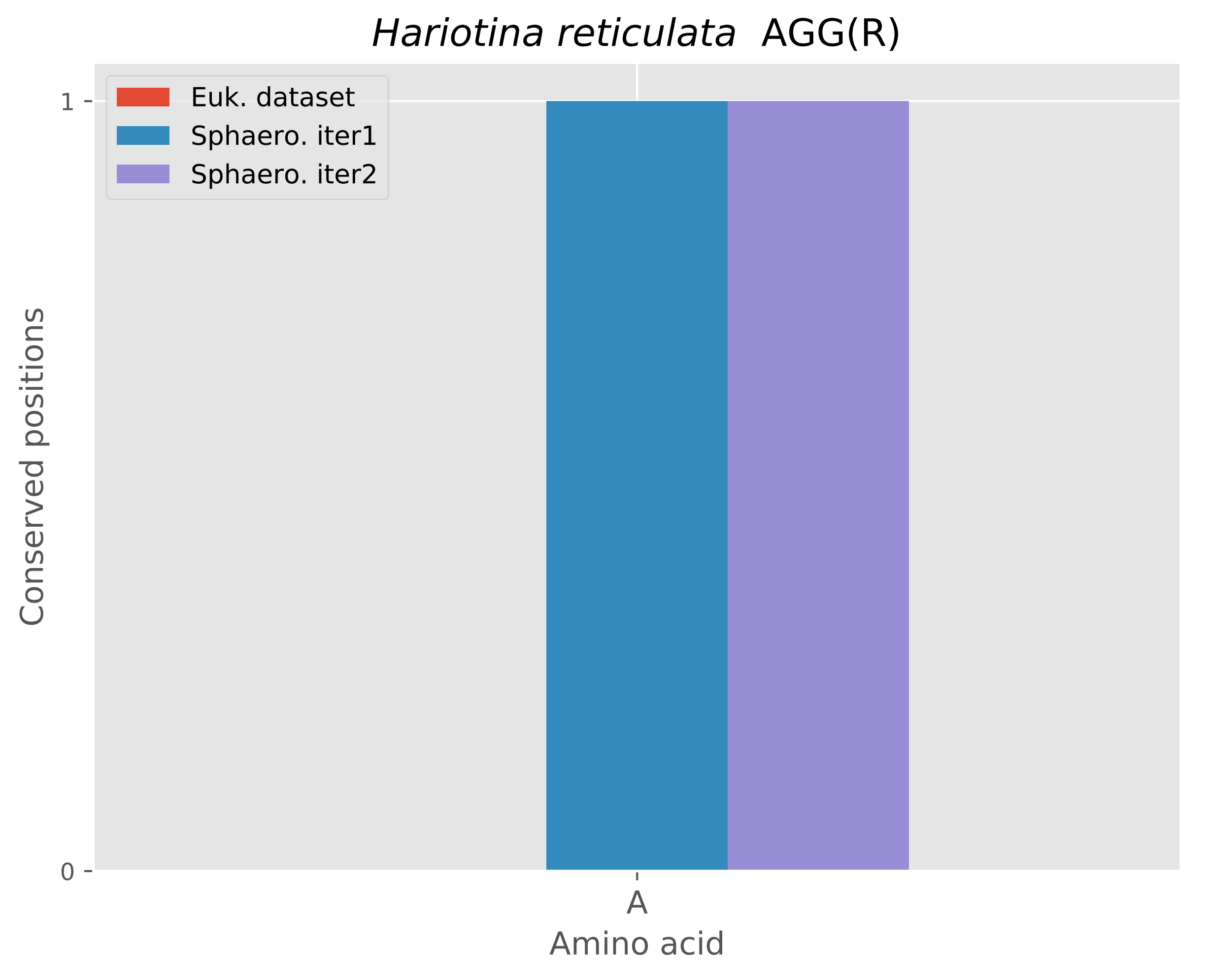

Hariotina reticulata AGG(R)
Euk. dataset
Sphaero. iter1
Sphaero. iter2
Conserved positions
1
0
A
Amino acid

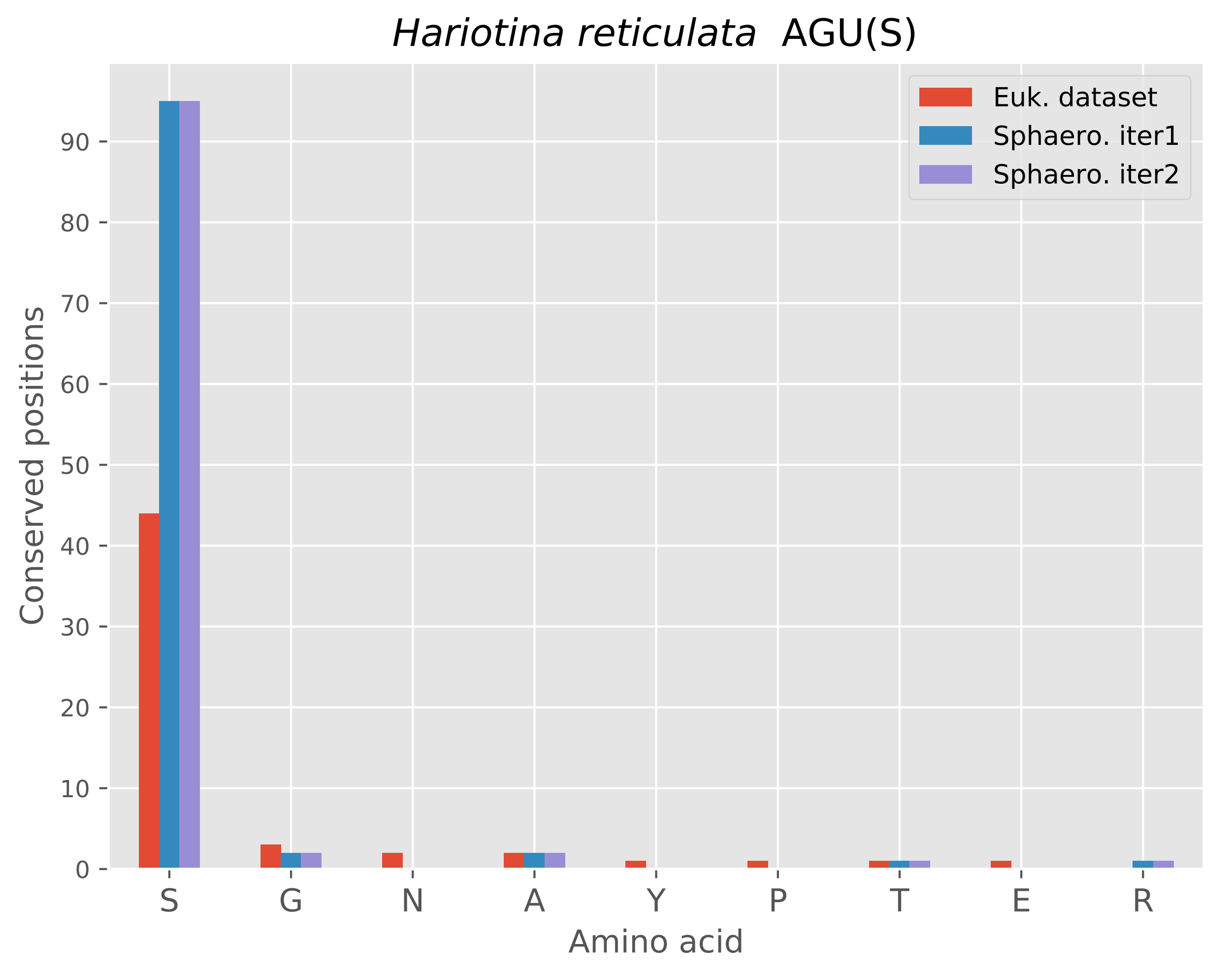

Hariotina reticulata AGU(S)
Euk. dataset
Sphaero. iter1
Sphaero. iter2
Conserved positions
0
10
20
30
40
50
60
70
80
90
S
G
N
A
Y
P
T
E
R
Amino acid

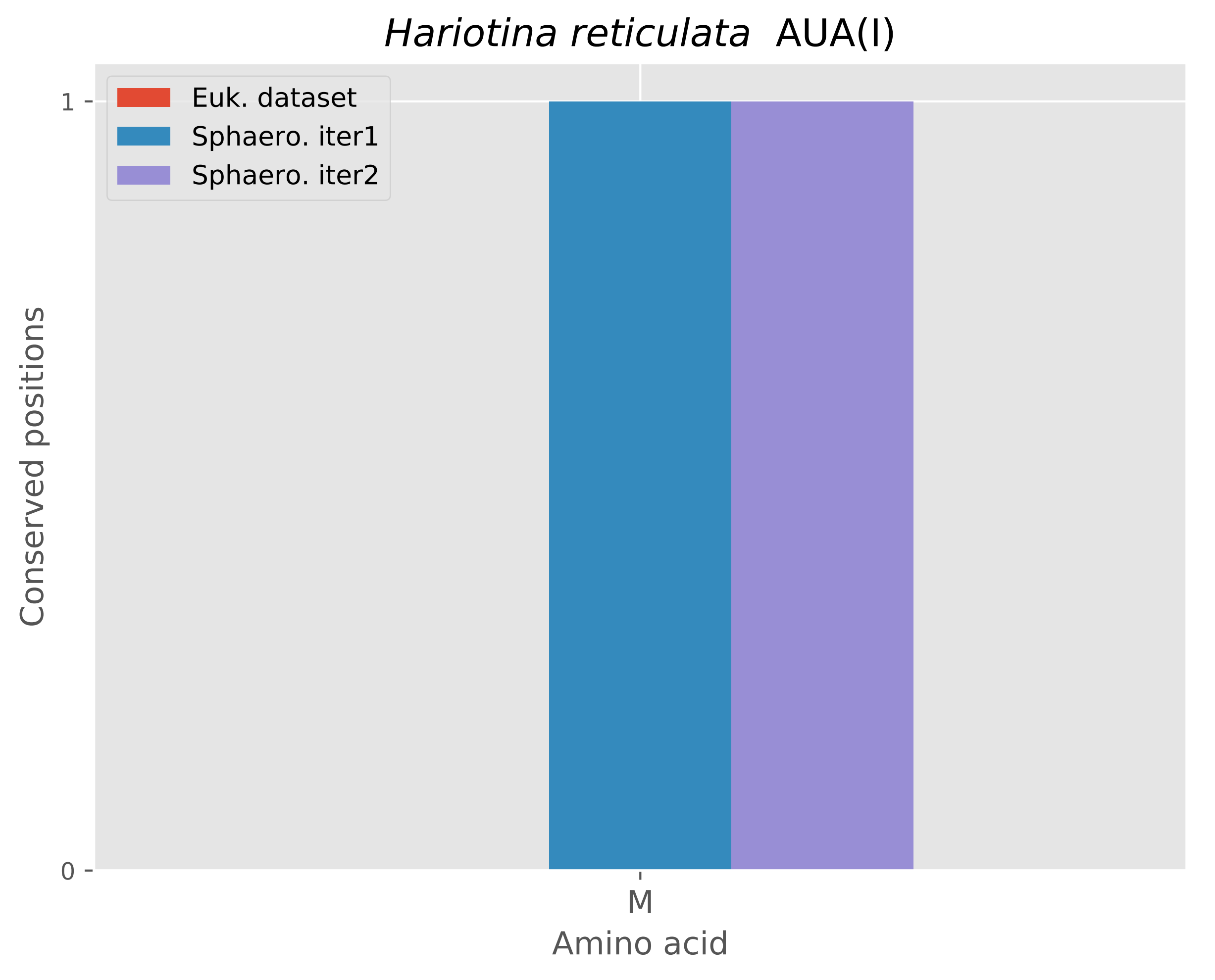

Hariotina reticulata AUA(I)
Euk. dataset
Sphaero. iter1
Sphaero. iter2
Conserved positions
1
0
M
Amino acid

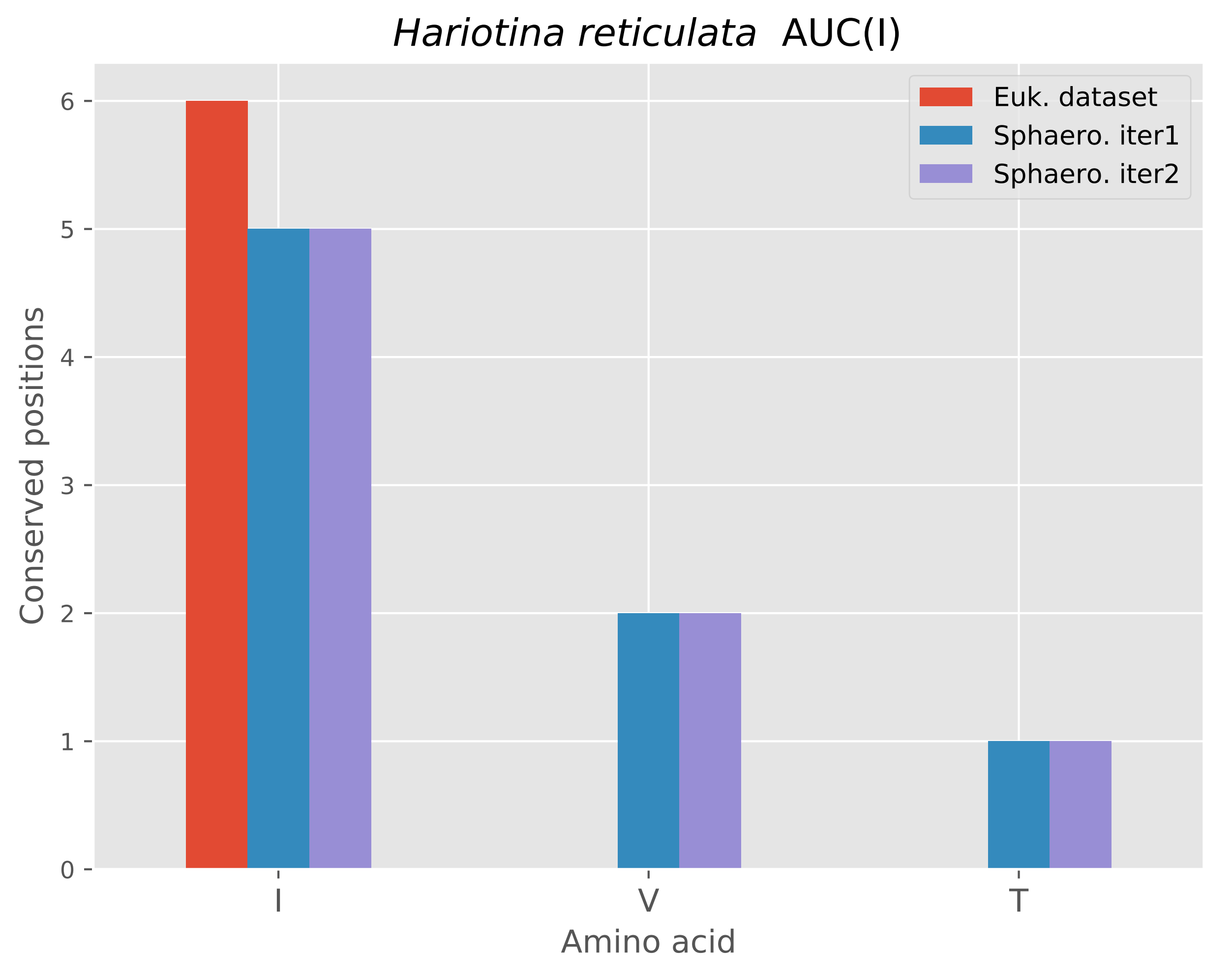
Hariotina reticulata AUC(I)
Euk. dataset
Sphaero. iter1
Sphaero. iter2
Conserved positions
0
1
2
3
4
5
6
I
V
T
Amino acid

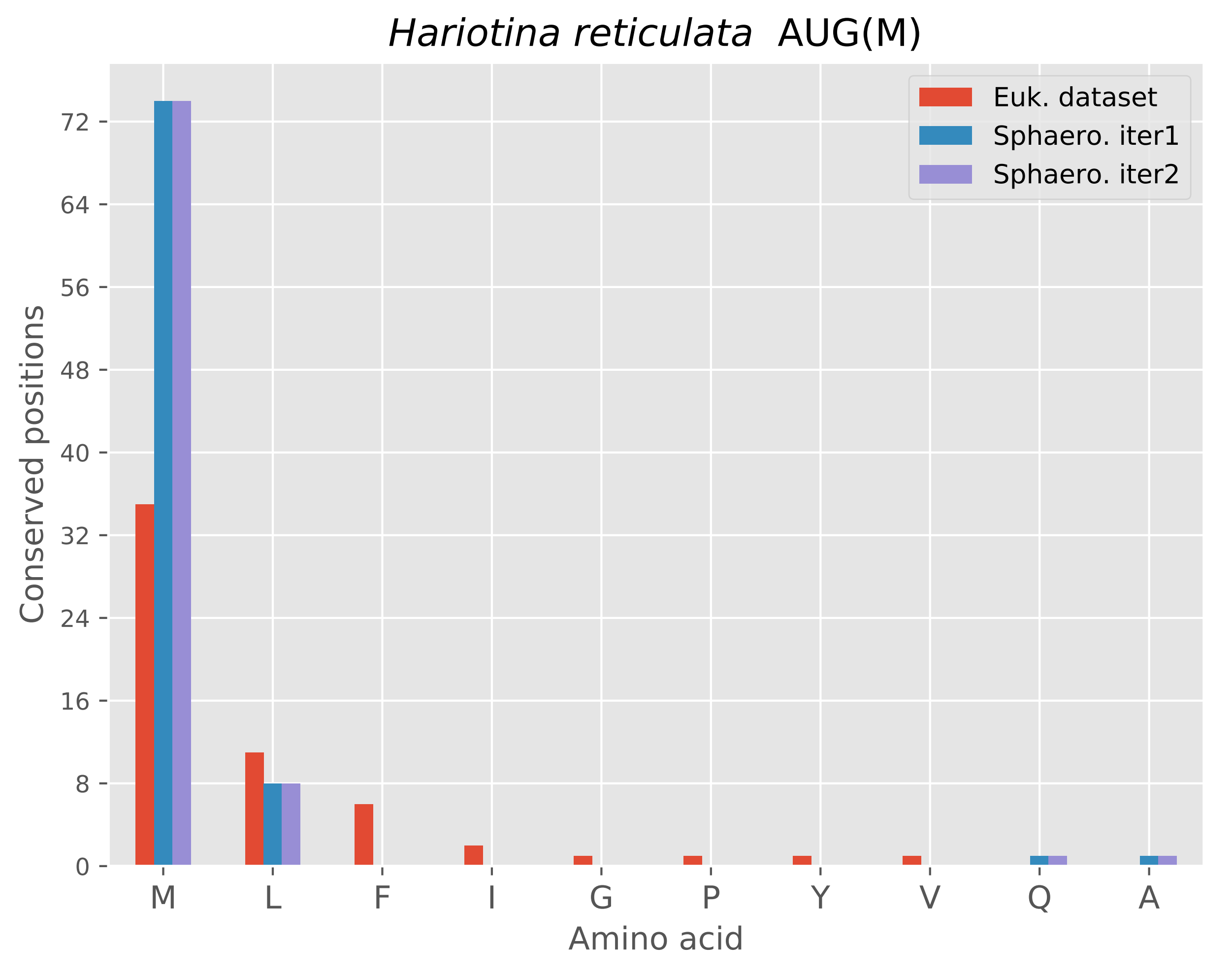

Hariotina reticulata AUG(M)
Euk. dataset
Sphaero. iter1
Sphaero. iter2
Conserved positions
0
8
16
24
32
40
48
56
64
72
M
L
F
I
G
P
Y
V
Q
A
Amino acid

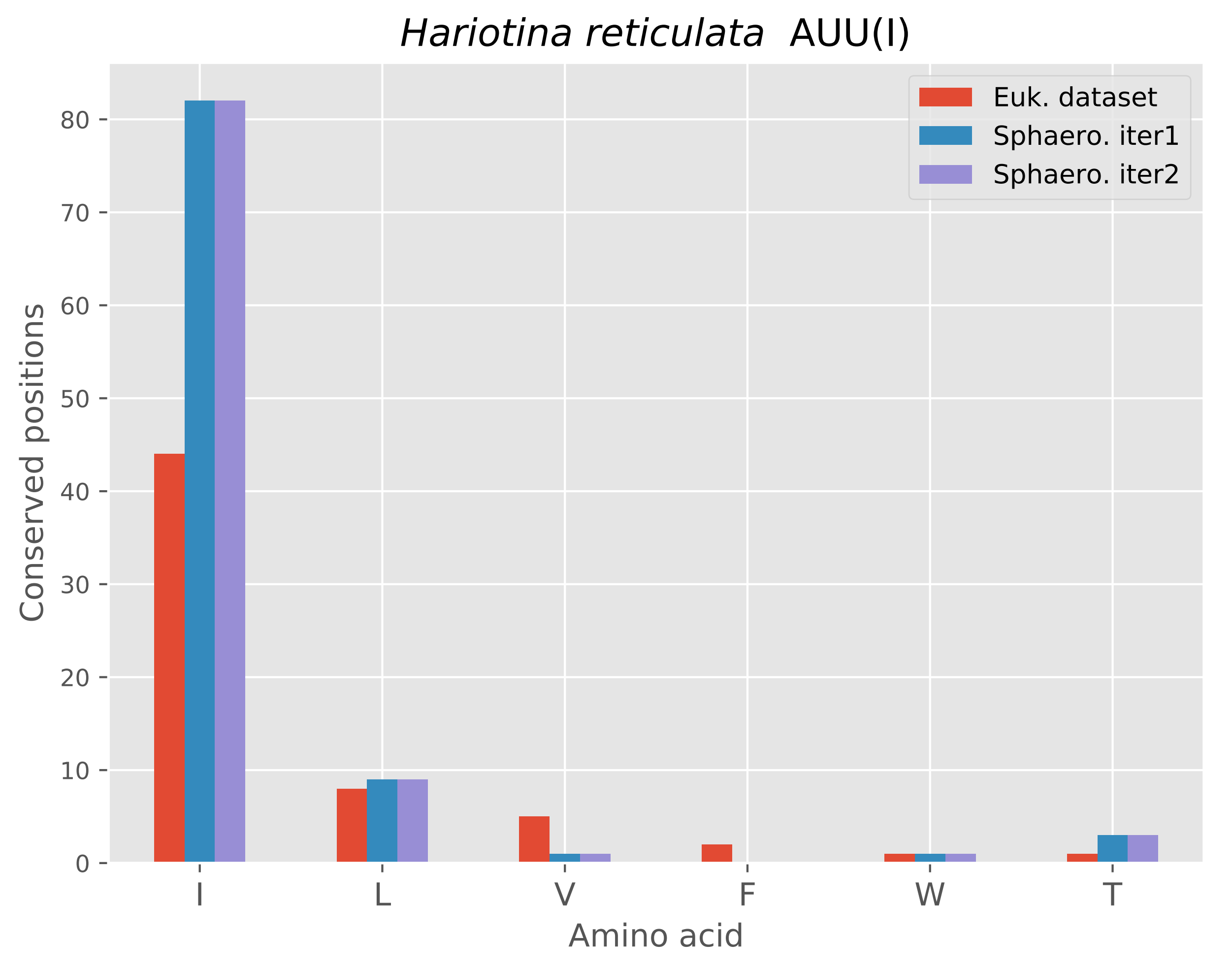

Hariotina reticulata AUU(I)
Euk. dataset
Sphaero. iter1
Sphaero. iter2
Conserved positions
0
10
20
30
40
50
60
70
80
I
L
V
F
W
T
Amino acid

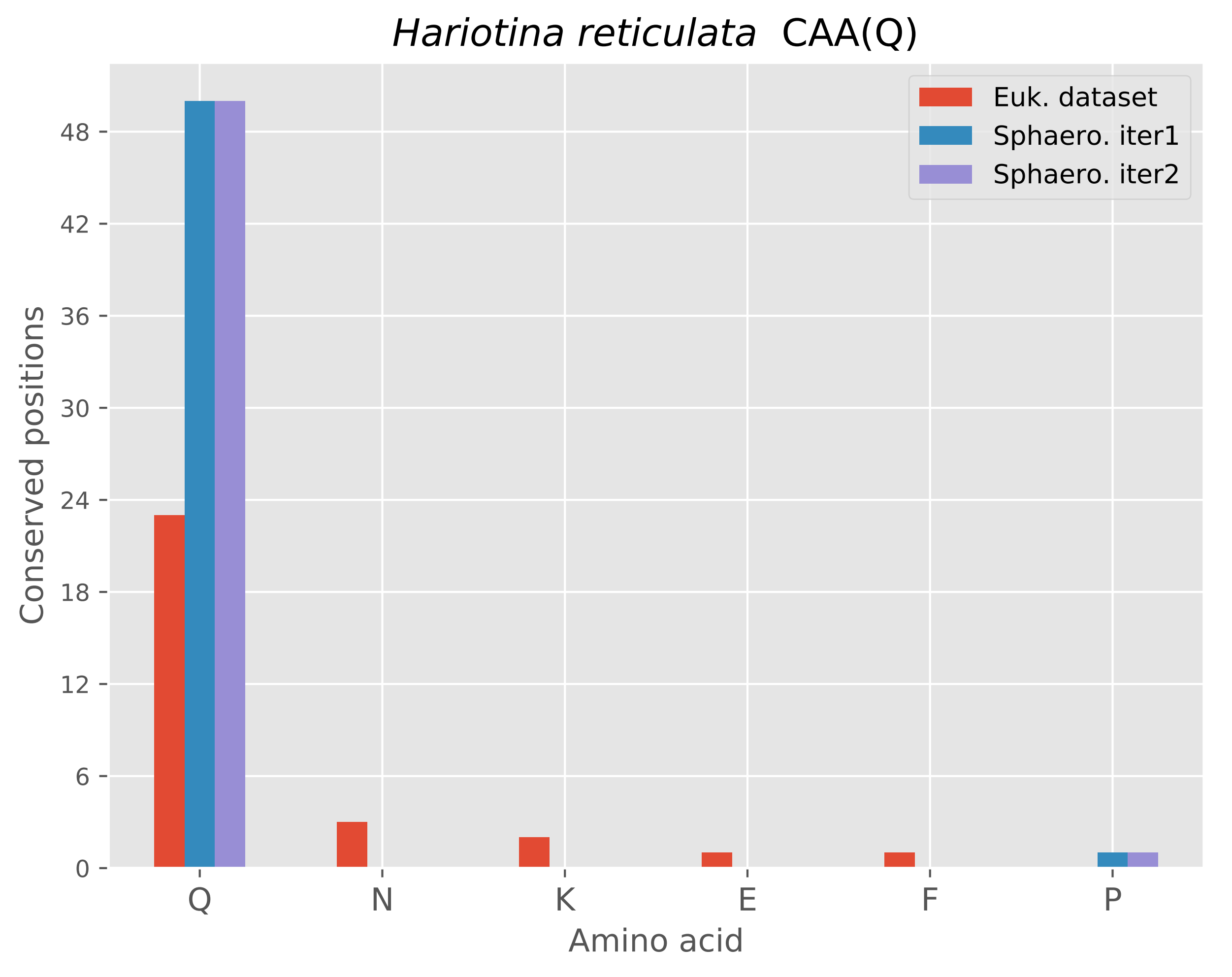

Hariotina reticulata CAA(Q)
Euk. dataset
Sphaero. iter1
Sphaero. iter2
Conserved positions
0
6
12
18
24
30
36
42
48
Q
N
K
E
F
P
Amino acid

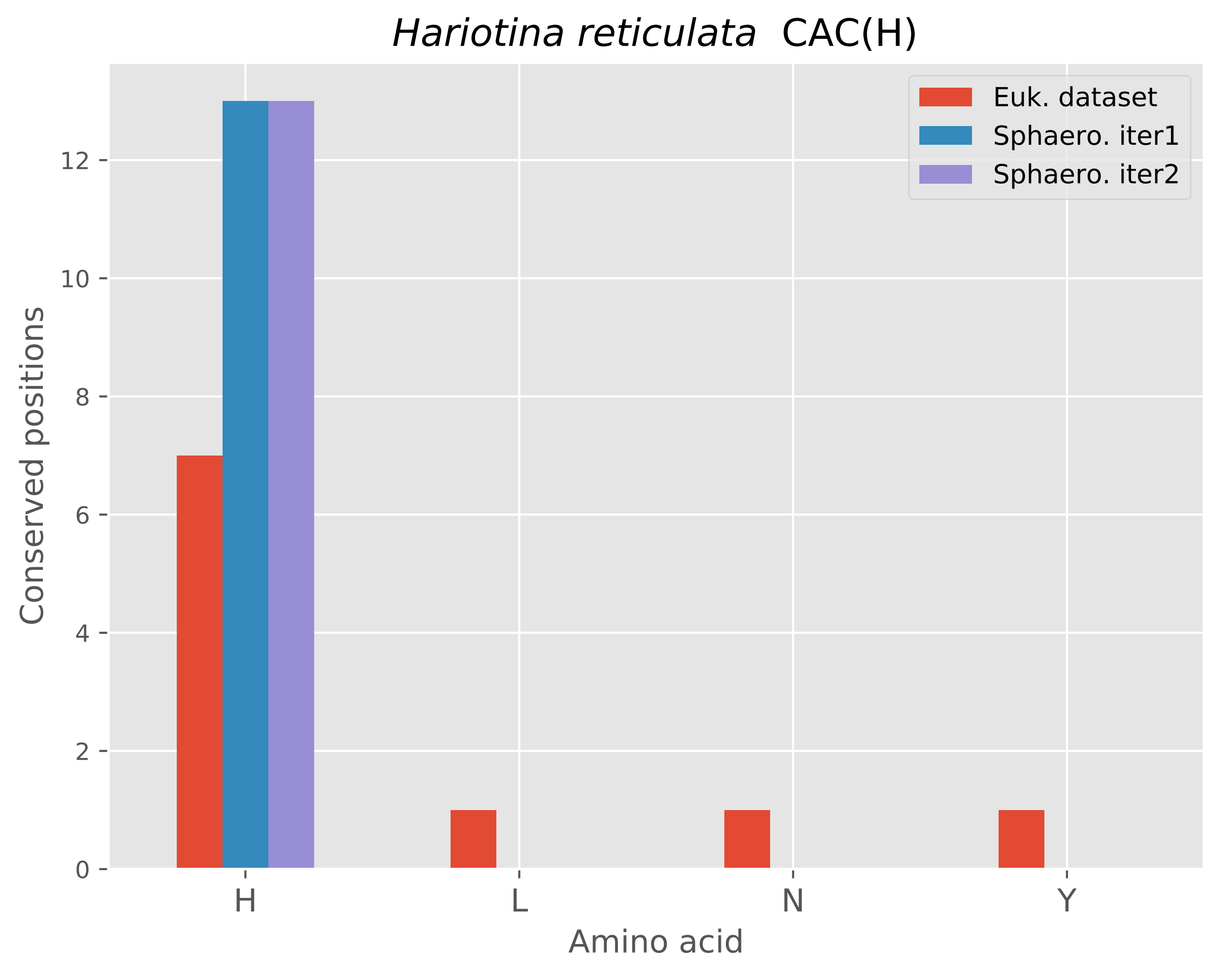

Hariotina reticulata CAC(H)
Euk. dataset
Sphaero. iter1
Sphaero. iter2
Conserved positions
0
2
4
6
8
10
12
H
L
N
Y
Amino acid

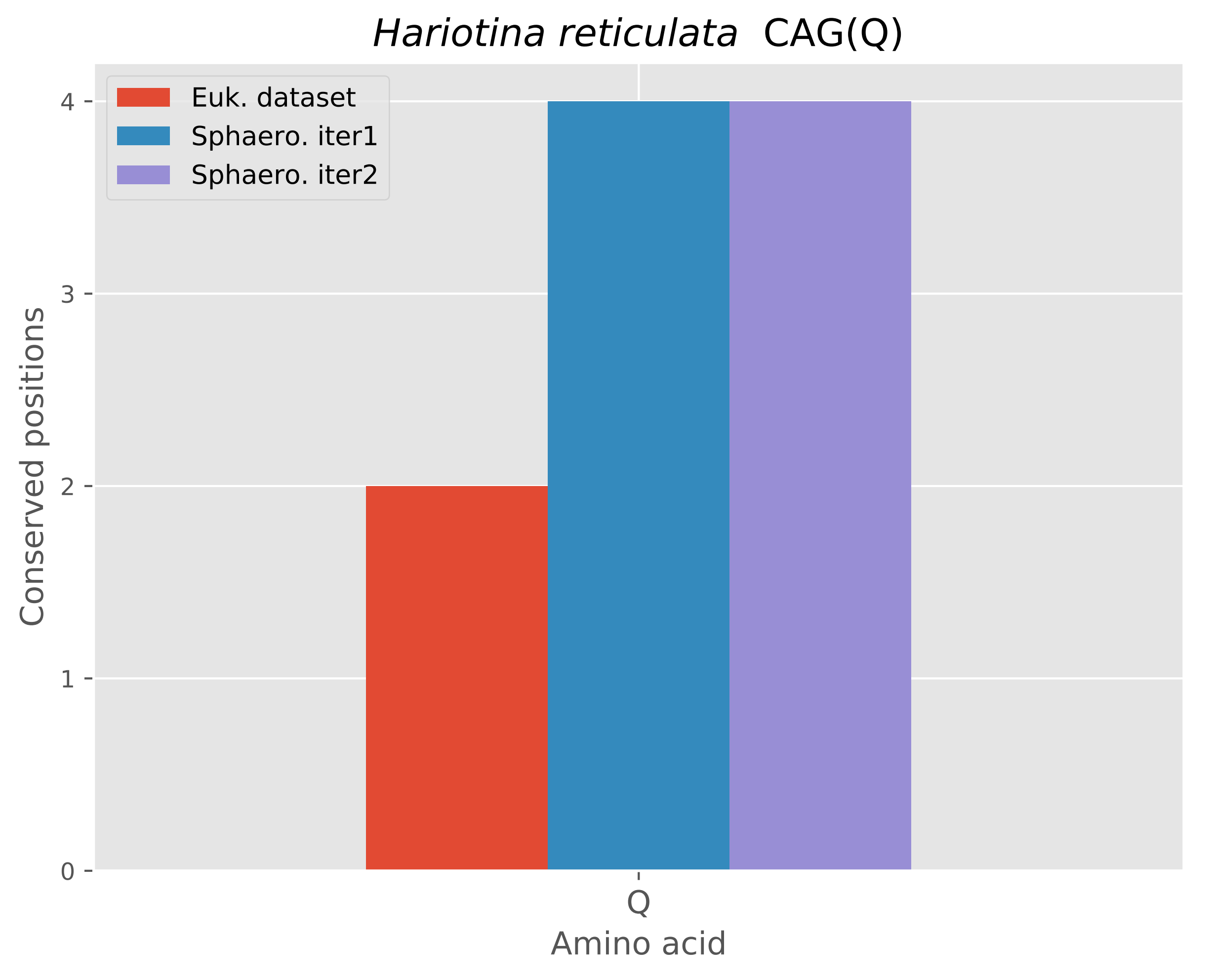

Hariotina reticulata CAG(Q)
Euk. dataset
Sphaero. iter1
Sphaero. iter2
Conserved positions
0
1
2
3
4
Q
Amino acid

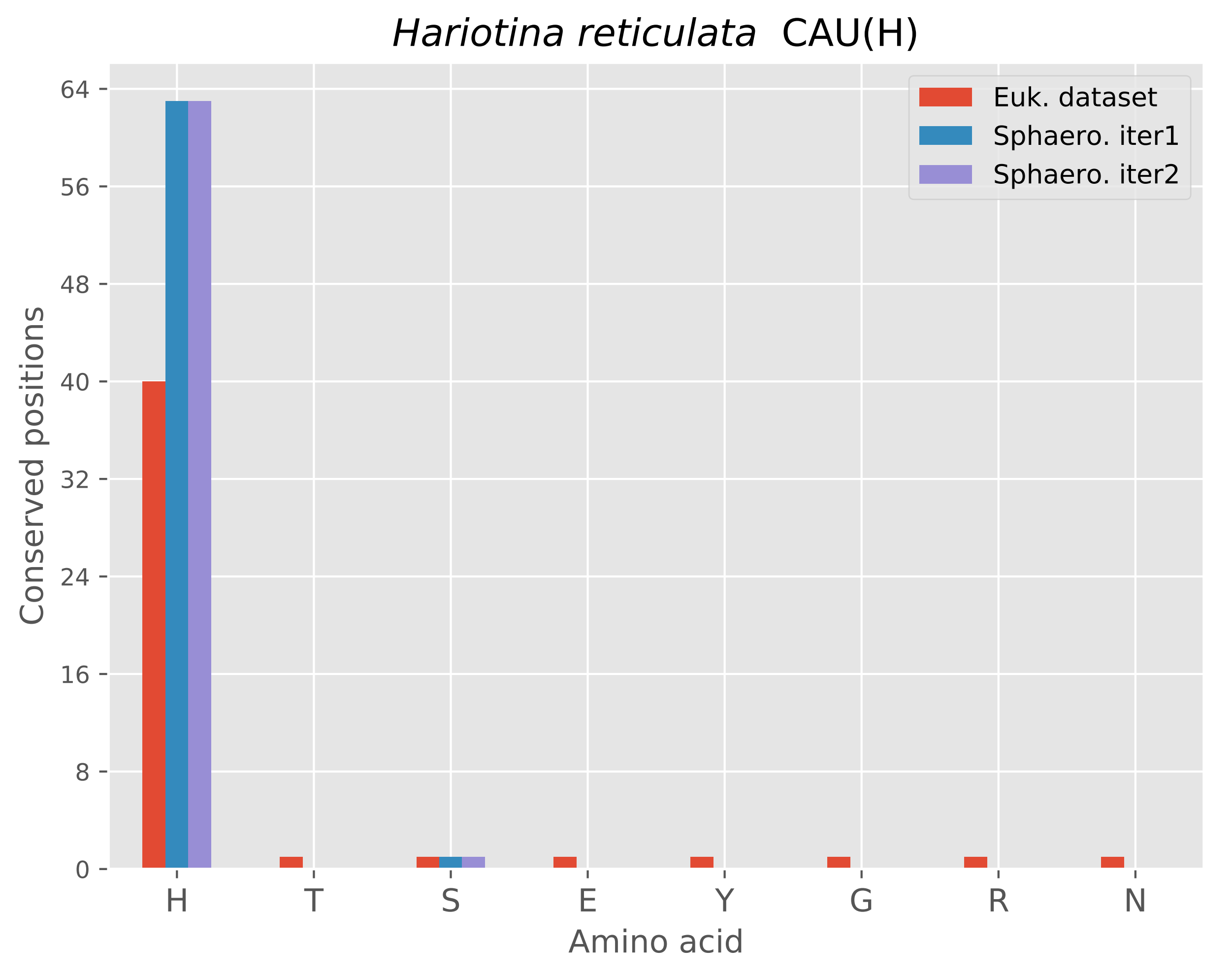

Hariotina reticulata CAU(H)
Euk. dataset
Sphaero. iter1
Sphaero. iter2
Conserved positions
0
8
16
24
32
40
48
56
64
H
T
S
E
Y
G
R
N
Amino acid

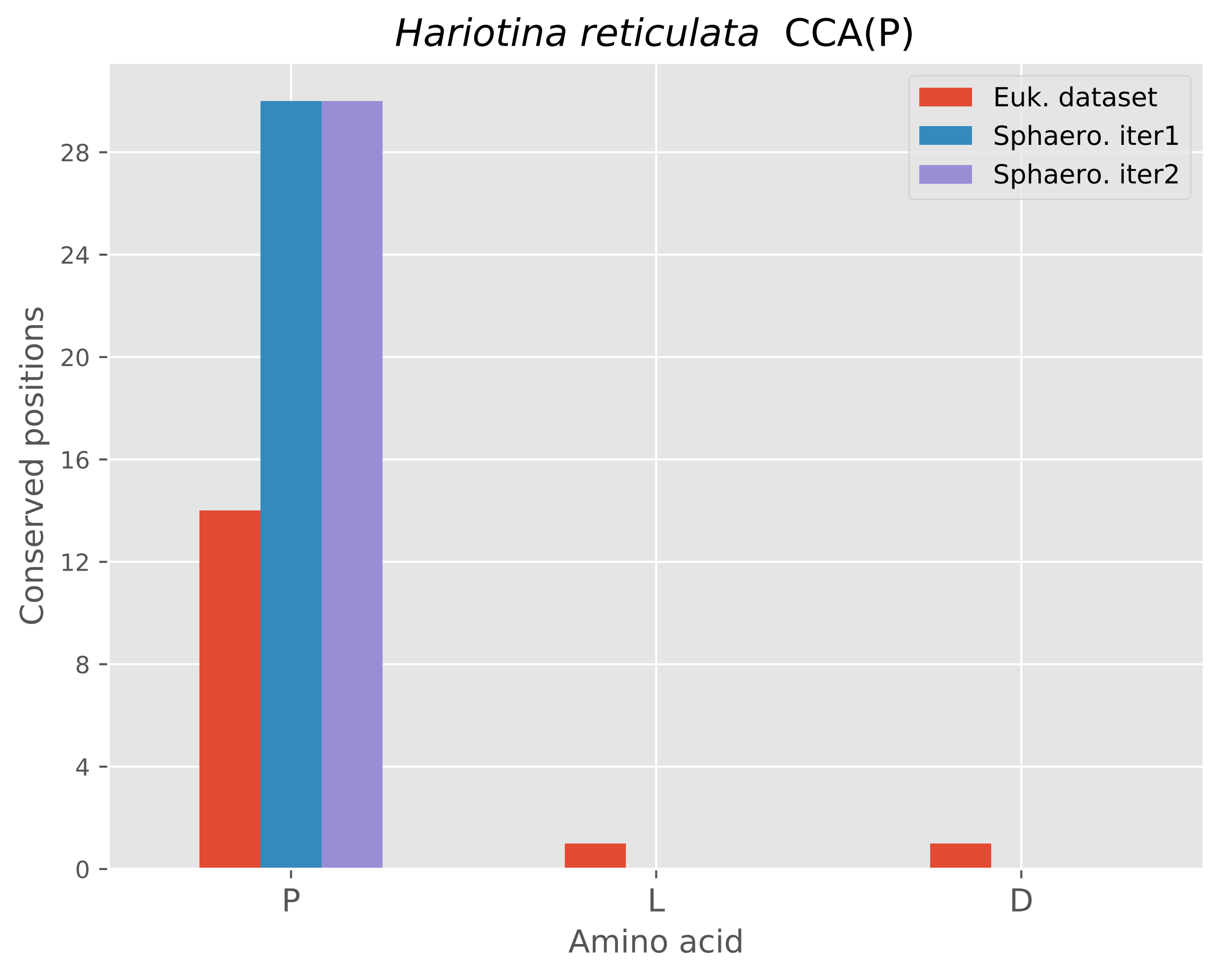

Hariotina reticulata CCA(P)
Euk. dataset
Sphaero. iter1
Sphaero. iter2
Conserved positions
0
4
8
12
16
20
24
28
P
L
D
Amino acid

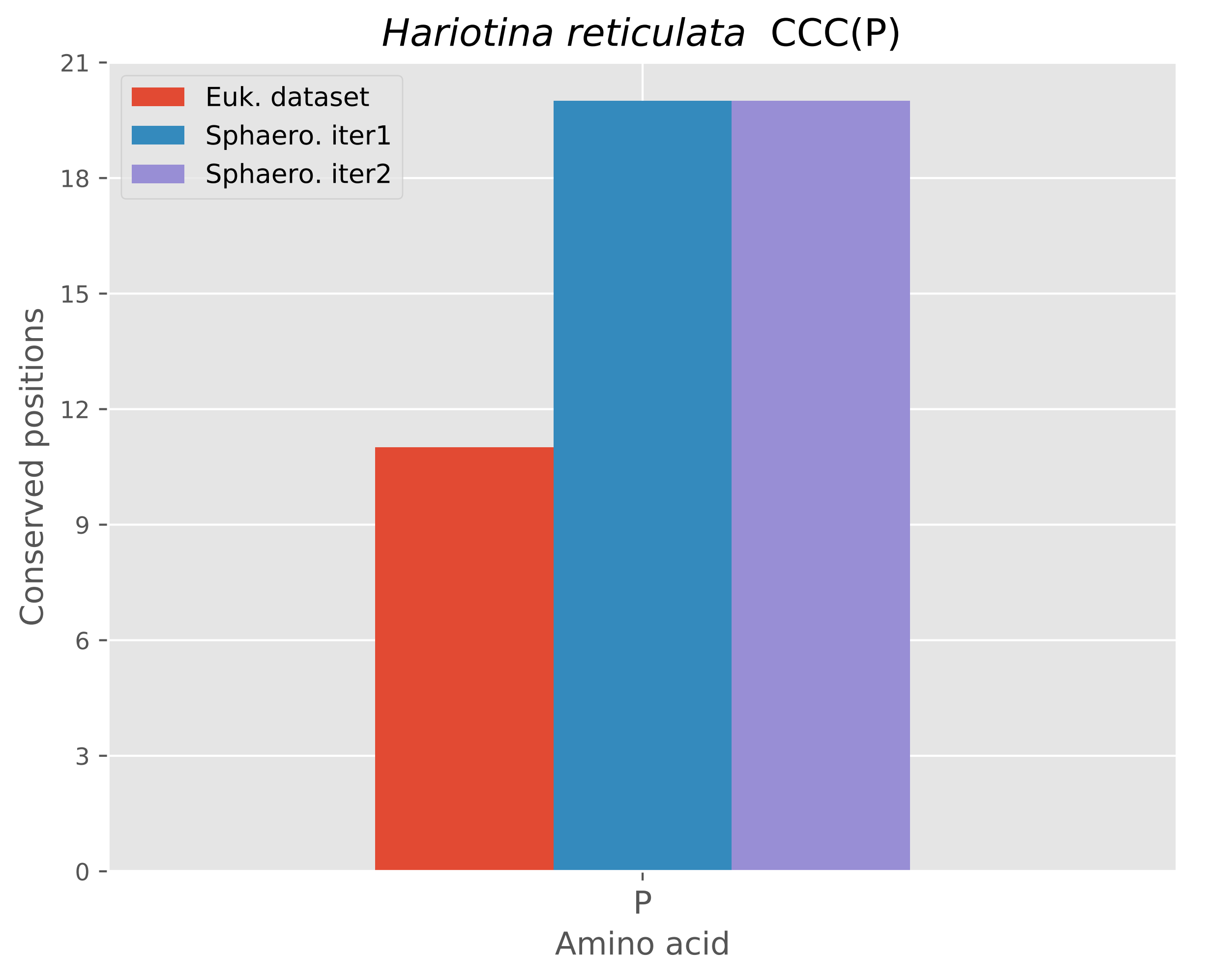

Hariotina reticulata CCC(P)
Euk. dataset
Sphaero. iter1
Sphaero. iter2
Conserved positions
0
3
6
9
12
15
18
21
P
Amino acid

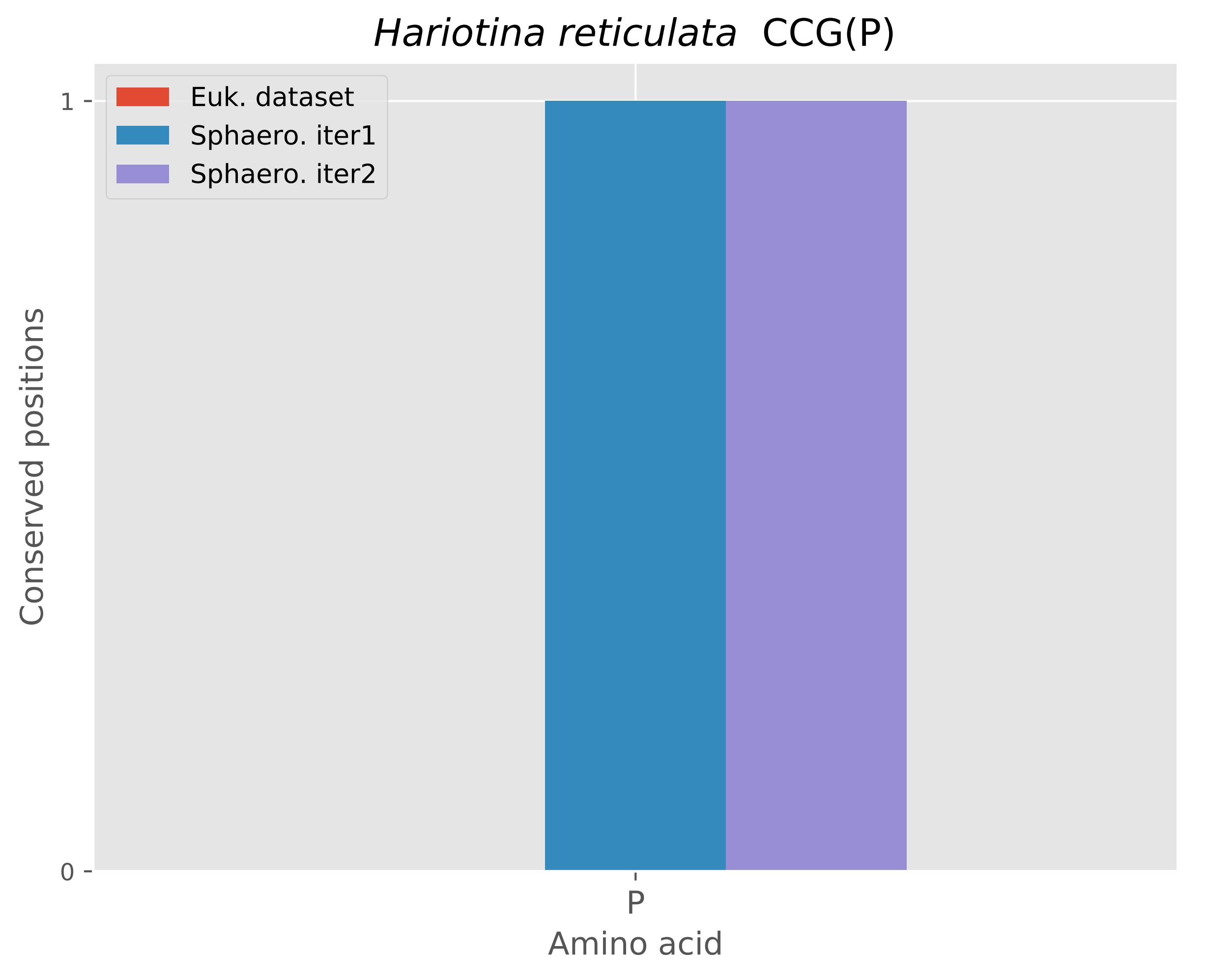
Hariotina reticulata CCG(P)
Euk. dataset
Sphaero. iter1
Sphaero. iter2
1
0
Conserved positions
P
Amino acid

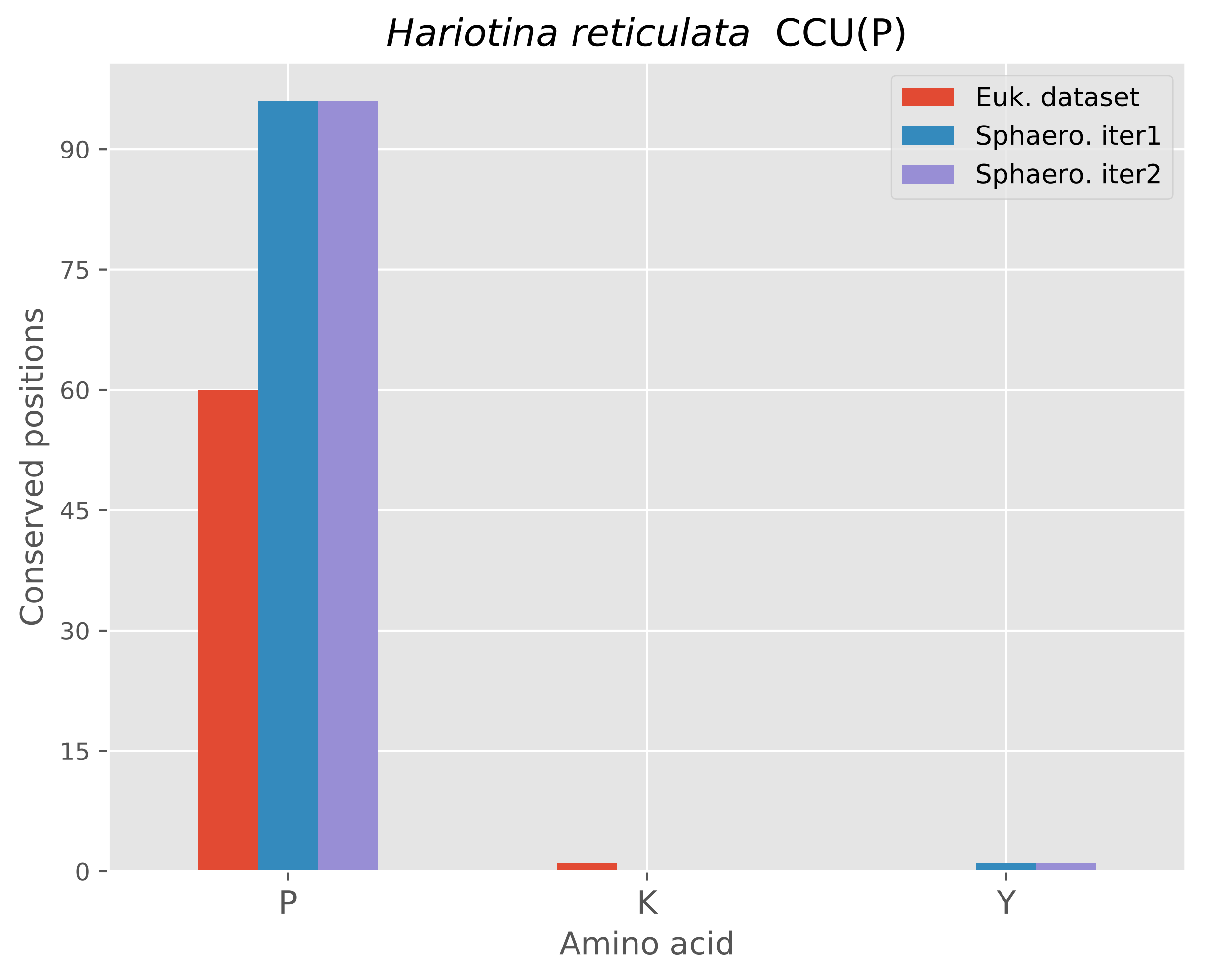

Hariotina reticulata CCU(P)
Euk. dataset
Sphaero. iter1
Sphaero. iter2
Conserved positions
0
15
30
45
60
75
90
P
K
Y
Amino acid

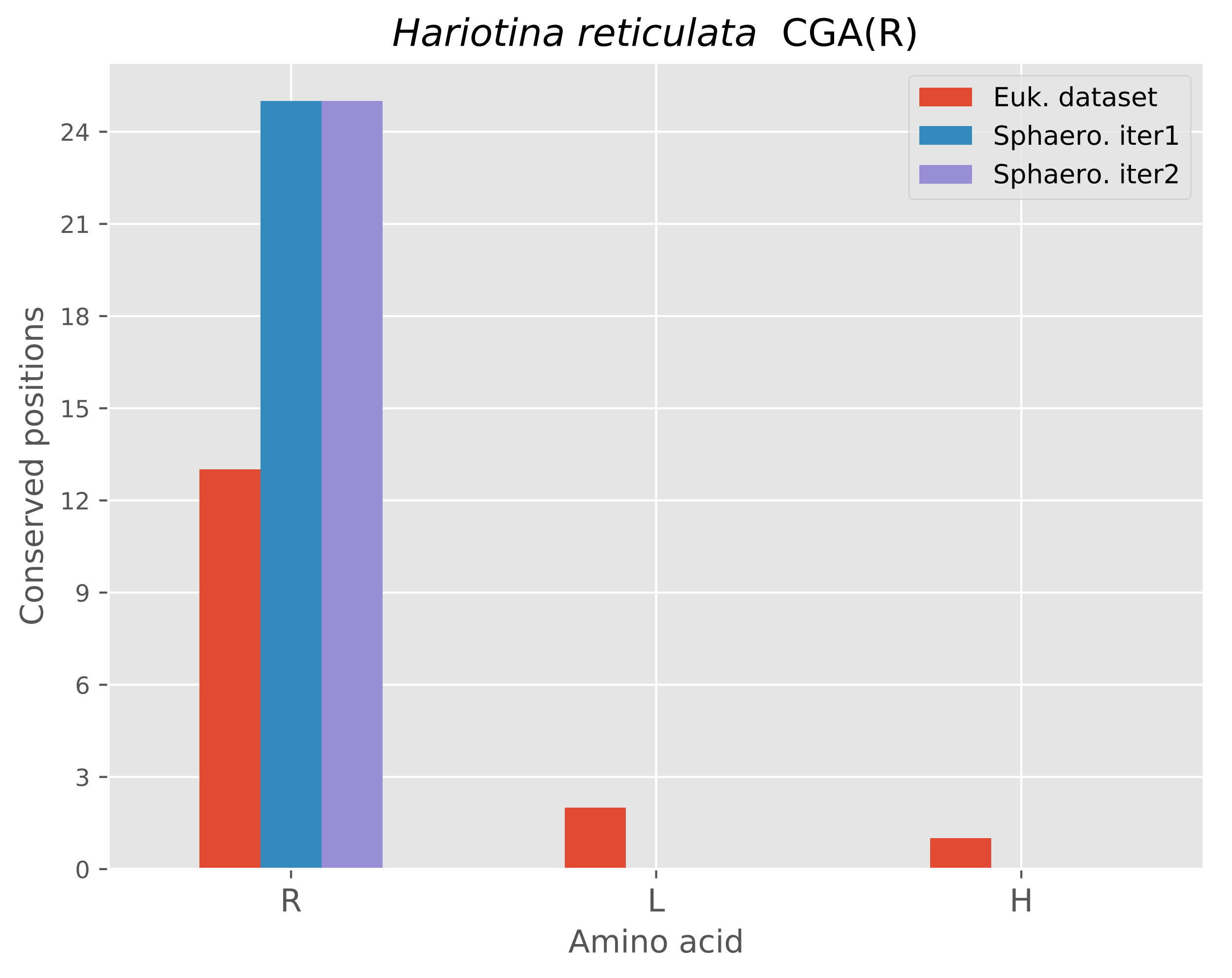

Hariotina reticulata CGA(R)
Euk. dataset
Sphaero. iter1
Sphaero. iter2
Conserved positions
0
3
6
9
12
15
18
21
24
R
L
H
Amino acid

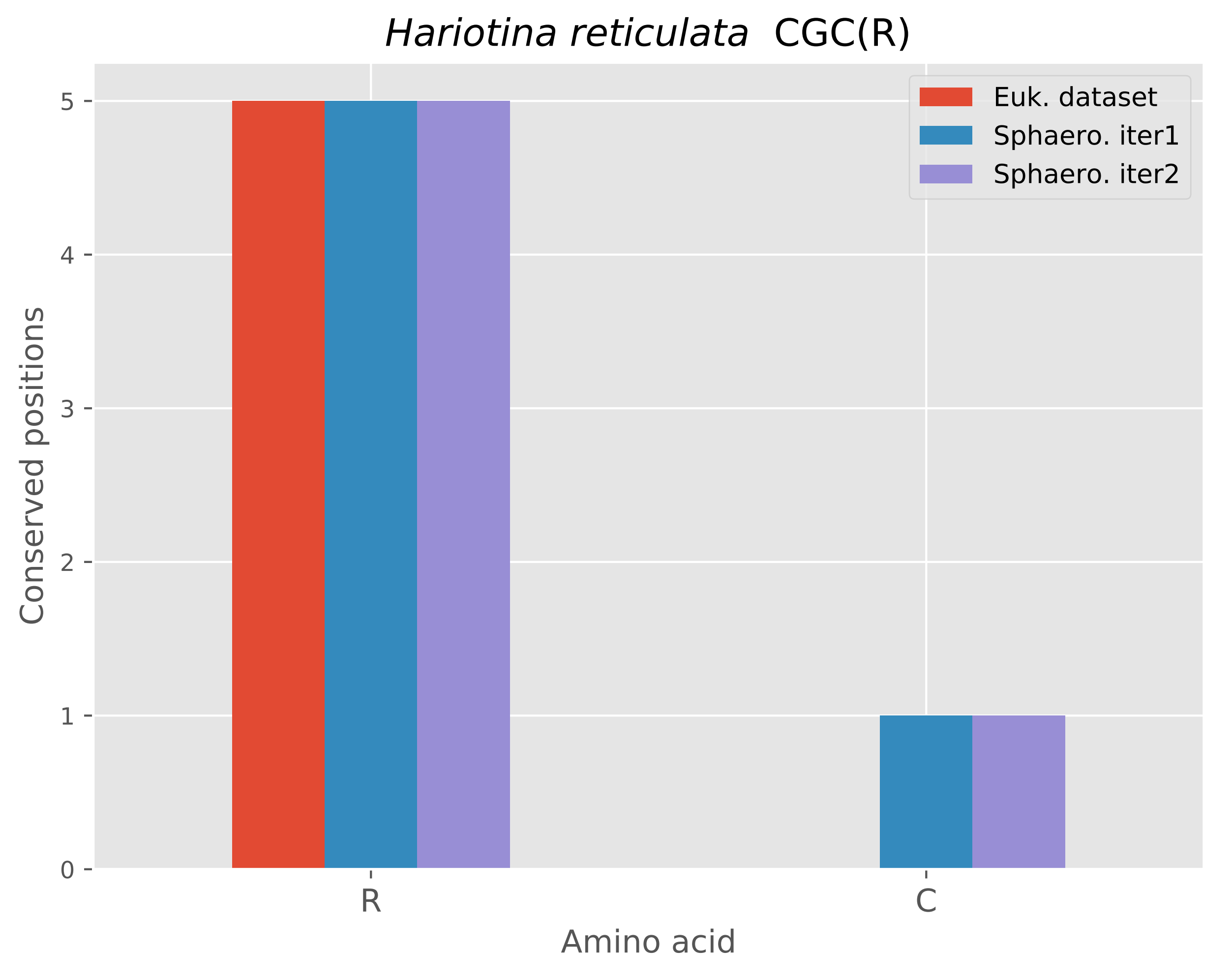

Hariotina reticulata CGC(R)
Euk. dataset
Sphaero. iter1
Sphaero. iter2
Conserved positions
0
1
2
3
4
5
R
C
Amino acid

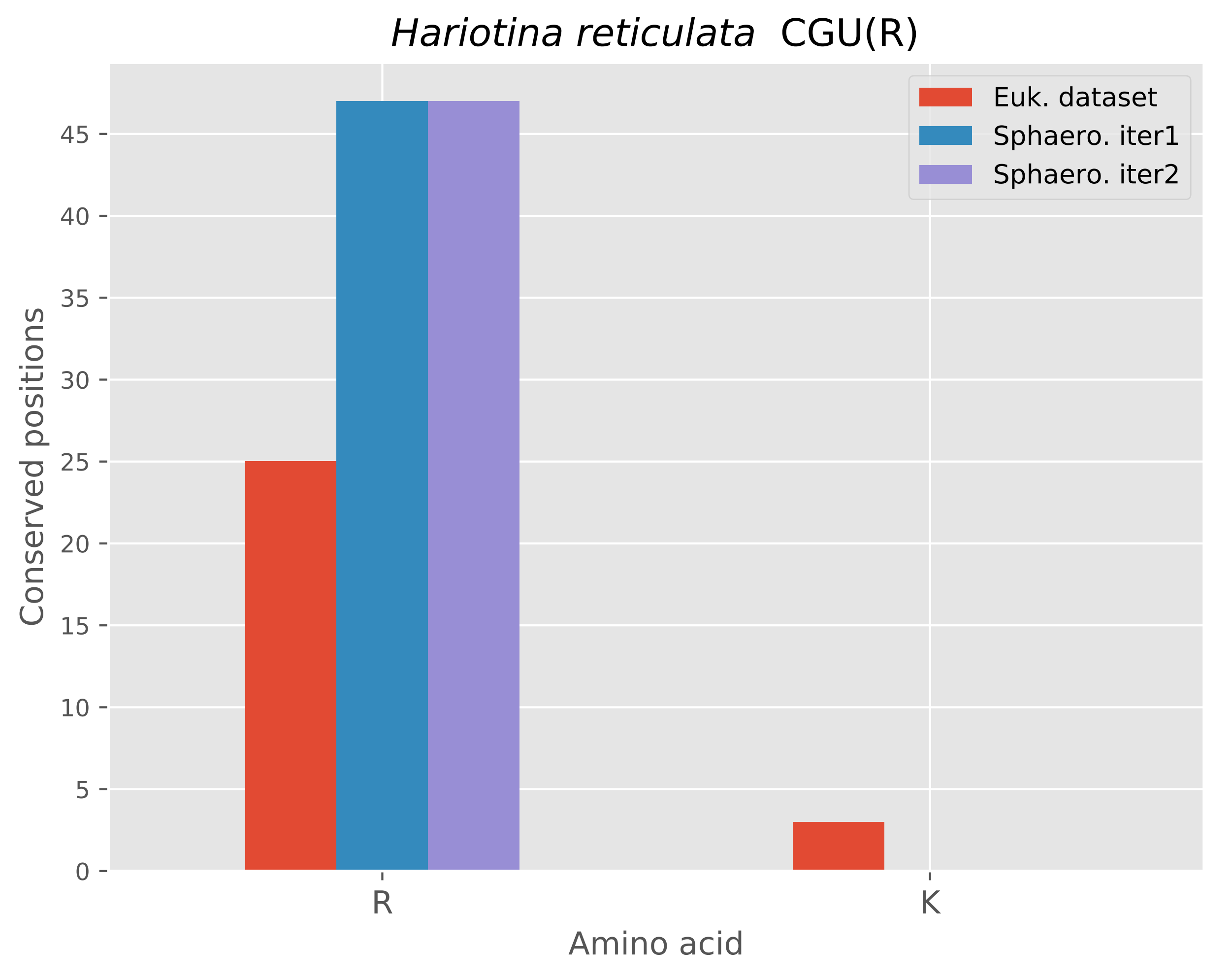

Hariotina reticulata CGU(R)
Euk. dataset
Sphaero. iter1
Sphaero. iter2
Conserved positions
0
5
10
15
20
25
30
35
40
45
R
K
Amino acid

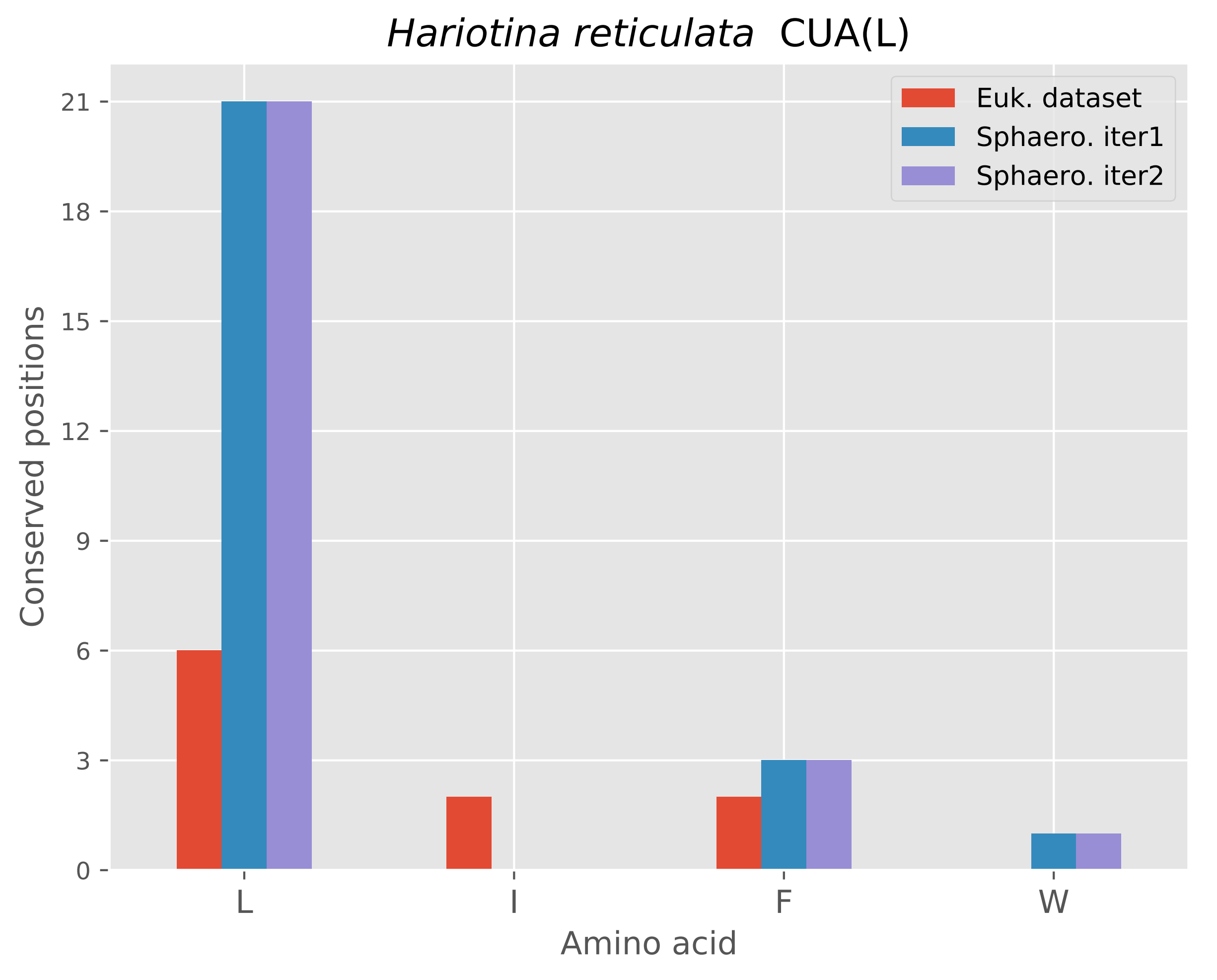

Hariotina reticulata CUA(L)
Euk. dataset
Sphaero. iter1
Sphaero. iter2
Conserved positions
0
3
6
9
12
15
18
21
L
I
F
W
Amino acid

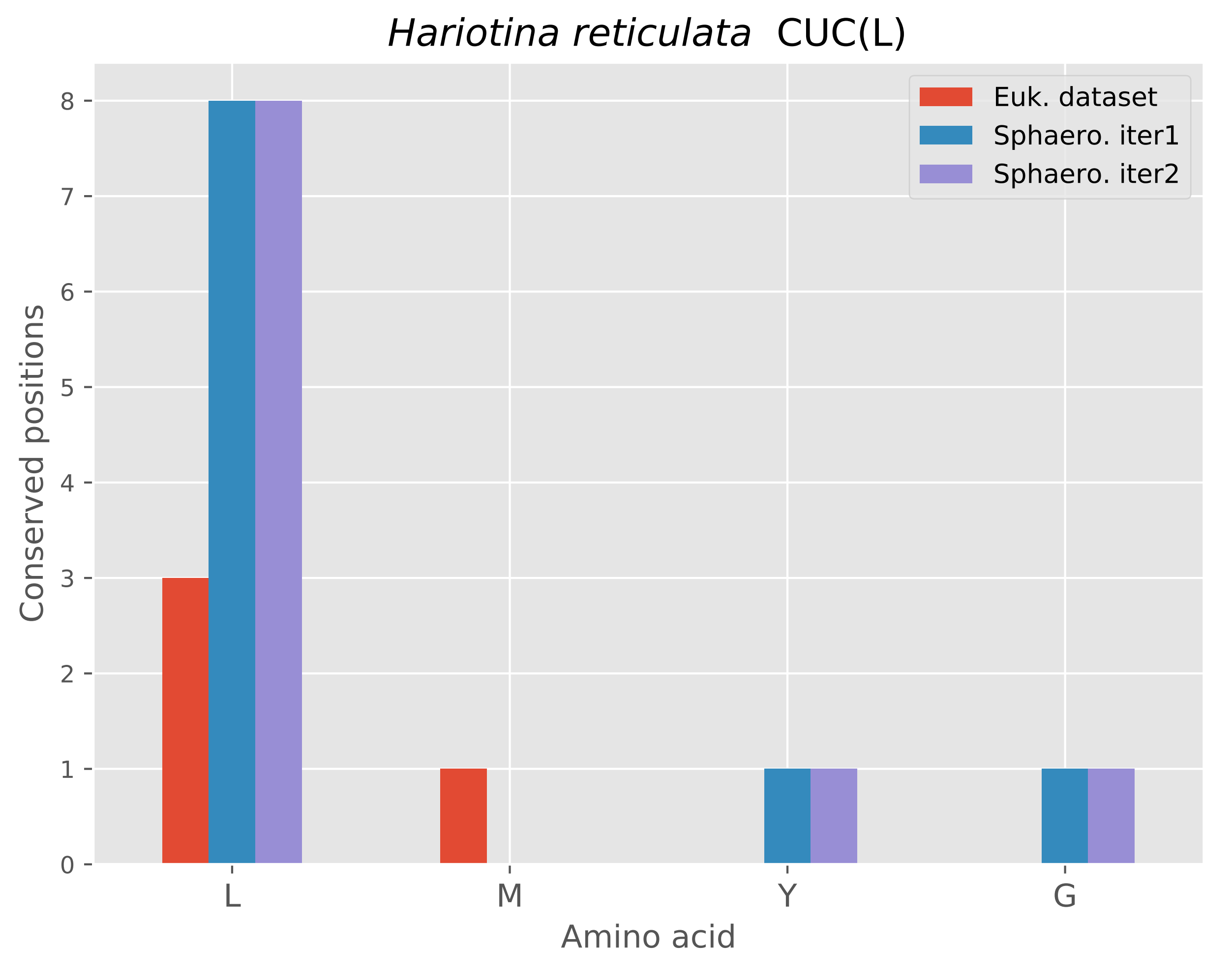

Hariotina reticulata CUC(L)
Euk. dataset
Sphaero. iter1
Sphaero. iter2
Conserved positions
0
1
2
3
4
5
6
7
8
L
M
Y
G
Amino acid

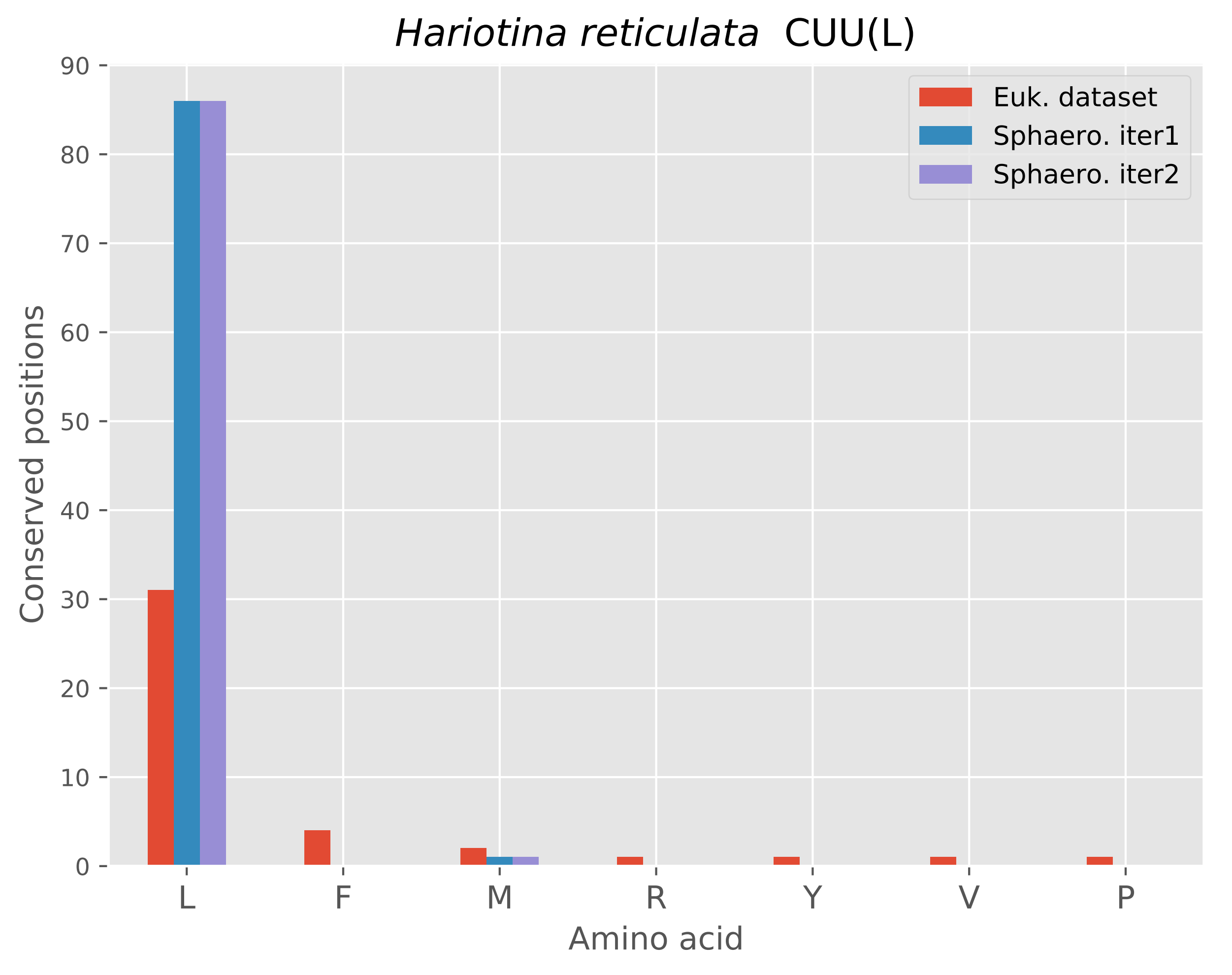

Hariotina reticulata CUU(L)
Conserved positions
Amino acid
Euk. dataset
Sphaero. iter1
Sphaero. iter2
0
10
20
30
40
50
60
70
80
90
L
F
M
R
Y
V
P

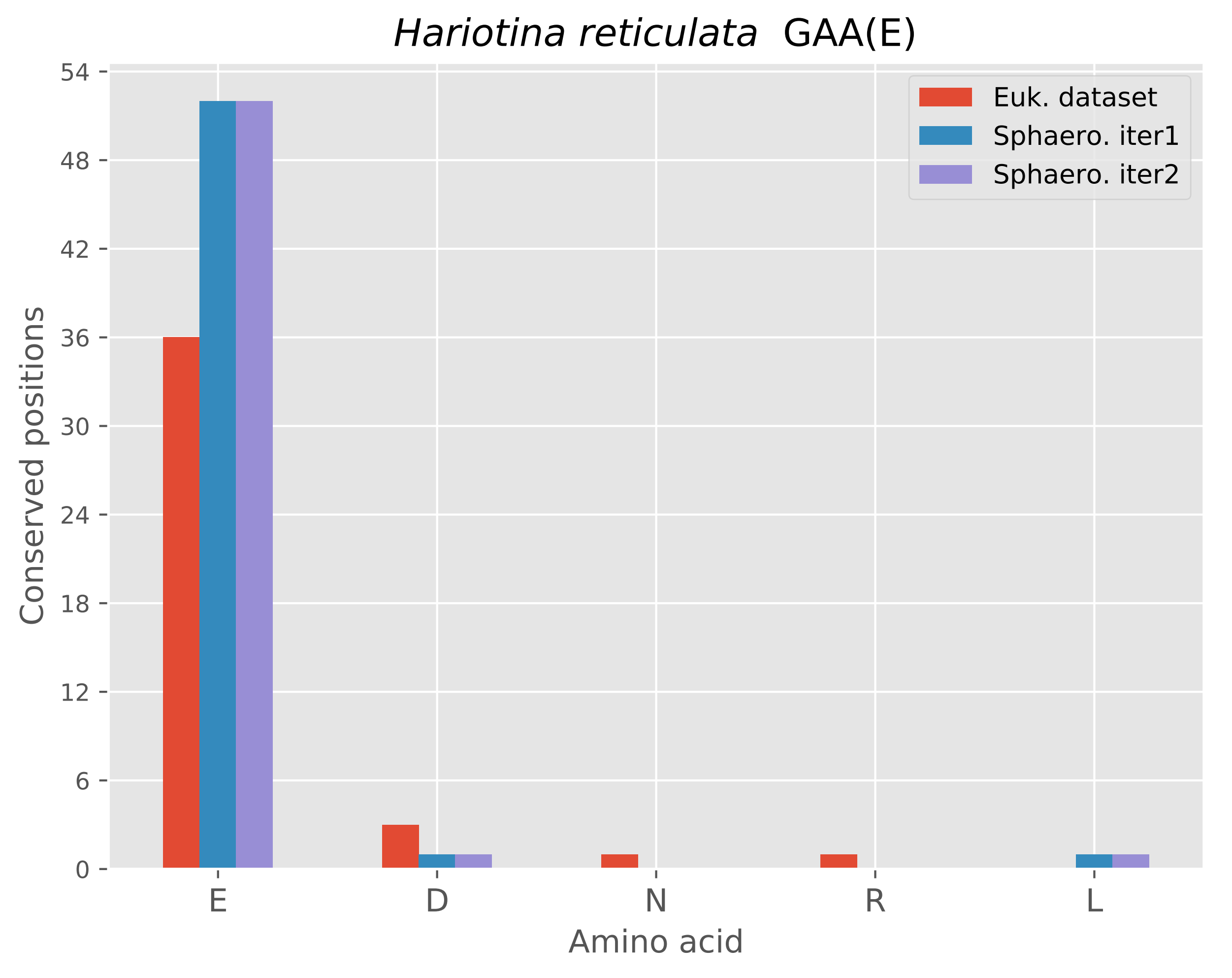

Hariotina reticulata GAA(E)
Euk. dataset
Sphaero. iter1
Sphaero. iter2
Conserved positions
0
6
12
18
24
30
36
42
48
54
E
D
N
R
L
Amino acid

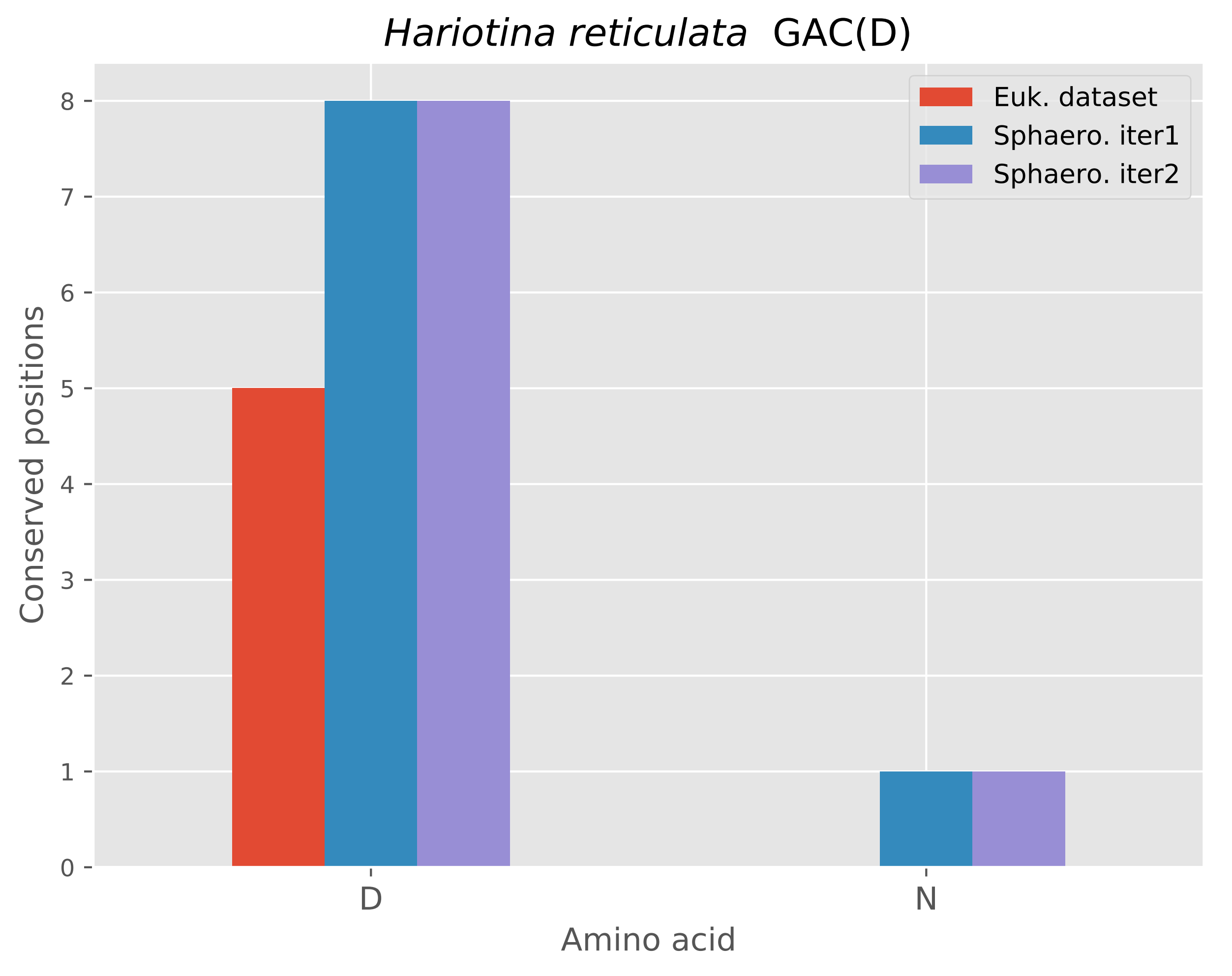

Hariotina reticulata GAC(D)
Euk. dataset
Sphaero. iter1
Sphaero. iter2
Conserved positions
0
1
2
3
4
5
6
7
8
D
N
Amino acid

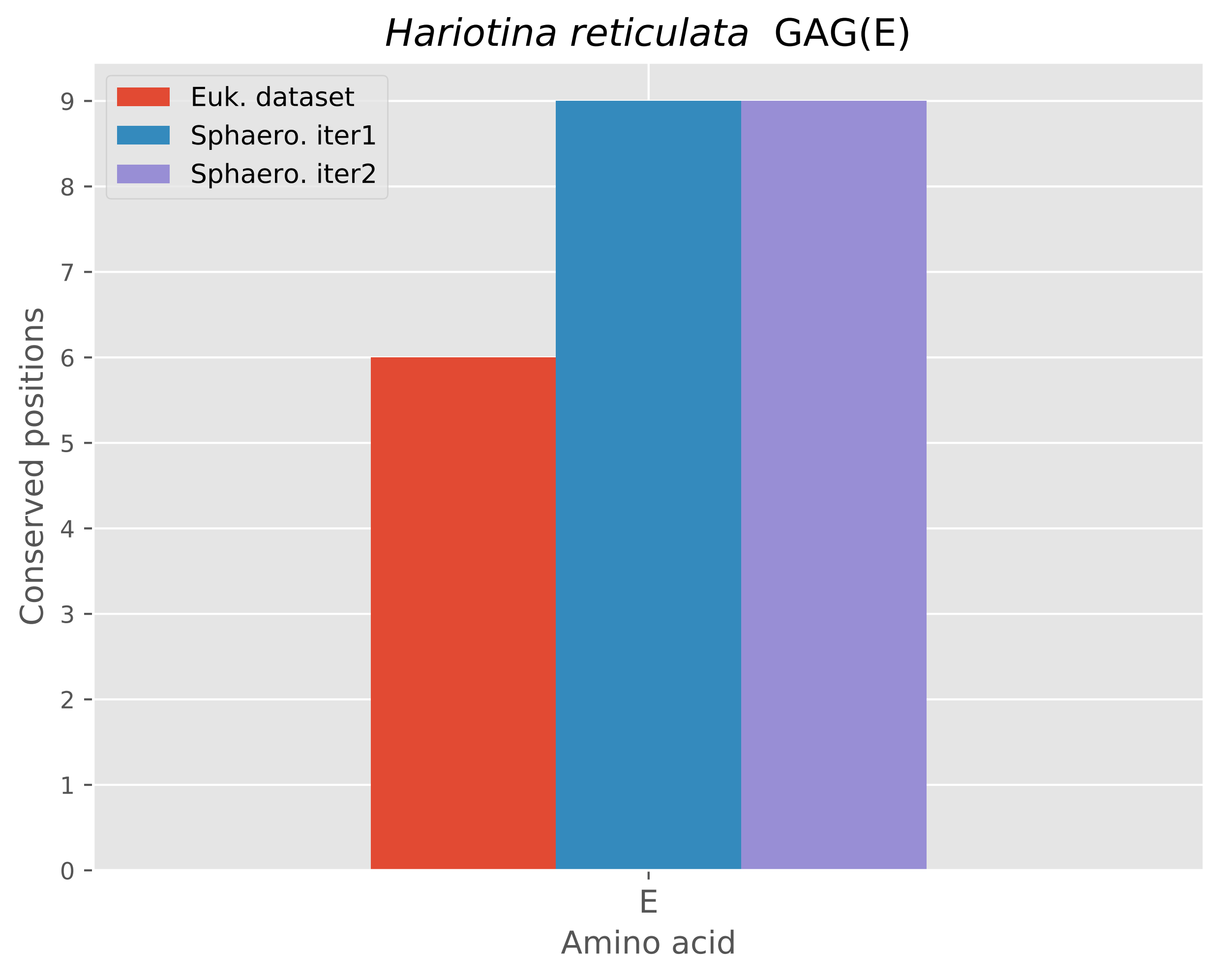

Hariotina reticulata GAG(E)
Euk. dataset
Sphaero. iter1
Sphaero. iter2
Conserved positions
0
1
2
3
4
5
6
7
8
9
E
Amino acid

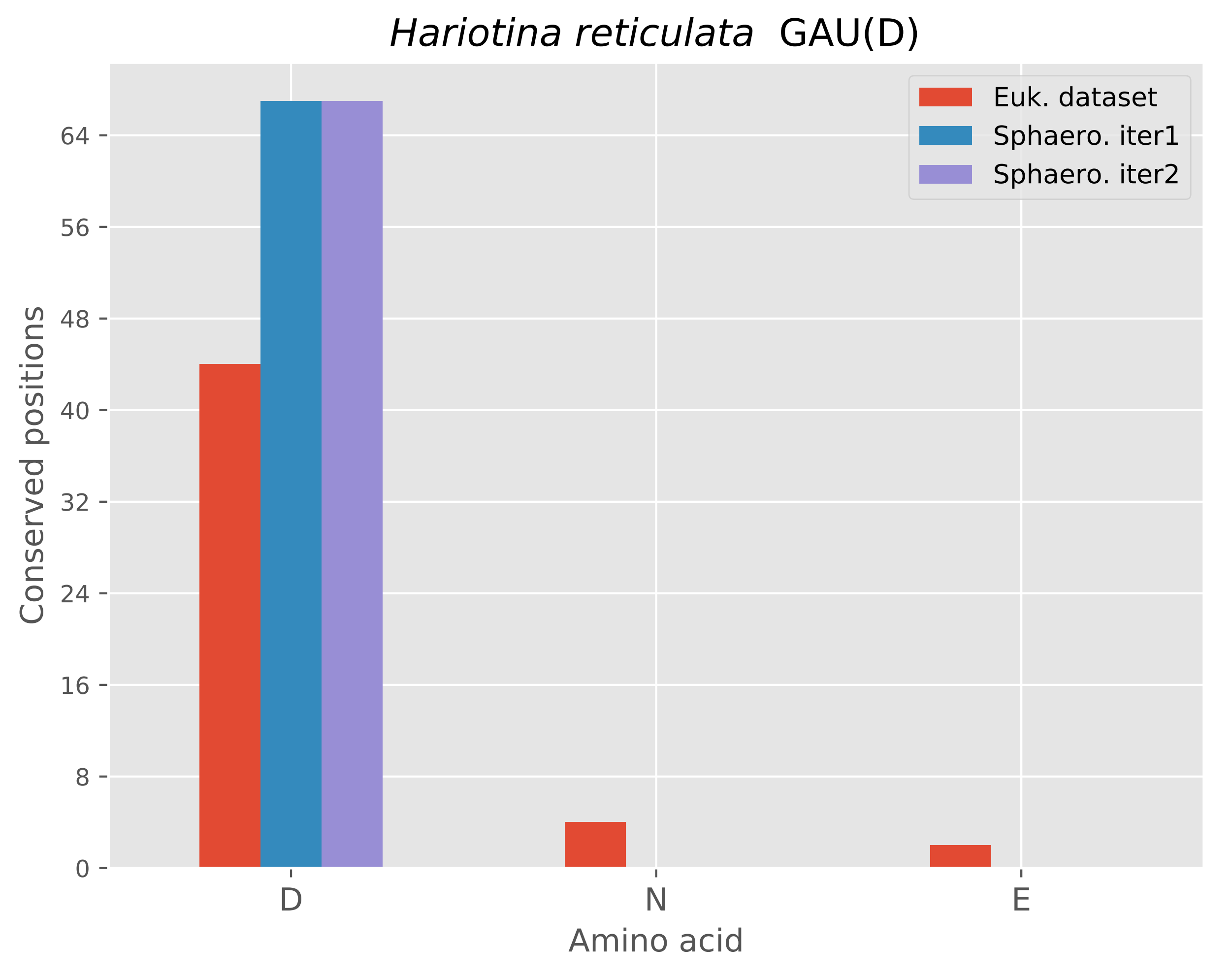

Hariotina reticulata GAU(D)
Euk. dataset
Sphaero. iter1
Sphaero. iter2
Conserved positions
0
8
16
24
32
40
48
56
64
D
N
E
Amino acid

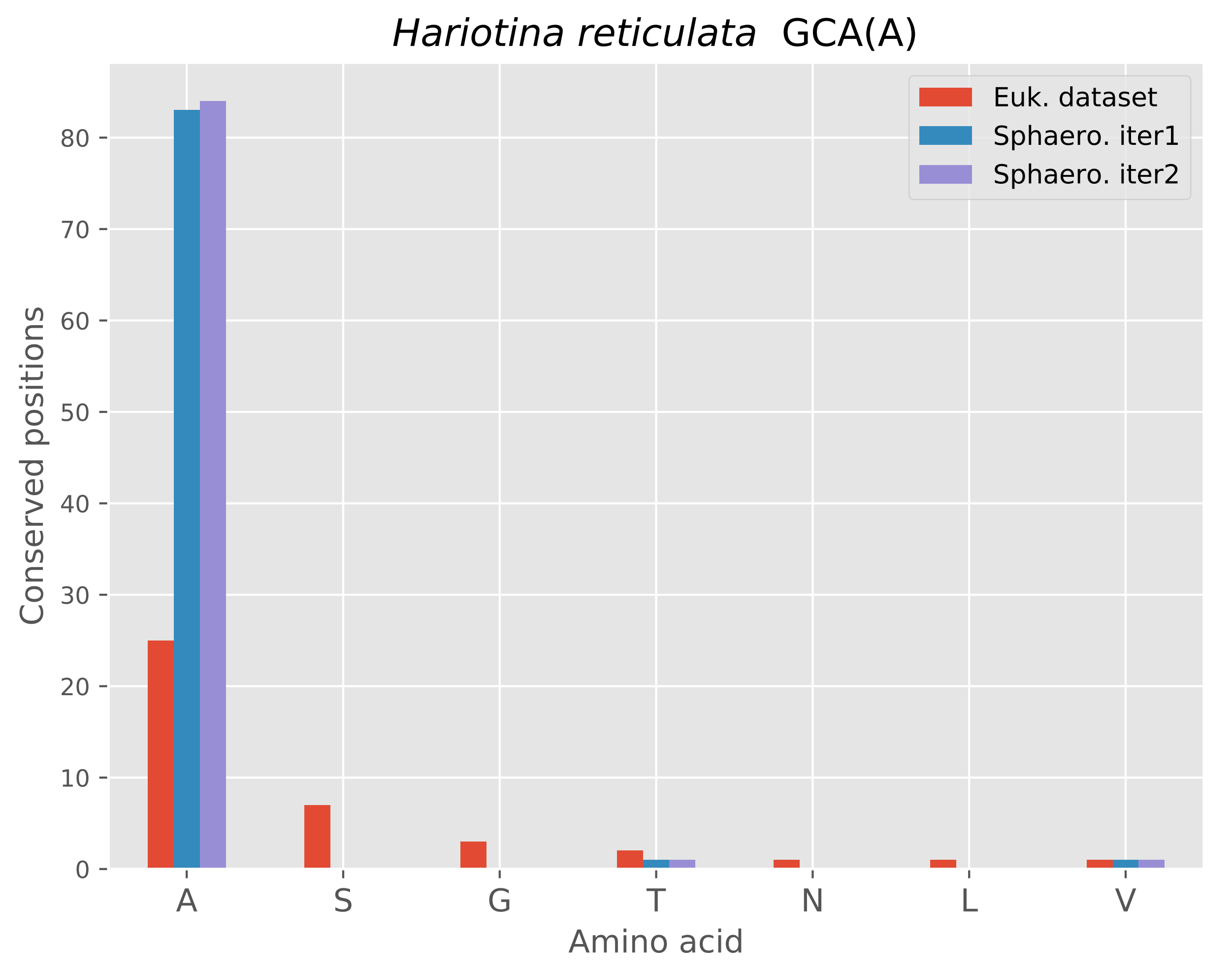

Hariotina reticulata GCA(A)
Euk. dataset
Sphaero. iter1
Sphaero. iter2
Conserved positions
0
10
20
30
40
50
60
70
80
A
S
G
T
N
L
V
Amino acid

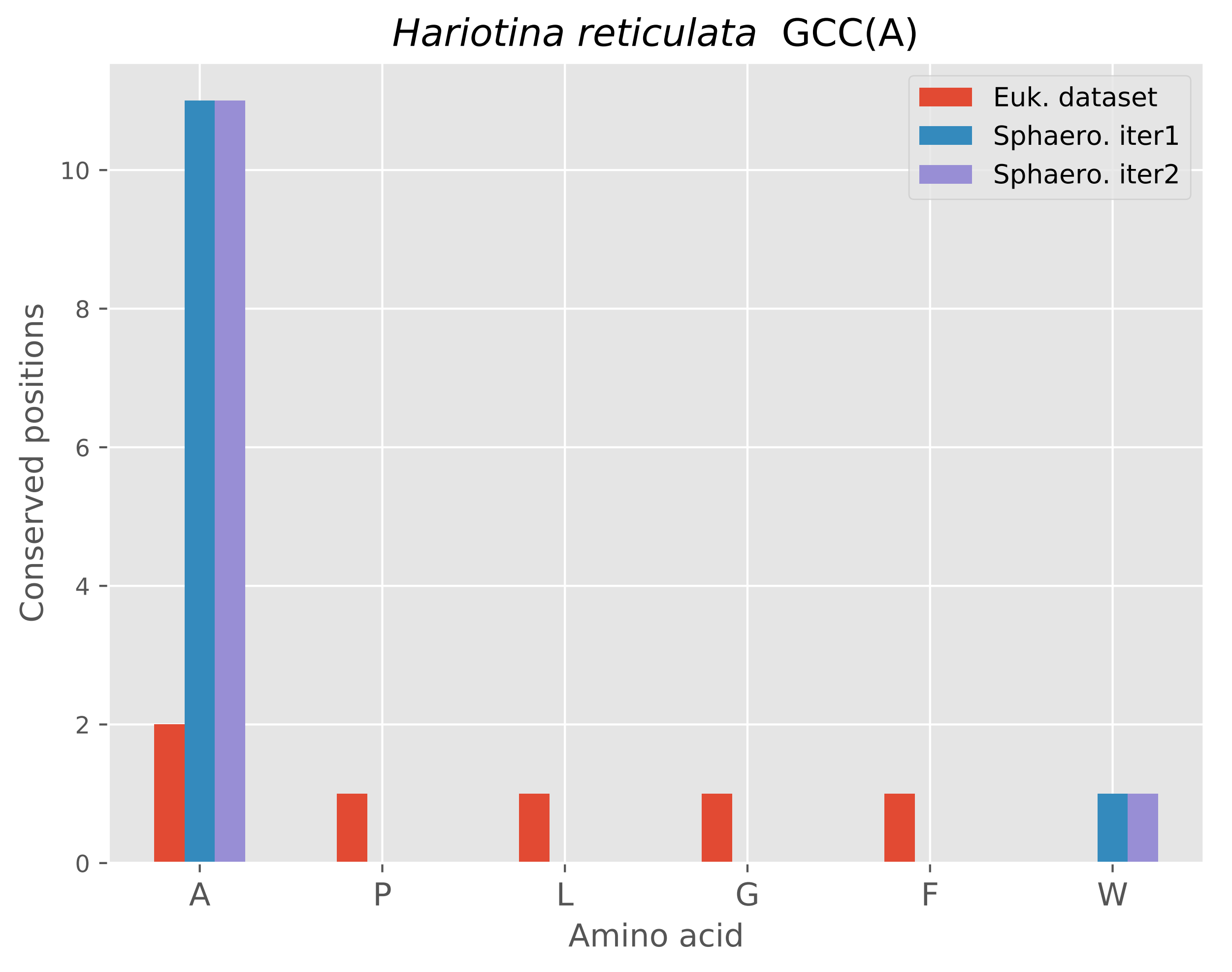
Hariotina reticulata GCC(A)
Euk. dataset
Sphaero. iter1
Sphaero. iter2
Conserved positions
0
2
4
6
8
10
A
P
L
G
F
W
Amino acid

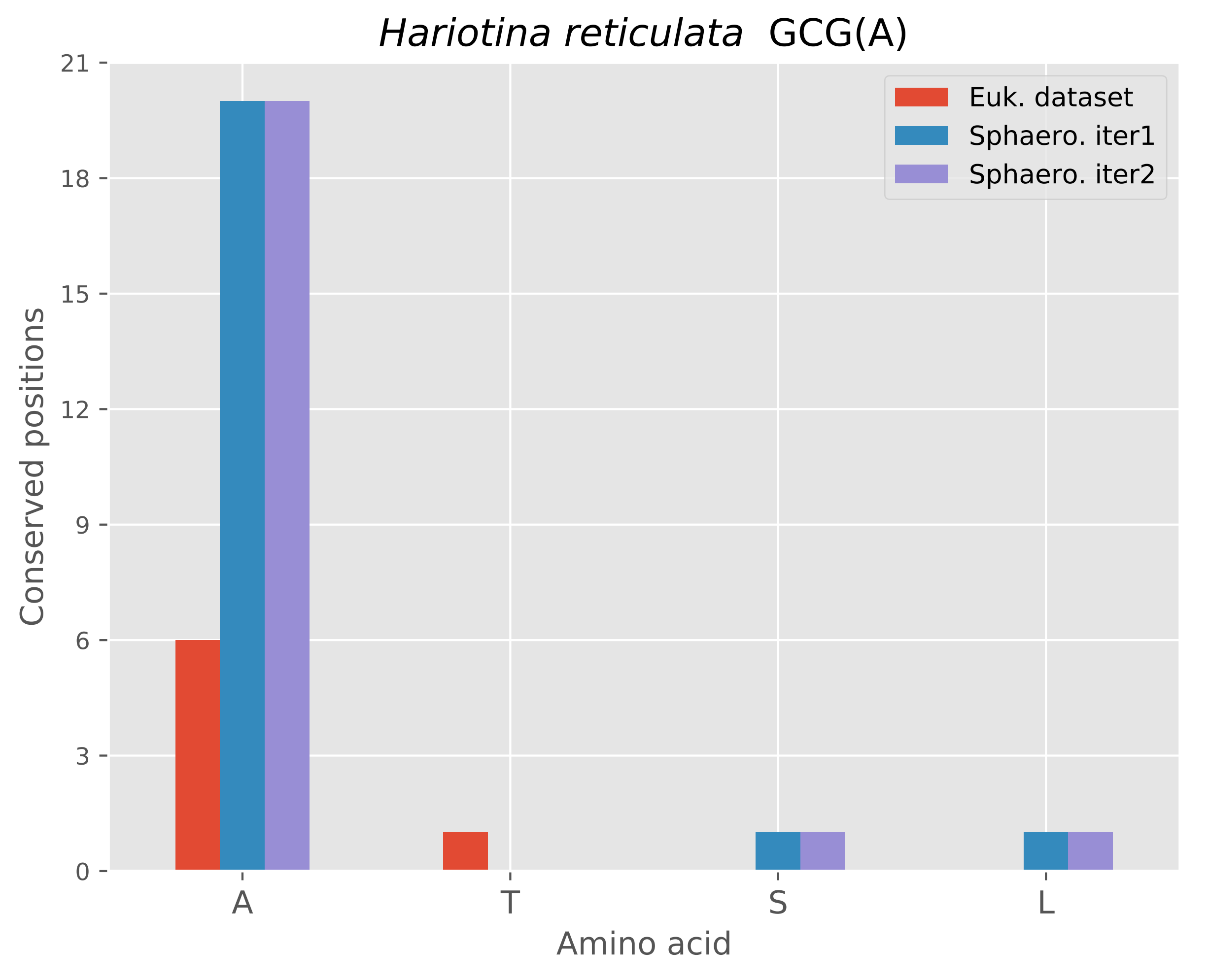

Hariotina reticulata GCG(A)
Euk. dataset
Sphaero. iter1
Sphaero. iter2
Conserved positions
Amino acid
0
3
6
9
12
15
18
21
A
T
S
L

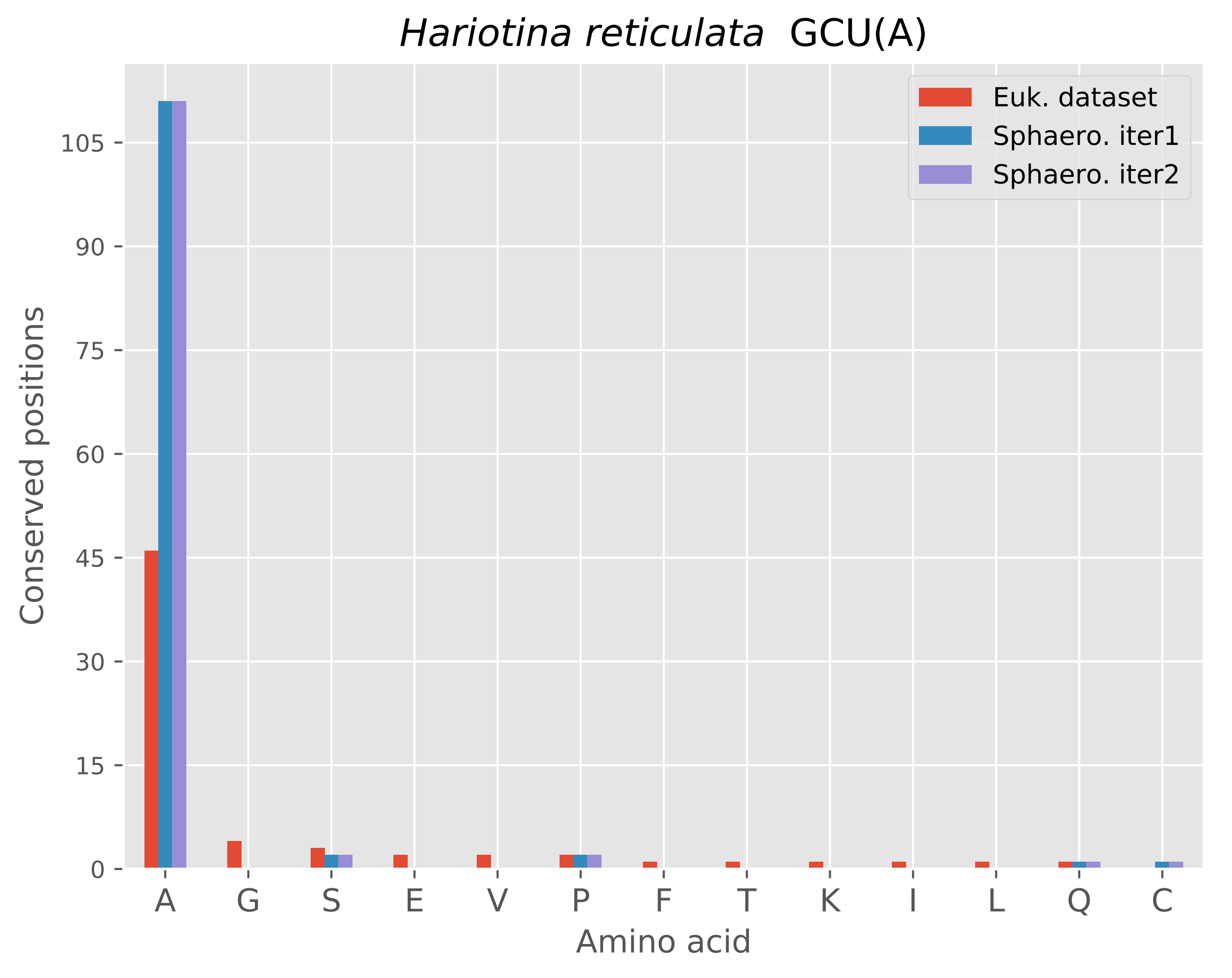
Hariotina reticulata GCU(A)
Euk. dataset
Sphaero. iter1
Sphaero. iter2
Conserved positions
0
15
30
45
60
75
90
105
A
G
S
E
V
P
F
T
K
I
L
Q
C
Amino acid

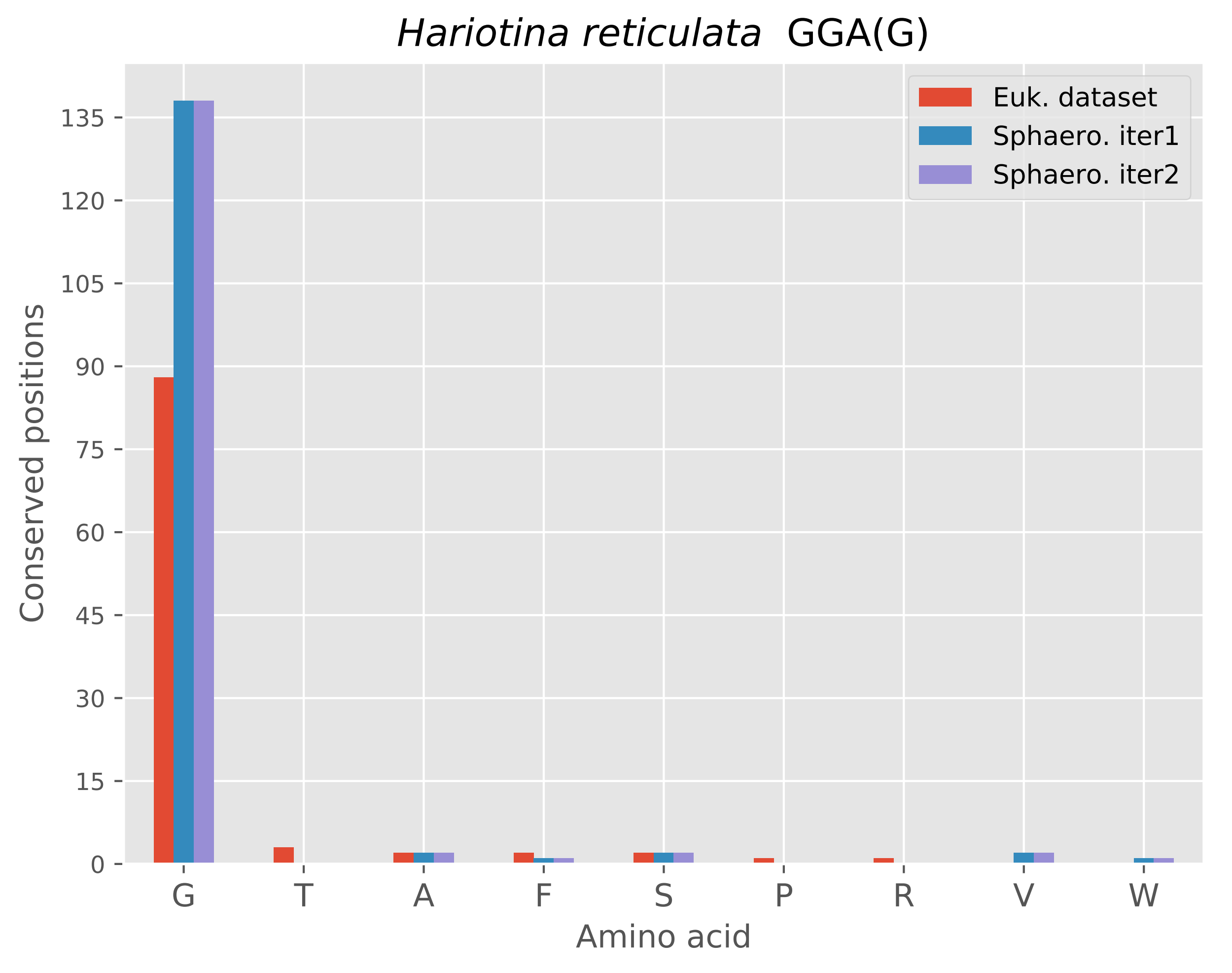

Hariotina reticulata GGA(G)
Euk. dataset
Sphaero. iter1
Sphaero. iter2
Conserved positions
0
15
30
45
60
75
90
105
120
135
G
T
A
F
S
P
R
V
W
Amino acid

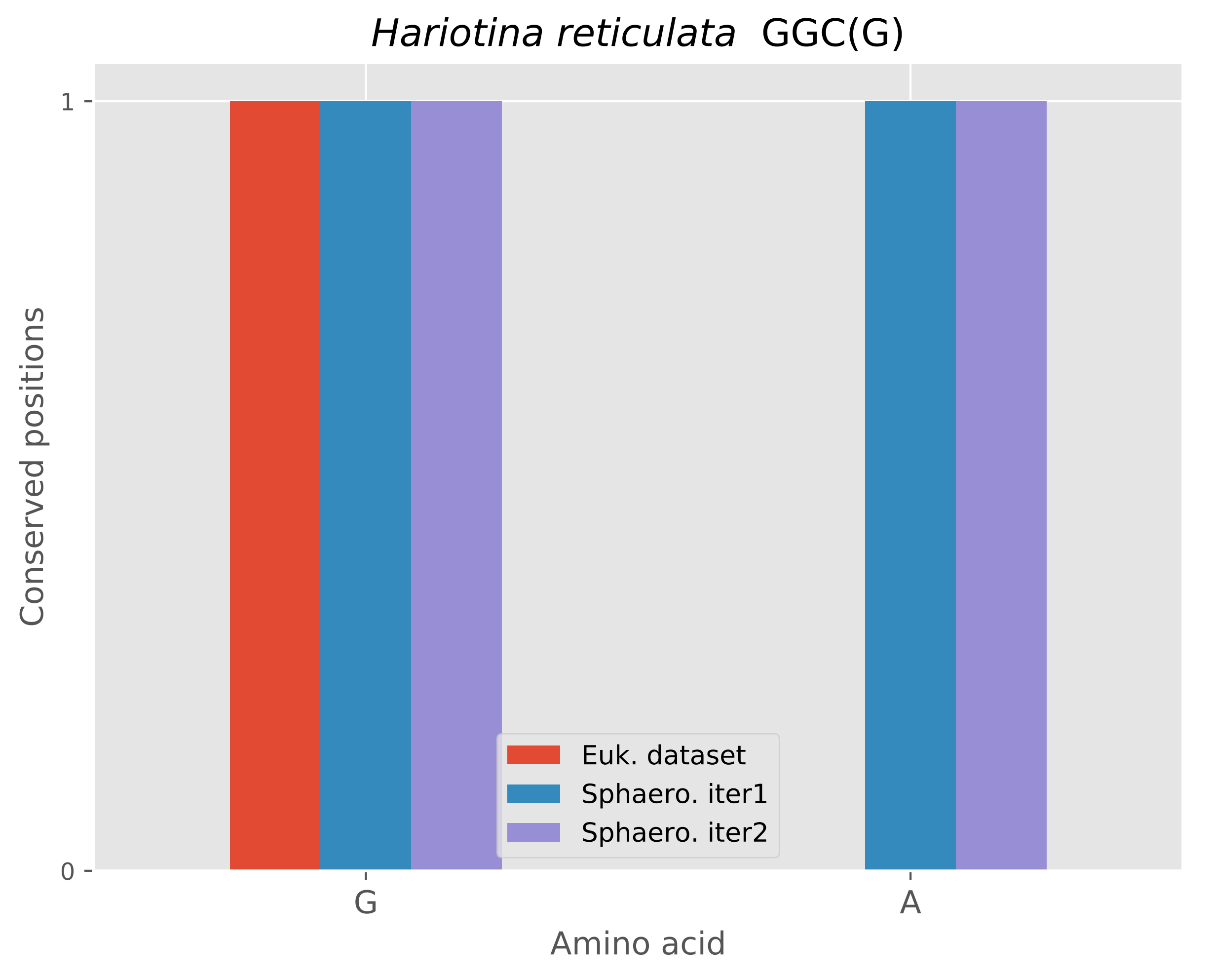

Hariotina reticulata GGC(G)
Conserved positions
1
0
G
A
Amino acid
Euk. dataset
Sphaero. iter1
Sphaero. iter2

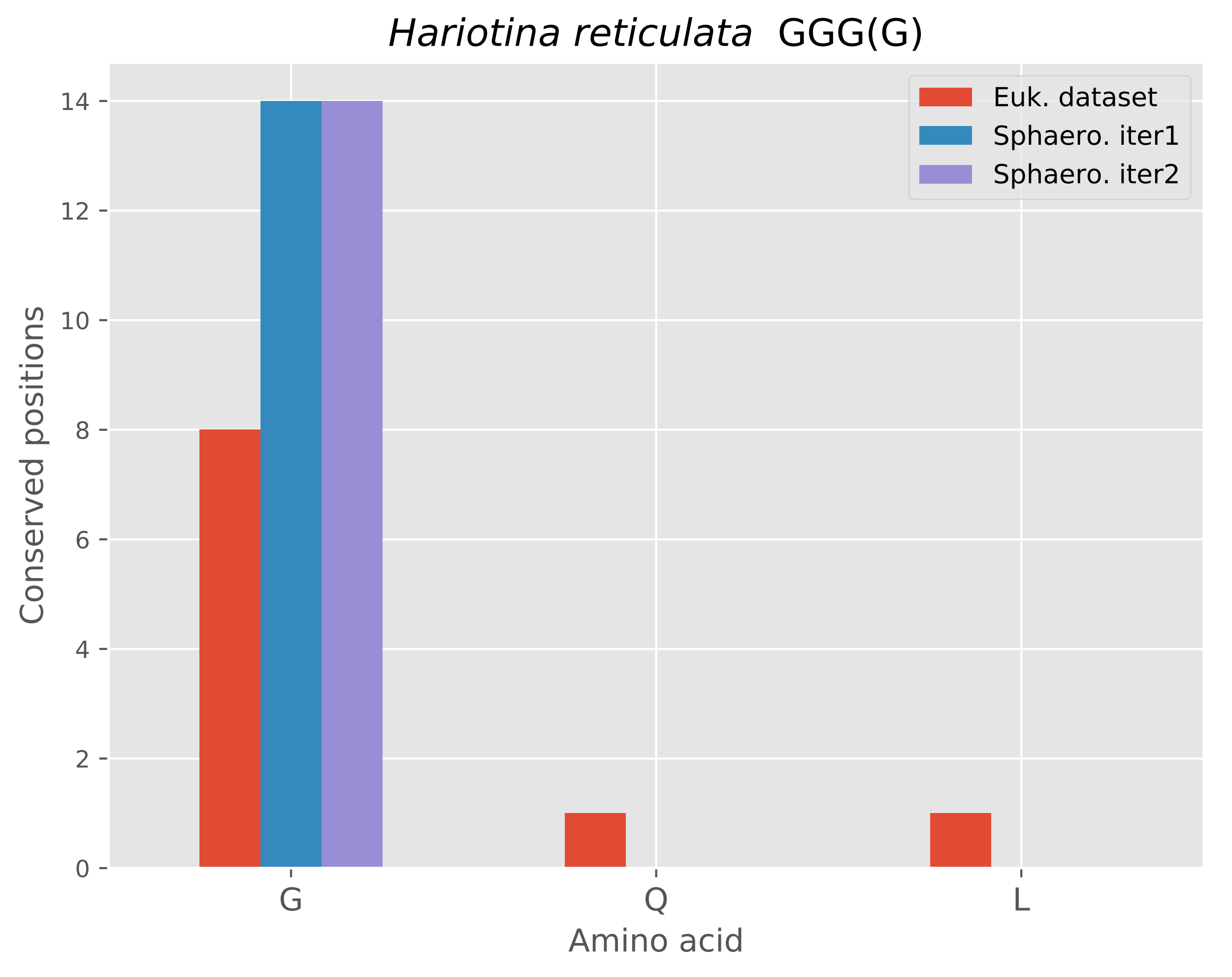

Hariotina reticulata GGG(G)
Euk. dataset
Sphaero. iter1
Sphaero. iter2
Conserved positions
0
2
4
6
8
10
12
14
G
Q
L
Amino acid

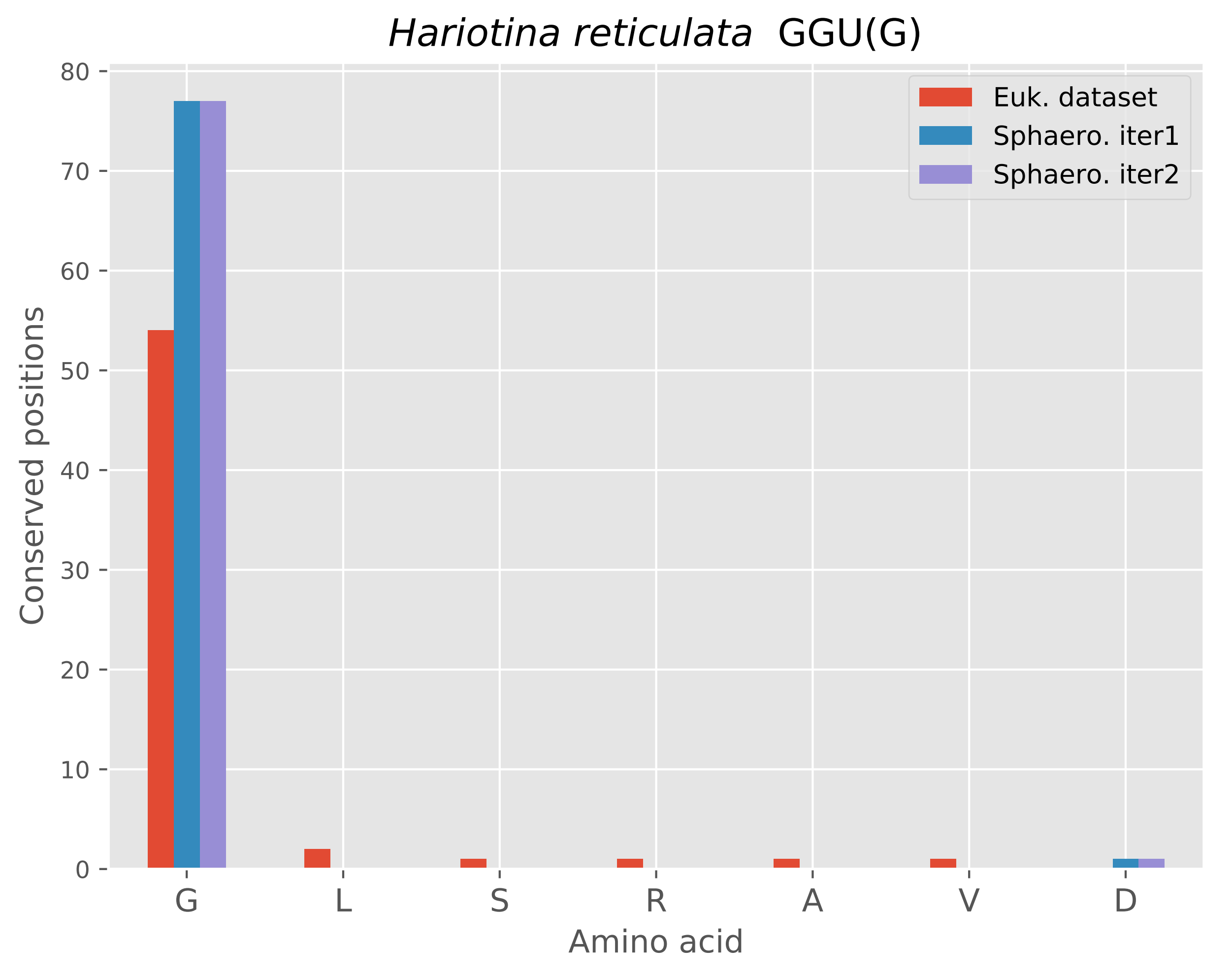

Hariotina reticulata GGU(G)
Conserved positions
Amino acid
0
10
20
30
40
50
60
70
80
G
L
S
R
A
V
D
Euk. dataset
Sphaero. iter1
Sphaero. iter2

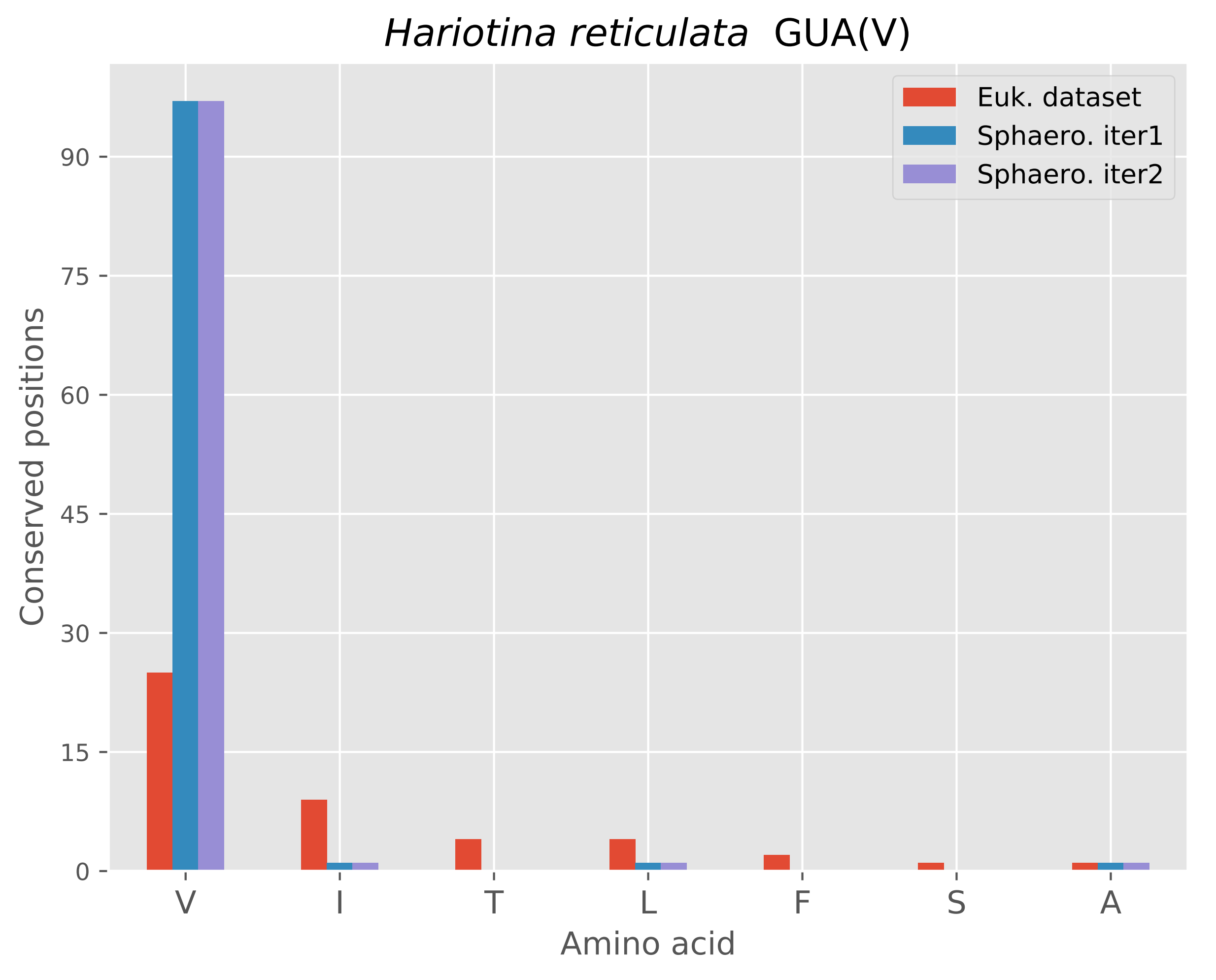
Hariotina reticulata GUA(V)
Euk. dataset
Sphaero. iter1
Sphaero. iter2
Conserved positions
0
15
30
45
60
75
90
V
I
T
L
F
S
A
Amino acid

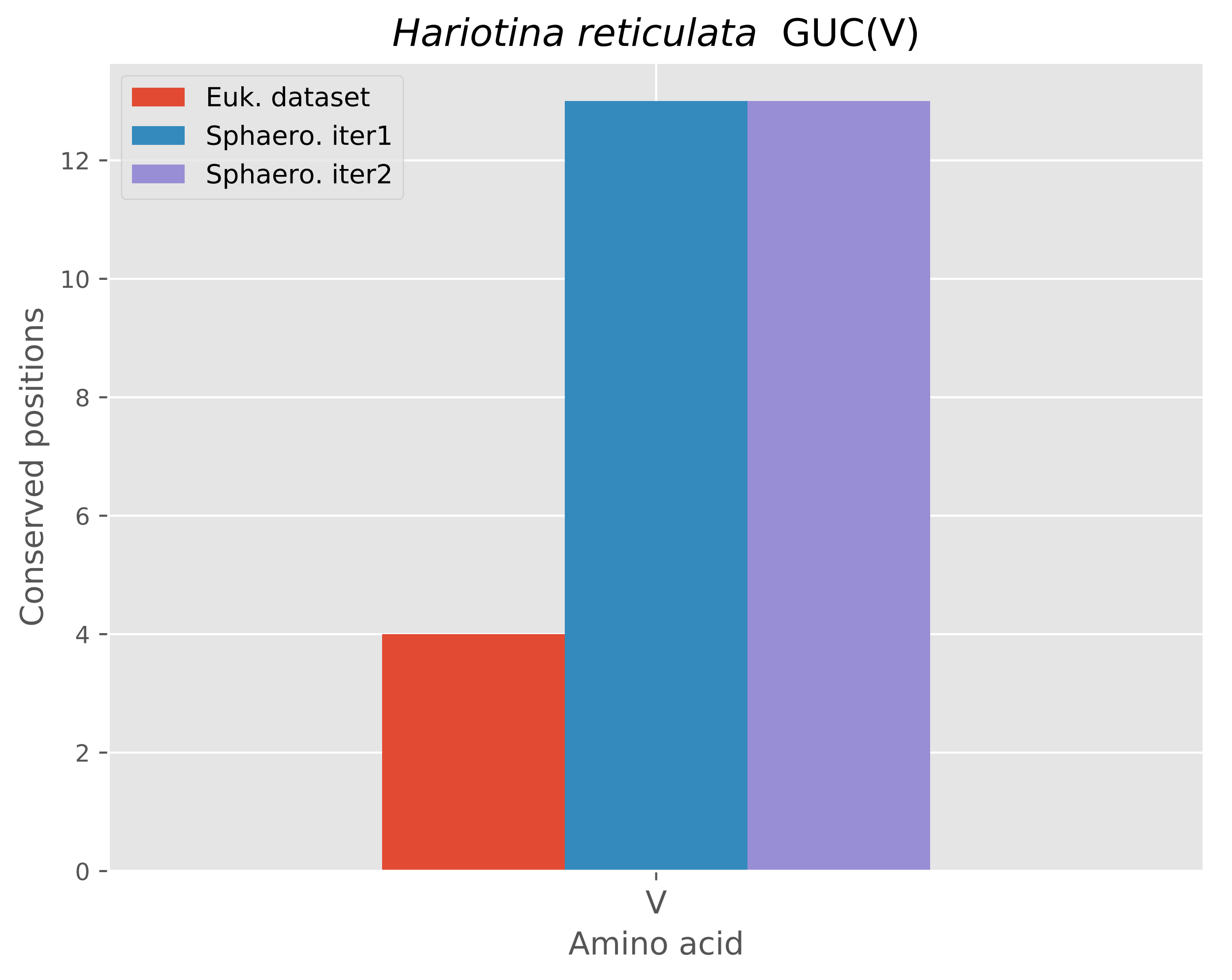

Hariotina reticulata GUC(V)
Euk. dataset
Sphaero. iter1
Sphaero. iter2
Conserved positions
0
2
4
6
8
10
12
V
Amino acid

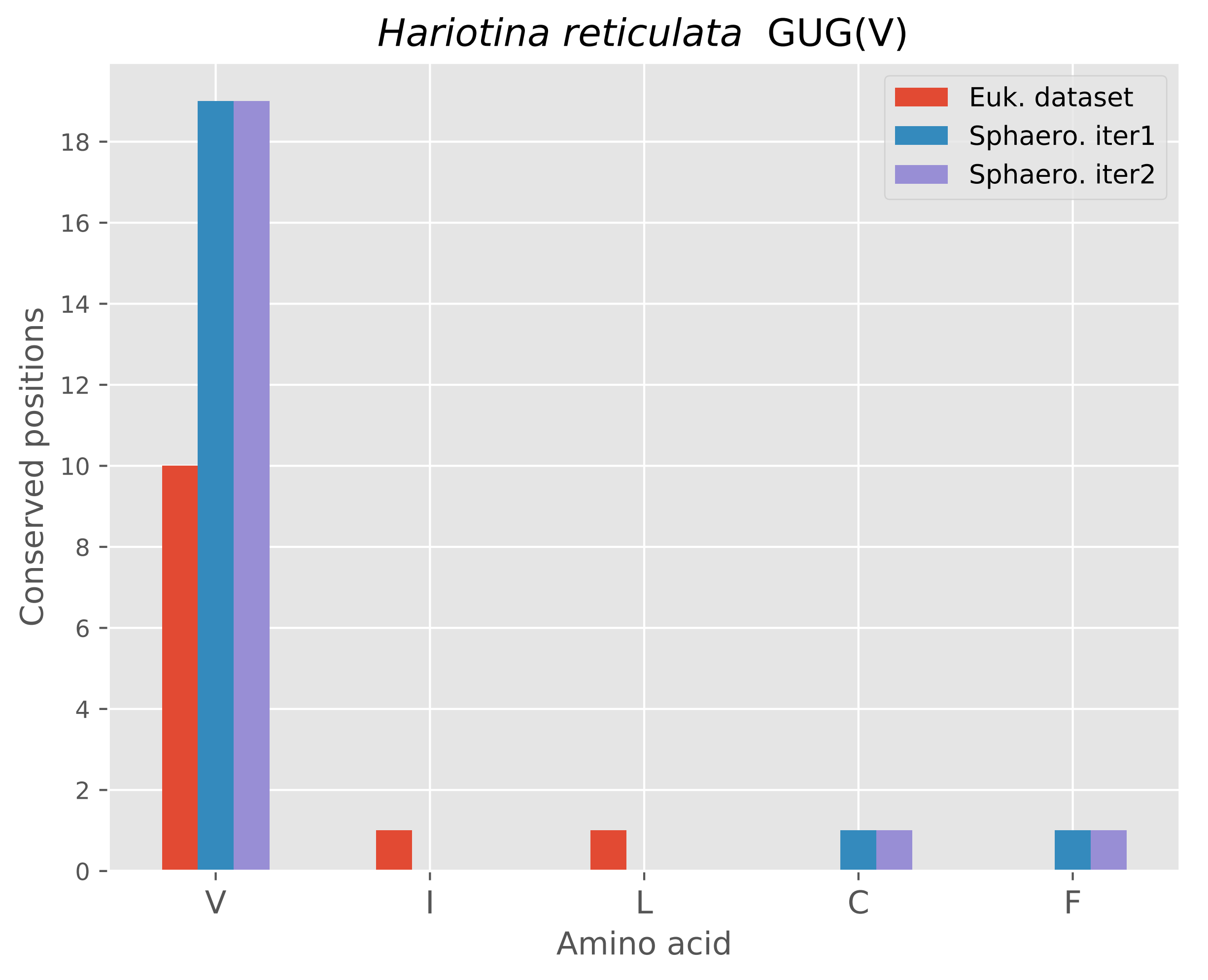

Hariotina reticulata GUG(V)
Euk. dataset
Sphaero. iter1
Sphaero. iter2
Conserved positions
0
2
4
6
8
10
12
14
16
18
V
I
L
C
F
Amino acid

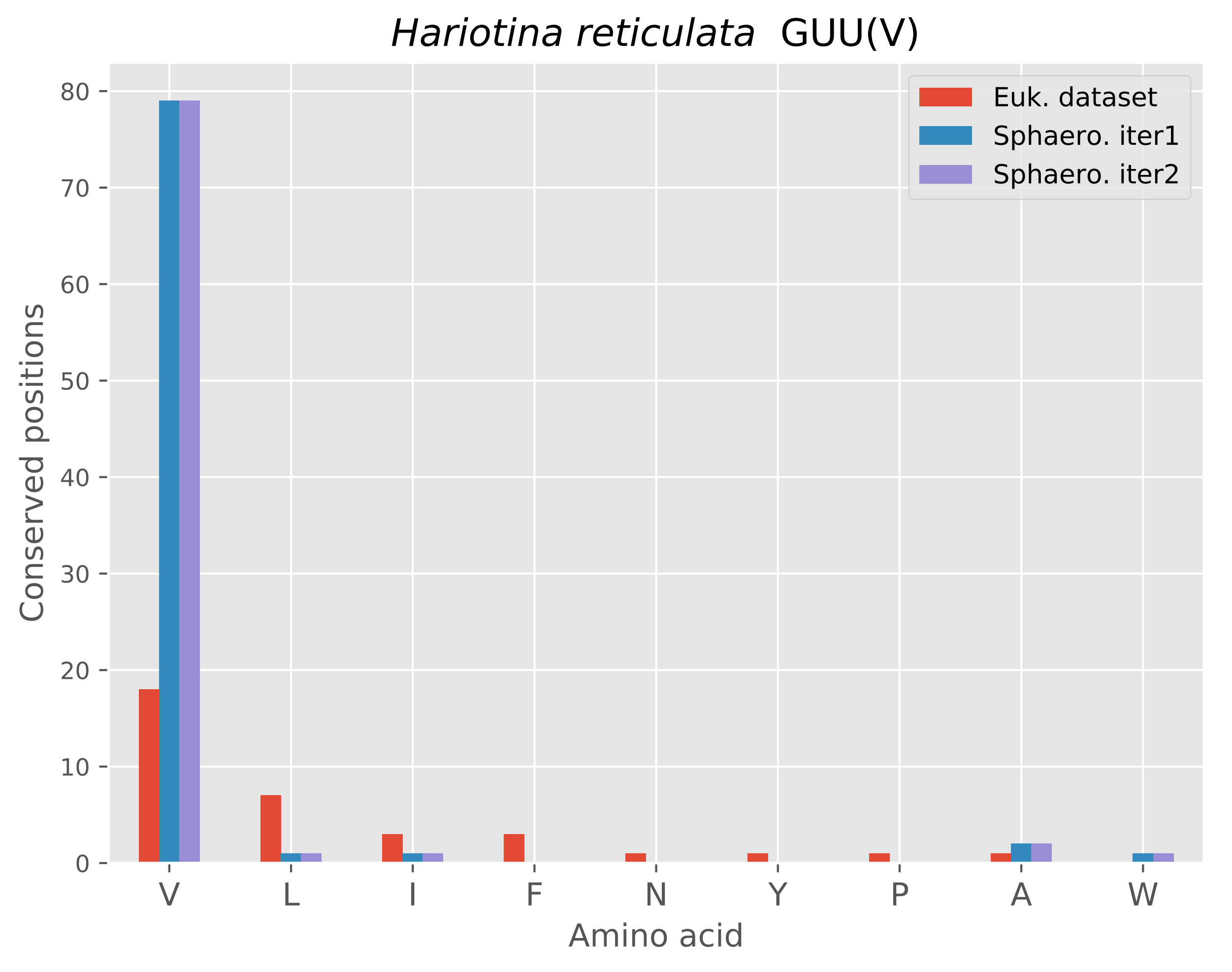
Hariotina reticulata GUU(V)
Euk. dataset
Sphaero. iter1
Sphaero. iter2
Conserved positions
0
10
20
30
40
50
60
70
80
V
L
I
F
N
Y
P
A
W
Amino acid

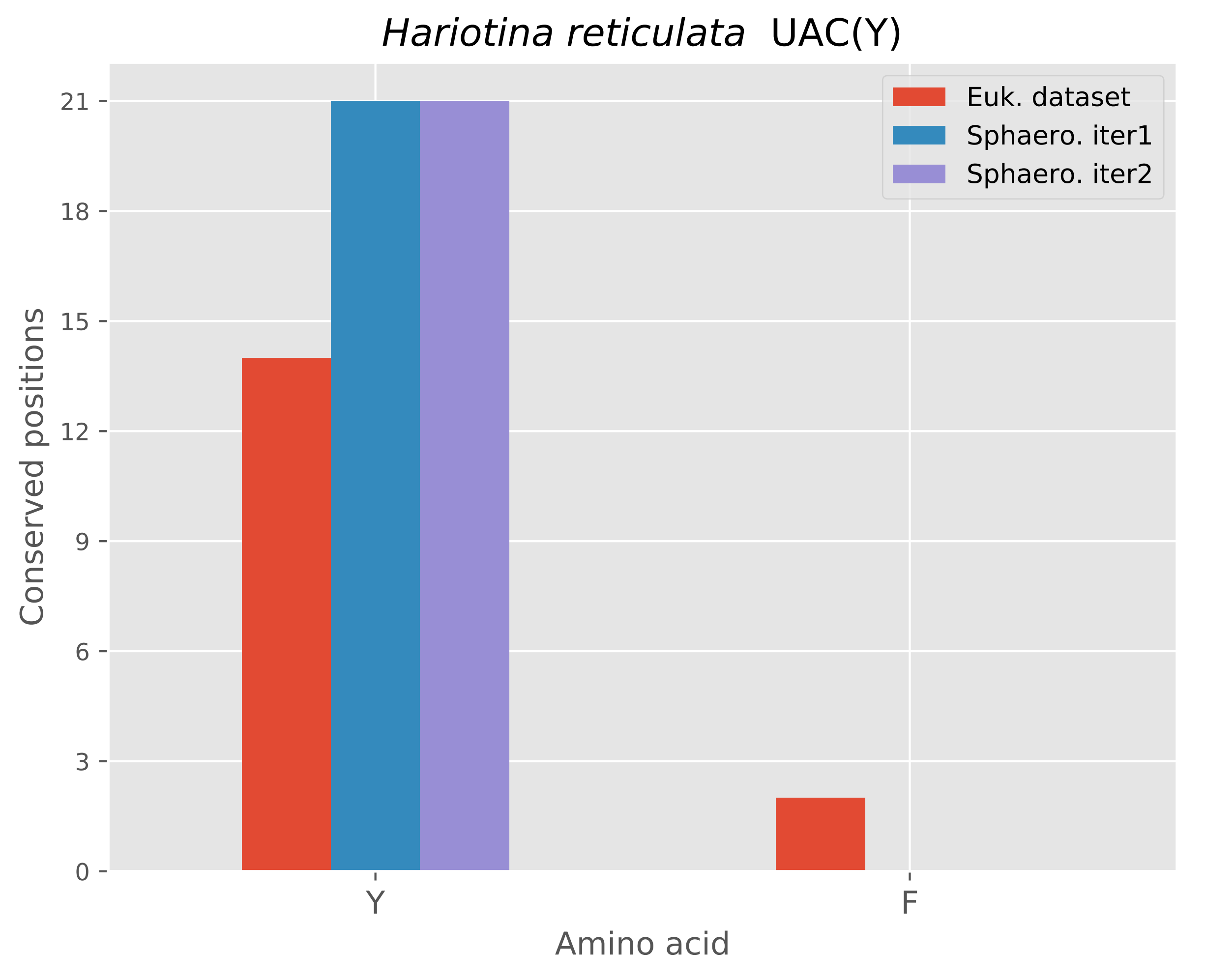

Hariotina reticulata UAC(Y)
Euk. dataset
Sphaero. iter1
Sphaero. iter2
Conserved positions
0
3
6
9
12
15
18
21
Y
F
Amino acid

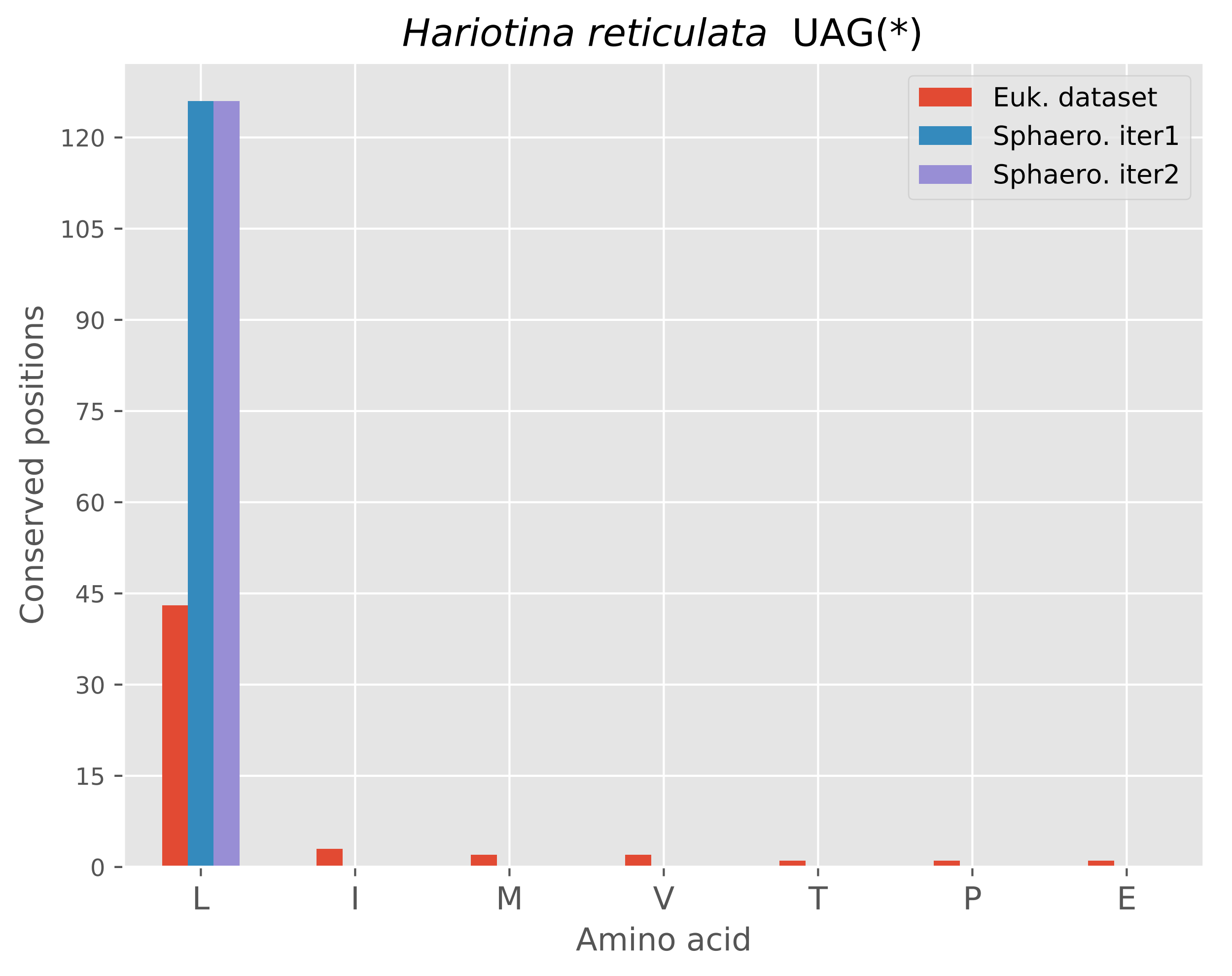
Hariotina reticulata UAG(*)
Euk. dataset
Sphaero. iter1
Sphaero. iter2
Conserved positions
0
15
30
45
60
75
90
105
120
L
I
M
V
T
P
E
Amino acid

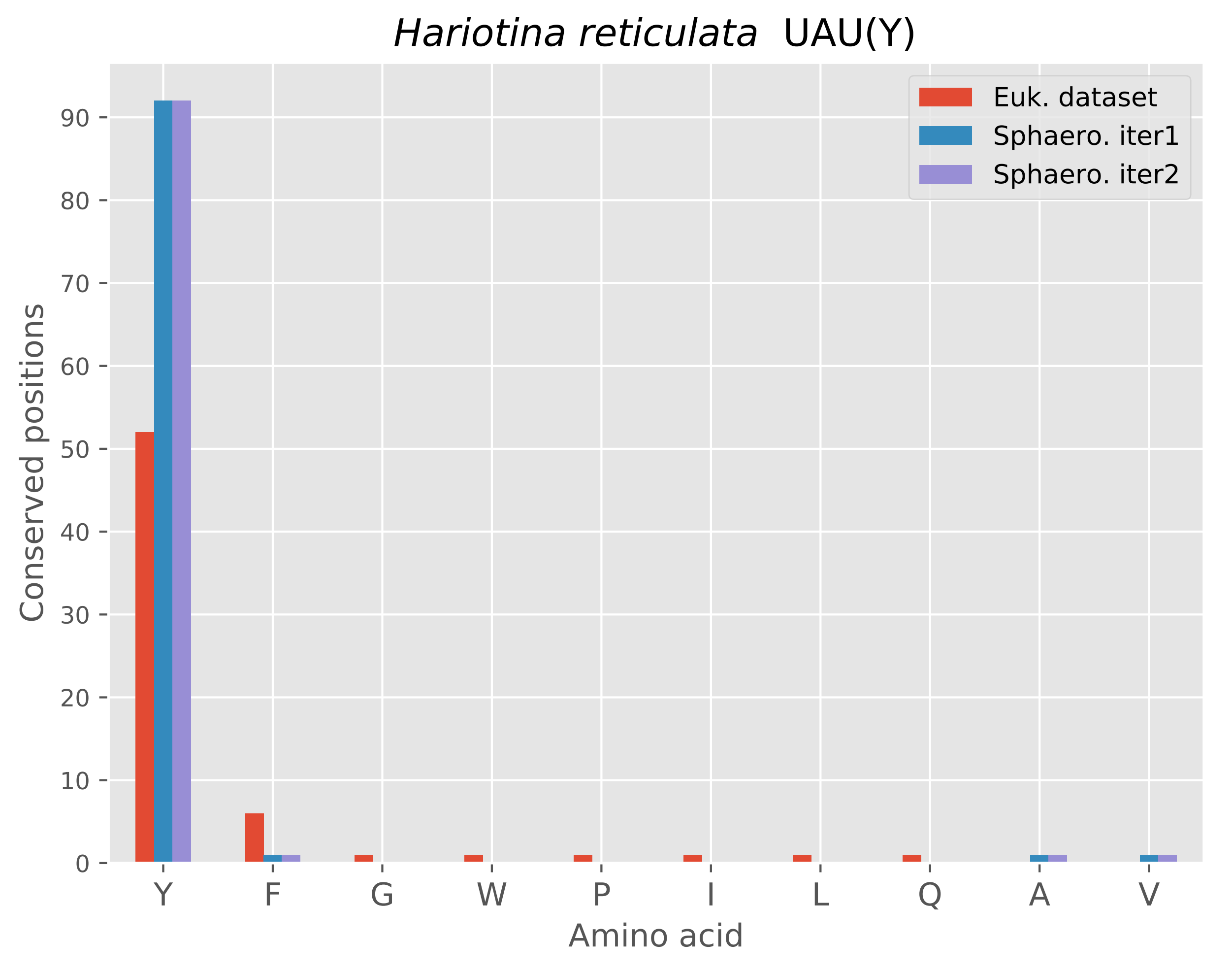

Hariotina reticulata UAU(Y)
Euk. dataset
Sphaero. iter1
Sphaero. iter2
Conserved positions
0
10
20
30
40
50
60
70
80
90
Y
F
G
W
P
I
L
Q
A
V
Amino acid

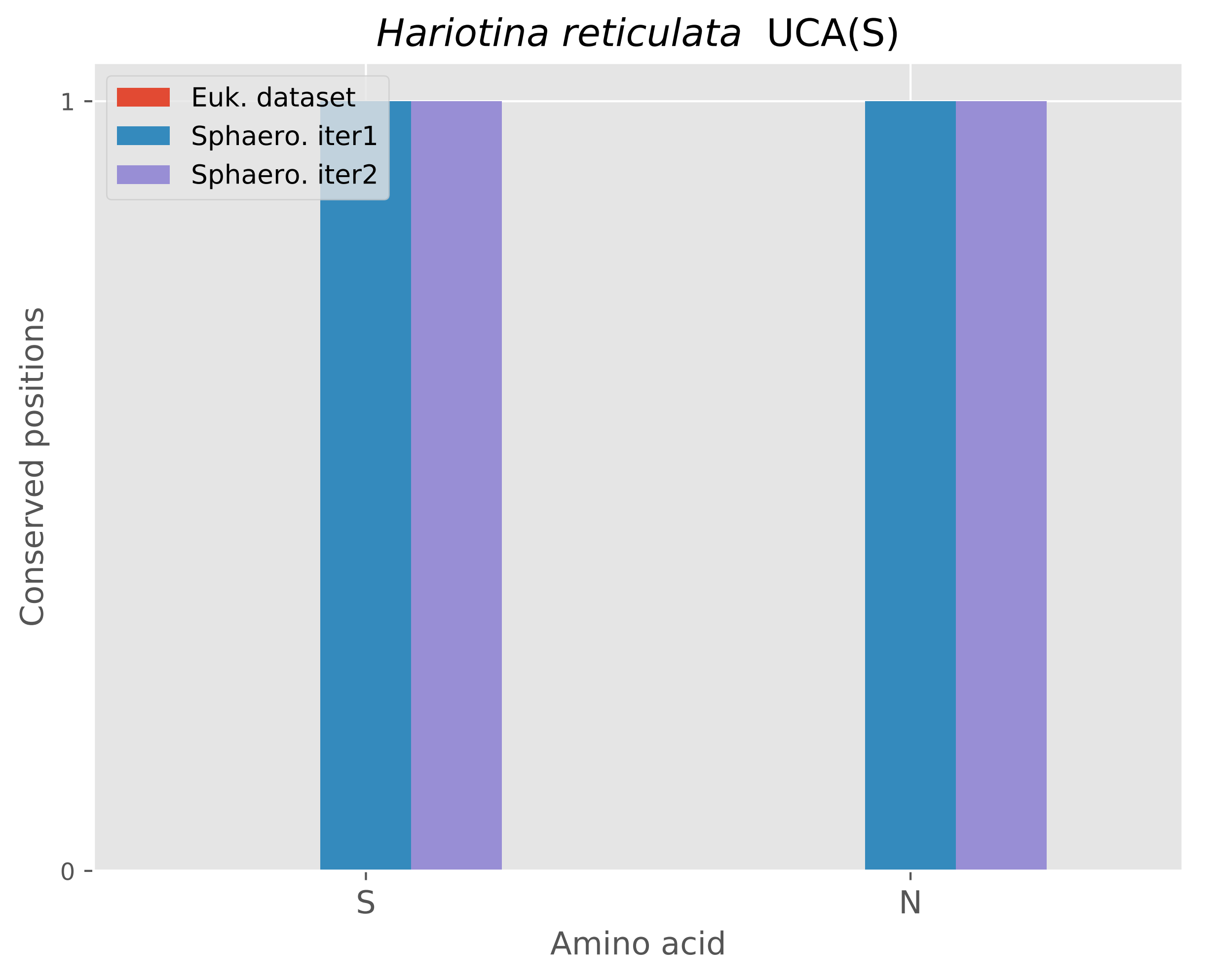

Hariotina reticulata UCA(S)
Euk. dataset
Sphaero. iter1
Sphaero. iter2
Conserved positions
1
0
S
N
Amino acid

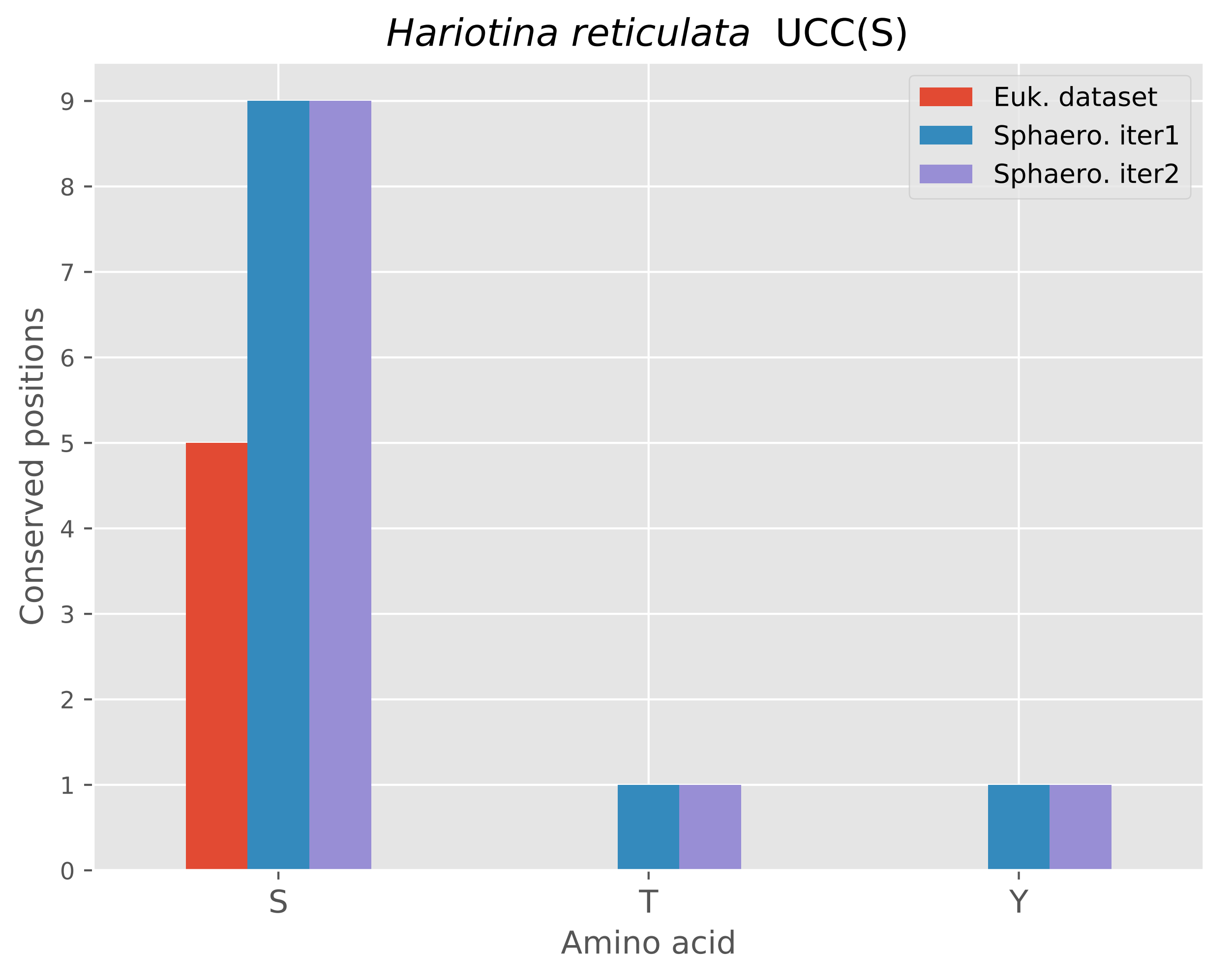
Hariotina reticulata UCC(S)
Euk. dataset
Sphaero. iter1
Sphaero. iter2
Conserved positions
0
1
2
3
4
5
6
7
8
9
S
T
Y
Amino acid

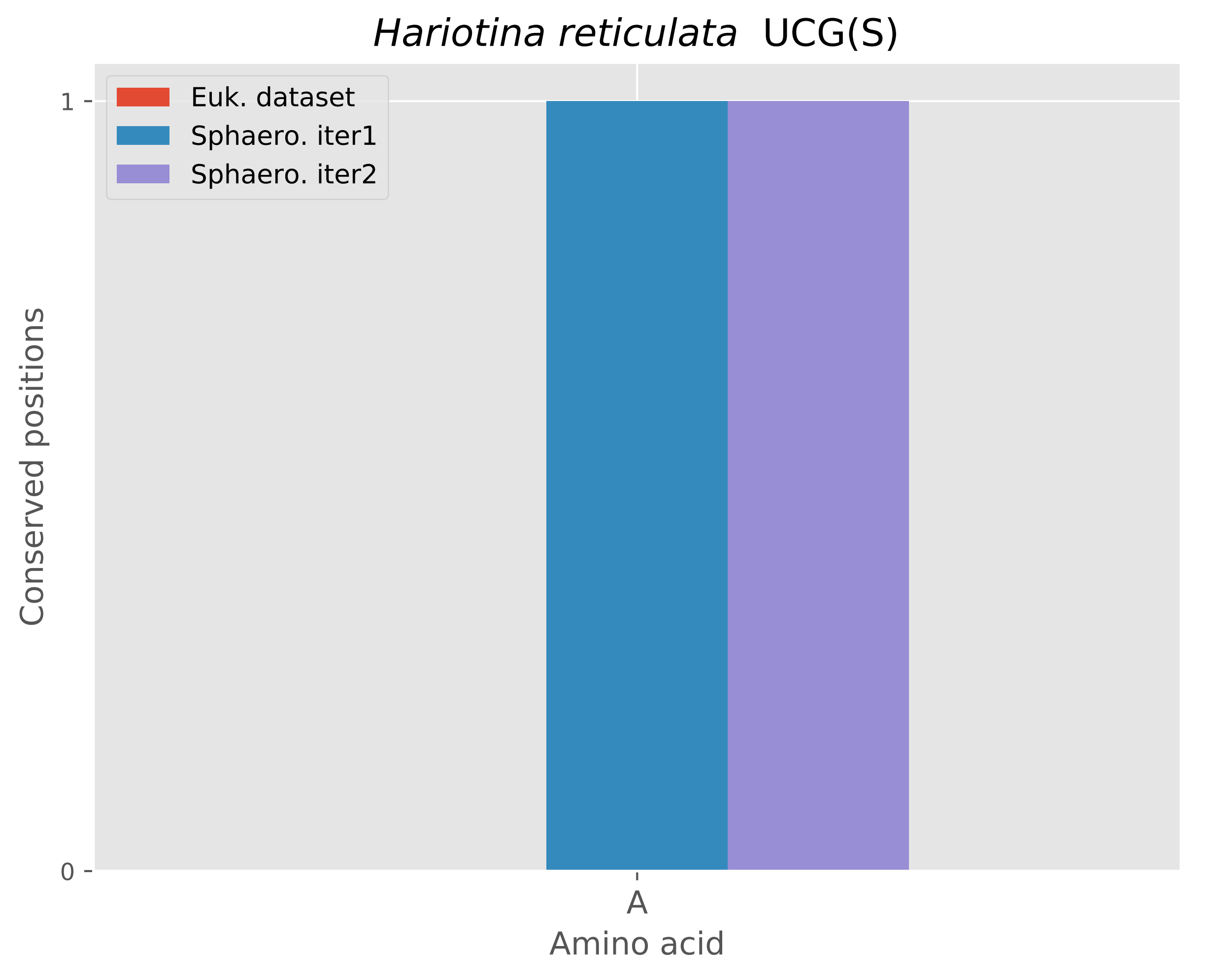

Hariotina reticulata UCG(S)
Euk. dataset
Sphaero. iter1
Sphaero. iter2
1
0
Conserved positions
A
Amino acid

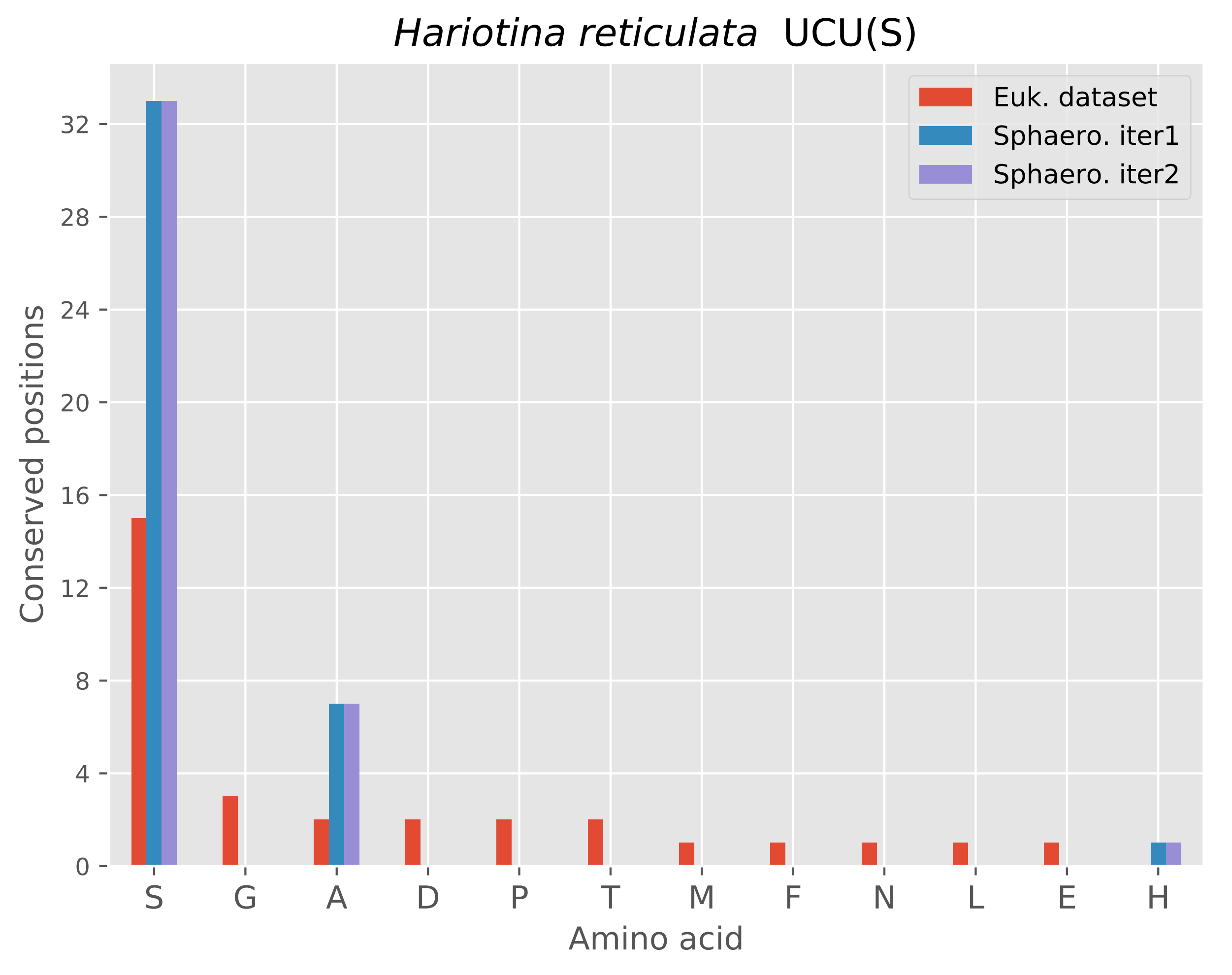

Hariotina reticulata UCU(S)
Euk. dataset
Sphaero. iter1
Sphaero. iter2
Conserved positions
0
4
8
12
16
20
24
28
32
S
G
A
D
P
T
M
F
N
L
E
H
Amino acid

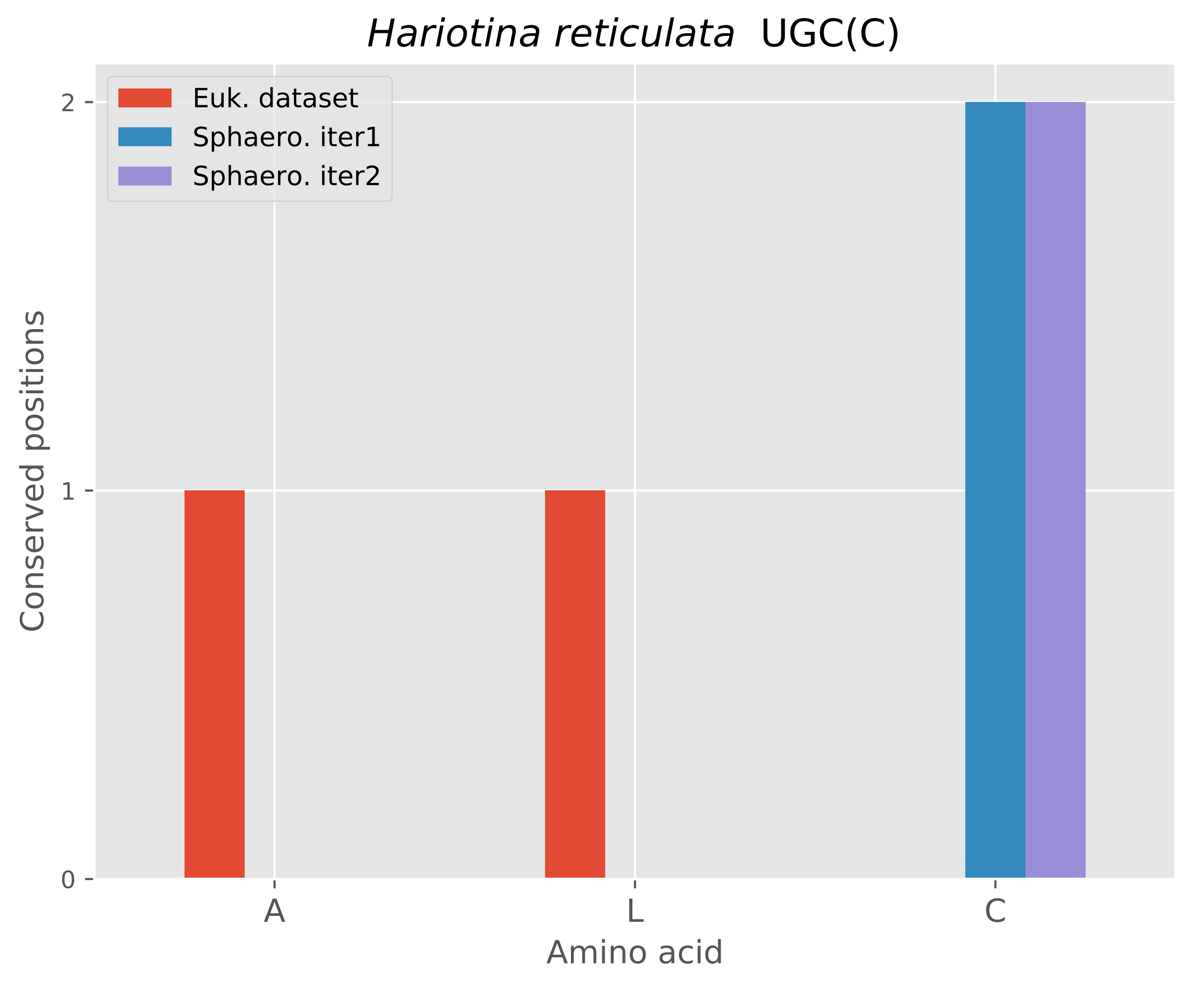

Hariotina reticulata UGC(C)
Euk. dataset
Sphaero. iter1
Sphaero. iter2
Conserved positions
0
1
2
A
L
C
Amino acid

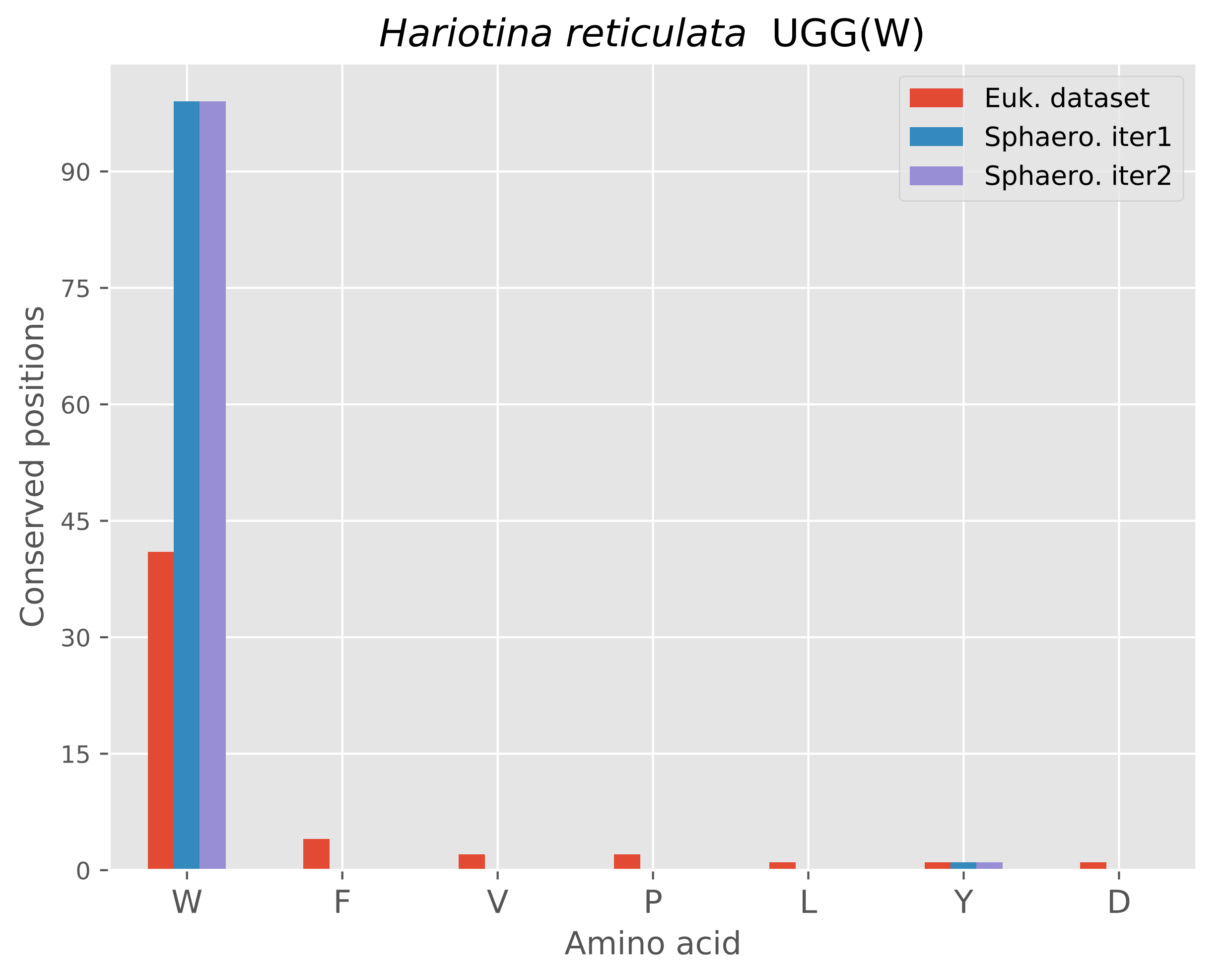

Hariotina reticulata UGG(W)
Euk. dataset
Sphaero. iter1
Sphaero. iter2
Conserved positions
0
15
30
45
60
75
90
W
F
V
P
L
Y
D
Amino acid

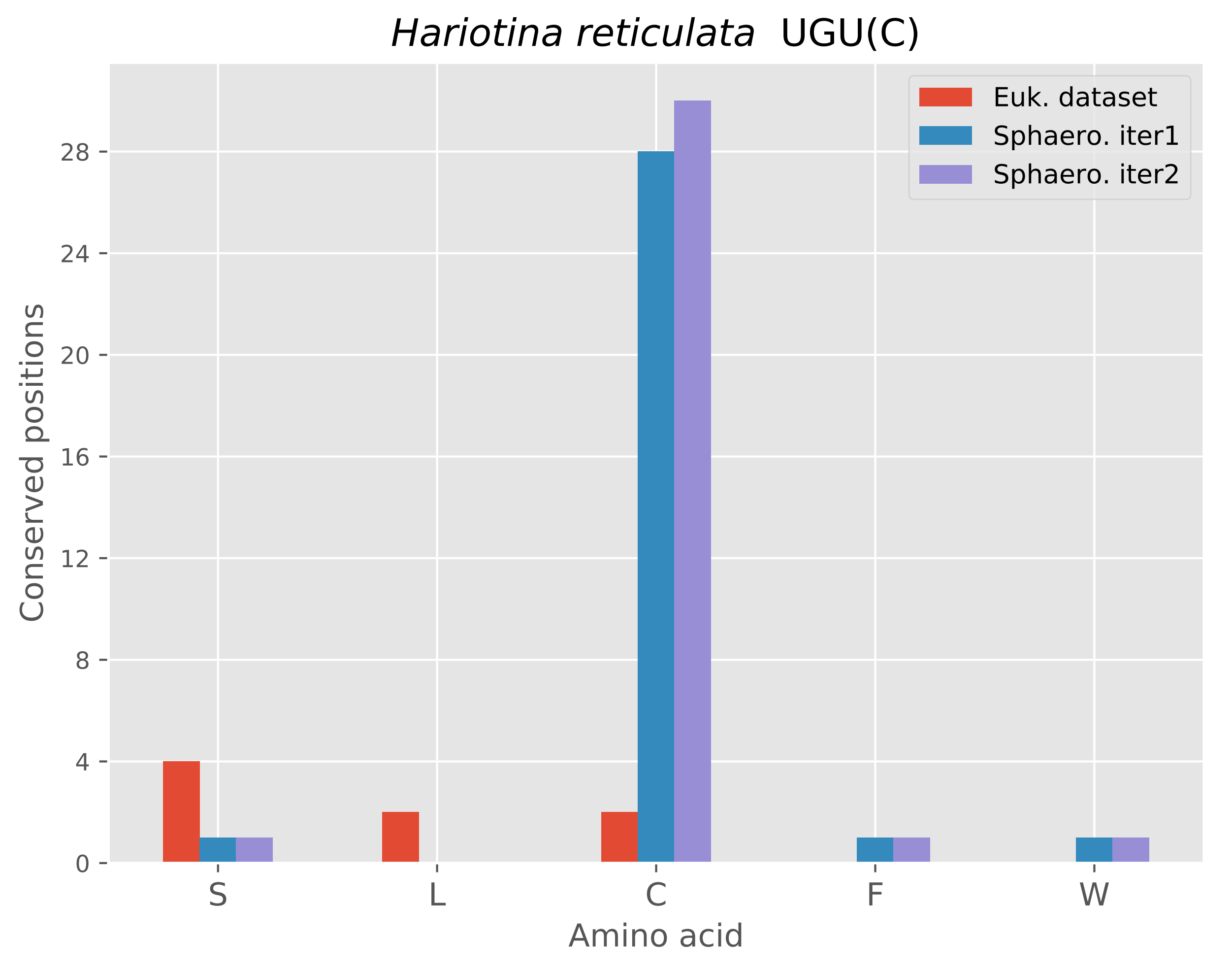

Hariotina reticulata UGU(C)
Euk. dataset
Sphaero. iter1
Sphaero. iter2
Conserved positions
0
4
8
12
16
20
24
28
S
L
C
F
W
Amino acid

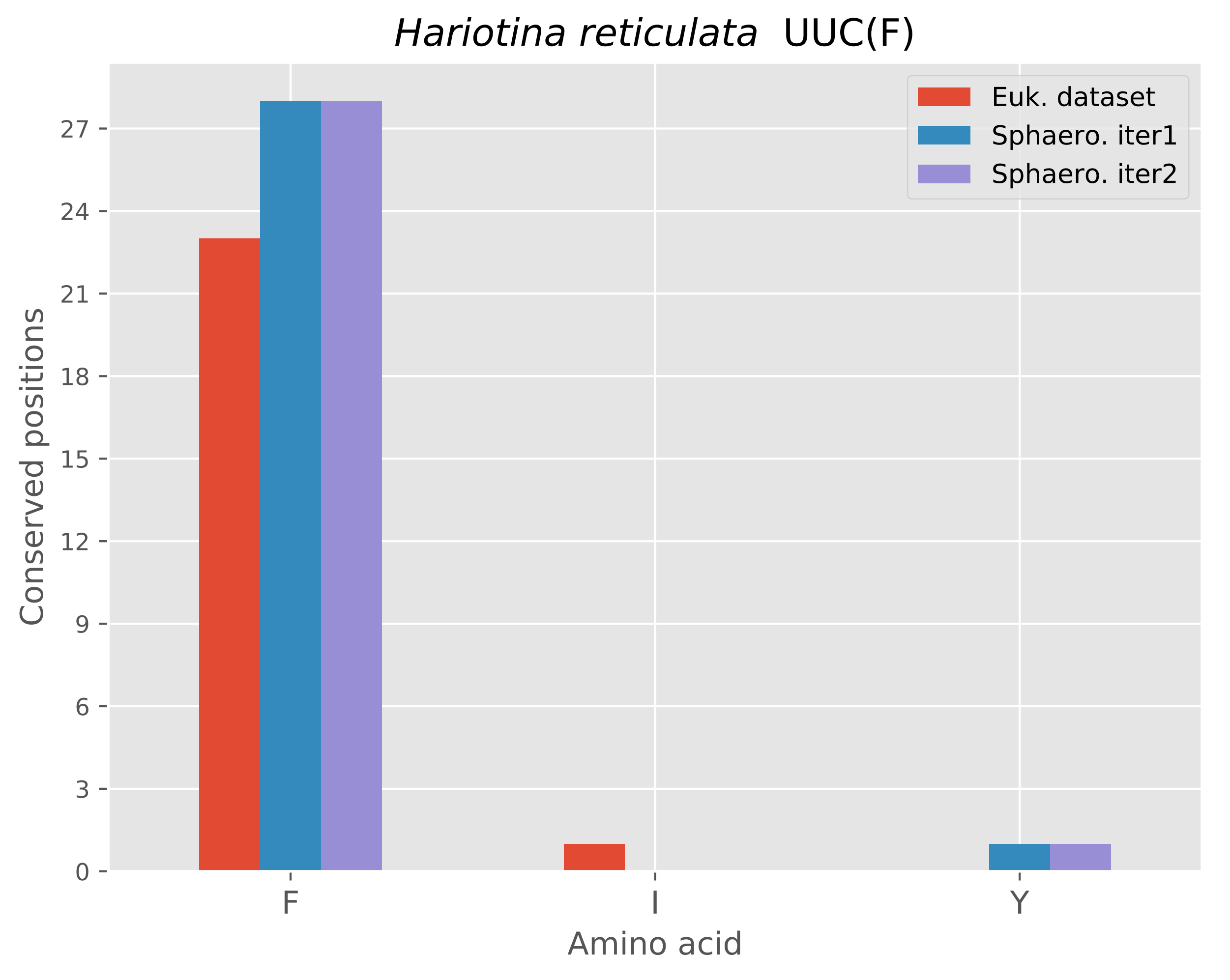
Hariotina reticulata UUC(F)
Euk. dataset
Sphaero. iter1
Sphaero. iter2
Conserved positions
0
3
6
9
12
15
18
21
24
27
F
I
Y
Amino acid

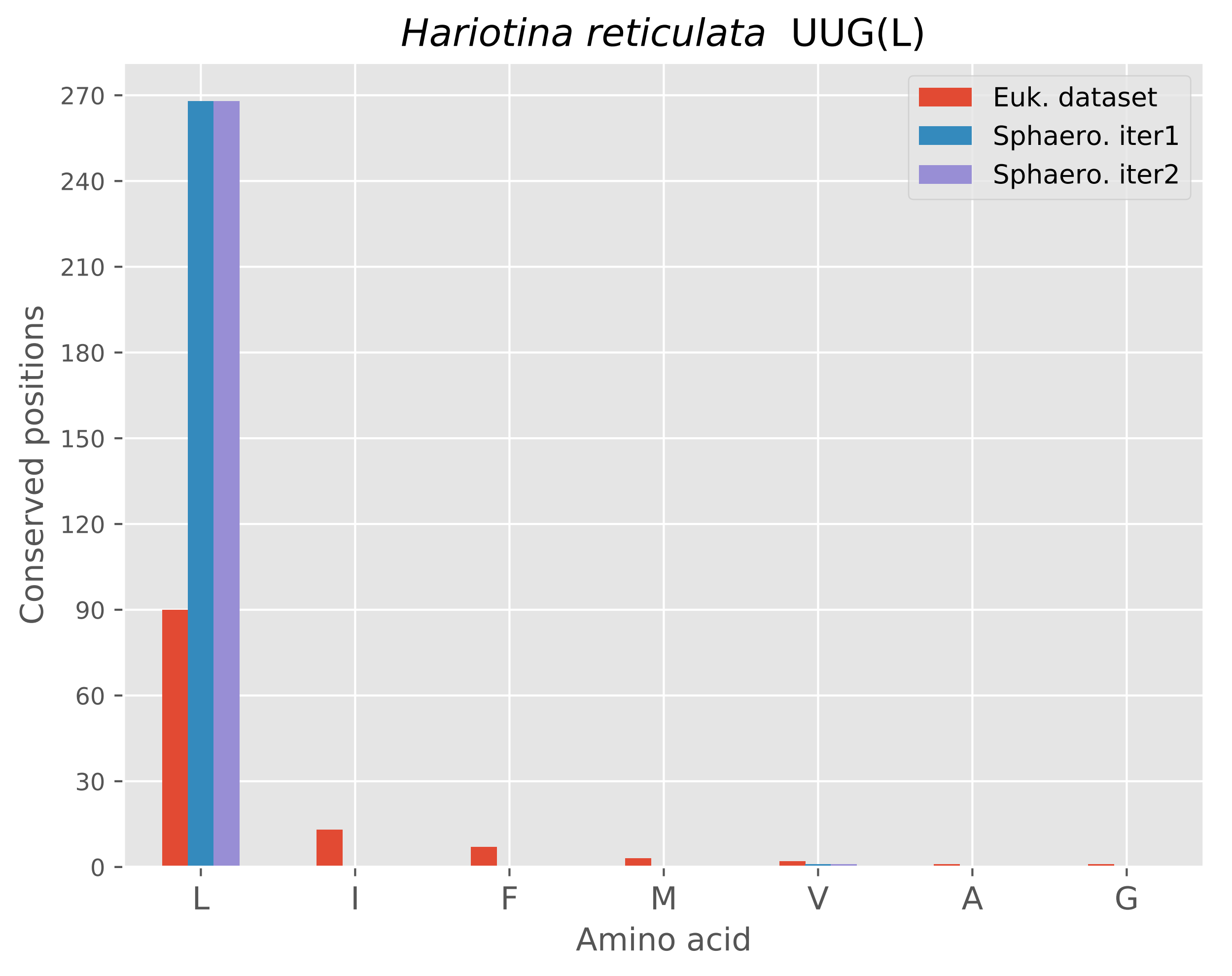

Hariotina reticulata UUG(L)
Euk. dataset
Sphaero. iter1
Sphaero. iter2
Conserved positions
0
30
60
90
120
150
180
210
240
270
L
I
F
M
V
A
G
Amino acid

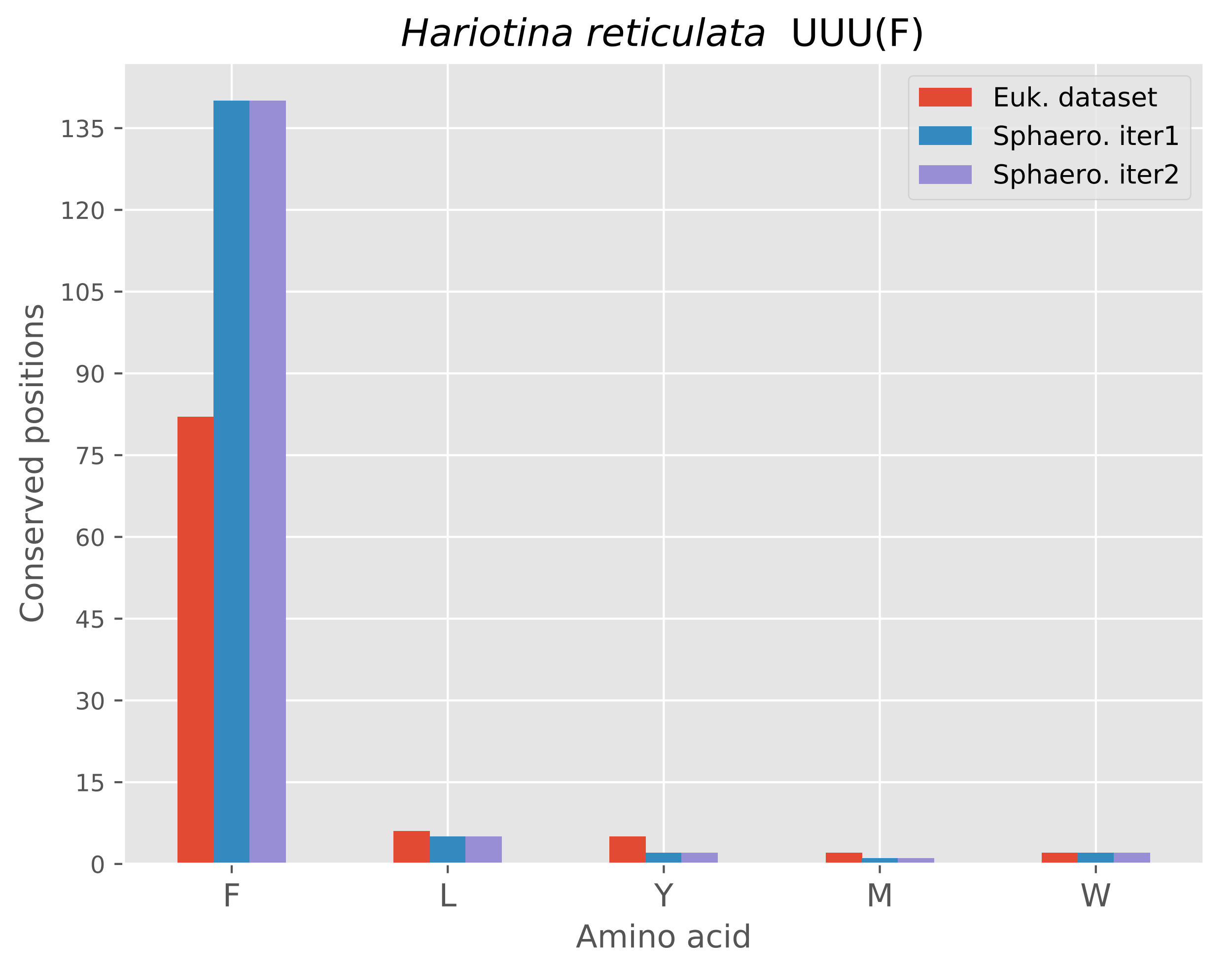
Hariotina reticulata UUU(F)
Euk. dataset
Sphaero. iter1
Sphaero. iter2
Conserved positions
0
15
30
45
60
75
90
105
120
135
F
L
Y
M
W
Amino acid

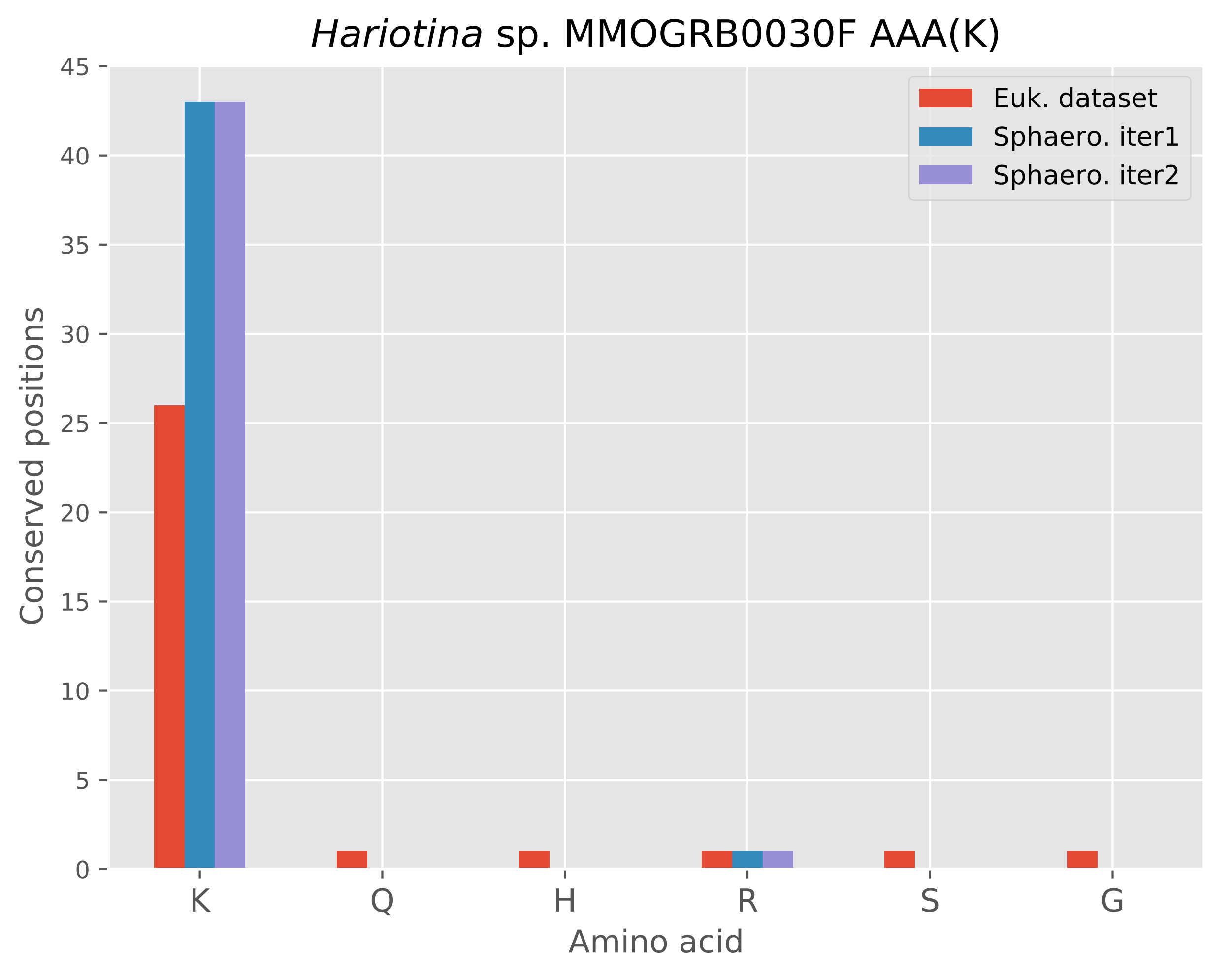

Hariotina sp. MMOGRB0030F AAA(K)
Euk. dataset
Sphaero. iter1
Sphaero. iter2
Conserved positions
0
5
10
15
20
25
30
35
40
45
K
Q
H
R
S
G
Amino acid

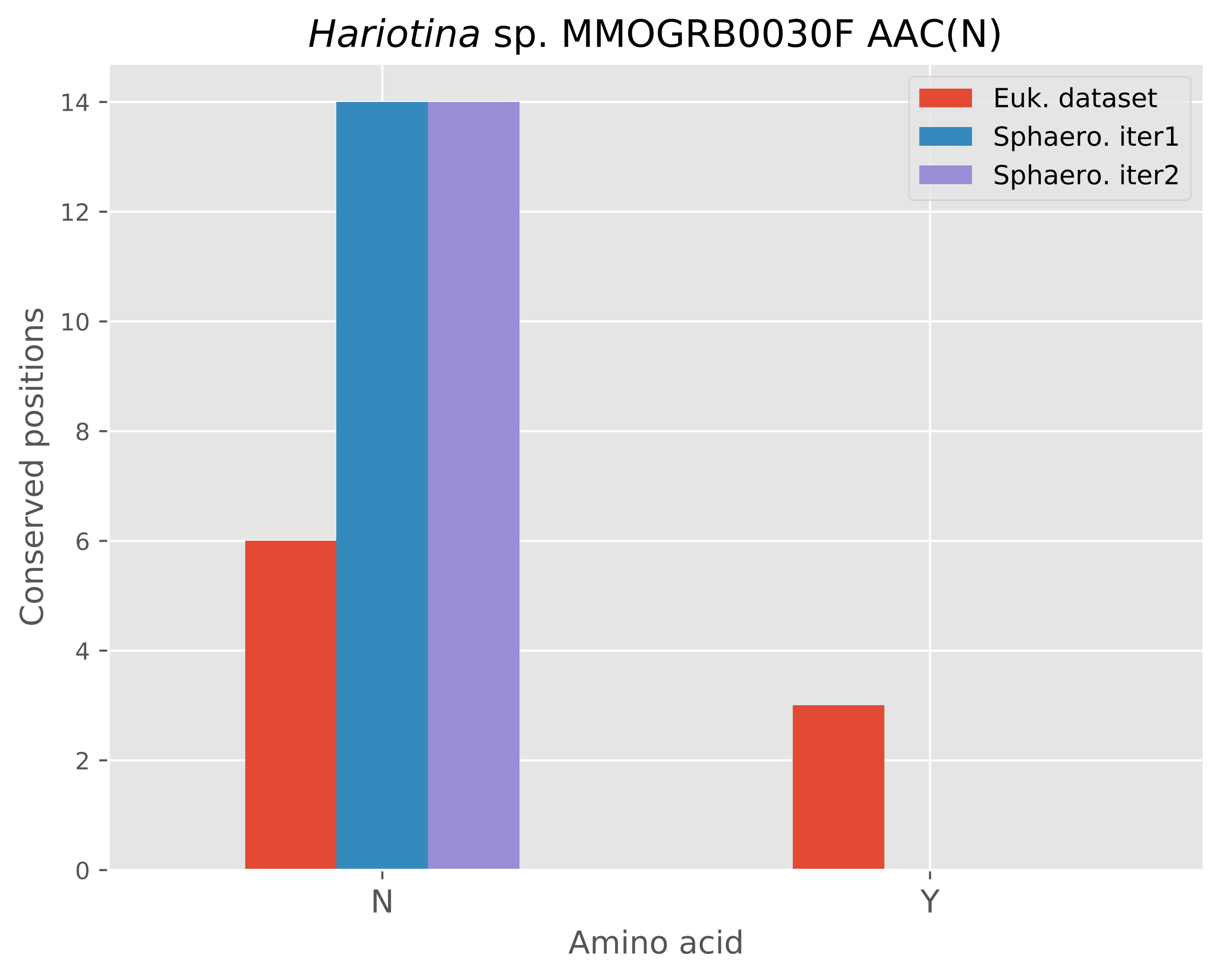

Hariotina sp. MMOGRB0030F AAC(N)
Euk. dataset
Sphaero. iter1
Sphaero. iter2
Conserved positions
0
2
4
6
8
10
12
14
N
Y
Amino acid

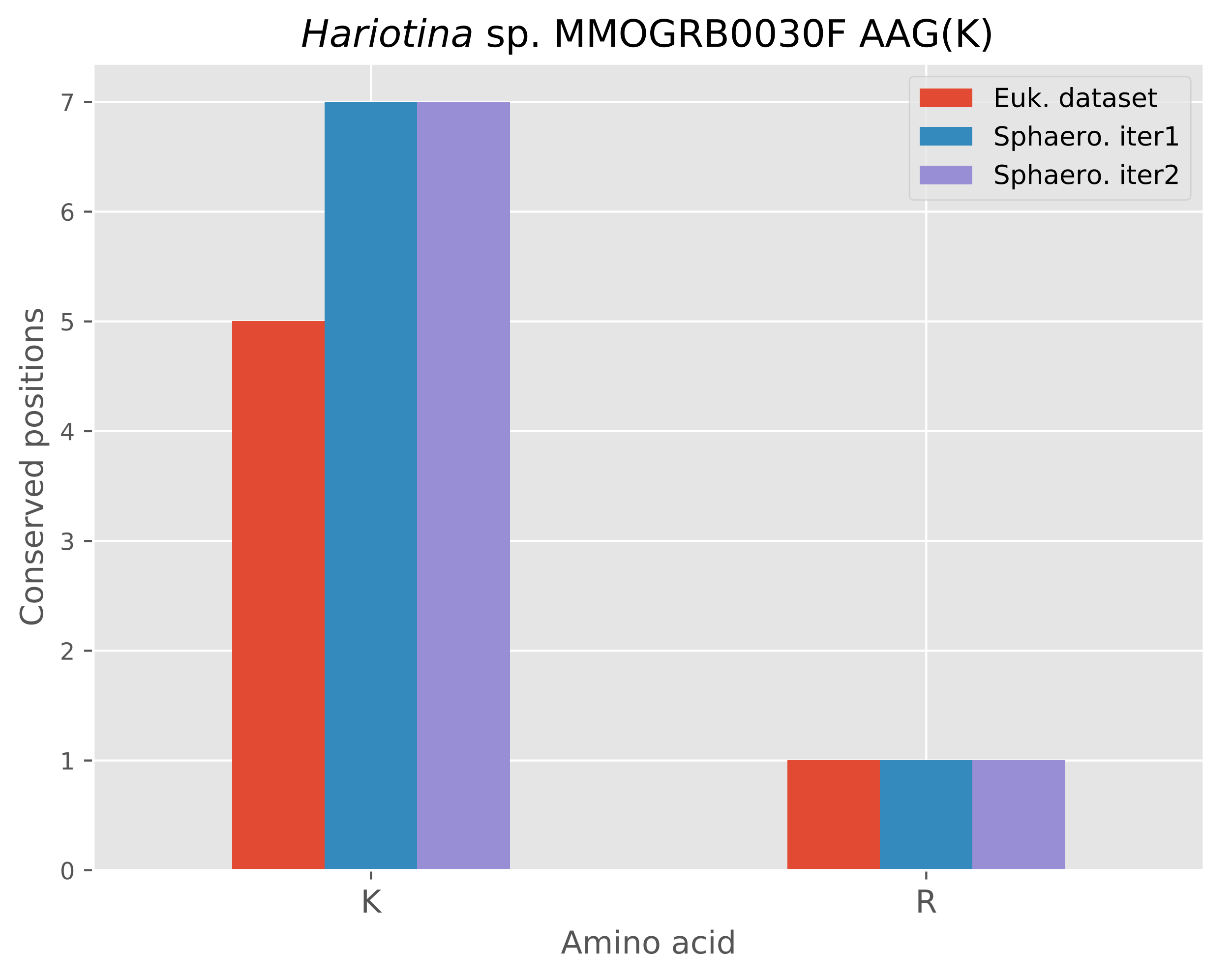
Hariotina sp. MMOGRB0030F AAG(K)
Euk. dataset
Sphaero. iter1
Sphaero. iter2
Conserved positions
0
1
2
3
4
5
6
7
K
R
Amino acid

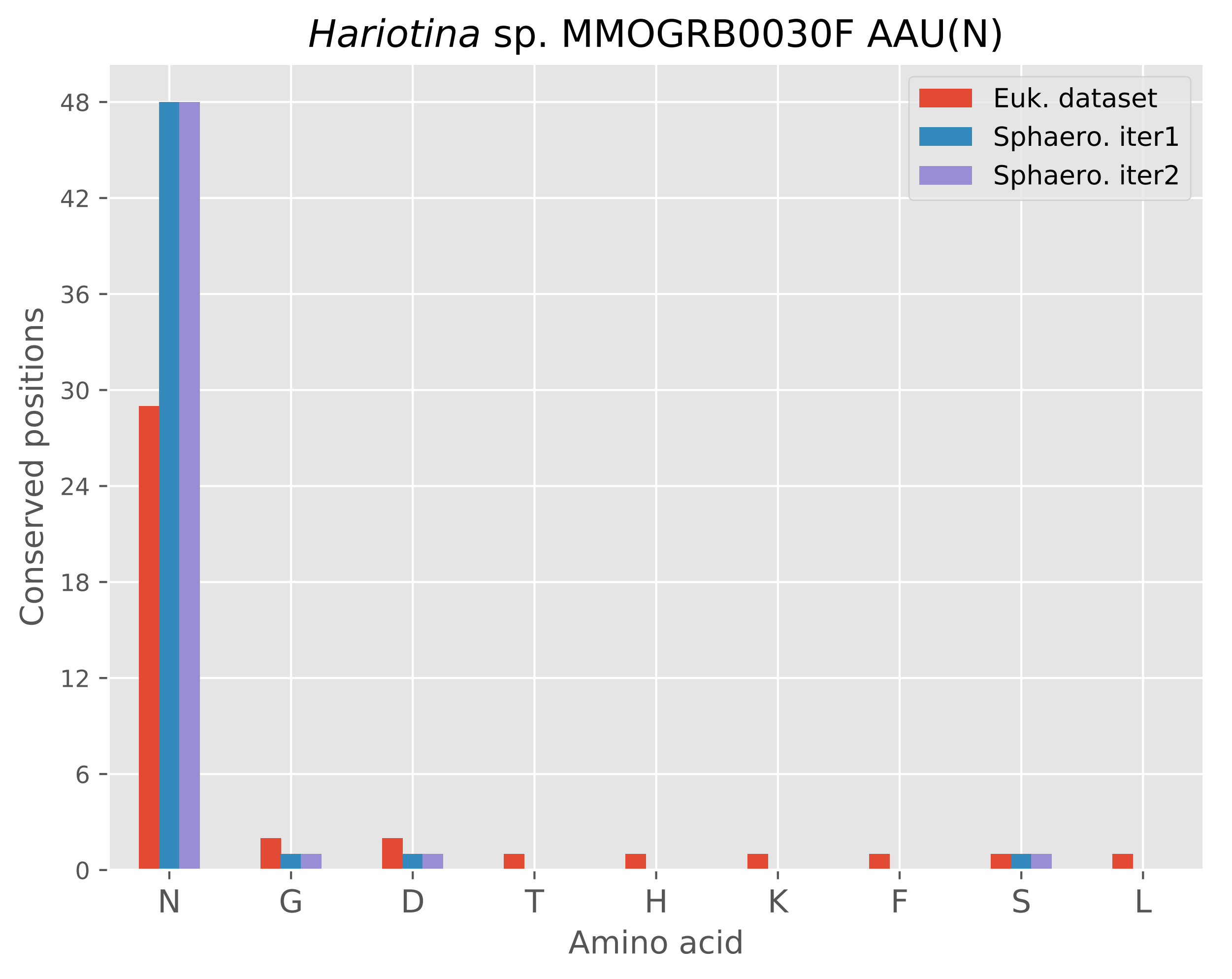

Hariotina sp. MMOGRB0030F AAU(N)
Euk. dataset
Sphaero. iter1
Sphaero. iter2
Conserved positions
0
6
12
18
24
30
36
42
48
N
G
D
T
H
K
F
S
L
Amino acid

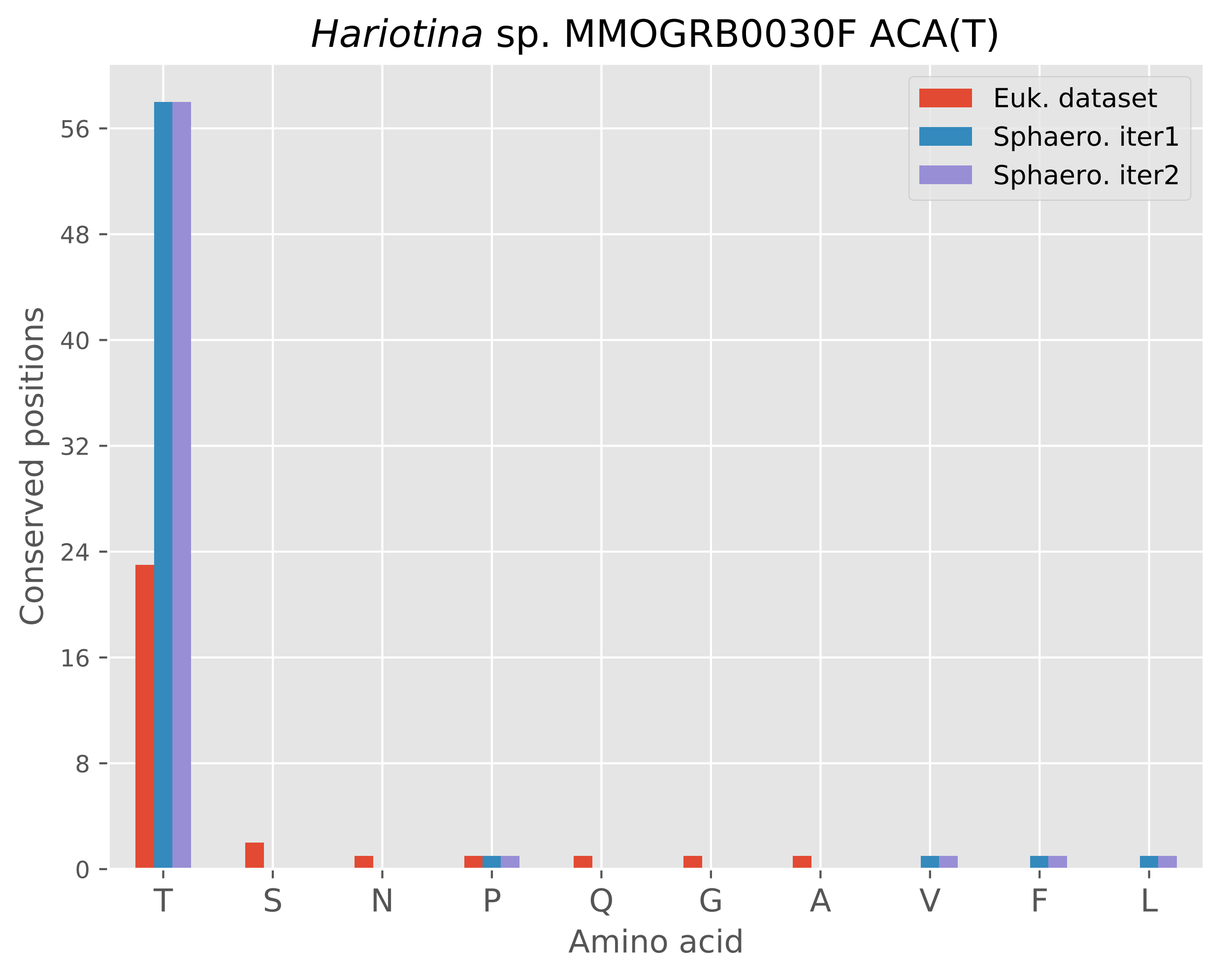
Hariotina sp. MMOGRB0030F ACA(T)
Euk. dataset
Sphaero. iter1
Sphaero. iter2
Conserved positions
0
8
16
24
32
40
48
56
T
S
N
P
Q
G
A
V
F
L
Amino acid

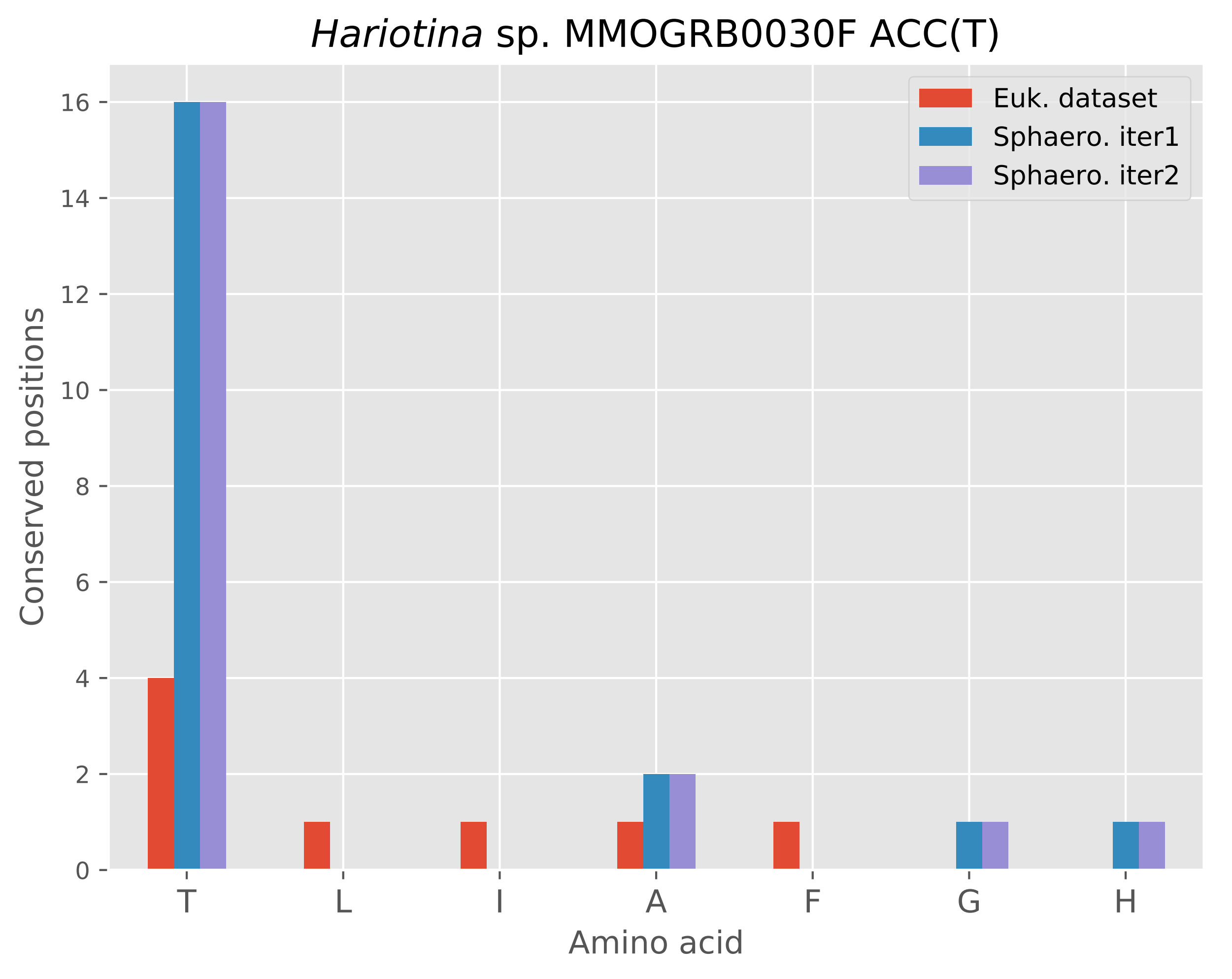

Hariotina sp. MMOGRB0030F ACC(T)
Conserved positions
0
2
4
6
8
10
12
14
16
T
L
I
A
F
G
H
Amino acid
Euk. dataset
Sphaero. iter1
Sphaero. iter2

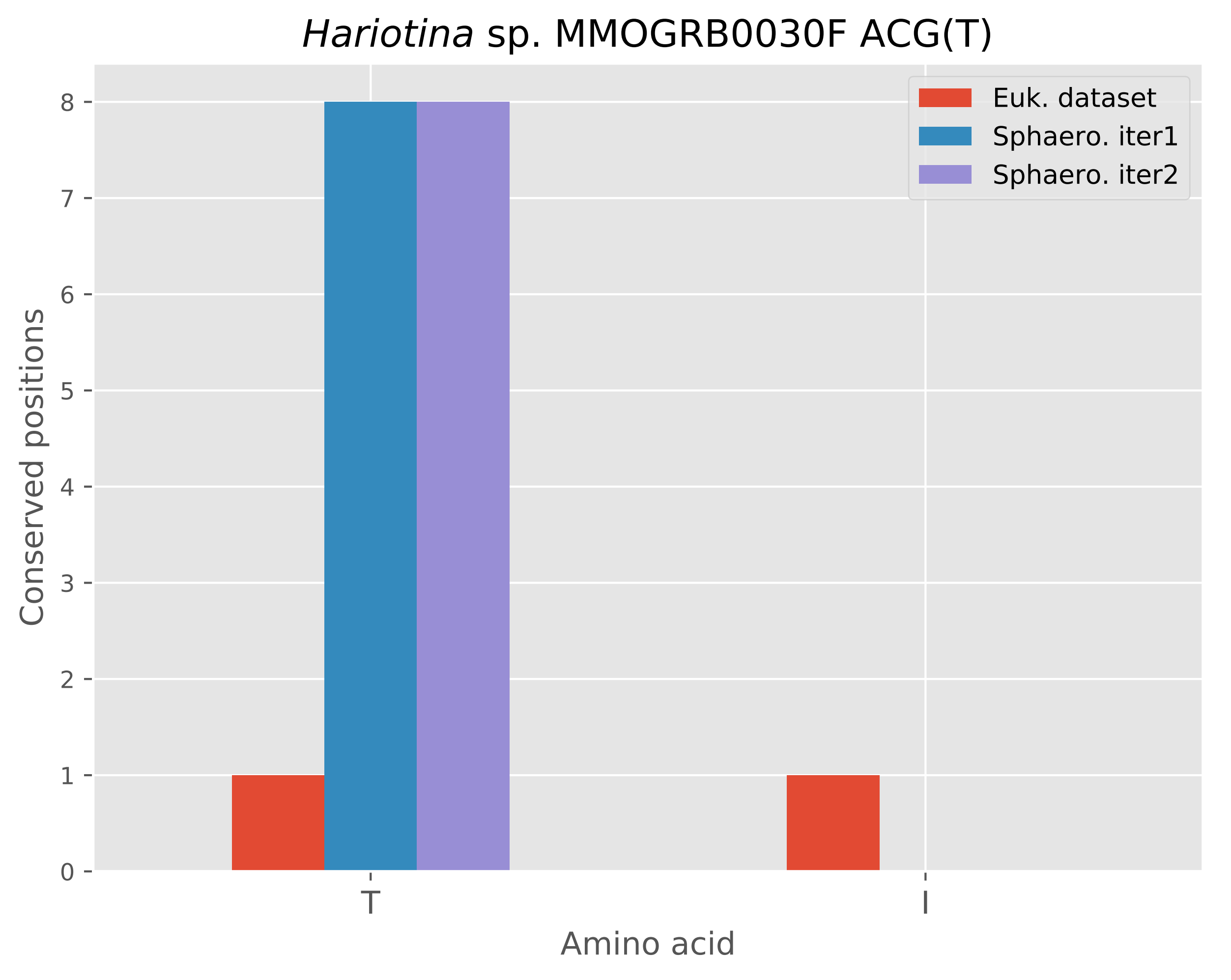
Hariotina sp. MMOGRB0030F ACG(T)
Euk. dataset
Sphaero. iter1
Sphaero. iter2
Conserved positions
0
1
2
3
4
5
6
7
8
T
I
Amino acid

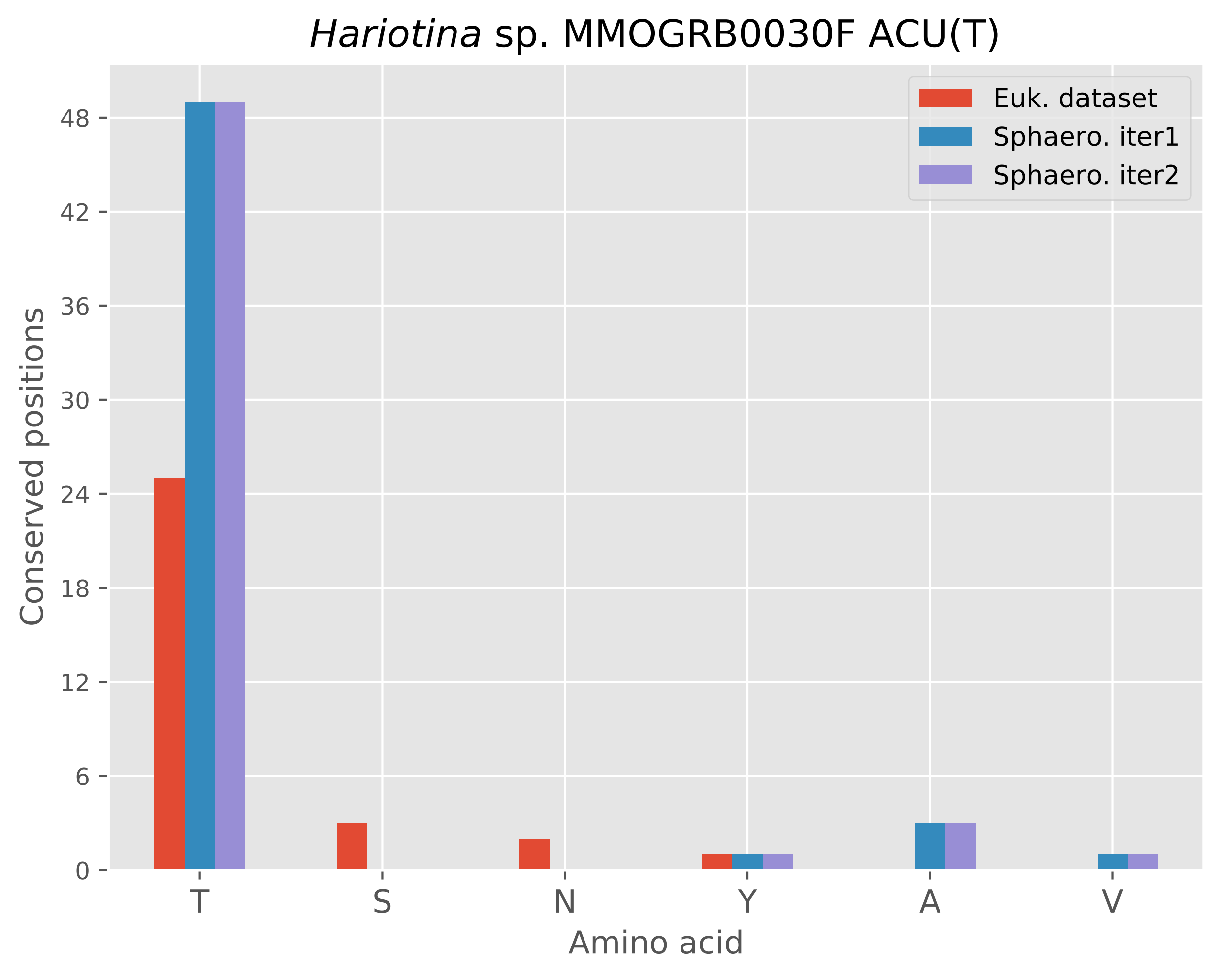

Hariotina sp. MMOGRB0030F ACU(T)
Euk. dataset
Sphaero. iter1
Sphaero. iter2
Conserved positions
0
6
12
18
24
30
36
42
48
T
S
N
Y
A
V
Amino acid

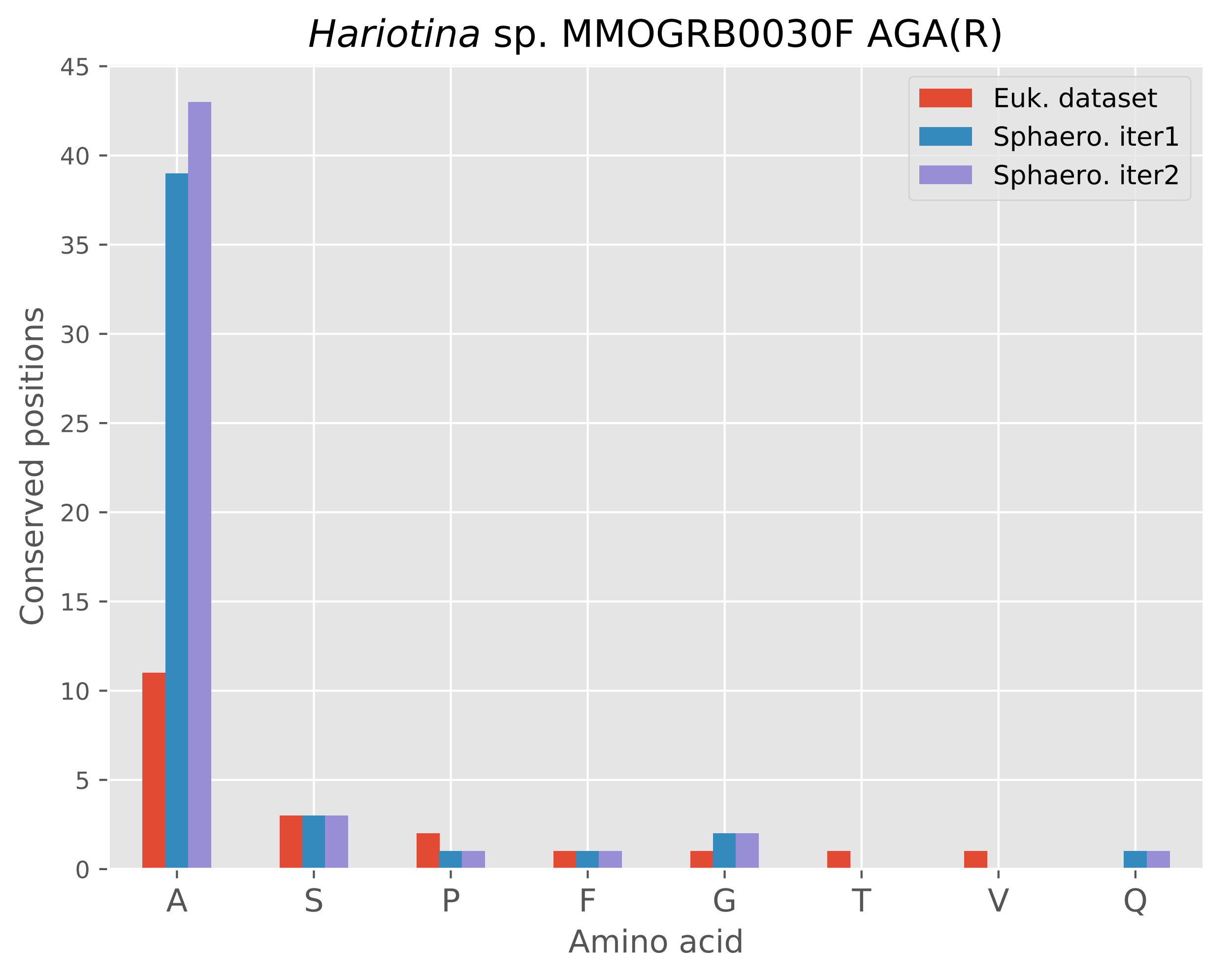

Hariotina sp. MMOGRB0030F AGA(R)
Conserved positions
Amino acid
Euk. dataset
Sphaero. iter1
Sphaero. iter2
0
5
10
15
20
25
30
35
40
45
A
S
P
F
G
T
V
Q

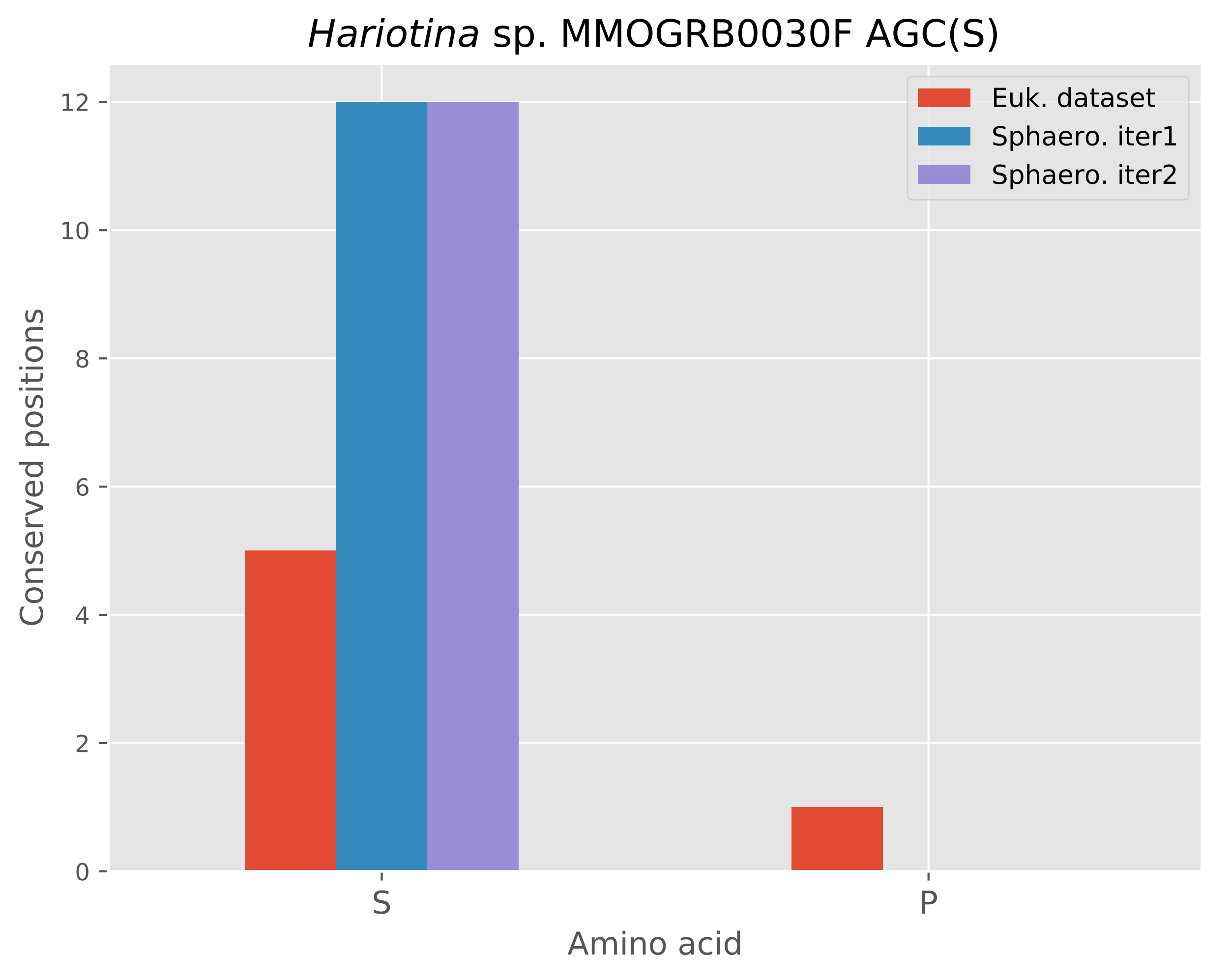
Hariotina sp. MMOGRB0030F AGC(S)
Euk. dataset
Sphaero. iter1
Sphaero. iter2
Conserved positions
0
2
4
6
8
10
12
S
P
Amino acid

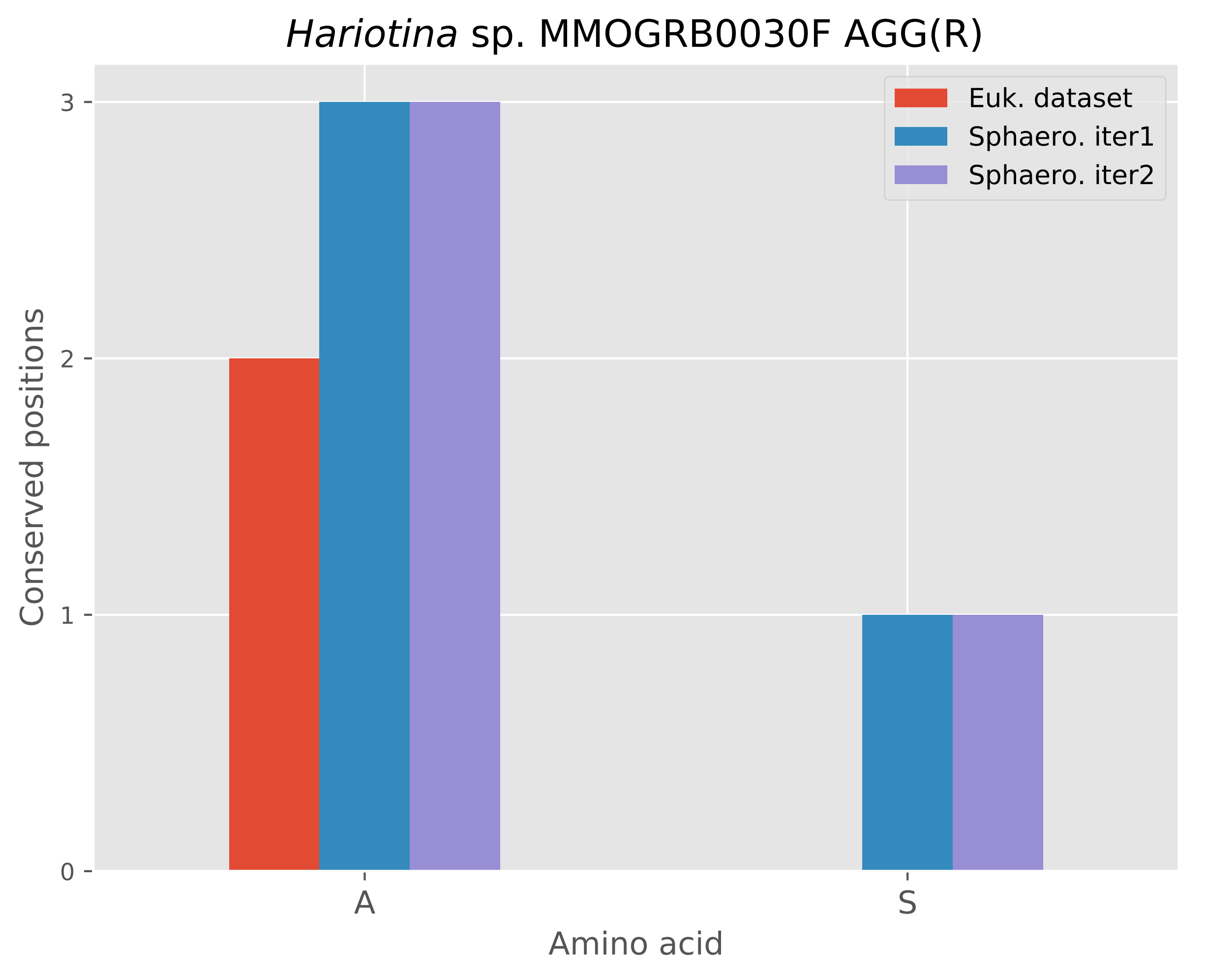
Hariotina sp. MMOGRB0030F AGG(R)
Euk. dataset
Sphaero. iter1
Sphaero. iter2
Conserved positions
0
1
2
3
A
S
Amino acid

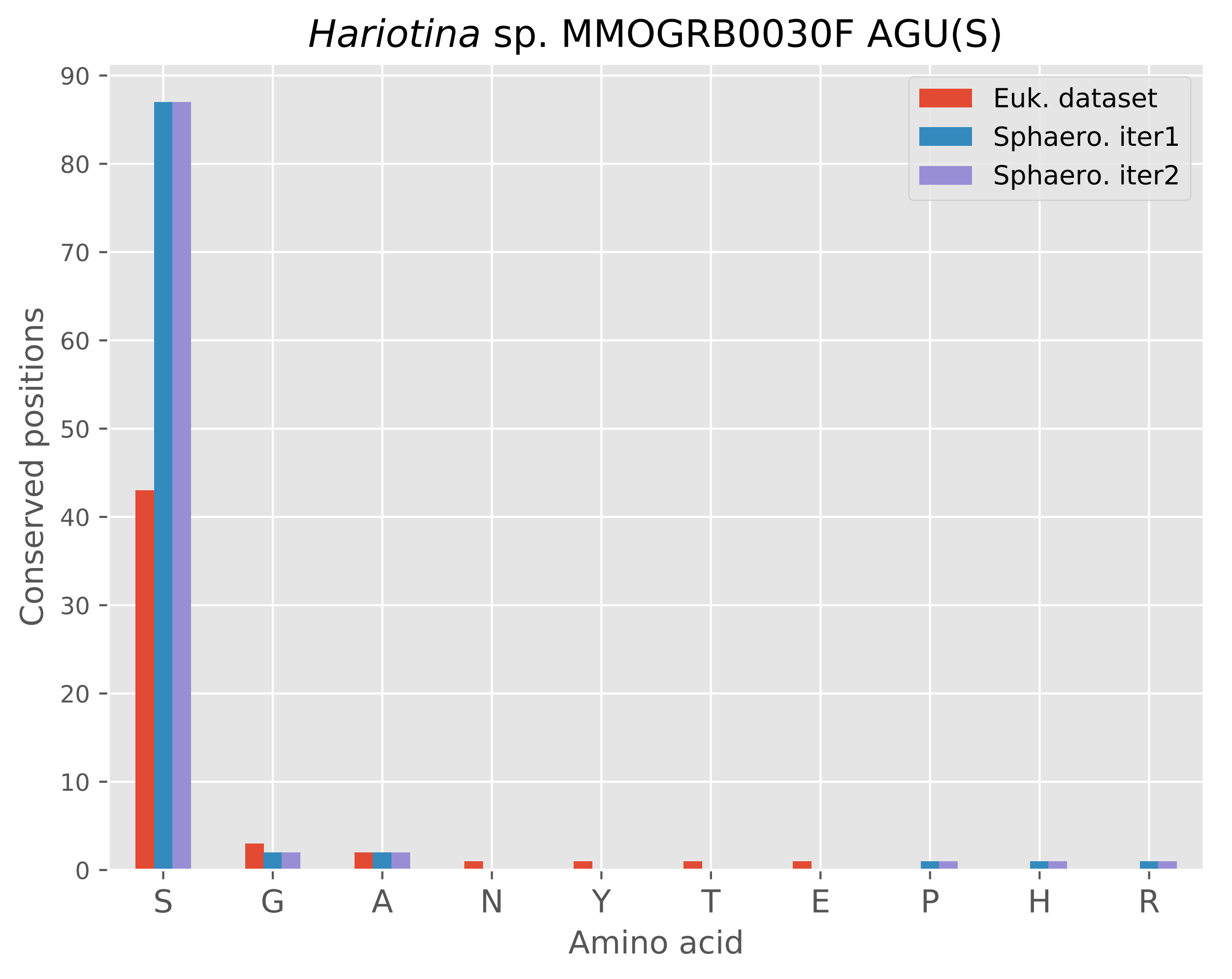

Hariotina sp. MMOGRB0030F AGU(S)
Euk. dataset
Sphaero. iter1
Sphaero. iter2
Conserved positions
Amino acid
0
10
20
30
40
50
60
70
80
90
S
G
A
N
Y
T
E
P
H
R

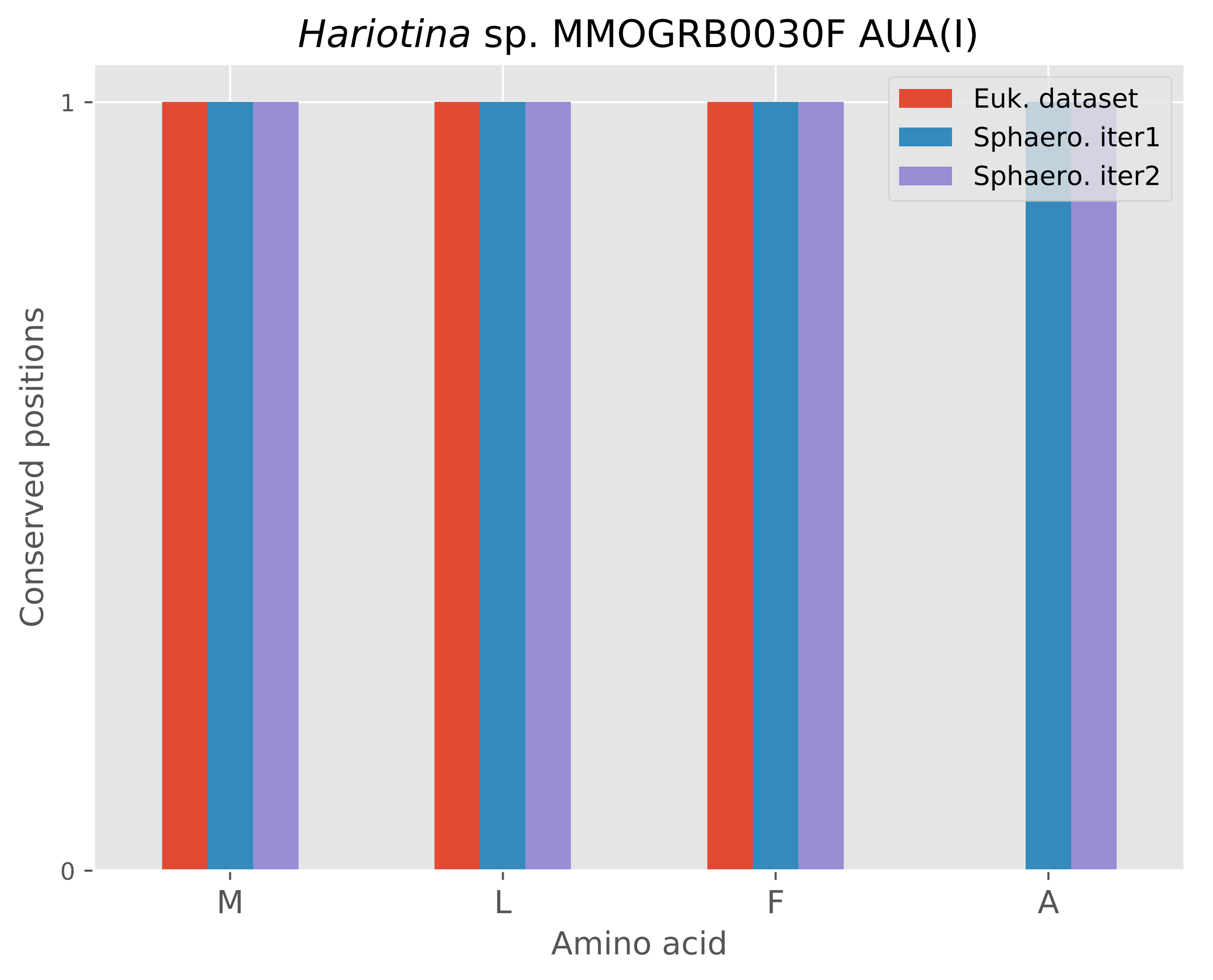

Hariotina sp. MMOGRB0030F AUA(I)
Euk. dataset
Sphaero. iter1
Sphaero. iter2
Conserved positions
1
0
M
L
F
A
Amino acid

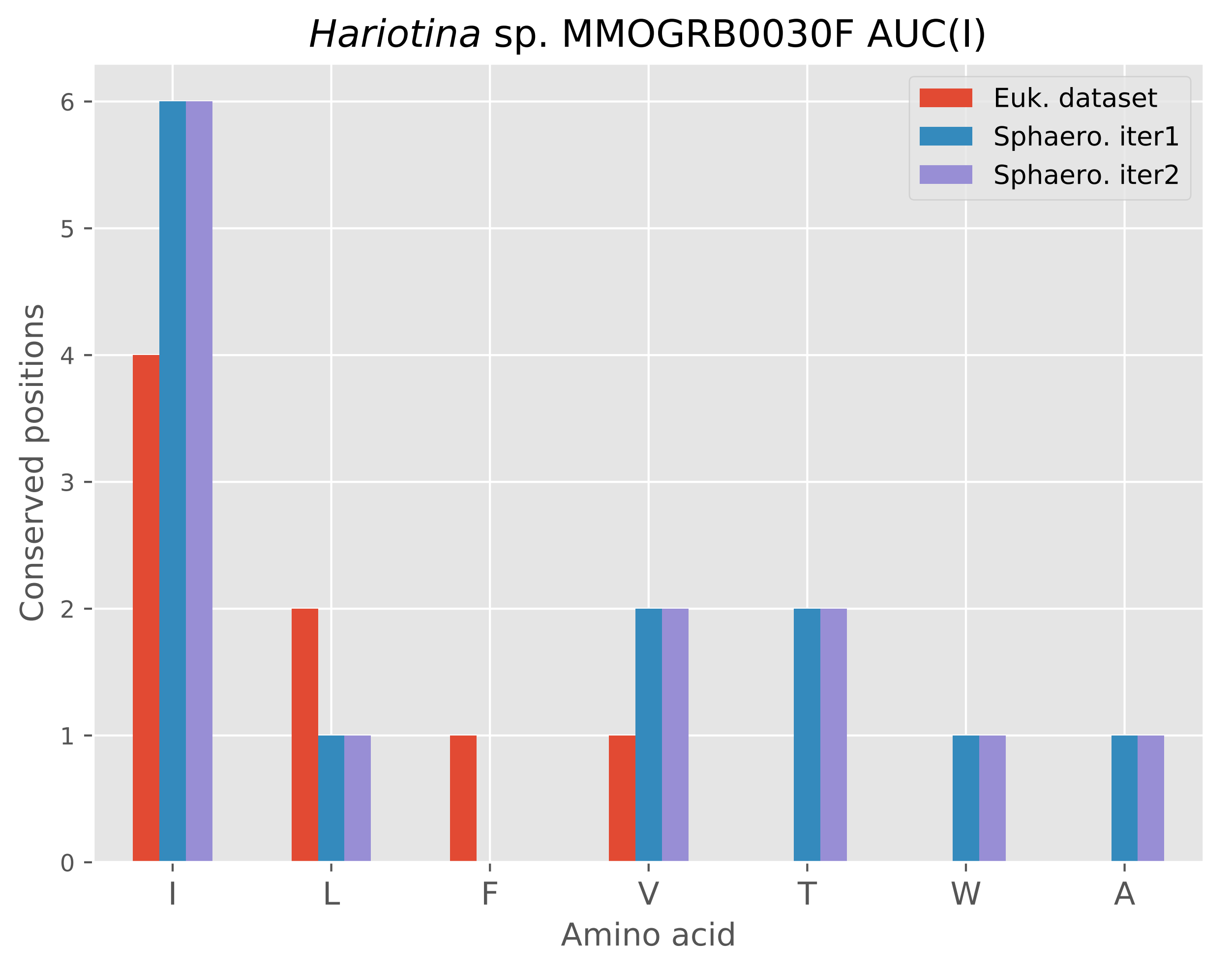
Hariotina sp. MMOGRB0030F AUC(I)
Euk. dataset
Sphaero. iter1
Sphaero. iter2
Conserved positions
0
1
2
3
4
5
6
I
L
F
V
T
W
A
Amino acid

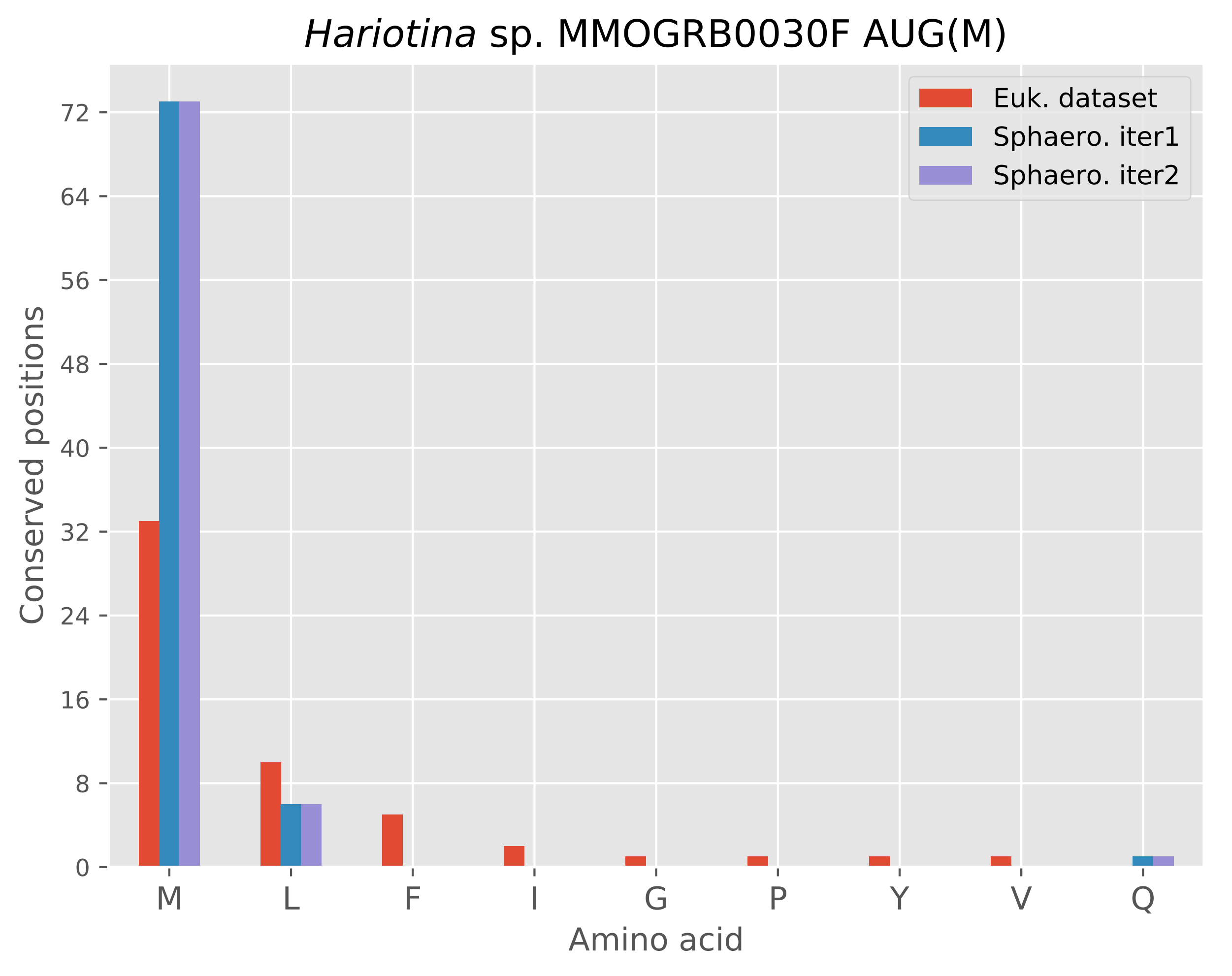
Hariotina sp. MMOGRB0030F AUG(M)
Euk. dataset
Sphaero. iter1
Sphaero. iter2
Conserved positions
72
64
56
48
40
32
24
16
8
0
M
L
F
I
G
P
Y
V
Q
Amino acid

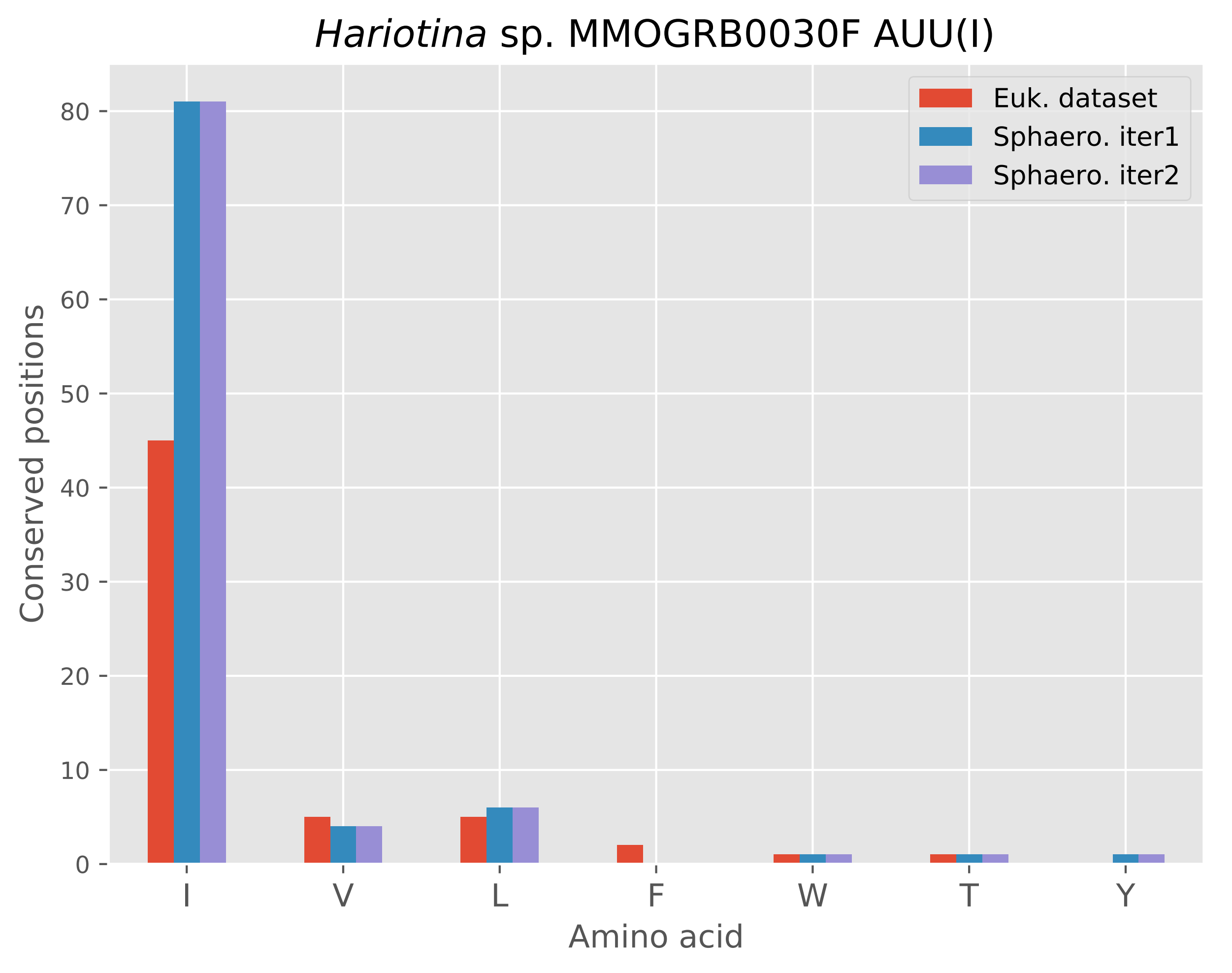

Hariotina sp. MMOGRB0030F AUU(I)
Euk. dataset
Sphaero. iter1
Sphaero. iter2
Conserved positions
0
10
20
30
40
50
60
70
80
I
V
L
F
W
T
Y
Amino acid

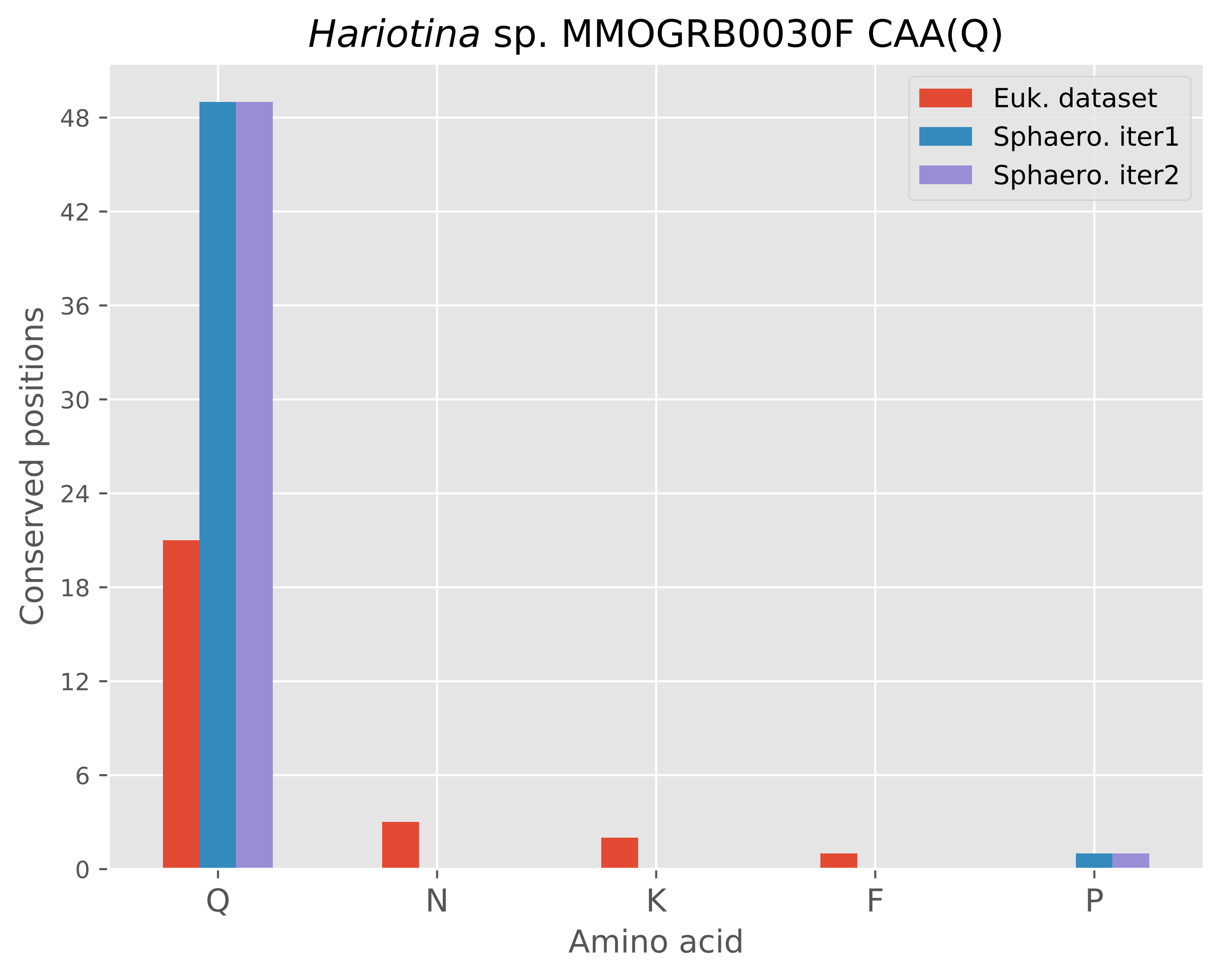

Hariotina sp. MMOGRB0030F CAA(Q)
Euk. dataset
Sphaero. iter1
Sphaero. iter2
Conserved positions
0
6
12
18
24
30
36
42
48
Q
N
K
F
P
Amino acid

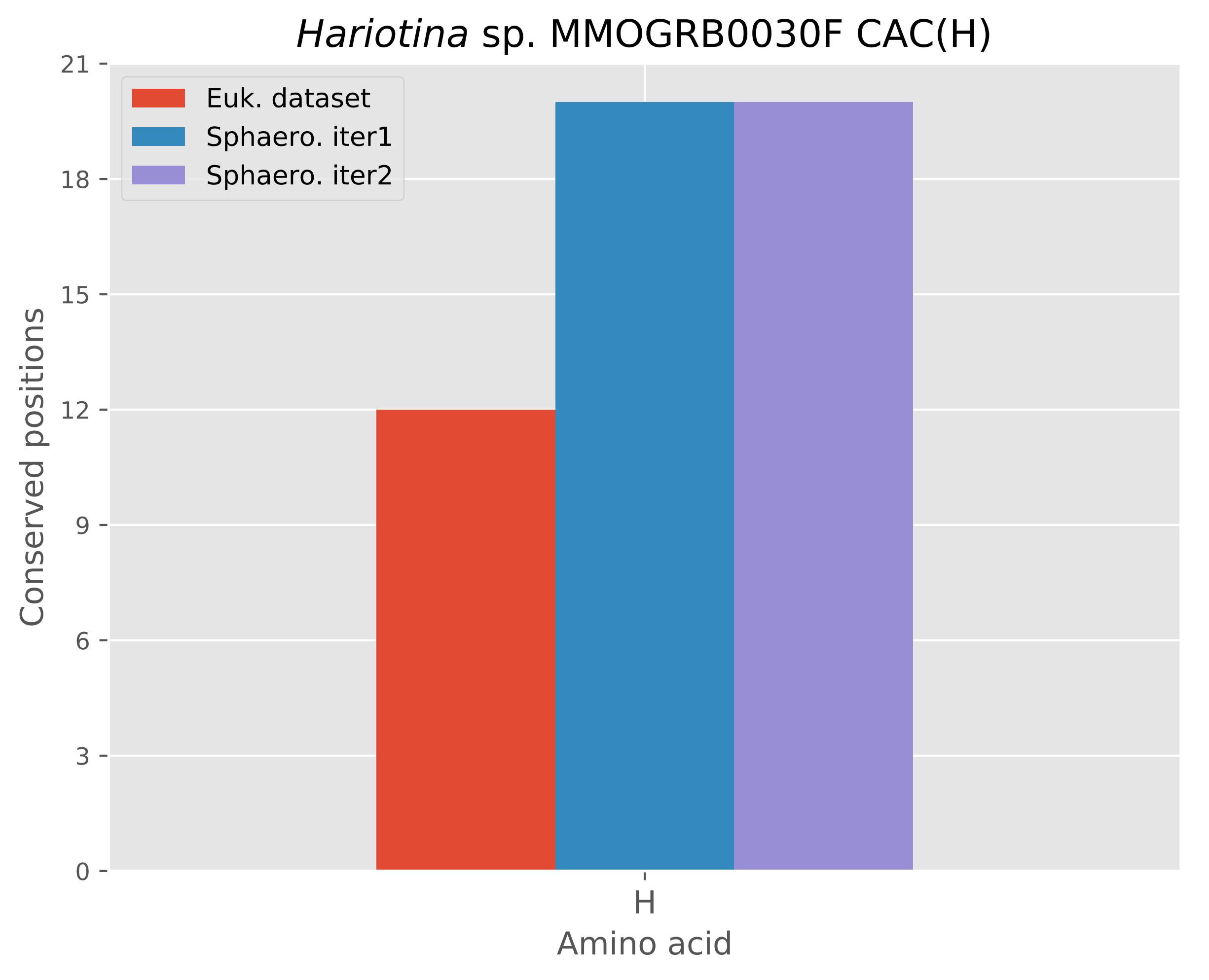

Hariotina sp. MMOGRB0030F CAC(H)
Euk. dataset
Sphaero. iter1
Sphaero. iter2
Conserved positions
Amino acid
H
0
3
6
9
12
15
18
21

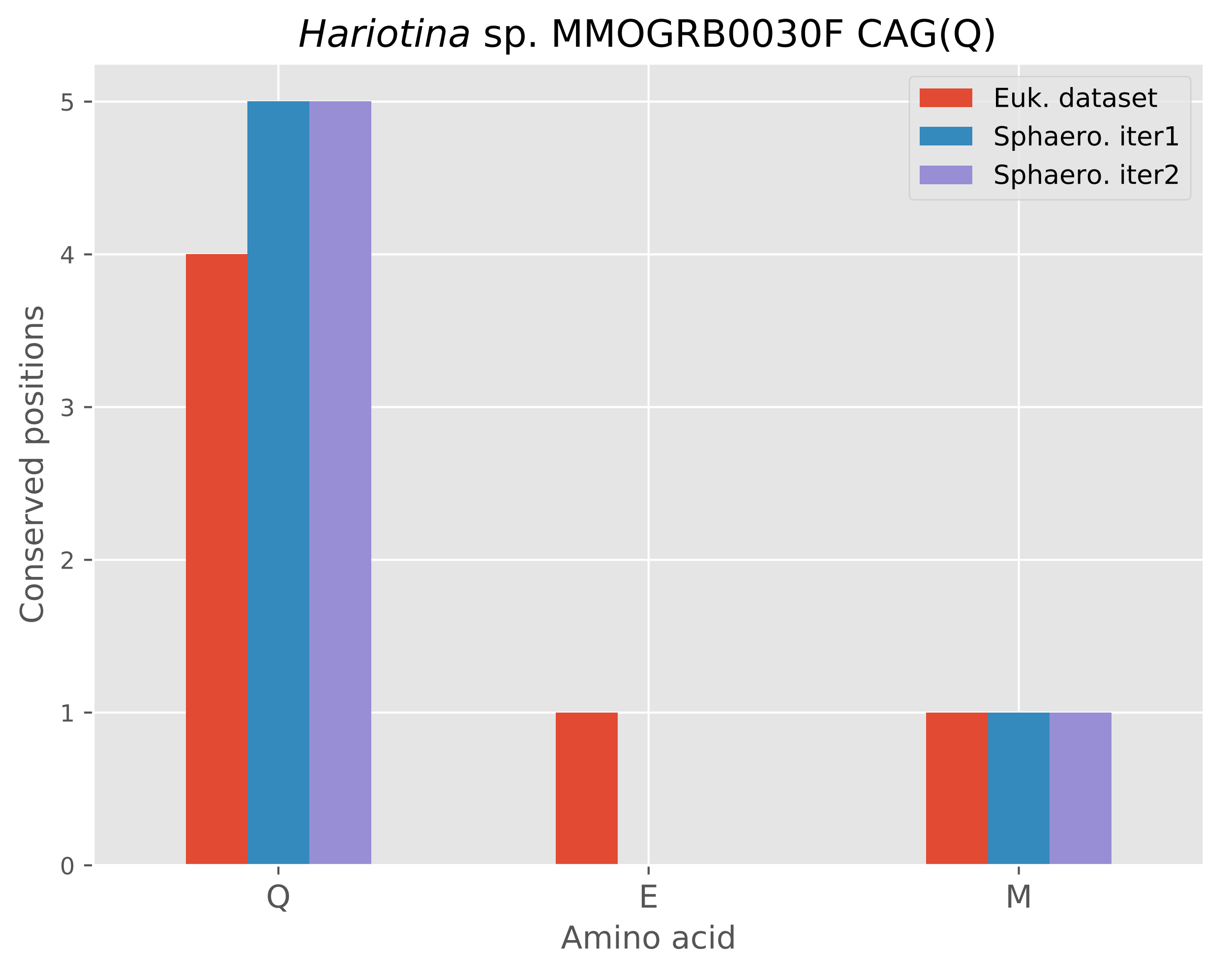
Hariotina sp. MMOGRB0030F CAG(Q)
Euk. dataset
Sphaero. iter1
Sphaero. iter2
Conserved positions
0
1
2
3
4
5
Q
E
M
Amino acid

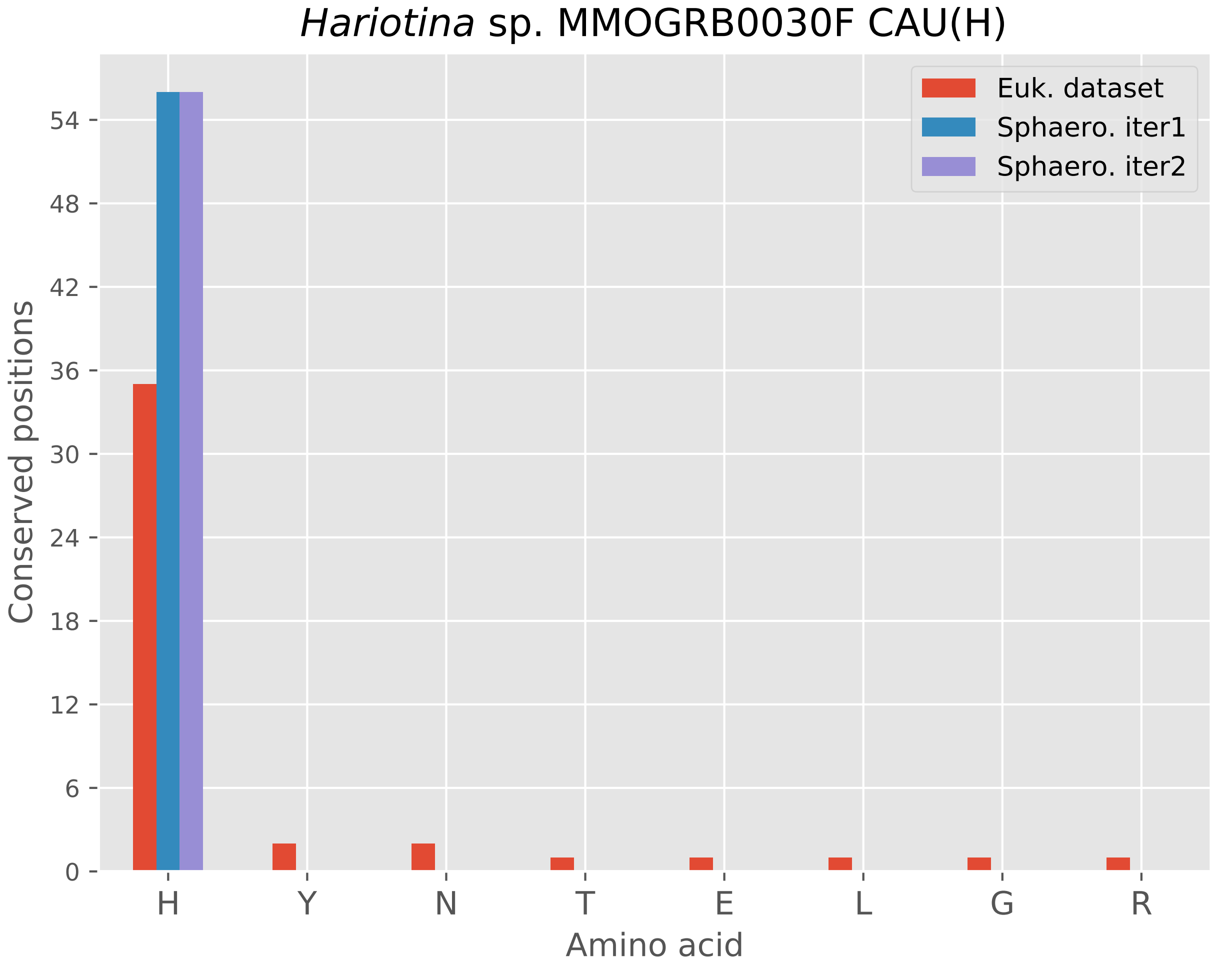

Hariotina sp. MMOGRB0030F CAU(H)
Euk. dataset
Sphaero. iter1
Sphaero. iter2
Conserved positions
0
6
12
18
24
30
36
42
48
54
H
Y
N
T
E
L
G
R
Amino acid

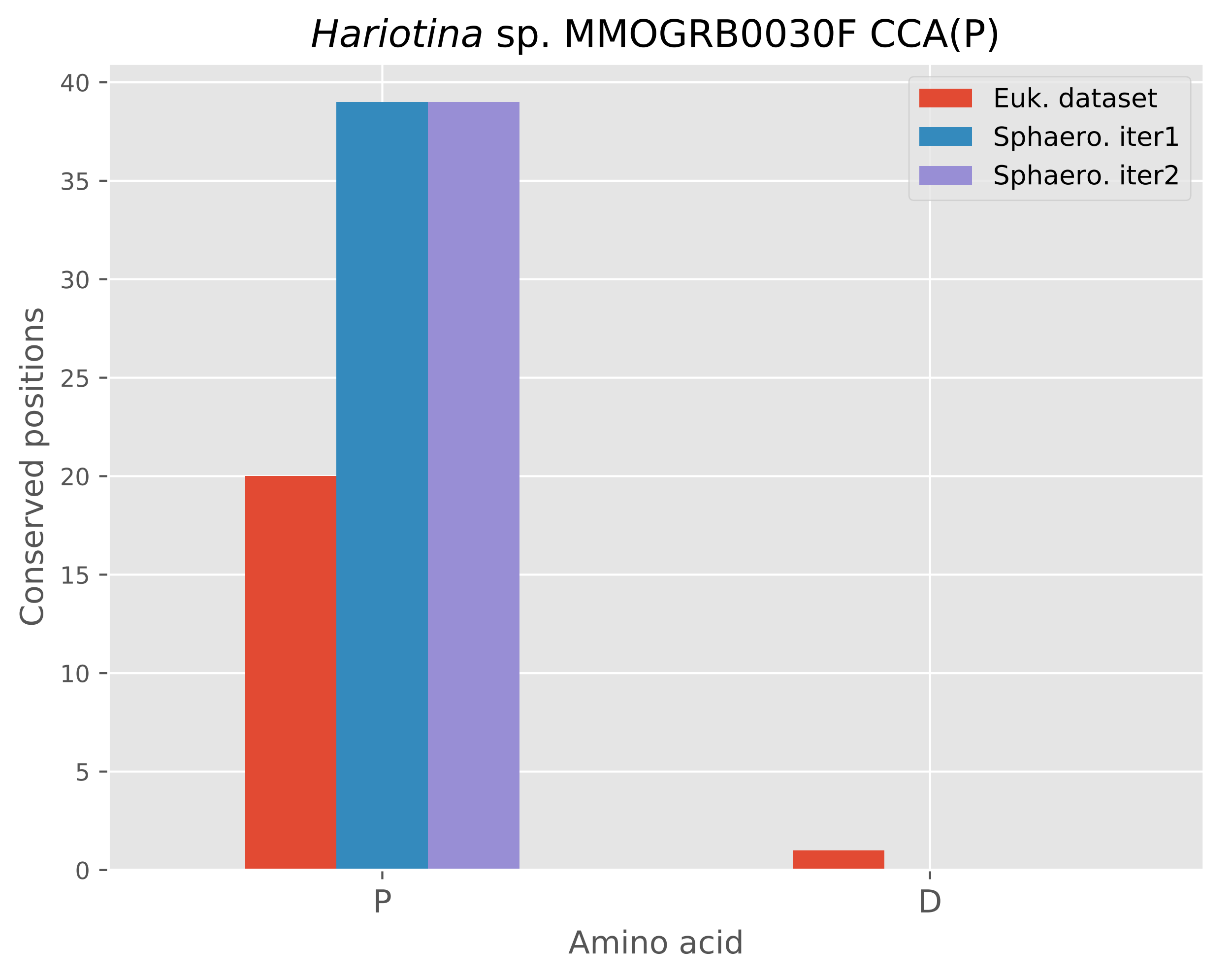

Hariotina sp. MMOGRB0030F CCA(P)
Euk. dataset
Sphaero. iter1
Sphaero. iter2
Conserved positions
0
5
10
15
20
25
30
35
40
P
D
Amino acid

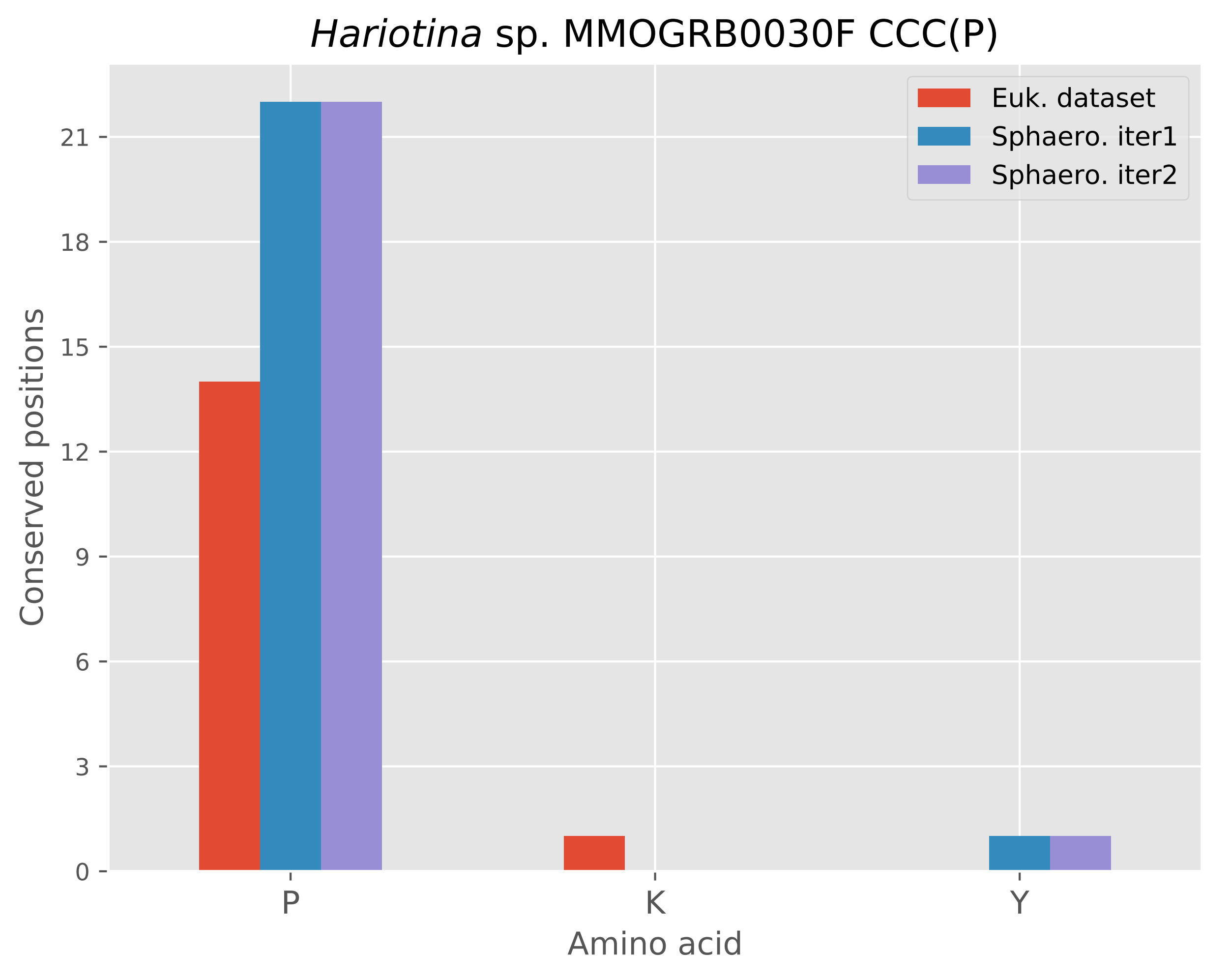
Hariotina sp. MMOGRB0030F CCC(P)
Euk. dataset
Sphaero. iter1
Sphaero. iter2
Conserved positions
0
3
6
9
12
15
18
21
P
K
Y
Amino acid

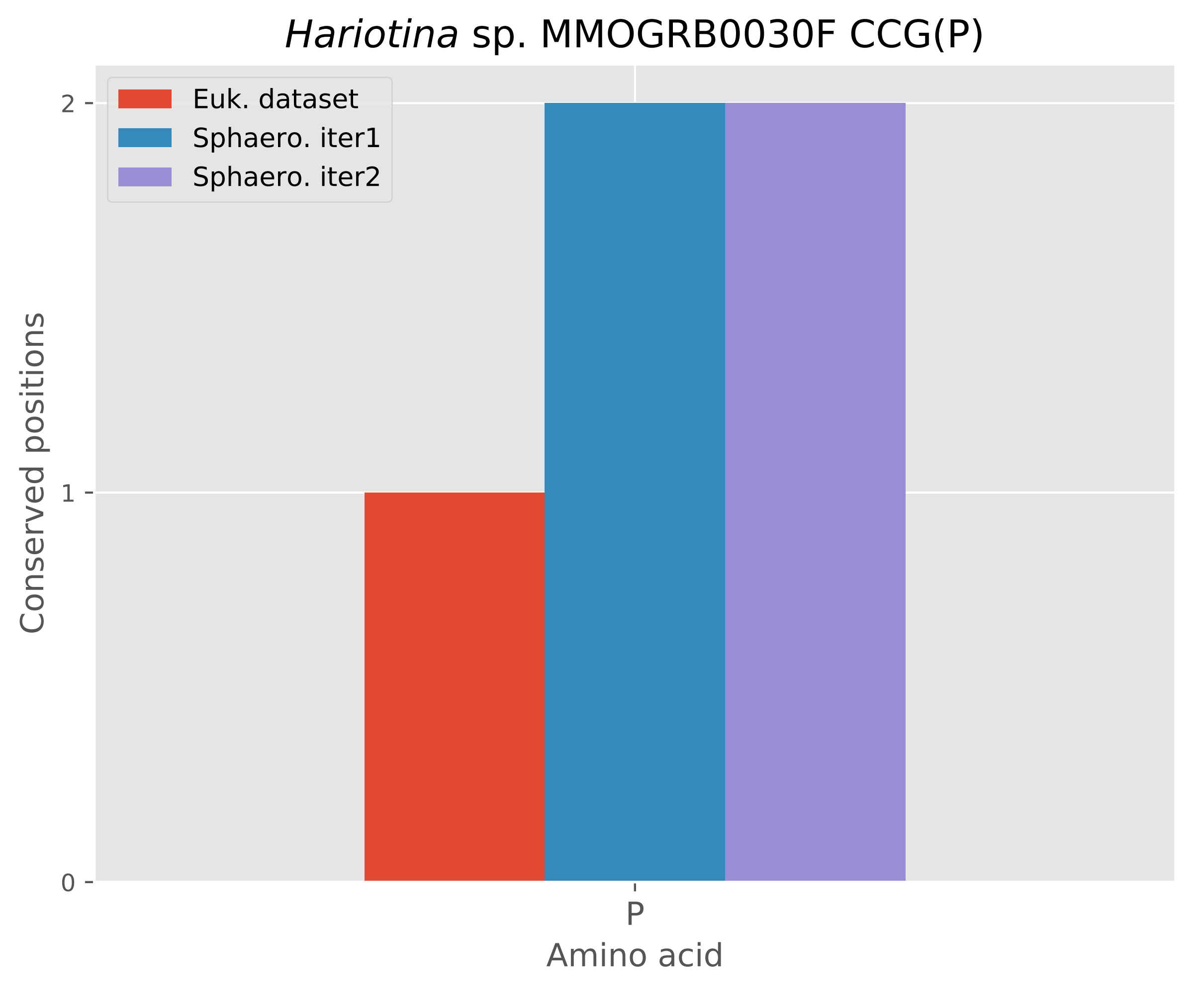

Hariotina sp. MMOGRB0030F CCG(P)
Euk. dataset
Sphaero. iter1
Sphaero. iter2
Conserved positions
0
1
2
P
Amino acid

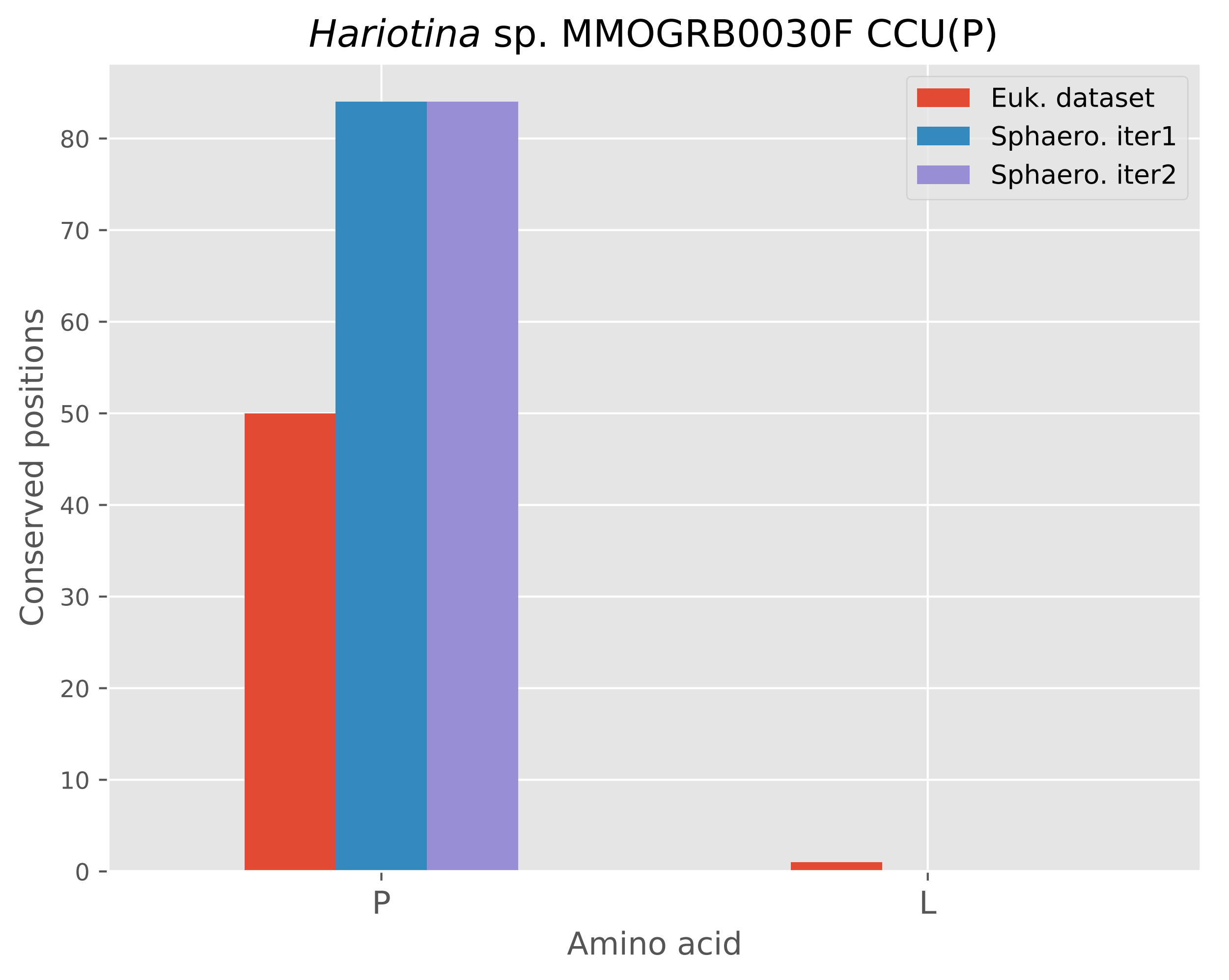
Hariotina sp. MMOGRB0030F CCU(P)
Euk. dataset
Sphaero. iter1
Sphaero. iter2
Conserved positions
0
10
20
30
40
50
60
70
80
P
L
Amino acid

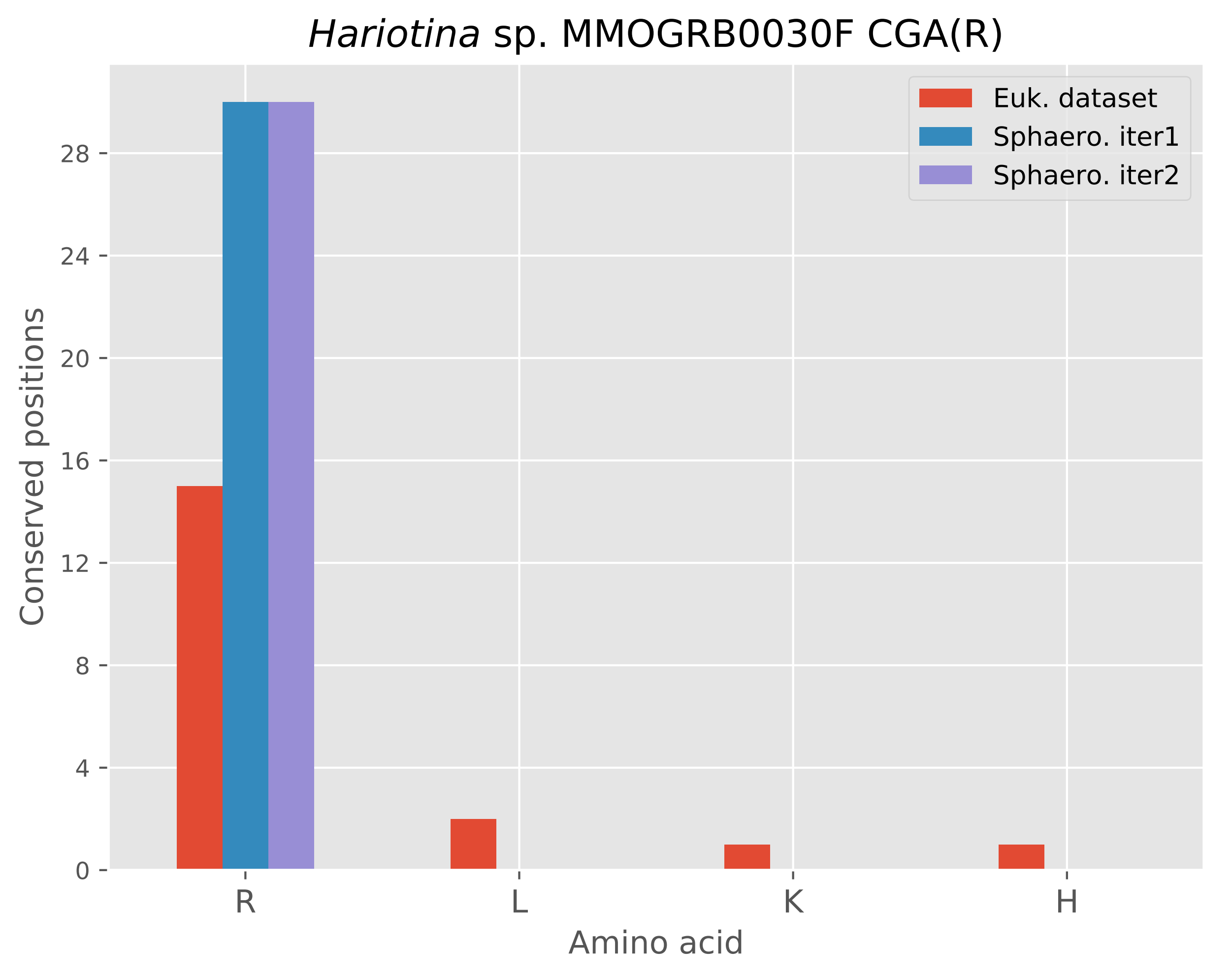

Hariotina sp. MMOGRB0030F CGA(R)
Euk. dataset
Sphaero. iter1
Sphaero. iter2
Conserved positions
0
4
8
12
16
20
24
28
R
L
K
H
Amino acid

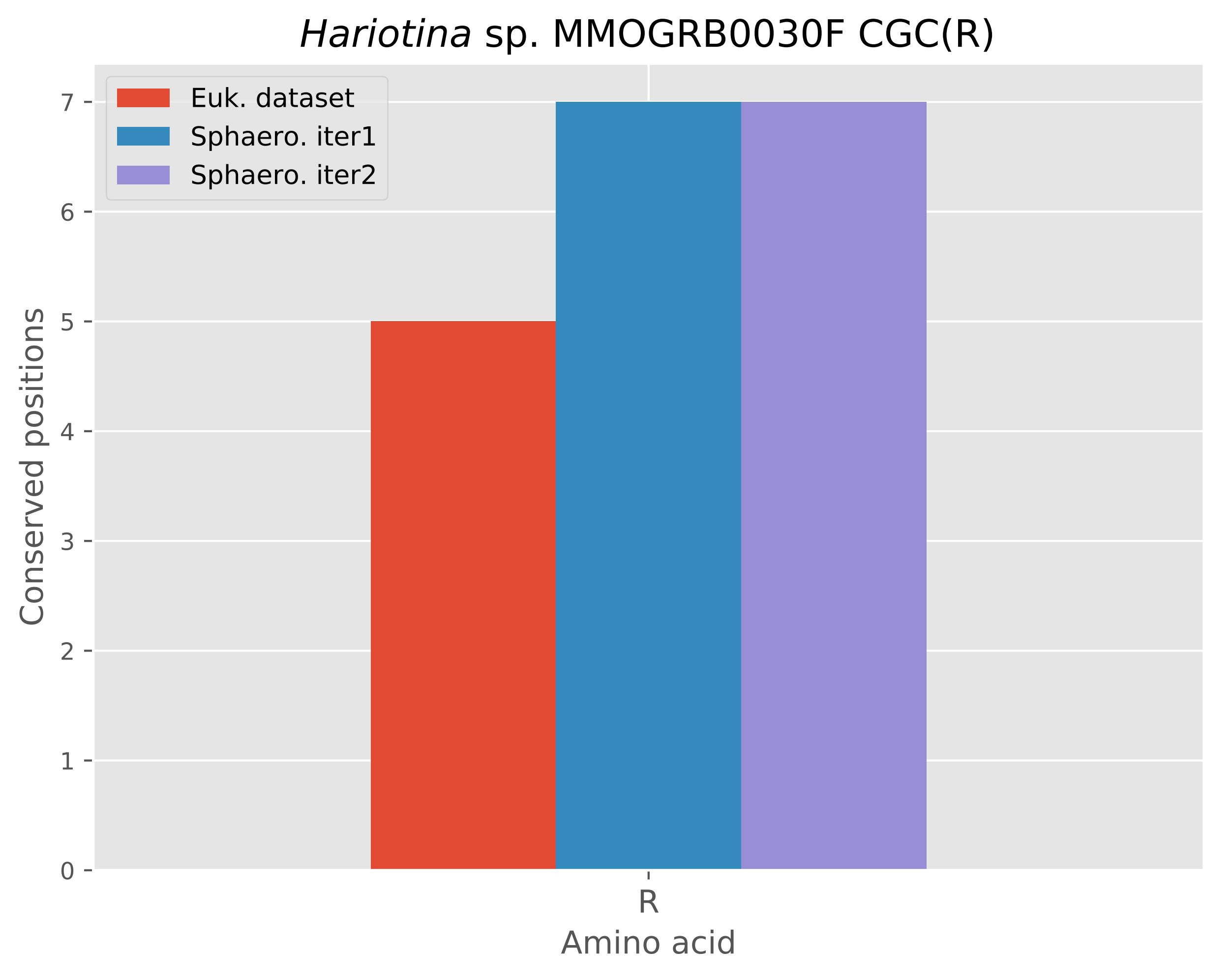

Hariotina sp. MMOGRB0030F CGC(R)
Euk. dataset
Sphaero. iter1
Sphaero. iter2
Conserved positions
0
1
2
3
4
5
6
7
R
Amino acid

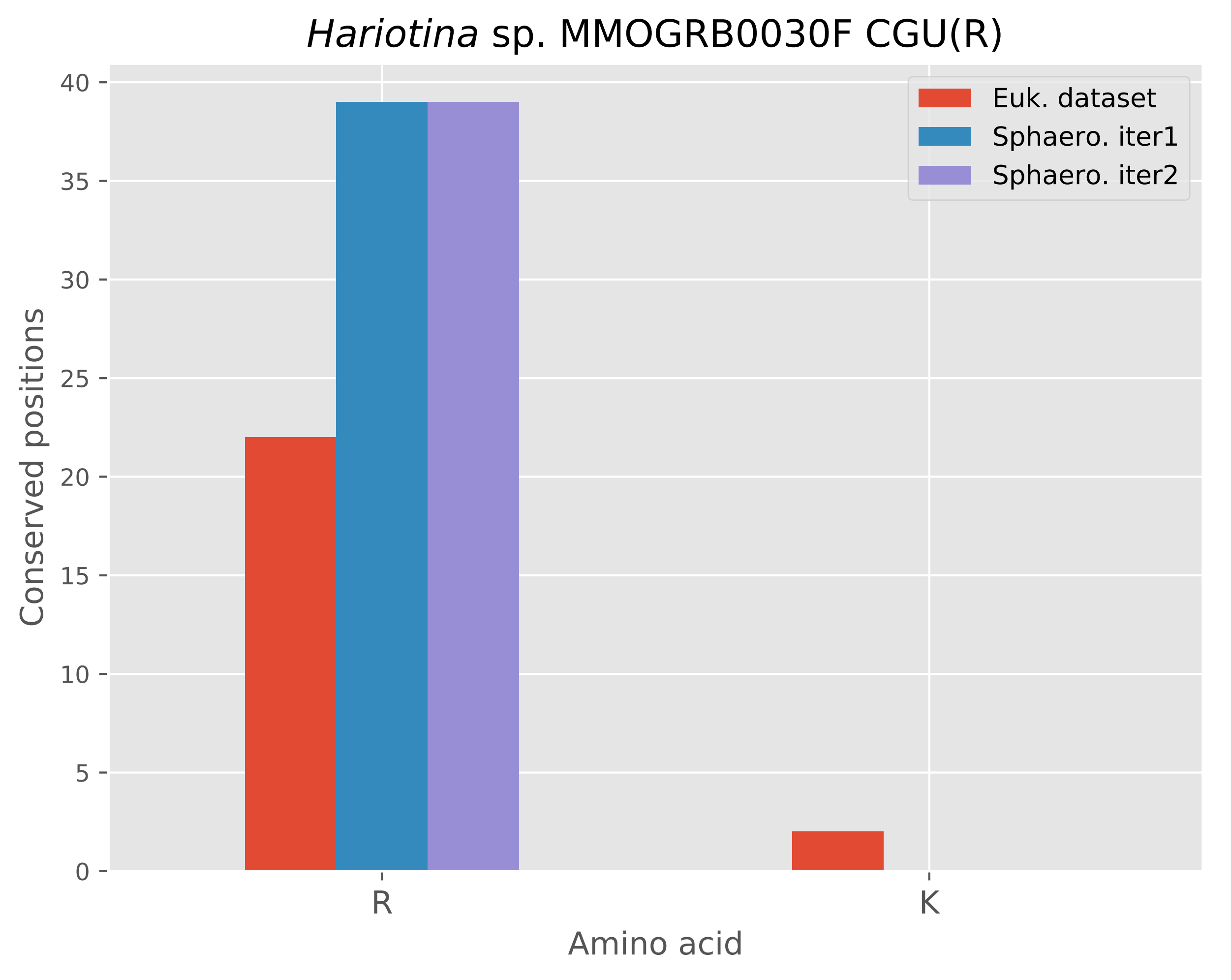
Hariotina sp. MMOGRB0030F CGU(R)
Euk. dataset
Sphaero. iter1
Sphaero. iter2
Conserved positions
0
5
10
15
20
25
30
35
40
R
K
Amino acid

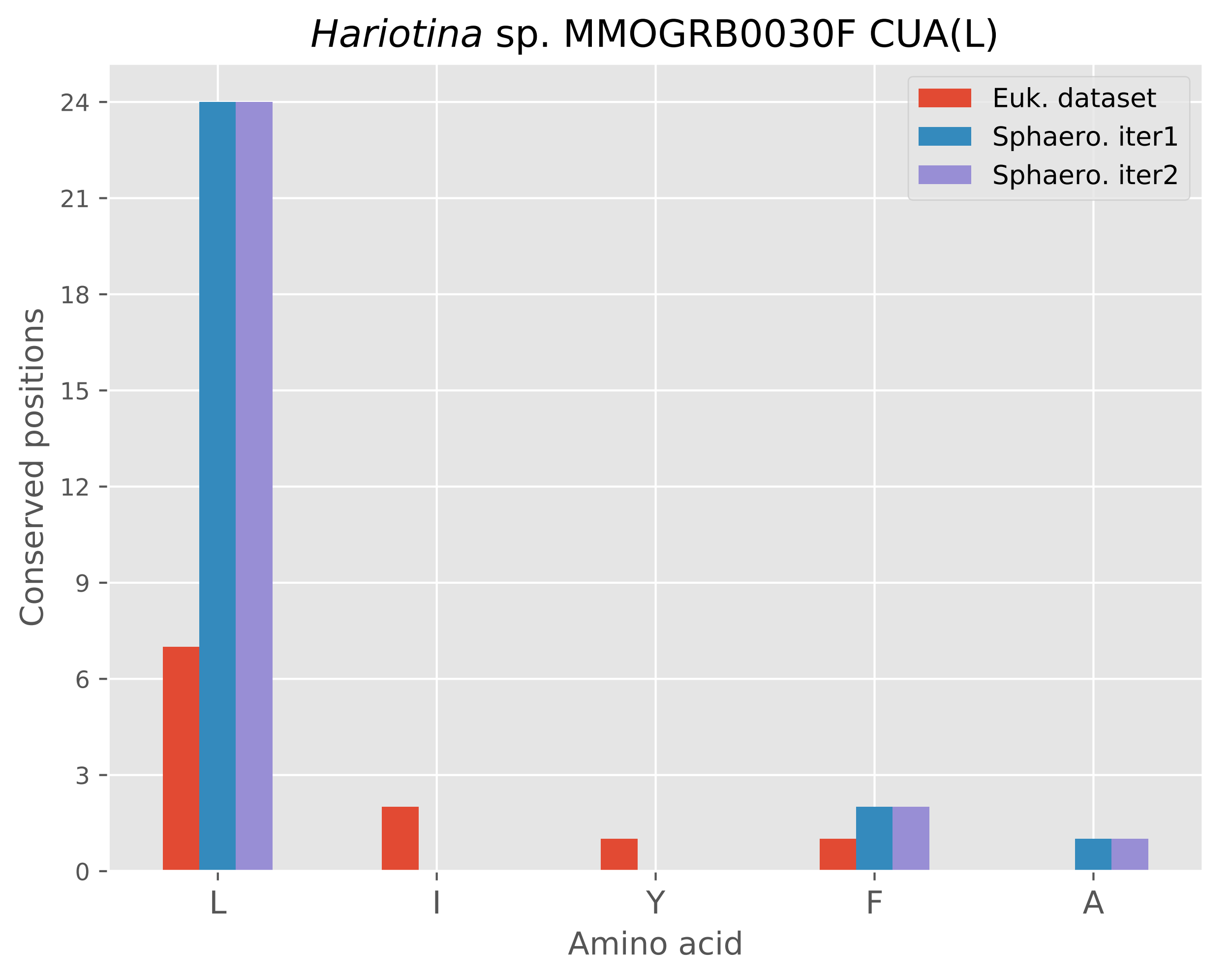
Hariotina sp. MMOGRB0030F CUA(L)
Euk. dataset
Sphaero. iter1
Sphaero. iter2
Conserved positions
0
3
6
9
12
15
18
21
24
L
I
Y
F
A
Amino acid

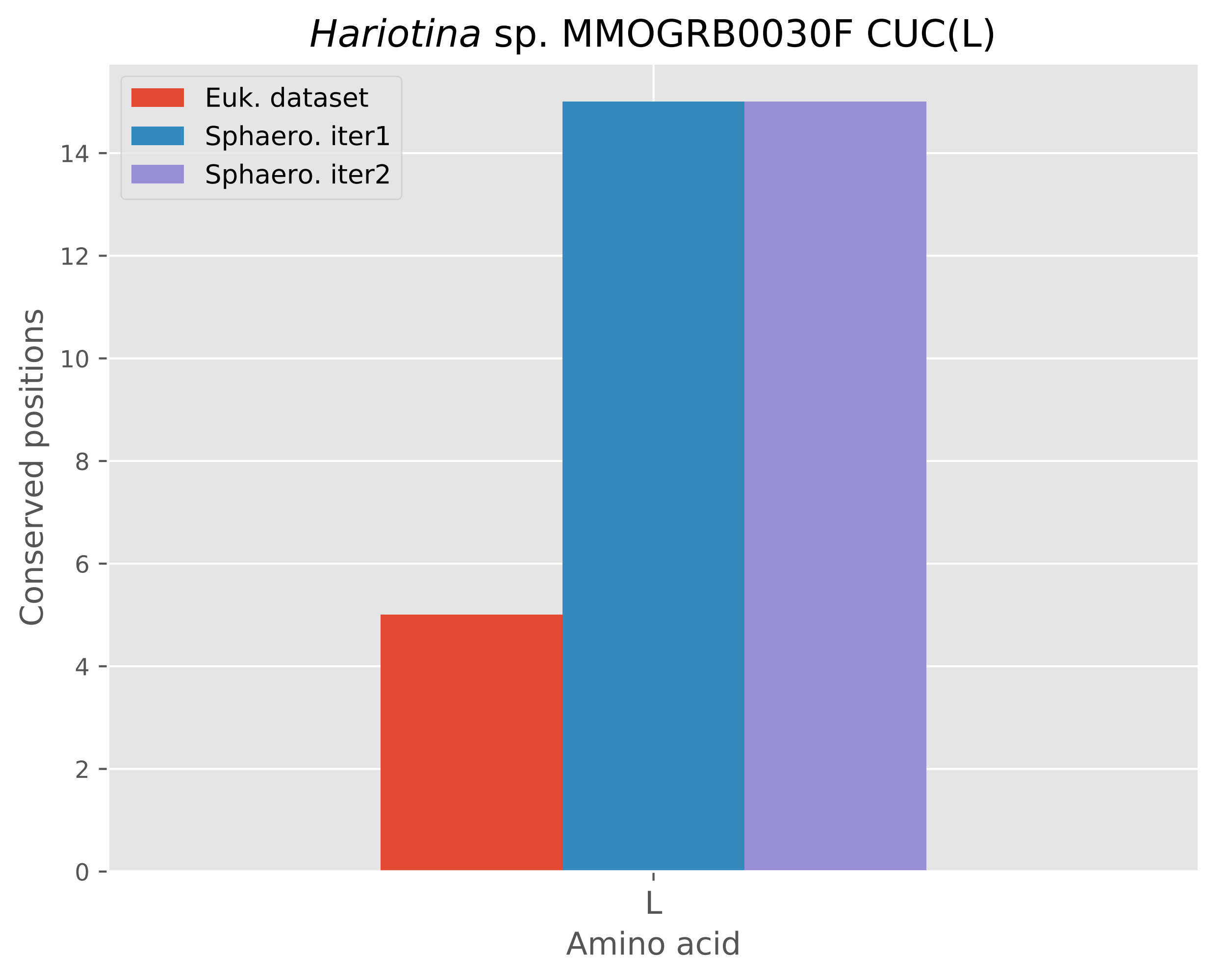

Hariotina sp. MMOGRB0030F CUC(L)
Euk. dataset
Sphaero. iter1
Sphaero. iter2
Conserved positions
0
2
4
6
8
10
12
14
L
Amino acid

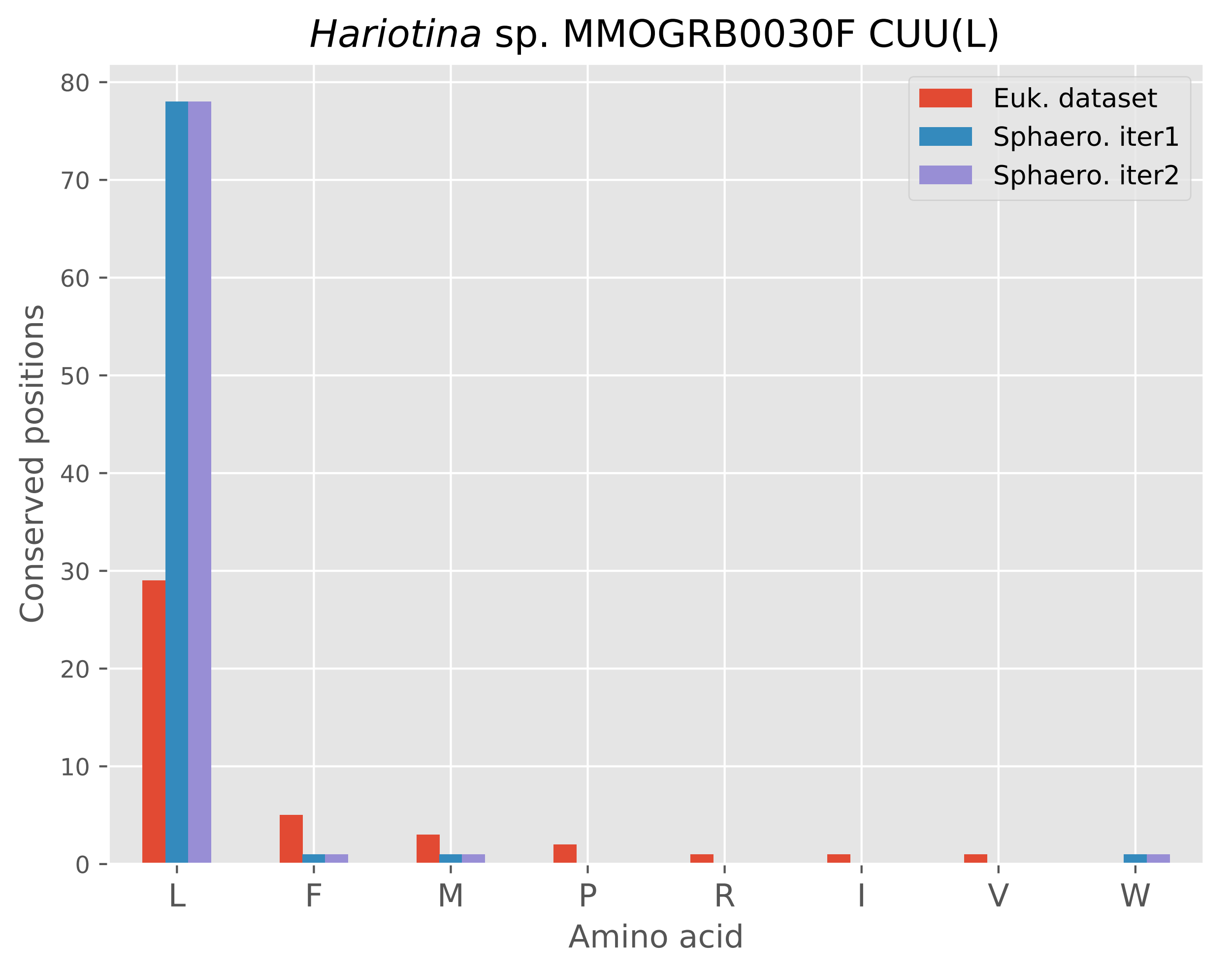

Hariotina sp. MMOGRB0030F CUU(L)
Euk. dataset
Sphaero. iter1
Sphaero. iter2
Conserved positions
Amino acid
0
10
20
30
40
50
60
70
80
L
F
M
P
R
I
V
W

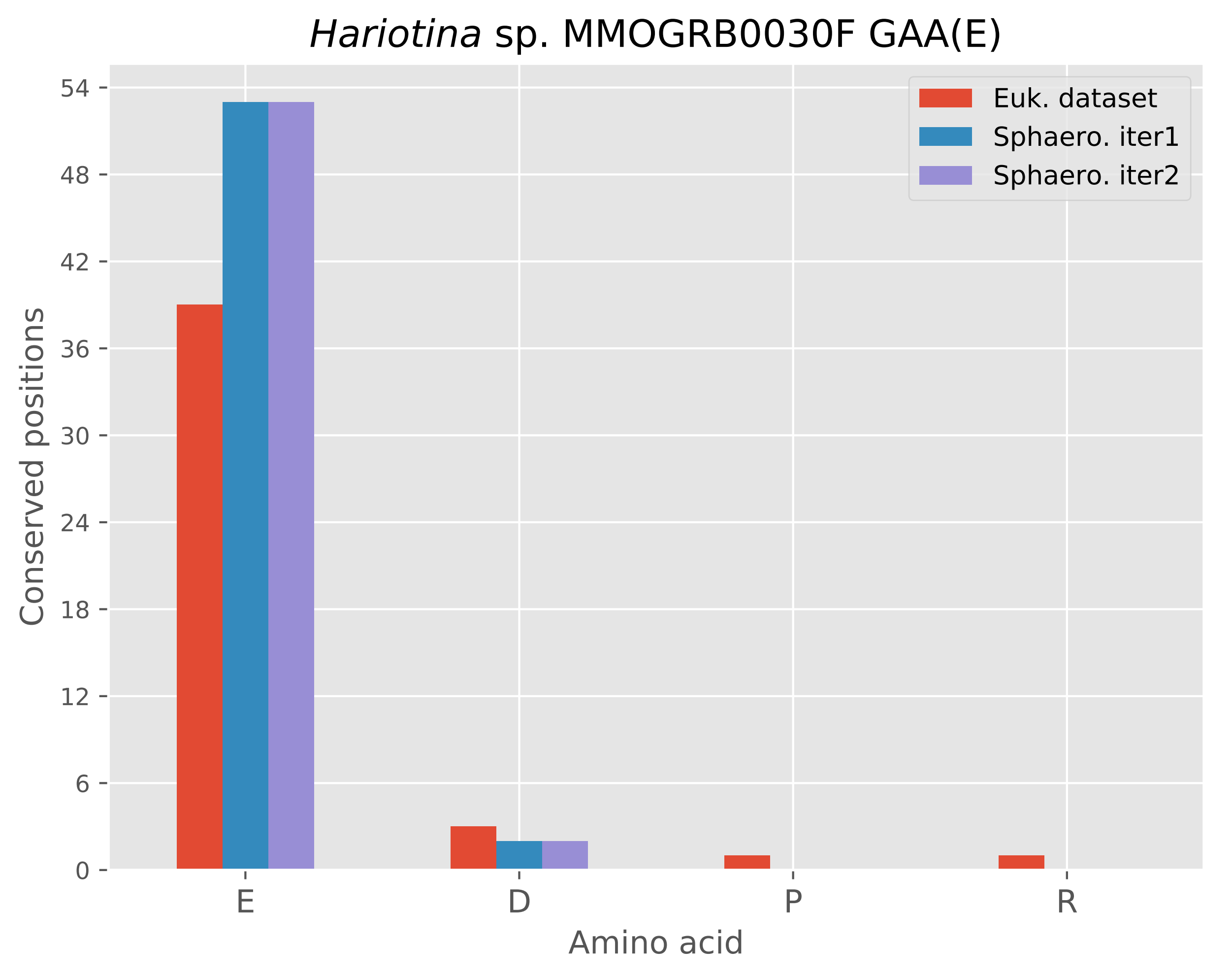

Hariotina sp. MMOGRB0030F GAA(E)
Euk. dataset
Sphaero. iter1
Sphaero. iter2
Conserved positions
0
6
12
18
24
30
36
42
48
54
E
D
P
R
Amino acid

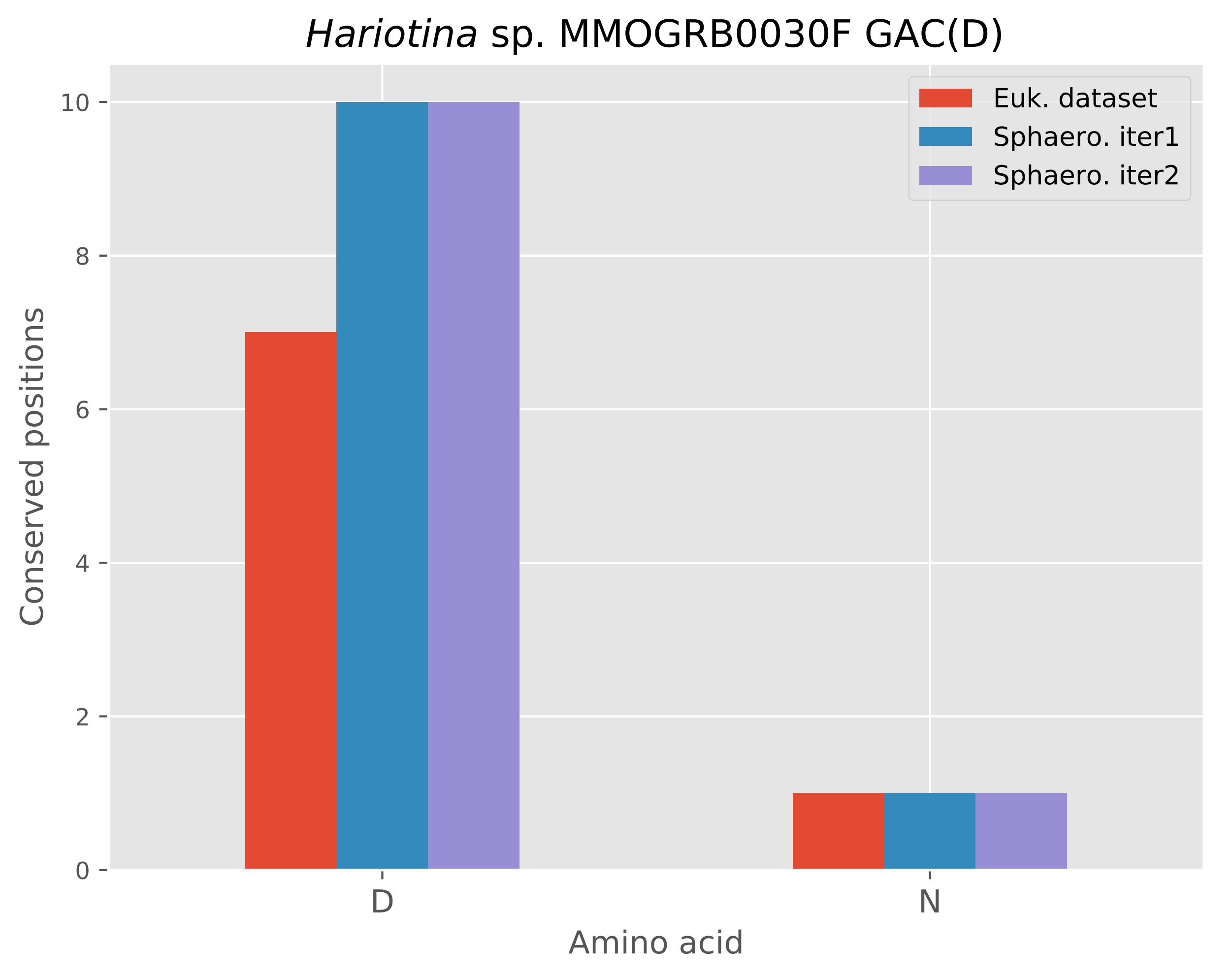
Hariotina sp. MMOGRB0030F GAC(D)
Euk. dataset
Sphaero. iter1
Sphaero. iter2
Conserved positions
0
2
4
6
8
10
D
N
Amino acid

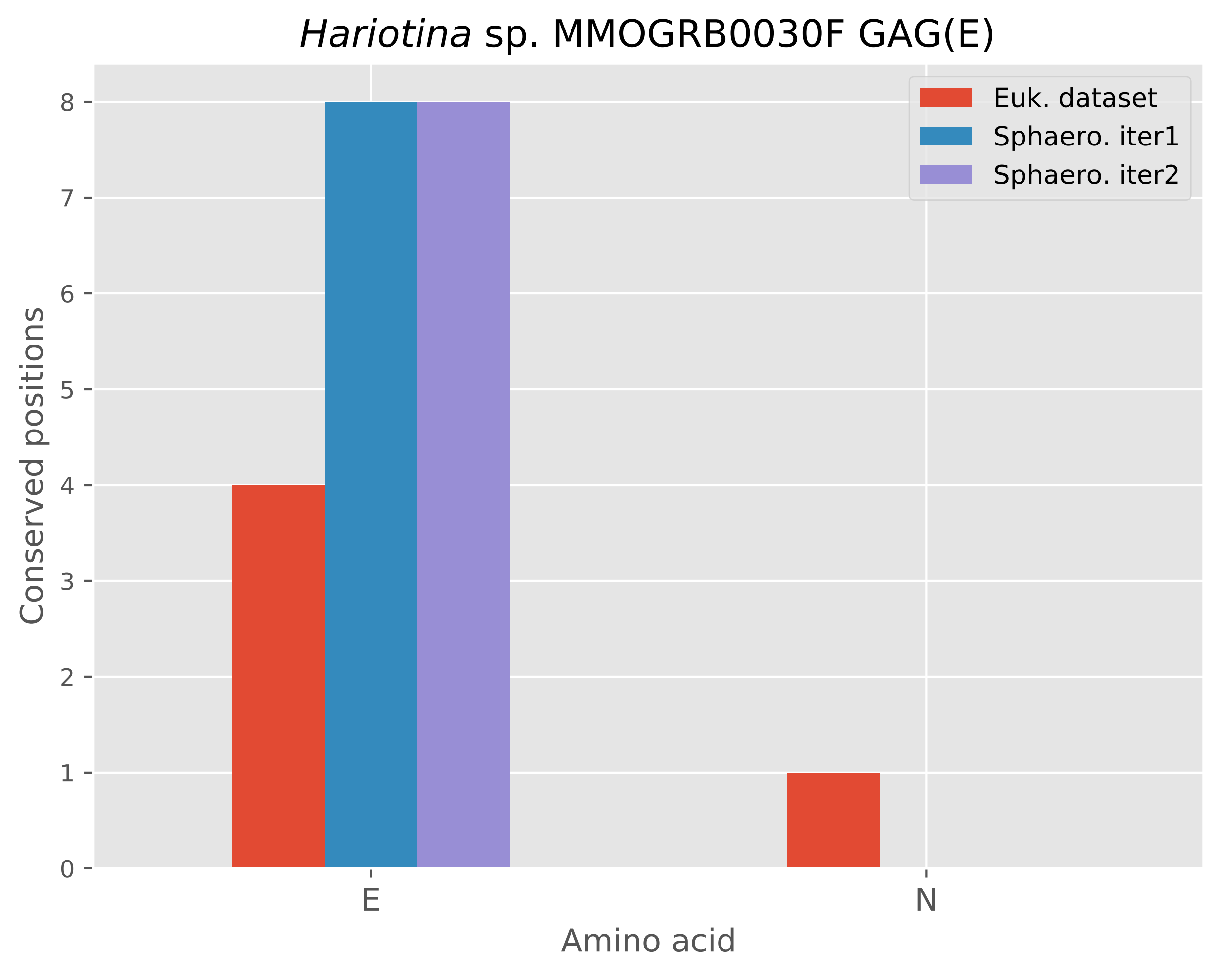

Hariotina sp. MMOGRB0030F GAG(E)
Euk. dataset
Sphaero. iter1
Sphaero. iter2
Conserved positions
0
1
2
3
4
5
6
7
8
E
N
Amino acid

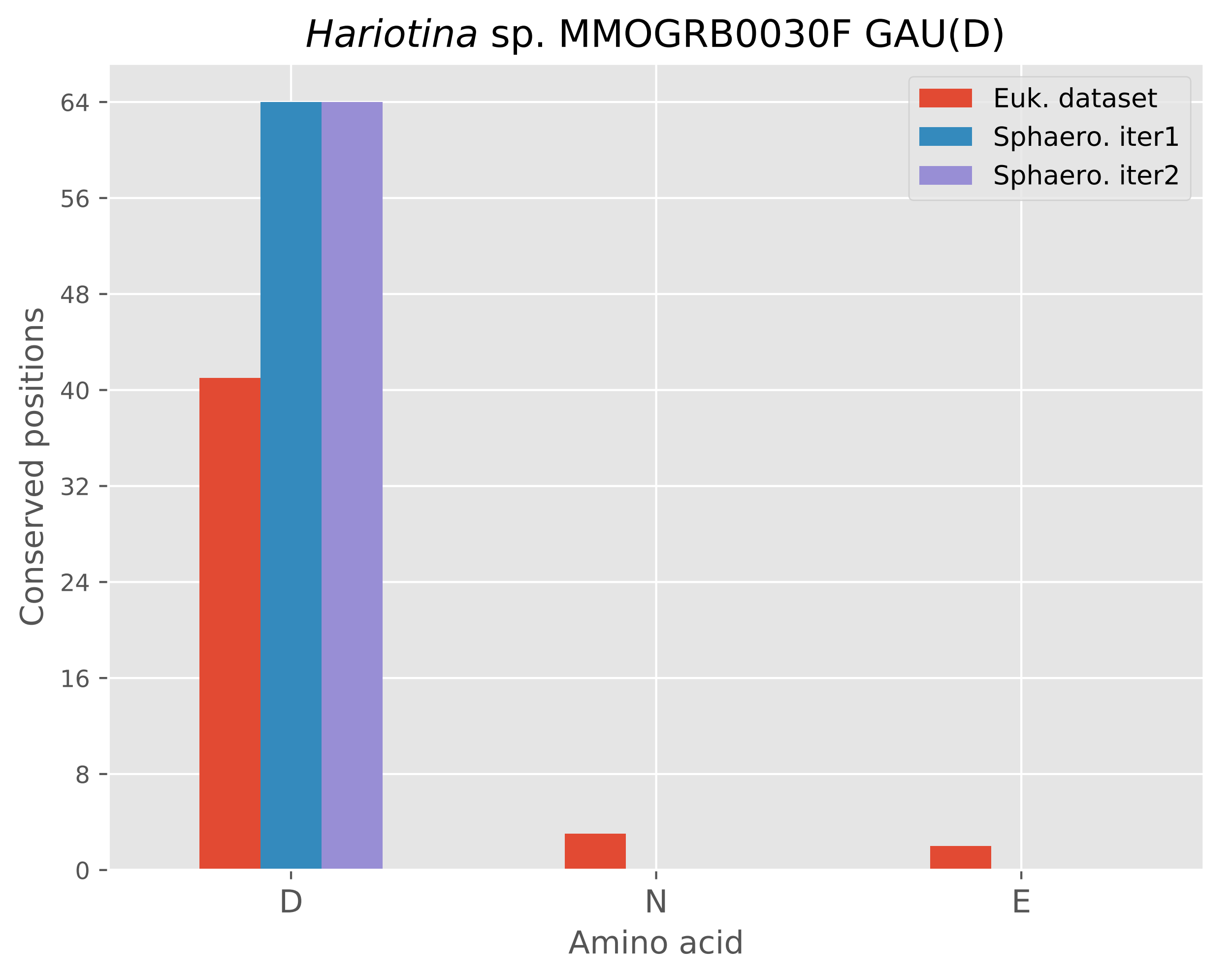
Hariotina sp. MMOGRB0030F GAU(D)
Euk. dataset
Sphaero. iter1
Sphaero. iter2
Conserved positions
0
8
16
24
32
40
48
56
64
D
N
E
Amino acid

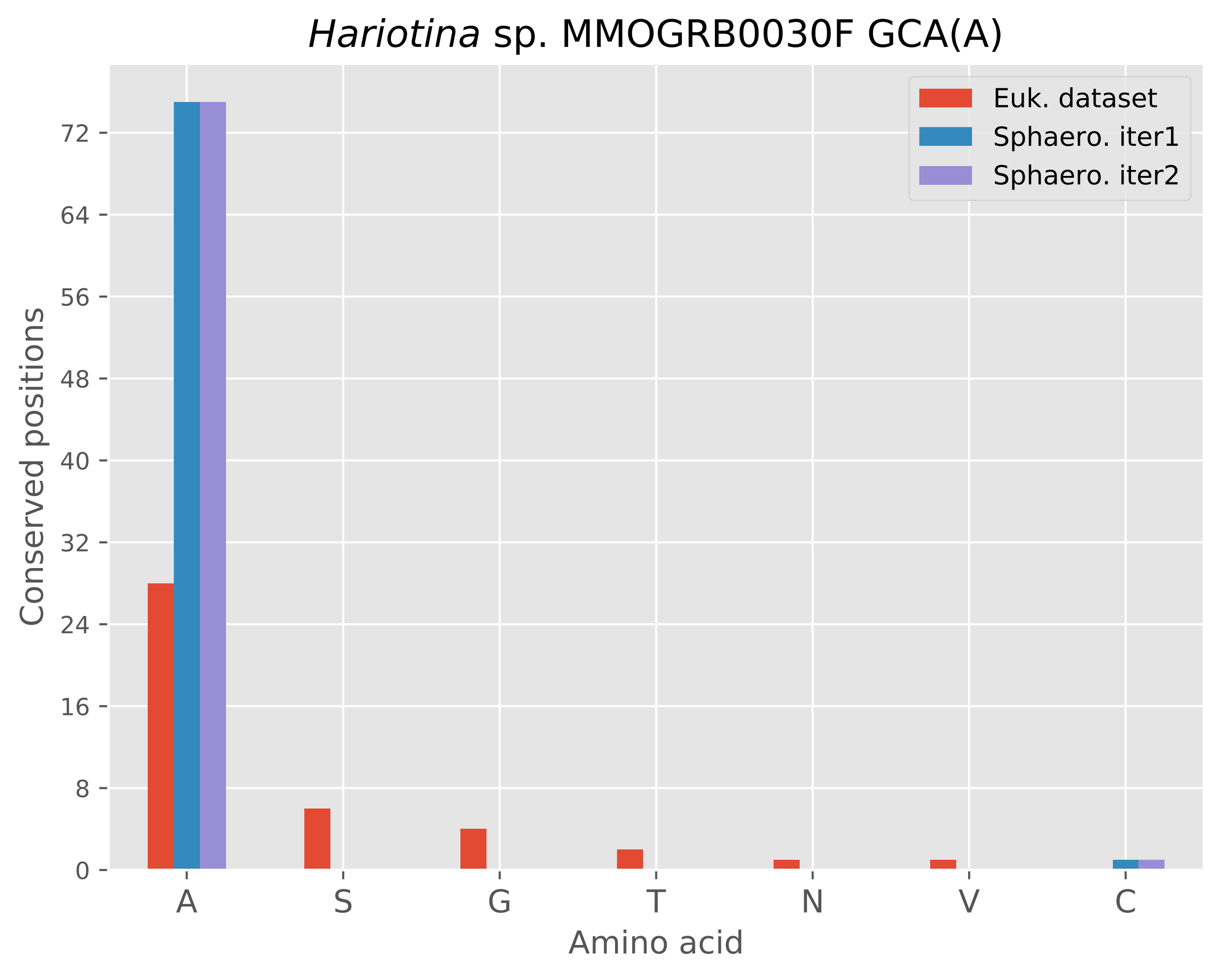
Hariotina sp. MMOGRB0030F GCA(A)
Euk. dataset
Sphaero. iter1
Sphaero. iter2
Conserved positions
0
8
16
24
32
40
48
56
64
72
A
S
G
T
N
V
C
Amino acid

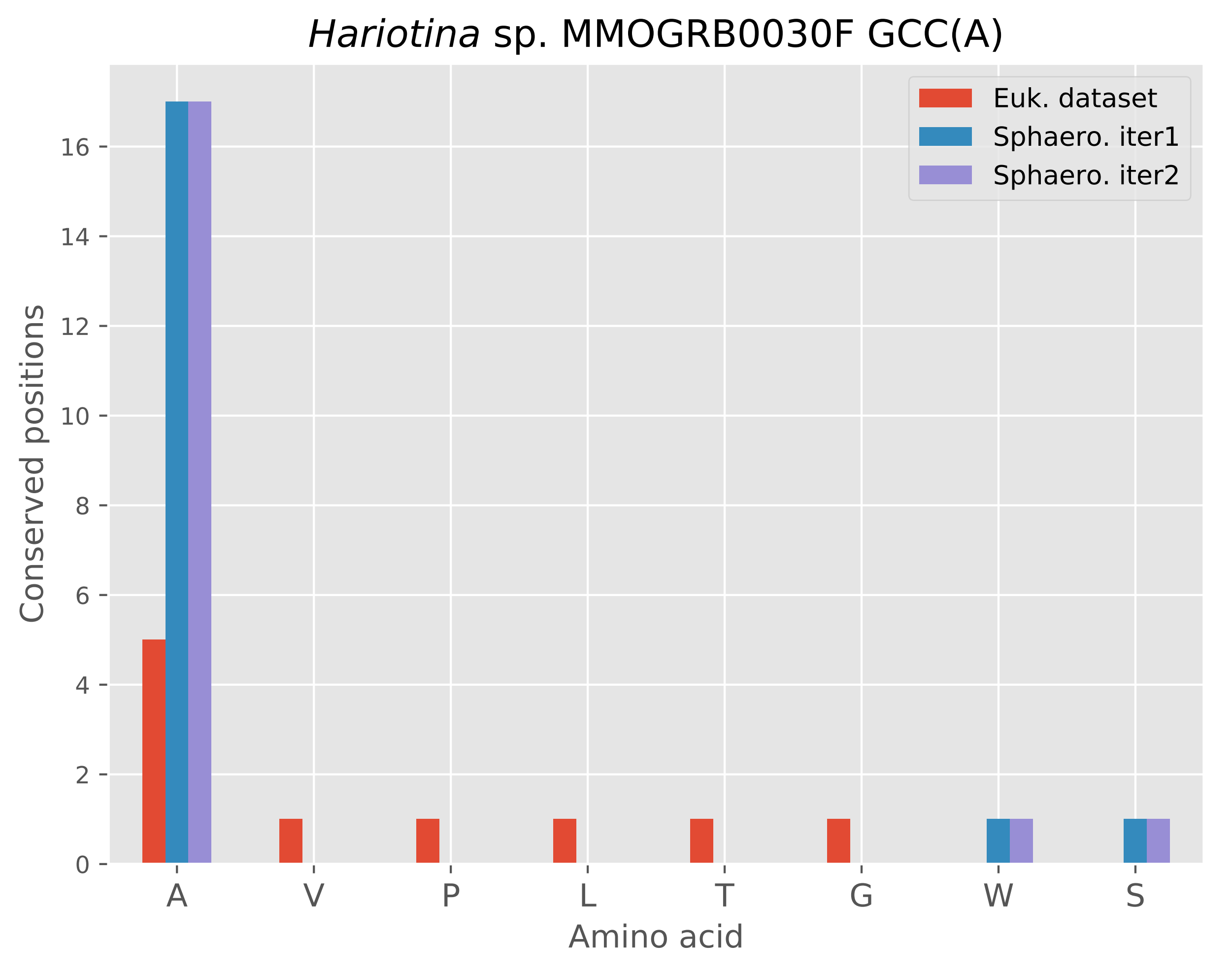

Hariotina sp. MMOGRB0030F GCC(A)
Euk. dataset
Sphaero. iter1
Sphaero. iter2
Conserved positions
0
2
4
6
8
10
12
14
16
A
V
P
L
T
G
W
S
Amino acid

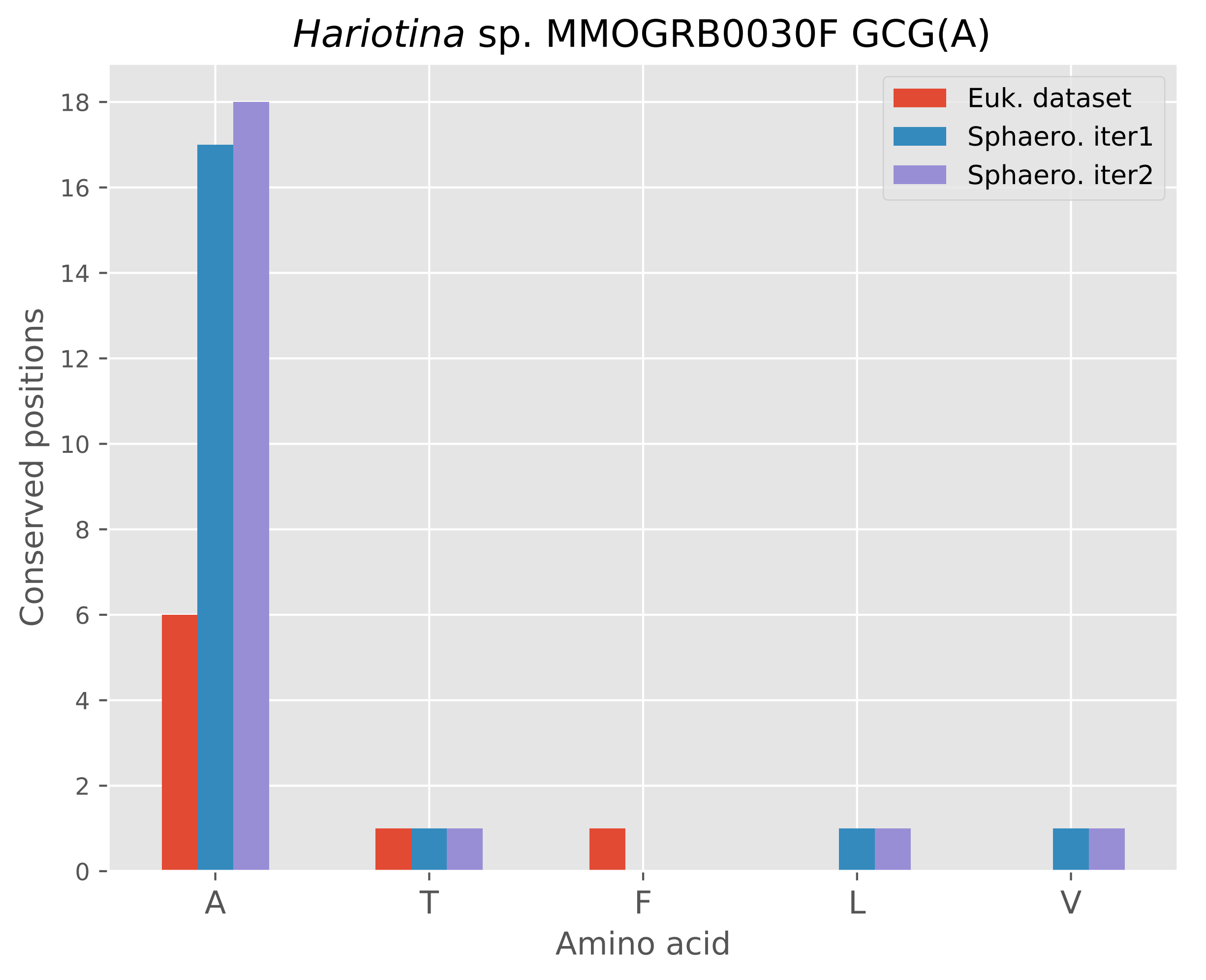

Hariotina sp. MMOGRB0030F GCG(A)
Euk. dataset
Sphaero. iter1
Sphaero. iter2
Conserved positions
0
2
4
6
8
10
12
14
16
18
A
T
F
L
V
Amino acid

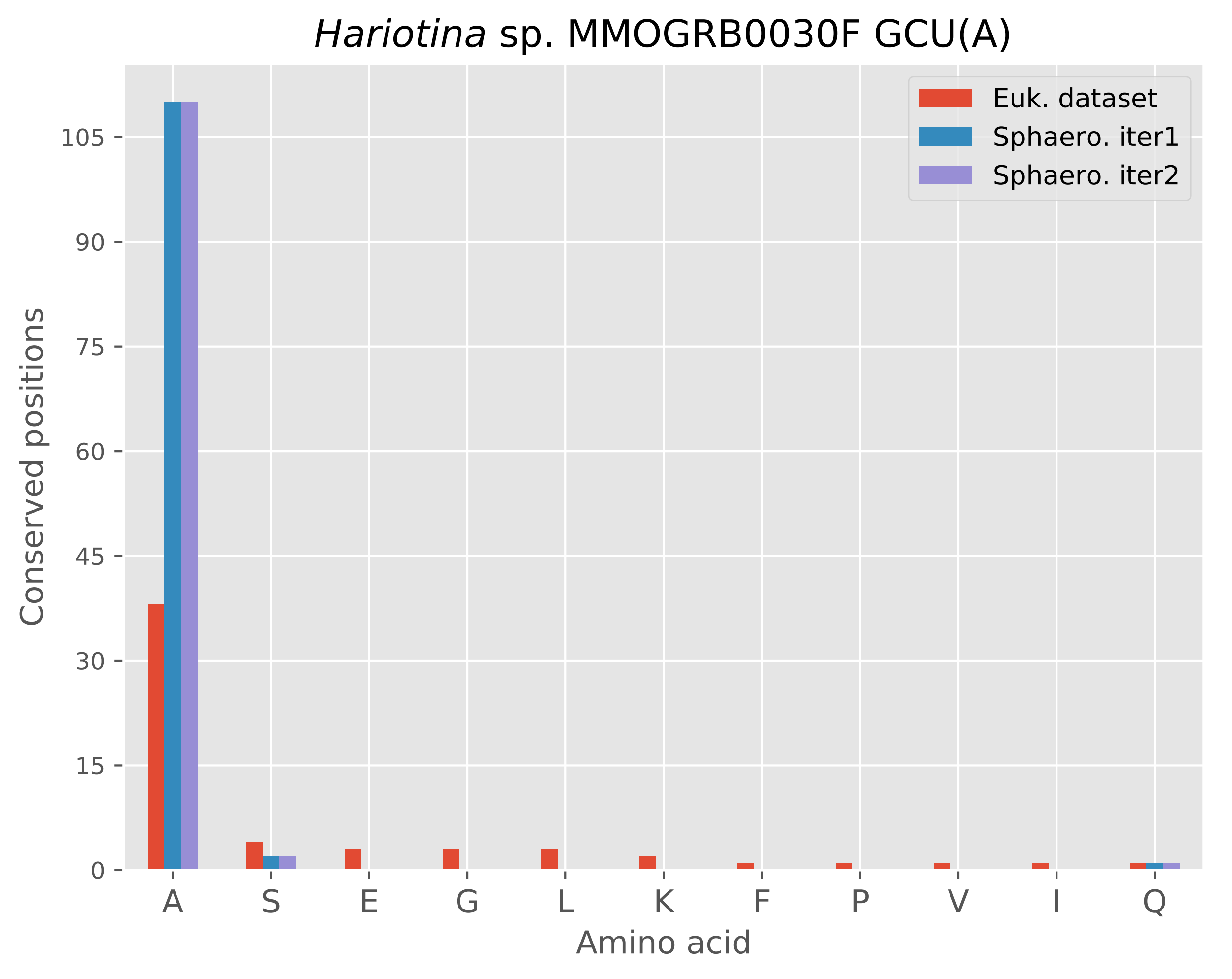
Hariotina sp. MMOGRB0030F GCU(A)
Euk. dataset
Sphaero. iter1
Sphaero. iter2
Conserved positions
0
15
30
45
60
75
90
105
A
S
E
G
L
K
F
P
V
I
Q
Amino acid

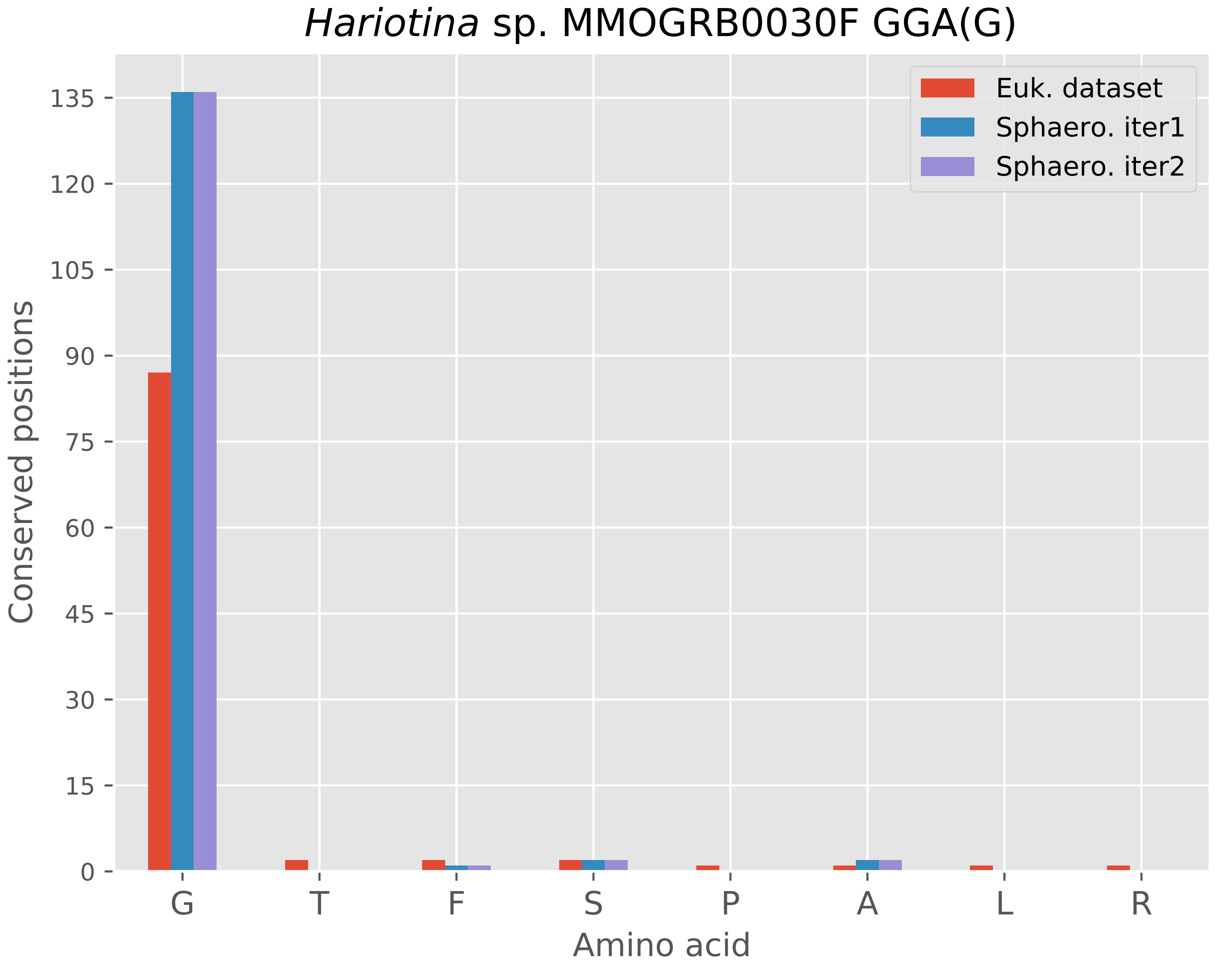

Hariotina sp. MMOGRB0030F GGA(G)
Euk. dataset
Sphaero. iter1
Sphaero. iter2
Conserved positions
0
15
30
45
60
75
90
105
120
135
G
T
F
S
P
A
L
R
Amino acid

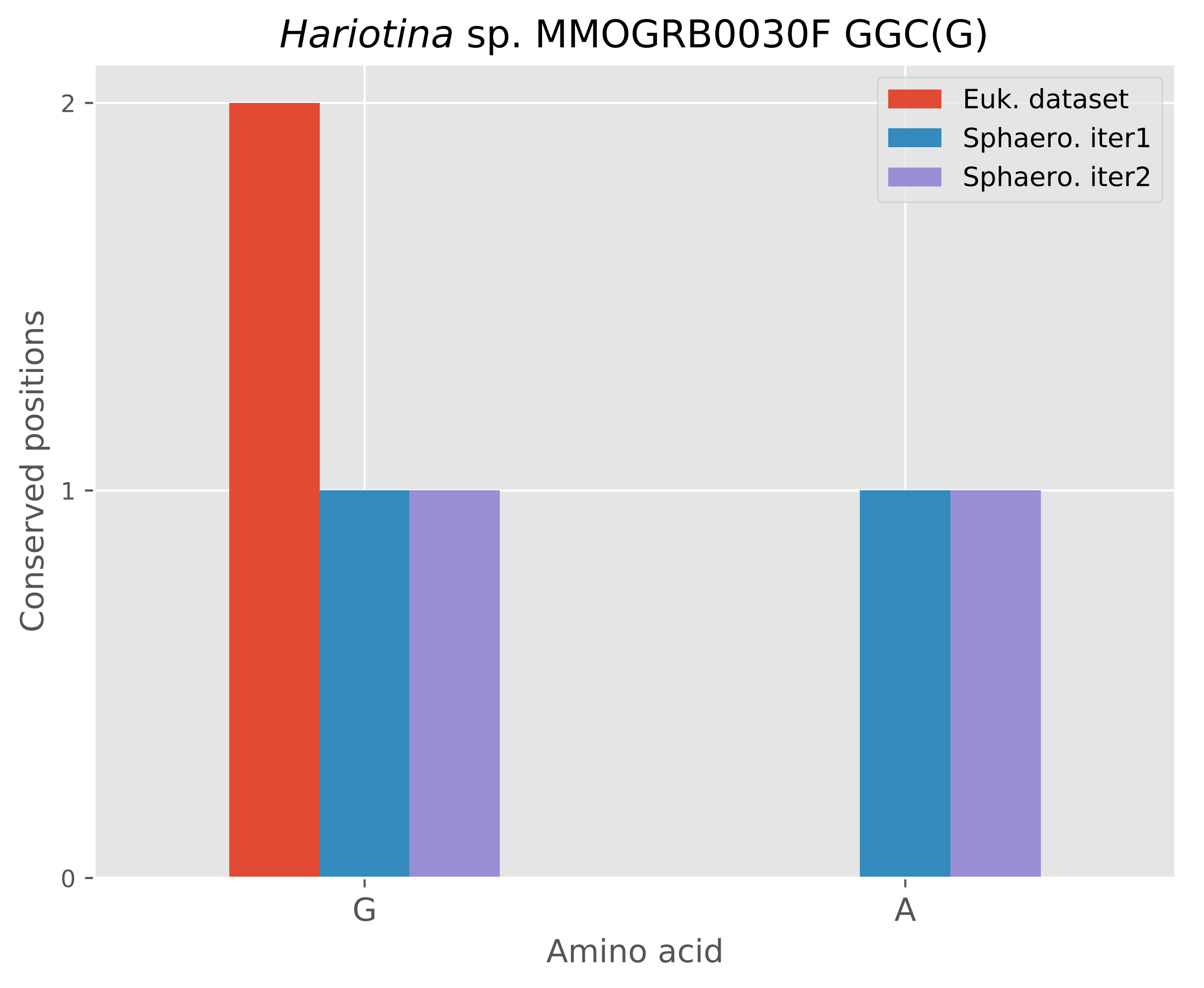

Hariotina sp. MMOGRB0030F GGC(G)
Euk. dataset
Sphaero. iter1
Sphaero. iter2
Conserved positions
2
1
0
G
A
Amino acid

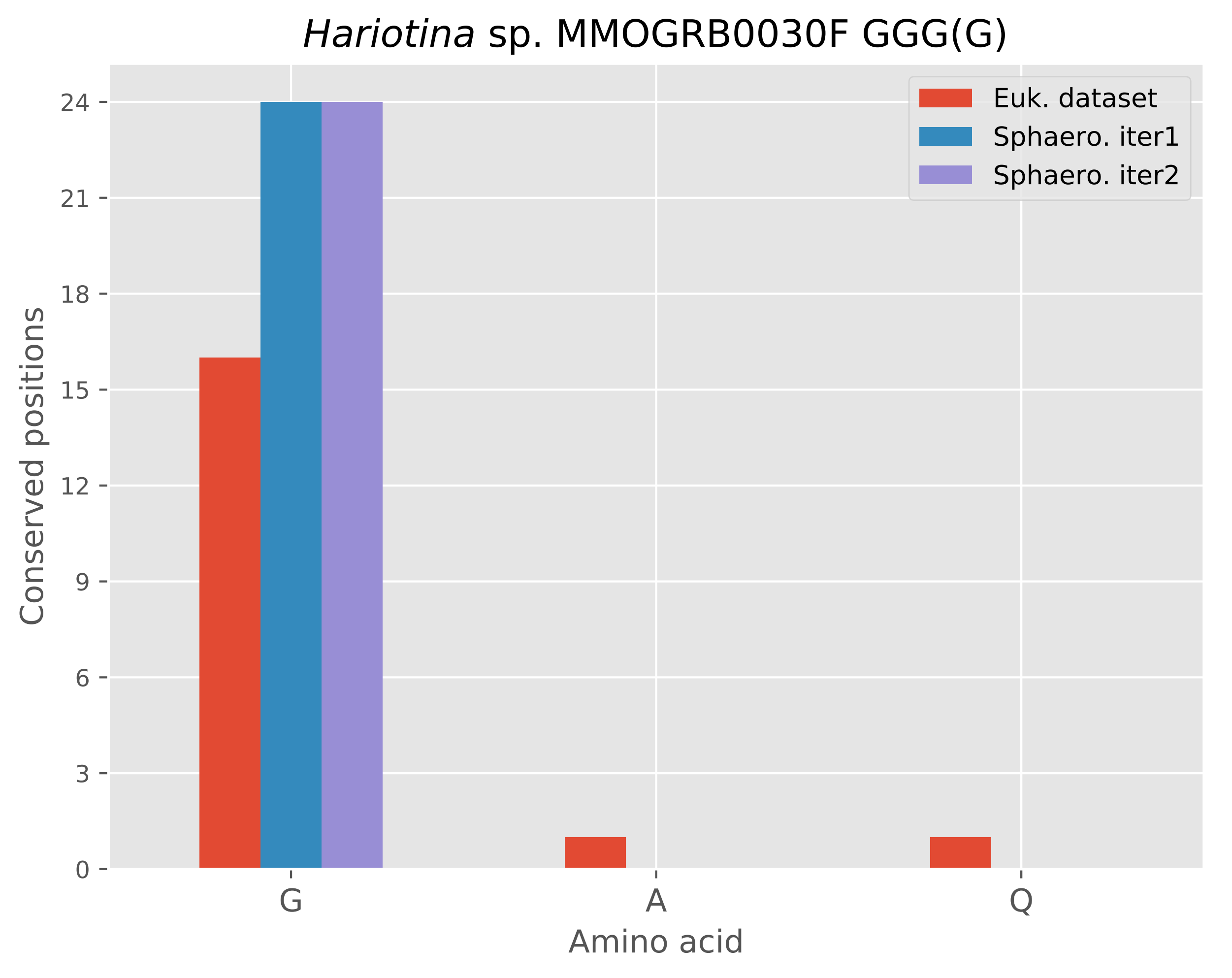
Hariotina sp. MMOGRB0030F GGG(G)
Euk. dataset
Sphaero. iter1
Sphaero. iter2
Conserved positions
0
3
6
9
12
15
18
21
24
G
A
Q
Amino acid

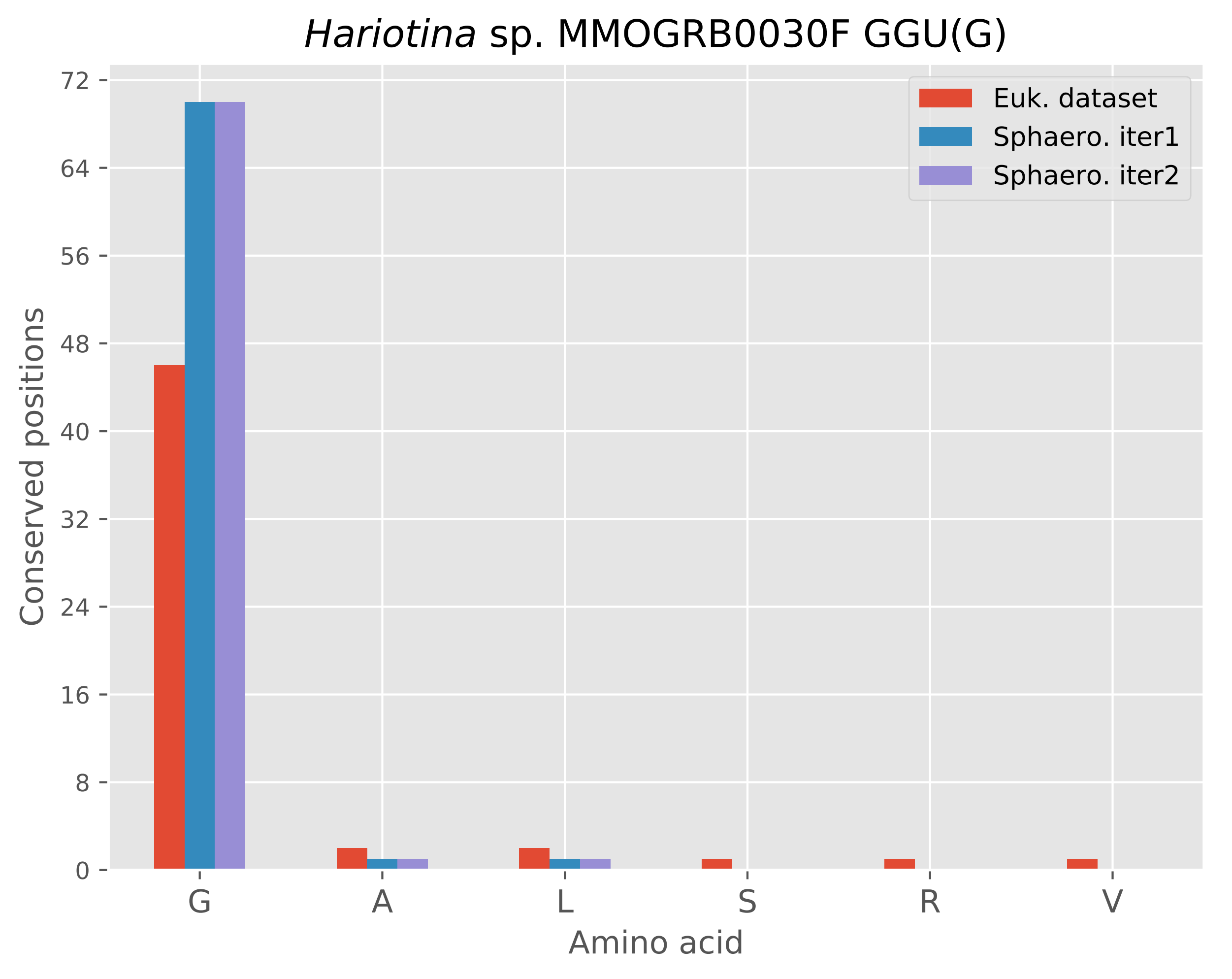

Hariotina sp. MMOGRB0030F GGU(G)
Euk. dataset
Sphaero. iter1
Sphaero. iter2
Conserved positions
0
8
16
24
32
40
48
56
64
72
G
A
L
S
R
V
Amino acid

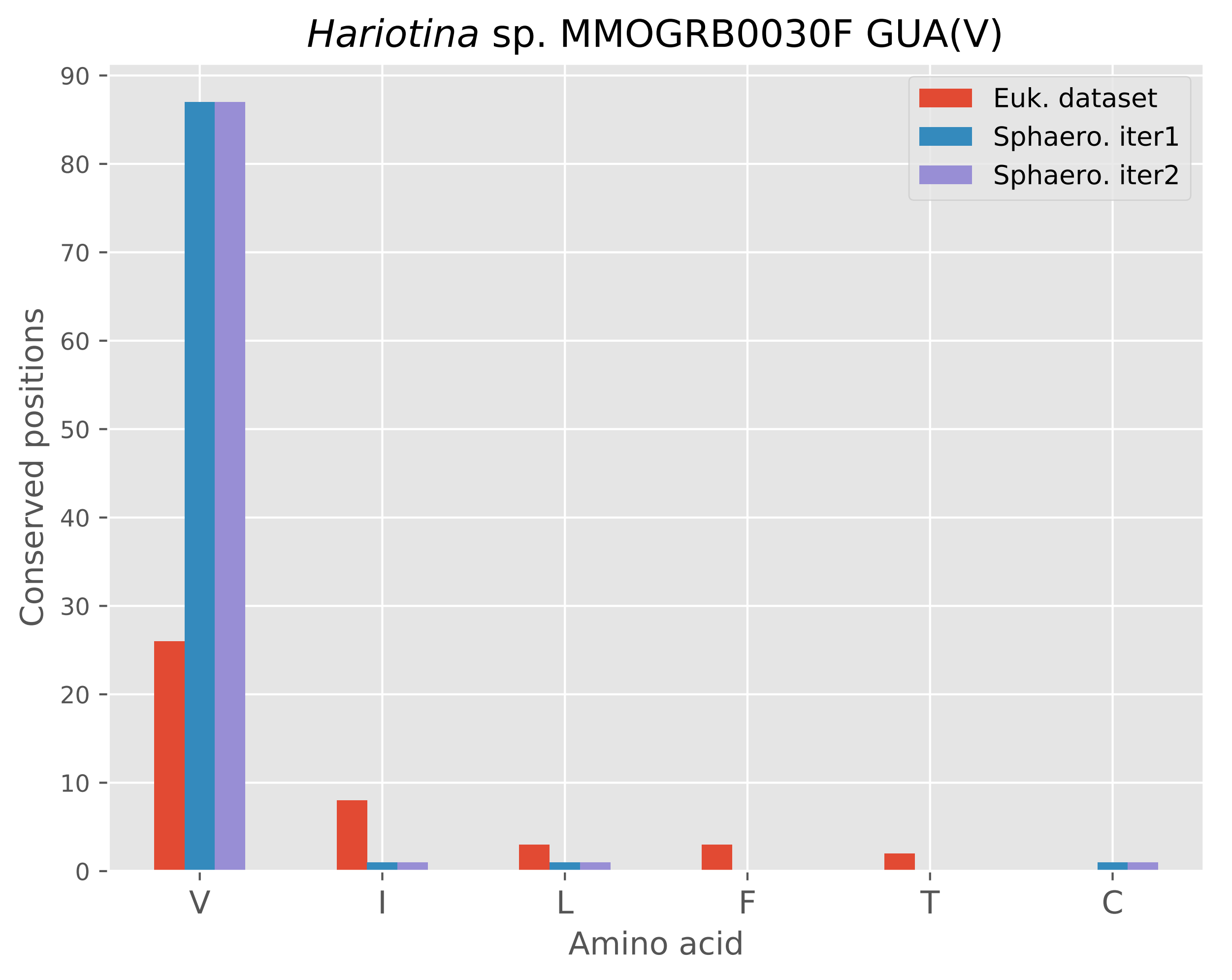

Hariotina sp. MMOGRB0030F GUA(V)
Euk. dataset
Sphaero. iter1
Sphaero. iter2
Conserved positions
Amino acid
0
10
20
30
40
50
60
70
80
90
V
I
L
F
T
C

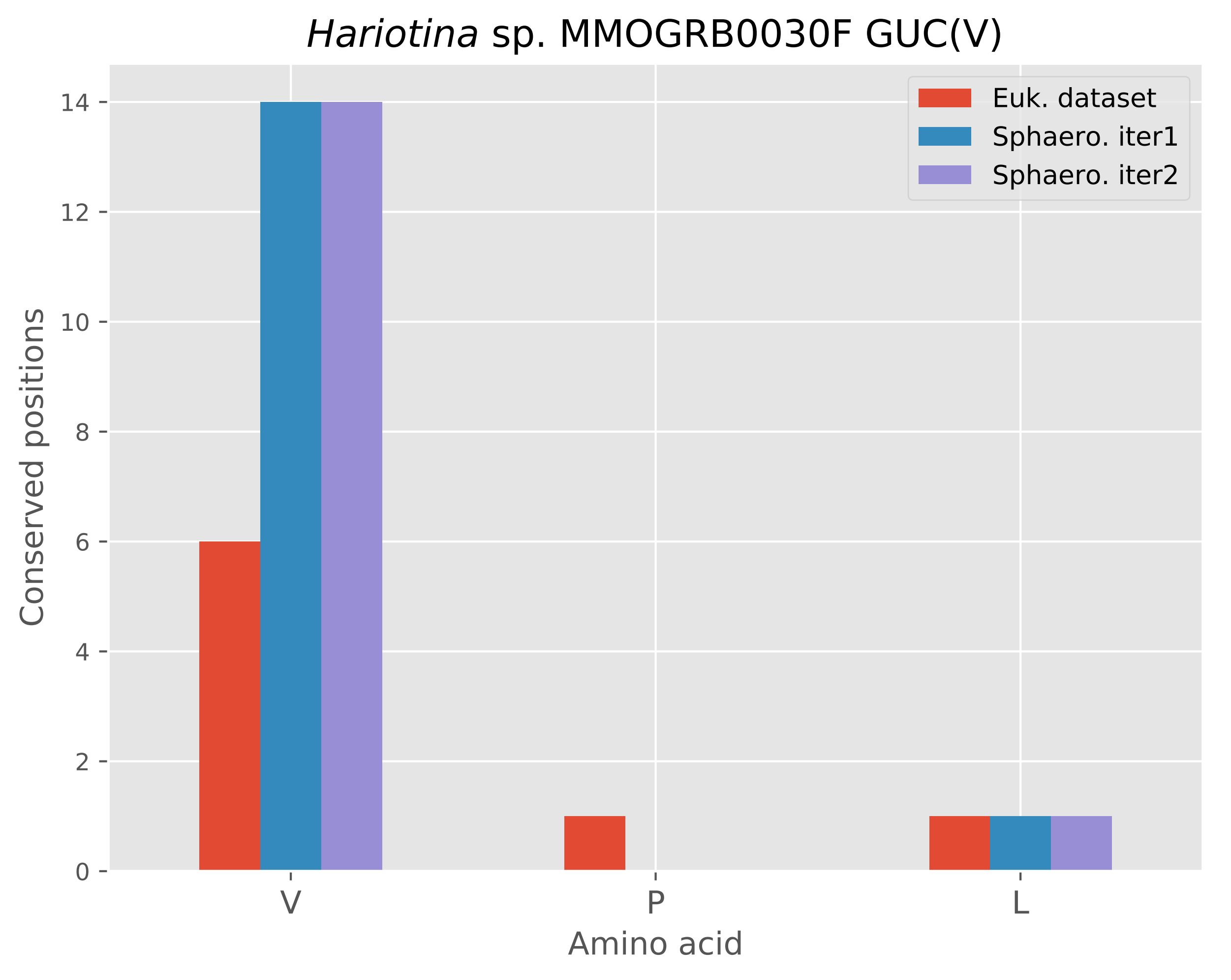

Hariotina sp. MMOGRB0030F GUC(V)
Euk. dataset
Sphaero. iter1
Sphaero. iter2
Conserved positions
0
2
4
6
8
10
12
14
V
P
L
Amino acid

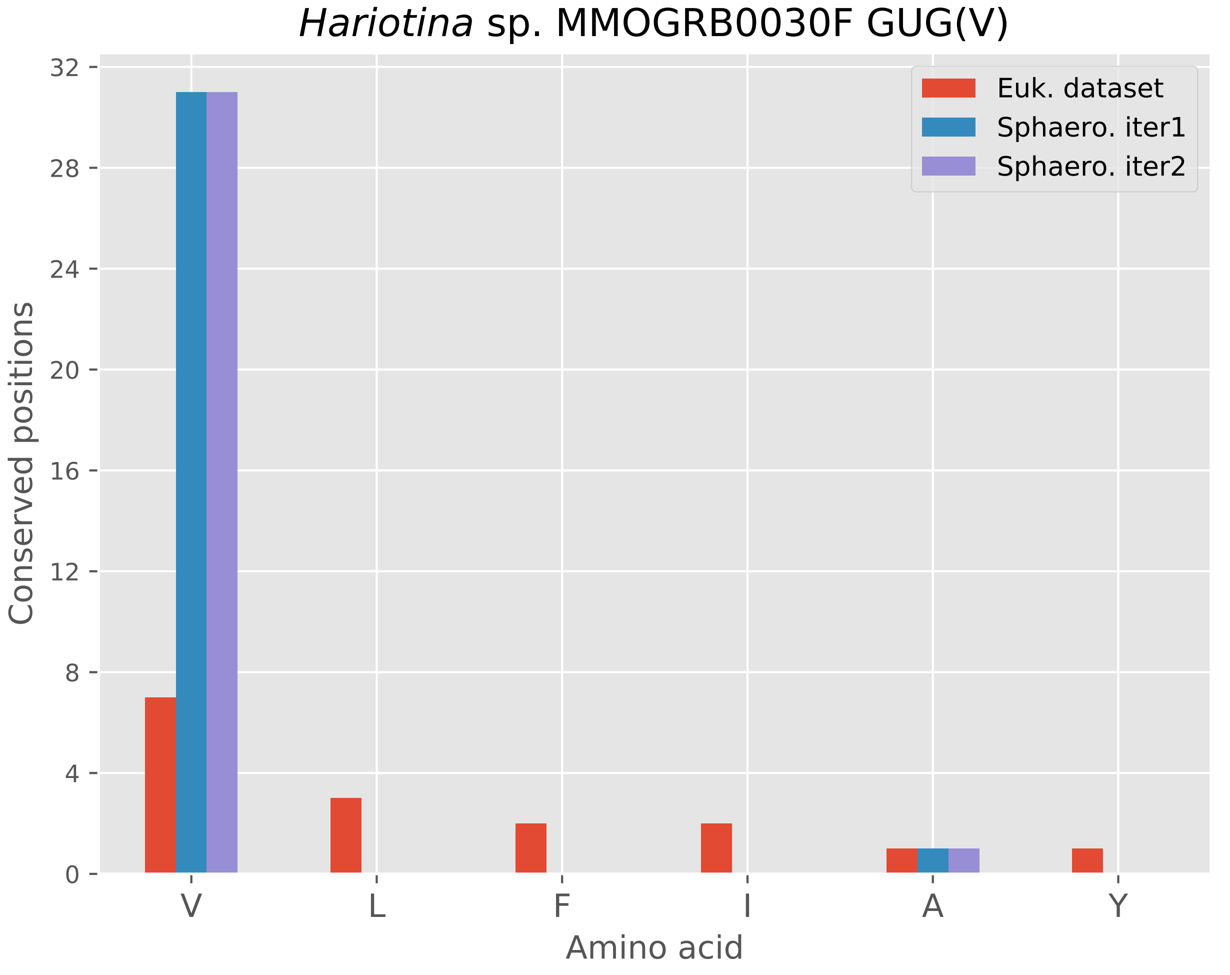

Hariotina sp. MMOGRB0030F GUG(V)
Euk. dataset
Sphaero. iter1
Sphaero. iter2
Conserved positions
0
4
8
12
16
20
24
28
32
V
L
F
I
A
Y
Amino acid

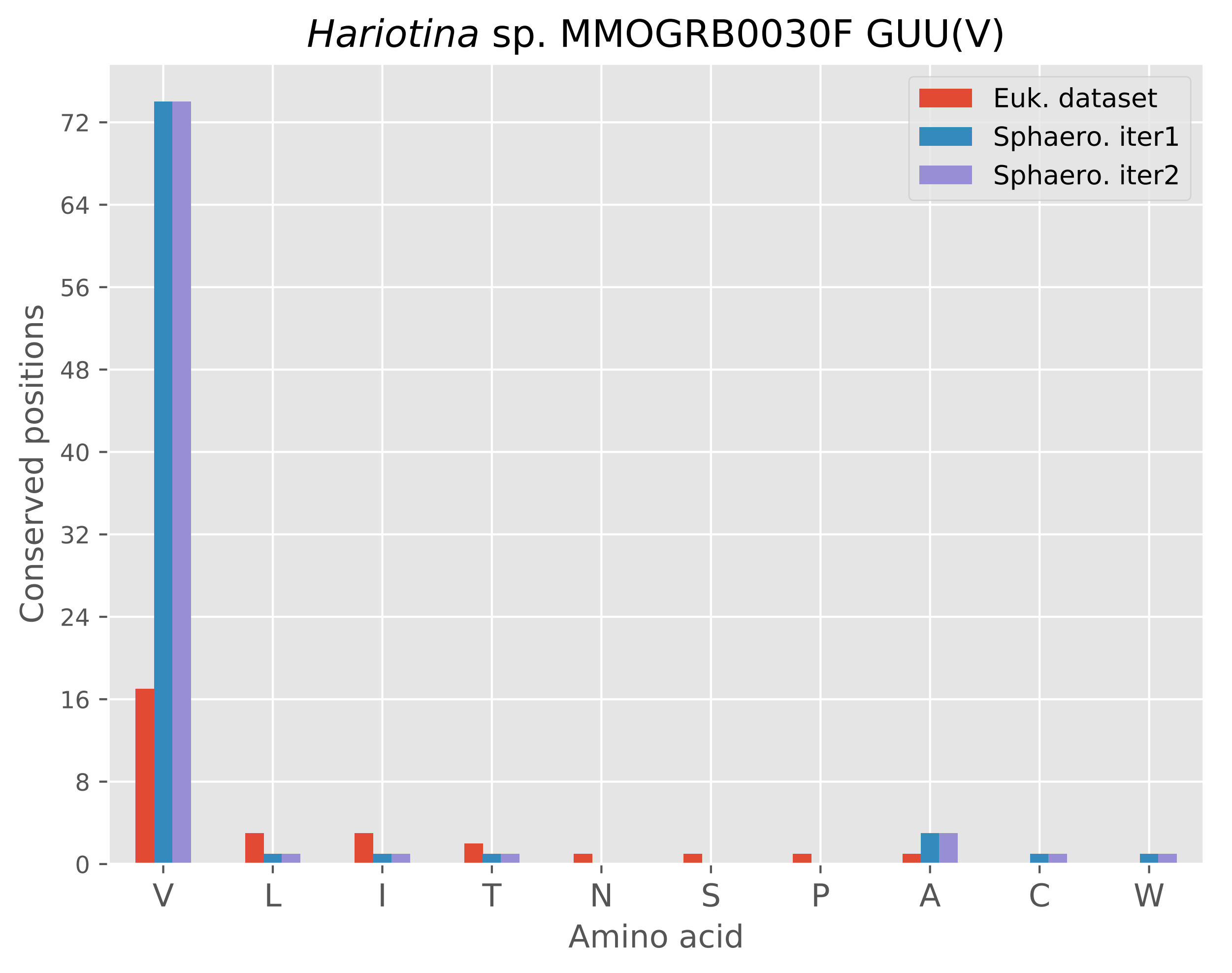
Hariotina sp. MMOGRB0030F GUU(V)
Conserved positions
Amino acid
Euk. dataset
Sphaero. iter1
Sphaero. iter2
0
8
16
24
32
40
48
56
64
72
V
L
I
T
N
S
P
A
C
W

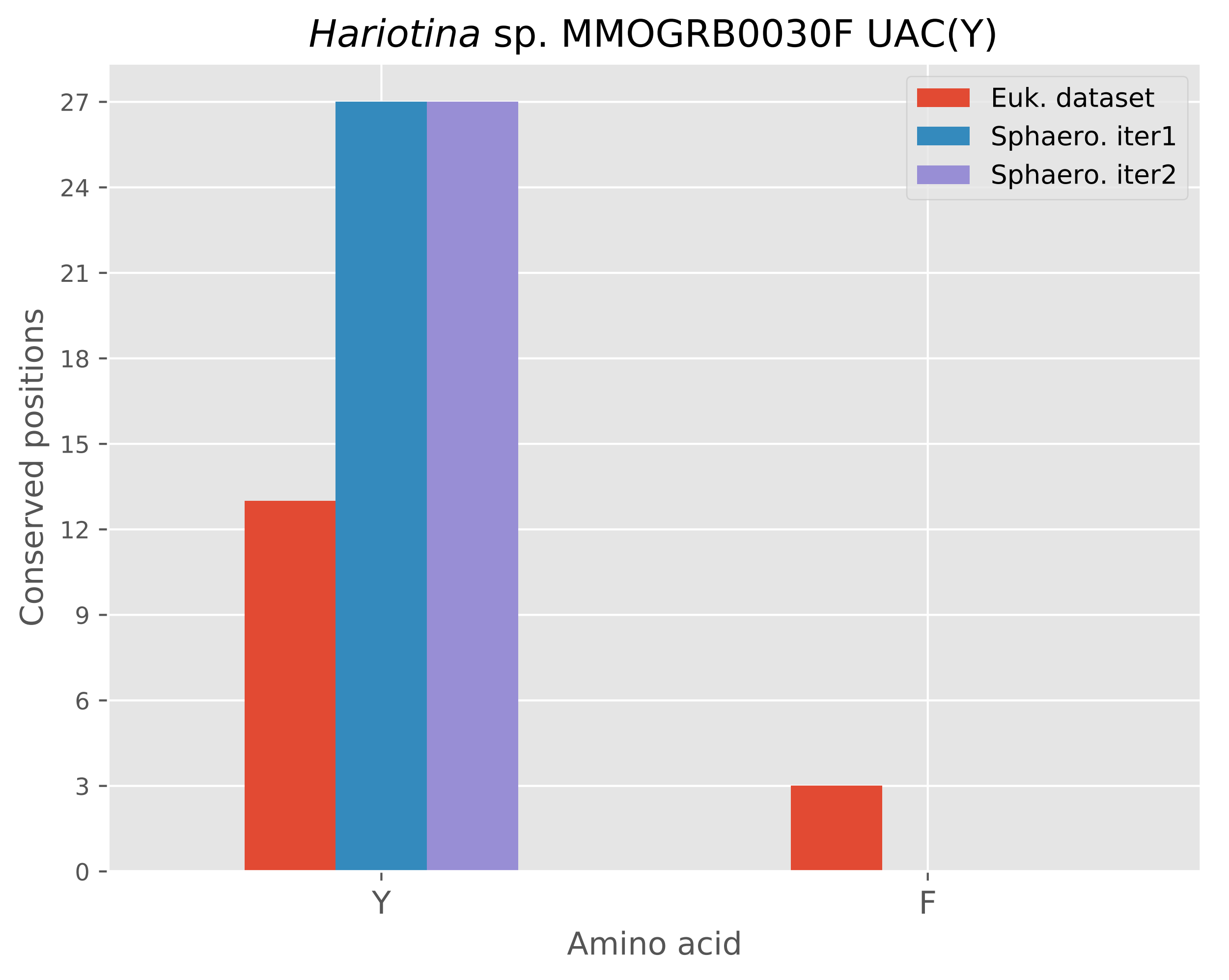

Hariotina sp. MMOGRB0030F UAC(Y)
Euk. dataset
Sphaero. iter1
Sphaero. iter2
Conserved positions
0
3
6
9
12
15
18
21
24
27
Y
F
Amino acid

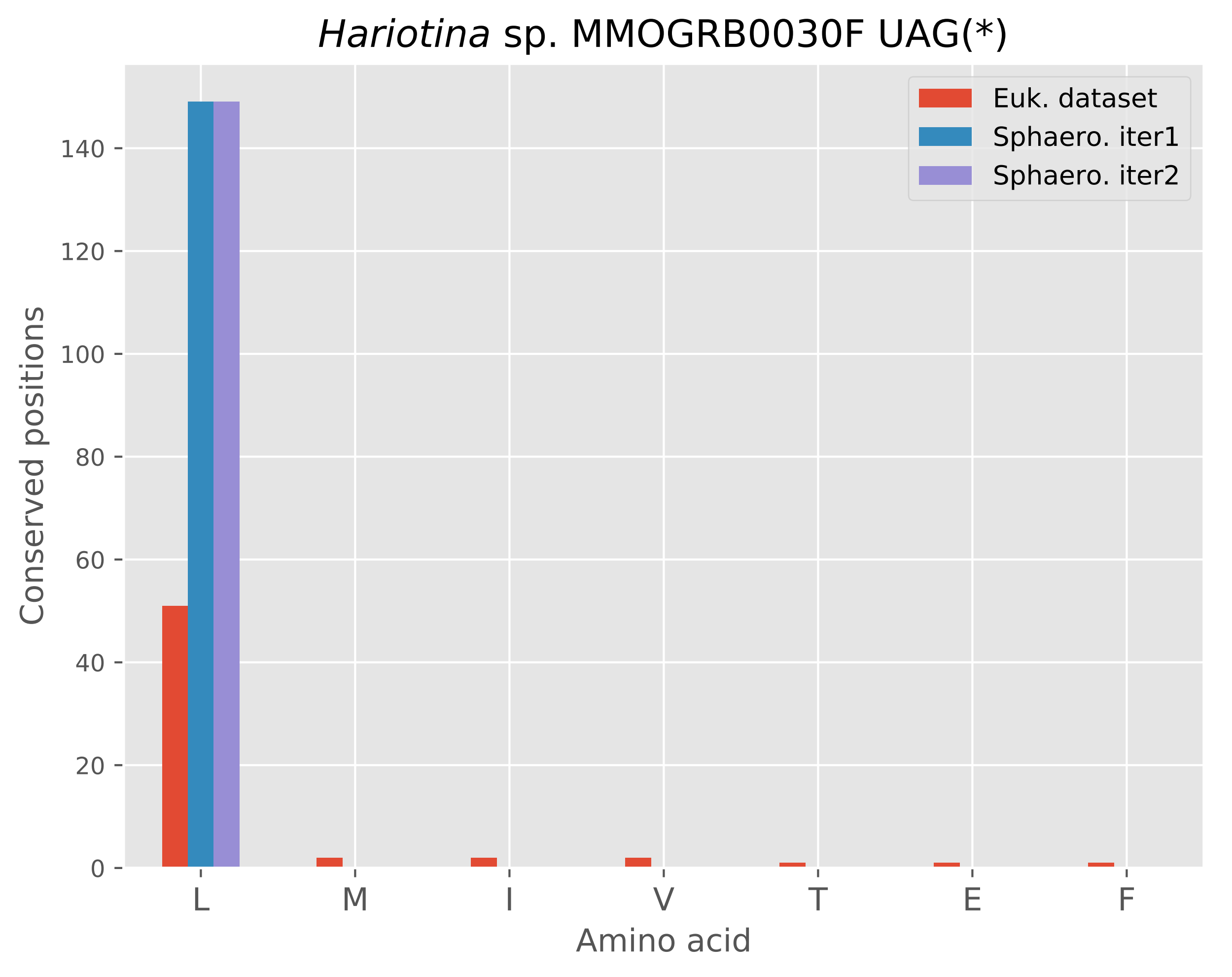

Hariotina sp. MMOGRB0030F UAG(*)
Euk. dataset
Sphaero. iter1
Sphaero. iter2
Conserved positions
0
20
40
60
80
100
120
140
L
M
I
V
T
E
F
Amino acid

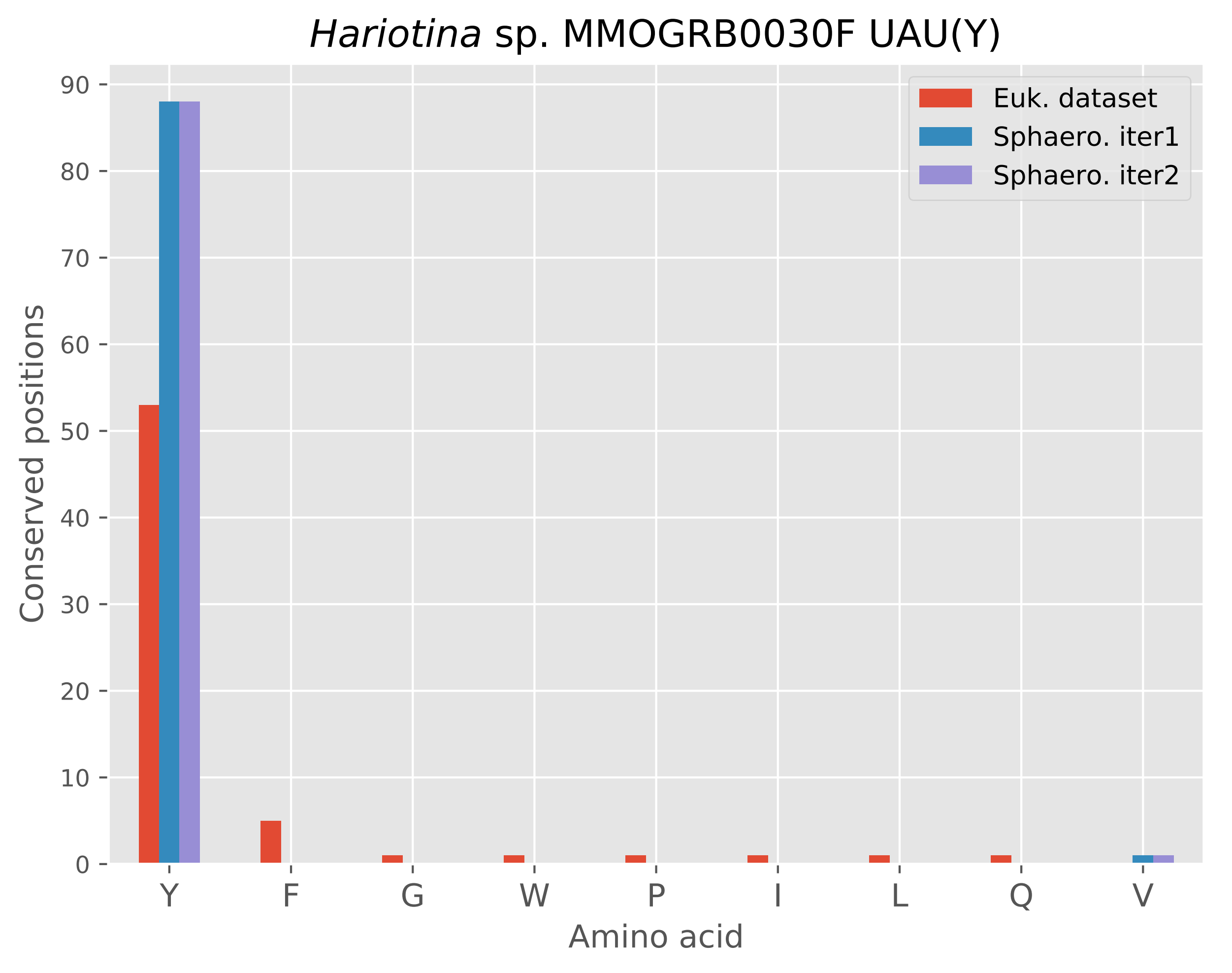

Hariotina sp. MMOGRB0030F UAU(Y)
Euk. dataset
Sphaero. iter1
Sphaero. iter2
Conserved positions
0
10
20
30
40
50
60
70
80
90
Y
F
G
W
P
I
L
Q
V
Amino acid

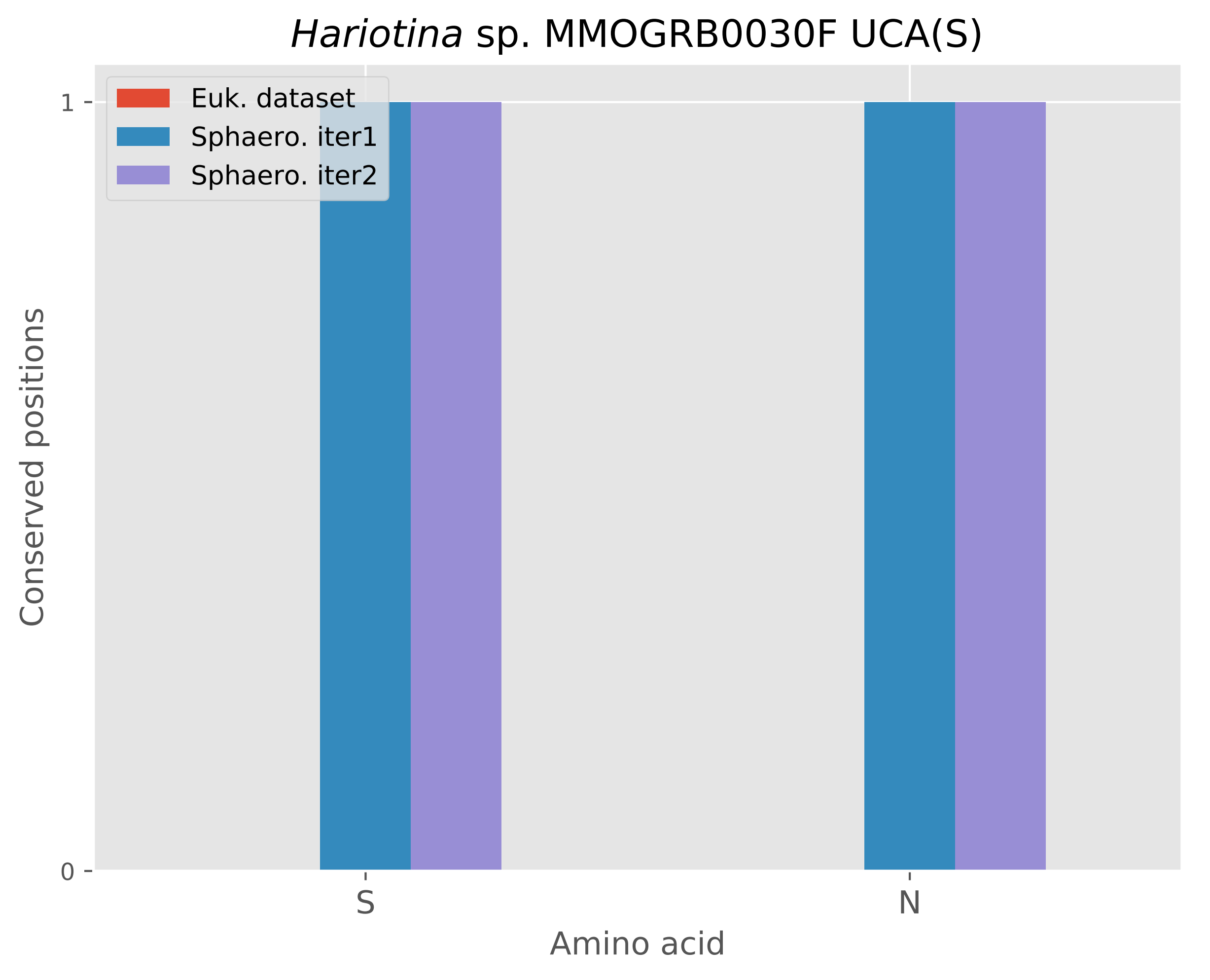
Hariotina sp. MMOGRB0030F UCA(S)
Euk. dataset
Sphaero. iter1
Sphaero. iter2
1
0
Conserved positions
S
N
Amino acid

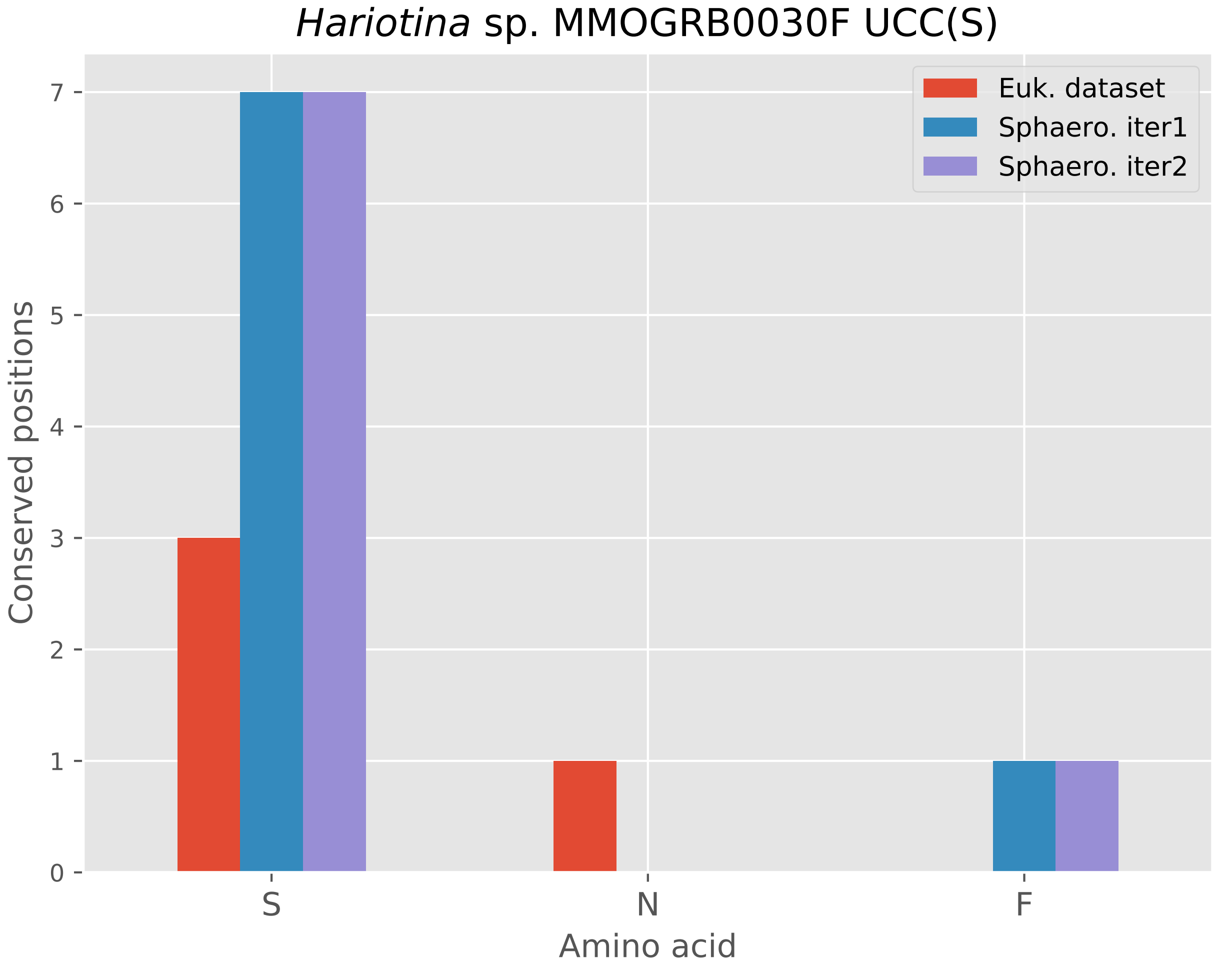
Hariotina sp. MMOGRB0030F UCC(S)
Euk. dataset
Sphaero. iter1
Sphaero. iter2
Conserved positions
0
1
2
3
4
5
6
7
S
N
F
Amino acid

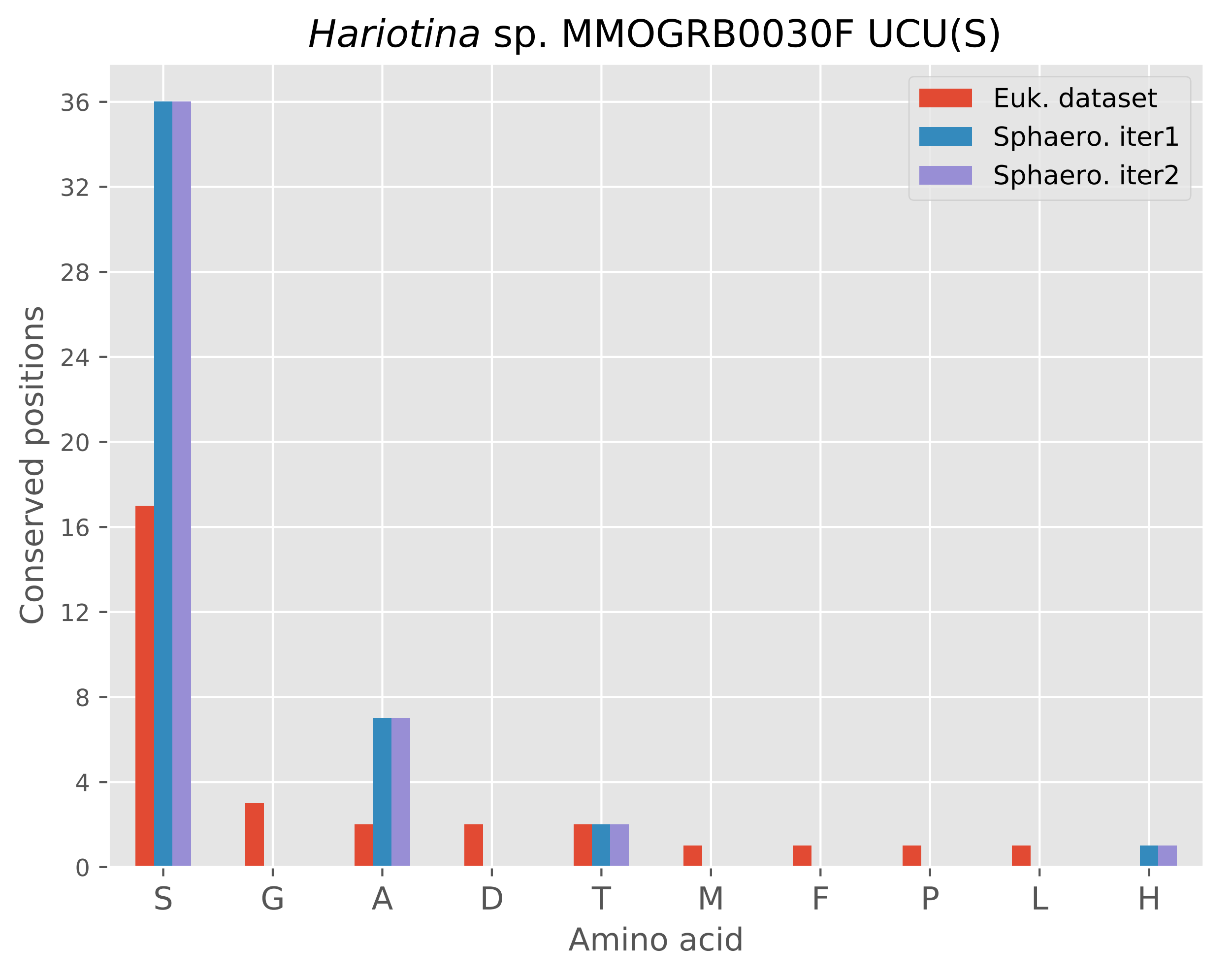

Hariotina sp. MMOGRB0030F UCU(S)
Euk. dataset
Sphaero. iter1
Sphaero. iter2
Conserved positions
0
4
8
12
16
20
24
28
32
36
S
G
A
D
T
M
F
P
L
H
Amino acid

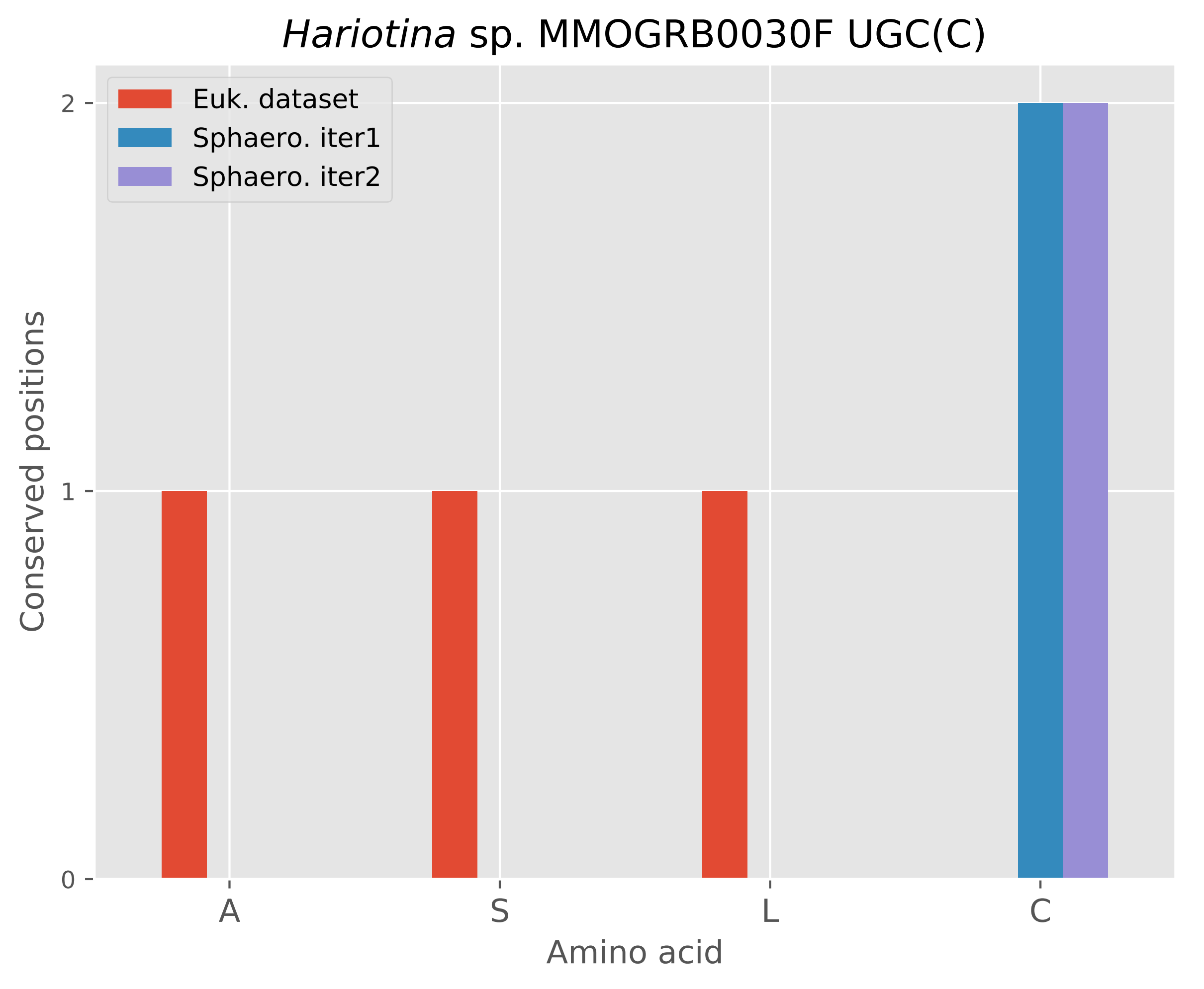

Hariotina sp. MMOGRB0030F UGC(C)
Euk. dataset
Sphaero. iter1
Sphaero. iter2
Conserved positions
0
1
2
A
S
L
C
Amino acid

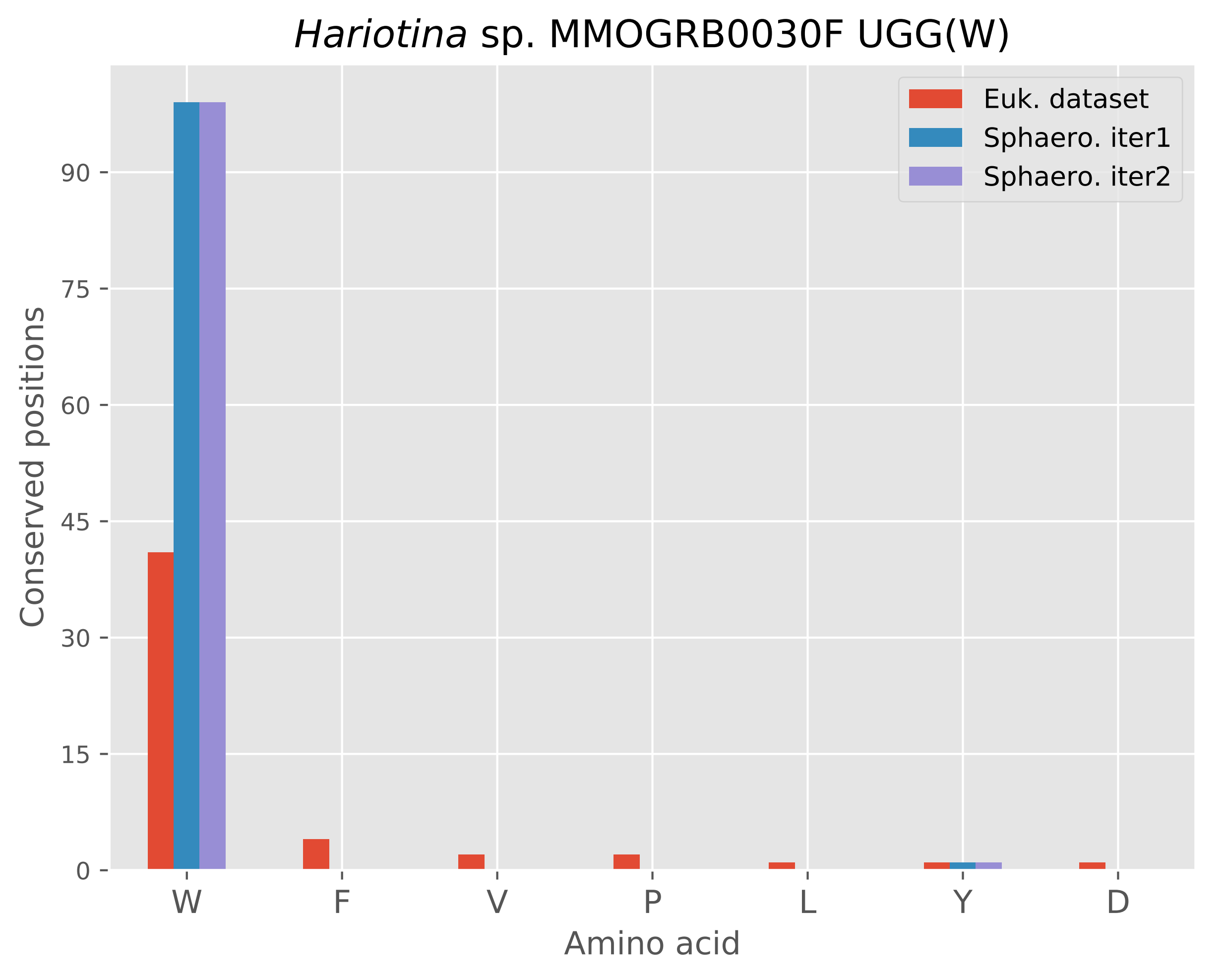

Hariotina sp. MMOGRB0030F UGG(W)
Euk. dataset
Sphaero. iter1
Sphaero. iter2
Conserved positions
0
15
30
45
60
75
90
W
F
V
P
L
Y
D
Amino acid

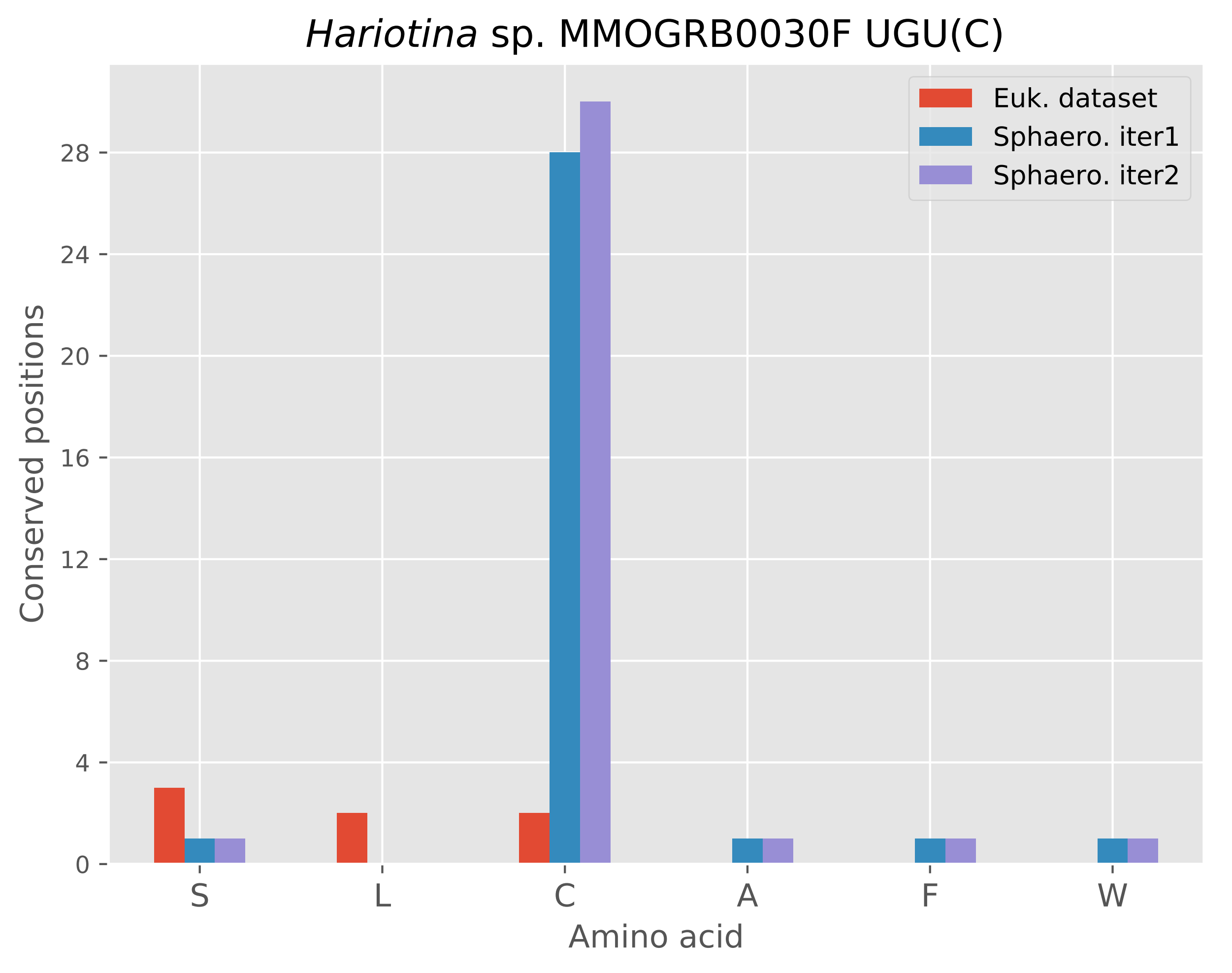

Hariotina sp. MMOGRB0030F UGU(C)
Euk. dataset
Sphaero. iter1
Sphaero. iter2
Conserved positions
0
4
8
12
16
20
24
28
S
L
C
A
F
W
Amino acid

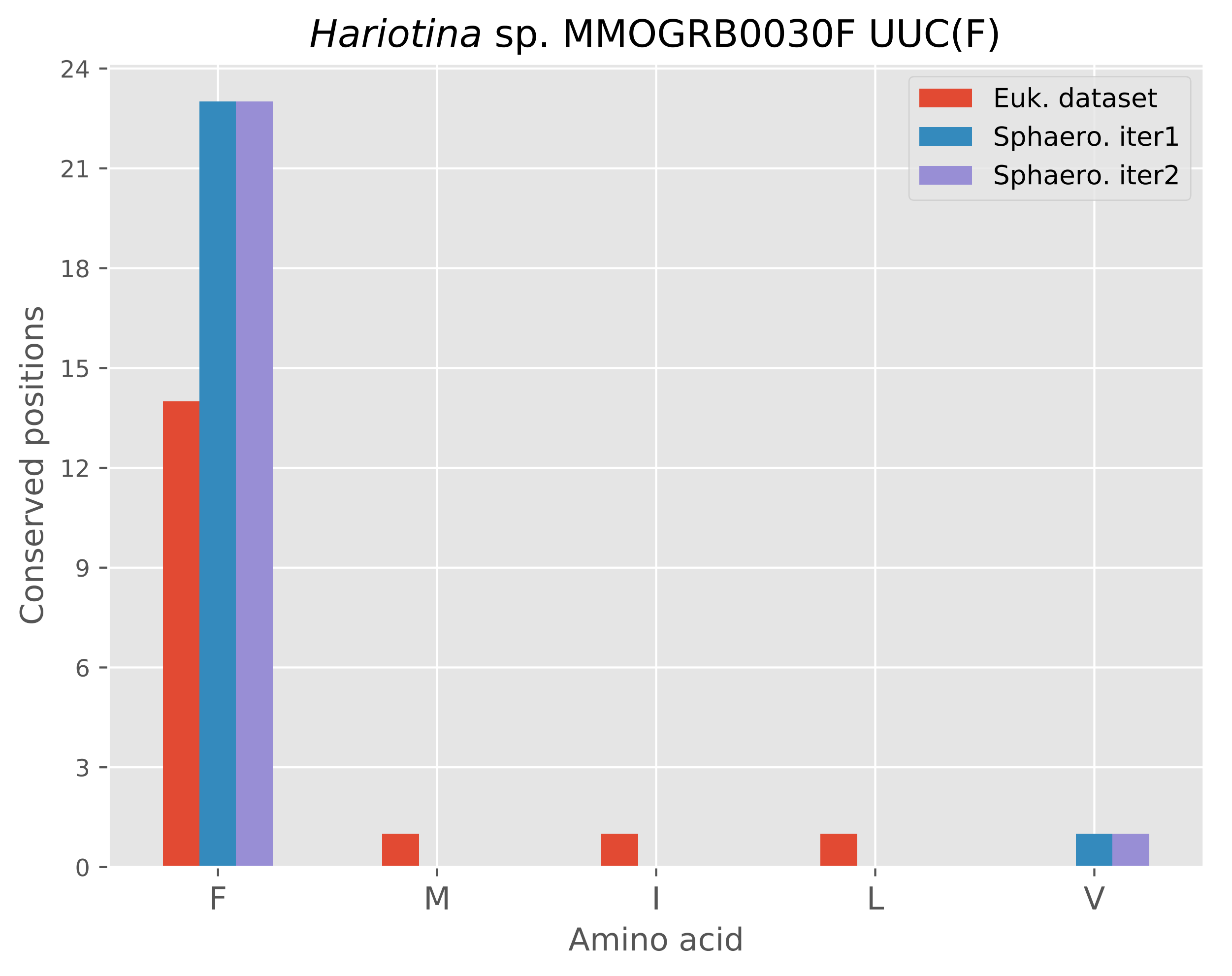

Hariotina sp. MMOGRB0030F UUC(F)
Conserved positions
Amino acid
Euk. dataset
Sphaero. iter1
Sphaero. iter2
0
3
6
9
12
15
18
21
24
F
M
I
L
V

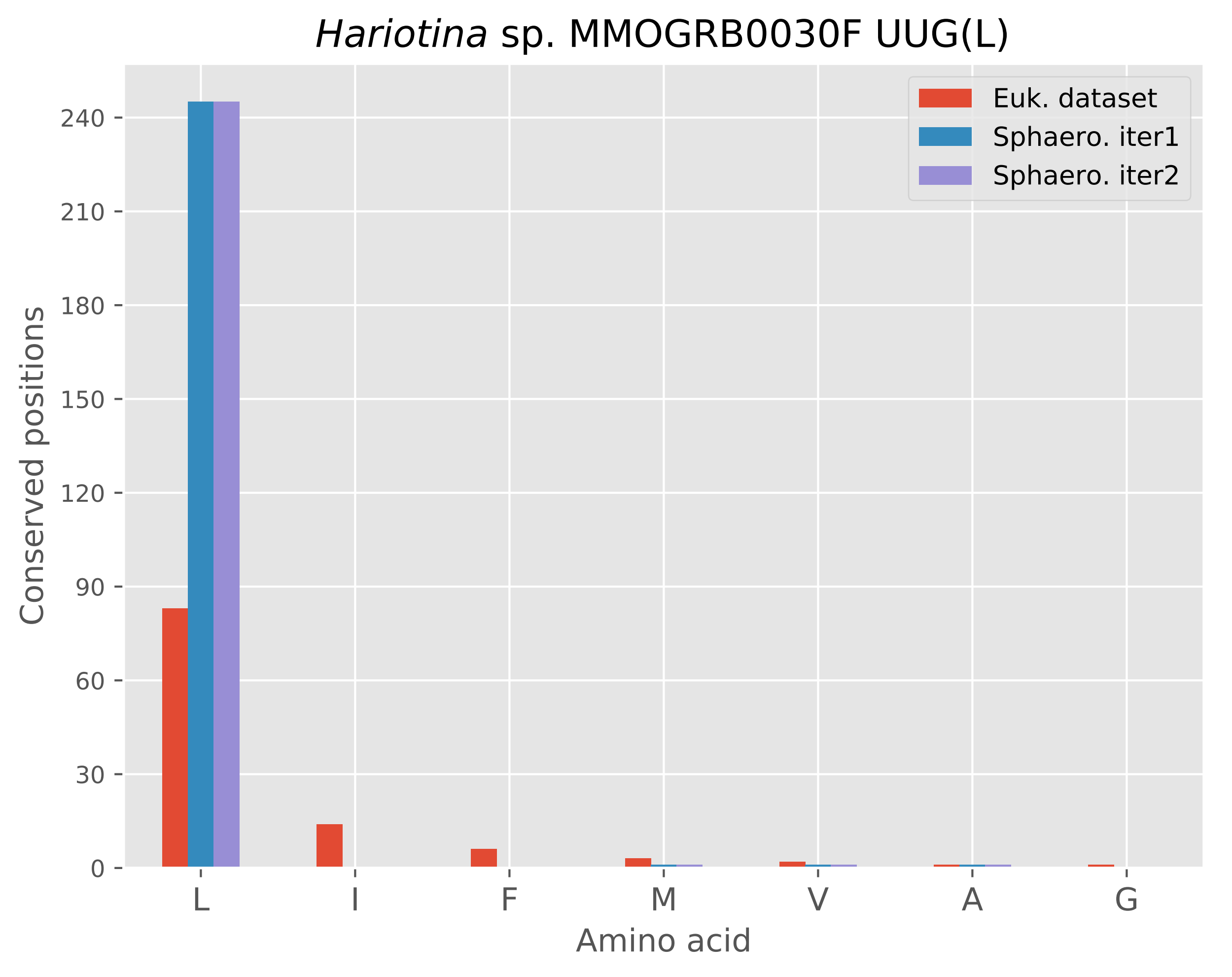

Hariotina sp. MMOGRB0030F UUG(L)
Euk. dataset
Sphaero. iter1
Sphaero. iter2
Conserved positions
0
30
60
90
120
150
180
210
240
L
I
F
M
V
A
G
Amino acid

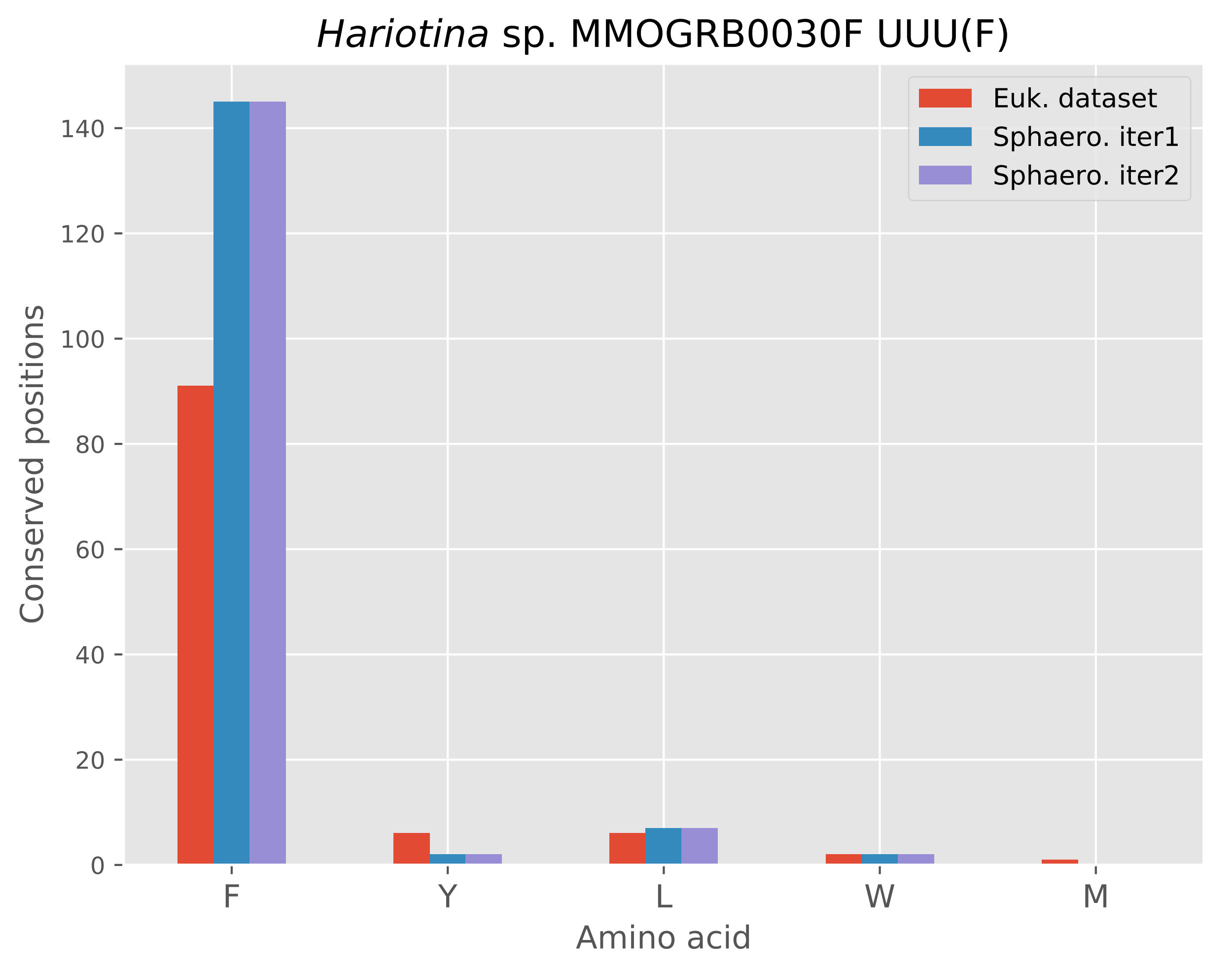

Hariotina sp. MMOGRB0030F UUU(F)
Euk. dataset
Sphaero. iter1
Sphaero. iter2
Conserved positions
0
20
40
60
80
100
120
140
F
Y
L
W
M
Amino acid

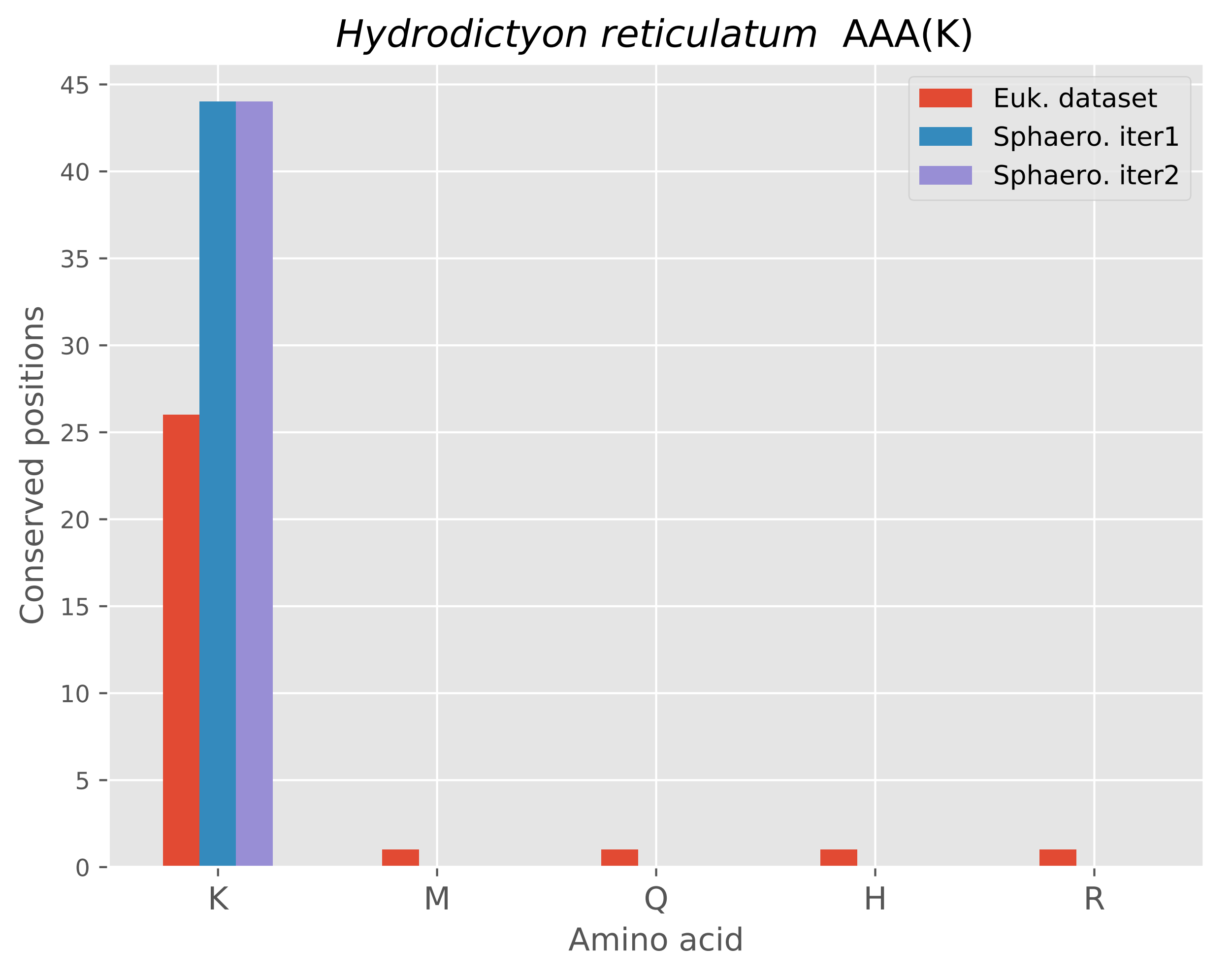

Hydrodictyon reticulatum AAA(K)
Euk. dataset
Sphaero. iter1
Sphaero. iter2
Conserved positions
0
5
10
15
20
25
30
35
40
45
K
M
Q
H
R
Amino acid

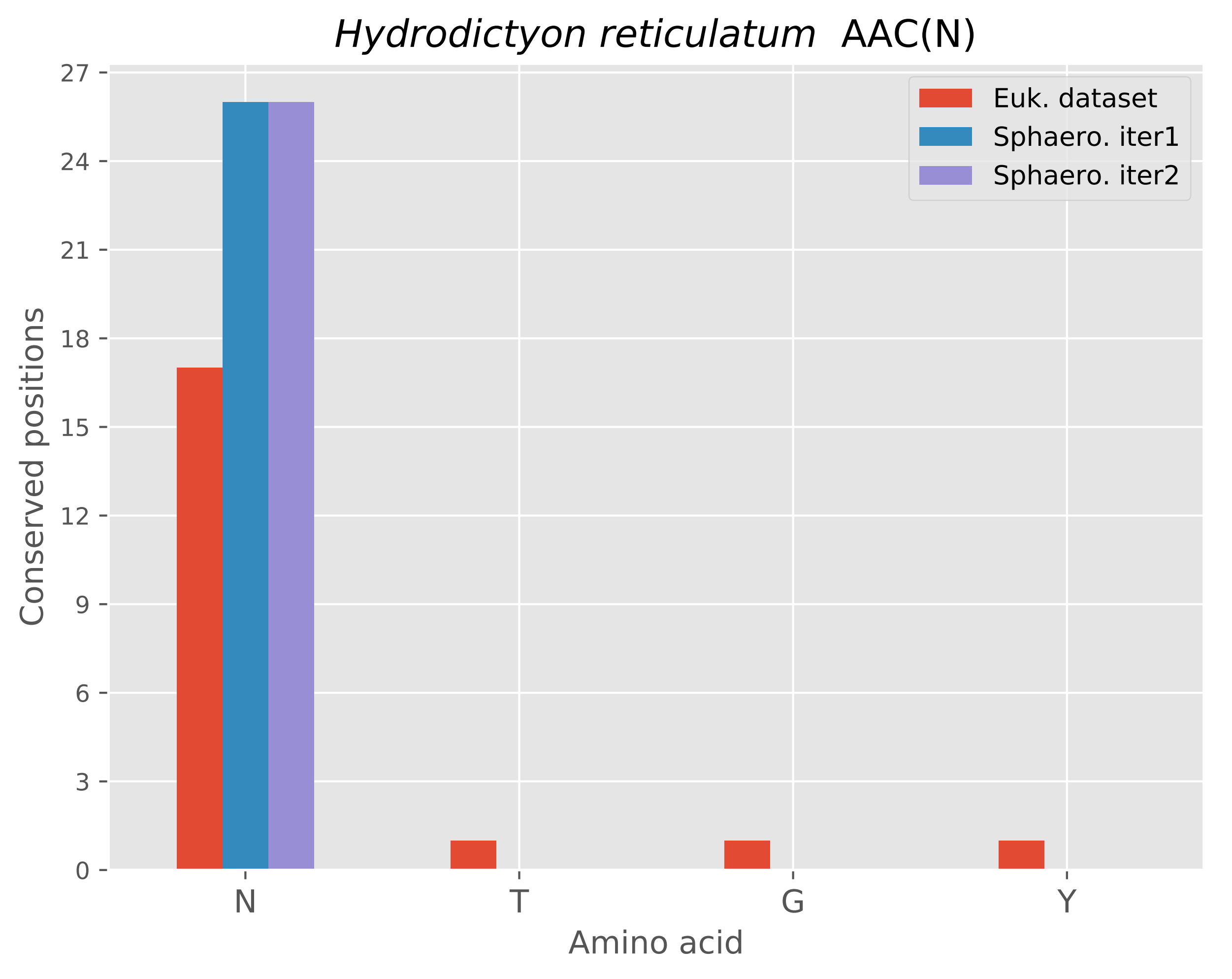

Hydrodictyon reticulatum AAC(N)
Euk. dataset
Sphaero. iter1
Sphaero. iter2
Conserved positions
0
3
6
9
12
15
18
21
24
27
N
T
G
Y
Amino acid

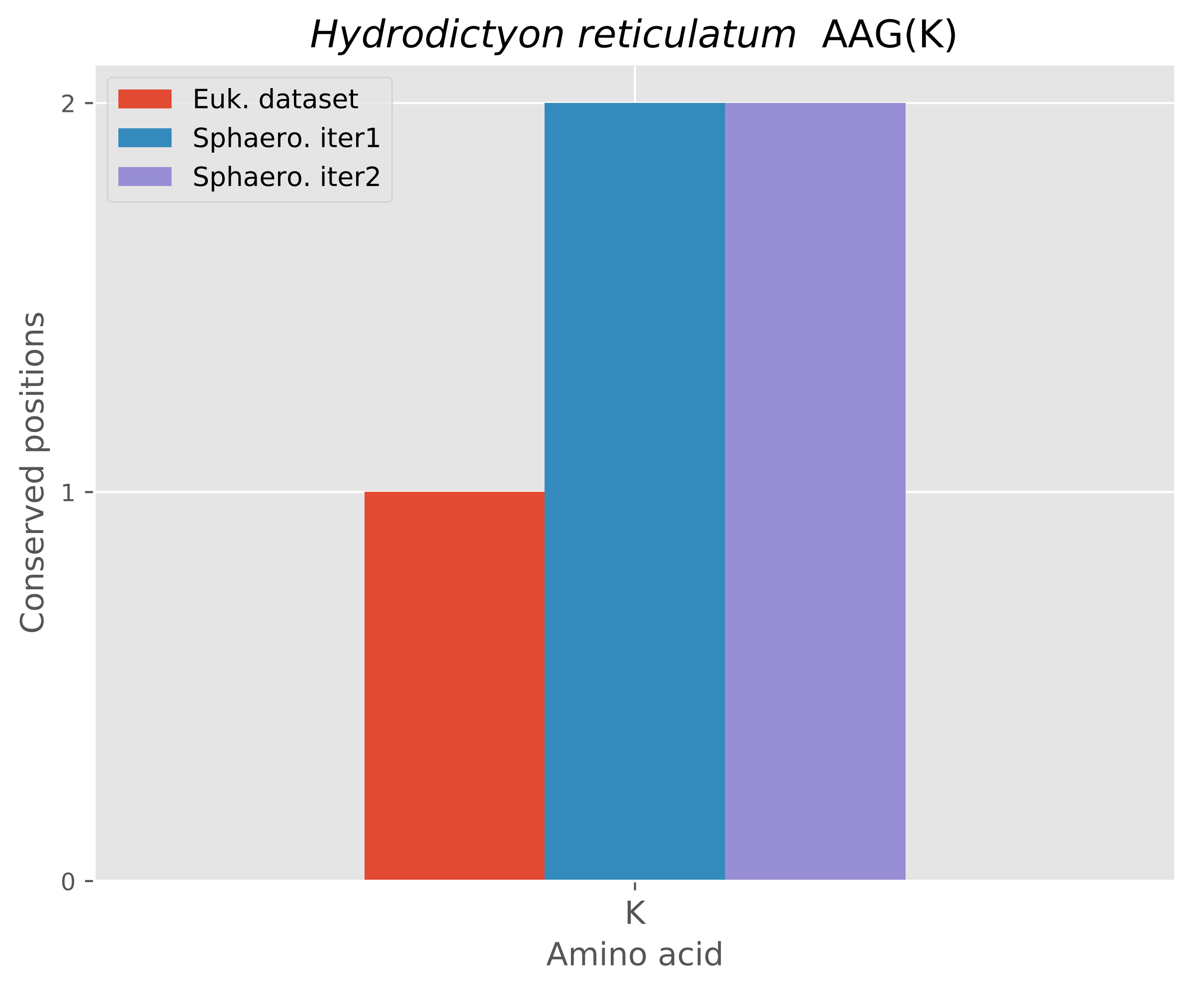
Hydrodictyon reticulatum AAG(K)
Euk. dataset
Sphaero. iter1
Sphaero. iter2
Conserved positions
0
1
2
K
Amino acid

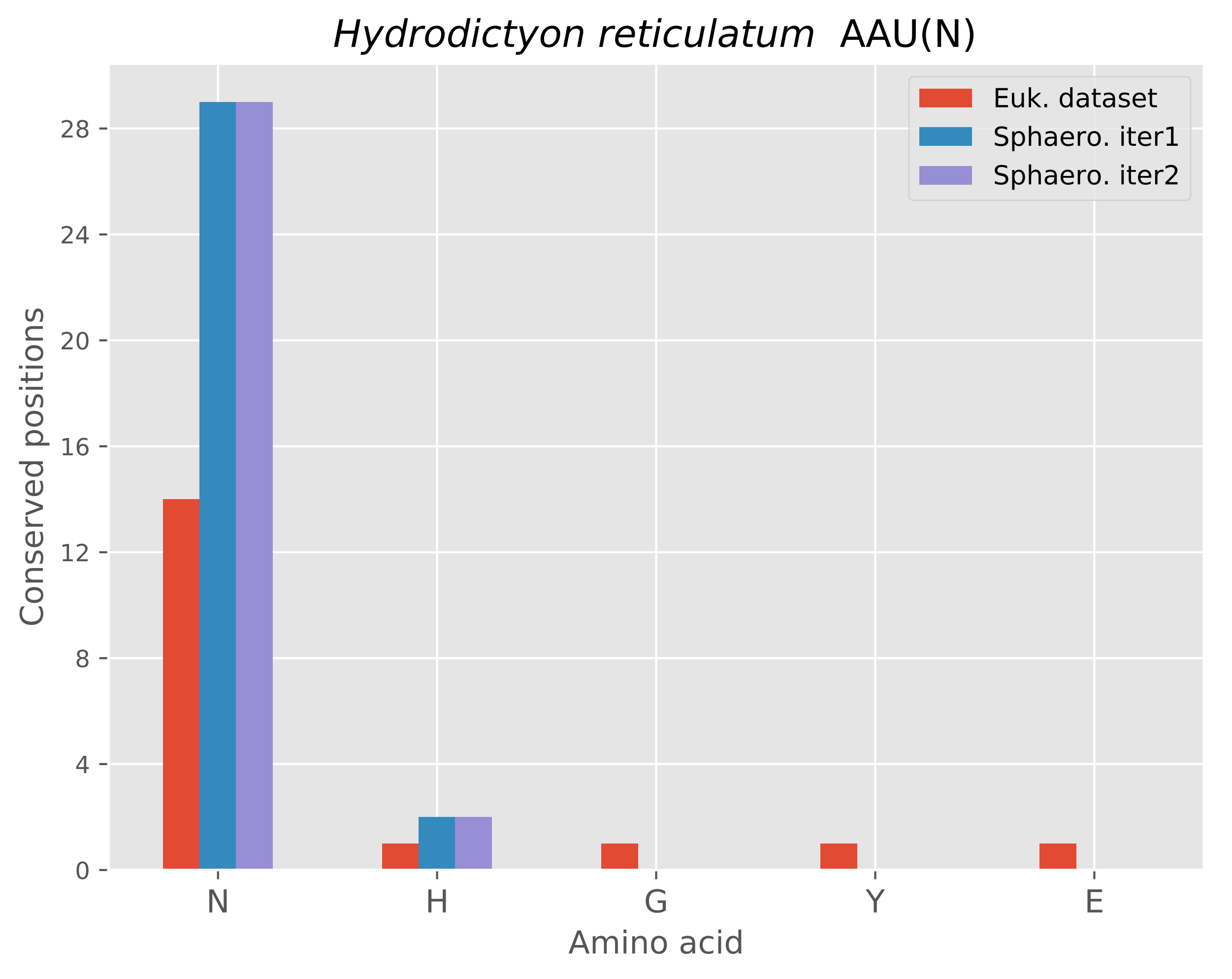

Hydrodictyon reticulatum AAU(N)
Euk. dataset
Sphaero. iter1
Sphaero. iter2
Conserved positions
0
4
8
12
16
20
24
28
N
H
G
Y
E
Amino acid

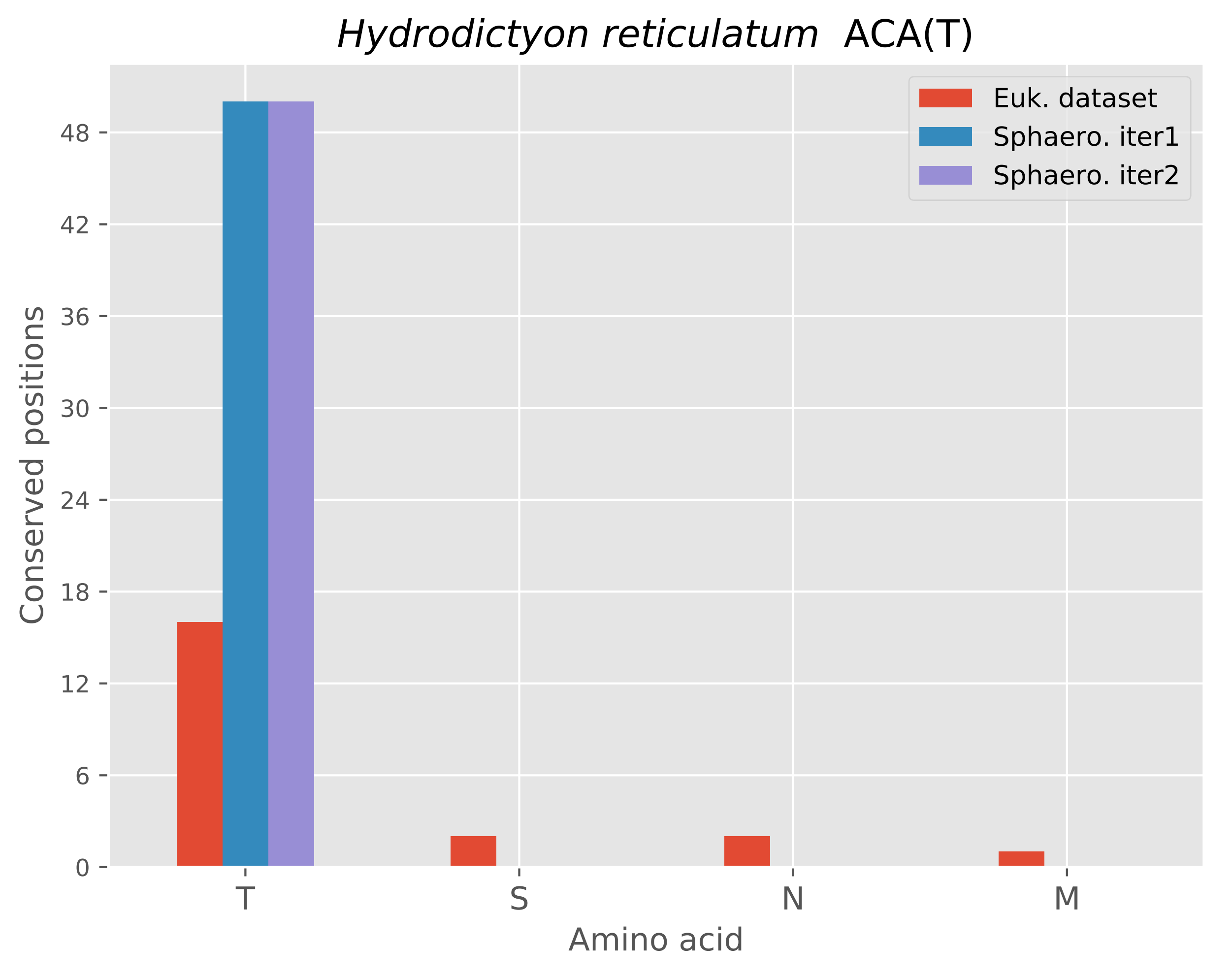

Hydrodictyon reticulatum ACA(T)
Euk. dataset
Sphaero. iter1
Sphaero. iter2
Conserved positions
0
6
12
18
24
30
36
42
48
T
S
N
M
Amino acid

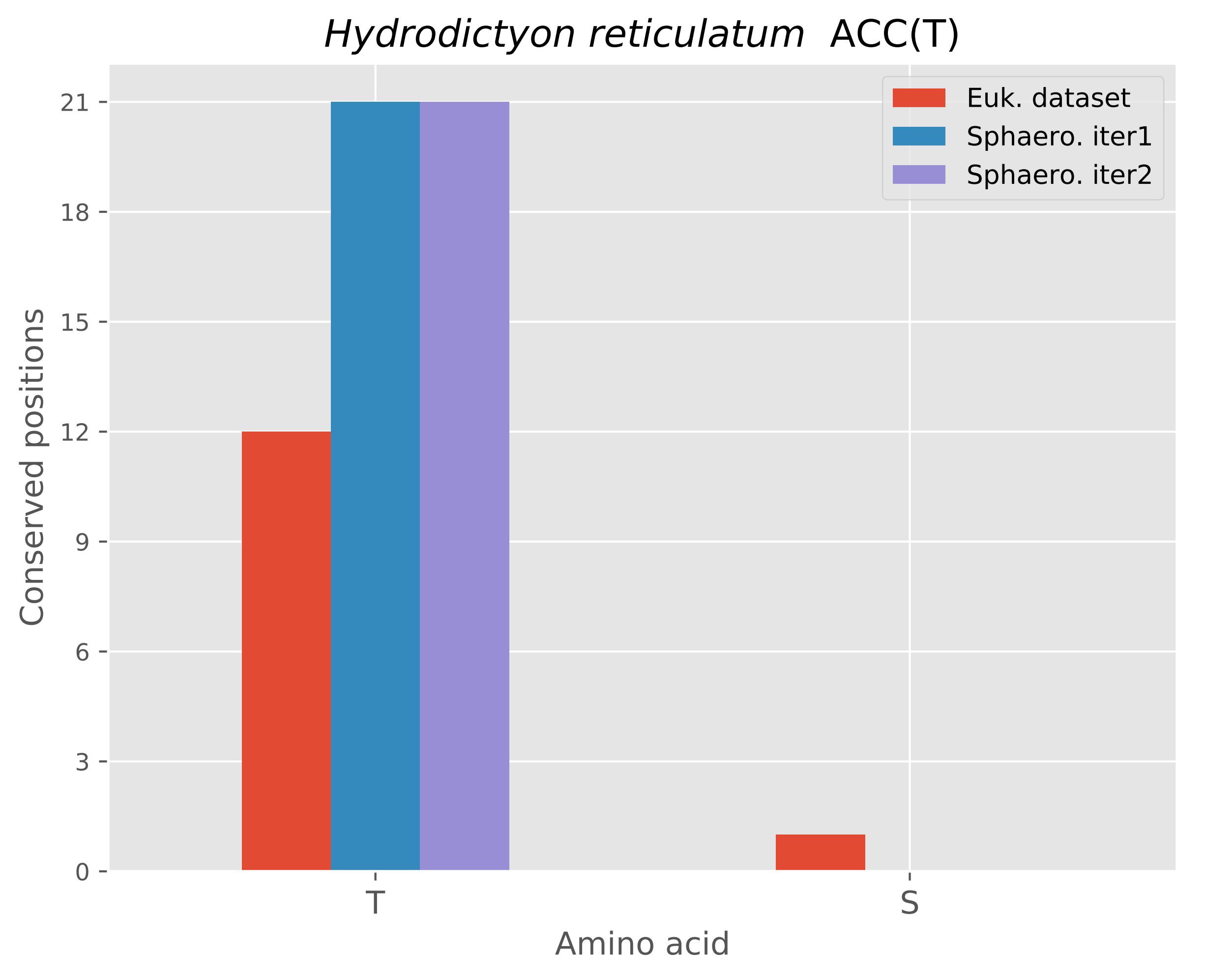

Hydrodictyon reticulatum ACC(T)
Euk. dataset
Sphaero. iter1
Sphaero. iter2
Conserved positions
0
3
6
9
12
15
18
21
T
S
Amino acid

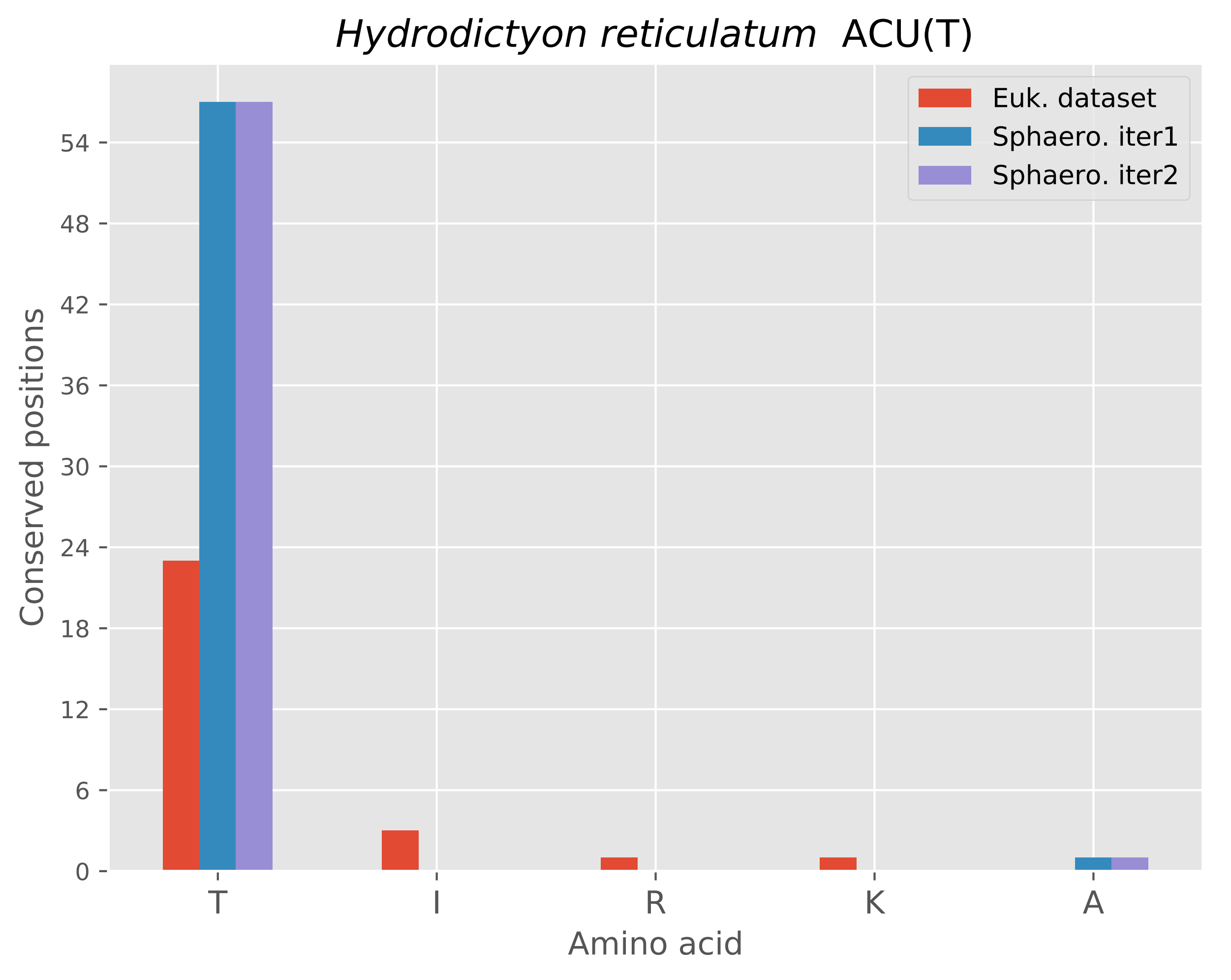

Hydrodictyon reticulatum ACU(T)
Euk. dataset
Sphaero. iter1
Sphaero. iter2
Conserved positions
0
6
12
18
24
30
36
42
48
54
T
I
R
K
A
Amino acid

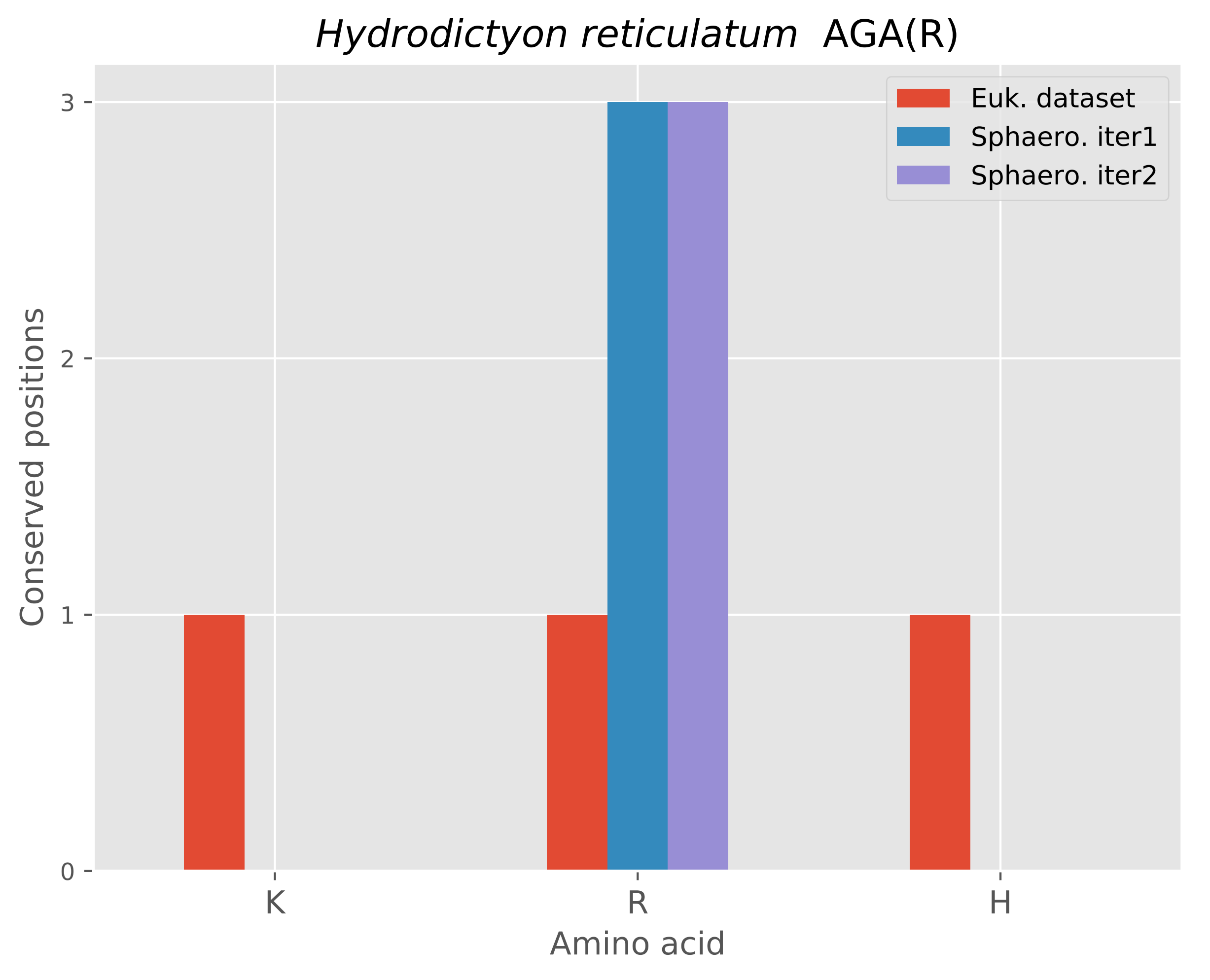
Hydrodictyon reticulatum AGA(R)
Euk. dataset
Sphaero. iter1
Sphaero. iter2
Conserved positions
0
1
2
3
K
R
H
Amino acid

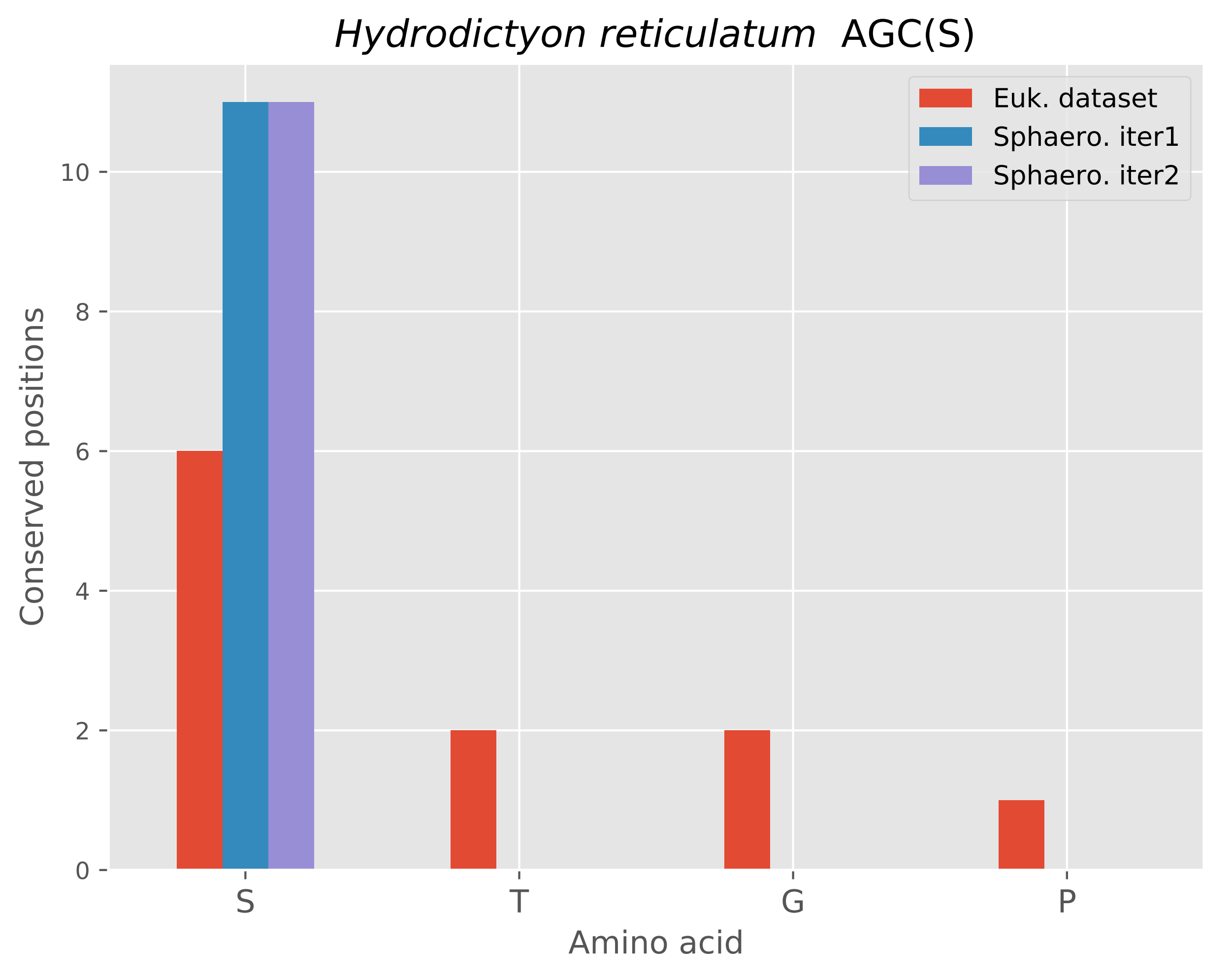

Hydrodictyon reticulatum AGC(S)
Euk. dataset
Sphaero. iter1
Sphaero. iter2
Conserved positions
0
2
4
6
8
10
S
T
G
P
Amino acid

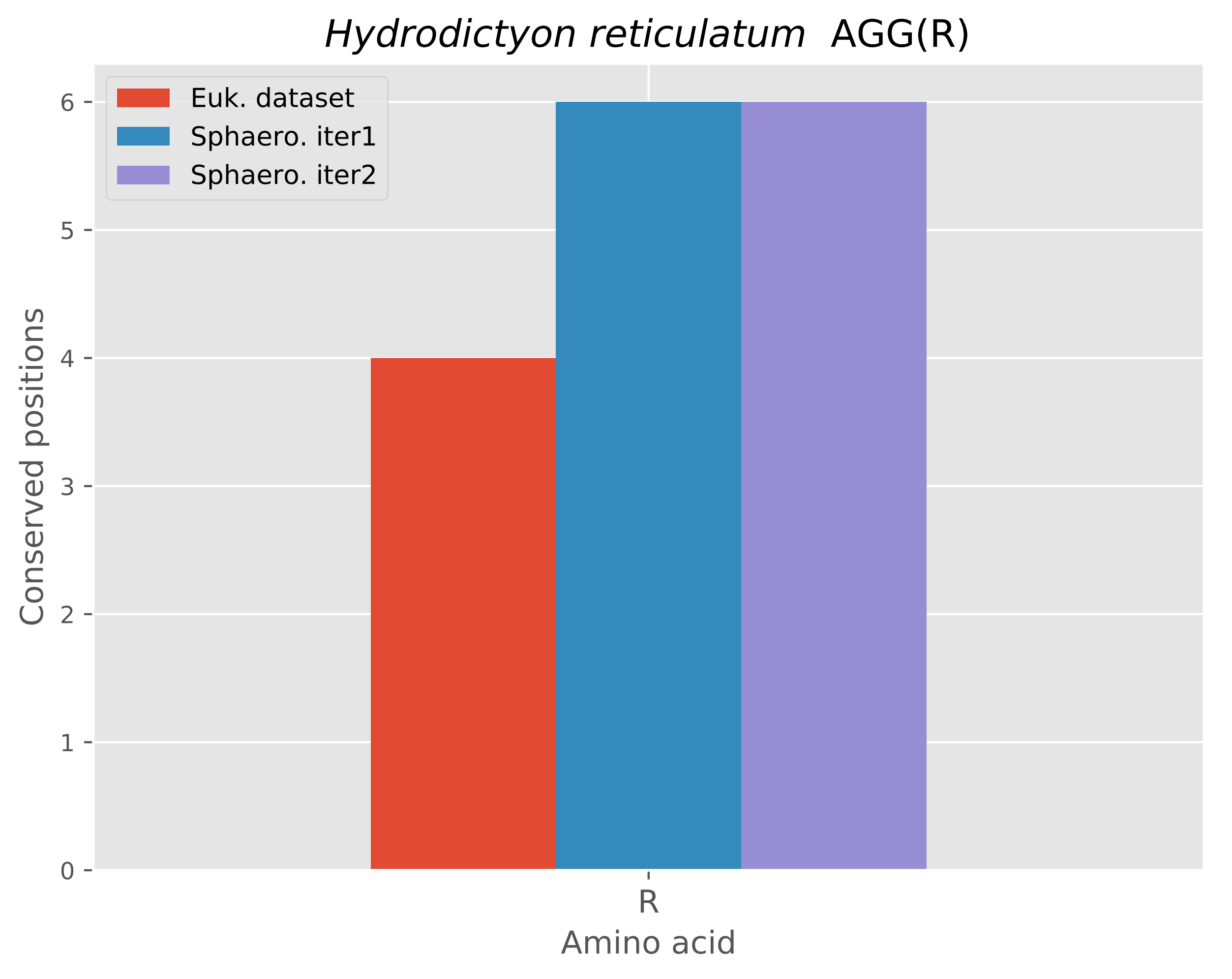

Hydrodictyon reticulatum AGG(R)
Euk. dataset
Sphaero. iter1
Sphaero. iter2
Conserved positions
0
1
2
3
4
5
6
R
Amino acid

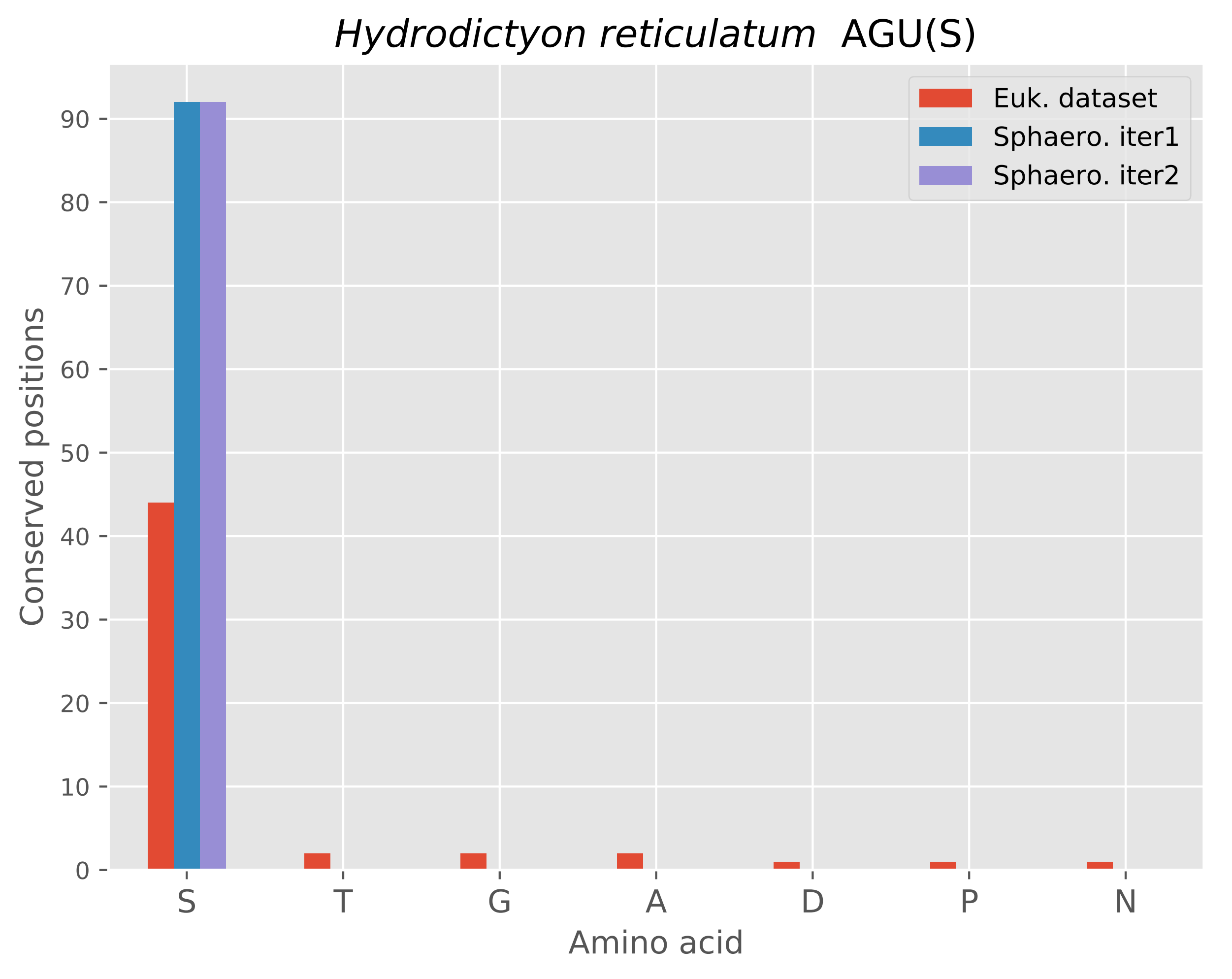

Hydrodictyon reticulatum AGU(S)
Euk. dataset
Sphaero. iter1
Sphaero. iter2
Conserved positions
0
10
20
30
40
50
60
70
80
90
S
T
G
A
D
P
N
Amino acid

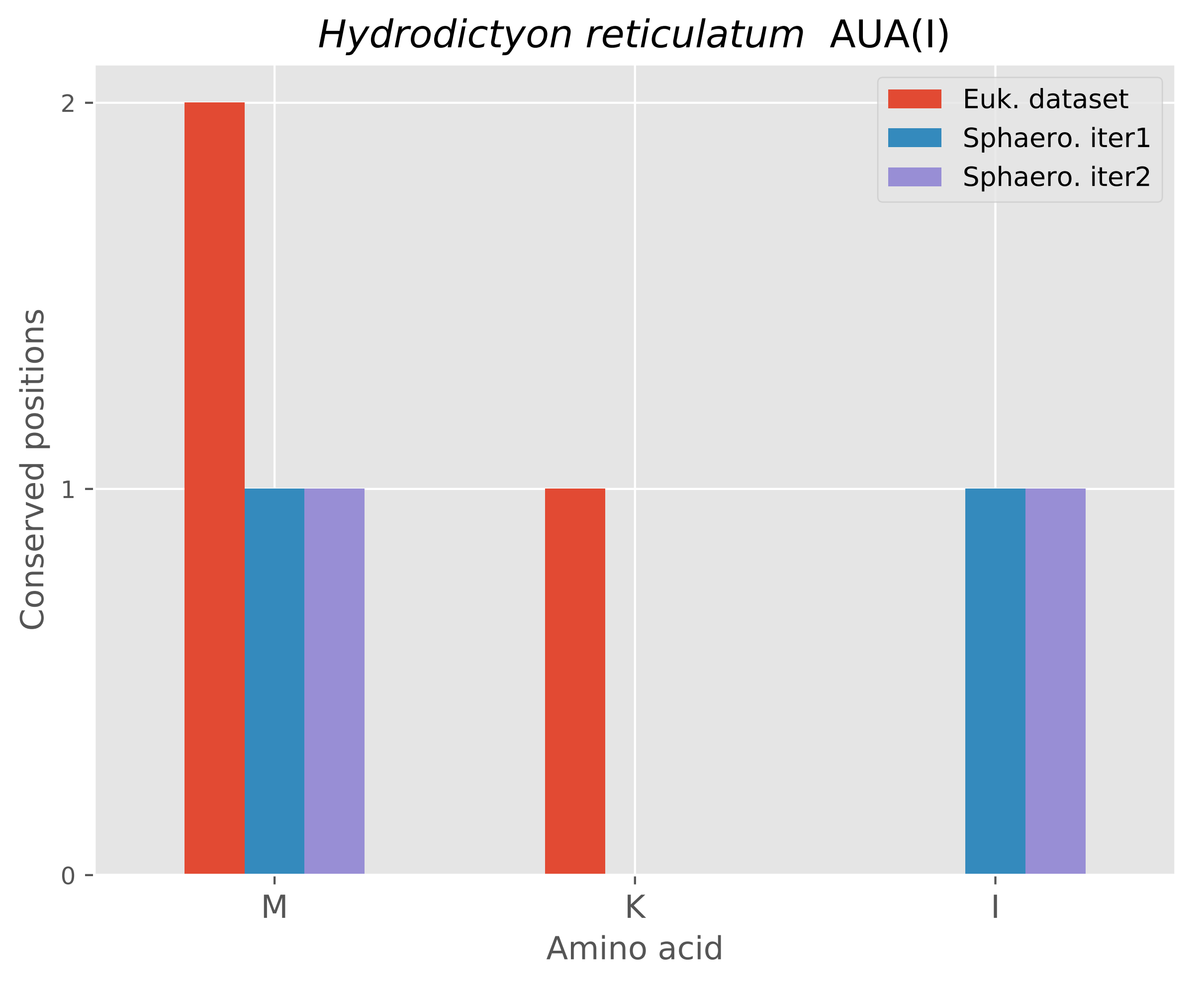

Hydrodictyon reticulatum AUA(I)
Euk. dataset
Sphaero. iter1
Sphaero. iter2
Conserved positions
2
1
0
M
K
I
Amino acid

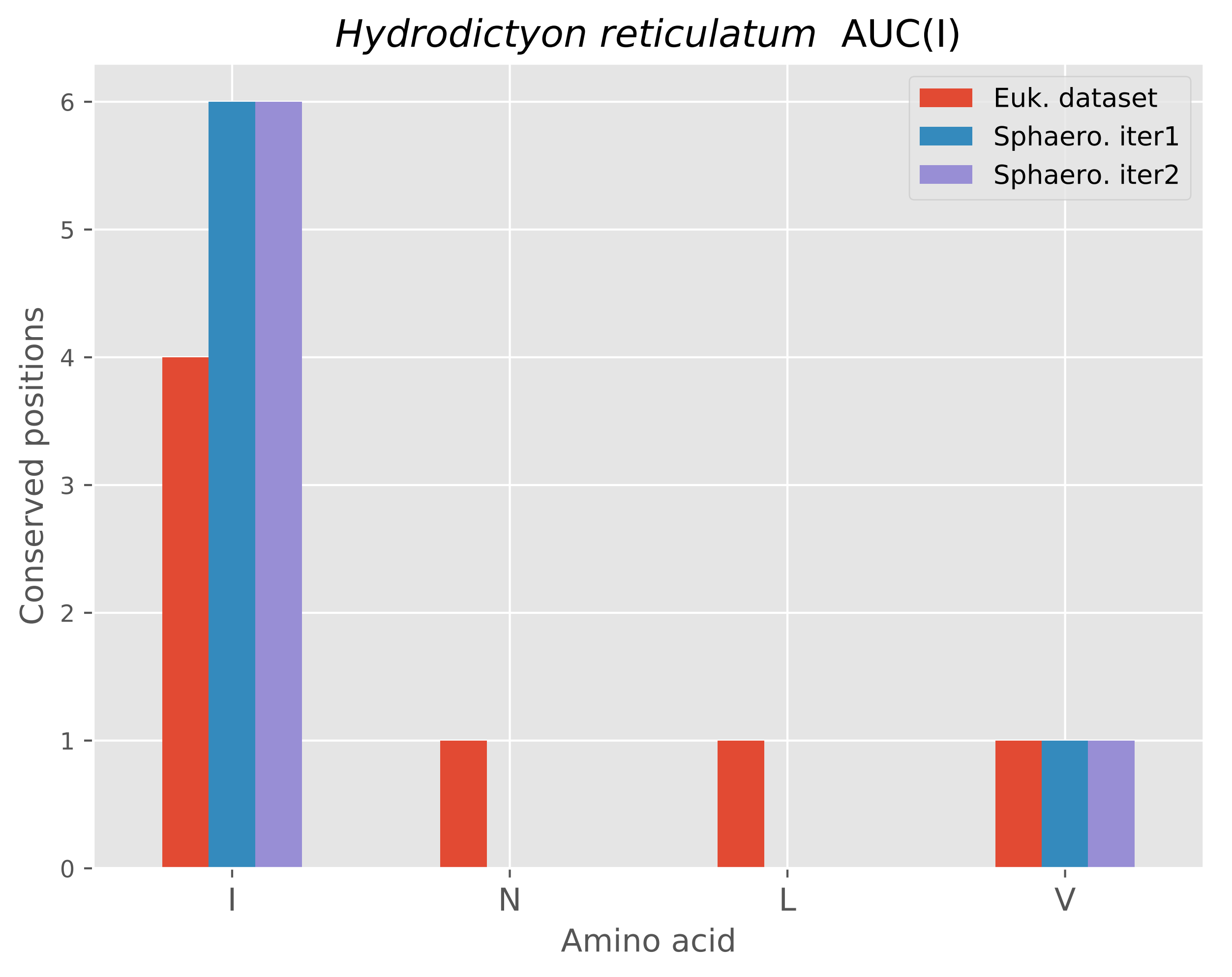
Hydrodictyon reticulatum AUC(I)
Euk. dataset
Sphaero. iter1
Sphaero. iter2
Conserved positions
0
1
2
3
4
5
6
I
N
L
V
Amino acid

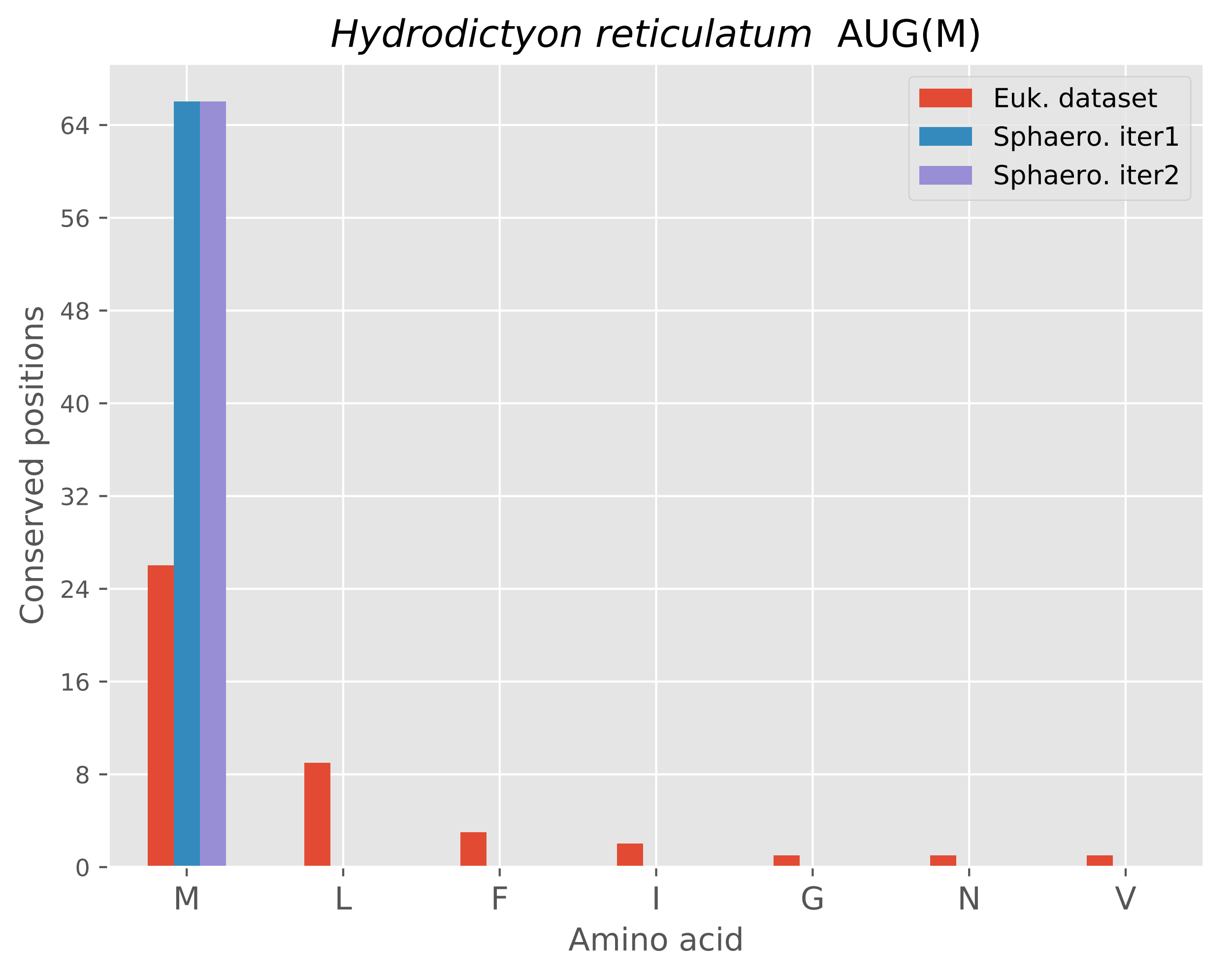

Hydrodictyon reticulatum AUG(M)
Euk. dataset
Sphaero. iter1
Sphaero. iter2
Conserved positions
64
56
48
40
32
24
16
8
0
M
L
F
I
G
N
V
Amino acid

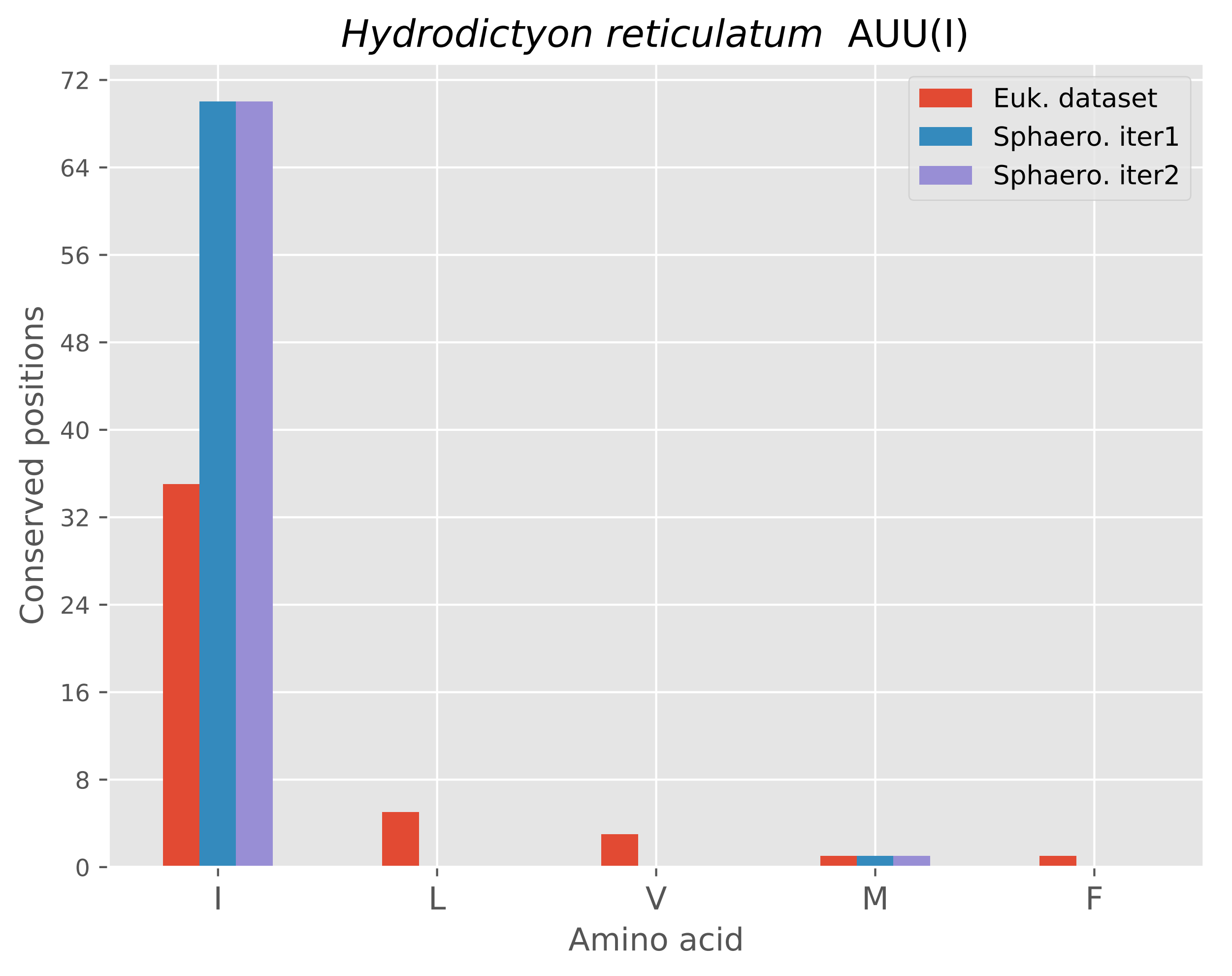
Hydrodictyon reticulatum AUU(I)
Euk. dataset
Sphaero. iter1
Sphaero. iter2
Conserved positions
0
8
16
24
32
40
48
56
64
72
I
L
V
M
F
Amino acid

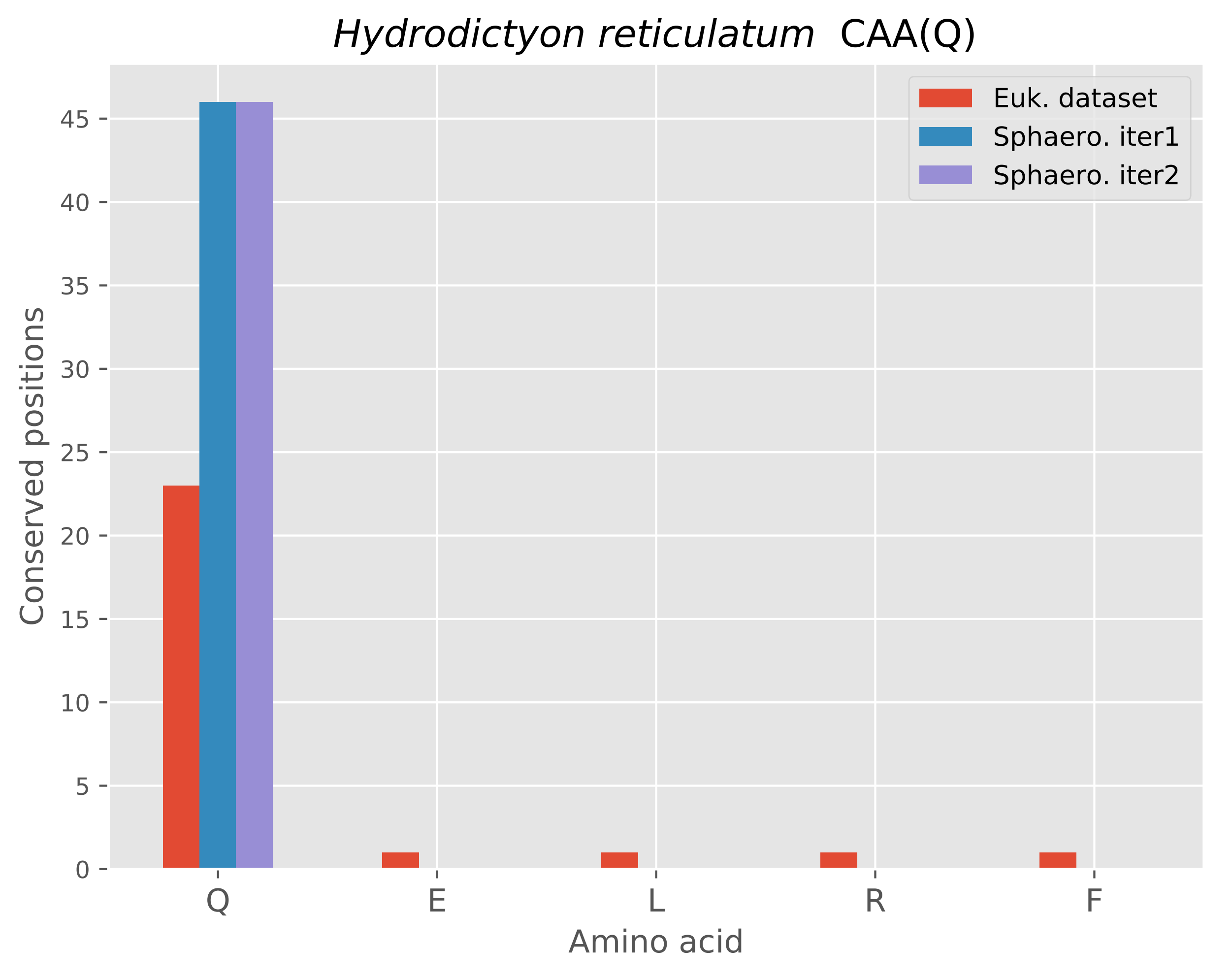

Hydrodictyon reticulatum CAA(Q)
Euk. dataset
Sphaero. iter1
Sphaero. iter2
Conserved positions
0
5
10
15
20
25
30
35
40
45
Q
E
L
R
F
Amino acid

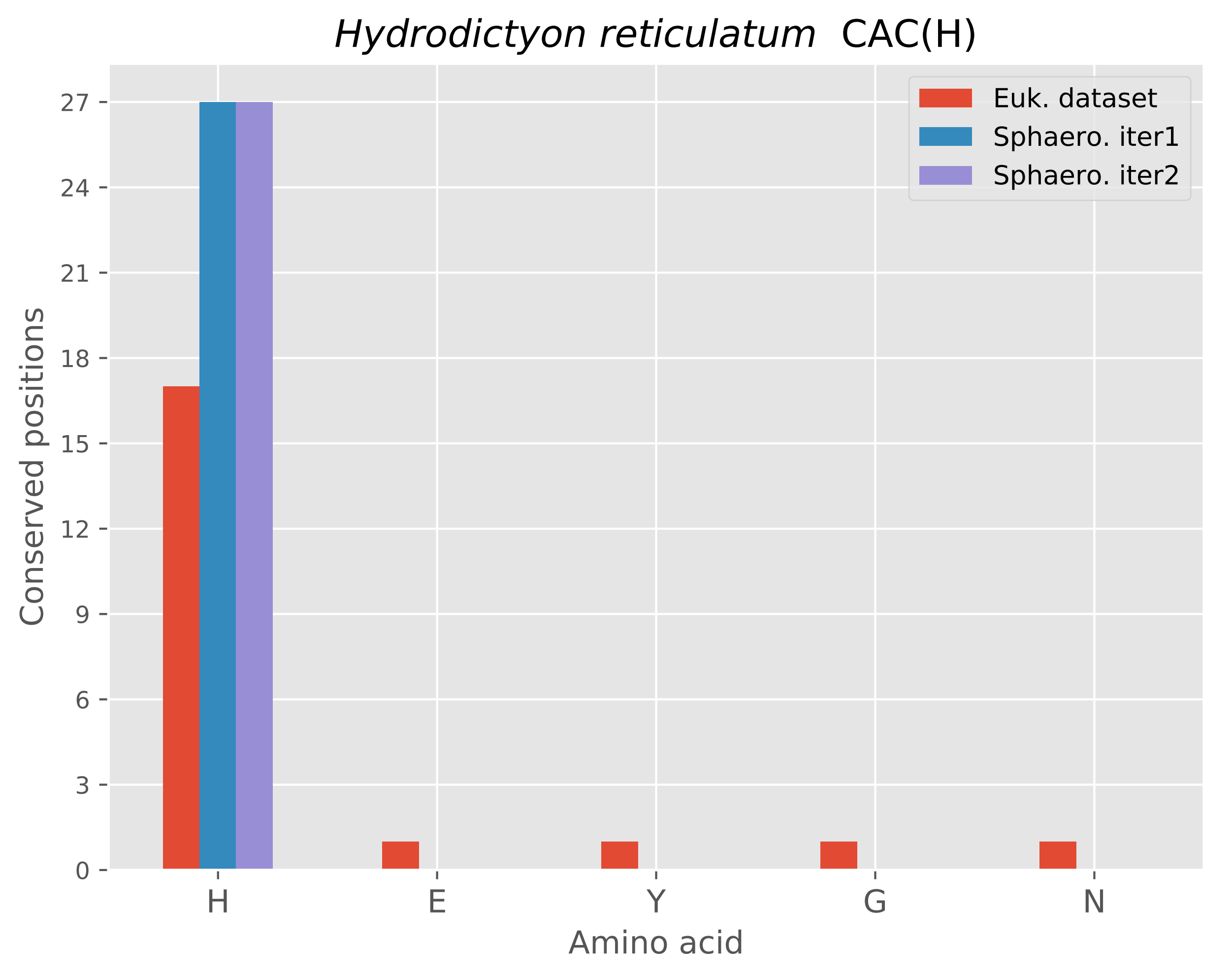

Hydrodictyon reticulatum CAC(H)
Euk. dataset
Sphaero. iter1
Sphaero. iter2
Conserved positions
0
3
6
9
12
15
18
21
24
27
H
E
Y
G
N
Amino acid

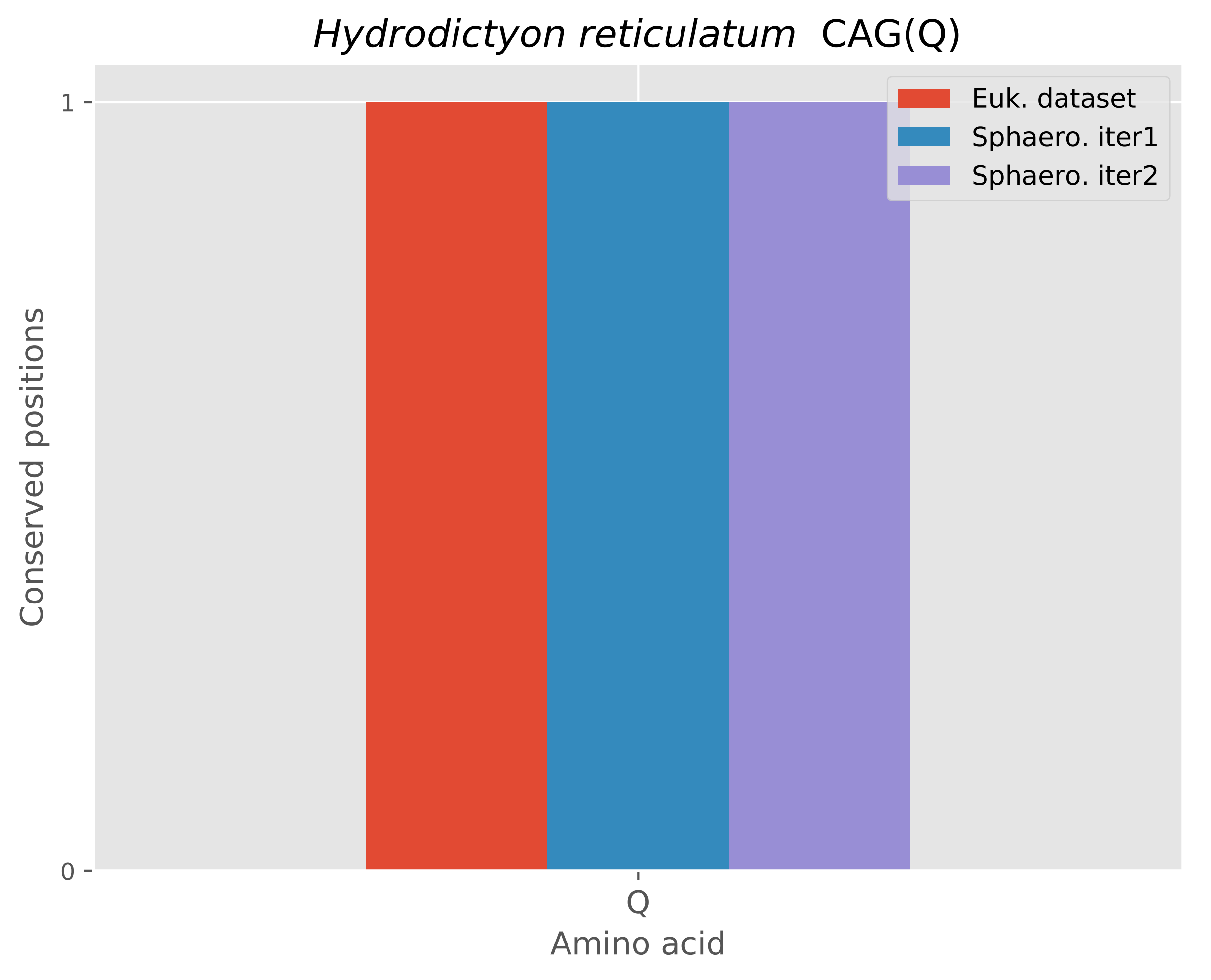

Hydrodictyon reticulatum CAG(Q)
Euk. dataset
Sphaero. iter1
Sphaero. iter2
Conserved positions
1
0
Q
Amino acid

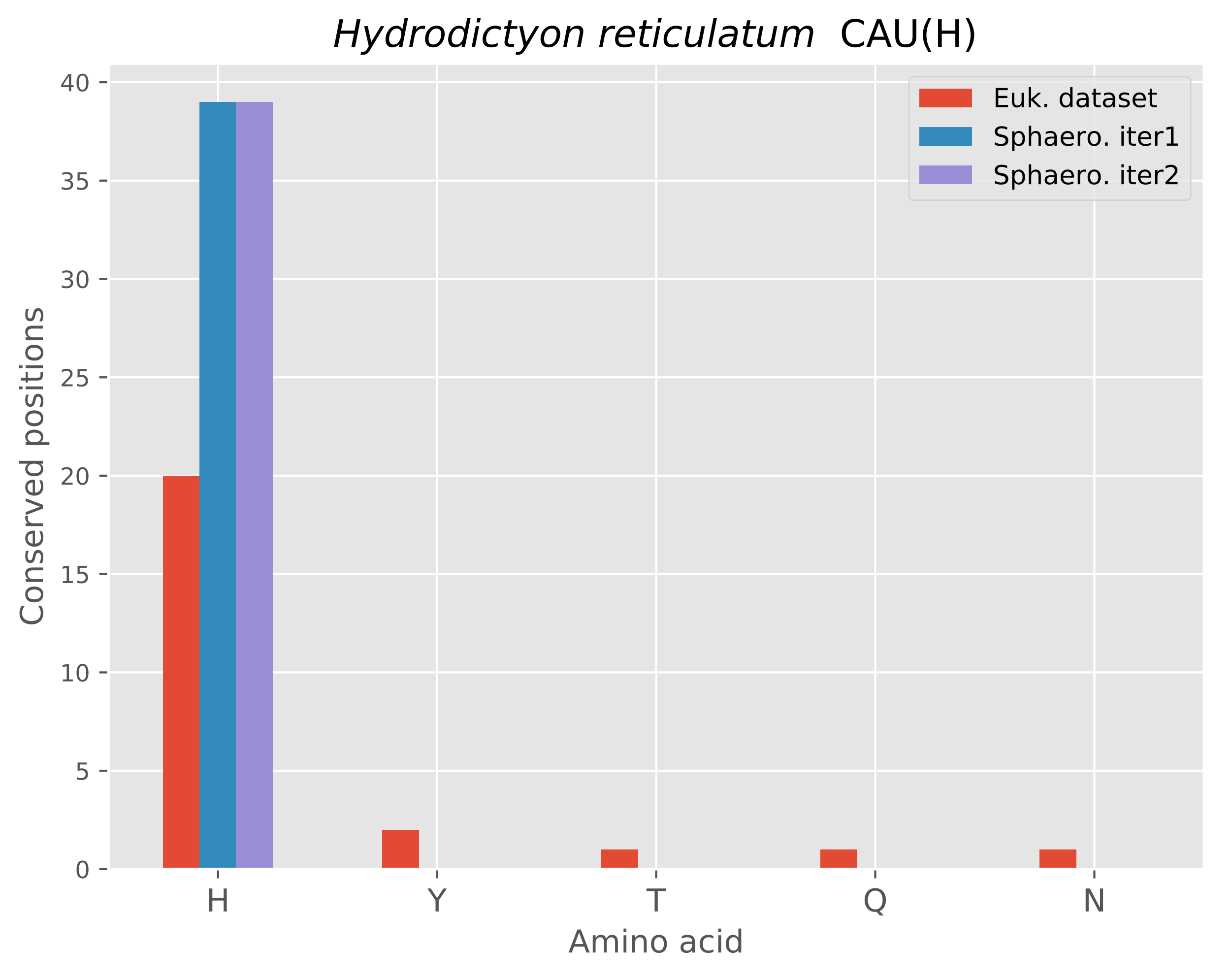

Hydrodictyon reticulatum CAU(H)
Euk. dataset
Sphaero. iter1
Sphaero. iter2
Conserved positions
0
5
10
15
20
25
30
35
40
H
Y
T
Q
N
Amino acid

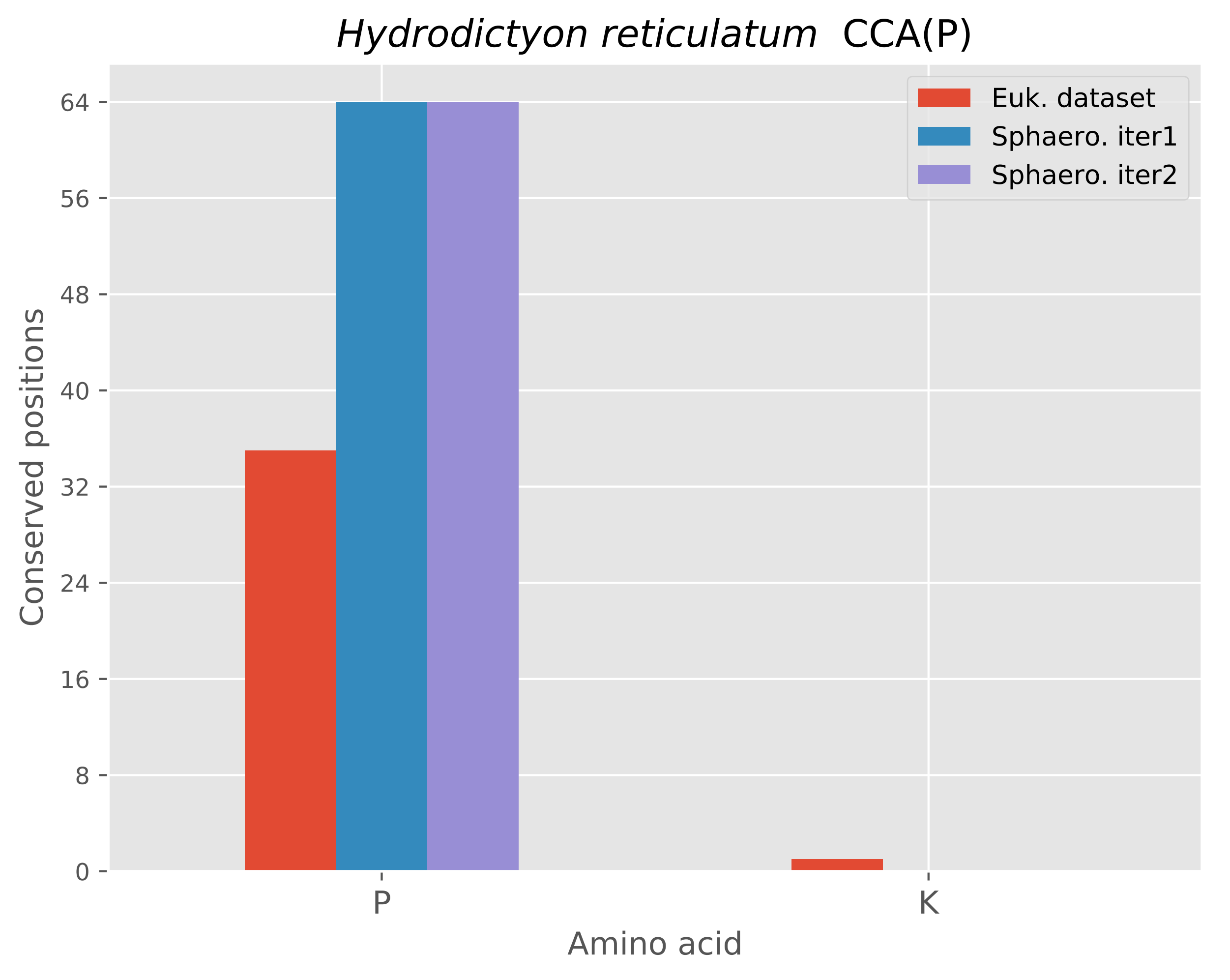

Hydrodictyon reticulatum CCA(P)
Euk. dataset
Sphaero. iter1
Sphaero. iter2
Conserved positions
0
8
16
24
32
40
48
56
64
P
K
Amino acid

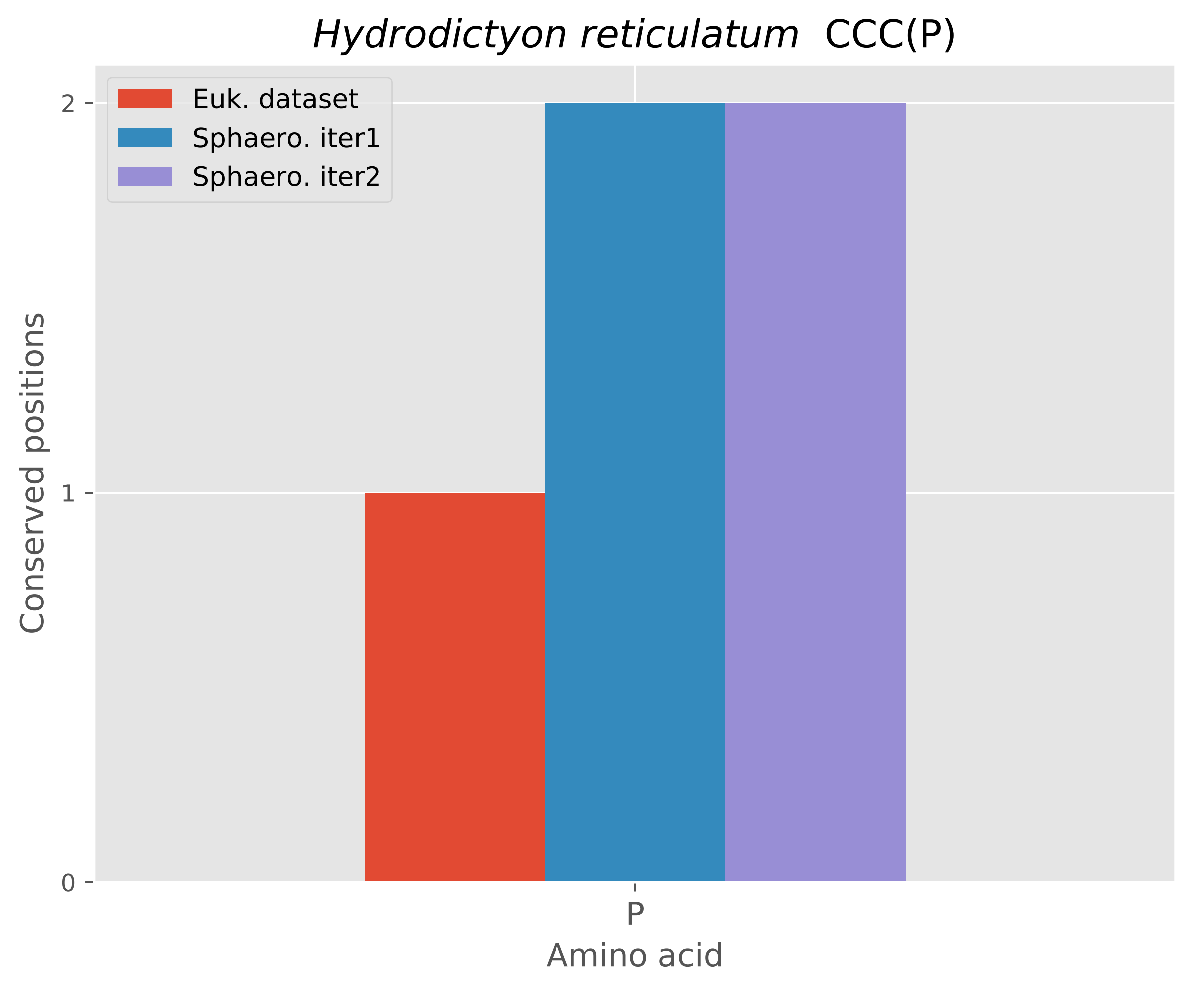

Hydrodictyon reticulatum CCC(P)
Euk. dataset
Sphaero. iter1
Sphaero. iter2
Conserved positions
2
1
0
P
Amino acid

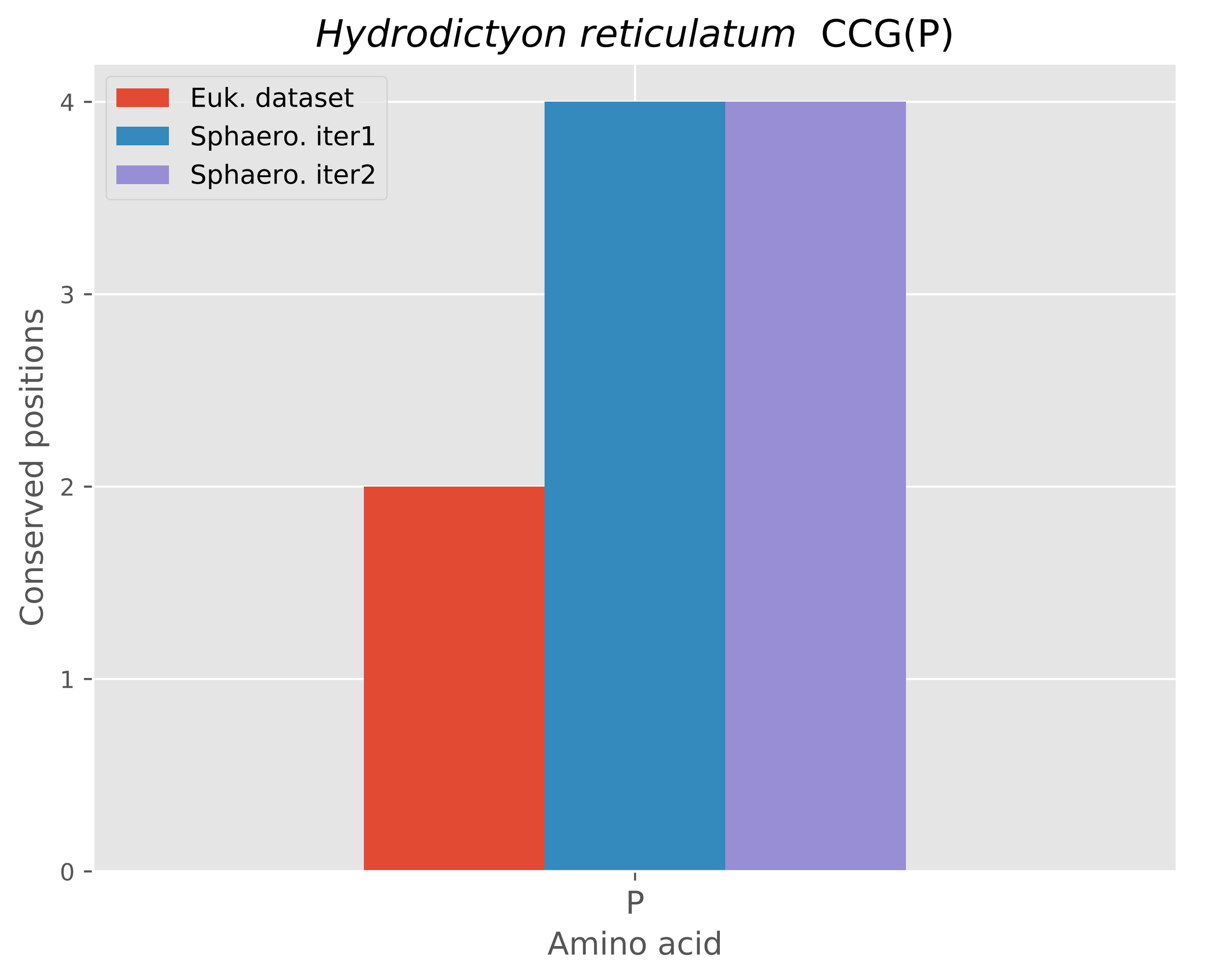
Hydrodictyon reticulatum CCG(P)
Euk. dataset
Sphaero. iter1
Sphaero. iter2
Conserved positions
0
1
2
3
4
P
Amino acid

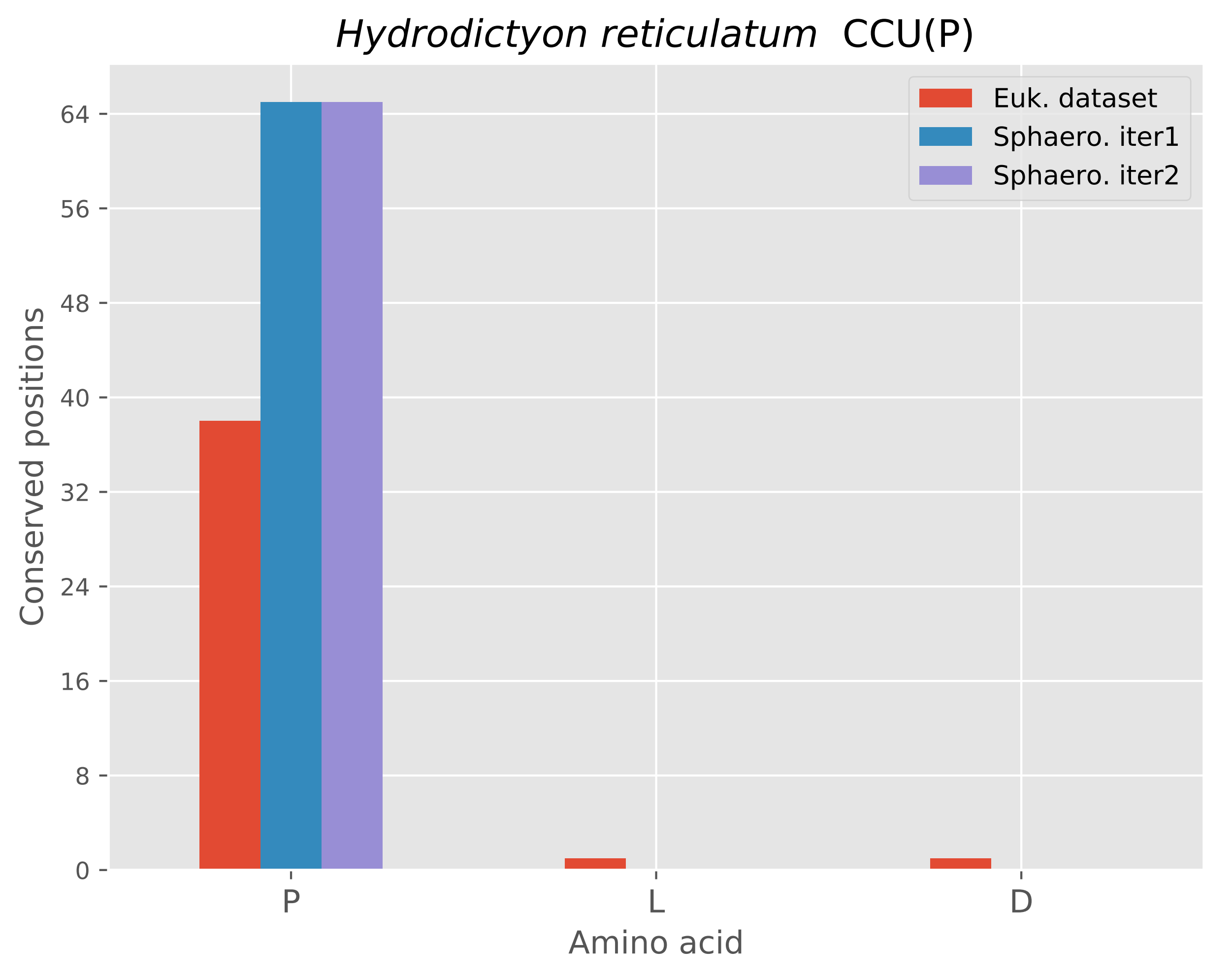

Hydrodictyon reticulatum CCU(P)
Euk. dataset
Sphaero. iter1
Sphaero. iter2
Conserved positions
0
8
16
24
32
40
48
56
64
P
L
D
Amino acid

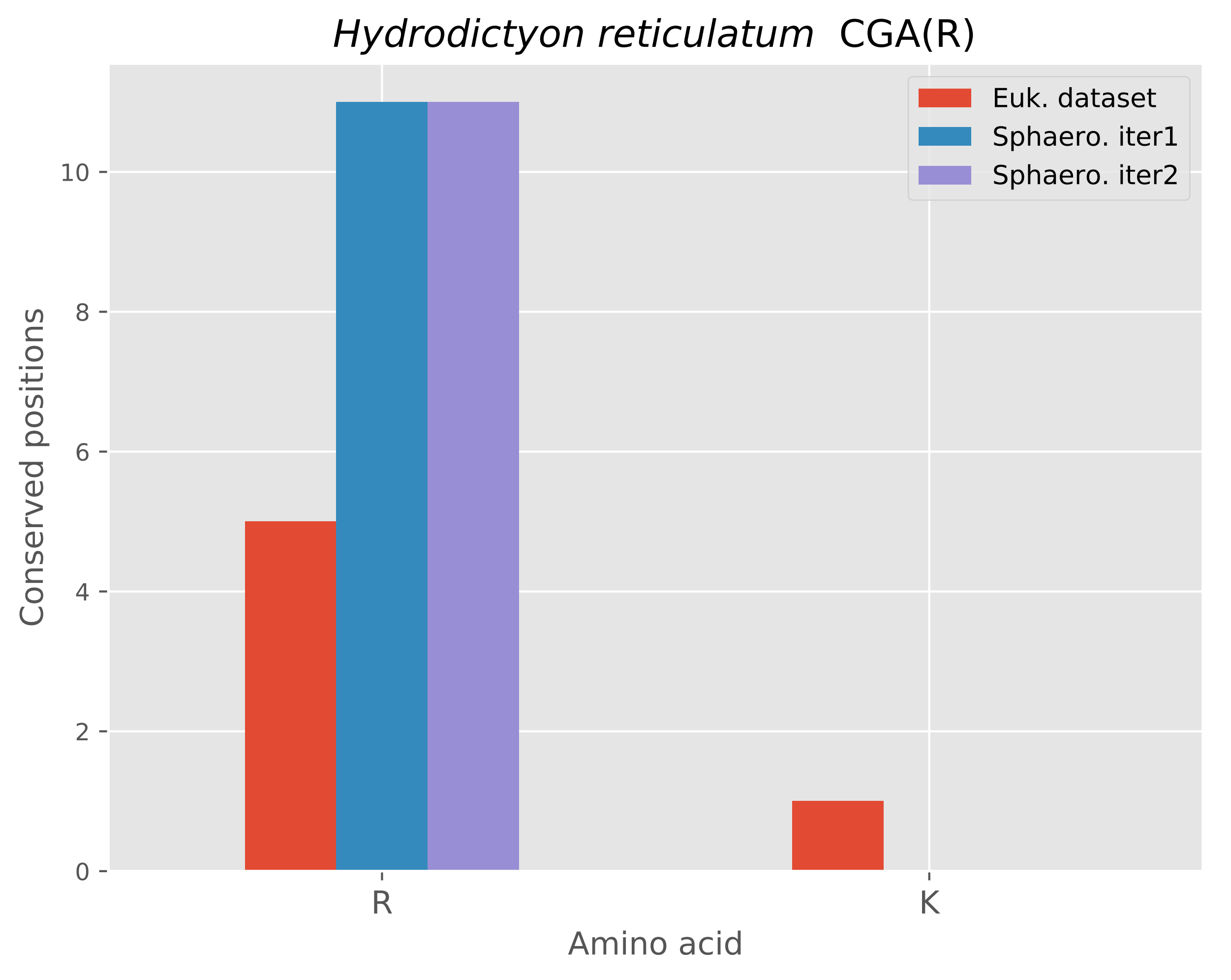

Hydrodictyon reticulatum CGA(R)
Euk. dataset
Sphaero. iter1
Sphaero. iter2
Conserved positions
0
2
4
6
8
10
R
K
Amino acid

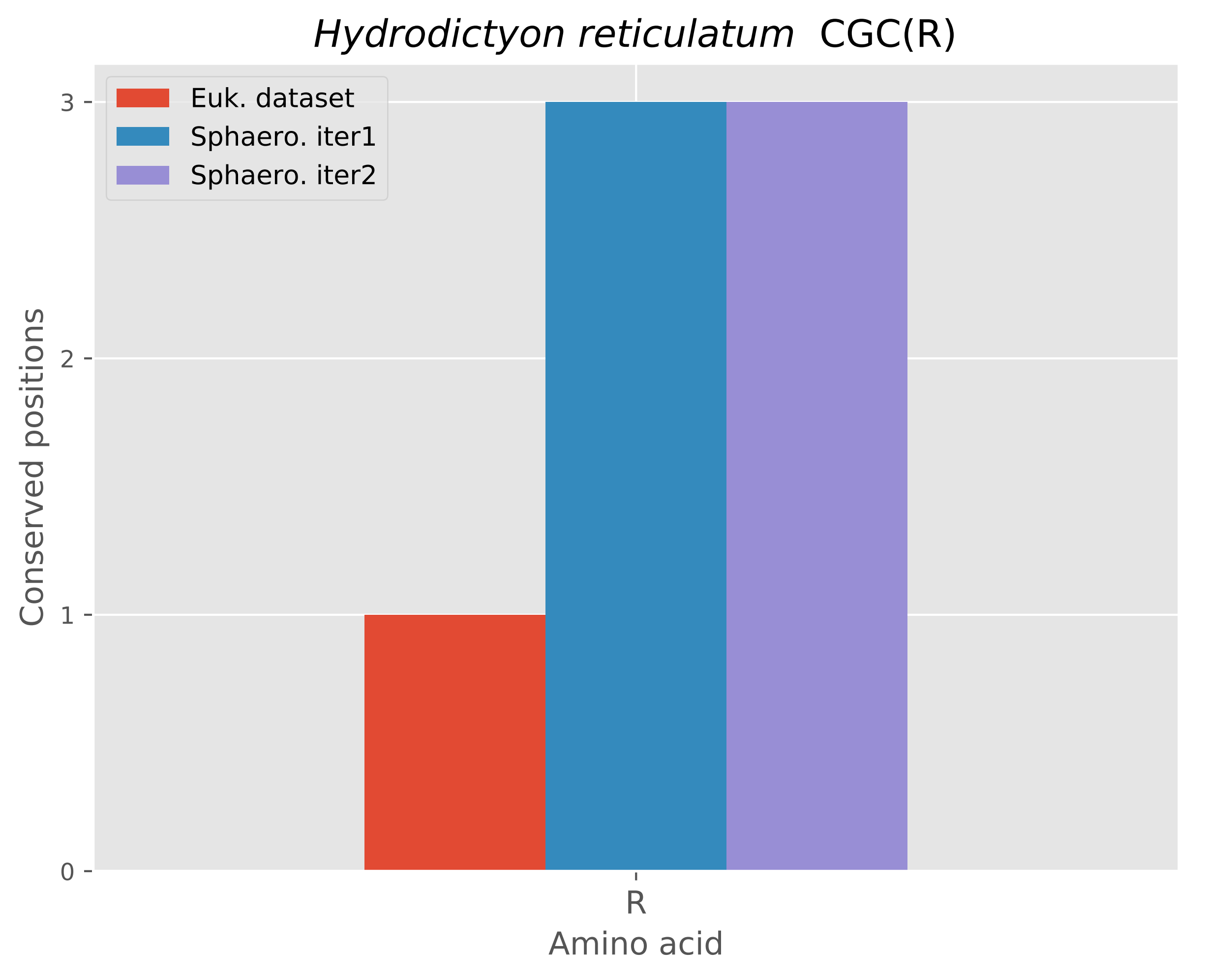

Hydrodictyon reticulatum CGC(R)
Euk. dataset
Sphaero. iter1
Sphaero. iter2
Conserved positions
0
1
2
3
R
Amino acid

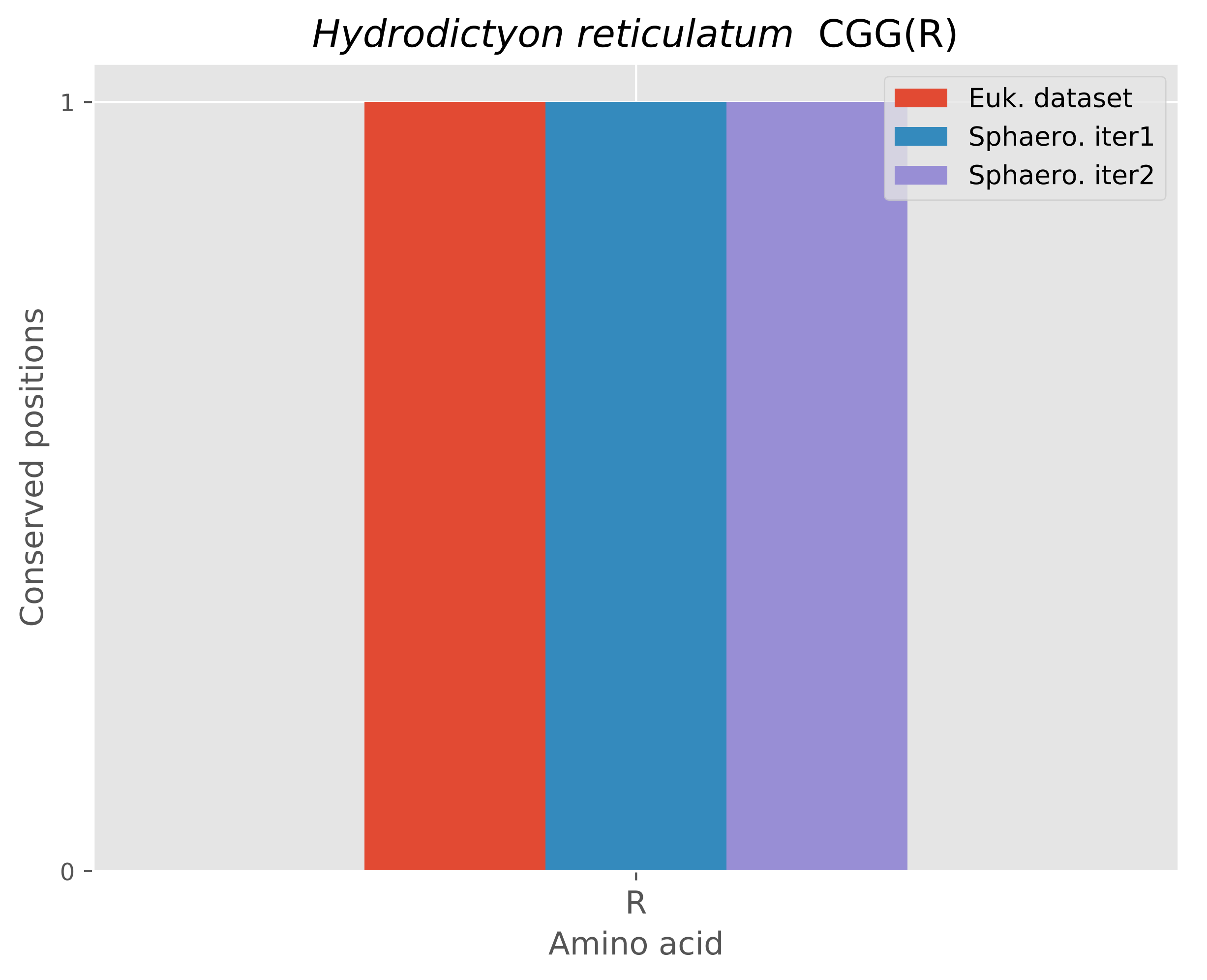

Hydrodictyon reticulatum CGG(R)
Euk. dataset
Sphaero. iter1
Sphaero. iter2
Conserved positions
1
0
R
Amino acid

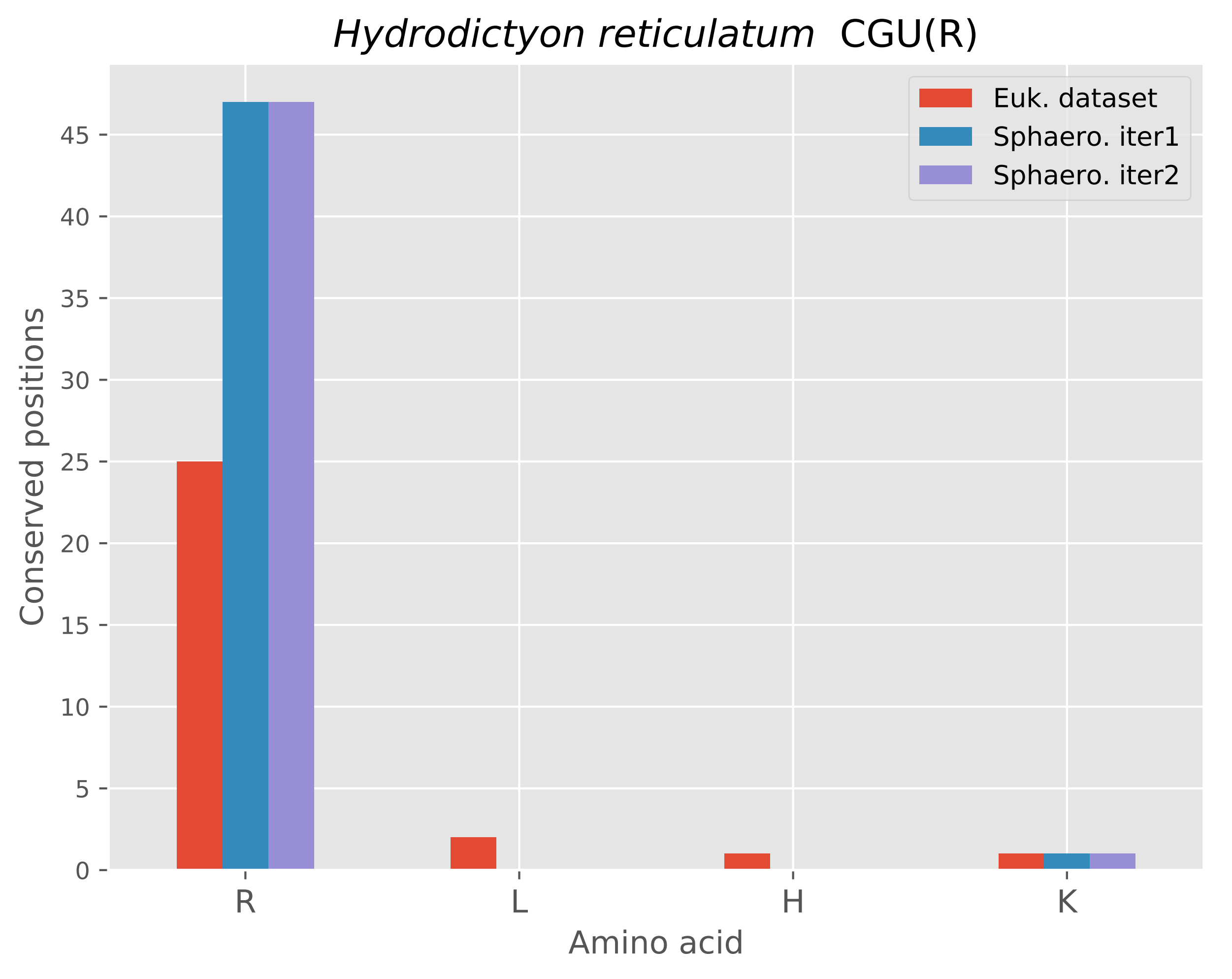

Hydrodictyon reticulatum CGU(R)
Euk. dataset
Sphaero. iter1
Sphaero. iter2
Conserved positions
0
5
10
15
20
25
30
35
40
45
R
L
H
K
Amino acid

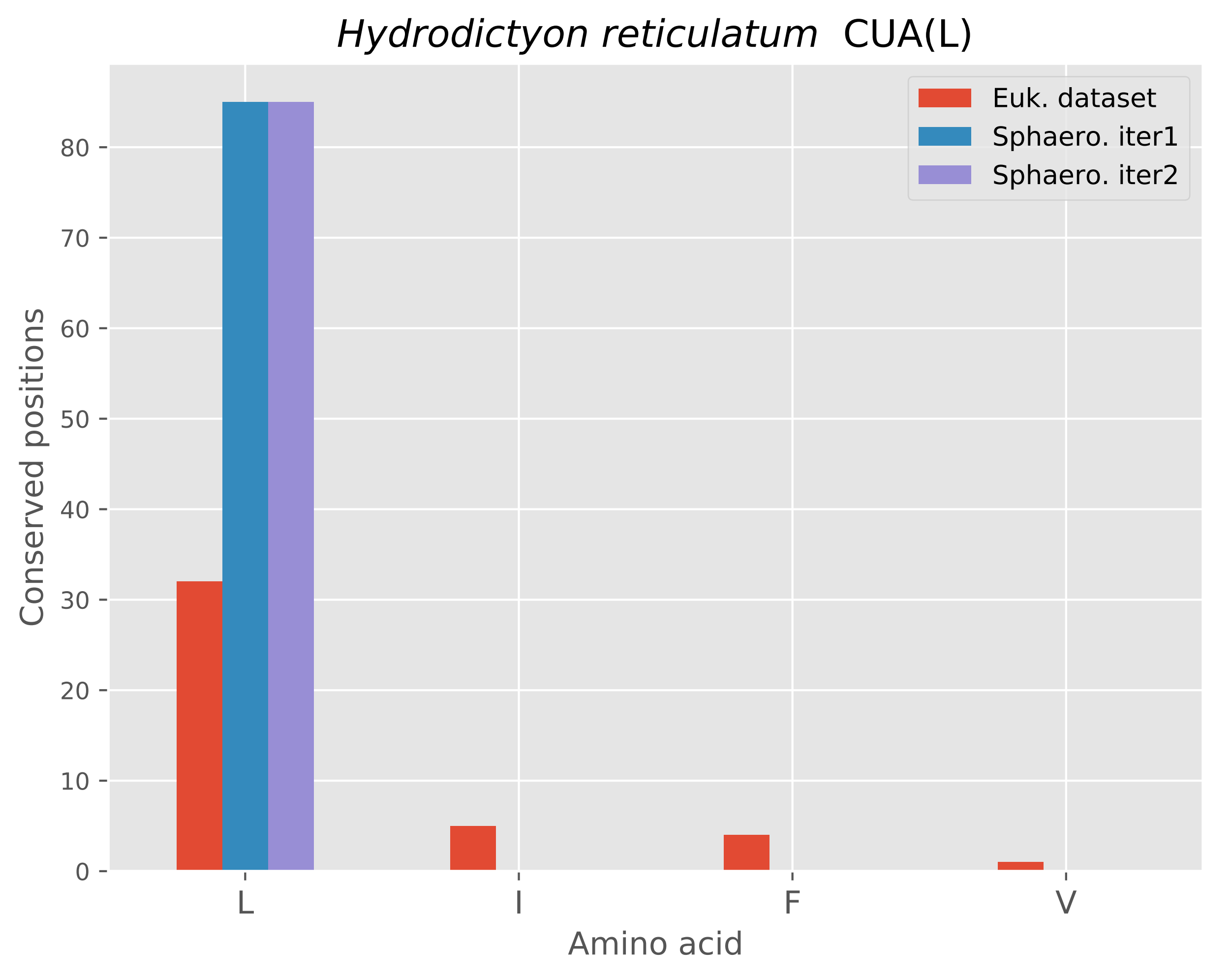

Hydrodictyon reticulatum CUA(L)
Euk. dataset
Sphaero. iter1
Sphaero. iter2
Conserved positions
0
10
20
30
40
50
60
70
80
L
I
F
V
Amino acid

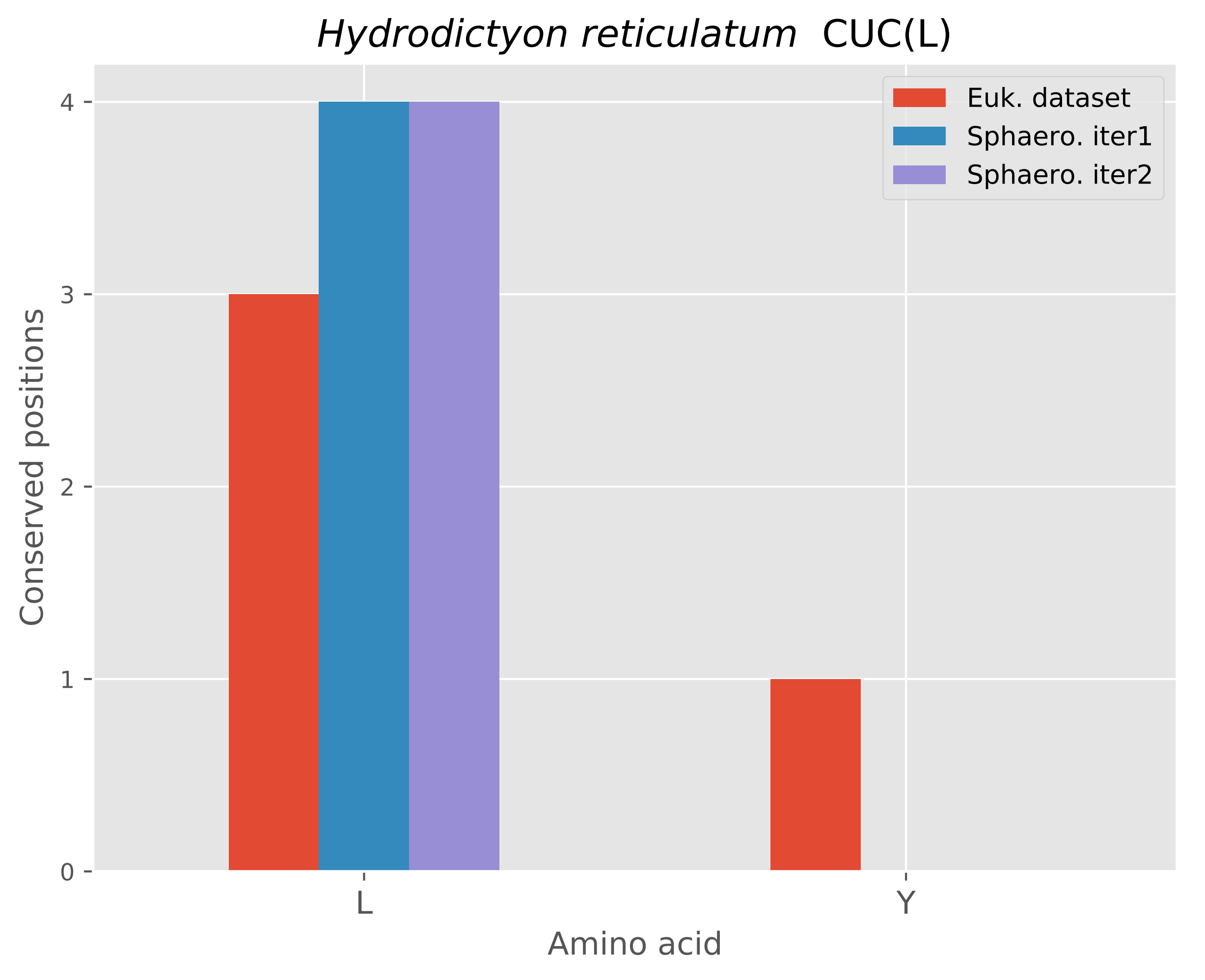

Hydrodictyon reticulatum CUC(L)
Euk. dataset
Sphaero. iter1
Sphaero. iter2
Conserved positions
0
1
2
3
4
L
Y
Amino acid

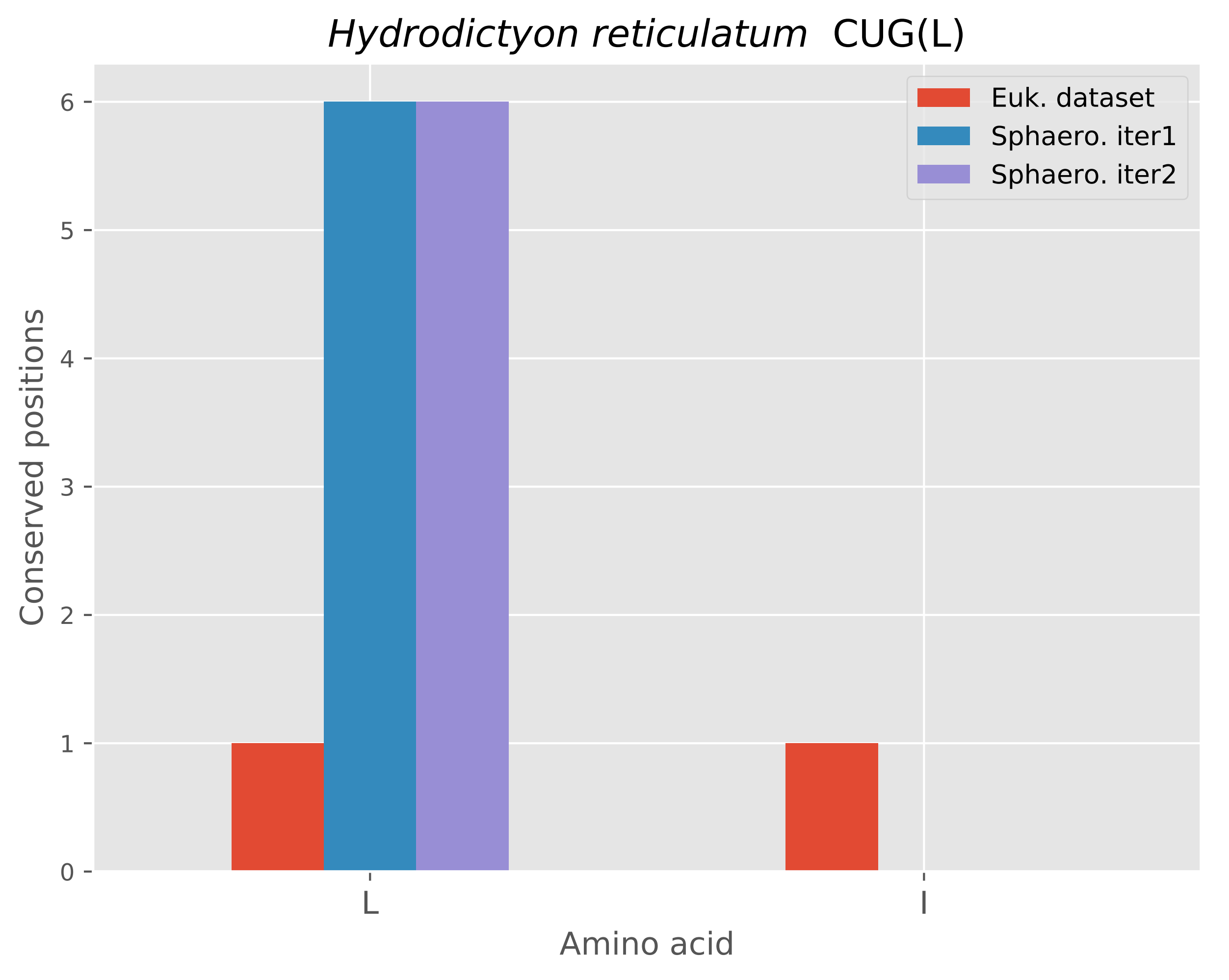

Hydrodictyon reticulatum CUG(L)
Euk. dataset
Sphaero. iter1
Sphaero. iter2
Conserved positions
0
1
2
3
4
5
6
L
I
Amino acid

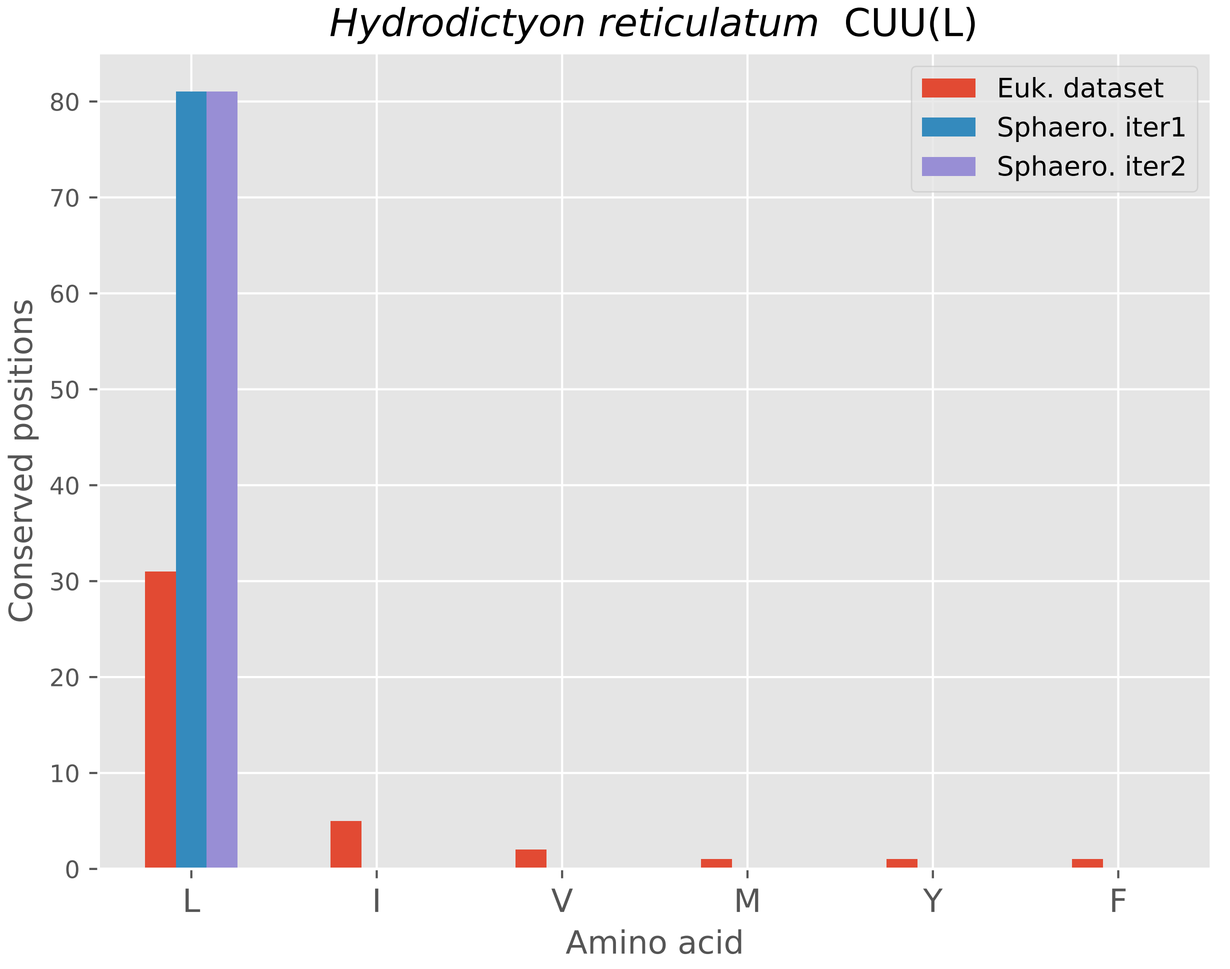

Hydrodictyon reticulatum CUU(L)
Euk. dataset
Sphaero. iter1
Sphaero. iter2
Conserved positions
0
10
20
30
40
50
60
70
80
L
I
V
M
Y
F
Amino acid

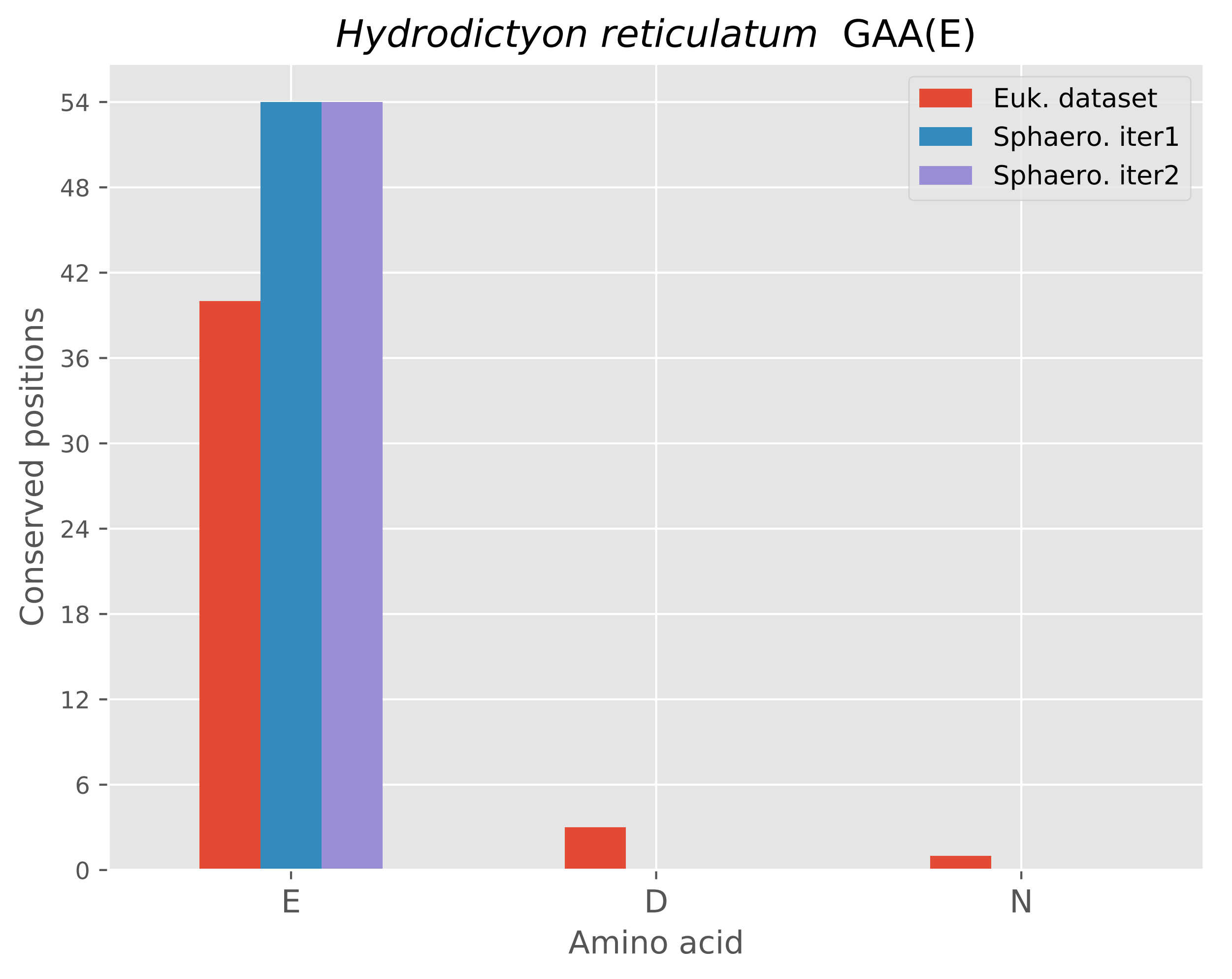
Hydrodictyon reticulatum GAA(E)
Euk. dataset
Sphaero. iter1
Sphaero. iter2
Conserved positions
0
6
12
18
24
30
36
42
48
54
E
D
N
Amino acid

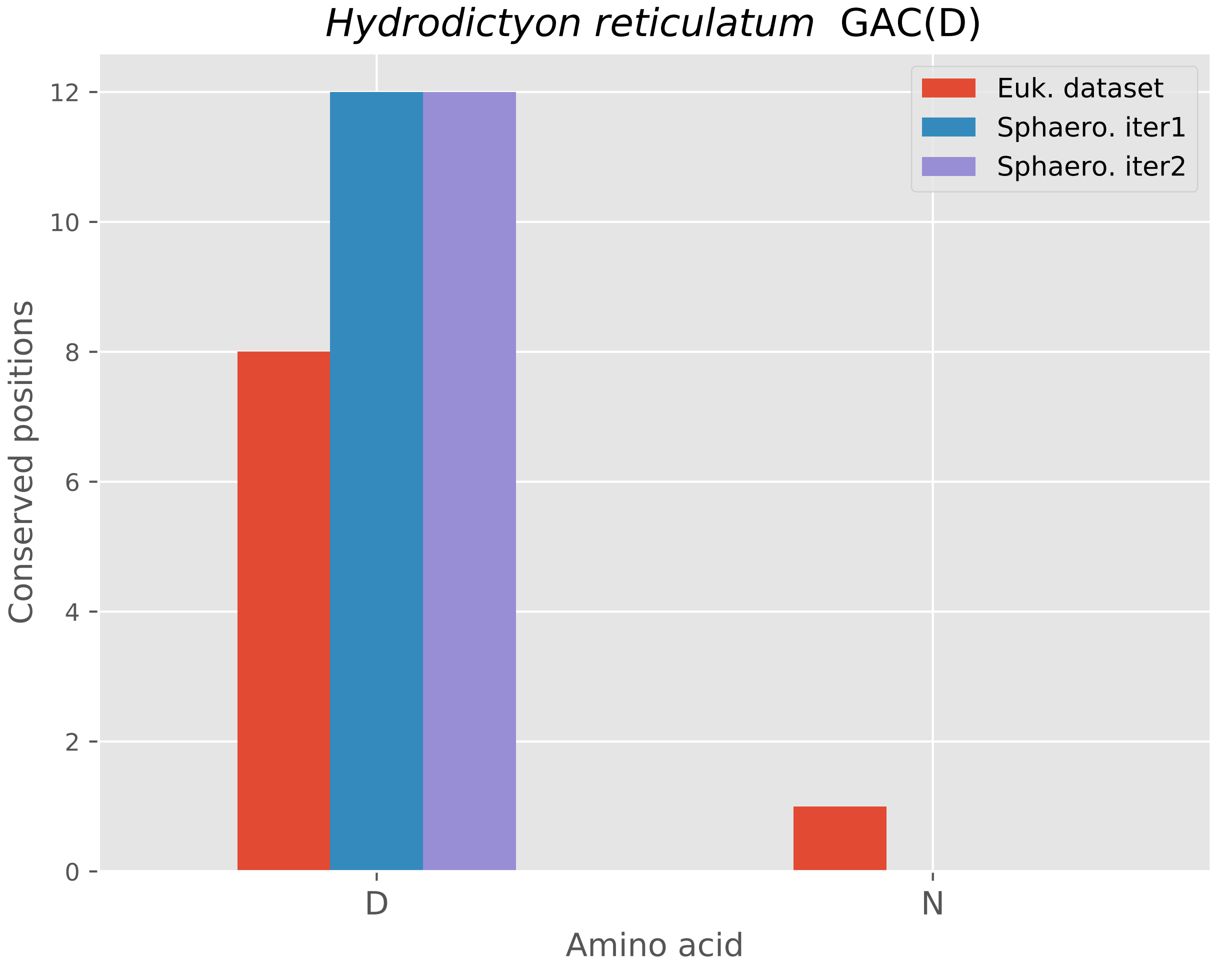
Hydrodictyon reticulatum GAC(D)
Euk. dataset
Sphaero. iter1
Sphaero. iter2
Conserved positions
0
2
4
6
8
10
12
D
N
Amino acid

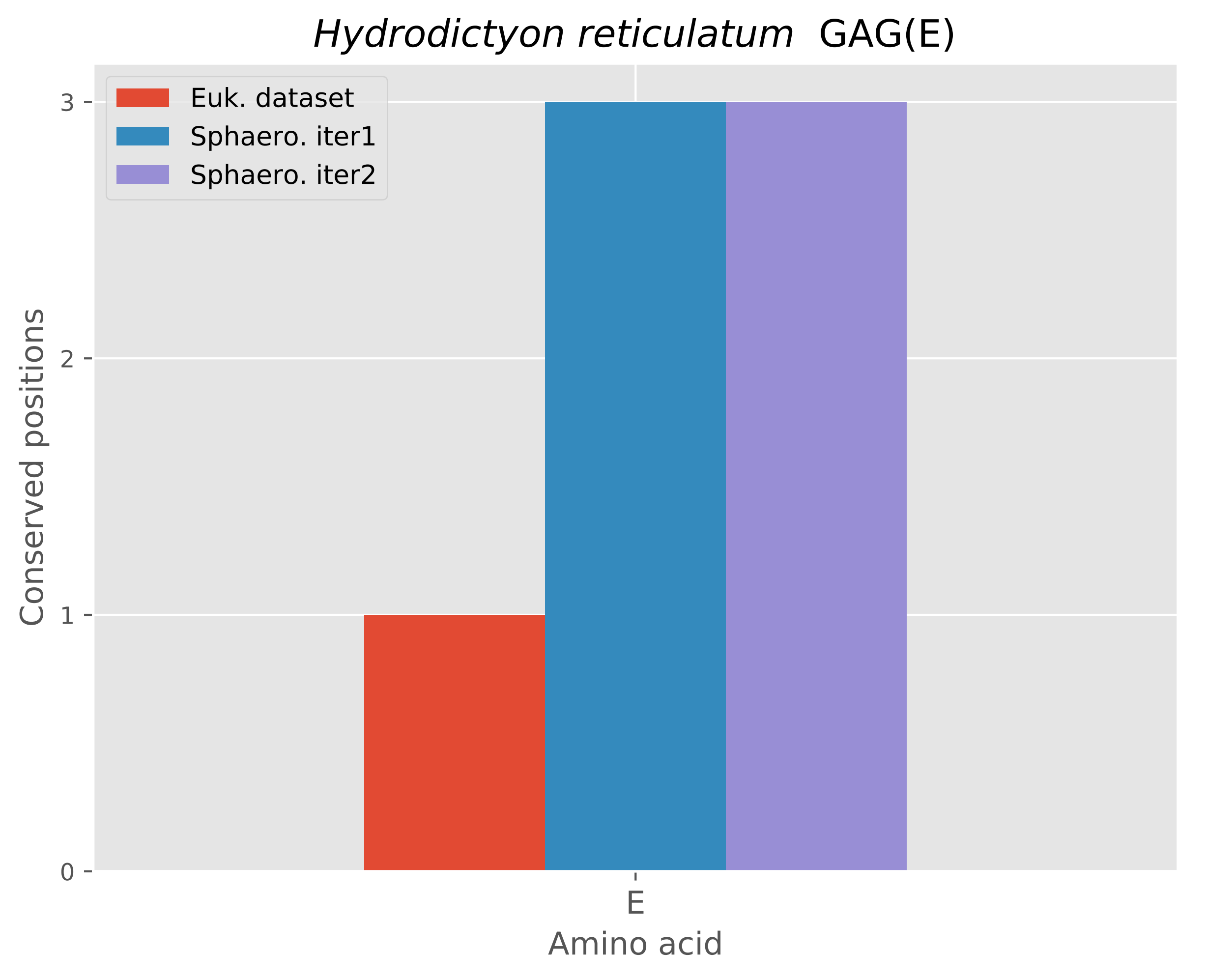

Hydrodictyon reticulatum GAG(E)
Euk. dataset
Sphaero. iter1
Sphaero. iter2
Conserved positions
0
1
2
3
E
Amino acid

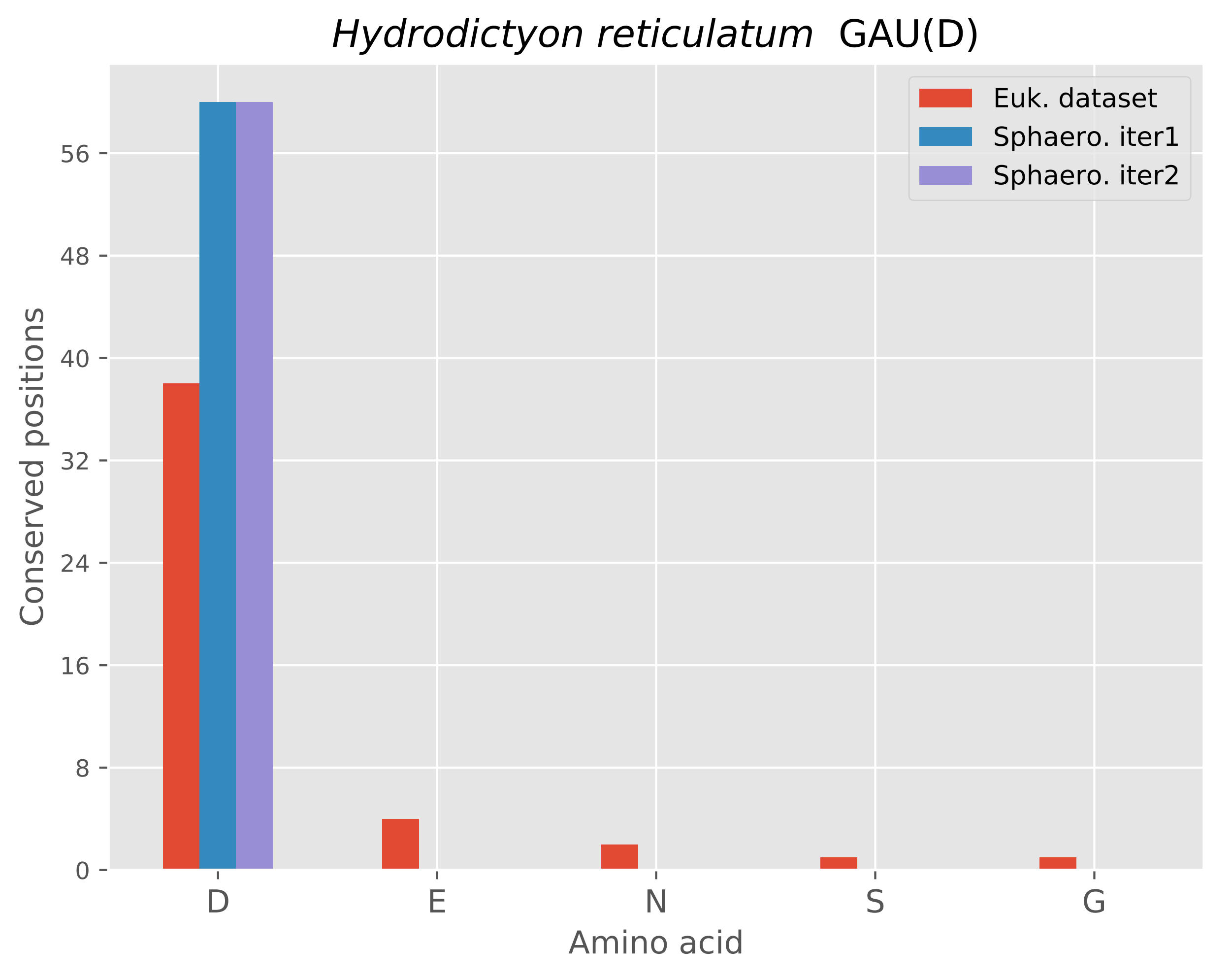

Hydrodictyon reticulatum GAU(D)
Euk. dataset
Sphaero. iter1
Sphaero. iter2
Conserved positions
0
8
16
24
32
40
48
56
D
E
N
S
G
Amino acid

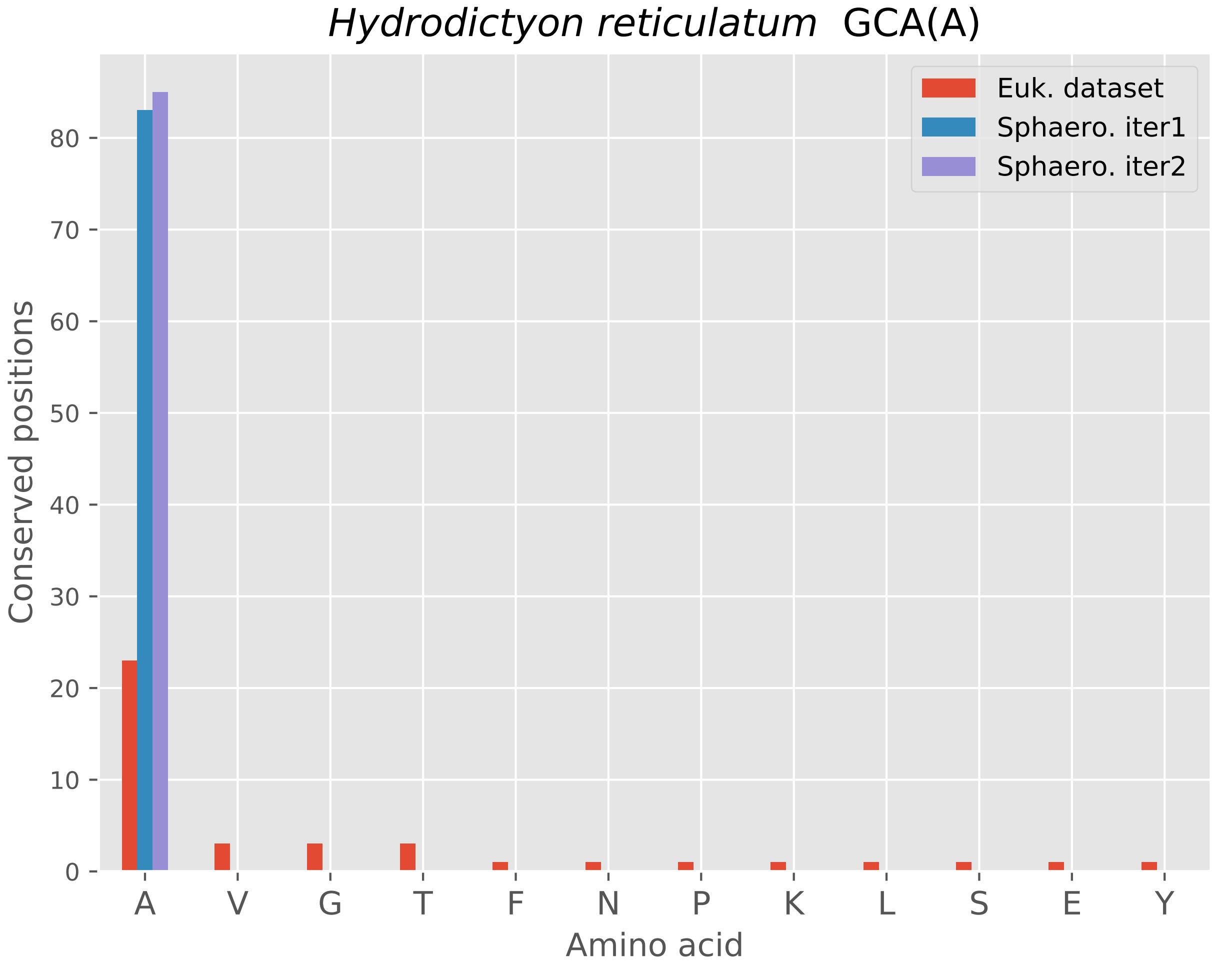

Hydrodictyon reticulatum GCA(A)
Euk. dataset
Sphaero. iter1
Sphaero. iter2
Conserved positions
0
10
20
30
40
50
60
70
80
A
V
G
T
F
N
P
K
L
S
E
Y
Amino acid

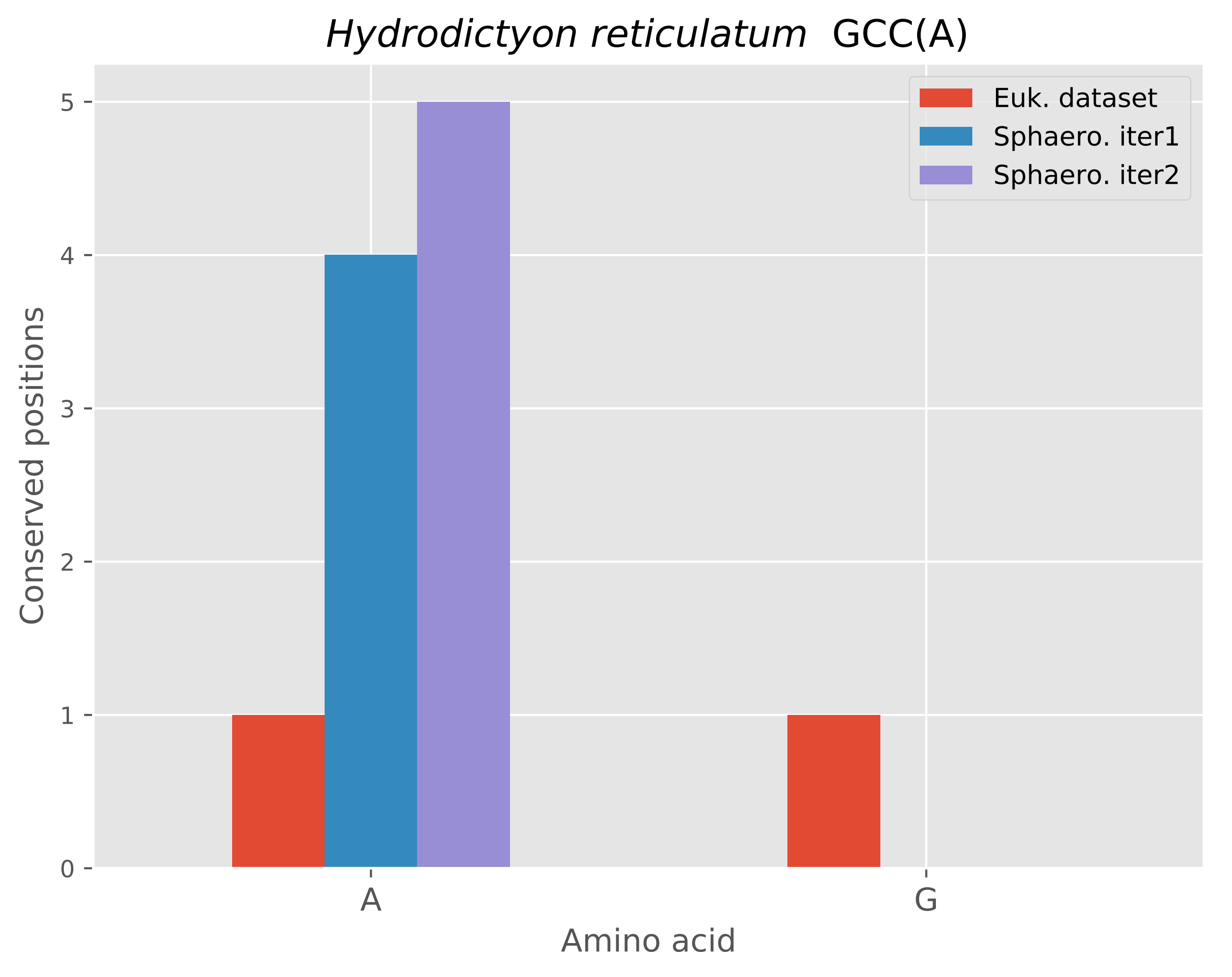

Hydrodictyon reticulatum GCC(A)
Euk. dataset
Sphaero. iter1
Sphaero. iter2
Conserved positions
0
1
2
3
4
5
A
G
Amino acid

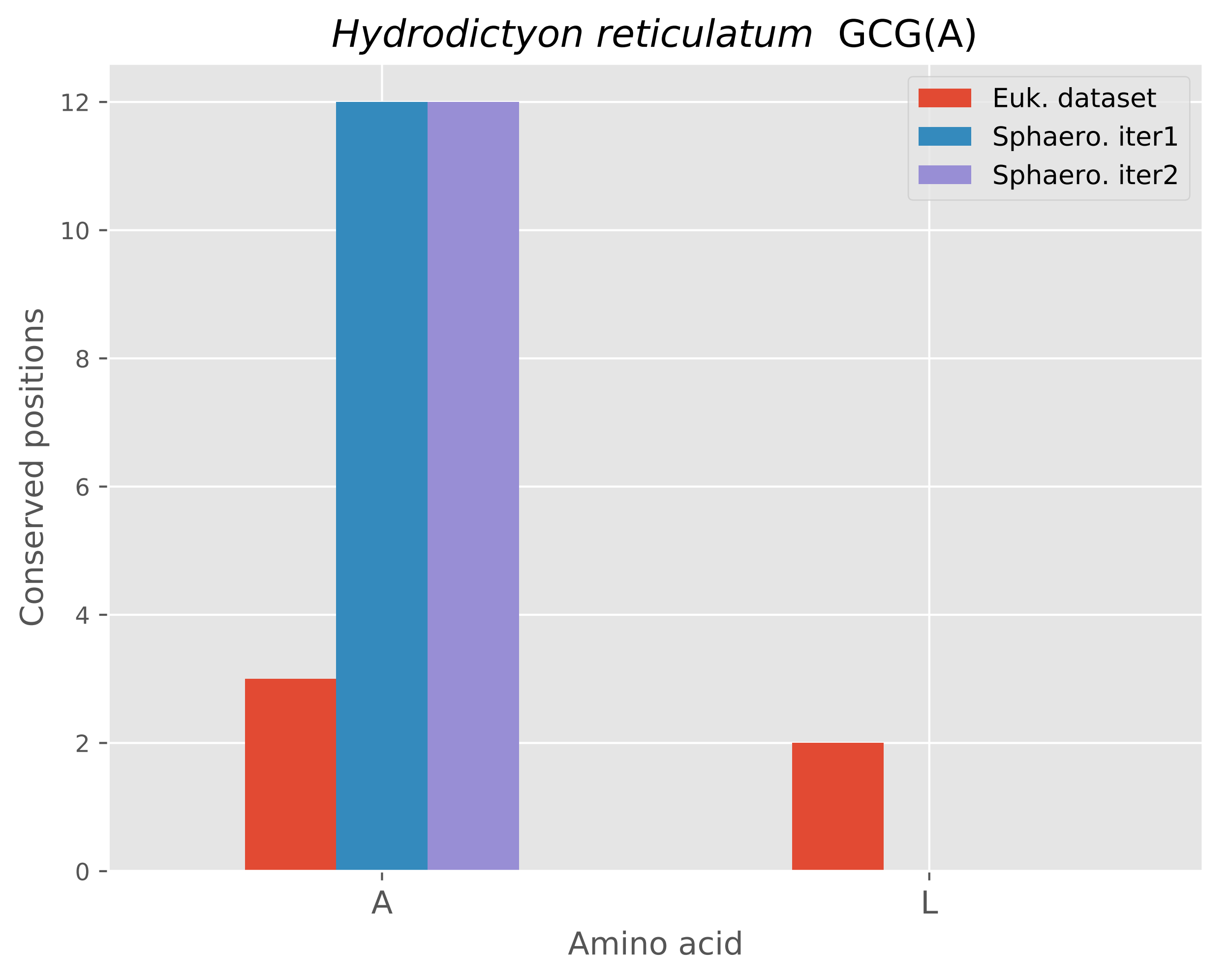

Hydrodictyon reticulatum GCG(A)
Euk. dataset
Sphaero. iter1
Sphaero. iter2
Conserved positions
0
2
4
6
8
10
12
A
L
Amino acid

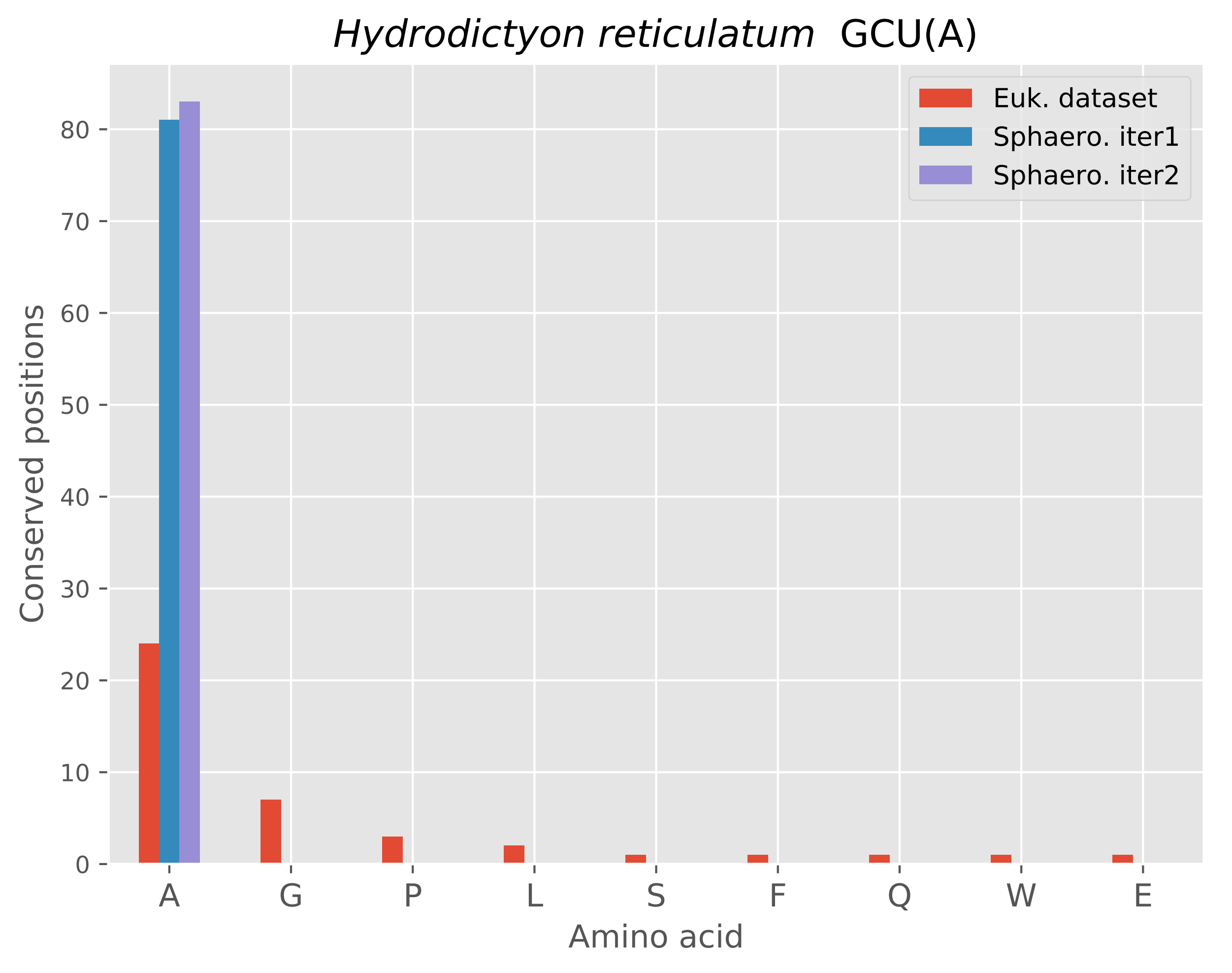

Hydrodictyon reticulatum GCU(A)
Euk. dataset
Sphaero. iter1
Sphaero. iter2
Conserved positions
0
10
20
30
40
50
60
70
80
A
G
P
L
S
F
Q
W
E
Amino acid

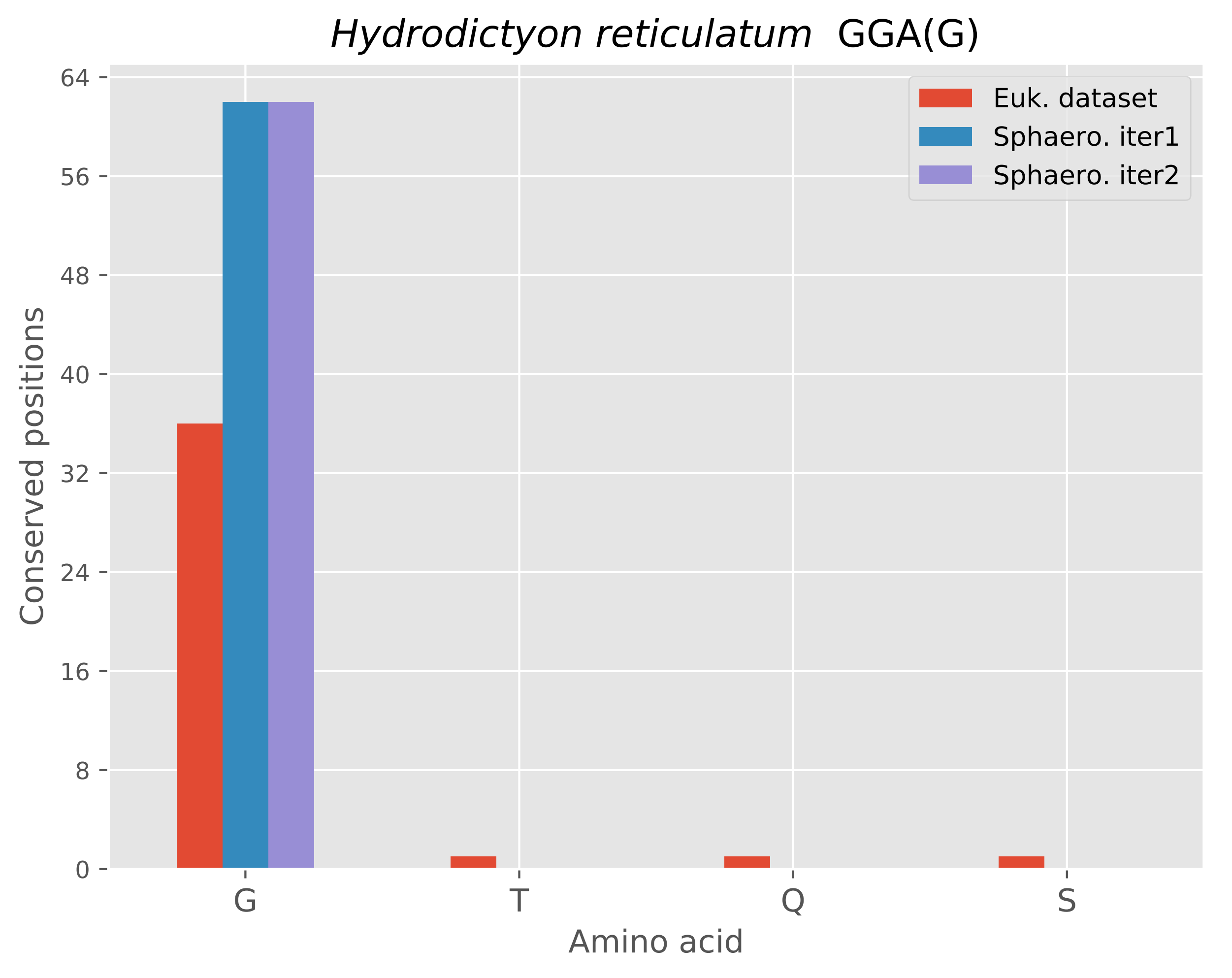

Hydrodictyon reticulatum GGA(G)
Euk. dataset
Sphaero. iter1
Sphaero. iter2
Conserved positions
0
8
16
24
32
40
48
56
64
G
T
Q
S
Amino acid

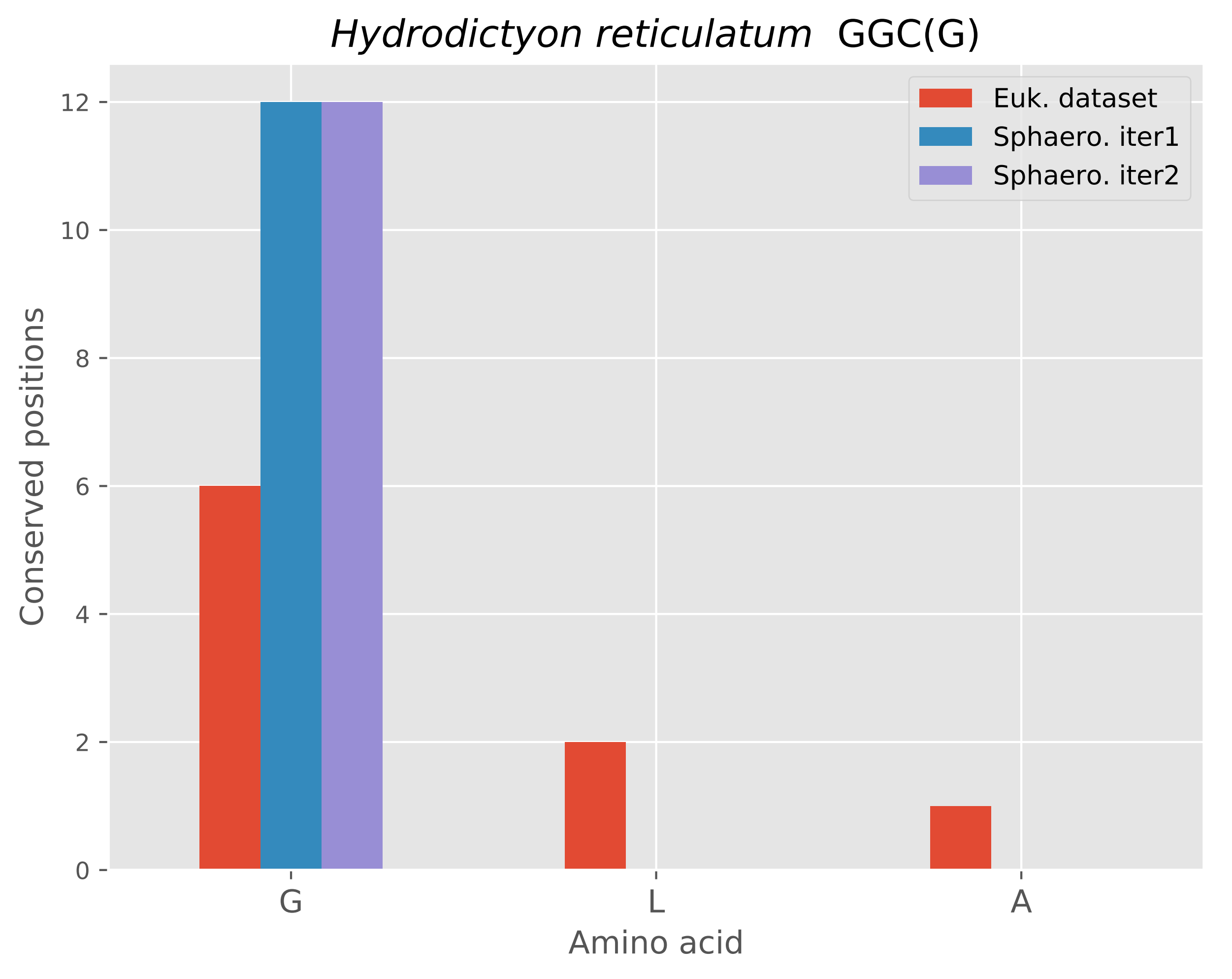

Hydrodictyon reticulatum GGC(G)
Euk. dataset
Sphaero. iter1
Sphaero. iter2
Conserved positions
0
2
4
6
8
10
12
G
L
A
Amino acid

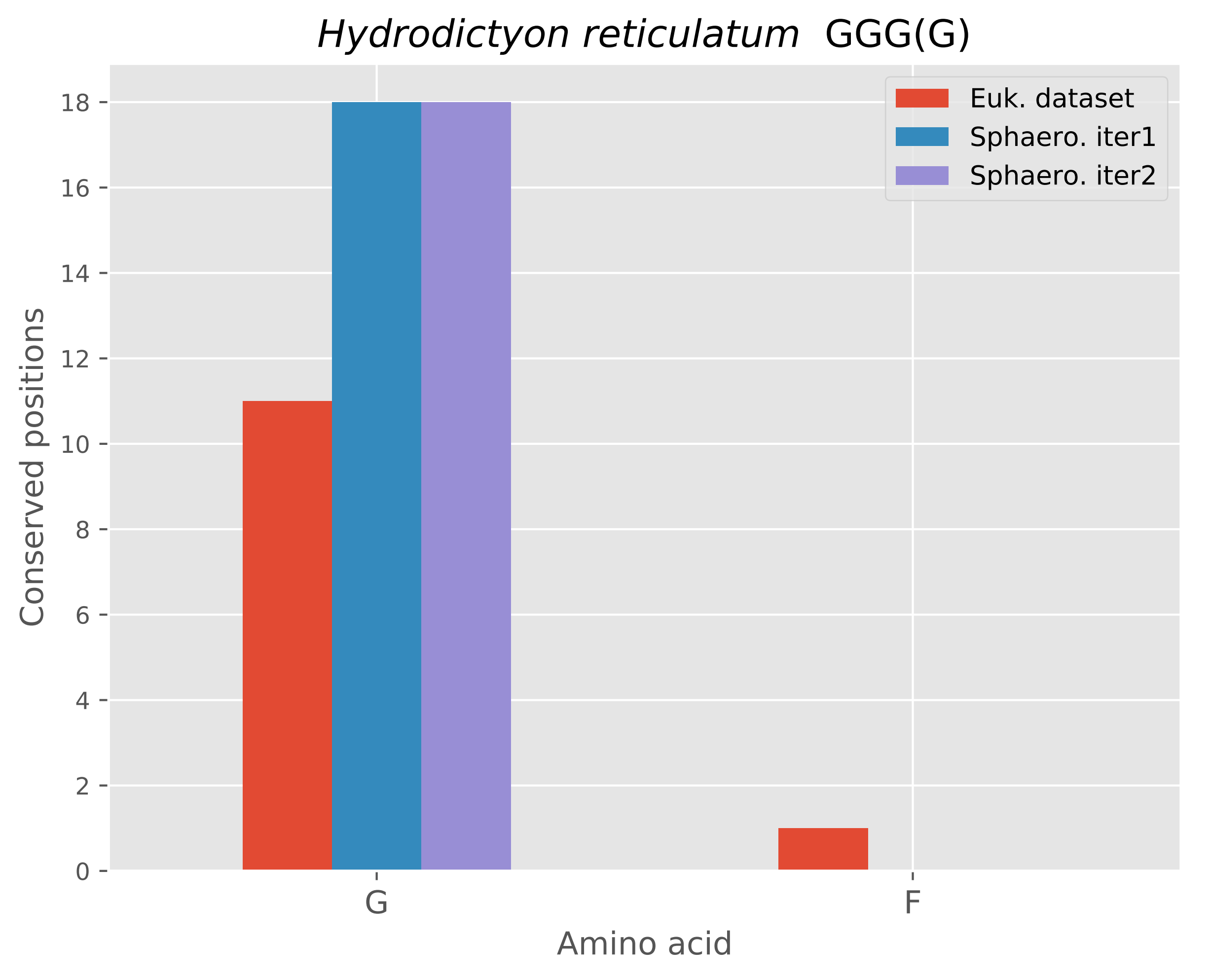

Hydrodictyon reticulatum GGG(G)
Euk. dataset
Sphaero. iter1
Sphaero. iter2
Conserved positions
0
2
4
6
8
10
12
14
16
18
G
F
Amino acid

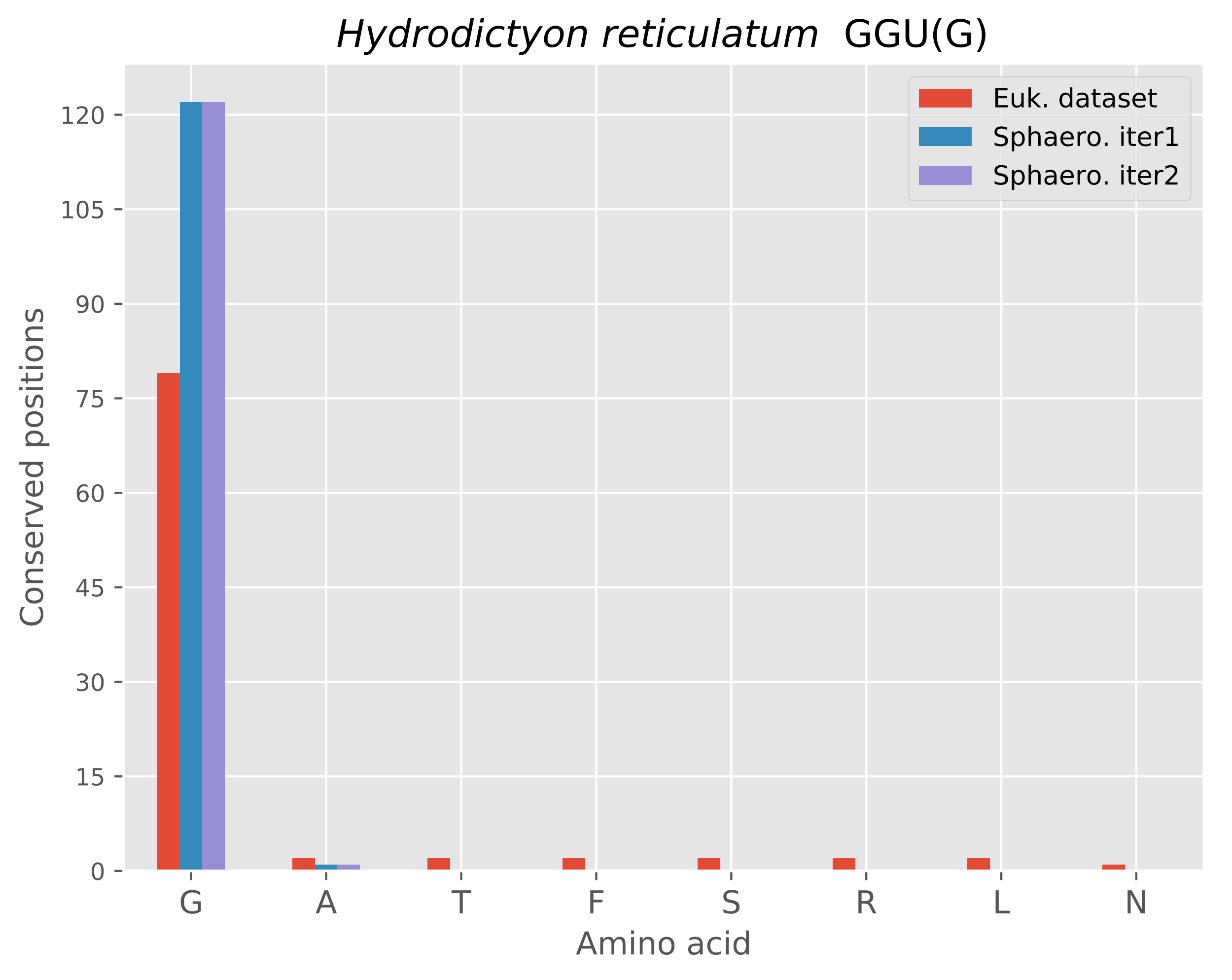

Hydrodictyon reticulatum GGU(G)
Euk. dataset
Sphaero. iter1
Sphaero. iter2
Conserved positions
0
15
30
45
60
75
90
105
120
G
A
T
F
S
R
L
N
Amino acid

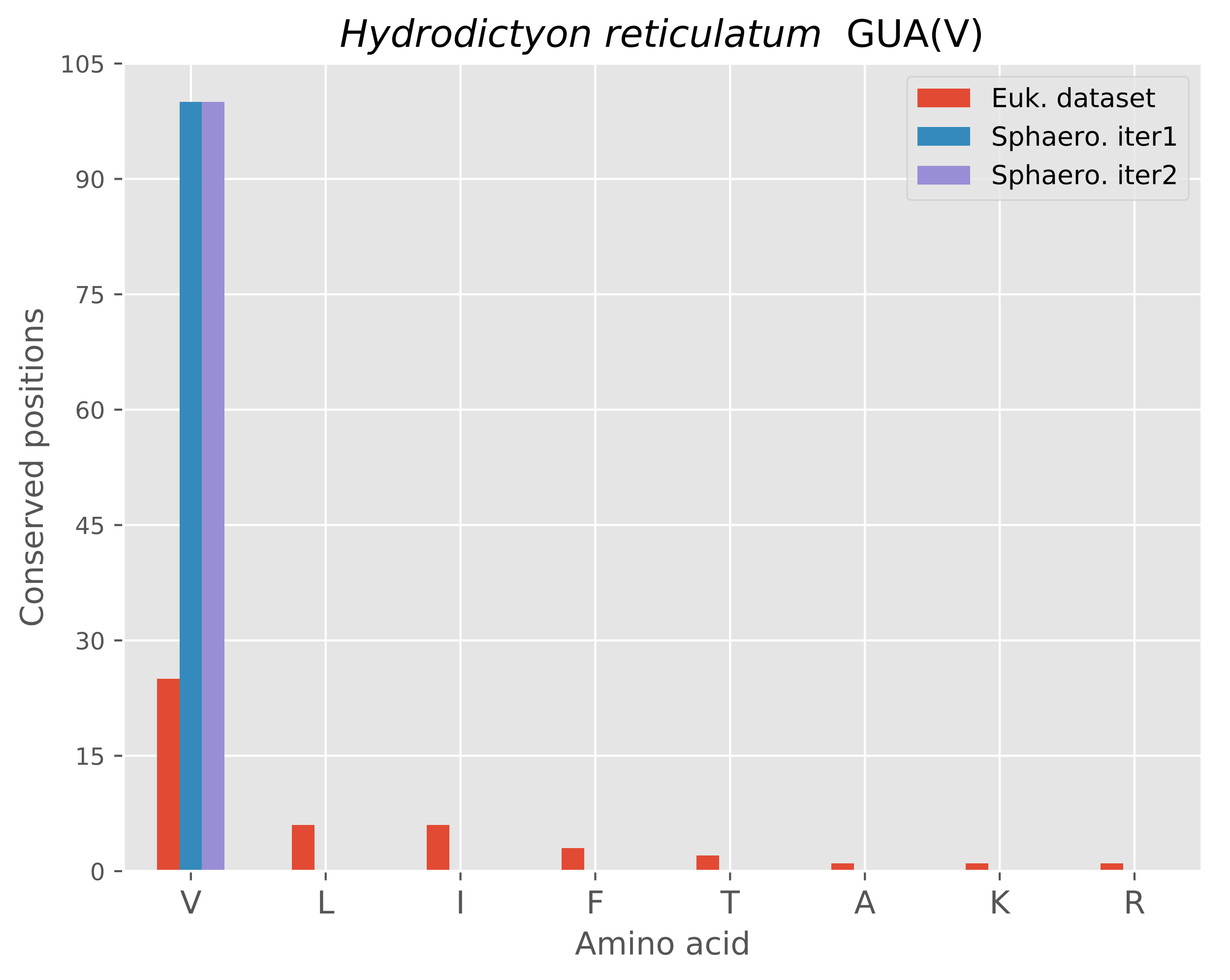

Hydrodictyon reticulatum GUA(V)
Euk. dataset
Sphaero. iter1
Sphaero. iter2
Conserved positions
105
90
75
60
45
30
15
0
V
L
I
F
T
A
K
R
Amino acid

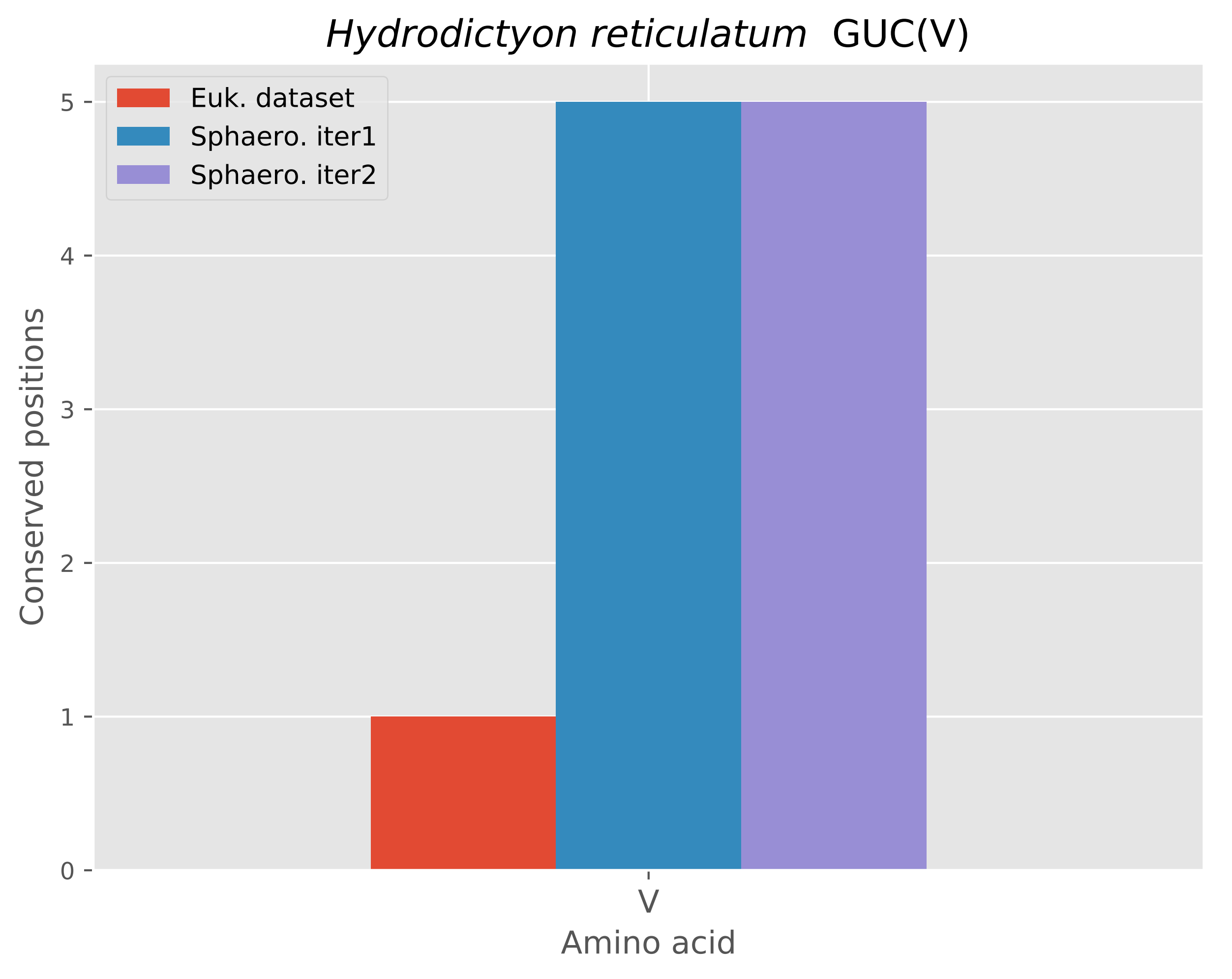

Hydrodictyon reticulatum GUC(V)
Euk. dataset
Sphaero. iter1
Sphaero. iter2
Conserved positions
0
1
2
3
4
5
V
Amino acid

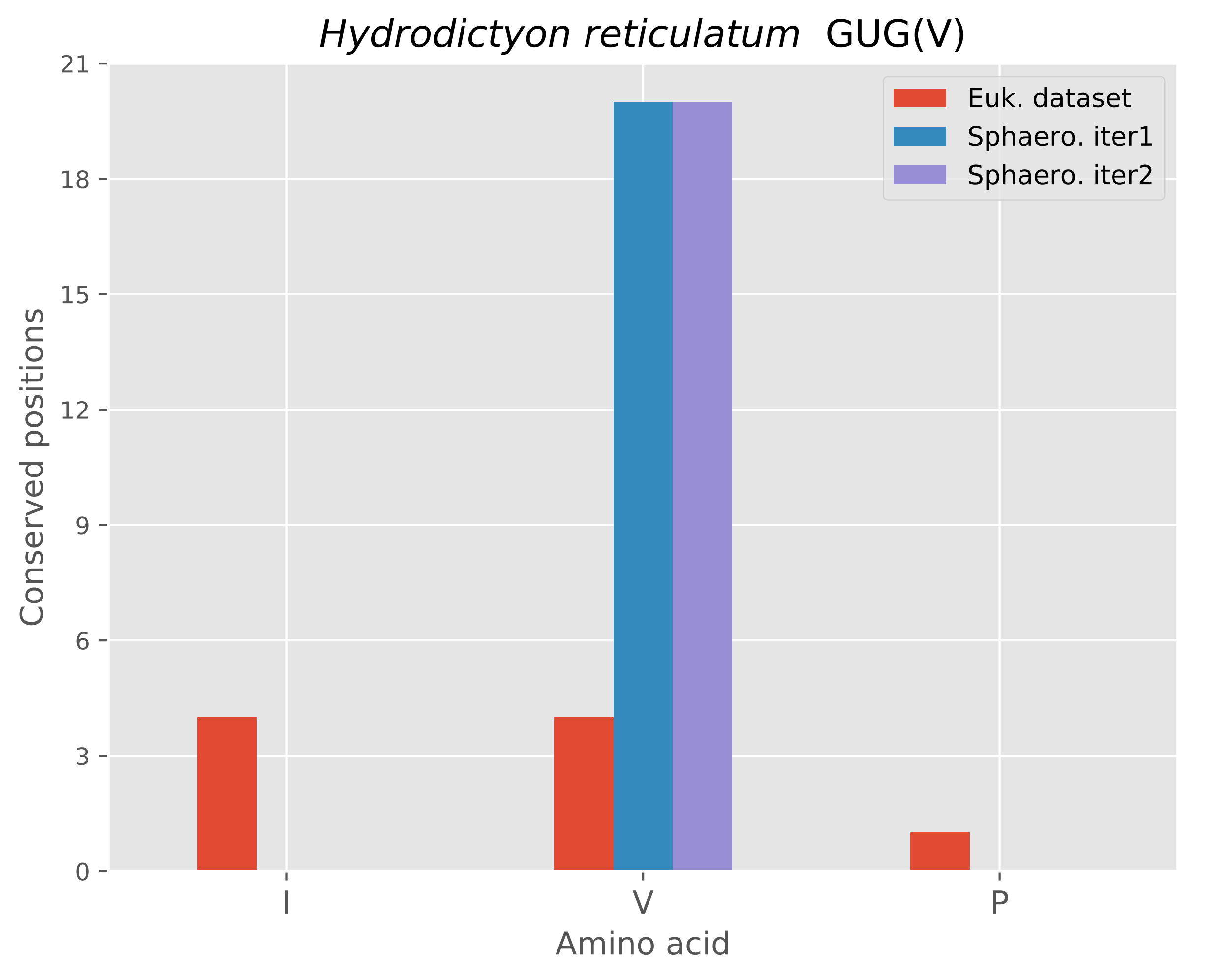

Hydrodictyon reticulatum GUG(V)
Euk. dataset
Sphaero. iter1
Sphaero. iter2
Conserved positions
0
3
6
9
12
15
18
21
I
V
P
Amino acid

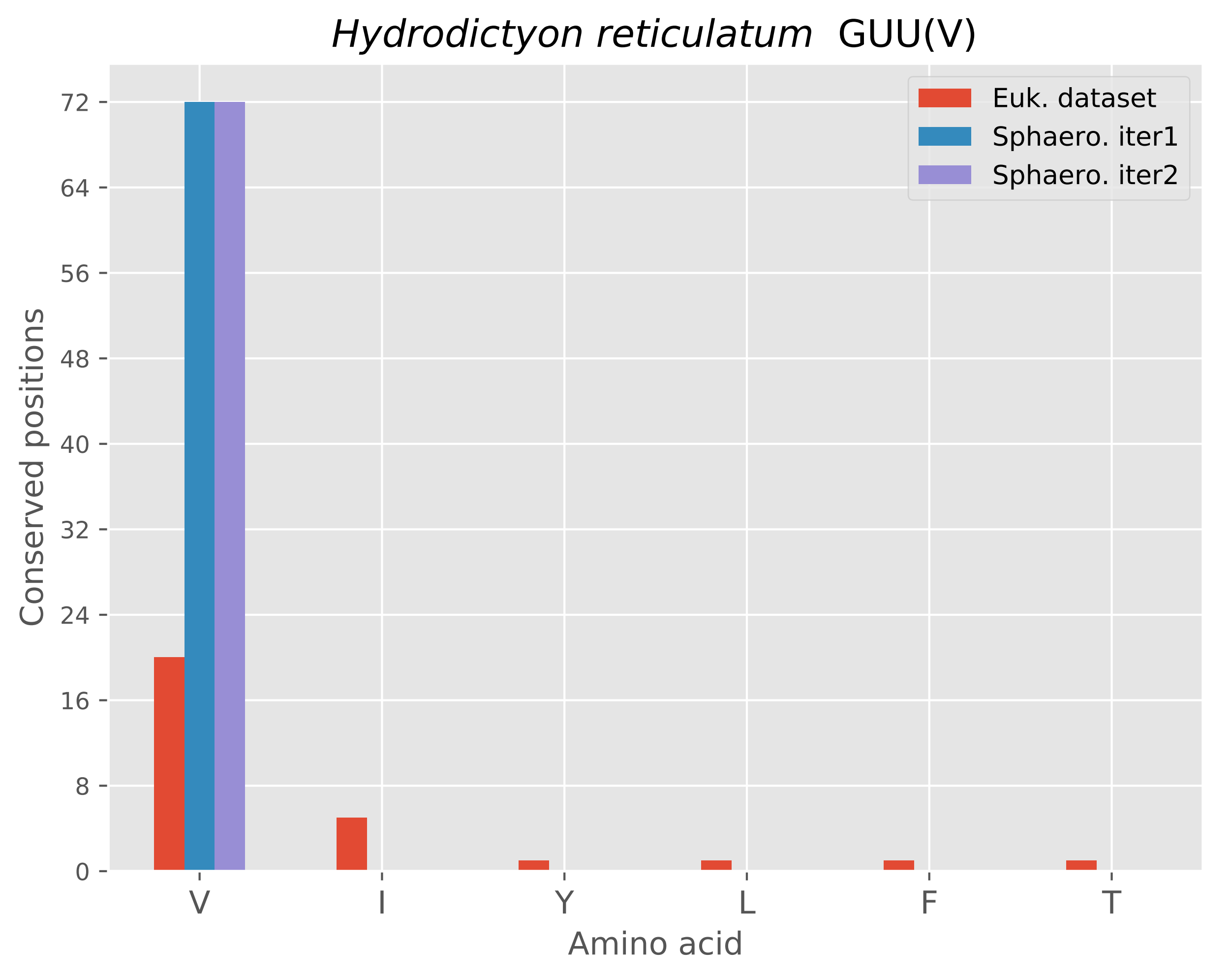

Hydrodictyon reticulatum GUU(V)
Euk. dataset
Sphaero. iter1
Sphaero. iter2
Conserved positions
Amino acid
0
8
16
24
32
40
48
56
64
72
V
I
Y
L
F
T

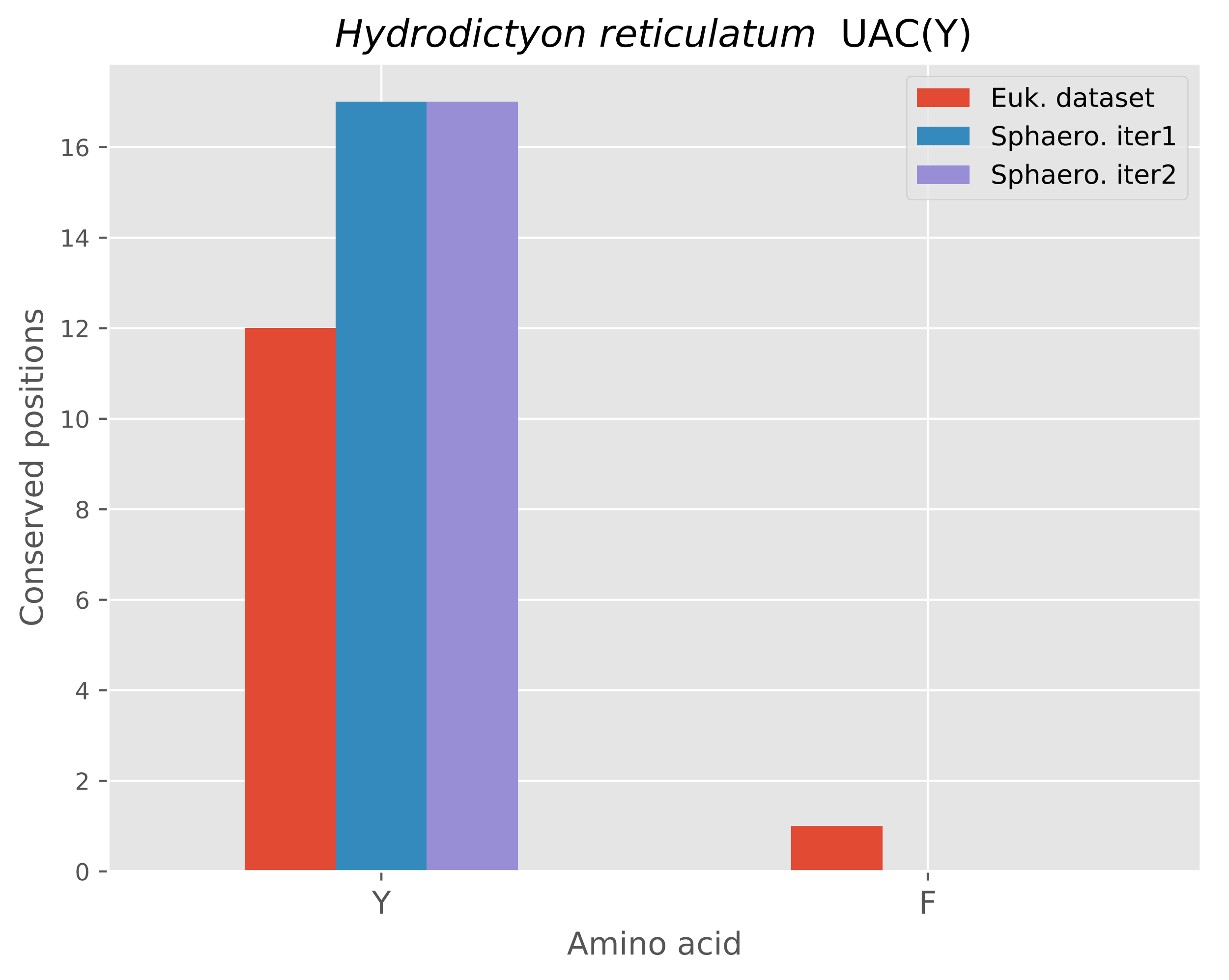

Hydrodictyon reticulatum UAC(Y)
Euk. dataset
Sphaero. iter1
Sphaero. iter2
Conserved positions
0
2
4
6
8
10
12
14
16
Y
F
Amino acid

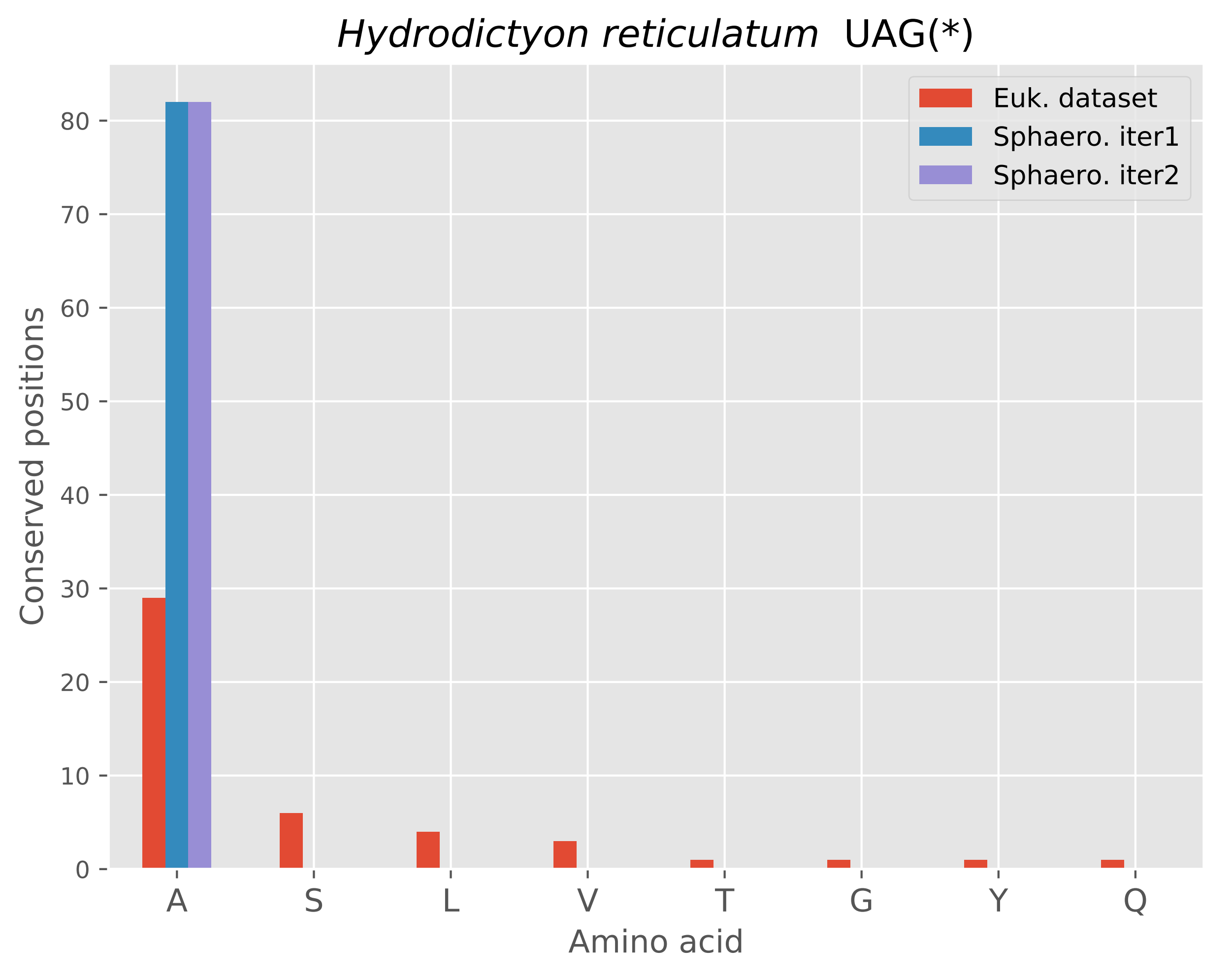

Hydrodictyon reticulatum UAG(*)
Conserved positions
Amino acid
Euk. dataset
Sphaero. iter1
Sphaero. iter2
0
10
20
30
40
50
60
70
80
A
S
L
V
T
G
Y
Q

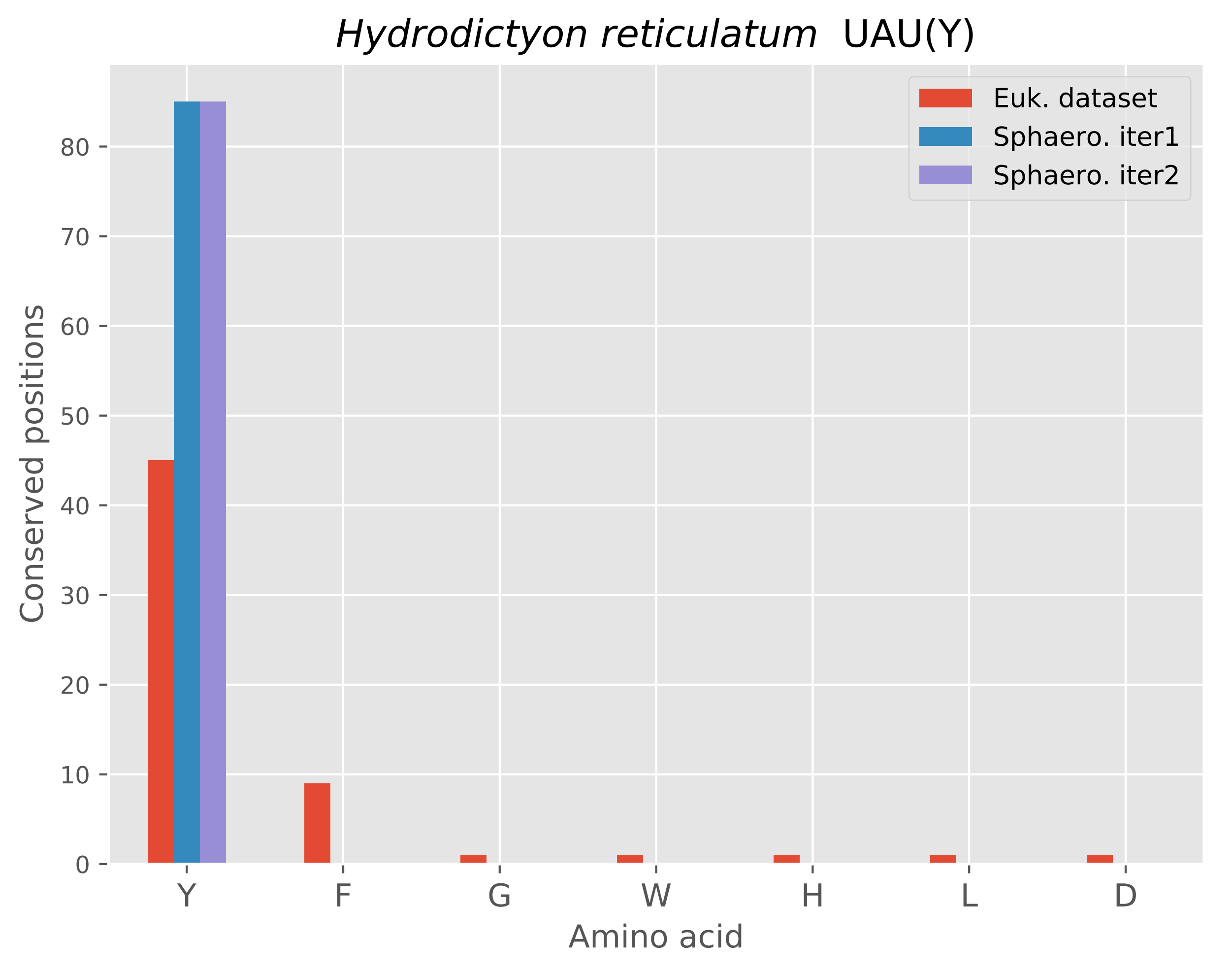

Hydrodictyon reticulatum UAU(Y)
Euk. dataset
Sphaero. iter1
Sphaero. iter2
Conserved positions
0
10
20
30
40
50
60
70
80
Y
F
G
W
H
L
D
Amino acid

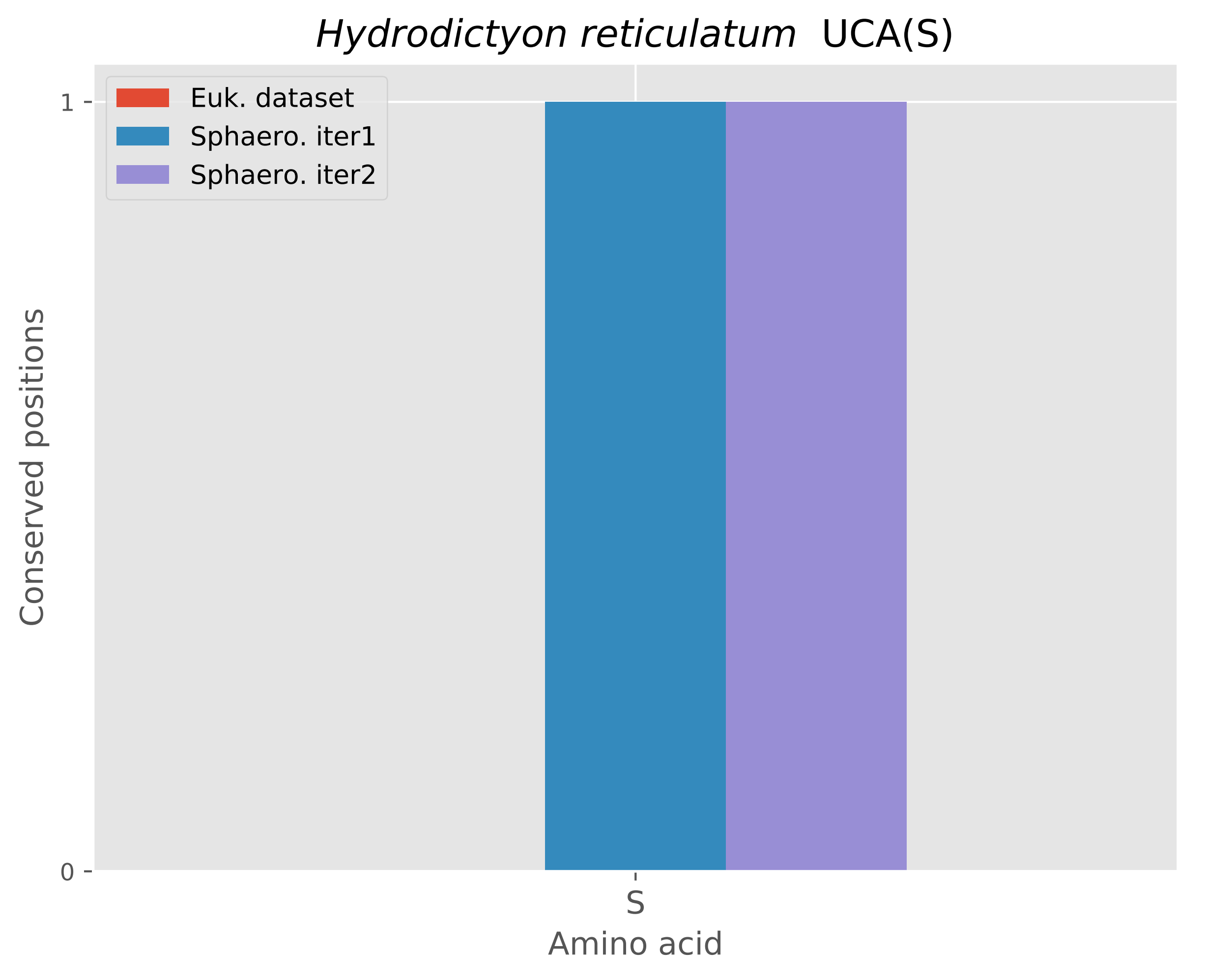
Hydrodictyon reticulatum UCA(S)
Euk. dataset
Sphaero. iter1
Sphaero. iter2
1
0
Conserved positions
S
Amino acid

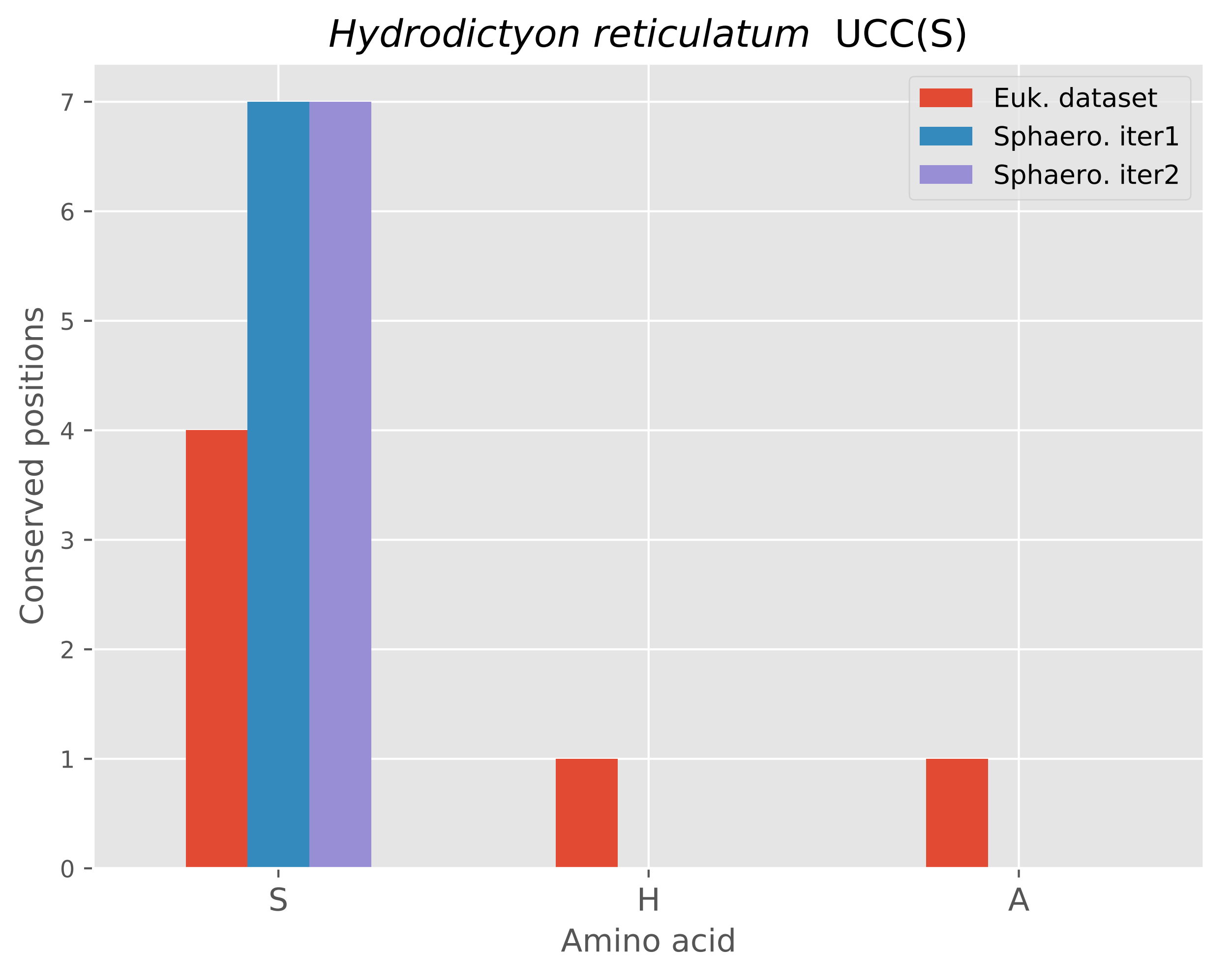
Hydrodictyon reticulatum UCC(S)
Euk. dataset
Sphaero. iter1
Sphaero. iter2
Conserved positions
0
1
2
3
4
5
6
7
S
H
A
Amino acid

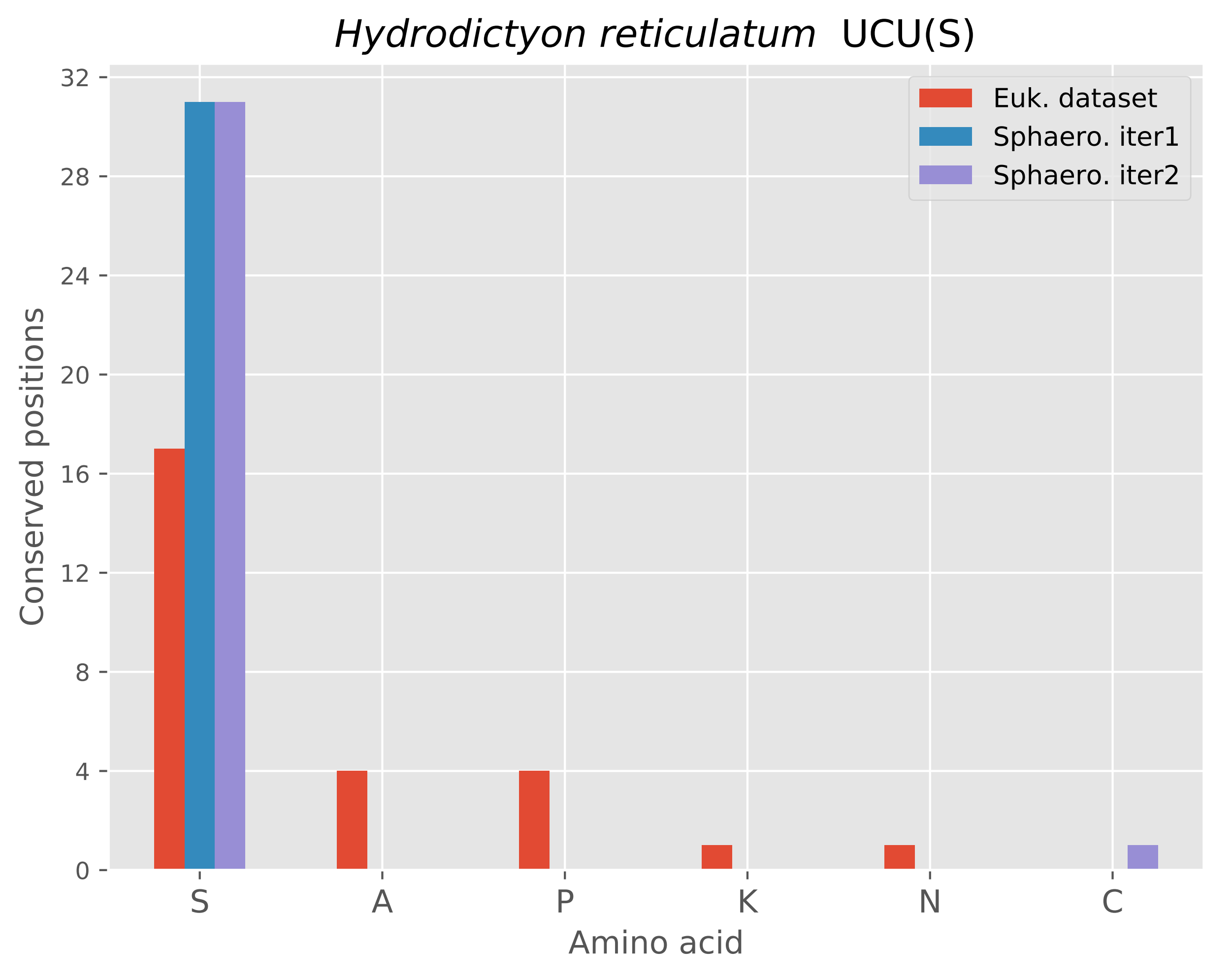

Hydrodictyon reticulatum UCU(S)
Euk. dataset
Sphaero. iter1
Sphaero. iter2
Conserved positions
0
4
8
12
16
20
24
28
32
S
A
P
K
N
C
Amino acid

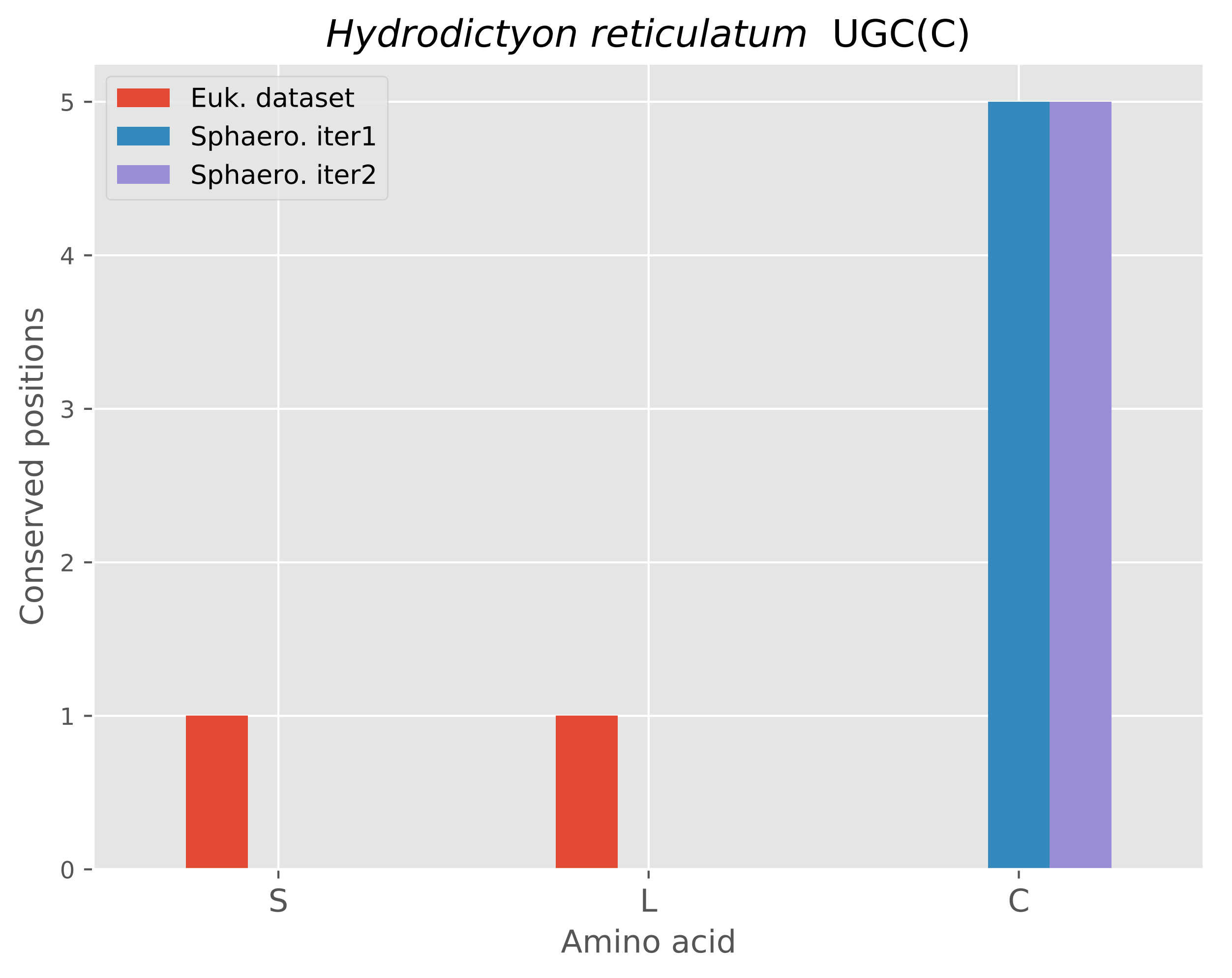

Hydrodictyon reticulatum UGC(C)
Euk. dataset
Sphaero. iter1
Sphaero. iter2
Conserved positions
0
1
2
3
4
5
S
L
C
Amino acid

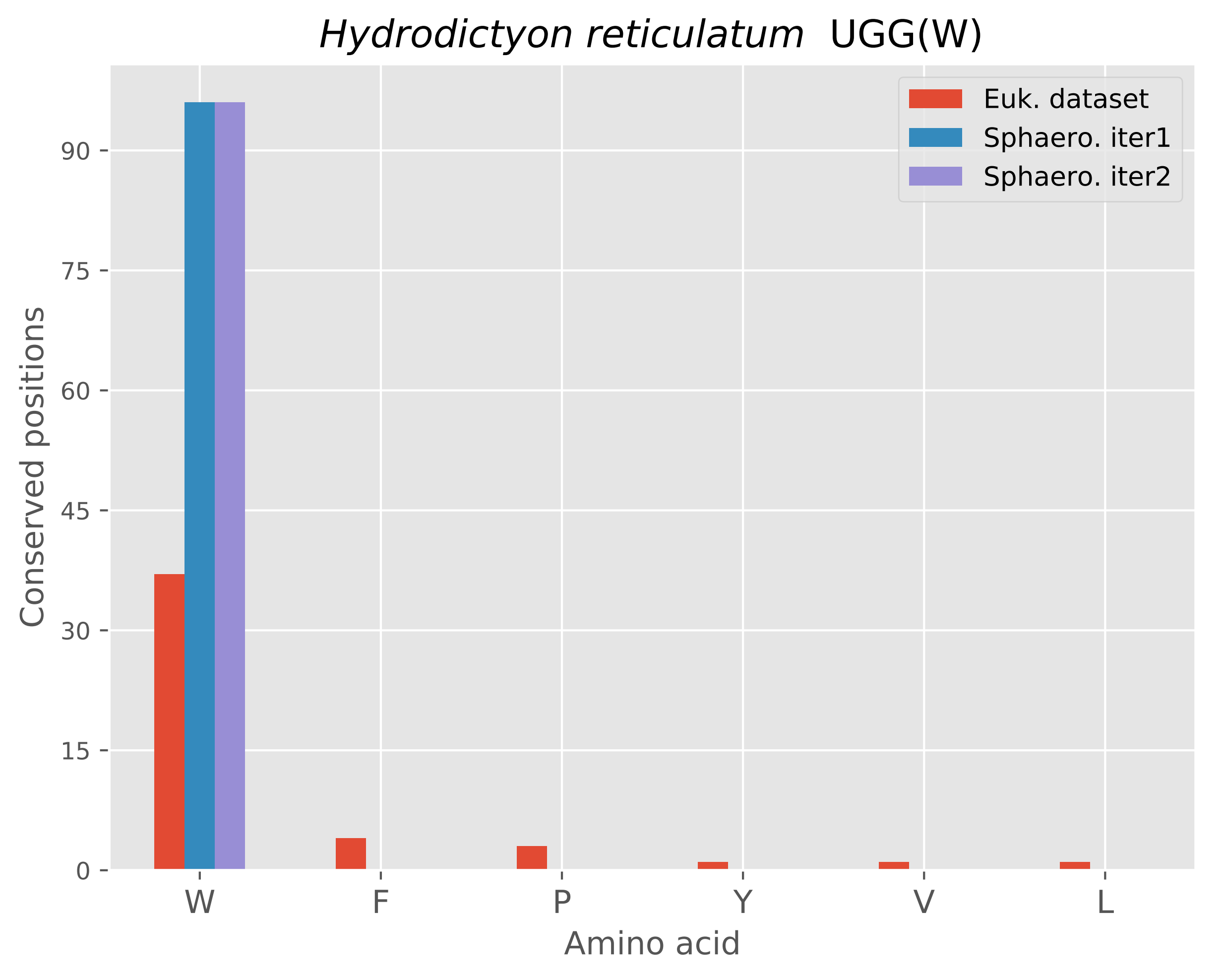

Hydrodictyon reticulatum UGG(W)
Euk. dataset
Sphaero. iter1
Sphaero. iter2
Conserved positions
0
15
30
45
60
75
90
W
F
P
Y
V
L
Amino acid

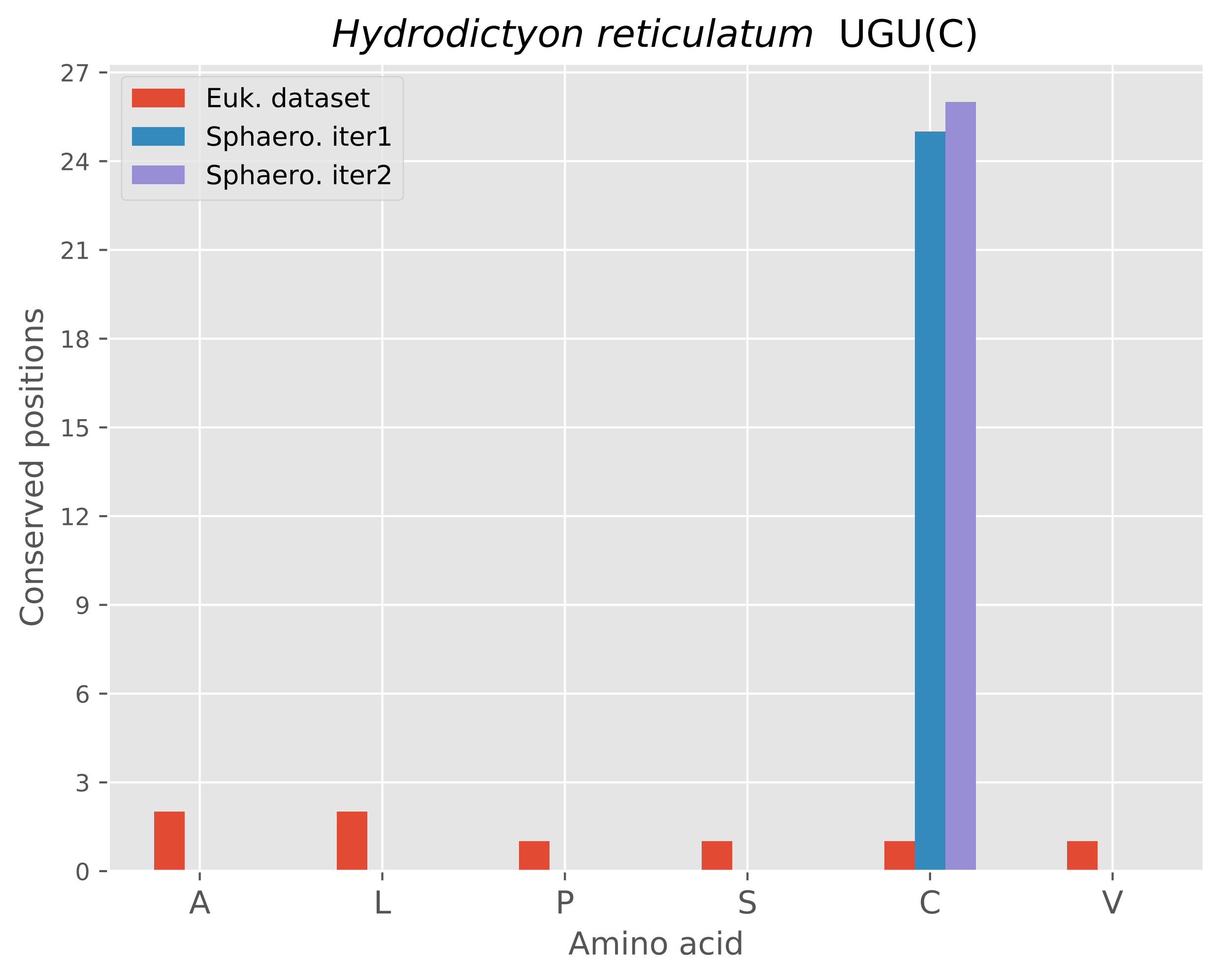

Hydrodictyon reticulatum UGU(C)
Euk. dataset
Sphaero. iter1
Sphaero. iter2
Conserved positions
Amino acid
0
3
6
9
12
15
18
21
24
27
A
L
P
S
C
V

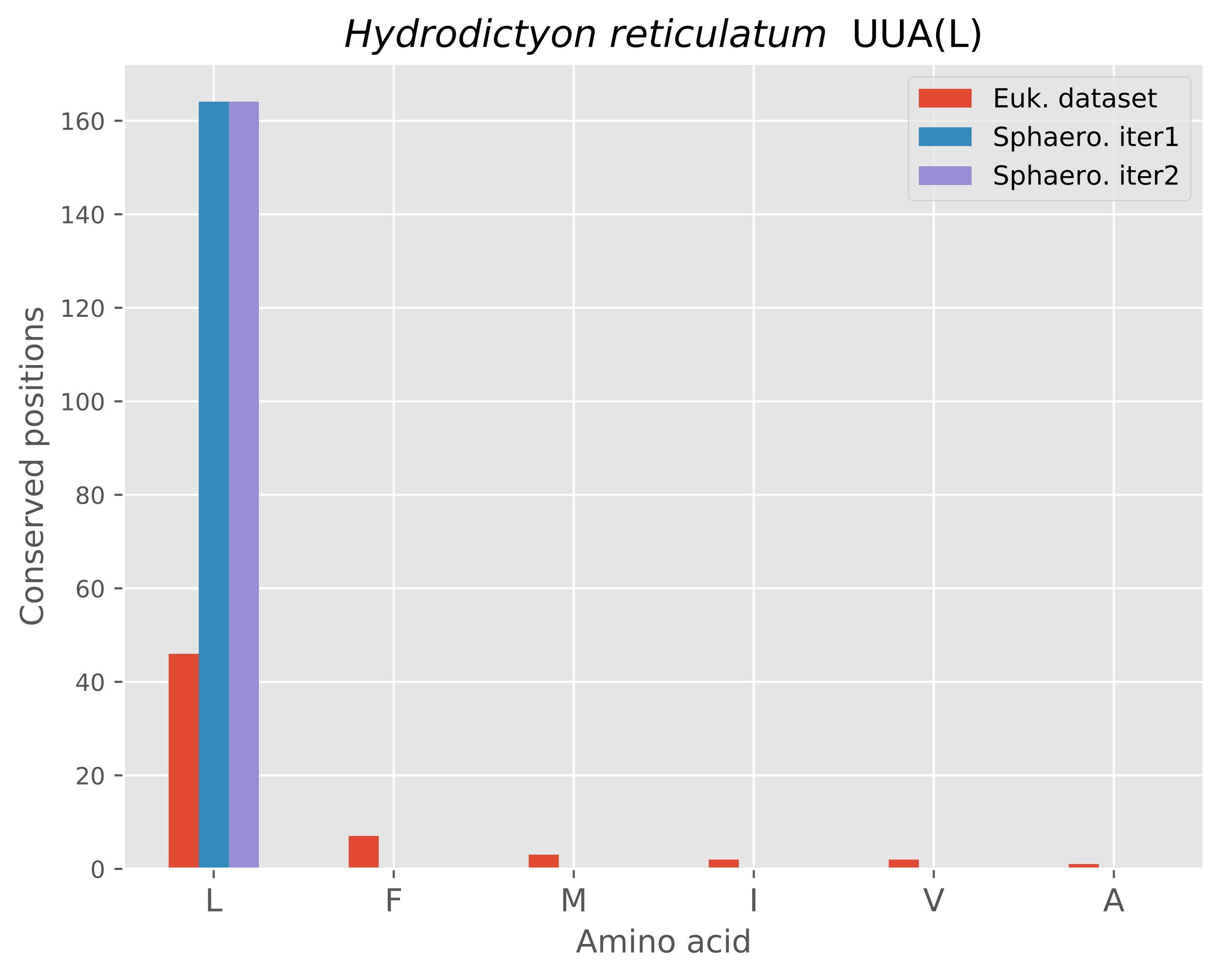

Hydrodictyon reticulatum UUA(L)
Euk. dataset
Sphaero. iter1
Sphaero. iter2
Conserved positions
0
20
40
60
80
100
120
140
160
L
F
M
I
V
A
Amino acid

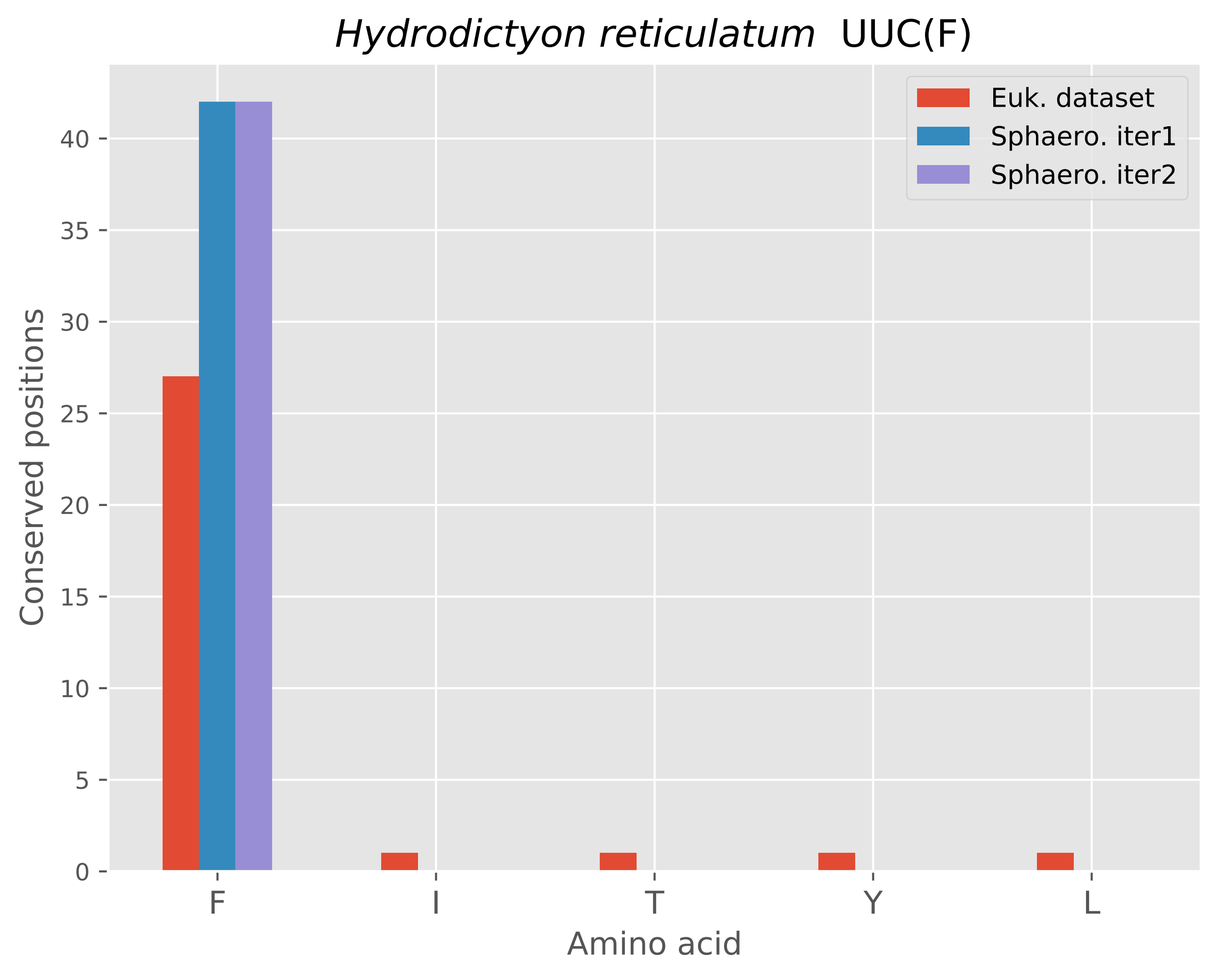
Hydrodictyon reticulatum UUC(F)
Euk. dataset
Sphaero. iter1
Sphaero. iter2
Conserved positions
0
5
10
15
20
25
30
35
40
F
I
T
Y
L
Amino acid

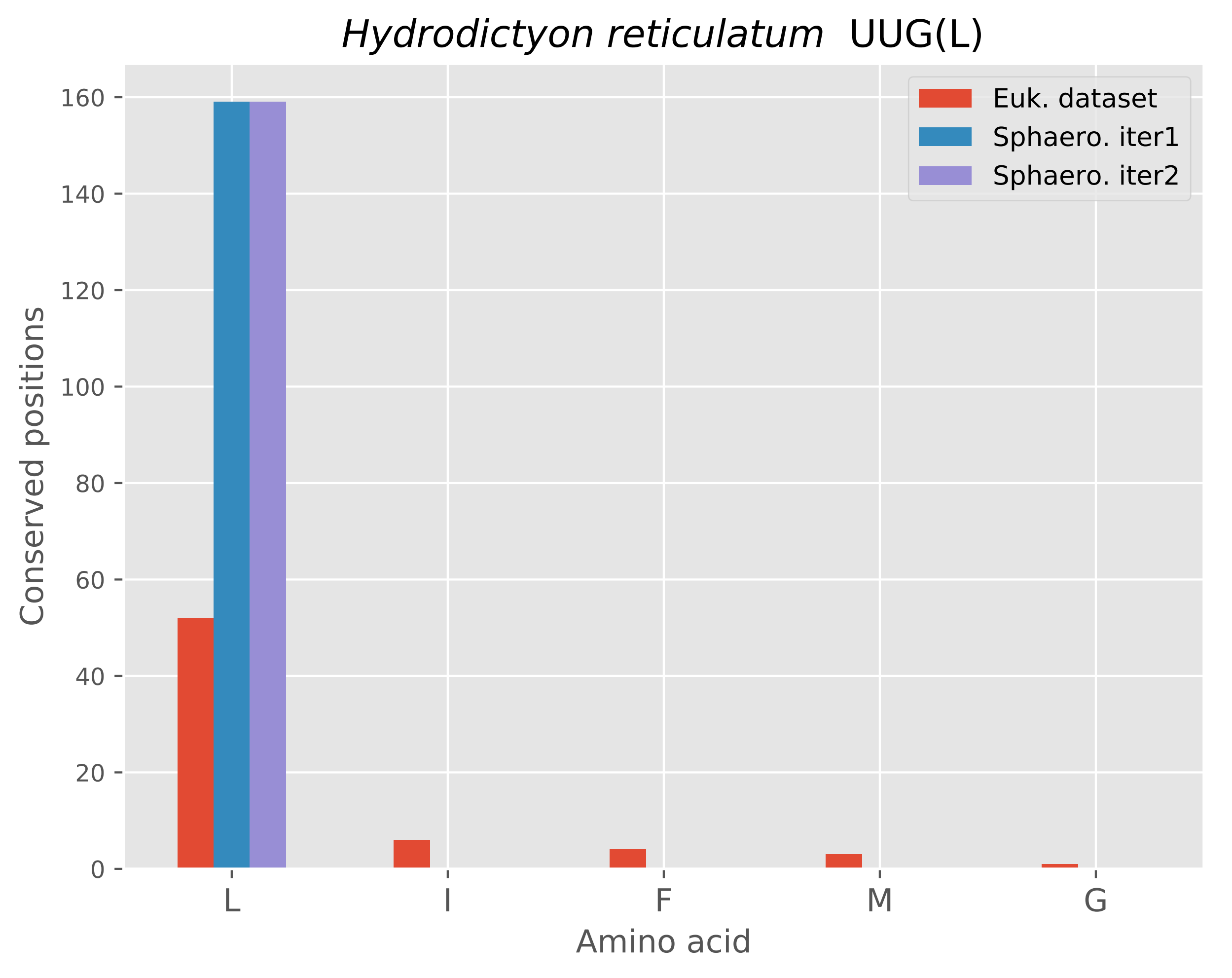

Hydrodictyon reticulatum UUG(L)
Euk. dataset
Sphaero. iter1
Sphaero. iter2
Conserved positions
0
20
40
60
80
100
120
140
160
L
I
F
M
G
Amino acid

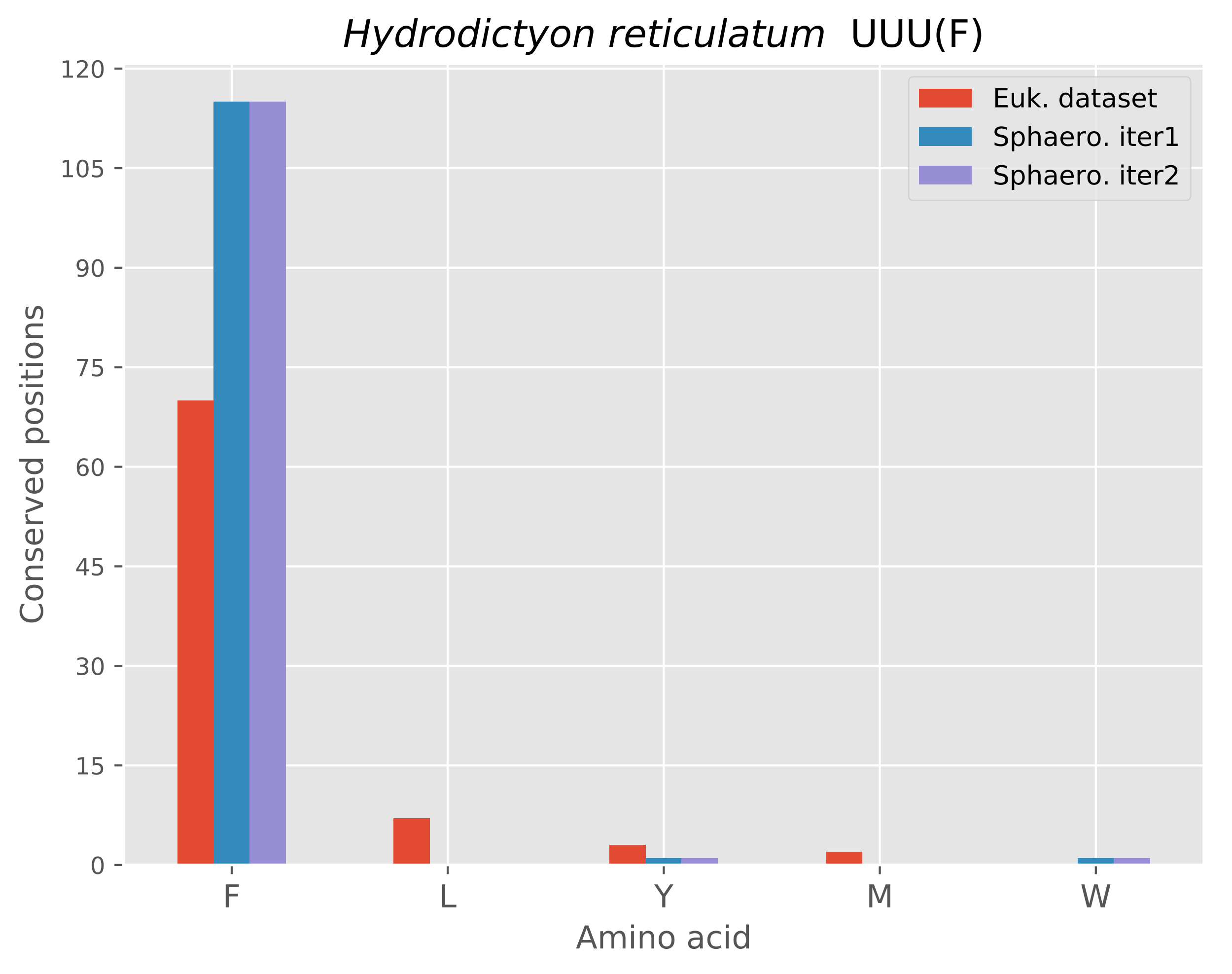

Hydrodictyon reticulatum UUU(F)
Euk. dataset
Sphaero. iter1
Sphaero. iter2
Conserved positions
0
15
30
45
60
75
90
105
120
F
L
Y
M
W
Amino acid

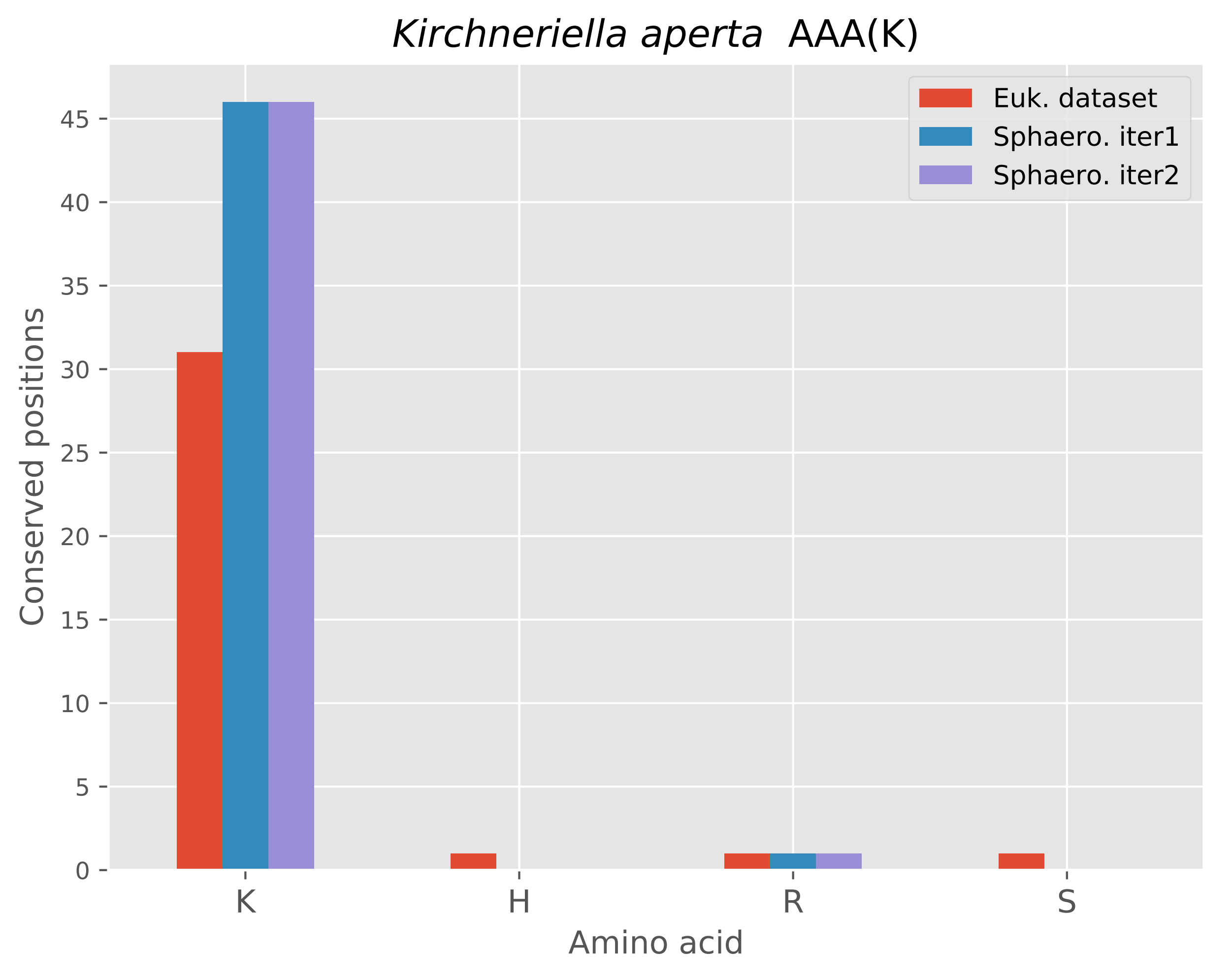

Kirchneriella aperta AAA(K)
Euk. dataset
Sphaero. iter1
Sphaero. iter2
Conserved positions
0
5
10
15
20
25
30
35
40
45
K
H
R
S
Amino acid

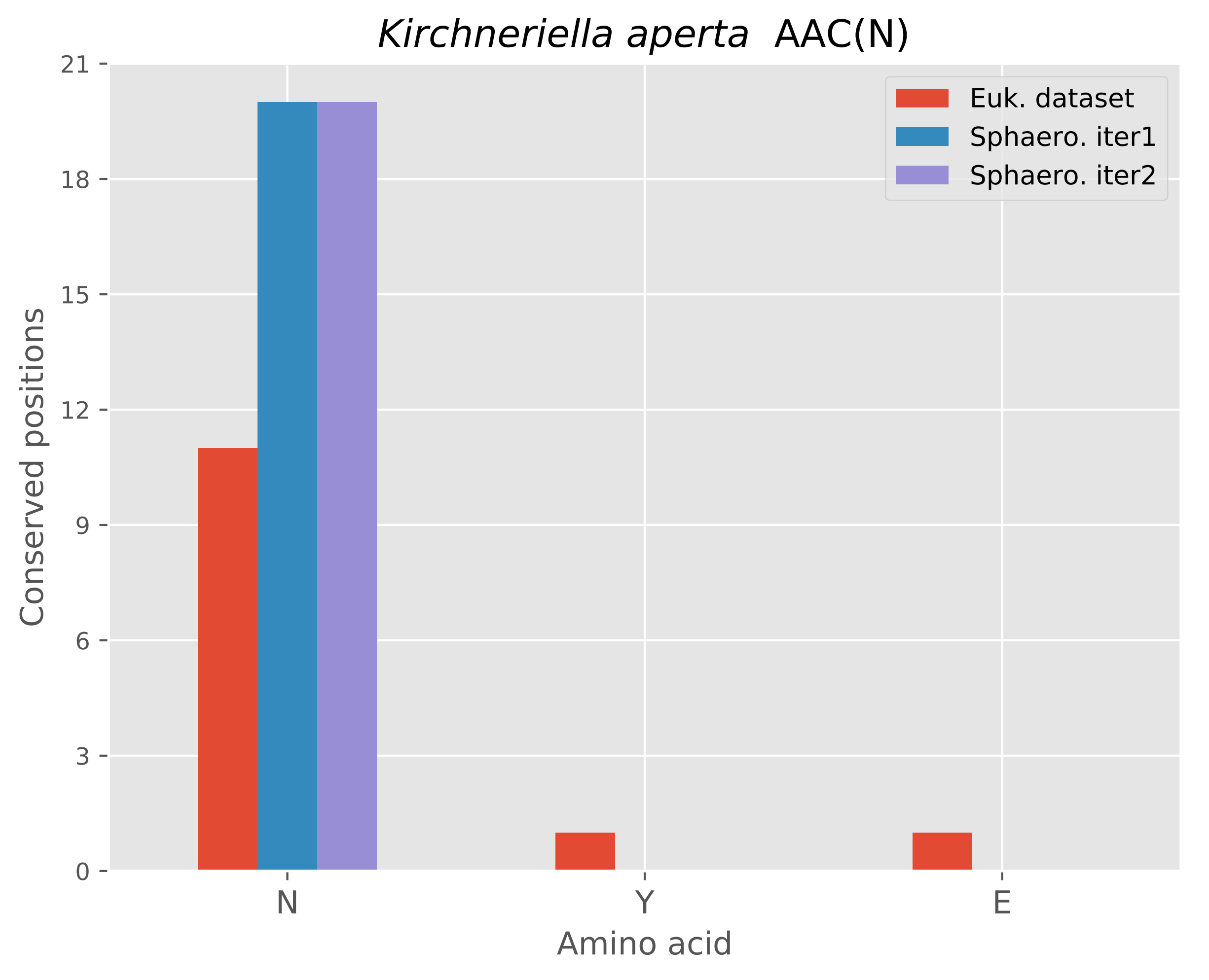

Kirchneriella aperta AAC(N)
Euk. dataset
Sphaero. iter1
Sphaero. iter2
Conserved positions
0
3
6
9
12
15
18
21
N
Y
E
Amino acid

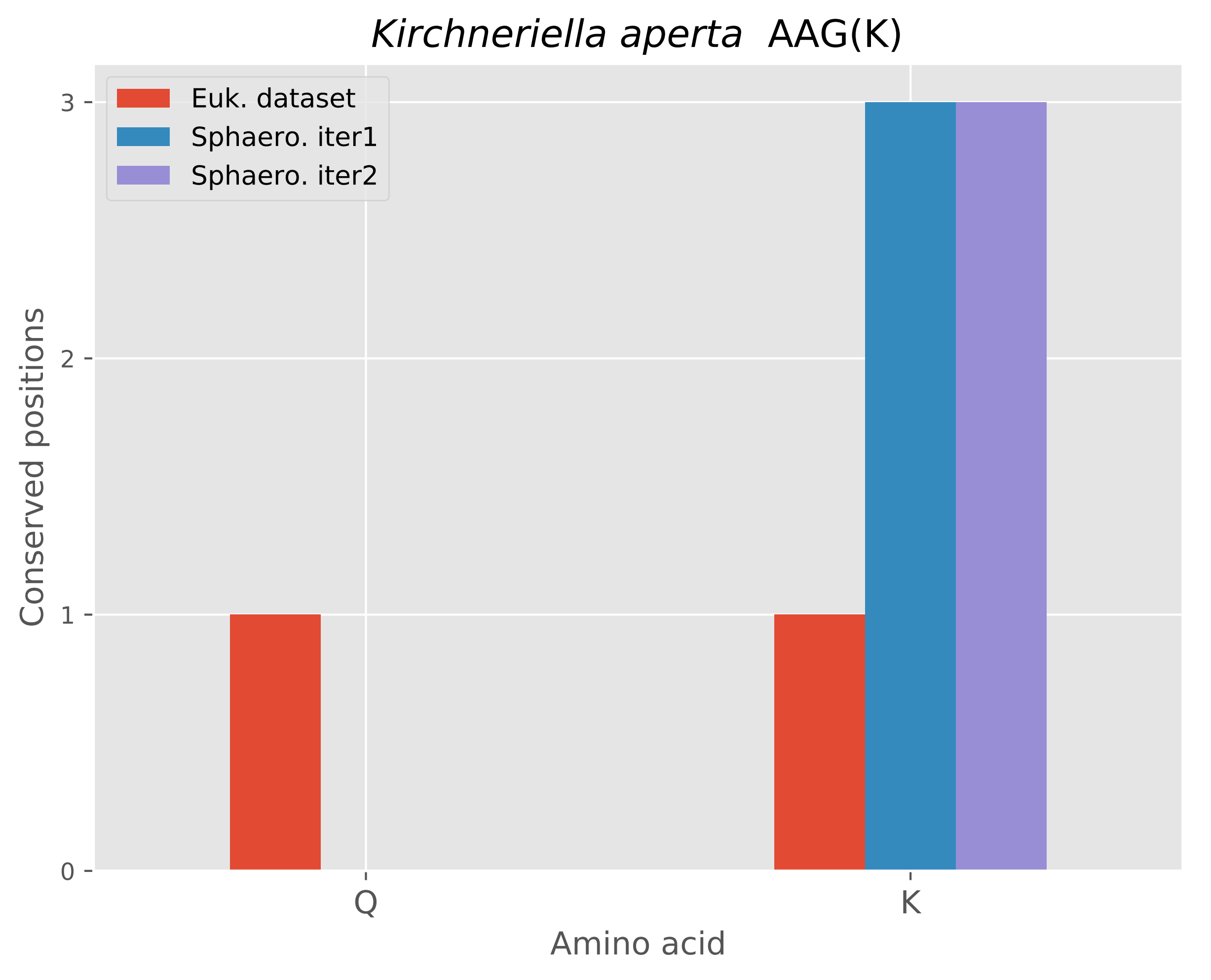

Kirchneriella aperta AAG(K)
Euk. dataset
Sphaero. iter1
Sphaero. iter2
Conserved positions
0
1
2
3
Q
K
Amino acid

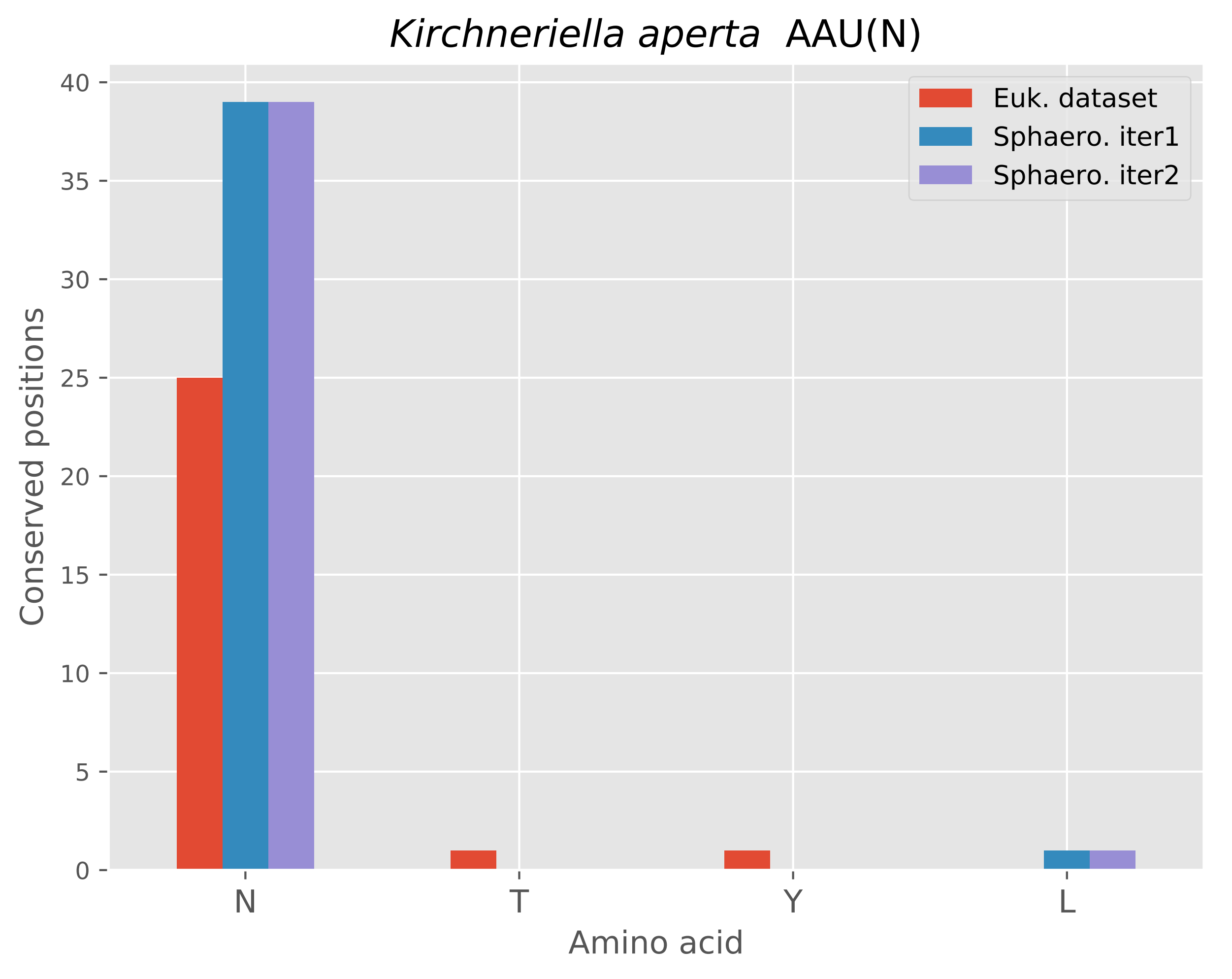

Kirchneriella aperta AAU(N)
Euk. dataset
Sphaero. iter1
Sphaero. iter2
Conserved positions
0
5
10
15
20
25
30
35
40
N
T
Y
L
Amino acid

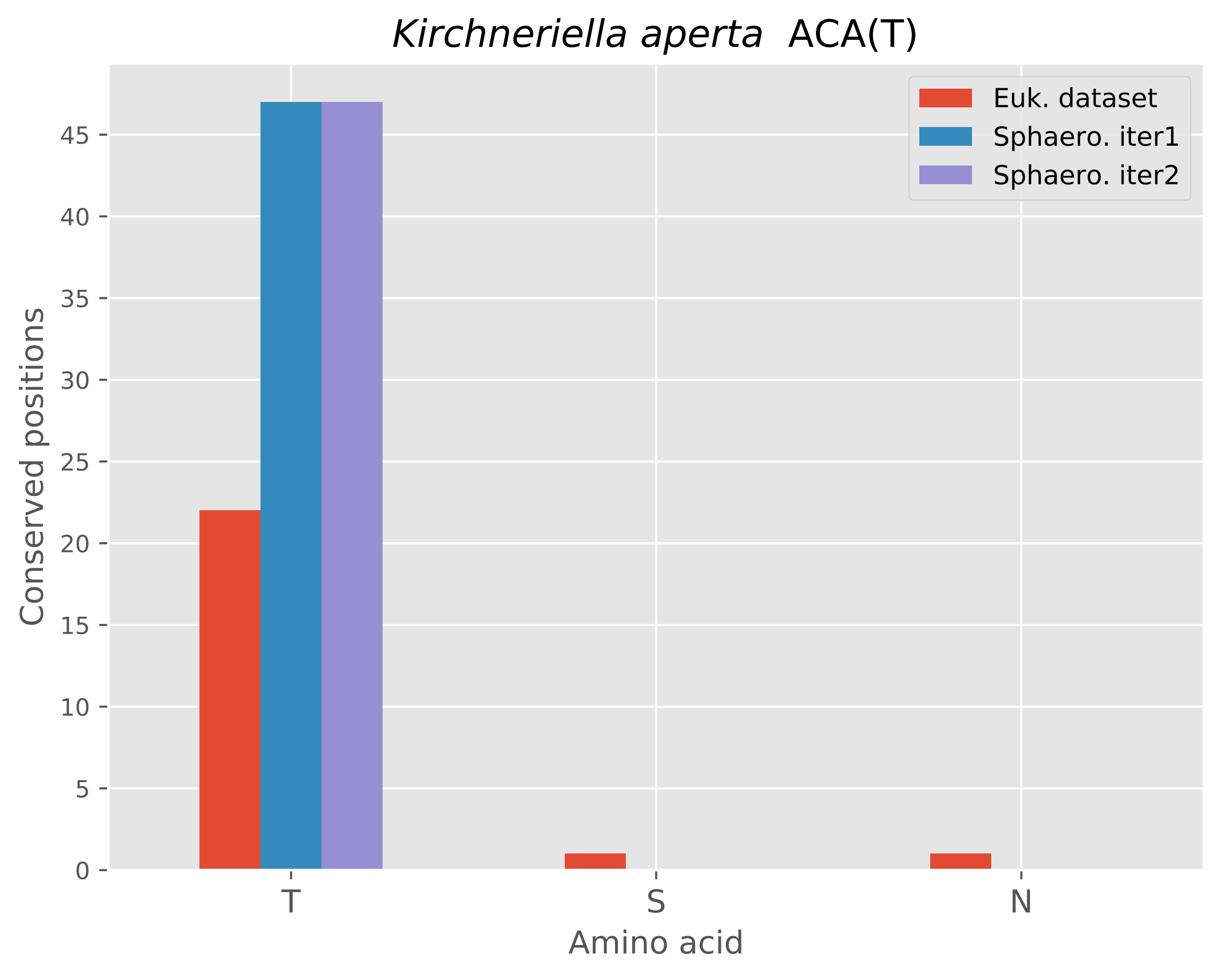

Kirchneriella aperta ACA(T)
Euk. dataset
Sphaero. iter1
Sphaero. iter2
Conserved positions
0
5
10
15
20
25
30
35
40
45
T
S
N
Amino acid

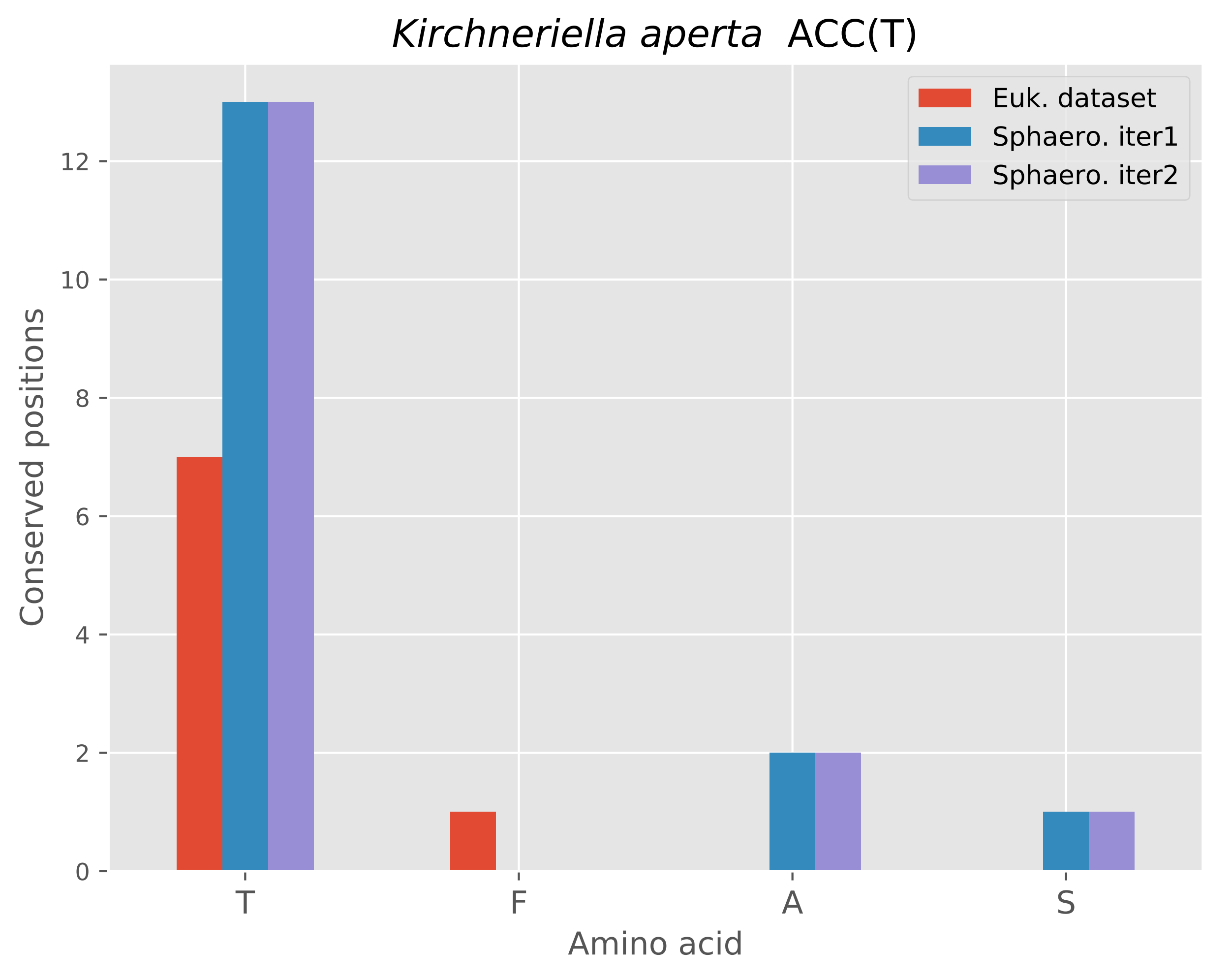

Kirchneriella aperta ACC(T)
Euk. dataset
Sphaero. iter1
Sphaero. iter2
Conserved positions
0
2
4
6
8
10
12
T
F
A
S
Amino acid

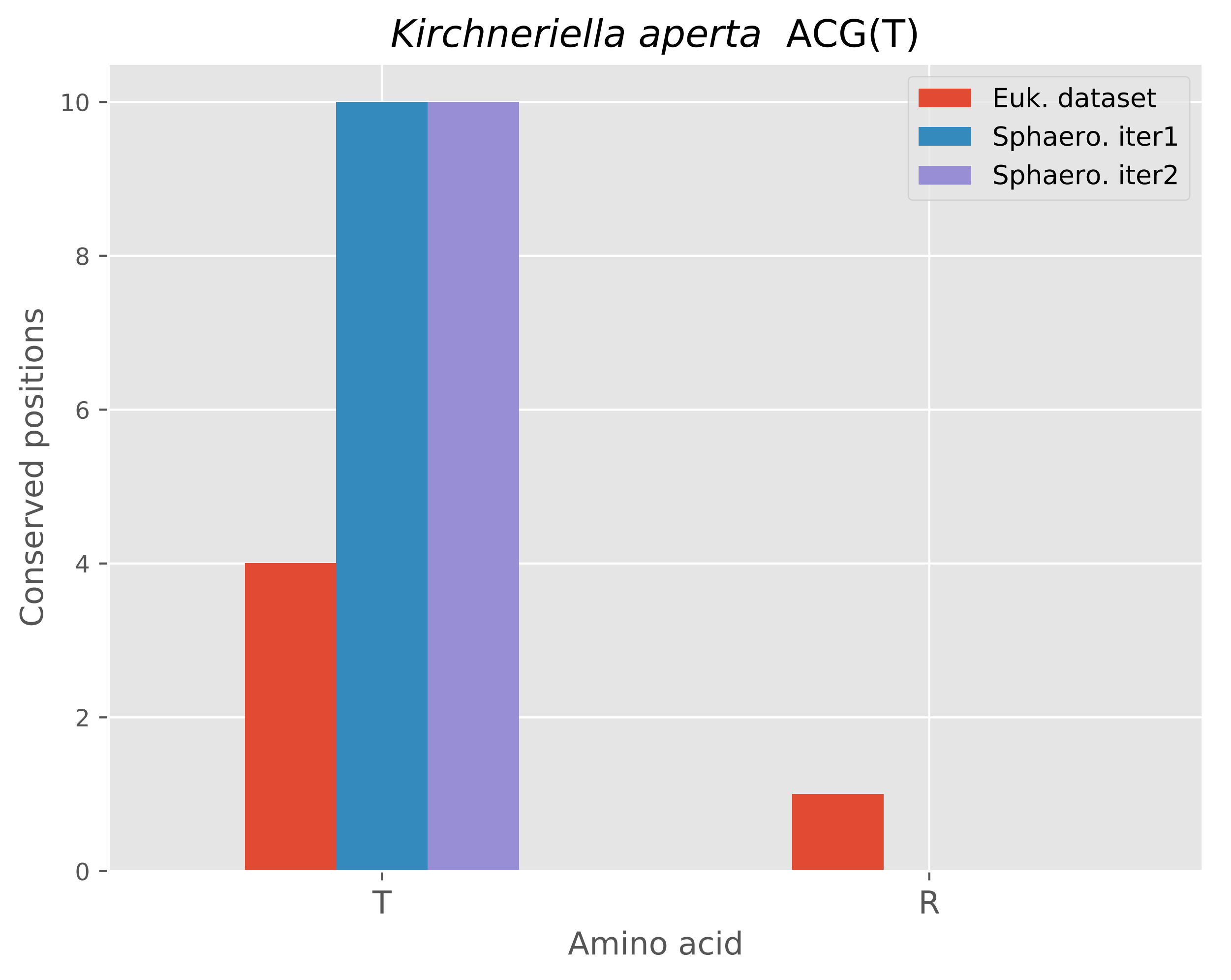

Kirchneriella aperta ACG(T)
Euk. dataset
Sphaero. iter1
Sphaero. iter2
Conserved positions
0
2
4
6
8
10
T
R
Amino acid

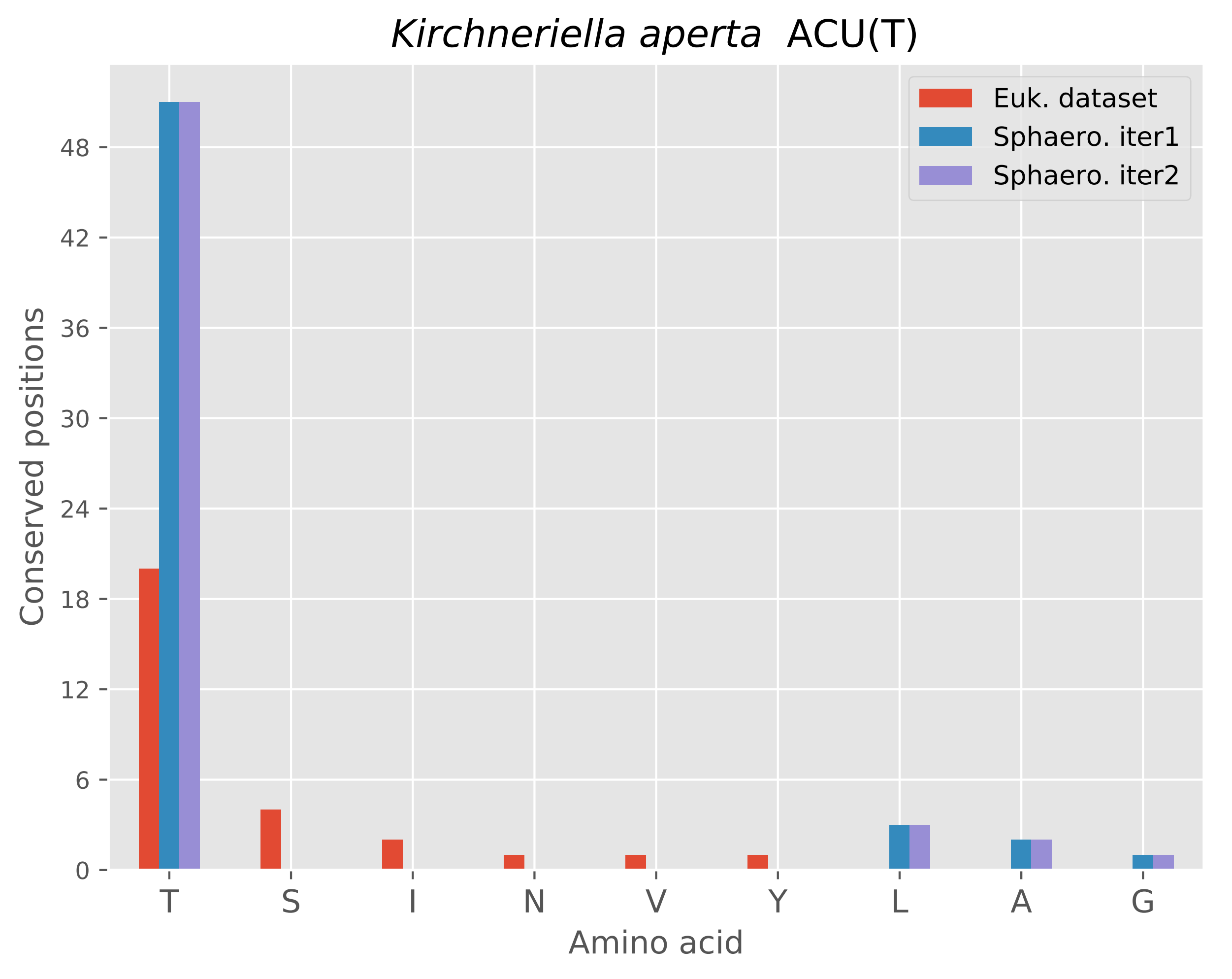

Kirchneriella aperta ACU(T)
Euk. dataset
Sphaero. iter1
Sphaero. iter2
Conserved positions
0
6
12
18
24
30
36
42
48
T
S
I
N
V
Y
L
A
G
Amino acid

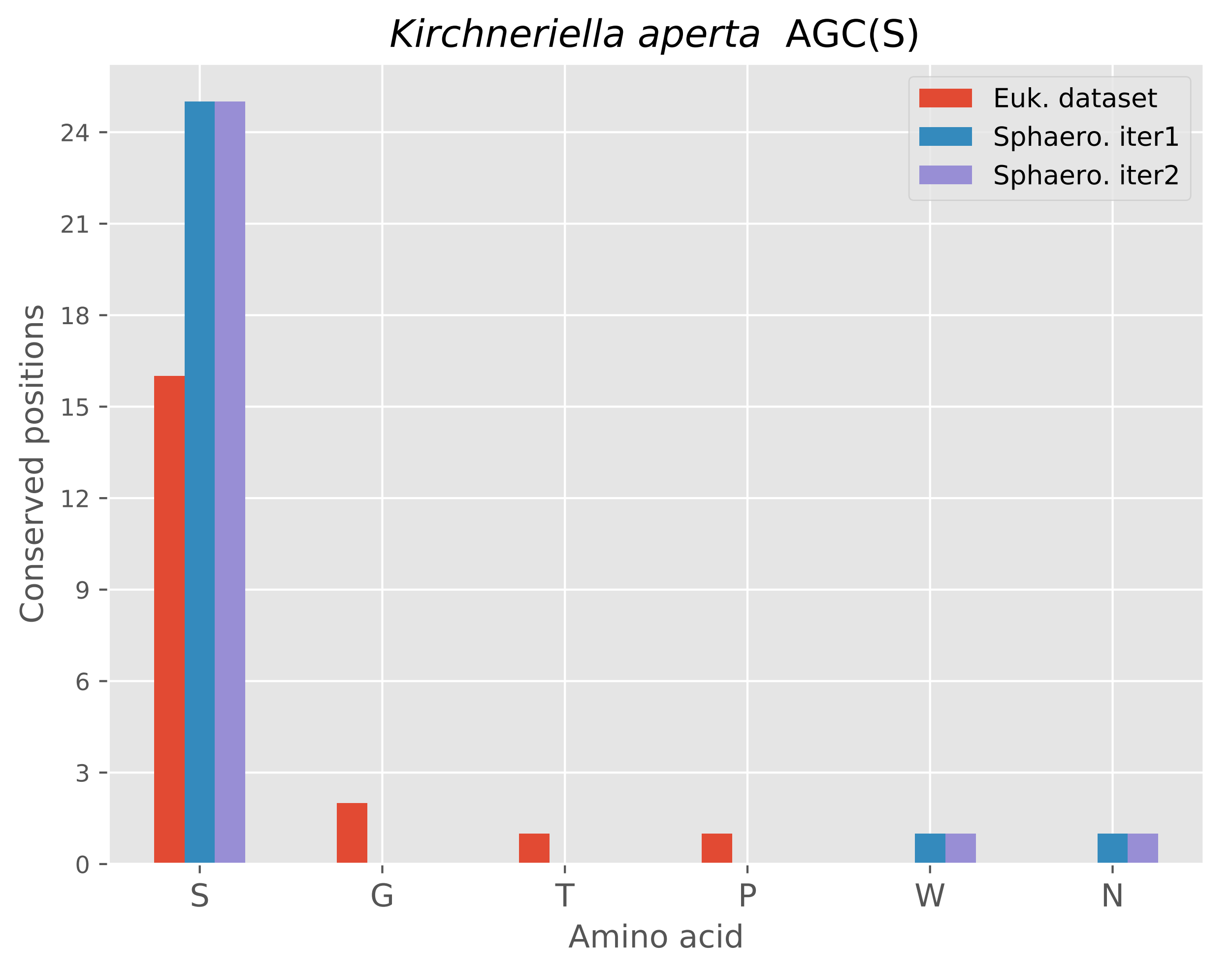
Kirchneriella aperta AGC(S)
Euk. dataset
Sphaero. iter1
Sphaero. iter2
Conserved positions
0
3
6
9
12
15
18
21
24
S
G
T
P
W
N
Amino acid

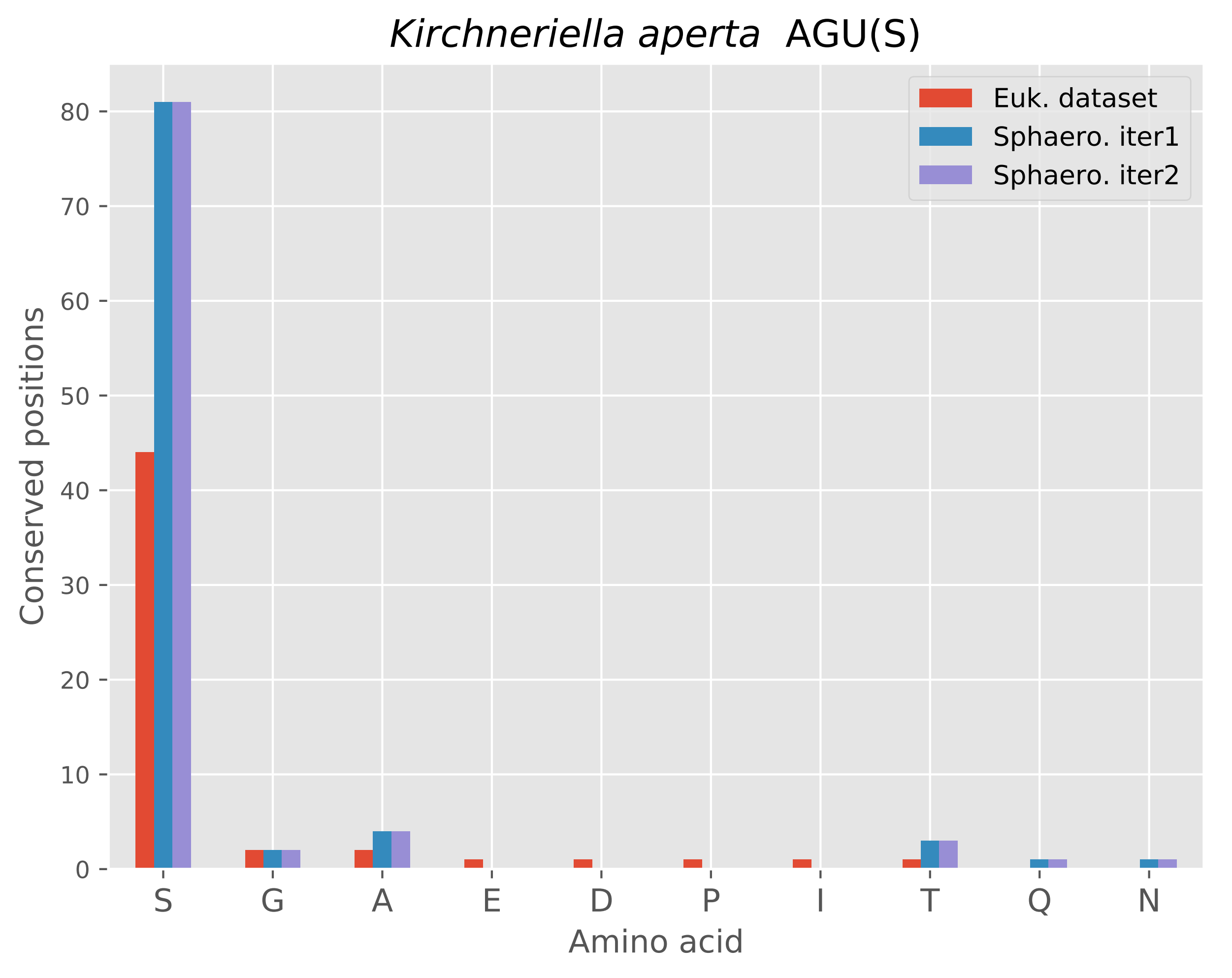
Kirchneriella aperta AGU(S)
Euk. dataset
Sphaero. iter1
Sphaero. iter2
Conserved positions
0
10
20
30
40
50
60
70
80
S
G
A
E
D
P
I
T
Q
N
Amino acid

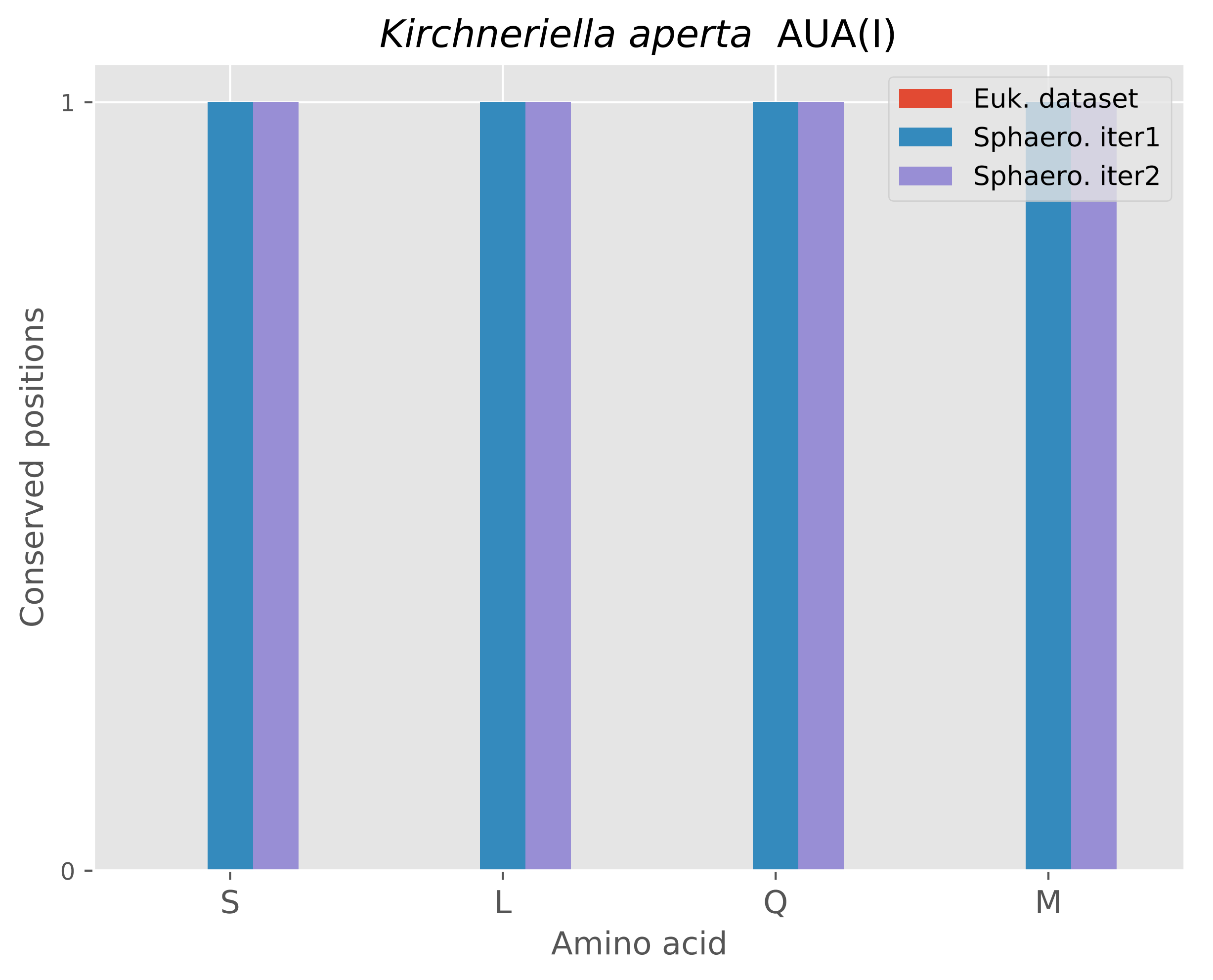

Kirchneriella aperta AUA(I)
Euk. dataset
Sphaero. iter1
Sphaero. iter2
Conserved positions
1
0
S
L
Q
M
Amino acid

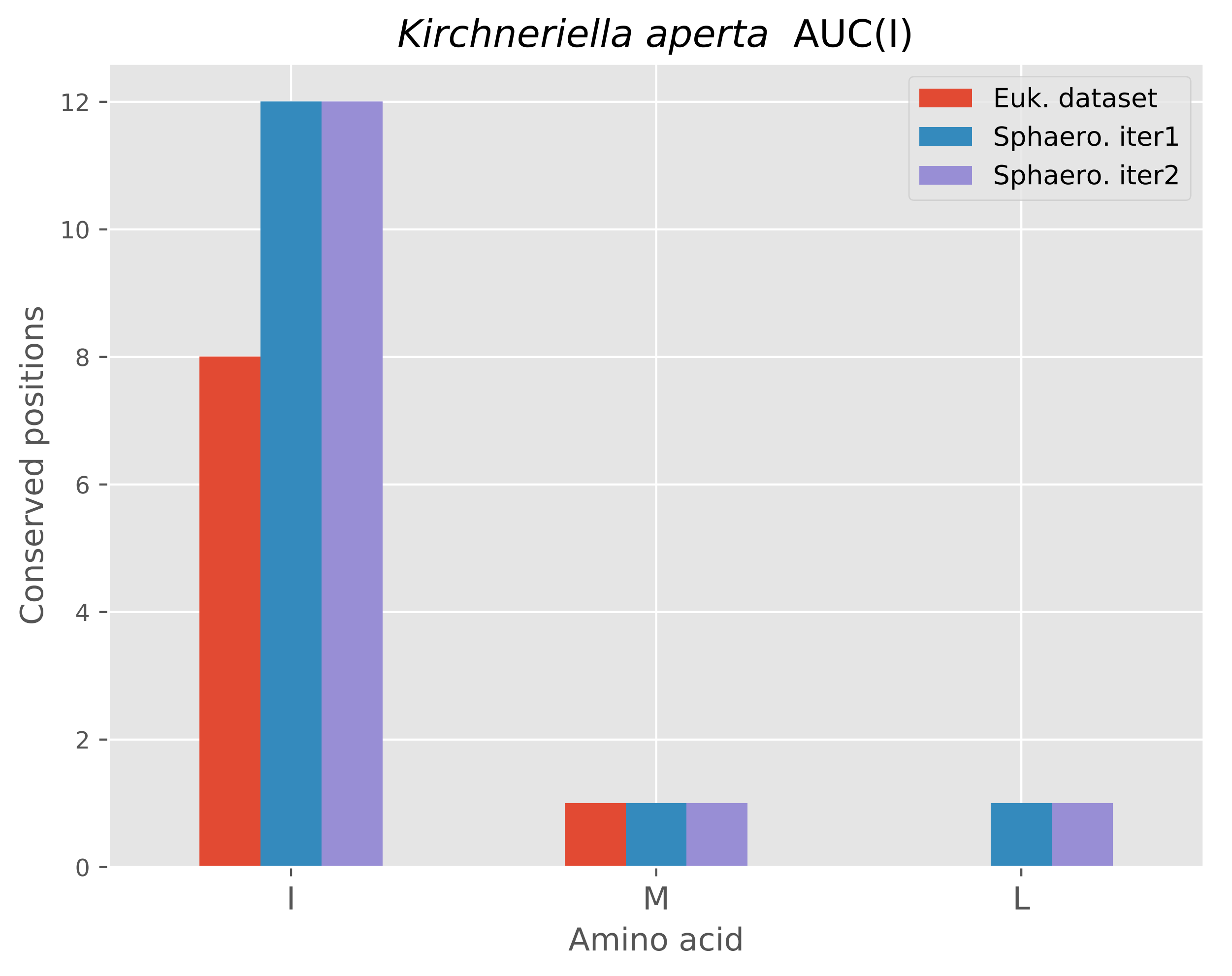

Kirchneriella aperta AUC(I)
Euk. dataset
Sphaero. iter1
Sphaero. iter2
Conserved positions
0
2
4
6
8
10
12
I
M
L
Amino acid

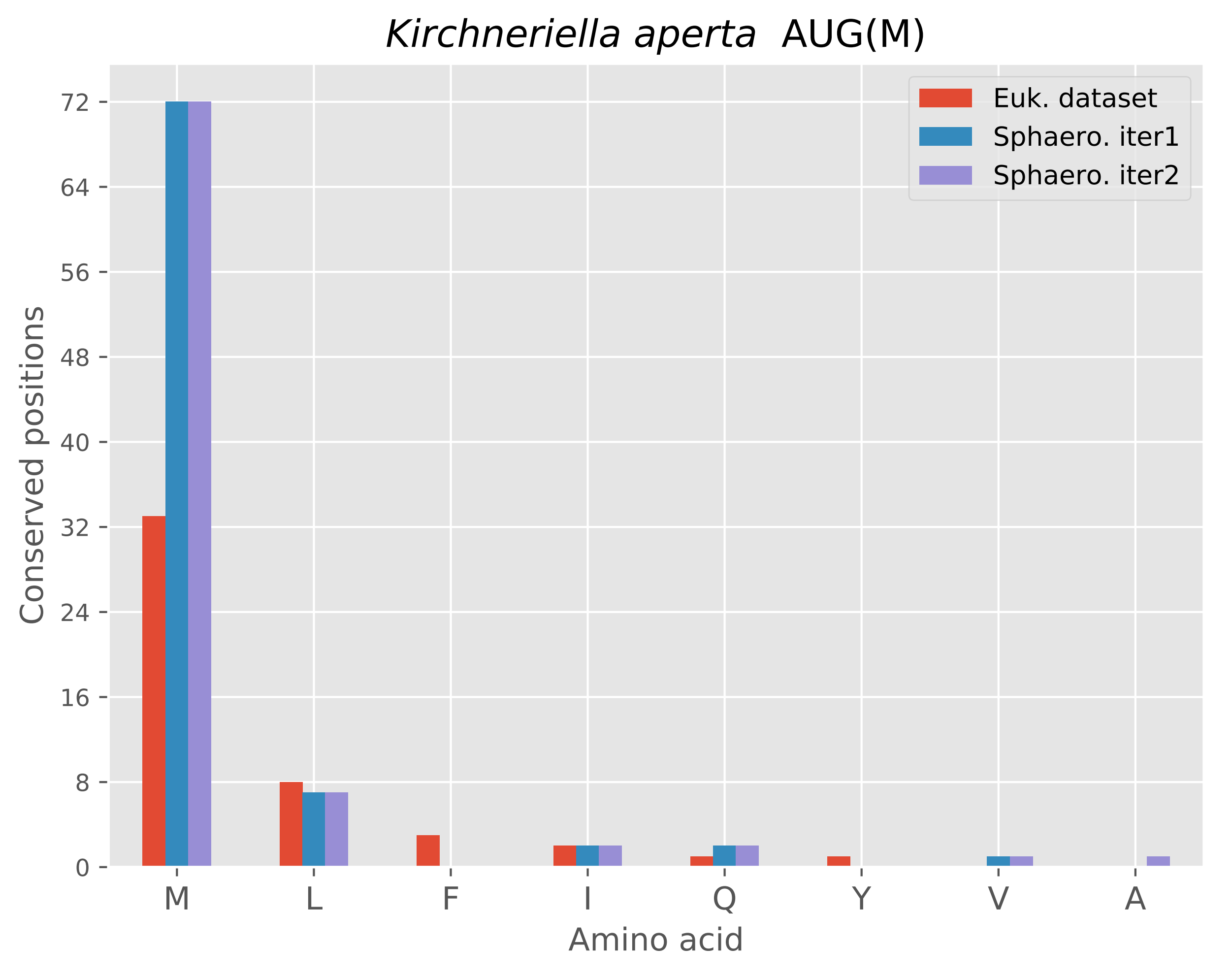

Kirchneriella aperta AUG(M)
Euk. dataset
Sphaero. iter1
Sphaero. iter2
Conserved positions
0
8
16
24
32
40
48
56
64
72
M
L
F
I
Q
Y
V
A
Amino acid

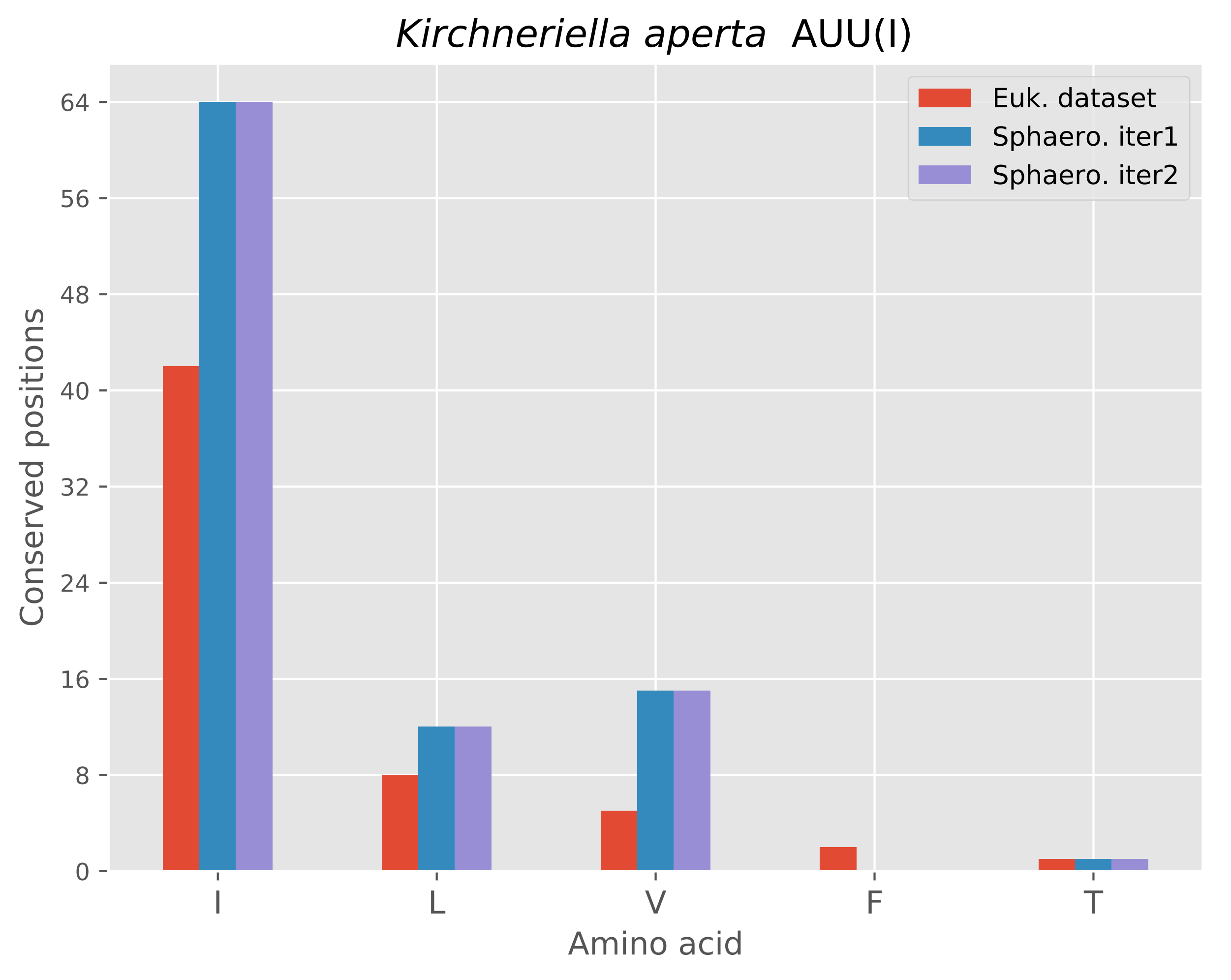
Kirchneriella aperta AUU(I)
Euk. dataset
Sphaero. iter1
Sphaero. iter2
Conserved positions
0
8
16
24
32
40
48
56
64
I
L
V
F
T
Amino acid

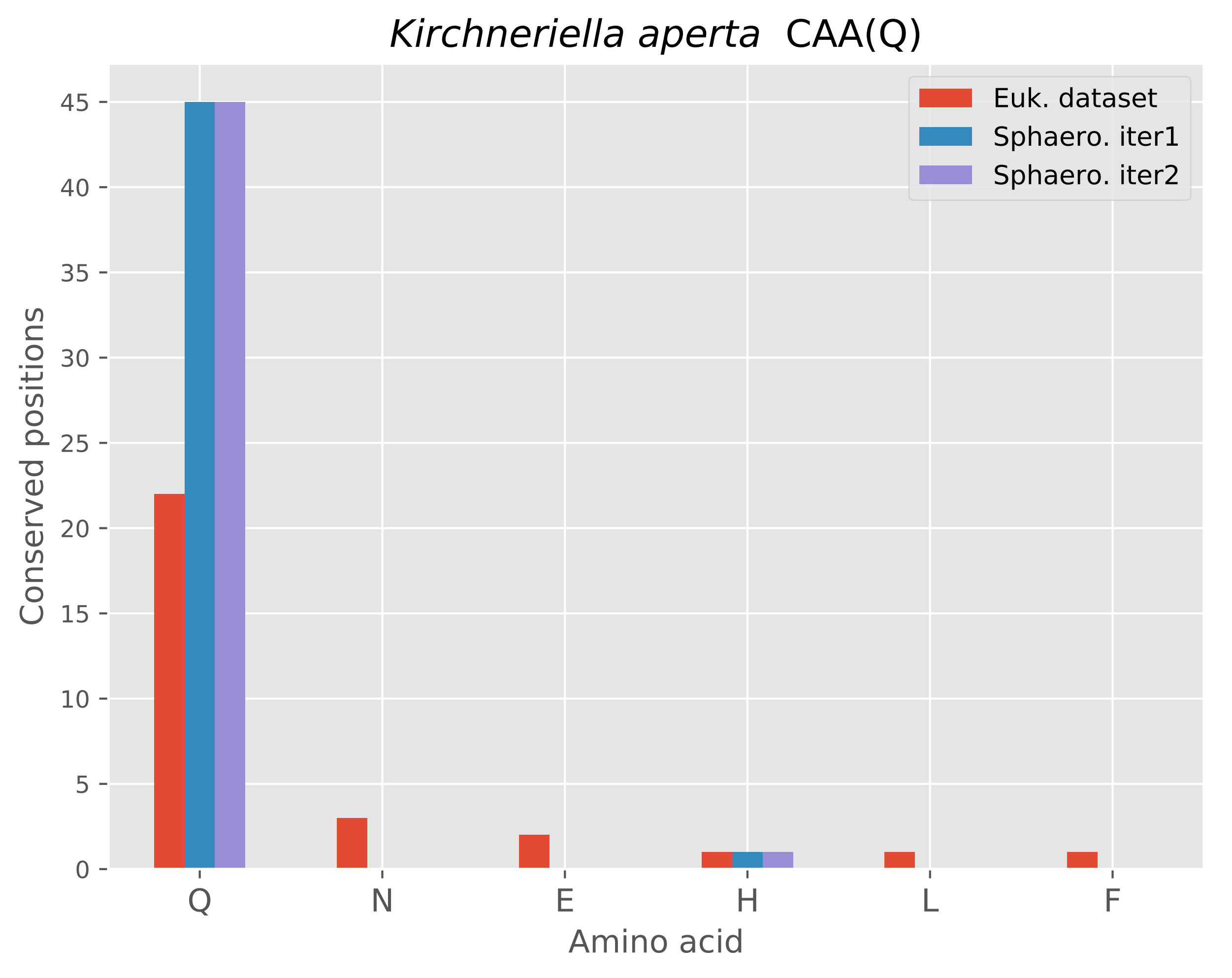

Kirchneriella aperta CAA(Q)
Euk. dataset
Sphaero. iter1
Sphaero. iter2
Conserved positions
0
5
10
15
20
25
30
35
40
45
Q
N
E
H
L
F
Amino acid

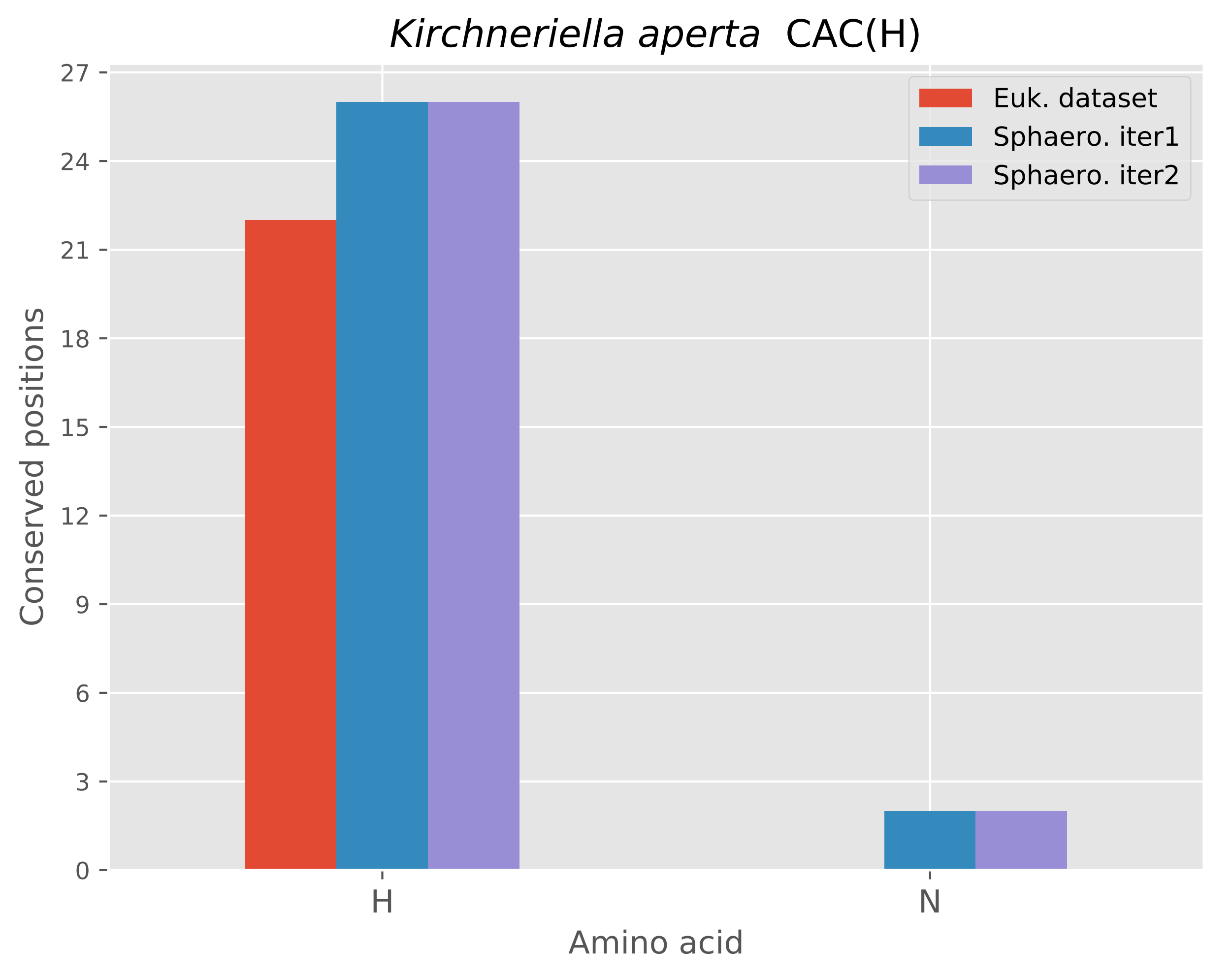

Kirchneriella aperta CAC(H)
Conserved positions
0
3
6
9
12
15
18
21
24
27
Euk. dataset
Sphaero. iter1
Sphaero. iter2
H
N
Amino acid

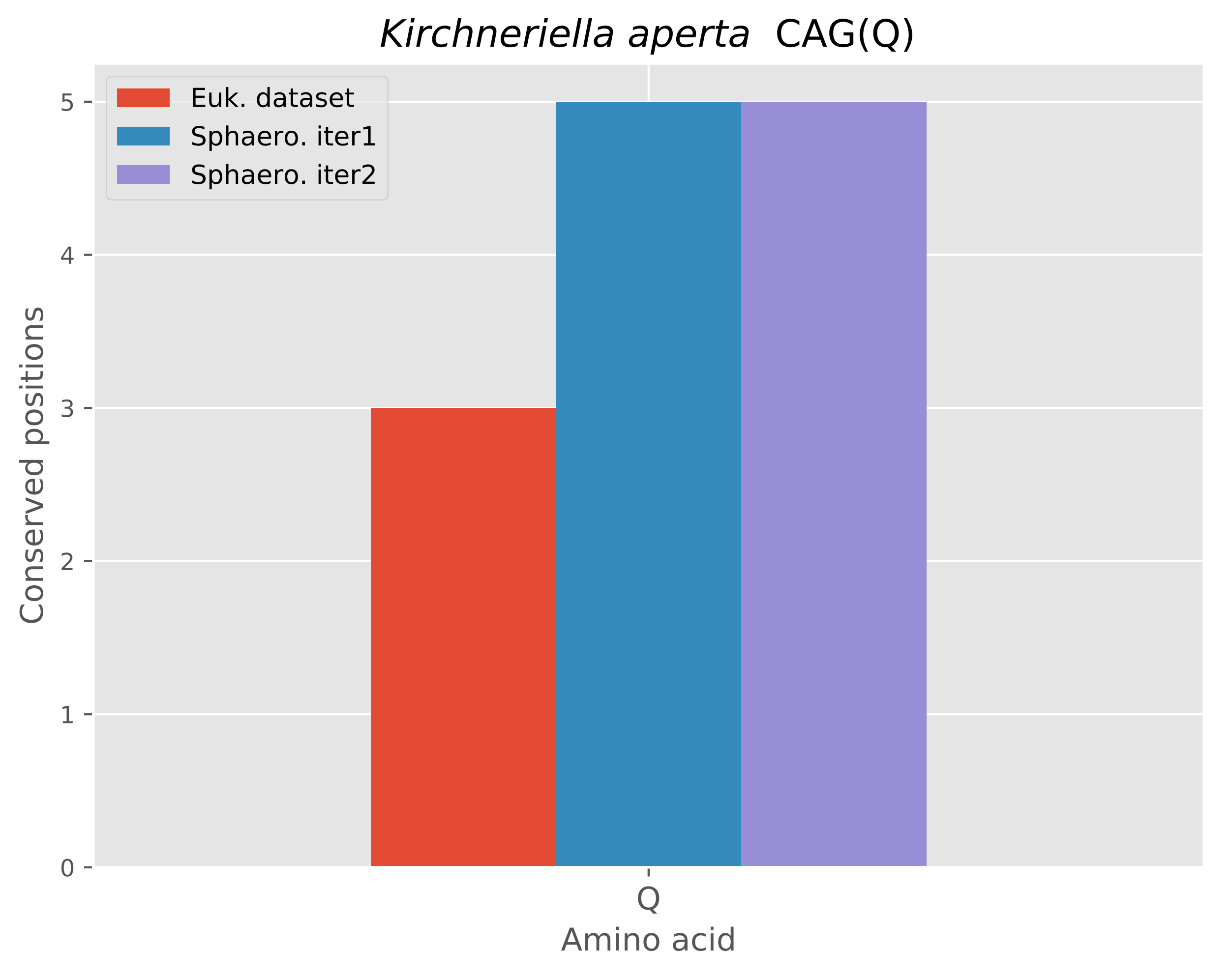

Kirchneriella aperta CAG(Q)
Euk. dataset
Sphaero. iter1
Sphaero. iter2
Conserved positions
0
1
2
3
4
5
Q
Amino acid

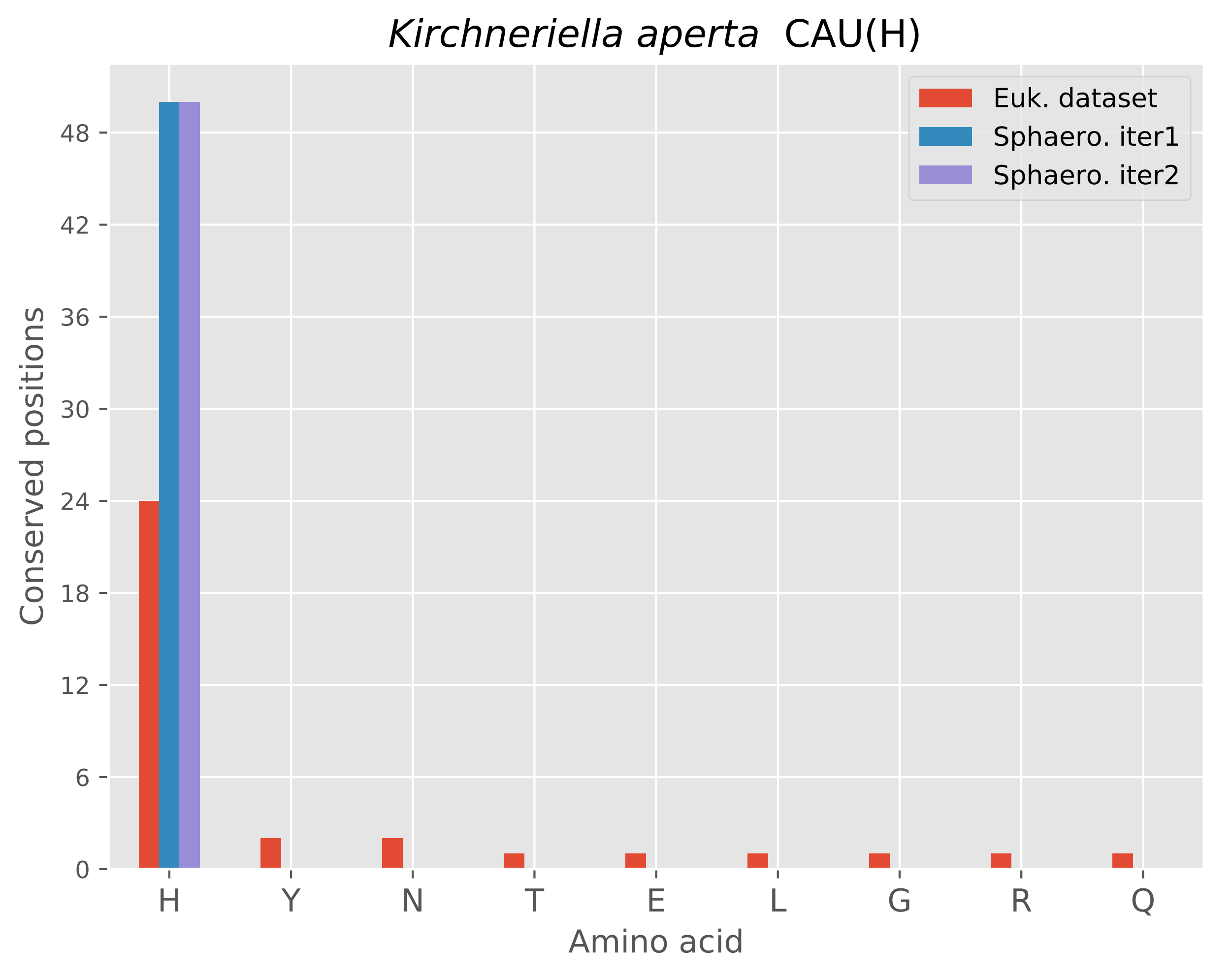

Kirchneriella aperta CAU(H)
Euk. dataset
Sphaero. iter1
Sphaero. iter2
Conserved positions
0
6
12
18
24
30
36
42
48
H
Y
N
T
E
L
G
R
Q
Amino acid

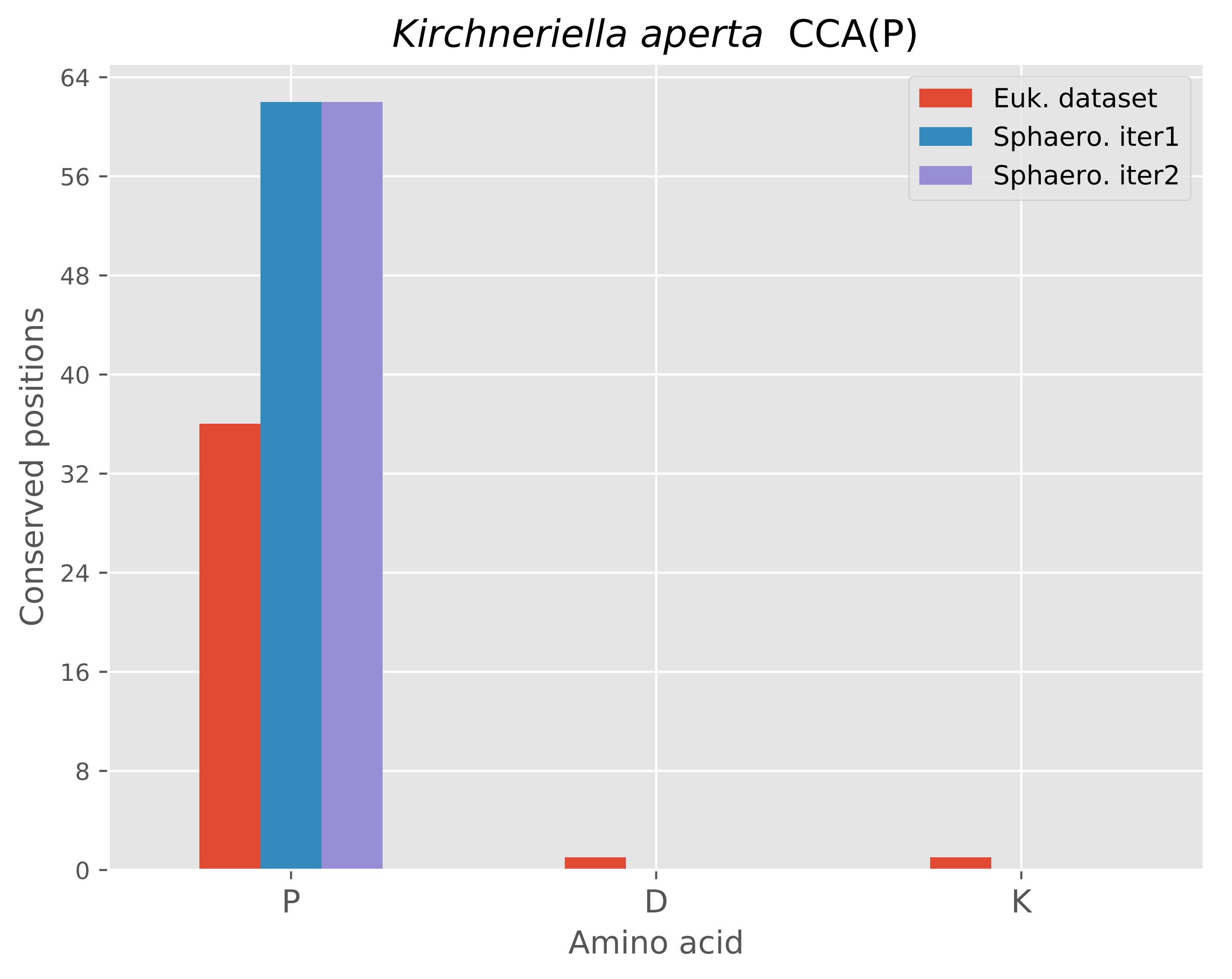

Kirchneriella aperta CCA(P)
Euk. dataset
Sphaero. iter1
Sphaero. iter2
Conserved positions
Amino acid
P
D
K
0
8
16
24
32
40
48
56
64

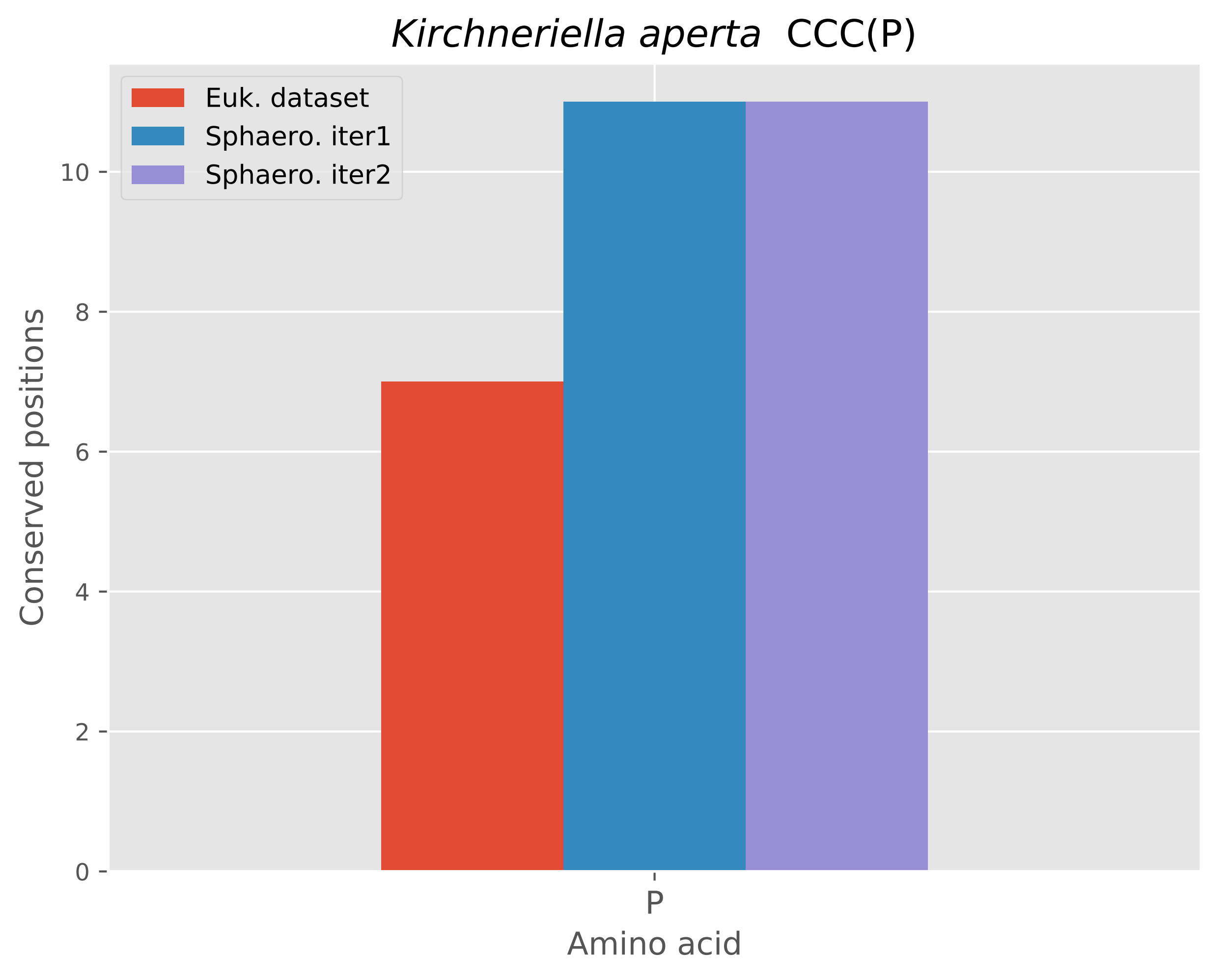

Kirchneriella aperta CCC(P)
Euk. dataset
Sphaero. iter1
Sphaero. iter2
Conserved positions
0
2
4
6
8
10
P
Amino acid

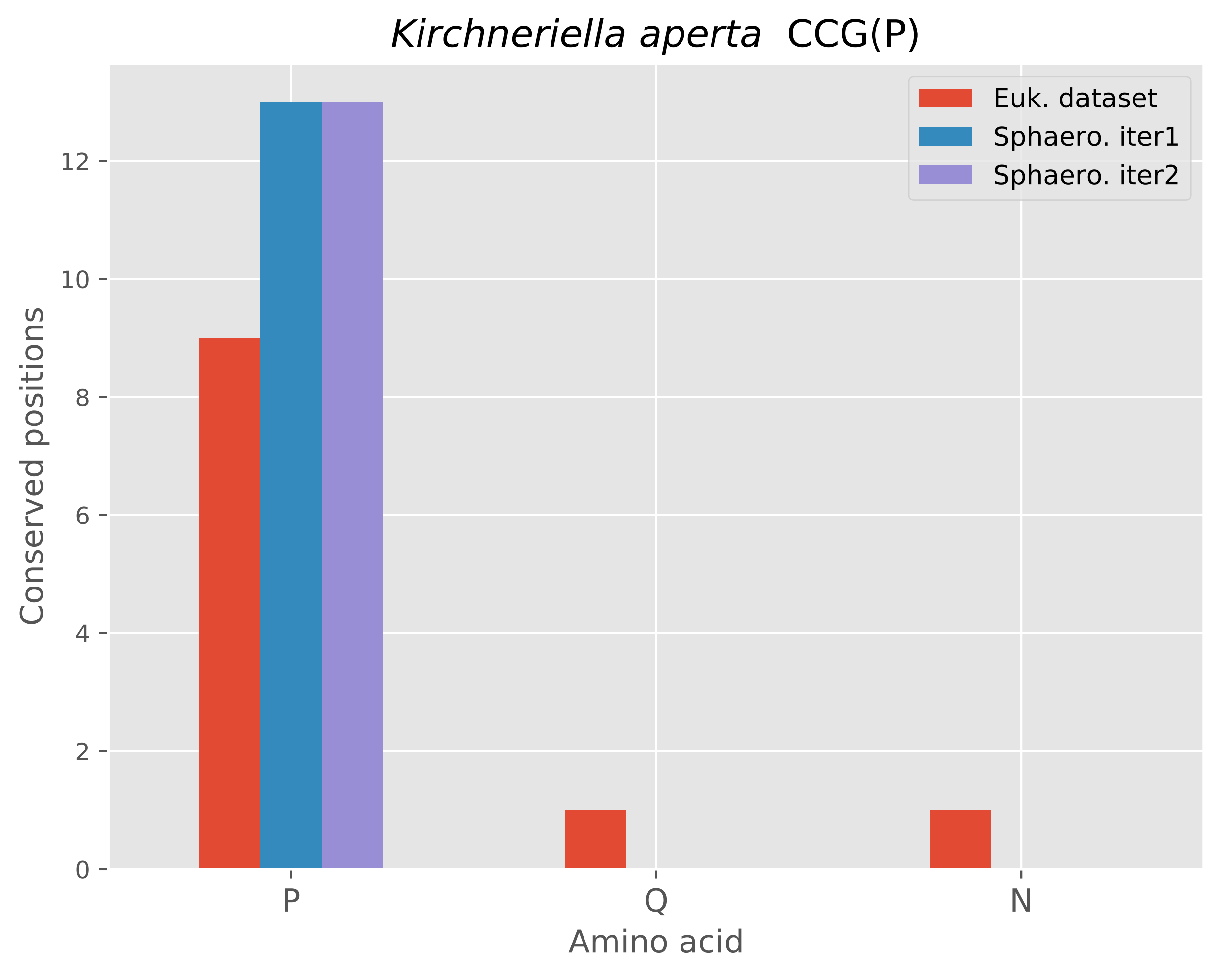

Kirchneriella aperta CCG(P)
Euk. dataset
Sphaero. iter1
Sphaero. iter2
Conserved positions
0
2
4
6
8
10
12
P
Q
N
Amino acid

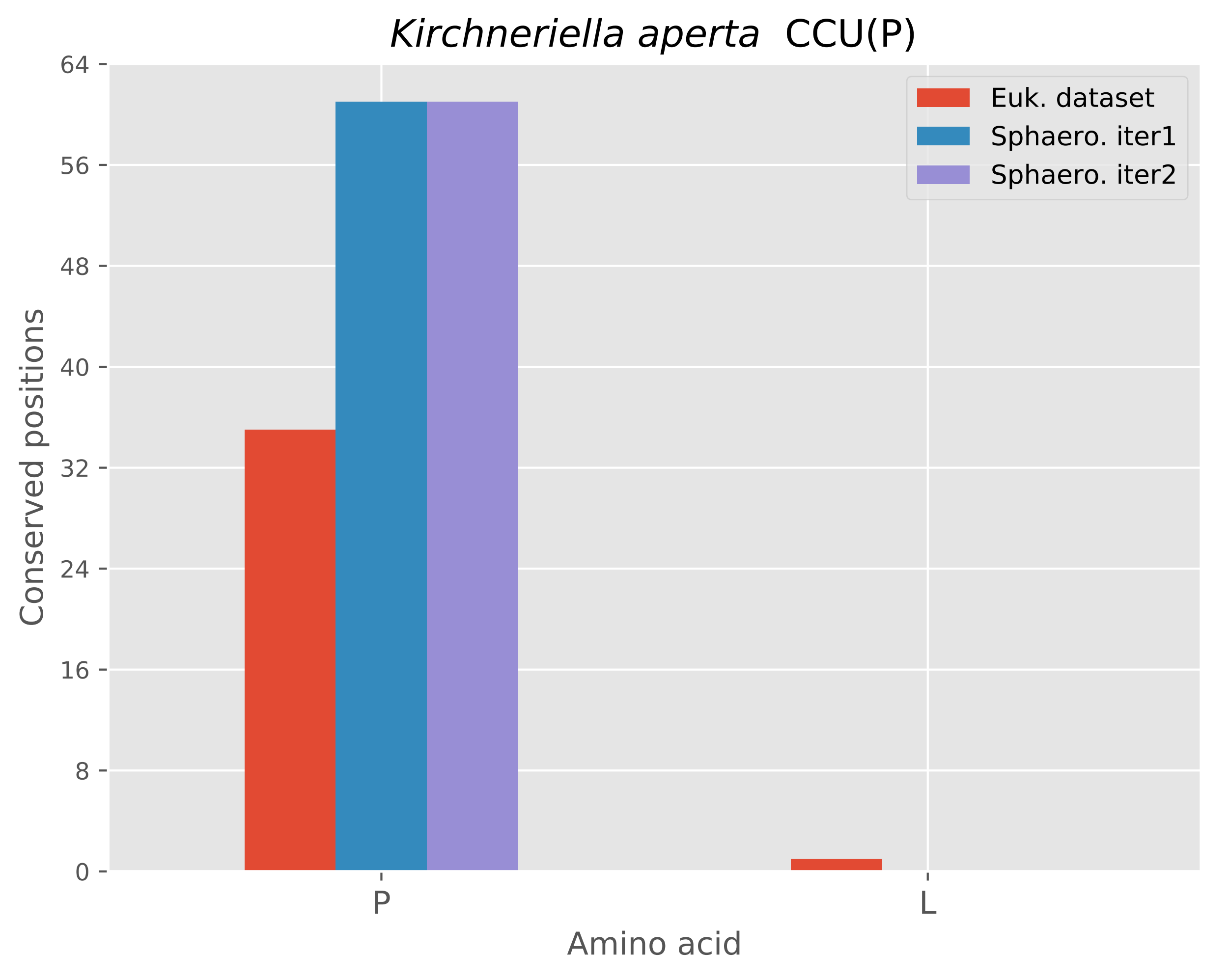

Kirchneriella aperta CCU(P)
Conserved positions
Amino acid
0
8
16
24
32
40
48
56
64
P
L
Euk. dataset
Sphaero. iter1
Sphaero. iter2

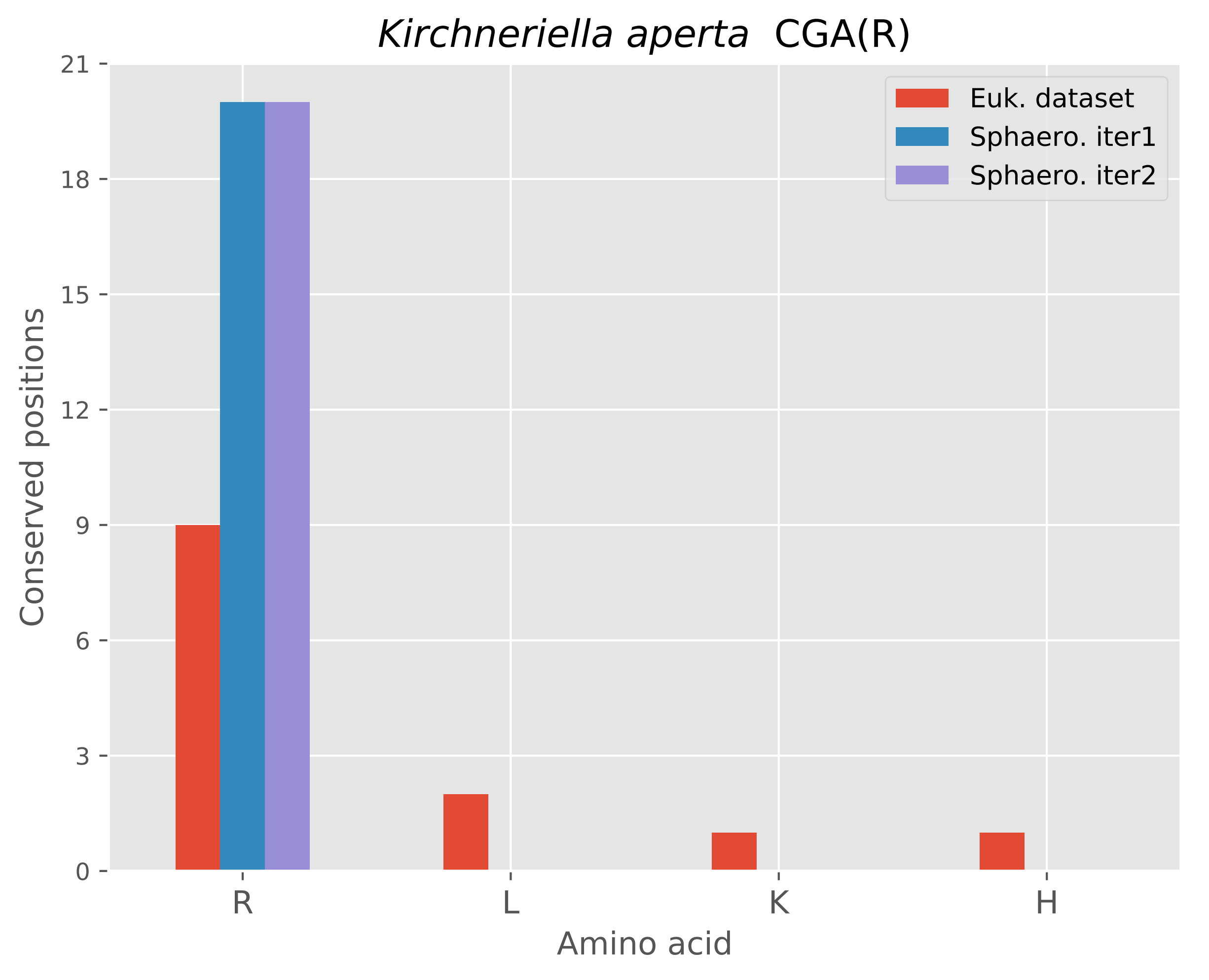
Kirchneriella aperta CGA(R)
Conserved positions
0
3
6
9
12
15
18
21
R
L
K
H
Amino acid
Euk. dataset
Sphaero. iter1
Sphaero. iter2

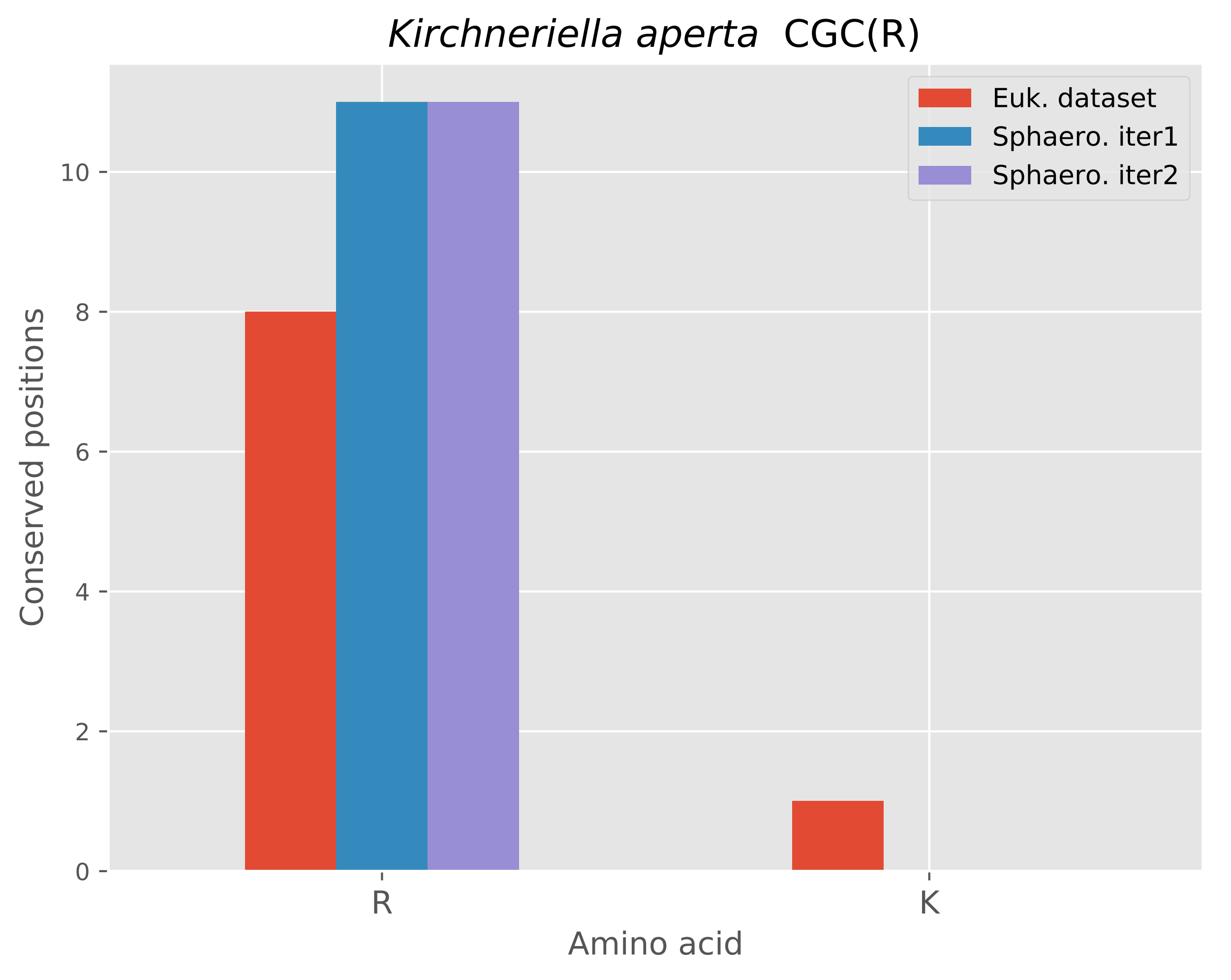

Kirchneriella aperta CGC(R)
Euk. dataset
Sphaero. iter1
Sphaero. iter2
Conserved positions
0
2
4
6
8
10
R
K
Amino acid

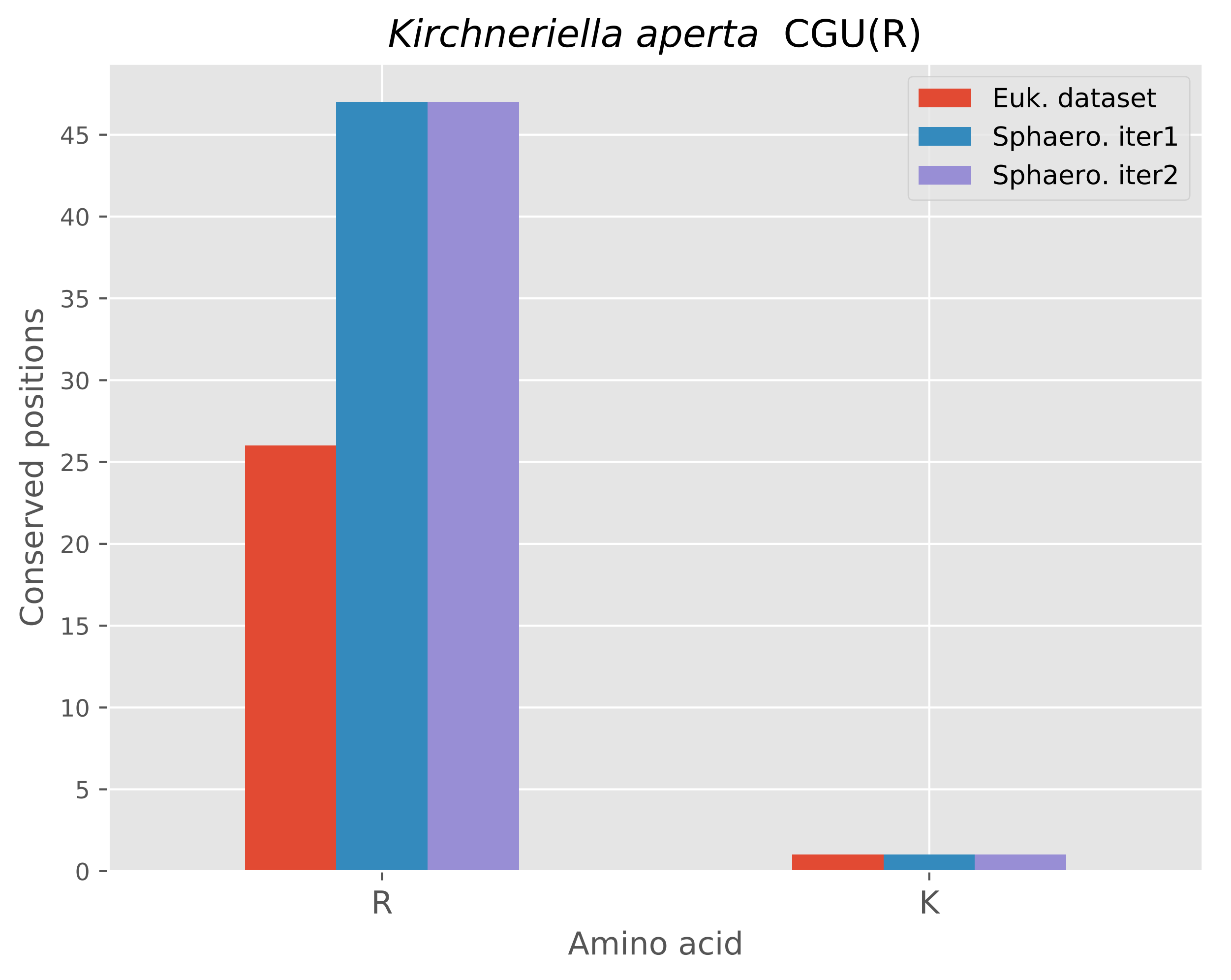

Kirchneriella aperta CGU(R)
Euk. dataset
Sphaero. iter1
Sphaero. iter2
Conserved positions
0
5
10
15
20
25
30
35
40
45
R
K
Amino acid

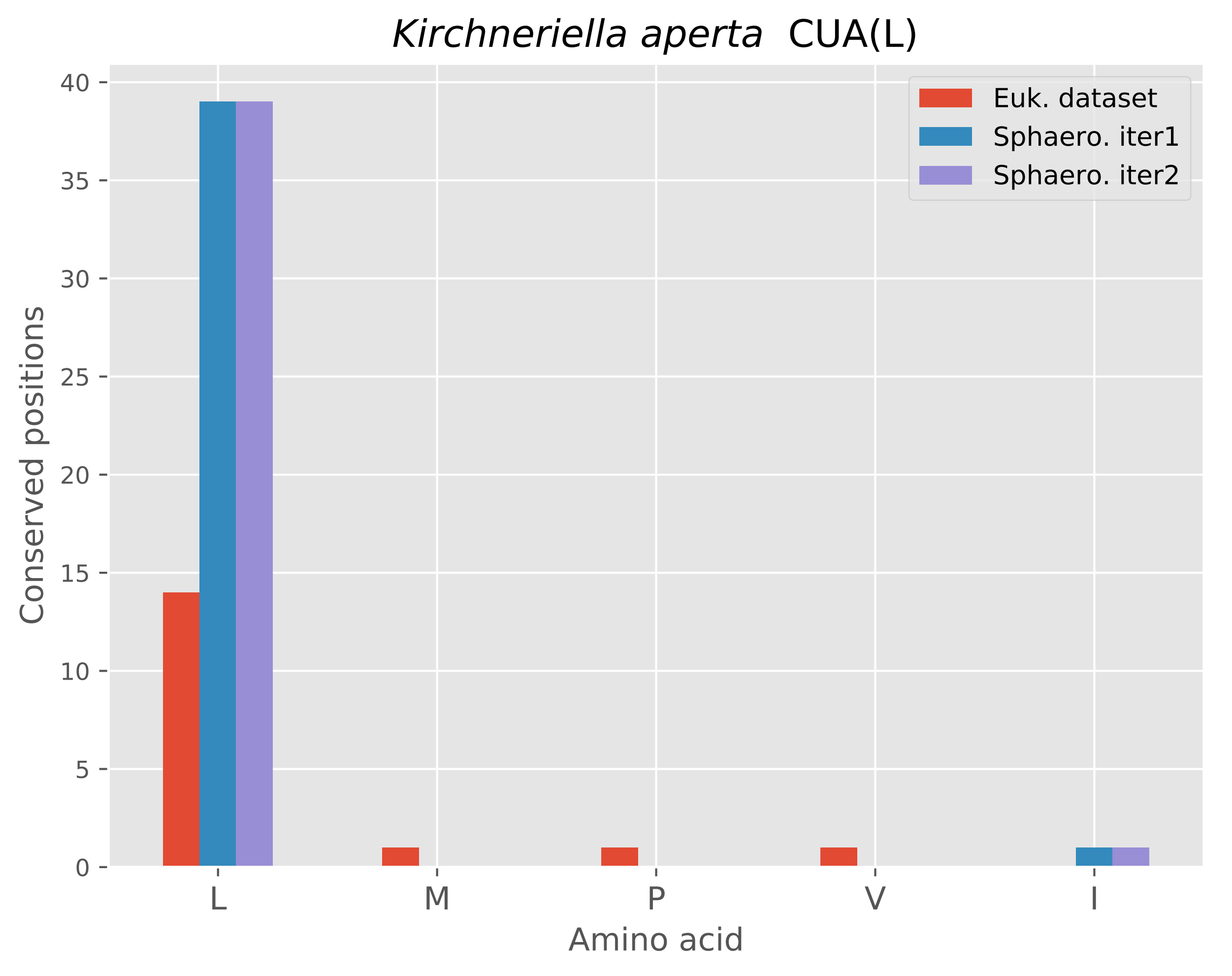

Kirchneriella aperta CUA(L)
Euk. dataset
Sphaero. iter1
Sphaero. iter2
Conserved positions
0
5
10
15
20
25
30
35
40
L
M
P
V
I
Amino acid

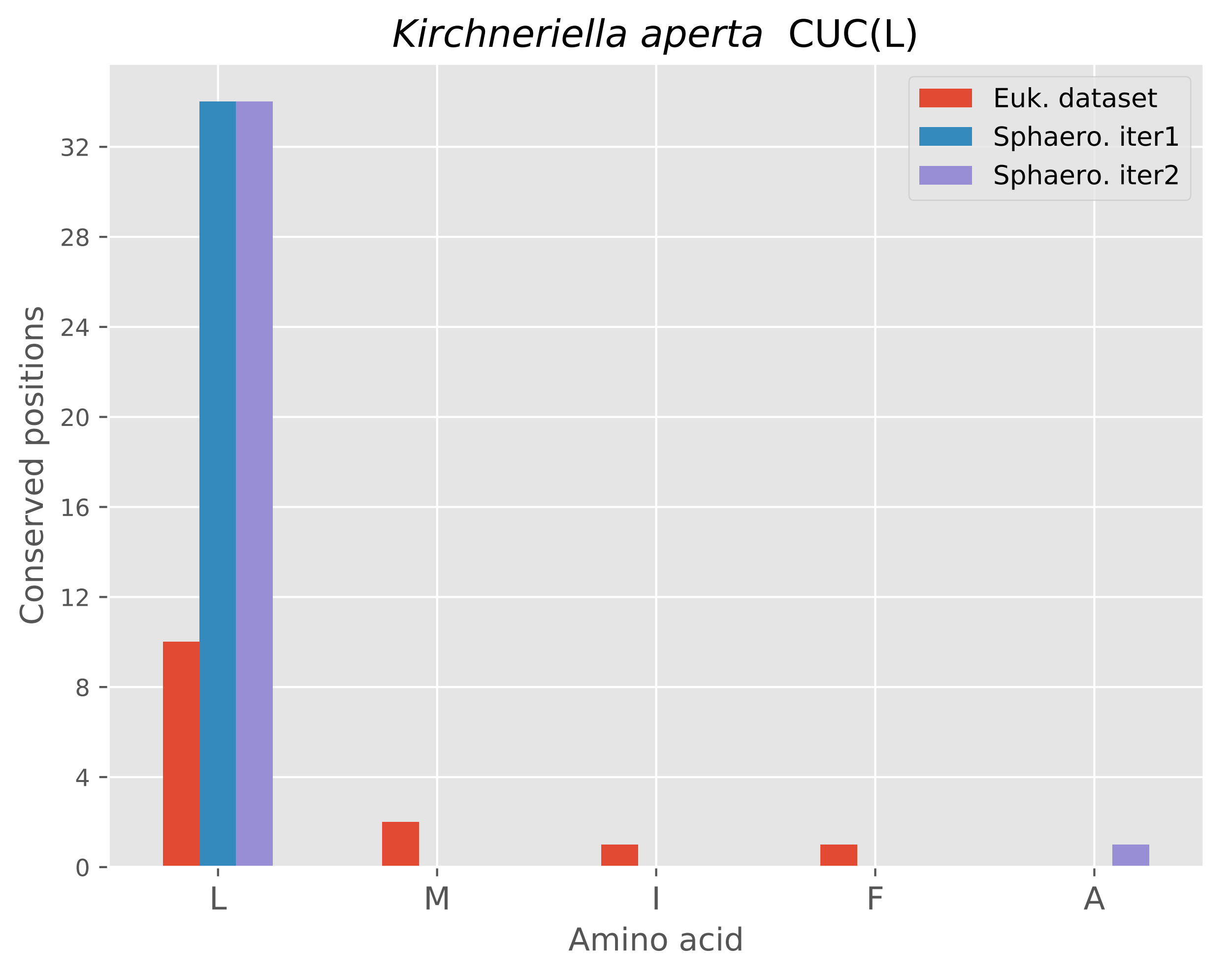

Kirchneriella aperta CUC(L)
Euk. dataset
Sphaero. iter1
Sphaero. iter2
Conserved positions
0
4
8
12
16
20
24
28
32
L
M
I
F
A
Amino acid

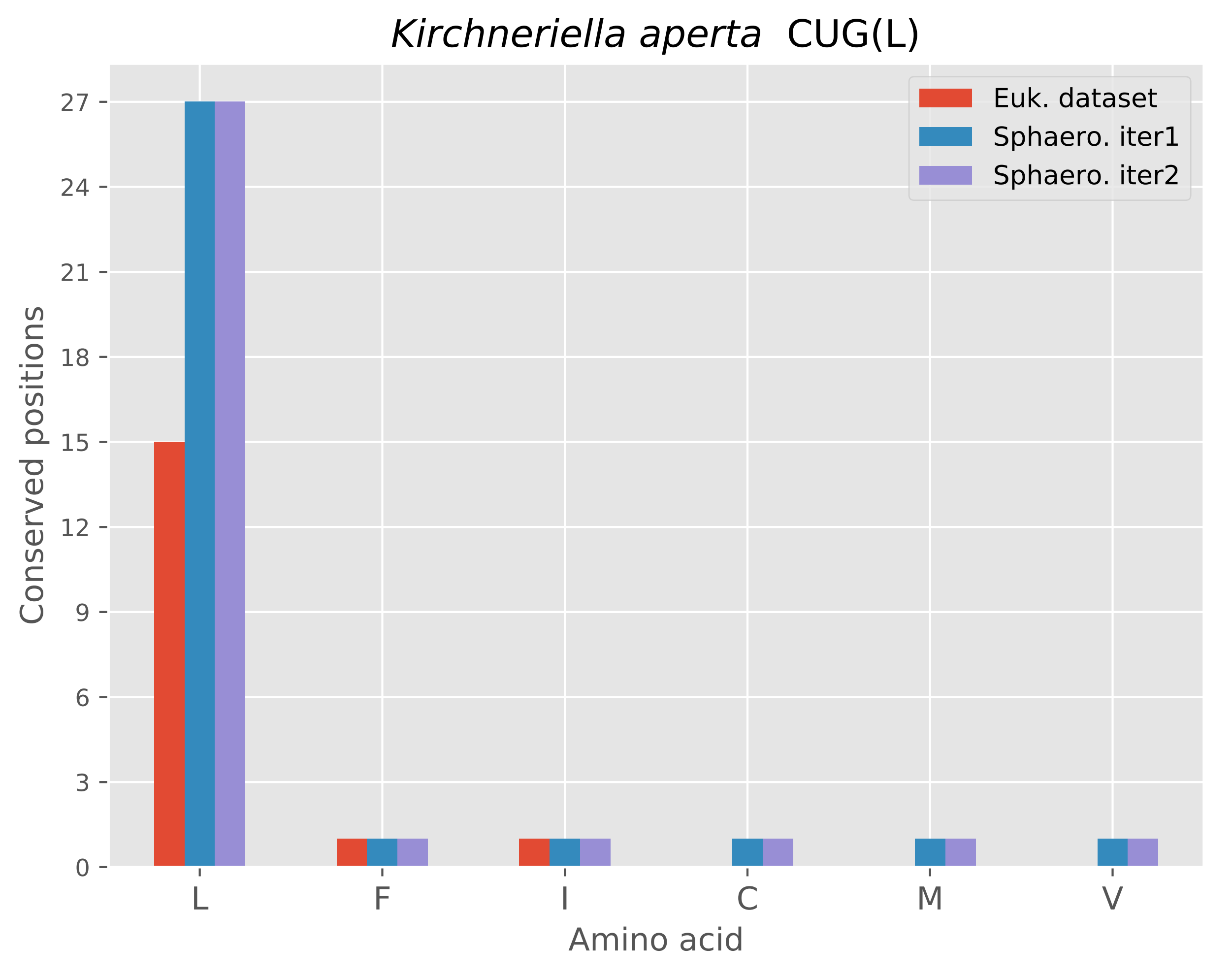

Kirchneriella aperta CUG(L)
Euk. dataset
Sphaero. iter1
Sphaero. iter2
Conserved positions
0
3
6
9
12
15
18
21
24
27
L
F
I
C
M
V
Amino acid

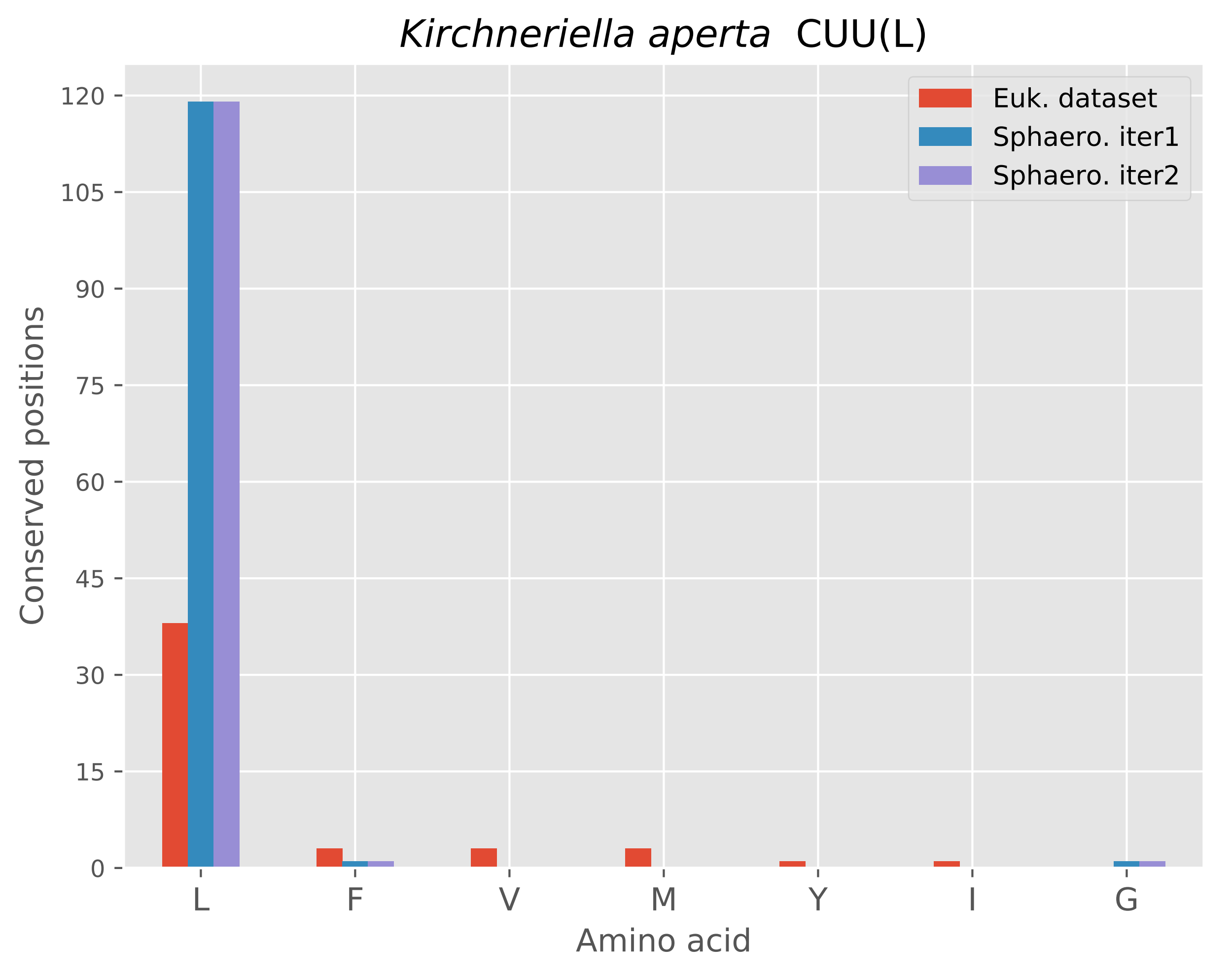

Kirchneriella aperta CUU(L)
Euk. dataset
Sphaero. iter1
Sphaero. iter2
Conserved positions
0
15
30
45
60
75
90
105
120
L
F
V
M
Y
I
G
Amino acid

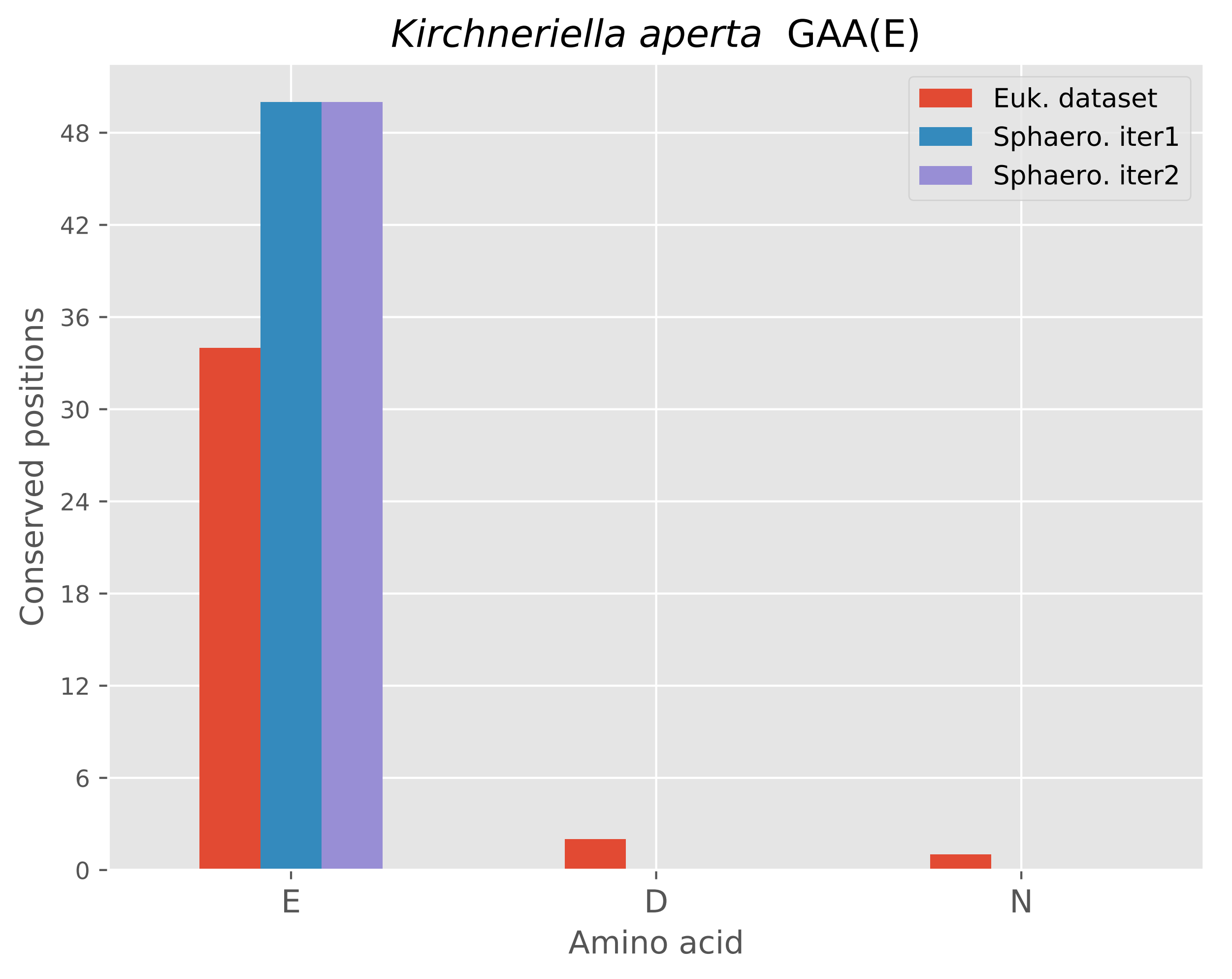

Kirchneriella aperta GAA(E)
Euk. dataset
Sphaero. iter1
Sphaero. iter2
Conserved positions
0
6
12
18
24
30
36
42
48
E
D
N
Amino acid

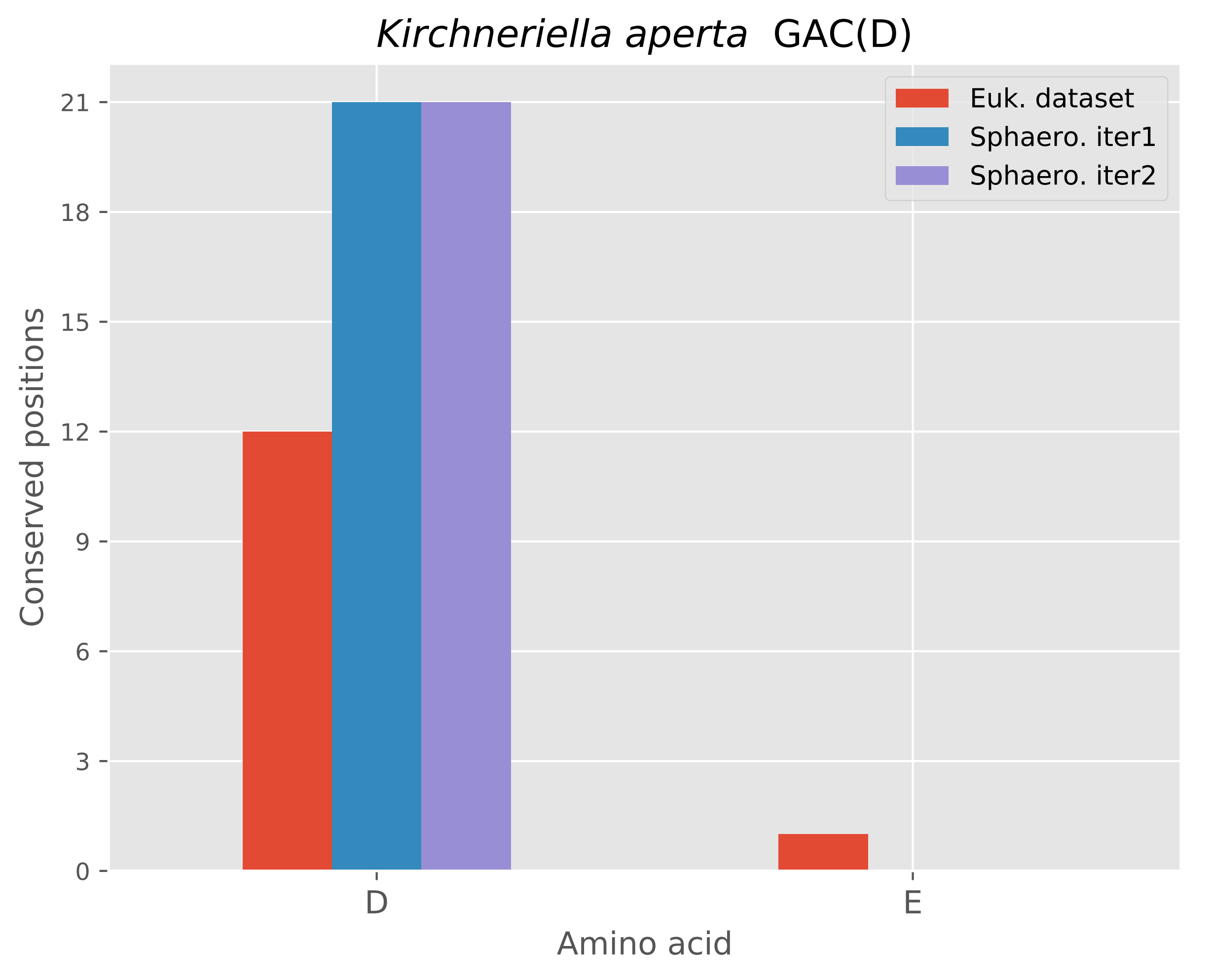

Kirchneriella aperta GAC(D)
Euk. dataset
Sphaero. iter1
Sphaero. iter2
Conserved positions
0
3
6
9
12
15
18
21
D
E
Amino acid

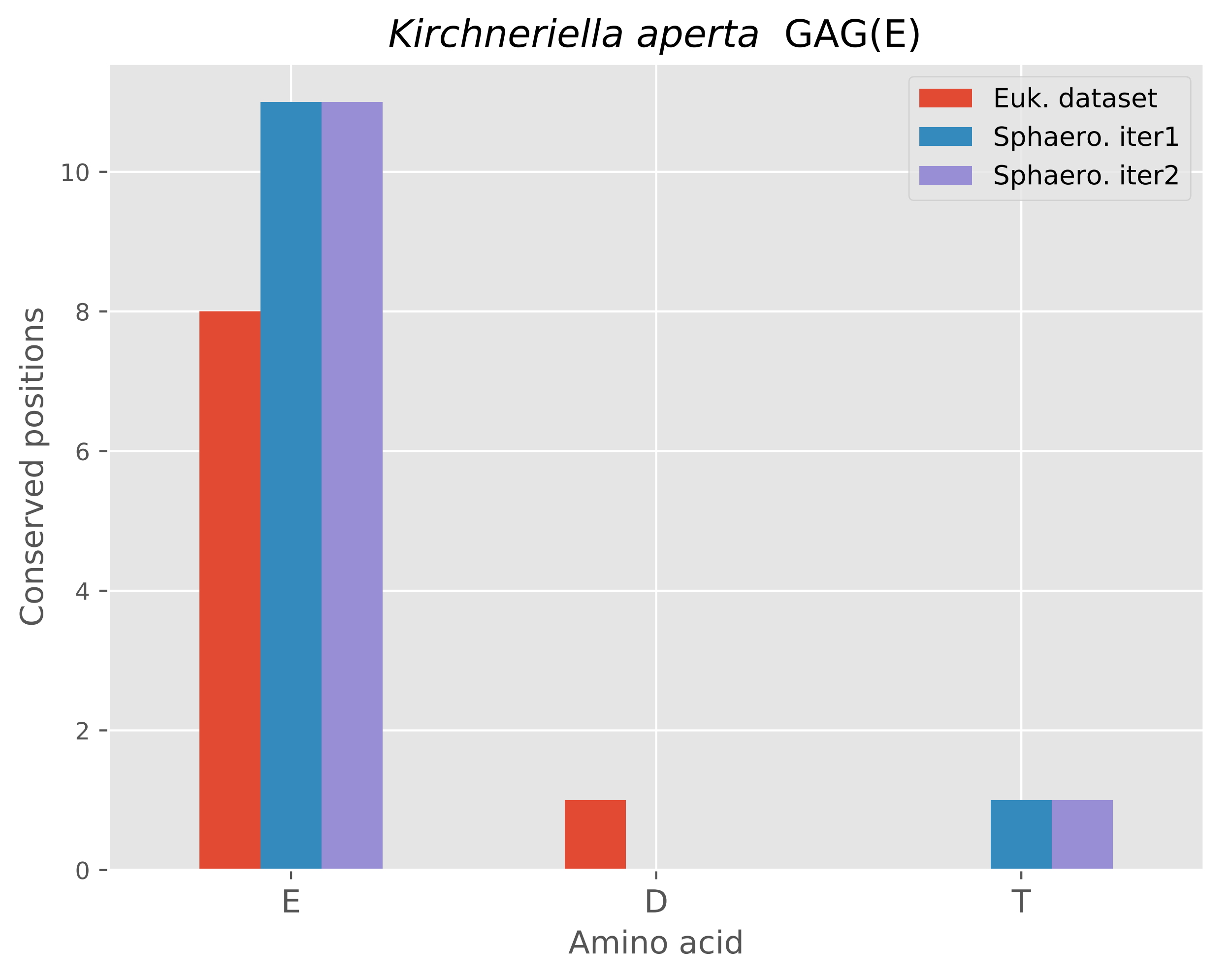
Kirchneriella aperta GAG(E)
Euk. dataset
Sphaero. iter1
Sphaero. iter2
Conserved positions
0
2
4
6
8
10
E
D
T
Amino acid

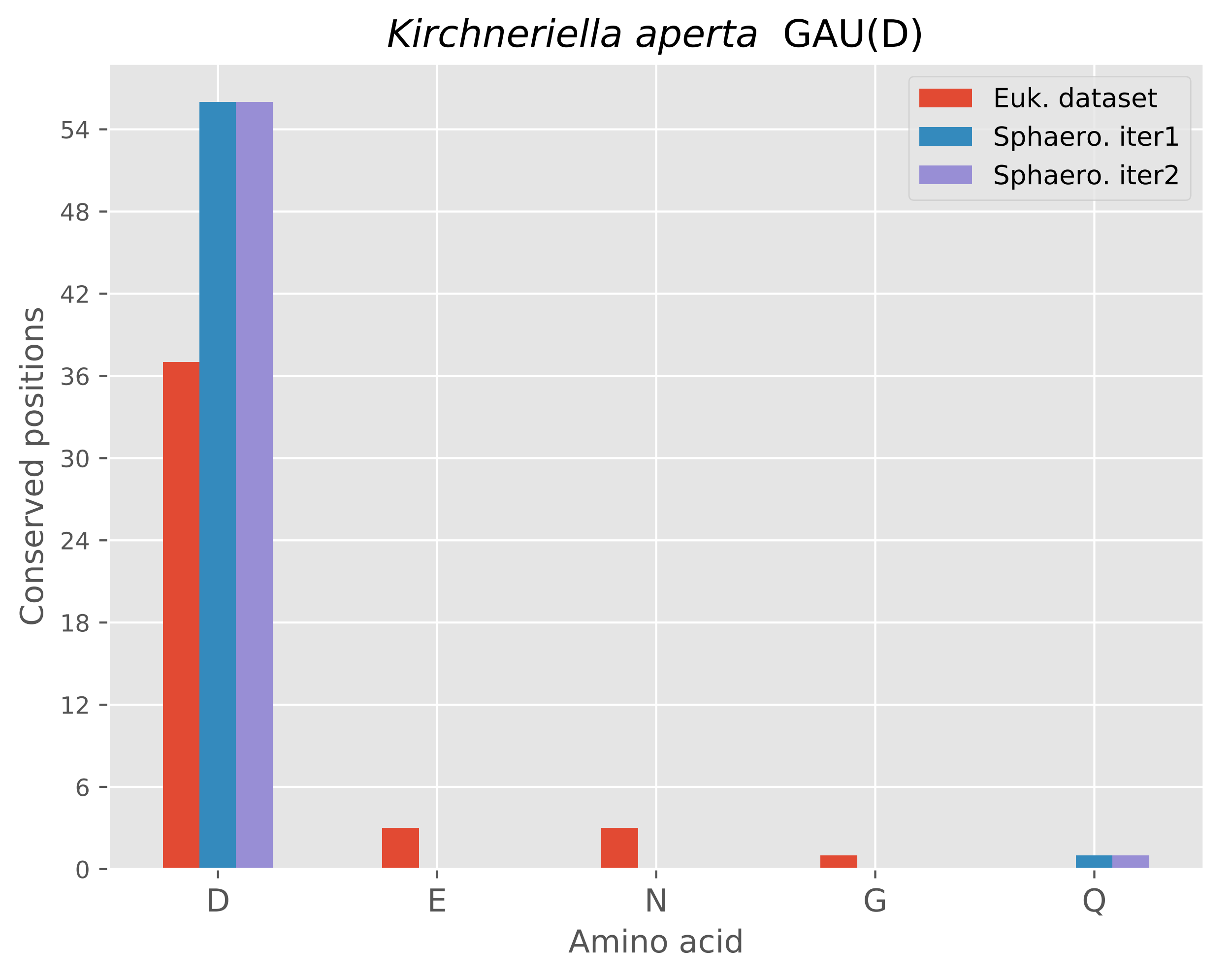
Kirchneriella aperta GAU(D)
Euk. dataset
Sphaero. iter1
Sphaero. iter2
Conserved positions
0
6
12
18
24
30
36
42
48
54
D
E
N
G
Q
Amino acid

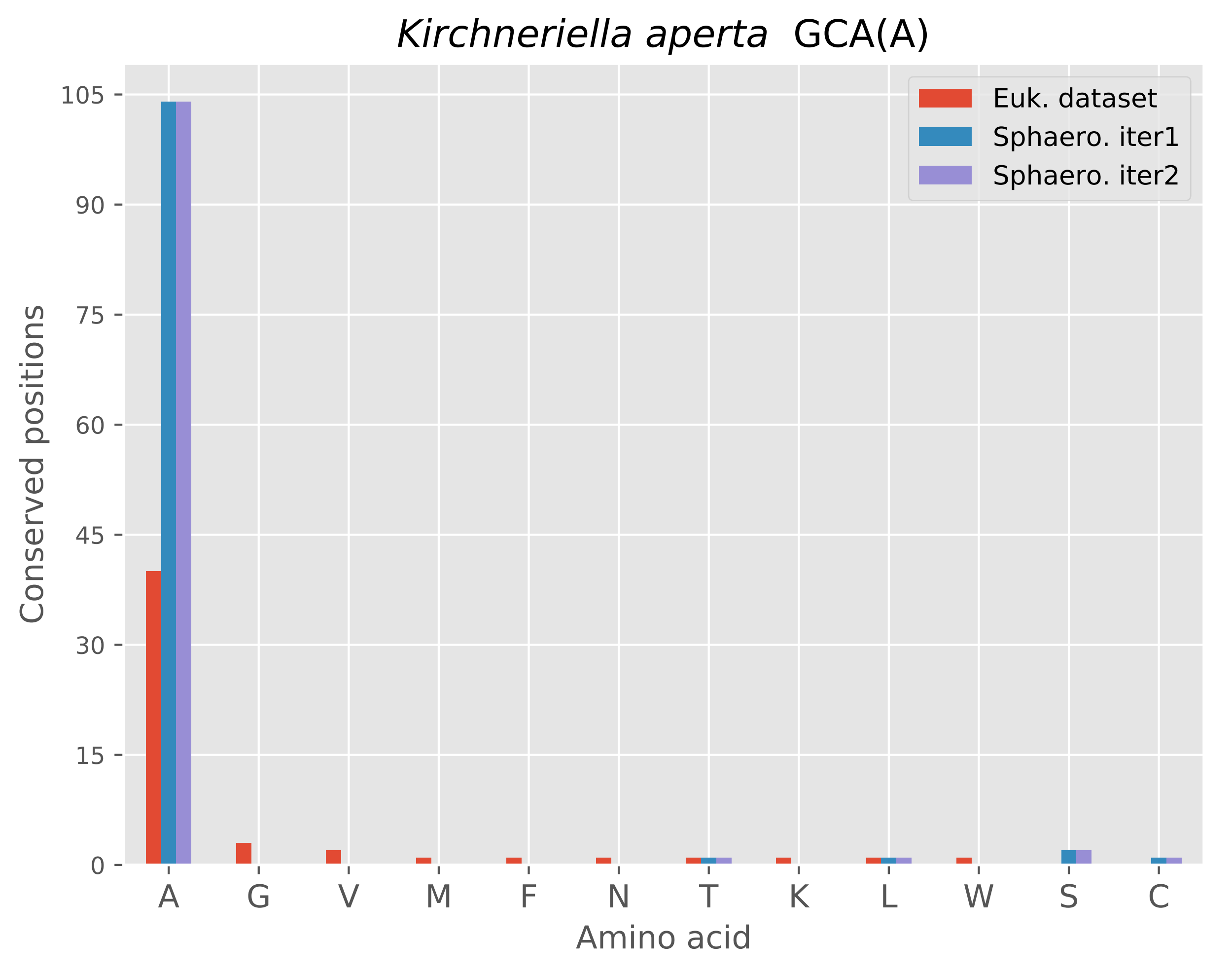

Kirchneriella aperta GCA(A)
Euk. dataset
Sphaero. iter1
Sphaero. iter2
Conserved positions
105
90
75
60
45
30
15
0
A
G
V
M
F
N
T
K
L
W
S
C
Amino acid

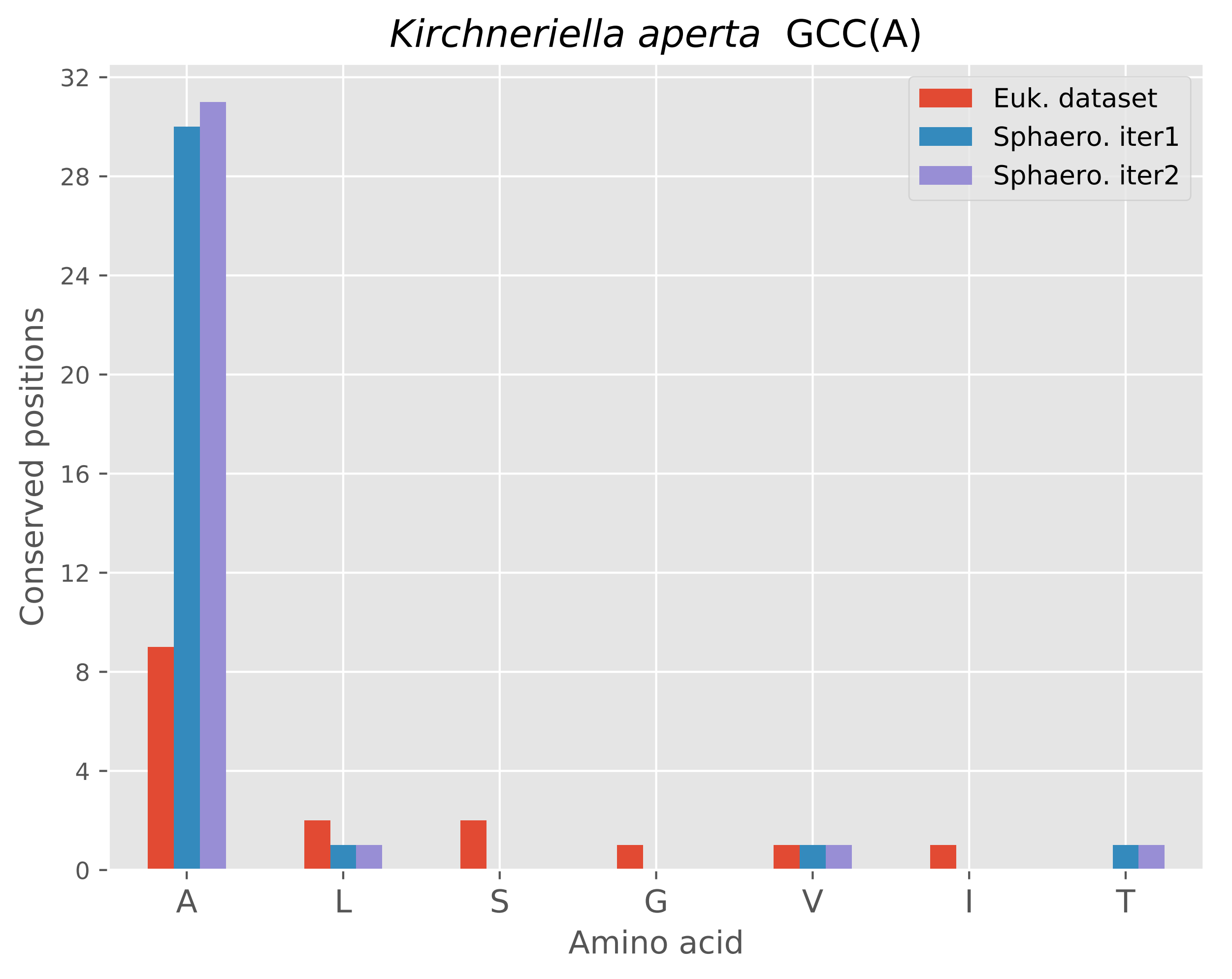
Kirchneriella aperta GCC(A)
Euk. dataset
Sphaero. iter1
Sphaero. iter2
Conserved positions
0
4
8
12
16
20
24
28
32
A
L
S
G
V
I
T
Amino acid

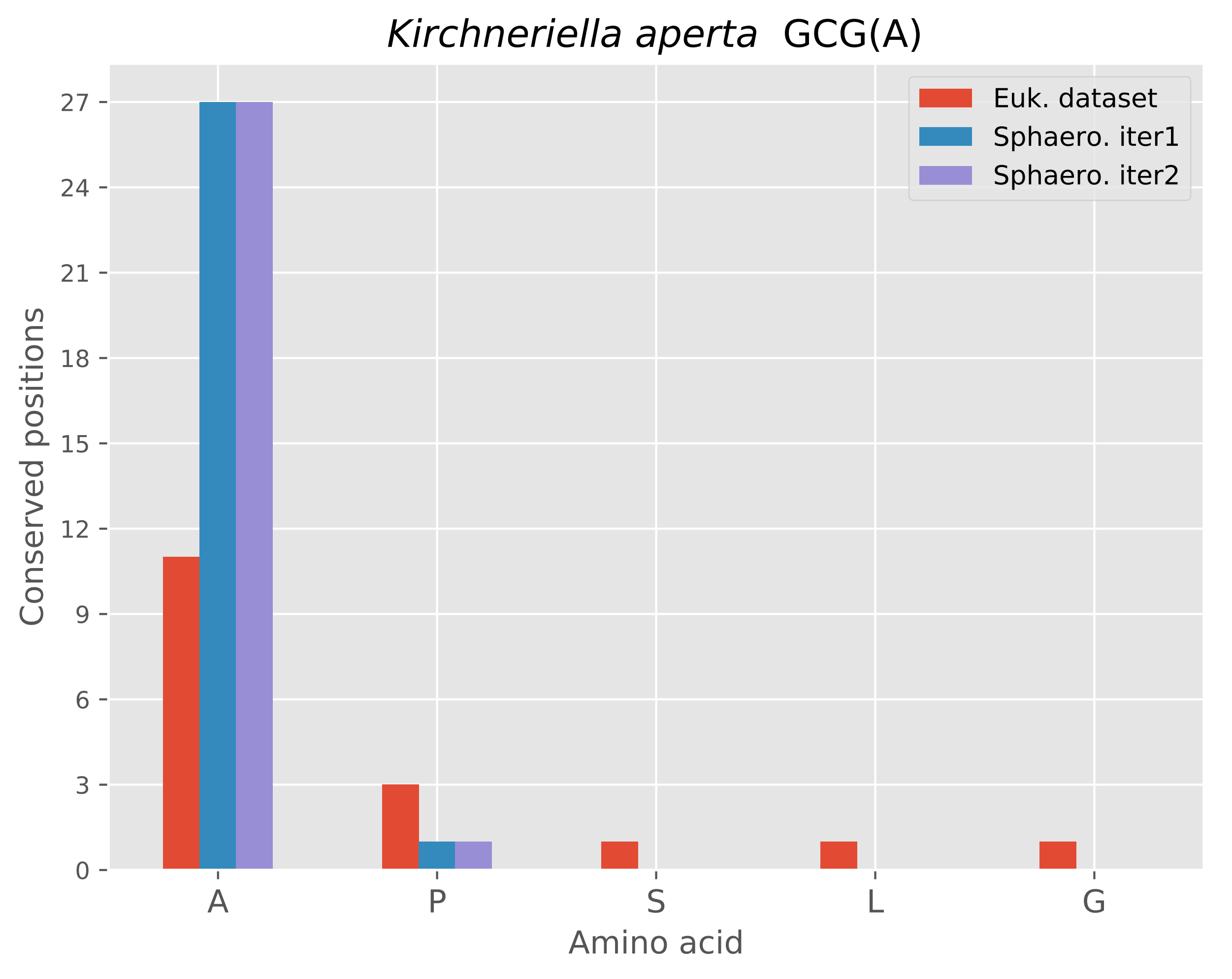

Kirchneriella aperta GCG(A)
Euk. dataset
Sphaero. iter1
Sphaero. iter2
Conserved positions
0
3
6
9
12
15
18
21
24
27
A
P
S
L
G
Amino acid

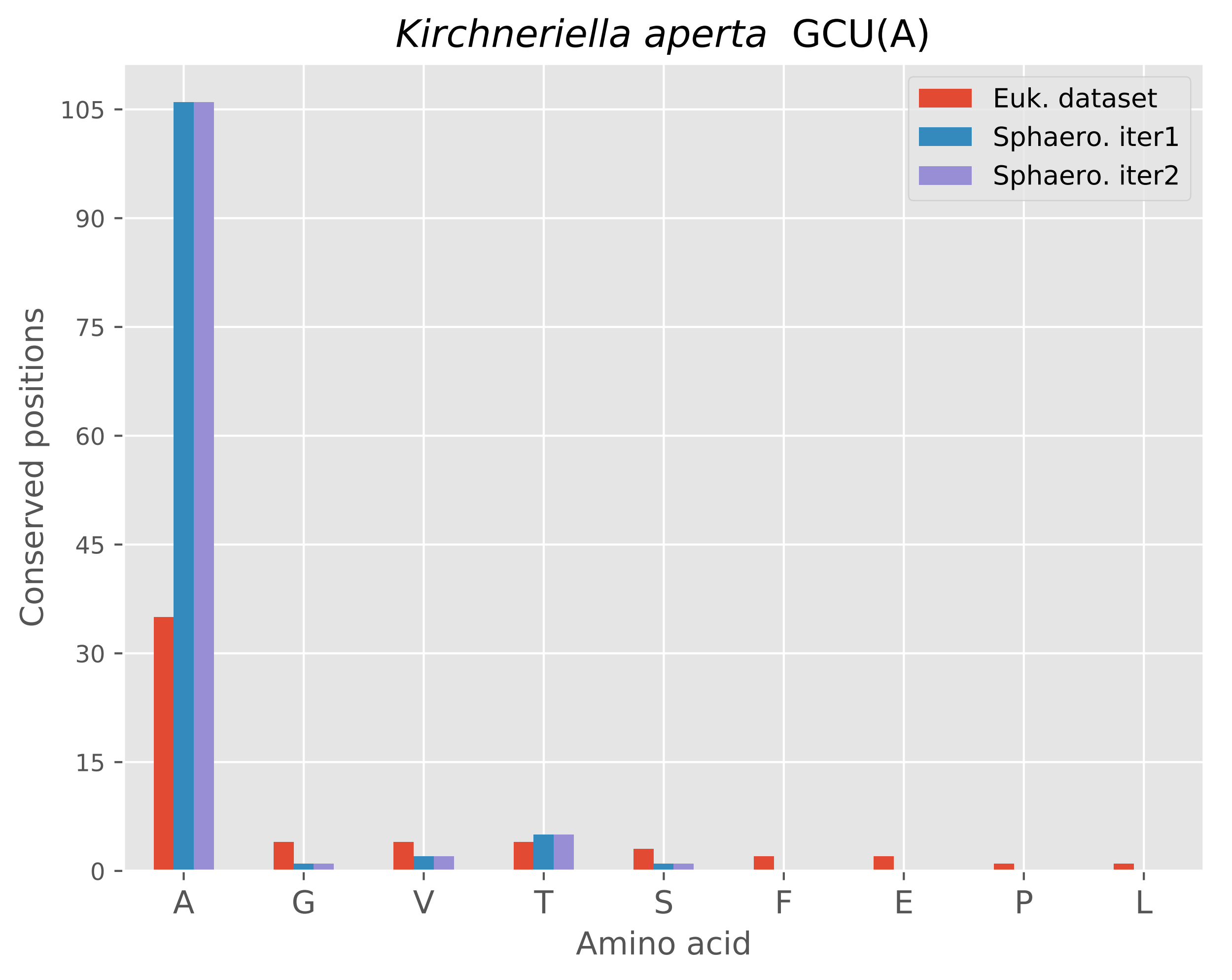

Kirchneriella aperta GCU(A)
Euk. dataset
Sphaero. iter1
Sphaero. iter2
Conserved positions
0
15
30
45
60
75
90
105
A
G
V
T
S
F
E
P
L
Amino acid

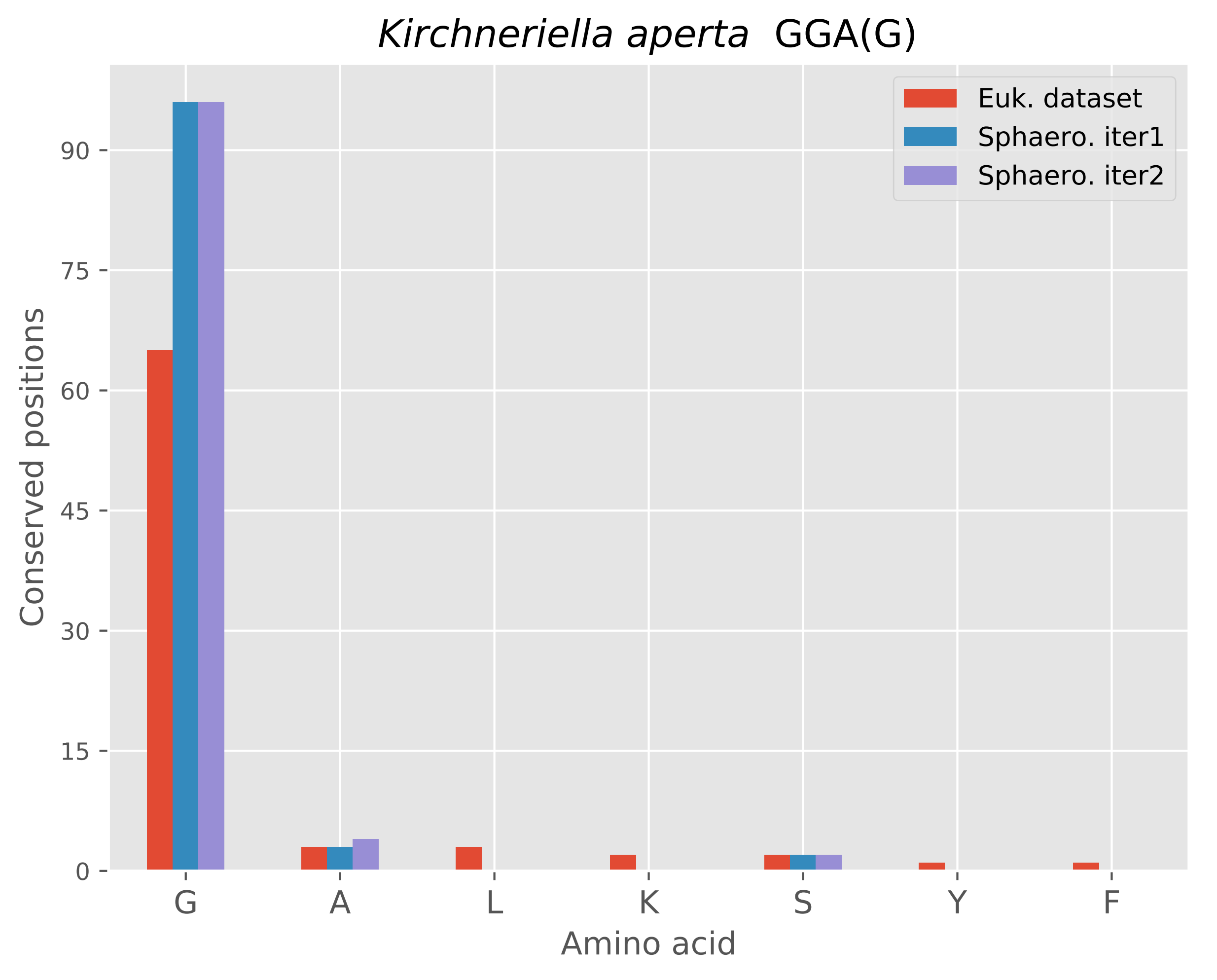

Kirchneriella aperta GGA(G)
Euk. dataset
Sphaero. iter1
Sphaero. iter2
Conserved positions
0
15
30
45
60
75
90
G
A
L
K
S
Y
F
Amino acid

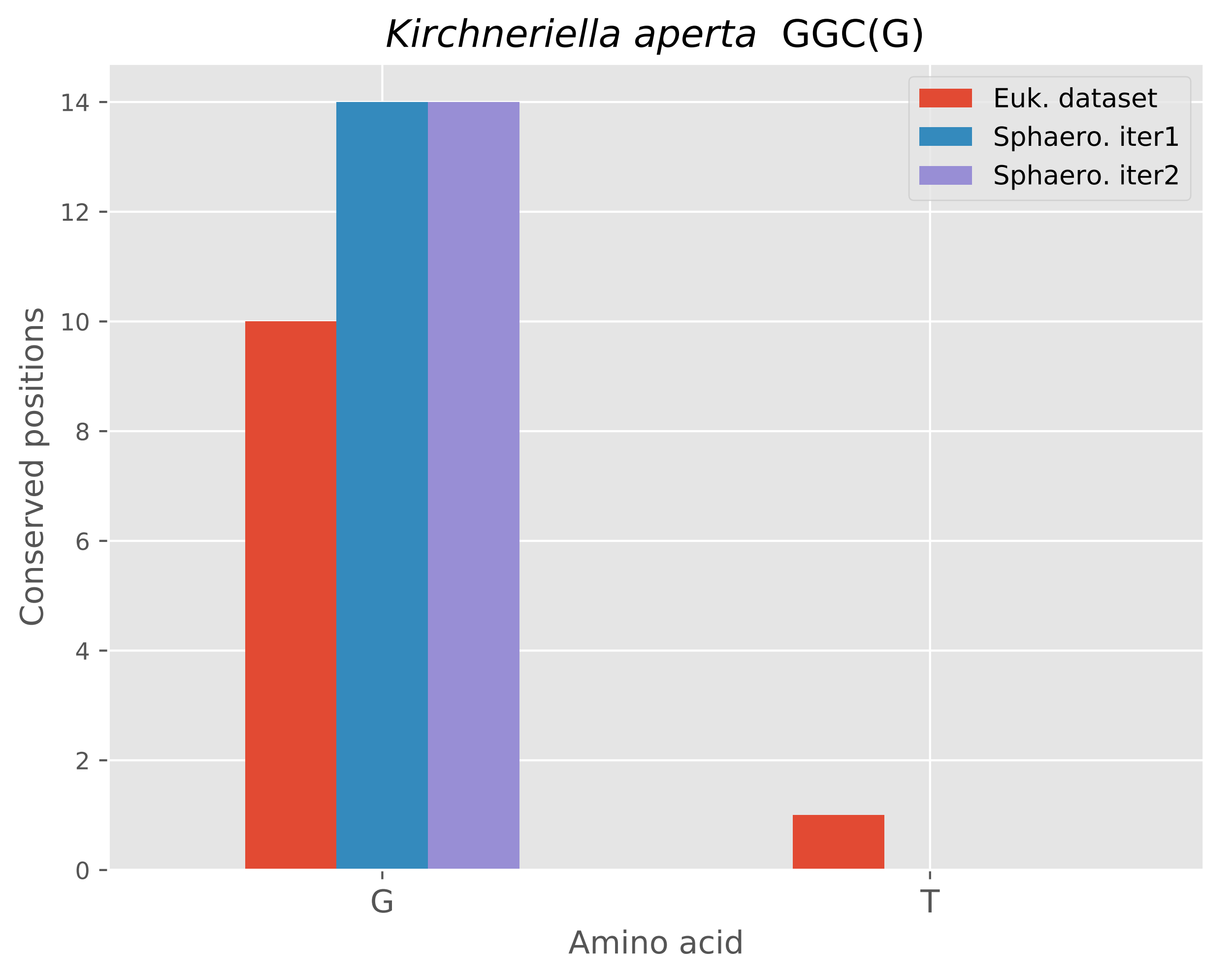

Kirchneriella aperta GGC(G)
Euk. dataset
Sphaero. iter1
Sphaero. iter2
Conserved positions
0
2
4
6
8
10
12
14
G
T
Amino acid

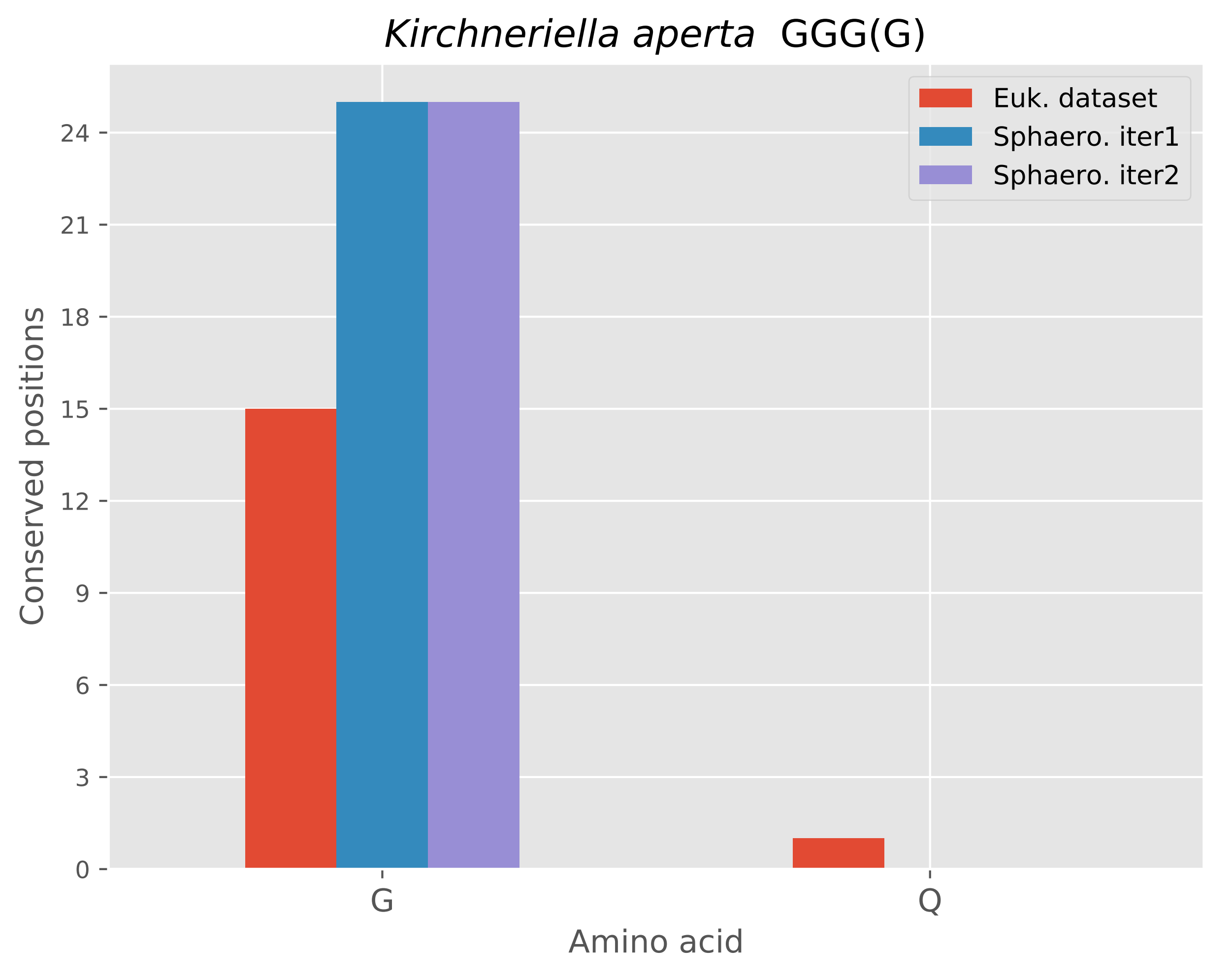

Kirchneriella aperta GGG(G)
Euk. dataset
Sphaero. iter1
Sphaero. iter2
Conserved positions
0
3
6
9
12
15
18
21
24
G
Q
Amino acid

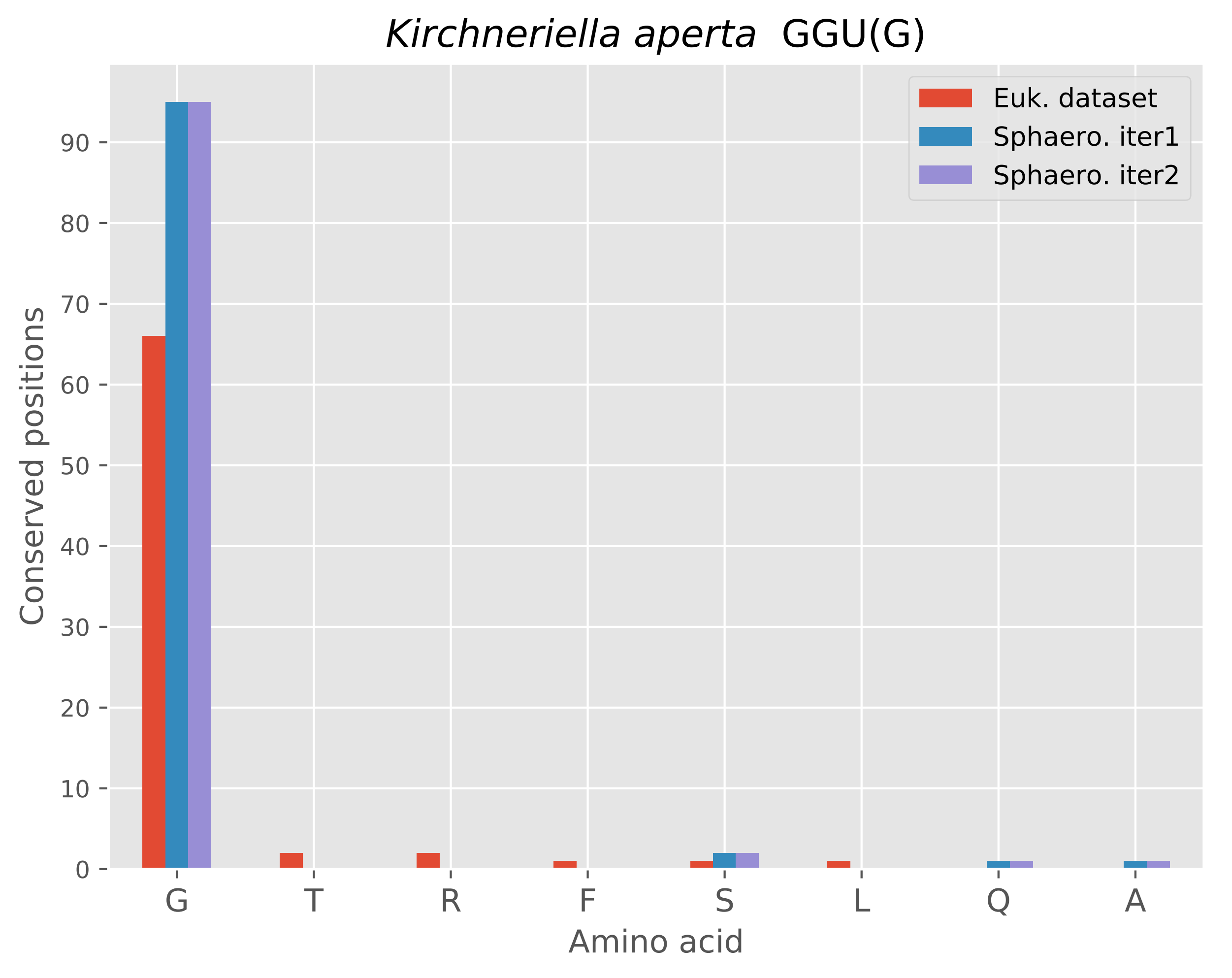

Kirchneriella aperta GGU(G)
Euk. dataset
Sphaero. iter1
Sphaero. iter2
Conserved positions
0
10
20
30
40
50
60
70
80
90
G
T
R
F
S
L
Q
A
Amino acid

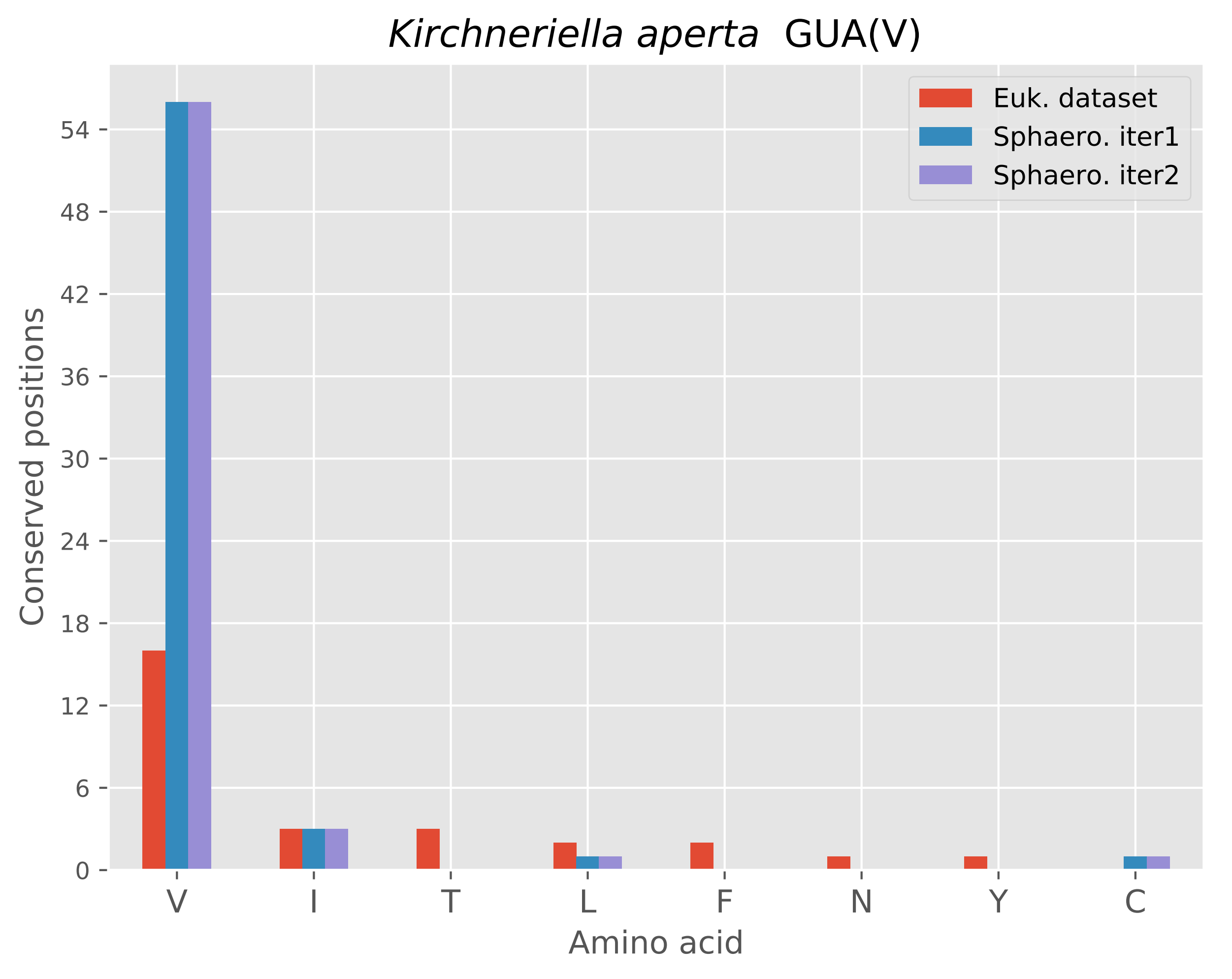

Kirchneriella aperta GUA(V)
Euk. dataset
Sphaero. iter1
Sphaero. iter2
Conserved positions
0
6
12
18
24
30
36
42
48
54
V
I
T
L
F
N
Y
C
Amino acid

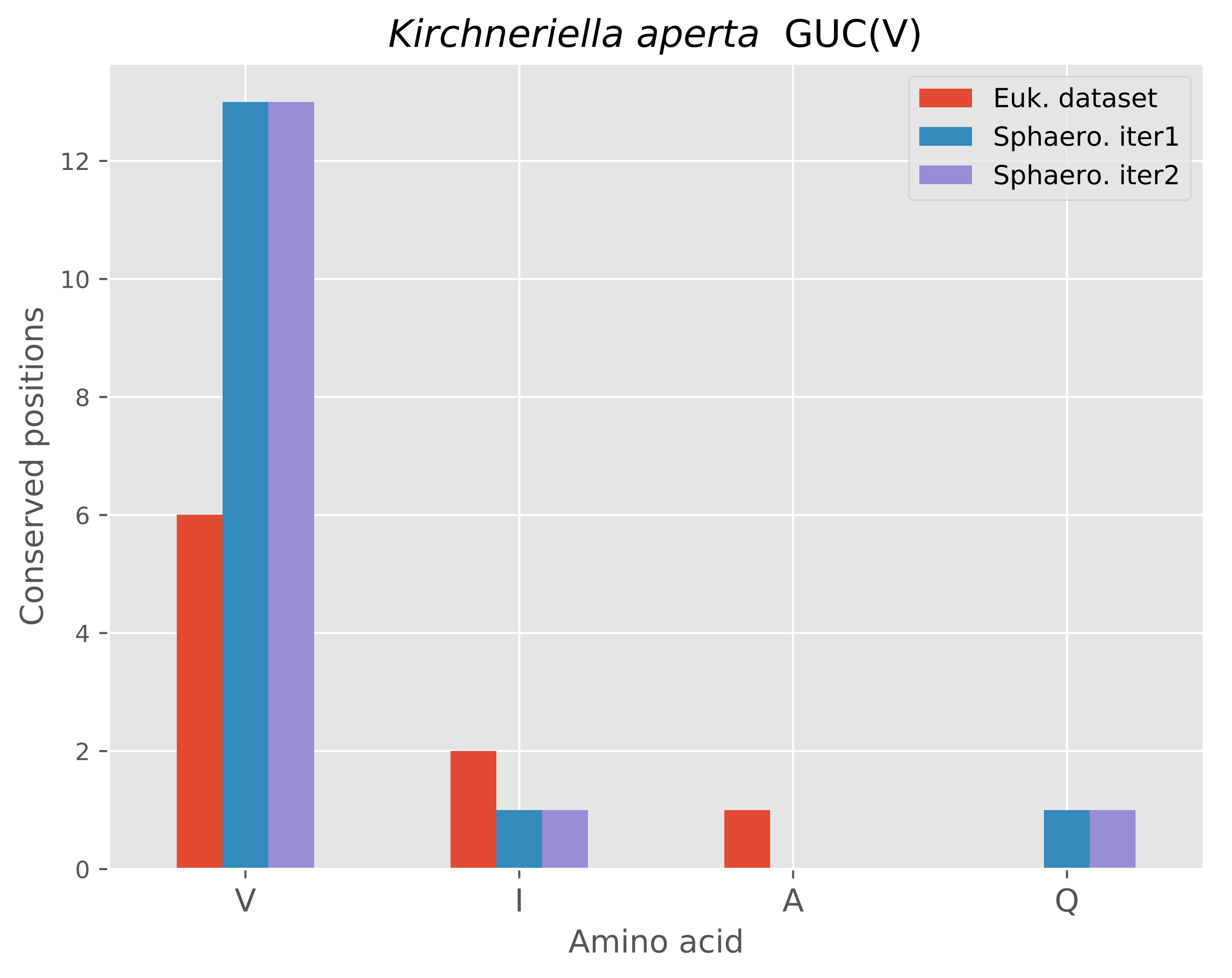

Kirchneriella aperta GUC(V)
Euk. dataset
Sphaero. iter1
Sphaero. iter2
Conserved positions
0
2
4
6
8
10
12
V
I
A
Q
Amino acid

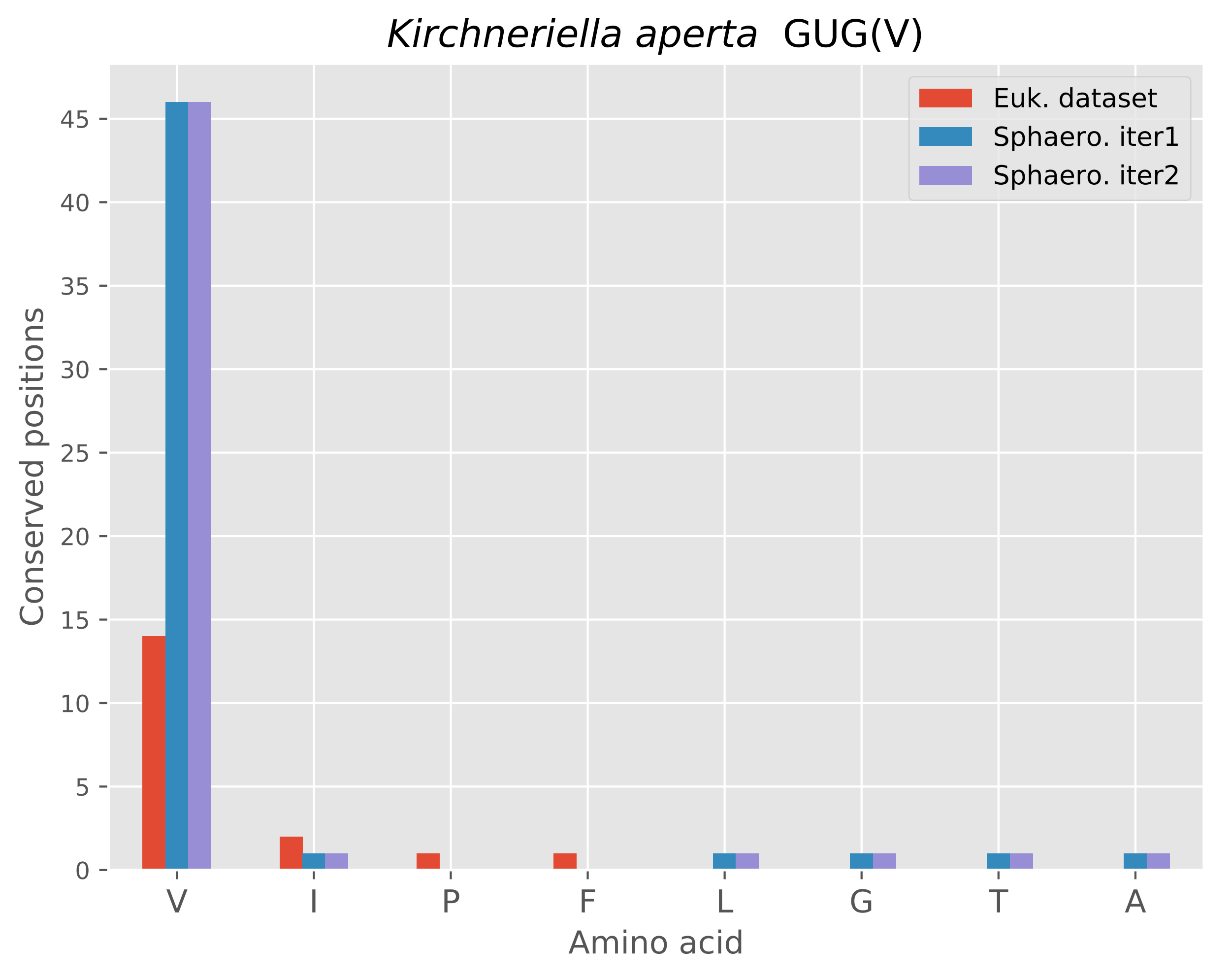

Kirchneriella aperta GUG(V)
Euk. dataset
Sphaero. iter1
Sphaero. iter2
Conserved positions
0
5
10
15
20
25
30
35
40
45
V
I
P
F
L
G
T
A
Amino acid

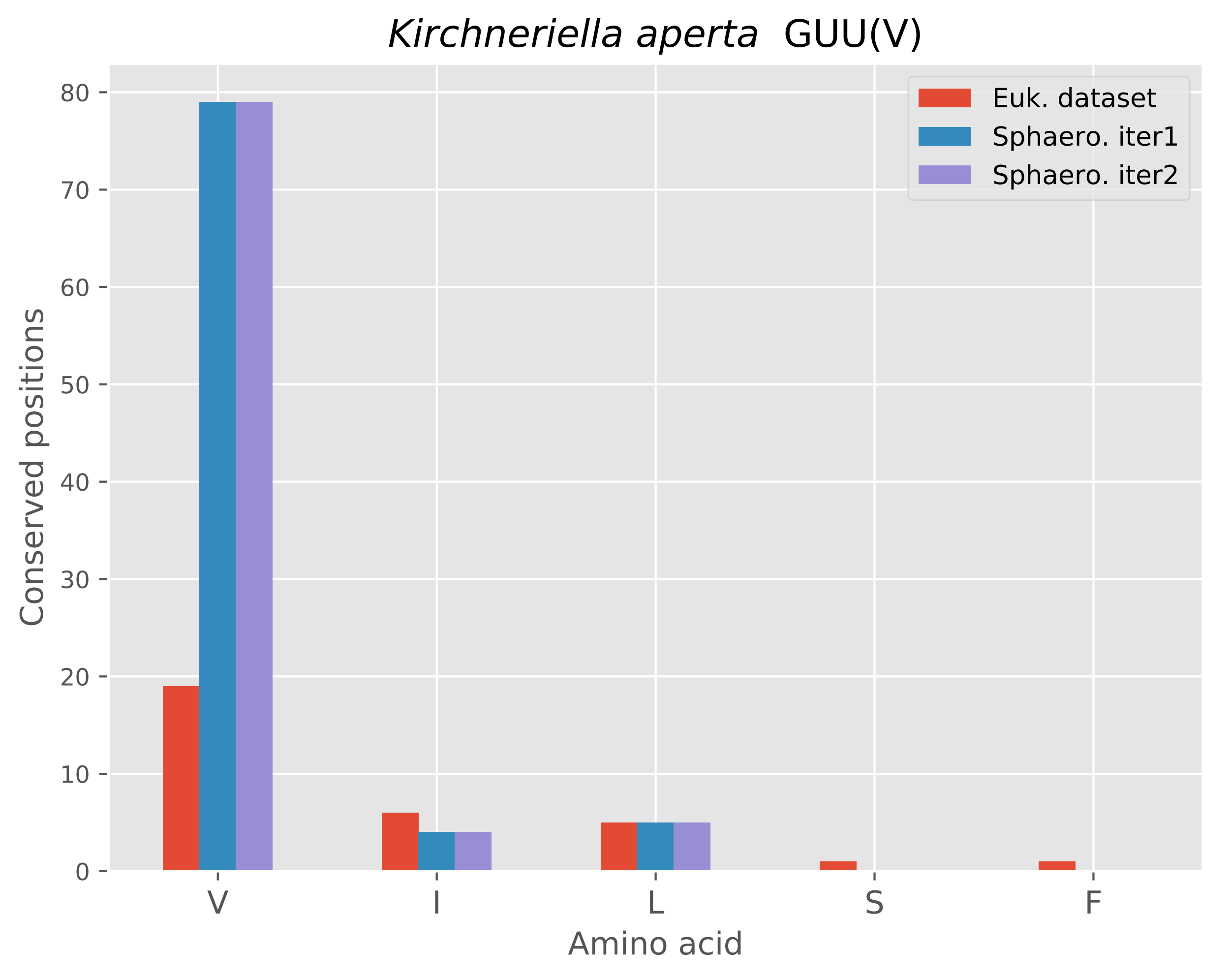

Kirchneriella aperta GUU(V)
Euk. dataset
Sphaero. iter1
Sphaero. iter2
Conserved positions
0
10
20
30
40
50
60
70
80
V
I
L
S
F
Amino acid

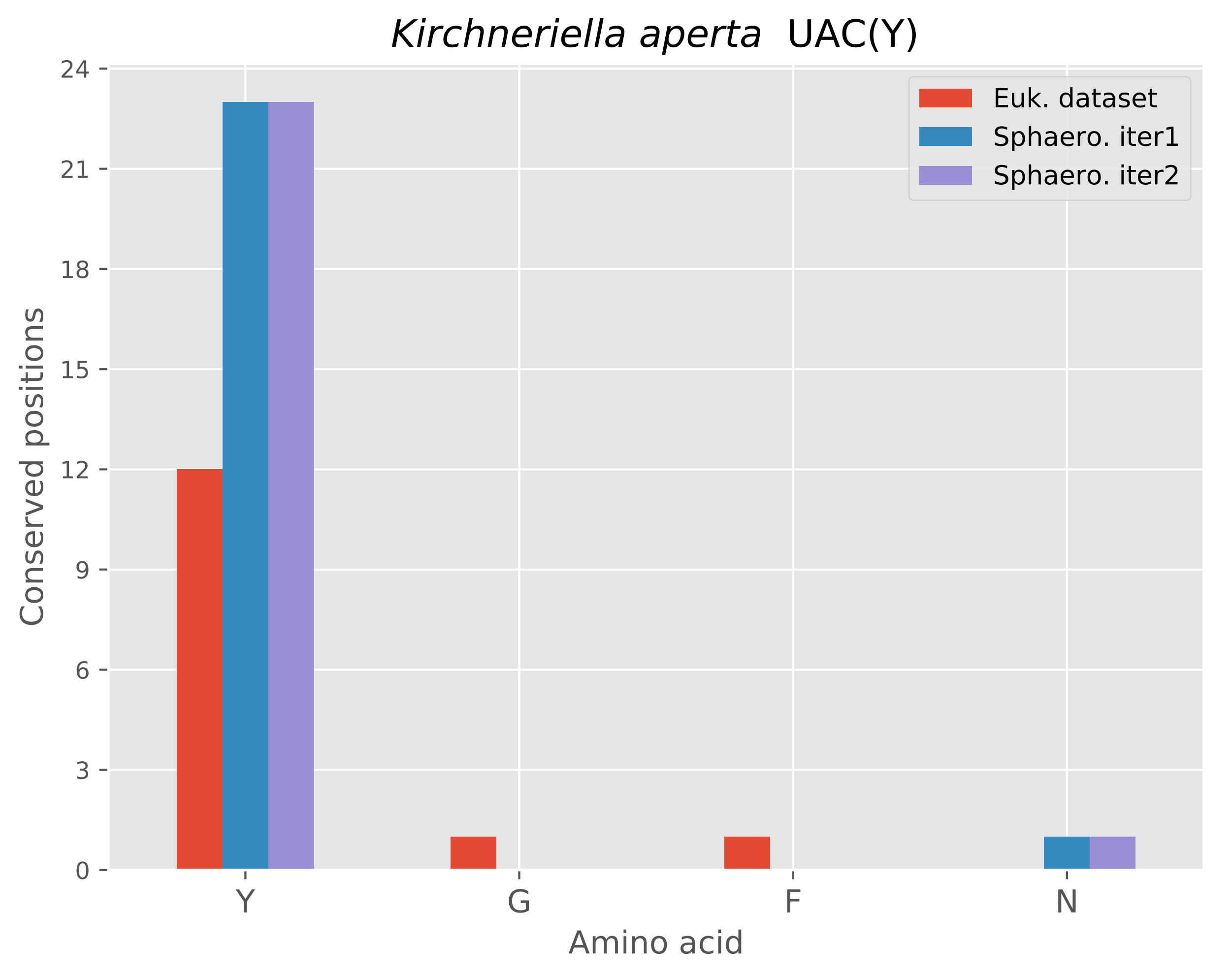

Kirchneriella aperta UAC(Y)
Euk. dataset
Sphaero. iter1
Sphaero. iter2
Conserved positions
0
3
6
9
12
15
18
21
24
Y
G
F
N
Amino acid

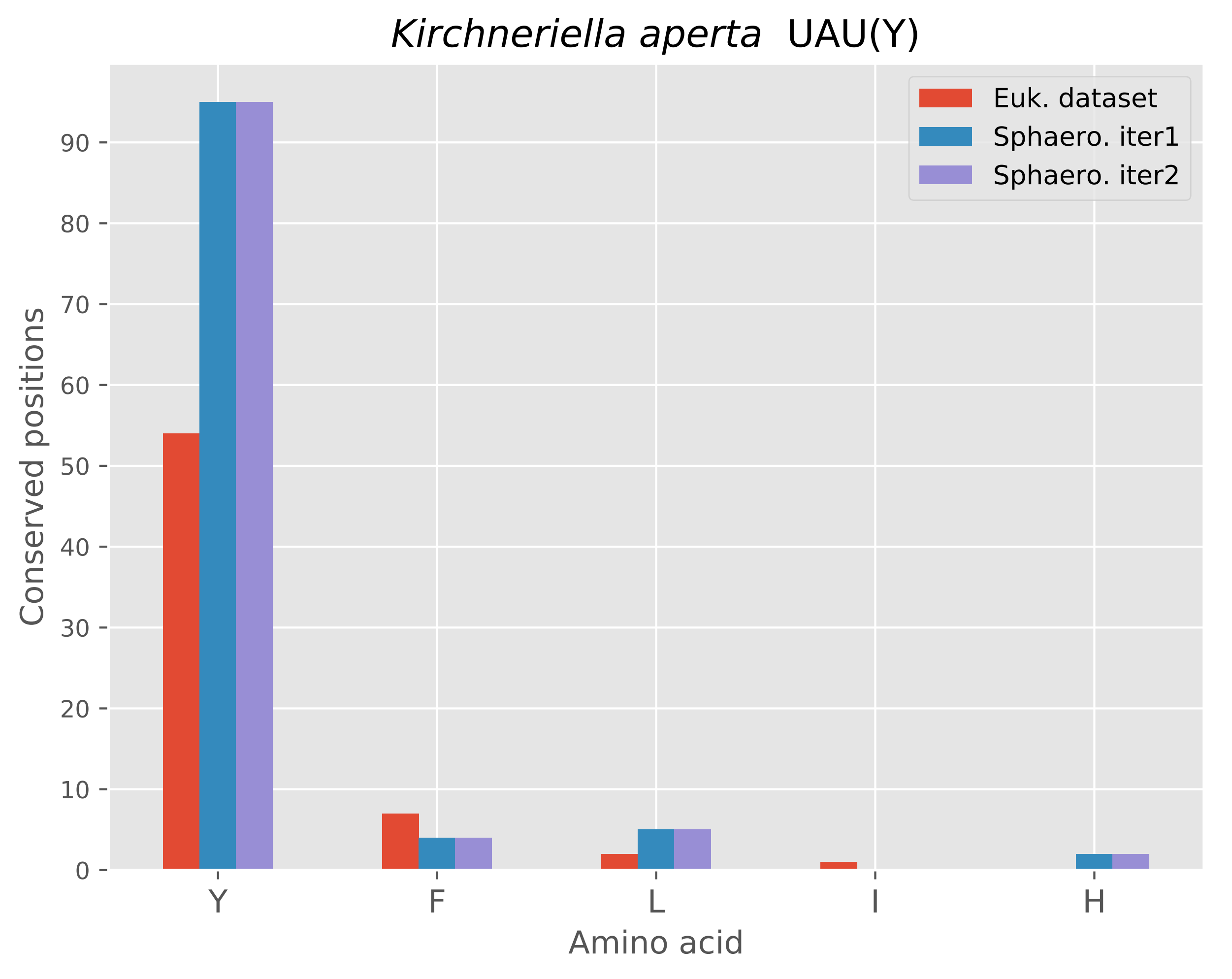

Kirchneriella aperta UAU(Y)
Euk. dataset
Sphaero. iter1
Sphaero. iter2
Conserved positions
0
10
20
30
40
50
60
70
80
90
Y
F
L
I
H
Amino acid

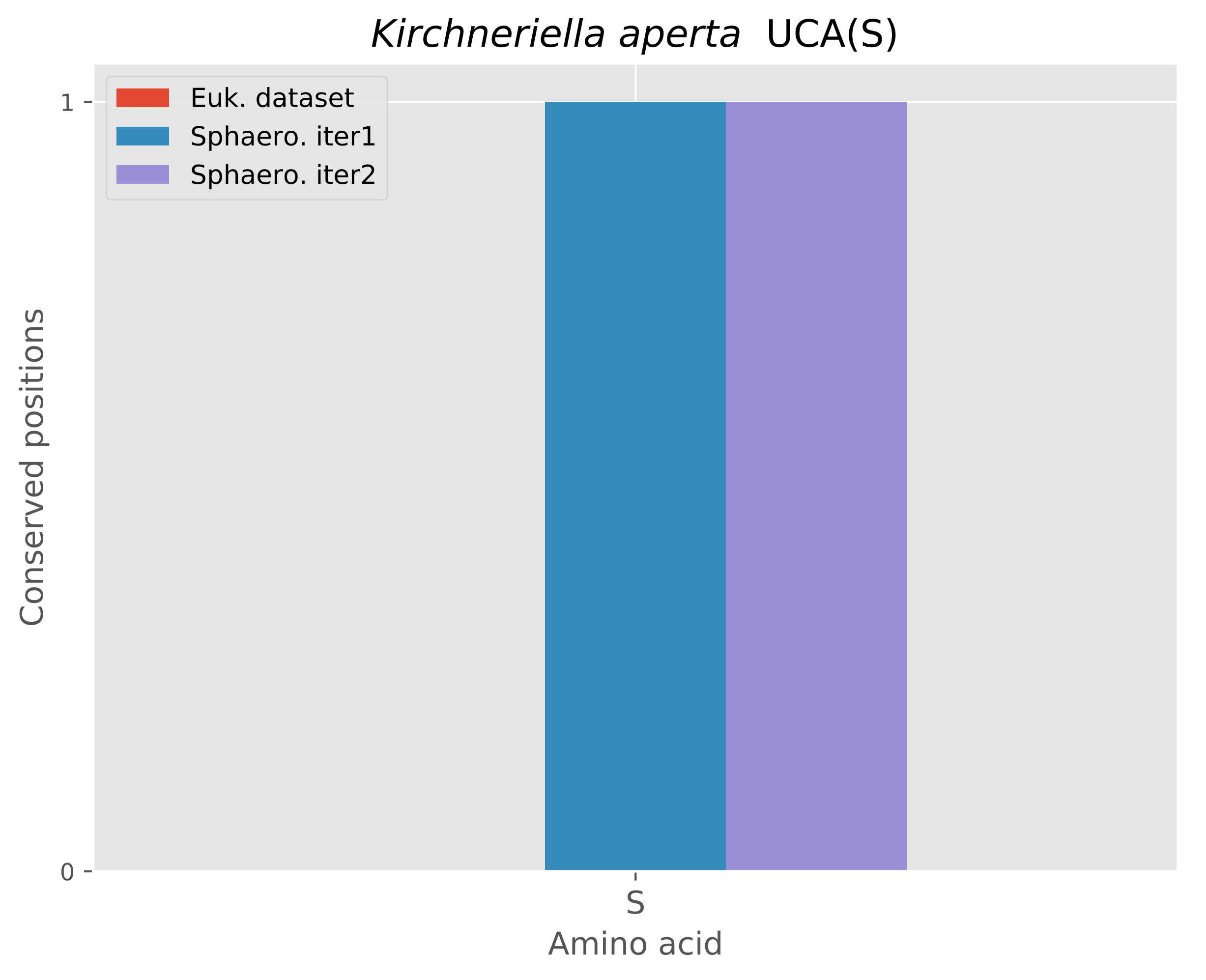
Kirchneriella aperta UCA(S)
Euk. dataset
Sphaero. iter1
Sphaero. iter2
Conserved positions
1
0
S
Amino acid

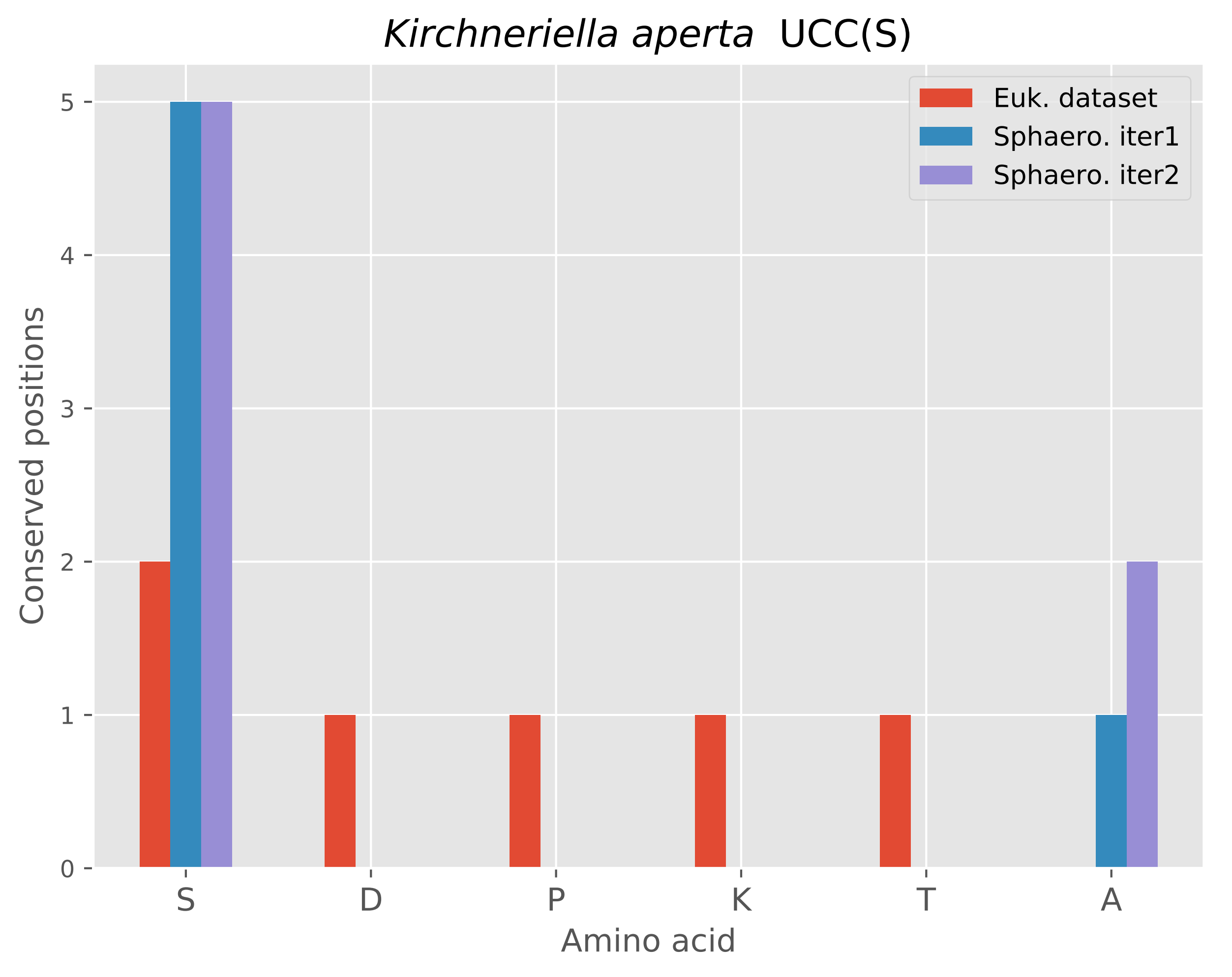
Kirchneriella aperta UCC(S)
Euk. dataset
Sphaero. iter1
Sphaero. iter2
Conserved positions
0
1
2
3
4
5
S
D
P
K
T
A
Amino acid

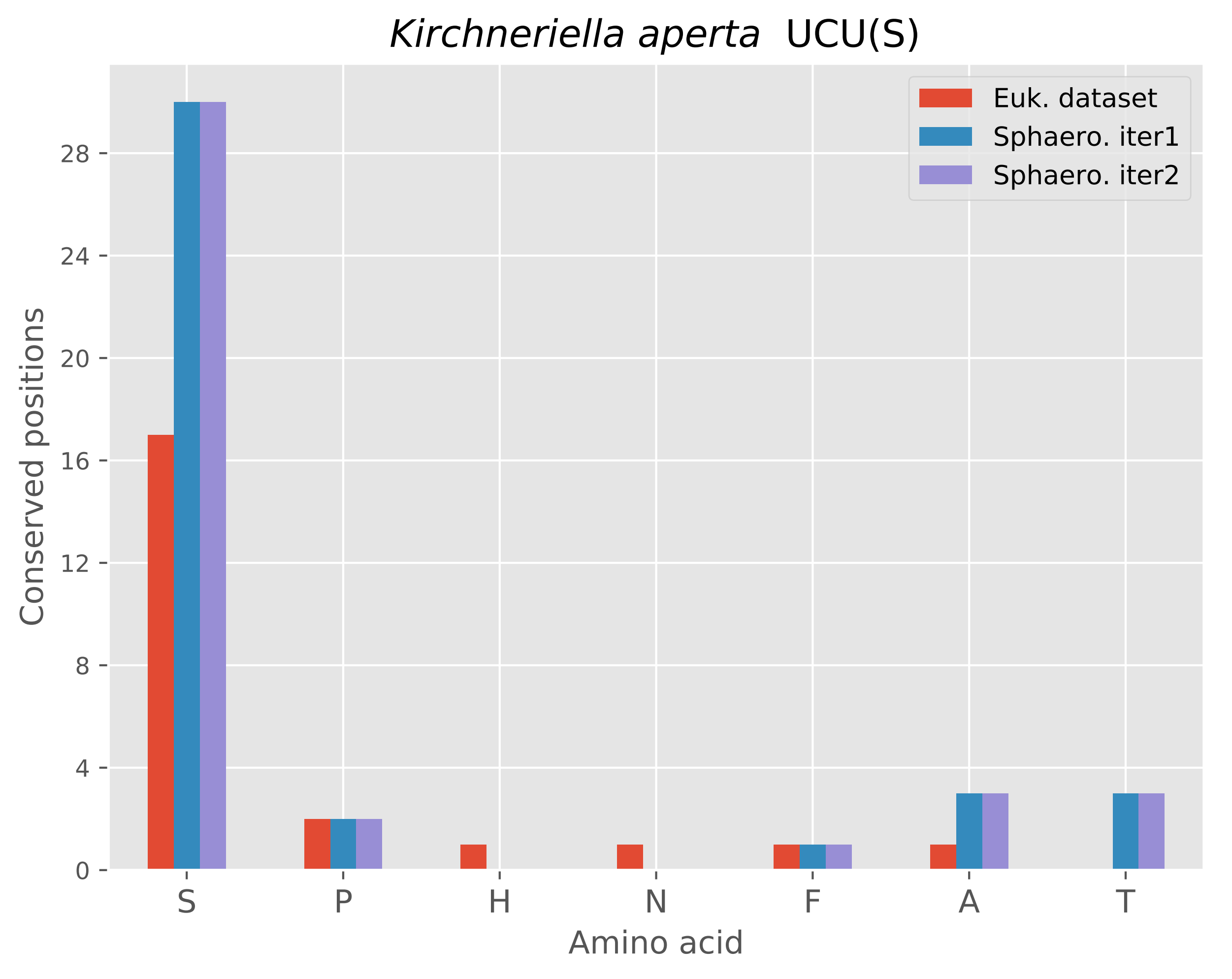
Kirchneriella aperta UCU(S)
Euk. dataset
Sphaero. iter1
Sphaero. iter2
Conserved positions
0
4
8
12
16
20
24
28
S
P
H
N
F
A
T
Amino acid

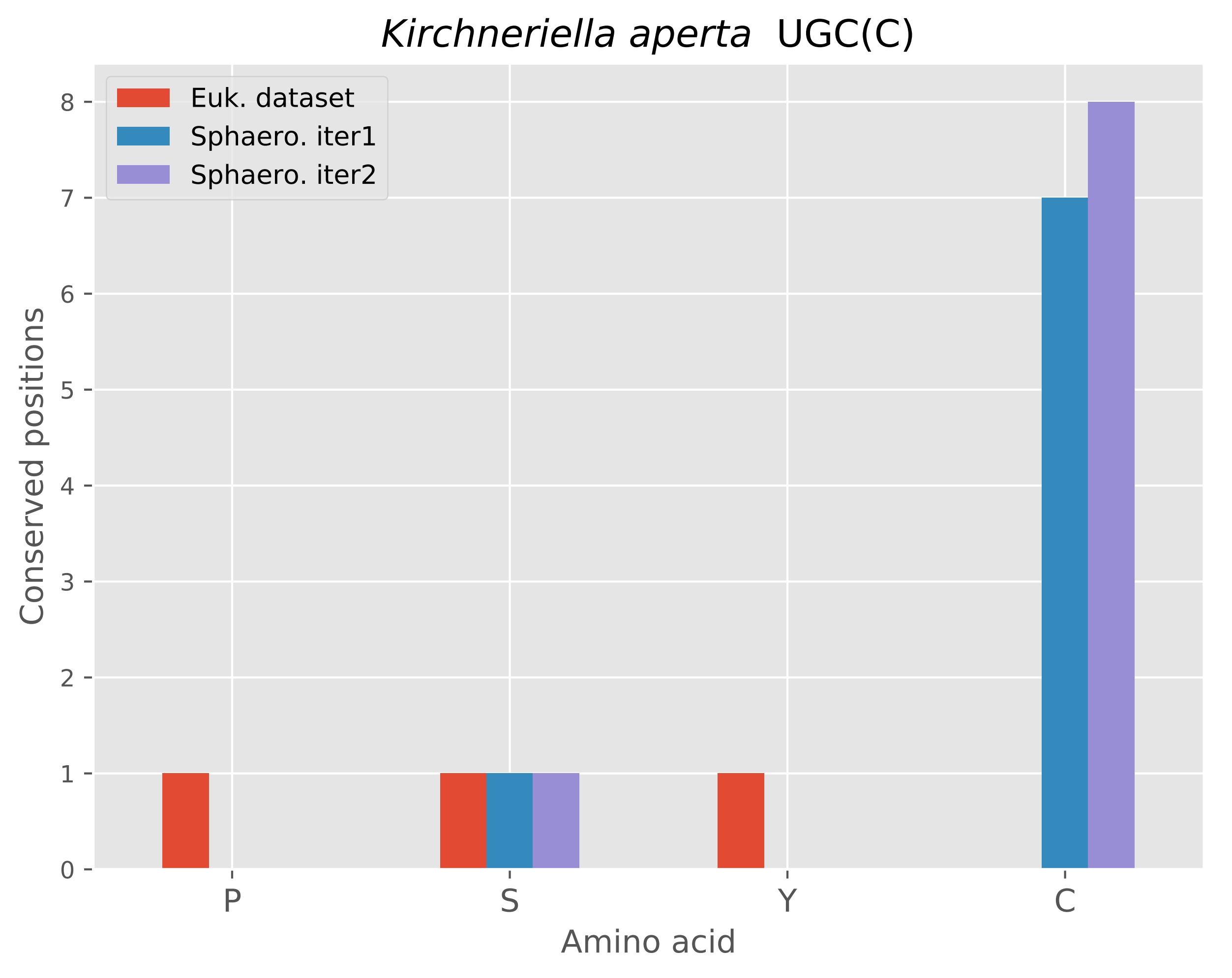

Kirchneriella aperta UGC(C)
Euk. dataset
Sphaero. iter1
Sphaero. iter2
Conserved positions
0
1
2
3
4
5
6
7
8
P
S
Y
C
Amino acid

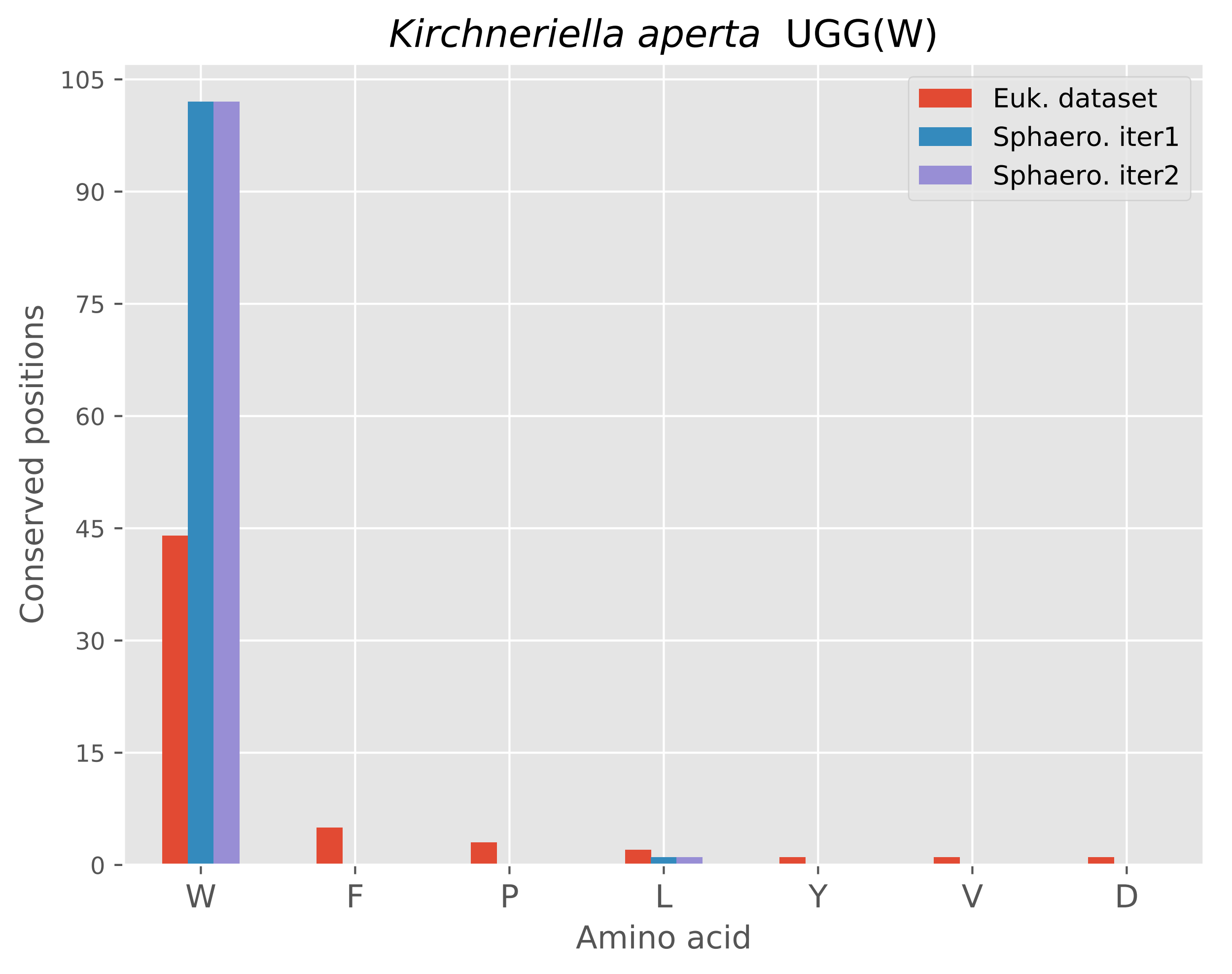

Kirchneriella aperta UGG(W)
Euk. dataset
Sphaero. iter1
Sphaero. iter2
Conserved positions
0
15
30
45
60
75
90
105
W
F
P
L
Y
V
D
Amino acid

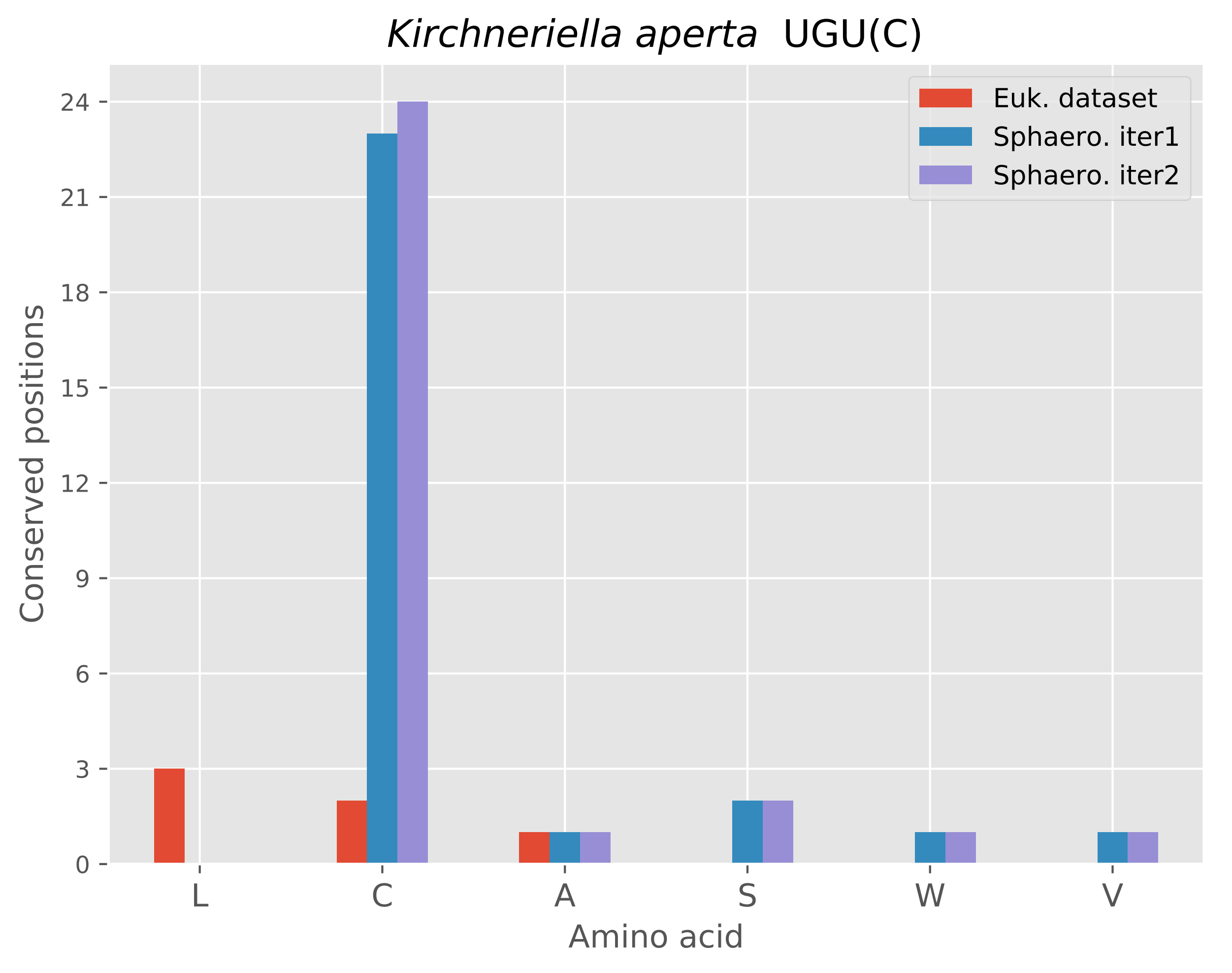

Kirchneriella aperta UGU(C)
Euk. dataset
Sphaero. iter1
Sphaero. iter2
Conserved positions
24
21
18
15
12
9
6
3
0
L
C
A
S
W
V
Amino acid

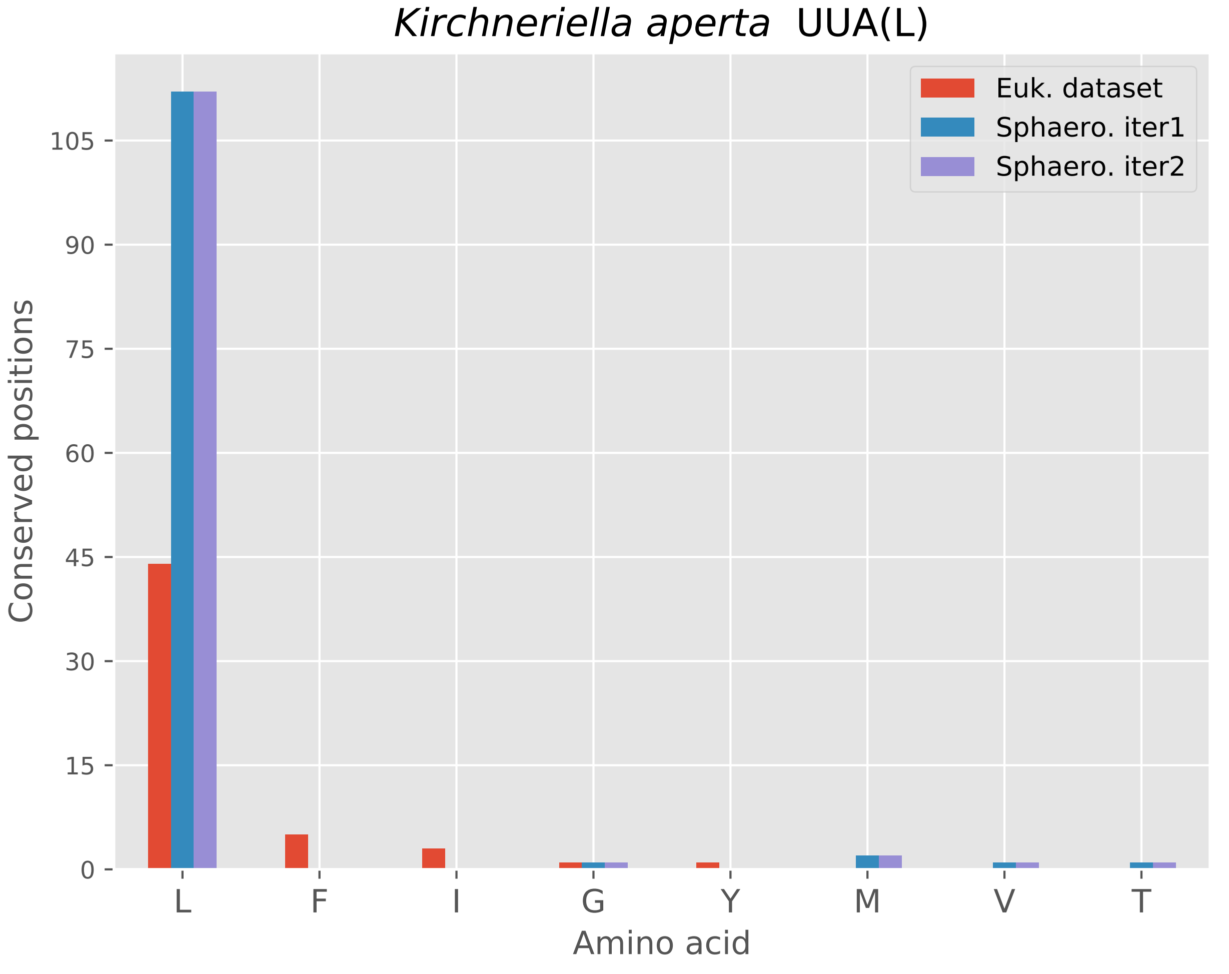

Kirchneriella aperta UUA(L)
Euk. dataset
Sphaero. iter1
Sphaero. iter2
Conserved positions
0
15
30
45
60
75
90
105
L
F
I
G
Y
M
V
T
Amino acid

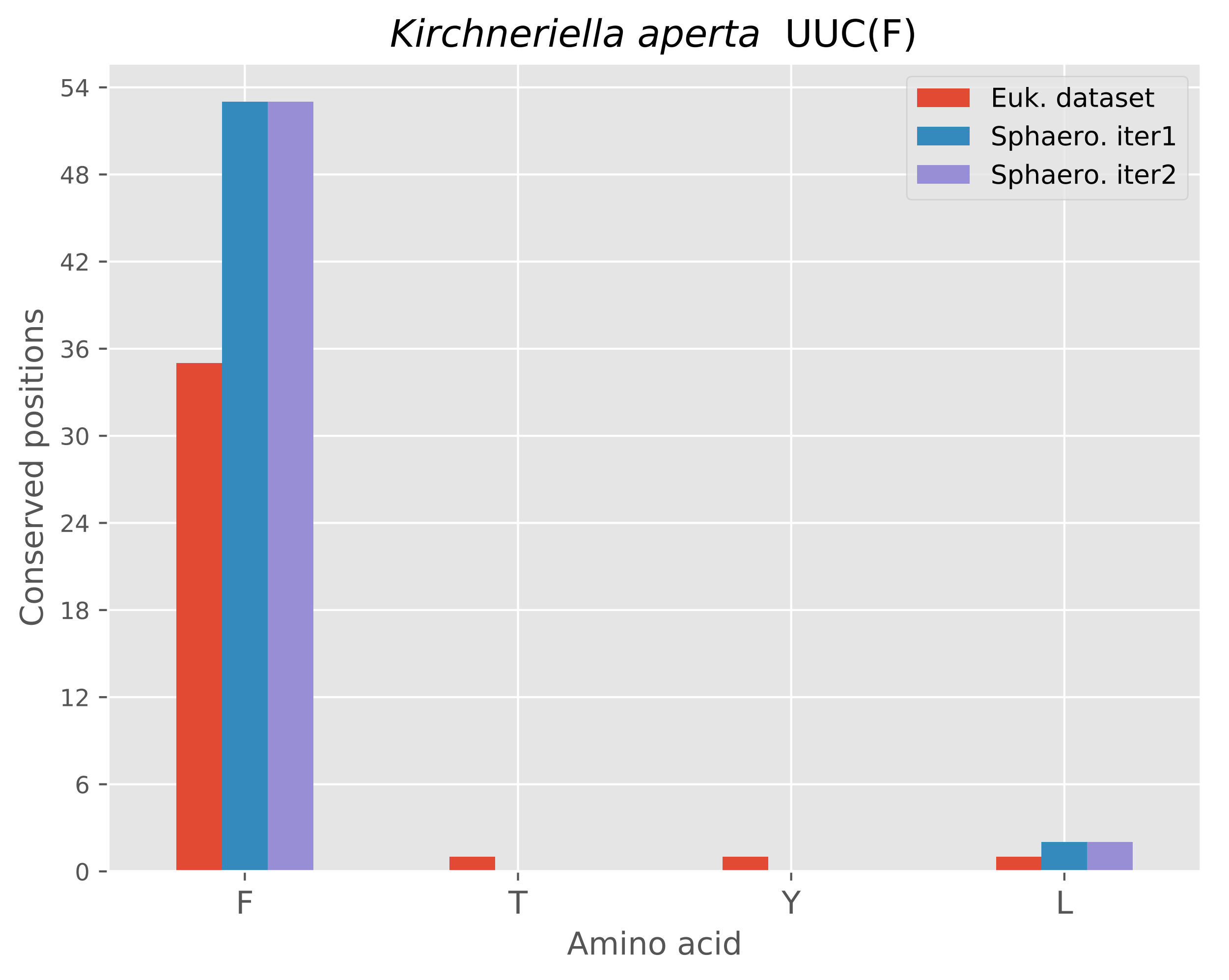
Kirchneriella aperta UUC(F)
Euk. dataset
Sphaero. iter1
Sphaero. iter2
Conserved positions
0
6
12
18
24
30
36
42
48
54
F
T
Y
L
Amino acid

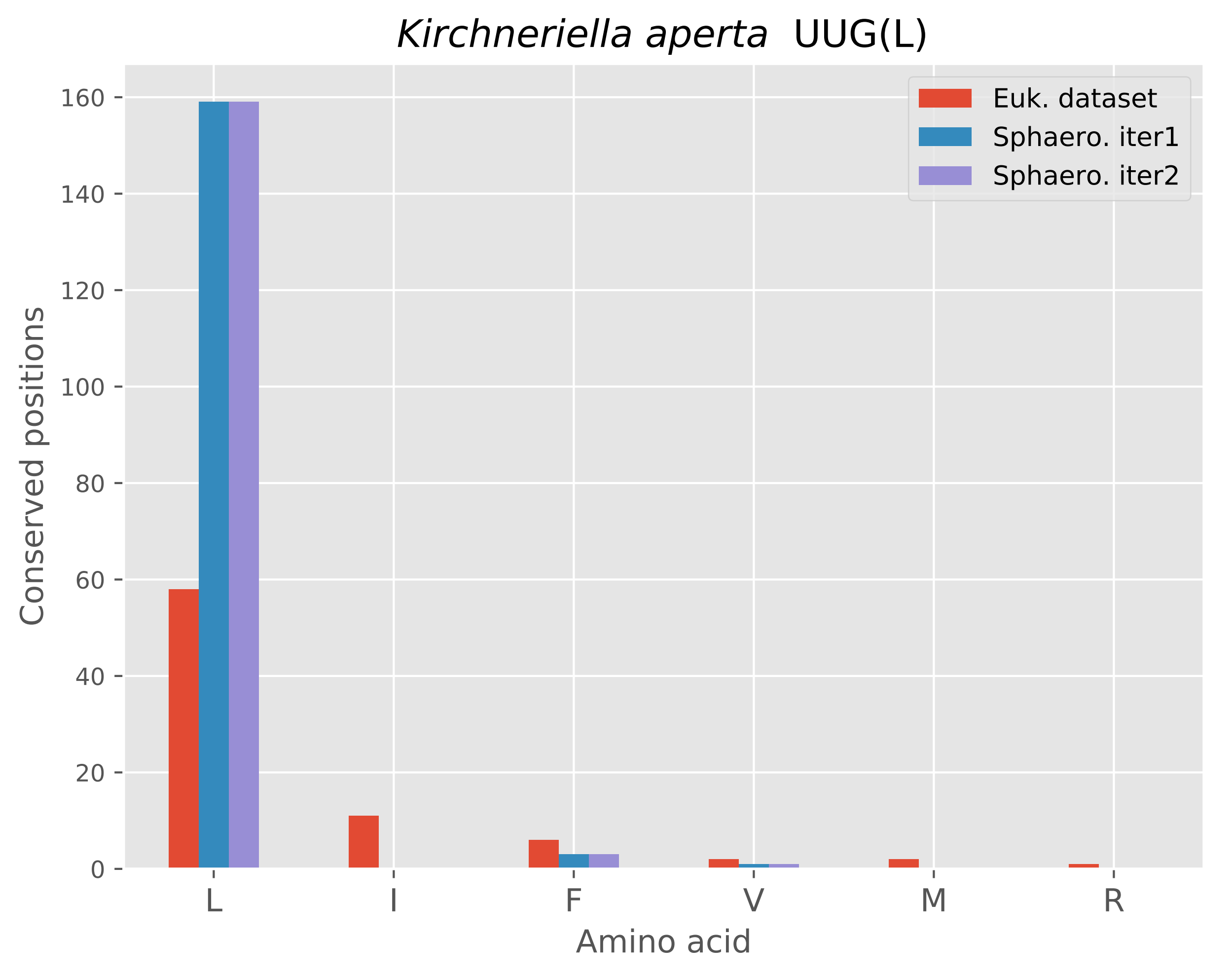

Kirchneriella aperta UUG(L)
Euk. dataset
Sphaero. iter1
Sphaero. iter2
Conserved positions
0
20
40
60
80
100
120
140
160
L
I
F
V
M
R
Amino acid

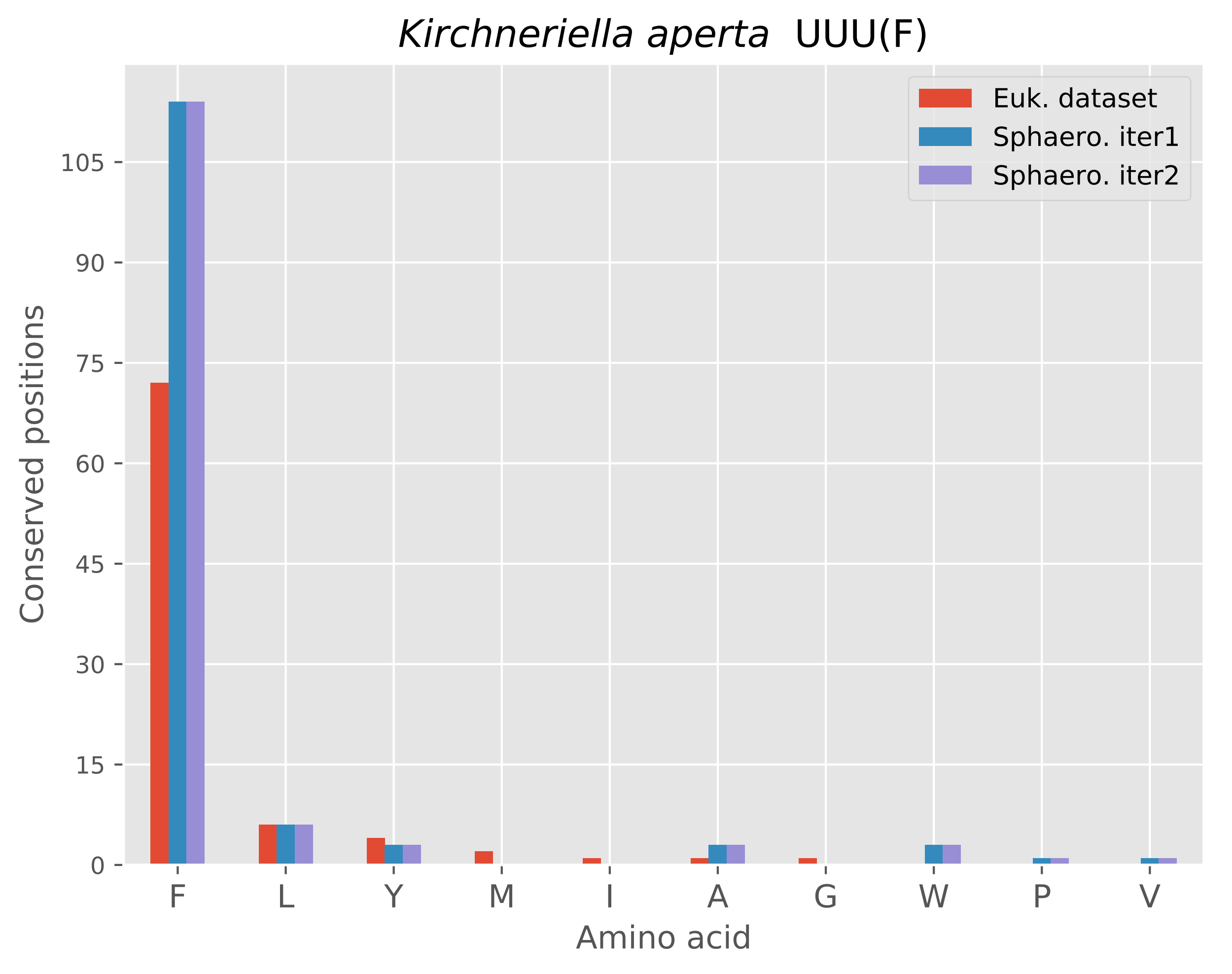

Kirchneriella aperta UUU(F)
Euk. dataset
Sphaero. iter1
Sphaero. iter2
Conserved positions
0
15
30
45
60
75
90
105
F
L
Y
M
I
A
G
W
P
V
Amino acid

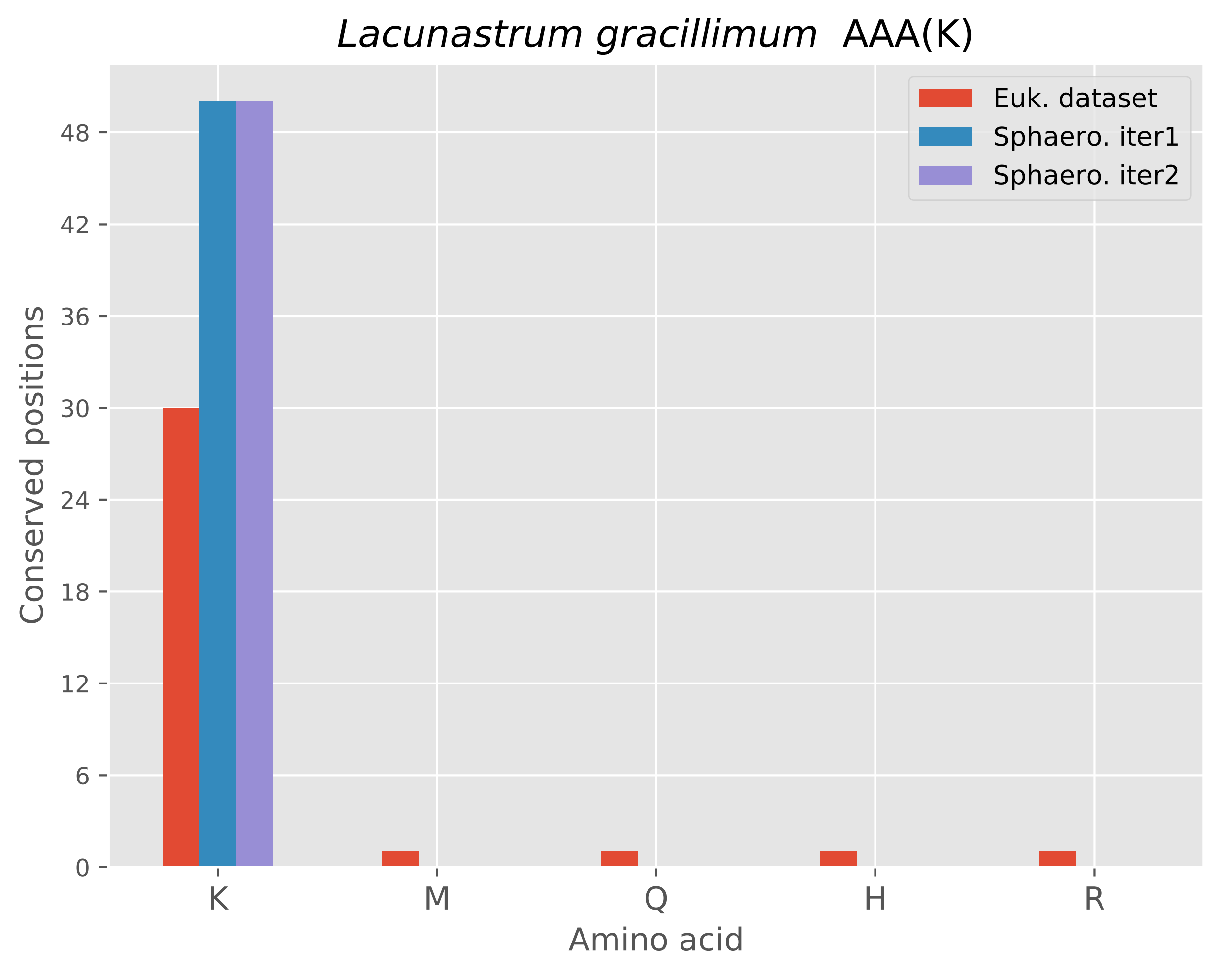

Lacunastrum gracillimum AAA(K)
Euk. dataset
Sphaero. iter1
Sphaero. iter2
Conserved positions
0
6
12
18
24
30
36
42
48
K
M
Q
H
R
Amino acid

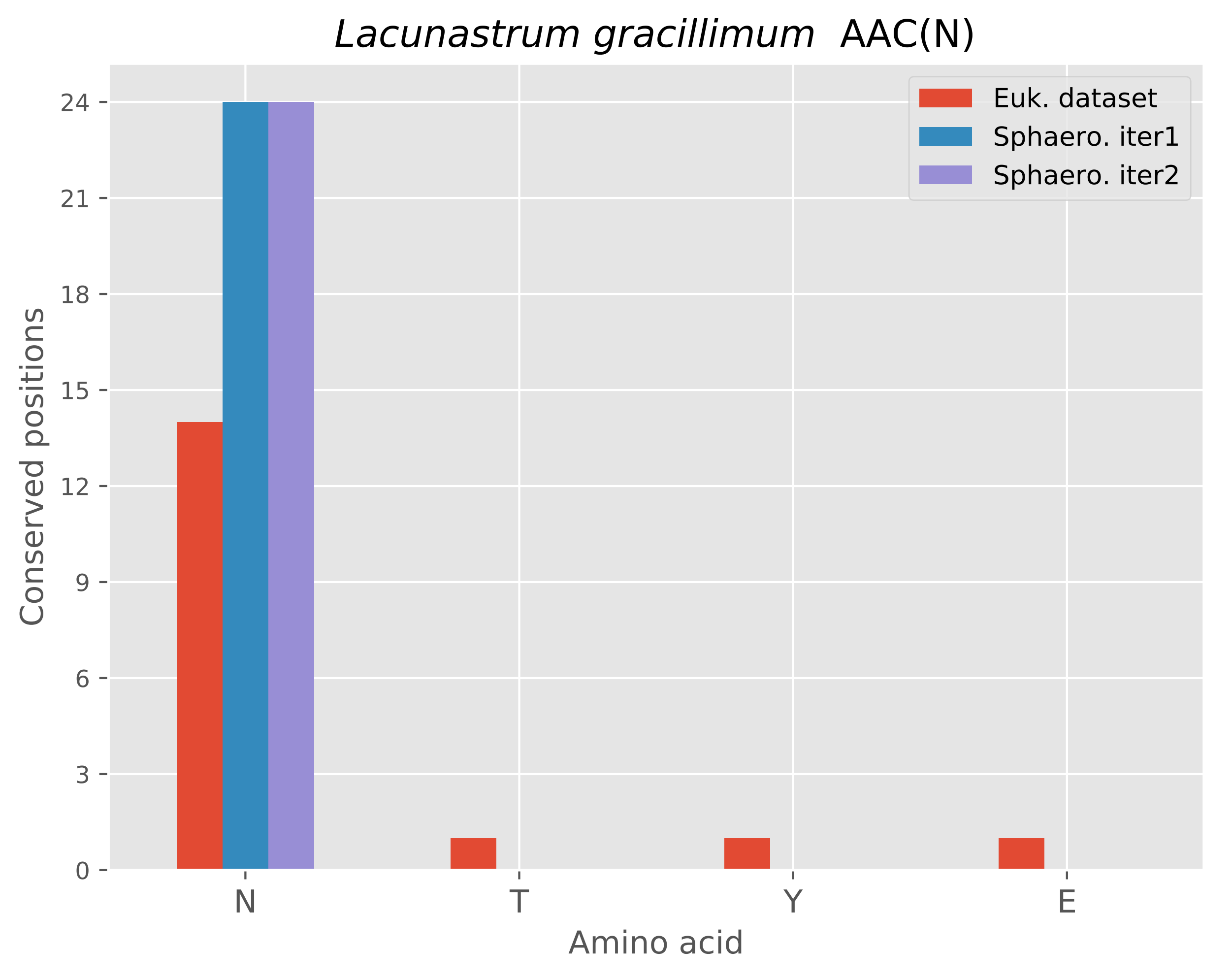

Lacunastrum gracillimum AAC(N)
Euk. dataset
Sphaero. iter1
Sphaero. iter2
Conserved positions
0
3
6
9
12
15
18
21
24
N
T
Y
E
Amino acid

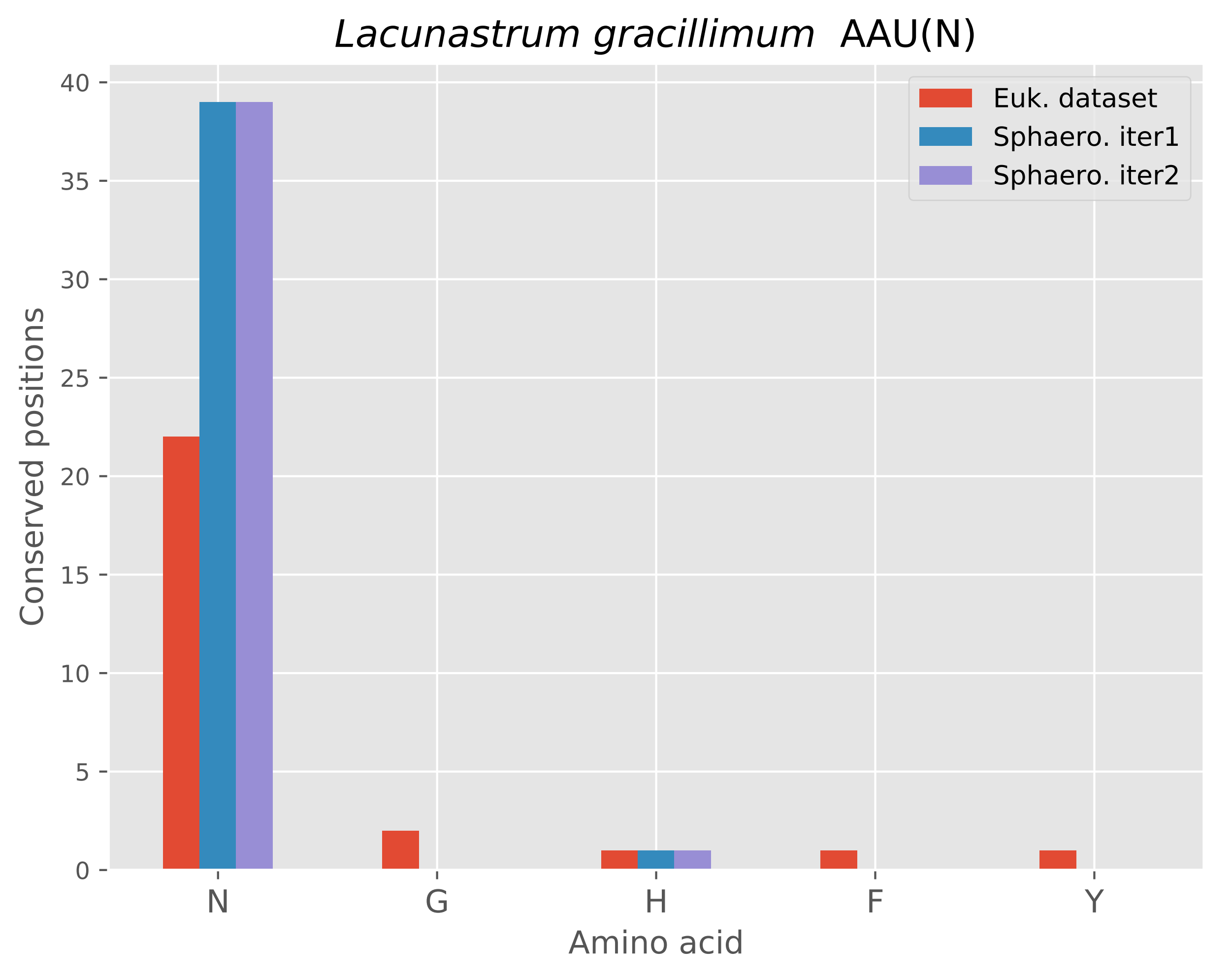

Lacunastrum gracillimum AAU(N)
Euk. dataset
Sphaero. iter1
Sphaero. iter2
Conserved positions
0
5
10
15
20
25
30
35
40
N
G
H
F
Y
Amino acid

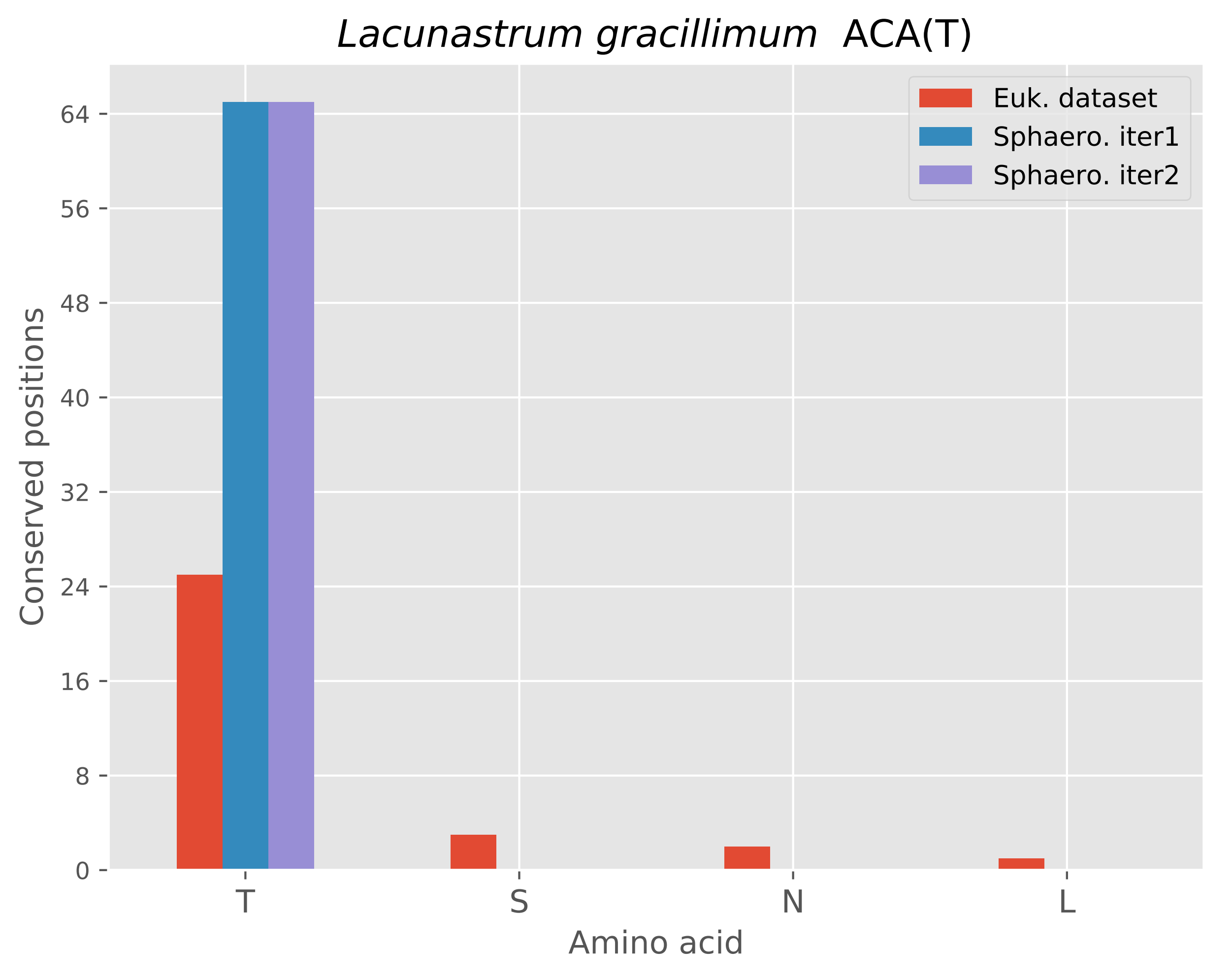

Lacunastrum gracillimum ACA(T)
Euk. dataset
Sphaero. iter1
Sphaero. iter2
Conserved positions
0
8
16
24
32
40
48
56
64
T
S
N
L
Amino acid

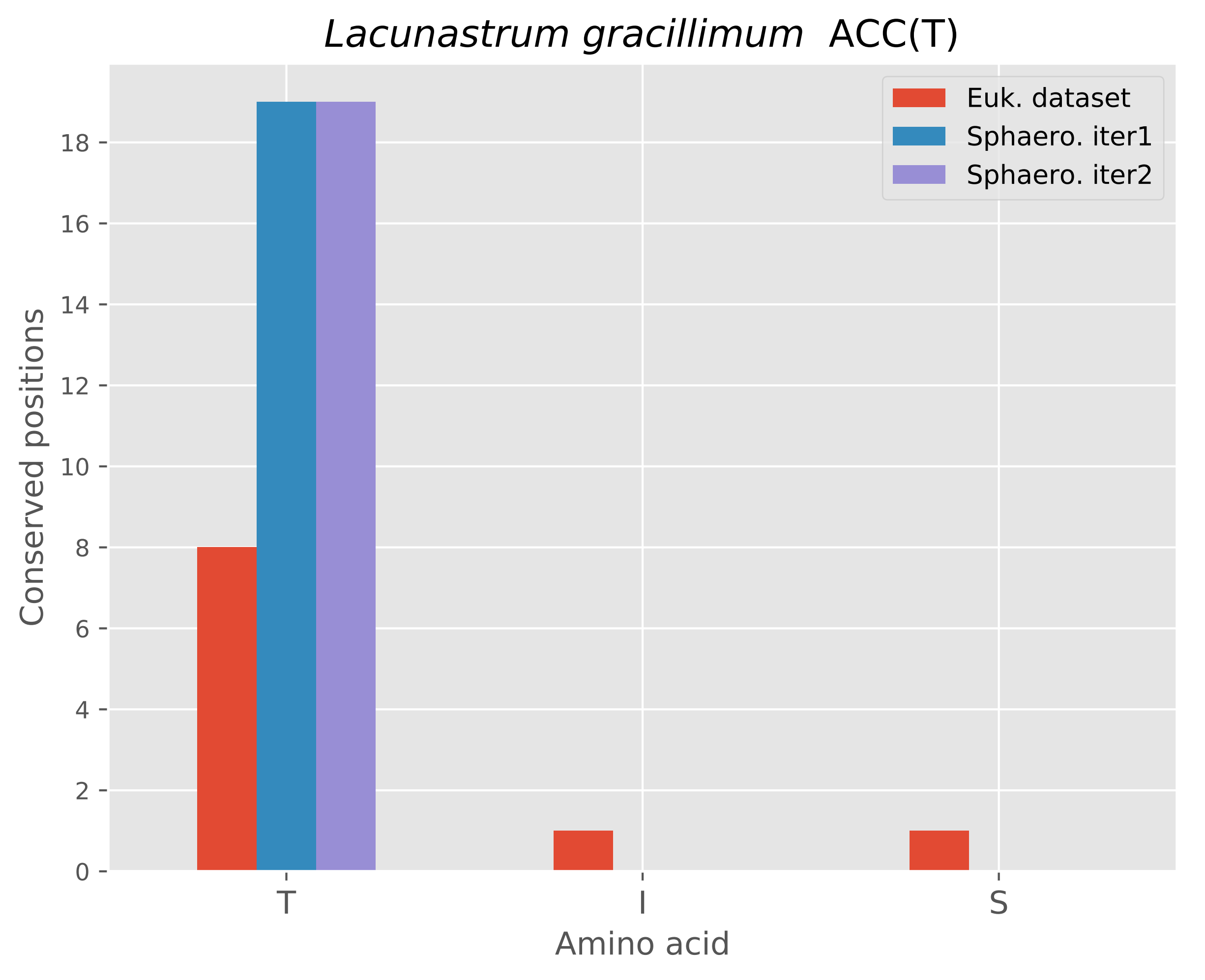

Lacunastrum gracillimum ACC(T)
Euk. dataset
Sphaero. iter1
Sphaero. iter2
Conserved positions
0
2
4
6
8
10
12
14
16
18
T
I
S
Amino acid

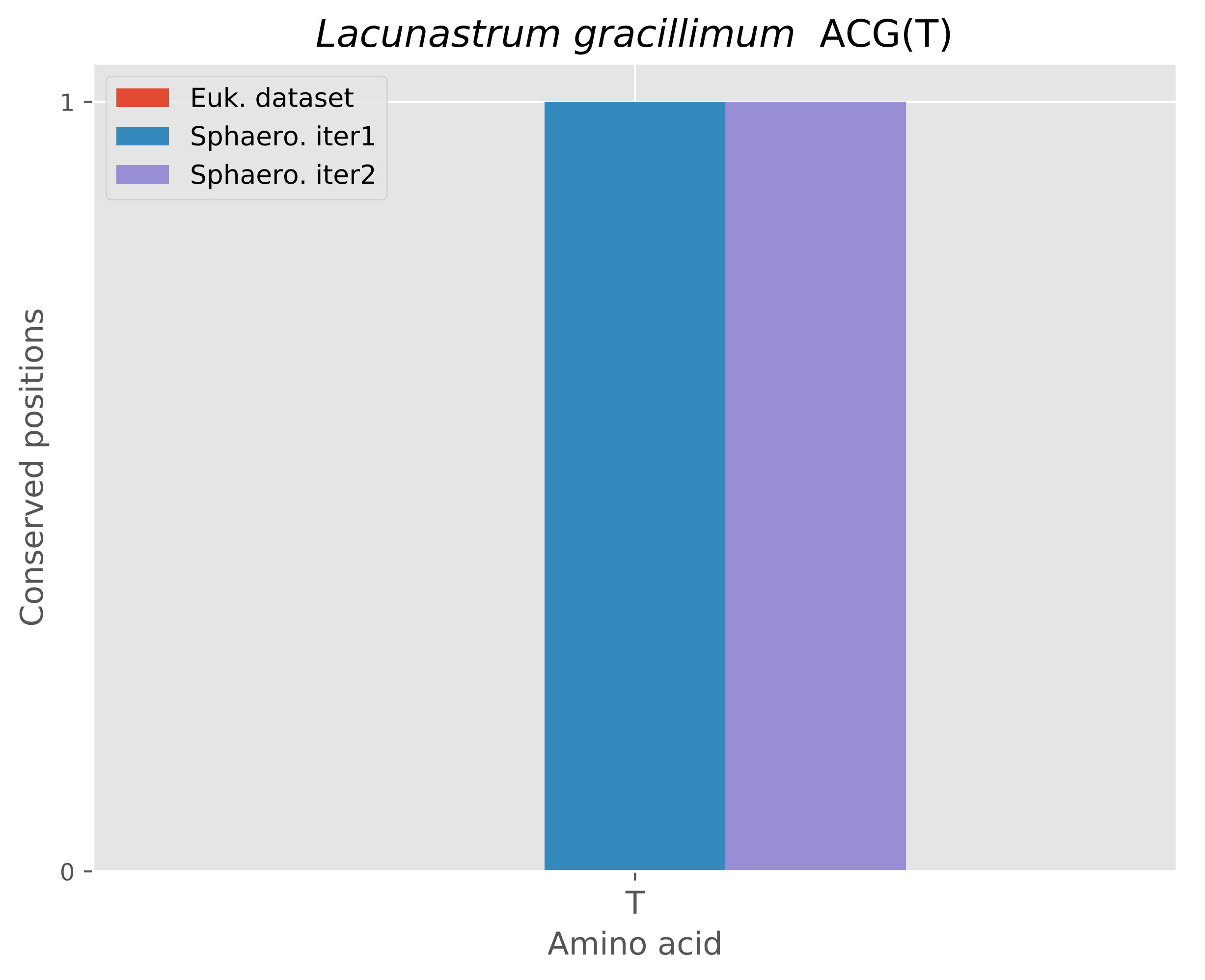

Lacunastrum gracillimum ACG(T)
Euk. dataset
Sphaero. iter1
Sphaero. iter2
1
0
Conserved positions
T
Amino acid

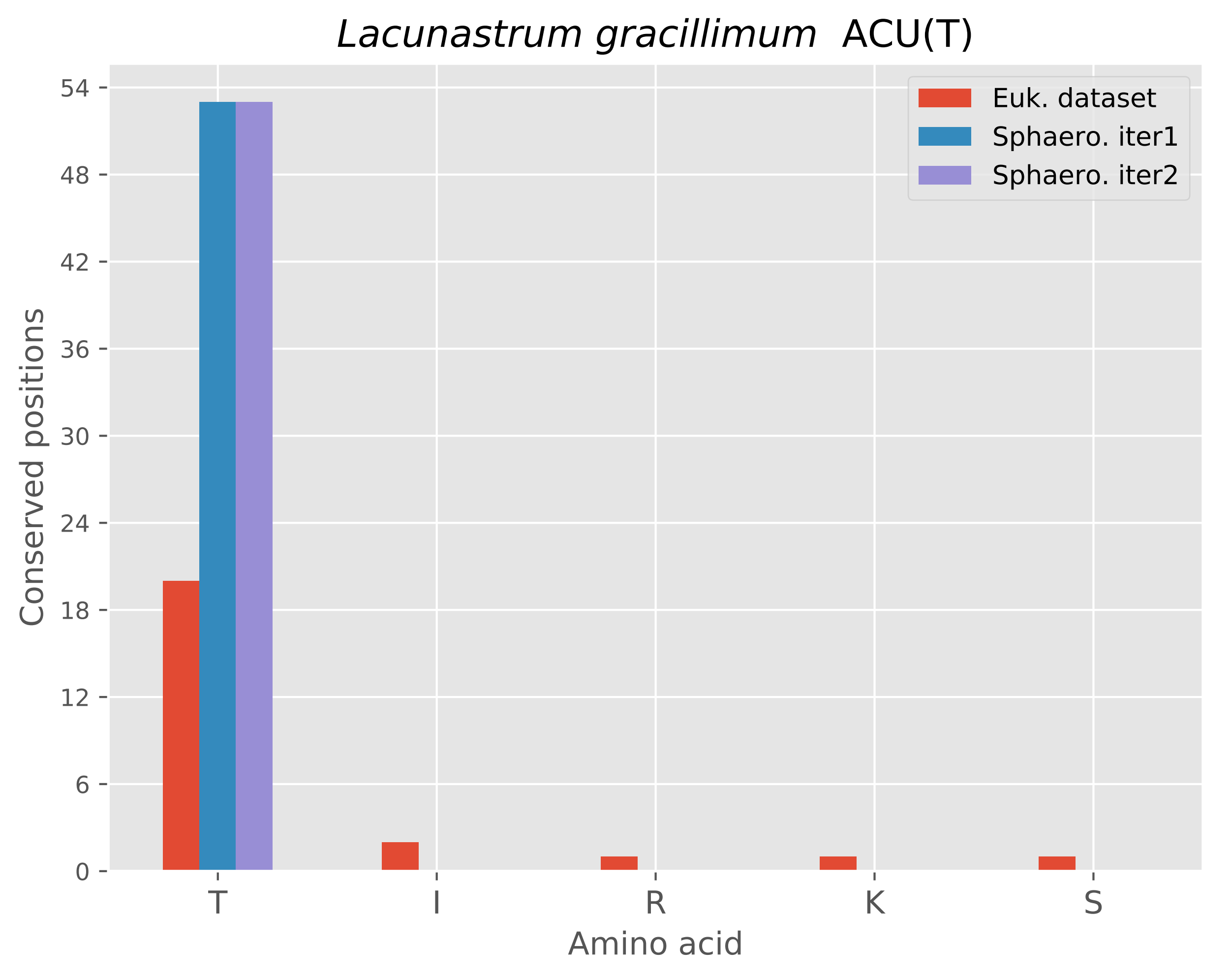

Lacunastrum gracillimum ACU(T)
Euk. dataset
Sphaero. iter1
Sphaero. iter2
Conserved positions
0
6
12
18
24
30
36
42
48
54
T
I
R
K
S
Amino acid

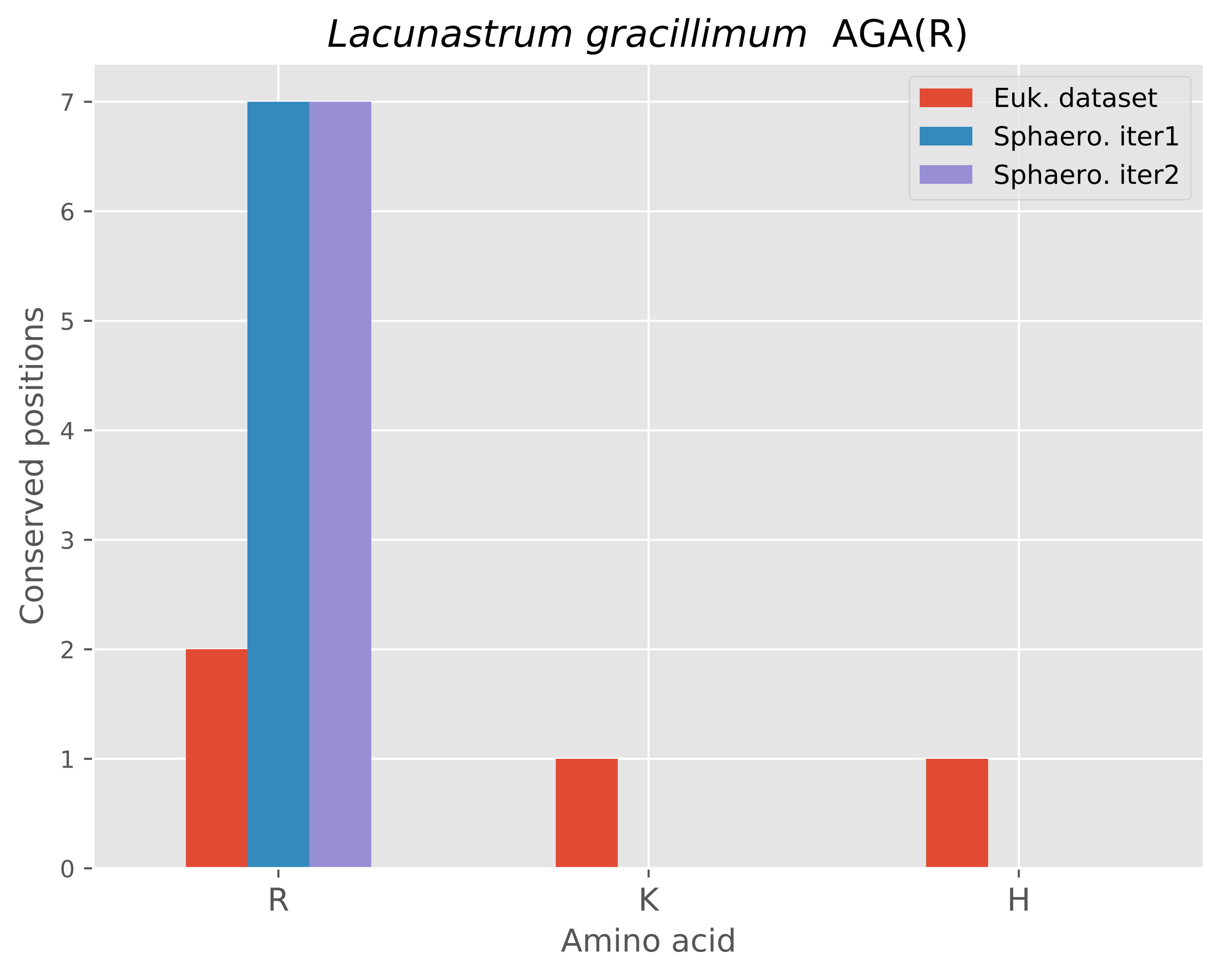
Lacunastrum gracillimum AGA(R)
Euk. dataset
Sphaero. iter1
Sphaero. iter2
Conserved positions
0
1
2
3
4
5
6
7
R
K
H
Amino acid

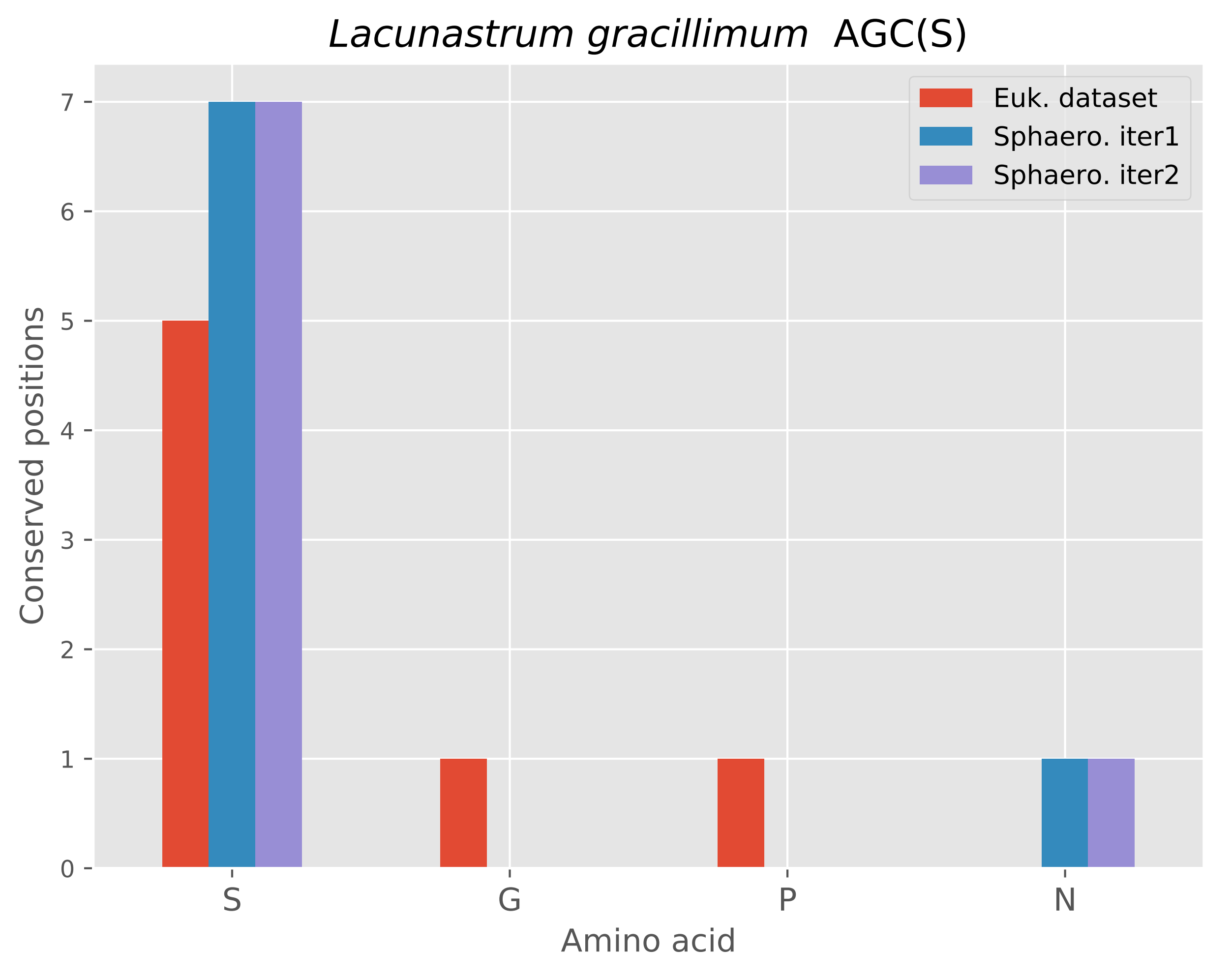

Lacunastrum gracillimum AGC(S)
Euk. dataset
Sphaero. iter1
Sphaero. iter2
Conserved positions
0
1
2
3
4
5
6
7
S
G
P
N
Amino acid

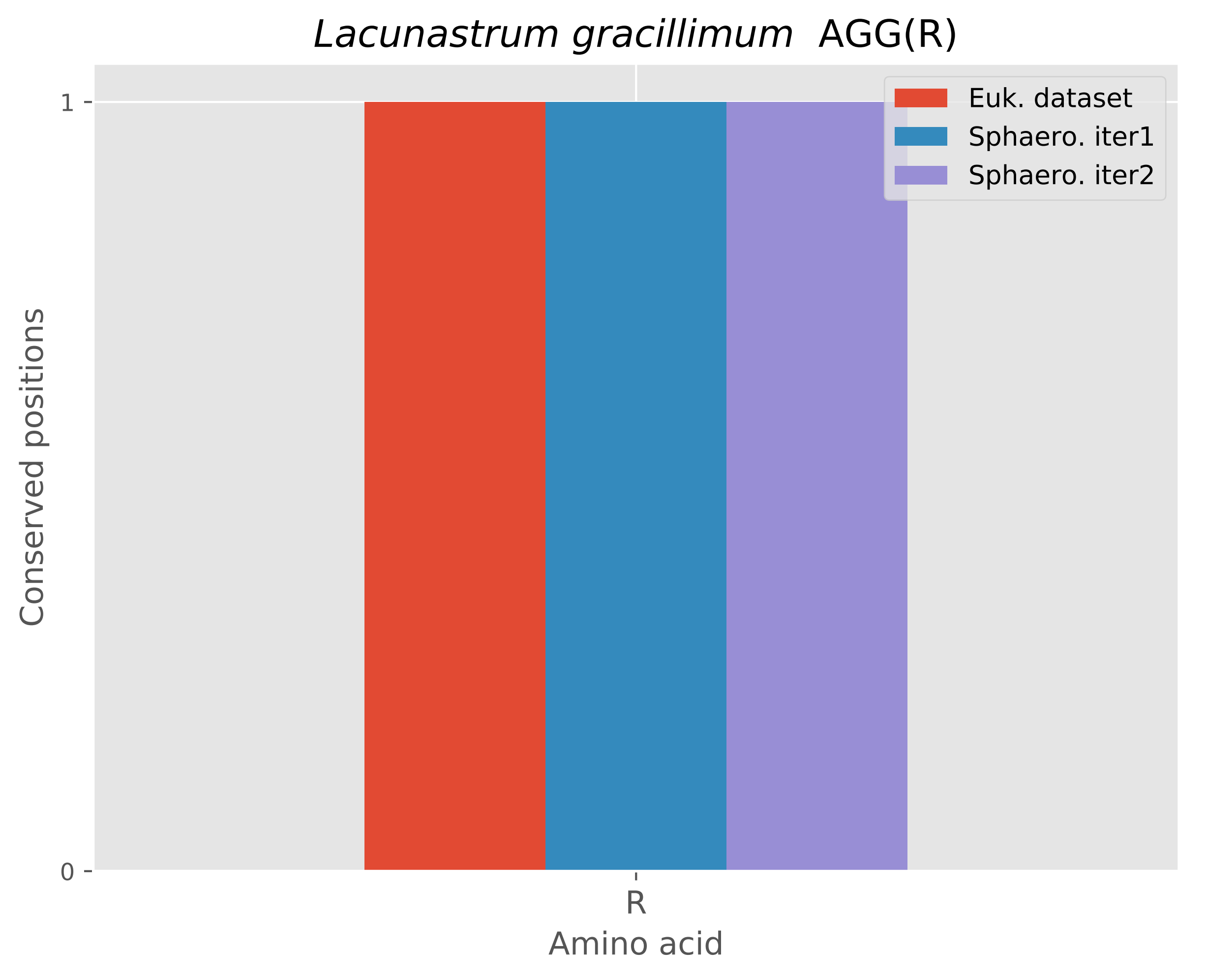

Lacunastrum gracillimum AGG(R)
Euk. dataset
Sphaero. iter1
Sphaero. iter2
Conserved positions
1
0
R
Amino acid

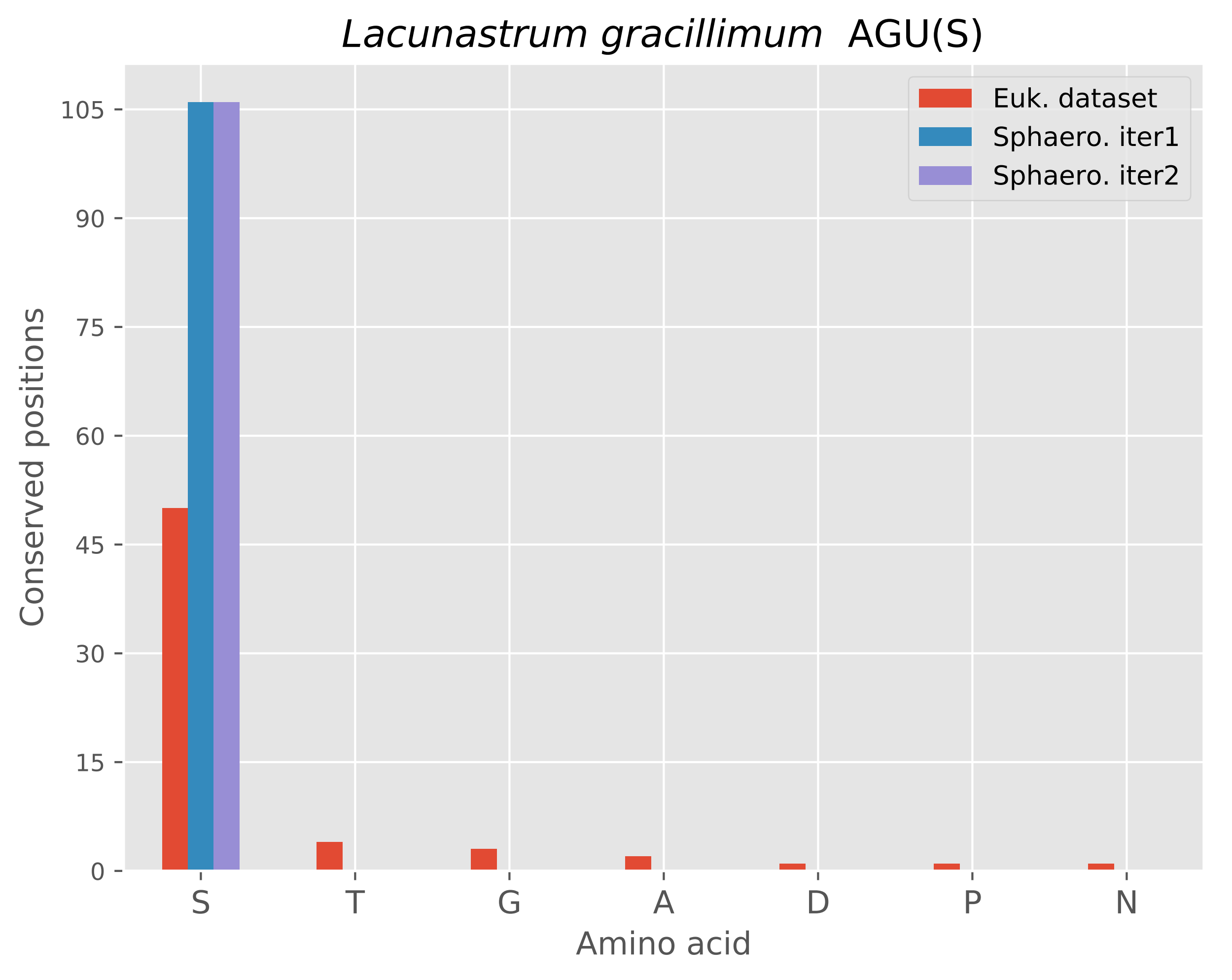

Lacunastrum gracillimum AGU(S)
Euk. dataset
Sphaero. iter1
Sphaero. iter2
Conserved positions
0
15
30
45
60
75
90
105
S
T
G
A
D
P
N
Amino acid

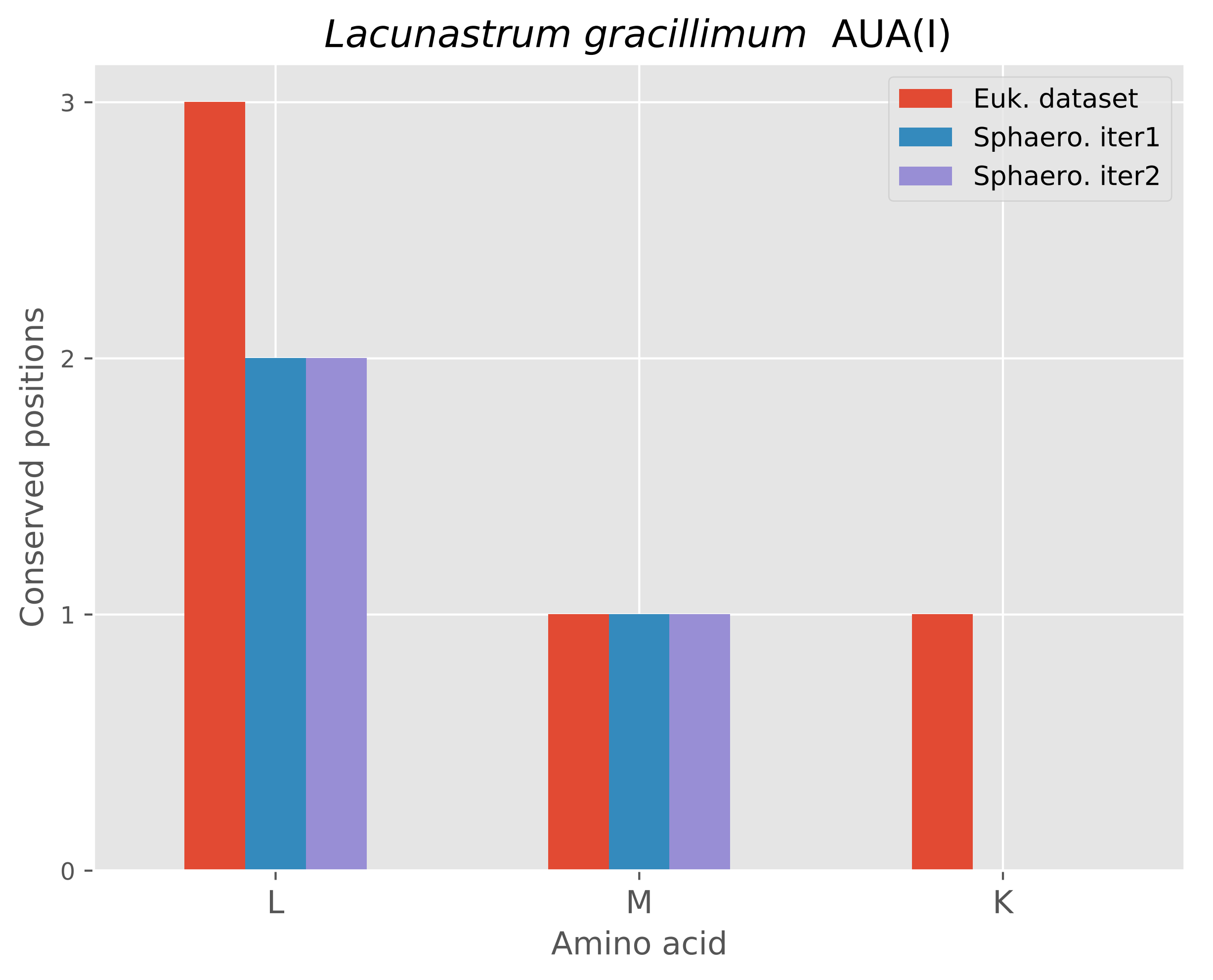

Lacunastrum gracillimum AUA(I)
Euk. dataset
Sphaero. iter1
Sphaero. iter2
Conserved positions
0
1
2
3
L
M
K
Amino acid

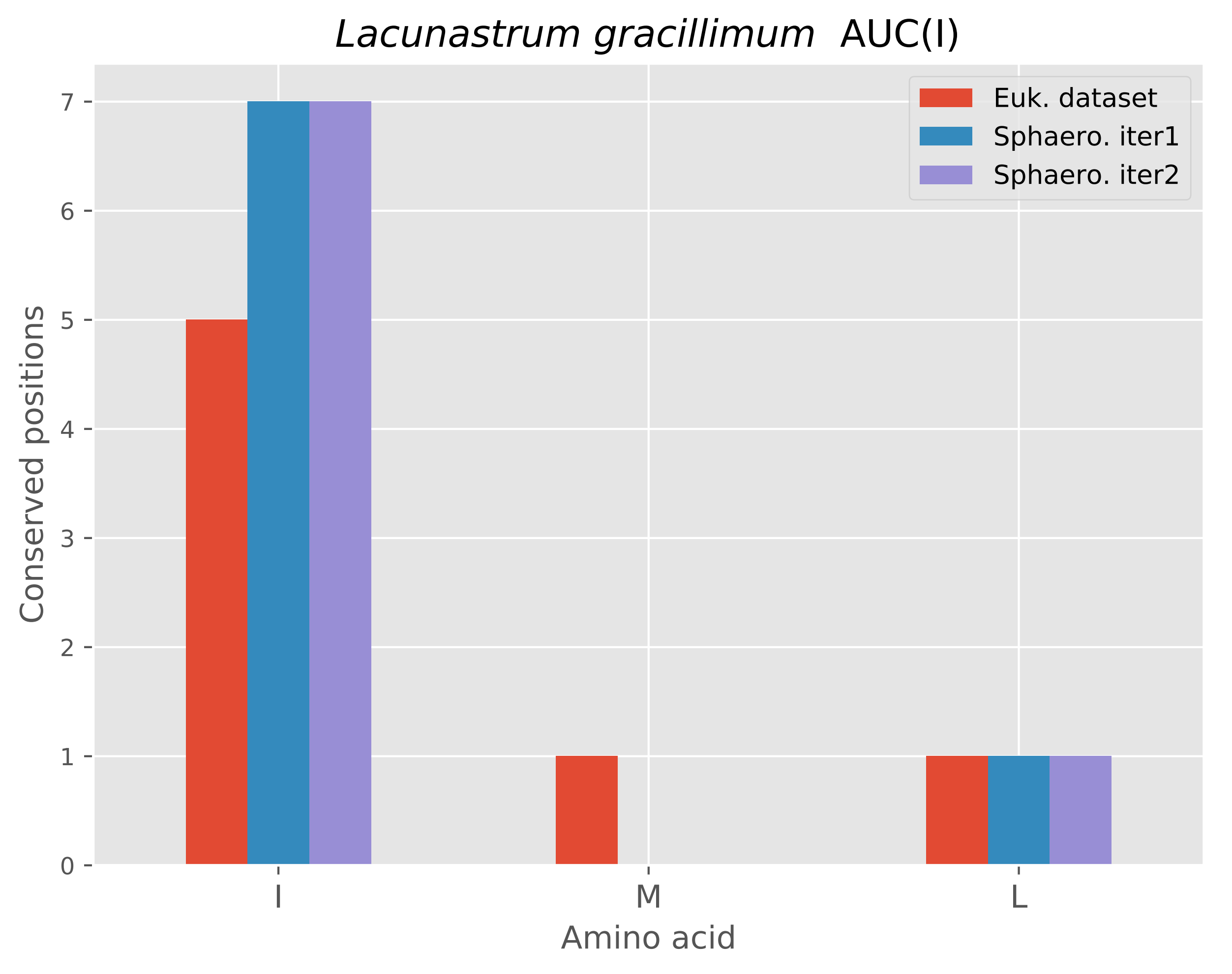

Lacunastrum gracillimum AUC(I)
Euk. dataset
Sphaero. iter1
Sphaero. iter2
Conserved positions
0
1
2
3
4
5
6
7
I
M
L
Amino acid

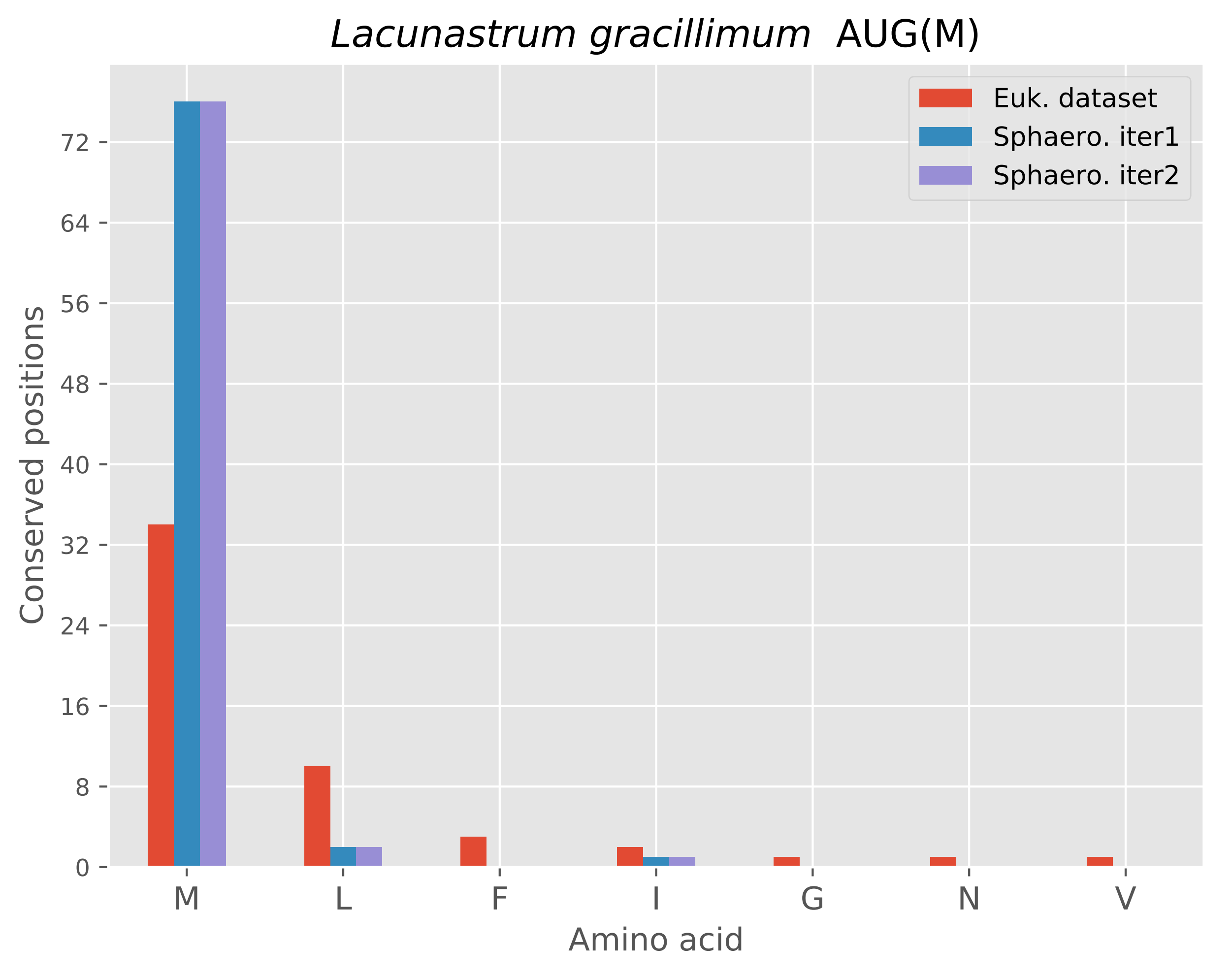

Lacunastrum gracillimum AUG(M)
Euk. dataset
Sphaero. iter1
Sphaero. iter2
Conserved positions
0
8
16
24
32
40
48
56
64
72
M
L
F
I
G
N
V
Amino acid

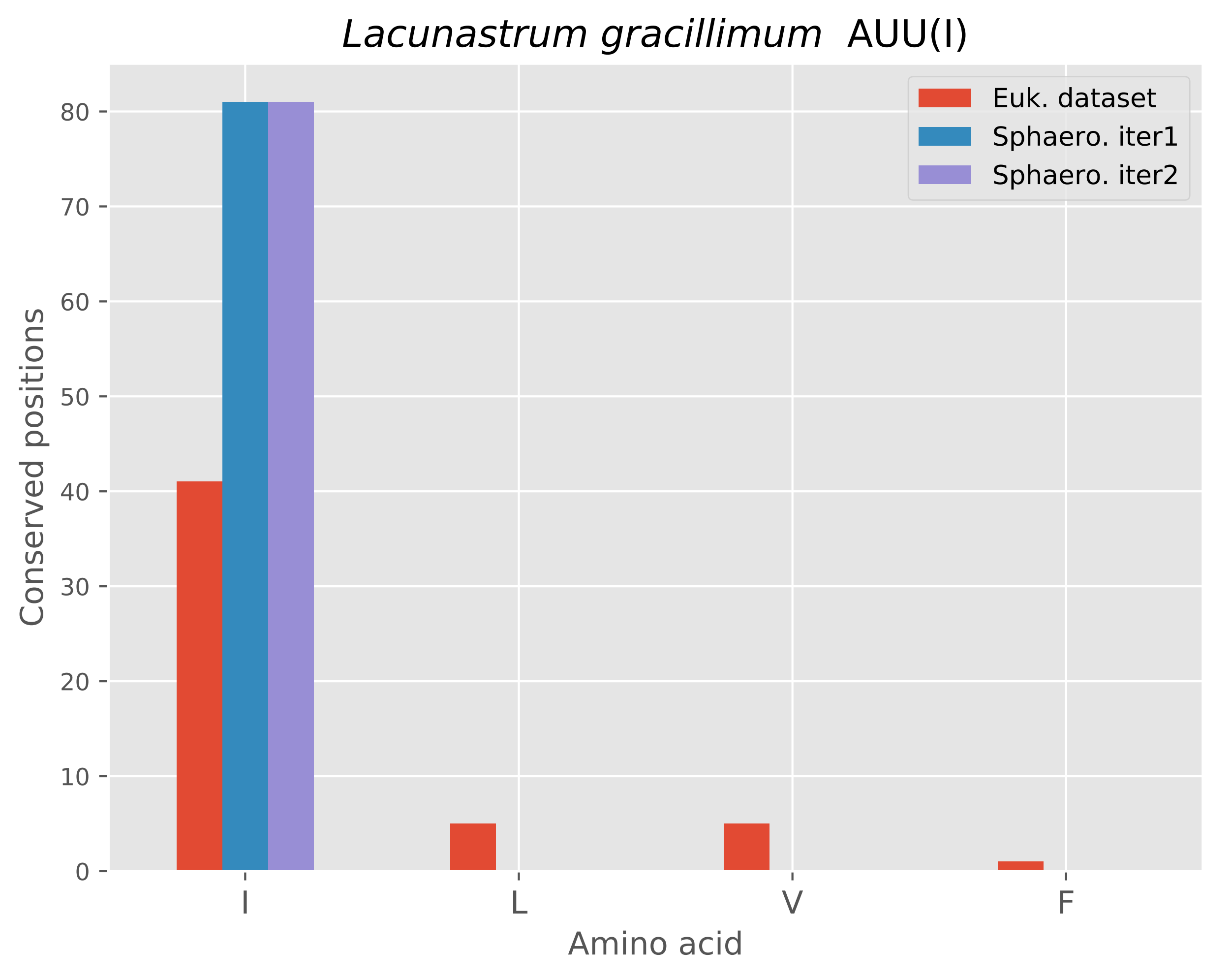

Lacunastrum gracillimum AUU(I)
Euk. dataset
Sphaero. iter1
Sphaero. iter2
Conserved positions
0
10
20
30
40
50
60
70
80
I
L
V
F
Amino acid

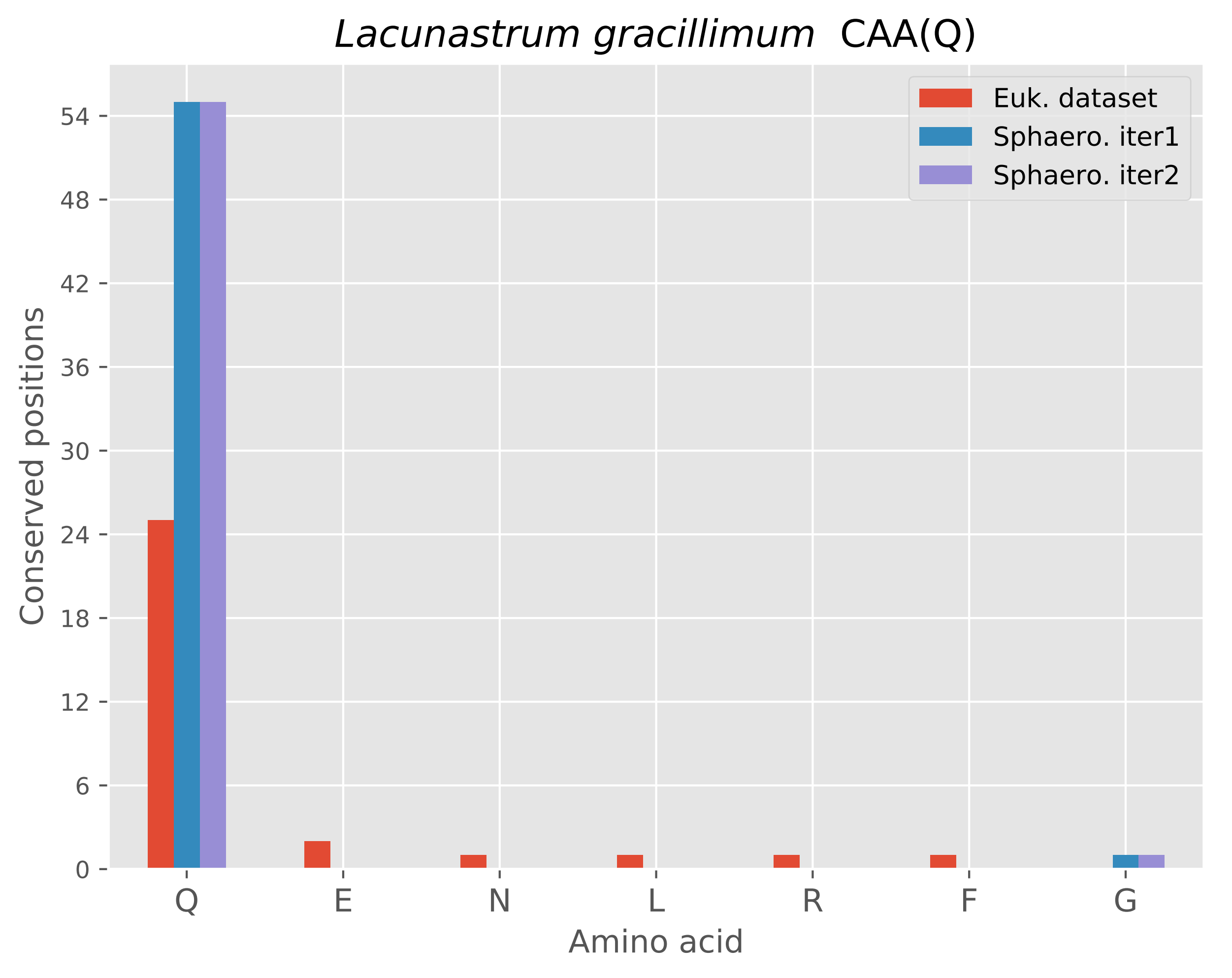

Lacunastrum gracillimum CAA(Q)
Euk. dataset
Sphaero. iter1
Sphaero. iter2
Conserved positions
0
6
12
18
24
30
36
42
48
54
Q
E
N
L
R
F
G
Amino acid

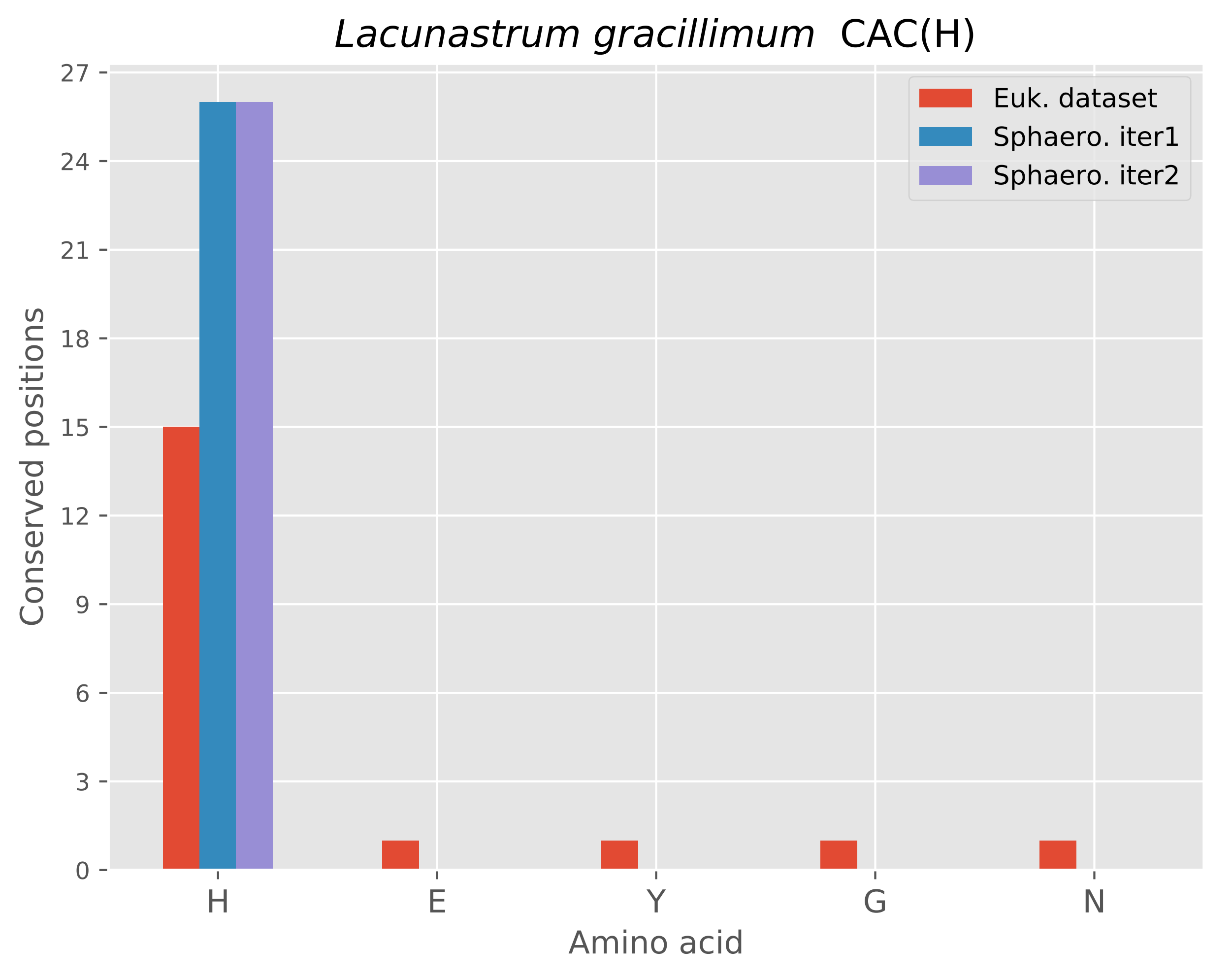

Lacunastrum gracillimum CAC(H)
Euk. dataset
Sphaero. iter1
Sphaero. iter2
Conserved positions
0
3
6
9
12
15
18
21
24
27
H
E
Y
G
N
Amino acid

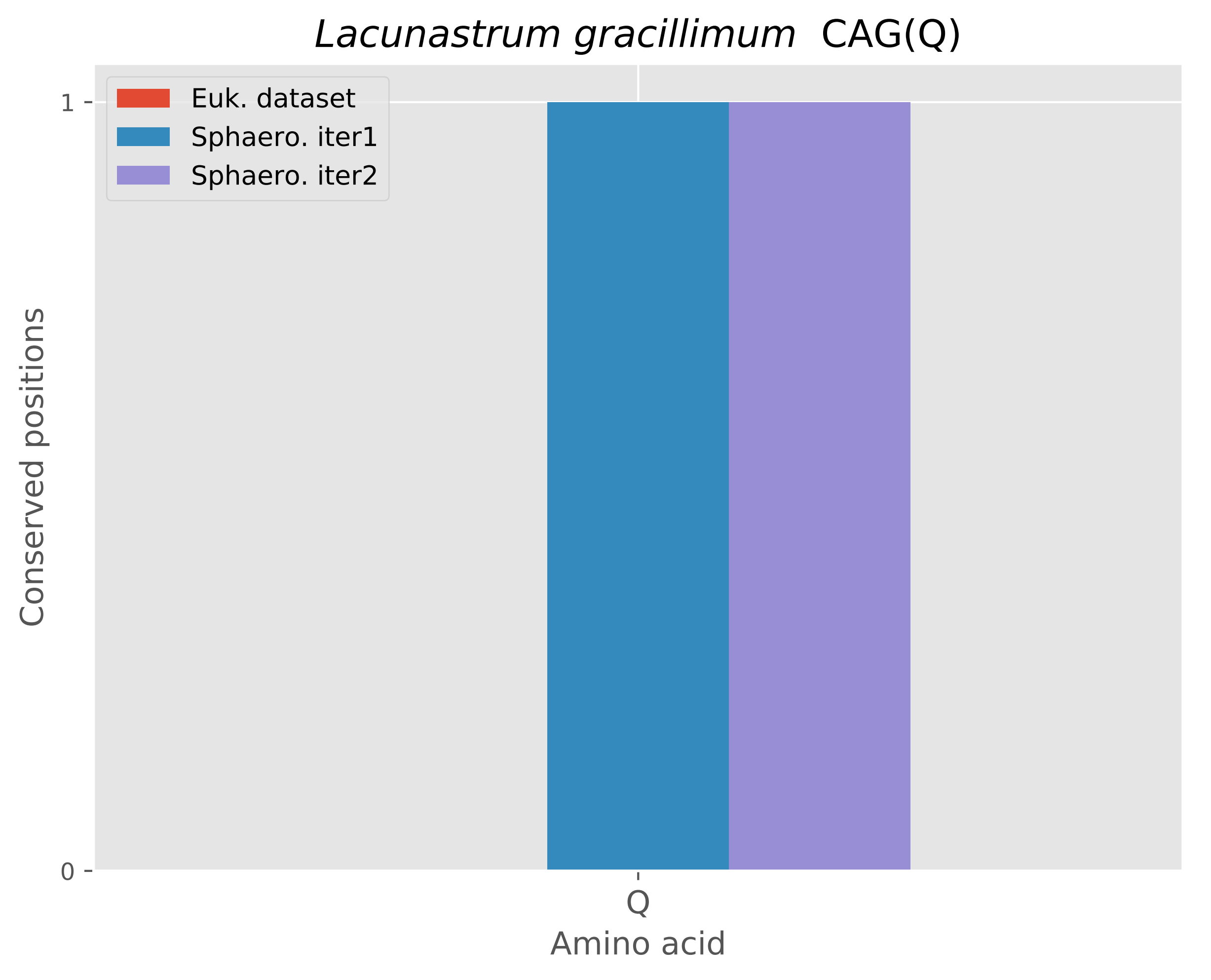
Lacunastrum gracillimum CAG(Q)
Euk. dataset
Sphaero. iter1
Sphaero. iter2
1
0
Conserved positions
Q
Amino acid

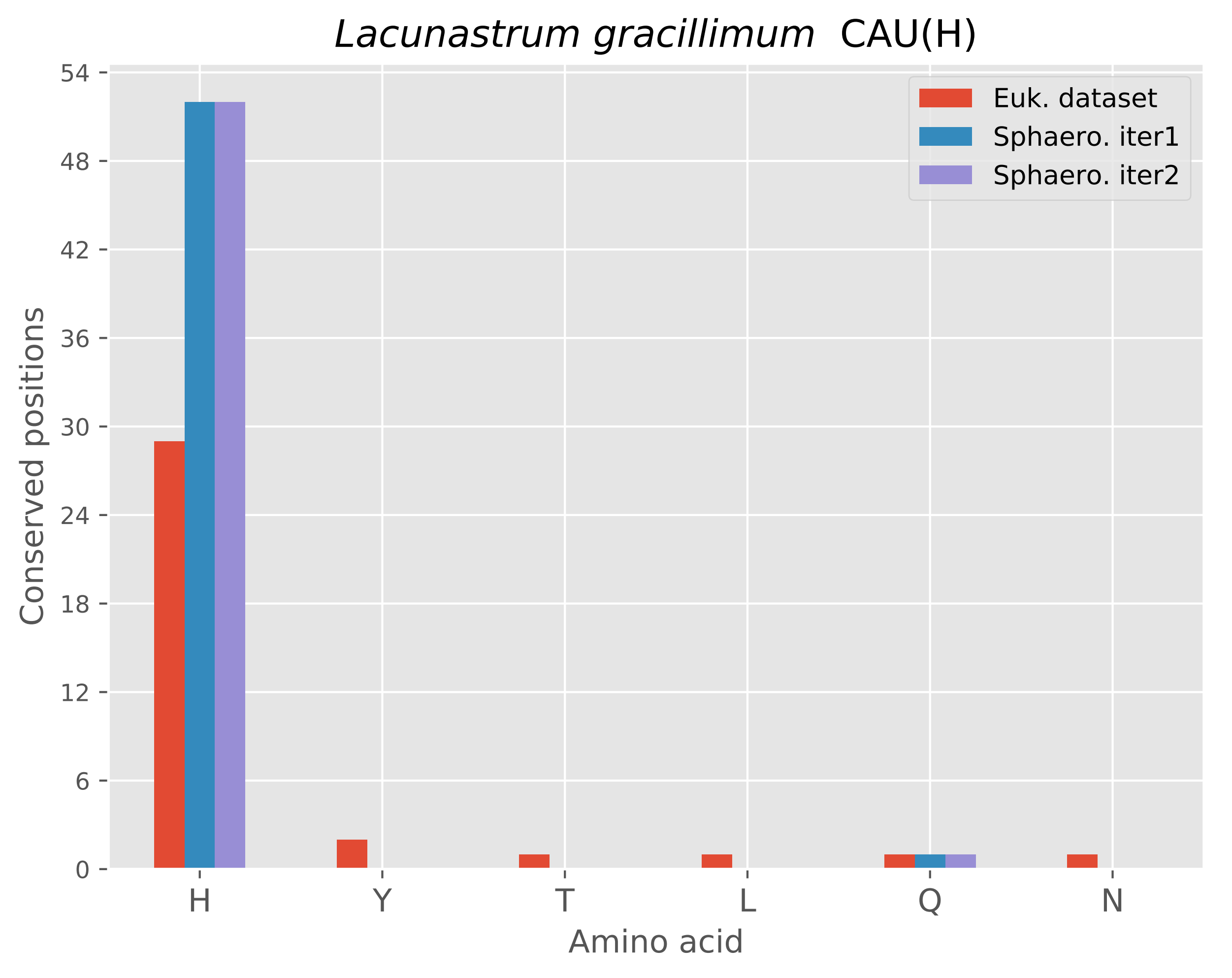

Lacunastrum gracillimum CAU(H)
Conserved positions
Amino acid
H
Y
T
L
Q
N
0
6
12
18
24
30
36
42
48
54
Euk. dataset
Sphaero. iter1
Sphaero. iter2

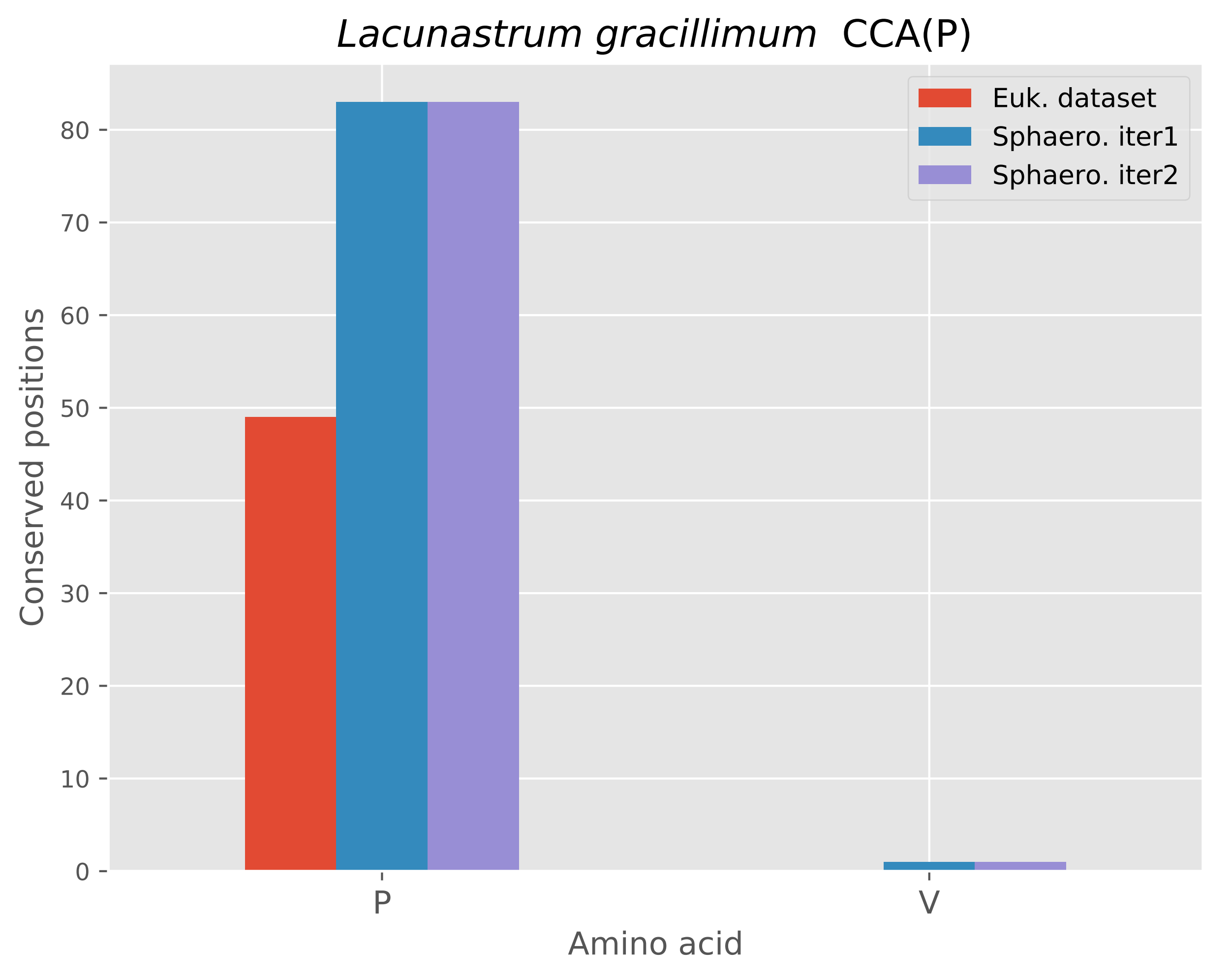

Lacunastrum gracillimum CCA(P)
Euk. dataset
Sphaero. iter1
Sphaero. iter2
Conserved positions
0
10
20
30
40
50
60
70
80
P
V
Amino acid

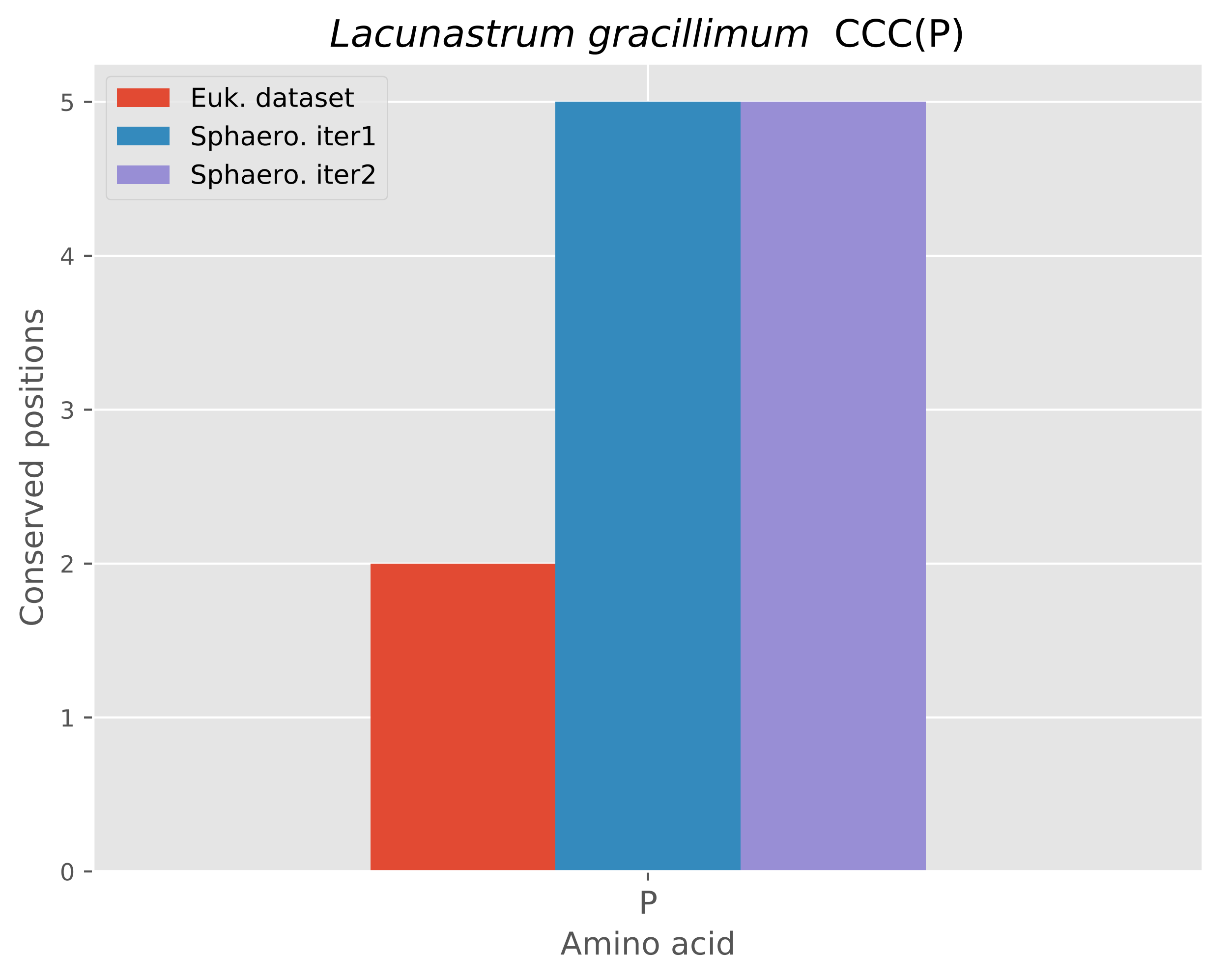
Lacunastrum gracillimum CCC(P)
Euk. dataset
Sphaero. iter1
Sphaero. iter2
Conserved positions
0
1
2
3
4
5
P
Amino acid

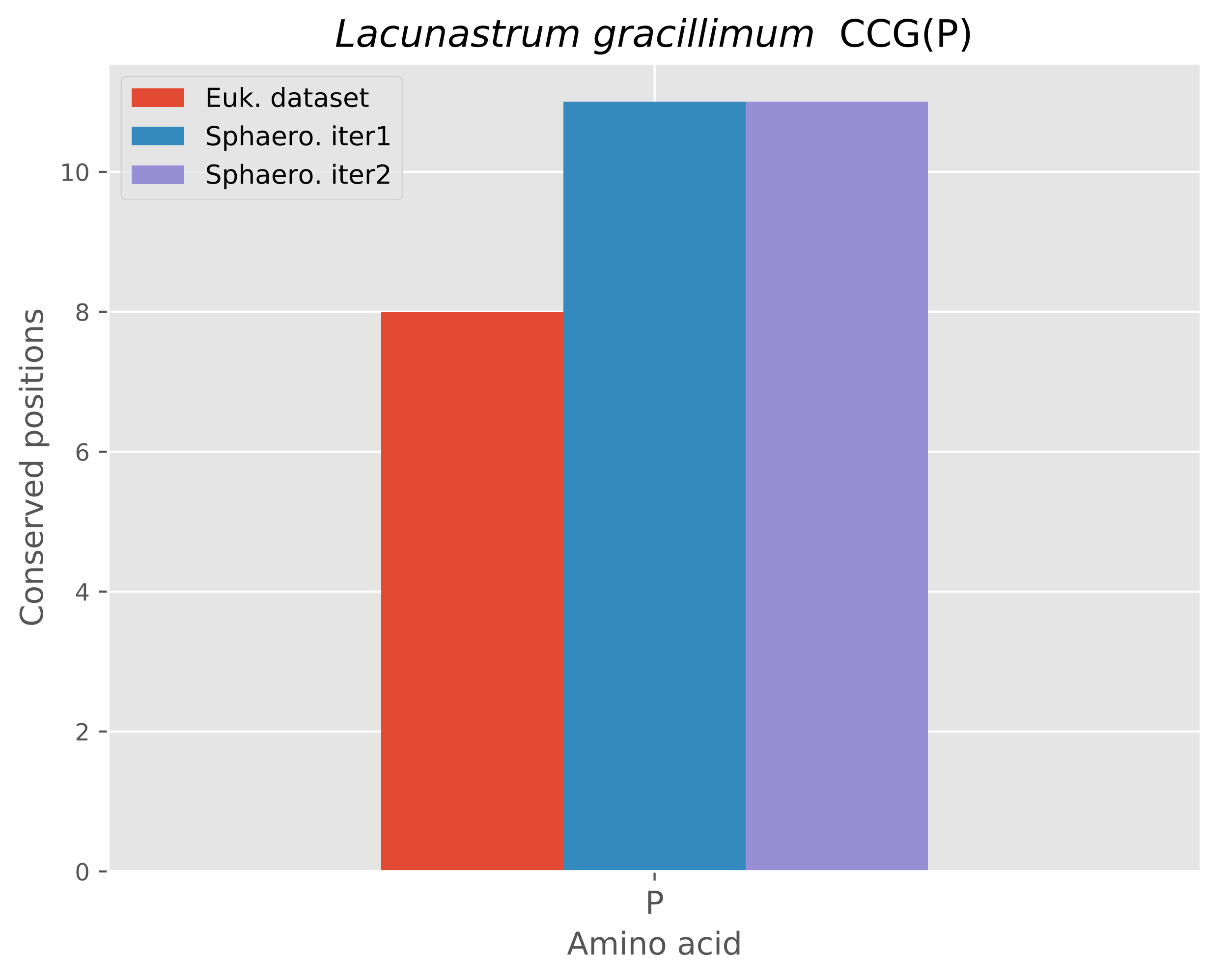

Lacunastrum gracillimum CCG(P)
Euk. dataset
Sphaero. iter1
Sphaero. iter2
Conserved positions
0
2
4
6
8
10
P
Amino acid

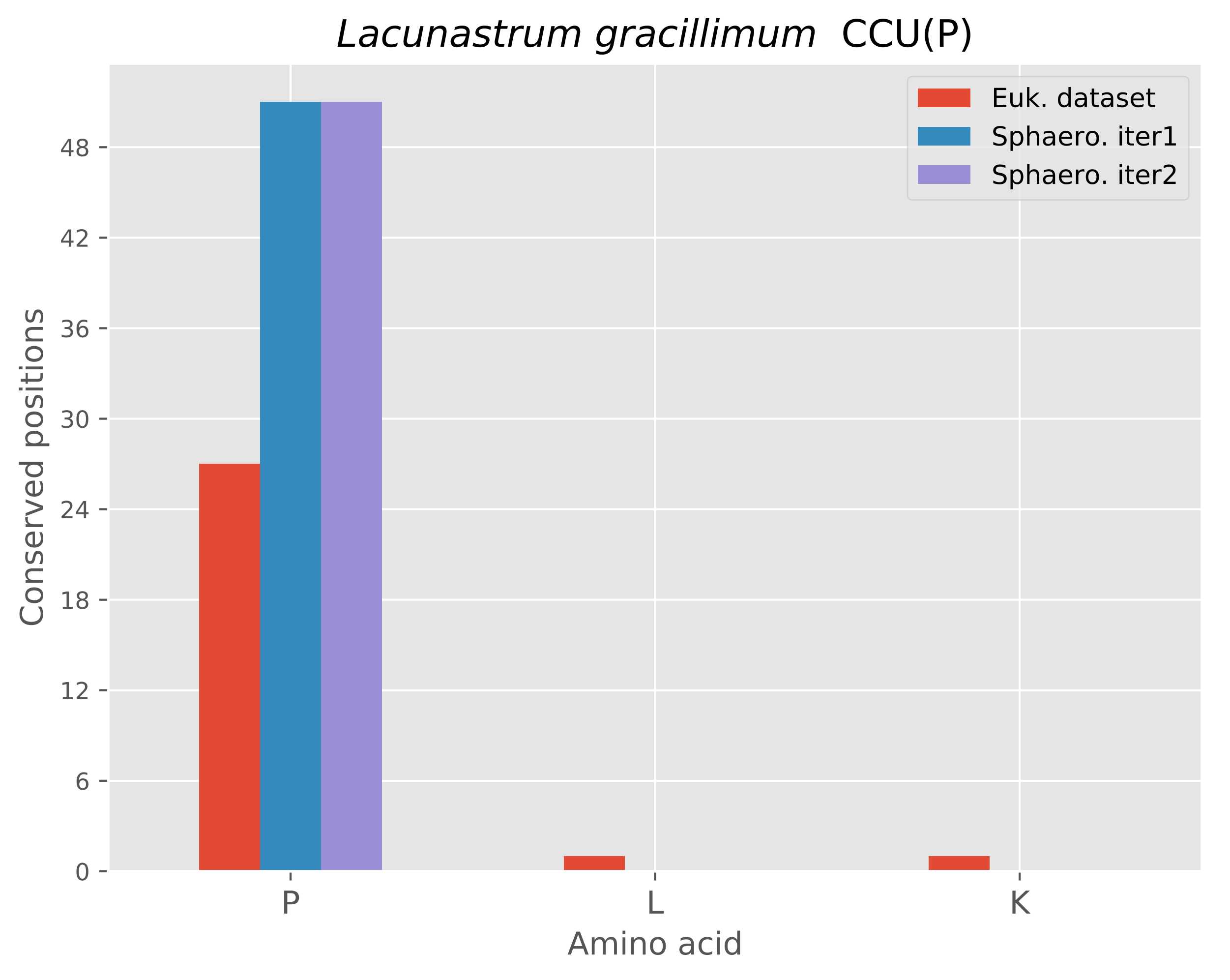

Lacunastrum gracillimum CCU(P)
Euk. dataset
Sphaero. iter1
Sphaero. iter2
Conserved positions
0
6
12
18
24
30
36
42
48
P
L
K
Amino acid

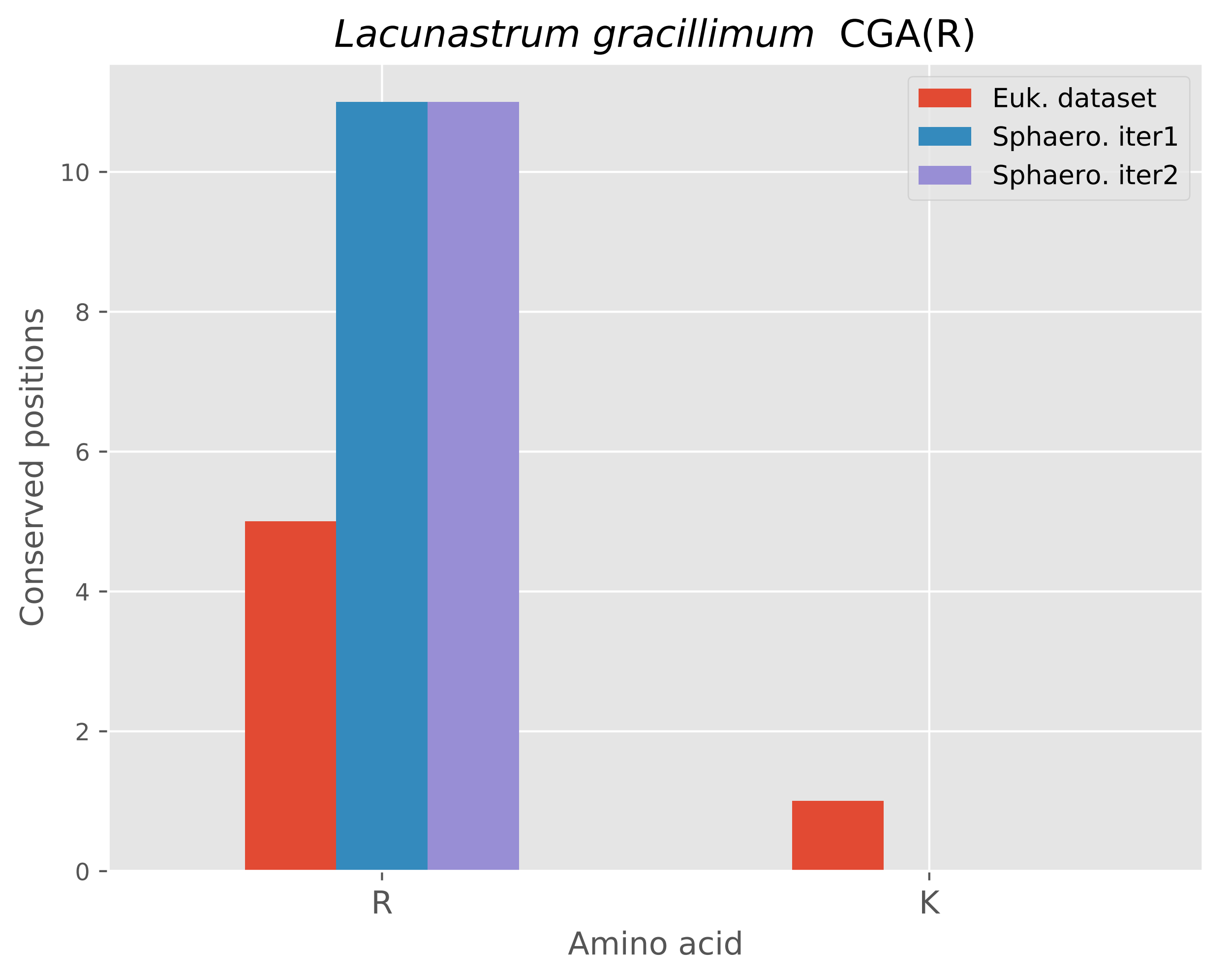

Lacunastrum gracillimum CGA(R)
Euk. dataset
Sphaero. iter1
Sphaero. iter2
Conserved positions
0
2
4
6
8
10
R
K
Amino acid

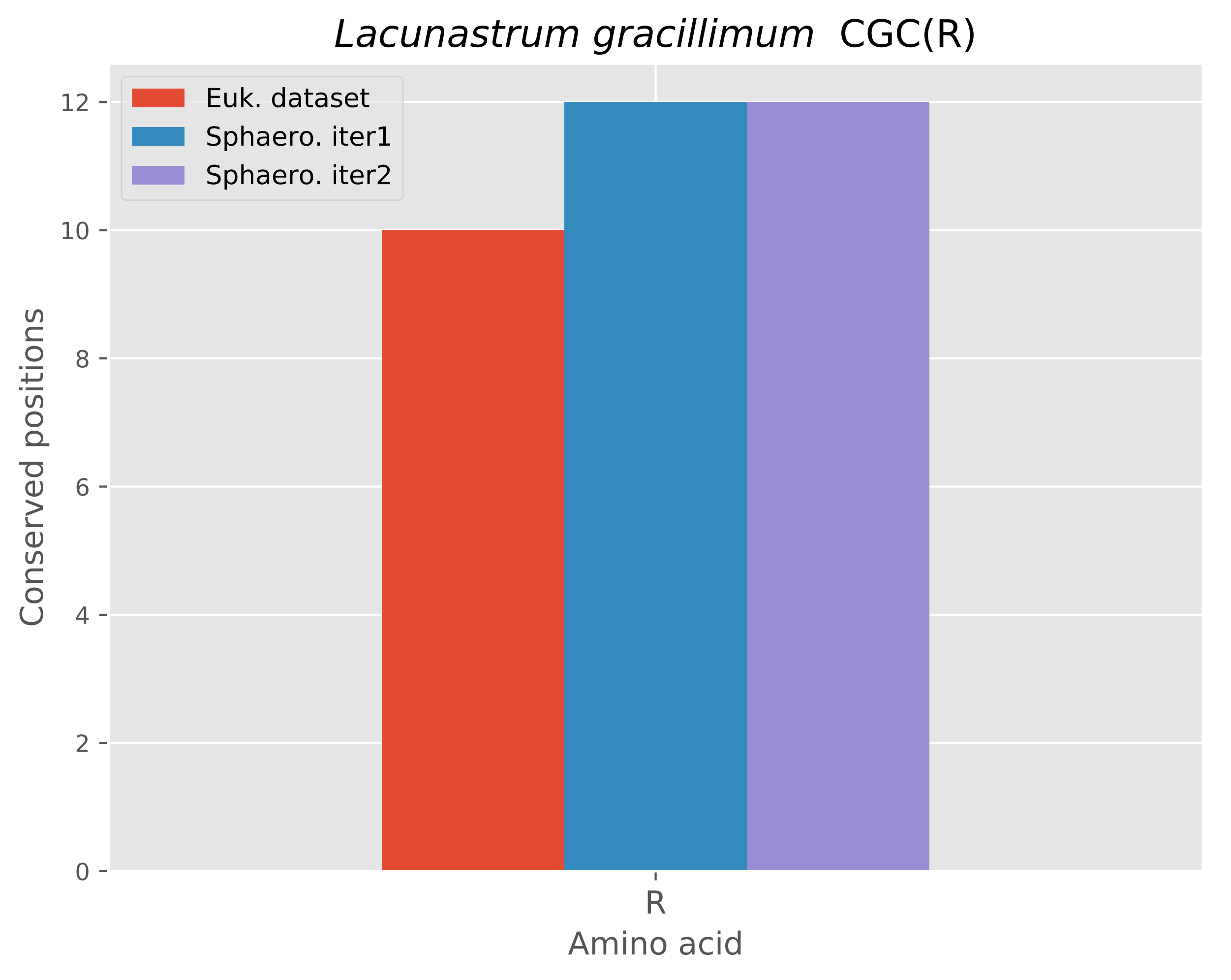
Lacunastrum gracillimum CGC(R)
Euk. dataset
Sphaero. iter1
Sphaero. iter2
Conserved positions
0
2
4
6
8
10
12
R
Amino acid

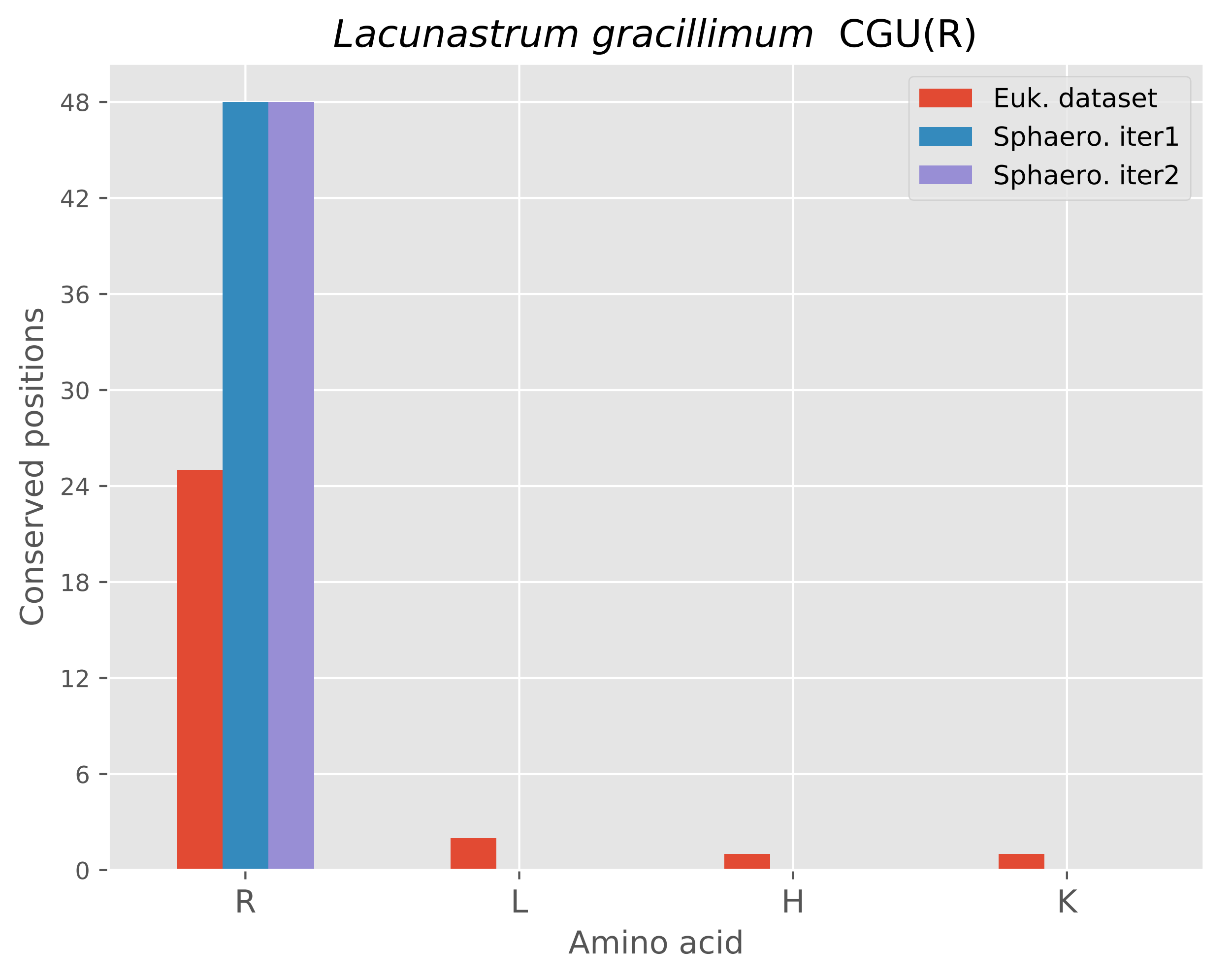
Lacunastrum gracillimum CGU(R)
Conserved positions
0
6
12
18
24
30
36
42
48
R
L
H
K
Amino acid
Euk. dataset
Sphaero. iter1
Sphaero. iter2

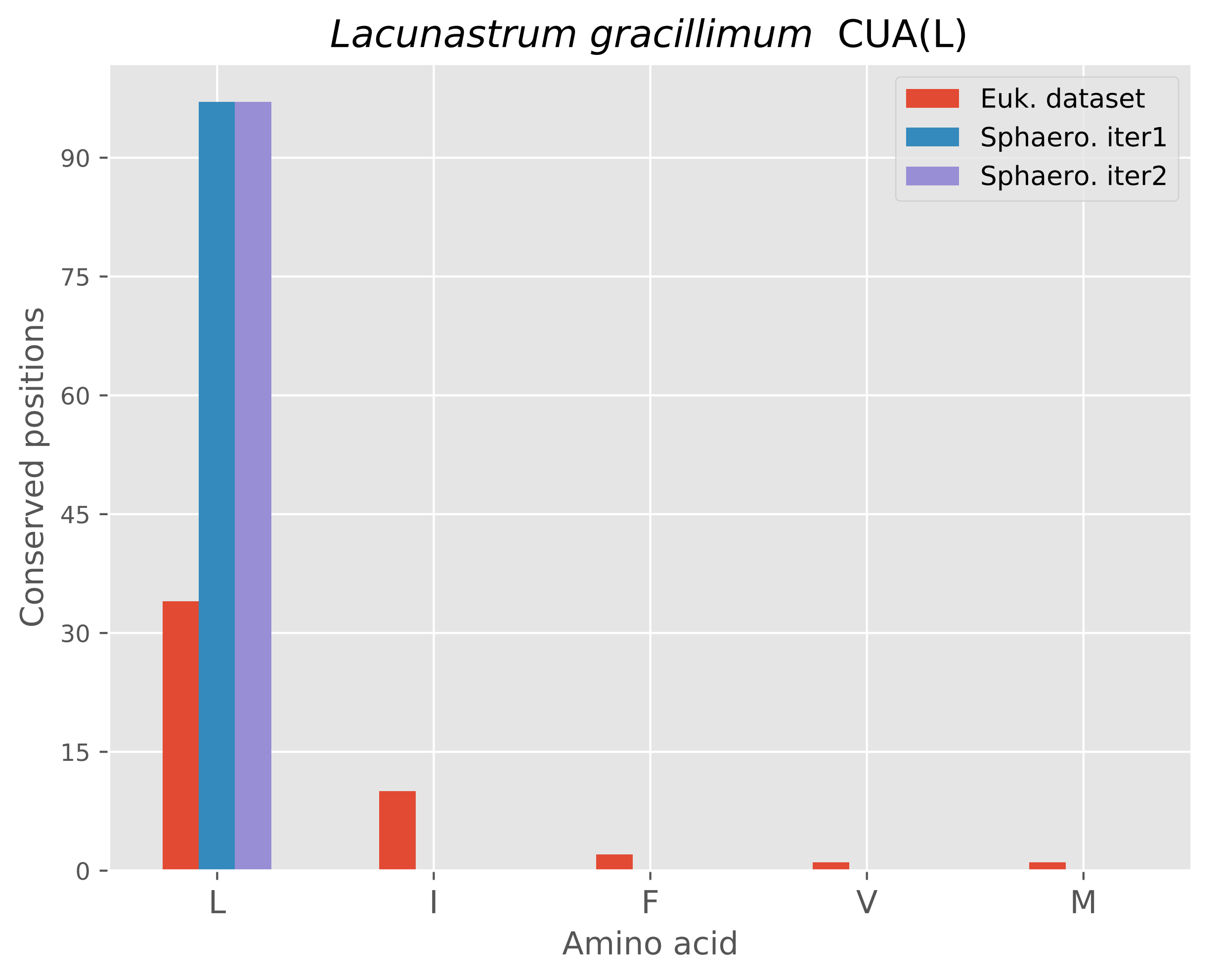

Lacunastrum gracillimum CUA(L)
Euk. dataset
Sphaero. iter1
Sphaero. iter2
Conserved positions
0
15
30
45
60
75
90
L
I
F
V
M
Amino acid

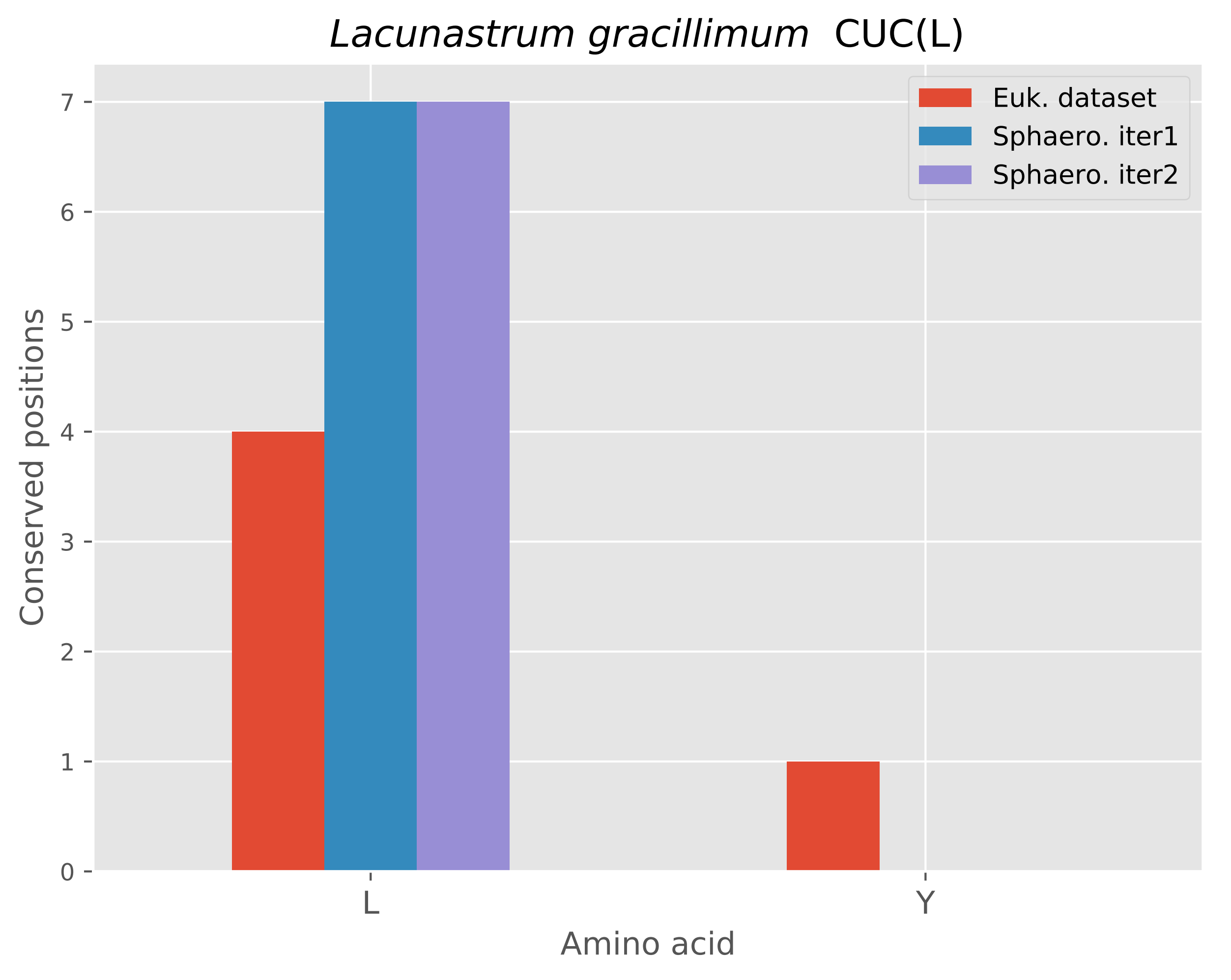

Lacunastrum gracillimum CUC(L)
Euk. dataset
Sphaero. iter1
Sphaero. iter2
Conserved positions
0
1
2
3
4
5
6
7
L
Y
Amino acid

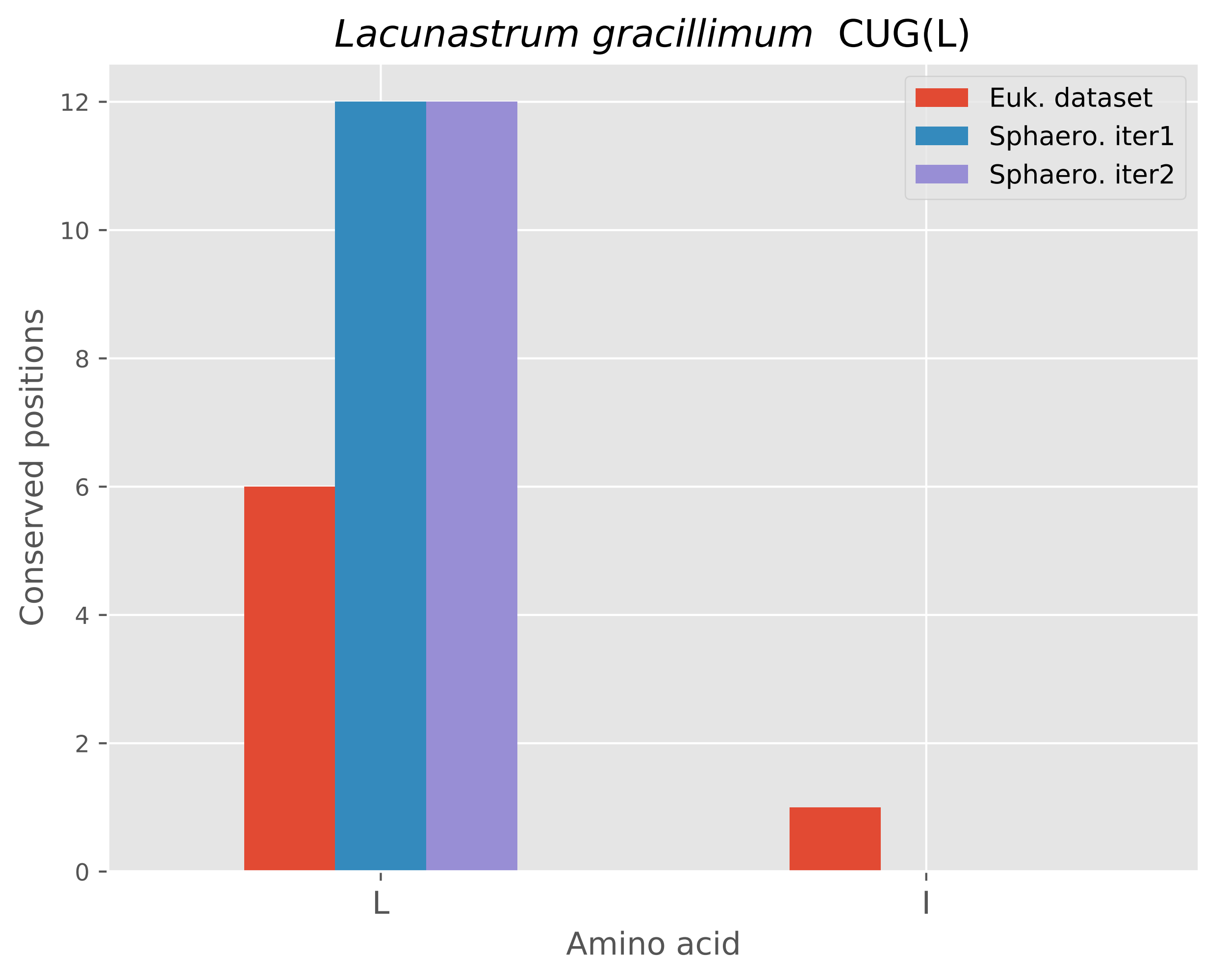

Lacunastrum gracillimum CUG(L)
Euk. dataset
Sphaero. iter1
Sphaero. iter2
Conserved positions
0
2
4
6
8
10
12
L
I
Amino acid

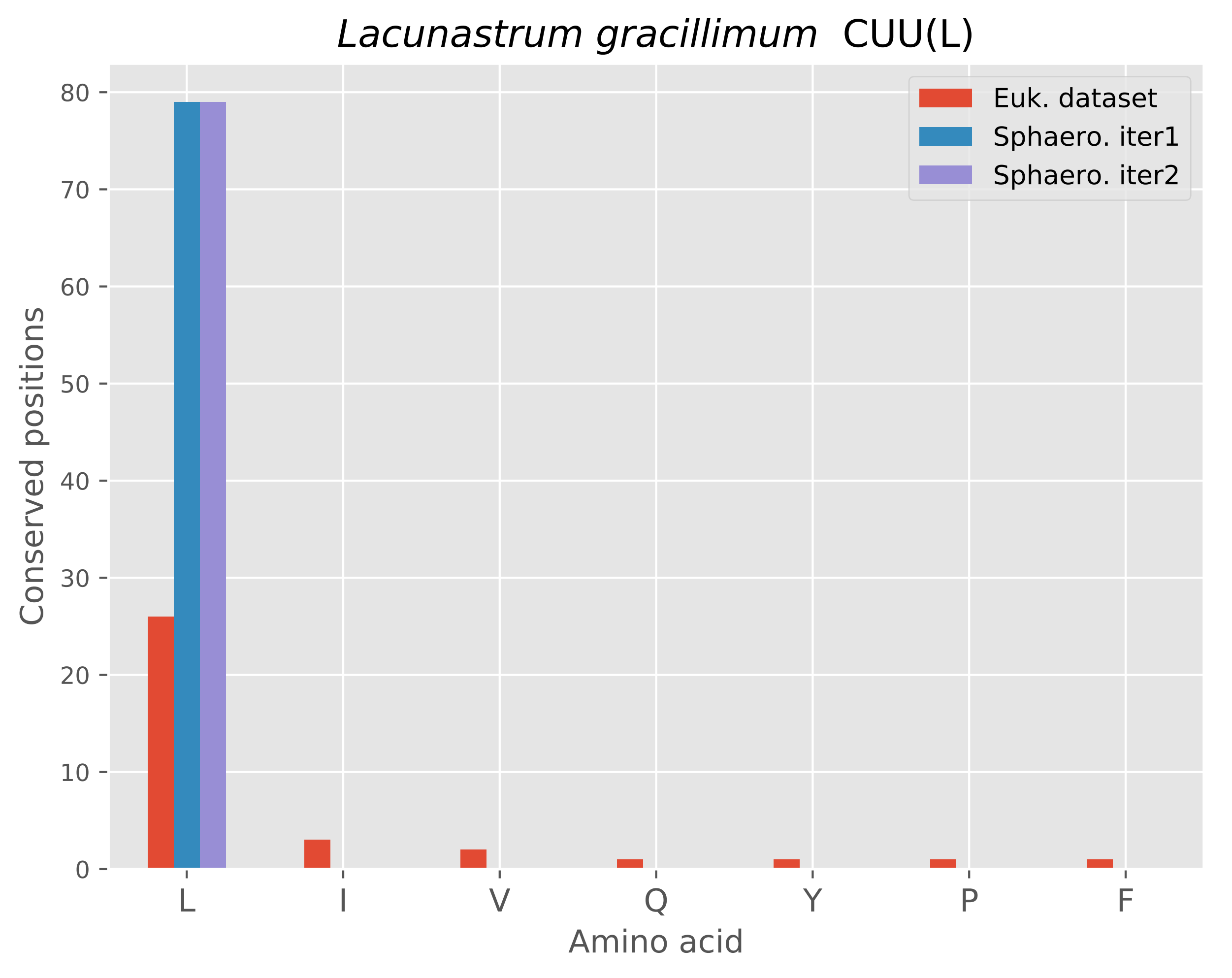
Lacunastrum gracillimum CUU(L)
Euk. dataset
Sphaero. iter1
Sphaero. iter2
Conserved positions
0
10
20
30
40
50
60
70
80
L
I
V
Q
Y
P
F
Amino acid

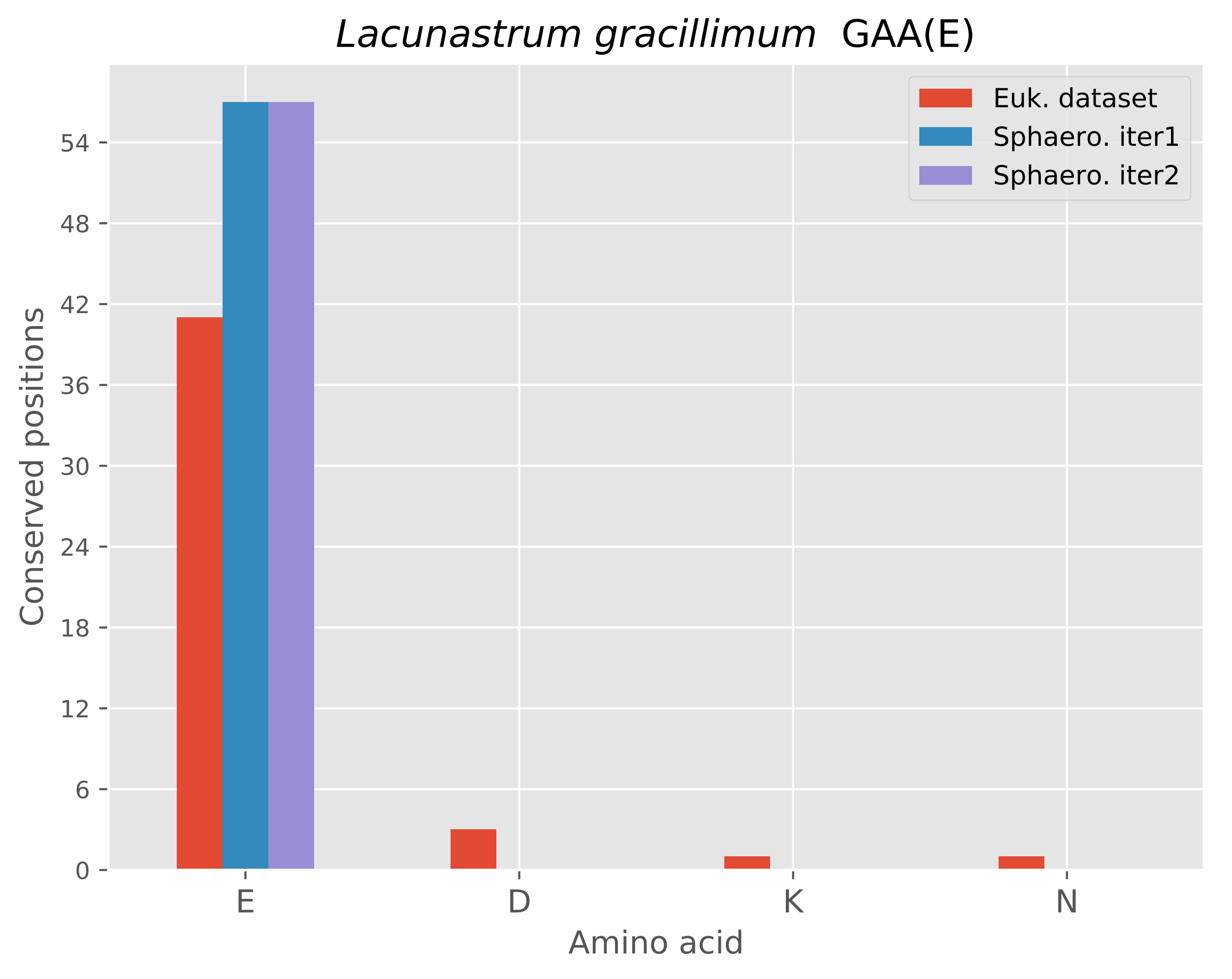

Lacunastrum gracillimum GAA(E)
Euk. dataset
Sphaero. iter1
Sphaero. iter2
Conserved positions
0
6
12
18
24
30
36
42
48
54
E
D
K
N
Amino acid

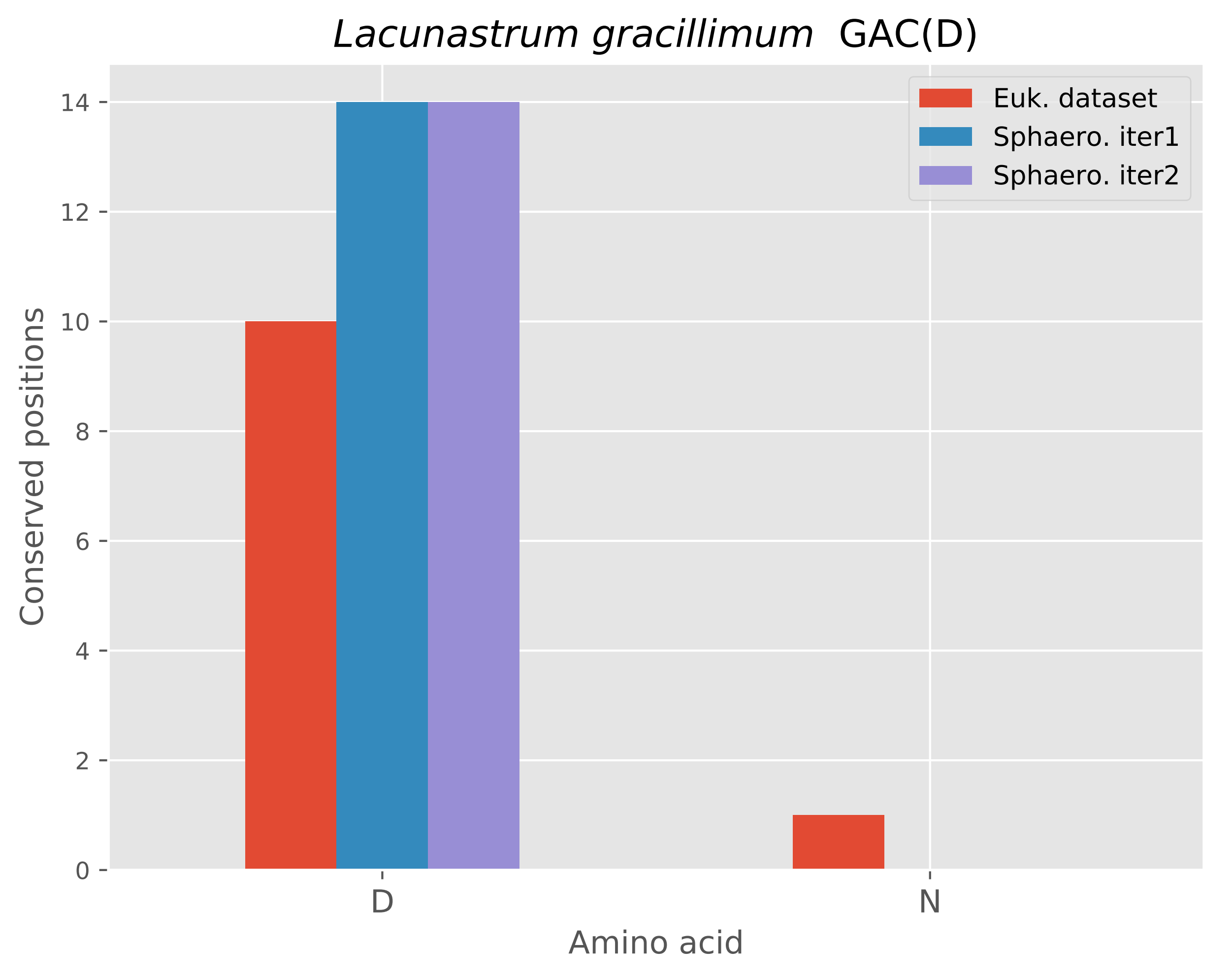

Lacunastrum gracillimum GAC(D)
Euk. dataset
Sphaero. iter1
Sphaero. iter2
Conserved positions
0
2
4
6
8
10
12
14
D
N
Amino acid

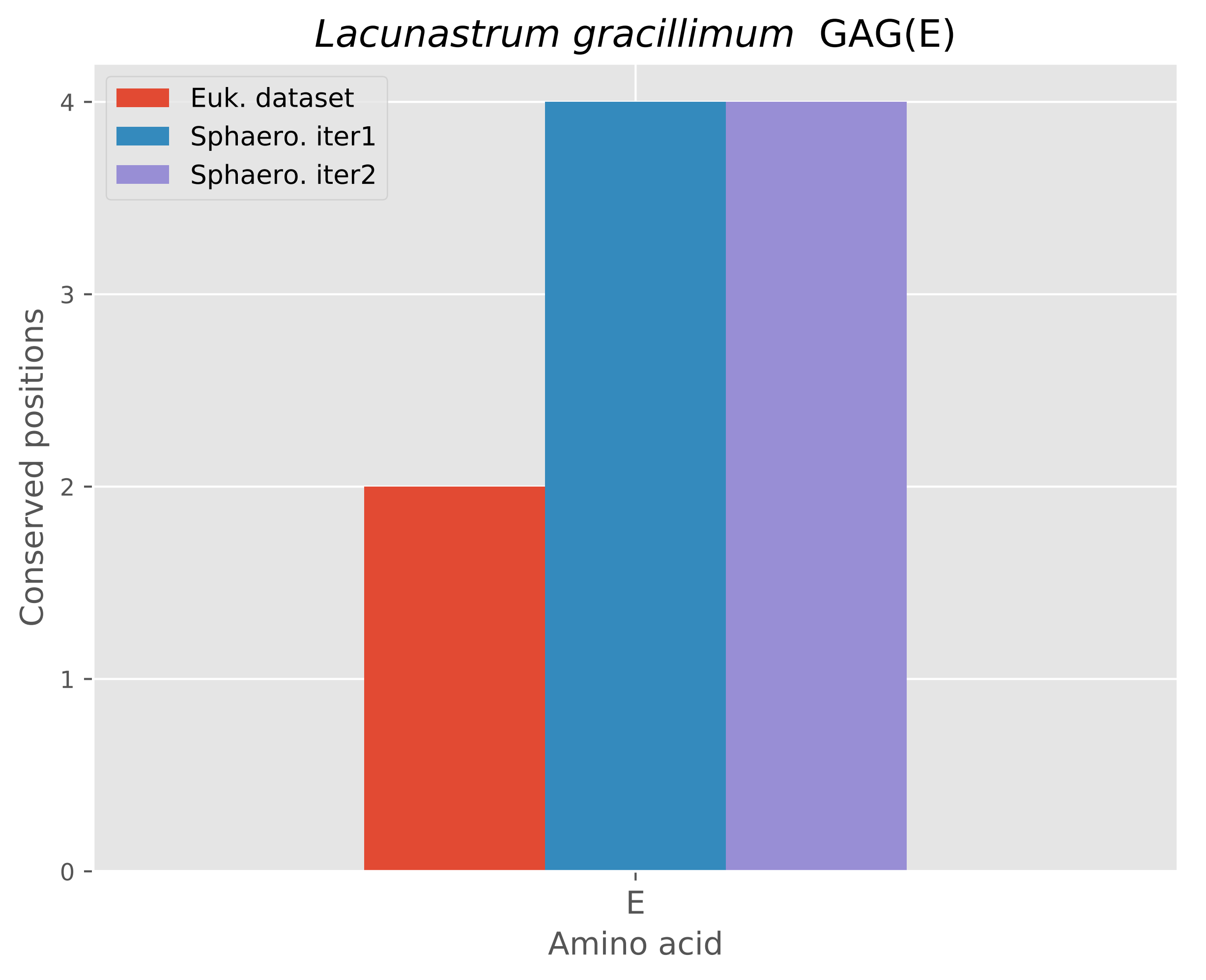

Lacunastrum gracillimum GAG(E)
Euk. dataset
Sphaero. iter1
Sphaero. iter2
Conserved positions
0
1
2
3
4
E
Amino acid

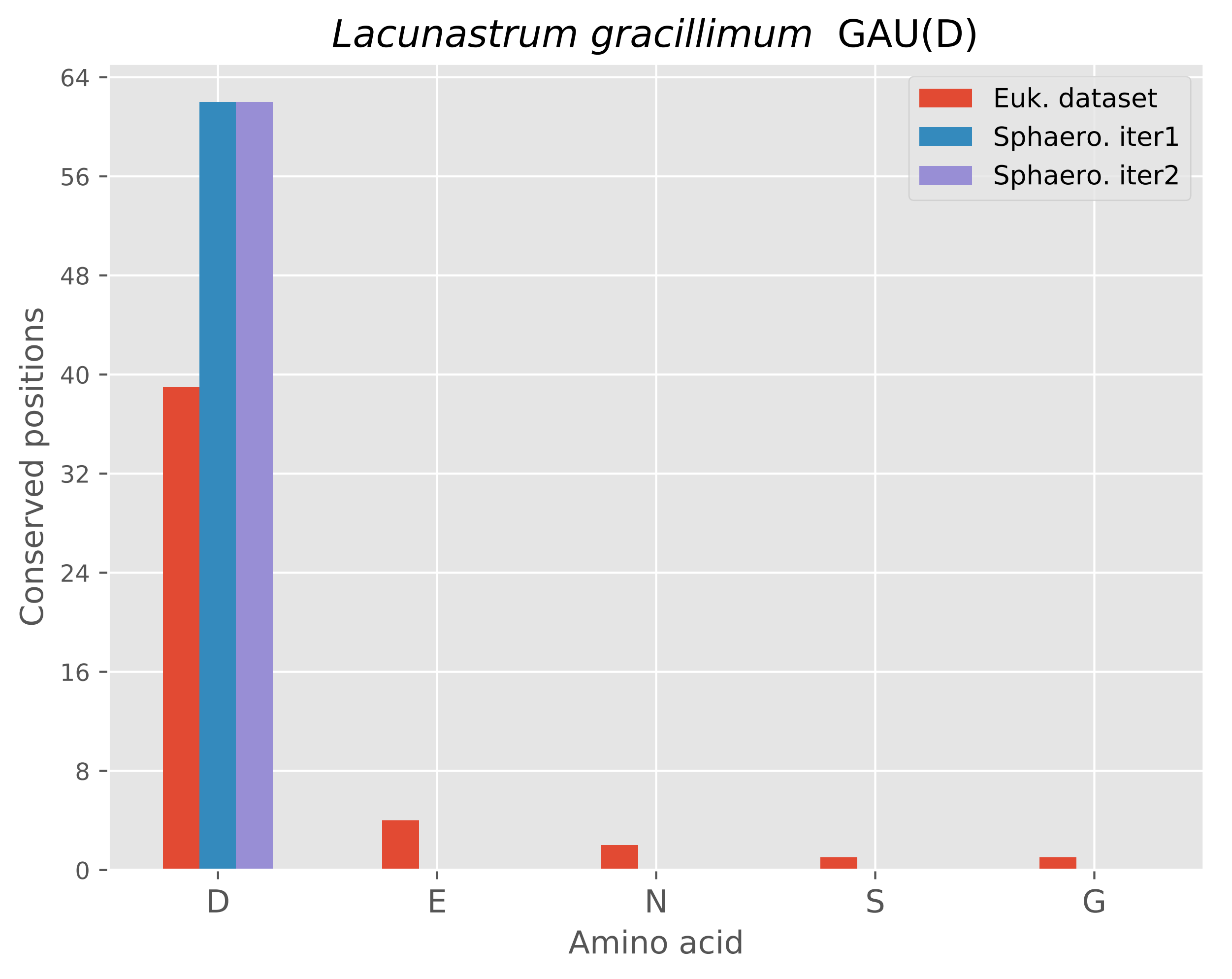

Lacunastrum gracillimum GAU(D)
Euk. dataset
Sphaero. iter1
Sphaero. iter2
Conserved positions
0
8
16
24
32
40
48
56
64
D
E
N
S
G
Amino acid

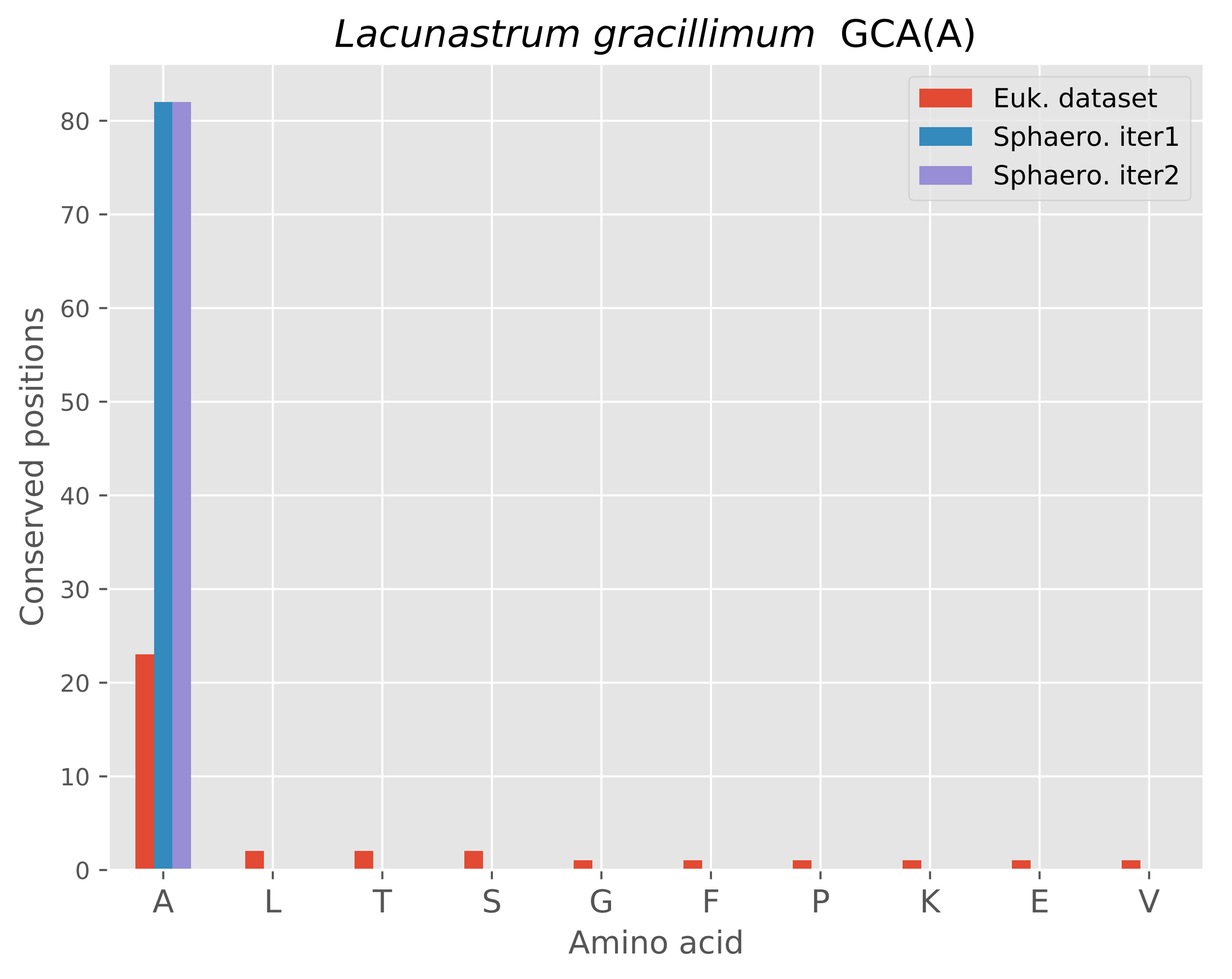

Lacunastrum gracillimum GCA(A)
Euk. dataset
Sphaero. iter1
Sphaero. iter2
Conserved positions
0
10
20
30
40
50
60
70
80
A
L
T
S
G
F
P
K
E
V
Amino acid

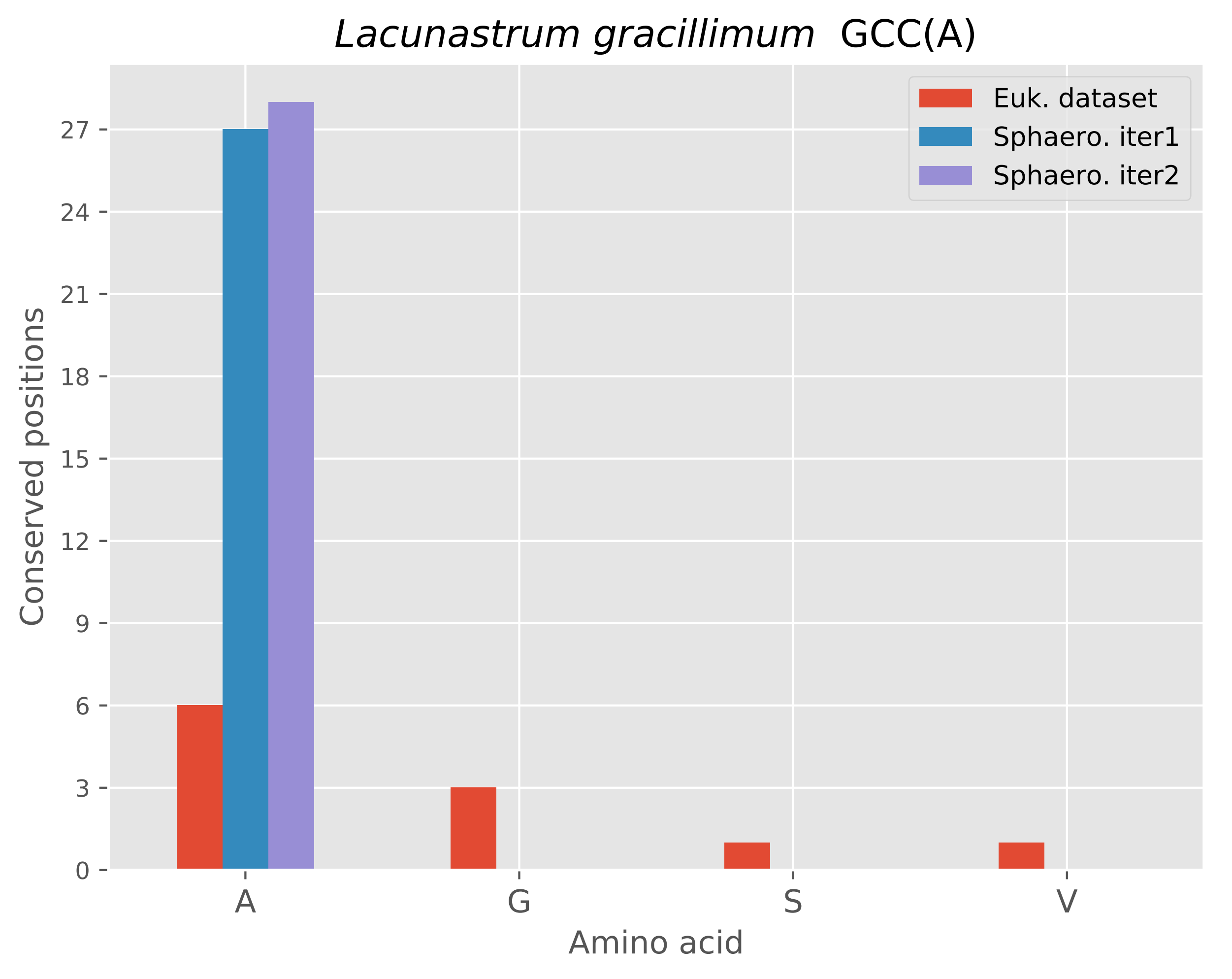
Lacunastrum gracillimum GCC(A)
Euk. dataset
Sphaero. iter1
Sphaero. iter2
Conserved positions
0
3
6
9
12
15
18
21
24
27
A
G
S
V
Amino acid

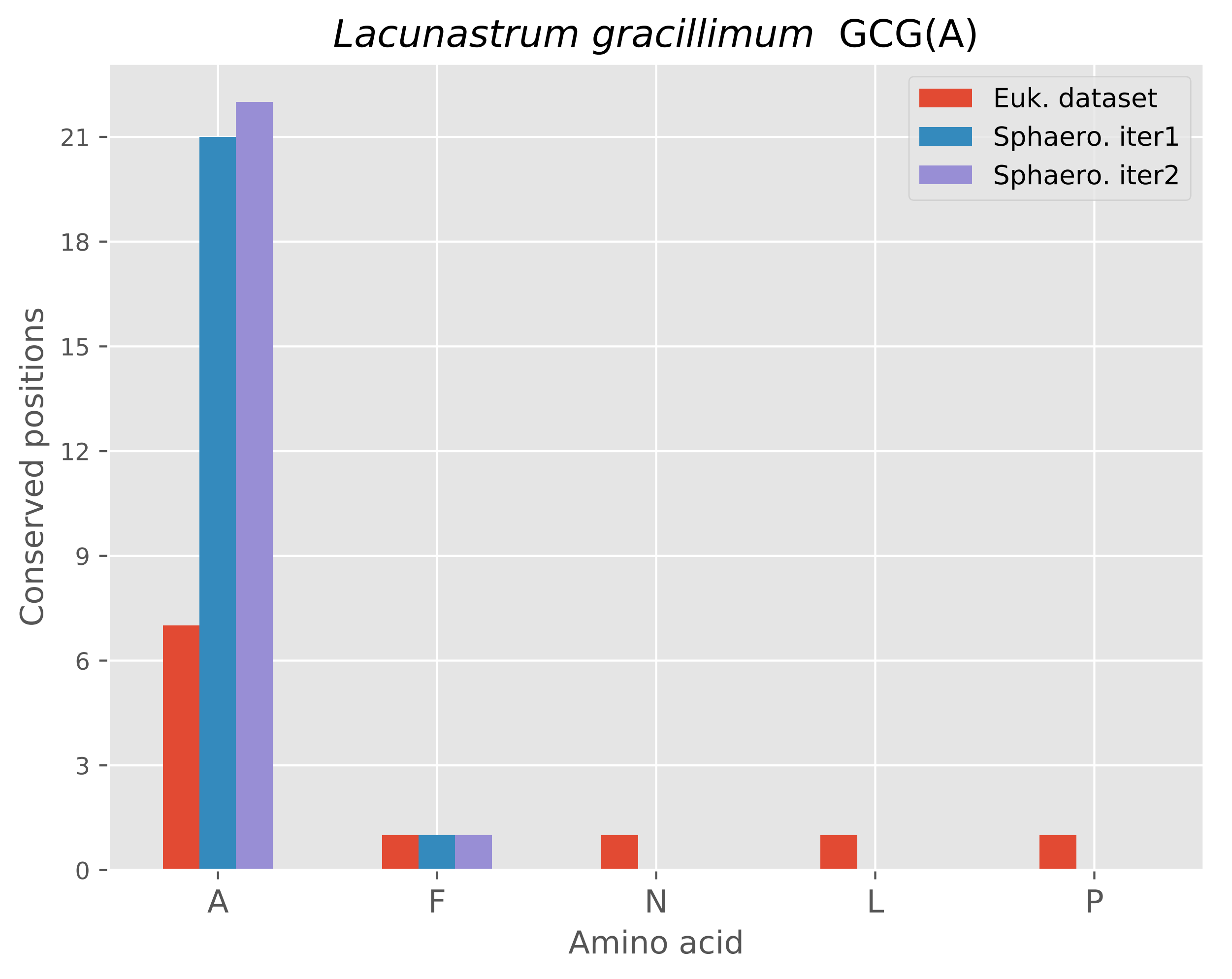

Lacunastrum gracillimum GCG(A)
Euk. dataset
Sphaero. iter1
Sphaero. iter2
Conserved positions
0
3
6
9
12
15
18
21
A
F
N
L
P
Amino acid

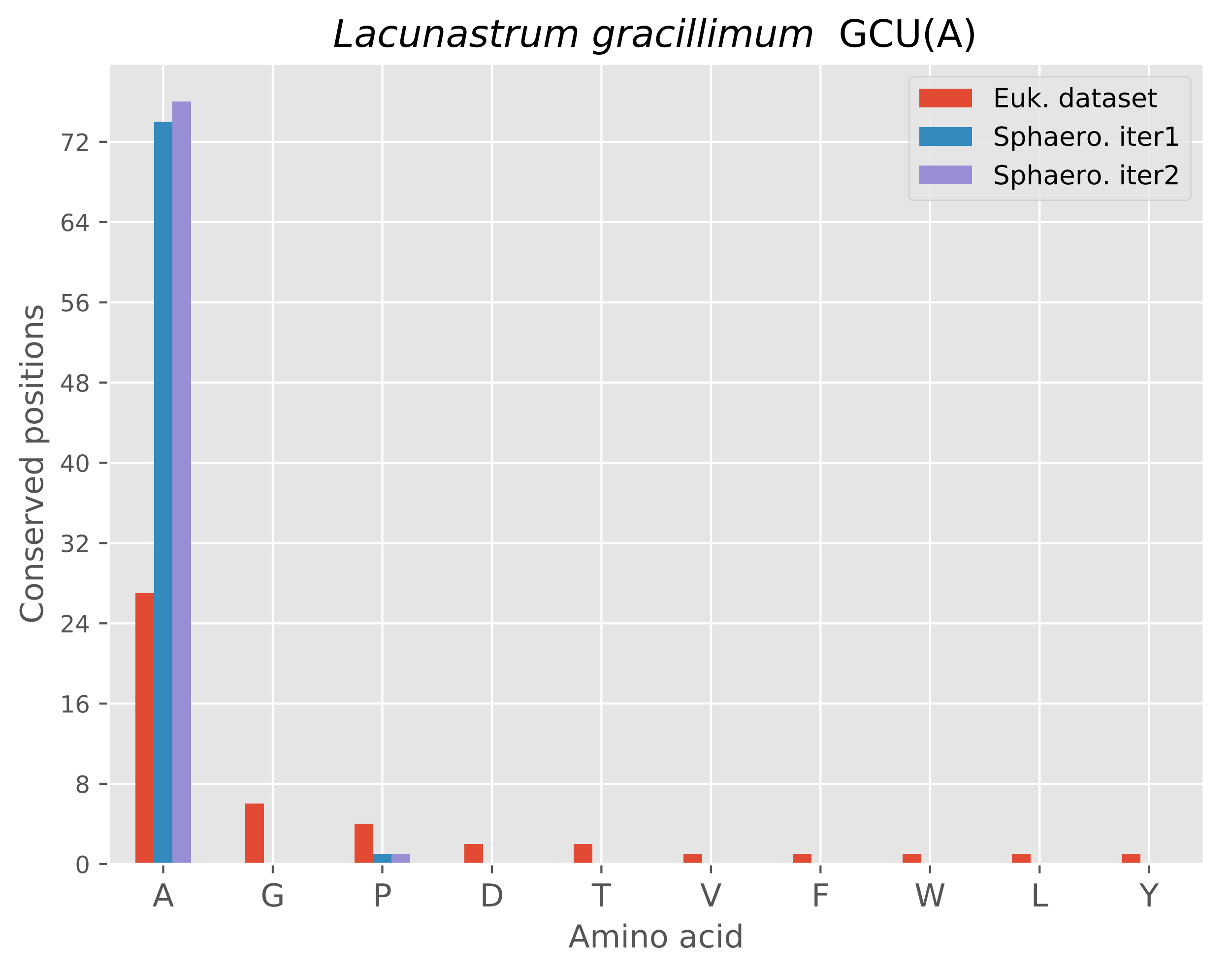

Lacunastrum gracillimum GCU(A)
Euk. dataset
Sphaero. iter1
Sphaero. iter2
Conserved positions
0
8
16
24
32
40
48
56
64
72
A
G
P
D
T
V
F
W
L
Y
Amino acid

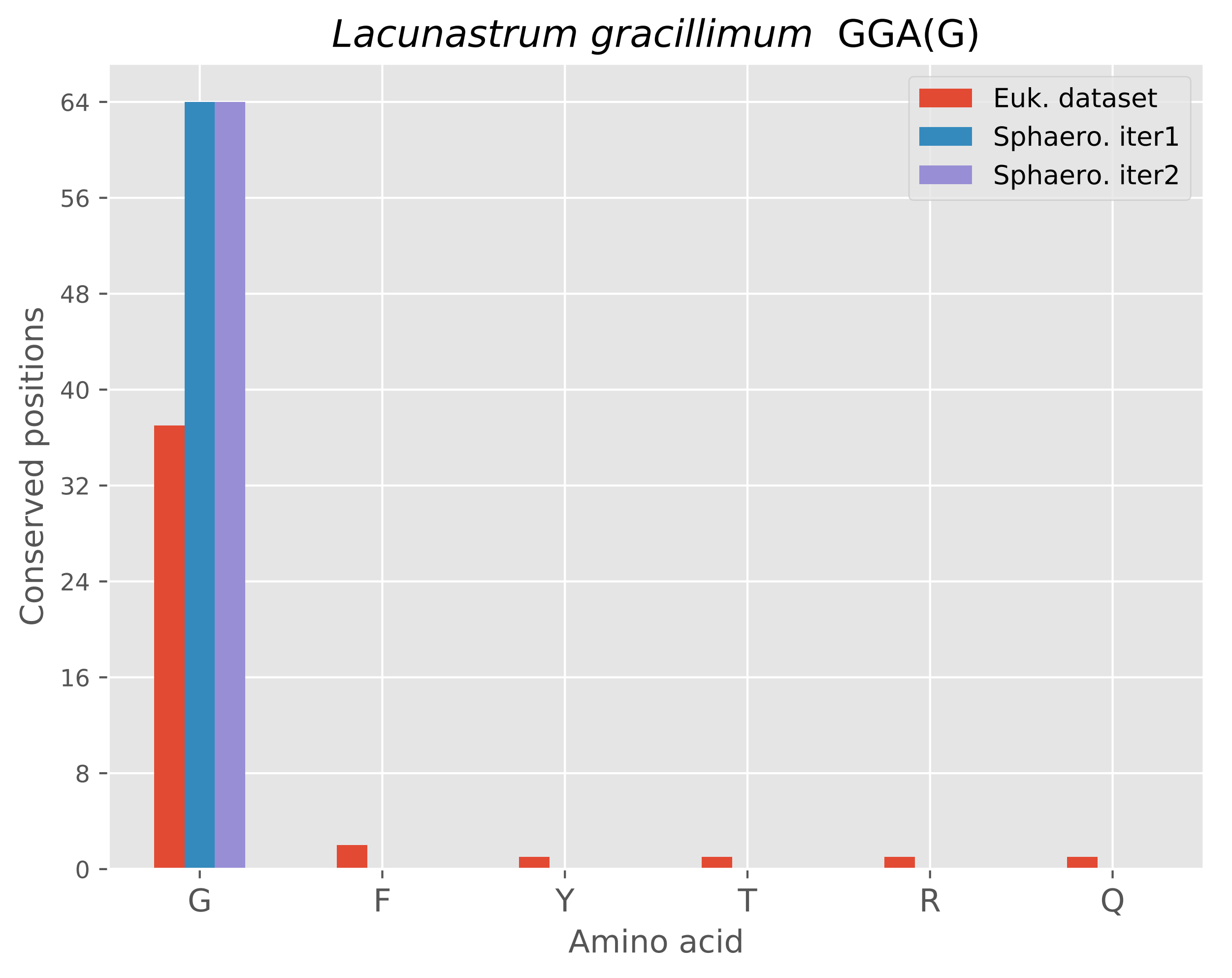

Lacunastrum gracillimum GGA(G)
Euk. dataset
Sphaero. iter1
Sphaero. iter2
Conserved positions
0
8
16
24
32
40
48
56
64
G
F
Y
T
R
Q
Amino acid

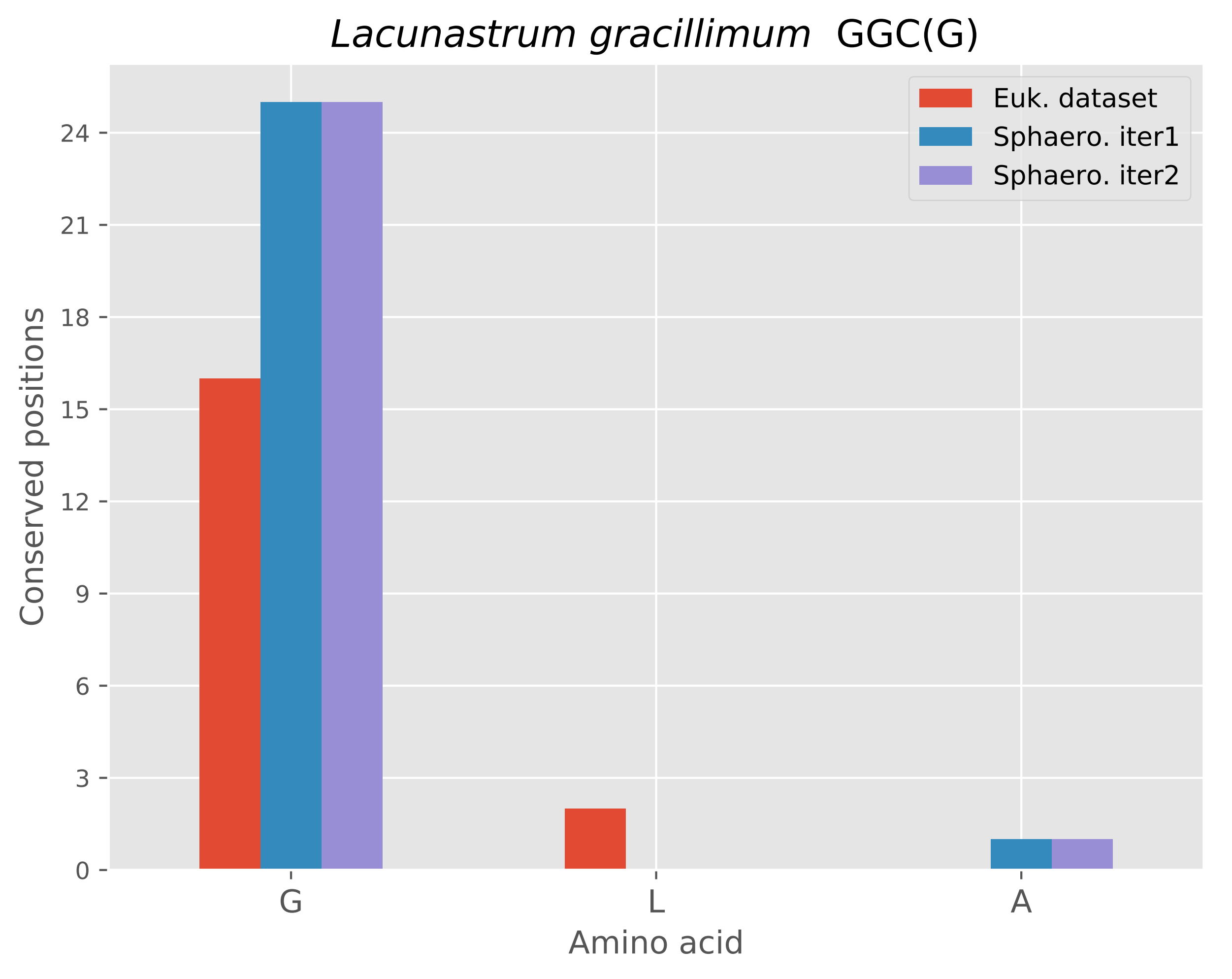

Lacunastrum gracillimum GGC(G)
Euk. dataset
Sphaero. iter1
Sphaero. iter2
Conserved positions
0
3
6
9
12
15
18
21
24
G
L
A
Amino acid

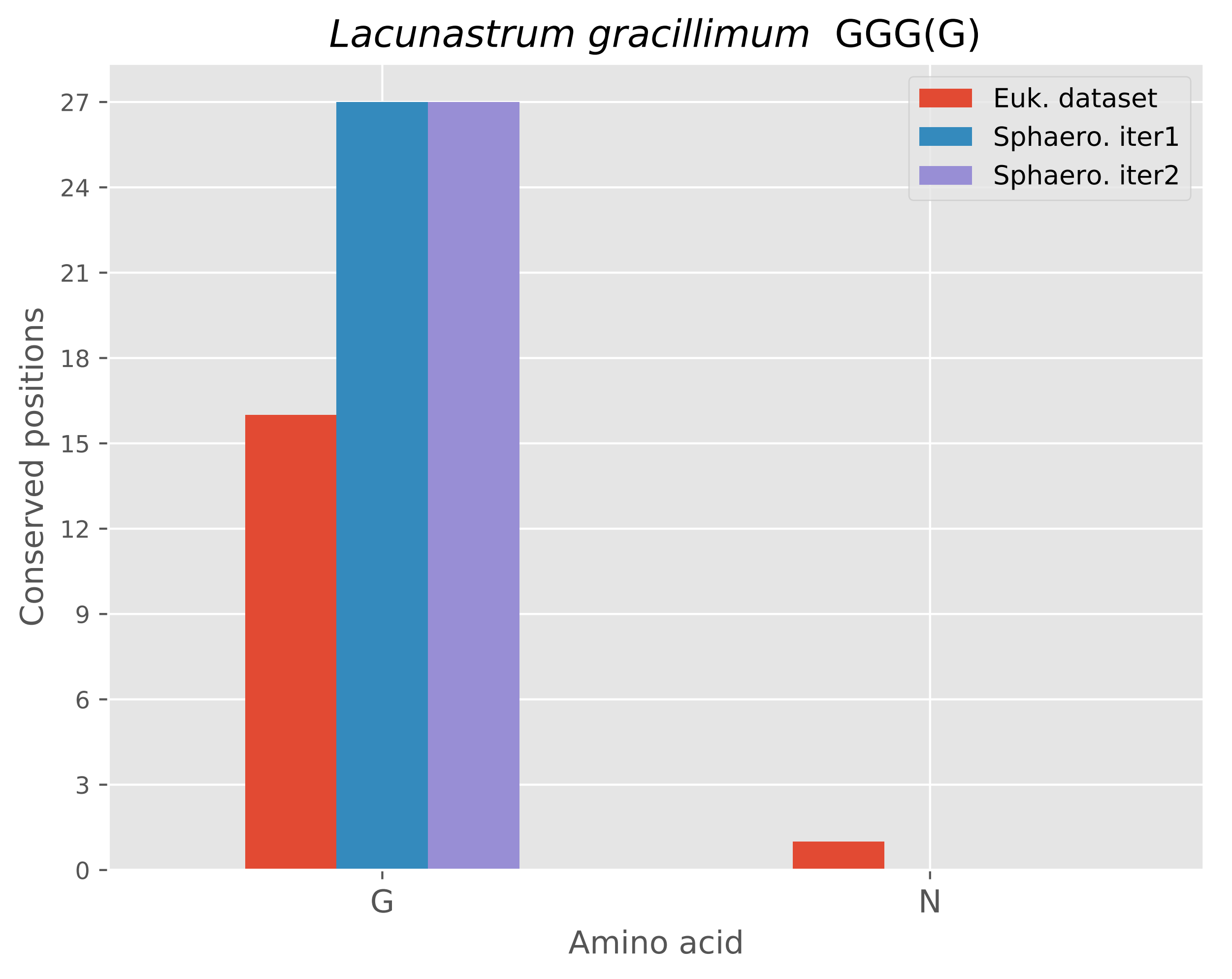

Lacunastrum gracillimum GGG(G)
Euk. dataset
Sphaero. iter1
Sphaero. iter2
Conserved positions
0
3
6
9
12
15
18
21
24
27
G
N
Amino acid

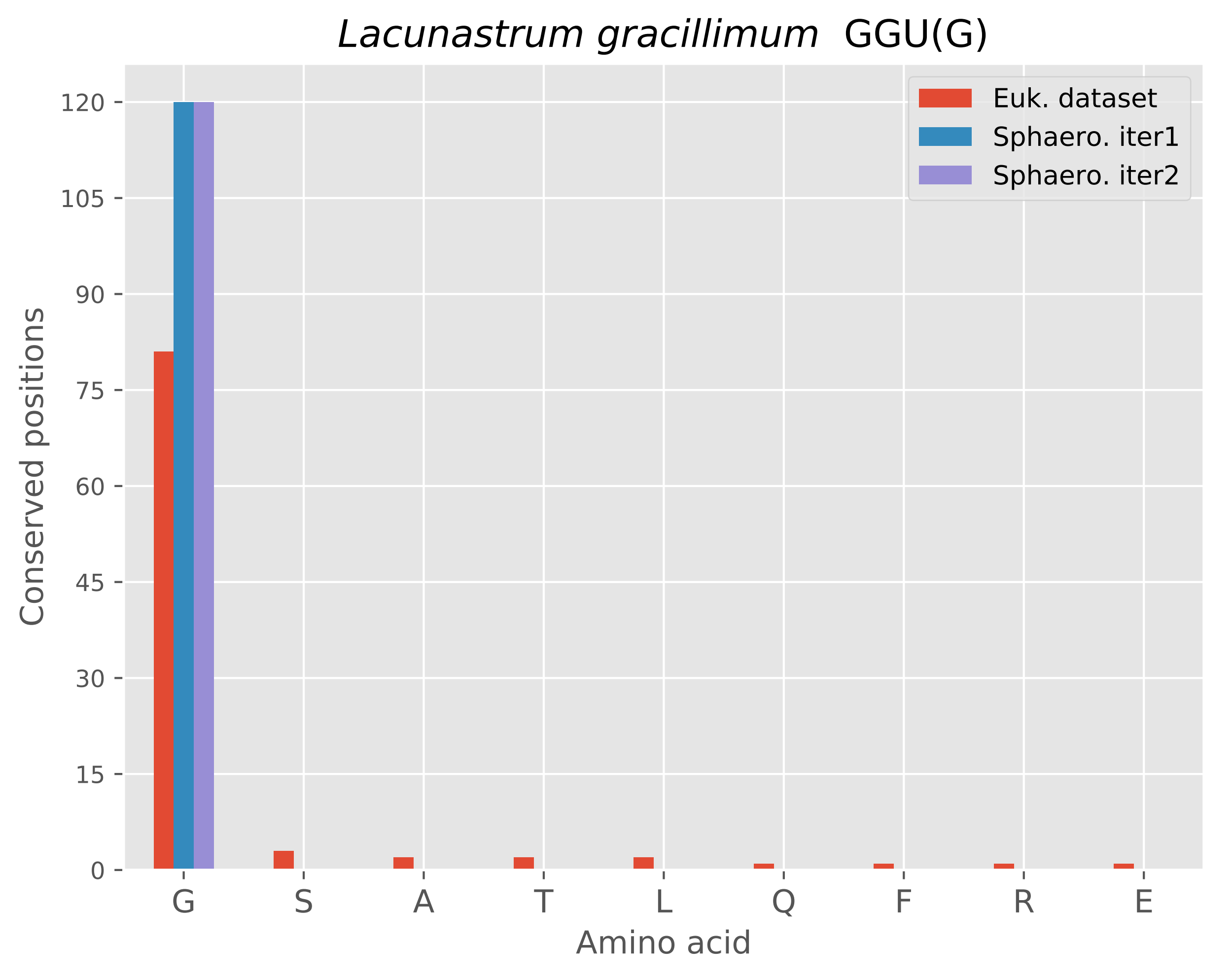

Lacunastrum gracillimum GGU(G)
Euk. dataset
Sphaero. iter1
Sphaero. iter2
Conserved positions
0
15
30
45
60
75
90
105
120
G
S
A
T
L
Q
F
R
E
Amino acid

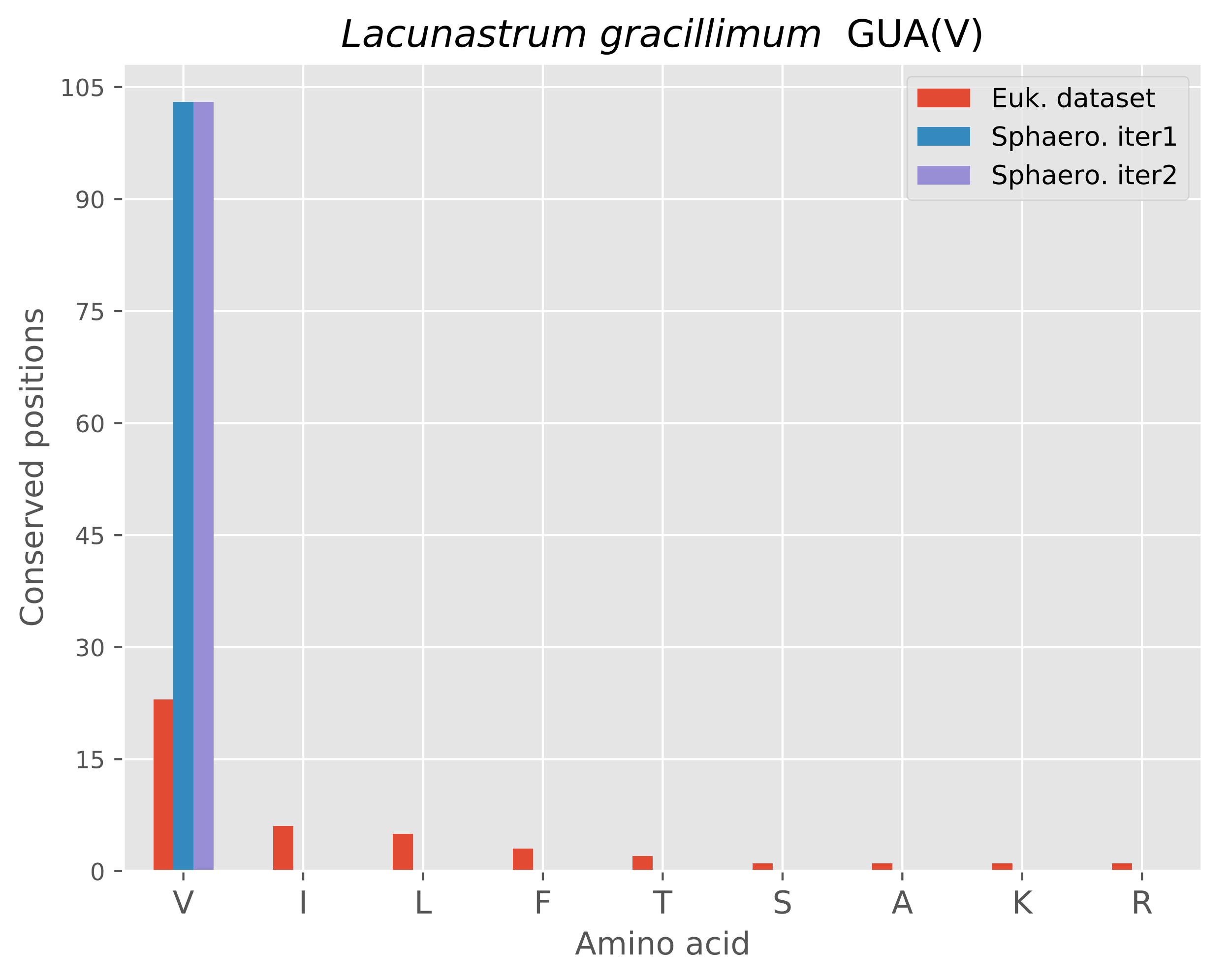

Lacunastrum gracillimum GUA(V)
Conserved positions
0
15
30
45
60
75
90
105
Amino acid
V
I
L
F
T
S
A
K
R
Euk. dataset
Sphaero. iter1
Sphaero. iter2

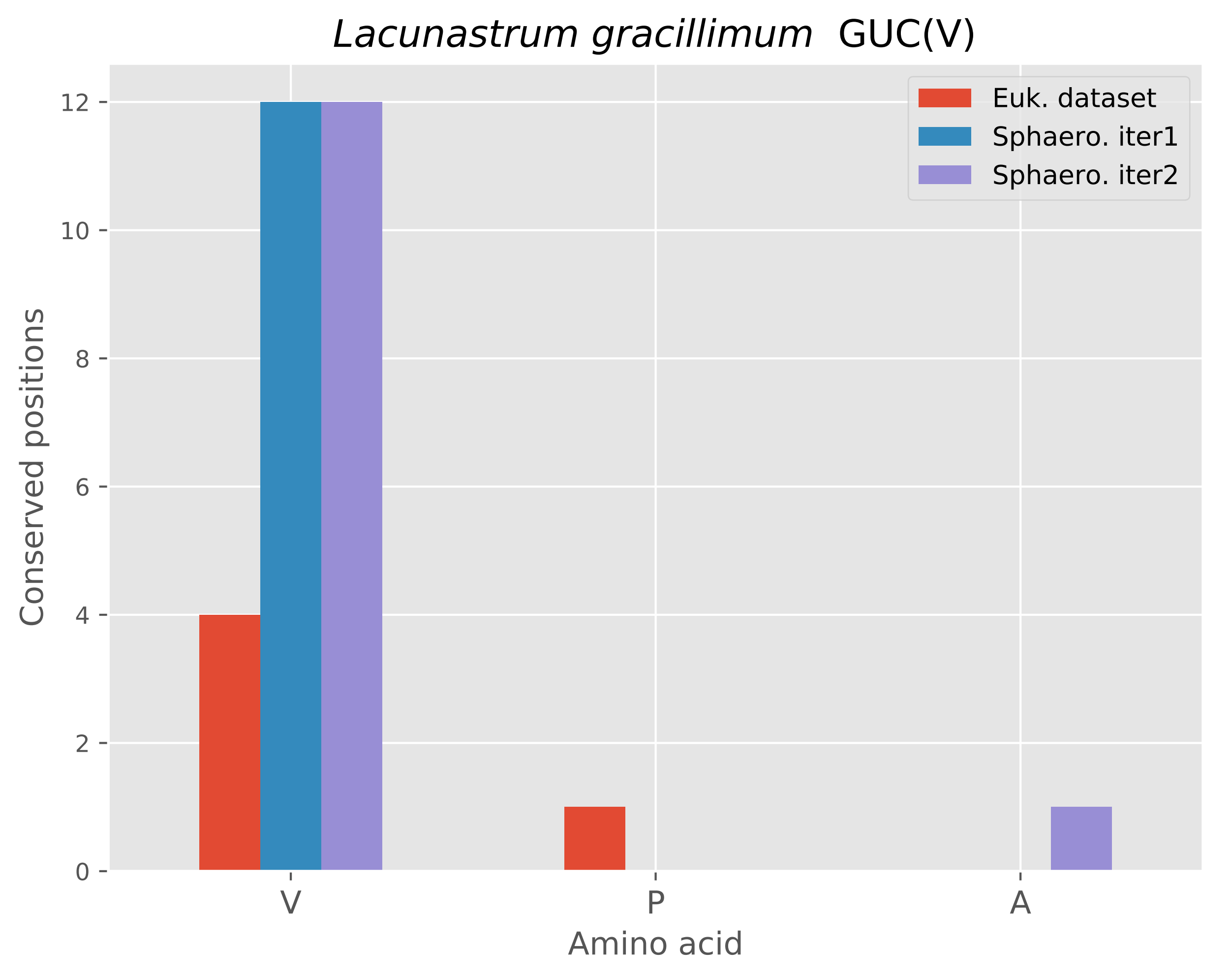

Lacunastrum gracillimum GUC(V)
Euk. dataset
Sphaero. iter1
Sphaero. iter2
Conserved positions
0
2
4
6
8
10
12
V
P
A
Amino acid

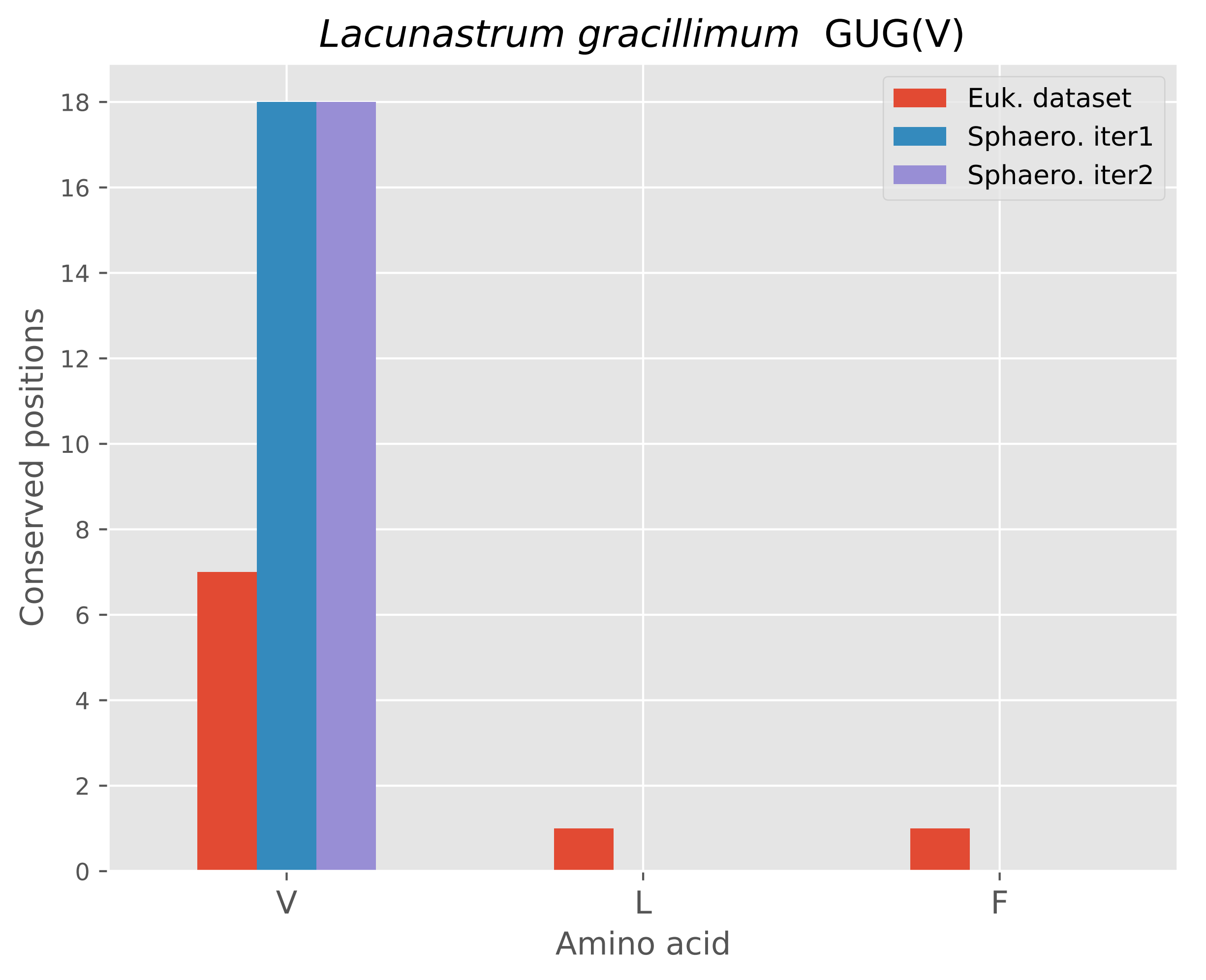
Lacunastrum gracillimum GUG(V)
Euk. dataset
Sphaero. iter1
Sphaero. iter2
Conserved positions
0
2
4
6
8
10
12
14
16
18
V
L
F
Amino acid

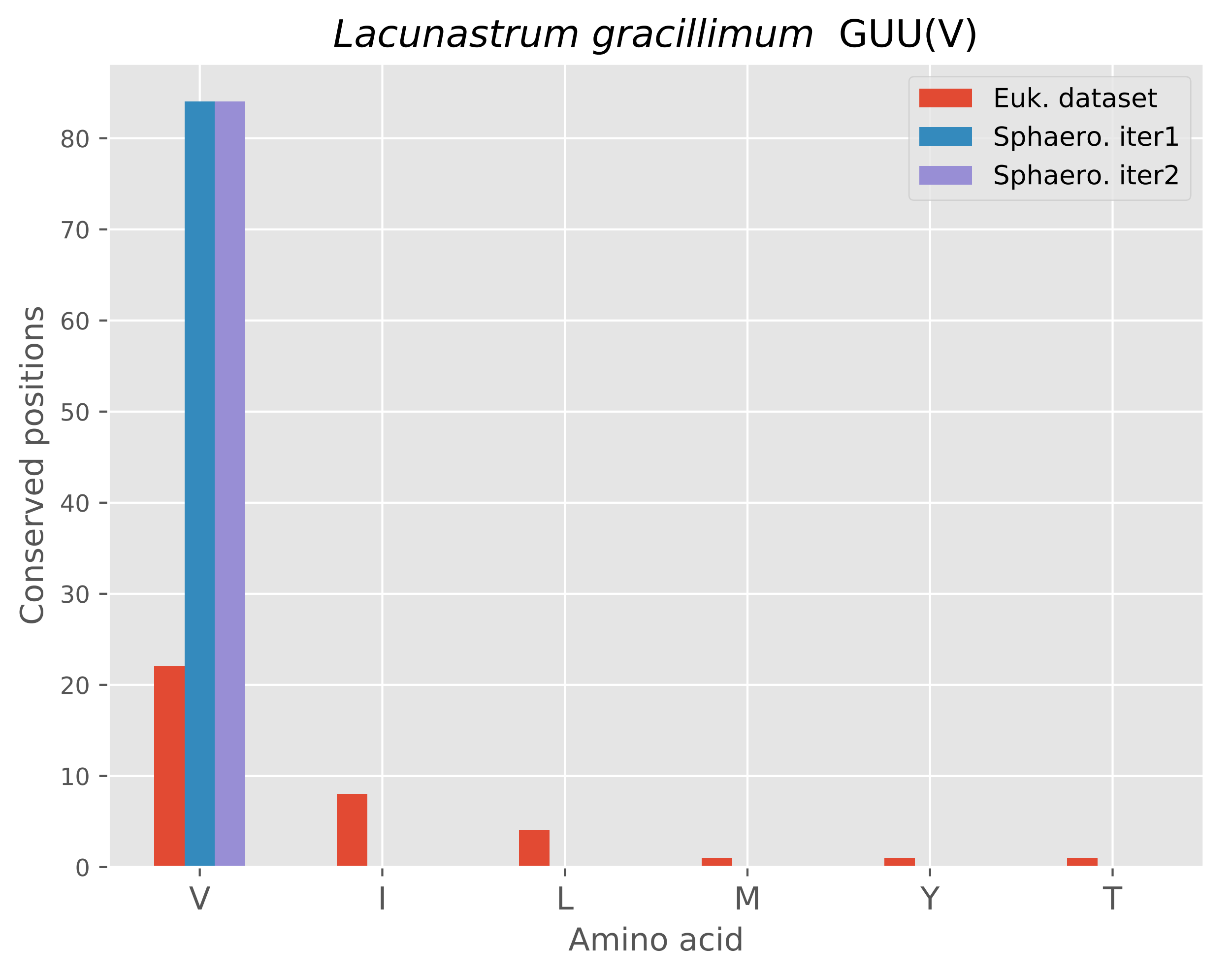

Lacunastrum gracillimum GUU(V)
Euk. dataset
Sphaero. iter1
Sphaero. iter2
Conserved positions
0
10
20
30
40
50
60
70
80
V
I
L
M
Y
T
Amino acid

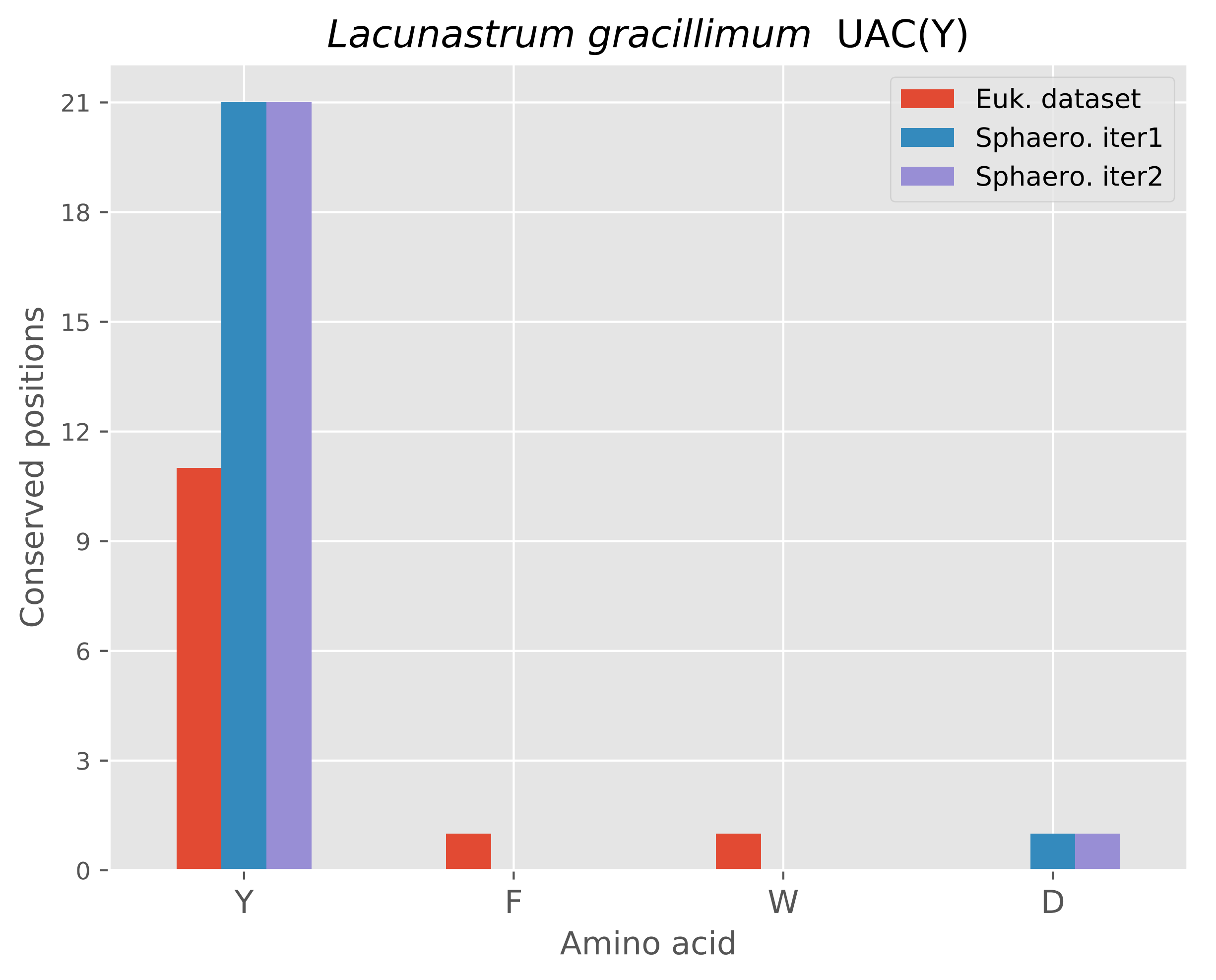

Lacunastrum gracillimum UAC(Y)
Euk. dataset
Sphaero. iter1
Sphaero. iter2
Conserved positions
0
3
6
9
12
15
18
21
Y
F
W
D
Amino acid

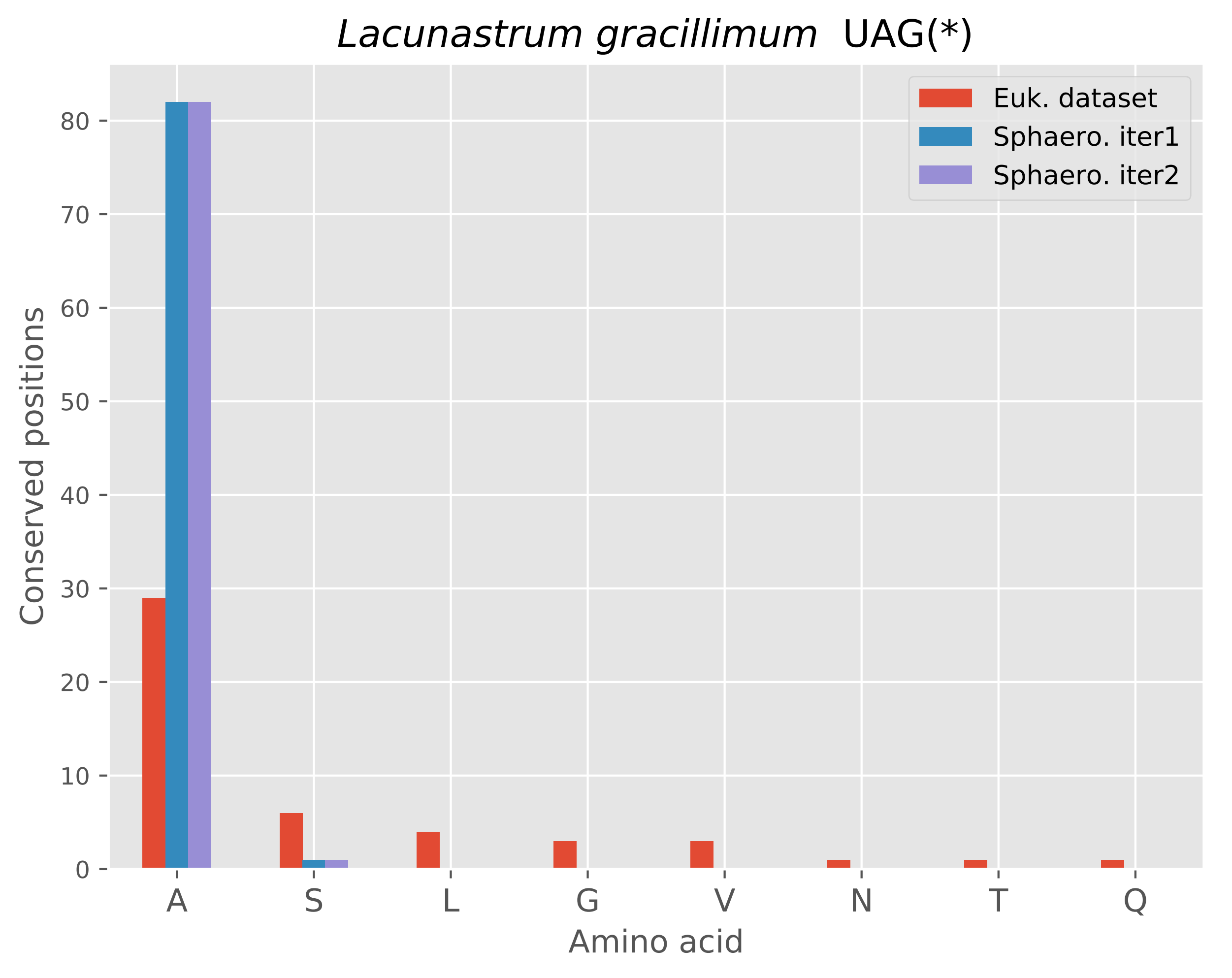
Lacunastrum gracillimum UAG(*)
Euk. dataset
Sphaero. iter1
Sphaero. iter2
Conserved positions
0
10
20
30
40
50
60
70
80
A
S
L
G
V
N
T
Q
Amino acid

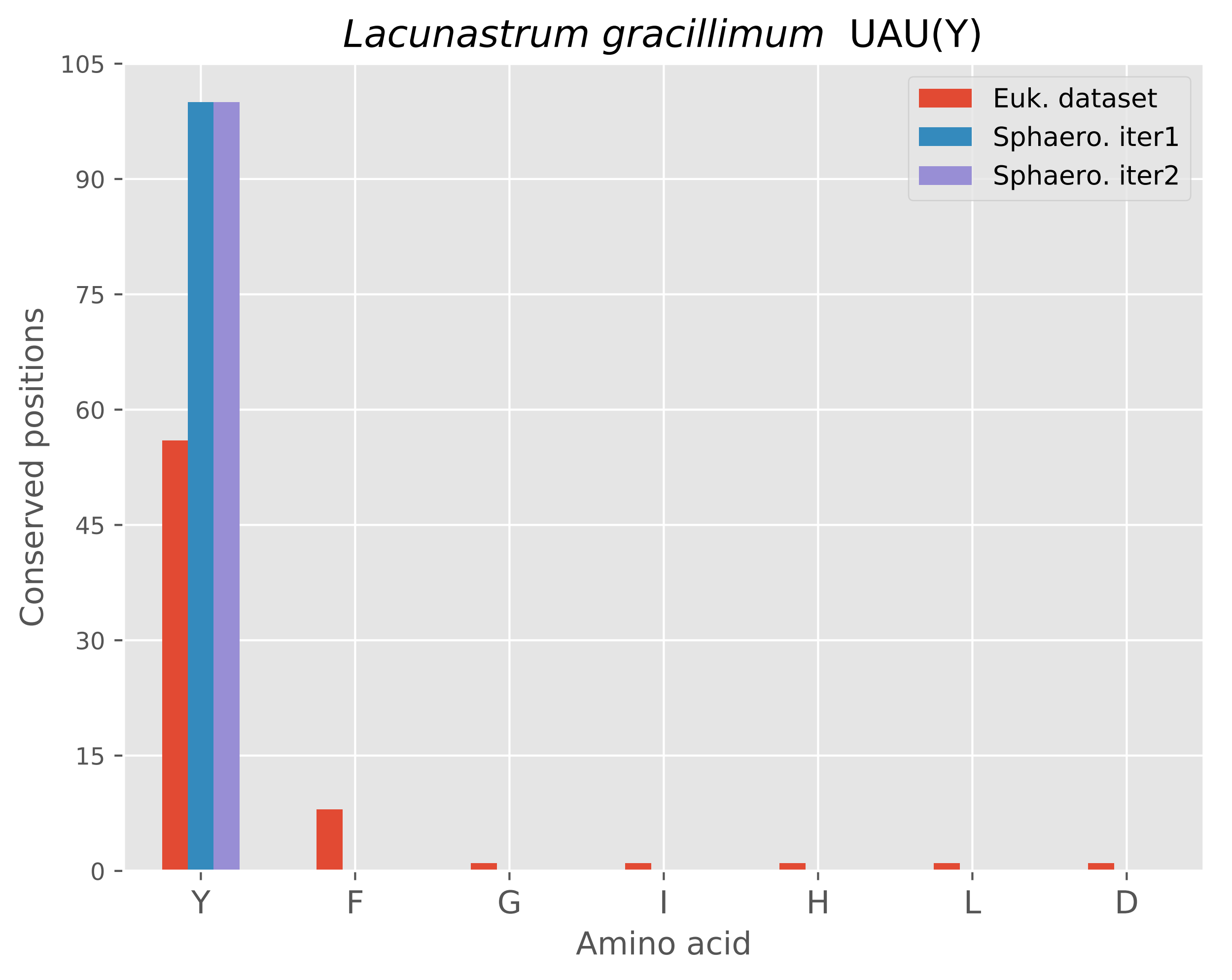
Lacunastrum gracillimum UAU(Y)
Conserved positions
Amino acid
Euk. dataset
Sphaero. iter1
Sphaero. iter2
0
15
30
45
60
75
90
105
Y
F
G
I
H
L
D

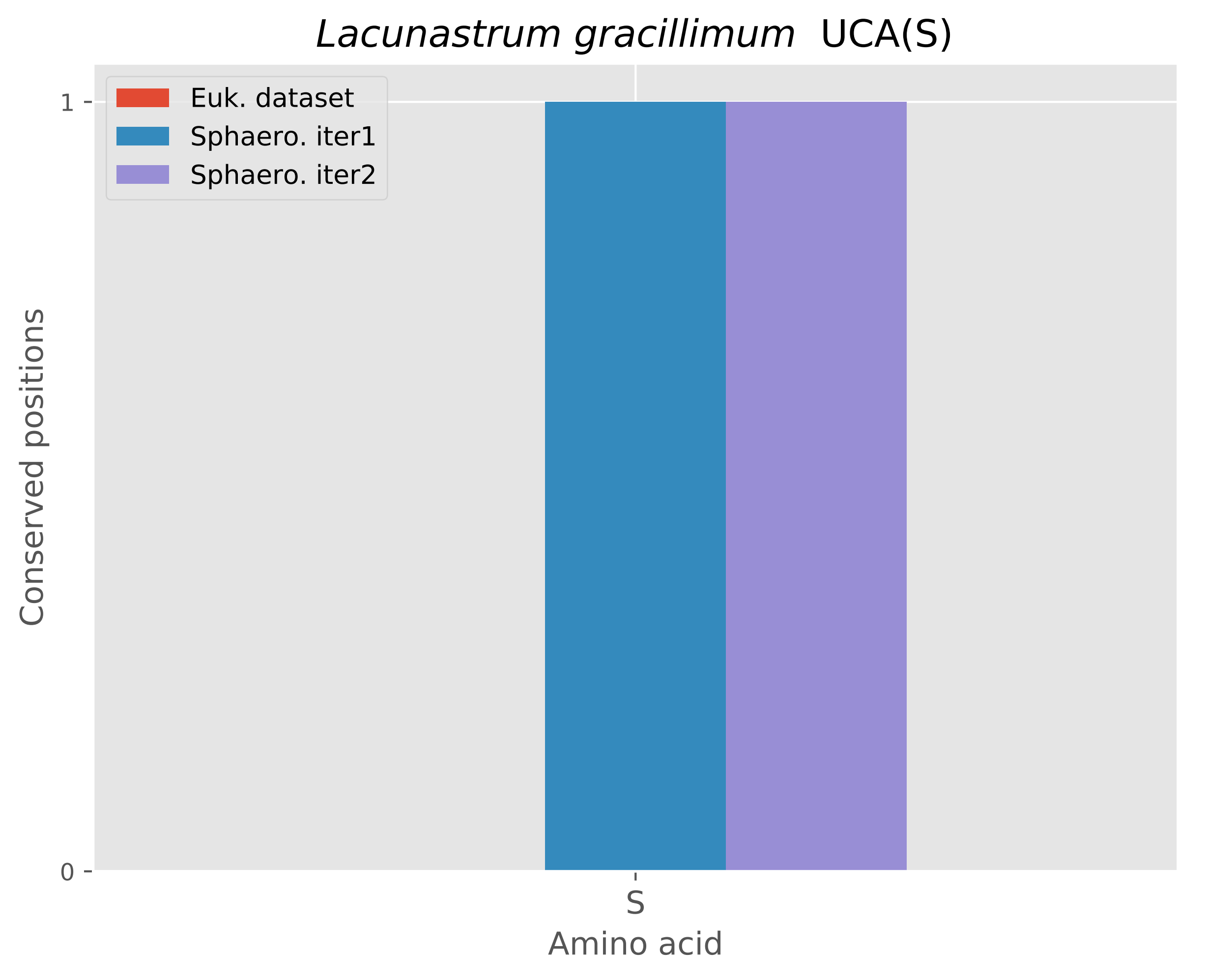

Lacunastrum gracillimum UCA(S)
Euk. dataset
Sphaero. iter1
Sphaero. iter2
1
0
Conserved positions
S
Amino acid

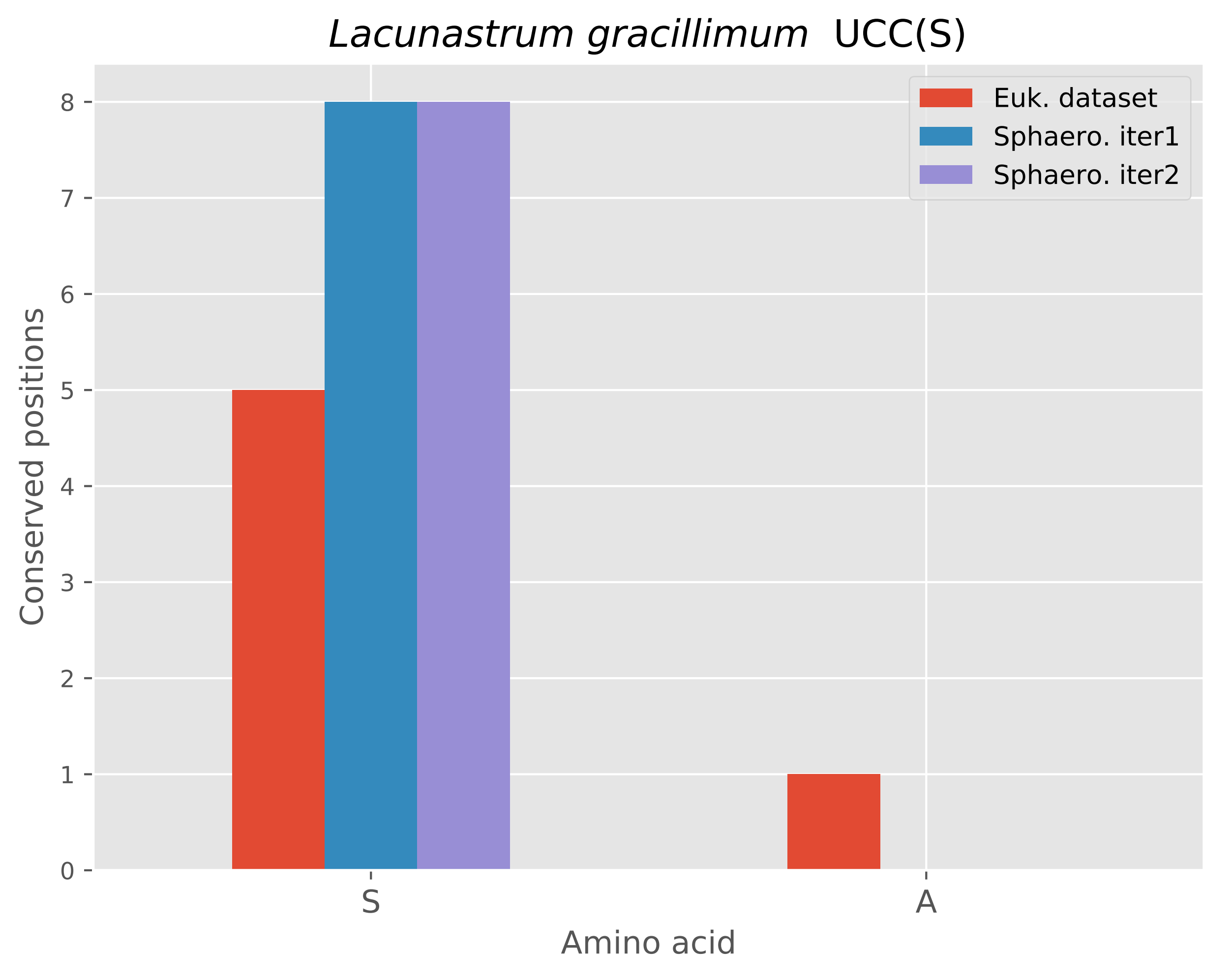

Lacunastrum gracillimum UCC(S)
Euk. dataset
Sphaero. iter1
Sphaero. iter2
Conserved positions
0
1
2
3
4
5
6
7
8
S
A
Amino acid

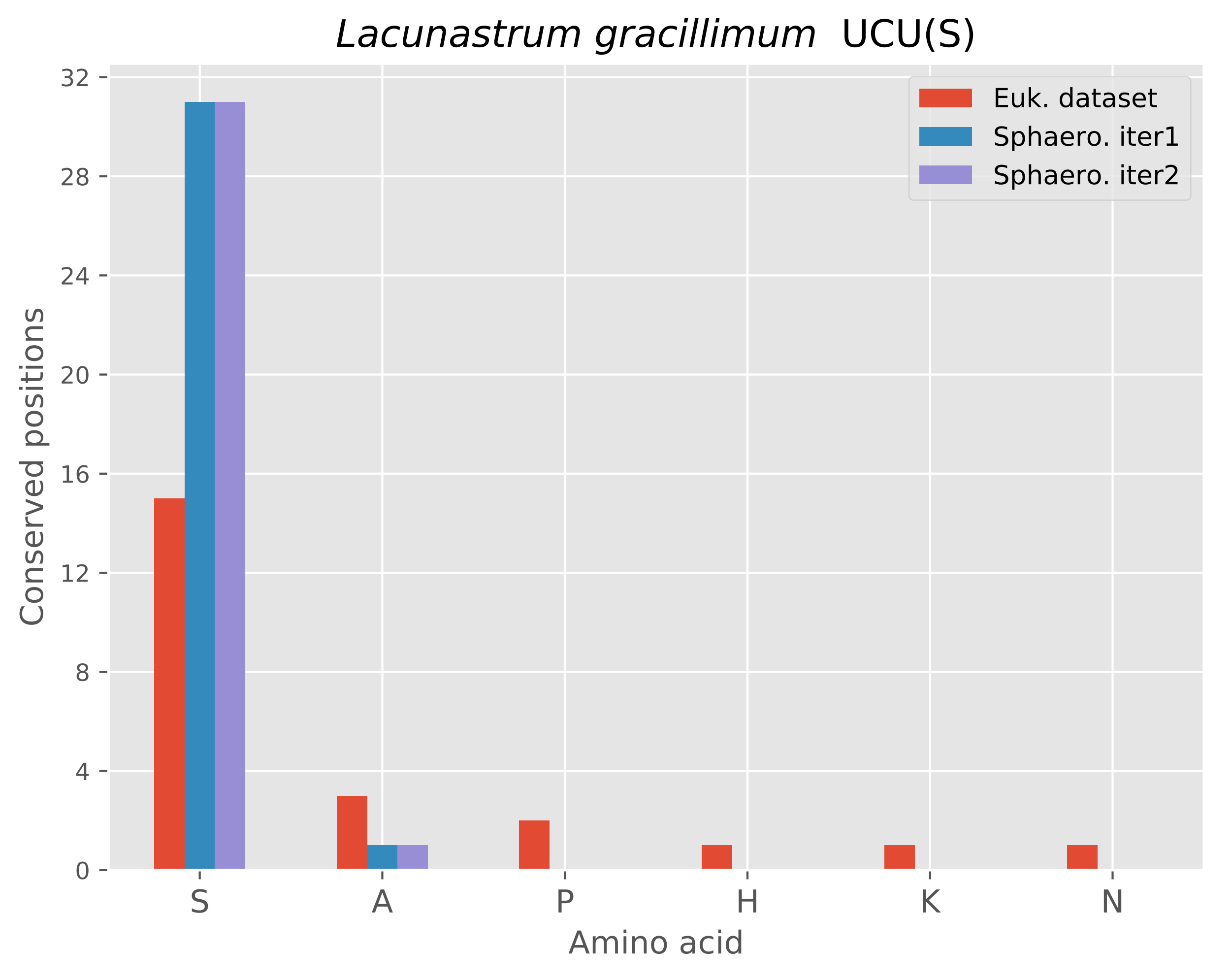

Lacunastrum gracillimum UCU(S)
Conserved positions
Amino acid
0
4
8
12
16
20
24
28
32
S
A
P
H
K
N
Euk. dataset
Sphaero. iter1
Sphaero. iter2

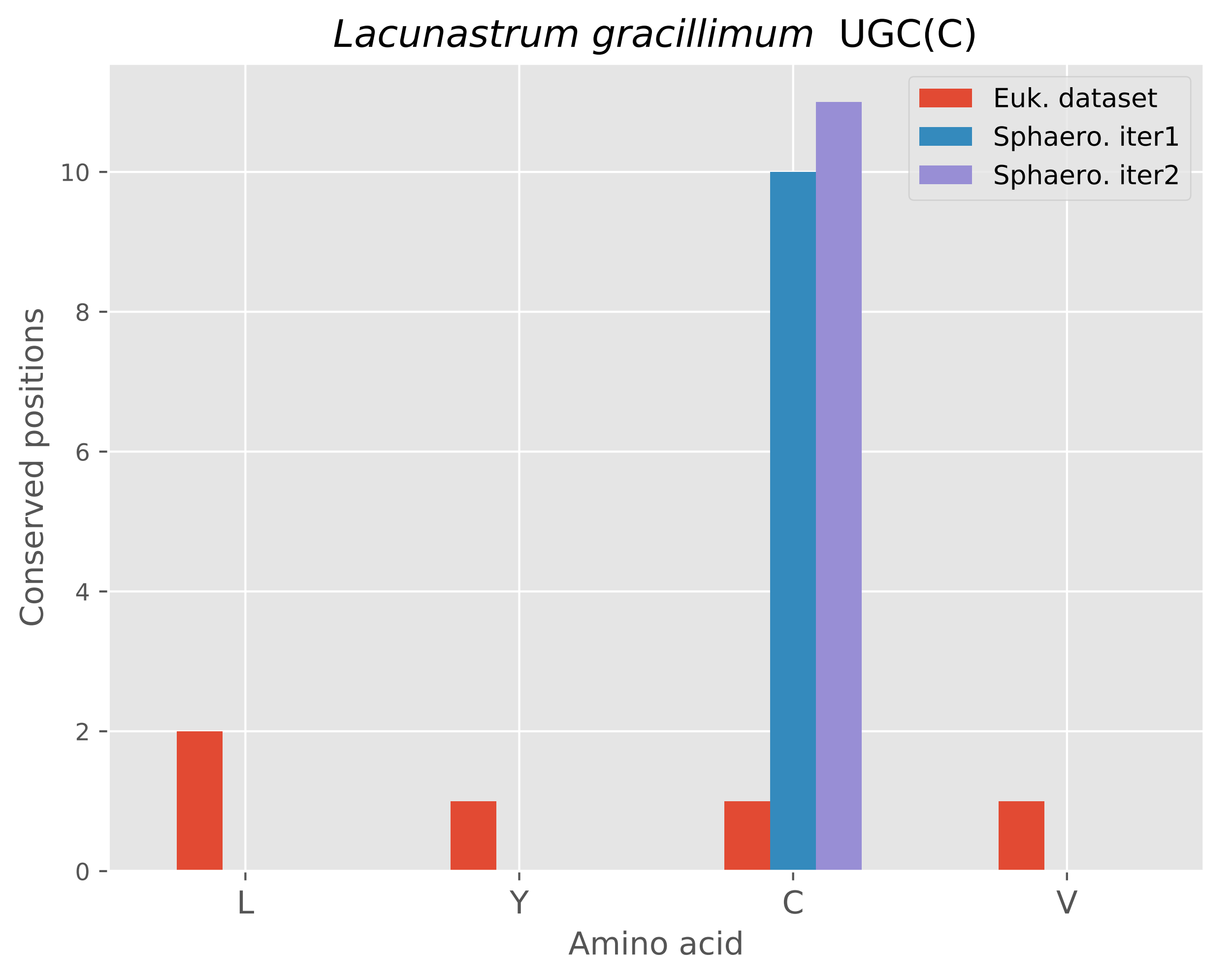

Lacunastrum gracillimum UGC(C)
Euk. dataset
Sphaero. iter1
Sphaero. iter2
Conserved positions
0
2
4
6
8
10
L
Y
C
V
Amino acid

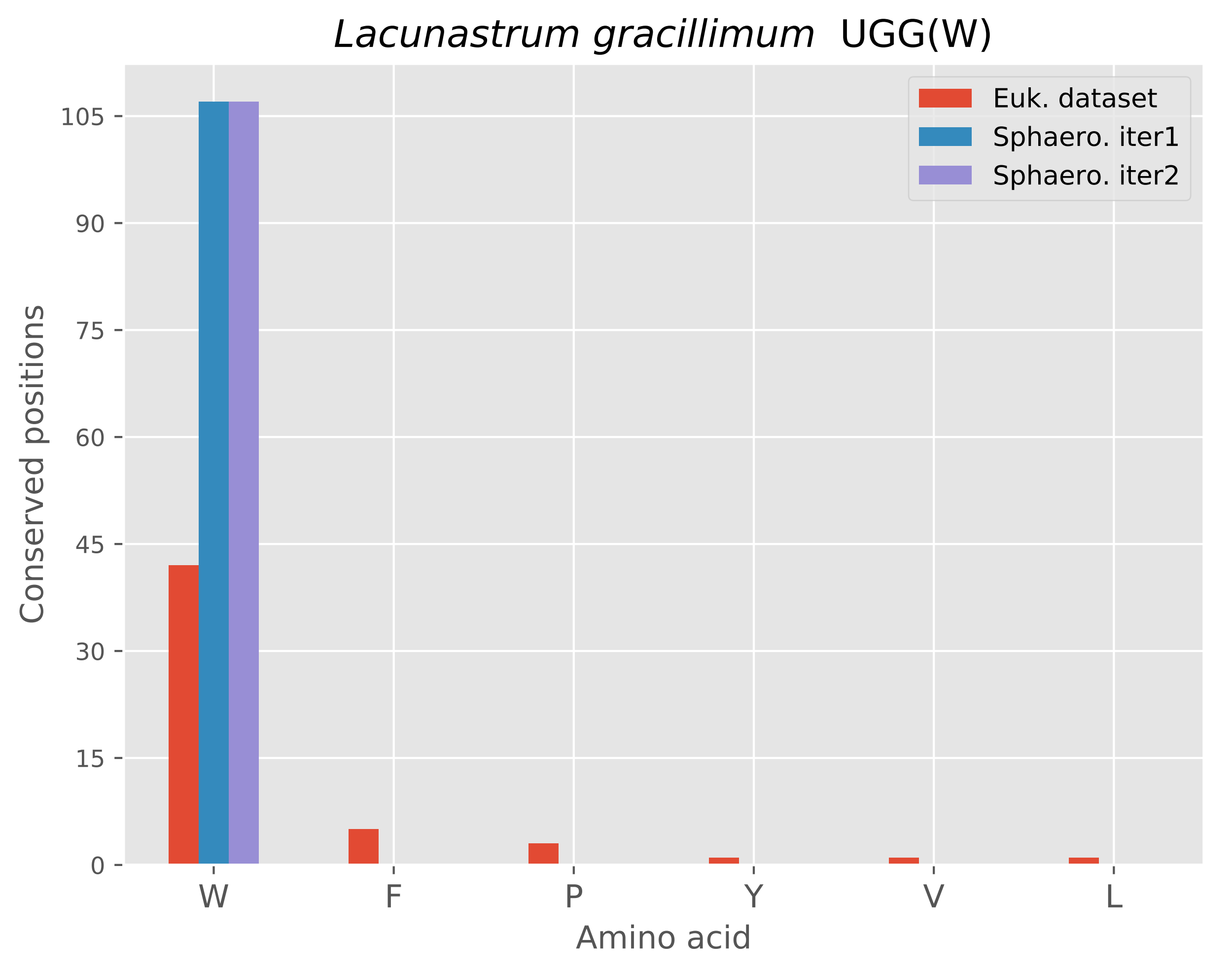

Lacunastrum gracillimum UGG(W)
Euk. dataset
Sphaero. iter1
Sphaero. iter2
Conserved positions
0
15
30
45
60
75
90
105
W
F
P
Y
V
L
Amino acid

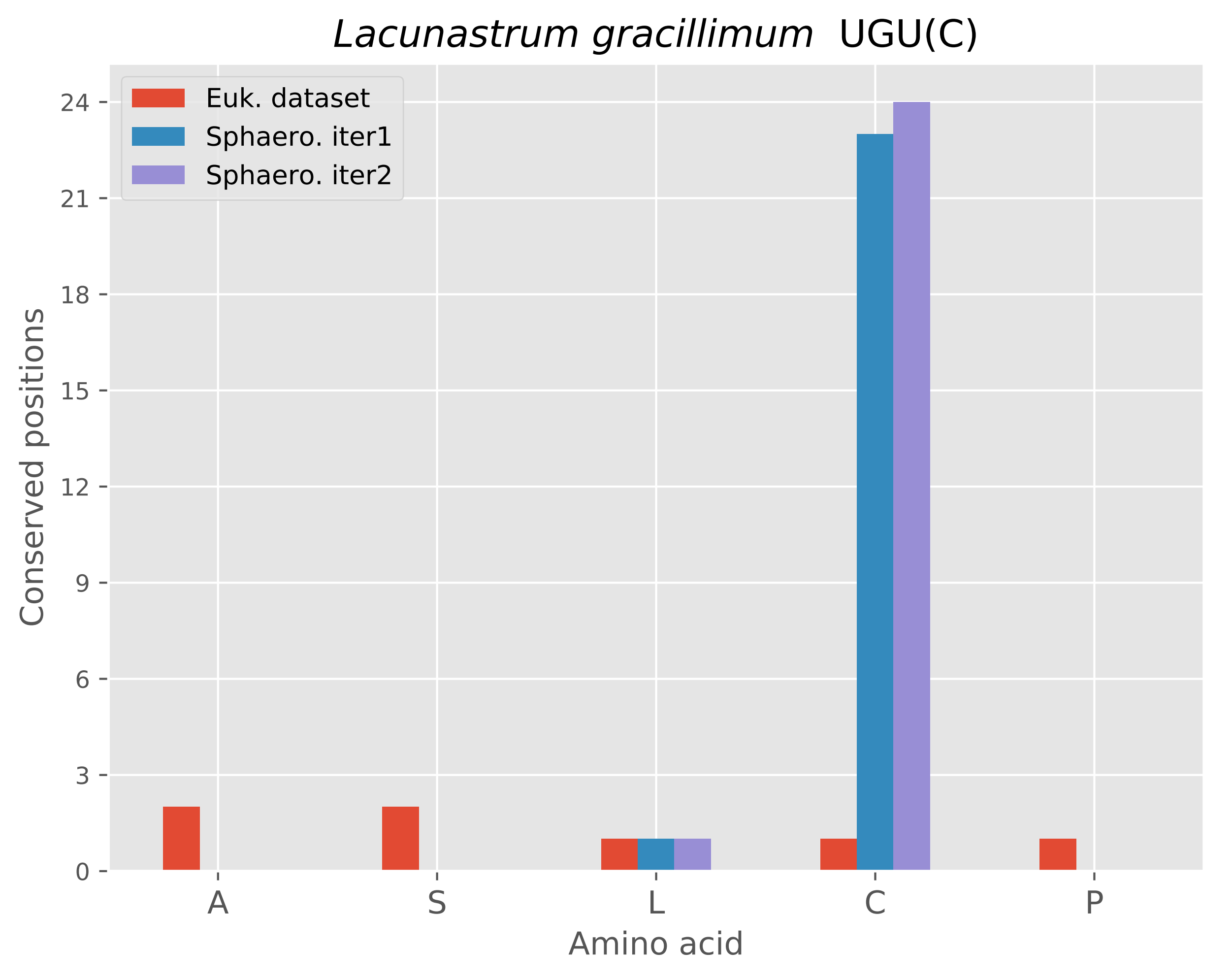

Lacunastrum gracillimum UGU(C)
Euk. dataset
Sphaero. iter1
Sphaero. iter2
Conserved positions
0
3
6
9
12
15
18
21
24
A
S
L
C
P
Amino acid

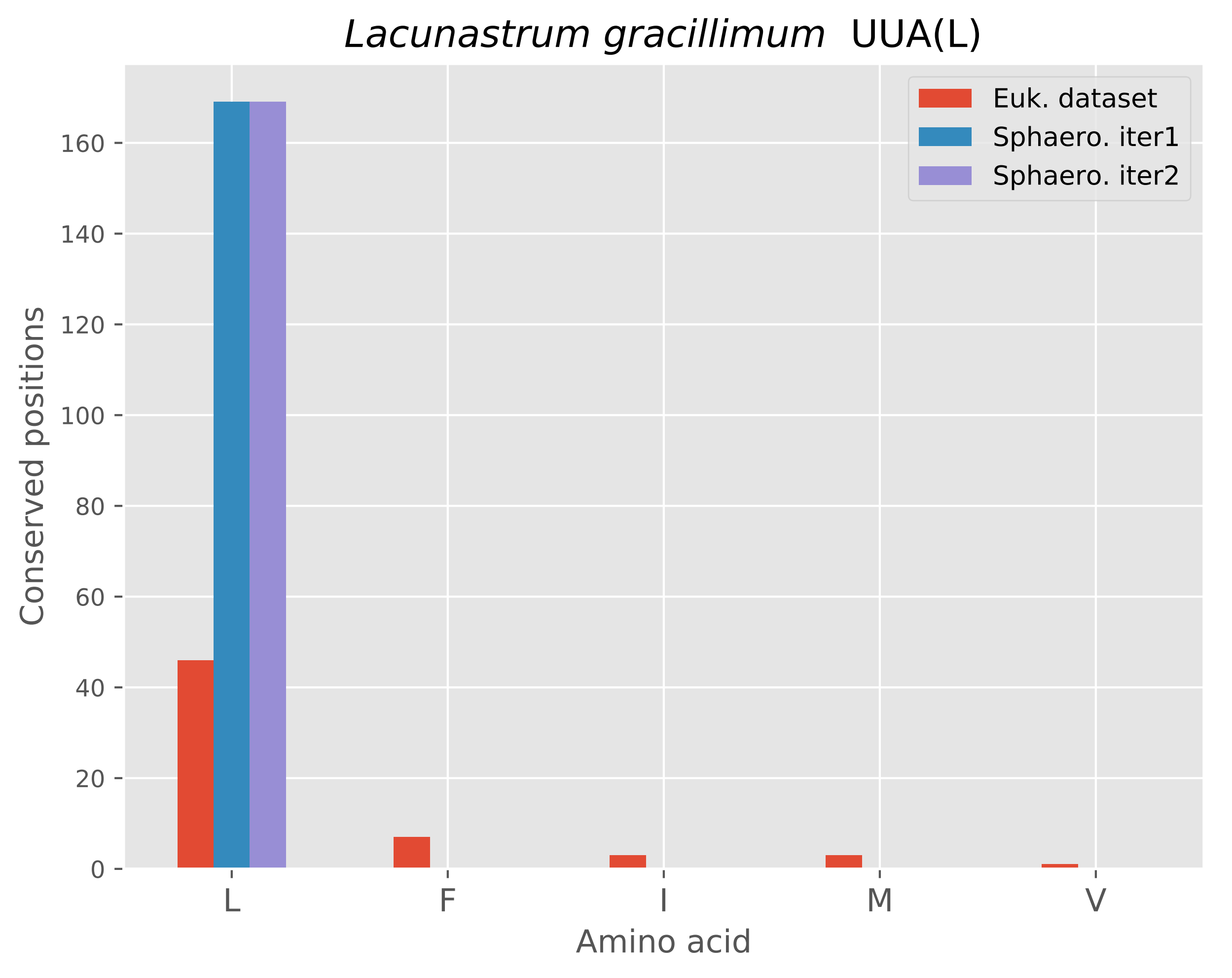

Lacunastrum gracillimum UUA(L)
Euk. dataset
Sphaero. iter1
Sphaero. iter2
Conserved positions
0
20
40
60
80
100
120
140
160
L
F
I
M
V
Amino acid

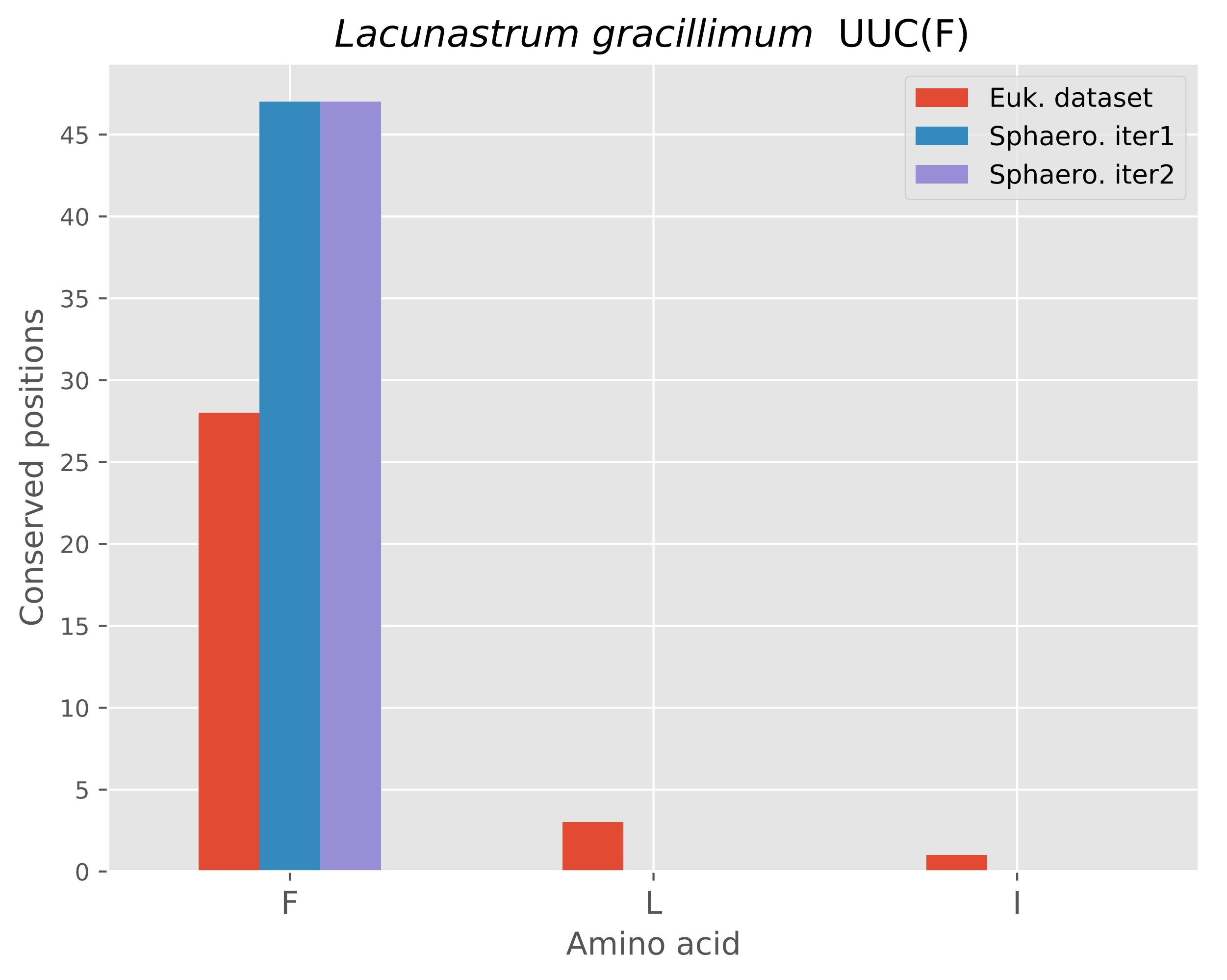

Lacunastrum gracillimum UUC(F)
Euk. dataset
Sphaero. iter1
Sphaero. iter2
Conserved positions
0
5
10
15
20
25
30
35
40
45
F
L
I
Amino acid

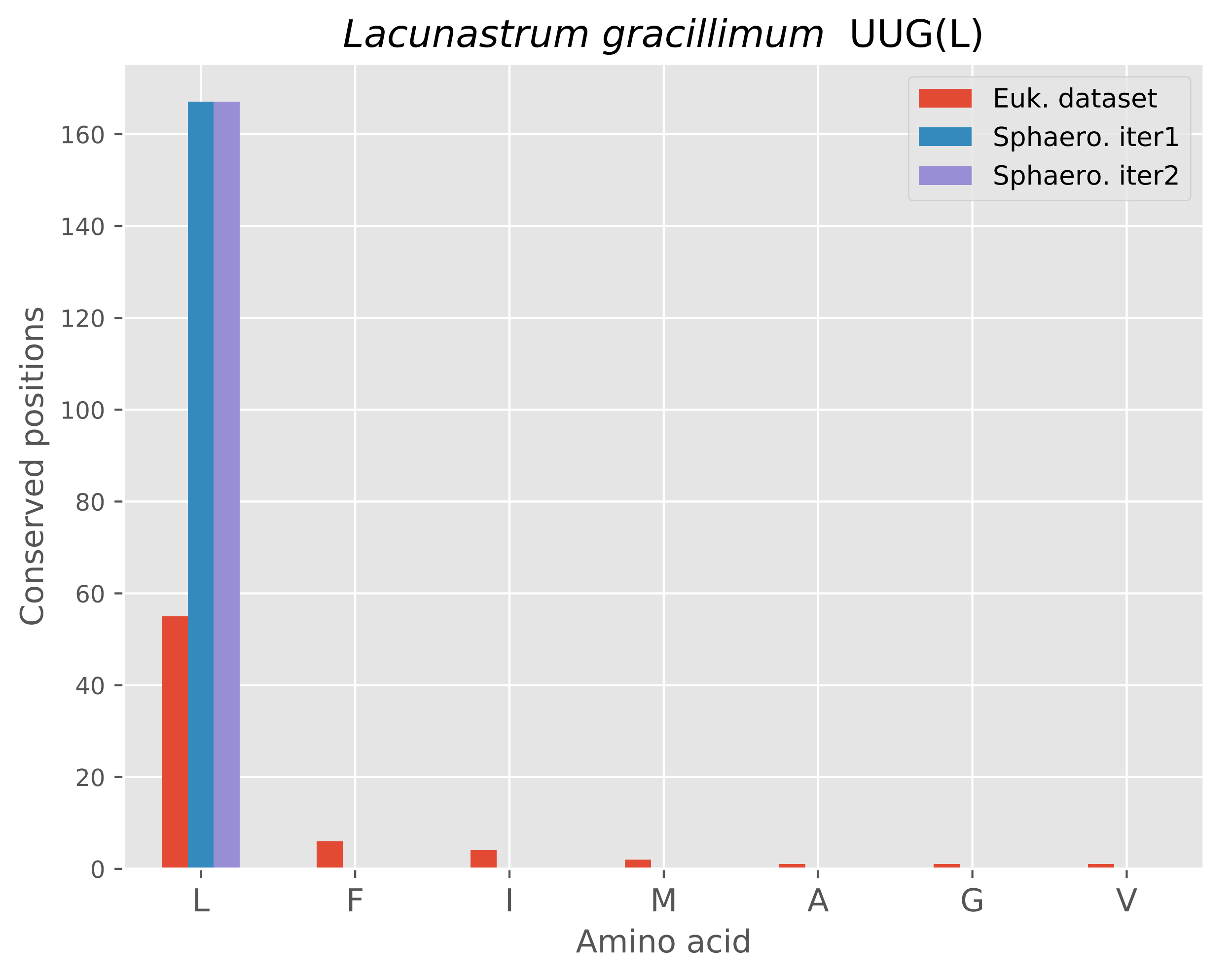

Lacunastrum gracillimum UUG(L)
Euk. dataset
Sphaero. iter1
Sphaero. iter2
Conserved positions
0
20
40
60
80
100
120
140
160
L
F
I
M
A
G
V
Amino acid

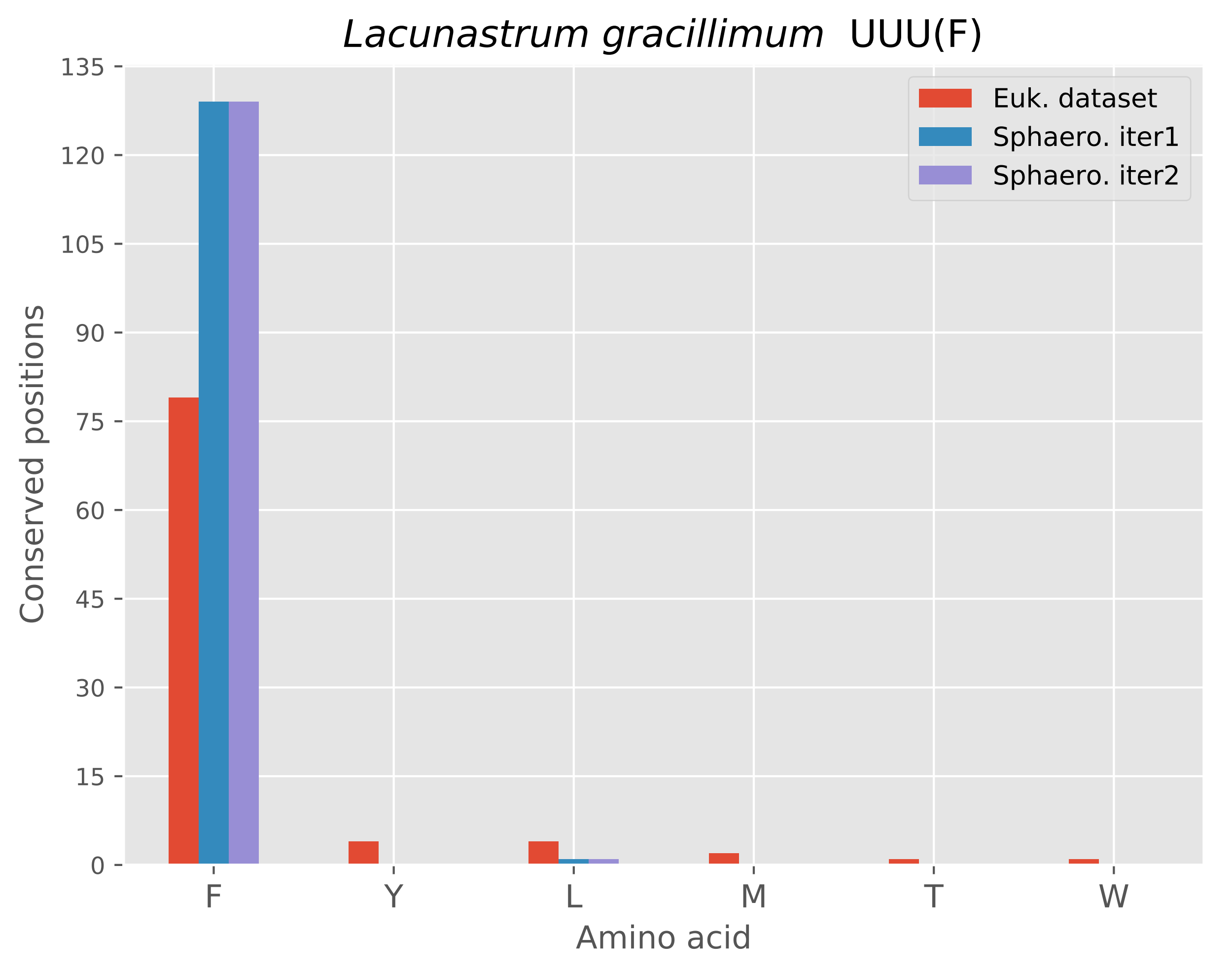

Lacunastrum gracillimum UUU(F)
Euk. dataset
Sphaero. iter1
Sphaero. iter2
Conserved positions
135
120
105
90
75
60
45
30
15
0
F
Y
L
M
T
W
Amino acid

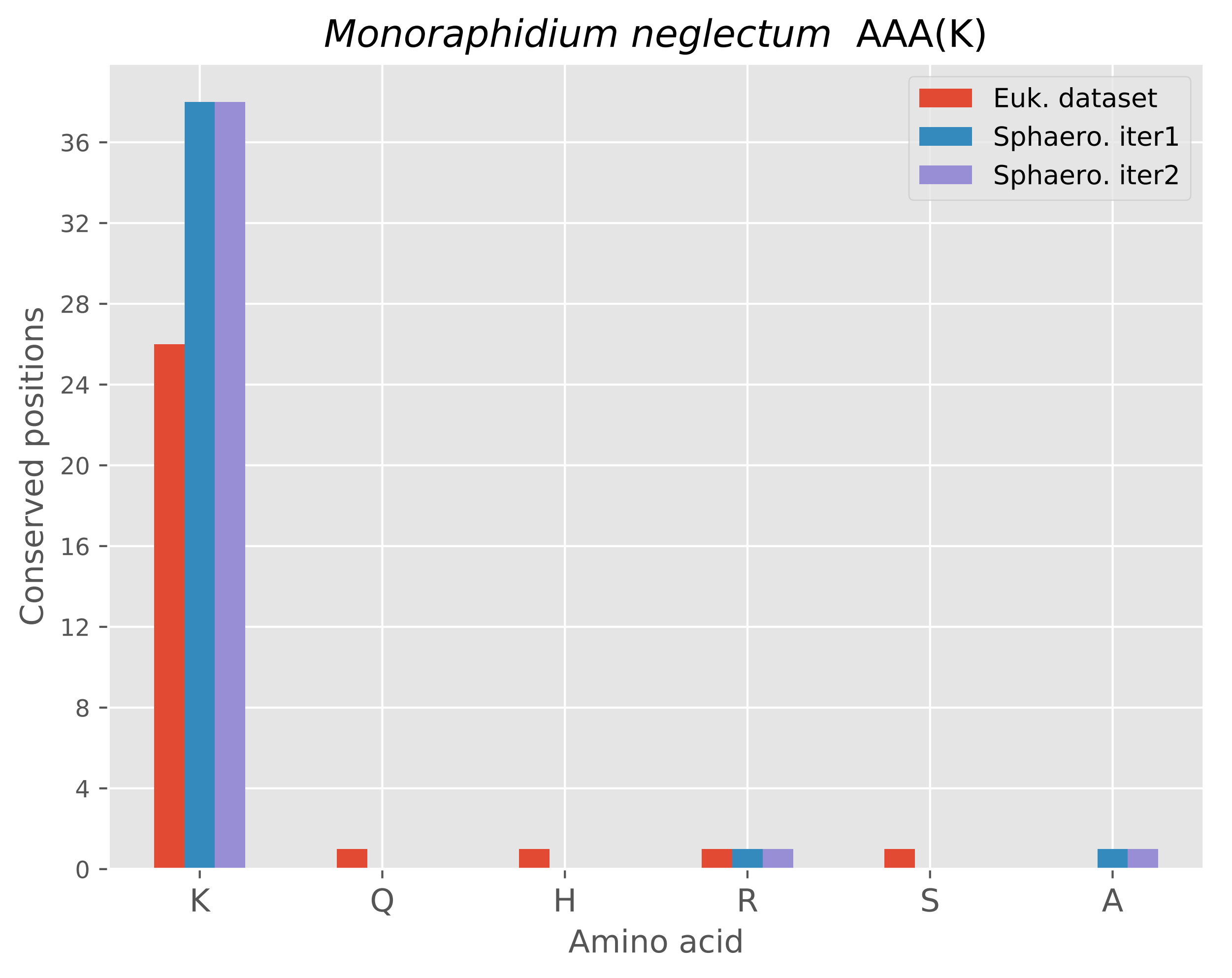

Monoraphidium neglectum AAA(K)
Euk. dataset
Sphaero. iter1
Sphaero. iter2
Conserved positions
0
4
8
12
16
20
24
28
32
36
K
Q
H
R
S
A
Amino acid

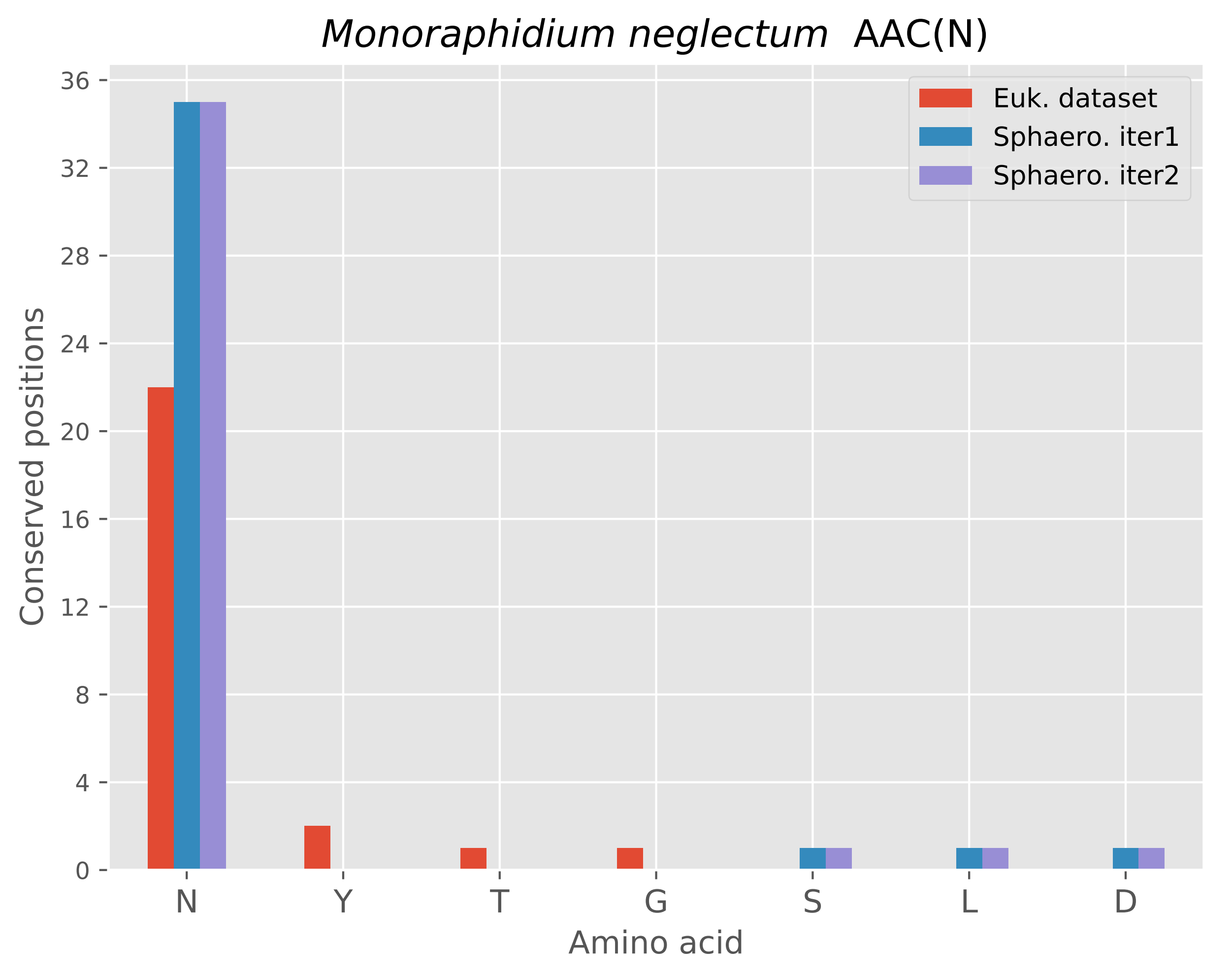

Monoraphidium neglectum AAC(N)
Euk. dataset
Sphaero. iter1
Sphaero. iter2
Conserved positions
0
4
8
12
16
20
24
28
32
36
N
Y
T
G
S
L
D
Amino acid

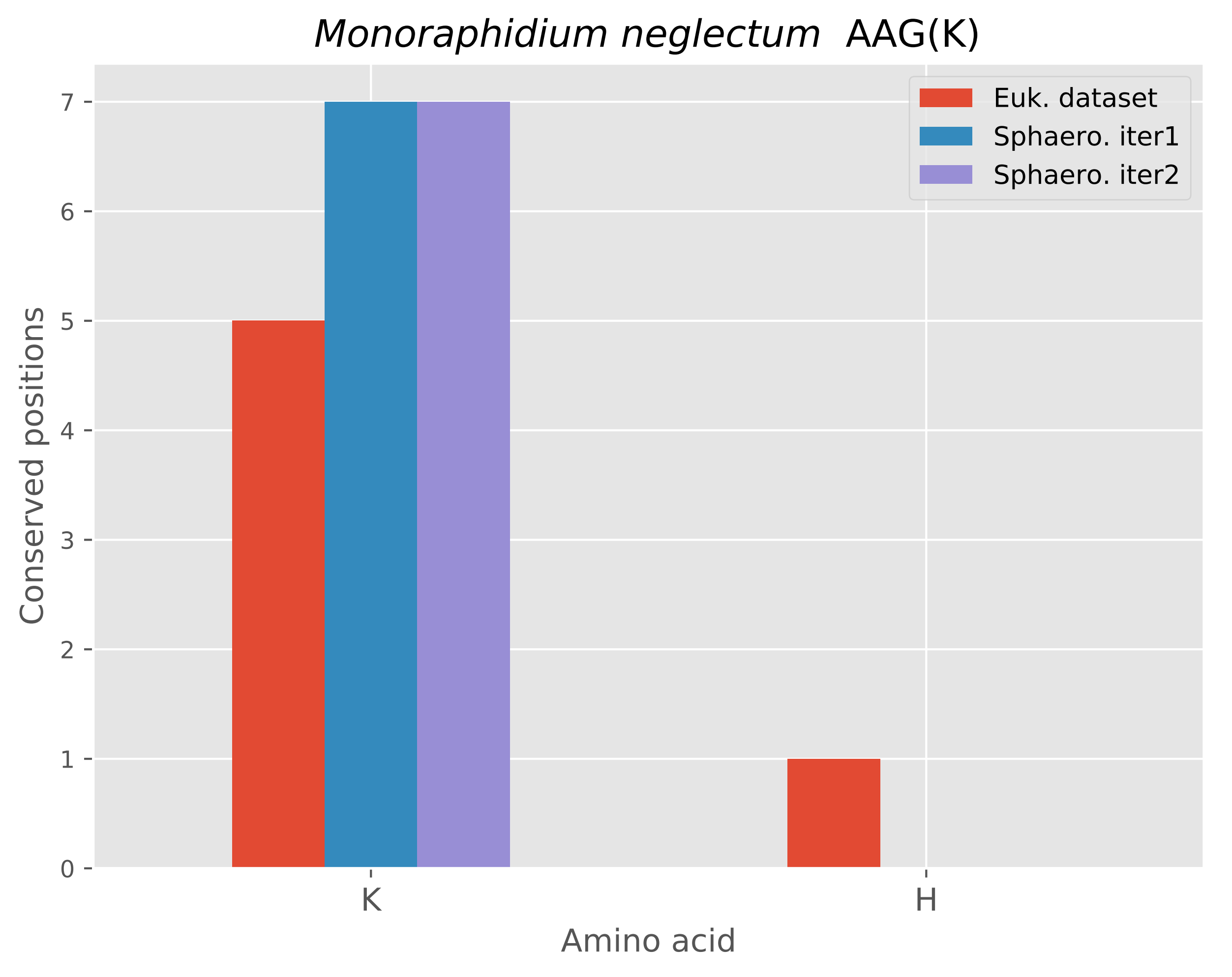

Monoraphidium neglectum AAG(K)
Euk. dataset
Sphaero. iter1
Sphaero. iter2
Conserved positions
0
1
2
3
4
5
6
7
K
H
Amino acid

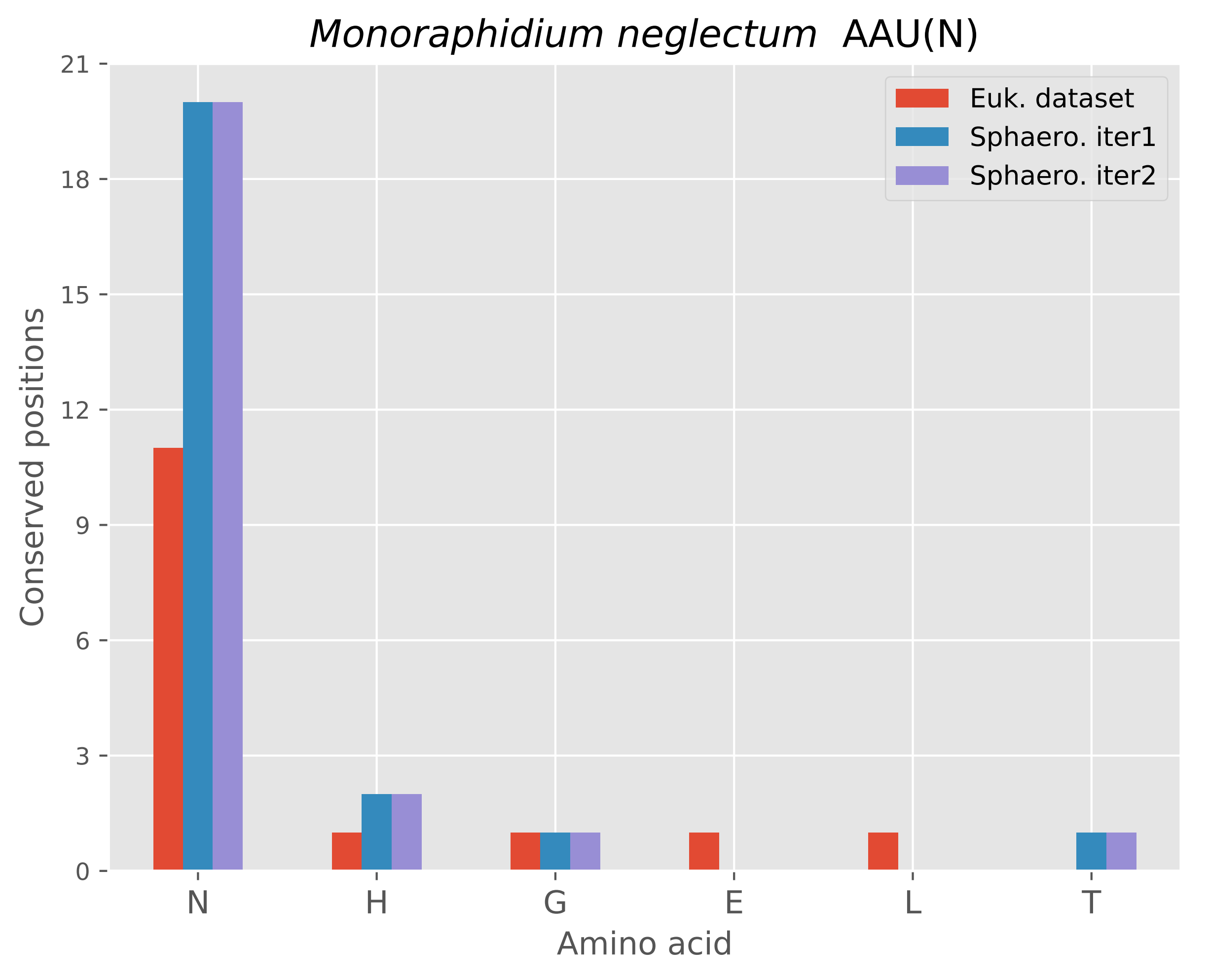

Monoraphidium neglectum AAU(N)
Euk. dataset
Sphaero. iter1
Sphaero. iter2
Conserved positions
Amino acid
0
3
6
9
12
15
18
21
N
H
G
E
L
T

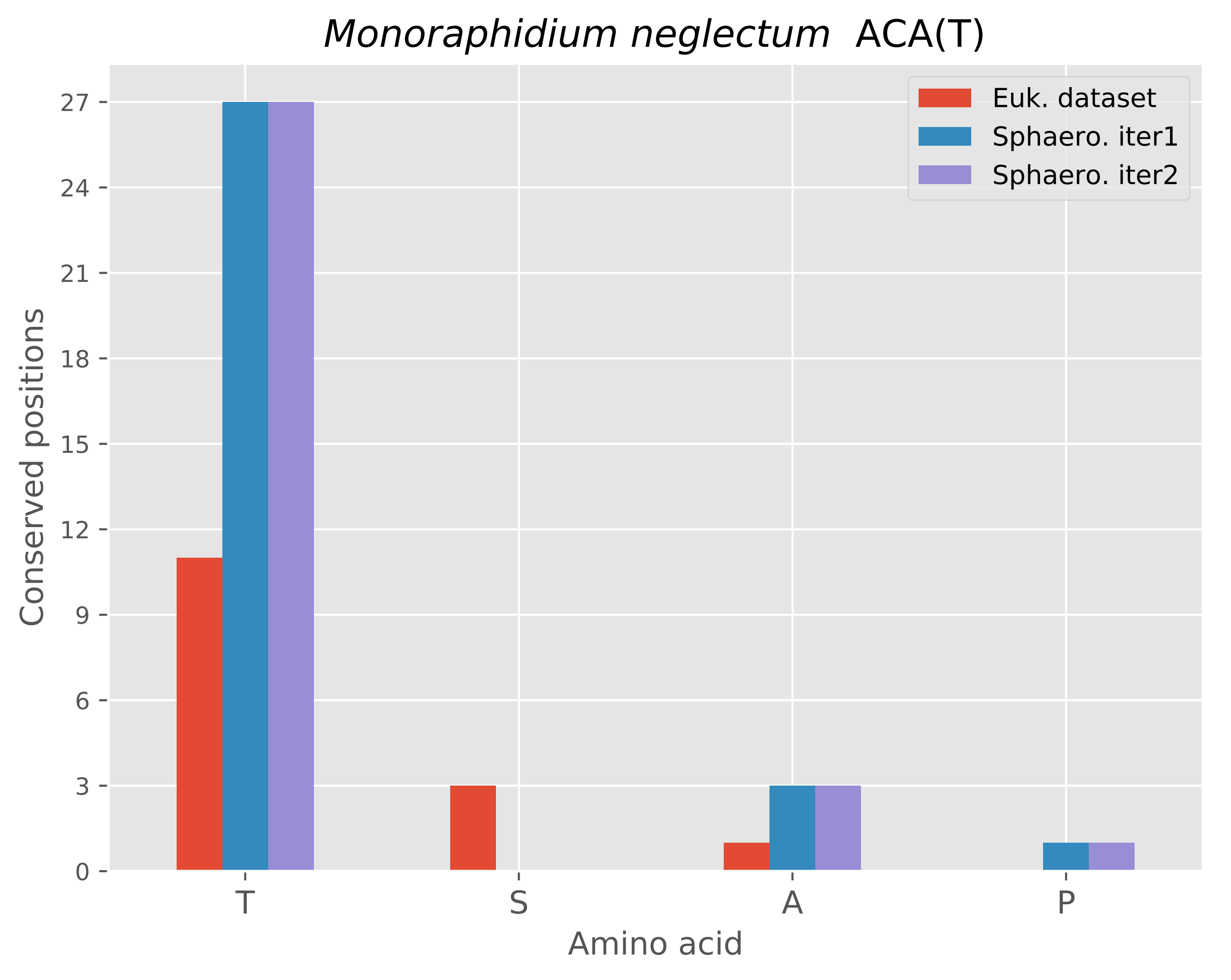

Monoraphidium neglectum ACA(T)
Euk. dataset
Sphaero. iter1
Sphaero. iter2
Conserved positions
0
3
6
9
12
15
18
21
24
27
T
S
A
P
Amino acid

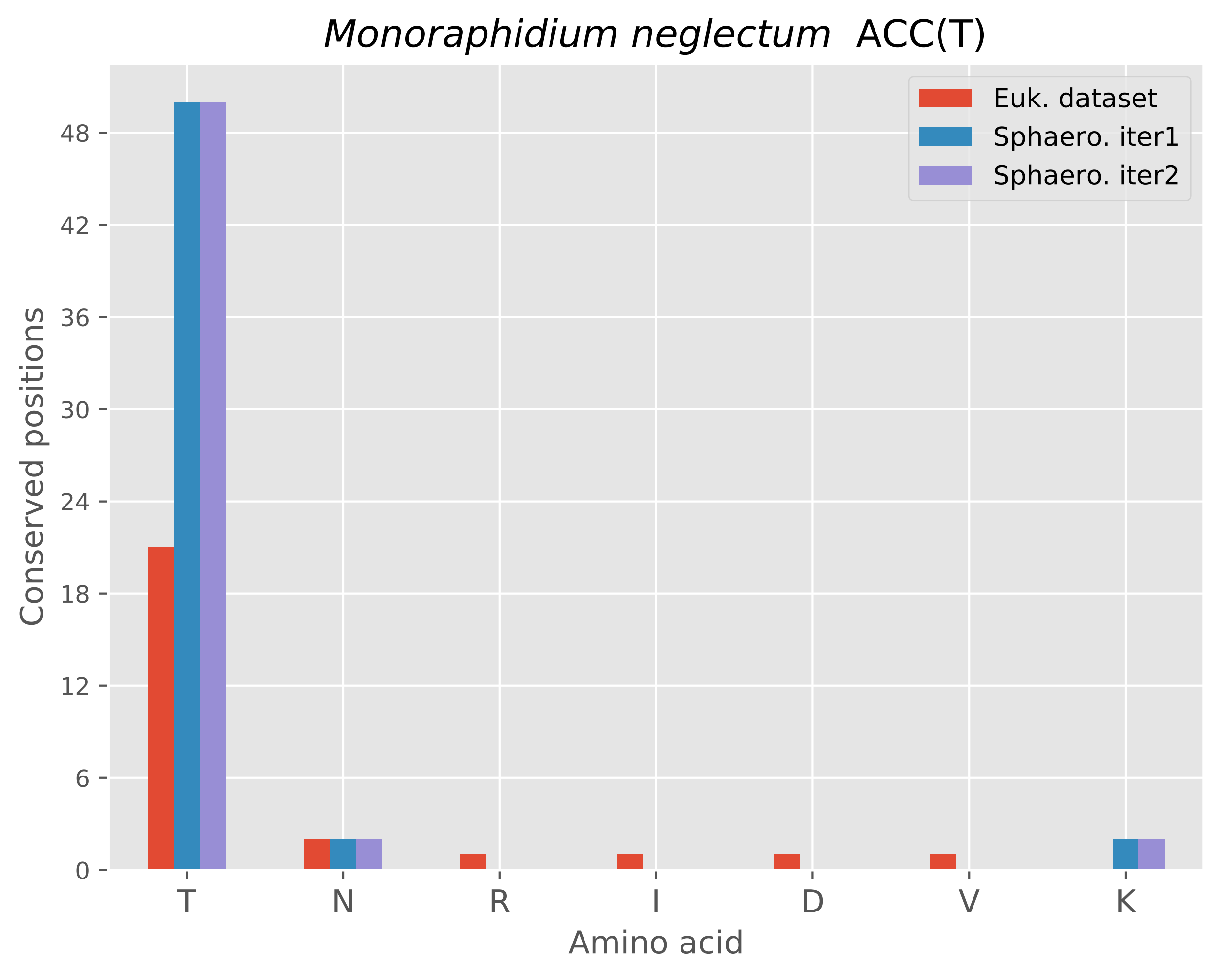

Monoraphidium neglectum ACC(T)
Euk. dataset
Sphaero. iter1
Sphaero. iter2
Conserved positions
0
6
12
18
24
30
36
42
48
T
N
R
I
D
V
K
Amino acid

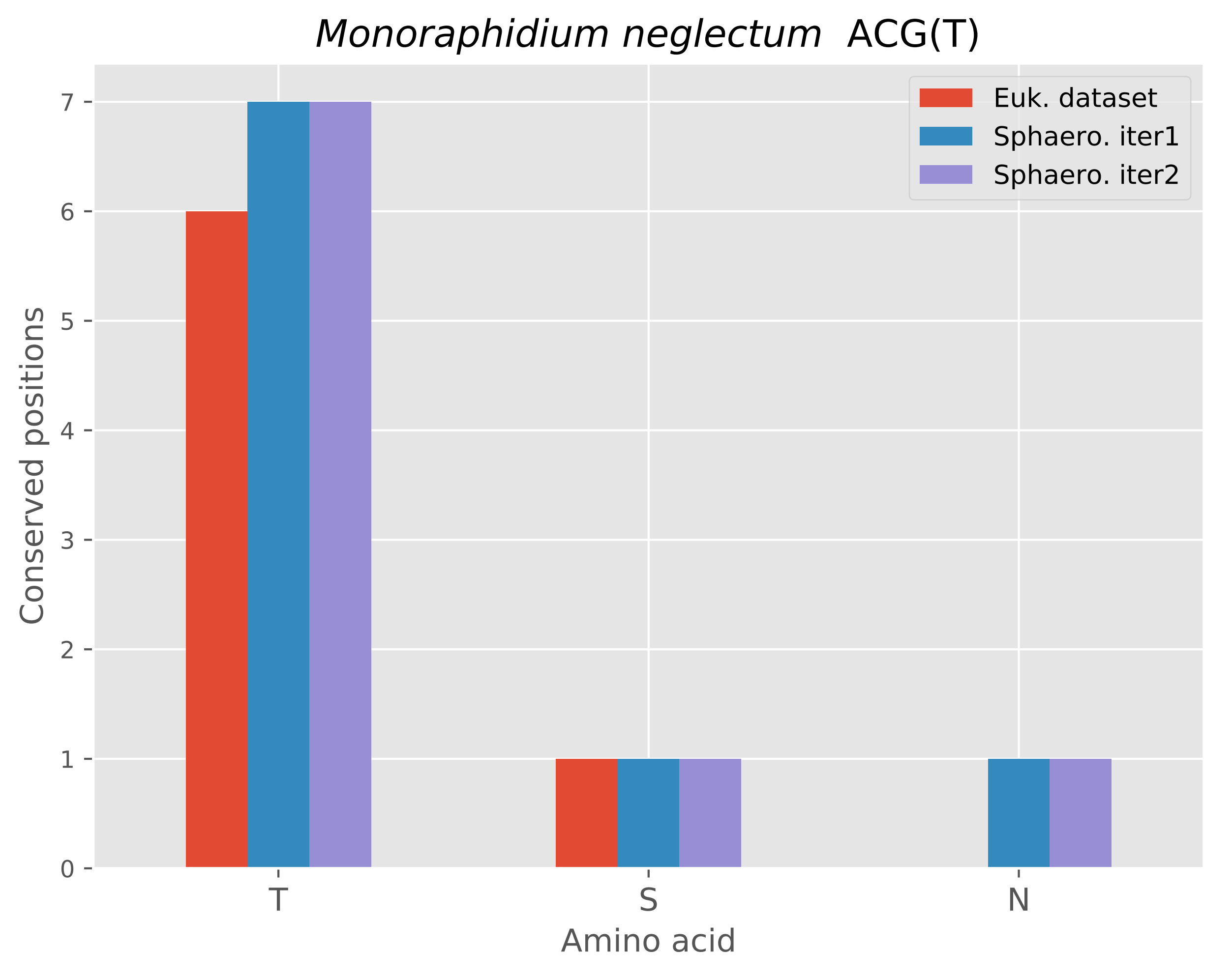

Monoraphidium neglectum ACG(T)
Euk. dataset
Sphaero. iter1
Sphaero. iter2
Conserved positions
0
1
2
3
4
5
6
7
T
S
N
Amino acid

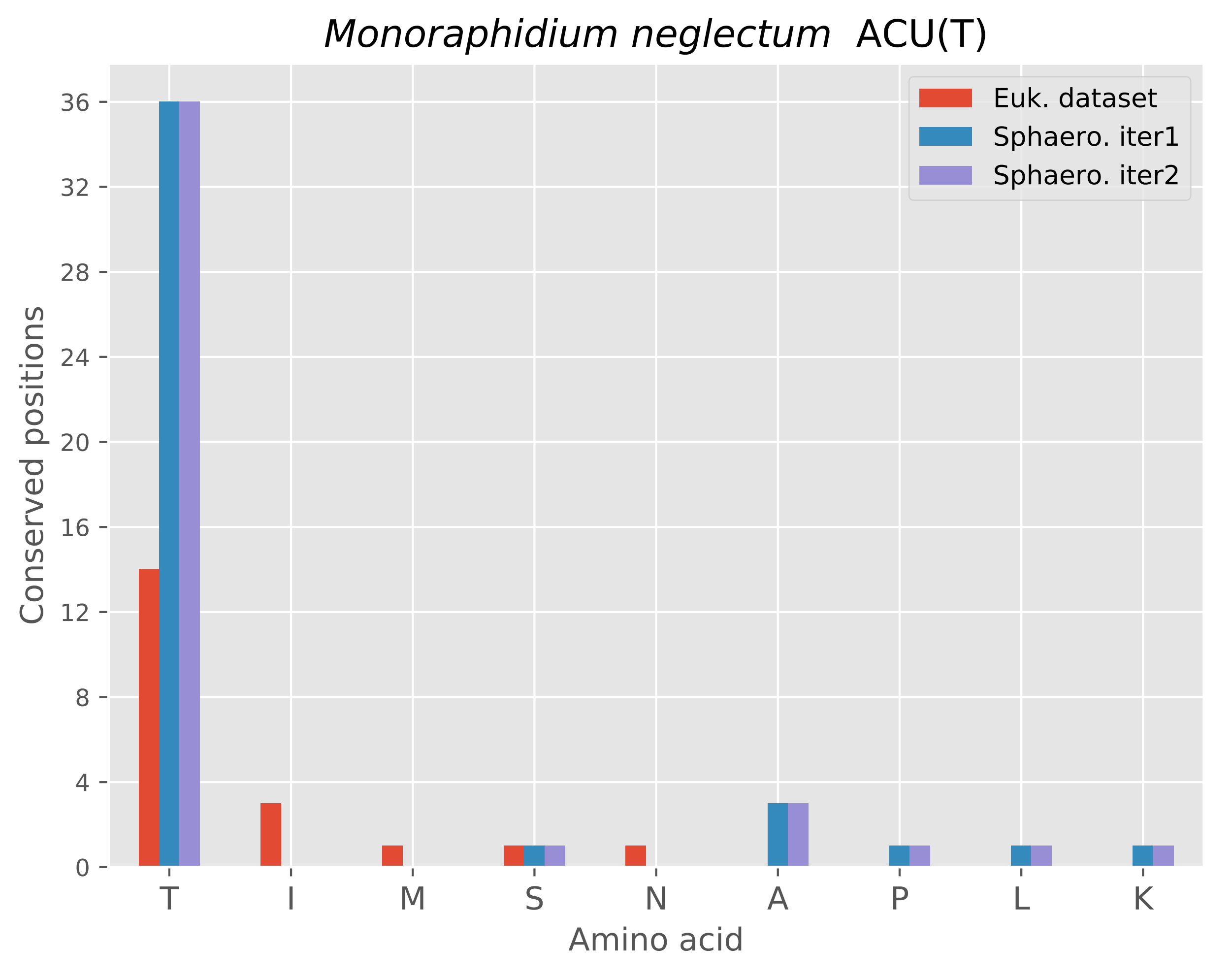

Monoraphidium neglectum ACU(T)
Euk. dataset
Sphaero. iter1
Sphaero. iter2
Conserved positions
0
4
8
12
16
20
24
28
32
36
T
I
M
S
N
A
P
L
K
Amino acid

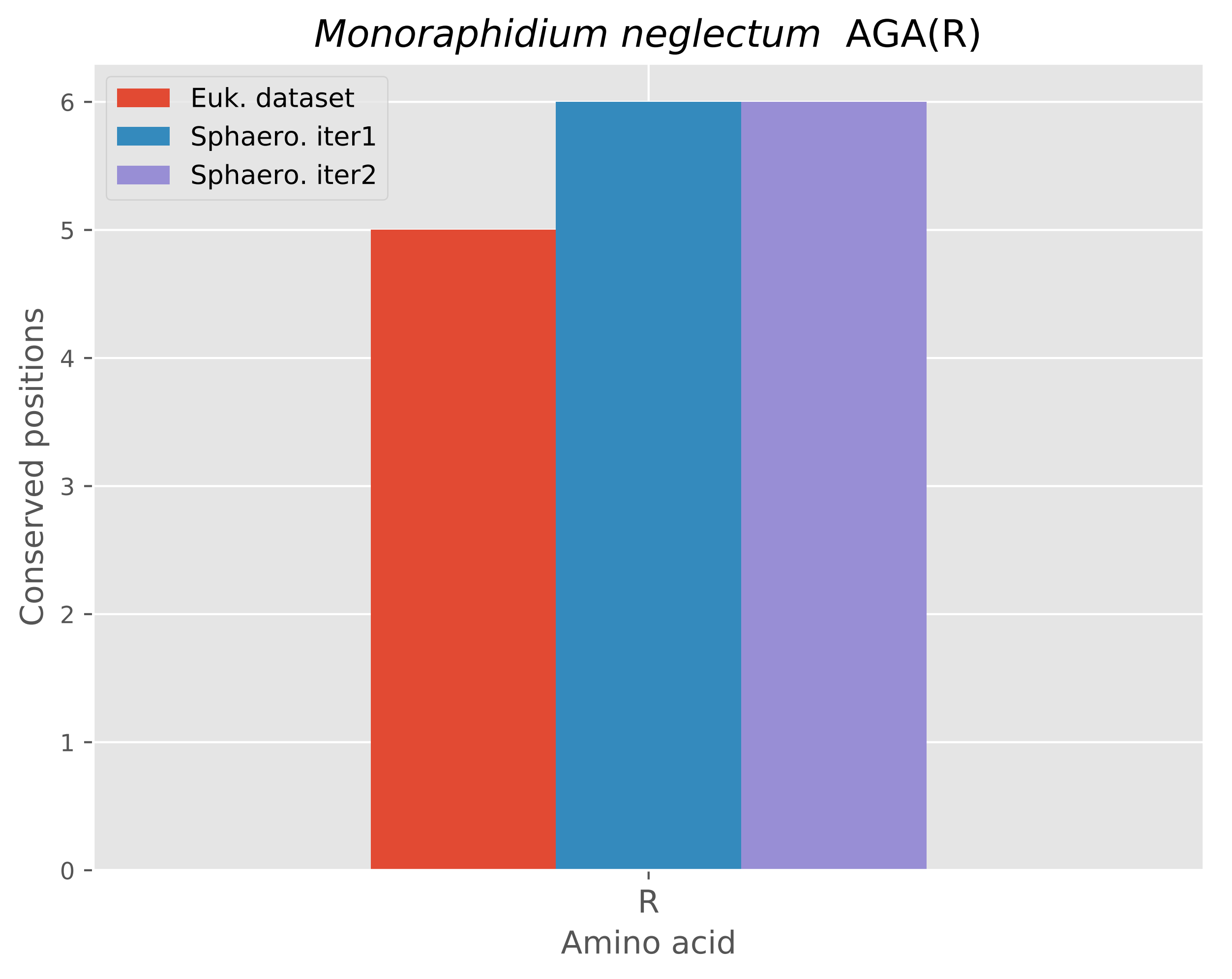

Monoraphidium neglectum AGA(R)
Euk. dataset
Sphaero. iter1
Sphaero. iter2
Conserved positions
0
1
2
3
4
5
6
R
Amino acid

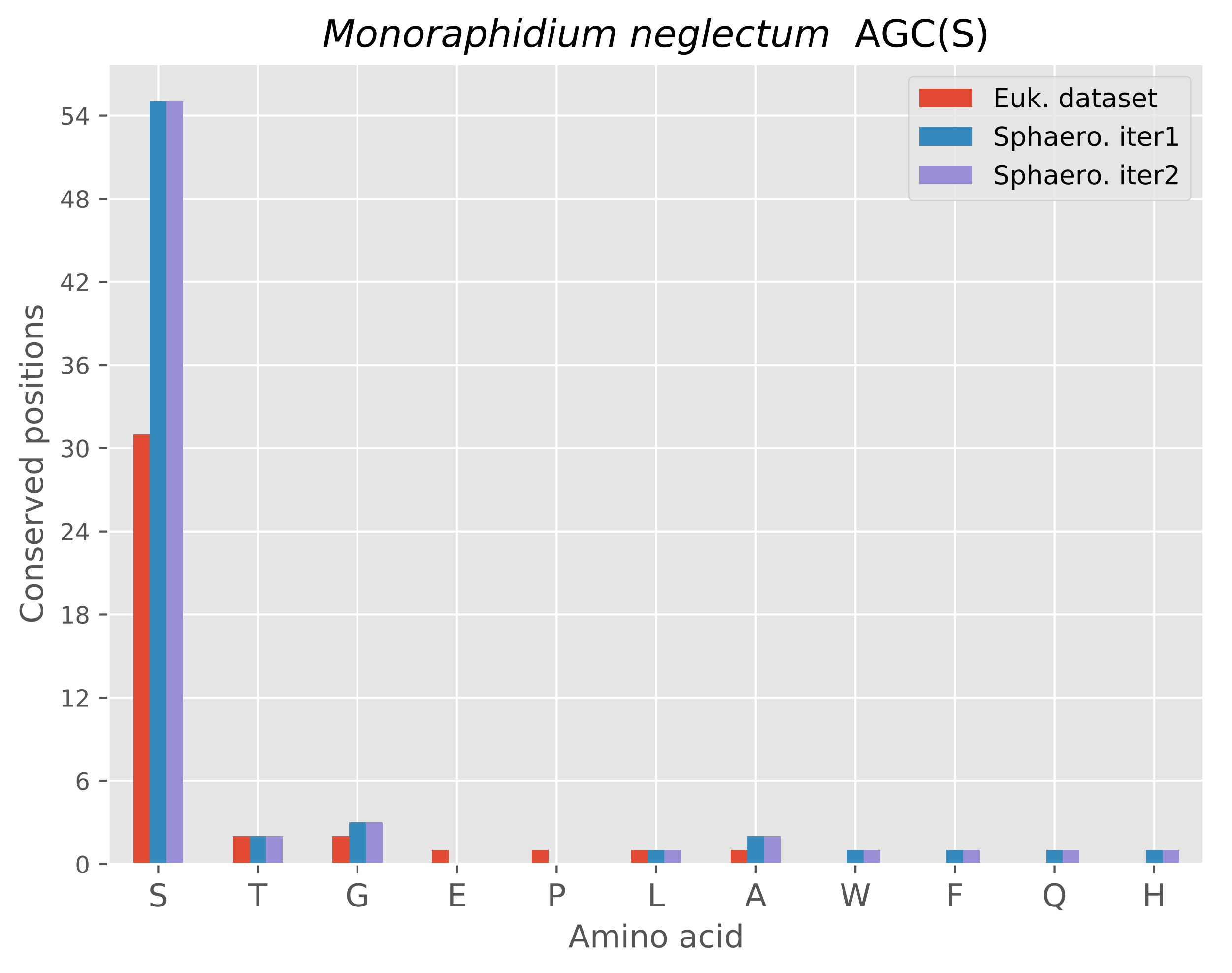
Monoraphidium neglectum AGC(S)
Euk. dataset
Sphaero. iter1
Sphaero. iter2
Conserved positions
0
6
12
18
24
30
36
42
48
54
S
T
G
E
P
L
A
W
F
Q
H
Amino acid

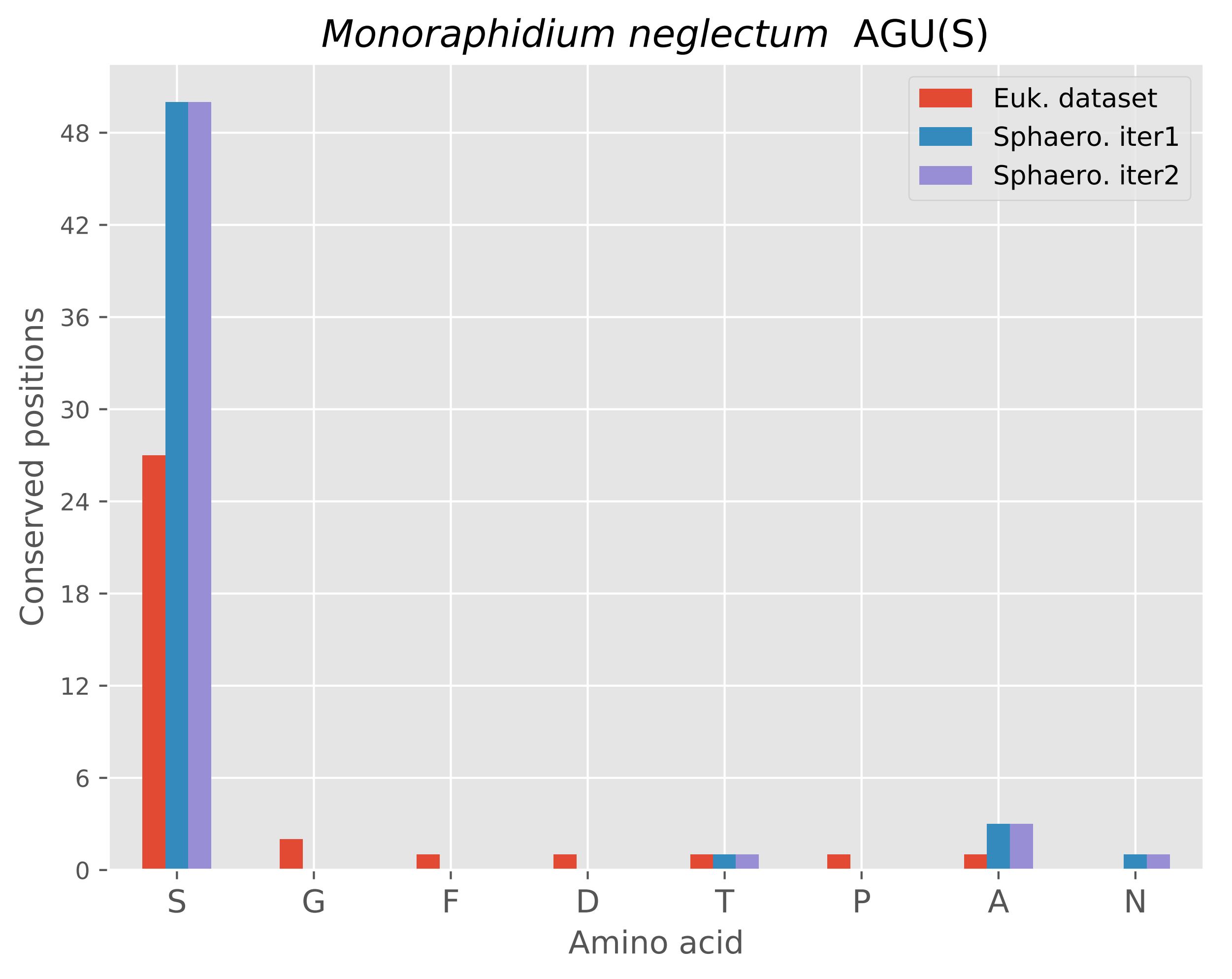

Monoraphidium neglectum AGU(S)
Euk. dataset
Sphaero. iter1
Sphaero. iter2
Conserved positions
0
6
12
18
24
30
36
42
48
S
G
F
D
T
P
A
N
Amino acid

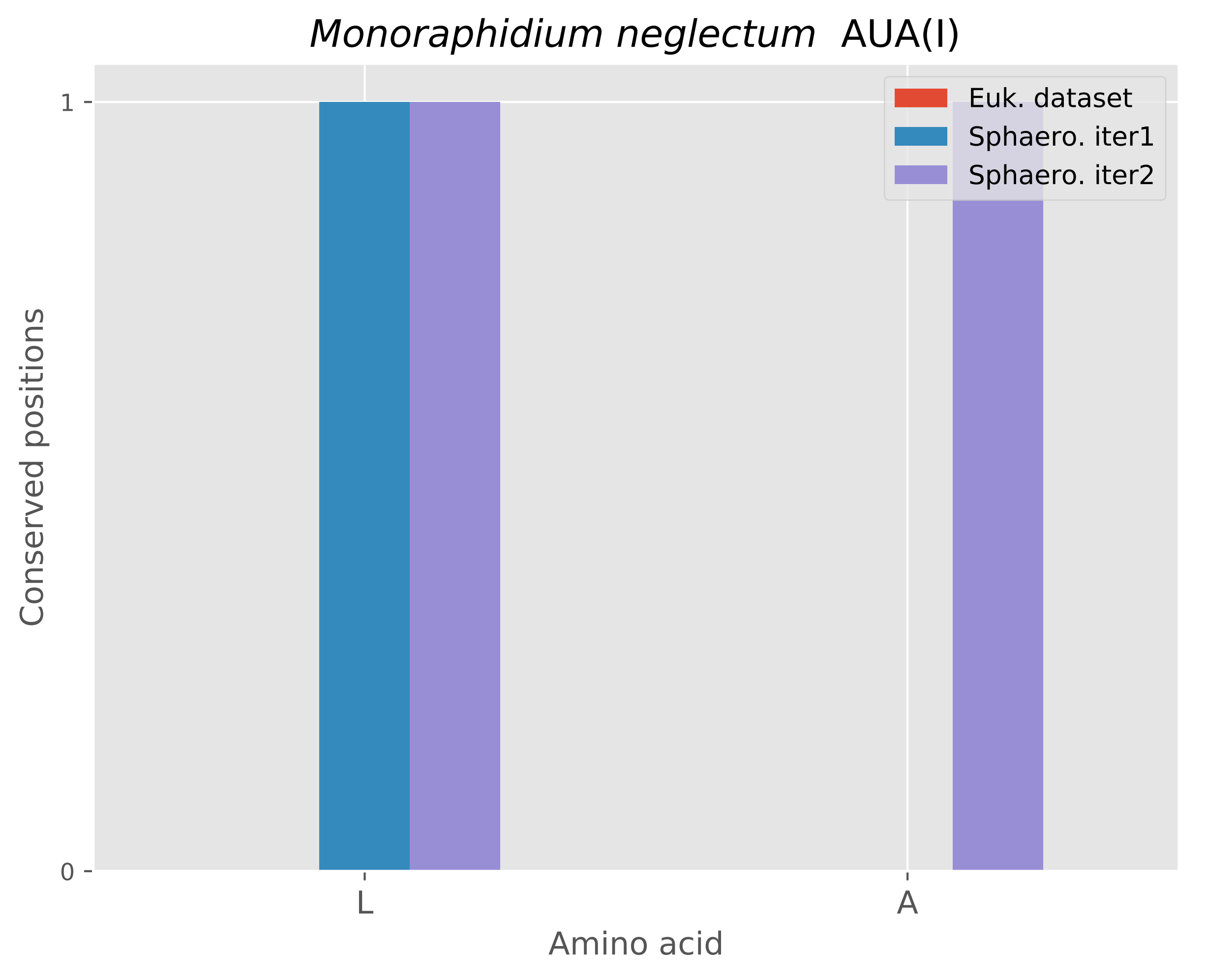

Monoraphidium neglectum AUA(I)
Euk. dataset
Sphaero. iter1
Sphaero. iter2
Conserved positions
1
0
L
A
Amino acid

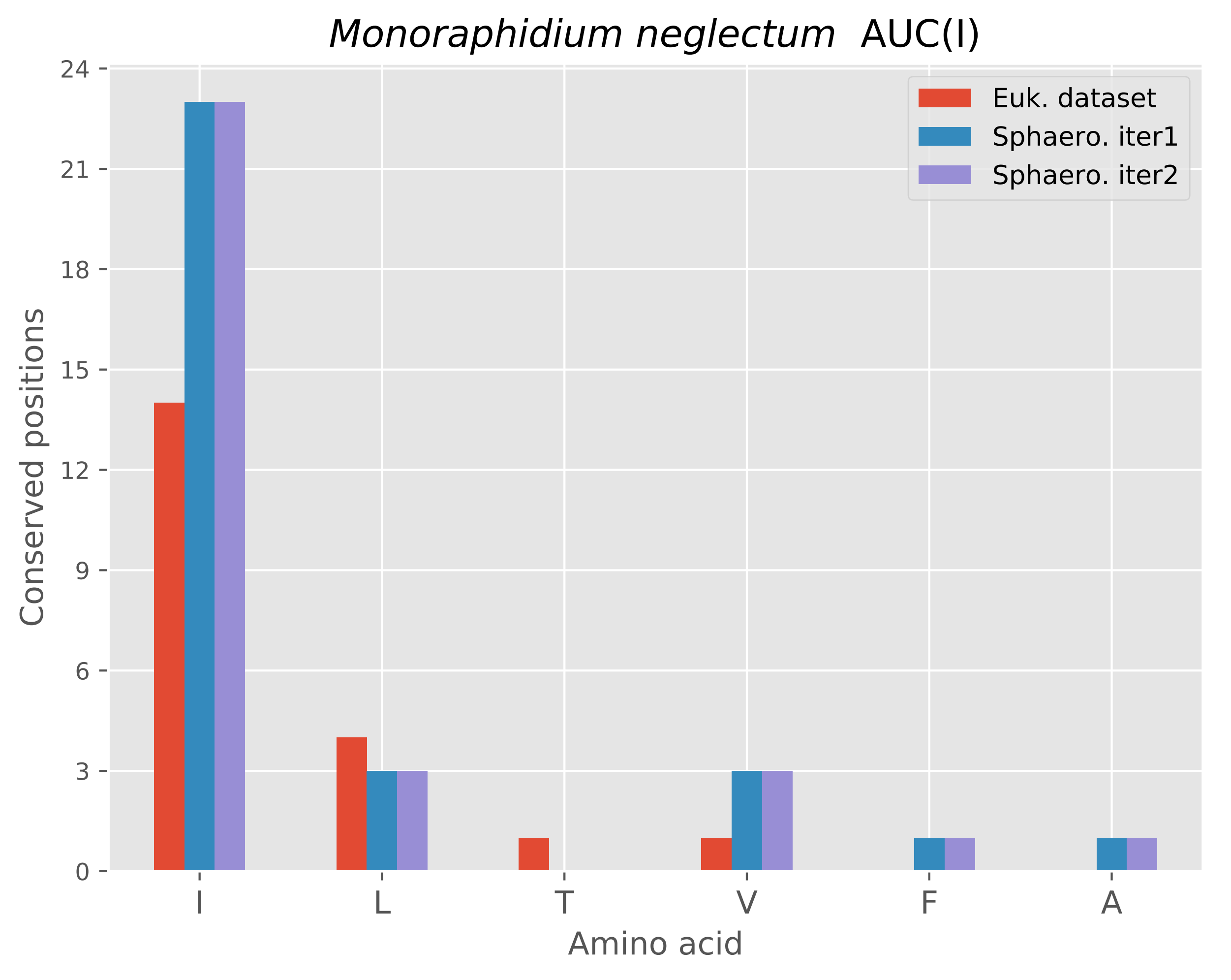
Monoraphidium neglectum AUC(I)
Euk. dataset
Sphaero. iter1
Sphaero. iter2
Conserved positions
0
3
6
9
12
15
18
21
24
I
L
T
V
F
A
Amino acid

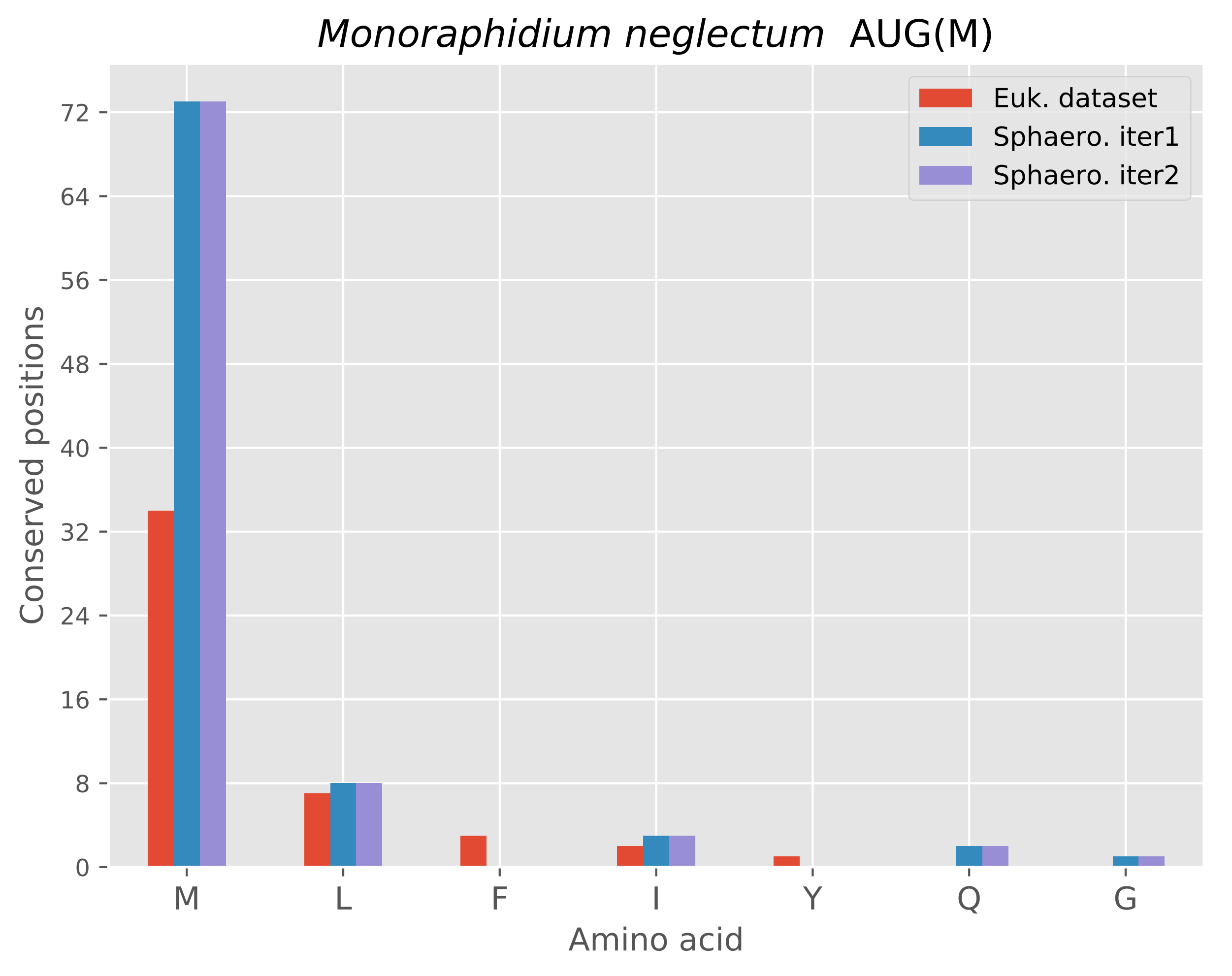

Monoraphidium neglectum AUG(M)
Euk. dataset
Sphaero. iter1
Sphaero. iter2
Conserved positions
0
8
16
24
32
40
48
56
64
72
M
L
F
I
Y
Q
G
Amino acid

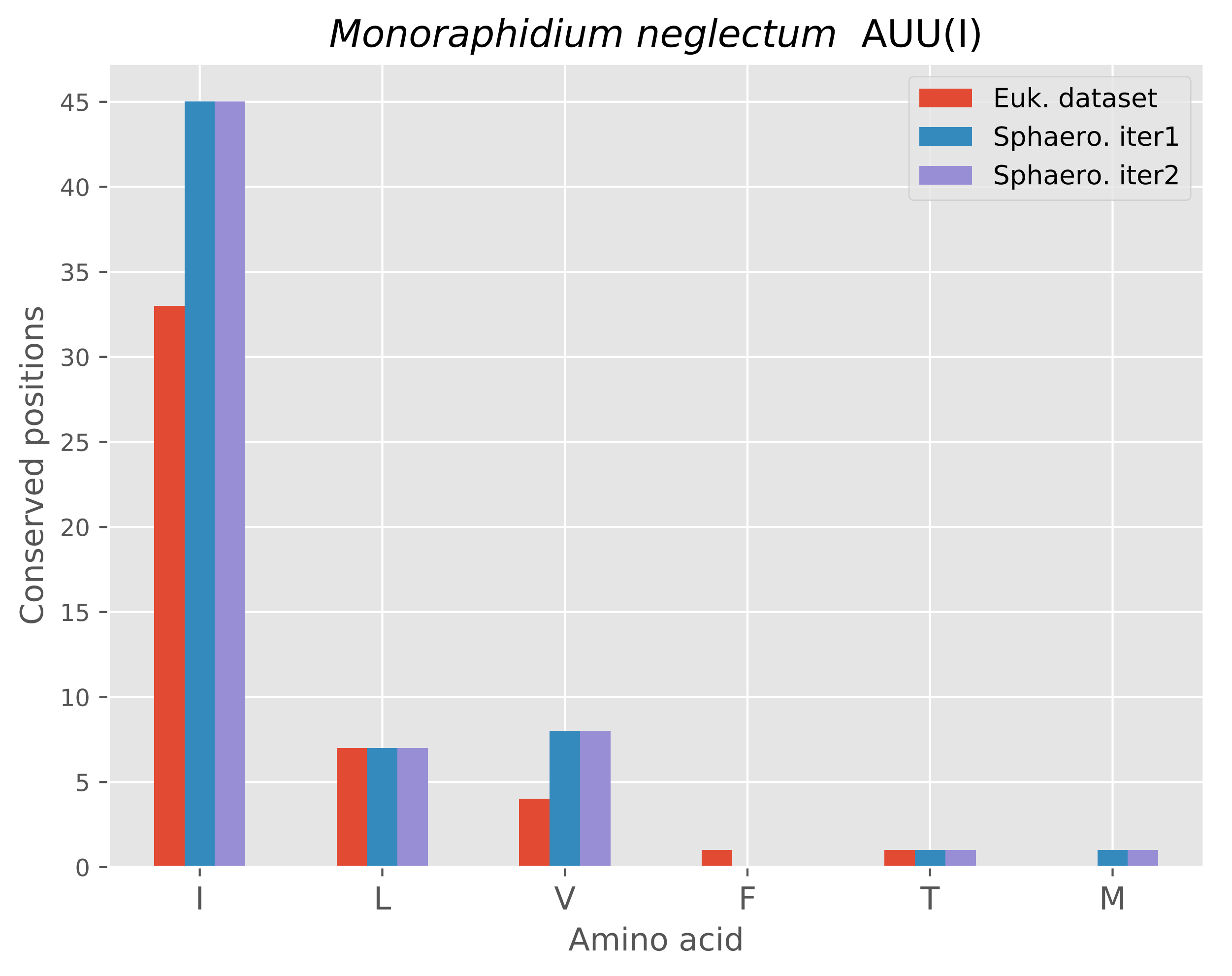

Monoraphidium neglectum AUU(I)
Euk. dataset
Sphaero. iter1
Sphaero. iter2
Conserved positions
0
5
10
15
20
25
30
35
40
45
I
L
V
F
T
M
Amino acid

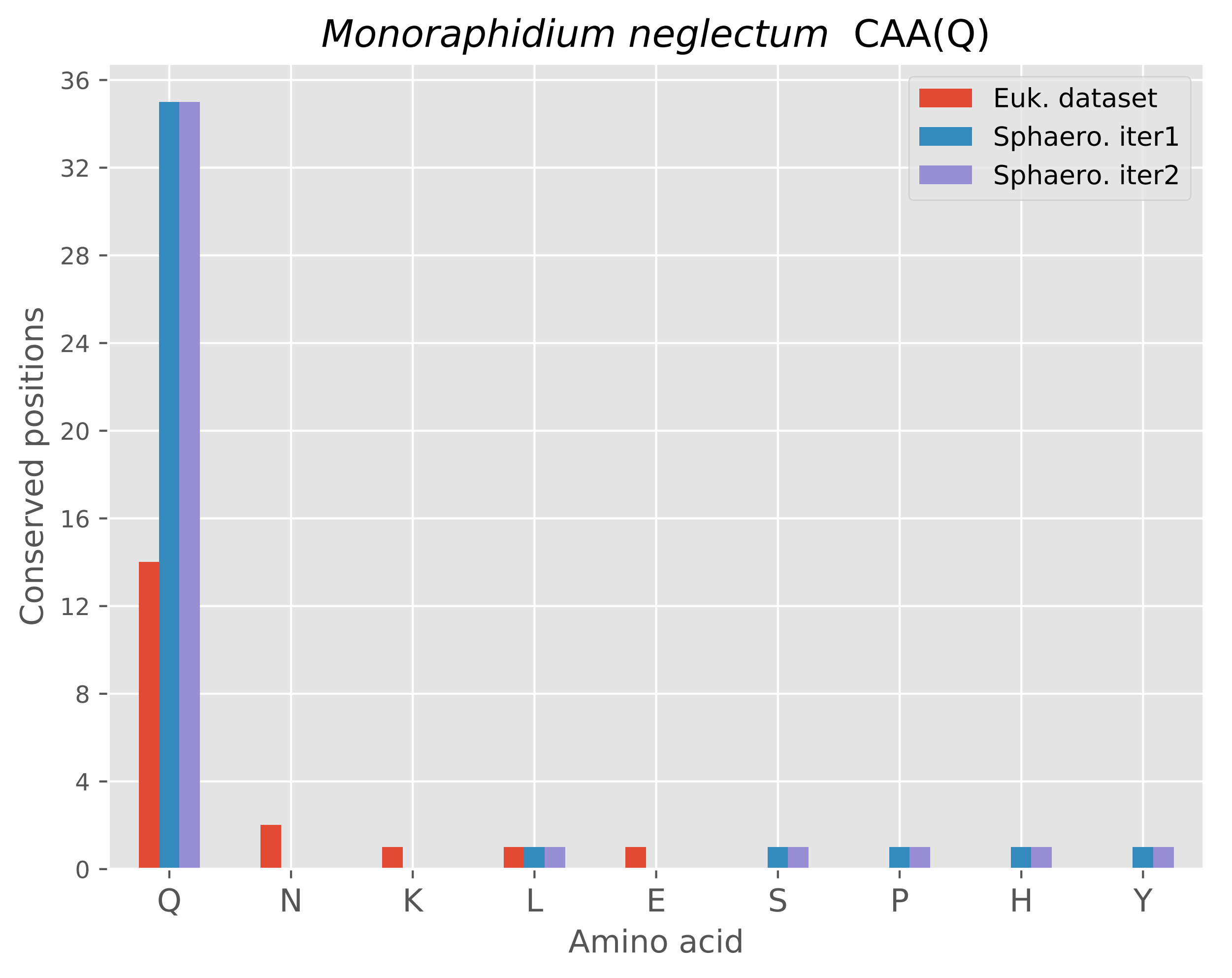

Monoraphidium neglectum CAA(Q)
Euk. dataset
Sphaero. iter1
Sphaero. iter2
Conserved positions
0
4
8
12
16
20
24
28
32
36
Q
N
K
L
E
S
P
H
Y
Amino acid

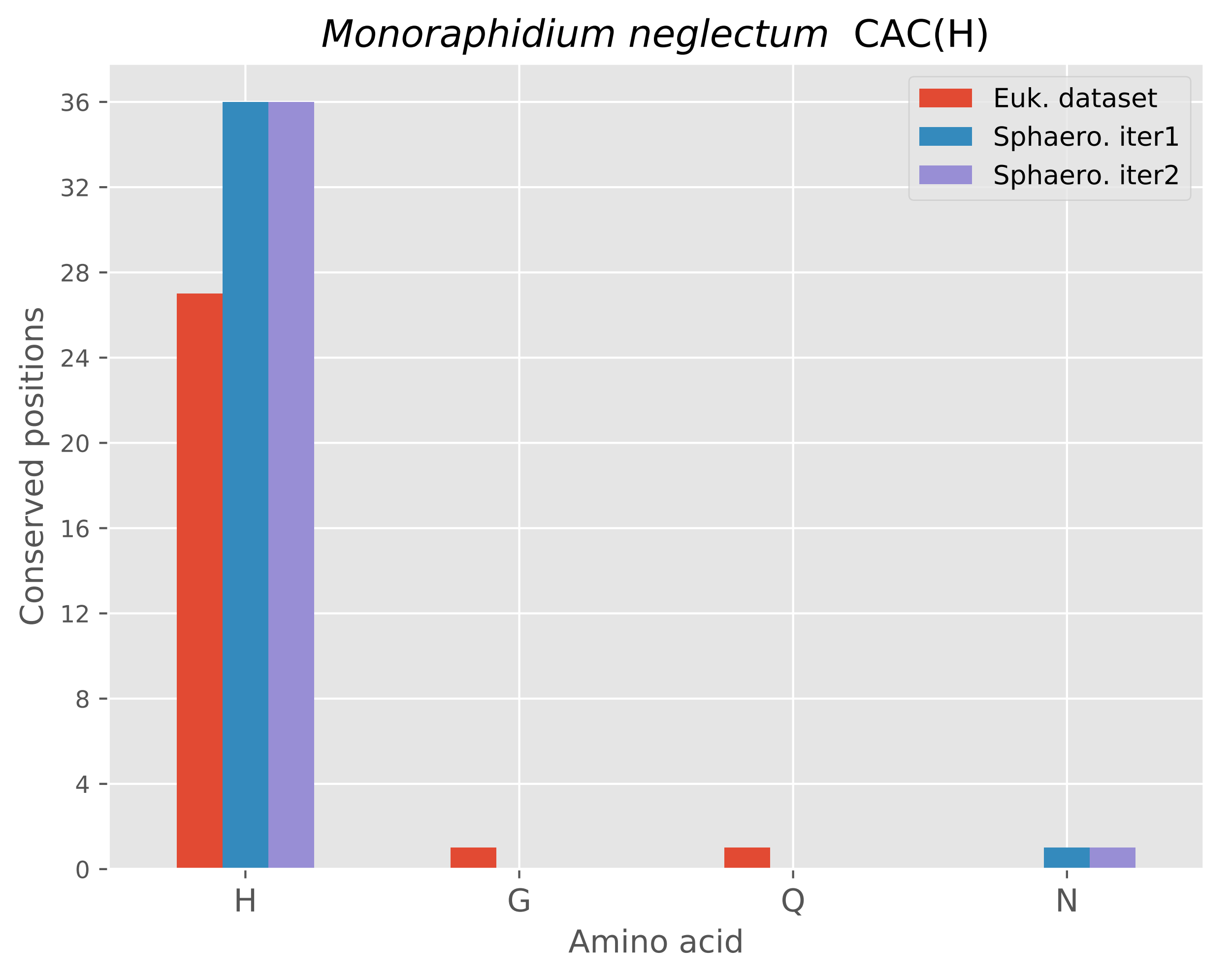

Monoraphidium neglectum CAC(H)
Euk. dataset
Sphaero. iter1
Sphaero. iter2
Conserved positions
0
4
8
12
16
20
24
28
32
36
H
G
Q
N
Amino acid

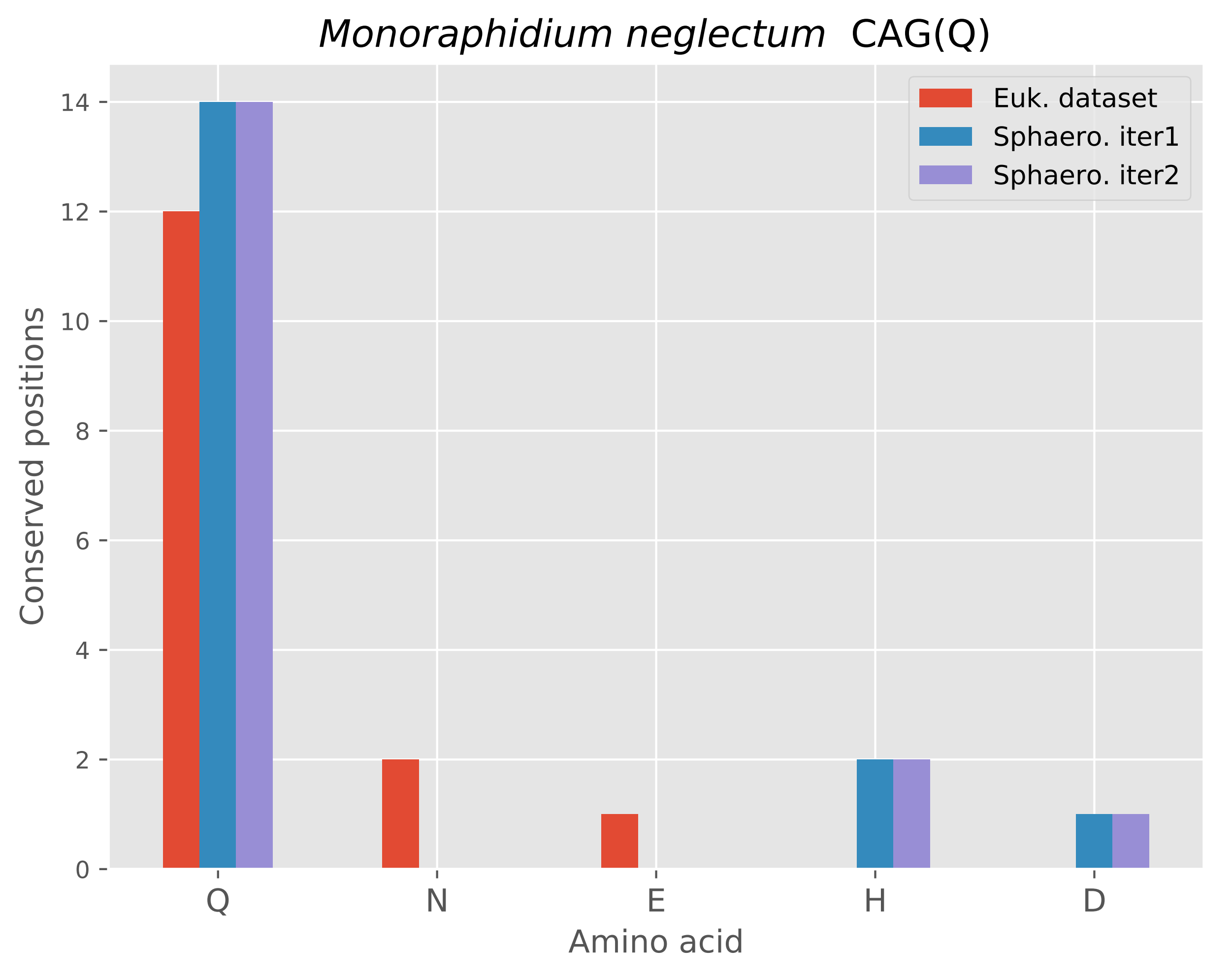

Monoraphidium neglectum CAG(Q)
Euk. dataset
Sphaero. iter1
Sphaero. iter2
Conserved positions
0
2
4
6
8
10
12
14
Q
N
E
H
D
Amino acid

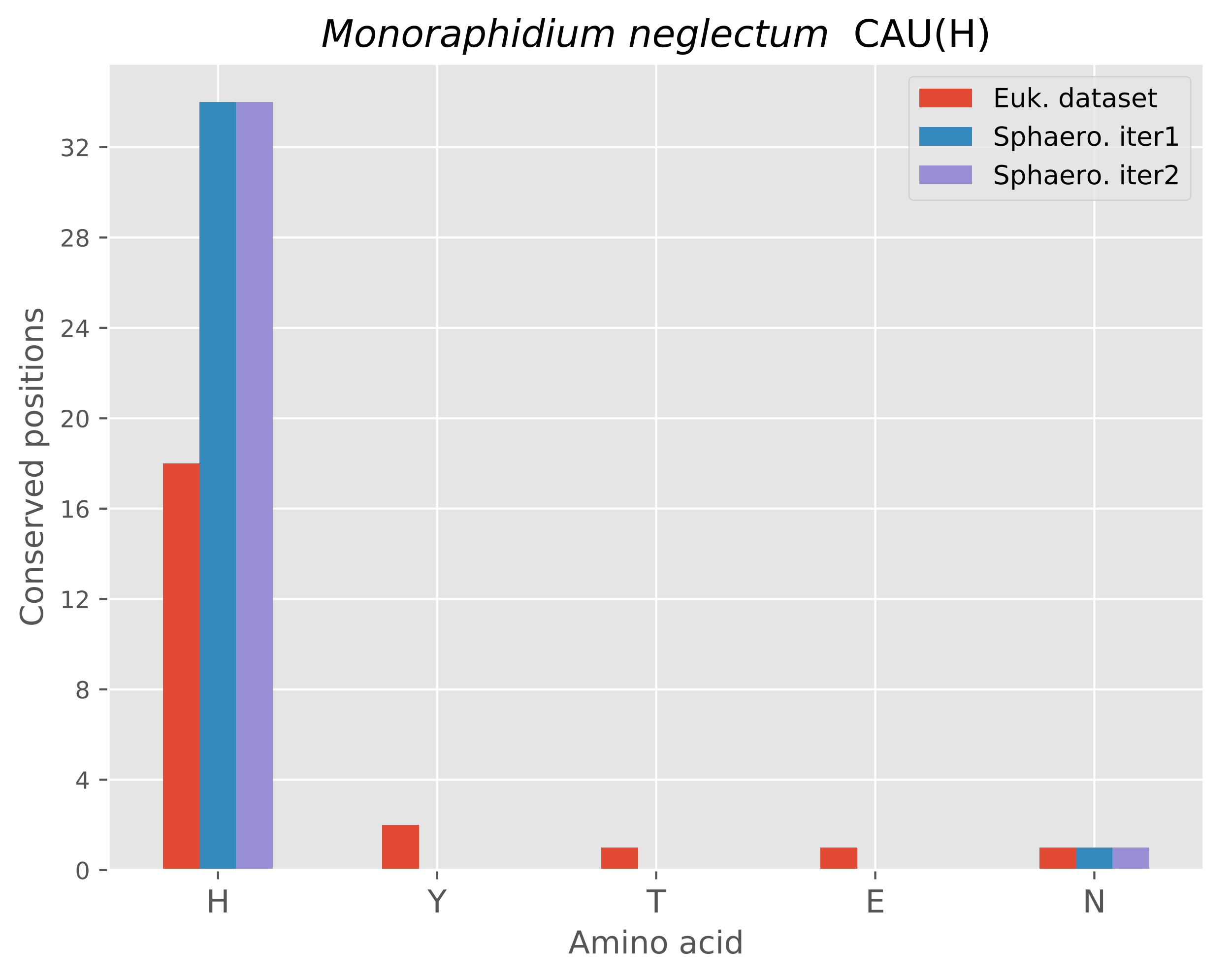
Monoraphidium neglectum CAU(H)
Euk. dataset
Sphaero. iter1
Sphaero. iter2
Conserved positions
0
4
8
12
16
20
24
28
32
H
Y
T
E
N
Amino acid

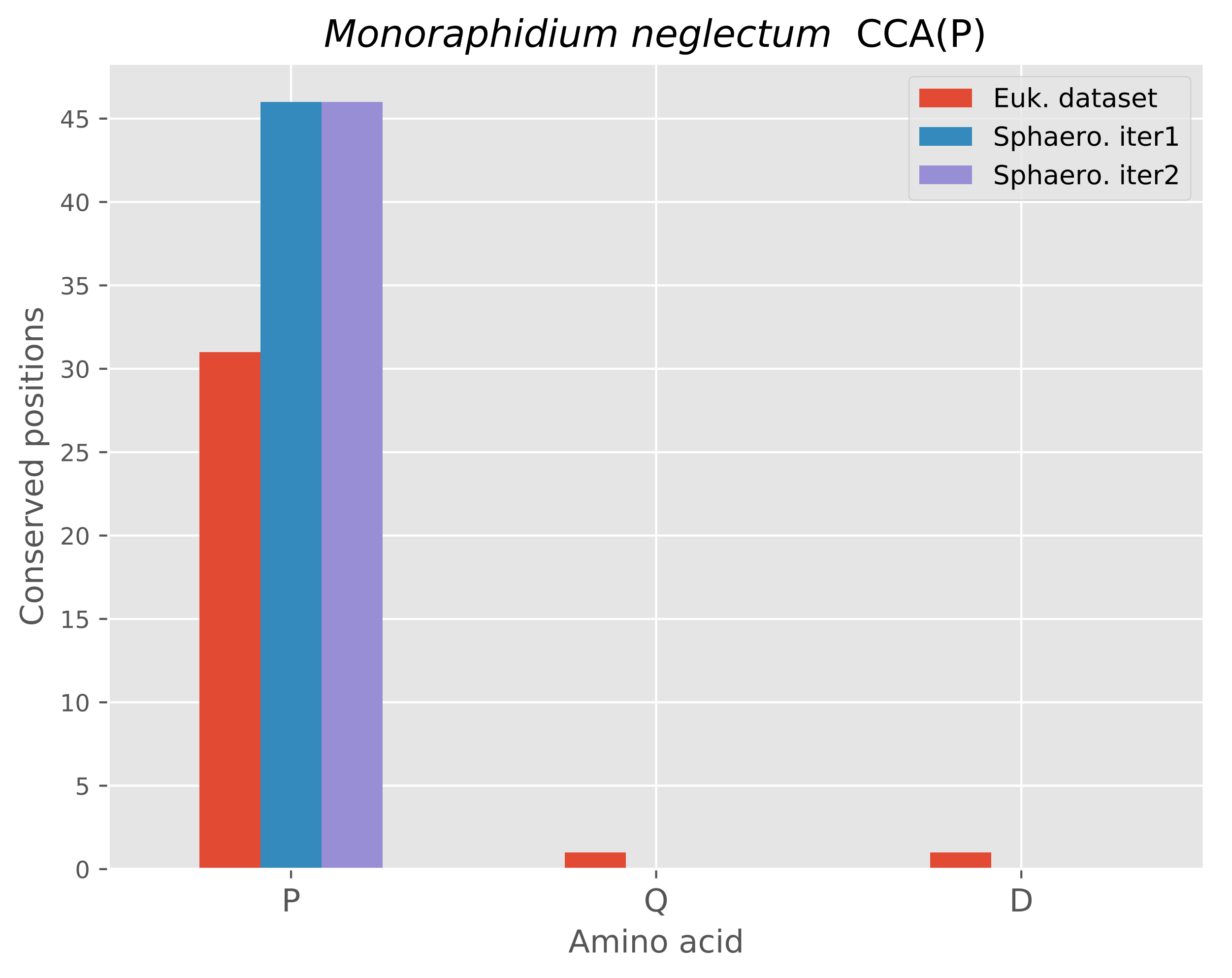

Monoraphidium neglectum CCA(P)
Euk. dataset
Sphaero. iter1
Sphaero. iter2
Conserved positions
0
5
10
15
20
25
30
35
40
45
P
Q
D
Amino acid

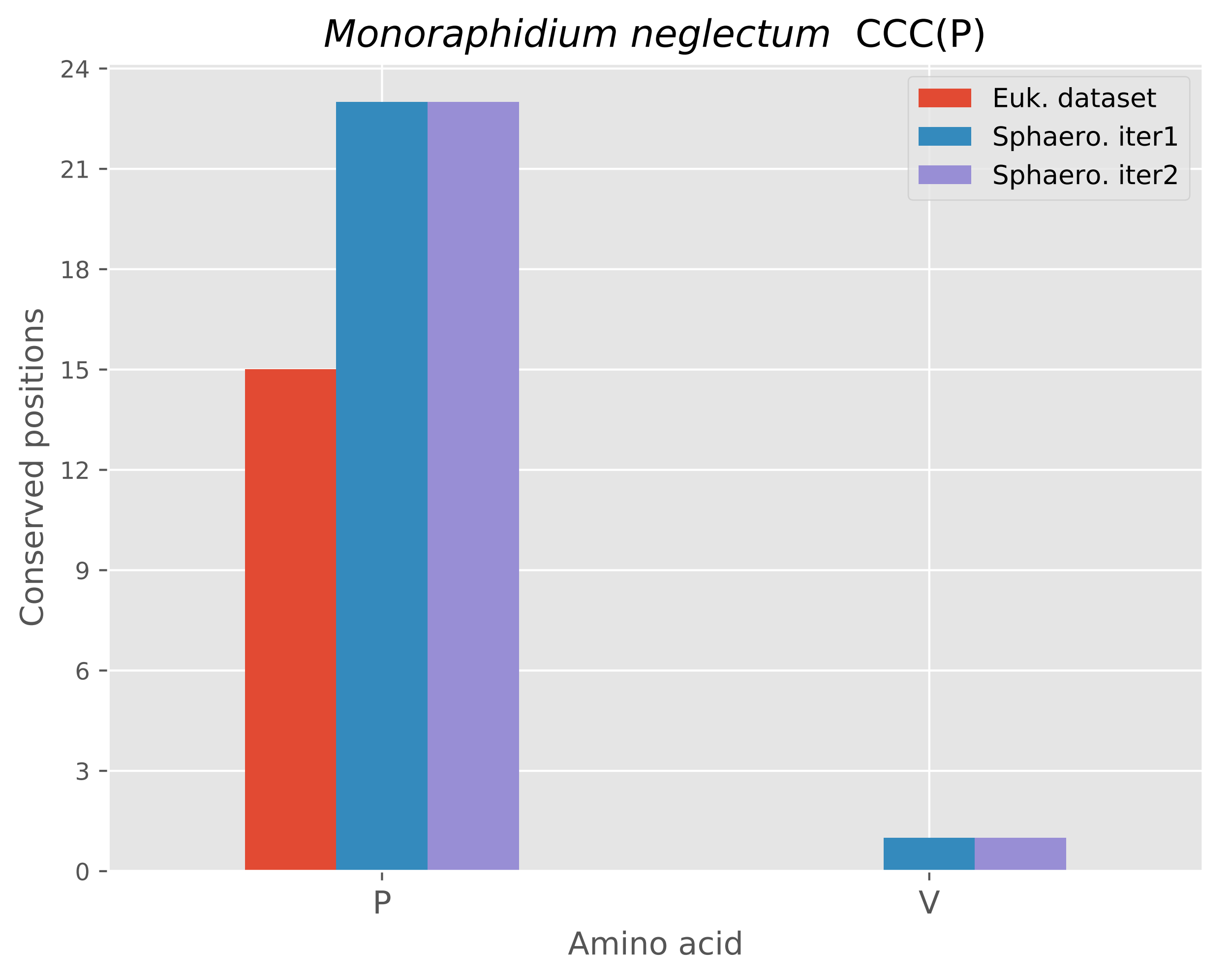
Monoraphidium neglectum CCC(P)
Euk. dataset
Sphaero. iter1
Sphaero. iter2
Conserved positions
Amino acid
P
V
0
3
6
9
12
15
18
21
24

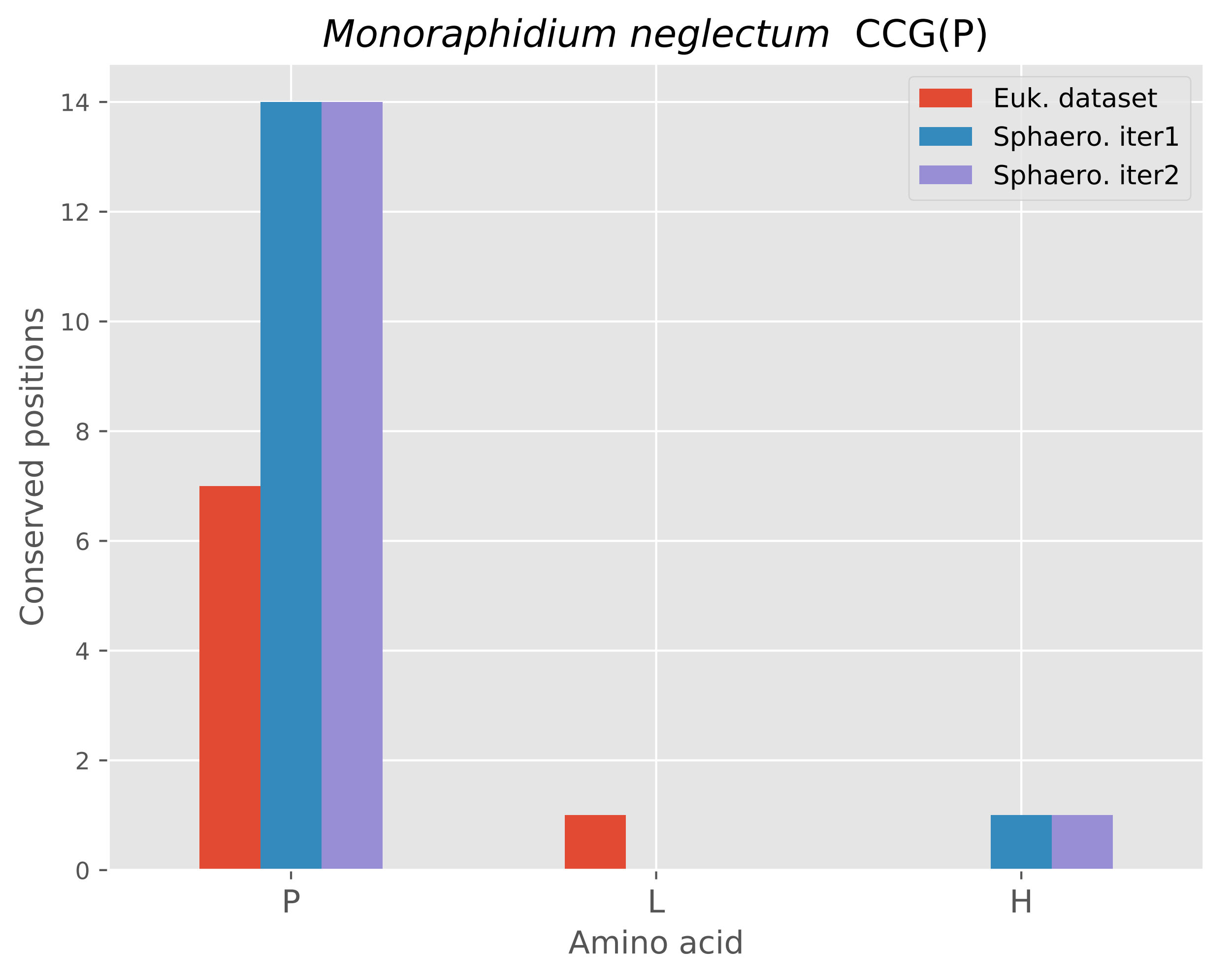

Monoraphidium neglectum CCG(P)
Euk. dataset
Sphaero. iter1
Sphaero. iter2
Conserved positions
0
2
4
6
8
10
12
14
P
L
H
Amino acid

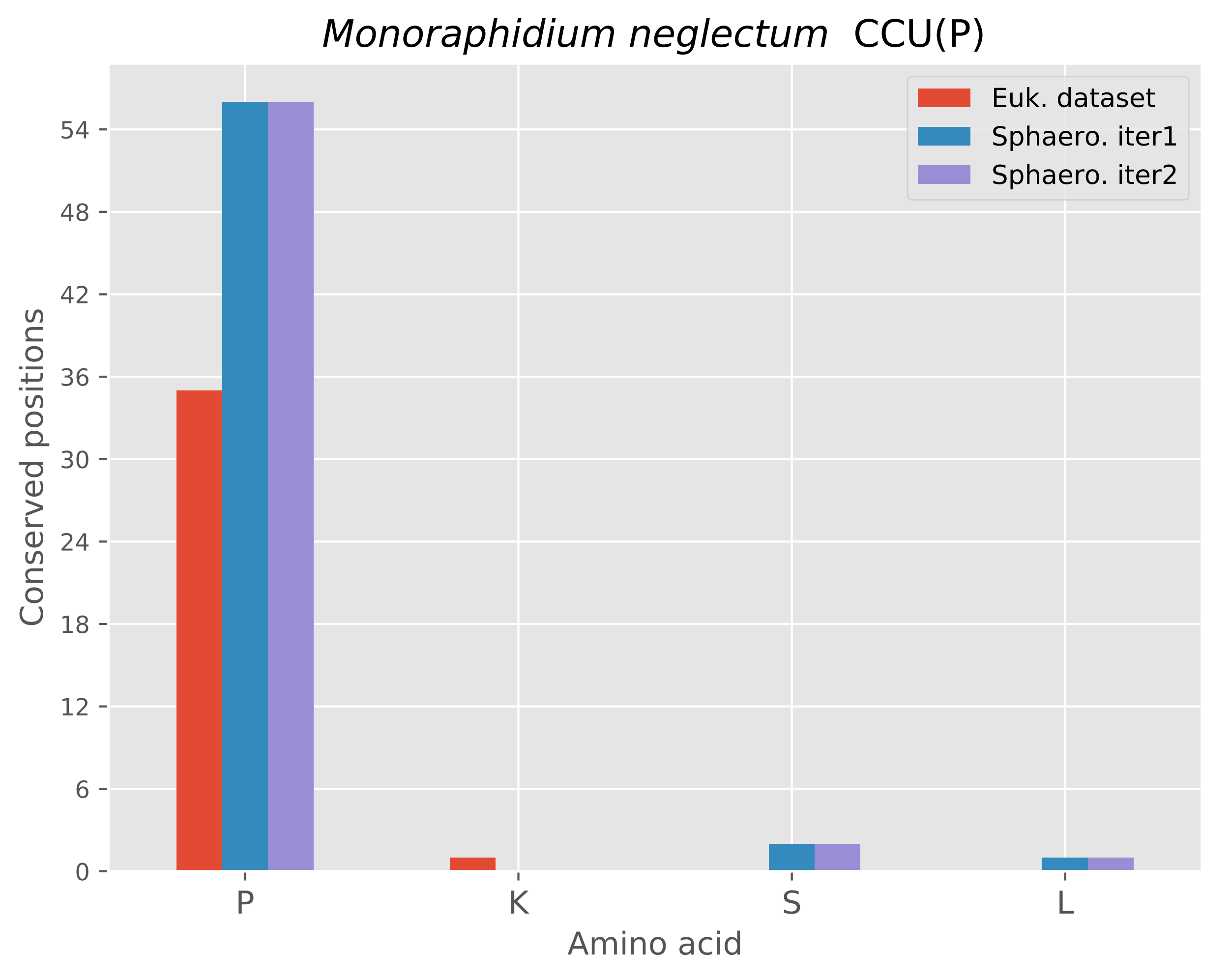

Monoraphidium neglectum CCU(P)
Euk. dataset
Sphaero. iter1
Sphaero. iter2
Conserved positions
0
6
12
18
24
30
36
42
48
54
P
K
S
L
Amino acid

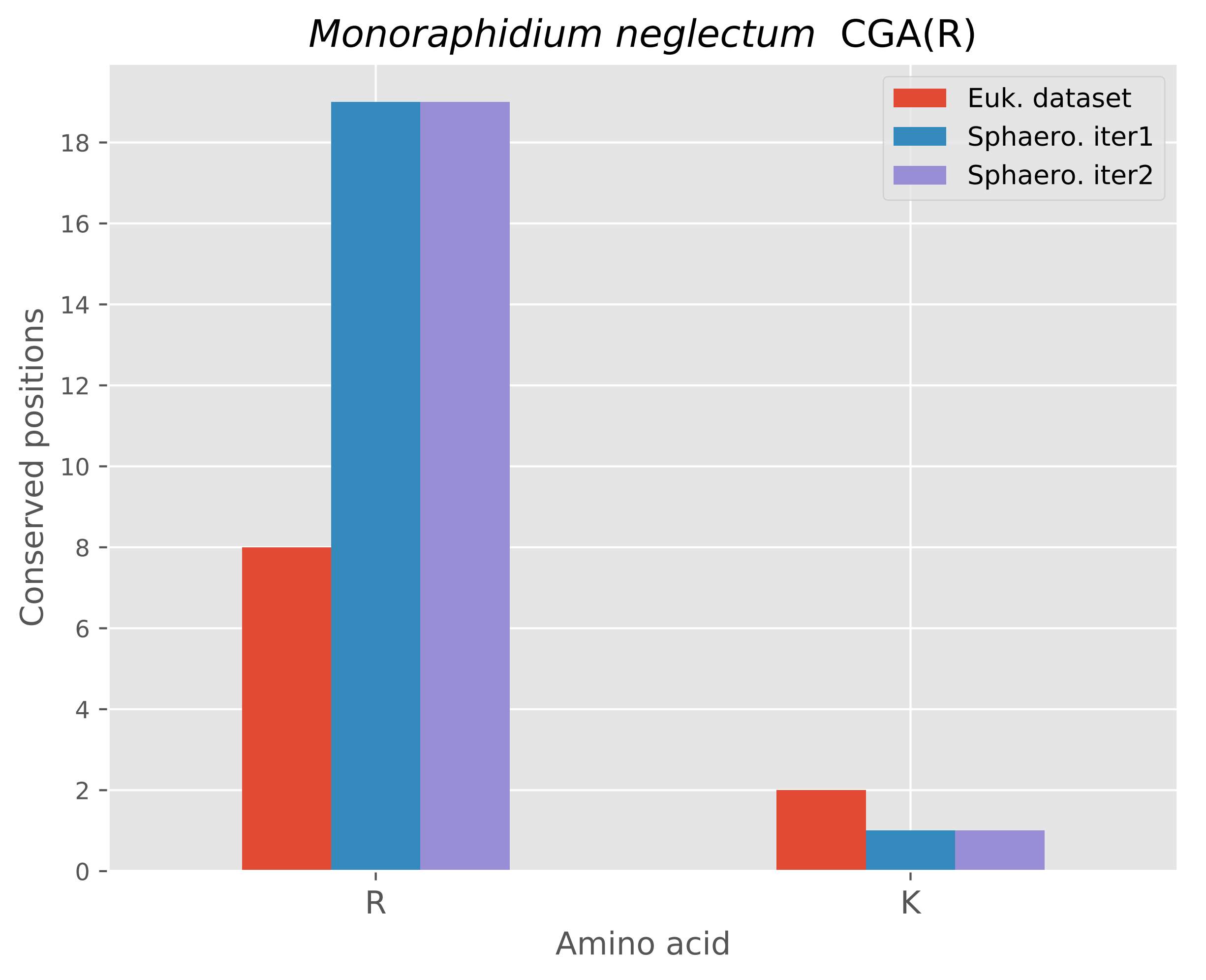

Monoraphidium neglectum CGA(R)
Euk. dataset
Sphaero. iter1
Sphaero. iter2
Conserved positions
0
2
4
6
8
10
12
14
16
18
R
K
Amino acid

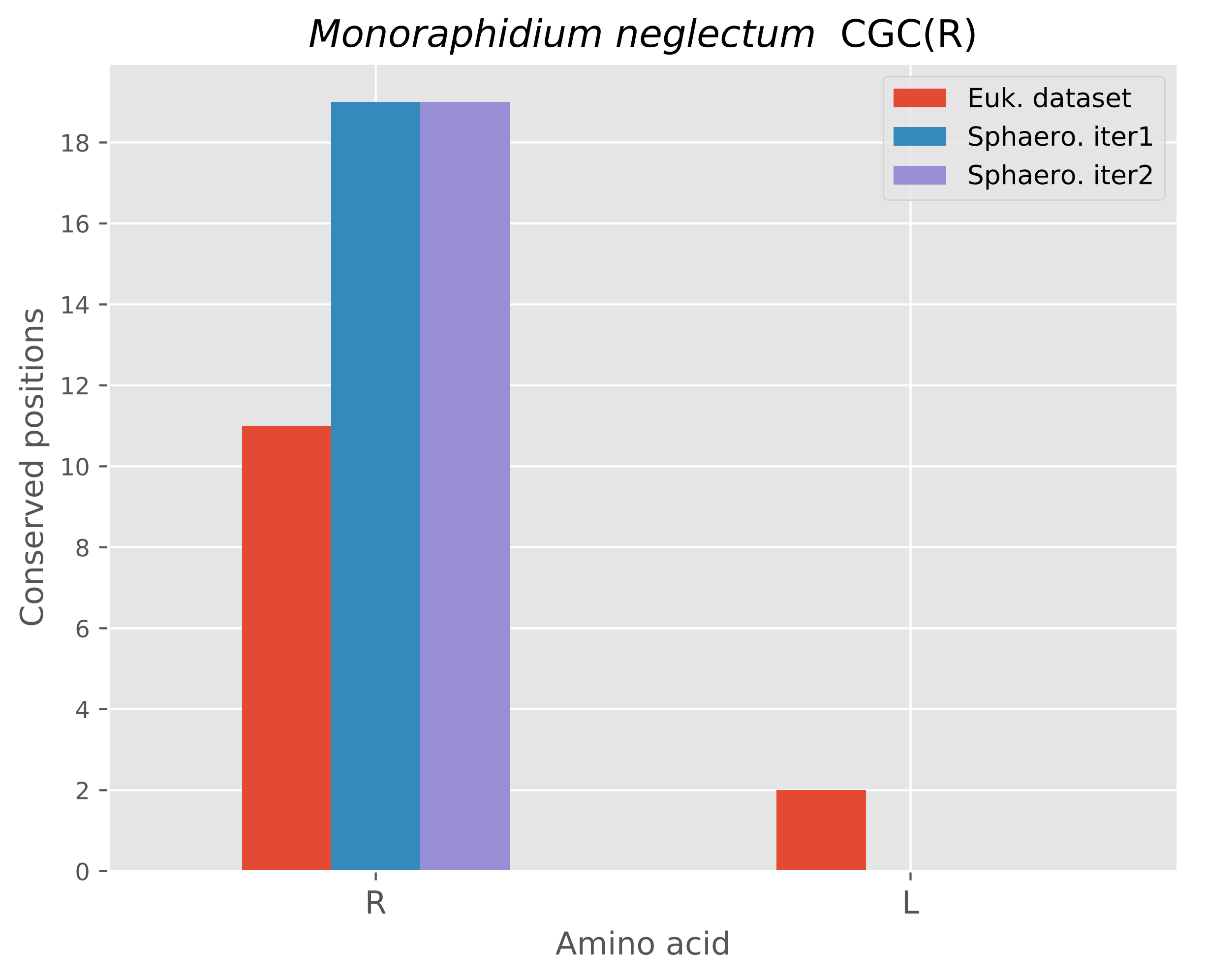

Monoraphidium neglectum CGC(R)
Euk. dataset
Sphaero. iter1
Sphaero. iter2
Conserved positions
0
2
4
6
8
10
12
14
16
18
R
L
Amino acid

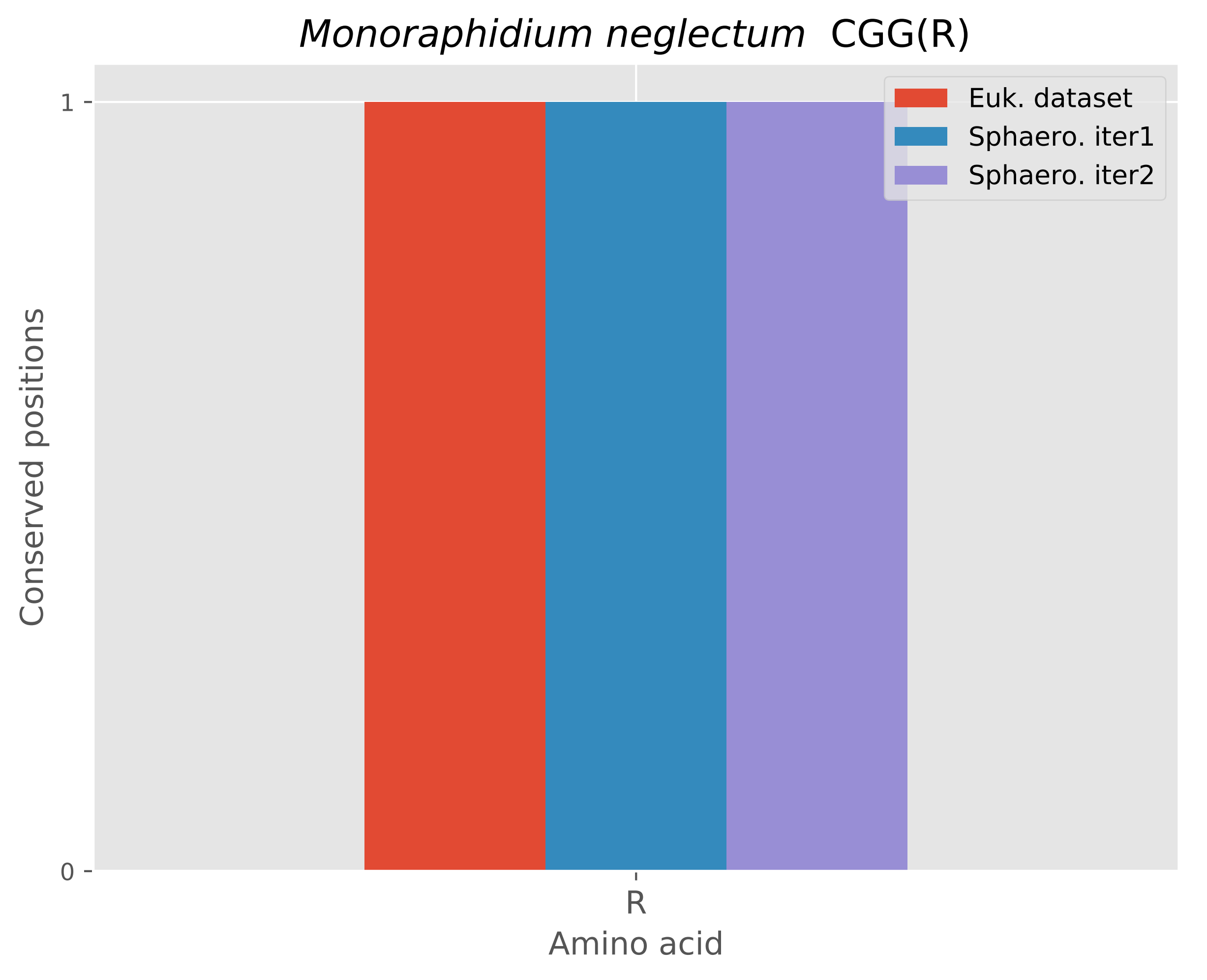

Monoraphidium neglectum CGG(R)
Conserved positions
1
0
R
Amino acid
Euk. dataset
Sphaero. iter1
Sphaero. iter2

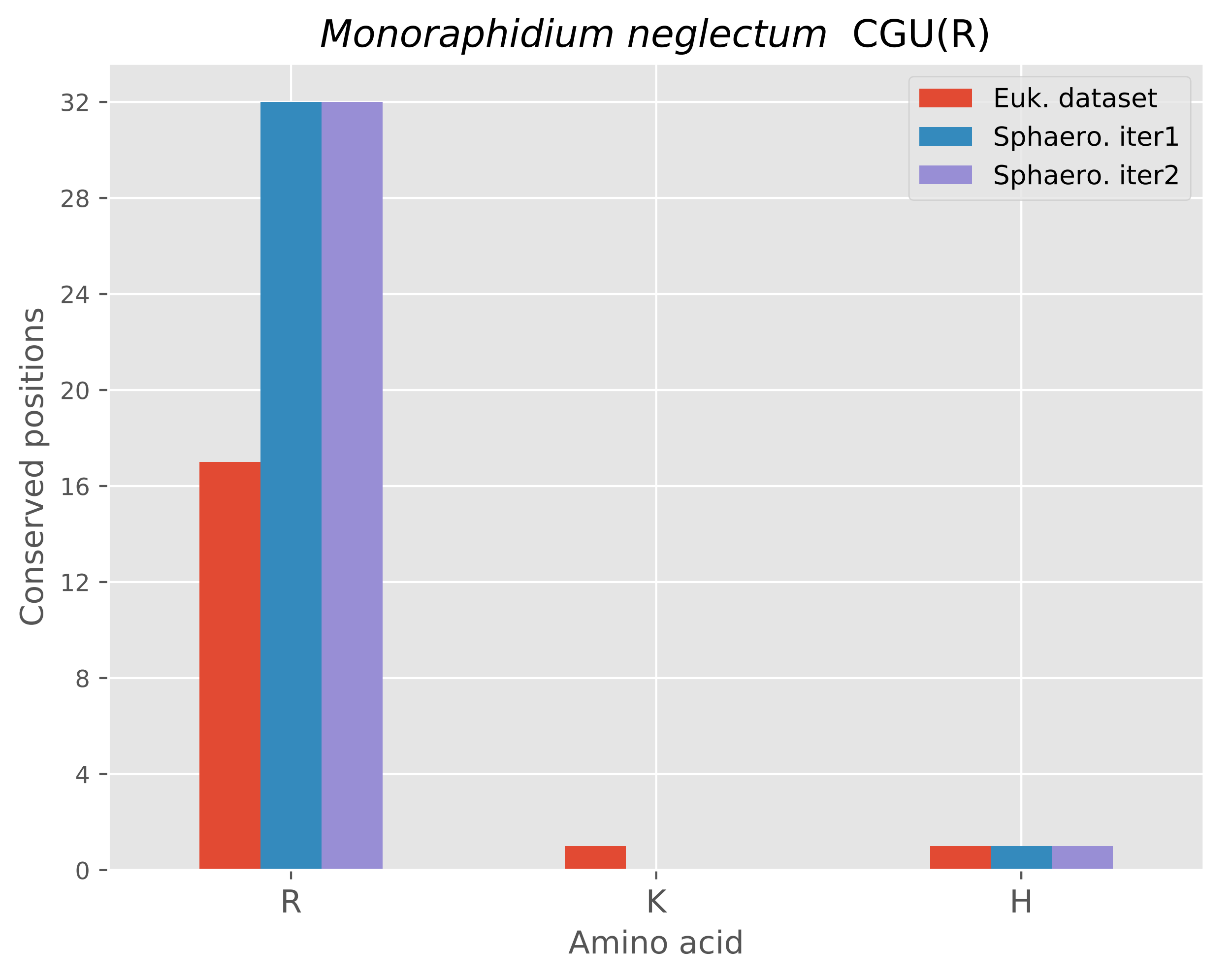

Monoraphidium neglectum CGU(R)
Euk. dataset
Sphaero. iter1
Sphaero. iter2
Conserved positions
0
4
8
12
16
20
24
28
32
R
K
H
Amino acid

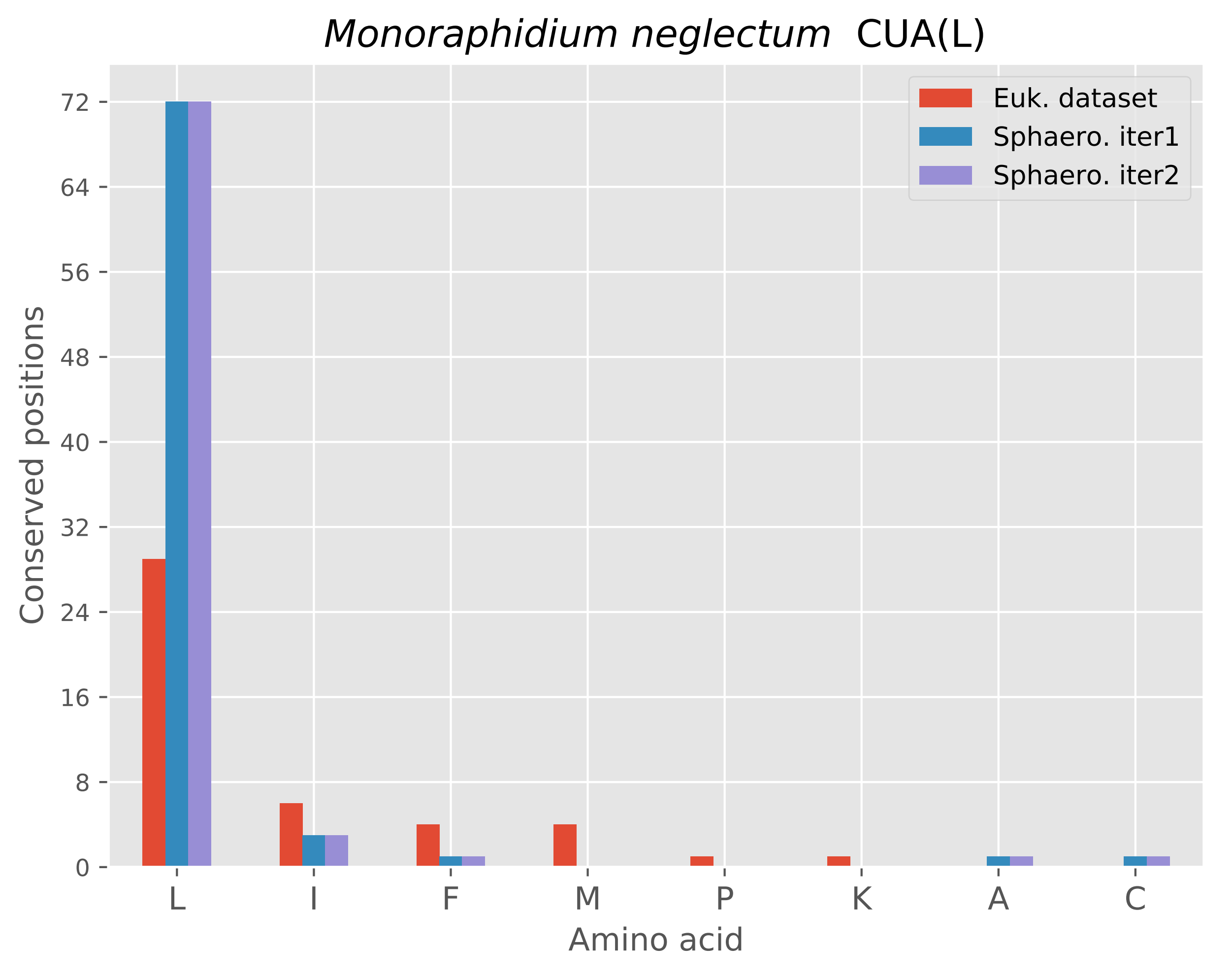
Monoraphidium neglectum CUA(L)
Euk. dataset
Sphaero. iter1
Sphaero. iter2
Conserved positions
0
8
16
24
32
40
48
56
64
72
L
I
F
M
P
K
A
C
Amino acid

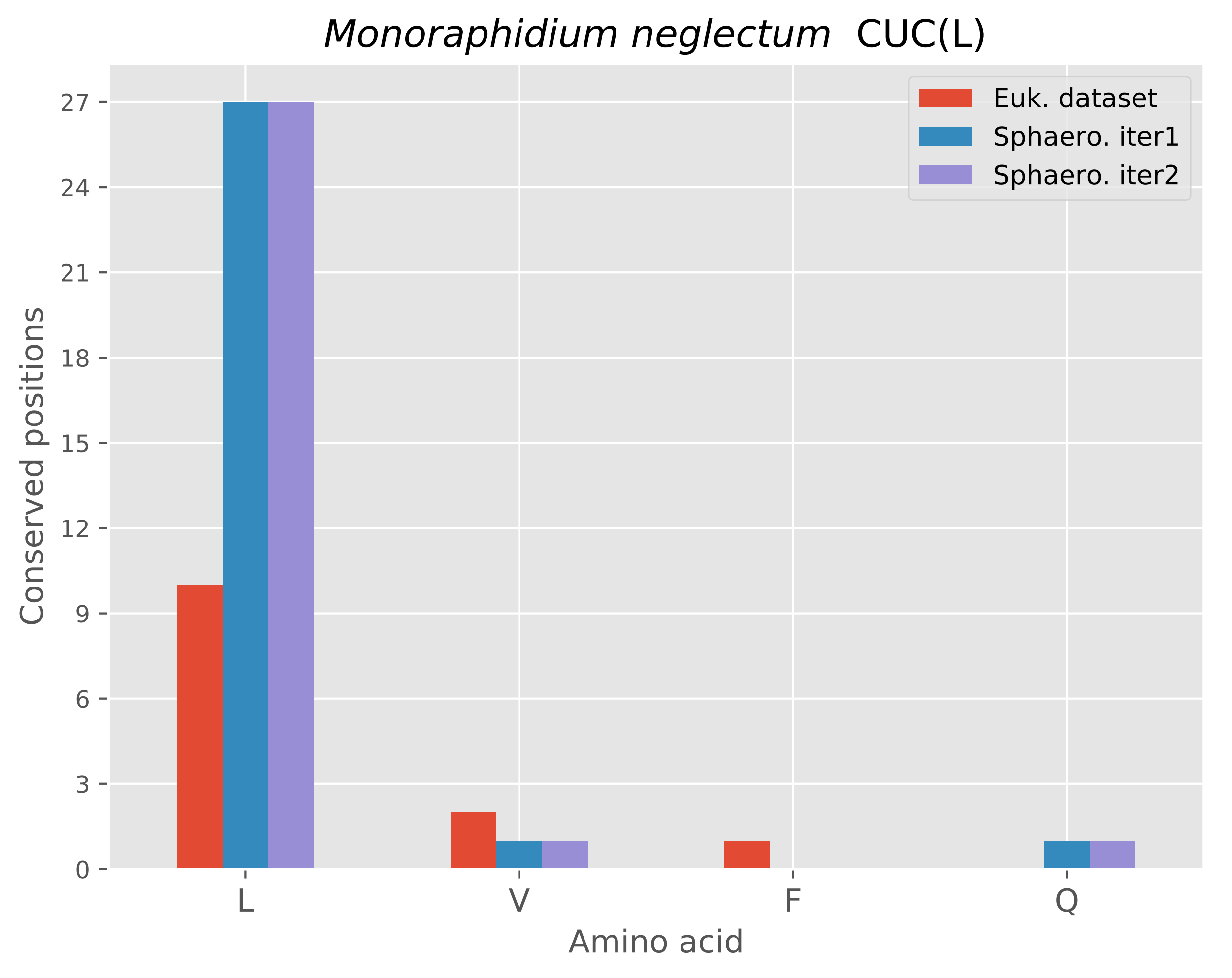

Monoraphidium neglectum CUC(L)
Euk. dataset
Sphaero. iter1
Sphaero. iter2
Conserved positions
0
3
6
9
12
15
18
21
24
27
L
V
F
Q
Amino acid

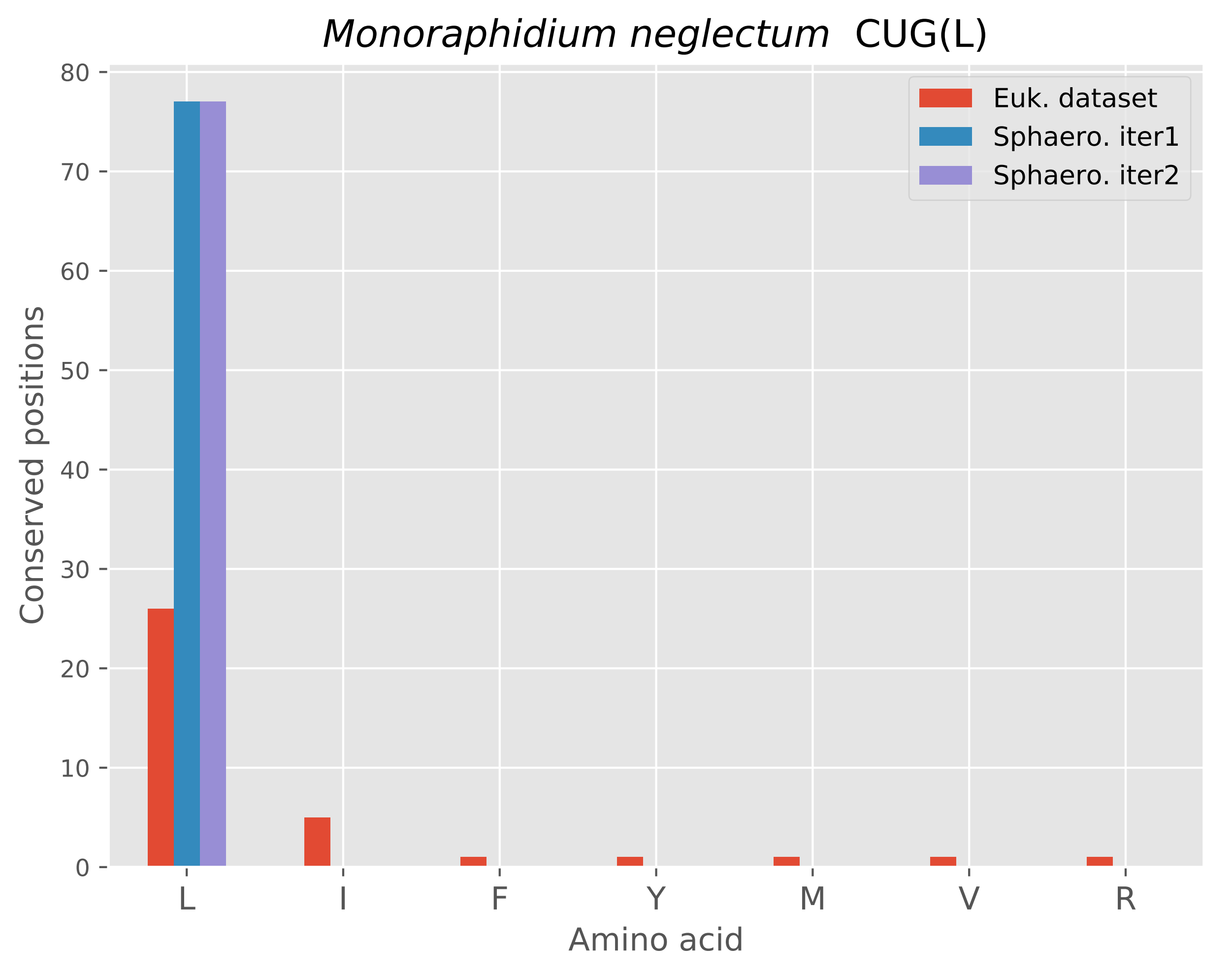

Monoraphidium neglectum CUG(L)
Euk. dataset
Sphaero. iter1
Sphaero. iter2
Conserved positions
0
10
20
30
40
50
60
70
80
L
I
F
Y
M
V
R
Amino acid

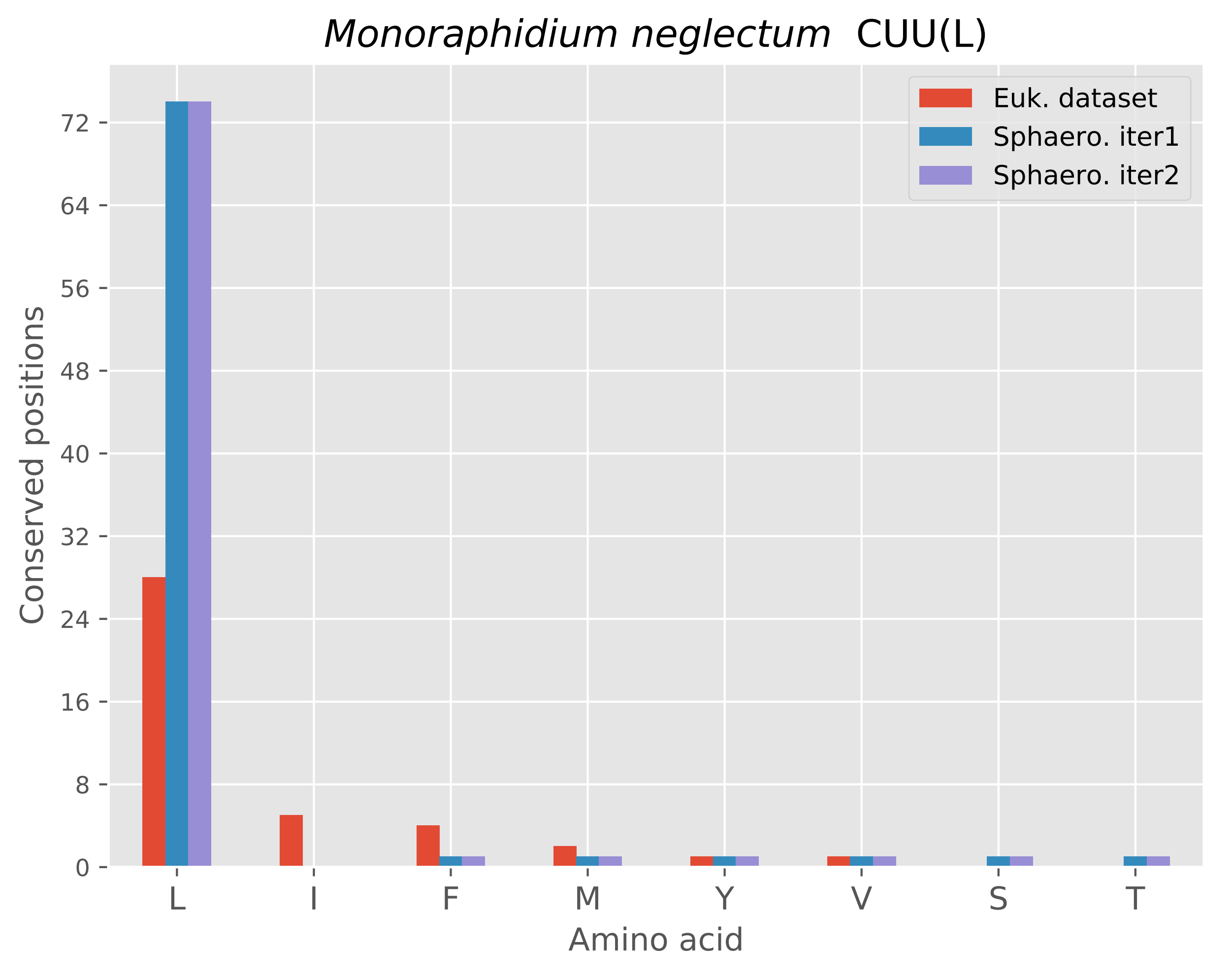

Monoraphidium neglectum CUU(L)
Euk. dataset
Sphaero. iter1
Sphaero. iter2
Conserved positions
0
8
16
24
32
40
48
56
64
72
L
I
F
M
Y
V
S
T
Amino acid

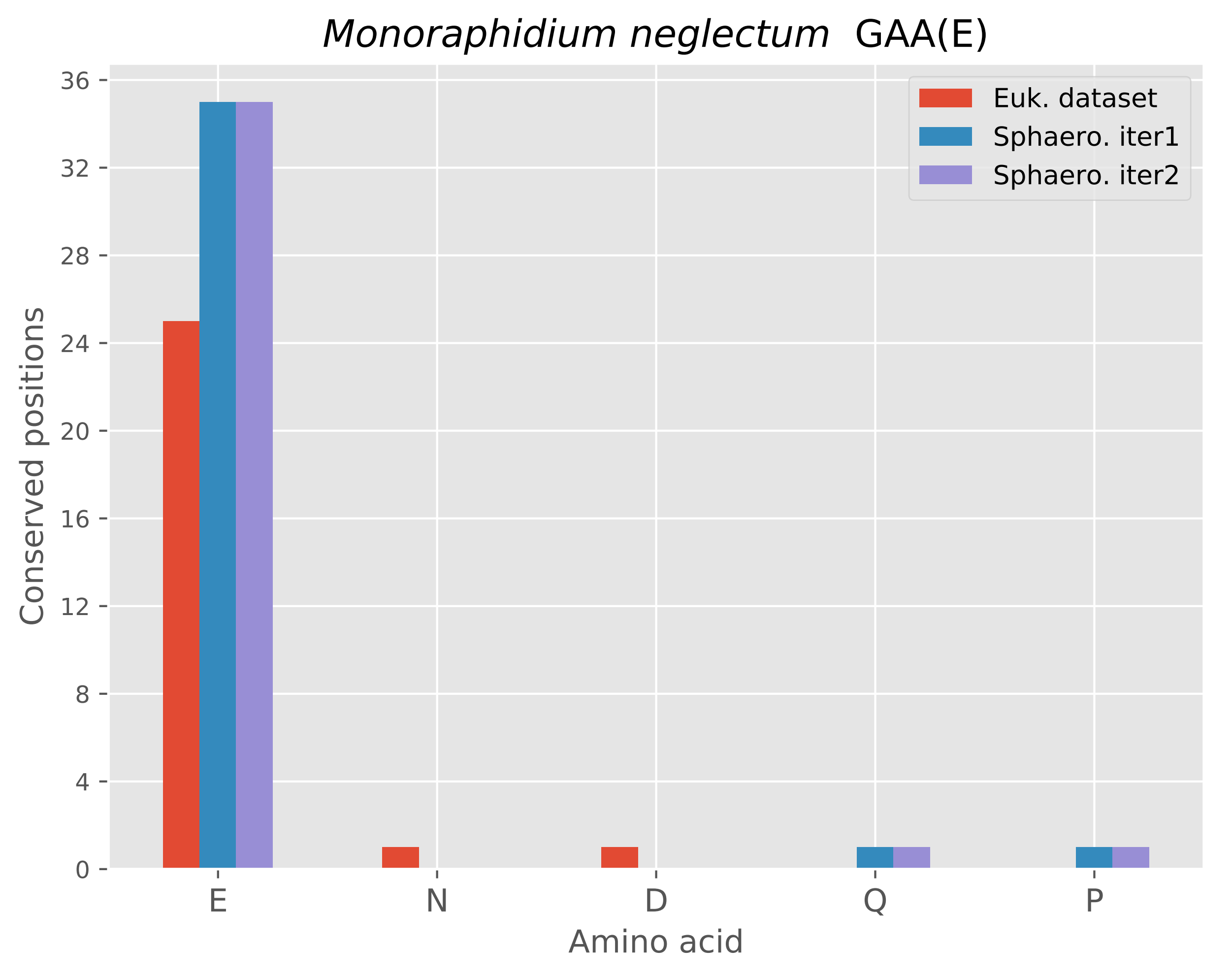
Monoraphidium neglectum GAA(E)
Euk. dataset
Sphaero. iter1
Sphaero. iter2
Conserved positions
0
4
8
12
16
20
24
28
32
36
E
N
D
Q
P
Amino acid

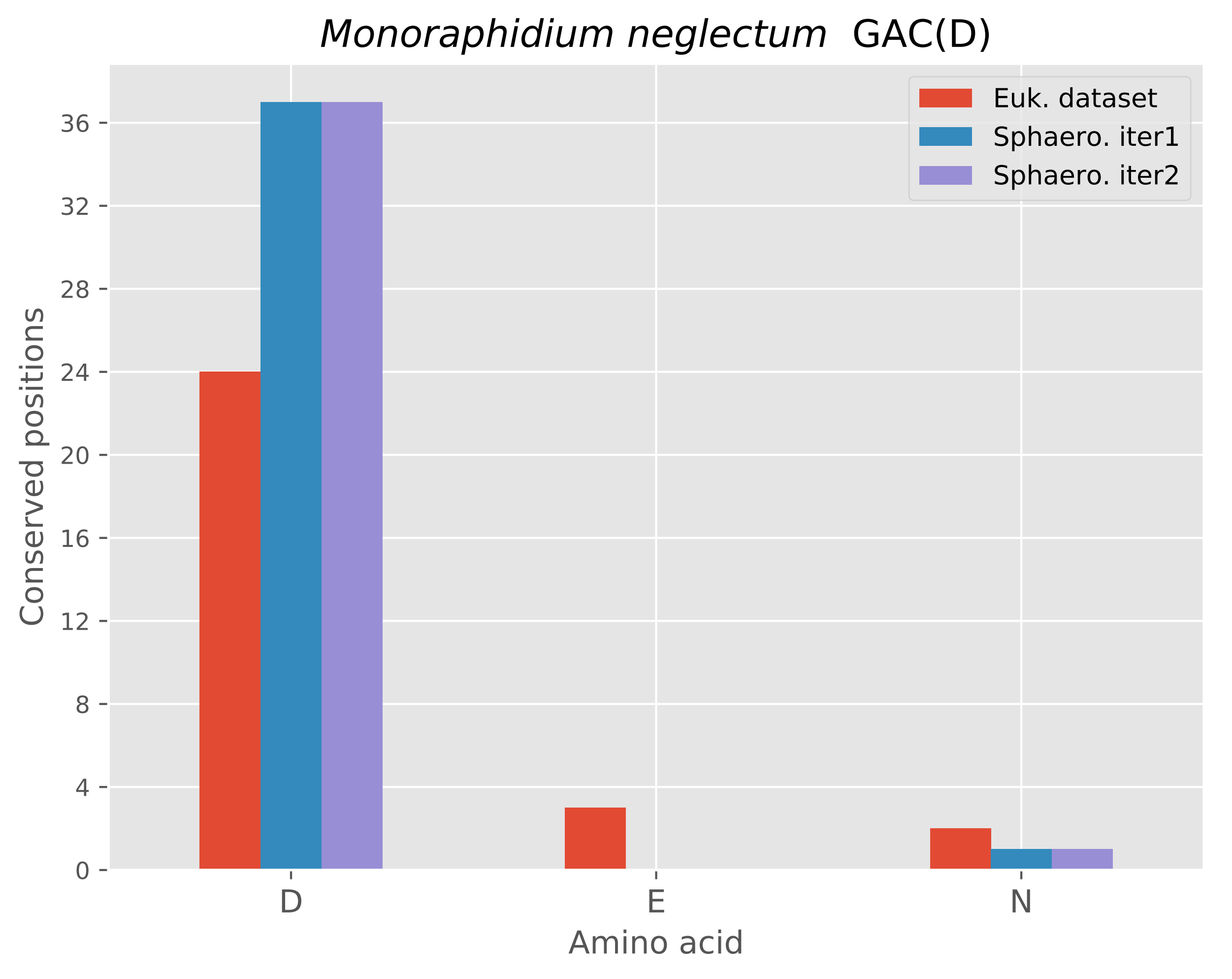

Monoraphidium neglectum GAC(D)
Euk. dataset
Sphaero. iter1
Sphaero. iter2
Conserved positions
0
4
8
12
16
20
24
28
32
36
D
E
N
Amino acid

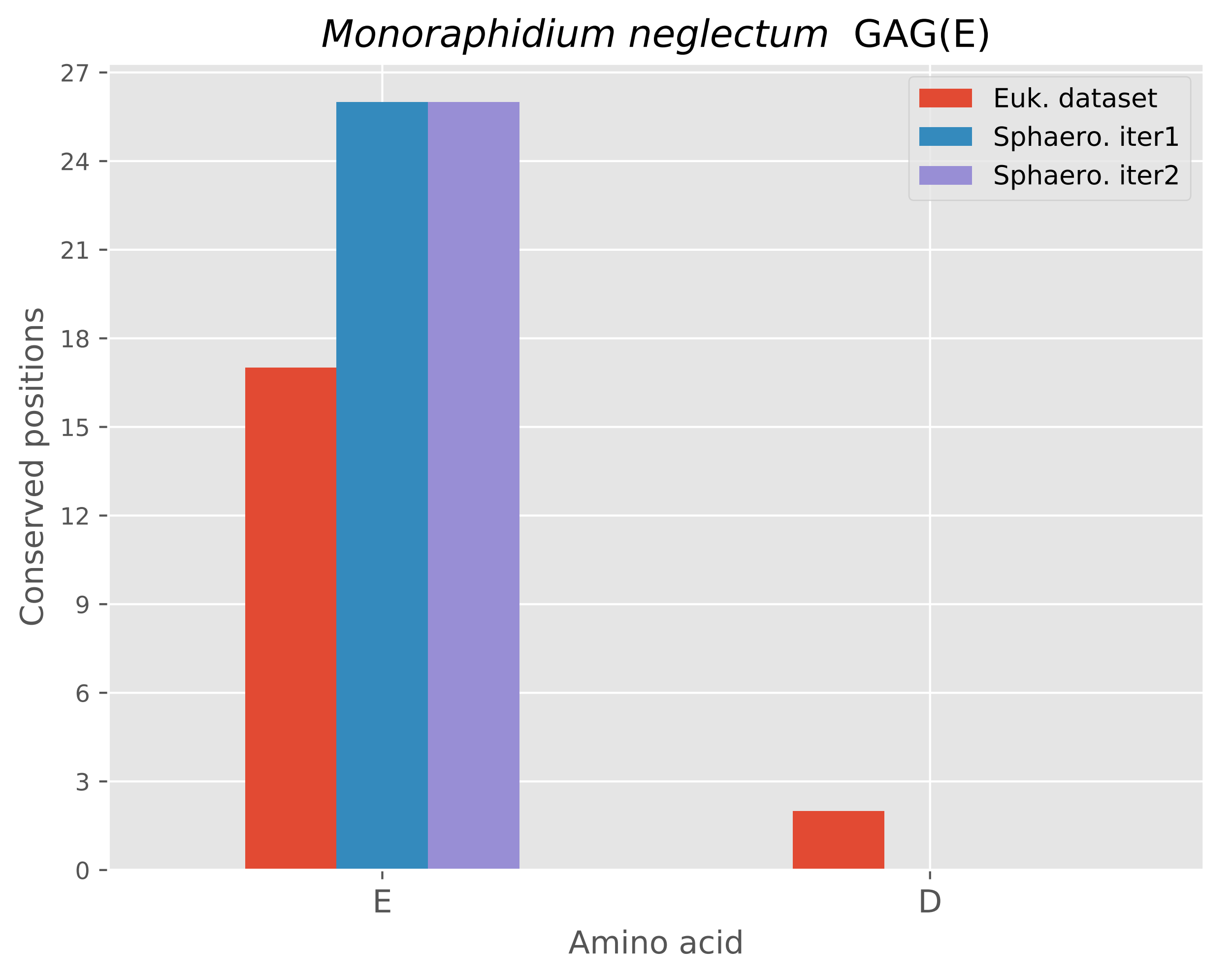

Monoraphidium neglectum GAG(E)
Euk. dataset
Sphaero. iter1
Sphaero. iter2
Conserved positions
0
3
6
9
12
15
18
21
24
27
E
D
Amino acid

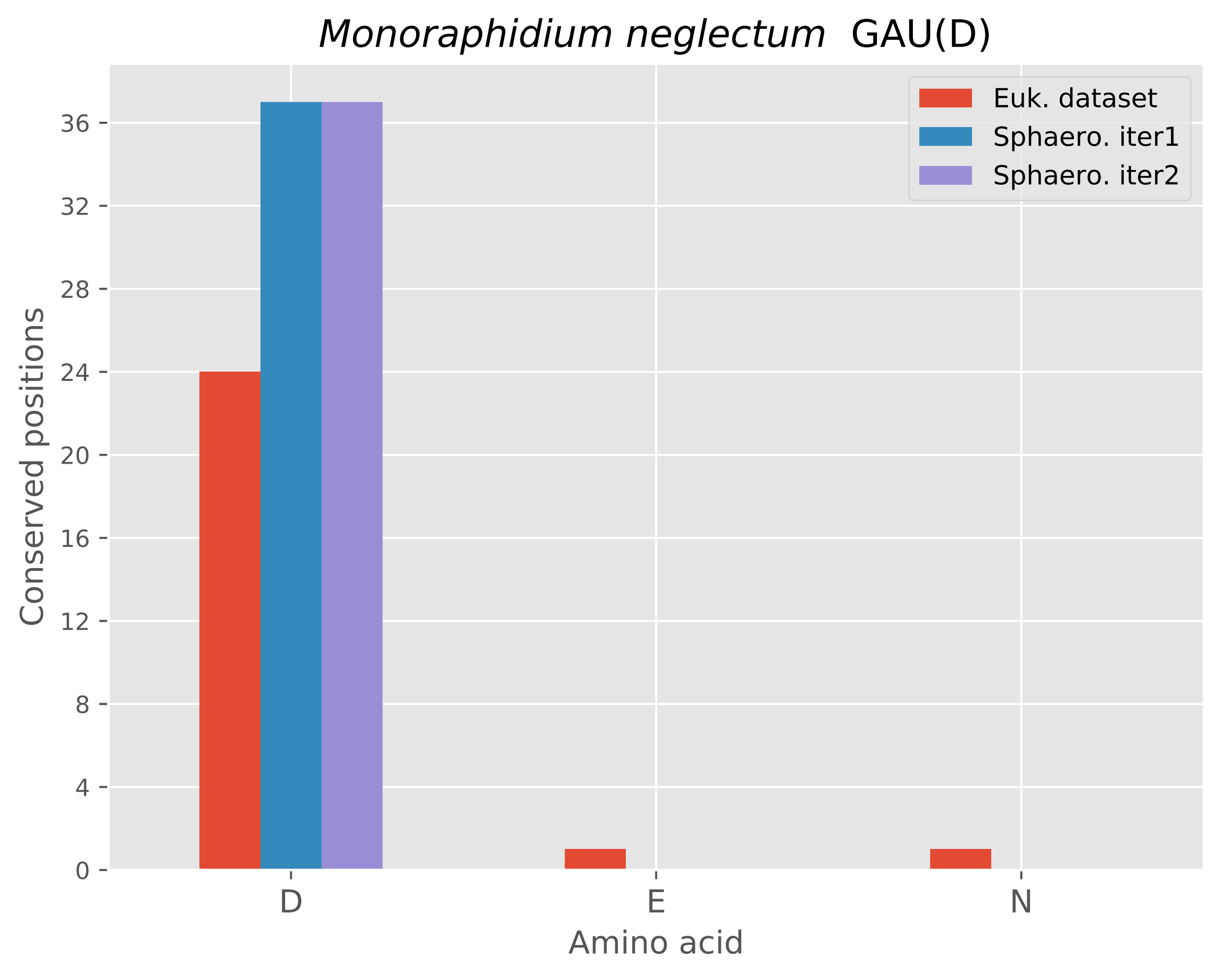

Monoraphidium neglectum GAU(D)
Euk. dataset
Sphaero. iter1
Sphaero. iter2
Conserved positions
0
4
8
12
16
20
24
28
32
36
D
E
N
Amino acid

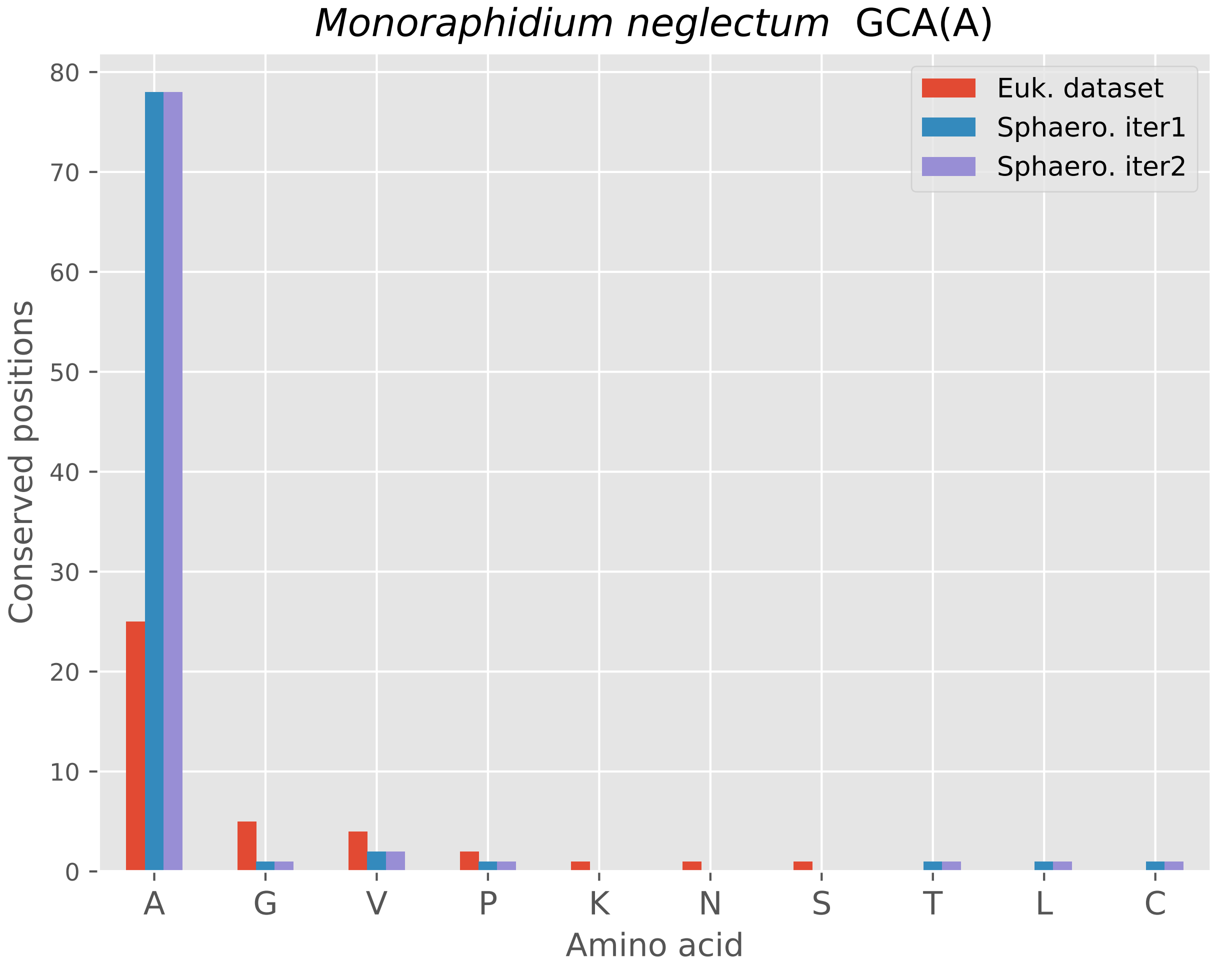

Monoraphidium neglectum GCA(A)
Euk. dataset
Sphaero. iter1
Sphaero. iter2
Conserved positions
0
10
20
30
40
50
60
70
80
A
G
V
P
K
N
S
T
L
C
Amino acid

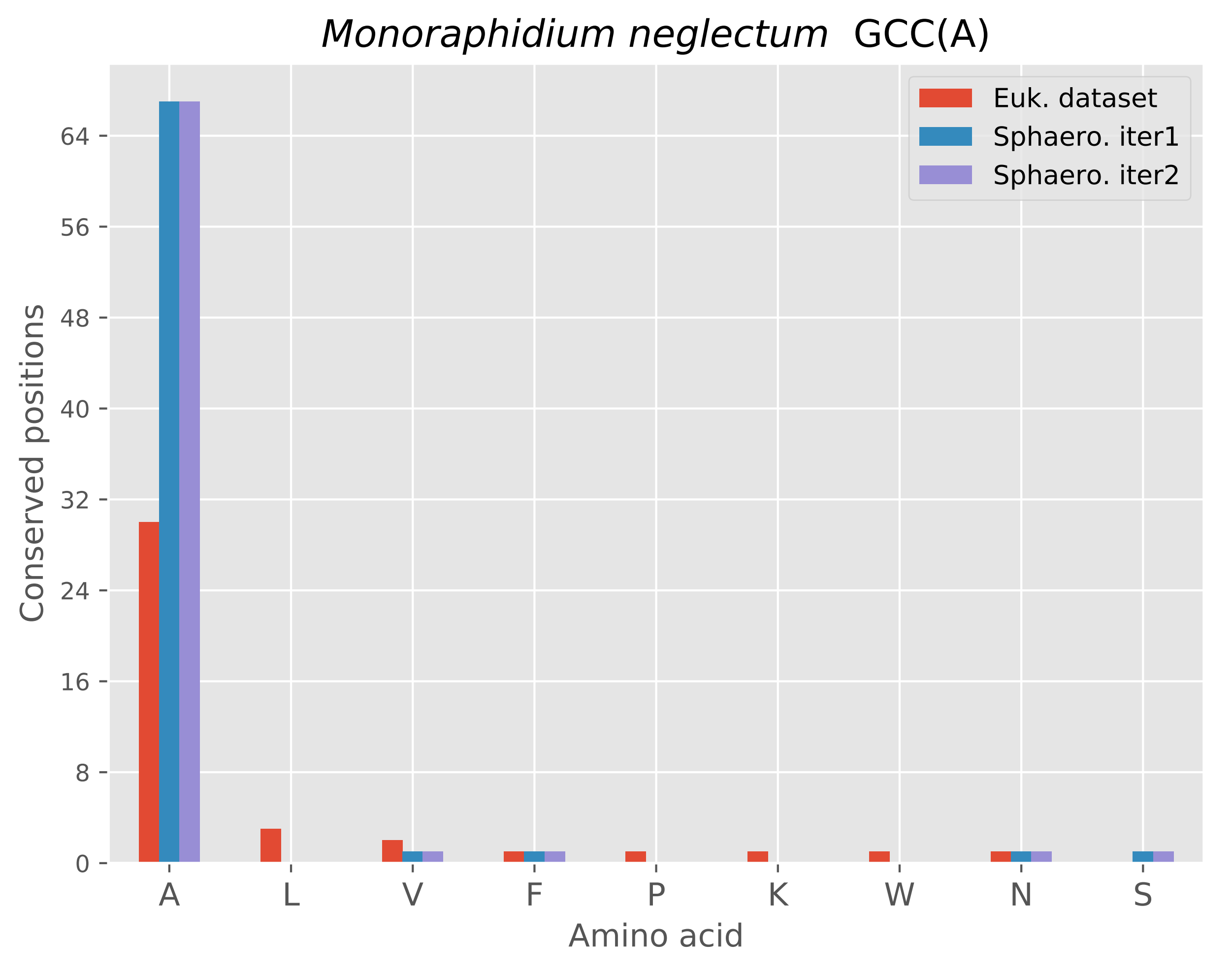

Monoraphidium neglectum GCC(A)
Euk. dataset
Sphaero. iter1
Sphaero. iter2
Conserved positions
0
8
16
24
32
40
48
56
64
A
L
V
F
P
K
W
N
S
Amino acid

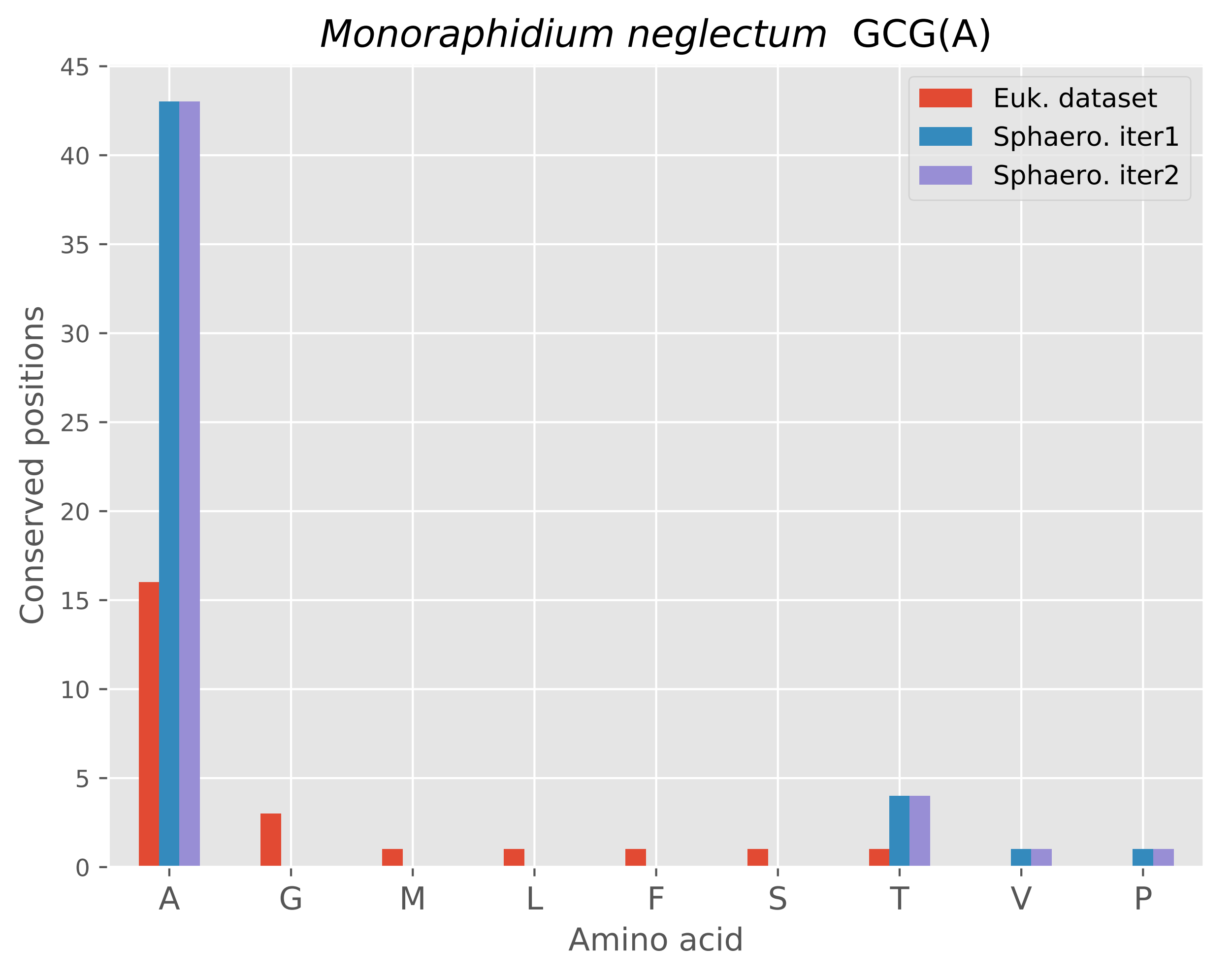

Monoraphidium neglectum GCG(A)
Euk. dataset
Sphaero. iter1
Sphaero. iter2
Conserved positions
0
5
10
15
20
25
30
35
40
45
A
G
M
L
F
S
T
V
P
Amino acid

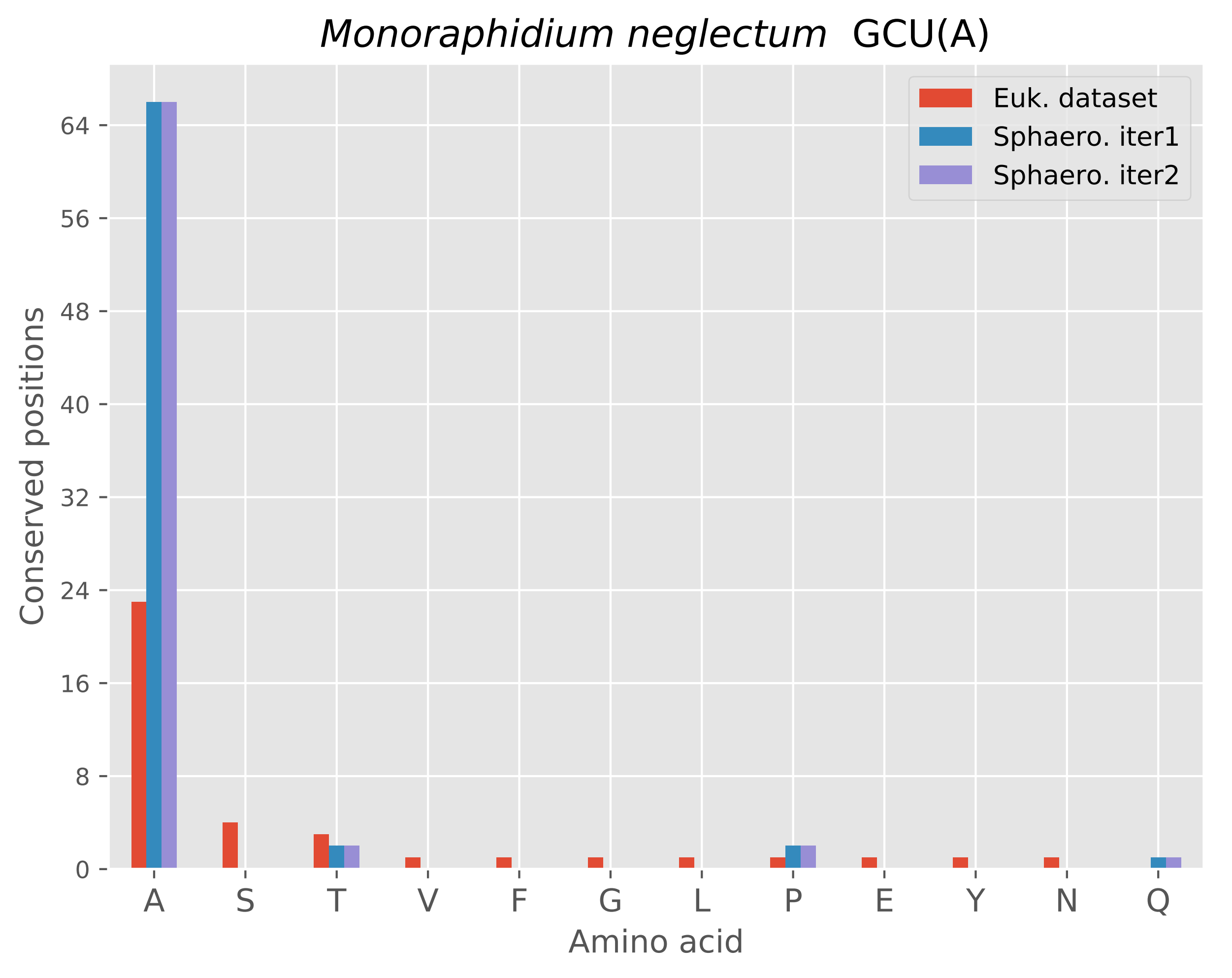

Monoraphidium neglectum GCU(A)
Euk. dataset
Sphaero. iter1
Sphaero. iter2
Conserved positions
0
8
16
24
32
40
48
56
64
A
S
T
V
F
G
L
P
E
Y
N
Q
Amino acid

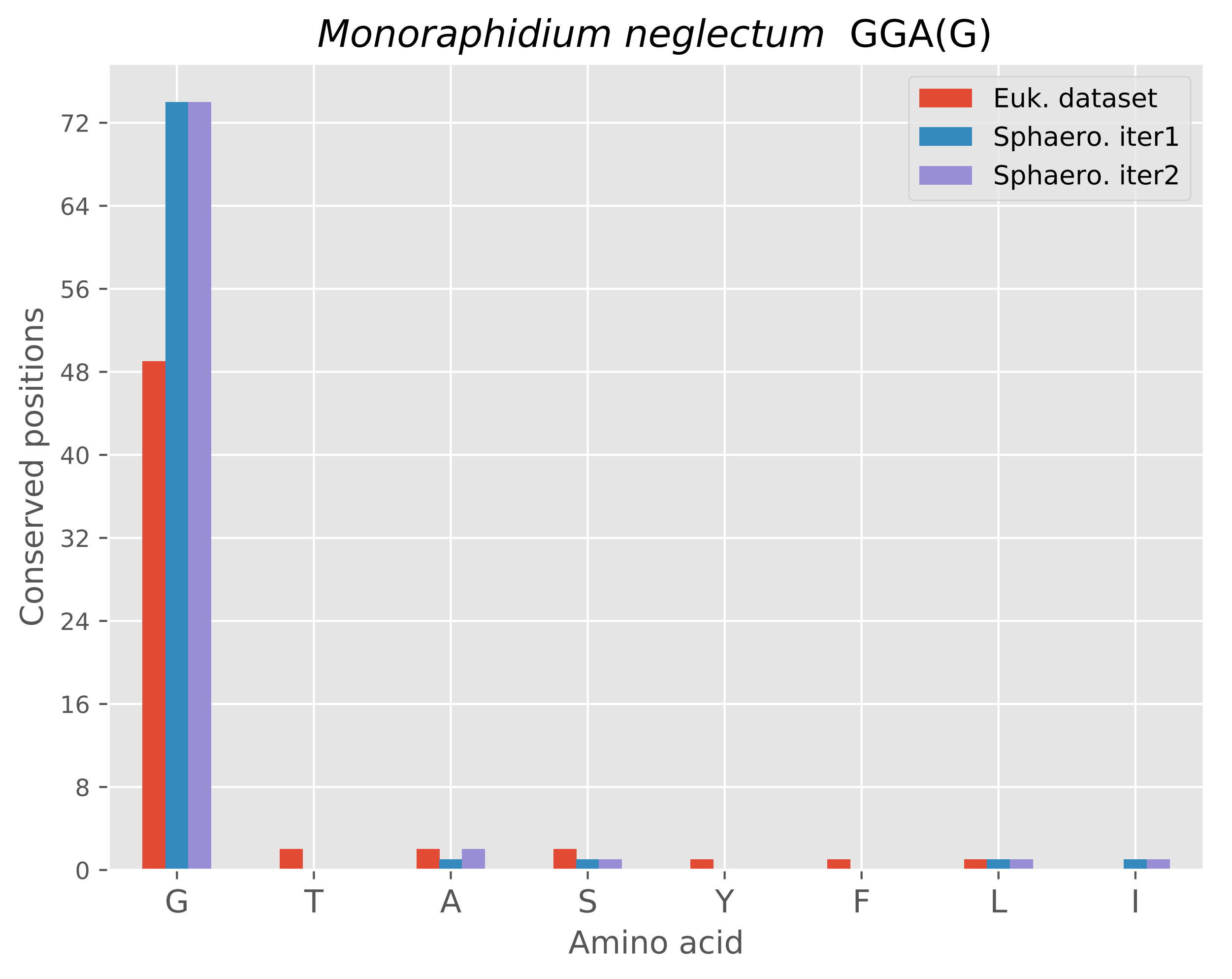
Monoraphidium neglectum GGA(G)
Euk. dataset
Sphaero. iter1
Sphaero. iter2
Conserved positions
0
8
16
24
32
40
48
56
64
72
G
T
A
S
Y
F
L
I
Amino acid

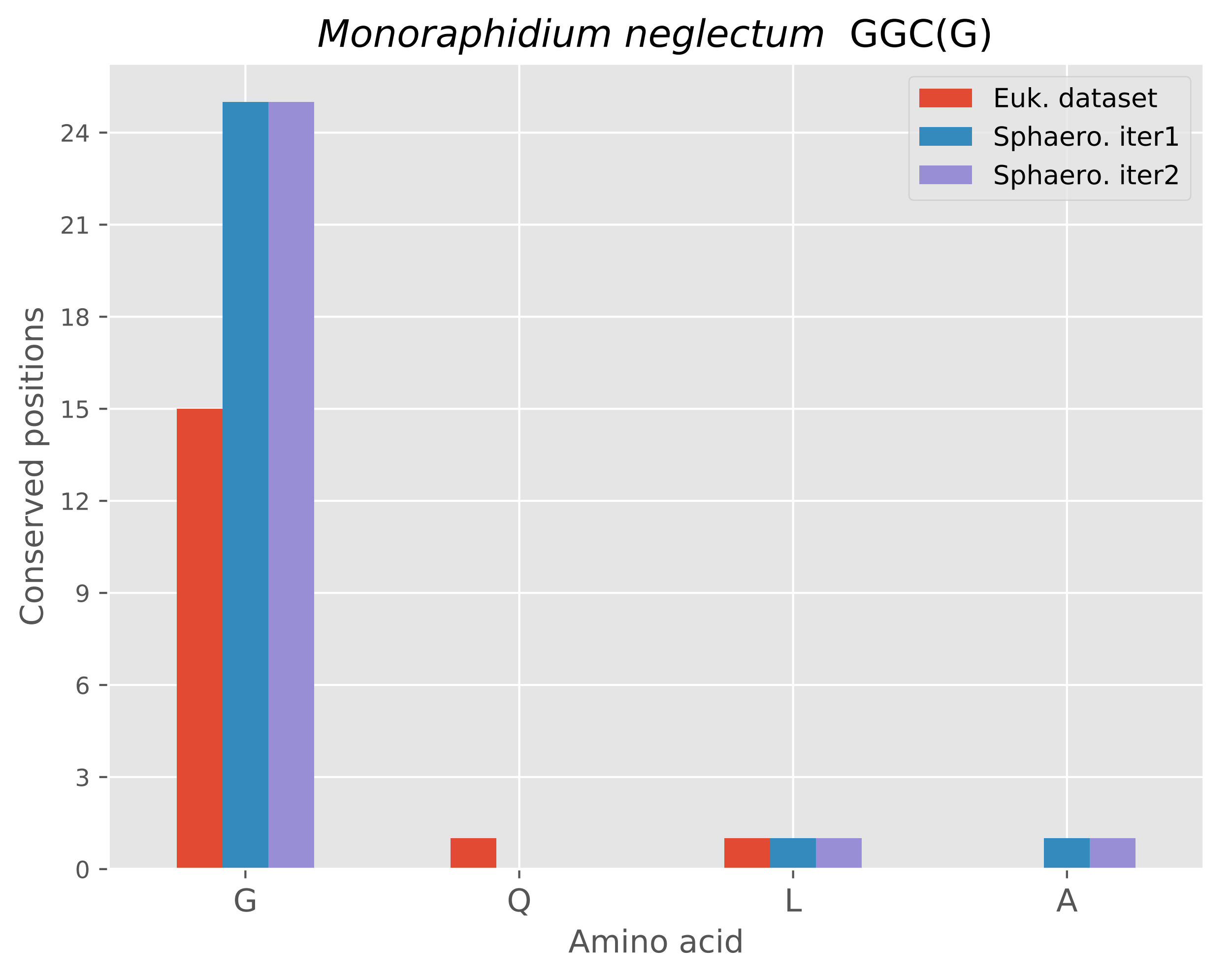

Monoraphidium neglectum GGC(G)
Euk. dataset
Sphaero. iter1
Sphaero. iter2
Conserved positions
0
3
6
9
12
15
18
21
24
G
Q
L
A
Amino acid

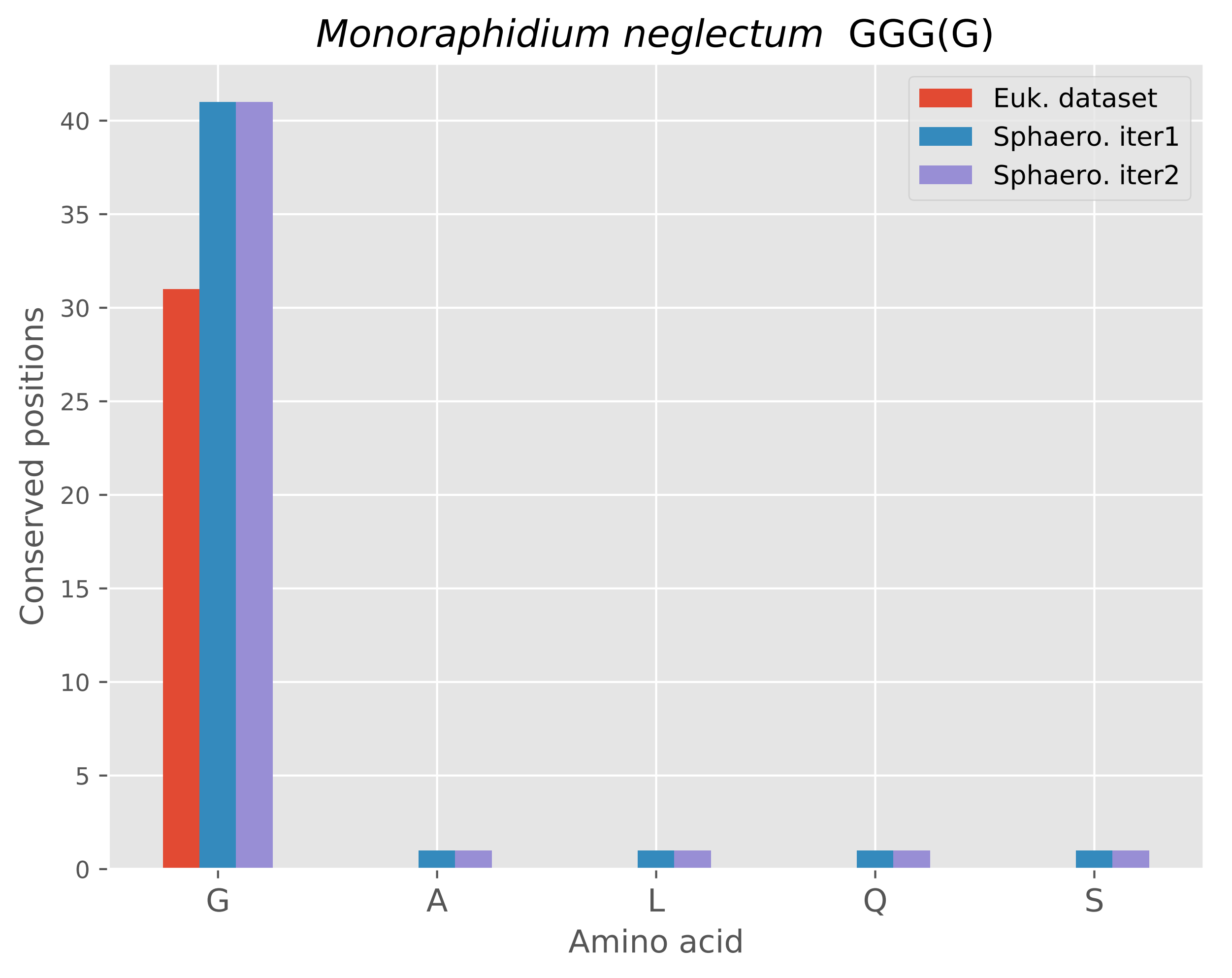
Monoraphidium neglectum GGG(G)
Euk. dataset
Sphaero. iter1
Sphaero. iter2
Conserved positions
0
5
10
15
20
25
30
35
40
G
A
L
Q
S
Amino acid

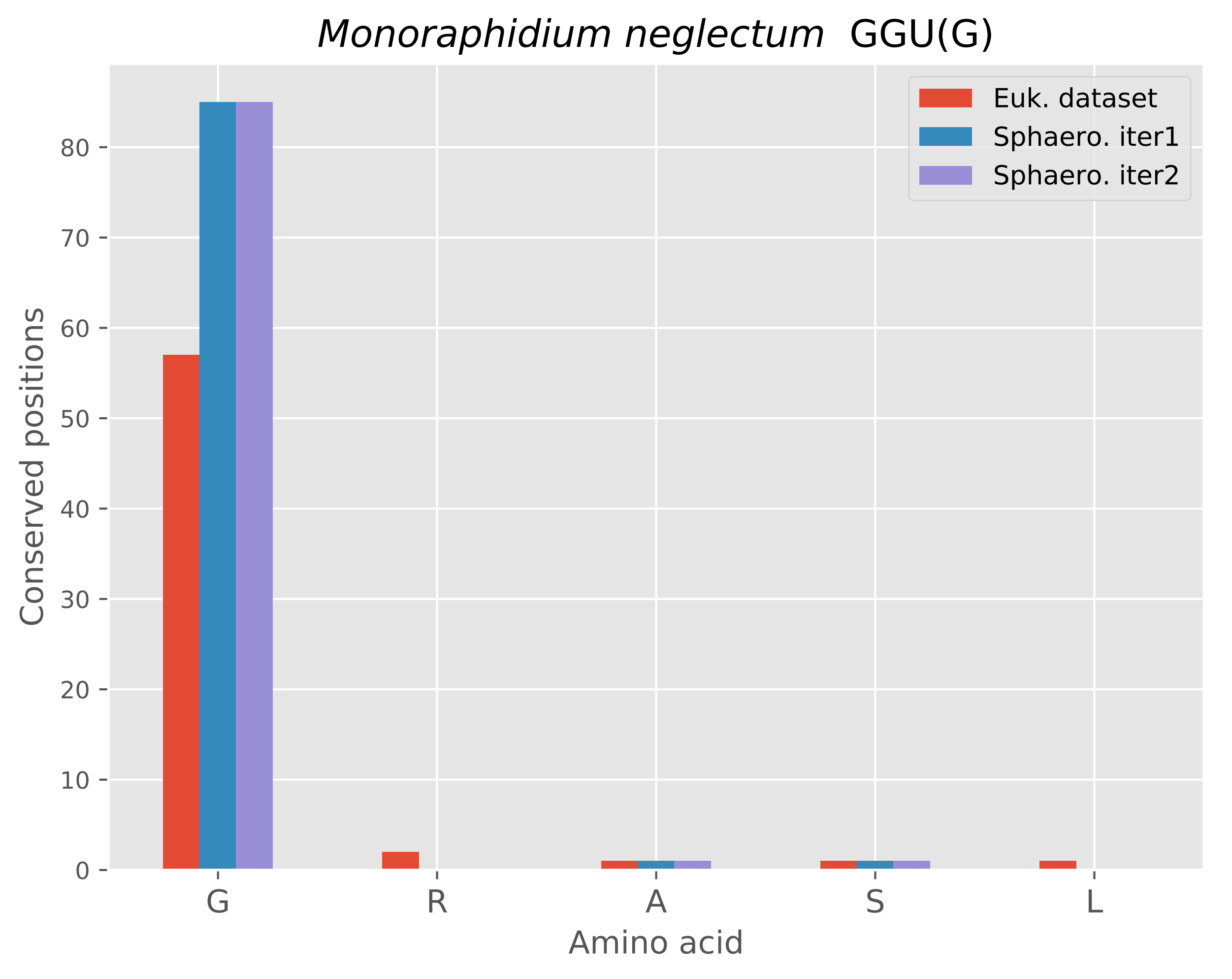
Monoraphidium neglectum GGU(G)
Euk. dataset
Sphaero. iter1
Sphaero. iter2
Conserved positions
0
10
20
30
40
50
60
70
80
G
R
A
S
L
Amino acid

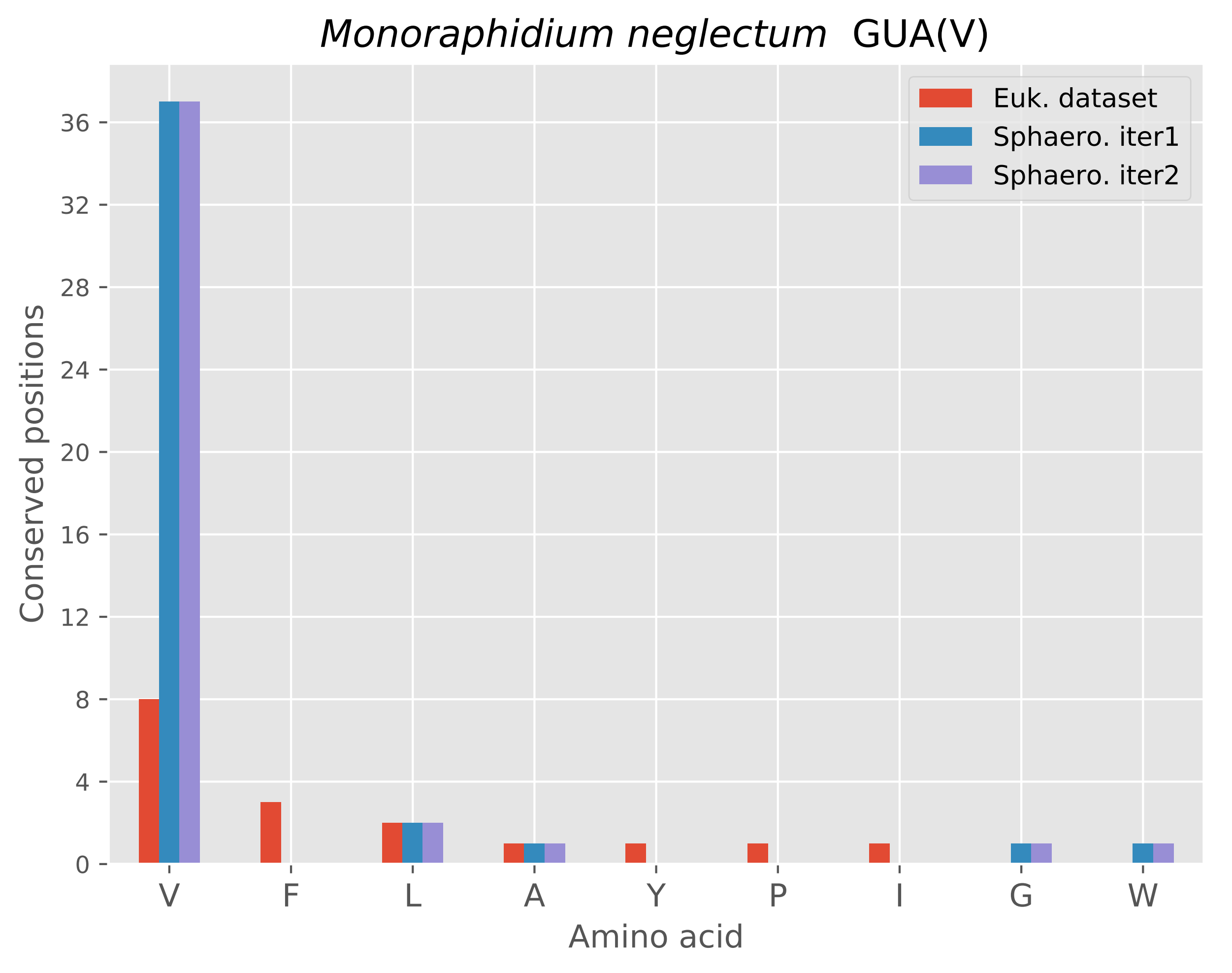

Monoraphidium neglectum GUA(V)
Euk. dataset
Sphaero. iter1
Sphaero. iter2
Conserved positions
0
4
8
12
16
20
24
28
32
36
V
F
L
A
Y
P
I
G
W
Amino acid

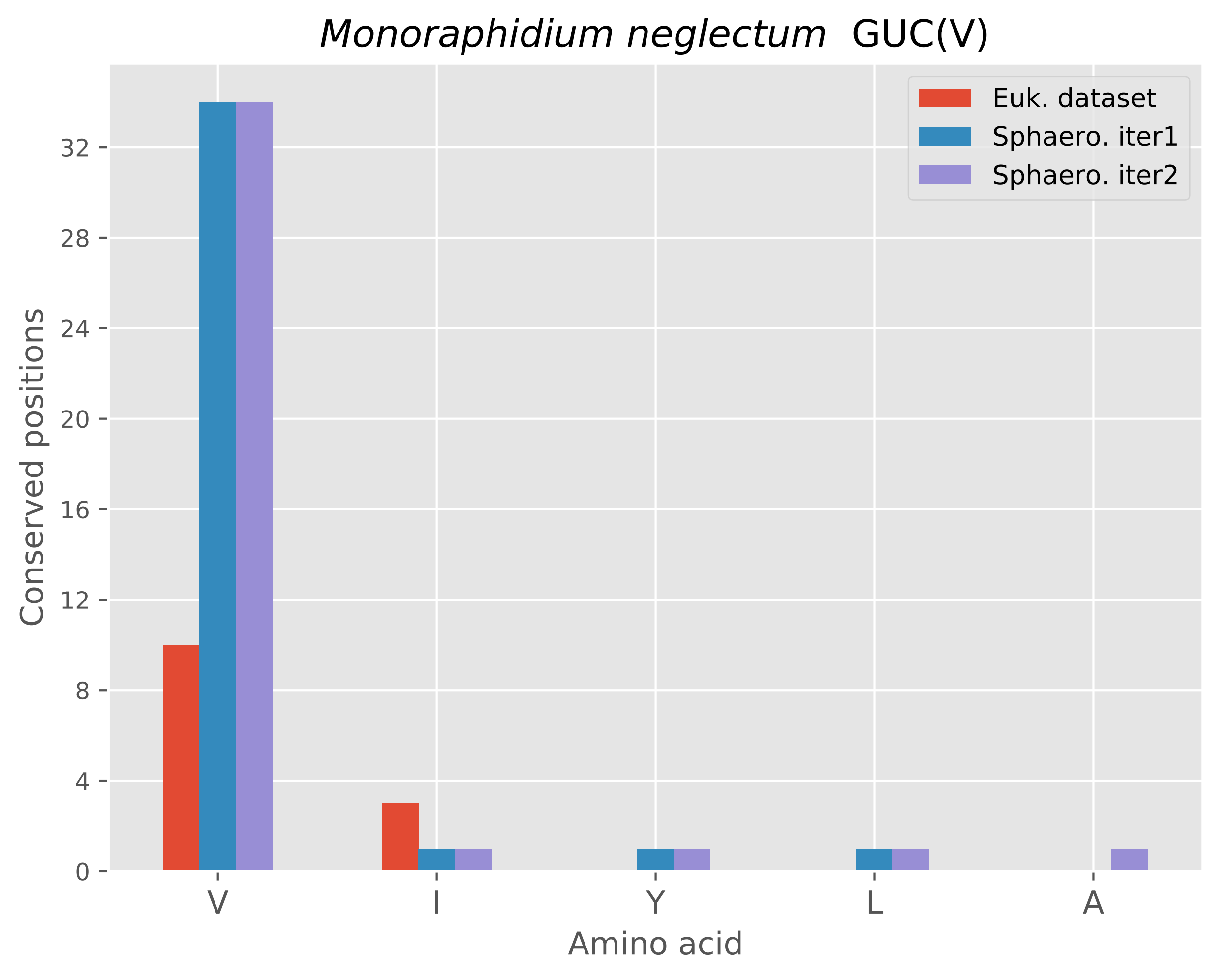

Monoraphidium neglectum GUC(V)
Euk. dataset
Sphaero. iter1
Sphaero. iter2
Conserved positions
0
4
8
12
16
20
24
28
32
V
I
Y
L
A
Amino acid

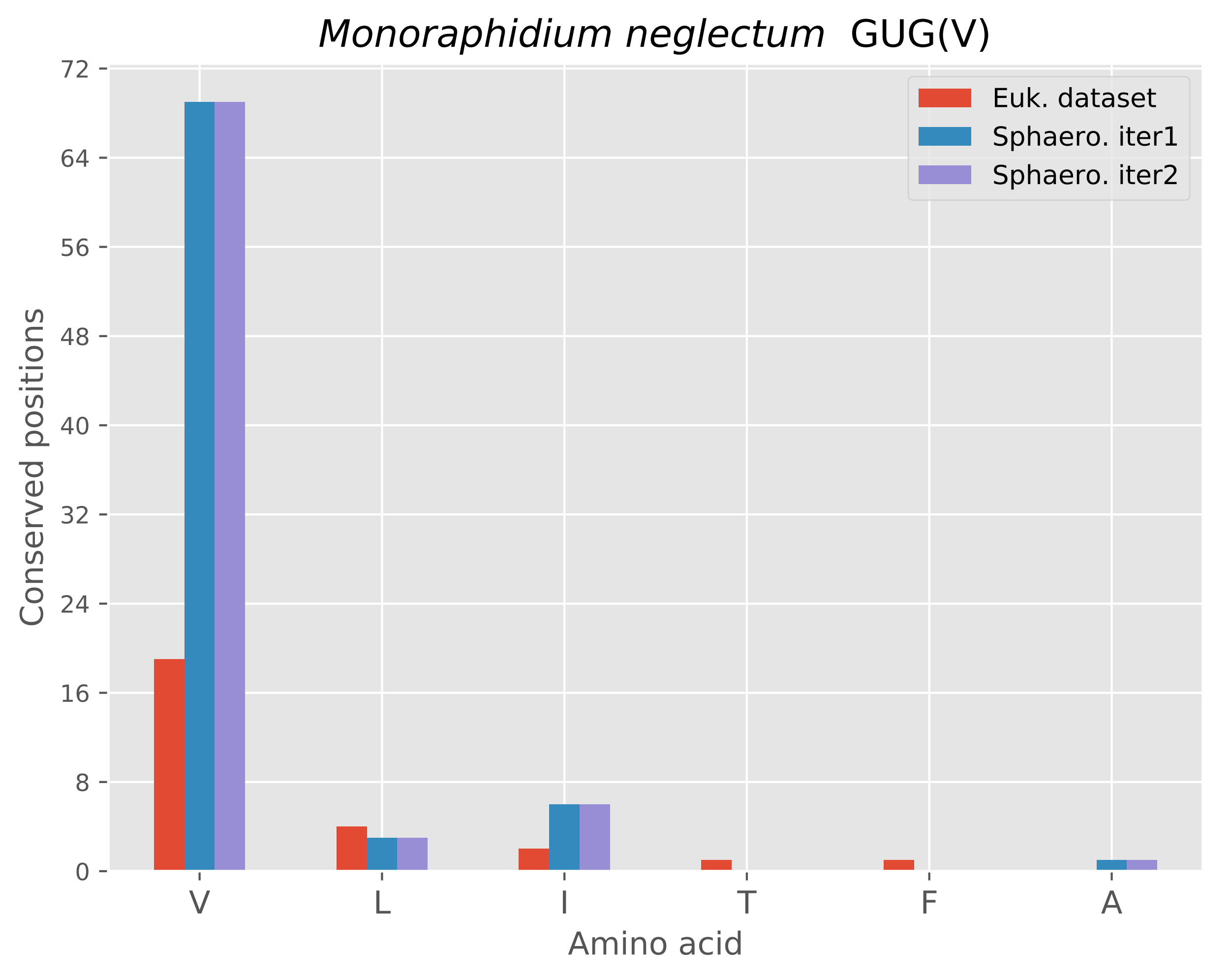
Monoraphidium neglectum GUG(V)
Euk. dataset
Sphaero. iter1
Sphaero. iter2
Conserved positions
0
8
16
24
32
40
48
56
64
72
V
L
I
T
F
A
Amino acid

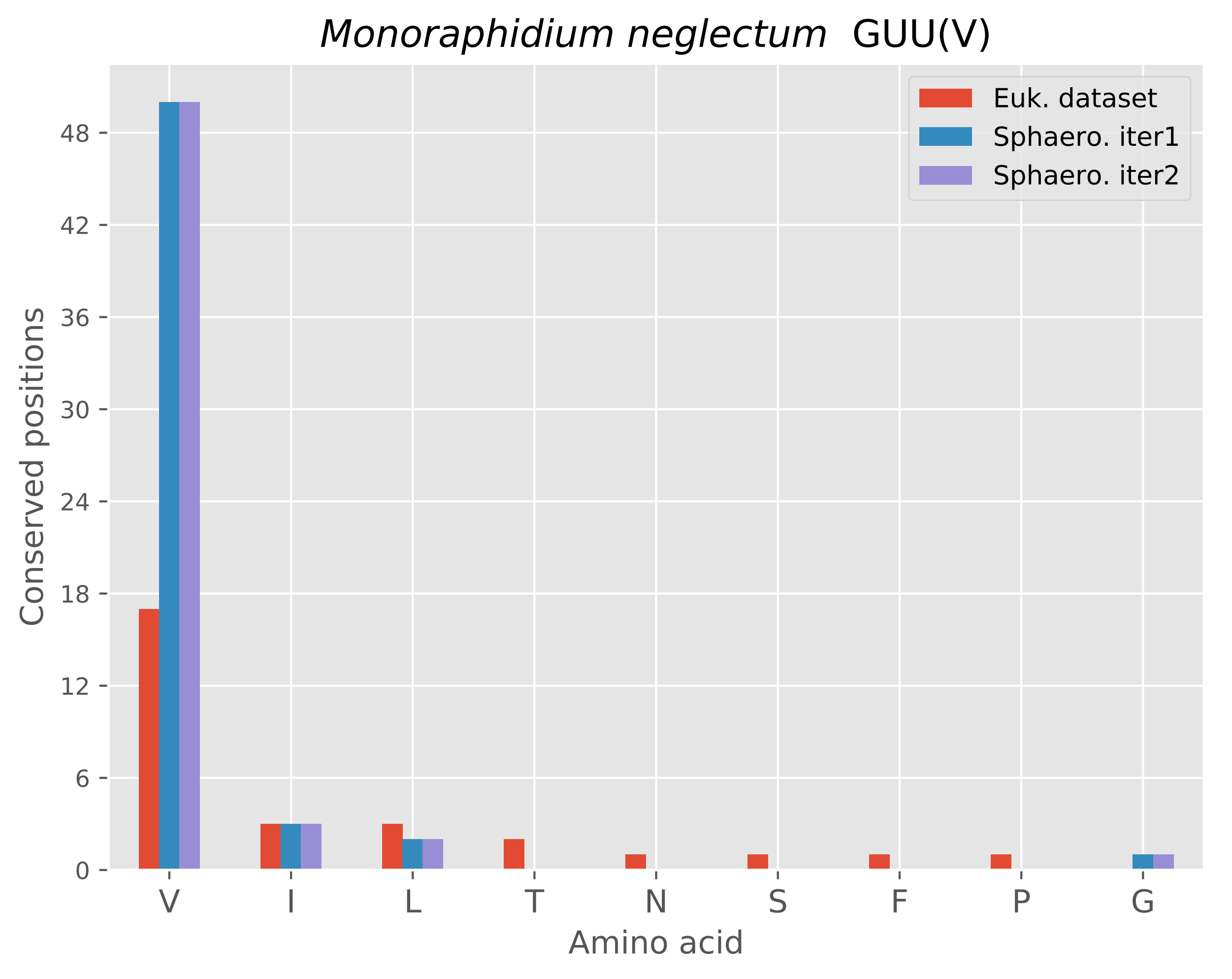

Monoraphidium neglectum GUU(V)
Euk. dataset
Sphaero. iter1
Sphaero. iter2
Conserved positions
0
6
12
18
24
30
36
42
48
V
I
L
T
N
S
F
P
G
Amino acid

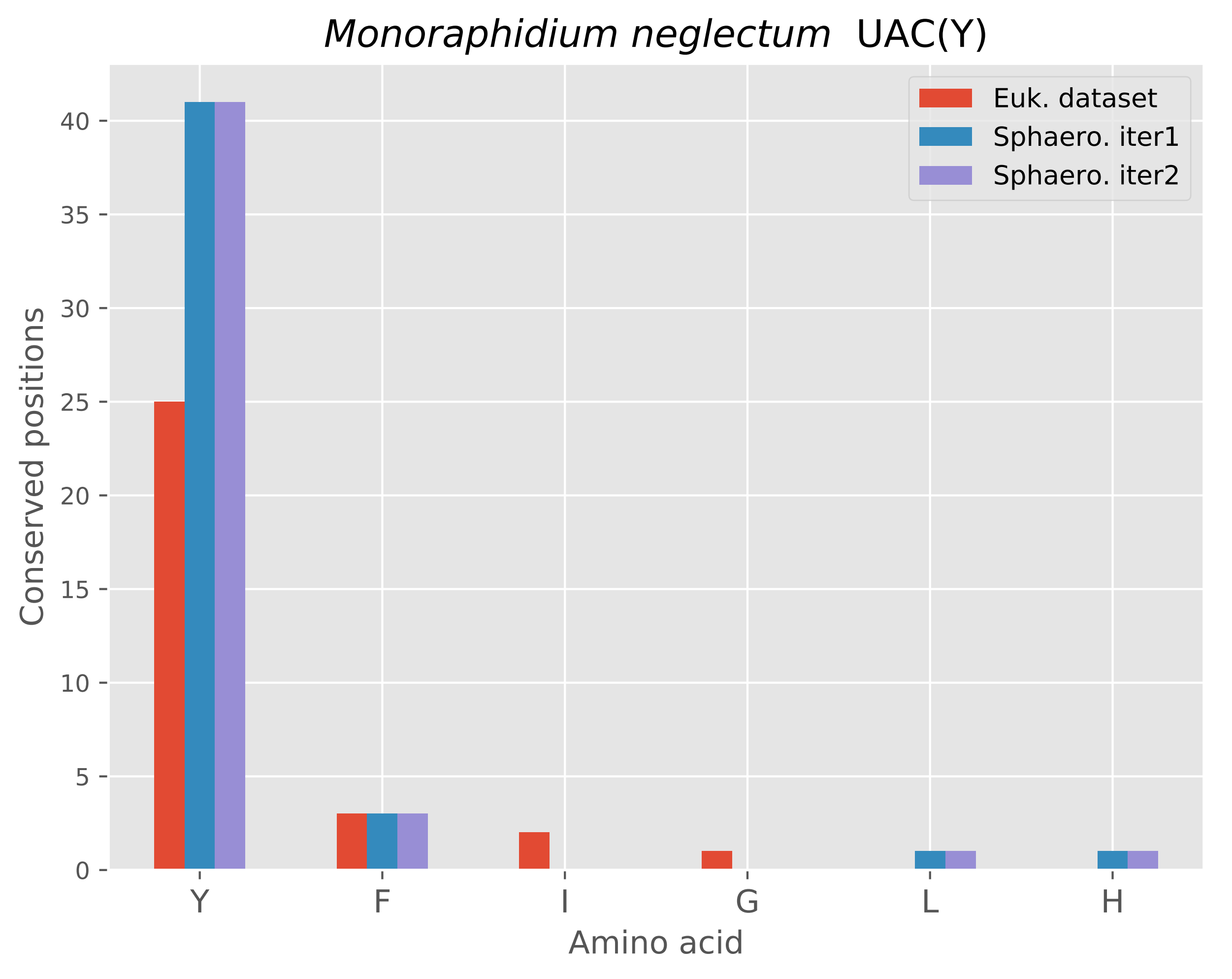

Monoraphidium neglectum UAC(Y)
Euk. dataset
Sphaero. iter1
Sphaero. iter2
Conserved positions
0
5
10
15
20
25
30
35
40
Y
F
I
G
L
H
Amino acid

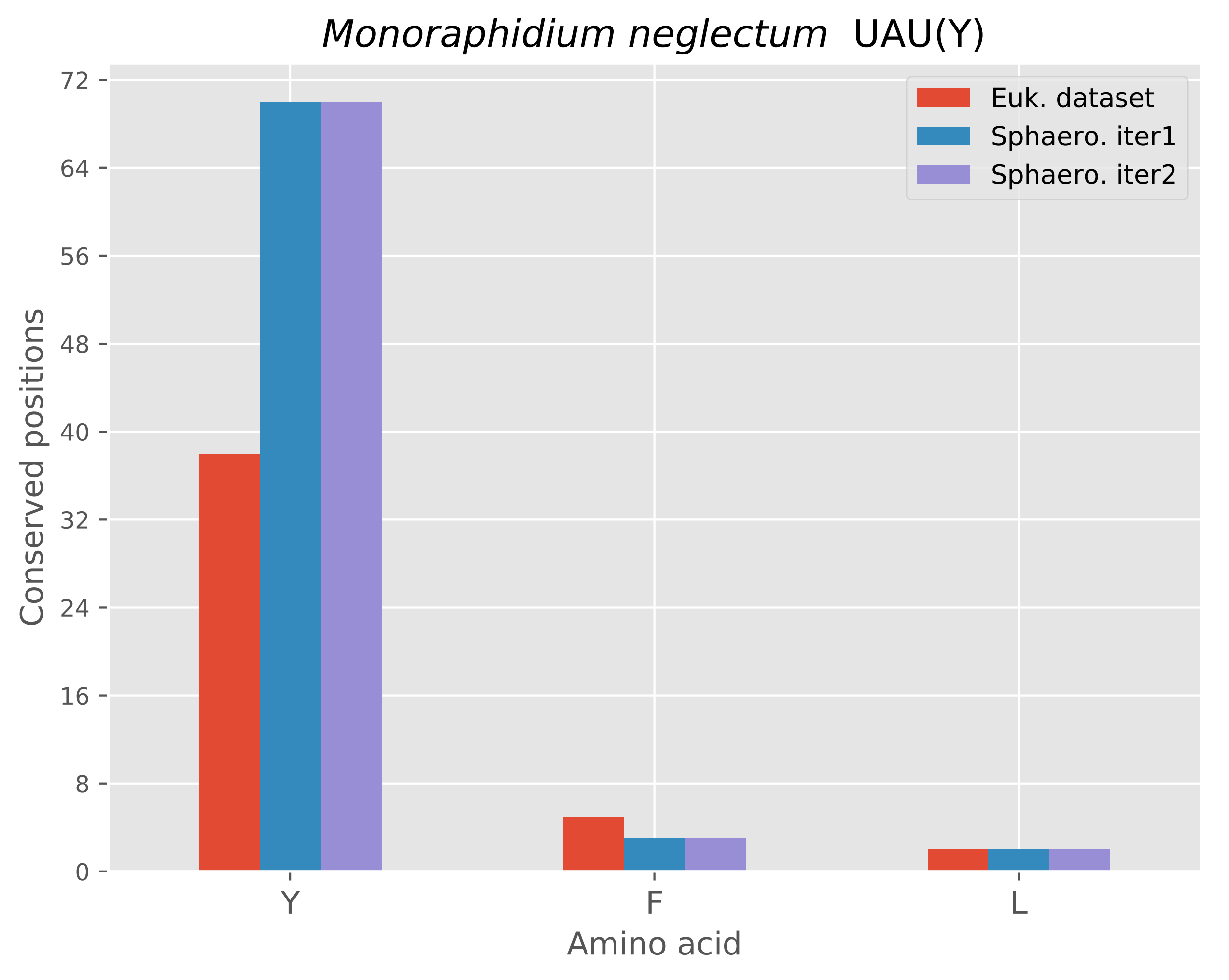

Monoraphidium neglectum UAU(Y)
Conserved positions
0
8
16
24
32
40
48
56
64
72
Y
F
L
Amino acid
Euk. dataset
Sphaero. iter1
Sphaero. iter2

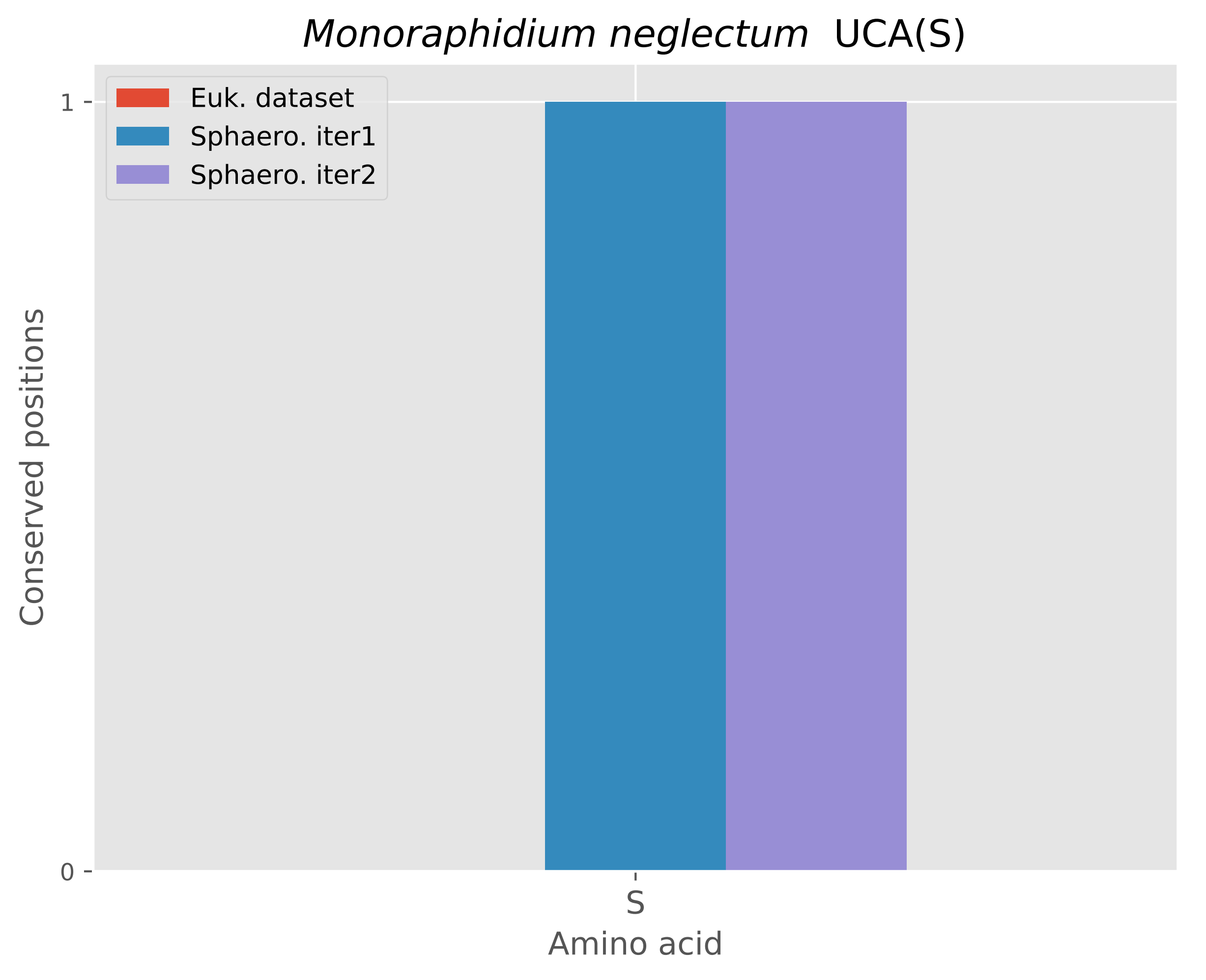

Monoraphidium neglectum UCA(S)
Euk. dataset
Sphaero. iter1
Sphaero. iter2
1
0
Conserved positions
S
Amino acid

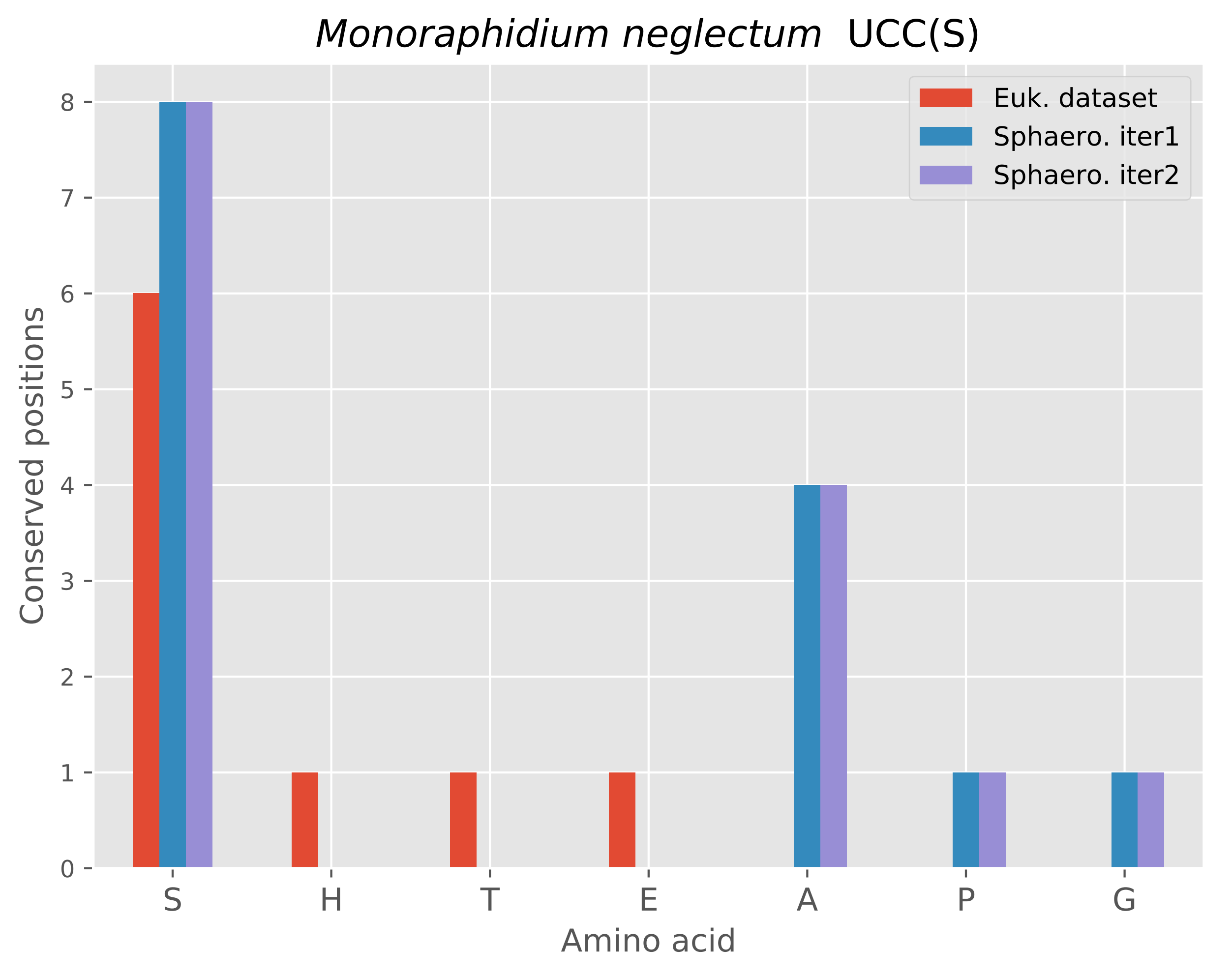

Monoraphidium neglectum UCC(S)
Euk. dataset
Sphaero. iter1
Sphaero. iter2
Conserved positions
0
1
2
3
4
5
6
7
8
S
H
T
E
A
P
G
Amino acid

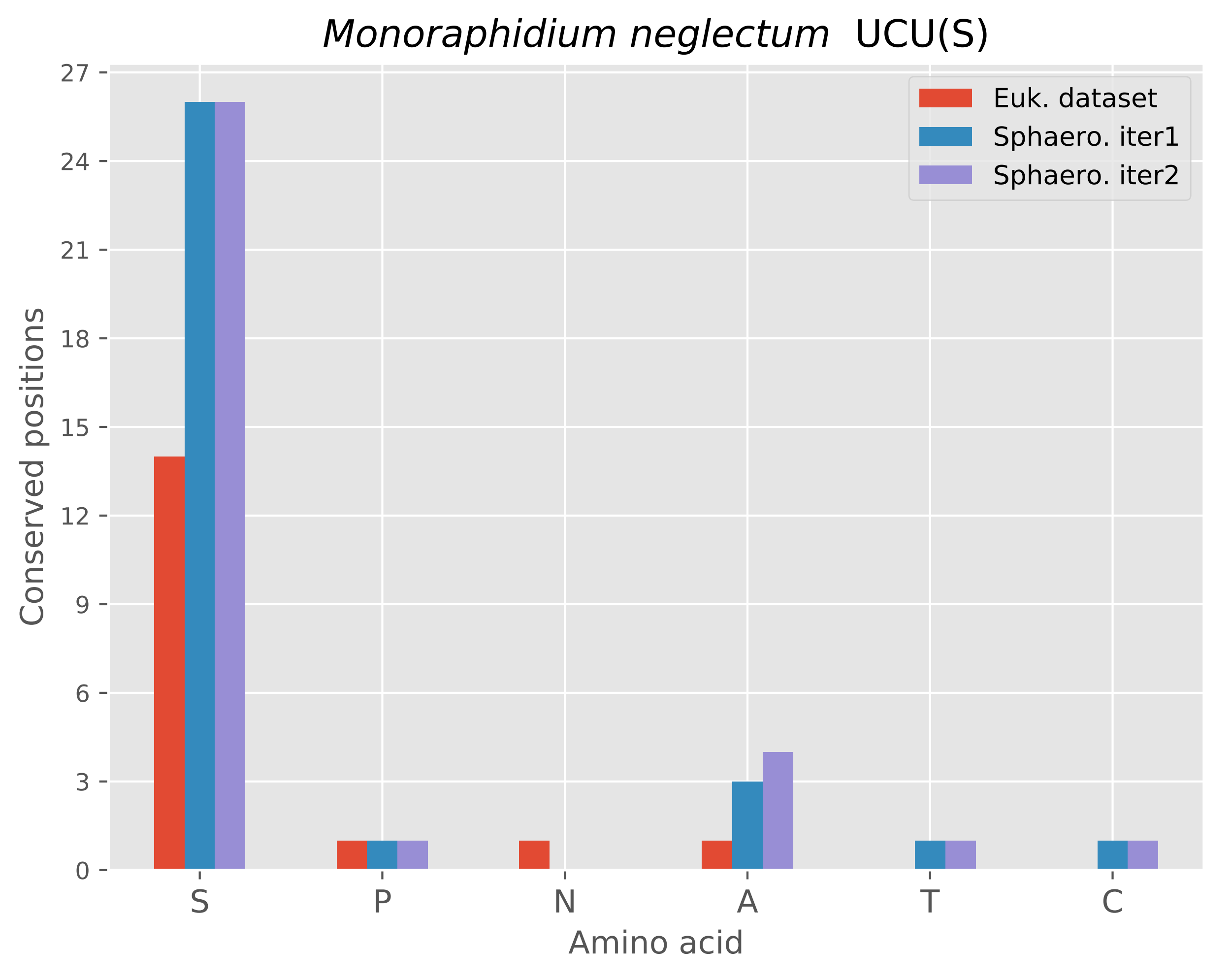

Monoraphidium neglectum UCU(S)
Euk. dataset
Sphaero. iter1
Sphaero. iter2
Conserved positions
0
3
6
9
12
15
18
21
24
27
S
P
N
A
T
C
Amino acid

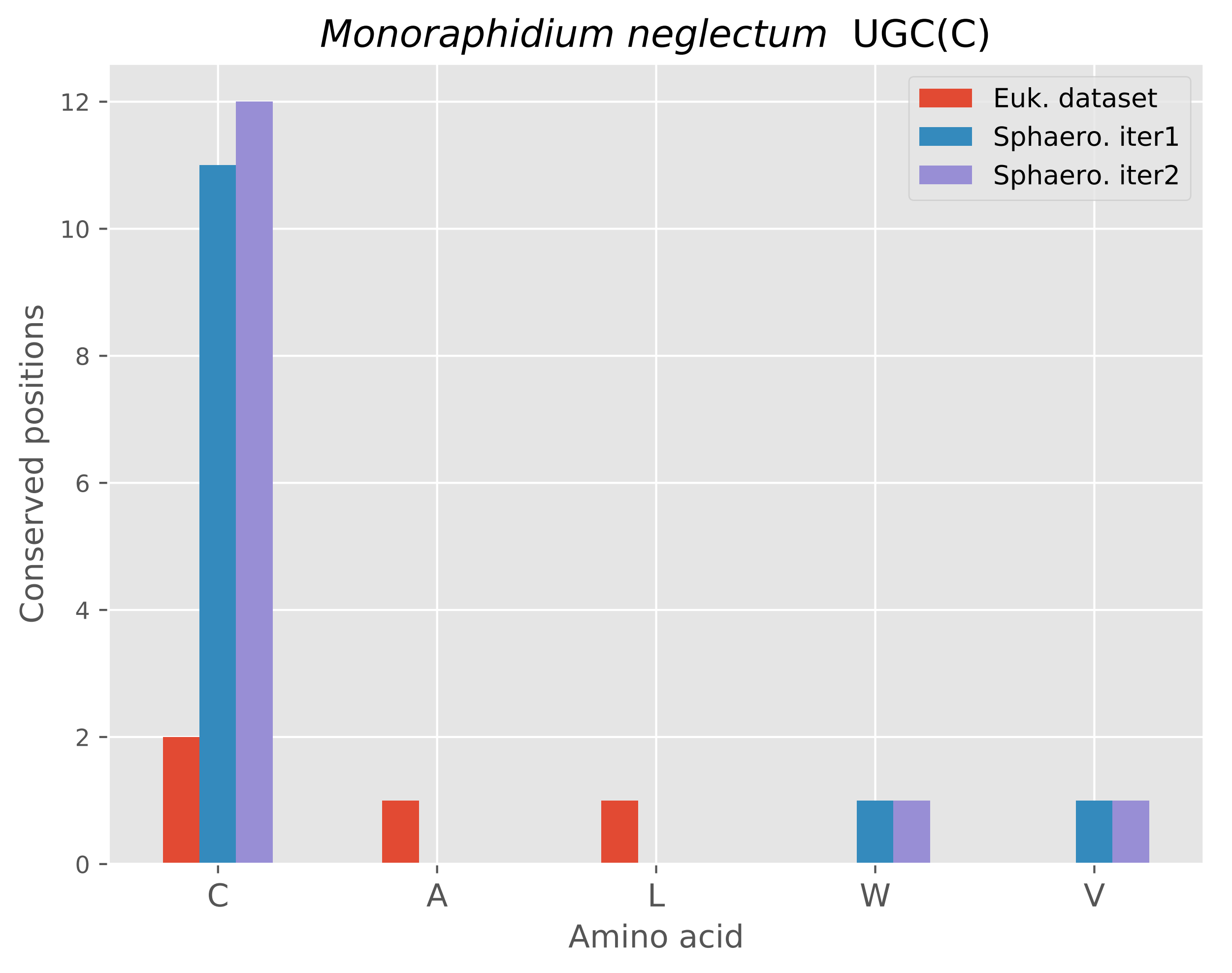
Monoraphidium neglectum UGC(C)
Euk. dataset
Sphaero. iter1
Sphaero. iter2
Conserved positions
0
2
4
6
8
10
12
C
A
L
W
V
Amino acid

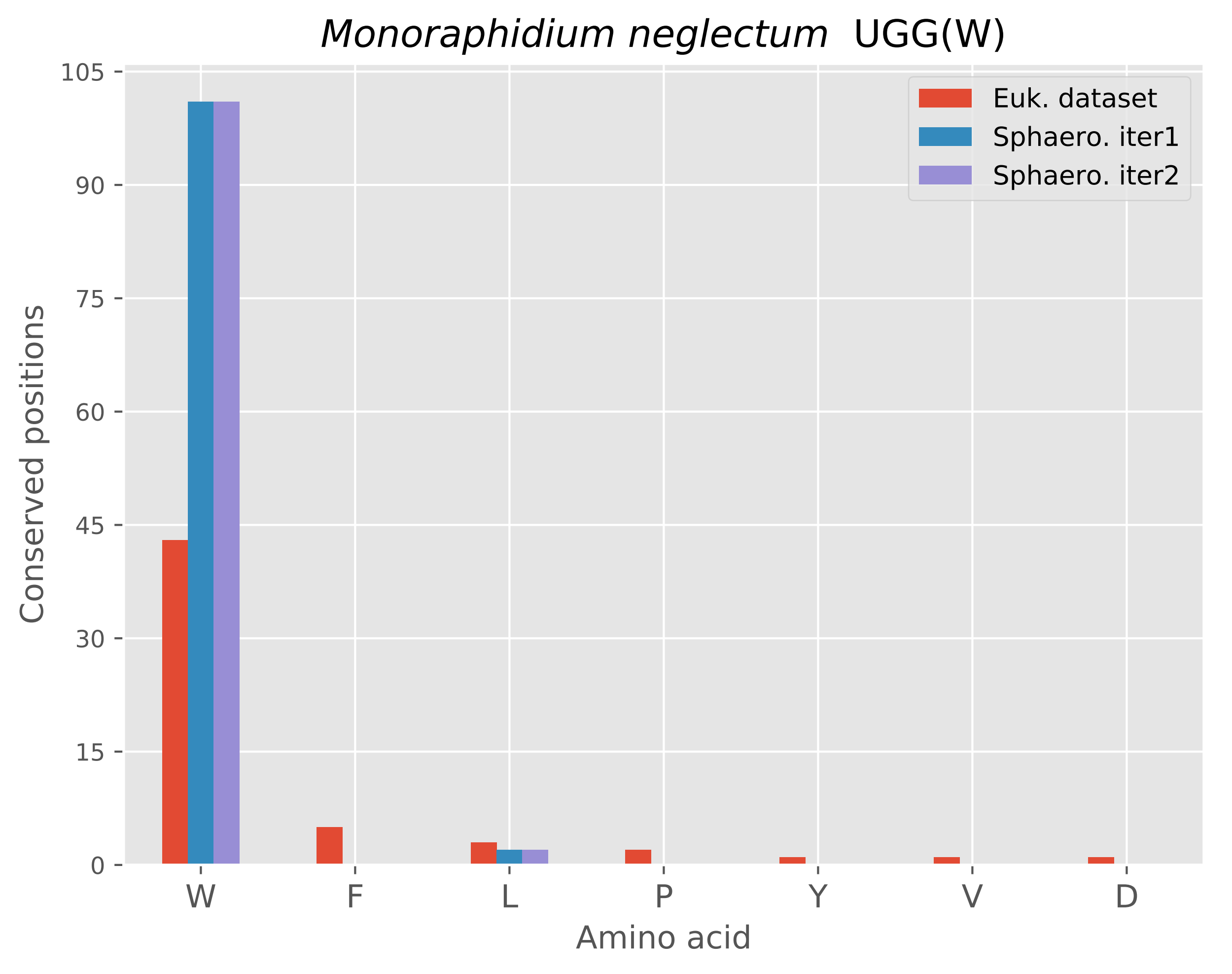
Monoraphidium neglectum UGG(W)
Euk. dataset
Sphaero. iter1
Sphaero. iter2
Conserved positions
0
15
30
45
60
75
90
105
W
F
L
P
Y
V
D
Amino acid

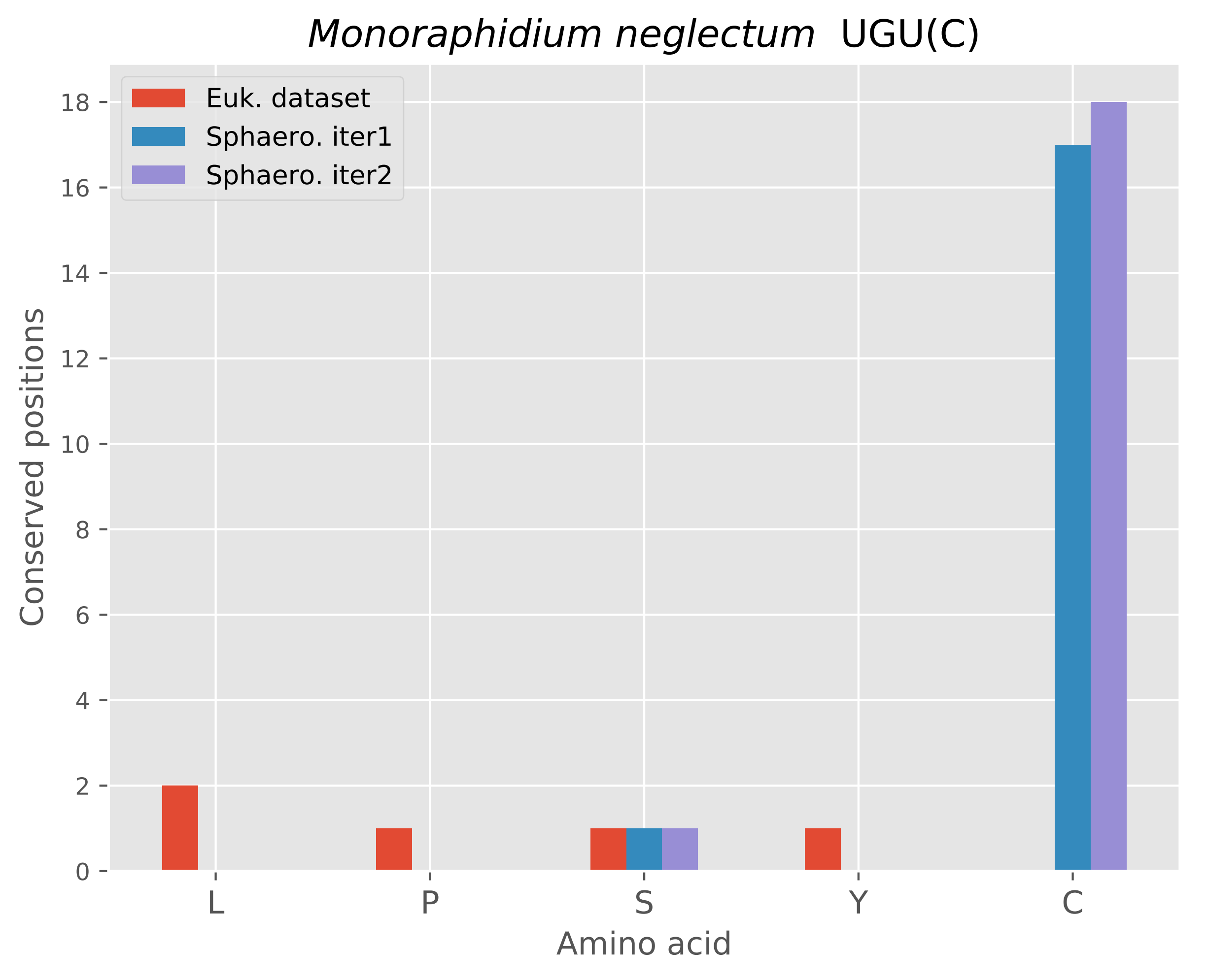
Monoraphidium neglectum UGU(C)
Euk. dataset
Sphaero. iter1
Sphaero. iter2
Conserved positions
0
2
4
6
8
10
12
14
16
18
L
P
S
Y
C
Amino acid

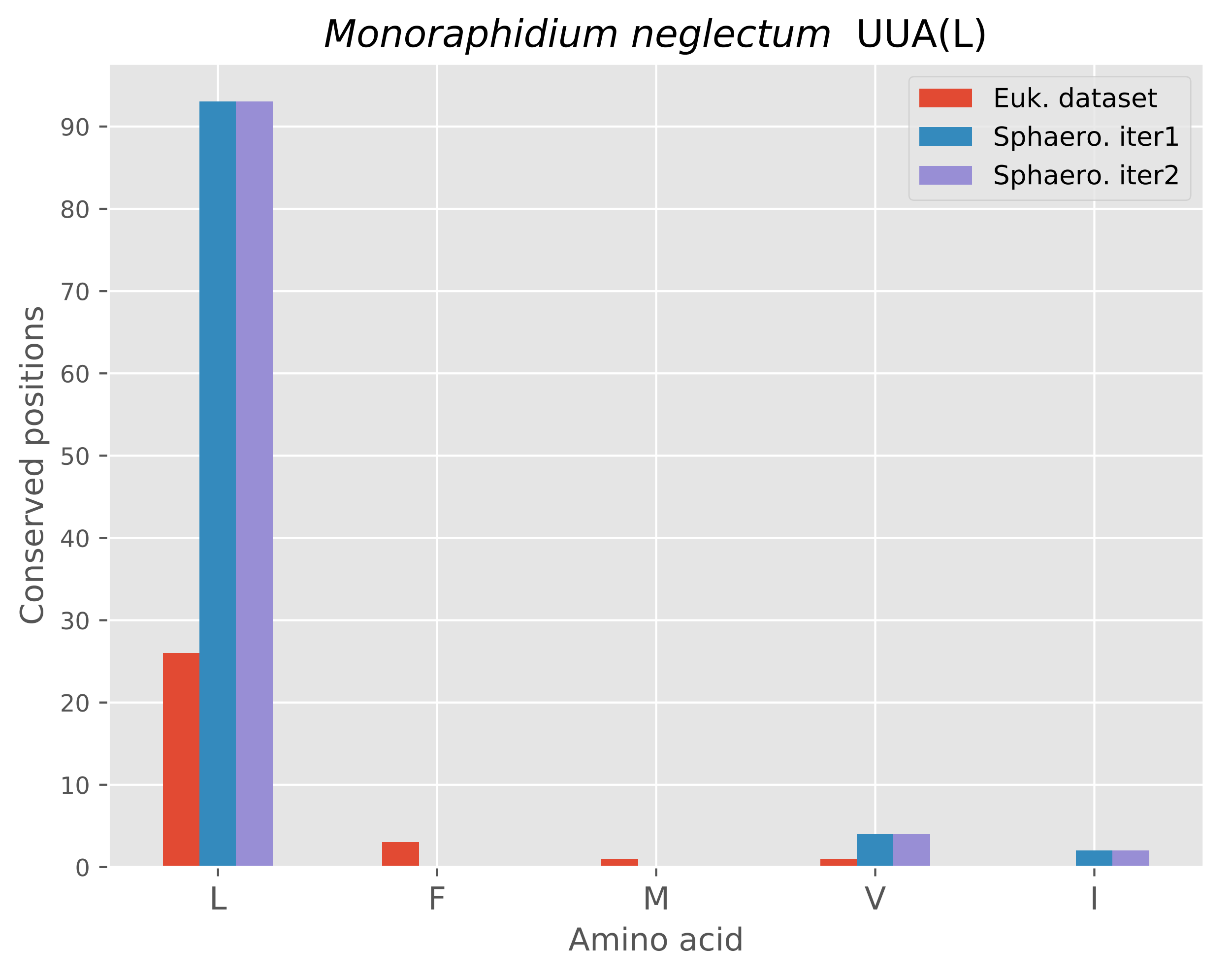

Monoraphidium neglectum UUA(L)
Euk. dataset
Sphaero. iter1
Sphaero. iter2
Conserved positions
0
10
20
30
40
50
60
70
80
90
L
F
M
V
I
Amino acid

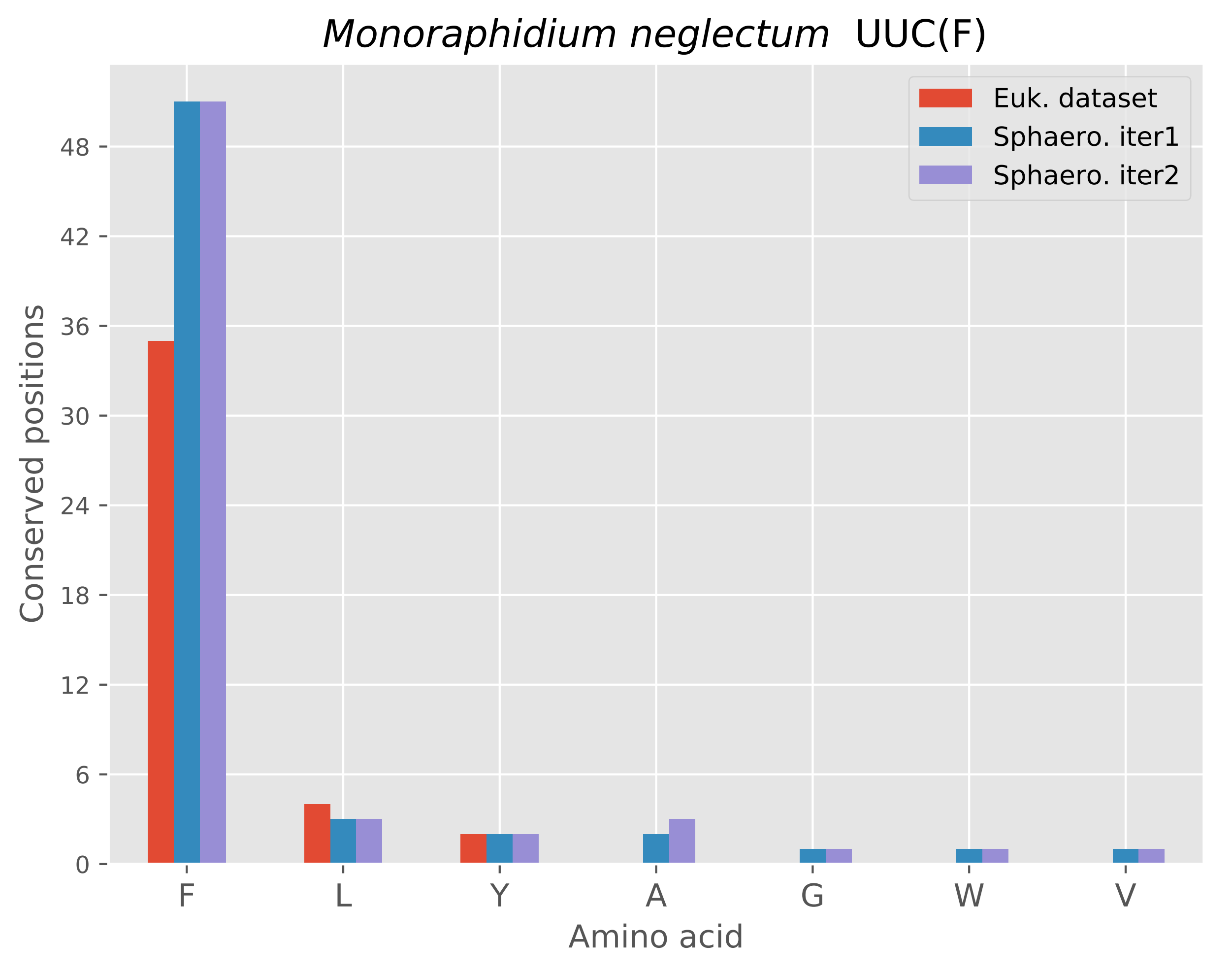

Monoraphidium neglectum UUC(F)
Euk. dataset
Sphaero. iter1
Sphaero. iter2
Conserved positions
0
6
12
18
24
30
36
42
48
F
L
Y
A
G
W
V
Amino acid

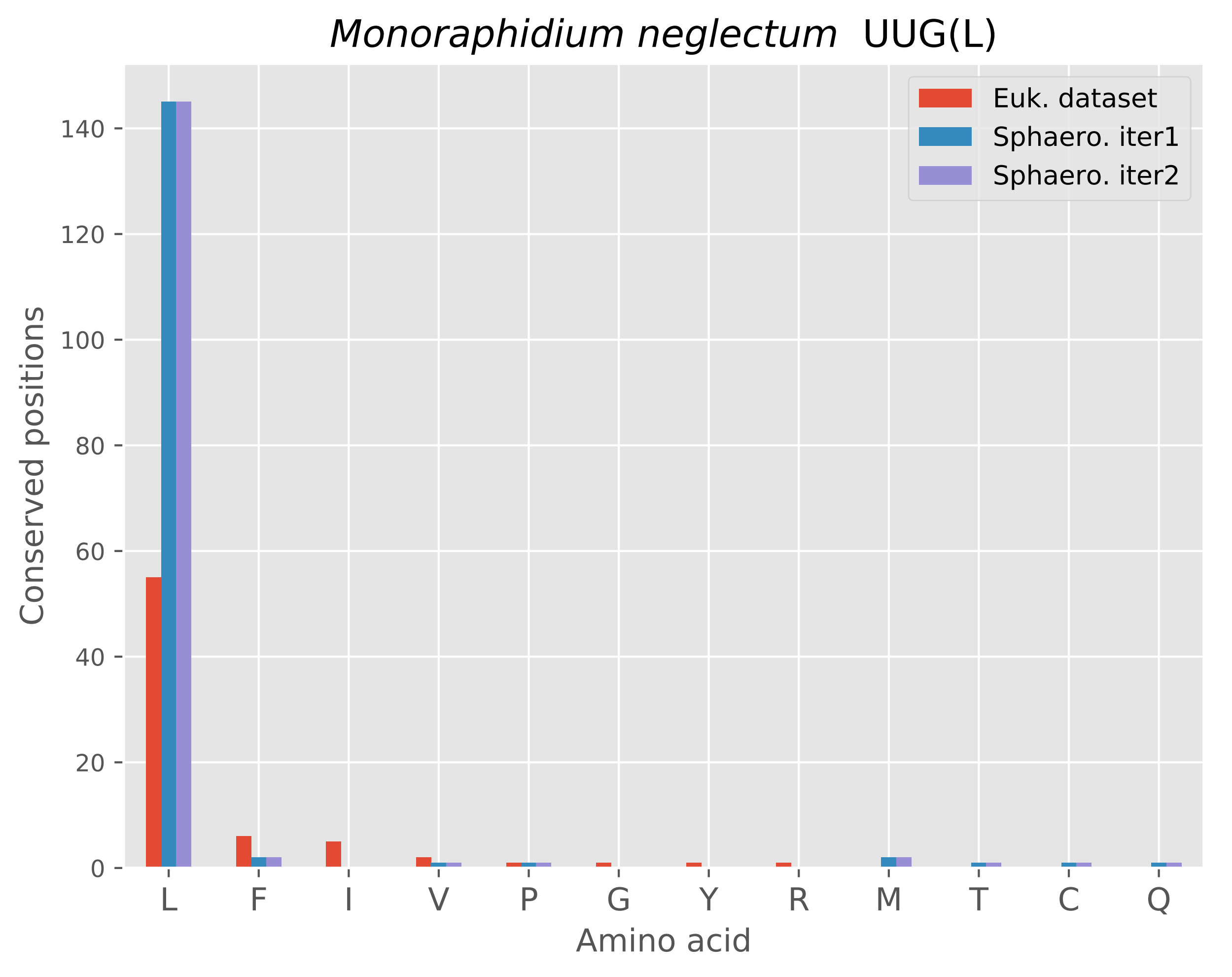

Monoraphidium neglectum UUG(L)
Euk. dataset
Sphaero. iter1
Sphaero. iter2
Conserved positions
0
20
40
60
80
100
120
140
L
F
I
V
P
G
Y
R
M
T
C
Q
Amino acid

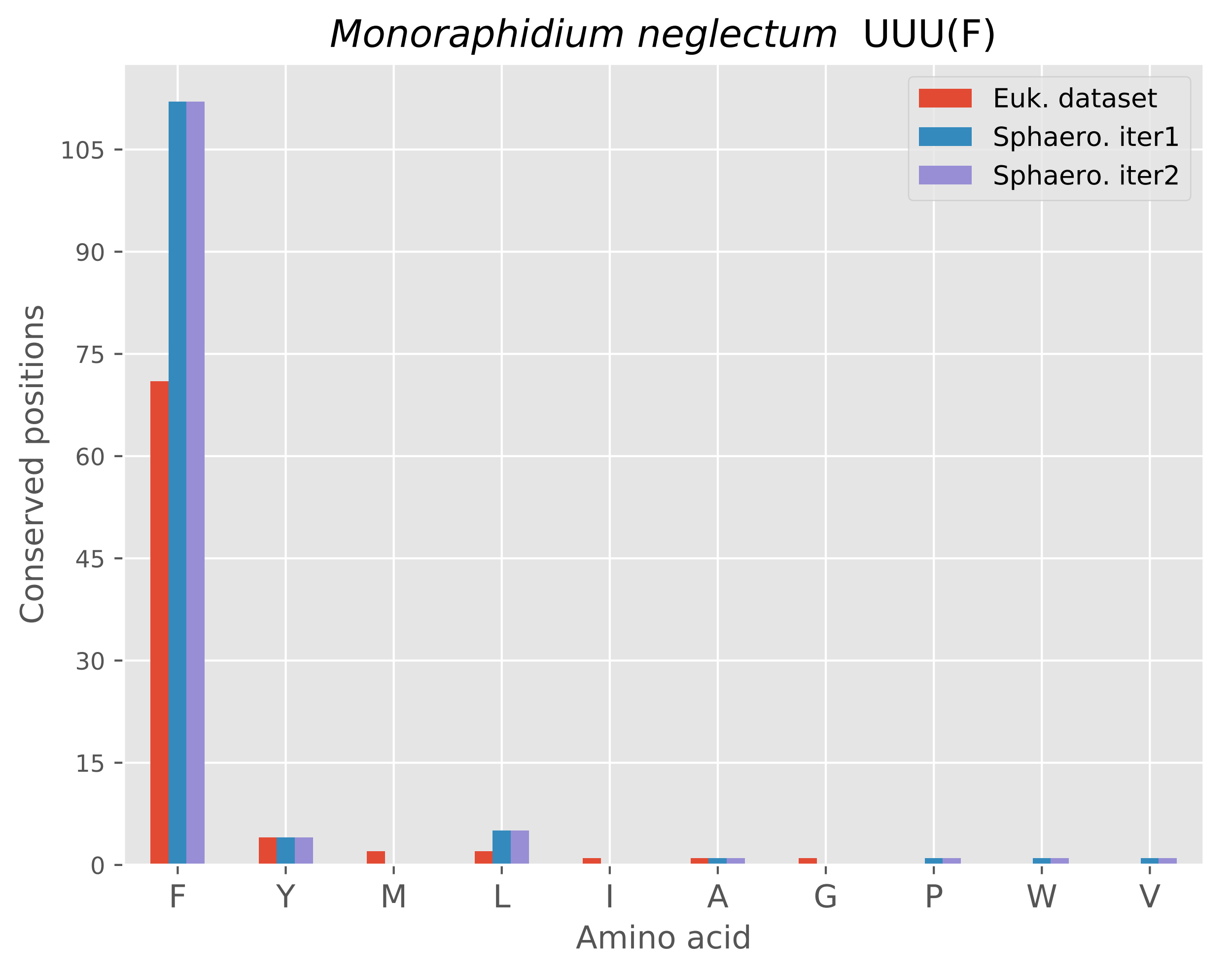

Monoraphidium neglectum UUU(F)
Euk. dataset
Sphaero. iter1
Sphaero. iter2
Conserved positions
0
15
30
45
60
75
90
105
F
Y
M
L
I
A
G
P
W
V
Amino acid

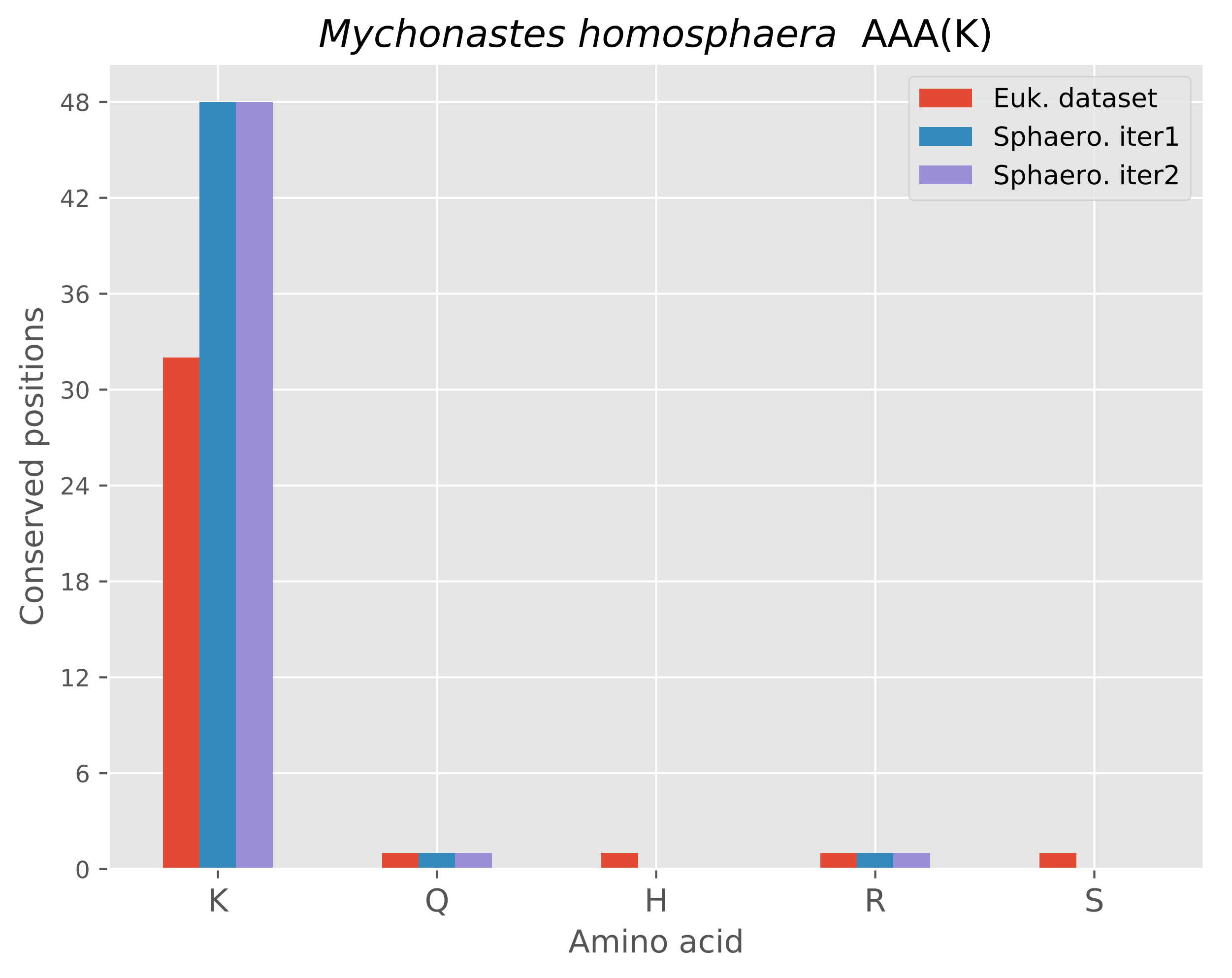

Mychonastes homosphaera AAA(K)
Euk. dataset
Sphaero. iter1
Sphaero. iter2
Conserved positions
0
6
12
18
24
30
36
42
48
K
Q
H
R
S
Amino acid

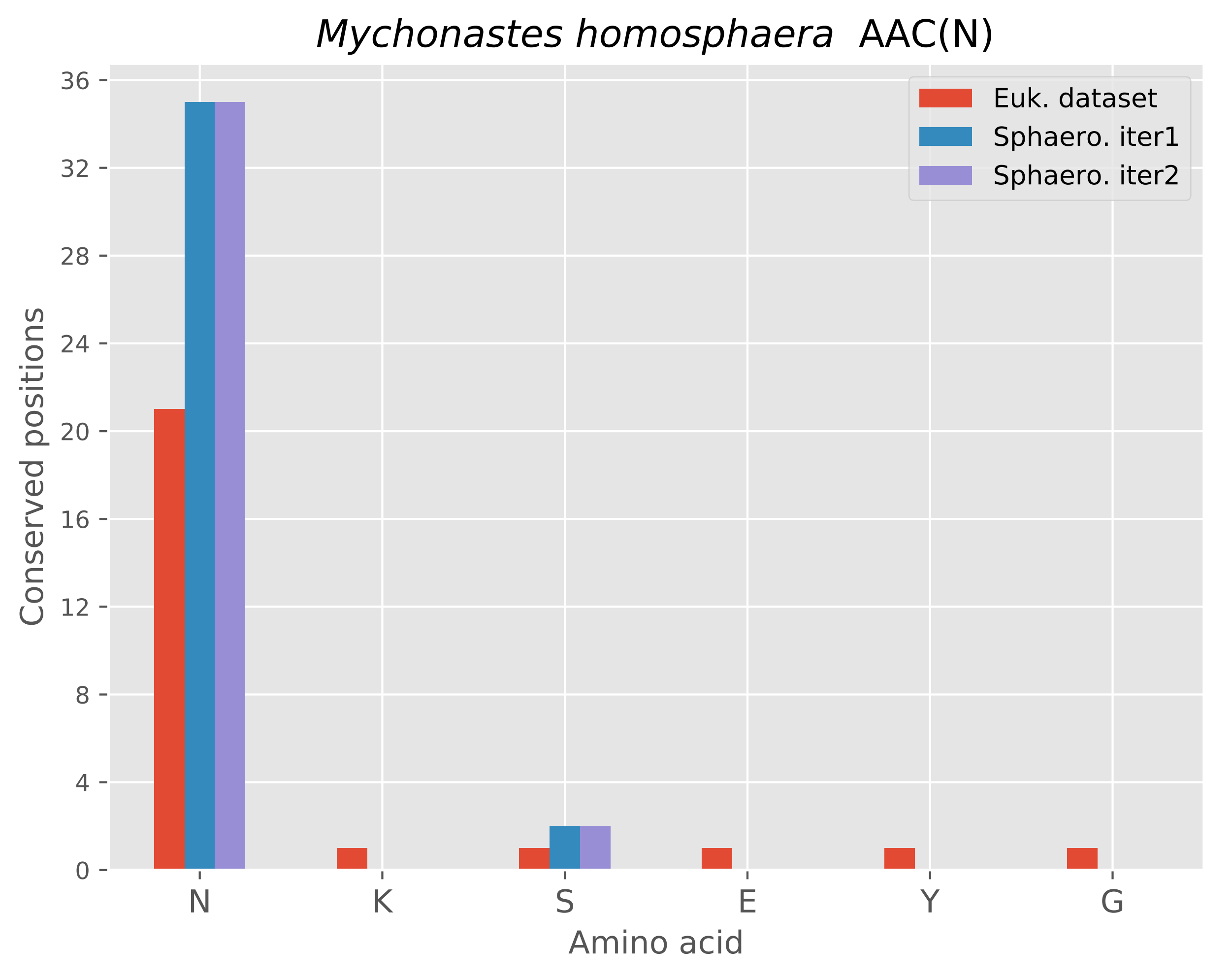

Mychonastes homosphaera AAC(N)
Euk. dataset
Sphaero. iter1
Sphaero. iter2
Conserved positions
0
4
8
12
16
20
24
28
32
36
N
K
S
E
Y
G
Amino acid

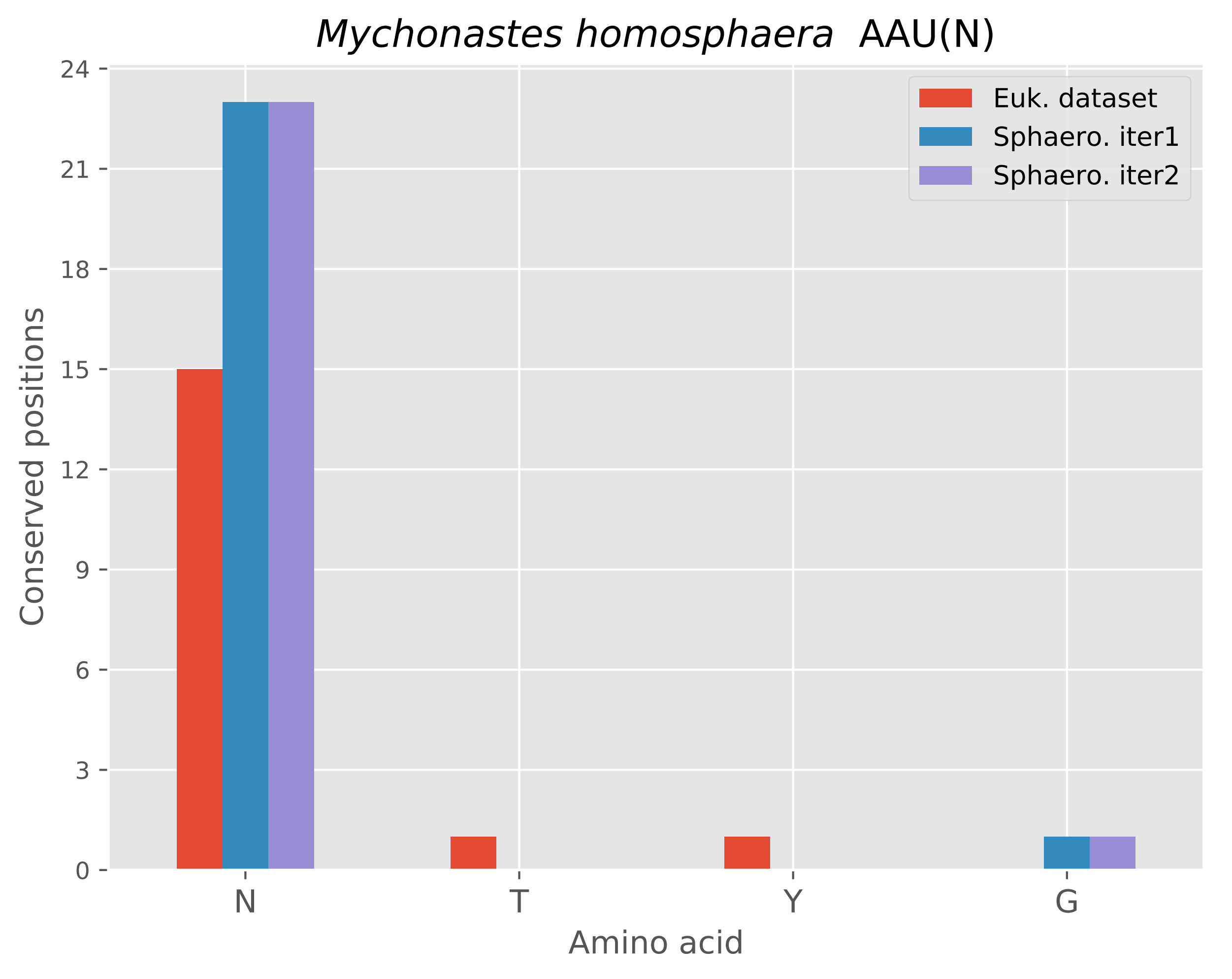

Mychonastes homosphaera AAU(N)
Euk. dataset
Sphaero. iter1
Sphaero. iter2
Conserved positions
Amino acid
0
3
6
9
12
15
18
21
24
N
T
Y
G

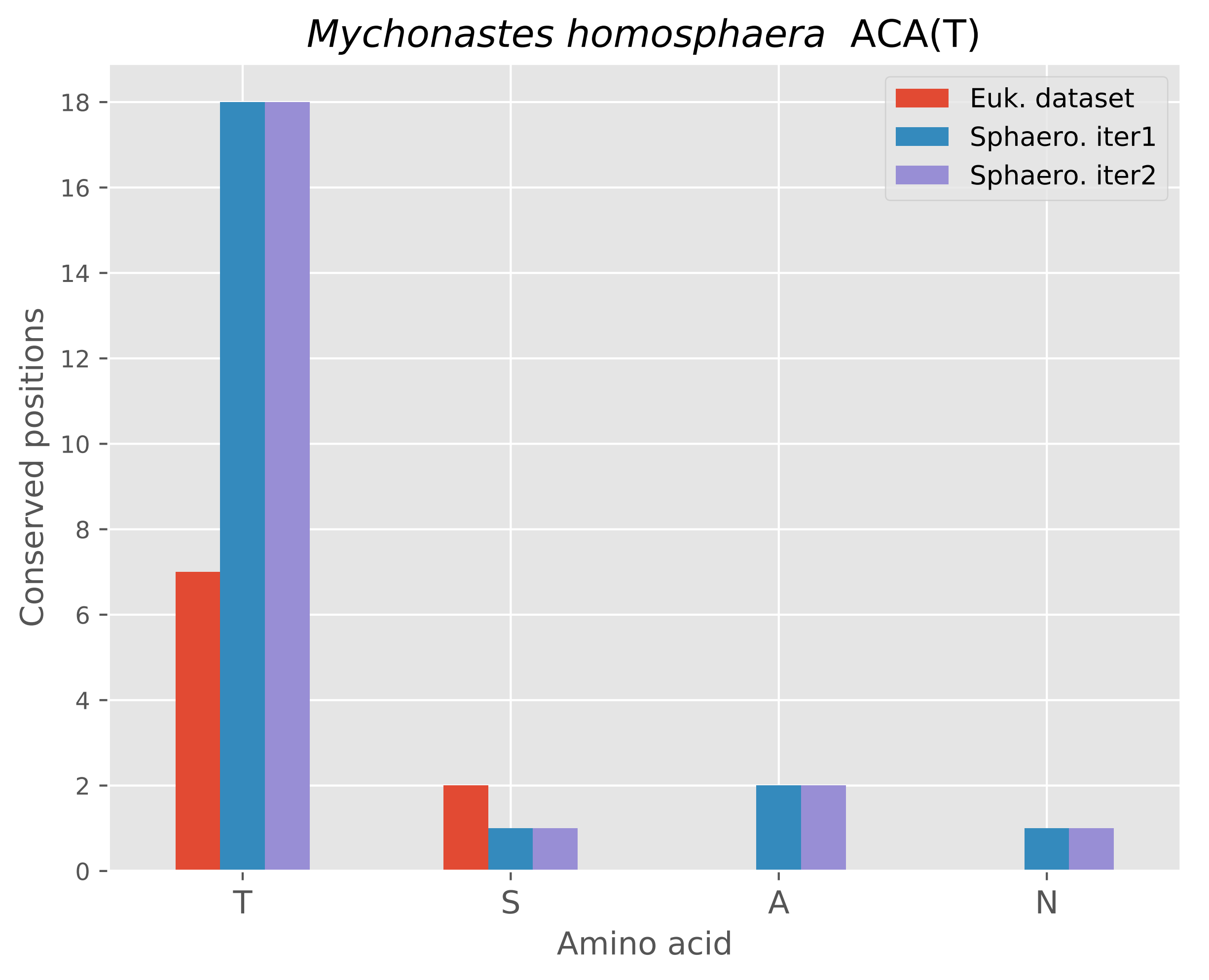

Mychonastes homosphaera ACA(T)
Euk. dataset
Sphaero. iter1
Sphaero. iter2
Conserved positions
0
2
4
6
8
10
12
14
16
18
T
S
A
N
Amino acid

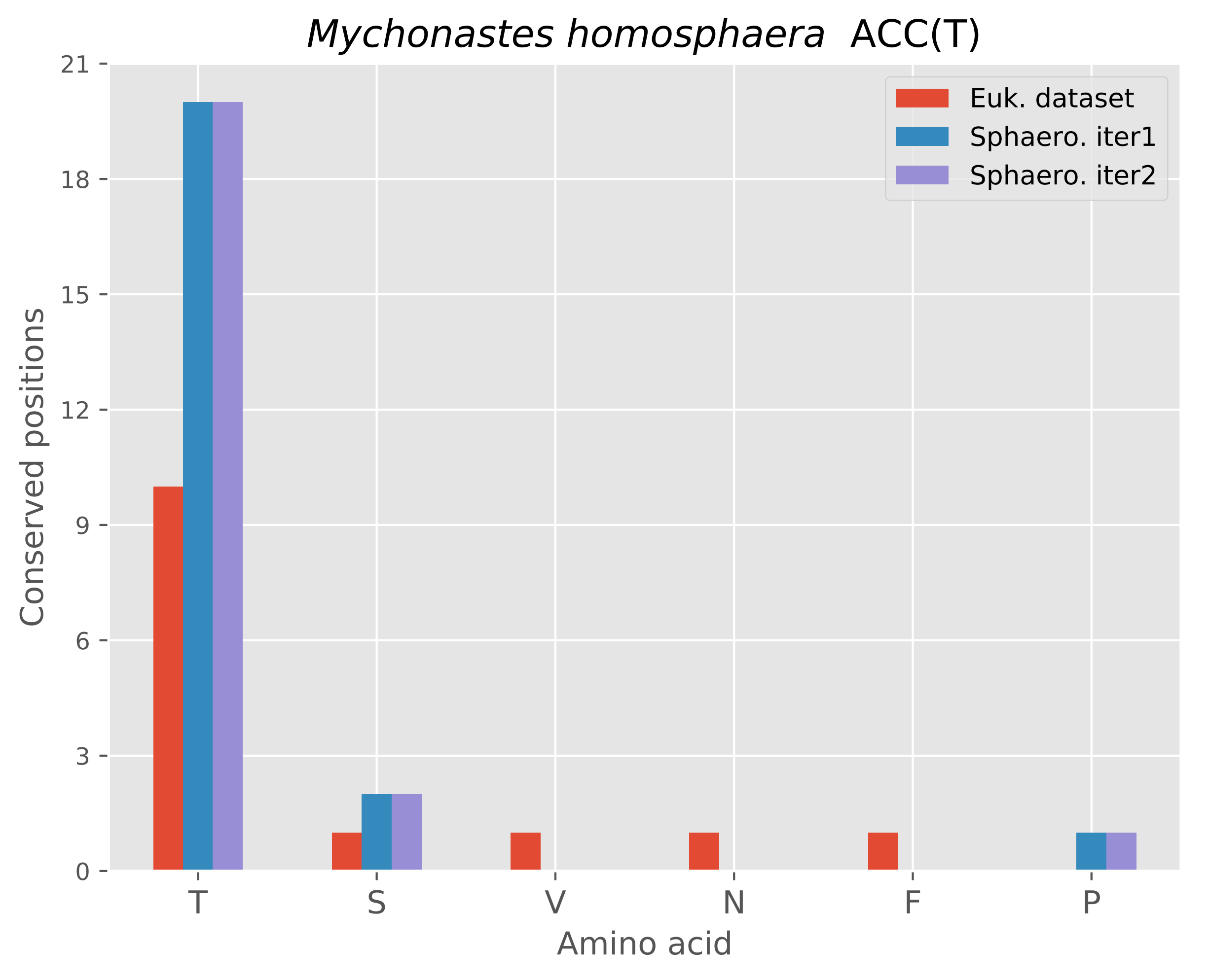

Mychonastes homosphaera ACC(T)
Euk. dataset
Sphaero. iter1
Sphaero. iter2
Conserved positions
0
3
6
9
12
15
18
21
T
S
V
N
F
P
Amino acid

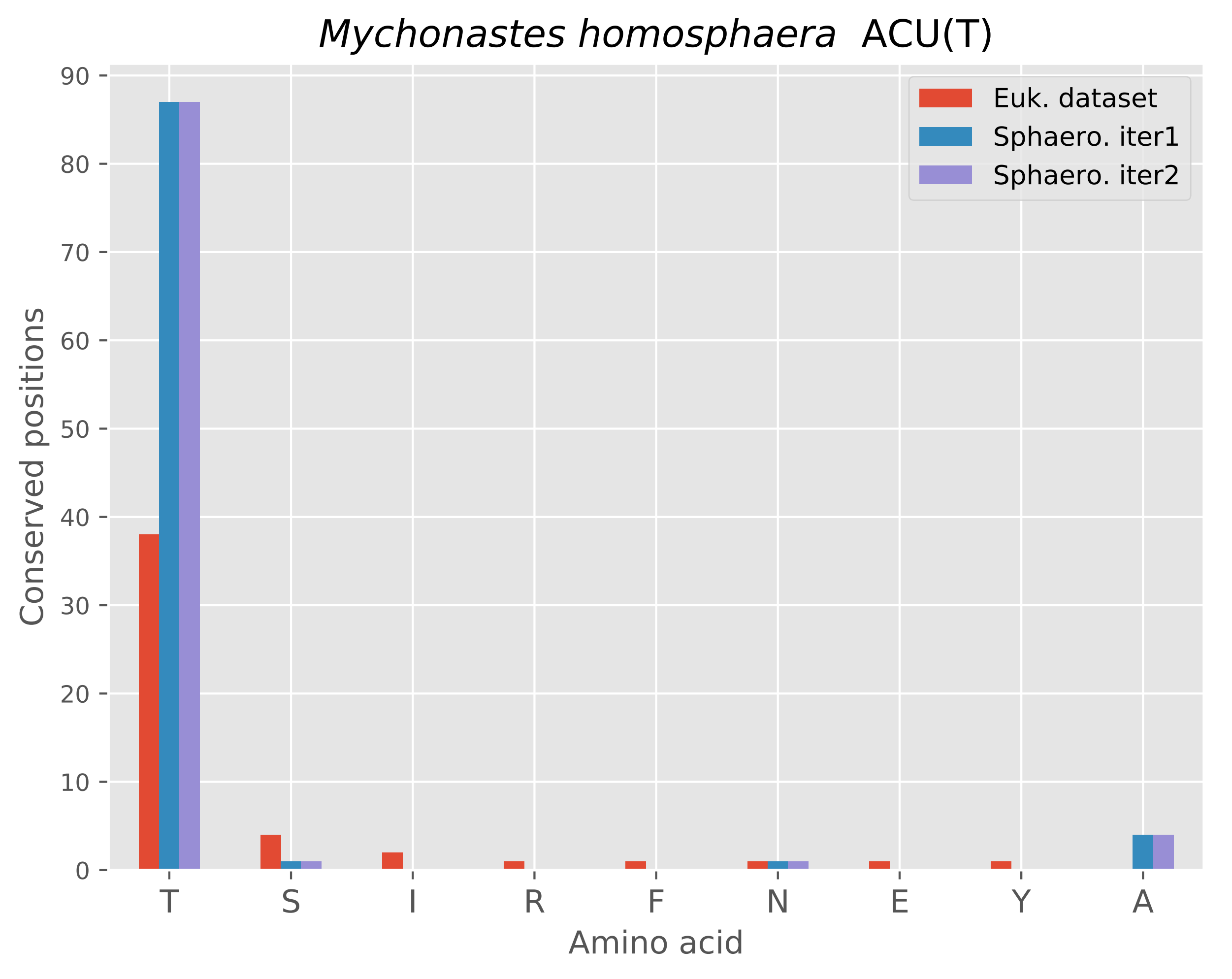

Mychonastes homosphaera ACU(T)
Conserved positions
Amino acid
Euk. dataset
Sphaero. iter1
Sphaero. iter2
0
10
20
30
40
50
60
70
80
90
T
S
I
R
F
N
E
Y
A

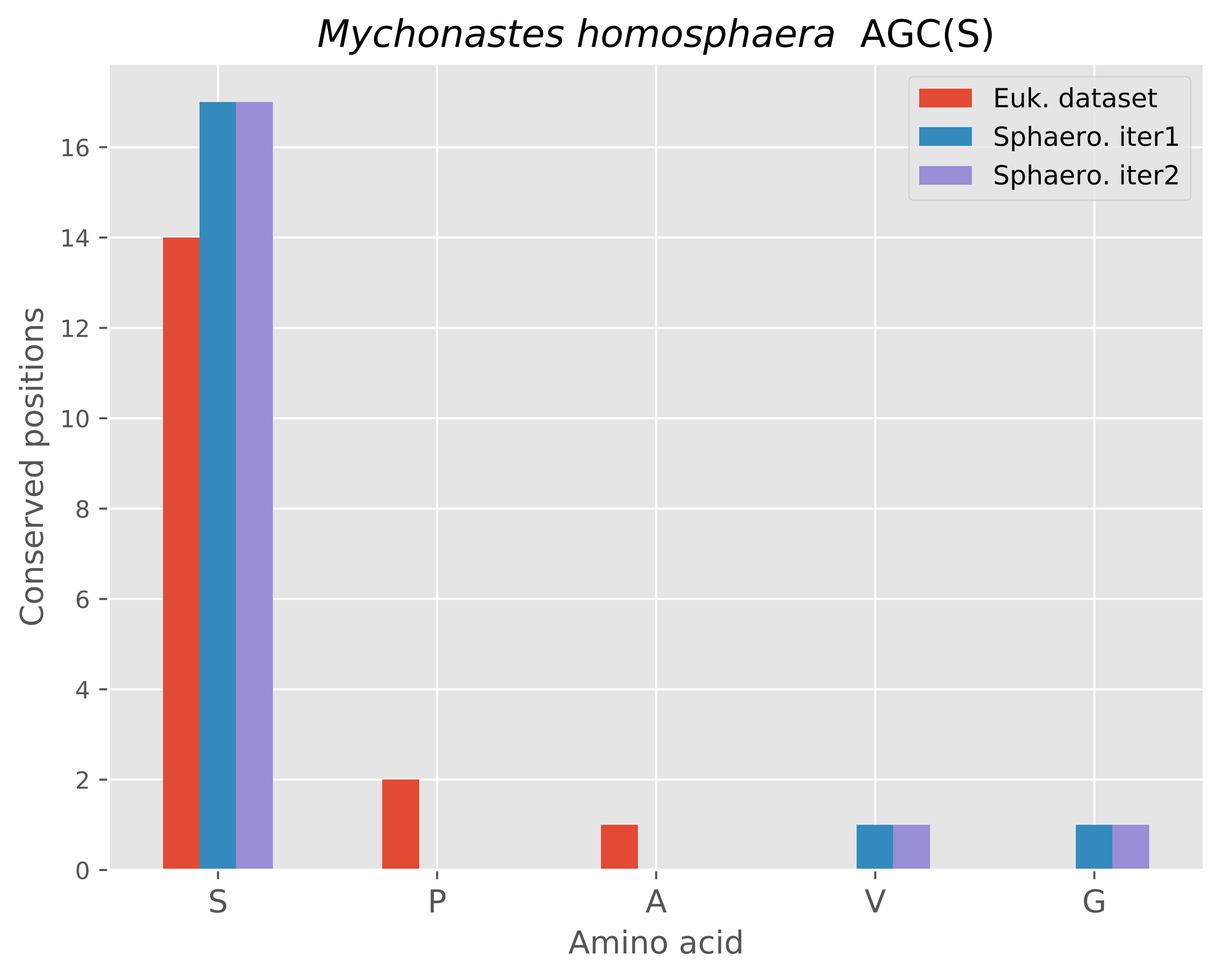

Mychonastes homosphaera AGC(S)
Euk. dataset
Sphaero. iter1
Sphaero. iter2
Conserved positions
0
2
4
6
8
10
12
14
16
S
P
A
V
G
Amino acid

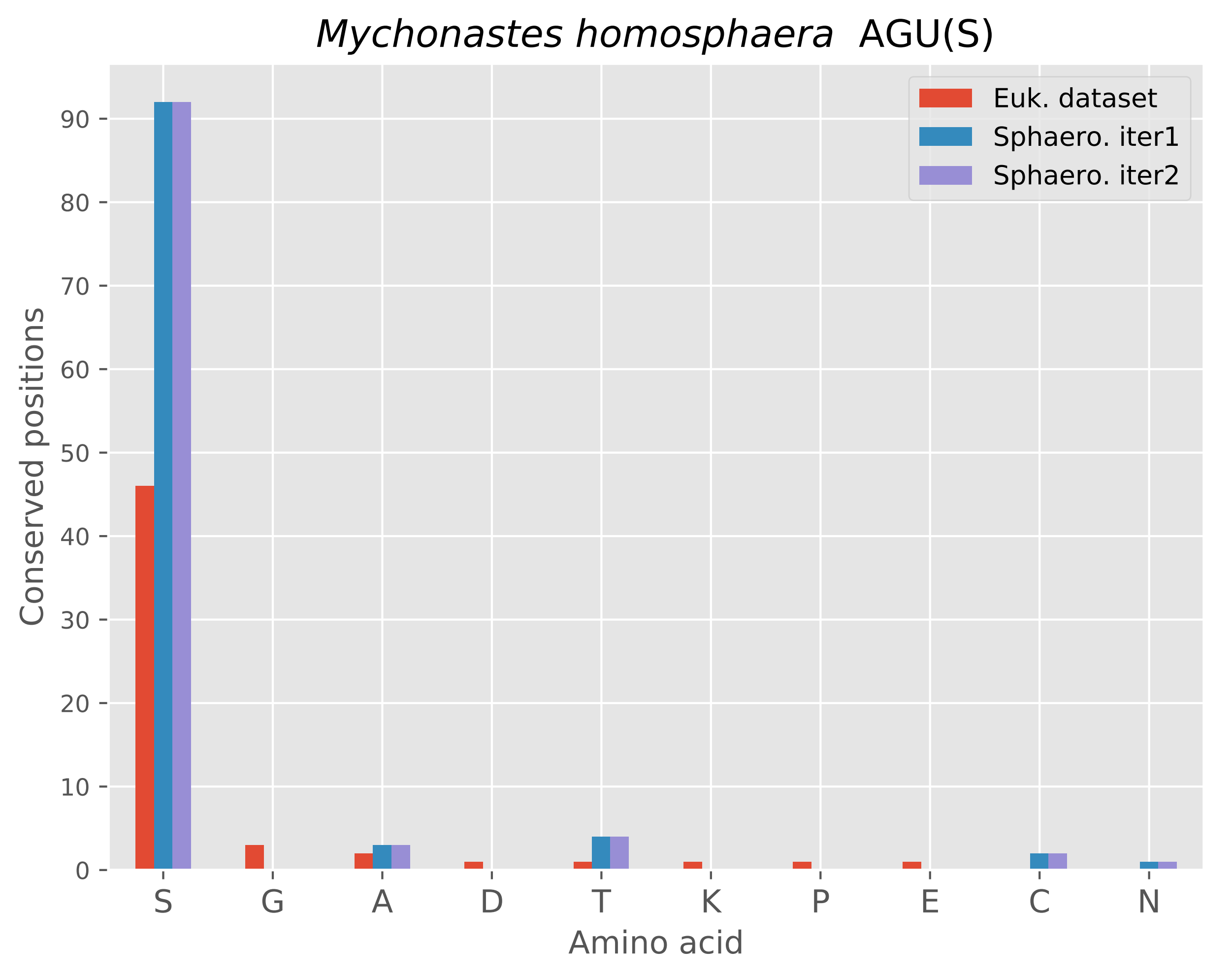

Mychonastes homosphaera AGU(S)
Euk. dataset
Sphaero. iter1
Sphaero. iter2
Conserved positions
0
10
20
30
40
50
60
70
80
90
S
G
A
D
T
K
P
E
C
N
Amino acid

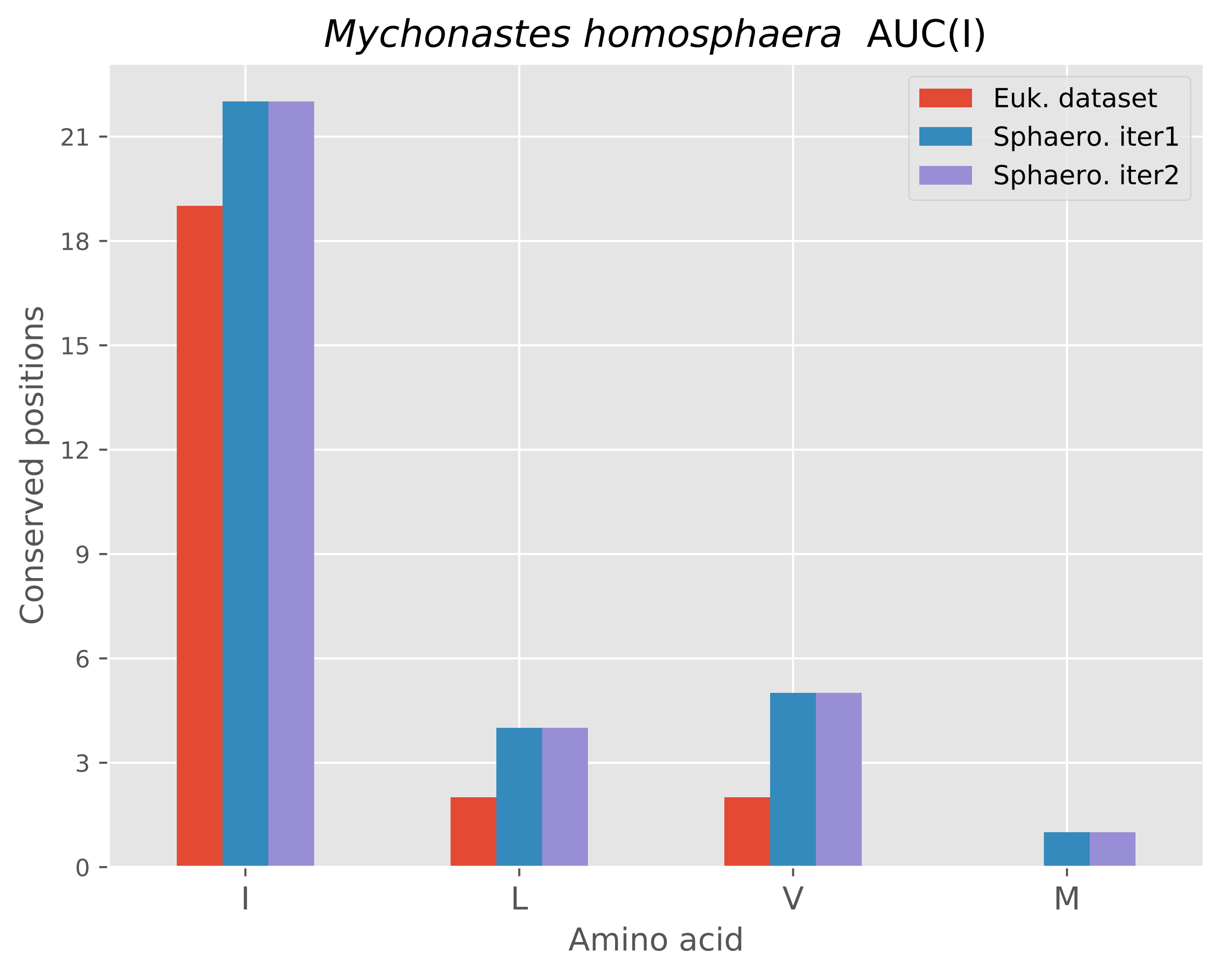

Mychonastes homosphaera AUC(I)
Euk. dataset
Sphaero. iter1
Sphaero. iter2
Conserved positions
0
3
6
9
12
15
18
21
I
L
V
M
Amino acid

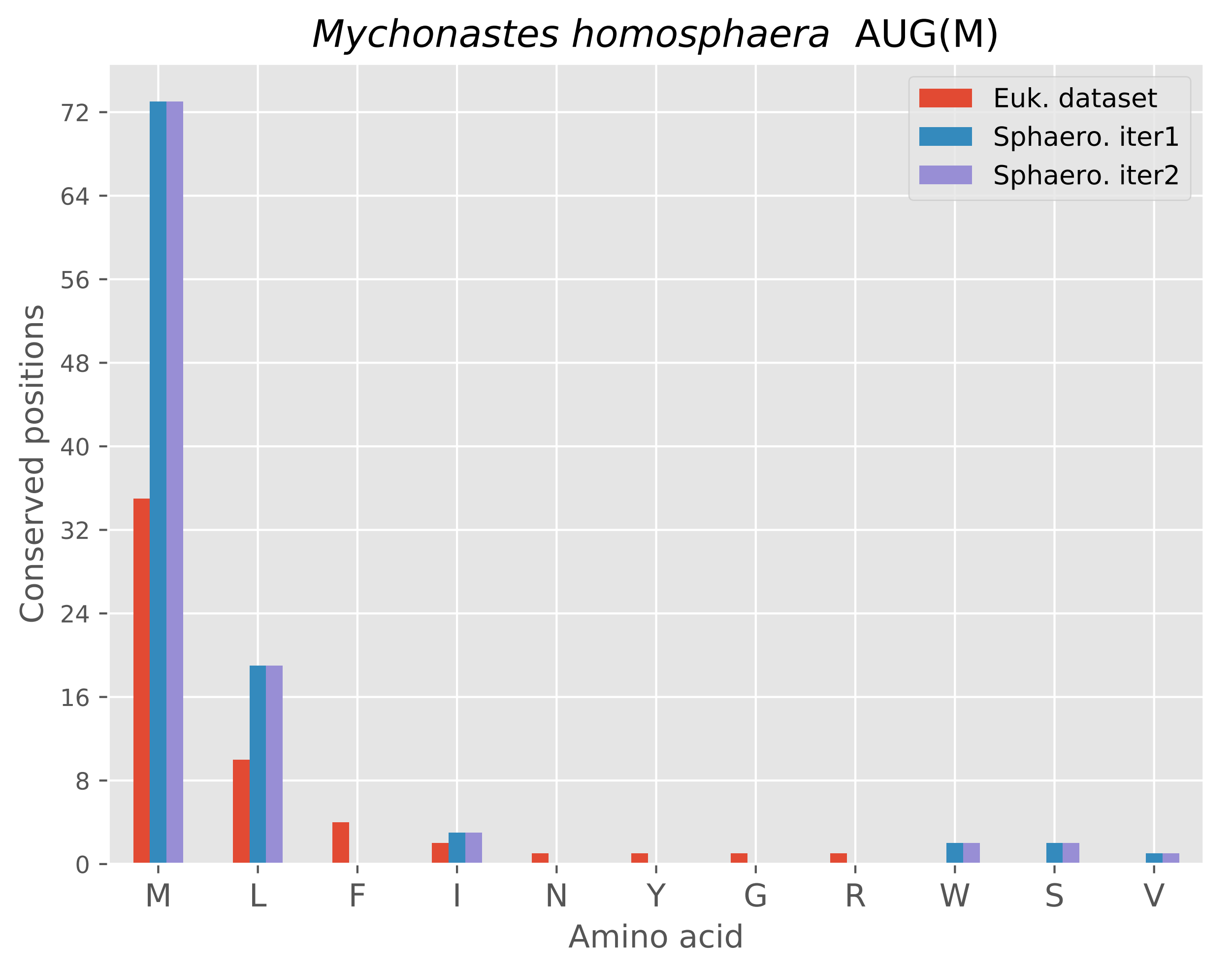
Mychonastes homosphaera AUG(M)
Euk. dataset
Sphaero. iter1
Sphaero. iter2
Conserved positions
0
8
16
24
32
40
48
56
64
72
M
L
F
I
N
Y
G
R
W
S
V
Amino acid

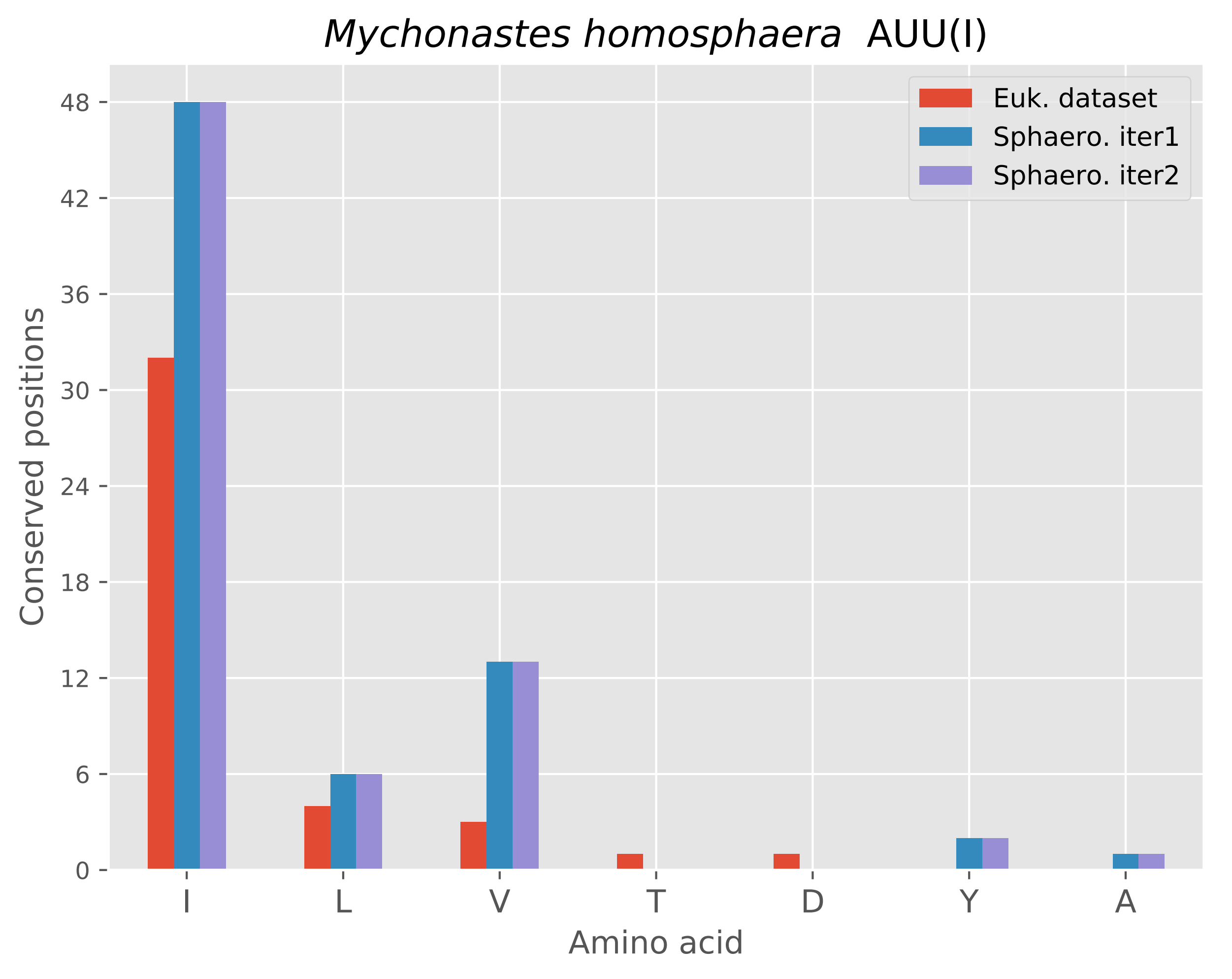

Mychonastes homosphaera AUU(I)
Euk. dataset
Sphaero. iter1
Sphaero. iter2
Conserved positions
0
6
12
18
24
30
36
42
48
I
L
V
T
D
Y
A
Amino acid

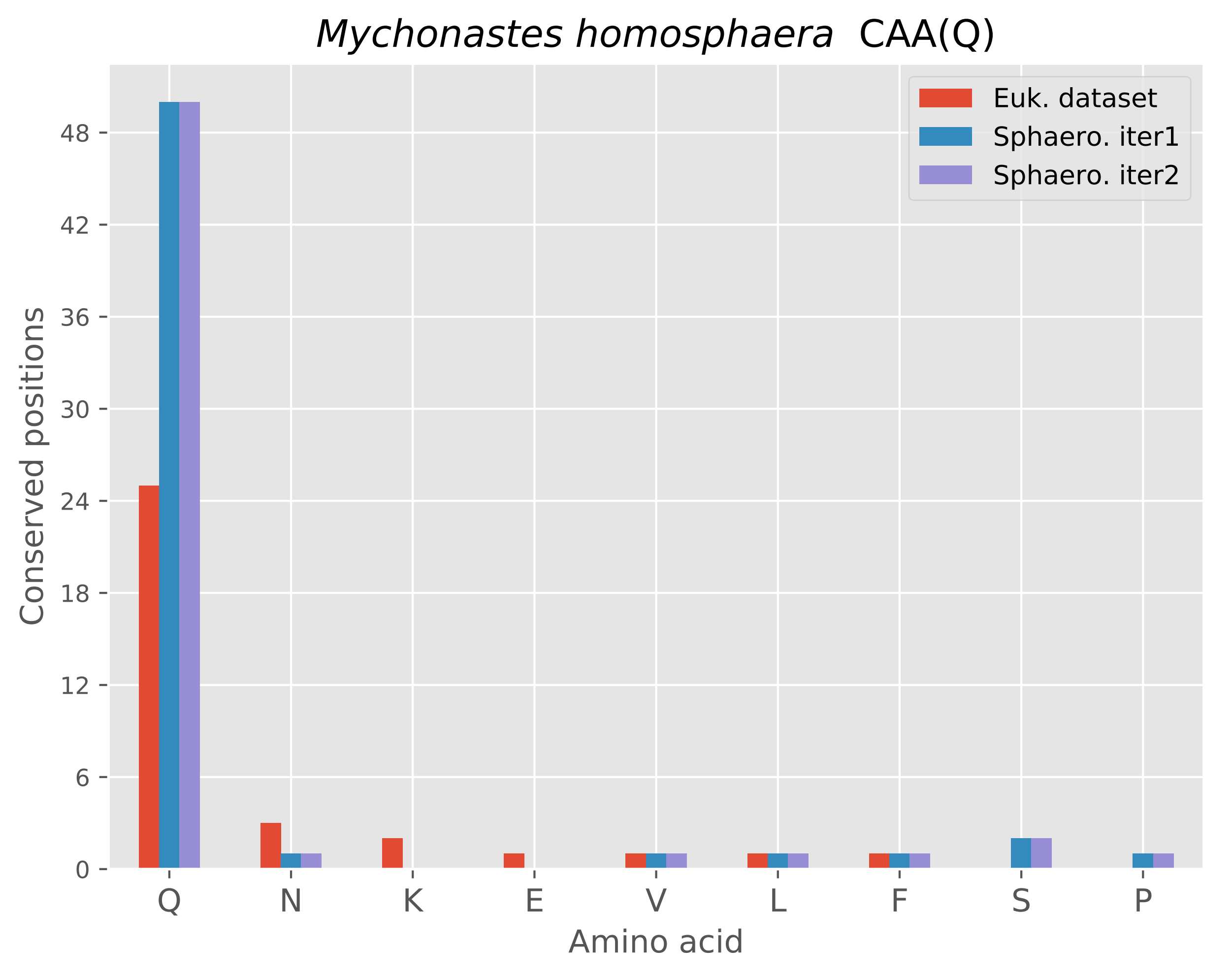

Mychonastes homosphaera CAA(Q)
Euk. dataset
Sphaero. iter1
Sphaero. iter2
Conserved positions
0
6
12
18
24
30
36
42
48
Q
N
K
E
V
L
F
S
P
Amino acid

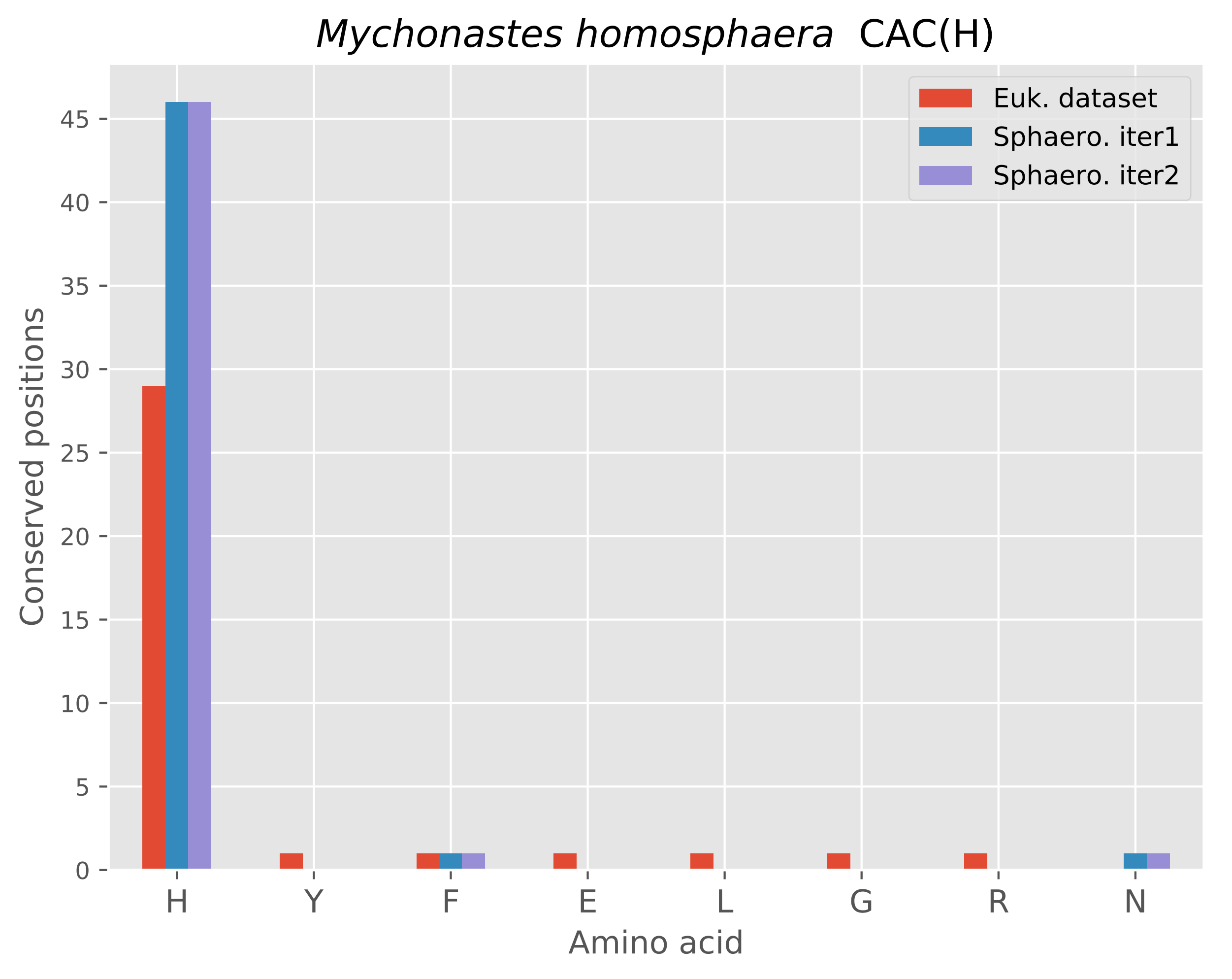

Mychonastes homosphaera CAC(H)
Euk. dataset
Sphaero. iter1
Sphaero. iter2
Conserved positions
0
5
10
15
20
25
30
35
40
45
H
Y
F
E
L
G
R
N
Amino acid

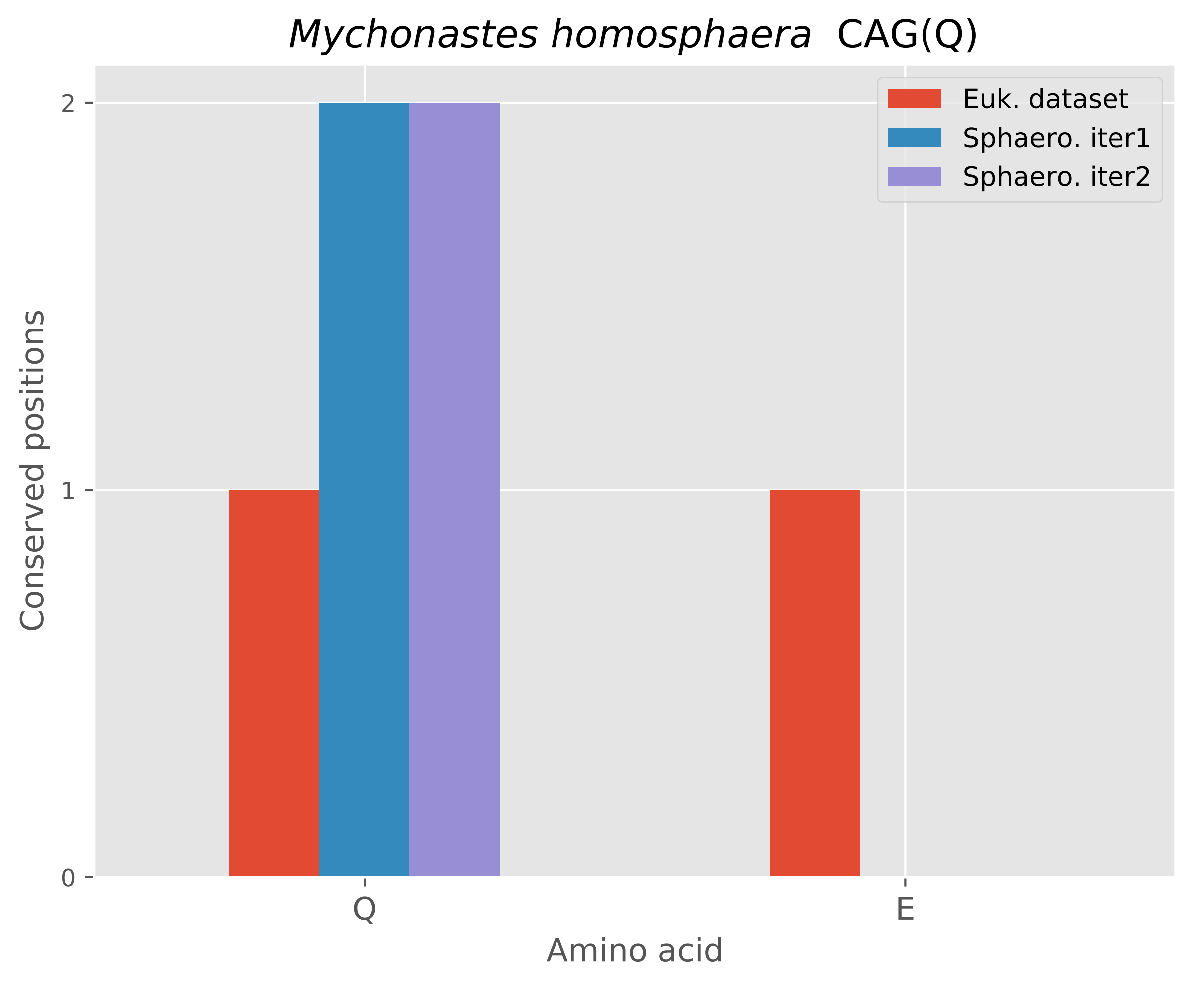

Mychonastes homosphaera CAG(Q)
Euk. dataset
Sphaero. iter1
Sphaero. iter2
Conserved positions
0
1
2
Q
E
Amino acid

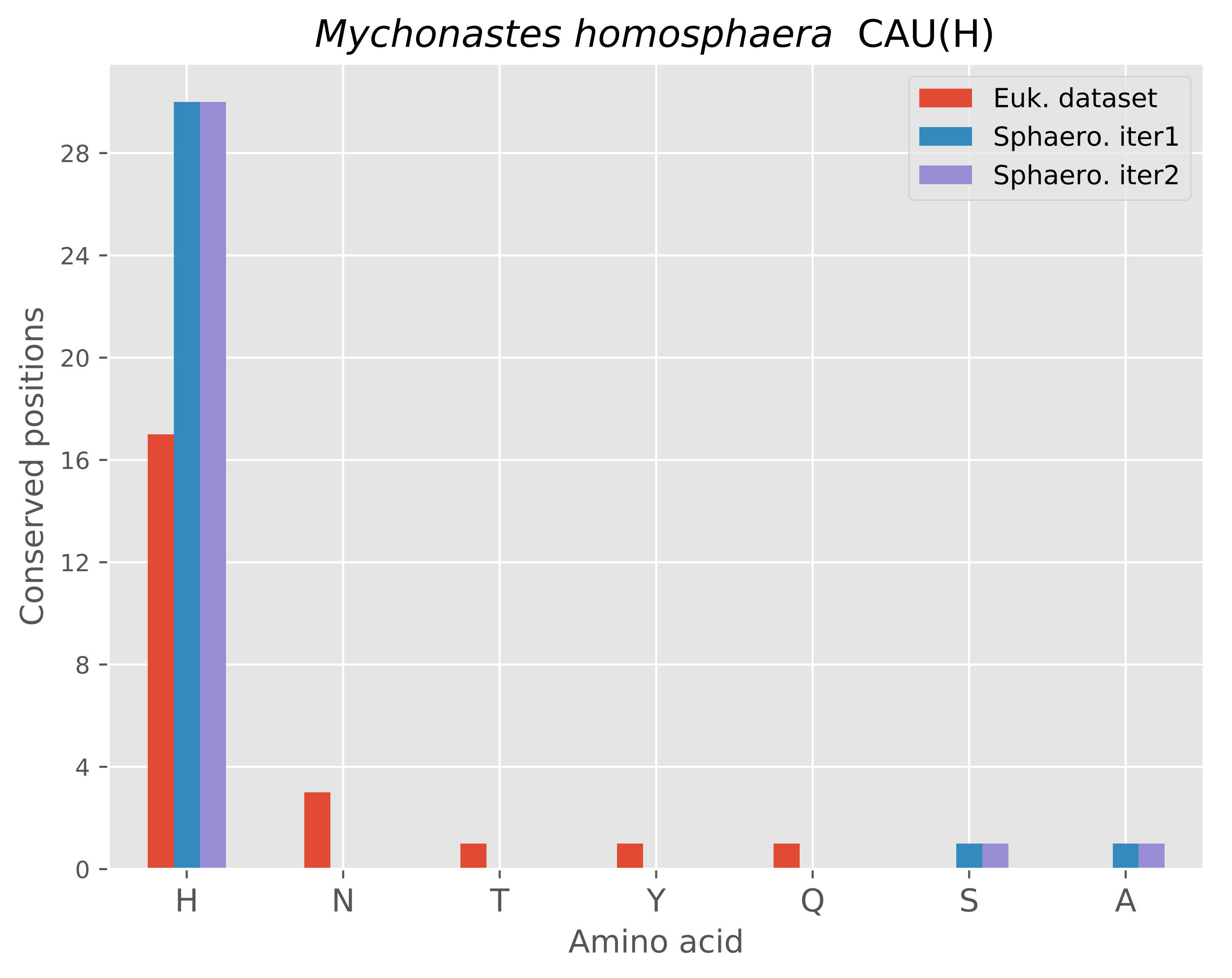

Mychonastes homosphaera CAU(H)
Euk. dataset
Sphaero. iter1
Sphaero. iter2
Conserved positions
0
4
8
12
16
20
24
28
H
N
T
Y
Q
S
A
Amino acid

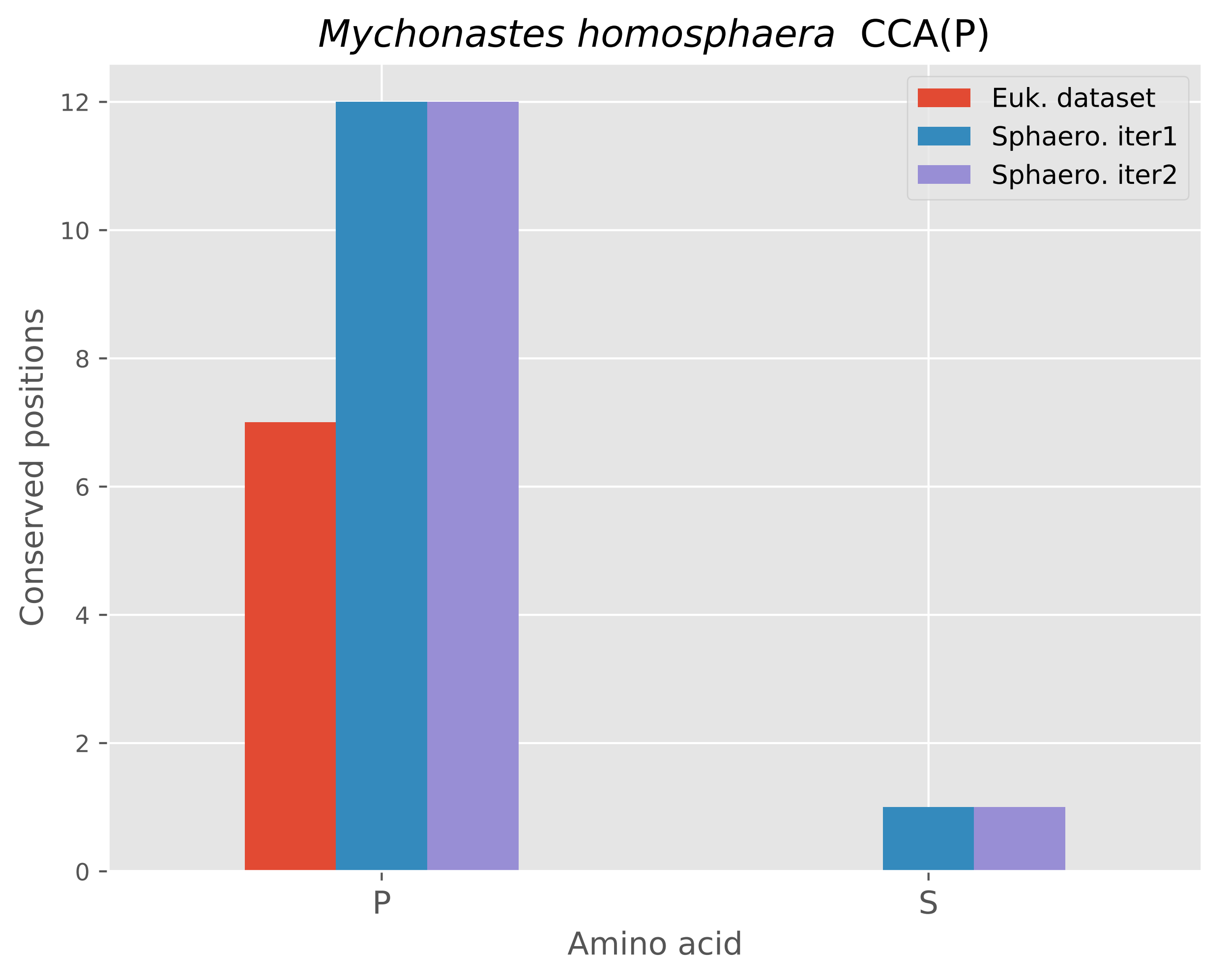

Mychonastes homosphaera CCA(P)
Euk. dataset
Sphaero. iter1
Sphaero. iter2
Conserved positions
0
2
4
6
8
10
12
P
S
Amino acid

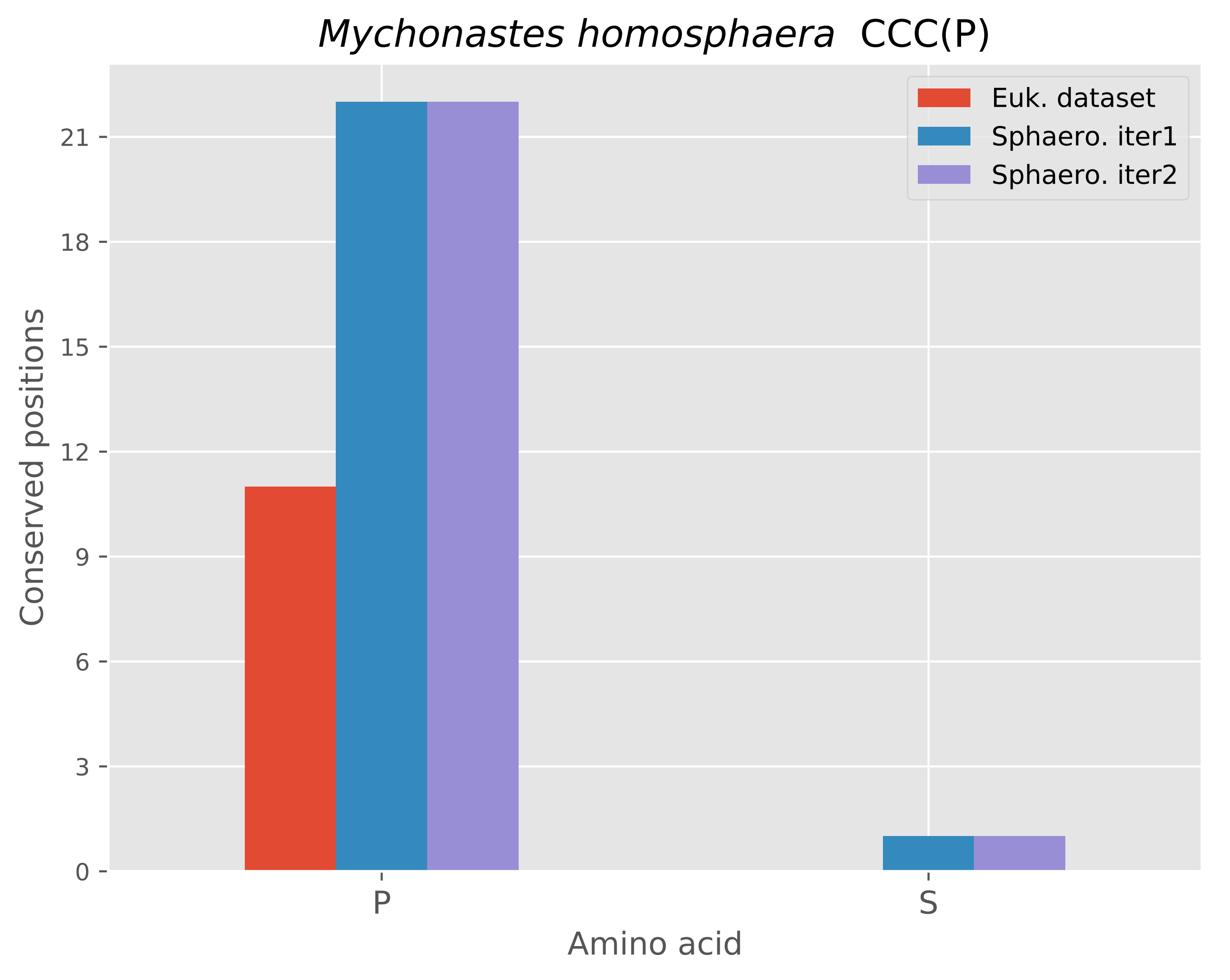

Mychonastes homosphaera CCC(P)
Euk. dataset
Sphaero. iter1
Sphaero. iter2
Conserved positions
0
3
6
9
12
15
18
21
P
S
Amino acid

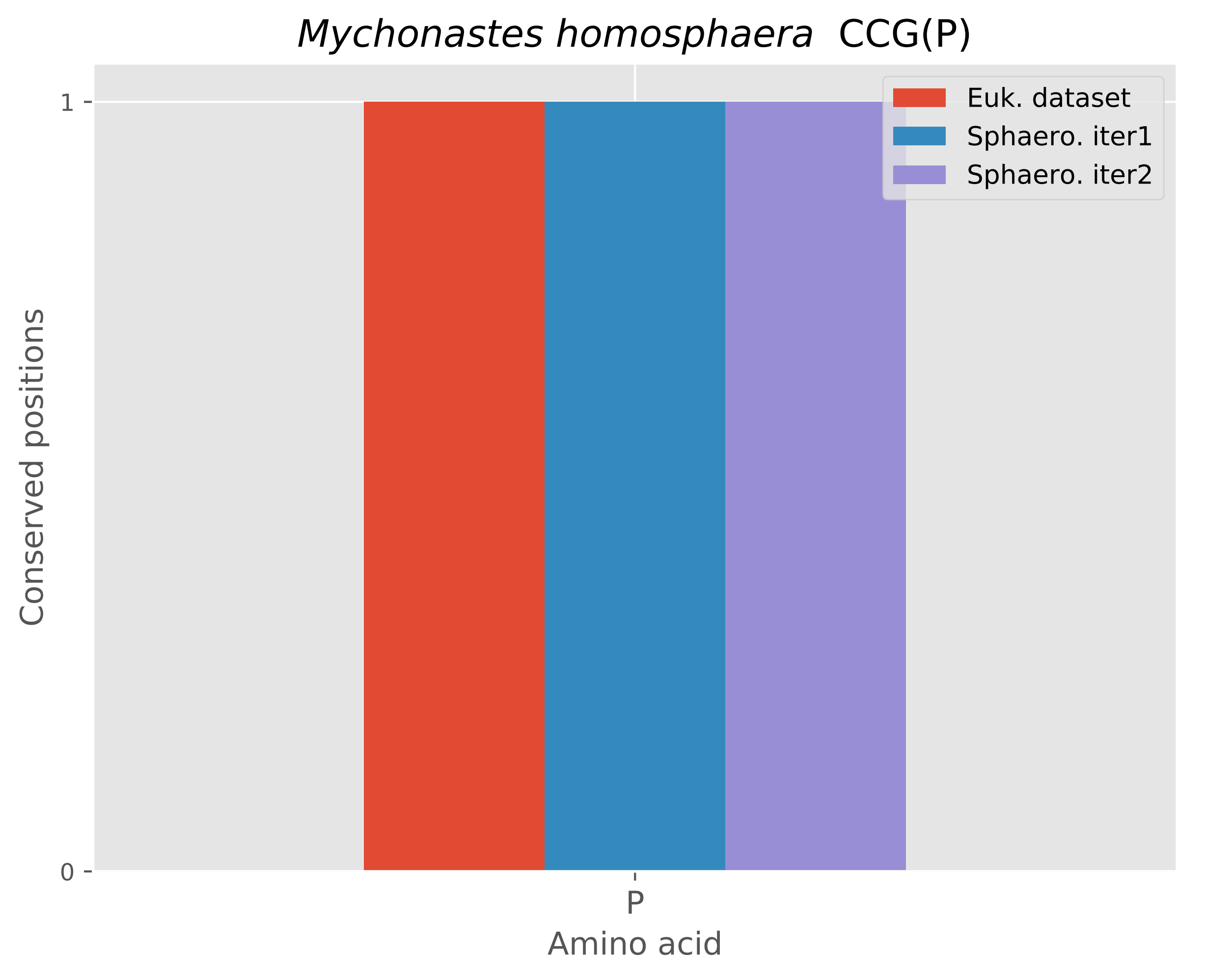

Mychonastes homosphaera CCG(P)
Euk. dataset
Sphaero. iter1
Sphaero. iter2
1
0
Conserved positions
P
Amino acid

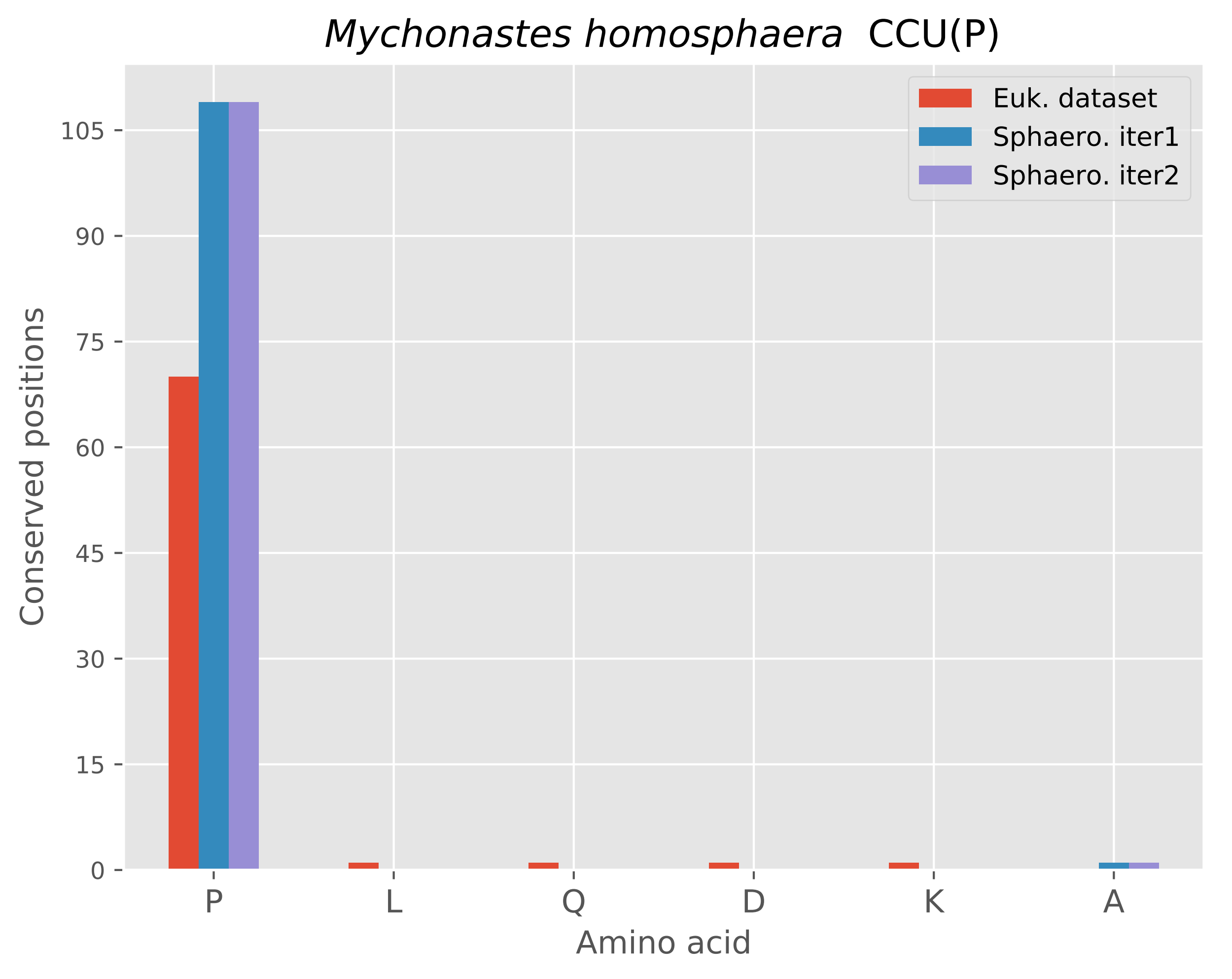

Mychonastes homosphaera CCU(P)
Euk. dataset
Sphaero. iter1
Sphaero. iter2
Conserved positions
0
15
30
45
60
75
90
105
P
L
Q
D
K
A
Amino acid

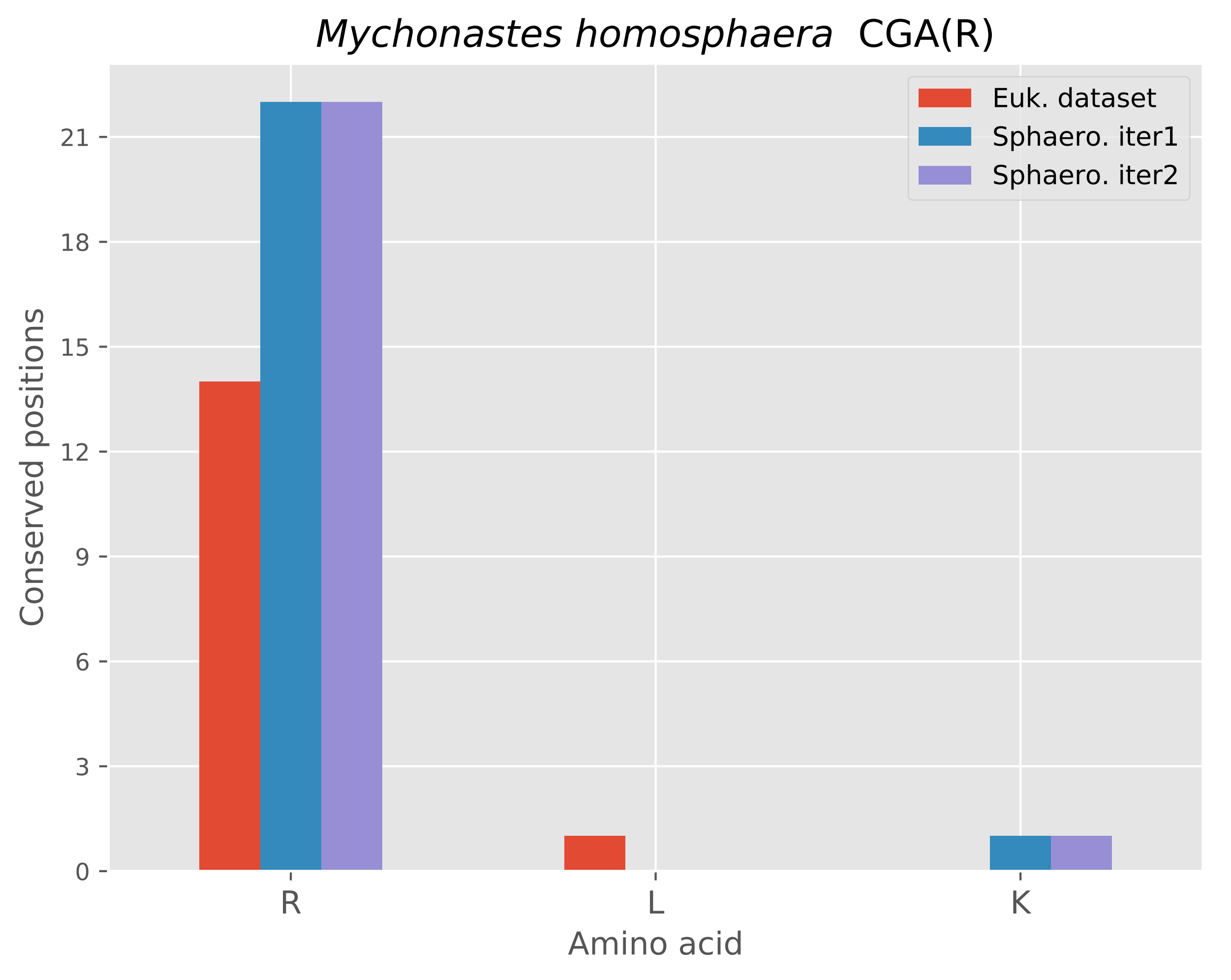

Mychonastes homosphaera CGA(R)
Euk. dataset
Sphaero. iter1
Sphaero. iter2
Conserved positions
0
3
6
9
12
15
18
21
R
L
K
Amino acid

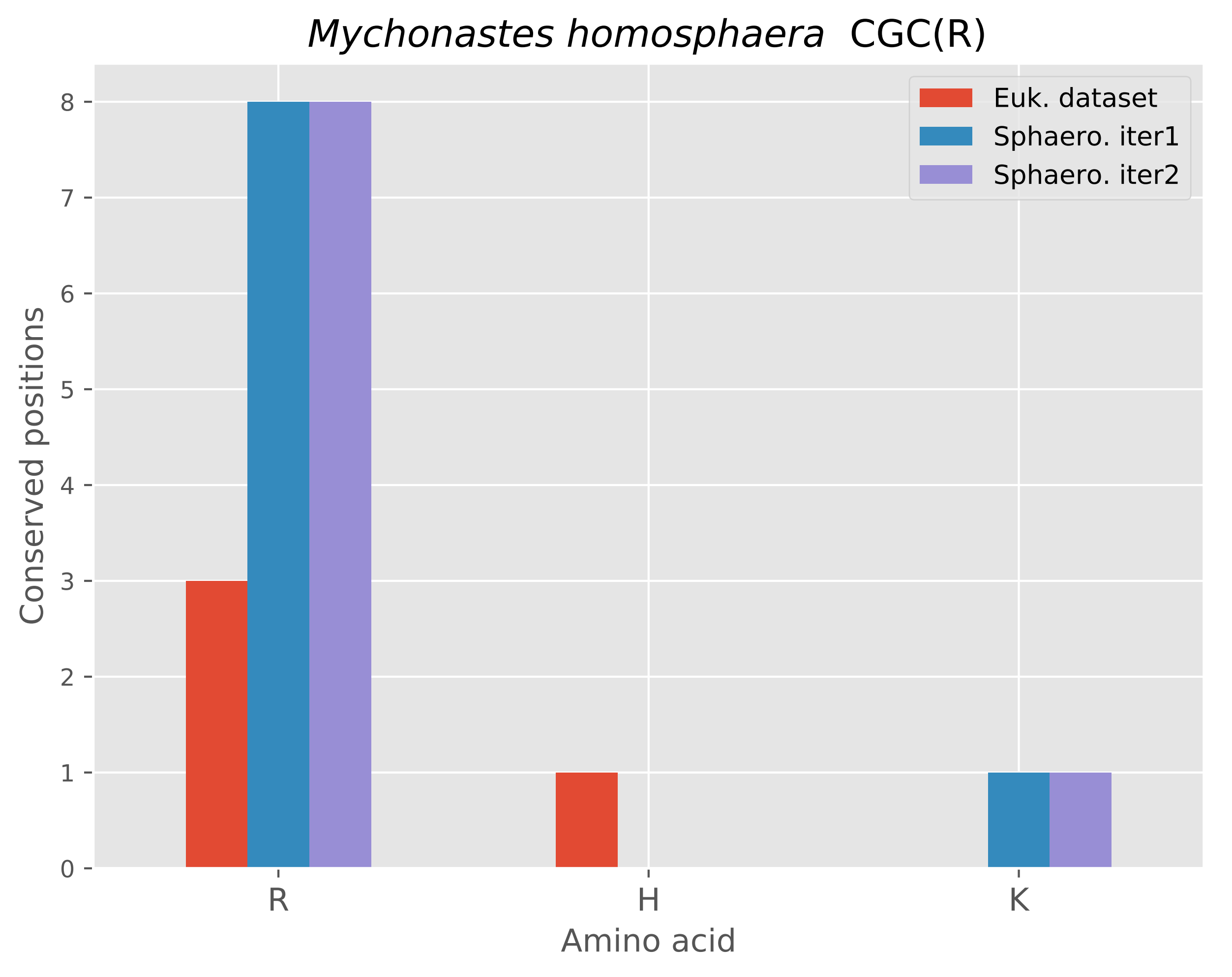

Mychonastes homosphaera CGC(R)
Euk. dataset
Sphaero. iter1
Sphaero. iter2
Conserved positions
0
1
2
3
4
5
6
7
8
R
H
K
Amino acid

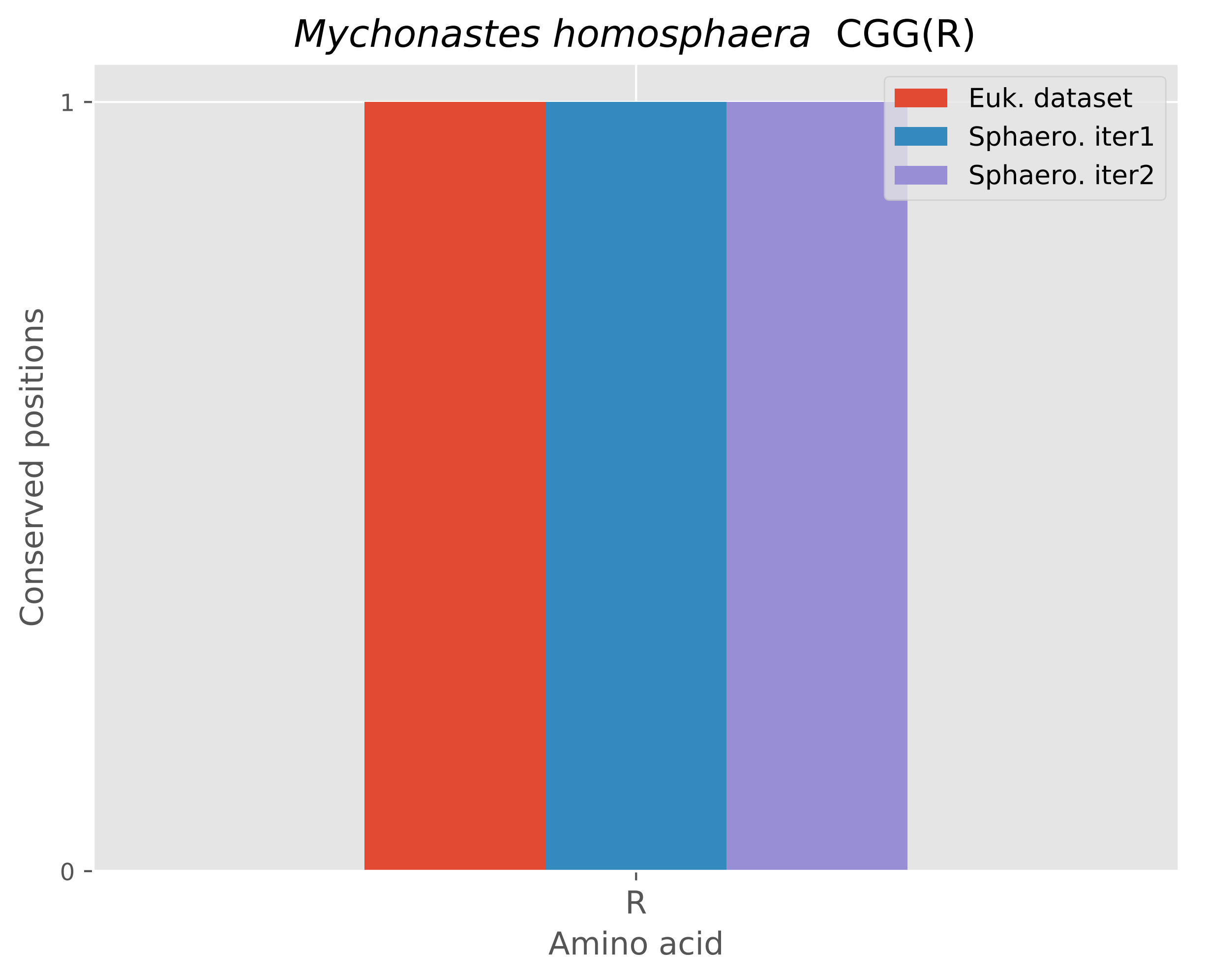

Mychonastes homosphaera CGG(R)
Euk. dataset
Sphaero. iter1
Sphaero. iter2
1
0
Conserved positions
R
Amino acid

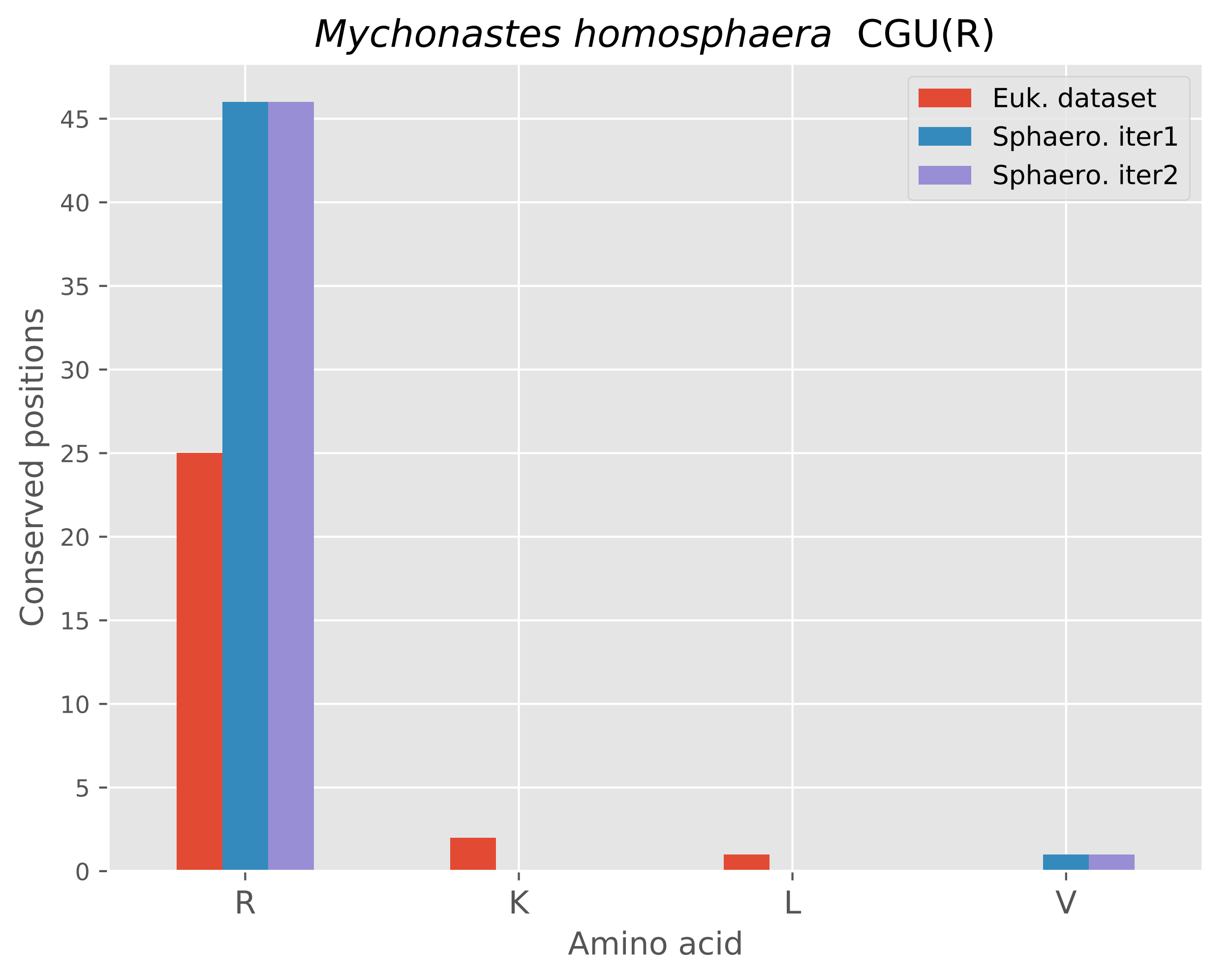
Mychonastes homosphaera CGU(R)
Euk. dataset
Sphaero. iter1
Sphaero. iter2
Conserved positions
0
5
10
15
20
25
30
35
40
45
R
K
L
V
Amino acid

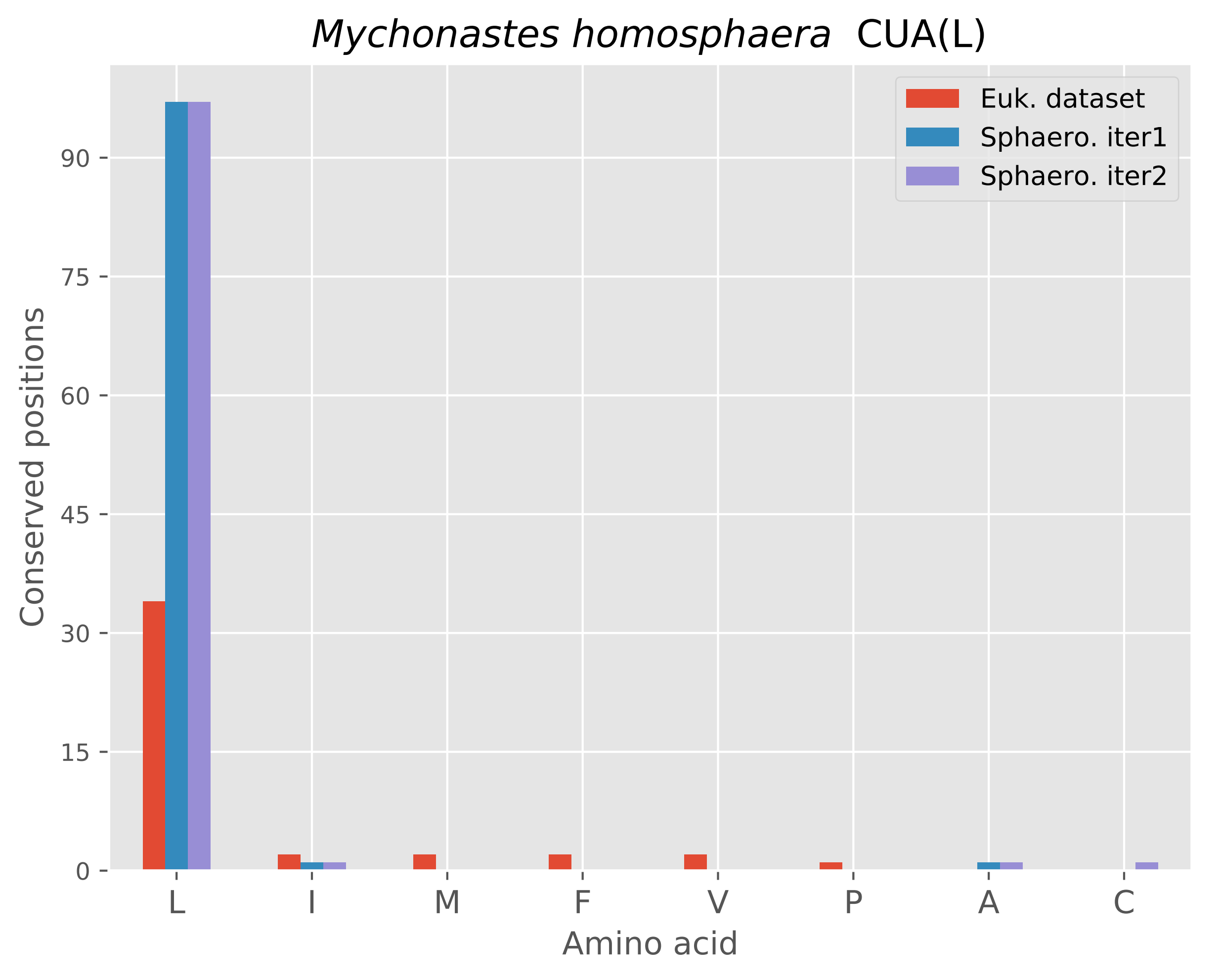

Mychonastes homosphaera CUA(L)
Euk. dataset
Sphaero. iter1
Sphaero. iter2
Conserved positions
0
15
30
45
60
75
90
L
I
M
F
V
P
A
C
Amino acid

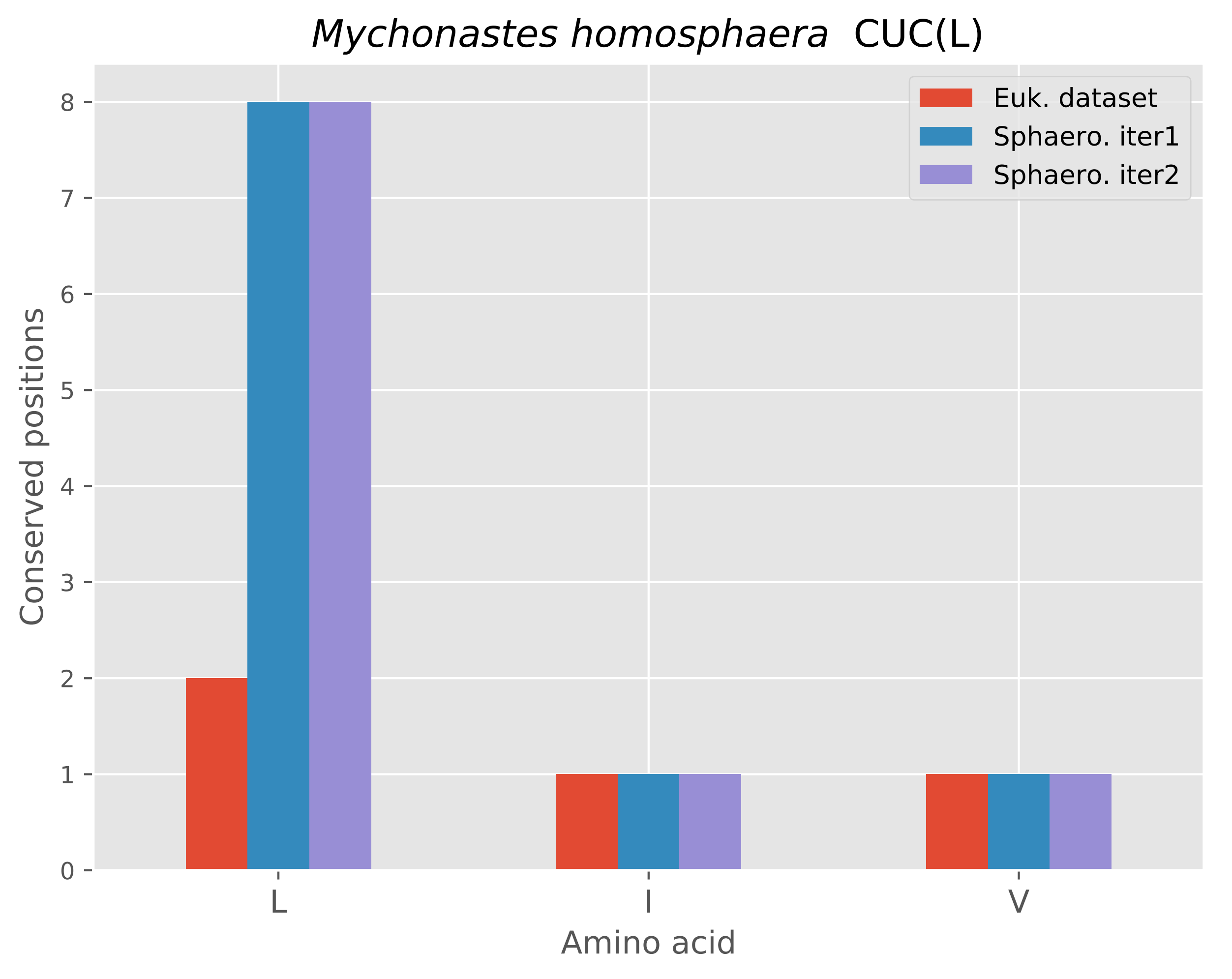

Mychonastes homosphaera CUC(L)
Euk. dataset
Sphaero. iter1
Sphaero. iter2
Conserved positions
0
1
2
3
4
5
6
7
8
L
I
V
Amino acid

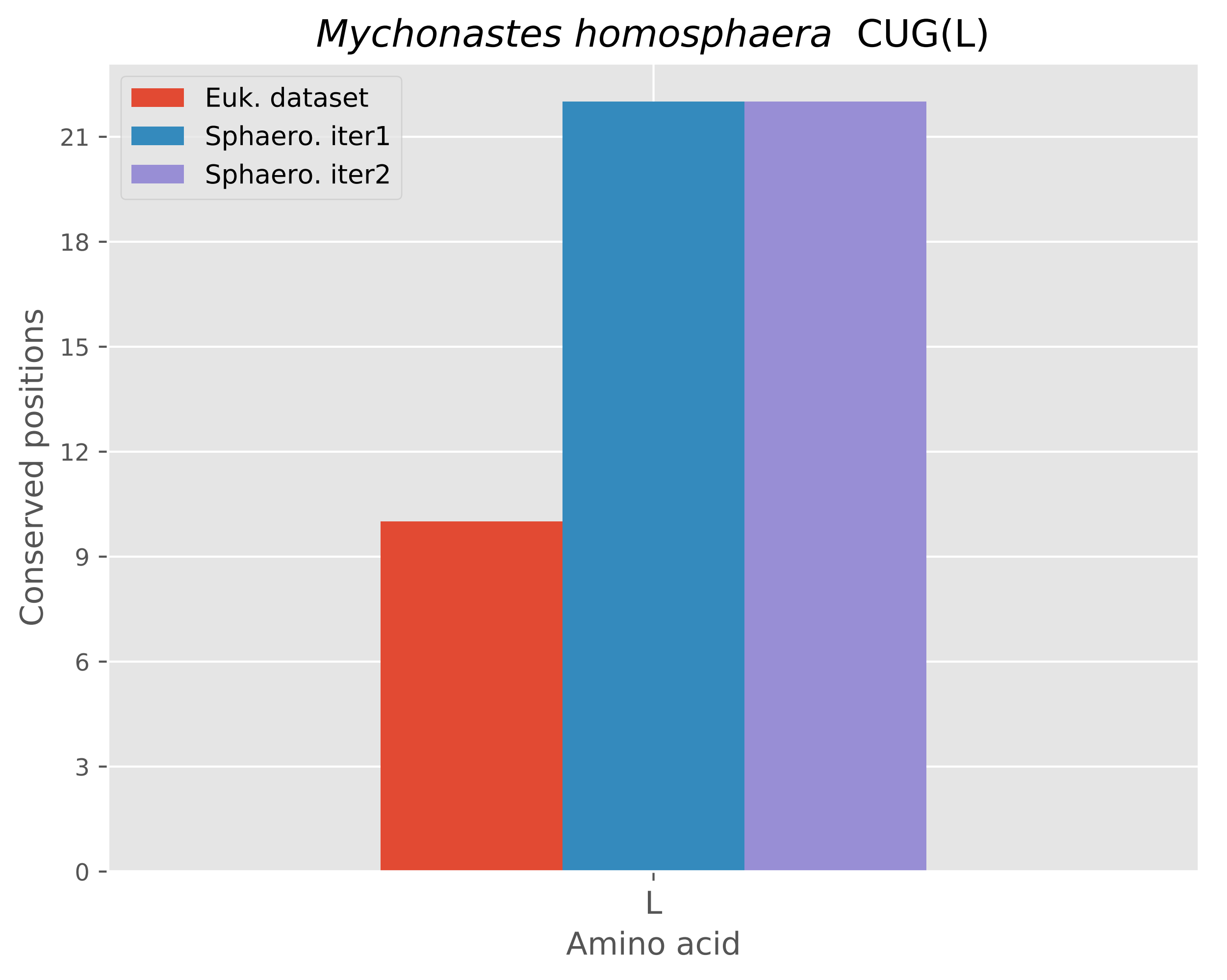
Mychonastes homosphaera CUG(L)
Euk. dataset
Sphaero. iter1
Sphaero. iter2
Conserved positions
0
3
6
9
12
15
18
21
L
Amino acid

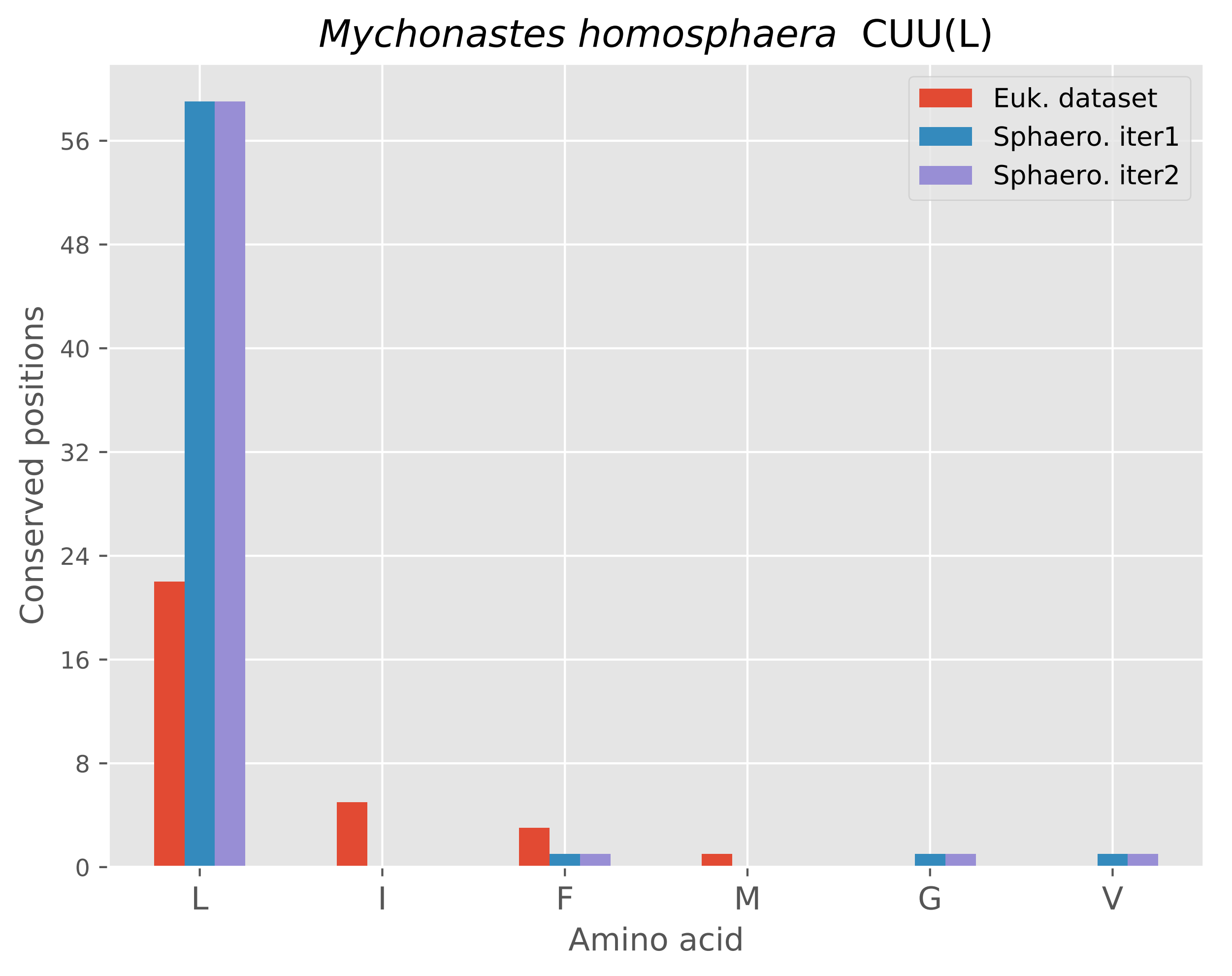

Mychonastes homosphaera CUU(L)
Euk. dataset
Sphaero. iter1
Sphaero. iter2
Conserved positions
0
8
16
24
32
40
48
56
L
I
F
M
G
V
Amino acid

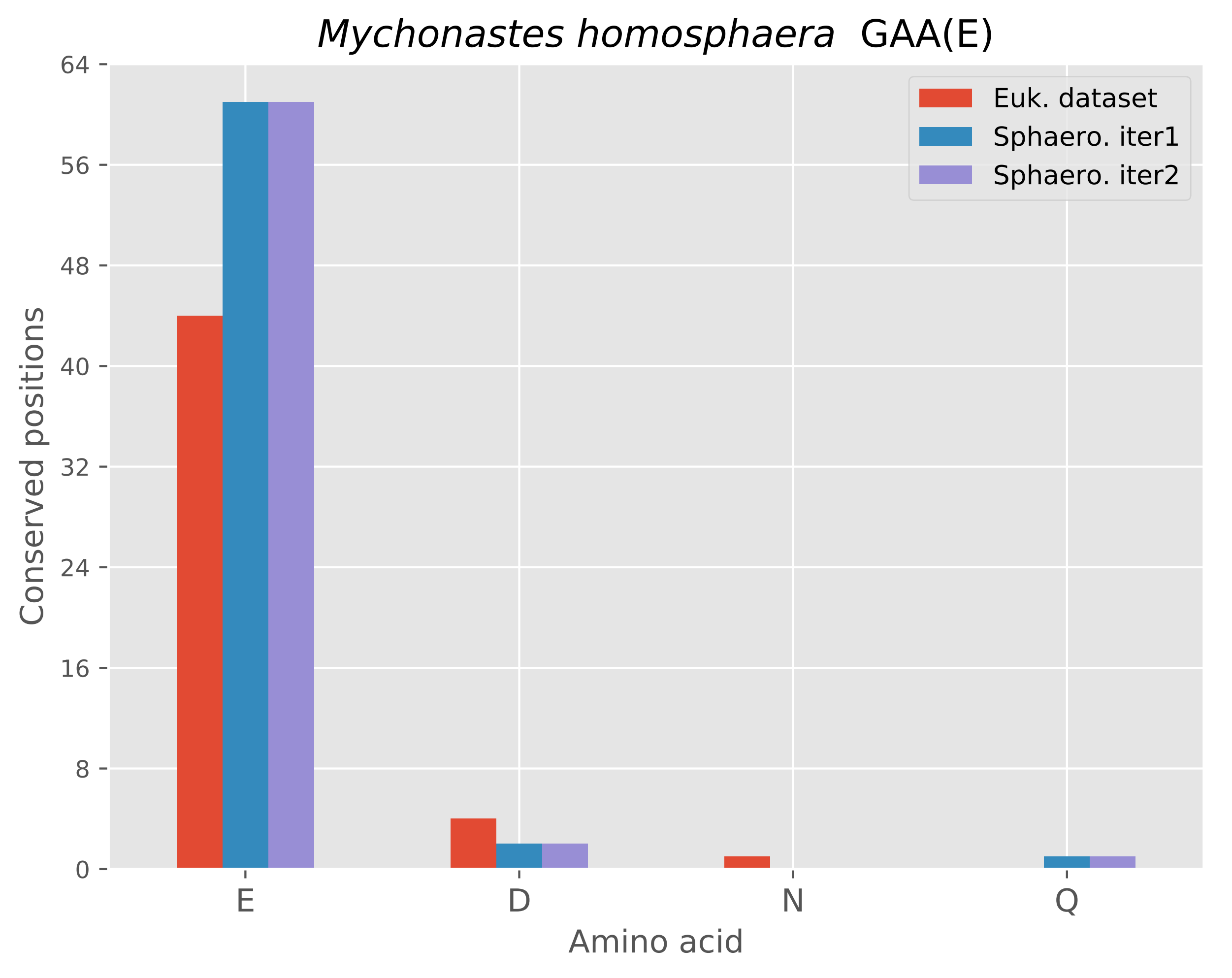

Mychonastes homosphaera GAA(E)
Conserved positions
0
8
16
24
32
40
48
56
64
E
D
N
Q
Amino acid
Euk. dataset
Sphaero. iter1
Sphaero. iter2

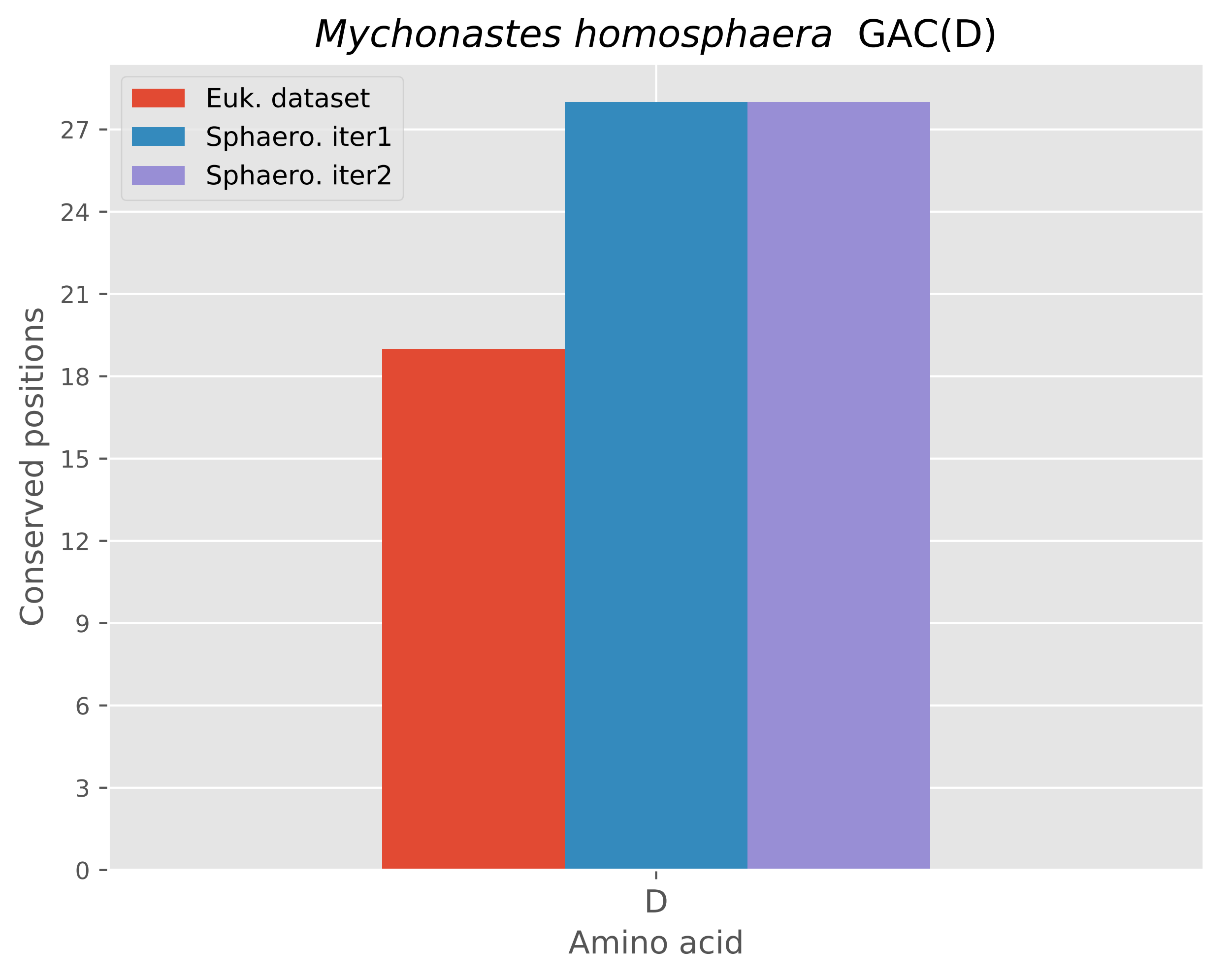

Mychonastes homosphaera GAC(D)
Euk. dataset
Sphaero. iter1
Sphaero. iter2
Conserved positions
0
3
6
9
12
15
18
21
24
27
D
Amino acid

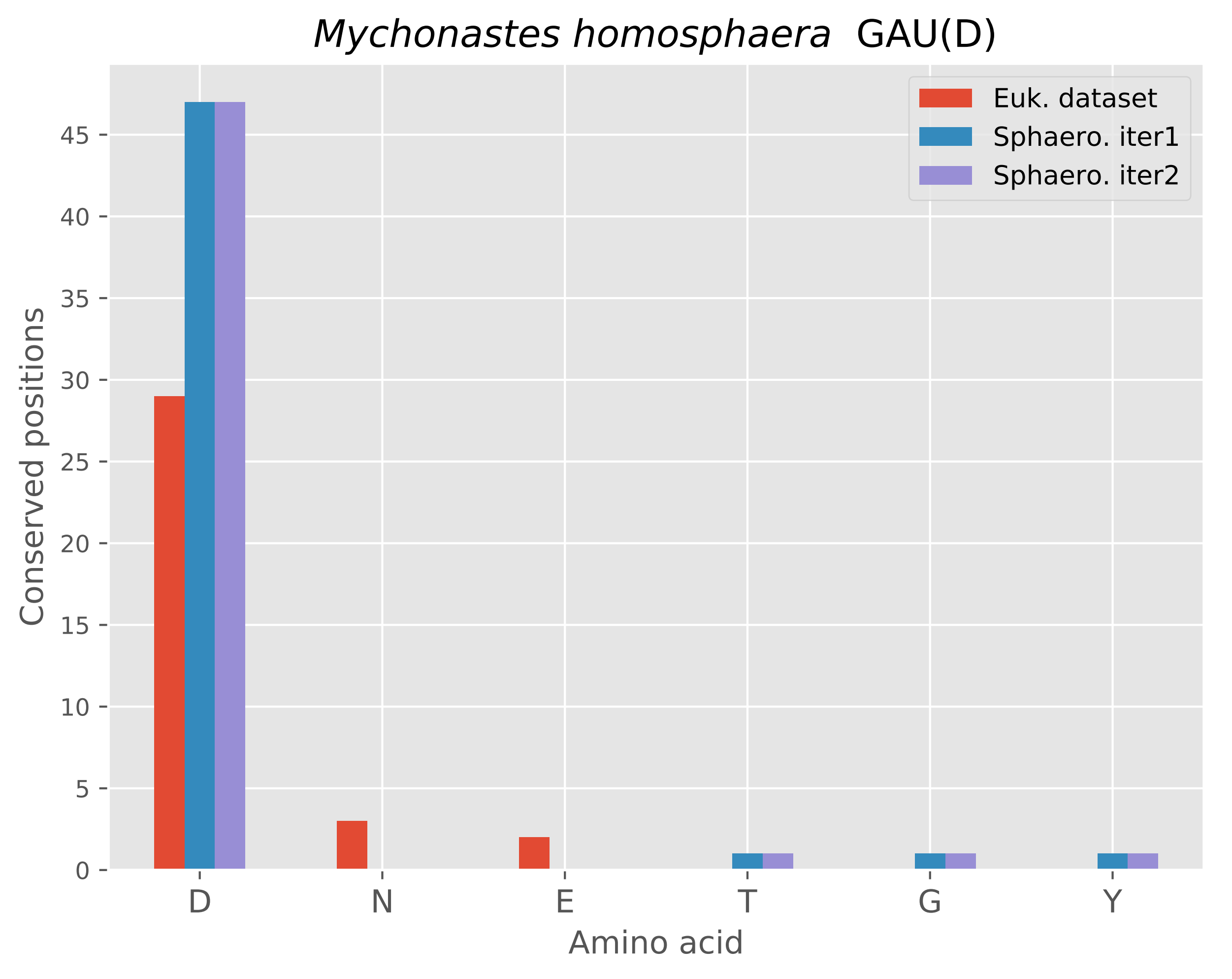

Mychonastes homosphaera GAU(D)
Euk. dataset
Sphaero. iter1
Sphaero. iter2
Conserved positions
0
5
10
15
20
25
30
35
40
45
D
N
E
T
G
Y
Amino acid

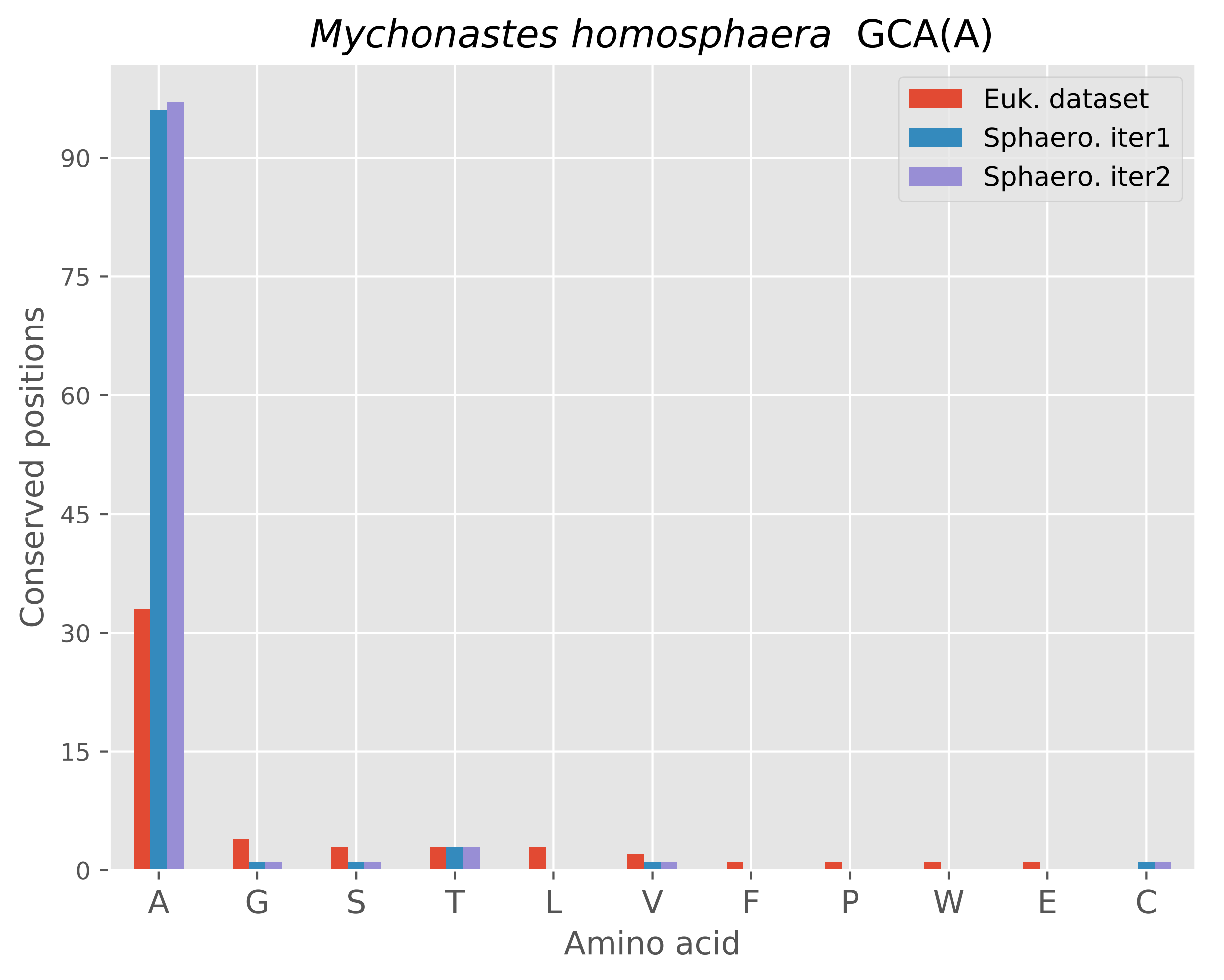

Mychonastes homosphaera GCA(A)
Euk. dataset
Sphaero. iter1
Sphaero. iter2
Conserved positions
0
15
30
45
60
75
90
A
G
S
T
L
V
F
P
W
E
C
Amino acid

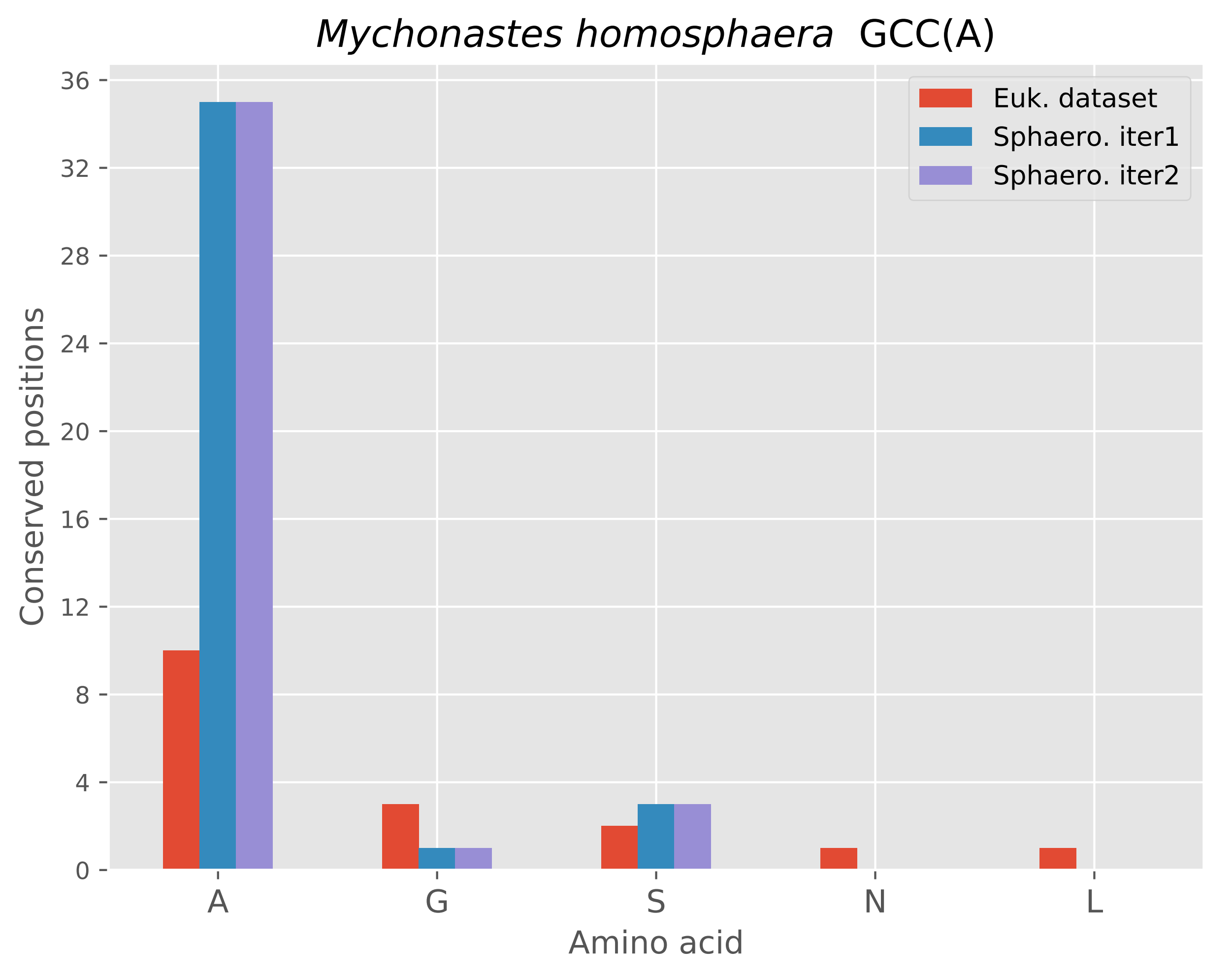
Mychonastes homosphaera GCC(A)
Euk. dataset
Sphaero. iter1
Sphaero. iter2
Conserved positions
0
4
8
12
16
20
24
28
32
36
A
G
S
N
L
Amino acid

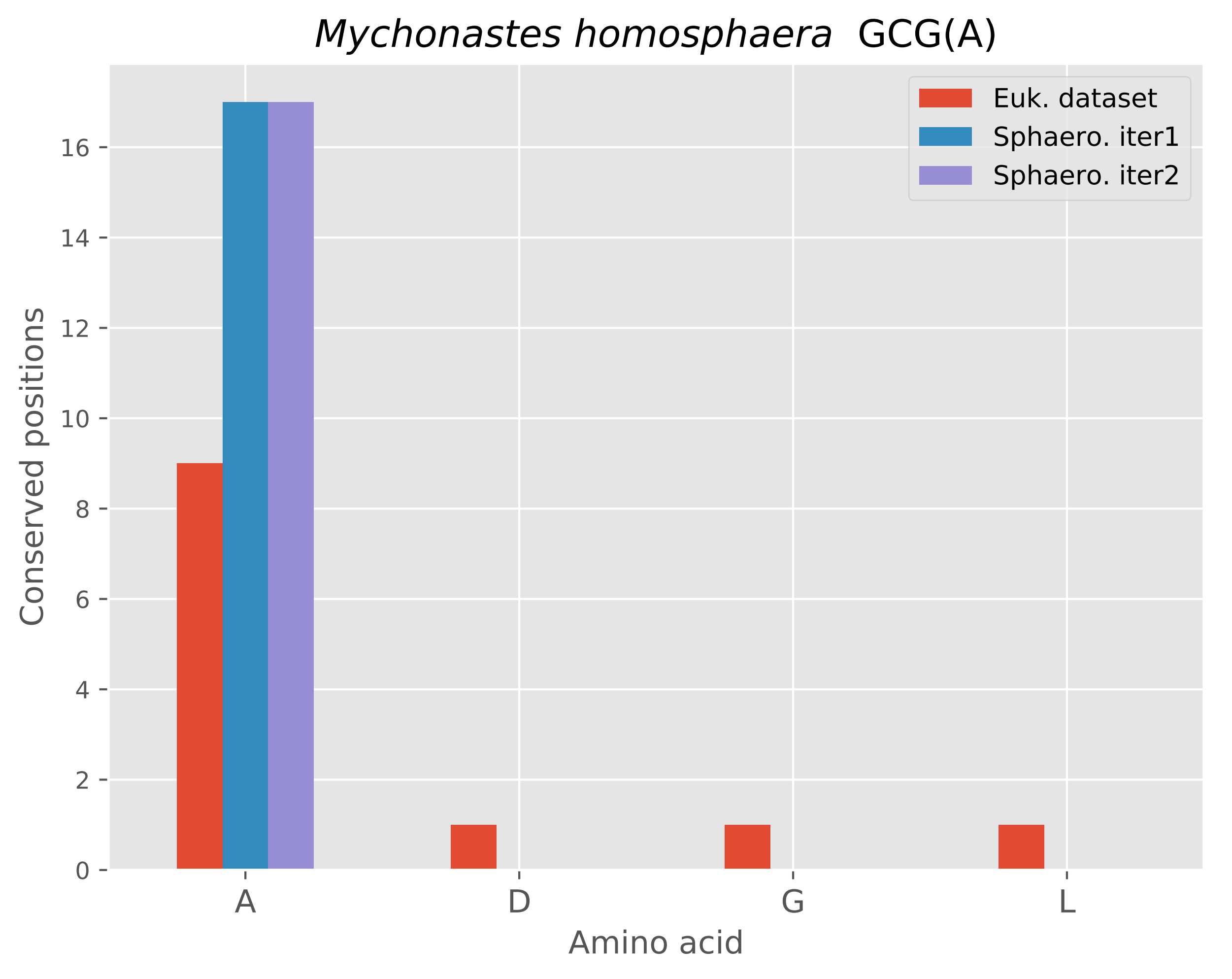

Mychonastes homosphaera GCG(A)
Euk. dataset
Sphaero. iter1
Sphaero. iter2
Conserved positions
0
2
4
6
8
10
12
14
16
A
D
G
L
Amino acid

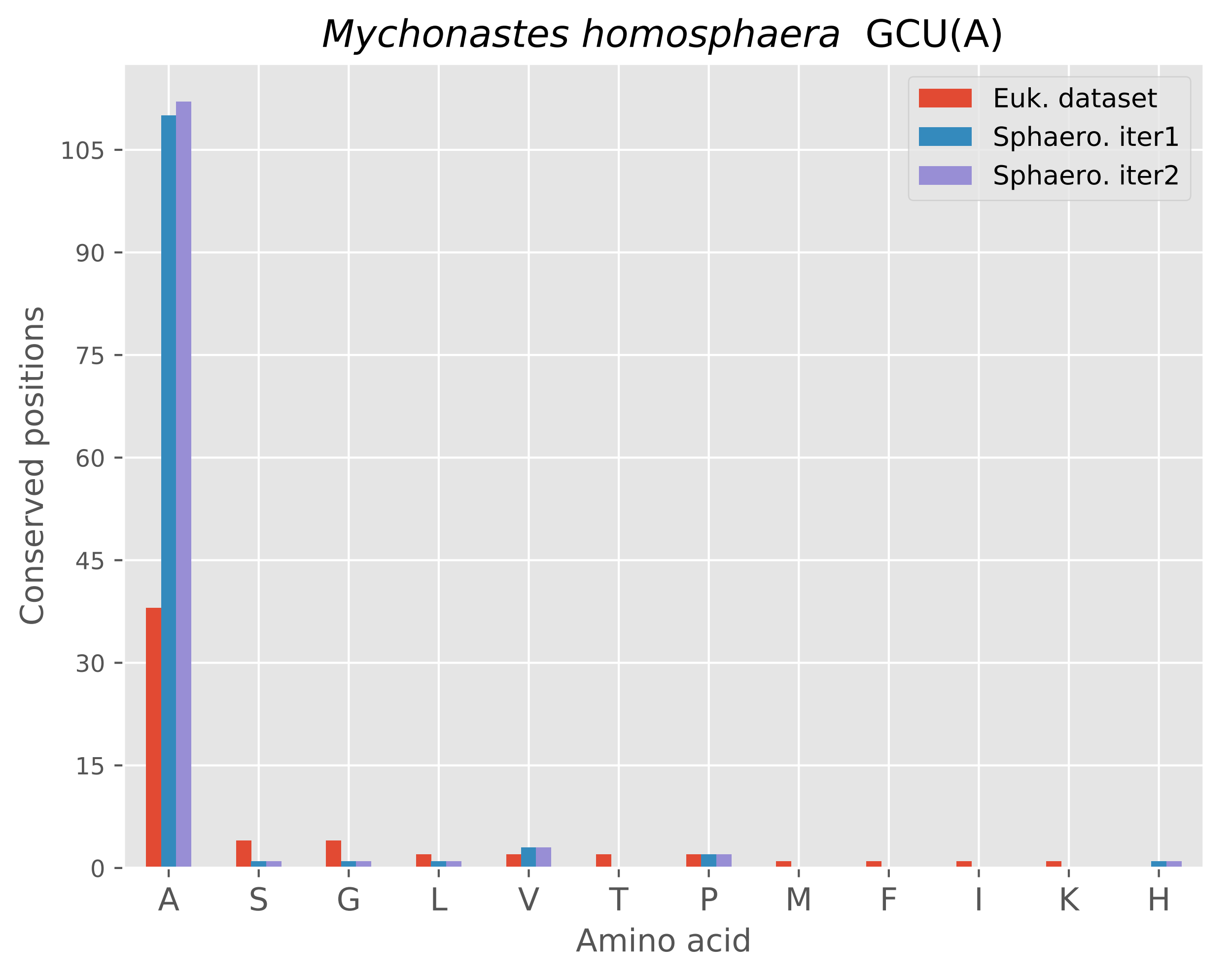

Mychonastes homosphaera GCU(A)
Euk. dataset
Sphaero. iter1
Sphaero. iter2
Conserved positions
0
15
30
45
60
75
90
105
A
S
G
L
V
T
P
M
F
I
K
H
Amino acid

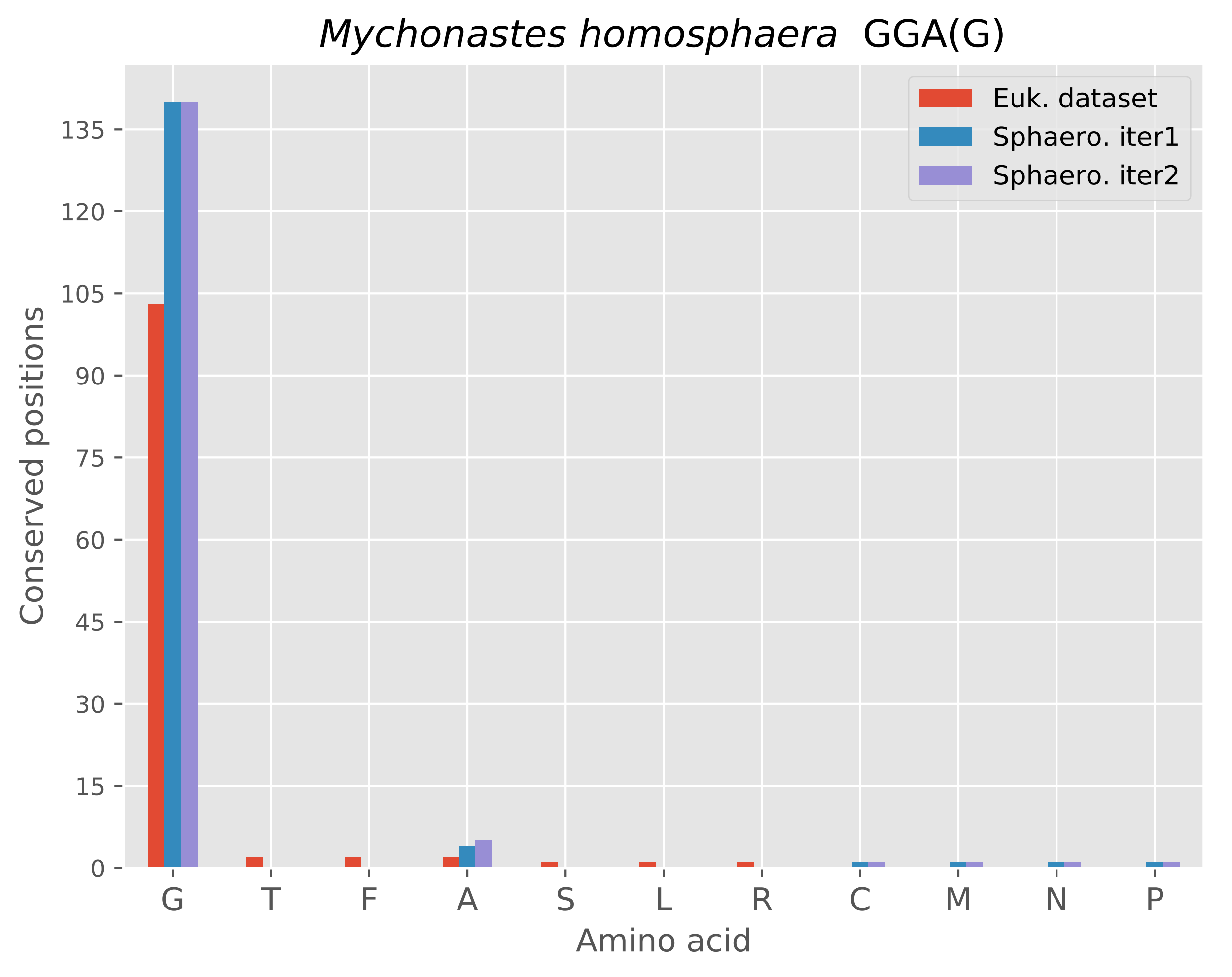

Mychonastes homosphaera GGA(G)
Euk. dataset
Sphaero. iter1
Sphaero. iter2
Conserved positions
0
15
30
45
60
75
90
105
120
135
G
T
F
A
S
L
R
C
M
N
P
Amino acid

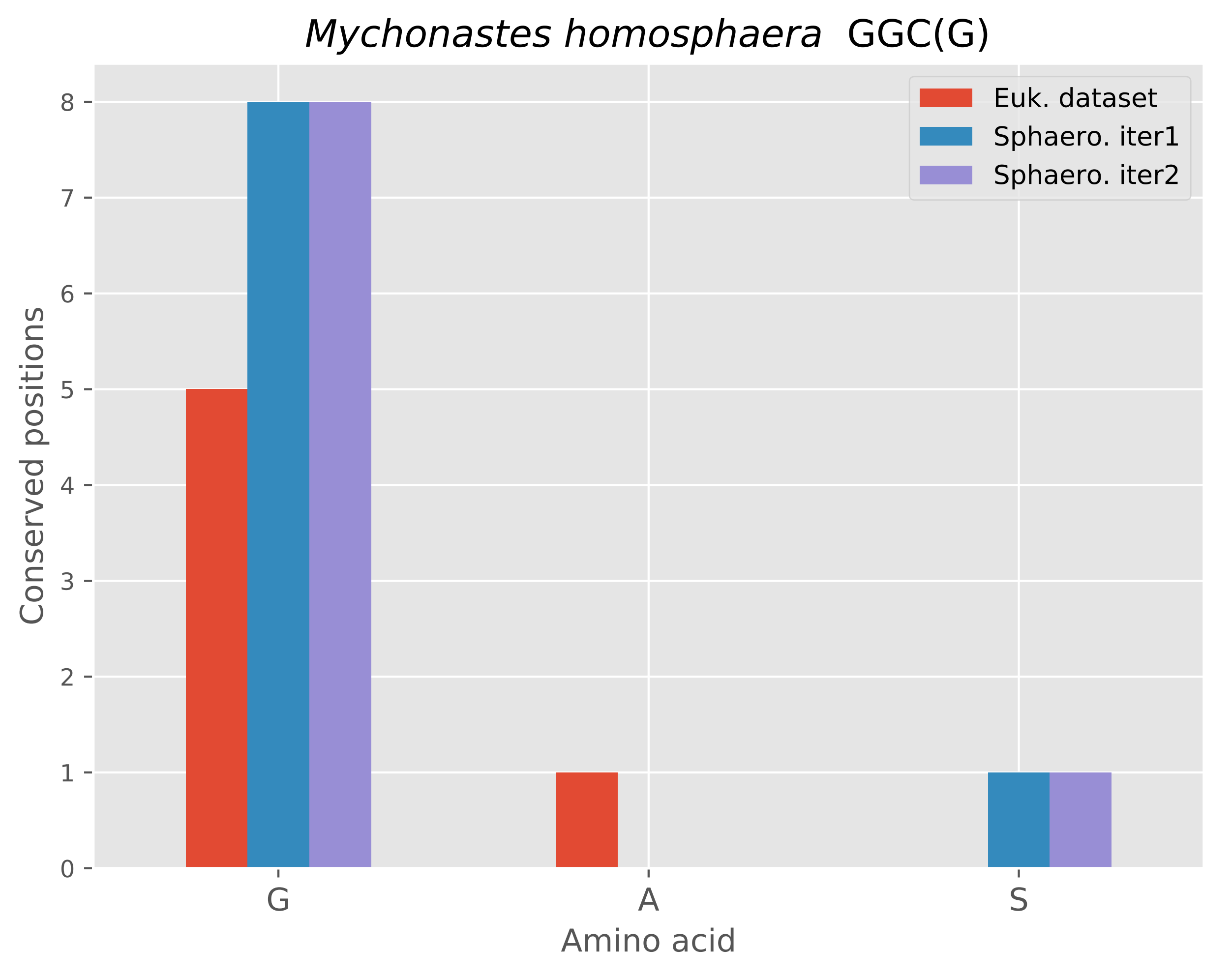
Mychonastes homosphaera GGC(G)
Euk. dataset
Sphaero. iter1
Sphaero. iter2
Conserved positions
0
1
2
3
4
5
6
7
8
G
A
S
Amino acid

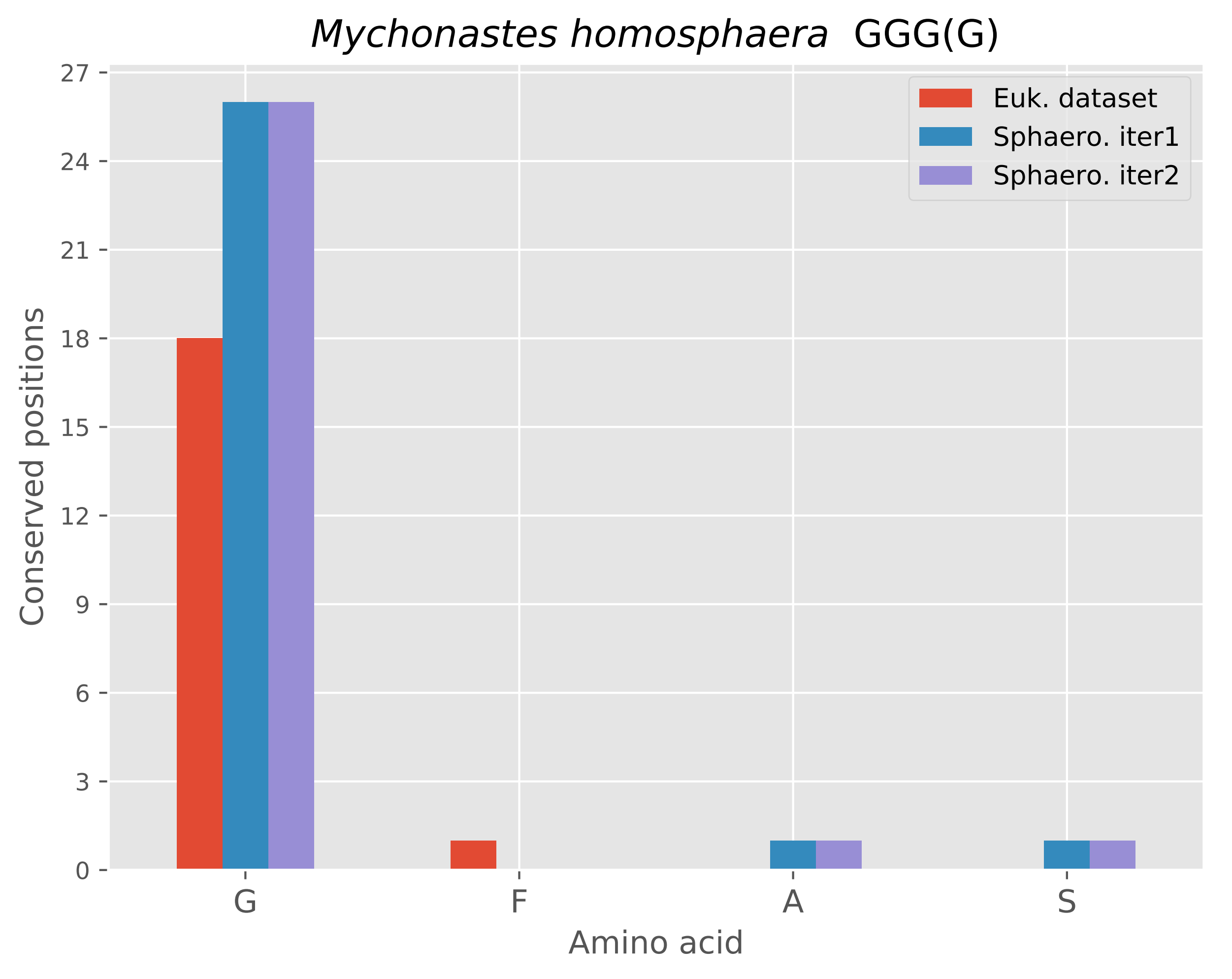
Mychonastes homosphaera GGG(G)
Euk. dataset
Sphaero. iter1
Sphaero. iter2
Conserved positions
0
3
6
9
12
15
18
21
24
27
G
F
A
S
Amino acid

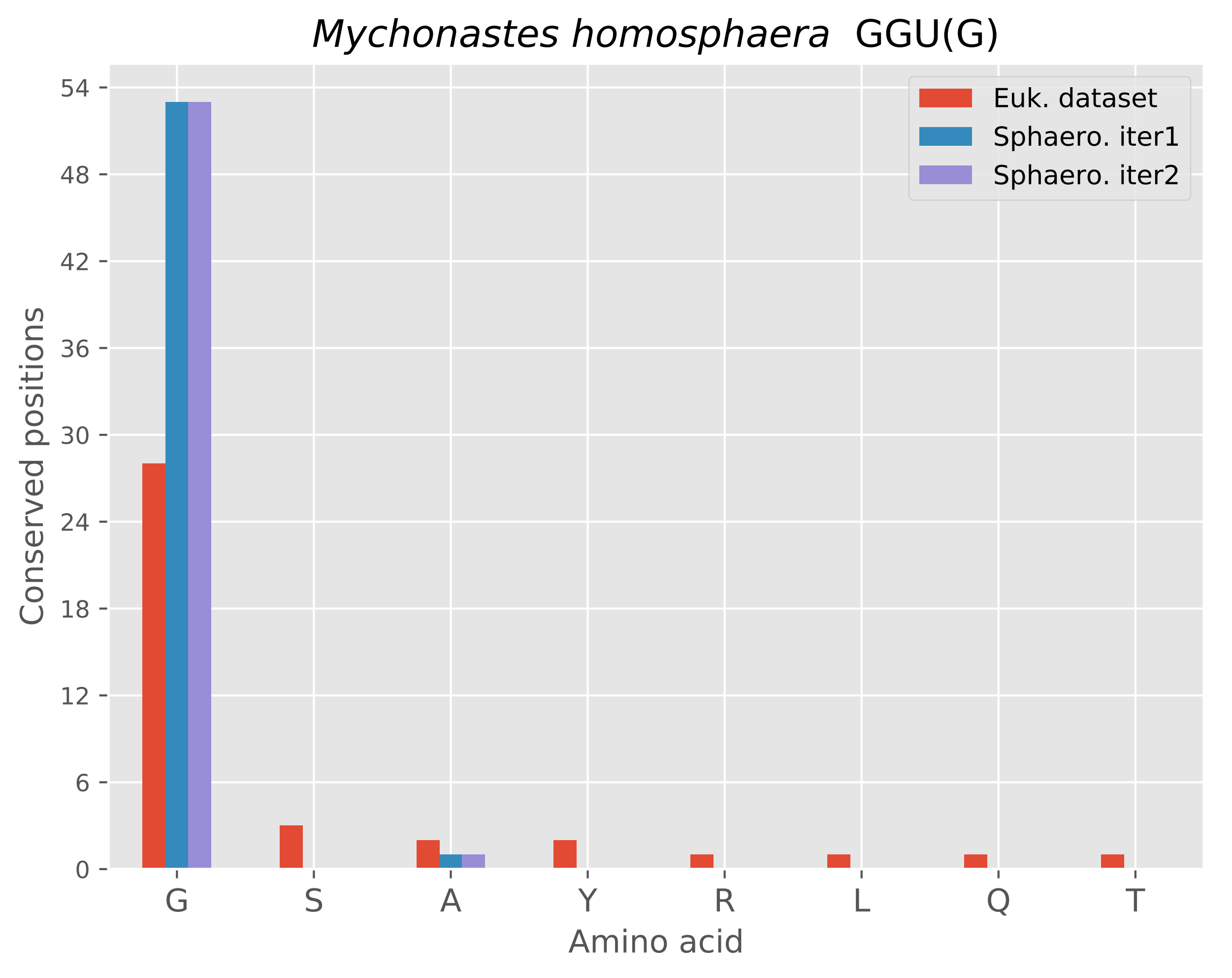

Mychonastes homosphaera GGU(G)
Euk. dataset
Sphaero. iter1
Sphaero. iter2
Conserved positions
54
48
42
36
30
24
18
12
6
0
G
S
A
Y
R
L
Q
T
Amino acid

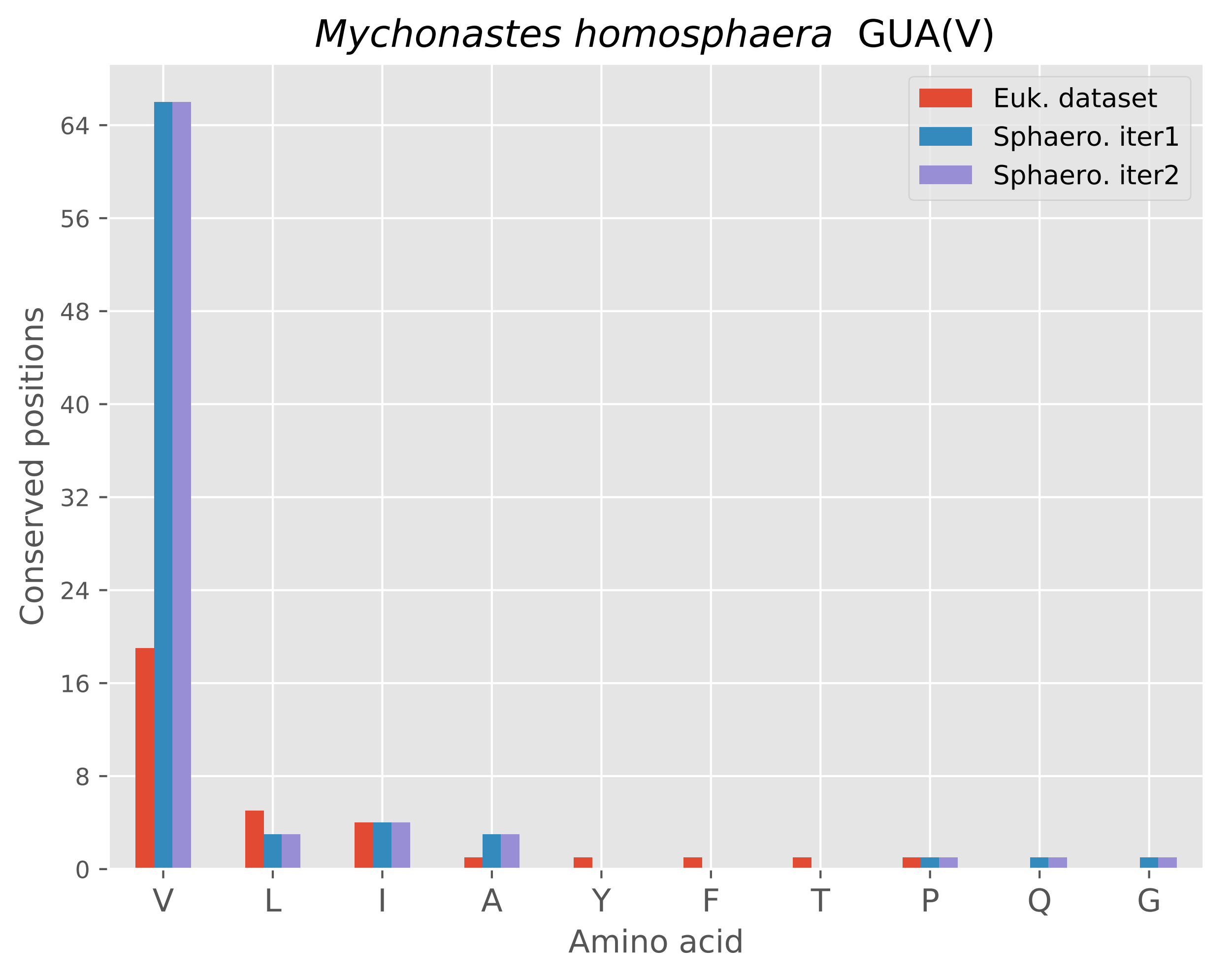

Mychonastes homosphaera GUA(V)
Euk. dataset
Sphaero. iter1
Sphaero. iter2
Conserved positions
0
8
16
24
32
40
48
56
64
Amino acid
V
L
I
A
Y
F
T
P
Q
G

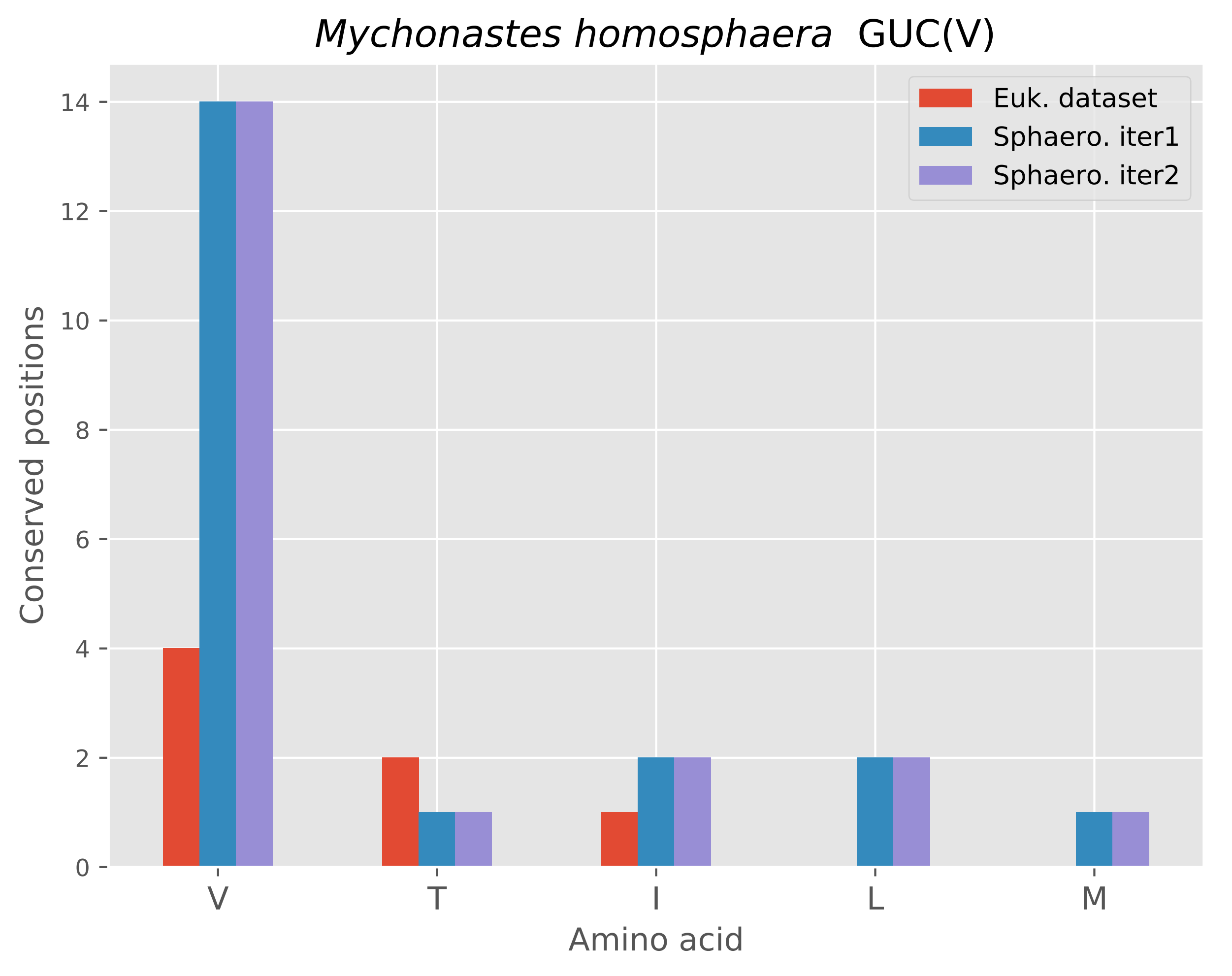

Mychonastes homosphaera GUC(V)
Conserved positions
0
2
4
6
8
10
12
14
Euk. dataset
Sphaero. iter1
Sphaero. iter2
V
T
I
L
M
Amino acid

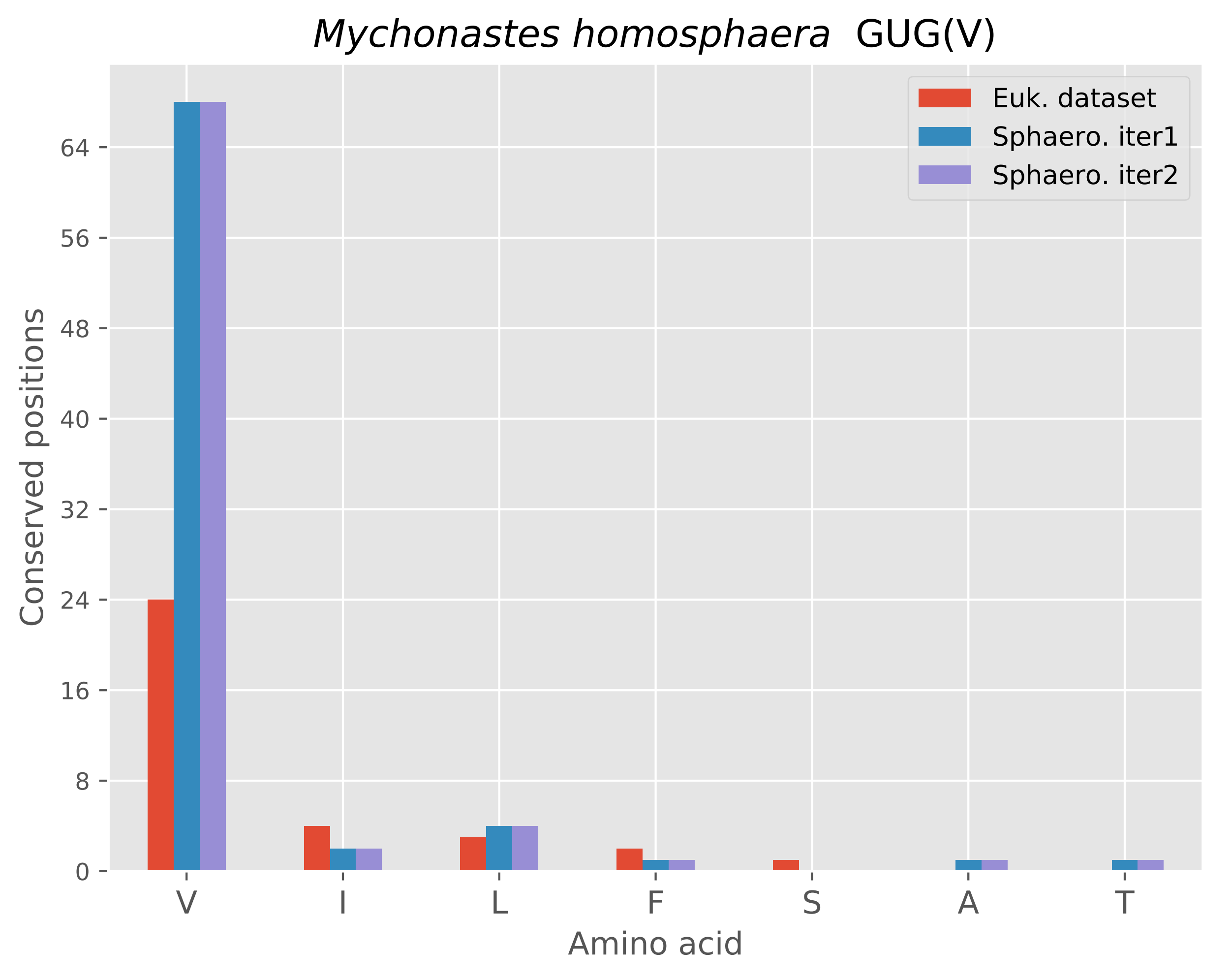

Mychonastes homosphaera GUG(V)
Euk. dataset
Sphaero. iter1
Sphaero. iter2
Conserved positions
0
8
16
24
32
40
48
56
64
V
I
L
F
S
A
T
Amino acid

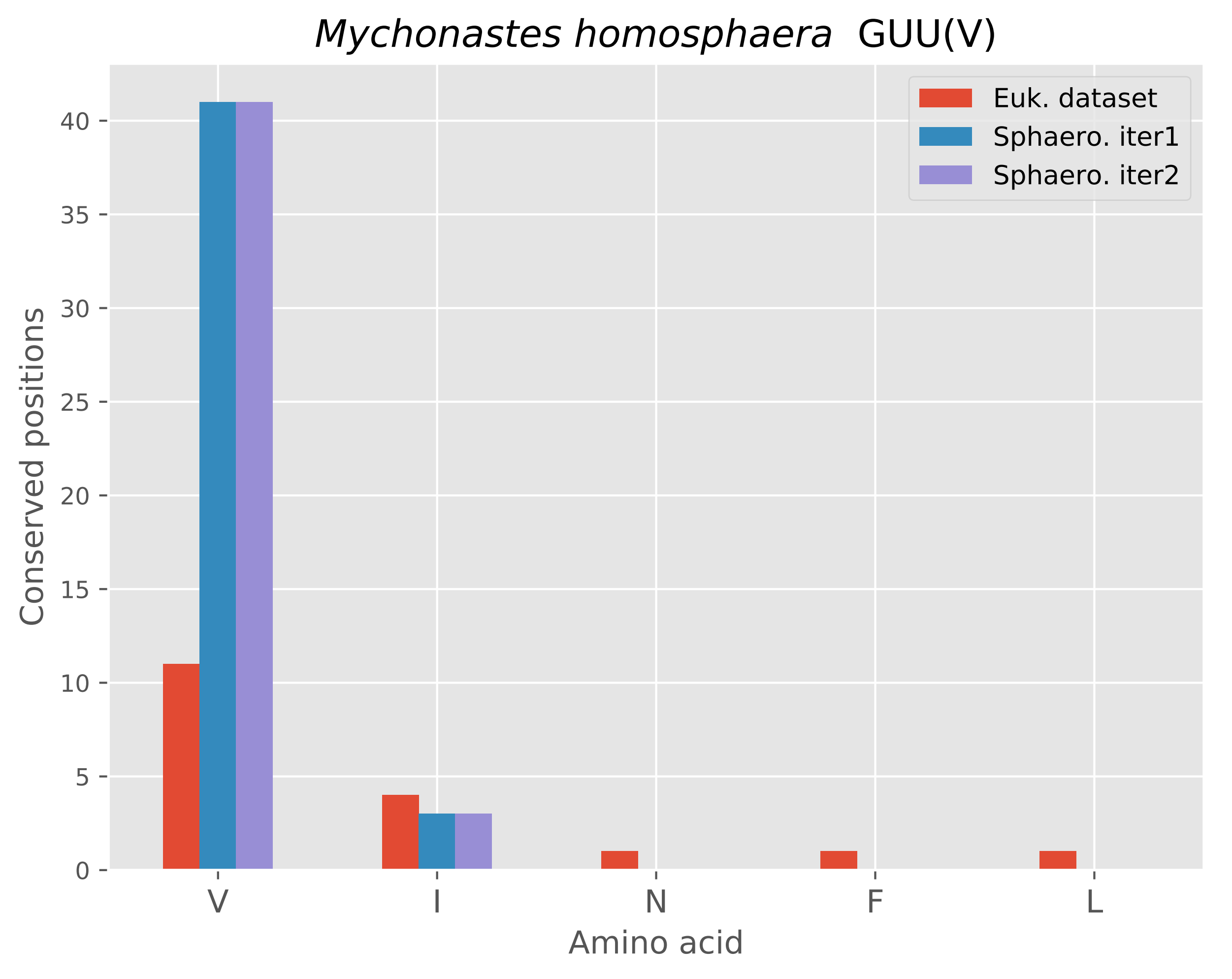

Mychonastes homosphaera GUU(V)
Euk. dataset
Sphaero. iter1
Sphaero. iter2
Conserved positions
0
5
10
15
20
25
30
35
40
V
I
N
F
L
Amino acid

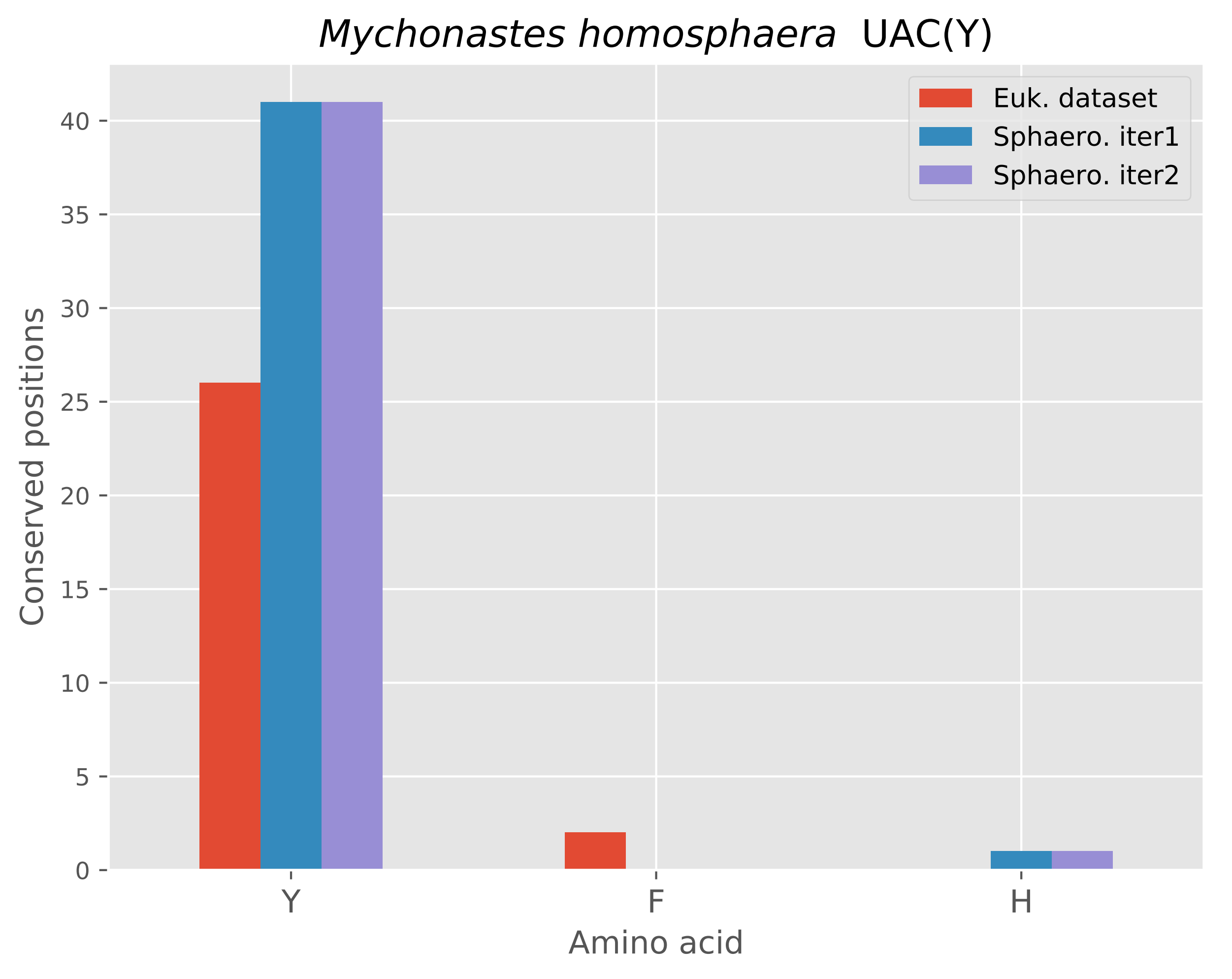

Mychonastes homosphaera UAC(Y)
Euk. dataset
Sphaero. iter1
Sphaero. iter2
Conserved positions
0
5
10
15
20
25
30
35
40
Y
F
H
Amino acid

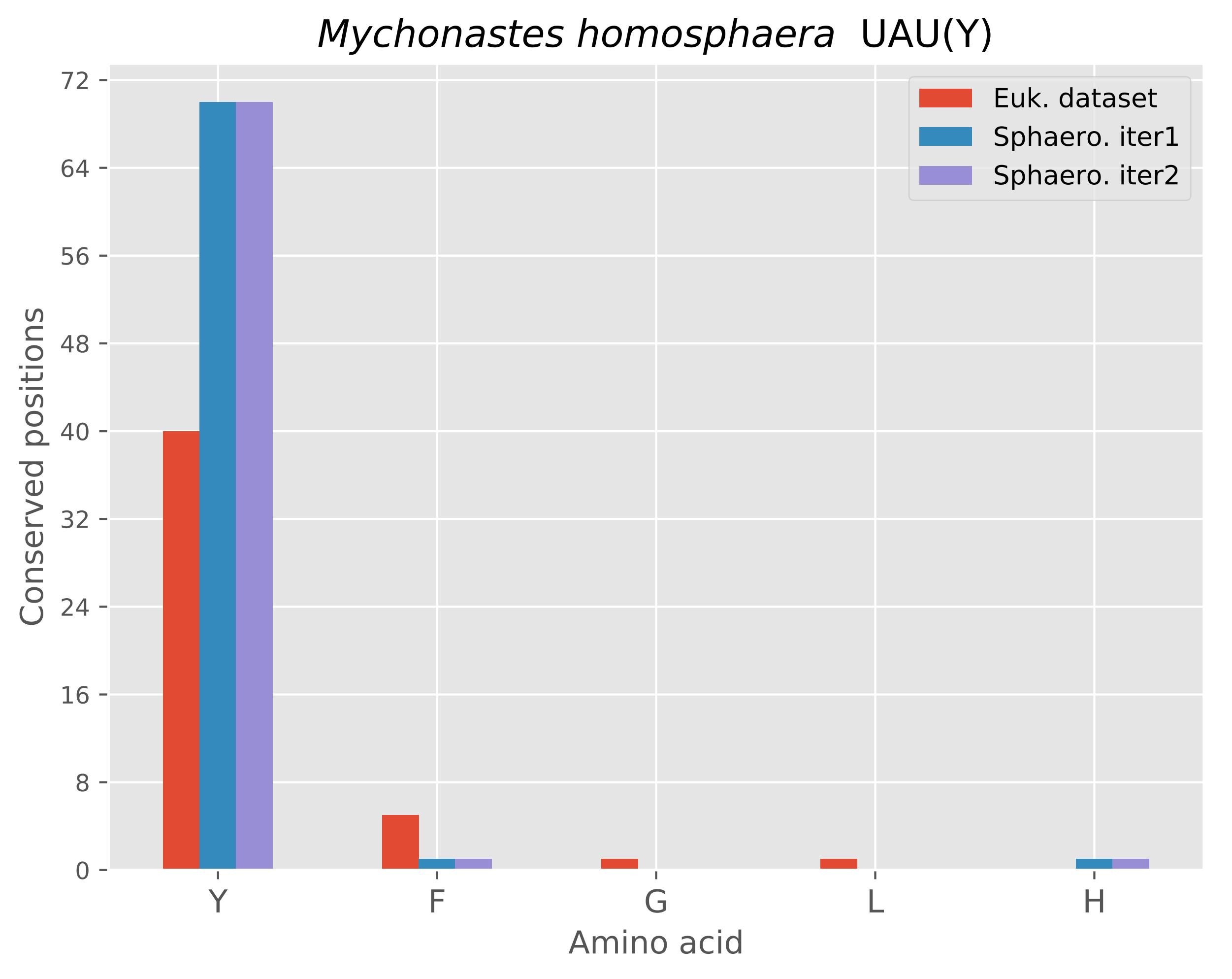

Mychonastes homosphaera UAU(Y)
Conserved positions
Amino acid
Euk. dataset
Sphaero. iter1
Sphaero. iter2
0
8
16
24
32
40
48
56
64
72
Y
F
G
L
H

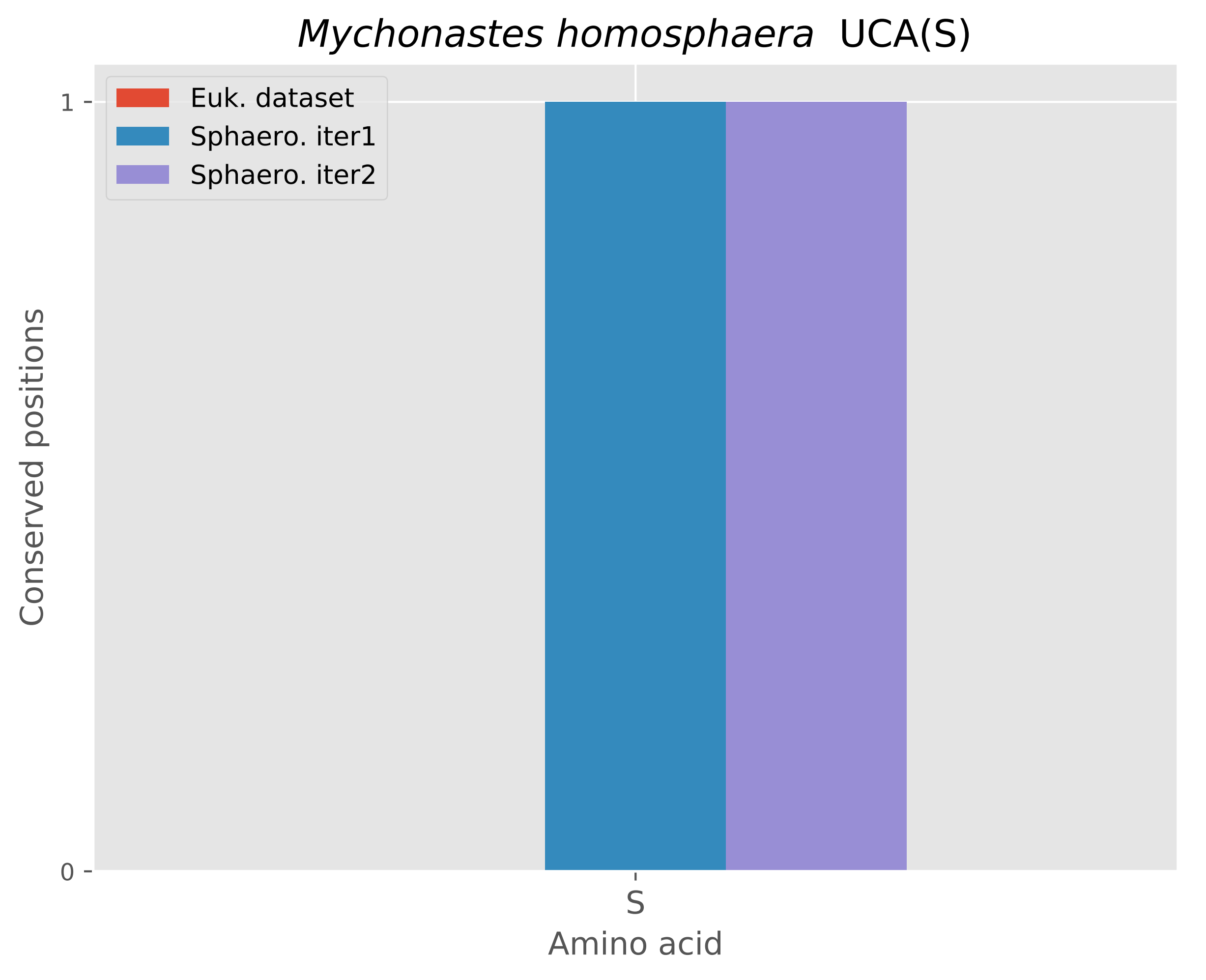
Mychonastes homosphaera UCA(S)
Euk. dataset
Sphaero. iter1
Sphaero. iter2
Conserved positions
1
0
S
Amino acid

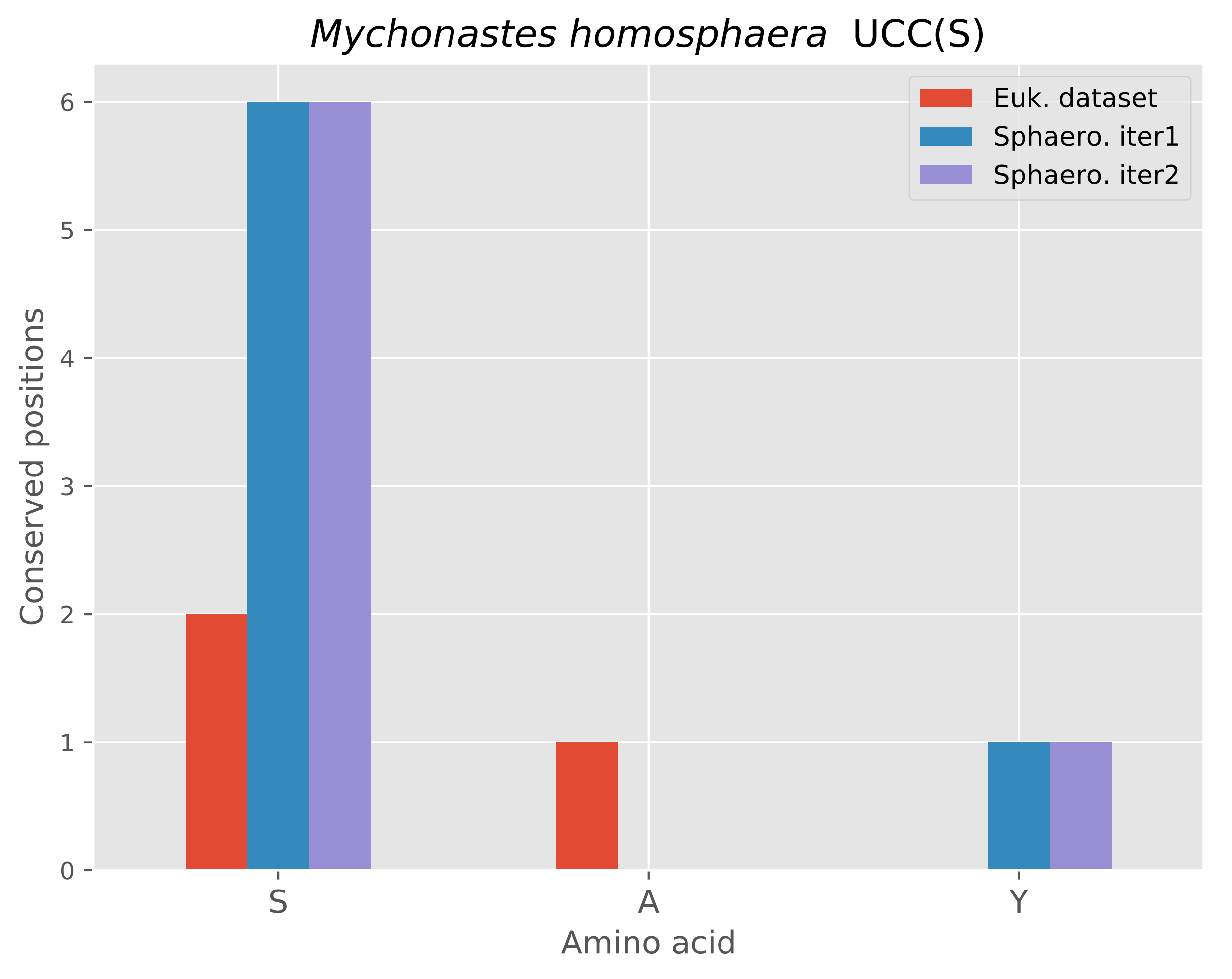
Mychonastes homosphaera UCC(S)
Euk. dataset
Sphaero. iter1
Sphaero. iter2
Conserved positions
0
1
2
3
4
5
6
S
A
Y
Amino acid

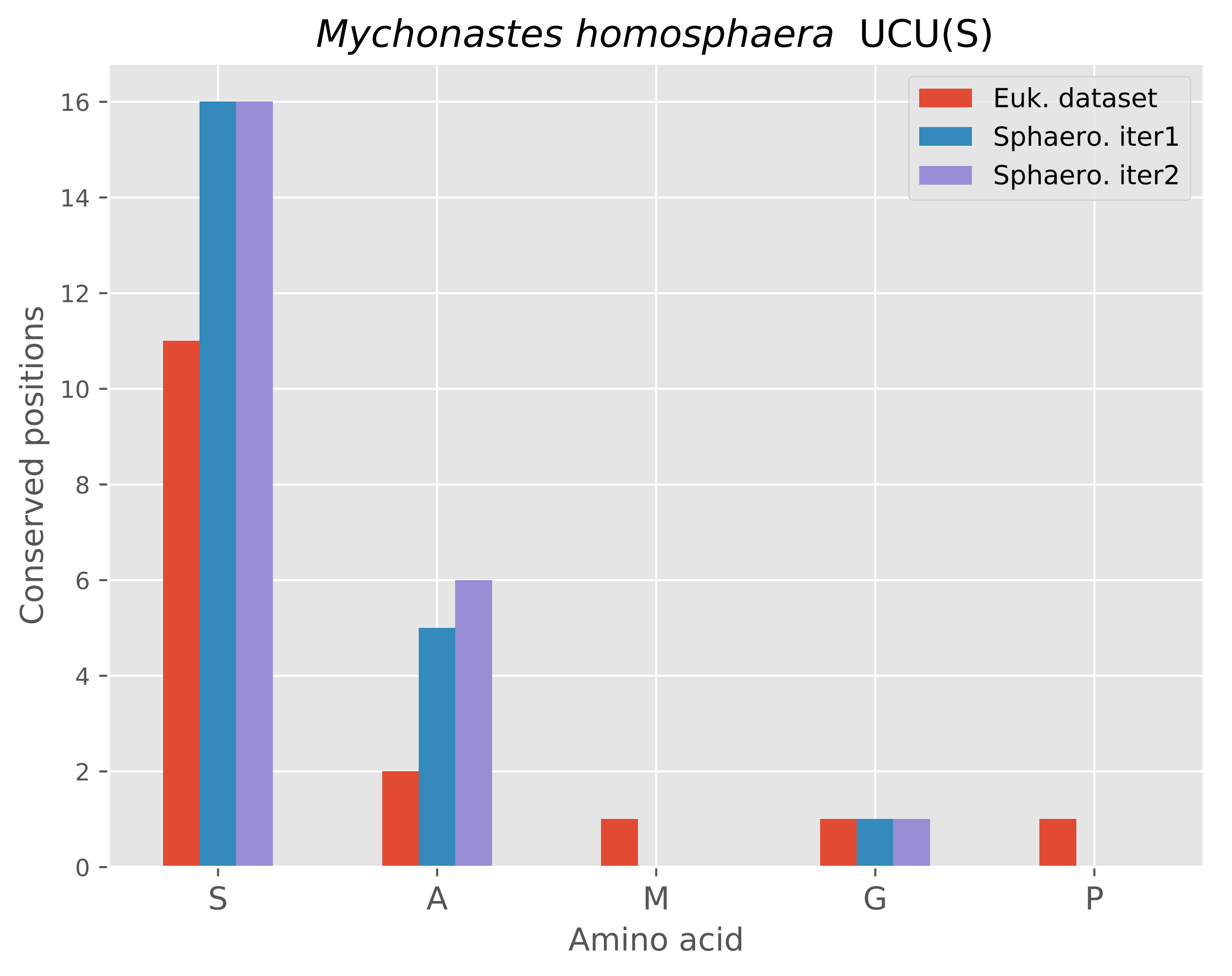

Mychonastes homosphaera UCU(S)
Euk. dataset
Sphaero. iter1
Sphaero. iter2
Conserved positions
0
2
4
6
8
10
12
14
16
S
A
M
G
P
Amino acid

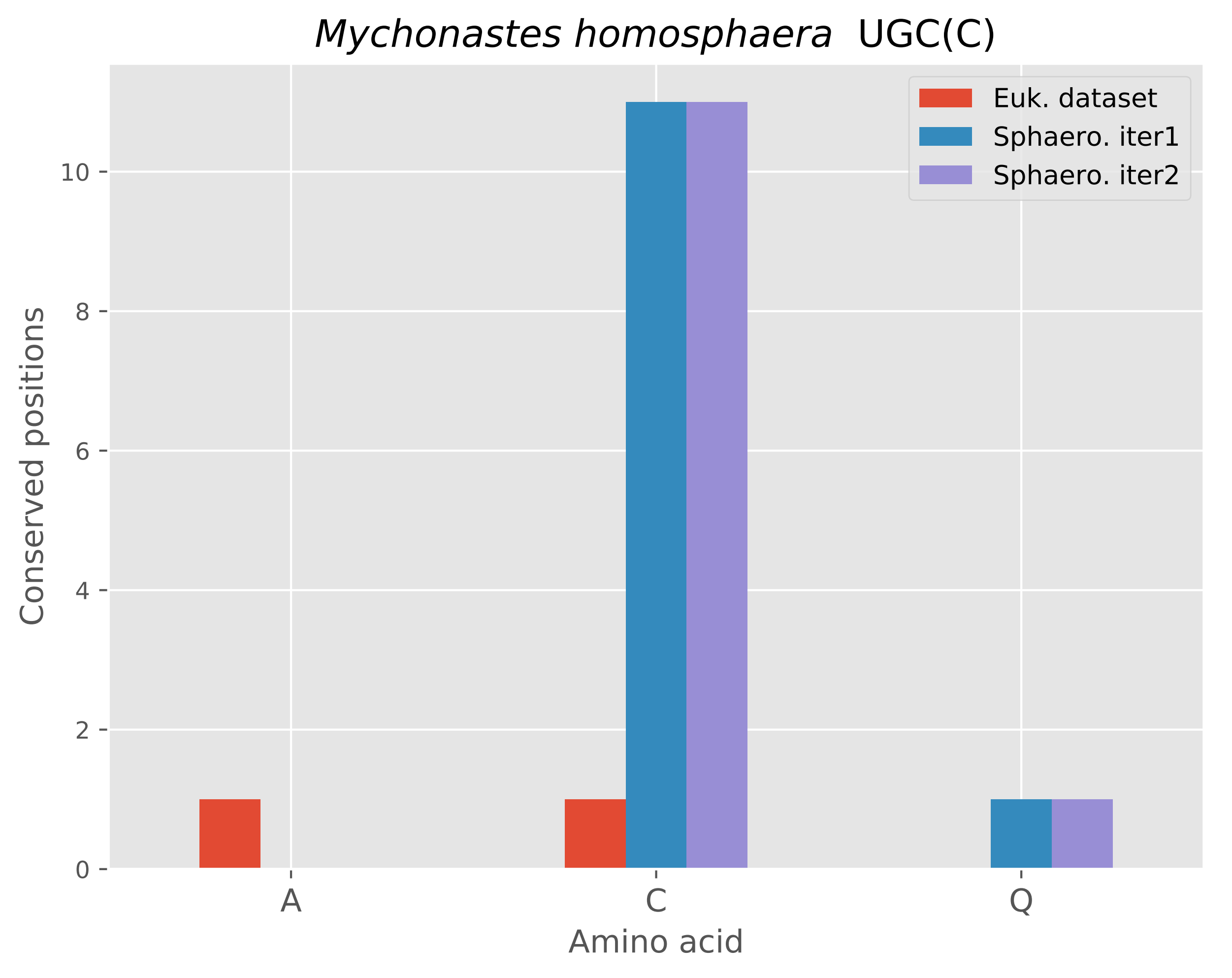

Mychonastes homosphaera UGC(C)
Euk. dataset
Sphaero. iter1
Sphaero. iter2
Conserved positions
0
2
4
6
8
10
A
C
Q
Amino acid

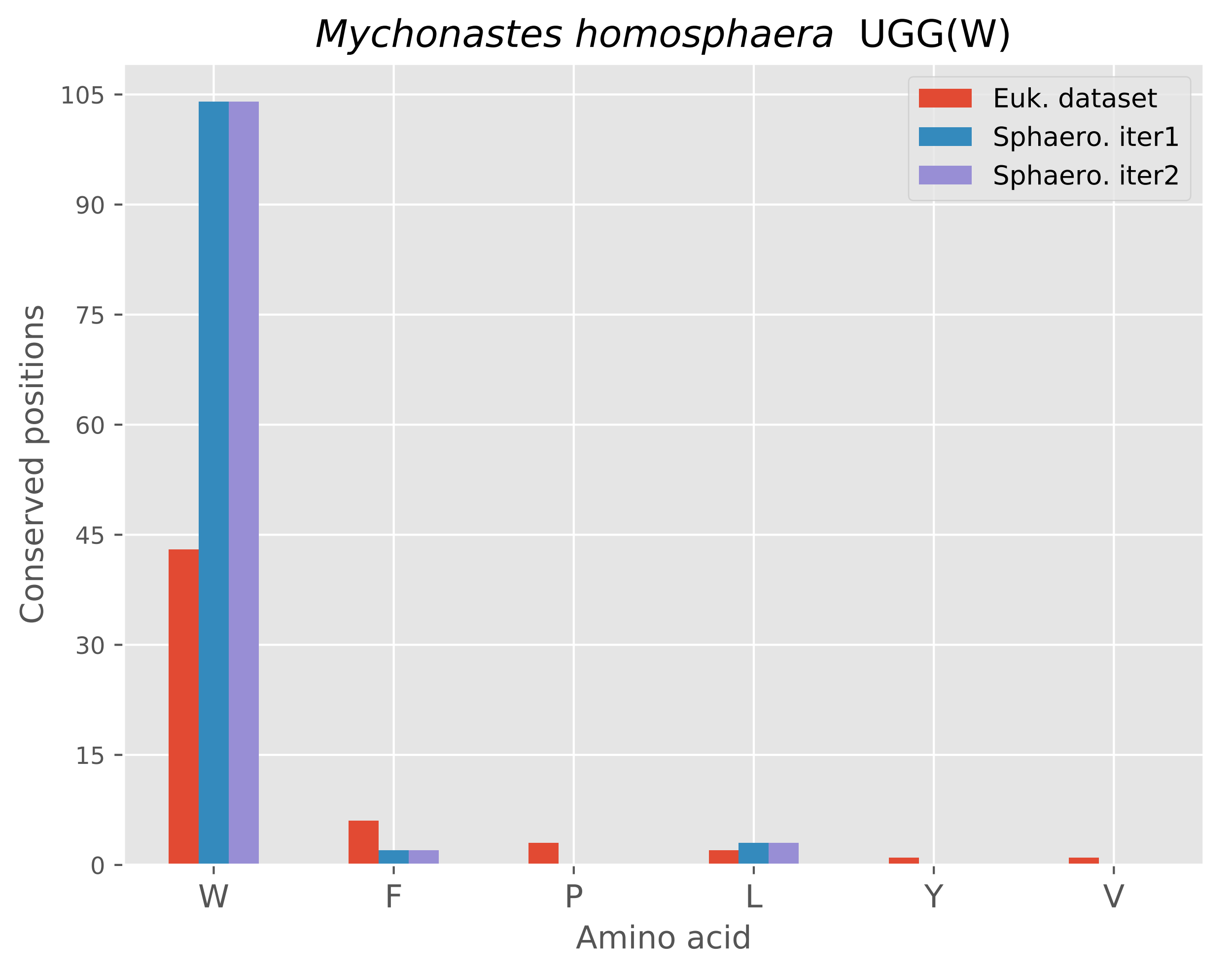

Mychonastes homosphaera UGG(W)
Euk. dataset
Sphaero. iter1
Sphaero. iter2
Conserved positions
105
90
75
60
45
30
15
0
W
F
P
L
Y
V
Amino acid

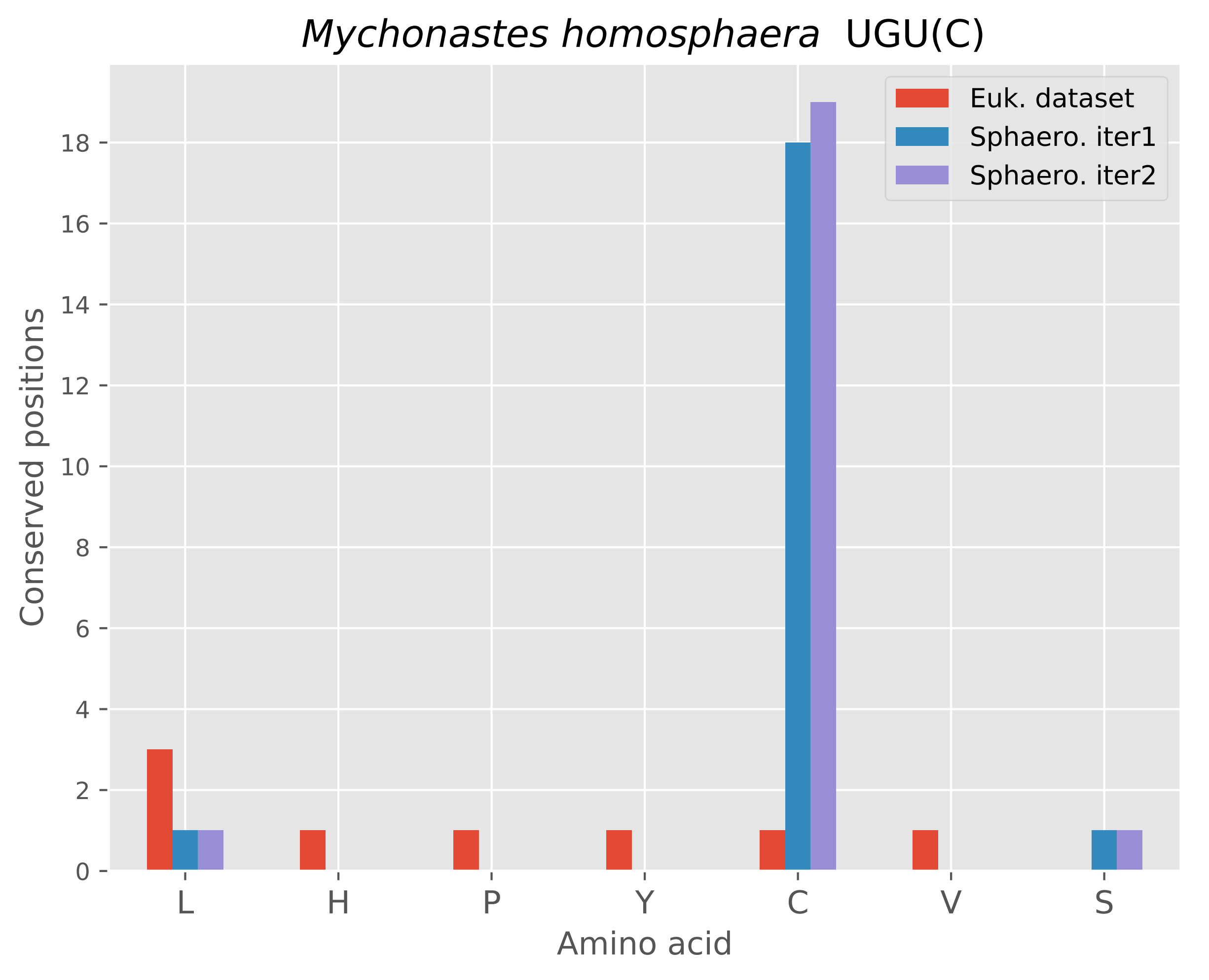
Mychonastes homosphaera UGU(C)
Euk. dataset
Sphaero. iter1
Sphaero. iter2
Conserved positions
0
2
4
6
8
10
12
14
16
18
L
H
P
Y
C
V
S
Amino acid

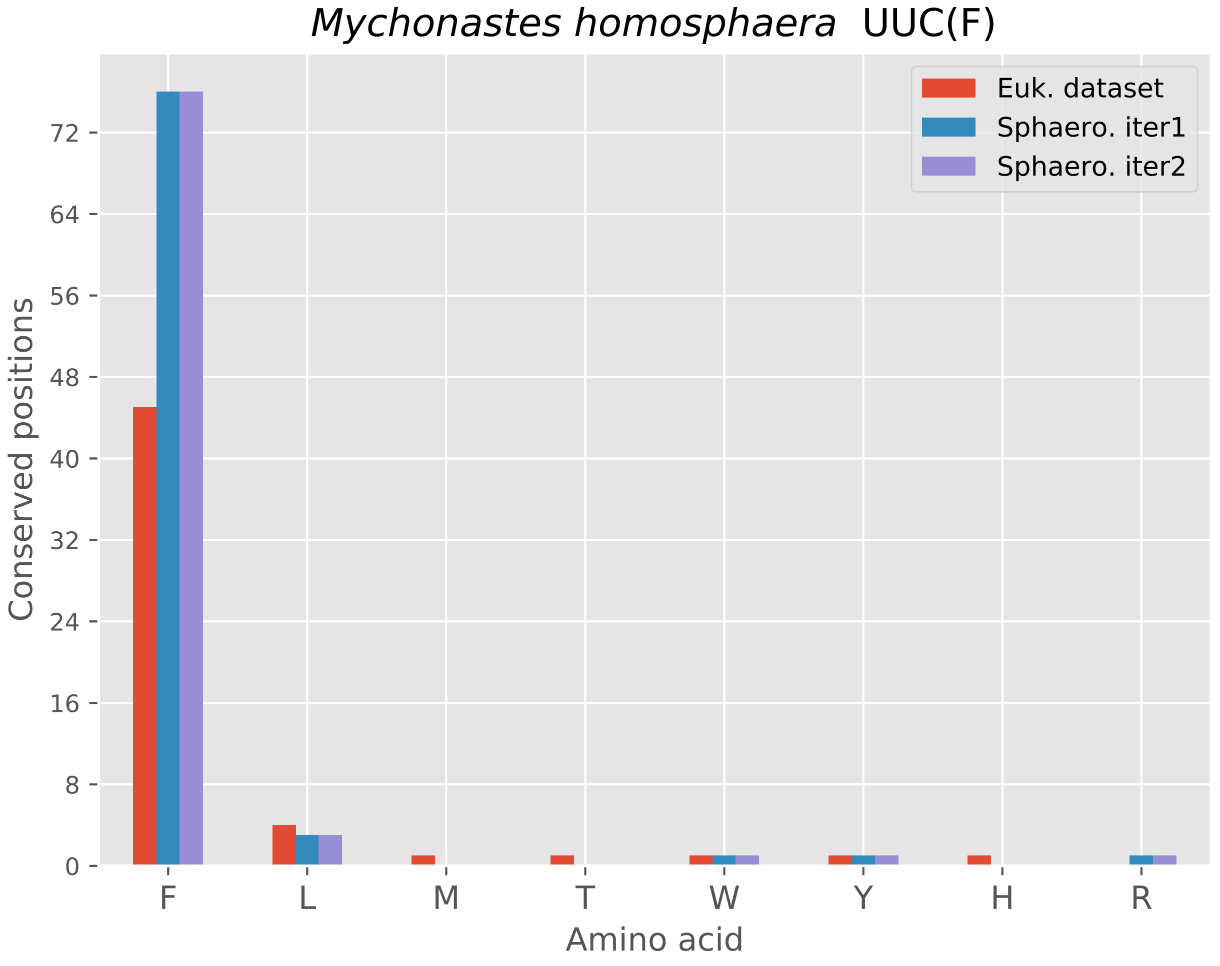

Mychonastes homosphaera UUC(F)
Euk. dataset
Sphaero. iter1
Sphaero. iter2
Conserved positions
0
8
16
24
32
40
48
56
64
72
F
L
M
T
W
Y
H
R
Amino acid

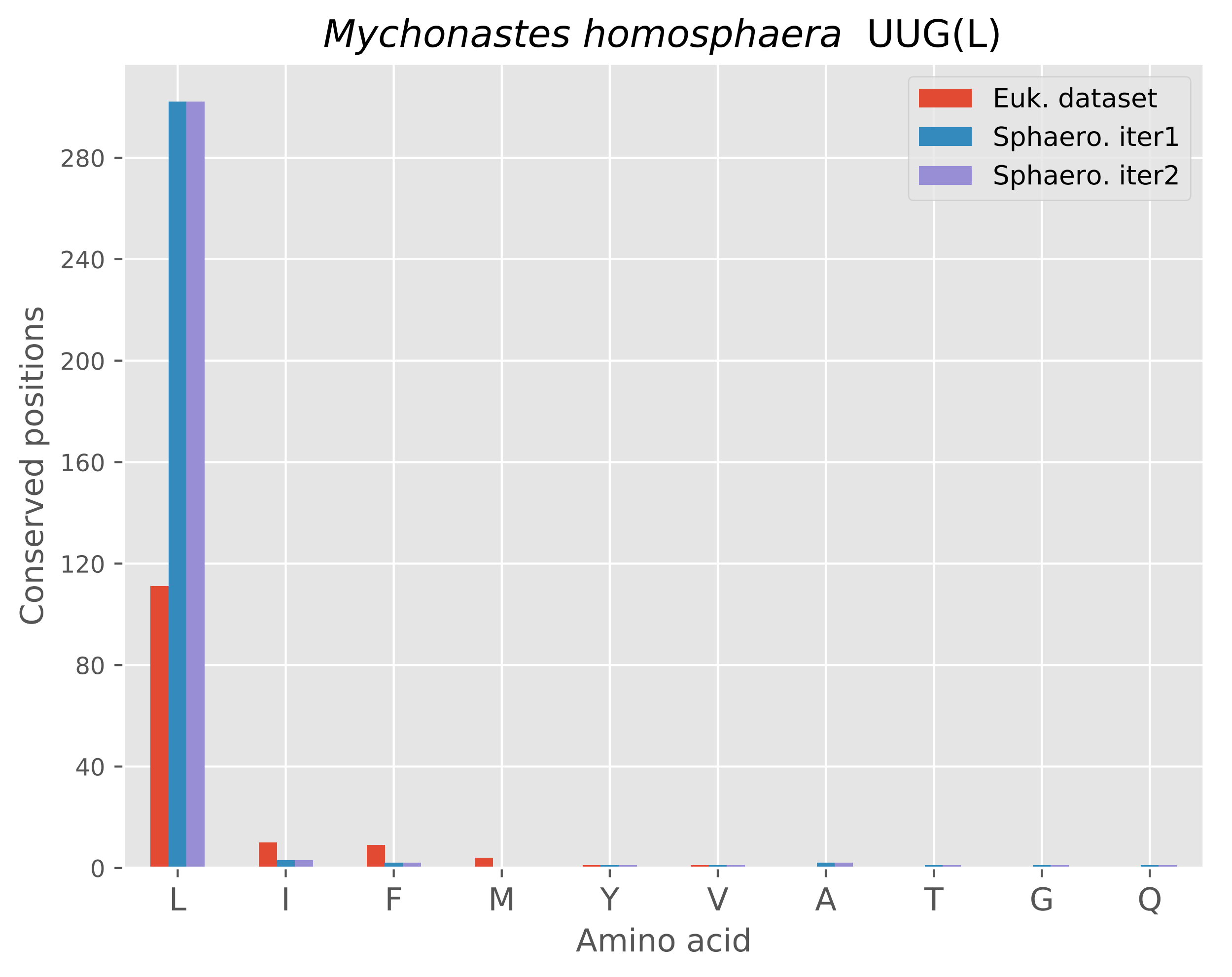

Mychonastes homosphaera UUG(L)
Euk. dataset
Sphaero. iter1
Sphaero. iter2
Conserved positions
0
40
80
120
160
200
240
280
L
I
F
M
Y
V
A
T
G
Q
Amino acid

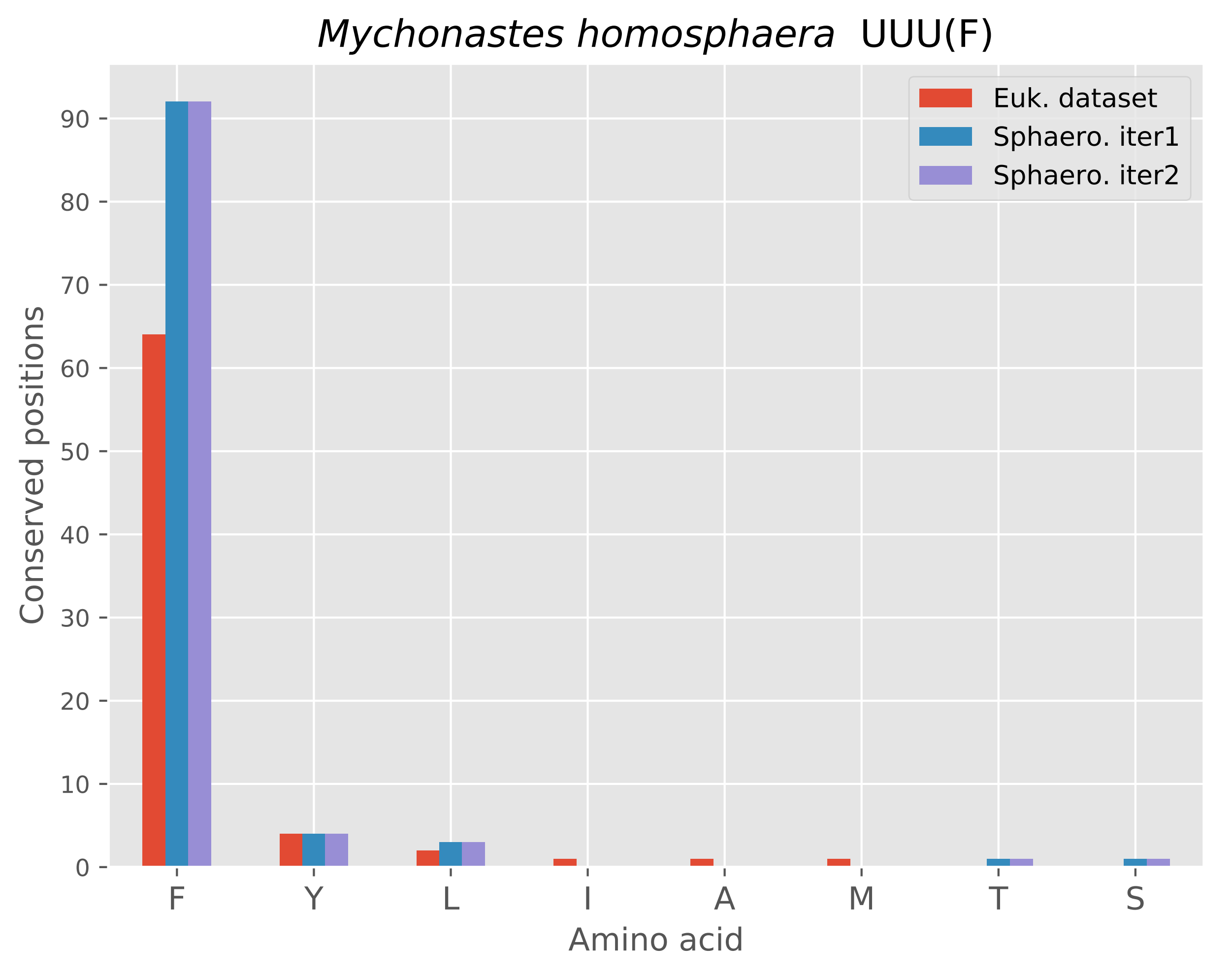

Mychonastes homosphaera UUU(F)
Euk. dataset
Sphaero. iter1
Sphaero. iter2
Conserved positions
0
10
20
30
40
50
60
70
80
90
F
Y
L
I
A
M
T
S
Amino acid

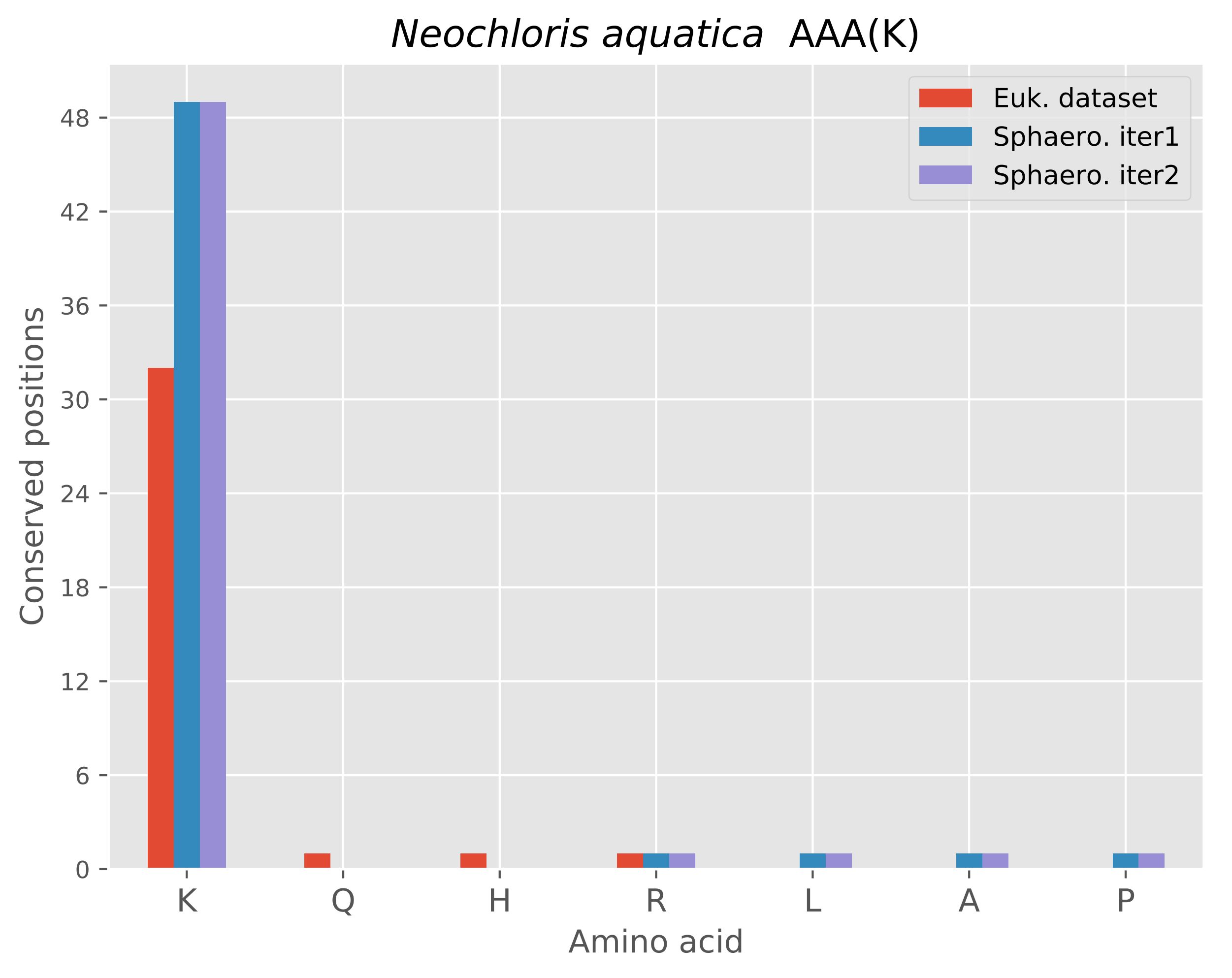
Neochloris aquatica AAA(K)
Euk. dataset
Sphaero. iter1
Sphaero. iter2
Conserved positions
0
6
12
18
24
30
36
42
48
K
Q
H
R
L
A
P
Amino acid

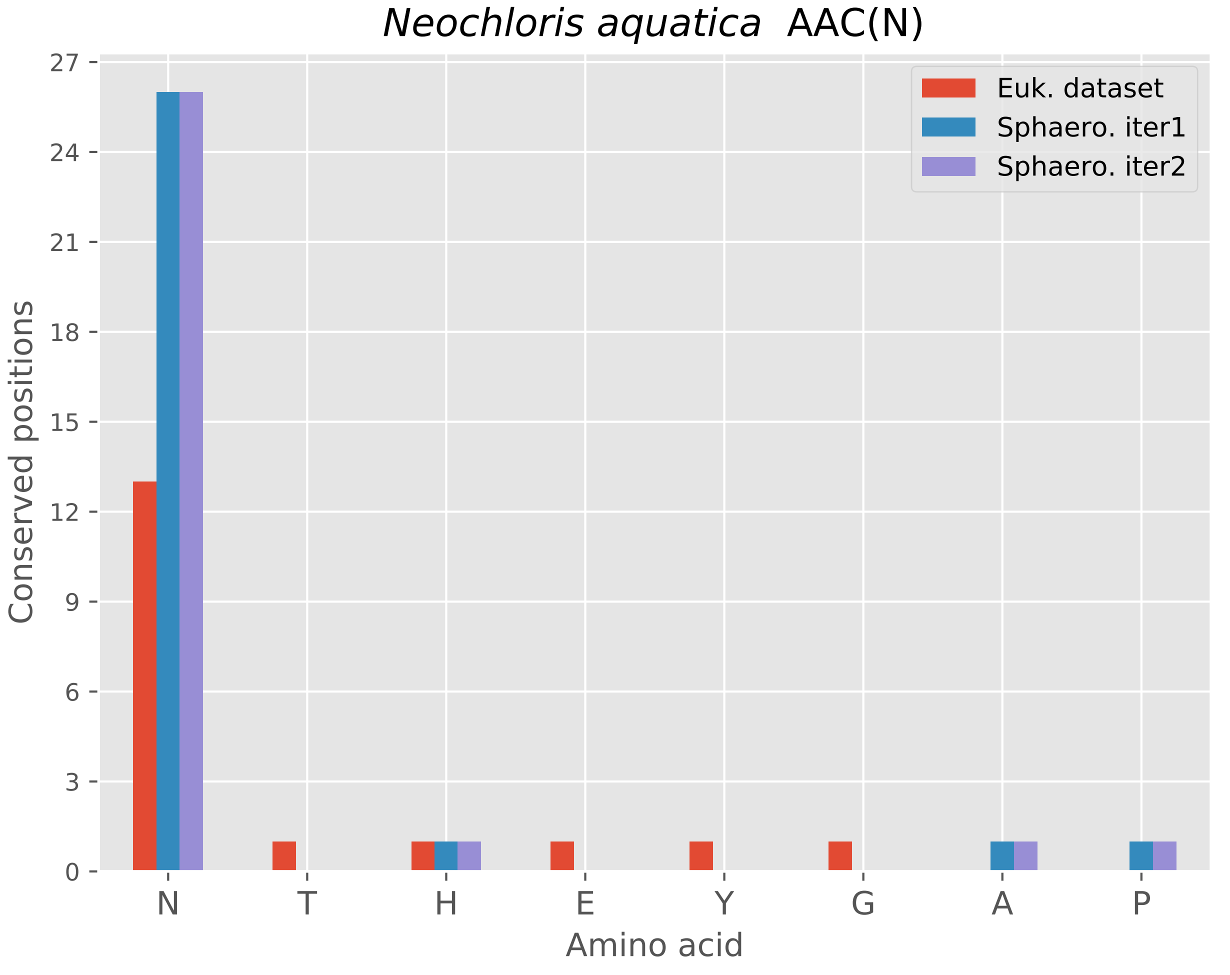

Neochloris aquatica AAC(N)
Euk. dataset
Sphaero. iter1
Sphaero. iter2
Conserved positions
0
3
6
9
12
15
18
21
24
27
N
T
H
E
Y
G
A
P
Amino acid

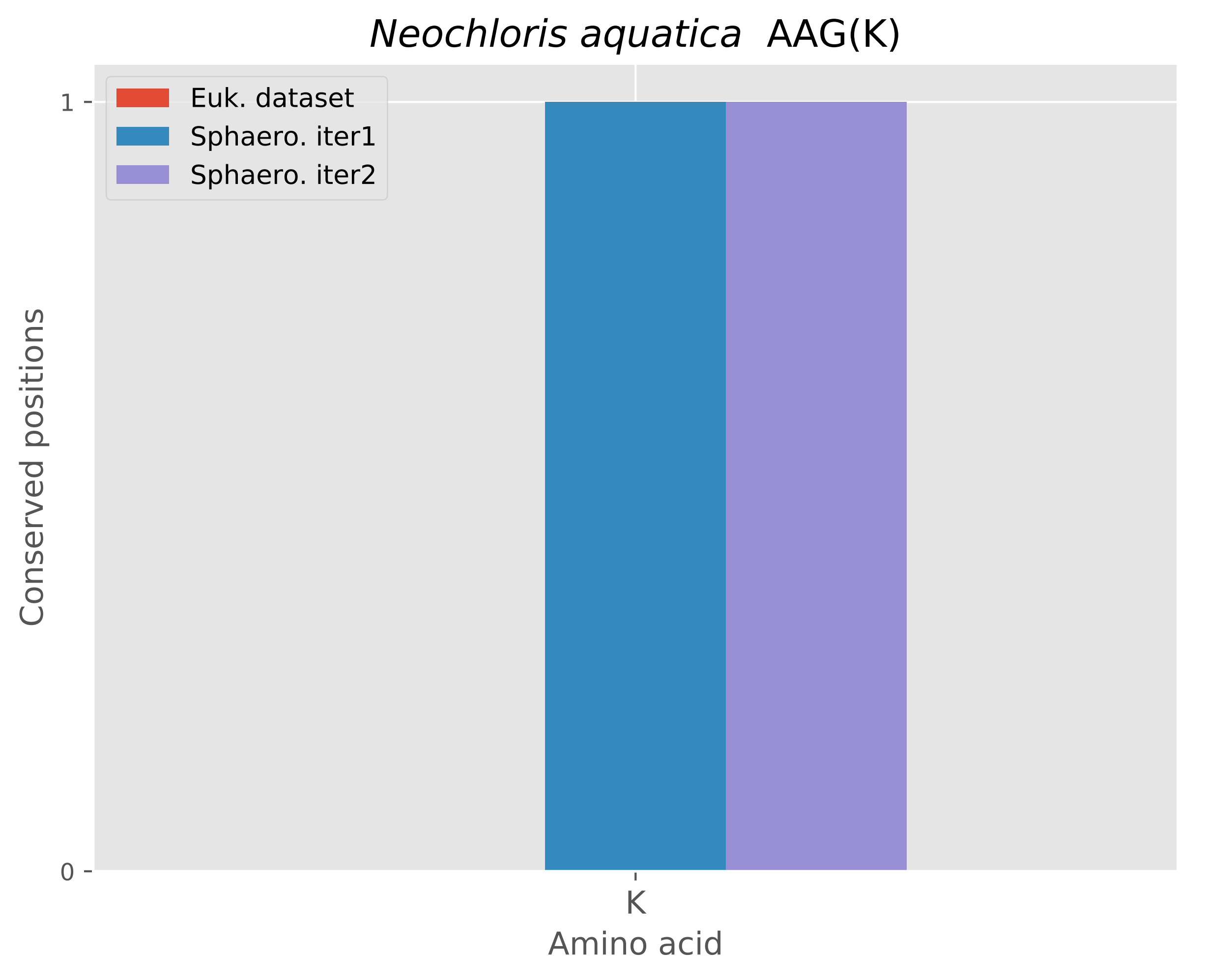

Neochloris aquatica AAG(K)
Euk. dataset
Sphaero. iter1
Sphaero. iter2
Conserved positions
1
0
K
Amino acid

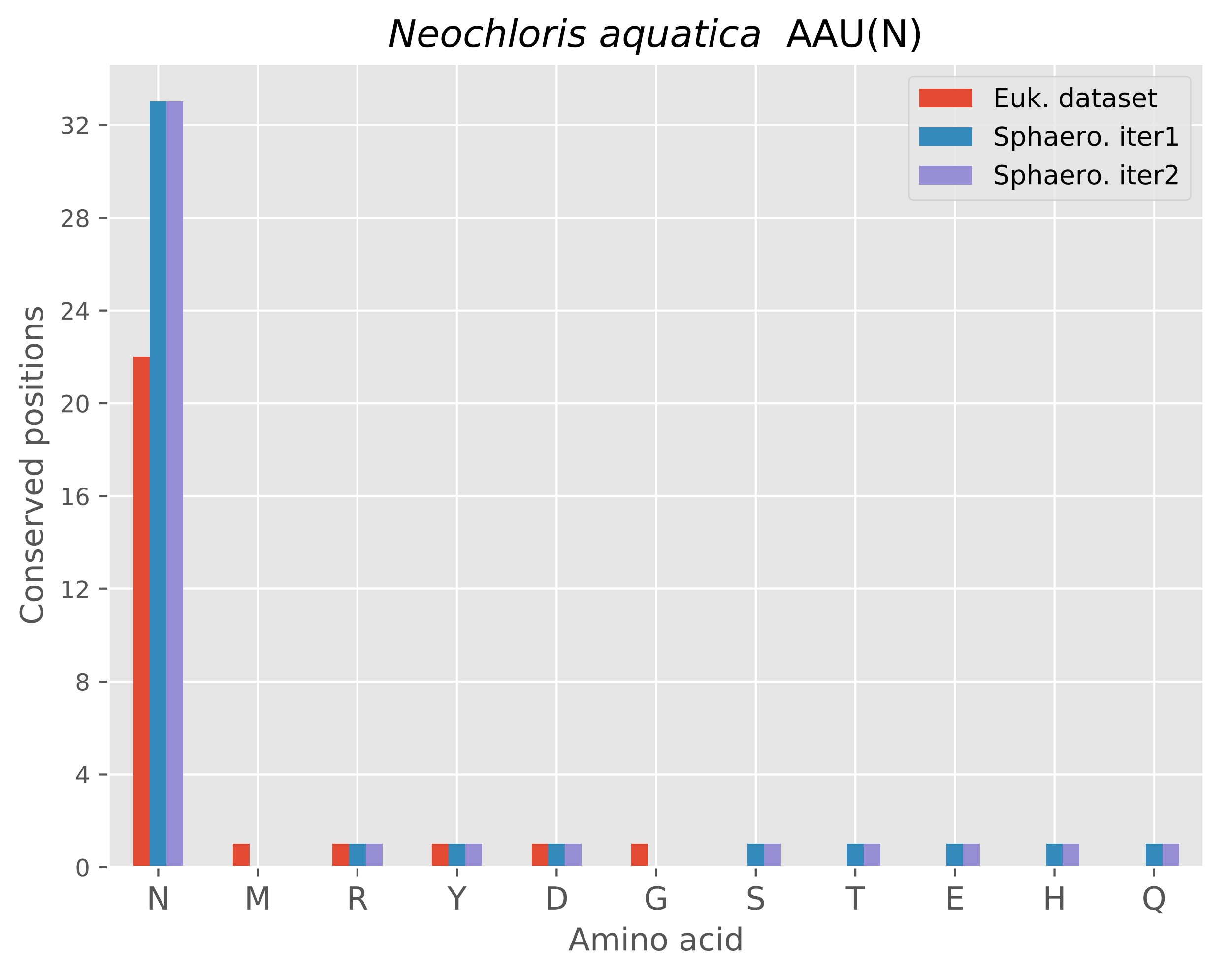

Neochloris aquatica AAU(N)
Conserved positions
Amino acid
0
4
8
12
16
20
24
28
32
N
M
R
Y
D
G
S
T
E
H
Q
Euk. dataset
Sphaero. iter1
Sphaero. iter2

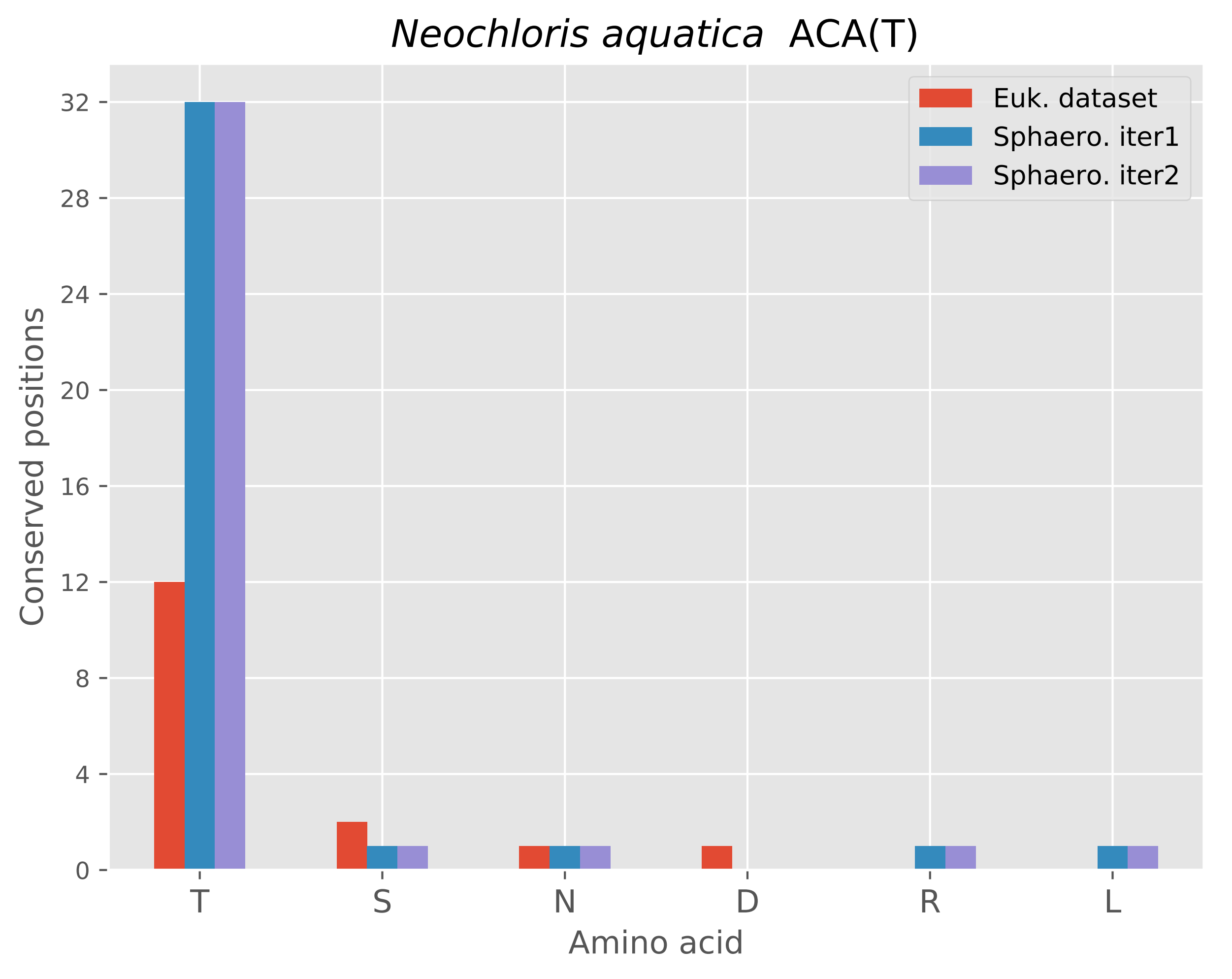

Neochloris aquatica ACA(T)
Euk. dataset
Sphaero. iter1
Sphaero. iter2
Conserved positions
0
4
8
12
16
20
24
28
32
T
S
N
D
R
L
Amino acid

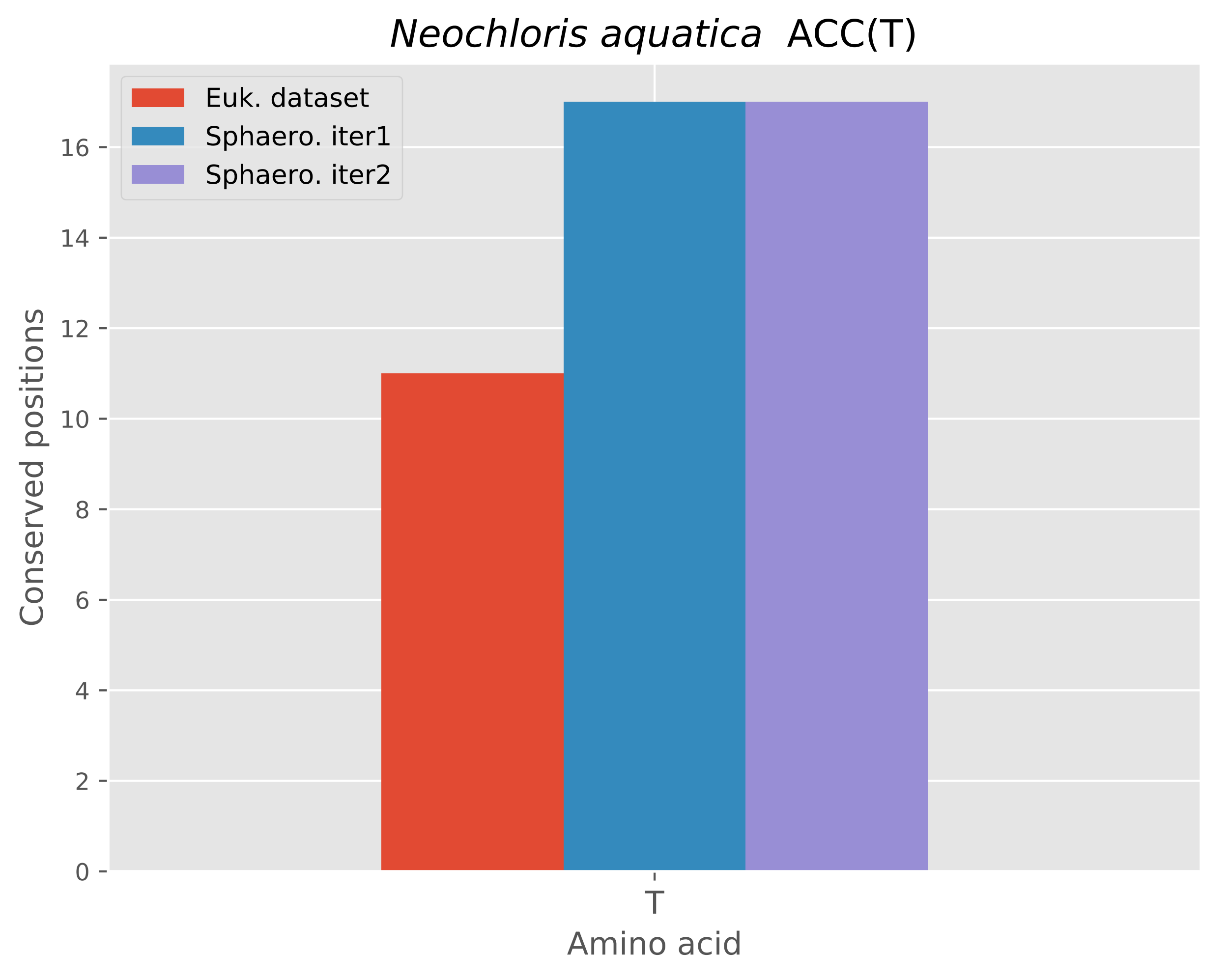

Neochloris aquatica ACC(T)
Euk. dataset
Sphaero. iter1
Sphaero. iter2
0
2
4
6
8
10
12
14
16
Conserved positions
T
Amino acid

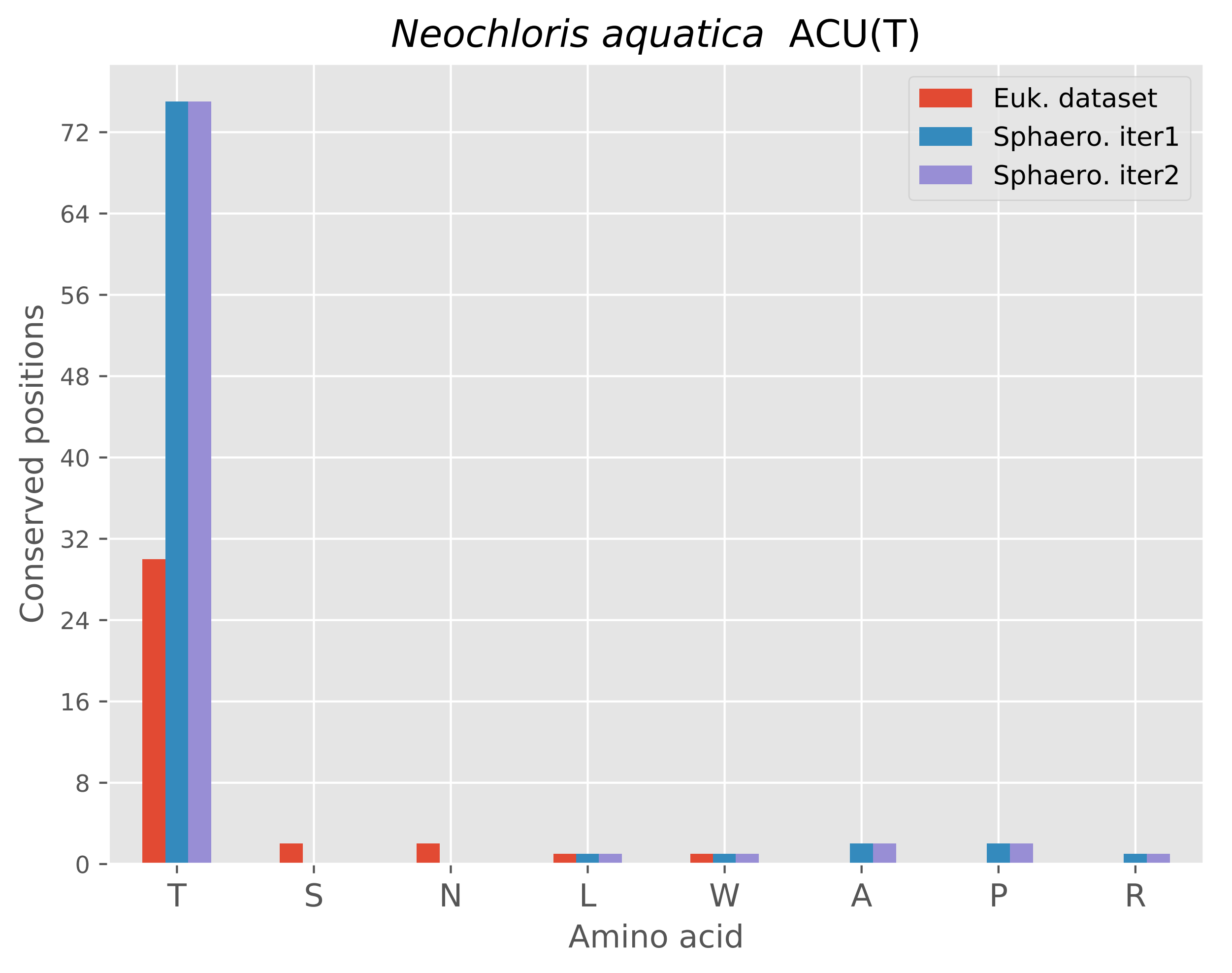

Neochloris aquatica ACU(T)
Euk. dataset
Sphaero. iter1
Sphaero. iter2
Conserved positions
0
8
16
24
32
40
48
56
64
72
T
S
N
L
W
A
P
R
Amino acid

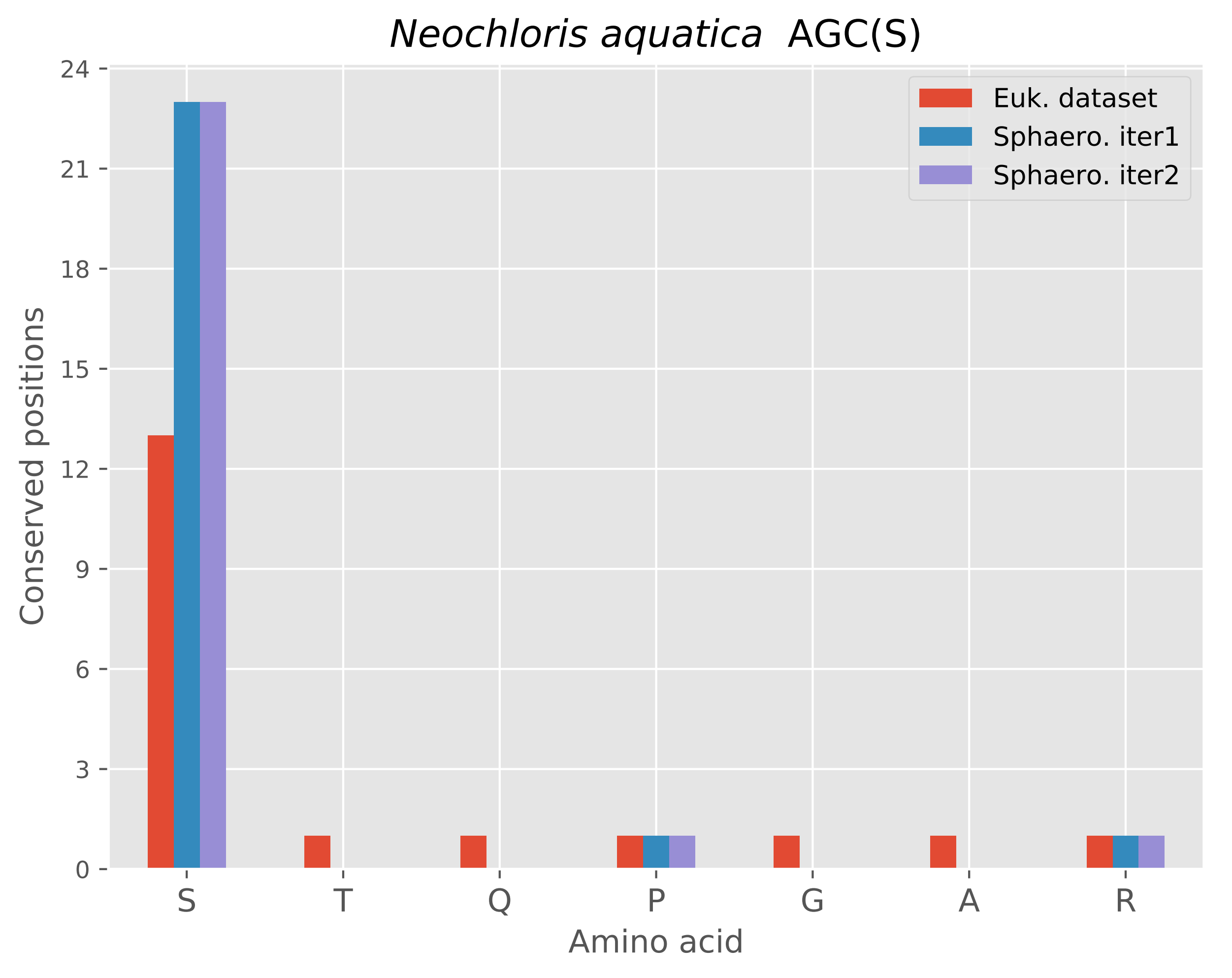

Neochloris aquatica AGC(S)
Conserved positions
Amino acid
S
T
Q
P
G
A
R
0
3
6
9
12
15
18
21
24
Euk. dataset
Sphaero. iter1
Sphaero. iter2

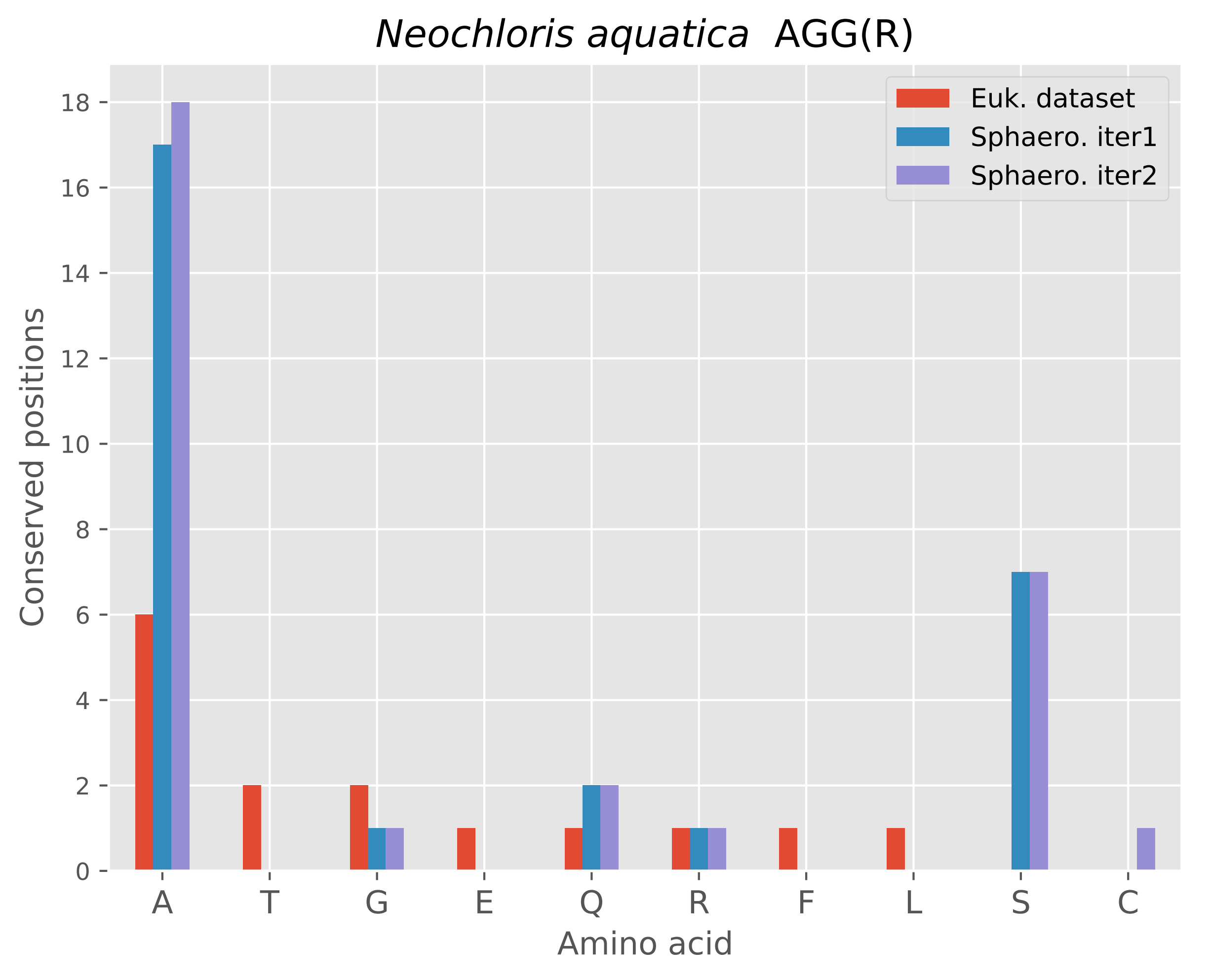

Neochloris aquatica AGG(R)
Euk. dataset
Sphaero. iter1
Sphaero. iter2
Conserved positions
0
2
4
6
8
10
12
14
16
18
A
T
G
E
Q
R
F
L
S
C
Amino acid

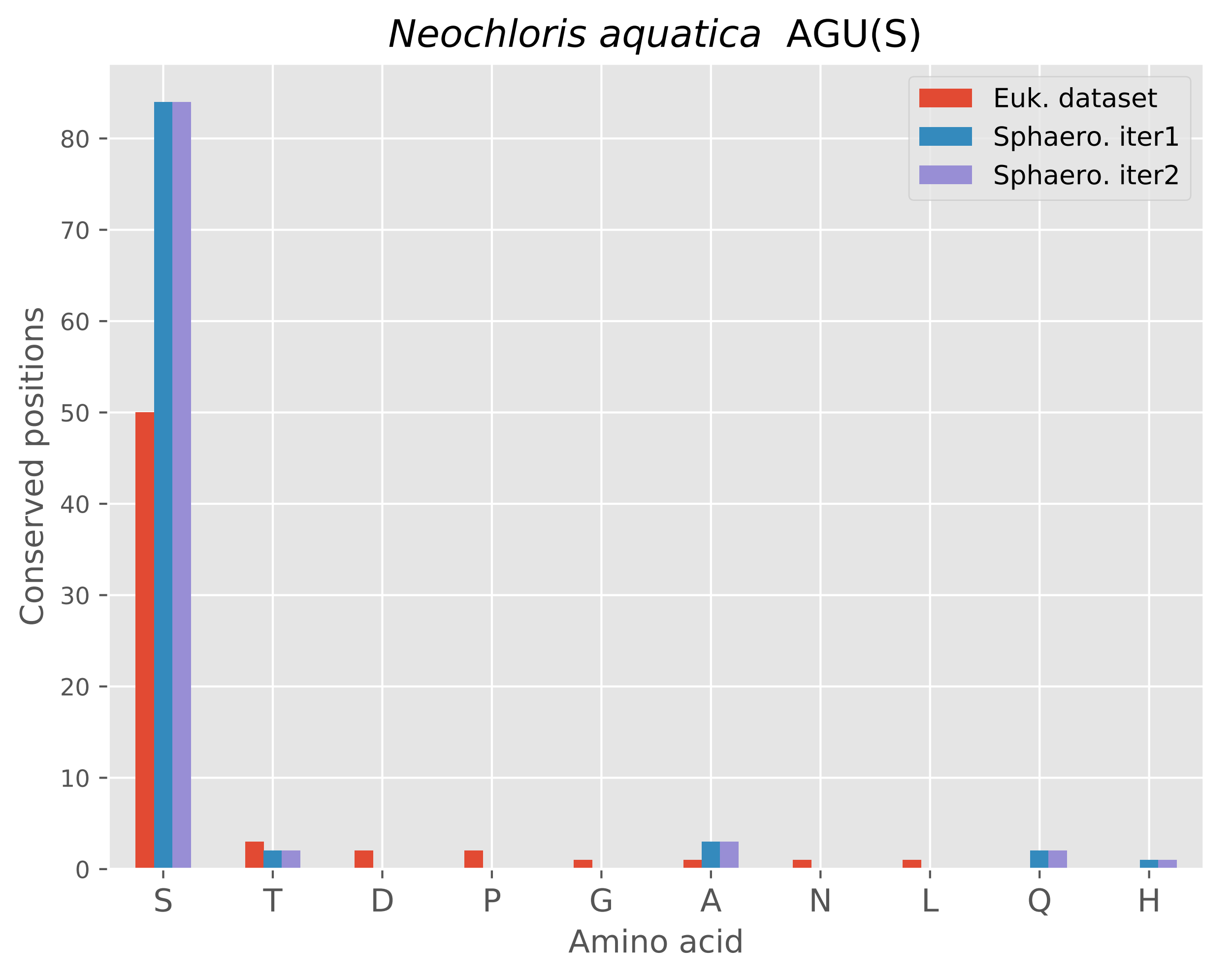

Neochloris aquatica AGU(S)
Euk. dataset
Sphaero. iter1
Sphaero. iter2
Conserved positions
0
10
20
30
40
50
60
70
80
S
T
D
P
G
A
N
L
Q
H
Amino acid

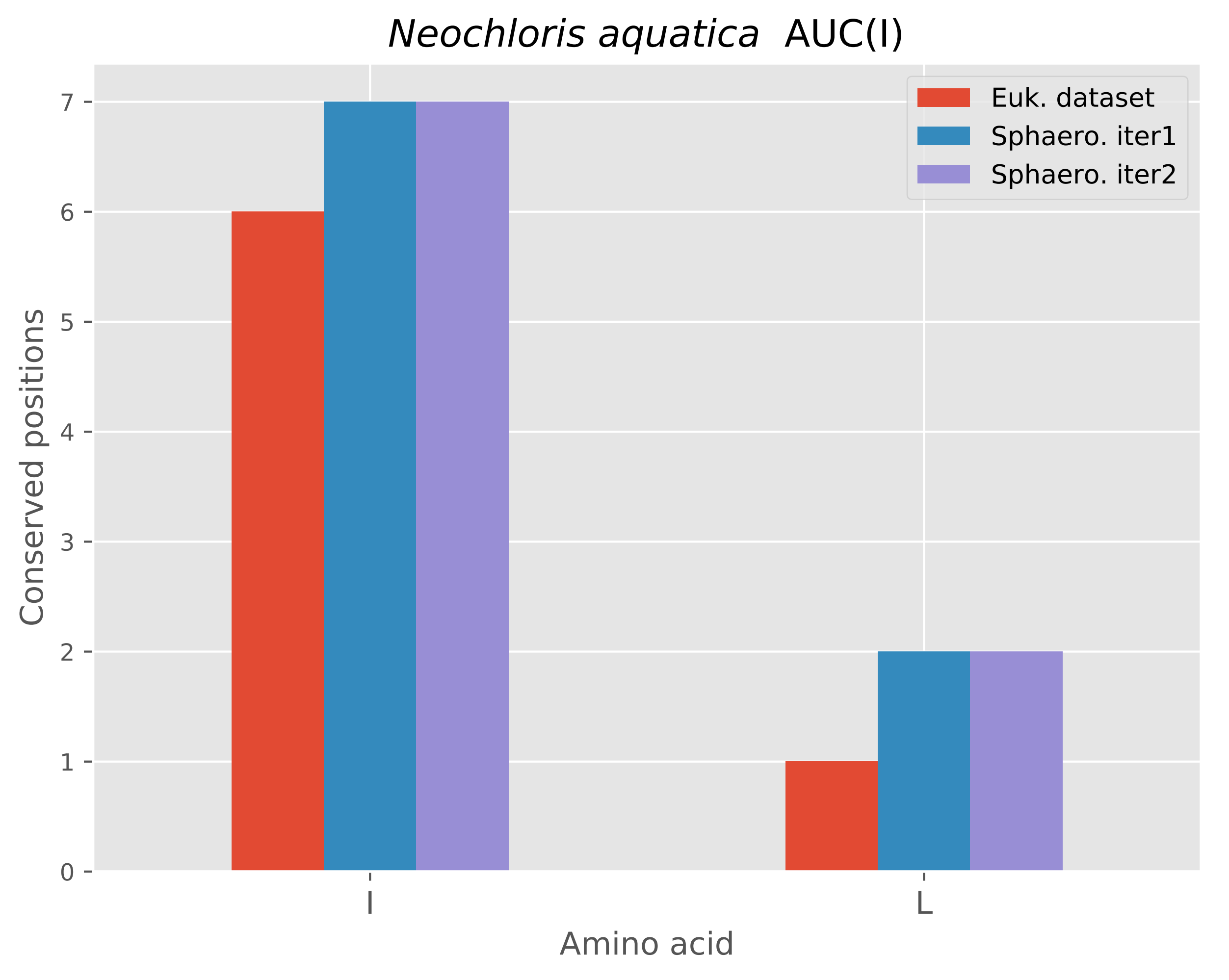

Neochloris aquatica AUC(I)
Euk. dataset
Sphaero. iter1
Sphaero. iter2
Conserved positions
0
1
2
3
4
5
6
7
I
L
Amino acid

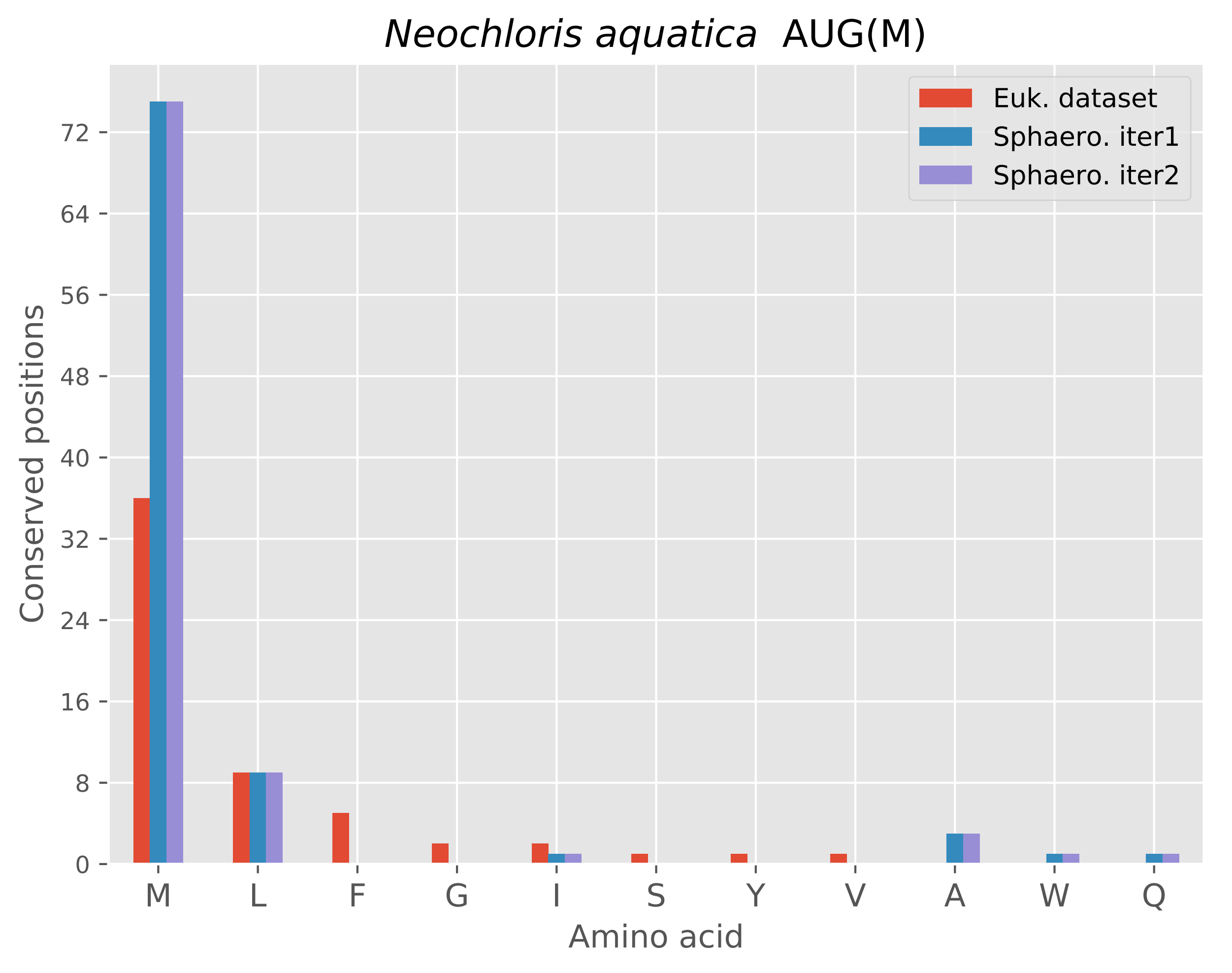

Neochloris aquatica AUG(M)
Euk. dataset
Sphaero. iter1
Sphaero. iter2
Conserved positions
0
8
16
24
32
40
48
56
64
72
M
L
F
G
I
S
Y
V
A
W
Q
Amino acid

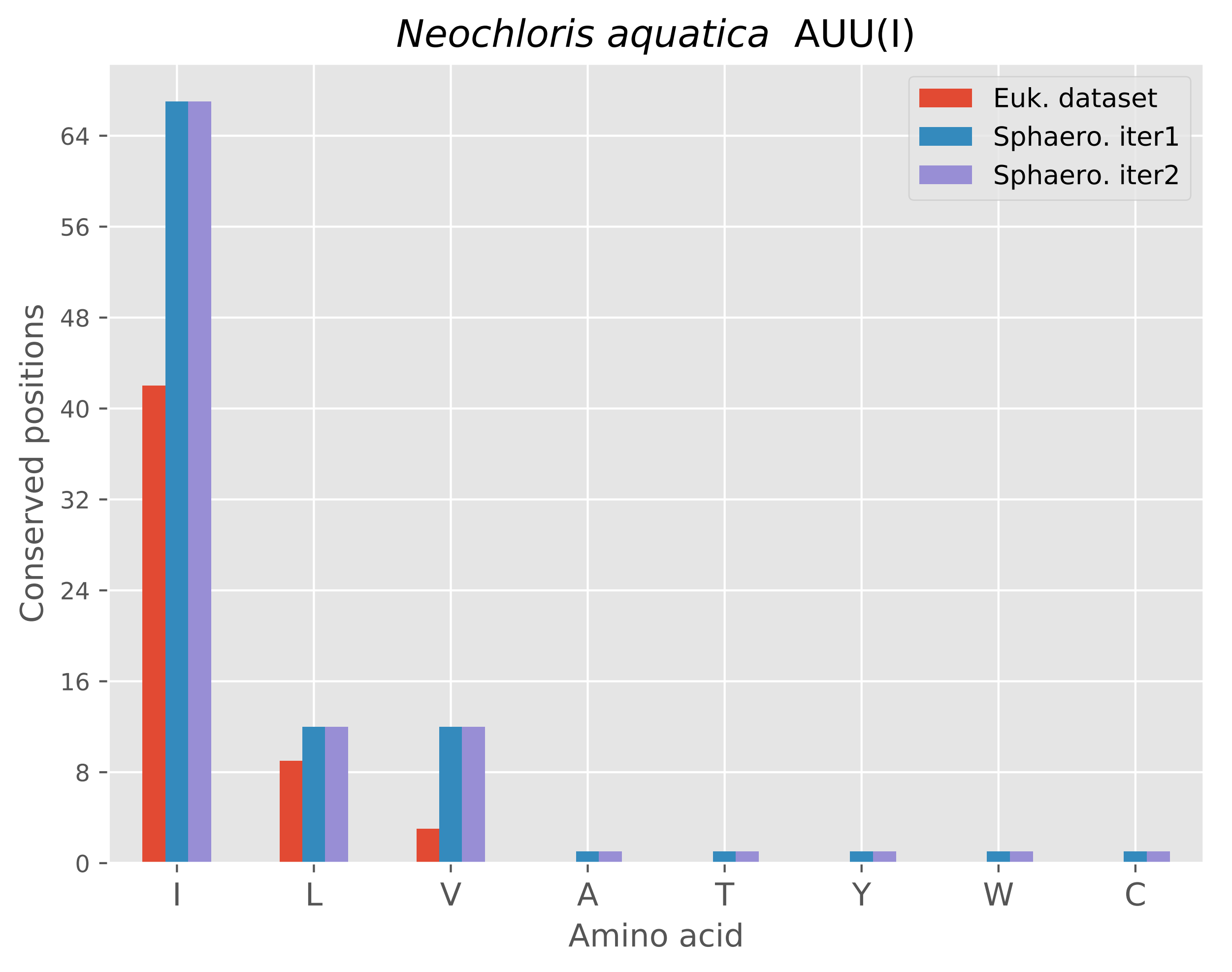

Neochloris aquatica AUU(I)
Euk. dataset
Sphaero. iter1
Sphaero. iter2
Conserved positions
0
8
16
24
32
40
48
56
64
I
L
V
A
T
Y
W
C
Amino acid

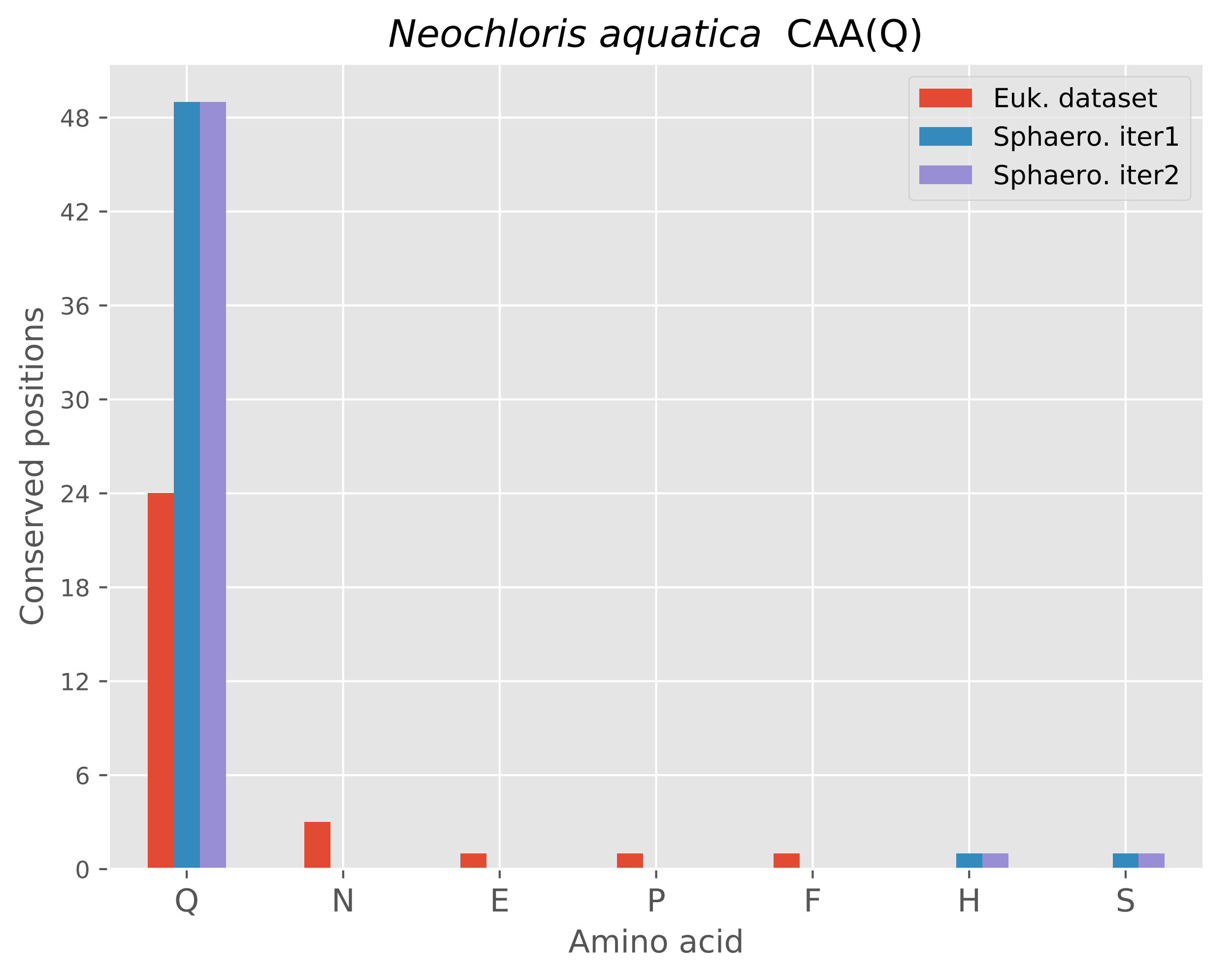

Neochloris aquatica CAA(Q)
Euk. dataset
Sphaero. iter1
Sphaero. iter2
Conserved positions
0
6
12
18
24
30
36
42
48
Q
N
E
P
F
H
S
Amino acid

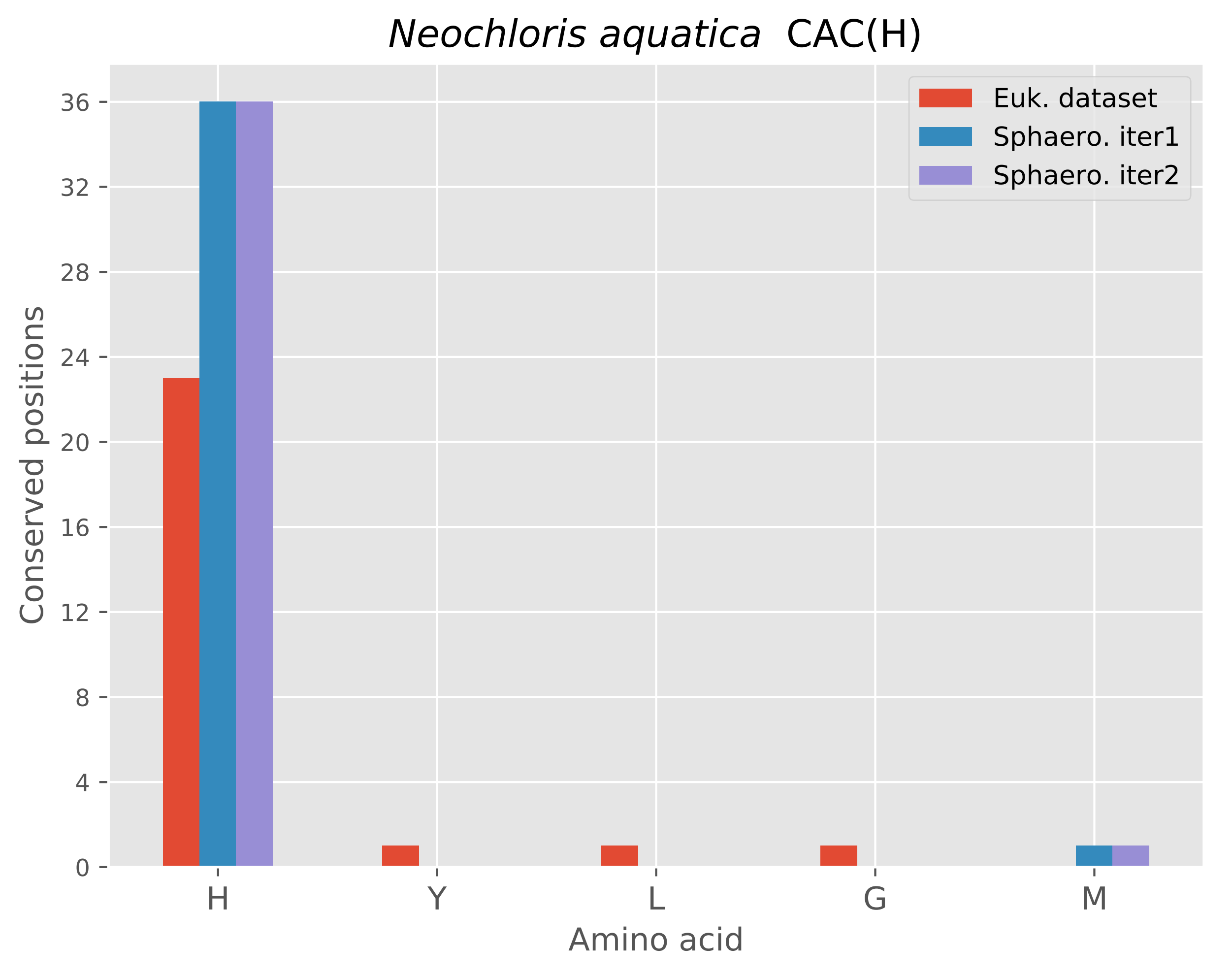

Neochloris aquatica CAC(H)
Euk. dataset
Sphaero. iter1
Sphaero. iter2
Conserved positions
0
4
8
12
16
20
24
28
32
36
H
Y
L
G
M
Amino acid

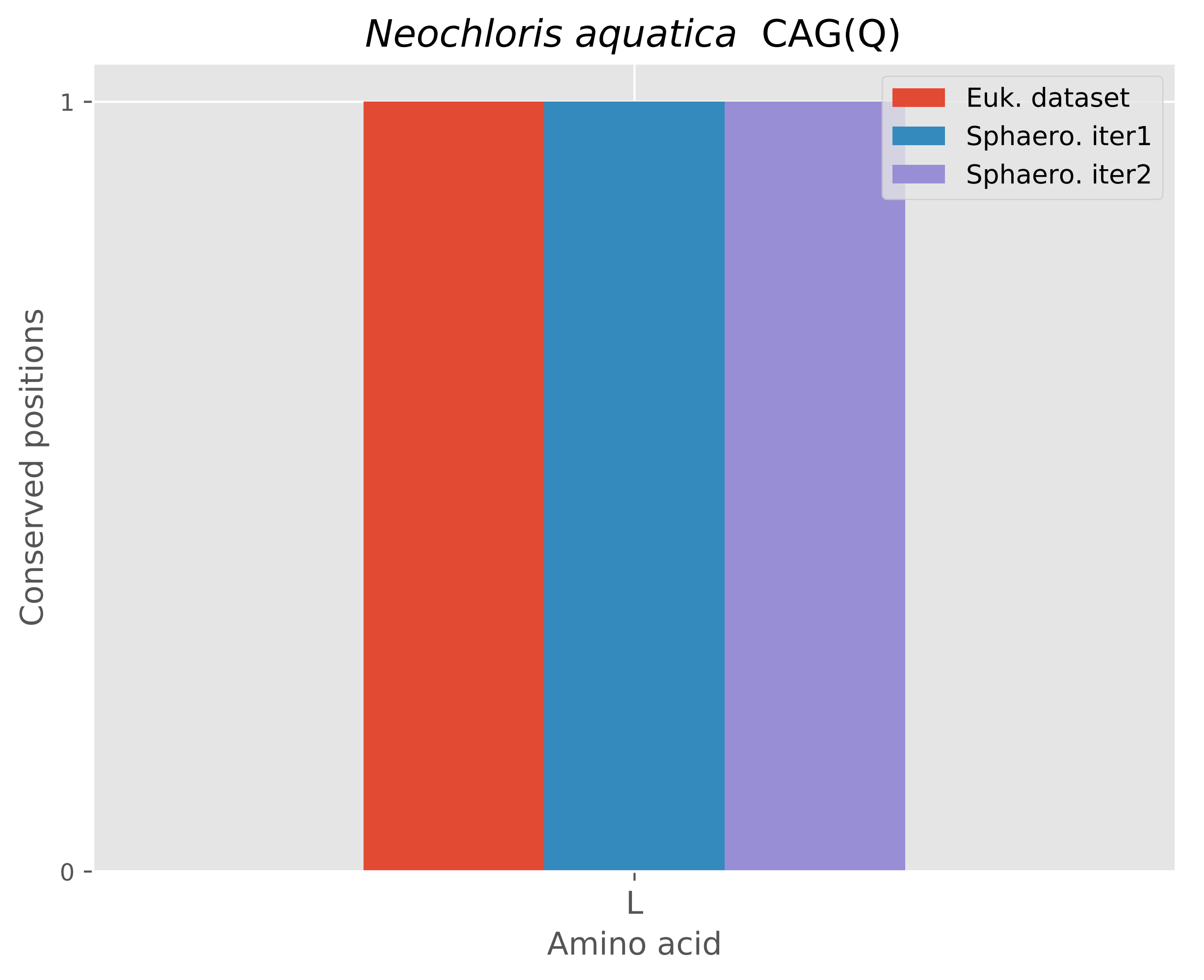

Neochloris aquatica CAG(Q)
Euk. dataset
Sphaero. iter1
Sphaero. iter2
1
0
Conserved positions
L
Amino acid

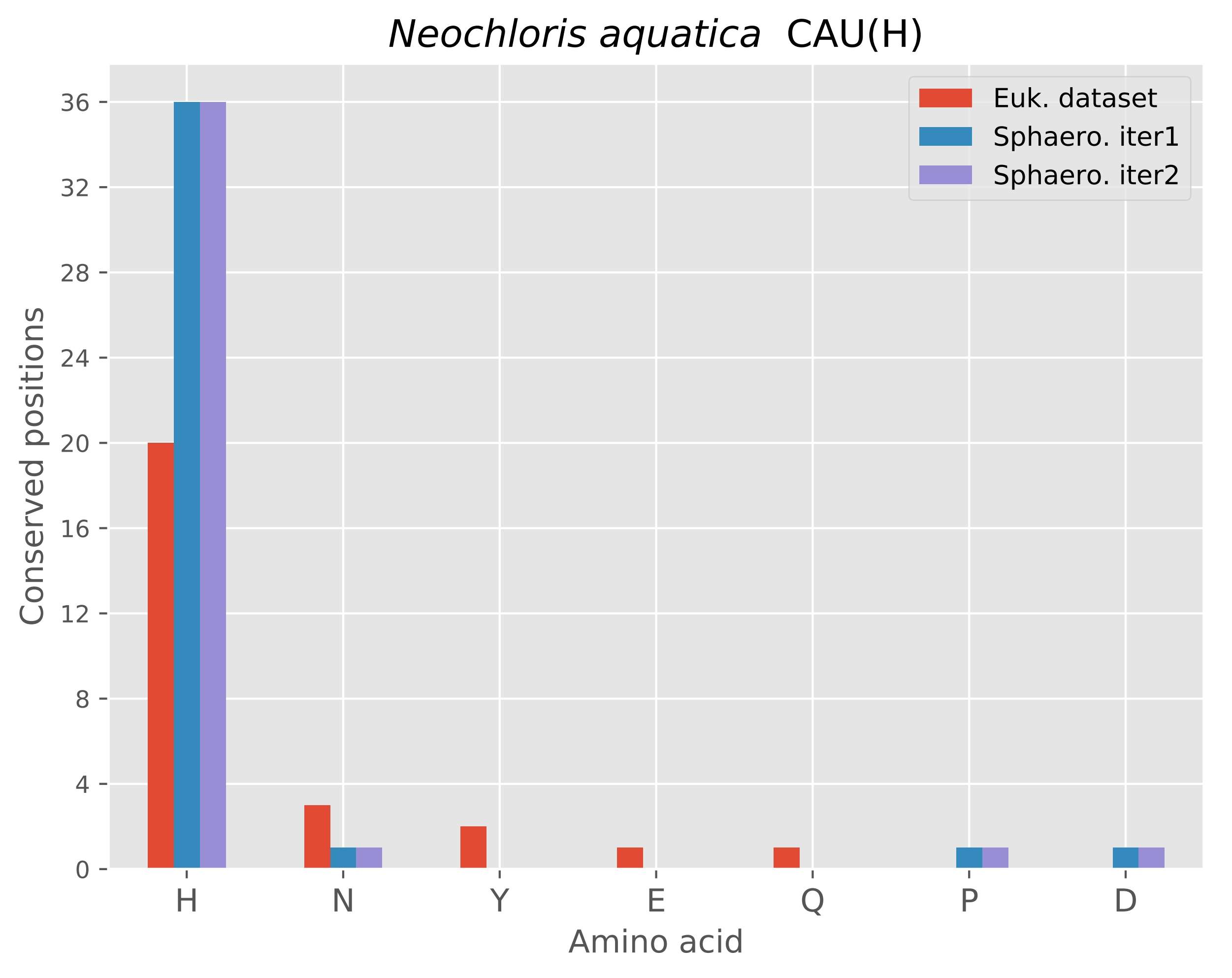

Neochloris aquatica CAU(H)
Euk. dataset
Sphaero. iter1
Sphaero. iter2
Conserved positions
0
4
8
12
16
20
24
28
32
36
H
N
Y
E
Q
P
D
Amino acid

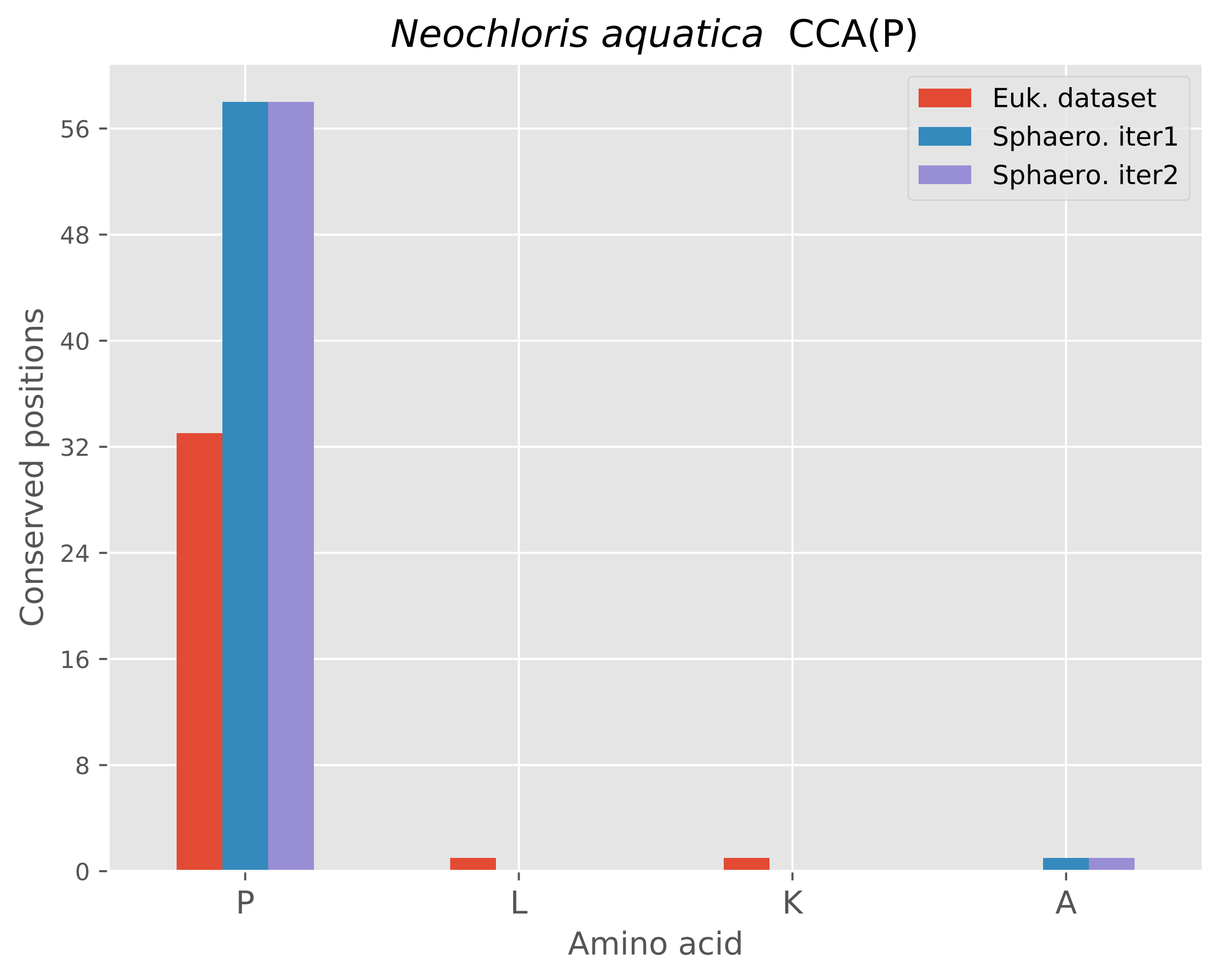
Neochloris aquatica CCA(P)
Conserved positions
0
8
16
24
32
40
48
56
Euk. dataset
Sphaero. iter1
Sphaero. iter2
P
L
K
A
Amino acid

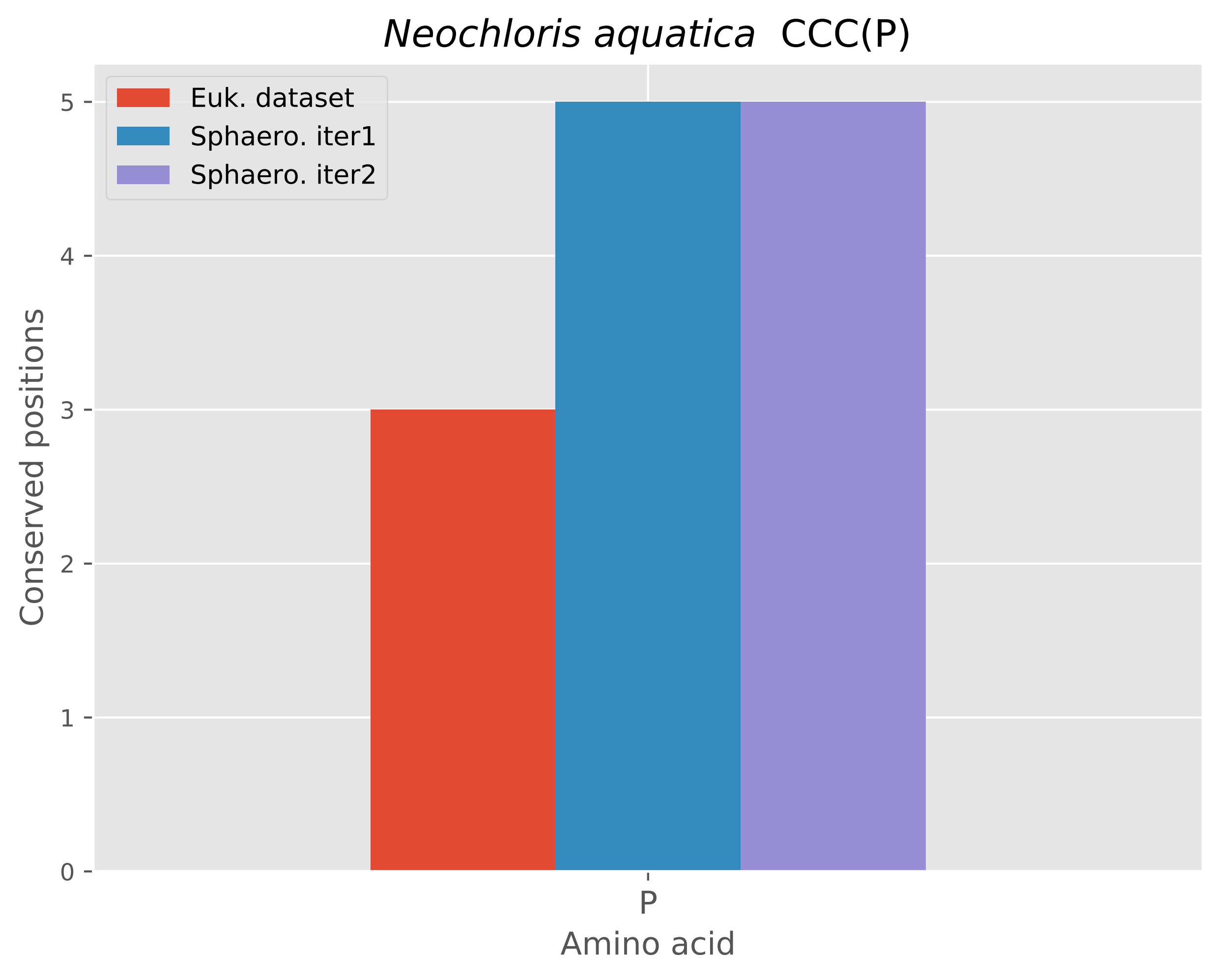

Neochloris aquatica CCC(P)
Euk. dataset
Sphaero. iter1
Sphaero. iter2
Conserved positions
0
1
2
3
4
5
P
Amino acid

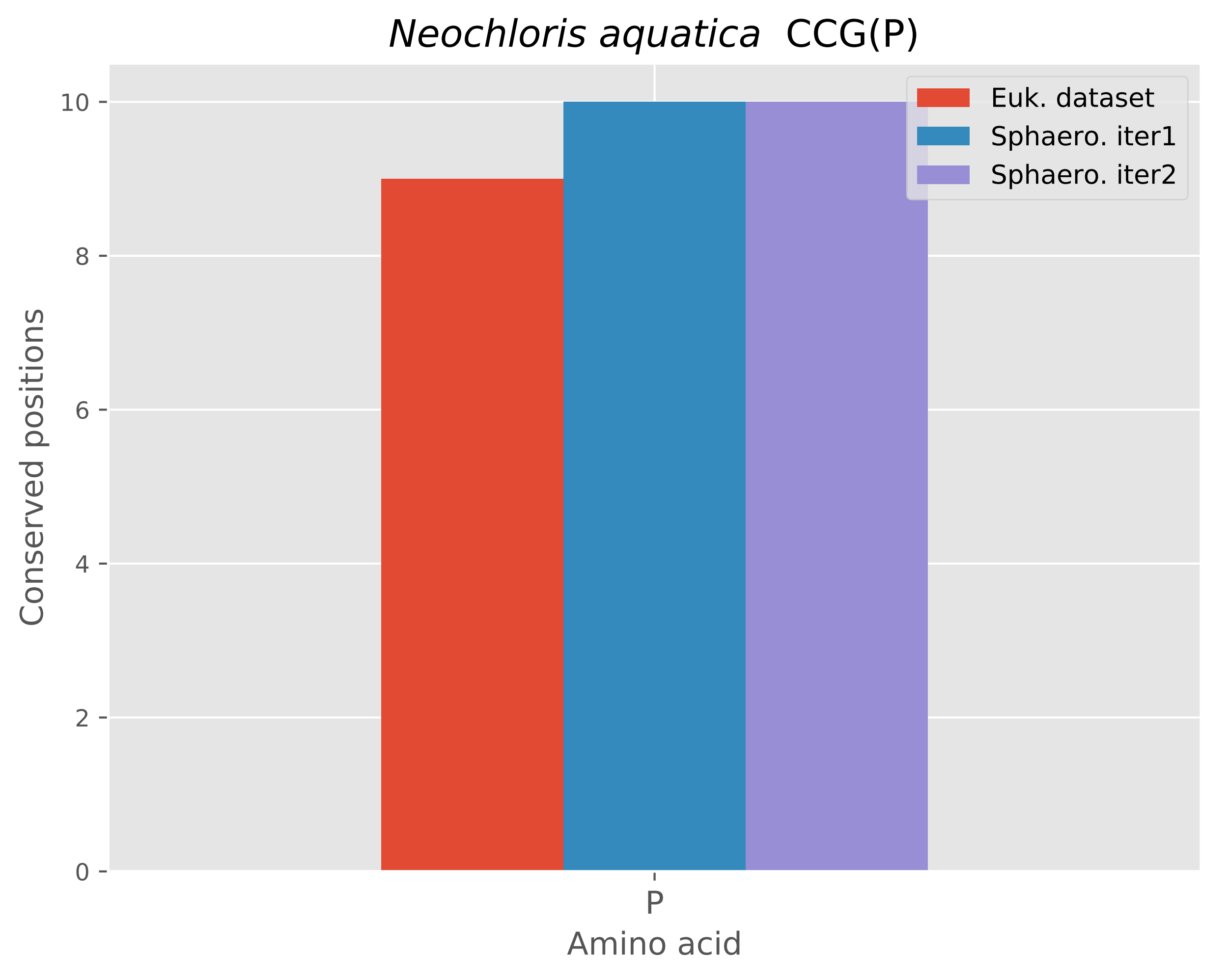
Neochloris aquatica CCG(P)
Conserved positions
Amino acid
P
0
2
4
6
8
10
Euk. dataset
Sphaero. iter1
Sphaero. iter2

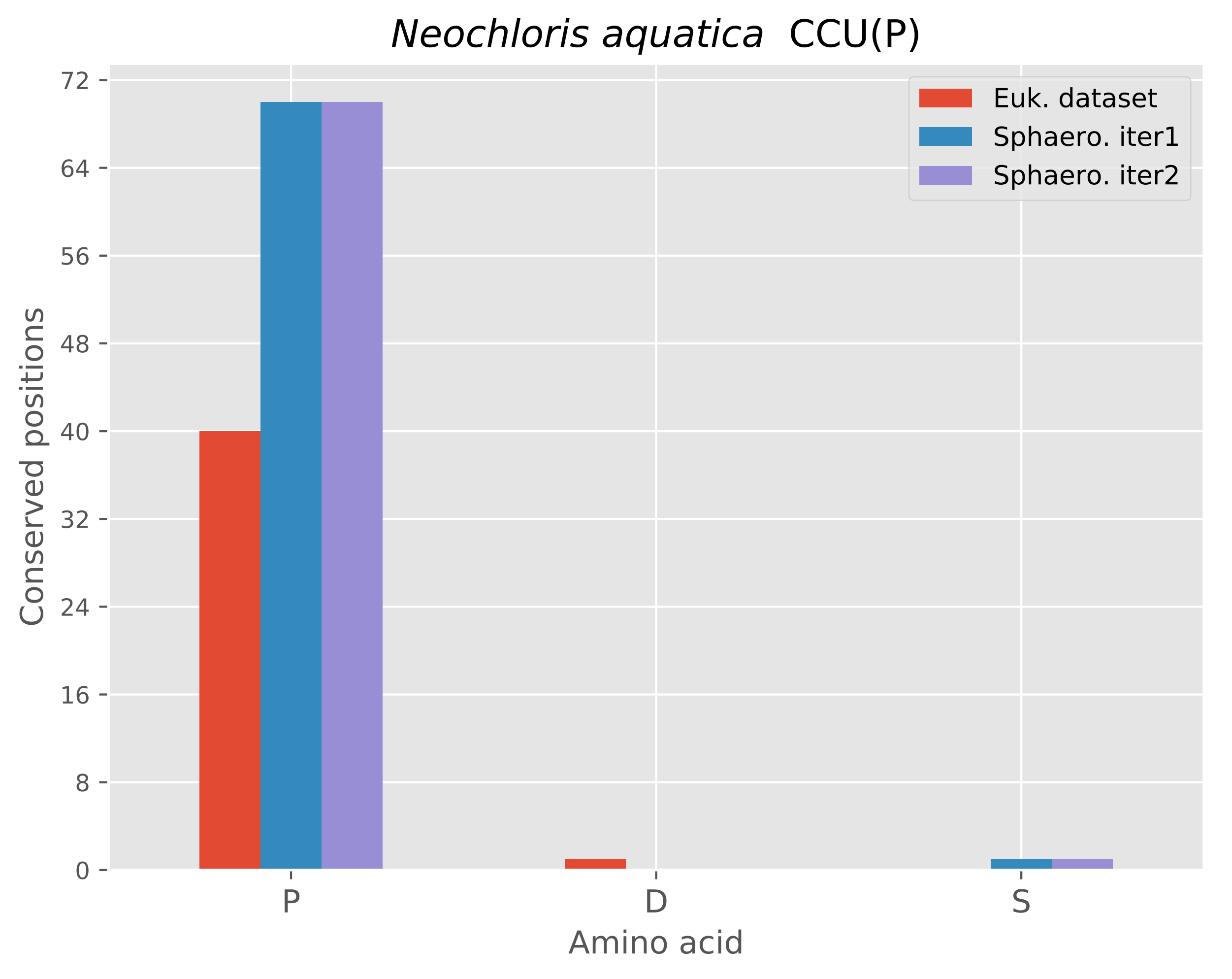

Neochloris aquatica CCU(P)
Euk. dataset
Sphaero. iter1
Sphaero. iter2
Conserved positions
0
8
16
24
32
40
48
56
64
72
P
D
S
Amino acid

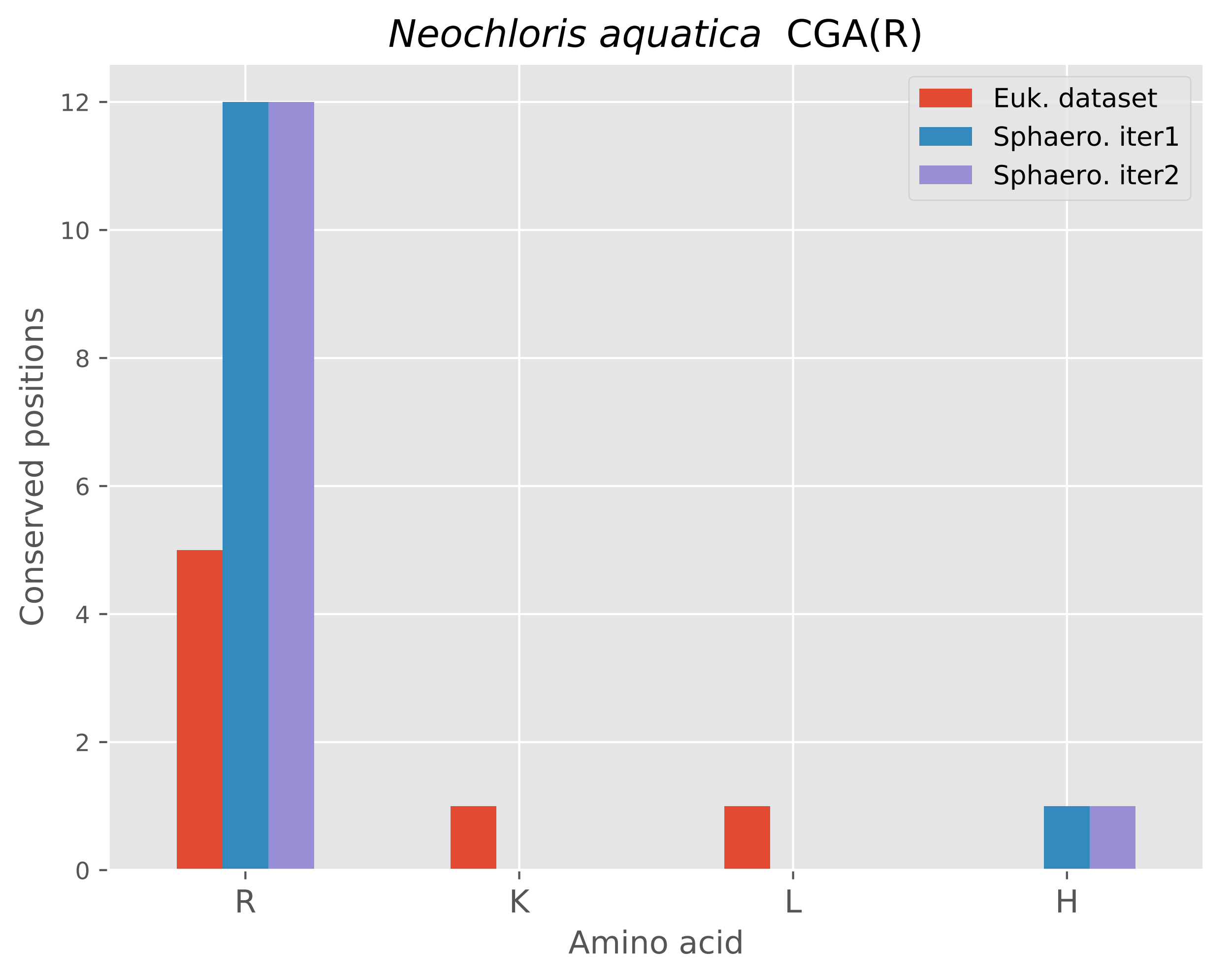
Neochloris aquatica CGA(R)
Euk. dataset
Sphaero. iter1
Sphaero. iter2
Conserved positions
0
2
4
6
8
10
12
R
K
L
H
Amino acid

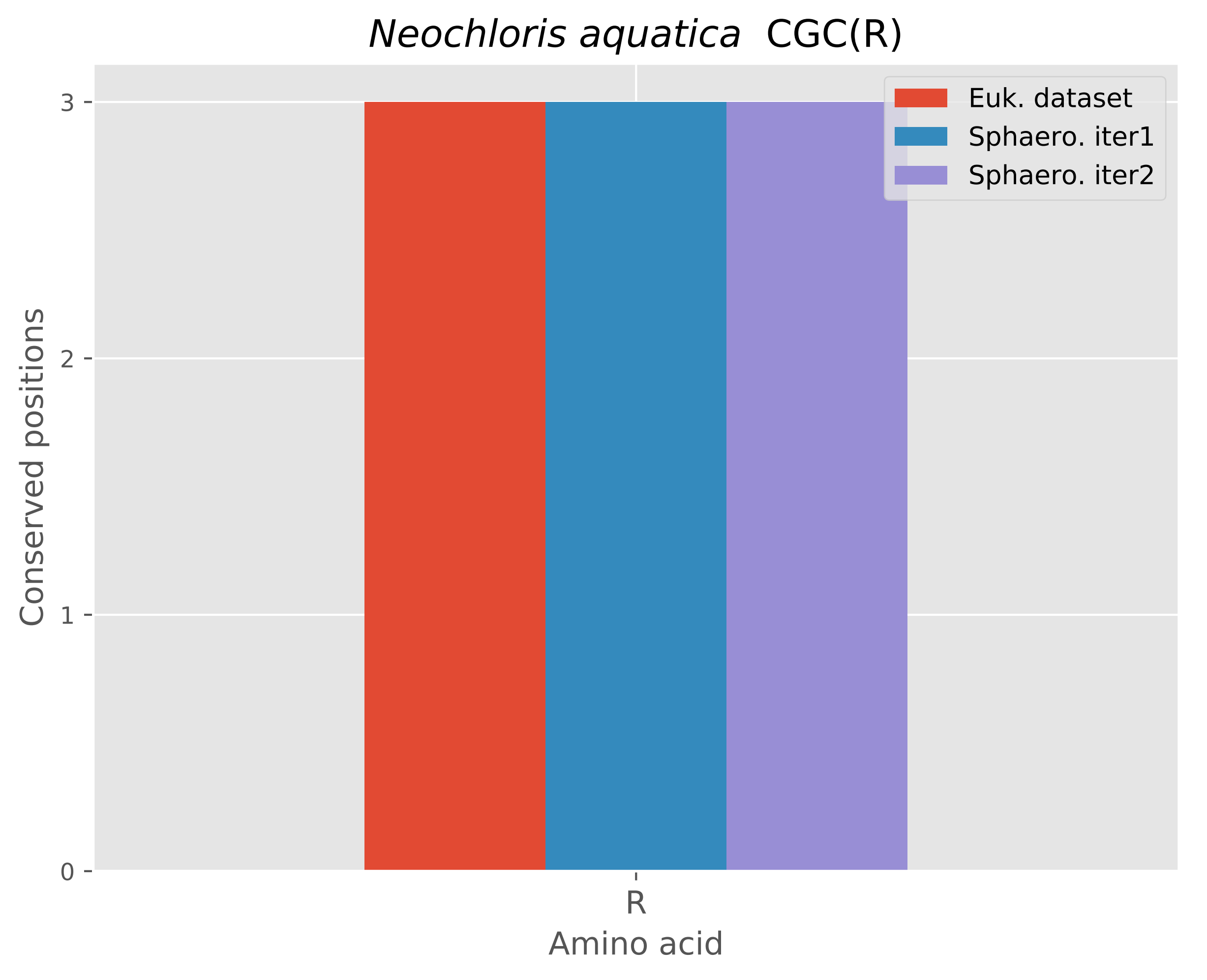
Neochloris aquatica CGC(R)
Euk. dataset
Sphaero. iter1
Sphaero. iter2
Conserved positions
3
2
1
0
R
Amino acid

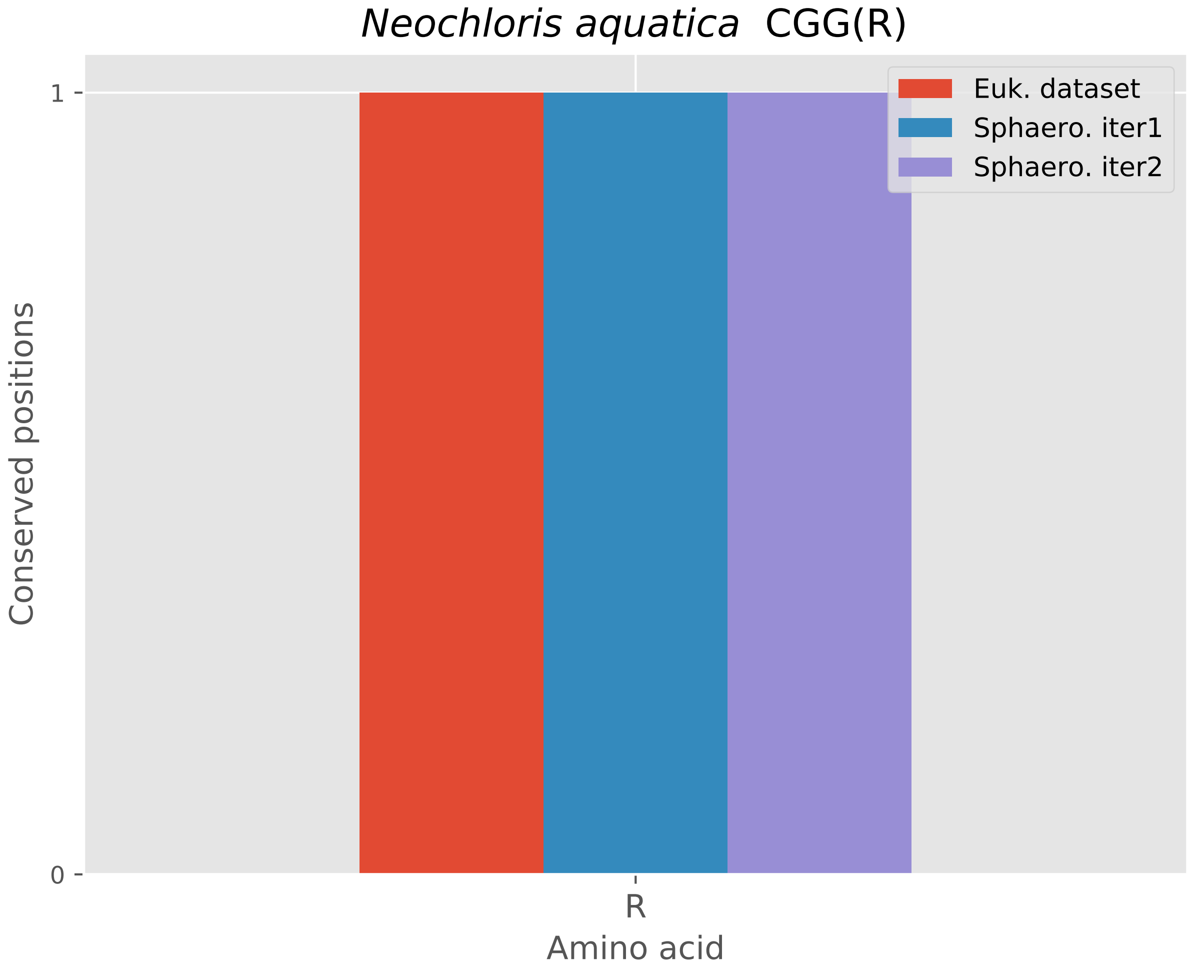

Neochloris aquatica CGG(R)
Conserved positions
1
0
R
Amino acid
Euk. dataset
Sphaero. iter1
Sphaero. iter2

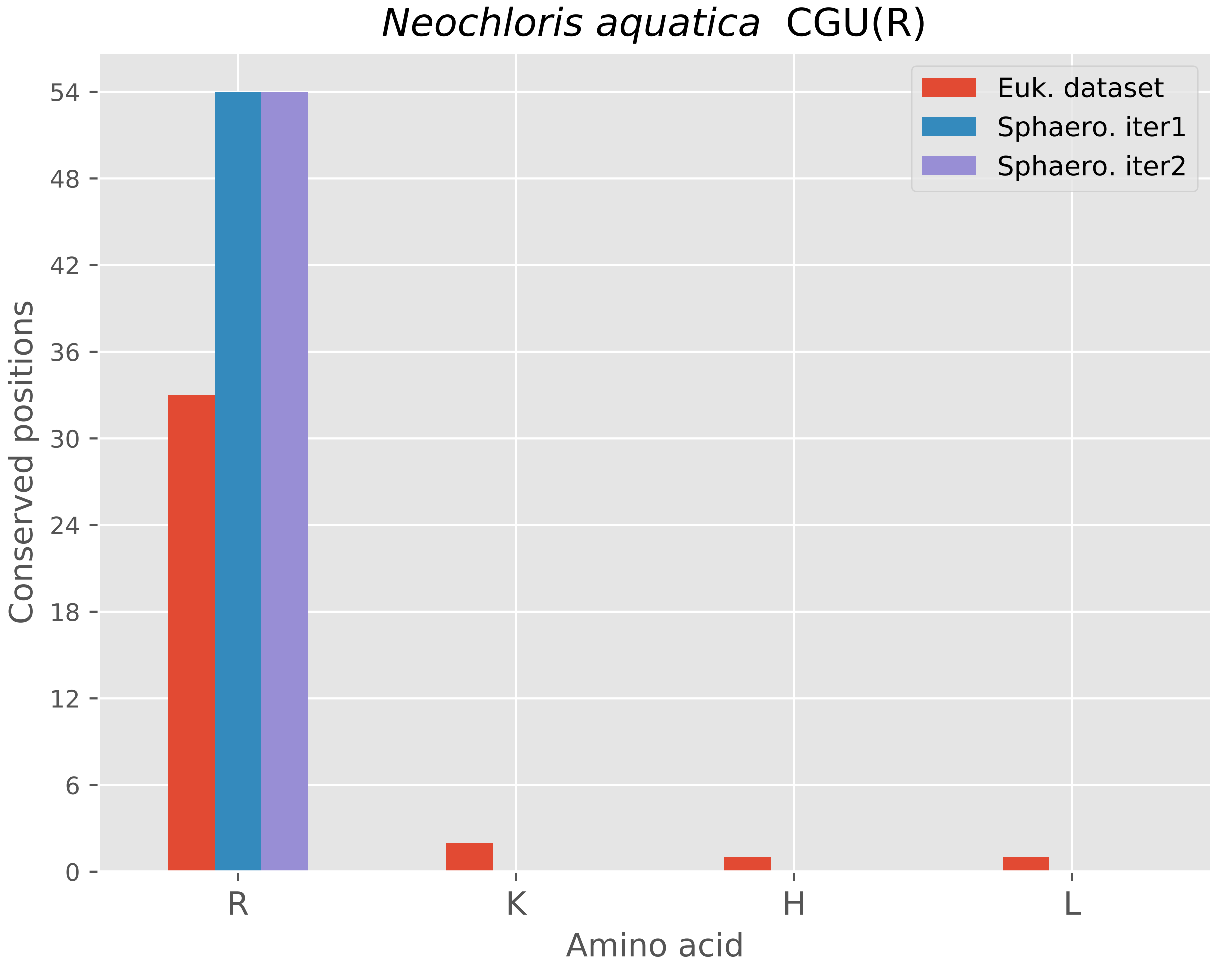

Neochloris aquatica CGU(R)
Euk. dataset
Sphaero. iter1
Sphaero. iter2
Conserved positions
0
6
12
18
24
30
36
42
48
54
R
K
H
L
Amino acid

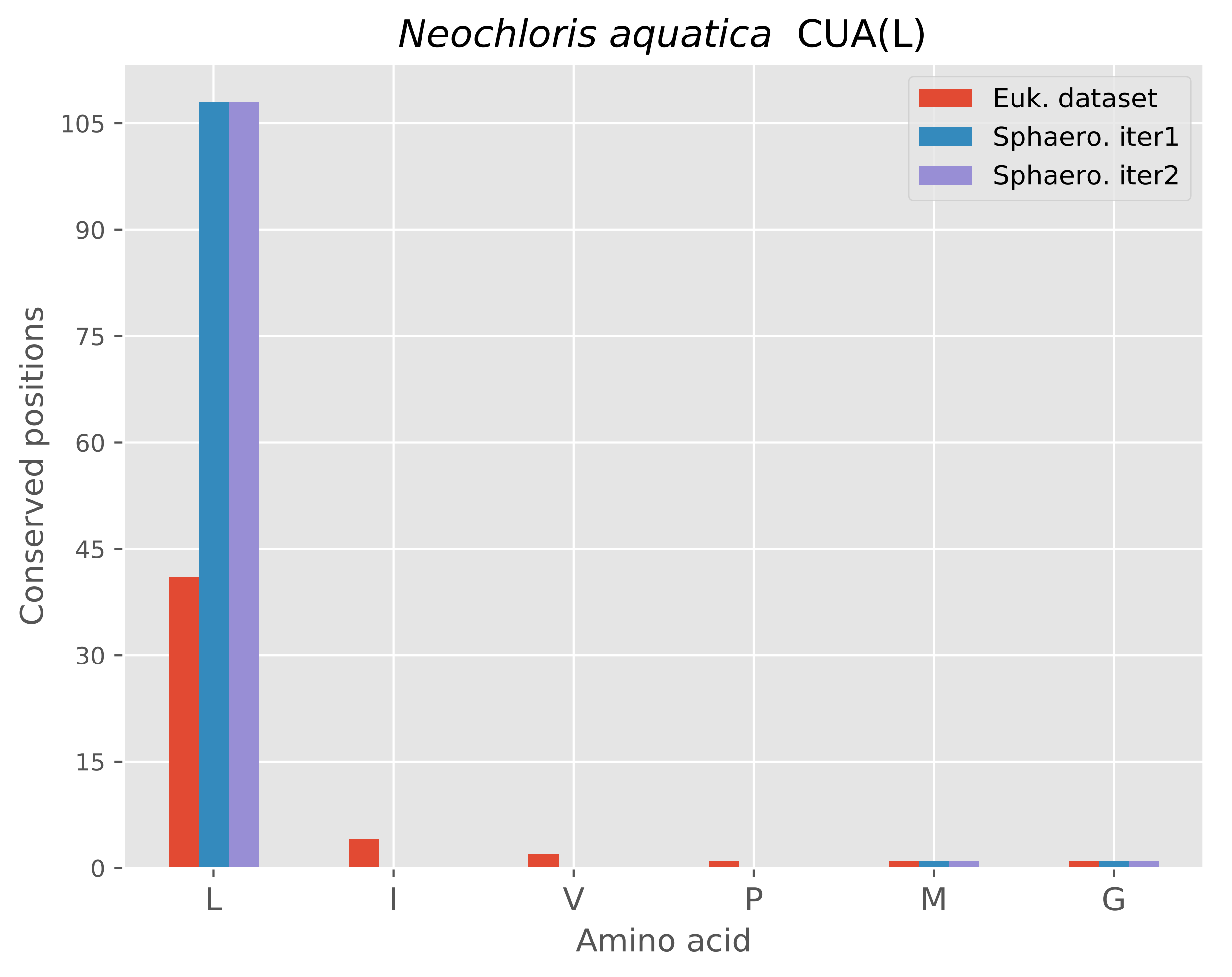

Neochloris aquatica CUA(L)
Euk. dataset
Sphaero. iter1
Sphaero. iter2
Conserved positions
0
15
30
45
60
75
90
105
L
I
V
P
M
G
Amino acid

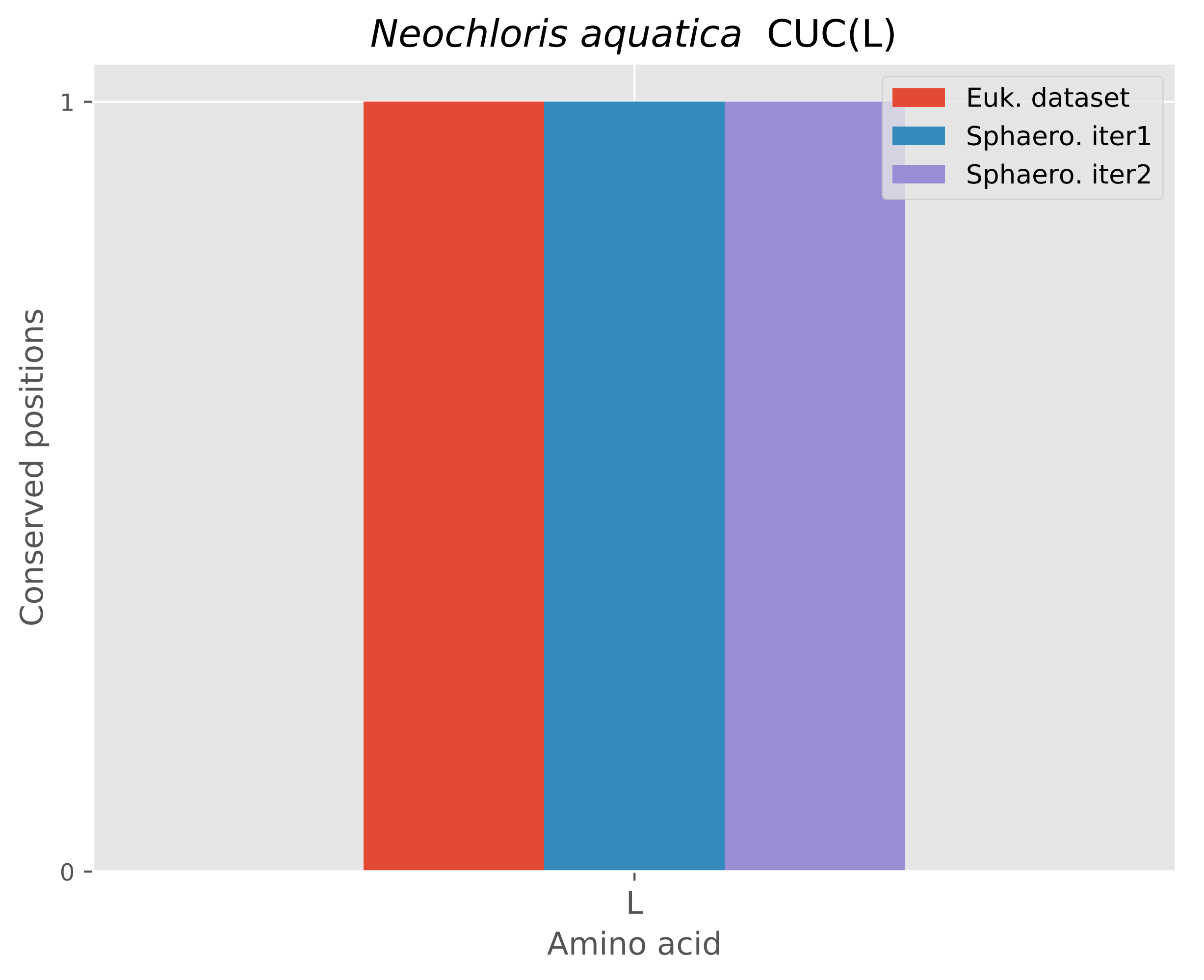

Neochloris aquatica CUC(L)
Euk. dataset
Sphaero. iter1
Sphaero. iter2
Conserved positions
1
0
L
Amino acid

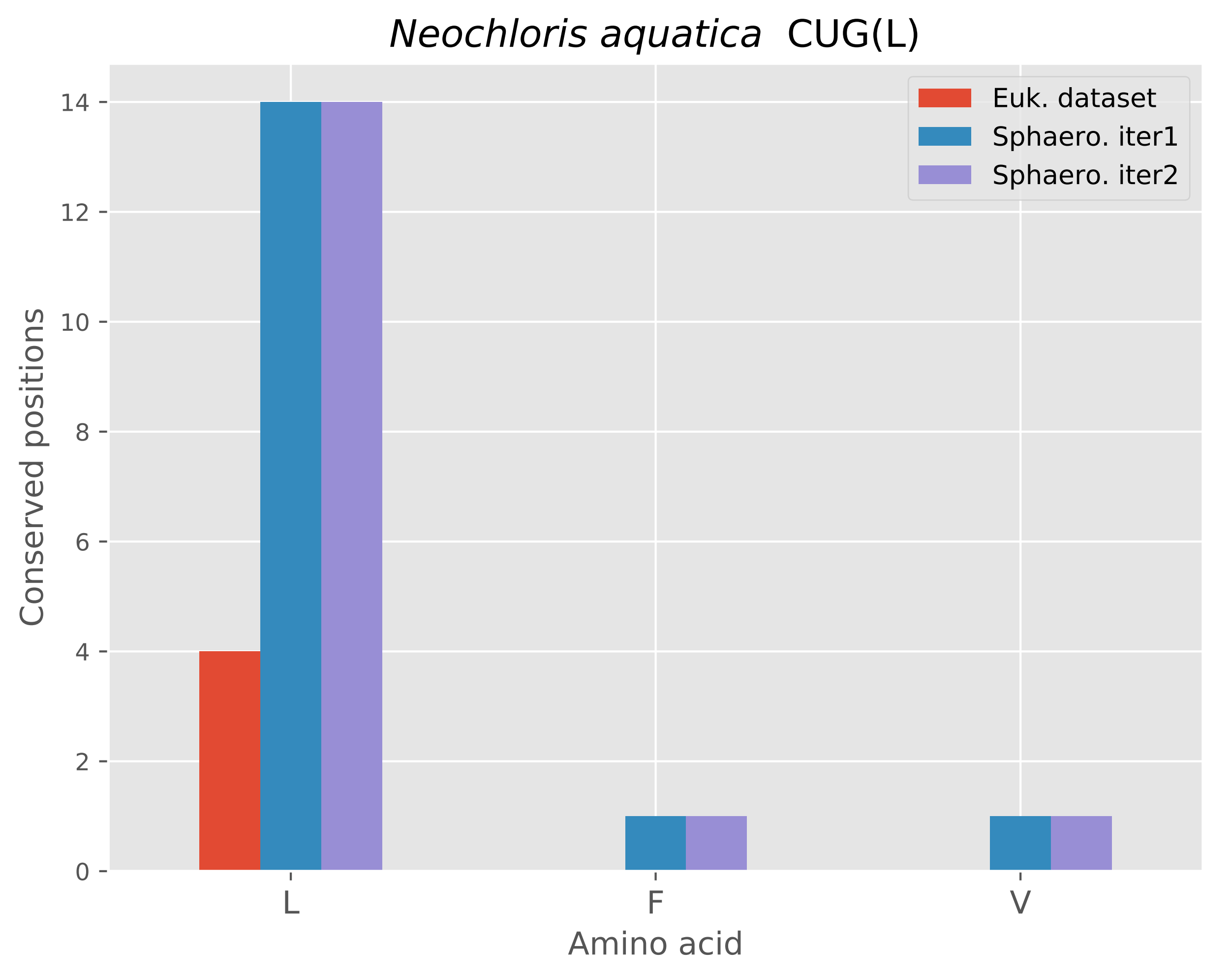

Neochloris aquatica CUG(L)
Euk. dataset
Sphaero. iter1
Sphaero. iter2
Conserved positions
0
2
4
6
8
10
12
14
L
F
V
Amino acid

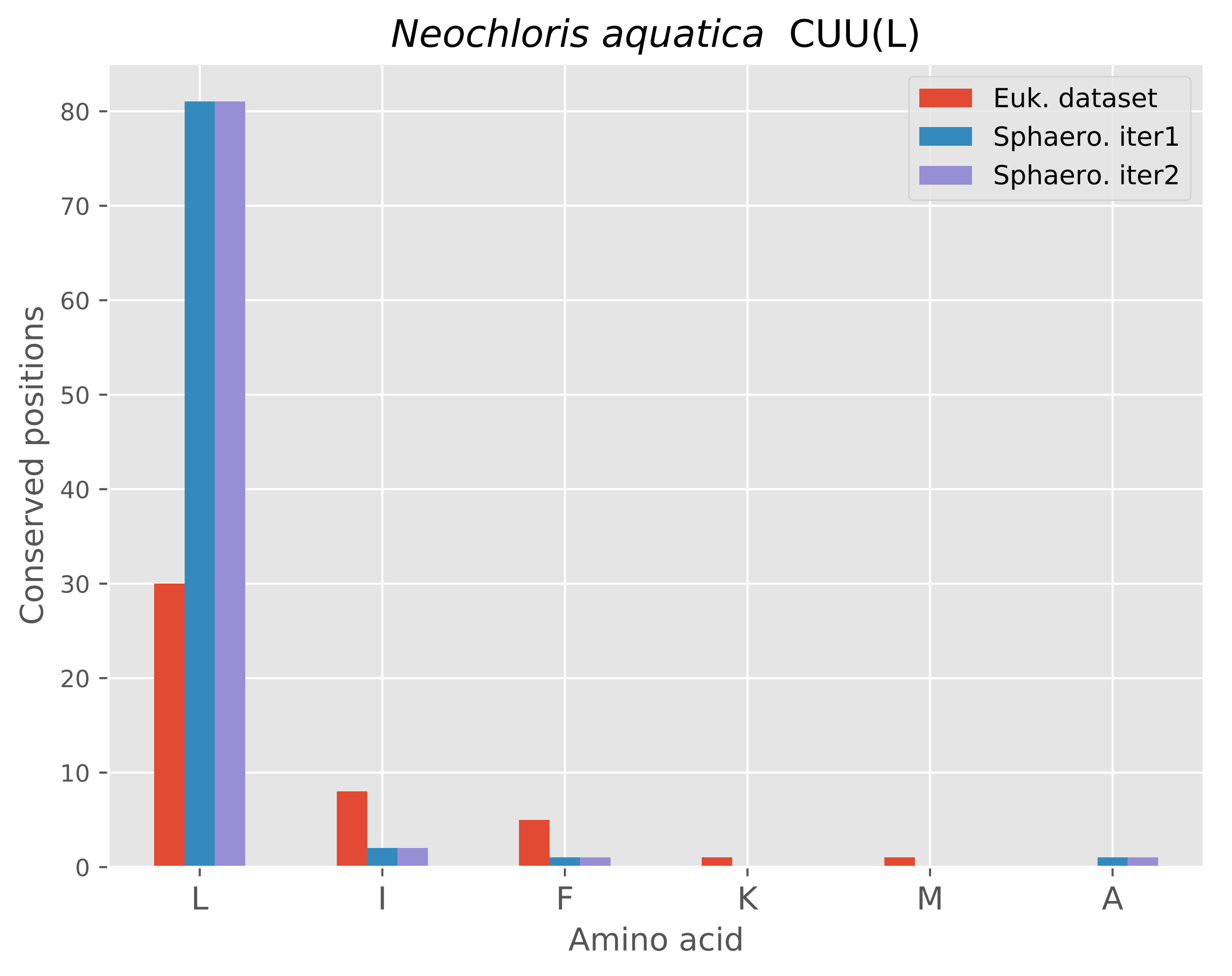

Neochloris aquatica CUU(L)
Euk. dataset
Sphaero. iter1
Sphaero. iter2
Conserved positions
0
10
20
30
40
50
60
70
80
L
I
F
K
M
A
Amino acid

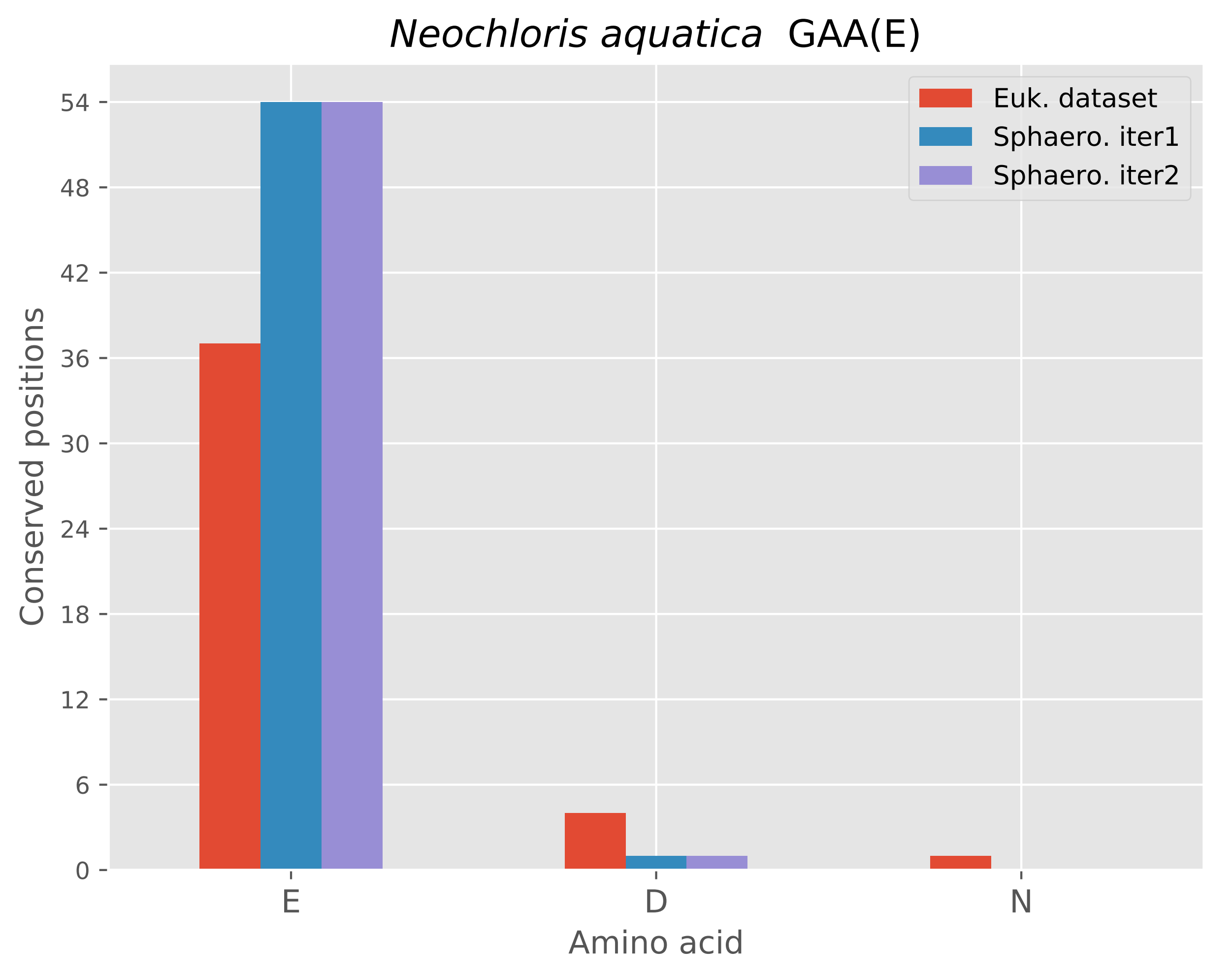

Neochloris aquatica GAA(E)
Euk. dataset
Sphaero. iter1
Sphaero. iter2
Conserved positions
0
6
12
18
24
30
36
42
48
54
E
D
N
Amino acid

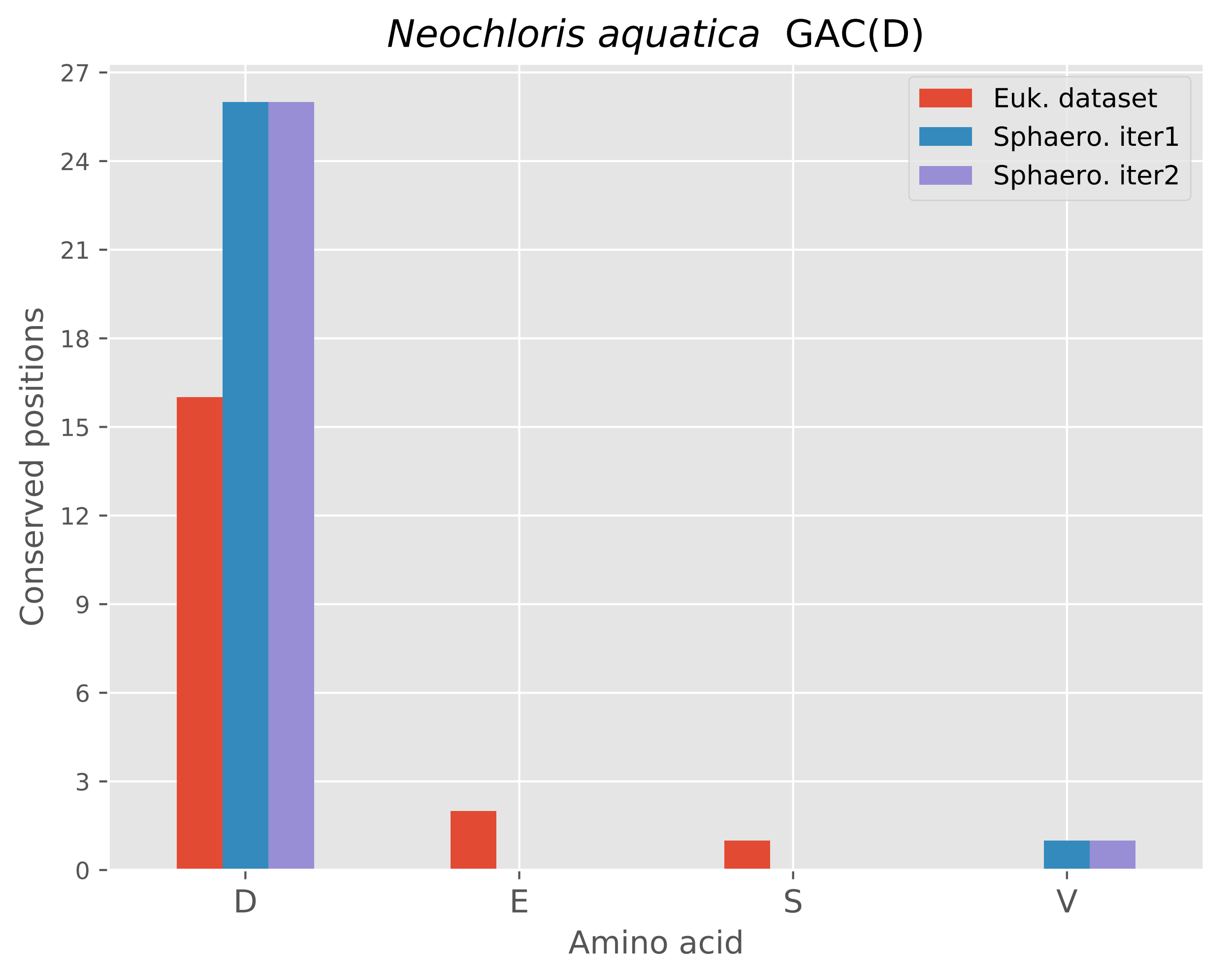

Neochloris aquatica GAC(D)
Euk. dataset
Sphaero. iter1
Sphaero. iter2
Conserved positions
0
3
6
9
12
15
18
21
24
27
D
E
S
V
Amino acid

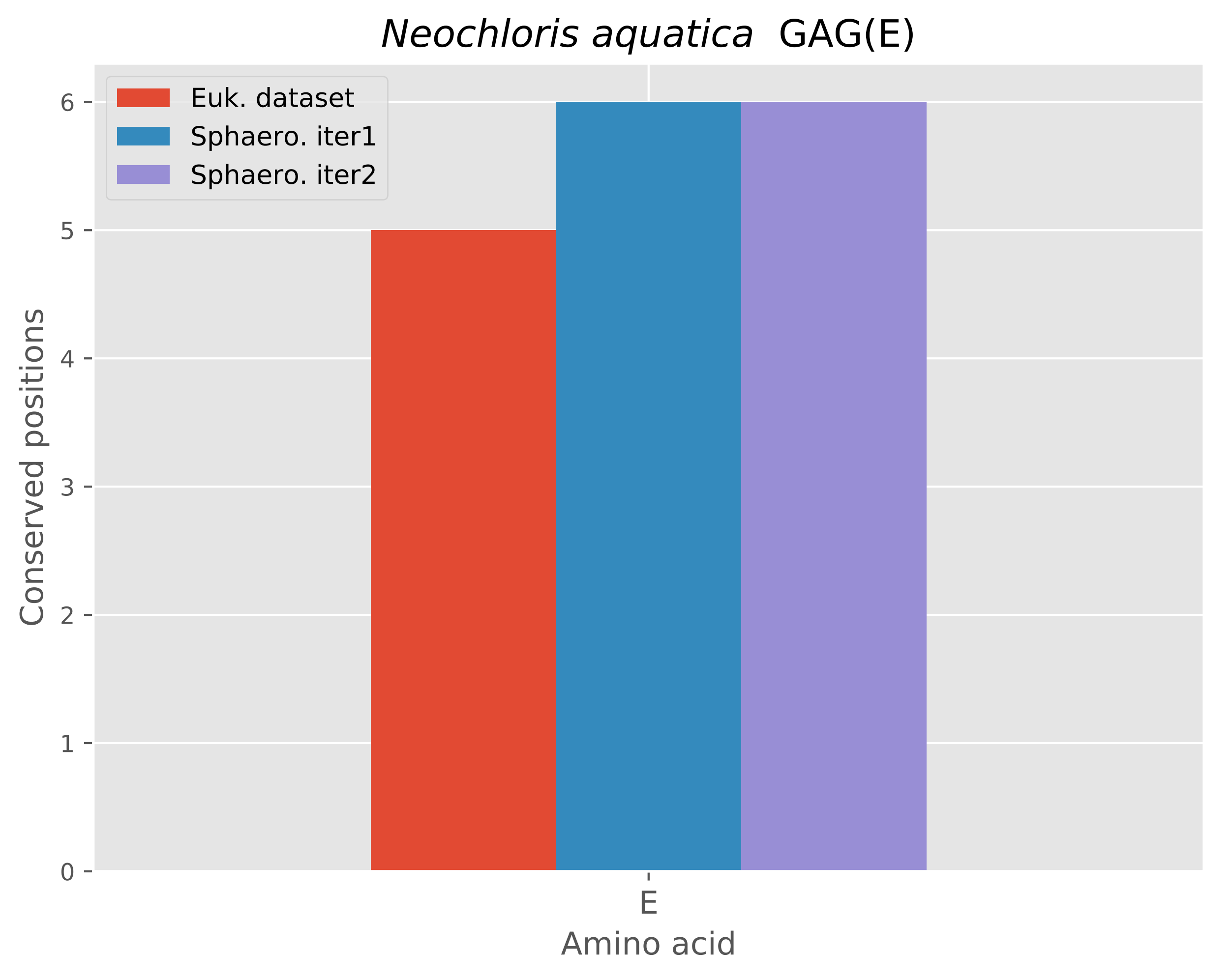

Neochloris aquatica GAG(E)
Euk. dataset
Sphaero. iter1
Sphaero. iter2
Conserved positions
0
1
2
3
4
5
6
E
Amino acid

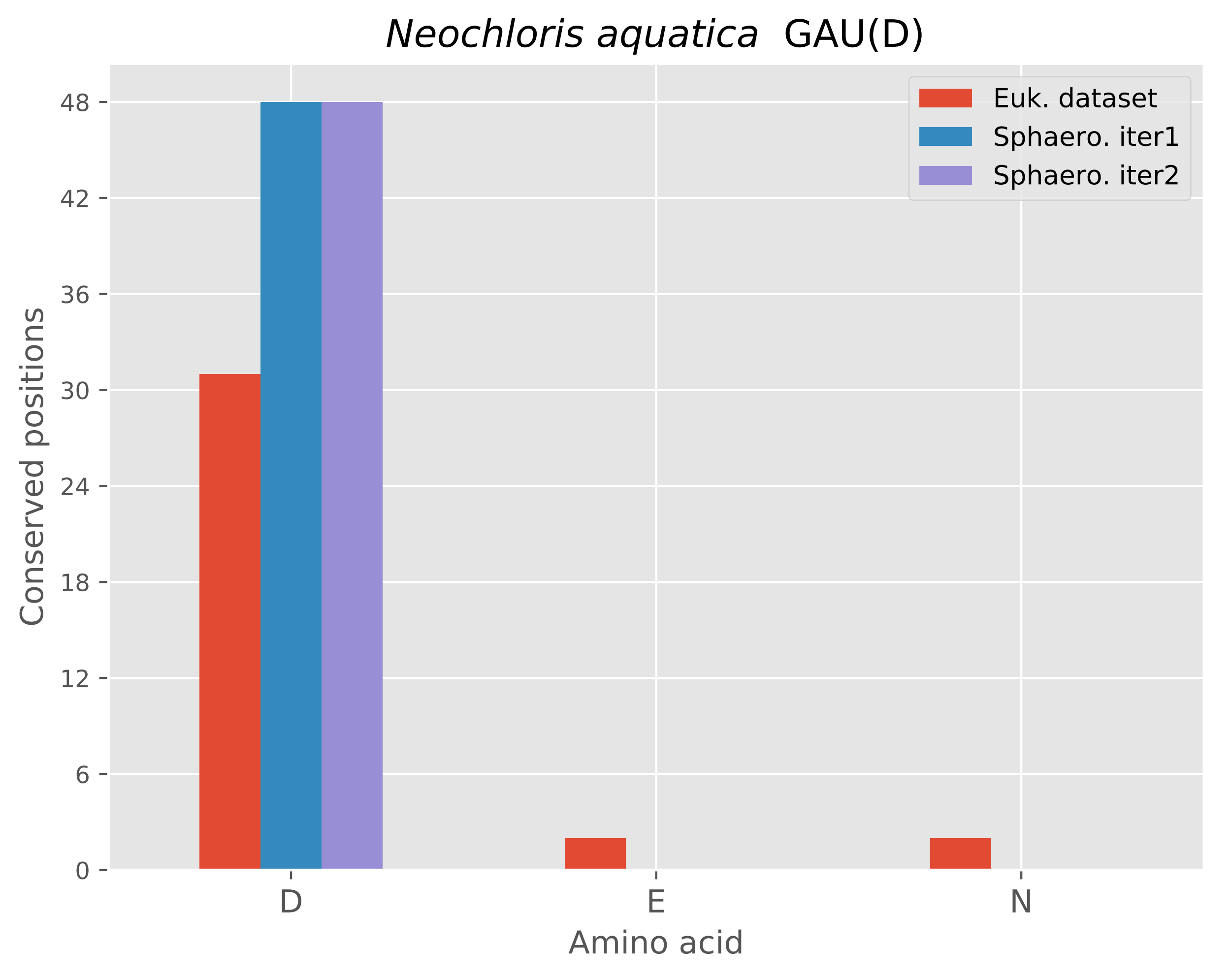

Neochloris aquatica GAU(D)
Euk. dataset
Sphaero. iter1
Sphaero. iter2
Conserved positions
0
6
12
18
24
30
36
42
48
D
E
N
Amino acid

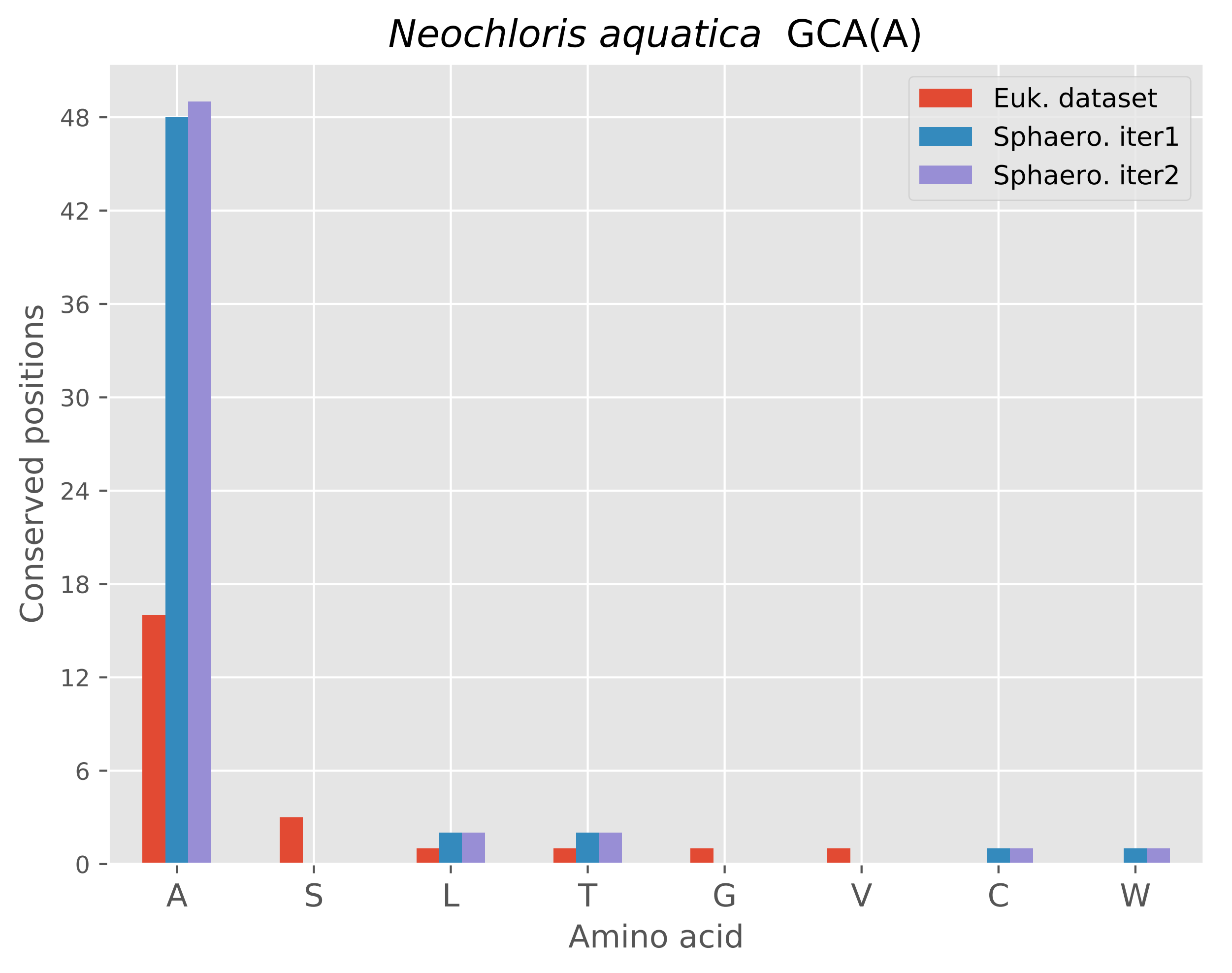

Neochloris aquatica GCA(A)
Euk. dataset
Sphaero. iter1
Sphaero. iter2
Conserved positions
0
6
12
18
24
30
36
42
48
A
S
L
T
G
V
C
W
Amino acid

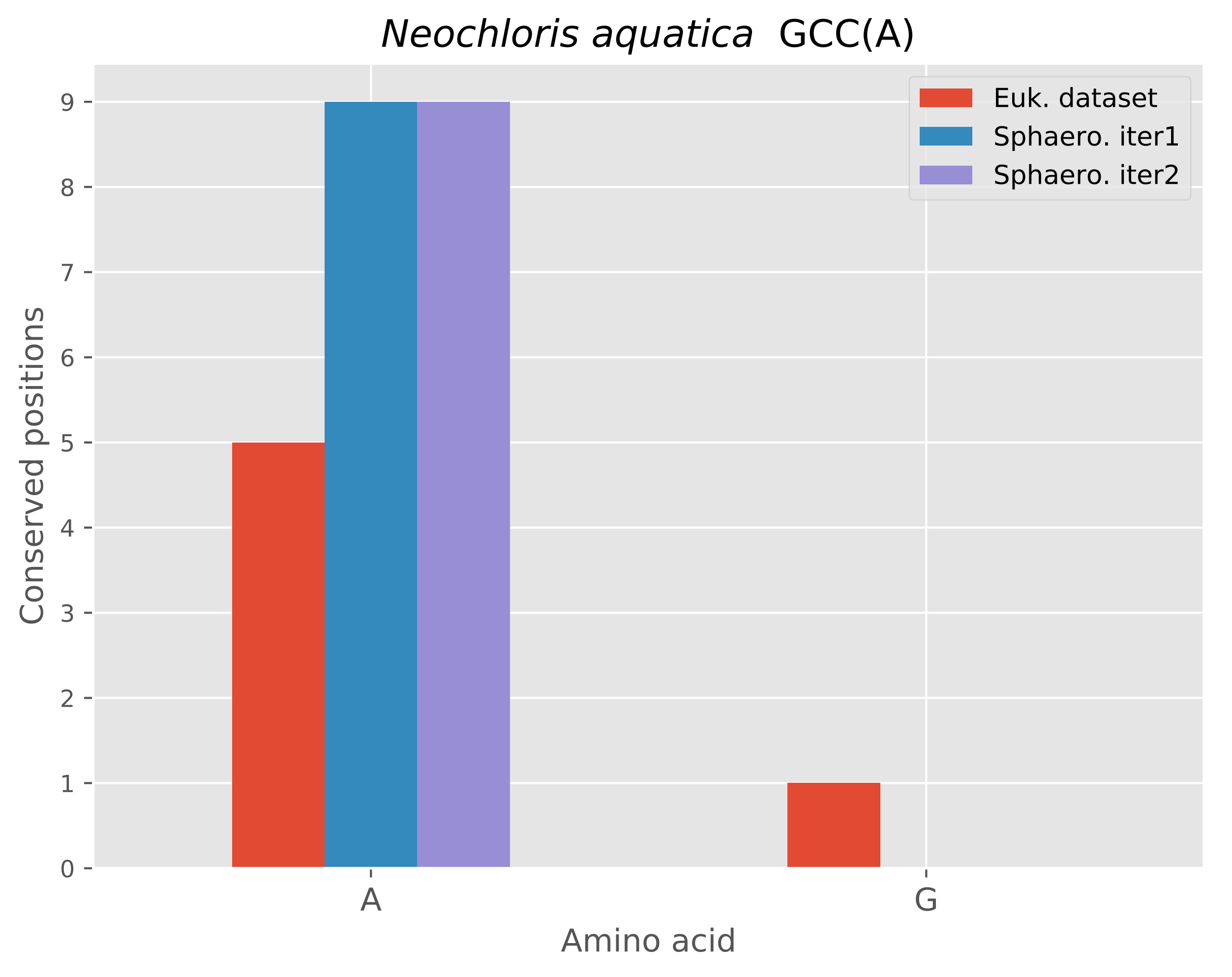

Neochloris aquatica GCC(A)
Euk. dataset
Sphaero. iter1
Sphaero. iter2
Conserved positions
0
1
2
3
4
5
6
7
8
9
A
G
Amino acid

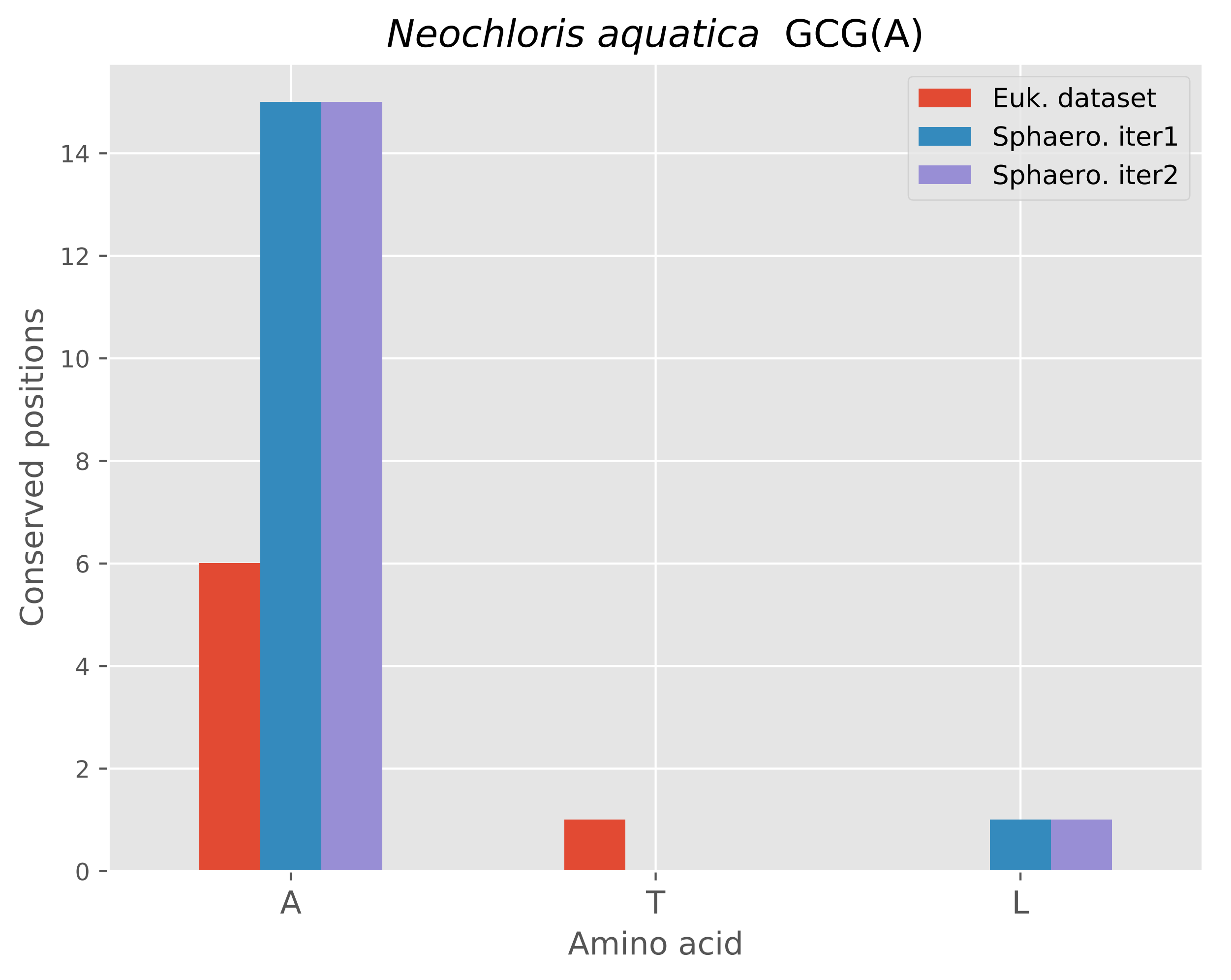

Neochloris aquatica GCG(A)
Euk. dataset
Sphaero. iter1
Sphaero. iter2
Conserved positions
0
2
4
6
8
10
12
14
A
T
L
Amino acid

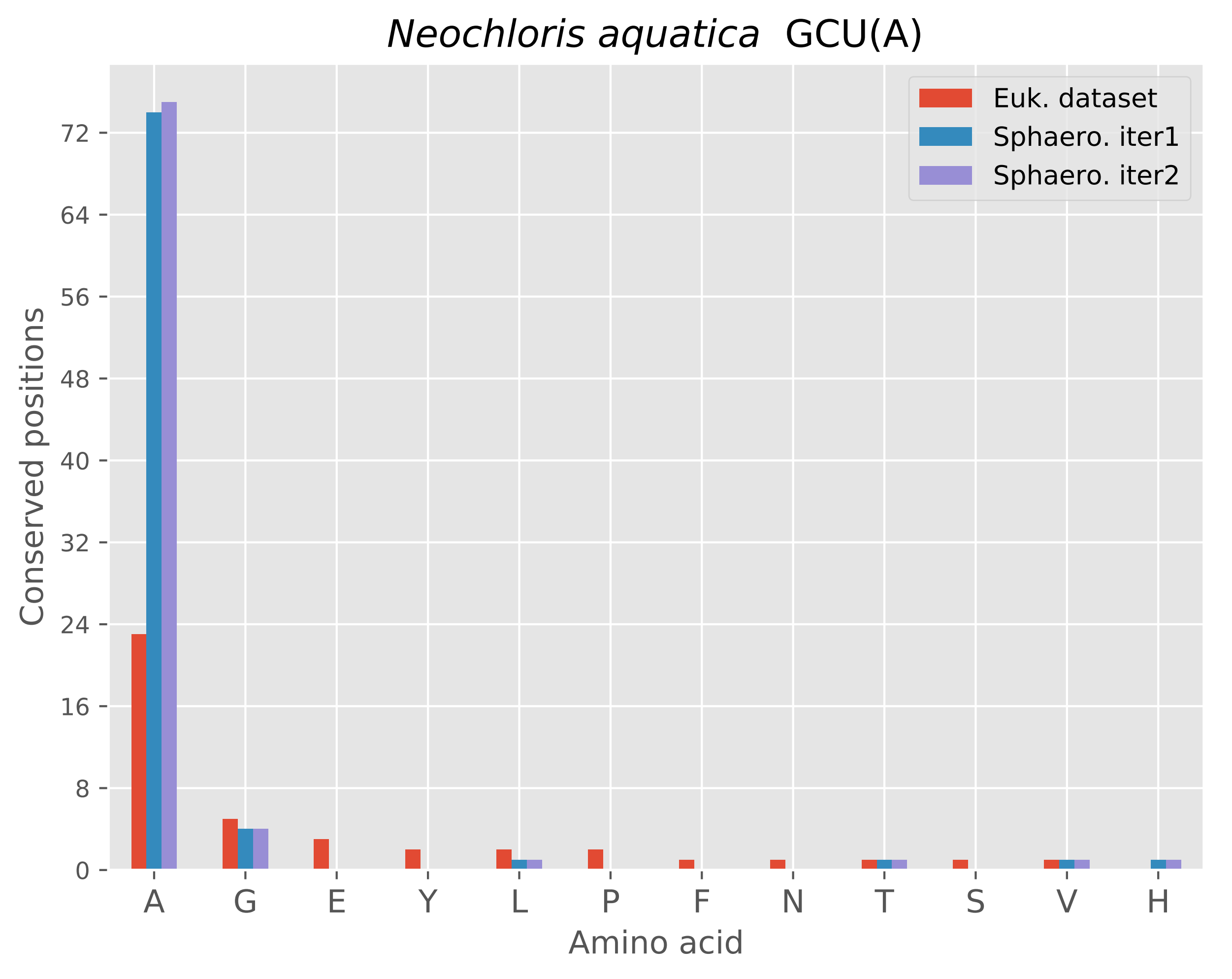

Neochloris aquatica GCU(A)
Euk. dataset
Sphaero. iter1
Sphaero. iter2
Conserved positions
0
8
16
24
32
40
48
56
64
72
A
G
E
Y
L
P
F
N
T
S
V
H
Amino acid

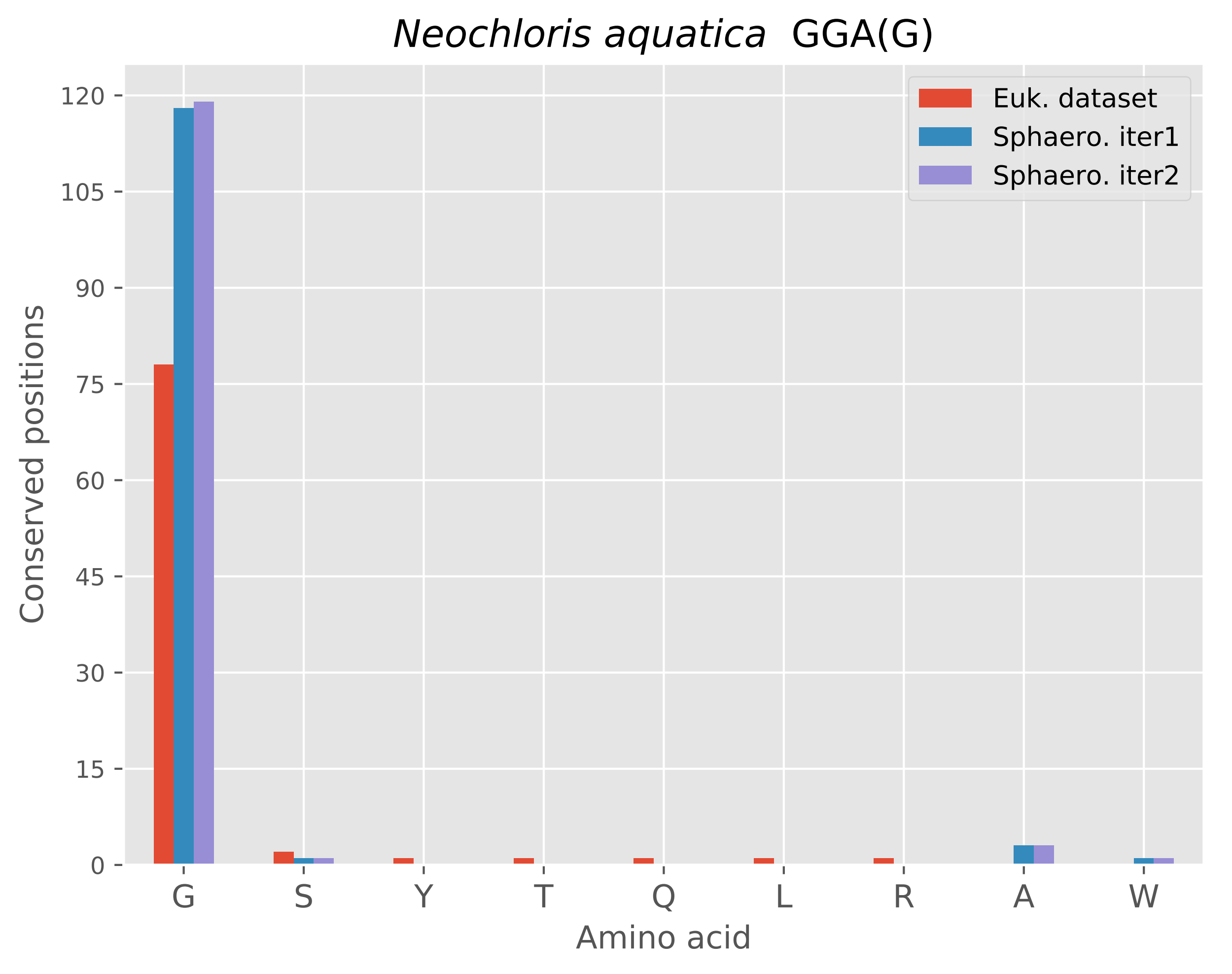

Neochloris aquatica GGA(G)
Euk. dataset
Sphaero. iter1
Sphaero. iter2
Conserved positions
0
15
30
45
60
75
90
105
120
G
S
Y
T
Q
L
R
A
W
Amino acid

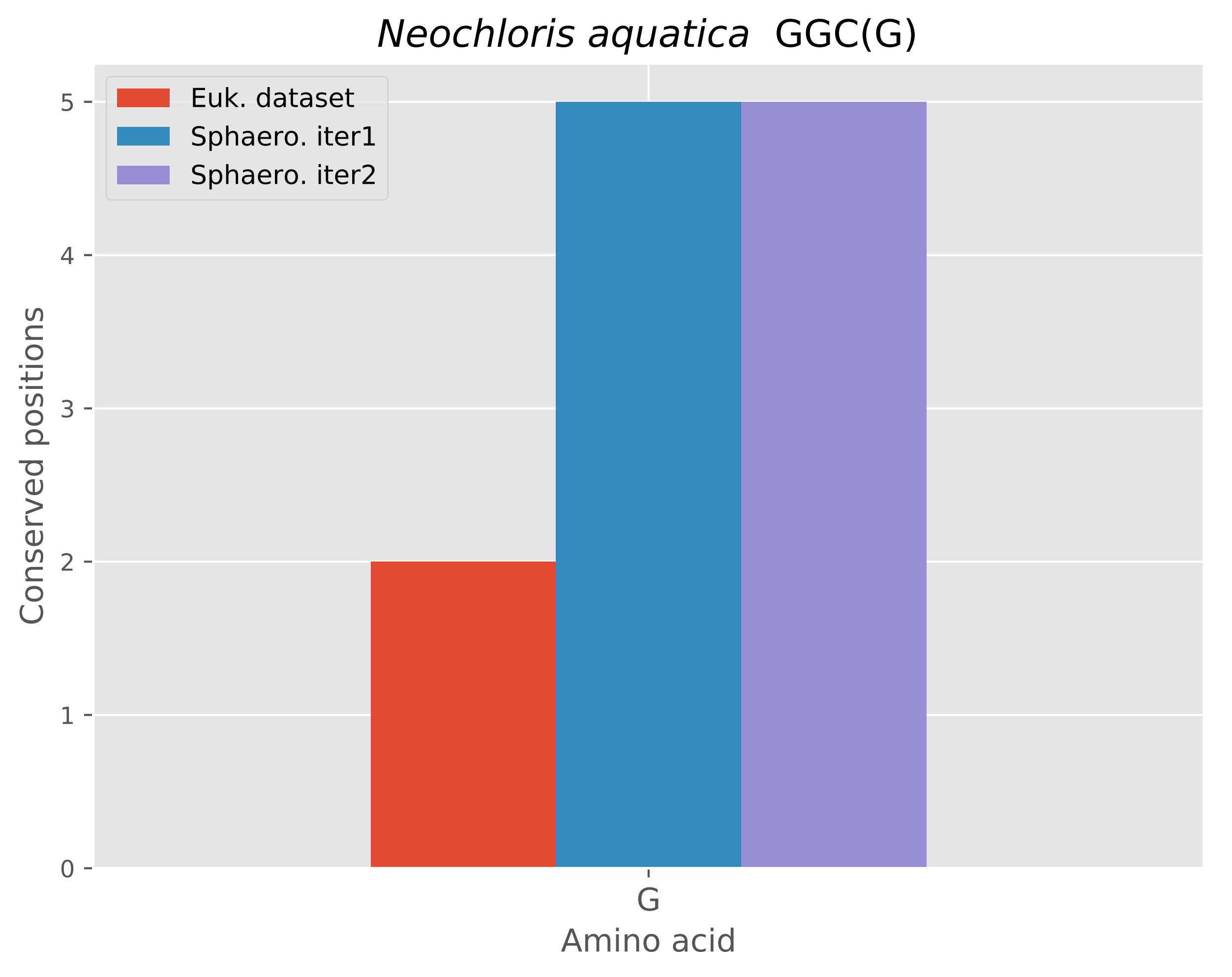

Neochloris aquatica GGC(G)
Euk. dataset
Sphaero. iter1
Sphaero. iter2
Conserved positions
0
1
2
3
4
5
G
Amino acid

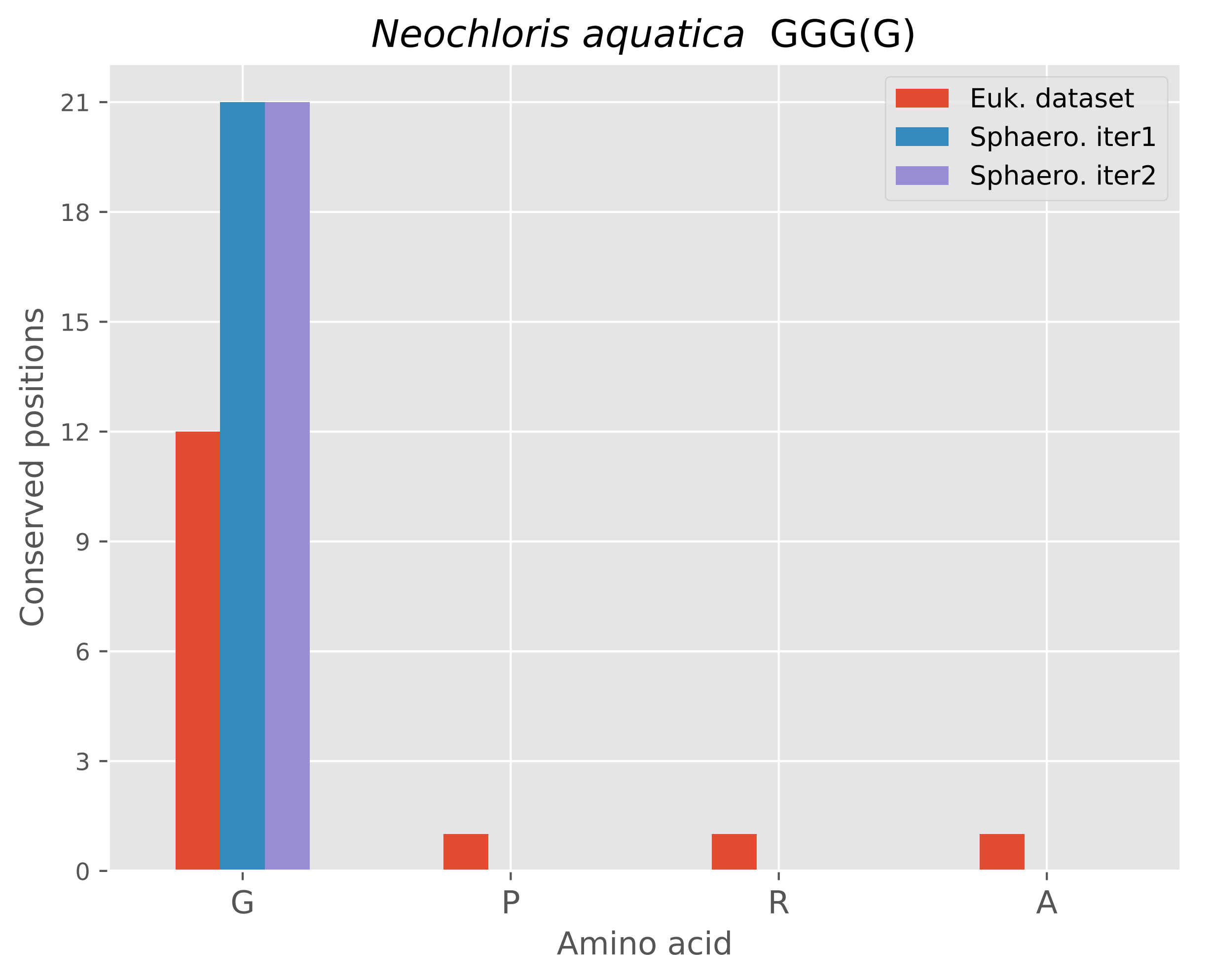

Neochloris aquatica GGG(G)
Euk. dataset
Sphaero. iter1
Sphaero. iter2
Conserved positions
0
3
6
9
12
15
18
21
G
P
R
A
Amino acid

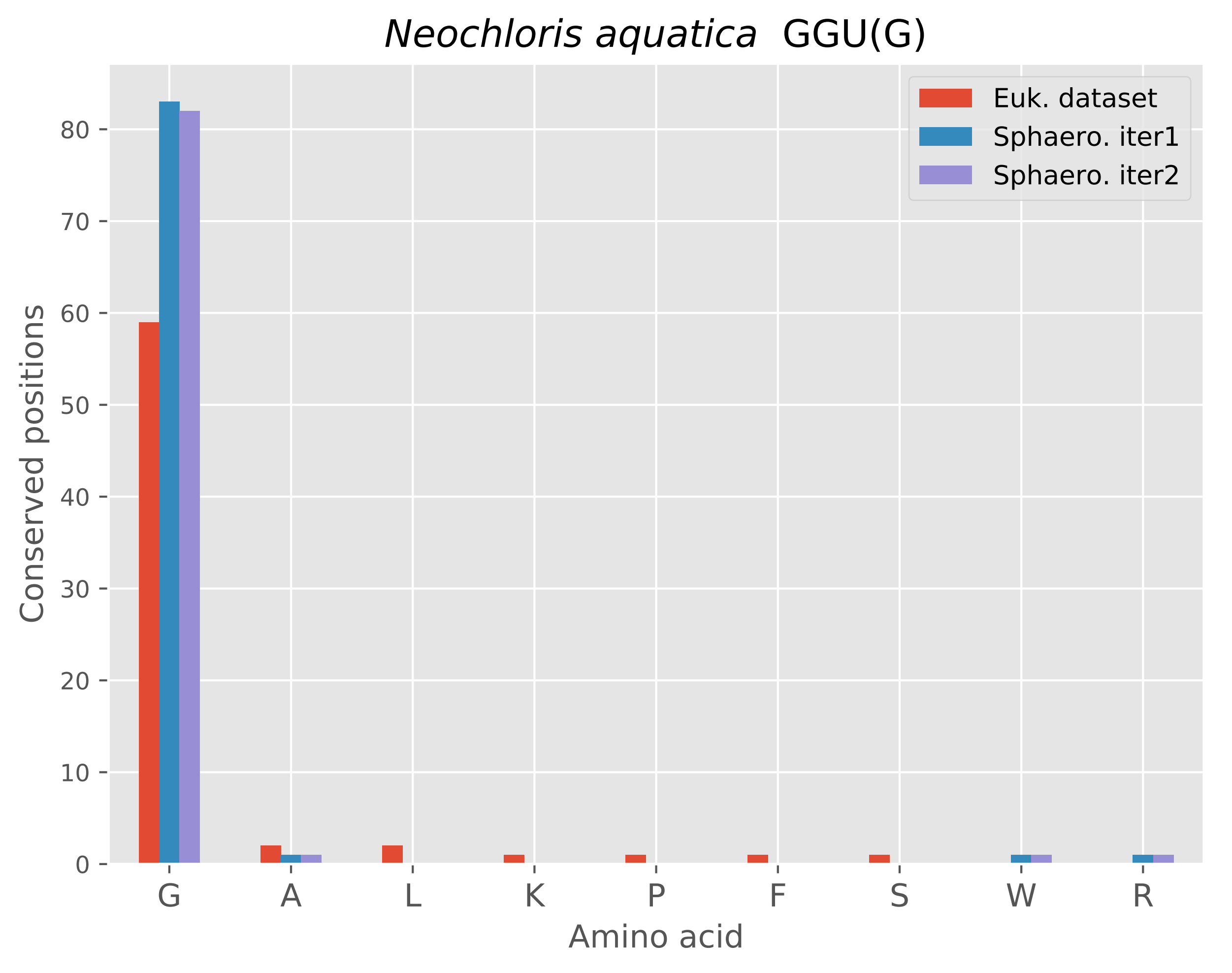

Neochloris aquatica GGU(G)
Euk. dataset
Sphaero. iter1
Sphaero. iter2
Conserved positions
0
10
20
30
40
50
60
70
80
G
A
L
K
P
F
S
W
R
Amino acid

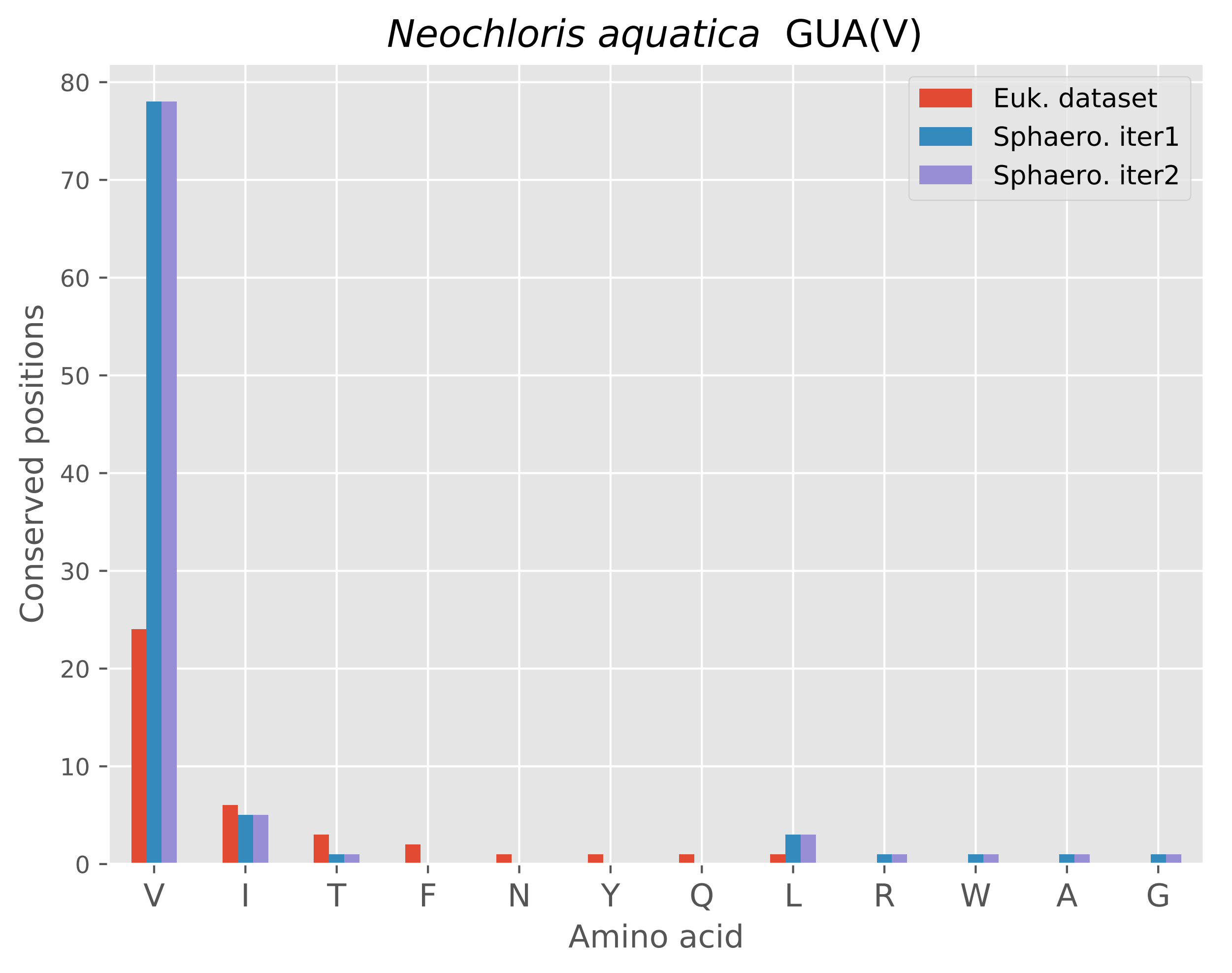

Neochloris aquatica GUA(V)
Euk. dataset
Sphaero. iter1
Sphaero. iter2
Conserved positions
0
10
20
30
40
50
60
70
80
V
I
T
F
N
Y
Q
L
R
W
A
G
Amino acid

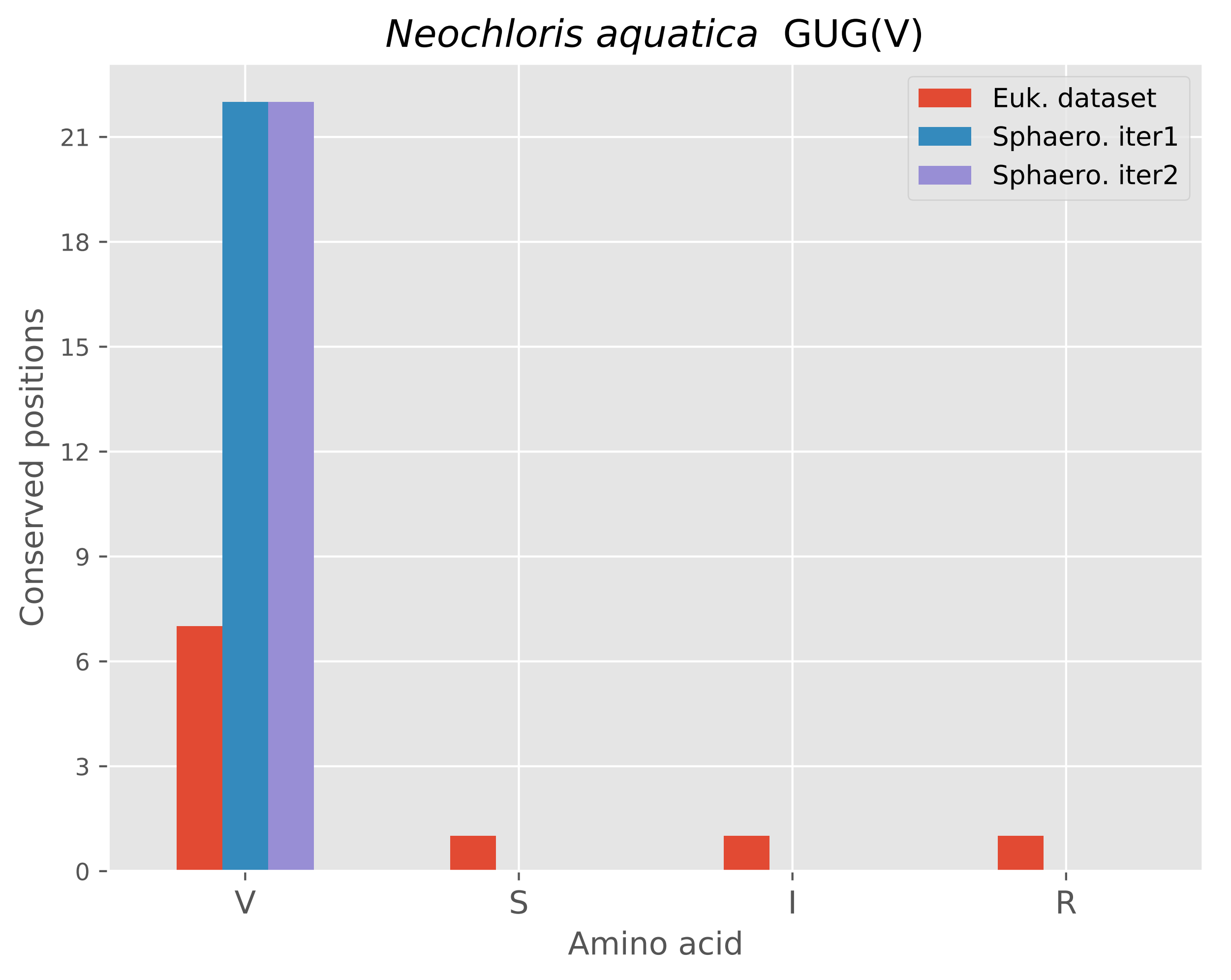

Neochloris aquatica GUG(V)
Euk. dataset
Sphaero. iter1
Sphaero. iter2
Conserved positions
0
3
6
9
12
15
18
21
V
S
I
R
Amino acid

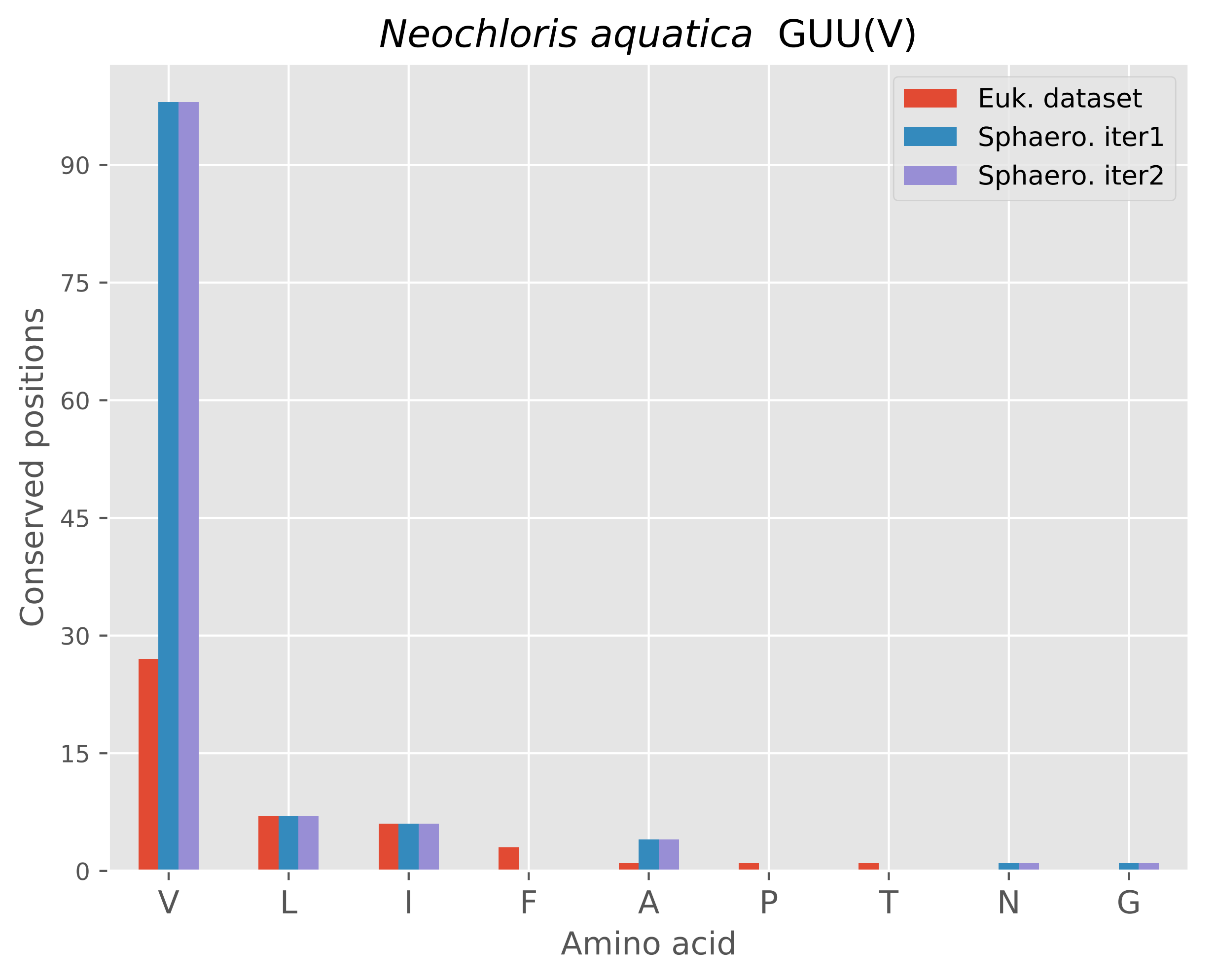

Neochloris aquatica GUU(V)
Euk. dataset
Sphaero. iter1
Sphaero. iter2
Conserved positions
0
15
30
45
60
75
90
V
L
I
F
A
P
T
N
G
Amino acid

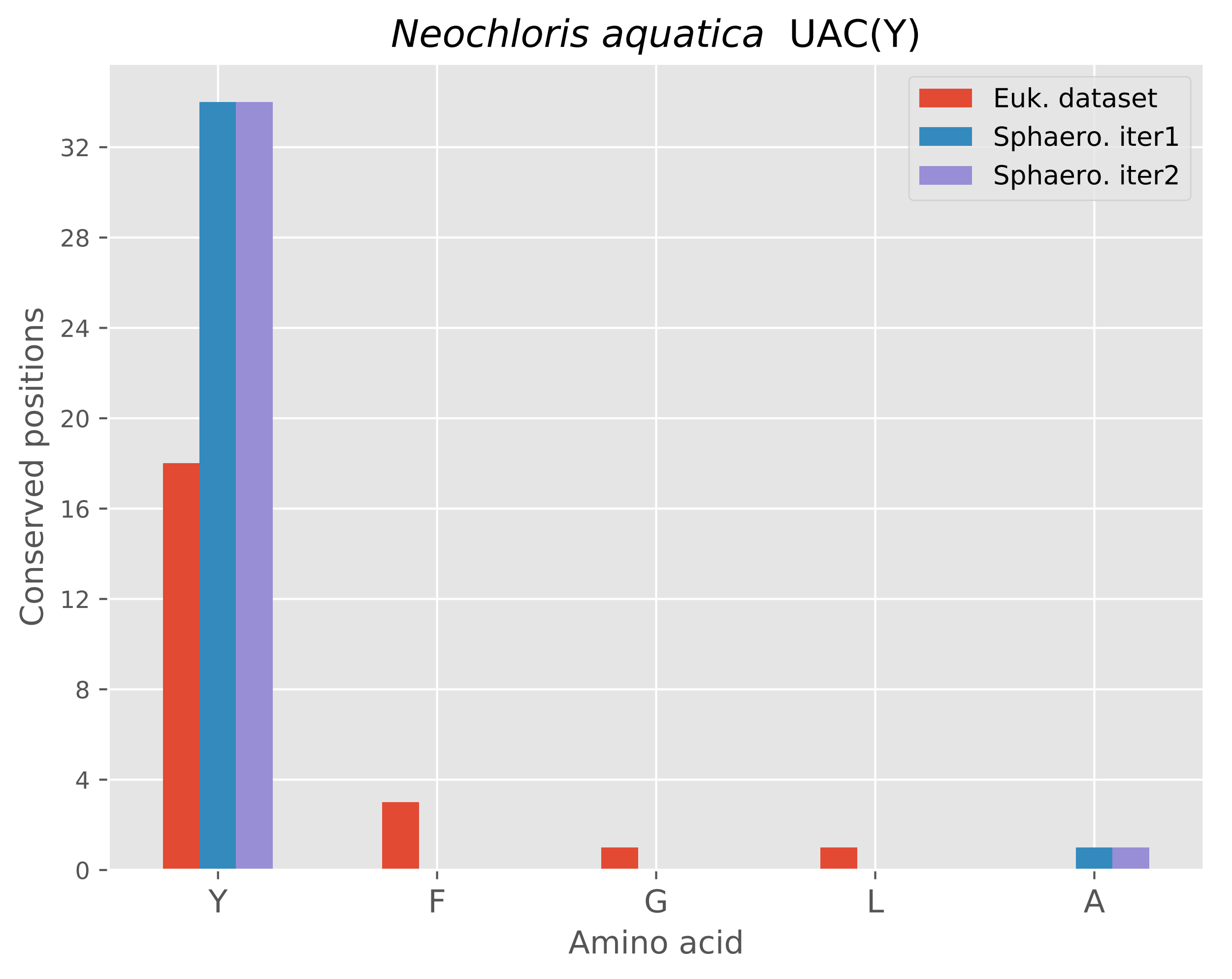

Neochloris aquatica UAC(Y)
Euk. dataset
Sphaero. iter1
Sphaero. iter2
Conserved positions
0
4
8
12
16
20
24
28
32
Y
F
G
L
A
Amino acid

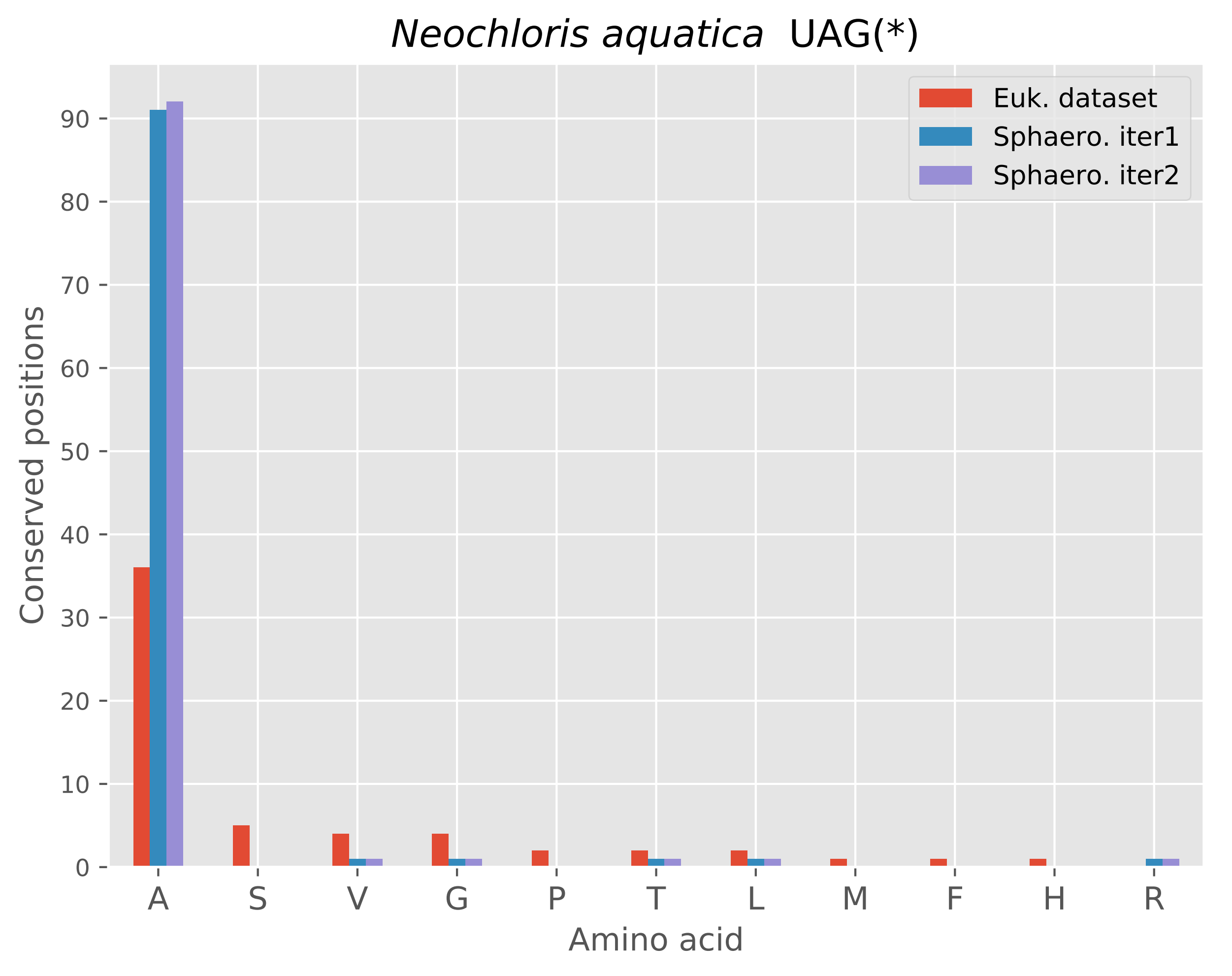

Neochloris aquatica UAG(*)
Euk. dataset
Sphaero. iter1
Sphaero. iter2
Conserved positions
0
10
20
30
40
50
60
70
80
90
A
S
V
G
P
T
L
M
F
H
R
Amino acid

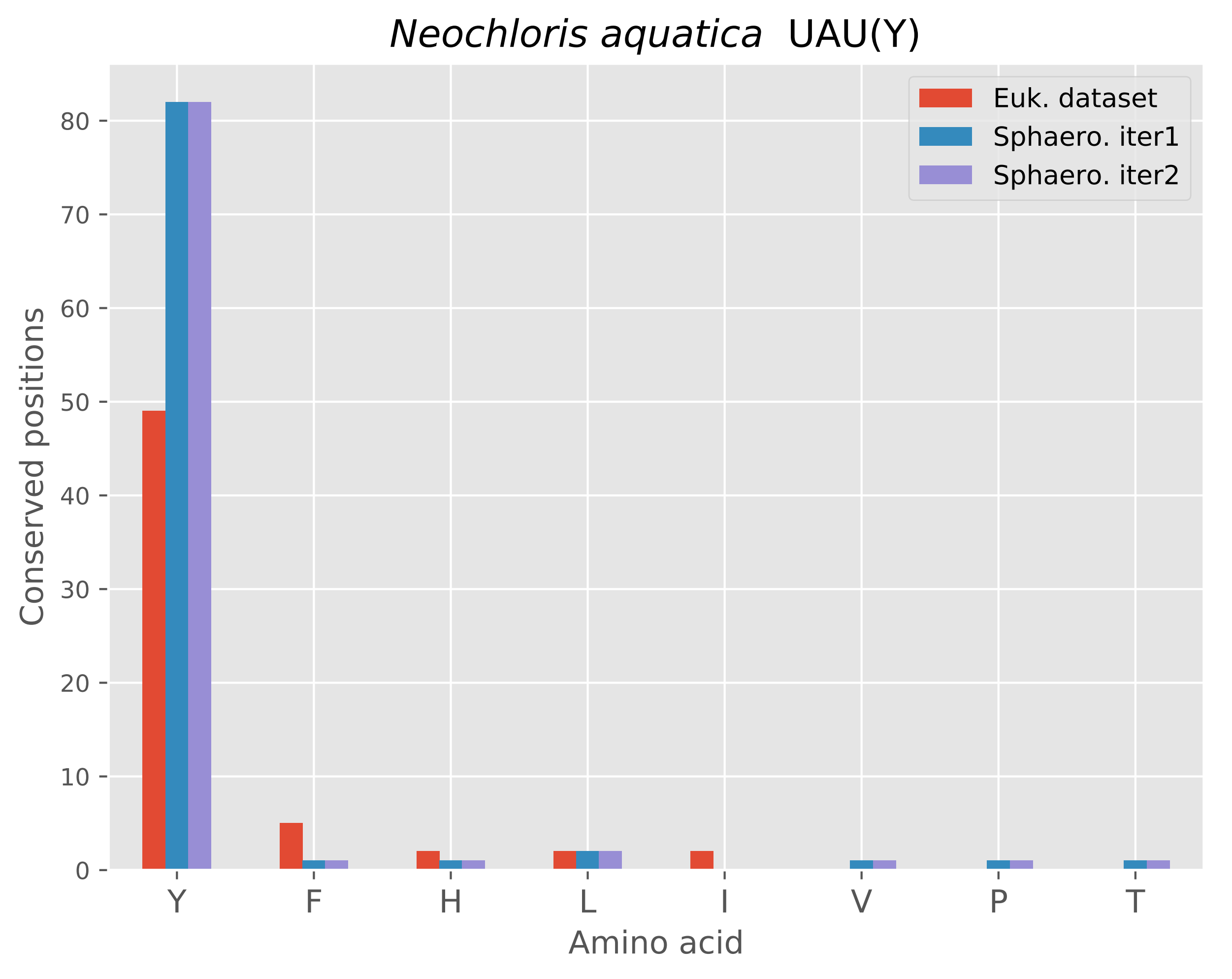

Neochloris aquatica UAU(Y)
Euk. dataset
Sphaero. iter1
Sphaero. iter2
Conserved positions
0
10
20
30
40
50
60
70
80
Y
F
H
L
I
V
P
T
Amino acid

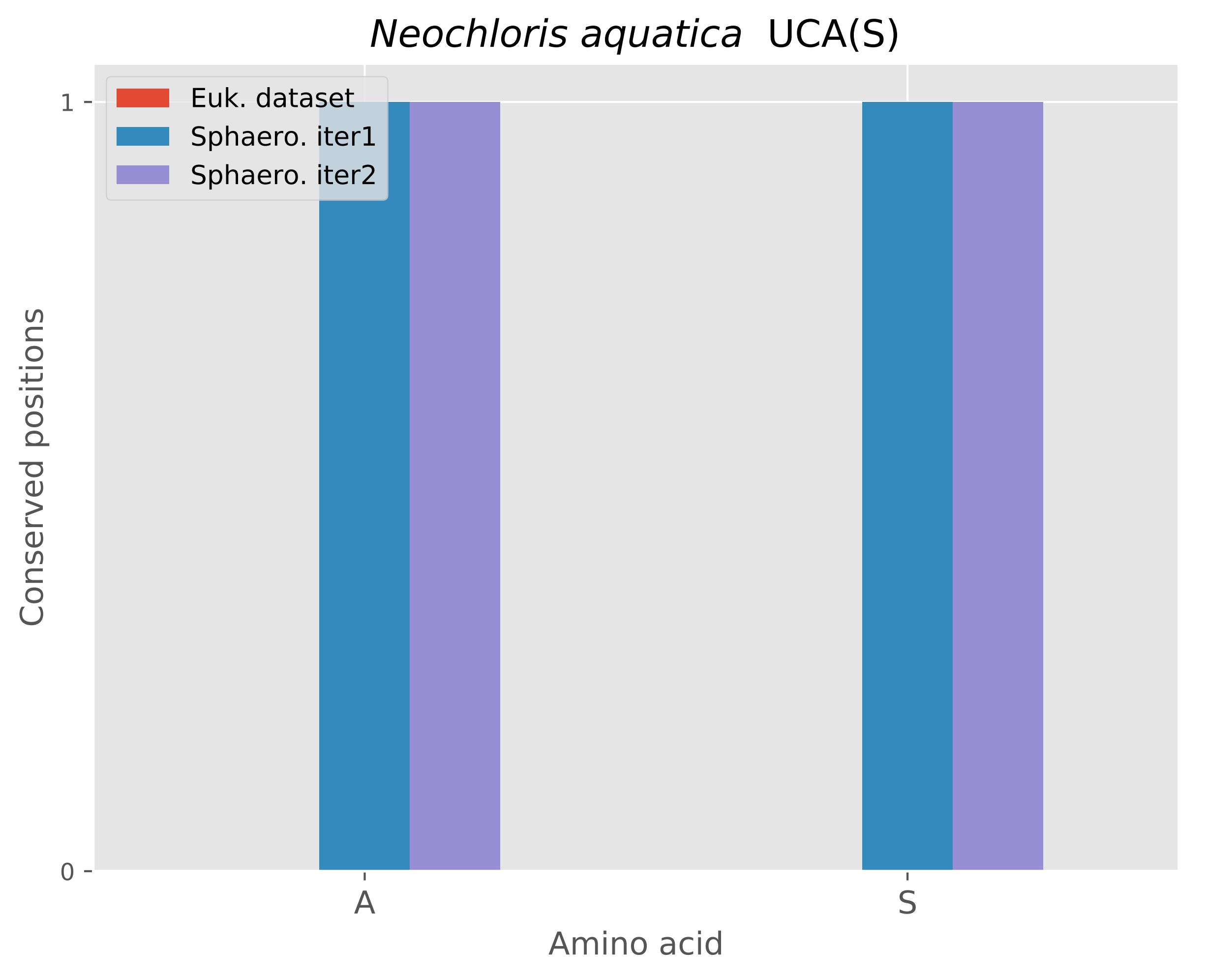

Neochloris aquatica UCA(S)
Euk. dataset
Sphaero. iter1
Sphaero. iter2
1
0
A
S
Amino acid
Conserved positions

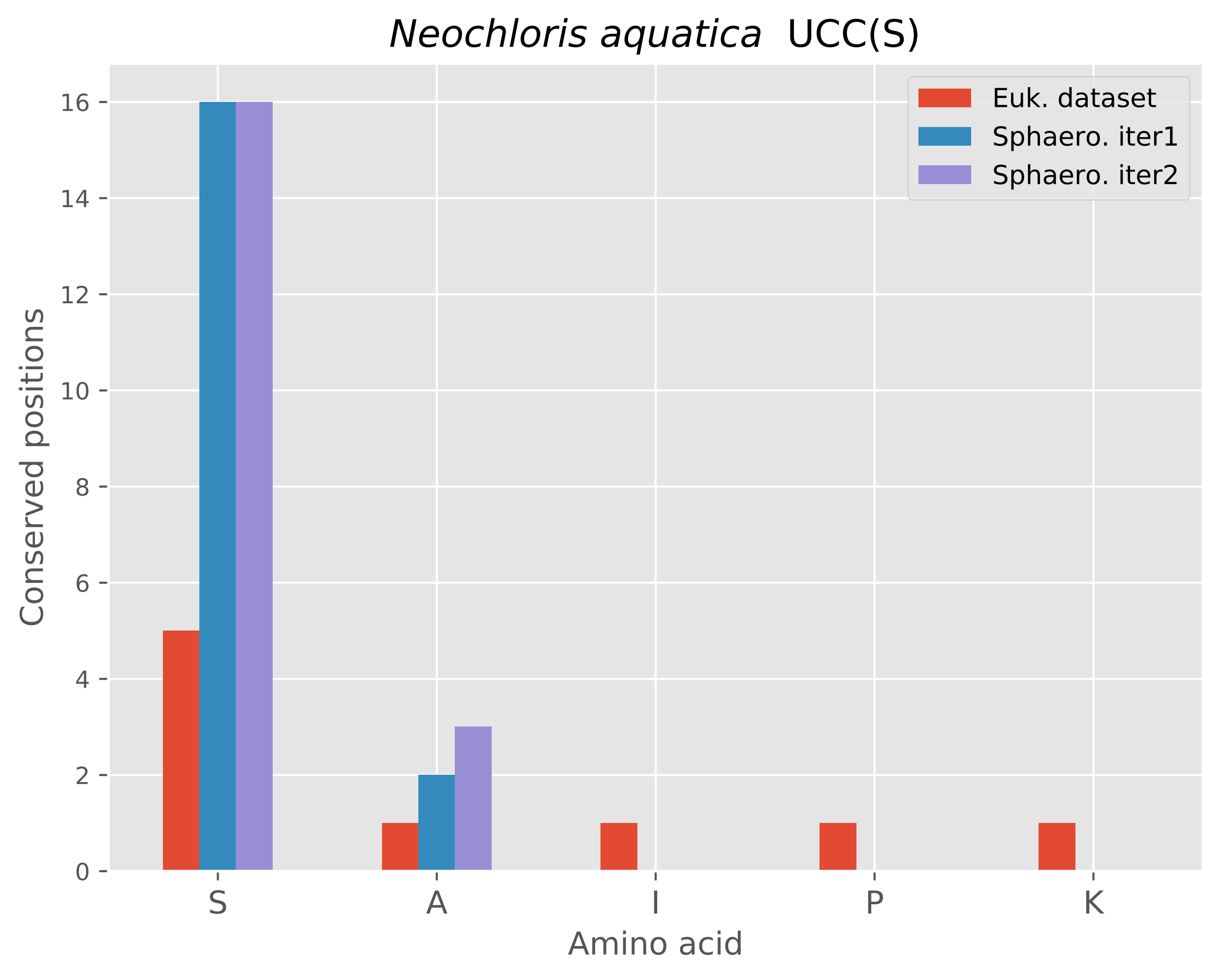

Neochloris aquatica UCC(S)
Euk. dataset
Sphaero. iter1
Sphaero. iter2
Conserved positions
0
2
4
6
8
10
12
14
16
S
A
I
P
K
Amino acid

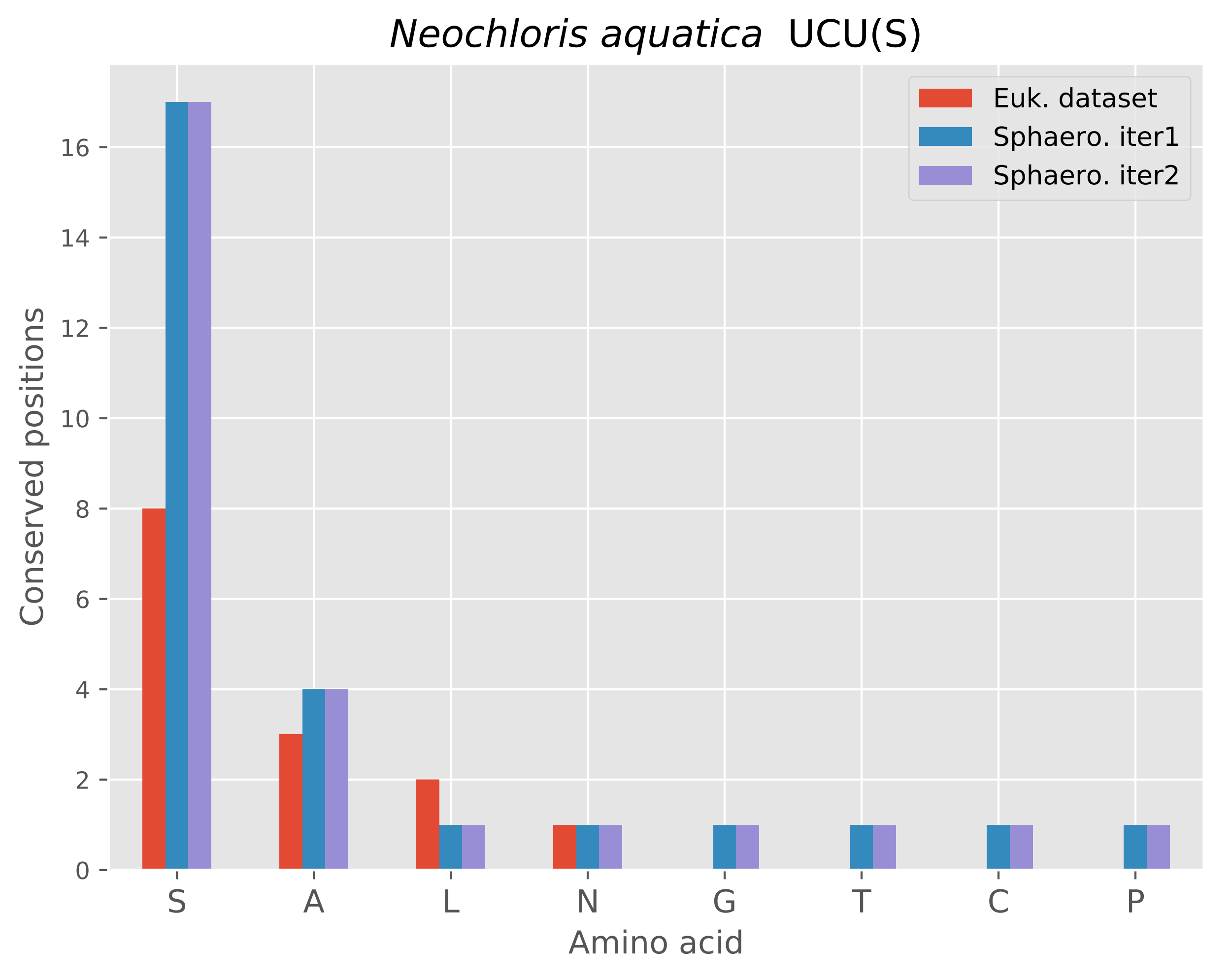

Neochloris aquatica UCU(S)
Euk. dataset
Sphaero. iter1
Sphaero. iter2
Conserved positions
0
2
4
6
8
10
12
14
16
S
A
L
N
G
T
C
P
Amino acid

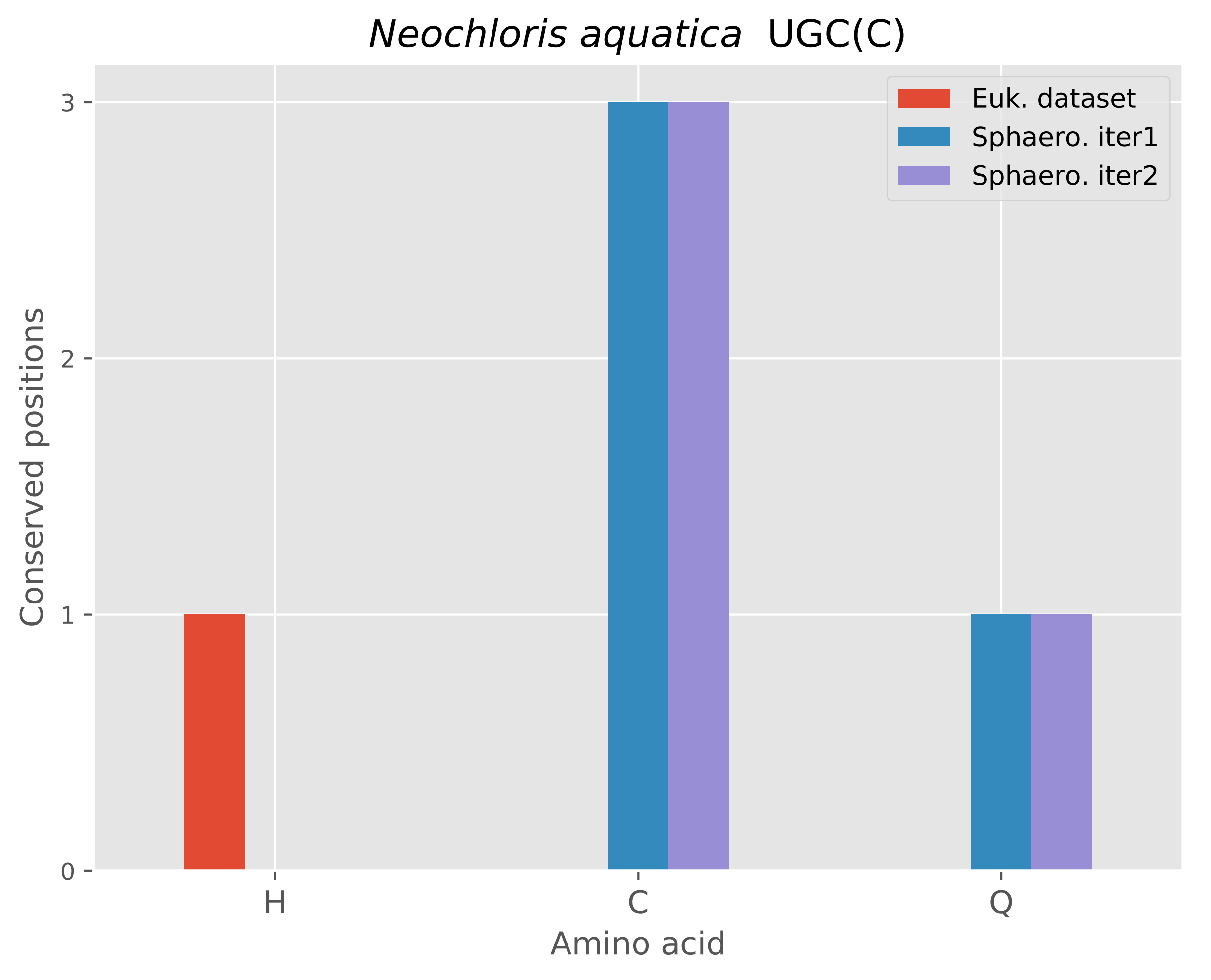

Neochloris aquatica UGC(C)
Euk. dataset
Sphaero. iter1
Sphaero. iter2
Conserved positions
0
1
2
3
H
C
Q
Amino acid

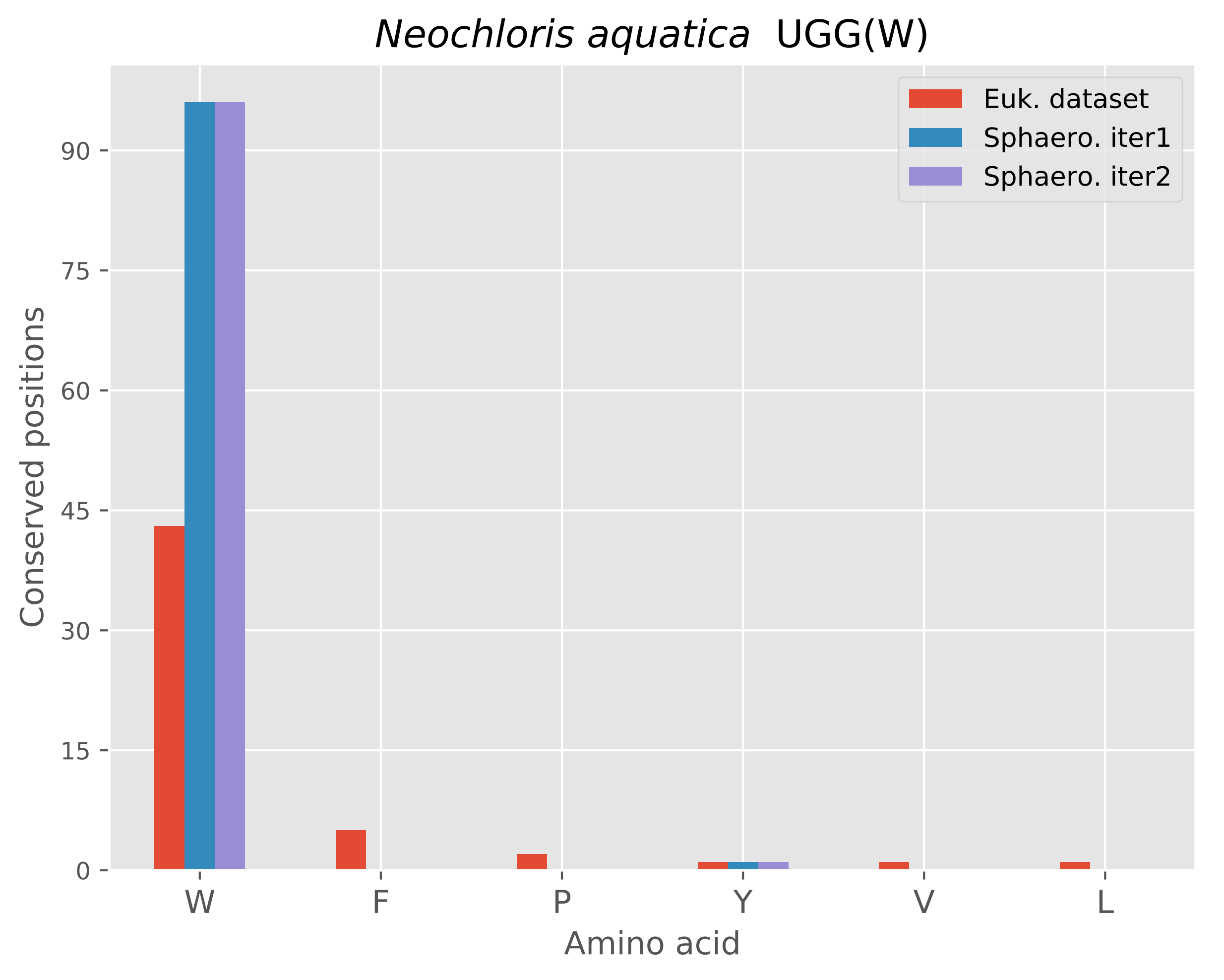

Neochloris aquatica UGG(W)
Euk. dataset
Sphaero. iter1
Sphaero. iter2
Conserved positions
0
15
30
45
60
75
90
W
F
P
Y
V
L
Amino acid

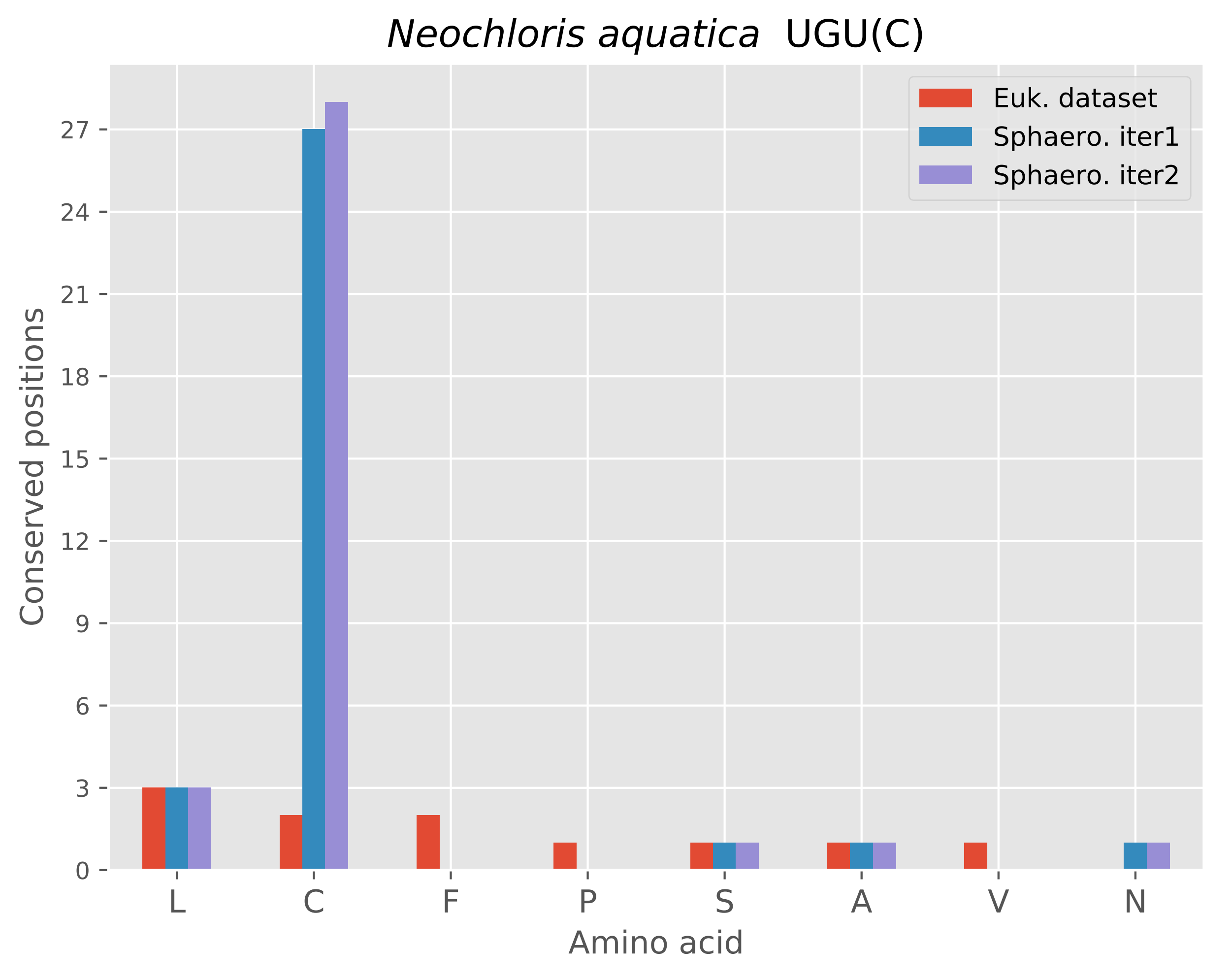
Neochloris aquatica UGU(C)
Euk. dataset
Sphaero. iter1
Sphaero. iter2
Conserved positions
0
3
6
9
12
15
18
21
24
27
L
C
F
P
S
A
V
N
Amino acid

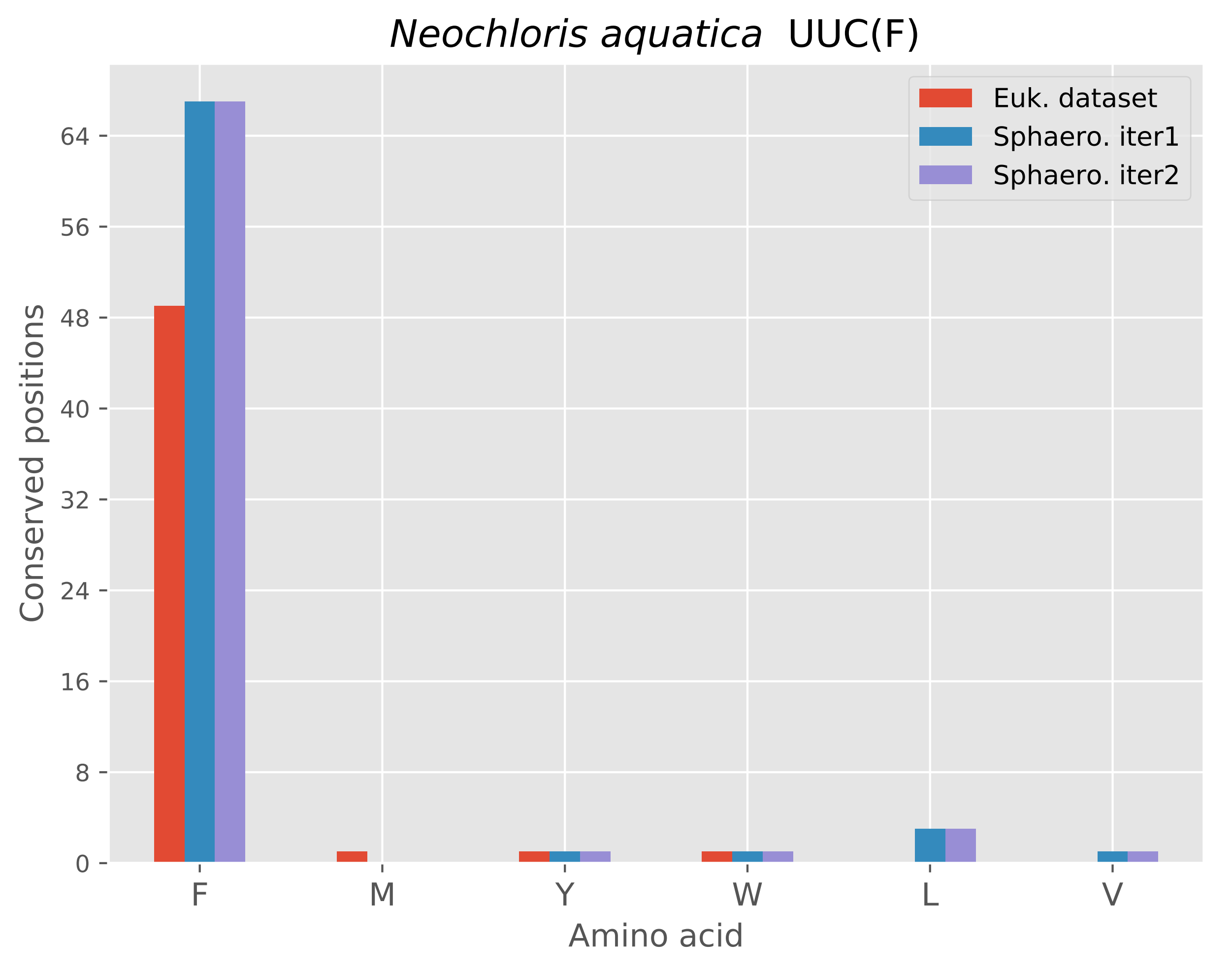

Neochloris aquatica UUC(F)
Euk. dataset
Sphaero. iter1
Sphaero. iter2
Conserved positions
0
8
16
24
32
40
48
56
64
F
M
Y
W
L
V
Amino acid

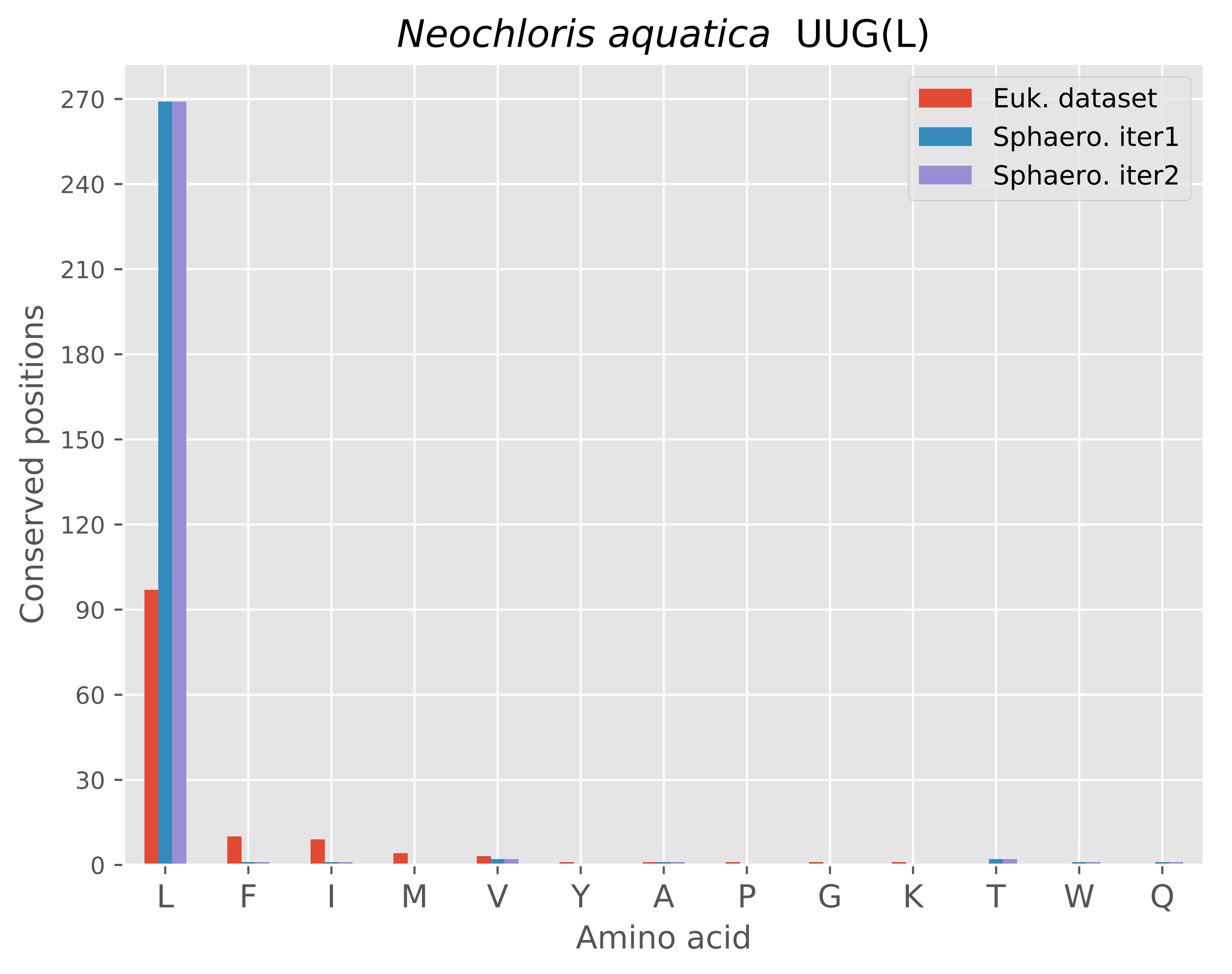
Neochloris aquatica UUG(L)
Euk. dataset
Sphaero. iter1
Sphaero. iter2
Conserved positions
0
30
60
90
120
150
180
210
240
270
L
F
I
M
V
Y
A
P
G
K
T
W
Q
Amino acid

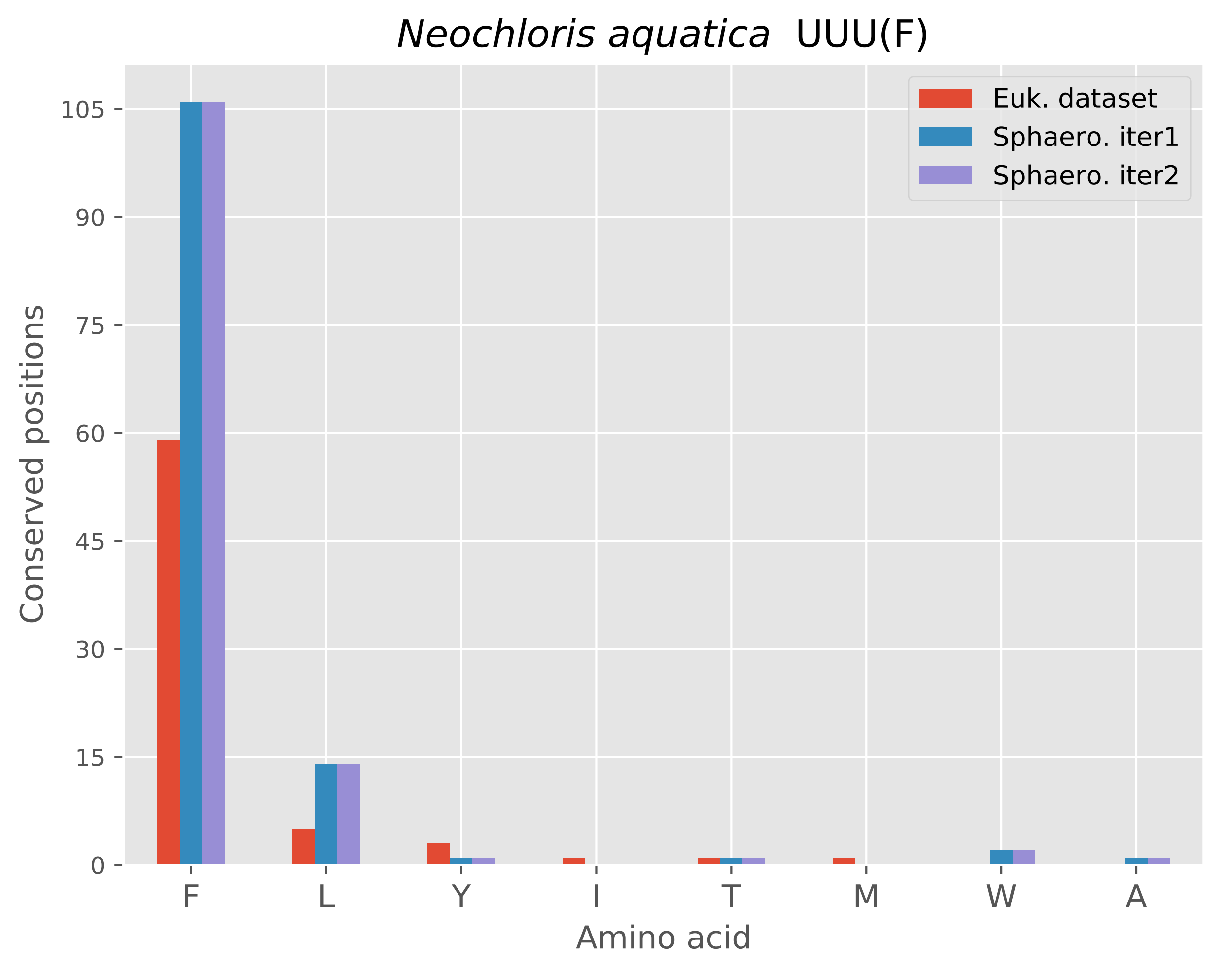

Neochloris aquatica UUU(F)
Conserved positions
Amino acid
0
15
30
45
60
75
90
105
F
L
Y
I
T
M
W
A
Euk. dataset
Sphaero. iter1
Sphaero. iter2

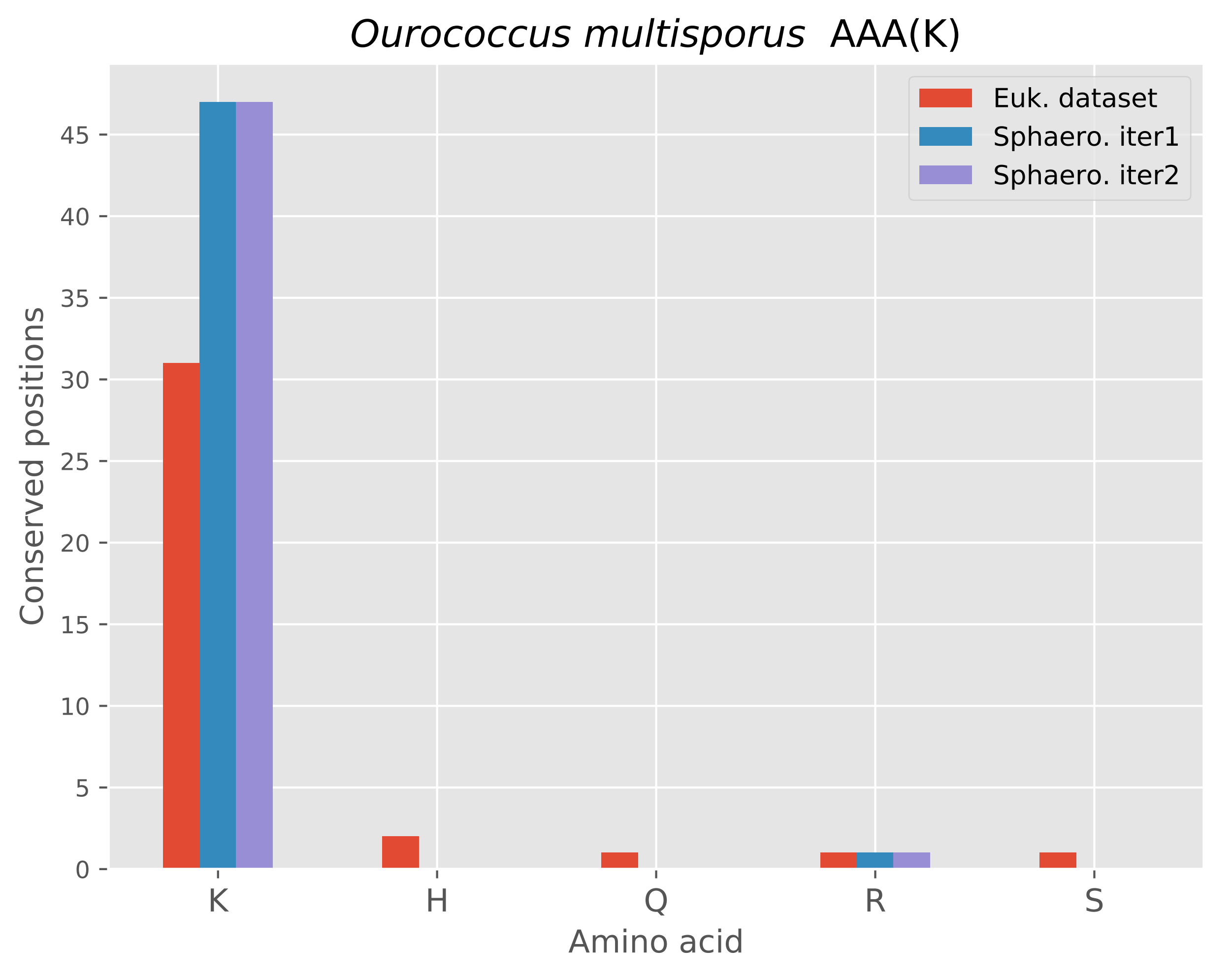

Ourococcus multisporus AAA(K)
Euk. dataset
Sphaero. iter1
Sphaero. iter2
Conserved positions
0
5
10
15
20
25
30
35
40
45
K
H
Q
R
S
Amino acid

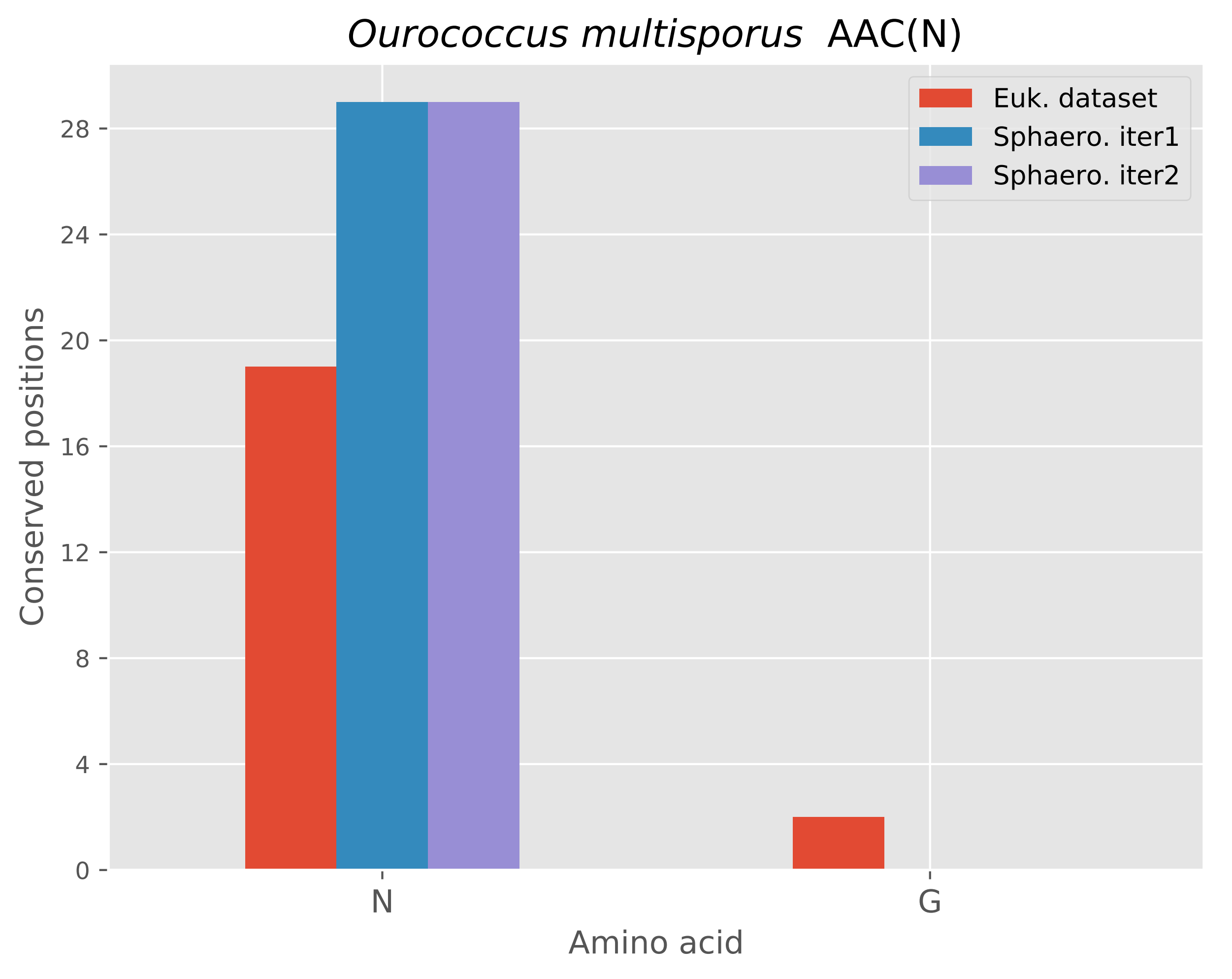

Ourococcus multisporus AAC(N)
Euk. dataset
Sphaero. iter1
Sphaero. iter2
Conserved positions
0
4
8
12
16
20
24
28
N
G
Amino acid

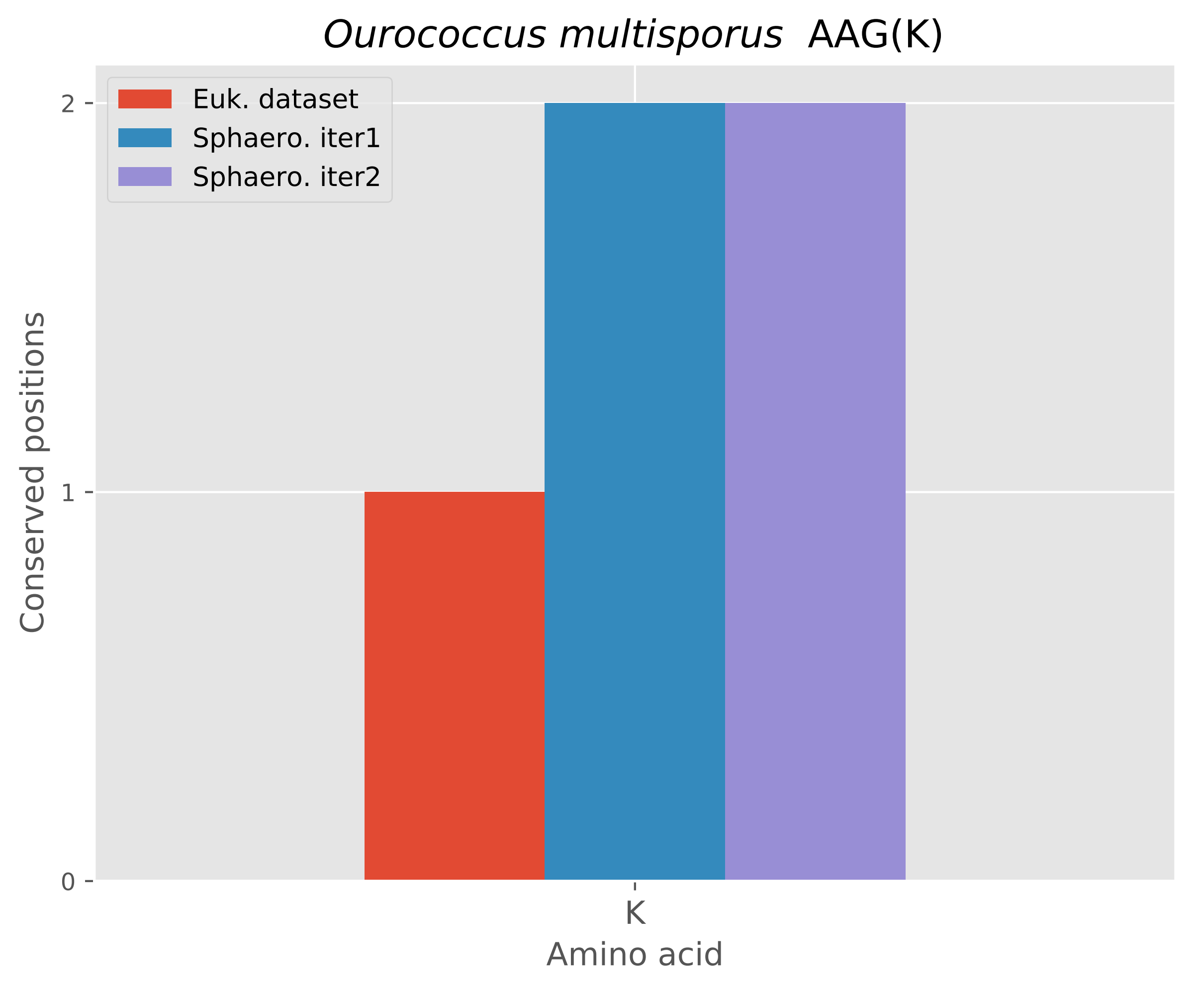

Ourococcus multisporus AAG(K)
Euk. dataset
Sphaero. iter1
Sphaero. iter2
Conserved positions
2
1
0
K
Amino acid

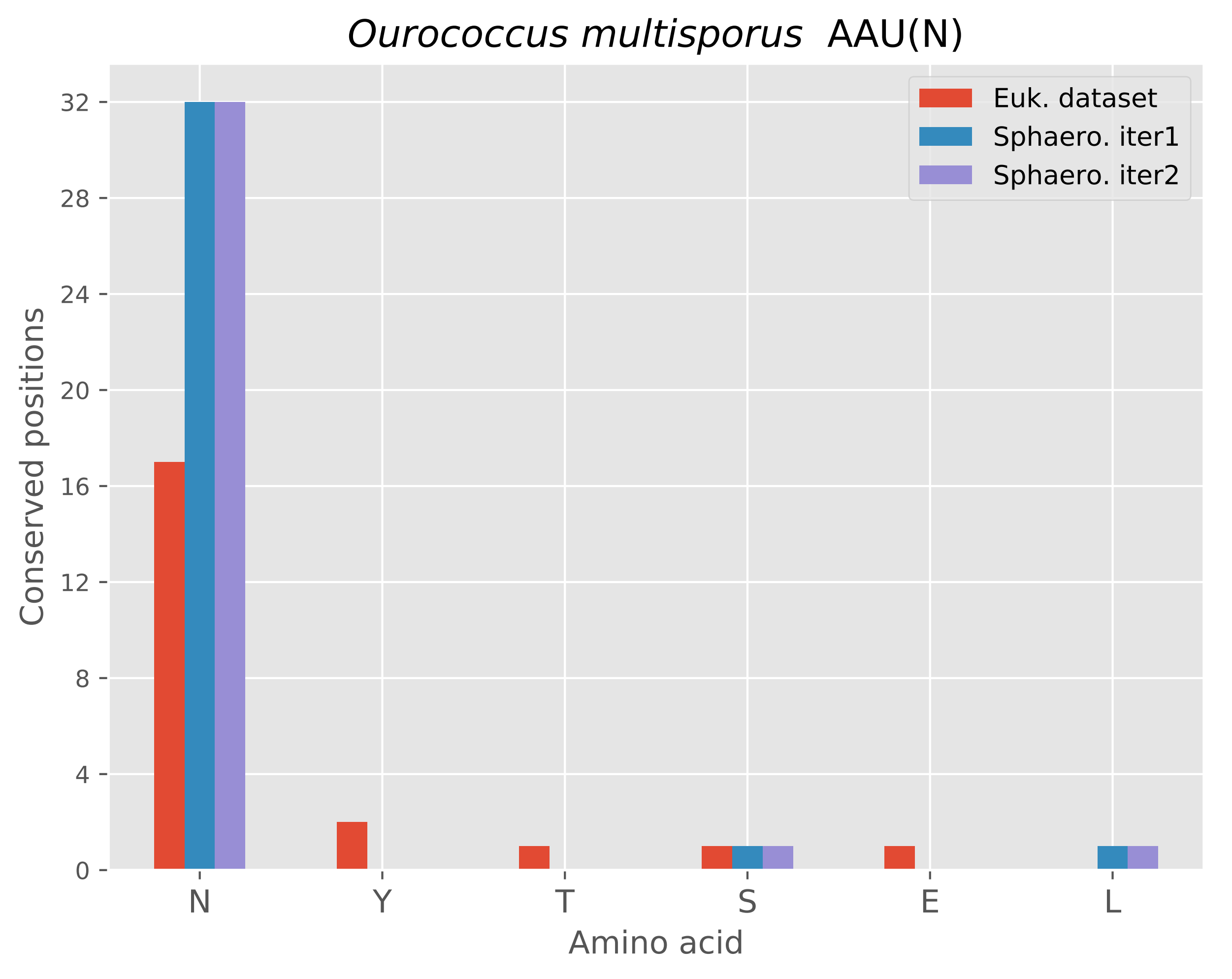
Ourococcus multisporus AAU(N)
Euk. dataset
Sphaero. iter1
Sphaero. iter2
Conserved positions
Amino acid
N
Y
T
S
E
L
0
4
8
12
16
20
24
28
32

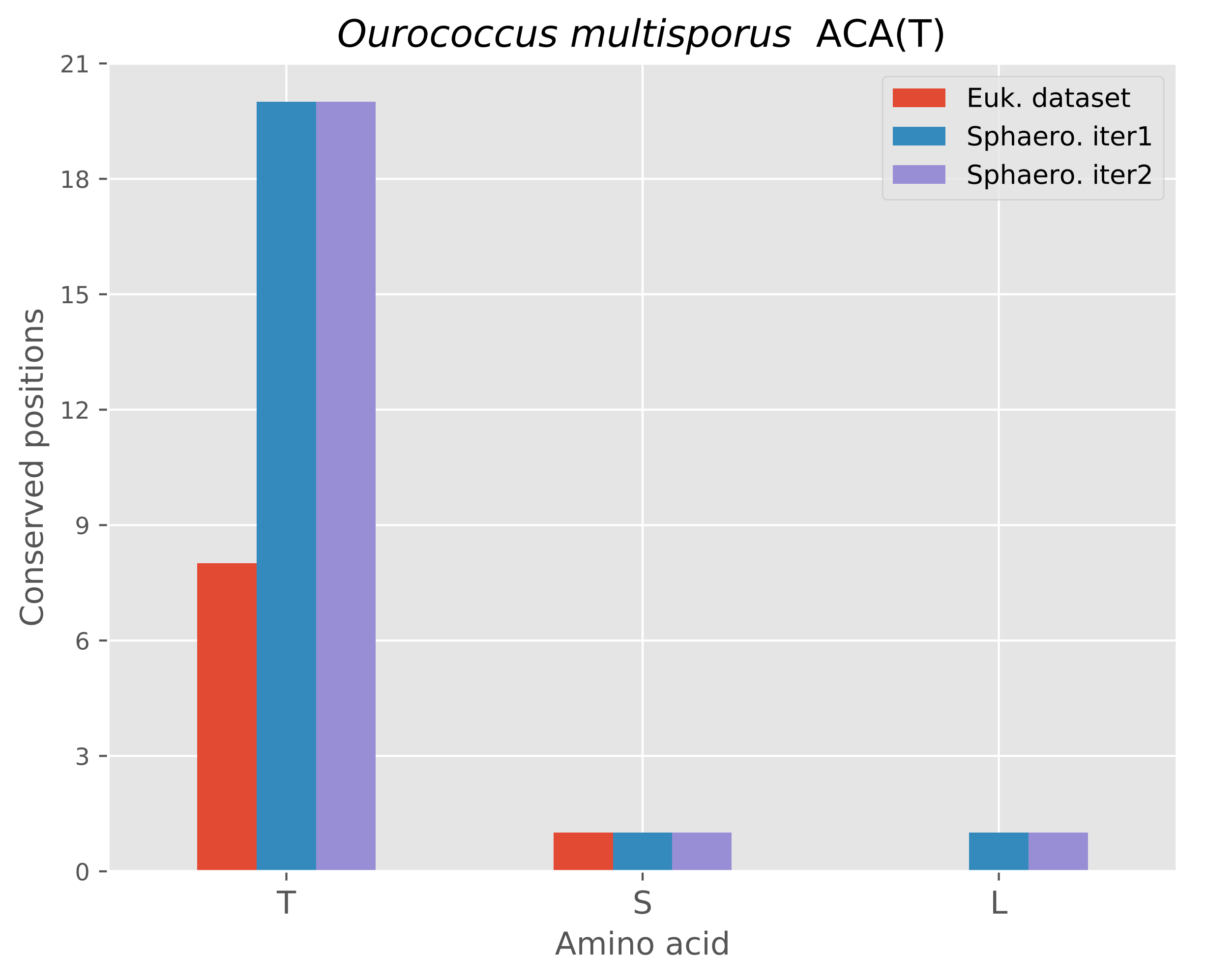
Ourococcus multisporus ACA(T)
Conserved positions
Amino acid
0
3
6
9
12
15
18
21
T
S
L
Euk. dataset
Sphaero. iter1
Sphaero. iter2

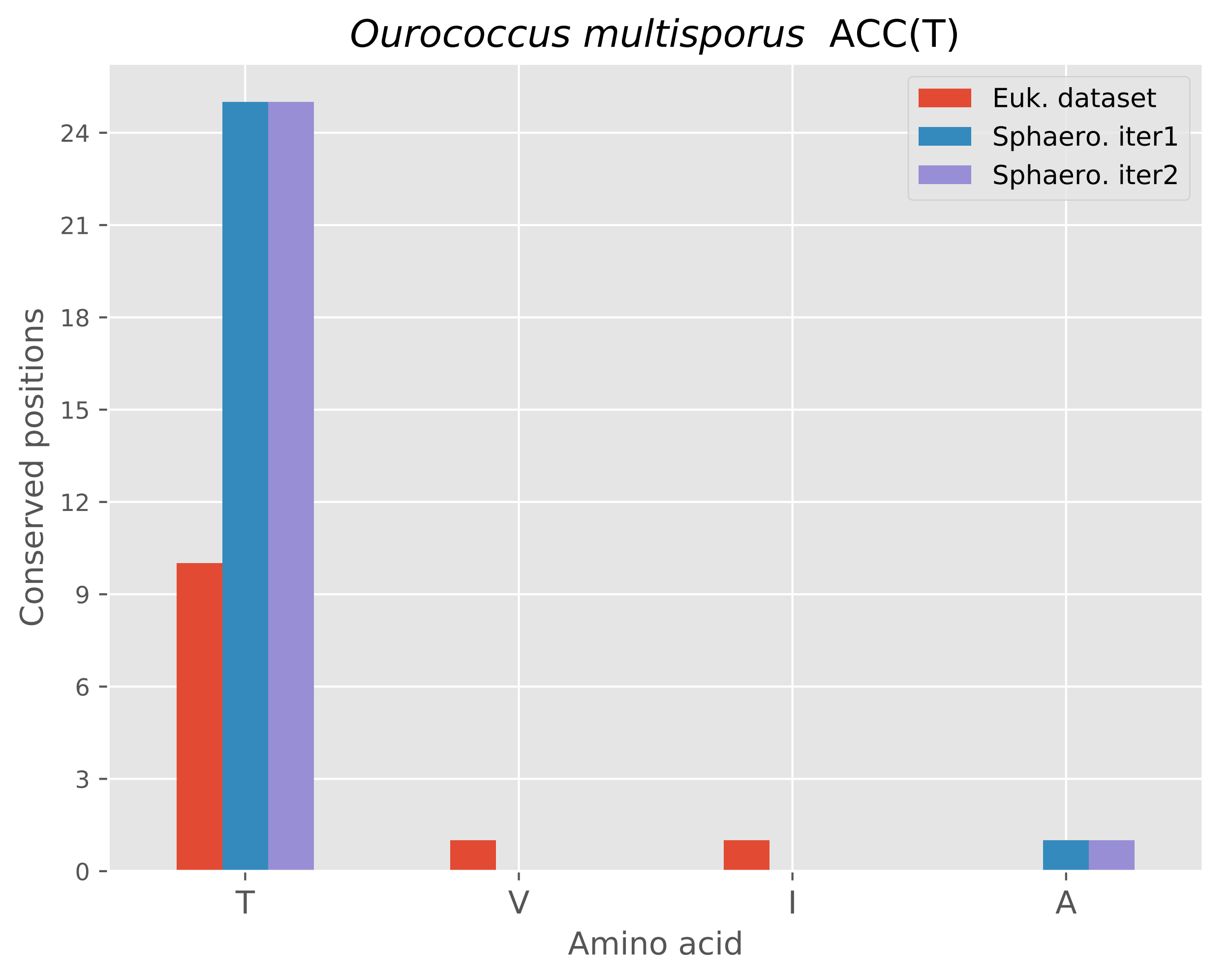

Ourococcus multisporus ACC(T)
Euk. dataset
Sphaero. iter1
Sphaero. iter2
Conserved positions
0
3
6
9
12
15
18
21
24
T
V
I
A
Amino acid

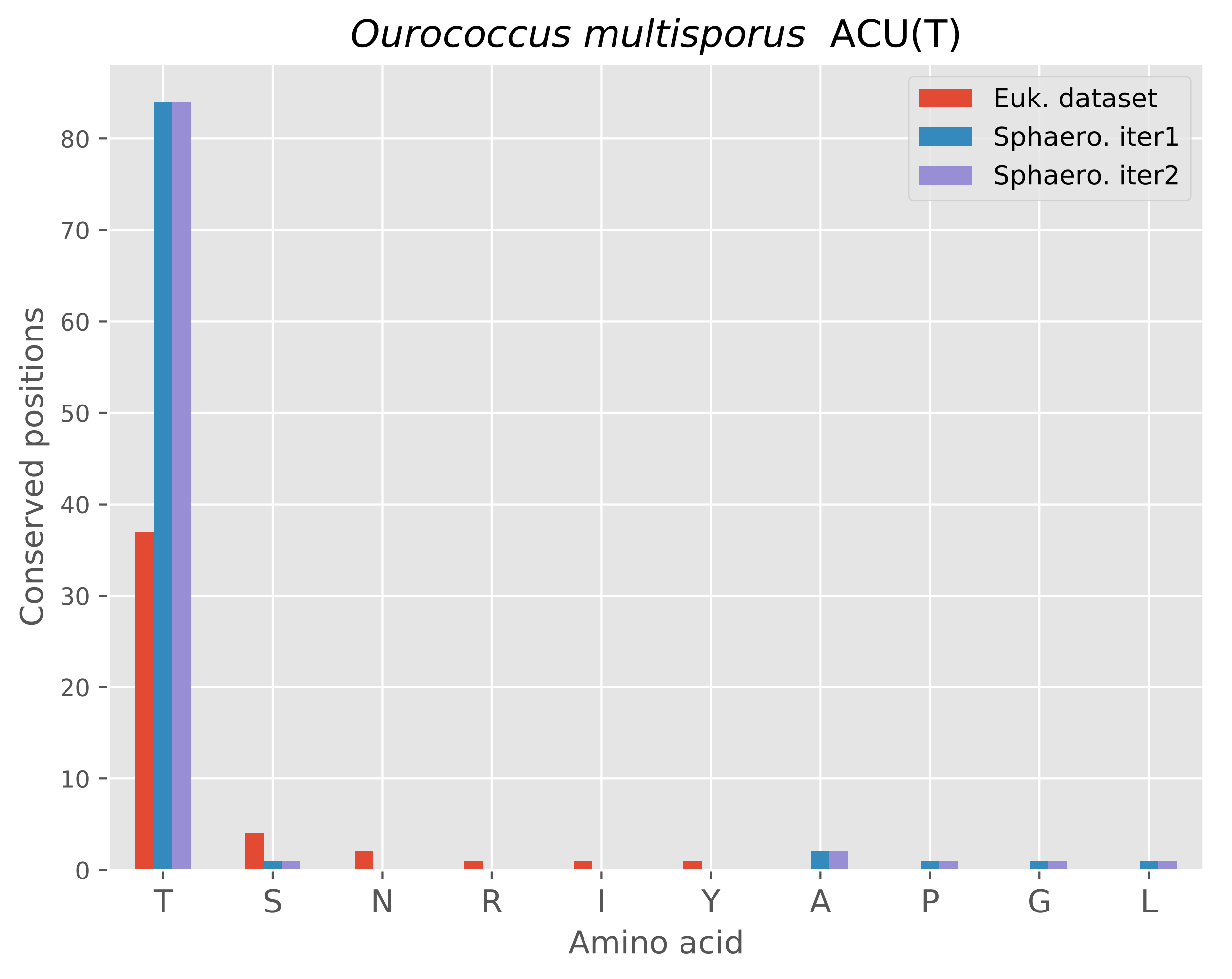
Ourococcus multisporus ACU(T)
Euk. dataset
Sphaero. iter1
Sphaero. iter2
Conserved positions
0
10
20
30
40
50
60
70
80
T
S
N
R
I
Y
A
P
G
L
Amino acid

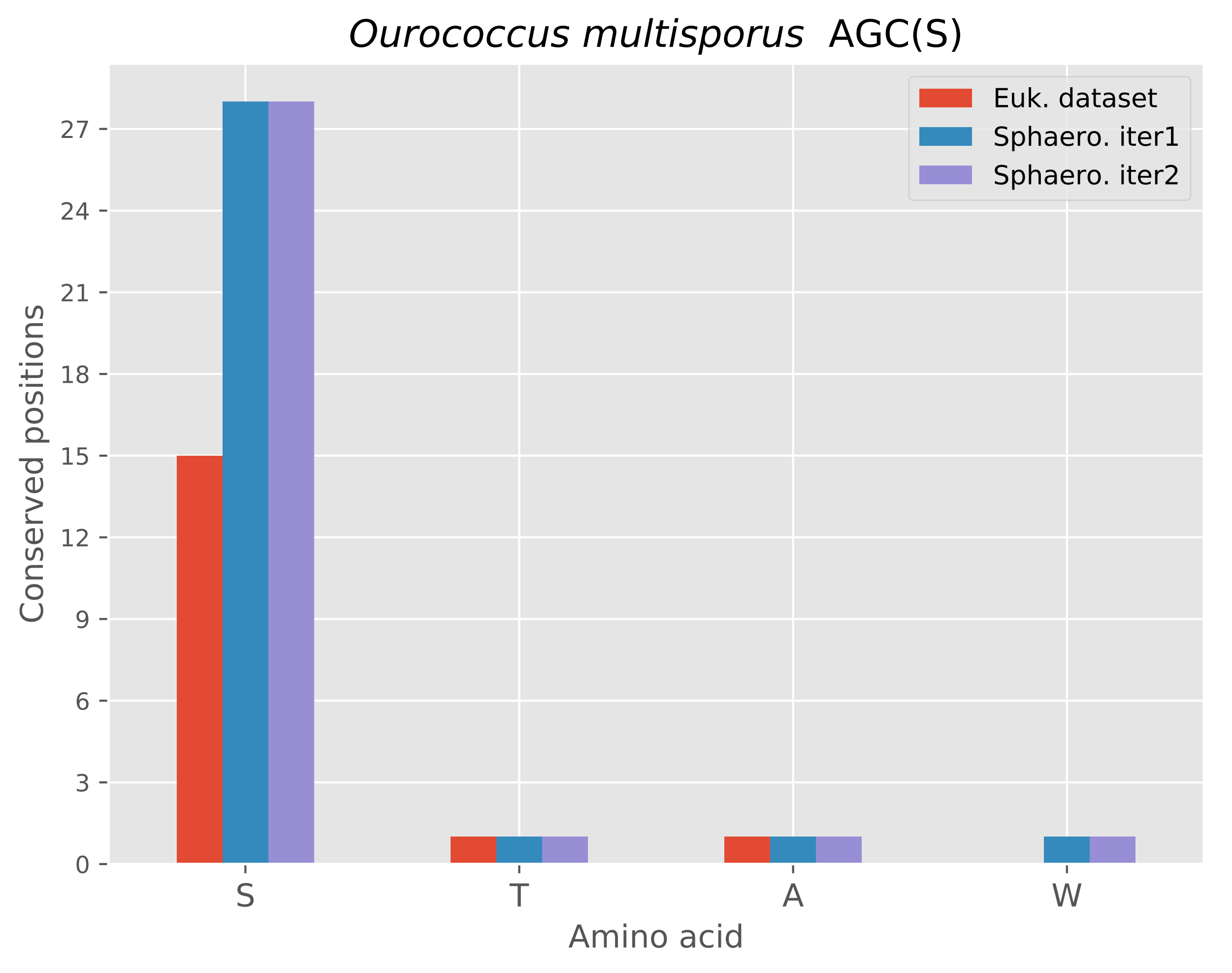

Ourococcus multisporus AGC(S)
Euk. dataset
Sphaero. iter1
Sphaero. iter2
Conserved positions
0
3
6
9
12
15
18
21
24
27
S
T
A
W
Amino acid

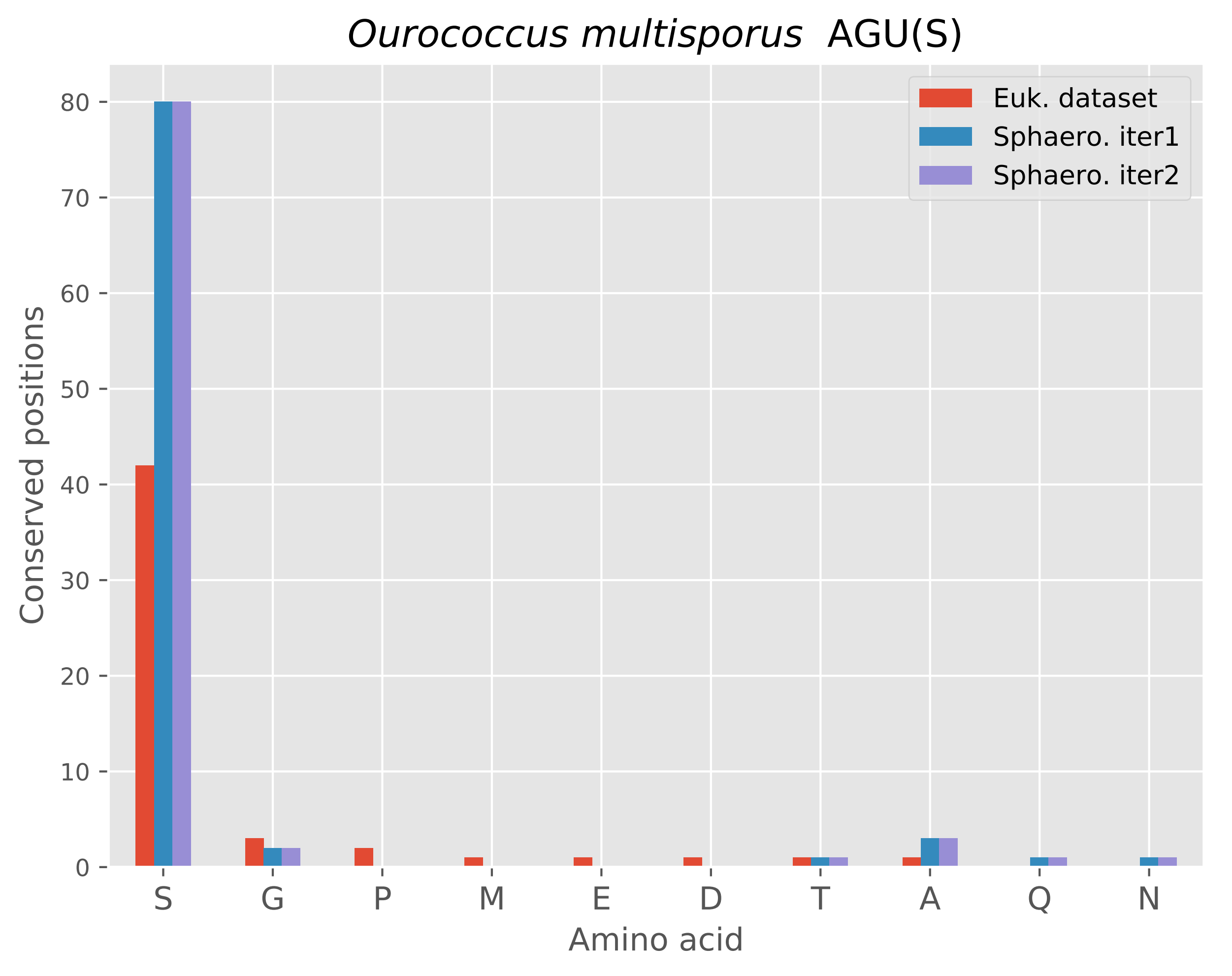
Ourococcus multisporus AGU(S)
Euk. dataset
Sphaero. iter1
Sphaero. iter2
Conserved positions
0
10
20
30
40
50
60
70
80
S
G
P
M
E
D
T
A
Q
N
Amino acid

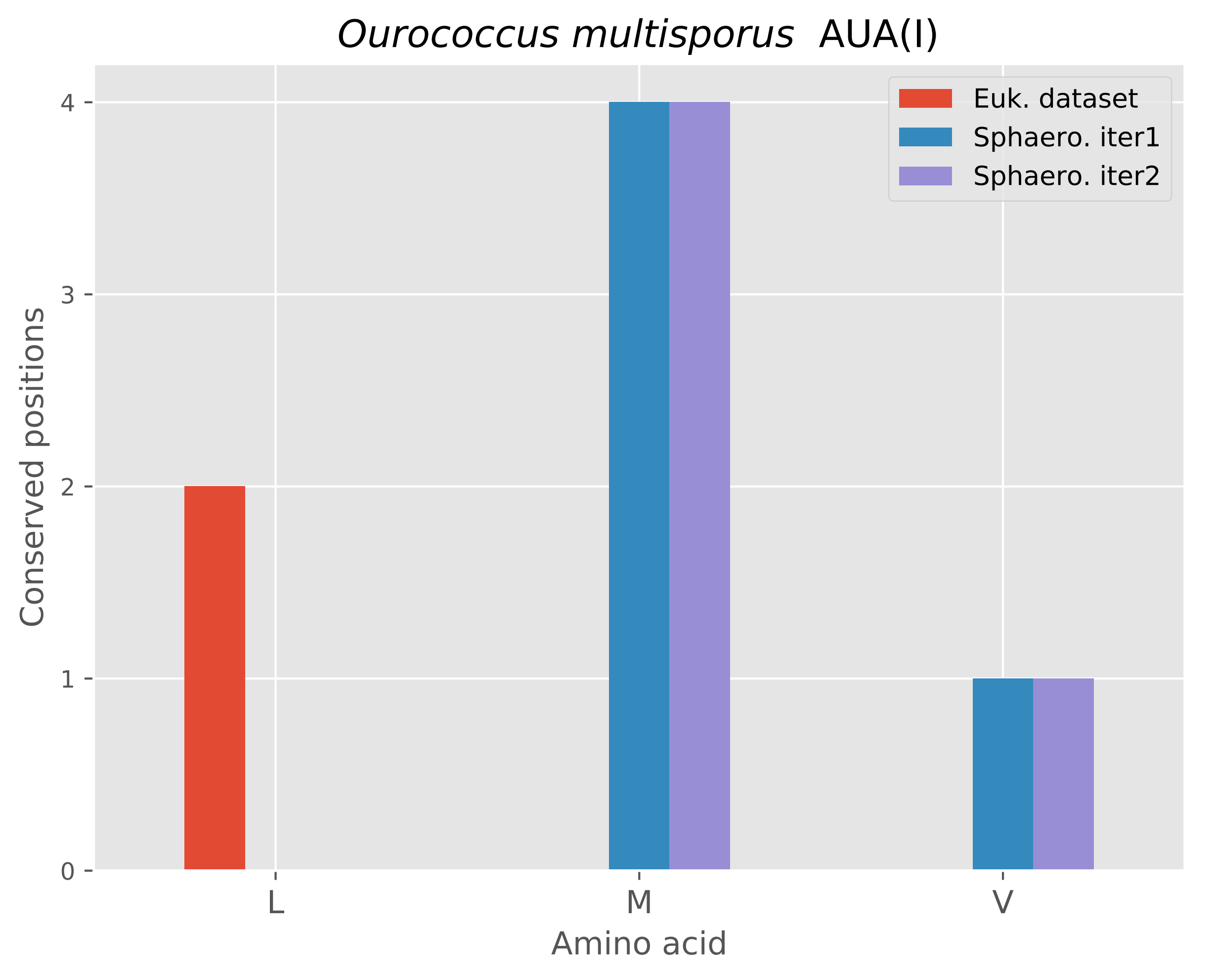
Ourococcus multisporus AUA(I)
Euk. dataset
Sphaero. iter1
Sphaero. iter2
Conserved positions
0
1
2
3
4
L
M
V
Amino acid

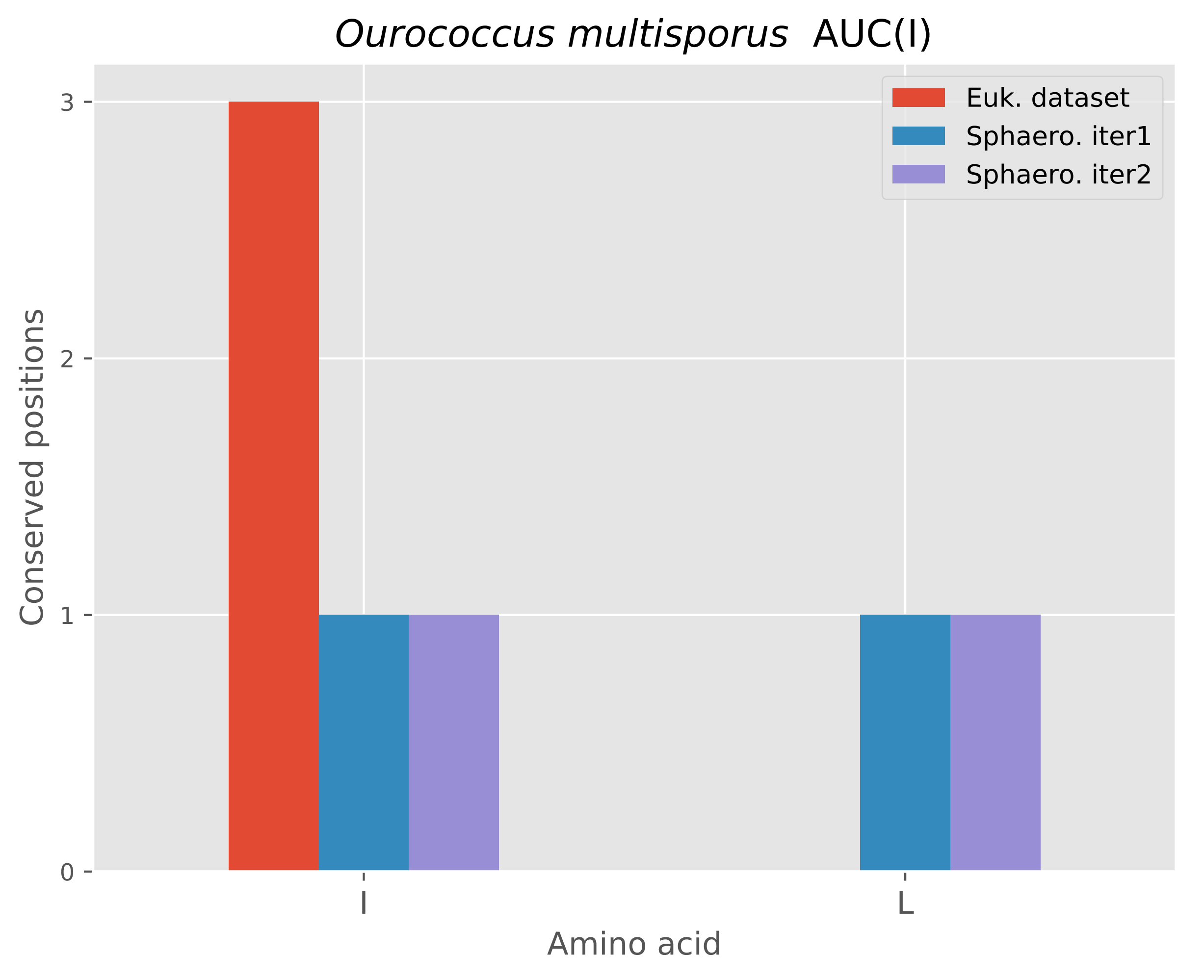

Ourococcus multisporus AUC(I)
Euk. dataset
Sphaero. iter1
Sphaero. iter2
Conserved positions
0
1
2
3
I
L
Amino acid

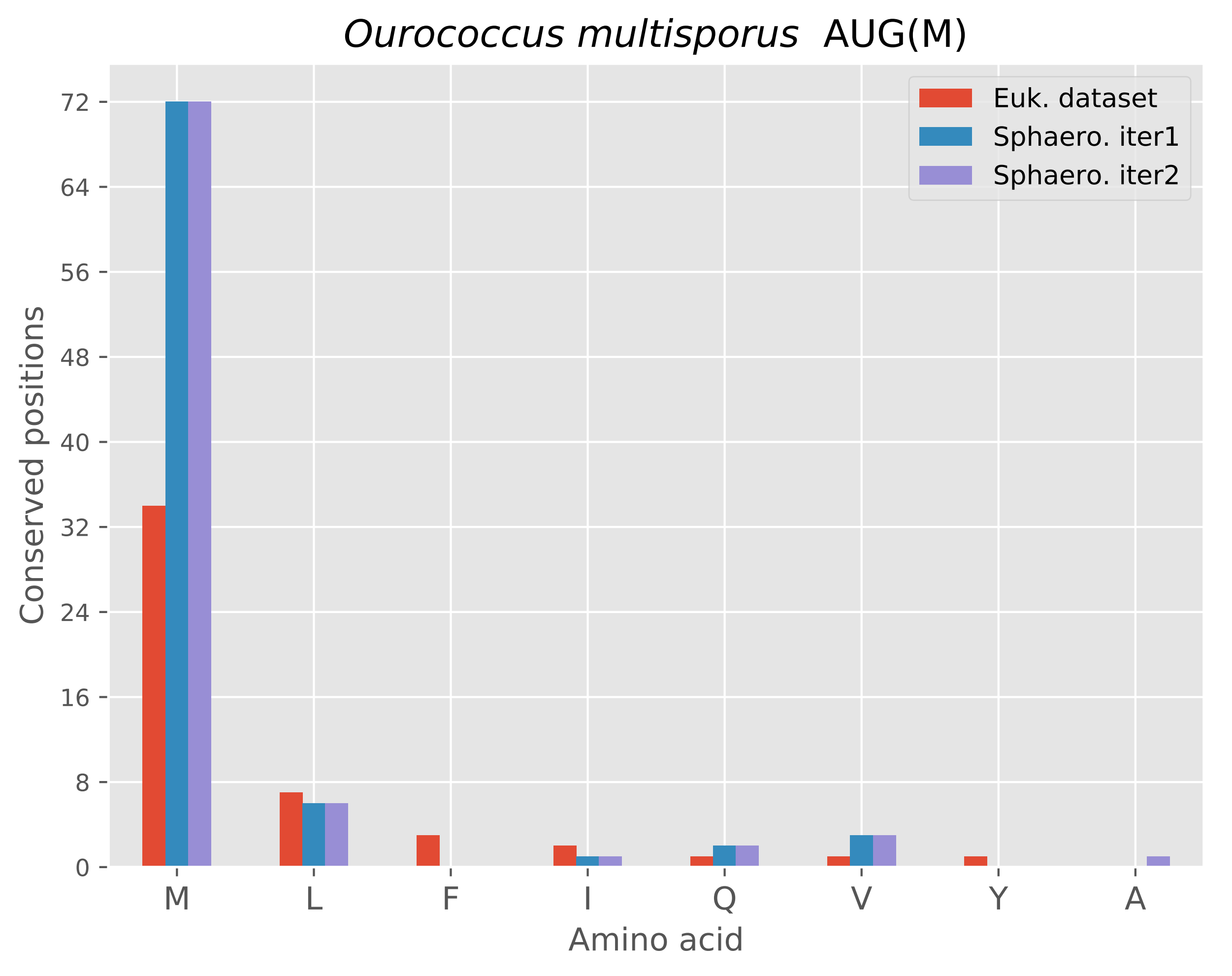
Ourococcus multisporus AUG(M)
Euk. dataset
Sphaero. iter1
Sphaero. iter2
Conserved positions
0
8
16
24
32
40
48
56
64
72
M
L
F
I
Q
V
Y
A
Amino acid

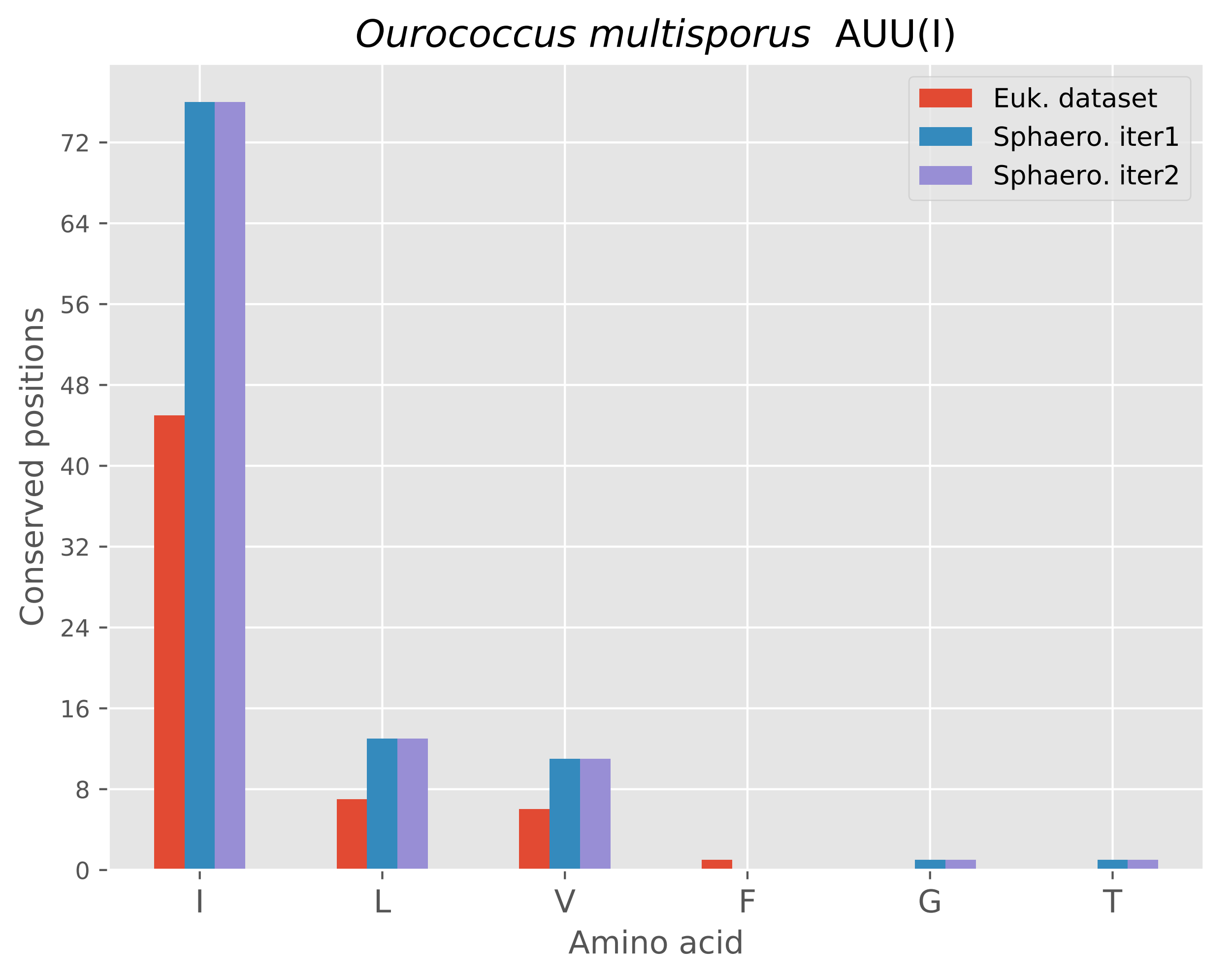

Ourococcus multisporus AUU(I)
Euk. dataset
Sphaero. iter1
Sphaero. iter2
Conserved positions
0
8
16
24
32
40
48
56
64
72
I
L
V
F
G
T
Amino acid

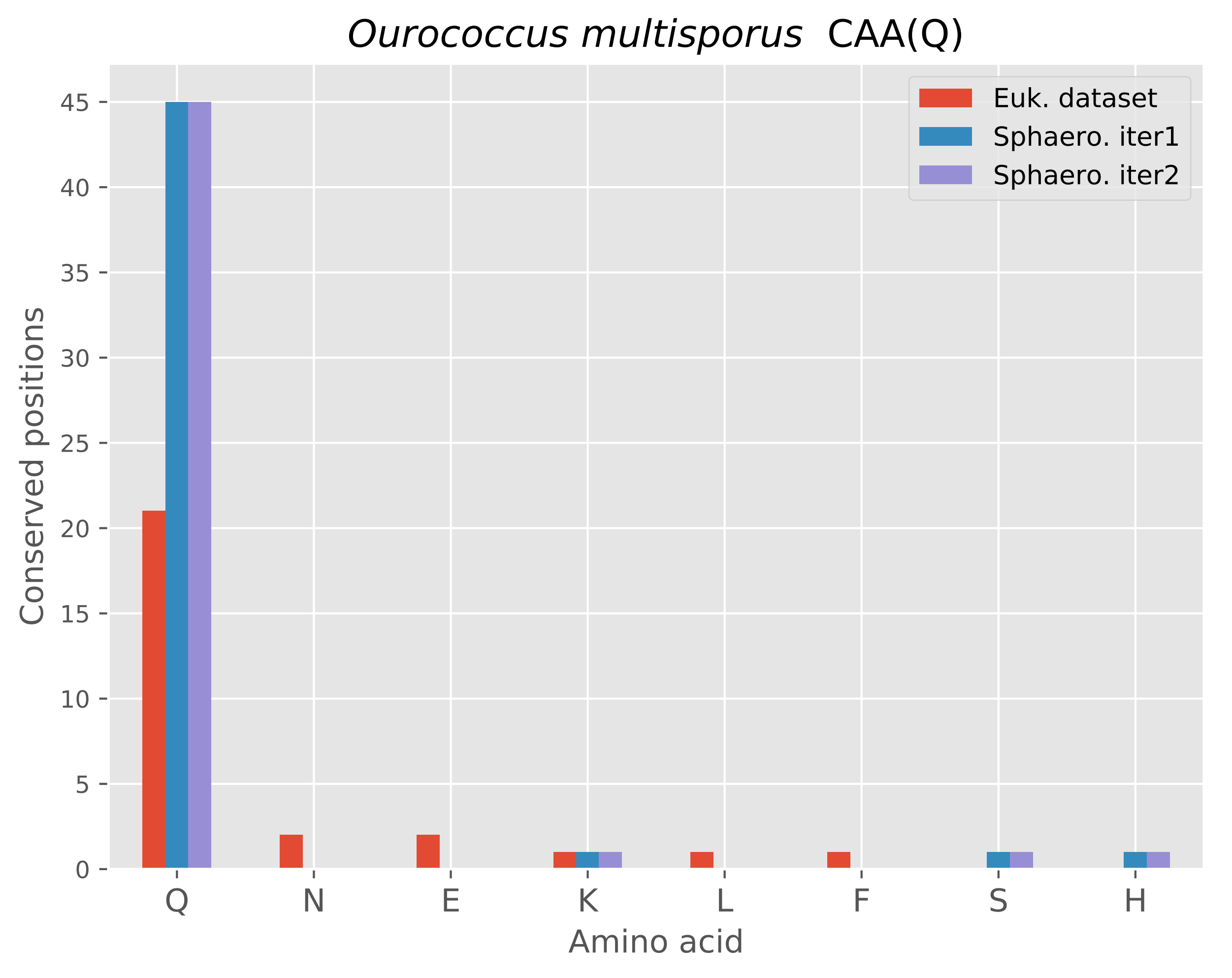

Ourococcus multisporus CAA(Q)
Euk. dataset
Sphaero. iter1
Sphaero. iter2
Conserved positions
0
5
10
15
20
25
30
35
40
45
Q
N
E
K
L
F
S
H
Amino acid

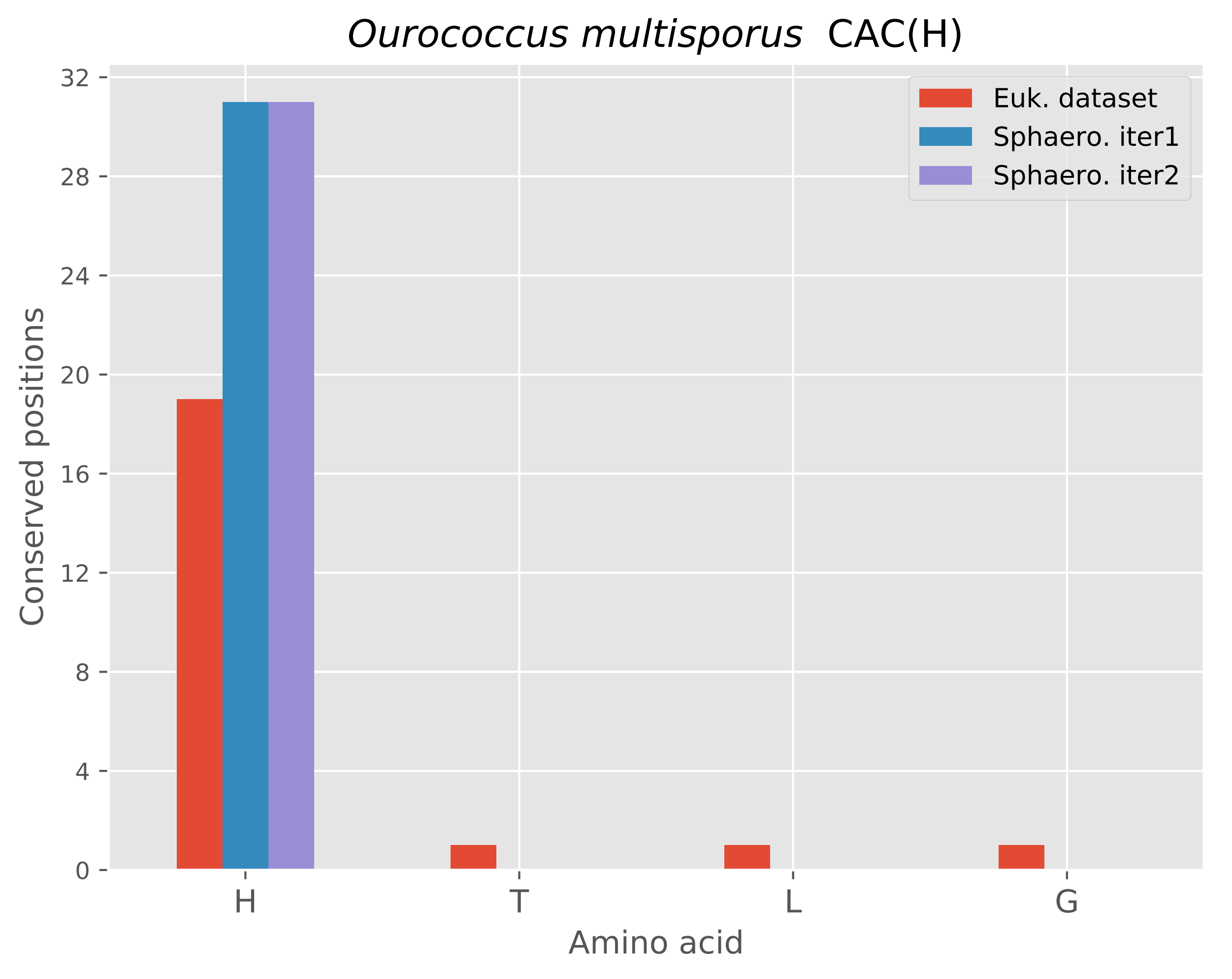

Ourococcus multisporus CAC(H)
Euk. dataset
Sphaero. iter1
Sphaero. iter2
Conserved positions
0
4
8
12
16
20
24
28
32
H
T
L
G
Amino acid

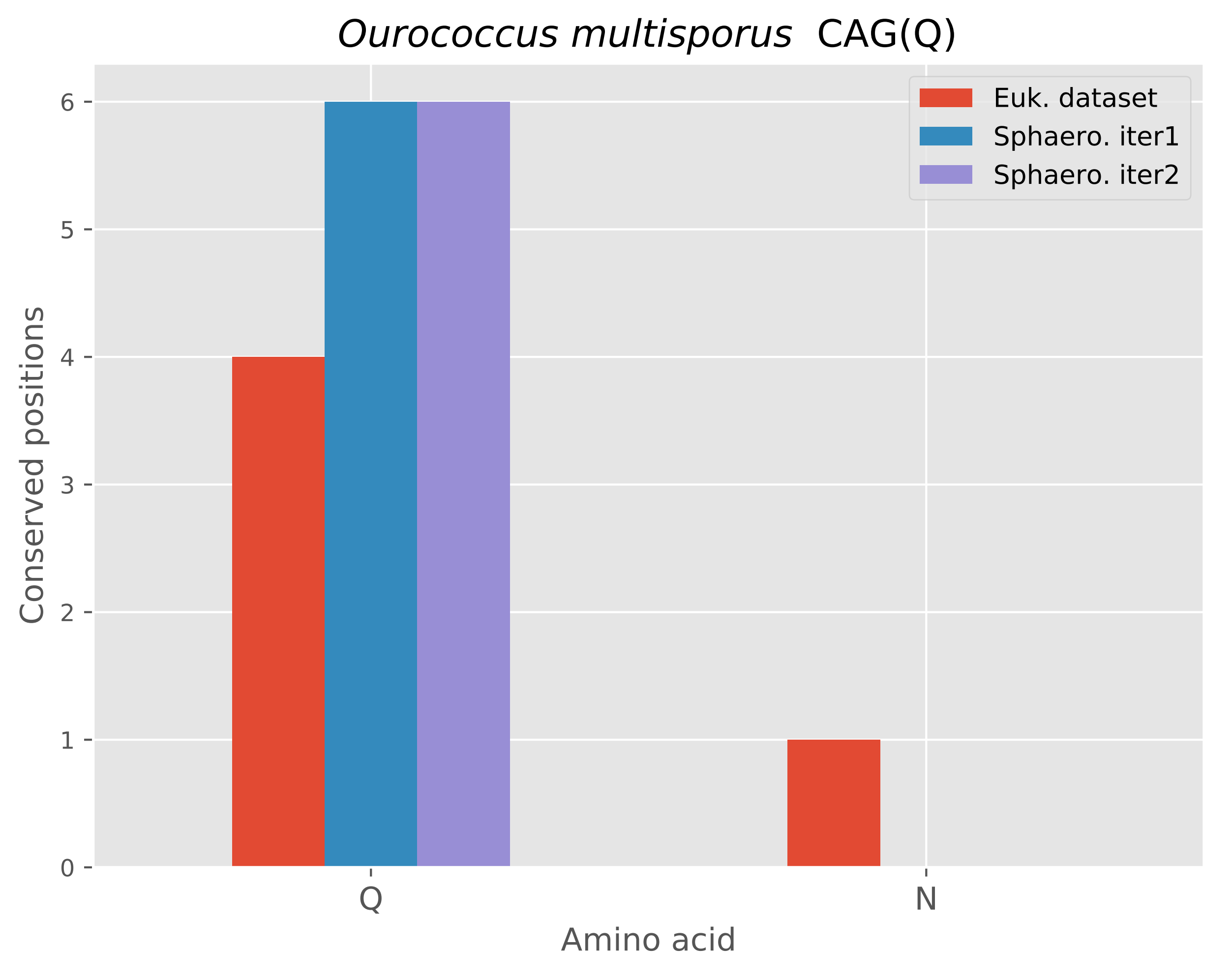

Ourococcus multisporus CAG(Q)
Euk. dataset
Sphaero. iter1
Sphaero. iter2
Conserved positions
0
1
2
3
4
5
6
Q
N
Amino acid

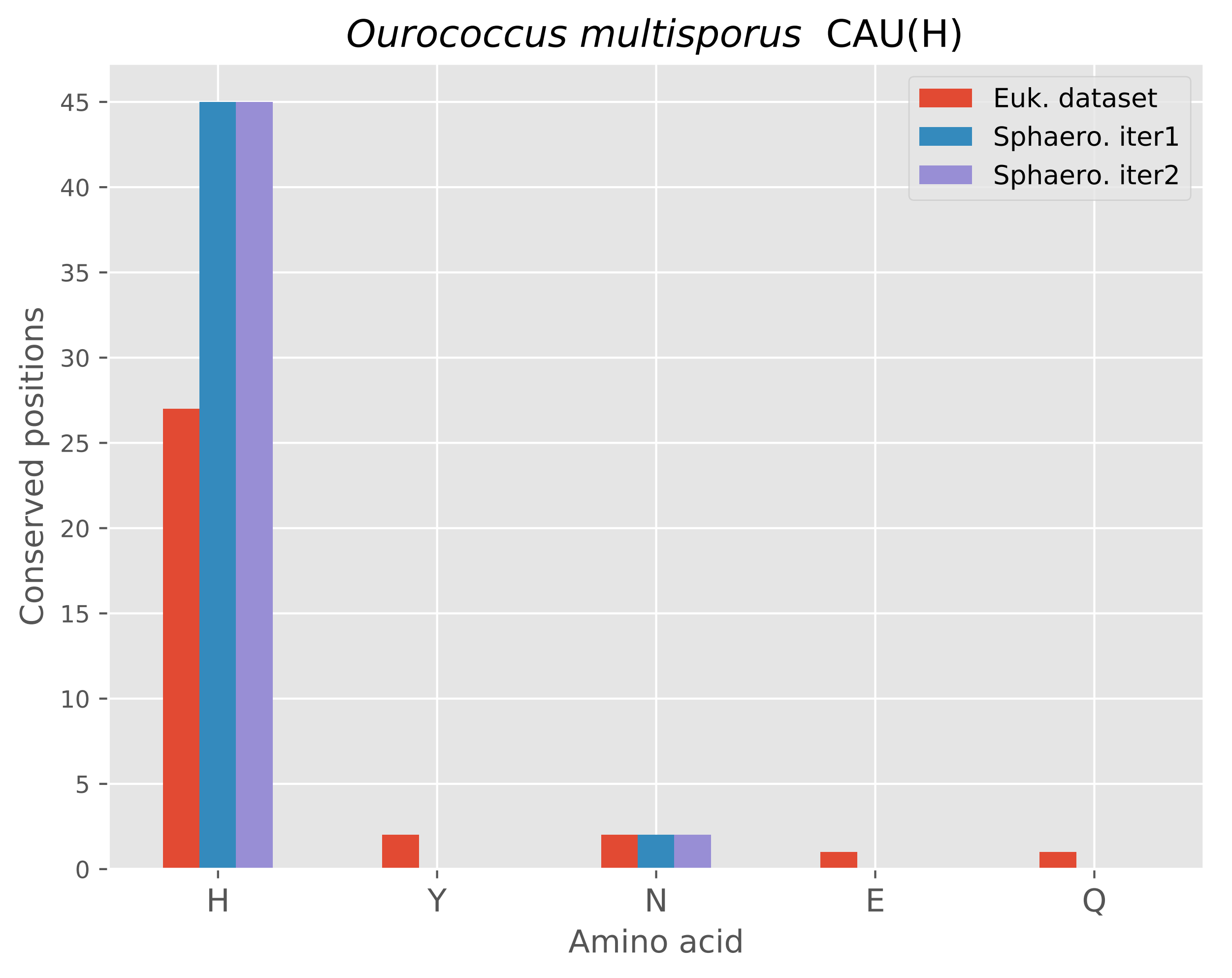

Ourococcus multisporus CAU(H)
Euk. dataset
Sphaero. iter1
Sphaero. iter2
Conserved positions
0
5
10
15
20
25
30
35
40
45
H
Y
N
E
Q
Amino acid

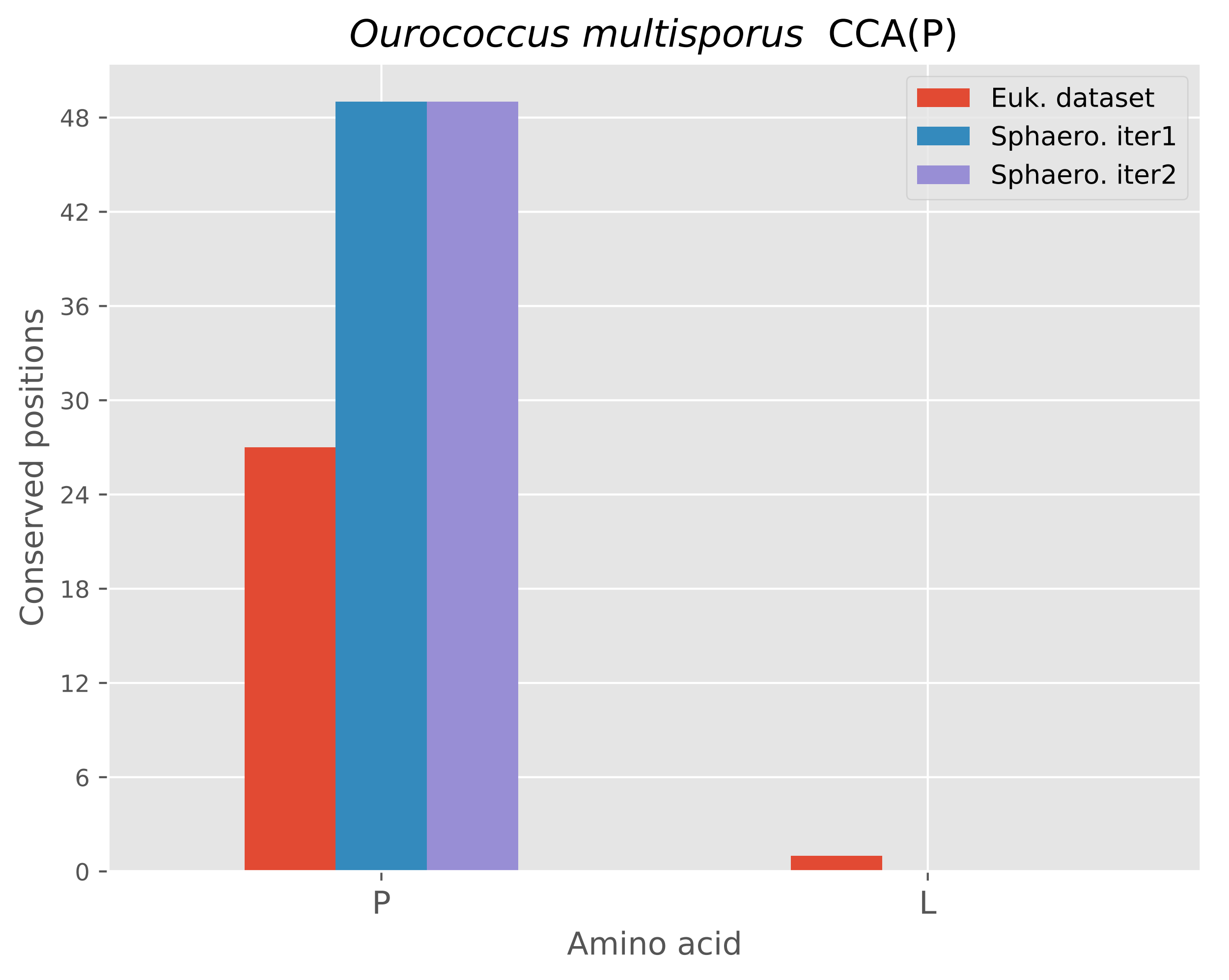

Ourococcus multisporus CCA(P)
Euk. dataset
Sphaero. iter1
Sphaero. iter2
Conserved positions
0
6
12
18
24
30
36
42
48
P
L
Amino acid

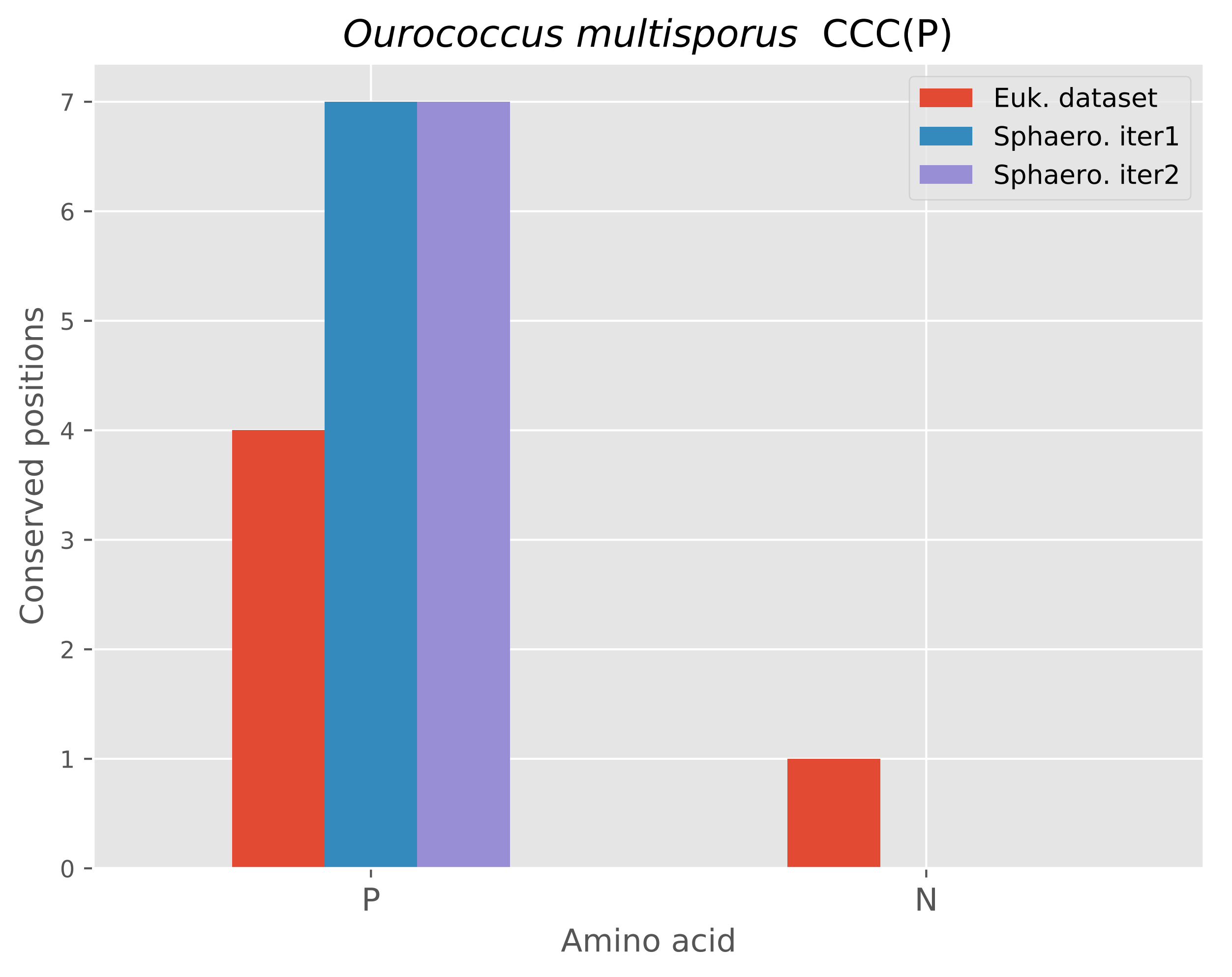
Ourococcus multisporus CCC(P)
Euk. dataset
Sphaero. iter1
Sphaero. iter2
Conserved positions
0
1
2
3
4
5
6
7
P
N
Amino acid

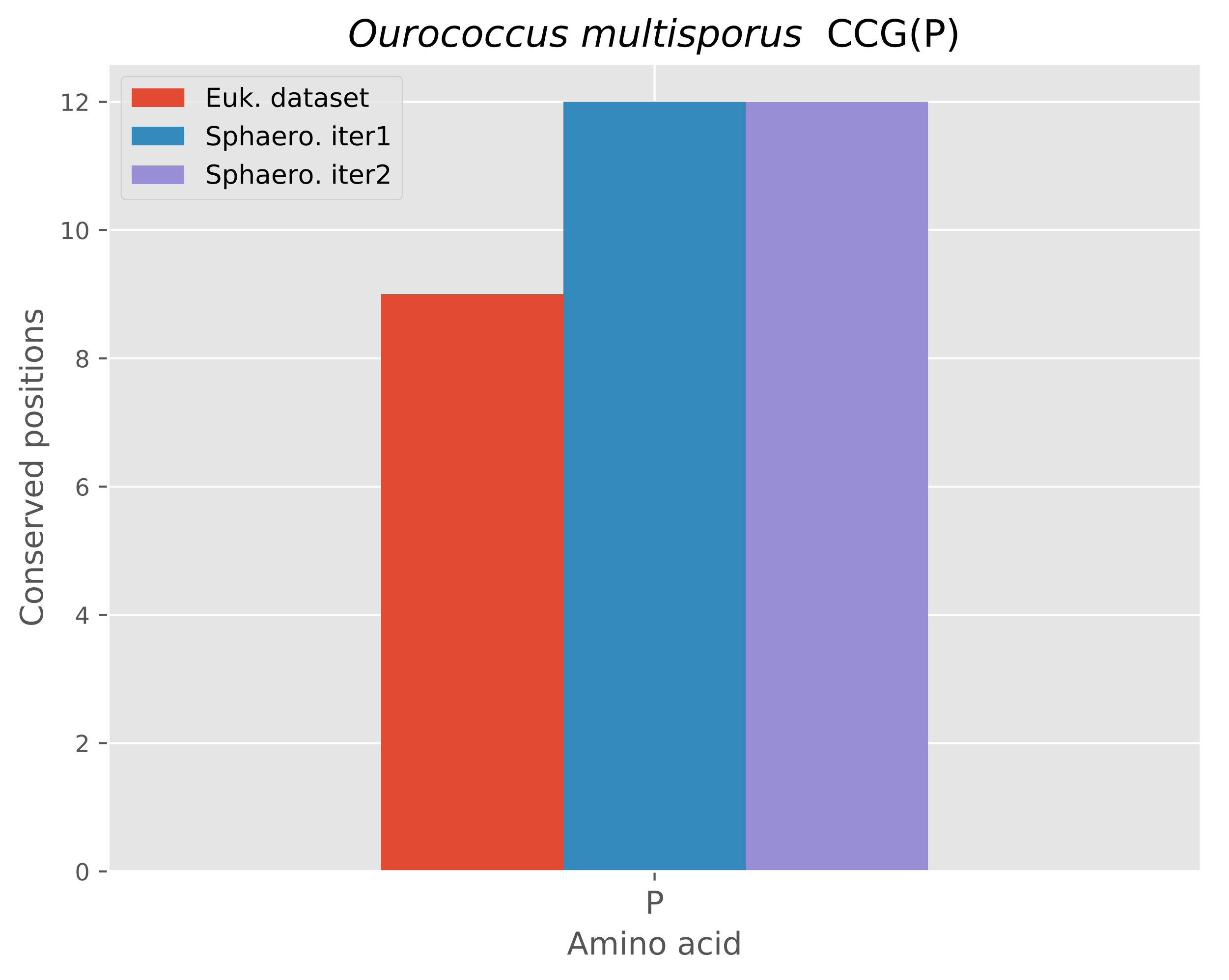

Ourococcus multisporus CCG(P)
Euk. dataset
Sphaero. iter1
Sphaero. iter2
Conserved positions
0
2
4
6
8
10
12
P
Amino acid

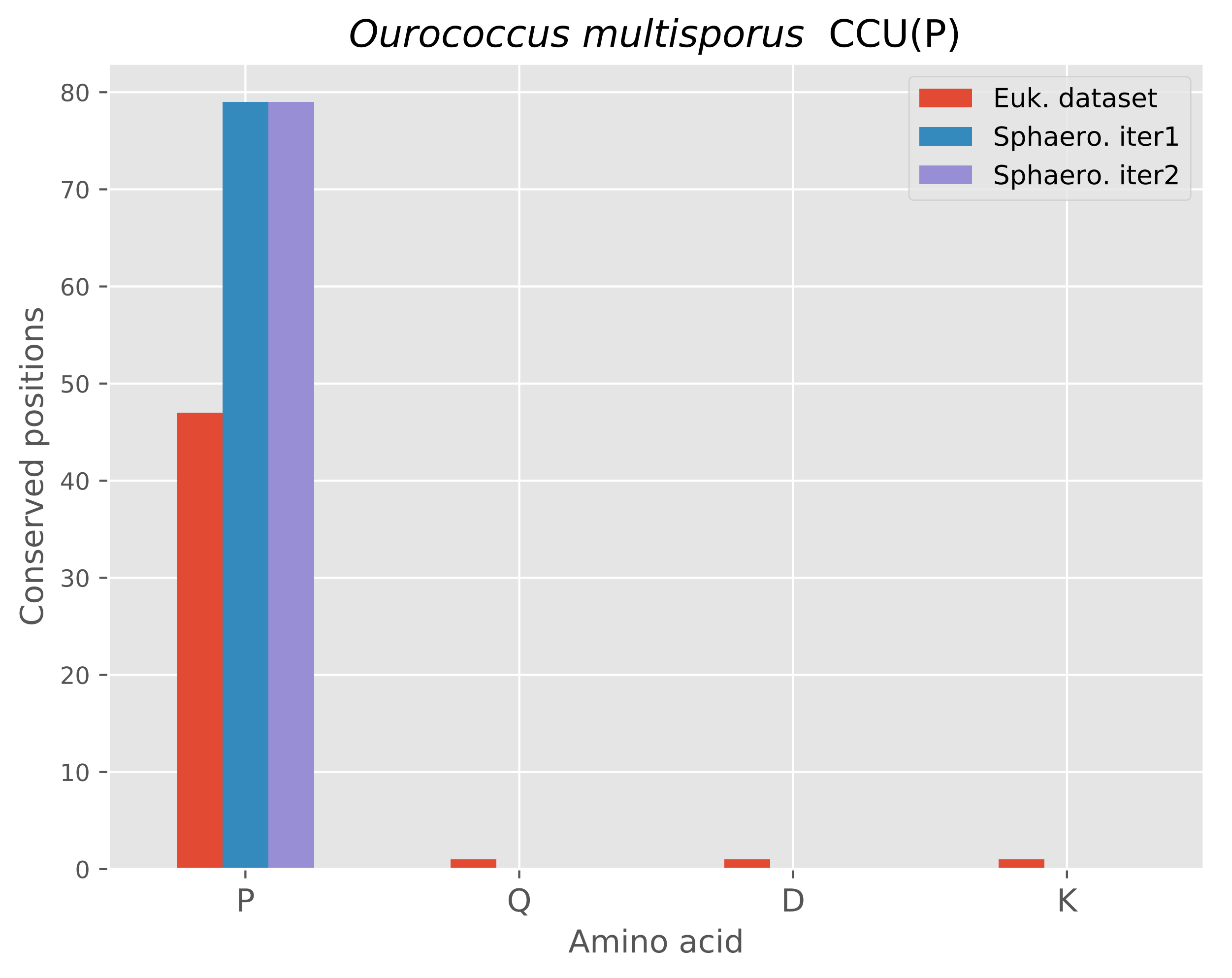

Ourococcus multisporus CCU(P)
Euk. dataset
Sphaero. iter1
Sphaero. iter2
Conserved positions
0
10
20
30
40
50
60
70
80
P
Q
D
K
Amino acid

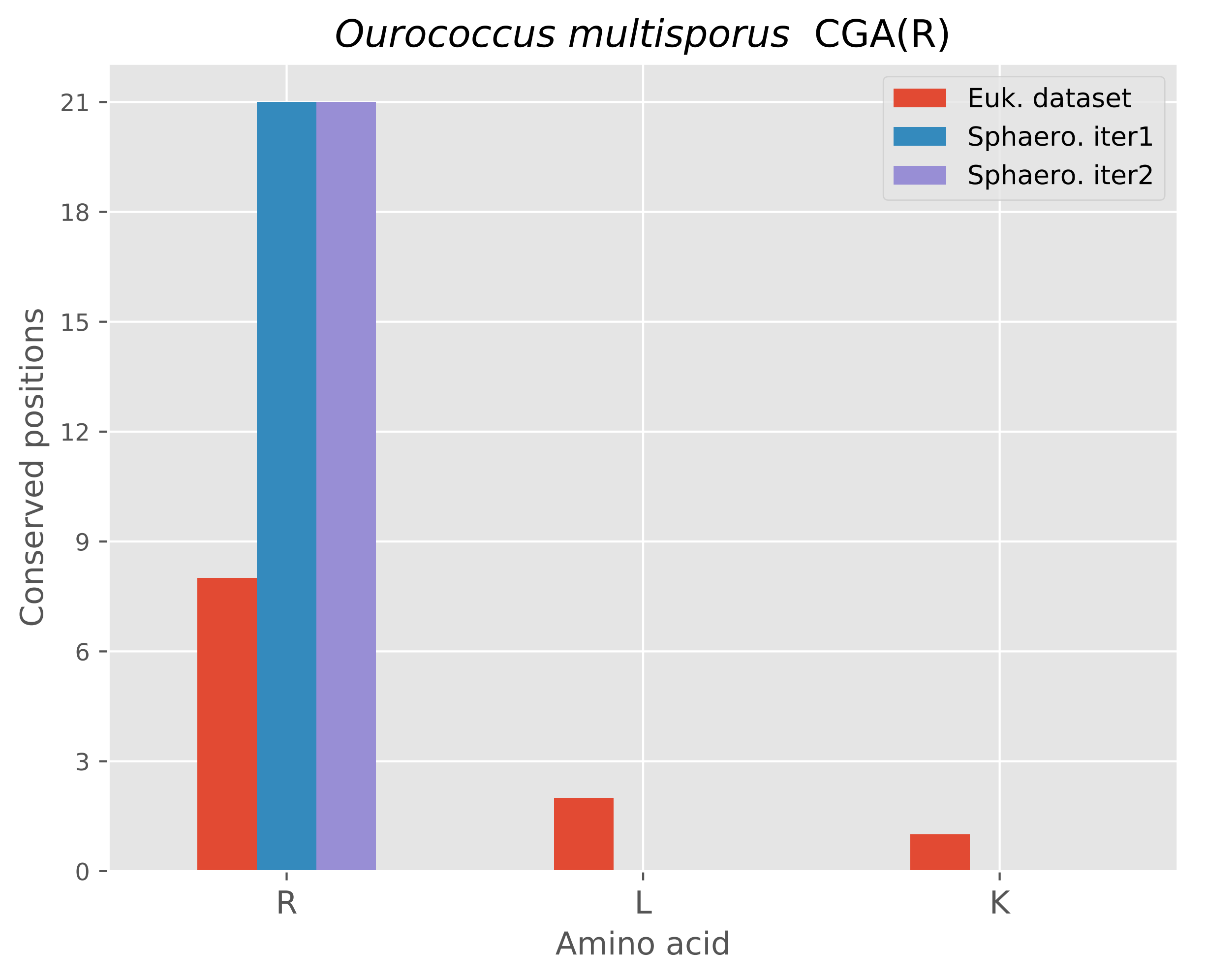

Ourococcus multisporus CGA(R)
Euk. dataset
Sphaero. iter1
Sphaero. iter2
Conserved positions
0
3
6
9
12
15
18
21
R
L
K
Amino acid

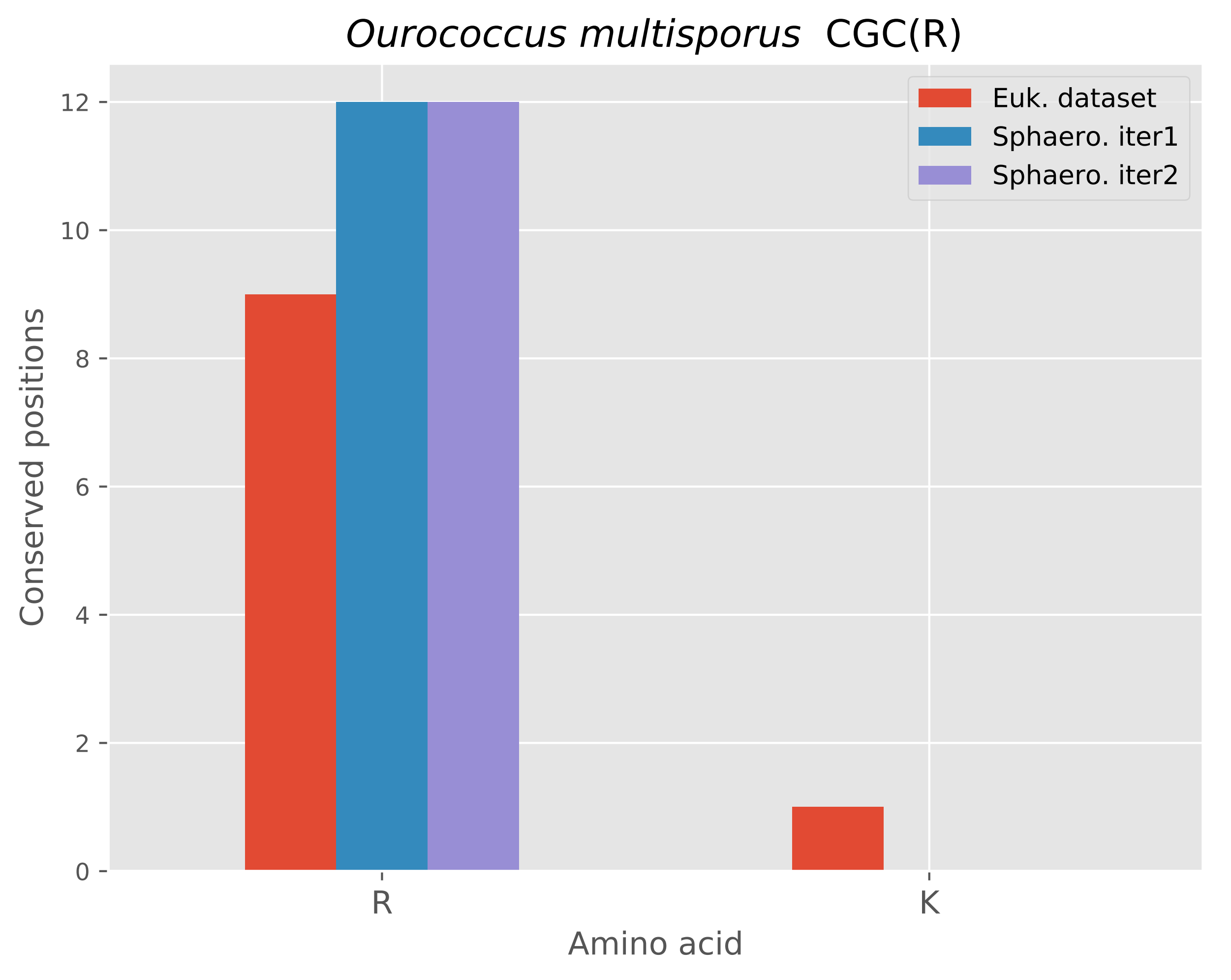

Ourococcus multisporus CGC(R)
Euk. dataset
Sphaero. iter1
Sphaero. iter2
Conserved positions
0
2
4
6
8
10
12
R
K
Amino acid

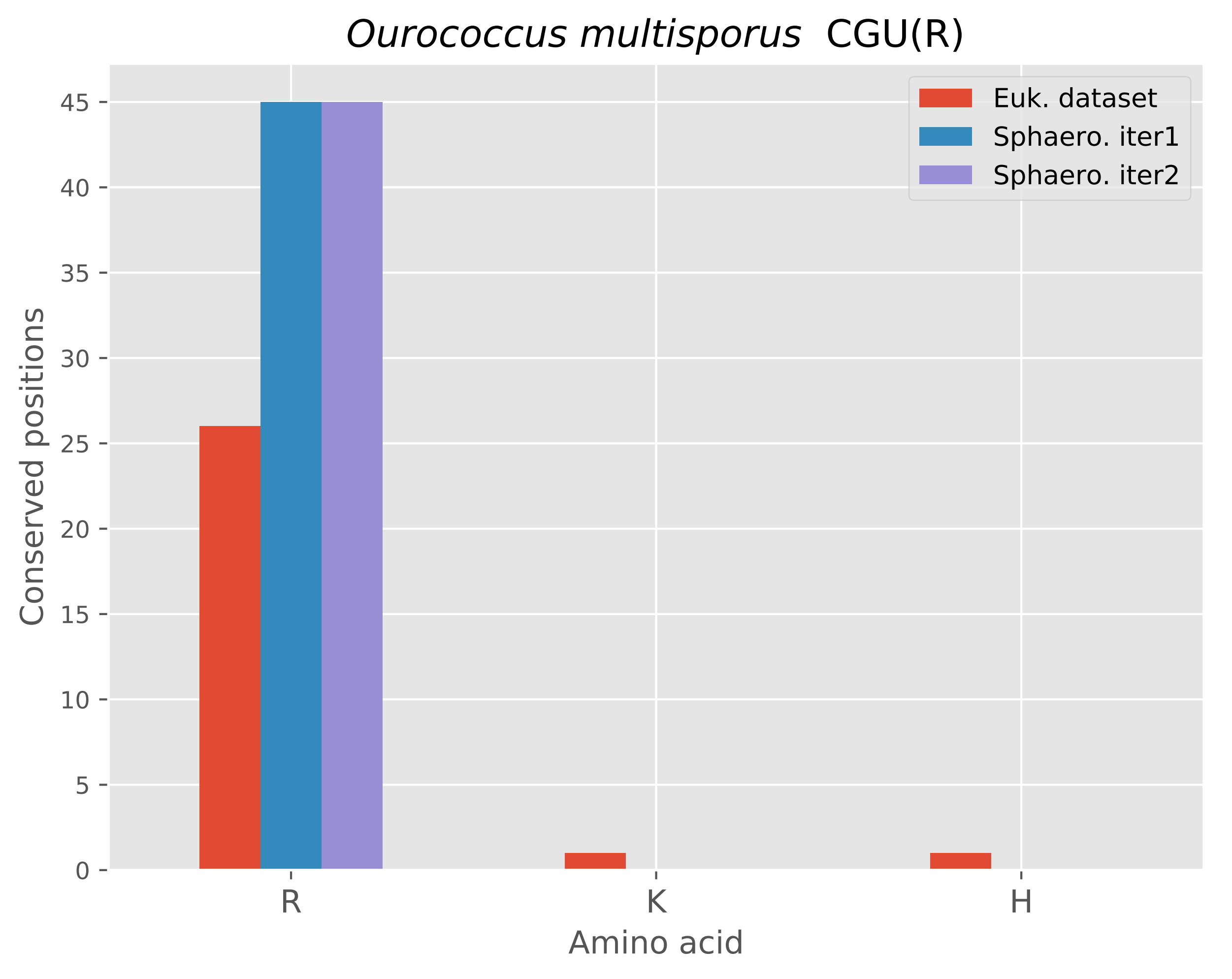

Ourococcus multisporus CGU(R)
Euk. dataset
Sphaero. iter1
Sphaero. iter2
Conserved positions
Amino acid
R
K
H
0
5
10
15
20
25
30
35
40
45

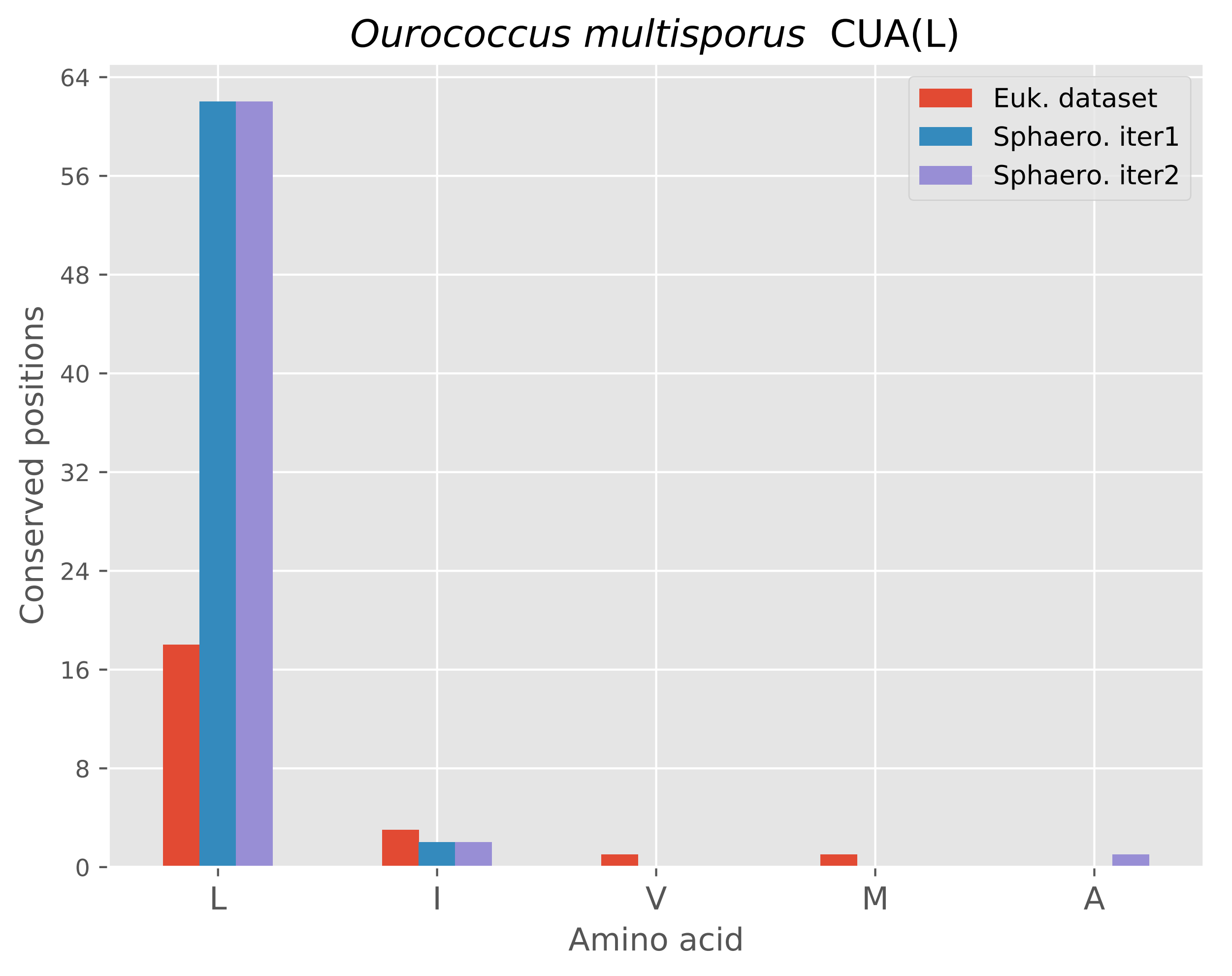

Ourococcus multisporus CUA(L)
Euk. dataset
Sphaero. iter1
Sphaero. iter2
Conserved positions
0
8
16
24
32
40
48
56
64
L
I
V
M
A
Amino acid

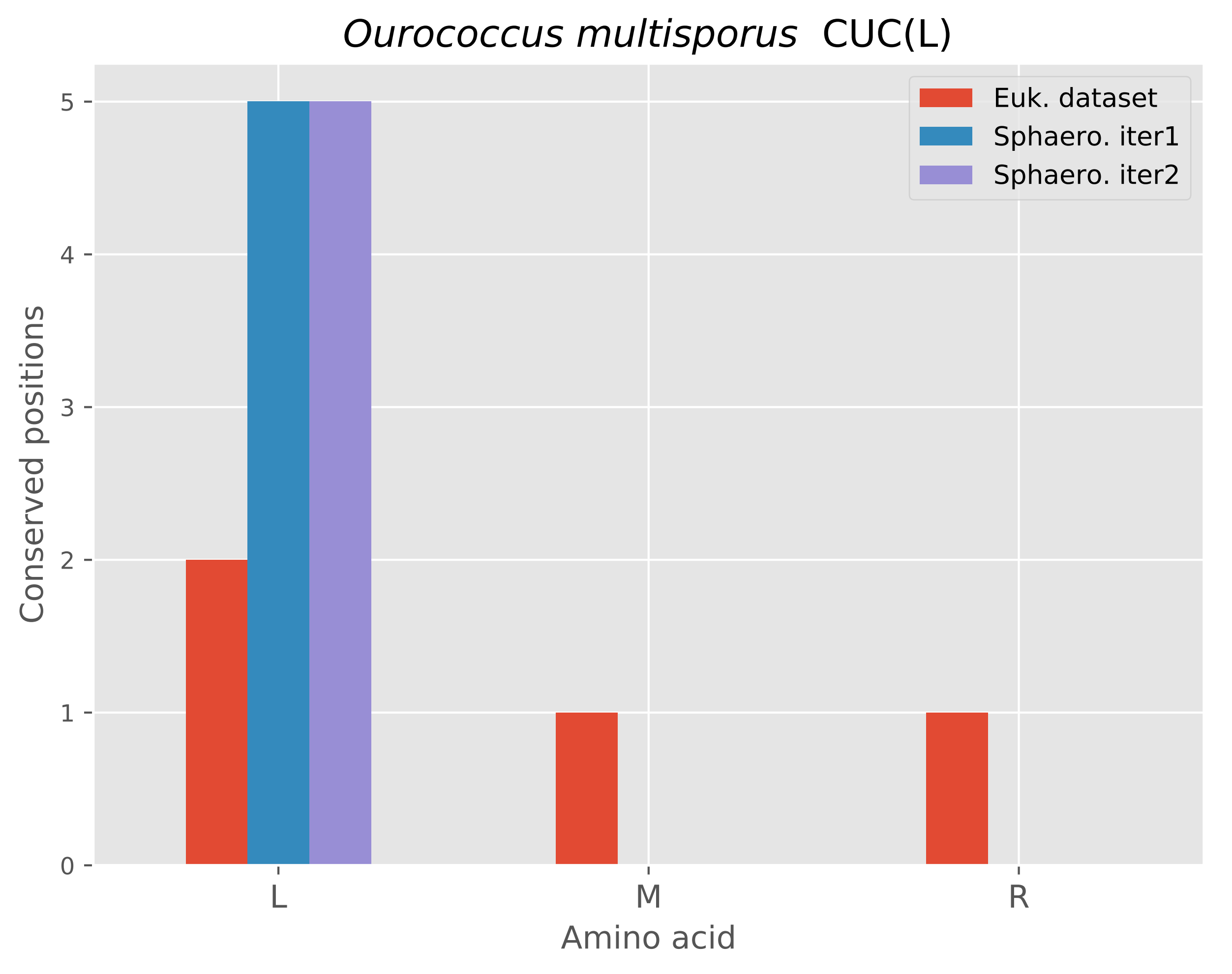

Ourococcus multisporus CUC(L)
Euk. dataset
Sphaero. iter1
Sphaero. iter2
Conserved positions
0
1
2
3
4
5
L
M
R
Amino acid

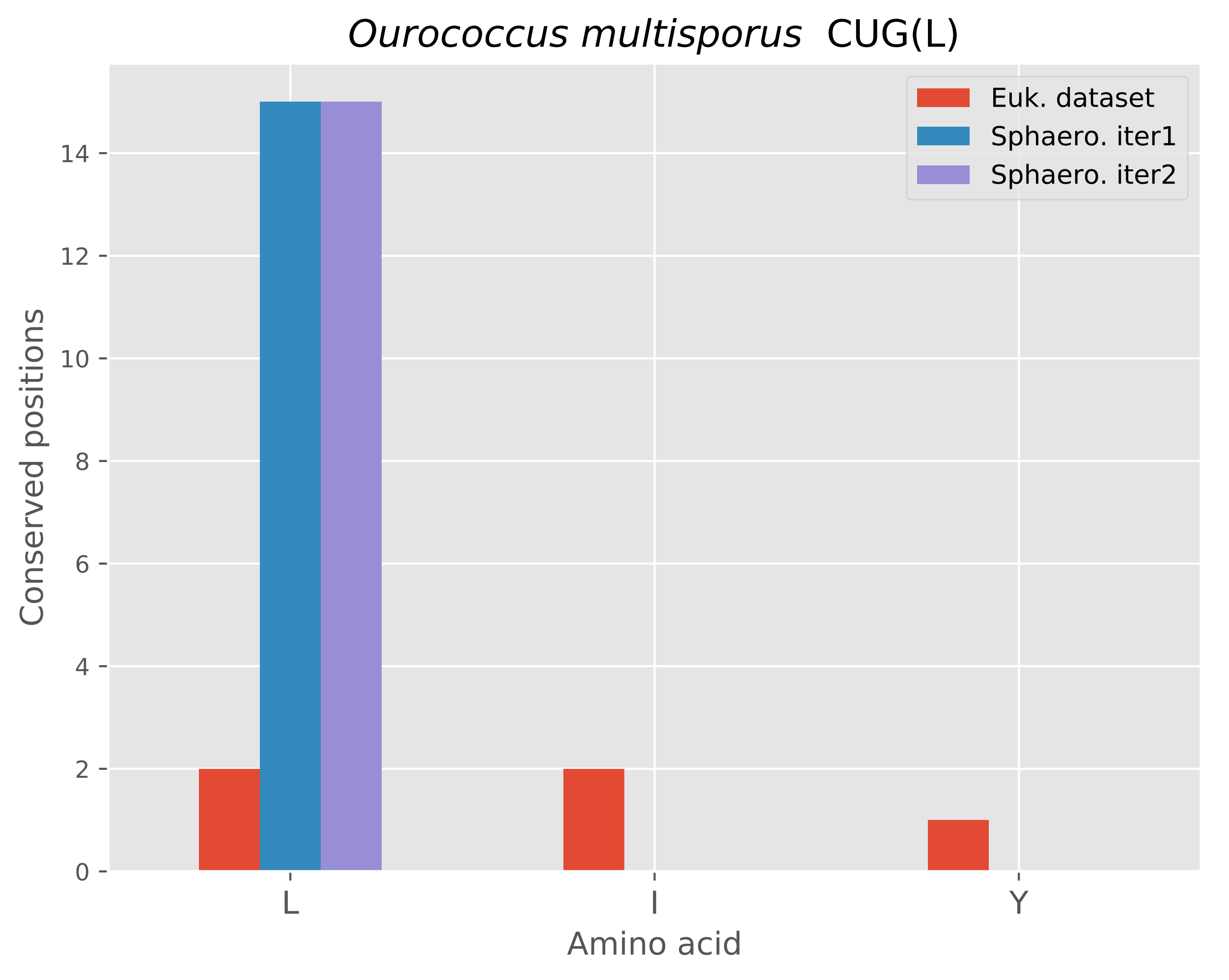

Ourococcus multisporus CUG(L)
Euk. dataset
Sphaero. iter1
Sphaero. iter2
Conserved positions
0
2
4
6
8
10
12
14
L
I
Y
Amino acid

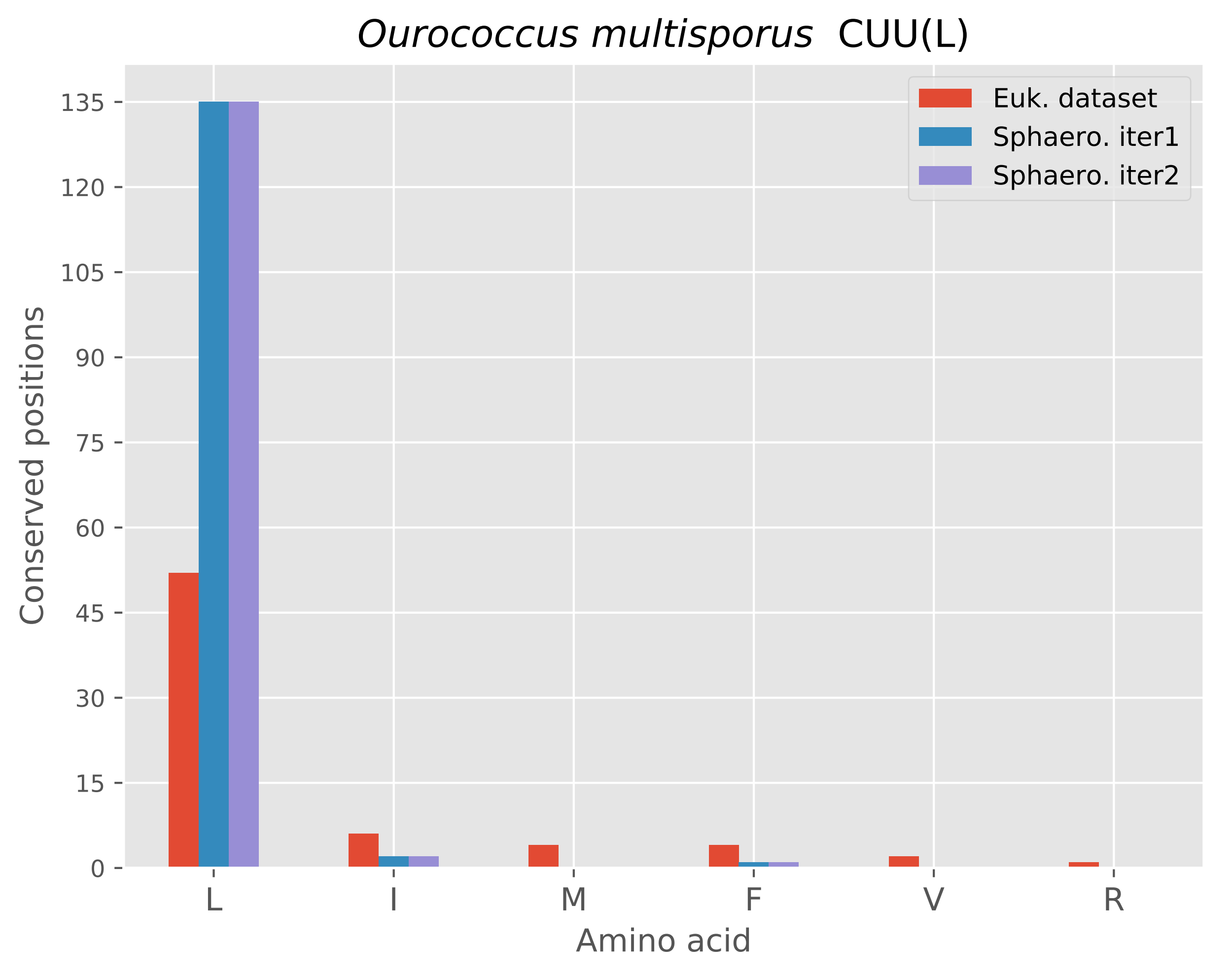

Ourococcus multisporus CUU(L)
Euk. dataset
Sphaero. iter1
Sphaero. iter2
Conserved positions
0
15
30
45
60
75
90
105
120
135
L
I
M
F
V
R
Amino acid

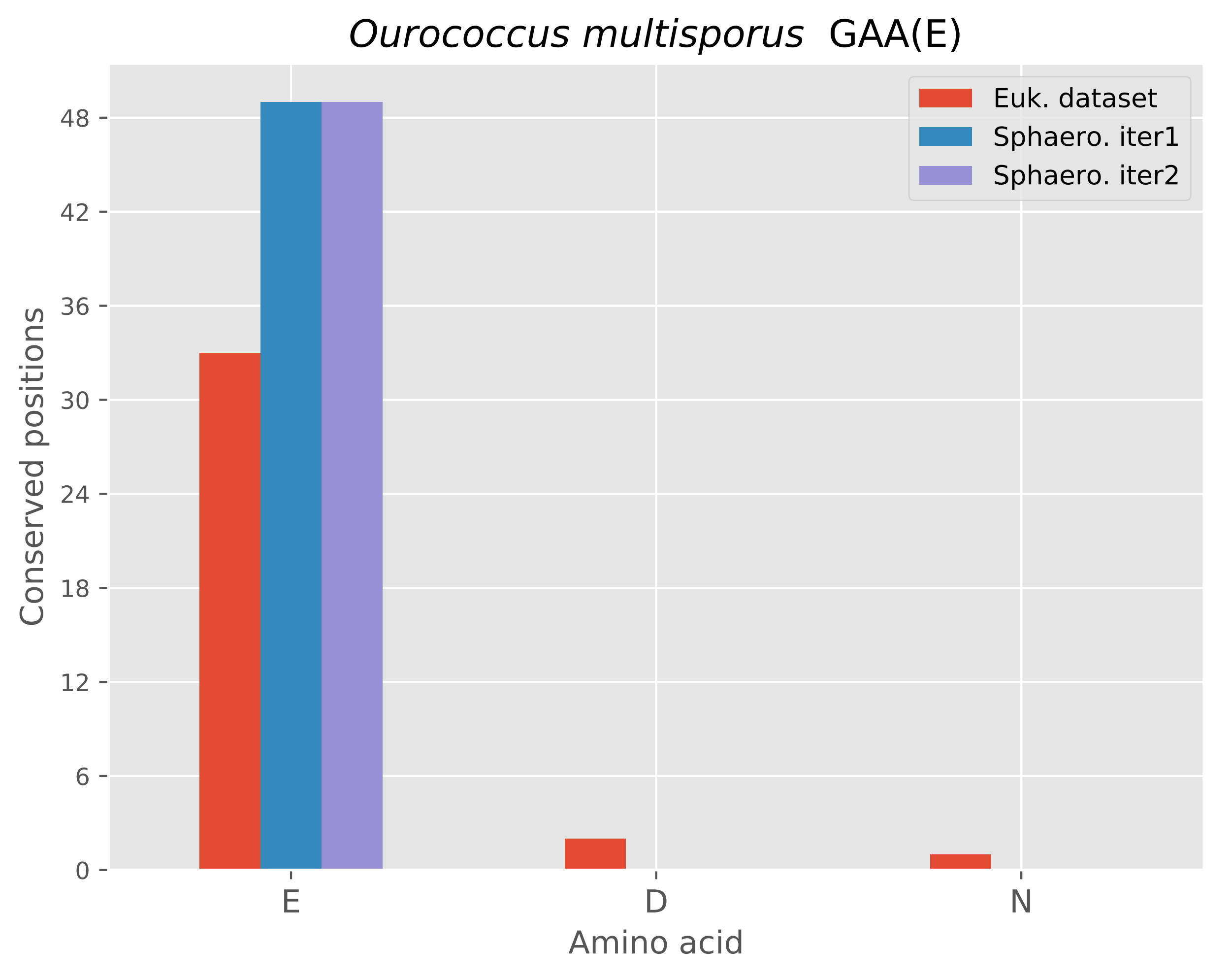

Ourococcus multisporus GAA(E)
Euk. dataset
Sphaero. iter1
Sphaero. iter2
Conserved positions
0
6
12
18
24
30
36
42
48
E
D
N
Amino acid

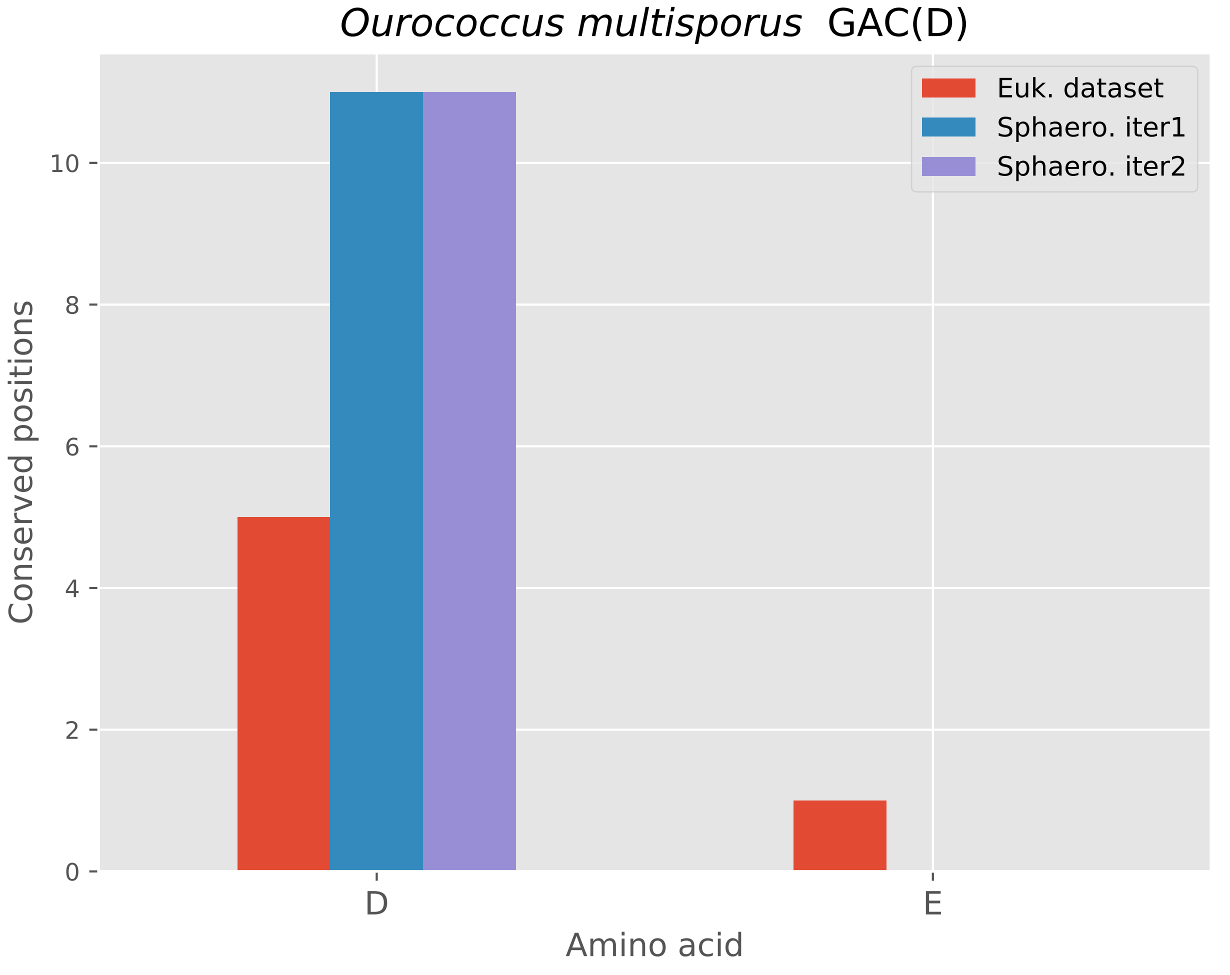

Ourococcus multisporus GAC(D)
Euk. dataset
Sphaero. iter1
Sphaero. iter2
Conserved positions
0
2
4
6
8
10
D
E
Amino acid

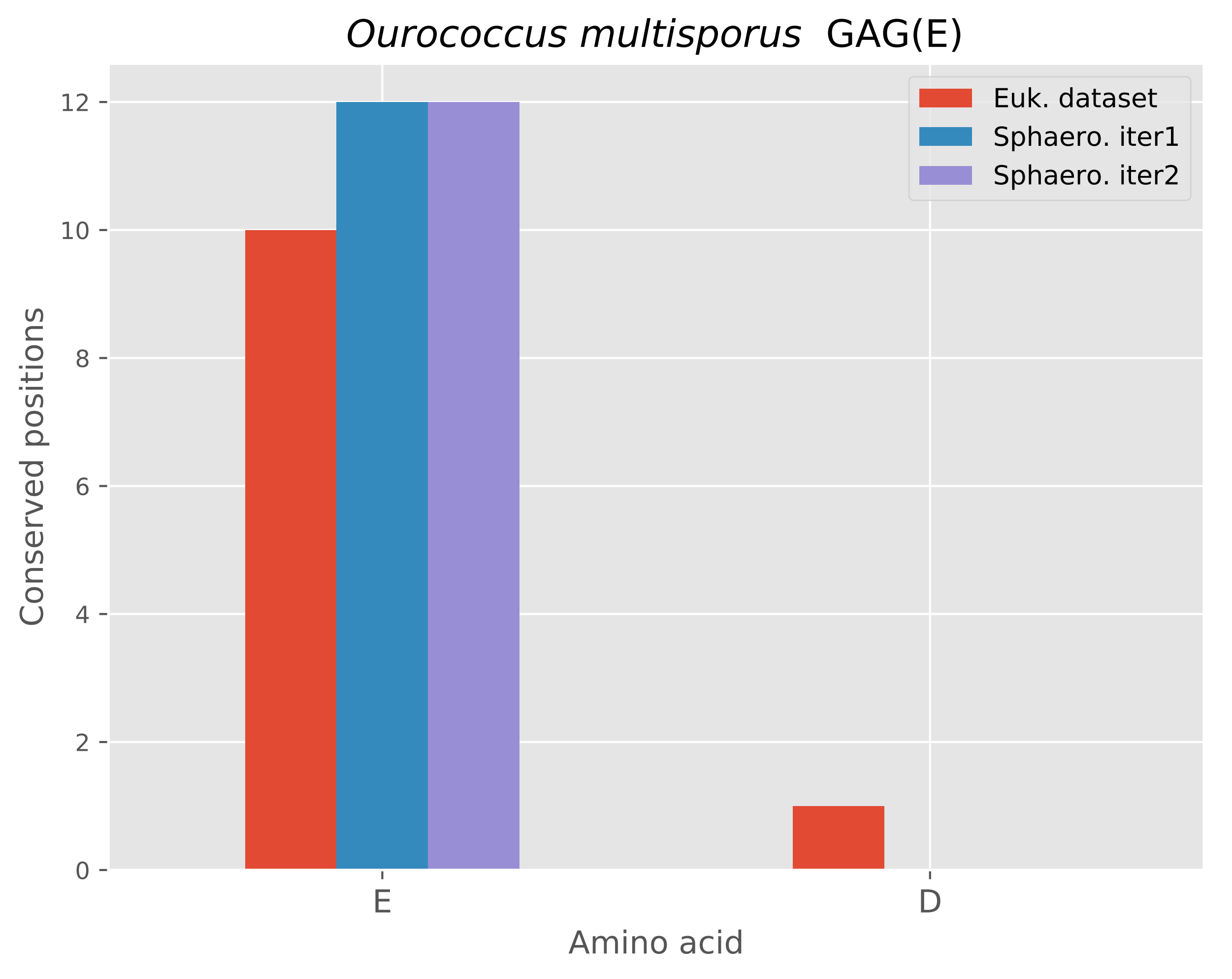

Ourococcus multisporus GAG(E)
Euk. dataset
Sphaero. iter1
Sphaero. iter2
Conserved positions
0
2
4
6
8
10
12
E
D
Amino acid

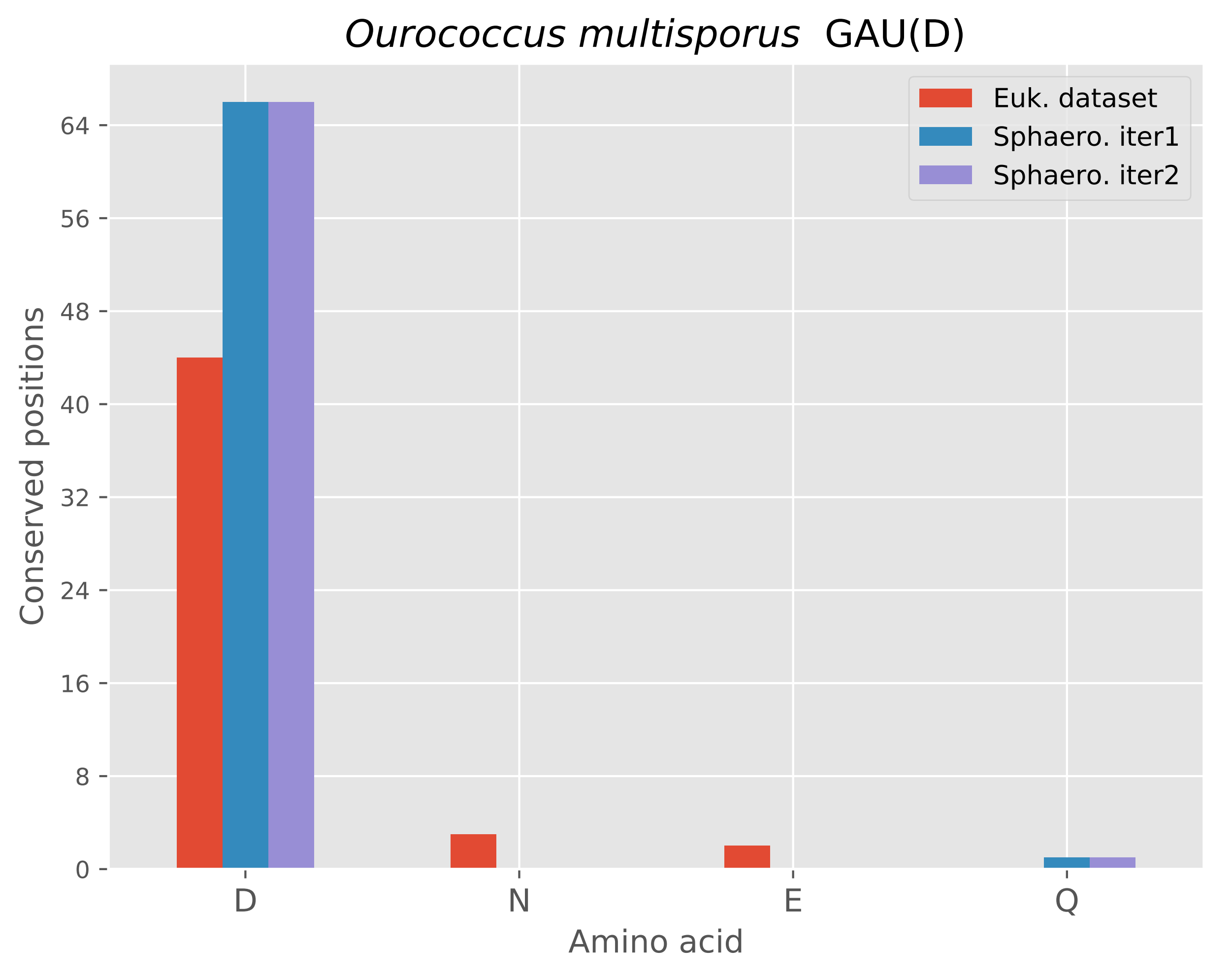

Ourococcus multisporus GAU(D)
Euk. dataset
Sphaero. iter1
Sphaero. iter2
Conserved positions
0
8
16
24
32
40
48
56
64
D
N
E
Q
Amino acid

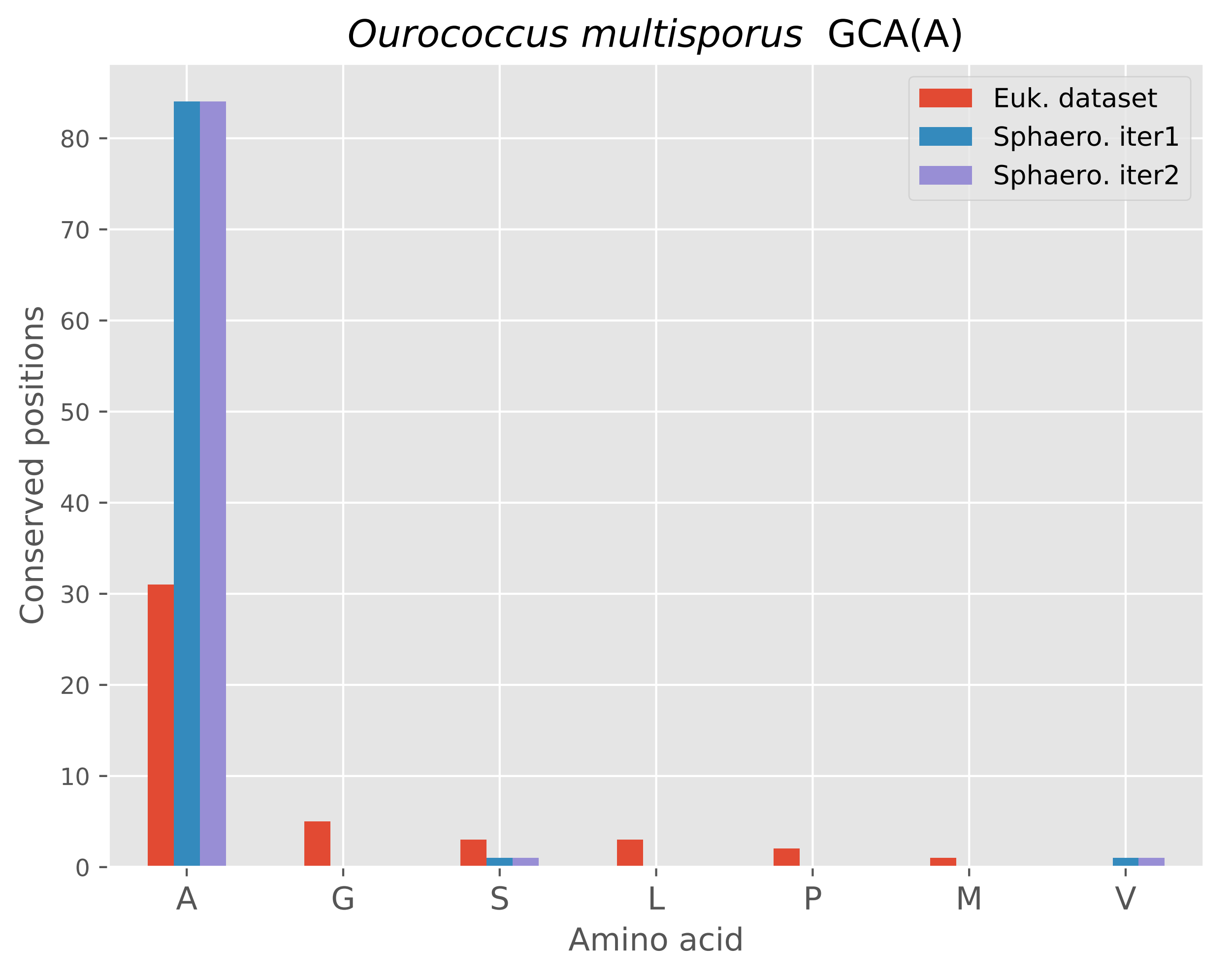
Ourococcus multisporus GCA(A)
Euk. dataset
Sphaero. iter1
Sphaero. iter2
Conserved positions
0
10
20
30
40
50
60
70
80
A
G
S
L
P
M
V
Amino acid

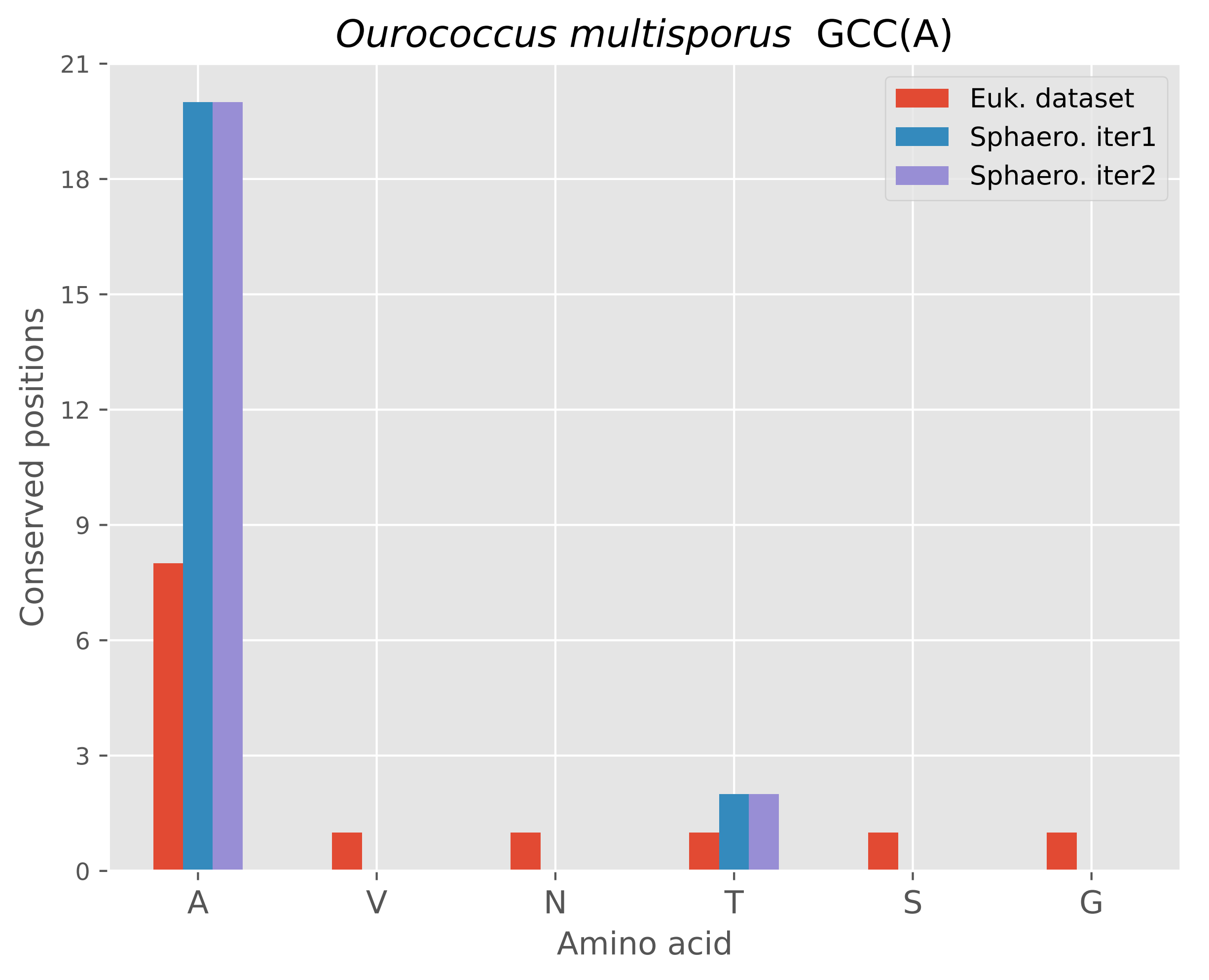
Ourococcus multisporus GCC(A)
Euk. dataset
Sphaero. iter1
Sphaero. iter2
Conserved positions
0
3
6
9
12
15
18
21
A
V
N
T
S
G
Amino acid

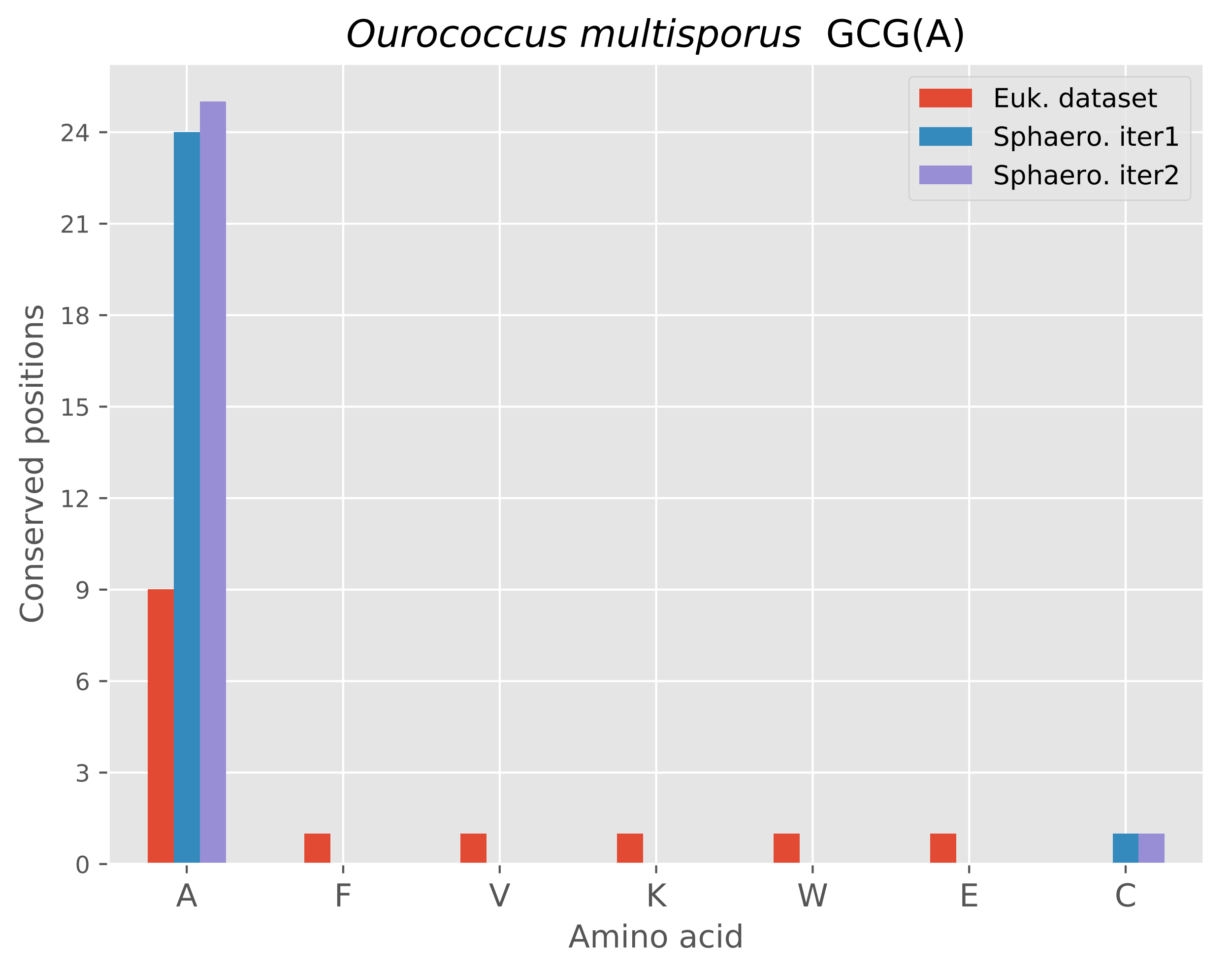

Ourococcus multisporus GCG(A)
Euk. dataset
Sphaero. iter1
Sphaero. iter2
Conserved positions
0
3
6
9
12
15
18
21
24
A
F
V
K
W
E
C
Amino acid

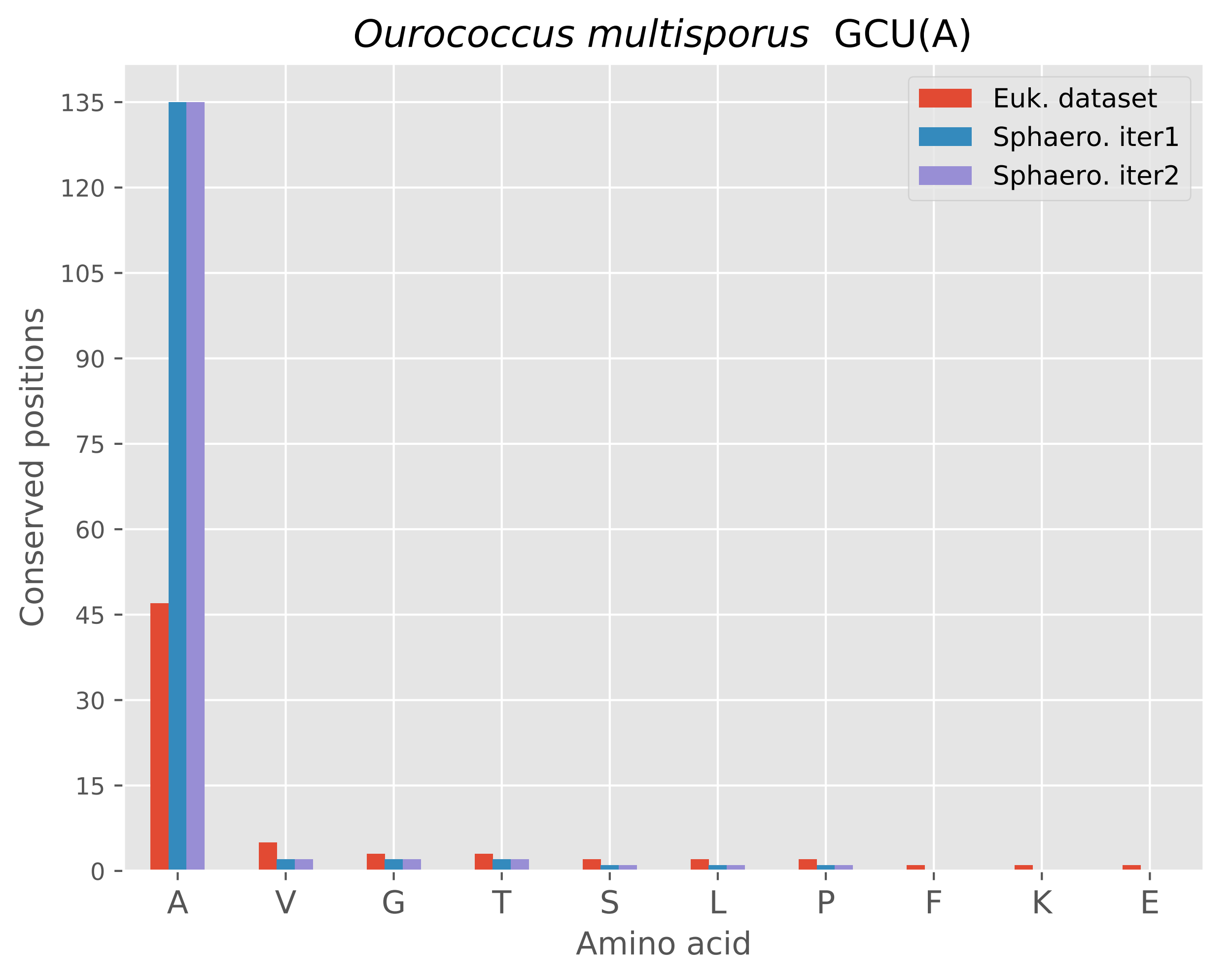

Ourococcus multisporus GCU(A)
Euk. dataset
Sphaero. iter1
Sphaero. iter2
Conserved positions
0
15
30
45
60
75
90
105
120
135
A
V
G
T
S
L
P
F
K
E
Amino acid

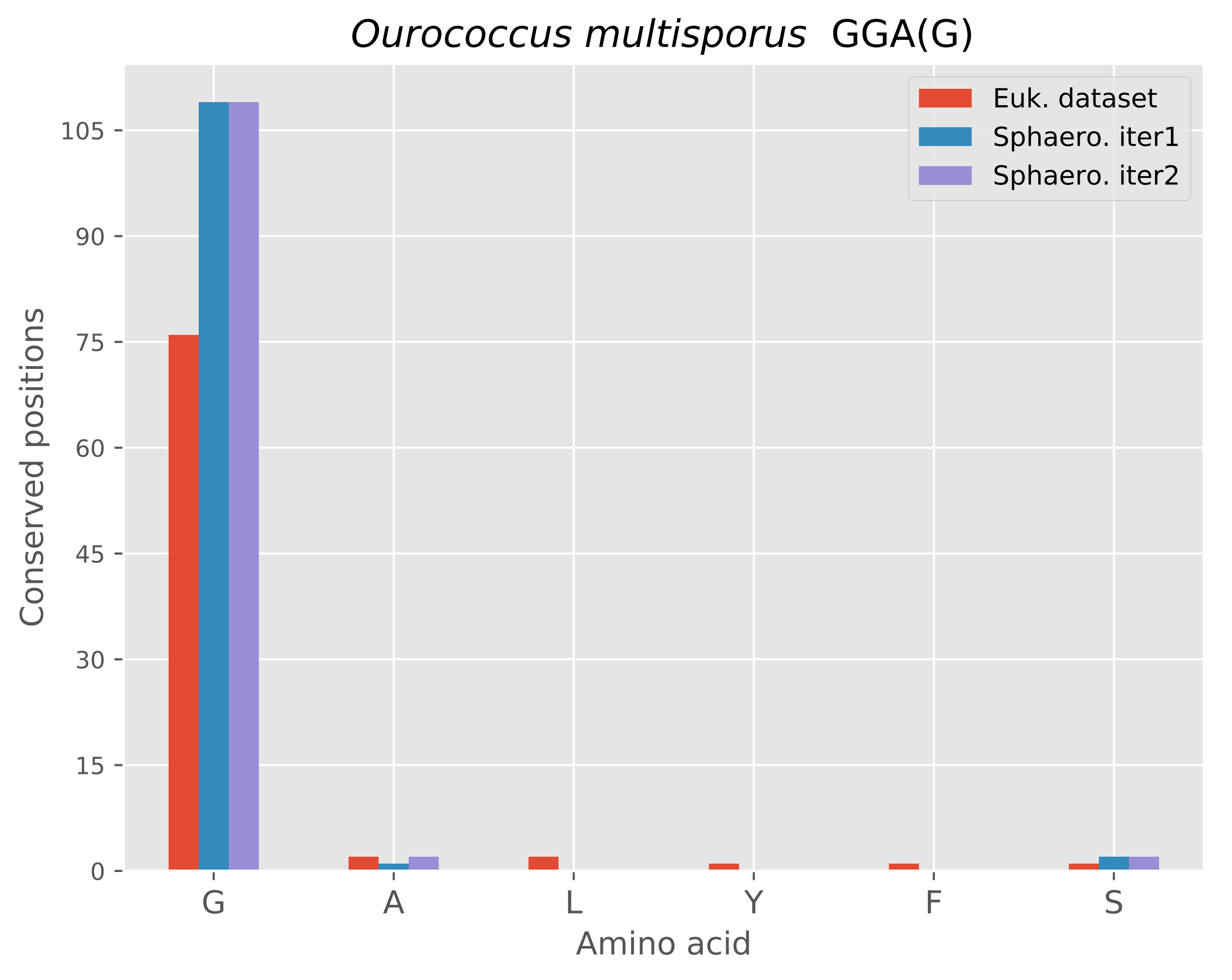

Ourococcus multisporus GGA(G)
Euk. dataset
Sphaero. iter1
Sphaero. iter2
Conserved positions
0
15
30
45
60
75
90
105
G
A
L
Y
F
S
Amino acid

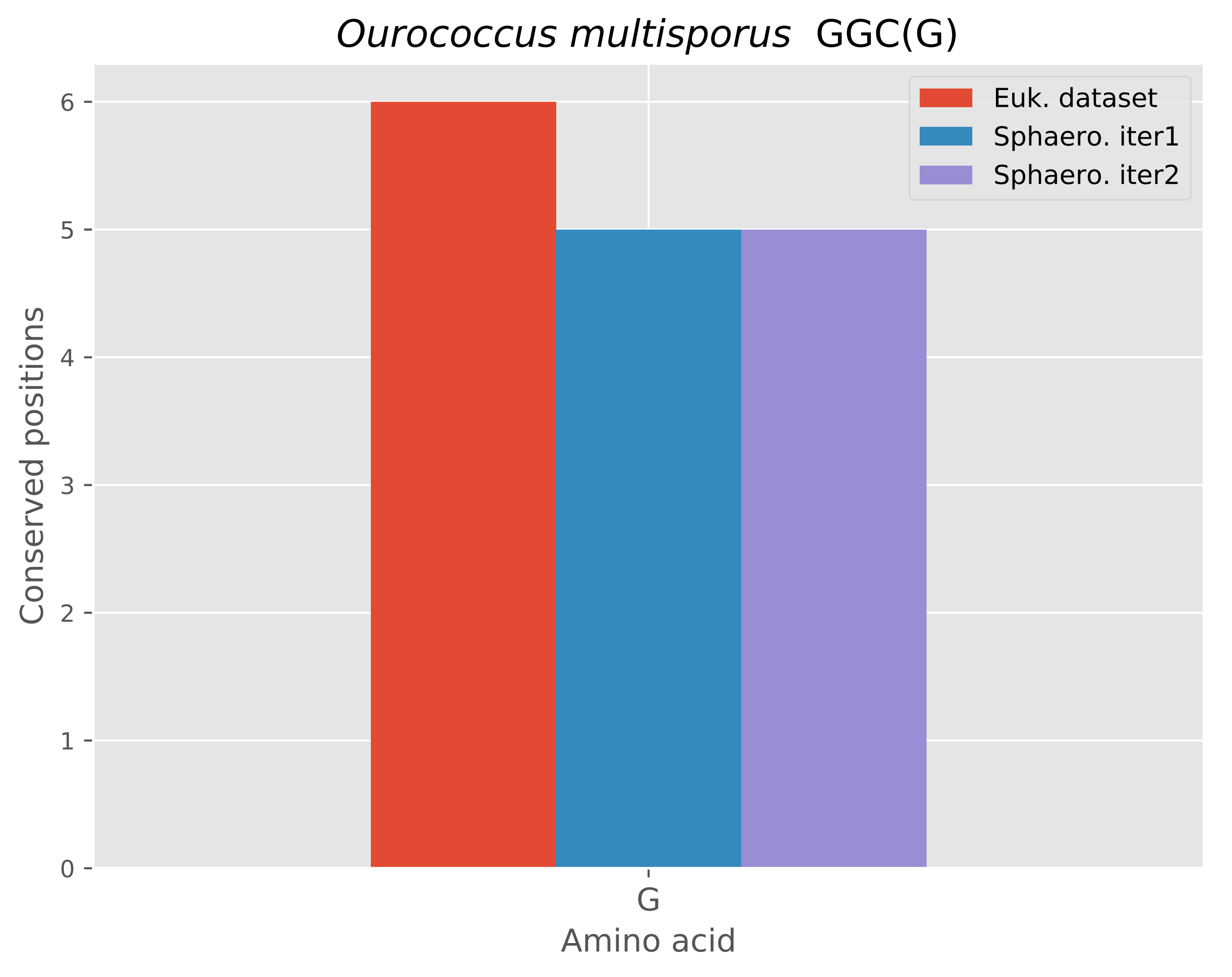

Ourococcus multisporus GGC(G)
Euk. dataset
Sphaero. iter1
Sphaero. iter2
Conserved positions
0
1
2
3
4
5
6
G
Amino acid

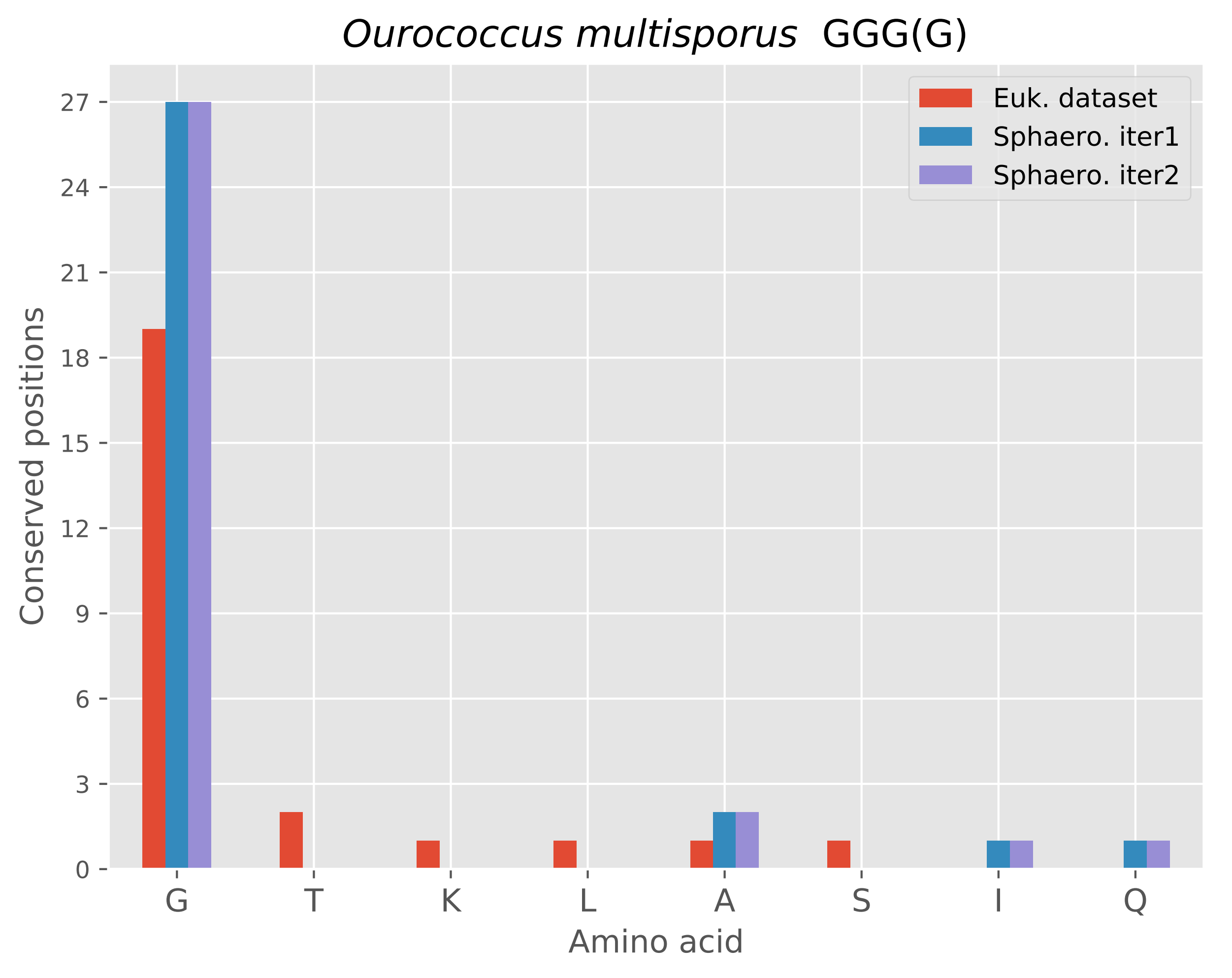

Ourococcus multisporus GGG(G)
Euk. dataset
Sphaero. iter1
Sphaero. iter2
Conserved positions
0
3
6
9
12
15
18
21
24
27
G
T
K
L
A
S
I
Q
Amino acid

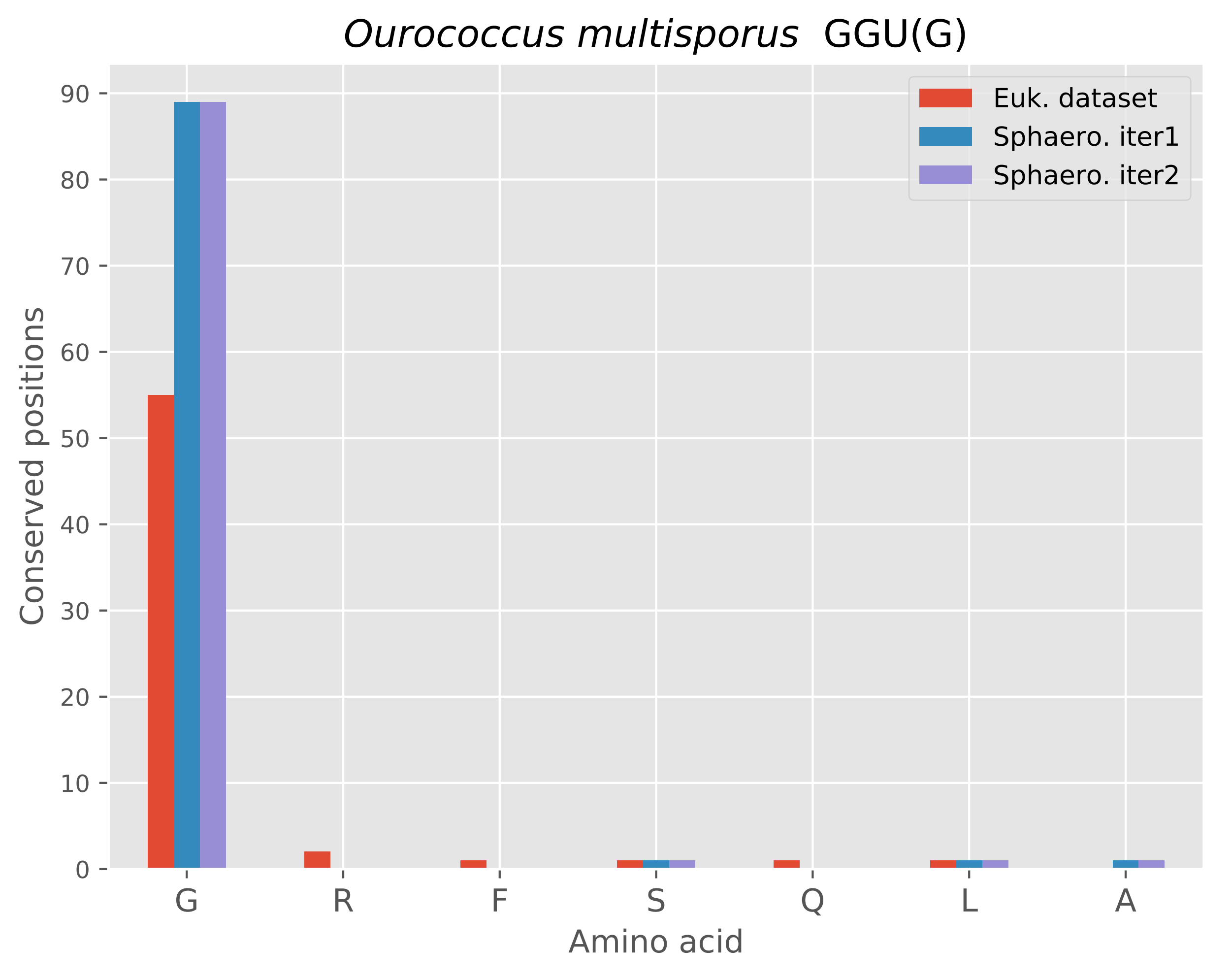
Ourococcus multisporus GGU(G)
Euk. dataset
Sphaero. iter1
Sphaero. iter2
Conserved positions
0
10
20
30
40
50
60
70
80
90
G
R
F
S
Q
L
A
Amino acid

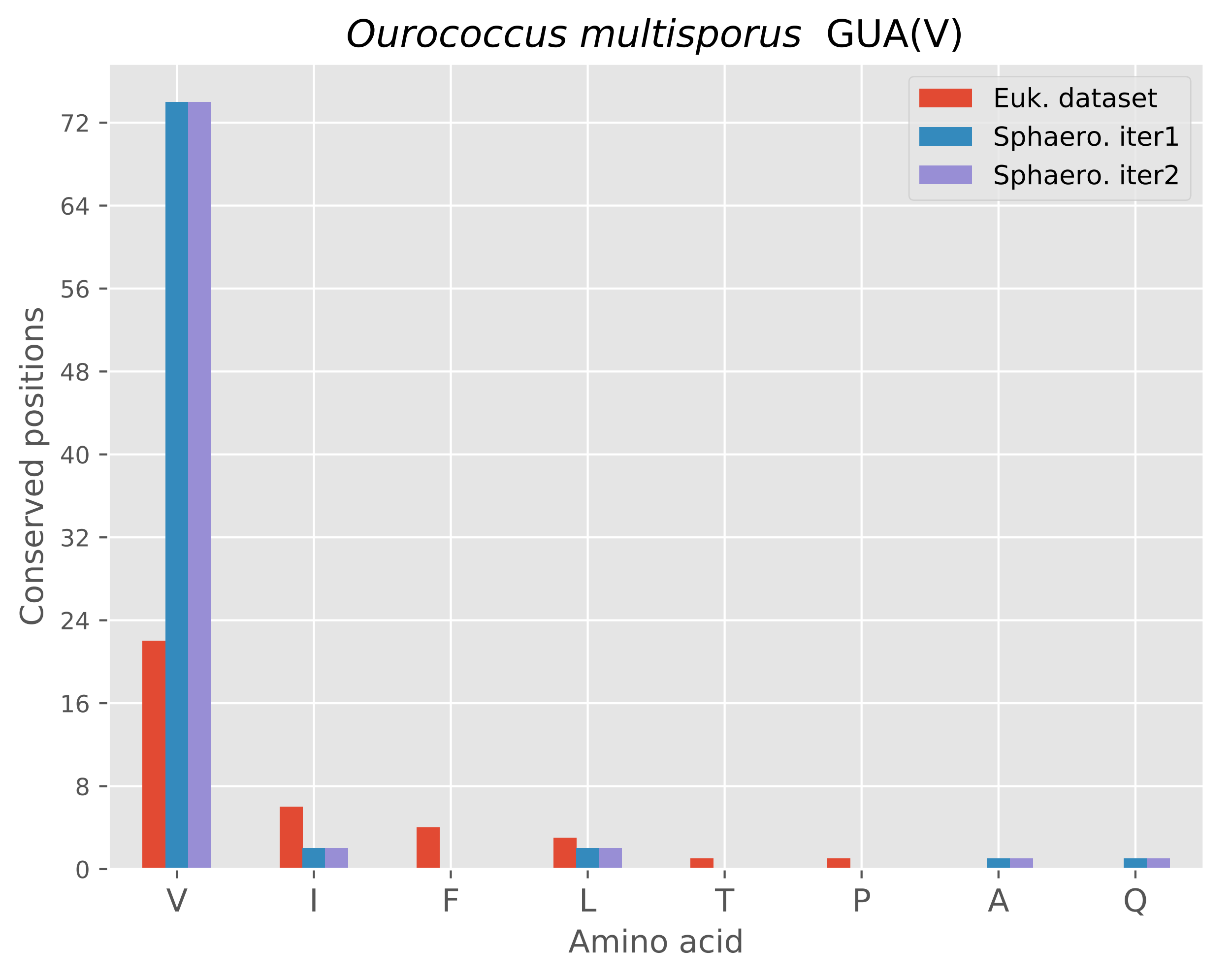

Ourococcus multisporus GUA(V)
Euk. dataset
Sphaero. iter1
Sphaero. iter2
Conserved positions
0
8
16
24
32
40
48
56
64
72
V
I
F
L
T
P
A
Q
Amino acid

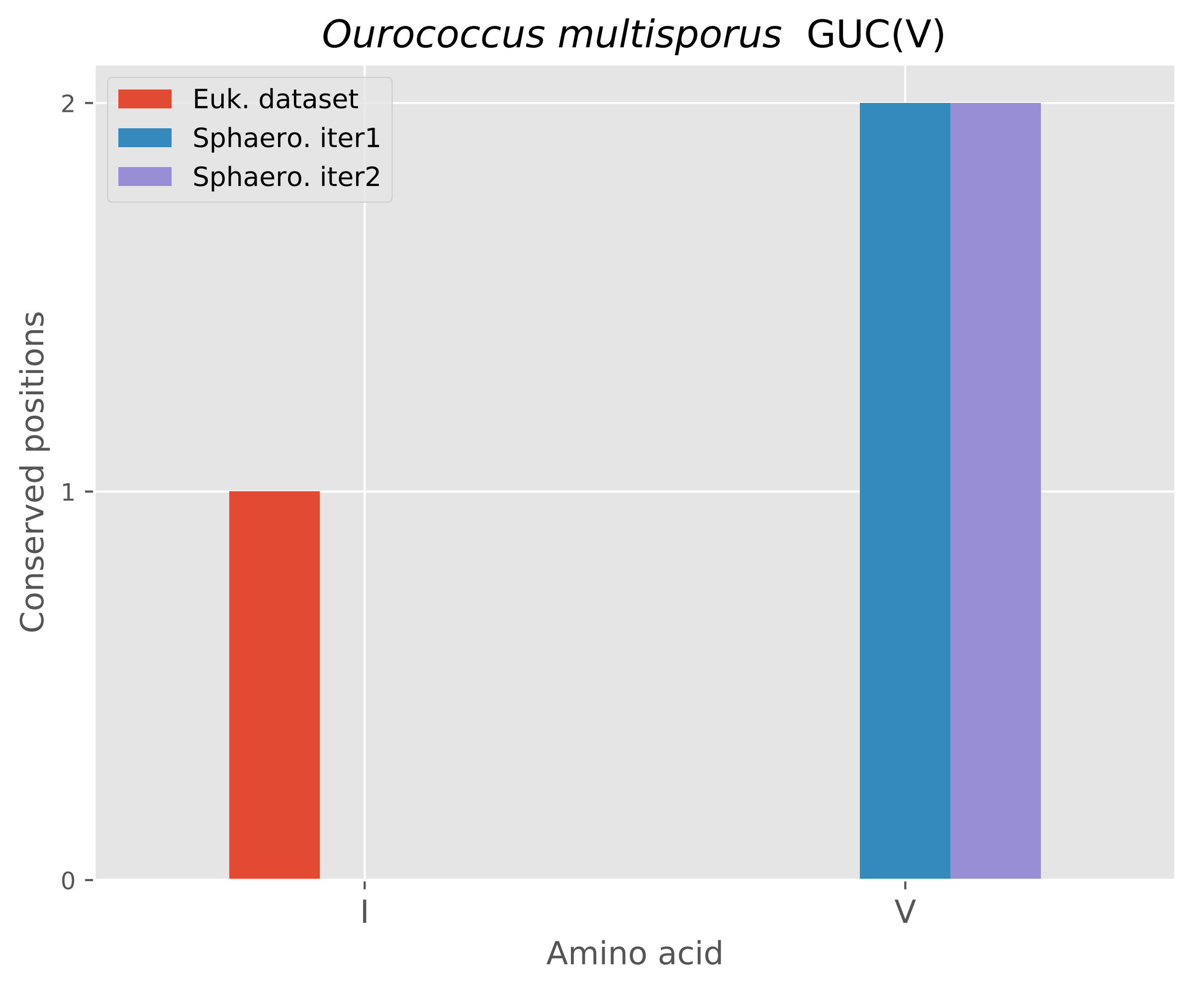
Ourococcus multisporus GUC(V)
Euk. dataset
Sphaero. iter1
Sphaero. iter2
Conserved positions
2
1
0
I
V
Amino acid

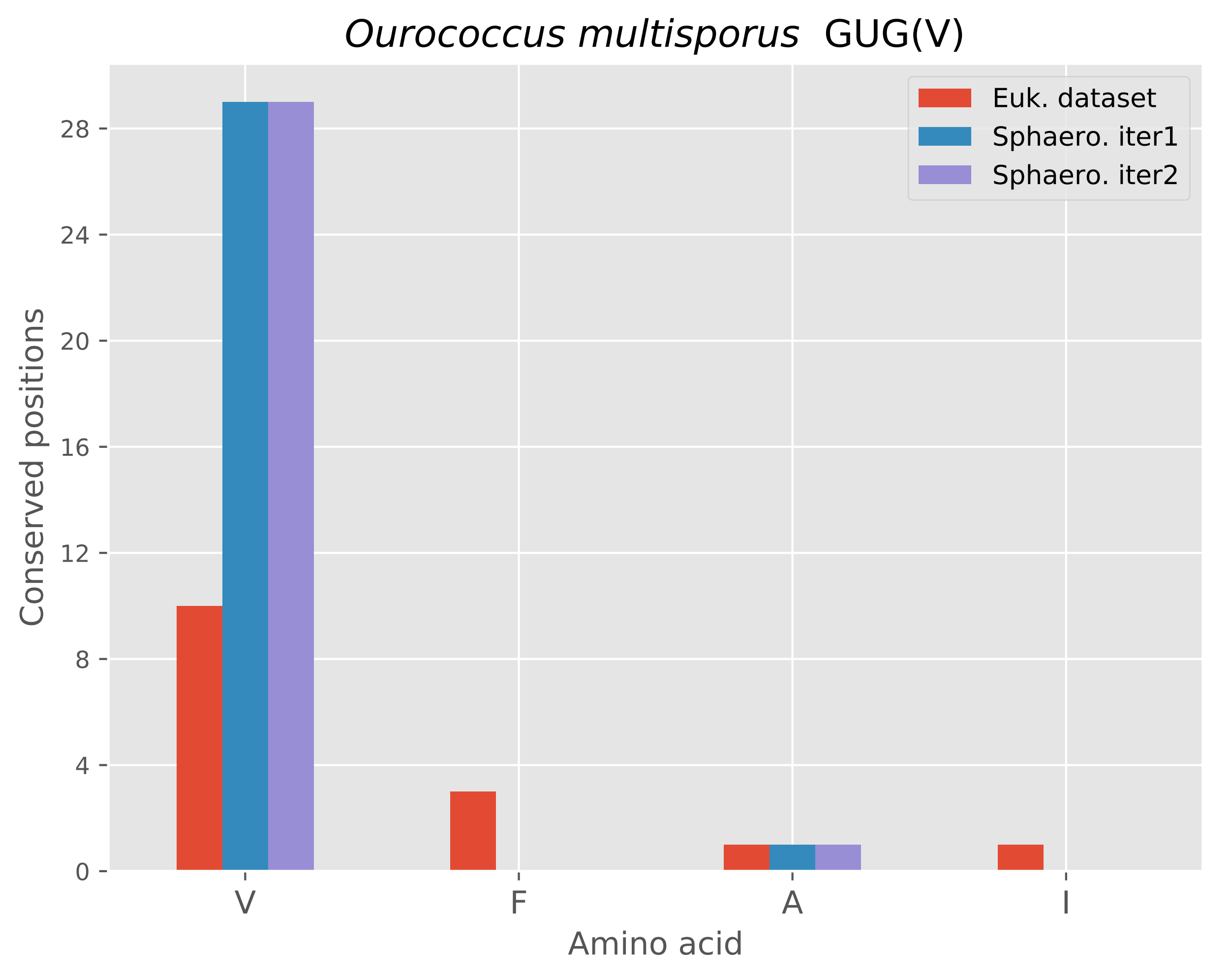

Ourococcus multisporus GUG(V)
Euk. dataset
Sphaero. iter1
Sphaero. iter2
Conserved positions
0
4
8
12
16
20
24
28
V
F
A
I
Amino acid

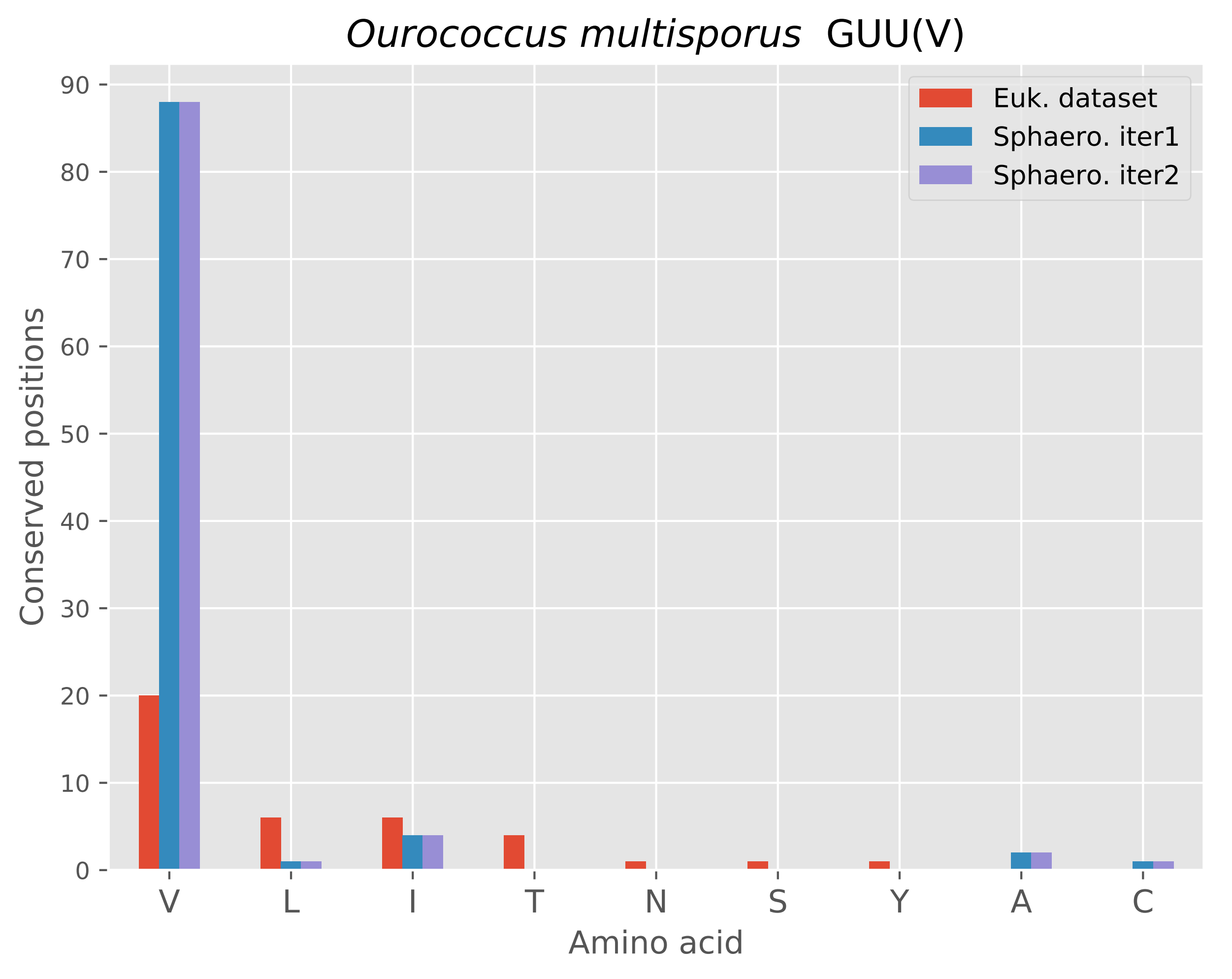

Ourococcus multisporus GUU(V)
Euk. dataset
Sphaero. iter1
Sphaero. iter2
Conserved positions
0
10
20
30
40
50
60
70
80
90
V
L
I
T
N
S
Y
A
C
Amino acid

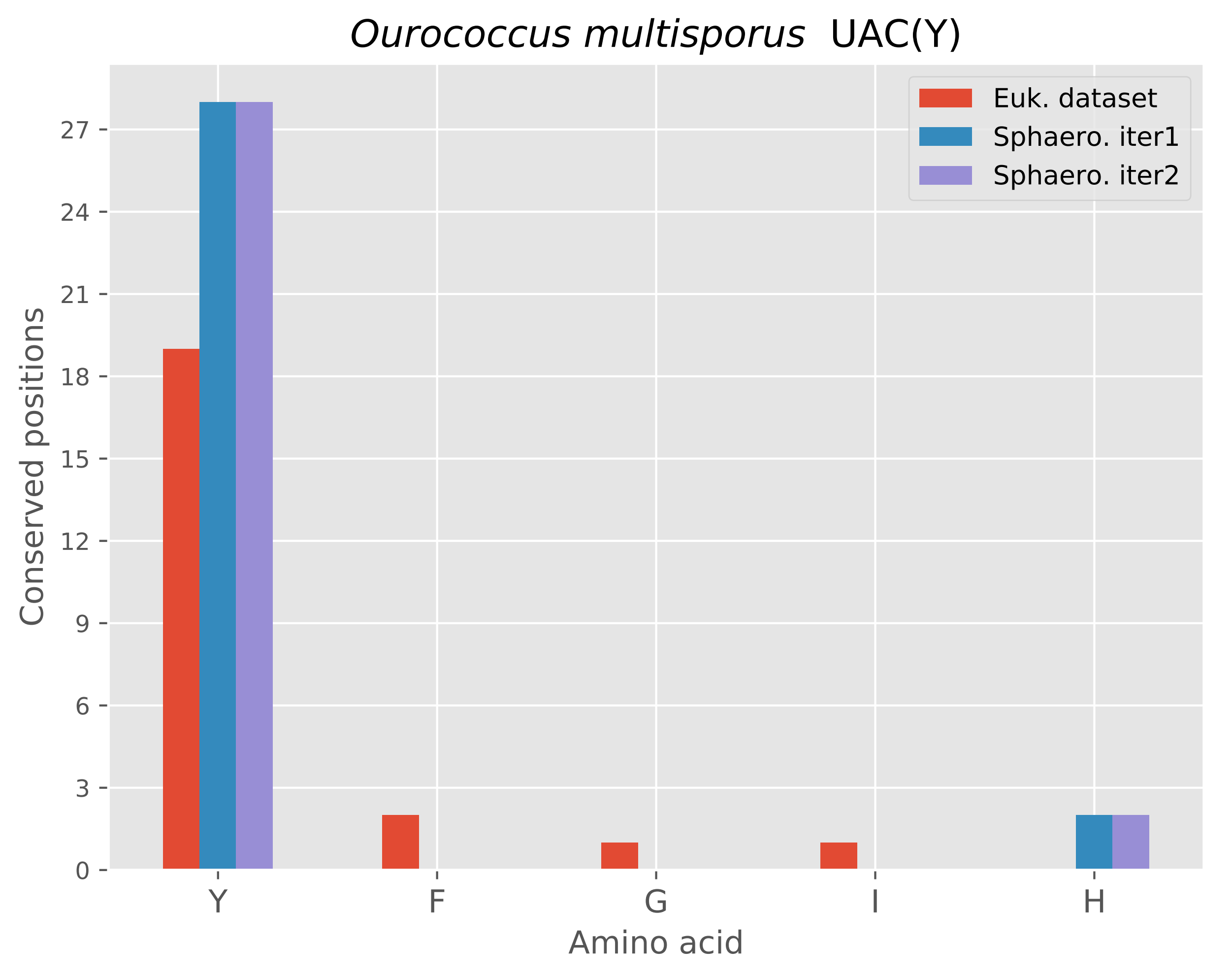
Ourococcus multisporus UAC(Y)
Euk. dataset
Sphaero. iter1
Sphaero. iter2
Conserved positions
0
3
6
9
12
15
18
21
24
27
Y
F
G
I
H
Amino acid

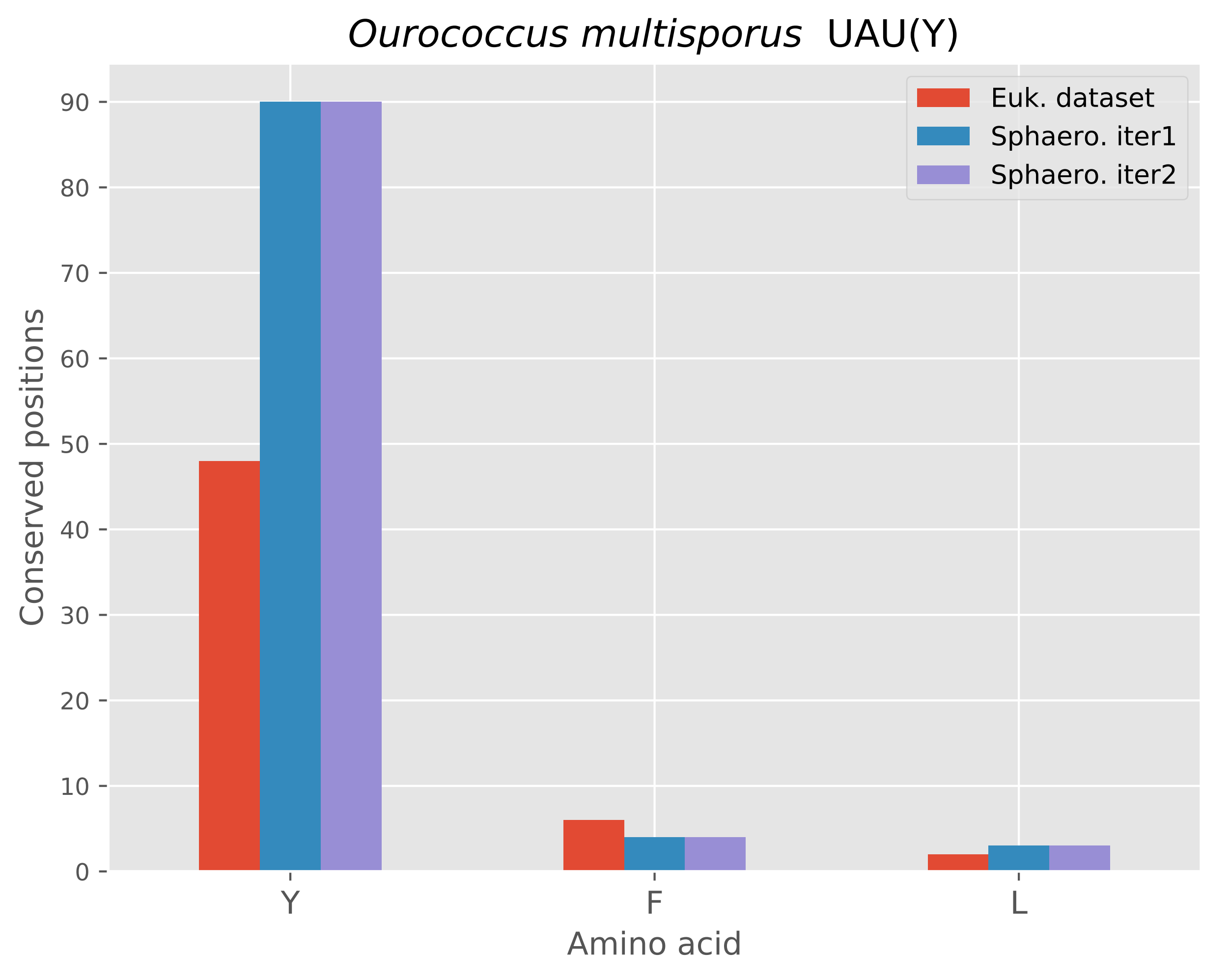

Ourococcus multisporus UAU(Y)
Euk. dataset
Sphaero. iter1
Sphaero. iter2
Conserved positions
0
10
20
30
40
50
60
70
80
90
Y
F
L
Amino acid

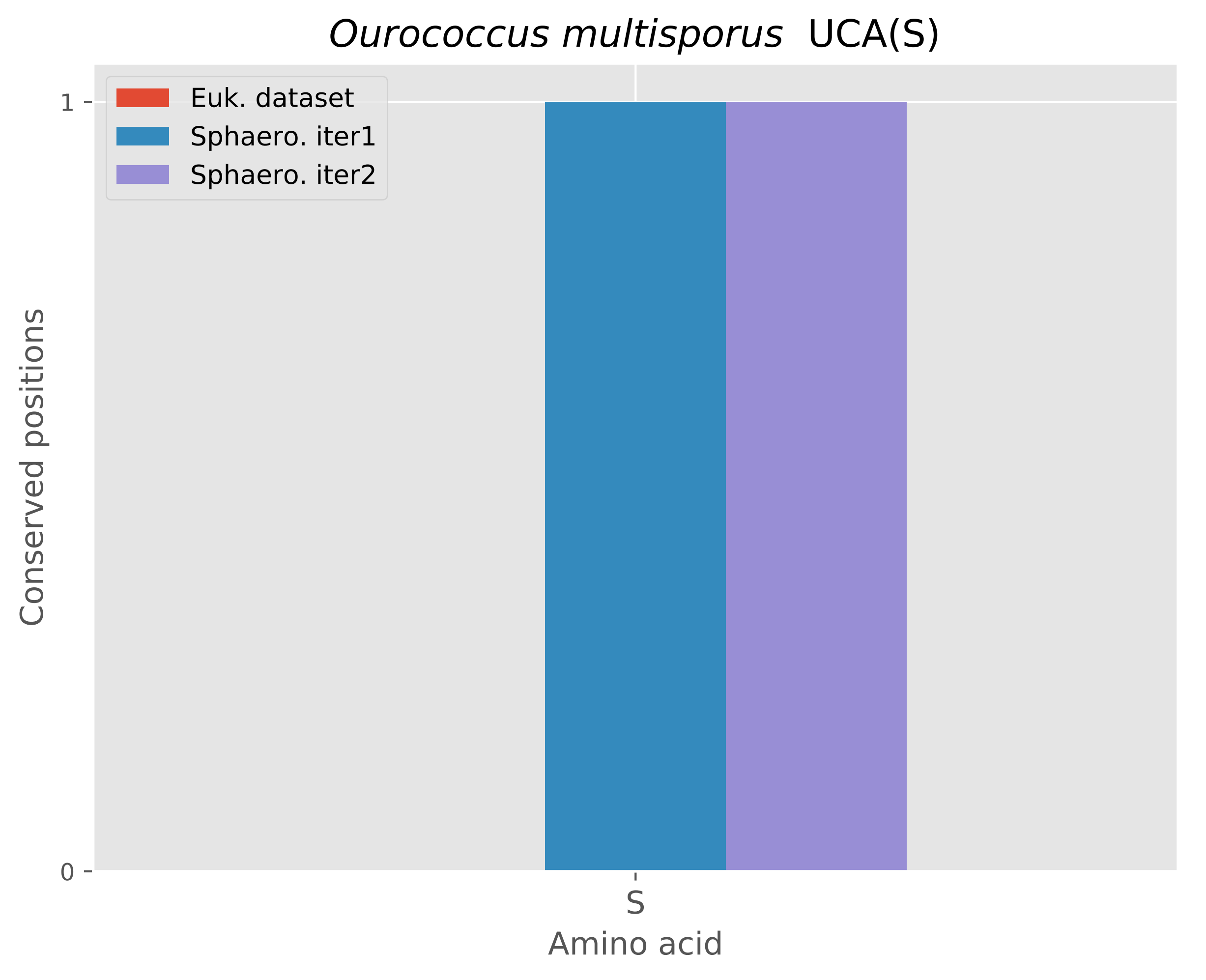

Ourococcus multisporus UCA(S)
Euk. dataset
Sphaero. iter1
Sphaero. iter2
1
0
Conserved positions
S
Amino acid

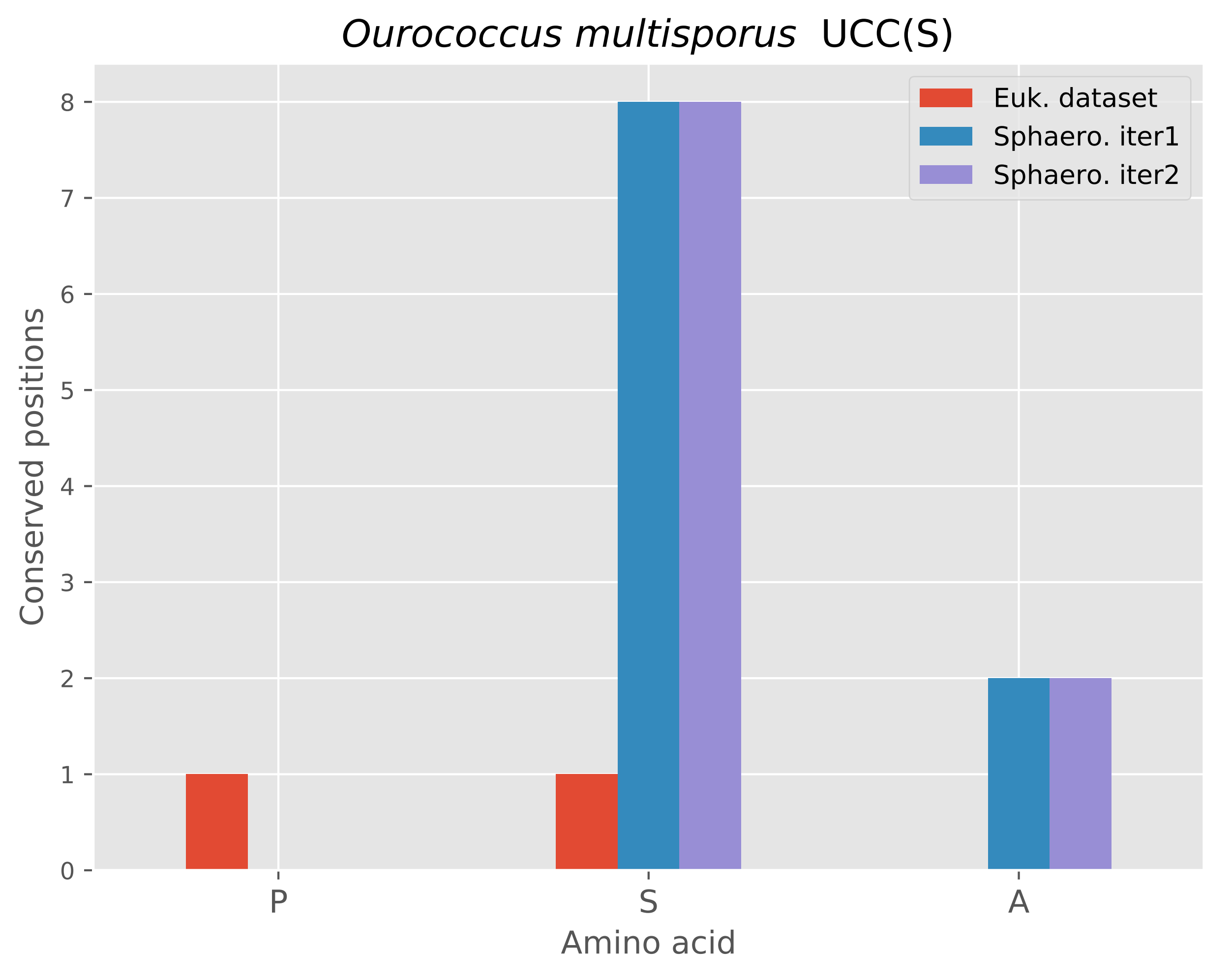
Ourococcus multisporus UCC(S)
Euk. dataset
Sphaero. iter1
Sphaero. iter2
Conserved positions
0
1
2
3
4
5
6
7
8
P
S
A
Amino acid

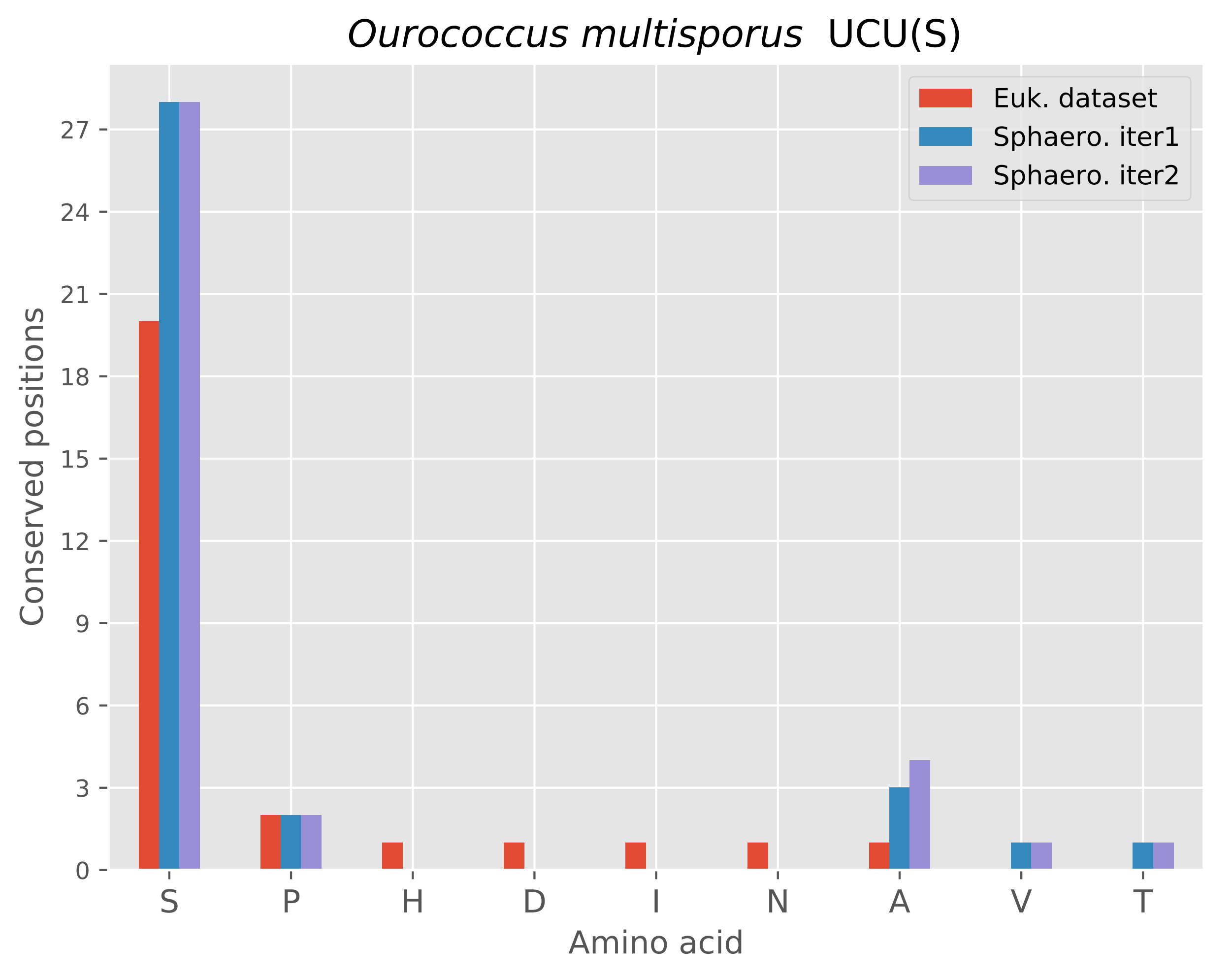

Ourococcus multisporus UCU(S)
Euk. dataset
Sphaero. iter1
Sphaero. iter2
Conserved positions
0
3
6
9
12
15
18
21
24
27
S
P
H
D
I
N
A
V
T
Amino acid

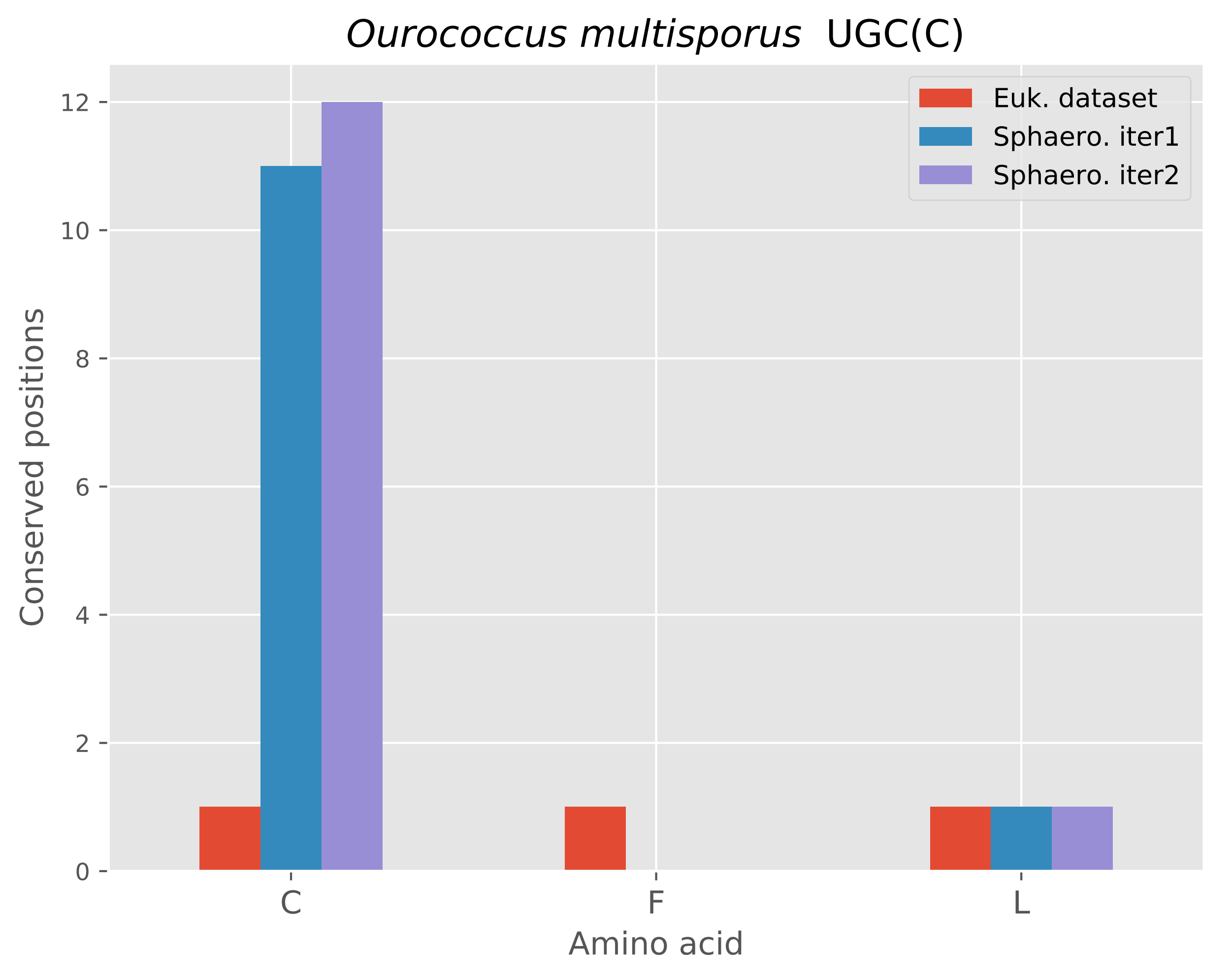

Ourococcus multisporus UGC(C)
Euk. dataset
Sphaero. iter1
Sphaero. iter2
Conserved positions
0
2
4
6
8
10
12
C
F
L
Amino acid

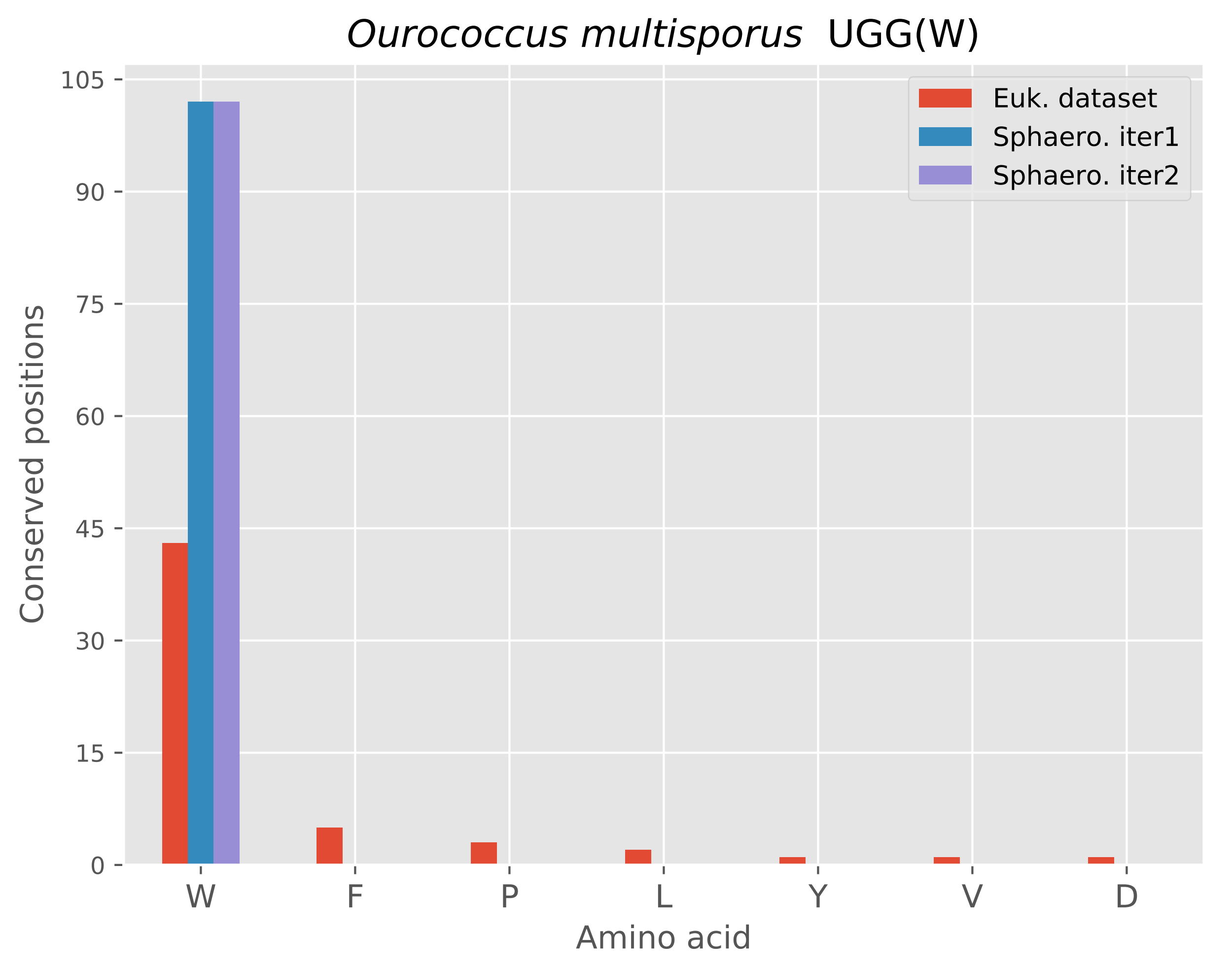
Ourococcus multisporus UGG(W)
Conserved positions
105
90
75
60
45
30
15
0
Euk. dataset
Sphaero. iter1
Sphaero. iter2
W
F
P
L
Y
V
D
Amino acid

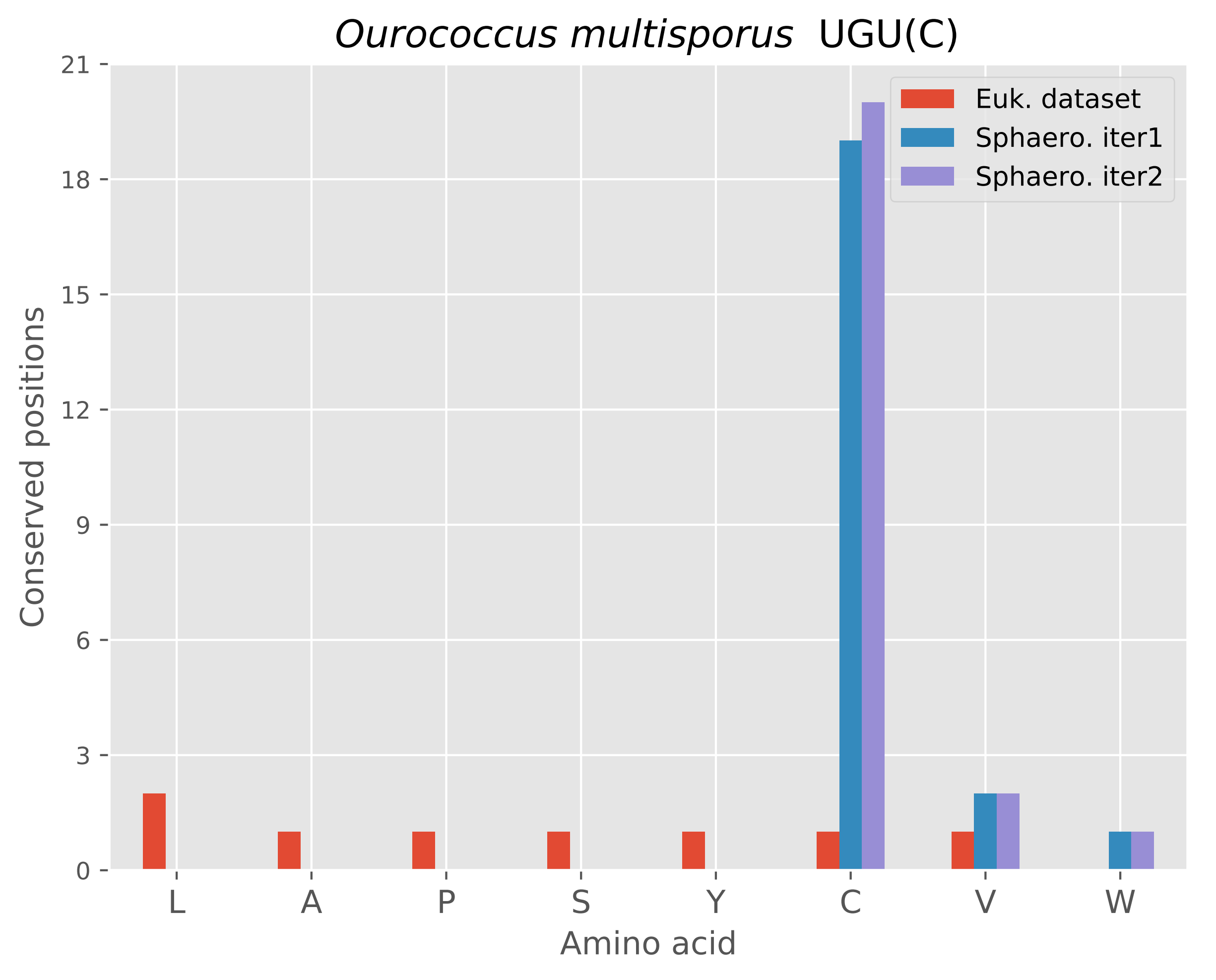
Ourococcus multisporus UGU(C)
Conserved positions
0
3
6
9
12
15
18
21
L
A
P
S
Y
C
V
W
Amino acid
Euk. dataset
Sphaero. iter1
Sphaero. iter2

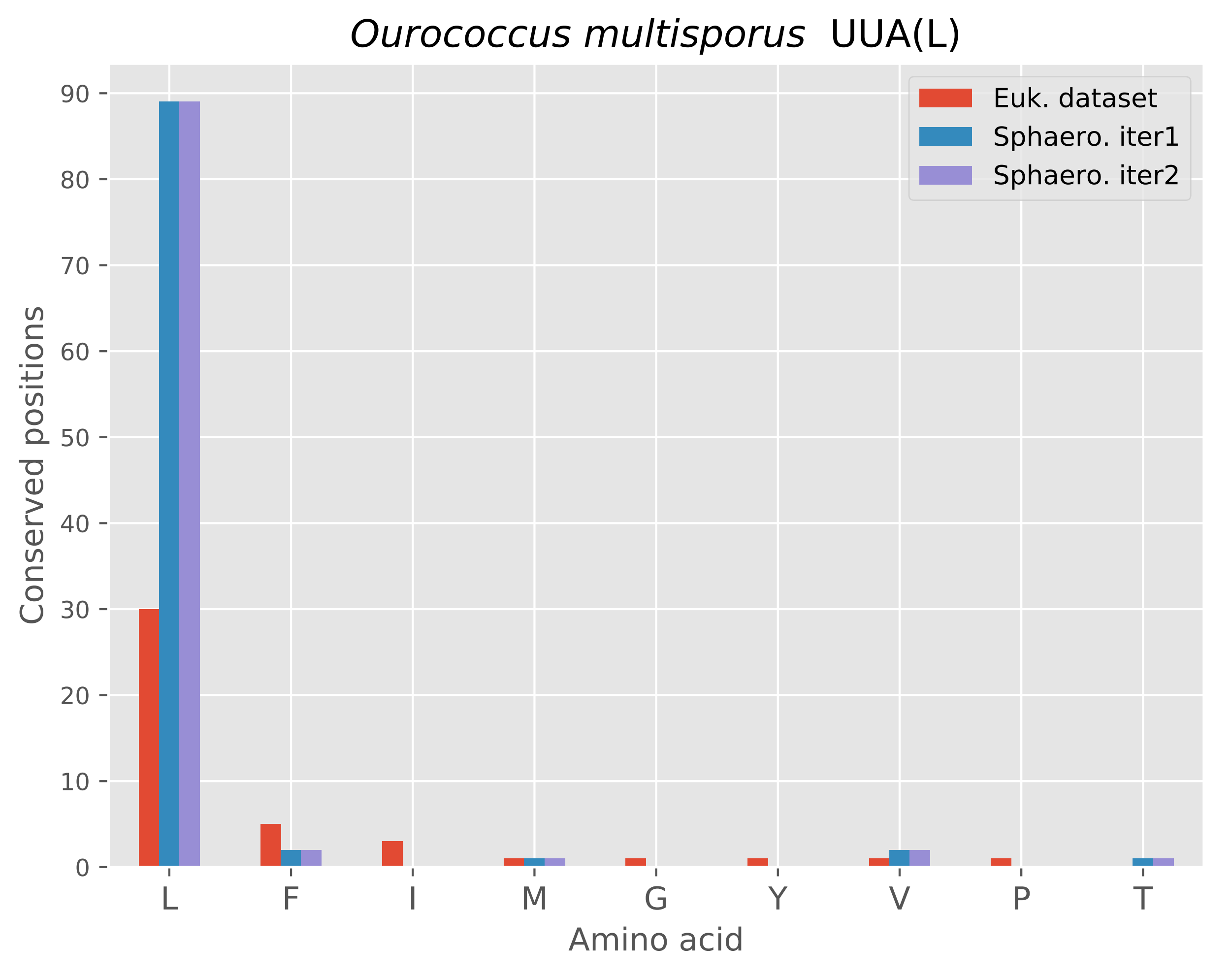

Ourococcus multisporus UUA(L)
Euk. dataset
Sphaero. iter1
Sphaero. iter2
Conserved positions
0
10
20
30
40
50
60
70
80
90
L
F
I
M
G
Y
V
P
T
Amino acid

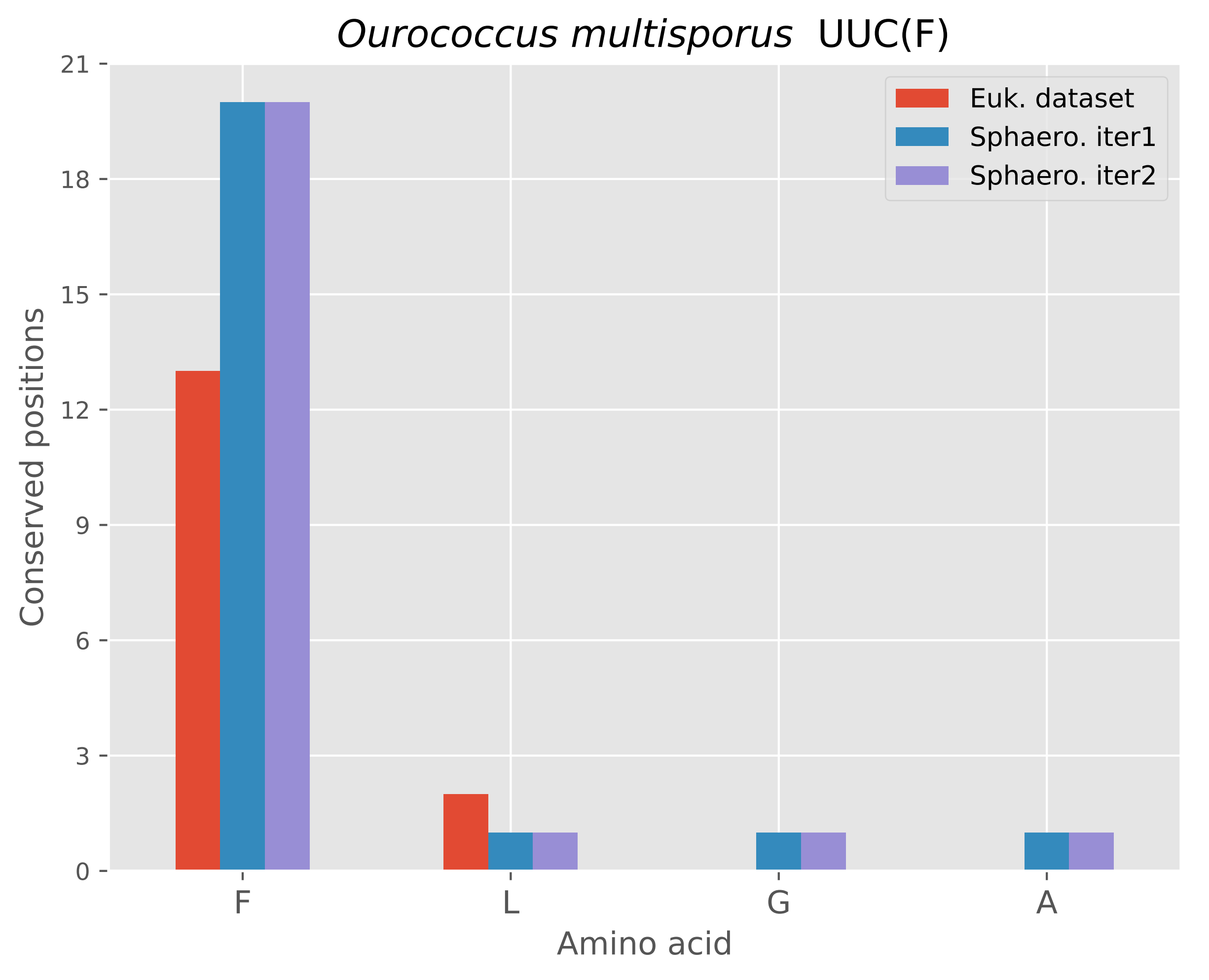

Ourococcus multisporus UUC(F)
Conserved positions
Amino acid
0
3
6
9
12
15
18
21
F
L
G
A
Euk. dataset
Sphaero. iter1
Sphaero. iter2

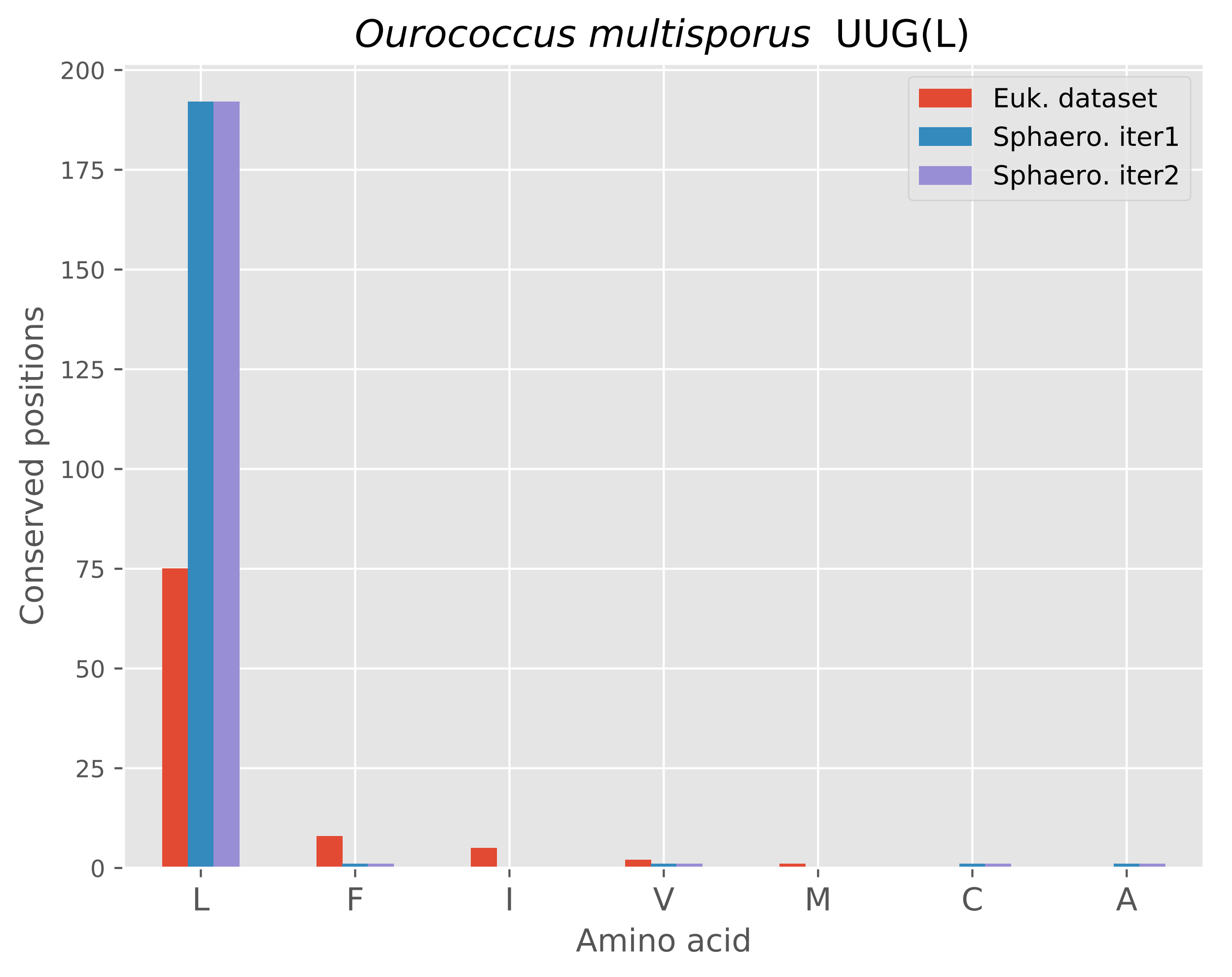

Ourococcus multisporus UUG(L)
Euk. dataset
Sphaero. iter1
Sphaero. iter2
Conserved positions
200
175
150
125
100
75
50
25
0
L
F
I
V
M
C
A
Amino acid

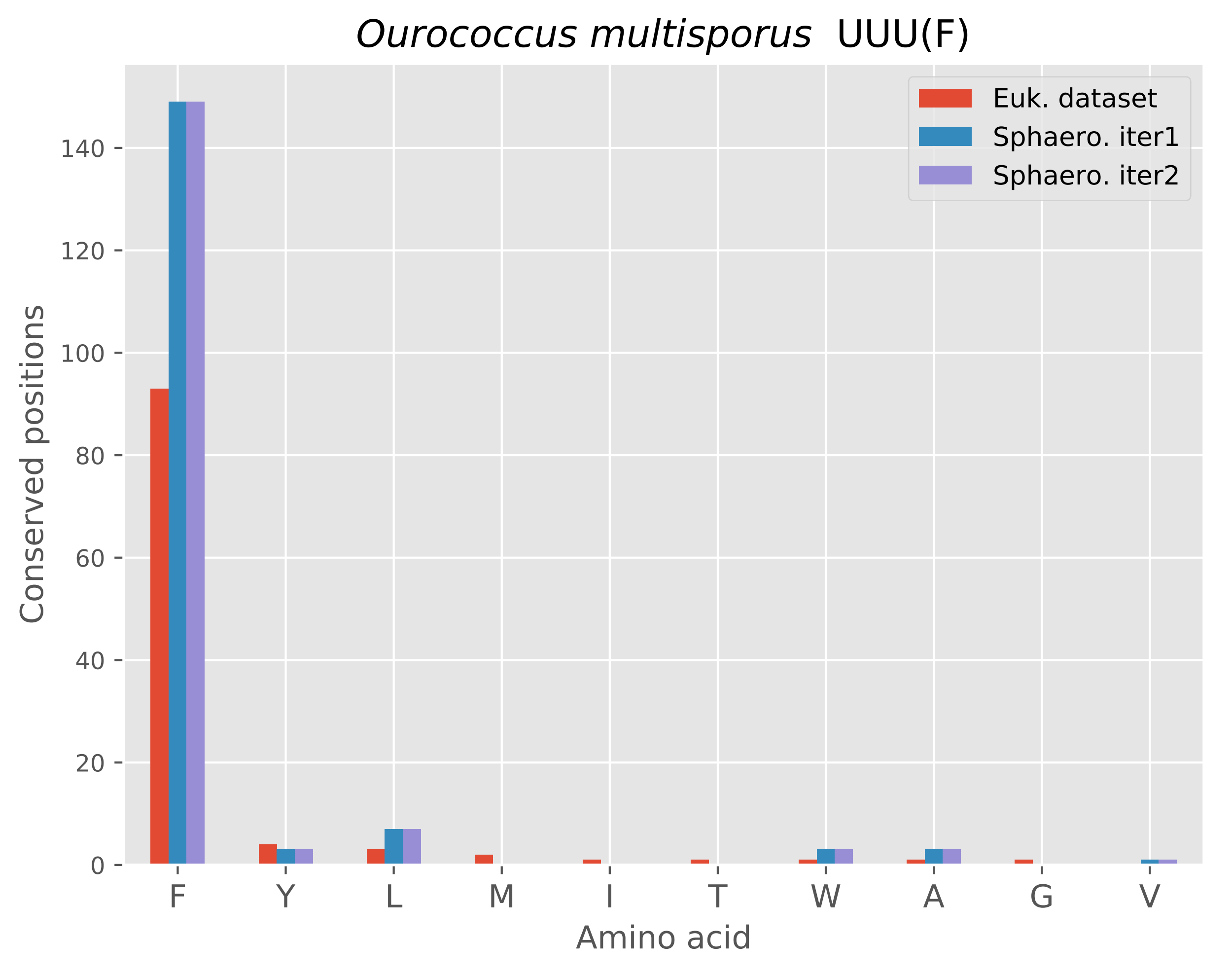

Ourococcus multisporus UUU(F)
Euk. dataset
Sphaero. iter1
Sphaero. iter2
Conserved positions
0
20
40
60
80
100
120
140
F
Y
L
M
I
T
W
A
G
V
Amino acid

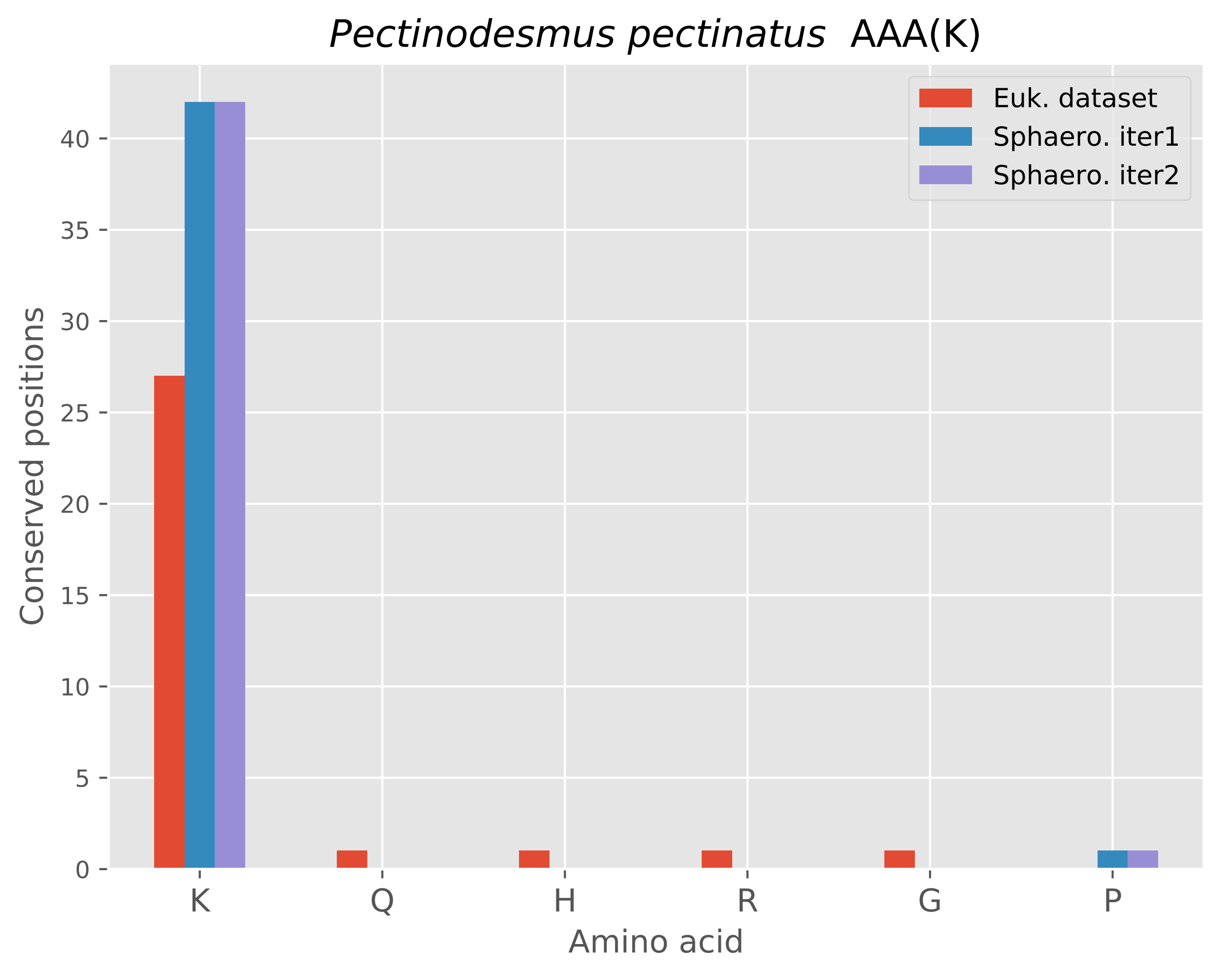

Pectinodesmus pectinatus AAA(K)
Euk. dataset
Sphaero. iter1
Sphaero. iter2
Conserved positions
0
5
10
15
20
25
30
35
40
K
Q
H
R
G
P
Amino acid

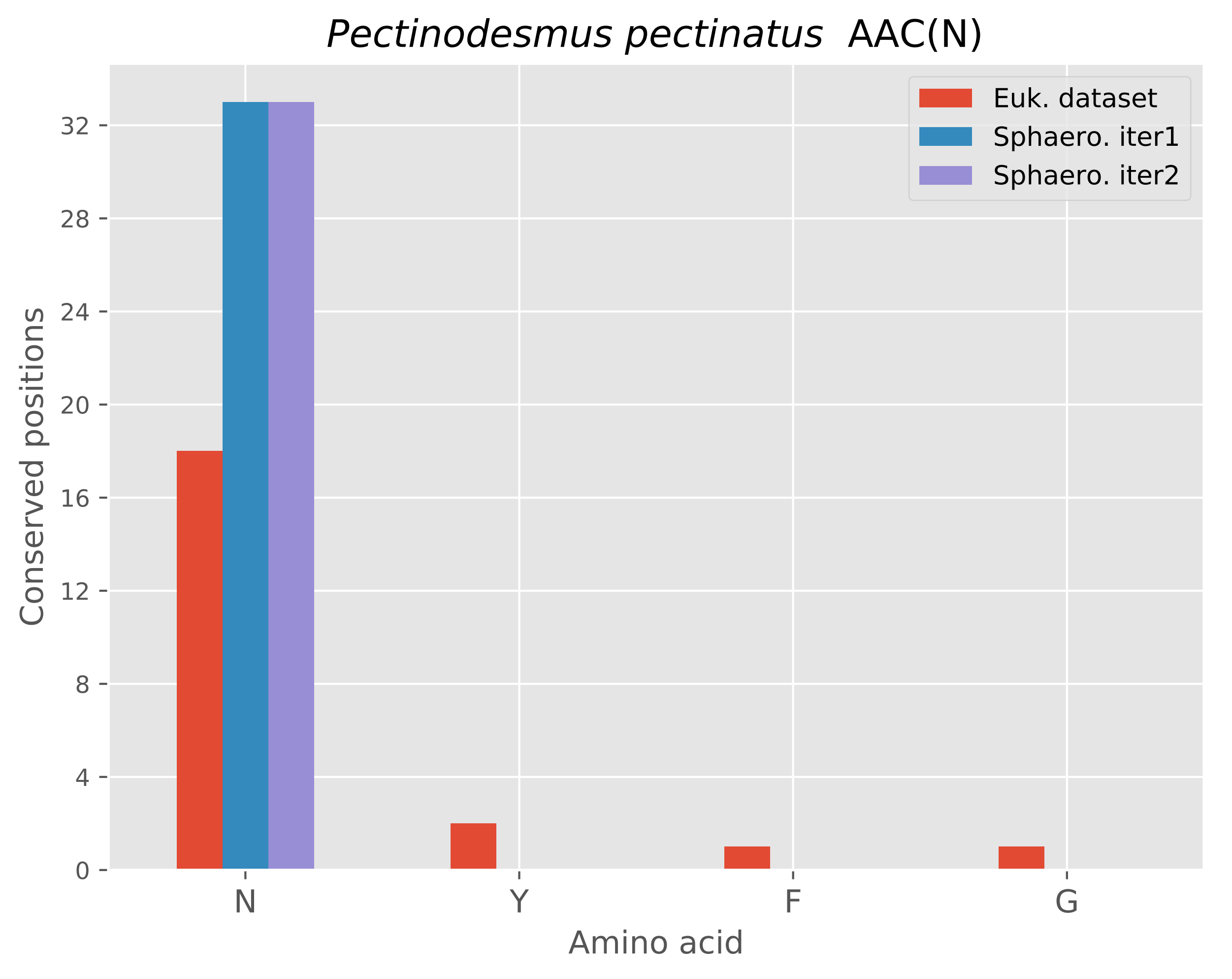

Pectinodesmus pectinatus AAC(N)
Euk. dataset
Sphaero. iter1
Sphaero. iter2
Conserved positions
0
4
8
12
16
20
24
28
32
N
Y
F
G
Amino acid

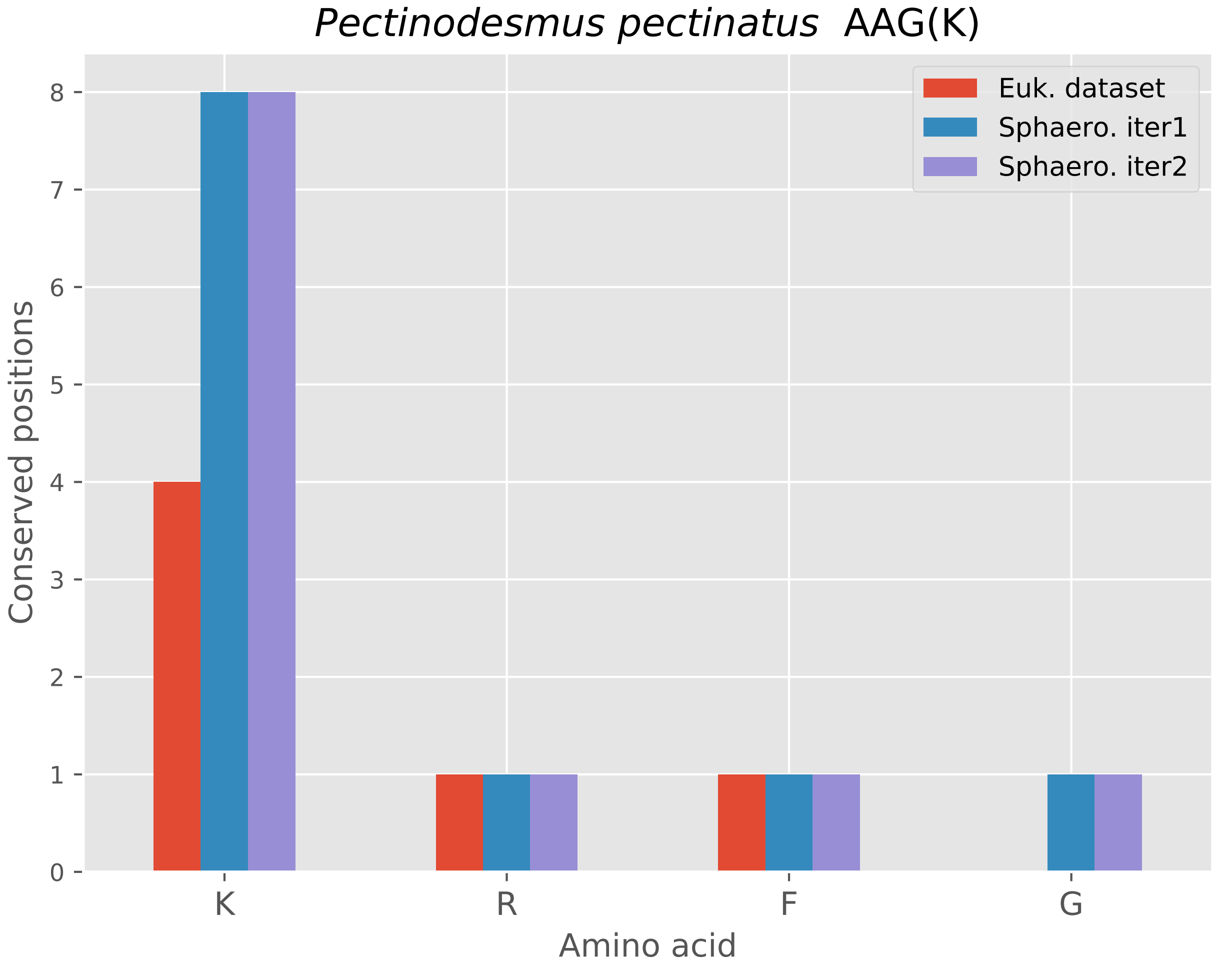

Pectinodesmus pectinatus AAG(K)
Euk. dataset
Sphaero. iter1
Sphaero. iter2
Conserved positions
0
1
2
3
4
5
6
7
8
K
R
F
G
Amino acid

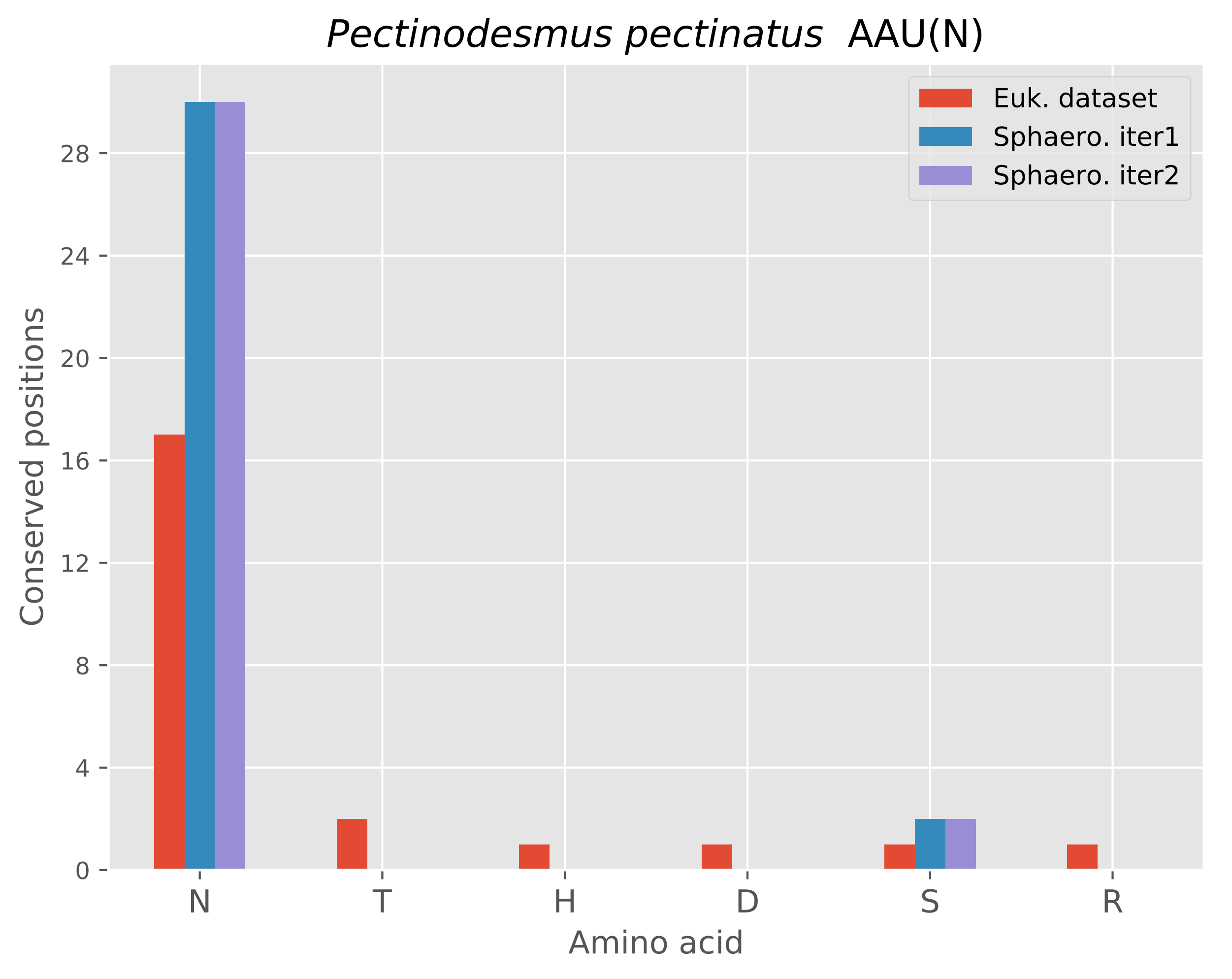

Pectinodesmus pectinatus AAU(N)
Euk. dataset
Sphaero. iter1
Sphaero. iter2
Conserved positions
0
4
8
12
16
20
24
28
N
T
H
D
S
R
Amino acid

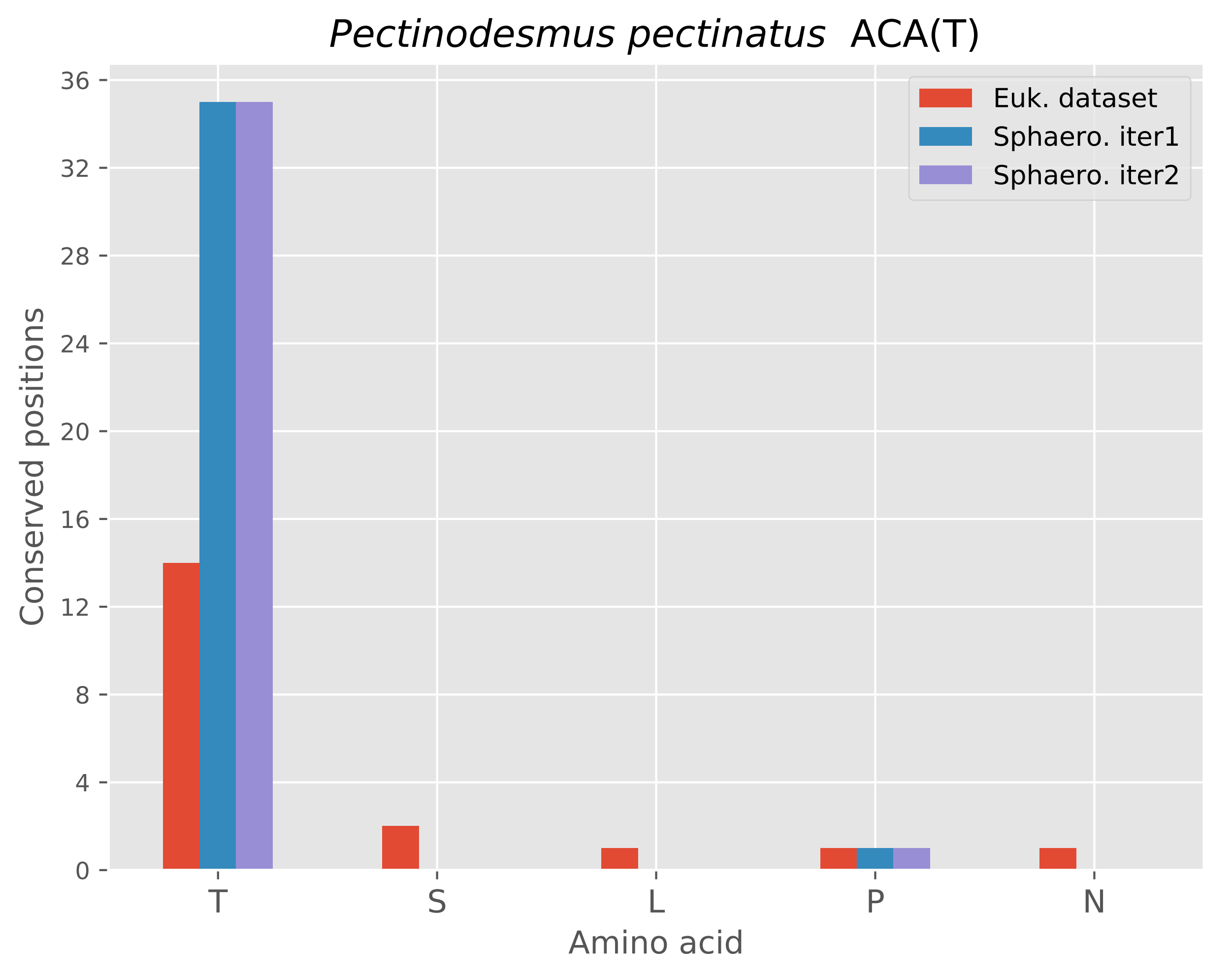

Pectinodesmus pectinatus ACA(T)
Euk. dataset
Sphaero. iter1
Sphaero. iter2
Conserved positions
0
4
8
12
16
20
24
28
32
36
T
S
L
P
N
Amino acid

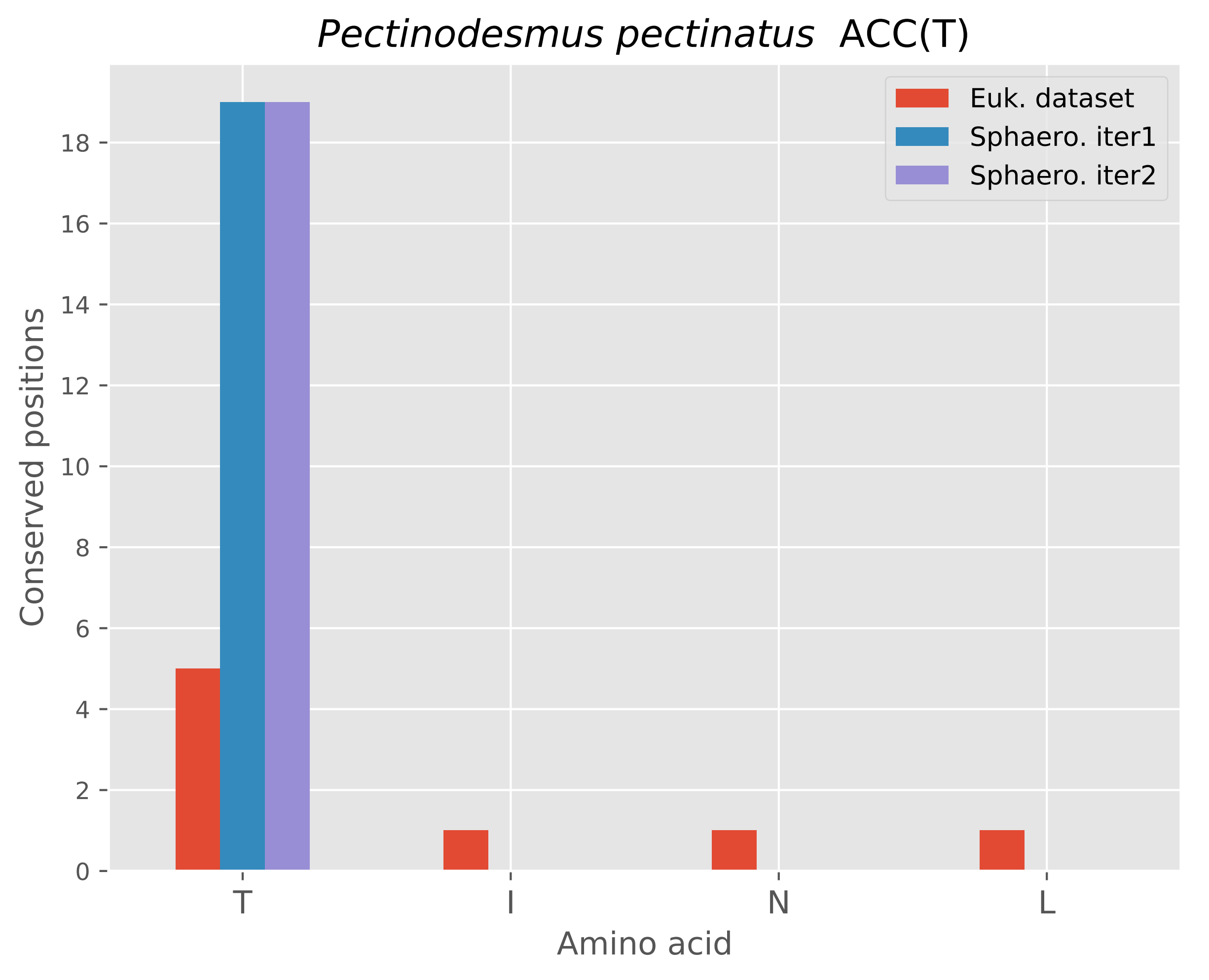

Pectinodesmus pectinatus ACC(T)
Euk. dataset
Sphaero. iter1
Sphaero. iter2
Conserved positions
0
2
4
6
8
10
12
14
16
18
T
I
N
L
Amino acid

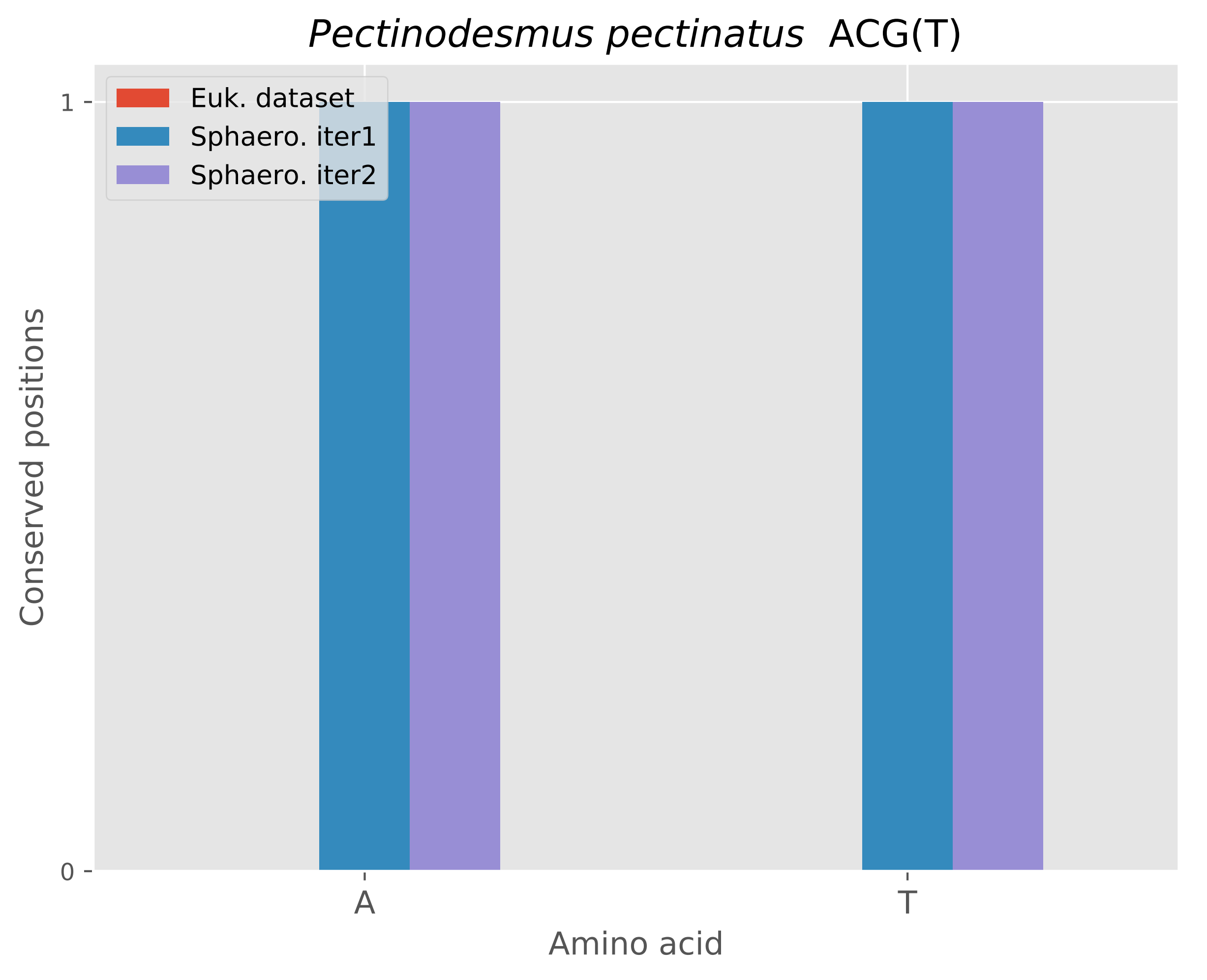

Pectinodesmus pectinatus ACG(T)
Euk. dataset
Sphaero. iter1
Sphaero. iter2
1
0
Conserved positions
A
T
Amino acid

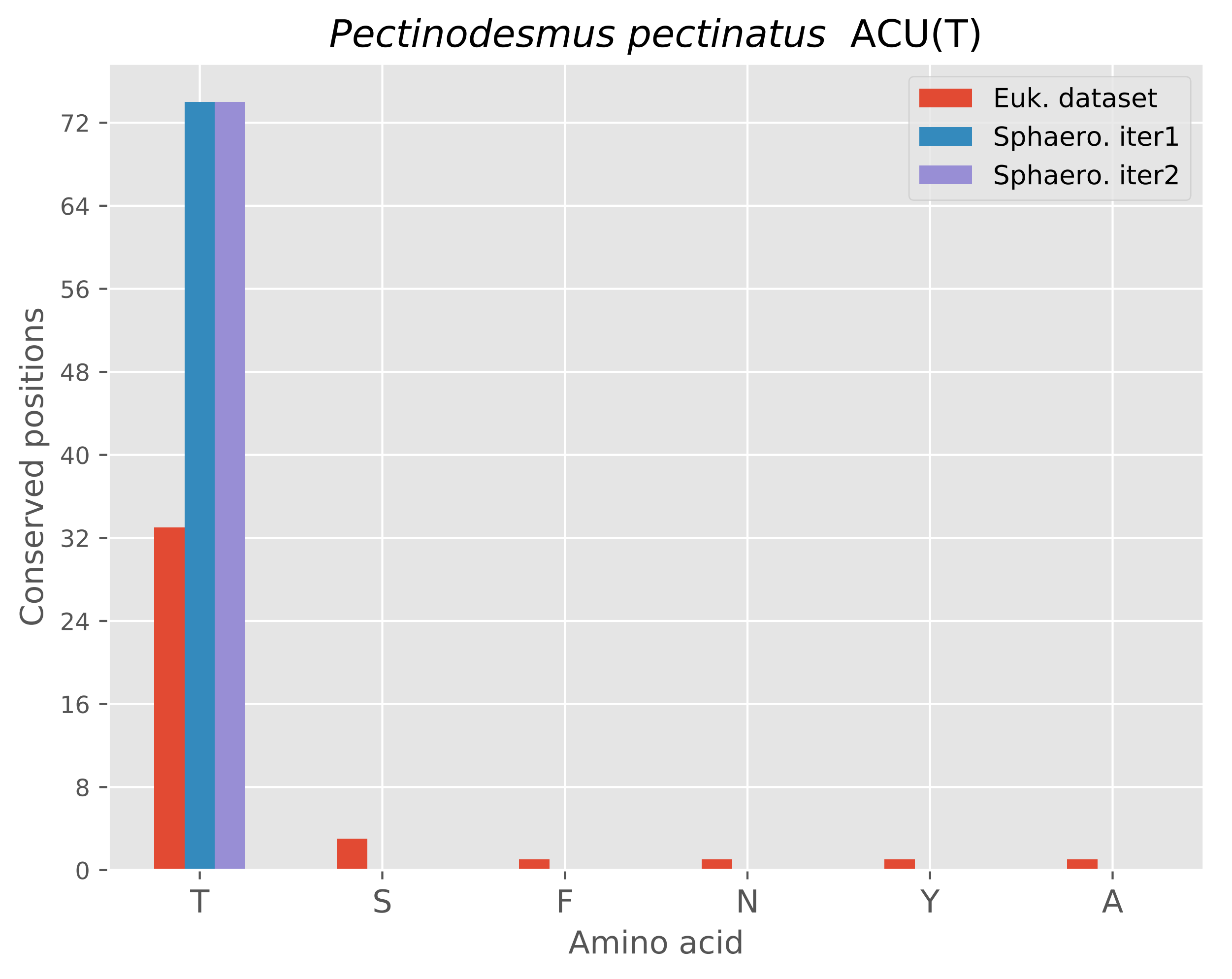

Pectinodesmus pectinatus ACU(T)
Euk. dataset
Sphaero. iter1
Sphaero. iter2
Conserved positions
0
8
16
24
32
40
48
56
64
72
T
S
F
N
Y
A
Amino acid

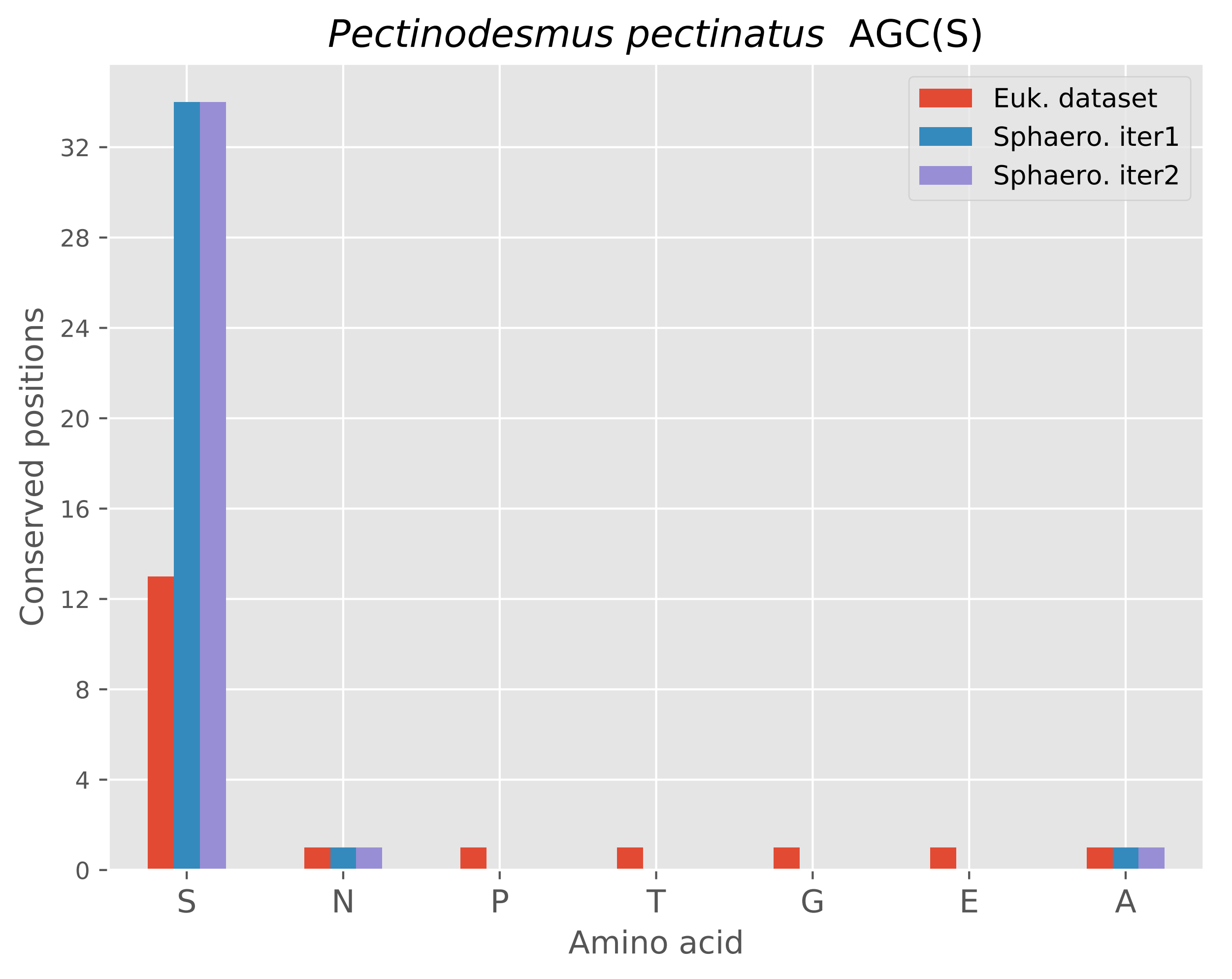
Pectinodesmus pectinatus AGC(S)
Euk. dataset
Sphaero. iter1
Sphaero. iter2
Conserved positions
0
4
8
12
16
20
24
28
32
S
N
P
T
G
E
A
Amino acid

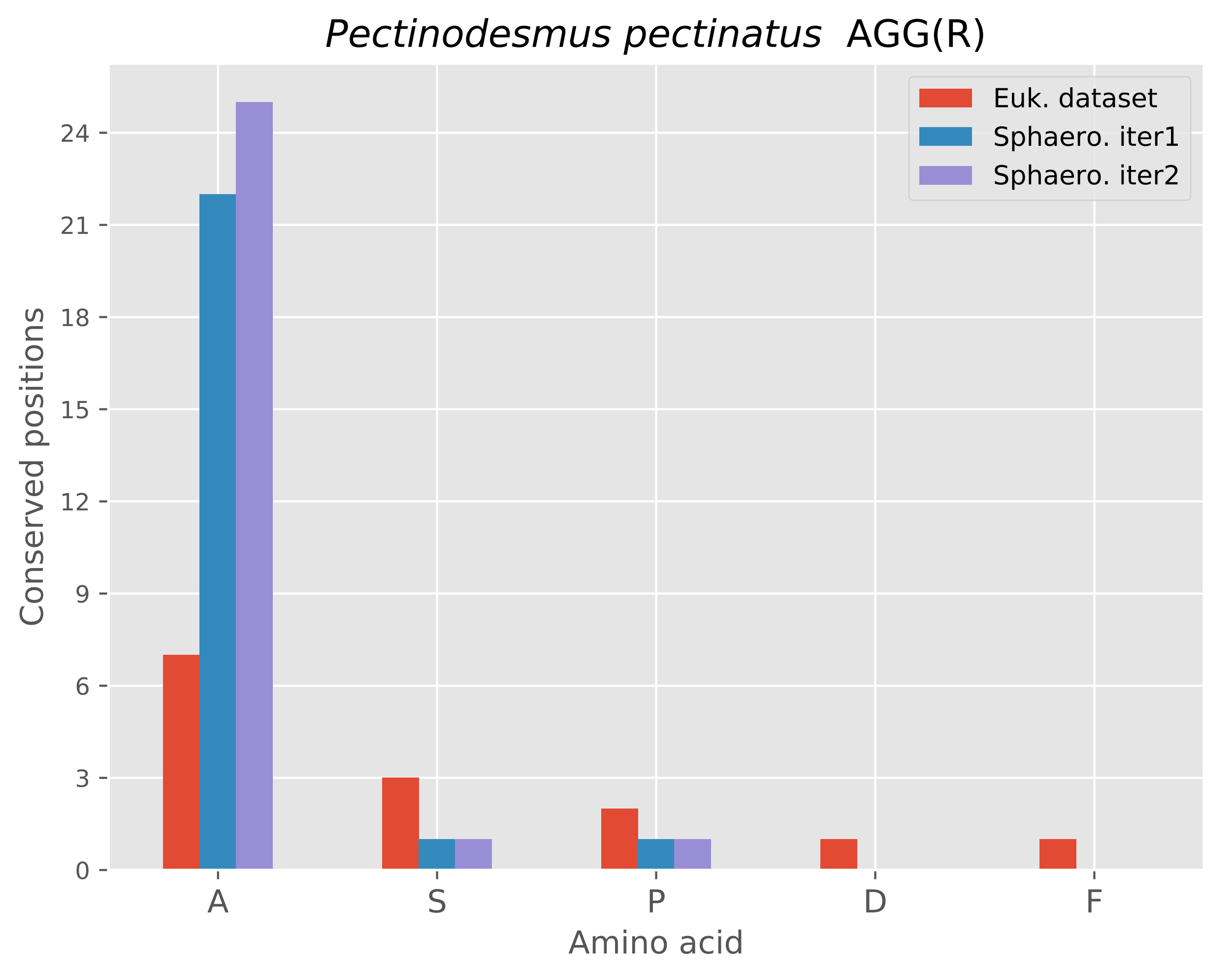

Pectinodesmus pectinatus AGG(R)
Euk. dataset
Sphaero. iter1
Sphaero. iter2
Conserved positions
0
3
6
9
12
15
18
21
24
A
S
P
D
F
Amino acid

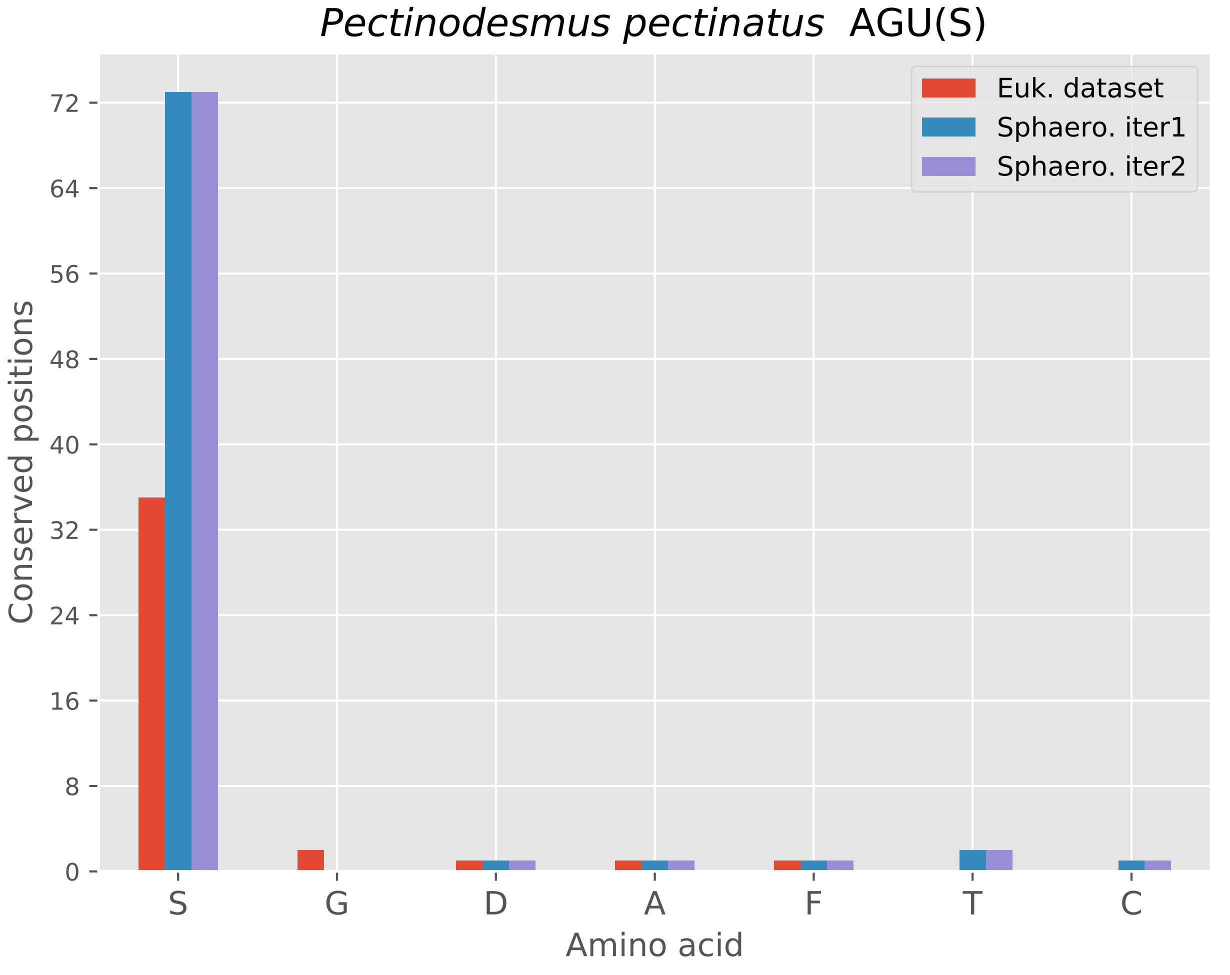

Pectinodesmus pectinatus AGU(S)
Euk. dataset
Sphaero. iter1
Sphaero. iter2
Conserved positions
0
8
16
24
32
40
48
56
64
72
S
G
D
A
F
T
C
Amino acid

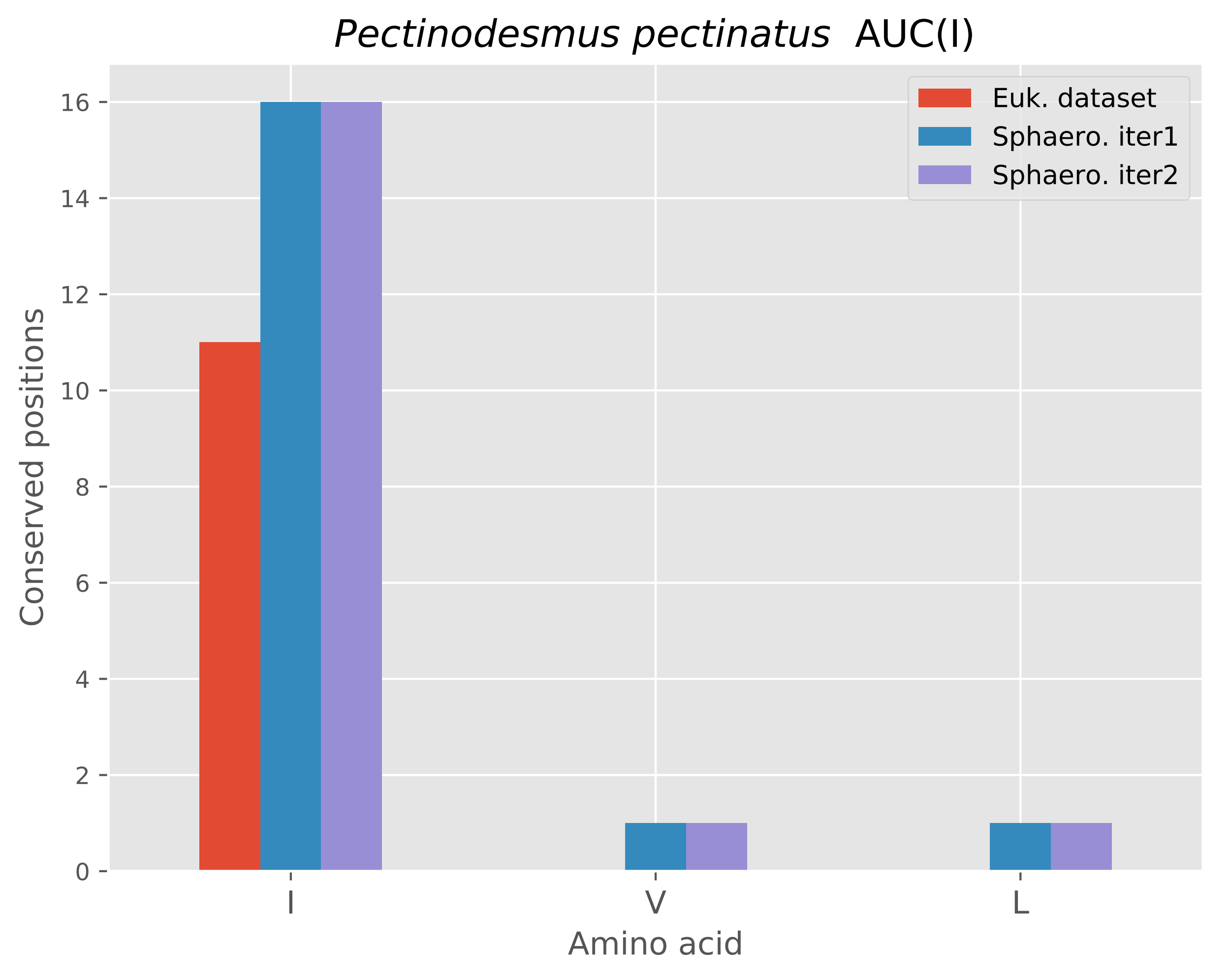

Pectinodesmus pectinatus AUC(I)
Euk. dataset
Sphaero. iter1
Sphaero. iter2
Conserved positions
0
2
4
6
8
10
12
14
16
I
V
L
Amino acid

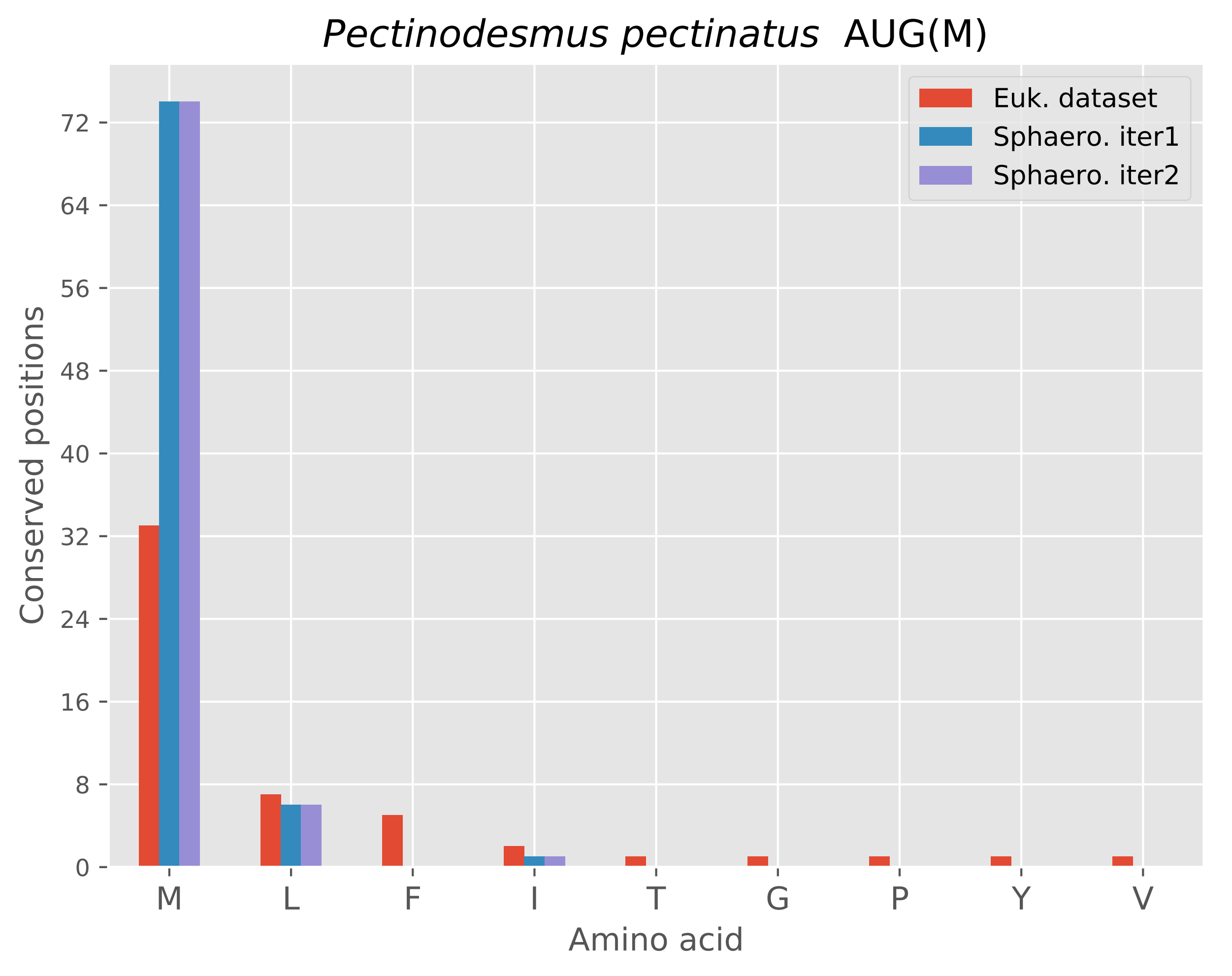
Pectinodesmus pectinatus AUG(M)
Euk. dataset
Sphaero. iter1
Sphaero. iter2
Conserved positions
0
8
16
24
32
40
48
56
64
72
M
L
F
I
T
G
P
Y
V
Amino acid

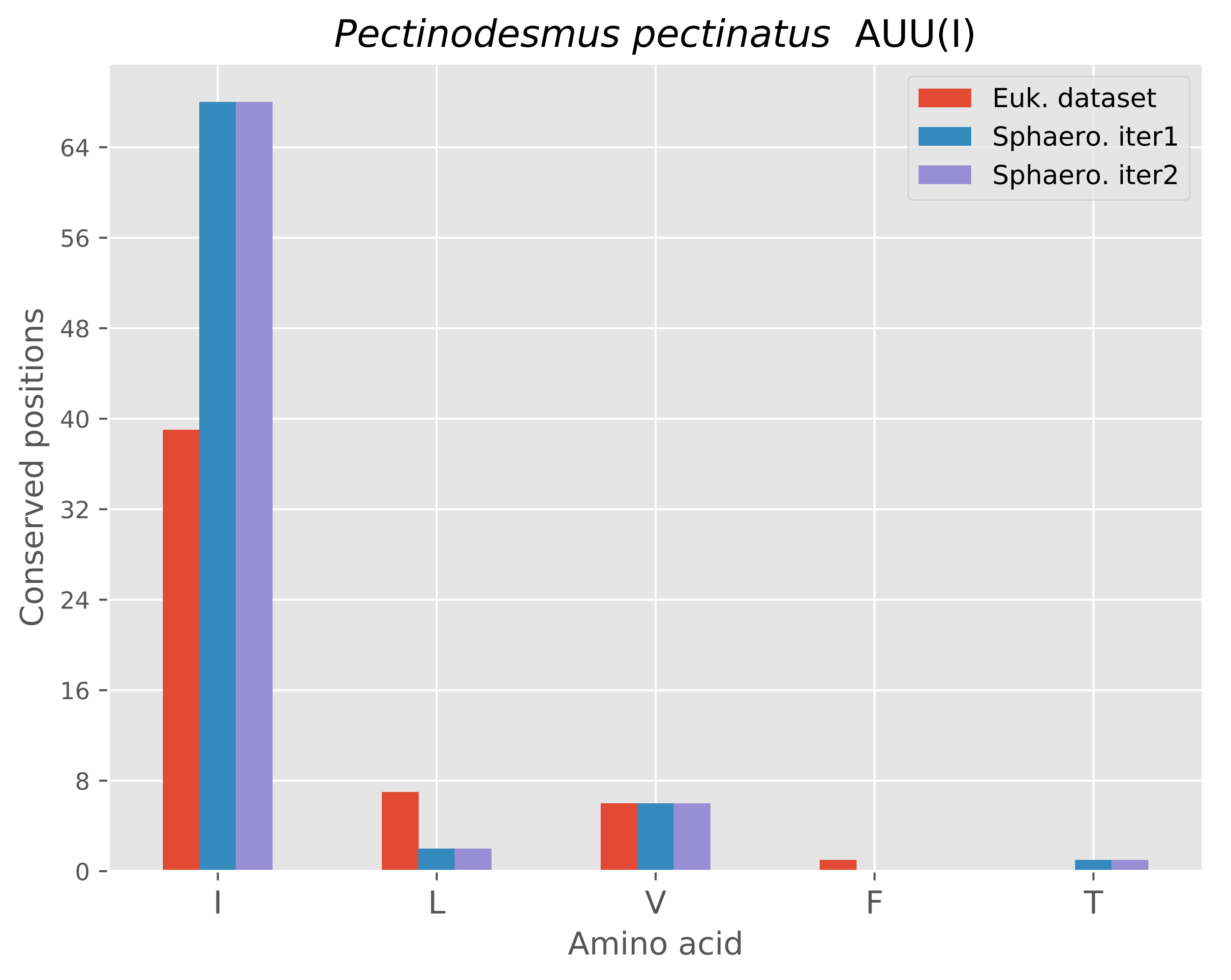
Pectinodesmus pectinatus AUU(I)
Euk. dataset
Sphaero. iter1
Sphaero. iter2
Conserved positions
0
8
16
24
32
40
48
56
64
I
L
V
F
T
Amino acid

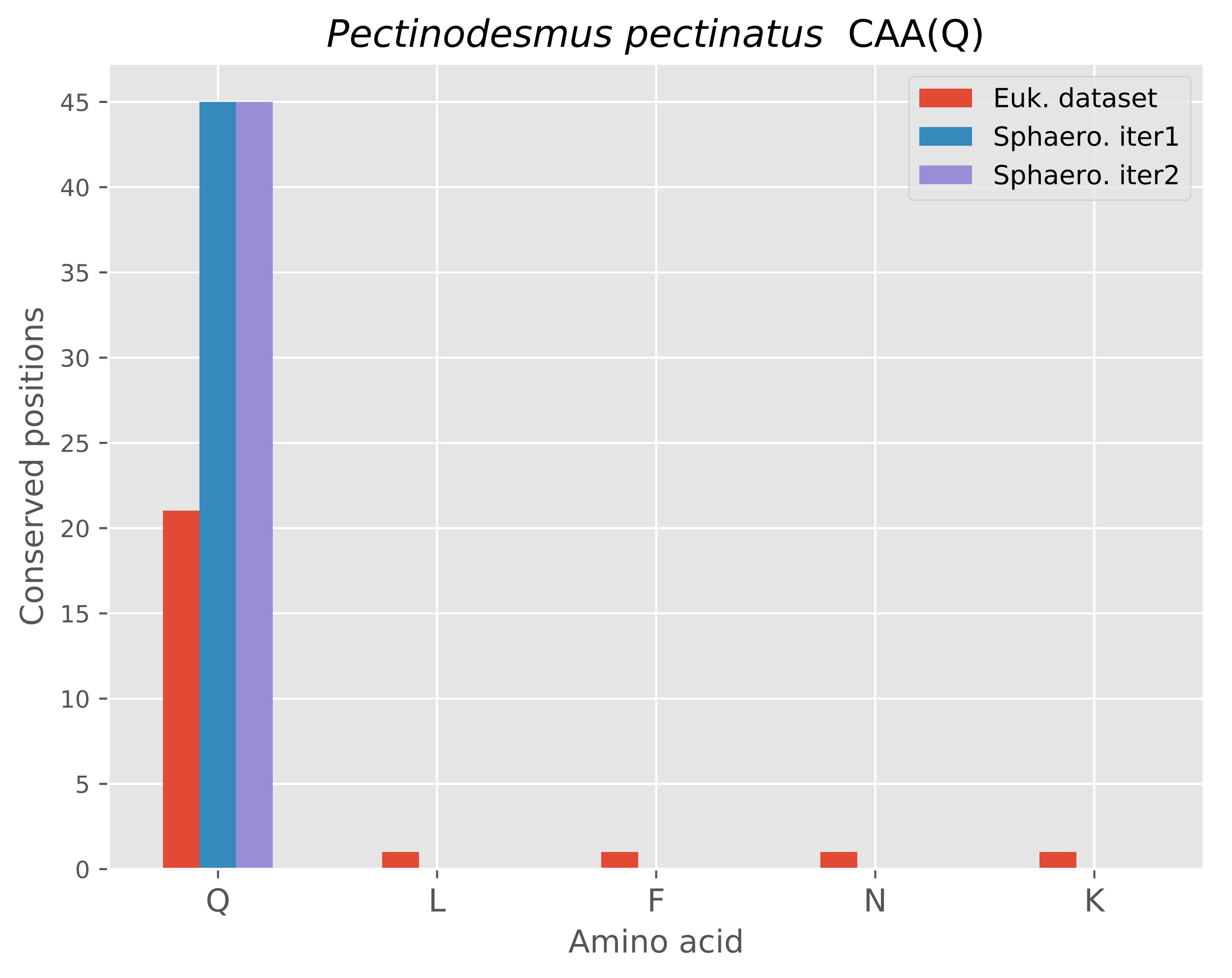

Pectinodesmus pectinatus CAA(Q)
Euk. dataset
Sphaero. iter1
Sphaero. iter2
Conserved positions
0
5
10
15
20
25
30
35
40
45
Q
L
F
N
K
Amino acid

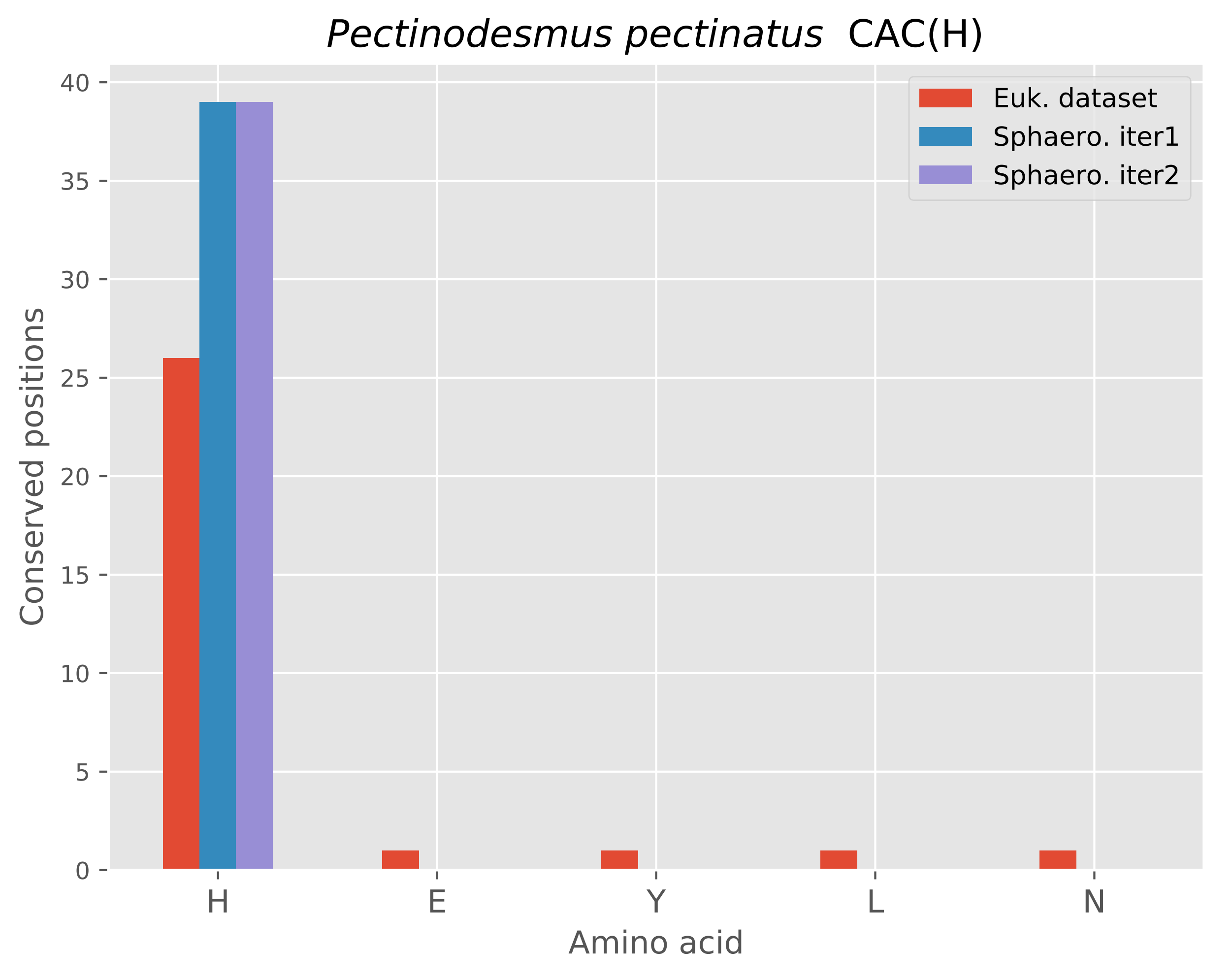

Pectinodesmus pectinatus CAC(H)
Euk. dataset
Sphaero. iter1
Sphaero. iter2
Conserved positions
0
5
10
15
20
25
30
35
40
H
E
Y
L
N
Amino acid

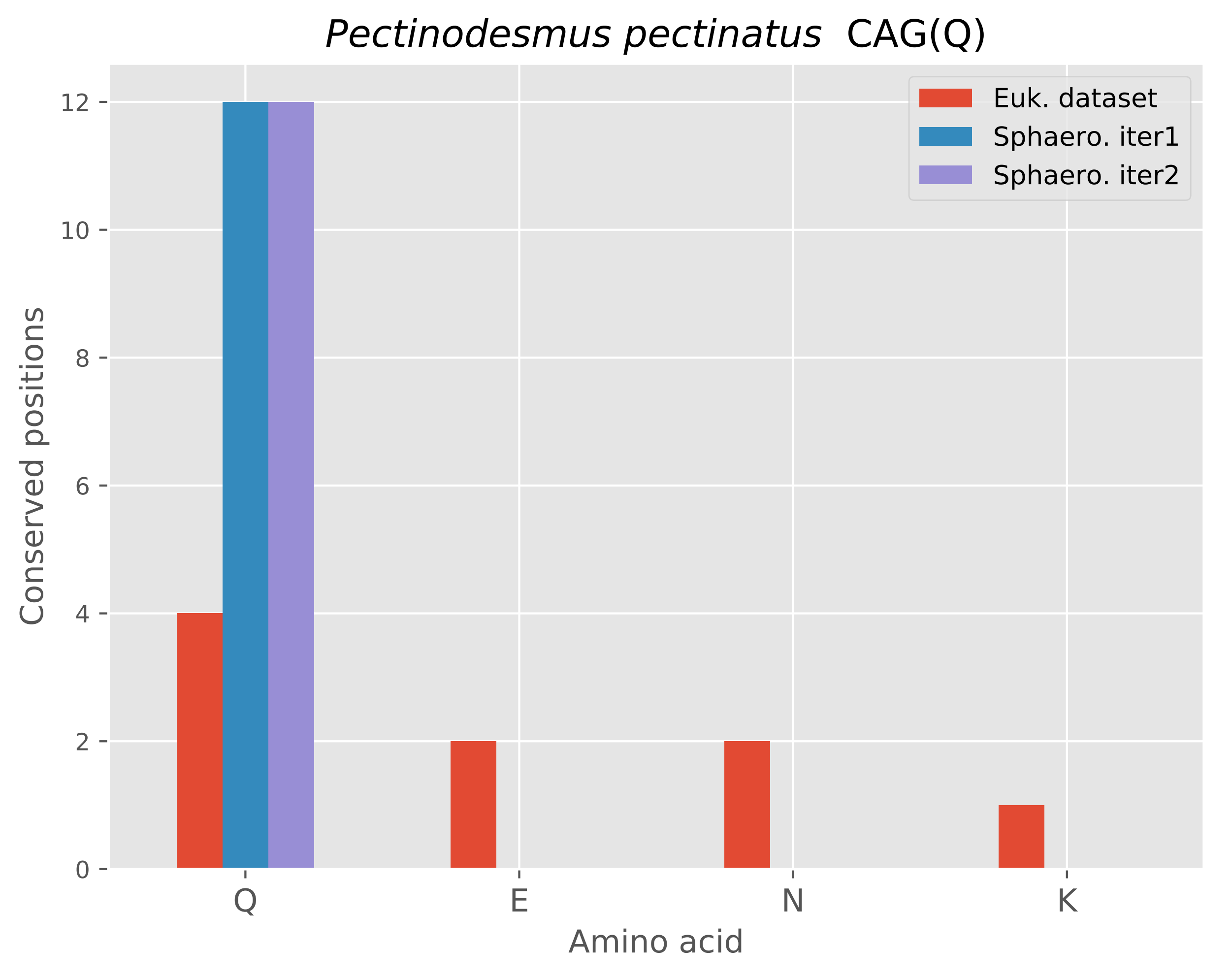

Pectinodesmus pectinatus CAG(Q)
Euk. dataset
Sphaero. iter1
Sphaero. iter2
Conserved positions
0
2
4
6
8
10
12
Q
E
N
K
Amino acid

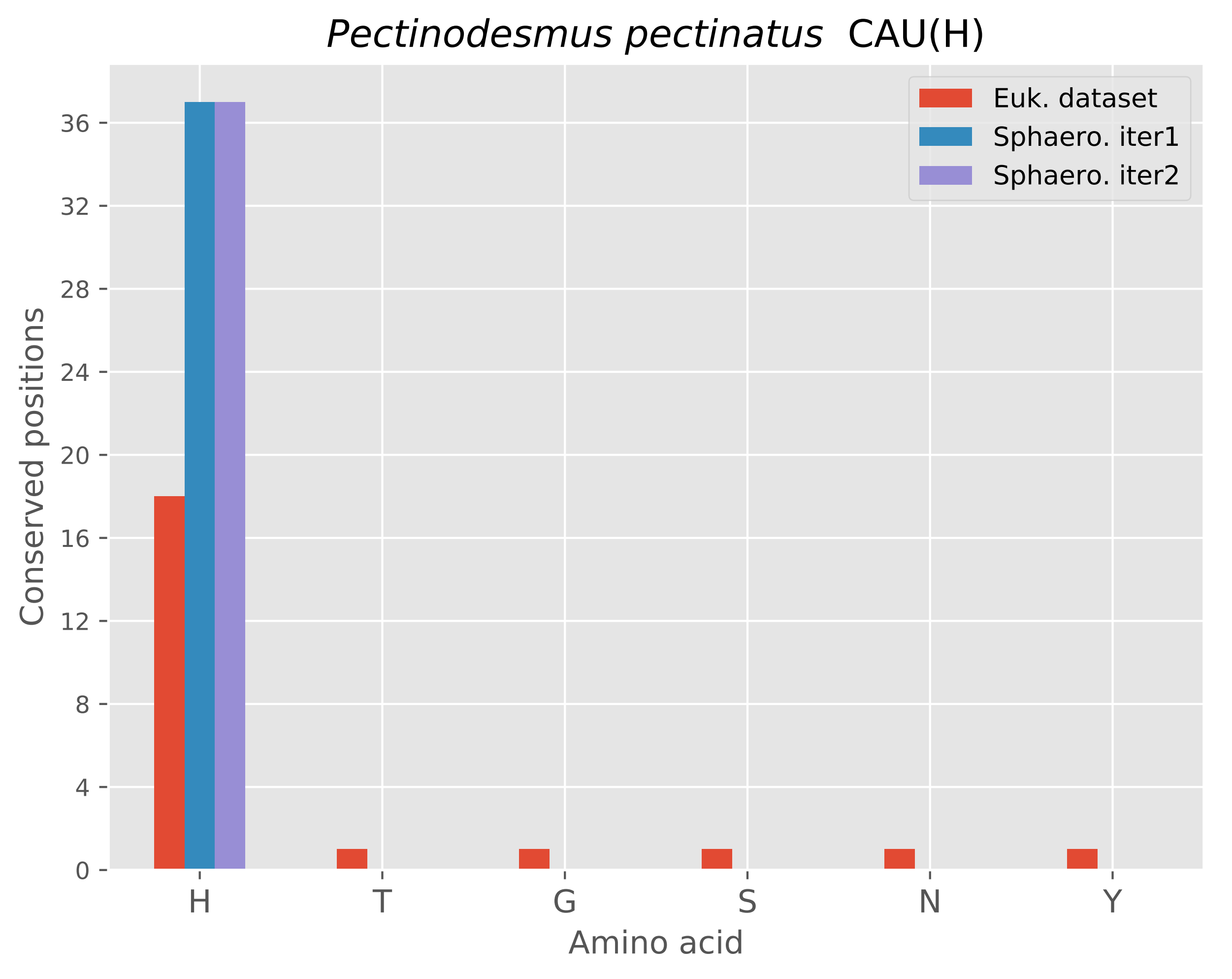

Pectinodesmus pectinatus CAU(H)
Euk. dataset
Sphaero. iter1
Sphaero. iter2
Conserved positions
0
4
8
12
16
20
24
28
32
36
H
T
G
S
N
Y
Amino acid

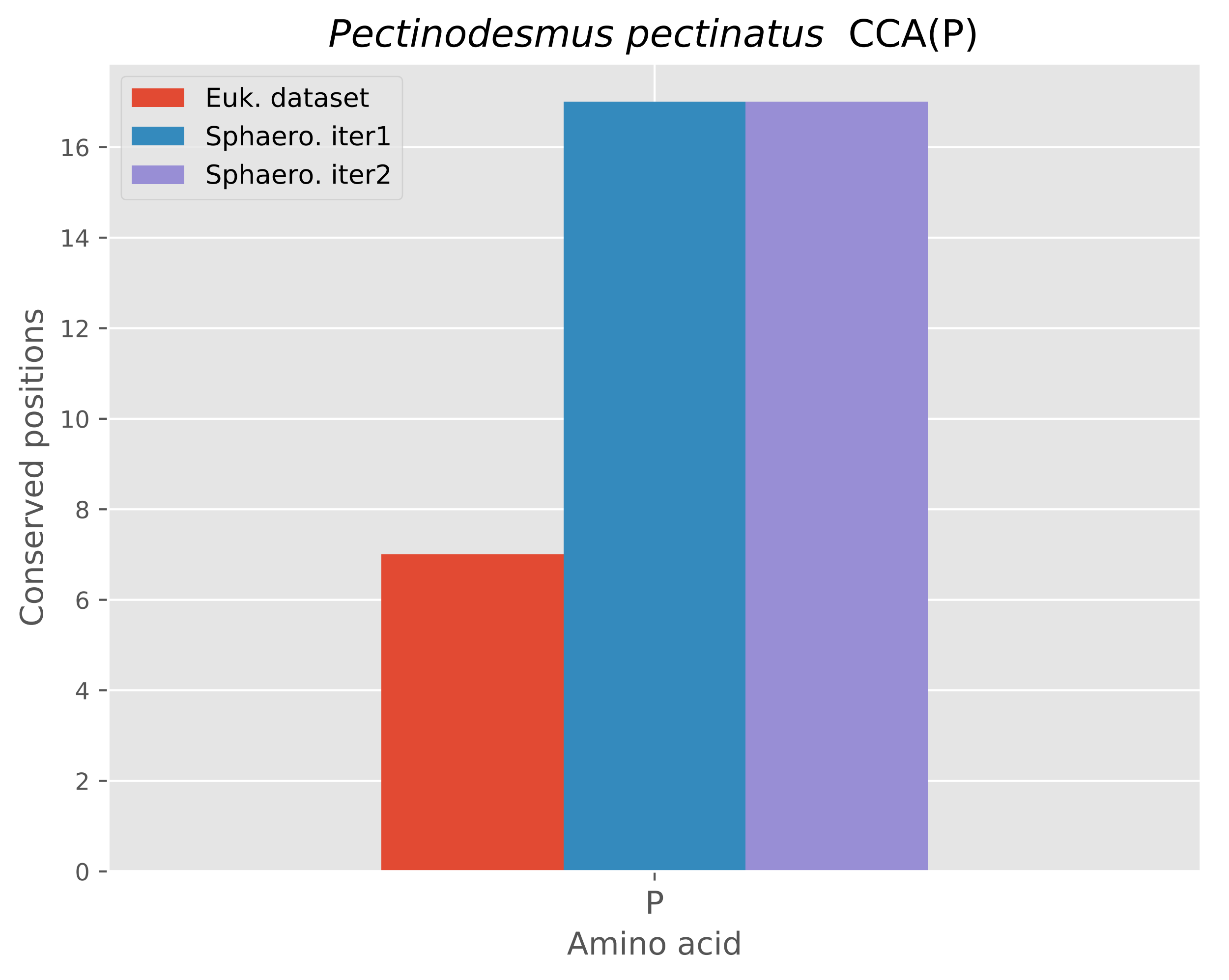

Pectinodesmus pectinatus CCA(P)
Euk. dataset
Sphaero. iter1
Sphaero. iter2
Conserved positions
0
2
4
6
8
10
12
14
16
P
Amino acid

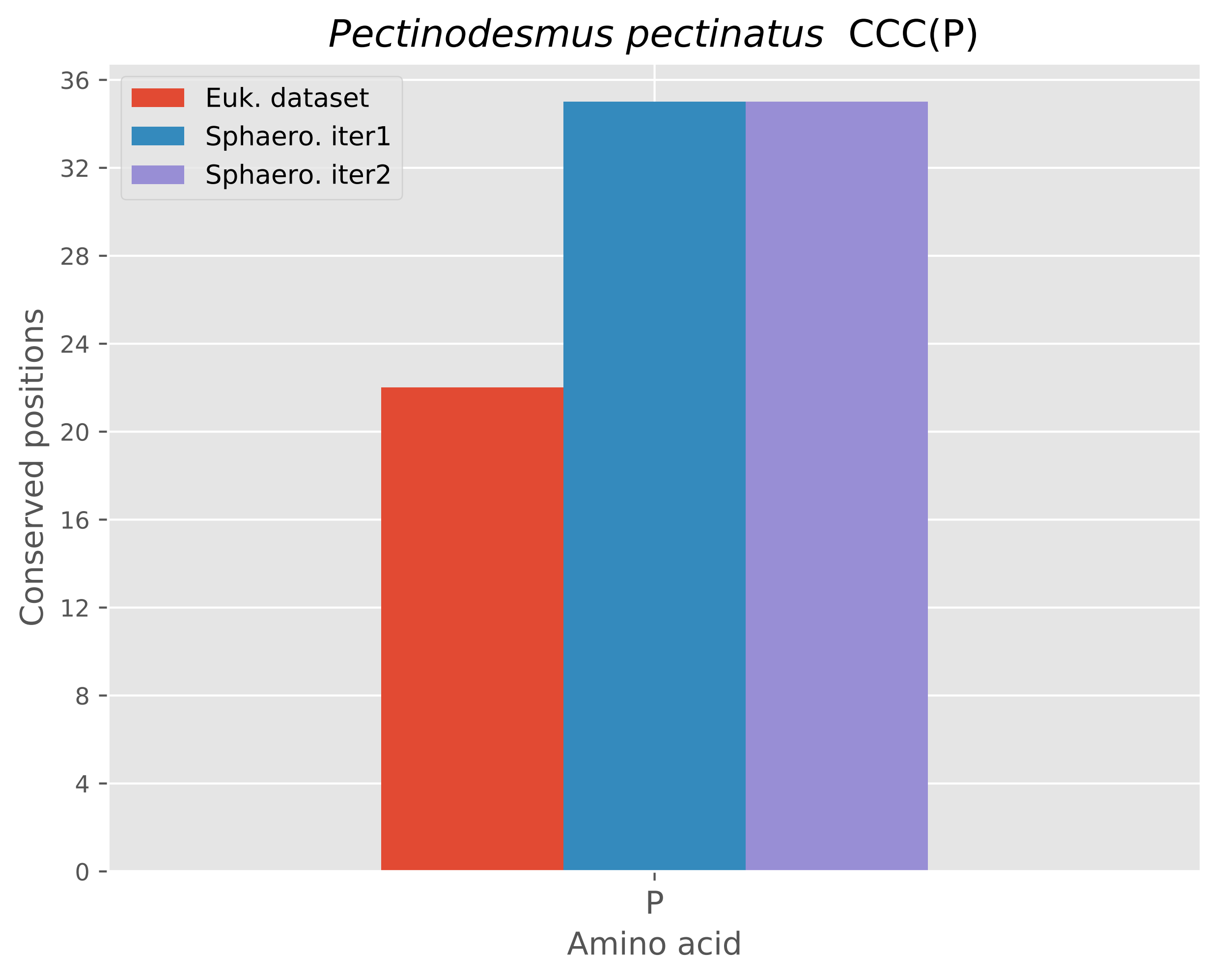

Pectinodesmus pectinatus CCC(P)
Euk. dataset
Sphaero. iter1
Sphaero. iter2
Conserved positions
0
4
8
12
16
20
24
28
32
36
P
Amino acid

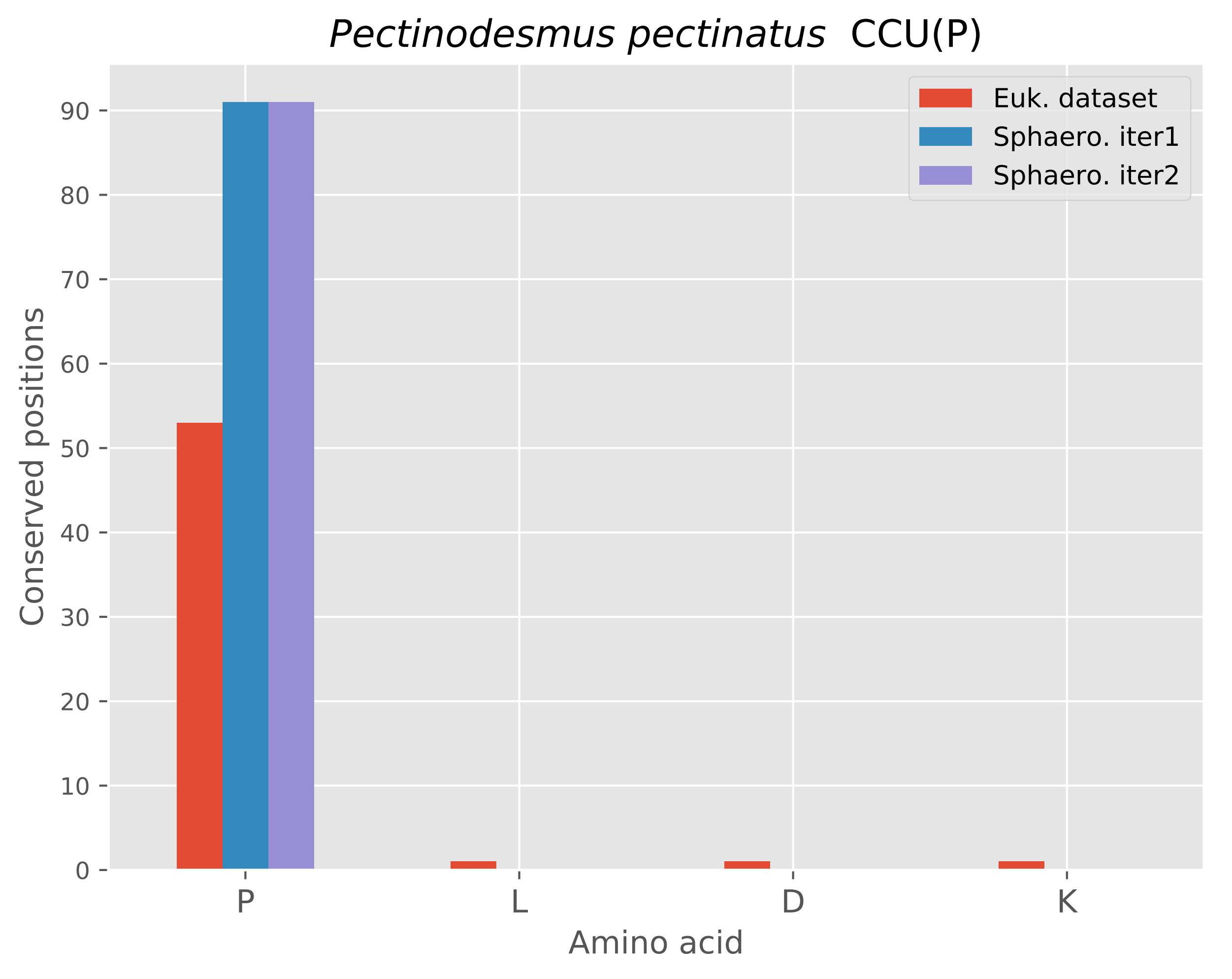

Pectinodesmus pectinatus CCU(P)
Euk. dataset
Sphaero. iter1
Sphaero. iter2
Conserved positions
0
10
20
30
40
50
60
70
80
90
P
L
D
K
Amino acid

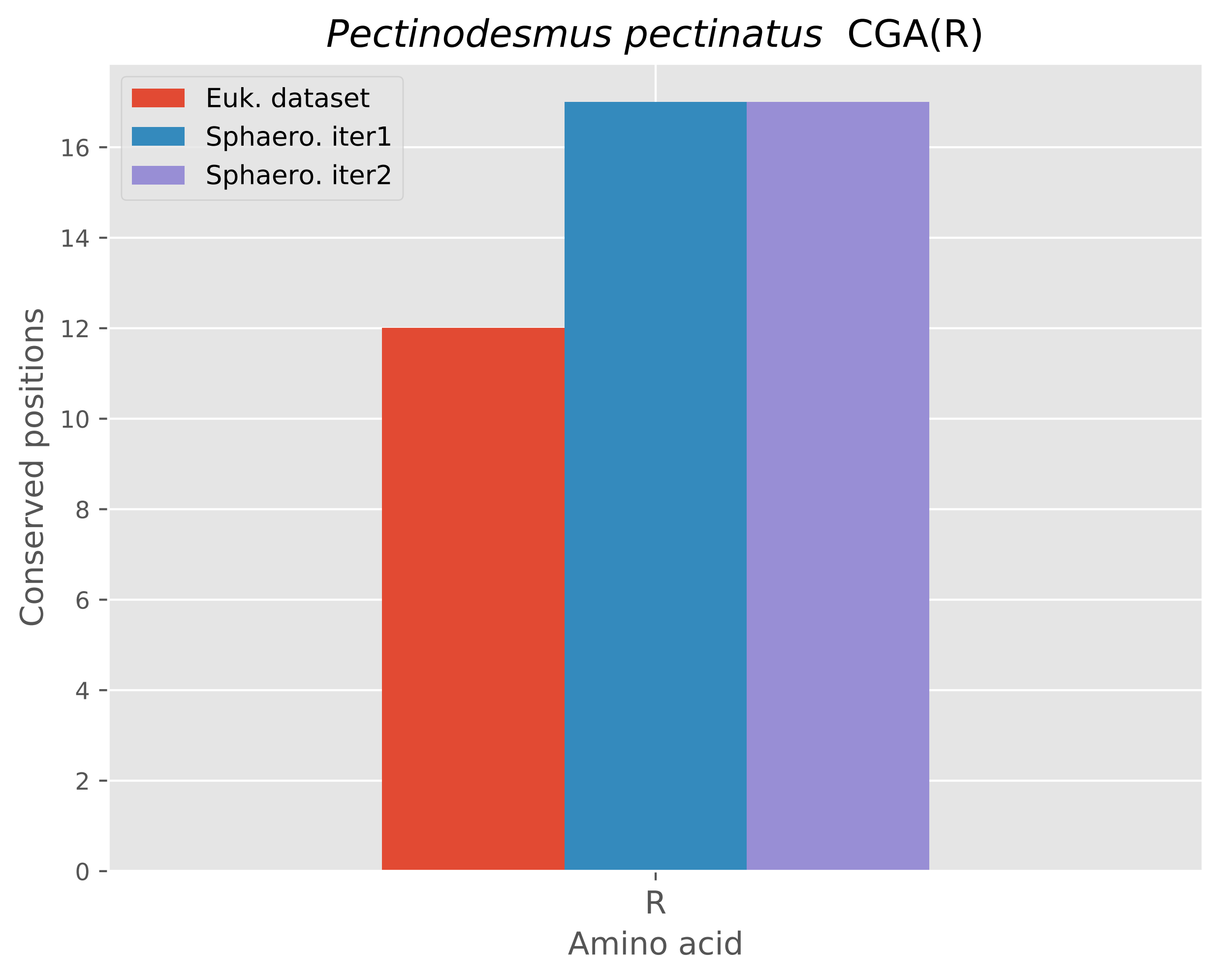
Pectinodesmus pectinatus CGA(R)
Euk. dataset
Sphaero. iter1
Sphaero. iter2
Conserved positions
0
2
4
6
8
10
12
14
16
R
Amino acid

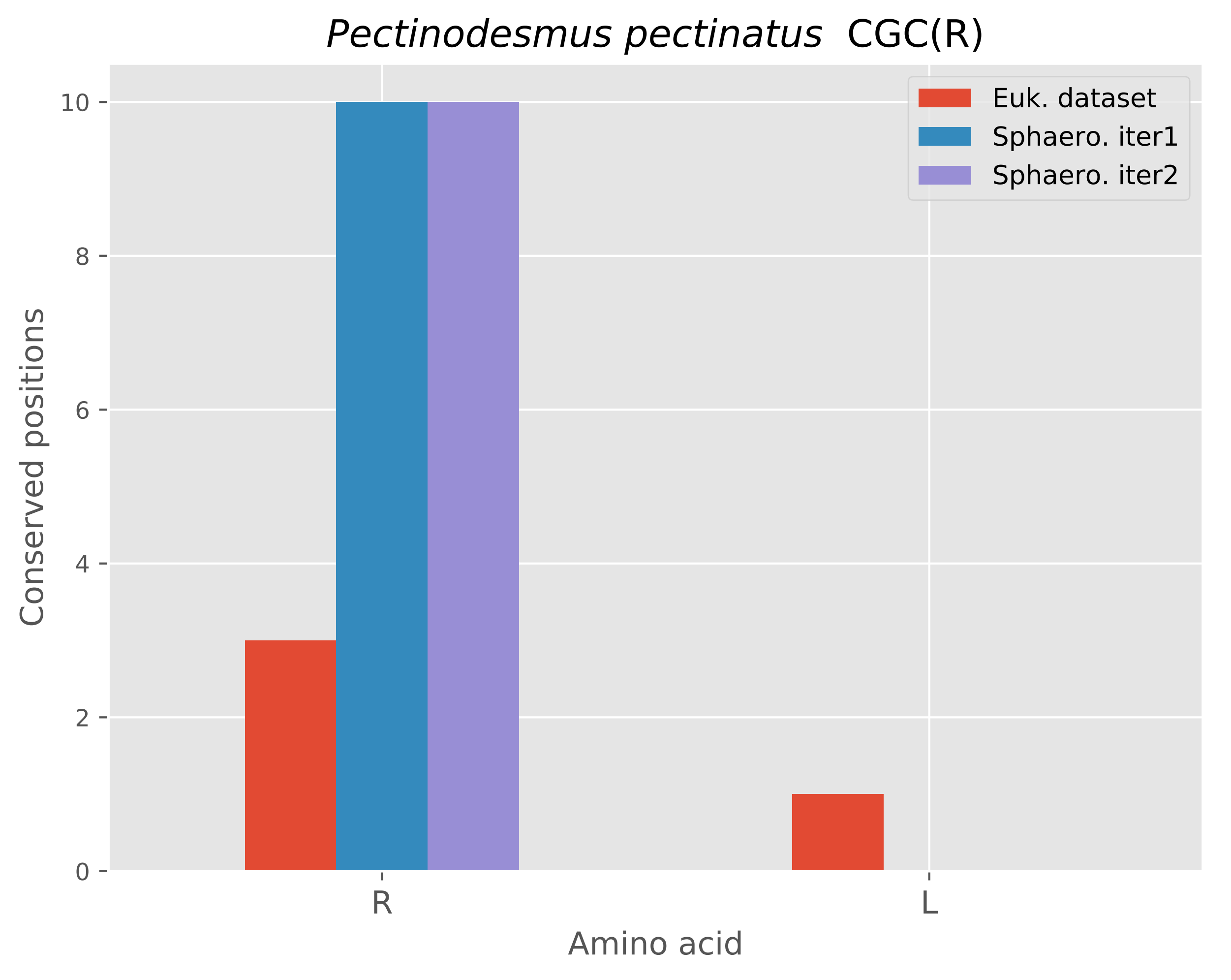

Pectinodesmus pectinatus CGC(R)
Conserved positions
Amino acid
0
2
4
6
8
10
R
L
Euk. dataset
Sphaero. iter1
Sphaero. iter2

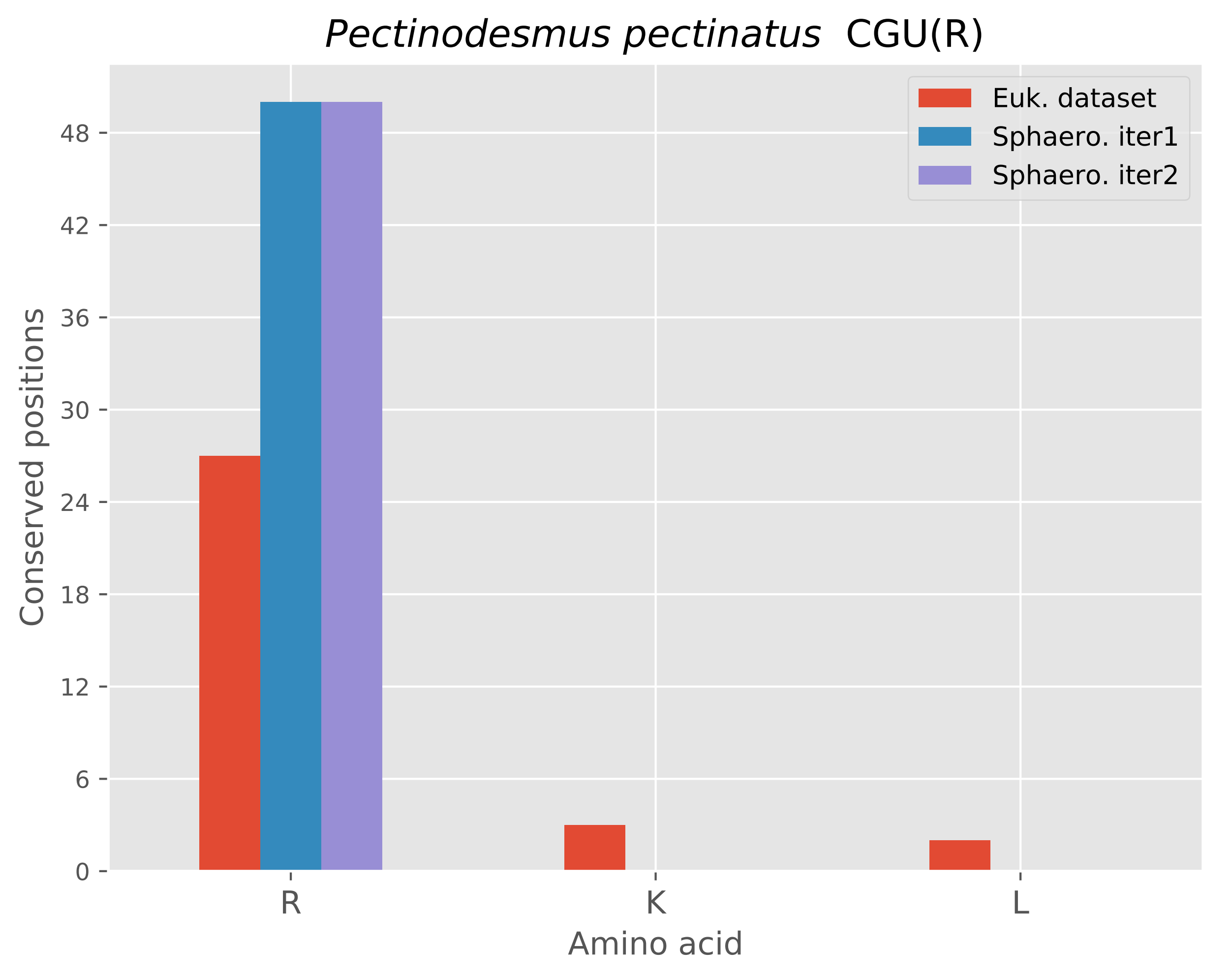

Pectinodesmus pectinatus CGU(R)
Euk. dataset
Sphaero. iter1
Sphaero. iter2
Conserved positions
0
6
12
18
24
30
36
42
48
R
K
L
Amino acid

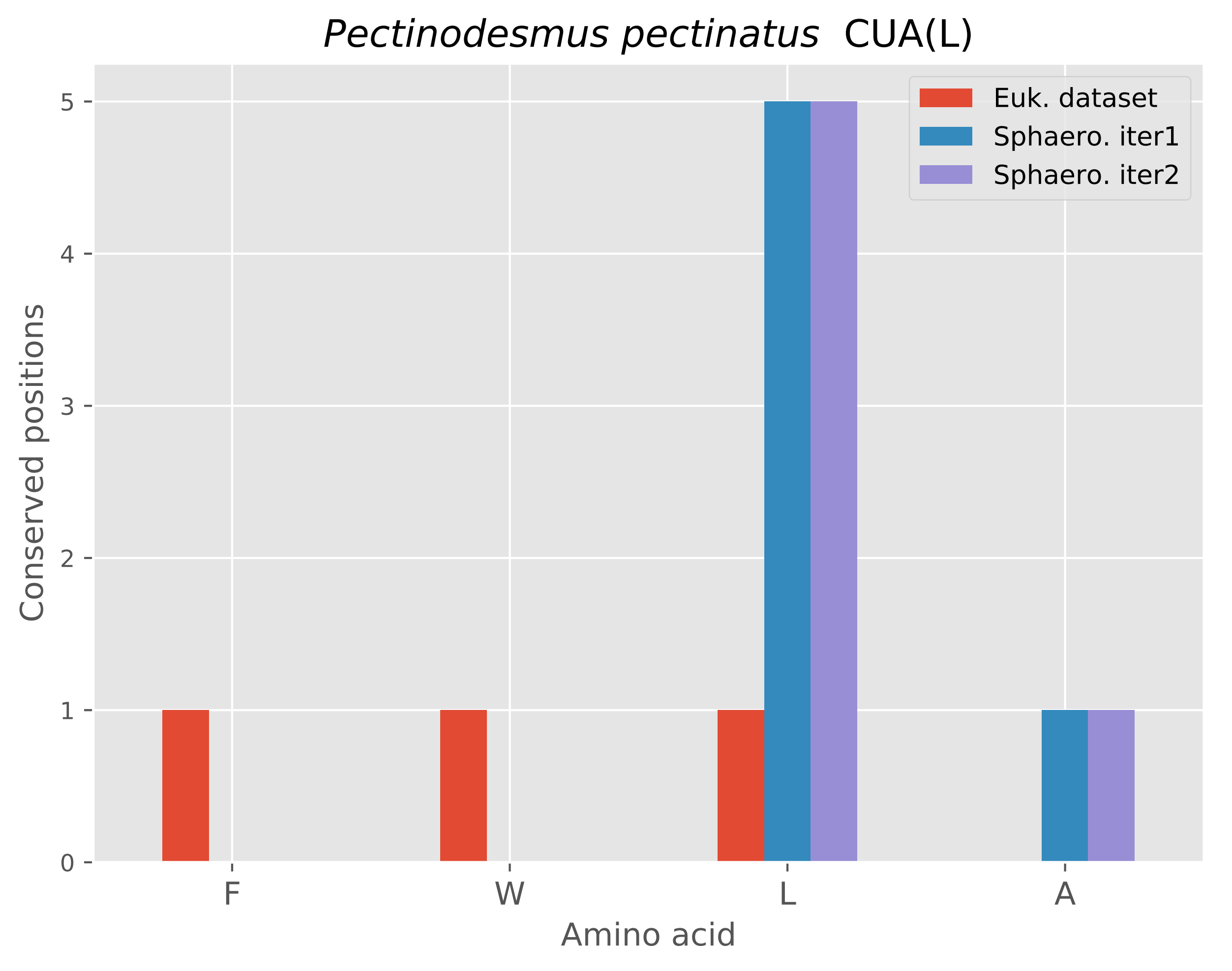
Pectinodesmus pectinatus CUA(L)
Euk. dataset
Sphaero. iter1
Sphaero. iter2
Conserved positions
0
1
2
3
4
5
F
W
L
A
Amino acid

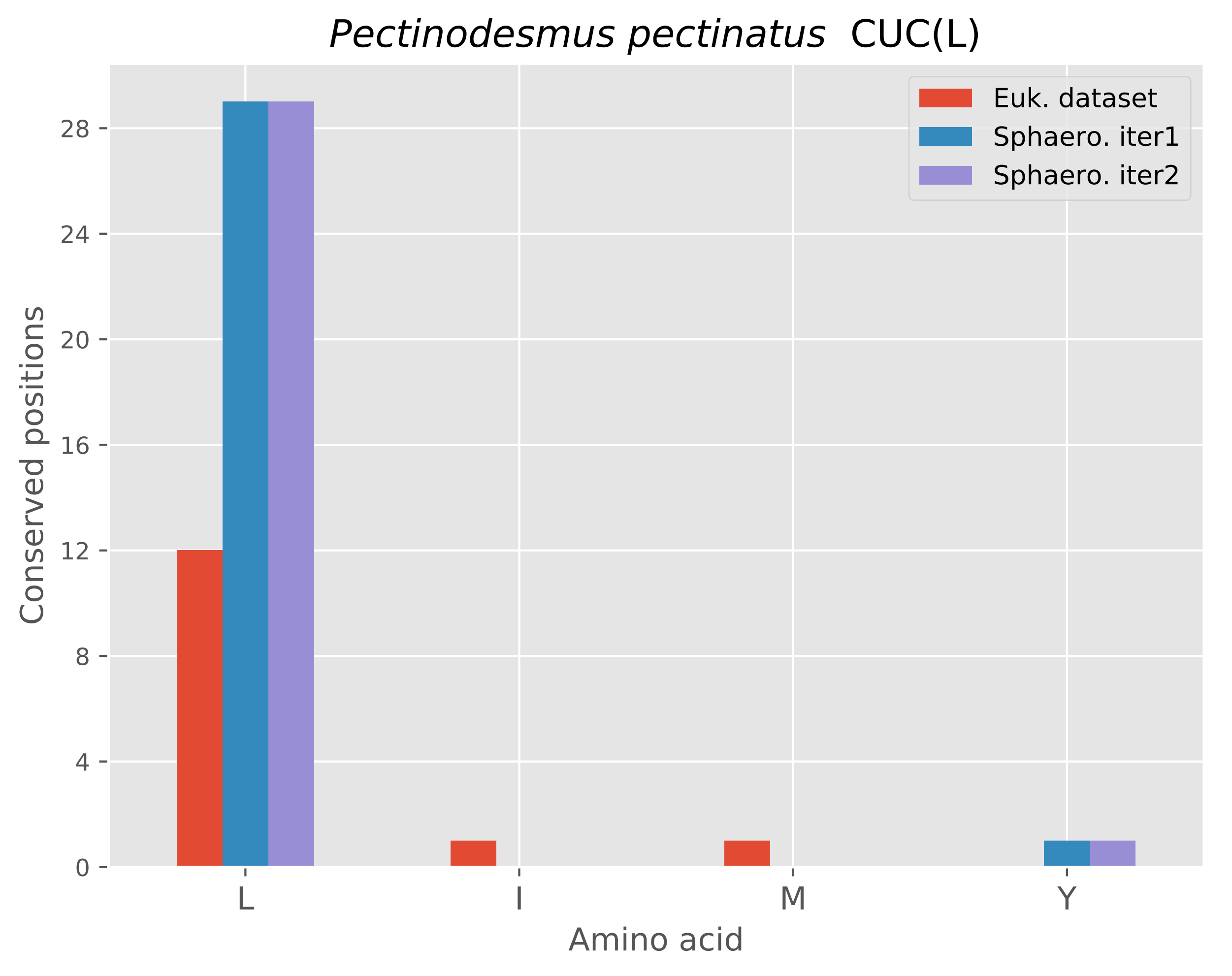
Pectinodesmus pectinatus CUC(L)
Euk. dataset
Sphaero. iter1
Sphaero. iter2
Conserved positions
0
4
8
12
16
20
24
28
L
I
M
Y
Amino acid

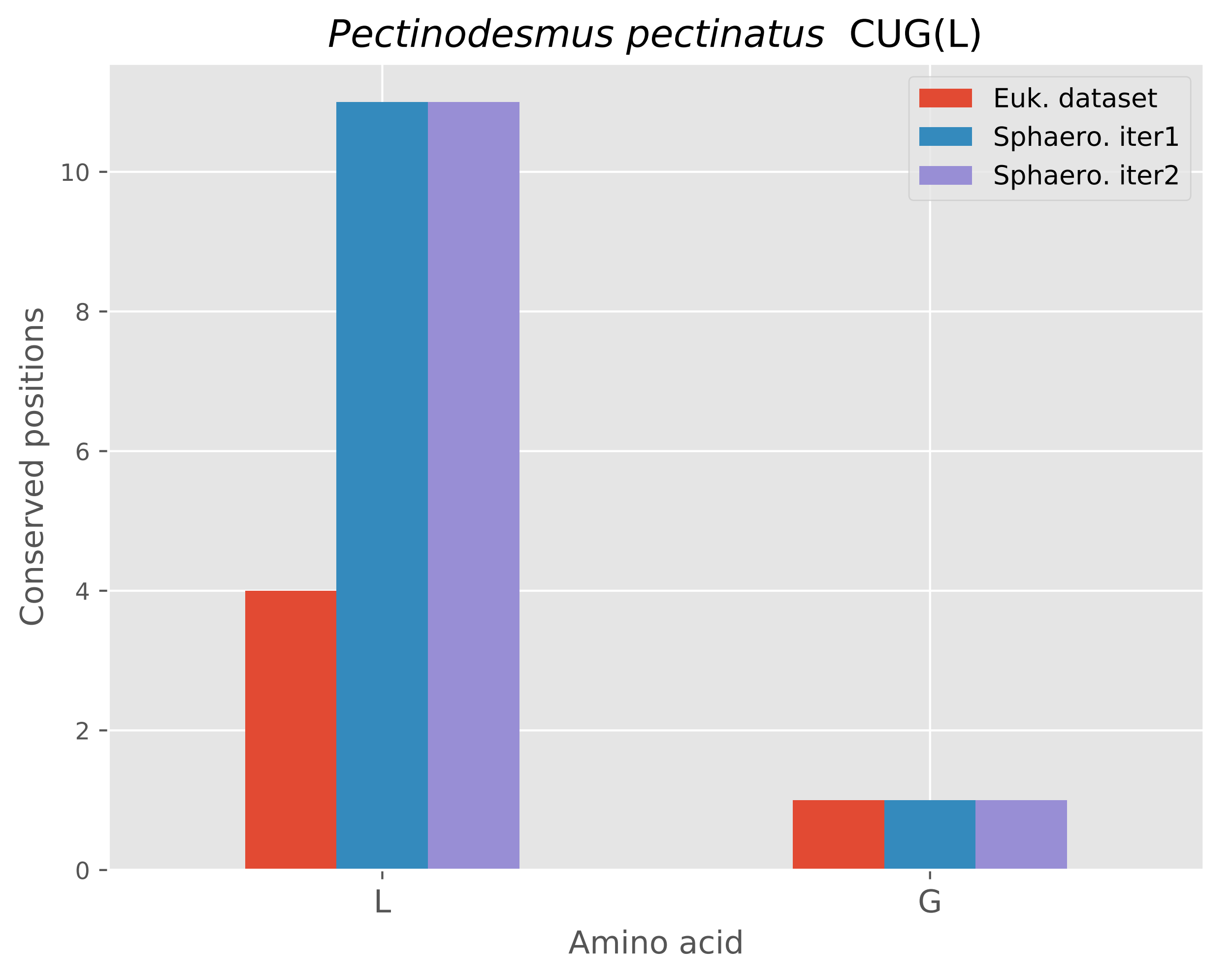

Pectinodesmus pectinatus CUG(L)
Euk. dataset
Sphaero. iter1
Sphaero. iter2
Conserved positions
0
2
4
6
8
10
L
G
Amino acid

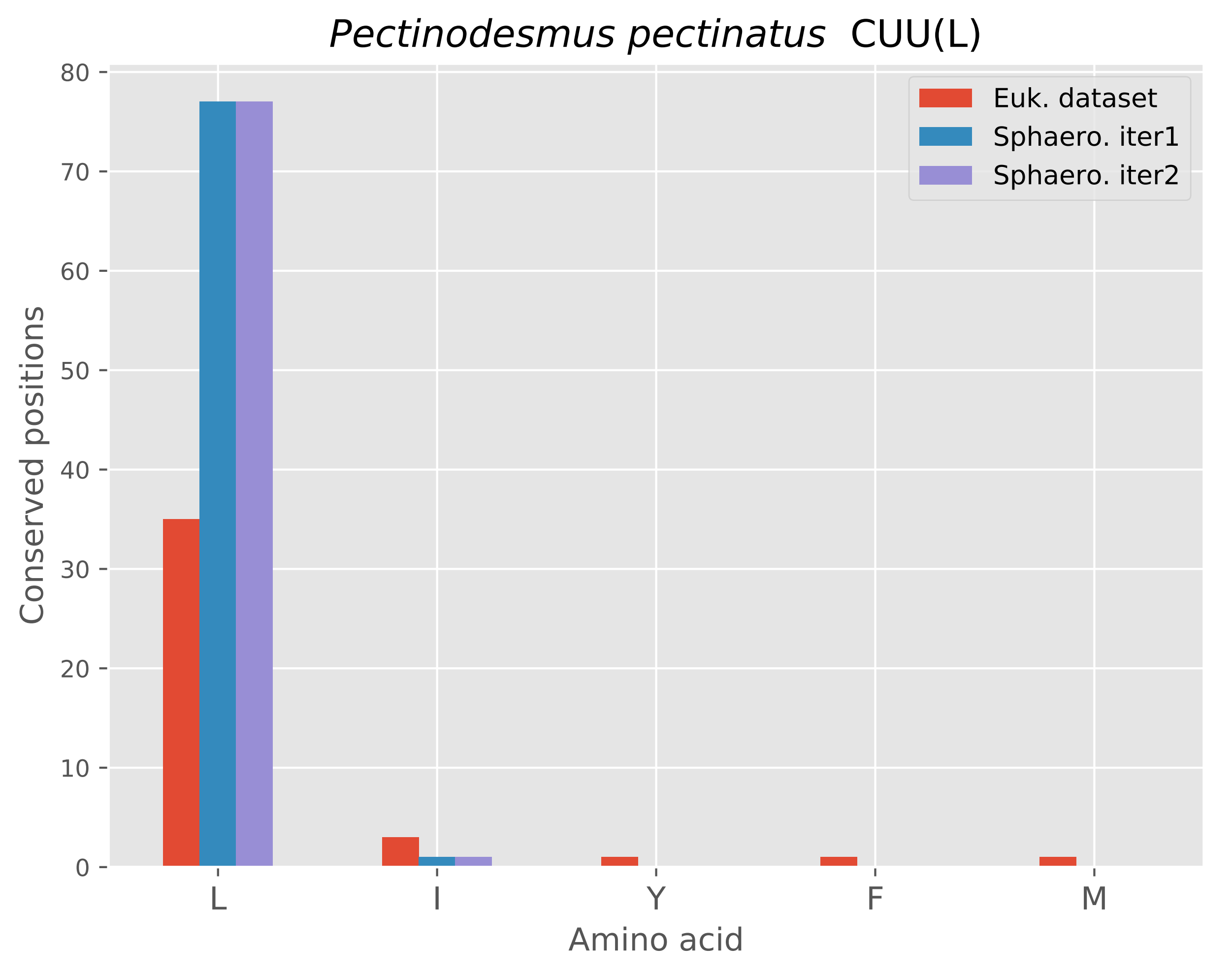

Pectinodesmus pectinatus CUU(L)
Euk. dataset
Sphaero. iter1
Sphaero. iter2
Conserved positions
0
10
20
30
40
50
60
70
80
L
I
Y
F
M
Amino acid

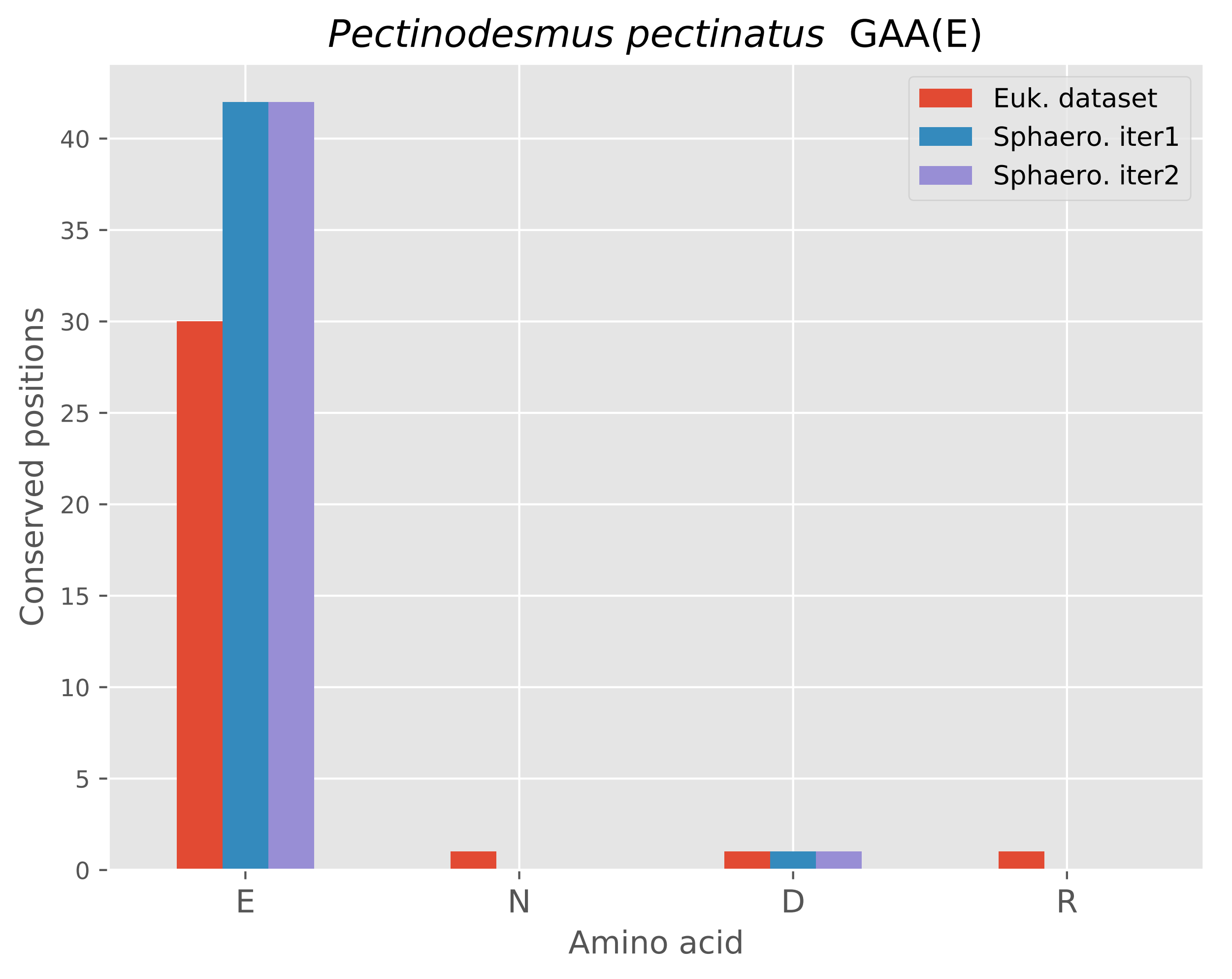

Pectinodesmus pectinatus GAA(E)
Euk. dataset
Sphaero. iter1
Sphaero. iter2
Conserved positions
0
5
10
15
20
25
30
35
40
E
N
D
R
Amino acid

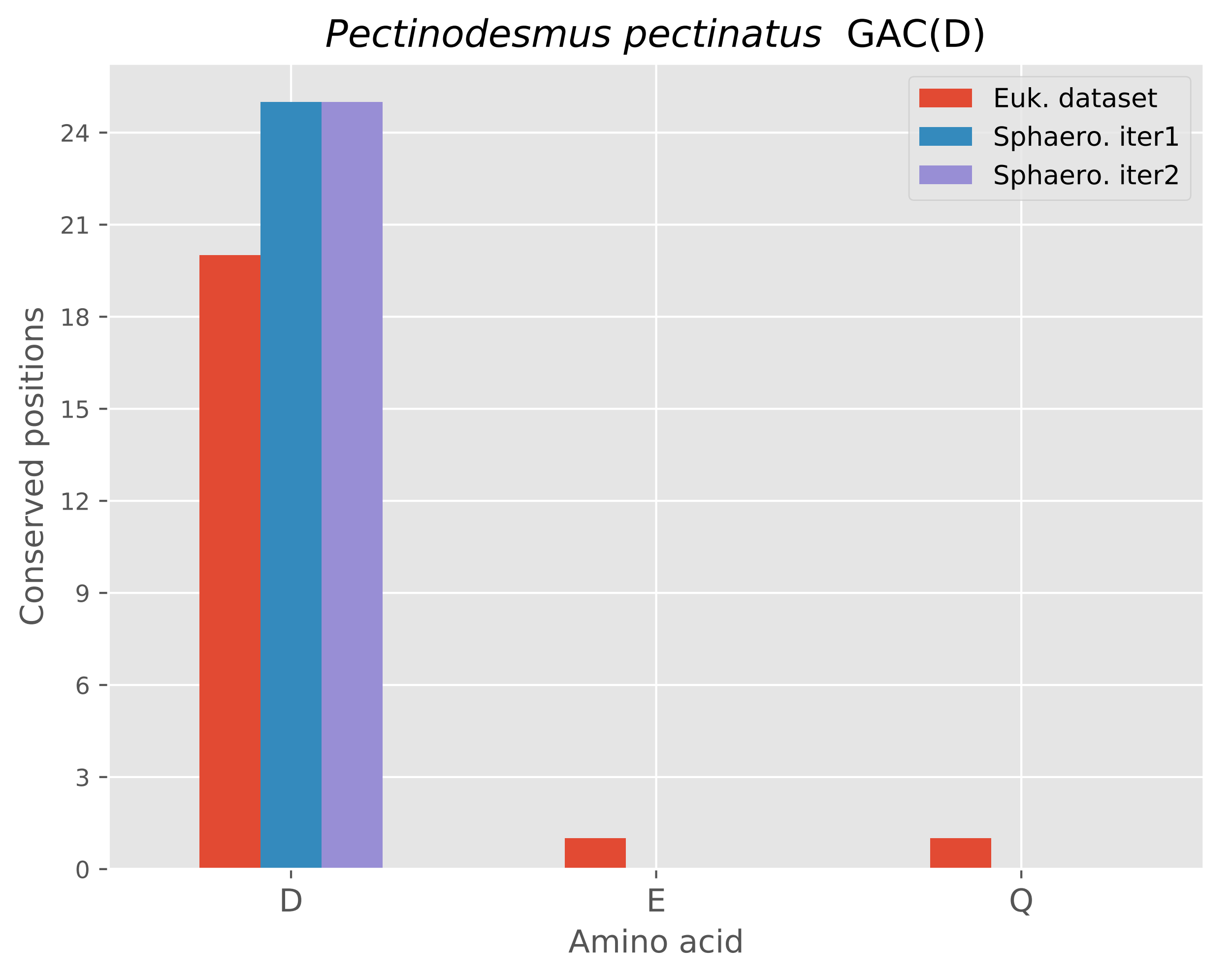

Pectinodesmus pectinatus GAC(D)
Euk. dataset
Sphaero. iter1
Sphaero. iter2
Conserved positions
0
3
6
9
12
15
18
21
24
D
E
Q
Amino acid

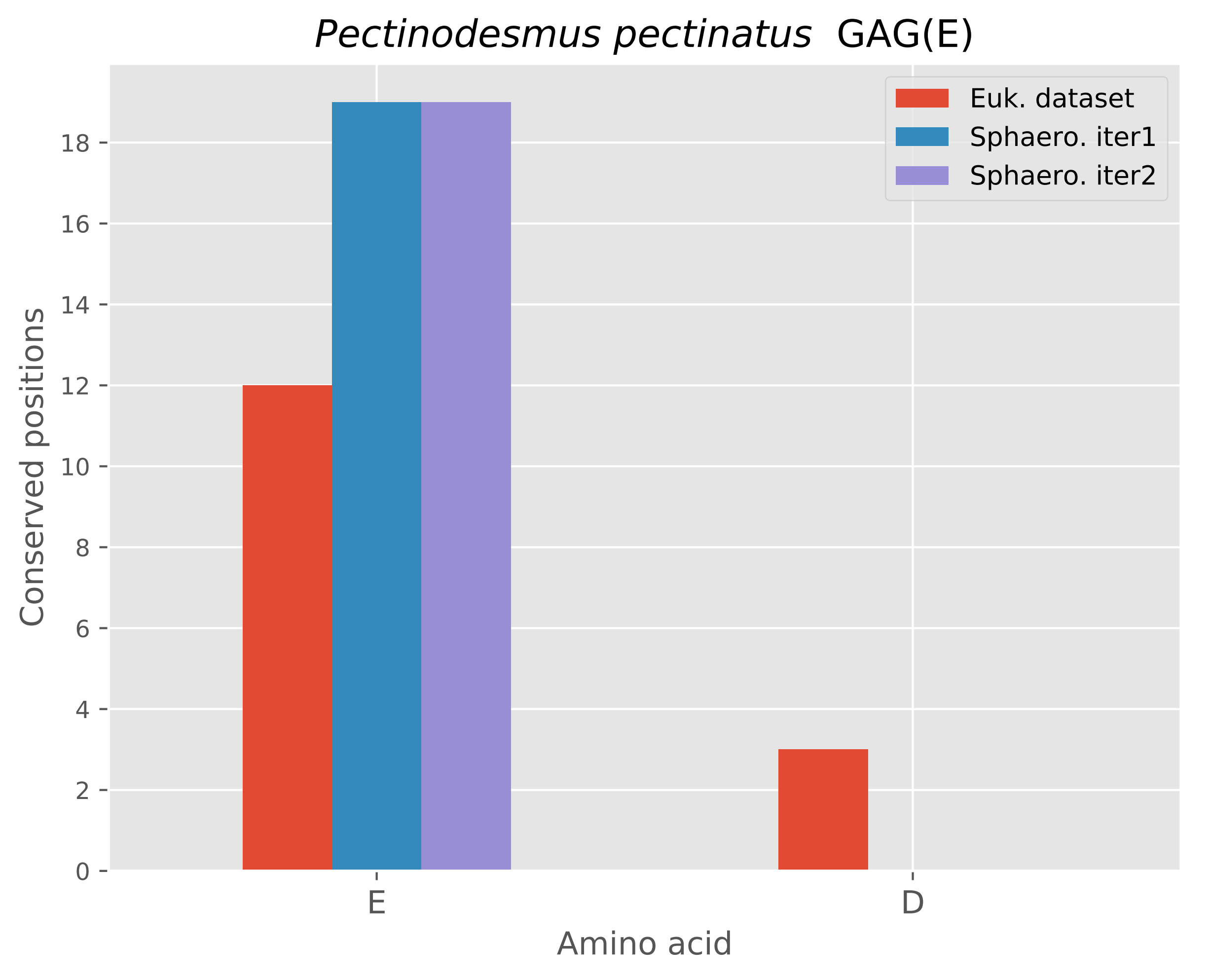

Pectinodesmus pectinatus GAG(E)
Euk. dataset
Sphaero. iter1
Sphaero. iter2
Conserved positions
0
2
4
6
8
10
12
14
16
18
E
D
Amino acid

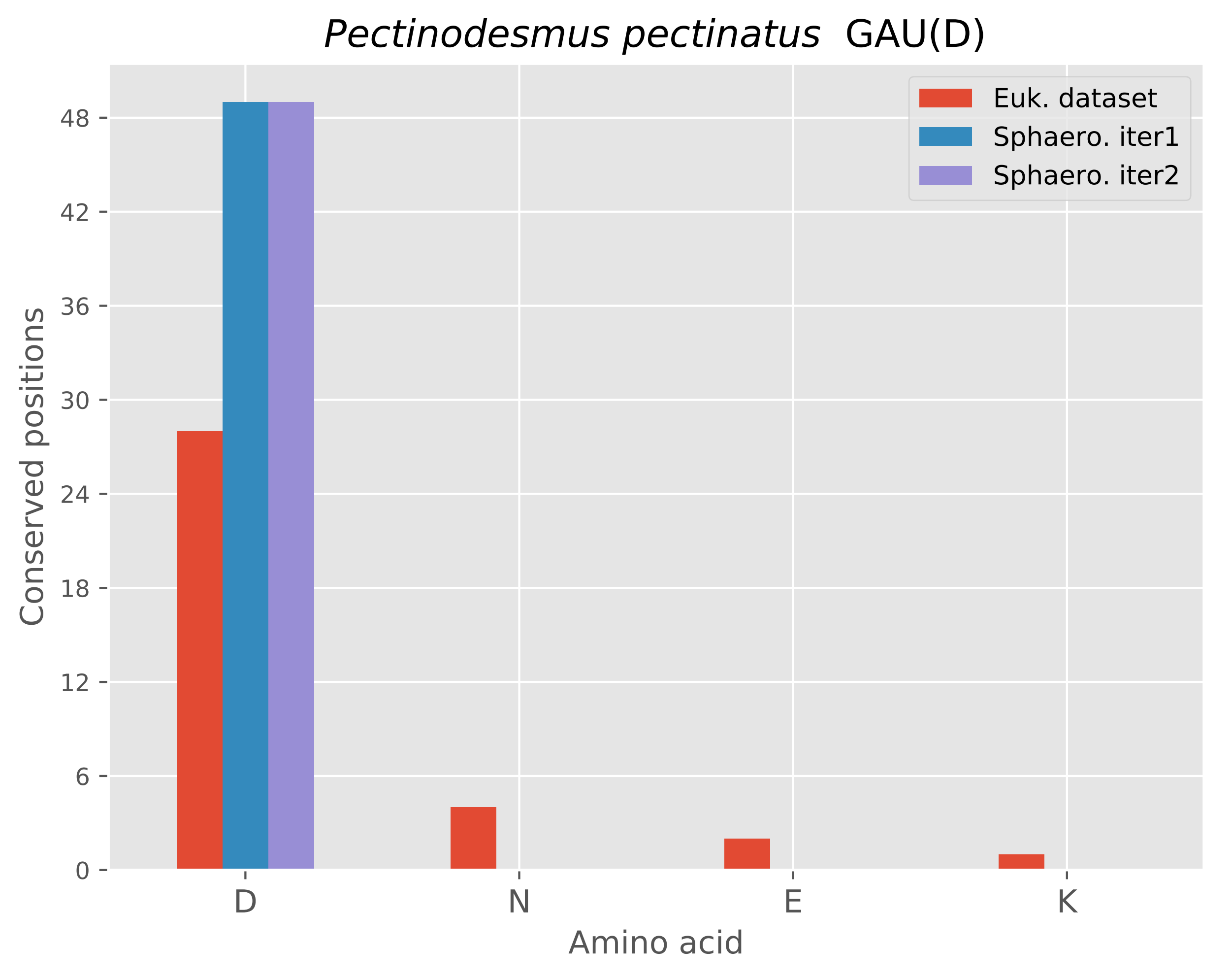

Pectinodesmus pectinatus GAU(D)
Euk. dataset
Sphaero. iter1
Sphaero. iter2
Conserved positions
0
6
12
18
24
30
36
42
48
D
N
E
K
Amino acid

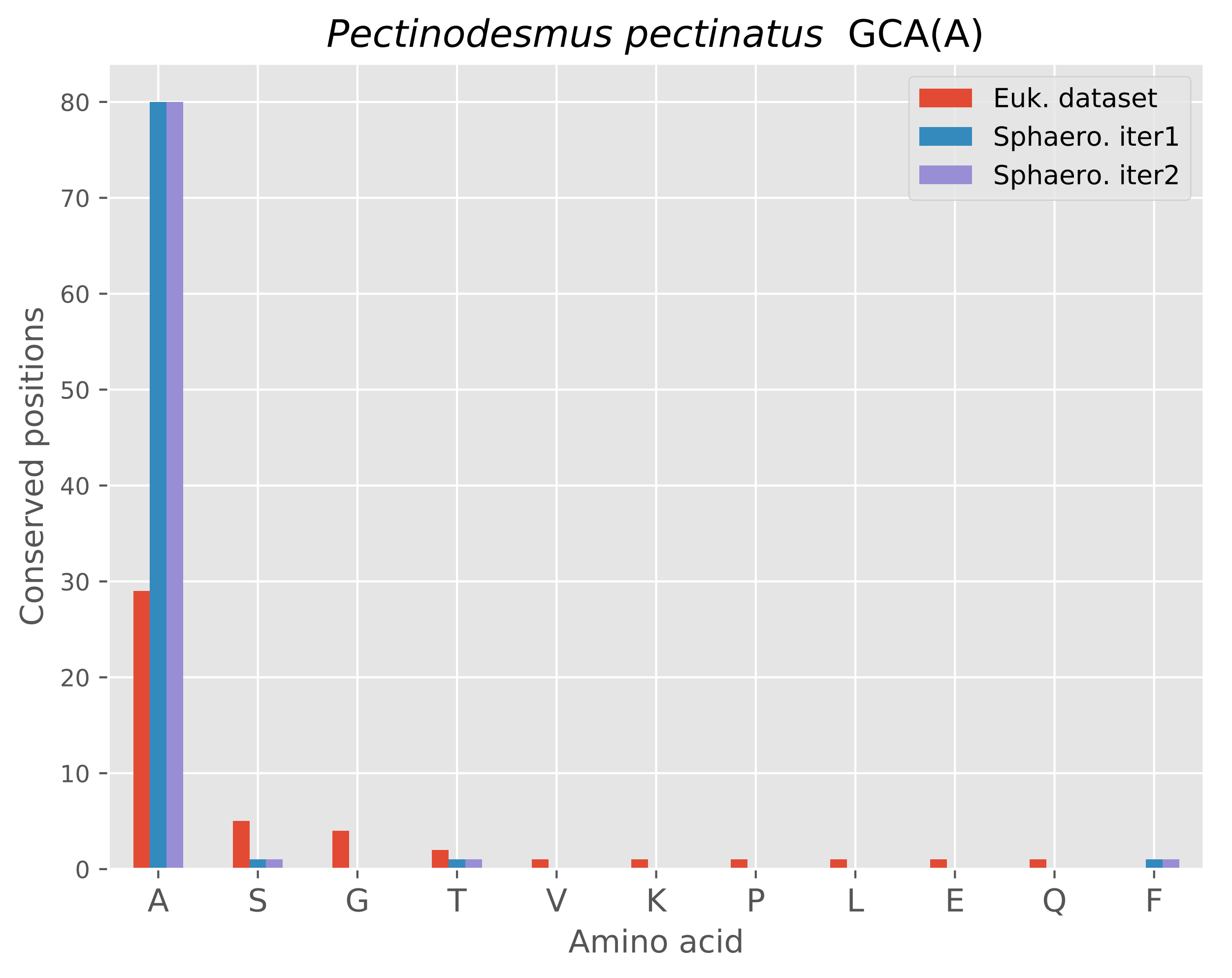

Pectinodesmus pectinatus GCA(A)
Euk. dataset
Sphaero. iter1
Sphaero. iter2
Conserved positions
0
10
20
30
40
50
60
70
80
A
S
G
T
V
K
P
L
E
Q
F
Amino acid

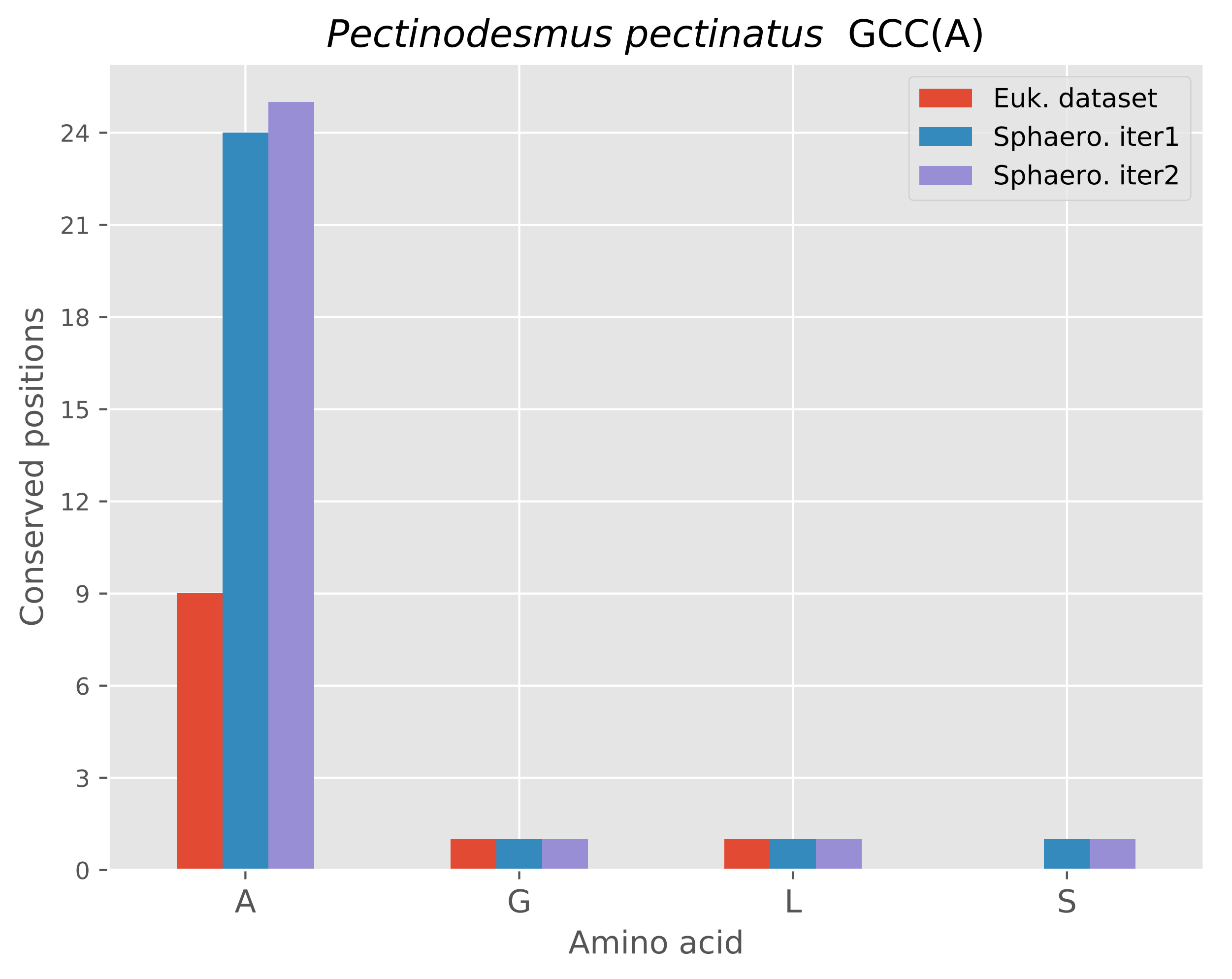

Pectinodesmus pectinatus GCC(A)
Euk. dataset
Sphaero. iter1
Sphaero. iter2
Conserved positions
0
3
6
9
12
15
18
21
24
A
G
L
S
Amino acid

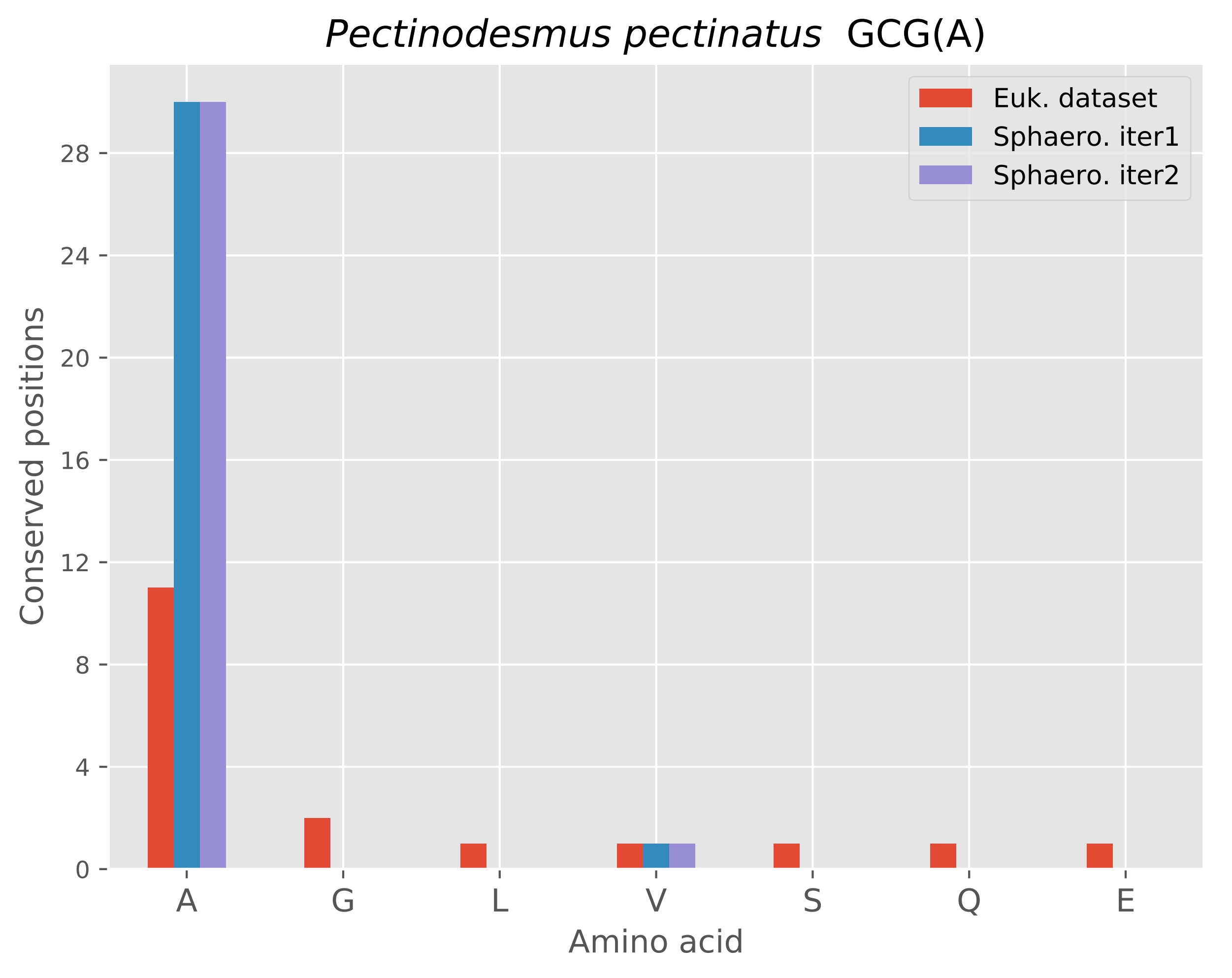

Pectinodesmus pectinatus GCG(A)
Euk. dataset
Sphaero. iter1
Sphaero. iter2
Conserved positions
0
4
8
12
16
20
24
28
A
G
L
V
S
Q
E
Amino acid

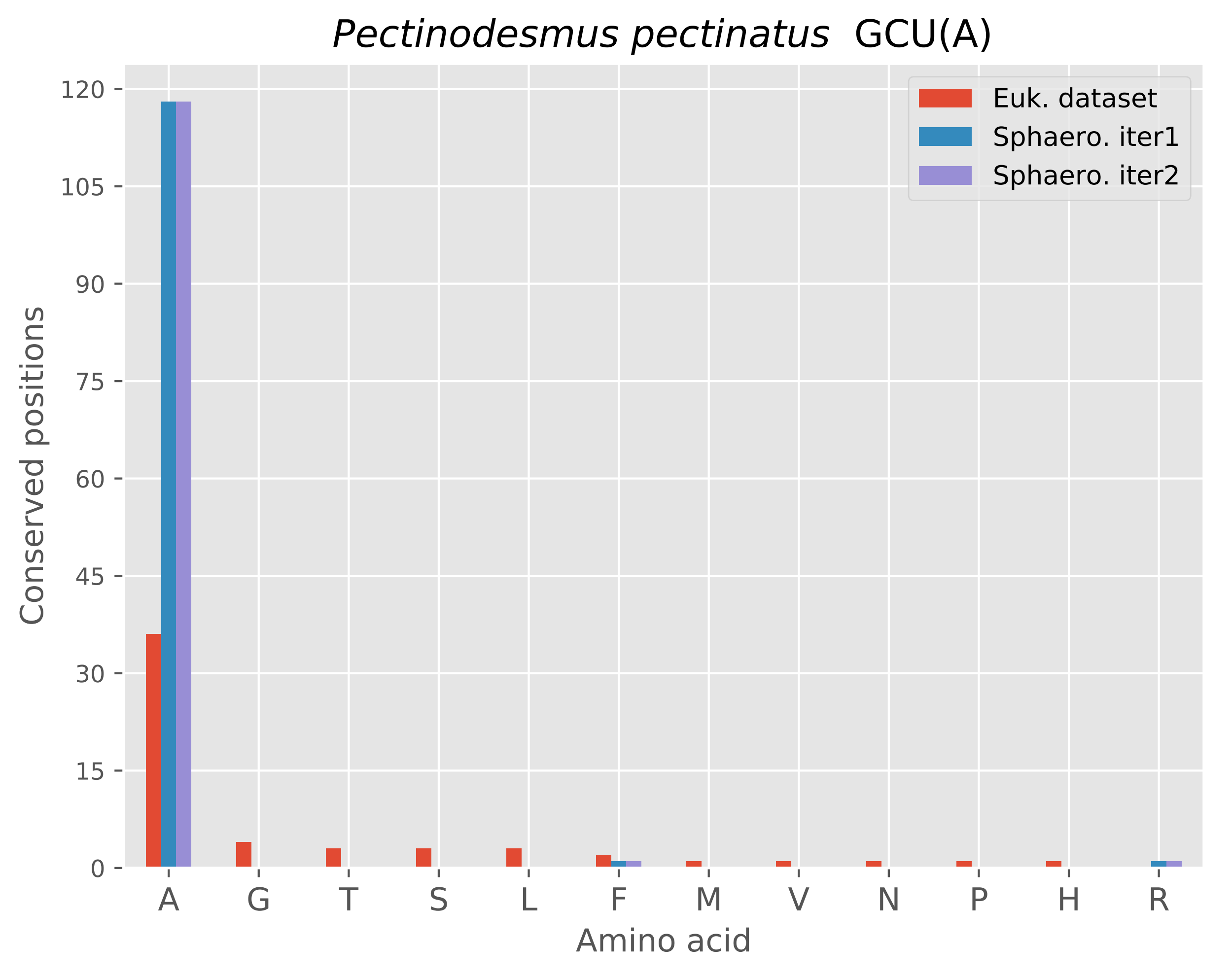

Pectinodesmus pectinatus GCU(A)
Euk. dataset
Sphaero. iter1
Sphaero. iter2
Conserved positions
0
15
30
45
60
75
90
105
120
A
G
T
S
L
F
M
V
N
P
H
R
Amino acid

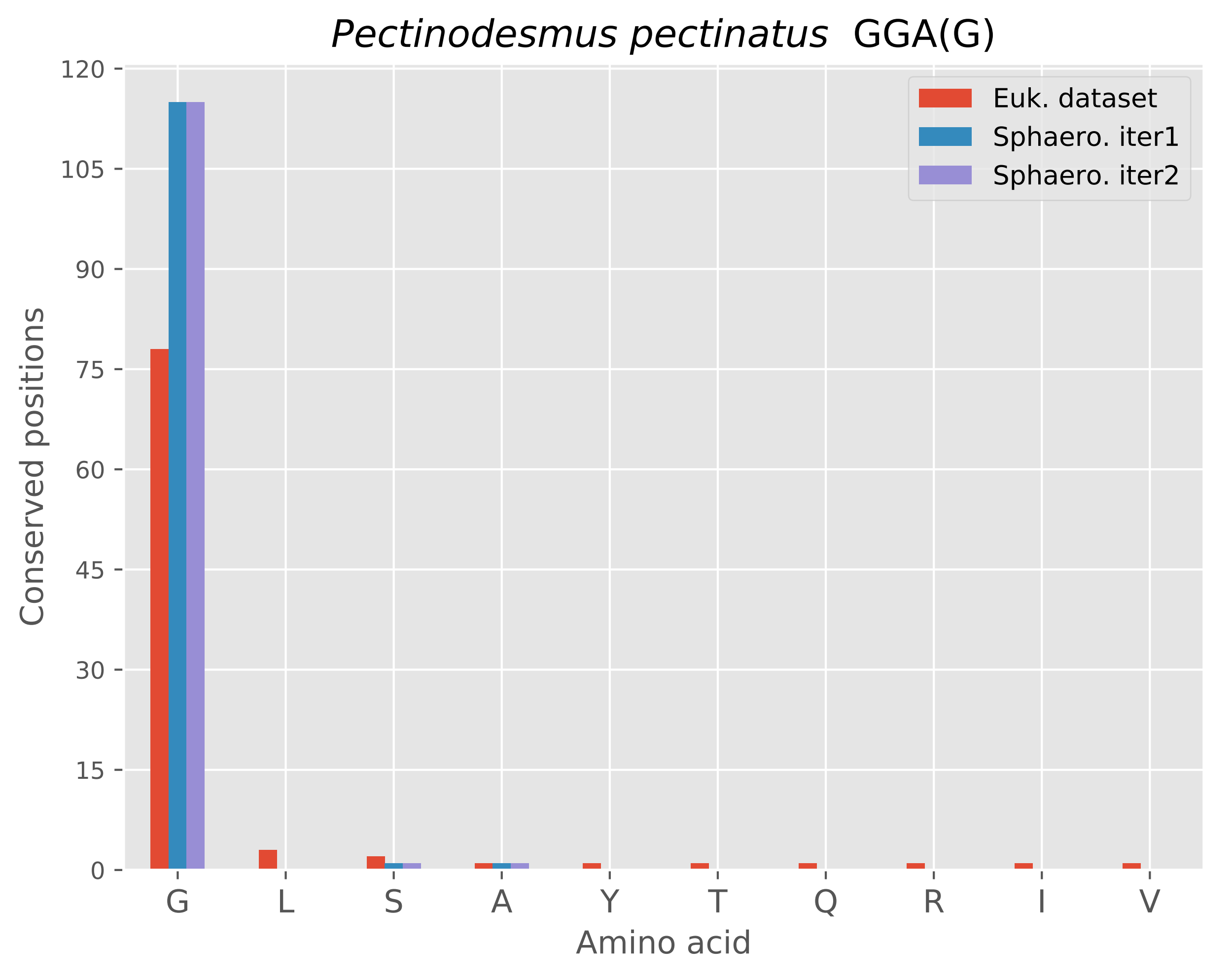

Pectinodesmus pectinatus GGA(G)
Euk. dataset
Sphaero. iter1
Sphaero. iter2
Conserved positions
0
15
30
45
60
75
90
105
120
G
L
S
A
Y
T
Q
R
I
V
Amino acid

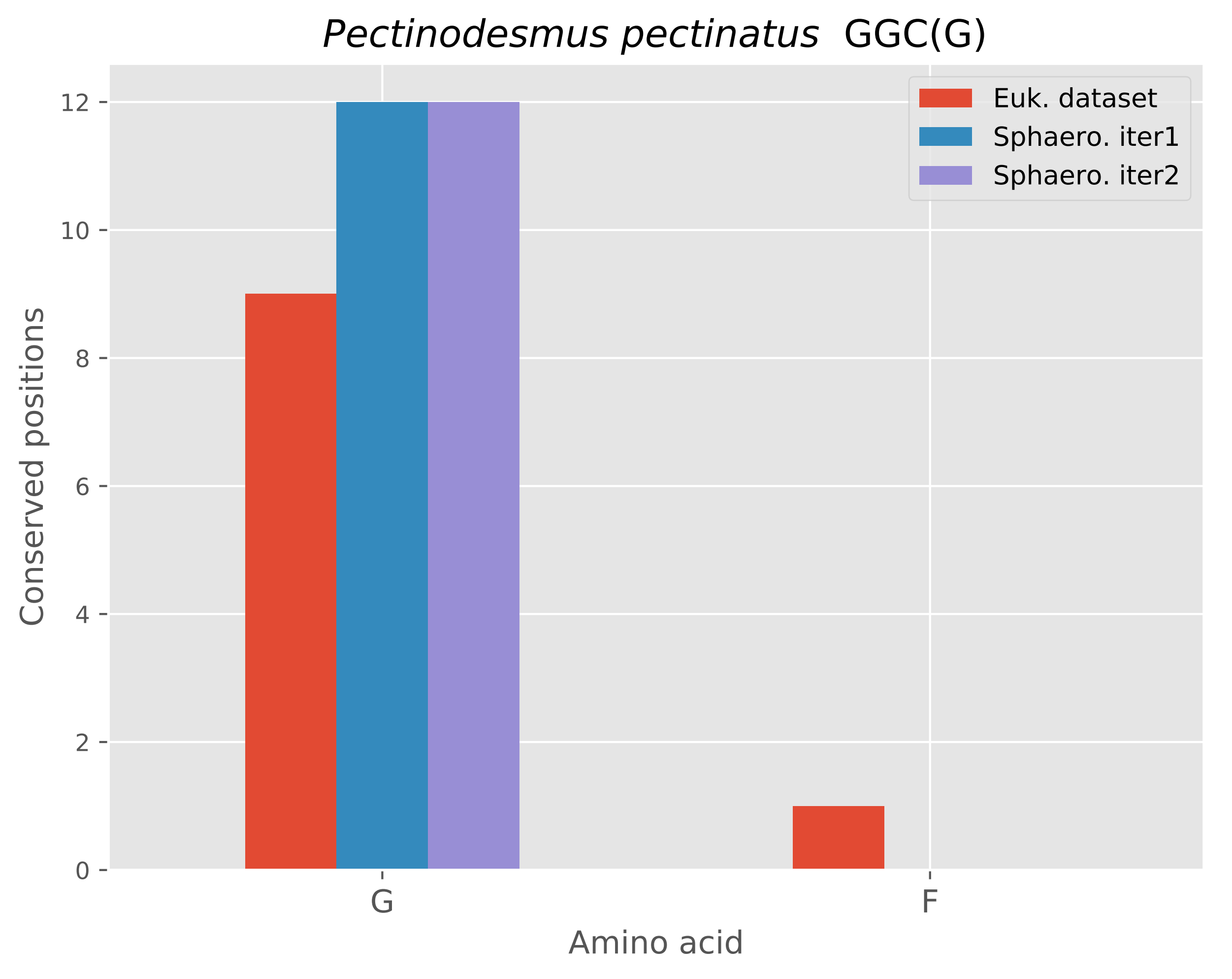

Pectinodesmus pectinatus GGC(G)
Euk. dataset
Sphaero. iter1
Sphaero. iter2
Conserved positions
0
2
4
6
8
10
12
G
F
Amino acid

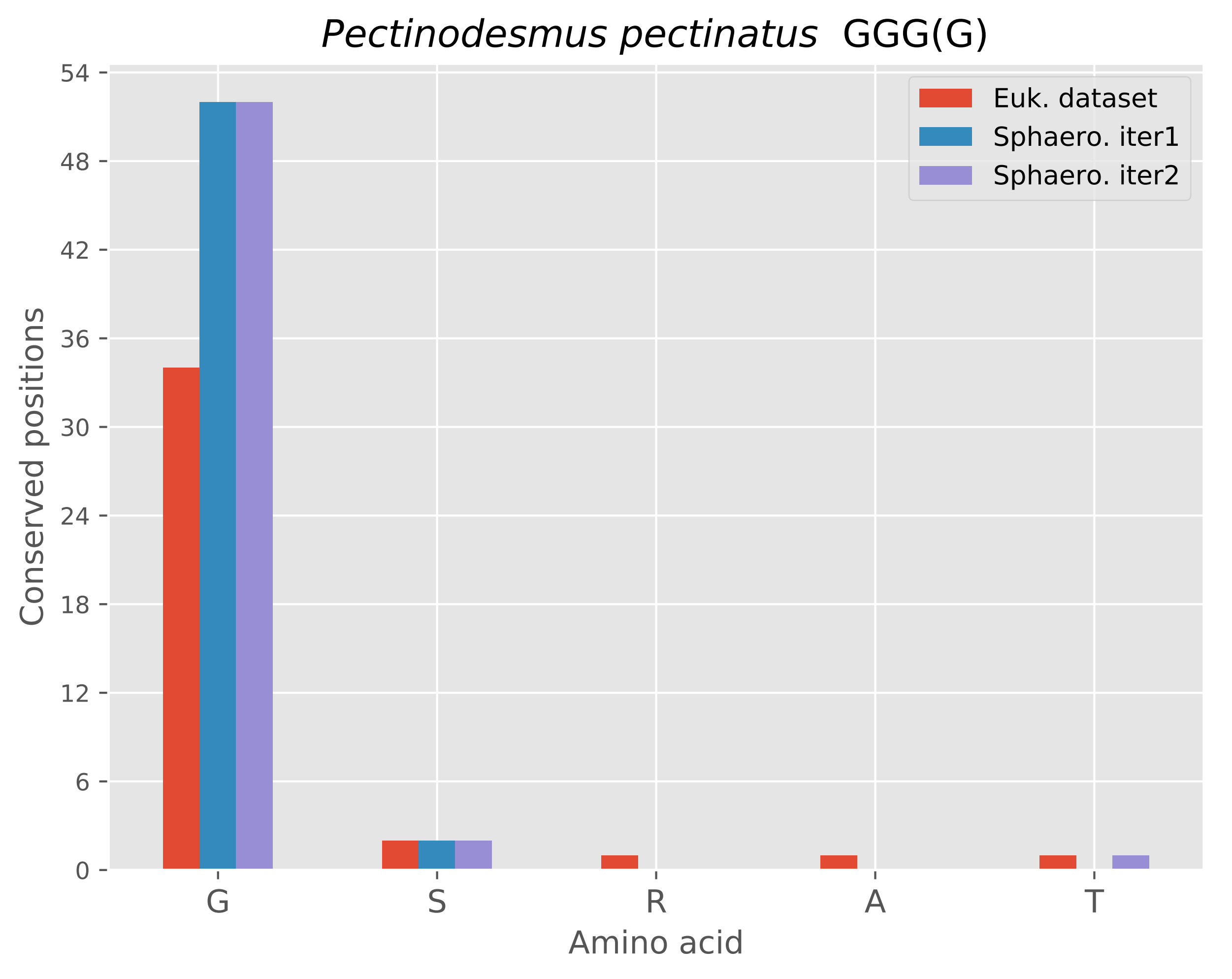

Pectinodesmus pectinatus GGG(G)
Conserved positions
Amino acid
0
6
12
18
24
30
36
42
48
54
G
S
R
A
T
Euk. dataset
Sphaero. iter1
Sphaero. iter2

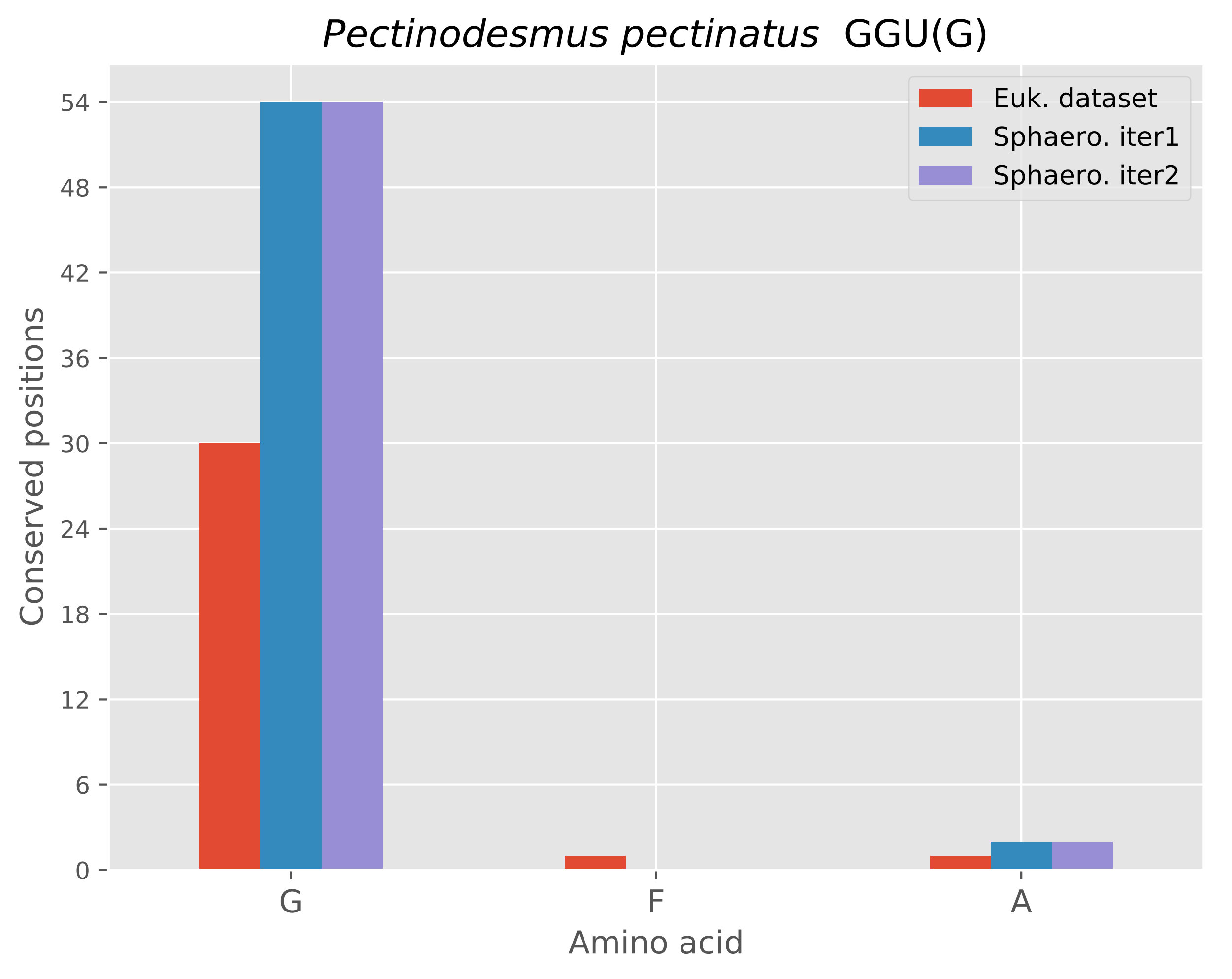
Pectinodesmus pectinatus GGU(G)
Euk. dataset
Sphaero. iter1
Sphaero. iter2
Conserved positions
0
6
12
18
24
30
36
42
48
54
G
F
A
Amino acid

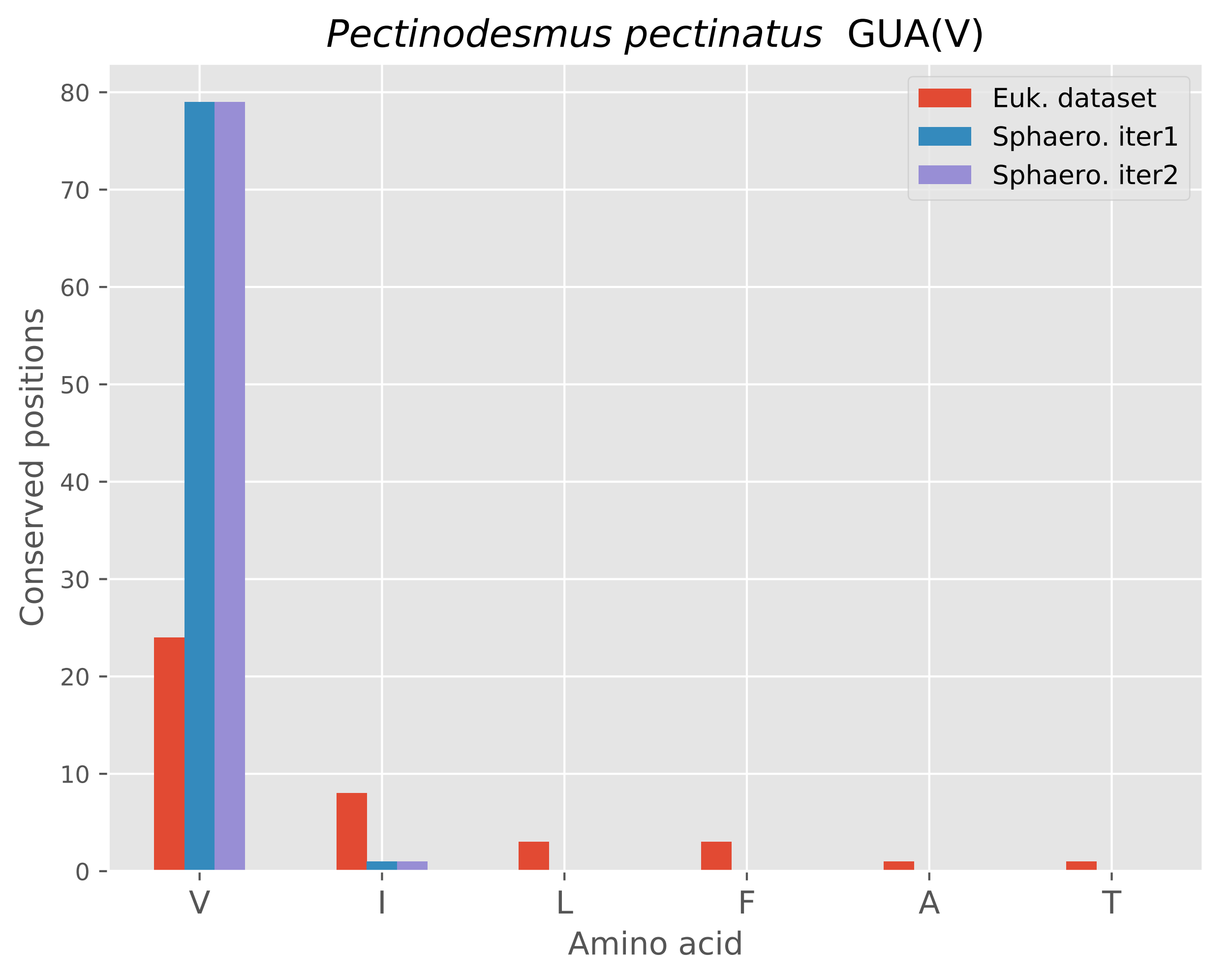
Pectinodesmus pectinatus GUA(V)
Euk. dataset
Sphaero. iter1
Sphaero. iter2
Conserved positions
0
10
20
30
40
50
60
70
80
V
I
L
F
A
T
Amino acid

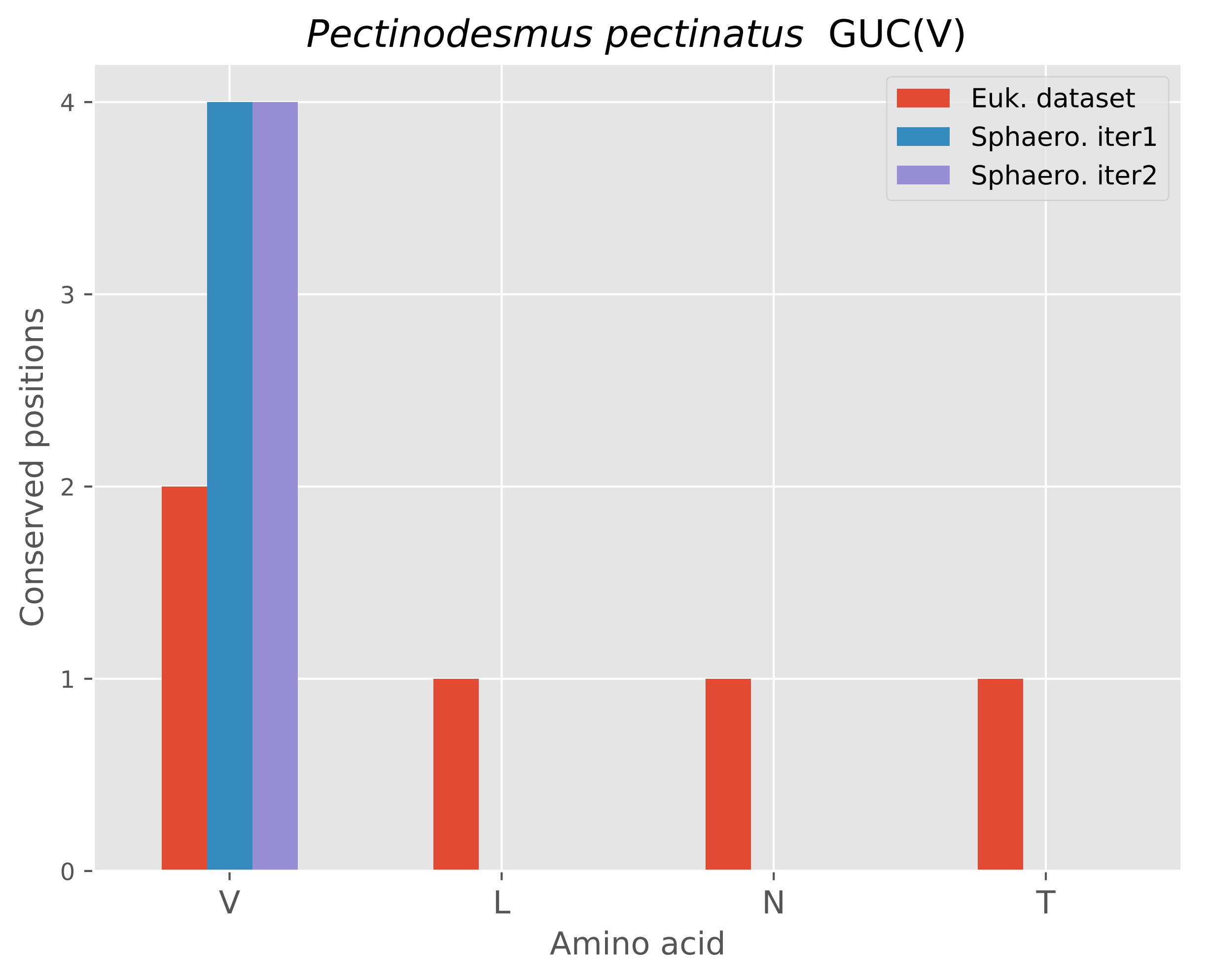

Pectinodesmus pectinatus GUC(V)
Euk. dataset
Sphaero. iter1
Sphaero. iter2
Conserved positions
0
1
2
3
4
V
L
N
T
Amino acid

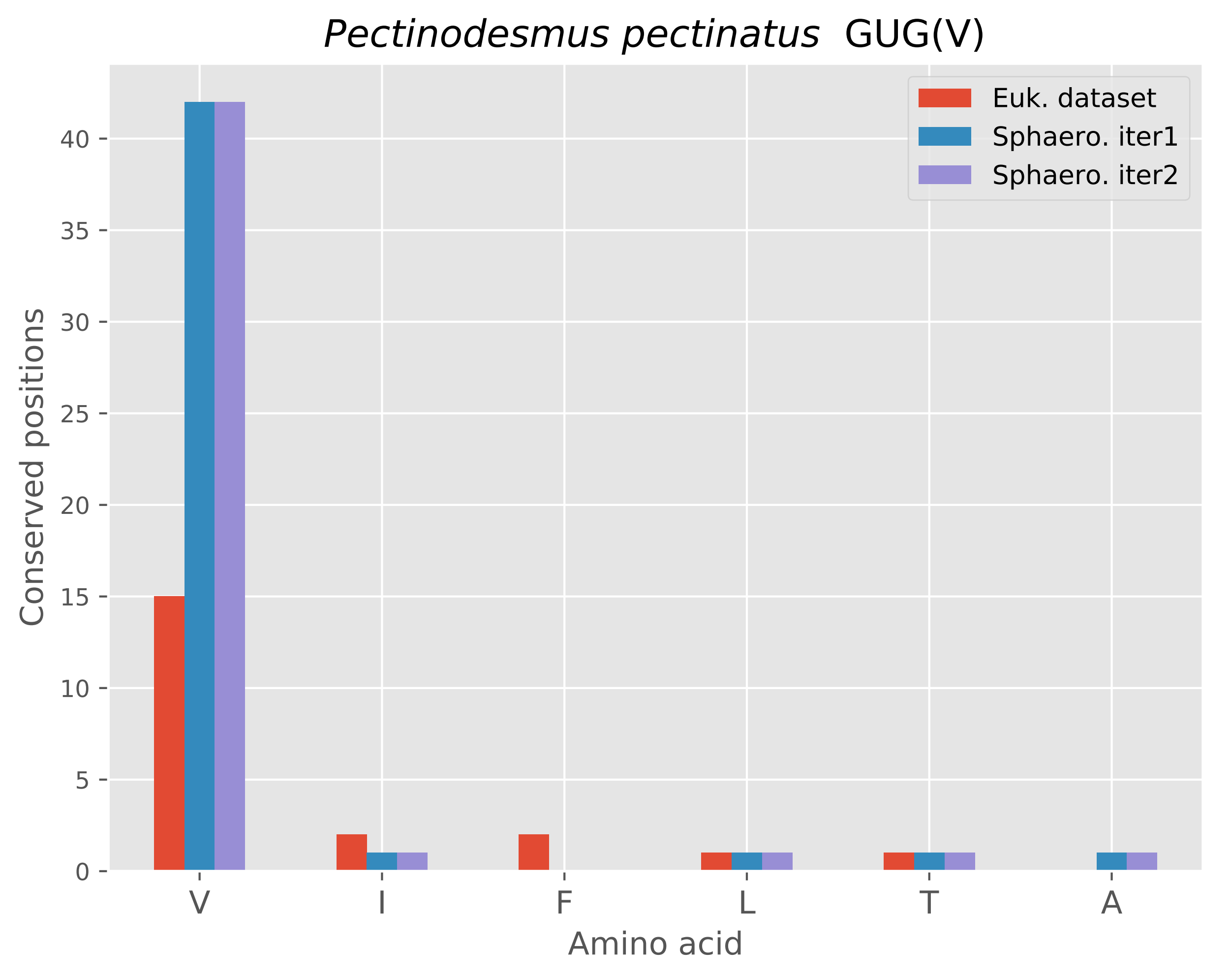

Pectinodesmus pectinatus GUG(V)
Euk. dataset
Sphaero. iter1
Sphaero. iter2
Conserved positions
0
5
10
15
20
25
30
35
40
V
I
F
L
T
A
Amino acid

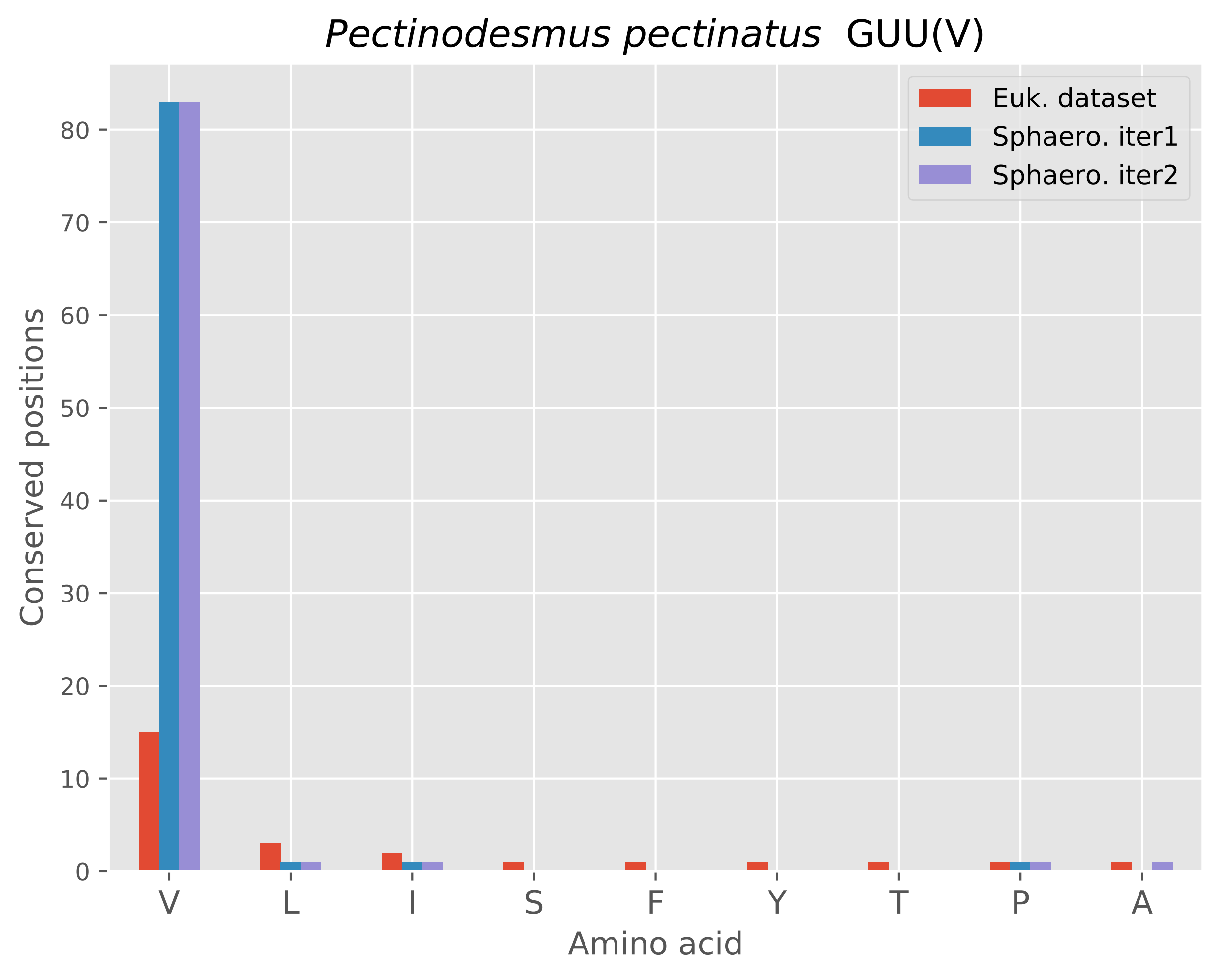

Pectinodesmus pectinatus GUU(V)
Euk. dataset
Sphaero. iter1
Sphaero. iter2
Conserved positions
0
10
20
30
40
50
60
70
80
V
L
I
S
F
Y
T
P
A
Amino acid

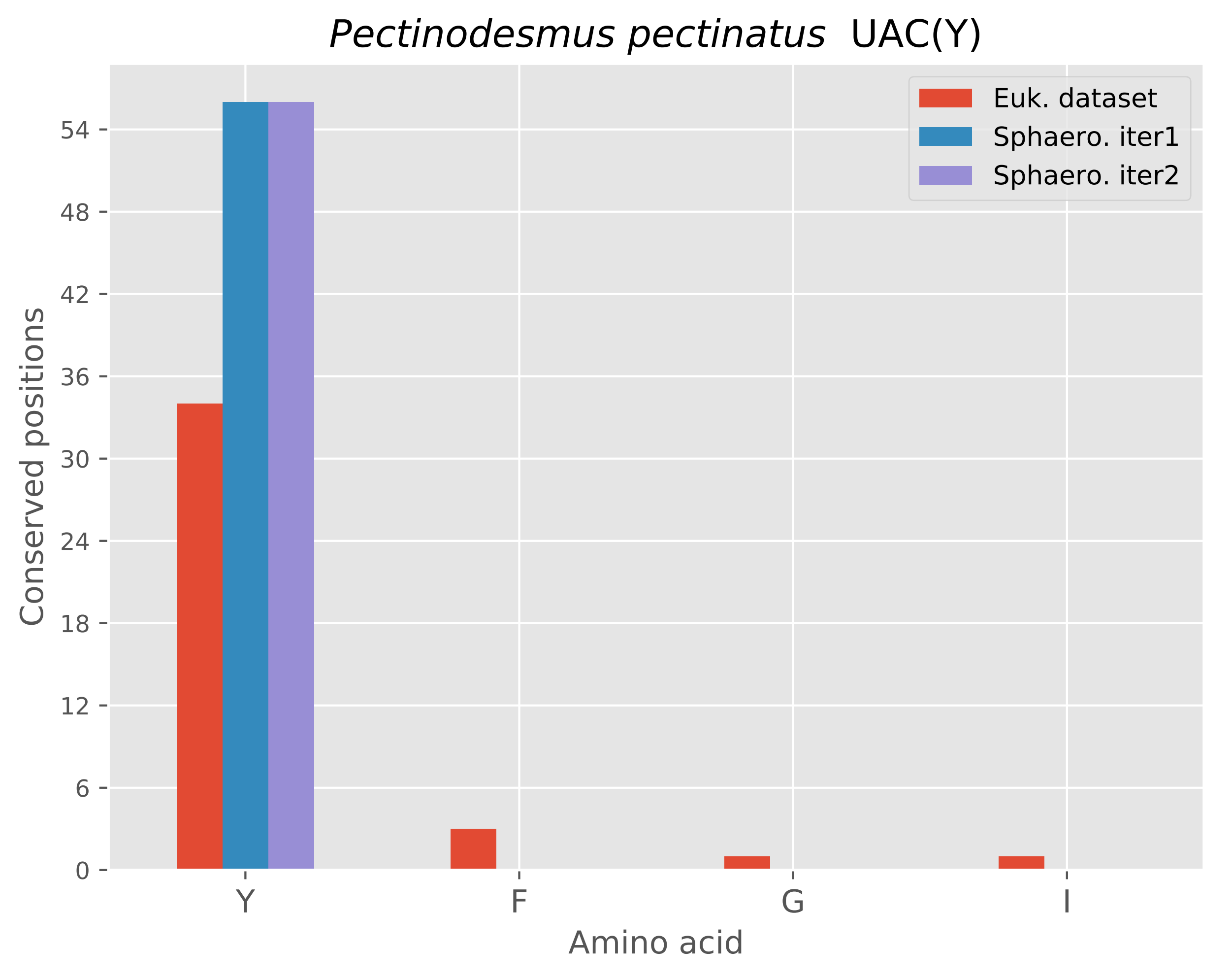

Pectinodesmus pectinatus UAC(Y)
Euk. dataset
Sphaero. iter1
Sphaero. iter2
Conserved positions
0
6
12
18
24
30
36
42
48
54
Y
F
G
I
Amino acid

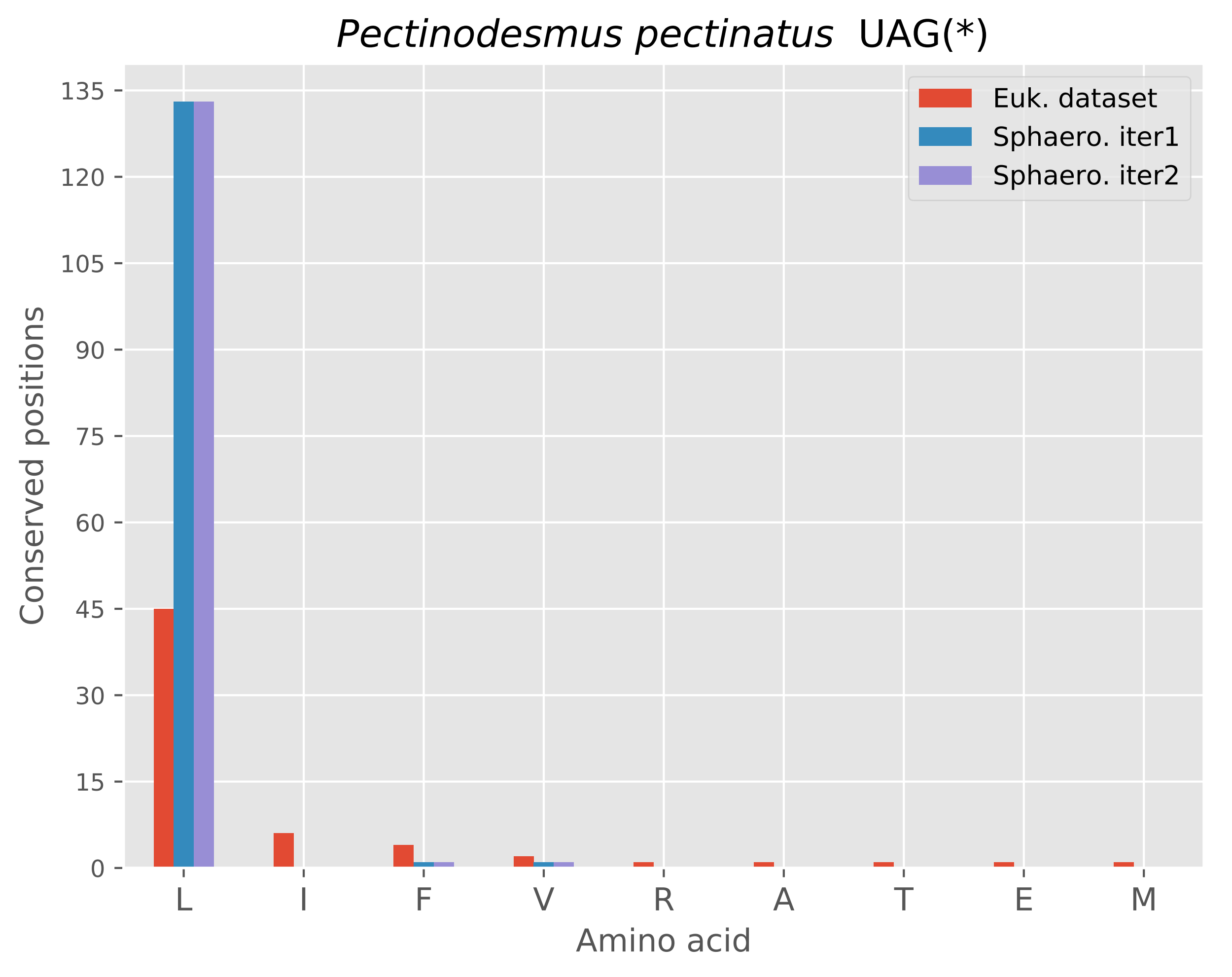

Pectinodesmus pectinatus UAG(*)
Euk. dataset
Sphaero. iter1
Sphaero. iter2
Conserved positions
135
120
105
90
75
60
45
30
15
0
L
I
F
V
R
A
T
E
M
Amino acid

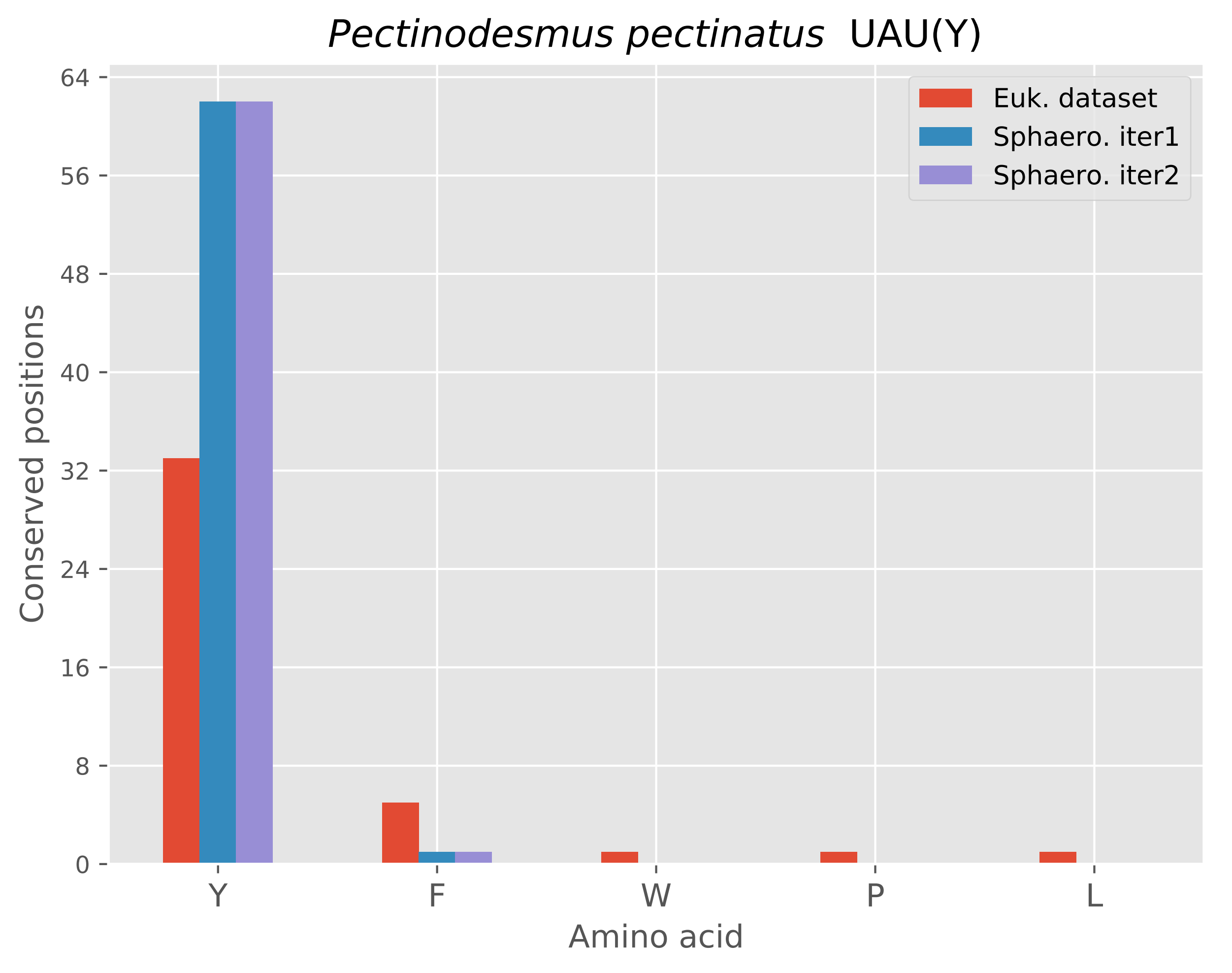

Pectinodesmus pectinatus UAU(Y)
Euk. dataset
Sphaero. iter1
Sphaero. iter2
Conserved positions
0
8
16
24
32
40
48
56
64
Y
F
W
P
L
Amino acid

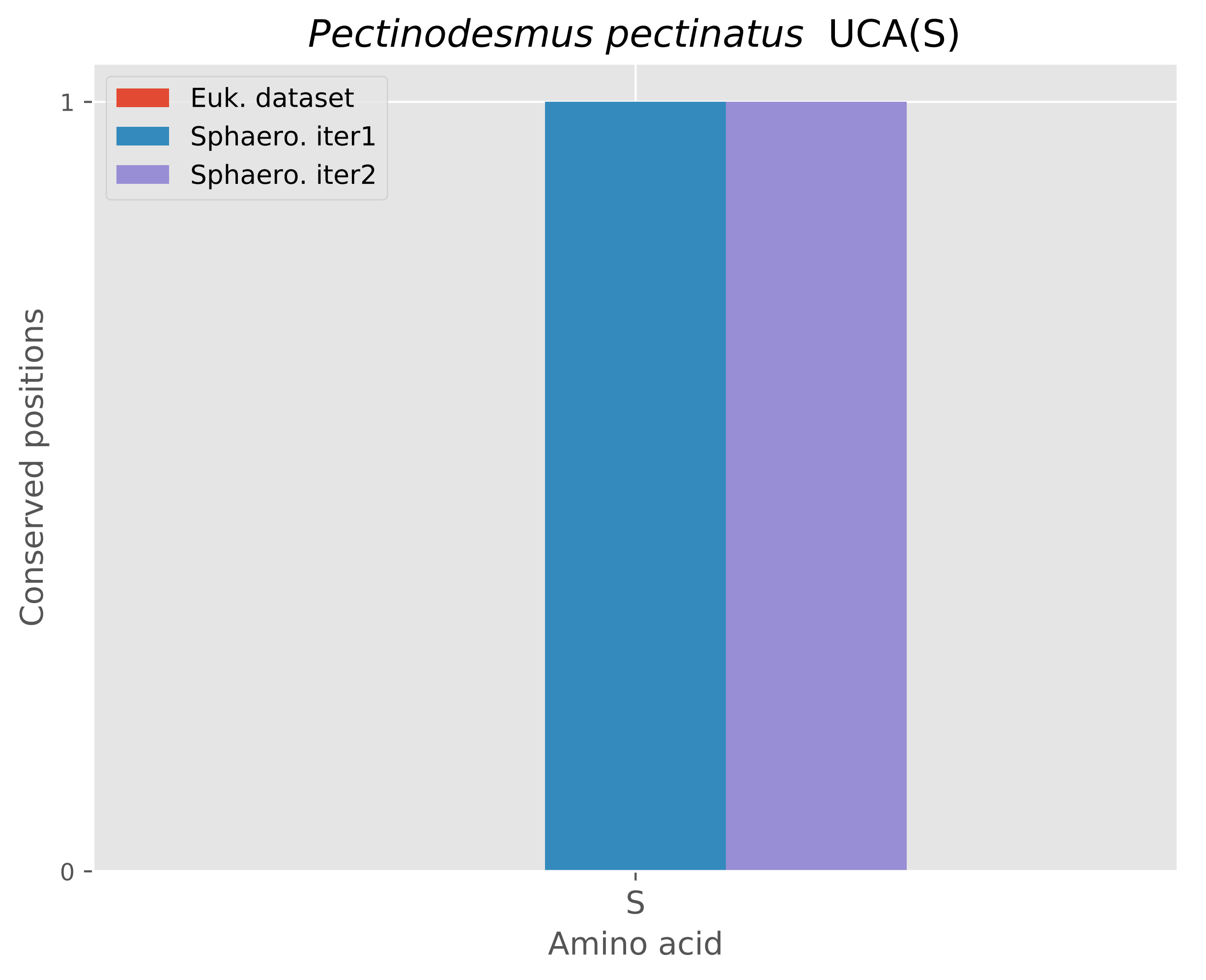

Pectinodesmus pectinatus UCA(S)
Euk. dataset
Sphaero. iter1
Sphaero. iter2
1
0
Conserved positions
S
Amino acid

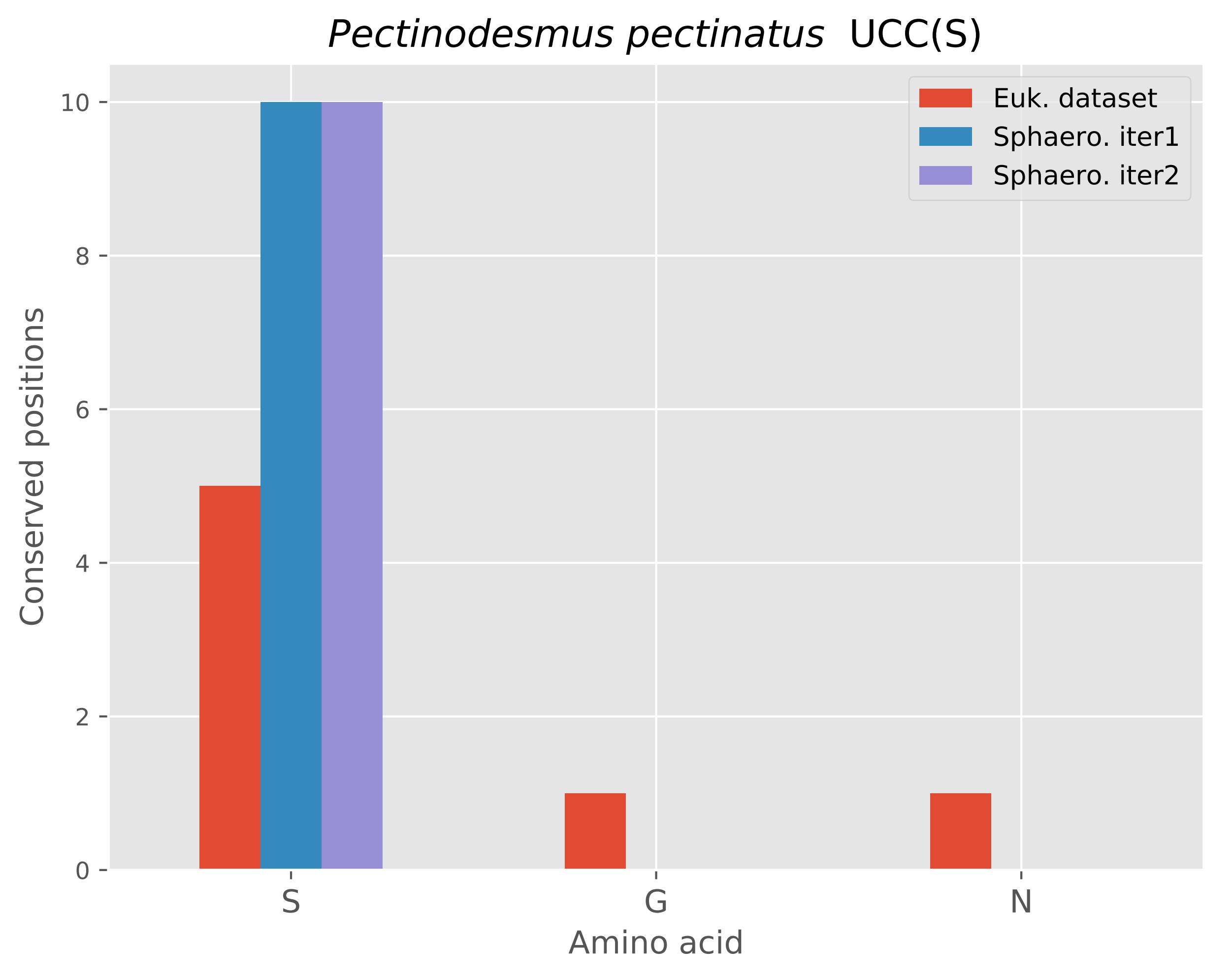

Pectinodesmus pectinatus UCC(S)
Euk. dataset
Sphaero. iter1
Sphaero. iter2
Conserved positions
0
2
4
6
8
10
S
G
N
Amino acid

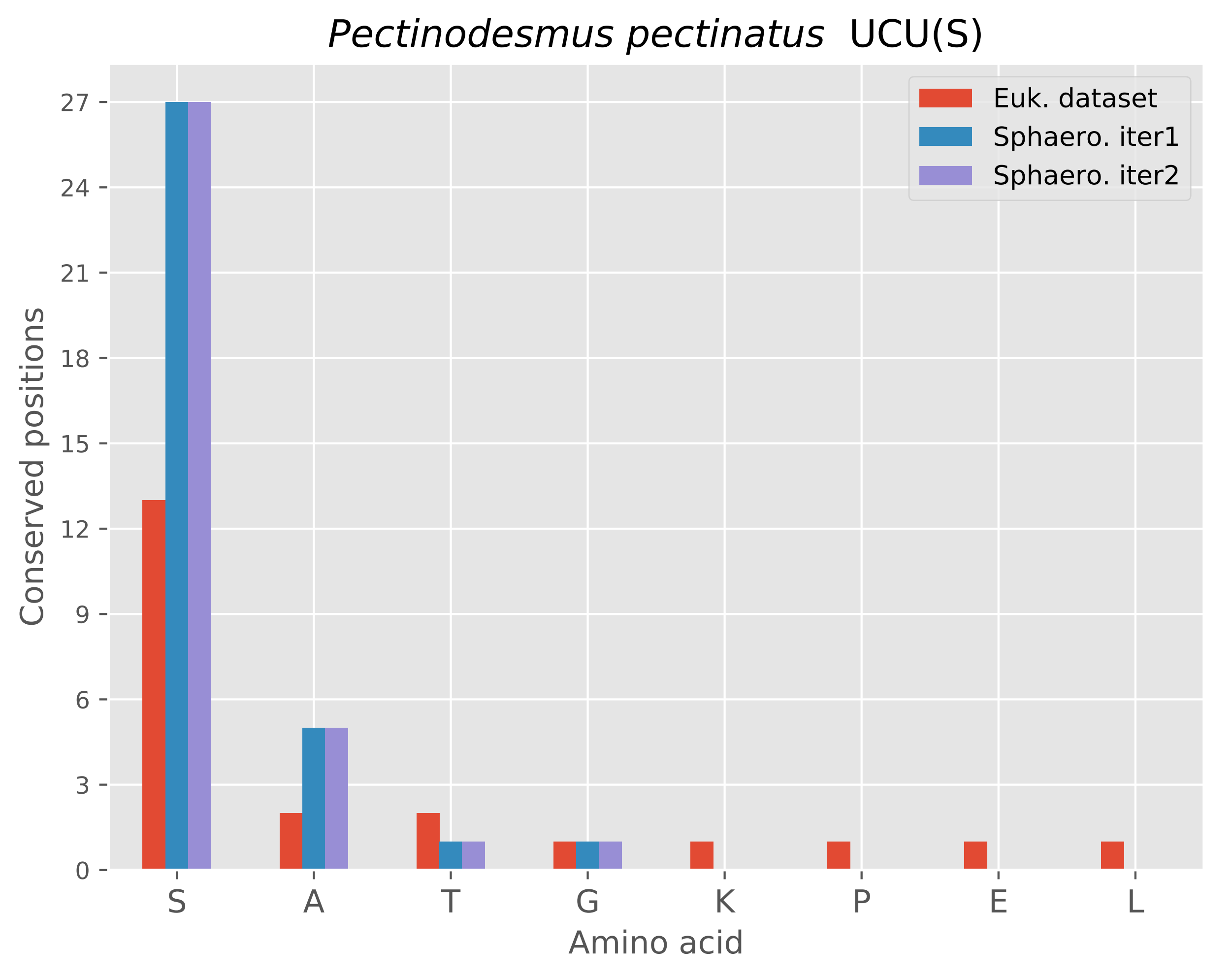

Pectinodesmus pectinatus UCU(S)
Euk. dataset
Sphaero. iter1
Sphaero. iter2
Conserved positions
0
3
6
9
12
15
18
21
24
27
S
A
T
G
K
P
E
L
Amino acid

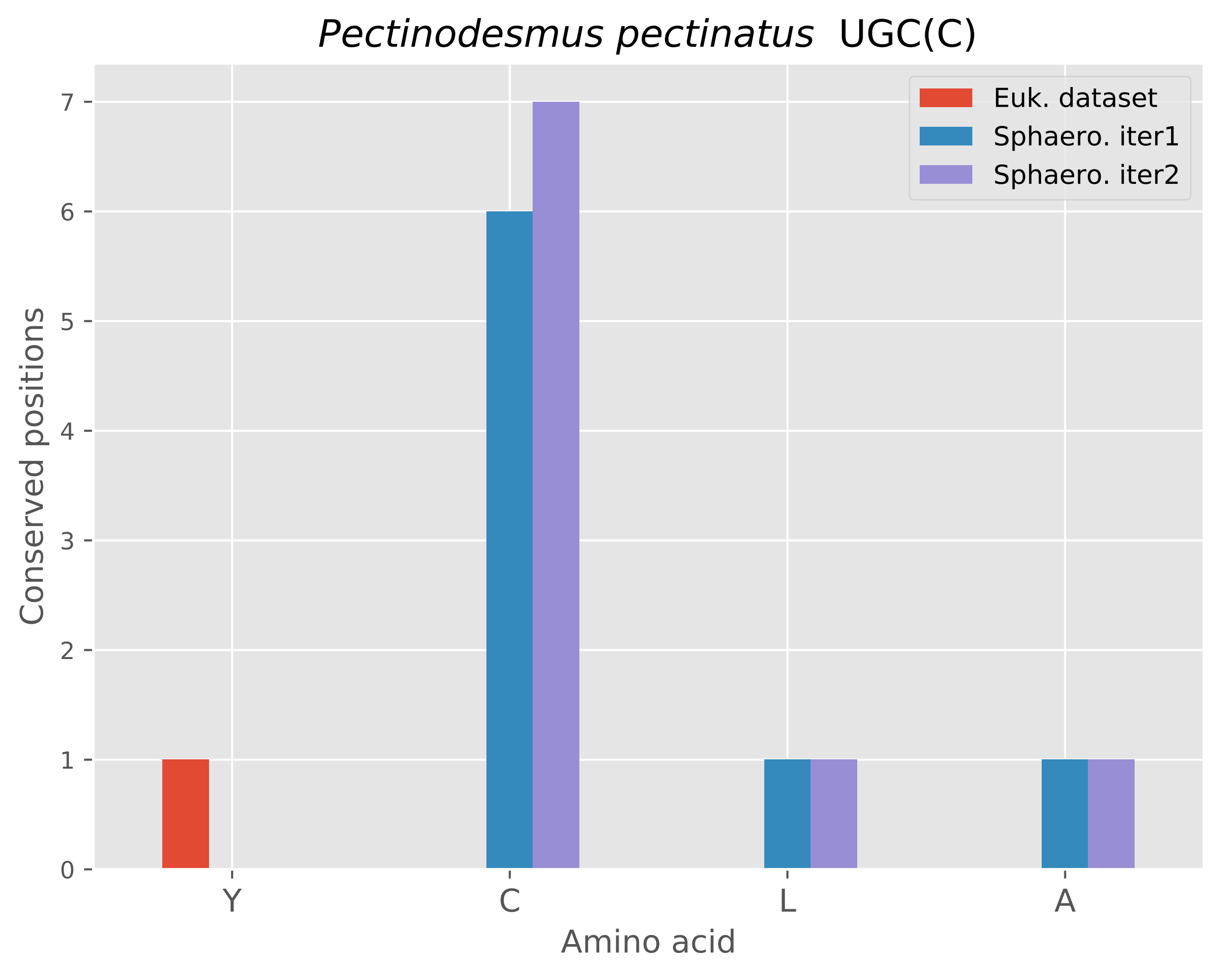

Pectinodesmus pectinatus UGC(C)
Euk. dataset
Sphaero. iter1
Sphaero. iter2
Conserved positions
0
1
2
3
4
5
6
7
Y
C
L
A
Amino acid

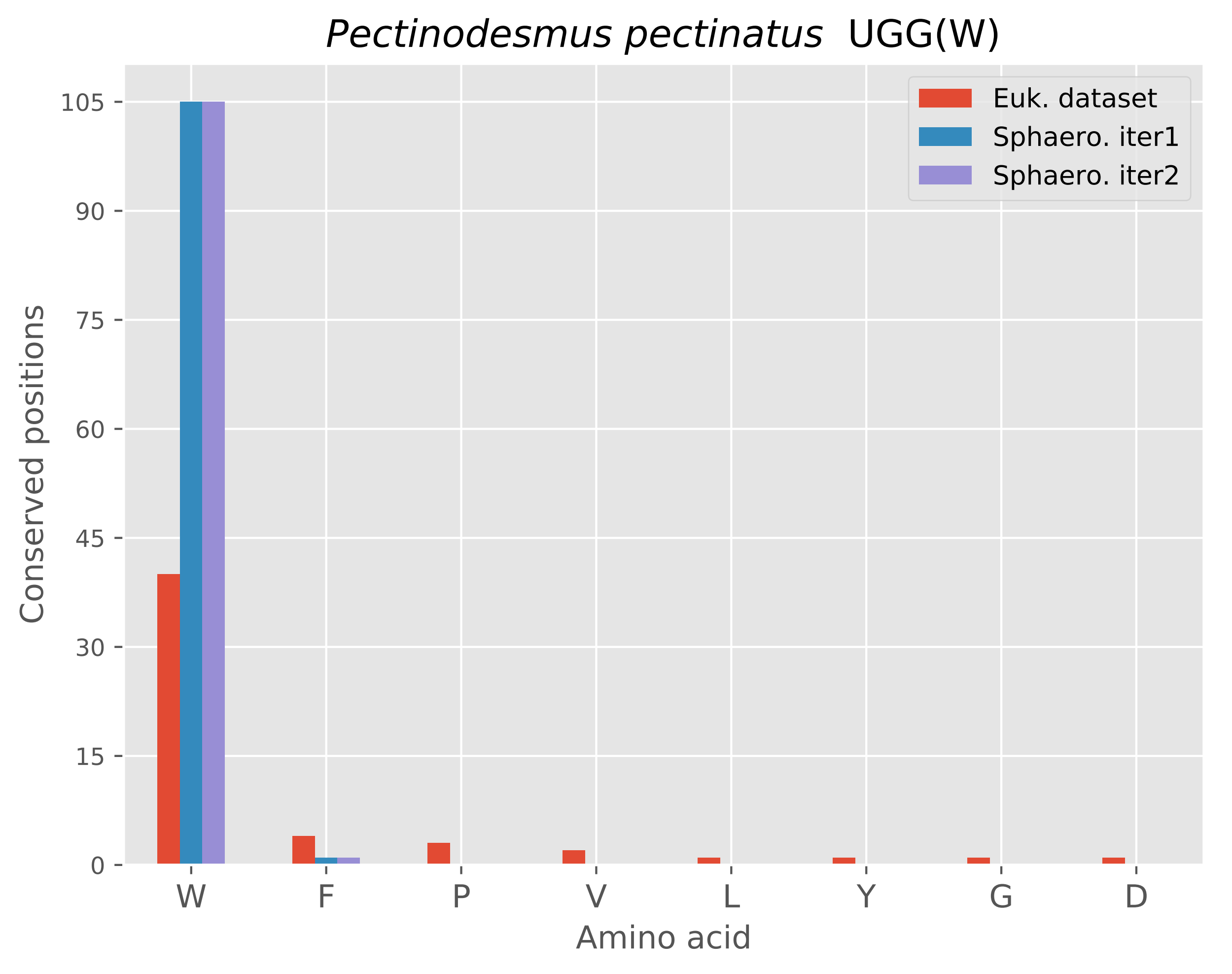

Pectinodesmus pectinatus UGG(W)
Euk. dataset
Sphaero. iter1
Sphaero. iter2
Conserved positions
0
15
30
45
60
75
90
105
W
F
P
V
L
Y
G
D
Amino acid

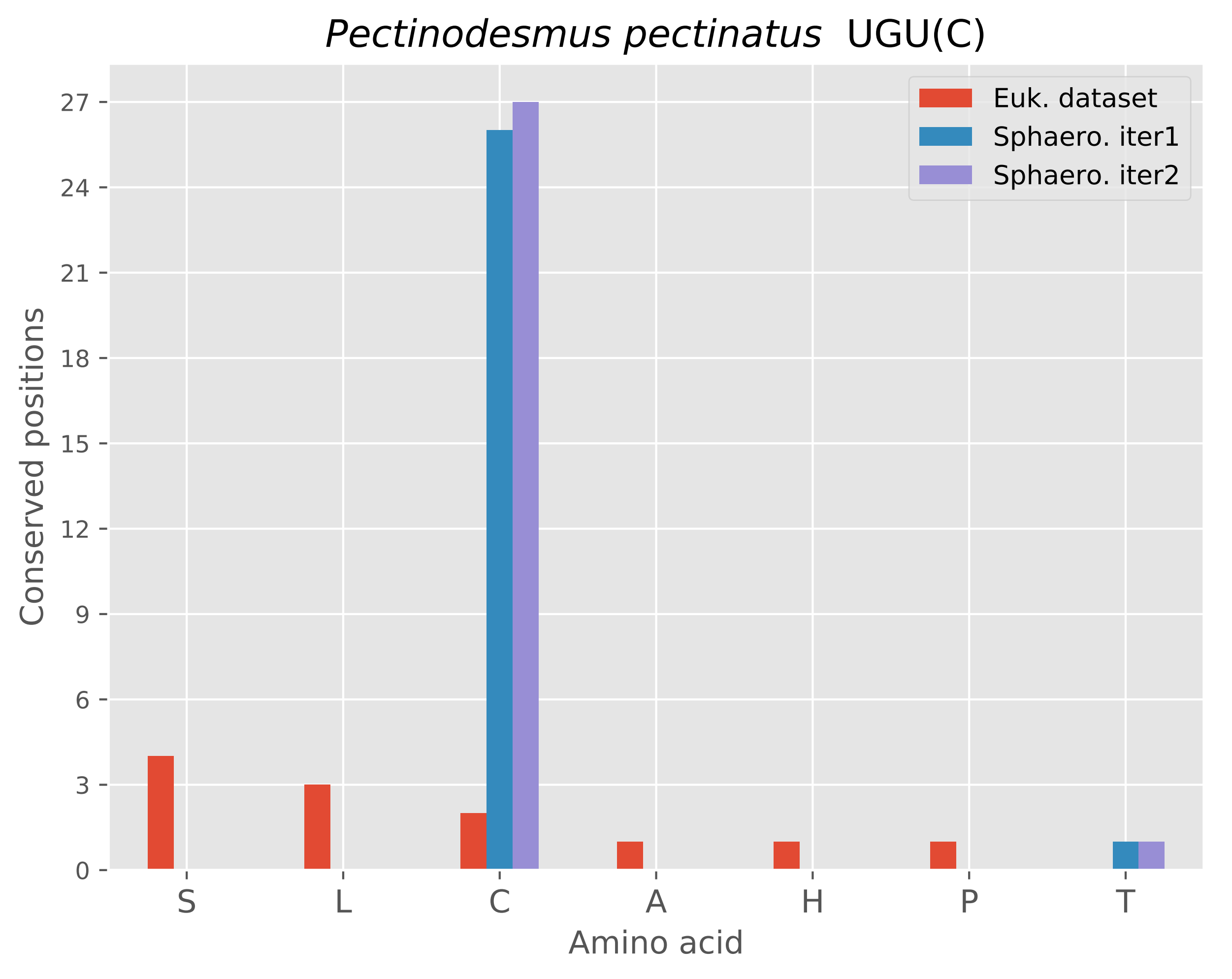

Pectinodesmus pectinatus UGU(C)
Euk. dataset
Sphaero. iter1
Sphaero. iter2
Conserved positions
0
3
6
9
12
15
18
21
24
27
S
L
C
A
H
P
T
Amino acid

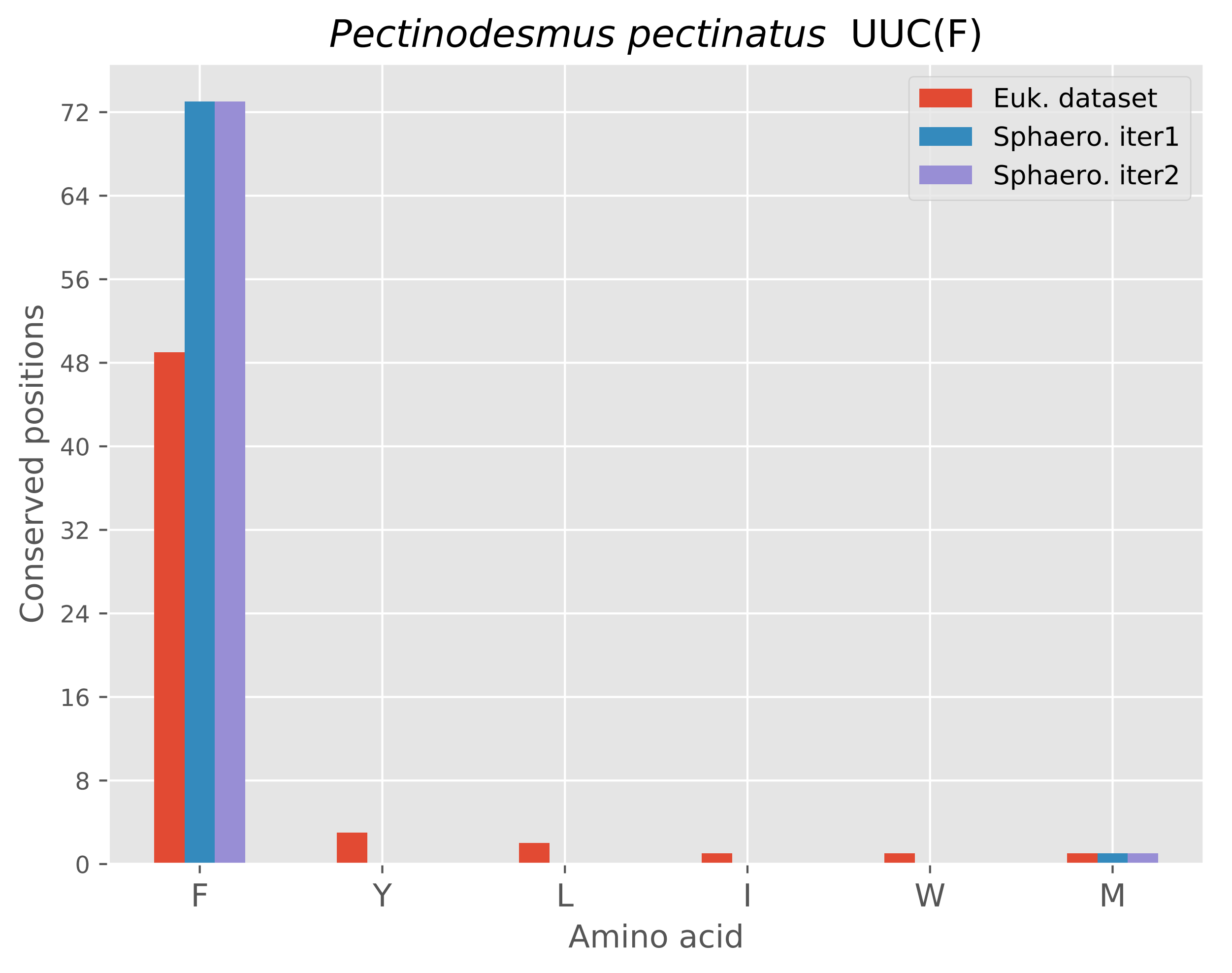

Pectinodesmus pectinatus UUC(F)
Euk. dataset
Sphaero. iter1
Sphaero. iter2
Conserved positions
0
8
16
24
32
40
48
56
64
72
F
Y
L
I
W
M
Amino acid

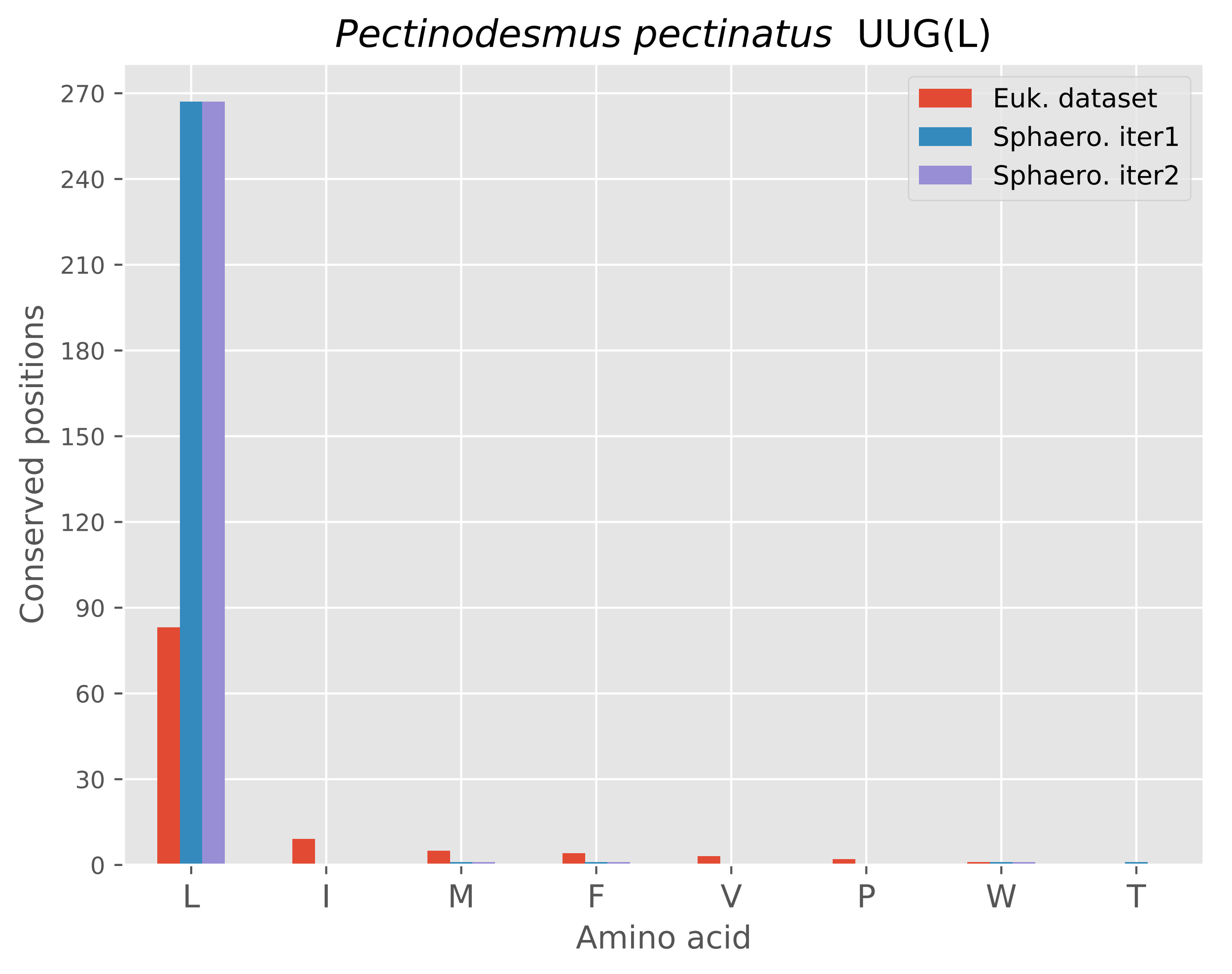

Pectinodesmus pectinatus UUG(L)
Euk. dataset
Sphaero. iter1
Sphaero. iter2
Conserved positions
0
30
60
90
120
150
180
210
240
270
L
I
M
F
V
P
W
T
Amino acid

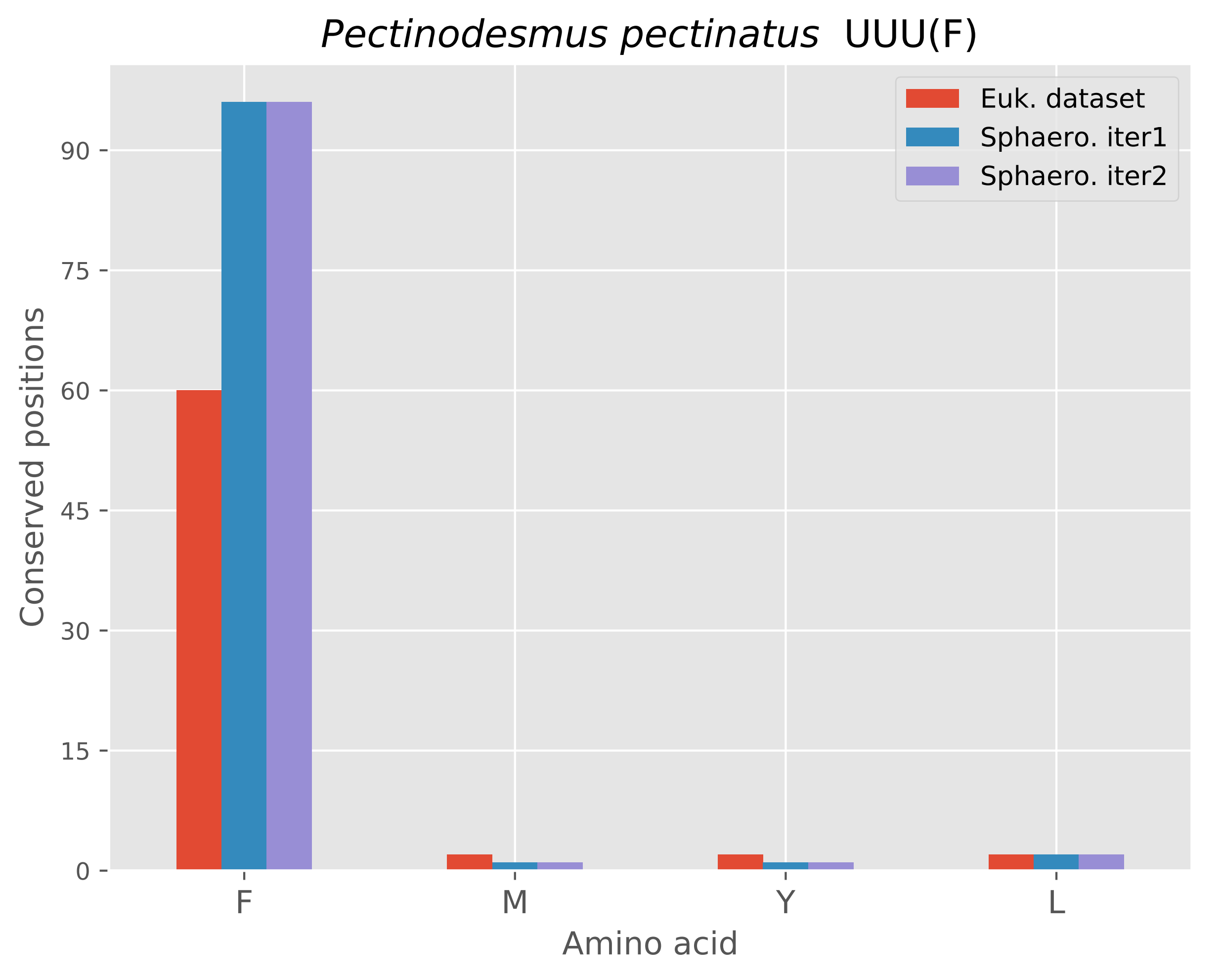

Pectinodesmus pectinatus UUU(F)
Euk. dataset
Sphaero. iter1
Sphaero. iter2
Conserved positions
0
15
30
45
60
75
90
F
M
Y
L
Amino acid

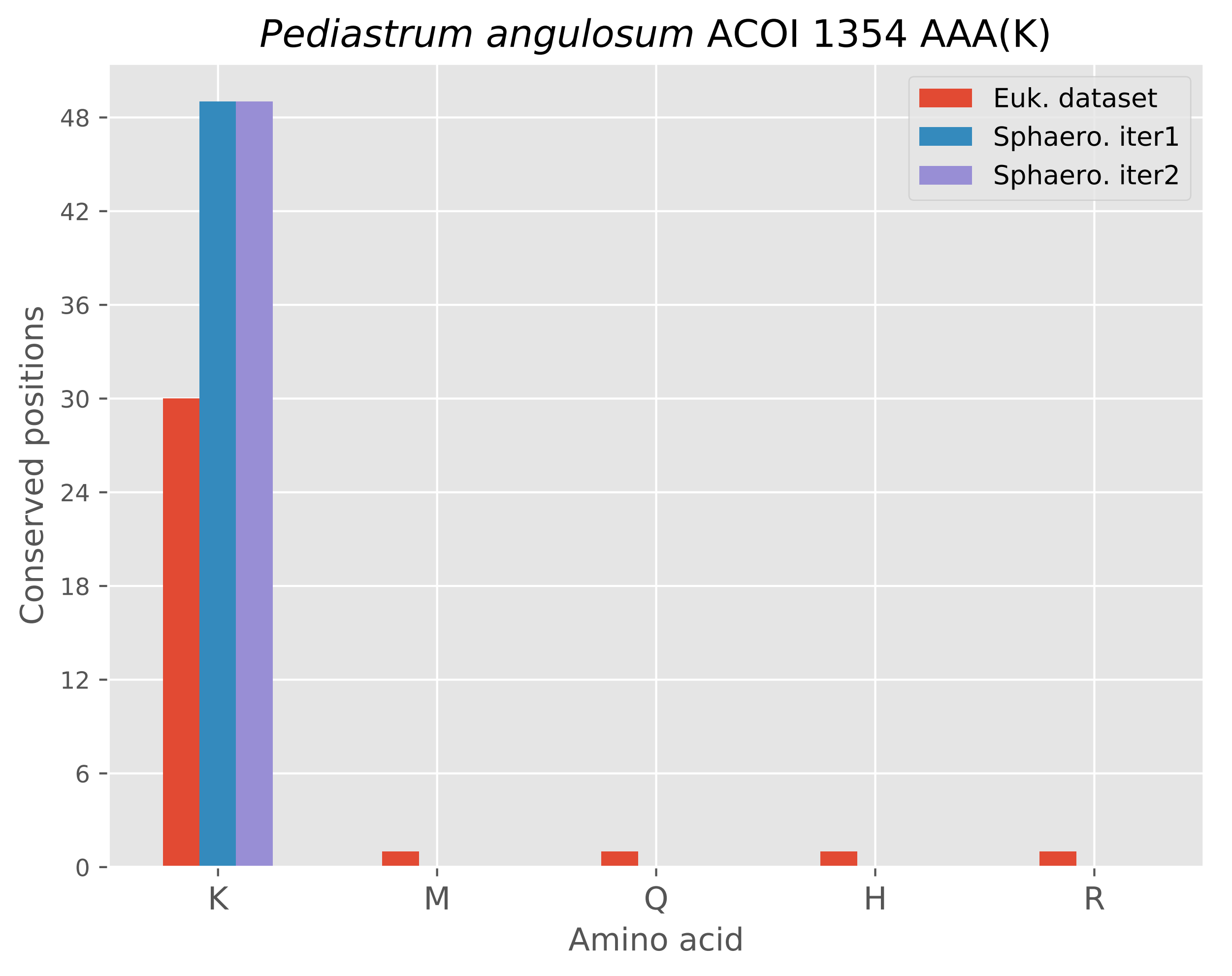

Pediastrum angulosum ACOI 1354 AAA(K)
Euk. dataset
Sphaero. iter1
Sphaero. iter2
Conserved positions
0
6
12
18
24
30
36
42
48
K
M
Q
H
R
Amino acid

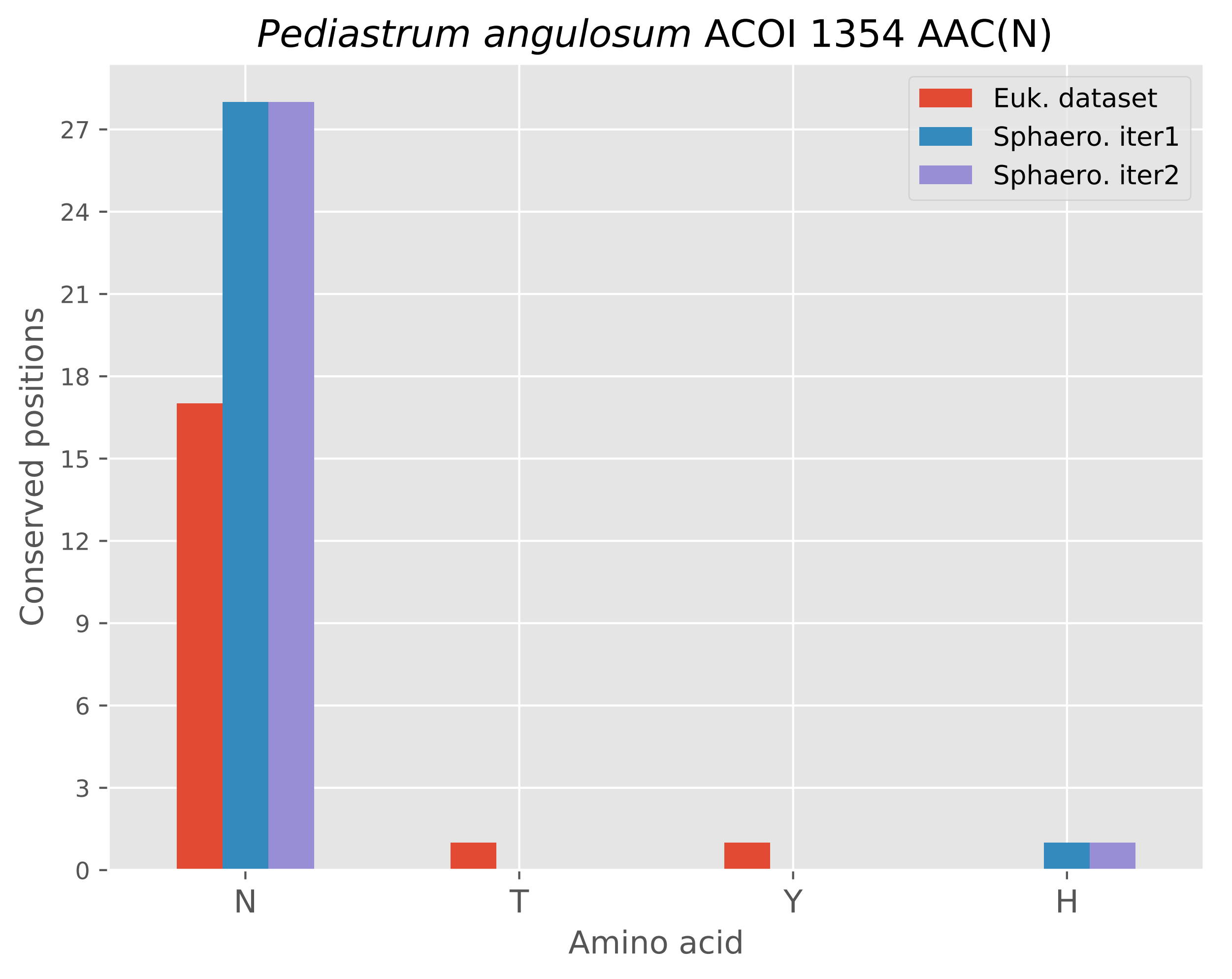

Pediastrum angulosum ACOI 1354 AAC(N)
Euk. dataset
Sphaero. iter1
Sphaero. iter2
Conserved positions
0
3
6
9
12
15
18
21
24
27
N
T
Y
H
Amino acid

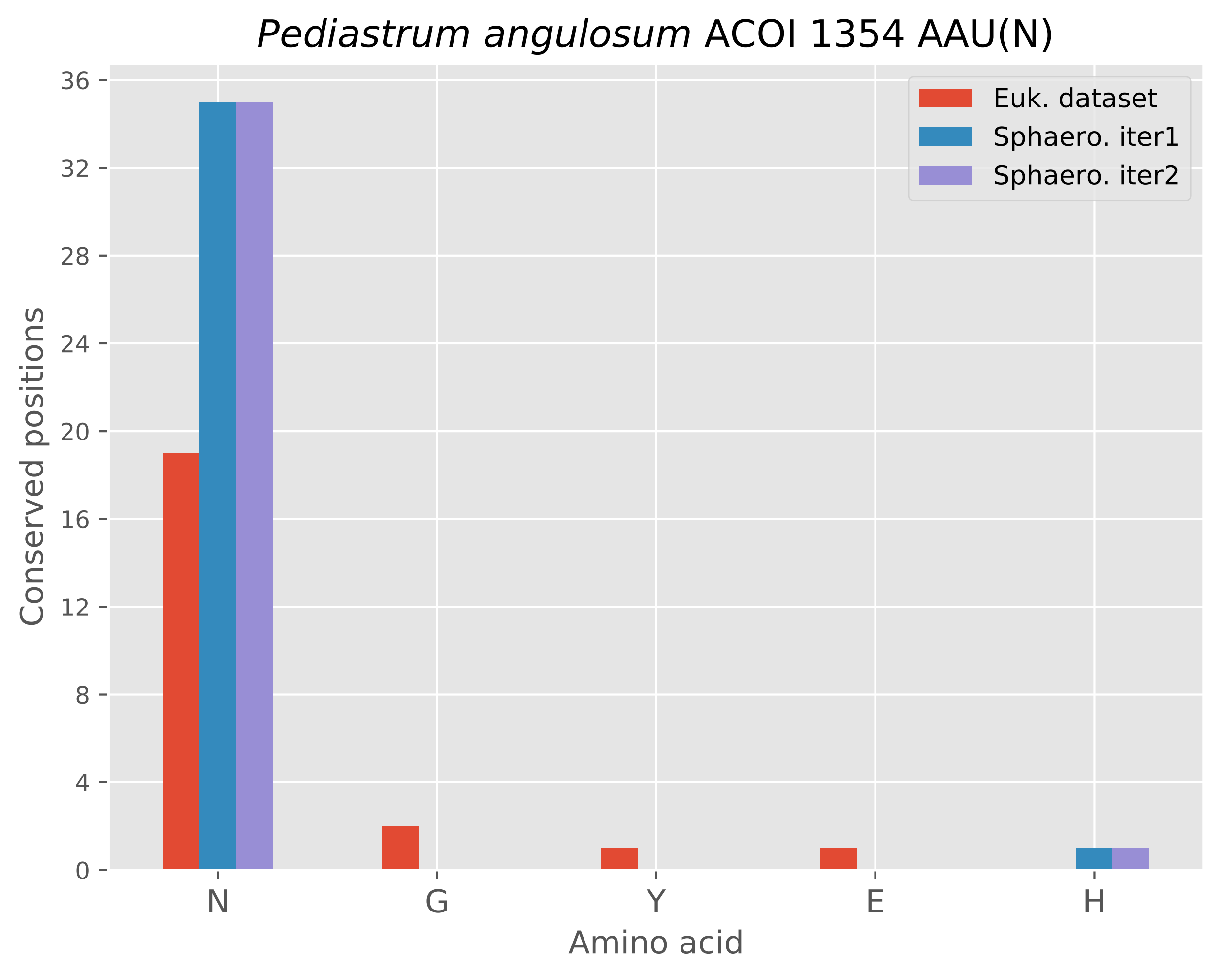

Pediastrum angulosum ACOI 1354 AAU(N)
Conserved positions
0
4
8
12
16
20
24
28
32
36
N
G
Y
E
H
Amino acid
Euk. dataset
Sphaero. iter1
Sphaero. iter2

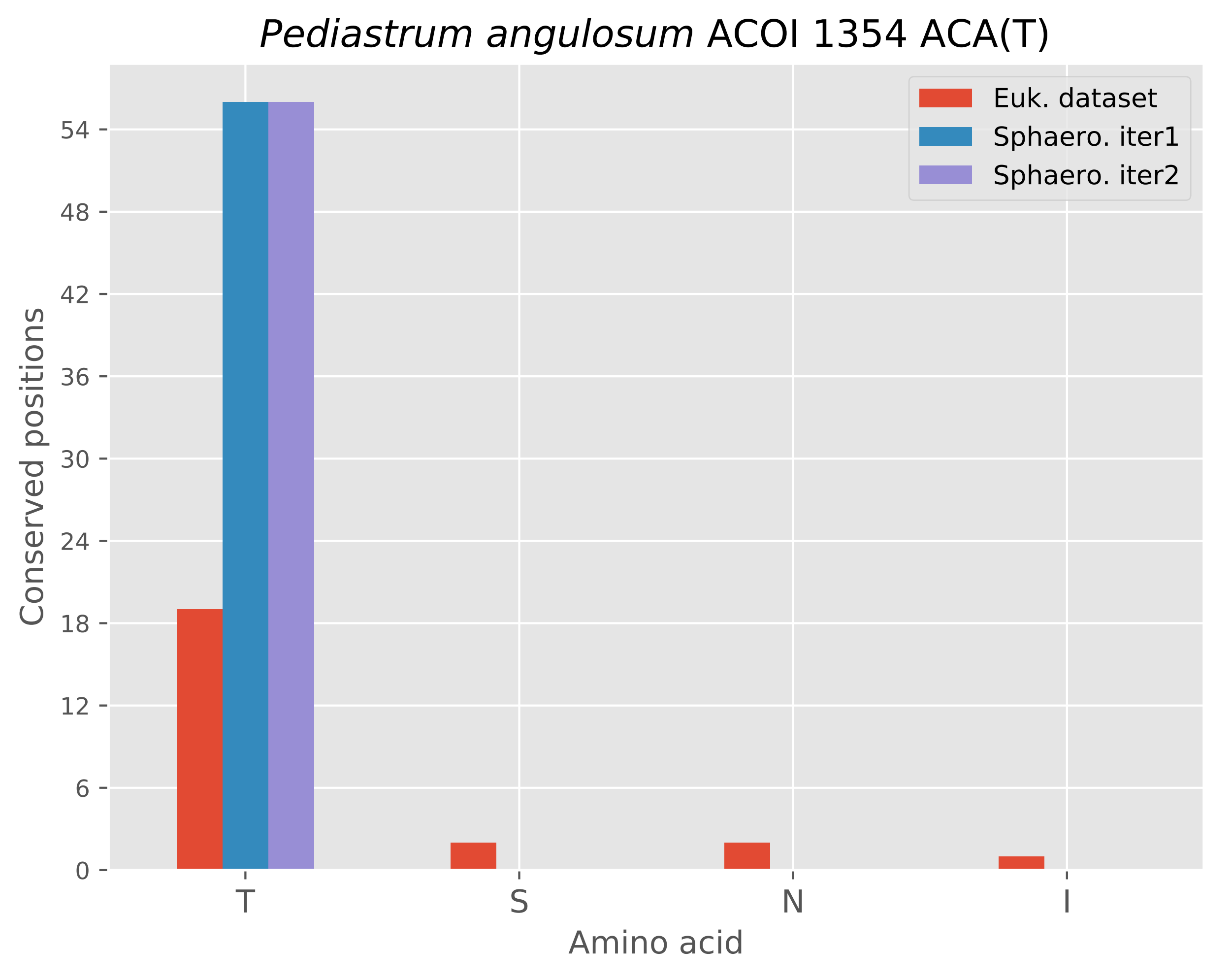

Pediastrum angulosum ACOI 1354 ACA(T)
Euk. dataset
Sphaero. iter1
Sphaero. iter2
Conserved positions
0
6
12
18
24
30
36
42
48
54
T
S
N
I
Amino acid

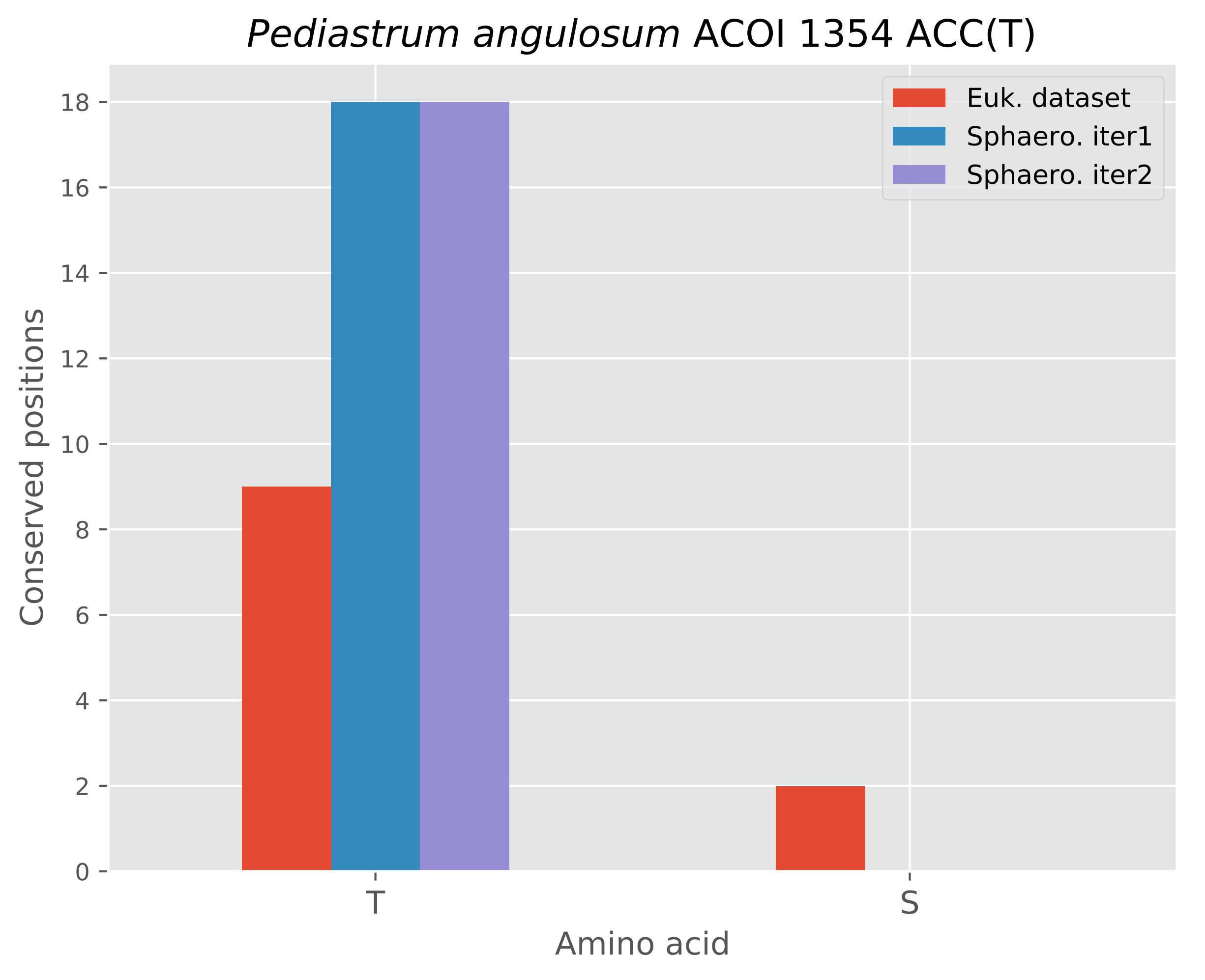

Pediastrum angulosum ACOI 1354 ACC(T)
Euk. dataset
Sphaero. iter1
Sphaero. iter2
Conserved positions
0
2
4
6
8
10
12
14
16
18
T
S
Amino acid

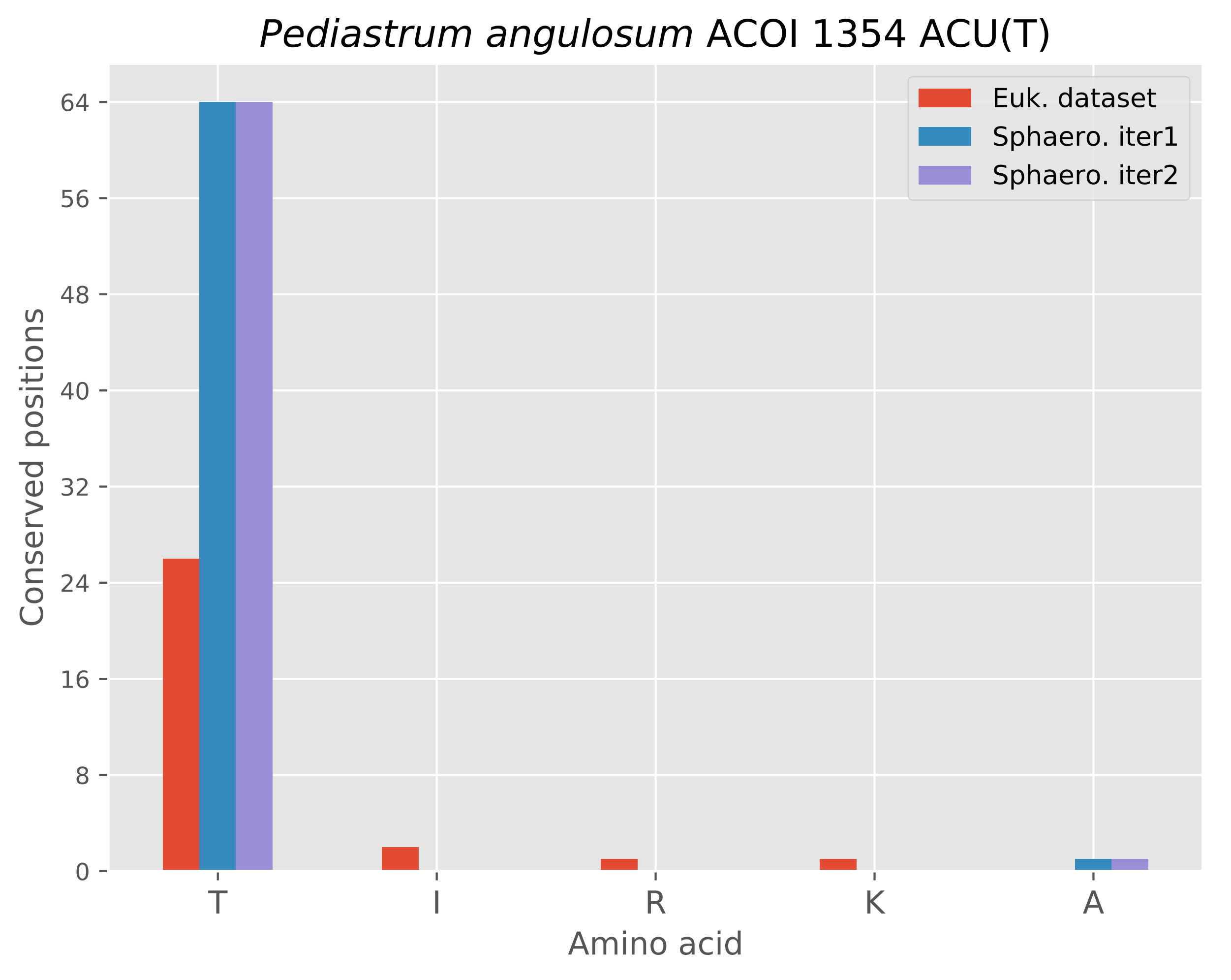

Pediastrum angulosum ACOI 1354 ACU(T)
Euk. dataset
Sphaero. iter1
Sphaero. iter2
Conserved positions
0
8
16
24
32
40
48
56
64
T
I
R
K
A
Amino acid

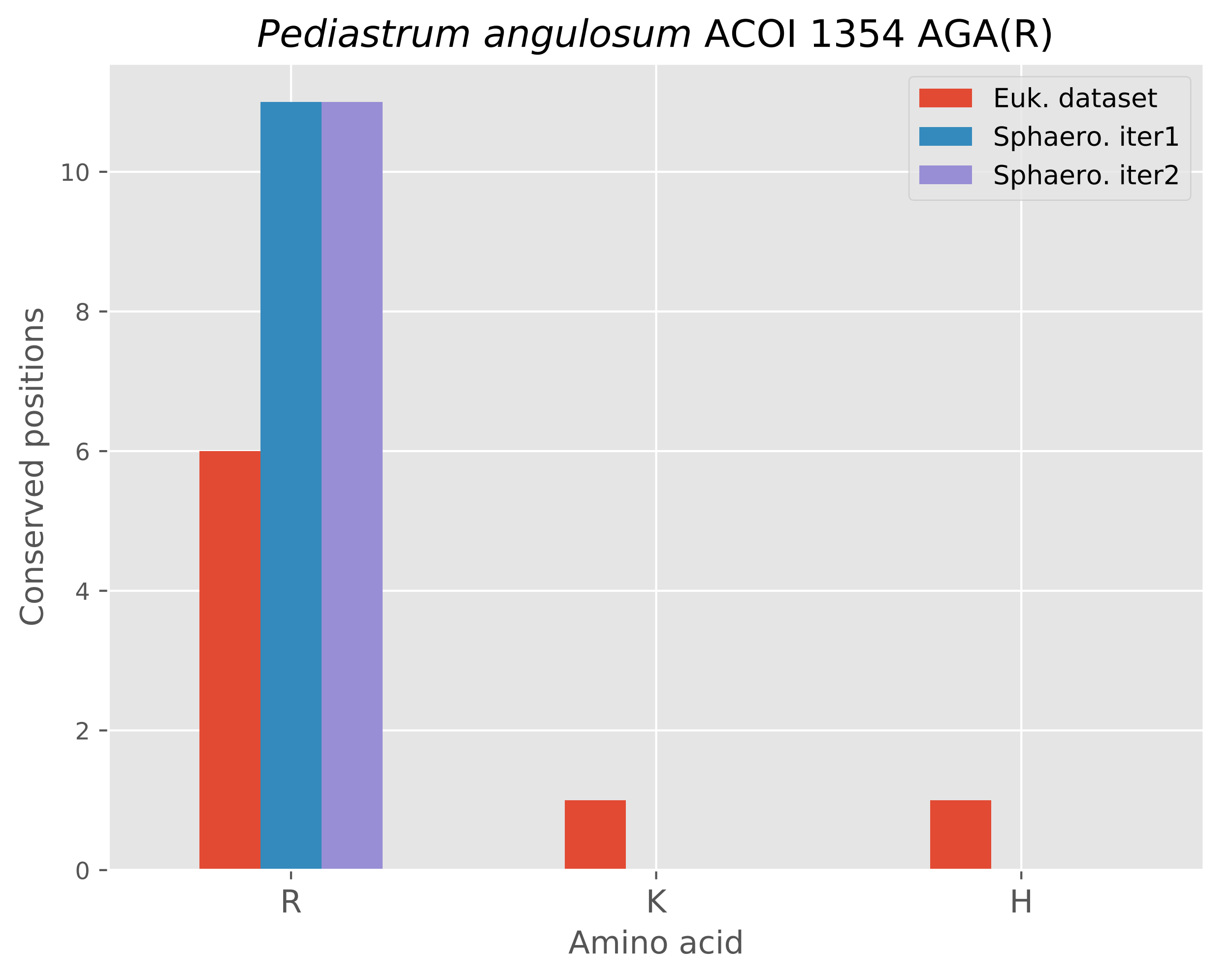

Pediastrum angulosum ACOI 1354 AGA(R)
Euk. dataset
Sphaero. iter1
Sphaero. iter2
Conserved positions
0
2
4
6
8
10
R
K
H
Amino acid

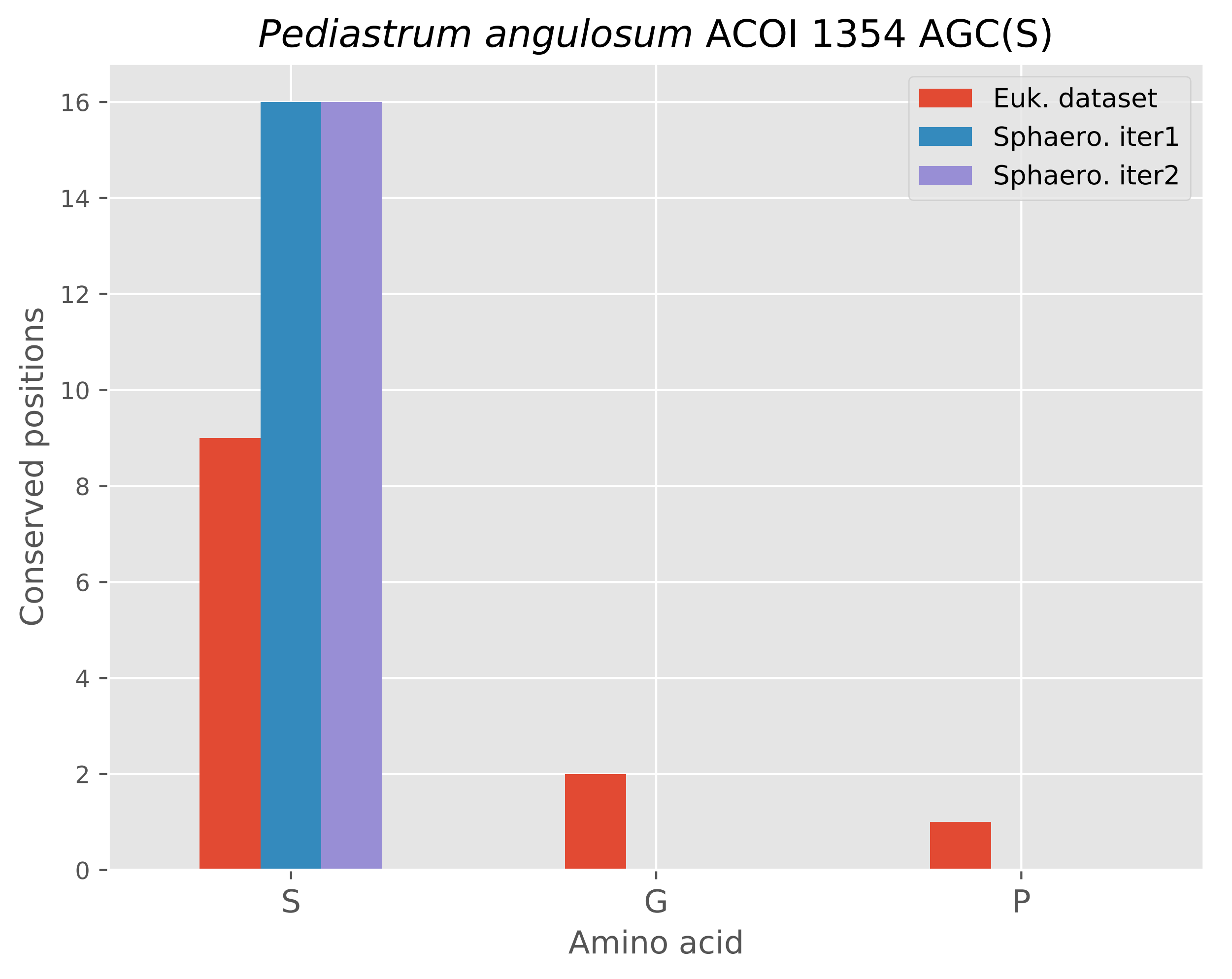

Pediastrum angulosum ACOI 1354 AGC(S)
Euk. dataset
Sphaero. iter1
Sphaero. iter2
Conserved positions
0
2
4
6
8
10
12
14
16
S
G
P
Amino acid

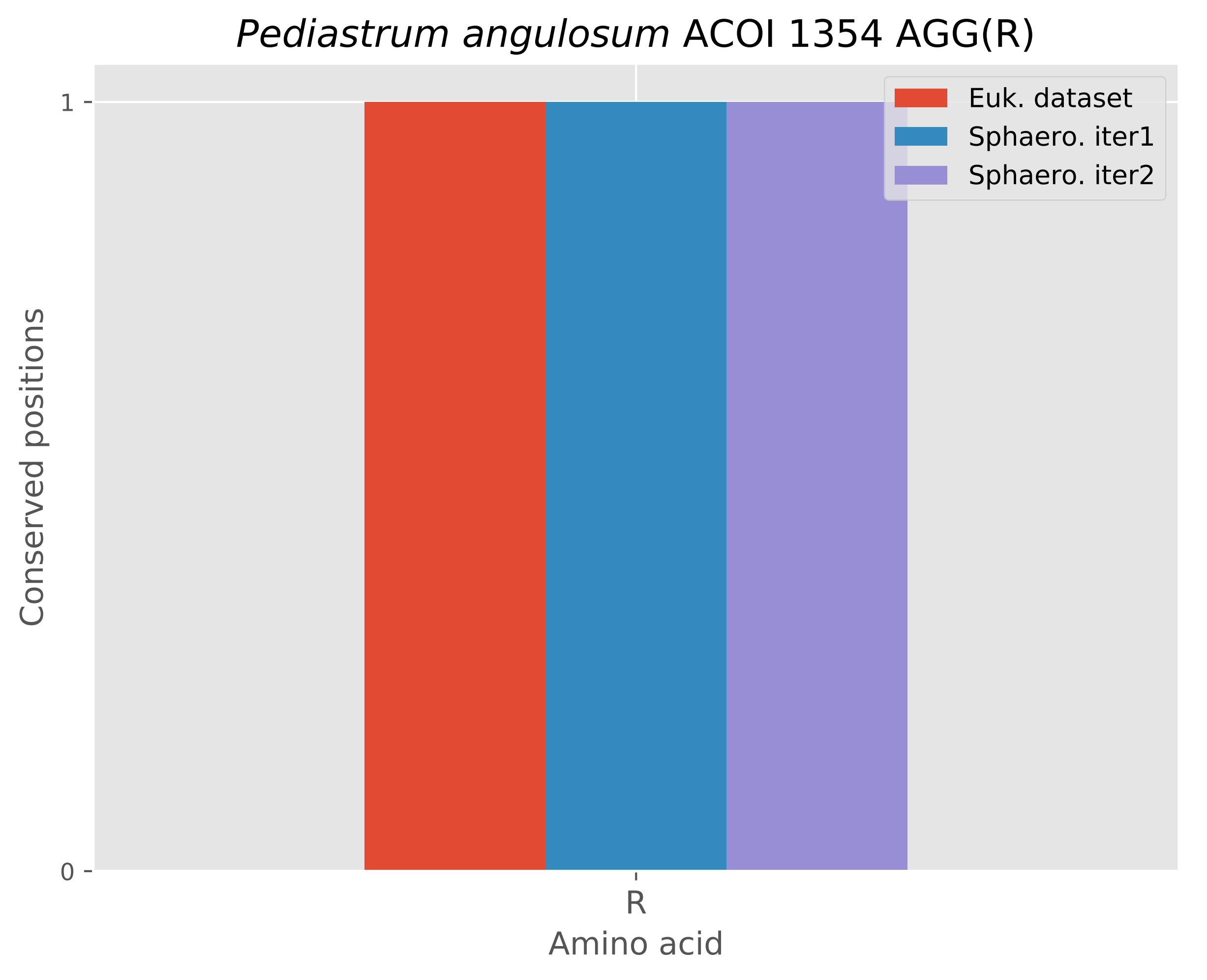

Pediastrum angulosum ACOI 1354 AGG(R)
Euk. dataset
Sphaero. iter1
Sphaero. iter2
1
0
Conserved positions
R
Amino acid

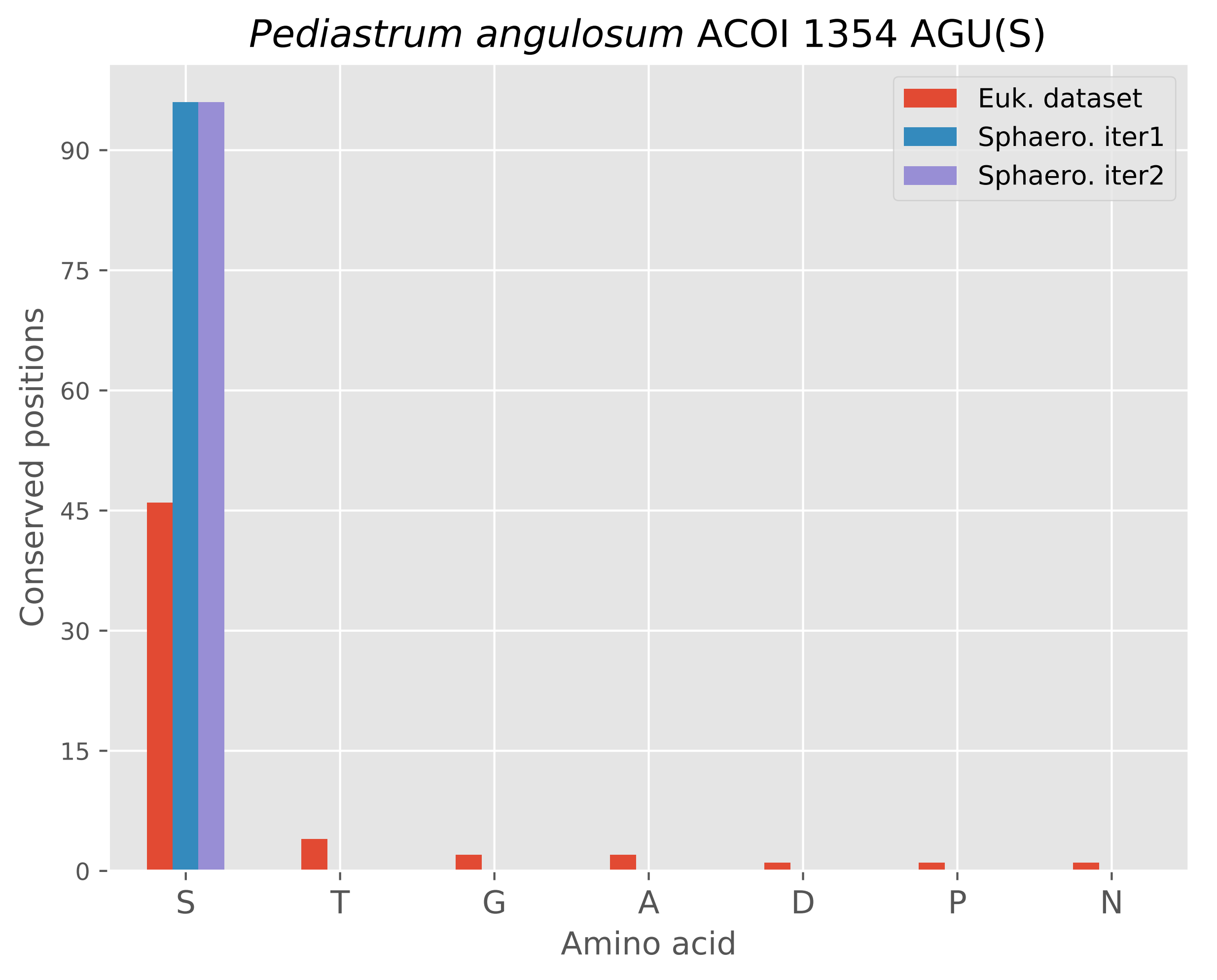

Pediastrum angulosum ACOI 1354 AGU(S)
Euk. dataset
Sphaero. iter1
Sphaero. iter2
Conserved positions
0
15
30
45
60
75
90
S
T
G
A
D
P
N
Amino acid

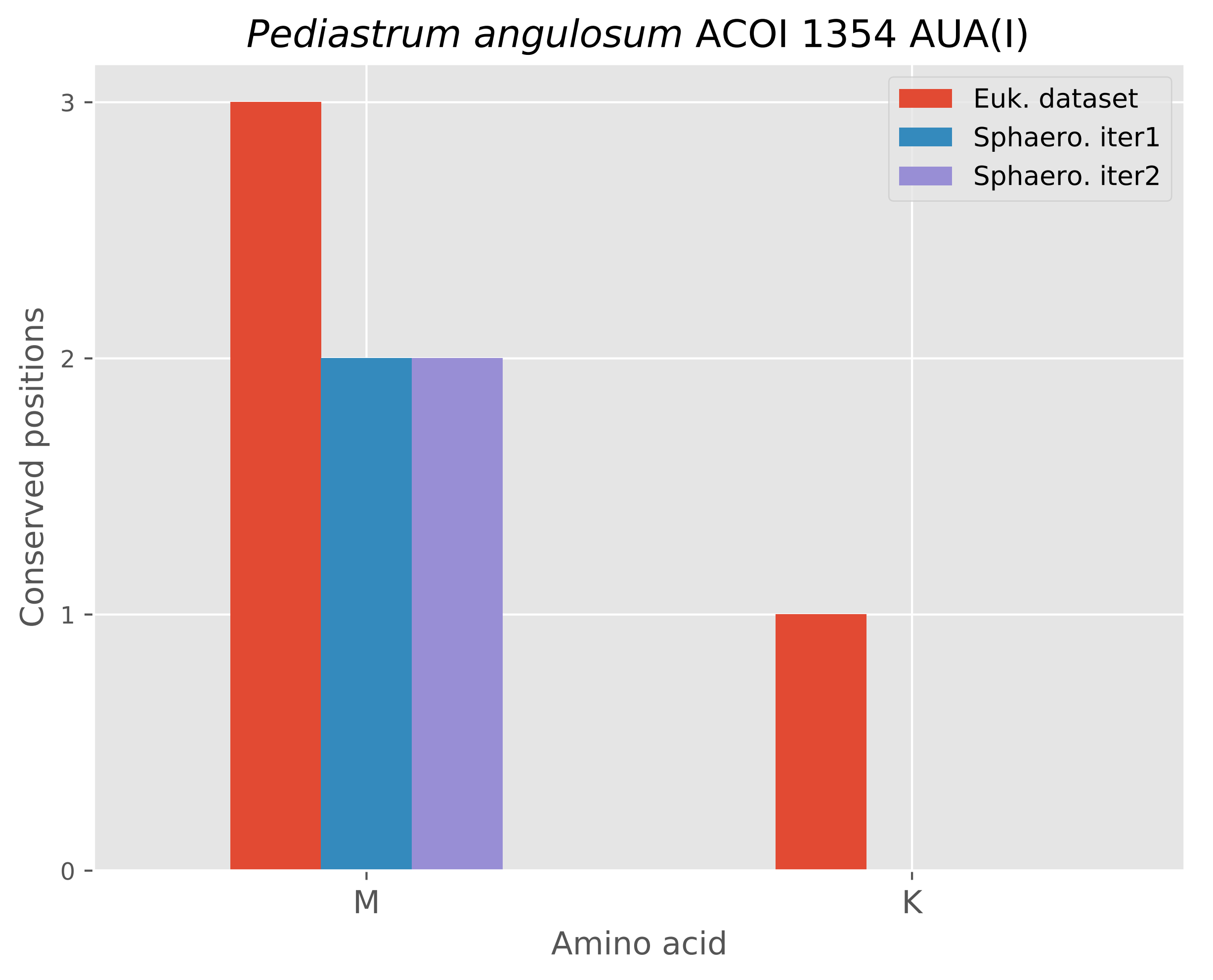

Pediastrum angulosum ACOI 1354 AUA(I)
Euk. dataset
Sphaero. iter1
Sphaero. iter2
Conserved positions
3
2
1
0
M
K
Amino acid

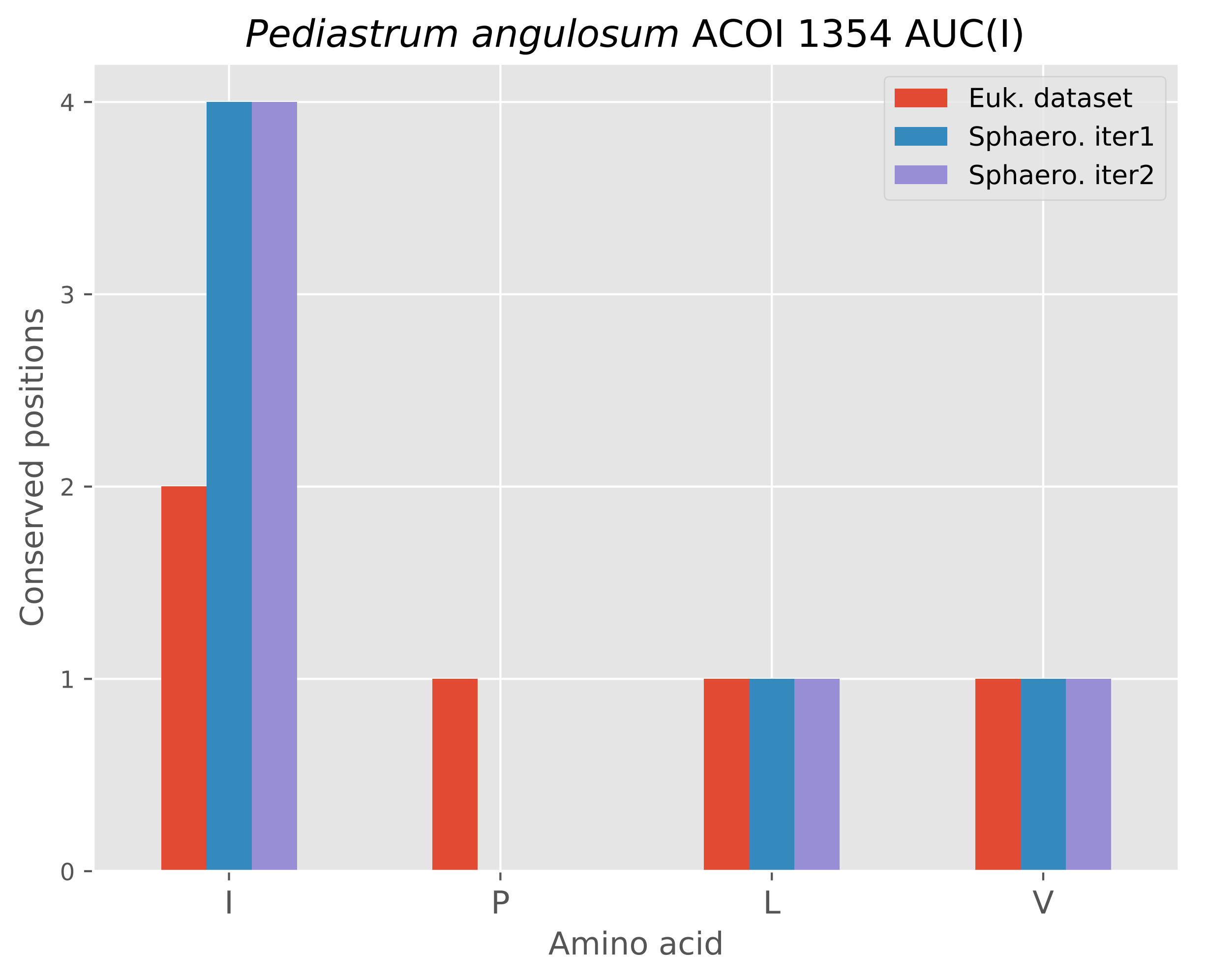
Pediastrum angulosum ACOI 1354 AUC(I)
Euk. dataset
Sphaero. iter1
Sphaero. iter2
Conserved positions
0
1
2
3
4
I
P
L
V
Amino acid

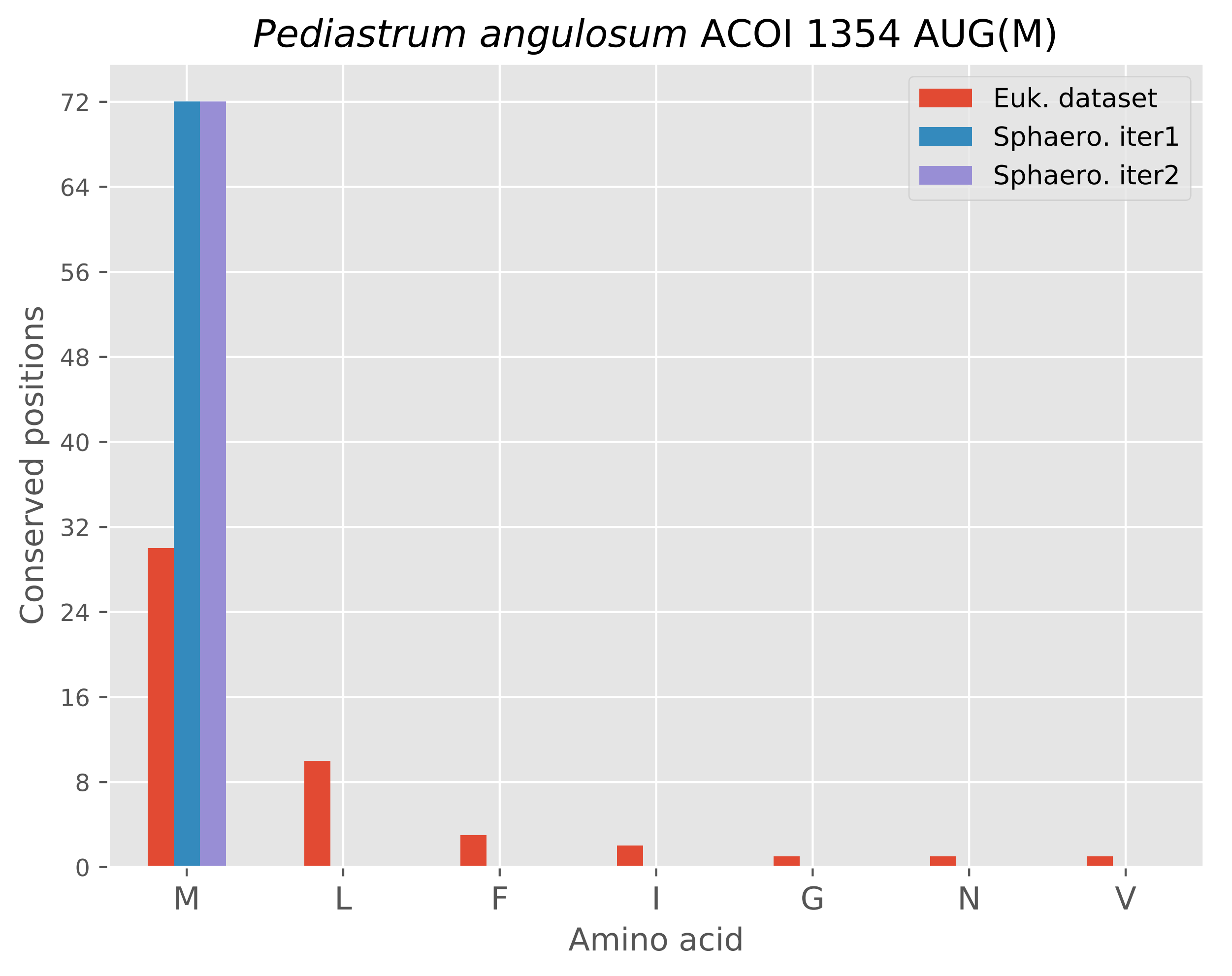

Pediastrum angulosum ACOI 1354 AUG(M)
Euk. dataset
Sphaero. iter1
Sphaero. iter2
Conserved positions
0
8
16
24
32
40
48
56
64
72
M
L
F
I
G
N
V
Amino acid

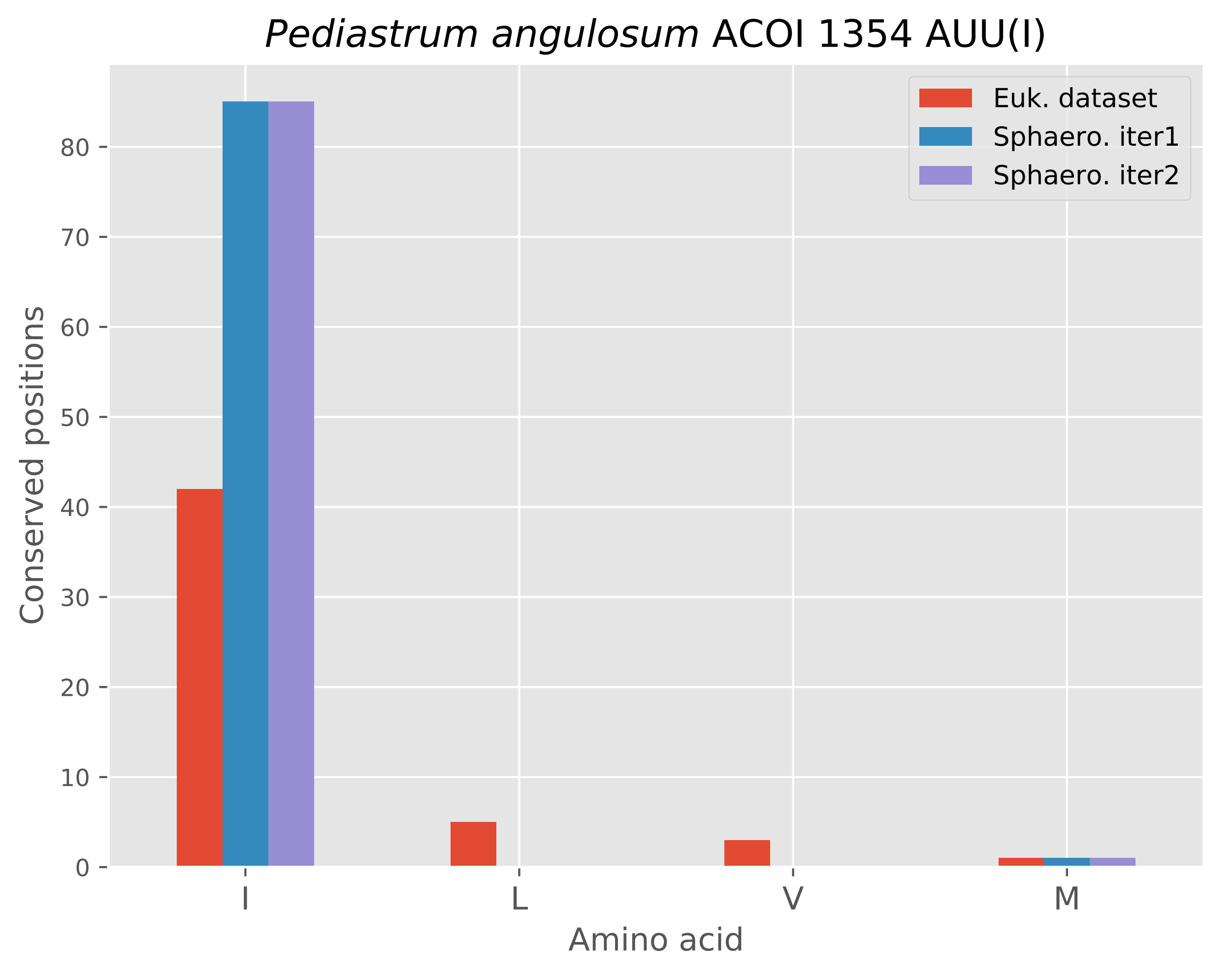

Pediastrum angulosum ACOI 1354 AUU(I)
Euk. dataset
Sphaero. iter1
Sphaero. iter2
Conserved positions
0
10
20
30
40
50
60
70
80
I
L
V
M
Amino acid

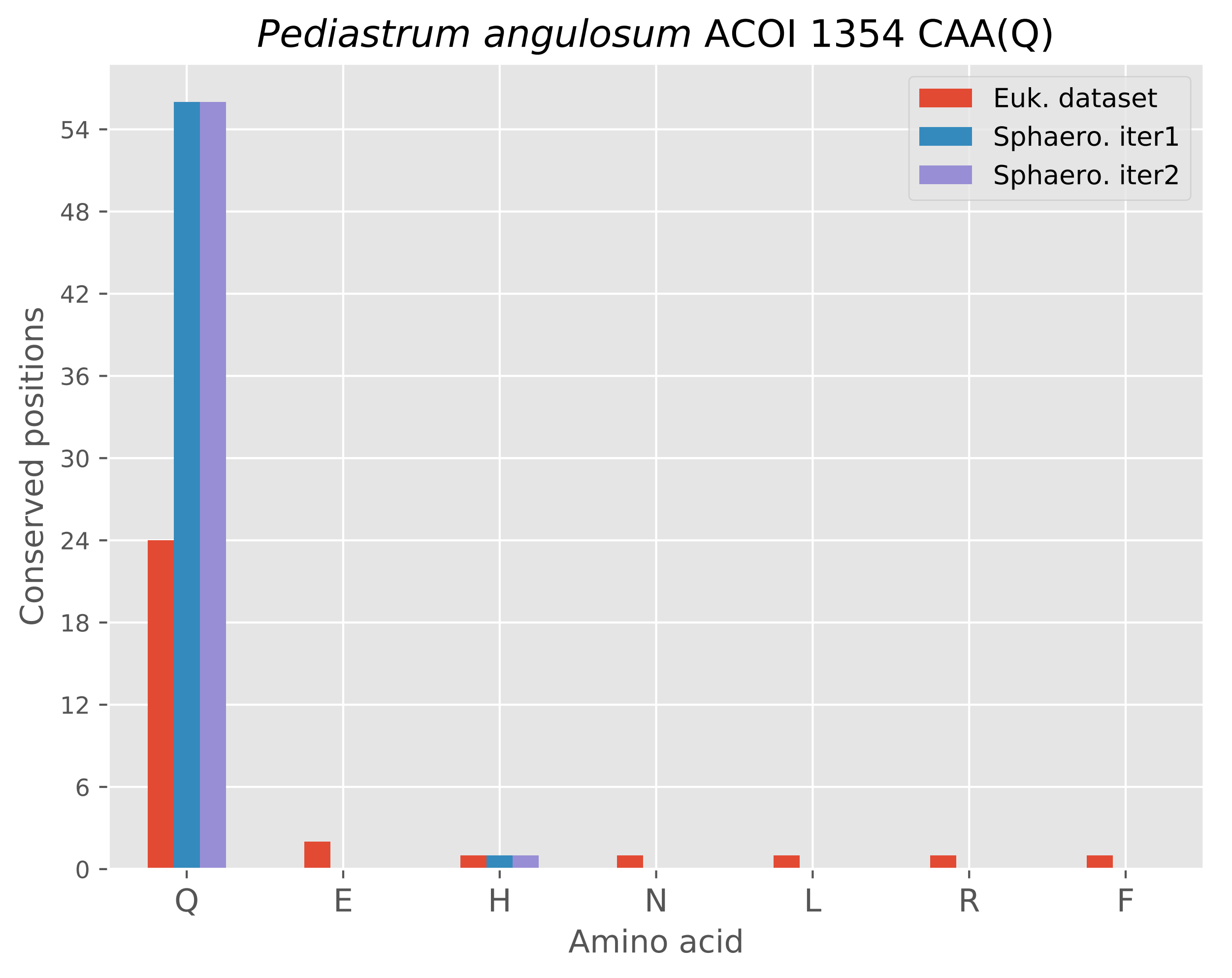

Pediastrum angulosum ACOI 1354 CAA(Q)
Euk. dataset
Sphaero. iter1
Sphaero. iter2
Conserved positions
0
6
12
18
24
30
36
42
48
54
Q
E
H
N
L
R
F
Amino acid

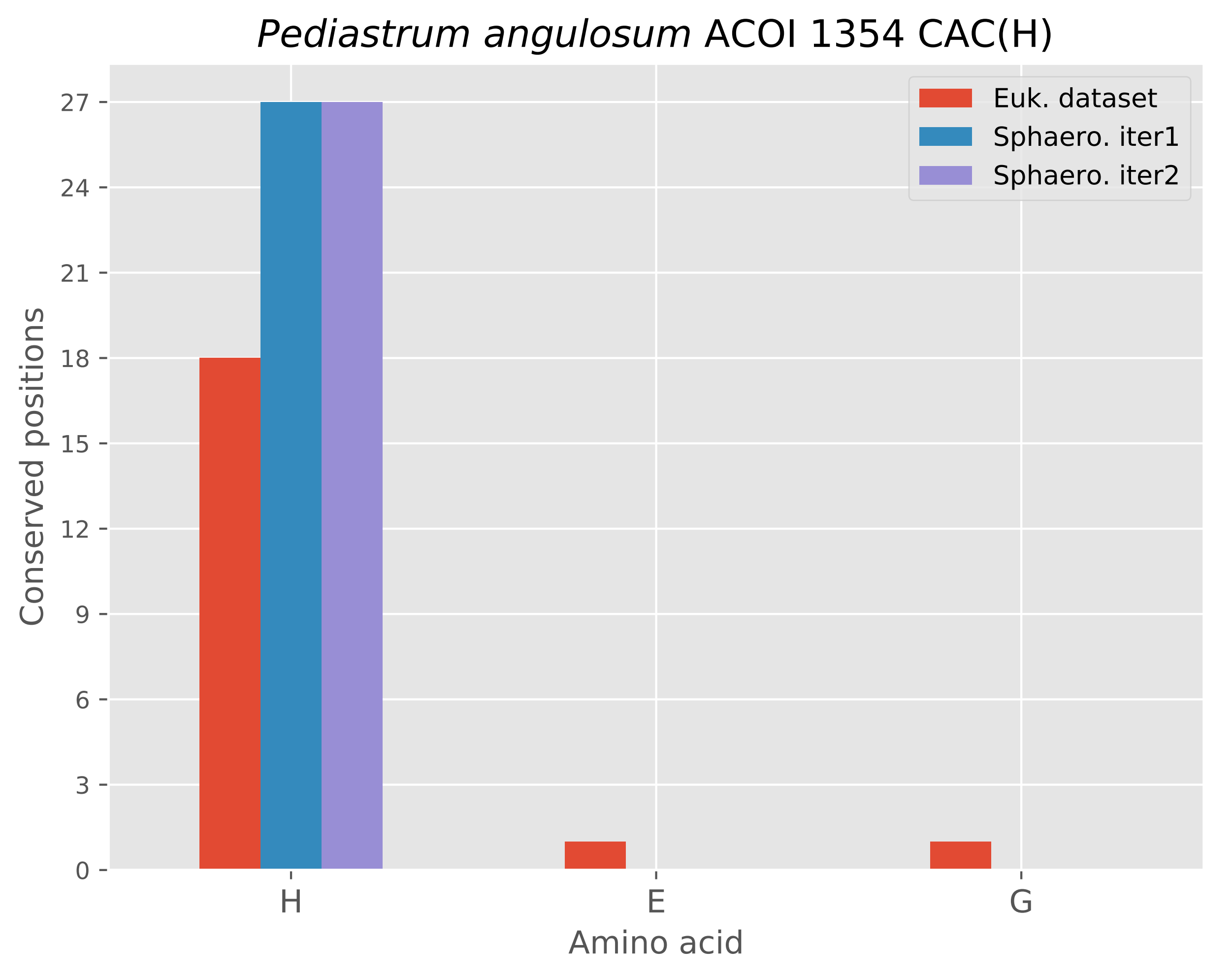

Pediastrum angulosum ACOI 1354 CAC(H)
Euk. dataset
Sphaero. iter1
Sphaero. iter2
Conserved positions
0
3
6
9
12
15
18
21
24
27
H
E
G
Amino acid

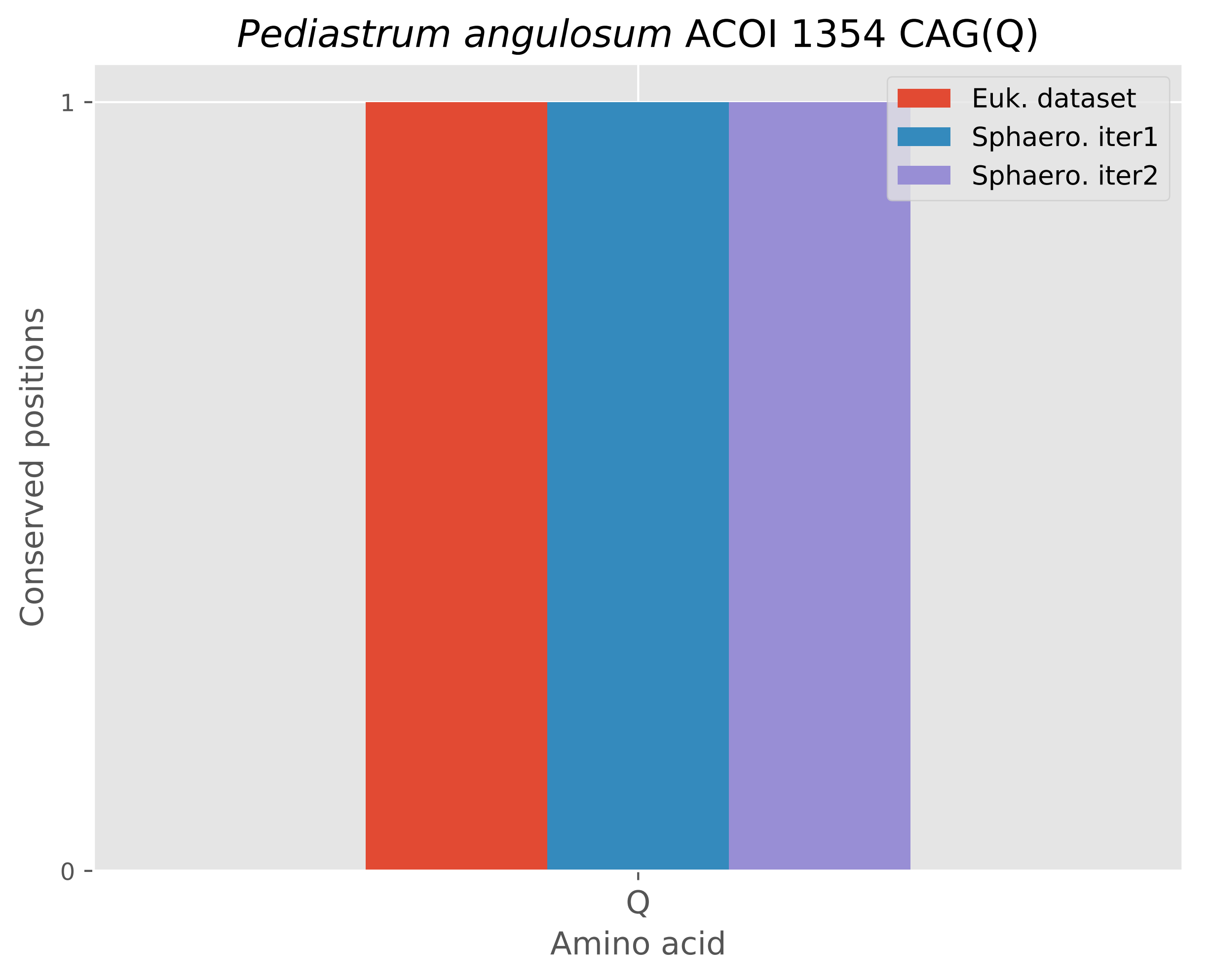

Pediastrum angulosum ACOI 1354 CAG(Q)
Euk. dataset
Sphaero. iter1
Sphaero. iter2
Conserved positions
1
0
Q
Amino acid

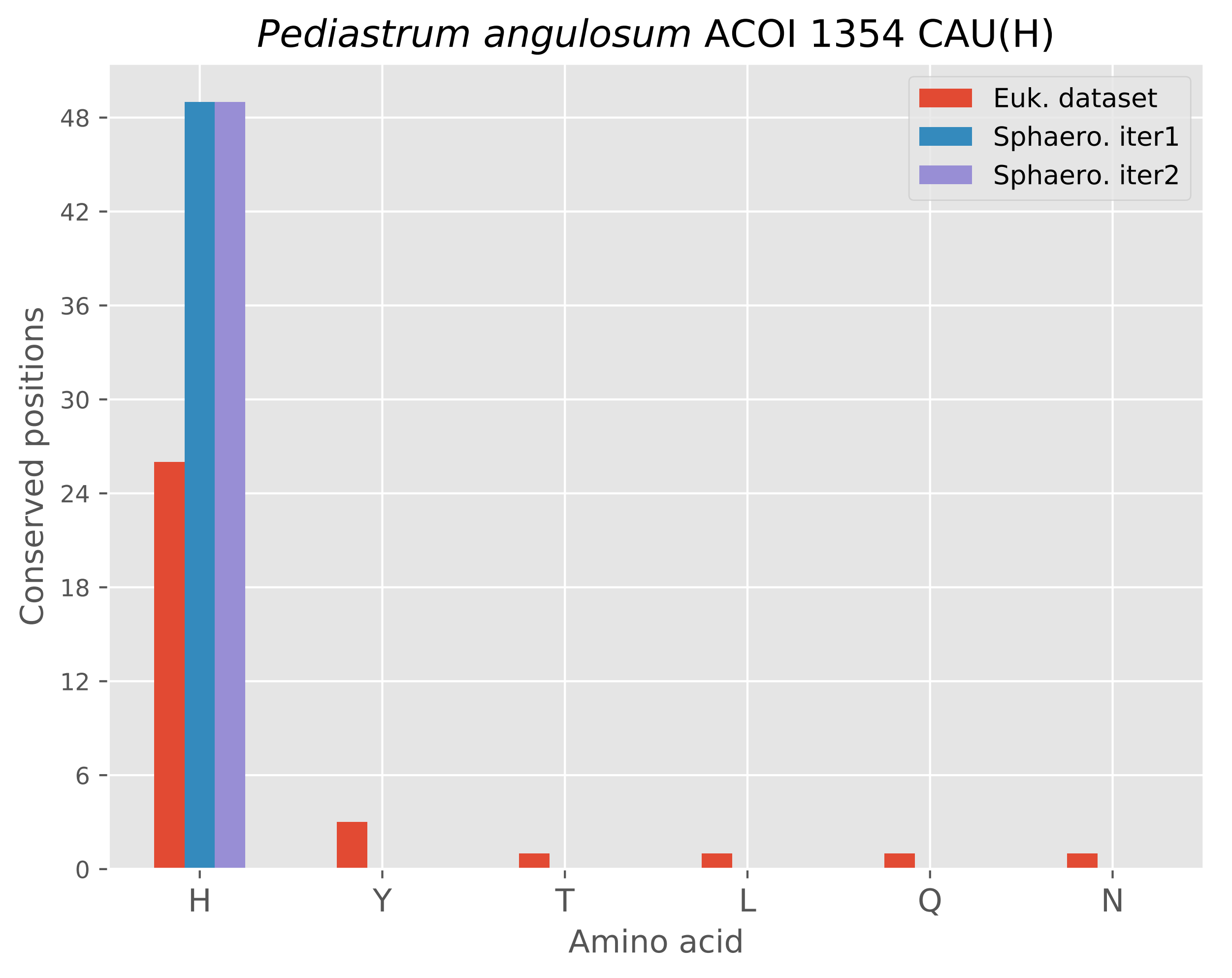

Pediastrum angulosum ACOI 1354 CAU(H)
Euk. dataset
Sphaero. iter1
Sphaero. iter2
Conserved positions
0
6
12
18
24
30
36
42
48
H
Y
T
L
Q
N
Amino acid

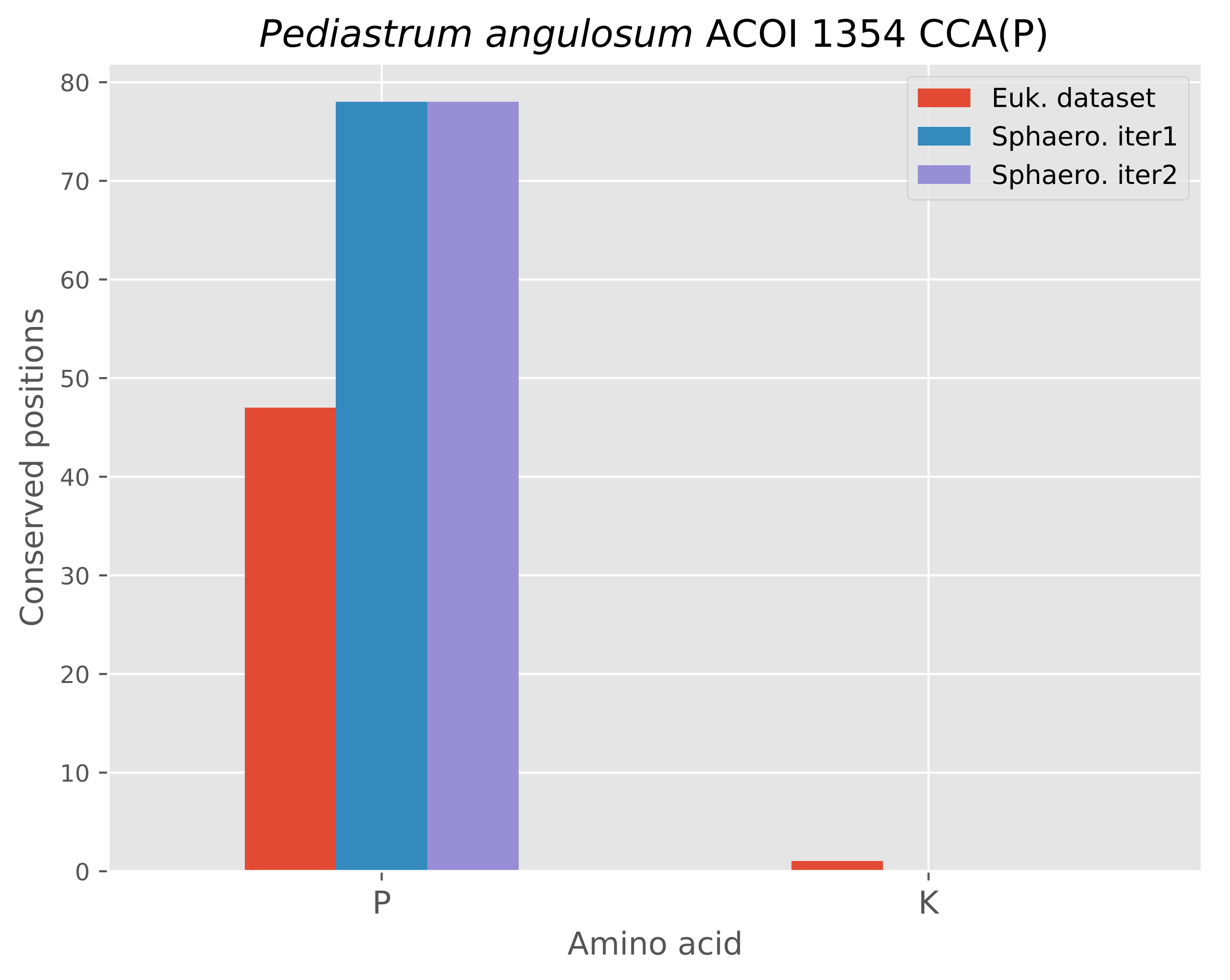

Pediastrum angulosum ACOI 1354 CCA(P)
Euk. dataset
Sphaero. iter1
Sphaero. iter2
Conserved positions
0
10
20
30
40
50
60
70
80
P
K
Amino acid

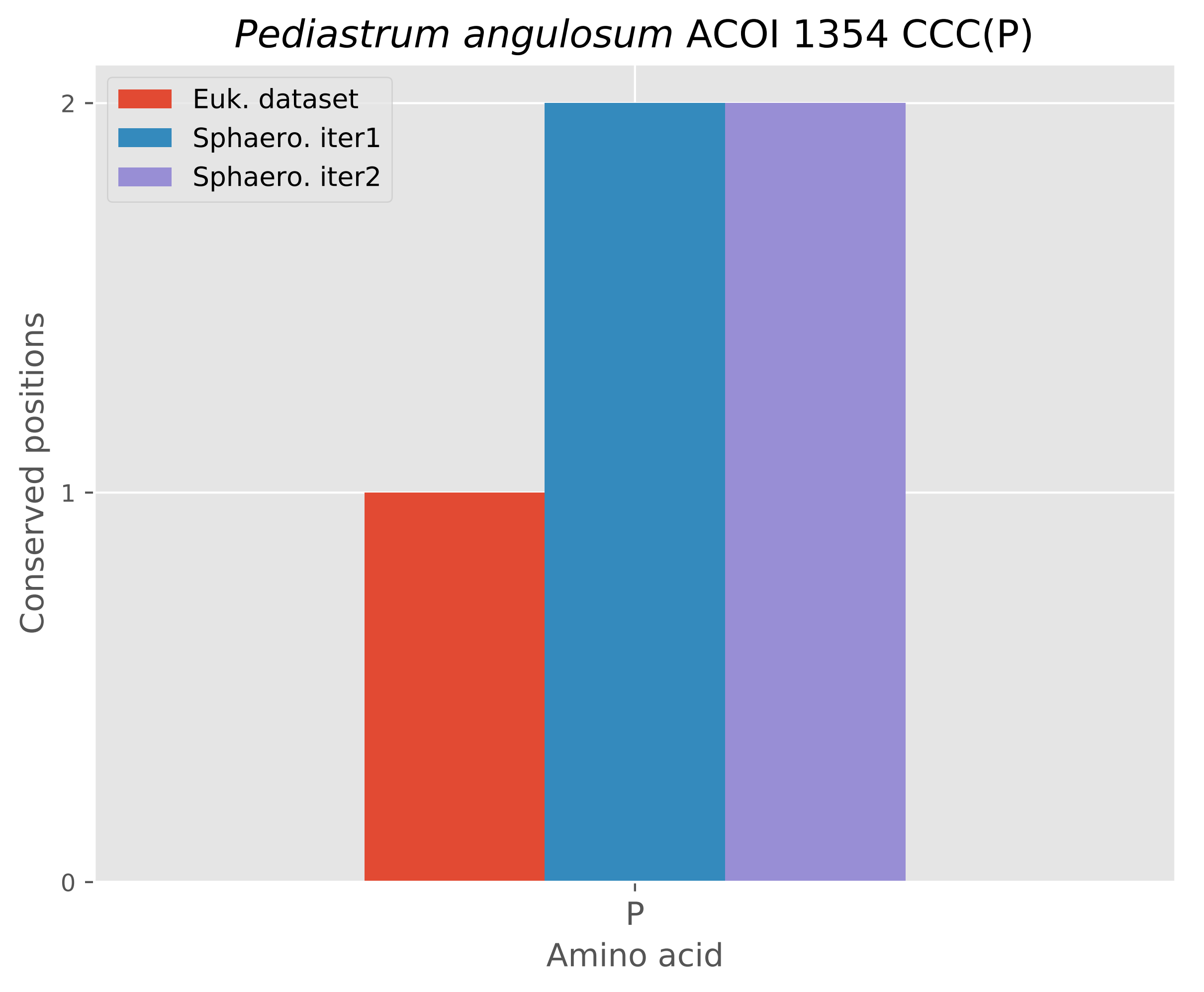

Pediastrum angulosum ACOI 1354 CCC(P)
Euk. dataset
Sphaero. iter1
Sphaero. iter2
Conserved positions
0
1
2
P
Amino acid

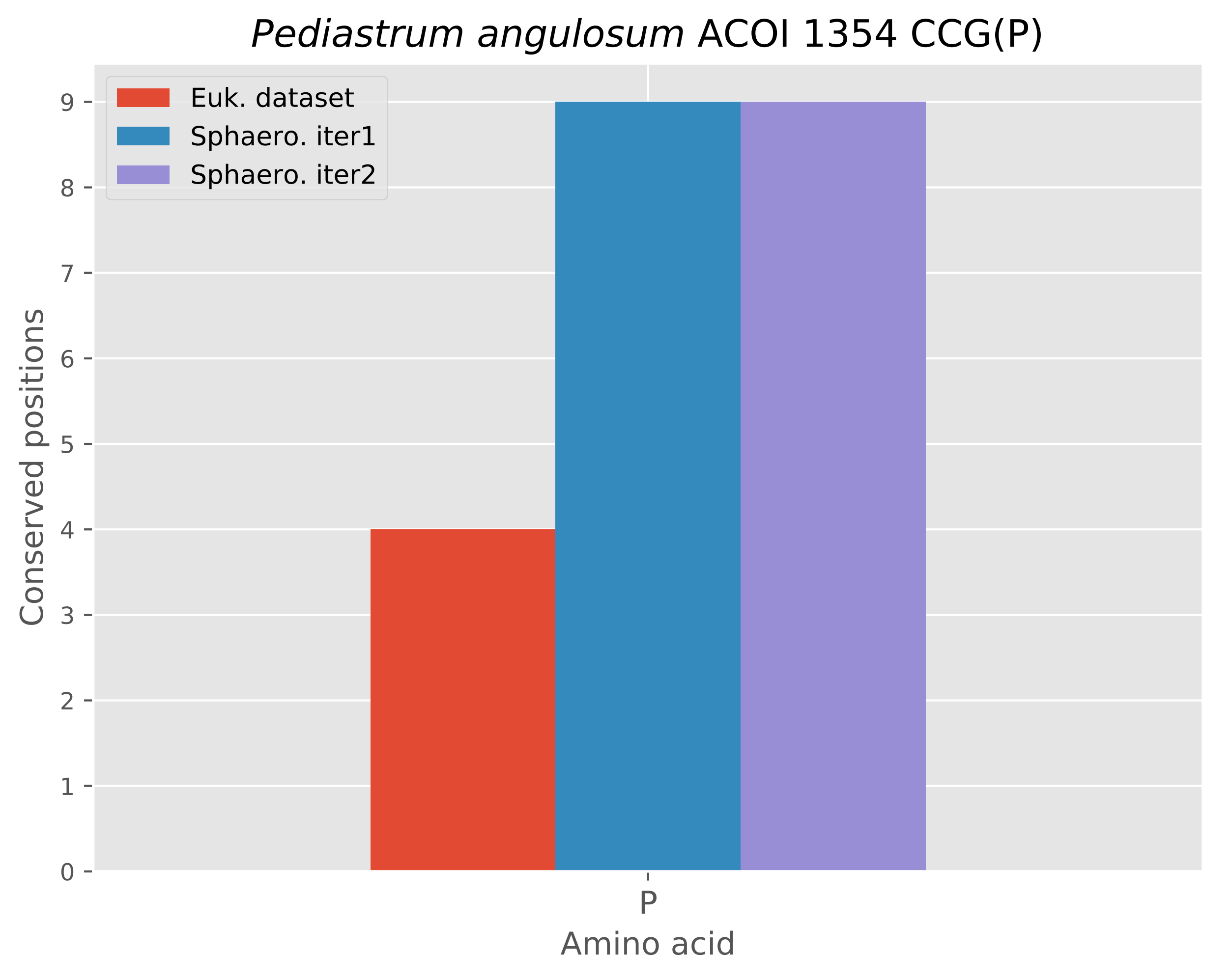

Pediastrum angulosum ACOI 1354 CCG(P)
Euk. dataset
Sphaero. iter1
Sphaero. iter2
Conserved positions
0
1
2
3
4
5
6
7
8
9
P
Amino acid

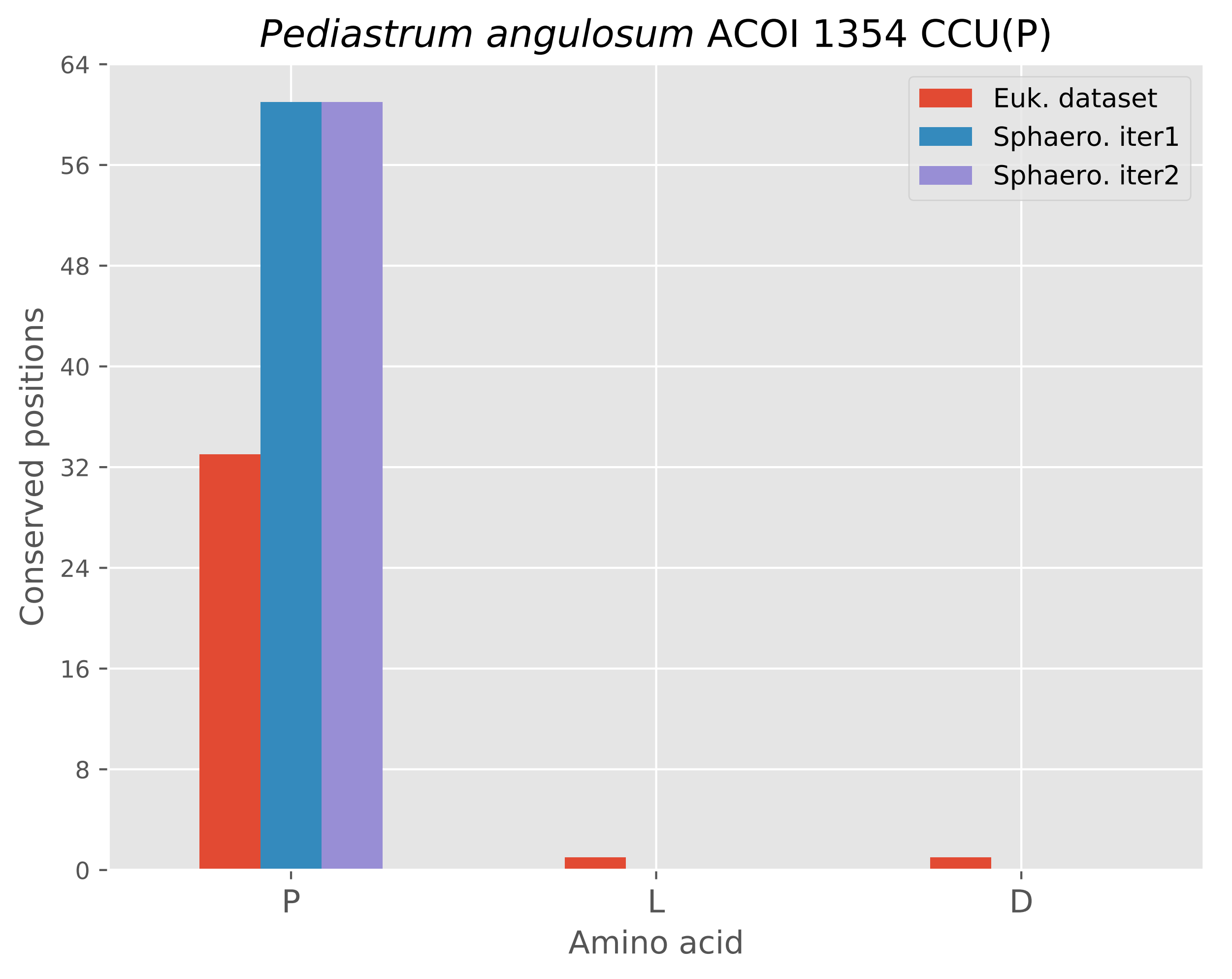

Pediastrum angulosum ACOI 1354 CCU(P)
Euk. dataset
Sphaero. iter1
Sphaero. iter2
Conserved positions
0
8
16
24
32
40
48
56
64
P
L
D
Amino acid

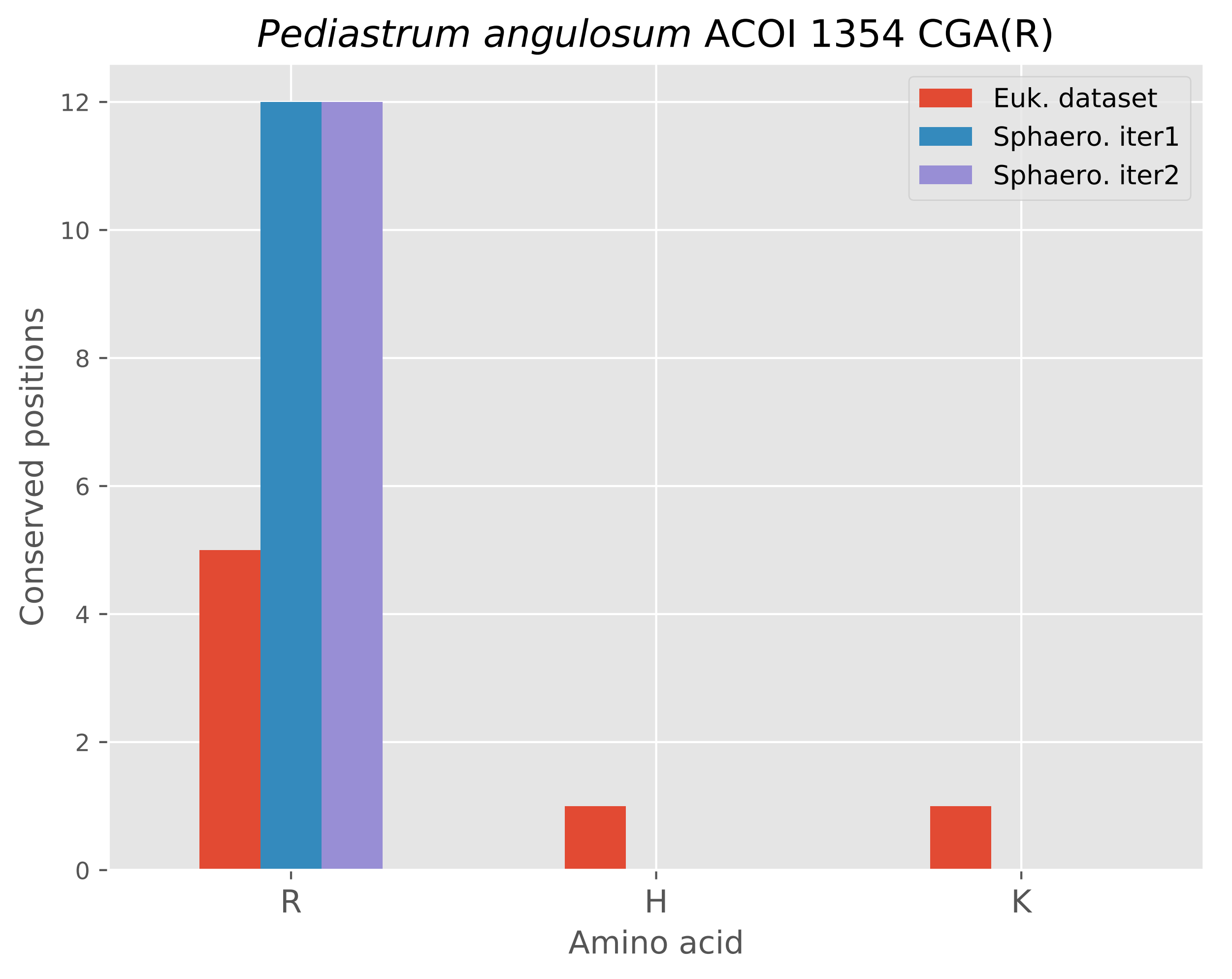

Pediastrum angulosum ACOI 1354 CGA(R)
Euk. dataset
Sphaero. iter1
Sphaero. iter2
Conserved positions
0
2
4
6
8
10
12
R
H
K
Amino acid

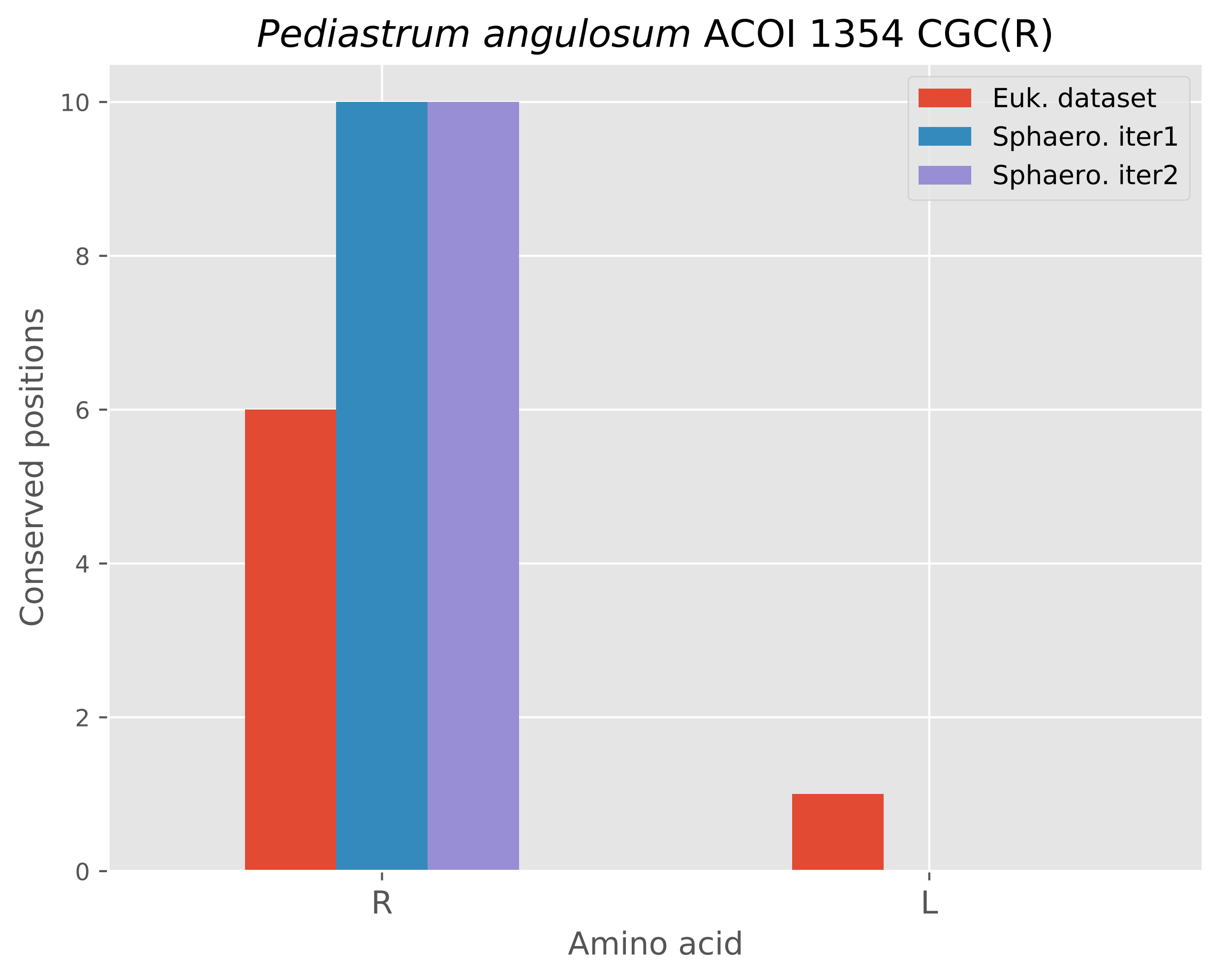
Pediastrum angulosum ACOI 1354 CGC(R)
Euk. dataset
Sphaero. iter1
Sphaero. iter2
Conserved positions
0
2
4
6
8
10
R
L
Amino acid

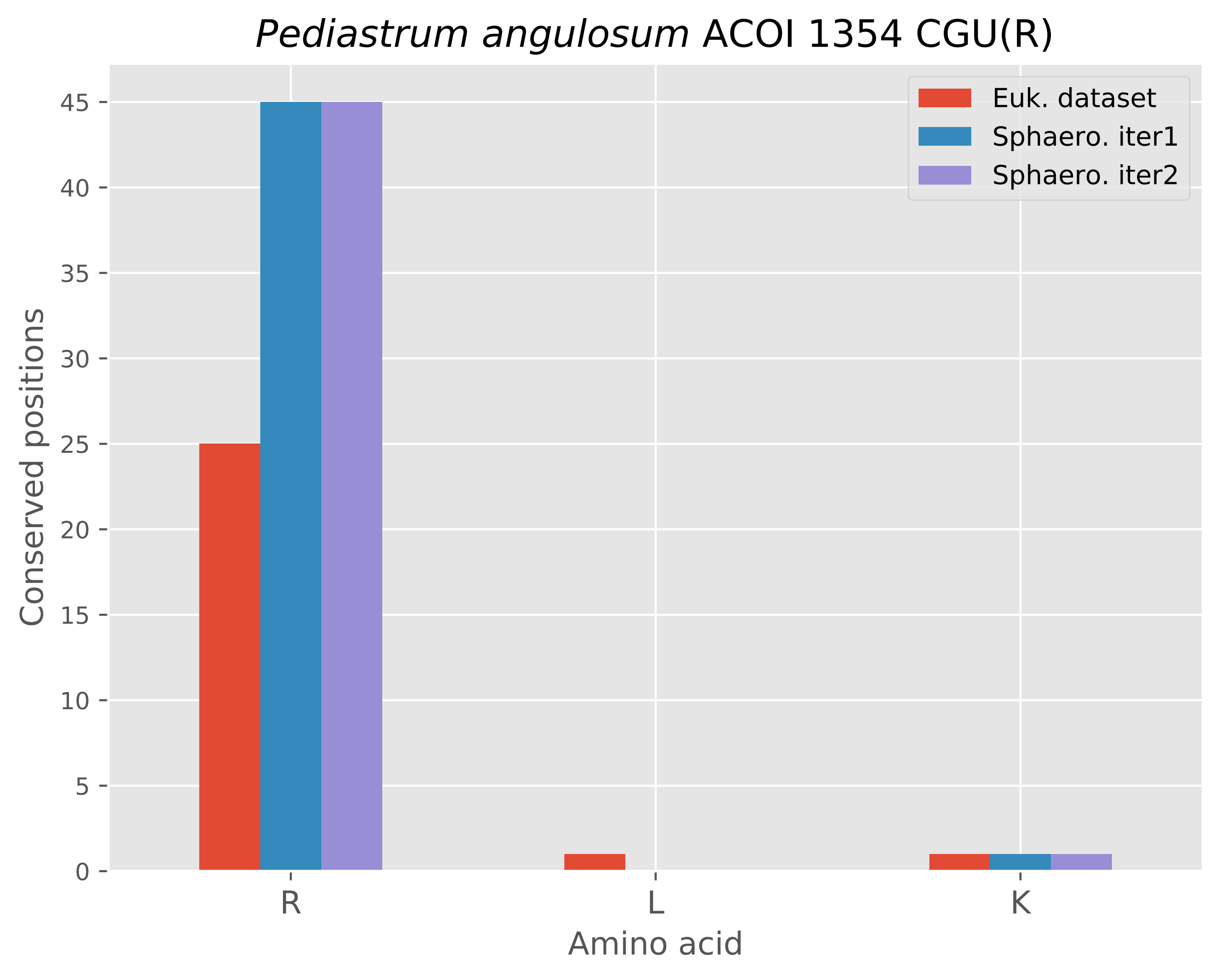

Pediastrum angulosum ACOI 1354 CGU(R)
Euk. dataset
Sphaero. iter1
Sphaero. iter2
Conserved positions
0
5
10
15
20
25
30
35
40
45
R
L
K
Amino acid

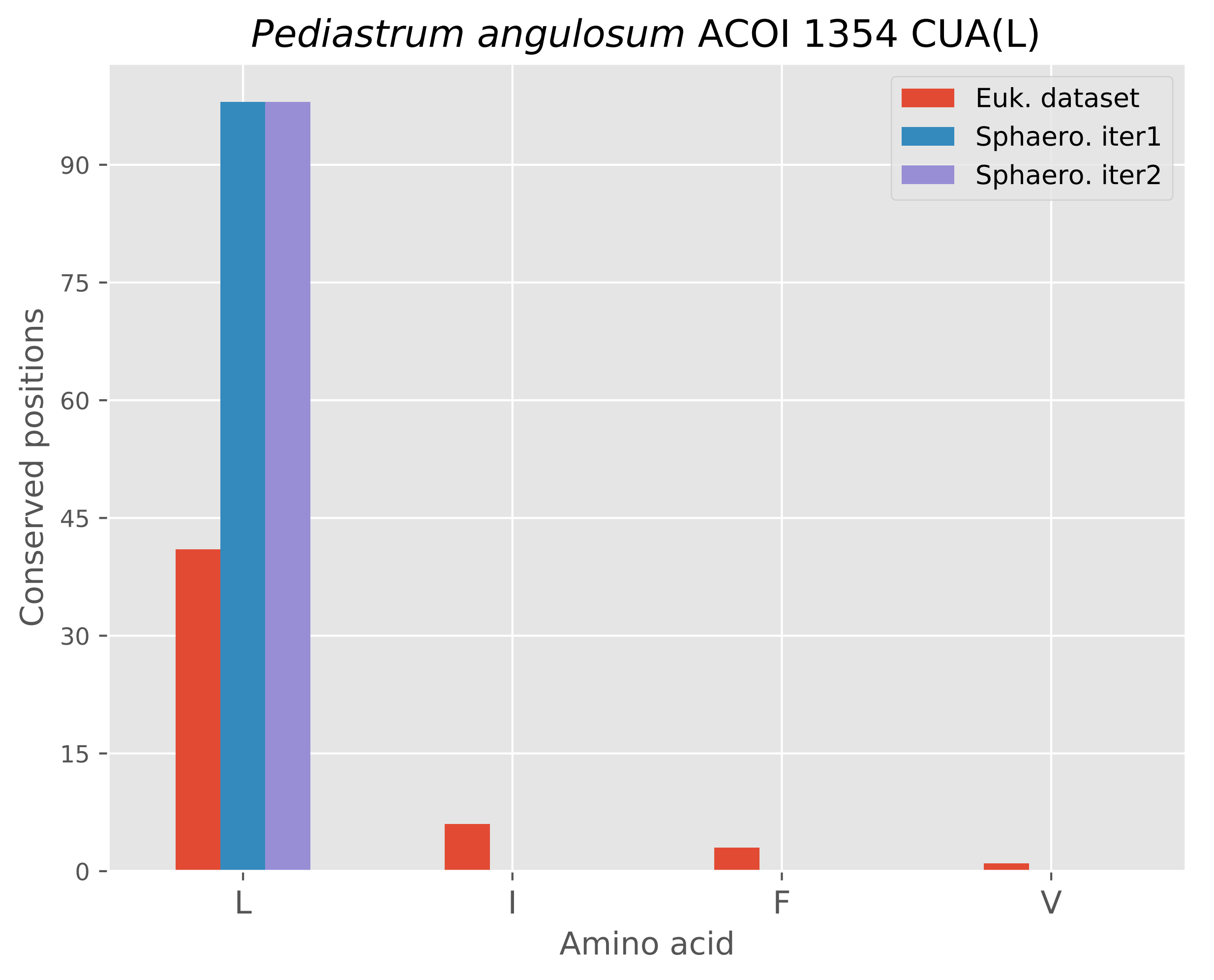

Pediastrum angulosum ACOI 1354 CUA(L)
Euk. dataset
Sphaero. iter1
Sphaero. iter2
Conserved positions
0
15
30
45
60
75
90
L
I
F
V
Amino acid

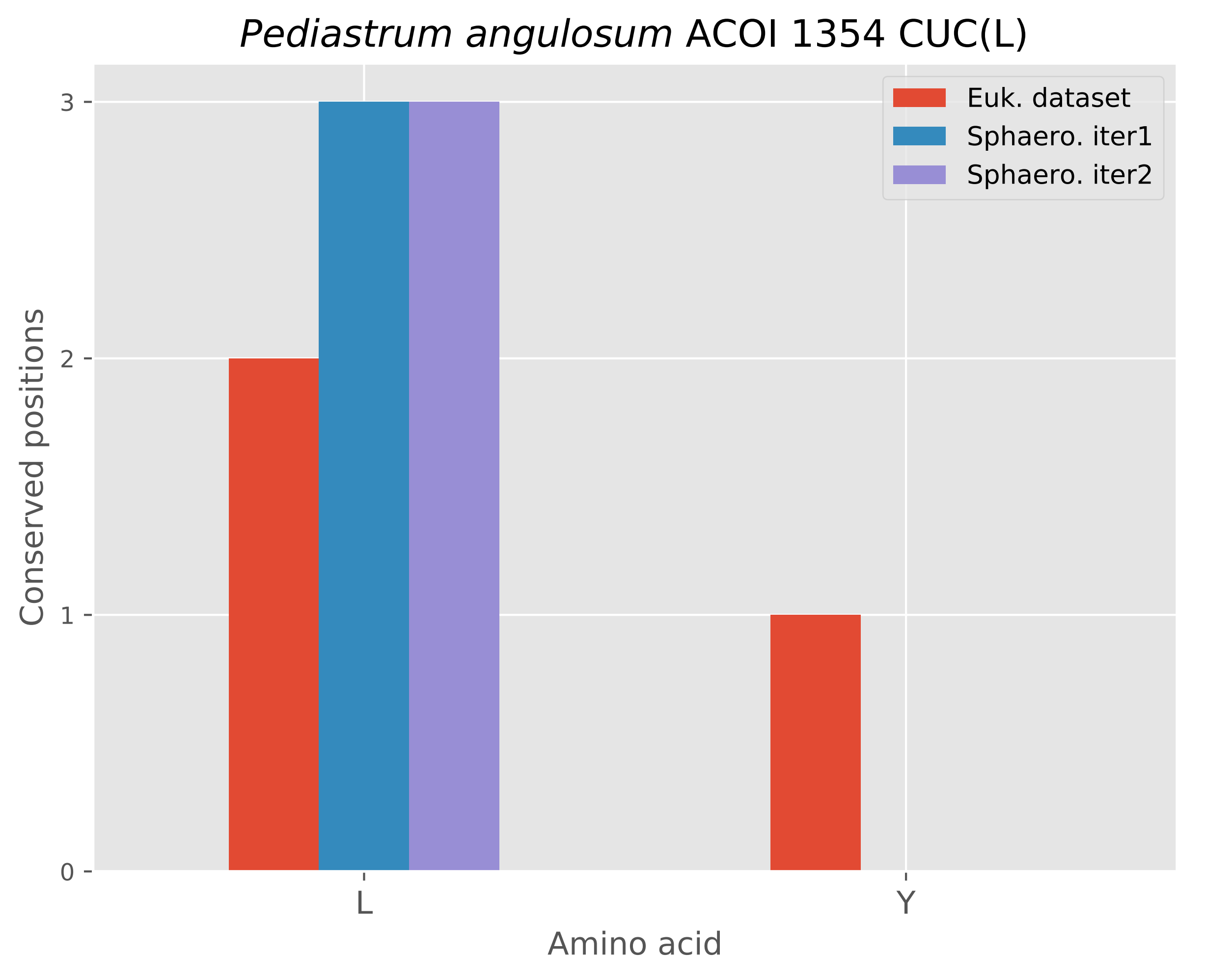

Pediastrum angulosum ACOI 1354 CUC(L)
Euk. dataset
Sphaero. iter1
Sphaero. iter2
Conserved positions
0
1
2
3
L
Y
Amino acid

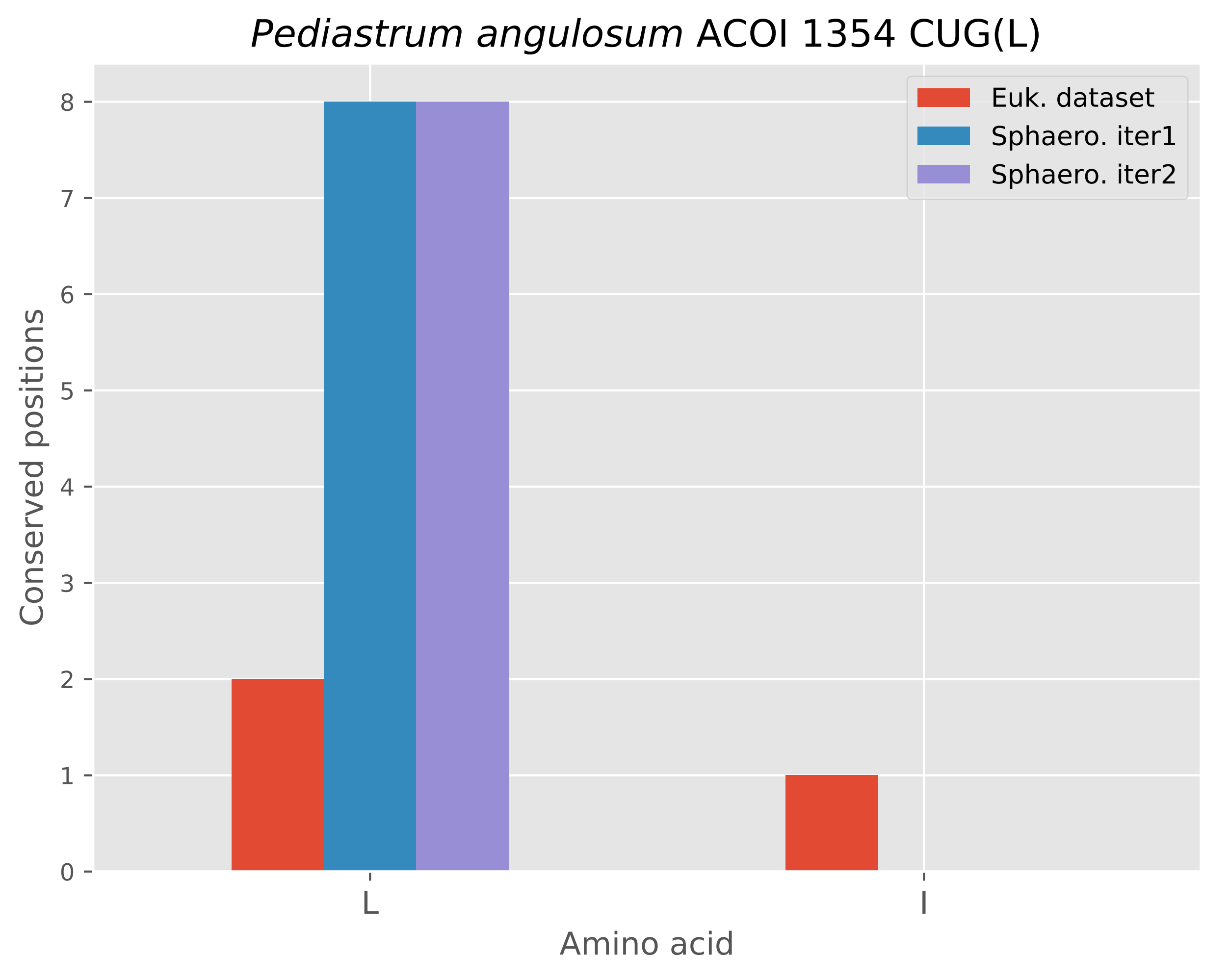

Pediastrum angulosum ACOI 1354 CUG(L)
Euk. dataset
Sphaero. iter1
Sphaero. iter2
Conserved positions
0
1
2
3
4
5
6
7
8
L
I
Amino acid

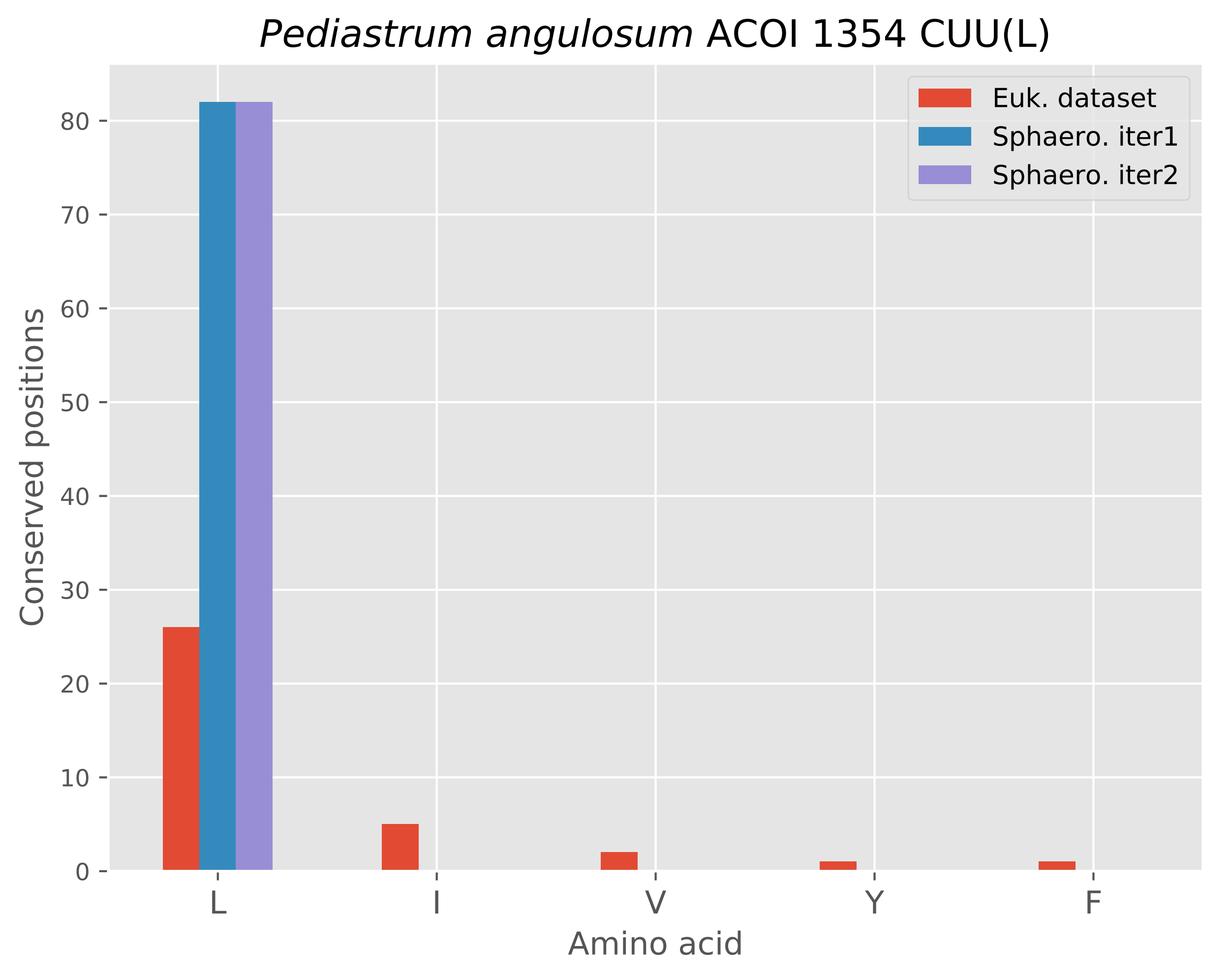

Pediastrum angulosum ACOI 1354 CUU(L)
Euk. dataset
Sphaero. iter1
Sphaero. iter2
Conserved positions
0
10
20
30
40
50
60
70
80
L
I
V
Y
F
Amino acid

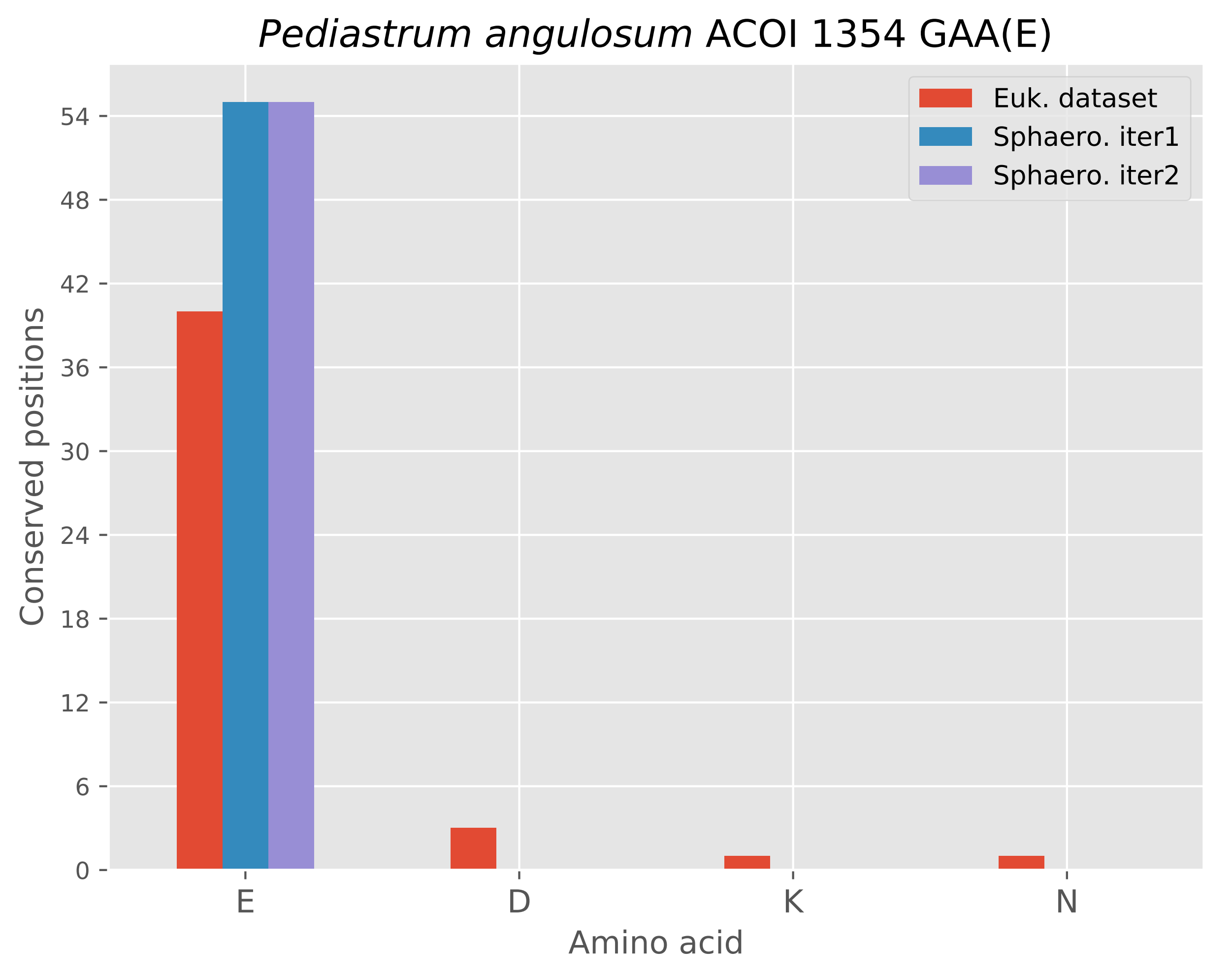

Pediastrum angulosum ACOI 1354 GAA(E)
Euk. dataset
Sphaero. iter1
Sphaero. iter2
Conserved positions
0
6
12
18
24
30
36
42
48
54
E
D
K
N
Amino acid

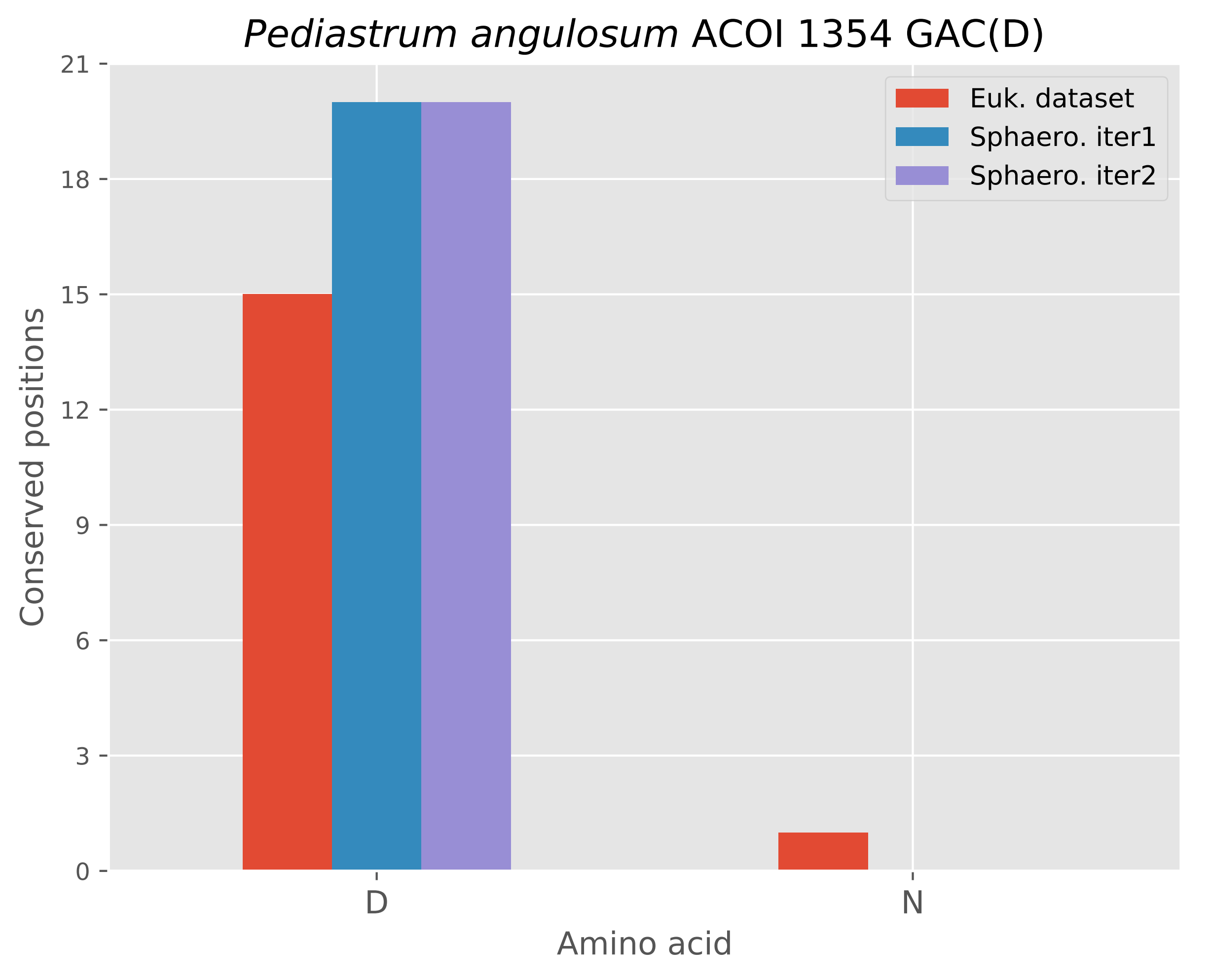

Pediastrum angulosum ACOI 1354 GAC(D)
Euk. dataset
Sphaero. iter1
Sphaero. iter2
Conserved positions
0
3
6
9
12
15
18
21
D
N
Amino acid

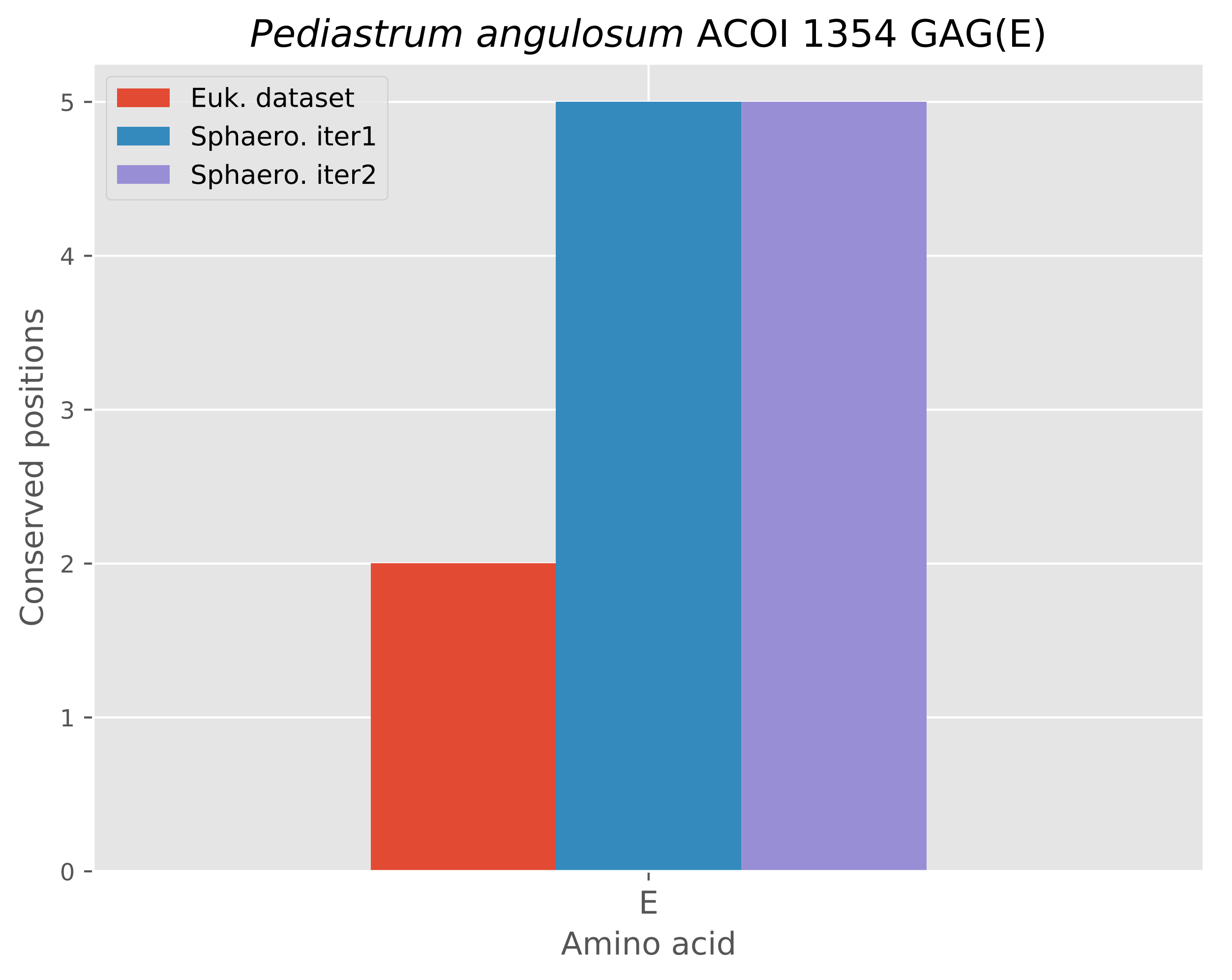

Pediastrum angulosum ACOI 1354 GAG(E)
Euk. dataset
Sphaero. iter1
Sphaero. iter2
Conserved positions
0
1
2
3
4
5
E
Amino acid

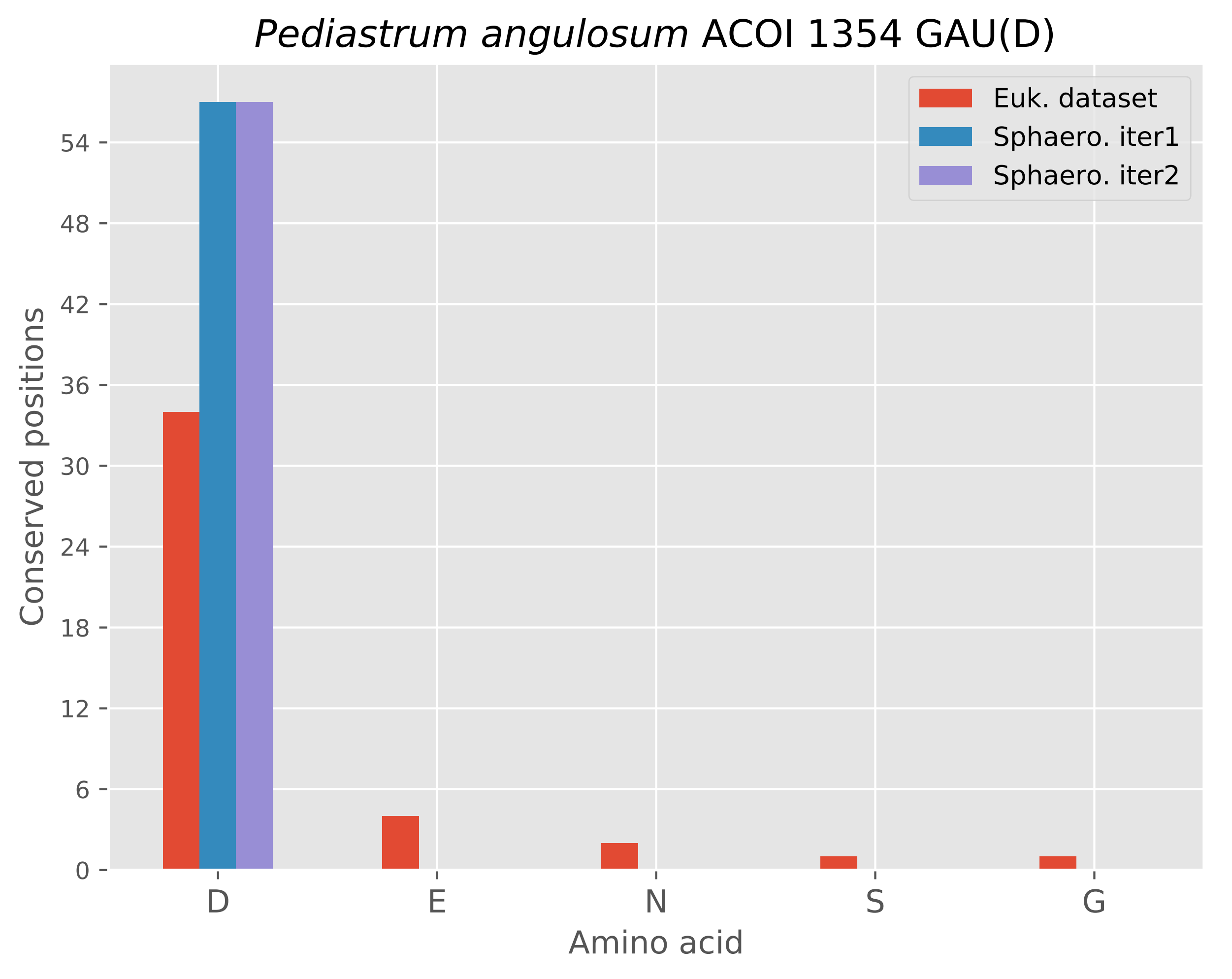

Pediastrum angulosum ACOI 1354 GAU(D)
Euk. dataset
Sphaero. iter1
Sphaero. iter2
Conserved positions
0
6
12
18
24
30
36
42
48
54
D
E
N
S
G
Amino acid

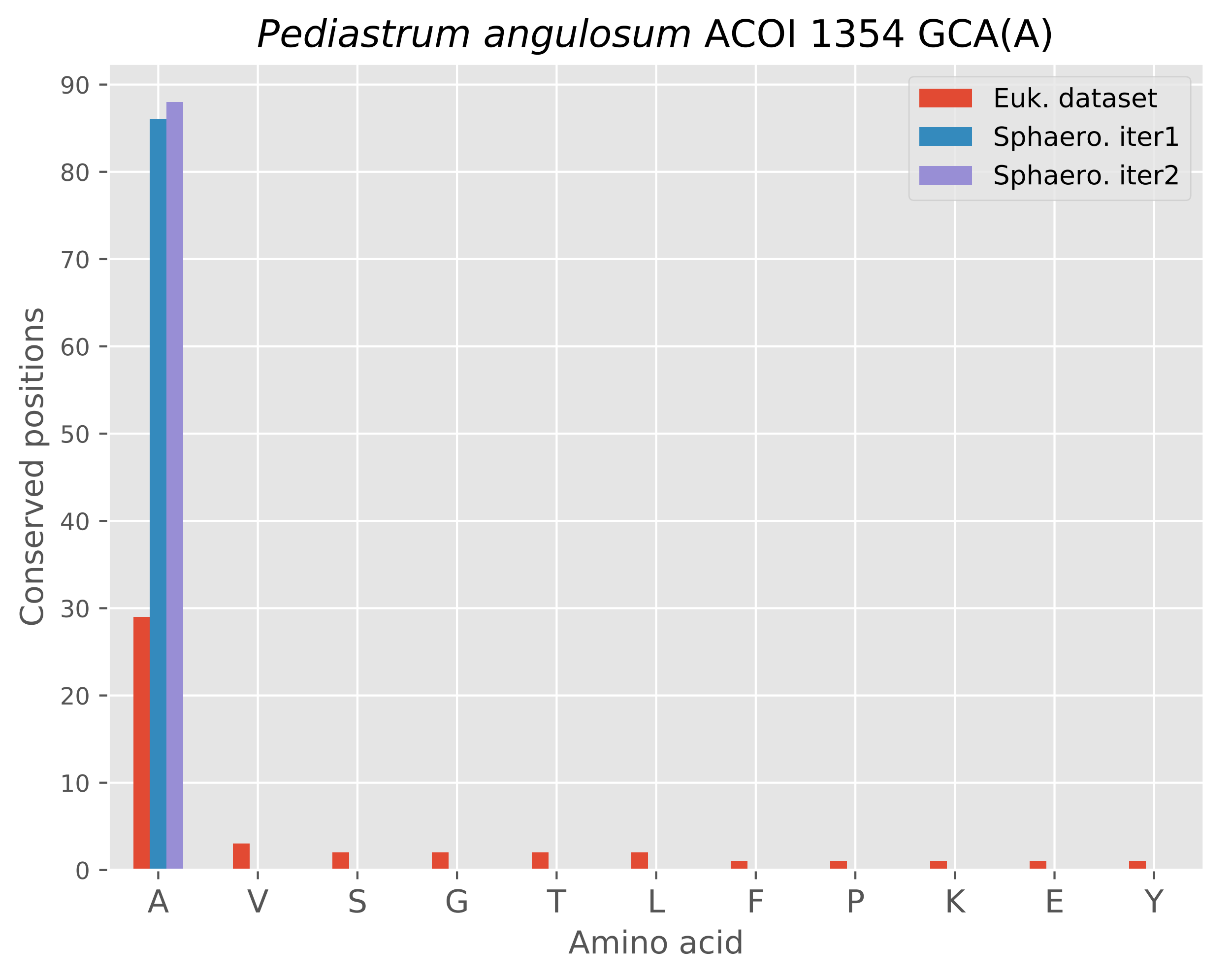

Pediastrum angulosum ACOI 1354 GCA(A)
Euk. dataset
Sphaero. iter1
Sphaero. iter2
Conserved positions
0
10
20
30
40
50
60
70
80
90
A
V
S
G
T
L
F
P
K
E
Y
Amino acid

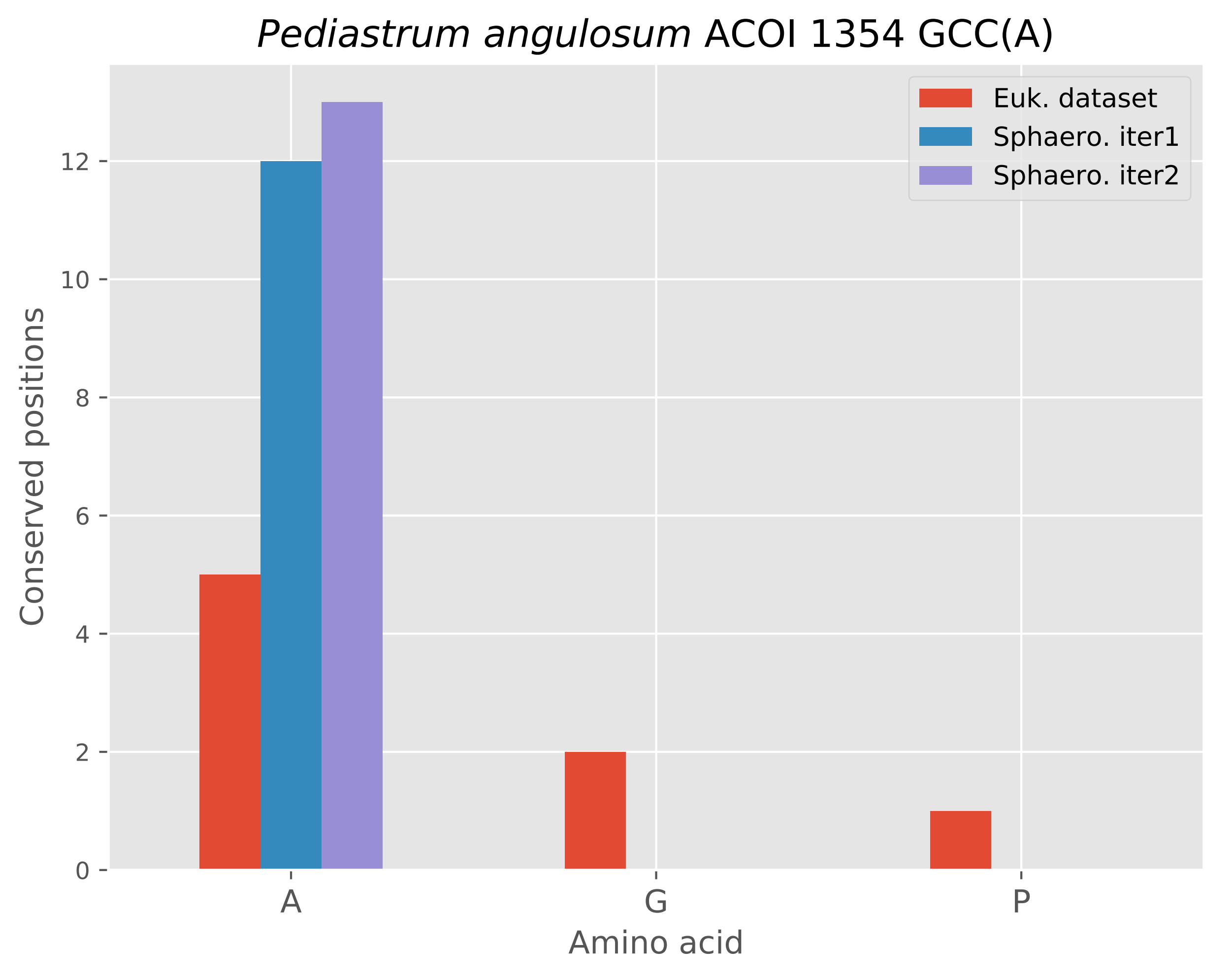
Pediastrum angulosum ACOI 1354 GCC(A)
Euk. dataset
Sphaero. iter1
Sphaero. iter2
Conserved positions
0
2
4
6
8
10
12
A
G
P
Amino acid

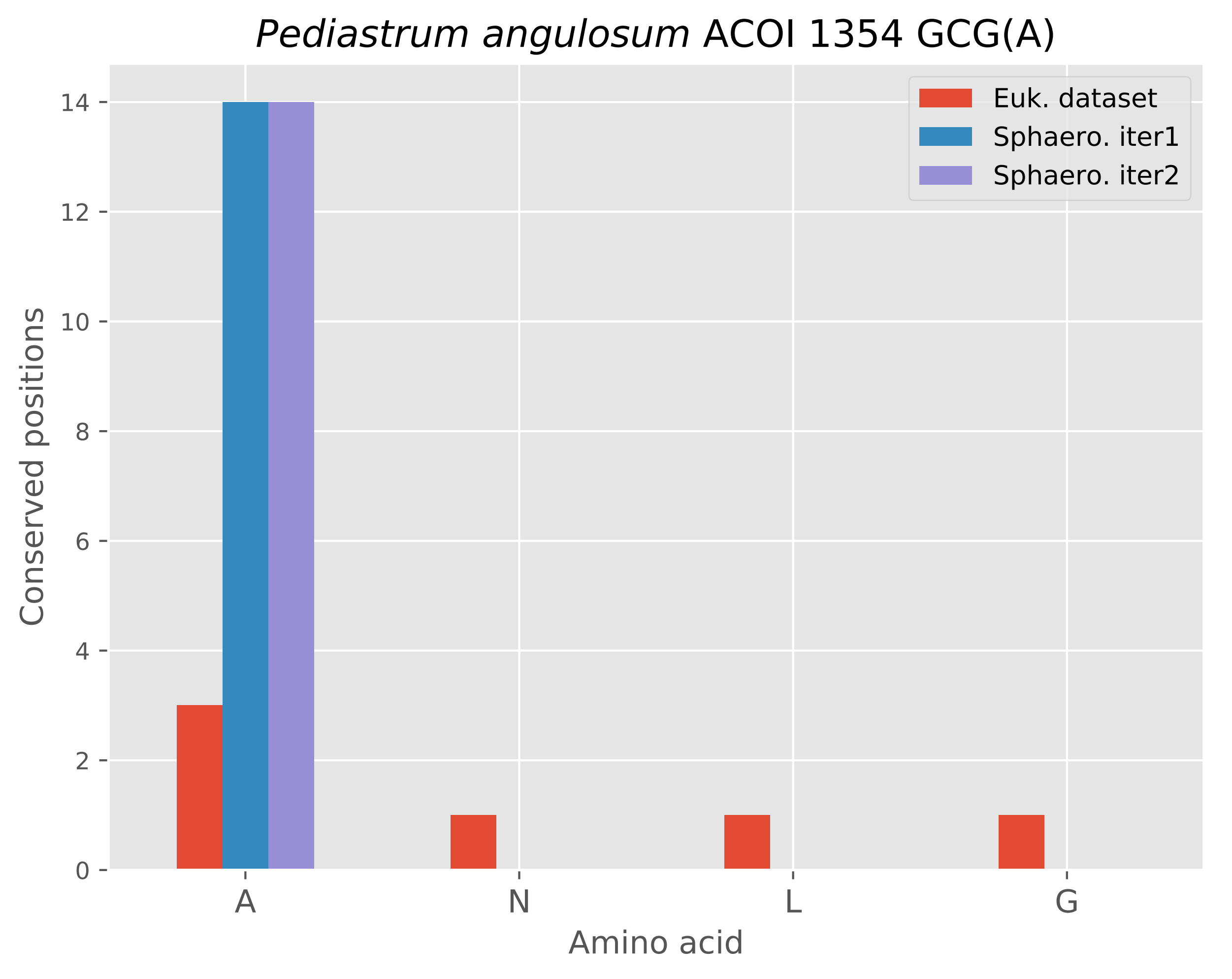

Pediastrum angulosum ACOI 1354 GCG(A)
Euk. dataset
Sphaero. iter1
Sphaero. iter2
Conserved positions
0
2
4
6
8
10
12
14
A
N
L
G
Amino acid

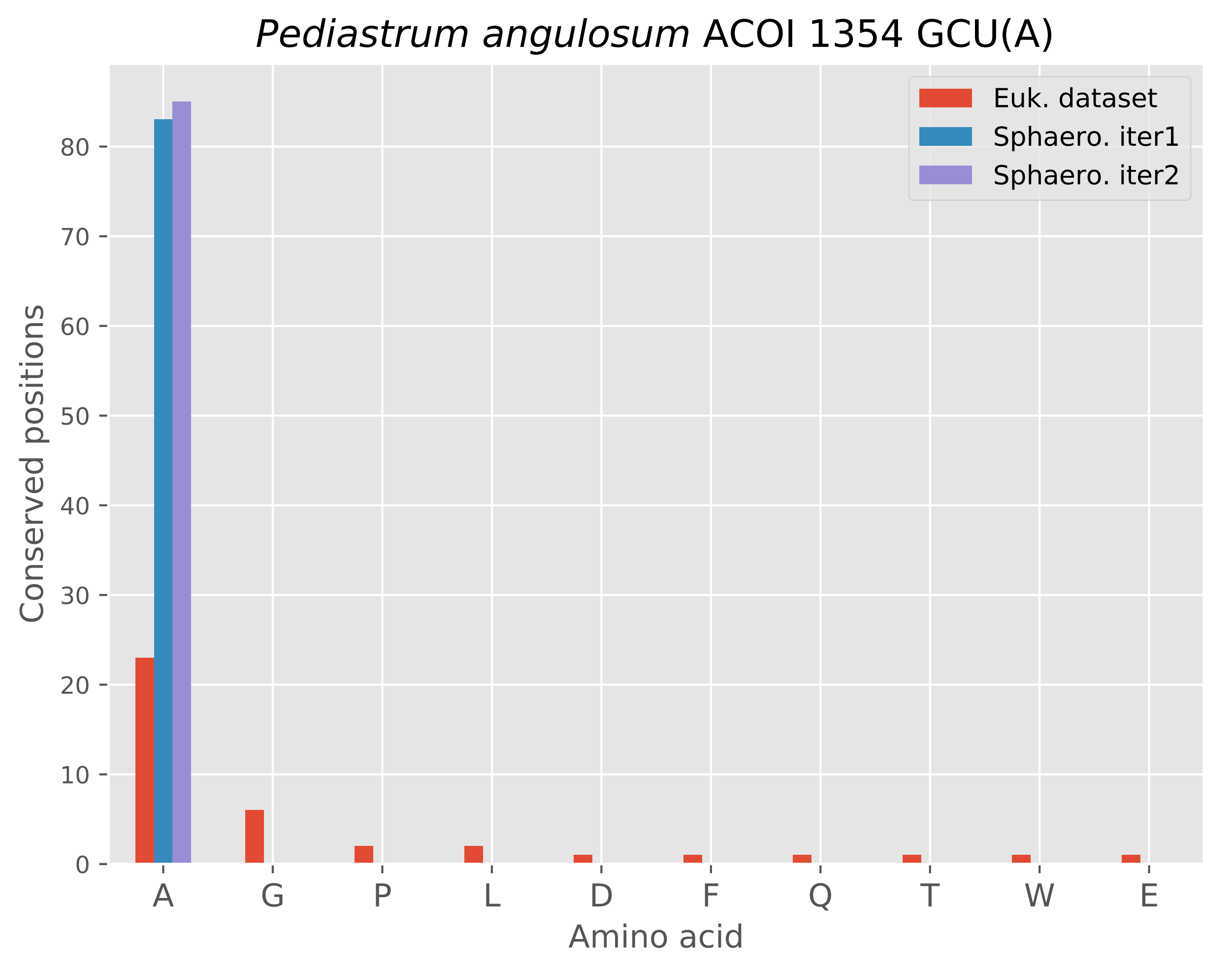
Pediastrum angulosum ACOI 1354 GCU(A)
Euk. dataset
Sphaero. iter1
Sphaero. iter2
Conserved positions
0
10
20
30
40
50
60
70
80
A
G
P
L
D
F
Q
T
W
E
Amino acid

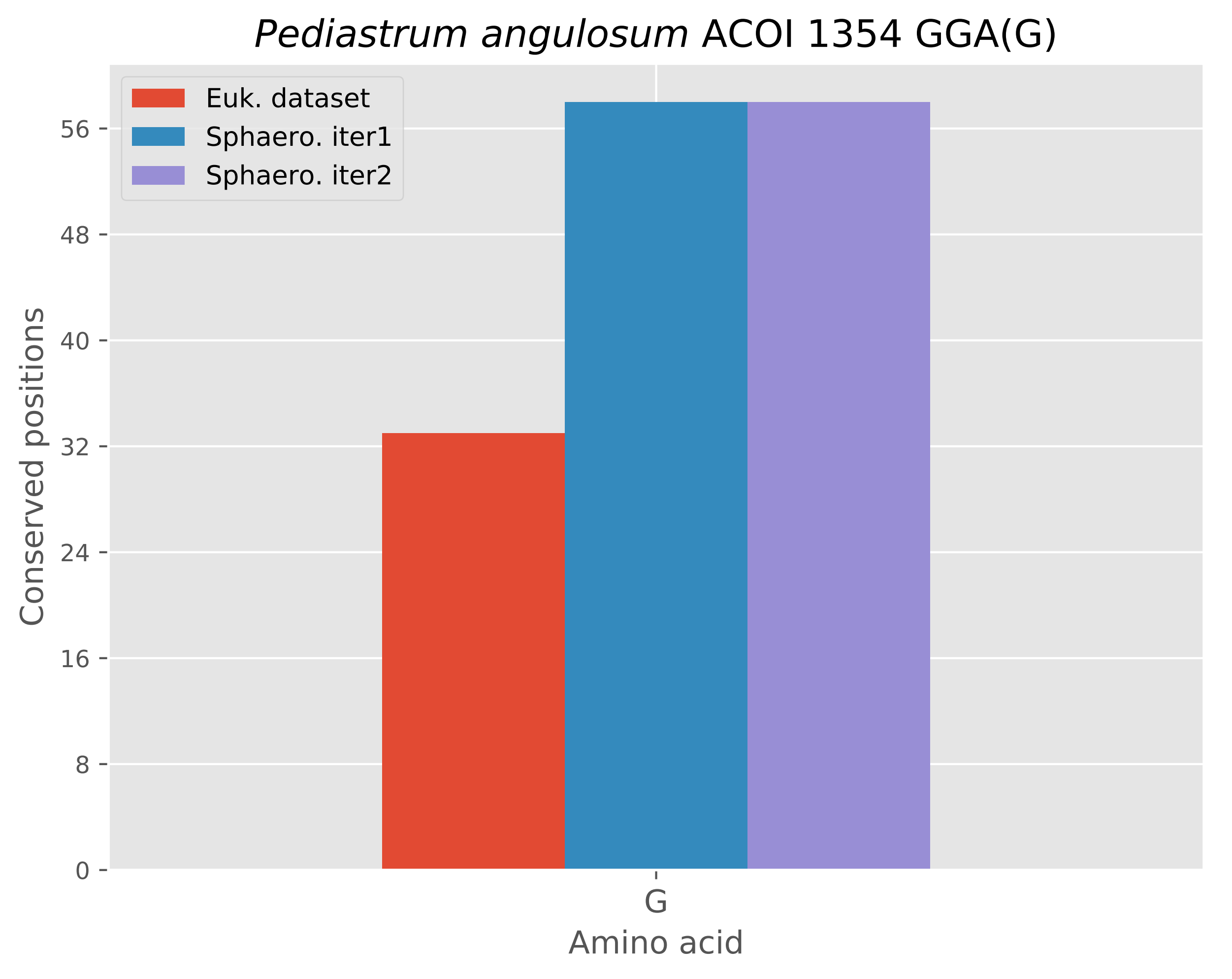

Pediastrum angulosum ACOI 1354 GGA(G)
Euk. dataset
Sphaero. iter1
Sphaero. iter2
Conserved positions
0
8
16
24
32
40
48
56
G
Amino acid

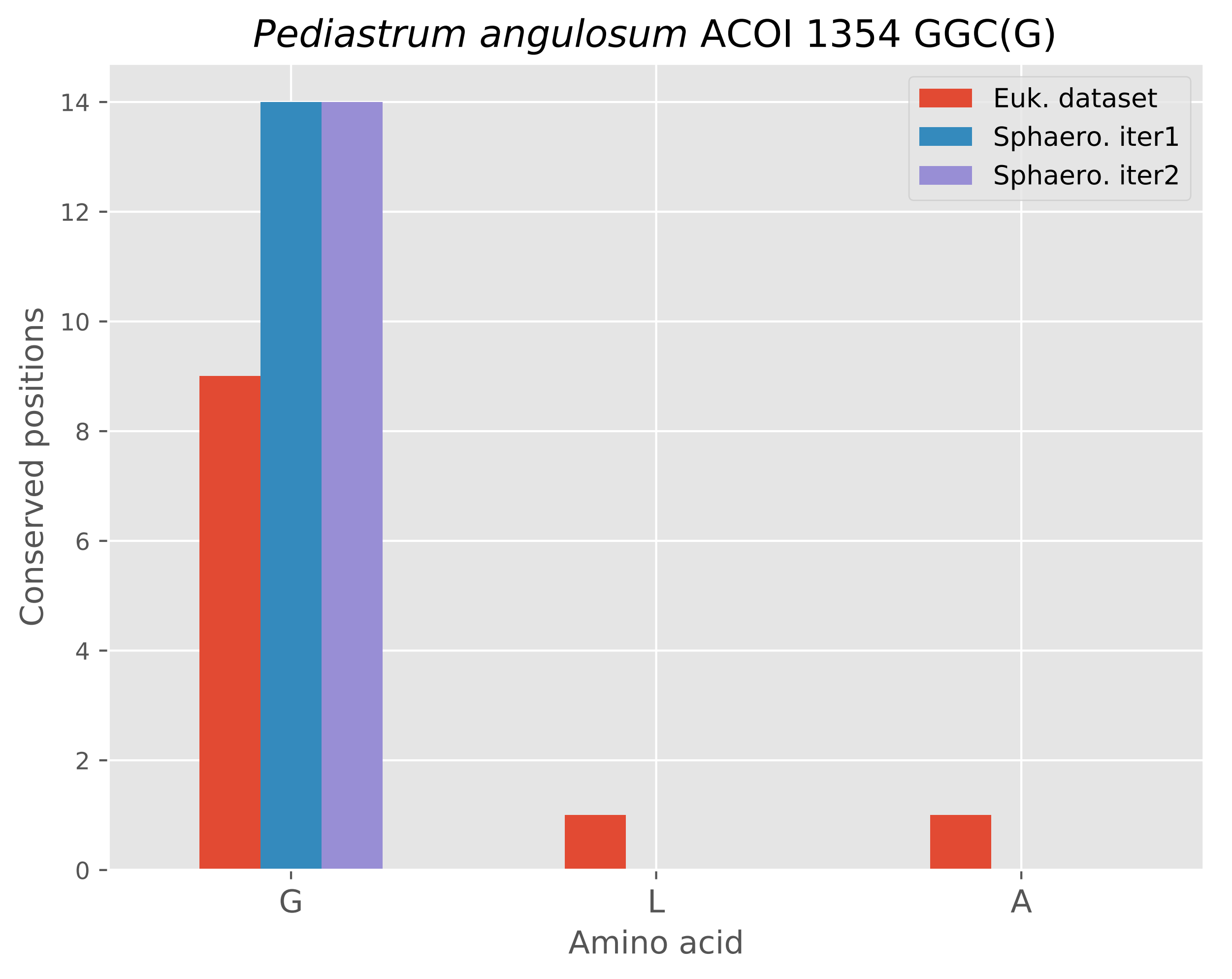

Pediastrum angulosum ACOI 1354 GGC(G)
Euk. dataset
Sphaero. iter1
Sphaero. iter2
Conserved positions
0
2
4
6
8
10
12
14
G
L
A
Amino acid

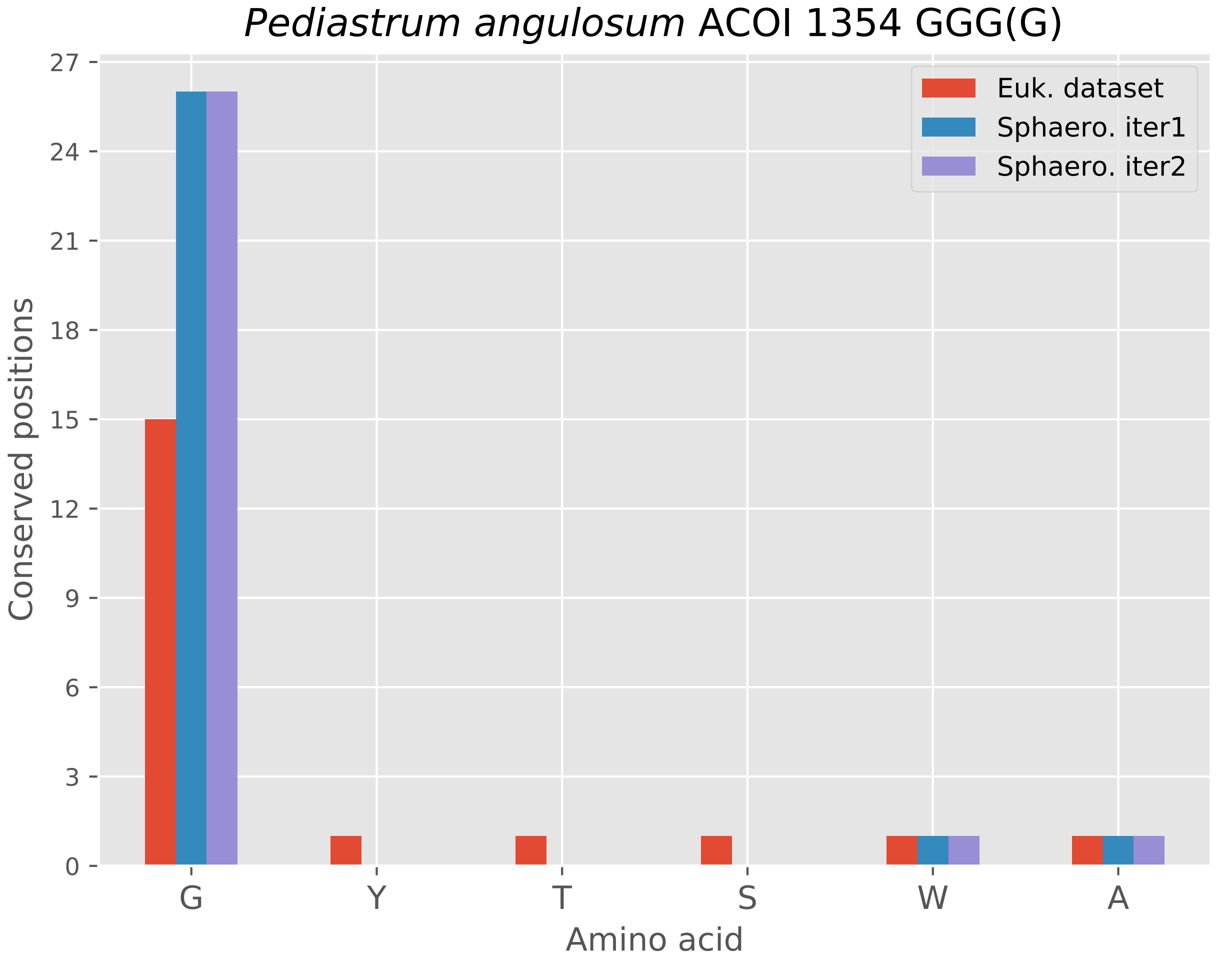

Pediastrum angulosum ACOI 1354 GGG(G)
Euk. dataset
Sphaero. iter1
Sphaero. iter2
Conserved positions
0
3
6
9
12
15
18
21
24
27
G
Y
T
S
W
A
Amino acid

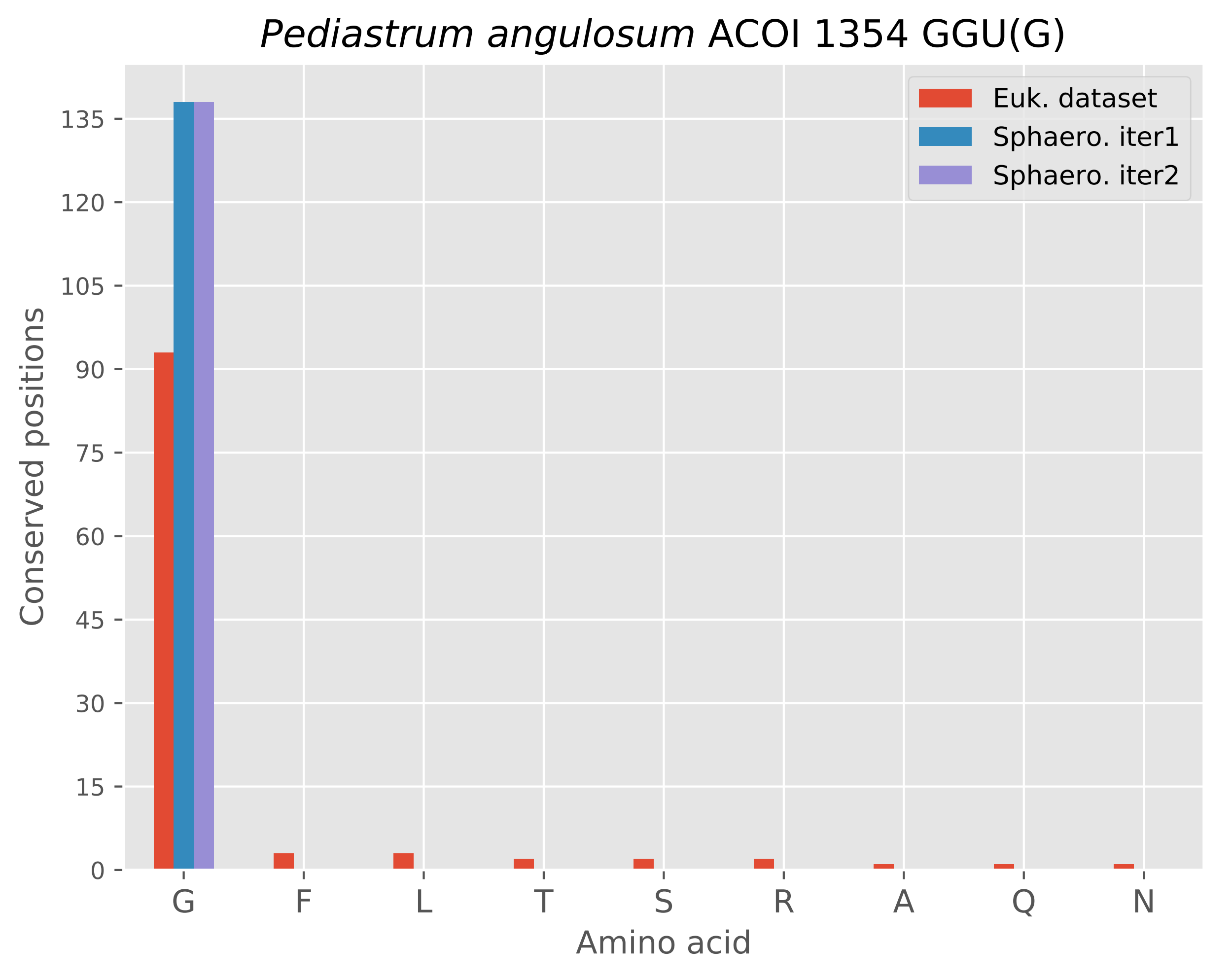

Pediastrum angulosum ACOI 1354 GGU(G)
Euk. dataset
Sphaero. iter1
Sphaero. iter2
Conserved positions
0
15
30
45
60
75
90
105
120
135
G
F
L
T
S
R
A
Q
N
Amino acid

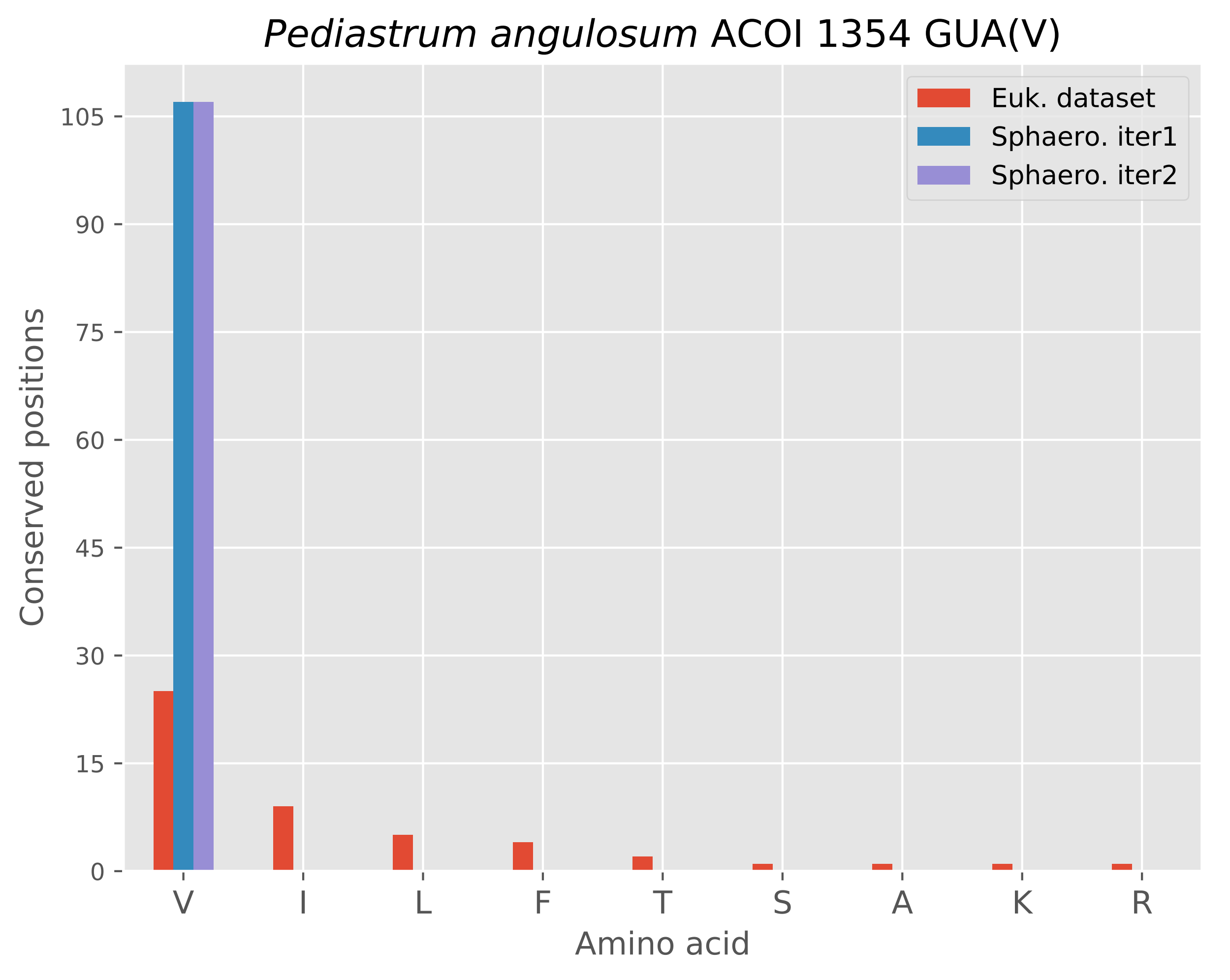

Pediastrum angulosum ACOI 1354 GUA(V)
Euk. dataset
Sphaero. iter1
Sphaero. iter2
Conserved positions
0
15
30
45
60
75
90
105
V
I
L
F
T
S
A
K
R
Amino acid

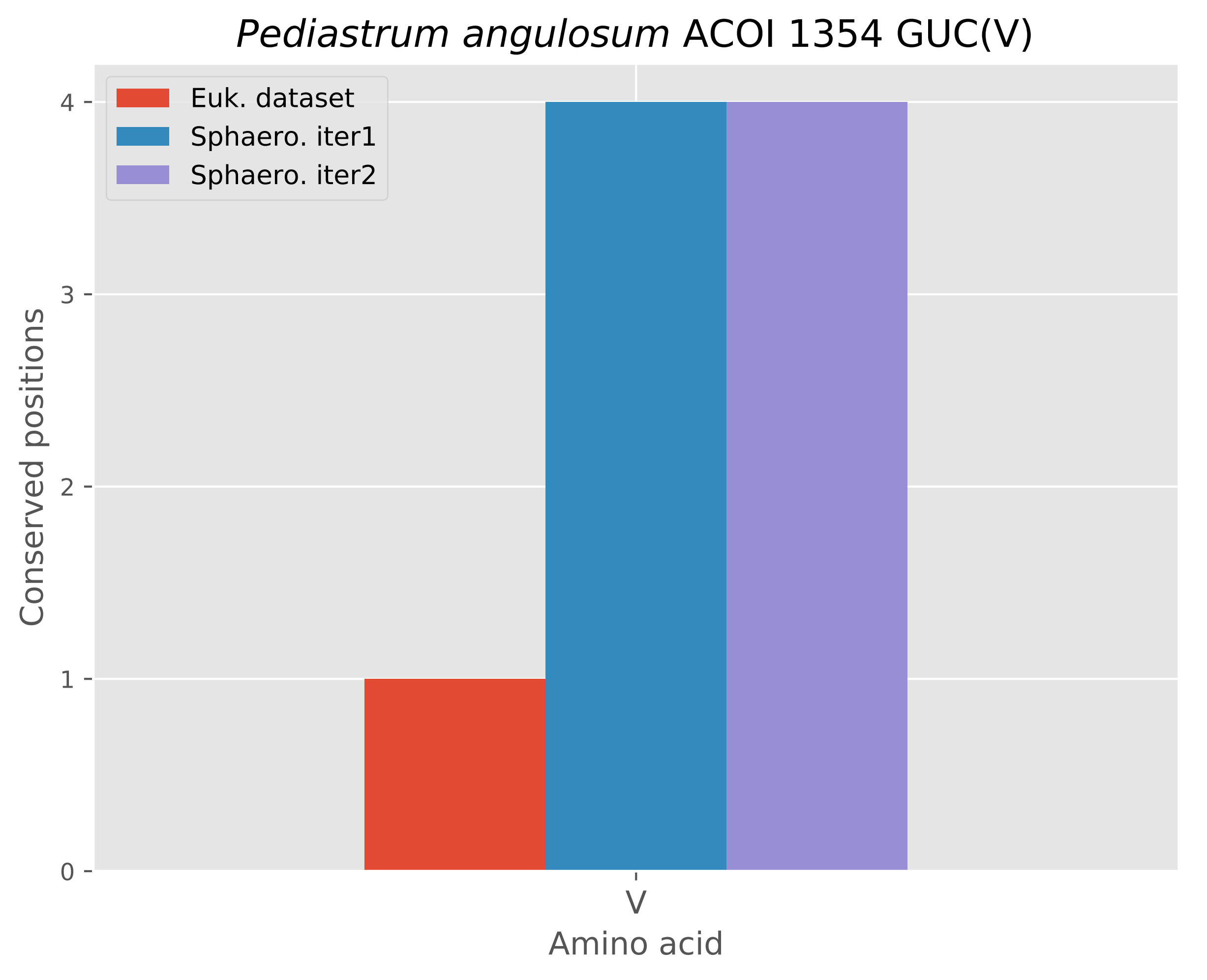

Pediastrum angulosum ACOI 1354 GUC(V)
Euk. dataset
Sphaero. iter1
Sphaero. iter2
Conserved positions
0
1
2
3
4
V
Amino acid

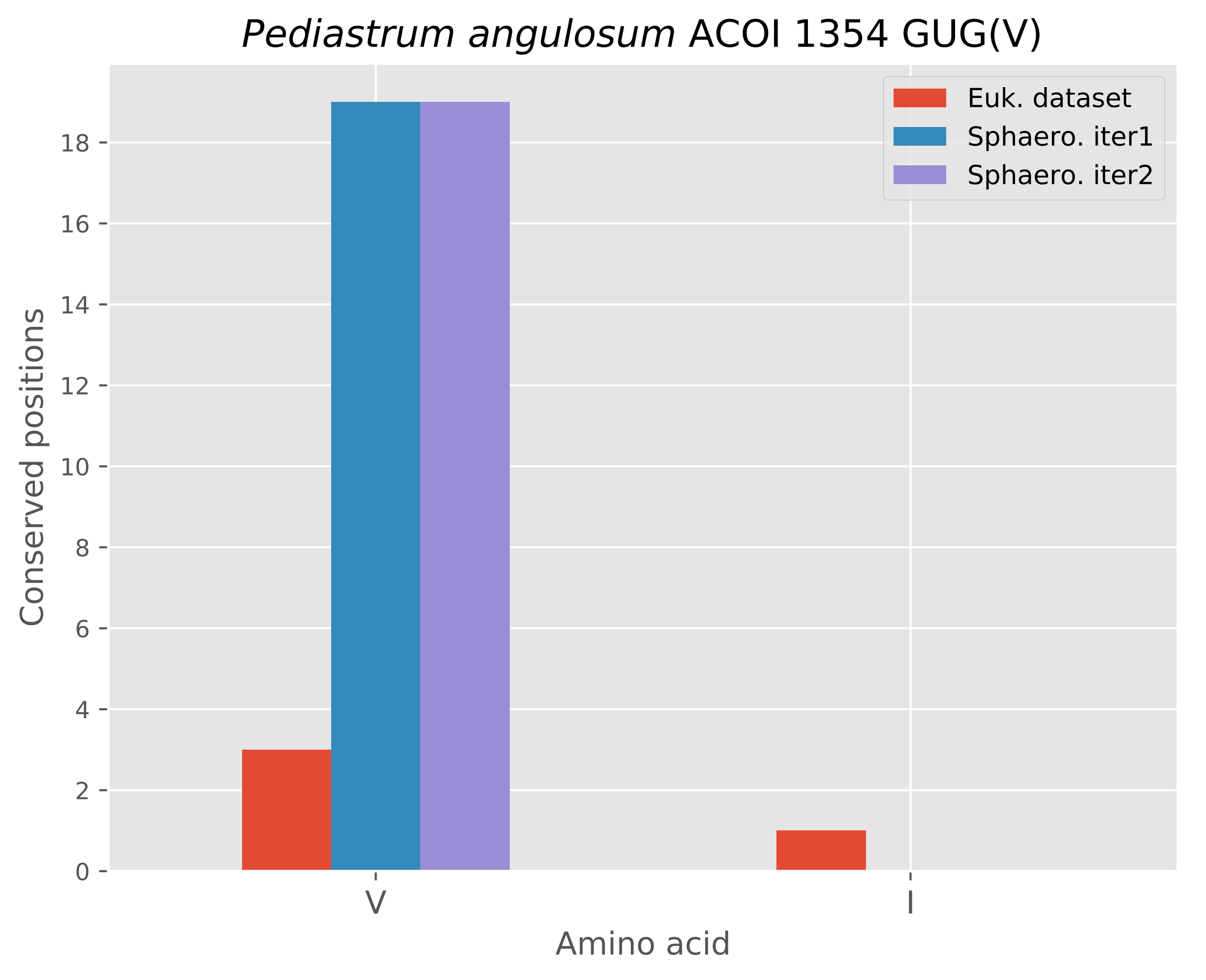

Pediastrum angulosum ACOI 1354 GUG(V)
Euk. dataset
Sphaero. iter1
Sphaero. iter2
Conserved positions
0
2
4
6
8
10
12
14
16
18
V
I
Amino acid

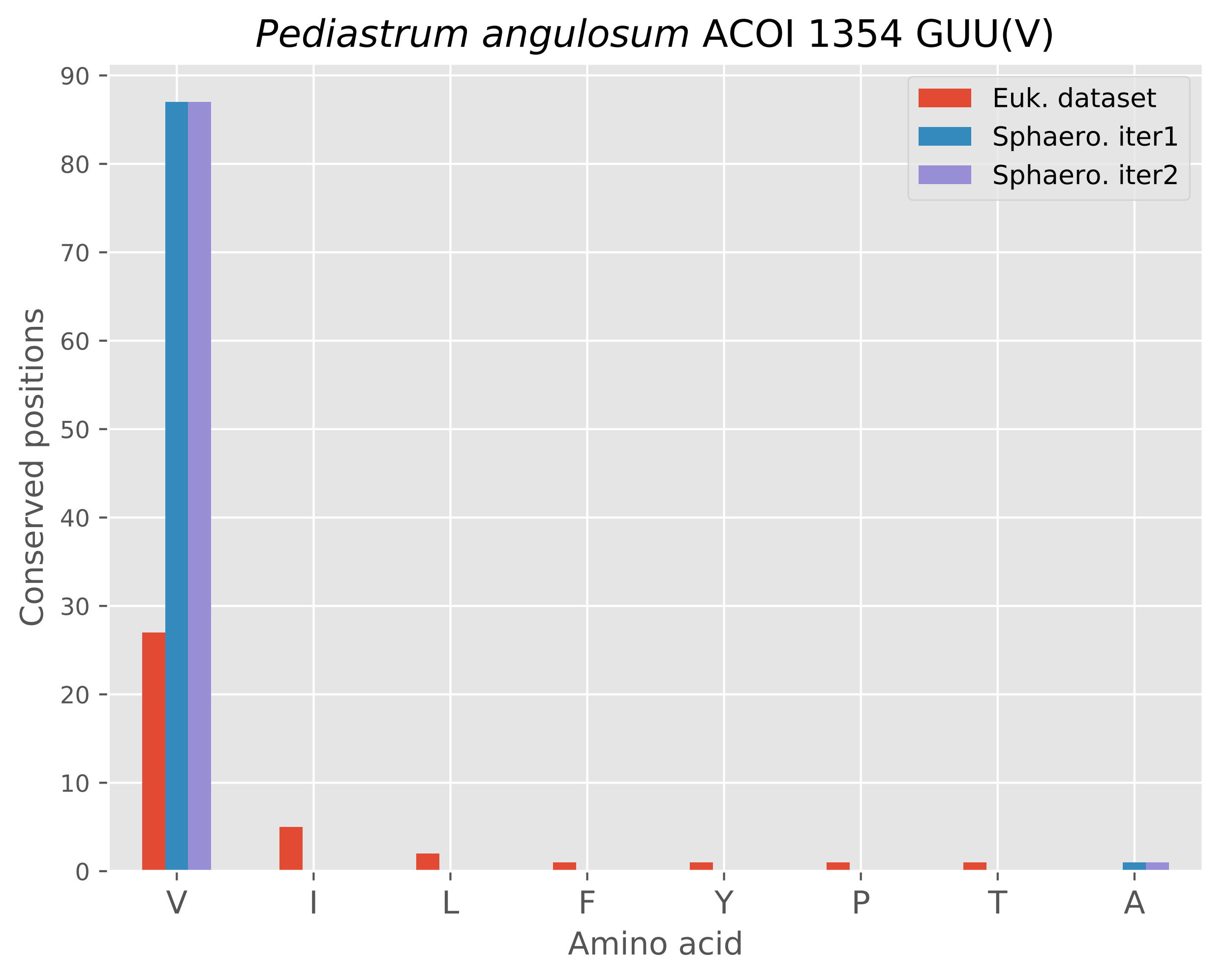
Pediastrum angulosum ACOI 1354 GUU(V)
Euk. dataset
Sphaero. iter1
Sphaero. iter2
Conserved positions
0
10
20
30
40
50
60
70
80
90
V
I
L
F
Y
P
T
A
Amino acid

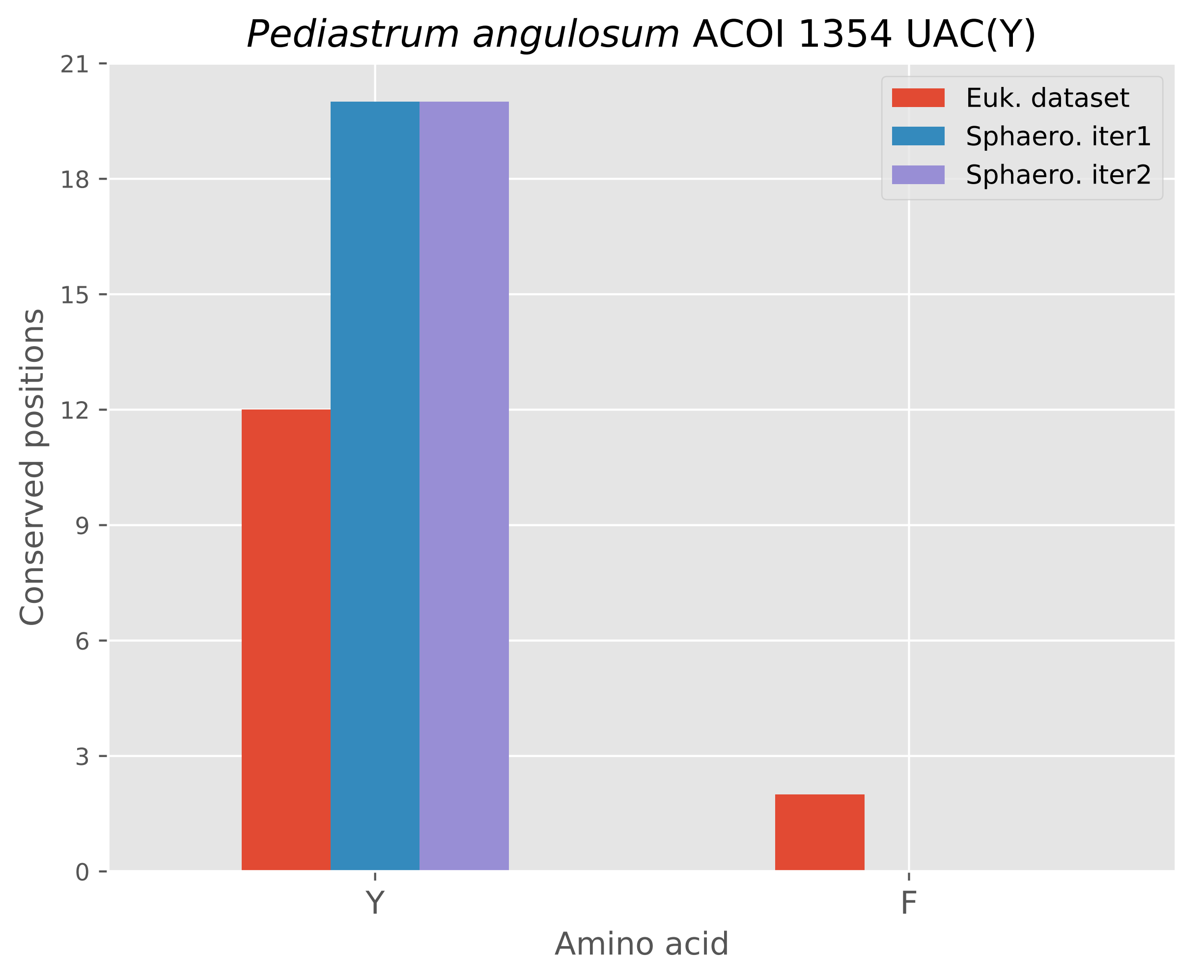

Pediastrum angulosum ACOI 1354 UAC(Y)
Euk. dataset
Sphaero. iter1
Sphaero. iter2
Conserved positions
Amino acid
Y
F
0
3
6
9
12
15
18
21

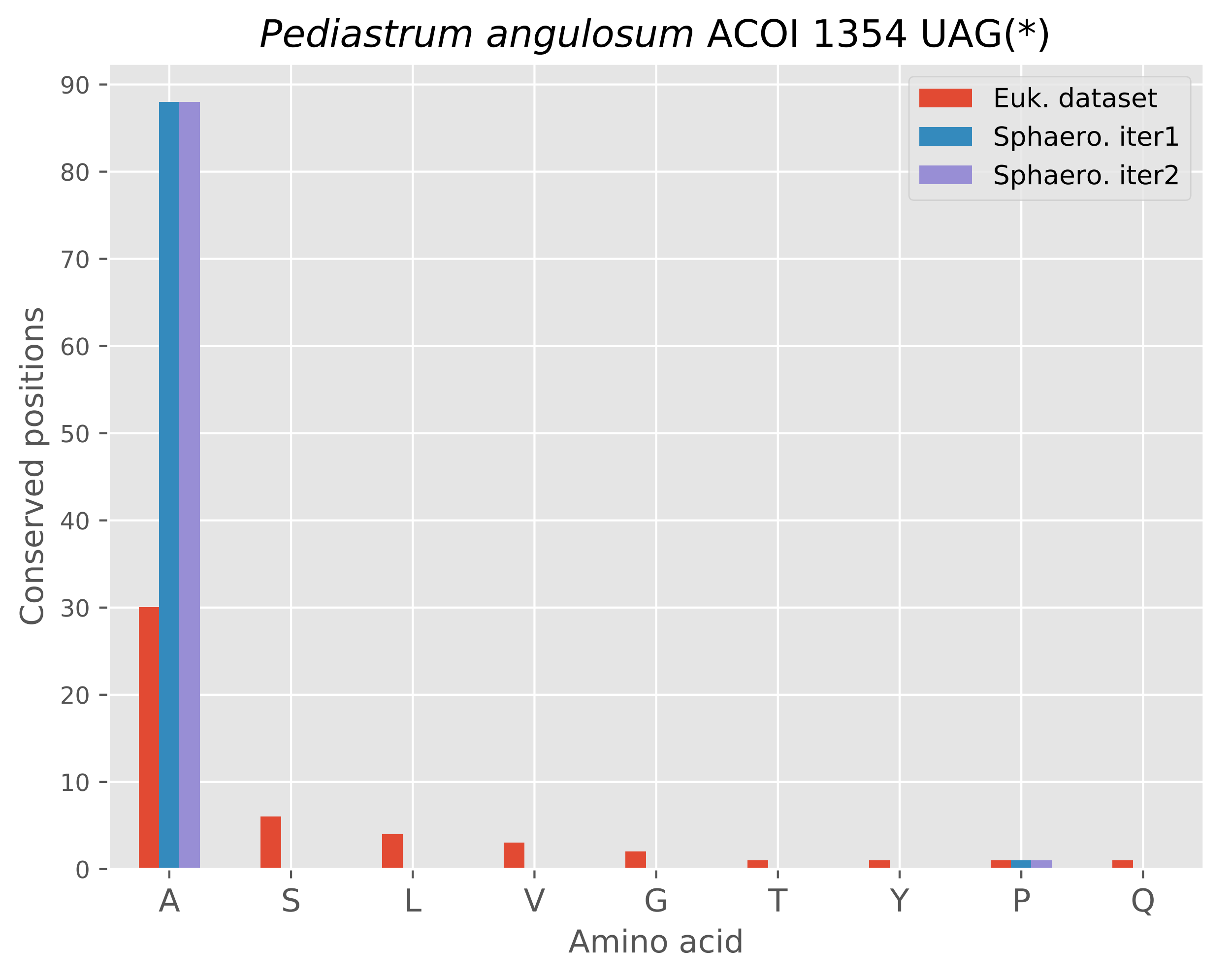
Pediastrum angulosum ACOI 1354 UAG(*)
Euk. dataset
Sphaero. iter1
Sphaero. iter2
Conserved positions
0
10
20
30
40
50
60
70
80
90
A
S
L
V
G
T
Y
P
Q
Amino acid

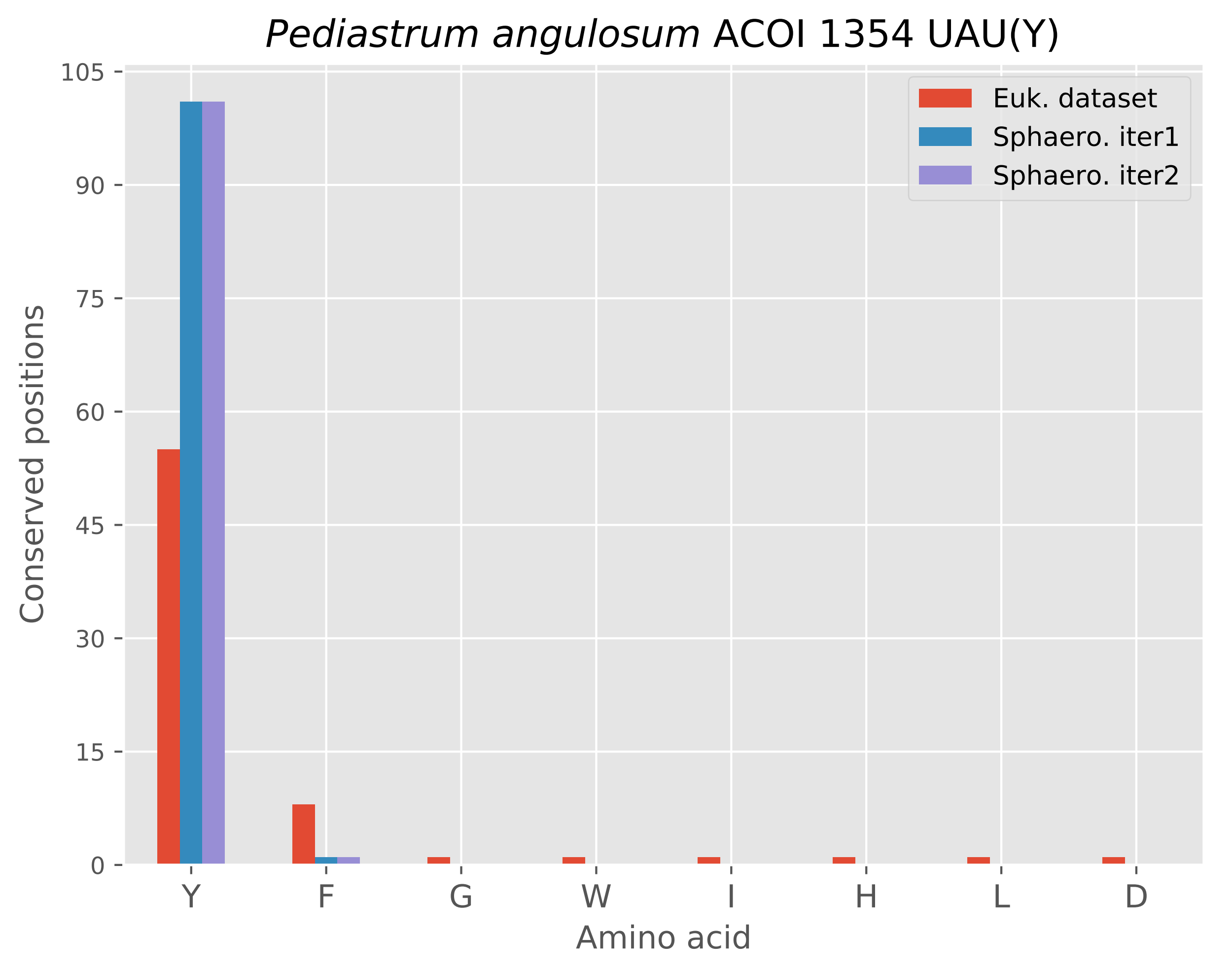

Pediastrum angulosum ACOI 1354 UAU(Y)
Euk. dataset
Sphaero. iter1
Sphaero. iter2
Conserved positions
105
90
75
60
45
30
15
0
Y
F
G
W
I
H
L
D
Amino acid

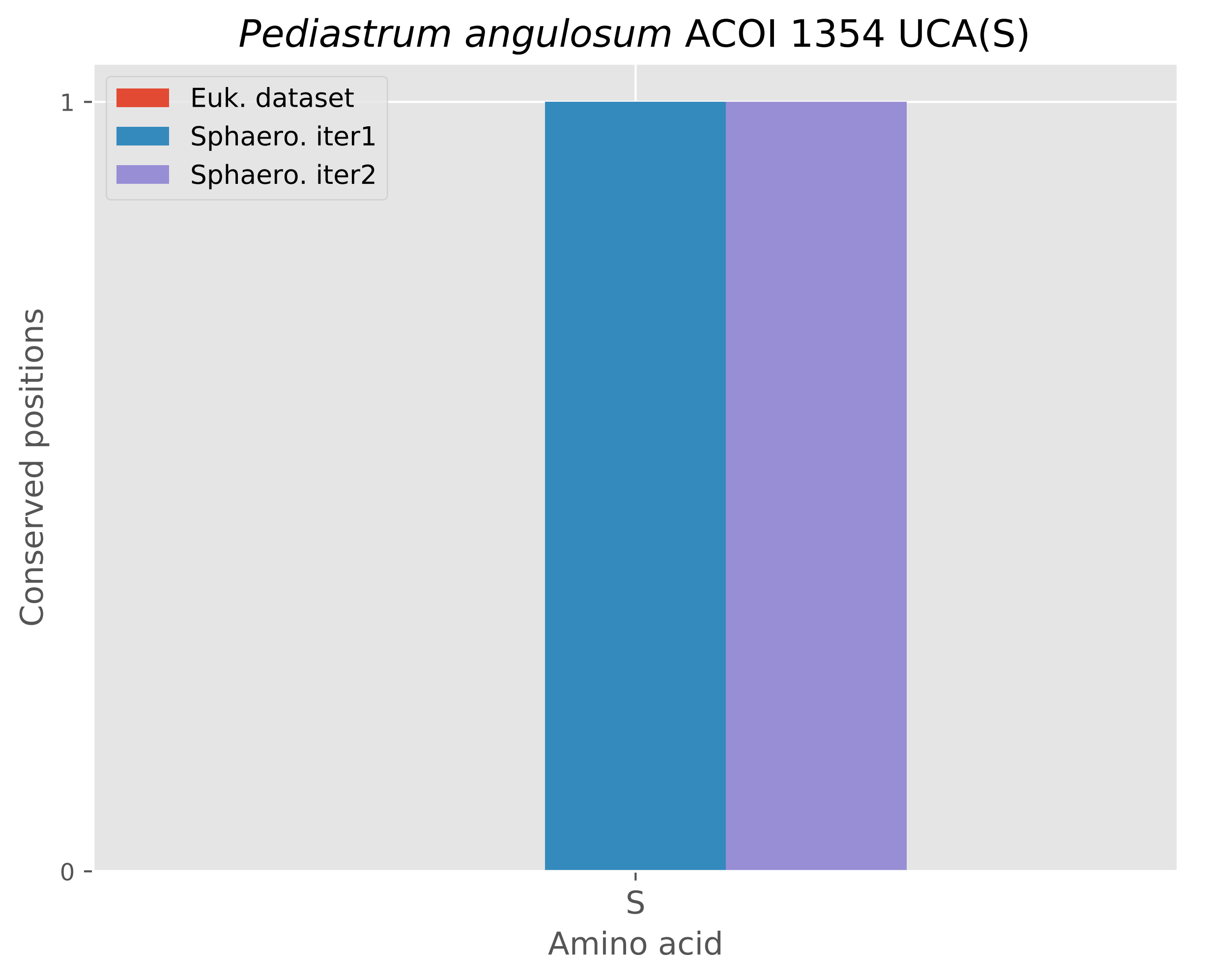

Pediastrum angulosum ACOI 1354 UCA(S)
Euk. dataset
Sphaero. iter1
Sphaero. iter2
Conserved positions
1
0
S
Amino acid

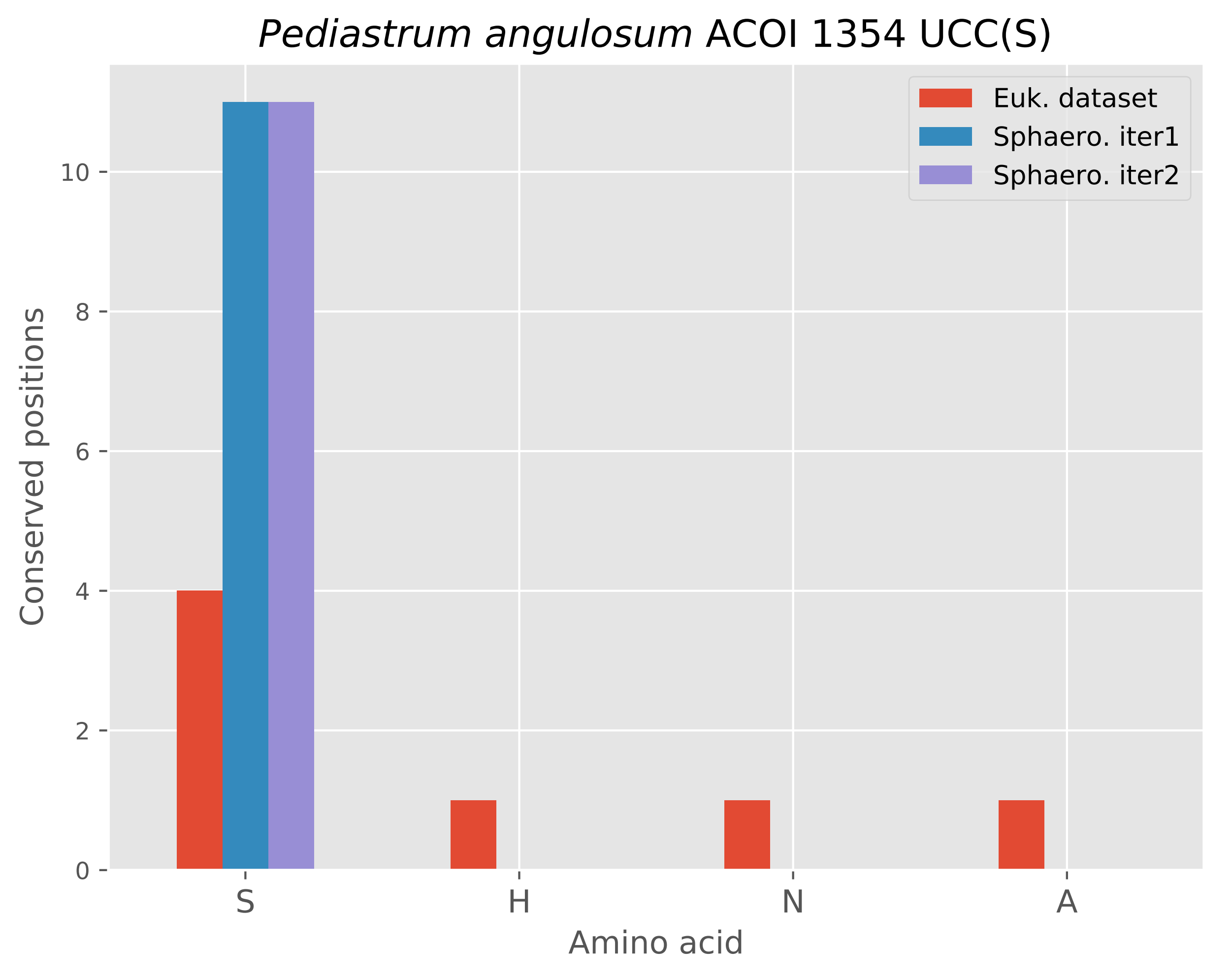

Pediastrum angulosum ACOI 1354 UCC(S)
Euk. dataset
Sphaero. iter1
Sphaero. iter2
Conserved positions
0
2
4
6
8
10
S
H
N
A
Amino acid

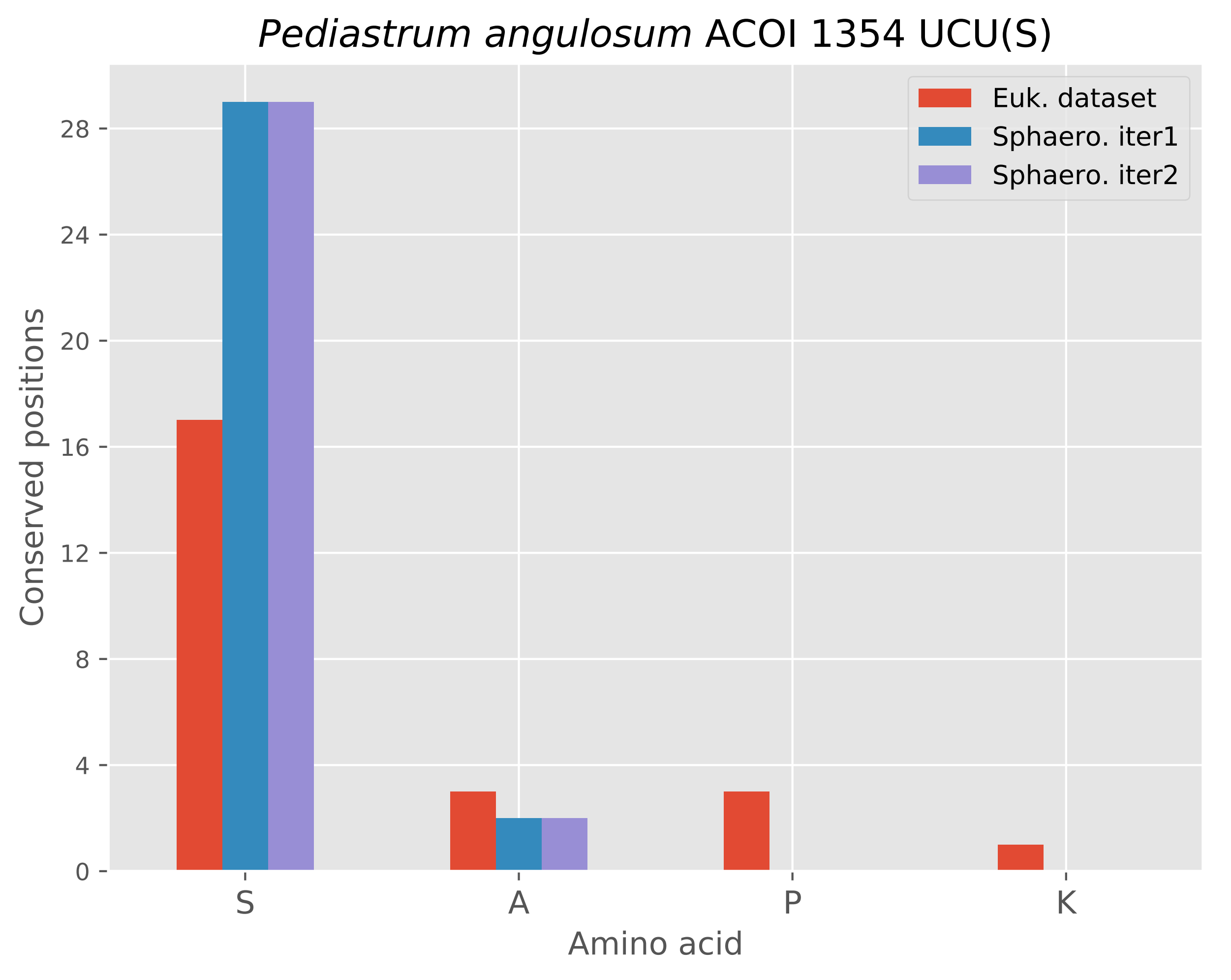

Pediastrum angulosum ACOI 1354 UCU(S)
Euk. dataset
Sphaero. iter1
Sphaero. iter2
Conserved positions
0
4
8
12
16
20
24
28
S
A
P
K
Amino acid

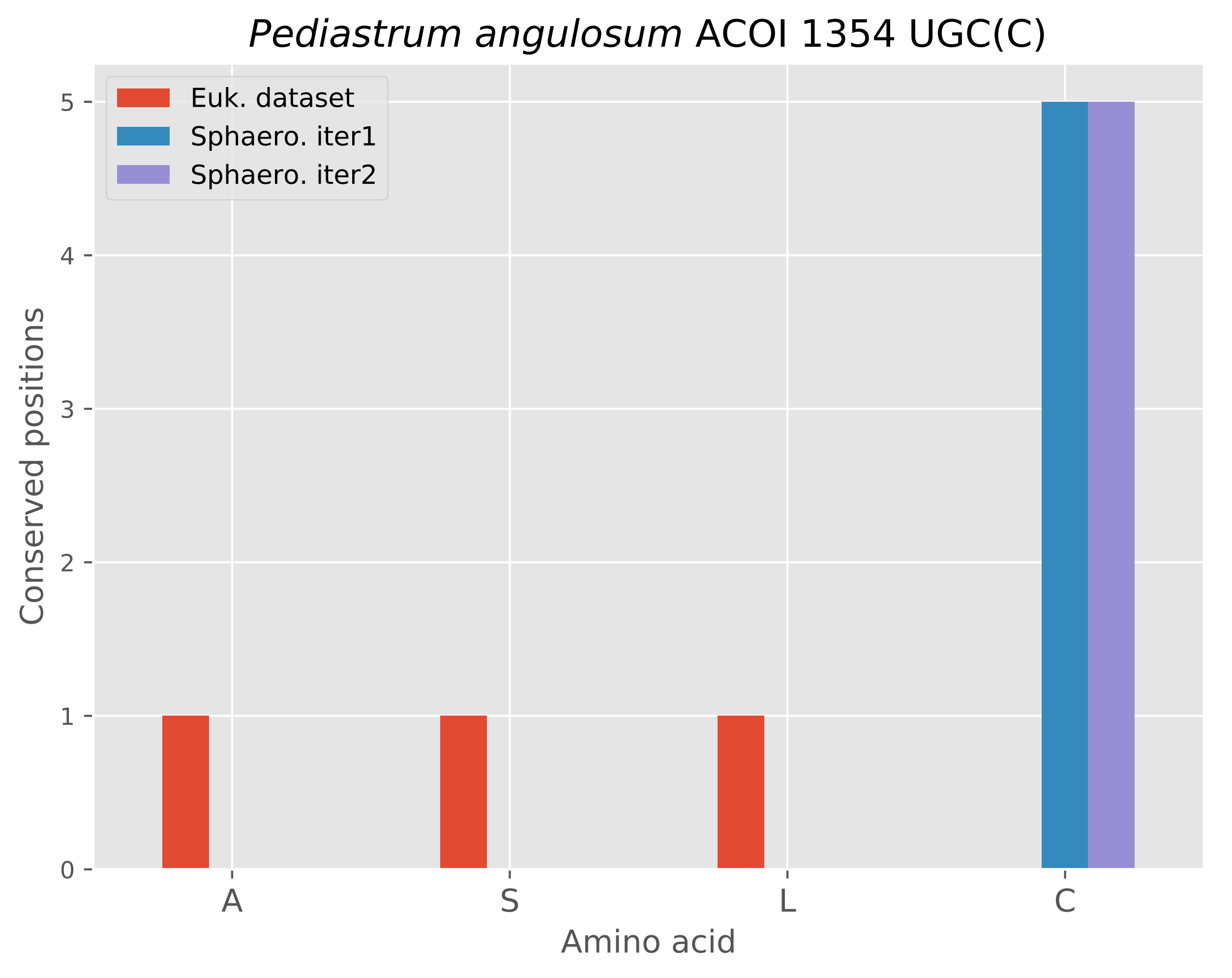

Pediastrum angulosum ACOI 1354 UGC(C)
Euk. dataset
Sphaero. iter1
Sphaero. iter2
Conserved positions
0
1
2
3
4
5
A
S
L
C
Amino acid

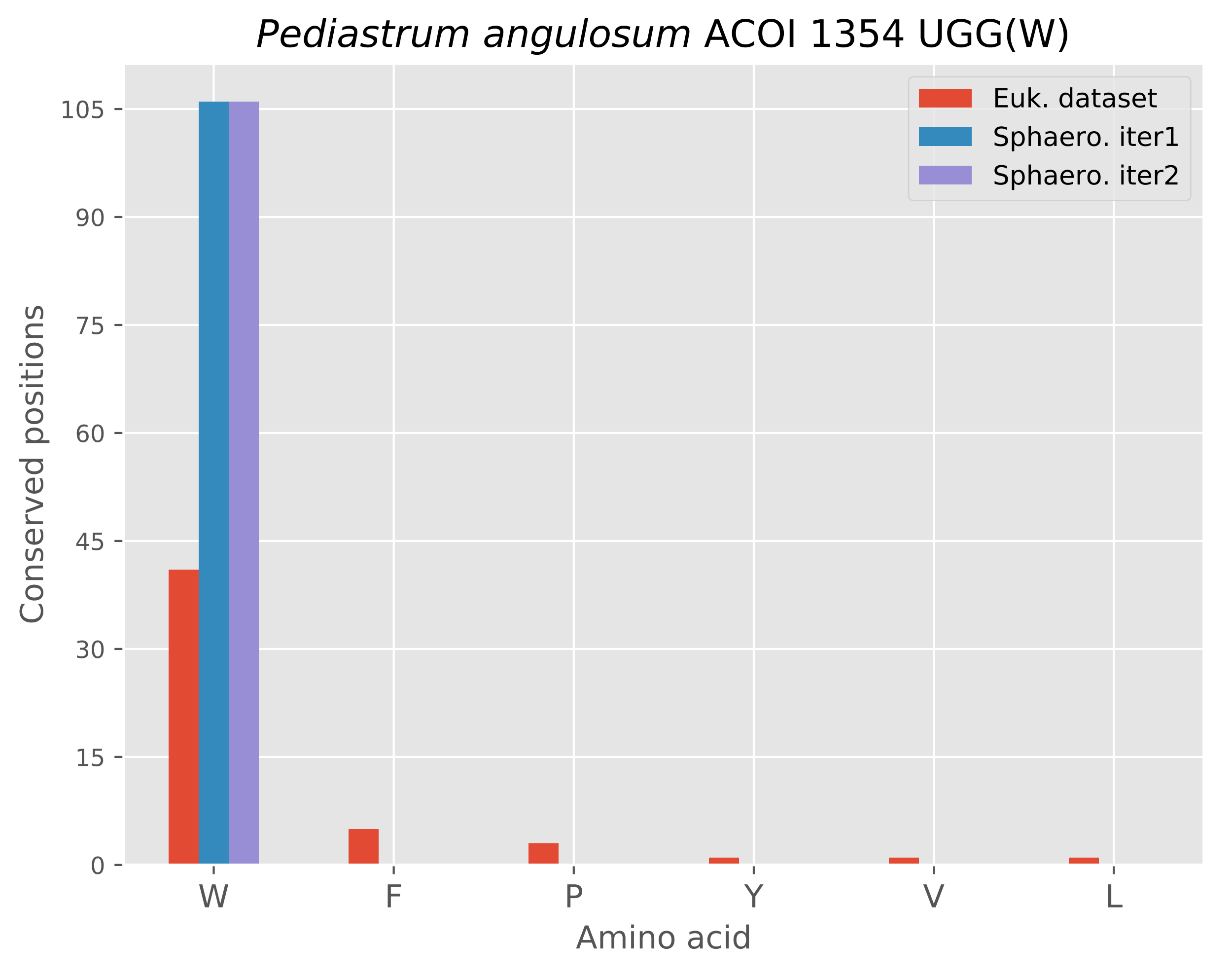

Pediastrum angulosum ACOI 1354 UGG(W)
Euk. dataset
Sphaero. iter1
Sphaero. iter2
Conserved positions
105
90
75
60
45
30
15
0
W
F
P
Y
V
L
Amino acid

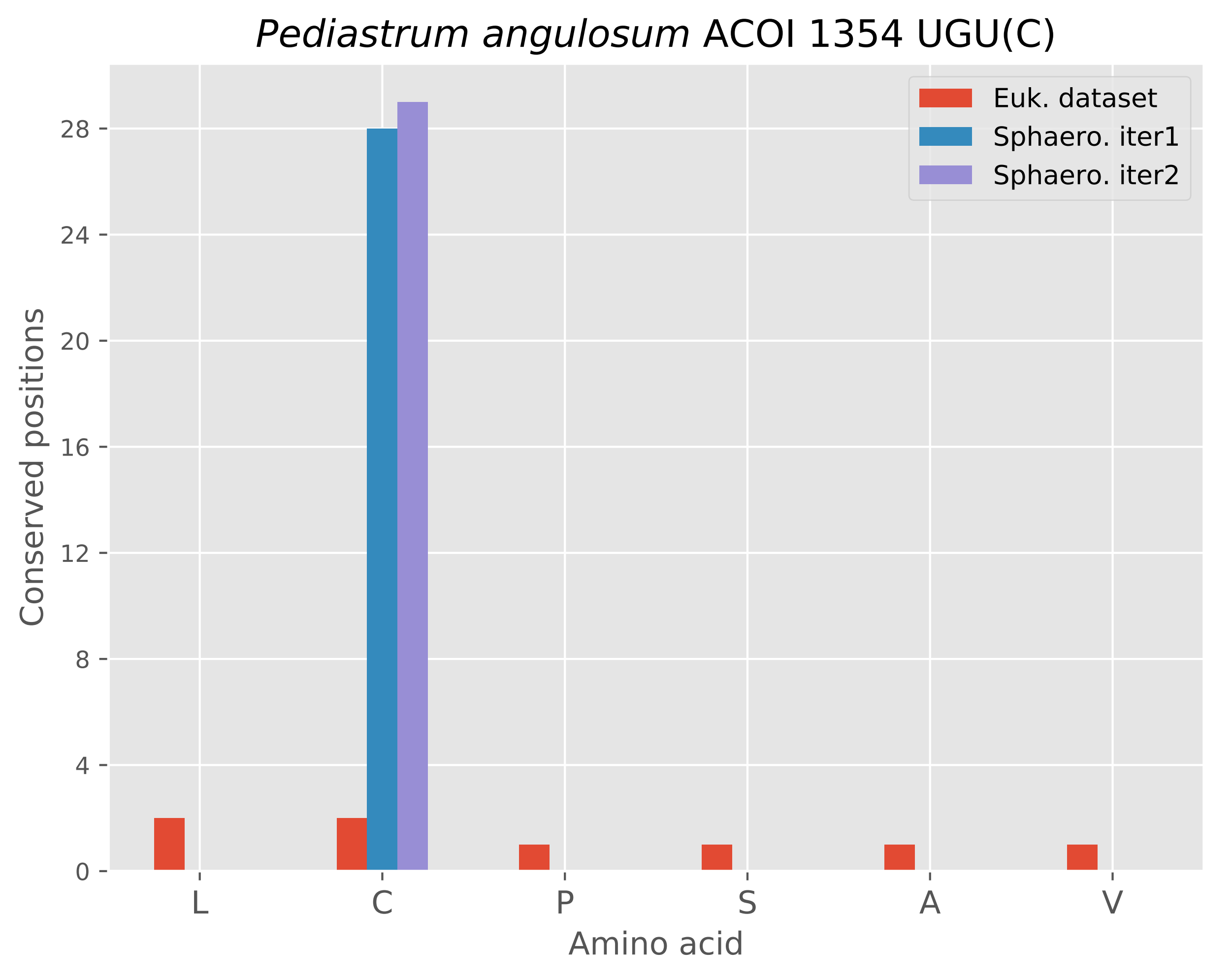

Pediastrum angulosum ACOI 1354 UGU(C)
Euk. dataset
Sphaero. iter1
Sphaero. iter2
Conserved positions
0
4
8
12
16
20
24
28
L
C
P
S
A
V
Amino acid

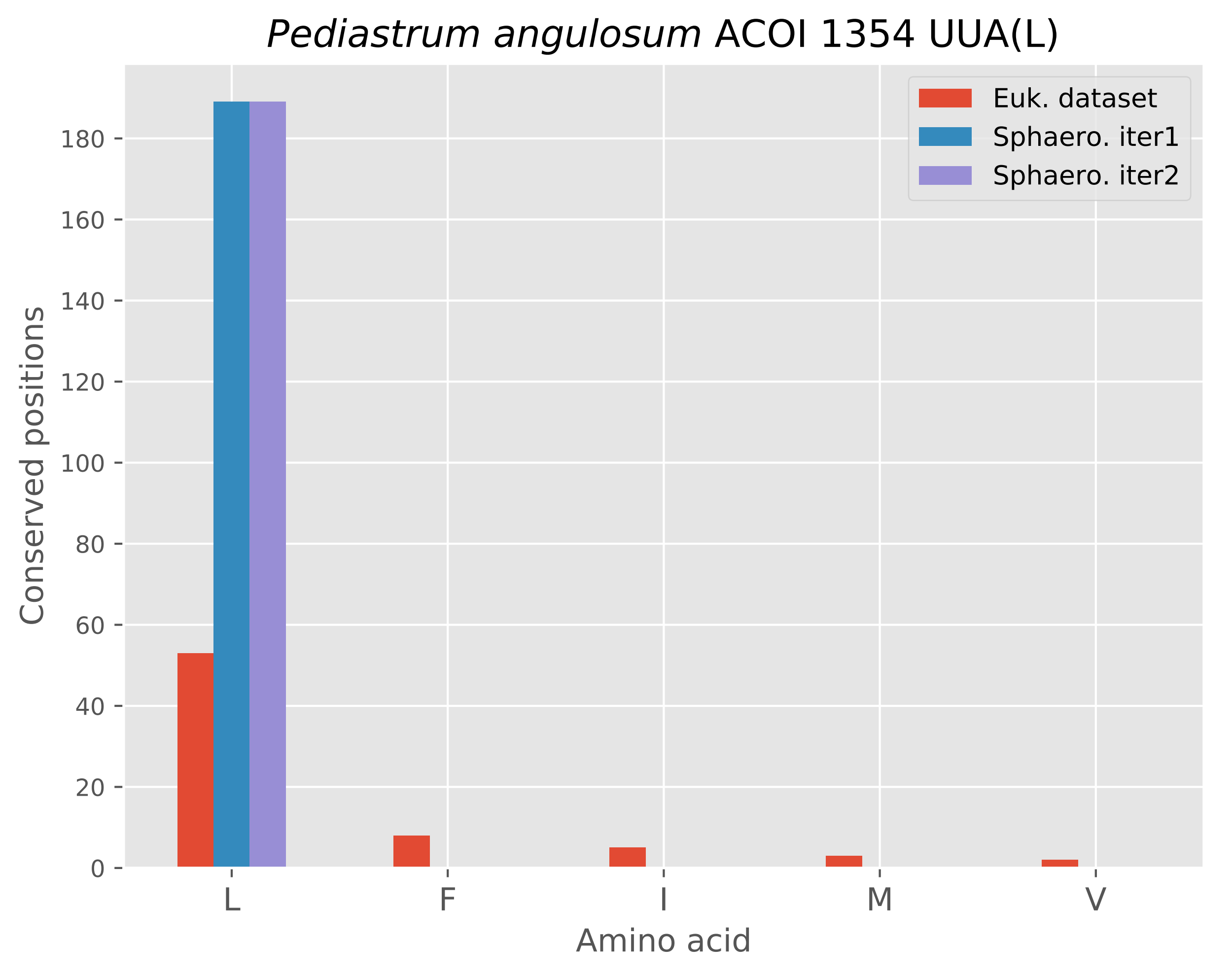
Pediastrum angulosum ACOI 1354 UUA(L)
Euk. dataset
Sphaero. iter1
Sphaero. iter2
Conserved positions
0
20
40
60
80
100
120
140
160
180
L
F
I
M
V
Amino acid

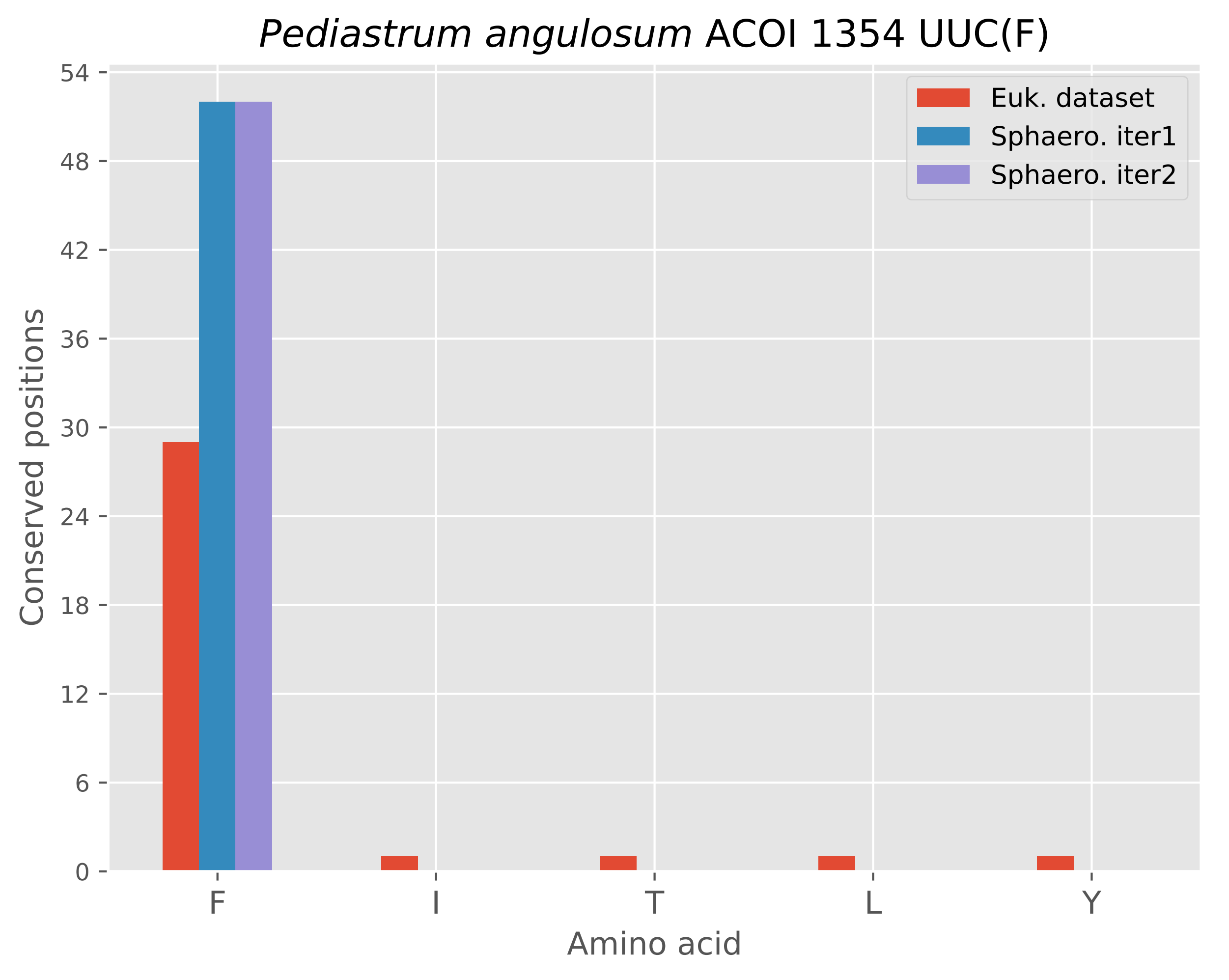

Pediastrum angulosum ACOI 1354 UUC(F)
Euk. dataset
Sphaero. iter1
Sphaero. iter2
Conserved positions
0
6
12
18
24
30
36
42
48
54
F
I
T
L
Y
Amino acid

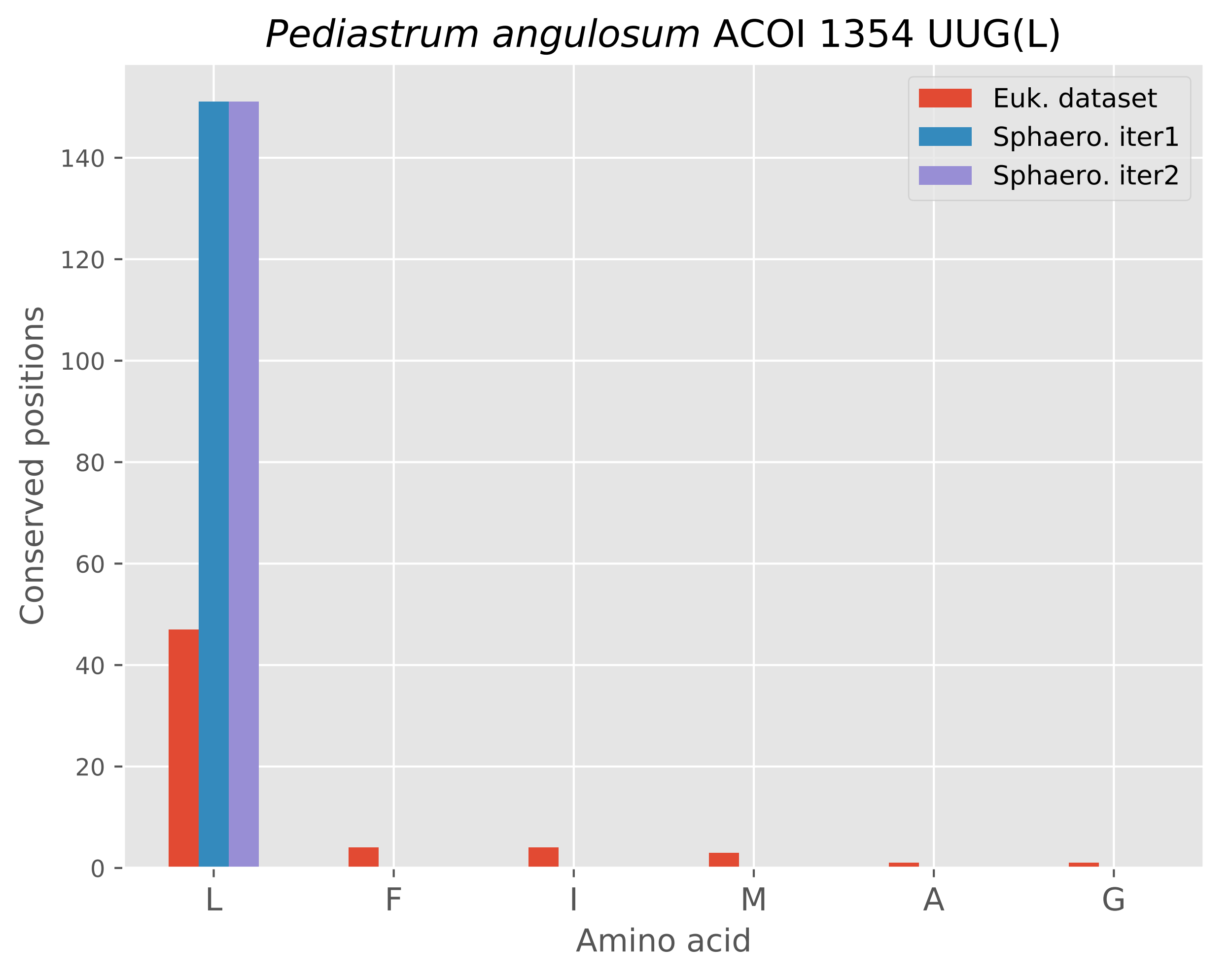

Pediastrum angulosum ACOI 1354 UUG(L)
Euk. dataset
Sphaero. iter1
Sphaero. iter2
Conserved positions
0
20
40
60
80
100
120
140
L
F
I
M
A
G
Amino acid

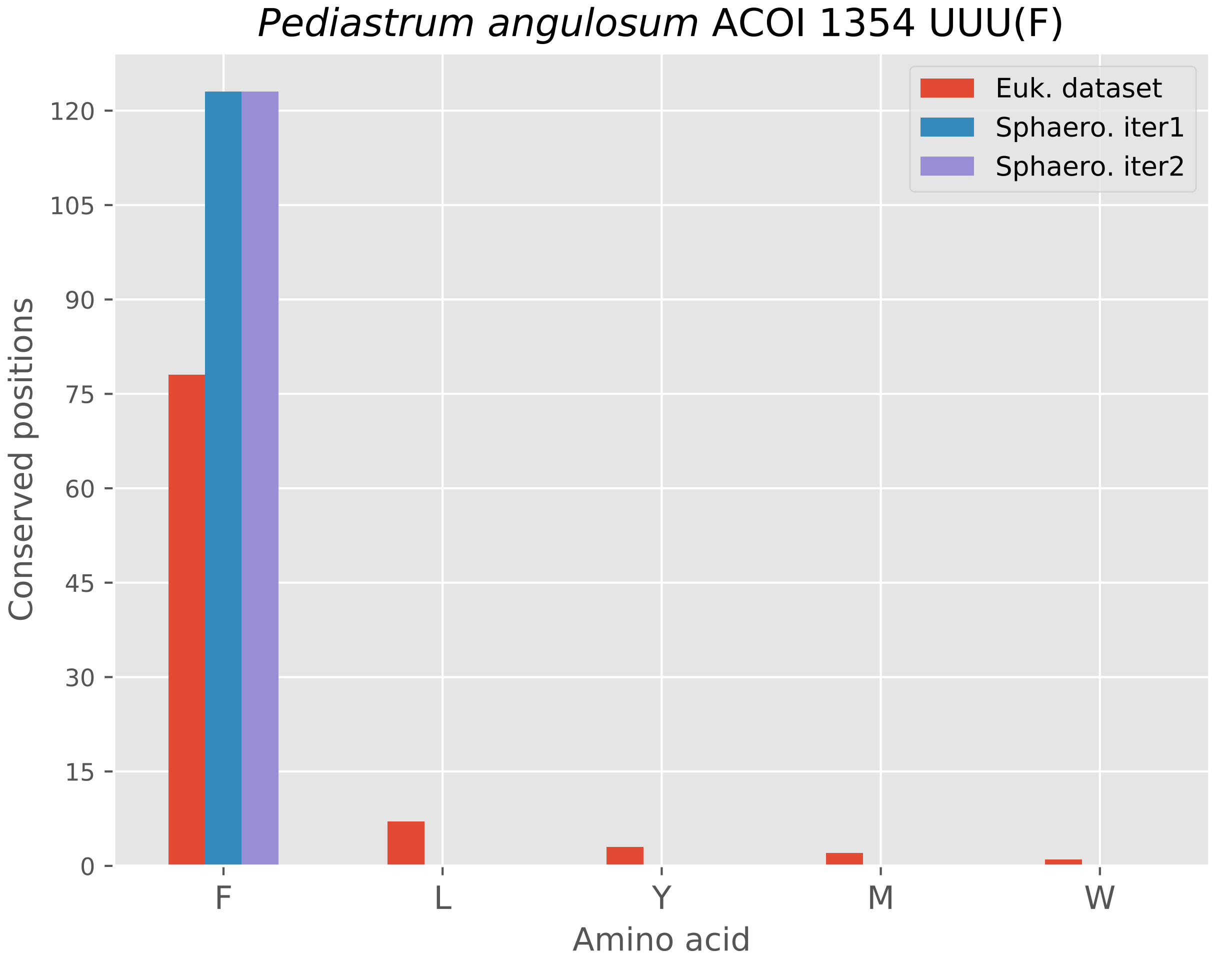

Pediastrum angulosum ACOI 1354 UUU(F)
Euk. dataset
Sphaero. iter1
Sphaero. iter2
Conserved positions
0
15
30
45
60
75
90
105
120
F
L
Y
M
W
Amino acid

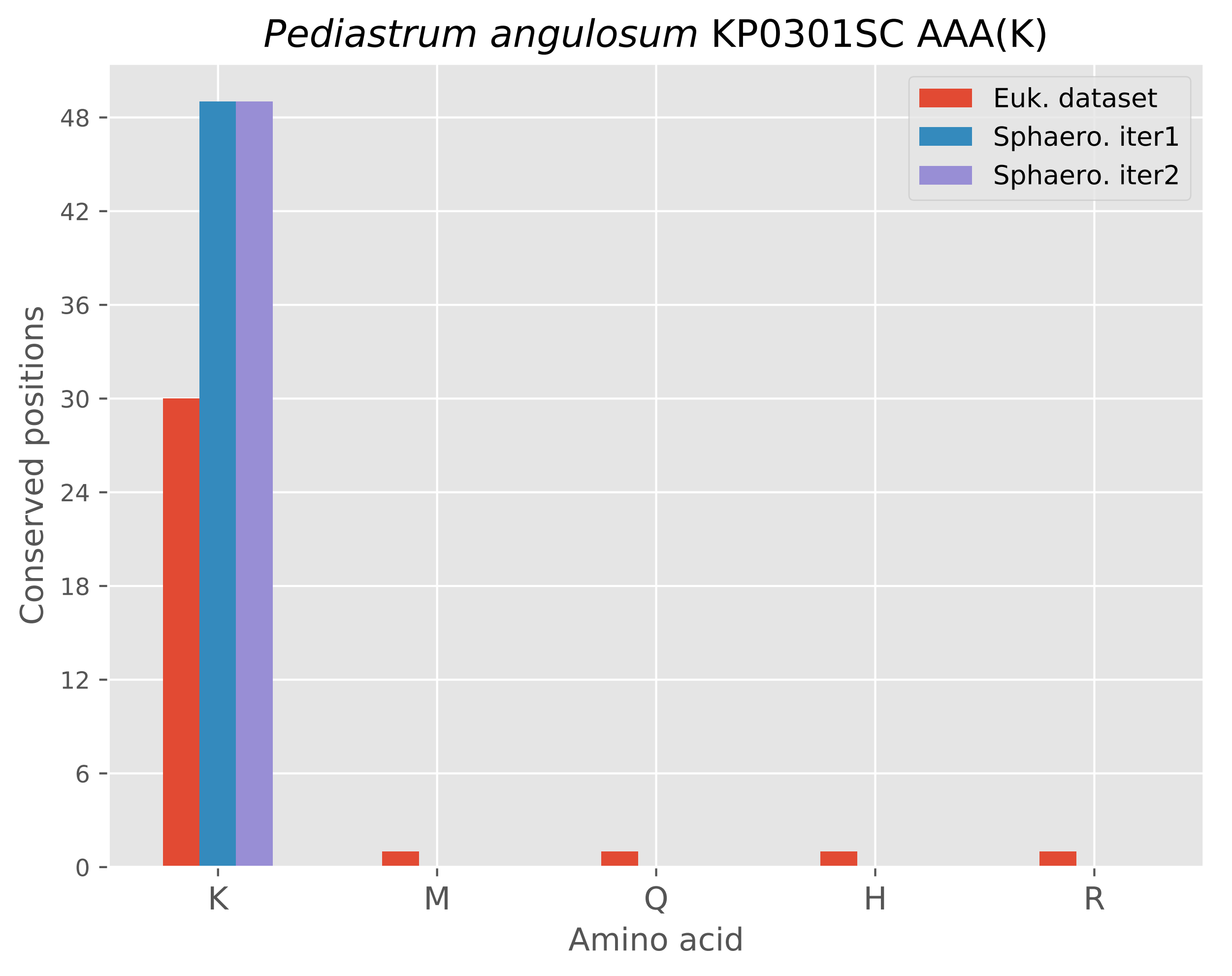

Pediastrum angulosum KP0301SC AAA(K)
Euk. dataset
Sphaero. iter1
Sphaero. iter2
Conserved positions
0
6
12
18
24
30
36
42
48
K
M
Q
H
R
Amino acid

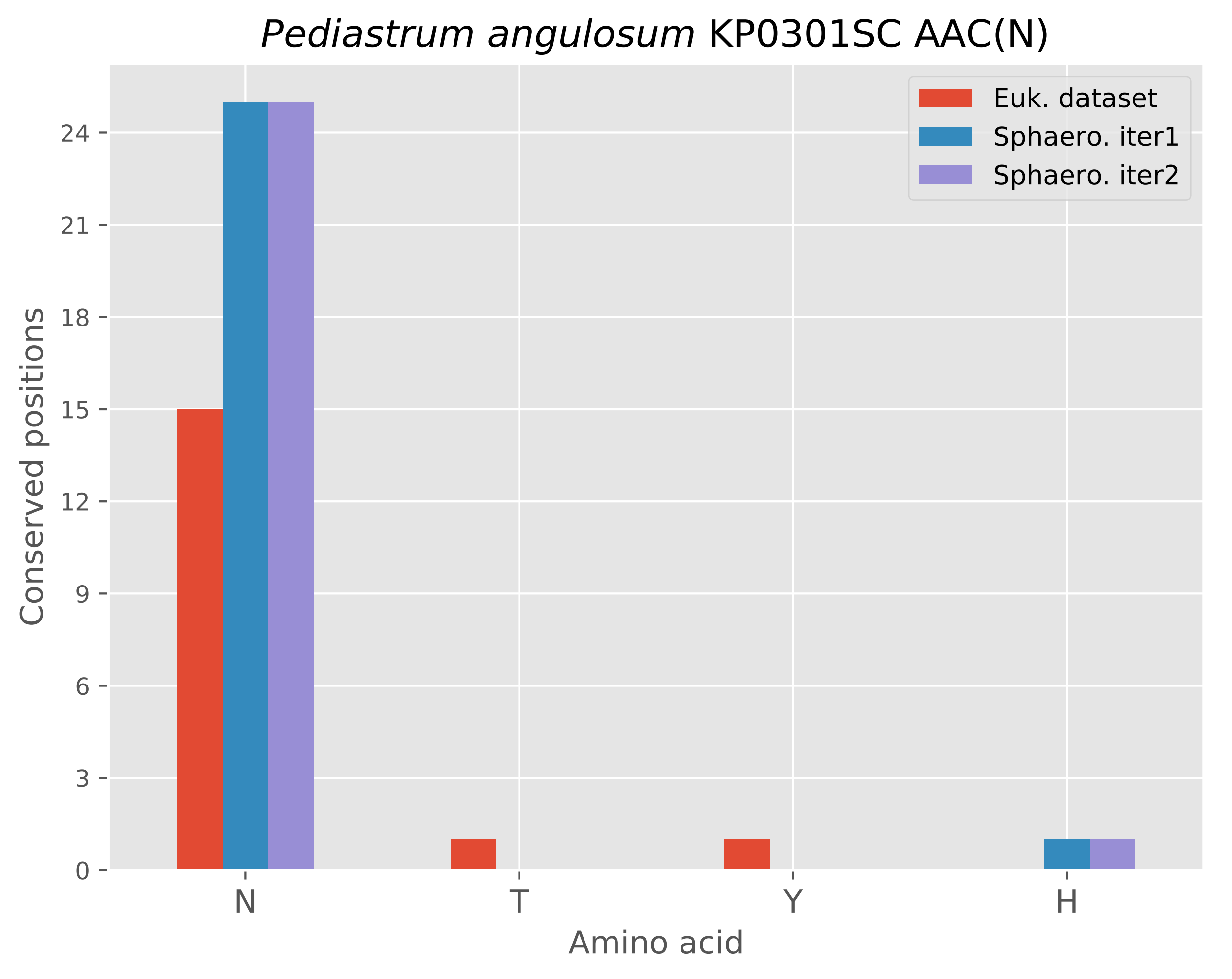

Pediastrum angulosum KP0301SC AAC(N)
Euk. dataset
Sphaero. iter1
Sphaero. iter2
Conserved positions
0
3
6
9
12
15
18
21
24
N
T
Y
H
Amino acid

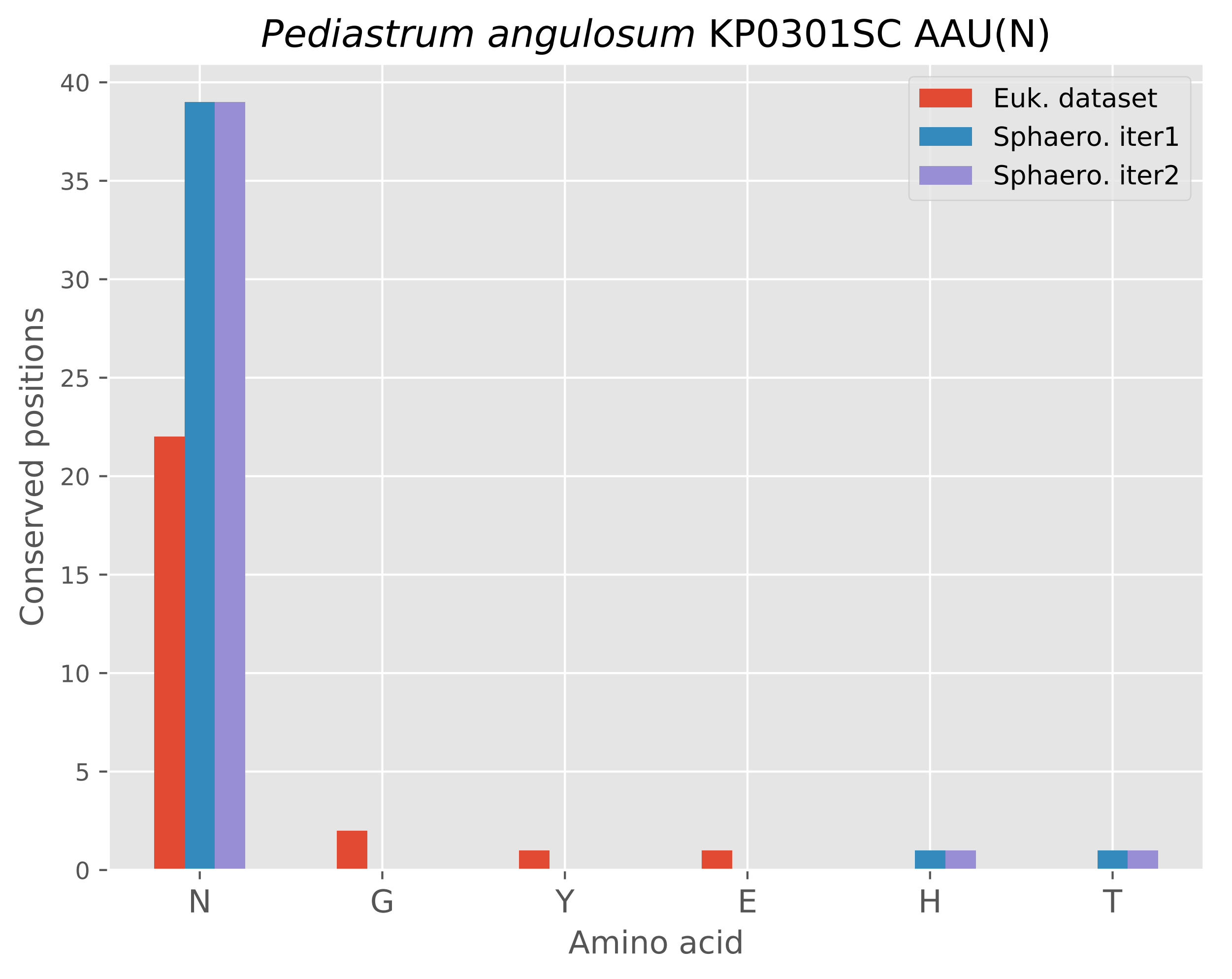
Pediastrum angulosum KP0301SC AAU(N)
Conserved positions
0
5
10
15
20
25
30
35
40
N
G
Y
E
H
T
Amino acid
Euk. dataset
Sphaero. iter1
Sphaero. iter2

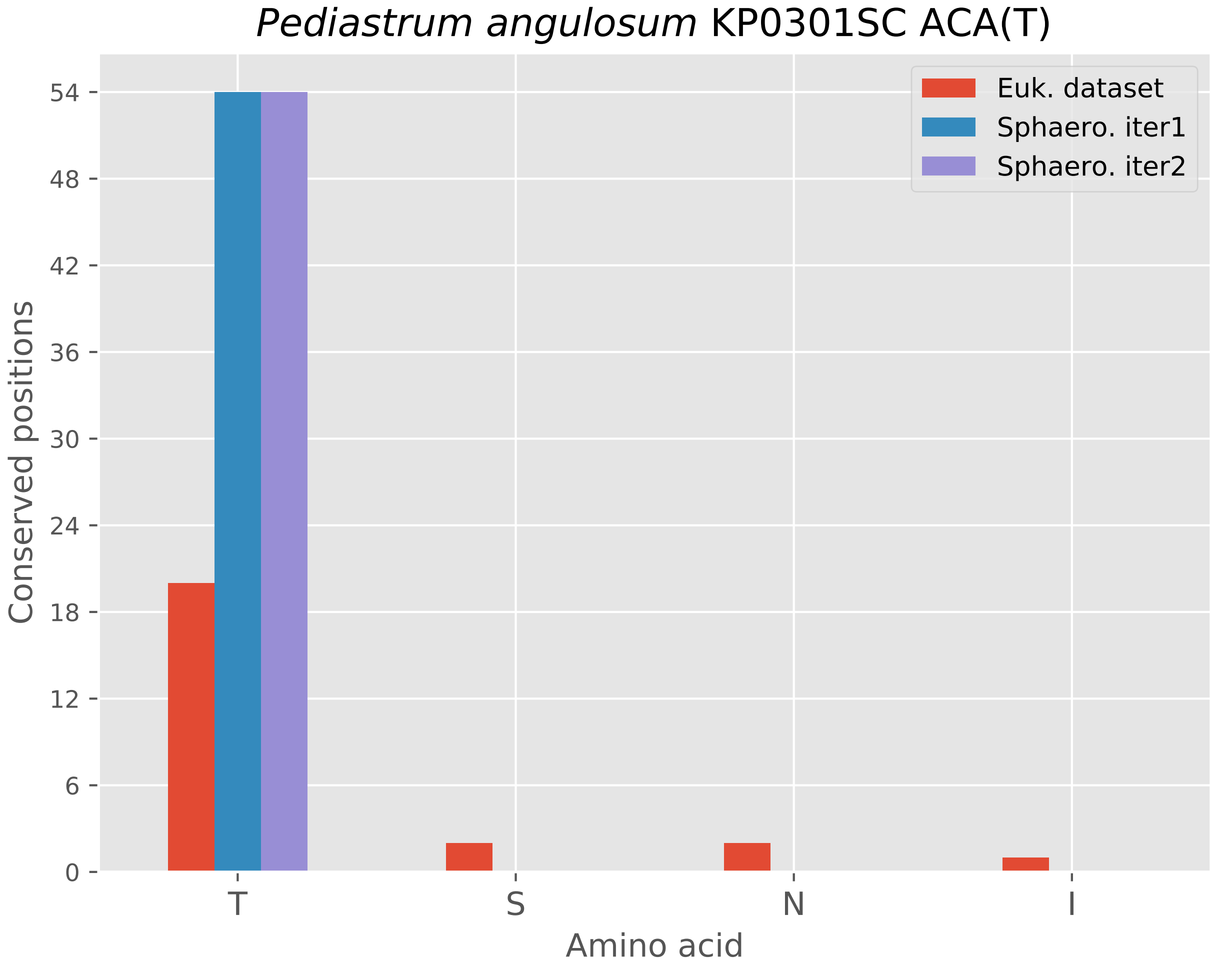

Pediastrum angulosum KP0301SC ACA(T)
Euk. dataset
Sphaero. iter1
Sphaero. iter2
Conserved positions
0
6
12
18
24
30
36
42
48
54
T
S
N
I
Amino acid

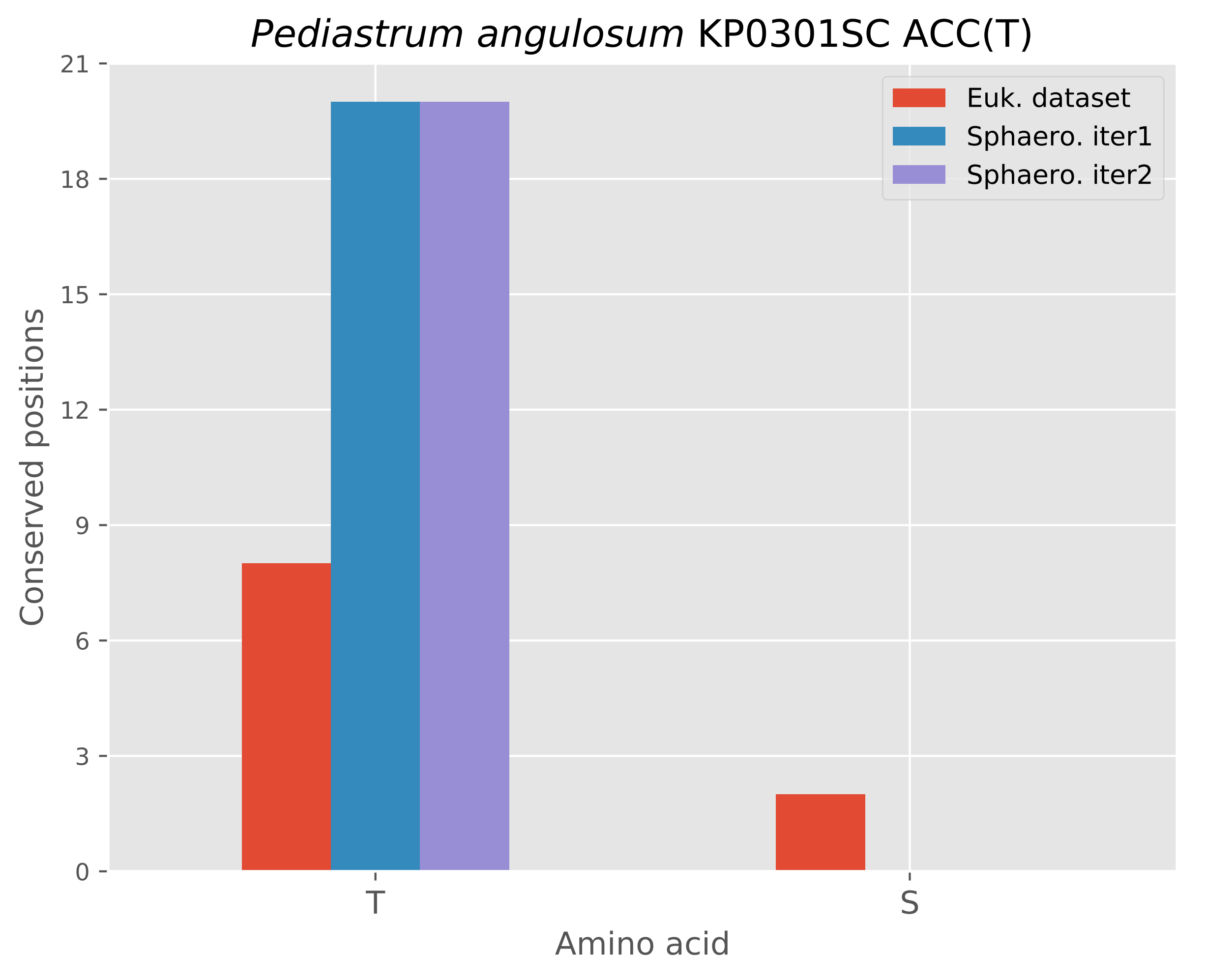

Pediastrum angulosum KP0301SC ACC(T)
Euk. dataset
Sphaero. iter1
Sphaero. iter2
Conserved positions
0
3
6
9
12
15
18
21
T
S
Amino acid

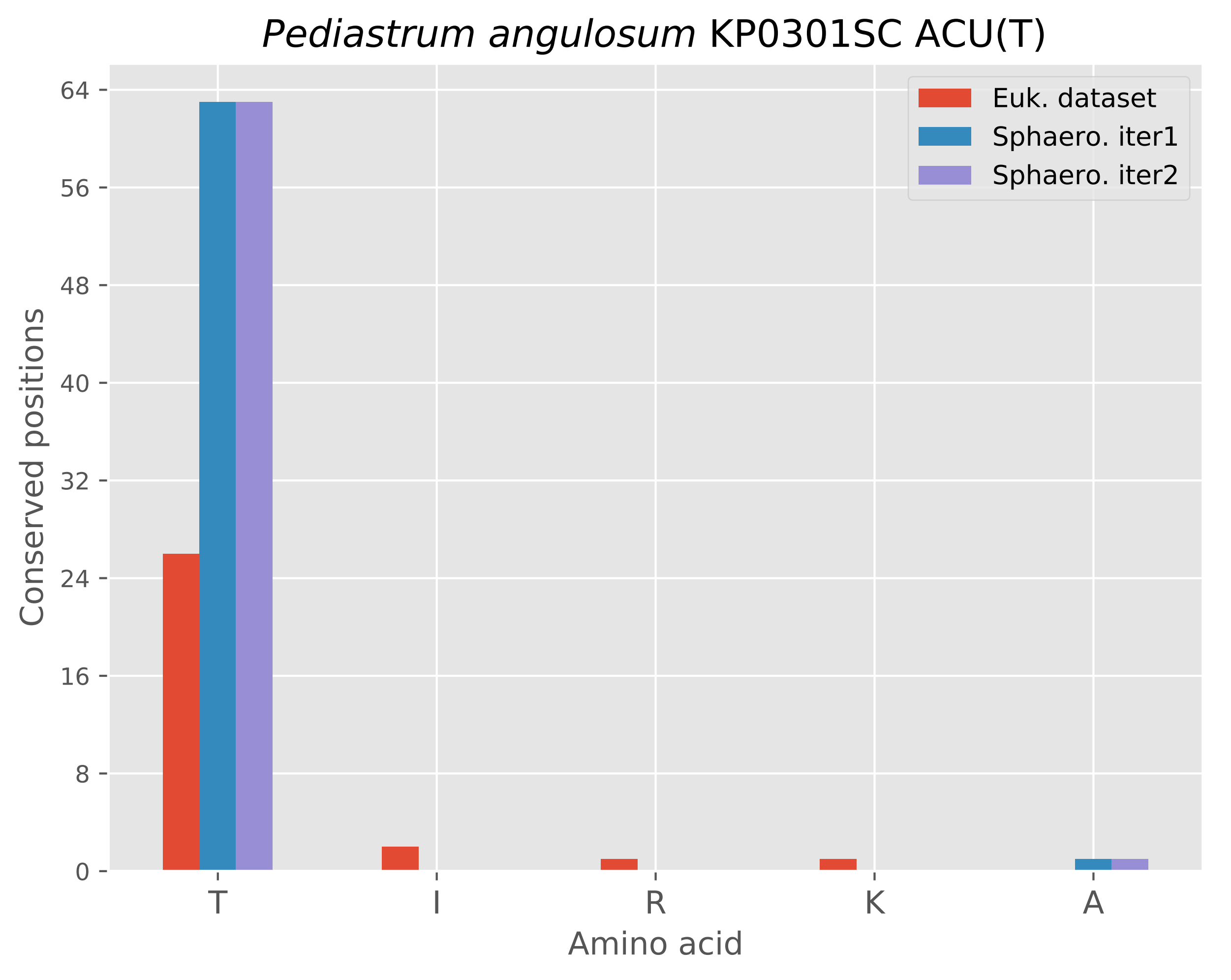

Pediastrum angulosum KP0301SC ACU(T)
Euk. dataset
Sphaero. iter1
Sphaero. iter2
Conserved positions
0
8
16
24
32
40
48
56
64
T
I
R
K
A
Amino acid

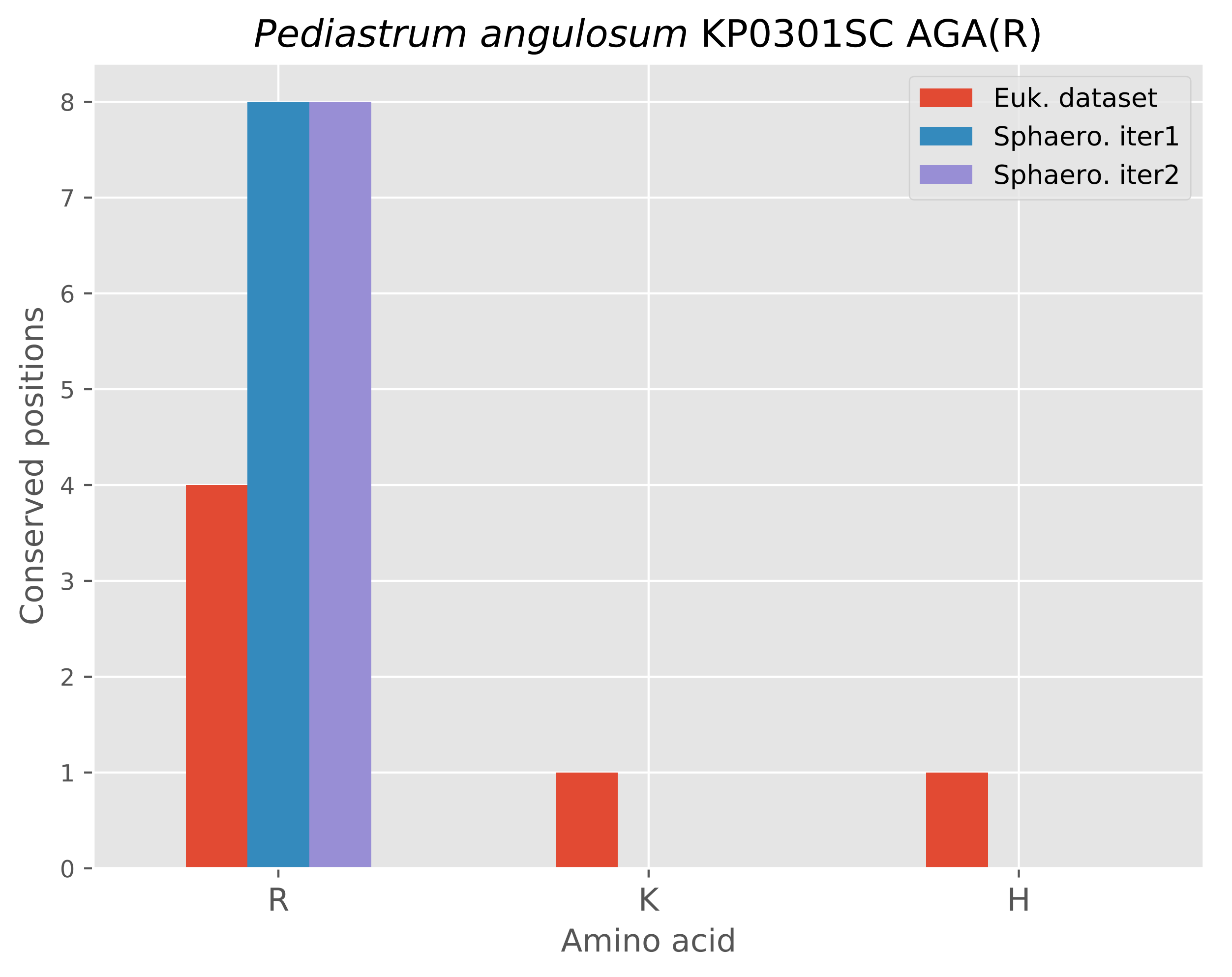

Pediastrum angulosum KP0301SC AGA(R)
Euk. dataset
Sphaero. iter1
Sphaero. iter2
Conserved positions
0
1
2
3
4
5
6
7
8
R
K
H
Amino acid

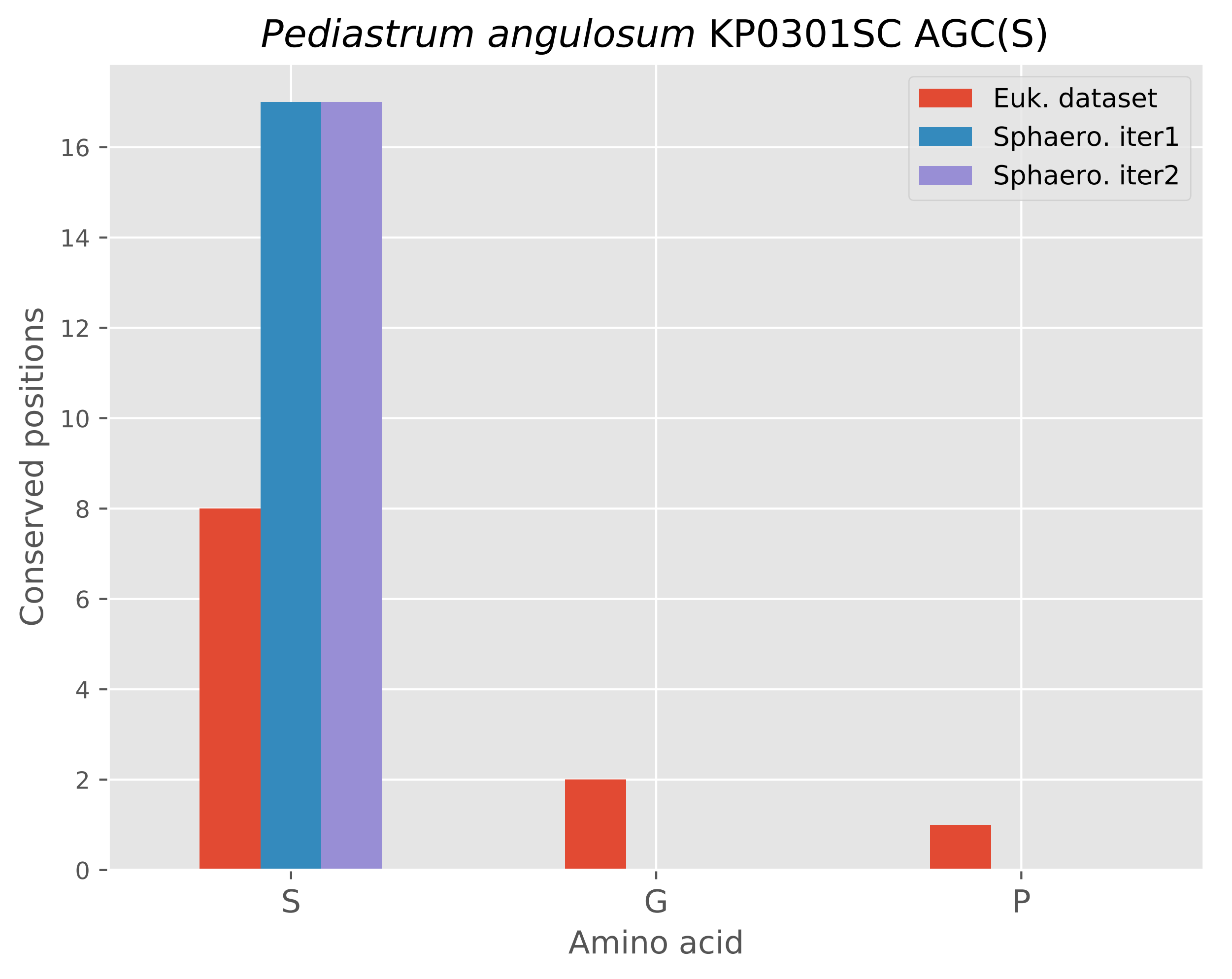

Pediastrum angulosum KP0301SC AGC(S)
Euk. dataset
Sphaero. iter1
Sphaero. iter2
Conserved positions
0
2
4
6
8
10
12
14
16
S
G
P
Amino acid

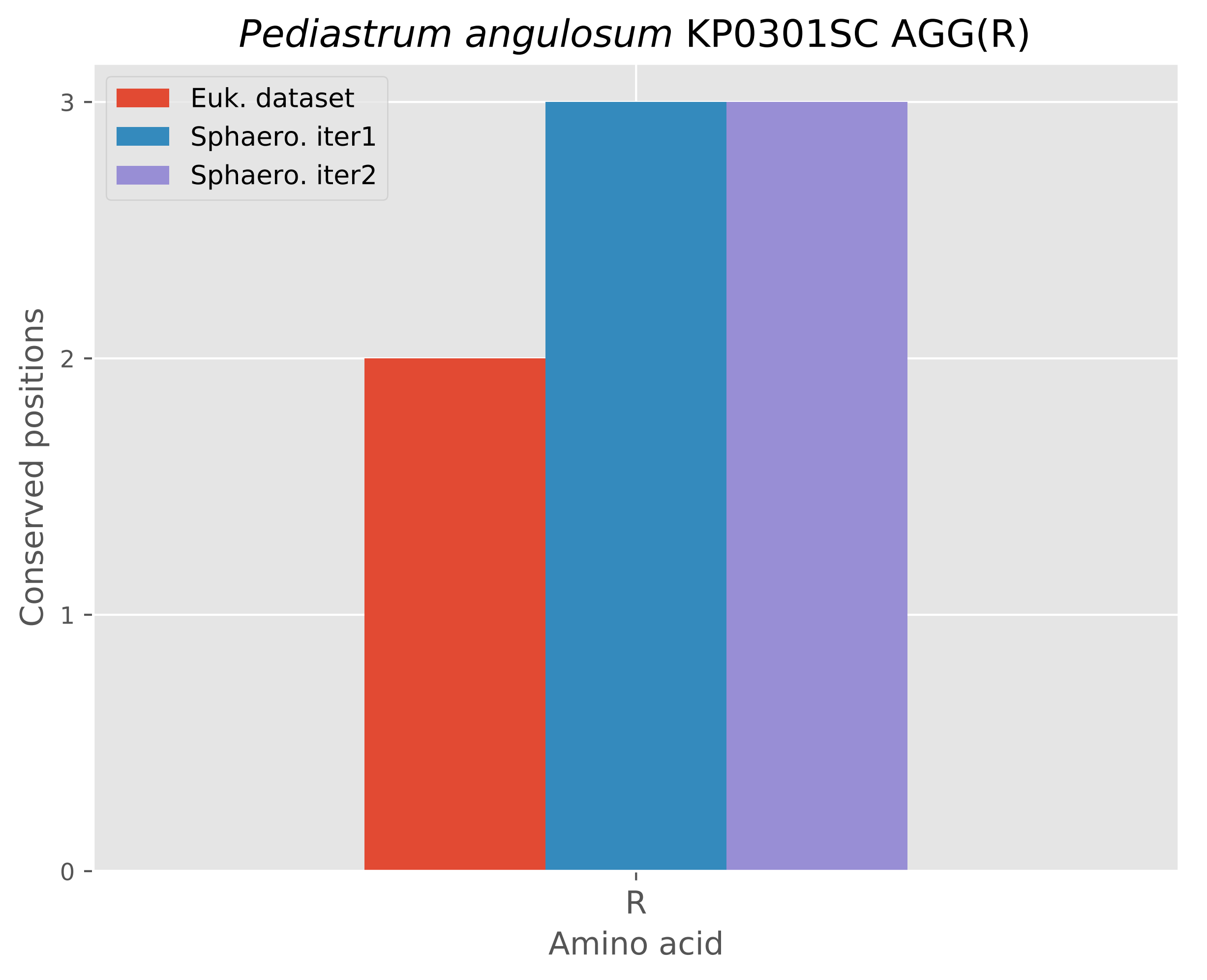

Pediastrum angulosum KP0301SC AGG(R)
Euk. dataset
Sphaero. iter1
Sphaero. iter2
Conserved positions
0
1
2
3
R
Amino acid

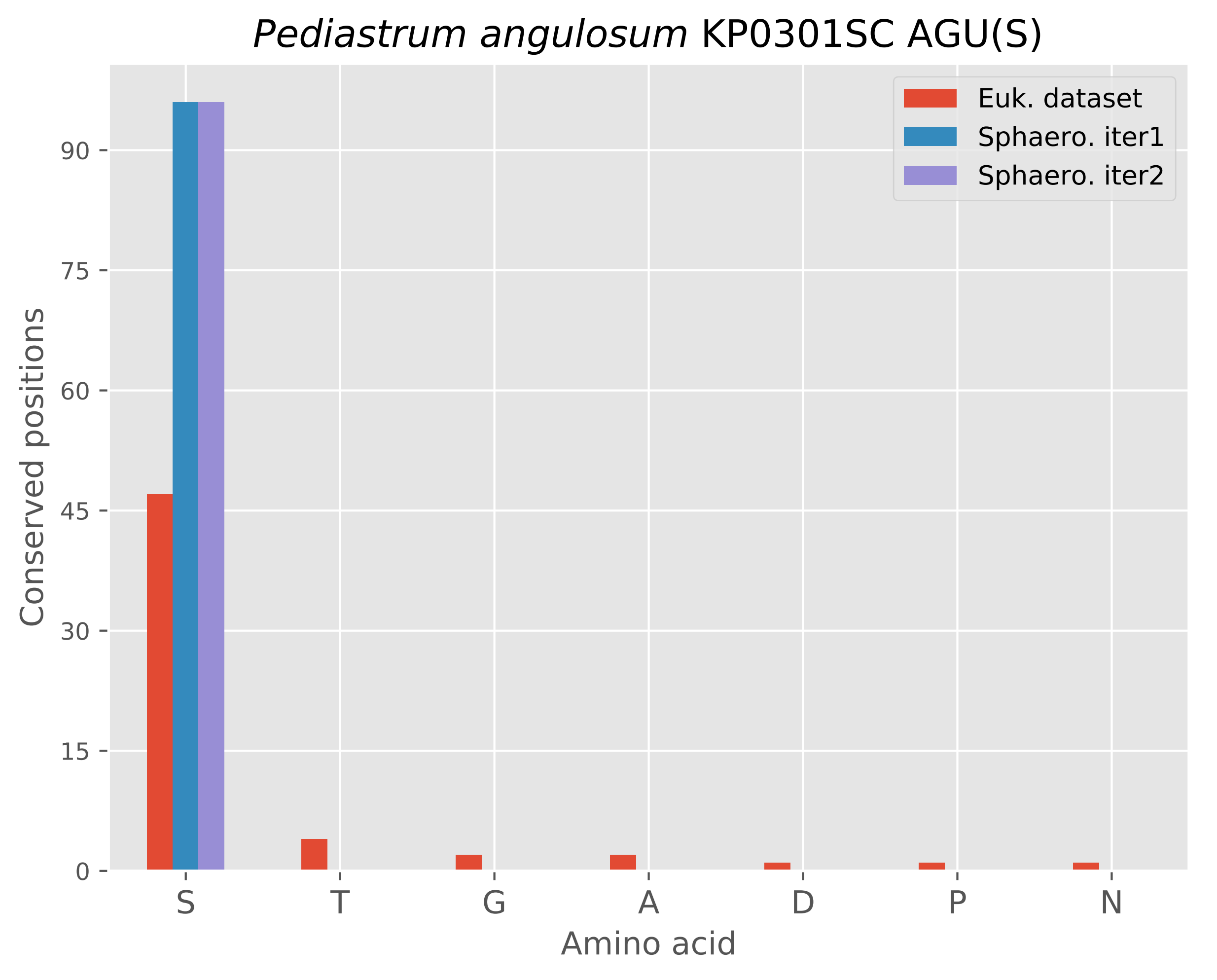

Pediastrum angulosum KP0301SC AGU(S)
Euk. dataset
Sphaero. iter1
Sphaero. iter2
Conserved positions
0
15
30
45
60
75
90
S
T
G
A
D
P
N
Amino acid

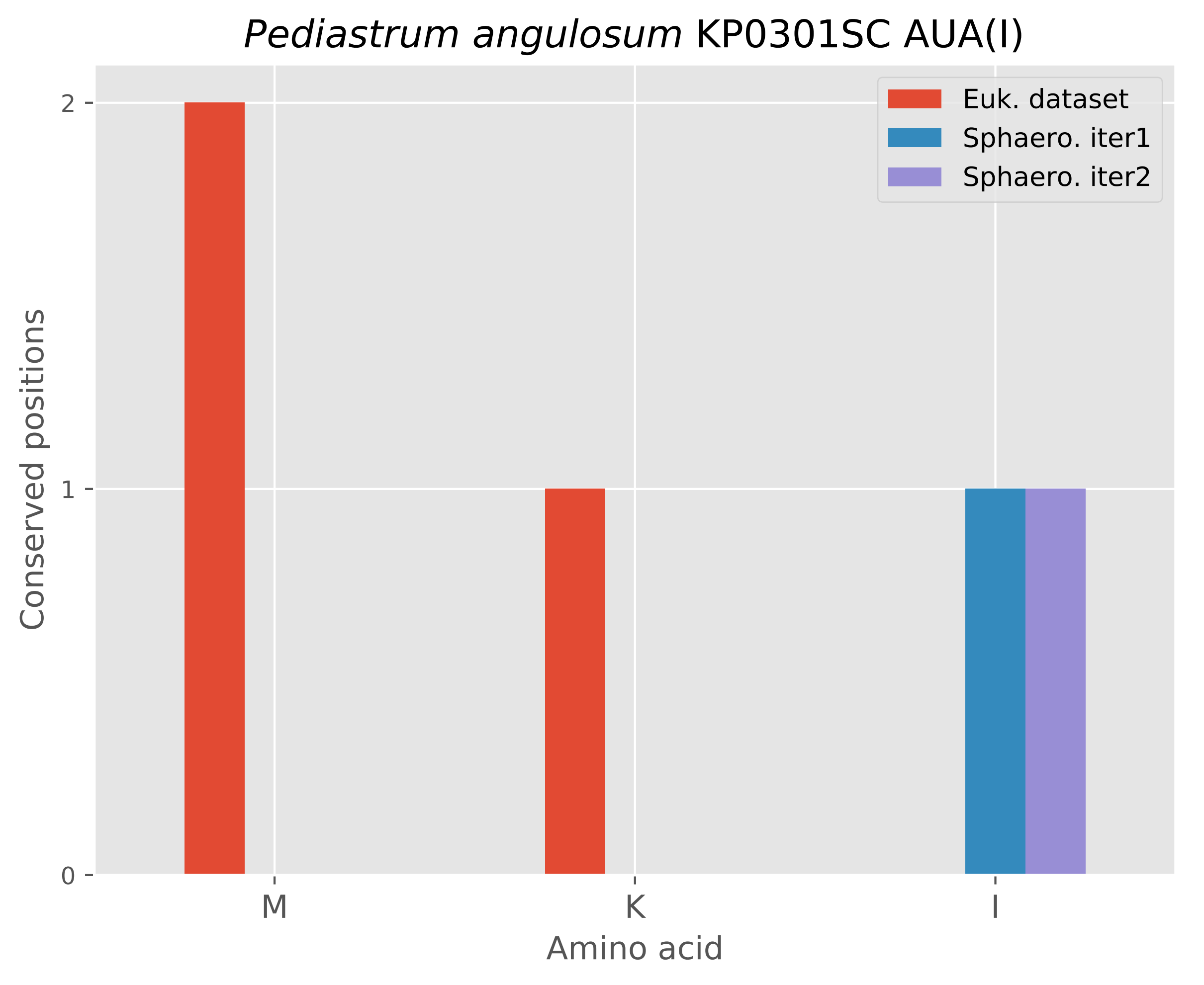

Pediastrum angulosum KP0301SC AUA(I)
Euk. dataset
Sphaero. iter1
Sphaero. iter2
Conserved positions
2
1
0
M
K
I
Amino acid

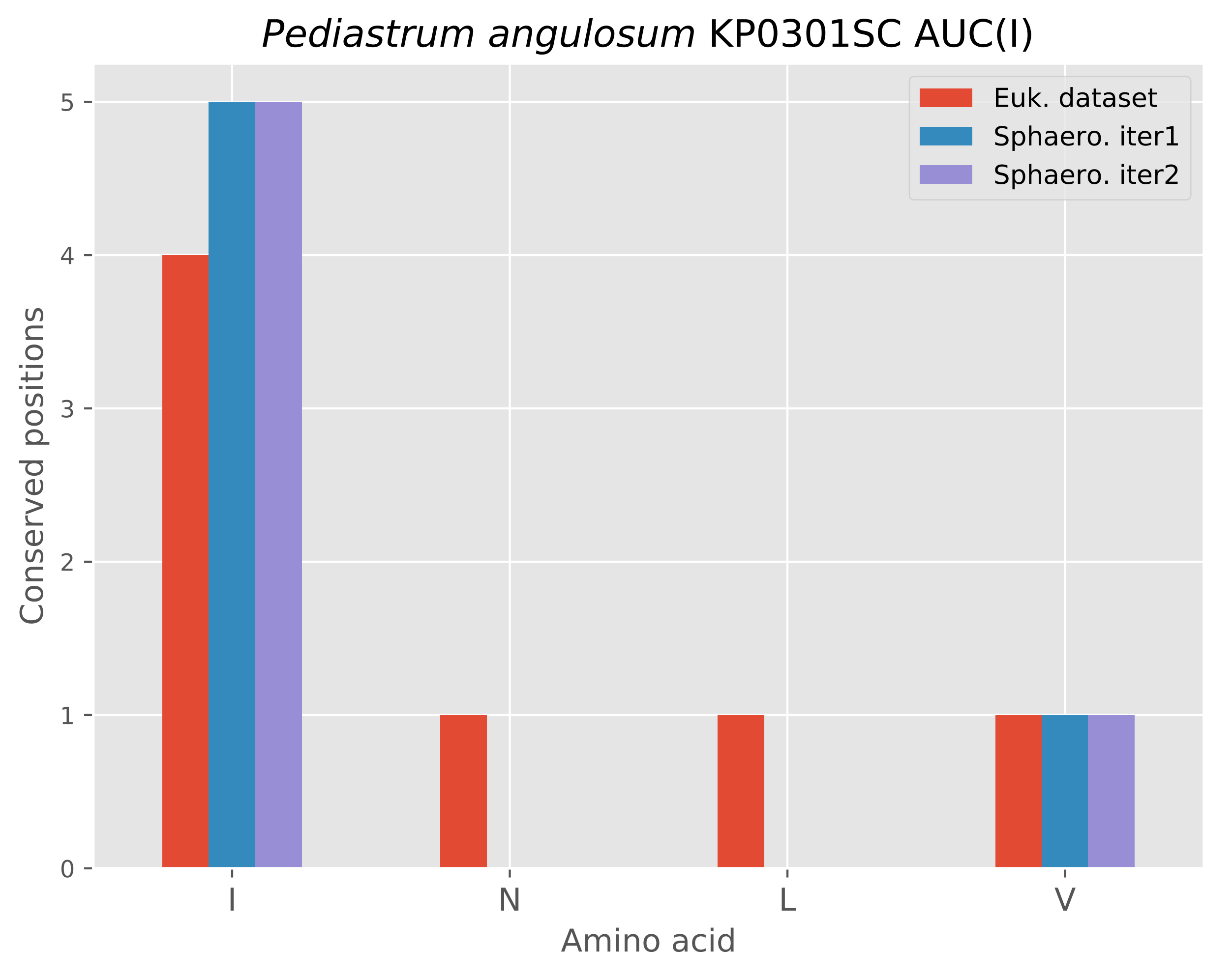

Pediastrum angulosum KP0301SC AUC(I)
Euk. dataset
Sphaero. iter1
Sphaero. iter2
Conserved positions
0
1
2
3
4
5
I
N
L
V
Amino acid

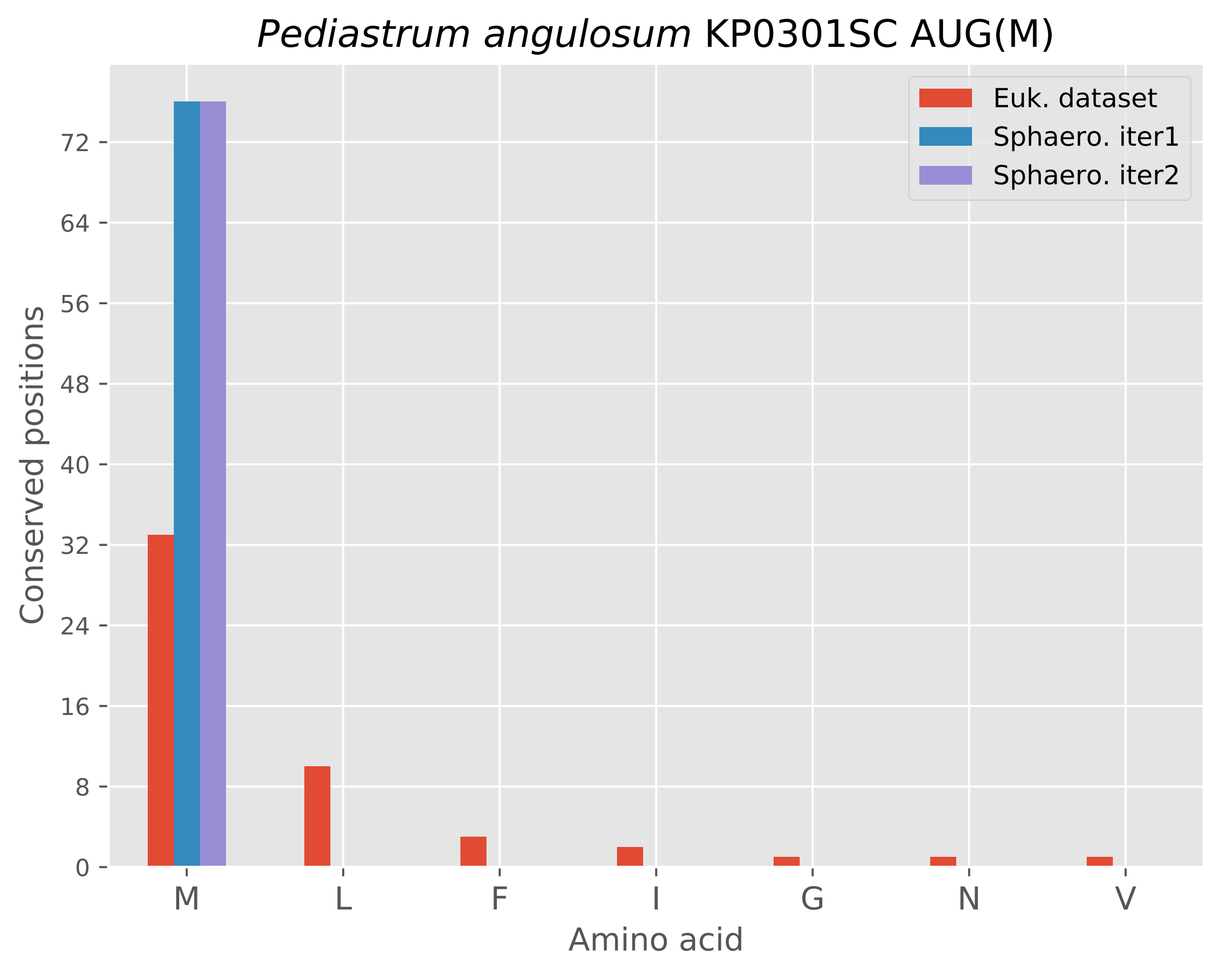

Pediastrum angulosum KP0301SC AUG(M)
Euk. dataset
Sphaero. iter1
Sphaero. iter2
Conserved positions
0
8
16
24
32
40
48
56
64
72
M
L
F
I
G
N
V
Amino acid

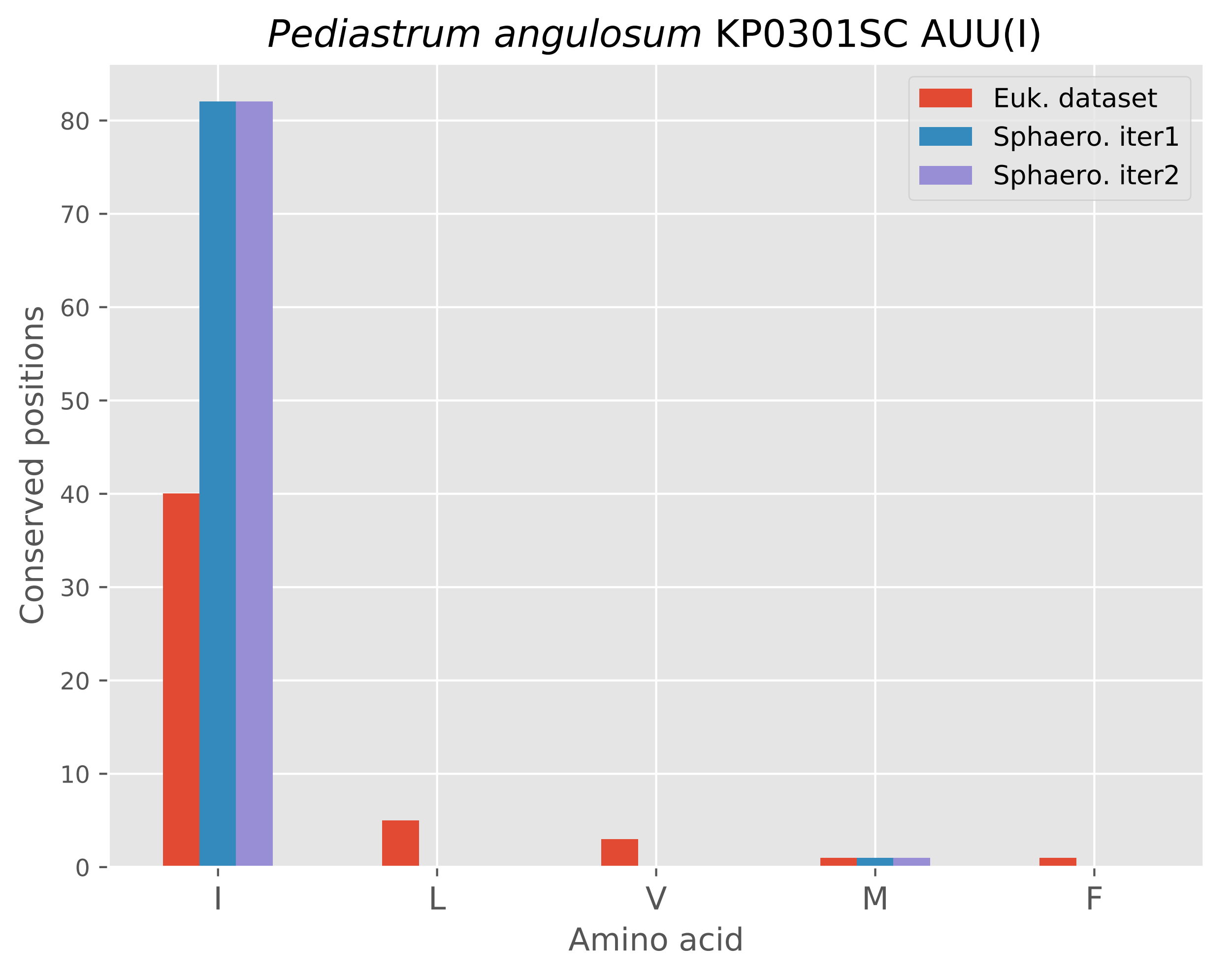

Pediastrum angulosum KP0301SC AUU(I)
Euk. dataset
Sphaero. iter1
Sphaero. iter2
Conserved positions
0
10
20
30
40
50
60
70
80
I
L
V
M
F
Amino acid

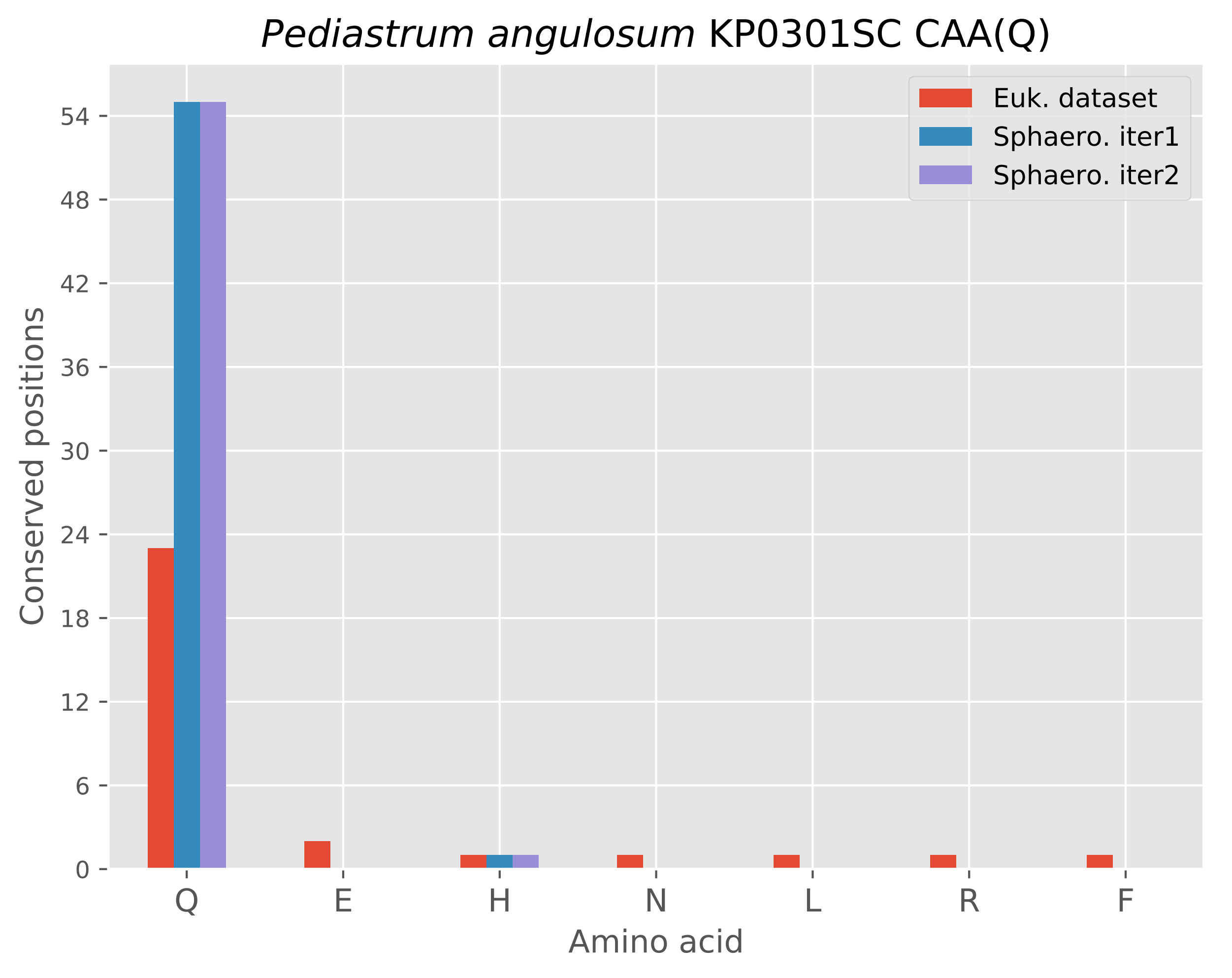

Pediastrum angulosum KP0301SC CAA(Q)
Euk. dataset
Sphaero. iter1
Sphaero. iter2
Conserved positions
0
6
12
18
24
30
36
42
48
54
Q
E
H
N
L
R
F
Amino acid

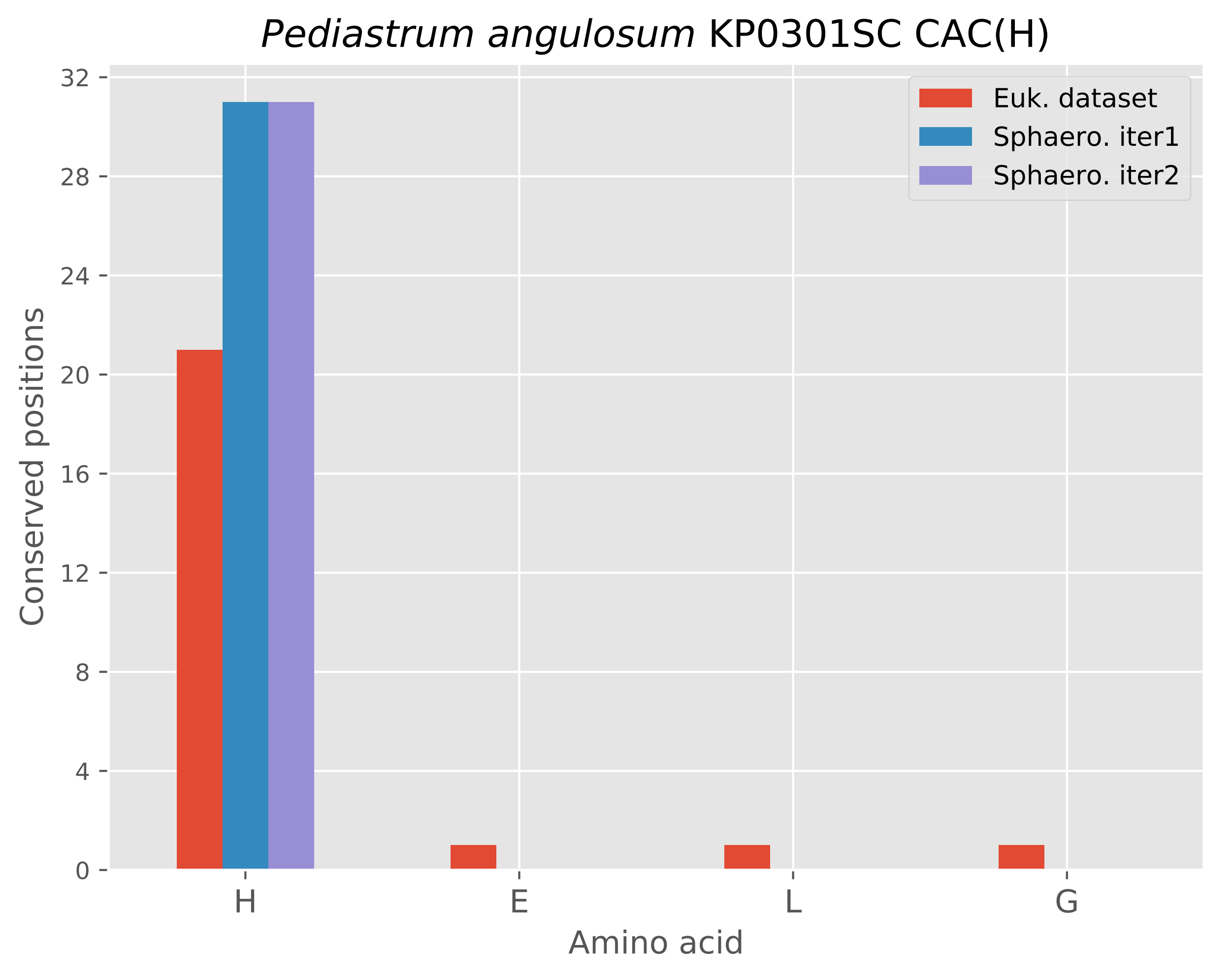
Pediastrum angulosum KP0301SC CAC(H)
Euk. dataset
Sphaero. iter1
Sphaero. iter2
Conserved positions
0
4
8
12
16
20
24
28
32
H
E
L
G
Amino acid

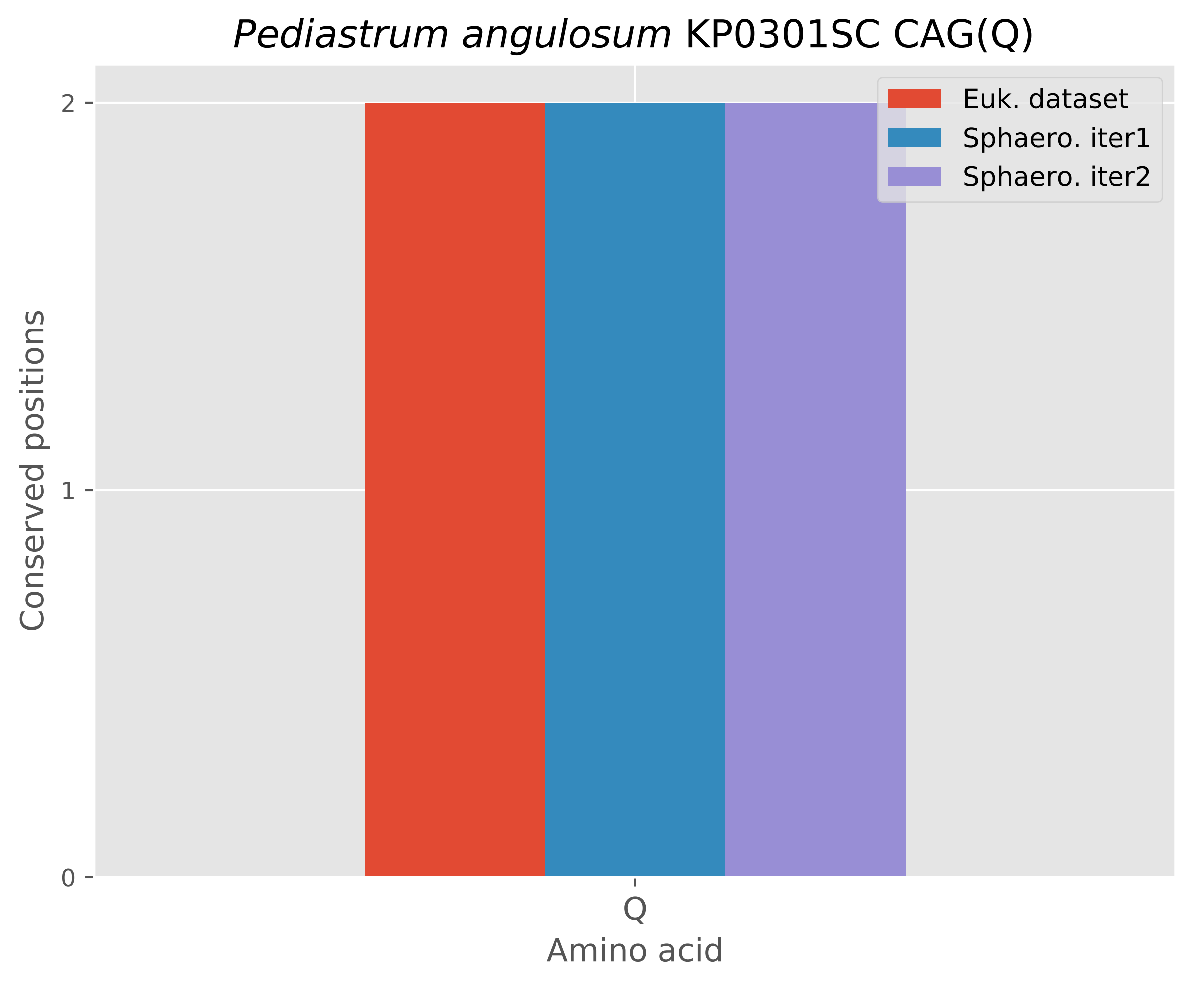

Pediastrum angulosum KP0301SC CAG(Q)
Conserved positions
0
1
2
Q
Amino acid
Euk. dataset
Sphaero. iter1
Sphaero. iter2

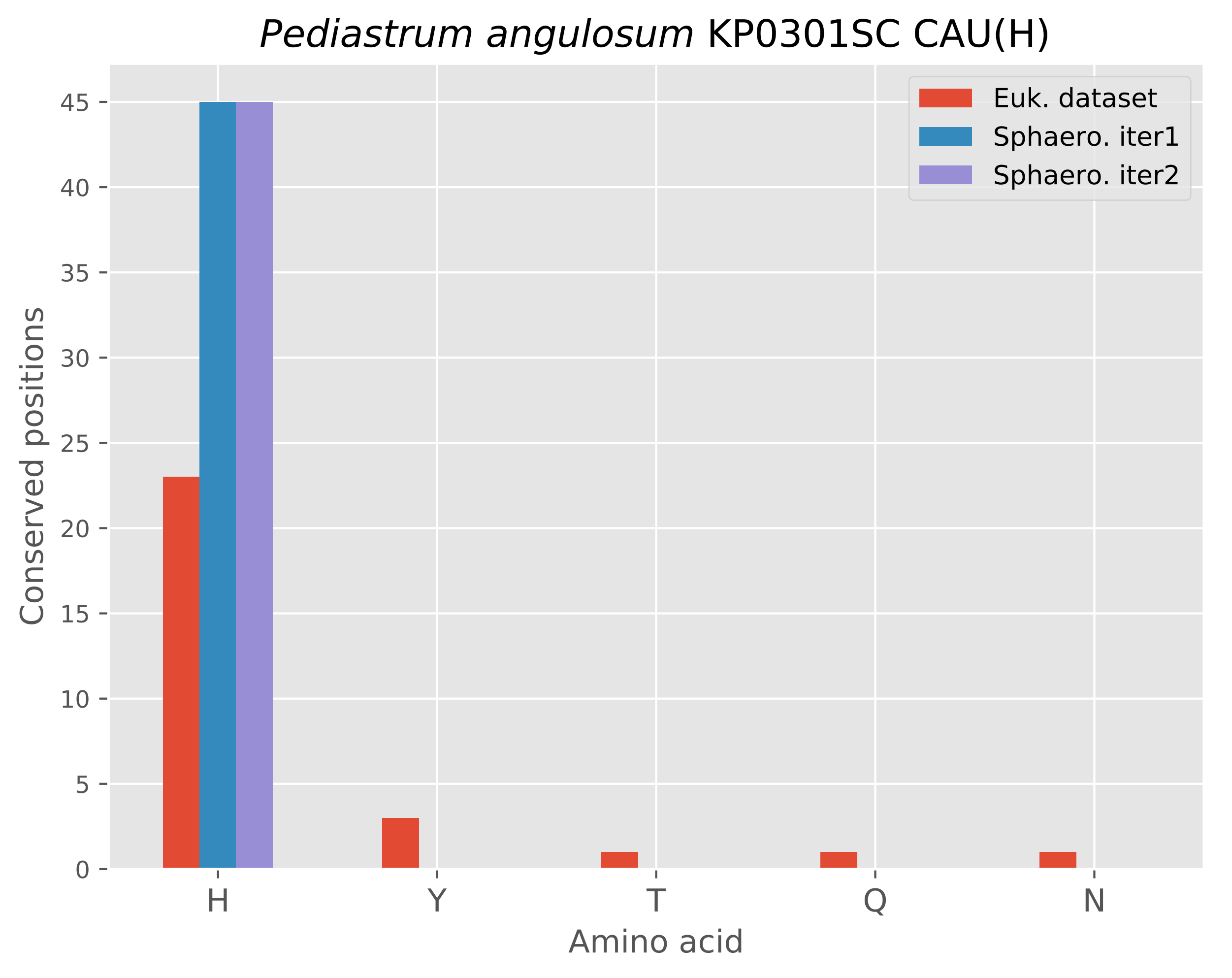

Pediastrum angulosum KP0301SC CAU(H)
Euk. dataset
Sphaero. iter1
Sphaero. iter2
Conserved positions
0
5
10
15
20
25
30
35
40
45
H
Y
T
Q
N
Amino acid

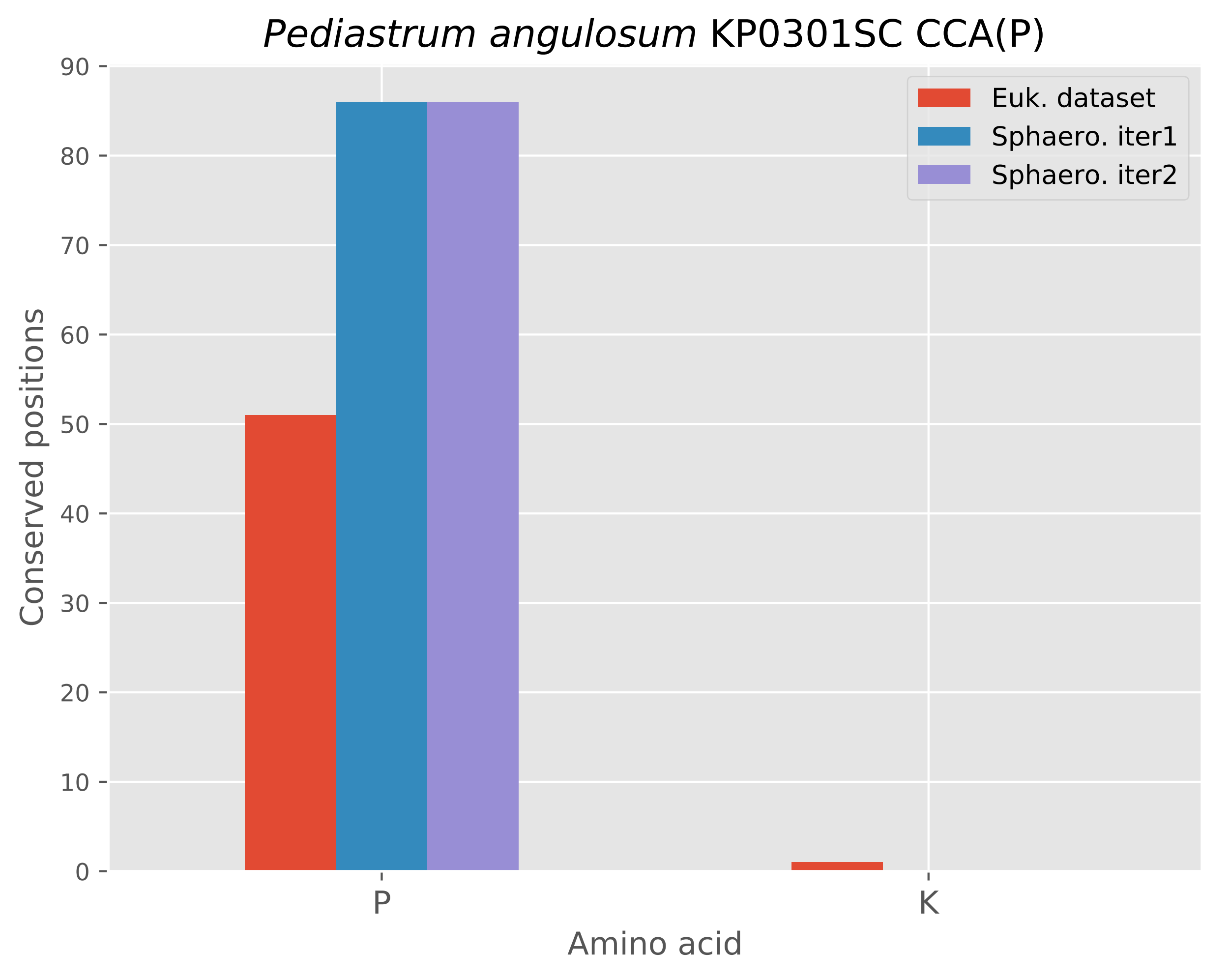

Pediastrum angulosum KP0301SC CCA(P)
Conserved positions
Amino acid
0
10
20
30
40
50
60
70
80
90
P
K
Euk. dataset
Sphaero. iter1
Sphaero. iter2

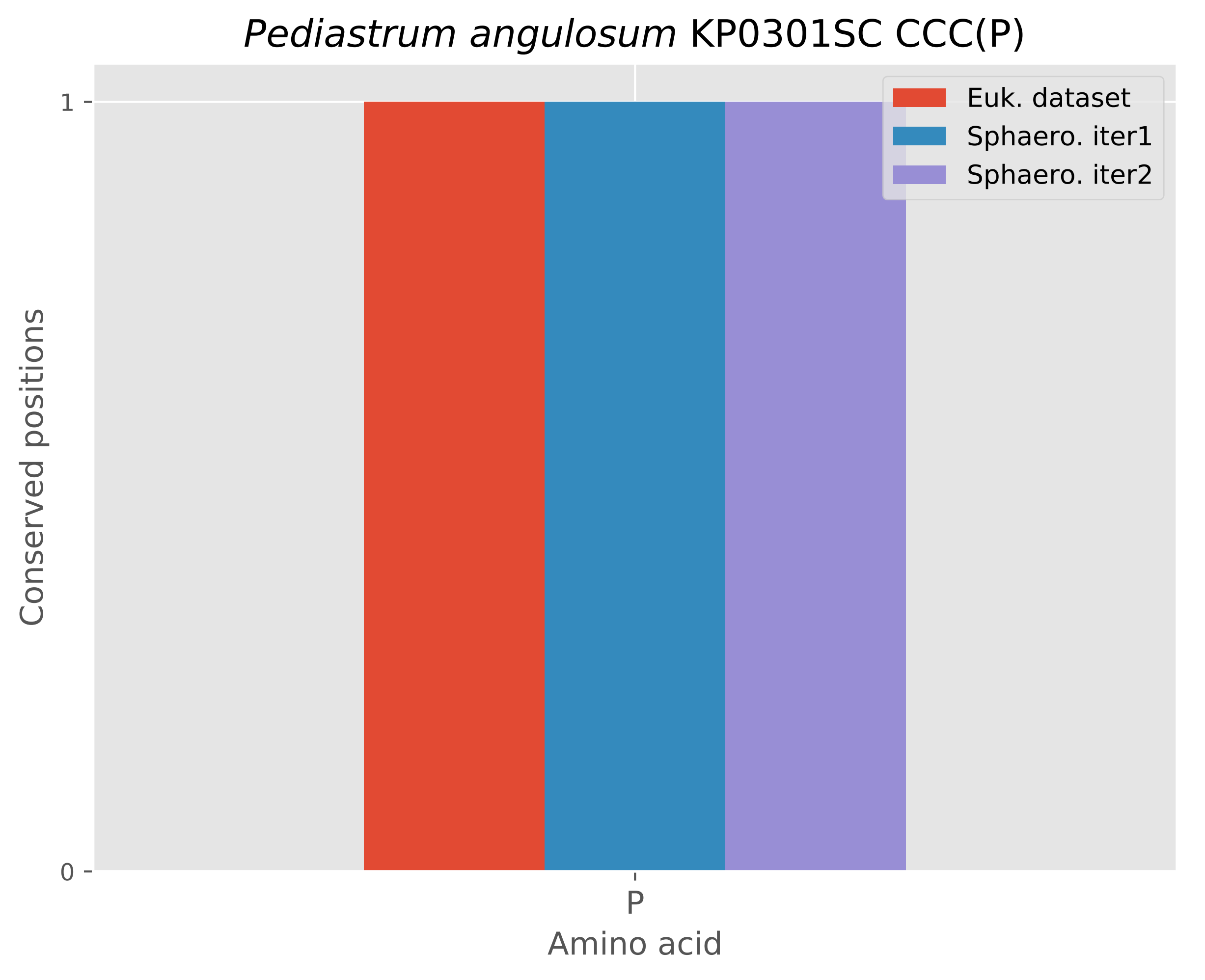

Pediastrum angulosum KP0301SC CCC(P)
Euk. dataset
Sphaero. iter1
Sphaero. iter2
Conserved positions
1
0
P
Amino acid

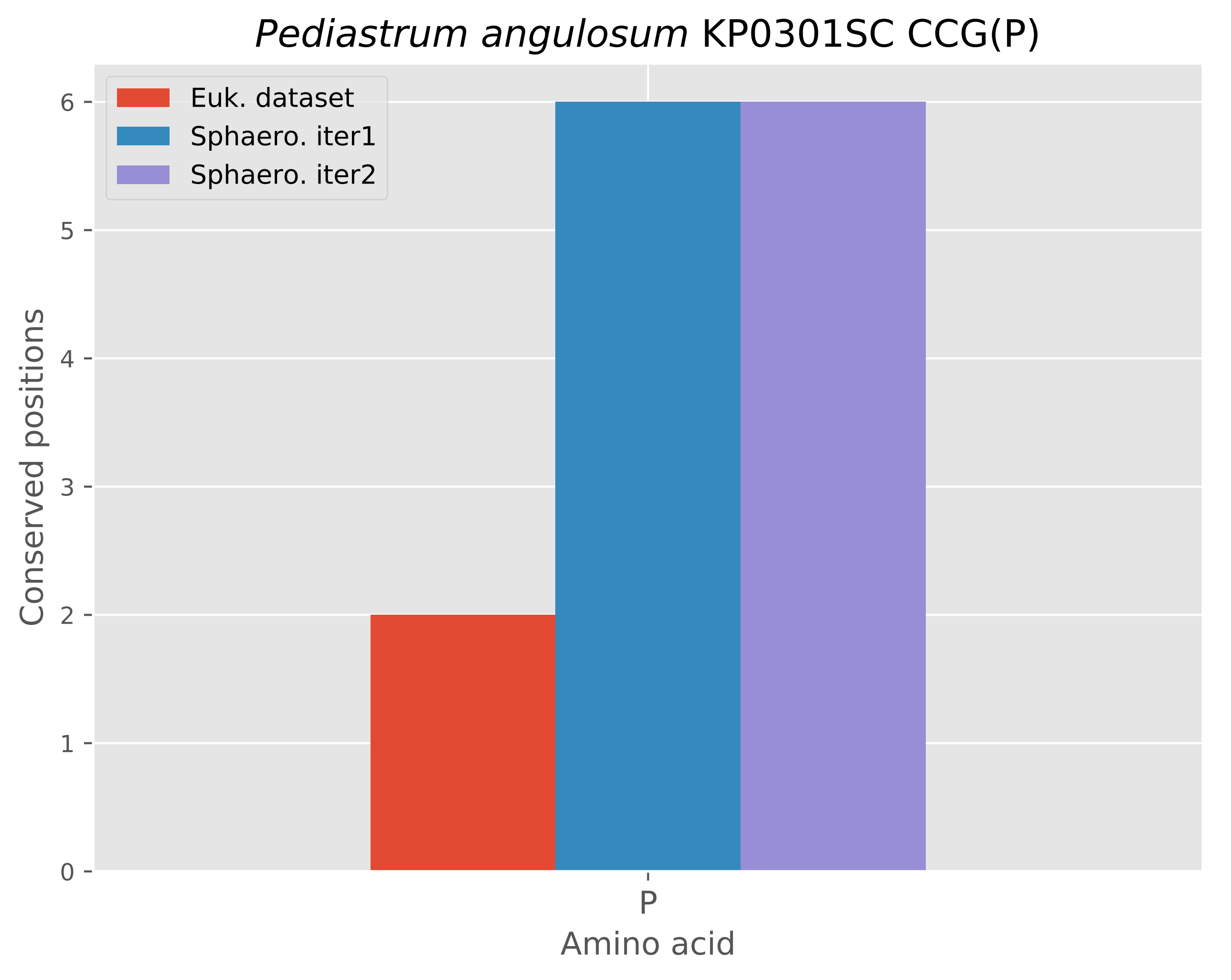
Pediastrum angulosum KP0301SC CCG(P)
Euk. dataset
Sphaero. iter1
Sphaero. iter2
Conserved positions
0
1
2
3
4
5
6
P
Amino acid

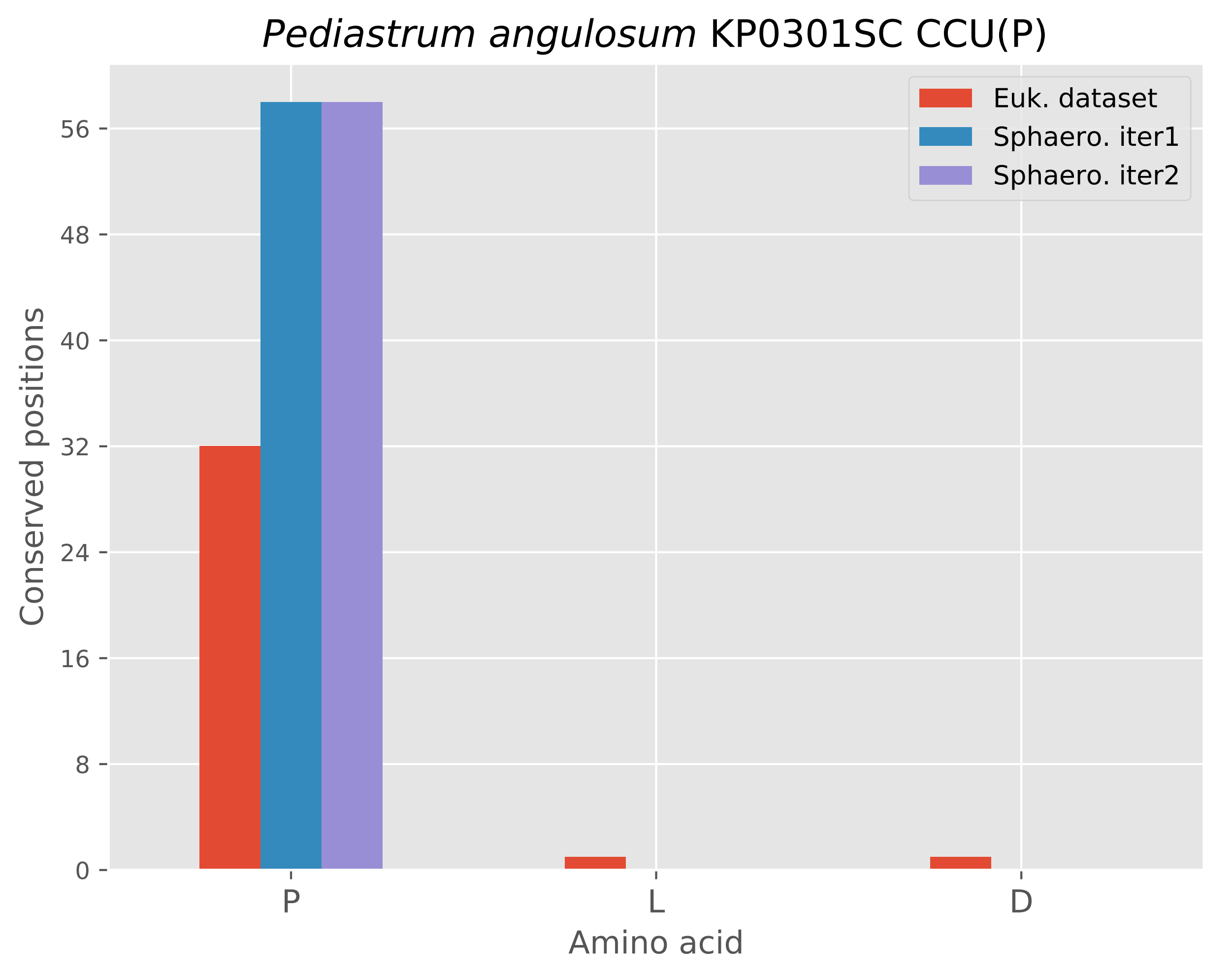

Pediastrum angulosum KP0301SC CCU(P)
Euk. dataset
Sphaero. iter1
Sphaero. iter2
Conserved positions
0
8
16
24
32
40
48
56
P
L
D
Amino acid

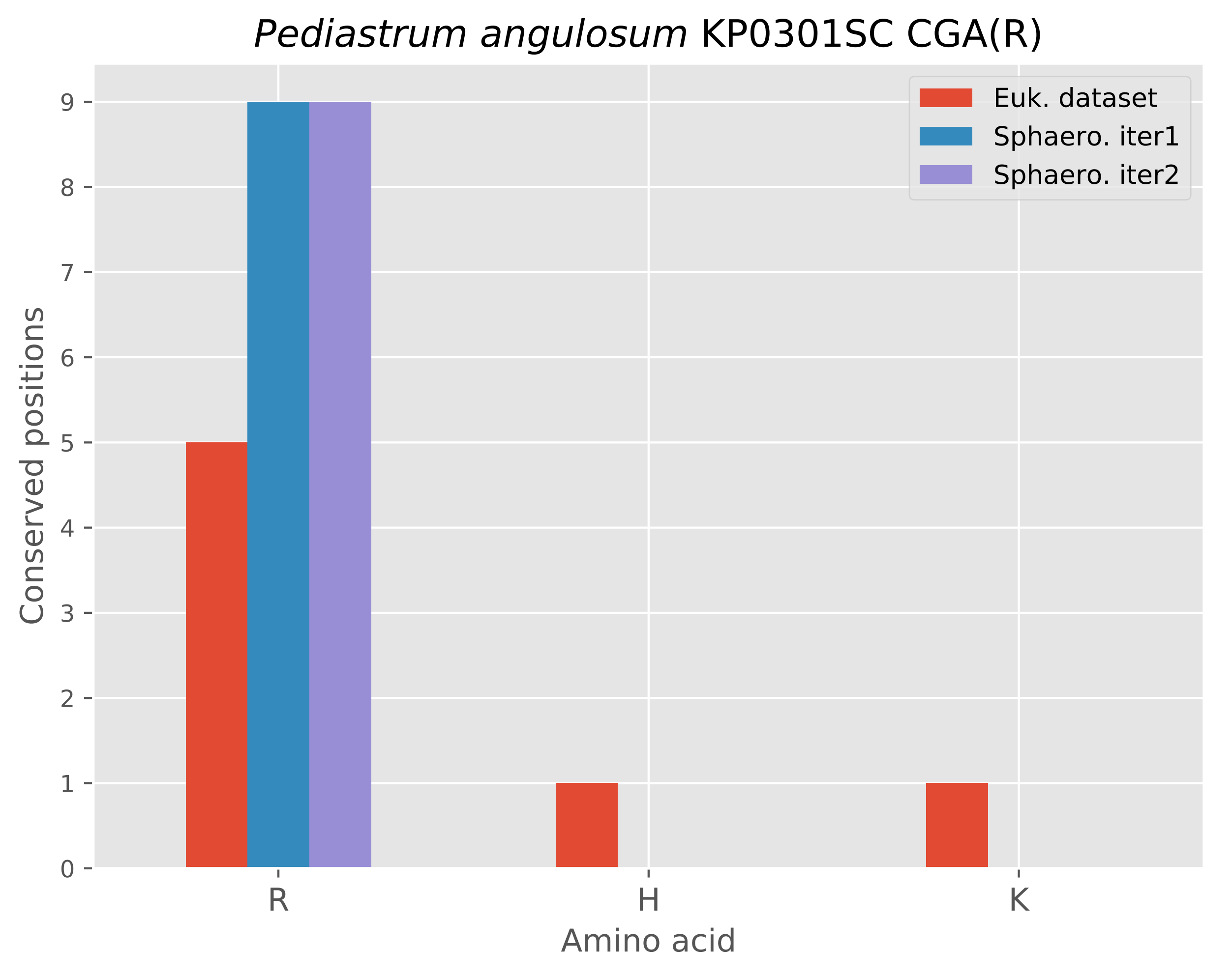

Pediastrum angulosum KP0301SC CGA(R)
Euk. dataset
Sphaero. iter1
Sphaero. iter2
Conserved positions
0
1
2
3
4
5
6
7
8
9
R
H
K
Amino acid

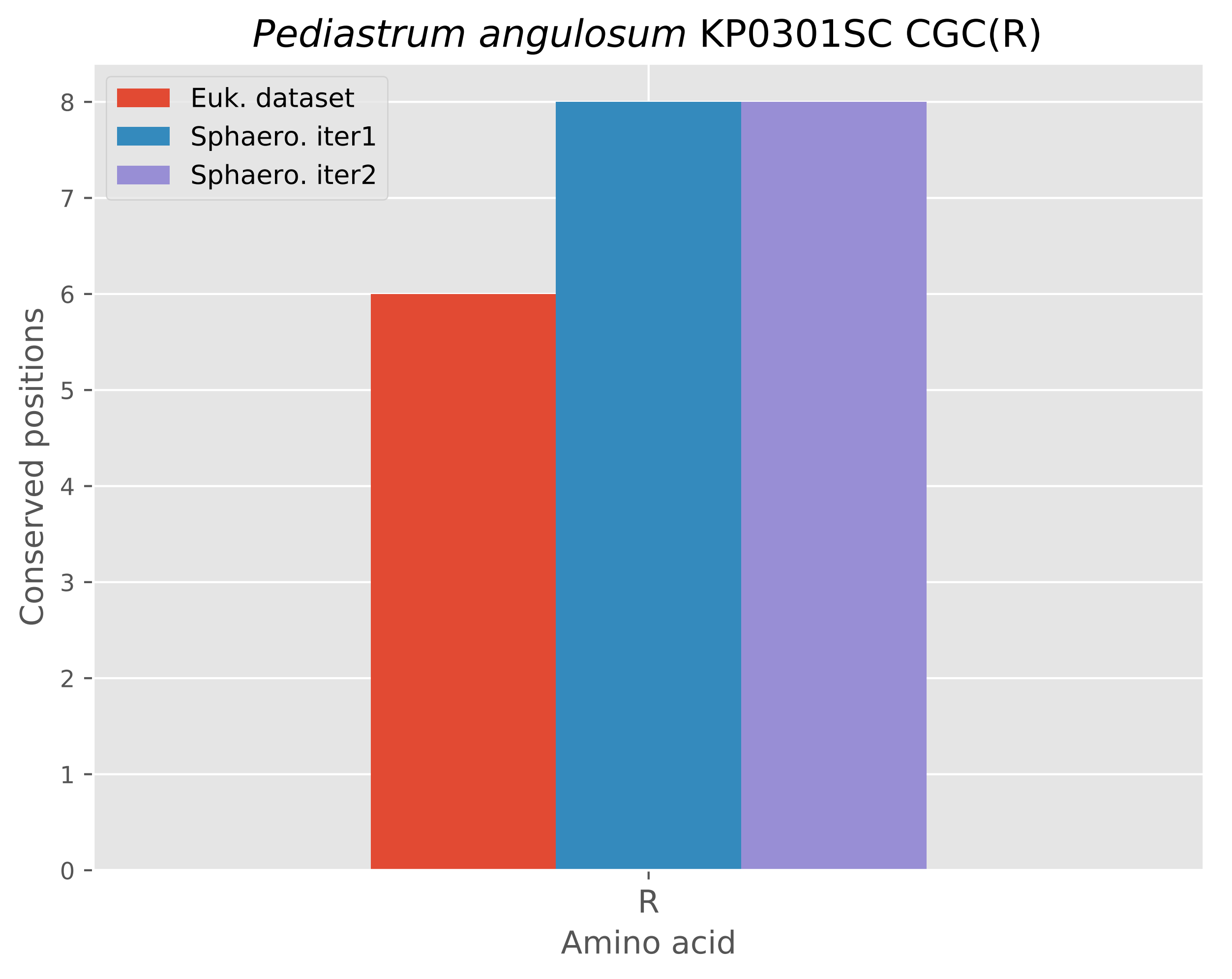

Pediastrum angulosum KP0301SC CGC(R)
Euk. dataset
Sphaero. iter1
Sphaero. iter2
Conserved positions
0
1
2
3
4
5
6
7
8
R
Amino acid

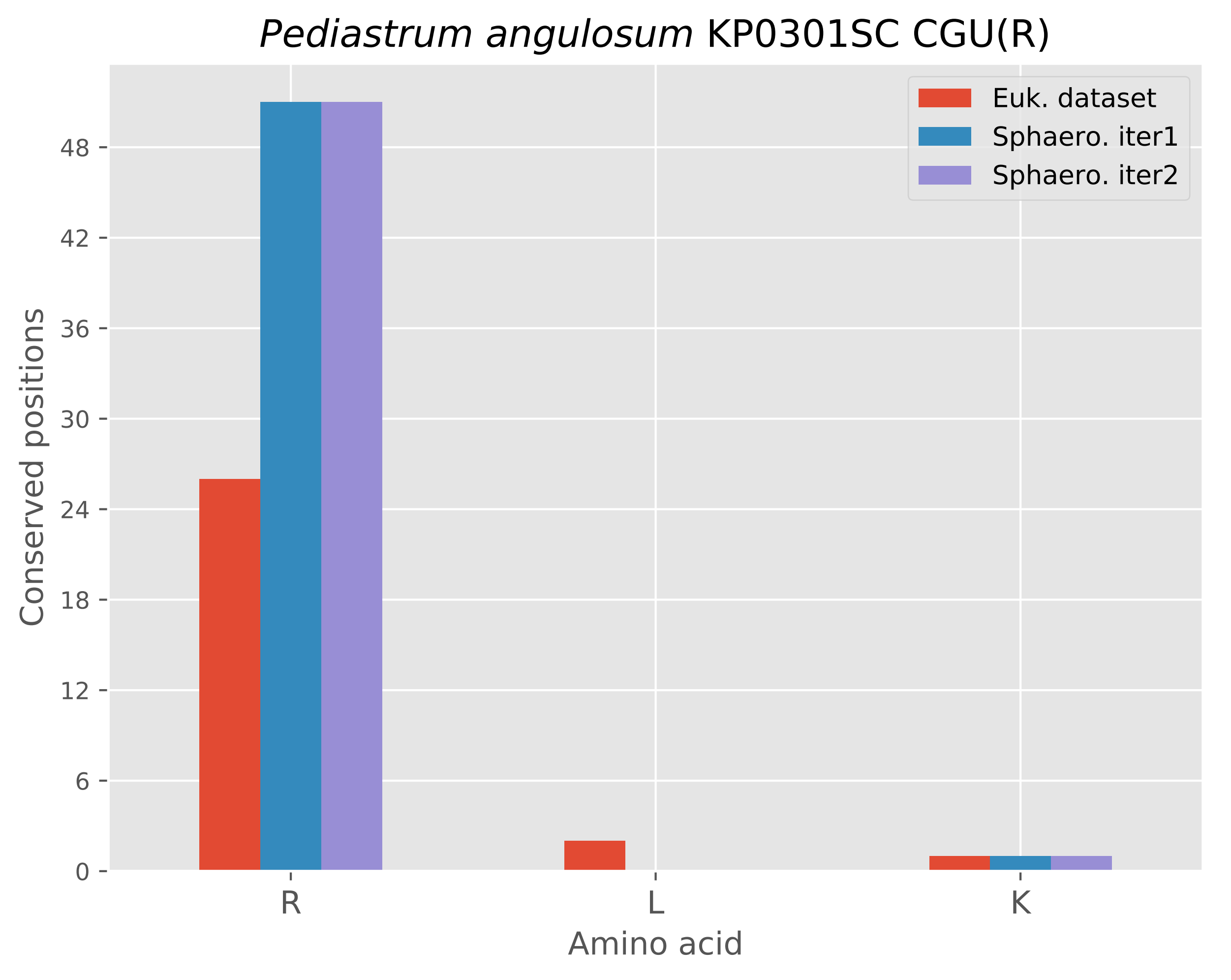

Pediastrum angulosum KP0301SC CGU(R)
Euk. dataset
Sphaero. iter1
Sphaero. iter2
Conserved positions
0
6
12
18
24
30
36
42
48
R
L
K
Amino acid

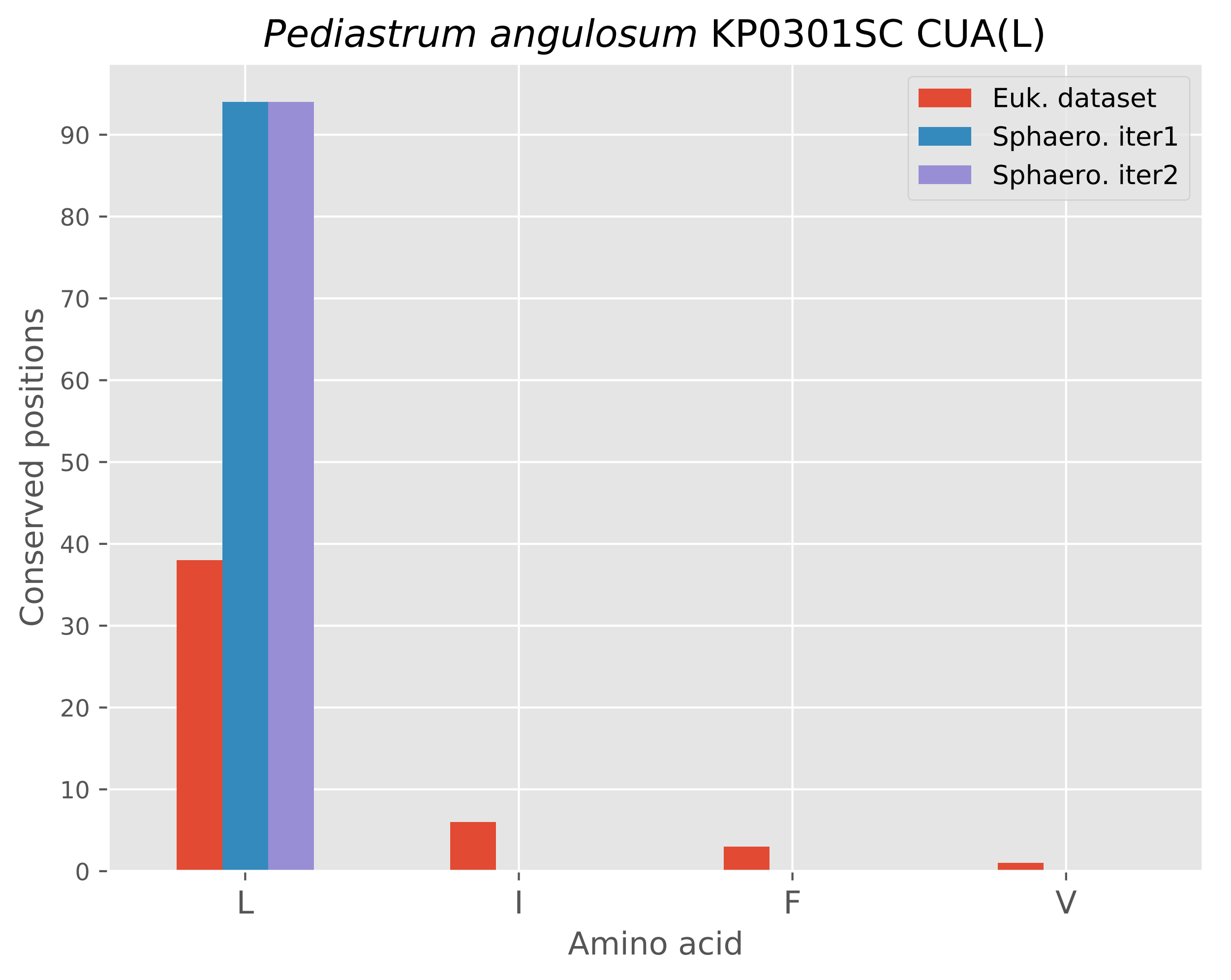

Pediastrum angulosum KP0301SC CUA(L)
Euk. dataset
Sphaero. iter1
Sphaero. iter2
Conserved positions
0
10
20
30
40
50
60
70
80
90
L
I
F
V
Amino acid

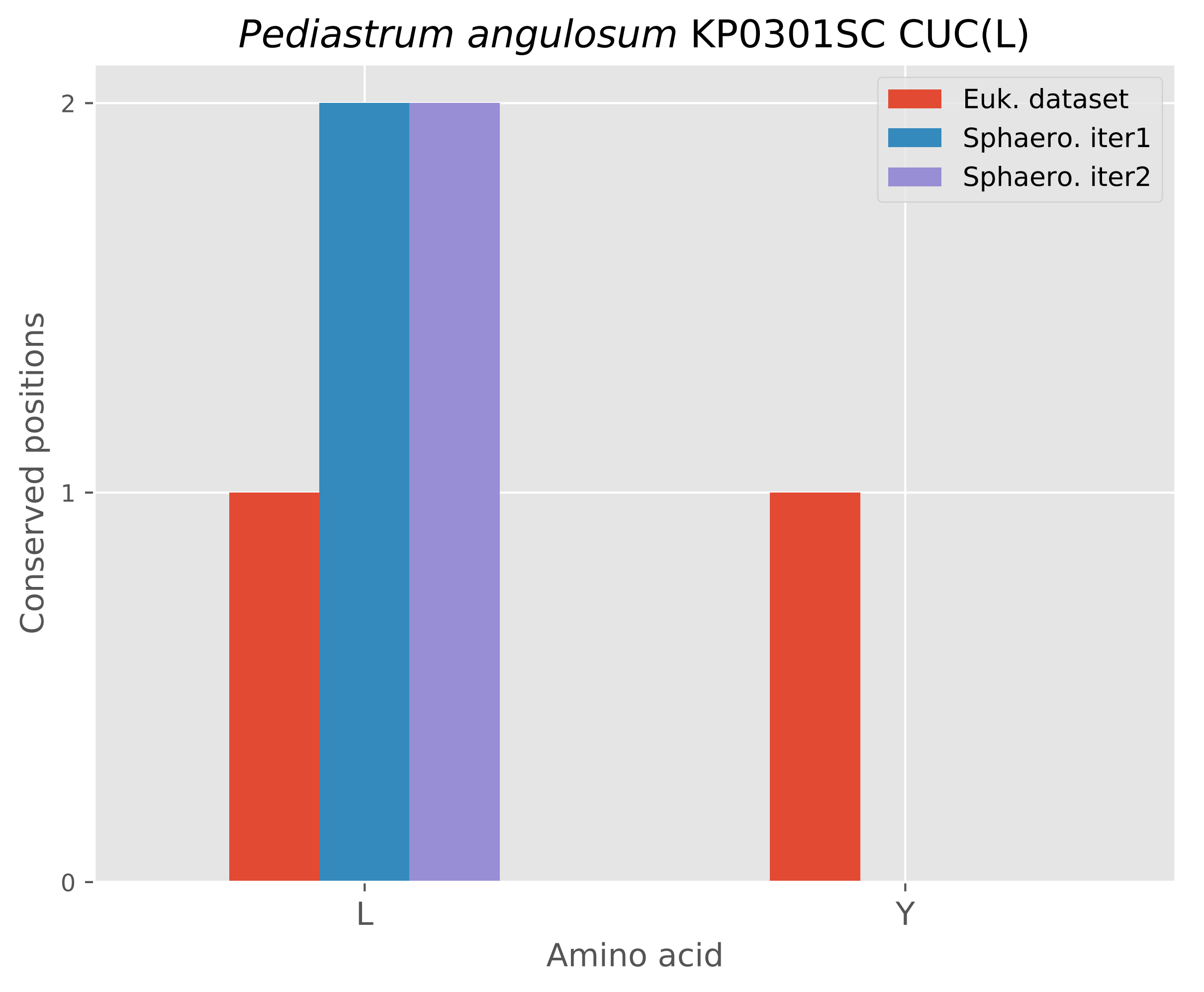

Pediastrum angulosum KP0301SC CUC(L)
Euk. dataset
Sphaero. iter1
Sphaero. iter2
Conserved positions
2
1
0
L
Y
Amino acid

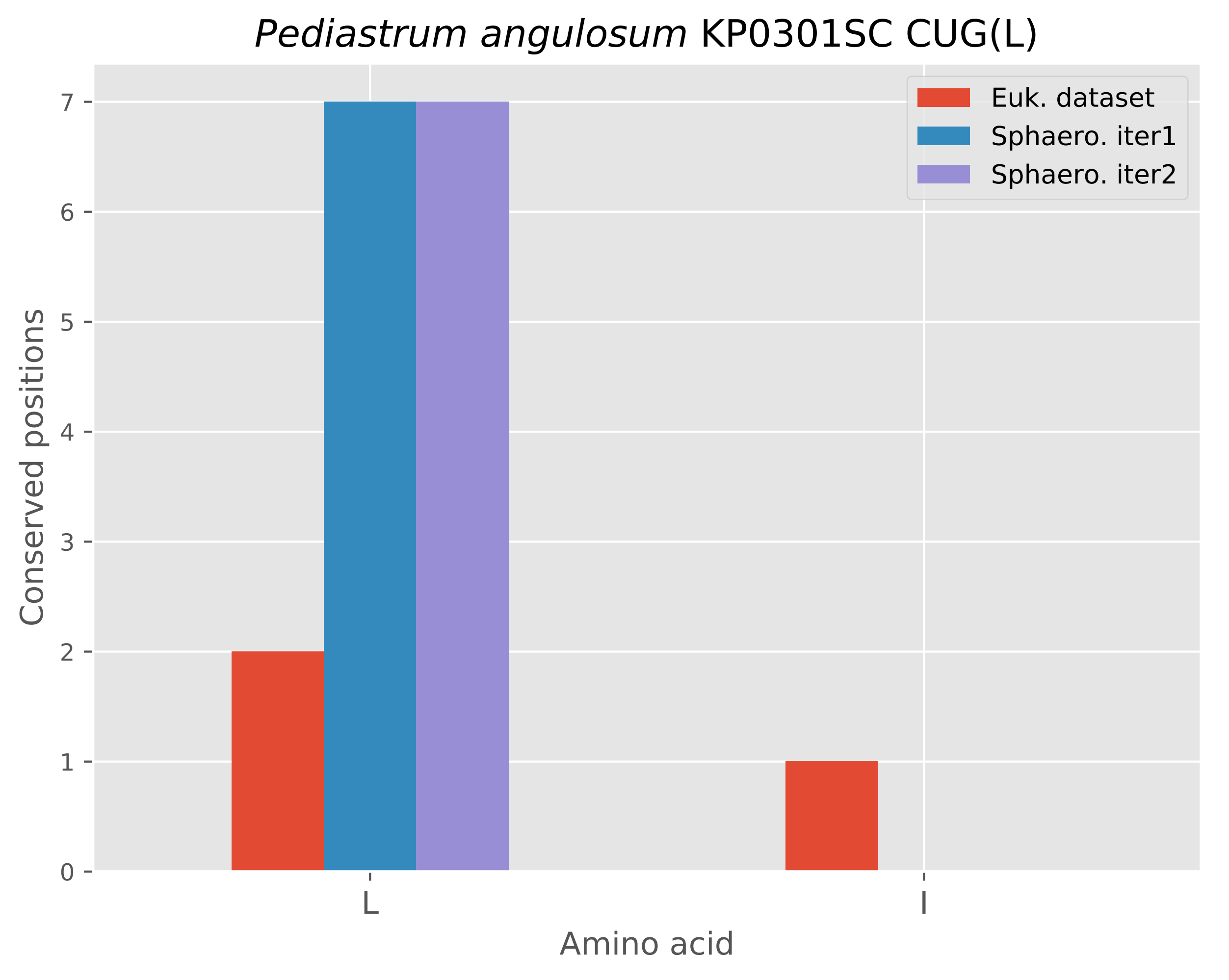

Pediastrum angulosum KP0301SC CUG(L)
Euk. dataset
Sphaero. iter1
Sphaero. iter2
Conserved positions
0
1
2
3
4
5
6
7
L
I
Amino acid

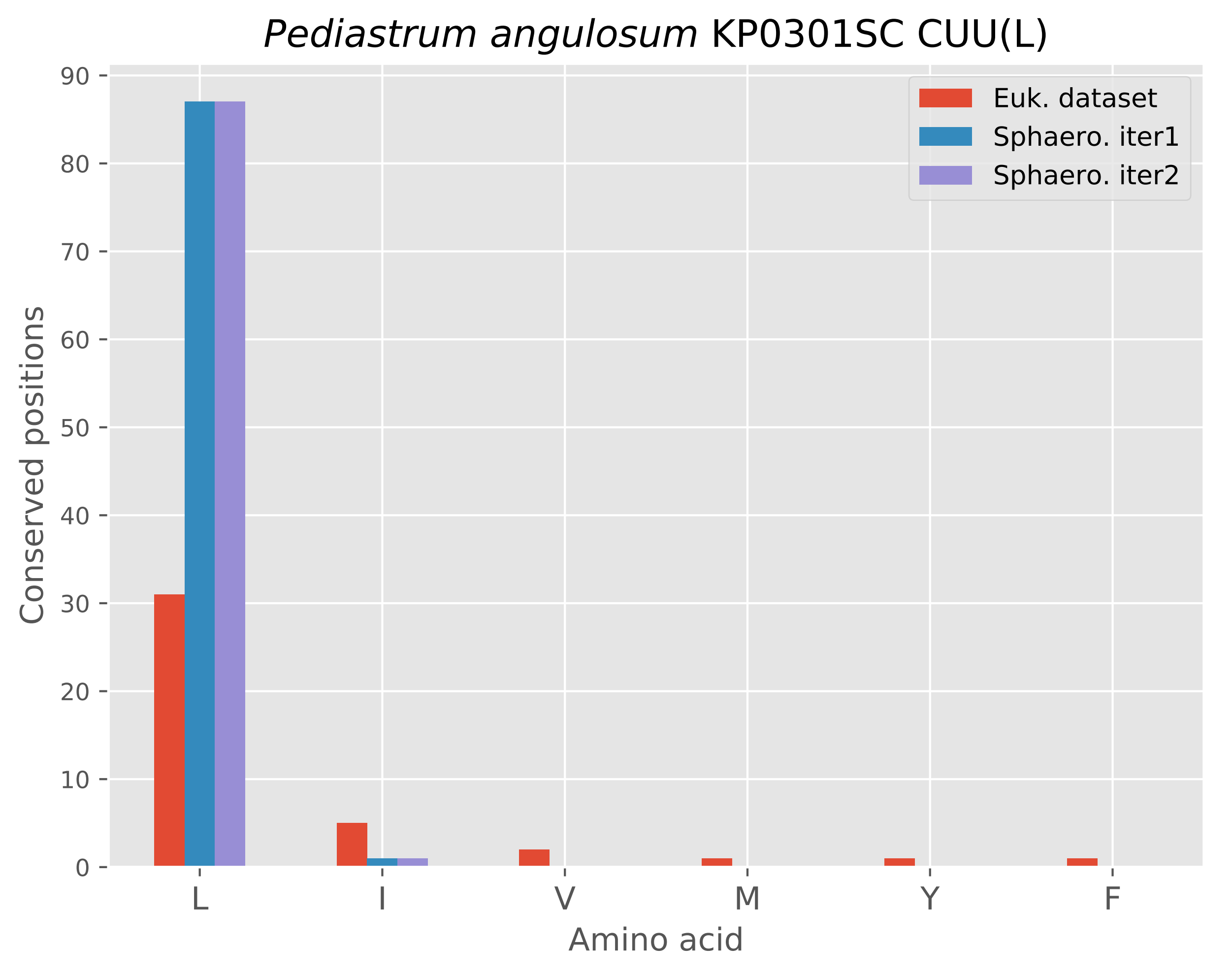

Pediastrum angulosum KP0301SC CUU(L)
Euk. dataset
Sphaero. iter1
Sphaero. iter2
Conserved positions
0
10
20
30
40
50
60
70
80
90
L
I
V
M
Y
F
Amino acid

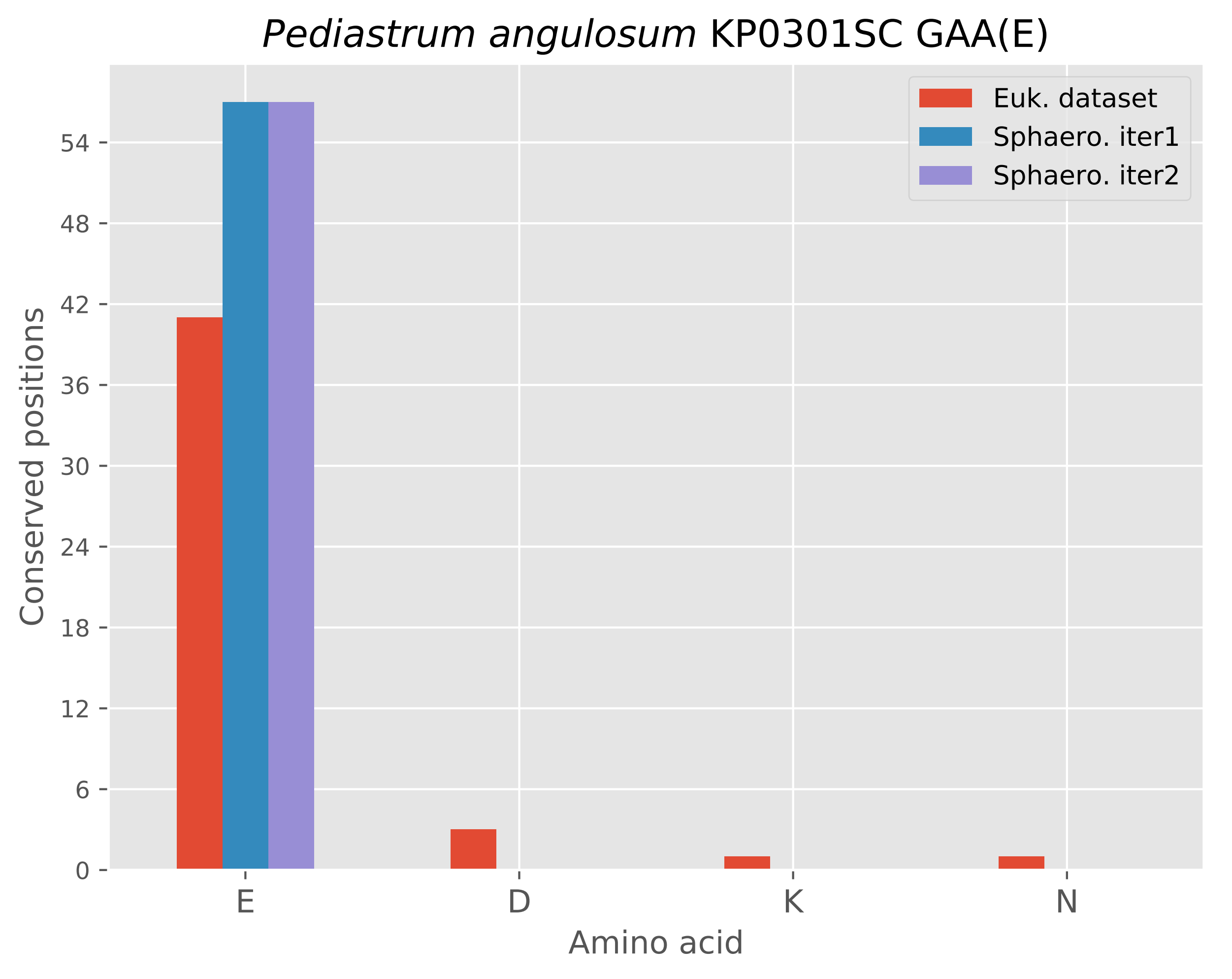

Pediastrum angulosum KP0301SC GAA(E)
Euk. dataset
Sphaero. iter1
Sphaero. iter2
Conserved positions
0
6
12
18
24
30
36
42
48
54
E
D
K
N
Amino acid

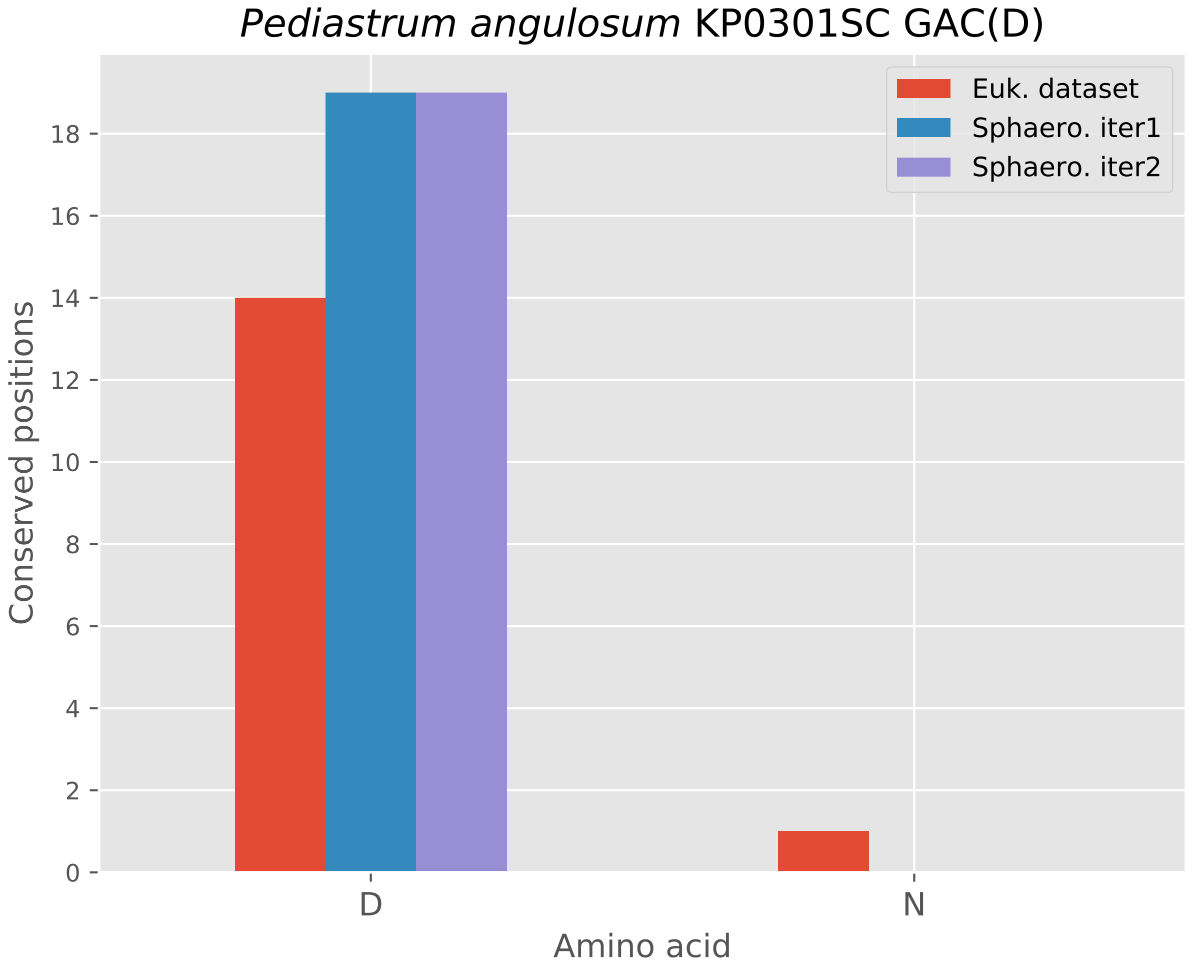

Pediastrum angulosum KP0301SC GAC(D)
Euk. dataset
Sphaero. iter1
Sphaero. iter2
Conserved positions
0
2
4
6
8
10
12
14
16
18
D
N
Amino acid

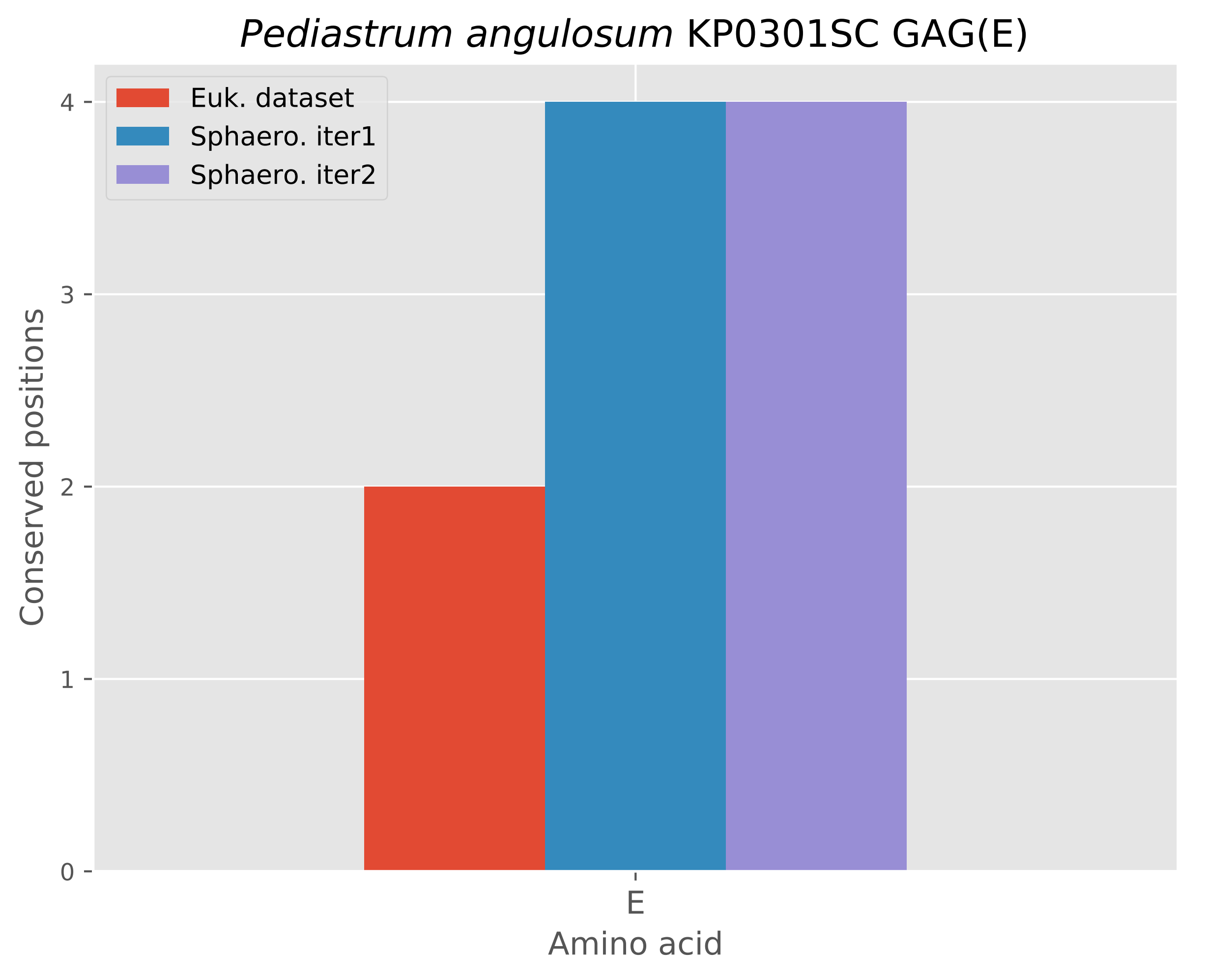

Pediastrum angulosum KP0301SC GAG(E)
Euk. dataset
Sphaero. iter1
Sphaero. iter2
Conserved positions
0
1
2
3
4
E
Amino acid

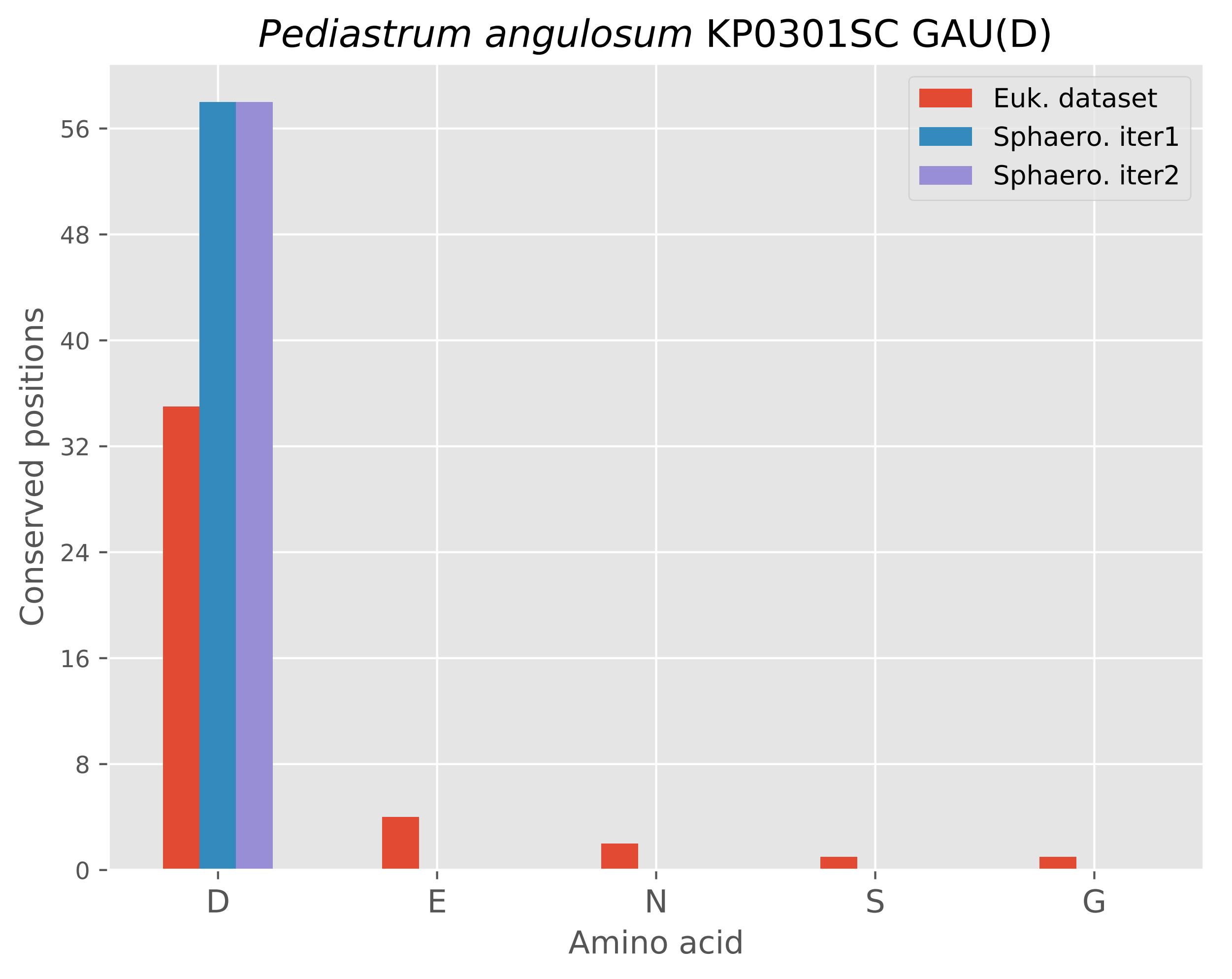

Pediastrum angulosum KP0301SC GAU(D)
Euk. dataset
Sphaero. iter1
Sphaero. iter2
Conserved positions
0
8
16
24
32
40
48
56
D
E
N
S
G
Amino acid

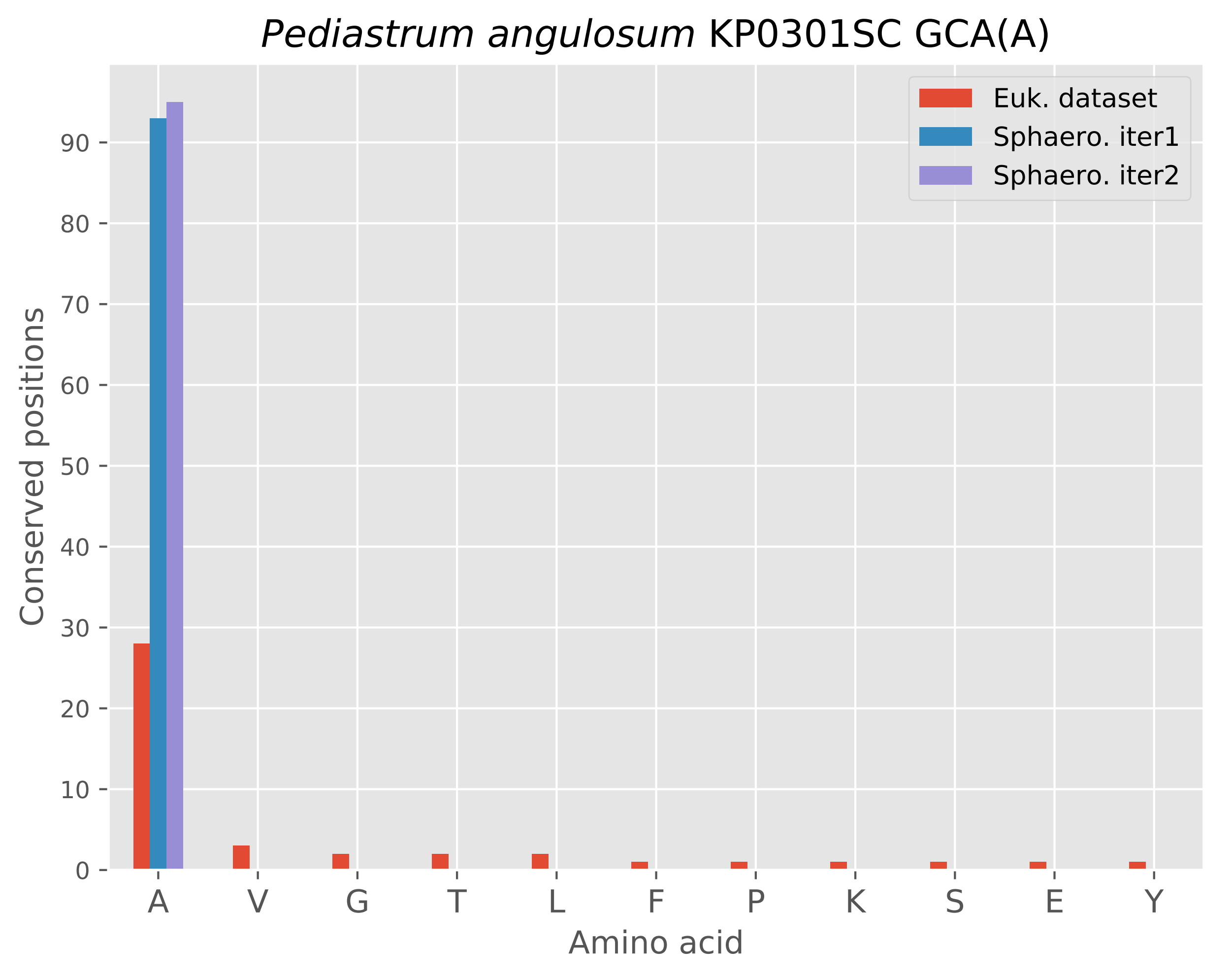

Pediastrum angulosum KP0301SC GCA(A)
Euk. dataset
Sphaero. iter1
Sphaero. iter2
Conserved positions
0
10
20
30
40
50
60
70
80
90
A
V
G
T
L
F
P
K
S
E
Y
Amino acid

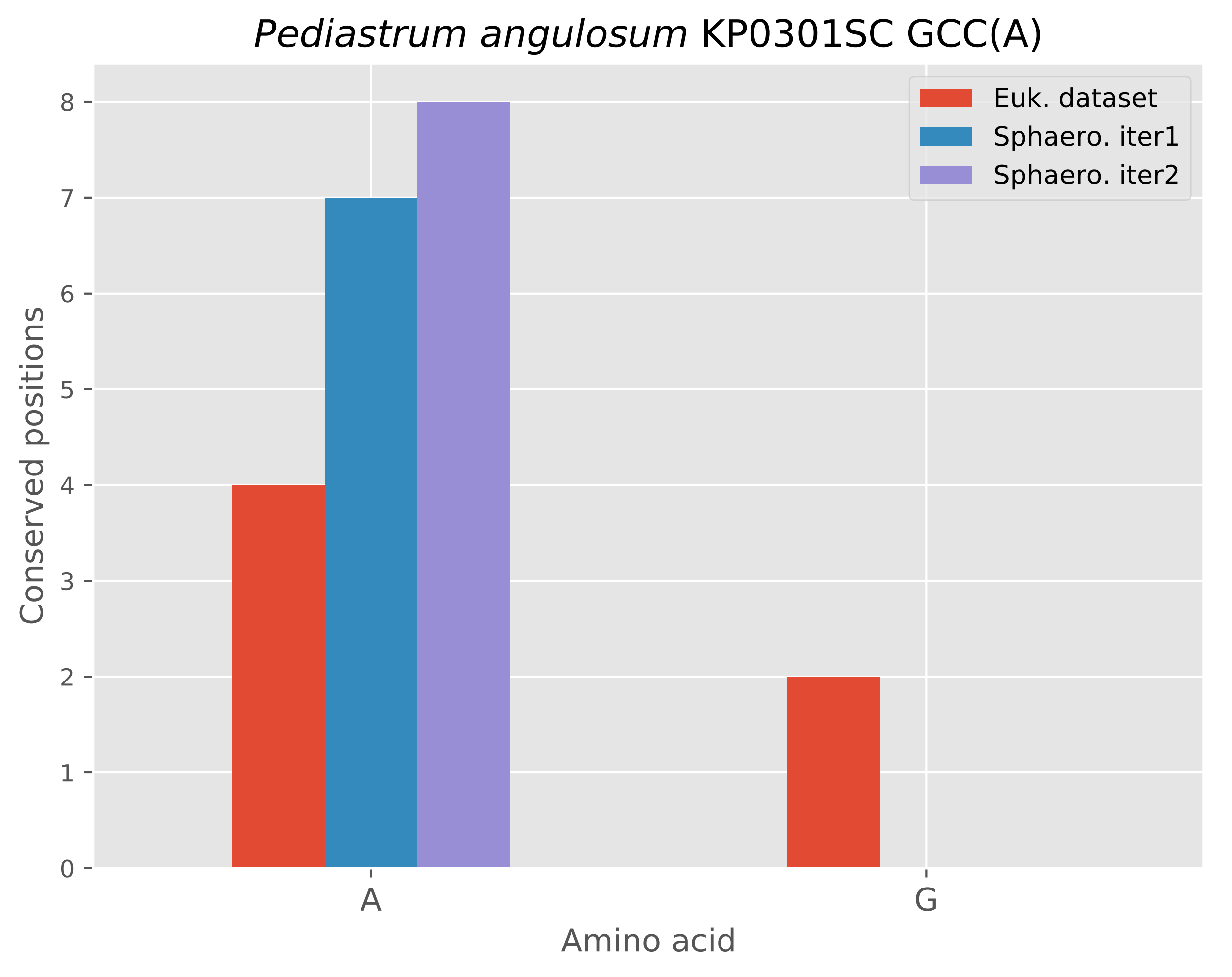
Pediastrum angulosum KP0301SC GCC(A)
Euk. dataset
Sphaero. iter1
Sphaero. iter2
Conserved positions
0
1
2
3
4
5
6
7
8
A
G
Amino acid

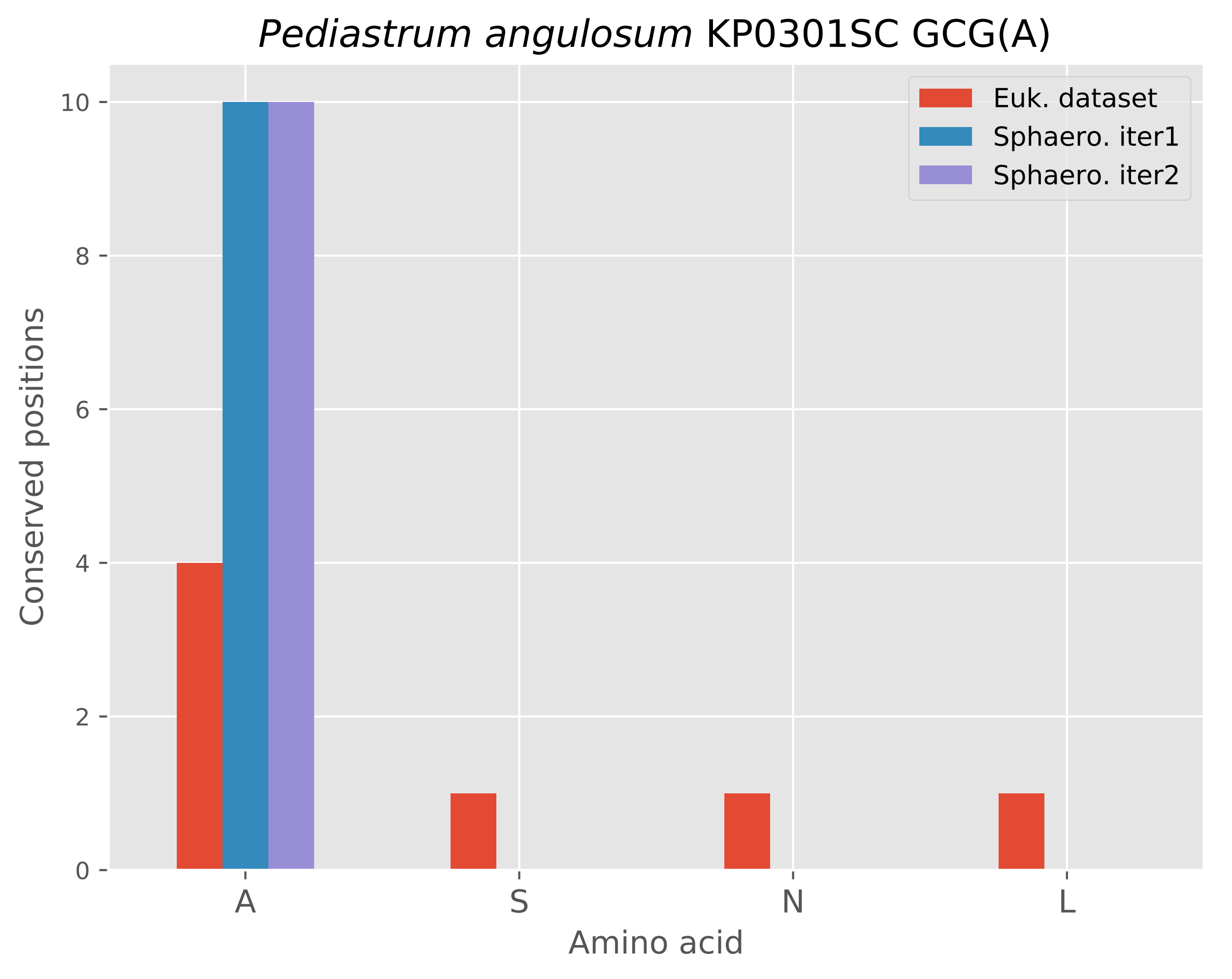

Pediastrum angulosum KP0301SC GCG(A)
Conserved positions
0
2
4
6
8
10
A
S
N
L
Amino acid
Euk. dataset
Sphaero. iter1
Sphaero. iter2

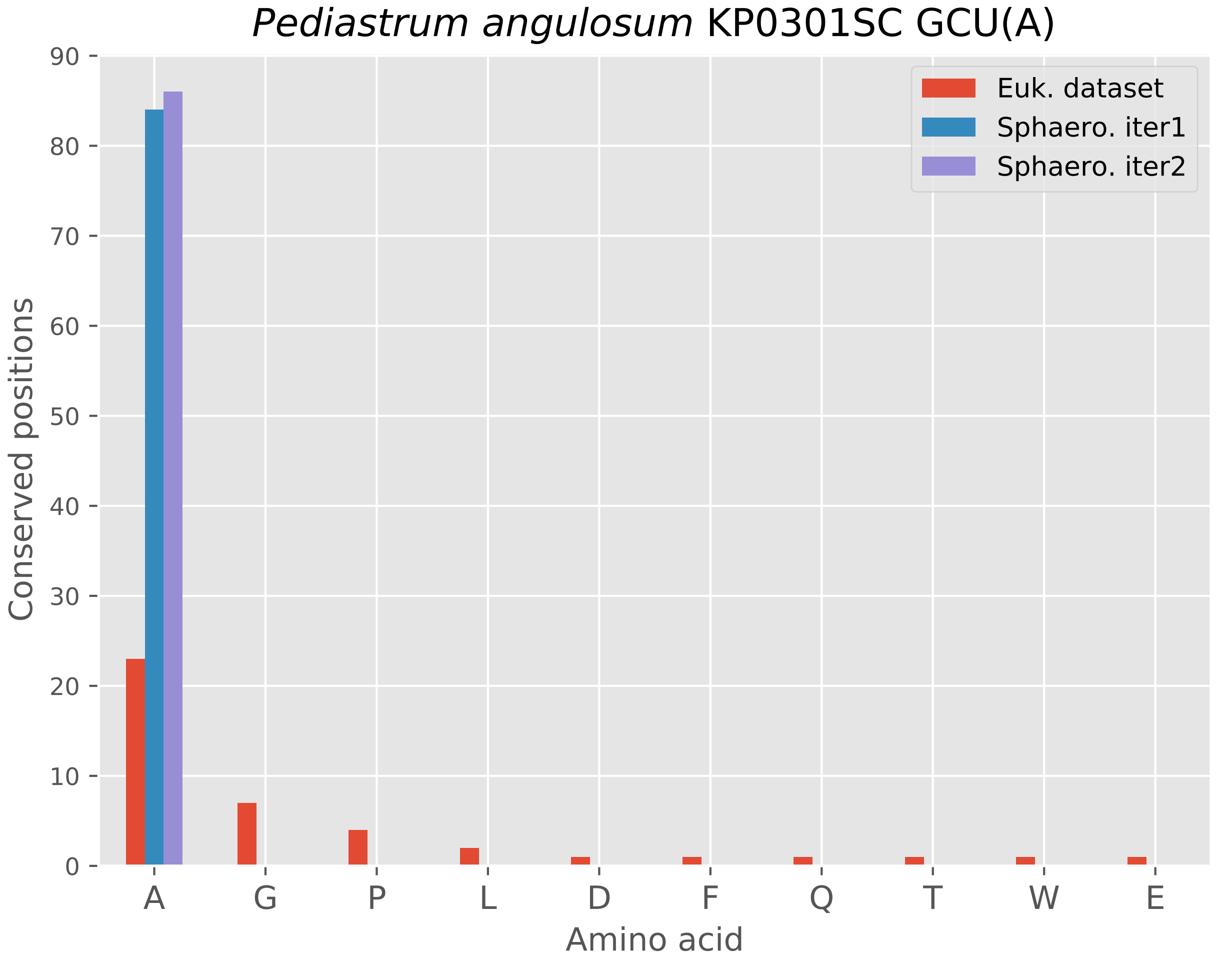

Pediastrum angulosum KP0301SC GCU(A)
Euk. dataset
Sphaero. iter1
Sphaero. iter2
Conserved positions
0
10
20
30
40
50
60
70
80
90
A
G
P
L
D
F
Q
T
W
E
Amino acid

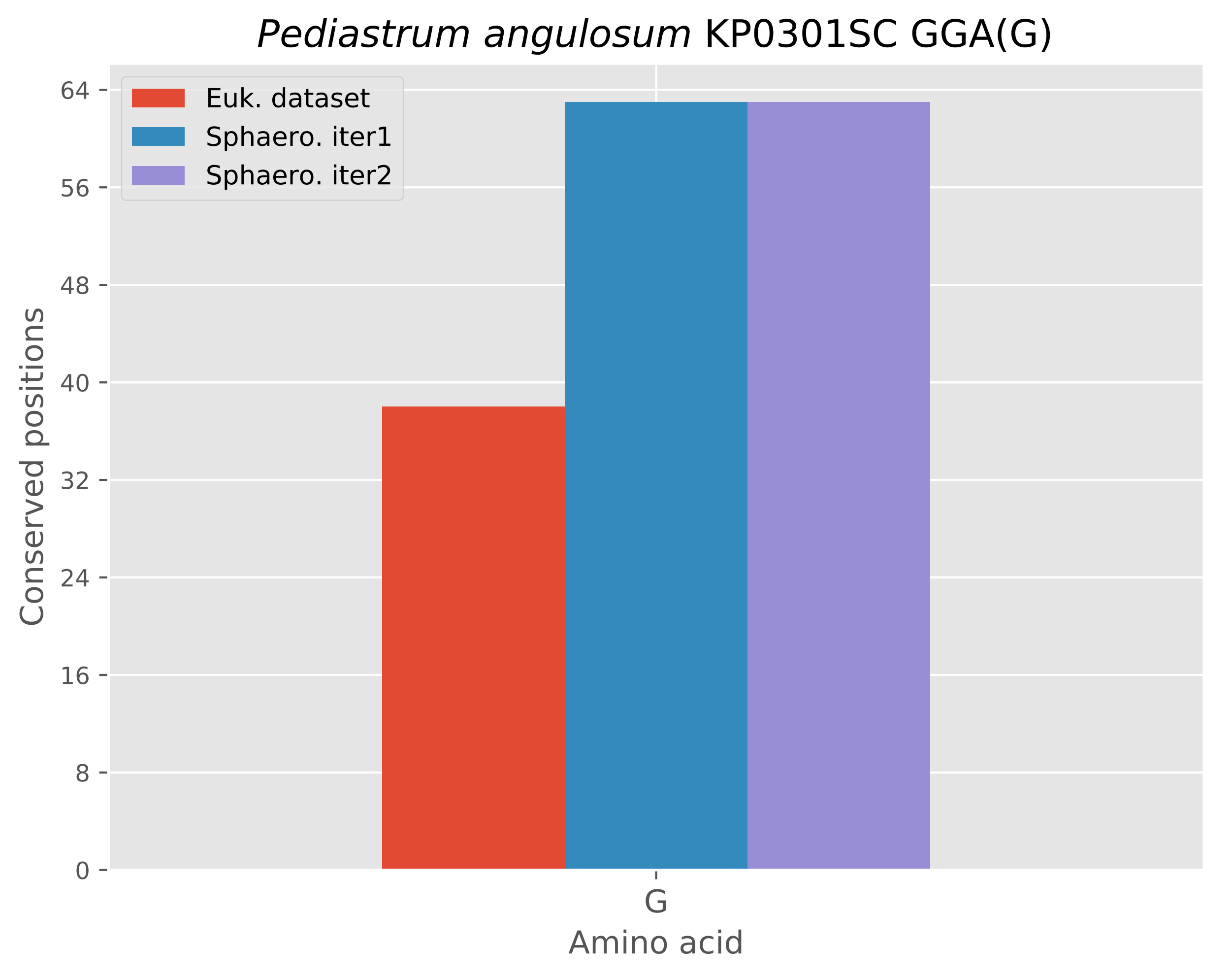

Pediastrum angulosum KP0301SC GGA(G)
Euk. dataset
Sphaero. iter1
Sphaero. iter2
Conserved positions
0
8
16
24
32
40
48
56
64
G
Amino acid

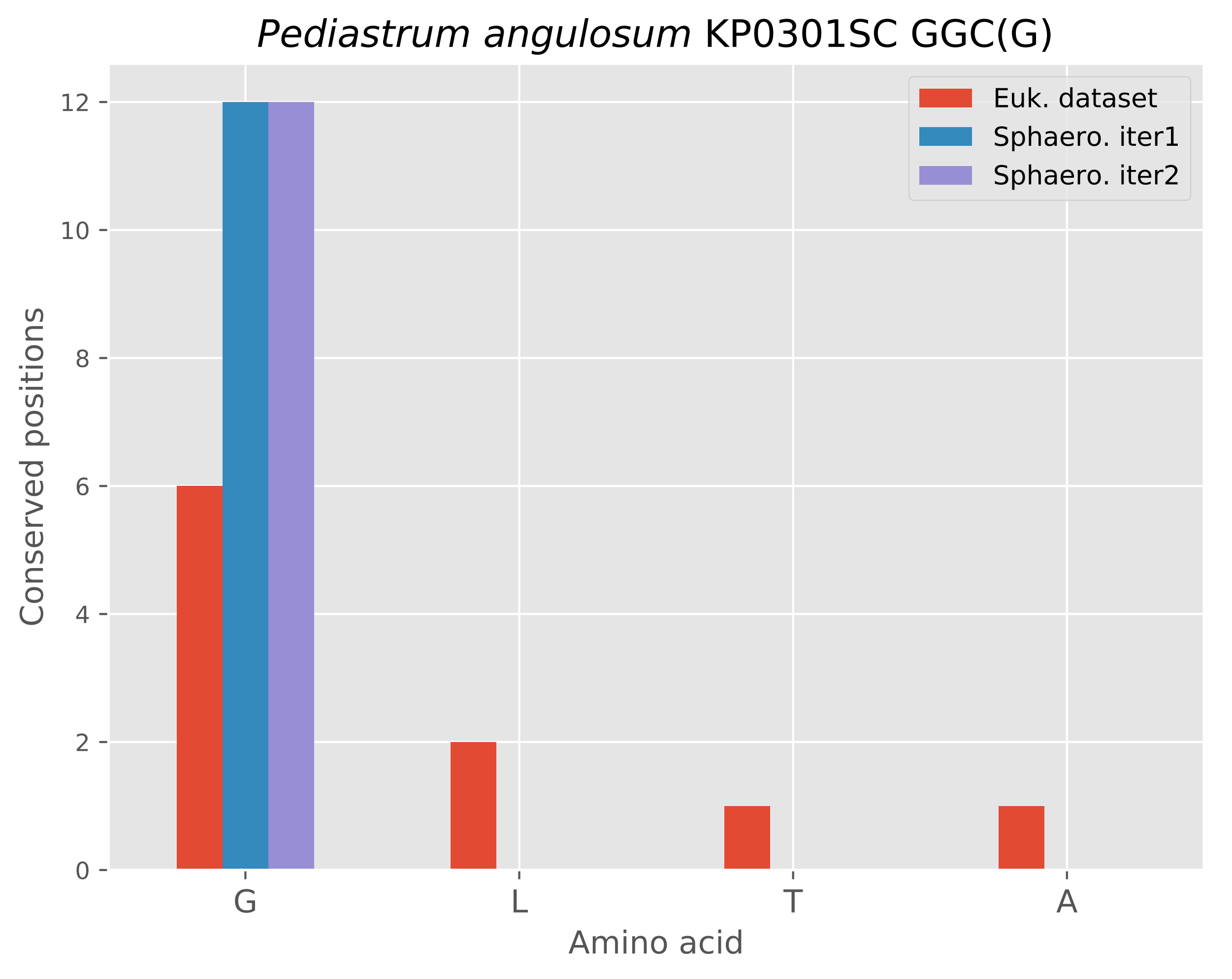

Pediastrum angulosum KP0301SC GGC(G)
Euk. dataset
Sphaero. iter1
Sphaero. iter2
Conserved positions
0
2
4
6
8
10
12
G
L
T
A
Amino acid

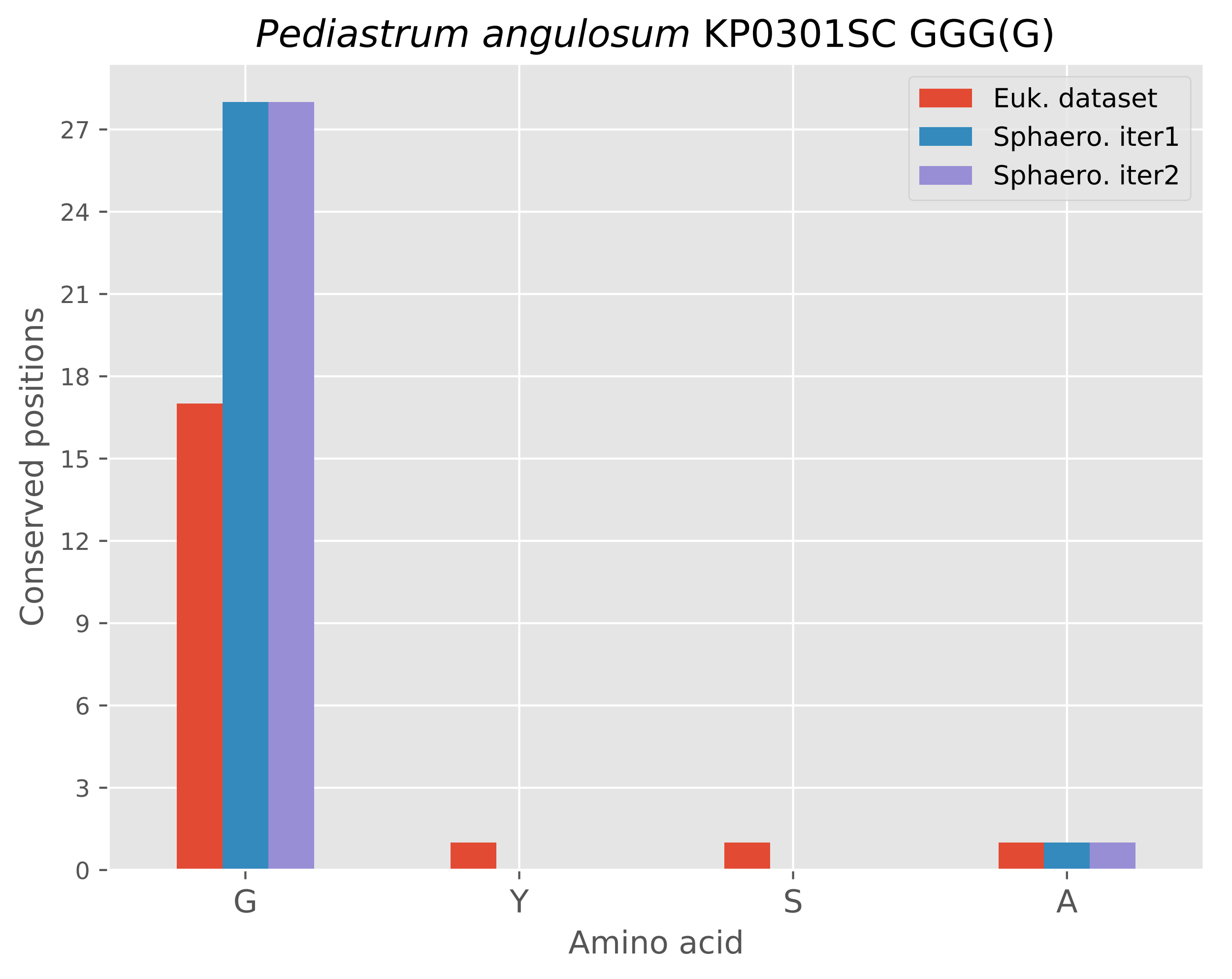

Pediastrum angulosum KP0301SC GGG(G)
Euk. dataset
Sphaero. iter1
Sphaero. iter2
Conserved positions
0
3
6
9
12
15
18
21
24
27
G
Y
S
A
Amino acid

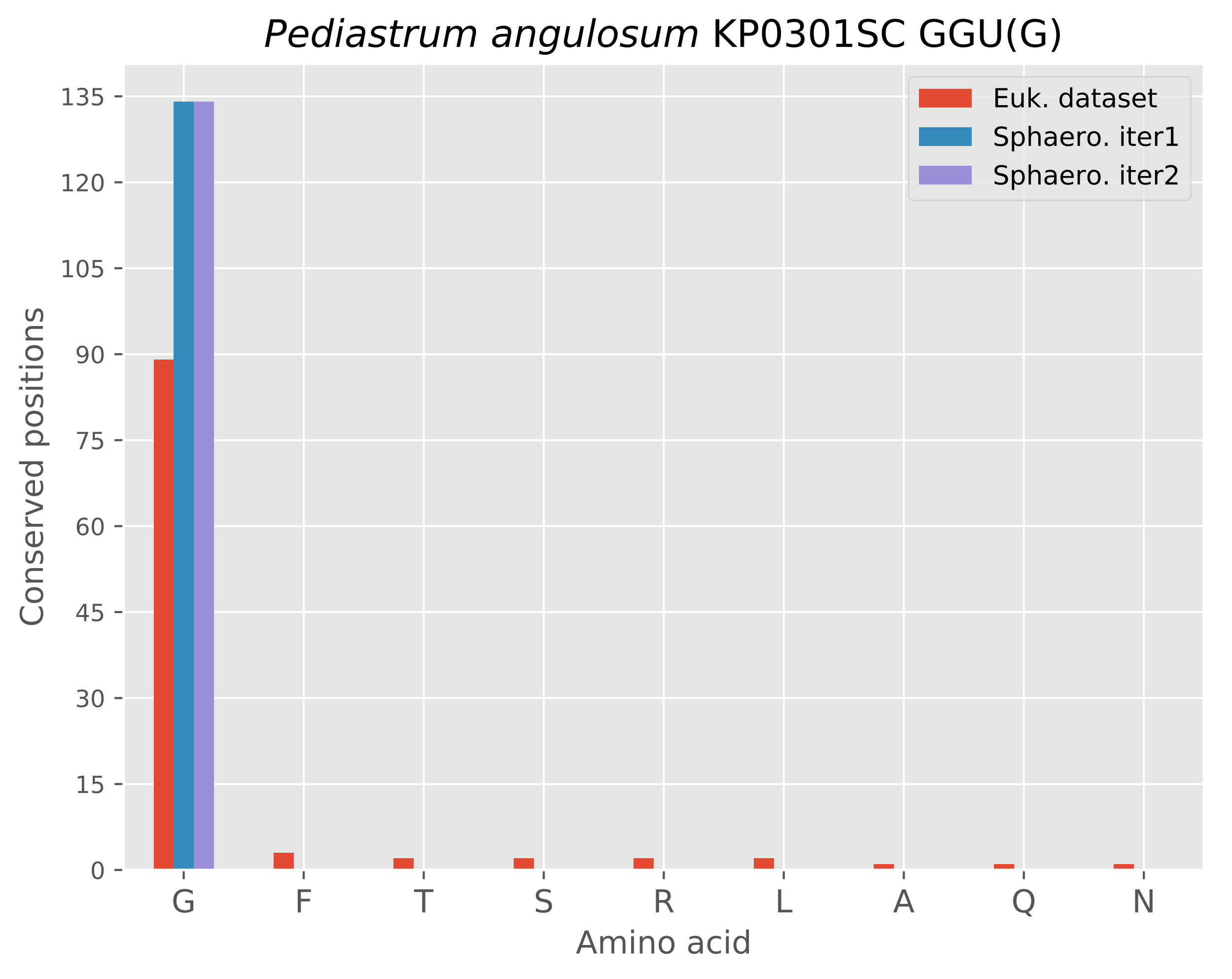

Pediastrum angulosum KP0301SC GGU(G)
Euk. dataset
Sphaero. iter1
Sphaero. iter2
Conserved positions
0
15
30
45
60
75
90
105
120
135
G
F
T
S
R
L
A
Q
N
Amino acid

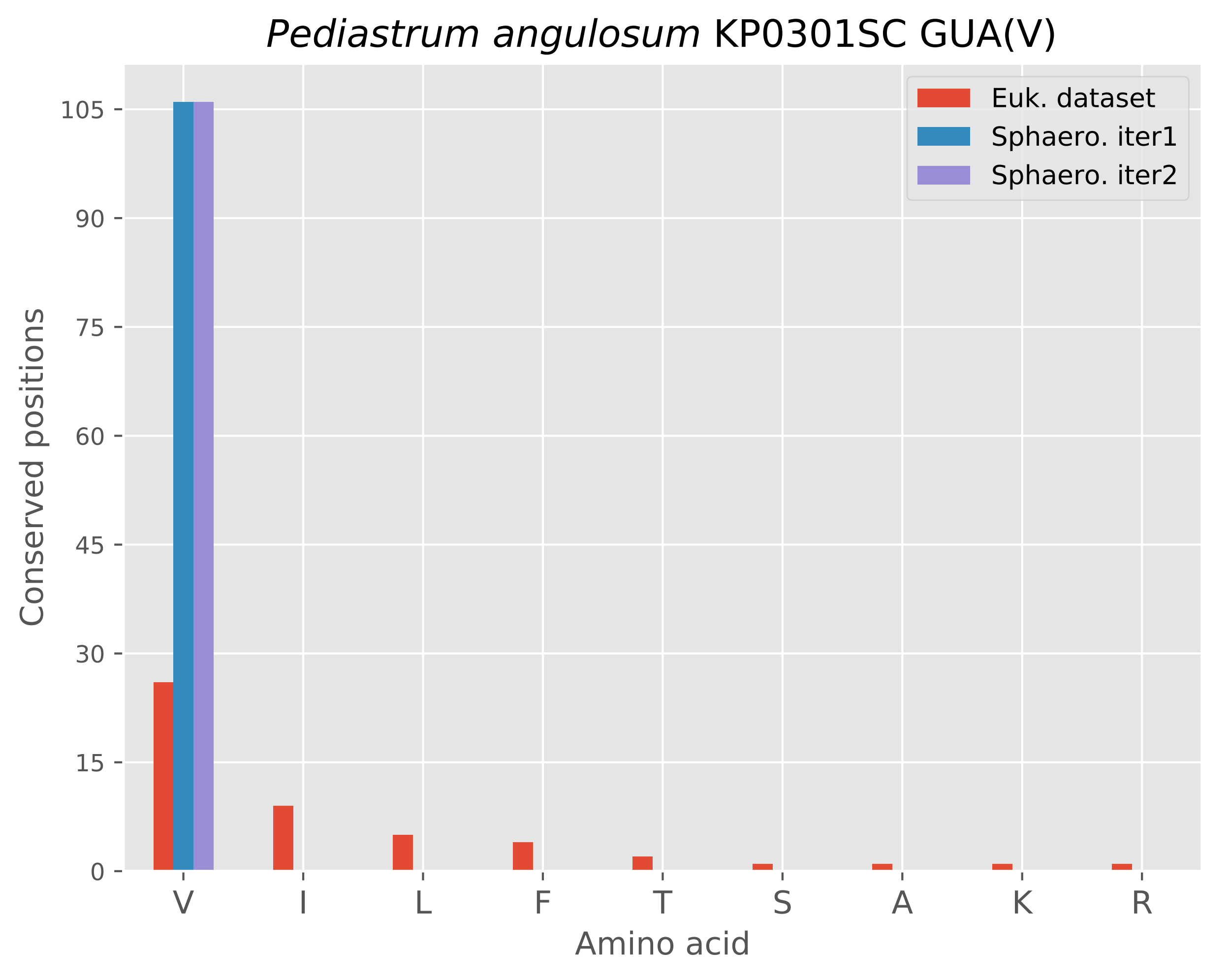

Pediastrum angulosum KP0301SC GUA(V)
Euk. dataset
Sphaero. iter1
Sphaero. iter2
Conserved positions
0
15
30
45
60
75
90
105
V
I
L
F
T
S
A
K
R
Amino acid

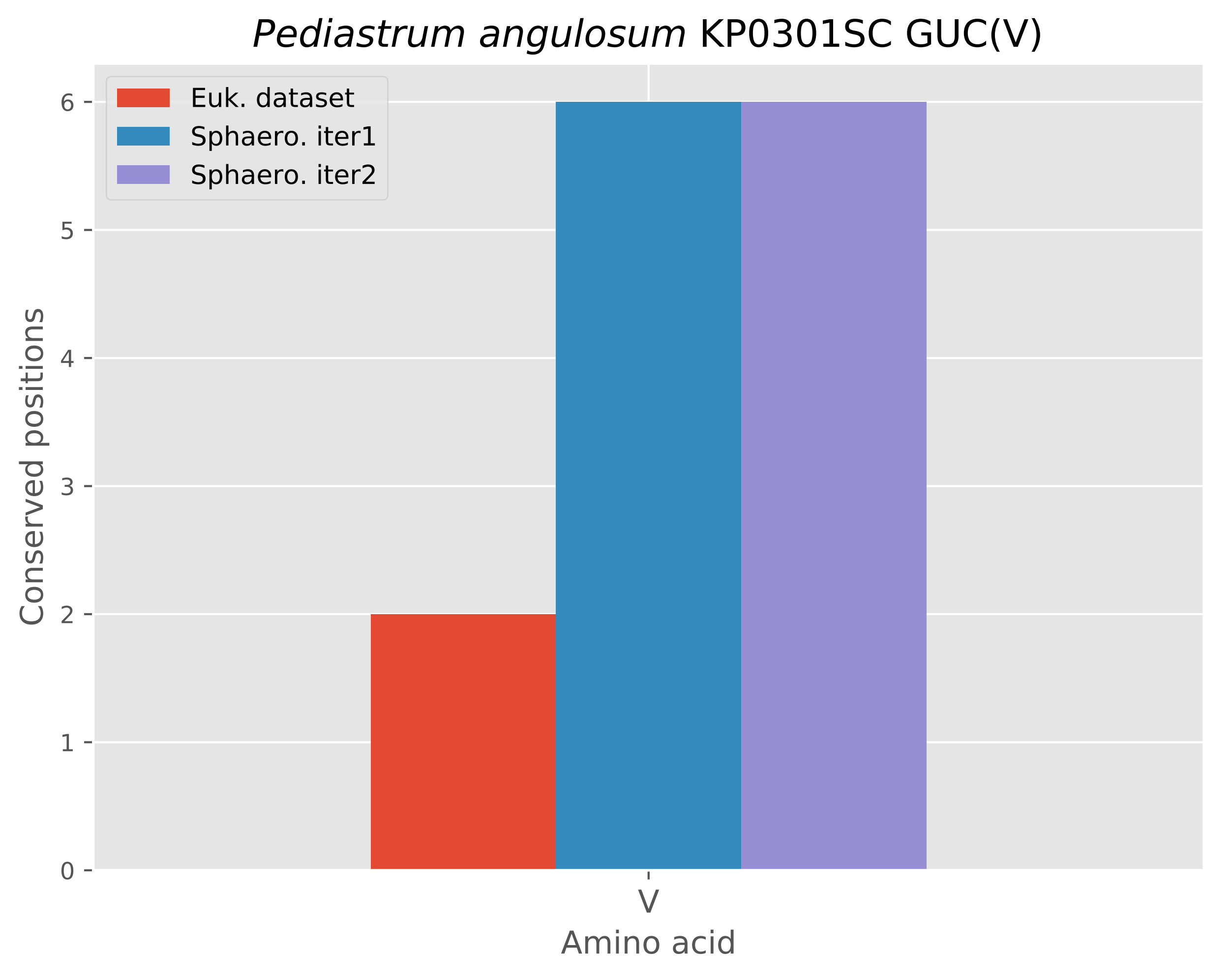

Pediastrum angulosum KP0301SC GUC(V)
Euk. dataset
Sphaero. iter1
Sphaero. iter2
Conserved positions
0
1
2
3
4
5
6
V
Amino acid

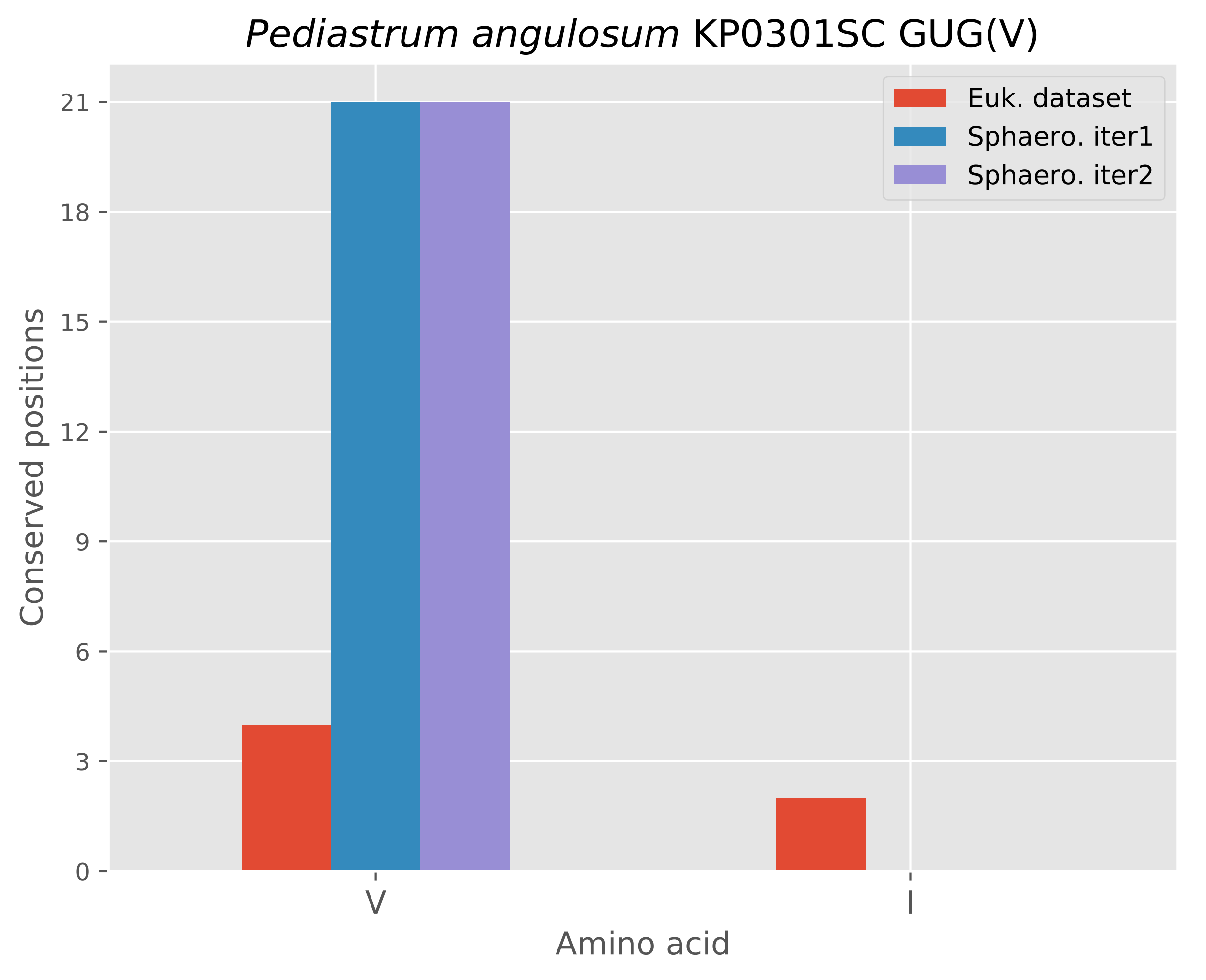

Pediastrum angulosum KP0301SC GUG(V)
Euk. dataset
Sphaero. iter1
Sphaero. iter2
Conserved positions
0
3
6
9
12
15
18
21
V
I
Amino acid

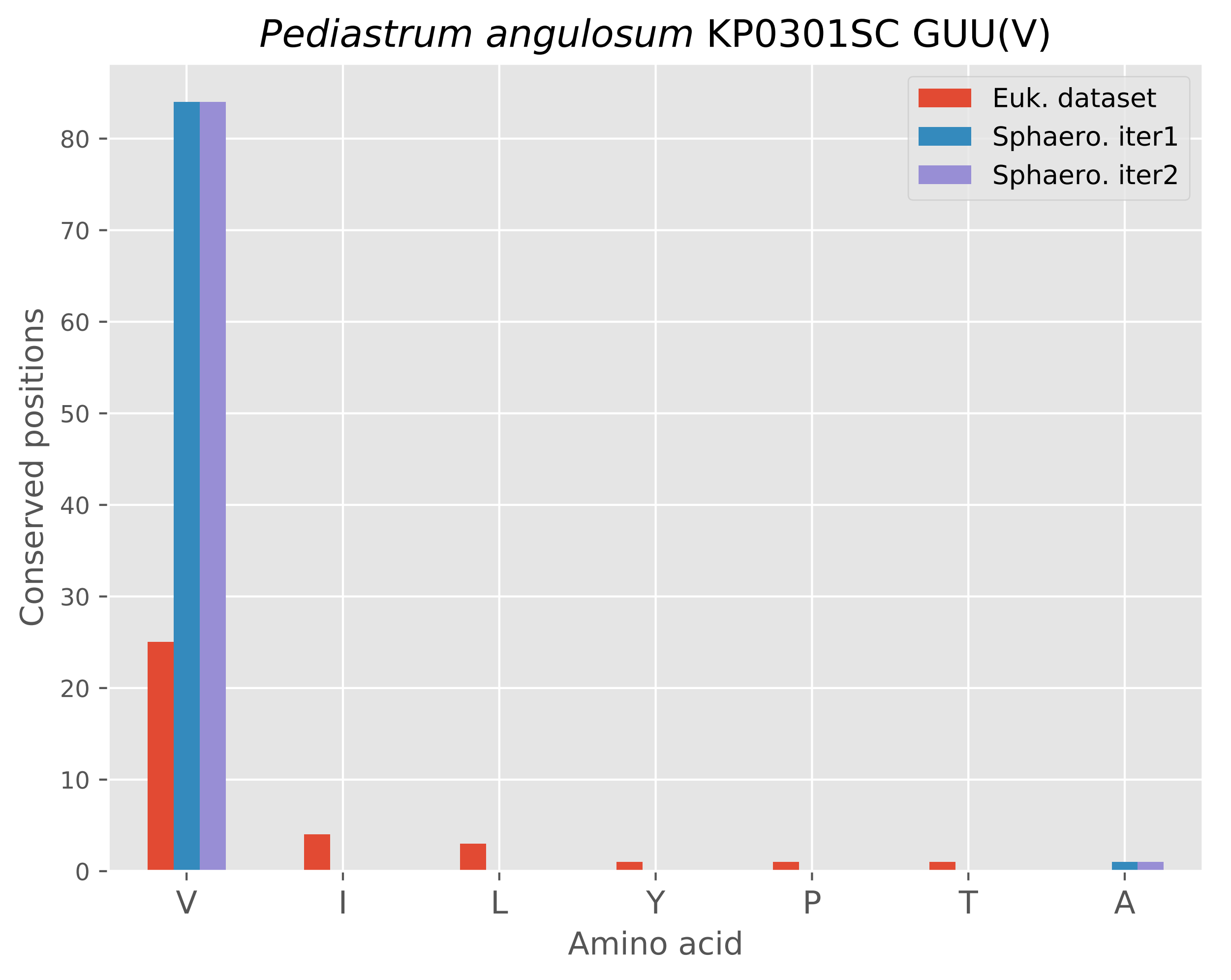
Pediastrum angulosum KP0301SC GUU(V)
Euk. dataset
Sphaero. iter1
Sphaero. iter2
Conserved positions
0
10
20
30
40
50
60
70
80
V
I
L
Y
P
T
A
Amino acid

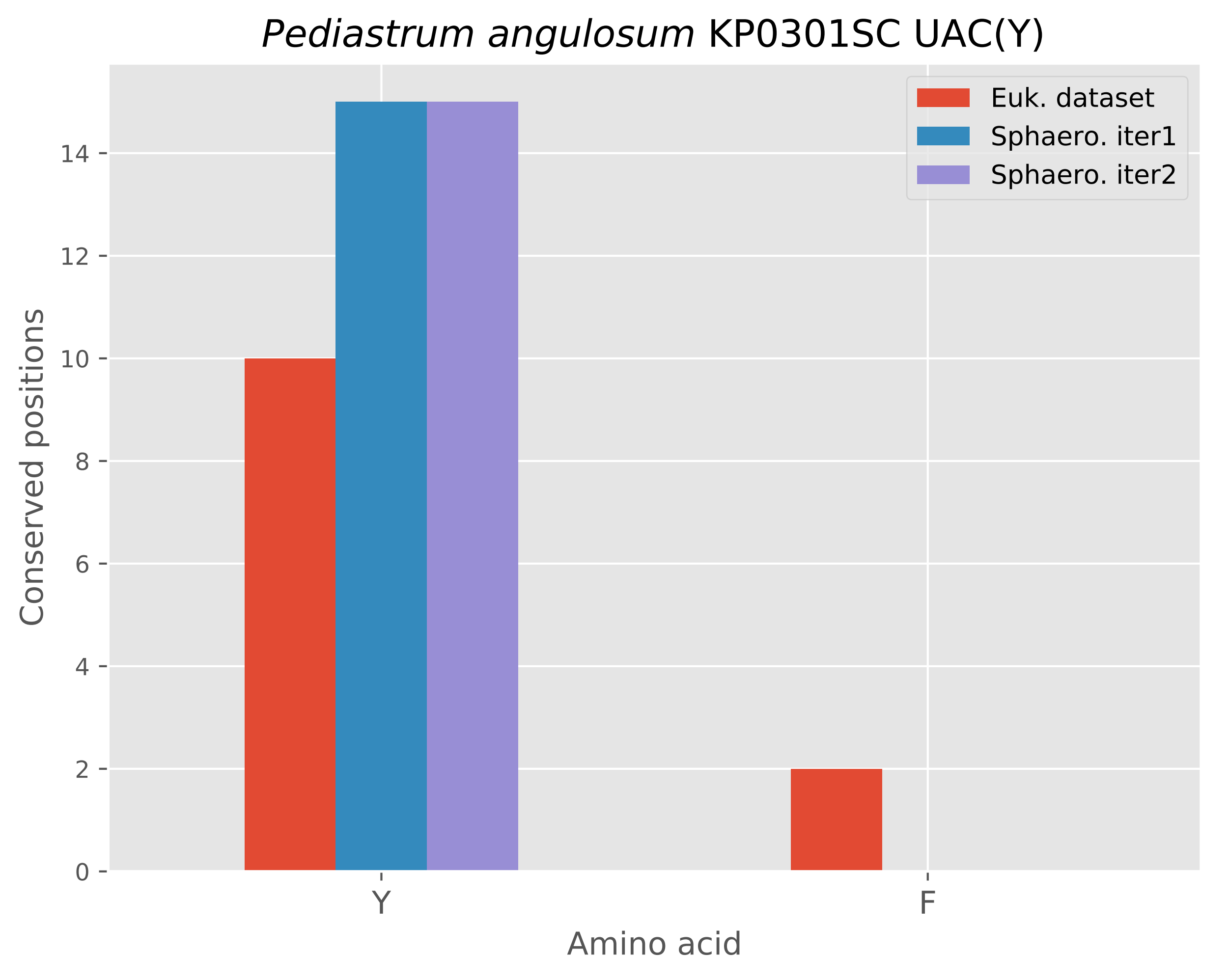

Pediastrum angulosum KP0301SC UAC(Y)
Euk. dataset
Sphaero. iter1
Sphaero. iter2
Conserved positions
0
2
4
6
8
10
12
14
Y
F
Amino acid

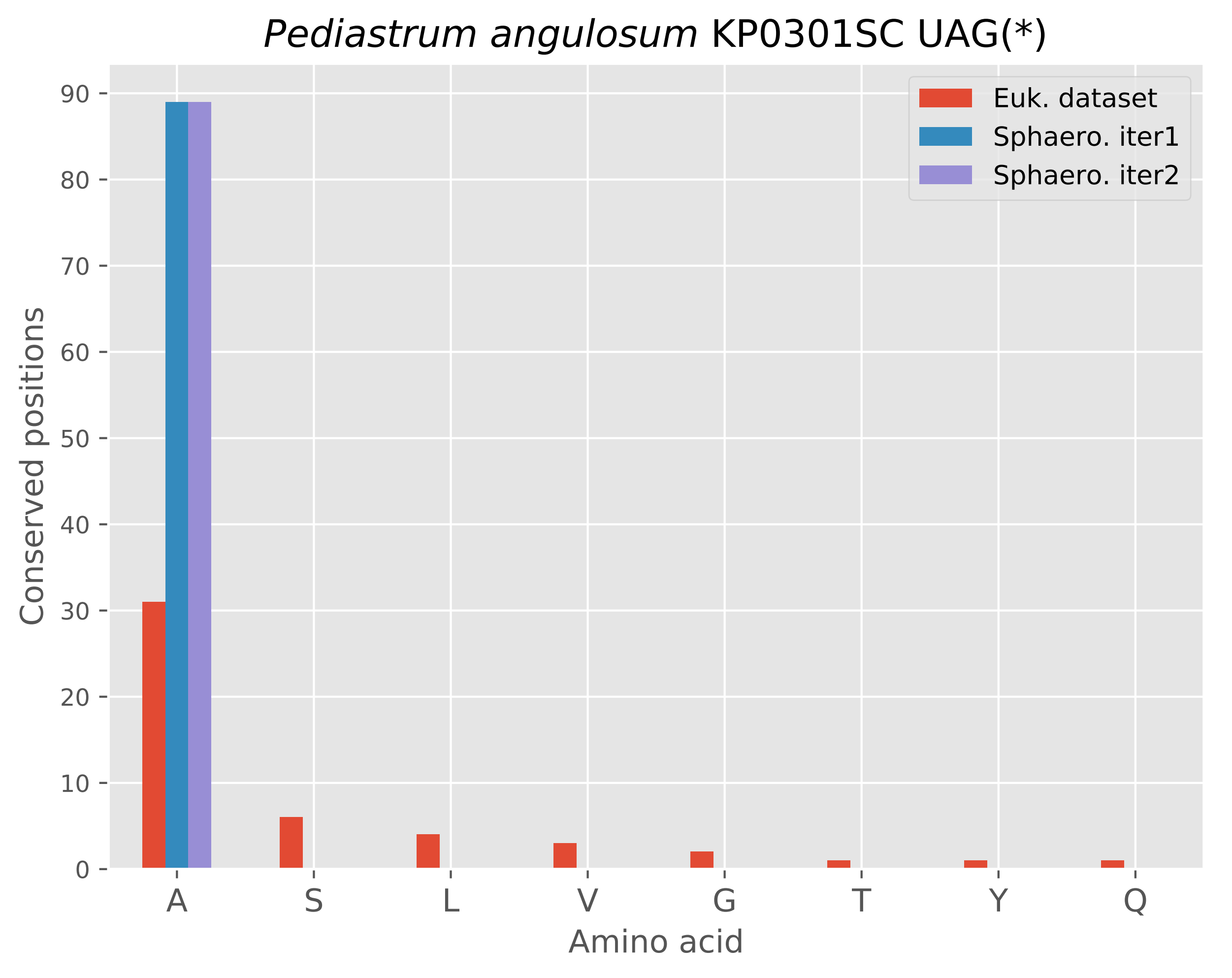

Pediastrum angulosum KP0301SC UAG(*)
Euk. dataset
Sphaero. iter1
Sphaero. iter2
Conserved positions
0
10
20
30
40
50
60
70
80
90
A
S
L
V
G
T
Y
Q
Amino acid

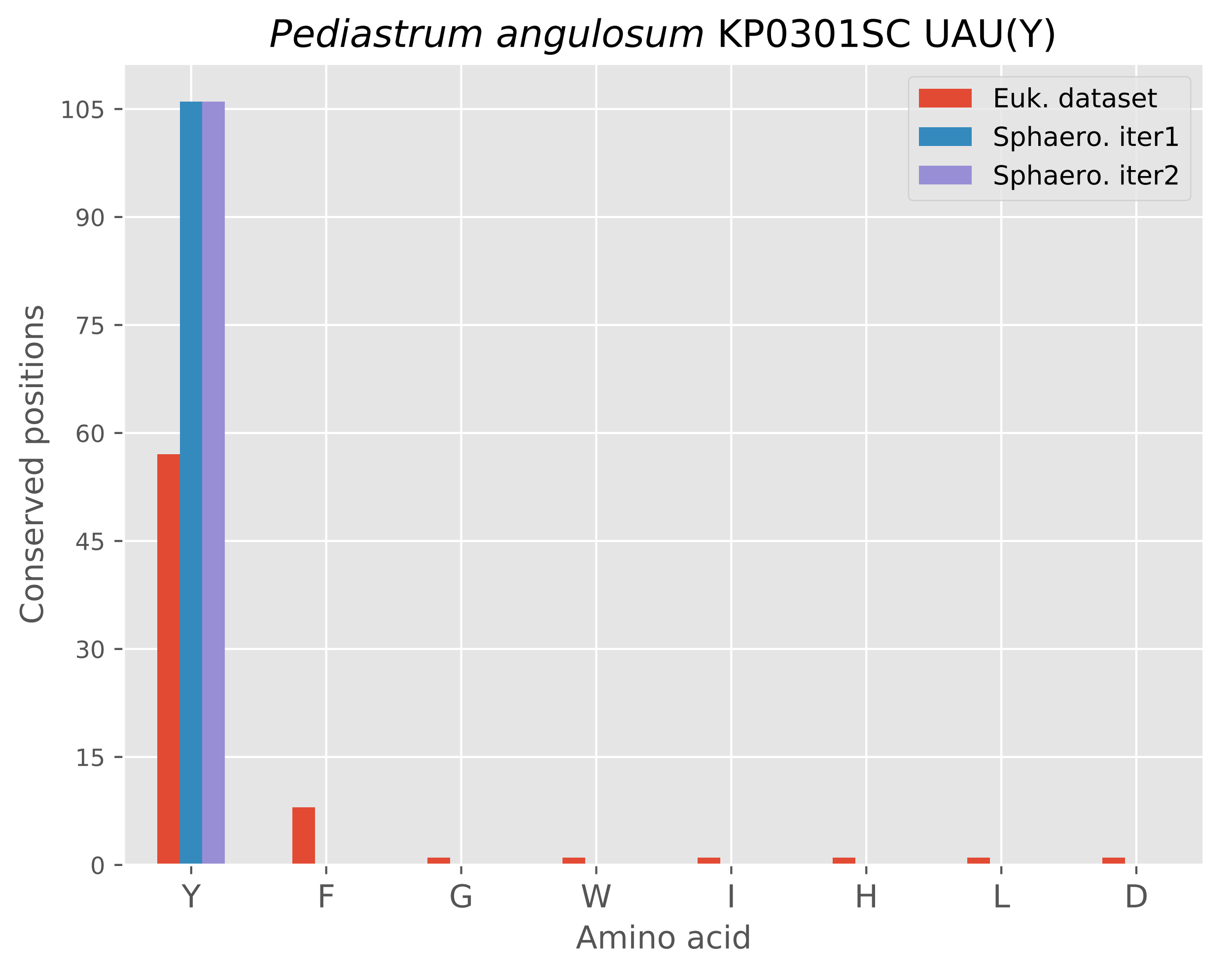

Pediastrum angulosum KP0301SC UAU(Y)
Euk. dataset
Sphaero. iter1
Sphaero. iter2
Conserved positions
0
15
30
45
60
75
90
105
Y
F
G
W
I
H
L
D
Amino acid

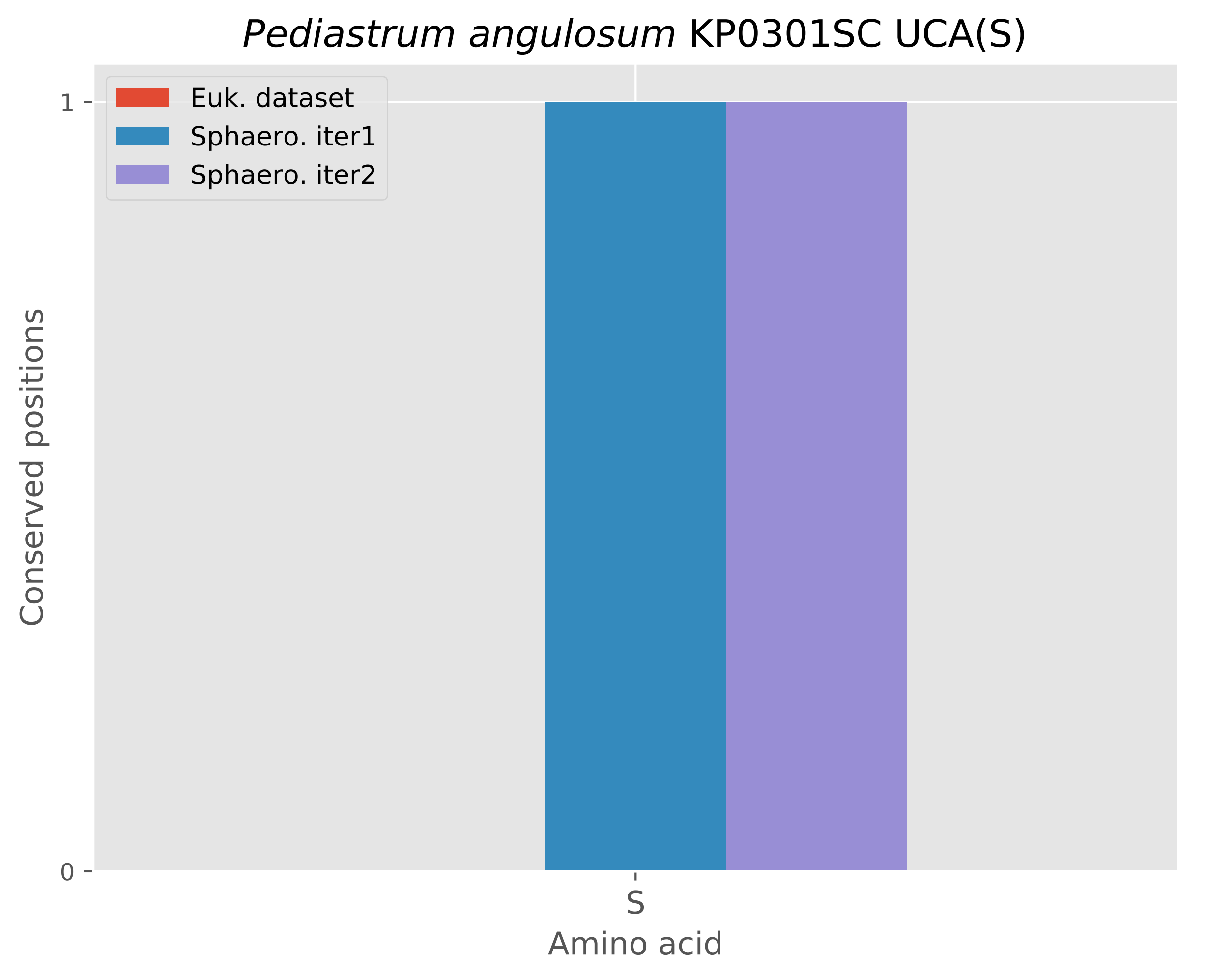

Pediastrum angulosum KP0301SC UCA(S)
Euk. dataset
Sphaero. iter1
Sphaero. iter2
Conserved positions
1
0
S
Amino acid

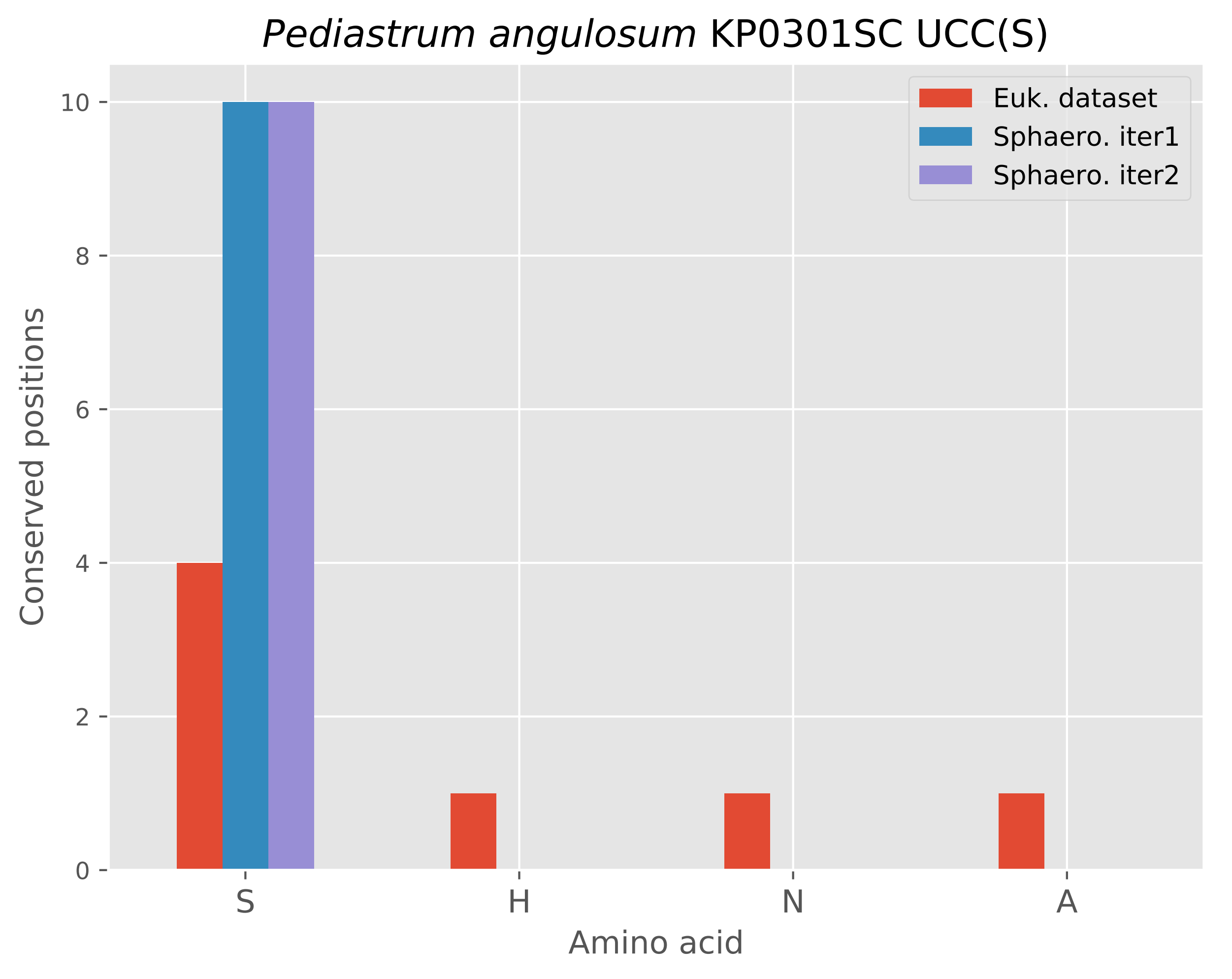

Pediastrum angulosum KP0301SC UCC(S)
Euk. dataset
Sphaero. iter1
Sphaero. iter2
Conserved positions
0
2
4
6
8
10
S
H
N
A
Amino acid

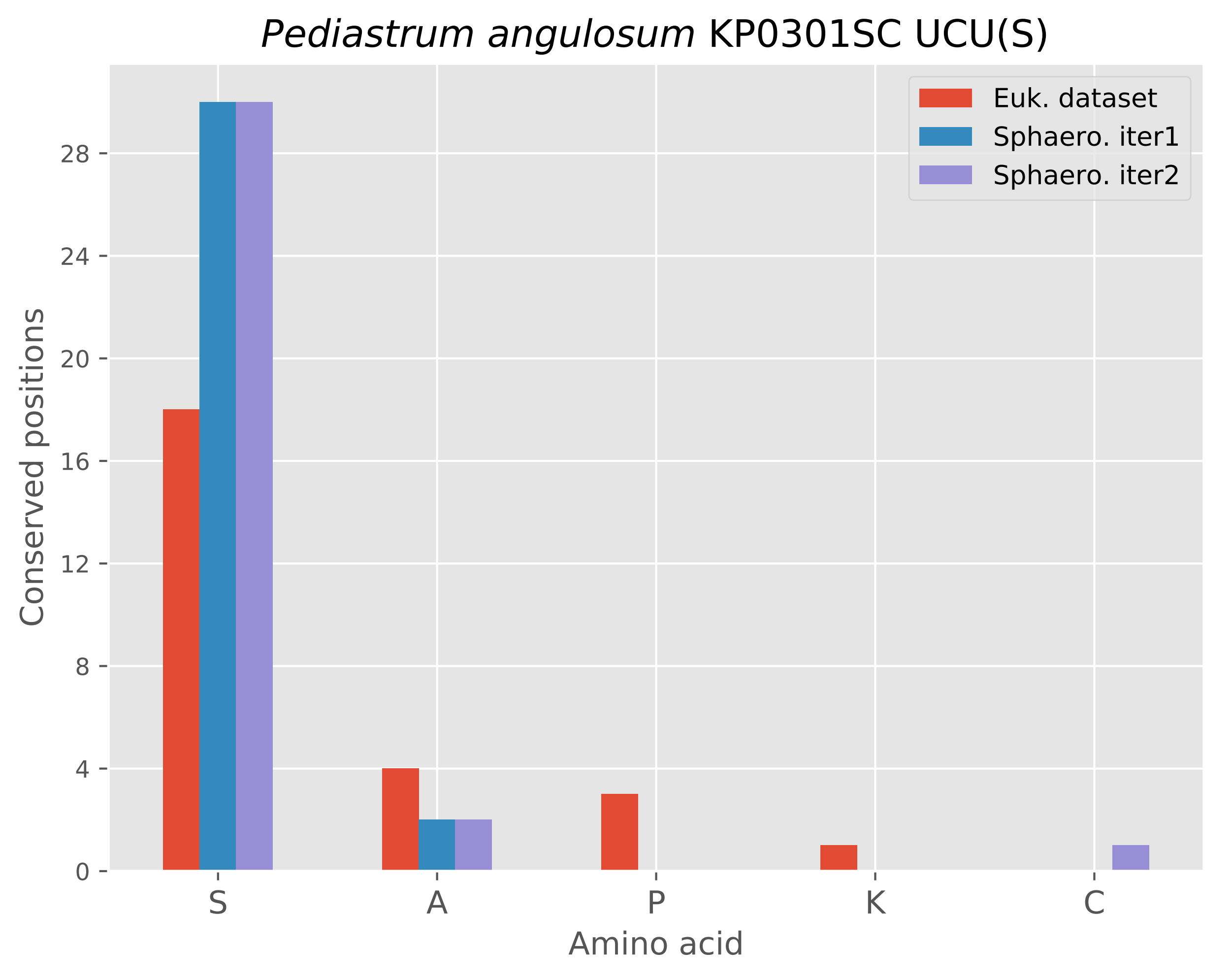

Pediastrum angulosum KP0301SC UCU(S)
Euk. dataset
Sphaero. iter1
Sphaero. iter2
Conserved positions
0
4
8
12
16
20
24
28
S
A
P
K
C
Amino acid

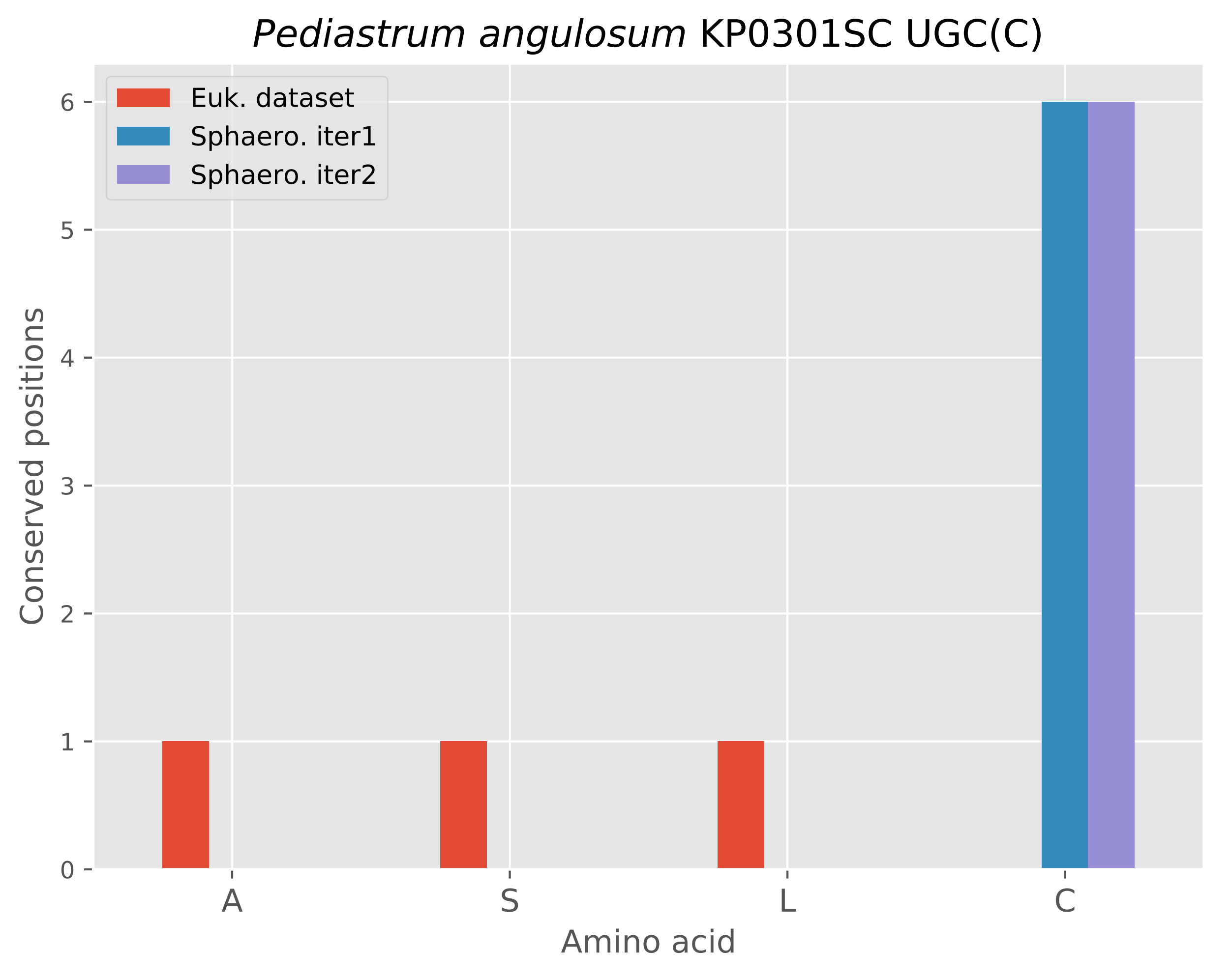

Pediastrum angulosum KP0301SC UGC(C)
Euk. dataset
Sphaero. iter1
Sphaero. iter2
Conserved positions
Amino acid
0
1
2
3
4
5
6
A
S
L
C

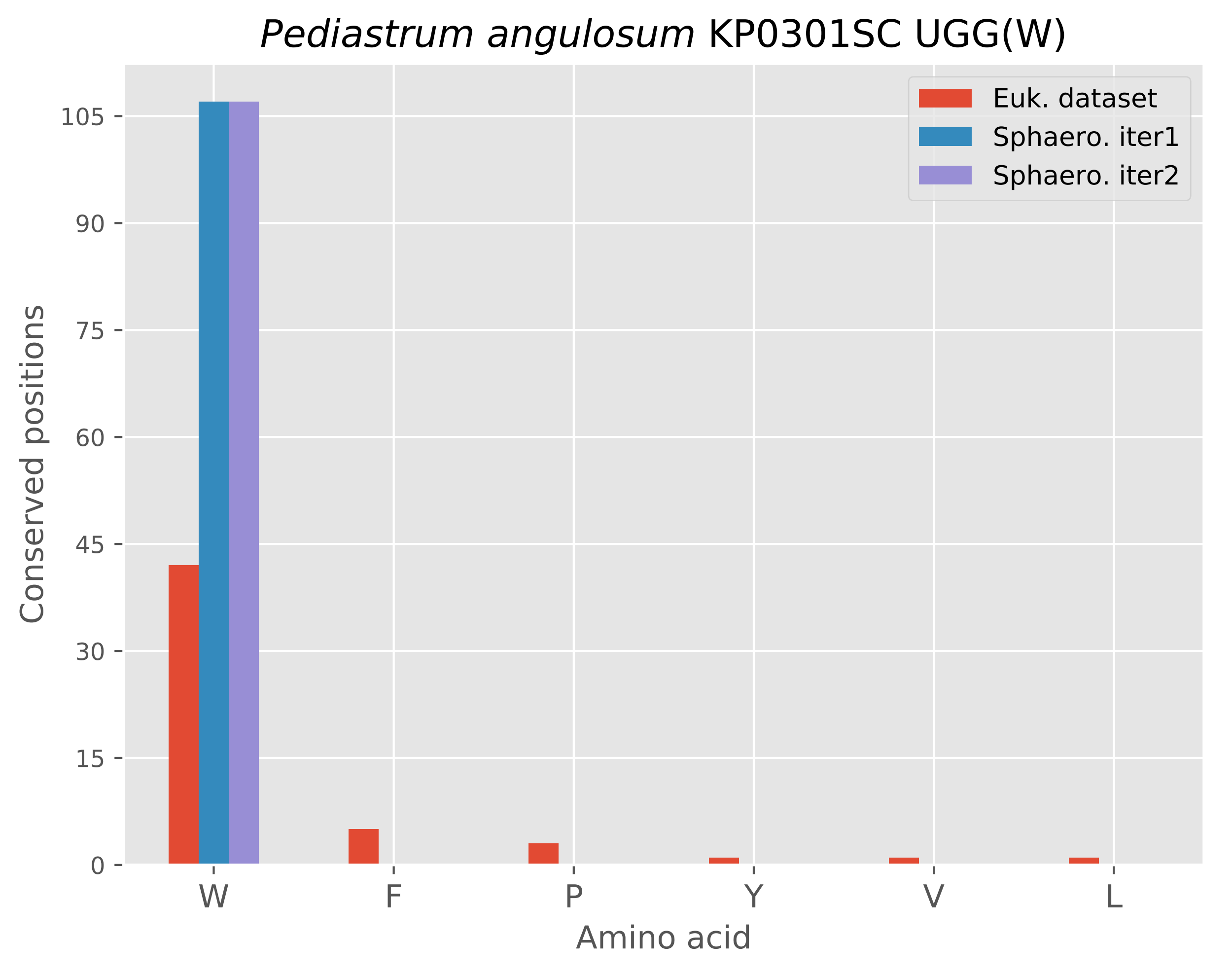

Pediastrum angulosum KP0301SC UGG(W)
Euk. dataset
Sphaero. iter1
Sphaero. iter2
Conserved positions
0
15
30
45
60
75
90
105
W
F
P
Y
V
L
Amino acid

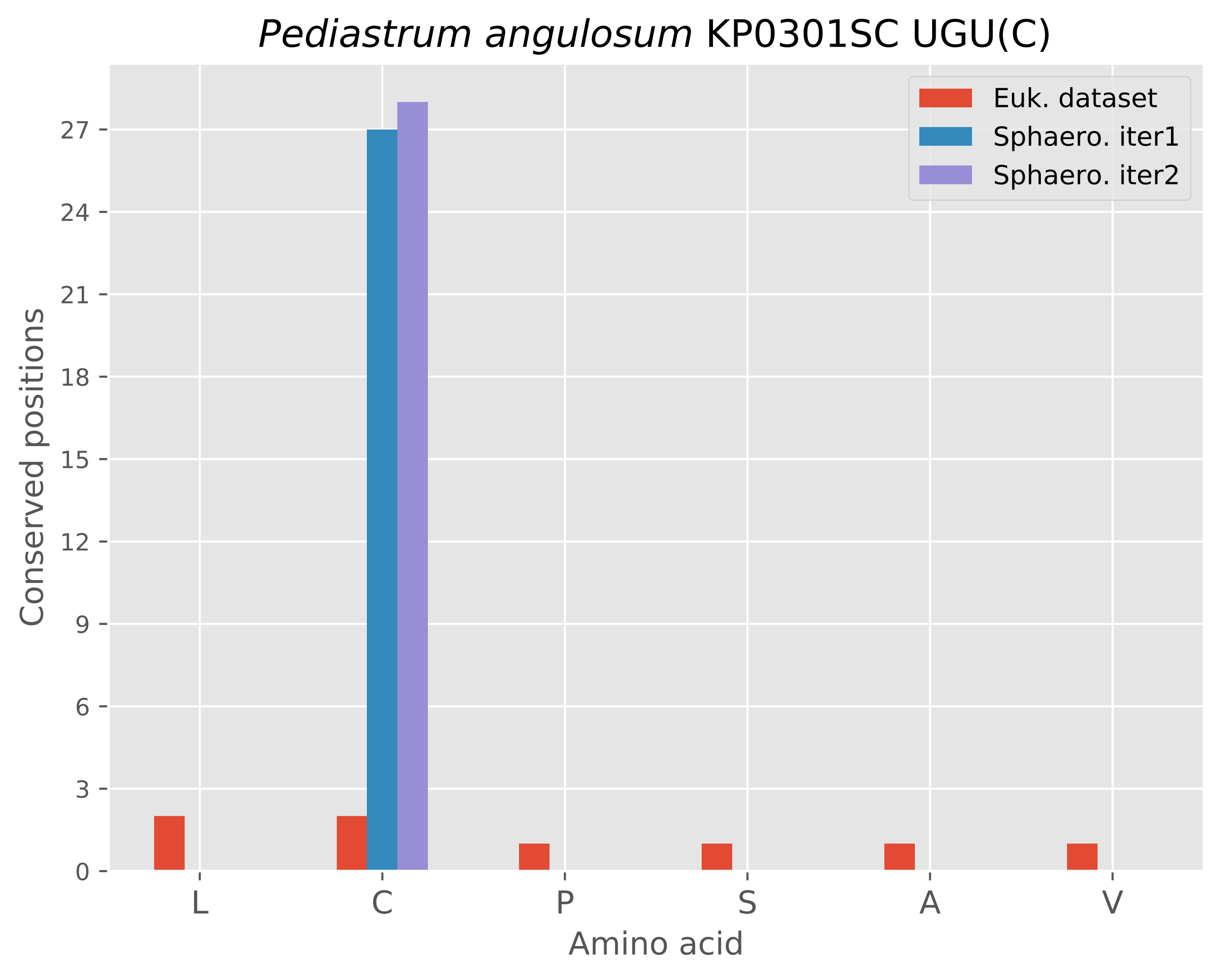

Pediastrum angulosum KP0301SC UGU(C)
Euk. dataset
Sphaero. iter1
Sphaero. iter2
Conserved positions
0
3
6
9
12
15
18
21
24
27
L
C
P
S
A
V
Amino acid

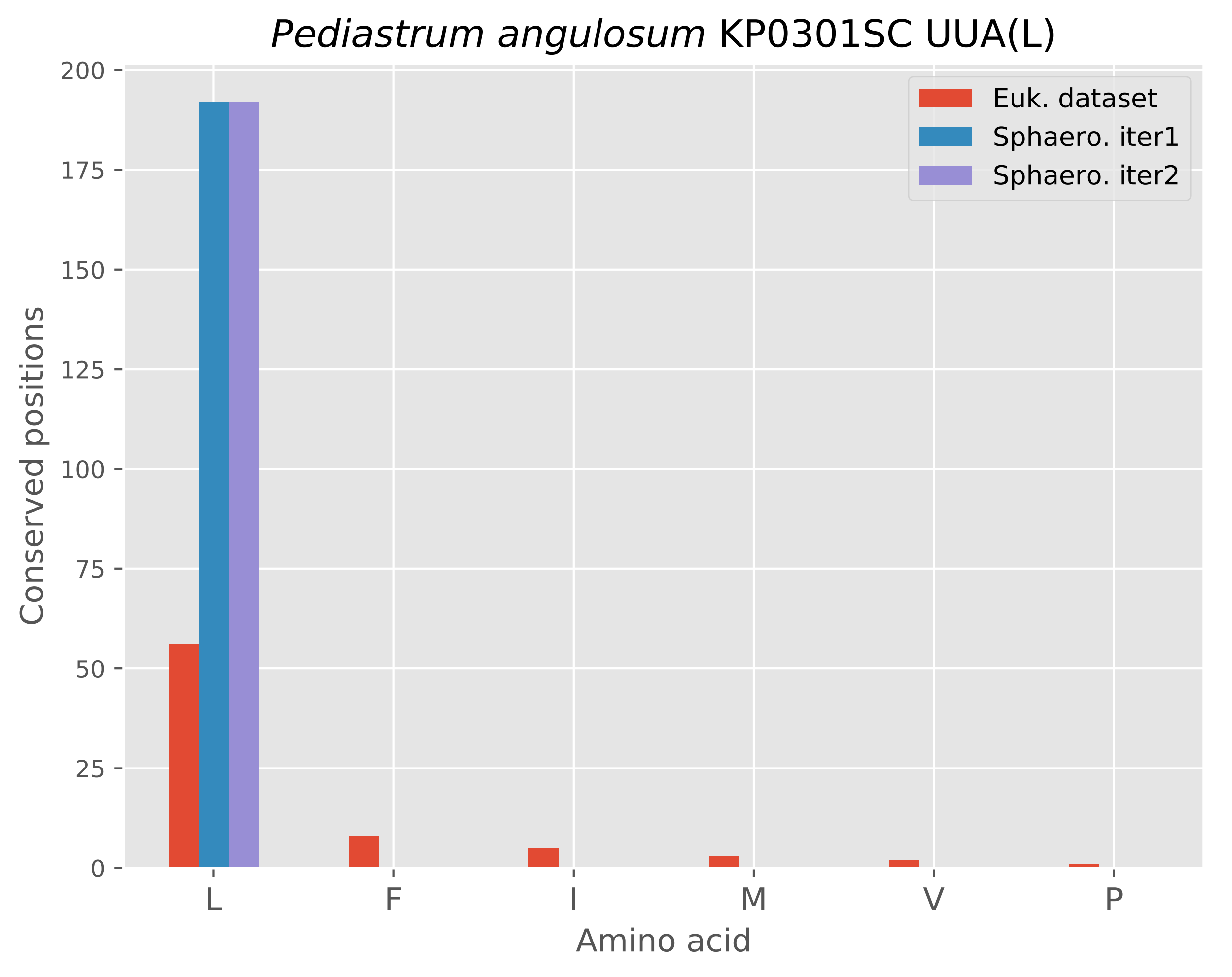
Pediastrum angulosum KP0301SC UUA(L)
Conserved positions
200
175
150
125
100
75
50
25
0
L
F
I
M
V
P
Amino acid
Euk. dataset
Sphaero. iter1
Sphaero. iter2

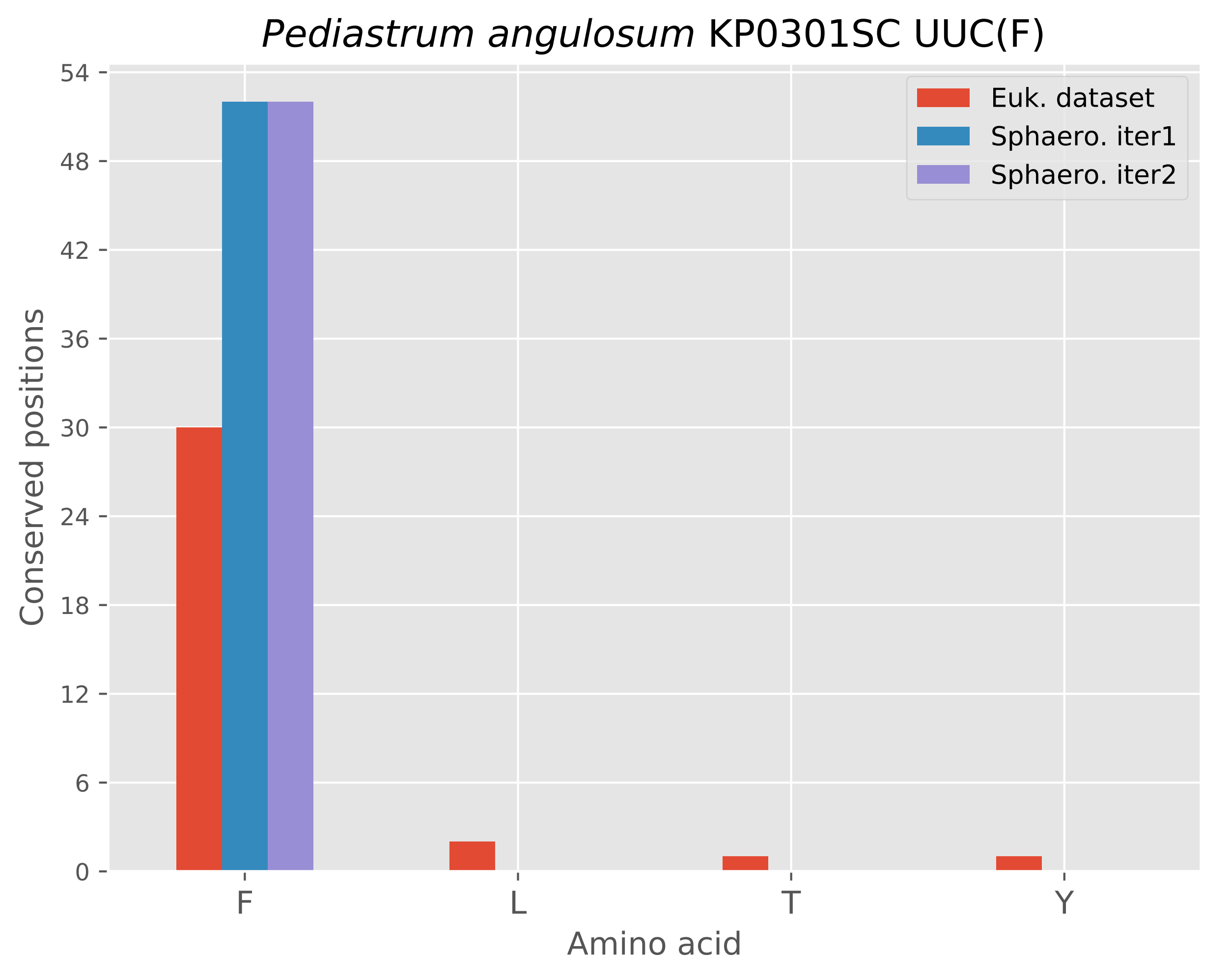

Pediastrum angulosum KP0301SC UUC(F)
Euk. dataset
Sphaero. iter1
Sphaero. iter2
Conserved positions
Amino acid
F
L
T
Y
0
6
12
18
24
30
36
42
48
54

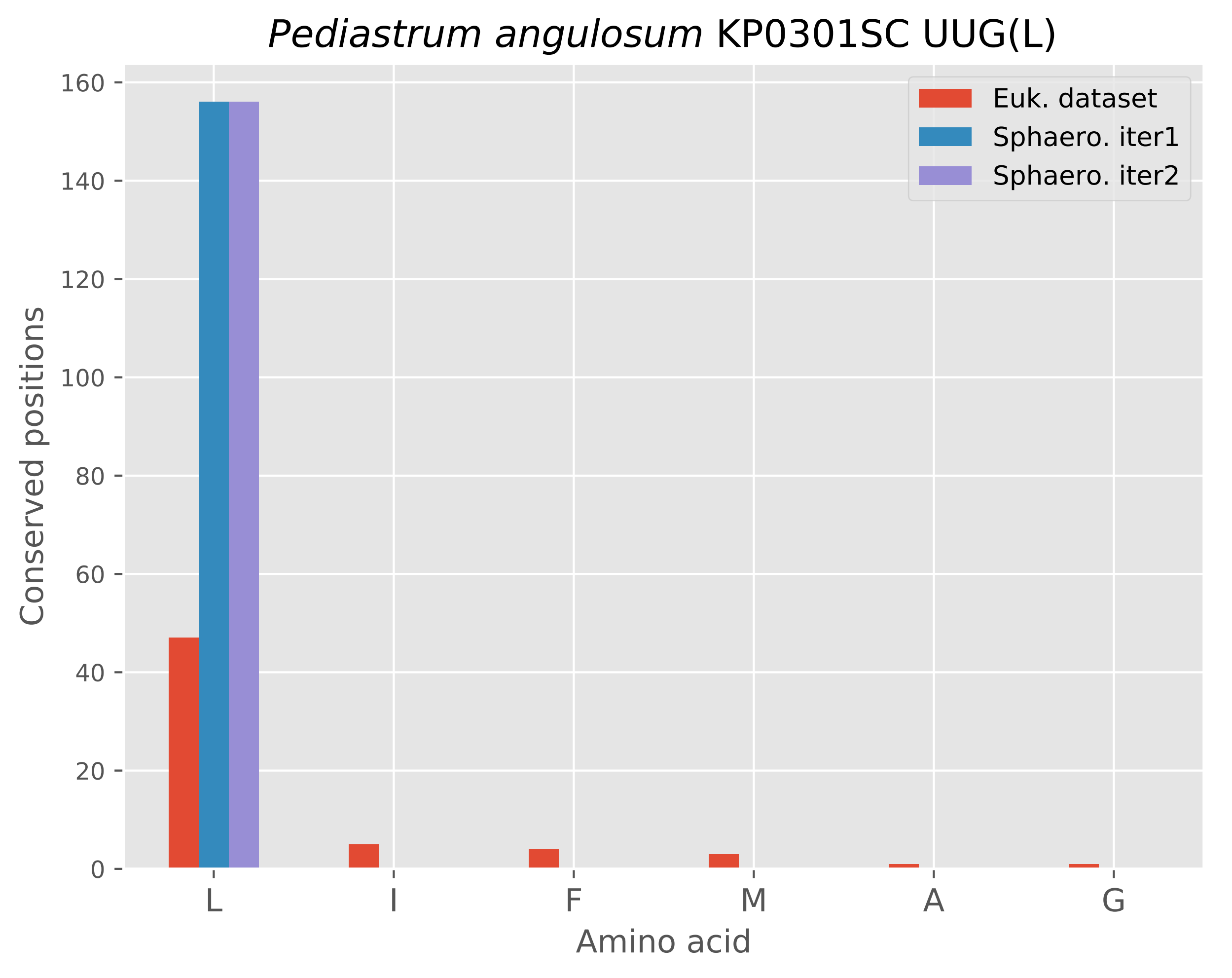

Pediastrum angulosum KP0301SC UUG(L)
Euk. dataset
Sphaero. iter1
Sphaero. iter2
Conserved positions
0
20
40
60
80
100
120
140
160
L
I
F
M
A
G
Amino acid

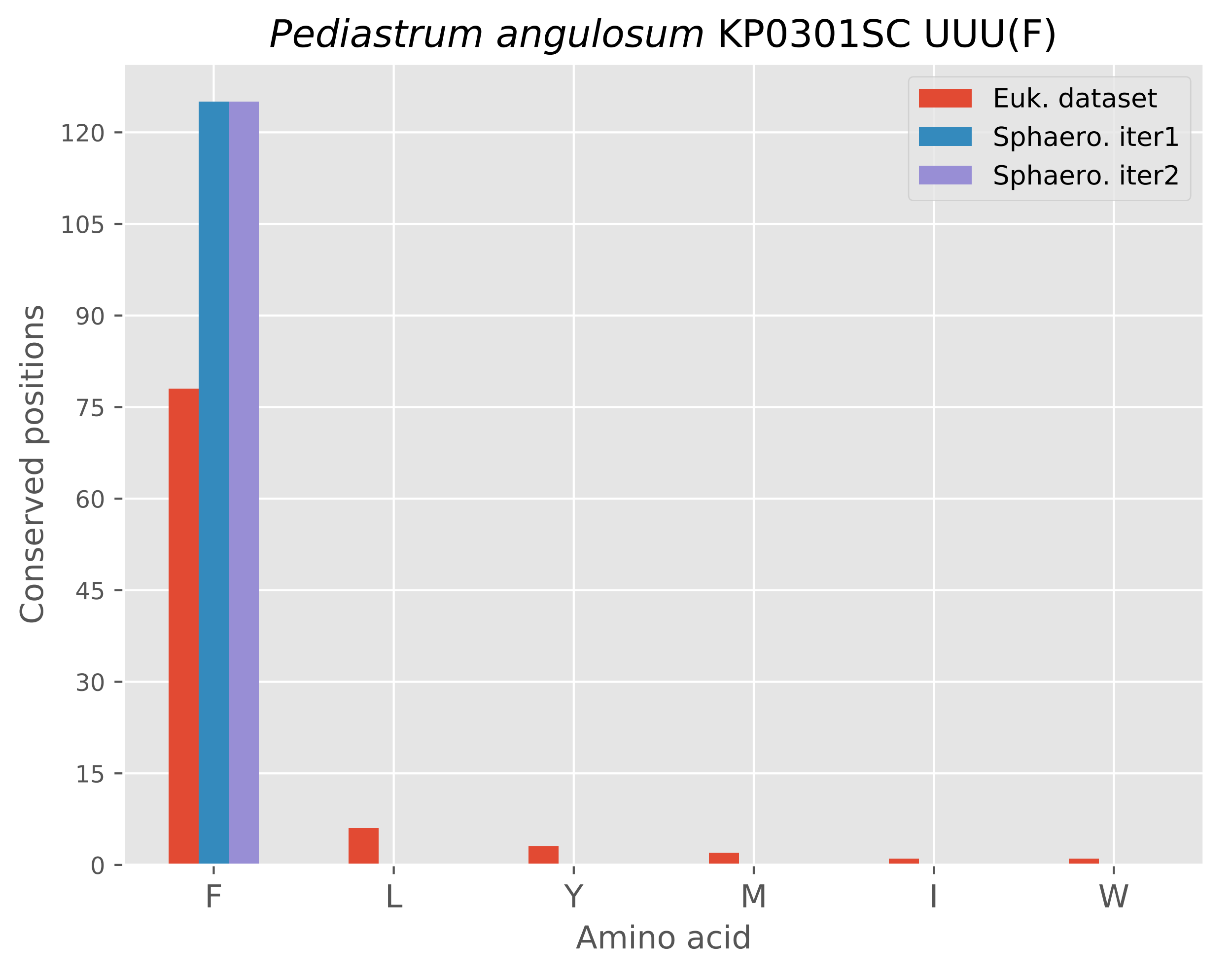

Pediastrum angulosum KP0301SC UUU(F)
Euk. dataset
Sphaero. iter1
Sphaero. iter2
Conserved positions
0
15
30
45
60
75
90
105
120
F
L
Y
M
I
W
Amino acid

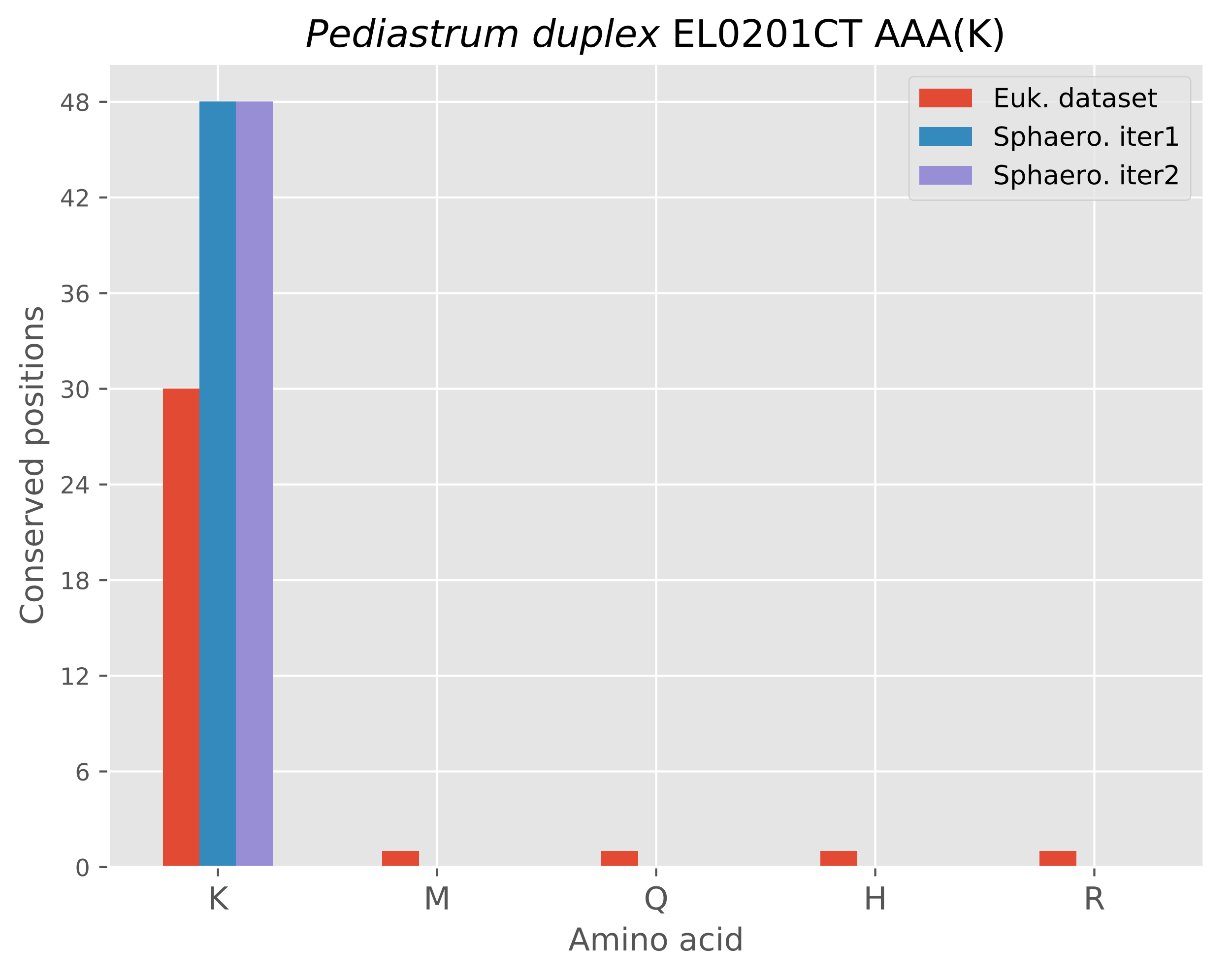

Pediastrum duplex EL0201CT AAA(K)
Euk. dataset
Sphaero. iter1
Sphaero. iter2
Conserved positions
0
6
12
18
24
30
36
42
48
K
M
Q
H
R
Amino acid

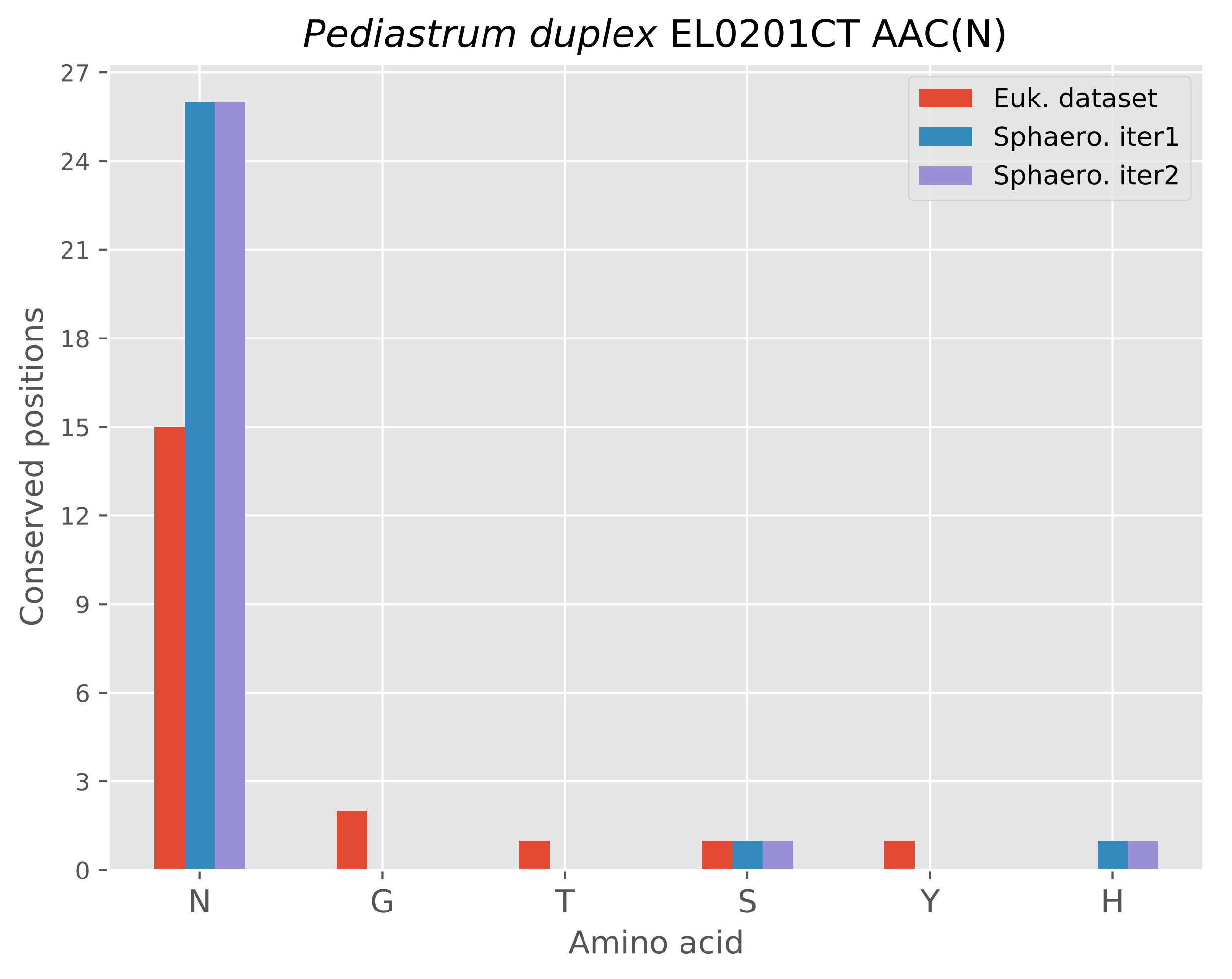

Pediastrum duplex EL0201CT AAC(N)
Euk. dataset
Sphaero. iter1
Sphaero. iter2
Conserved positions
0
3
6
9
12
15
18
21
24
27
N
G
T
S
Y
H
Amino acid

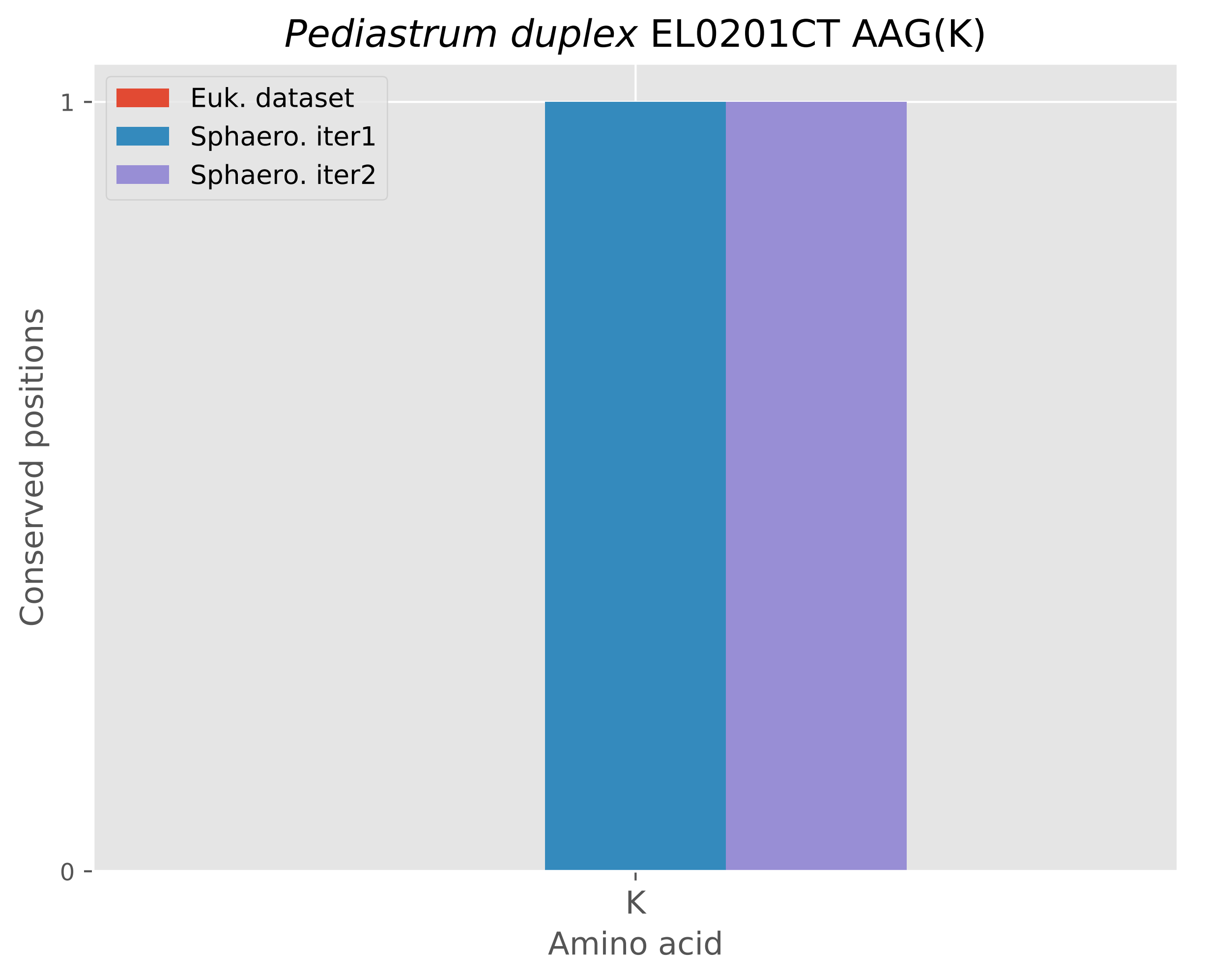

Pediastrum duplex EL0201CT AAG(K)
Euk. dataset
Sphaero. iter1
Sphaero. iter2
1
0
Conserved positions
K
Amino acid

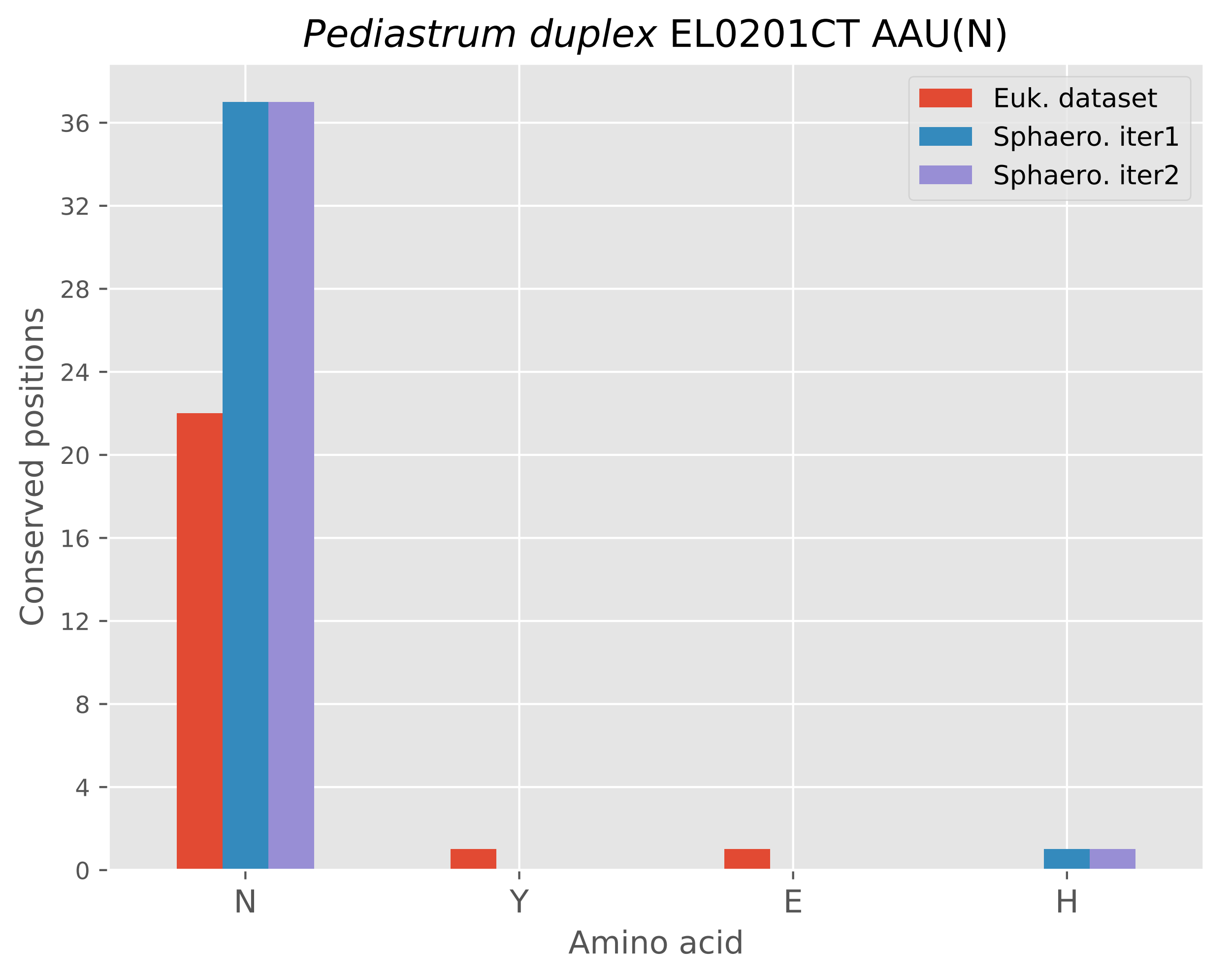

Pediastrum duplex EL0201CT AAU(N)
Euk. dataset
Sphaero. iter1
Sphaero. iter2
Conserved positions
0
4
8
12
16
20
24
28
32
36
N
Y
E
H
Amino acid

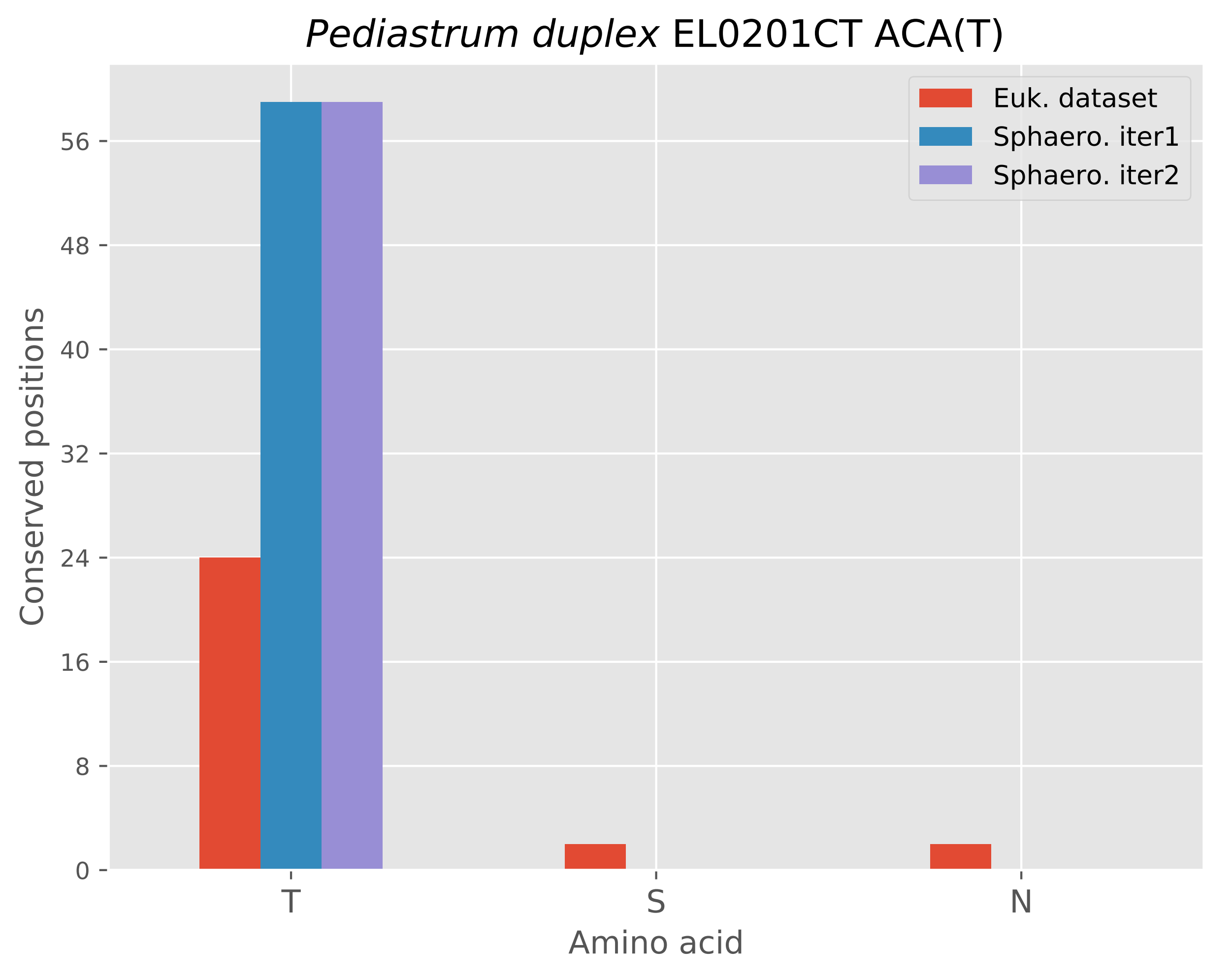

Pediastrum duplex EL0201CT ACA(T)
Euk. dataset
Sphaero. iter1
Sphaero. iter2
Conserved positions
0
8
16
24
32
40
48
56
T
S
N
Amino acid

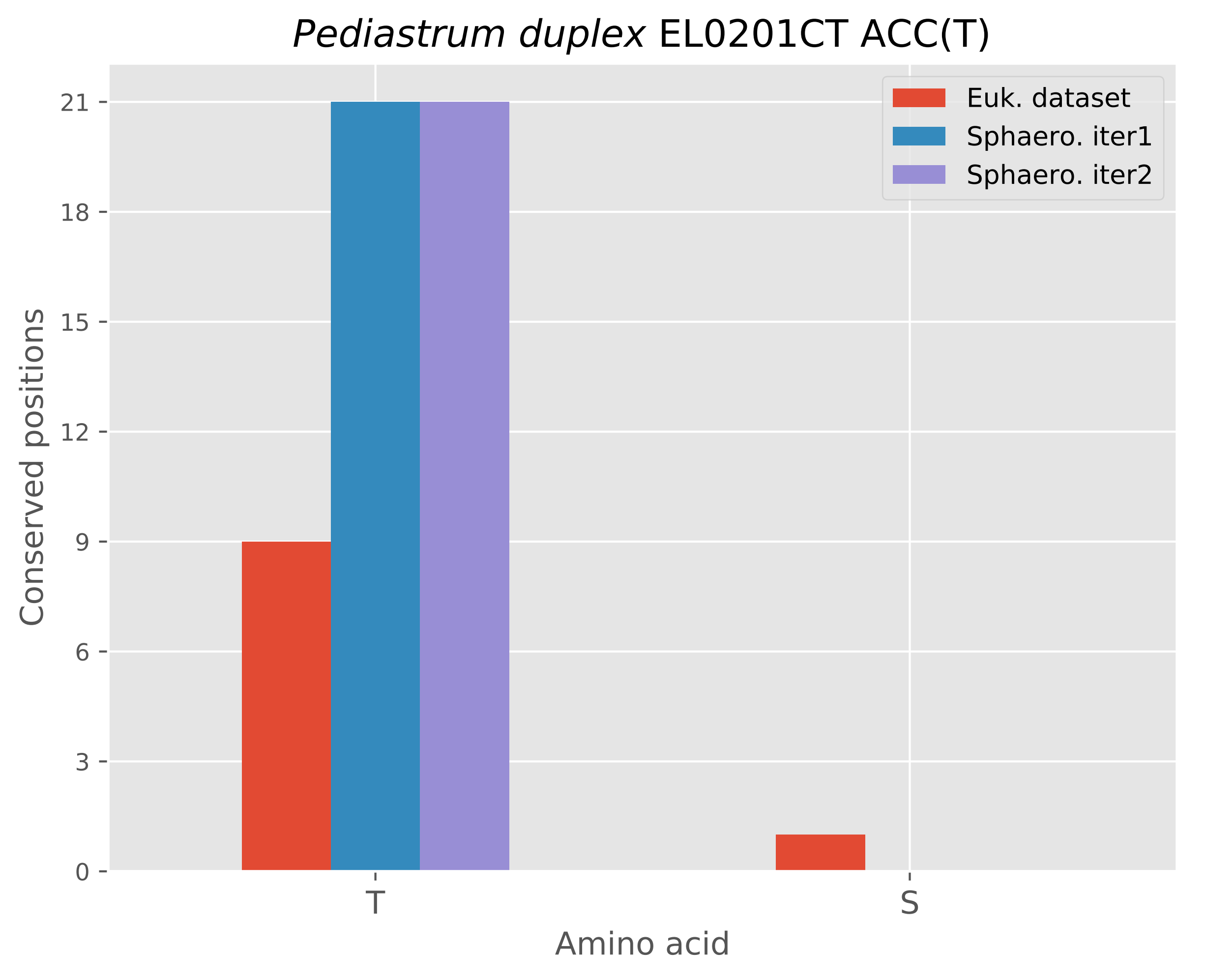

Pediastrum duplex EL0201CT ACC(T)
Euk. dataset
Sphaero. iter1
Sphaero. iter2
Conserved positions
0
3
6
9
12
15
18
21
T
S
Amino acid

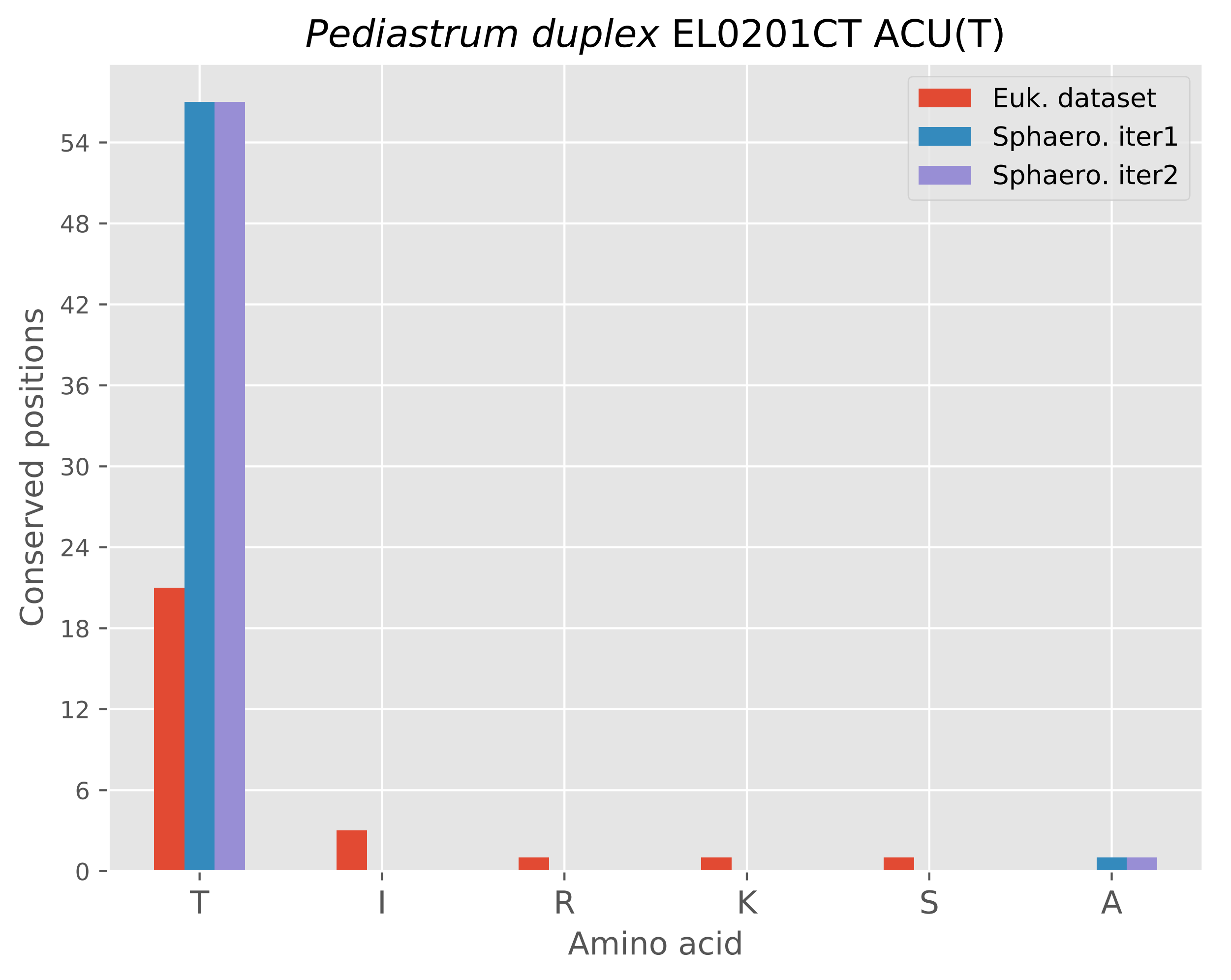

Pediastrum duplex EL0201CT ACU(T)
Euk. dataset
Sphaero. iter1
Sphaero. iter2
Conserved positions
0
6
12
18
24
30
36
42
48
54
T
I
R
K
S
A
Amino acid

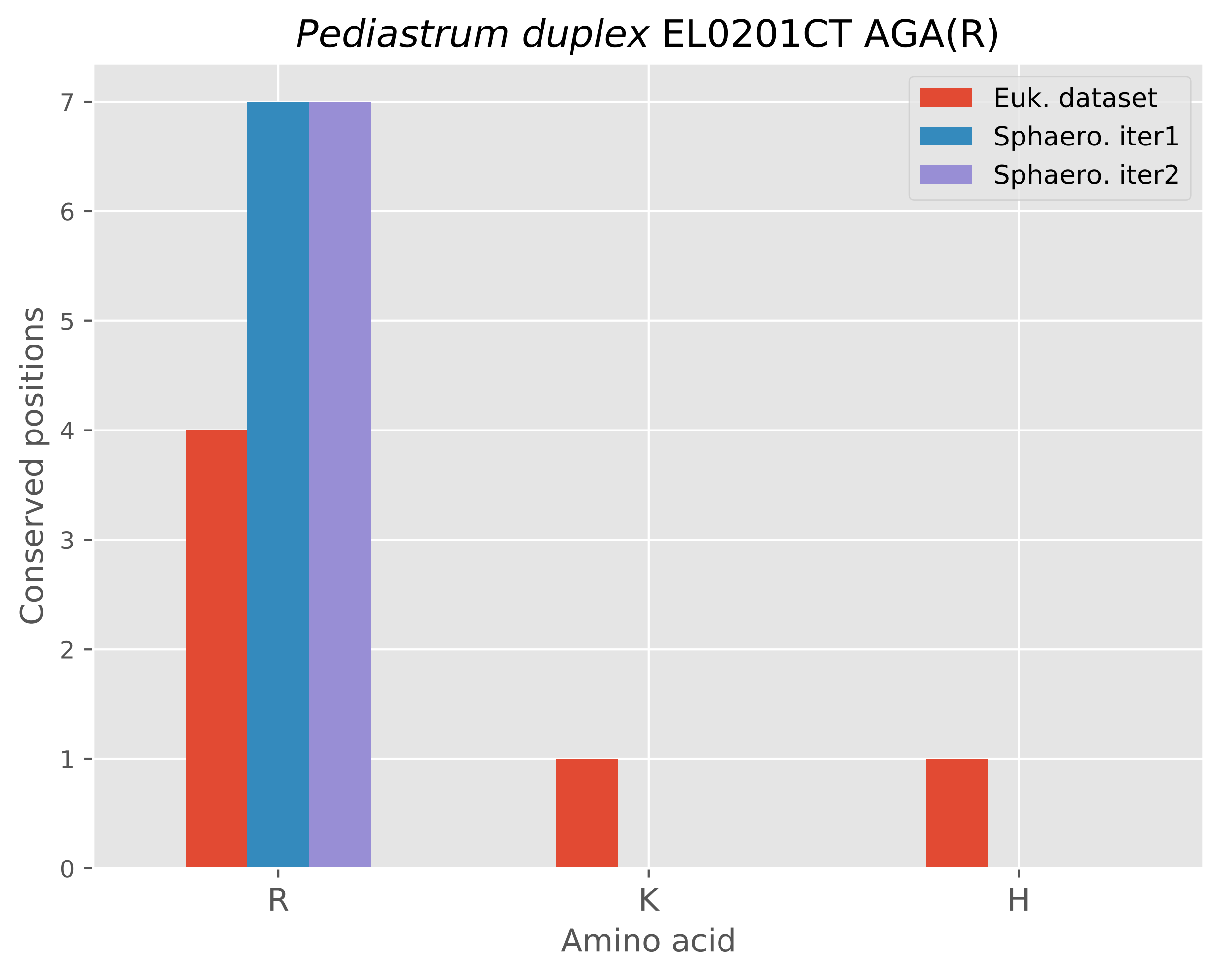

Pediastrum duplex EL0201CT AGA(R)
Euk. dataset
Sphaero. iter1
Sphaero. iter2
Conserved positions
0
1
2
3
4
5
6
7
R
K
H
Amino acid

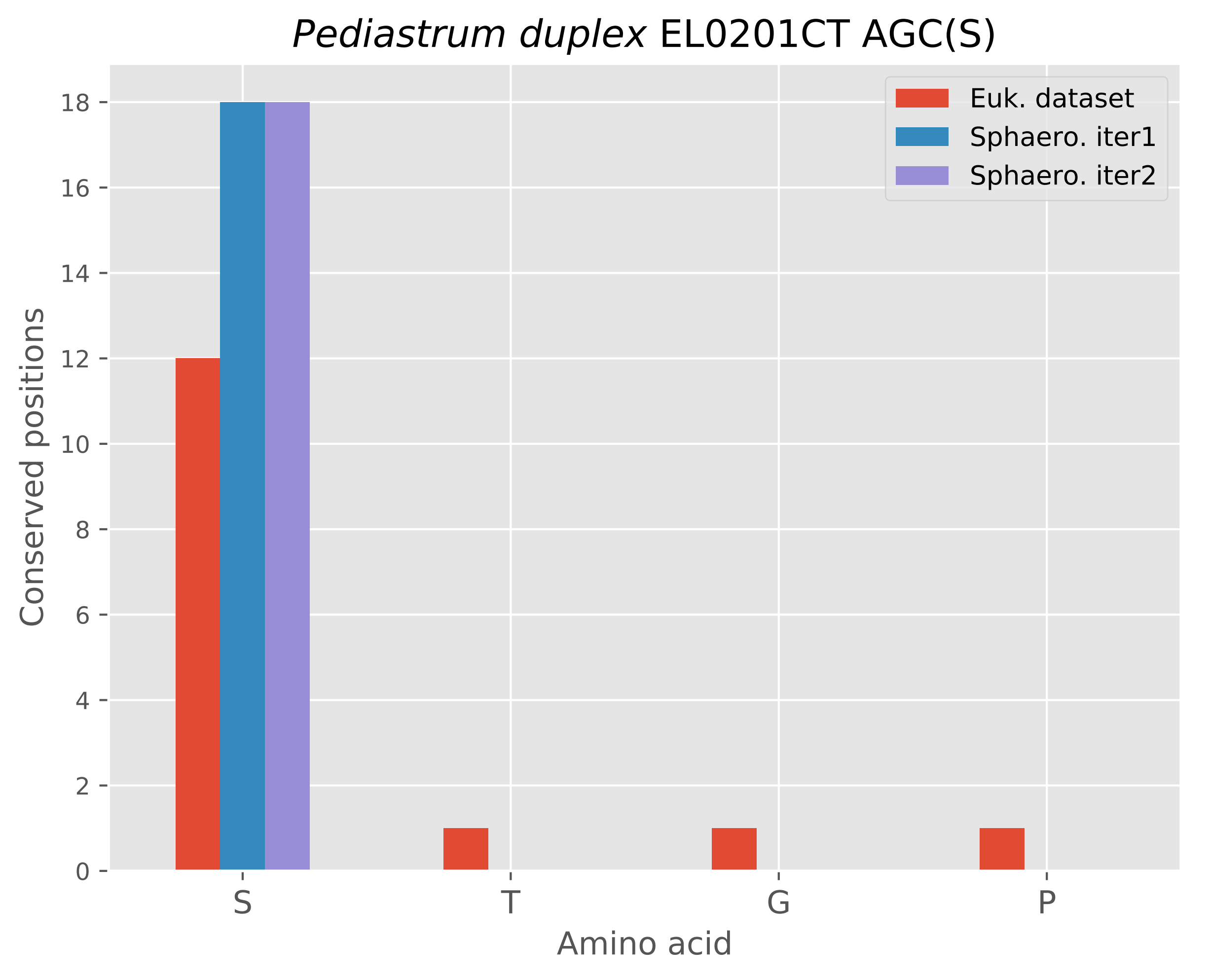

Pediastrum duplex EL0201CT AGC(S)
Euk. dataset
Sphaero. iter1
Sphaero. iter2
Conserved positions
0
2
4
6
8
10
12
14
16
18
S
T
G
P
Amino acid

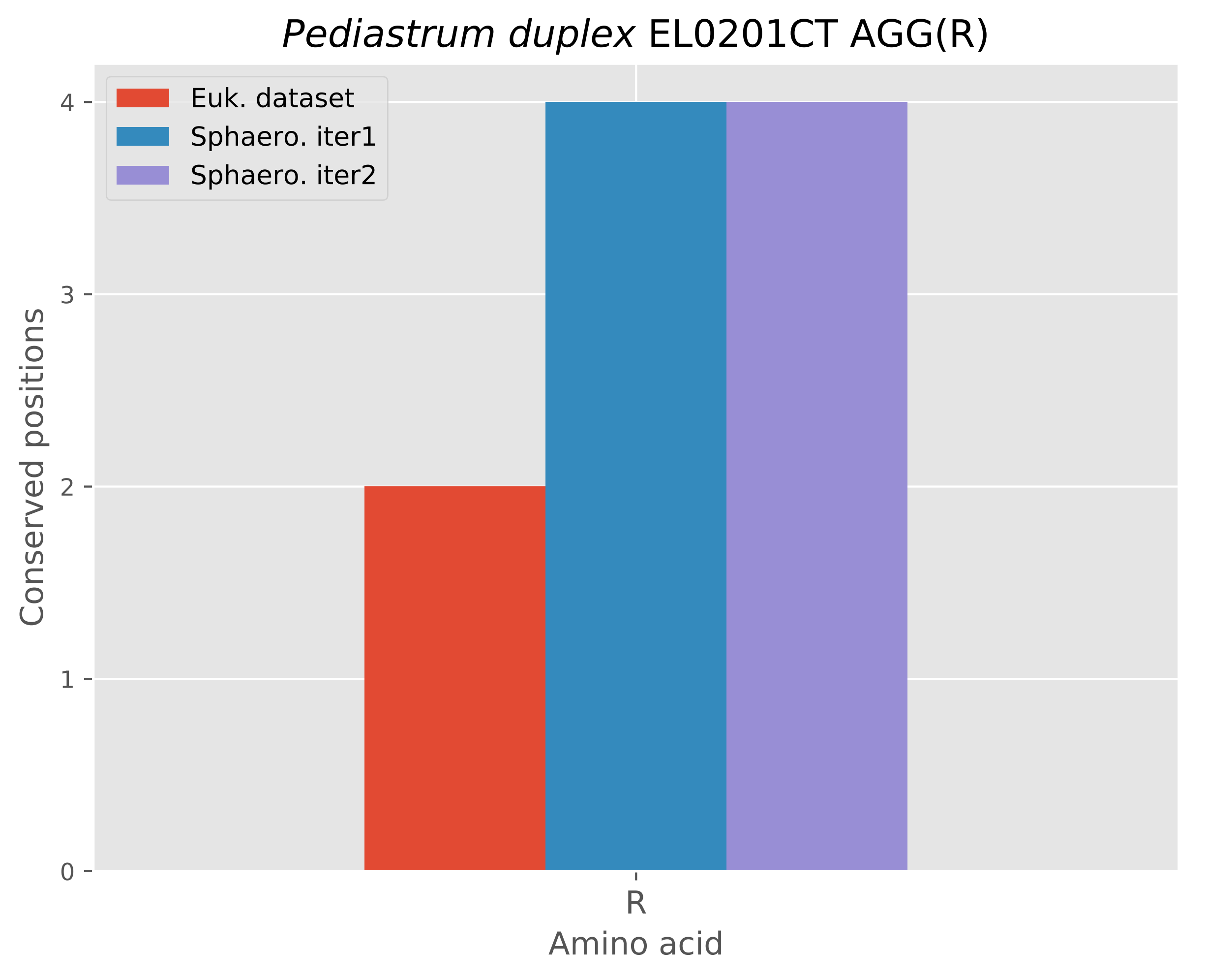

Pediastrum duplex EL0201CT AGG(R)
Euk. dataset
Sphaero. iter1
Sphaero. iter2
Conserved positions
0
1
2
3
4
R
Amino acid

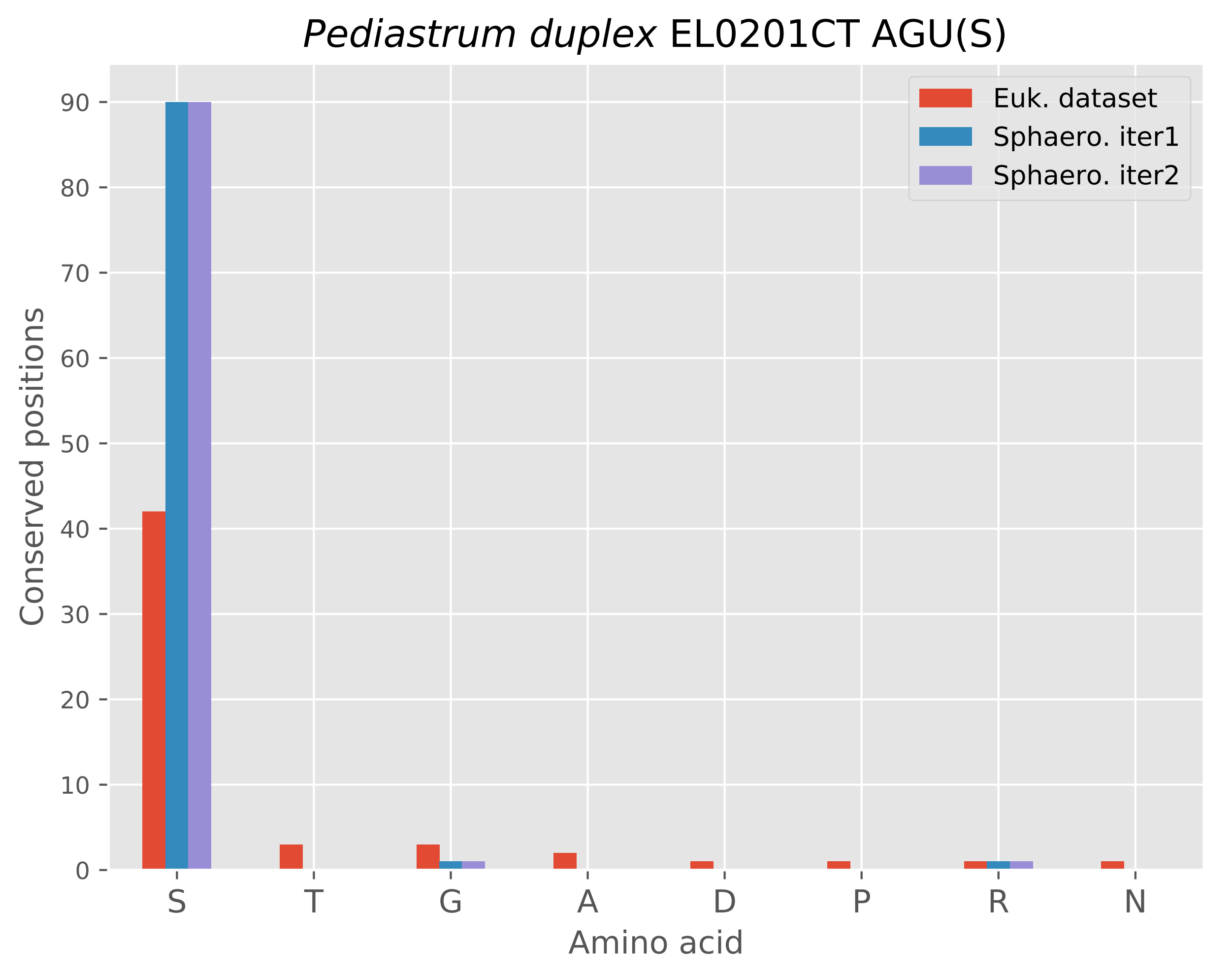

Pediastrum duplex EL0201CT AGU(S)
Euk. dataset
Sphaero. iter1
Sphaero. iter2
Conserved positions
0
10
20
30
40
50
60
70
80
90
S
T
G
A
D
P
R
N
Amino acid

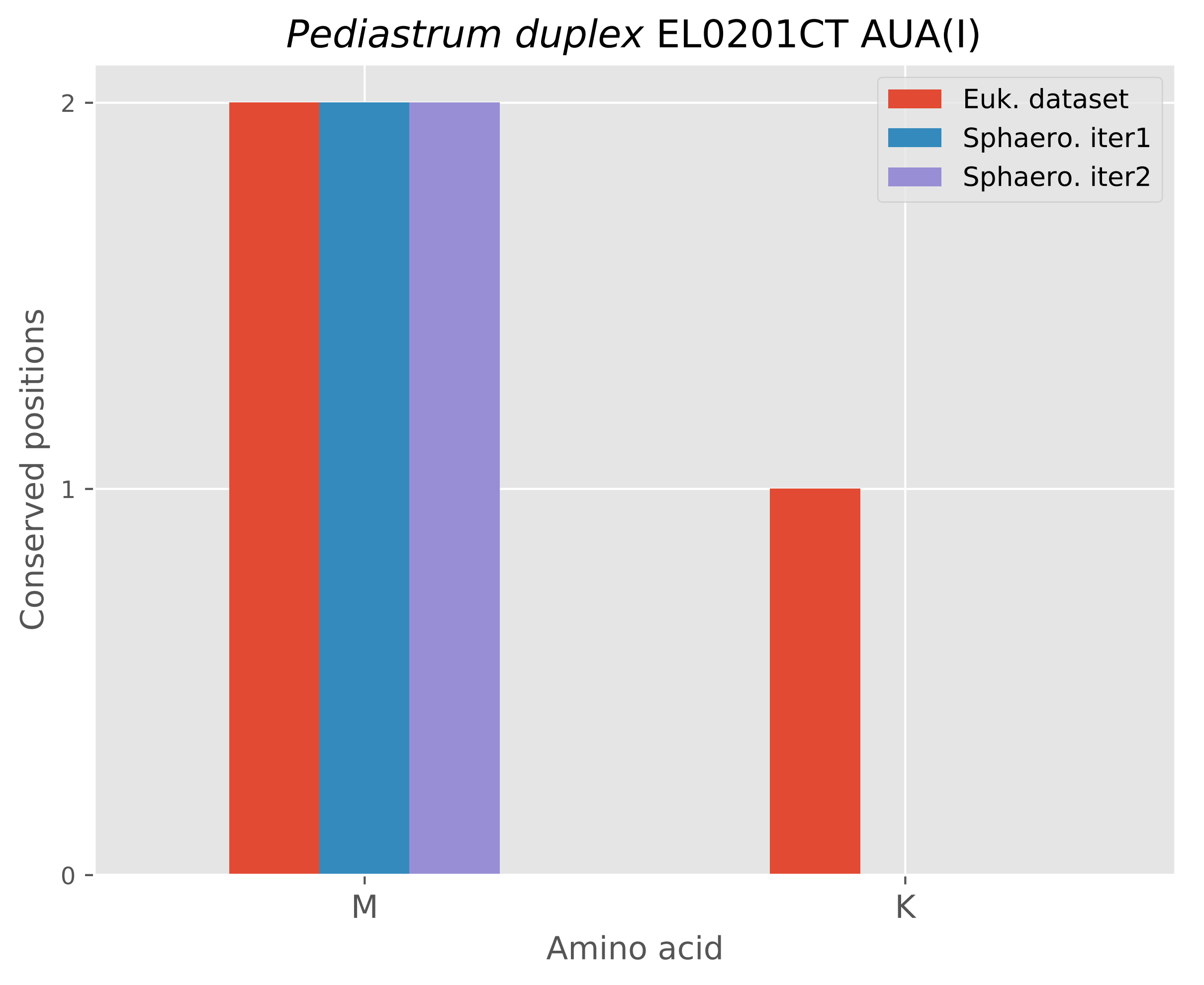

Pediastrum duplex EL0201CT AUA(I)
Euk. dataset
Sphaero. iter1
Sphaero. iter2
Conserved positions
0
1
2
M
K
Amino acid

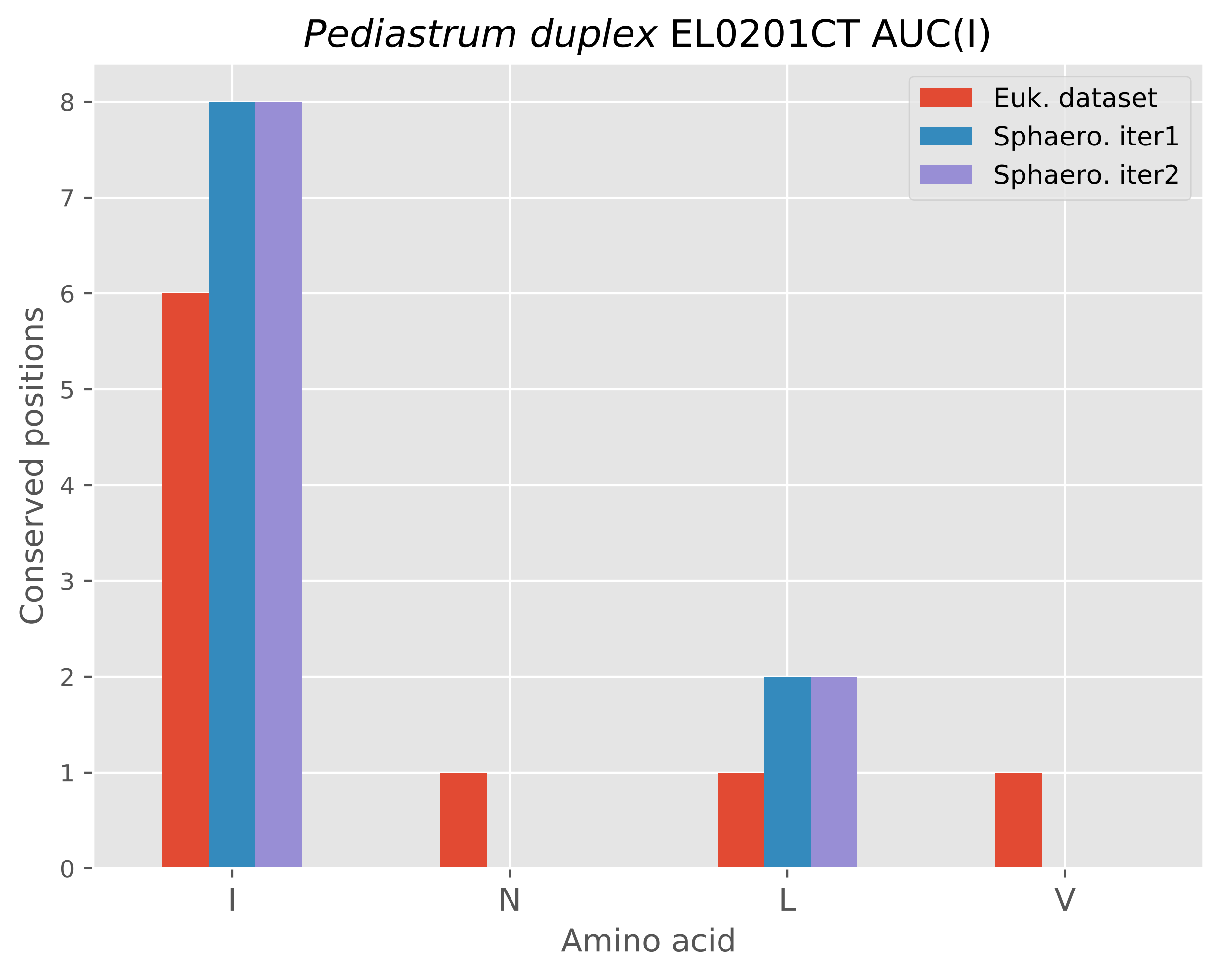

Pediastrum duplex EL0201CT AUC(I)
Euk. dataset
Sphaero. iter1
Sphaero. iter2
Conserved positions
0
1
2
3
4
5
6
7
8
I
N
L
V
Amino acid

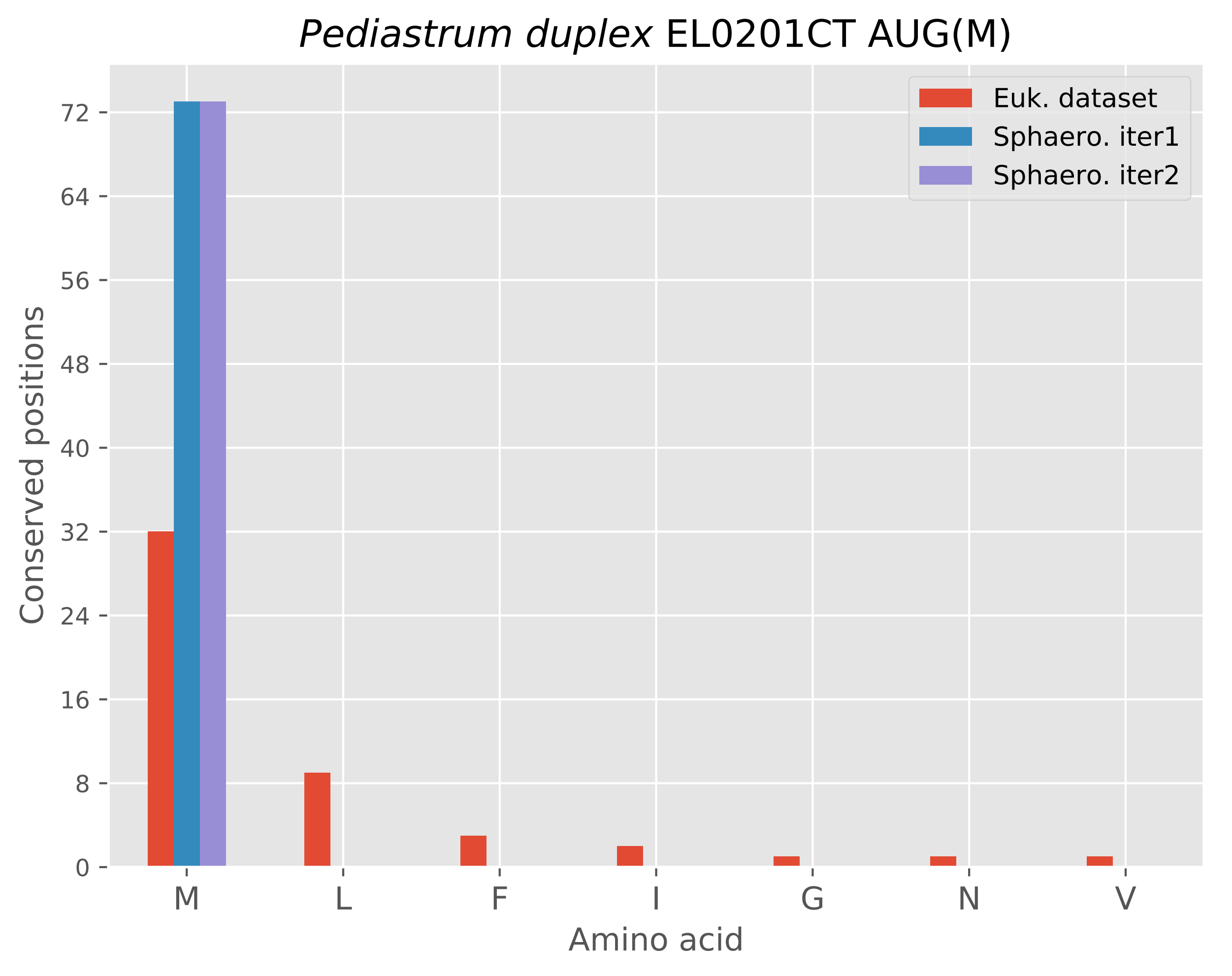

Pediastrum duplex EL0201CT AUG(M)
Euk. dataset
Sphaero. iter1
Sphaero. iter2
Conserved positions
0
8
16
24
32
40
48
56
64
72
M
L
F
I
G
N
V
Amino acid

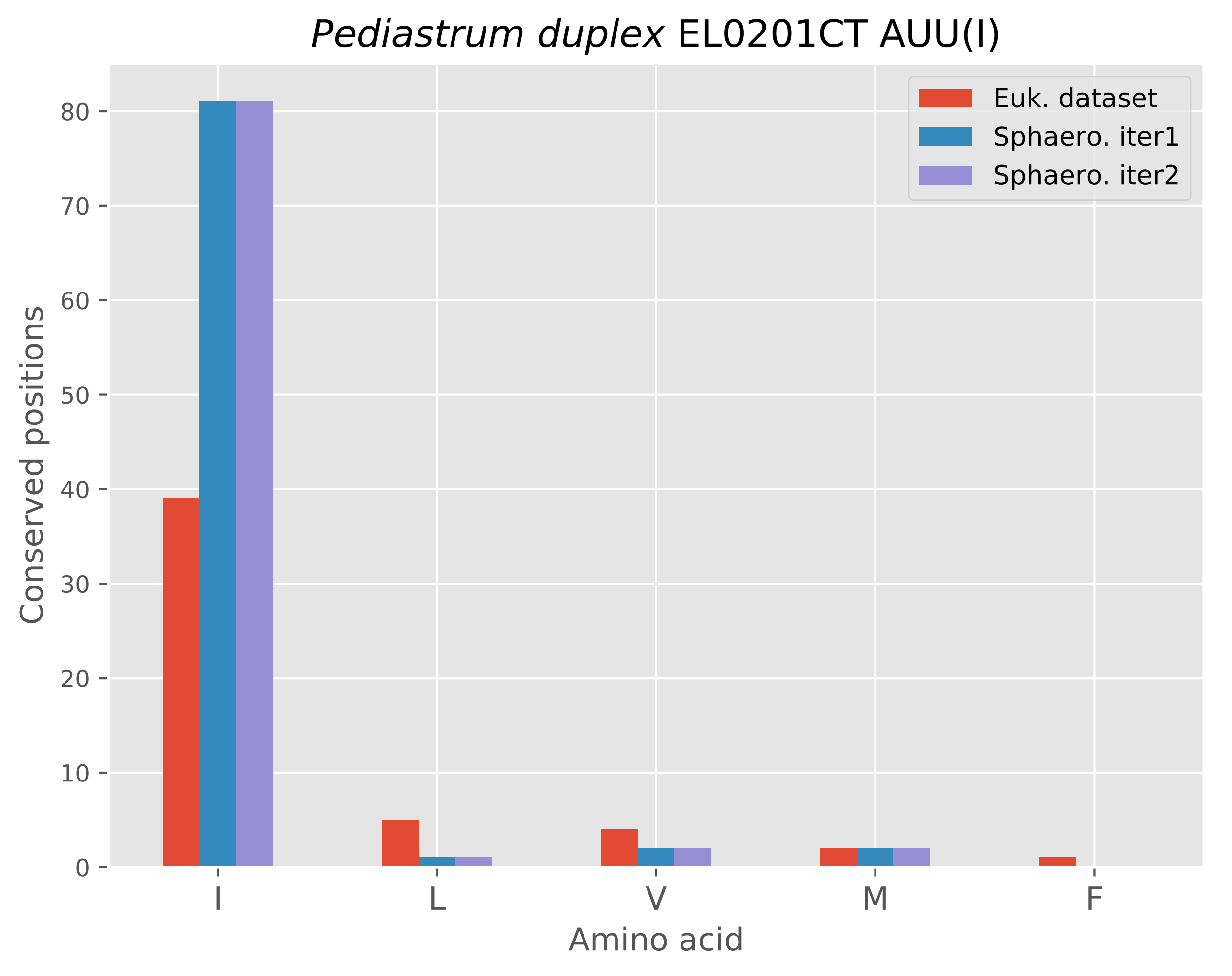

Pediastrum duplex EL0201CT AUU(I)
Euk. dataset
Sphaero. iter1
Sphaero. iter2
Conserved positions
0
10
20
30
40
50
60
70
80
I
L
V
M
F
Amino acid

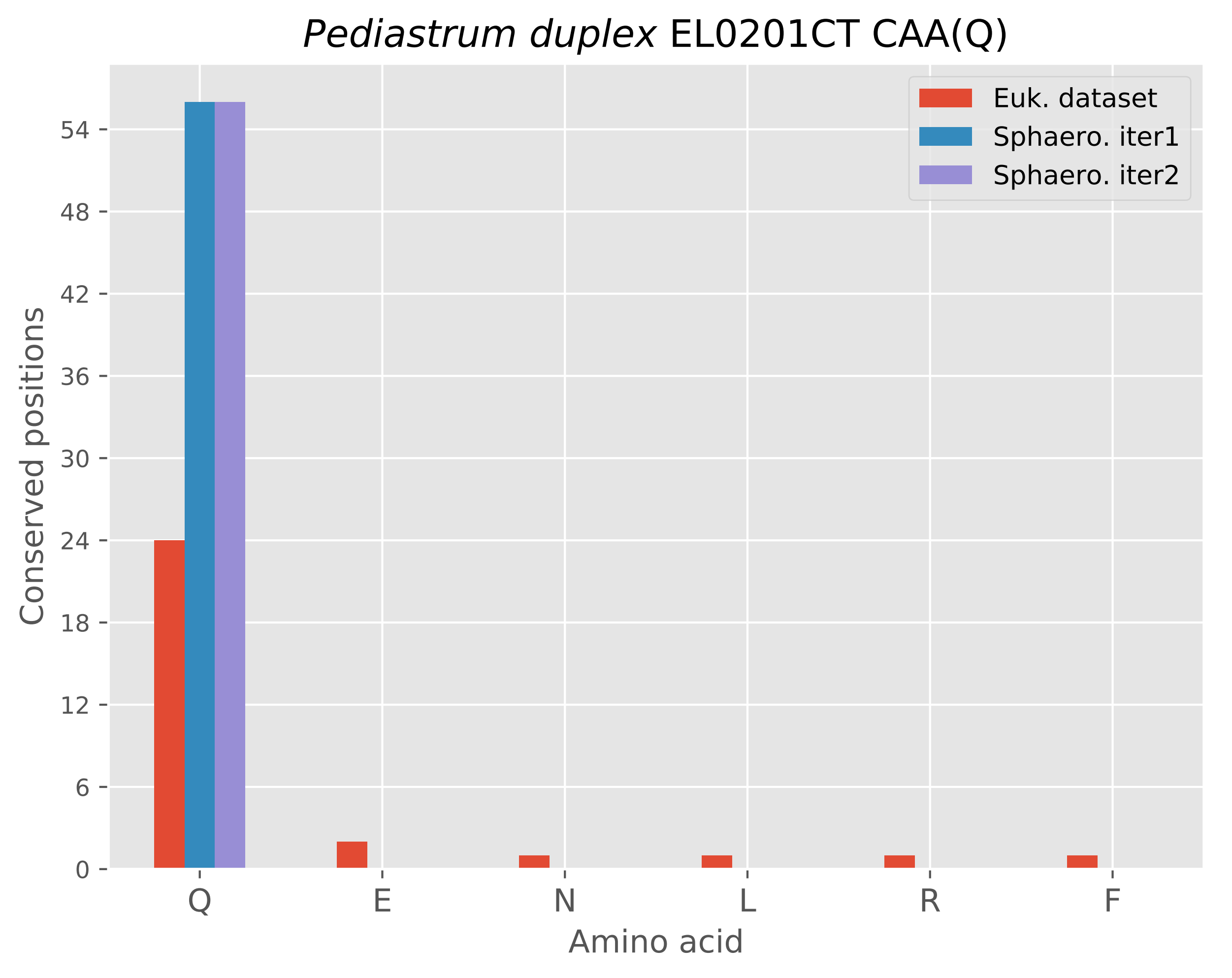

Pediastrum duplex EL0201CT CAA(Q)
Euk. dataset
Sphaero. iter1
Sphaero. iter2
Conserved positions
0
6
12
18
24
30
36
42
48
54
Q
E
N
L
R
F
Amino acid

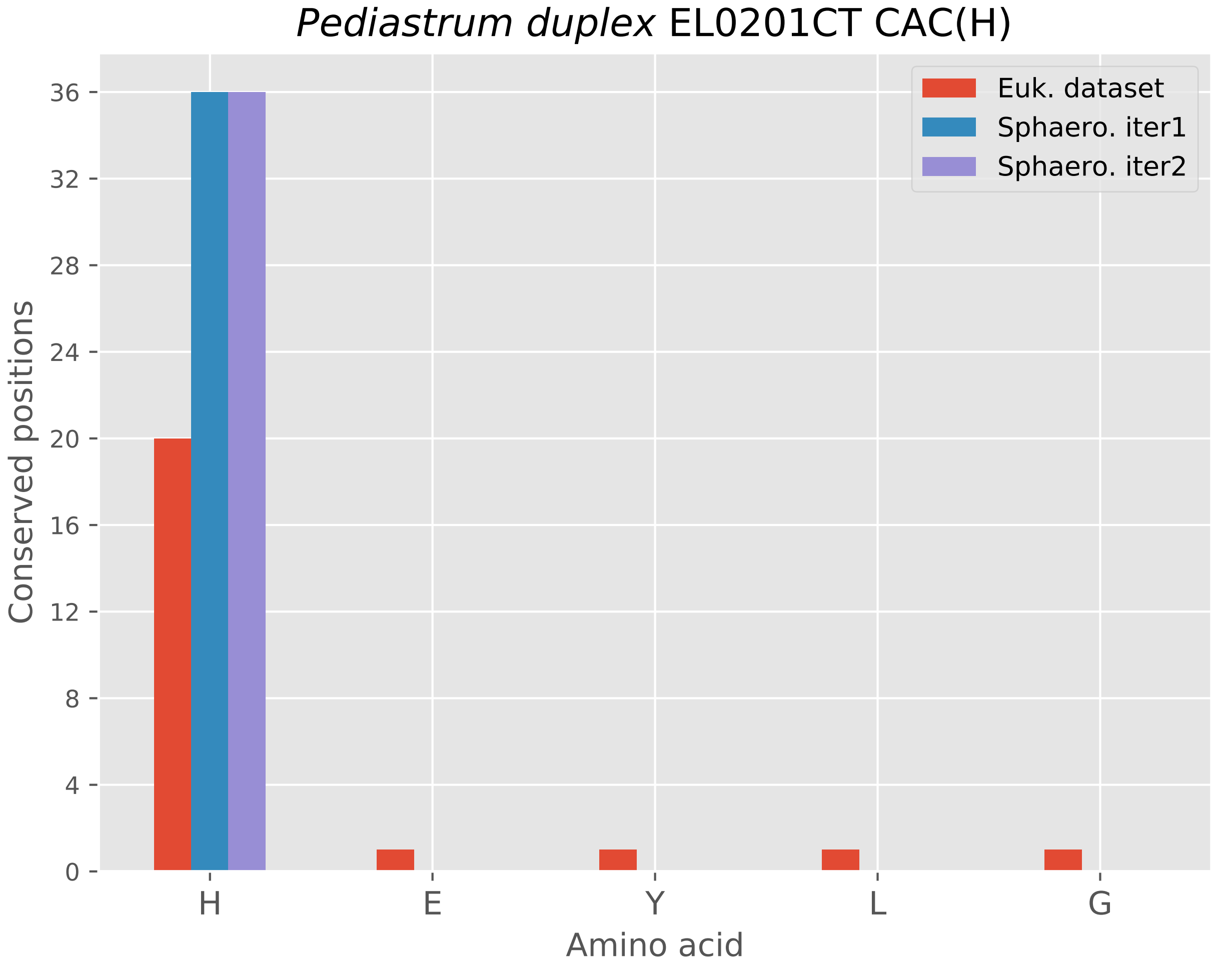

Pediastrum duplex EL0201CT CAC(H)
Euk. dataset
Sphaero. iter1
Sphaero. iter2
Conserved positions
0
4
8
12
16
20
24
28
32
36
H
E
Y
L
G
Amino acid

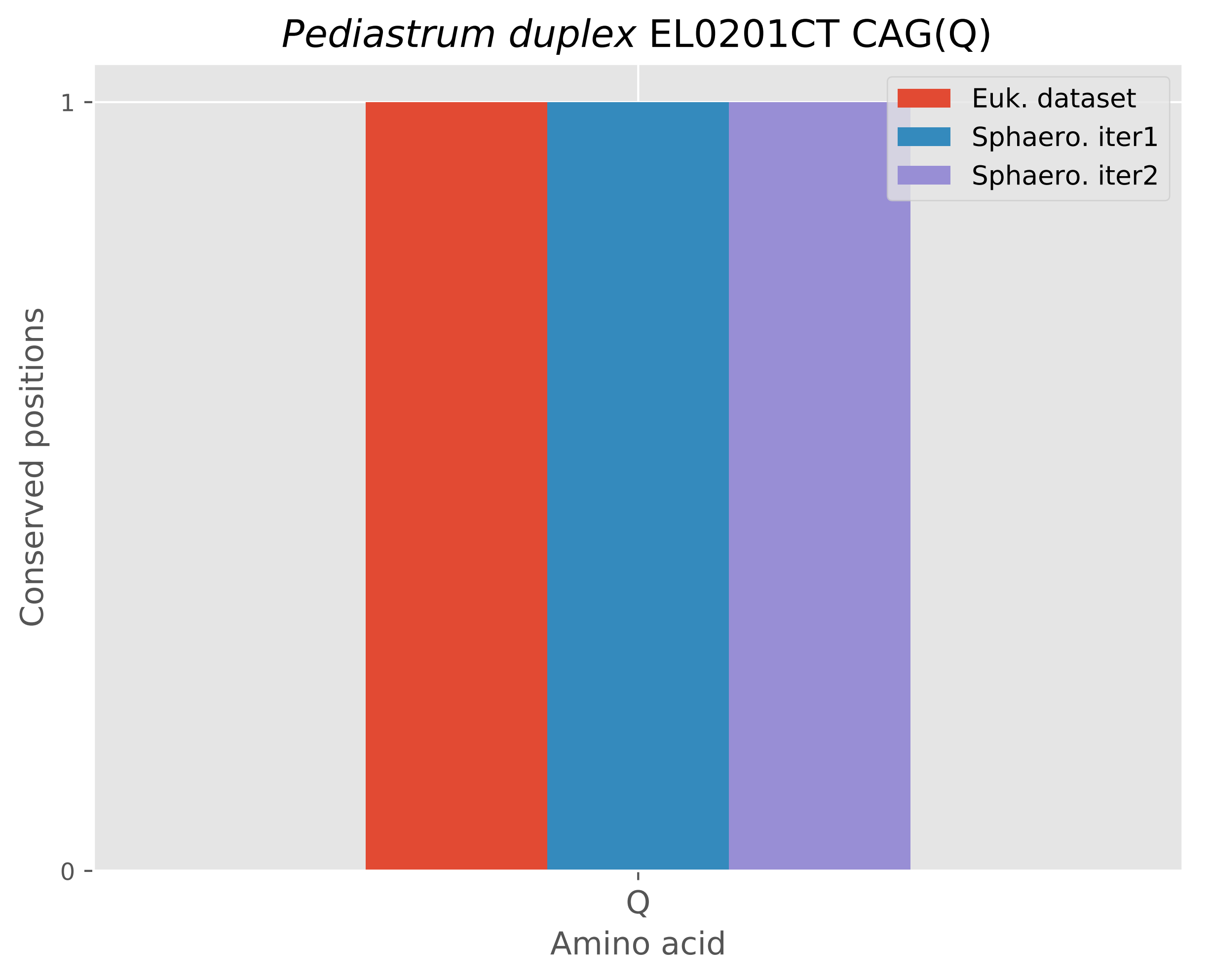

Pediastrum duplex EL0201CT CAG(Q)
Euk. dataset
Sphaero. iter1
Sphaero. iter2
1
0
Conserved positions
Q
Amino acid

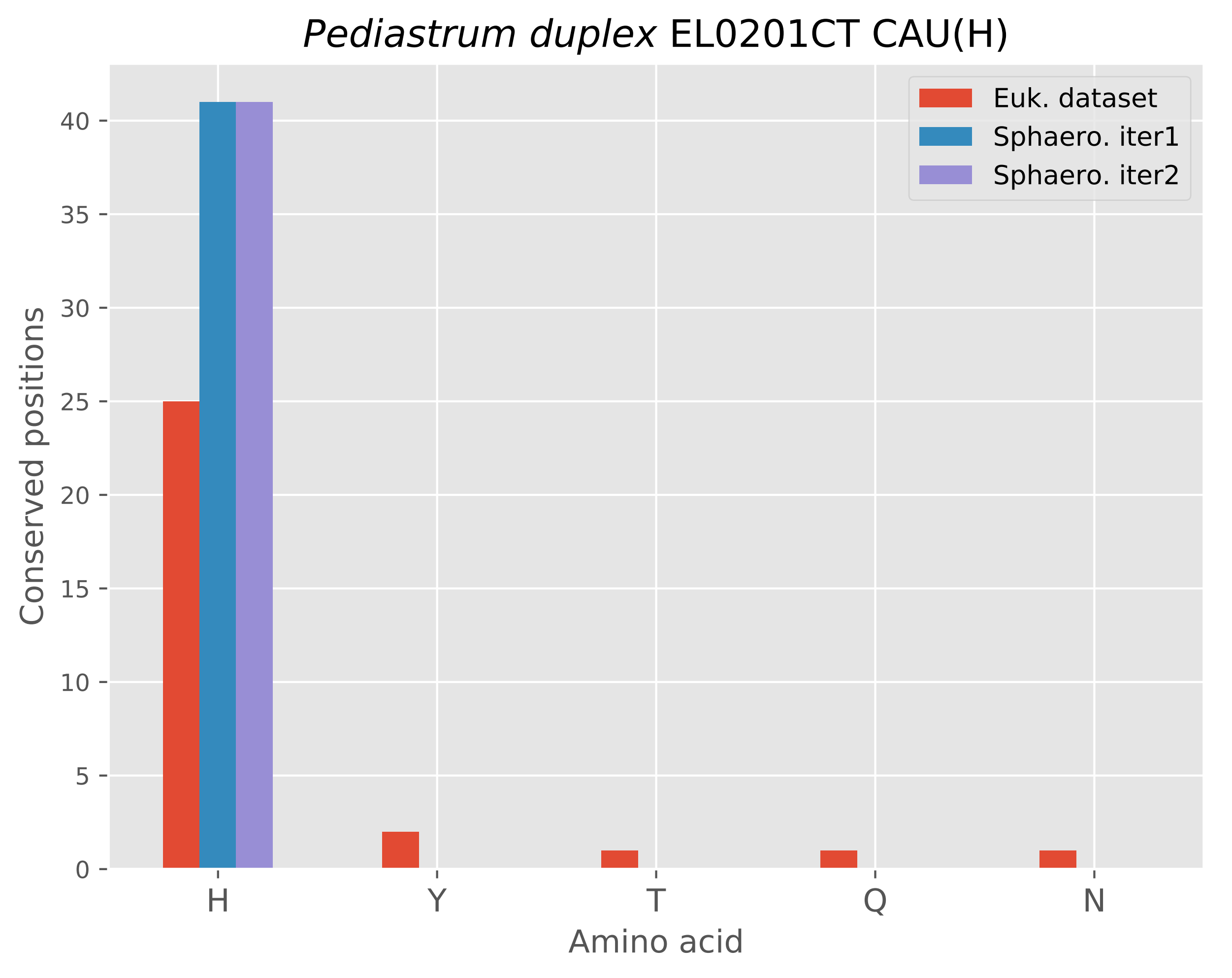

Pediastrum duplex EL0201CT CAU(H)
Euk. dataset
Sphaero. iter1
Sphaero. iter2
Conserved positions
0
5
10
15
20
25
30
35
40
H
Y
T
Q
N
Amino acid

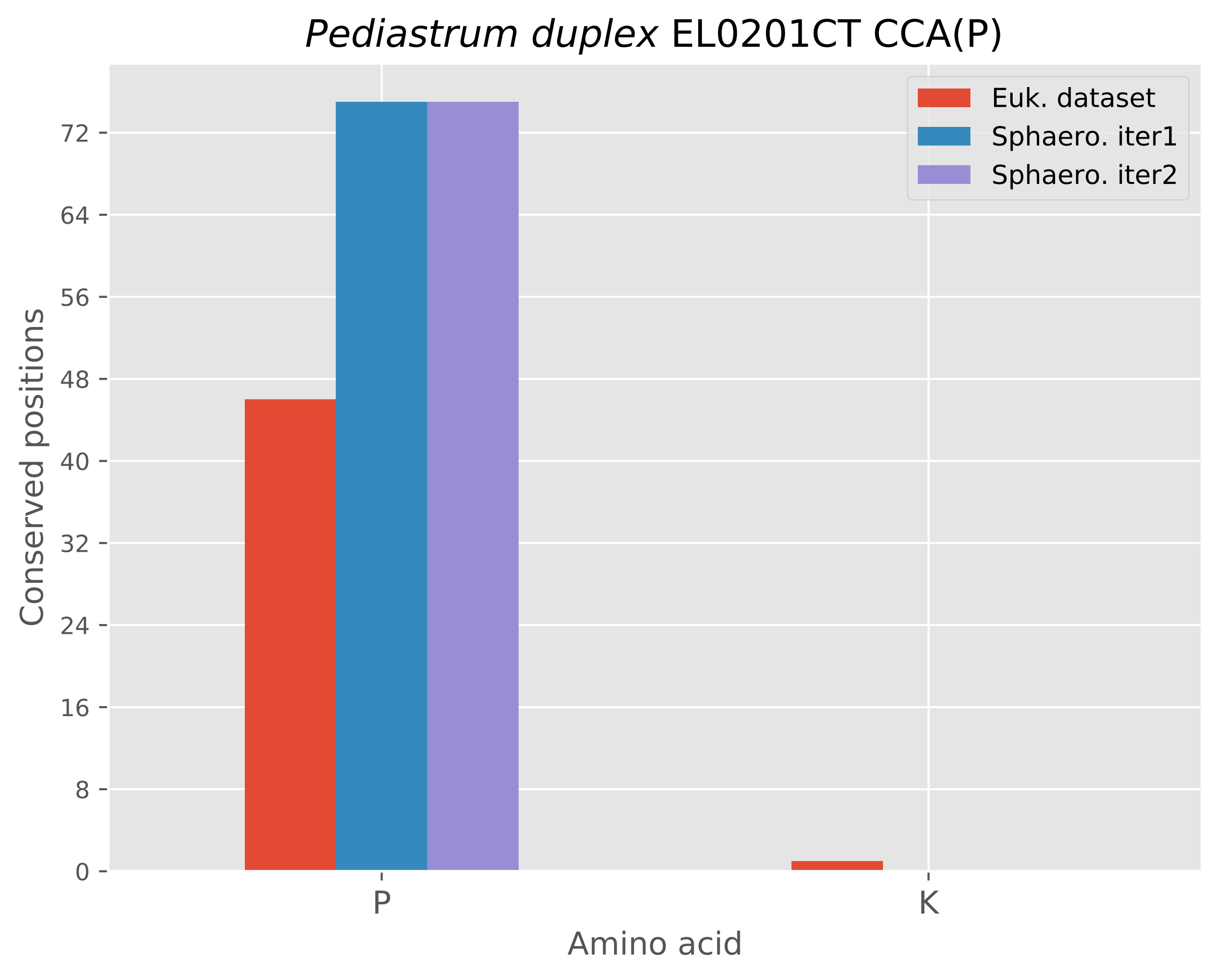

Pediastrum duplex EL0201CT CCA(P)
Euk. dataset
Sphaero. iter1
Sphaero. iter2
Conserved positions
0
8
16
24
32
40
48
56
64
72
P
K
Amino acid

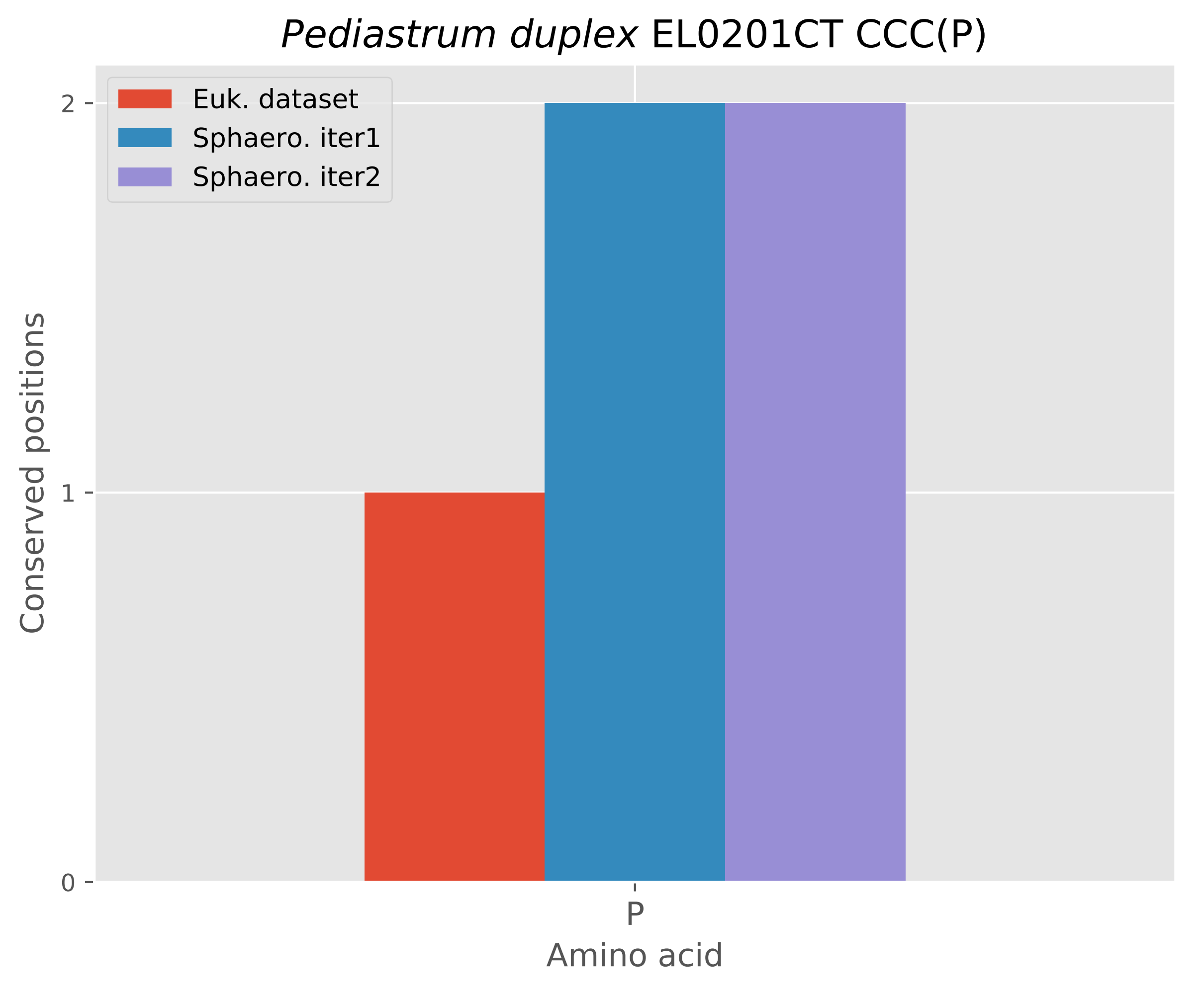
Pediastrum duplex EL0201CT CCC(P)
Euk. dataset
Sphaero. iter1
Sphaero. iter2
2
1
0
Conserved positions
P
Amino acid

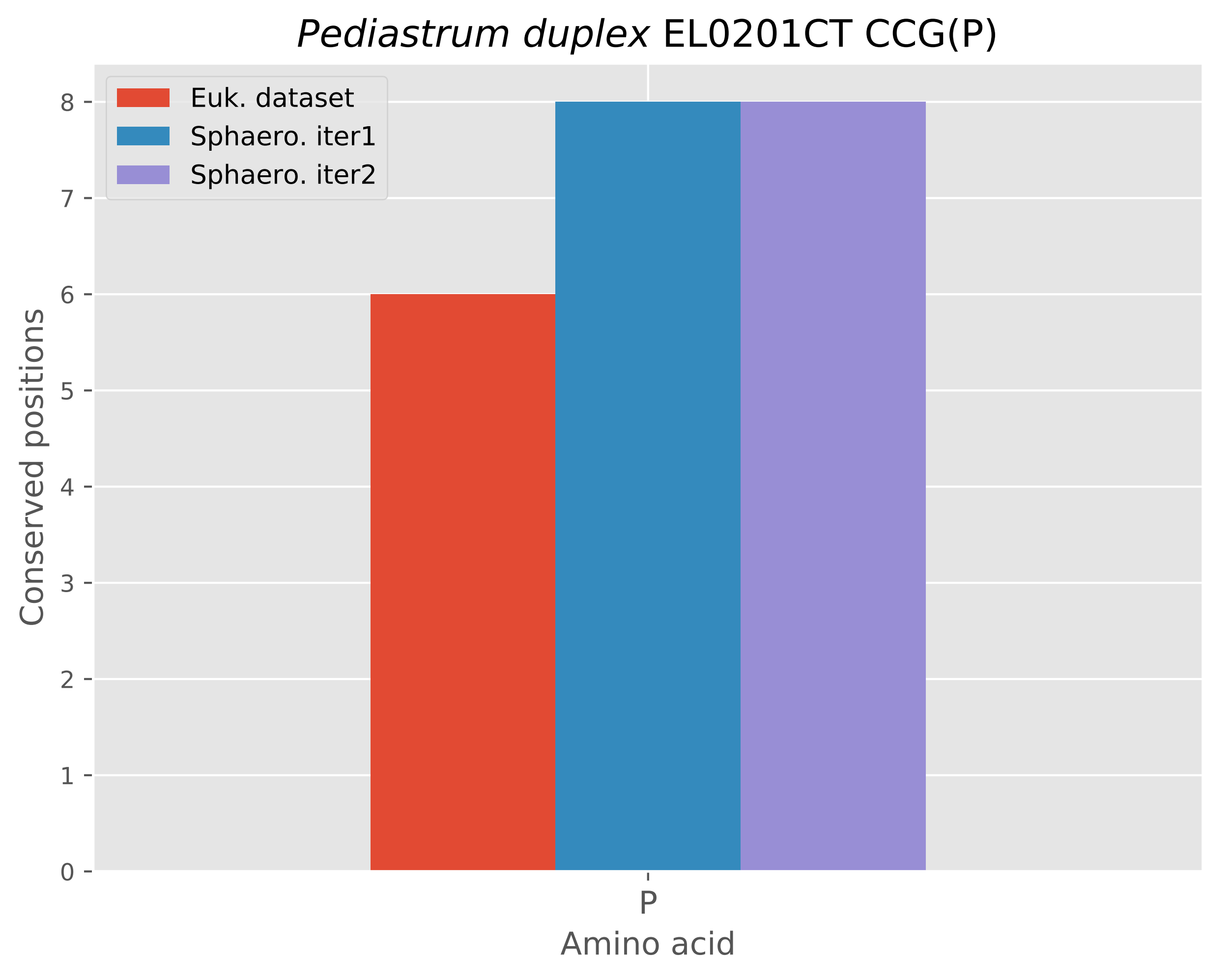
Pediastrum duplex EL0201CT CCG(P)
Euk. dataset
Sphaero. iter1
Sphaero. iter2
Conserved positions
0
1
2
3
4
5
6
7
8
P
Amino acid

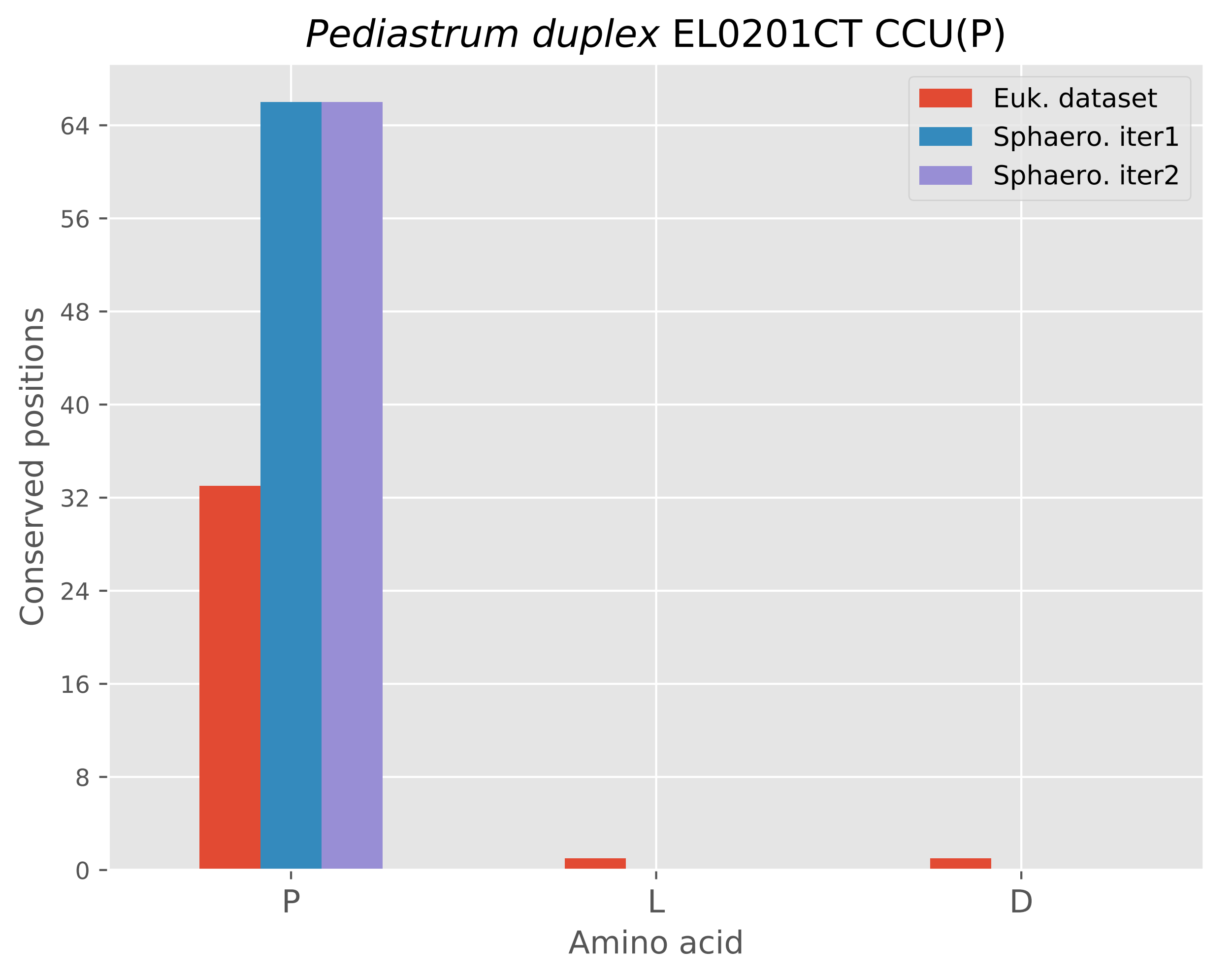

Pediastrum duplex EL0201CT CCU(P)
Euk. dataset
Sphaero. iter1
Sphaero. iter2
Conserved positions
0
8
16
24
32
40
48
56
64
P
L
D
Amino acid

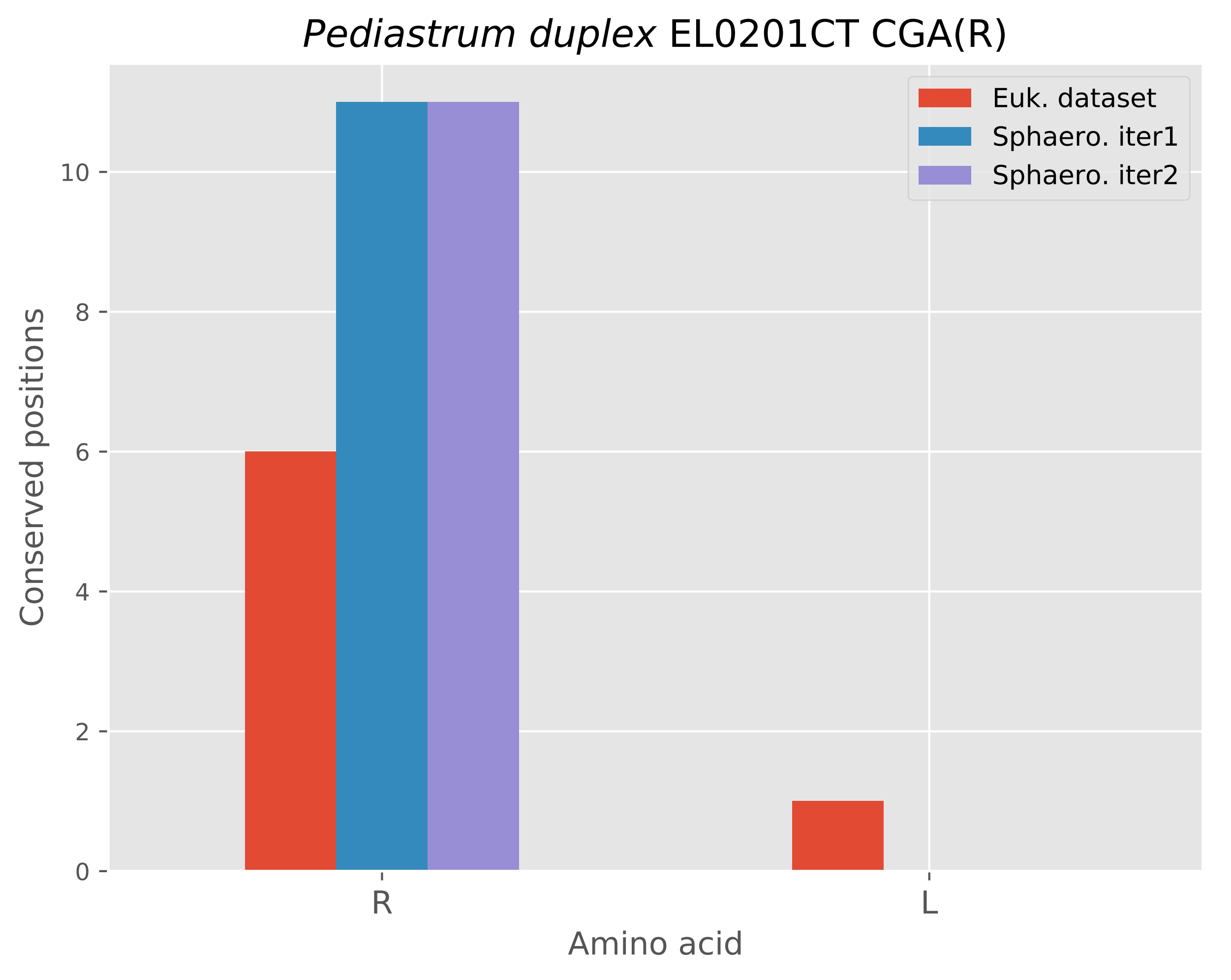

Pediastrum duplex EL0201CT CGA(R)
Euk. dataset
Sphaero. iter1
Sphaero. iter2
Conserved positions
0
2
4
6
8
10
R
L
Amino acid

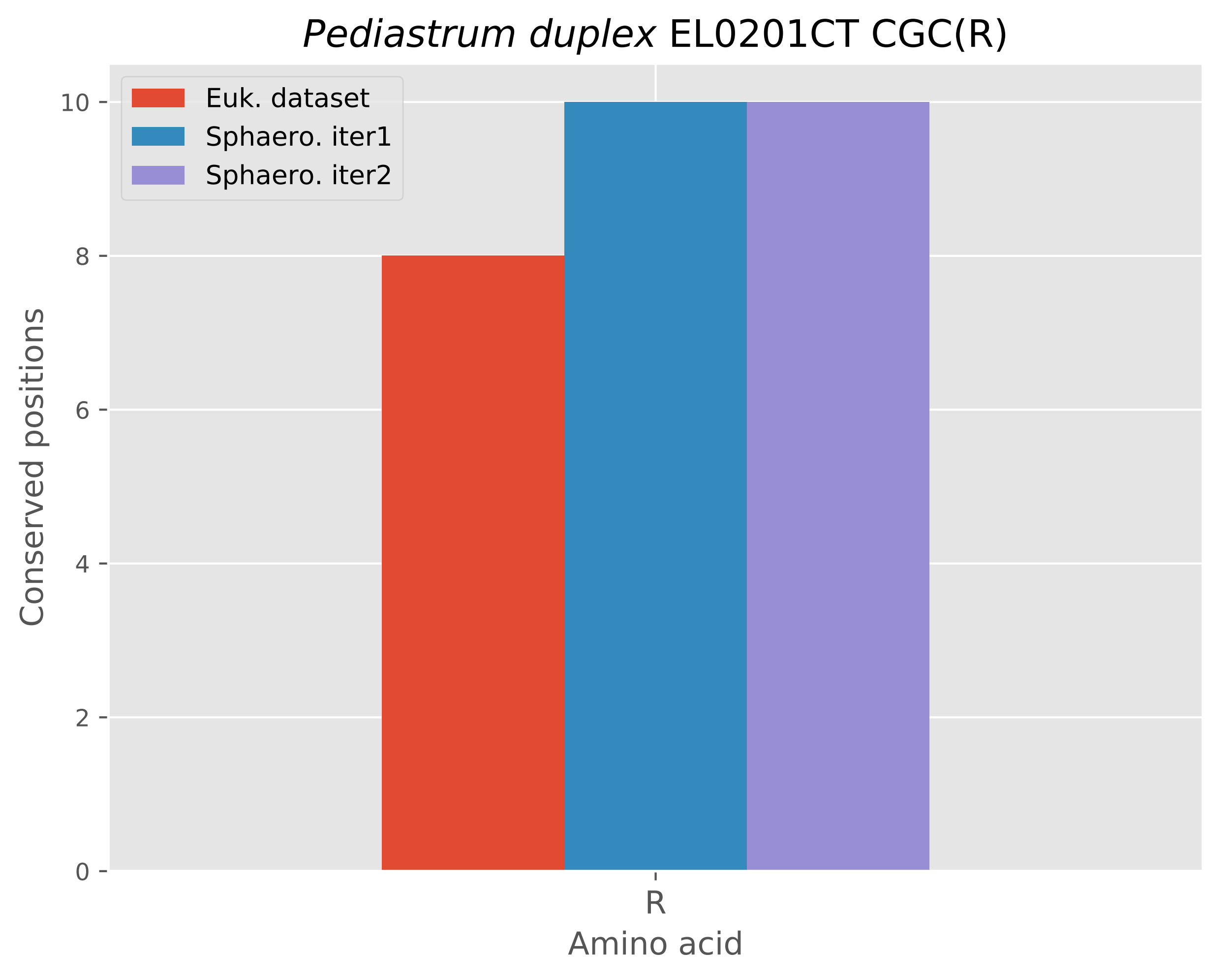

Pediastrum duplex EL0201CT CGC(R)
Euk. dataset
Sphaero. iter1
Sphaero. iter2
Conserved positions
0
2
4
6
8
10
R
Amino acid

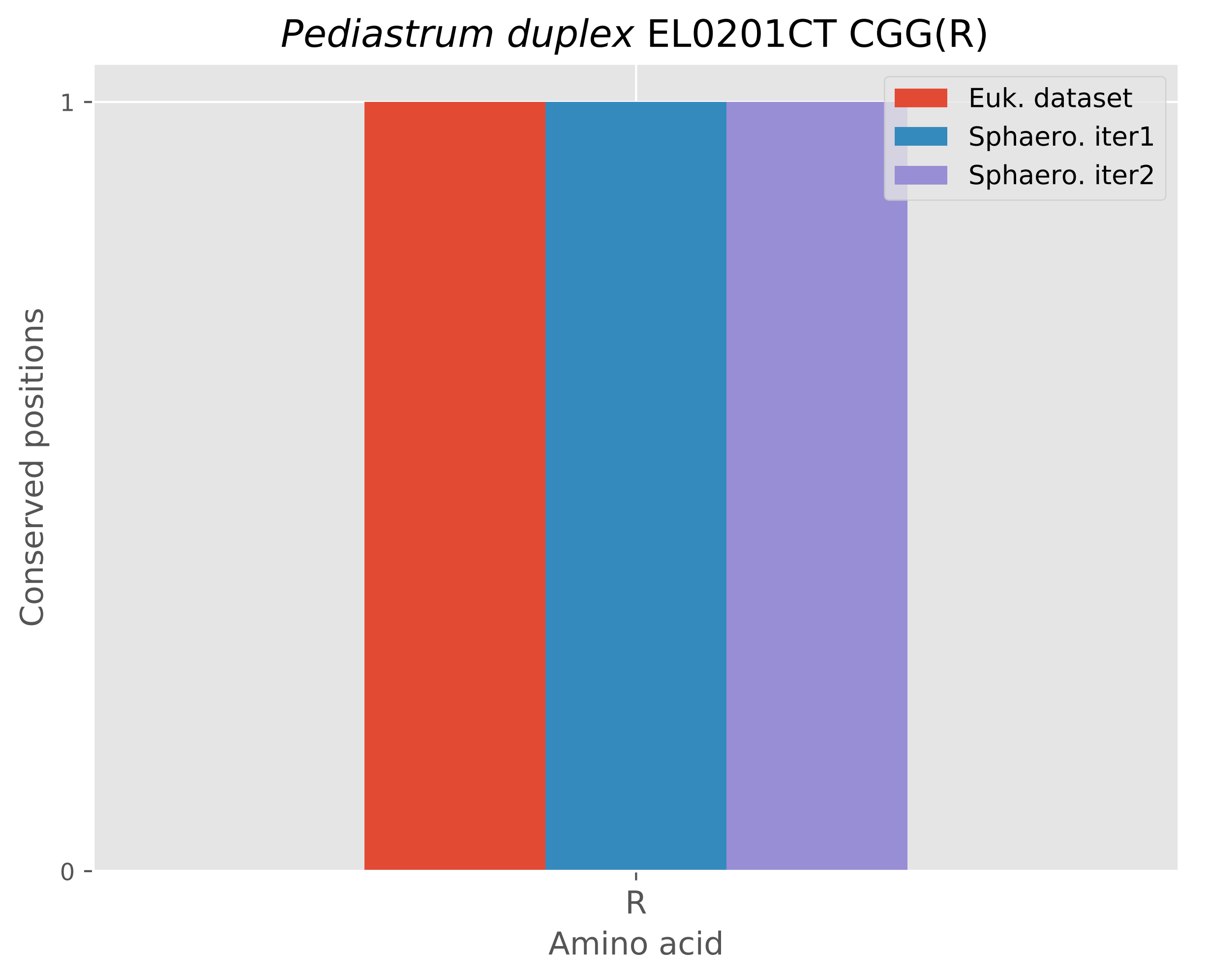

Pediastrum duplex EL0201CT CGG(R)
Euk. dataset
Sphaero. iter1
Sphaero. iter2
1
0
Conserved positions
R
Amino acid

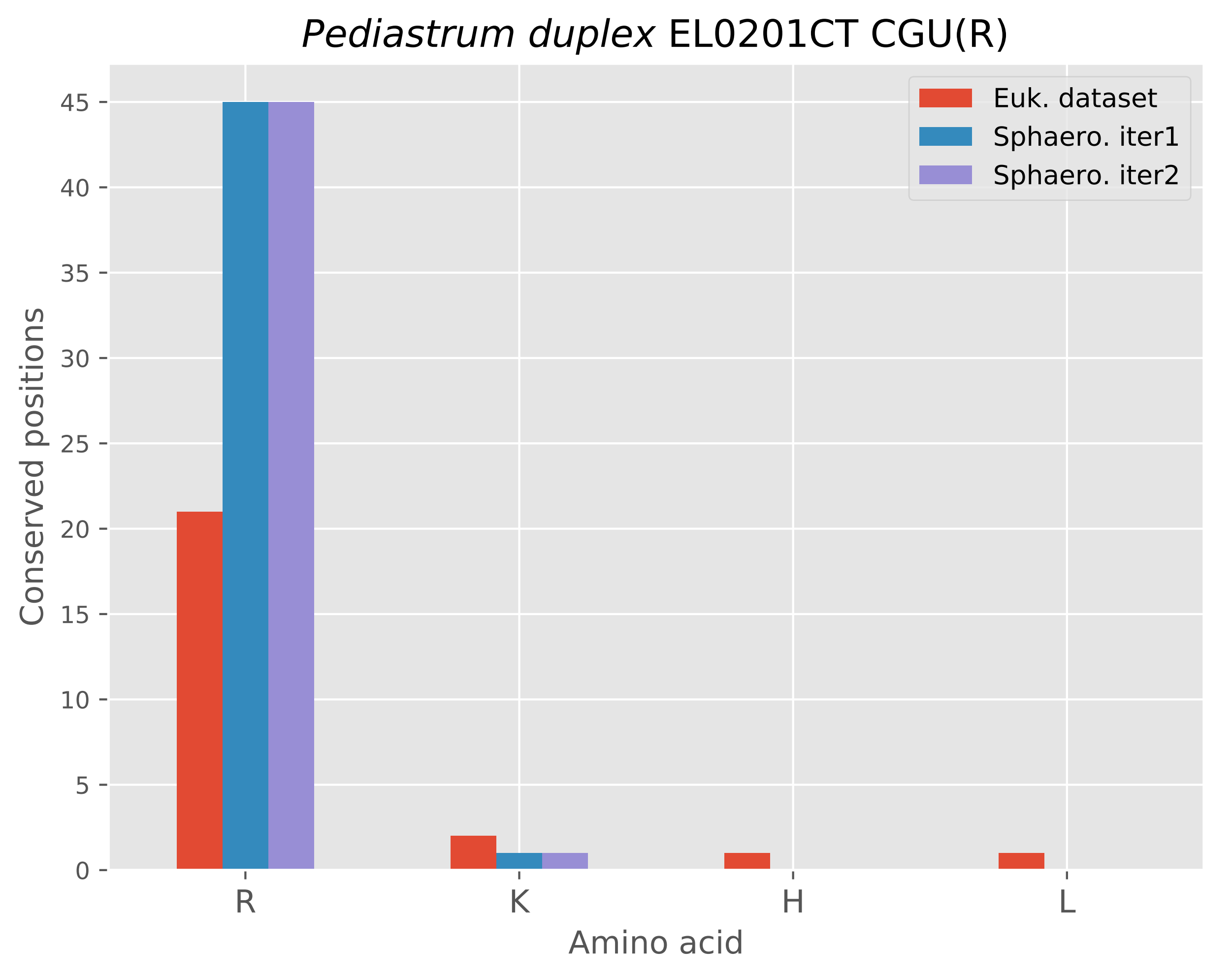
Pediastrum duplex EL0201CT CGU(R)
Euk. dataset
Sphaero. iter1
Sphaero. iter2
Conserved positions
0
5
10
15
20
25
30
35
40
45
R
K
H
L
Amino acid

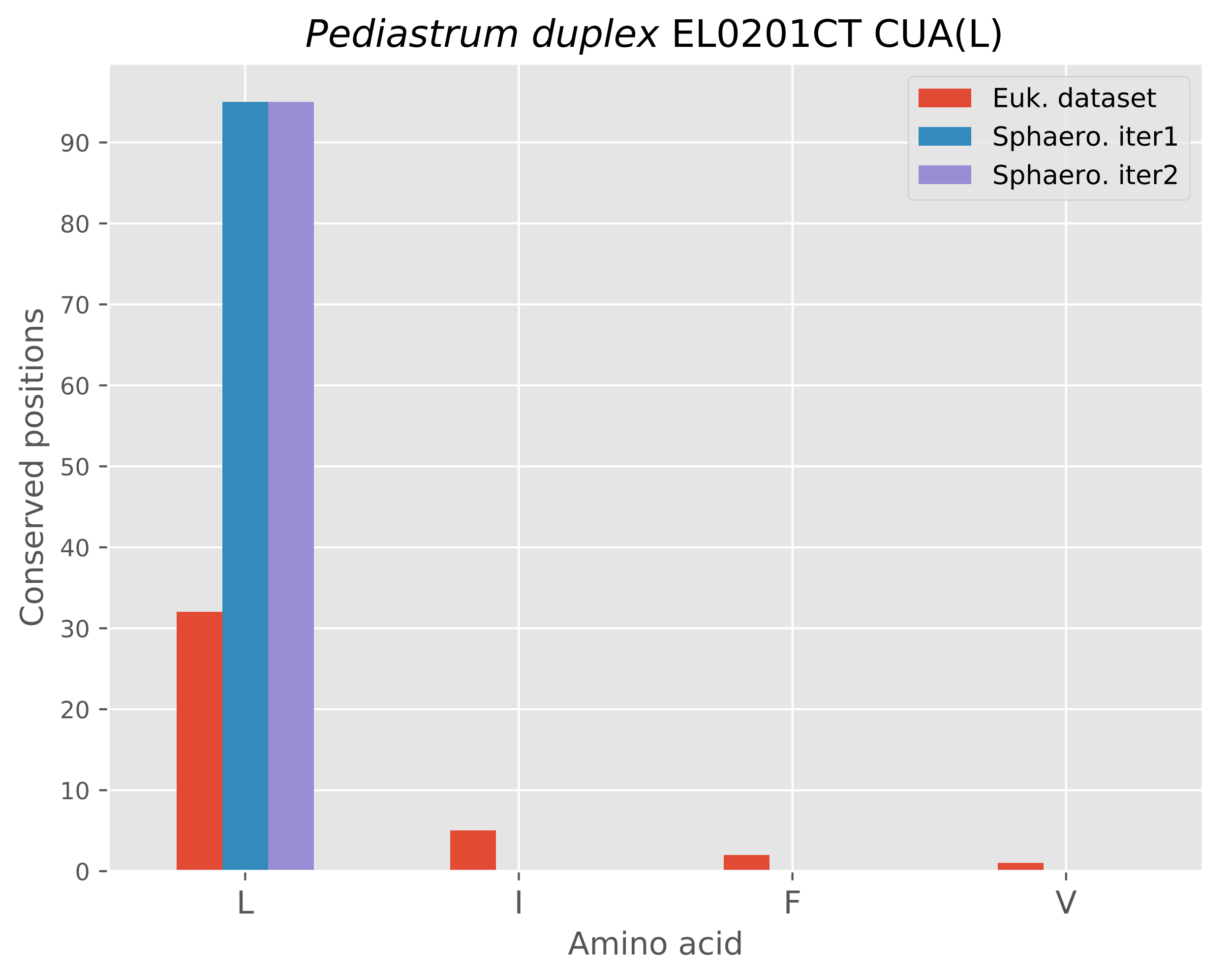

Pediastrum duplex EL0201CT CUA(L)
Euk. dataset
Sphaero. iter1
Sphaero. iter2
Conserved positions
0
10
20
30
40
50
60
70
80
90
L
I
F
V
Amino acid

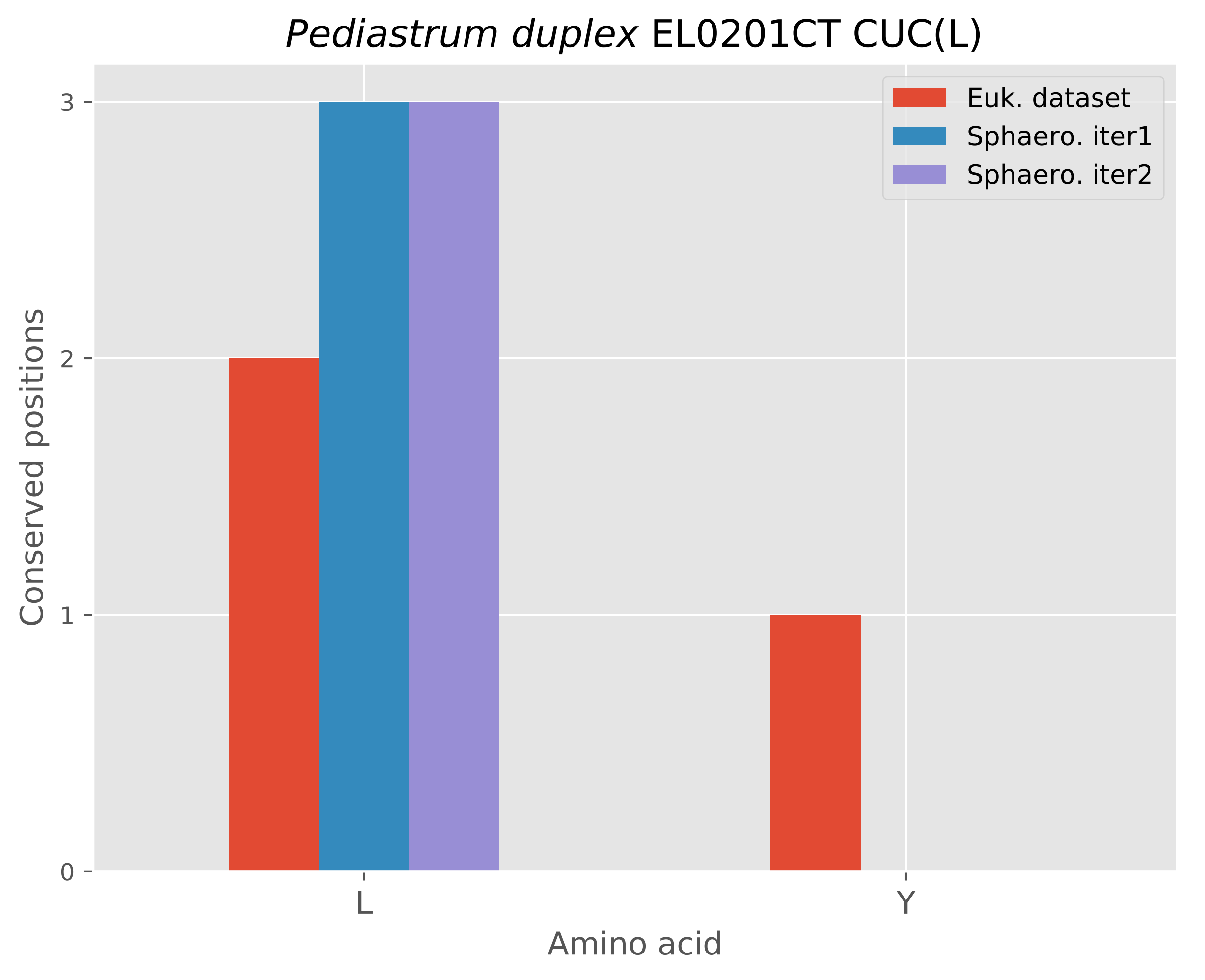

Pediastrum duplex EL0201CT CUC(L)
Euk. dataset
Sphaero. iter1
Sphaero. iter2
Conserved positions
0
1
2
3
L
Y
Amino acid

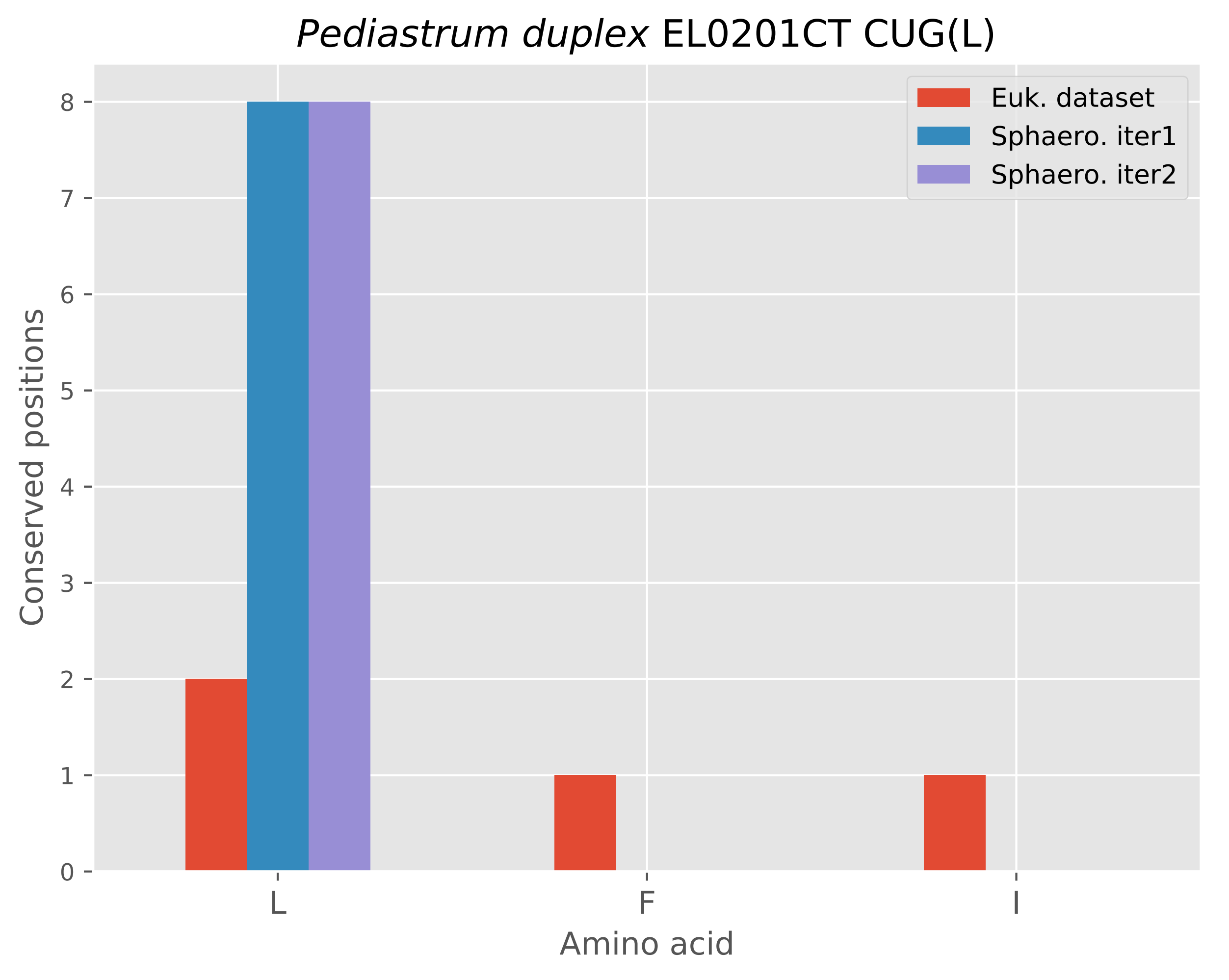

Pediastrum duplex EL0201CT CUG(L)
Euk. dataset
Sphaero. iter1
Sphaero. iter2
Conserved positions
0
1
2
3
4
5
6
7
8
L
F
I
Amino acid

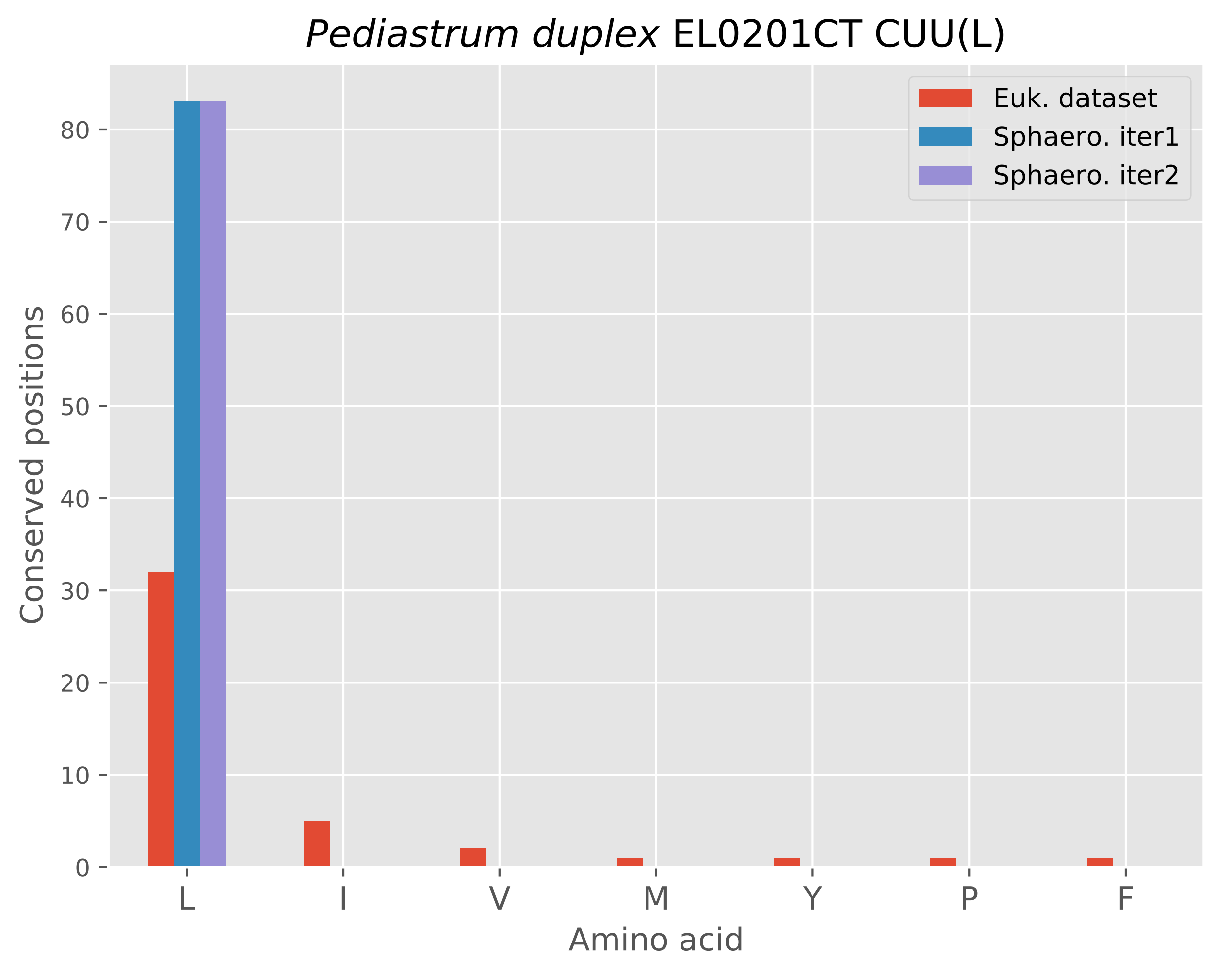

Pediastrum duplex EL0201CT CUU(L)
Euk. dataset
Sphaero. iter1
Sphaero. iter2
Conserved positions
0
10
20
30
40
50
60
70
80
L
I
V
M
Y
P
F
Amino acid

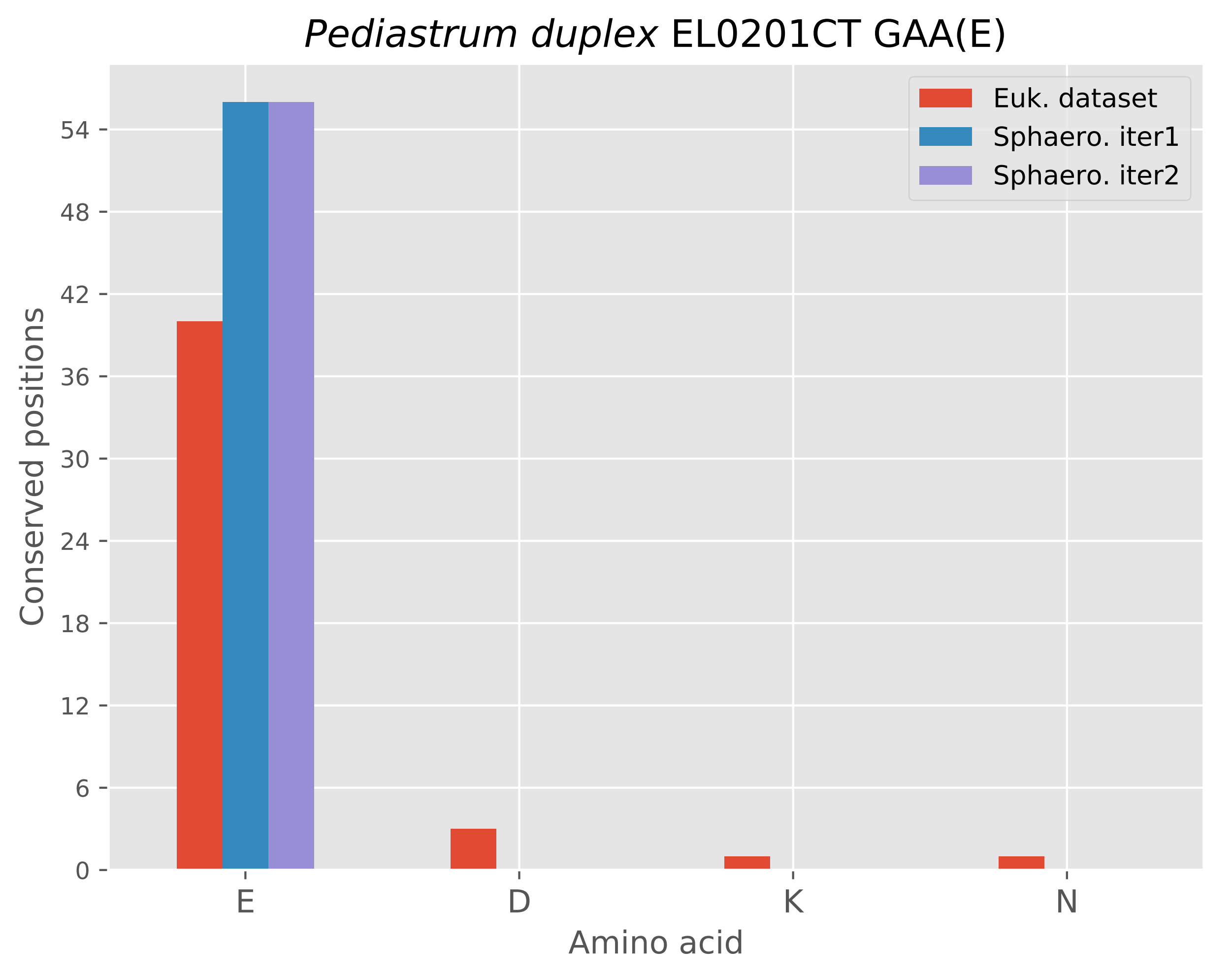
Pediastrum duplex EL0201CT GAA(E)
Euk. dataset
Sphaero. iter1
Sphaero. iter2
Conserved positions
0
6
12
18
24
30
36
42
48
54
E
D
K
N
Amino acid

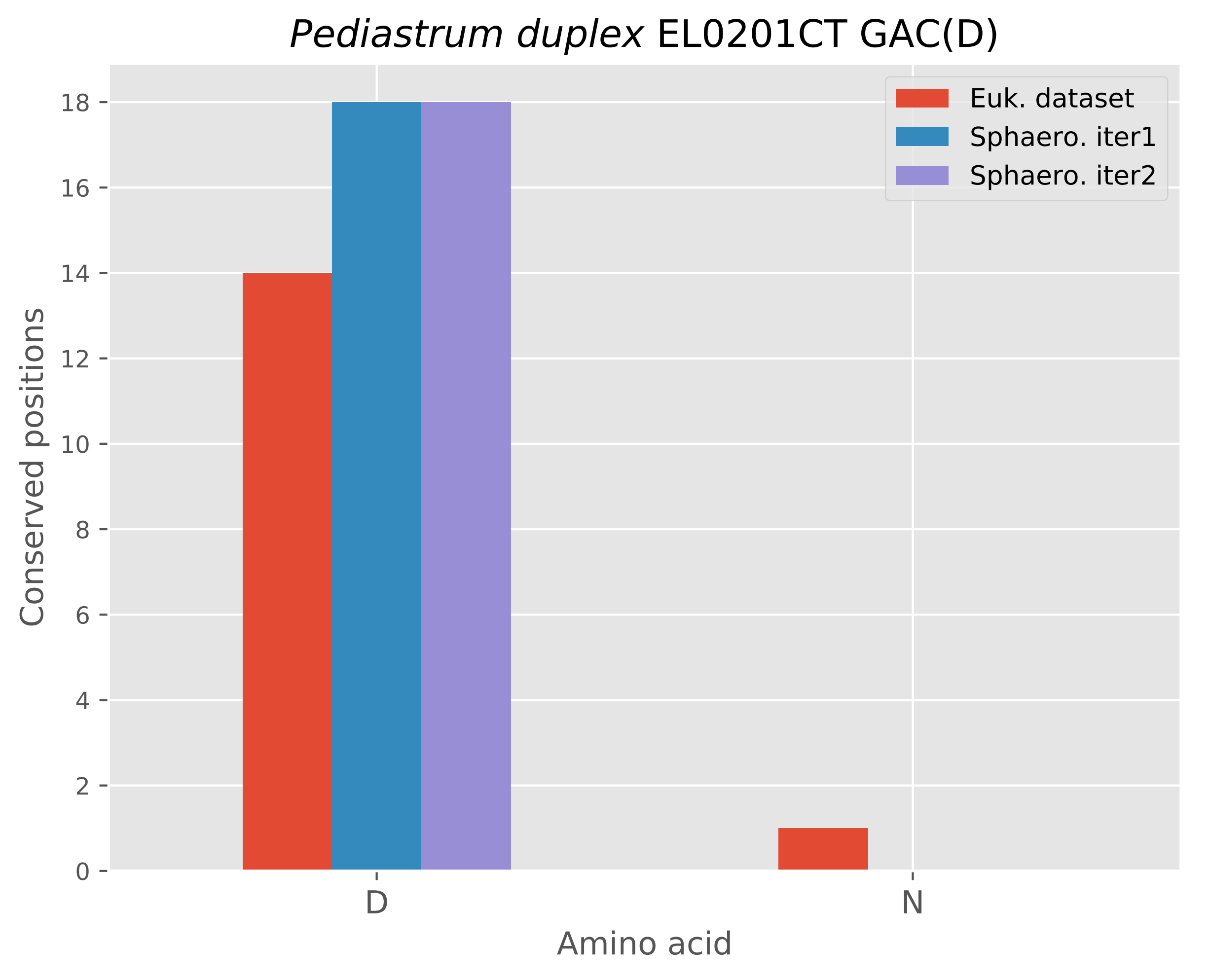

Pediastrum duplex EL0201CT GAC(D)
Euk. dataset
Sphaero. iter1
Sphaero. iter2
Conserved positions
0
2
4
6
8
10
12
14
16
18
D
N
Amino acid

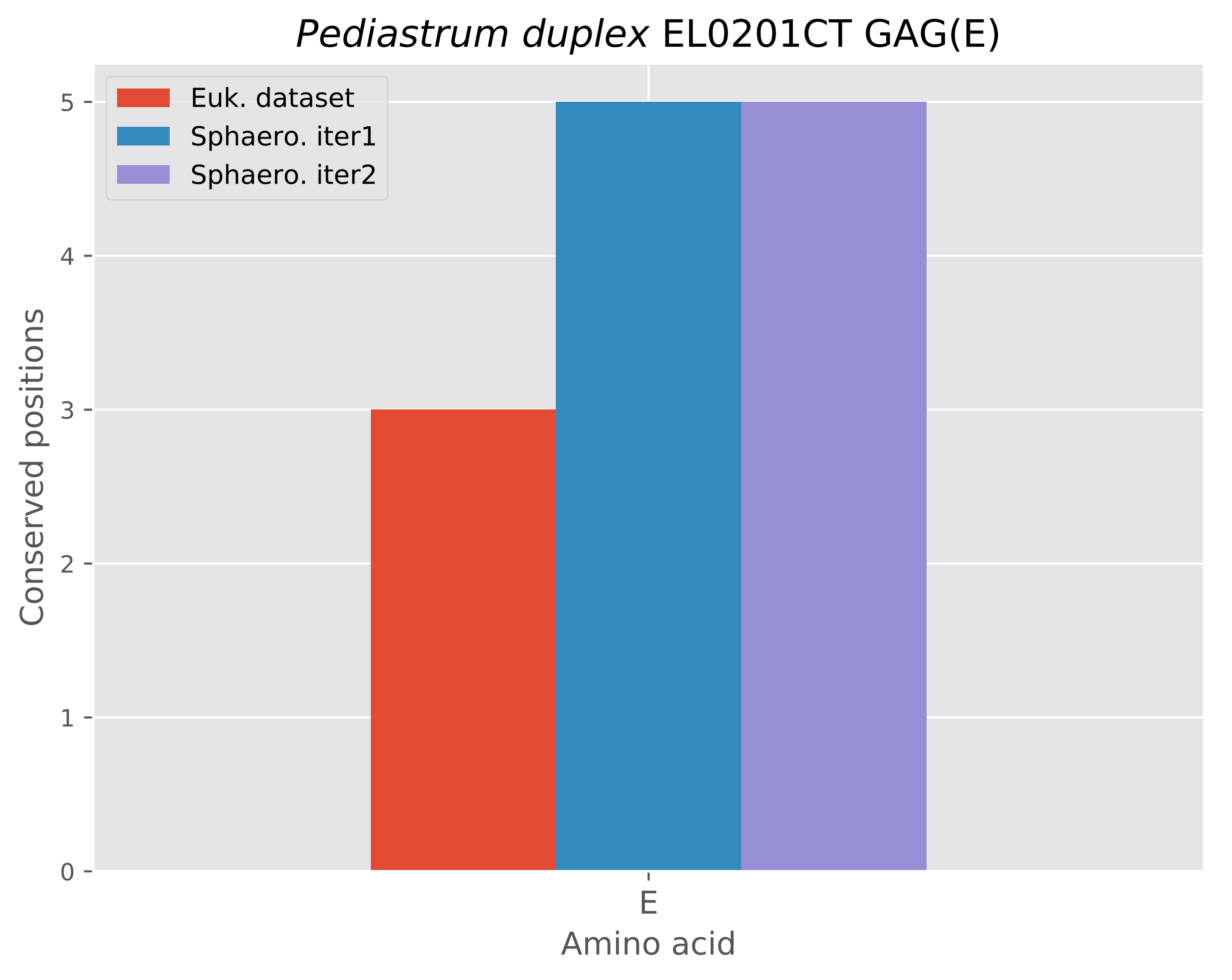
Pediastrum duplex EL0201CT GAG(E)
Euk. dataset
Sphaero. iter1
Sphaero. iter2
Conserved positions
0
1
2
3
4
5
E
Amino acid

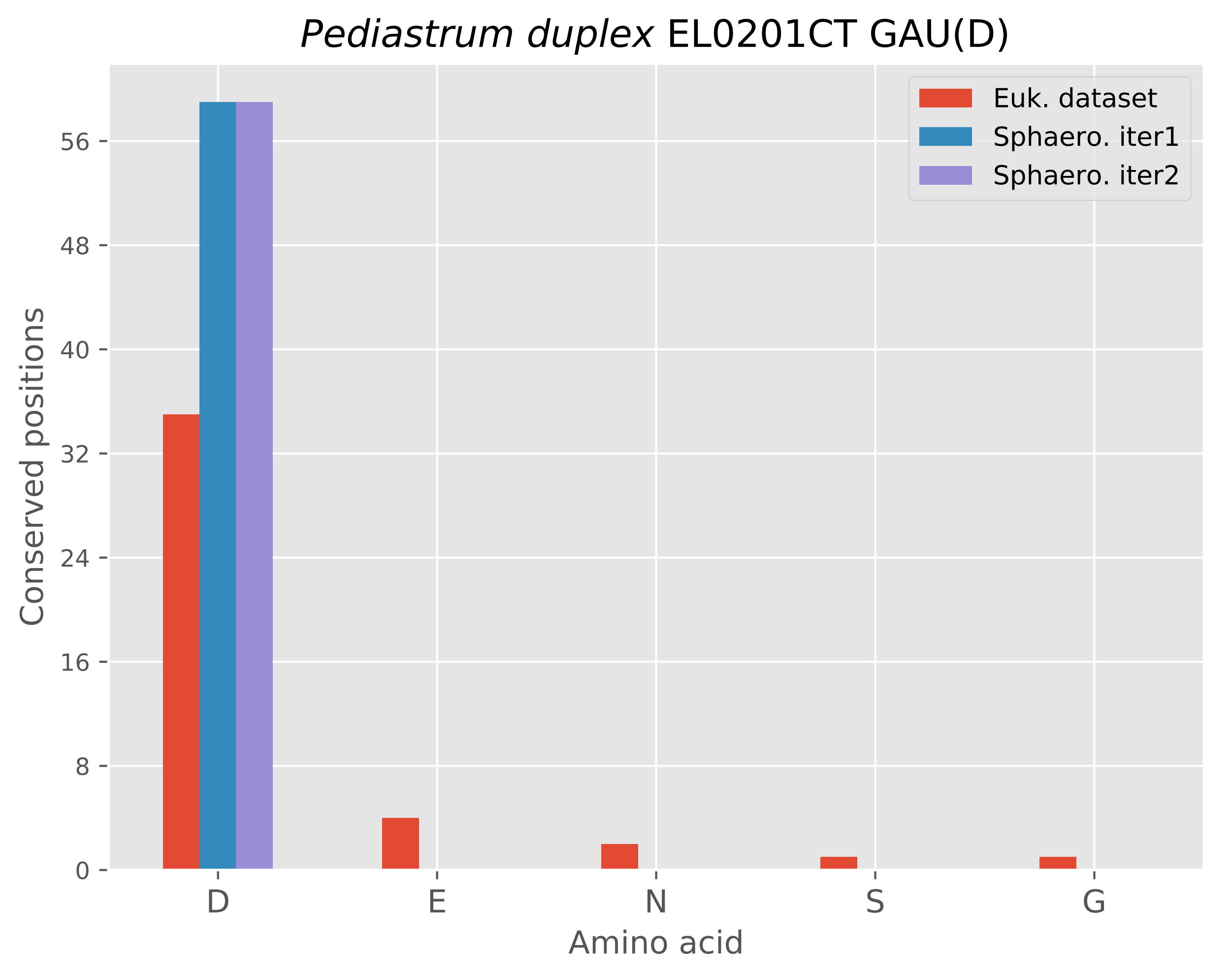

Pediastrum duplex EL0201CT GAU(D)
Euk. dataset
Sphaero. iter1
Sphaero. iter2
Conserved positions
0
8
16
24
32
40
48
56
D
E
N
S
G
Amino acid

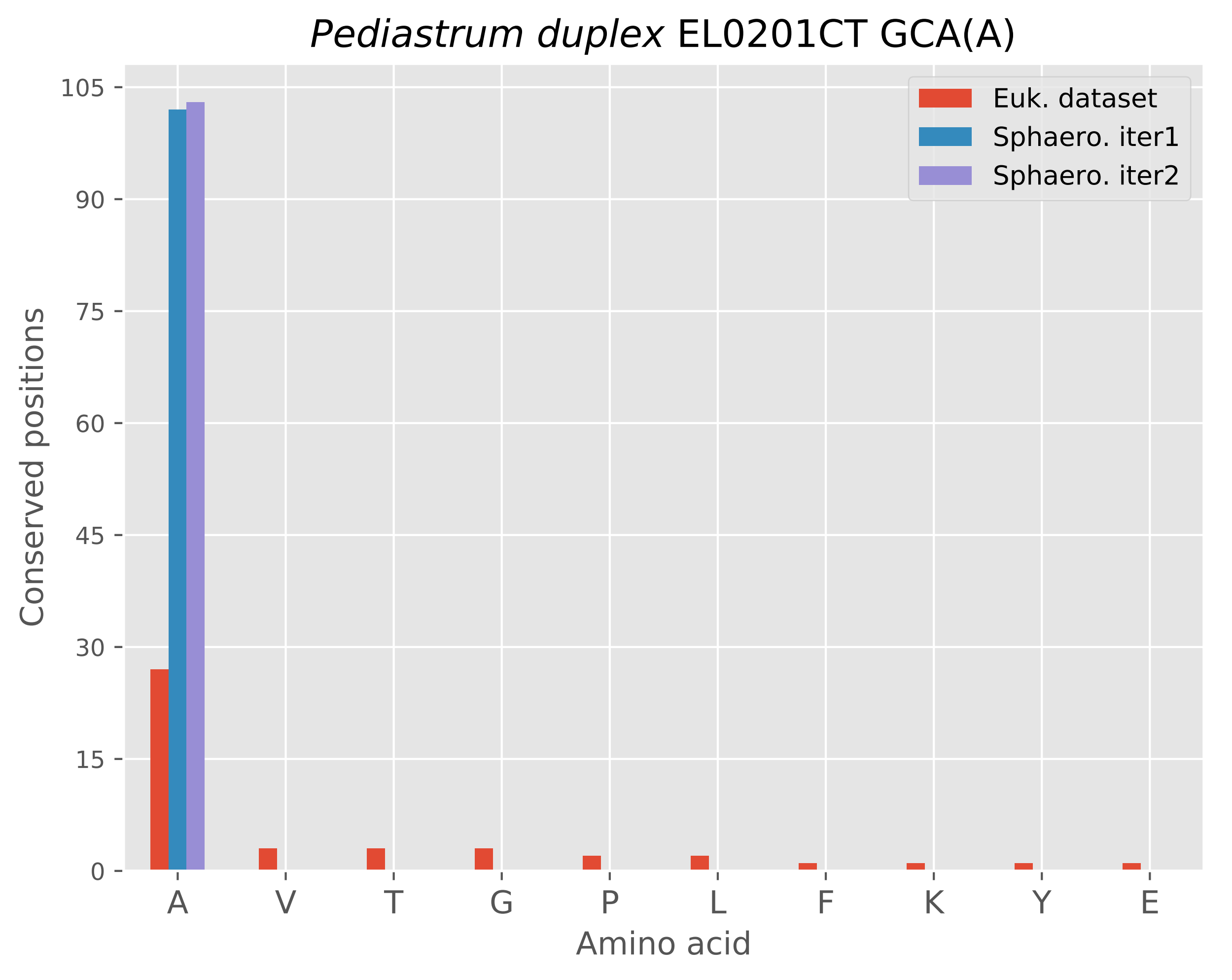

Pediastrum duplex EL0201CT GCA(A)
Euk. dataset
Sphaero. iter1
Sphaero. iter2
Conserved positions
105
90
75
60
45
30
15
0
A
V
T
G
P
L
F
K
Y
E
Amino acid

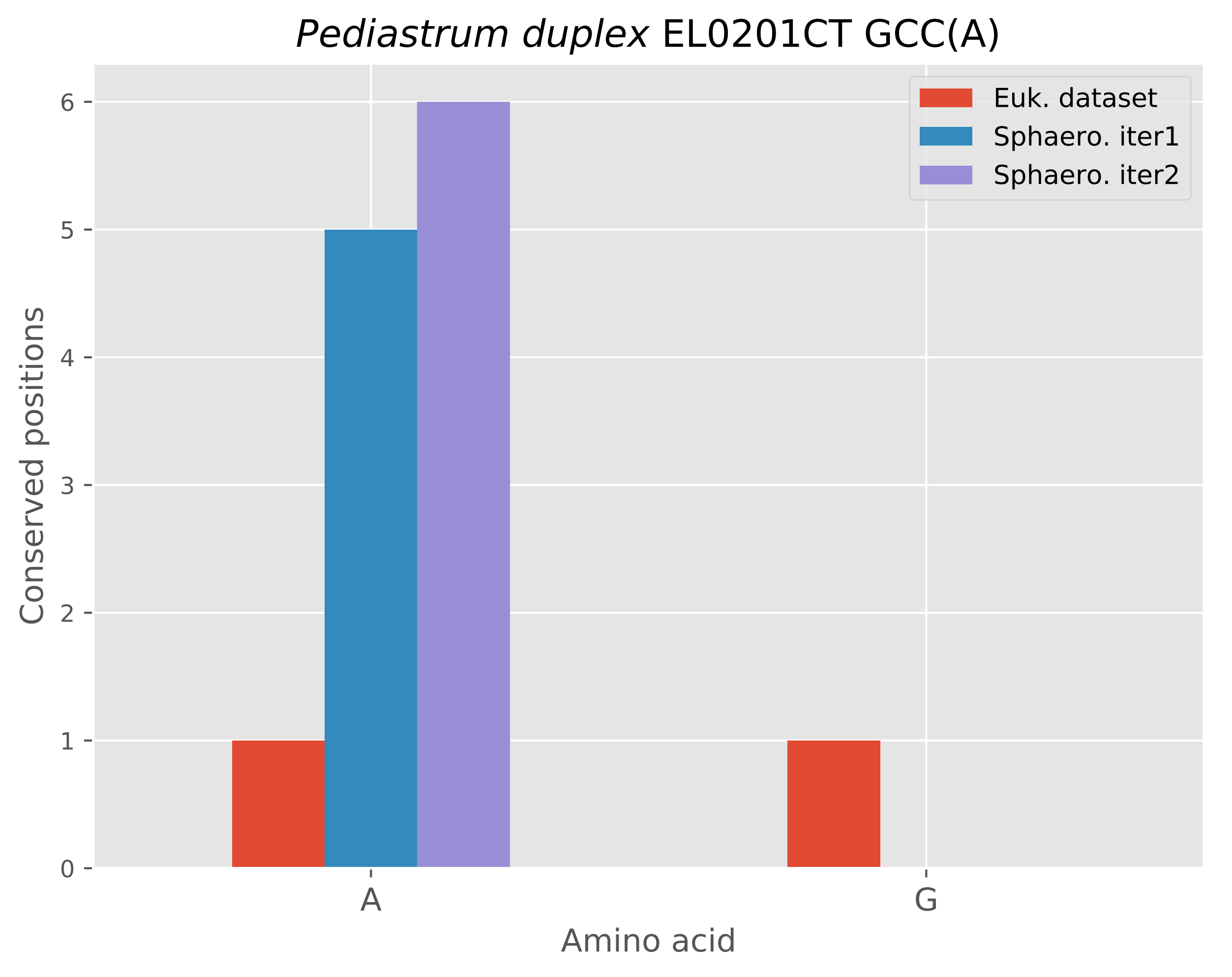
Pediastrum duplex EL0201CT GCC(A)
Euk. dataset
Sphaero. iter1
Sphaero. iter2
Conserved positions
0
1
2
3
4
5
6
A
G
Amino acid

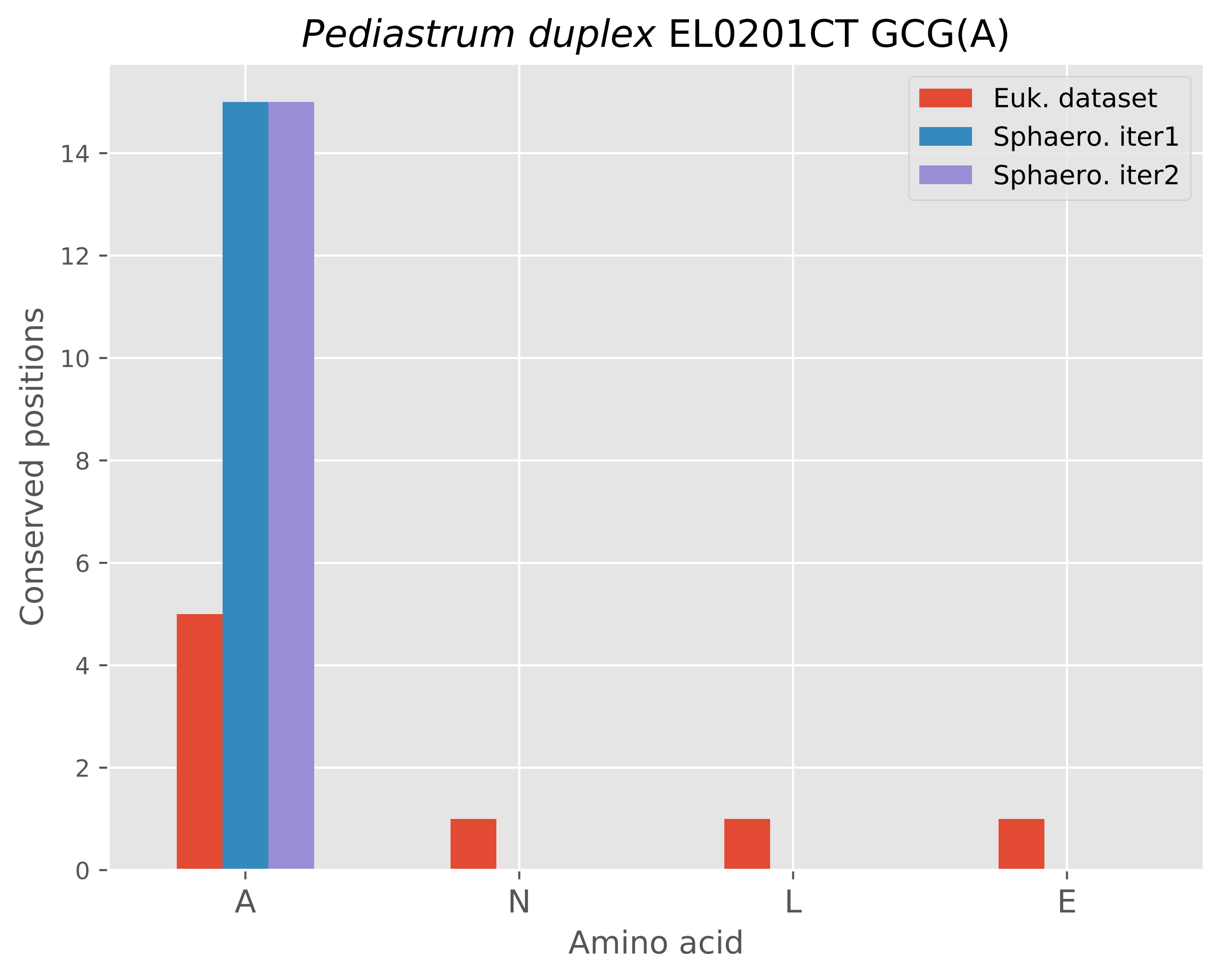

Pediastrum duplex EL0201CT GCG(A)
Euk. dataset
Sphaero. iter1
Sphaero. iter2
Conserved positions
0
2
4
6
8
10
12
14
A
N
L
E
Amino acid

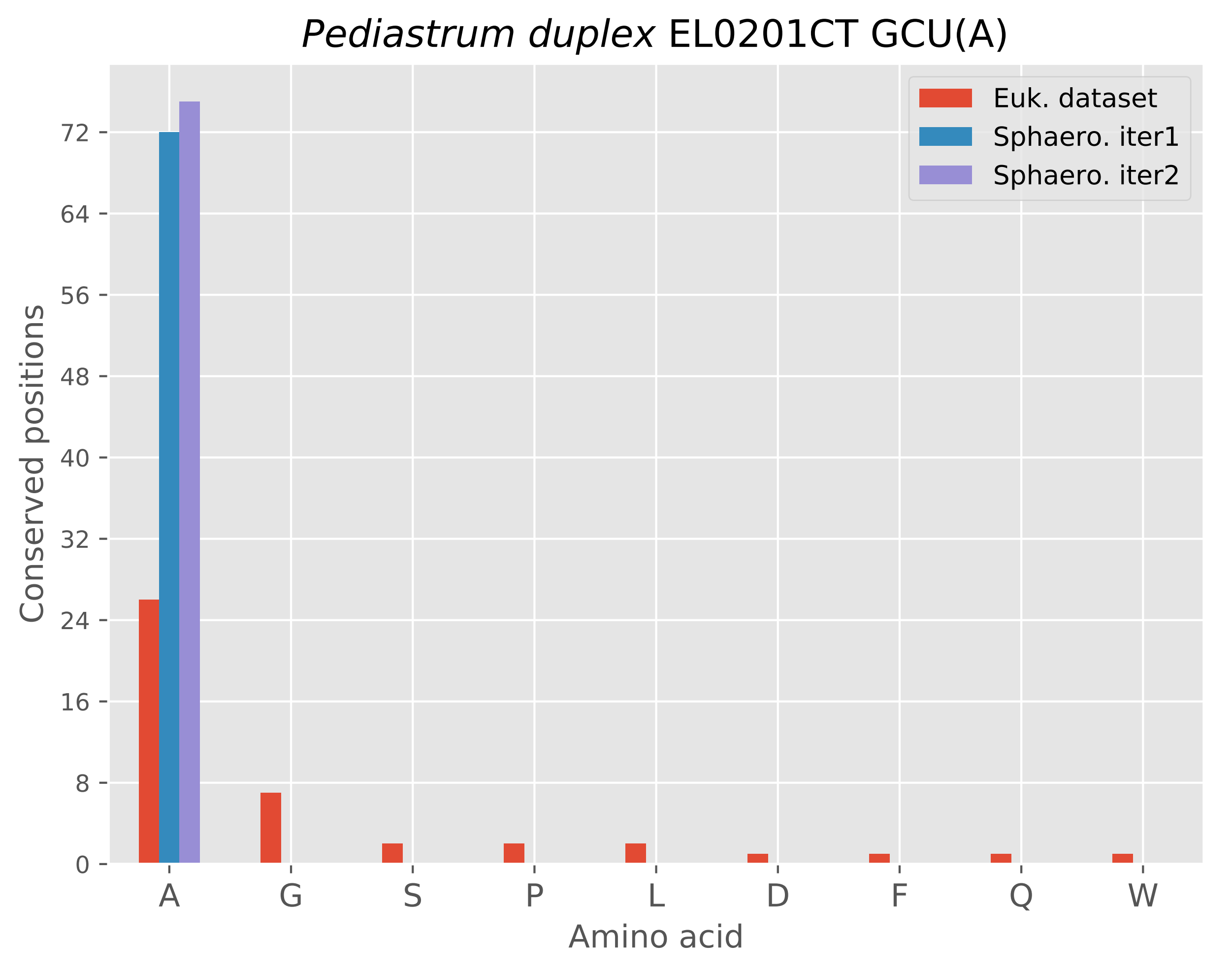

Pediastrum duplex EL0201CT GCU(A)
Euk. dataset
Sphaero. iter1
Sphaero. iter2
Conserved positions
0
8
16
24
32
40
48
56
64
72
A
G
S
P
L
D
F
Q
W
Amino acid

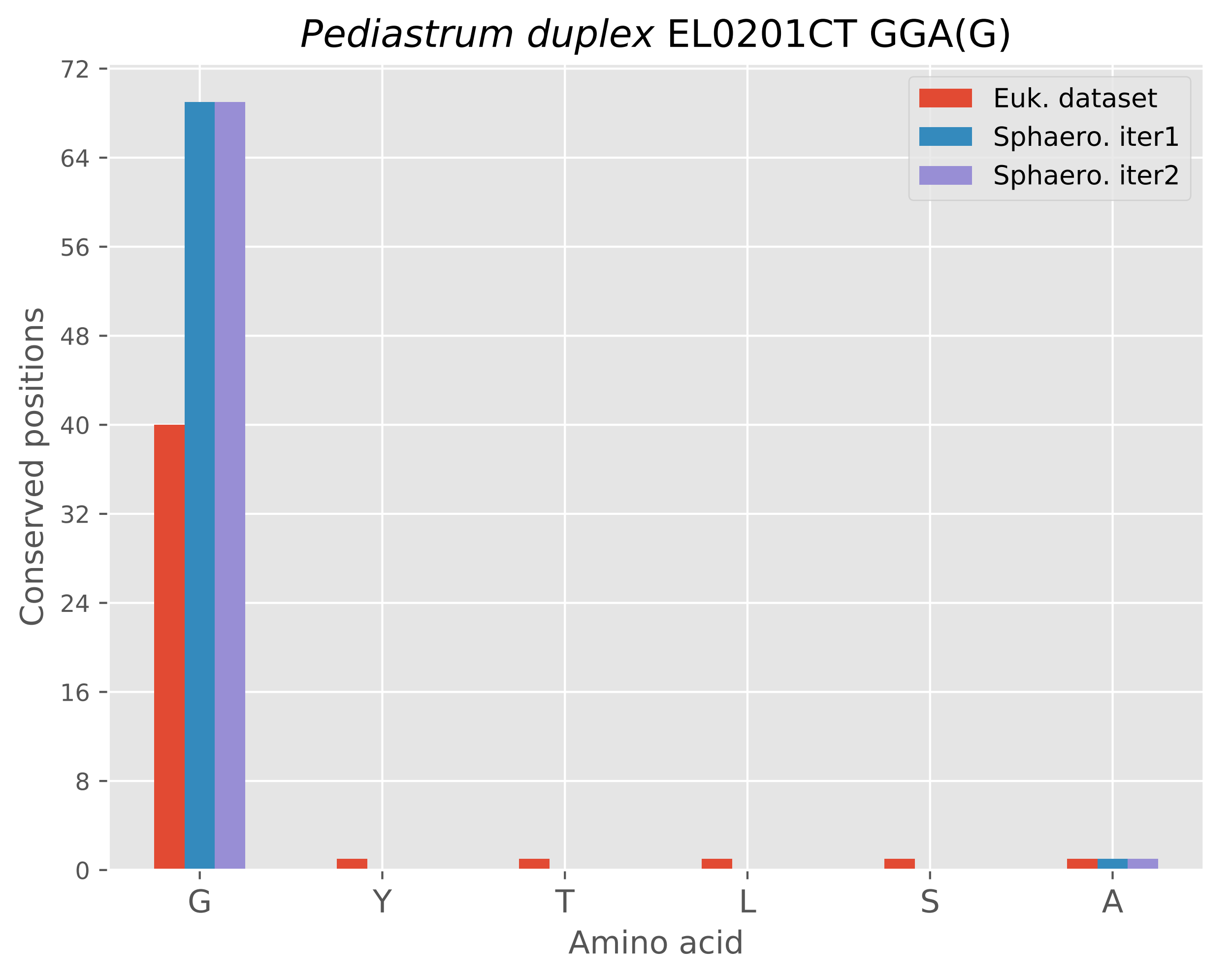

Pediastrum duplex EL0201CT GGA(G)
Euk. dataset
Sphaero. iter1
Sphaero. iter2
Conserved positions
Amino acid
0
8
16
24
32
40
48
56
64
72
G
Y
T
L
S
A

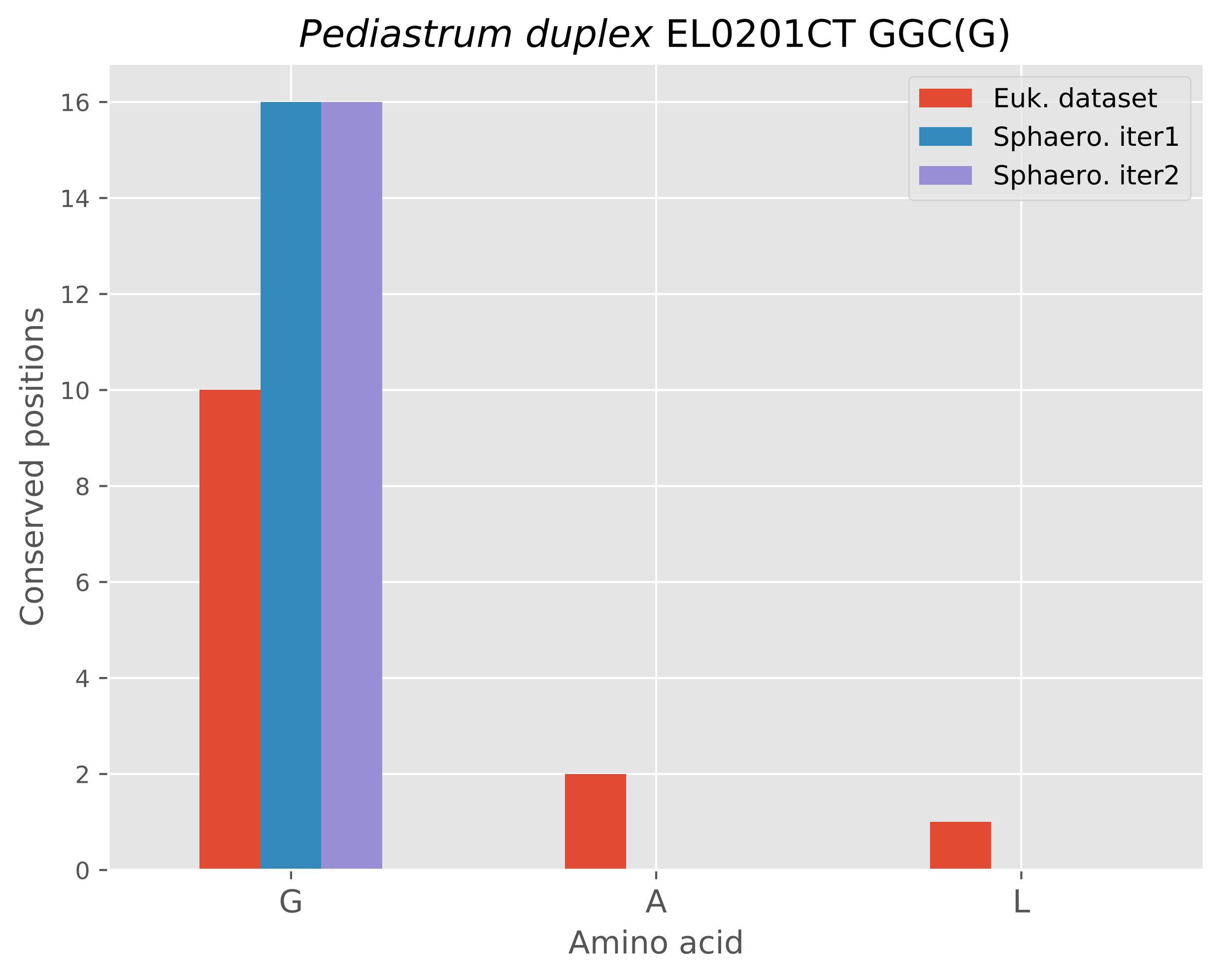
Pediastrum duplex EL0201CT GGC(G)
Conserved positions
Amino acid
Euk. dataset
Sphaero. iter1
Sphaero. iter2
G
A
L
0
2
4
6
8
10
12
14
16

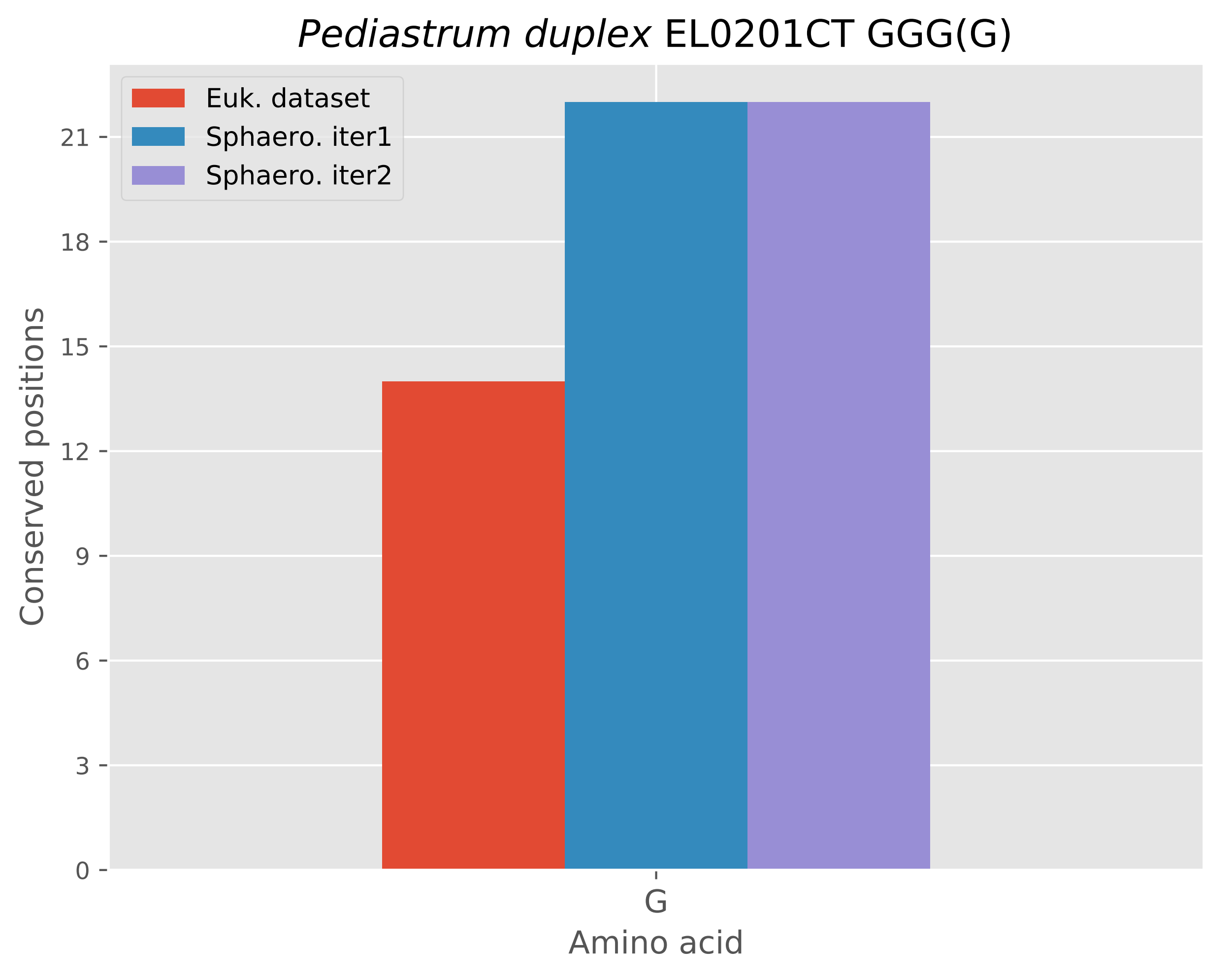

Pediastrum duplex EL0201CT GGG(G)
Euk. dataset
Sphaero. iter1
Sphaero. iter2
Conserved positions
0
3
6
9
12
15
18
21
G
Amino acid

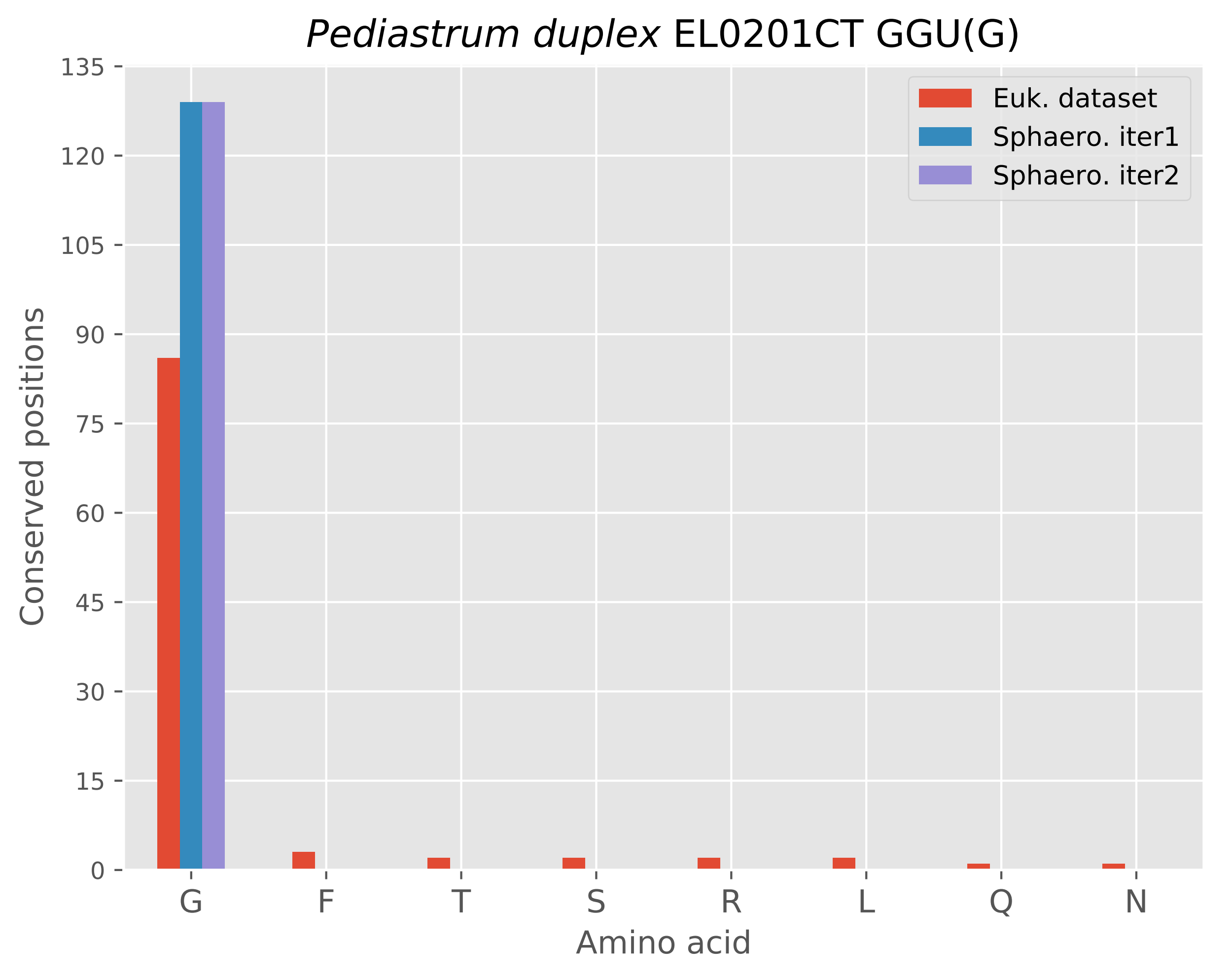

Pediastrum duplex EL0201CT GGU(G)
Euk. dataset
Sphaero. iter1
Sphaero. iter2
Conserved positions
0
15
30
45
60
75
90
105
120
135
G
F
T
S
R
L
Q
N
Amino acid

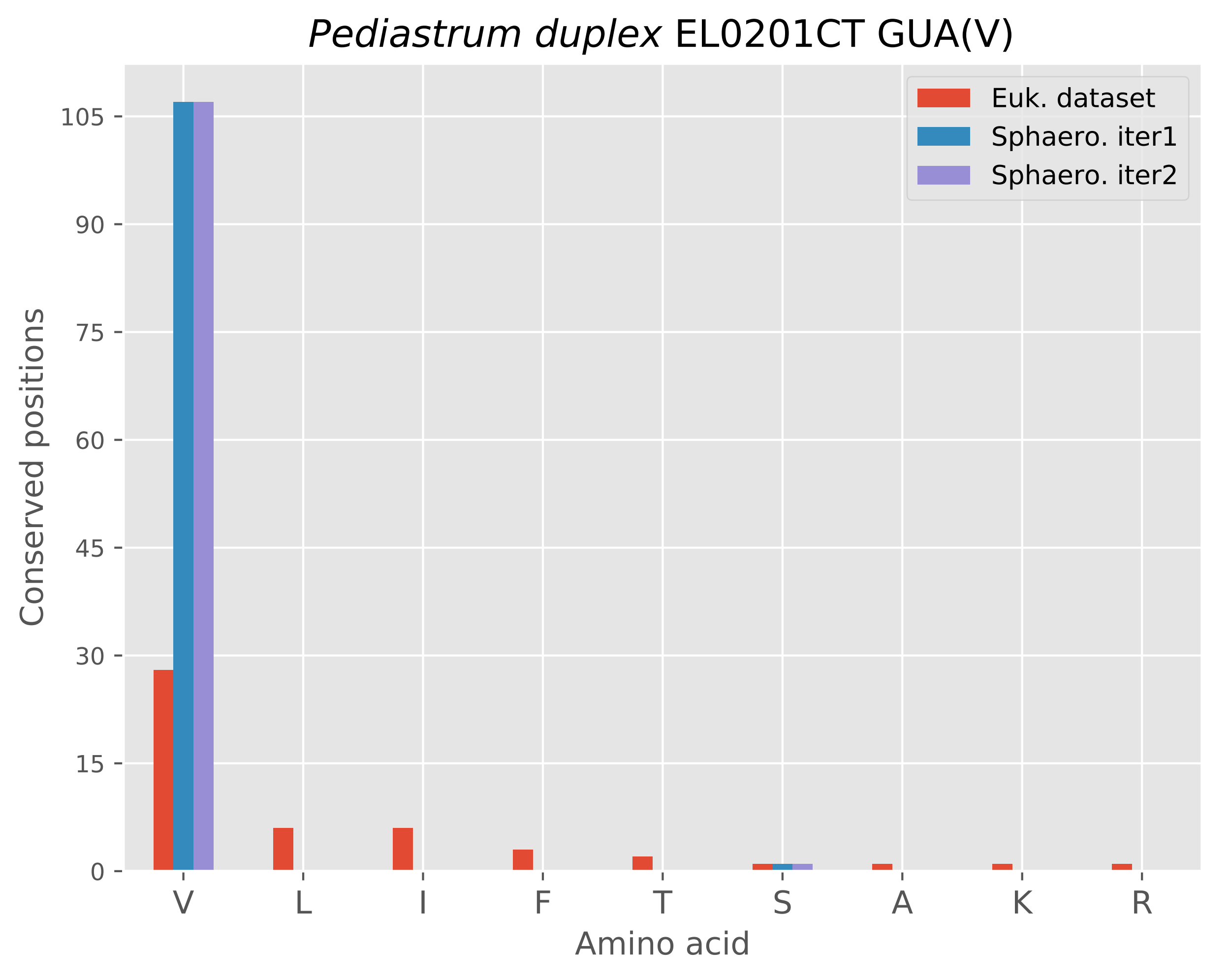

Pediastrum duplex EL0201CT GUA(V)
Euk. dataset
Sphaero. iter1
Sphaero. iter2
Conserved positions
0
15
30
45
60
75
90
105
V
L
I
F
T
S
A
K
R
Amino acid

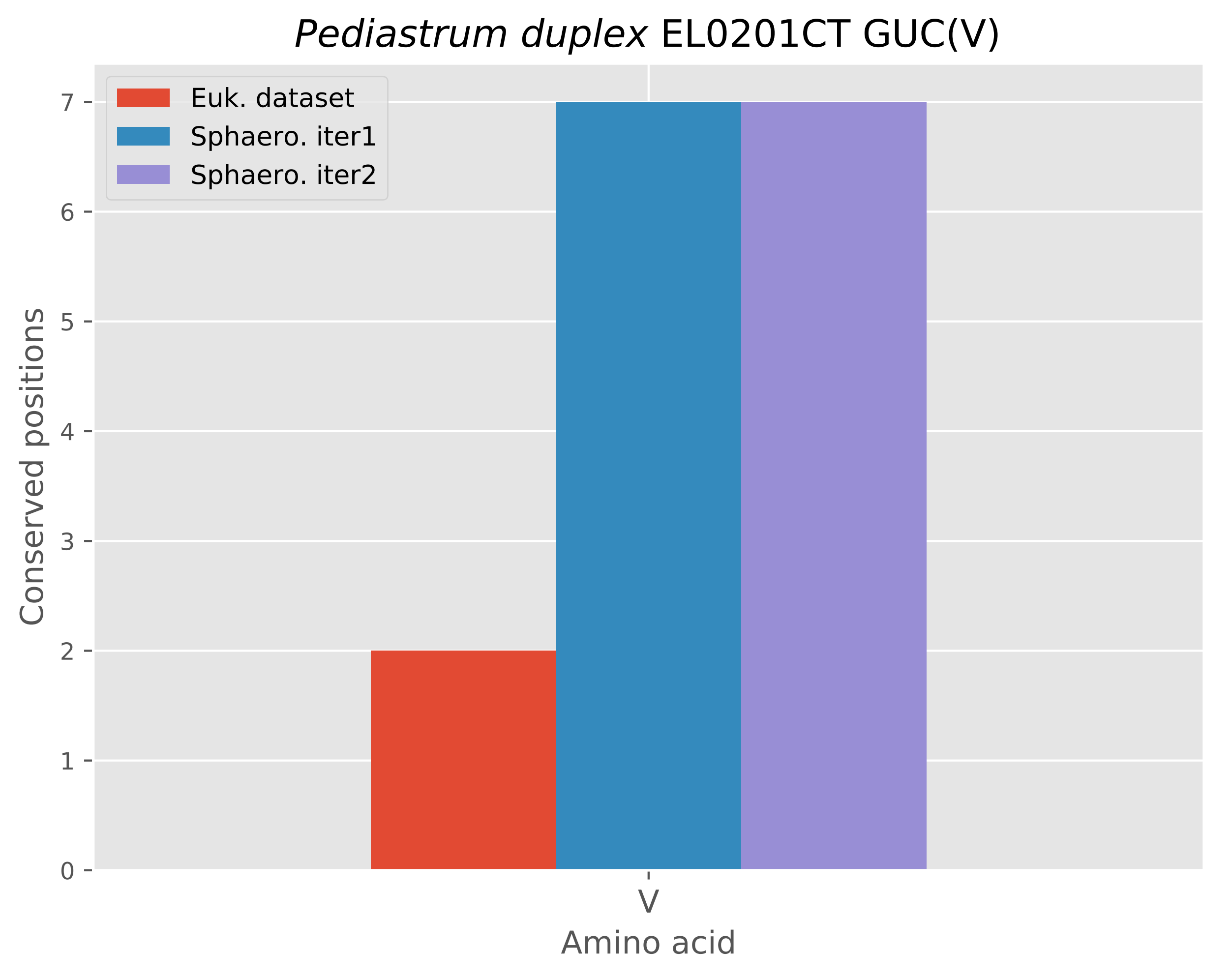

Pediastrum duplex EL0201CT GUC(V)
Euk. dataset
Sphaero. iter1
Sphaero. iter2
Conserved positions
0
1
2
3
4
5
6
7
V
Amino acid

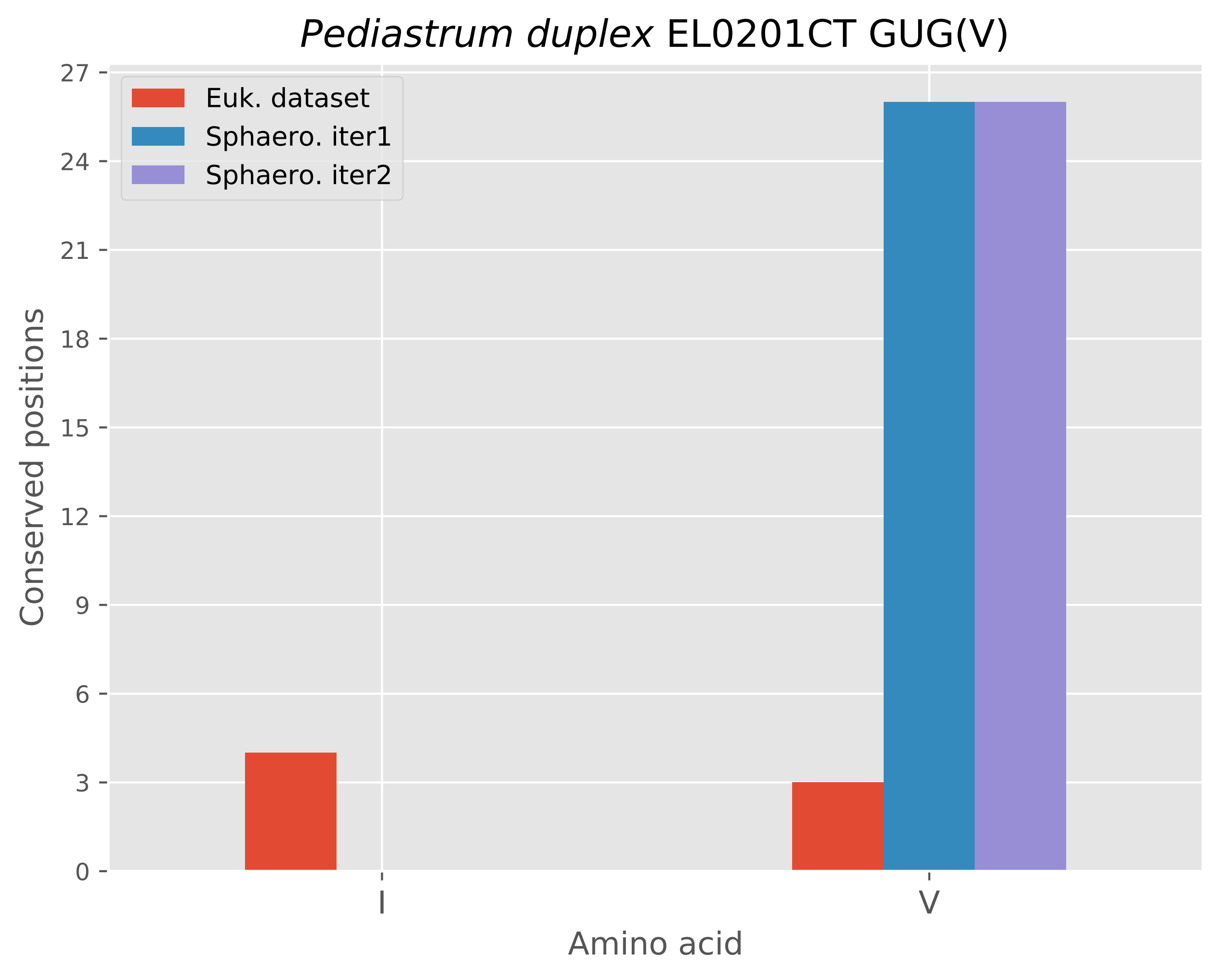

Pediastrum duplex EL0201CT GUG(V)
Euk. dataset
Sphaero. iter1
Sphaero. iter2
Conserved positions
0
3
6
9
12
15
18
21
24
27
I
V
Amino acid

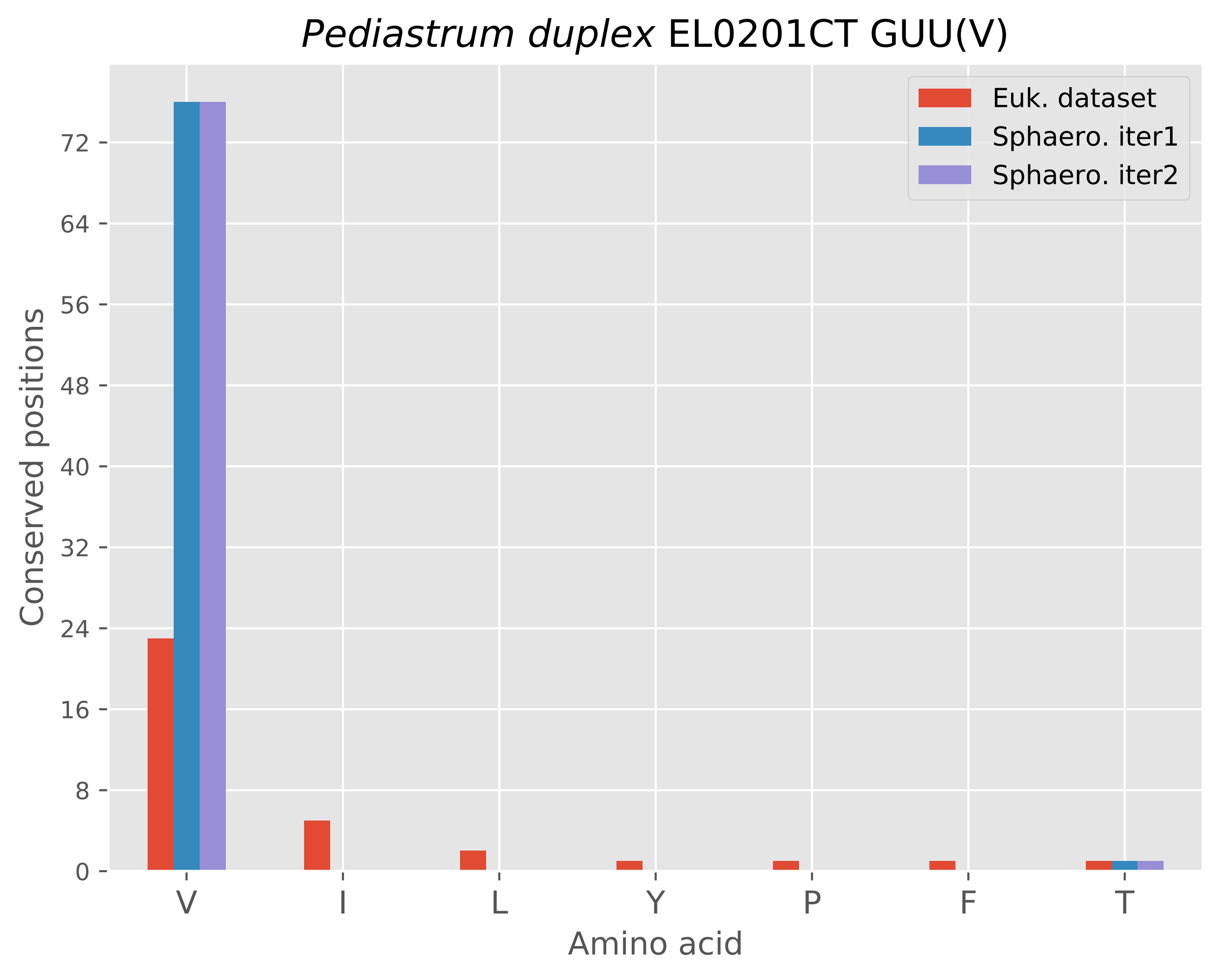

Pediastrum duplex EL0201CT GUU(V)
Euk. dataset
Sphaero. iter1
Sphaero. iter2
Conserved positions
0
8
16
24
32
40
48
56
64
72
V
I
L
Y
P
F
T
Amino acid

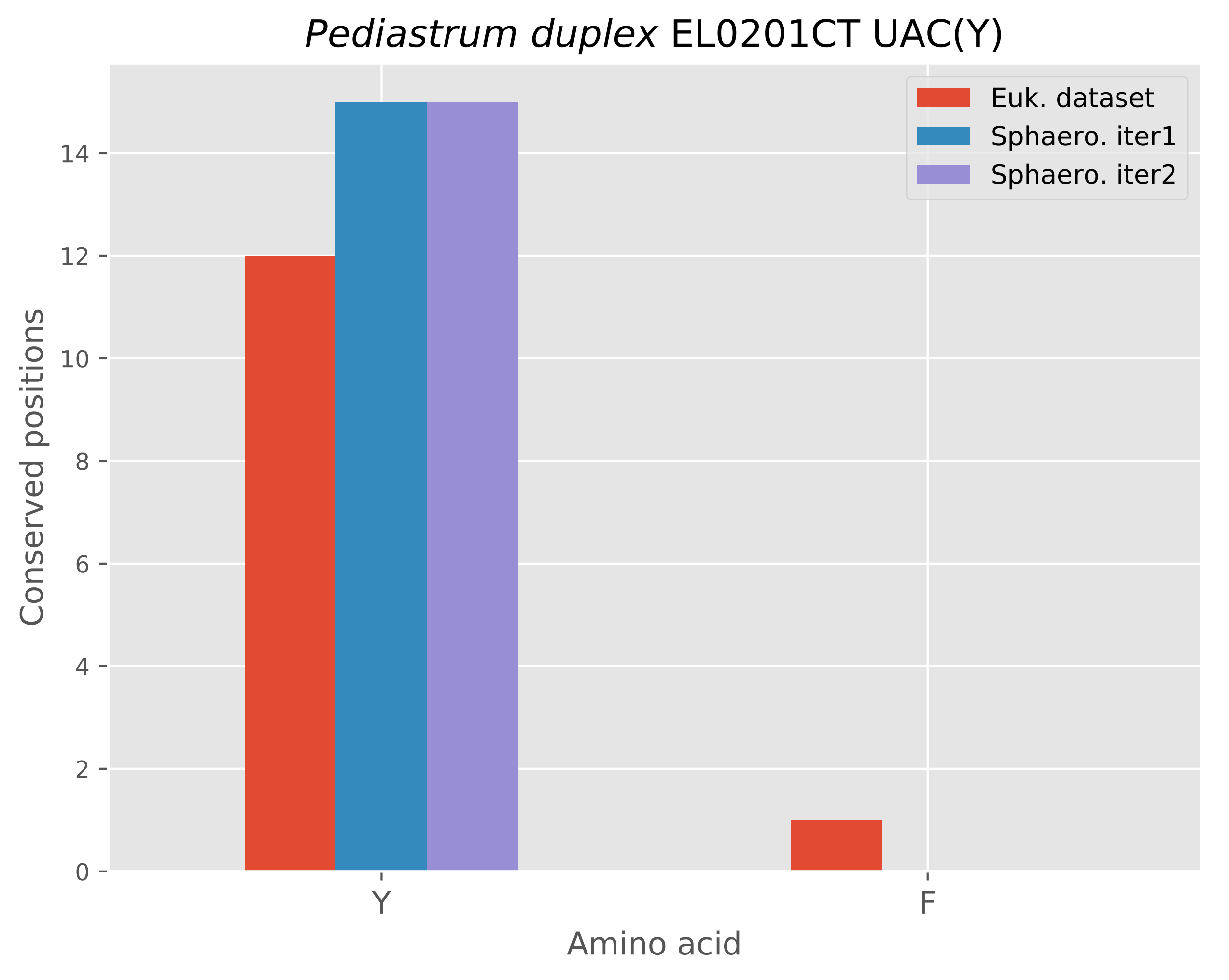

Pediastrum duplex EL0201CT UAC(Y)
Euk. dataset
Sphaero. iter1
Sphaero. iter2
Conserved positions
0
2
4
6
8
10
12
14
Y
F
Amino acid

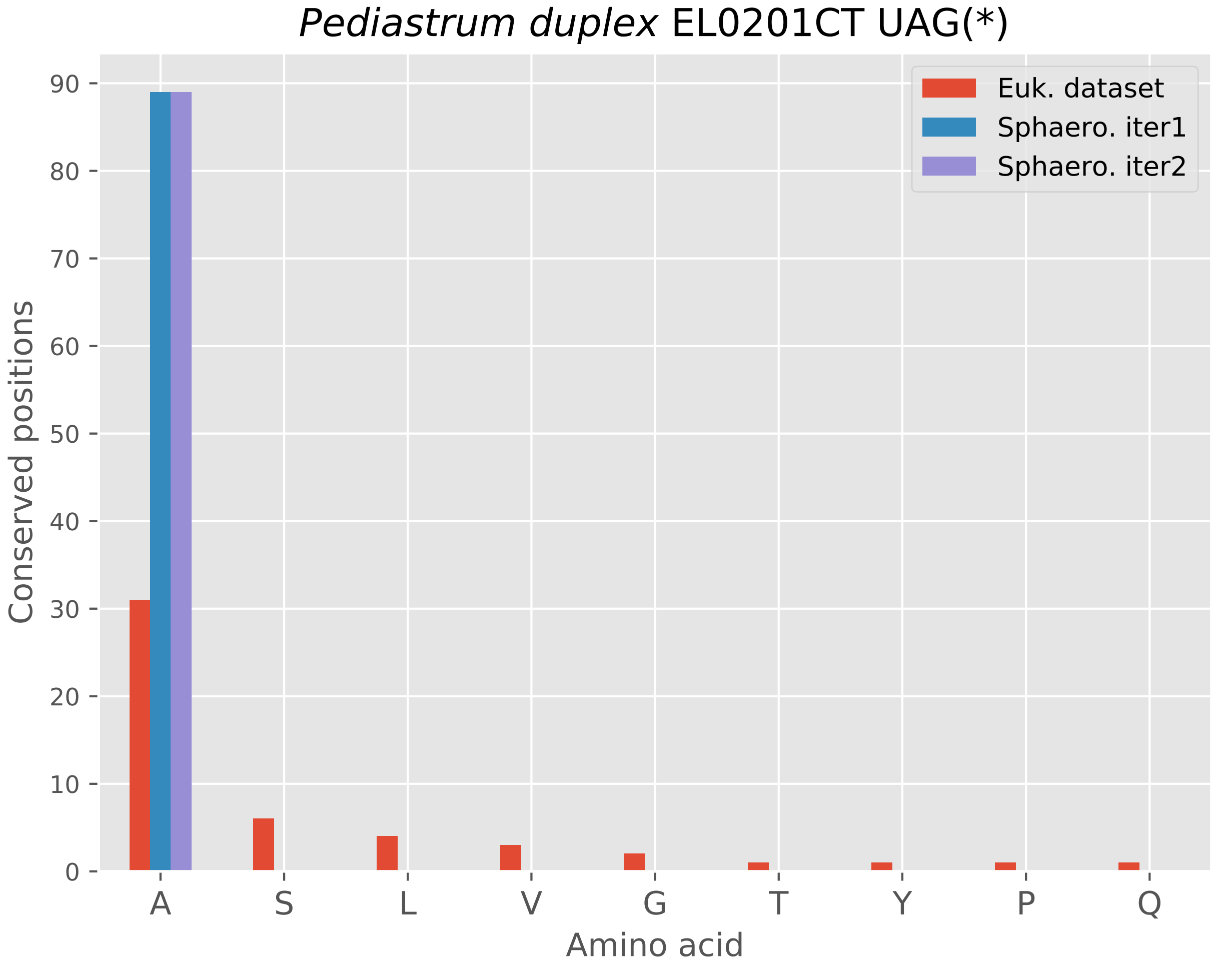
Pediastrum duplex EL0201CT UAG(*)
Euk. dataset
Sphaero. iter1
Sphaero. iter2
Conserved positions
0
10
20
30
40
50
60
70
80
90
A
S
L
V
G
T
Y
P
Q
Amino acid

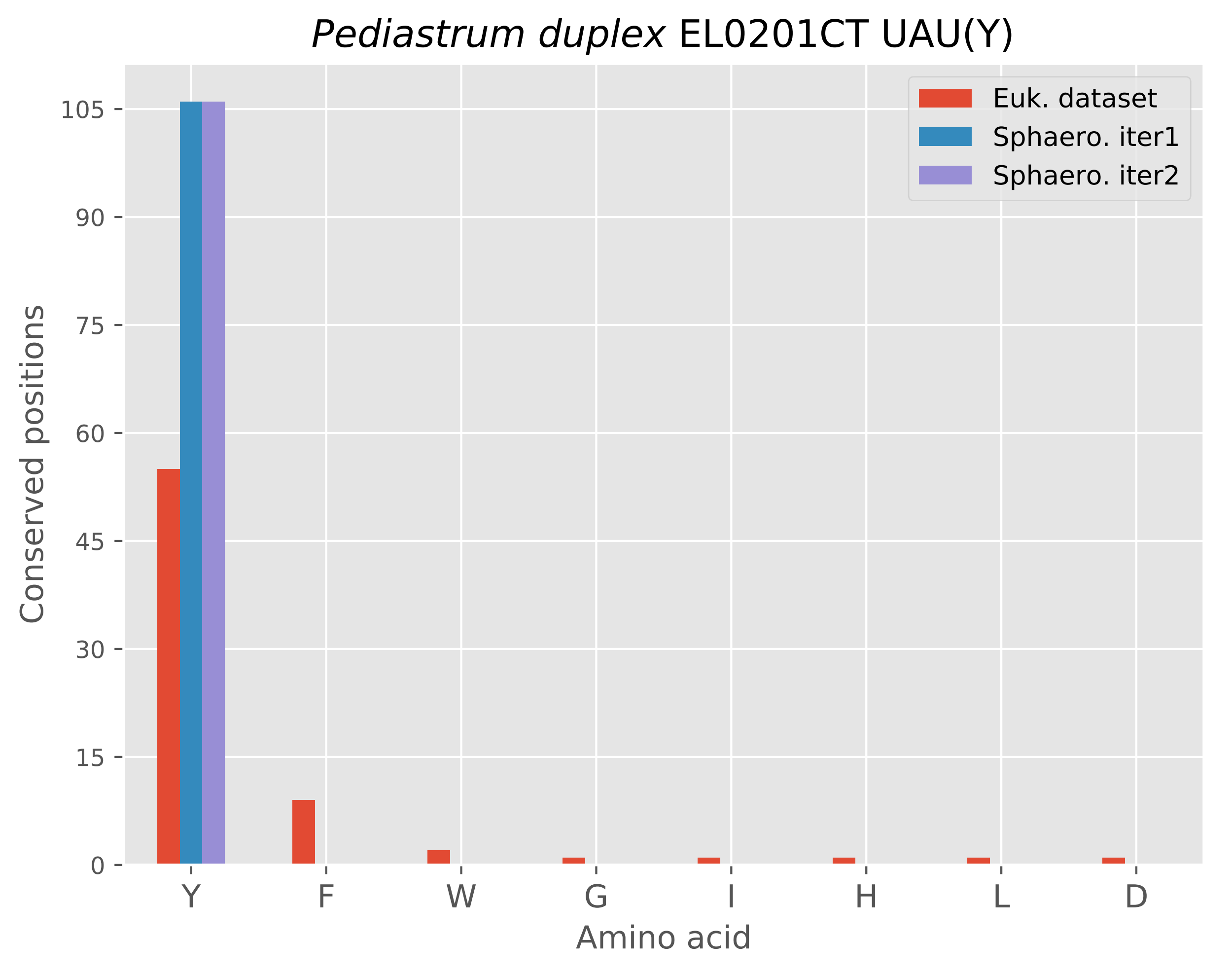

Pediastrum duplex EL0201CT UAU(Y)
Euk. dataset
Sphaero. iter1
Sphaero. iter2
Conserved positions
105
90
75
60
45
30
15
0
Y
F
W
G
I
H
L
D
Amino acid

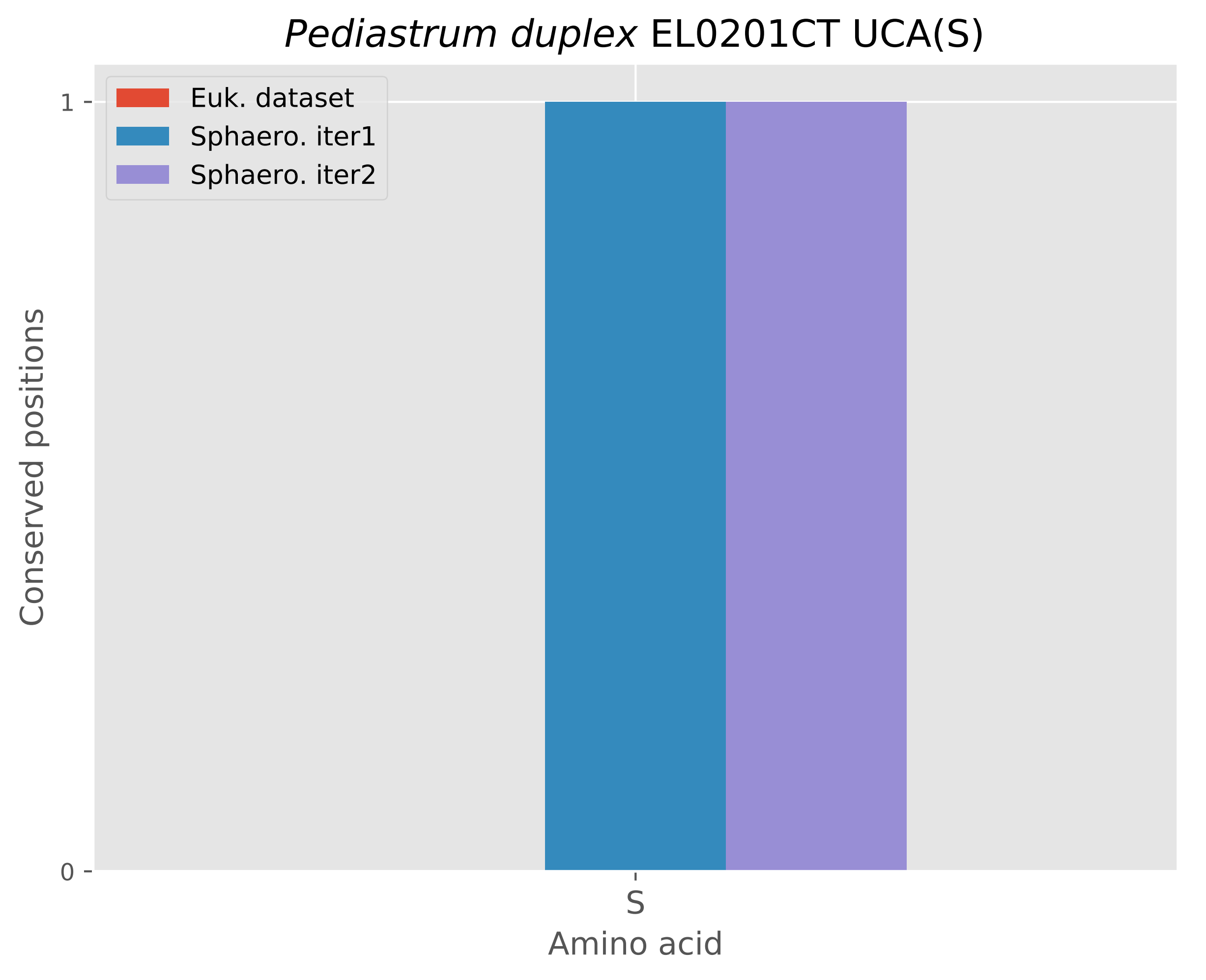

Pediastrum duplex EL0201CT UCA(S)
Euk. dataset
Sphaero. iter1
Sphaero. iter2
1
0
Conserved positions
S
Amino acid

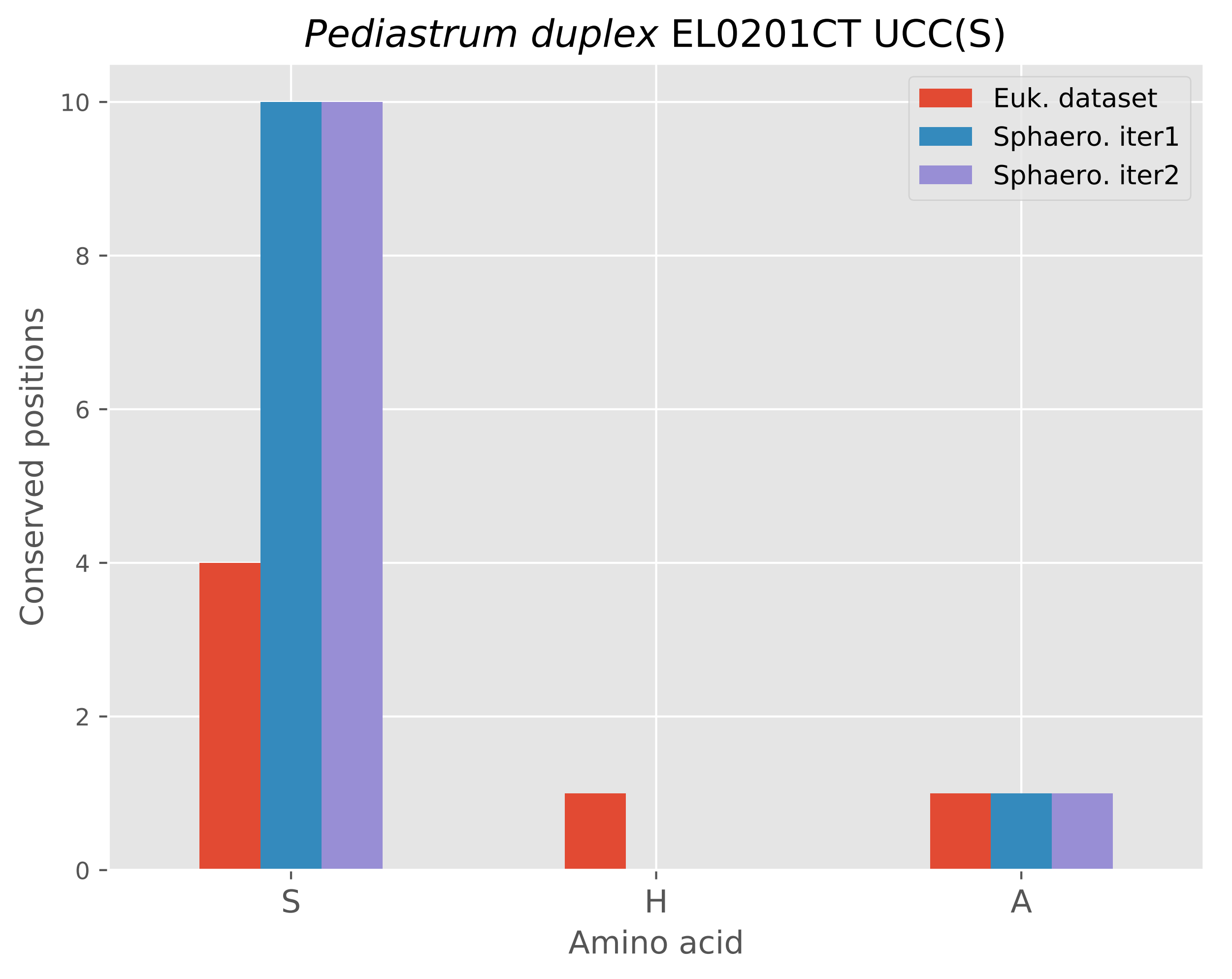

Pediastrum duplex EL0201CT UCC(S)
Conserved positions
0
2
4
6
8
10
S
H
A
Amino acid
Euk. dataset
Sphaero. iter1
Sphaero. iter2

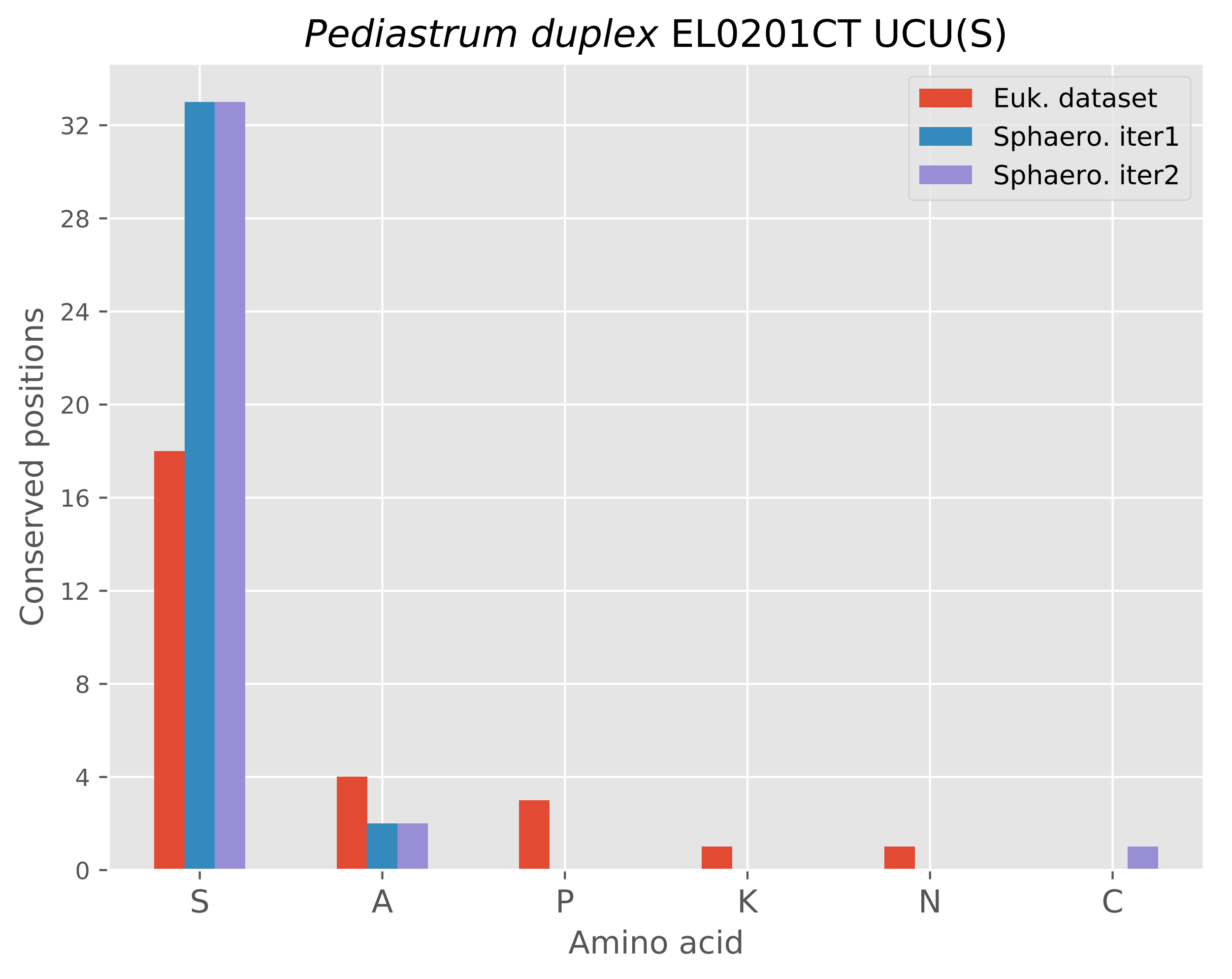

Pediastrum duplex EL0201CT UCU(S)
Euk. dataset
Sphaero. iter1
Sphaero. iter2
Conserved positions
0
4
8
12
16
20
24
28
32
S
A
P
K
N
C
Amino acid

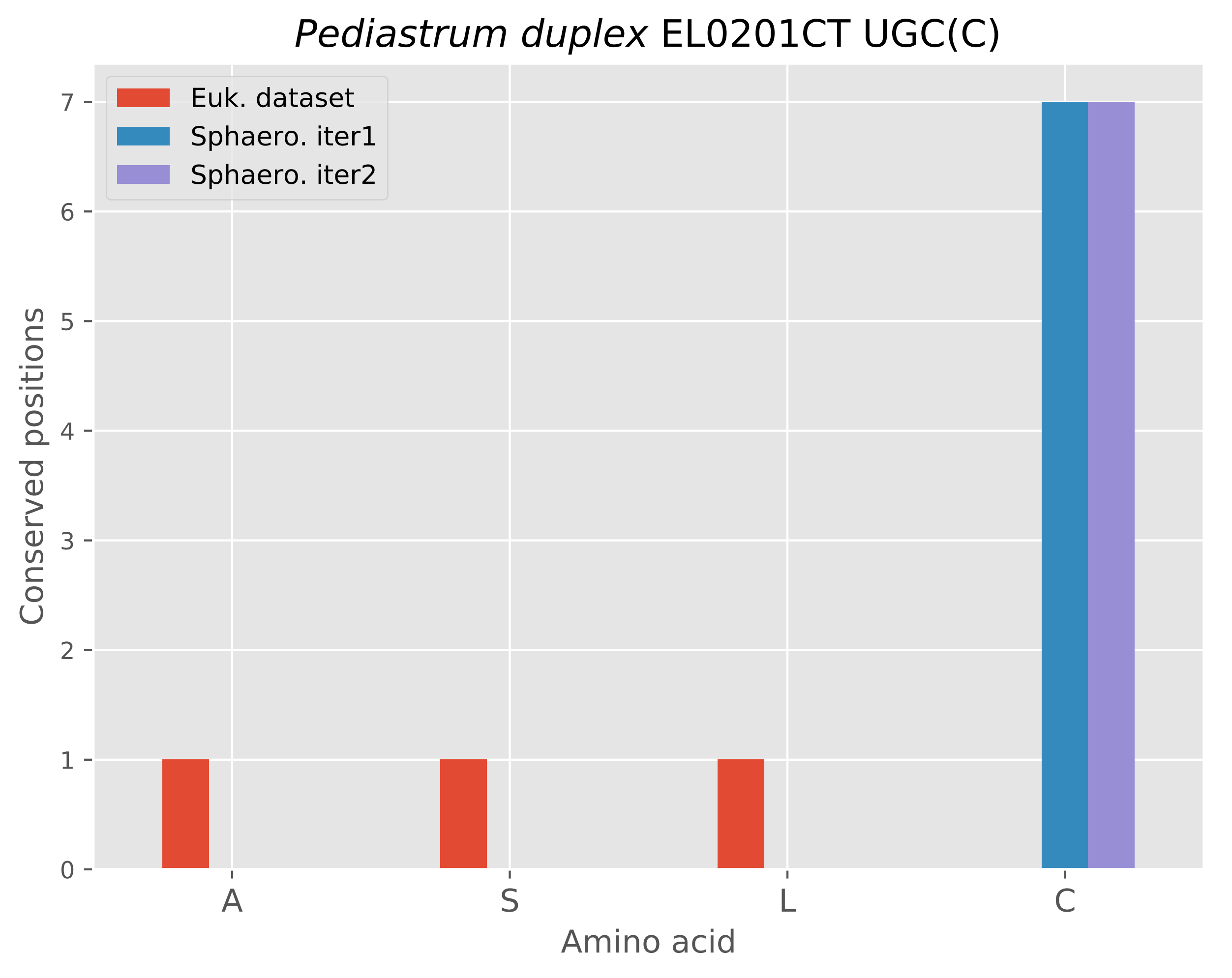

Pediastrum duplex EL0201CT UGC(C)
Euk. dataset
Sphaero. iter1
Sphaero. iter2
Conserved positions
0
1
2
3
4
5
6
7
A
S
L
C
Amino acid

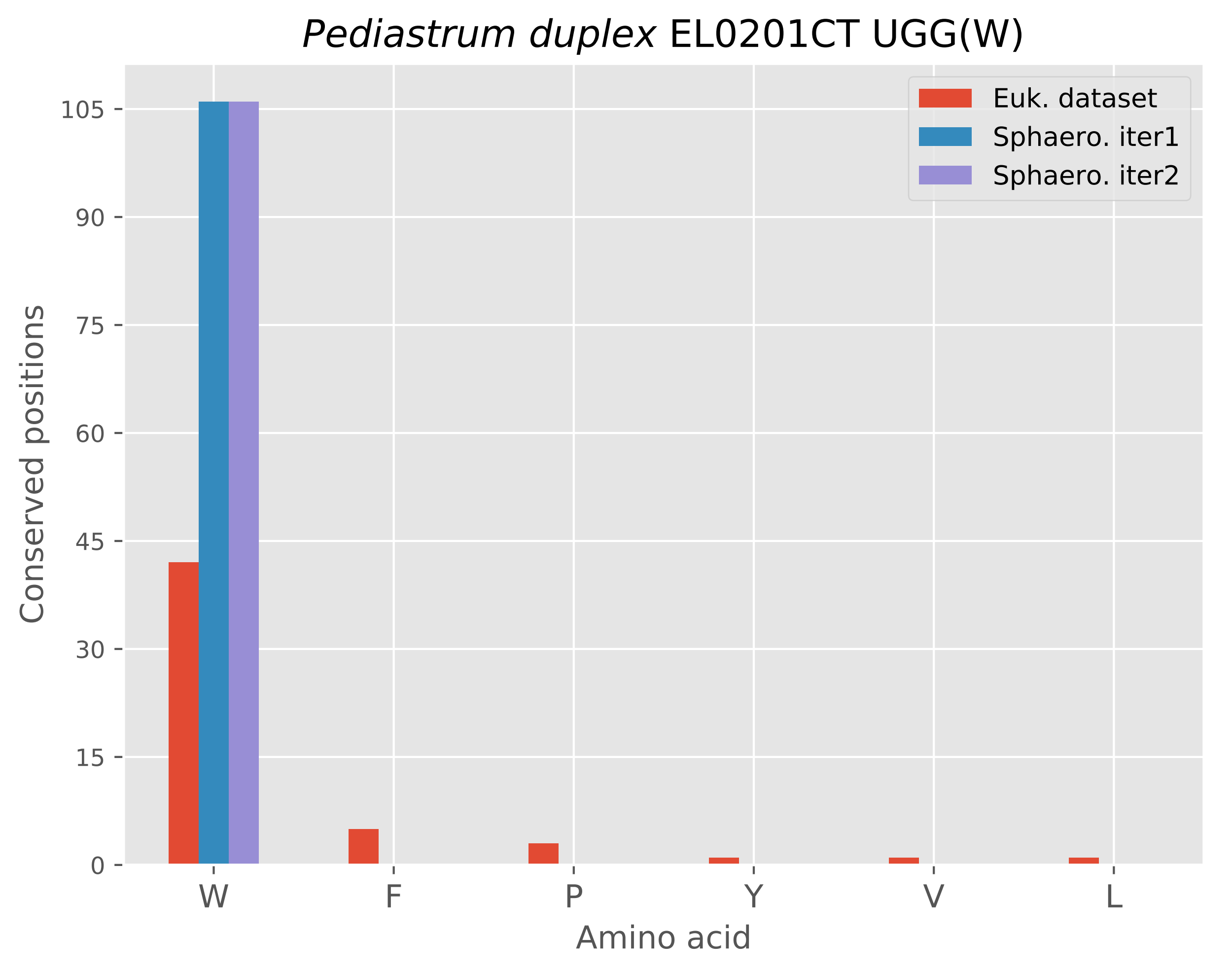

Pediastrum duplex EL0201CT UGG(W)
Euk. dataset
Sphaero. iter1
Sphaero. iter2
Conserved positions
0
15
30
45
60
75
90
105
W
F
P
Y
V
L
Amino acid

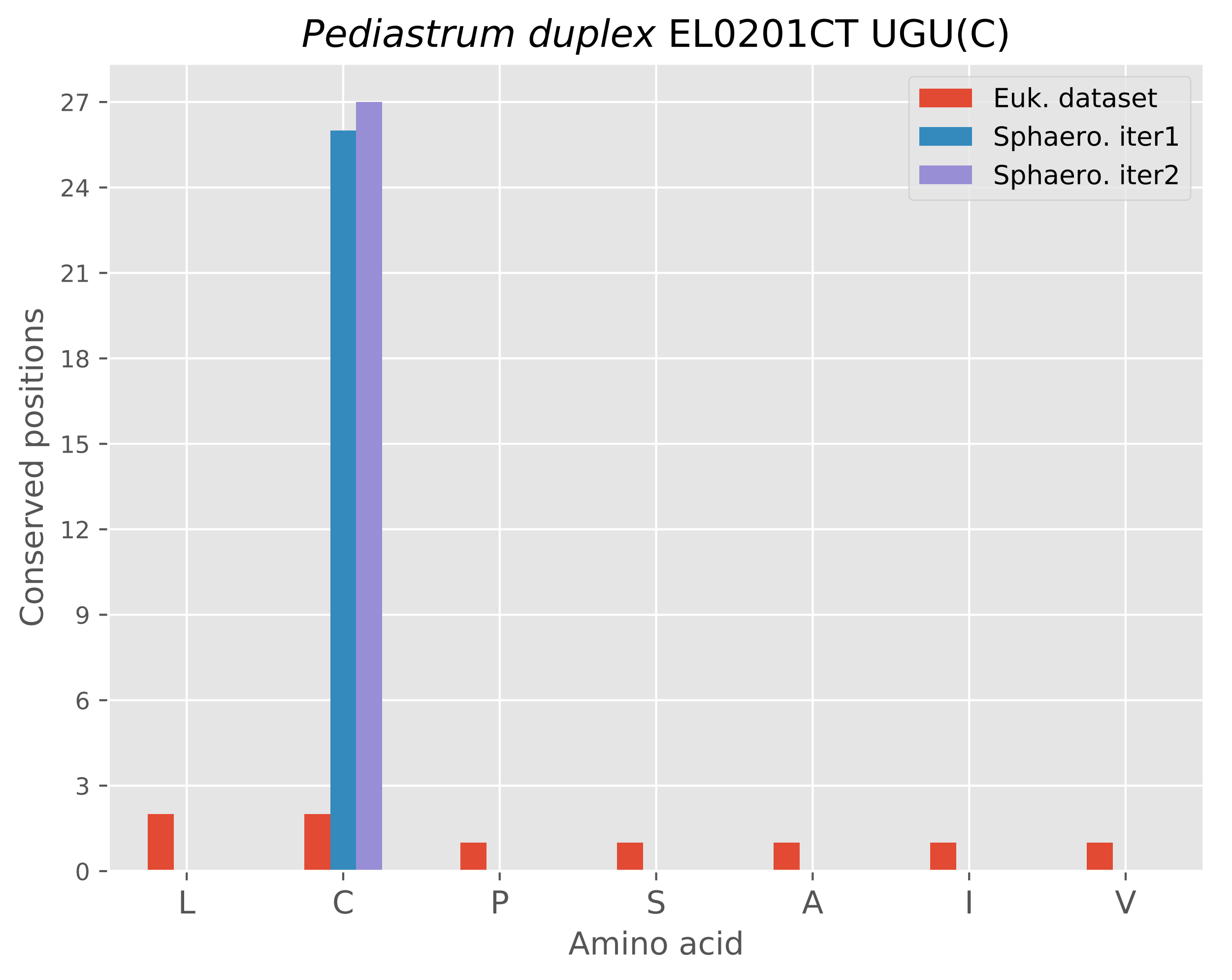

Pediastrum duplex EL0201CT UGU(C)
Euk. dataset
Sphaero. iter1
Sphaero. iter2
Conserved positions
0
3
6
9
12
15
18
21
24
27
L
C
P
S
A
I
V
Amino acid

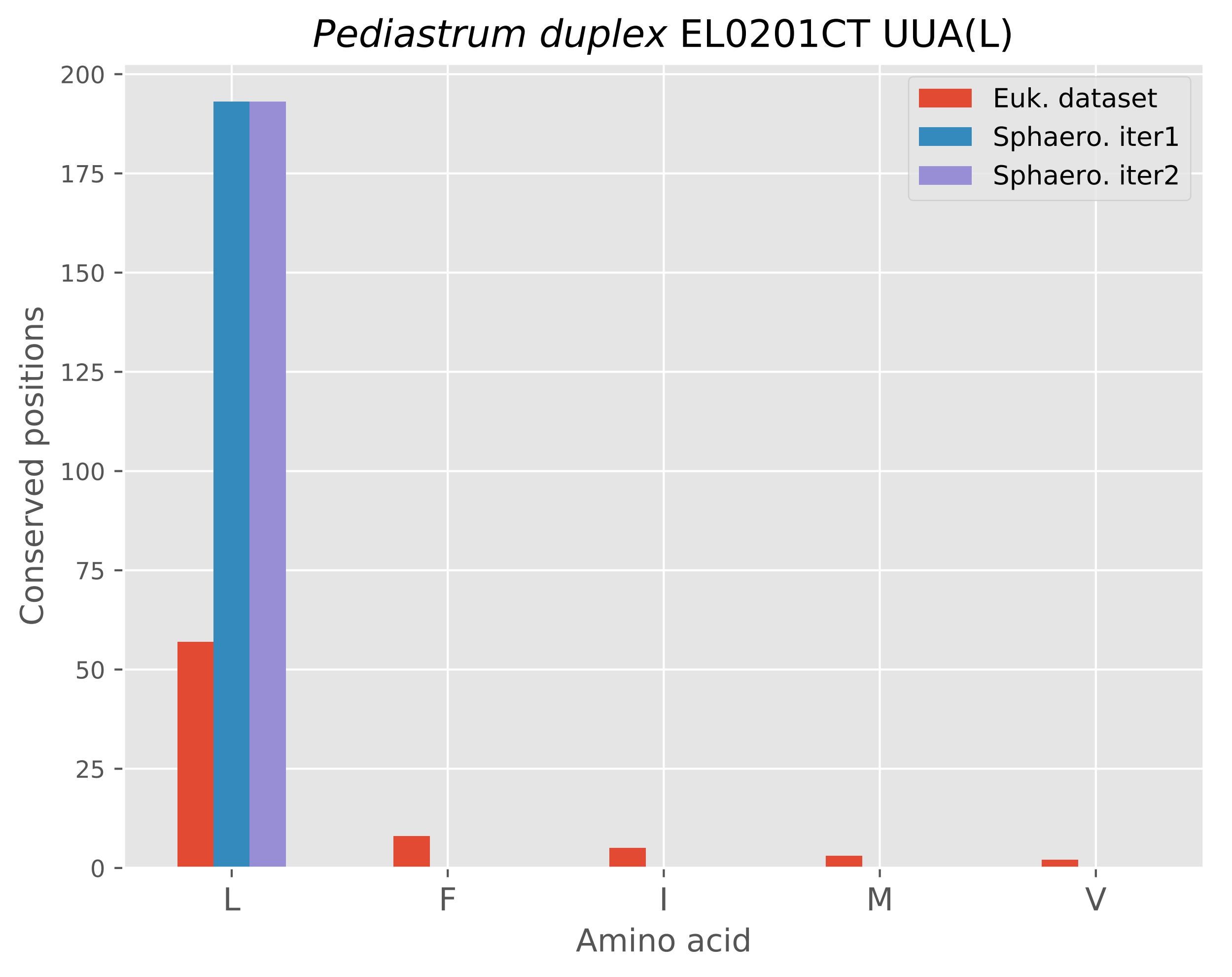

Pediastrum duplex EL0201CT UUA(L)
Euk. dataset
Sphaero. iter1
Sphaero. iter2
Conserved positions
0
25
50
75
100
125
150
175
200
L
F
I
M
V
Amino acid

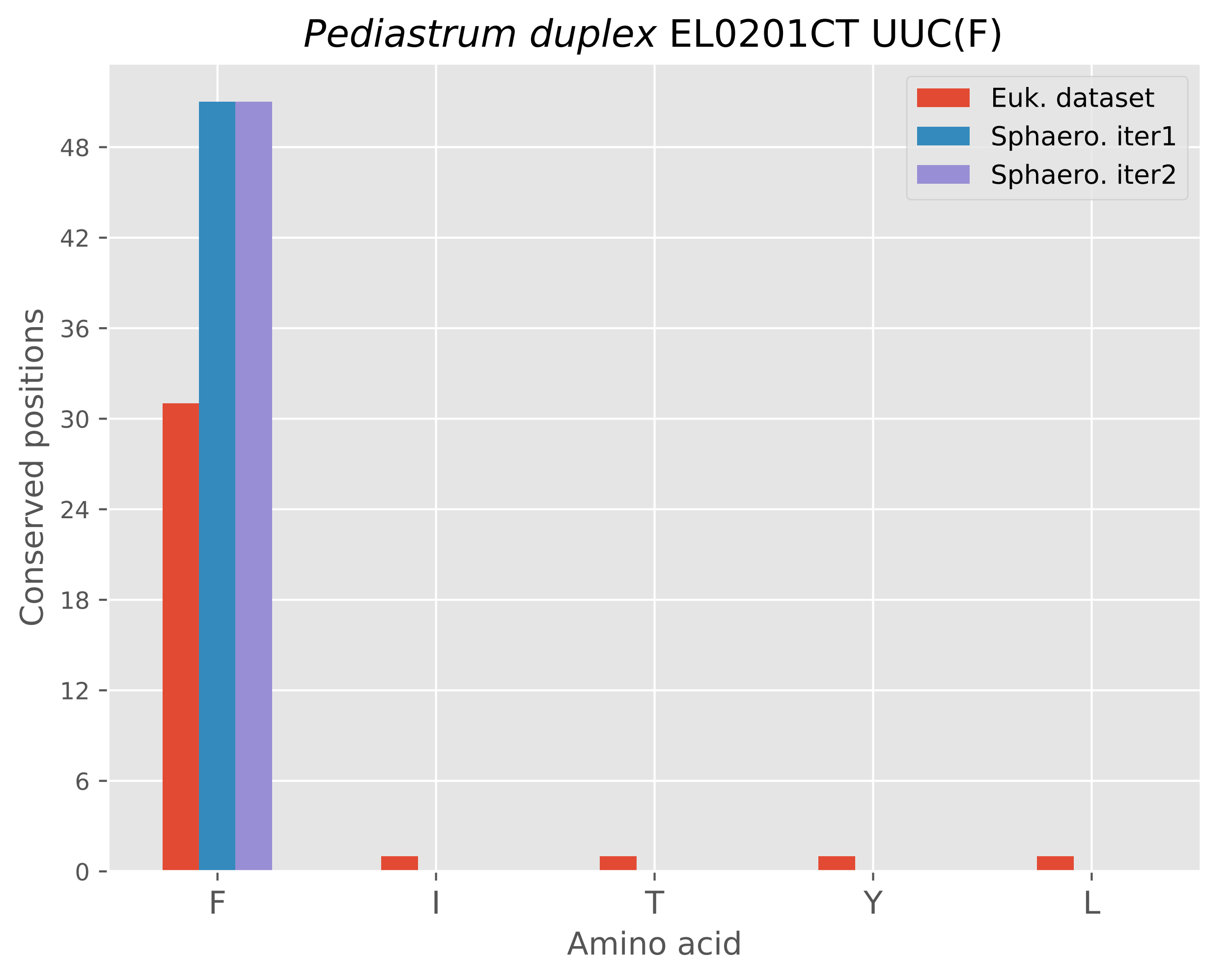

Pediastrum duplex EL0201CT UUC(F)
Euk. dataset
Sphaero. iter1
Sphaero. iter2
Conserved positions
0
6
12
18
24
30
36
42
48
F
I
T
Y
L
Amino acid

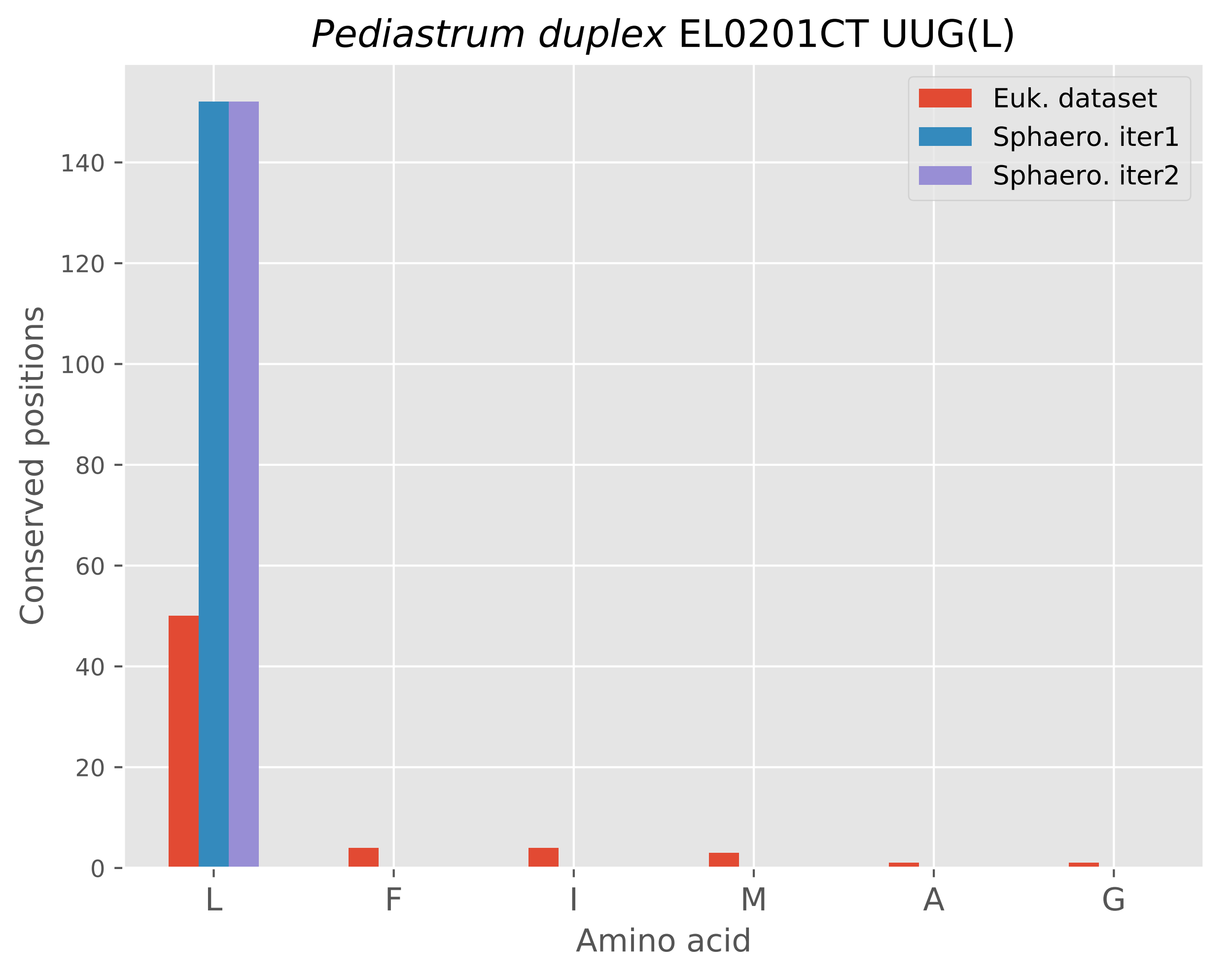

Pediastrum duplex EL0201CT UUG(L)
Euk. dataset
Sphaero. iter1
Sphaero. iter2
Conserved positions
0
20
40
60
80
100
120
140
L
F
I
M
A
G
Amino acid

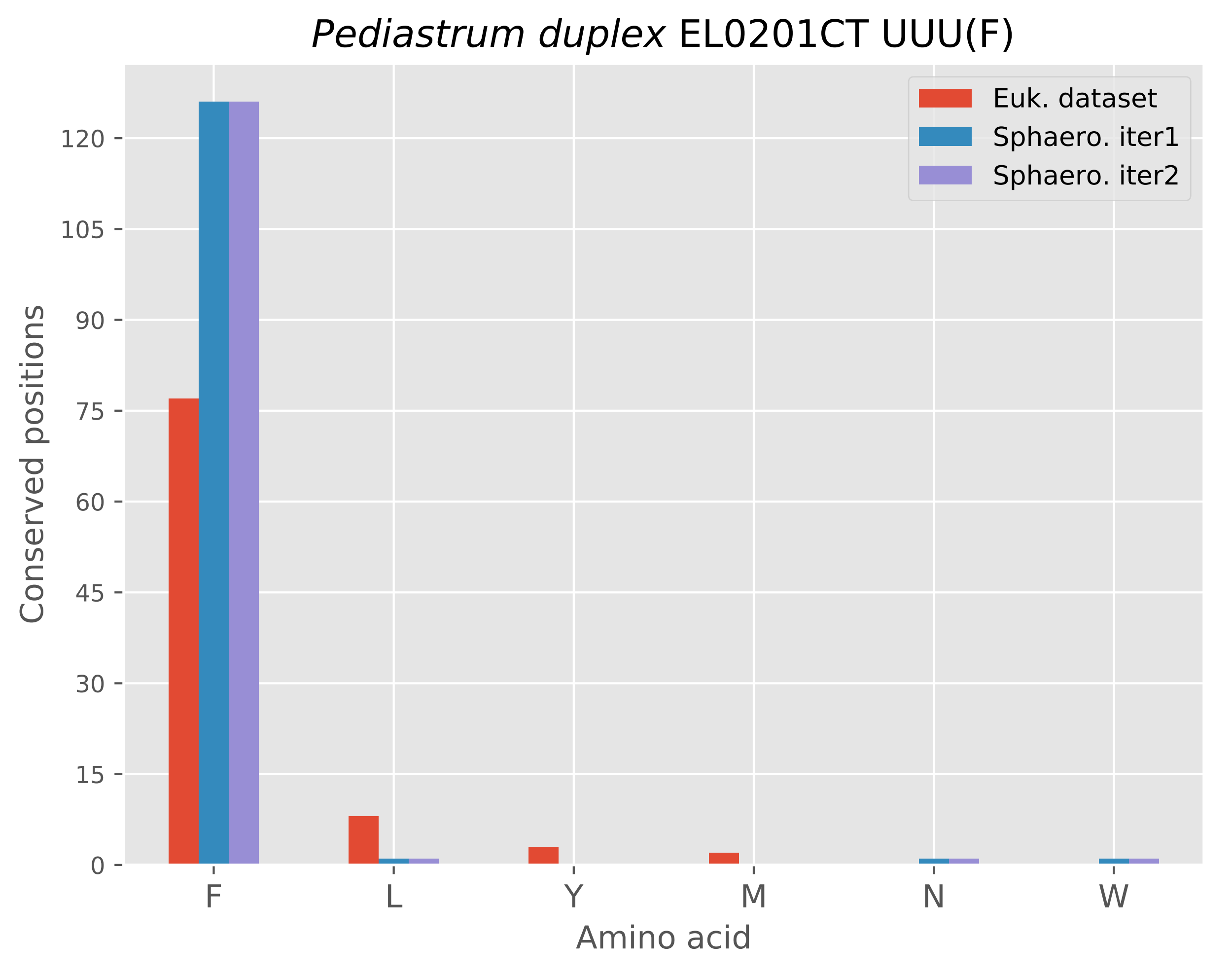

Pediastrum duplex EL0201CT UUU(F)
Euk. dataset
Sphaero. iter1
Sphaero. iter2
Conserved positions
0
15
30
45
60
75
90
105
120
F
L
Y
M
N
W
Amino acid

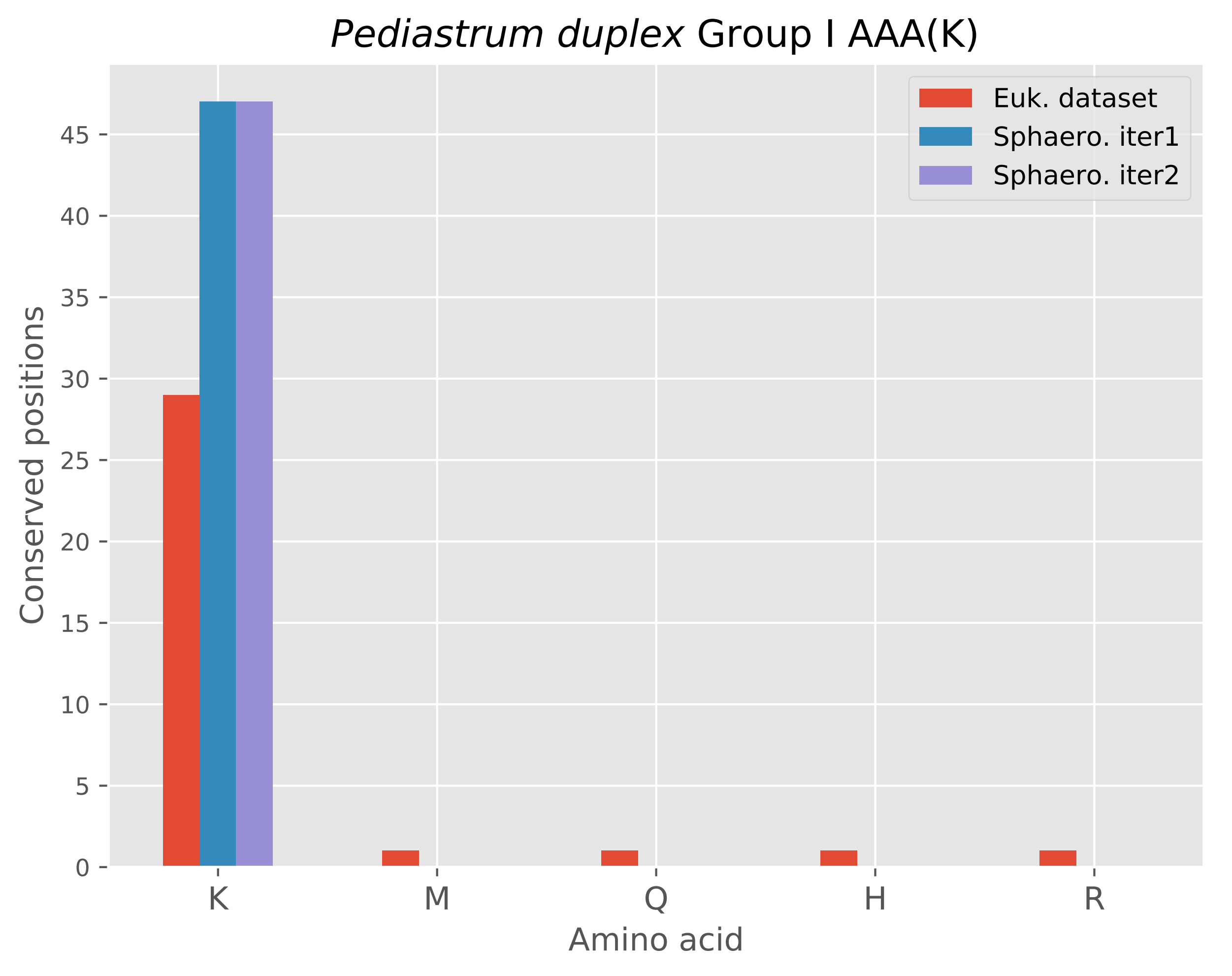
Pediastrum duplex Group I AAA(K)
Euk. dataset
Sphaero. iter1
Sphaero. iter2
Conserved positions
0
5
10
15
20
25
30
35
40
45
K
M
Q
H
R
Amino acid

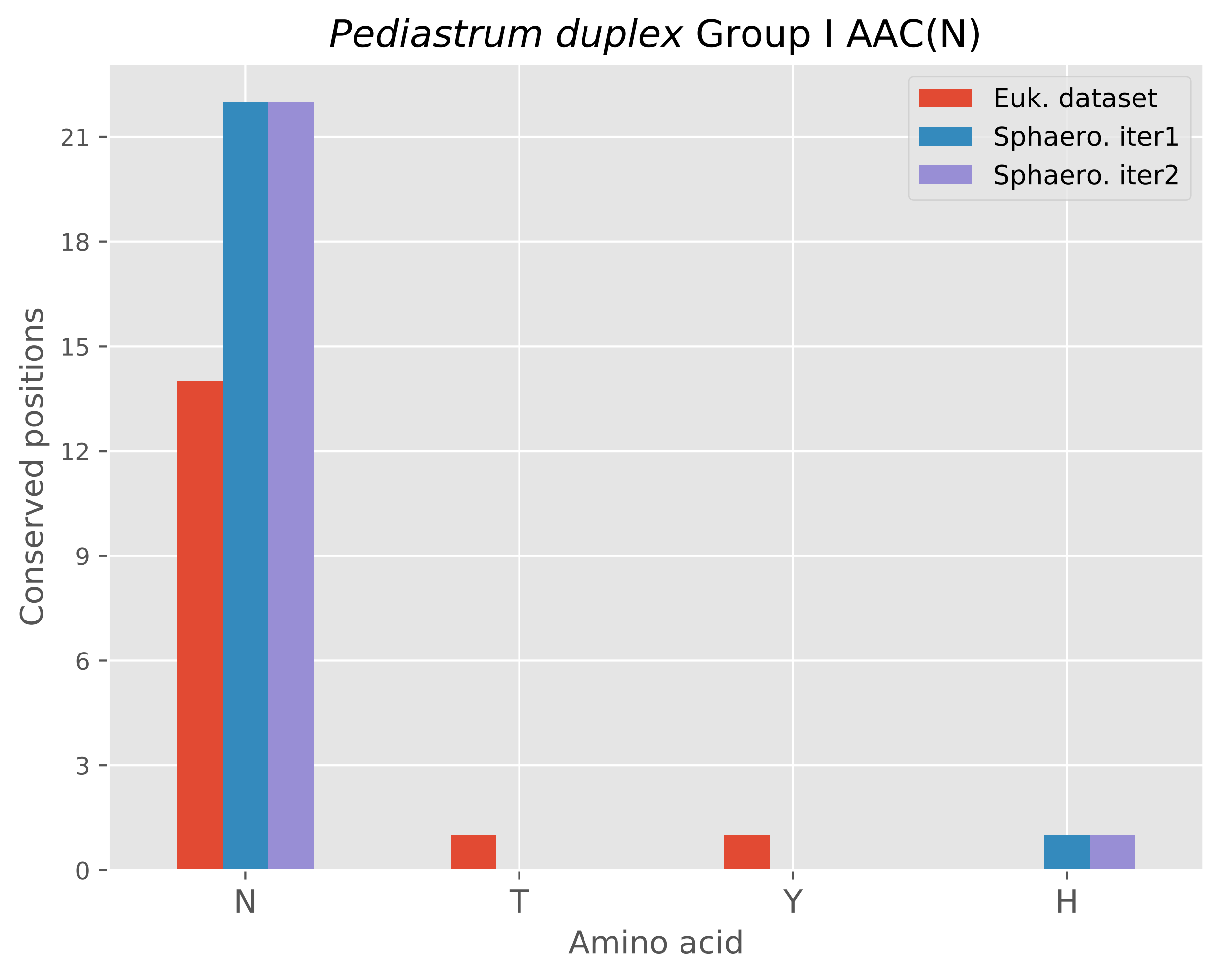

Pediastrum duplex Group I AAC(N)
Euk. dataset
Sphaero. iter1
Sphaero. iter2
Conserved positions
0
3
6
9
12
15
18
21
N
T
Y
H
Amino acid

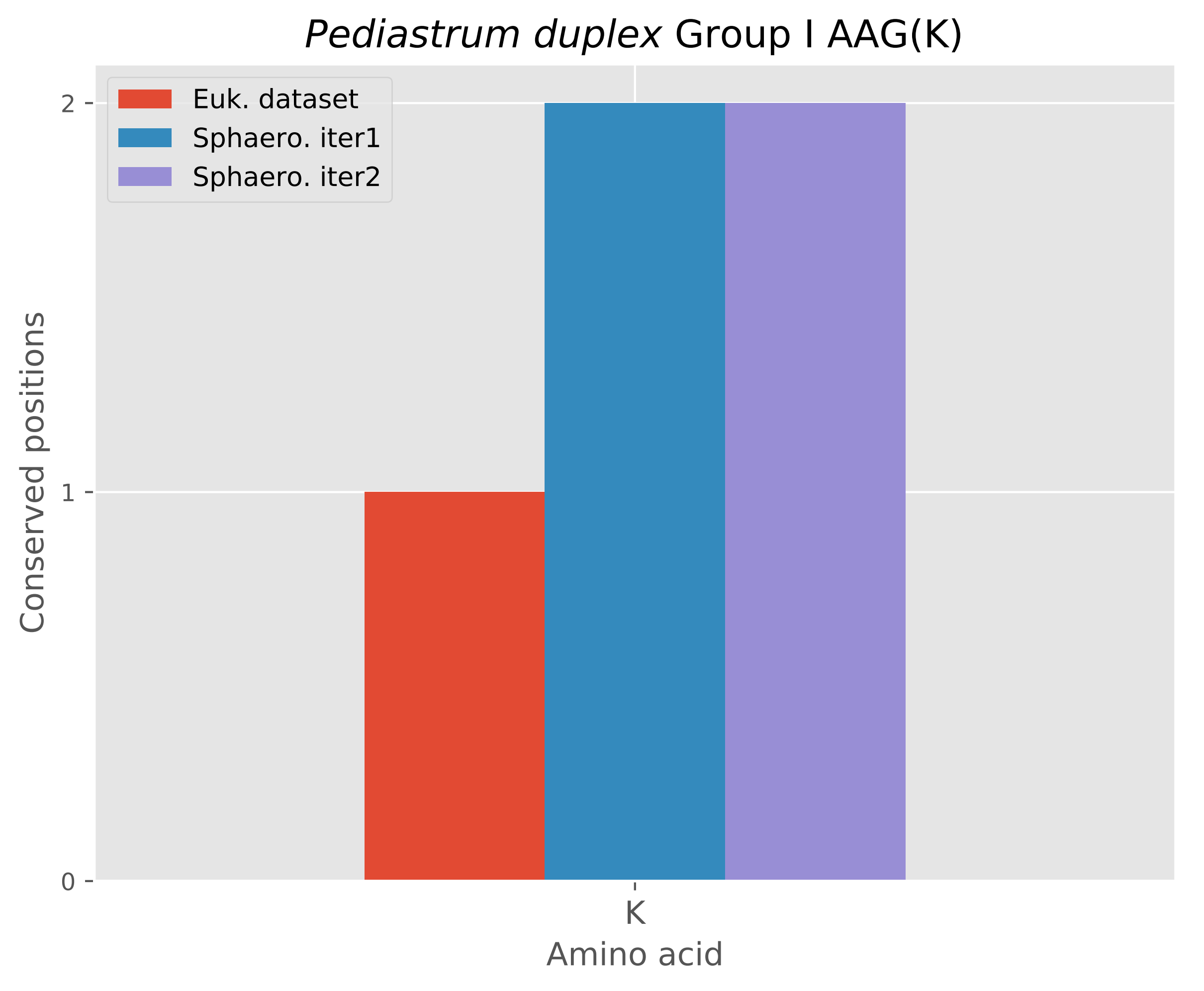
Pediastrum duplex Group I AAG(K)
Euk. dataset
Sphaero. iter1
Sphaero. iter2
Conserved positions
2
1
0
K
Amino acid

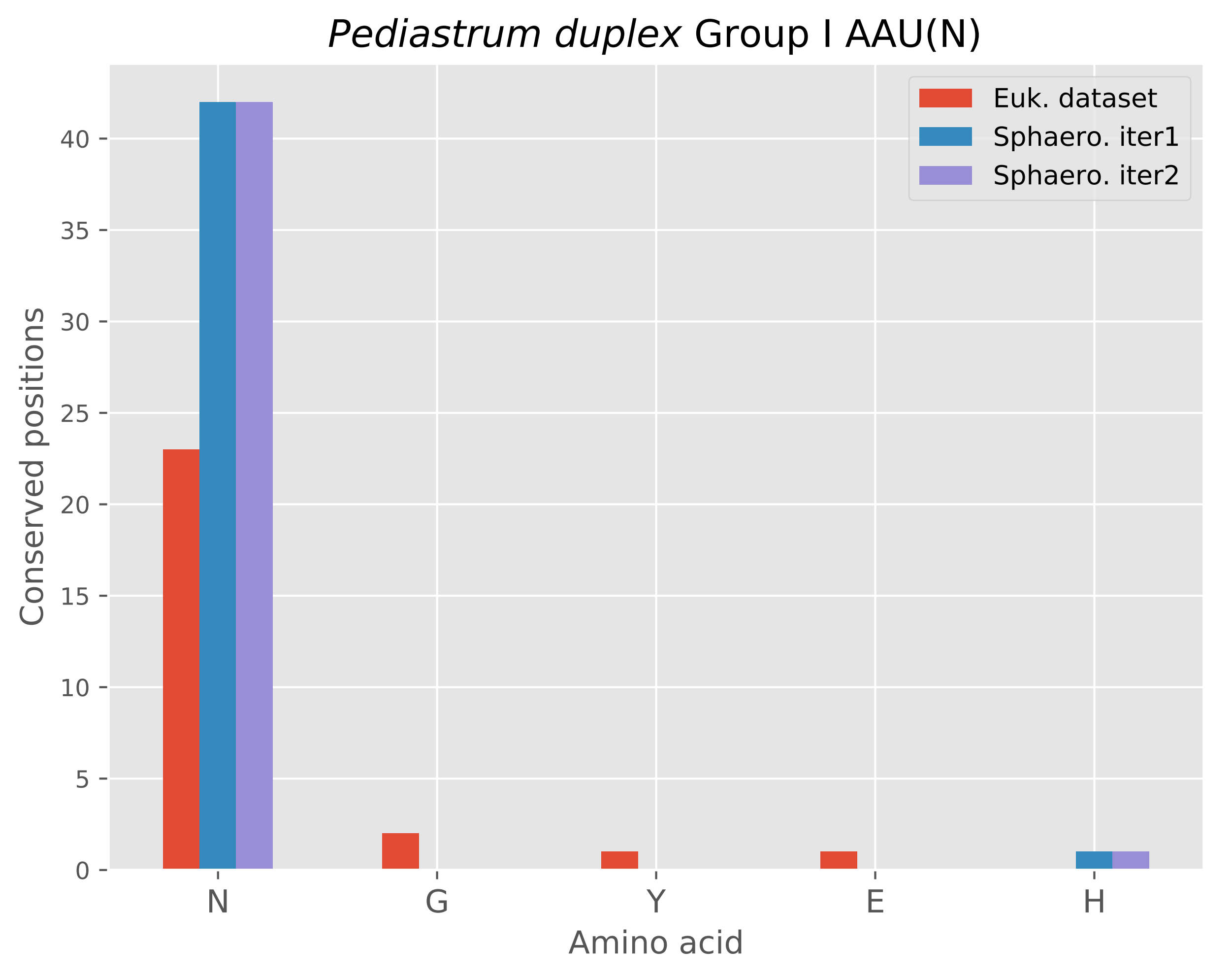

Pediastrum duplex Group I AAU(N)
Euk. dataset
Sphaero. iter1
Sphaero. iter2
Conserved positions
0
5
10
15
20
25
30
35
40
N
G
Y
E
H
Amino acid

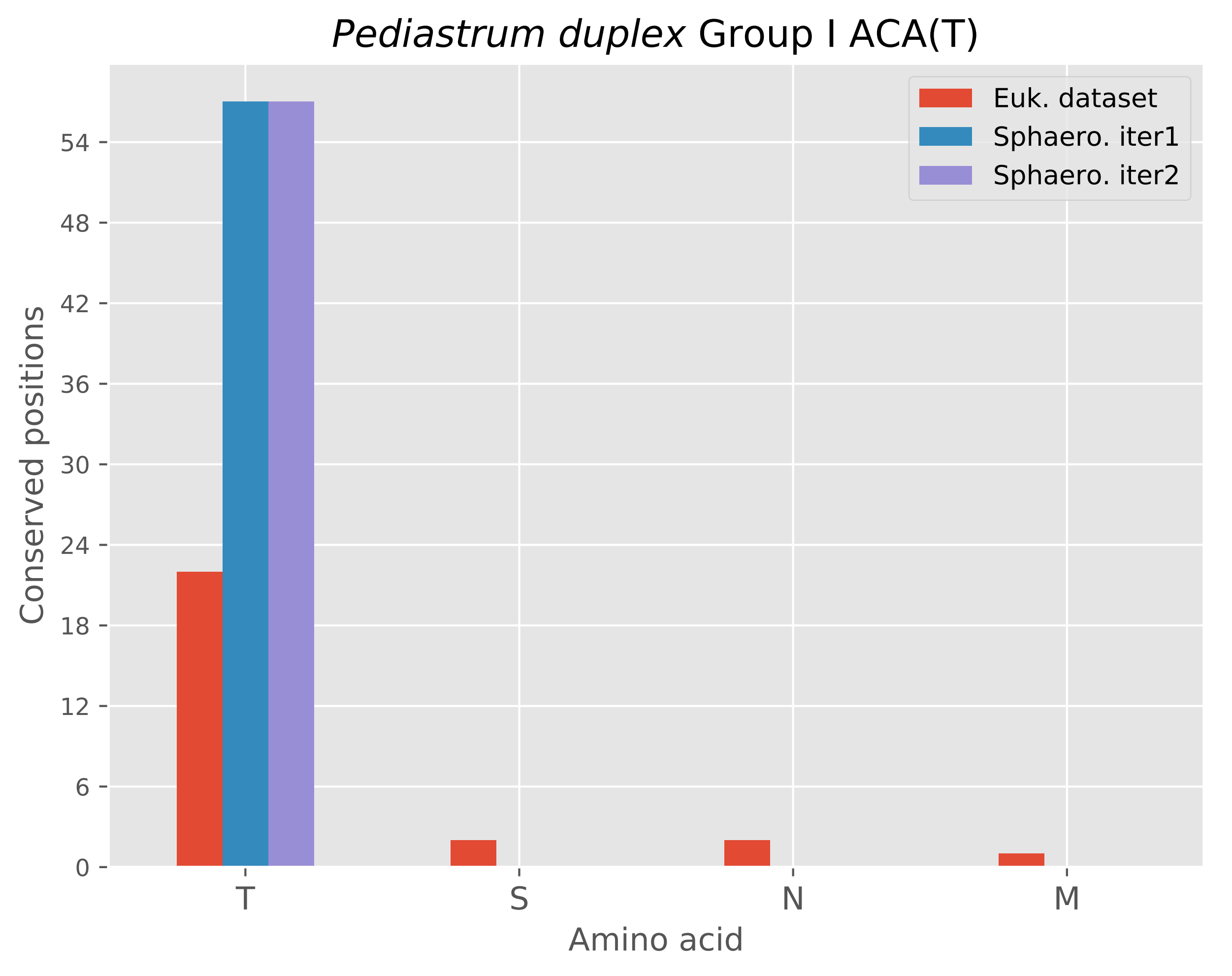

Pediastrum duplex Group I ACA(T)
Euk. dataset
Sphaero. iter1
Sphaero. iter2
Conserved positions
0
6
12
18
24
30
36
42
48
54
T
S
N
M
Amino acid

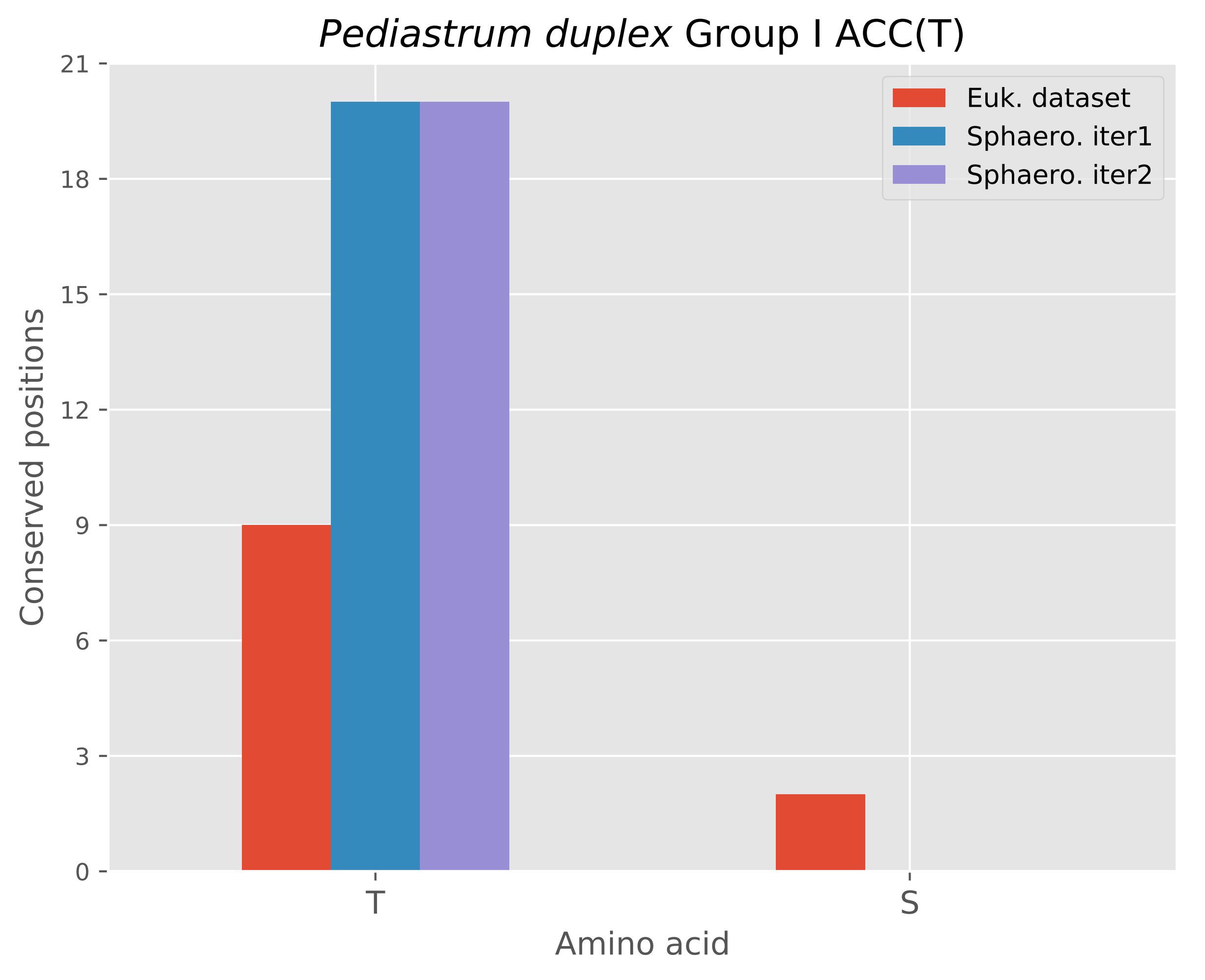

Pediastrum duplex Group I ACC(T)
Conserved positions
Amino acid
T
S
0
3
6
9
12
15
18
21
Euk. dataset
Sphaero. iter1
Sphaero. iter2

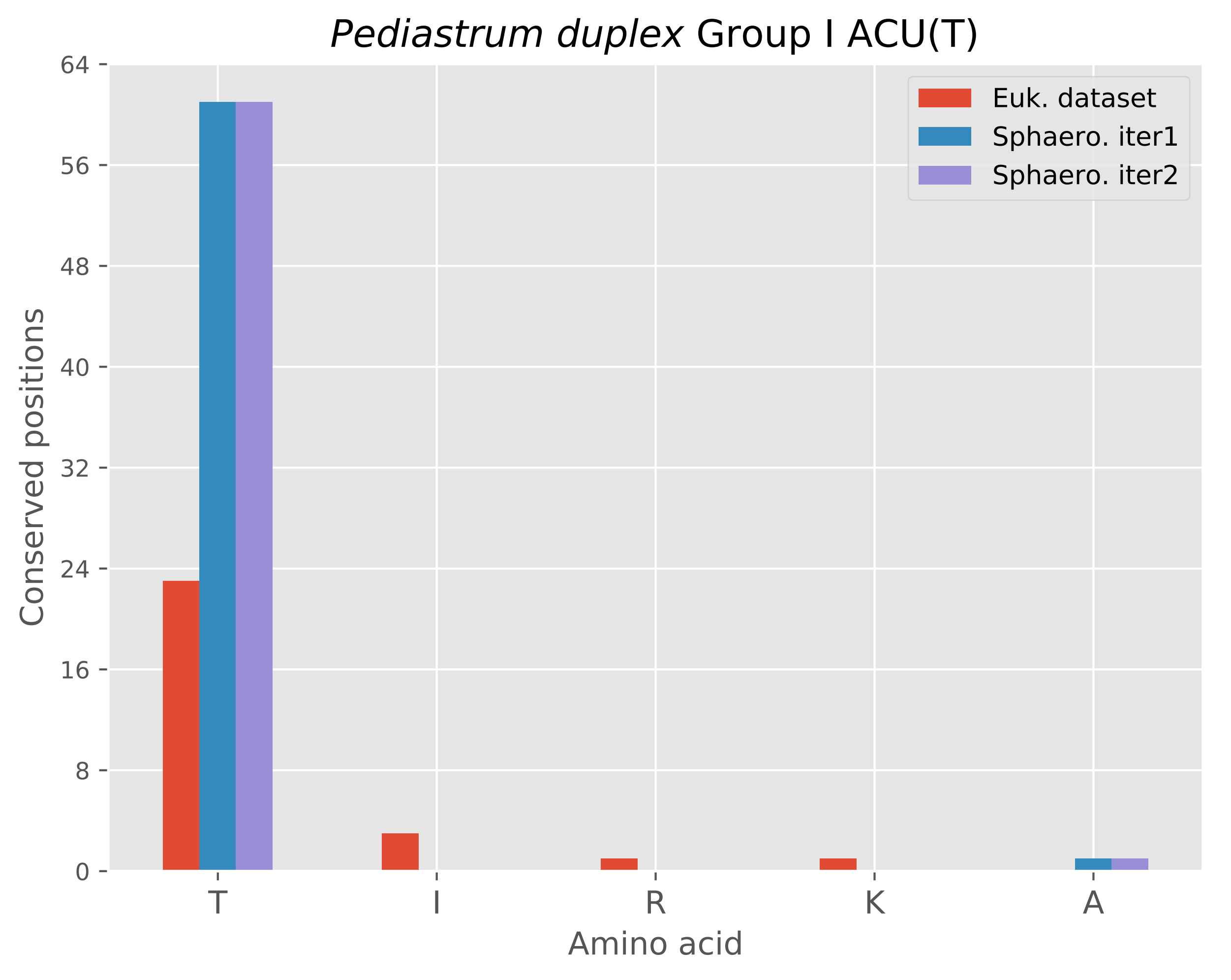

Pediastrum duplex Group I ACU(T)
Euk. dataset
Sphaero. iter1
Sphaero. iter2
Conserved positions
Amino acid
64
56
48
40
32
24
16
8
0
T
I
R
K
A

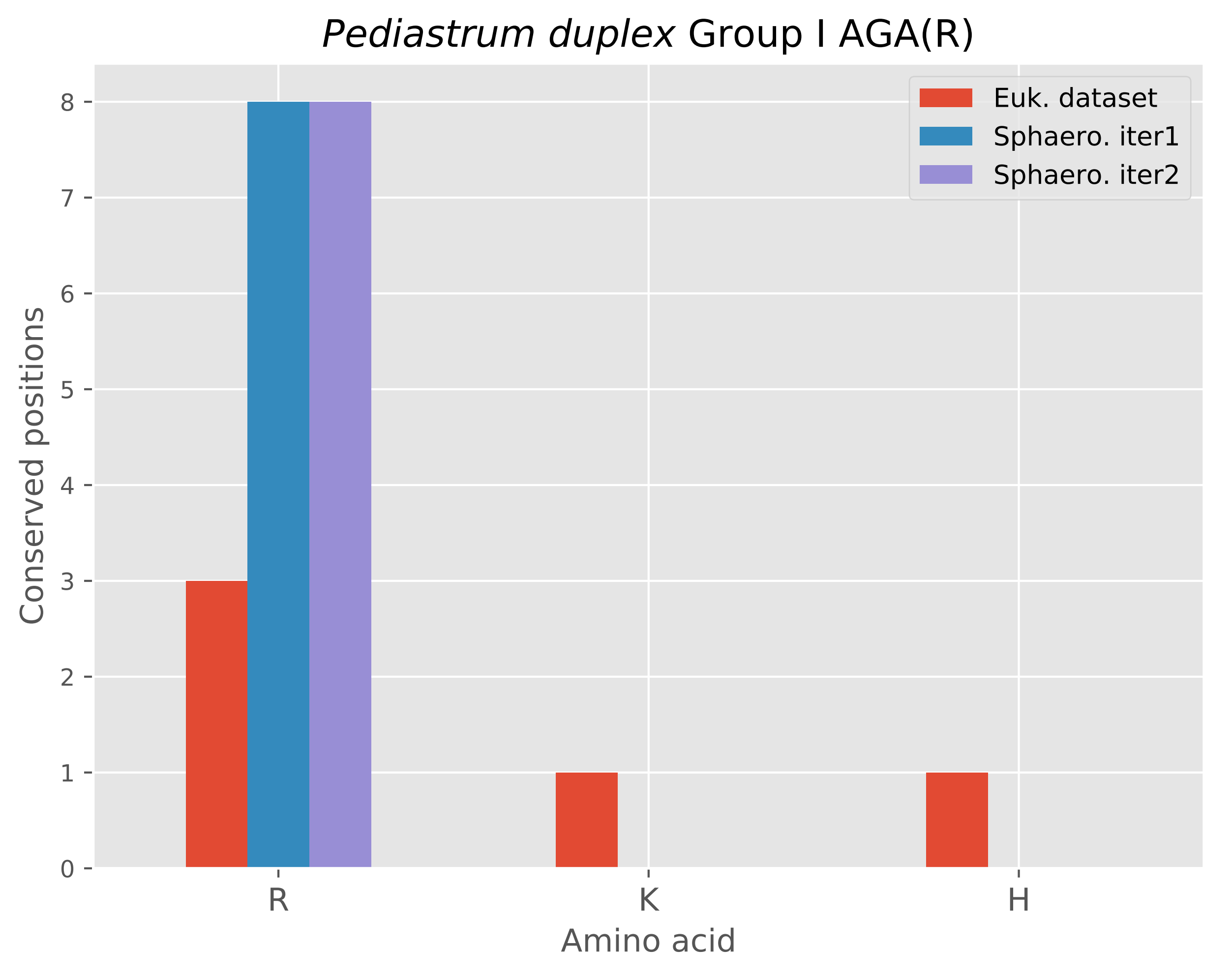

Pediastrum duplex Group I AGA(R)
Conserved positions
0
1
2
3
4
5
6
7
8
R
K
H
Amino acid
Euk. dataset
Sphaero. iter1
Sphaero. iter2

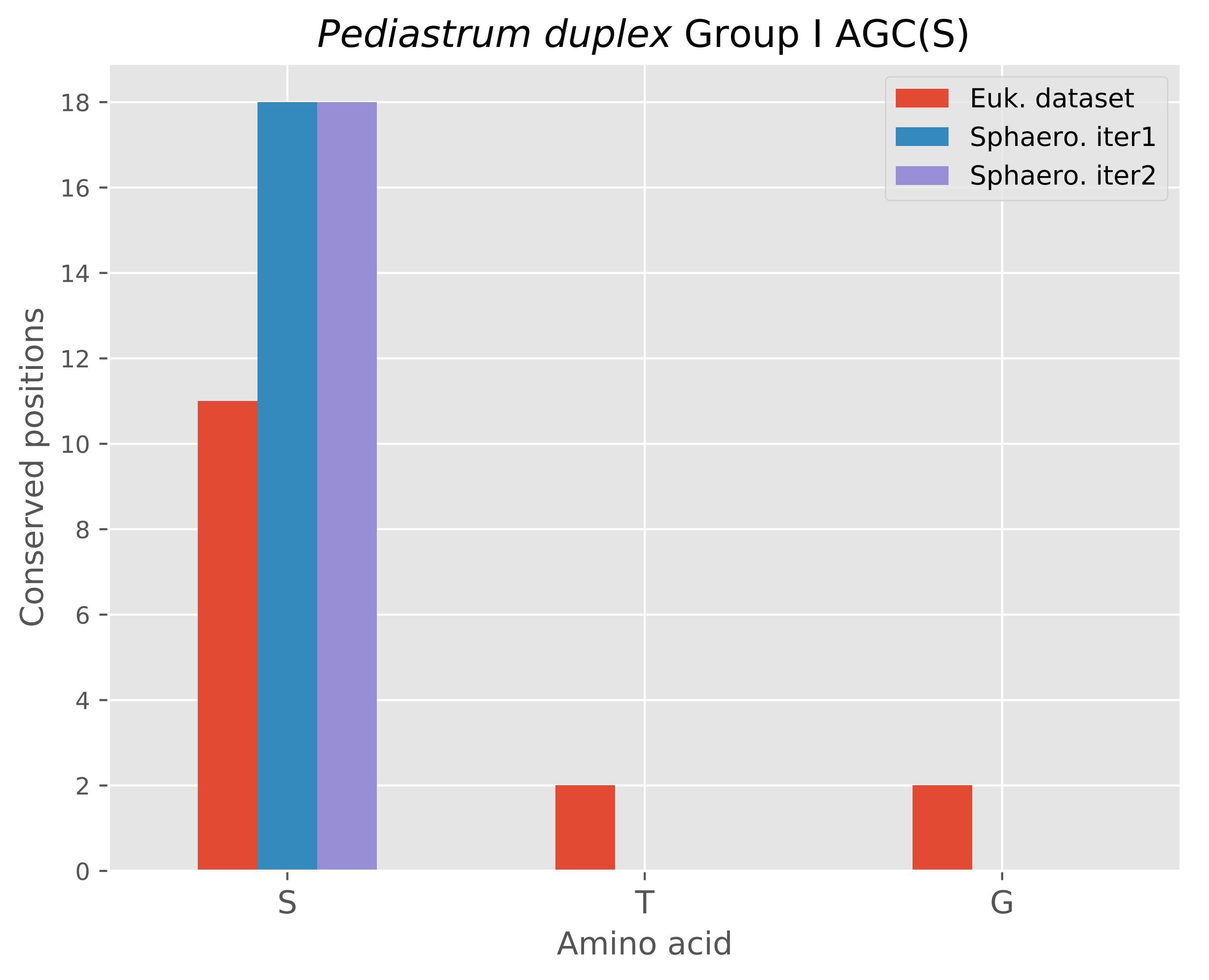

Pediastrum duplex Group I AGC(S)
Euk. dataset
Sphaero. iter1
Sphaero. iter2
Conserved positions
0
2
4
6
8
10
12
14
16
18
S
T
G
Amino acid

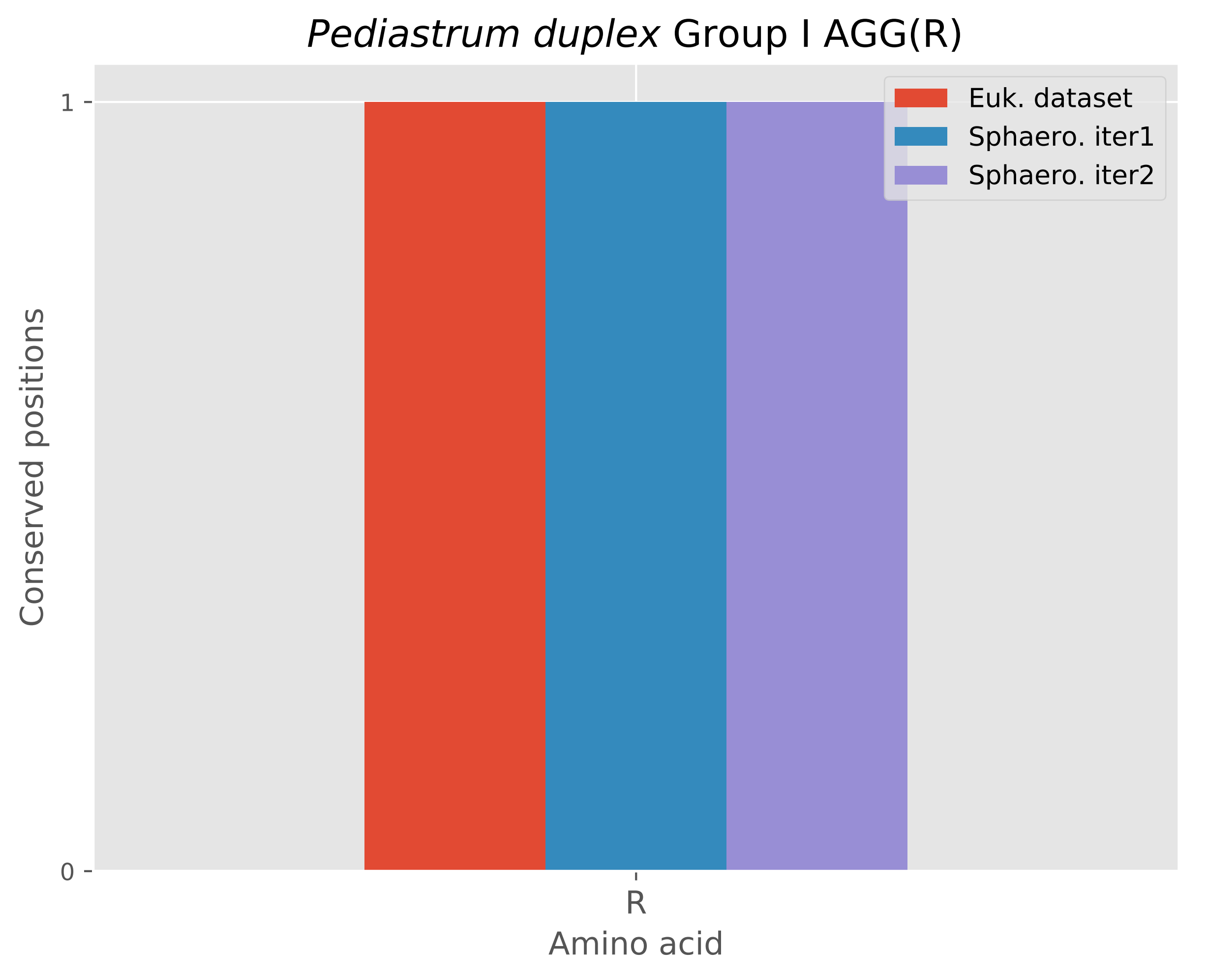

Pediastrum duplex Group I AGG(R)
Euk. dataset
Sphaero. iter1
Sphaero. iter2
1
0
Conserved positions
R
Amino acid

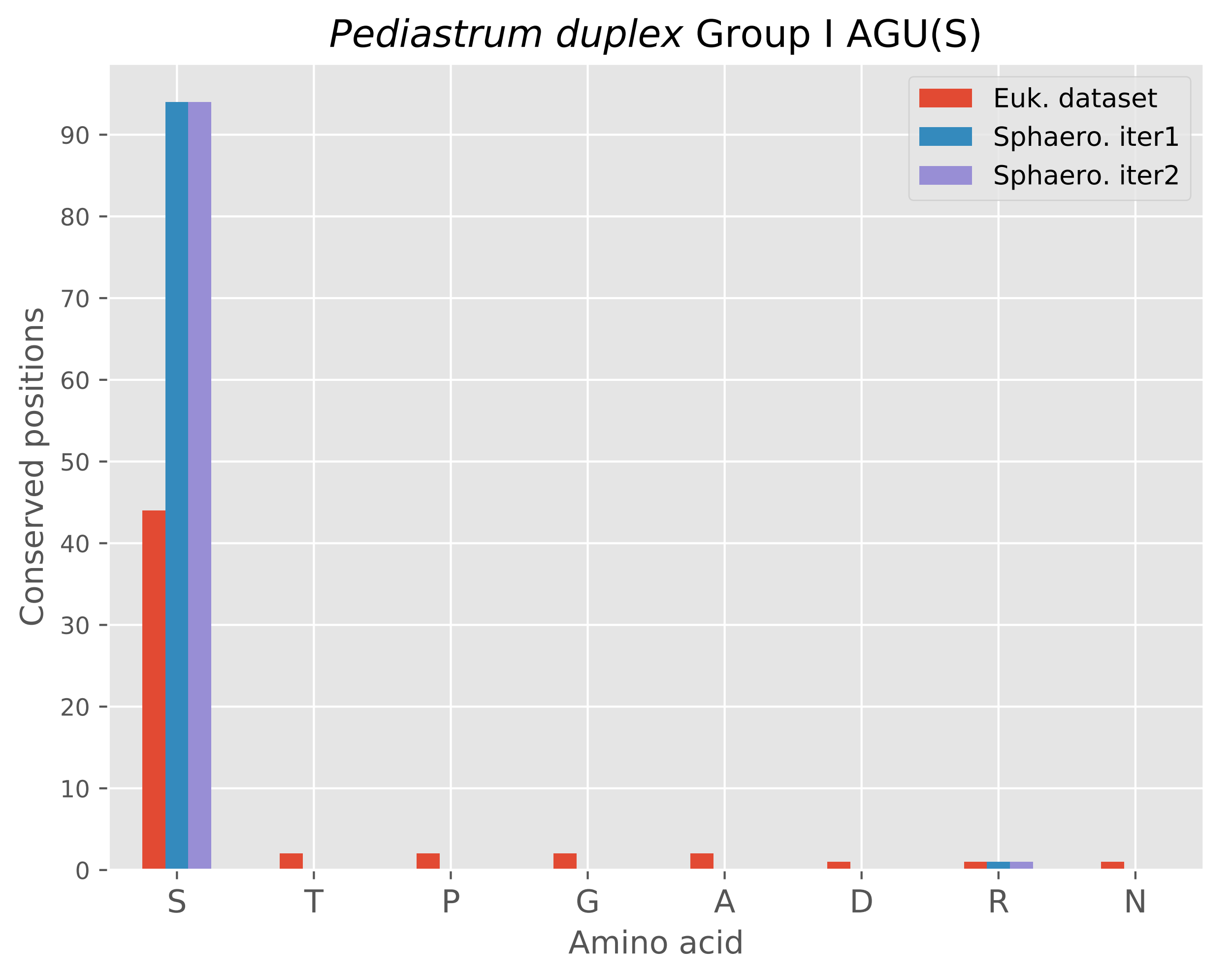

Pediastrum duplex Group I AGU(S)
Euk. dataset
Sphaero. iter1
Sphaero. iter2
Conserved positions
0
10
20
30
40
50
60
70
80
90
S
T
P
G
A
D
R
N
Amino acid

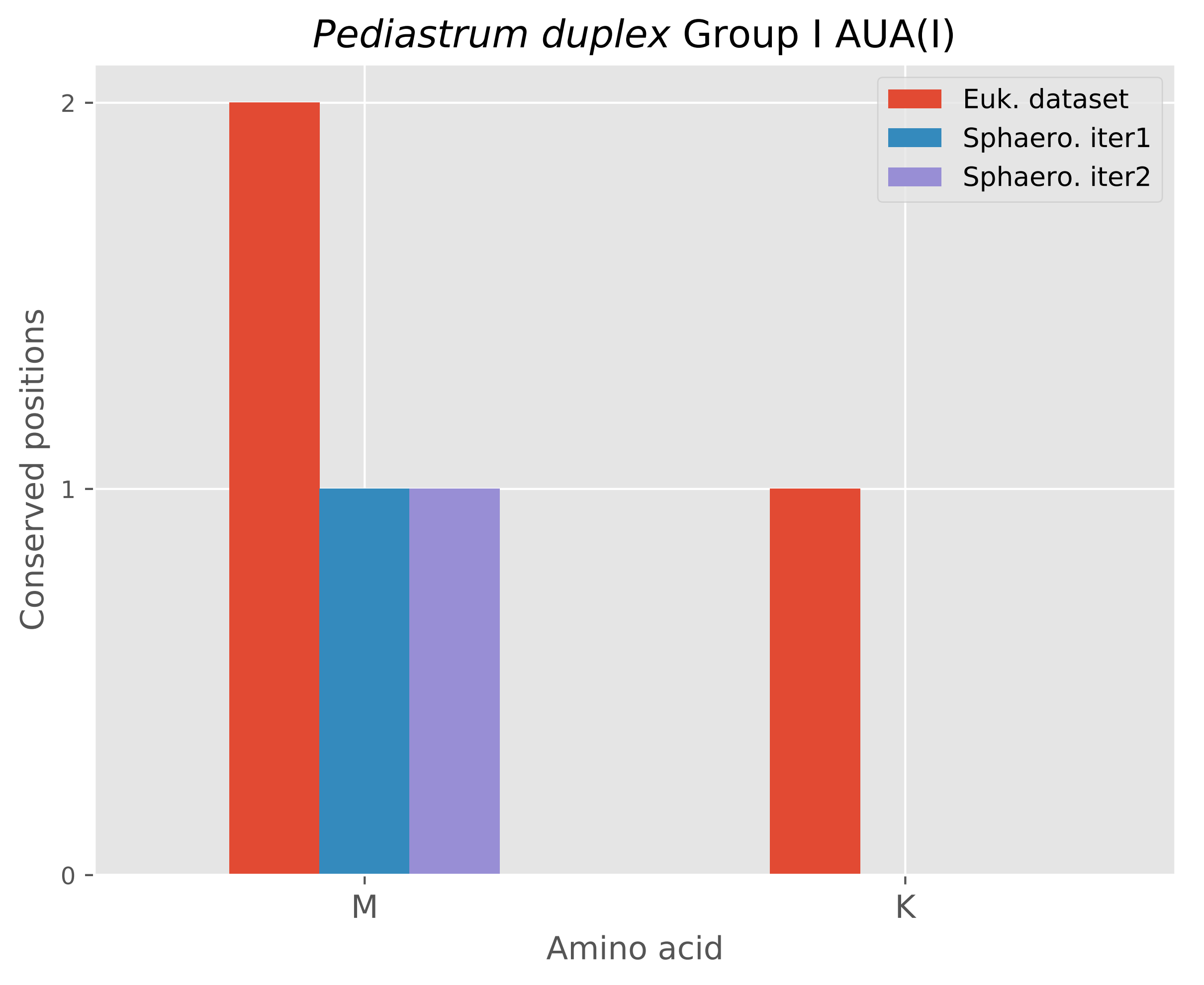

Pediastrum duplex Group I AUA(I)
Euk. dataset
Sphaero. iter1
Sphaero. iter2
Conserved positions
2
1
0
M
K
Amino acid

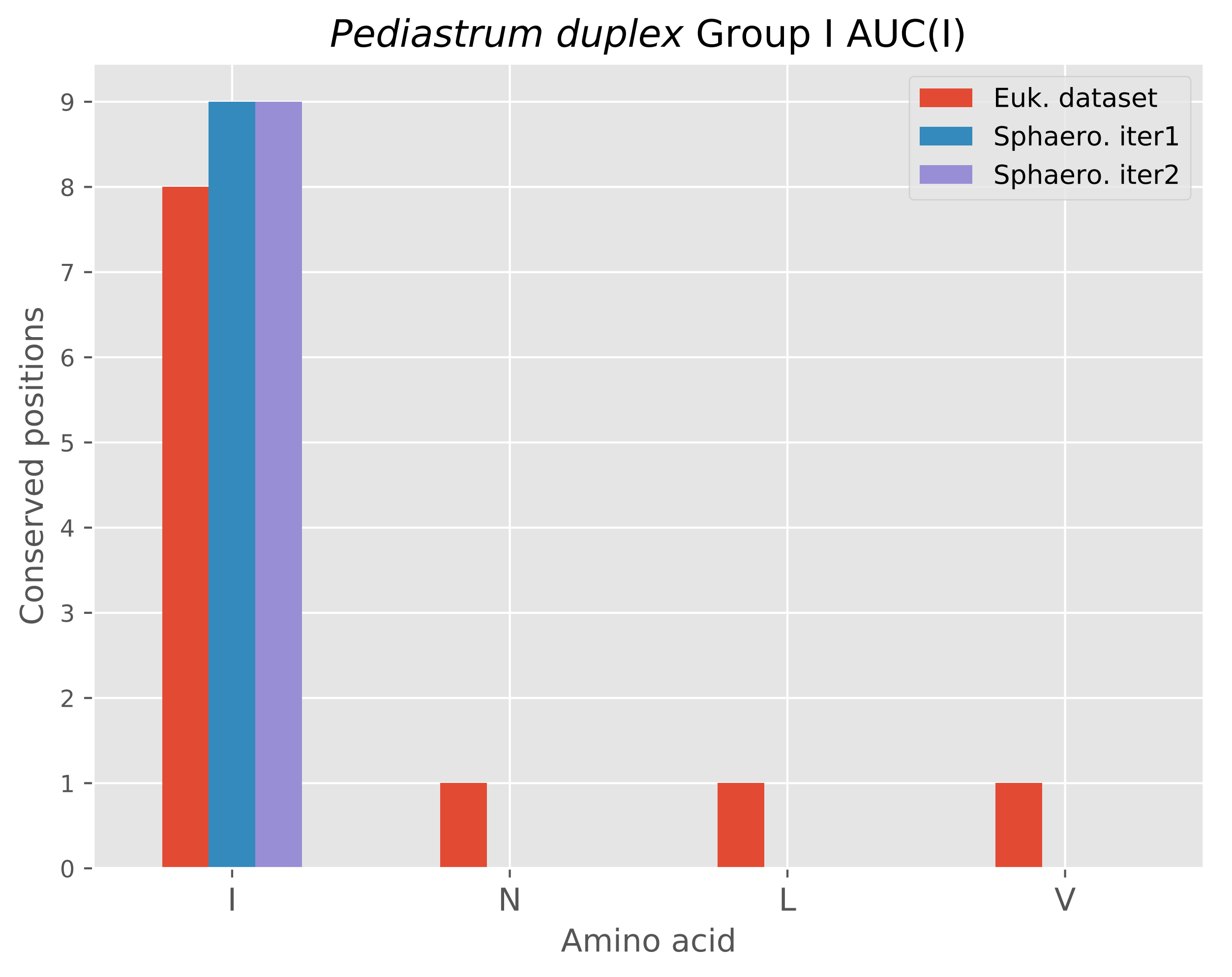

Pediastrum duplex Group I AUC(I)
Euk. dataset
Sphaero. iter1
Sphaero. iter2
Conserved positions
0
1
2
3
4
5
6
7
8
9
I
N
L
V
Amino acid

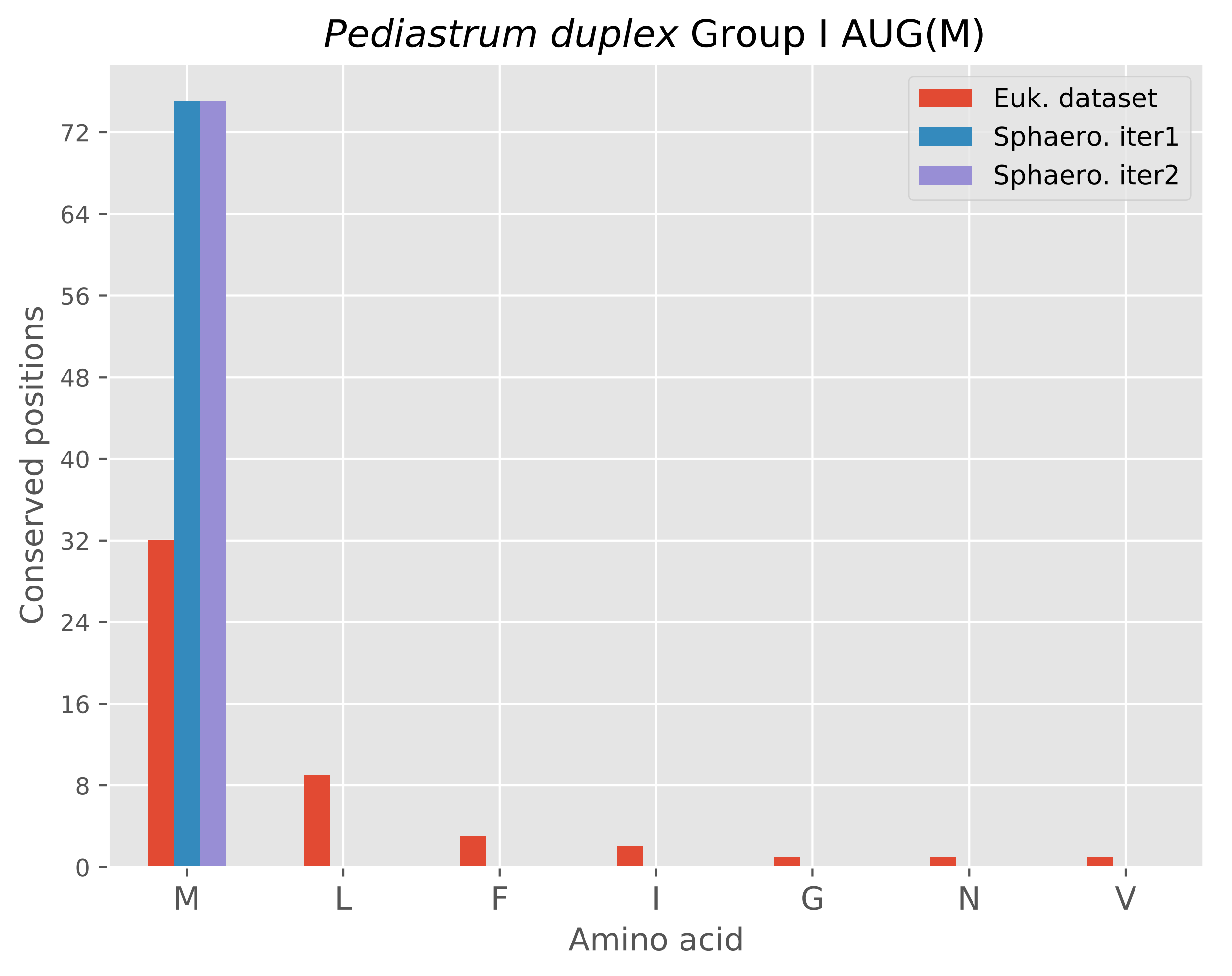

Pediastrum duplex Group I AUG(M)
Euk. dataset
Sphaero. iter1
Sphaero. iter2
Conserved positions
0
8
16
24
32
40
48
56
64
72
M
L
F
I
G
N
V
Amino acid

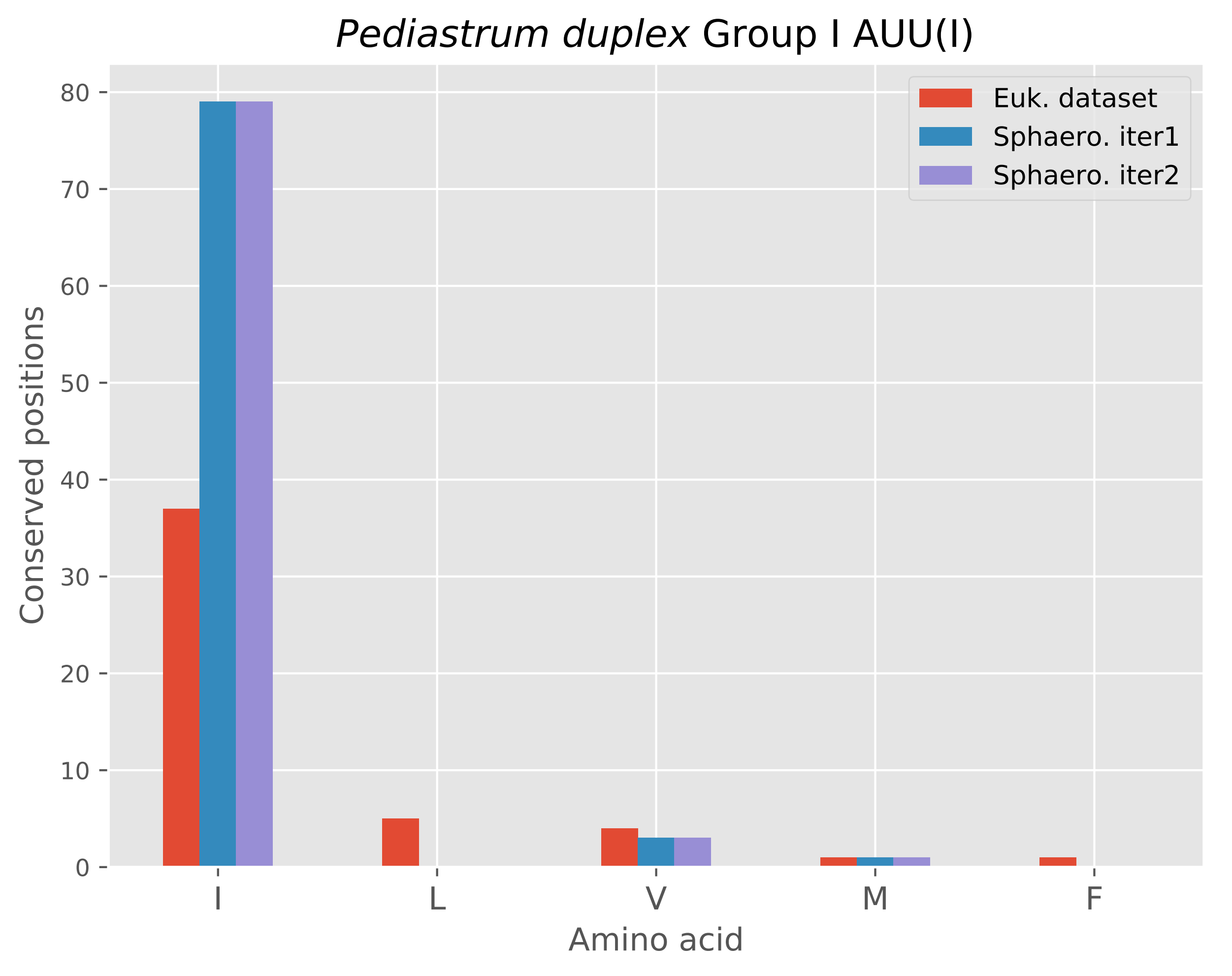

Pediastrum duplex Group I AUU(I)
Euk. dataset
Sphaero. iter1
Sphaero. iter2
Conserved positions
0
10
20
30
40
50
60
70
80
I
L
V
M
F
Amino acid

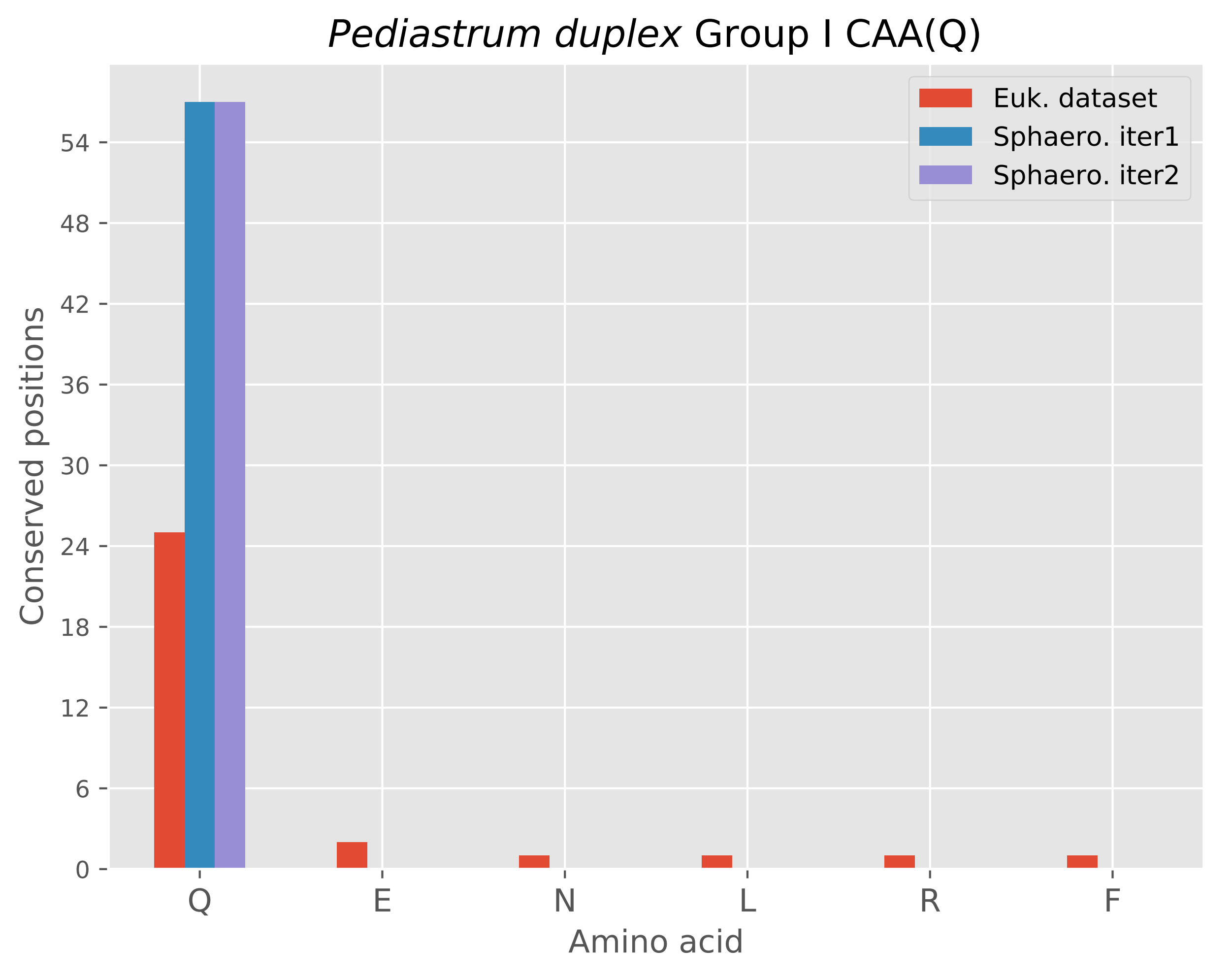

Pediastrum duplex Group I CAA(Q)
Euk. dataset
Sphaero. iter1
Sphaero. iter2
Conserved positions
0
6
12
18
24
30
36
42
48
54
Q
E
N
L
R
F
Amino acid

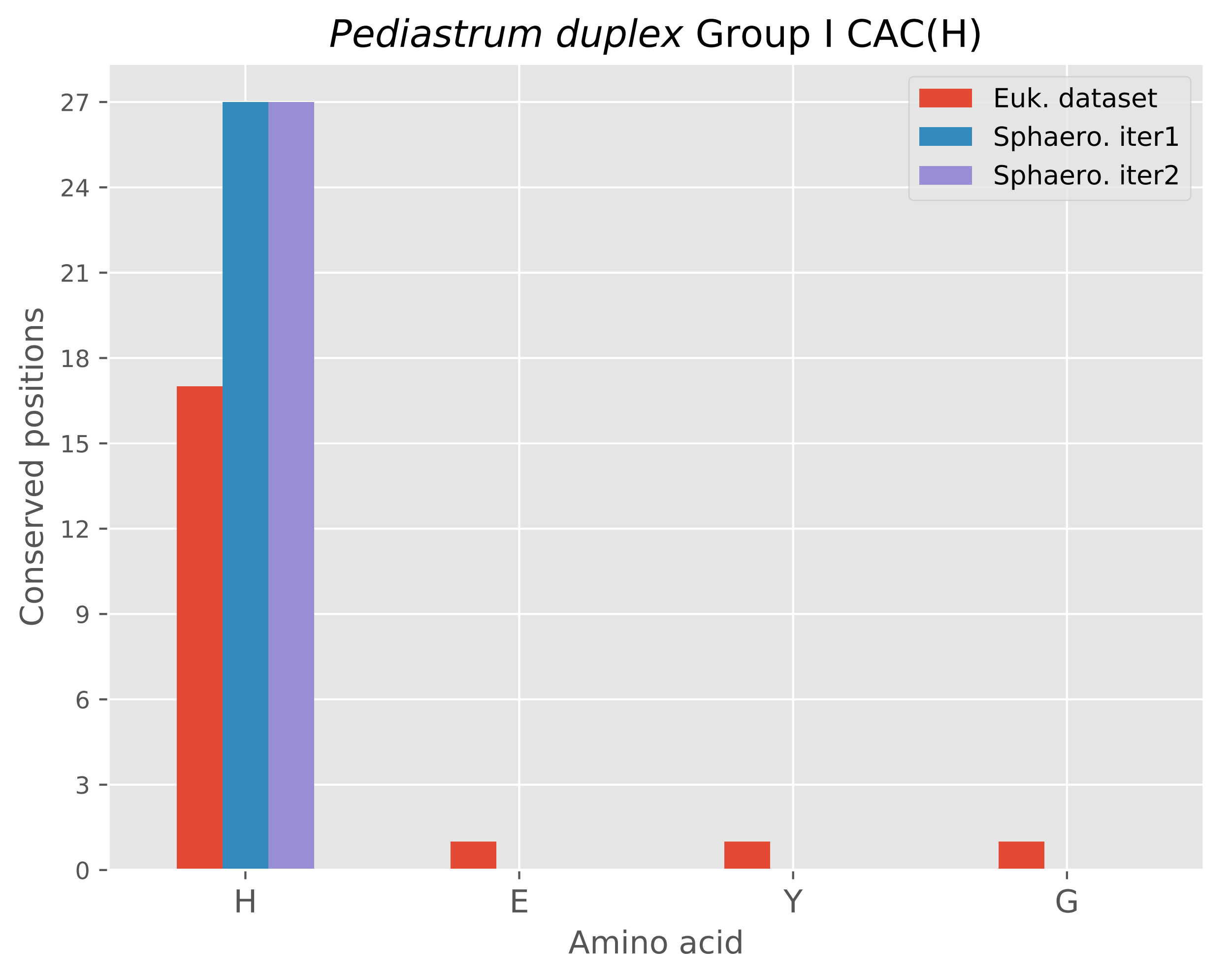

Pediastrum duplex Group I CAC(H)
Euk. dataset
Sphaero. iter1
Sphaero. iter2
Conserved positions
0
3
6
9
12
15
18
21
24
27
H
E
Y
G
Amino acid

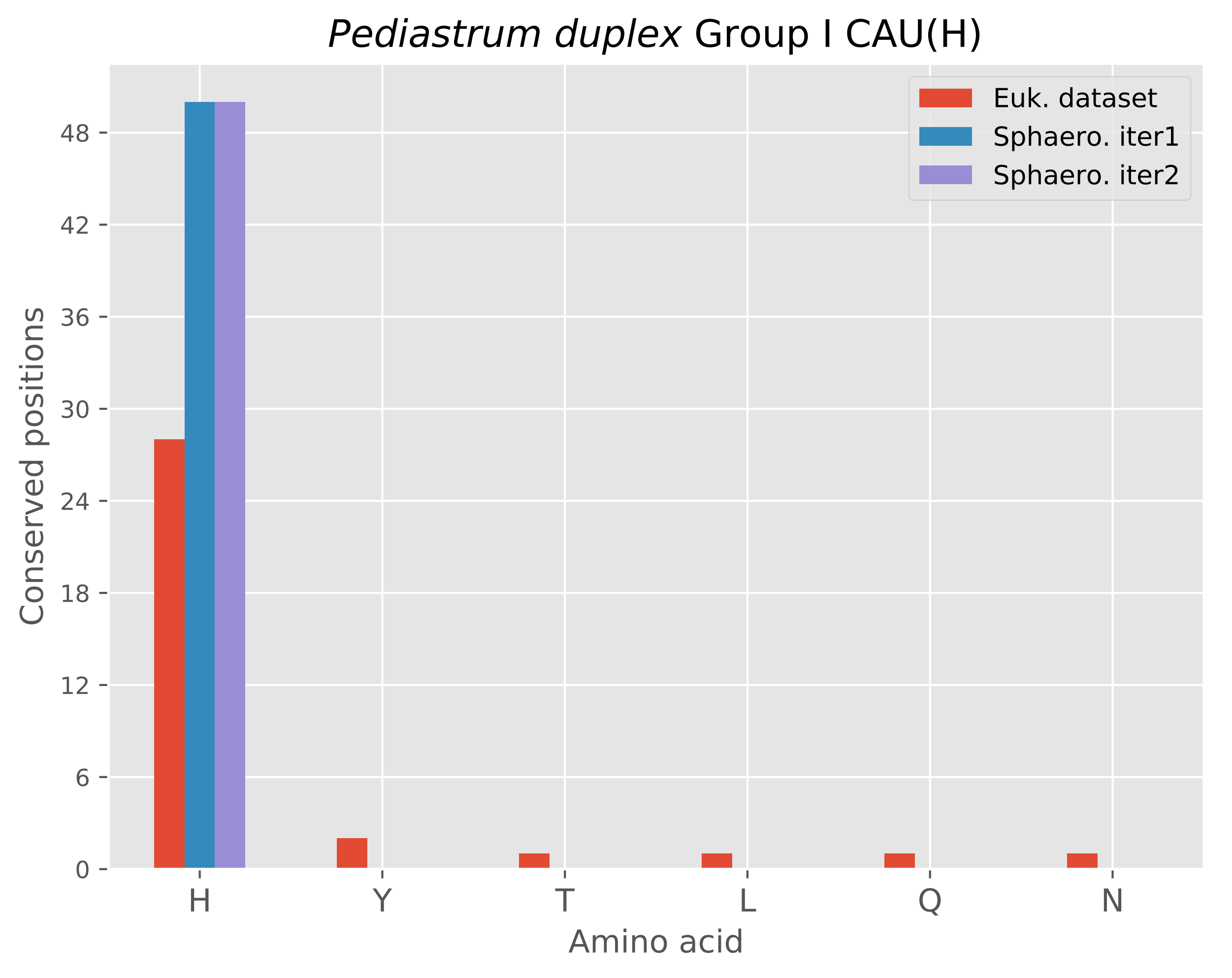
Pediastrum duplex Group I CAU(H)
Euk. dataset
Sphaero. iter1
Sphaero. iter2
Conserved positions
0
6
12
18
24
30
36
42
48
H
Y
T
L
Q
N
Amino acid

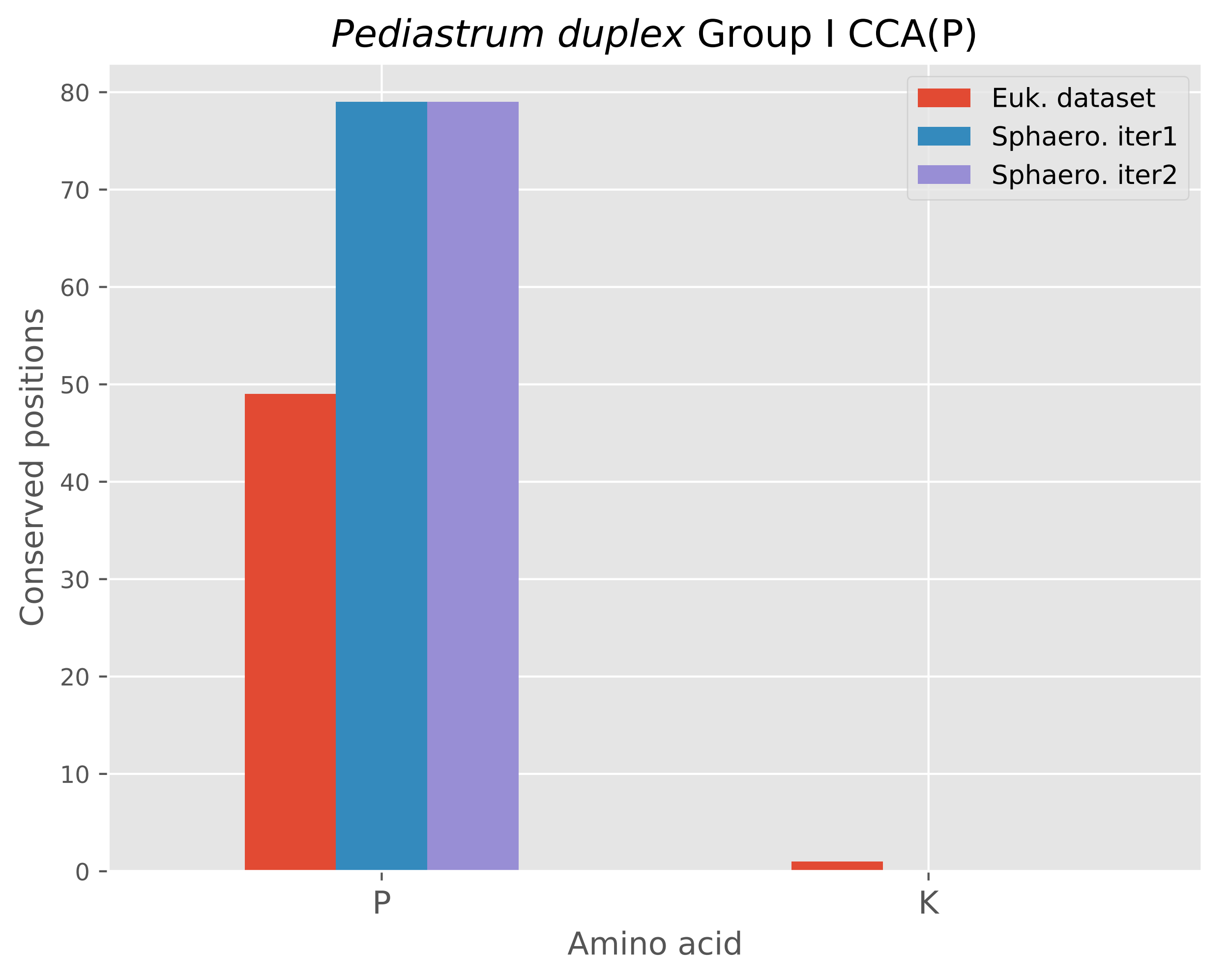
Pediastrum duplex Group I CCA(P)
Euk. dataset
Sphaero. iter1
Sphaero. iter2
Conserved positions
0
10
20
30
40
50
60
70
80
P
K
Amino acid

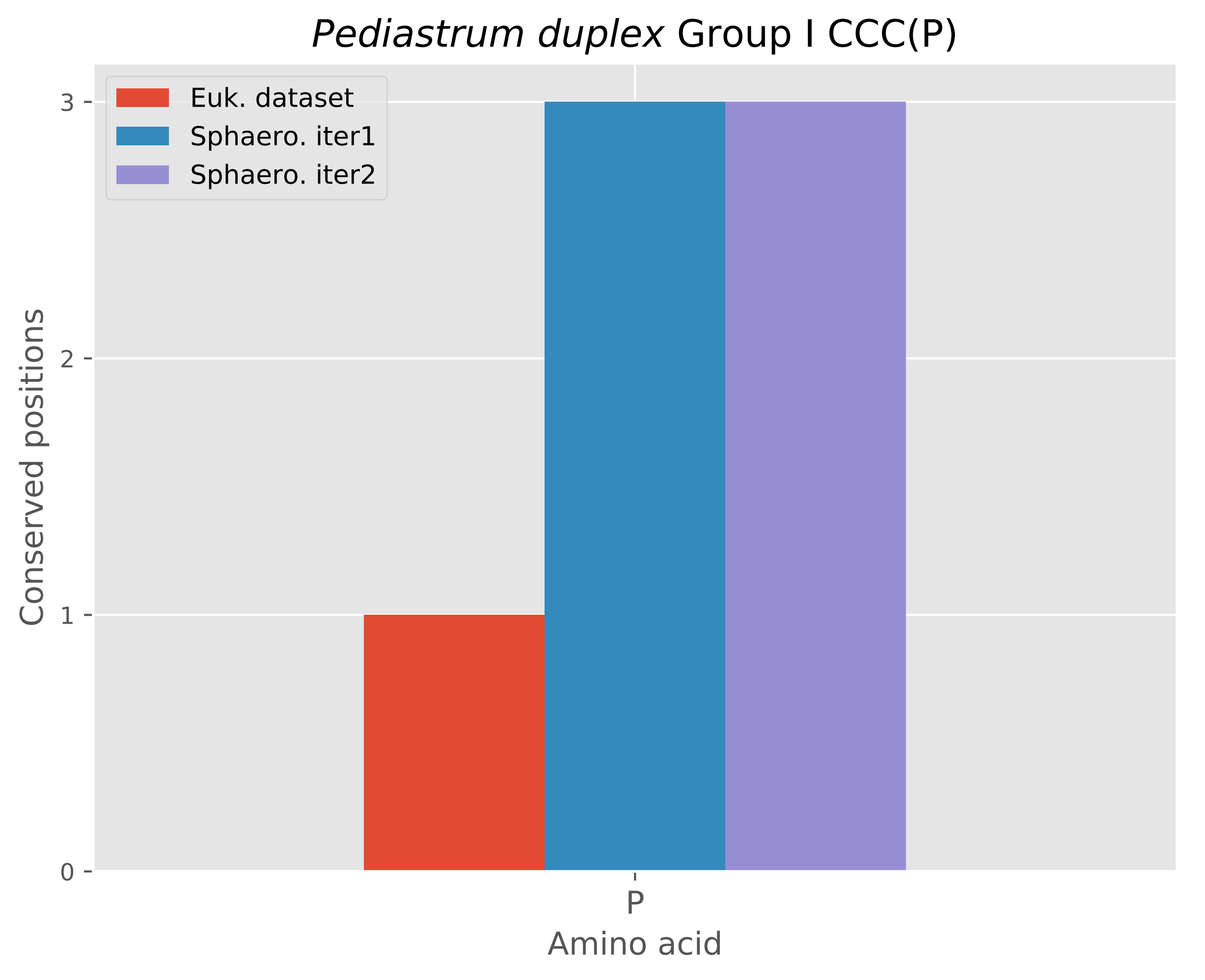

Pediastrum duplex Group I CCC(P)
Euk. dataset
Sphaero. iter1
Sphaero. iter2
Conserved positions
0
1
2
3
P
Amino acid

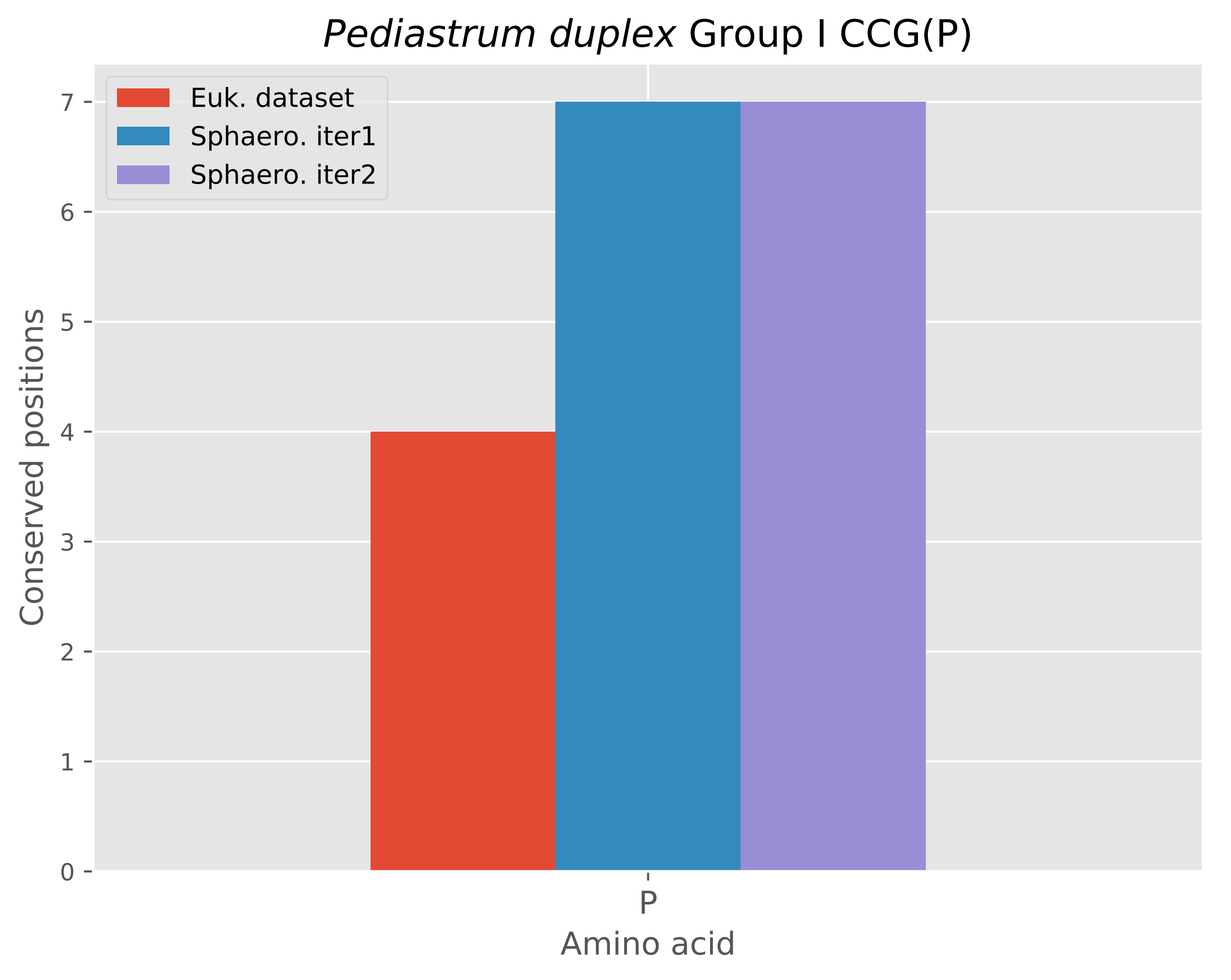

Pediastrum duplex Group I CCG(P)
Euk. dataset
Sphaero. iter1
Sphaero. iter2
Conserved positions
0
1
2
3
4
5
6
7
P
Amino acid

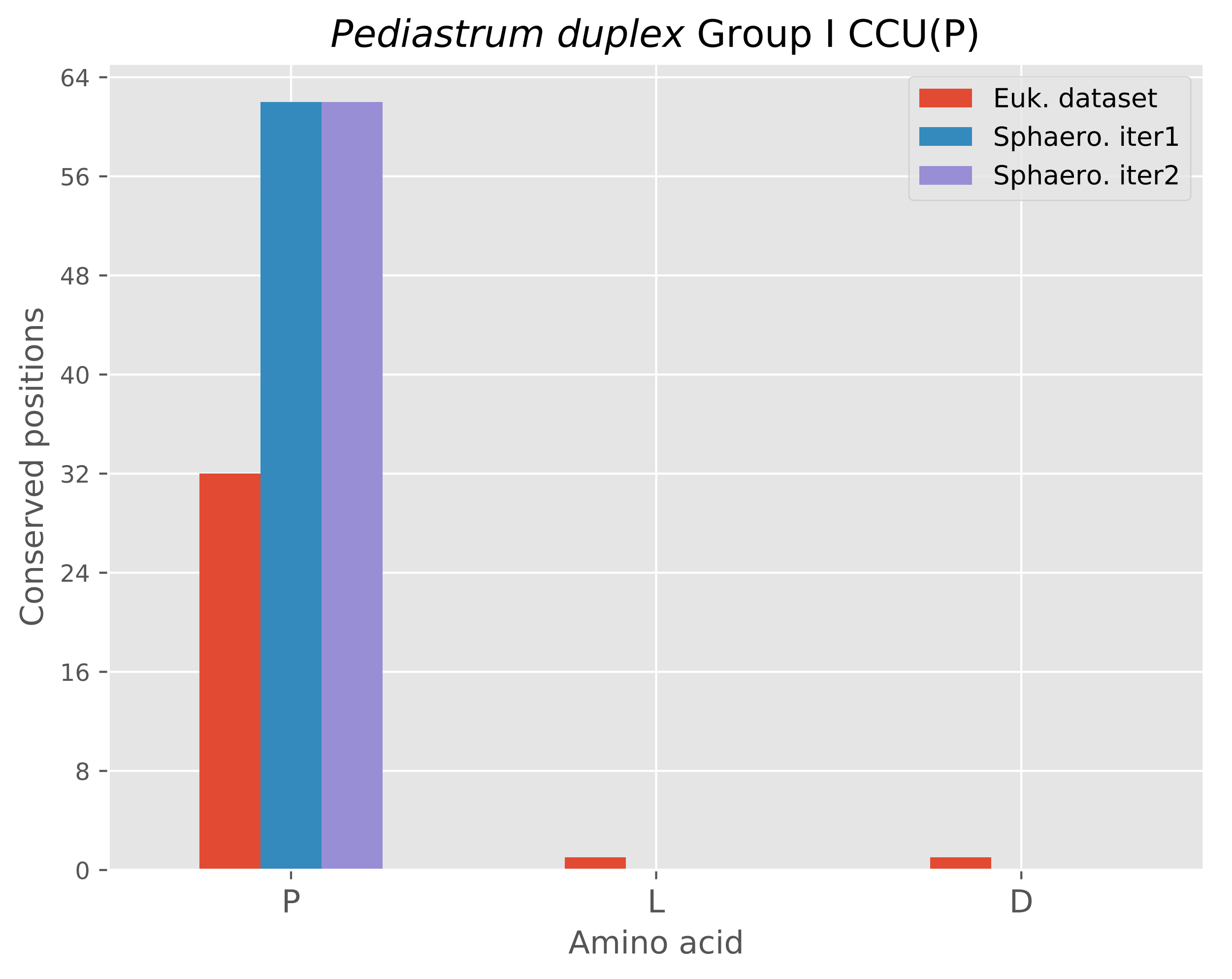

Pediastrum duplex Group I CCU(P)
Euk. dataset
Sphaero. iter1
Sphaero. iter2
Conserved positions
0
8
16
24
32
40
48
56
64
P
L
D
Amino acid

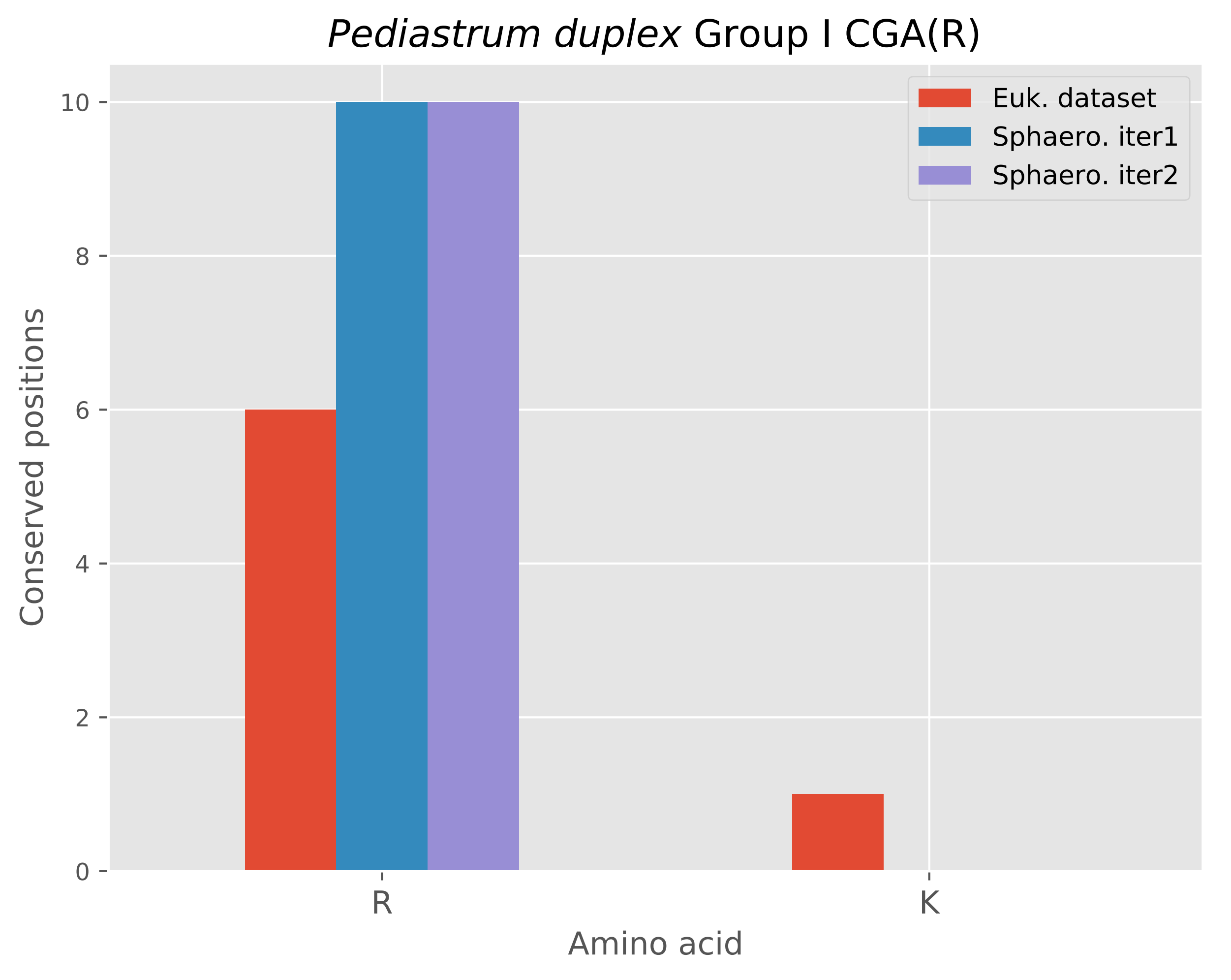

Pediastrum duplex Group I CGA(R)
Conserved positions
0
2
4
6
8
10
R
K
Amino acid
Euk. dataset
Sphaero. iter1
Sphaero. iter2

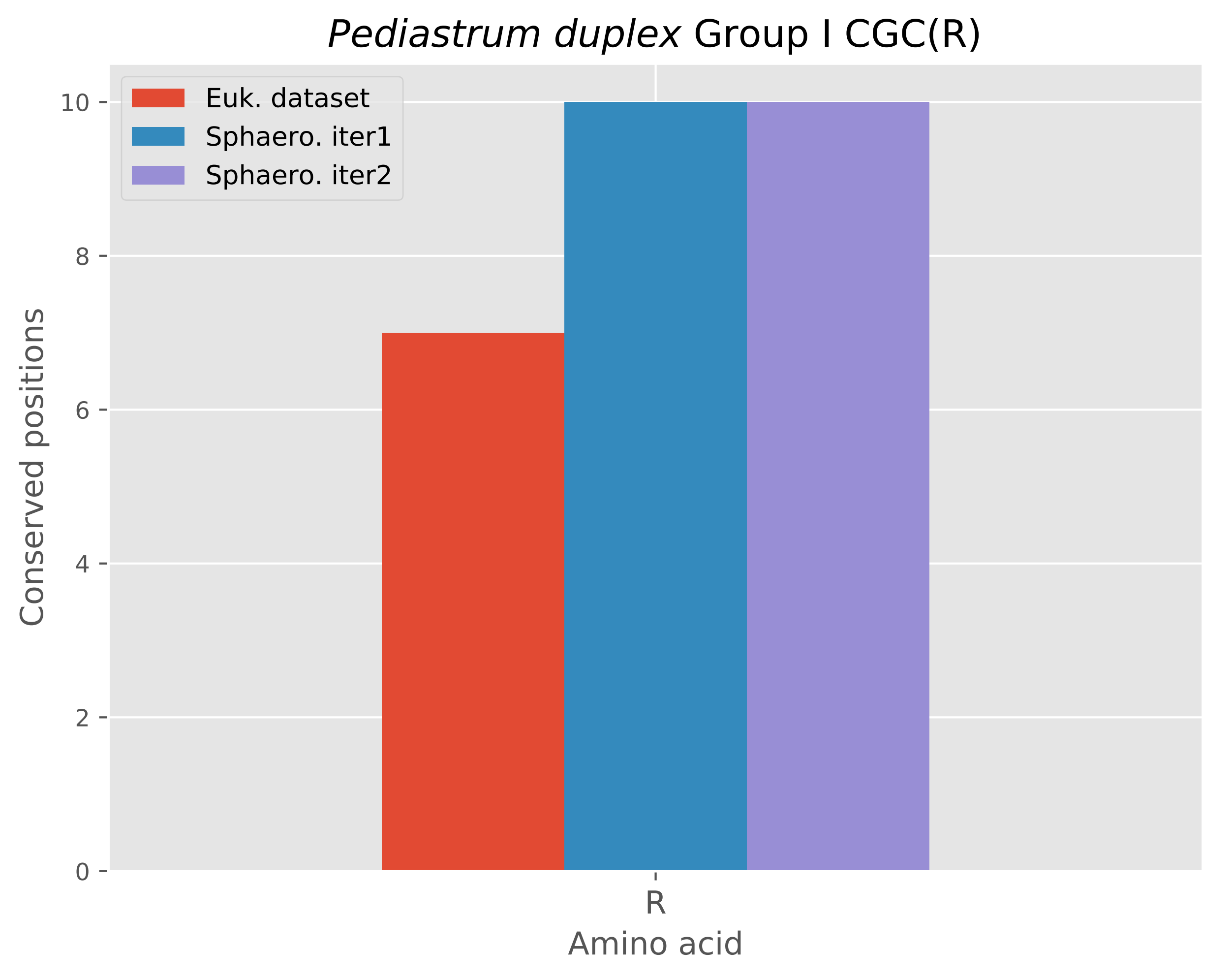

Pediastrum duplex Group I CGC(R)
Euk. dataset
Sphaero. iter1
Sphaero. iter2
Conserved positions
0
2
4
6
8
10
R
Amino acid

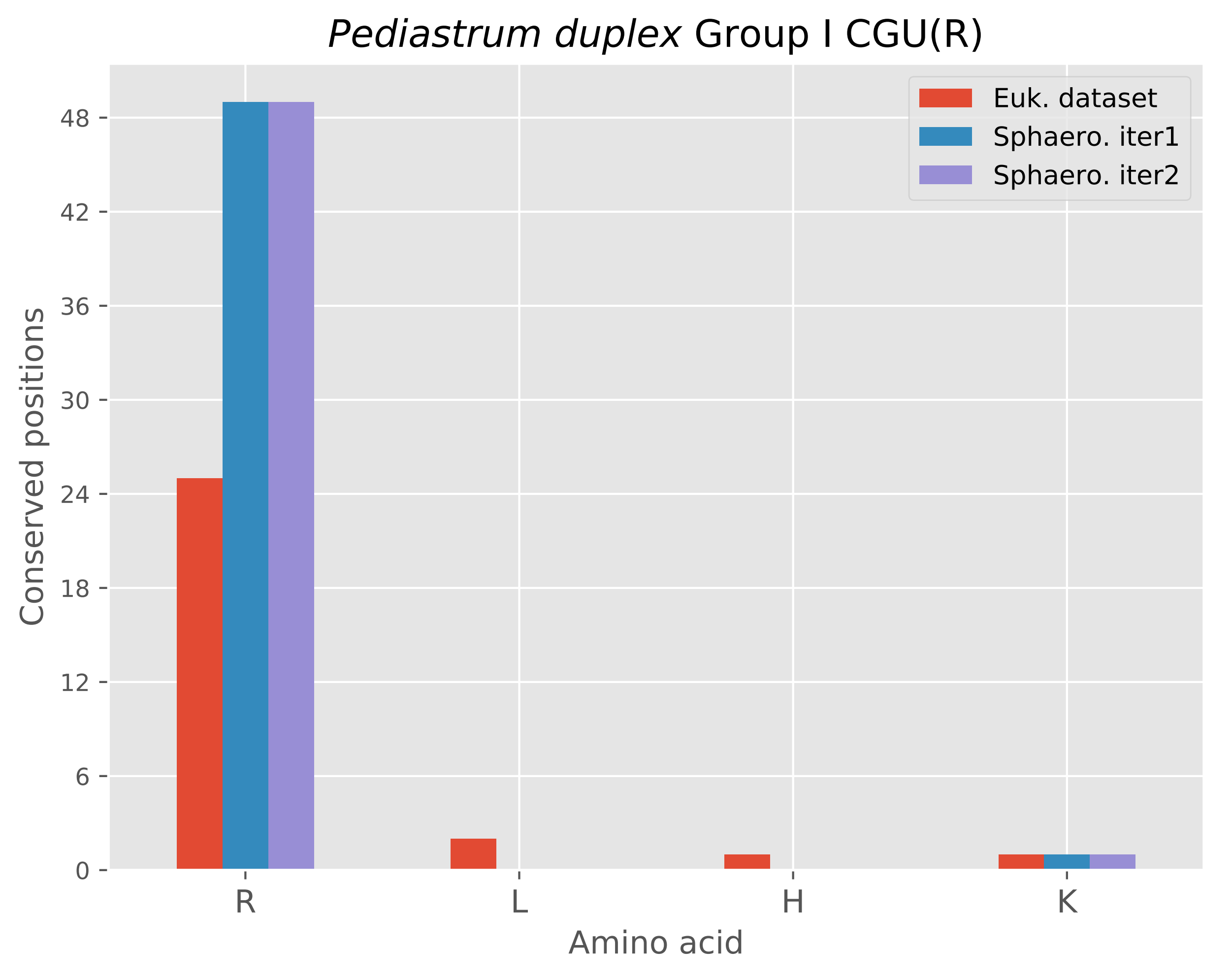
Pediastrum duplex Group I CGU(R)
Euk. dataset
Sphaero. iter1
Sphaero. iter2
Conserved positions
0
6
12
18
24
30
36
42
48
R
L
H
K
Amino acid

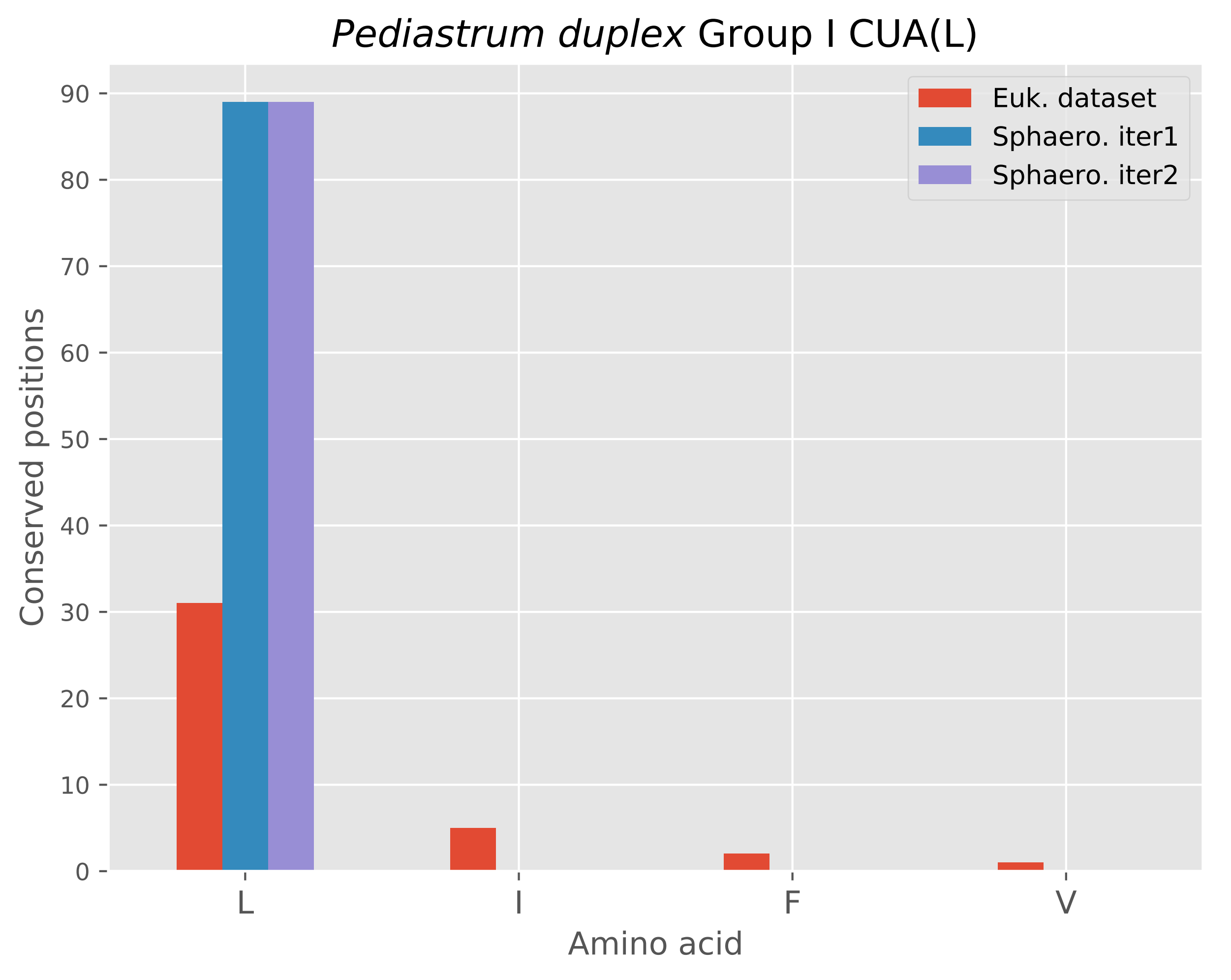

Pediastrum duplex Group I CUA(L)
Euk. dataset
Sphaero. iter1
Sphaero. iter2
Conserved positions
0
10
20
30
40
50
60
70
80
90
L
I
F
V
Amino acid

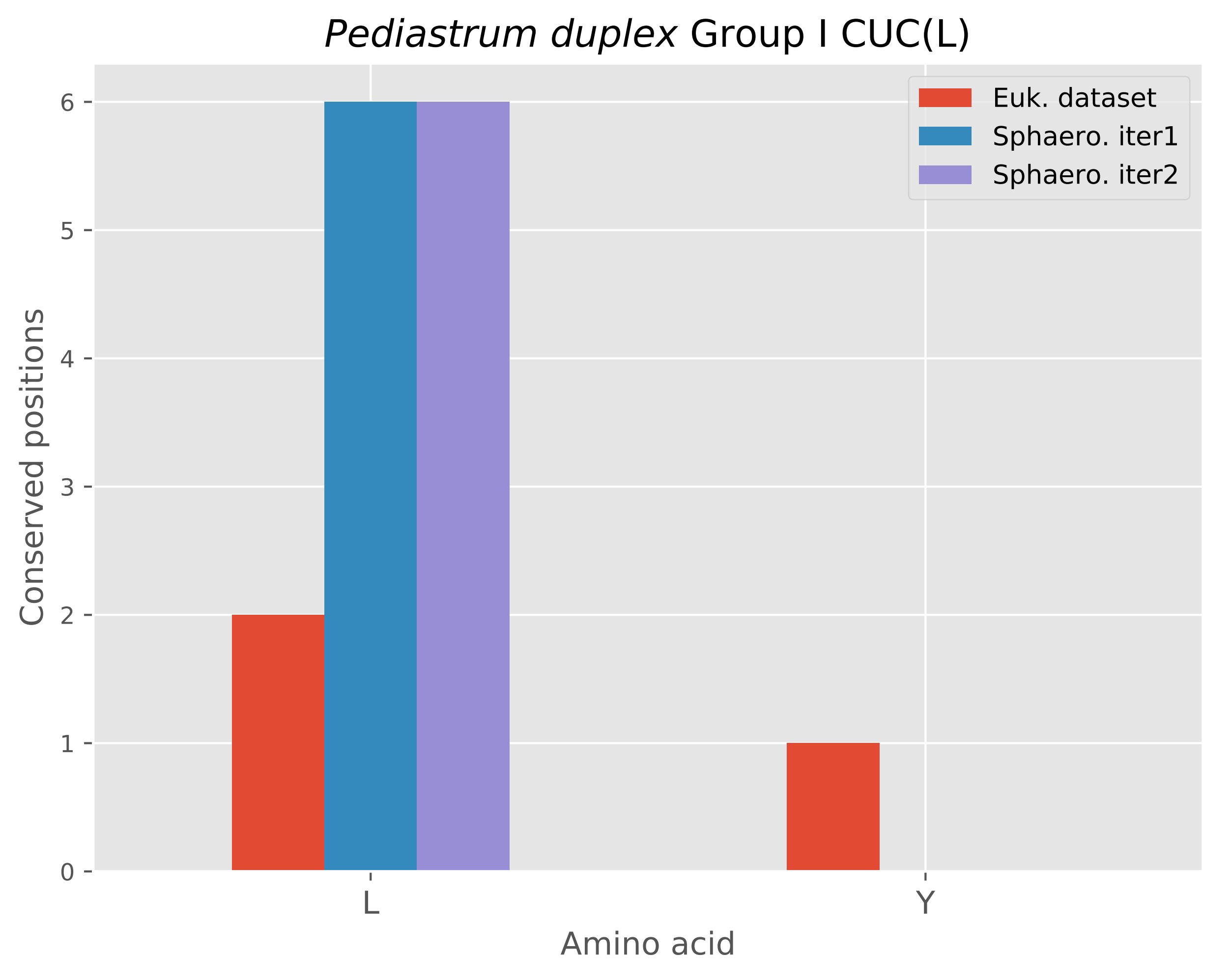

Pediastrum duplex Group I CUC(L)
Euk. dataset
Sphaero. iter1
Sphaero. iter2
Conserved positions
0
1
2
3
4
5
6
L
Y
Amino acid

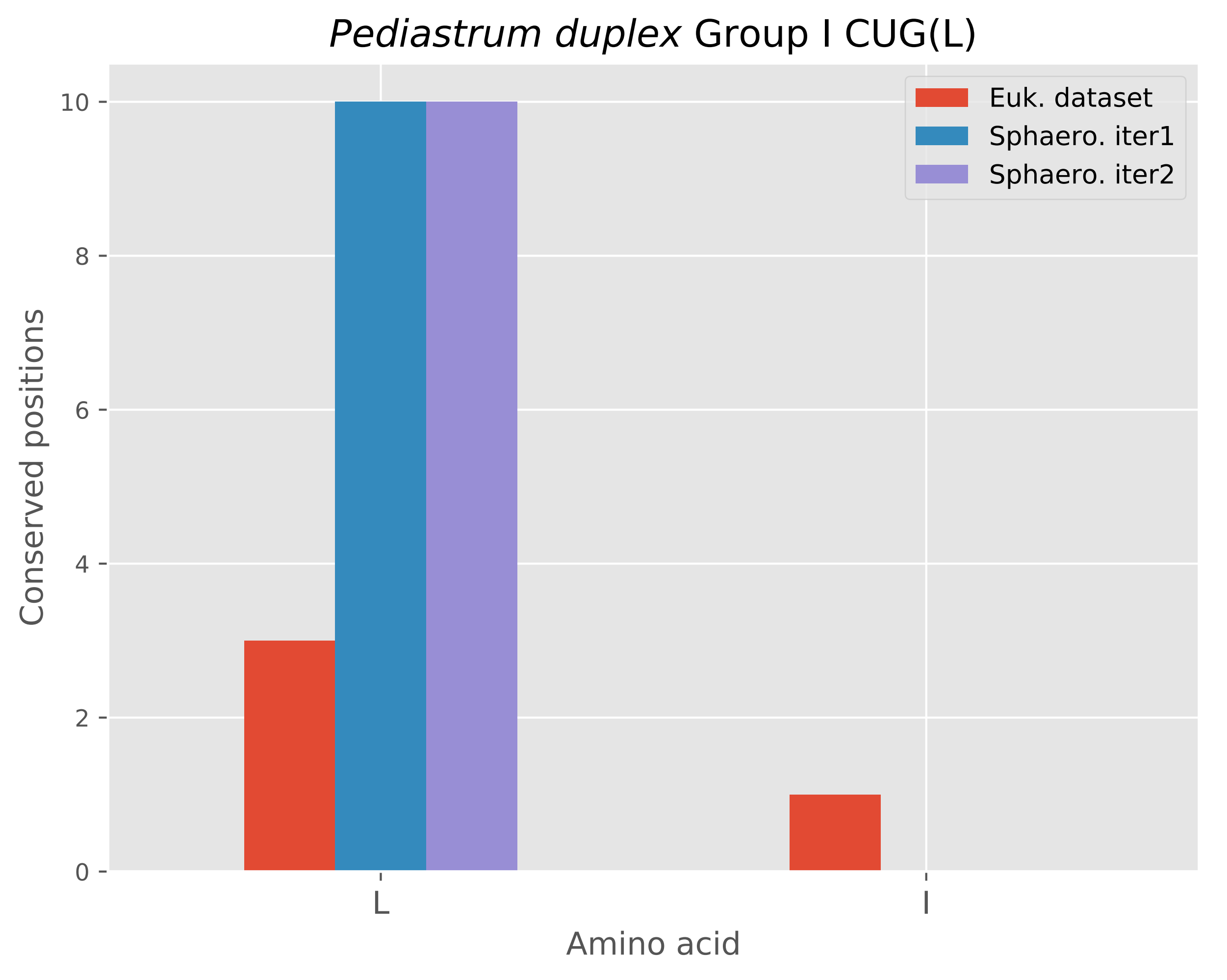

Pediastrum duplex Group I CUG(L)
Euk. dataset
Sphaero. iter1
Sphaero. iter2
Conserved positions
0
2
4
6
8
10
L
I
Amino acid

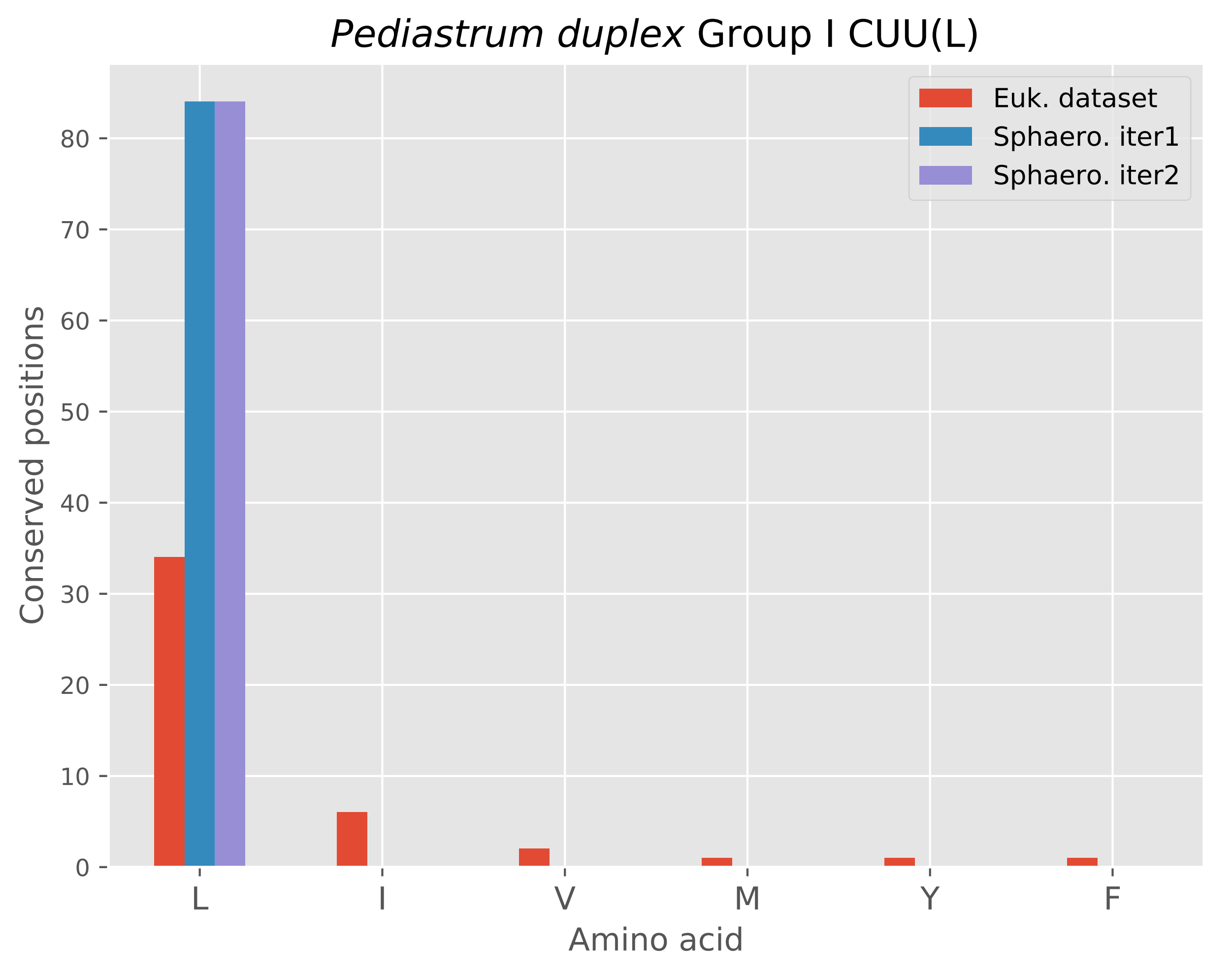
Pediastrum duplex Group I CUU(L)
Euk. dataset
Sphaero. iter1
Sphaero. iter2
Conserved positions
0
10
20
30
40
50
60
70
80
L
I
V
M
Y
F
Amino acid

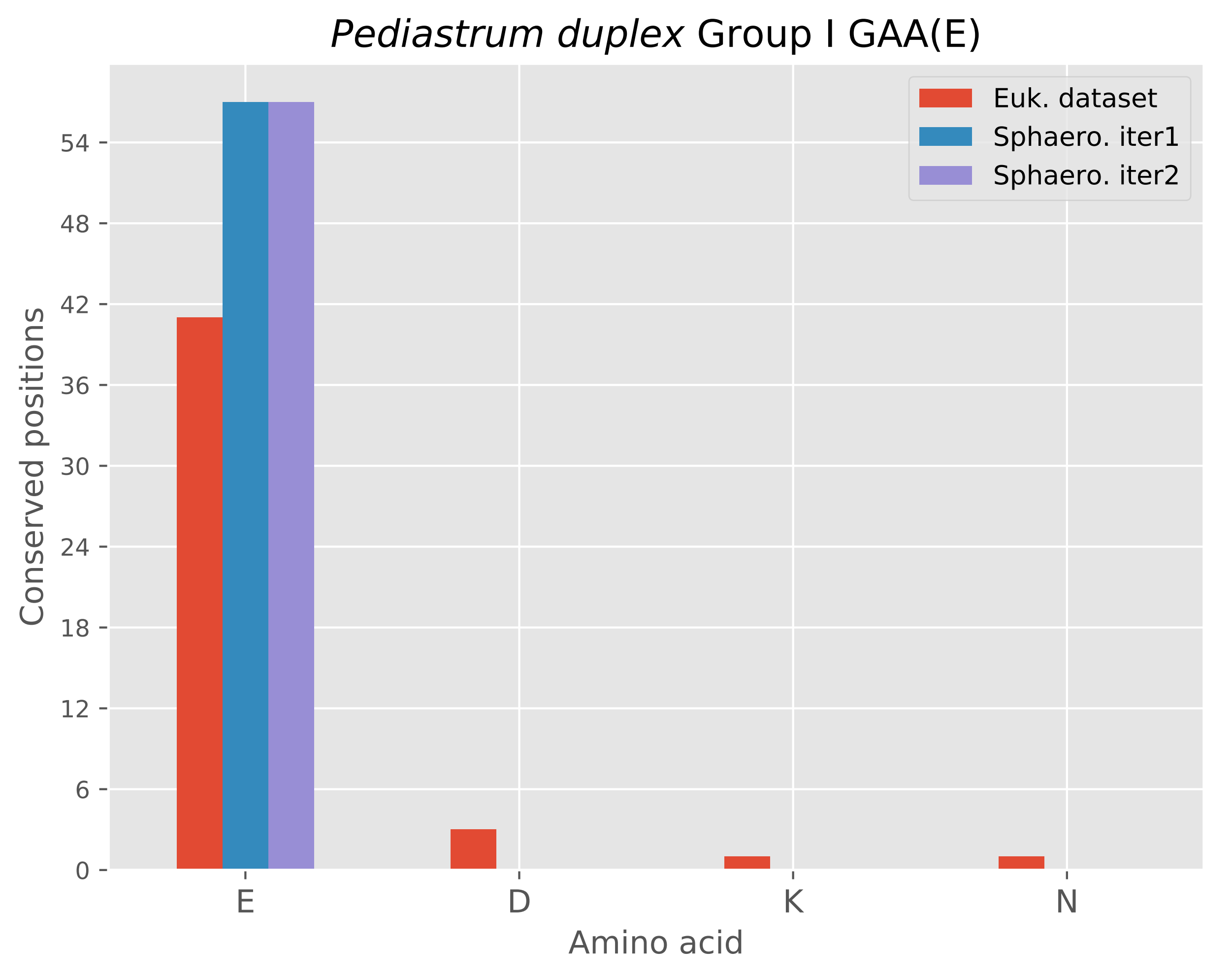

Pediastrum duplex Group I GAA(E)
Euk. dataset
Sphaero. iter1
Sphaero. iter2
Conserved positions
0
6
12
18
24
30
36
42
48
54
E
D
K
N
Amino acid

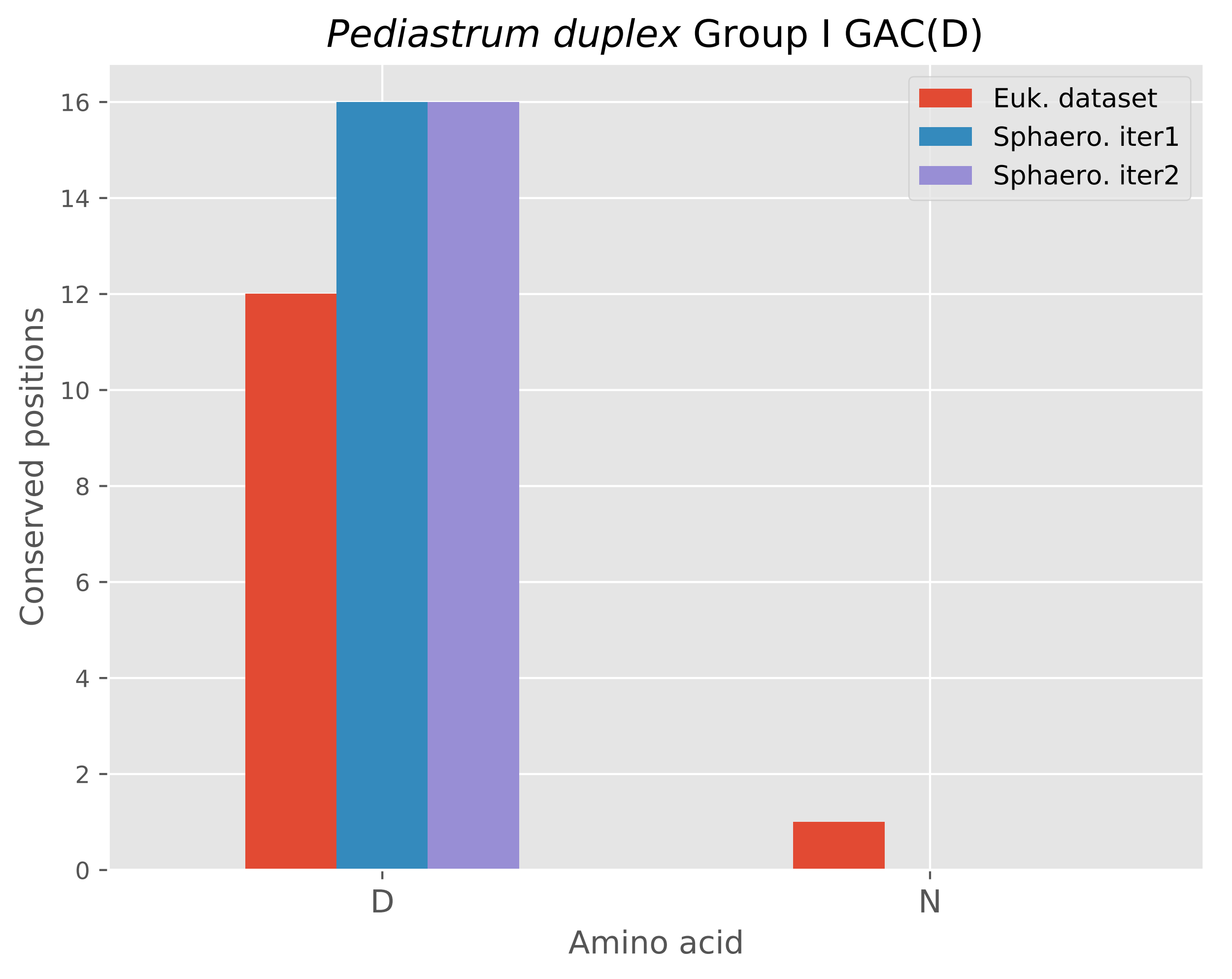
Pediastrum duplex Group I GAC(D)
Euk. dataset
Sphaero. iter1
Sphaero. iter2
Conserved positions
0
2
4
6
8
10
12
14
16
D
N
Amino acid

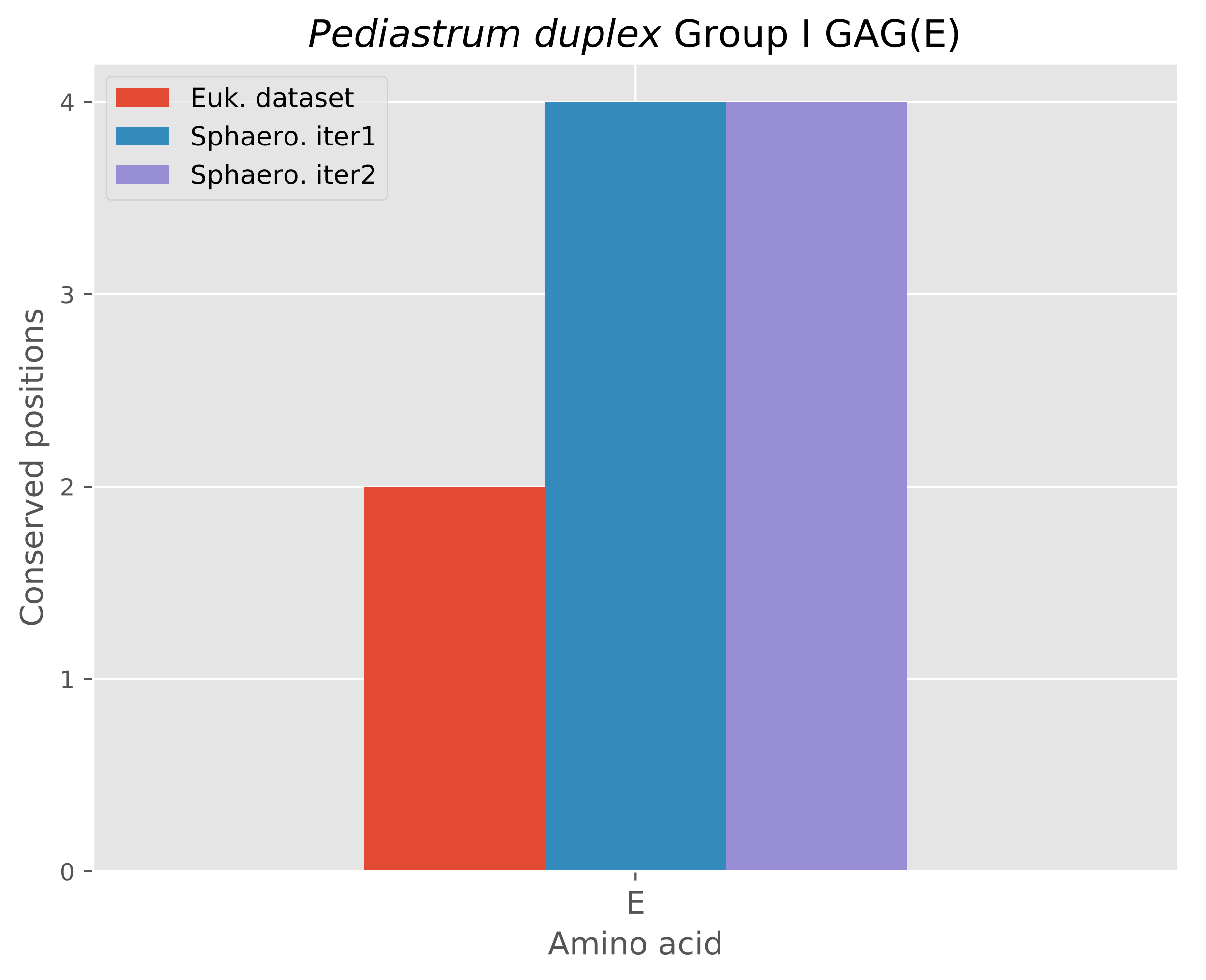

Pediastrum duplex Group I GAG(E)
Euk. dataset
Sphaero. iter1
Sphaero. iter2
Conserved positions
0
1
2
3
4
E
Amino acid

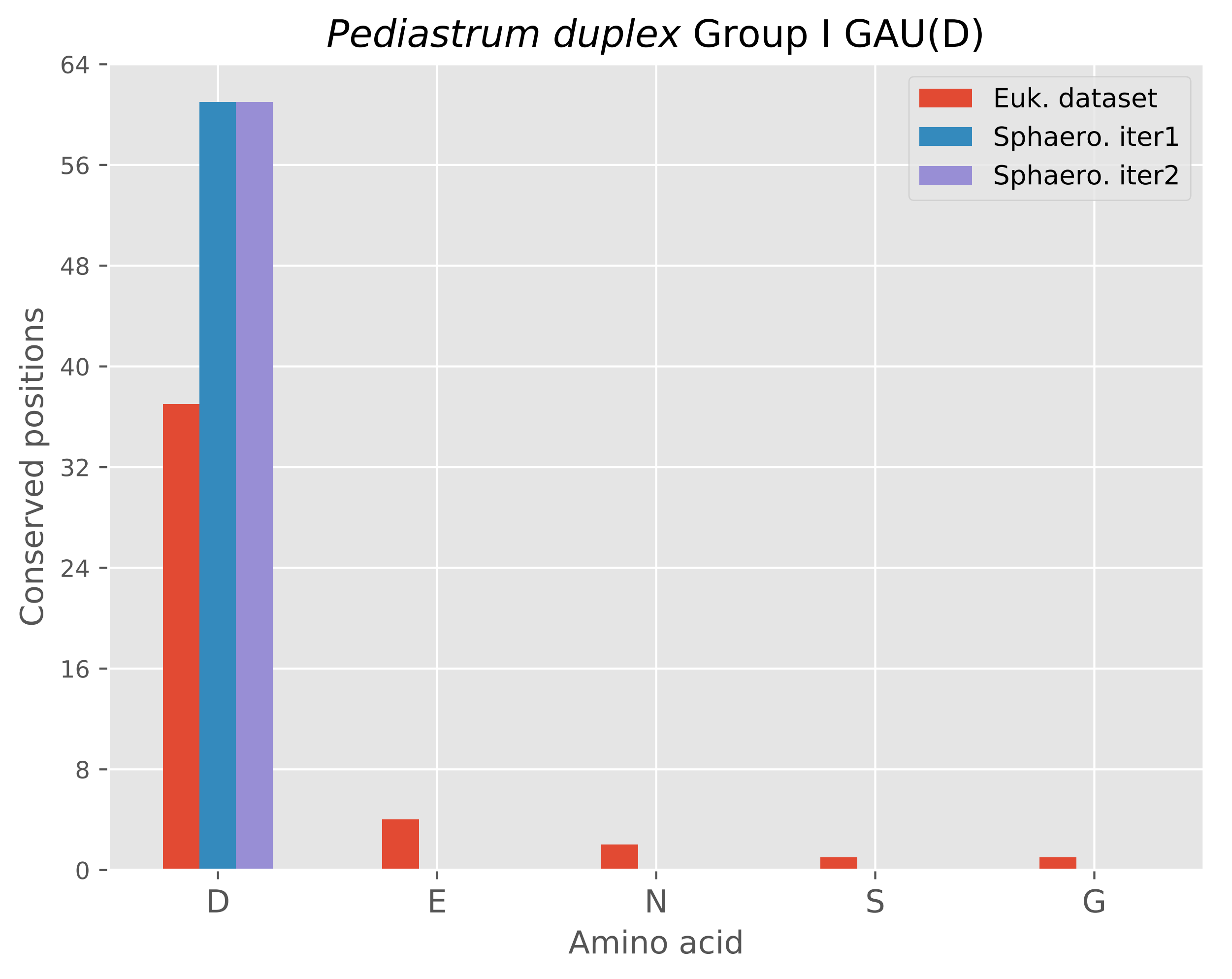
Pediastrum duplex Group I GAU(D)
Conserved positions
Amino acid
0
8
16
24
32
40
48
56
64
D
E
N
S
G
Euk. dataset
Sphaero. iter1
Sphaero. iter2

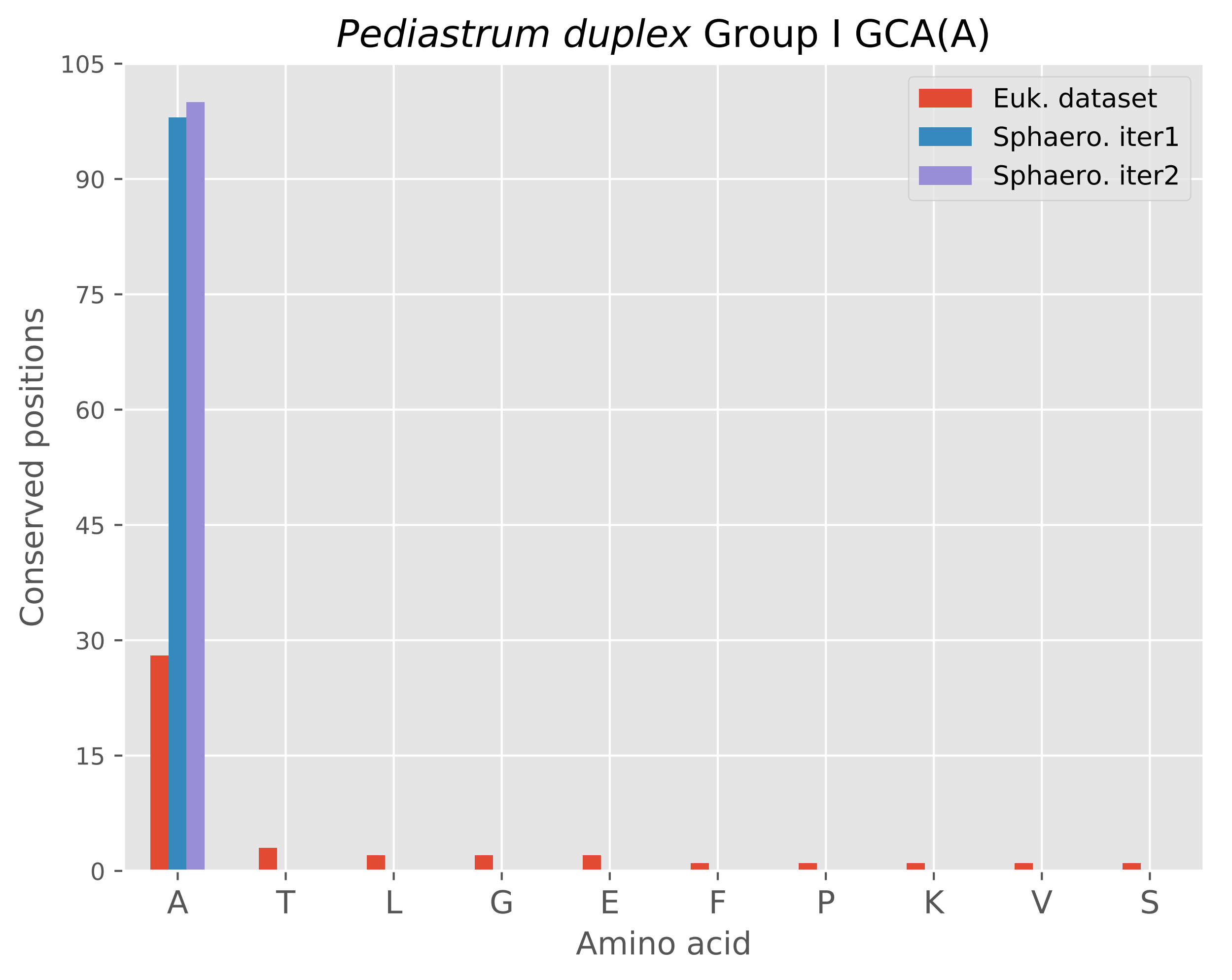

Pediastrum duplex Group I GCA(A)
Euk. dataset
Sphaero. iter1
Sphaero. iter2
Conserved positions
0
15
30
45
60
75
90
105
A
T
L
G
E
F
P
K
V
S
Amino acid

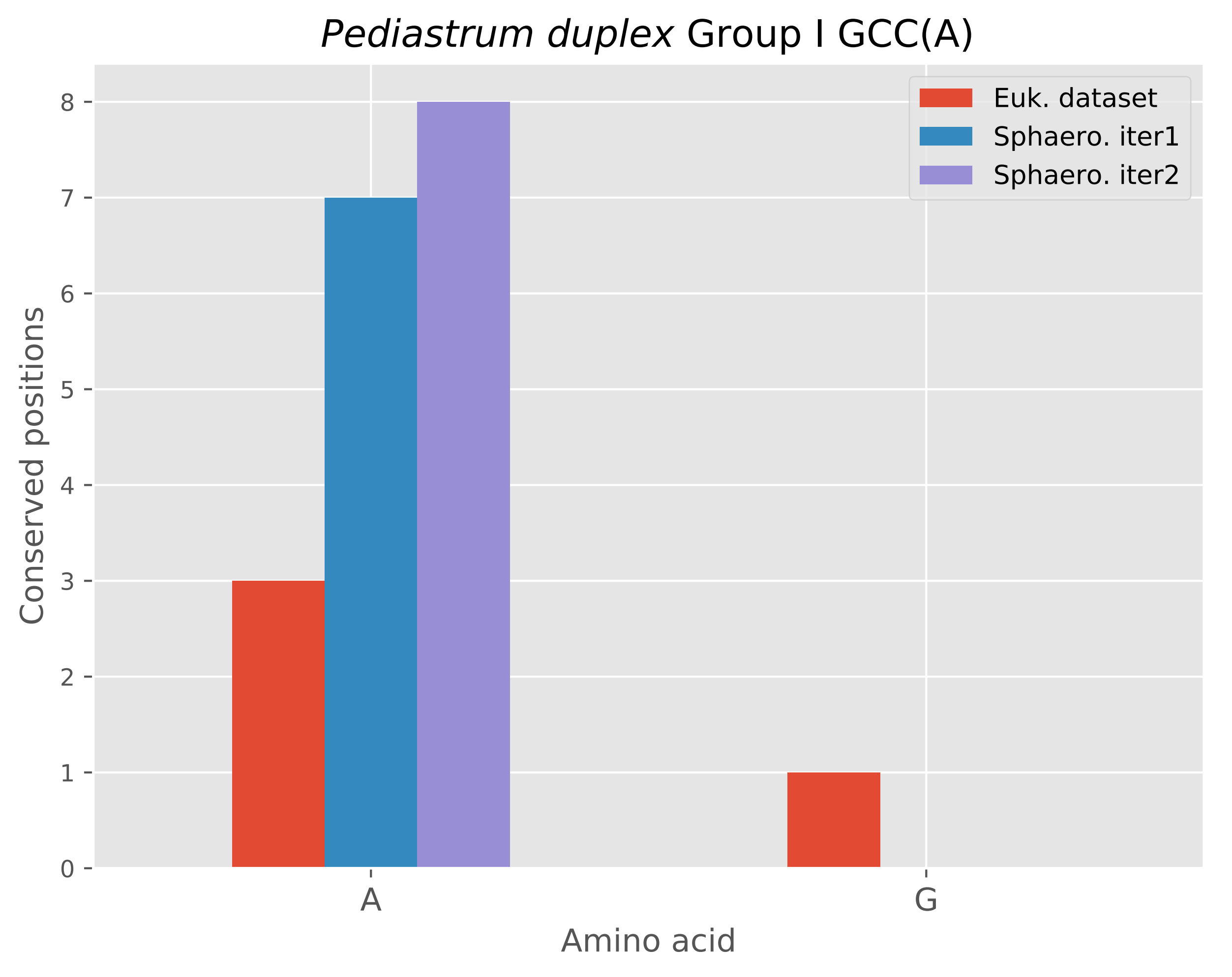

Pediastrum duplex Group I GCC(A)
Euk. dataset
Sphaero. iter1
Sphaero. iter2
Conserved positions
0
1
2
3
4
5
6
7
8
A
G
Amino acid

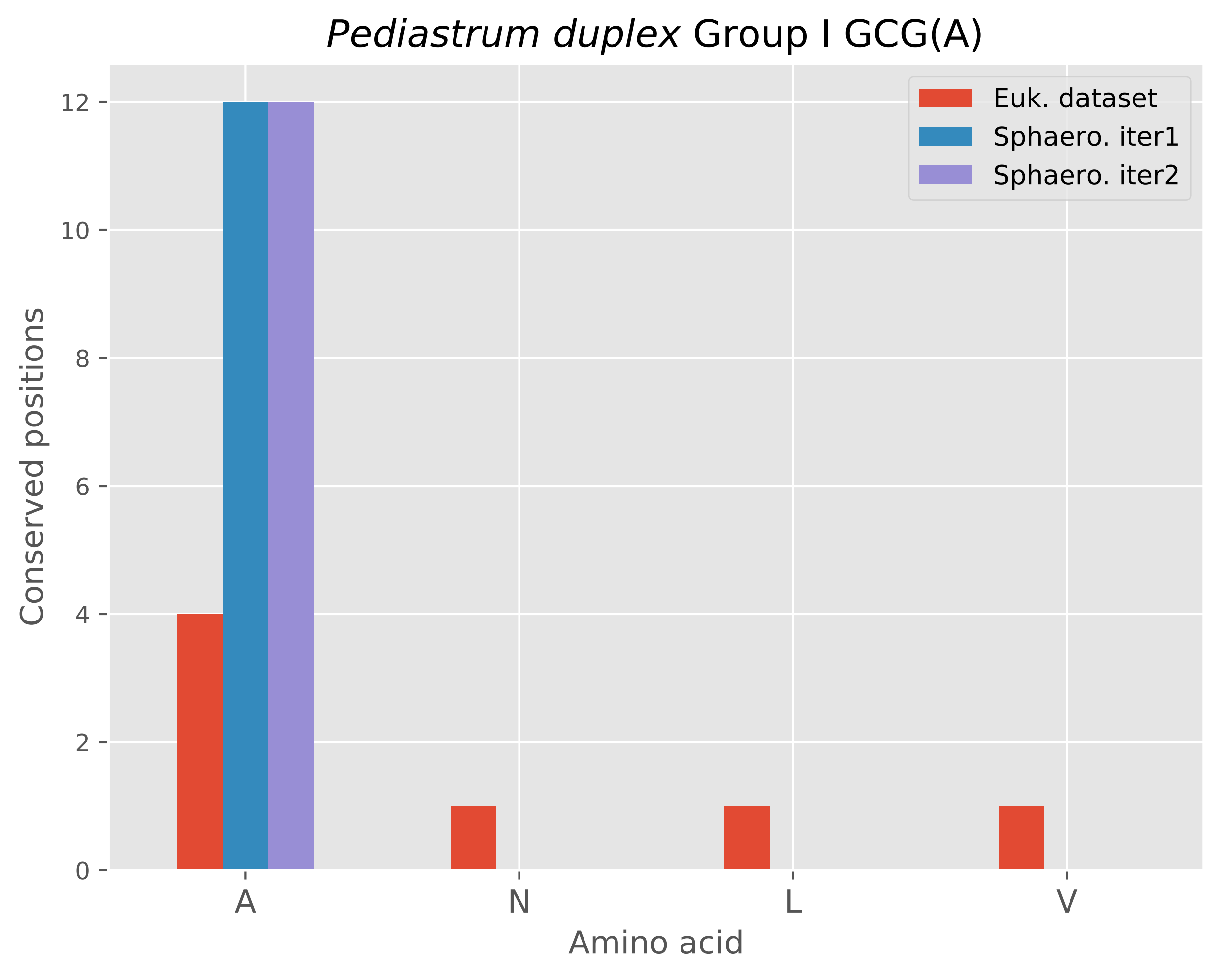

Pediastrum duplex Group I GCG(A)
Euk. dataset
Sphaero. iter1
Sphaero. iter2
Conserved positions
0
2
4
6
8
10
12
A
N
L
V
Amino acid

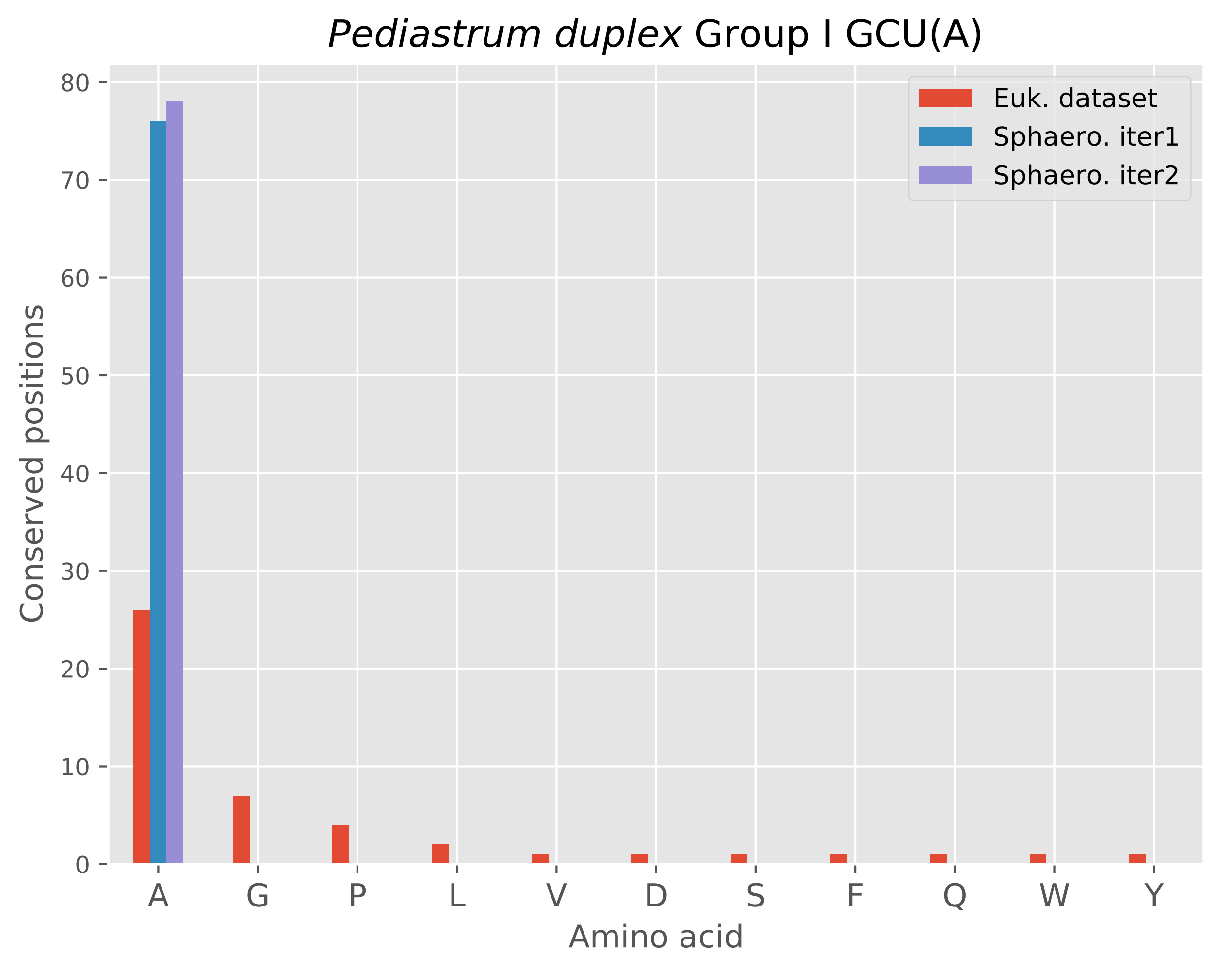
Pediastrum duplex Group I GCU(A)
Conserved positions
0
10
20
30
40
50
60
70
80
Amino acid
A
G
P
L
V
D
S
F
Q
W
Y
Euk. dataset
Sphaero. iter1
Sphaero. iter2

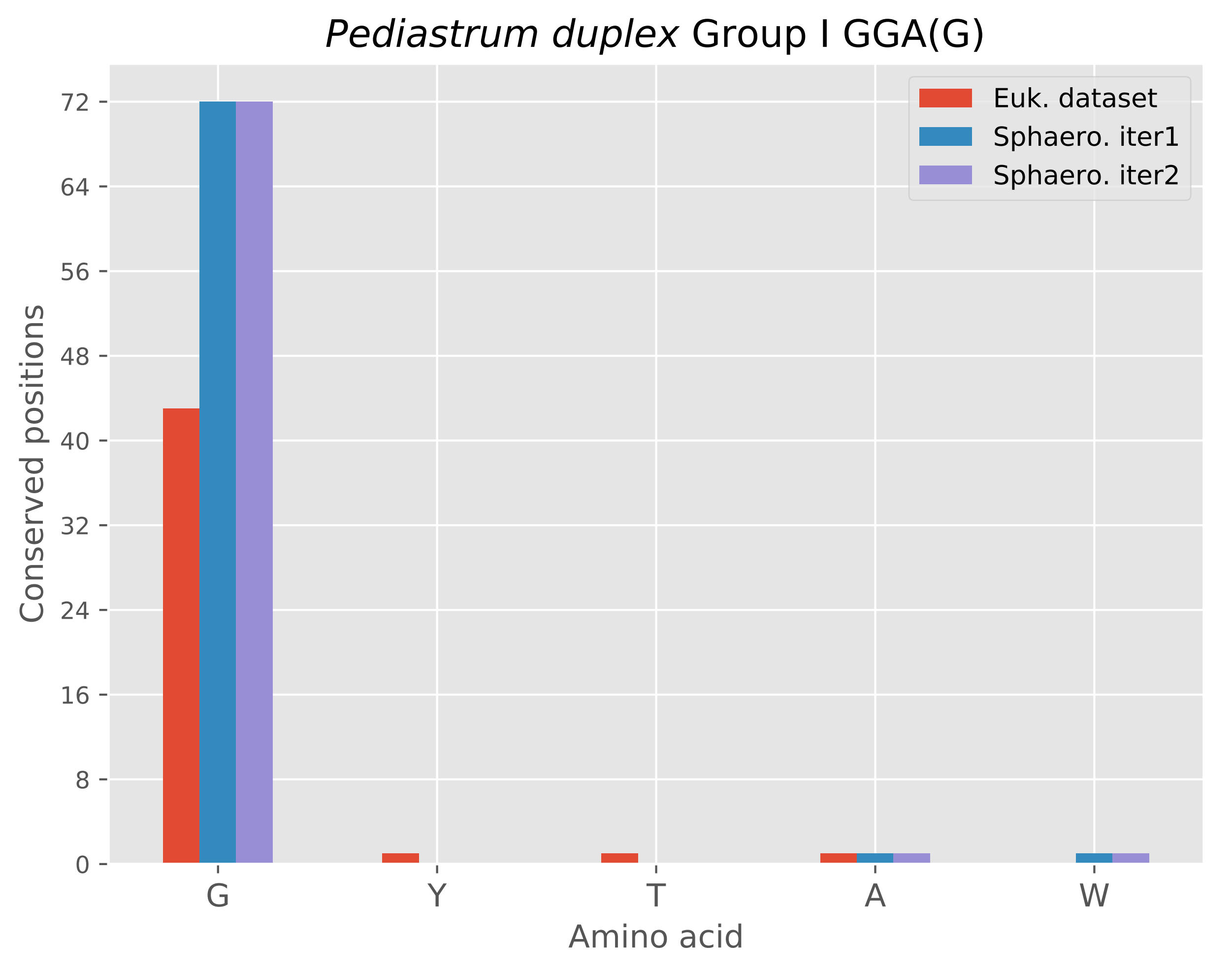
Pediastrum duplex Group I GGA(G)
Conserved positions
0
8
16
24
32
40
48
56
64
72
G
Y
T
A
W
Amino acid
Euk. dataset
Sphaero. iter1
Sphaero. iter2

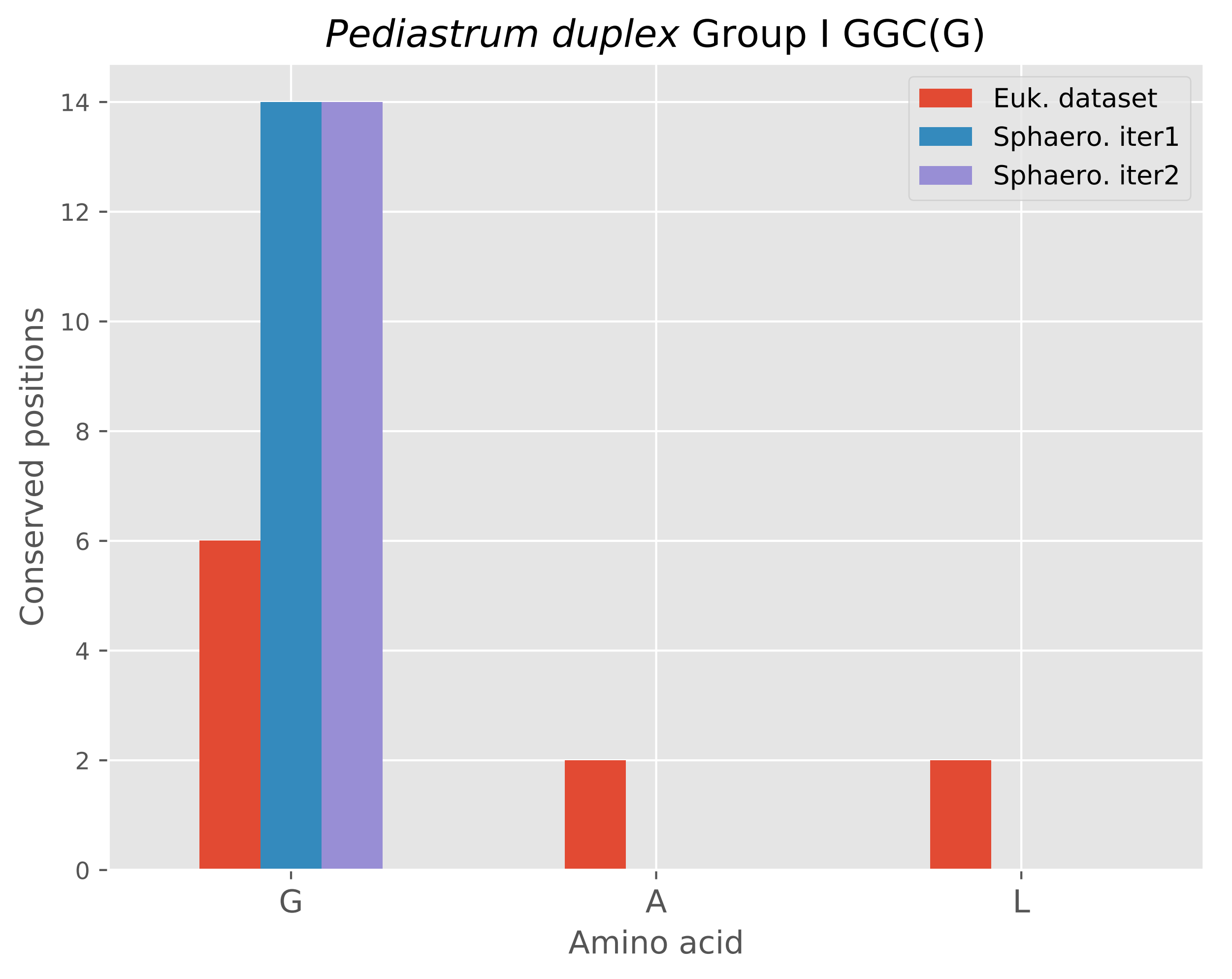
Pediastrum duplex Group I GGC(G)
Euk. dataset
Sphaero. iter1
Sphaero. iter2
Conserved positions
0
2
4
6
8
10
12
14
G
A
L
Amino acid

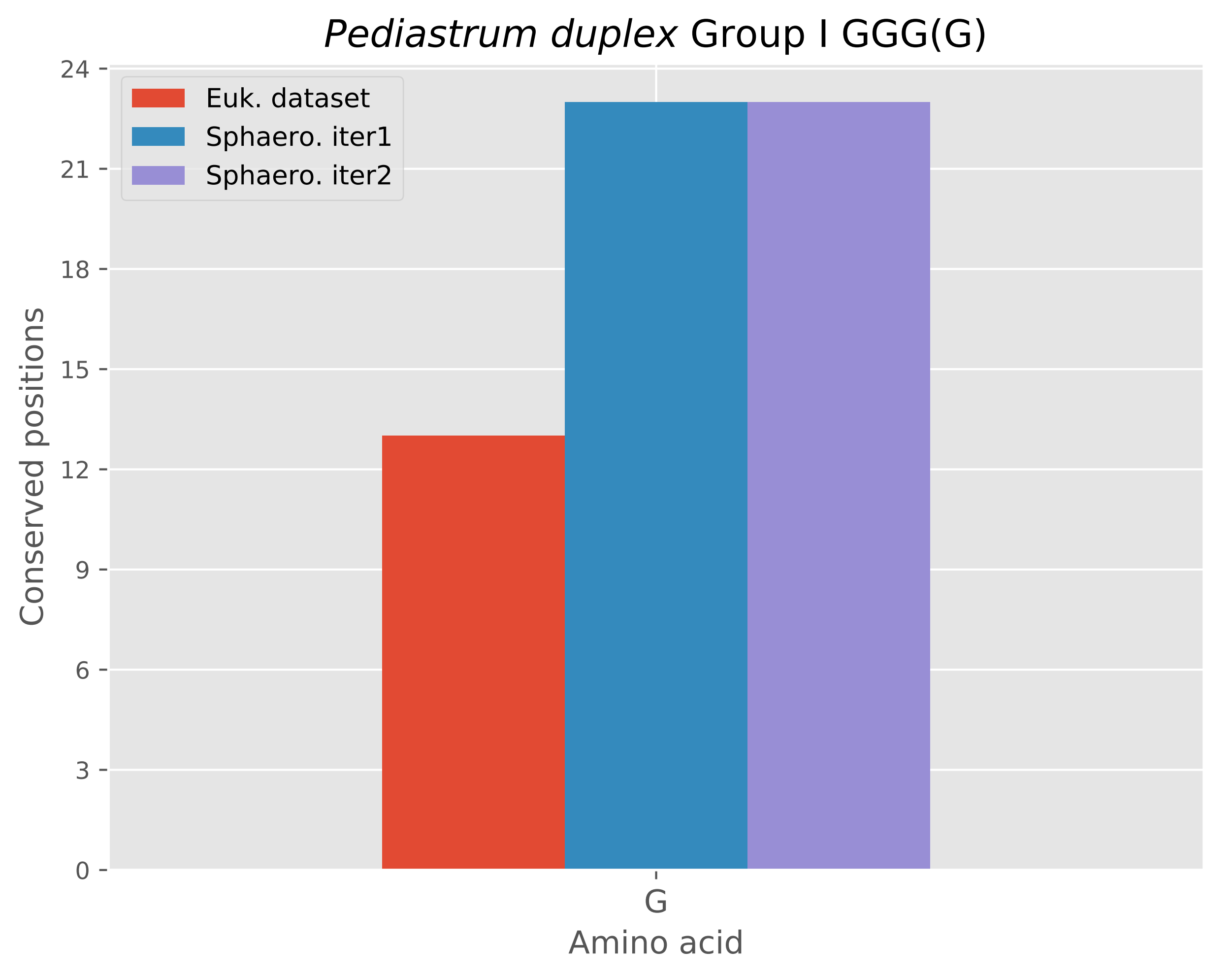

Pediastrum duplex Group I GGG(G)
Euk. dataset
Sphaero. iter1
Sphaero. iter2
Conserved positions
0
3
6
9
12
15
18
21
24
G
Amino acid

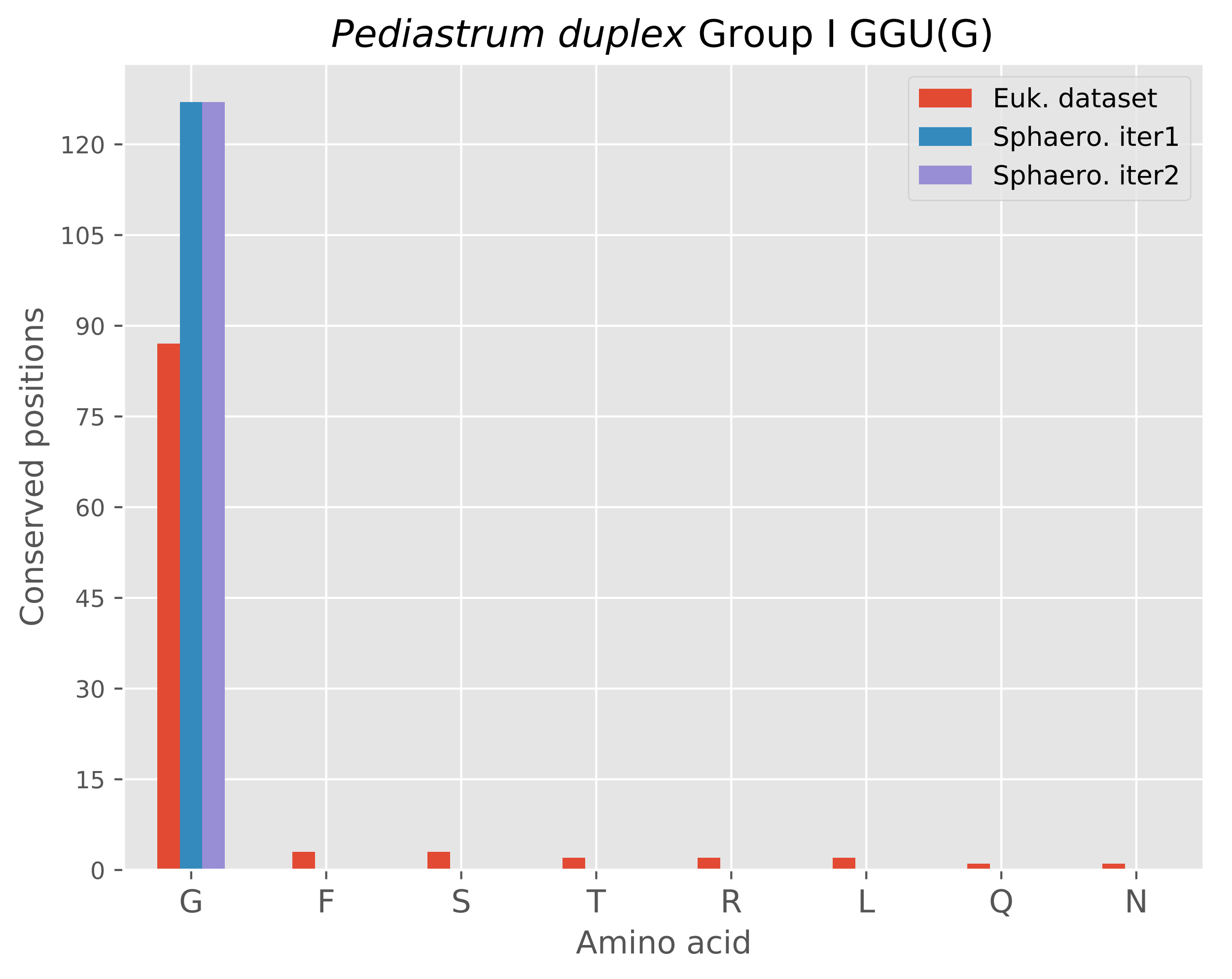

Pediastrum duplex Group I GGU(G)
Euk. dataset
Sphaero. iter1
Sphaero. iter2
Conserved positions
0
15
30
45
60
75
90
105
120
G
F
S
T
R
L
Q
N
Amino acid

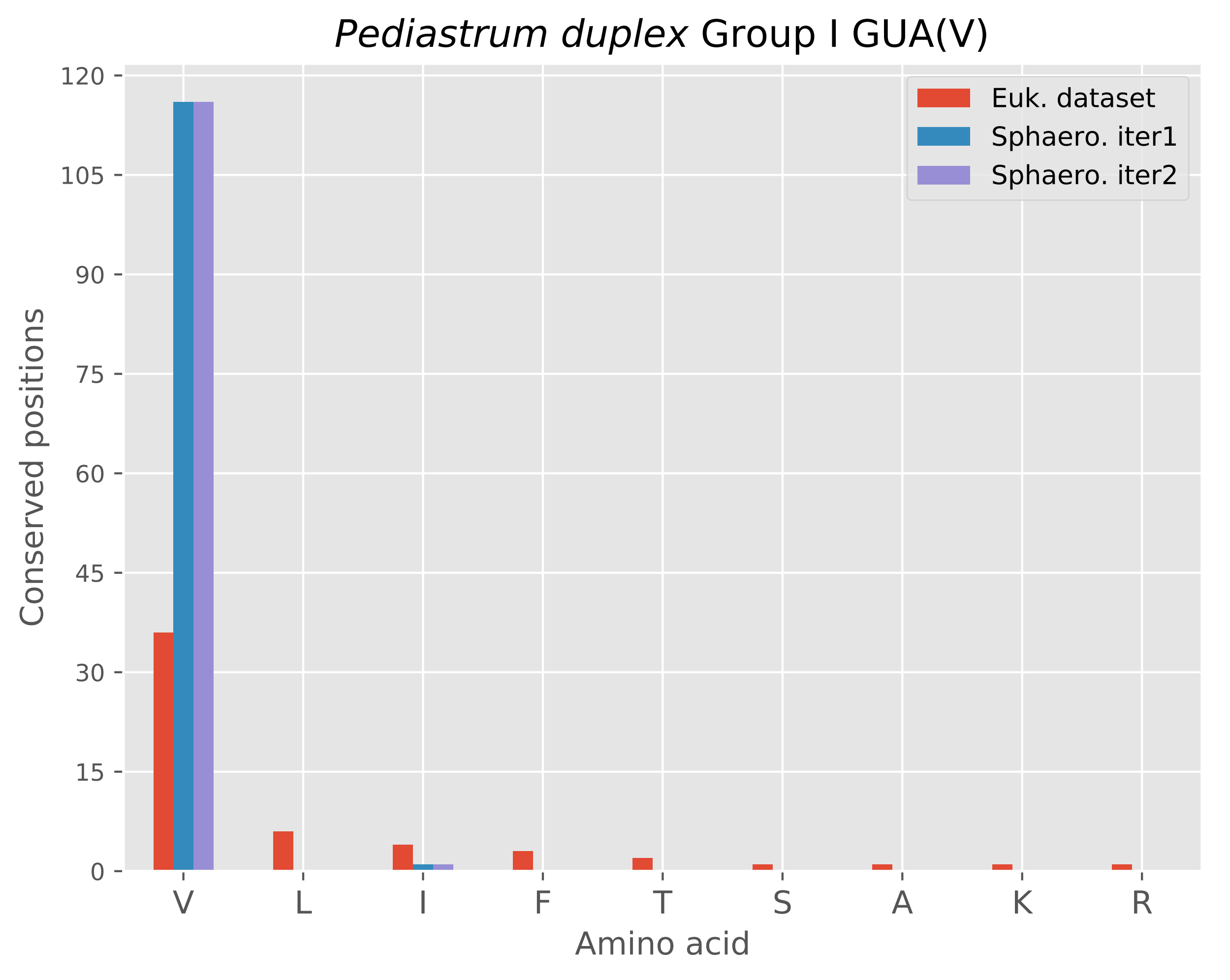

Pediastrum duplex Group I GUA(V)
Euk. dataset
Sphaero. iter1
Sphaero. iter2
Conserved positions
0
15
30
45
60
75
90
105
120
V
L
I
F
T
S
A
K
R
Amino acid

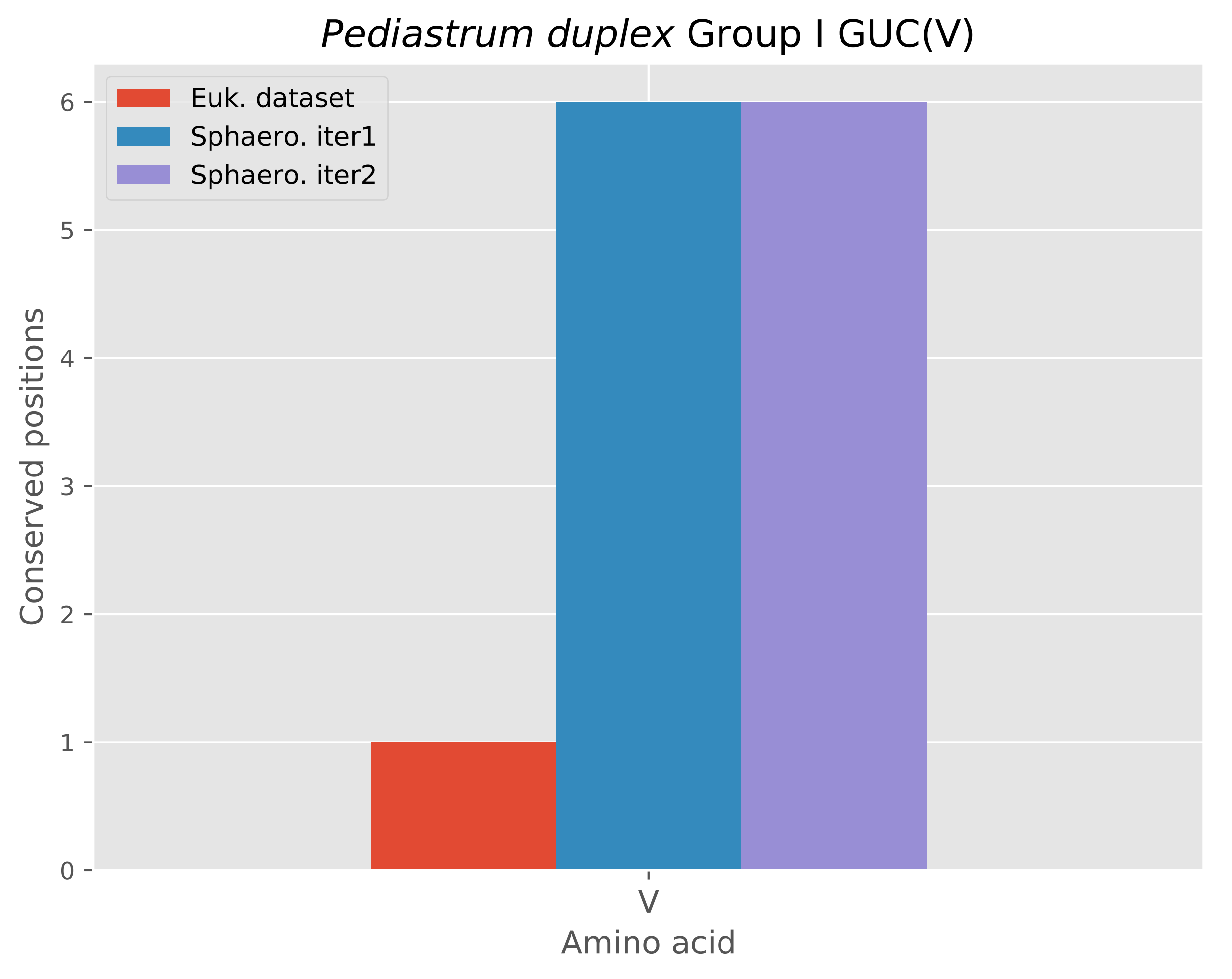

Pediastrum duplex Group I GUC(V)
Euk. dataset
Sphaero. iter1
Sphaero. iter2
Conserved positions
0
1
2
3
4
5
6
V
Amino acid

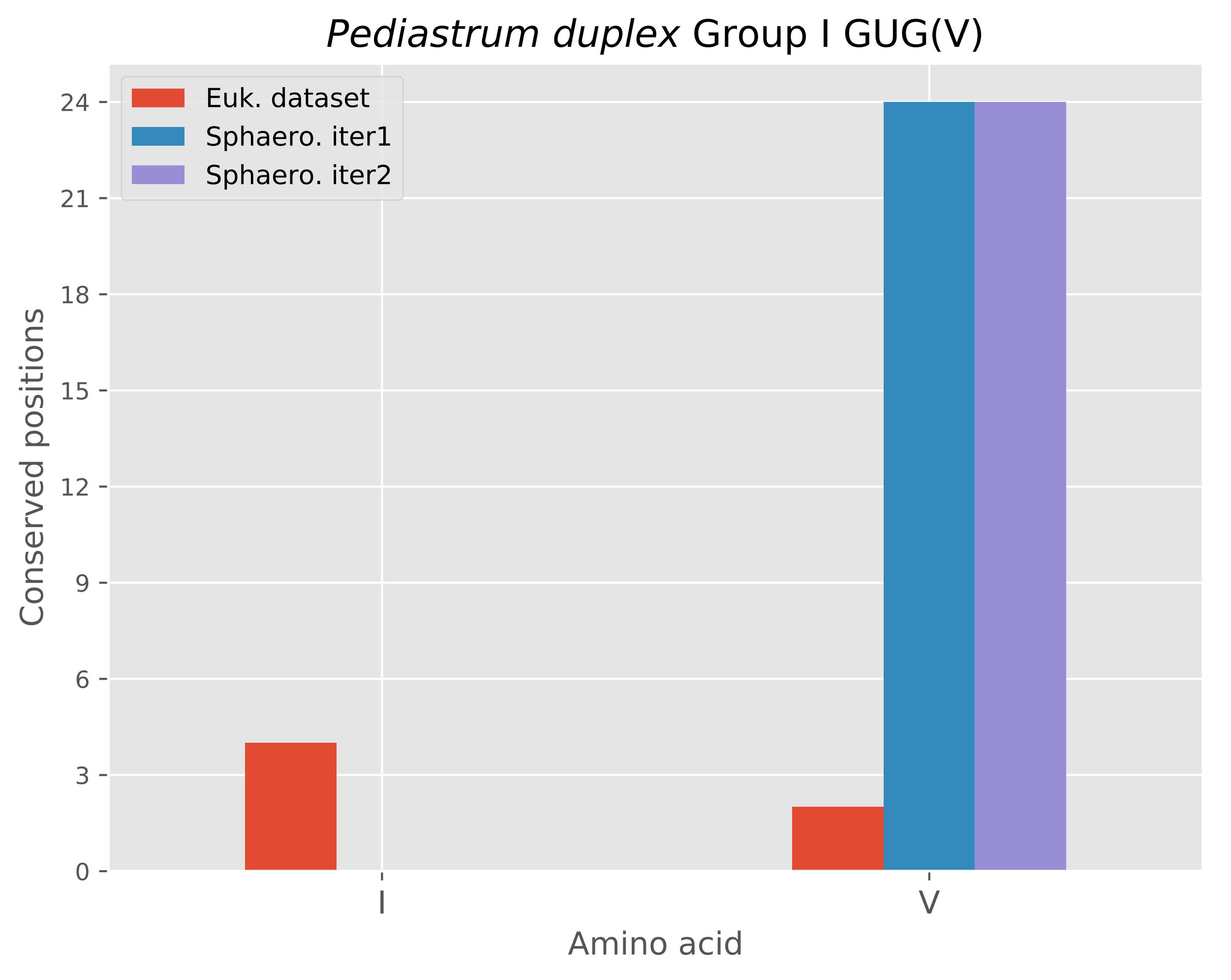

Pediastrum duplex Group I GUG(V)
Euk. dataset
Sphaero. iter1
Sphaero. iter2
Conserved positions
0
3
6
9
12
15
18
21
24
I
V
Amino acid

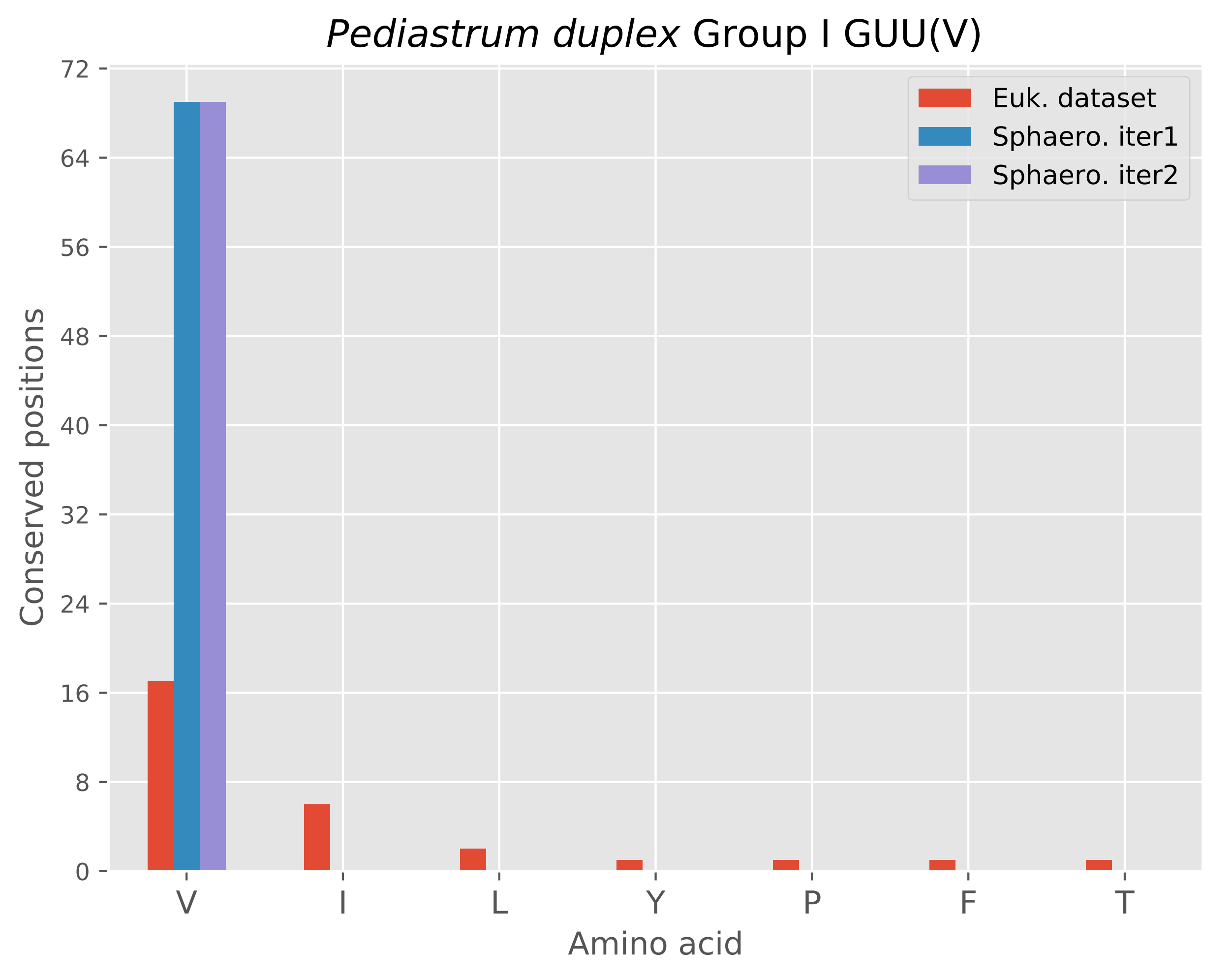

Pediastrum duplex Group I GUU(V)
Euk. dataset
Sphaero. iter1
Sphaero. iter2
Conserved positions
0
8
16
24
32
40
48
56
64
72
V
I
L
Y
P
F
T
Amino acid

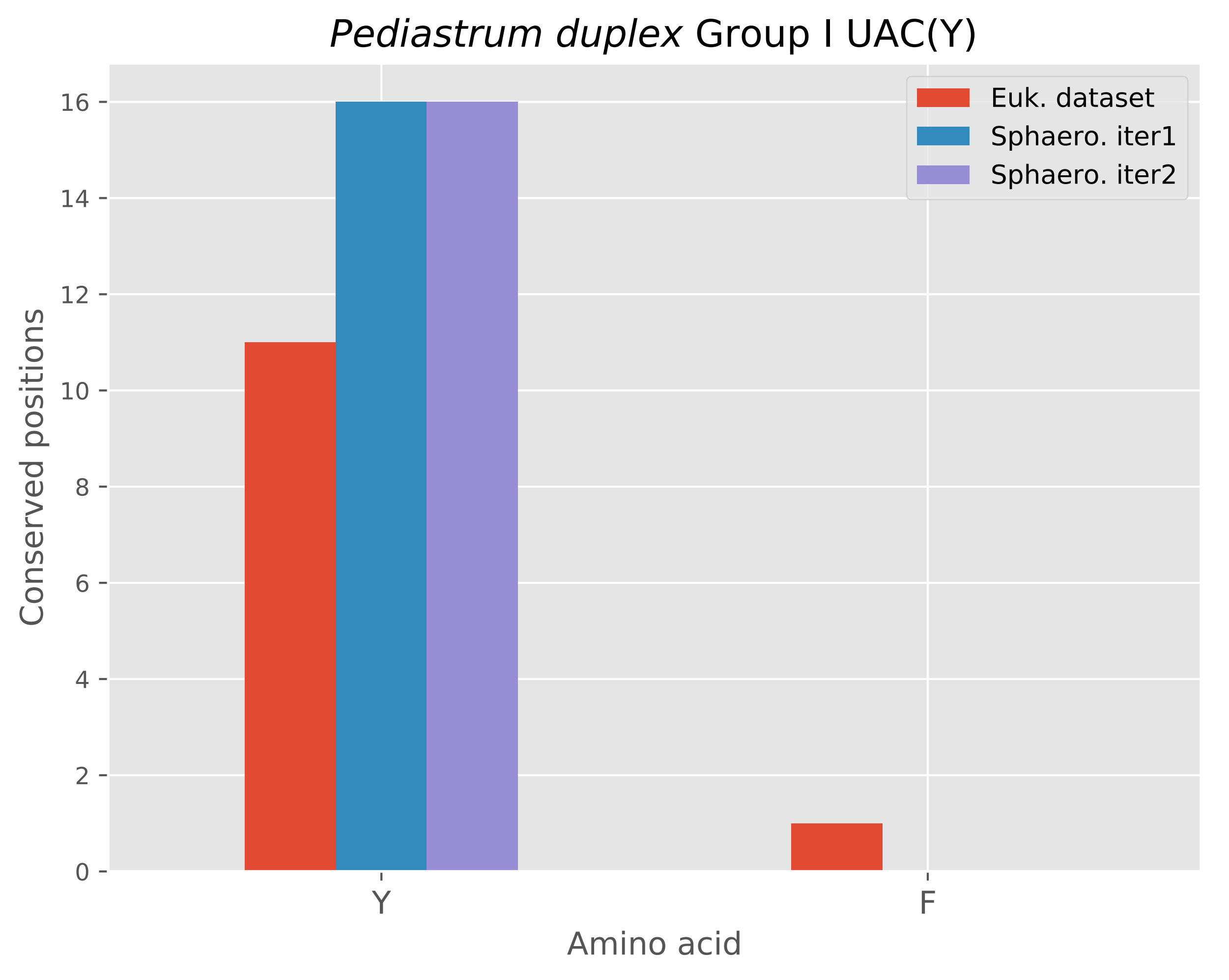
Pediastrum duplex Group I UAC(Y)
Euk. dataset
Sphaero. iter1
Sphaero. iter2
Conserved positions
0
2
4
6
8
10
12
14
16
Y
F
Amino acid

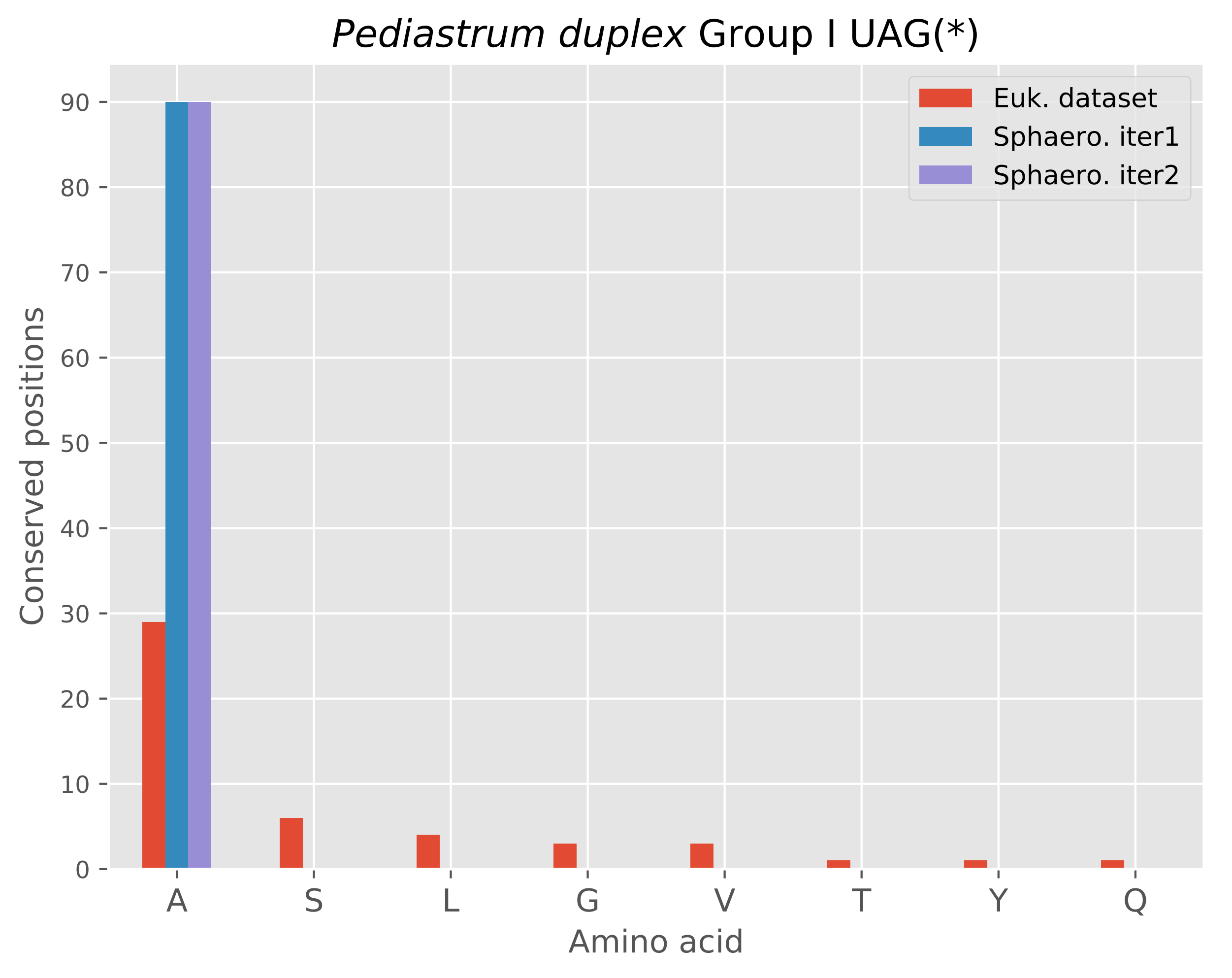

Pediastrum duplex Group I UAG(*)
Euk. dataset
Sphaero. iter1
Sphaero. iter2
Conserved positions
0
10
20
30
40
50
60
70
80
90
A
S
L
G
V
T
Y
Q
Amino acid

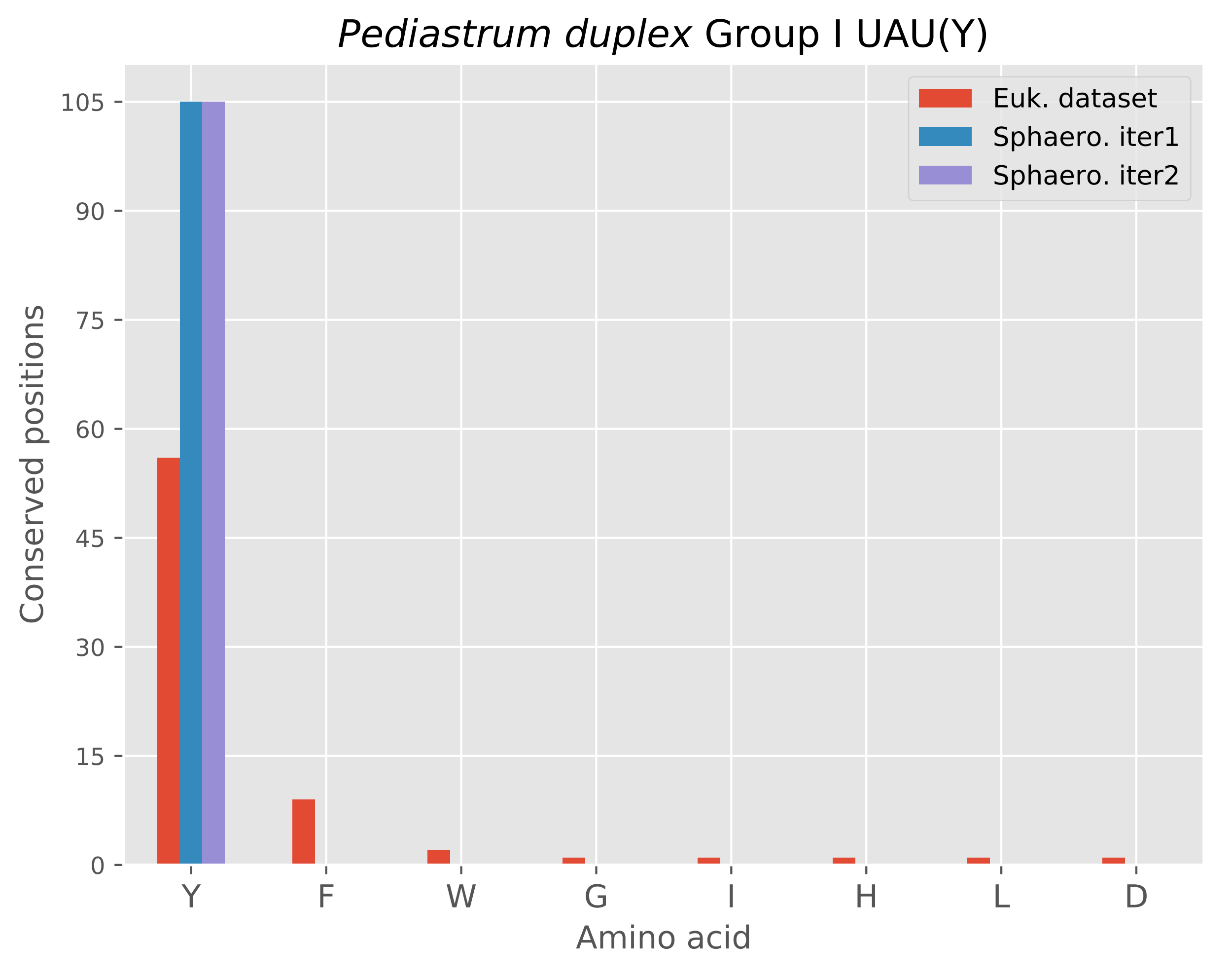

Pediastrum duplex Group I UAU(Y)
Euk. dataset
Sphaero. iter1
Sphaero. iter2
Conserved positions
0
15
30
45
60
75
90
105
Y
F
W
G
I
H
L
D
Amino acid

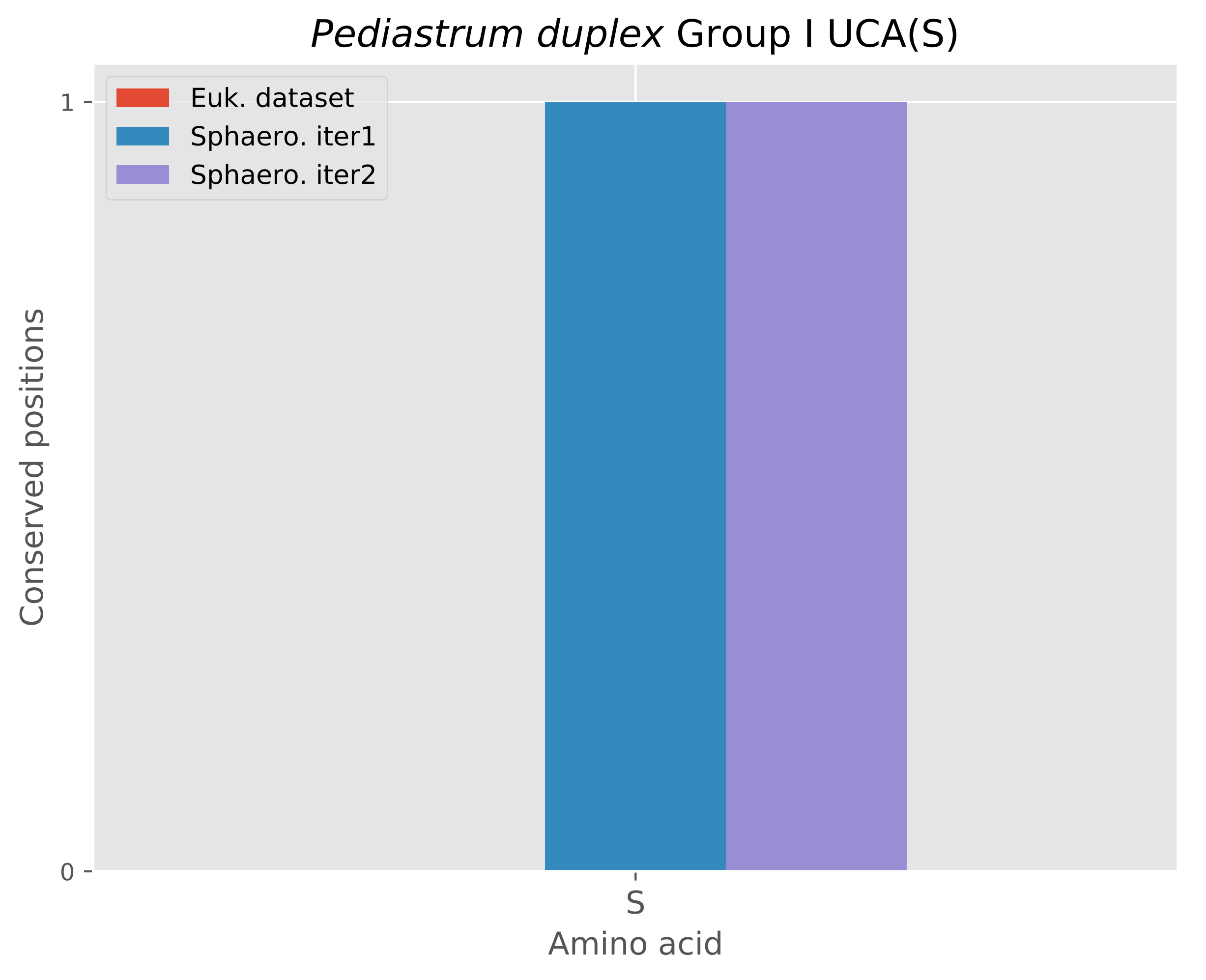

Pediastrum duplex Group I UCA(S)
Euk. dataset
Sphaero. iter1
Sphaero. iter2
Conserved positions
1
0
S
Amino acid

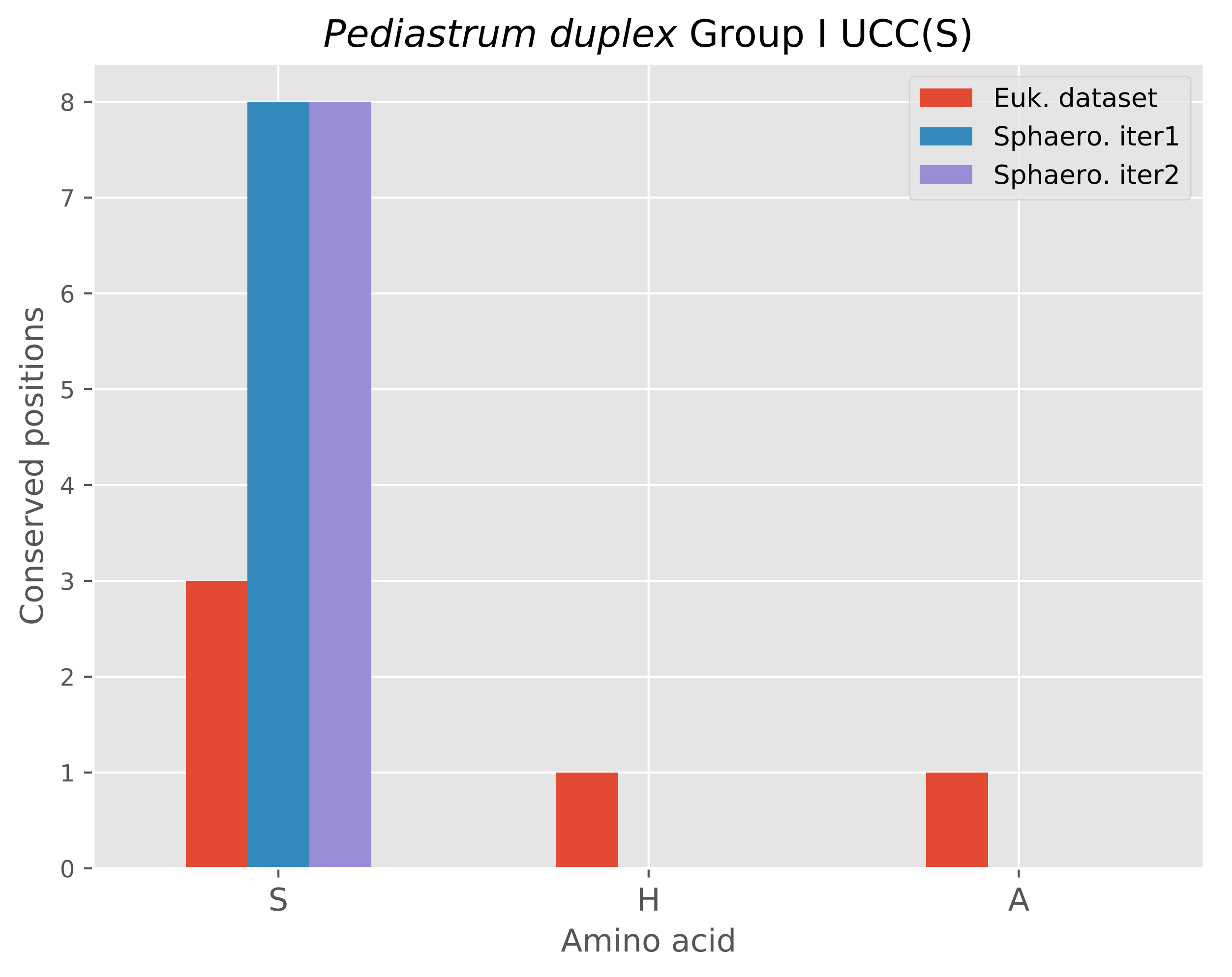
Pediastrum duplex Group I UCC(S)
Euk. dataset
Sphaero. iter1
Sphaero. iter2
Conserved positions
0
1
2
3
4
5
6
7
8
S
H
A
Amino acid

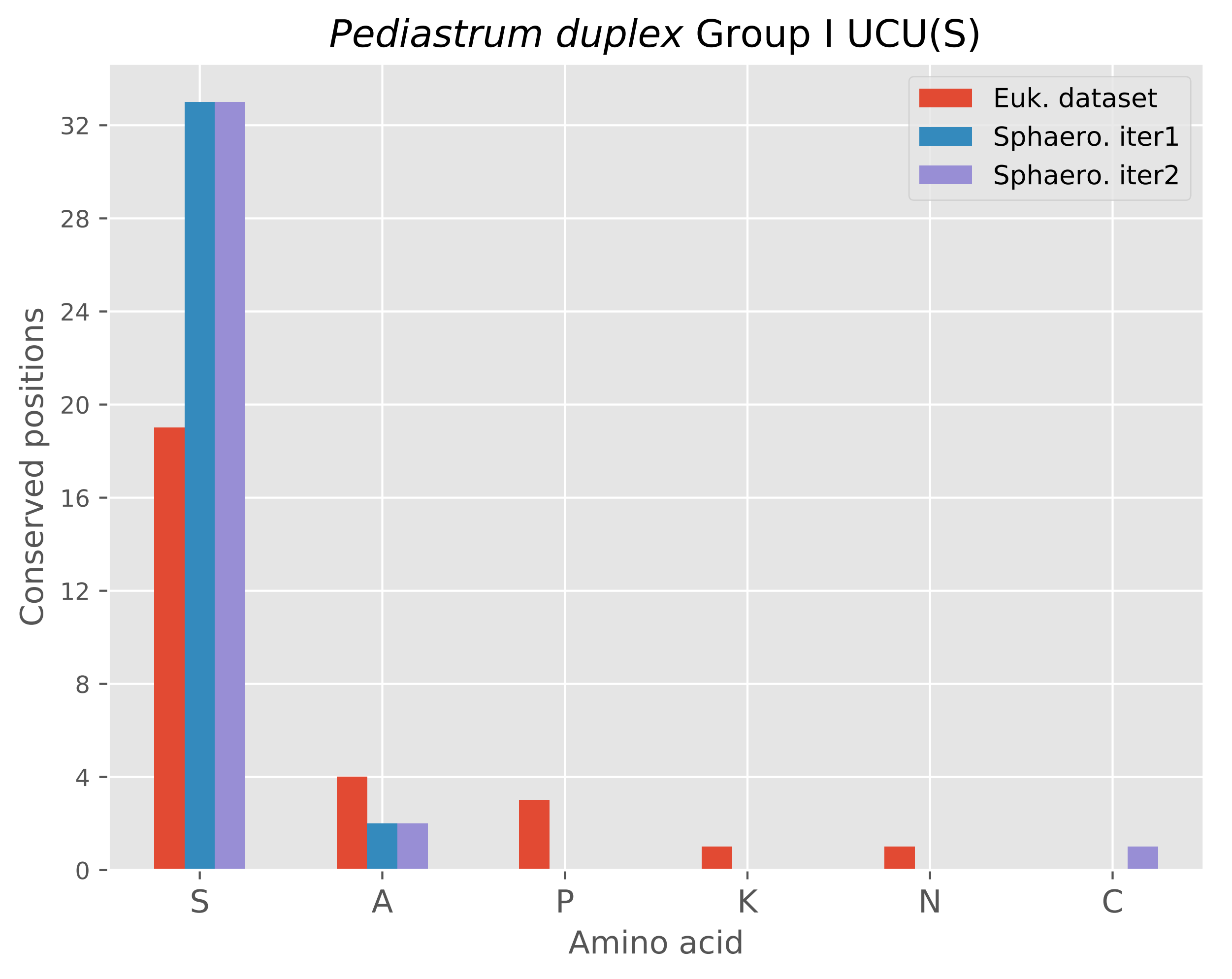

Pediastrum duplex Group I UCU(S)
Euk. dataset
Sphaero. iter1
Sphaero. iter2
Conserved positions
0
4
8
12
16
20
24
28
32
S
A
P
K
N
C
Amino acid

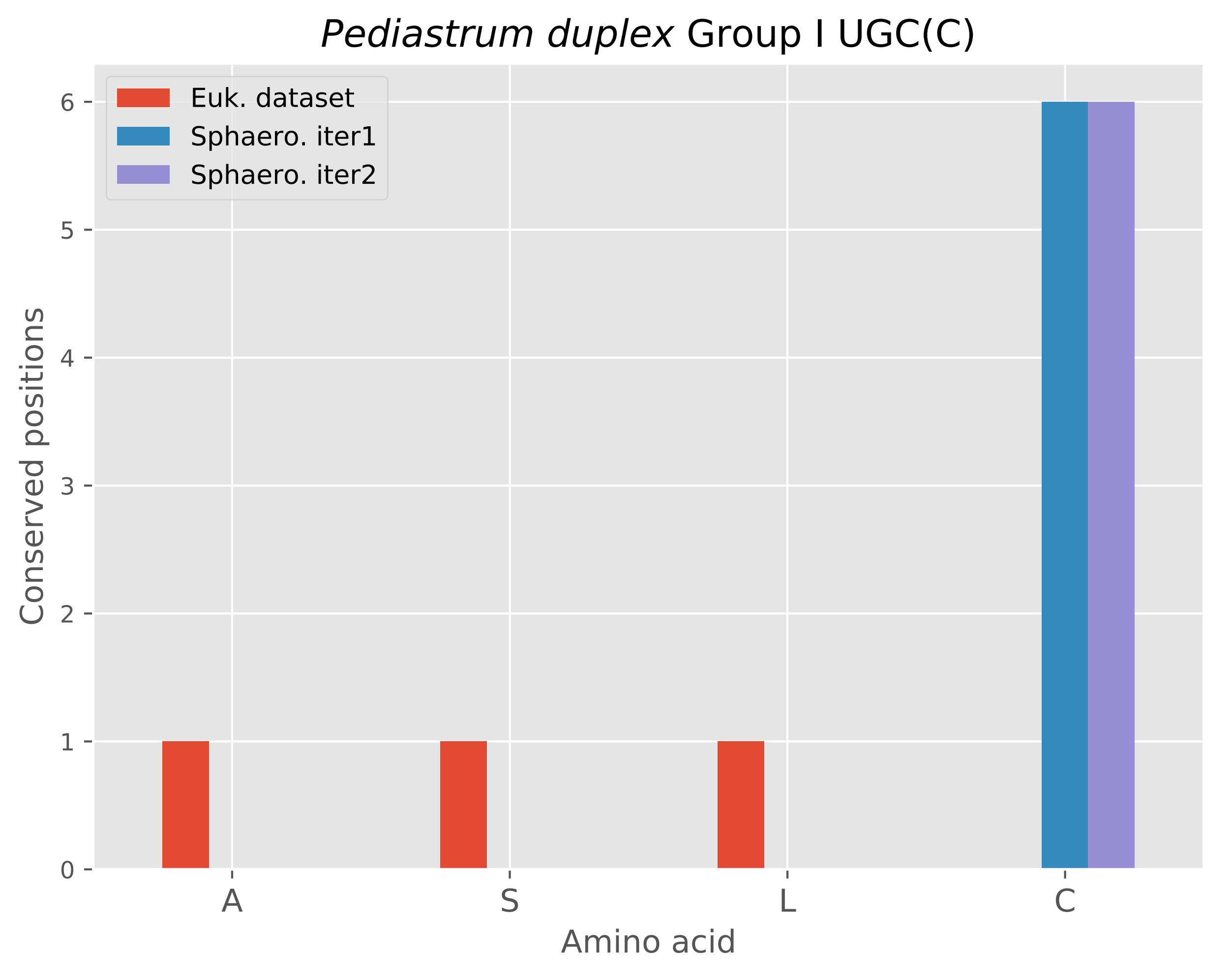
Pediastrum duplex Group I UGC(C)
Euk. dataset
Sphaero. iter1
Sphaero. iter2
Conserved positions
0
1
2
3
4
5
6
A
S
L
C
Amino acid

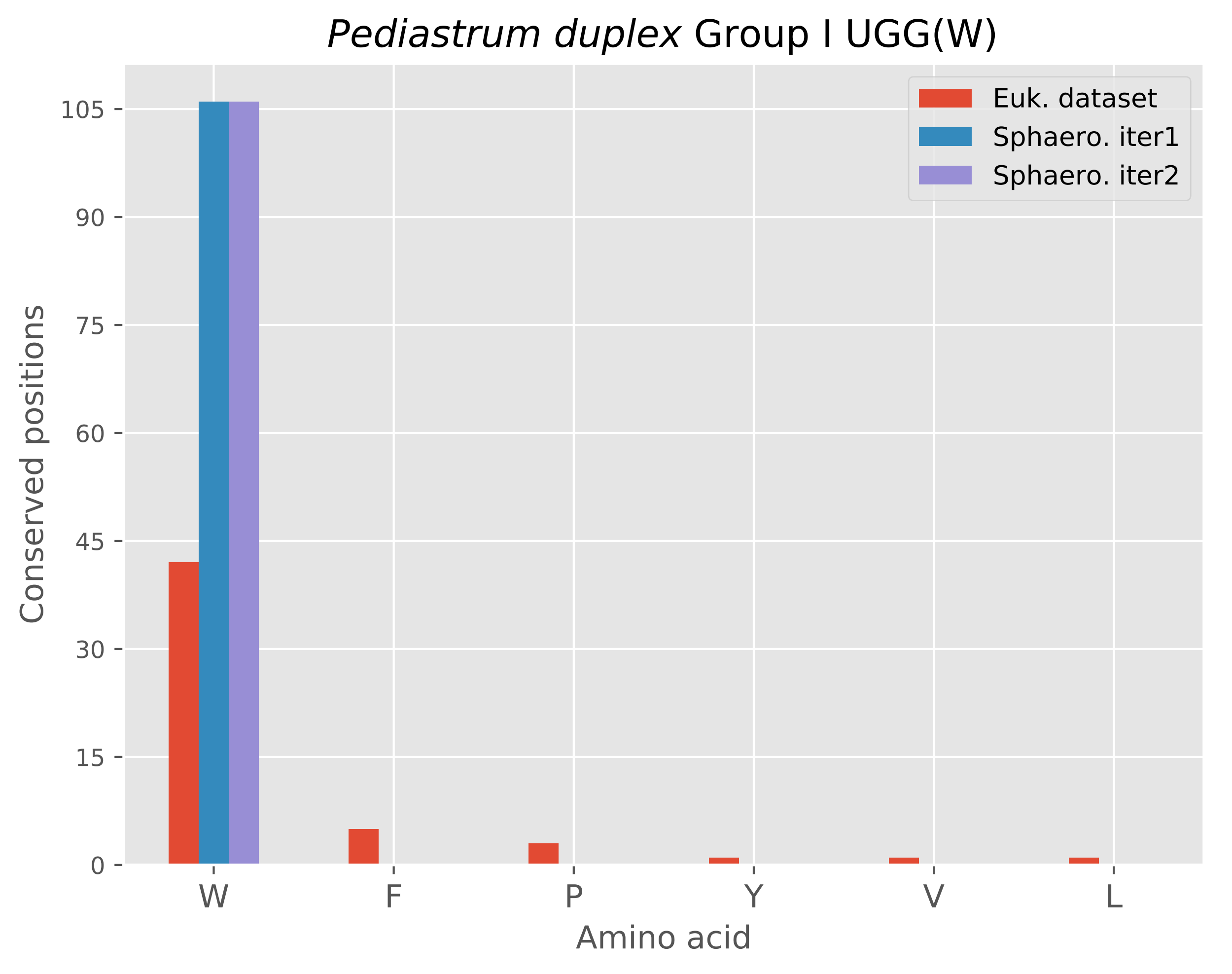
Pediastrum duplex Group I UGG(W)
Euk. dataset
Sphaero. iter1
Sphaero. iter2
Conserved positions
0
15
30
45
60
75
90
105
W
F
P
Y
V
L
Amino acid

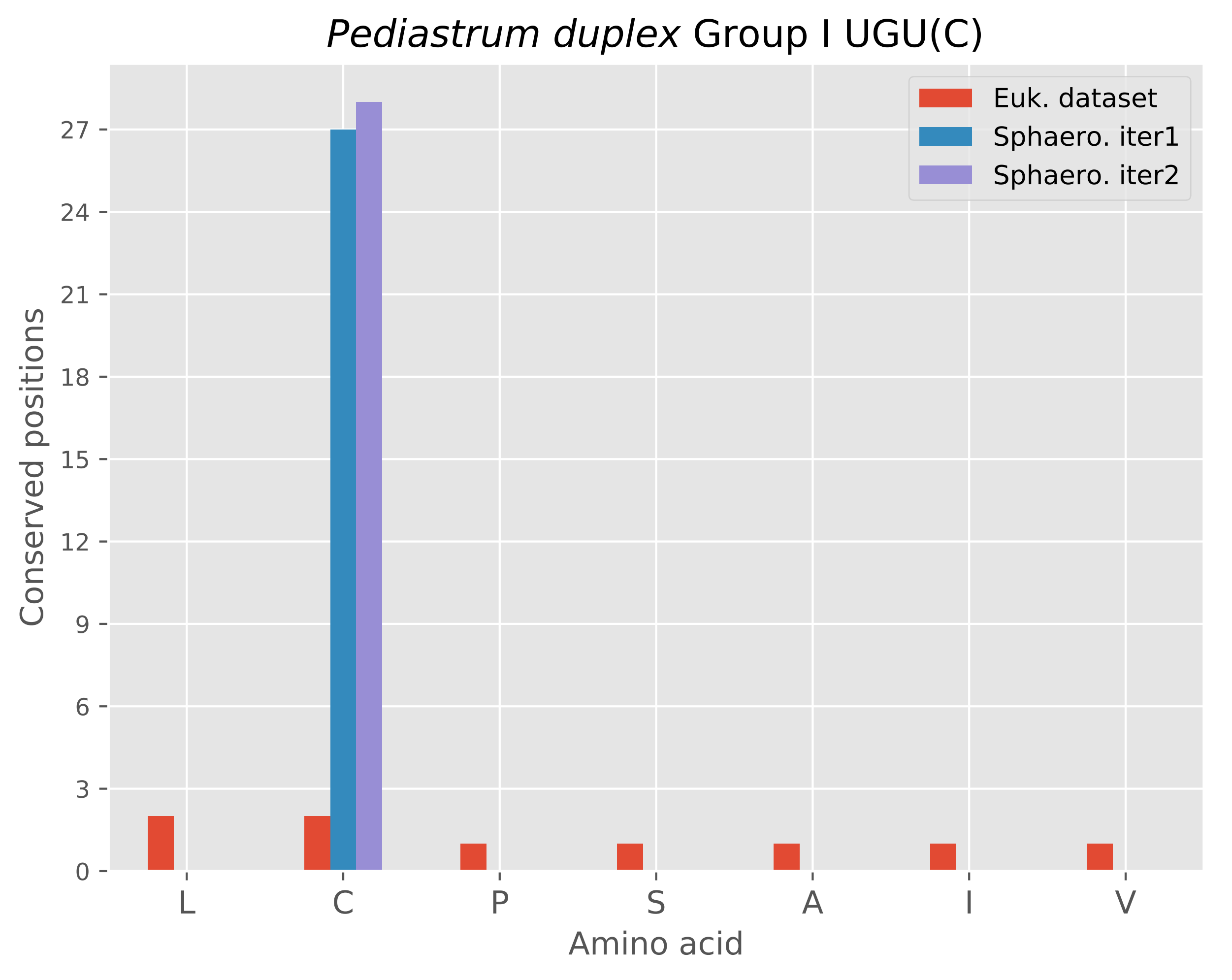

Pediastrum duplex Group I UGU(C)
Euk. dataset
Sphaero. iter1
Sphaero. iter2
Conserved positions
0
3
6
9
12
15
18
21
24
27
L
C
P
S
A
I
V
Amino acid

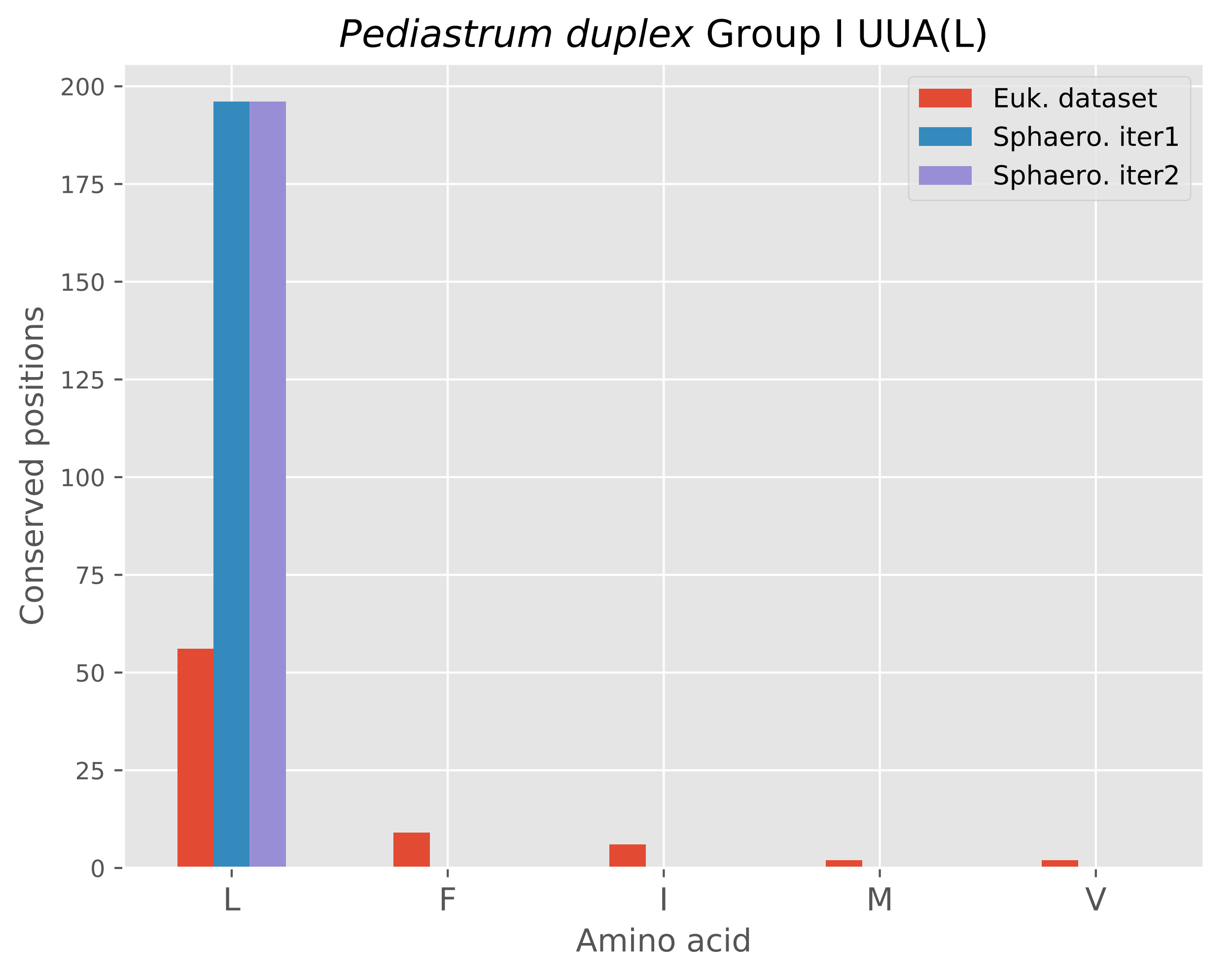

Pediastrum duplex Group I UUA(L)
Euk. dataset
Sphaero. iter1
Sphaero. iter2
Conserved positions
0
25
50
75
100
125
150
175
200
L
F
I
M
V
Amino acid

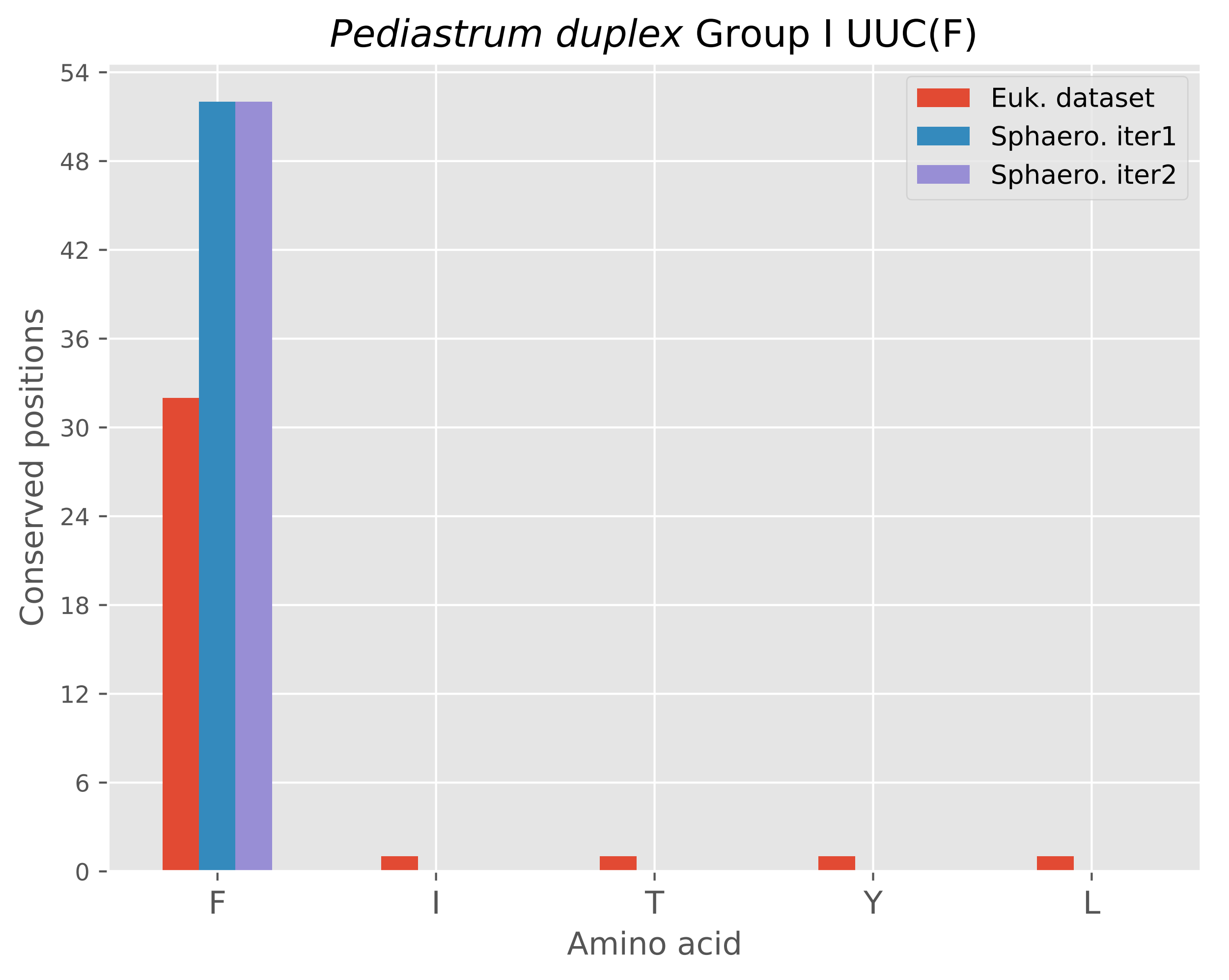

Pediastrum duplex Group I UUC(F)
Conserved positions
Amino acid
0
6
12
18
24
30
36
42
48
54
F
I
T
Y
L
Euk. dataset
Sphaero. iter1
Sphaero. iter2

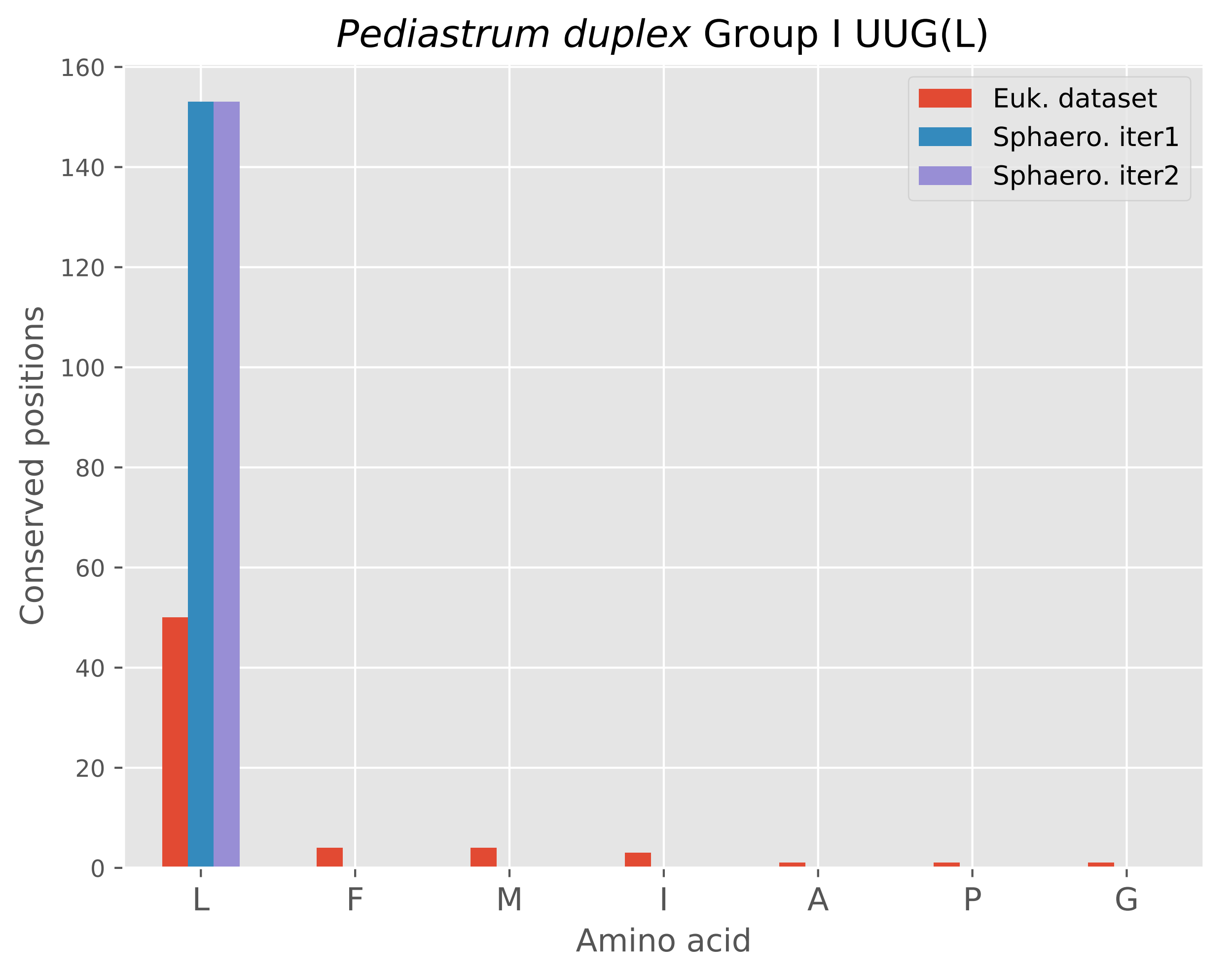

Pediastrum duplex Group I UUG(L)
Conserved positions
Amino acid
0
20
40
60
80
100
120
140
160
L
F
M
I
A
P
G
Euk. dataset
Sphaero. iter1
Sphaero. iter2

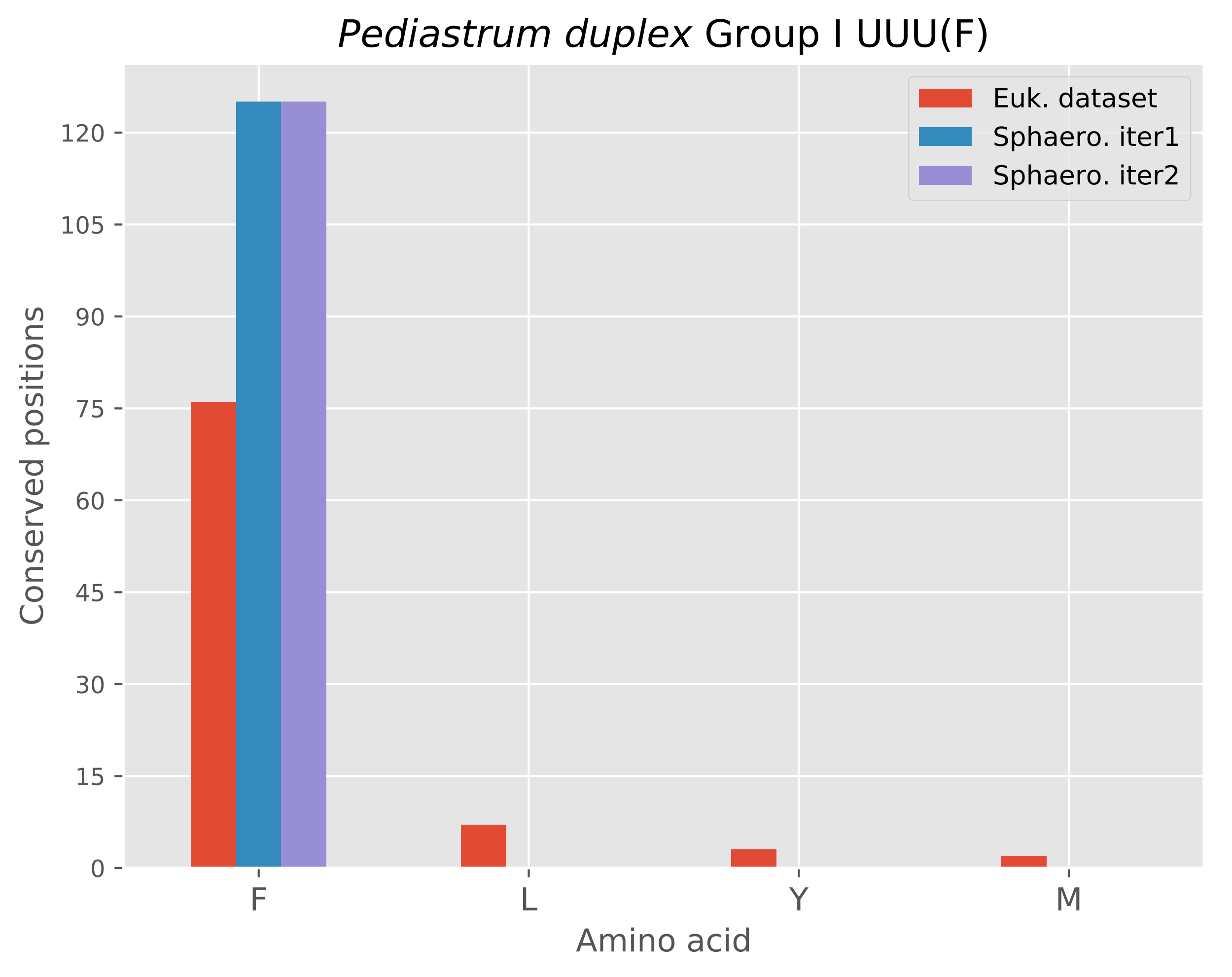

Pediastrum duplex Group I UUU(F)
Euk. dataset
Sphaero. iter1
Sphaero. iter2
Conserved positions
0
15
30
45
60
75
90
105
120
F
L
Y
M
Amino acid

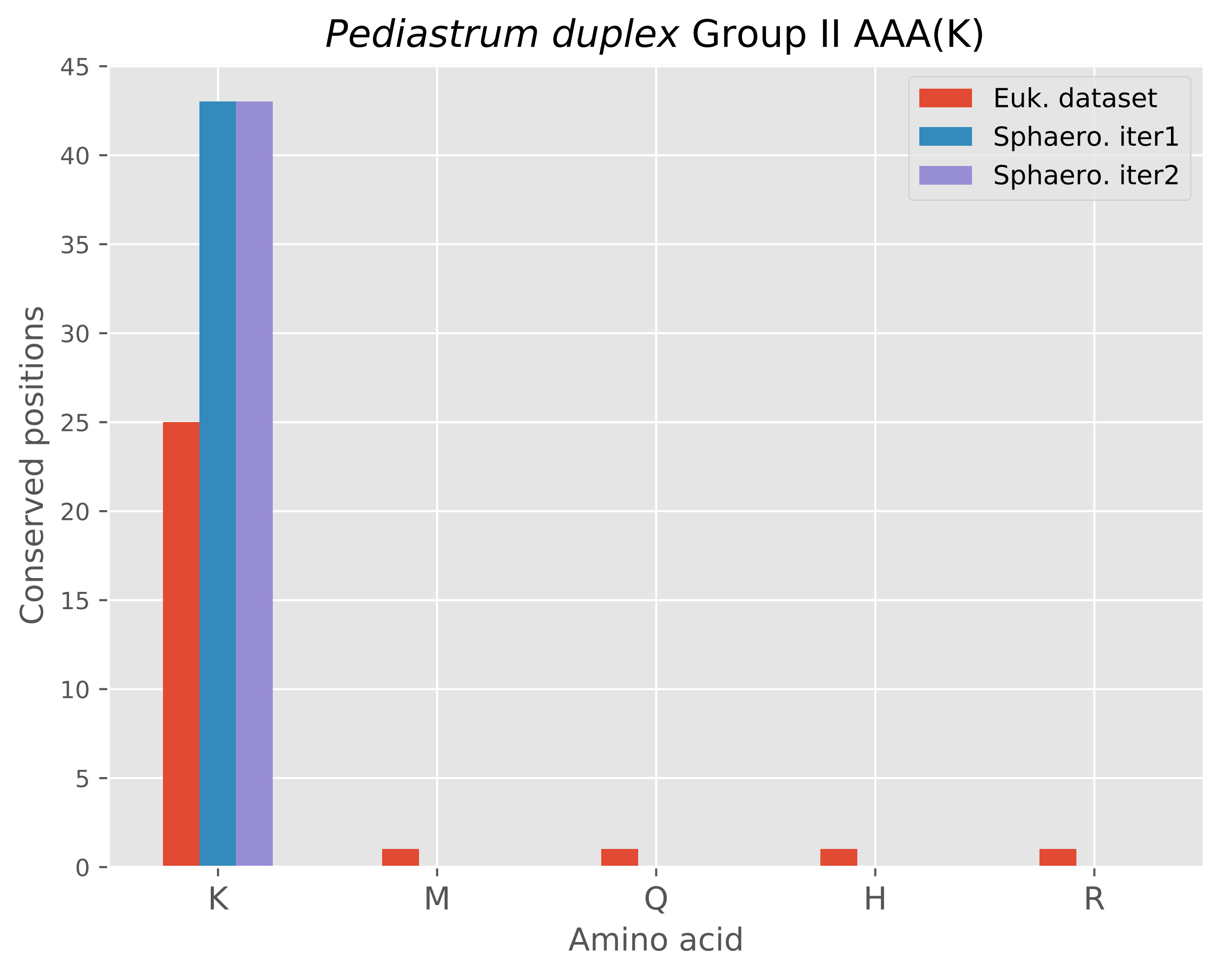
Pediastrum duplex Group II AAA(K)
Euk. dataset
Sphaero. iter1
Sphaero. iter2
Conserved positions
0
5
10
15
20
25
30
35
40
45
K
M
Q
H
R
Amino acid

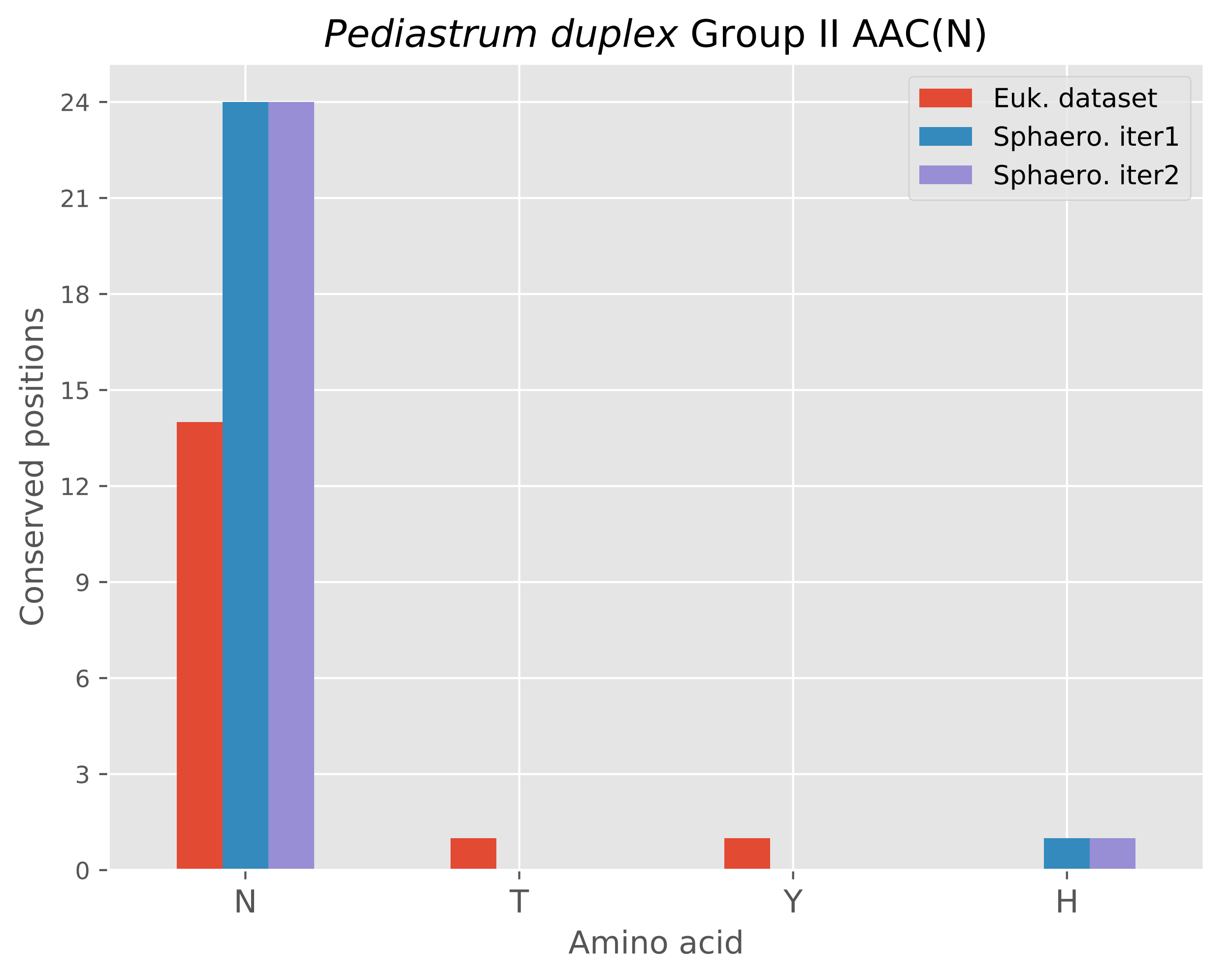

Pediastrum duplex Group II AAC(N)
Euk. dataset
Sphaero. iter1
Sphaero. iter2
Conserved positions
0
3
6
9
12
15
18
21
24
N
T
Y
H
Amino acid

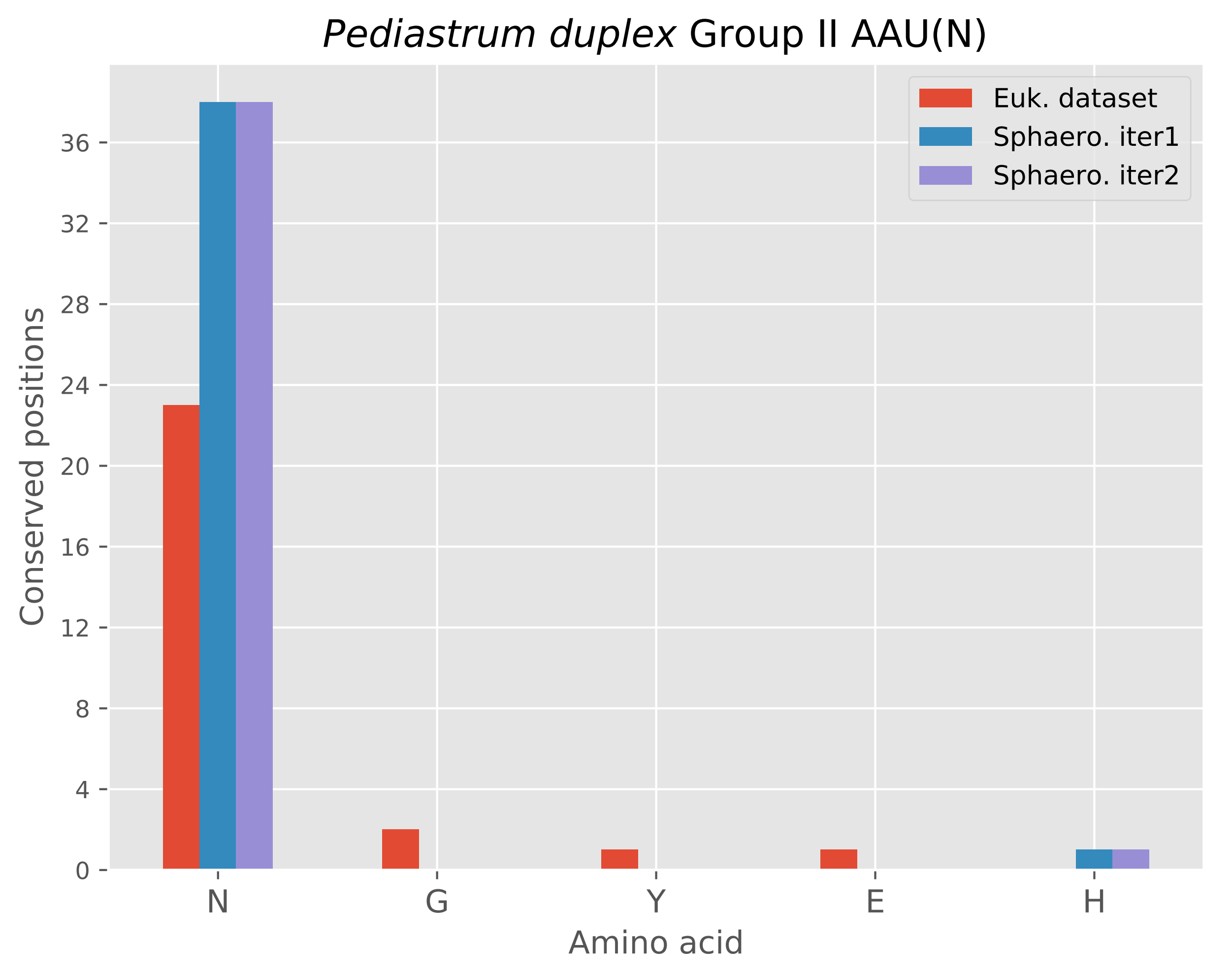

Pediastrum duplex Group II AAU(N)
Euk. dataset
Sphaero. iter1
Sphaero. iter2
Conserved positions
0
4
8
12
16
20
24
28
32
36
N
G
Y
E
H
Amino acid

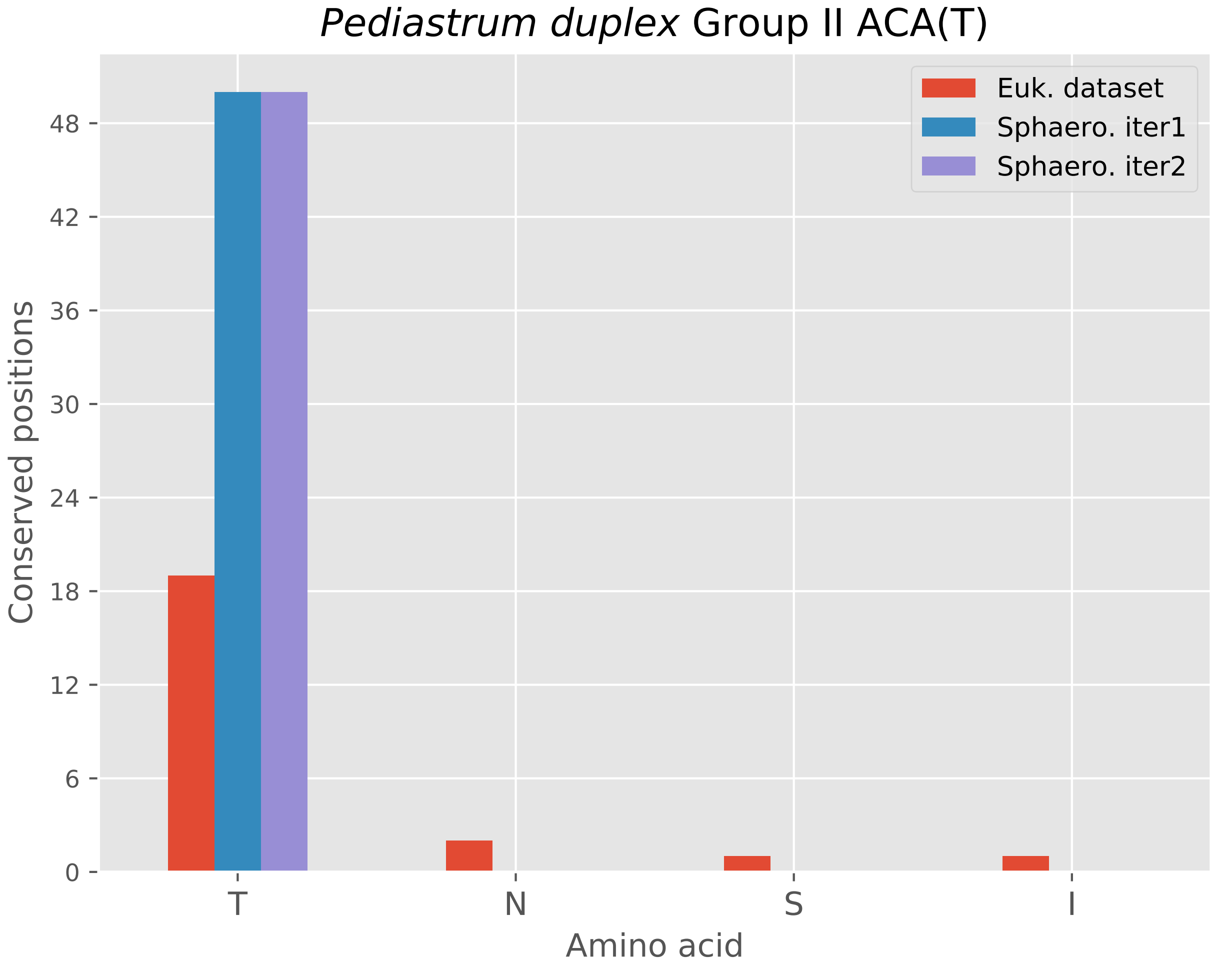

Pediastrum duplex Group II ACA(T)
Euk. dataset
Sphaero. iter1
Sphaero. iter2
Conserved positions
0
6
12
18
24
30
36
42
48
T
N
S
I
Amino acid

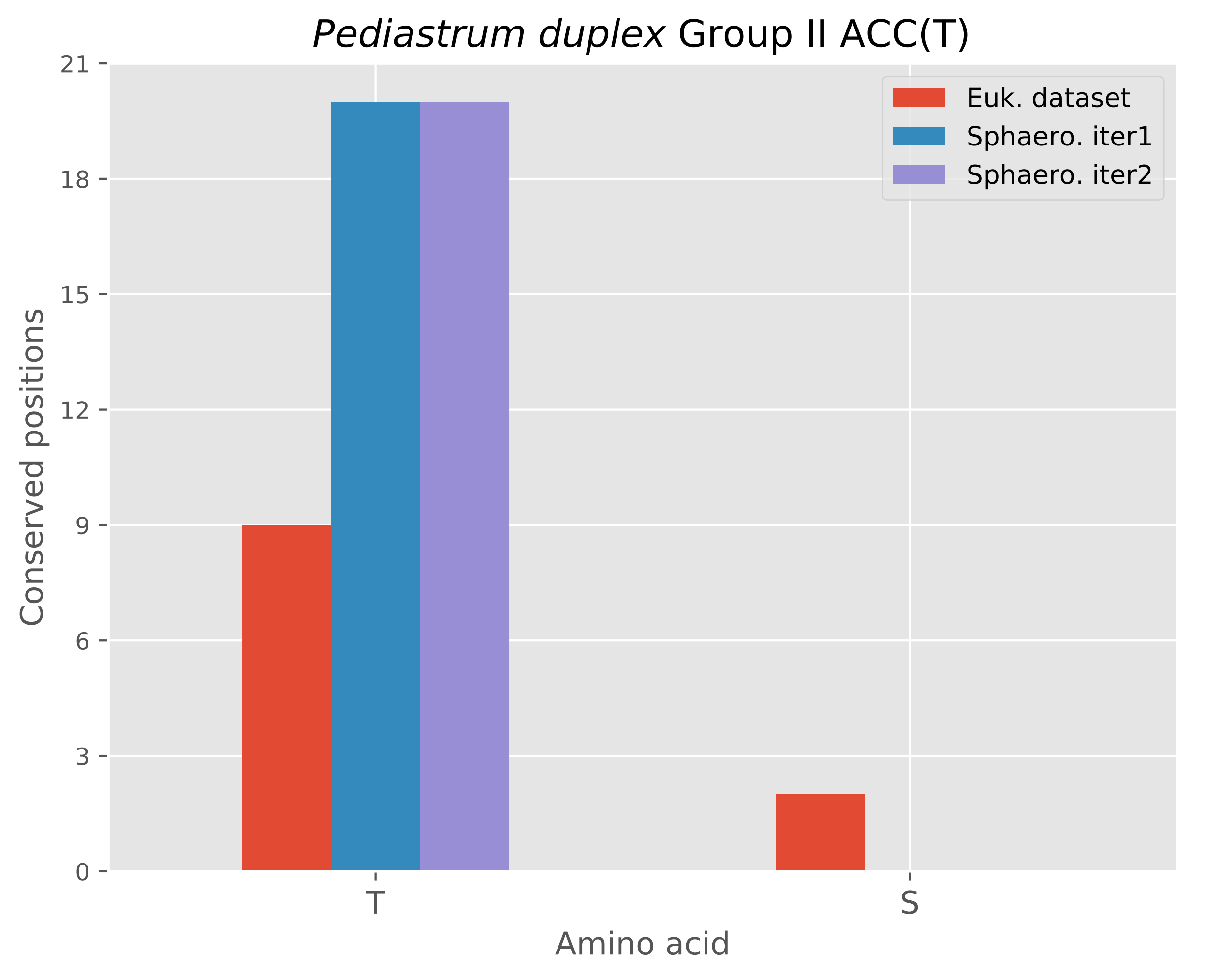

Pediastrum duplex Group II ACC(T)
Euk. dataset
Sphaero. iter1
Sphaero. iter2
Conserved positions
0
3
6
9
12
15
18
21
T
S
Amino acid

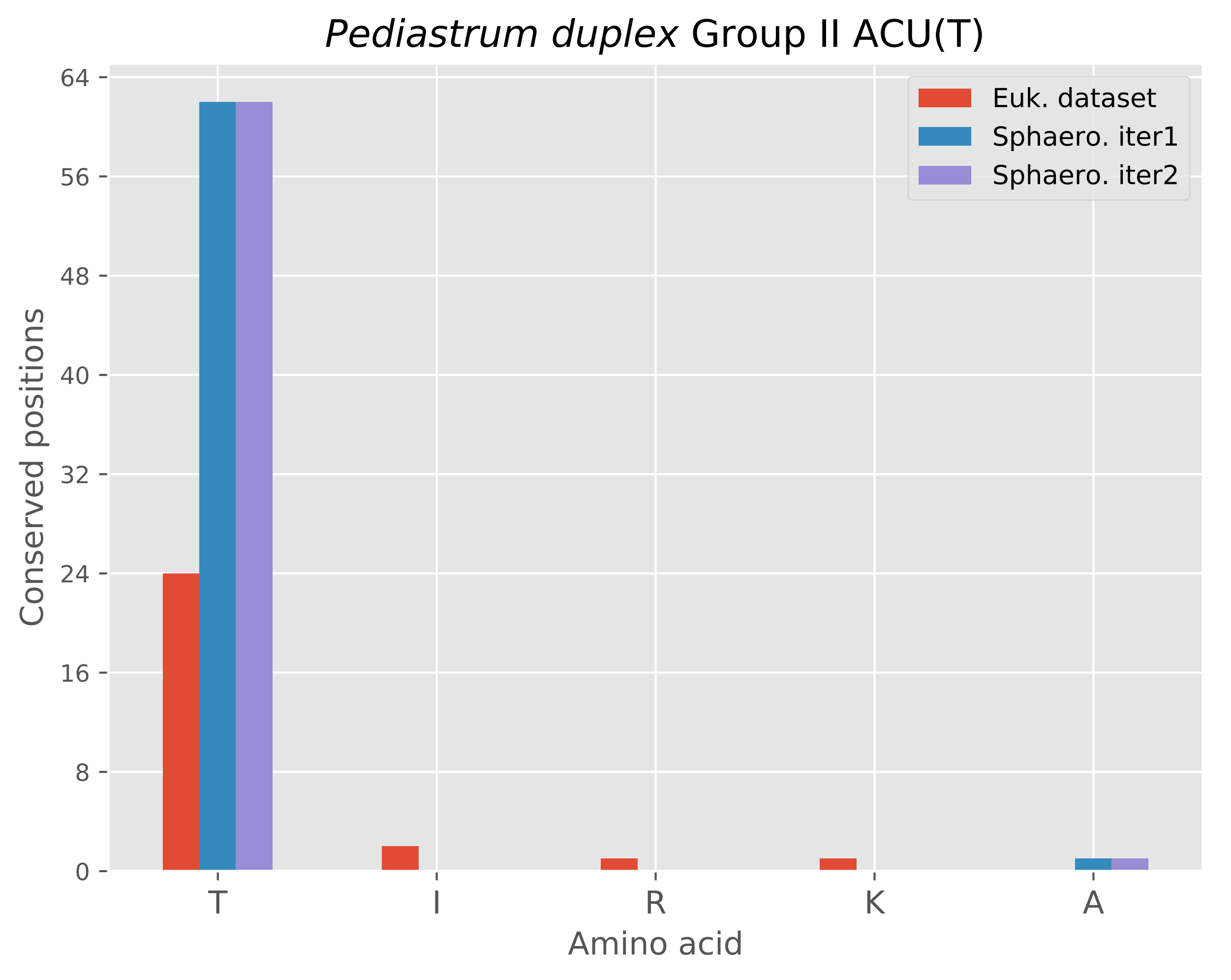
Pediastrum duplex Group II ACU(T)
Euk. dataset
Sphaero. iter1
Sphaero. iter2
Conserved positions
Amino acid
0
8
16
24
32
40
48
56
64
T
I
R
K
A

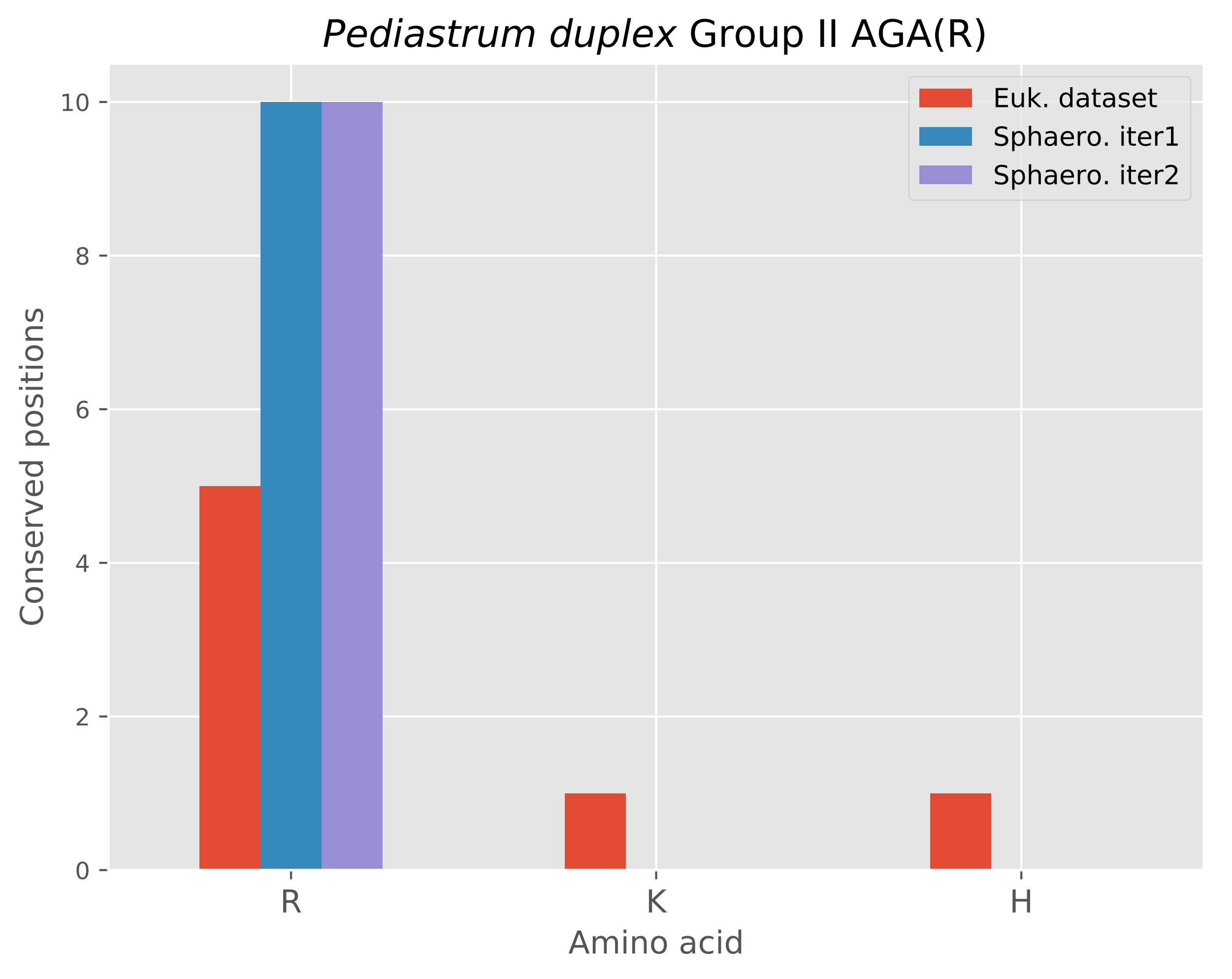

Pediastrum duplex Group II AGA(R)
Euk. dataset
Sphaero. iter1
Sphaero. iter2
Conserved positions
0
2
4
6
8
10
R
K
H
Amino acid

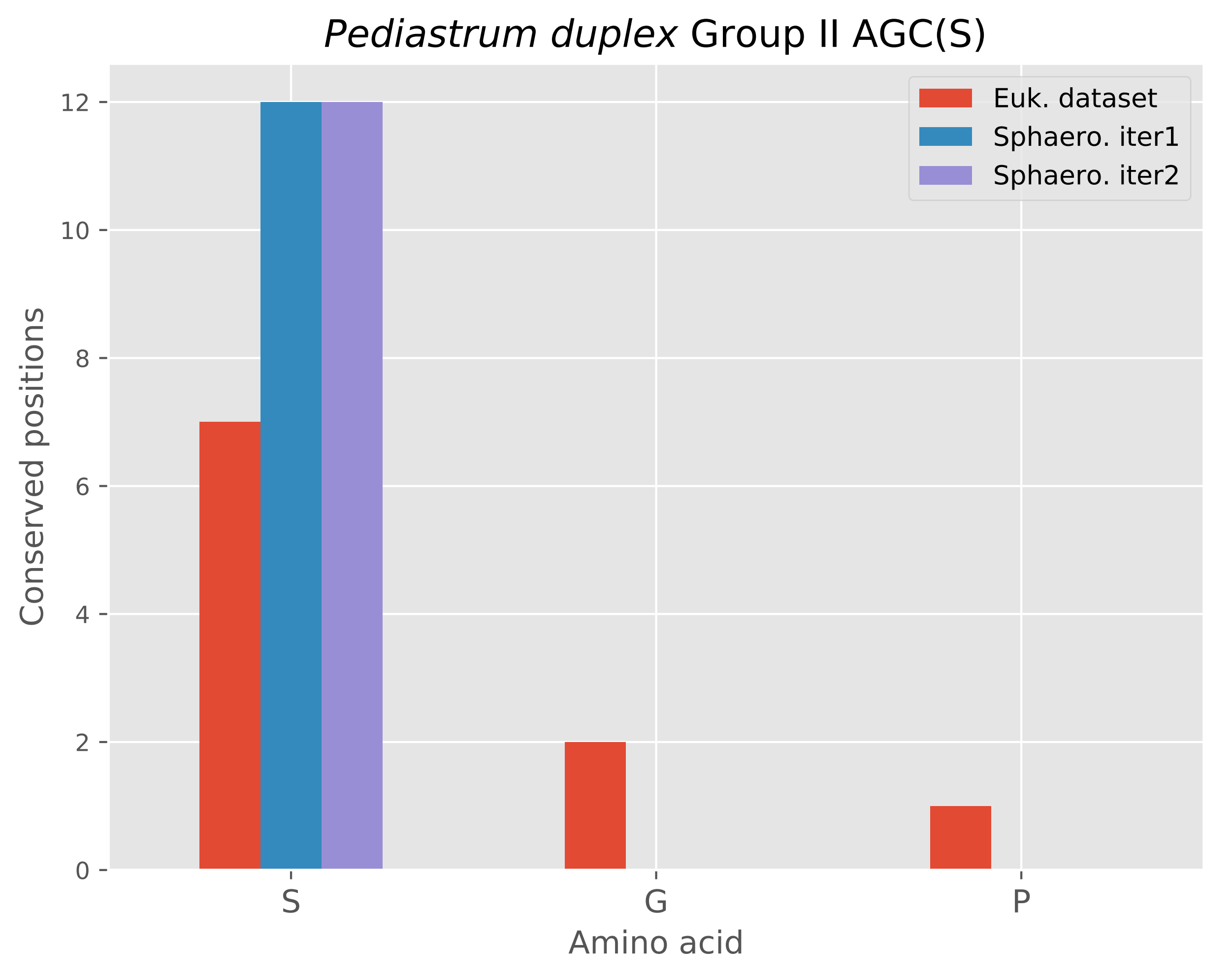

Pediastrum duplex Group II AGC(S)
Euk. dataset
Sphaero. iter1
Sphaero. iter2
Conserved positions
0
2
4
6
8
10
12
S
G
P
Amino acid

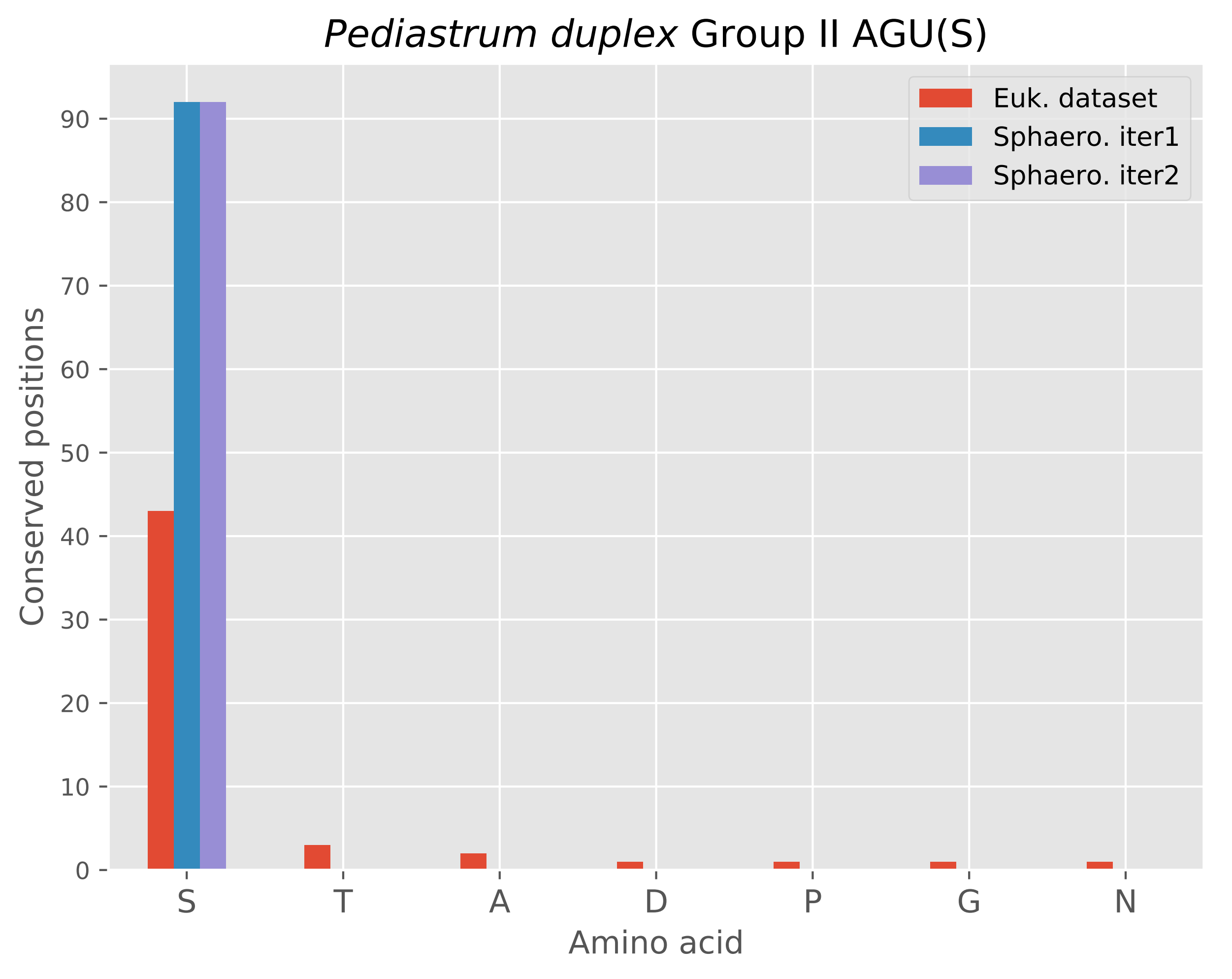

Pediastrum duplex Group II AGU(S)
Euk. dataset
Sphaero. iter1
Sphaero. iter2
Conserved positions
0
10
20
30
40
50
60
70
80
90
S
T
A
D
P
G
N
Amino acid

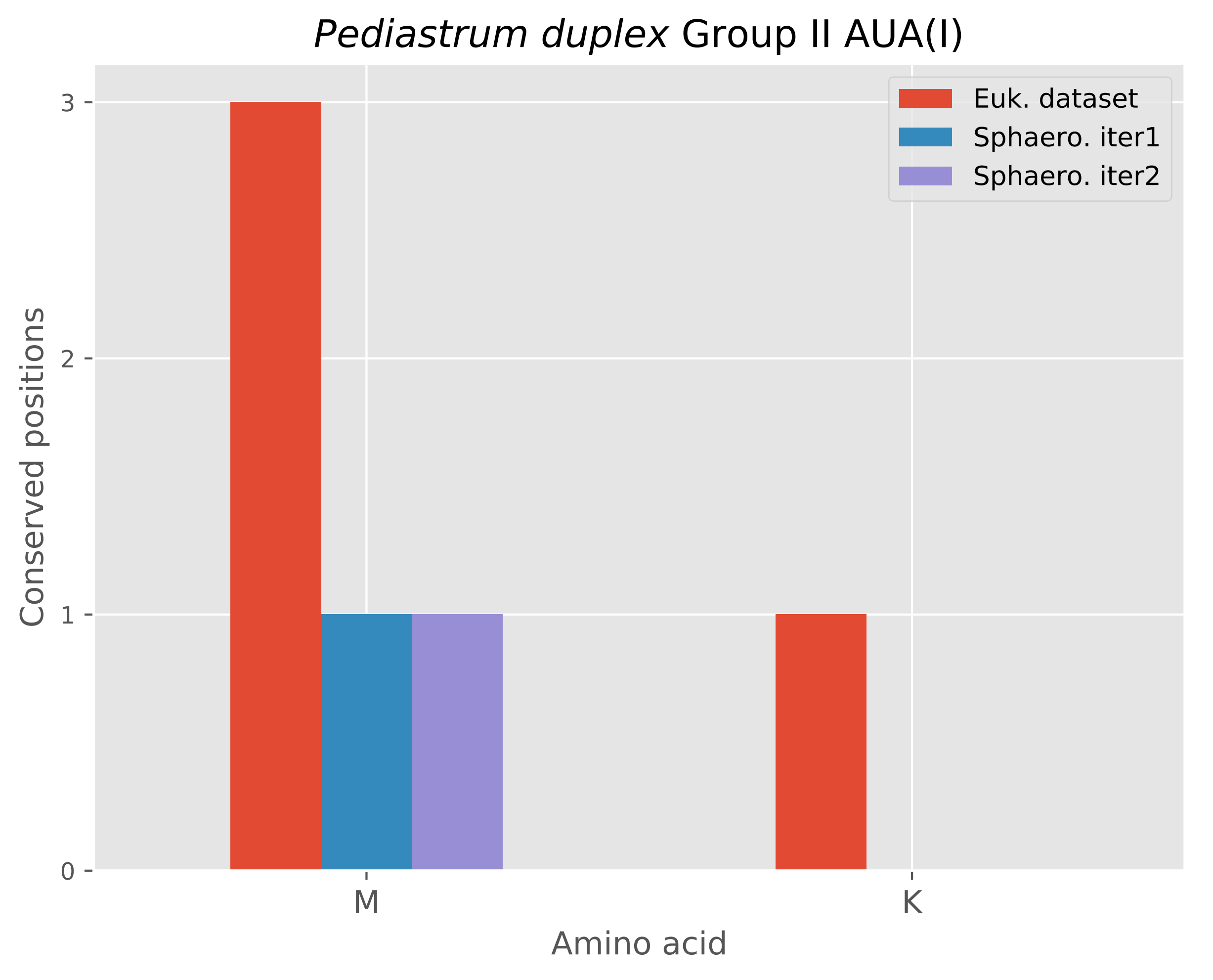

Pediastrum duplex Group II AUA(I)
Euk. dataset
Sphaero. iter1
Sphaero. iter2
Conserved positions
0
1
2
3
M
K
Amino acid

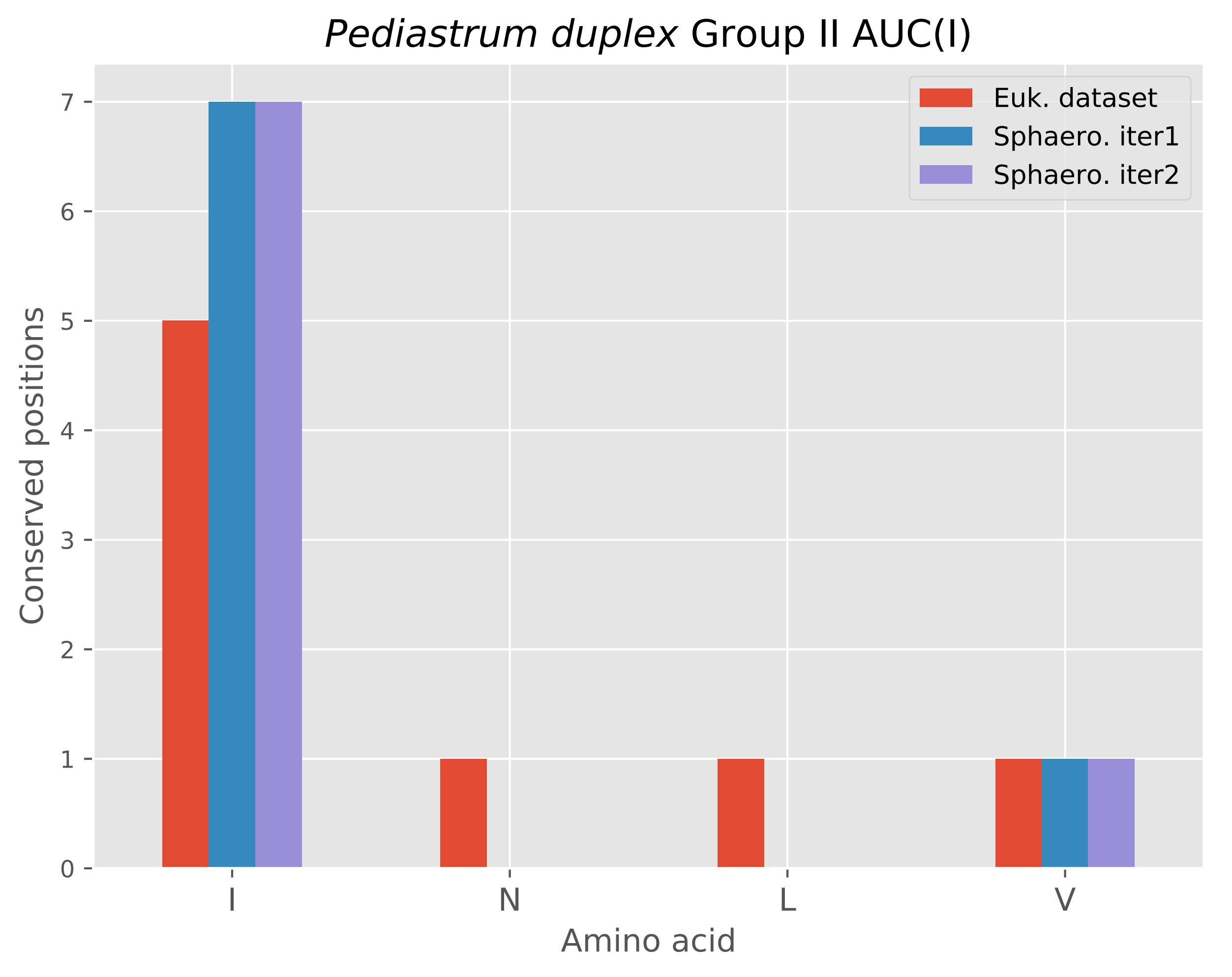

Pediastrum duplex Group II AUC(I)
Euk. dataset
Sphaero. iter1
Sphaero. iter2
Conserved positions
0
1
2
3
4
5
6
7
I
N
L
V
Amino acid

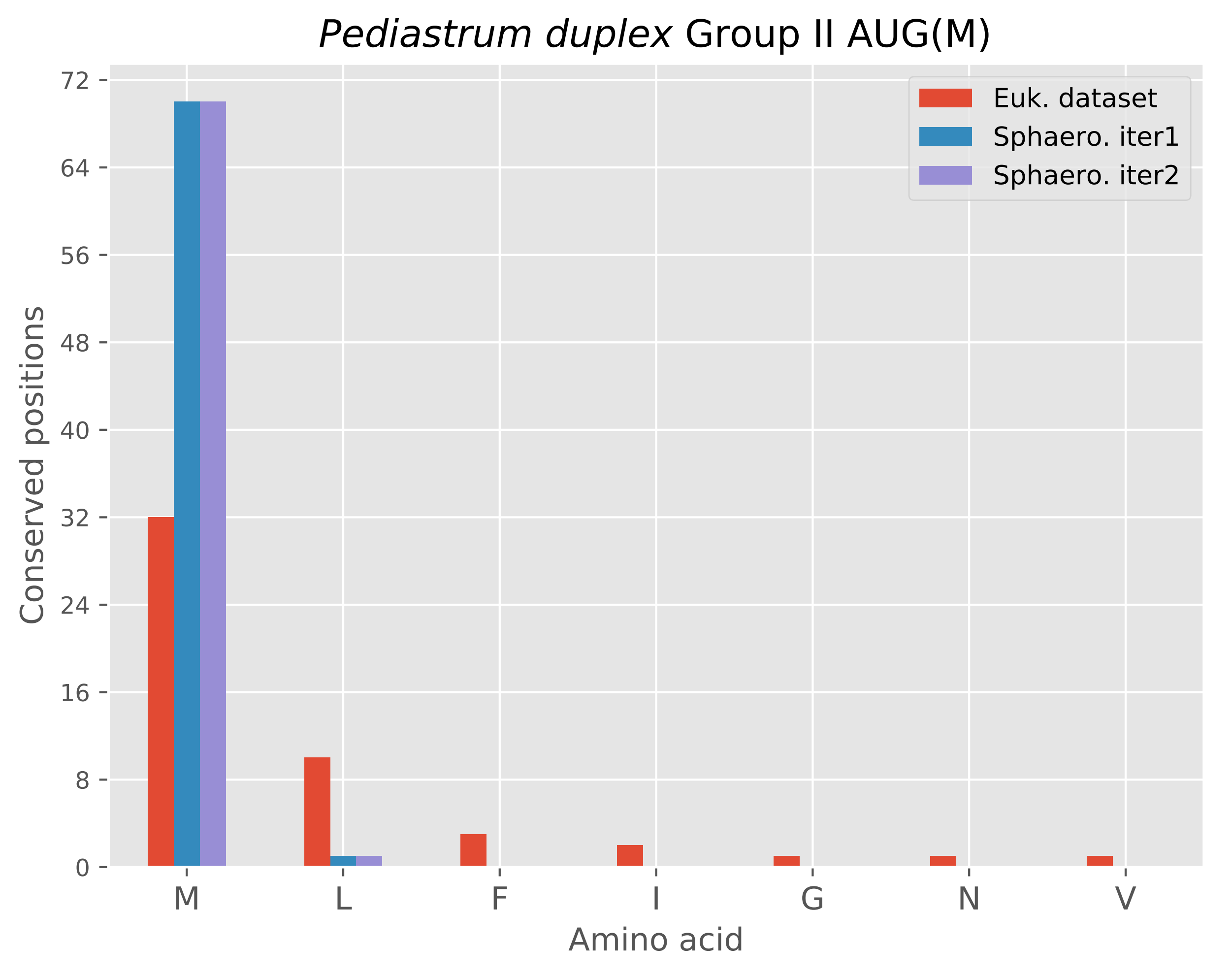
Pediastrum duplex Group II AUG(M)
Euk. dataset
Sphaero. iter1
Sphaero. iter2
Conserved positions
0
8
16
24
32
40
48
56
64
72
M
L
F
I
G
N
V
Amino acid

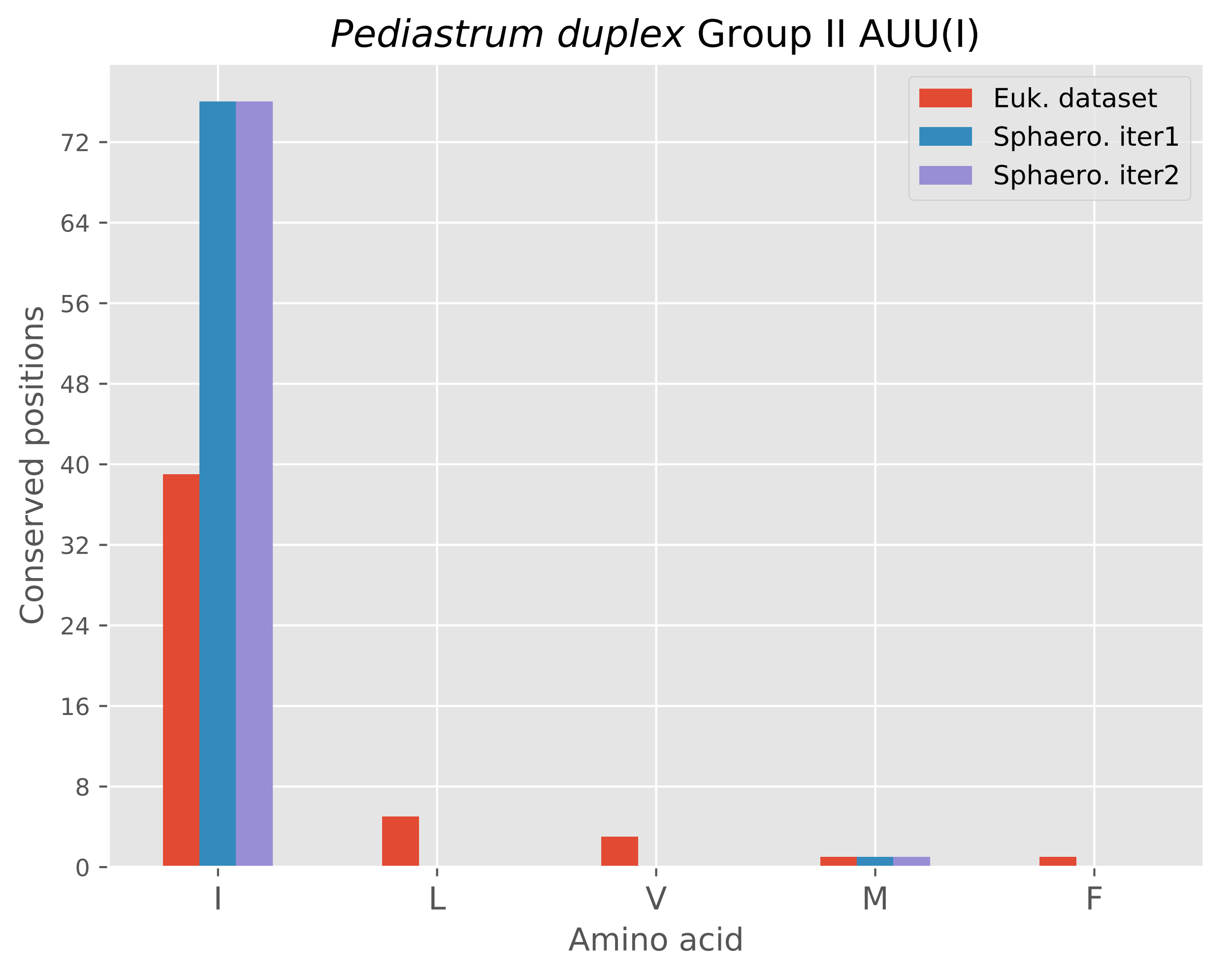

Pediastrum duplex Group II AUU(I)
Euk. dataset
Sphaero. iter1
Sphaero. iter2
Conserved positions
72
64
56
48
40
32
24
16
8
0
I
L
V
M
F
Amino acid

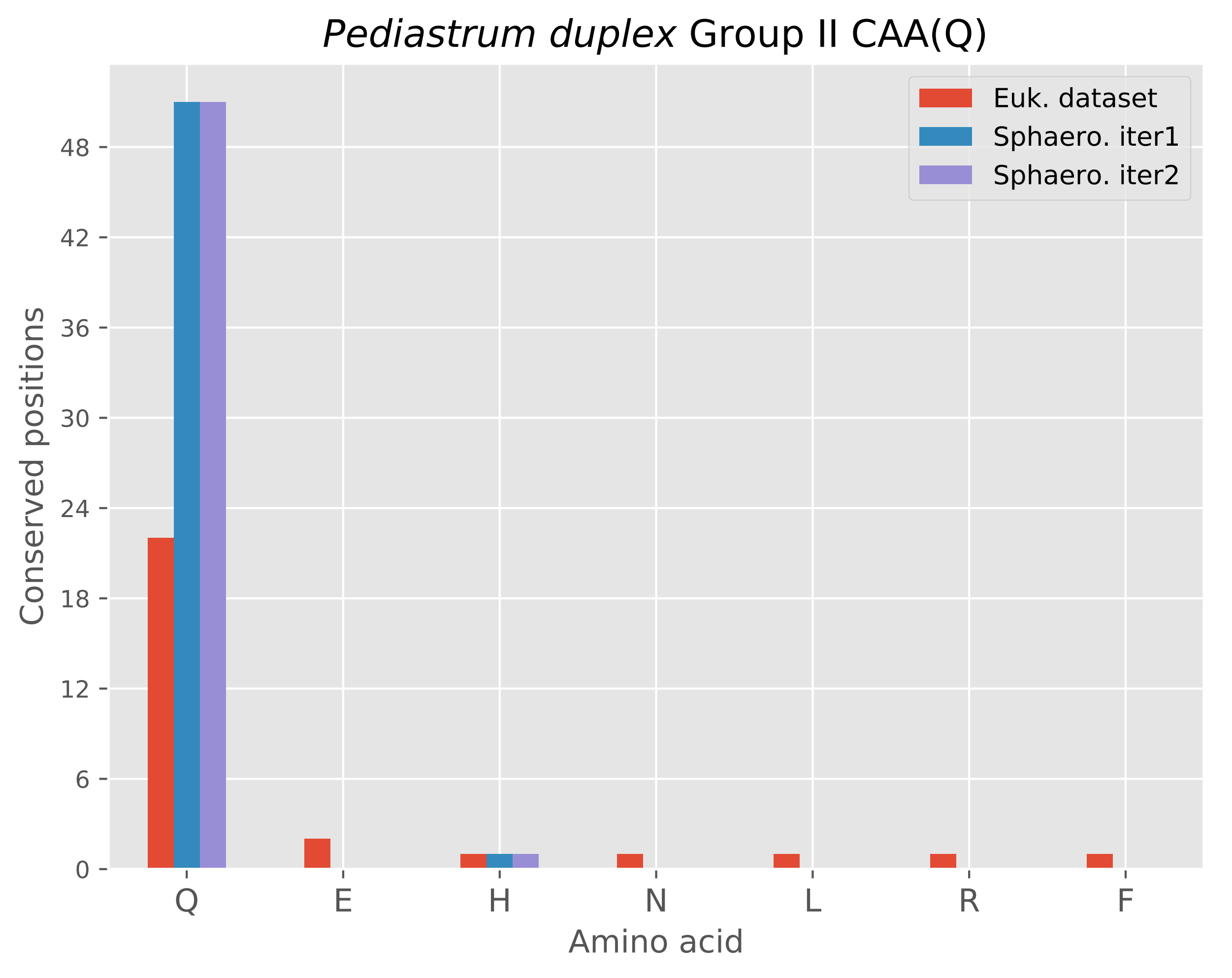

Pediastrum duplex Group II CAA(Q)
Euk. dataset
Sphaero. iter1
Sphaero. iter2
Conserved positions
0
6
12
18
24
30
36
42
48
Q
E
H
N
L
R
F
Amino acid

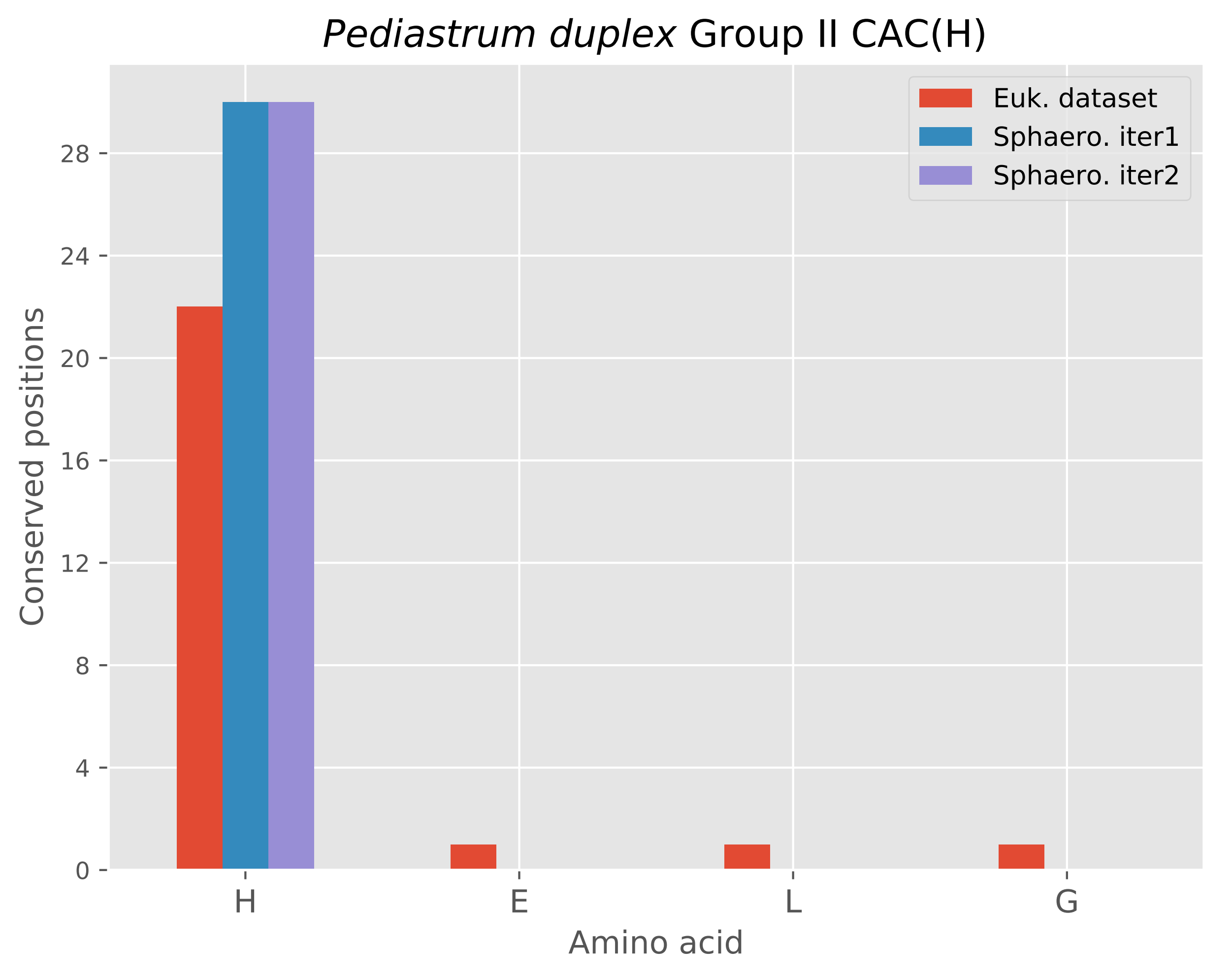

Pediastrum duplex Group II CAC(H)
Euk. dataset
Sphaero. iter1
Sphaero. iter2
Conserved positions
0
4
8
12
16
20
24
28
H
E
L
G
Amino acid

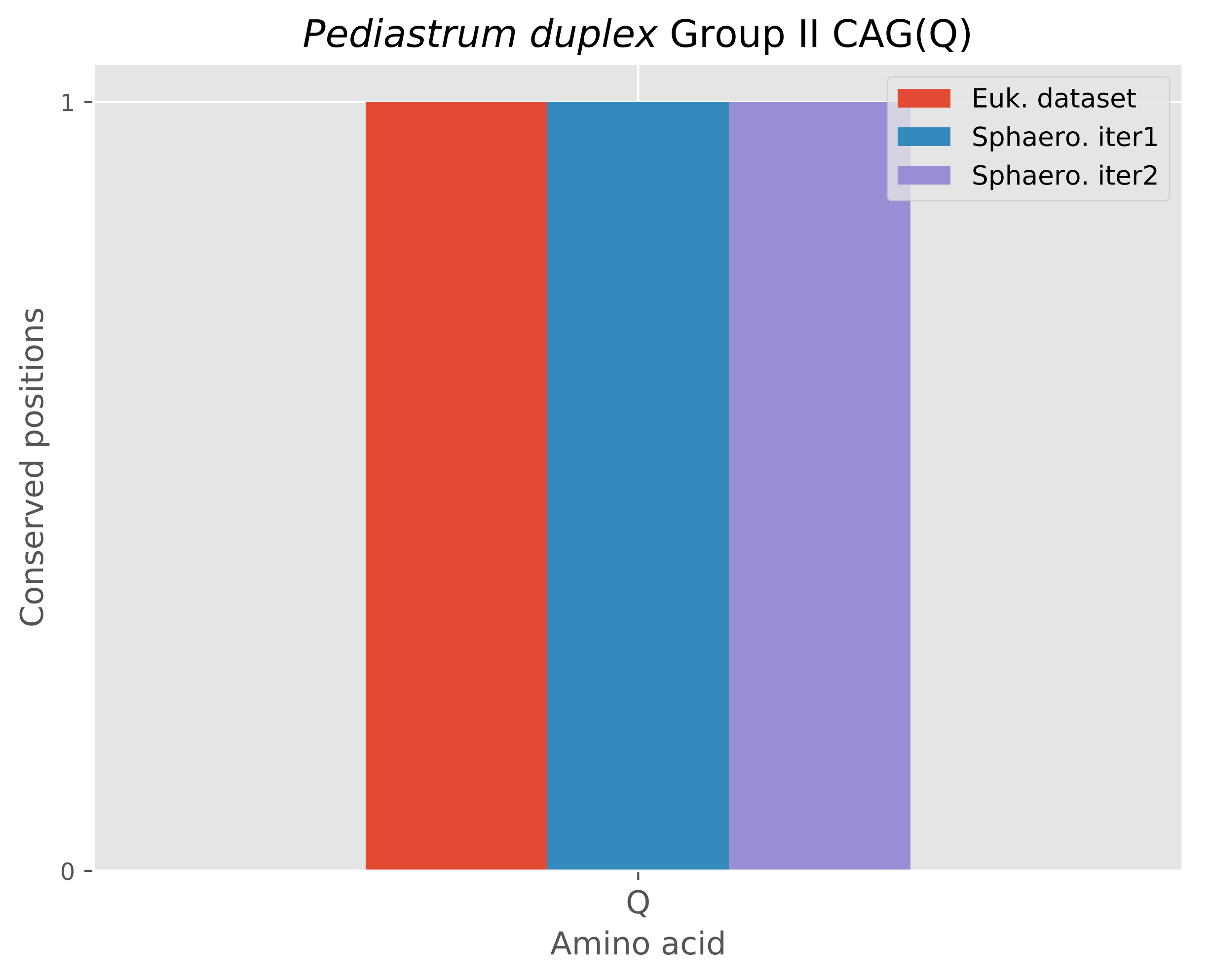
Pediastrum duplex Group II CAG(Q)
Euk. dataset
Sphaero. iter1
Sphaero. iter2
Conserved positions
1
0
Q
Amino acid

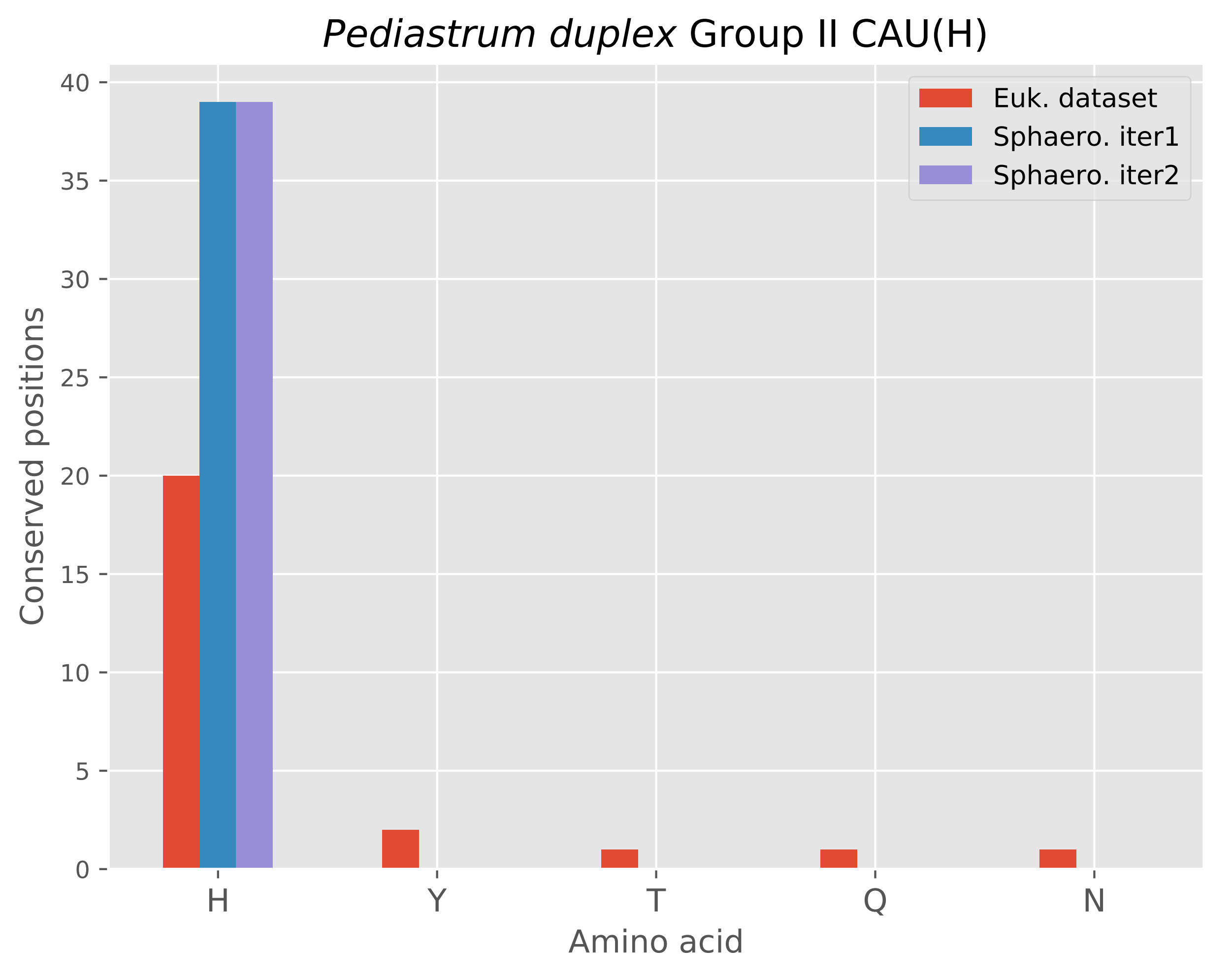

Pediastrum duplex Group II CAU(H)
Euk. dataset
Sphaero. iter1
Sphaero. iter2
Conserved positions
0
5
10
15
20
25
30
35
40
H
Y
T
Q
N
Amino acid

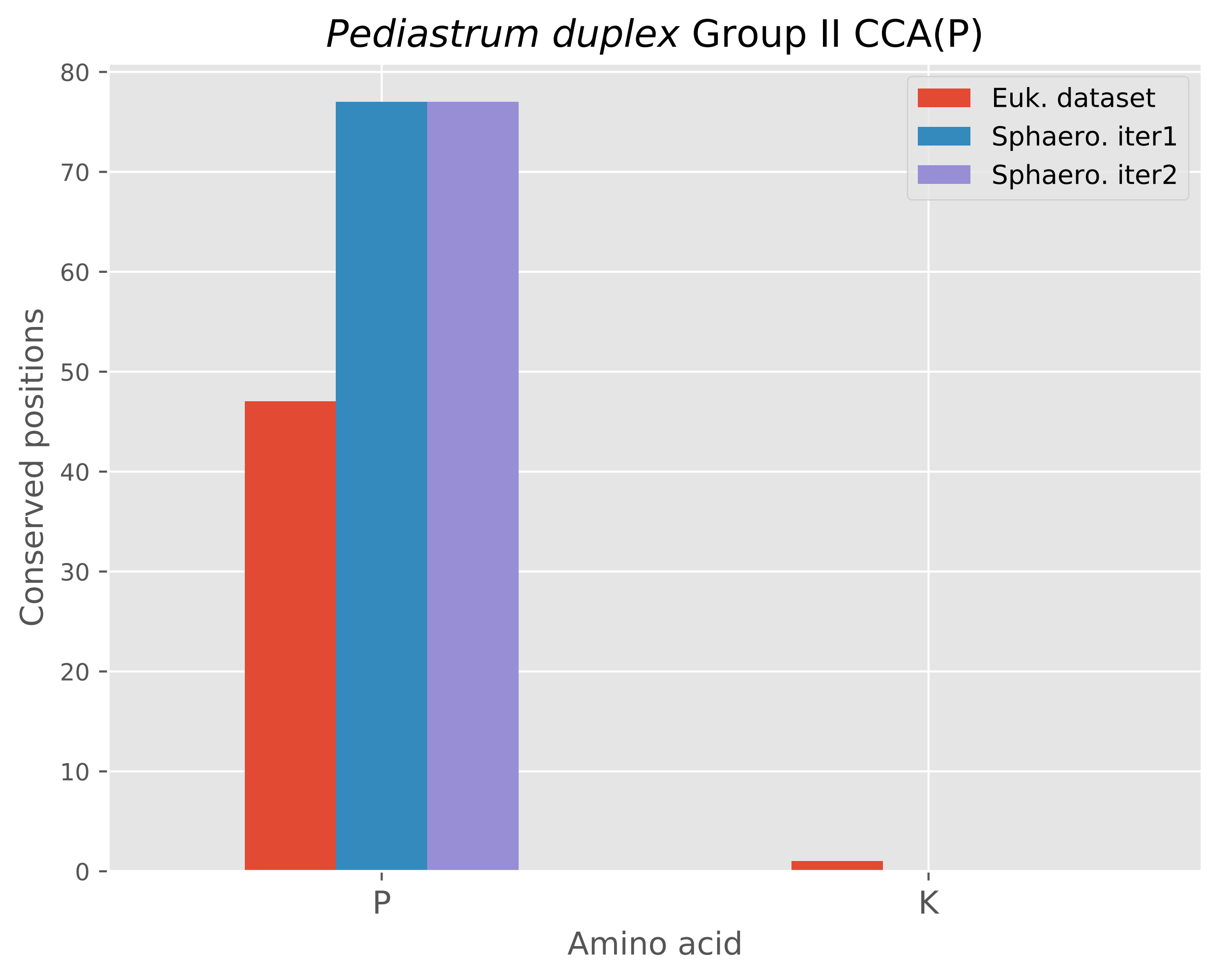

Pediastrum duplex Group II CCA(P)
Conserved positions
0
10
20
30
40
50
60
70
80
P
K
Amino acid
Euk. dataset
Sphaero. iter1
Sphaero. iter2

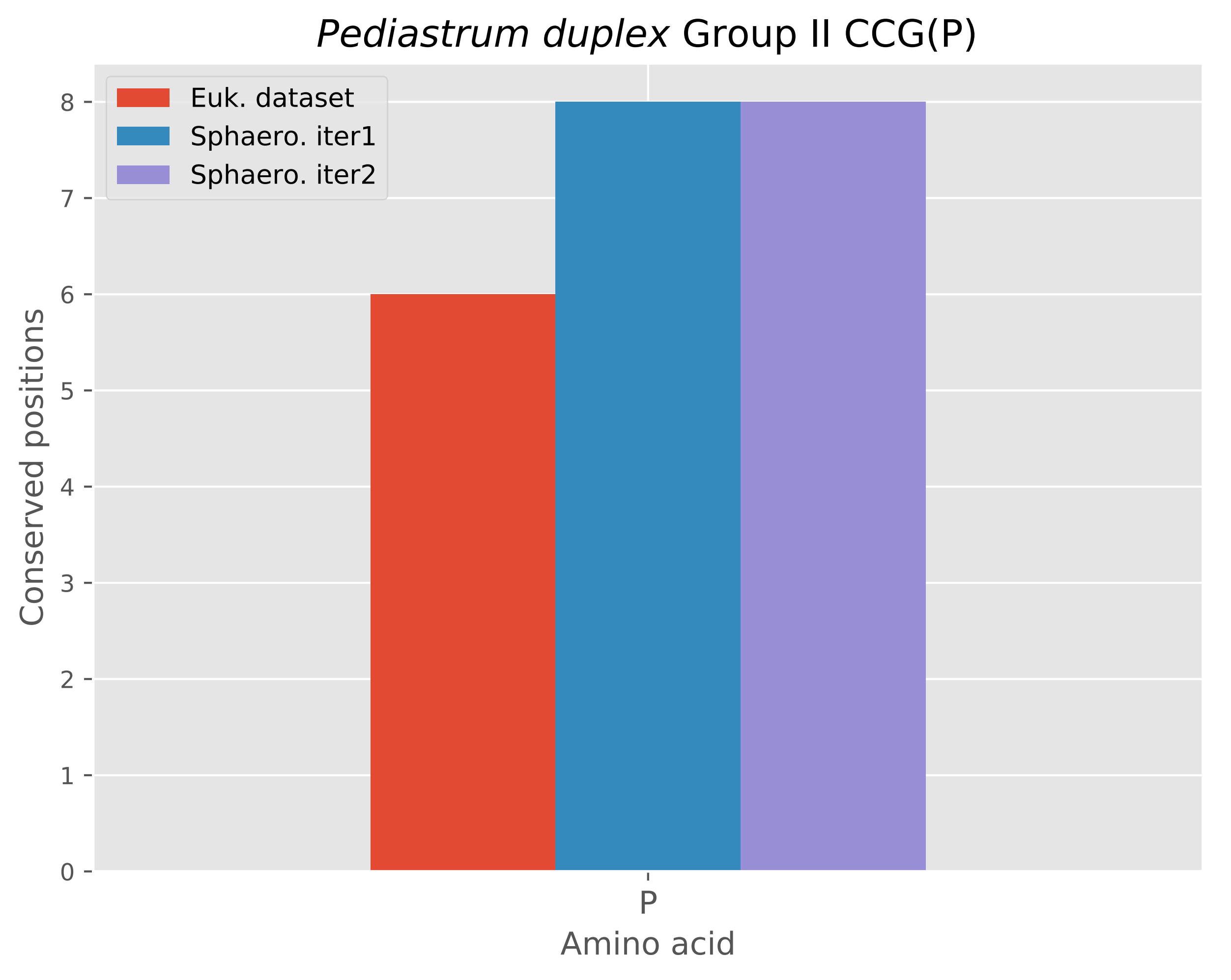

Pediastrum duplex Group II CCG(P)
Euk. dataset
Sphaero. iter1
Sphaero. iter2
Conserved positions
0
1
2
3
4
5
6
7
8
P
Amino acid

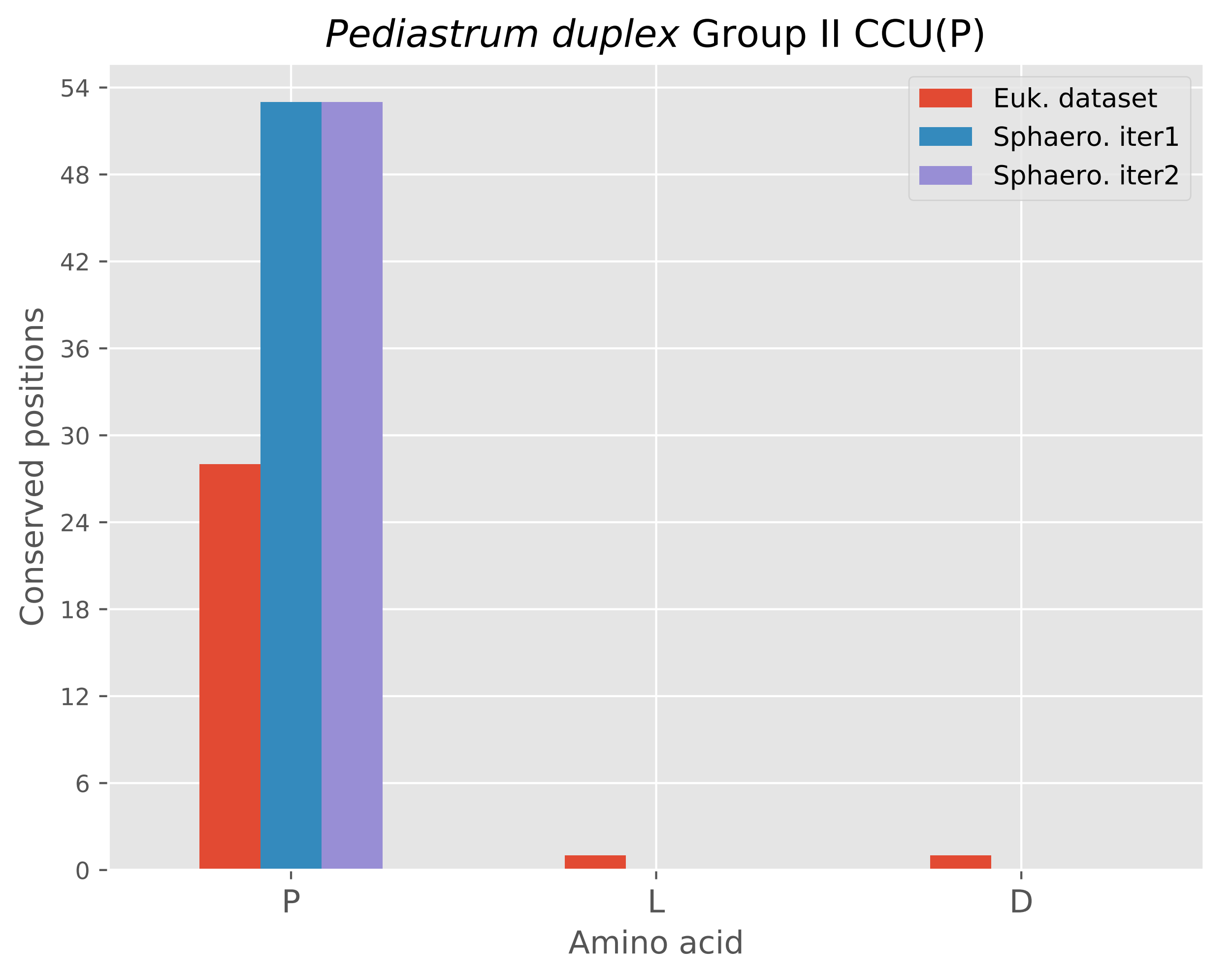

Pediastrum duplex Group II CCU(P)
Conserved positions
0
6
12
18
24
30
36
42
48
54
P
L
D
Amino acid
Euk. dataset
Sphaero. iter1
Sphaero. iter2

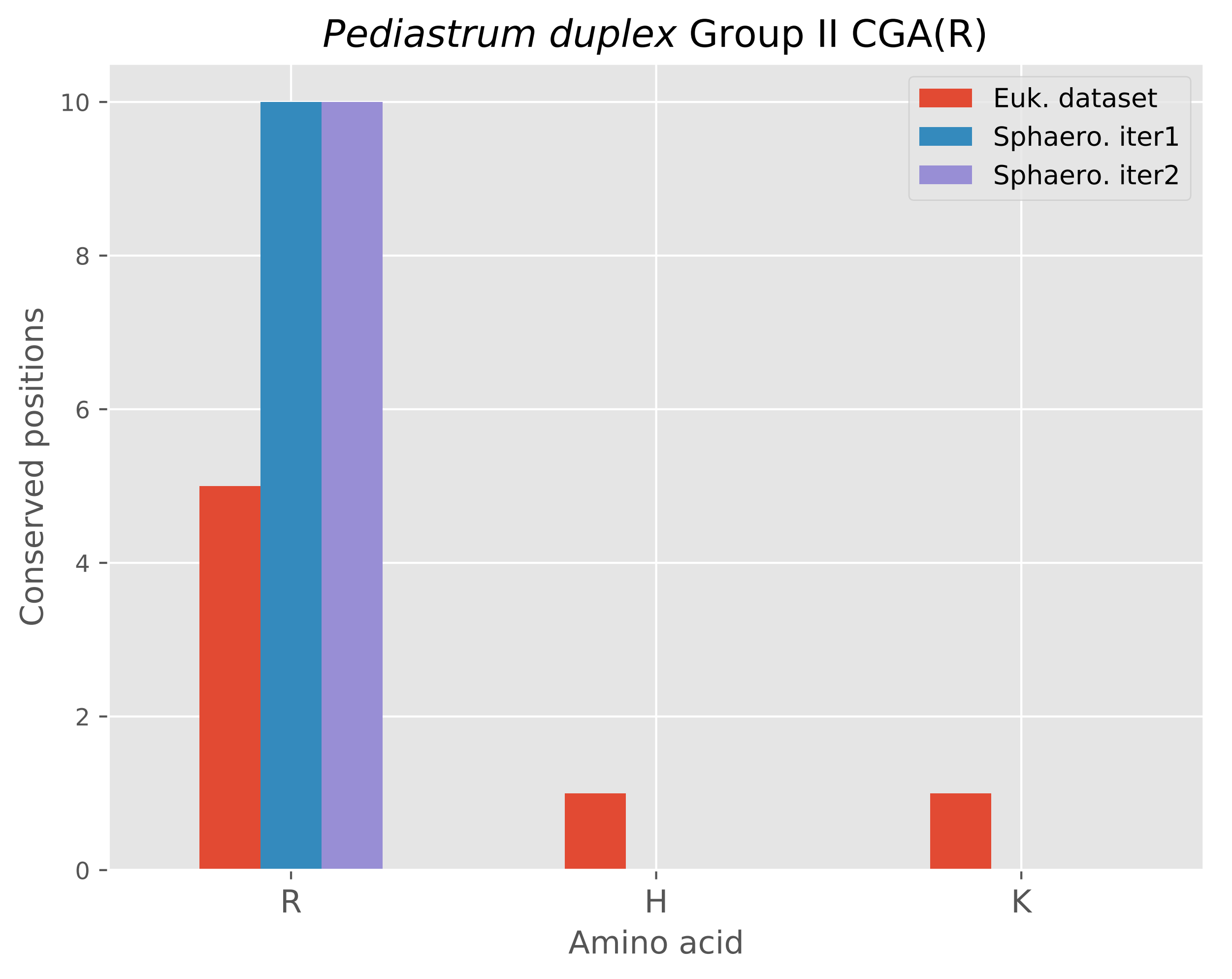

Pediastrum duplex Group II CGA(R)
Euk. dataset
Sphaero. iter1
Sphaero. iter2
Conserved positions
0
2
4
6
8
10
R
H
K
Amino acid

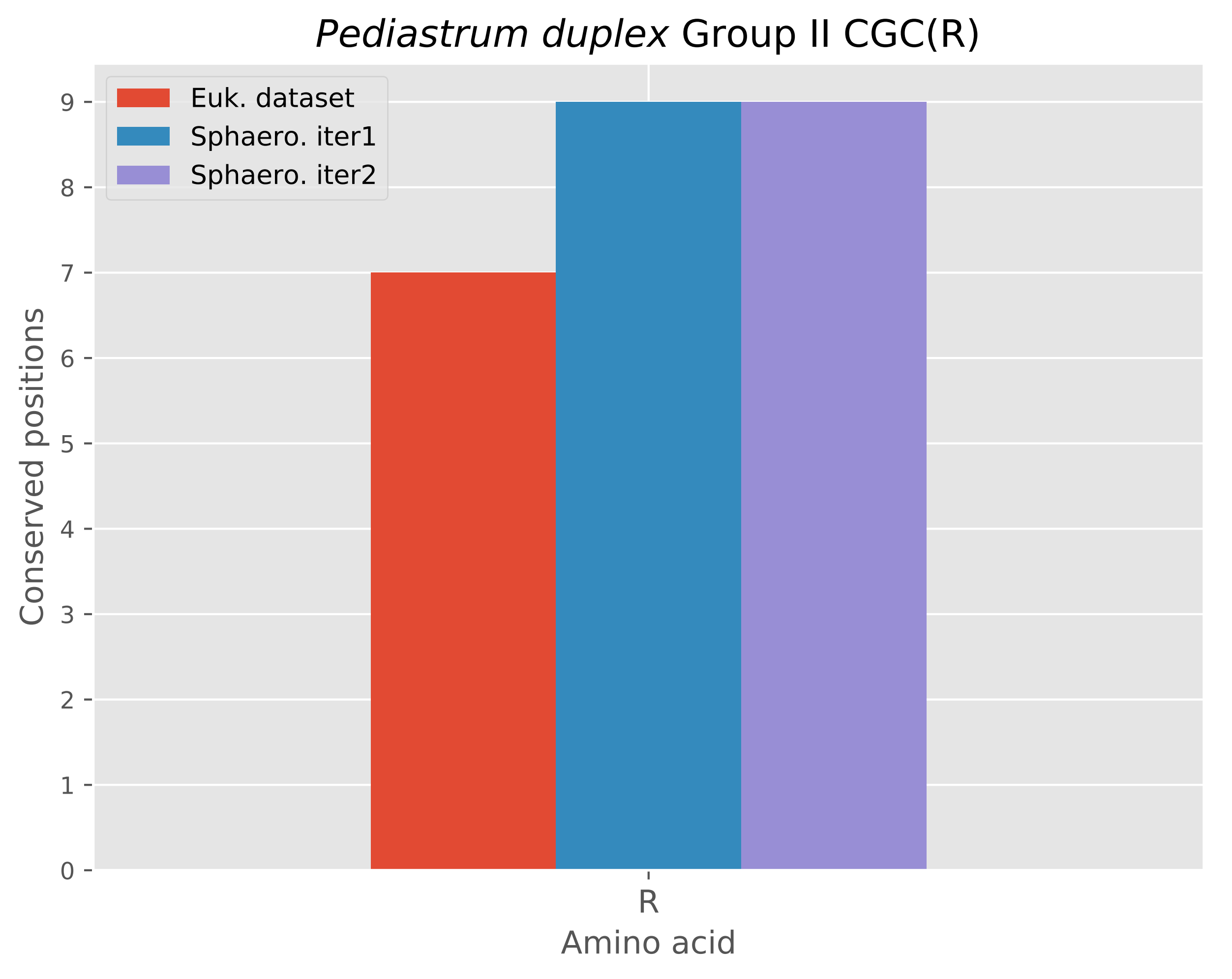
Pediastrum duplex Group II CGC(R)
Euk. dataset
Sphaero. iter1
Sphaero. iter2
Conserved positions
0
1
2
3
4
5
6
7
8
9
R
Amino acid

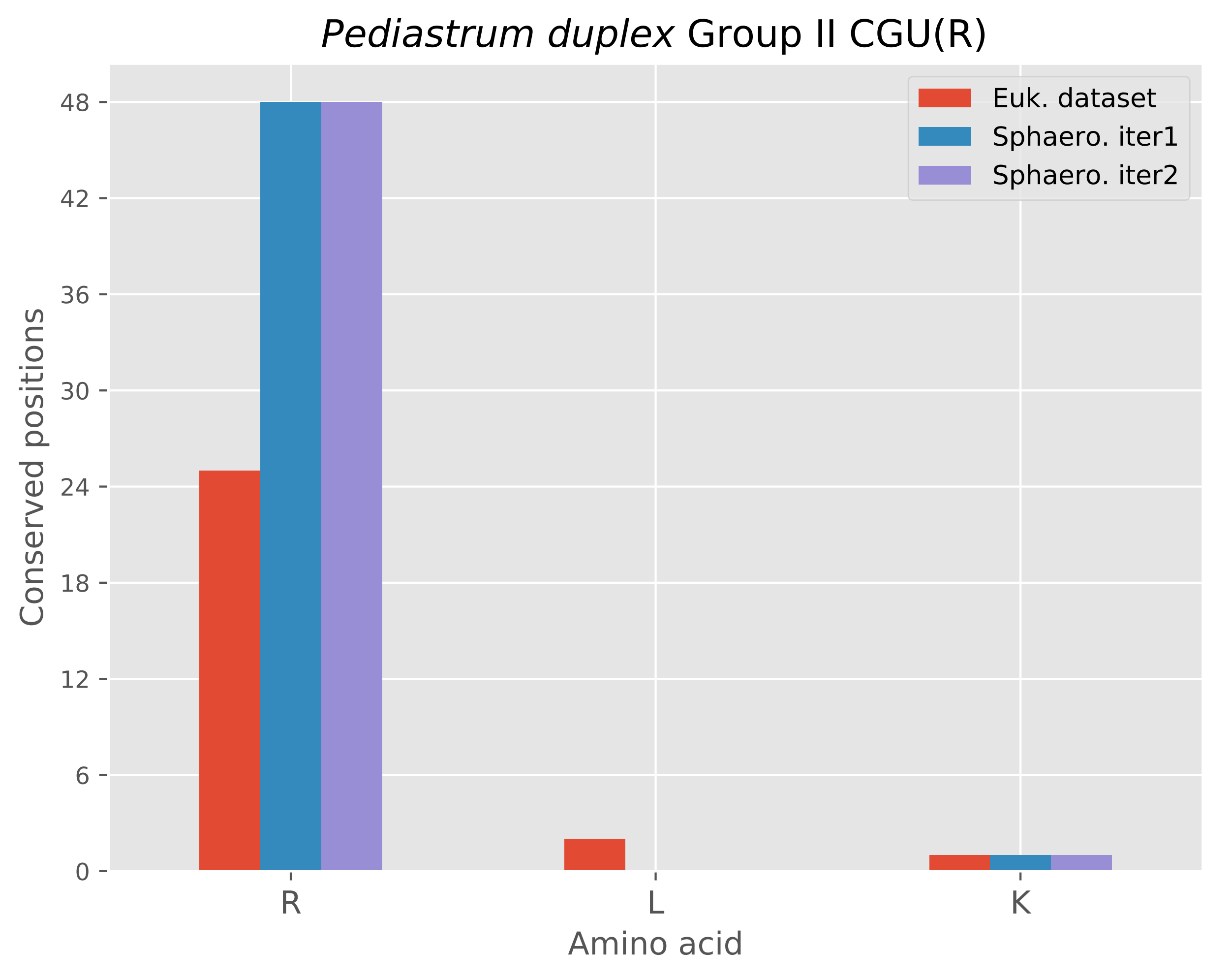

Pediastrum duplex Group II CGU(R)
Euk. dataset
Sphaero. iter1
Sphaero. iter2
Conserved positions
48
42
36
30
24
18
12
6
0
R
L
K
Amino acid

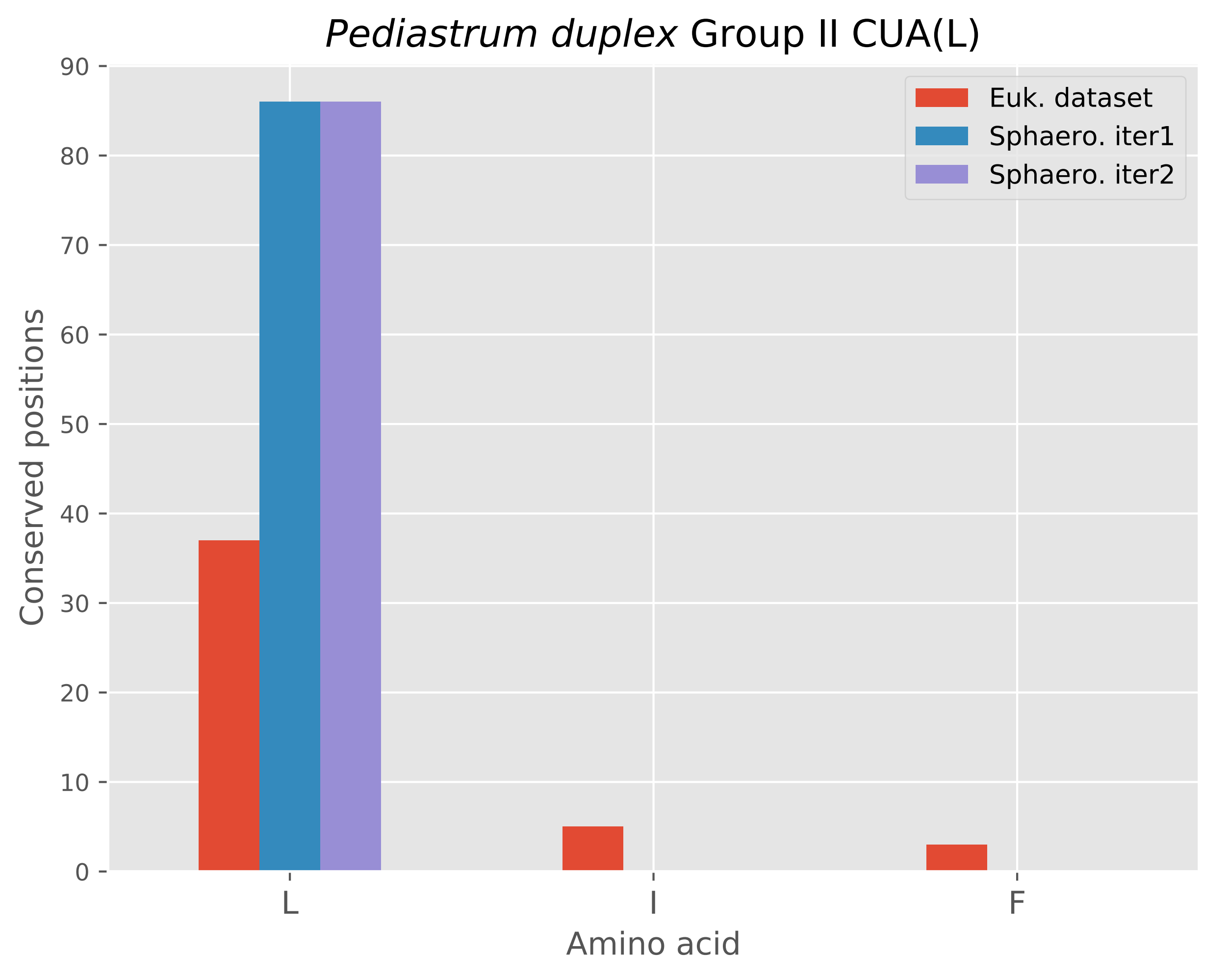

Pediastrum duplex Group II CUA(L)
Conserved positions
0
10
20
30
40
50
60
70
80
90
L
I
F
Amino acid
Euk. dataset
Sphaero. iter1
Sphaero. iter2

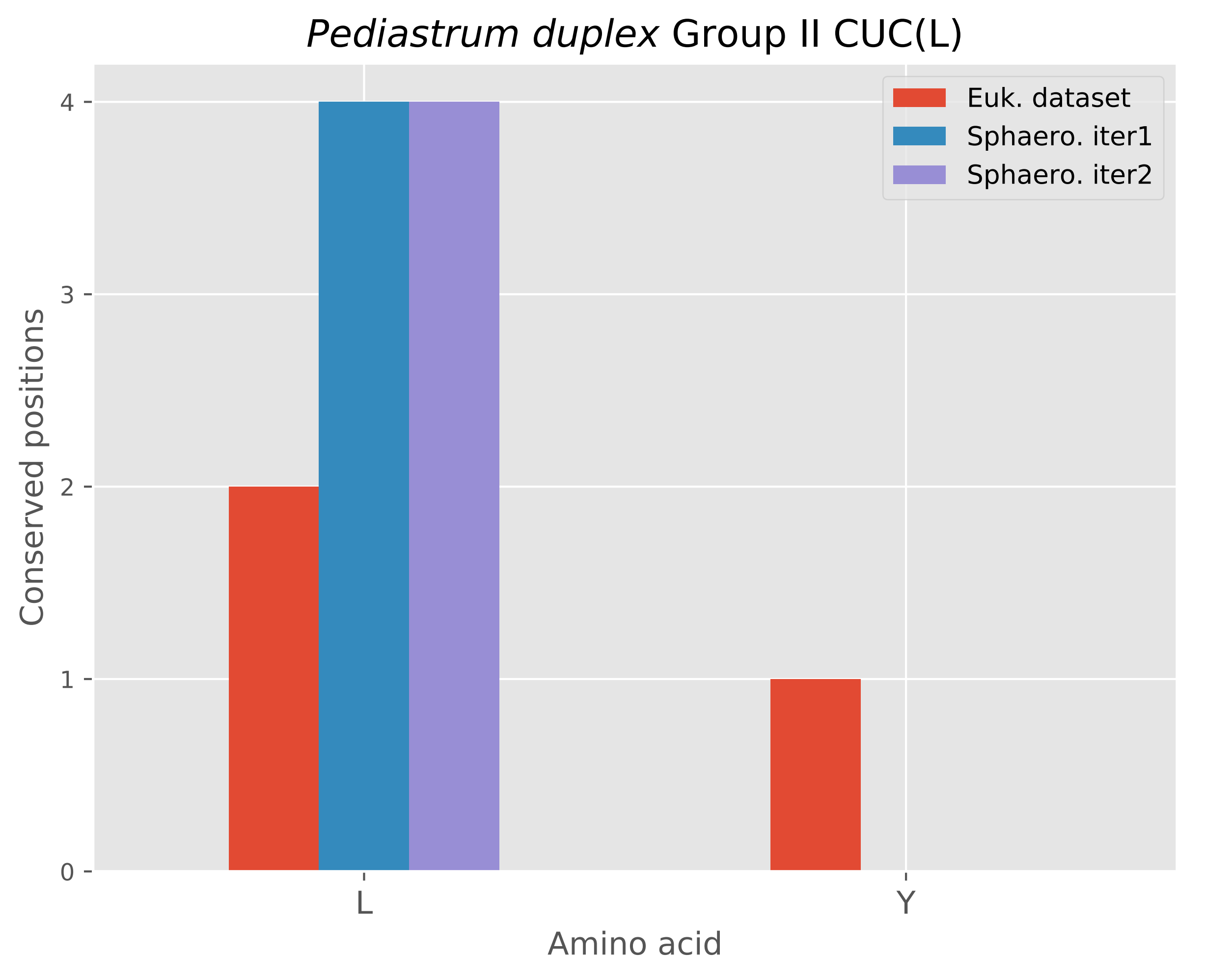

Pediastrum duplex Group II CUC(L)
Euk. dataset
Sphaero. iter1
Sphaero. iter2
Conserved positions
0
1
2
3
4
L
Y
Amino acid

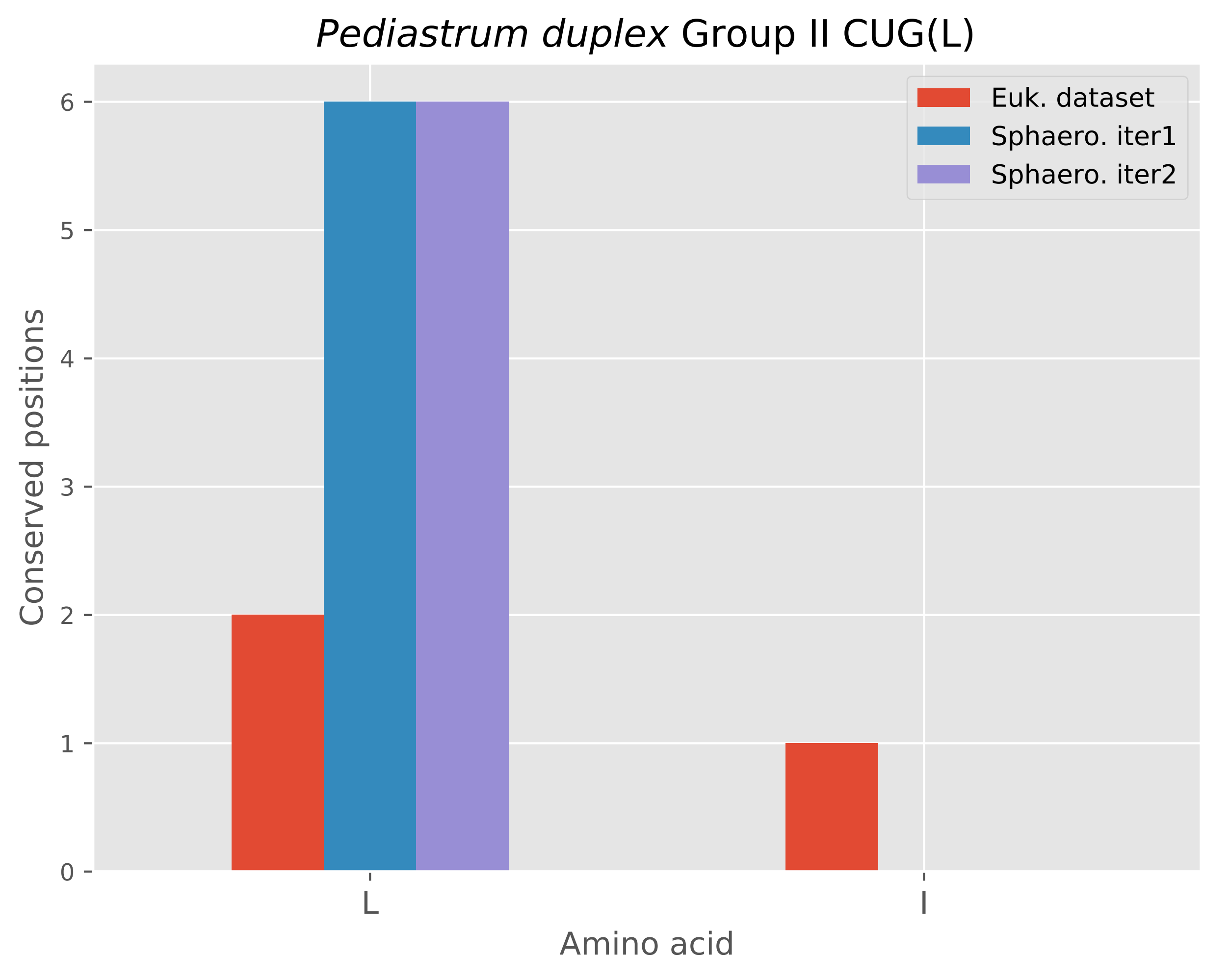

Pediastrum duplex Group II CUG(L)
Euk. dataset
Sphaero. iter1
Sphaero. iter2
Conserved positions
0
1
2
3
4
5
6
L
I
Amino acid

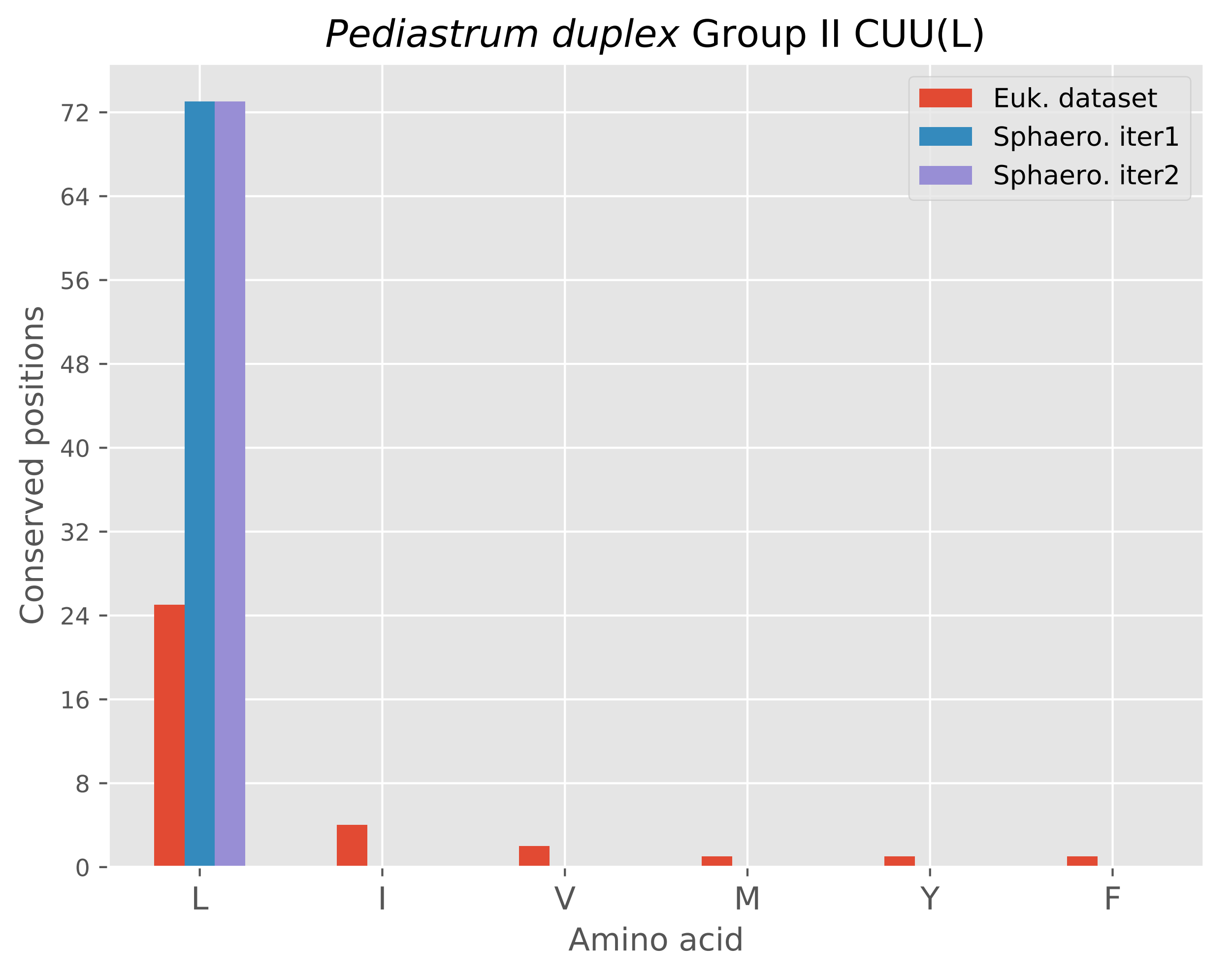

Pediastrum duplex Group II CUU(L)
Euk. dataset
Sphaero. iter1
Sphaero. iter2
Conserved positions
0
8
16
24
32
40
48
56
64
72
L
I
V
M
Y
F
Amino acid

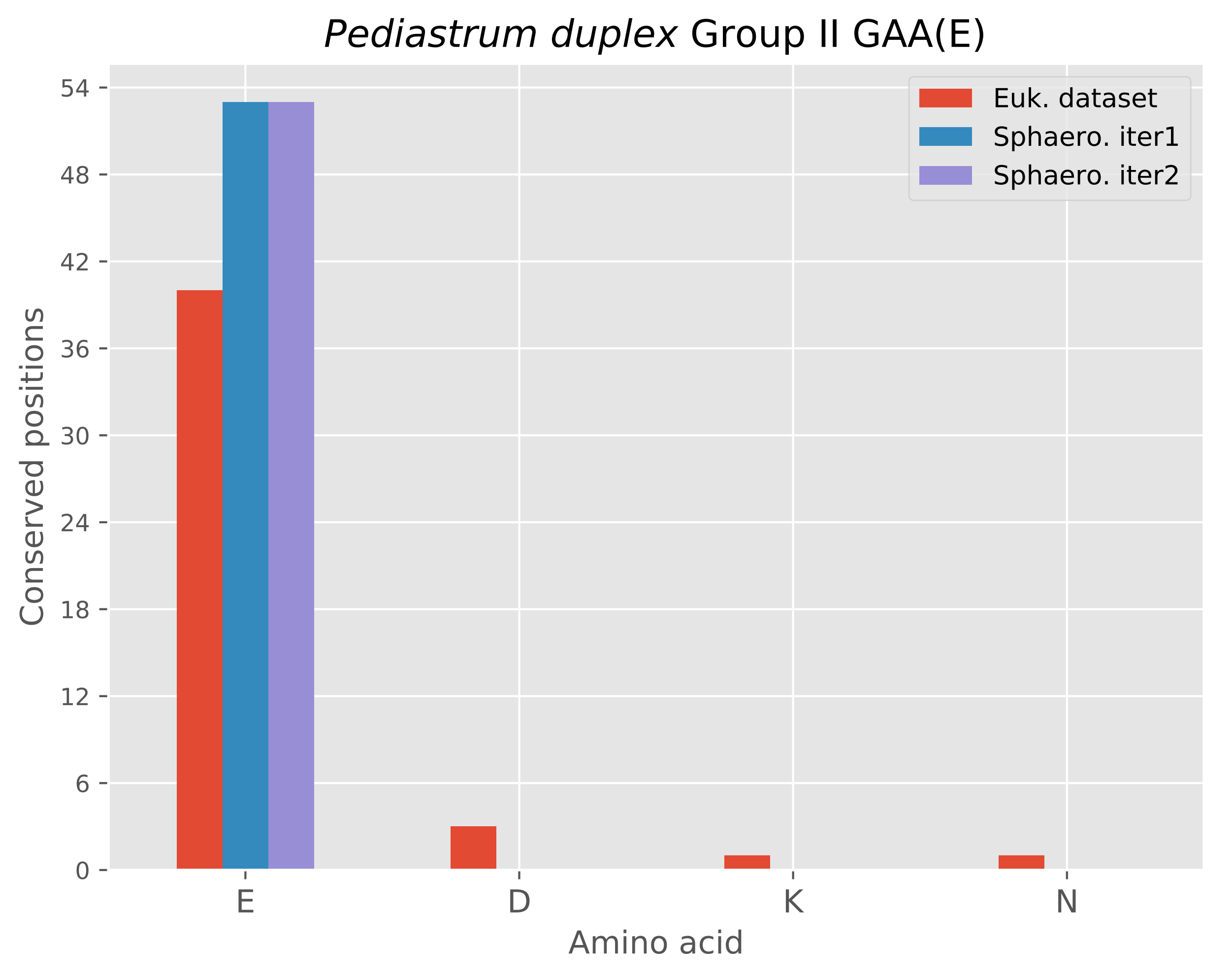

Pediastrum duplex Group II GAA(E)
Euk. dataset
Sphaero. iter1
Sphaero. iter2
Conserved positions
54
48
42
36
30
24
18
12
6
0
E
D
K
N
Amino acid

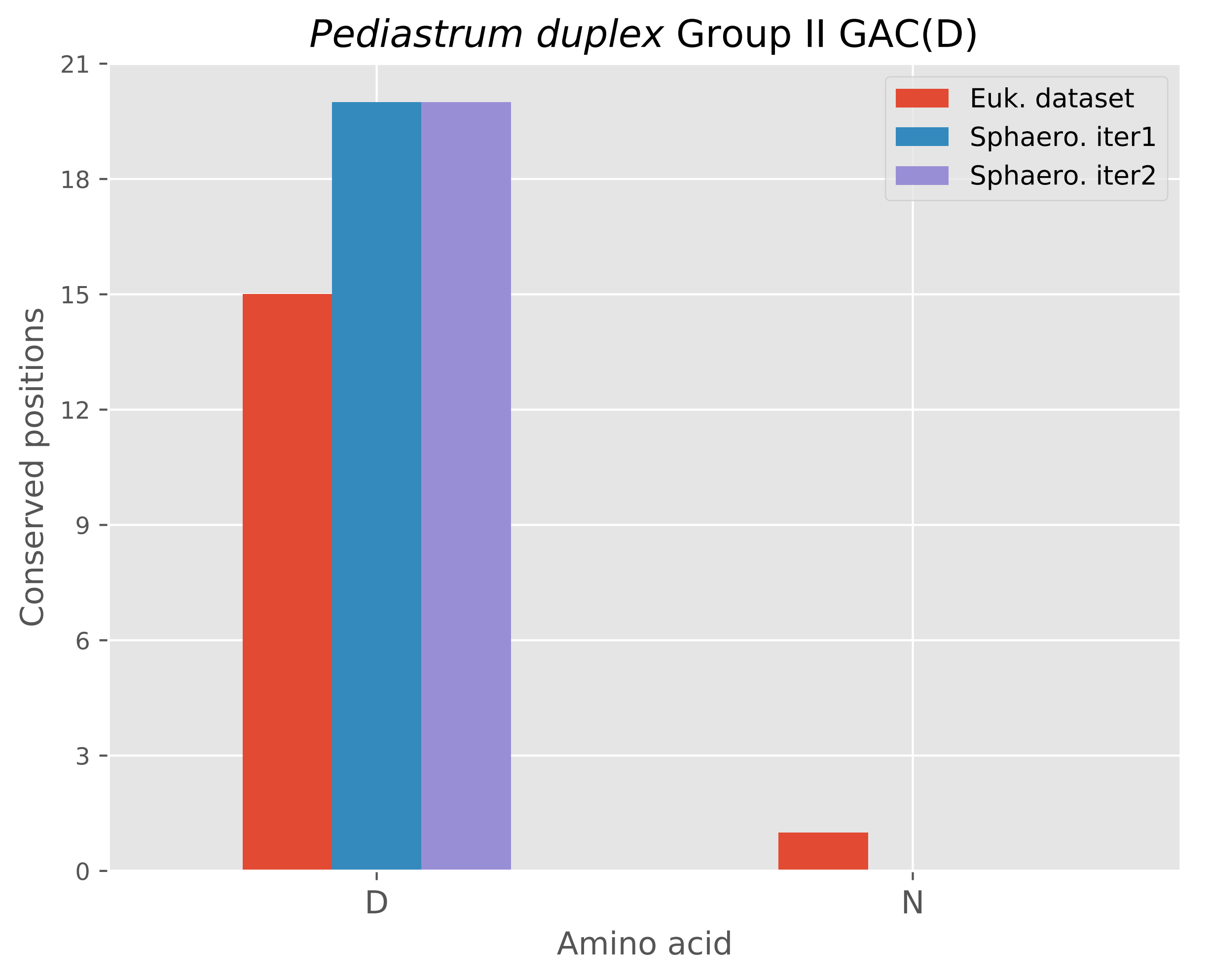
Pediastrum duplex Group II GAC(D)
Conserved positions
0
3
6
9
12
15
18
21
D
N
Amino acid
Euk. dataset
Sphaero. iter1
Sphaero. iter2

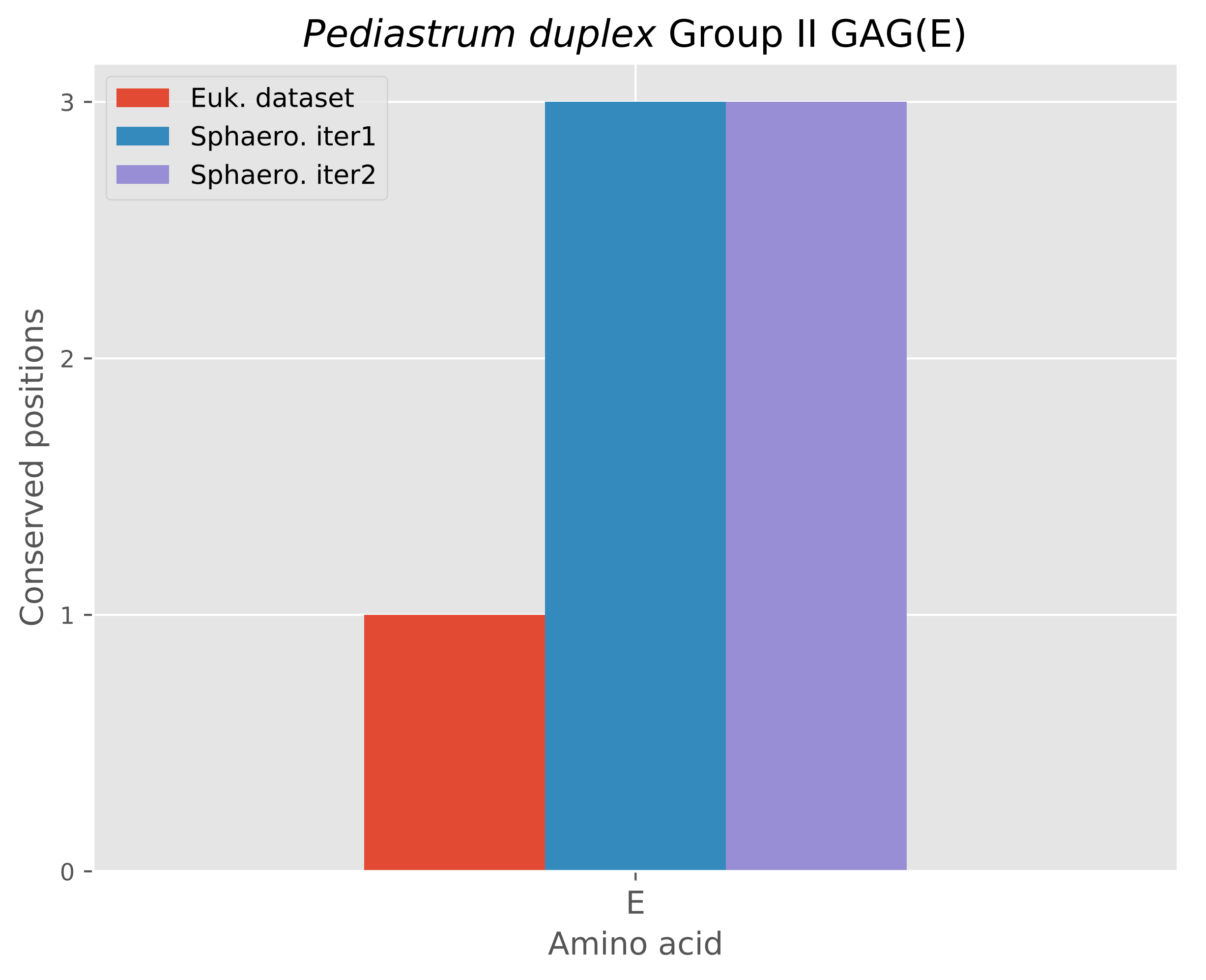
Pediastrum duplex Group II GAG(E)
Euk. dataset
Sphaero. iter1
Sphaero. iter2
Conserved positions
0
1
2
3
E
Amino acid

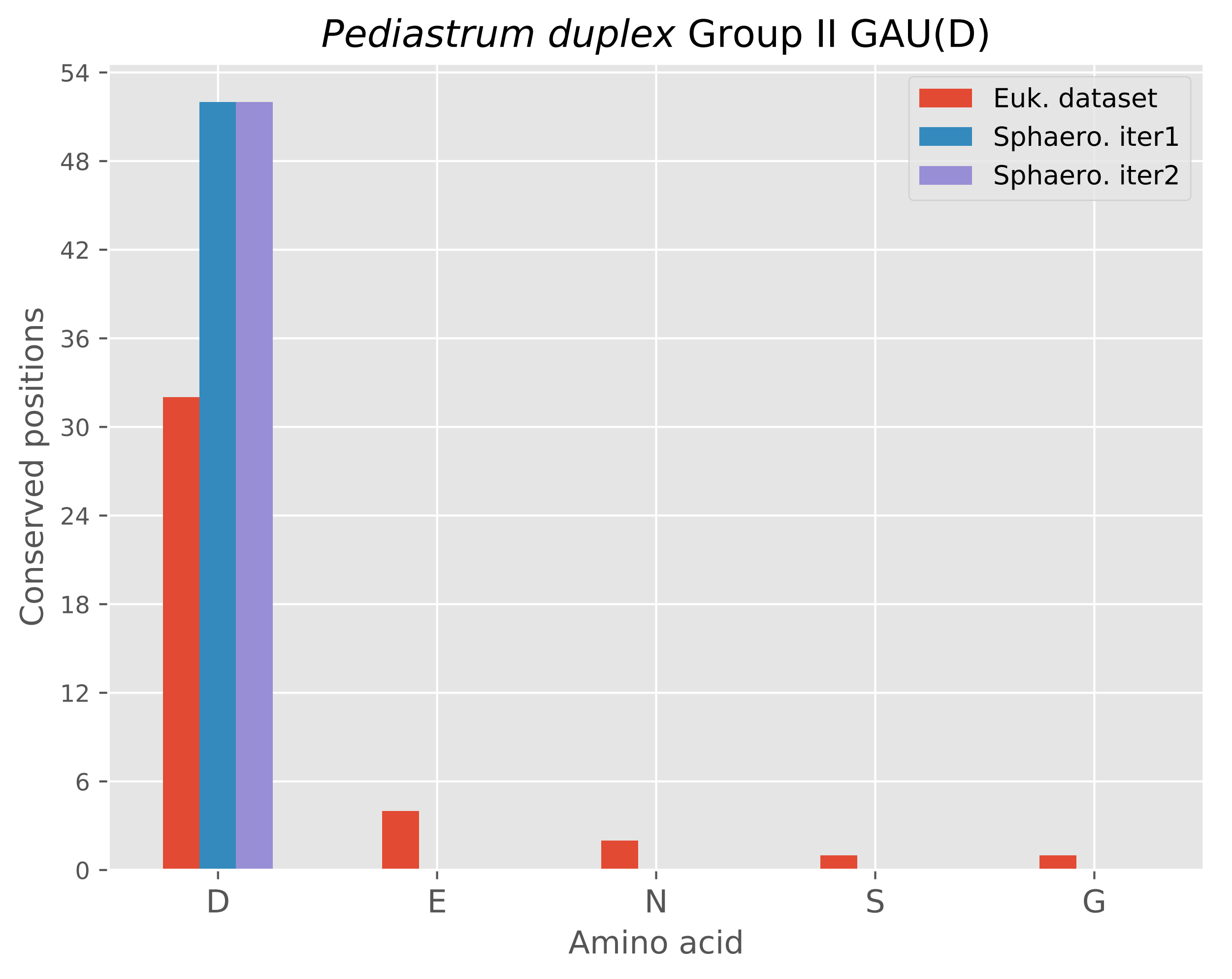

Pediastrum duplex Group II GAU(D)
Euk. dataset
Sphaero. iter1
Sphaero. iter2
Conserved positions
0
6
12
18
24
30
36
42
48
54
D
E
N
S
G
Amino acid

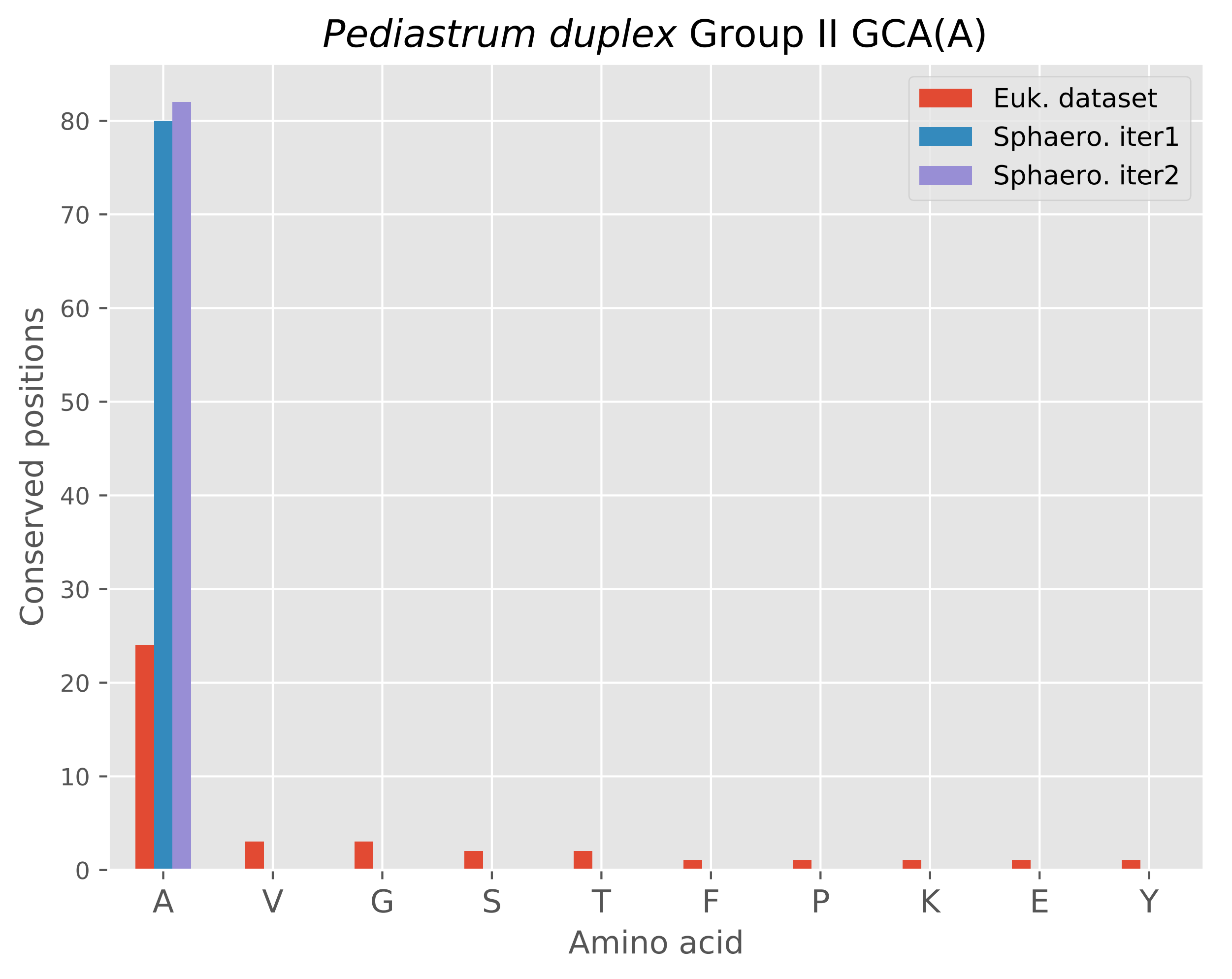

Pediastrum duplex Group II GCA(A)
Euk. dataset
Sphaero. iter1
Sphaero. iter2
Conserved positions
0
10
20
30
40
50
60
70
80
A
V
G
S
T
F
P
K
E
Y
Amino acid

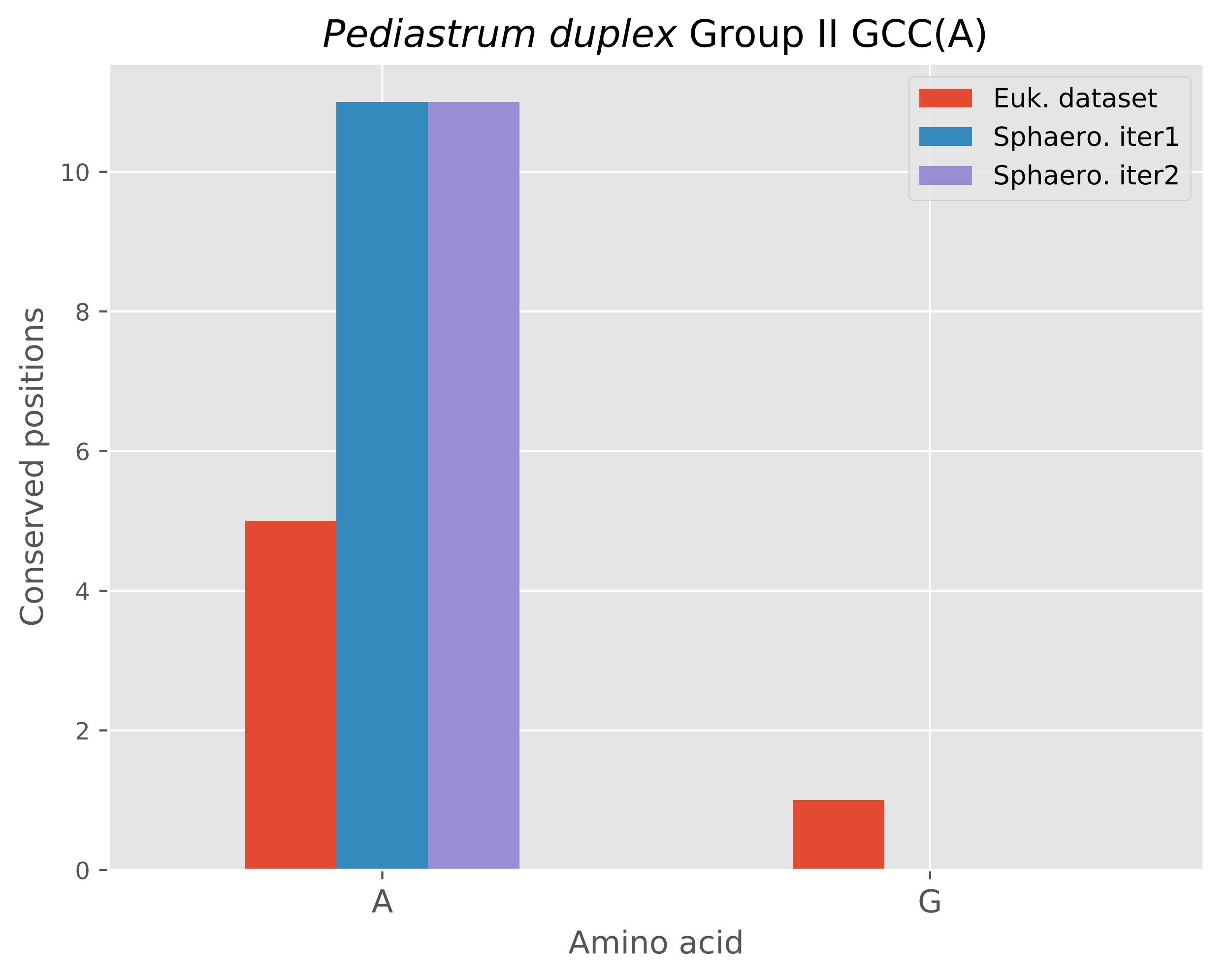
Pediastrum duplex Group II GCC(A)
Euk. dataset
Sphaero. iter1
Sphaero. iter2
Conserved positions
0
2
4
6
8
10
A
G
Amino acid

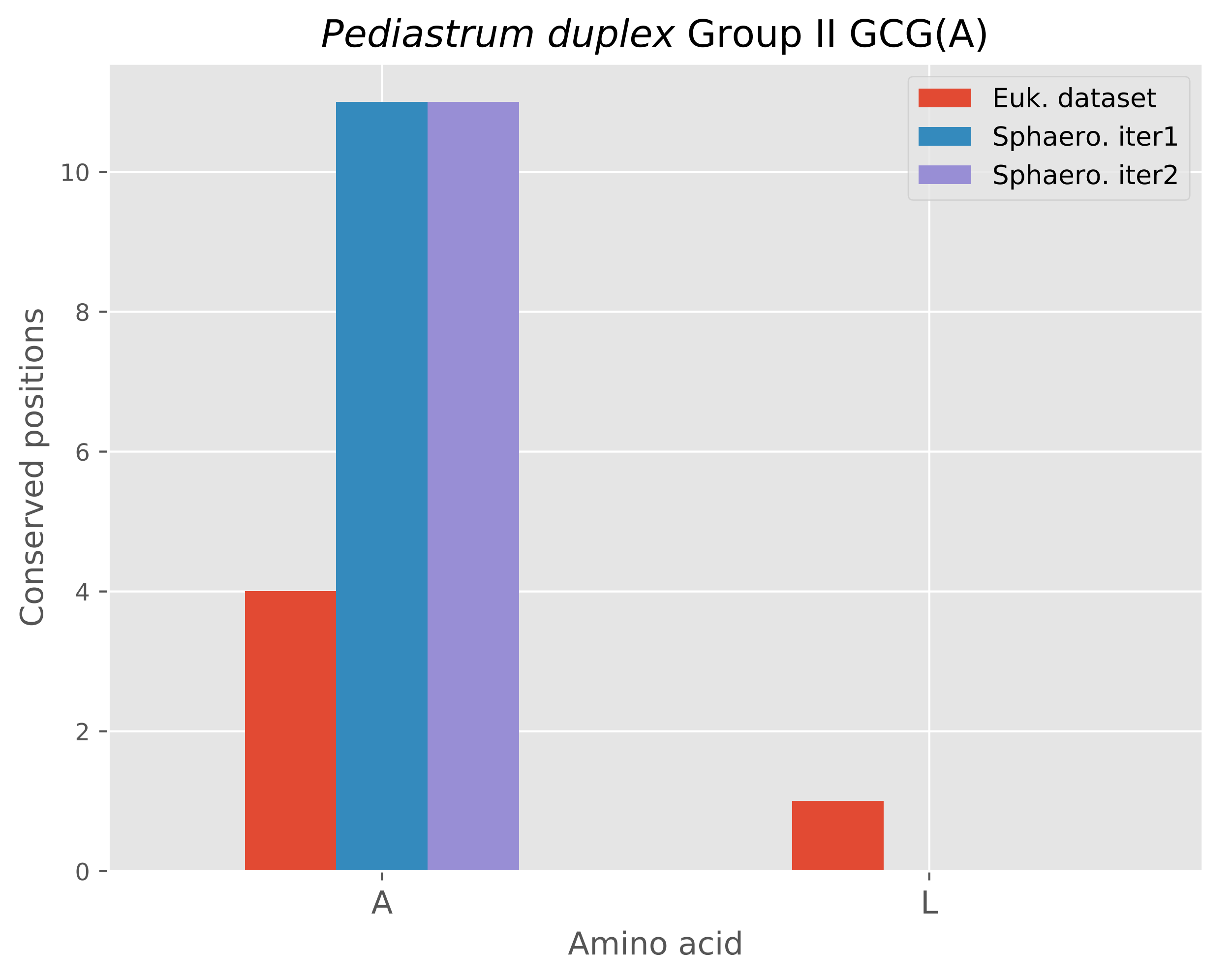
Pediastrum duplex Group II GCG(A)
Euk. dataset
Sphaero. iter1
Sphaero. iter2
Conserved positions
0
2
4
6
8
10
A
L
Amino acid

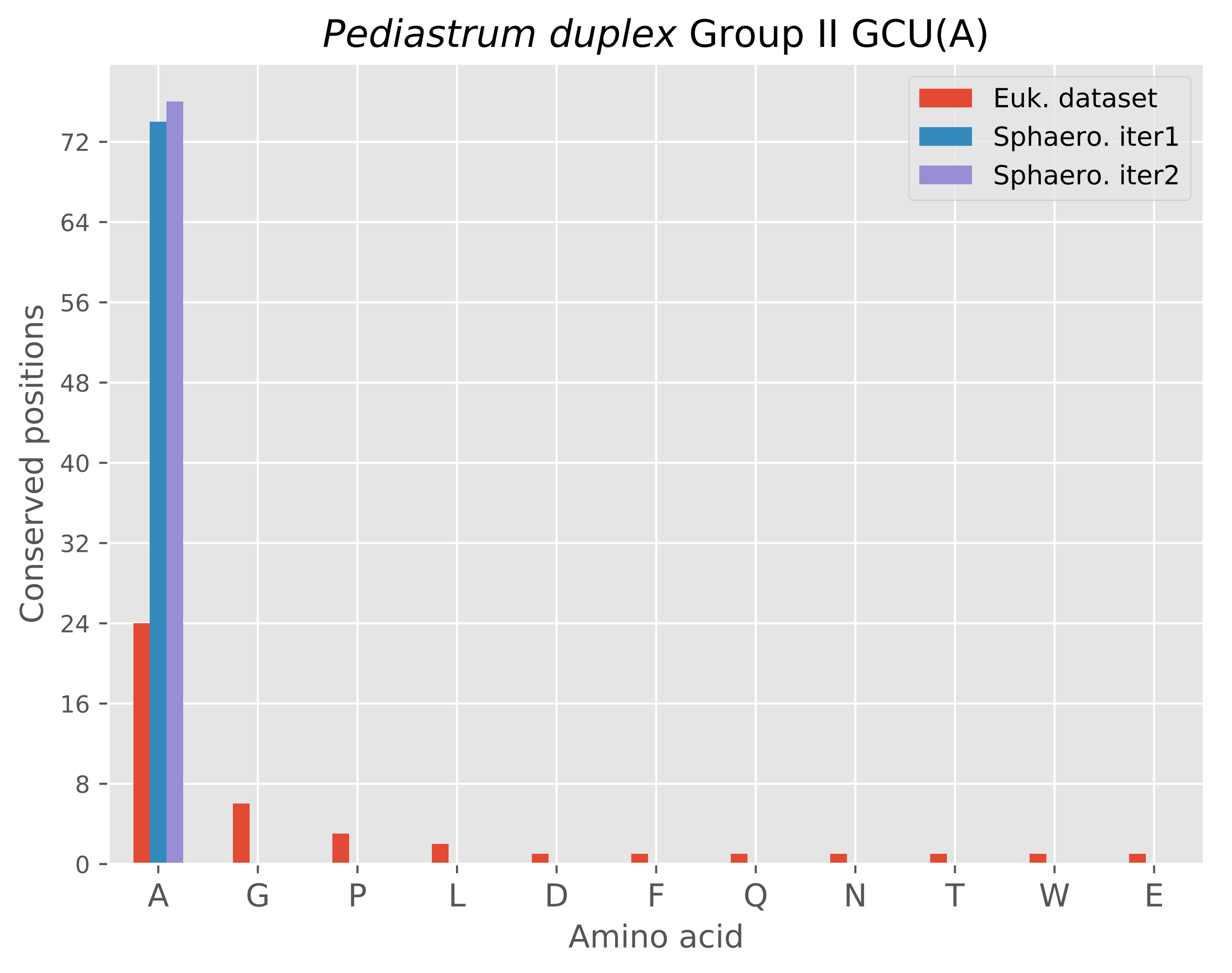

Pediastrum duplex Group II GCU(A)
Euk. dataset
Sphaero. iter1
Sphaero. iter2
Conserved positions
0
8
16
24
32
40
48
56
64
72
A
G
P
L
D
F
Q
N
T
W
E
Amino acid

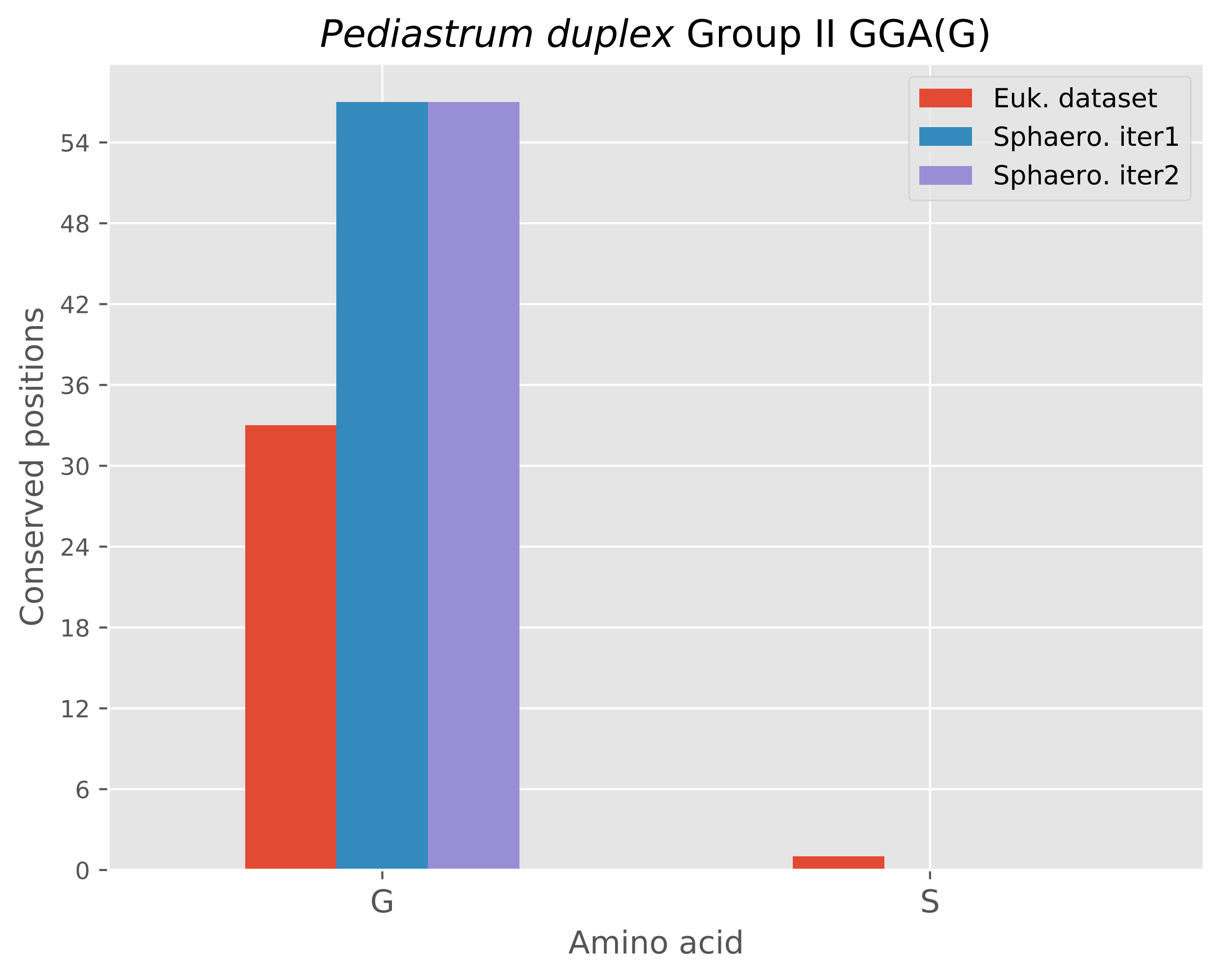

Pediastrum duplex Group II GGA(G)
Euk. dataset
Sphaero. iter1
Sphaero. iter2
Conserved positions
0
6
12
18
24
30
36
42
48
54
G
S
Amino acid

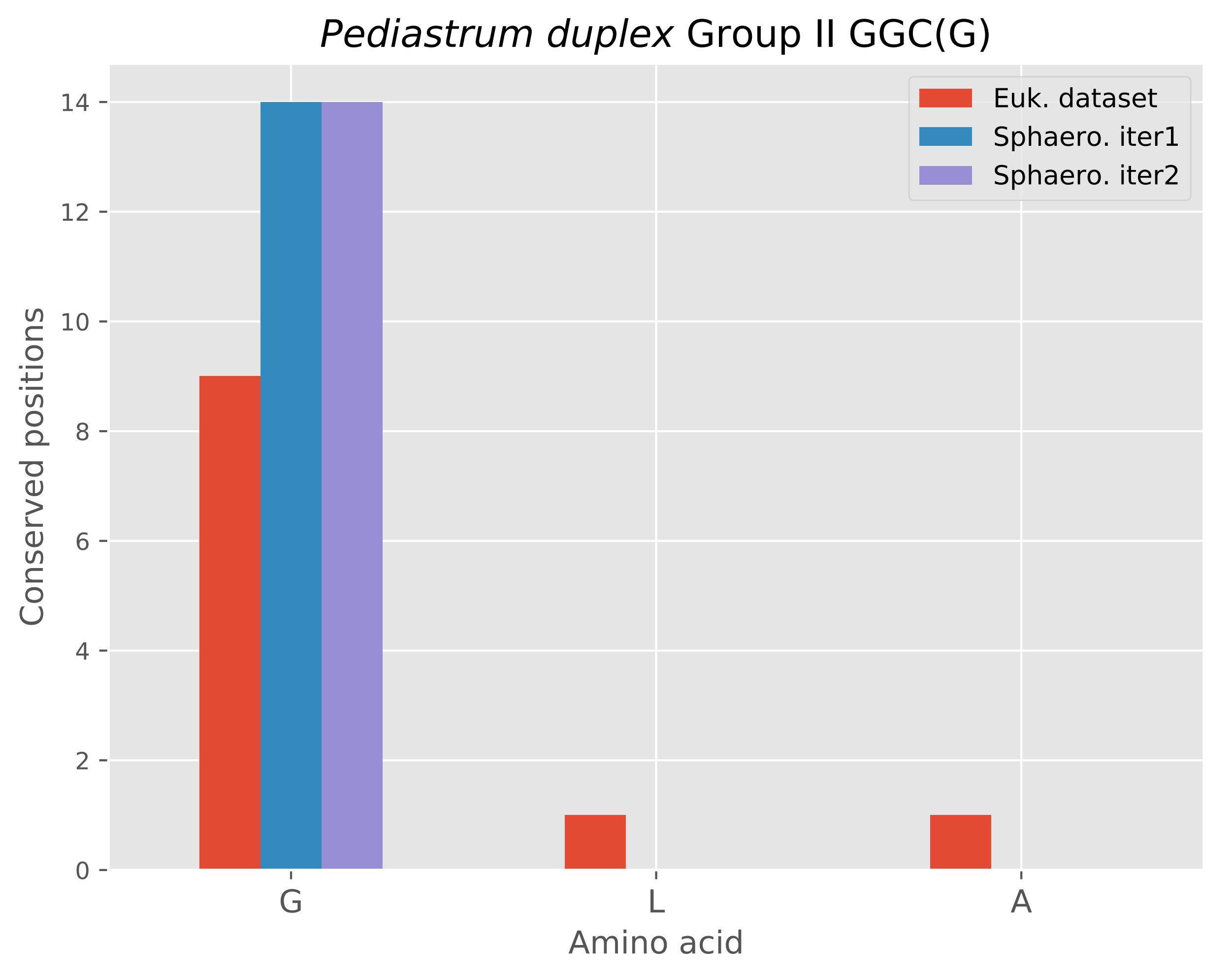

Pediastrum duplex Group II GGC(G)
Euk. dataset
Sphaero. iter1
Sphaero. iter2
Conserved positions
0
2
4
6
8
10
12
14
G
L
A
Amino acid

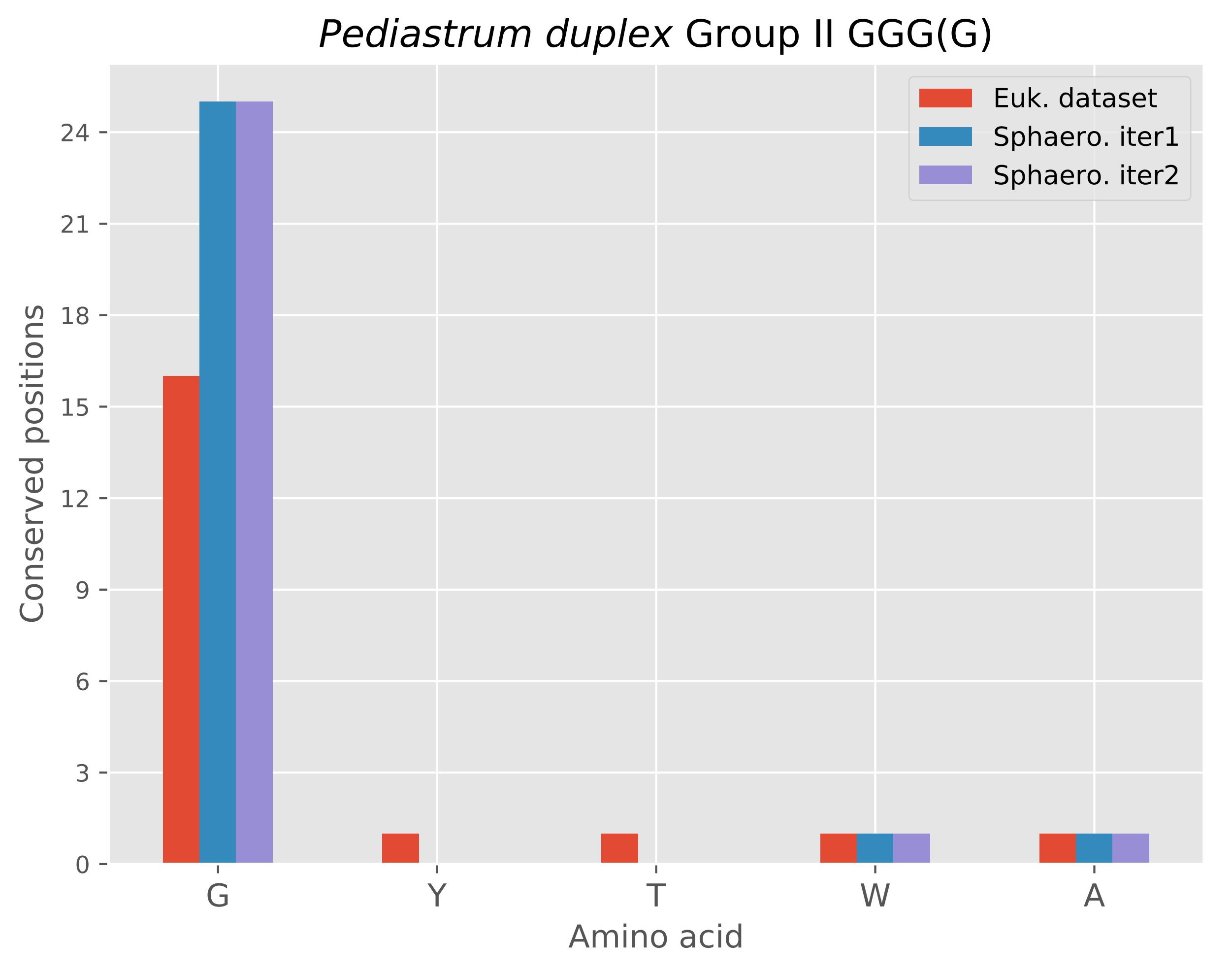
Pediastrum duplex Group II GGG(G)
Euk. dataset
Sphaero. iter1
Sphaero. iter2
Conserved positions
0
3
6
9
12
15
18
21
24
G
Y
T
W
A
Amino acid

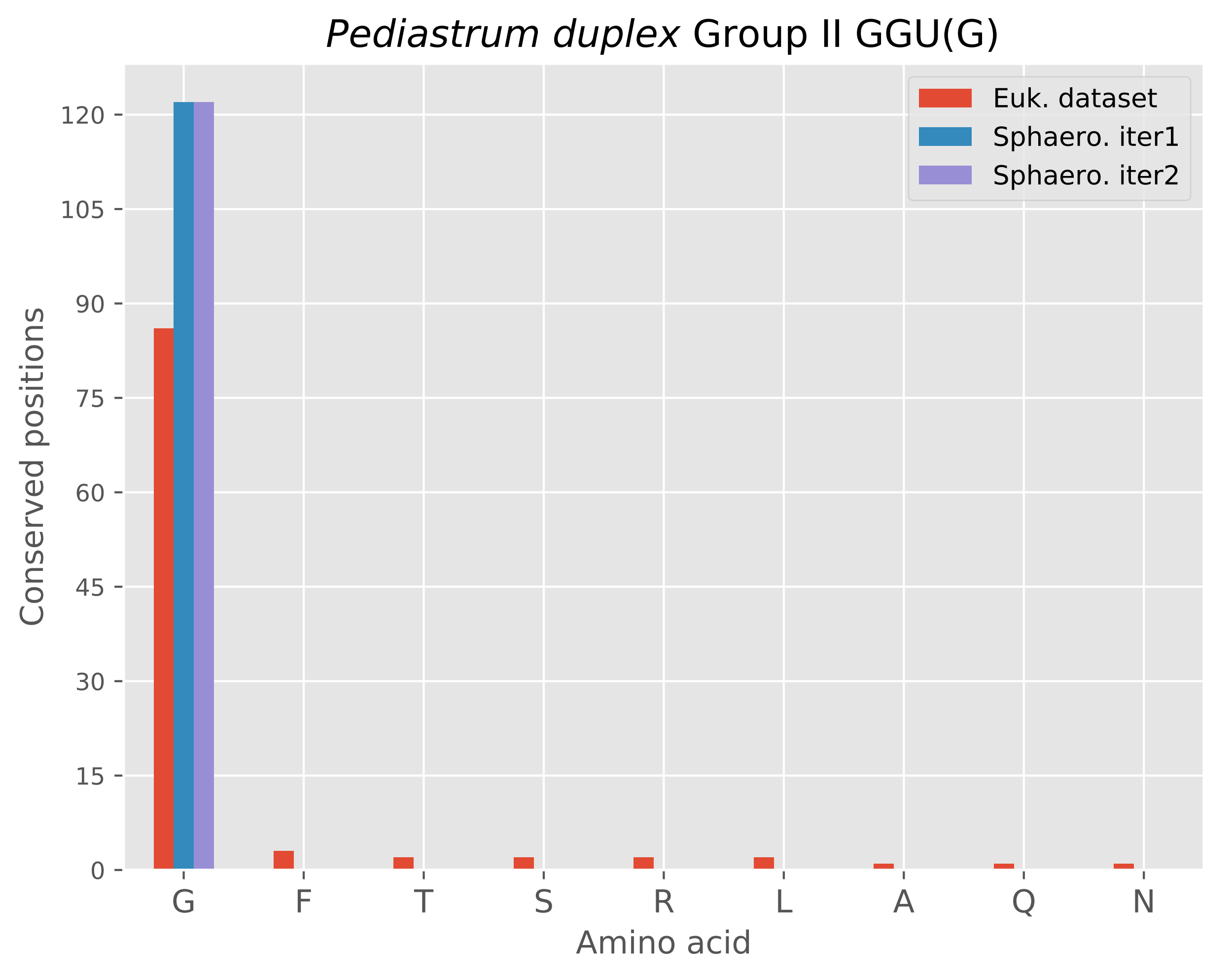

Pediastrum duplex Group II GGU(G)
Euk. dataset
Sphaero. iter1
Sphaero. iter2
Conserved positions
0
15
30
45
60
75
90
105
120
G
F
T
S
R
L
A
Q
N
Amino acid

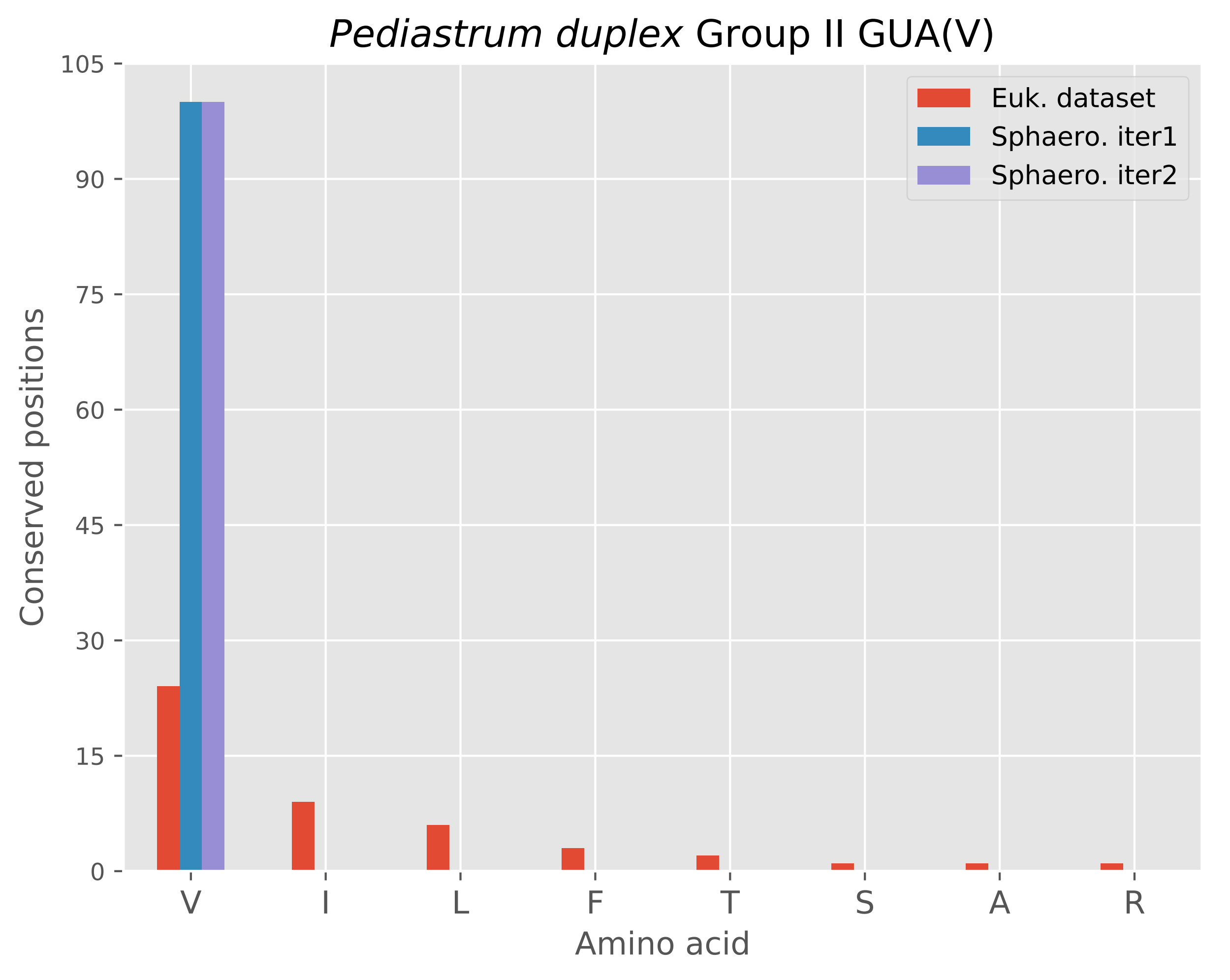

Pediastrum duplex Group II GUA(V)
Conserved positions
105
90
75
60
45
30
15
0
Euk. dataset
Sphaero. iter1
Sphaero. iter2
V
I
L
F
T
S
A
R
Amino acid

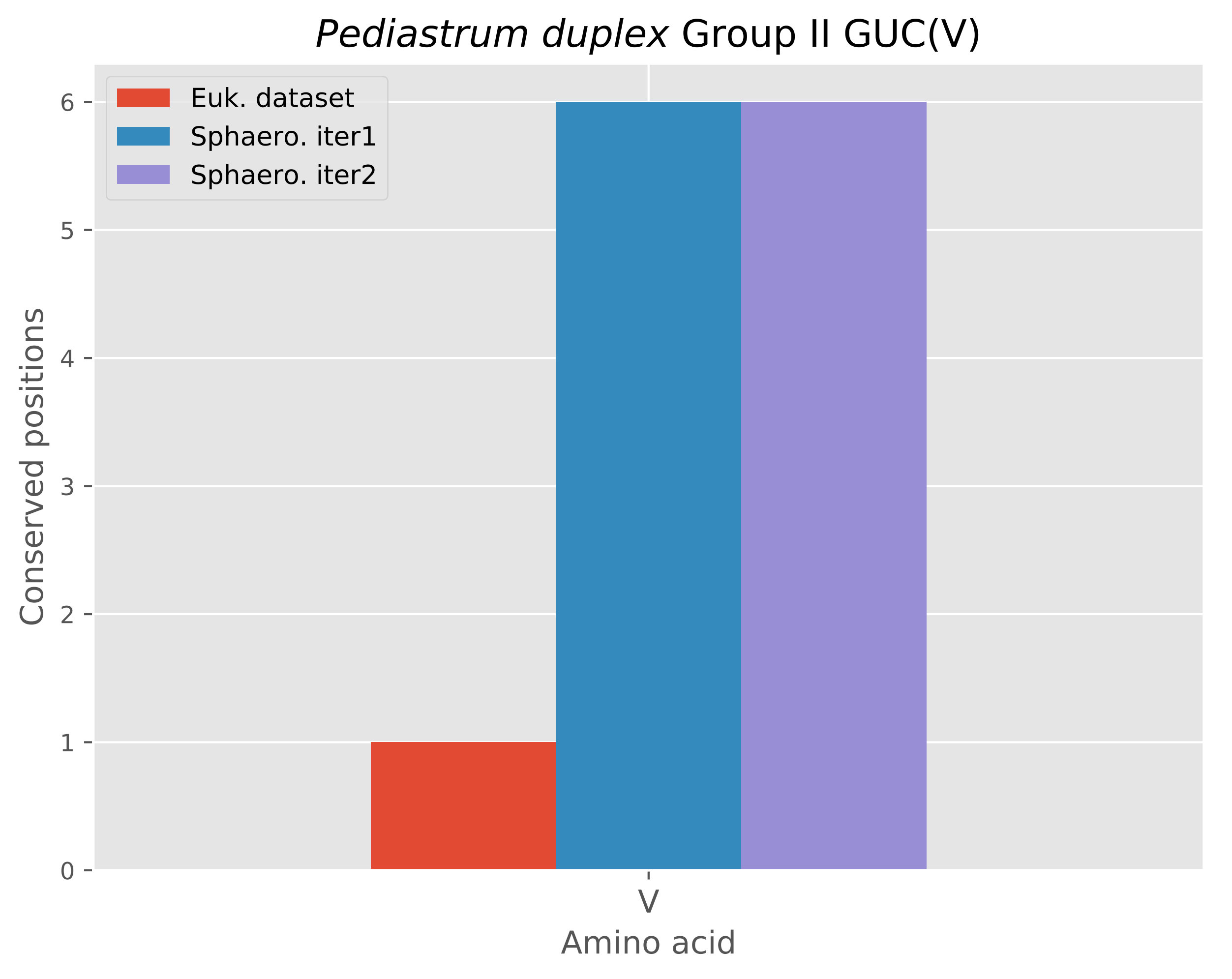

Pediastrum duplex Group II GUC(V)
Euk. dataset
Sphaero. iter1
Sphaero. iter2
Conserved positions
0
1
2
3
4
5
6
V
Amino acid

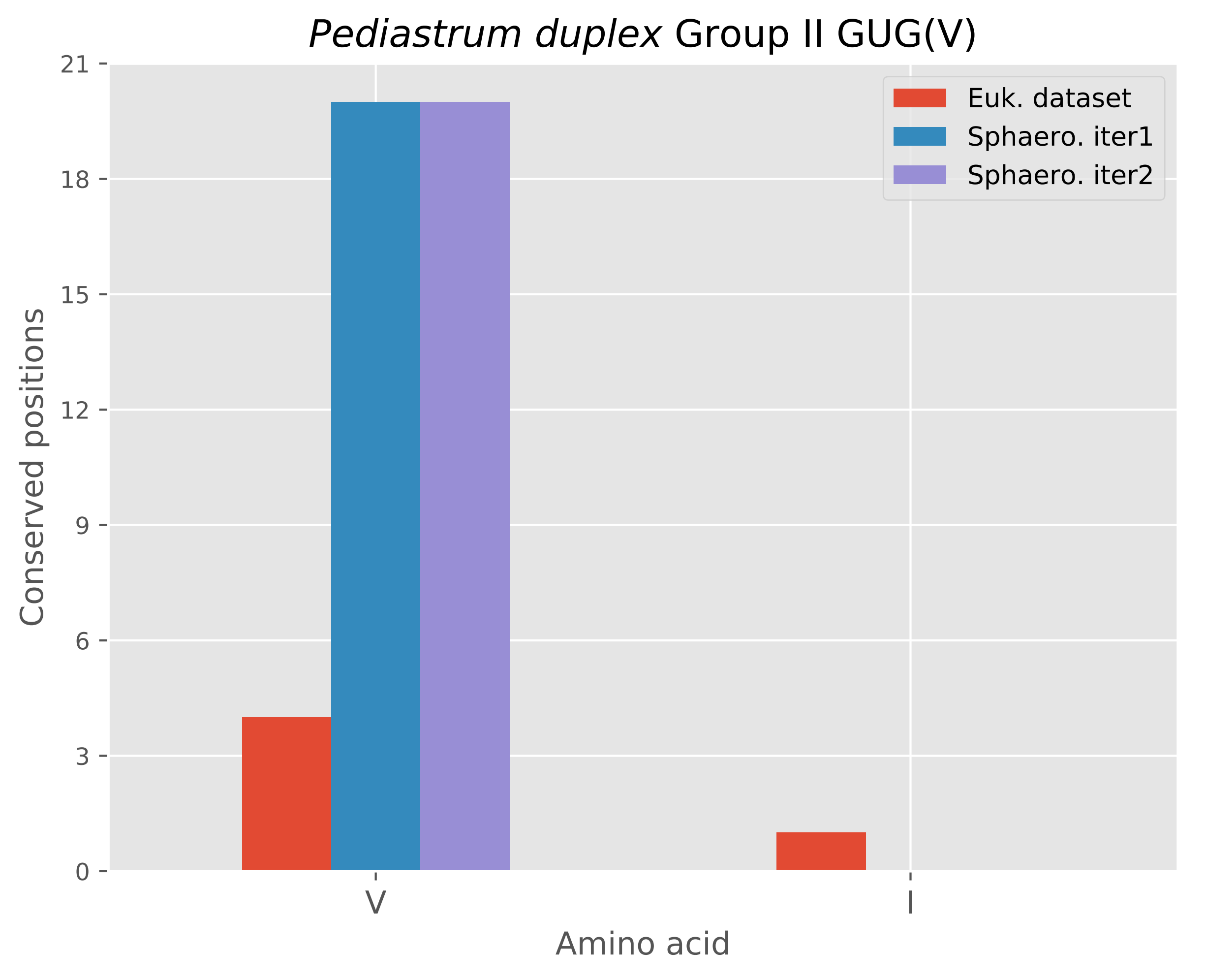
Pediastrum duplex Group II GUG(V)
Euk. dataset
Sphaero. iter1
Sphaero. iter2
Conserved positions
0
3
6
9
12
15
18
21
V
I
Amino acid

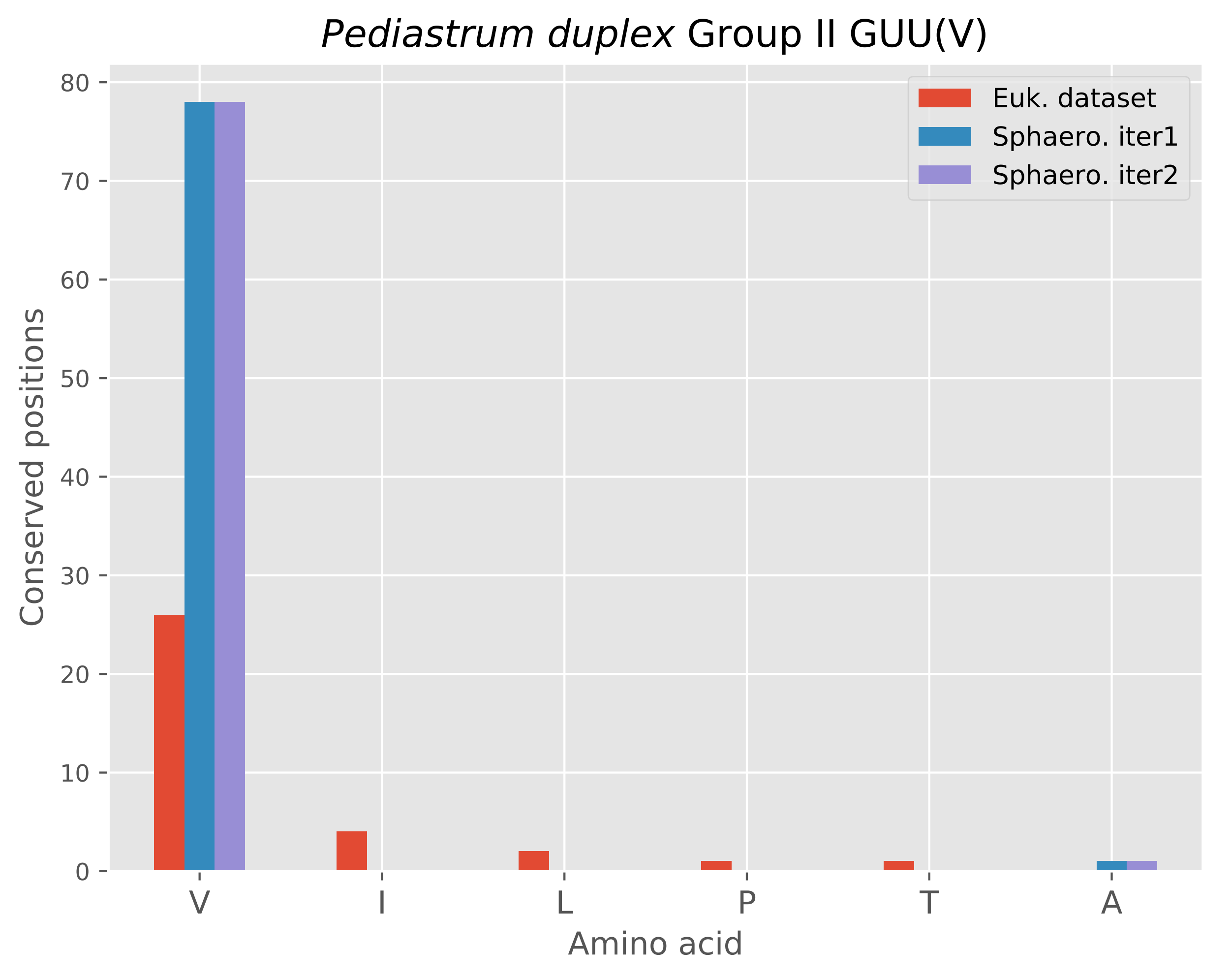

Pediastrum duplex Group II GUU(V)
Euk. dataset
Sphaero. iter1
Sphaero. iter2
Conserved positions
0
10
20
30
40
50
60
70
80
V
I
L
P
T
A
Amino acid

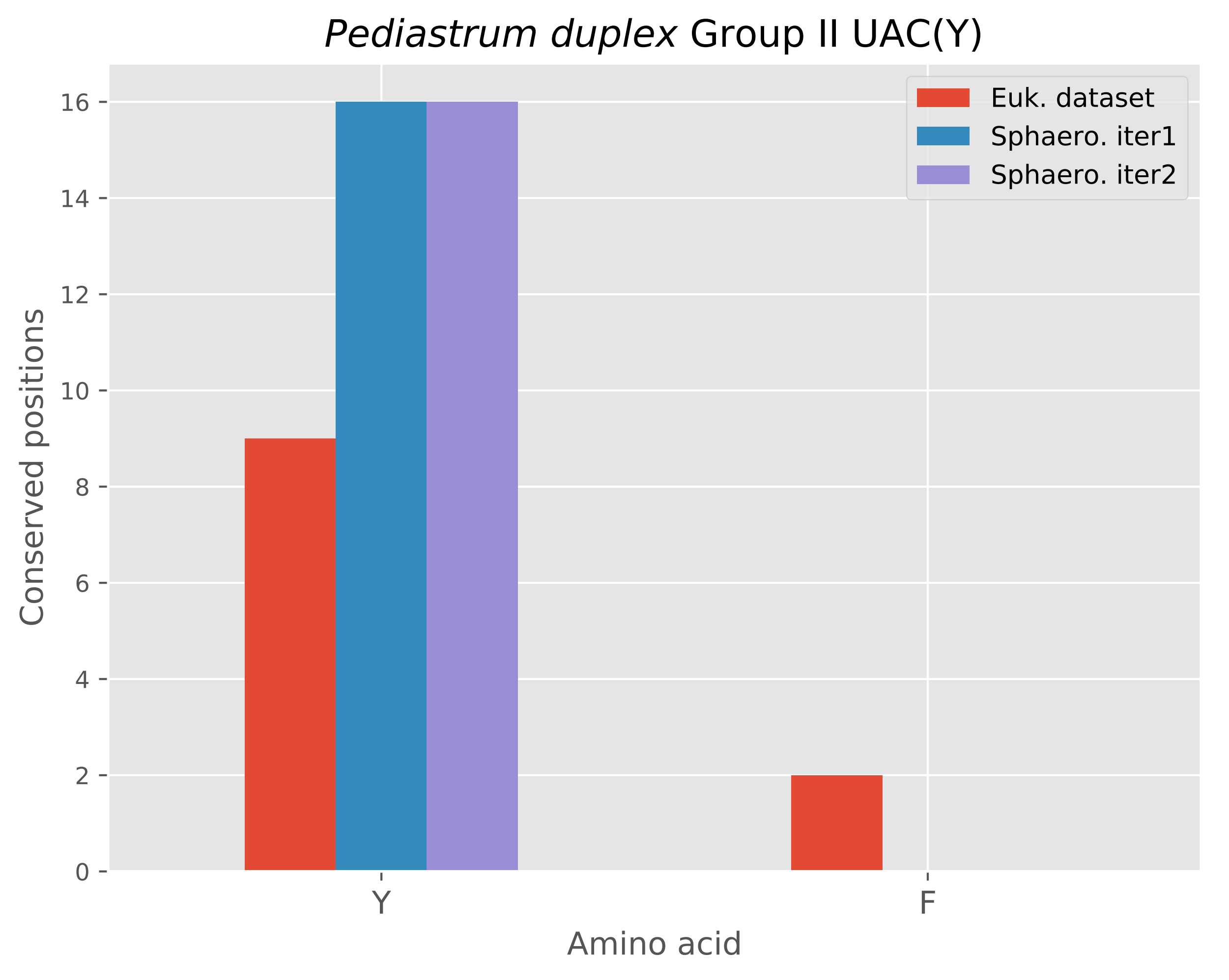
Pediastrum duplex Group II UAC(Y)
Euk. dataset
Sphaero. iter1
Sphaero. iter2
Conserved positions
0
2
4
6
8
10
12
14
16
Y
F
Amino acid

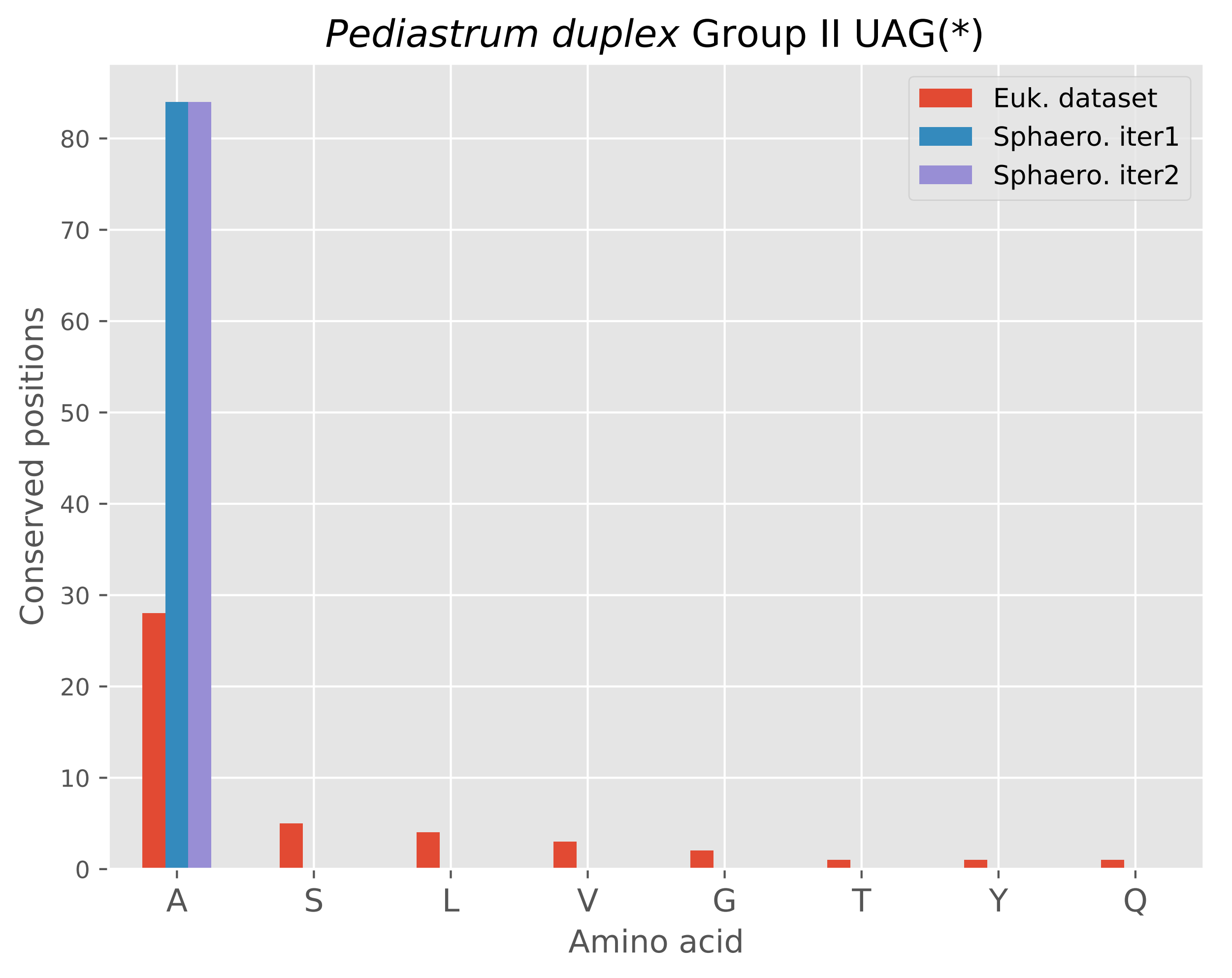

Pediastrum duplex Group II UAG(*)
Euk. dataset
Sphaero. iter1
Sphaero. iter2
Conserved positions
0
10
20
30
40
50
60
70
80
A
S
L
V
G
T
Y
Q
Amino acid

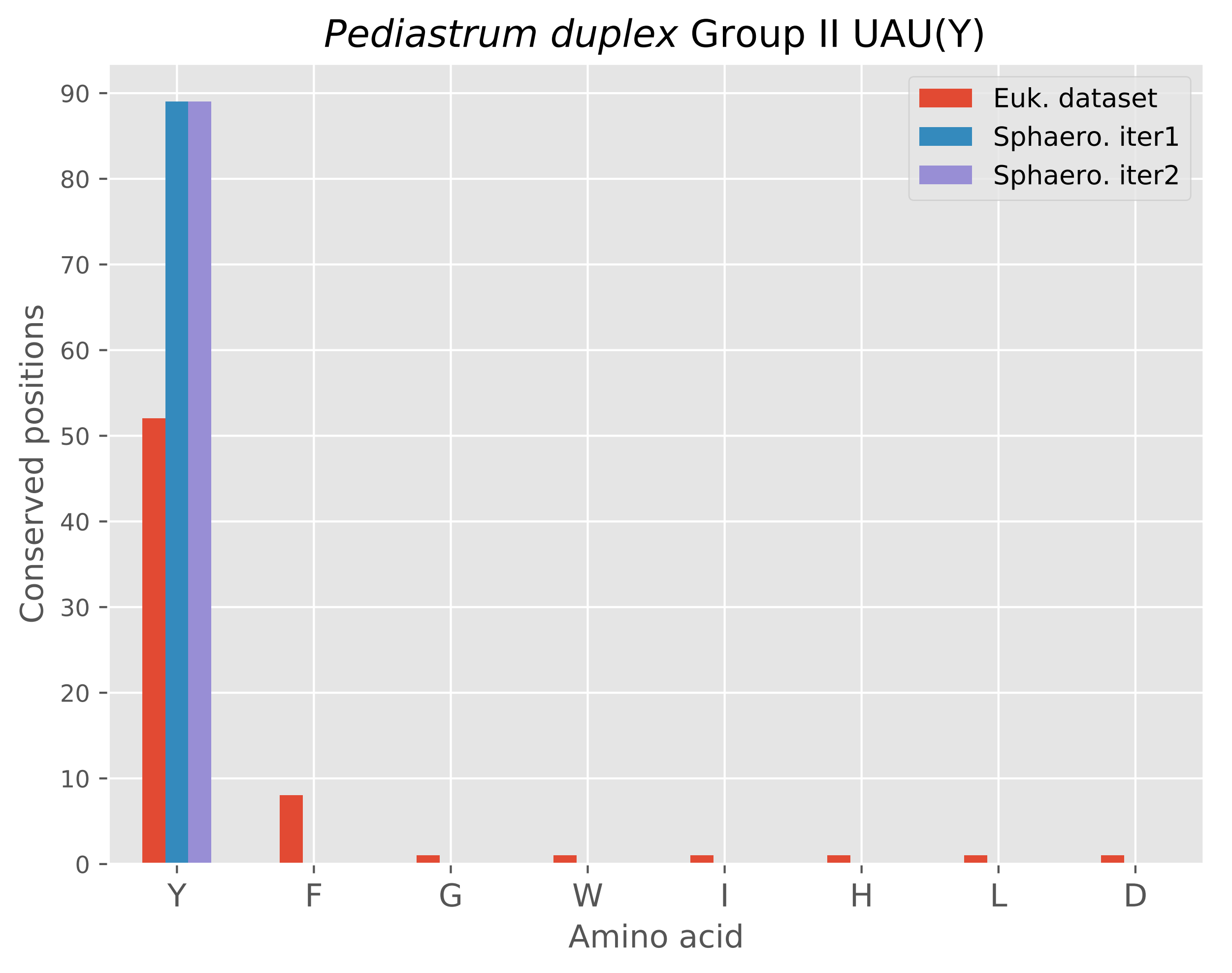
Pediastrum duplex Group II UAU(Y)
Euk. dataset
Sphaero. iter1
Sphaero. iter2
Conserved positions
0
10
20
30
40
50
60
70
80
90
Y
F
G
W
I
H
L
D
Amino acid

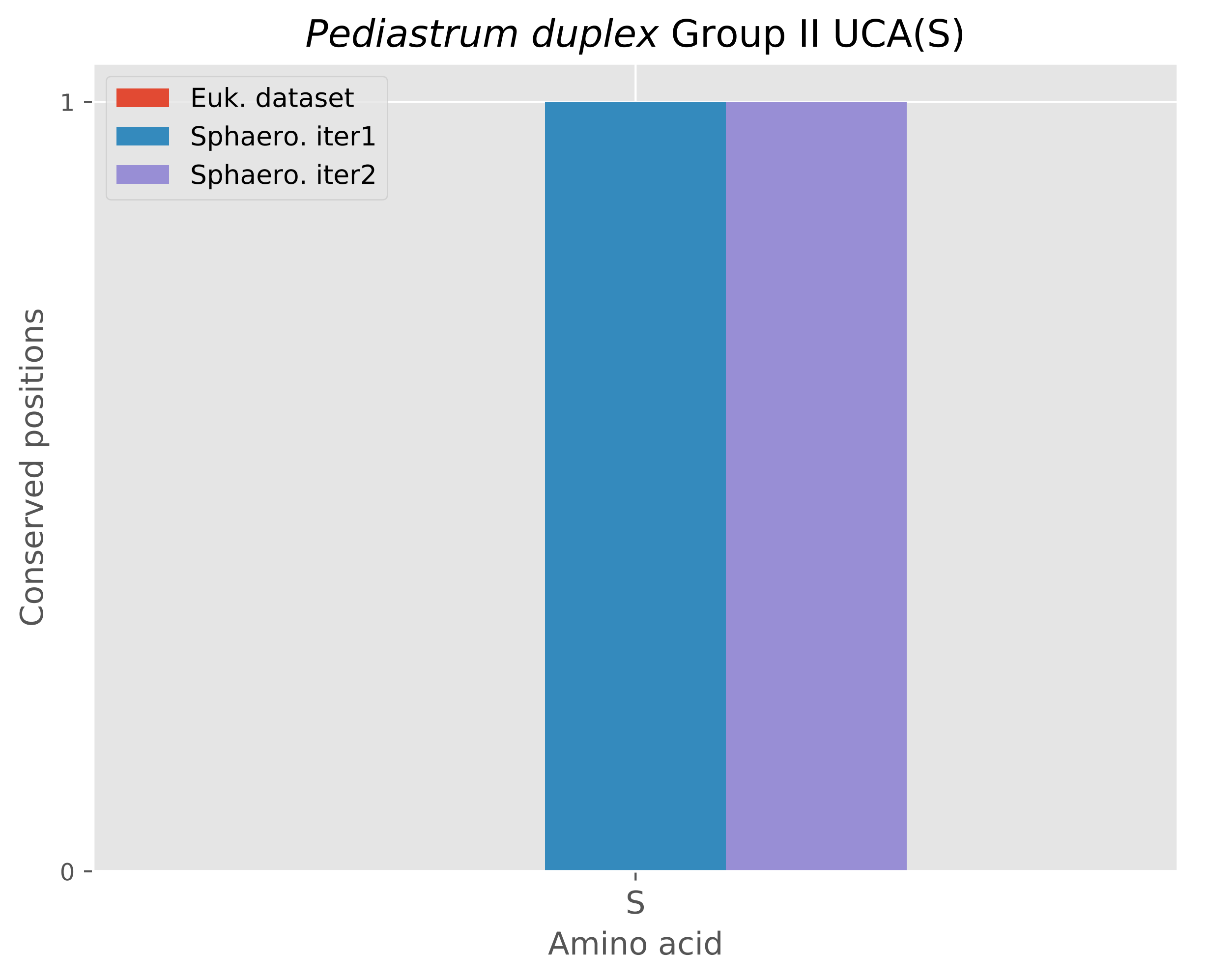

Pediastrum duplex Group II UCA(S)
Euk. dataset
Sphaero. iter1
Sphaero. iter2
1
0
Conserved positions
S
Amino acid

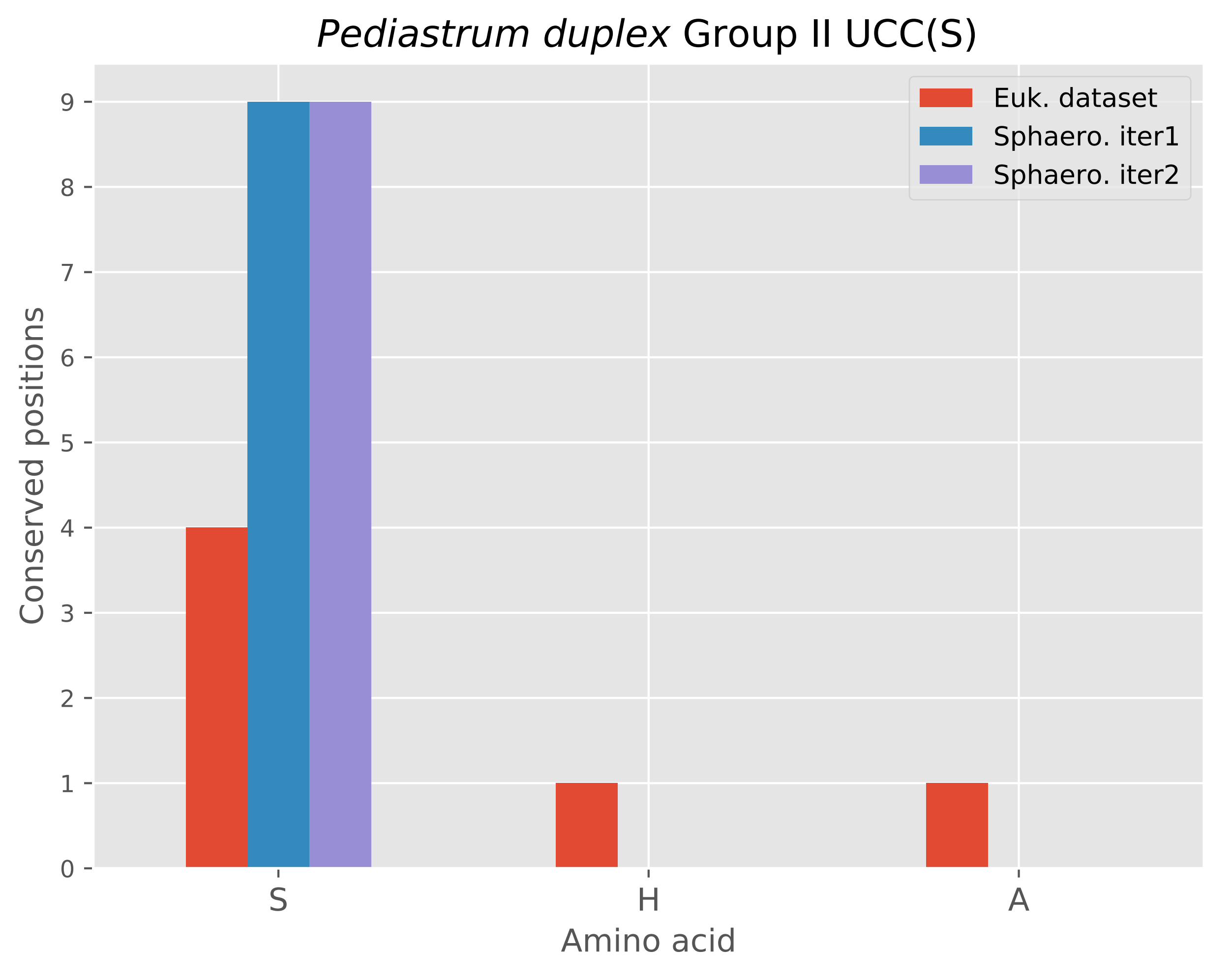
Pediastrum duplex Group II UCC(S)
Euk. dataset
Sphaero. iter1
Sphaero. iter2
Conserved positions
0
1
2
3
4
5
6
7
8
9
S
H
A
Amino acid

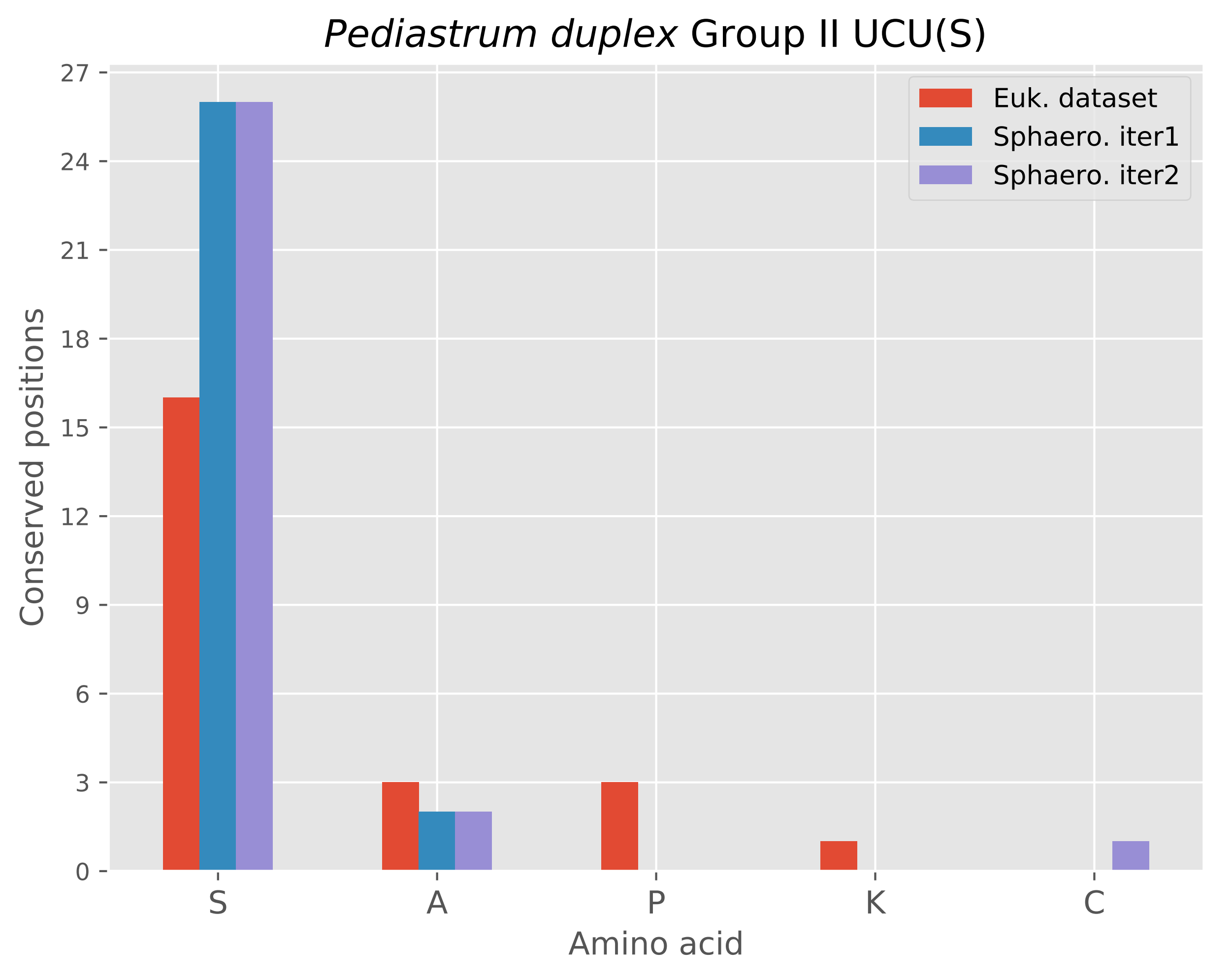

Pediastrum duplex Group II UCU(S)
Euk. dataset
Sphaero. iter1
Sphaero. iter2
Conserved positions
0
3
6
9
12
15
18
21
24
27
S
A
P
K
C
Amino acid

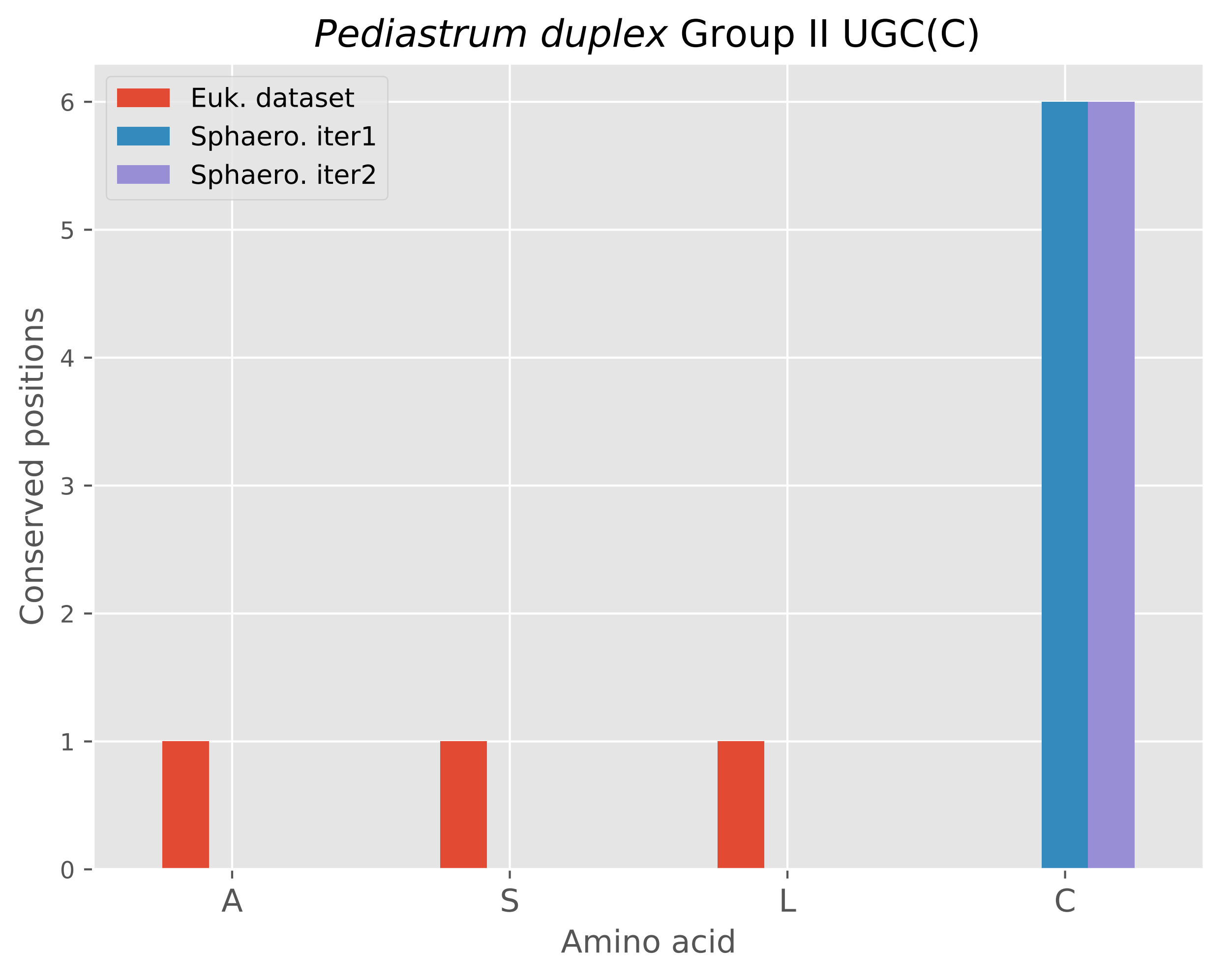

Pediastrum duplex Group II UGC(C)
Euk. dataset
Sphaero. iter1
Sphaero. iter2
Conserved positions
0
1
2
3
4
5
6
A
S
L
C
Amino acid

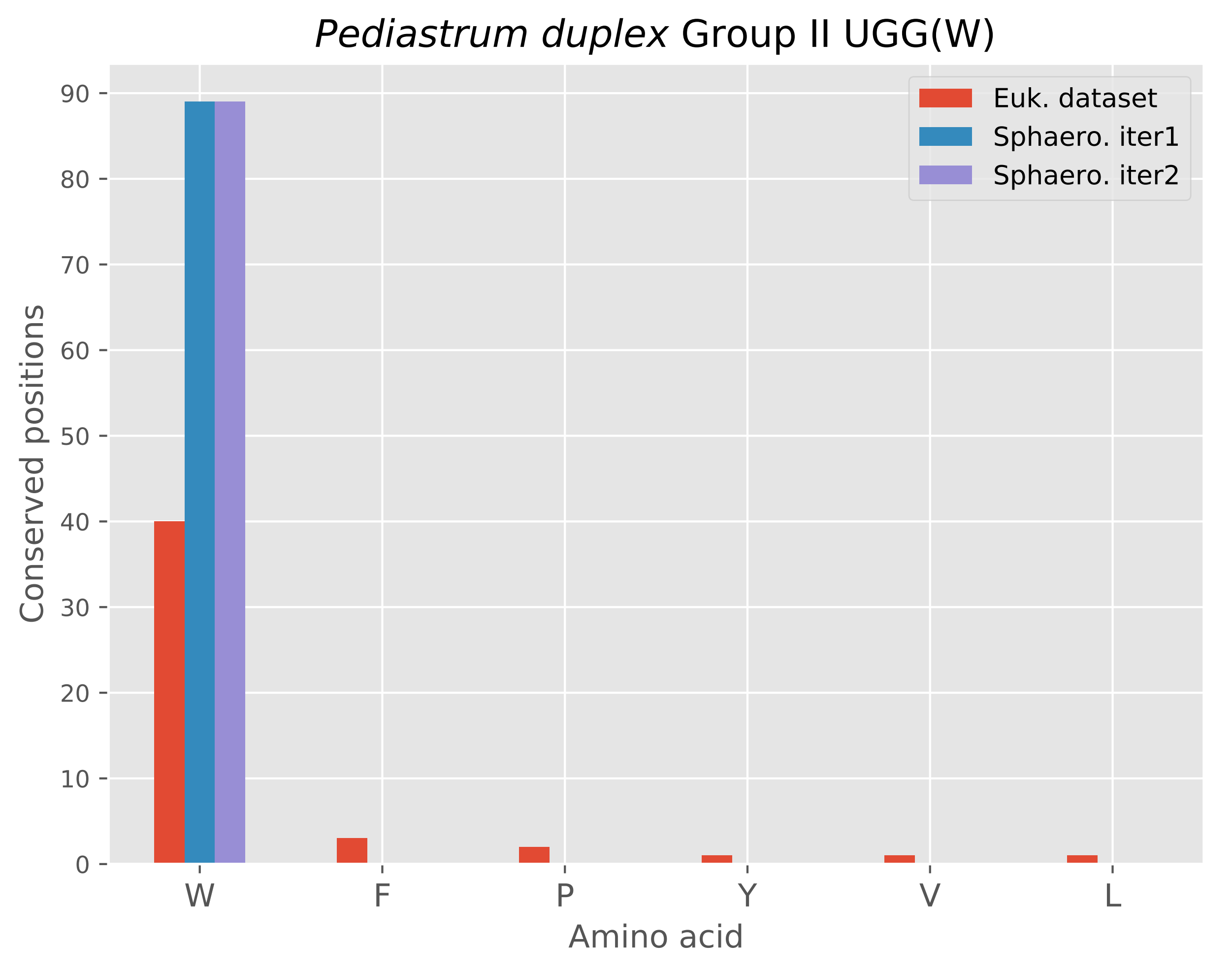
Pediastrum duplex Group II UGG(W)
Euk. dataset
Sphaero. iter1
Sphaero. iter2
Conserved positions
Amino acid
W
F
P
Y
V
L
0
10
20
30
40
50
60
70
80
90

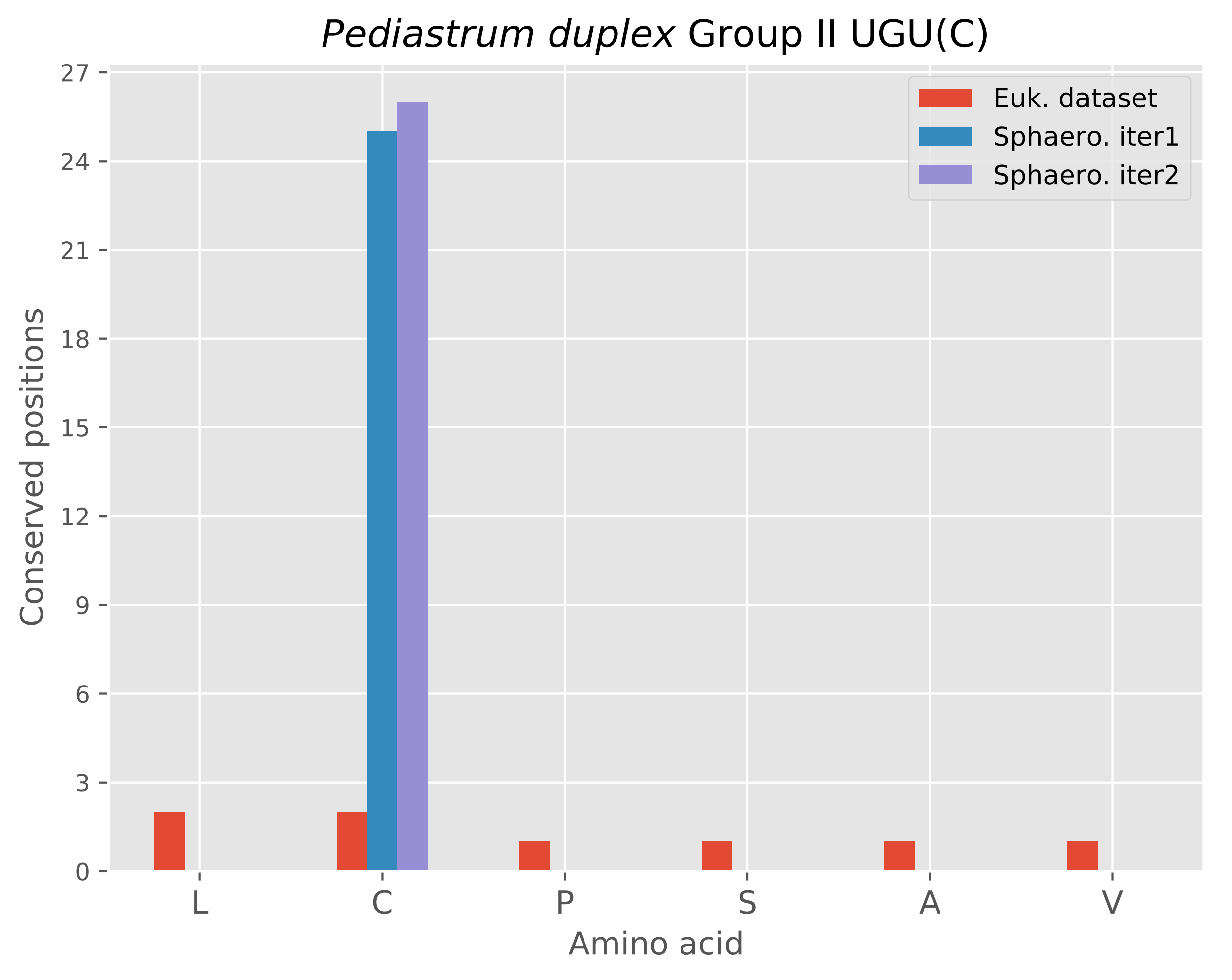

Pediastrum duplex Group II UGU(C)
Euk. dataset
Sphaero. iter1
Sphaero. iter2
Conserved positions
0
3
6
9
12
15
18
21
24
27
L
C
P
S
A
V
Amino acid

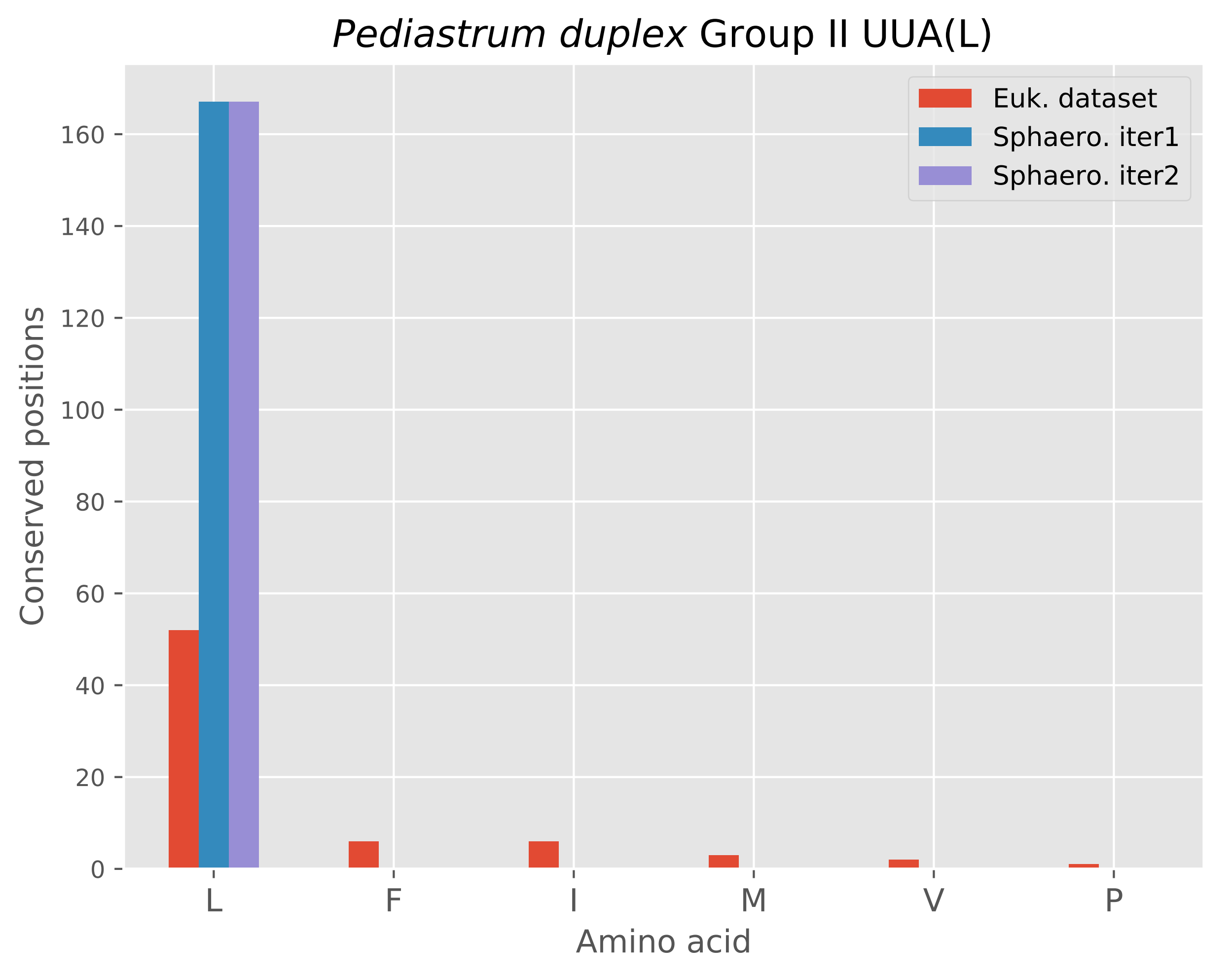
Pediastrum duplex Group II UUA(L)
Euk. dataset
Sphaero. iter1
Sphaero. iter2
Conserved positions
0
20
40
60
80
100
120
140
160
L
F
I
M
V
P
Amino acid

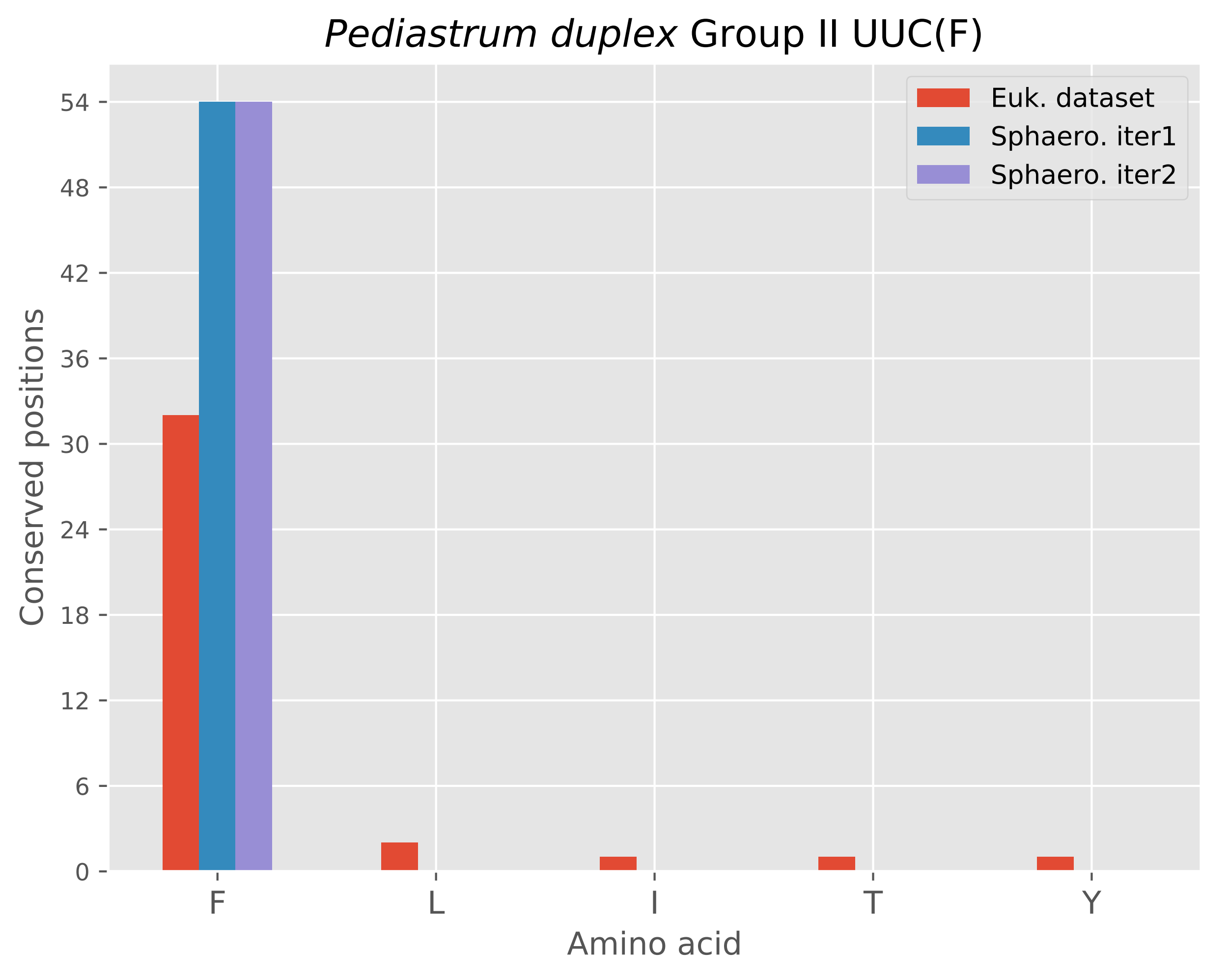
Pediastrum duplex Group II UUC(F)
Euk. dataset
Sphaero. iter1
Sphaero. iter2
Conserved positions
0
6
12
18
24
30
36
42
48
54
F
L
I
T
Y
Amino acid

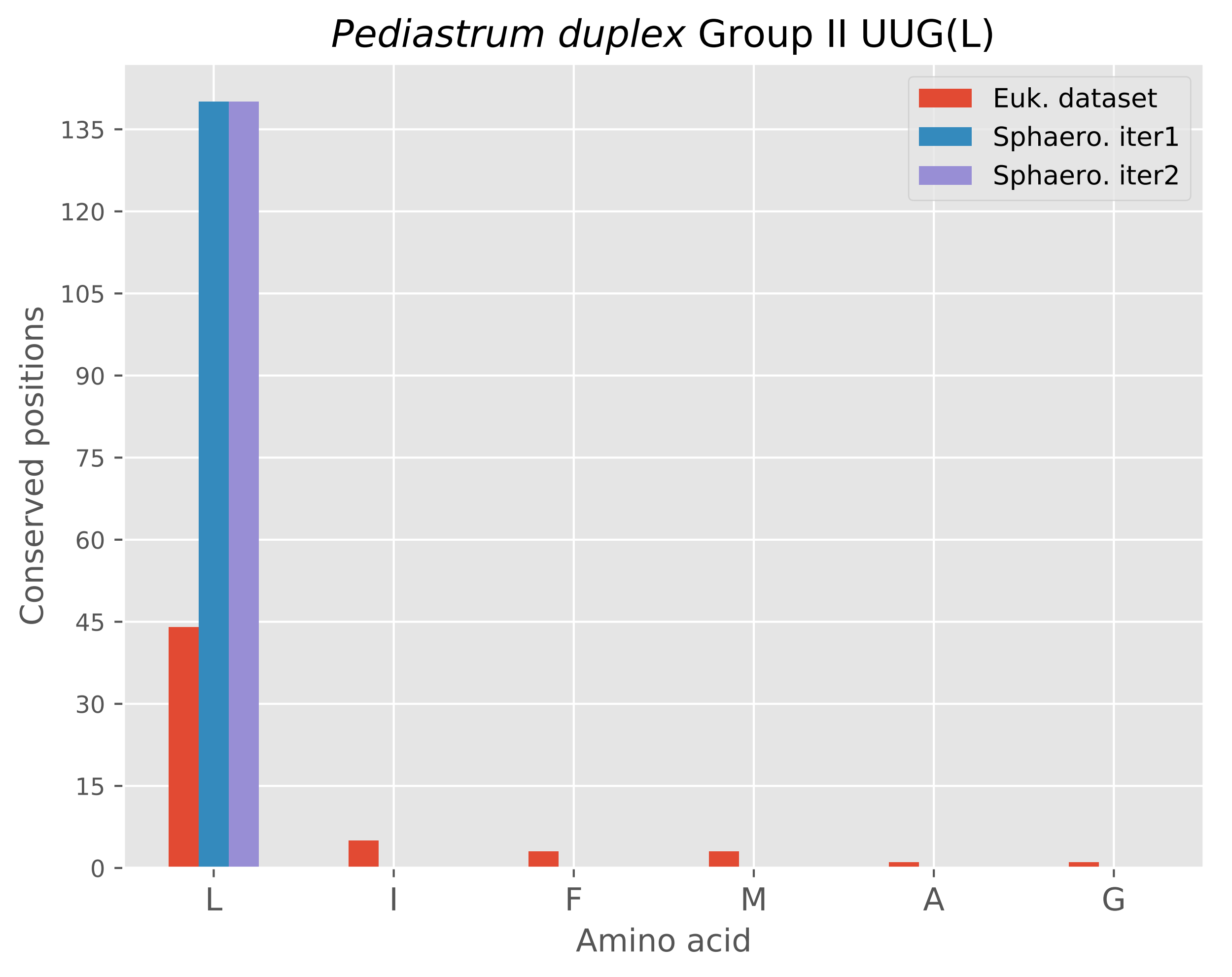

Pediastrum duplex Group II UUG(L)
Conserved positions
0
15
30
45
60
75
90
105
120
135
L
I
F
M
A
G
Amino acid
Euk. dataset
Sphaero. iter1
Sphaero. iter2

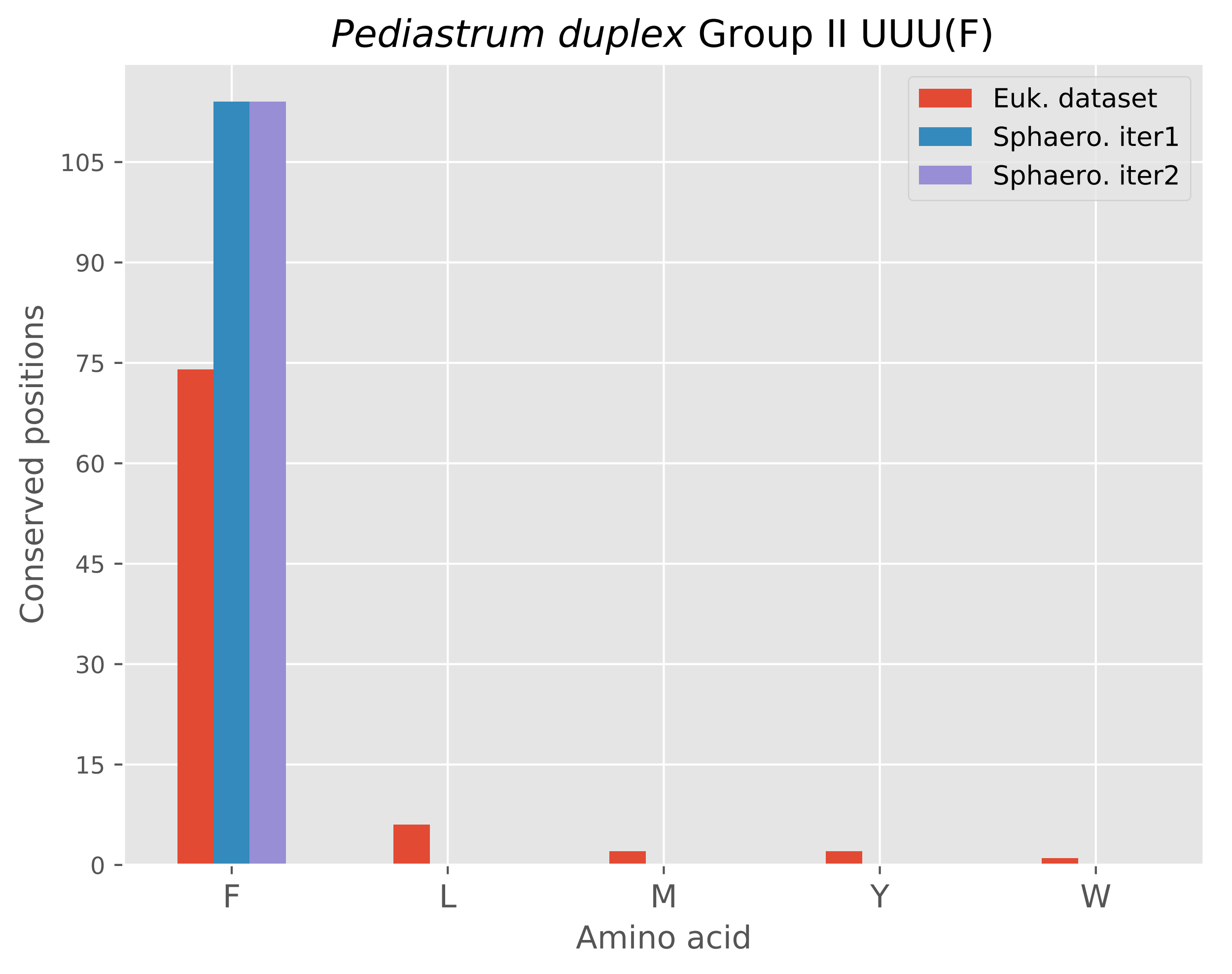

Pediastrum duplex Group II UUU(F)
Euk. dataset
Sphaero. iter1
Sphaero. iter2
Conserved positions
0
15
30
45
60
75
90
105
F
L
M
Y
W
Amino acid

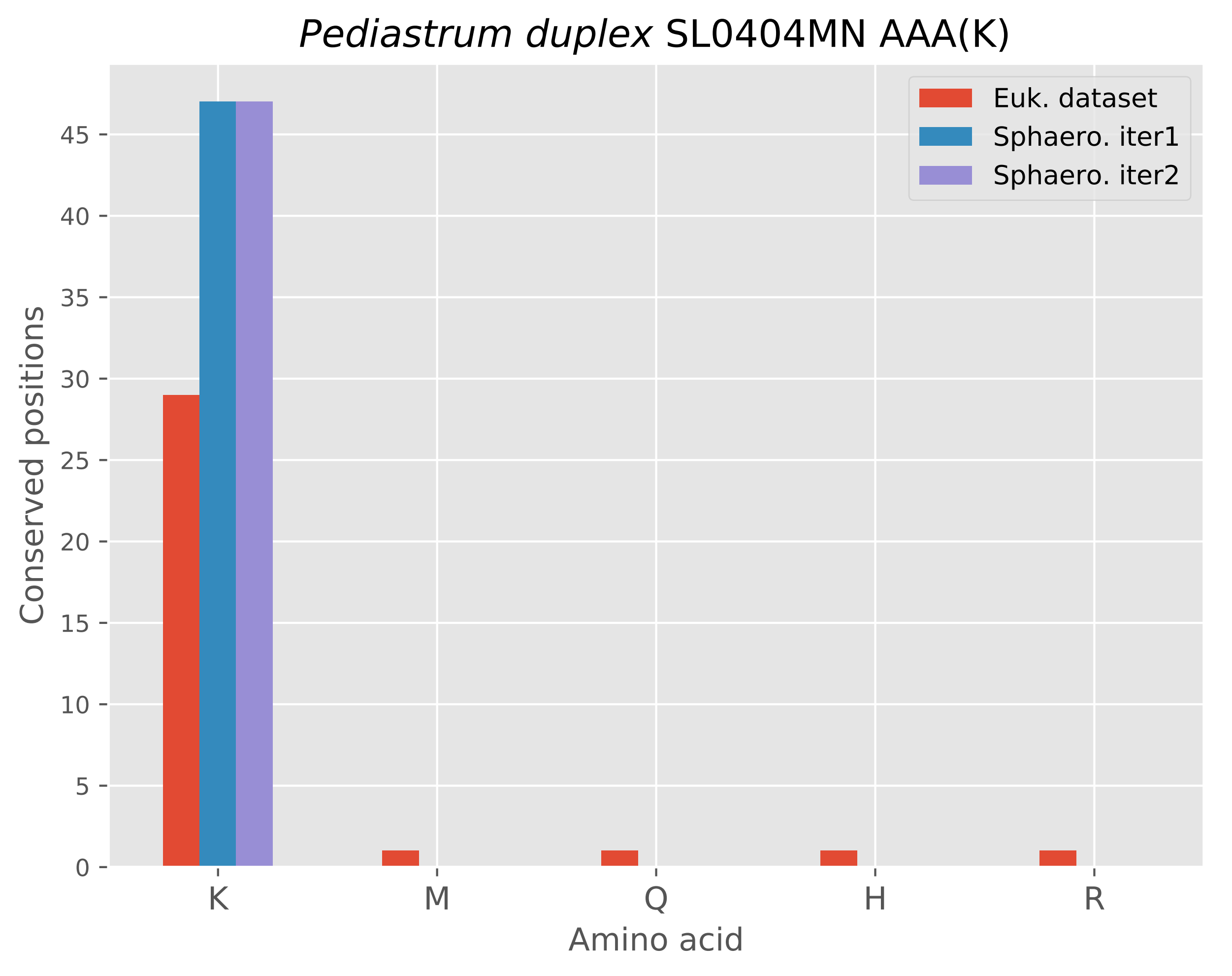

Pediastrum duplex SL0404MN AAA(K)
Euk. dataset
Sphaero. iter1
Sphaero. iter2
Conserved positions
0
5
10
15
20
25
30
35
40
45
K
M
Q
H
R
Amino acid

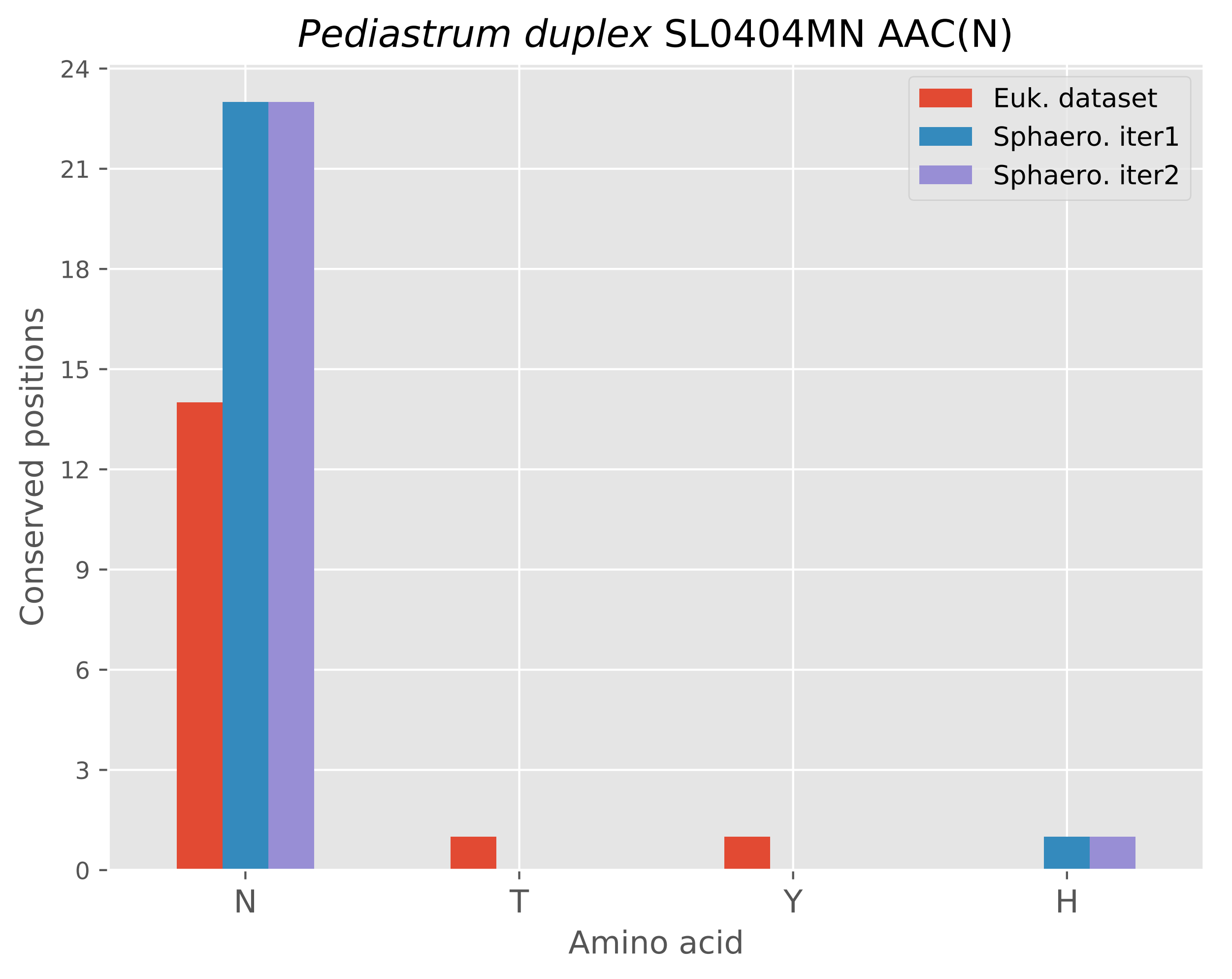
Pediastrum duplex SL0404MN AAC(N)
Euk. dataset
Sphaero. iter1
Sphaero. iter2
Conserved positions
0
3
6
9
12
15
18
21
24
N
T
Y
H
Amino acid

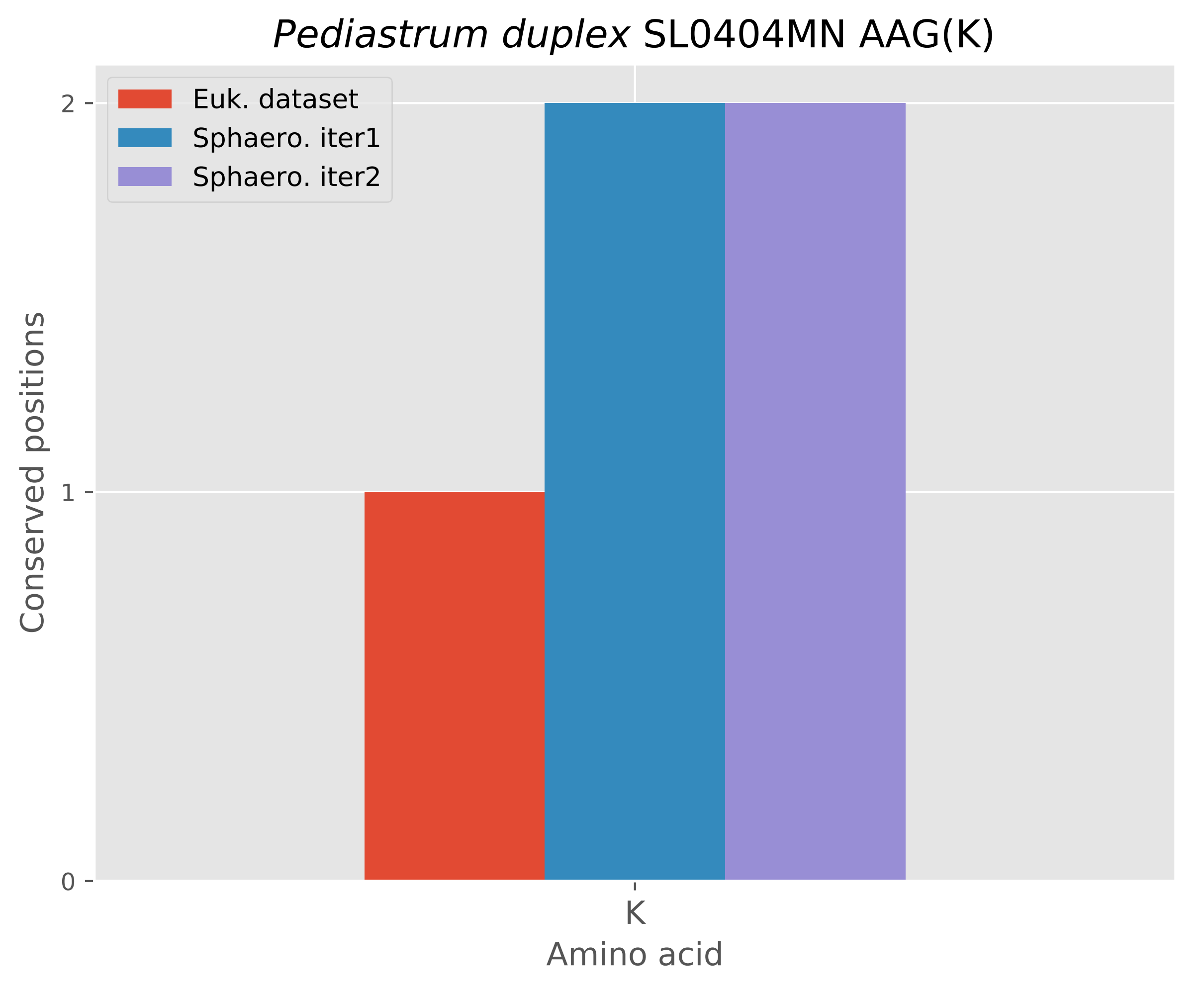

Pediastrum duplex SL0404MN AAG(K)
Euk. dataset
Sphaero. iter1
Sphaero. iter2
Conserved positions
0
1
2
K
Amino acid

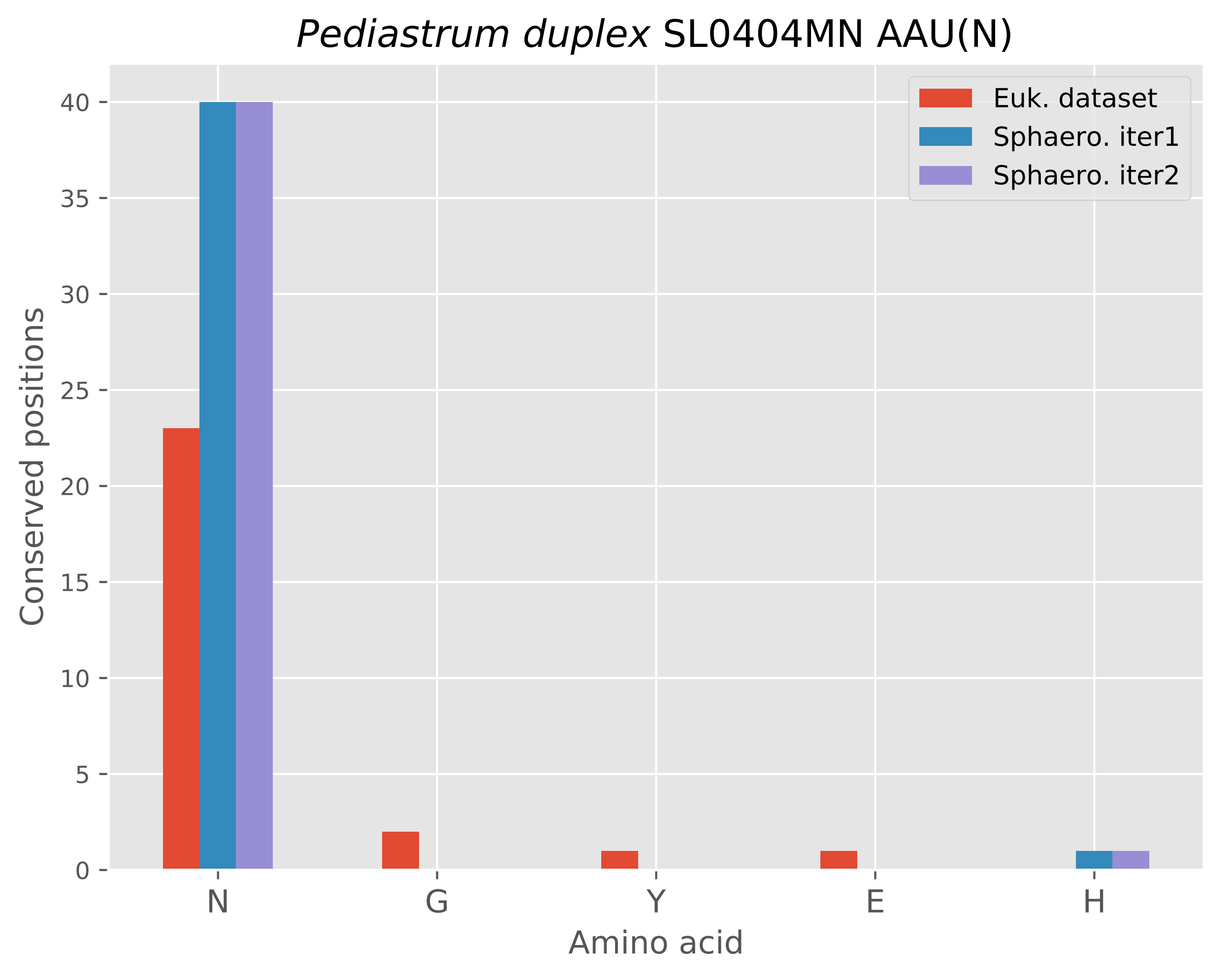

Pediastrum duplex SL0404MN AAU(N)
Euk. dataset
Sphaero. iter1
Sphaero. iter2
Conserved positions
0
5
10
15
20
25
30
35
40
N
G
Y
E
H
Amino acid

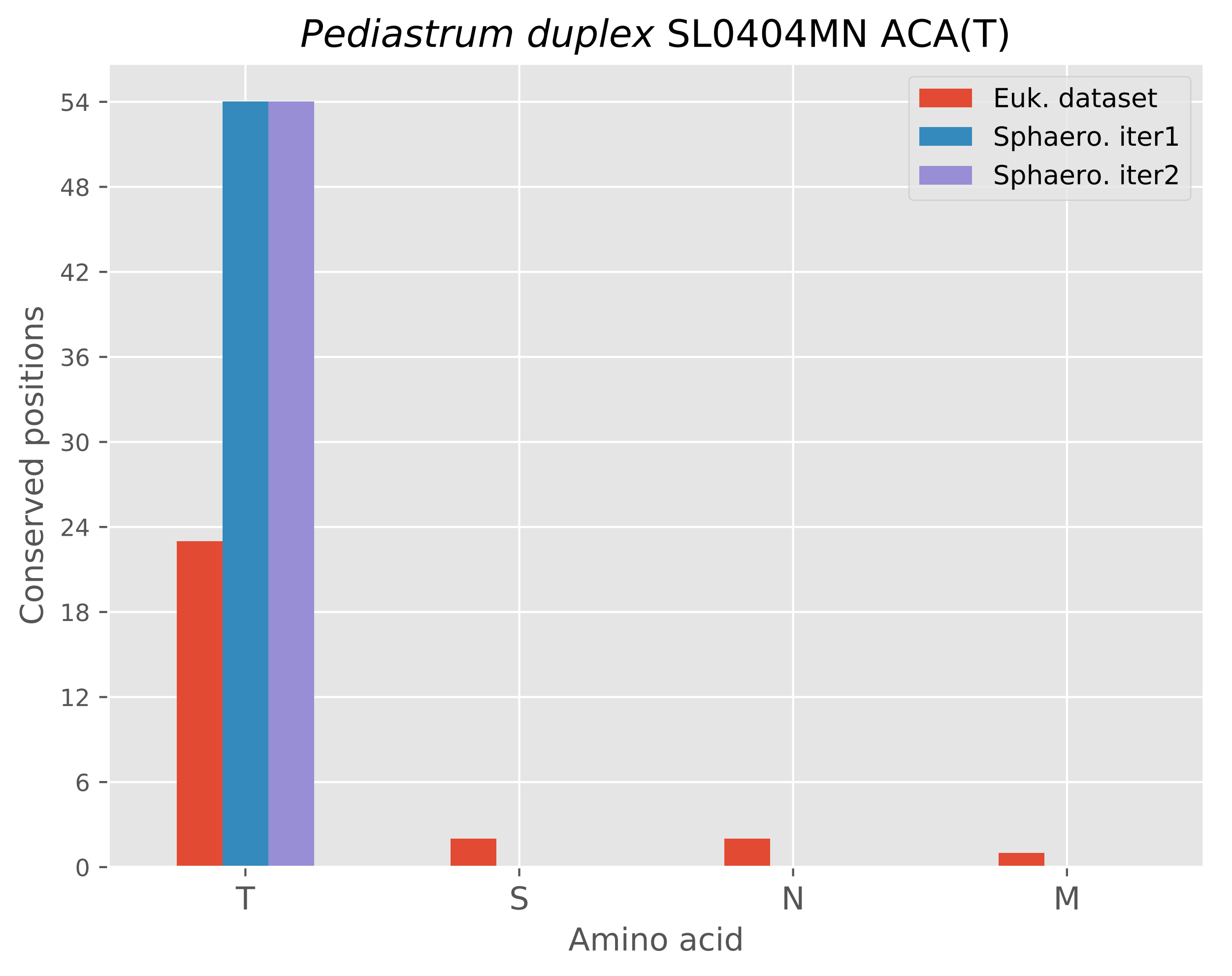

Pediastrum duplex SL0404MN ACA(T)
Euk. dataset
Sphaero. iter1
Sphaero. iter2
Conserved positions
0
6
12
18
24
30
36
42
48
54
T
S
N
M
Amino acid

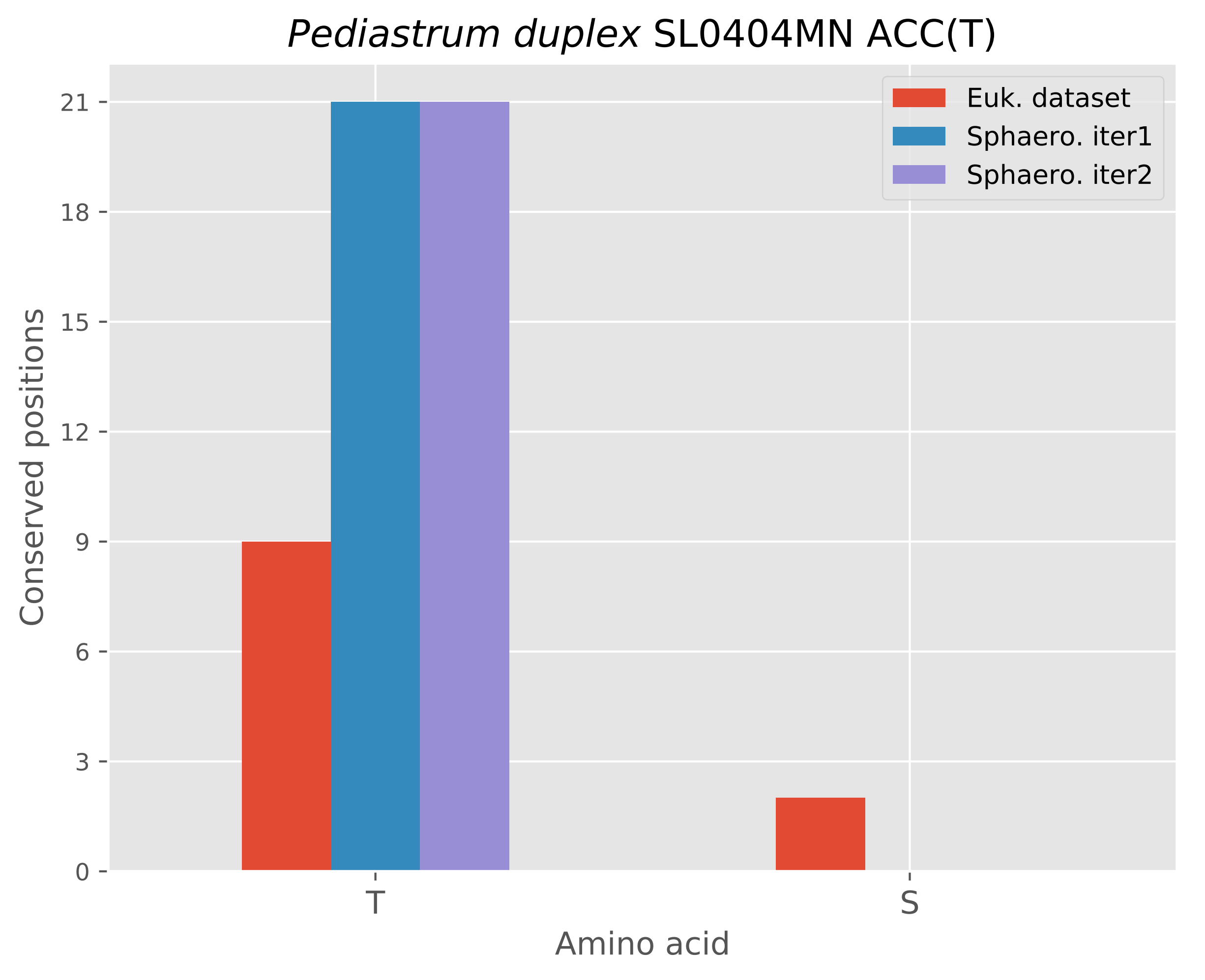

Pediastrum duplex SL0404MN ACC(T)
Euk. dataset
Sphaero. iter1
Sphaero. iter2
Conserved positions
0
3
6
9
12
15
18
21
T
S
Amino acid

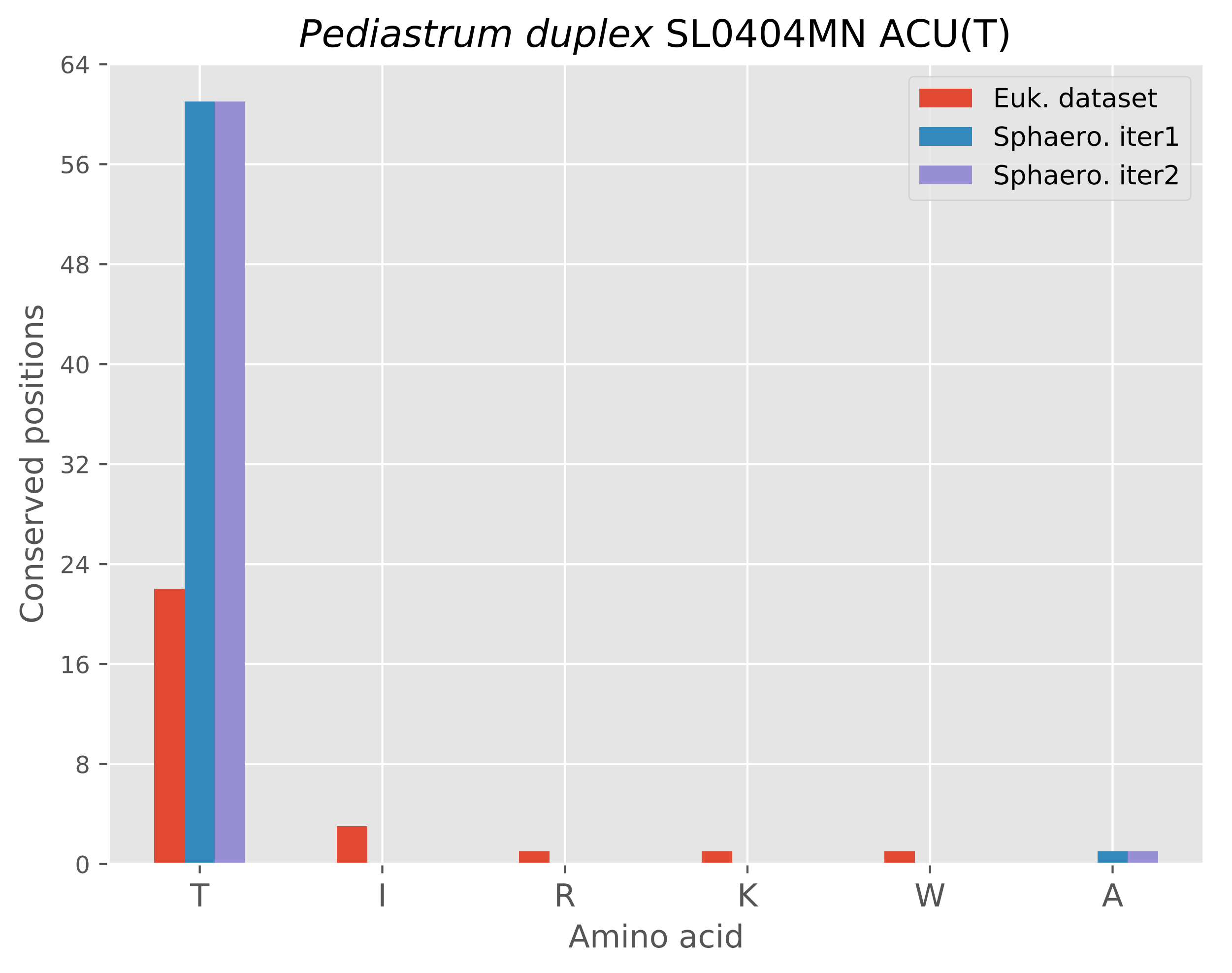

Pediastrum duplex SL0404MN ACU(T)
Conserved positions
Amino acid
0
8
16
24
32
40
48
56
64
T
I
R
K
W
A
Euk. dataset
Sphaero. iter1
Sphaero. iter2

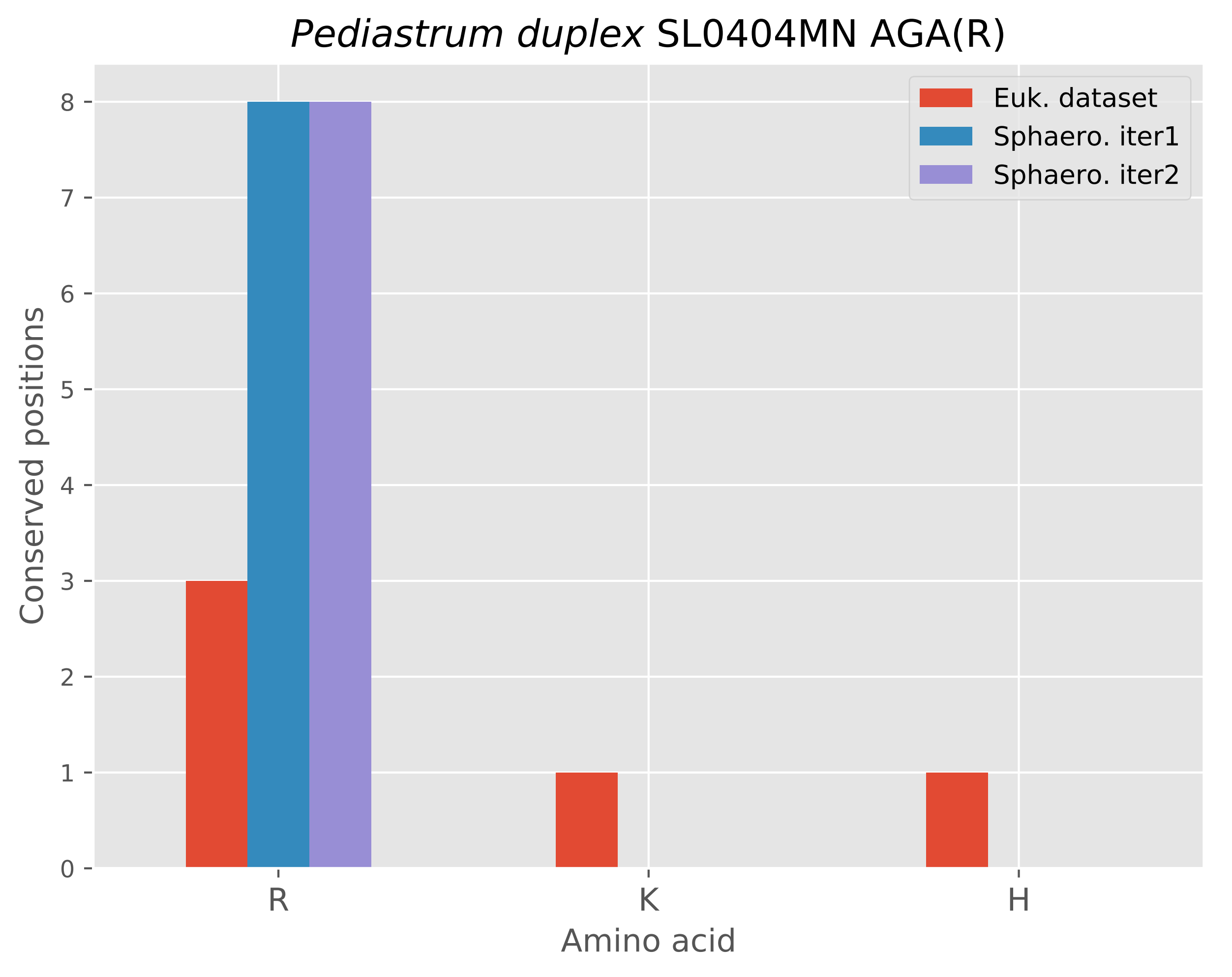

Pediastrum duplex SL0404MN AGA(R)
Euk. dataset
Sphaero. iter1
Sphaero. iter2
Conserved positions
0
1
2
3
4
5
6
7
8
R
K
H
Amino acid

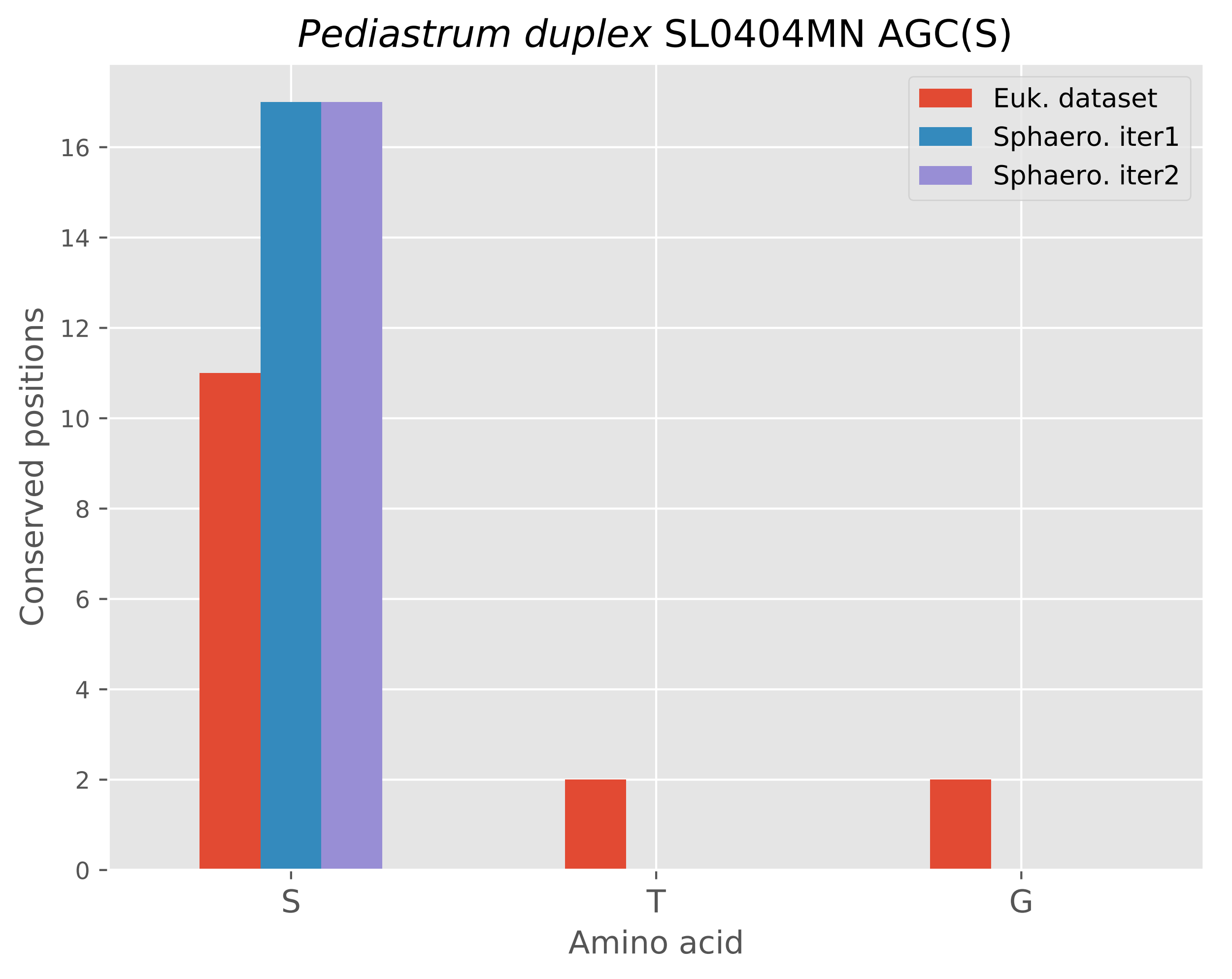

Pediastrum duplex SL0404MN AGC(S)
Euk. dataset
Sphaero. iter1
Sphaero. iter2
Conserved positions
0
2
4
6
8
10
12
14
16
S
T
G
Amino acid

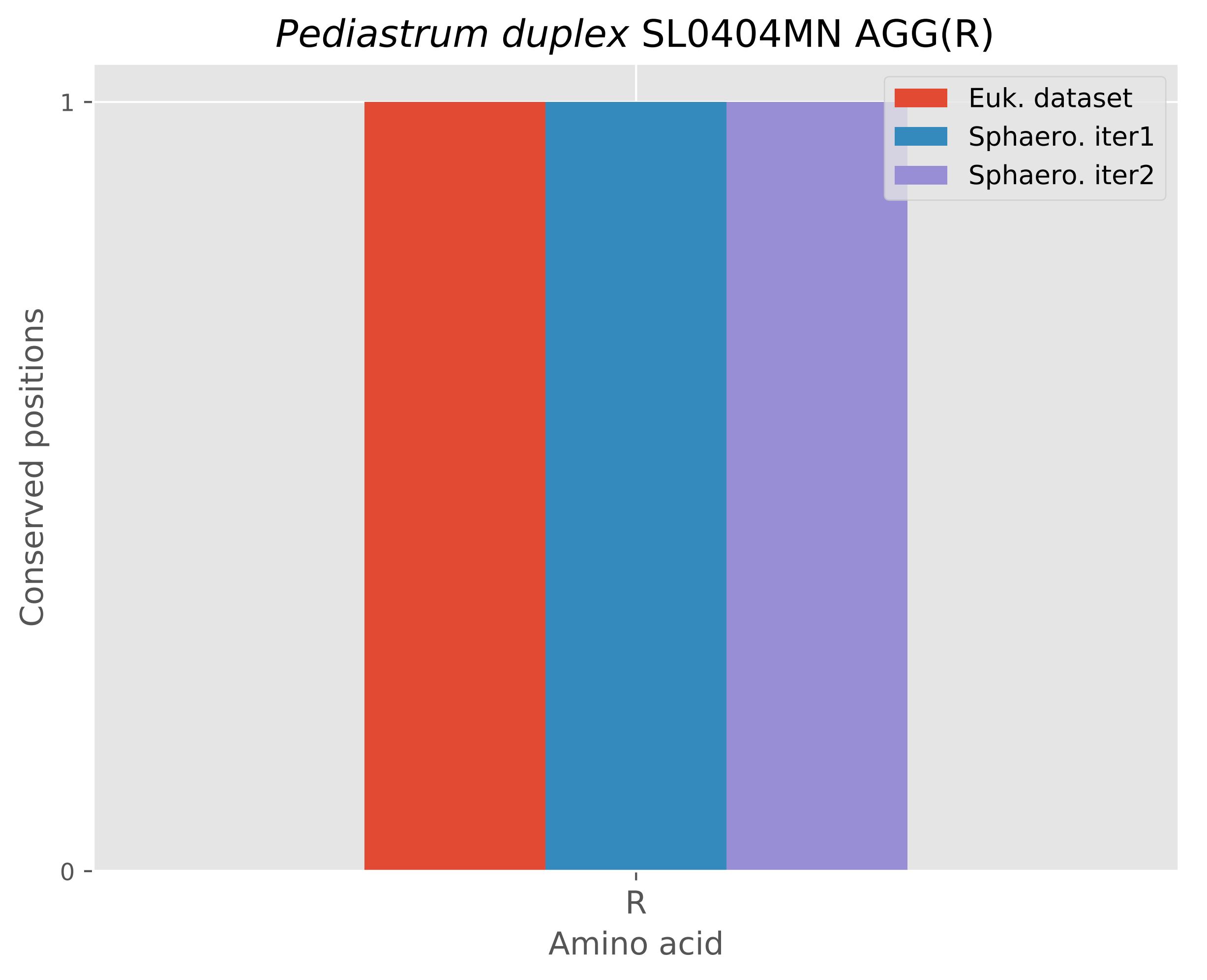

Pediastrum duplex SL0404MN AGG(R)
Euk. dataset
Sphaero. iter1
Sphaero. iter2
1
0
Conserved positions
R
Amino acid

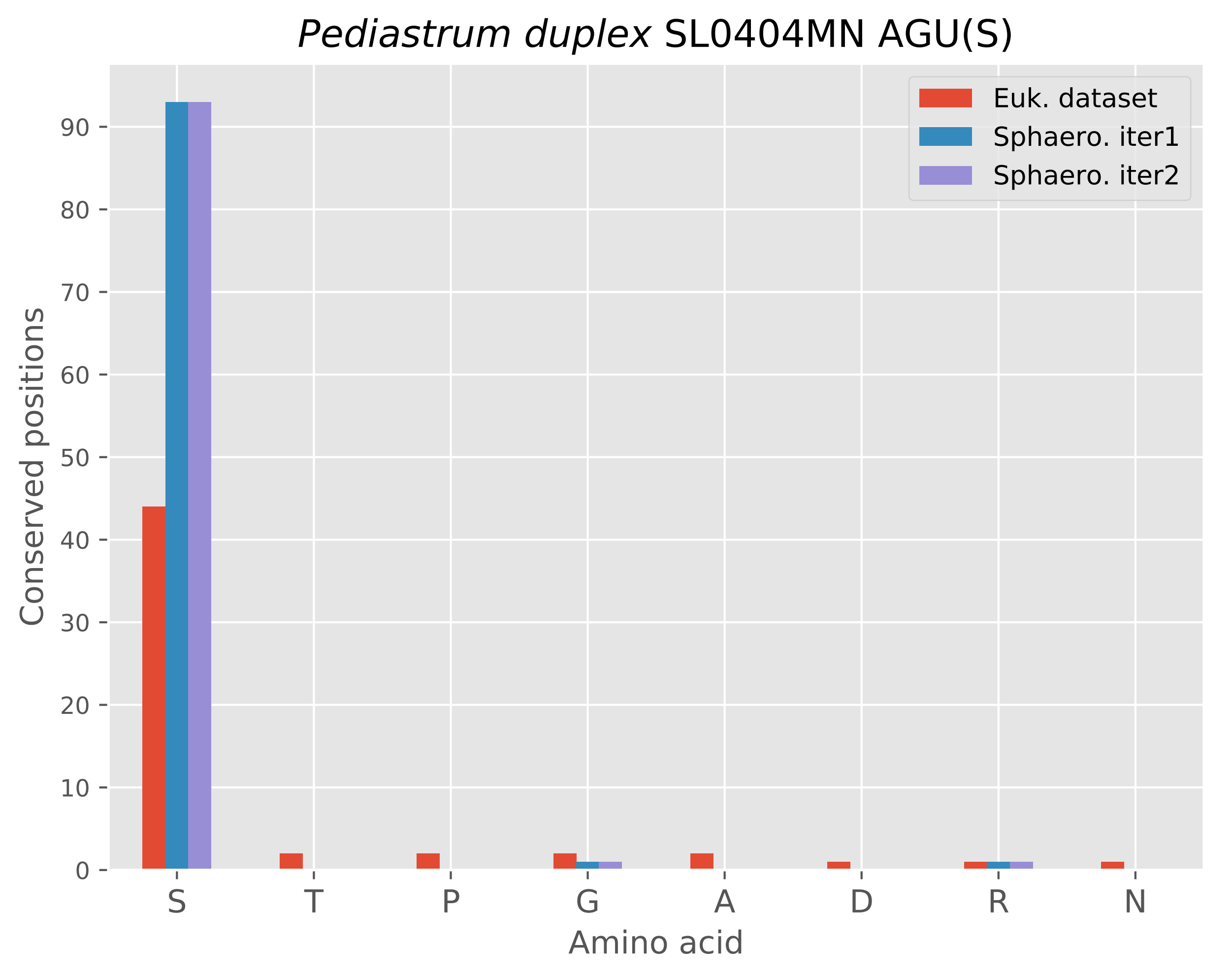

Pediastrum duplex SL0404MN AGU(S)
Euk. dataset
Sphaero. iter1
Sphaero. iter2
Conserved positions
0
10
20
30
40
50
60
70
80
90
S
T
P
G
A
D
R
N
Amino acid

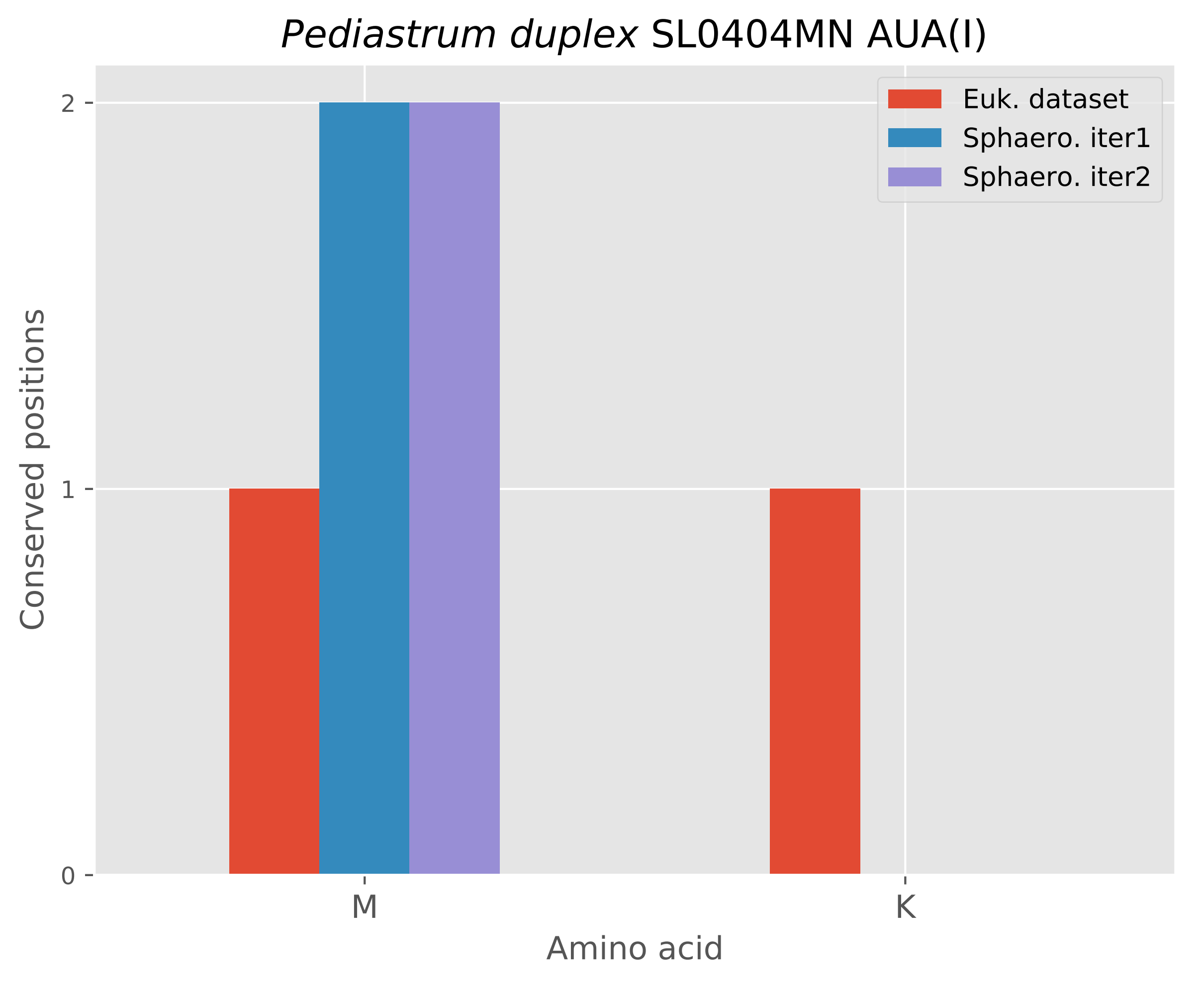

Pediastrum duplex SL0404MN AUA(I)
Euk. dataset
Sphaero. iter1
Sphaero. iter2
Conserved positions
0
1
2
M
K
Amino acid

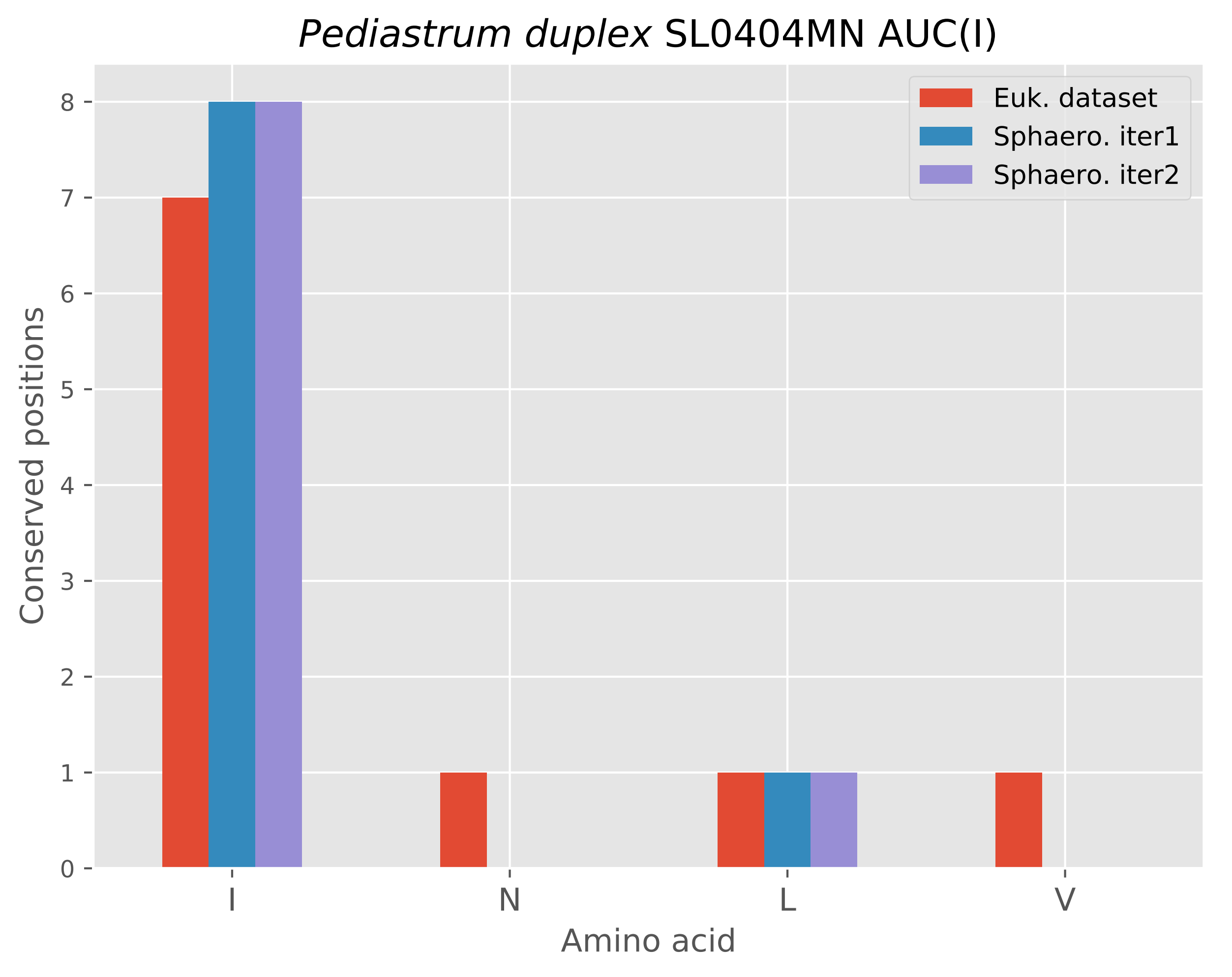

Pediastrum duplex SL0404MN AUC(I)
Euk. dataset
Sphaero. iter1
Sphaero. iter2
Conserved positions
0
1
2
3
4
5
6
7
8
I
N
L
V
Amino acid

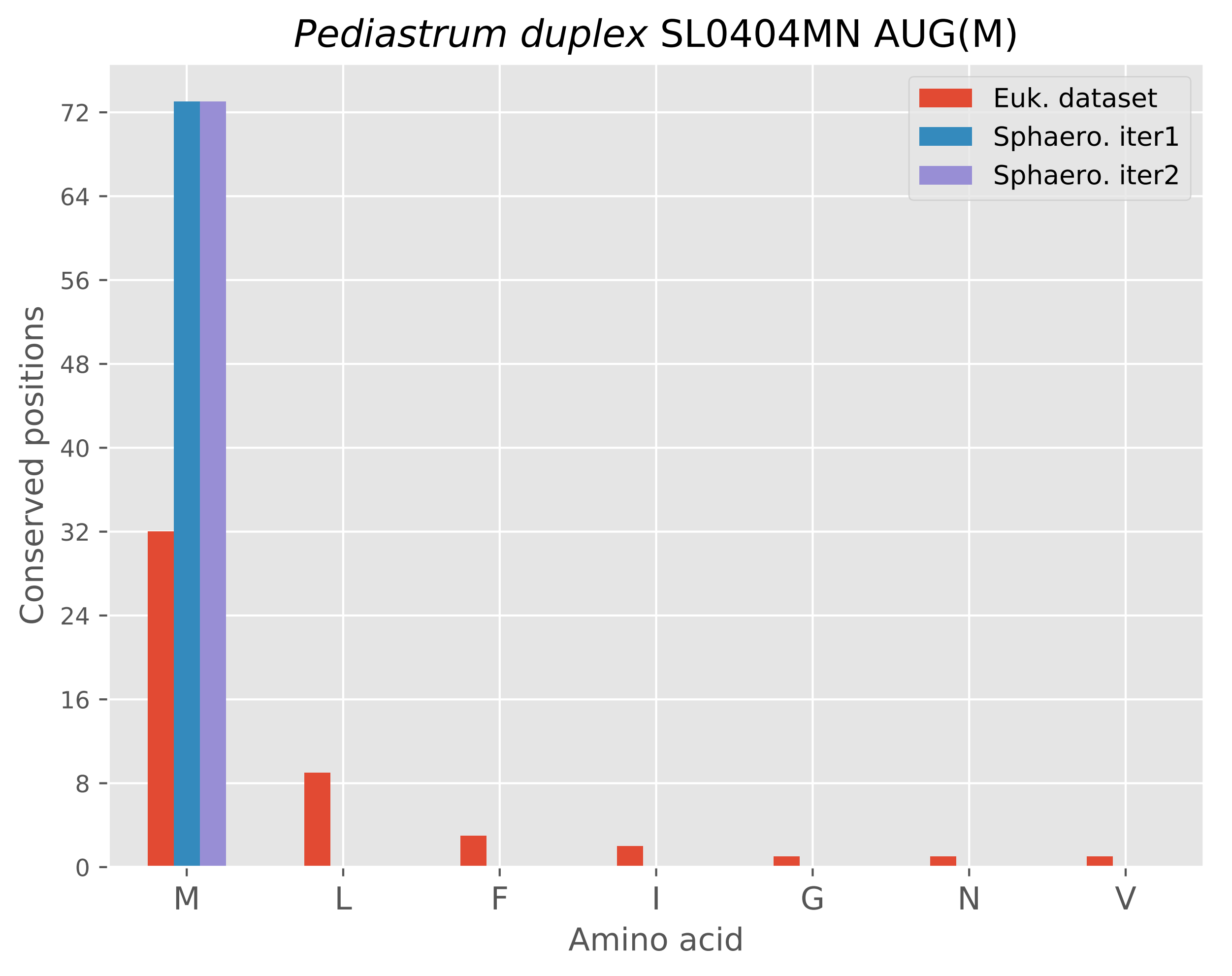

Pediastrum duplex SL0404MN AUG(M)
Euk. dataset
Sphaero. iter1
Sphaero. iter2
Conserved positions
0
8
16
24
32
40
48
56
64
72
M
L
F
I
G
N
V
Amino acid

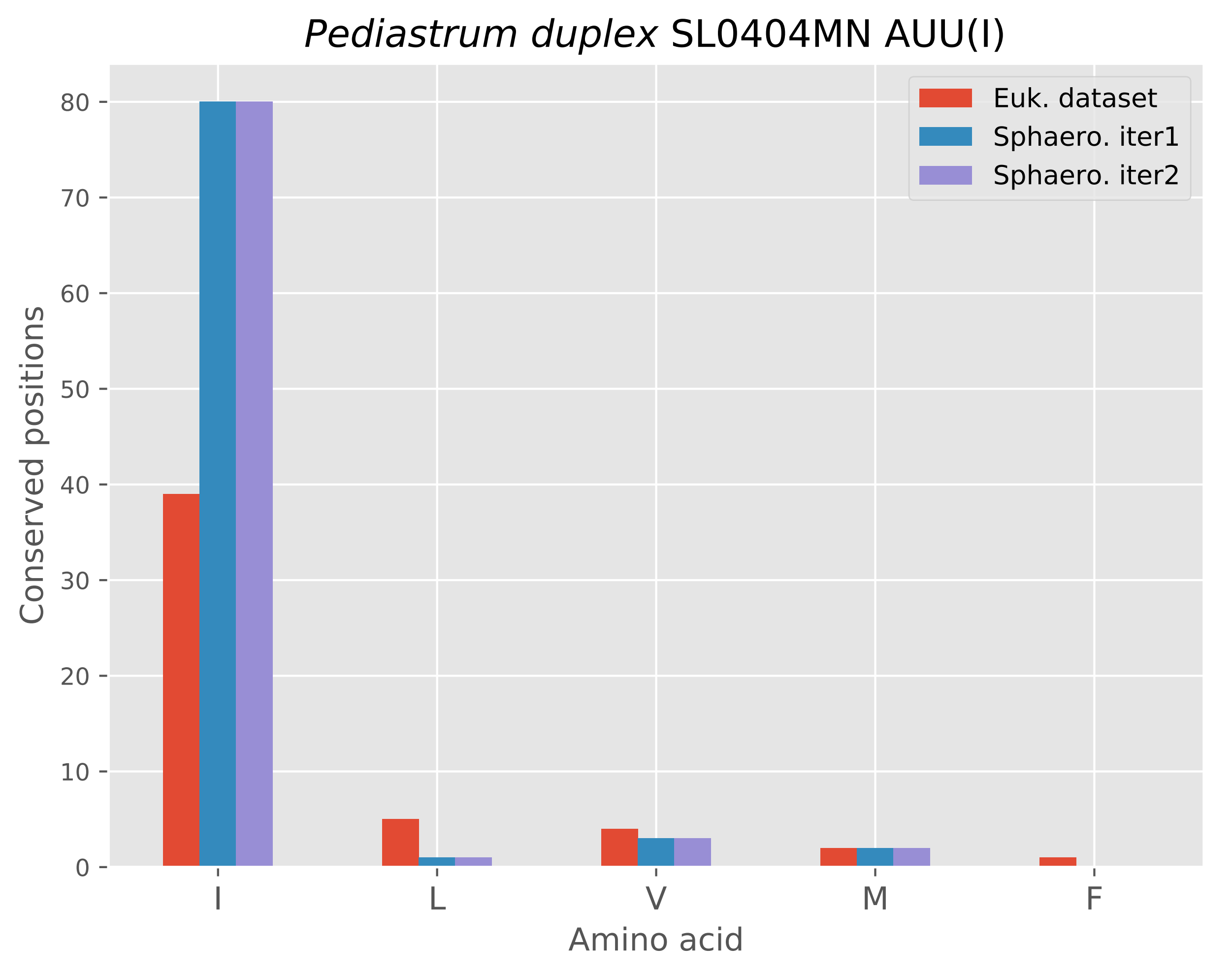
Pediastrum duplex SL0404MN AUU(I)
Euk. dataset
Sphaero. iter1
Sphaero. iter2
Conserved positions
0
10
20
30
40
50
60
70
80
I
L
V
M
F
Amino acid

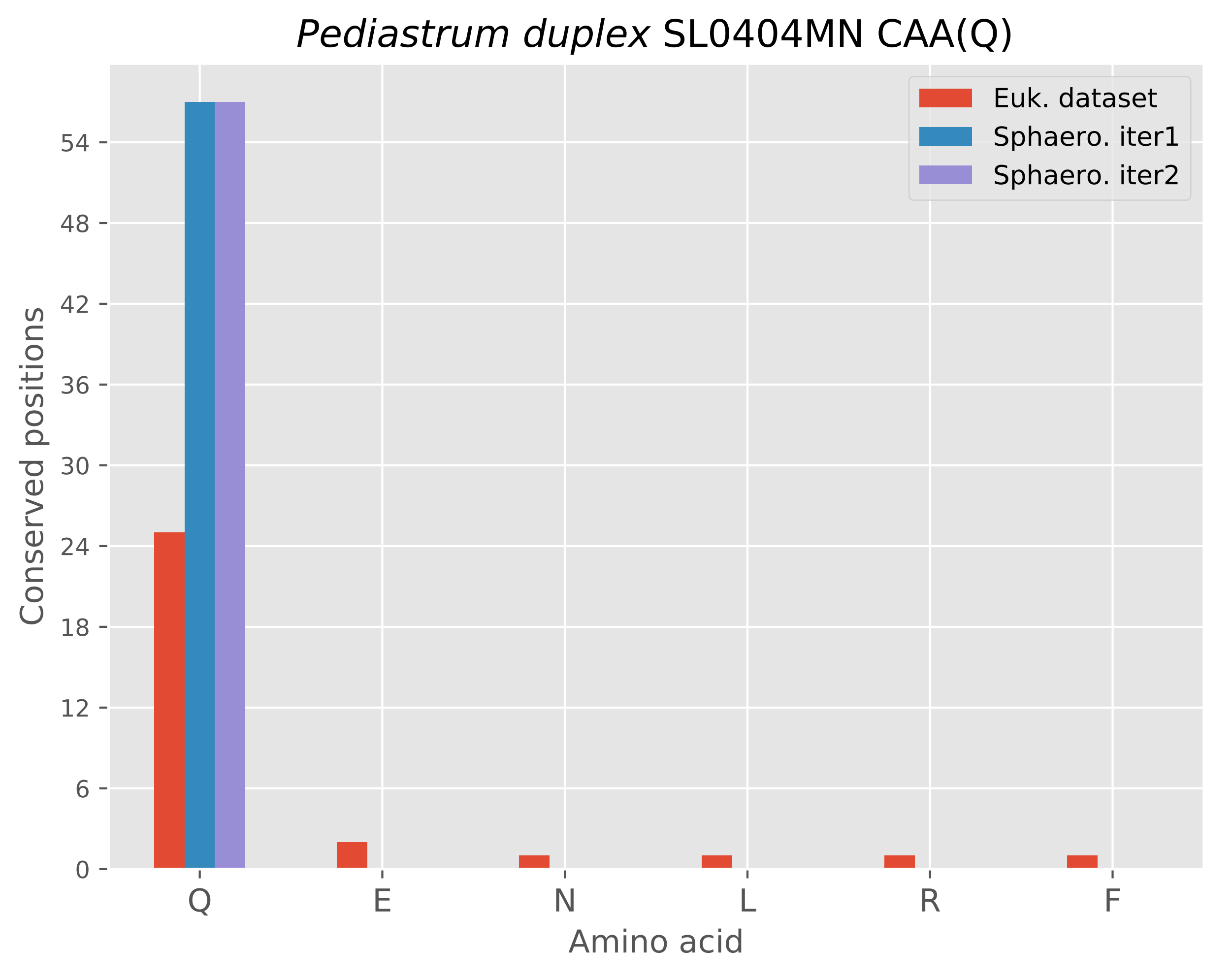

Pediastrum duplex SL0404MN CAA(Q)
Euk. dataset
Sphaero. iter1
Sphaero. iter2
Conserved positions
0
6
12
18
24
30
36
42
48
54
Q
E
N
L
R
F
Amino acid

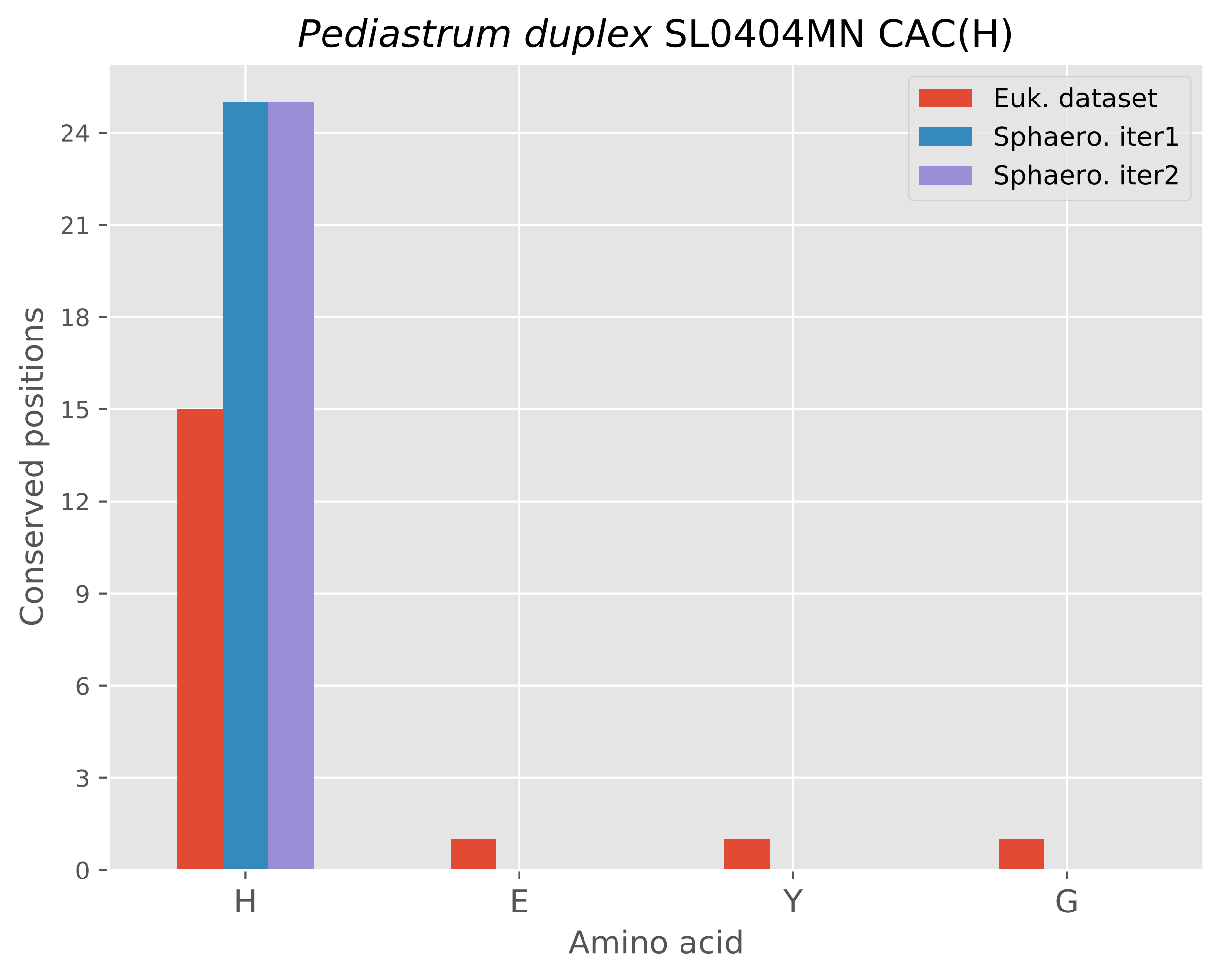

Pediastrum duplex SL0404MN CAC(H)
Euk. dataset
Sphaero. iter1
Sphaero. iter2
Conserved positions
0
3
6
9
12
15
18
21
24
H
E
Y
G
Amino acid

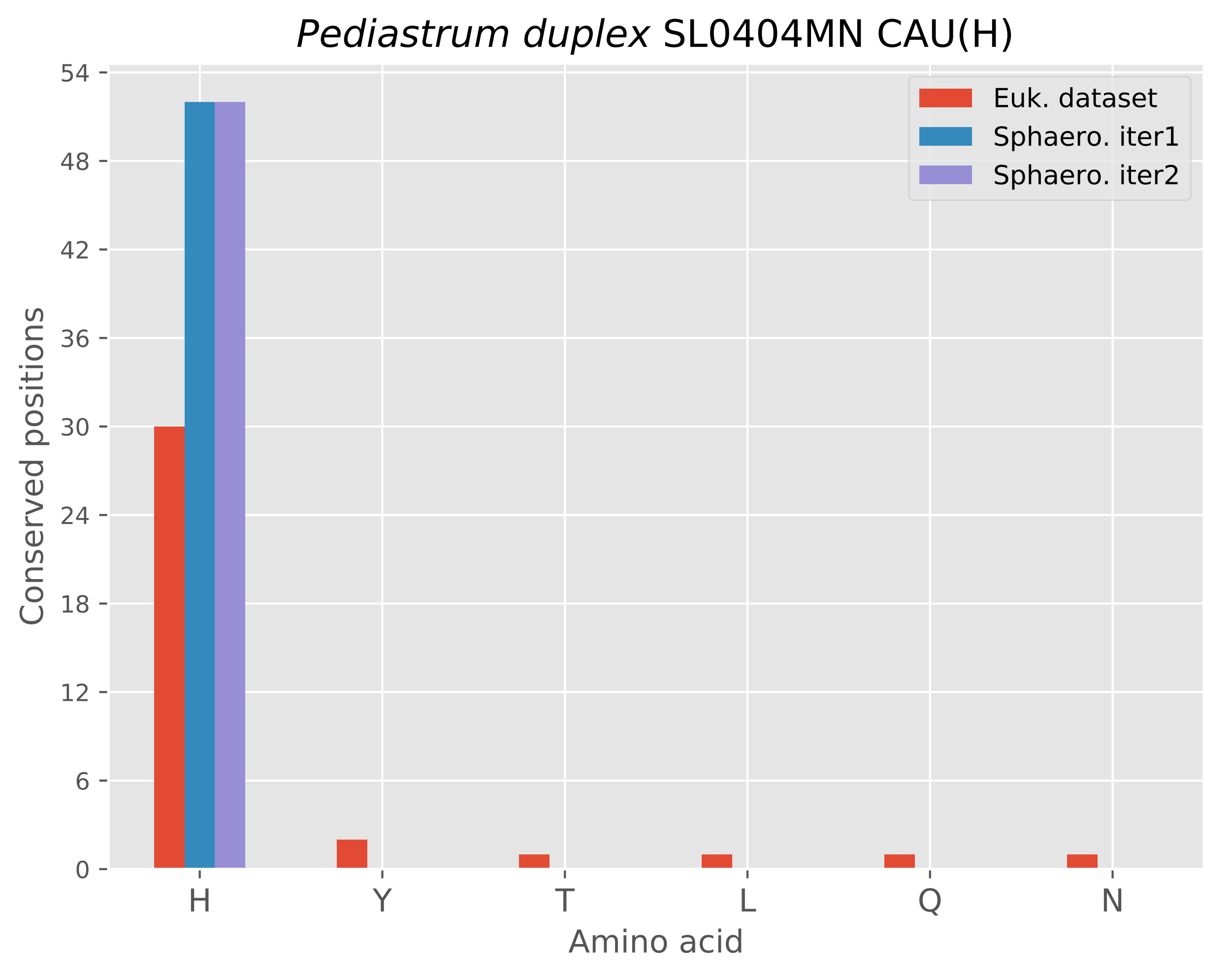

Pediastrum duplex SL0404MN CAU(H)
Conserved positions
Amino acid
0
6
12
18
24
30
36
42
48
54
H
Y
T
L
Q
N
Euk. dataset
Sphaero. iter1
Sphaero. iter2

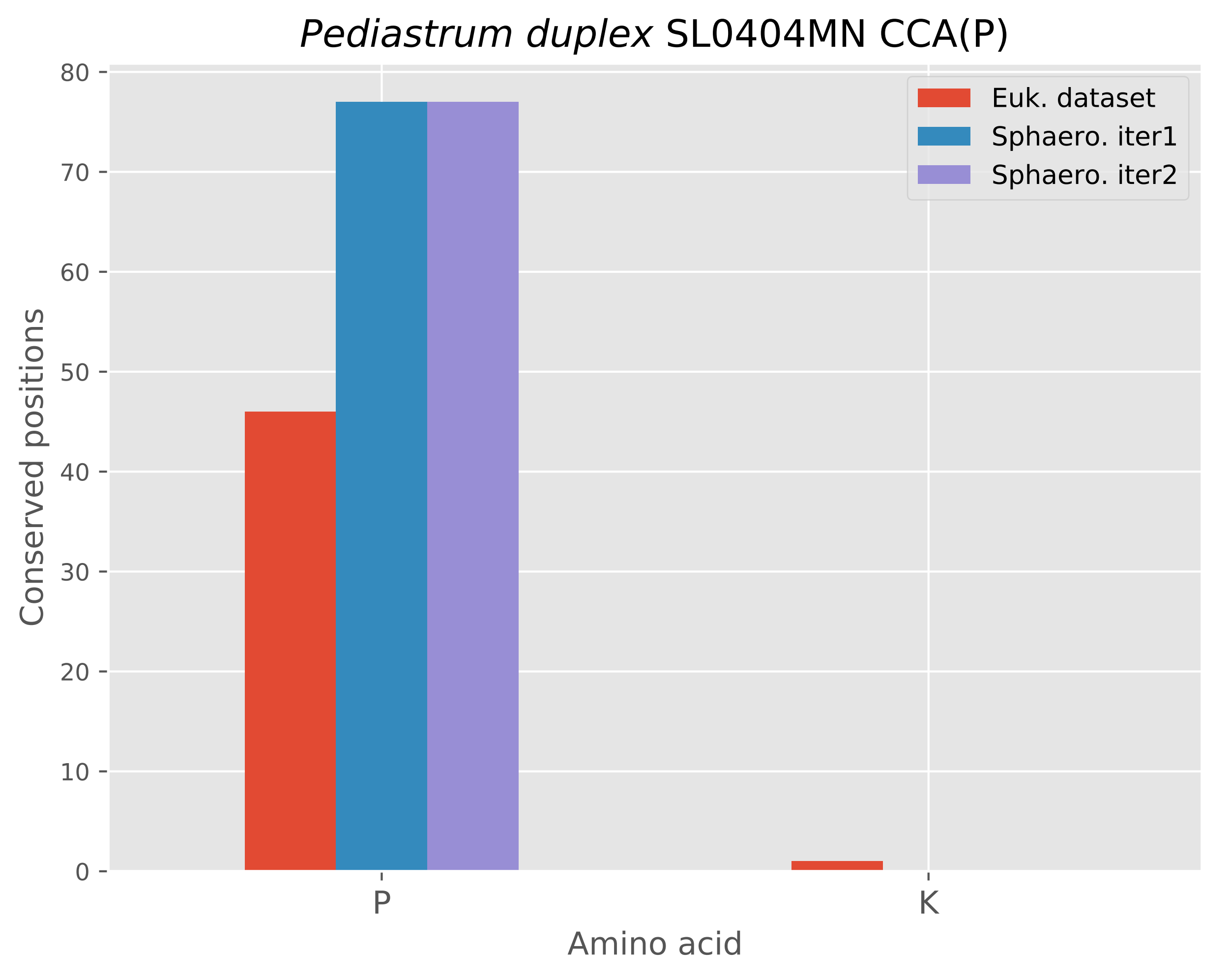

Pediastrum duplex SL0404MN CCA(P)
Conserved positions
0
10
20
30
40
50
60
70
80
P
K
Amino acid
Euk. dataset
Sphaero. iter1
Sphaero. iter2

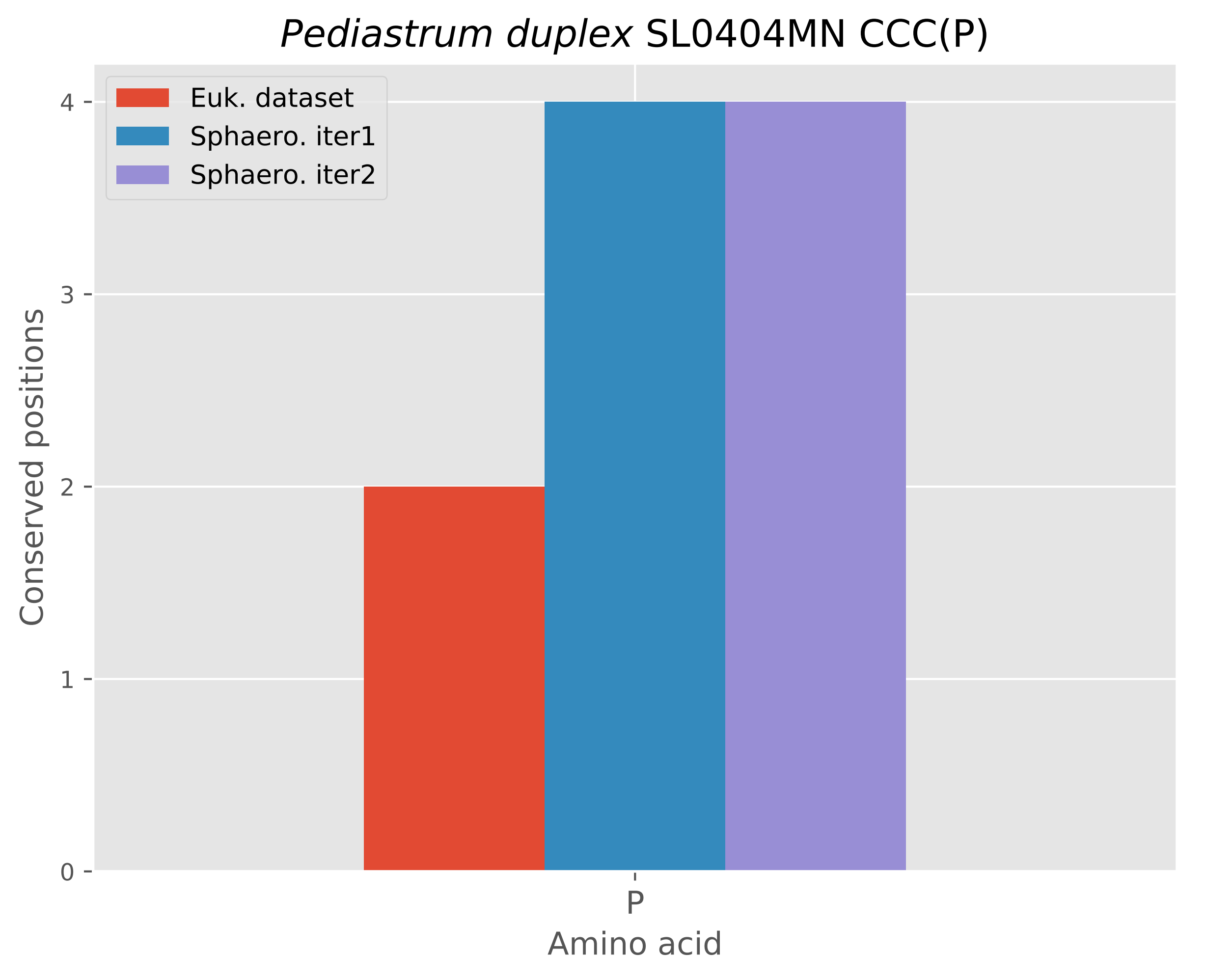

Pediastrum duplex SL0404MN CCC(P)
Euk. dataset
Sphaero. iter1
Sphaero. iter2
Conserved positions
0
1
2
3
4
P
Amino acid

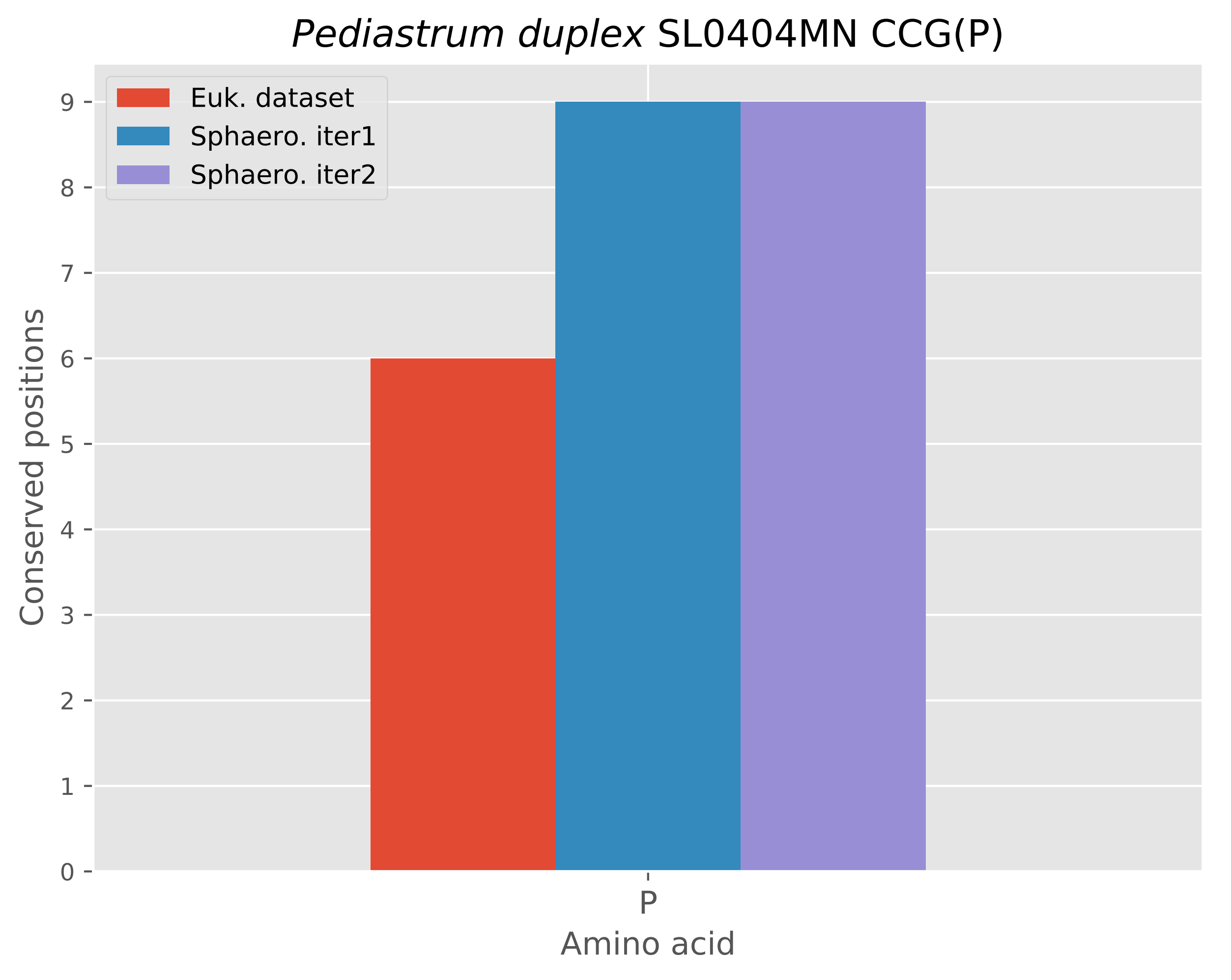

Pediastrum duplex SL0404MN CCG(P)
Euk. dataset
Sphaero. iter1
Sphaero. iter2
Conserved positions
0
1
2
3
4
5
6
7
8
9
P
Amino acid

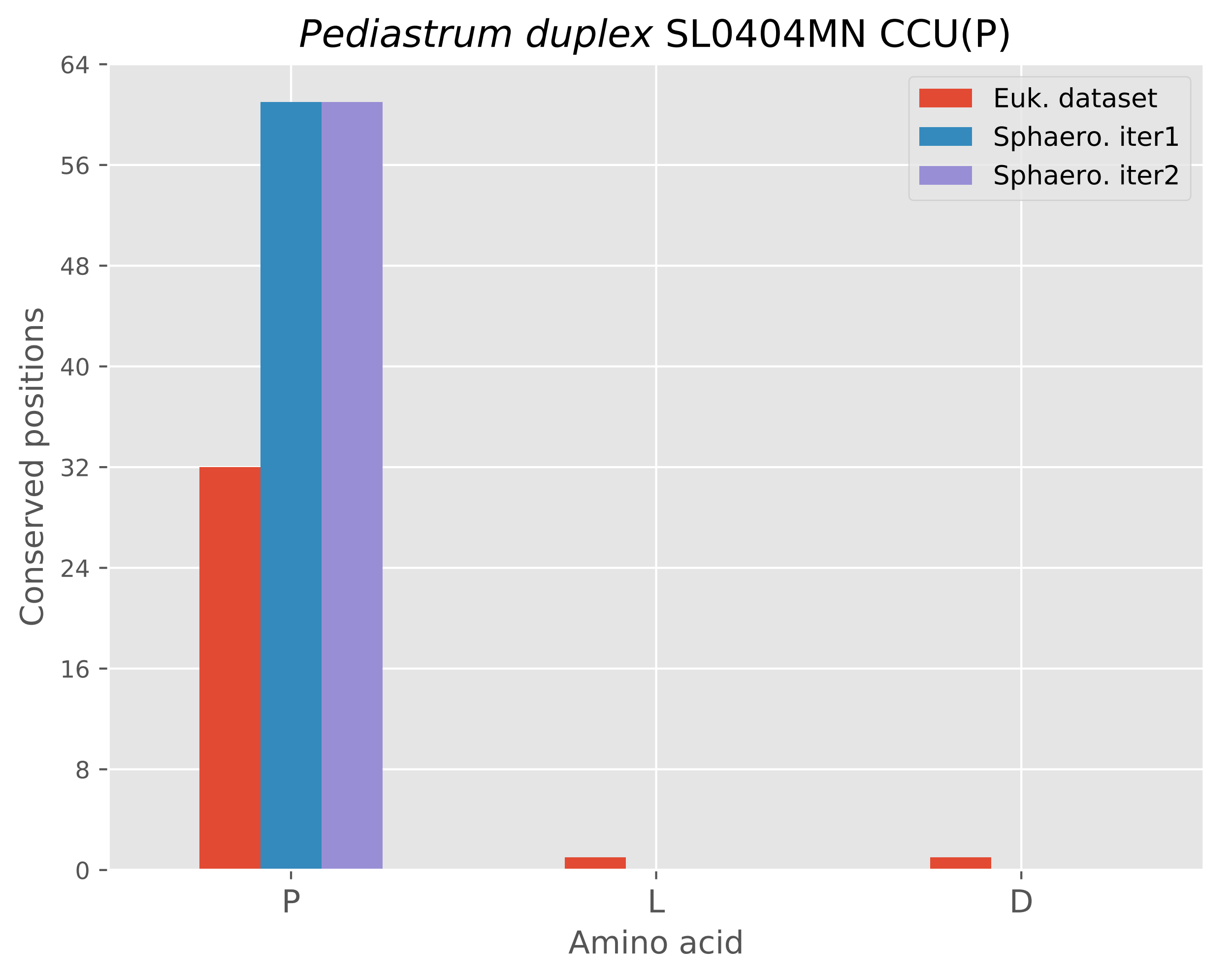
Pediastrum duplex SL0404MN CCU(P)
Conserved positions
Amino acid
P
L
D
Euk. dataset
Sphaero. iter1
Sphaero. iter2
0
8
16
24
32
40
48
56
64

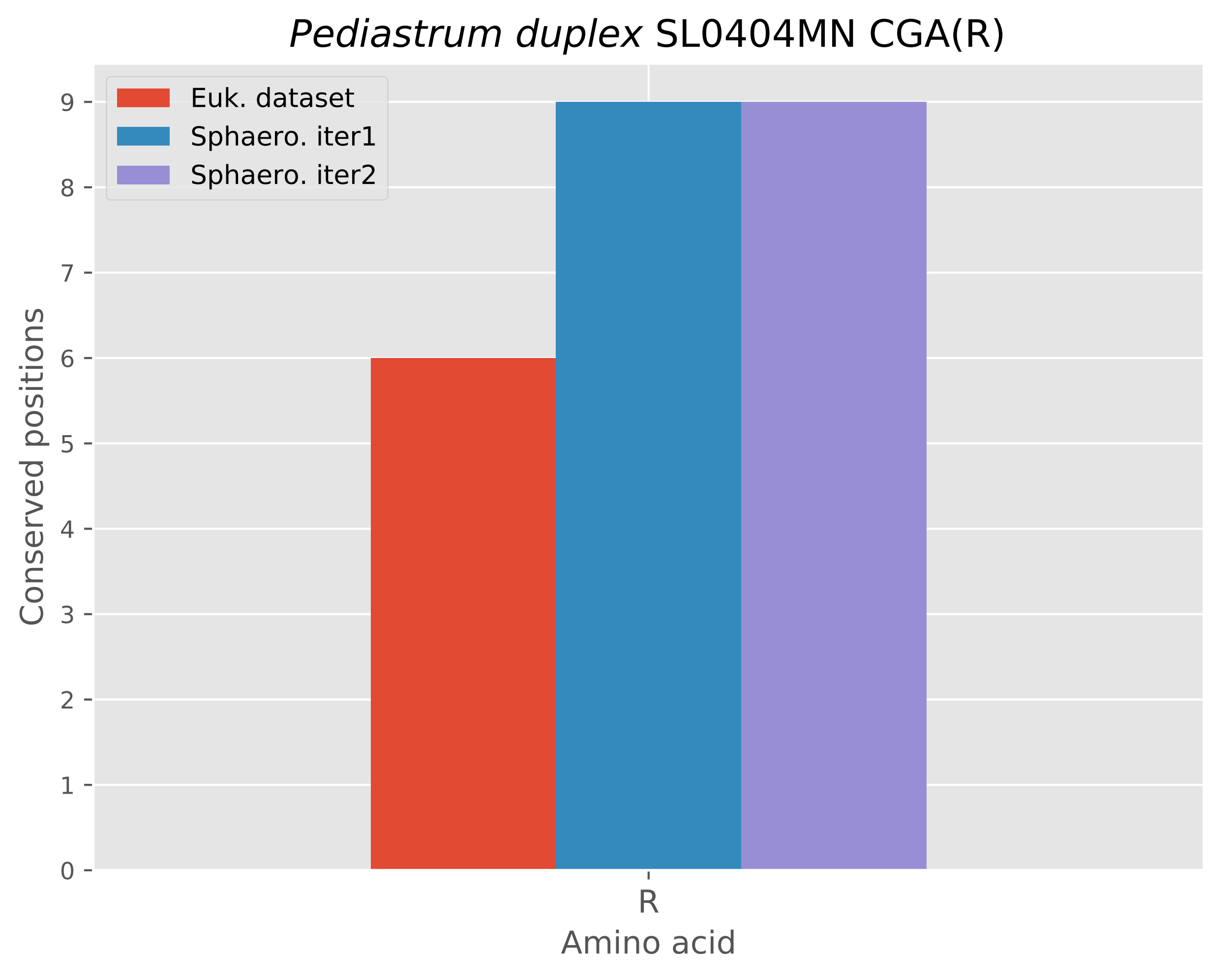

Pediastrum duplex SL0404MN CGA(R)
Euk. dataset
Sphaero. iter1
Sphaero. iter2
Conserved positions
0
1
2
3
4
5
6
7
8
9
R
Amino acid

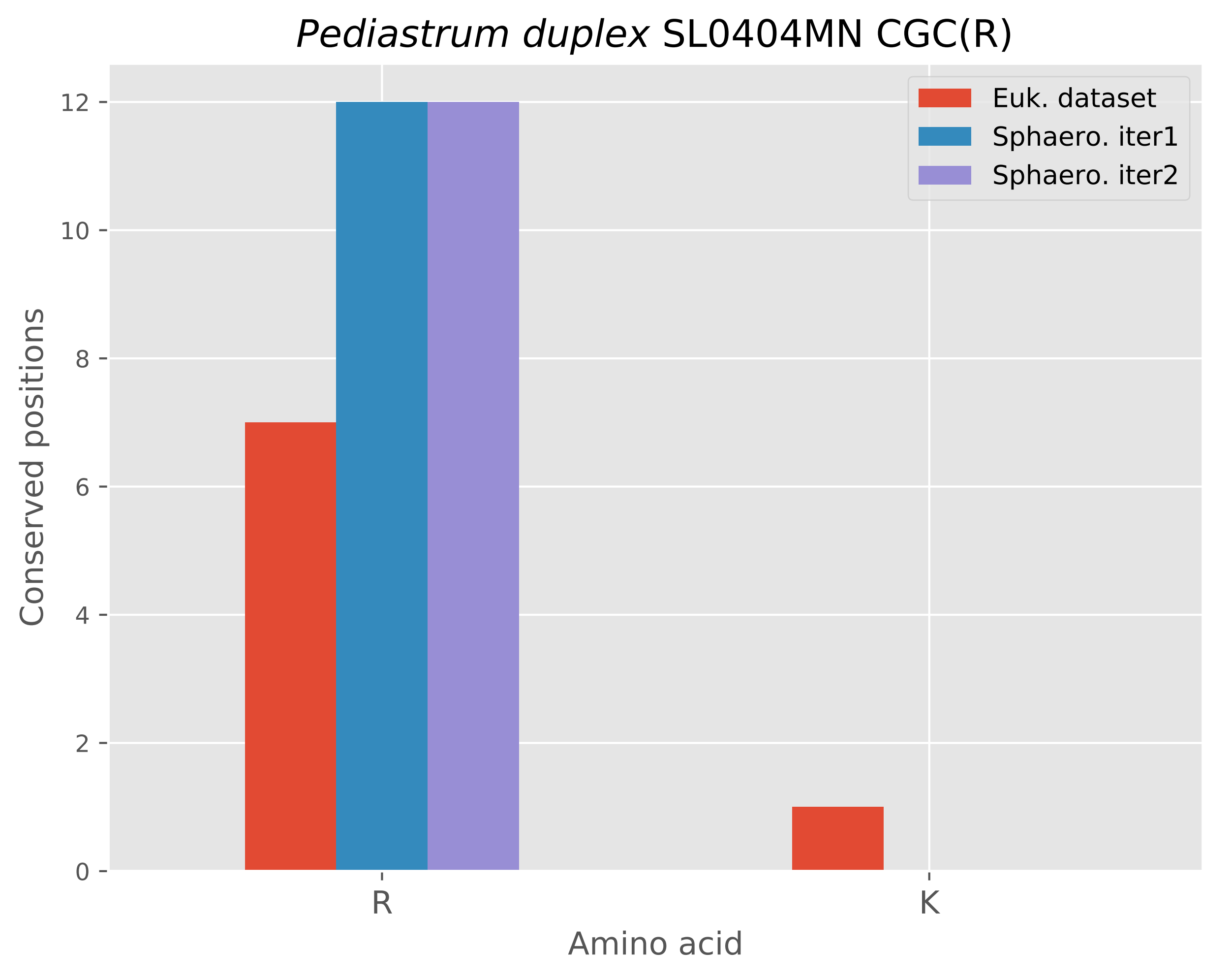

Pediastrum duplex SL0404MN CGC(R)
Euk. dataset
Sphaero. iter1
Sphaero. iter2
Conserved positions
0
2
4
6
8
10
12
R
K
Amino acid

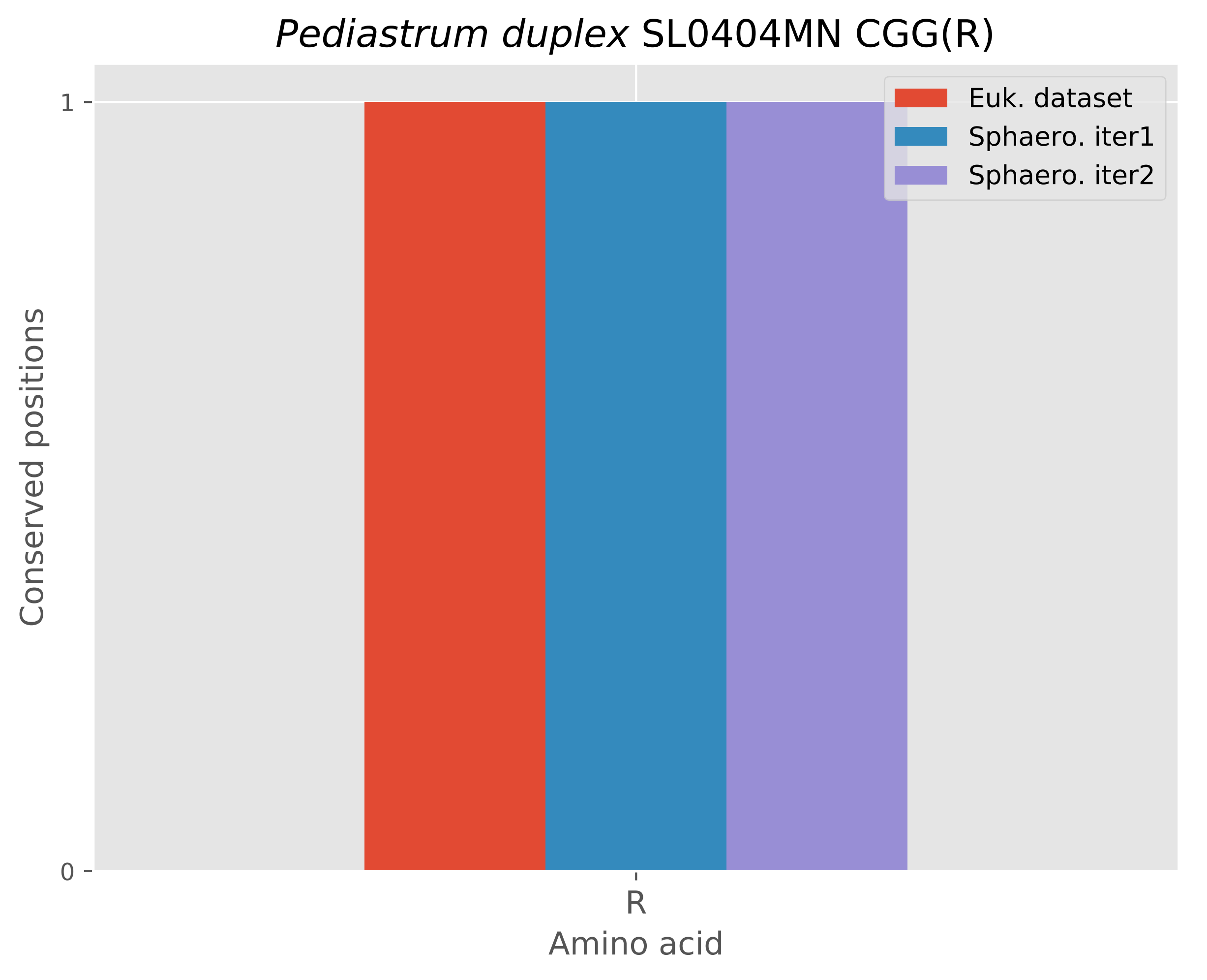

Pediastrum duplex SL0404MN CGG(R)
Euk. dataset
Sphaero. iter1
Sphaero. iter2
Conserved positions
1
0
R
Amino acid

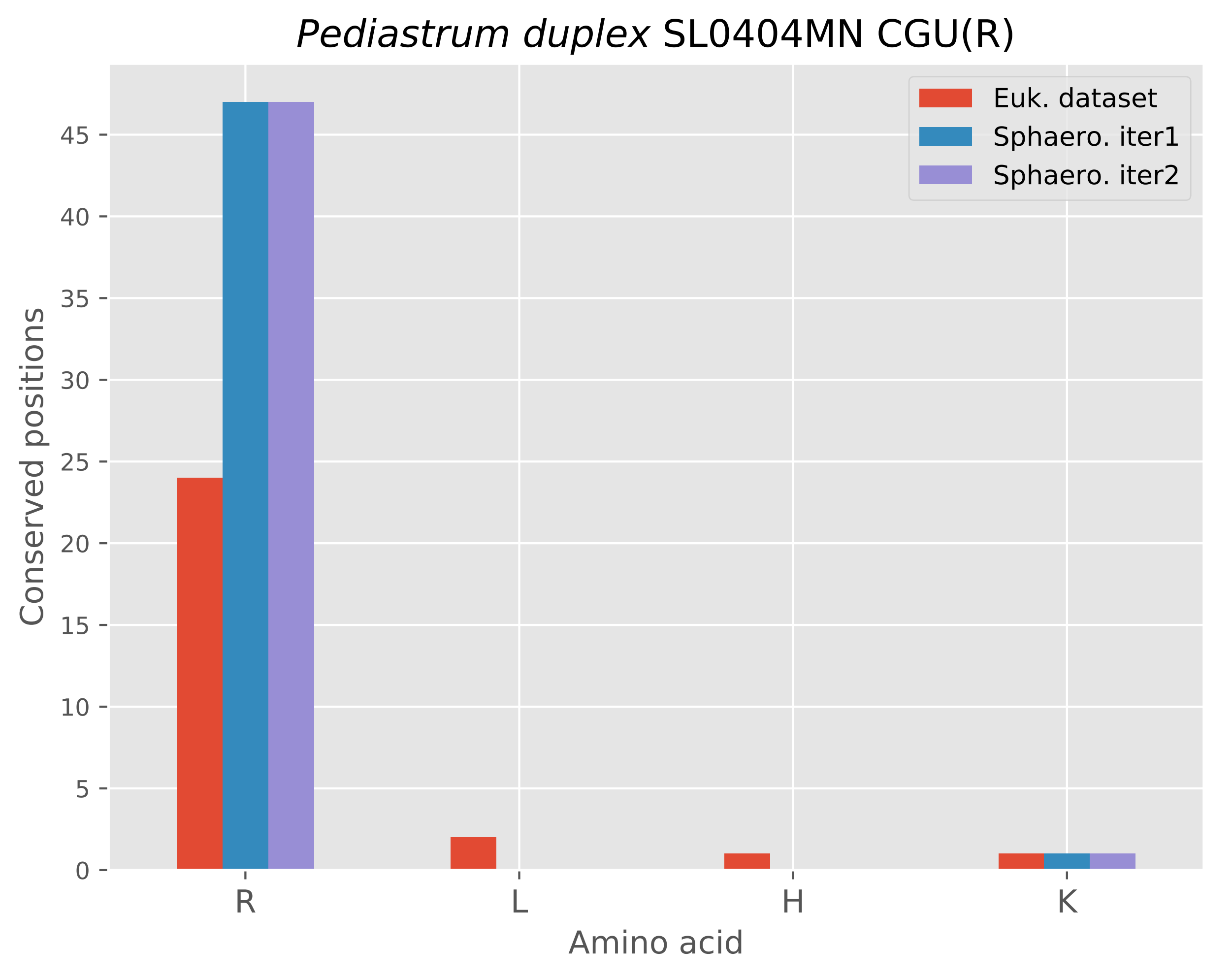

Pediastrum duplex SL0404MN CGU(R)
Euk. dataset
Sphaero. iter1
Sphaero. iter2
Conserved positions
0
5
10
15
20
25
30
35
40
45
R
L
H
K
Amino acid

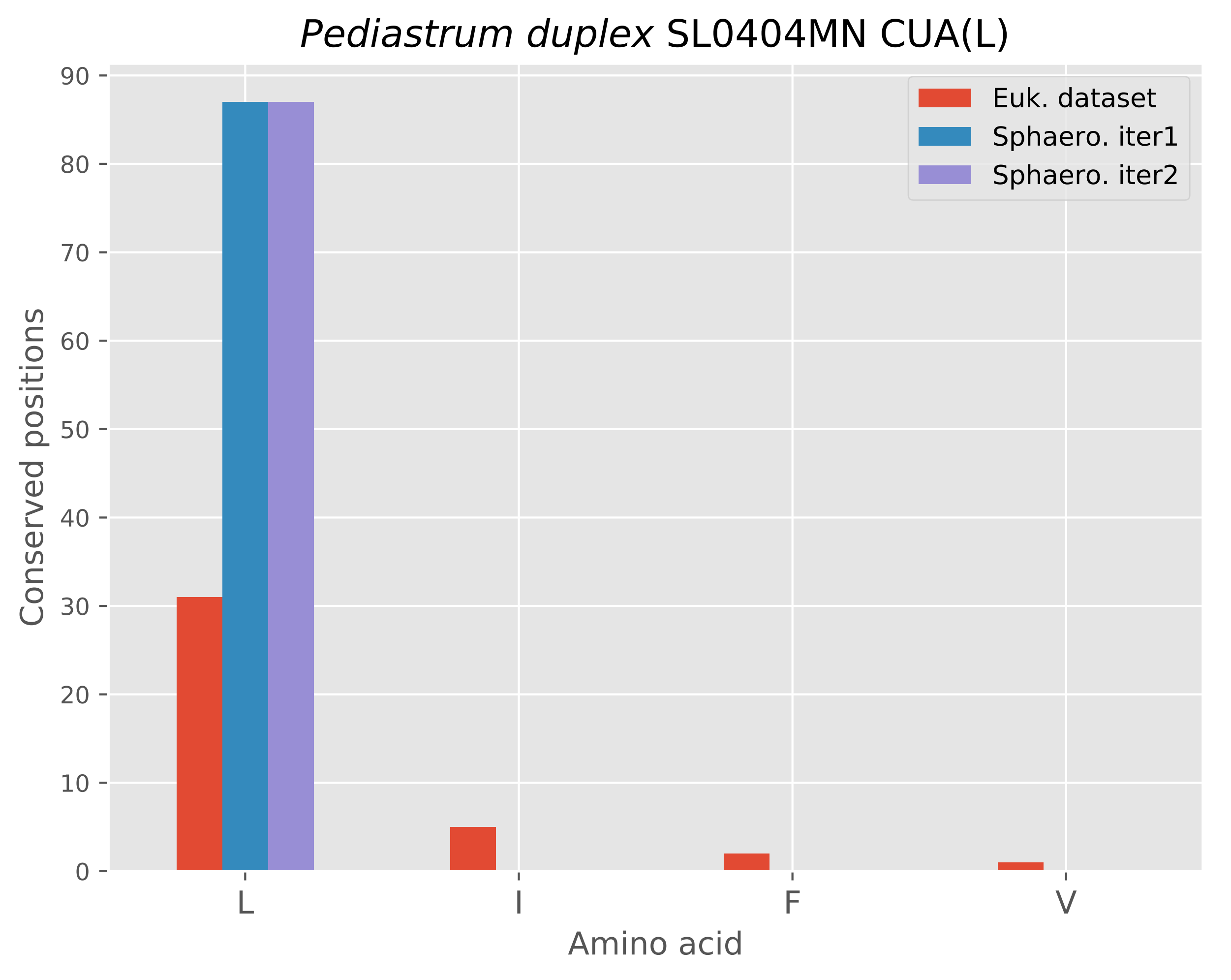

Pediastrum duplex SL0404MN CUA(L)
Euk. dataset
Sphaero. iter1
Sphaero. iter2
Conserved positions
0
10
20
30
40
50
60
70
80
90
L
I
F
V
Amino acid

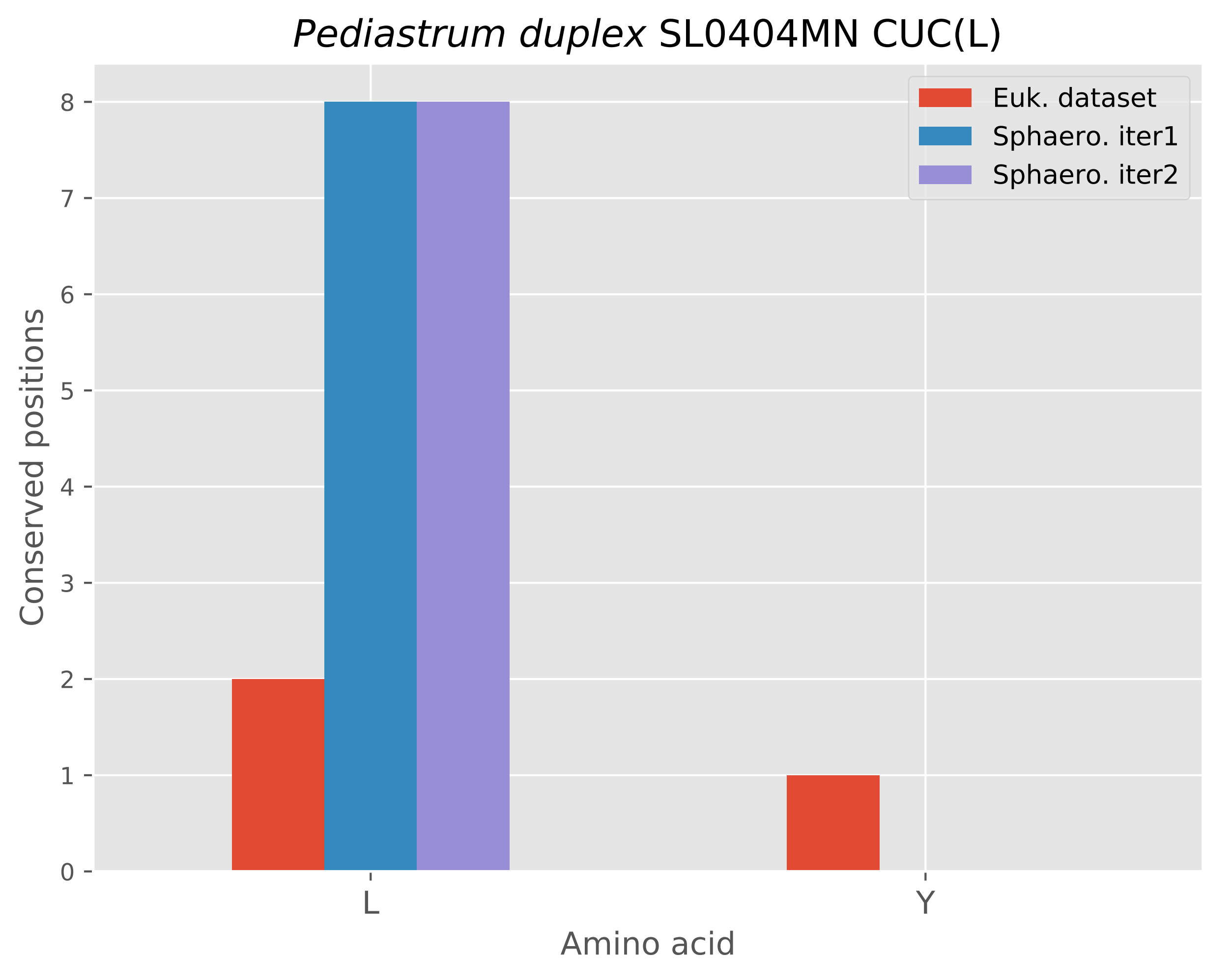

Pediastrum duplex SL0404MN CUC(L)
Euk. dataset
Sphaero. iter1
Sphaero. iter2
Conserved positions
0
1
2
3
4
5
6
7
8
L
Y
Amino acid

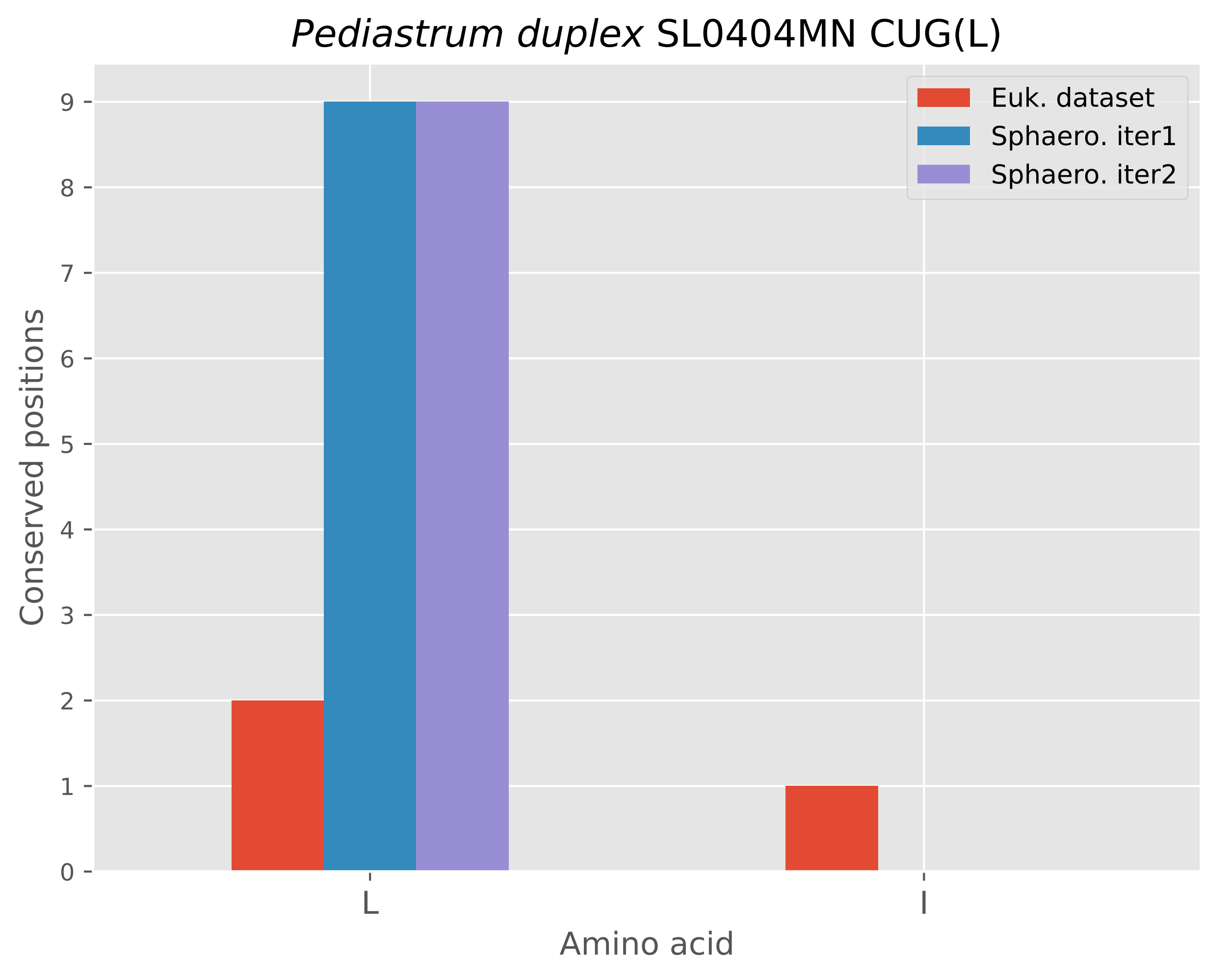

Pediastrum duplex SL0404MN CUG(L)
Euk. dataset
Sphaero. iter1
Sphaero. iter2
Conserved positions
0
1
2
3
4
5
6
7
8
9
L
I
Amino acid

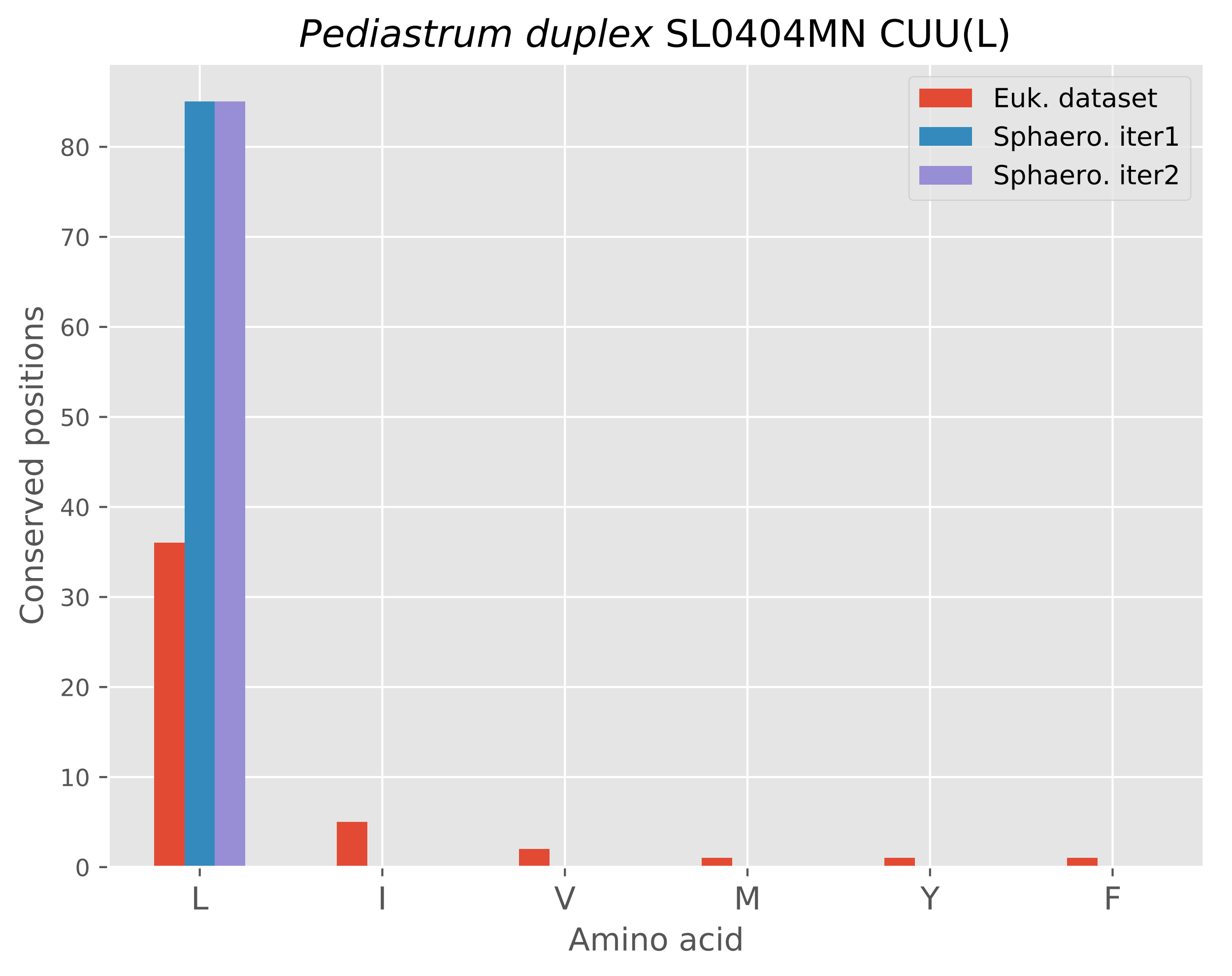

Pediastrum duplex SL0404MN CUU(L)
Euk. dataset
Sphaero. iter1
Sphaero. iter2
Conserved positions
0
10
20
30
40
50
60
70
80
L
I
V
M
Y
F
Amino acid

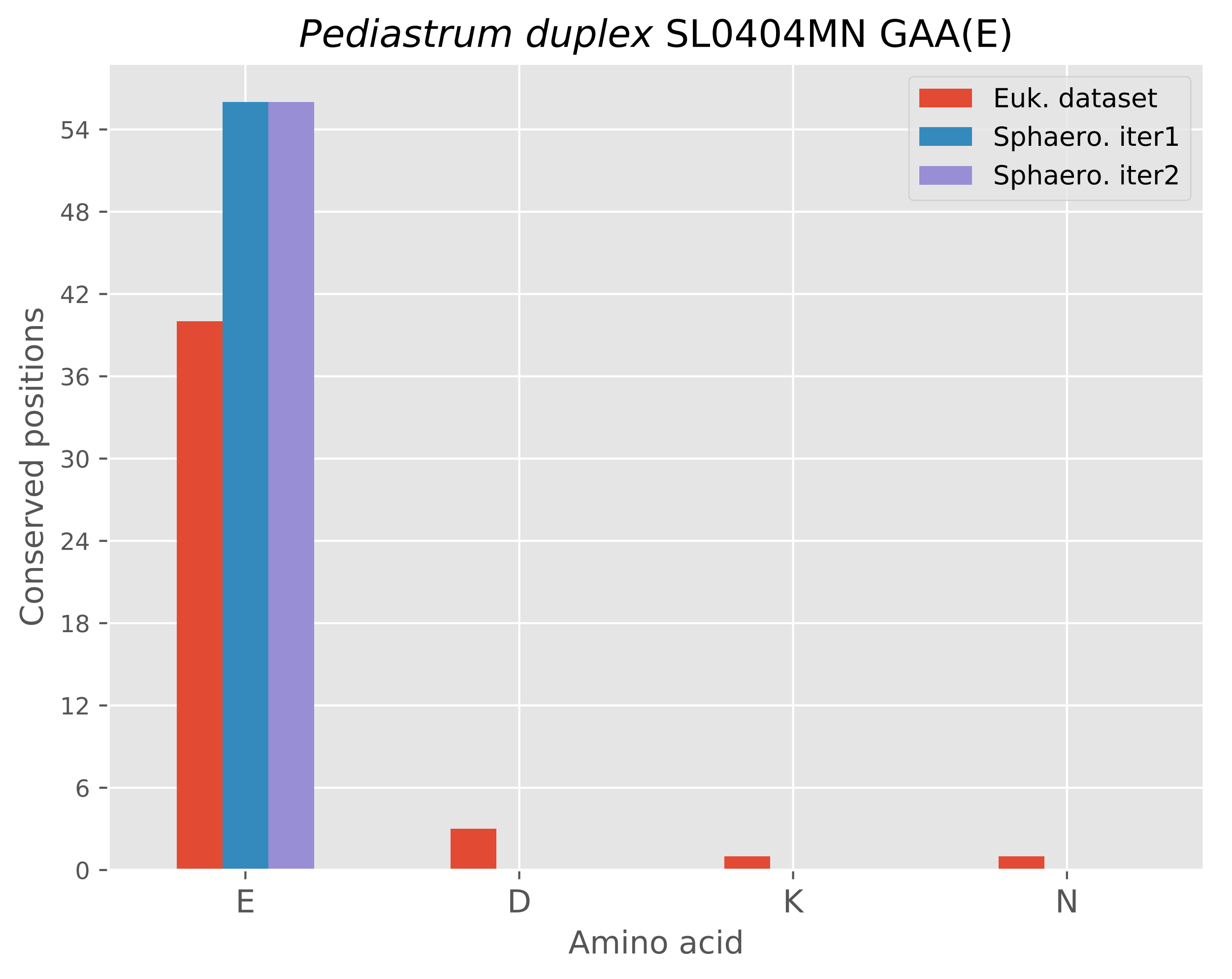

Pediastrum duplex SL0404MN GAA(E)
Euk. dataset
Sphaero. iter1
Sphaero. iter2
Conserved positions
0
6
12
18
24
30
36
42
48
54
E
D
K
N
Amino acid

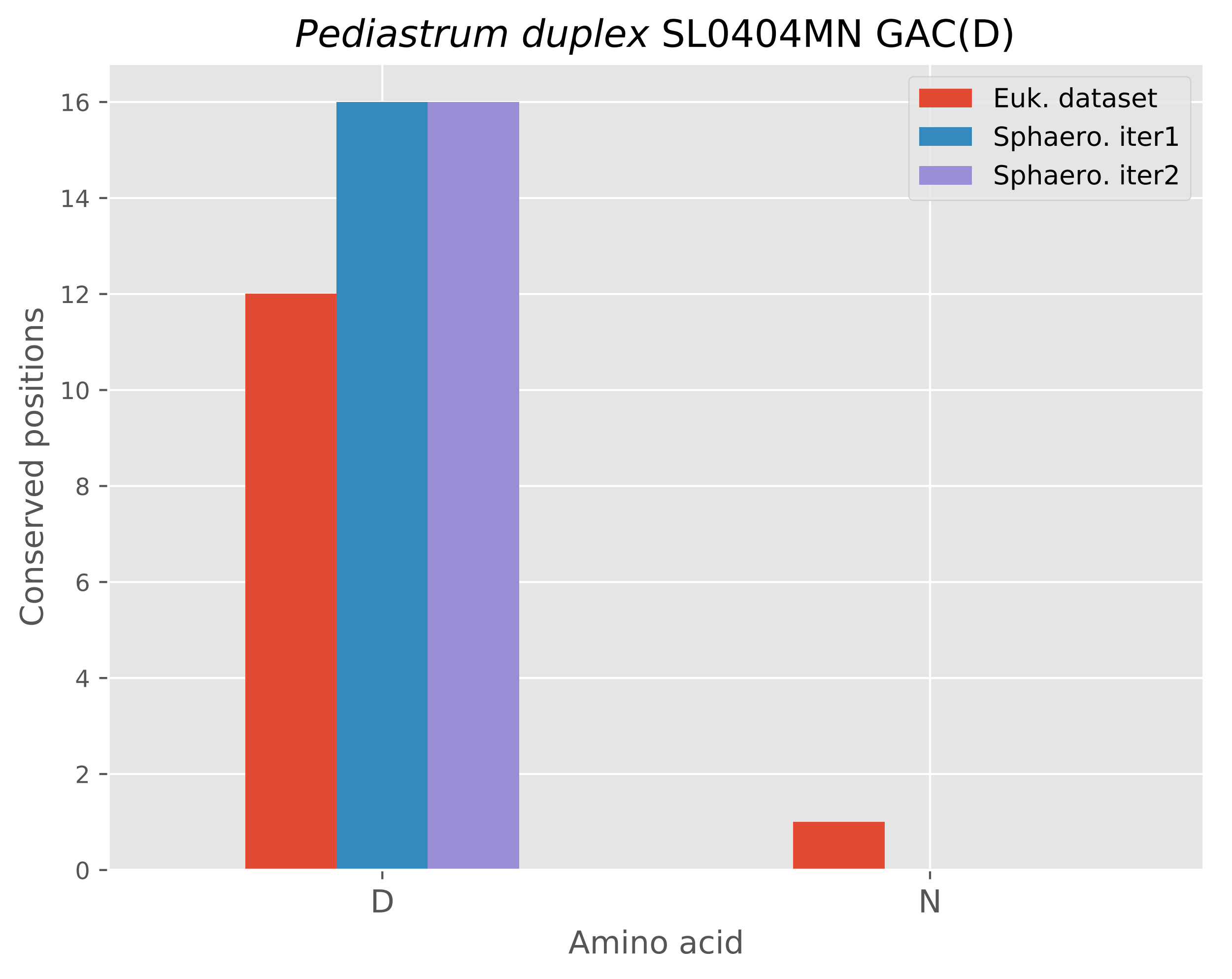

Pediastrum duplex SL0404MN GAC(D)
Euk. dataset
Sphaero. iter1
Sphaero. iter2
Conserved positions
0
2
4
6
8
10
12
14
16
D
N
Amino acid

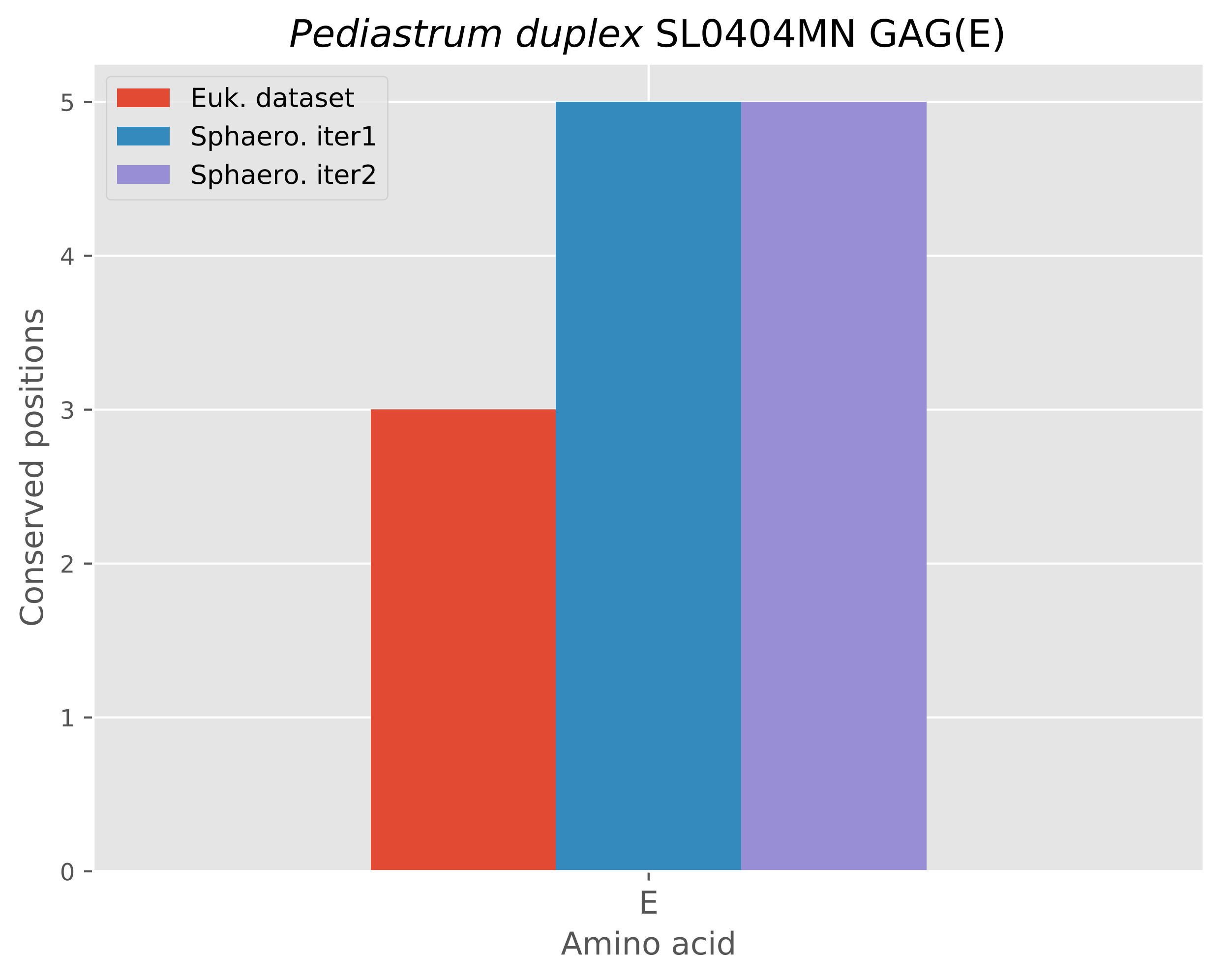

Pediastrum duplex SL0404MN GAG(E)
Euk. dataset
Sphaero. iter1
Sphaero. iter2
Conserved positions
0
1
2
3
4
5
E
Amino acid

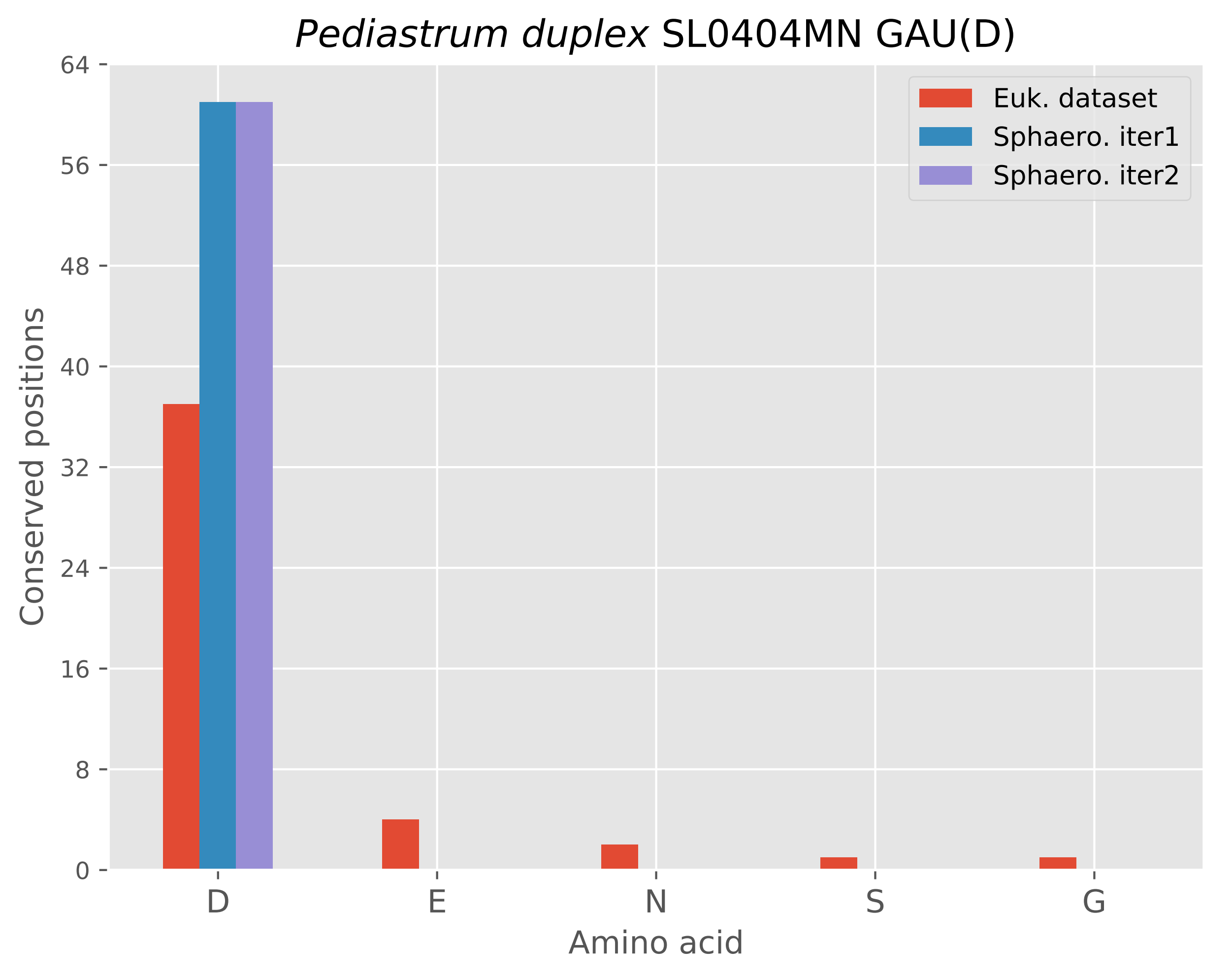

Pediastrum duplex SL0404MN GAU(D)
Euk. dataset
Sphaero. iter1
Sphaero. iter2
Conserved positions
0
8
16
24
32
40
48
56
64
D
E
N
S
G
Amino acid

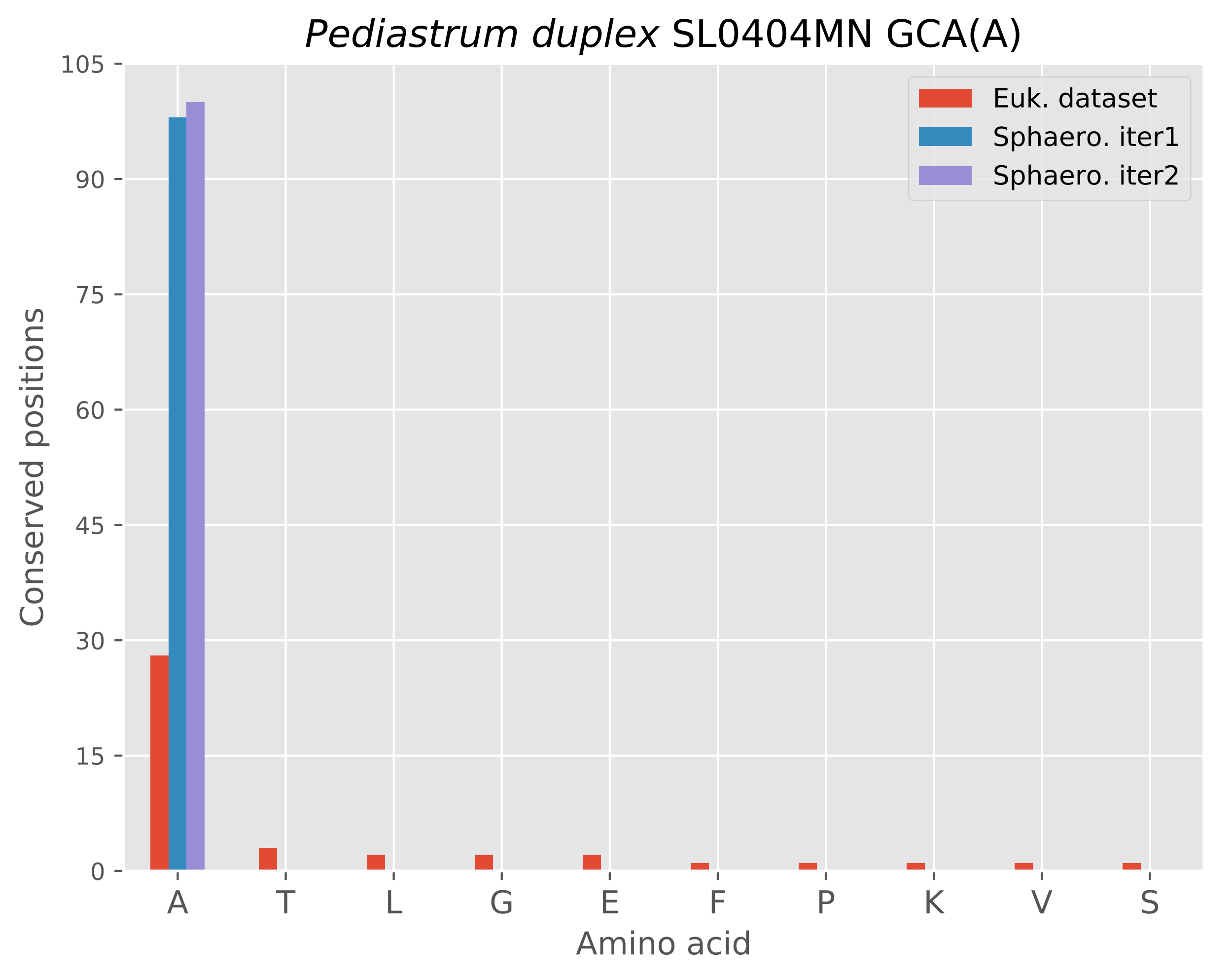

Pediastrum duplex SL0404MN GCA(A)
Conserved positions
Amino acid
Euk. dataset
Sphaero. iter1
Sphaero. iter2
0
15
30
45
60
75
90
105
A
T
L
G
E
F
P
K
V
S

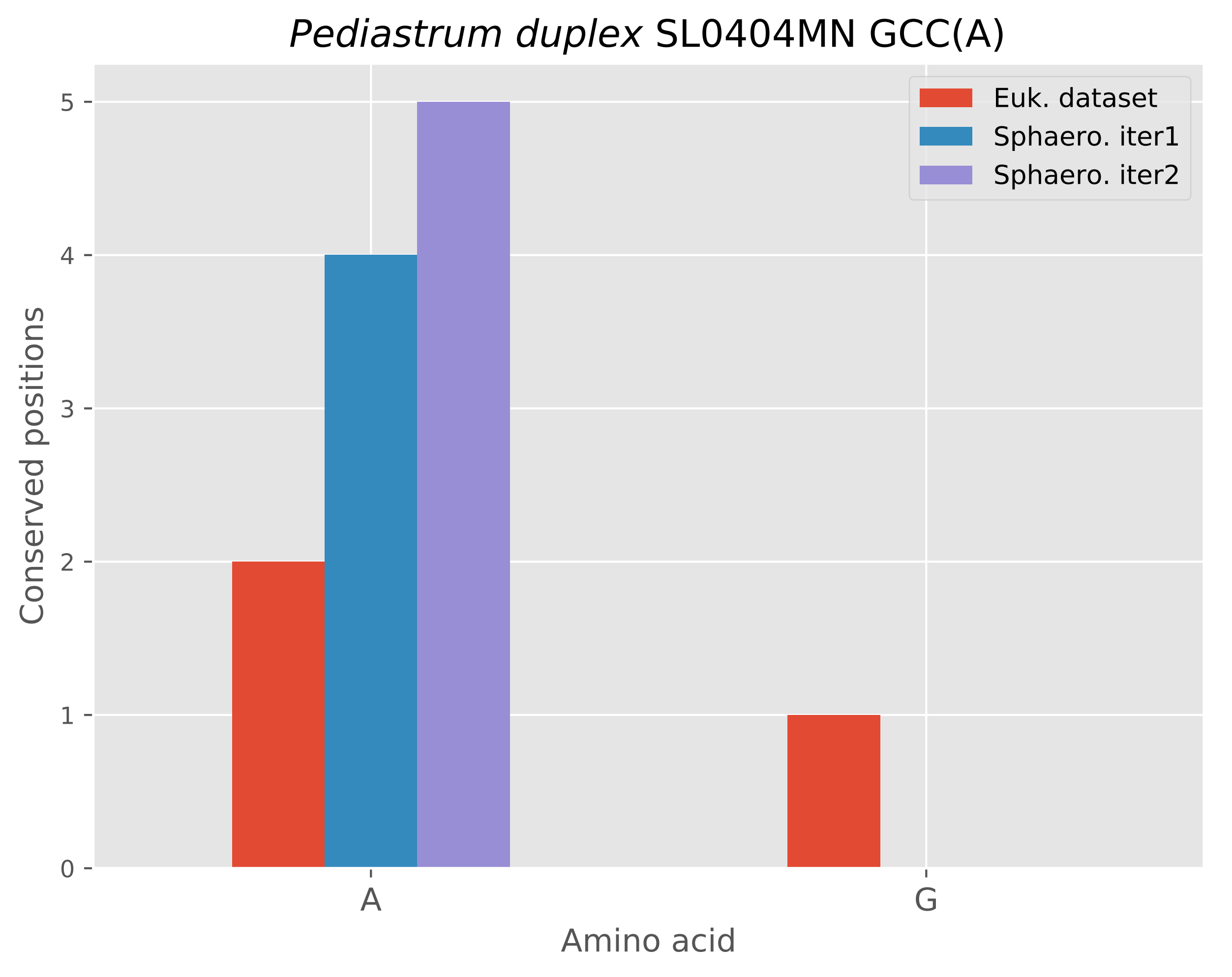
Pediastrum duplex SL0404MN GCC(A)
Euk. dataset
Sphaero. iter1
Sphaero. iter2
Conserved positions
0
1
2
3
4
5
A
G
Amino acid

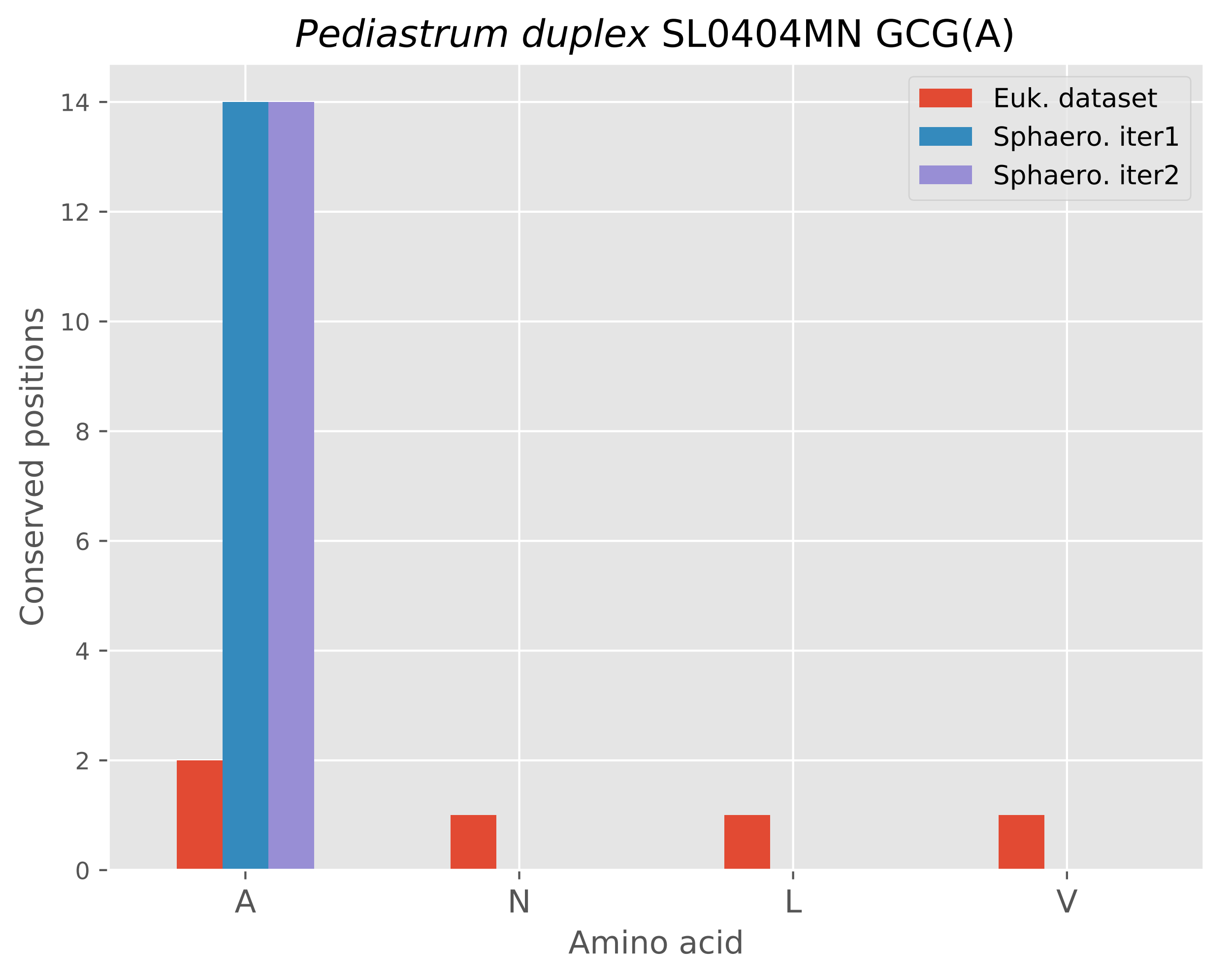

Pediastrum duplex SL0404MN GCG(A)
Euk. dataset
Sphaero. iter1
Sphaero. iter2
Conserved positions
0
2
4
6
8
10
12
14
A
N
L
V
Amino acid

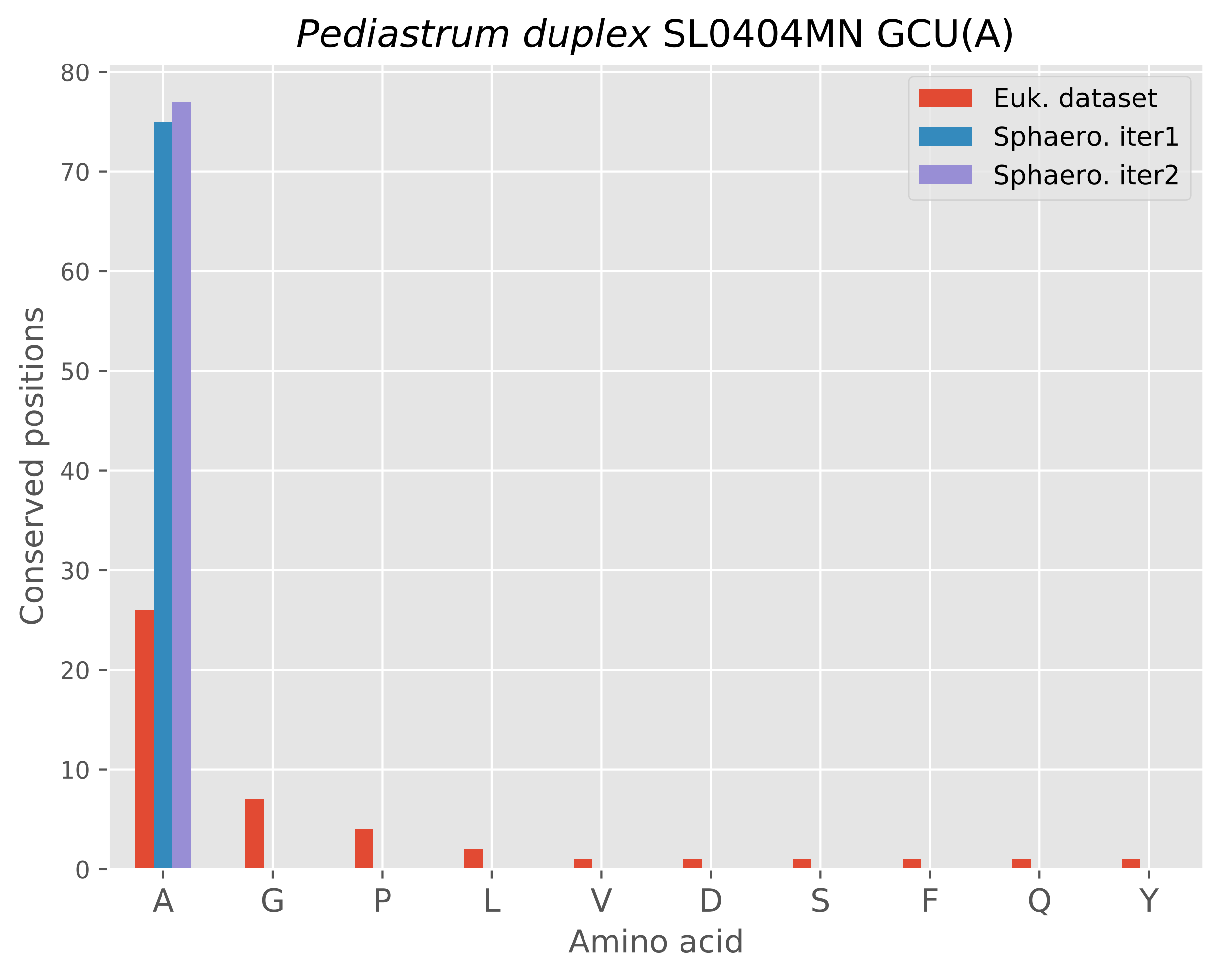

Pediastrum duplex SL0404MN GCU(A)
Euk. dataset
Sphaero. iter1
Sphaero. iter2
Conserved positions
0
10
20
30
40
50
60
70
80
A
G
P
L
V
D
S
F
Q
Y
Amino acid

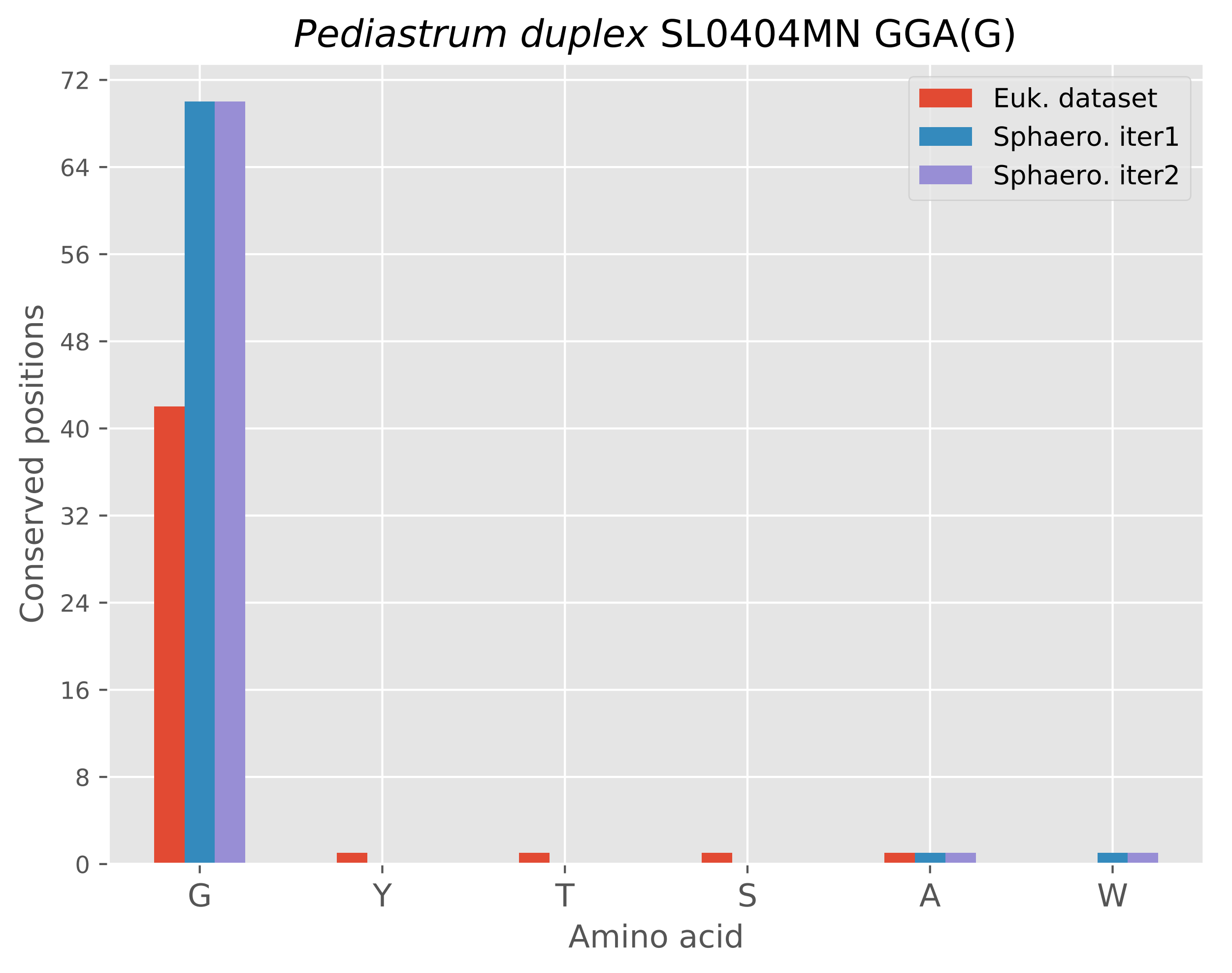

Pediastrum duplex SL0404MN GGA(G)
Euk. dataset
Sphaero. iter1
Sphaero. iter2
Conserved positions
0
8
16
24
32
40
48
56
64
72
G
Y
T
S
A
W
Amino acid

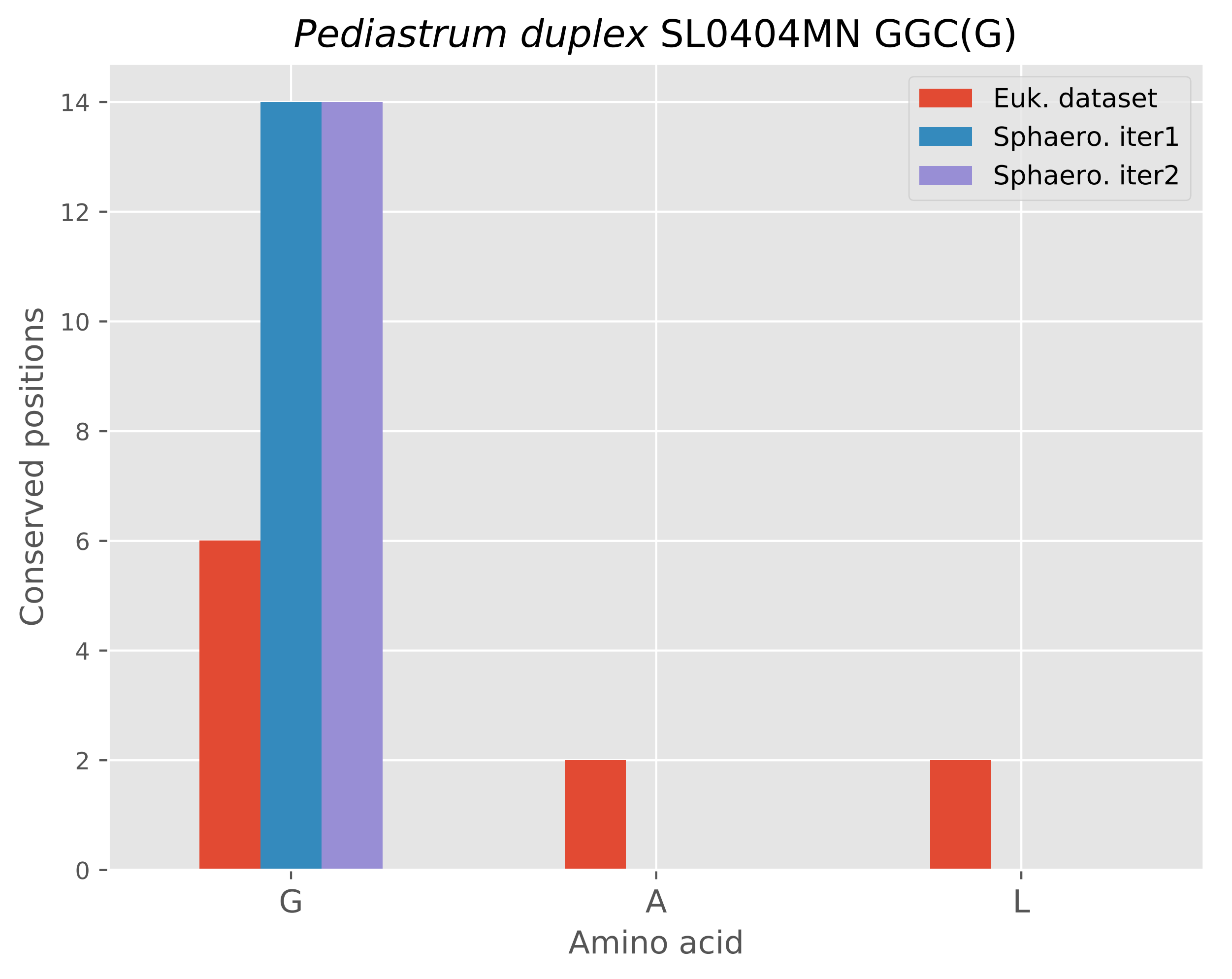

Pediastrum duplex SL0404MN GGC(G)
Euk. dataset
Sphaero. iter1
Sphaero. iter2
Conserved positions
0
2
4
6
8
10
12
14
G
A
L
Amino acid

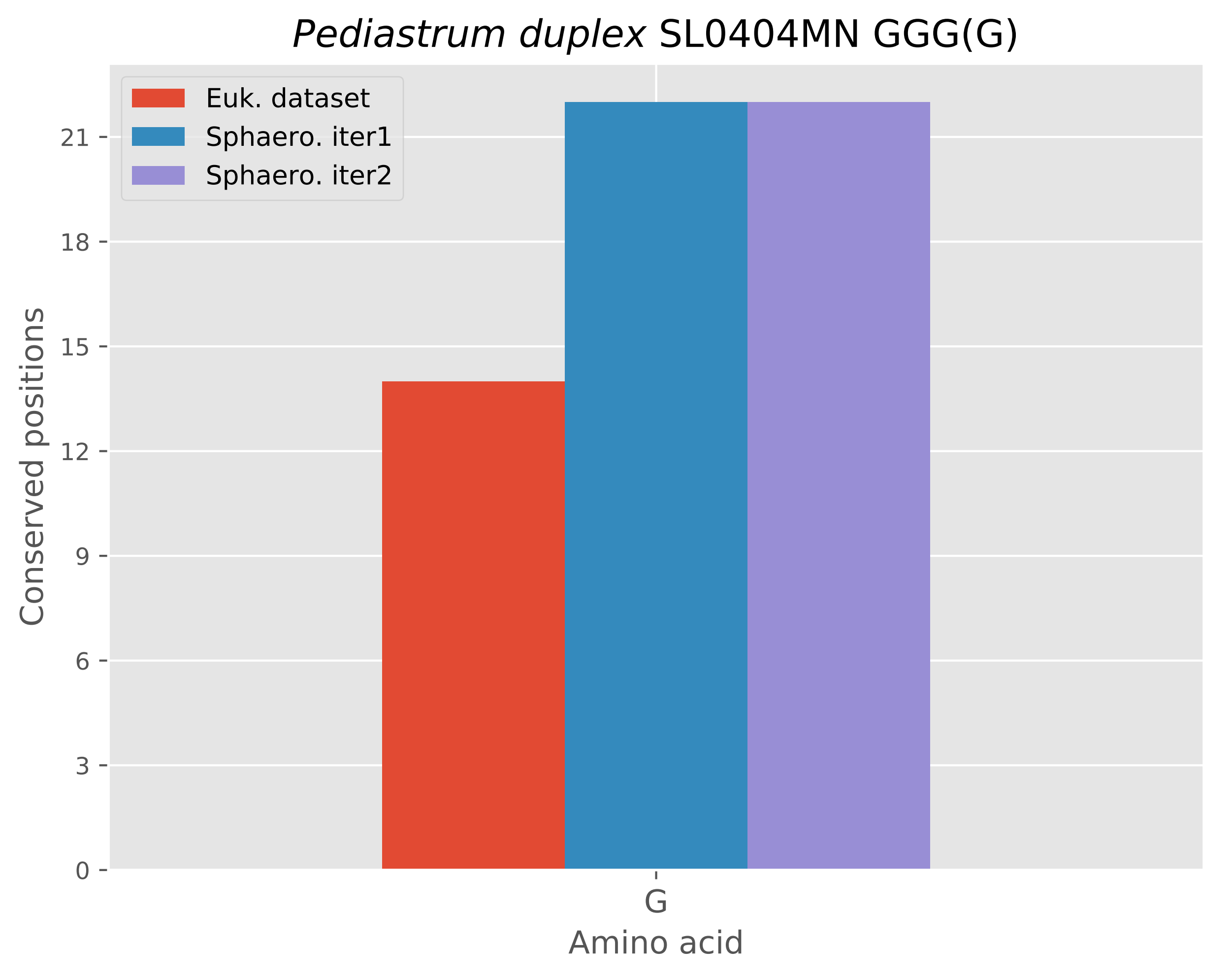

Pediastrum duplex SL0404MN GGG(G)
Euk. dataset
Sphaero. iter1
Sphaero. iter2
Conserved positions
0
3
6
9
12
15
18
21
G
Amino acid

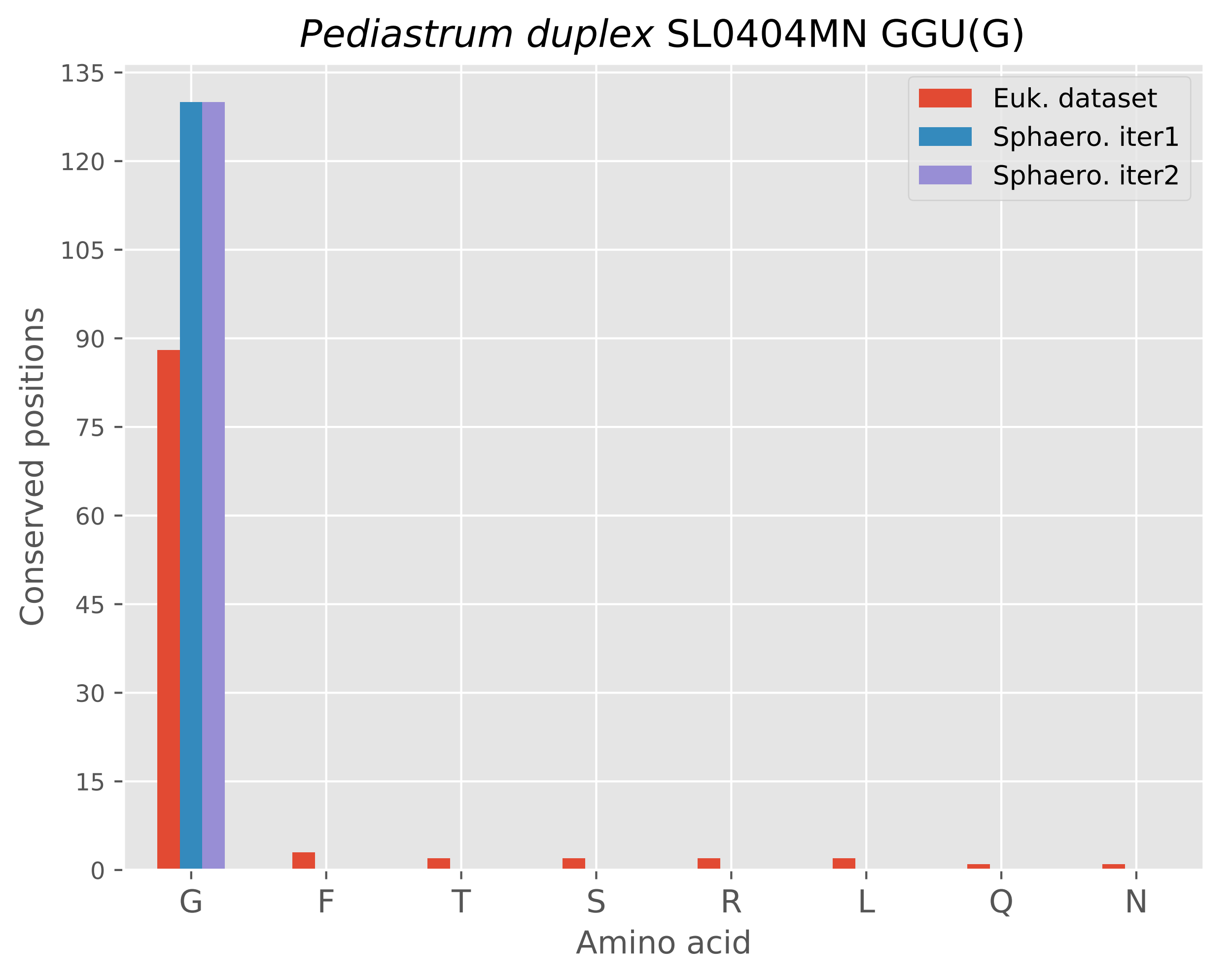

Pediastrum duplex SL0404MN GGU(G)
Euk. dataset
Sphaero. iter1
Sphaero. iter2
Conserved positions
0
15
30
45
60
75
90
105
120
135
G
F
T
S
R
L
Q
N
Amino acid

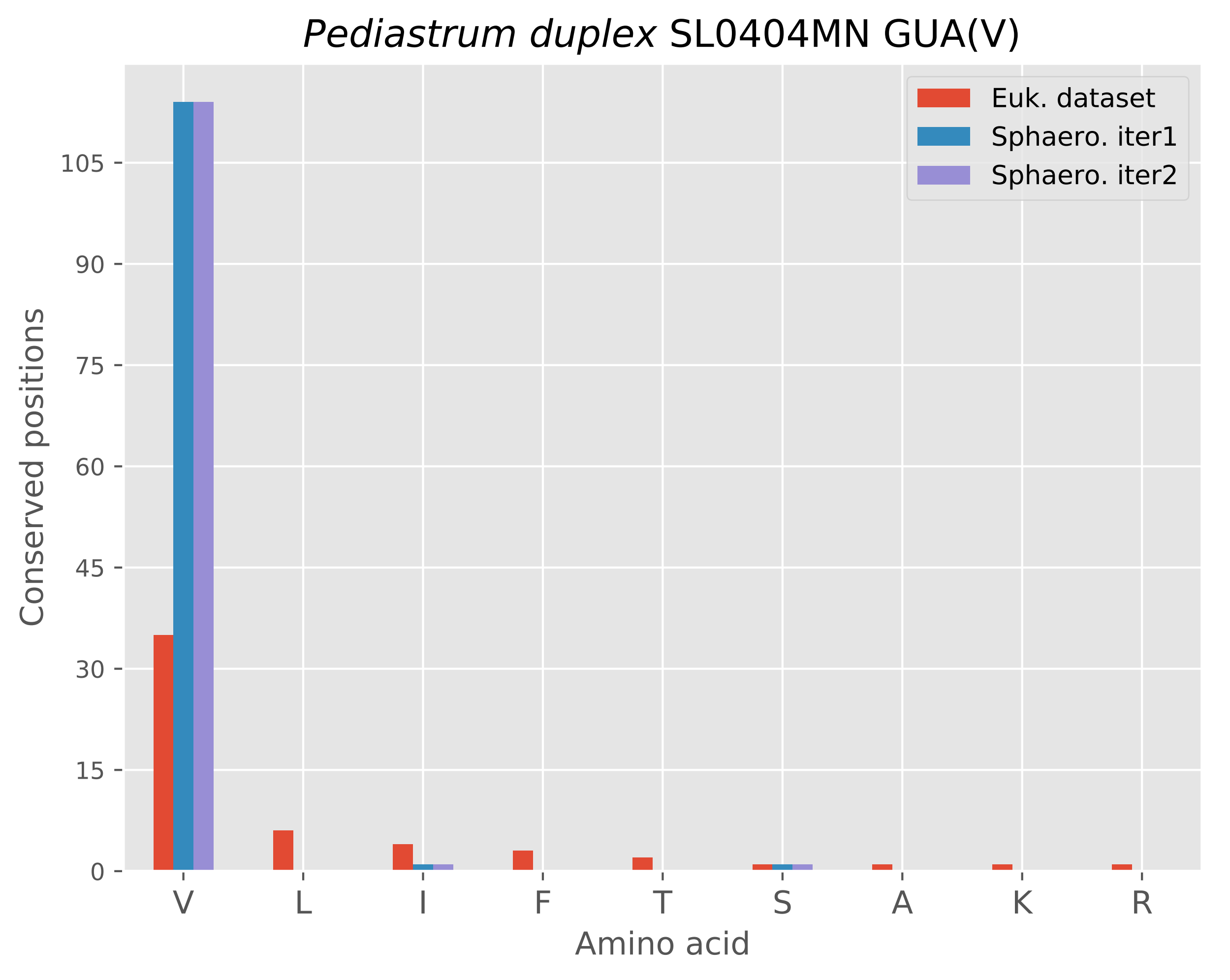

Pediastrum duplex SL0404MN GUA(V)
Euk. dataset
Sphaero. iter1
Sphaero. iter2
Conserved positions
0
15
30
45
60
75
90
105
V
L
I
F
T
S
A
K
R
Amino acid

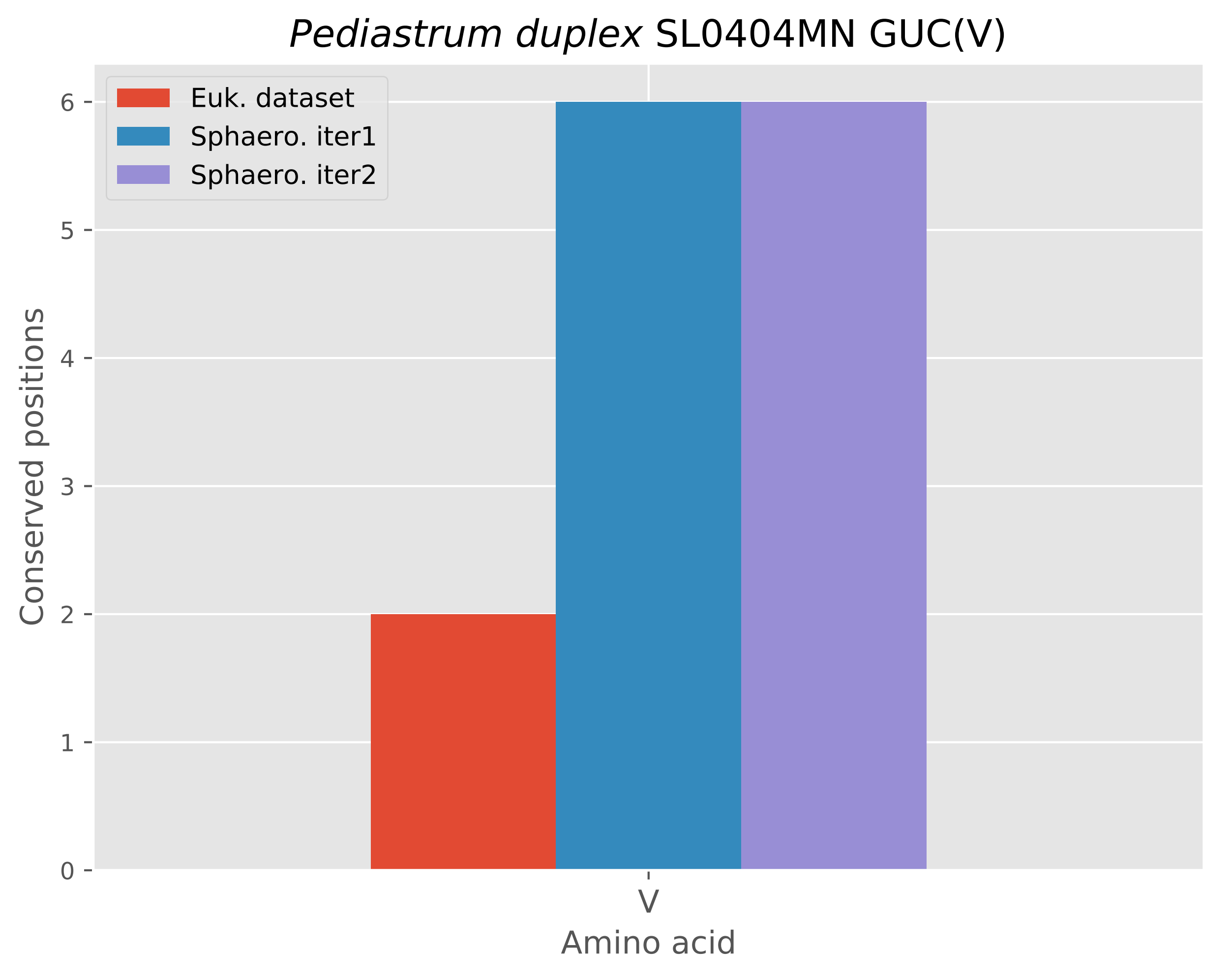

Pediastrum duplex SL0404MN GUC(V)
Euk. dataset
Sphaero. iter1
Sphaero. iter2
Conserved positions
0
1
2
3
4
5
6
V
Amino acid

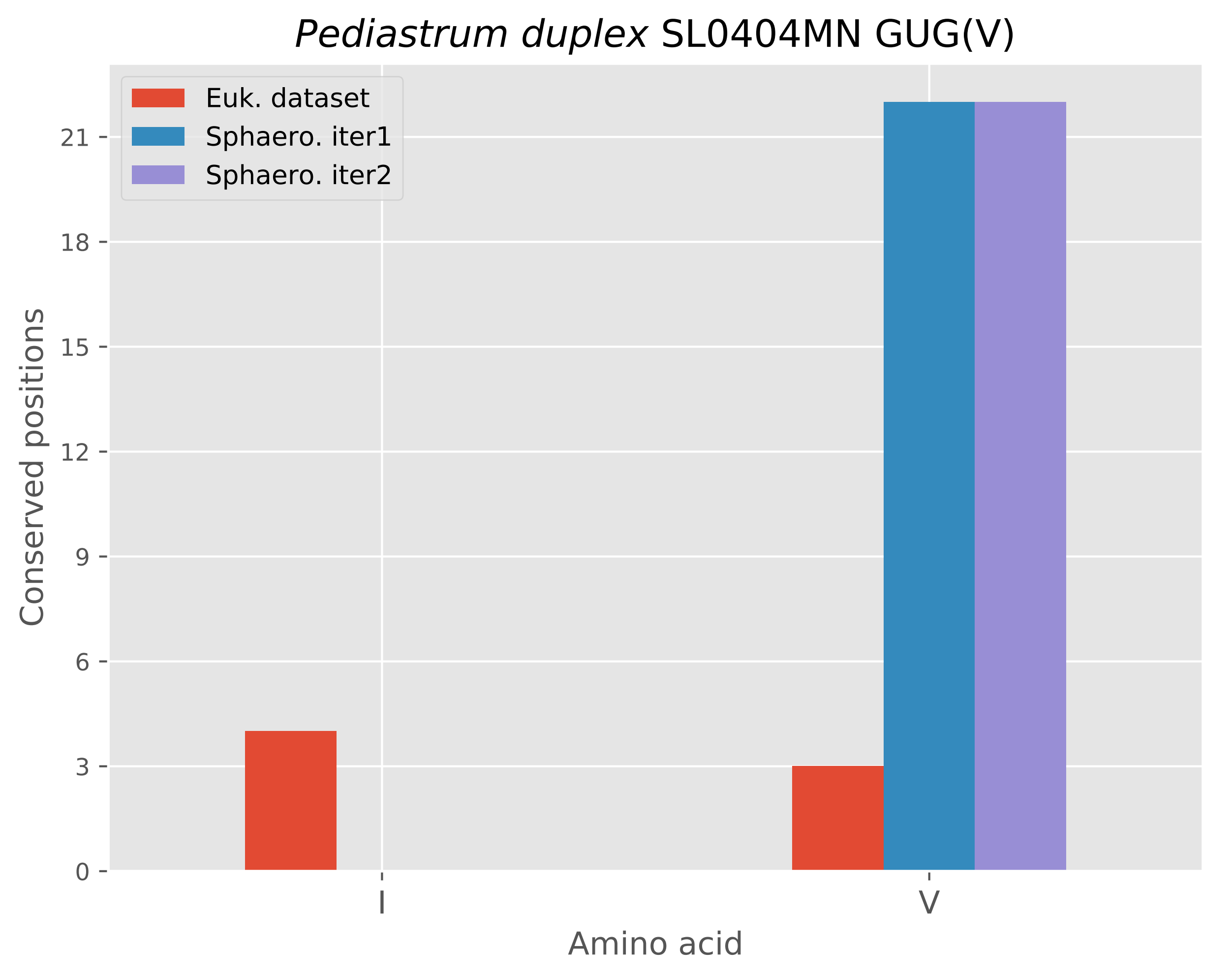

Pediastrum duplex SL0404MN GUG(V)
Euk. dataset
Sphaero. iter1
Sphaero. iter2
Conserved positions
0
3
6
9
12
15
18
21
I
V
Amino acid

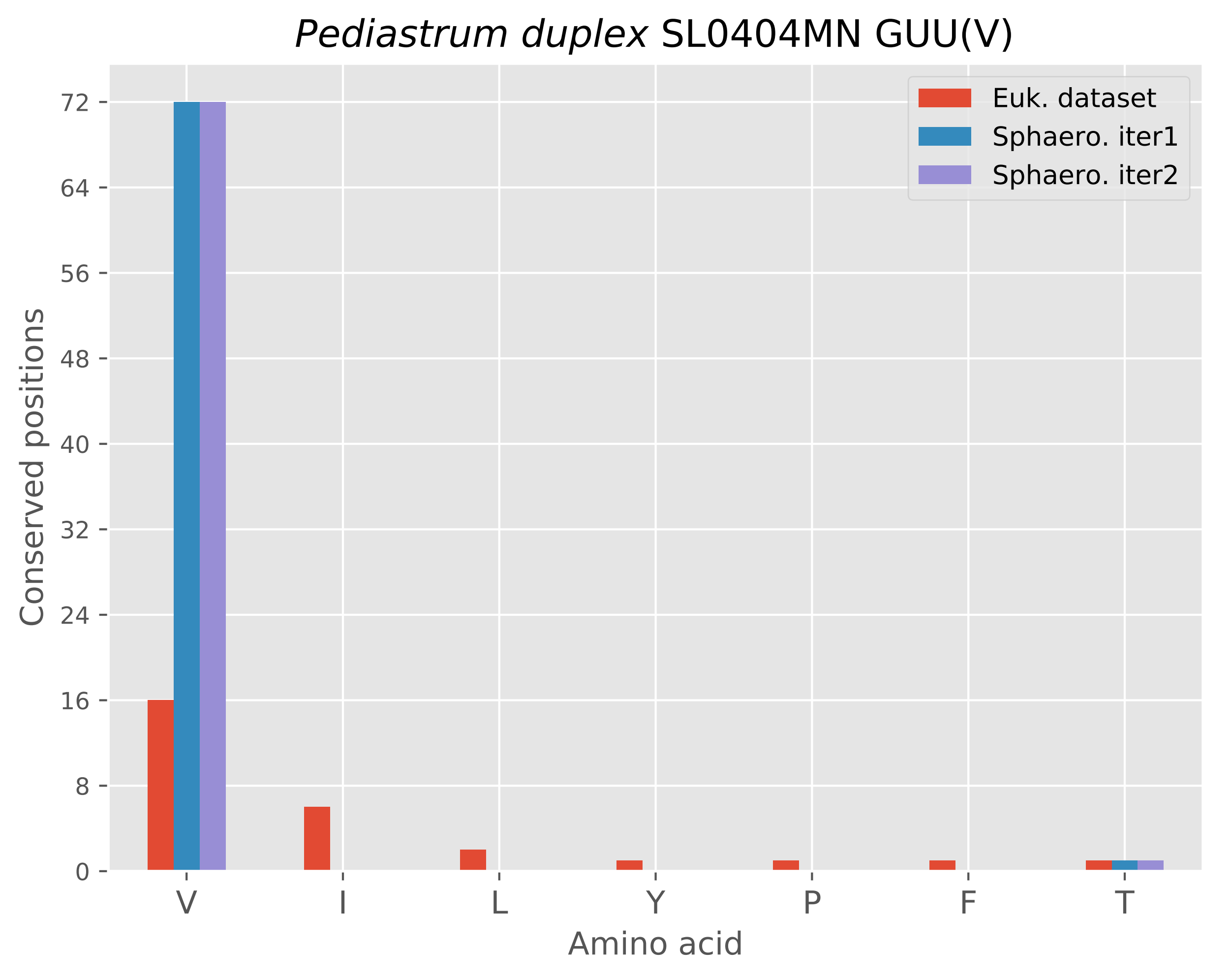

Pediastrum duplex SL0404MN GUU(V)
Euk. dataset
Sphaero. iter1
Sphaero. iter2
Conserved positions
0
8
16
24
32
40
48
56
64
72
V
I
L
Y
P
F
T
Amino acid

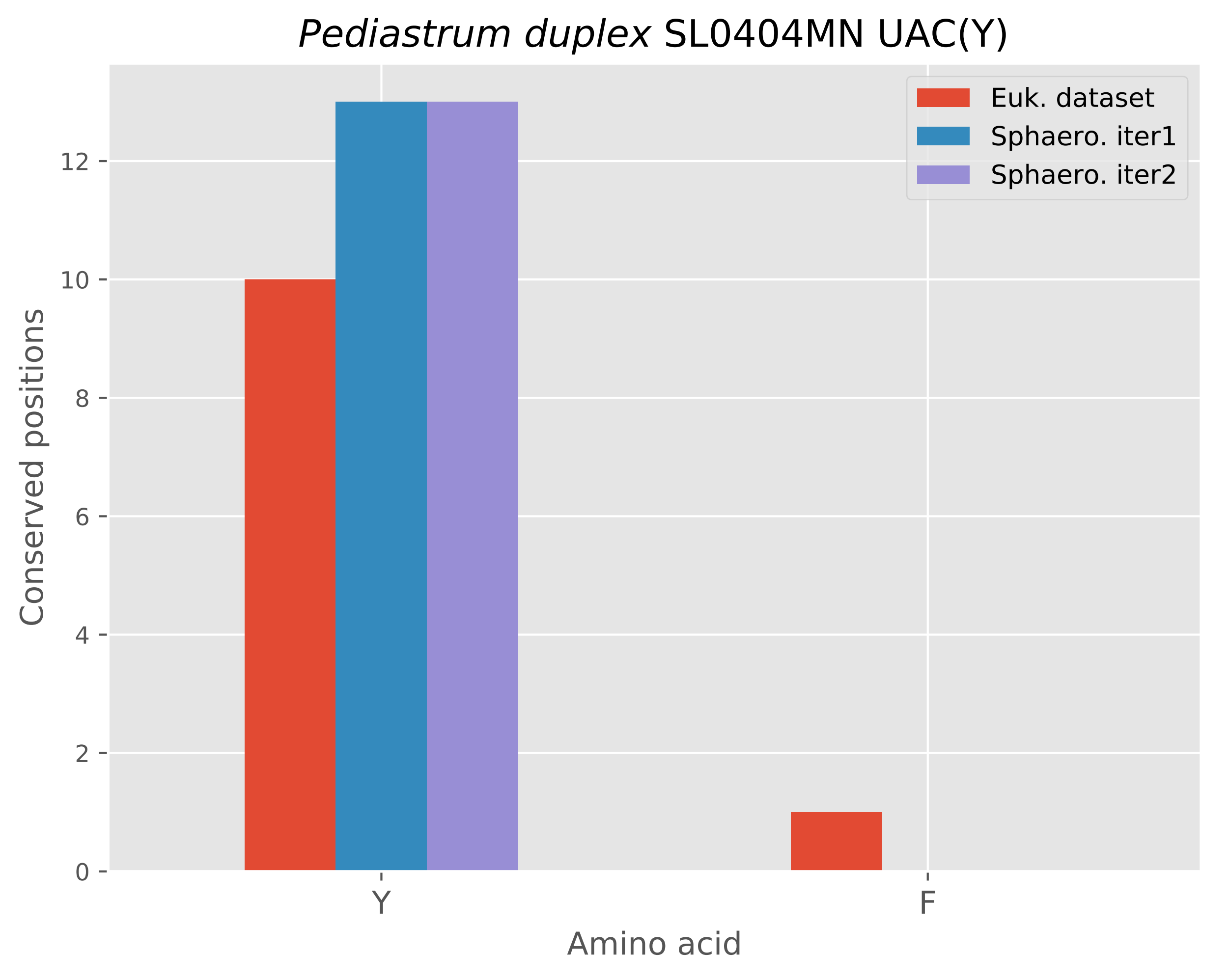

Pediastrum duplex SL0404MN UAC(Y)
Euk. dataset
Sphaero. iter1
Sphaero. iter2
Conserved positions
0
2
4
6
8
10
12
Y
F
Amino acid

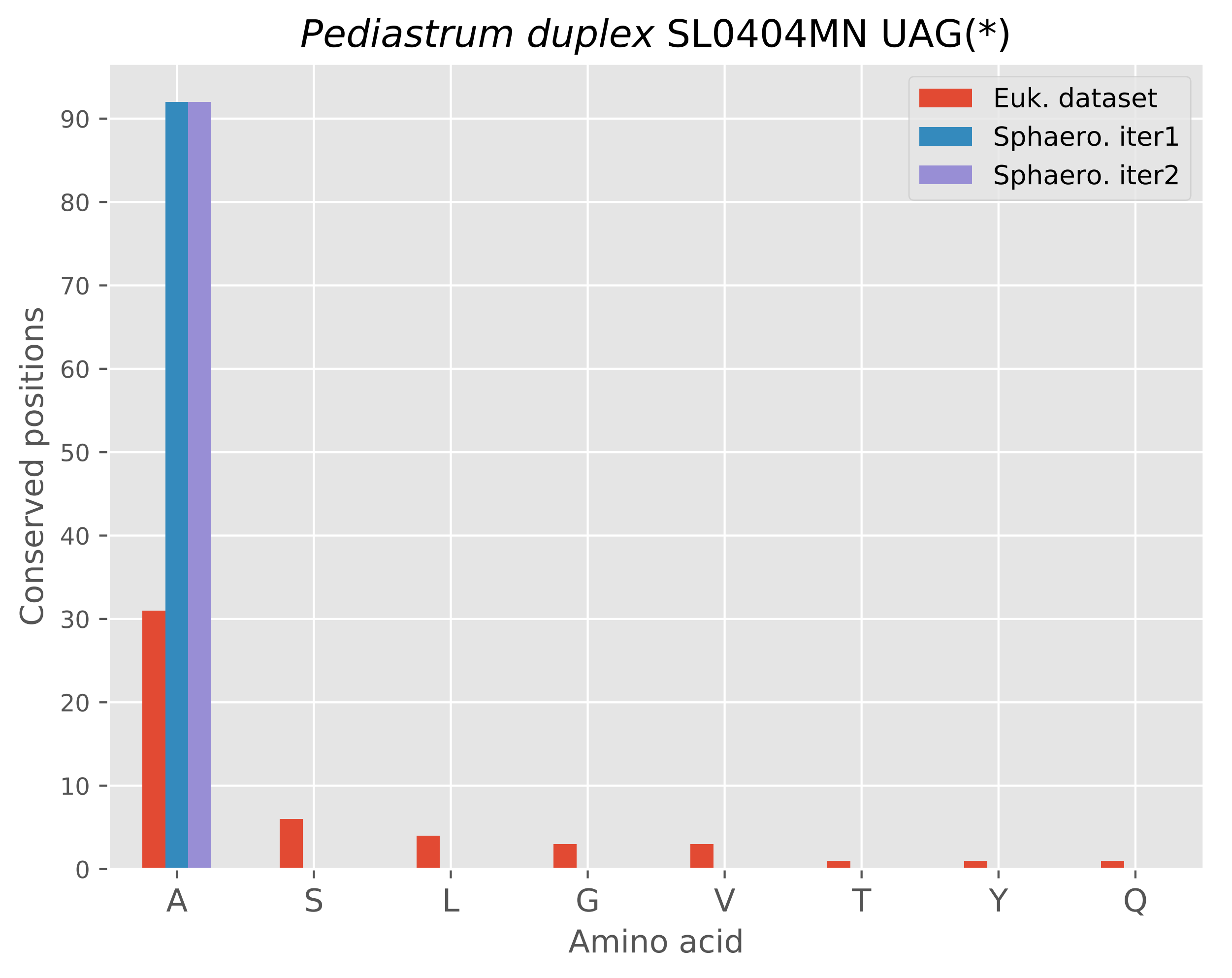

Pediastrum duplex SL0404MN UAG(*)
Euk. dataset
Sphaero. iter1
Sphaero. iter2
Conserved positions
0
10
20
30
40
50
60
70
80
90
A
S
L
G
V
T
Y
Q
Amino acid

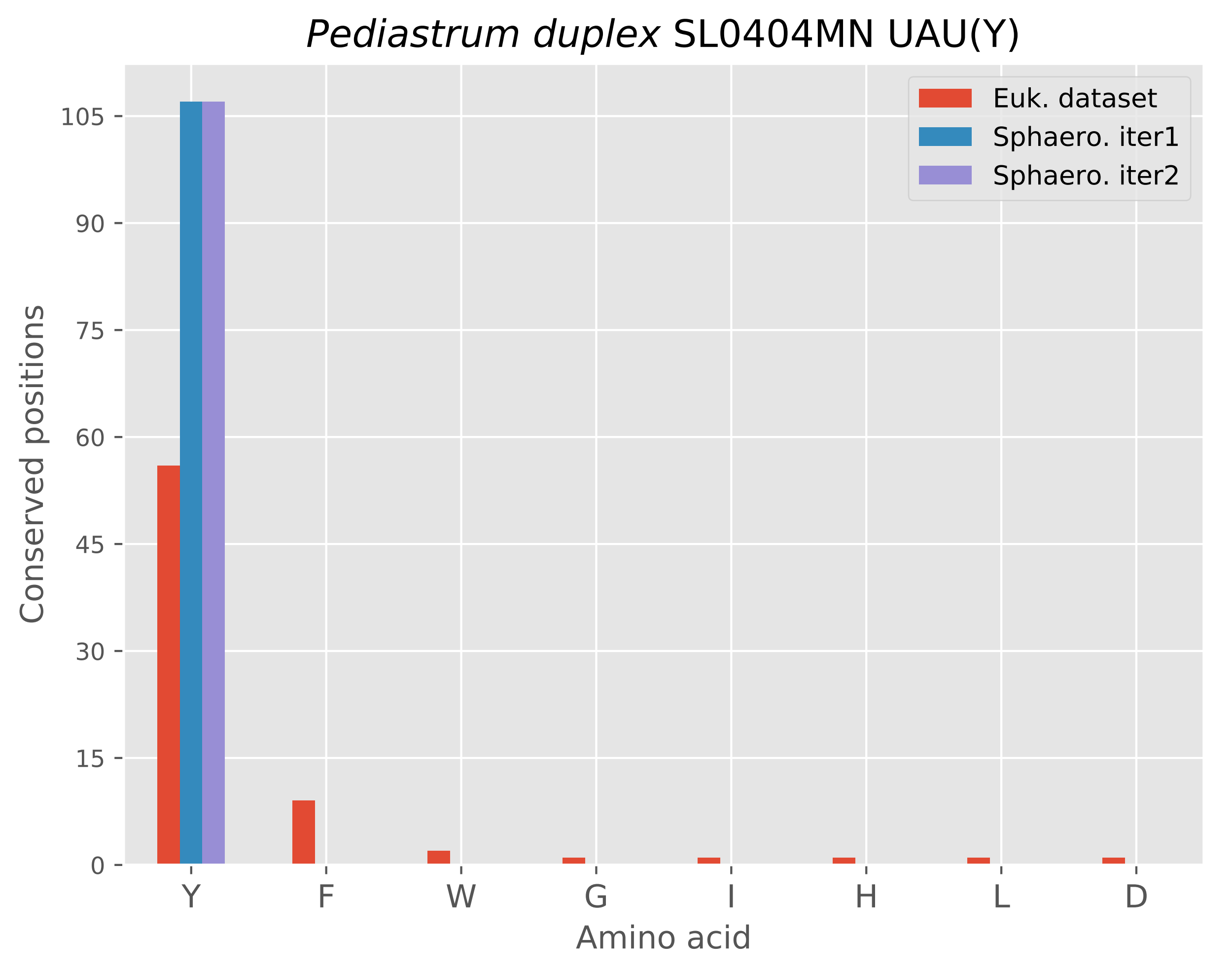

Pediastrum duplex SL0404MN UAU(Y)
Euk. dataset
Sphaero. iter1
Sphaero. iter2
Conserved positions
0
15
30
45
60
75
90
105
Y
F
W
G
I
H
L
D
Amino acid

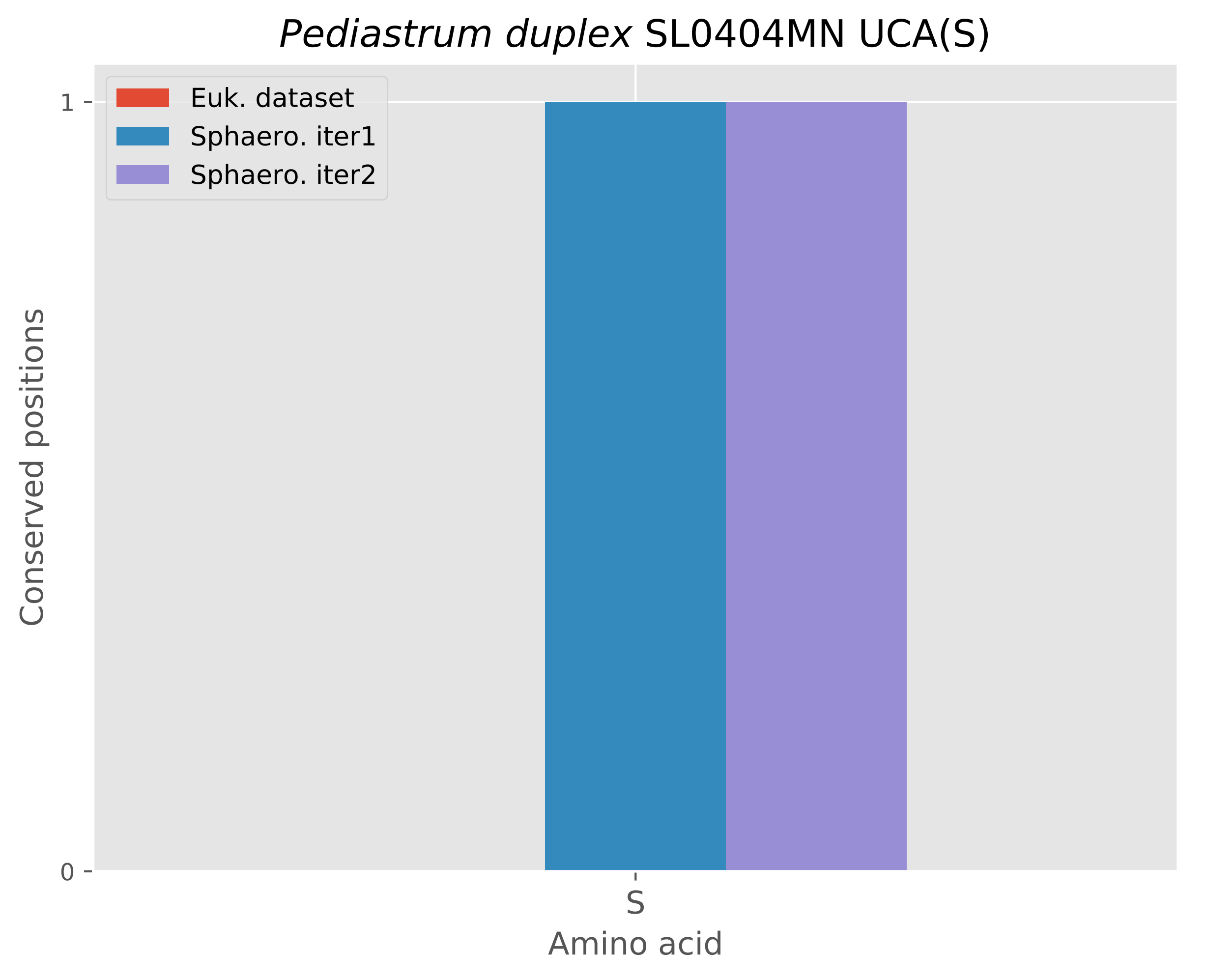

Pediastrum duplex SL0404MN UCA(S)
Euk. dataset
Sphaero. iter1
Sphaero. iter2
1
0
Conserved positions
S
Amino acid

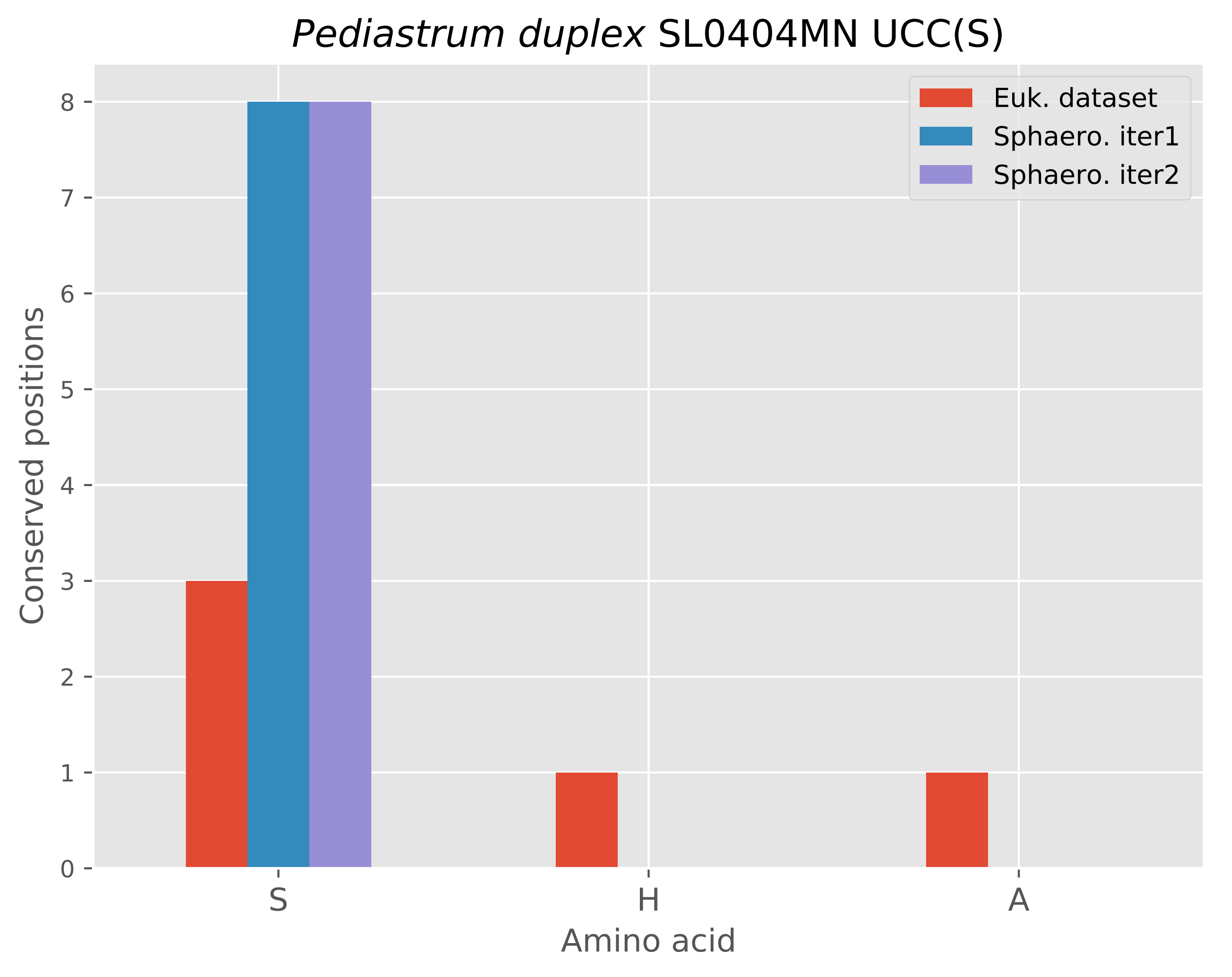

Pediastrum duplex SL0404MN UCC(S)
Euk. dataset
Sphaero. iter1
Sphaero. iter2
Conserved positions
0
1
2
3
4
5
6
7
8
S
H
A
Amino acid

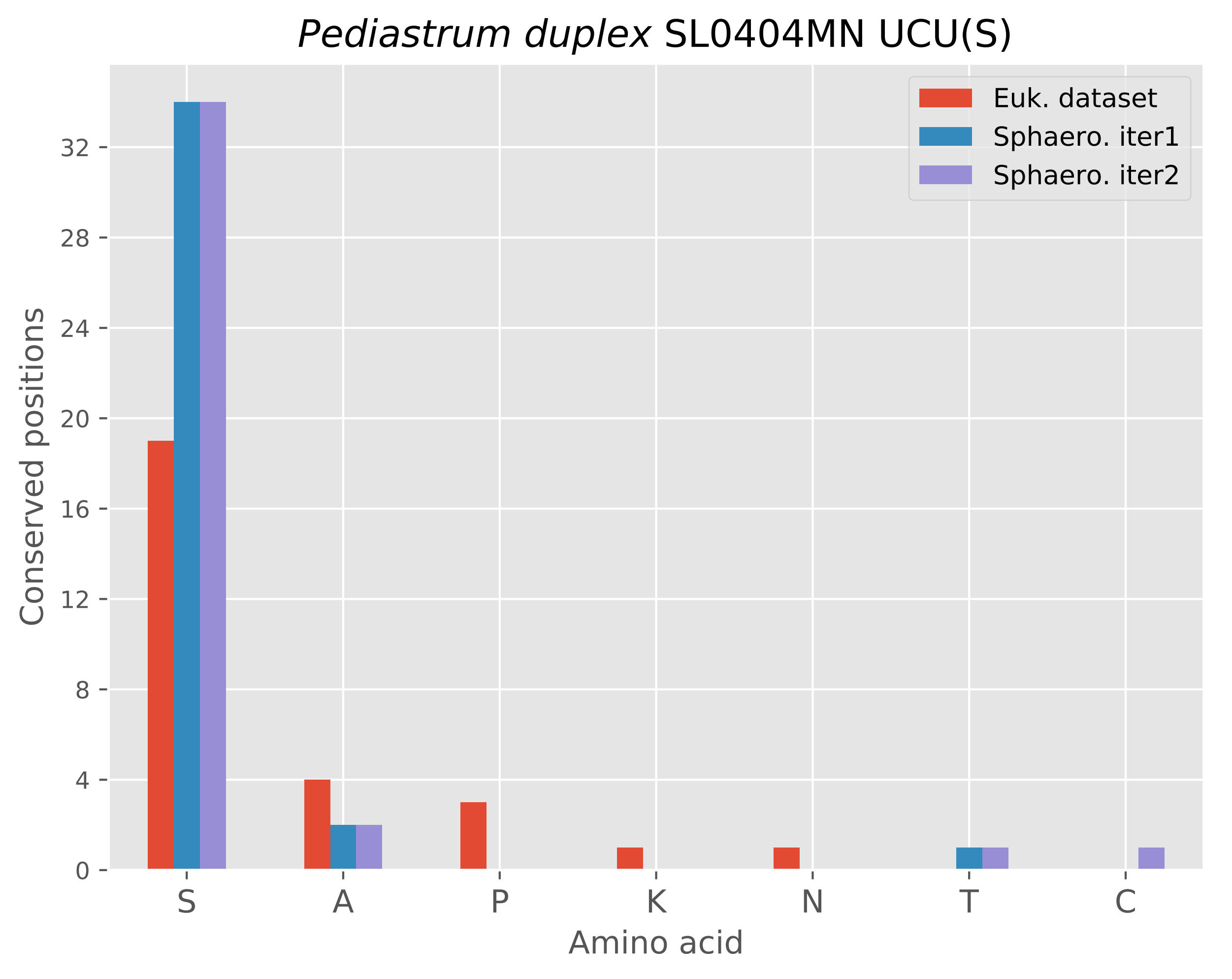

Pediastrum duplex SL0404MN UCU(S)
Euk. dataset
Sphaero. iter1
Sphaero. iter2
Conserved positions
0
4
8
12
16
20
24
28
32
S
A
P
K
N
T
C
Amino acid

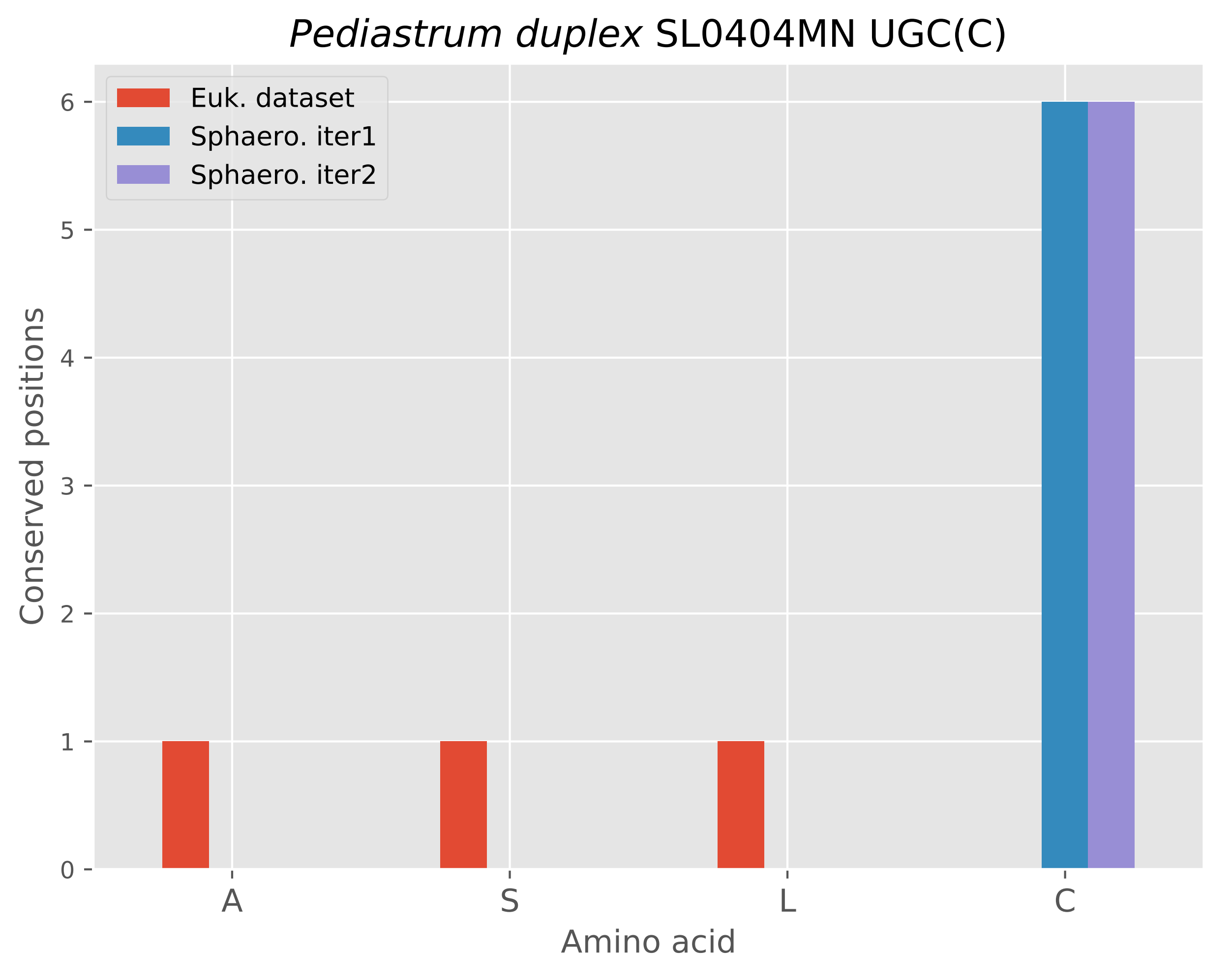

Pediastrum duplex SL0404MN UGC(C)
Euk. dataset
Sphaero. iter1
Sphaero. iter2
Conserved positions
0
1
2
3
4
5
6
A
S
L
C
Amino acid

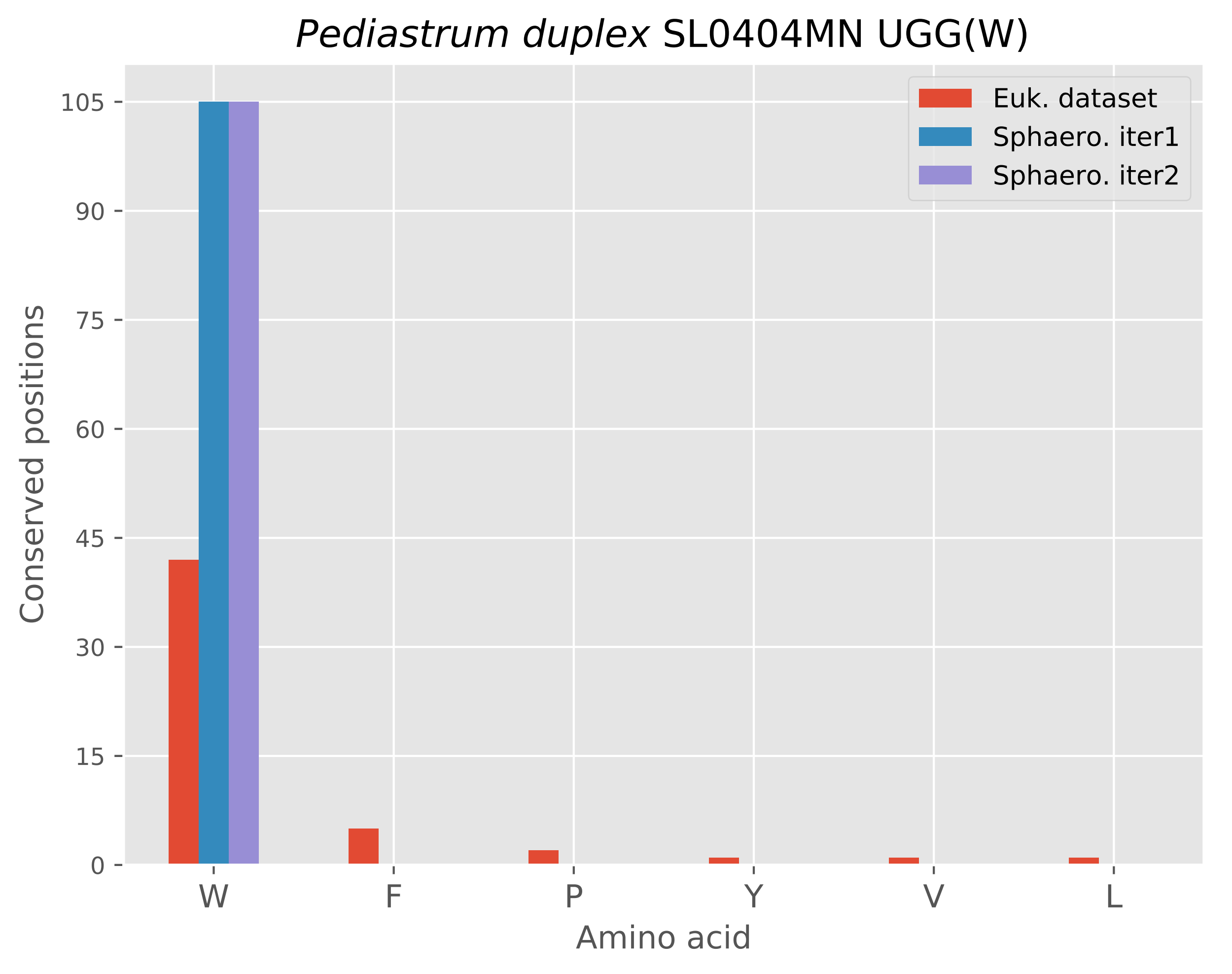

Pediastrum duplex SL0404MN UGG(W)
Euk. dataset
Sphaero. iter1
Sphaero. iter2
Conserved positions
0
15
30
45
60
75
90
105
W
F
P
Y
V
L
Amino acid

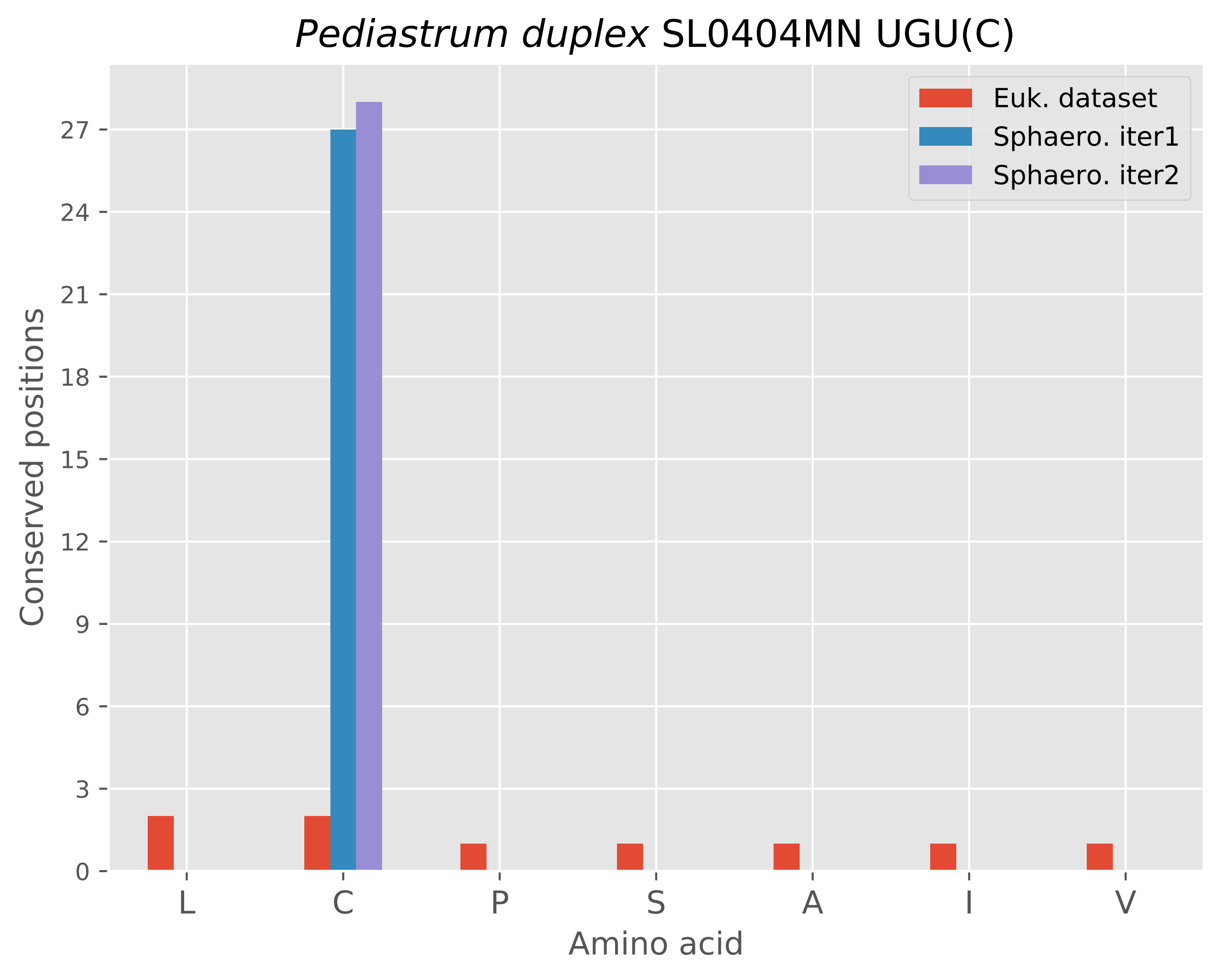

Pediastrum duplex SL0404MN UGU(C)
Euk. dataset
Sphaero. iter1
Sphaero. iter2
Conserved positions
0
3
6
9
12
15
18
21
24
27
L
C
P
S
A
I
V
Amino acid

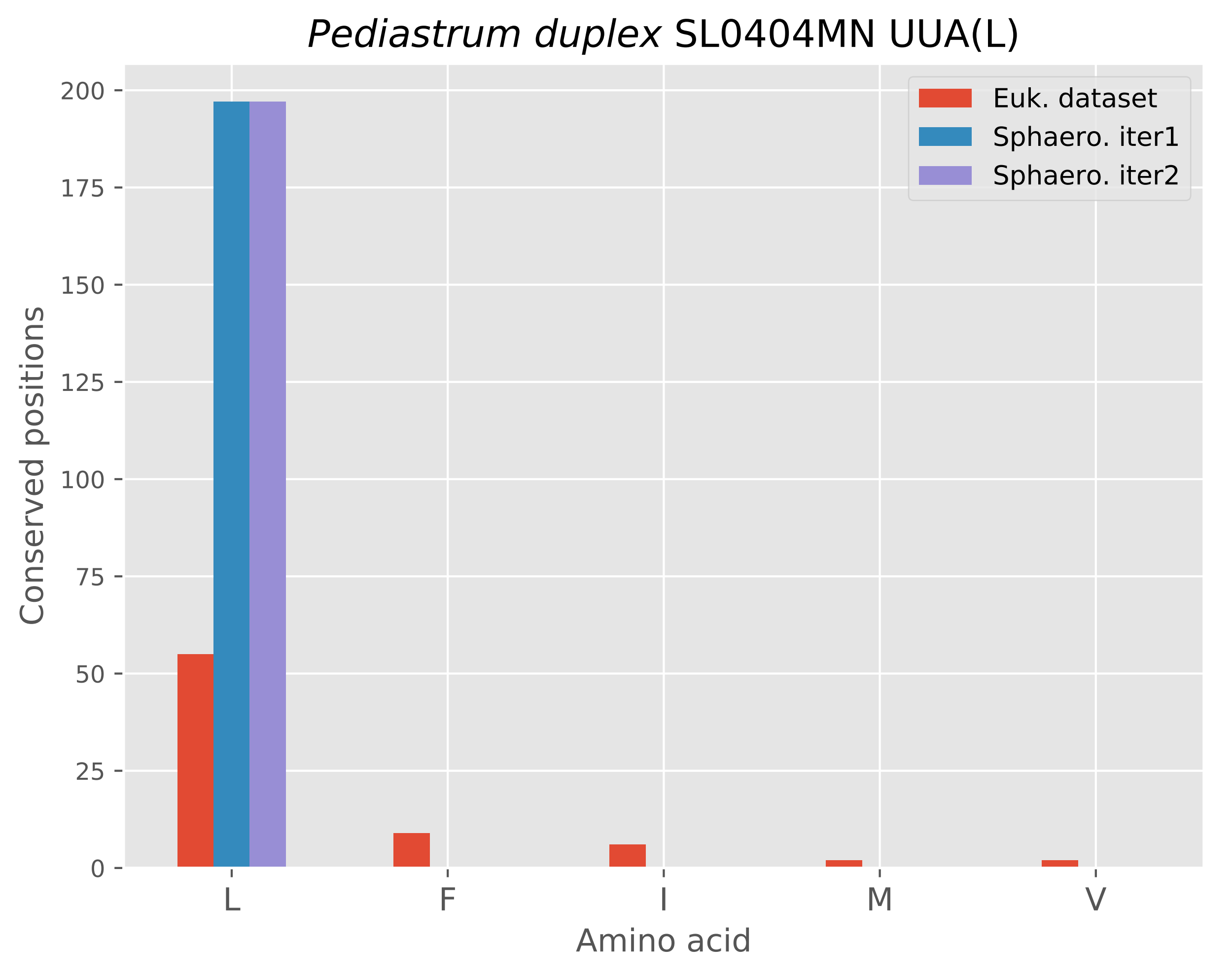
Pediastrum duplex SL0404MN UUA(L)
Euk. dataset
Sphaero. iter1
Sphaero. iter2
Conserved positions
0
25
50
75
100
125
150
175
200
L
F
I
M
V
Amino acid

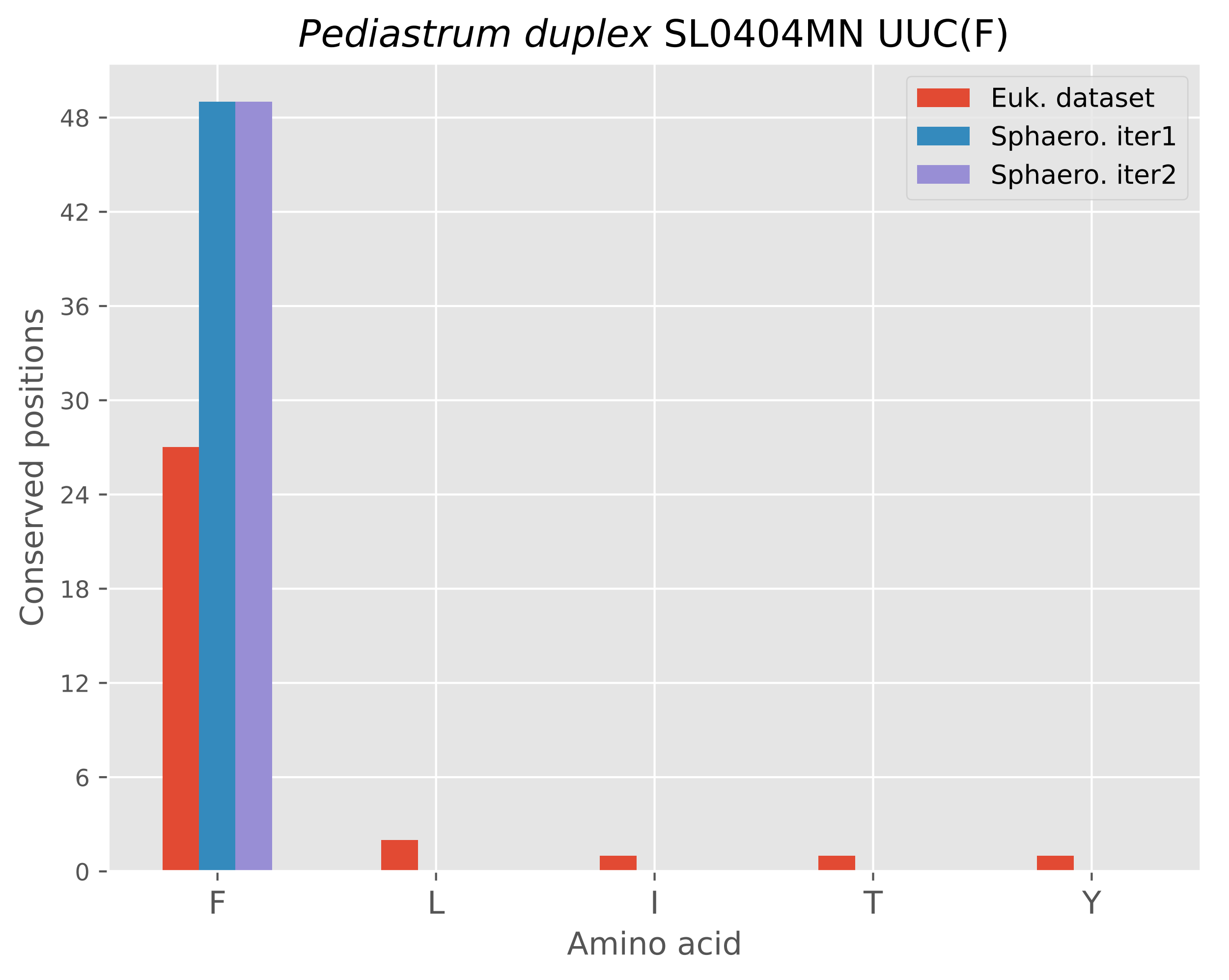
Pediastrum duplex SL0404MN UUC(F)
Euk. dataset
Sphaero. iter1
Sphaero. iter2
Conserved positions
0
6
12
18
24
30
36
42
48
F
L
I
T
Y
Amino acid

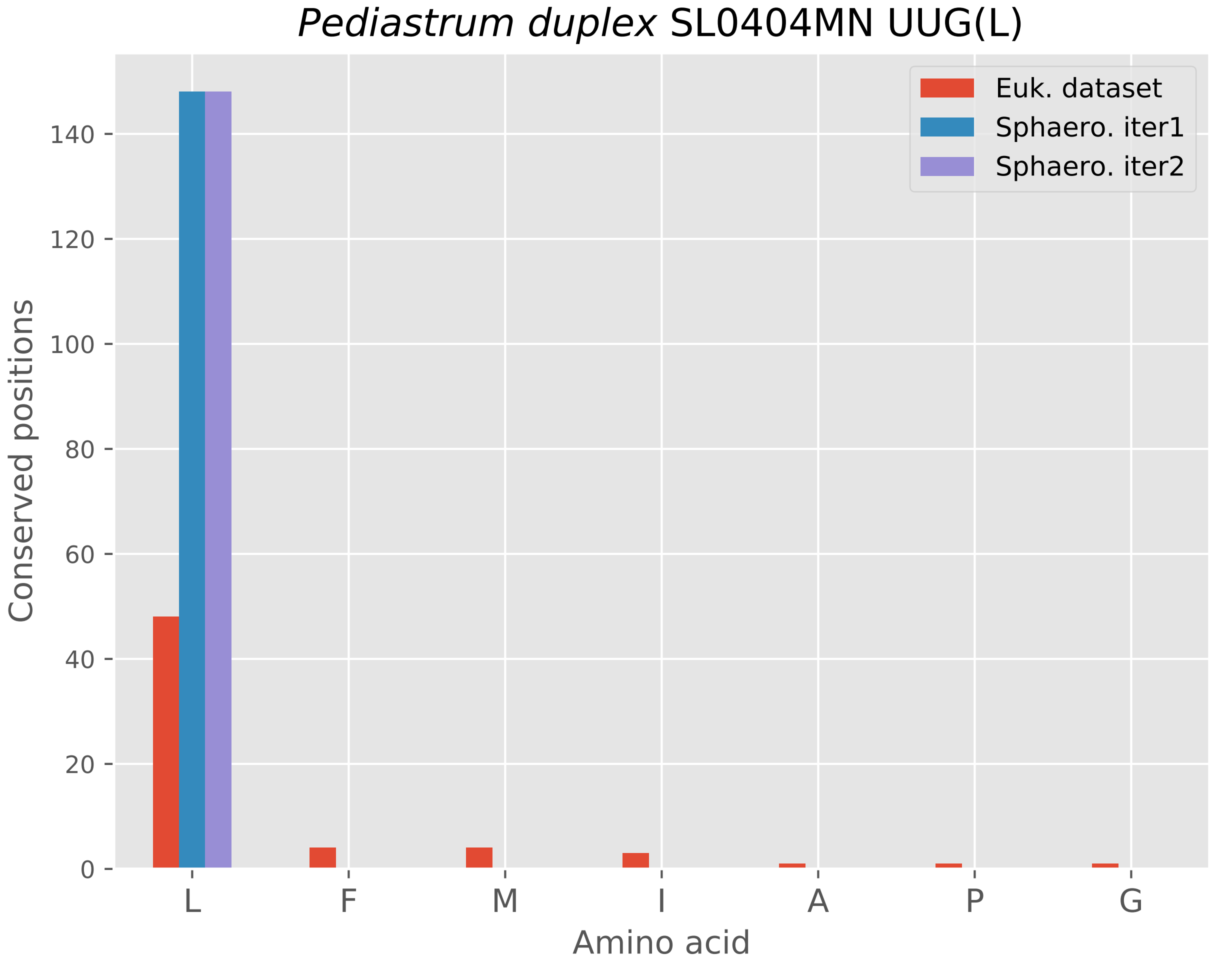

Pediastrum duplex SL0404MN UUG(L)
Euk. dataset
Sphaero. iter1
Sphaero. iter2
Conserved positions
0
20
40
60
80
100
120
140
L
F
M
I
A
P
G
Amino acid

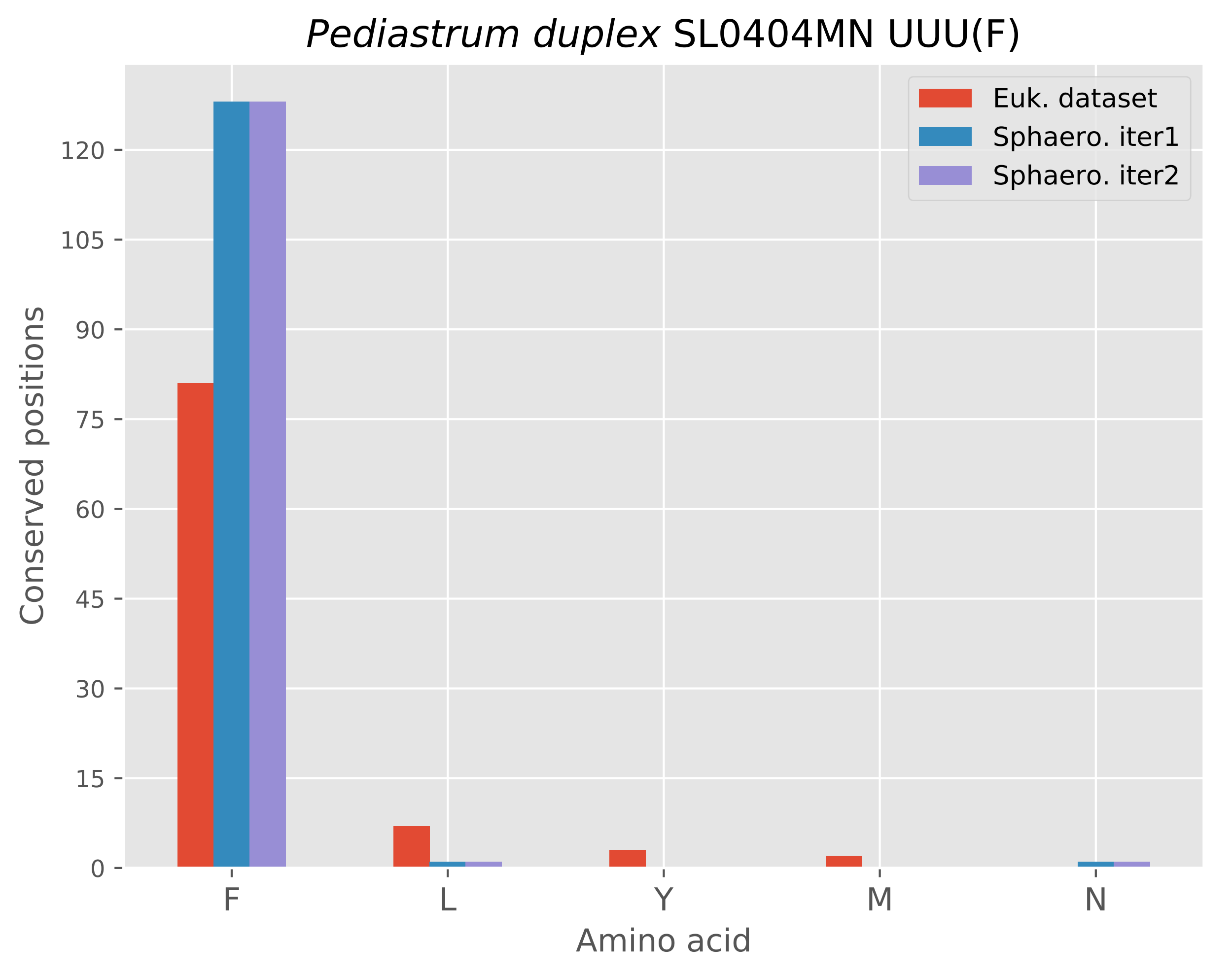

Pediastrum duplex SL0404MN UUU(F)
Euk. dataset
Sphaero. iter1
Sphaero. iter2
Conserved positions
0
15
30
45
60
75
90
105
120
F
L
Y
M
N
Amino acid

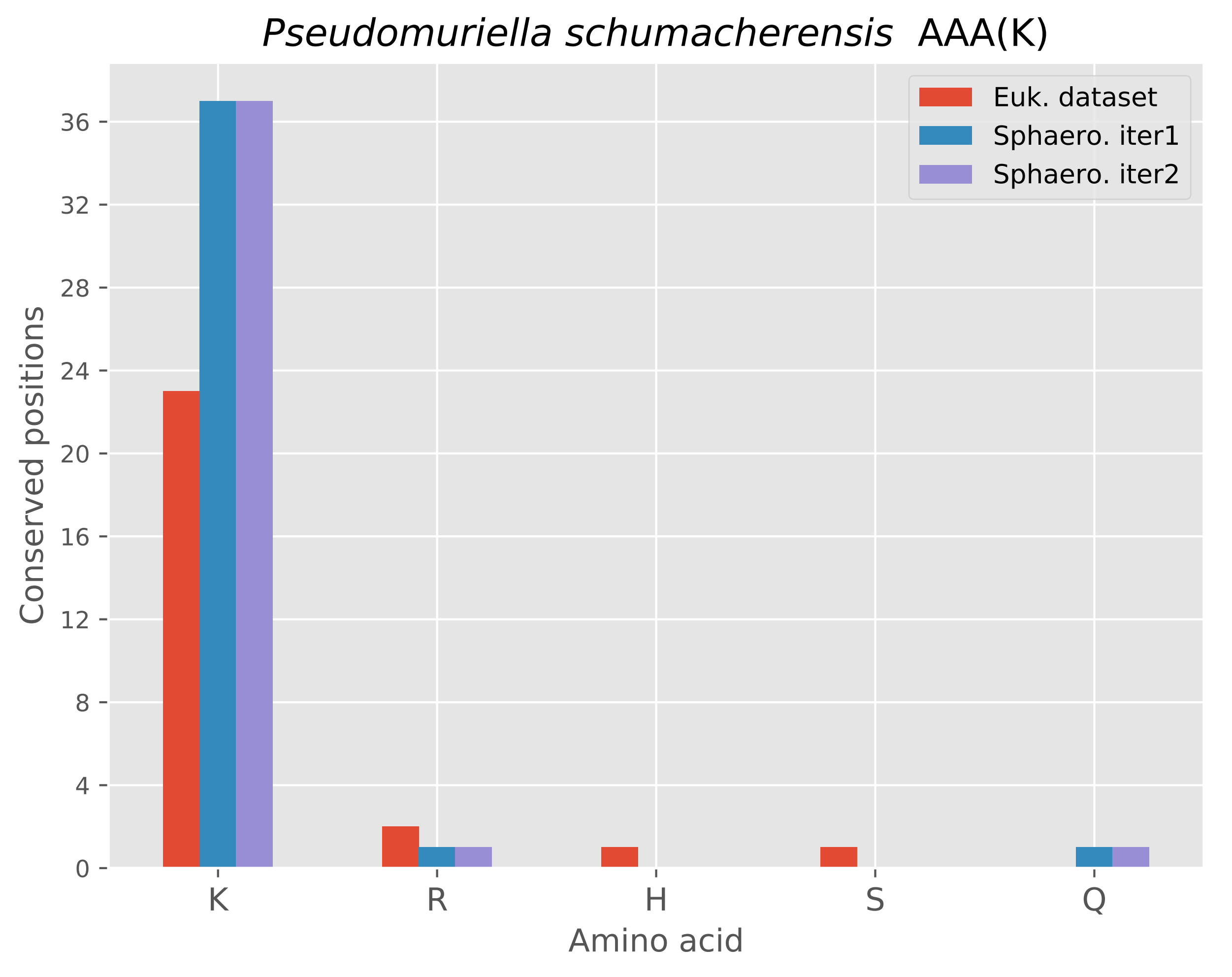
Pseudomuriella schumacherensis AAA(K)
Euk. dataset
Sphaero. iter1
Sphaero. iter2
Conserved positions
0
4
8
12
16
20
24
28
32
36
K
R
H
S
Q
Amino acid

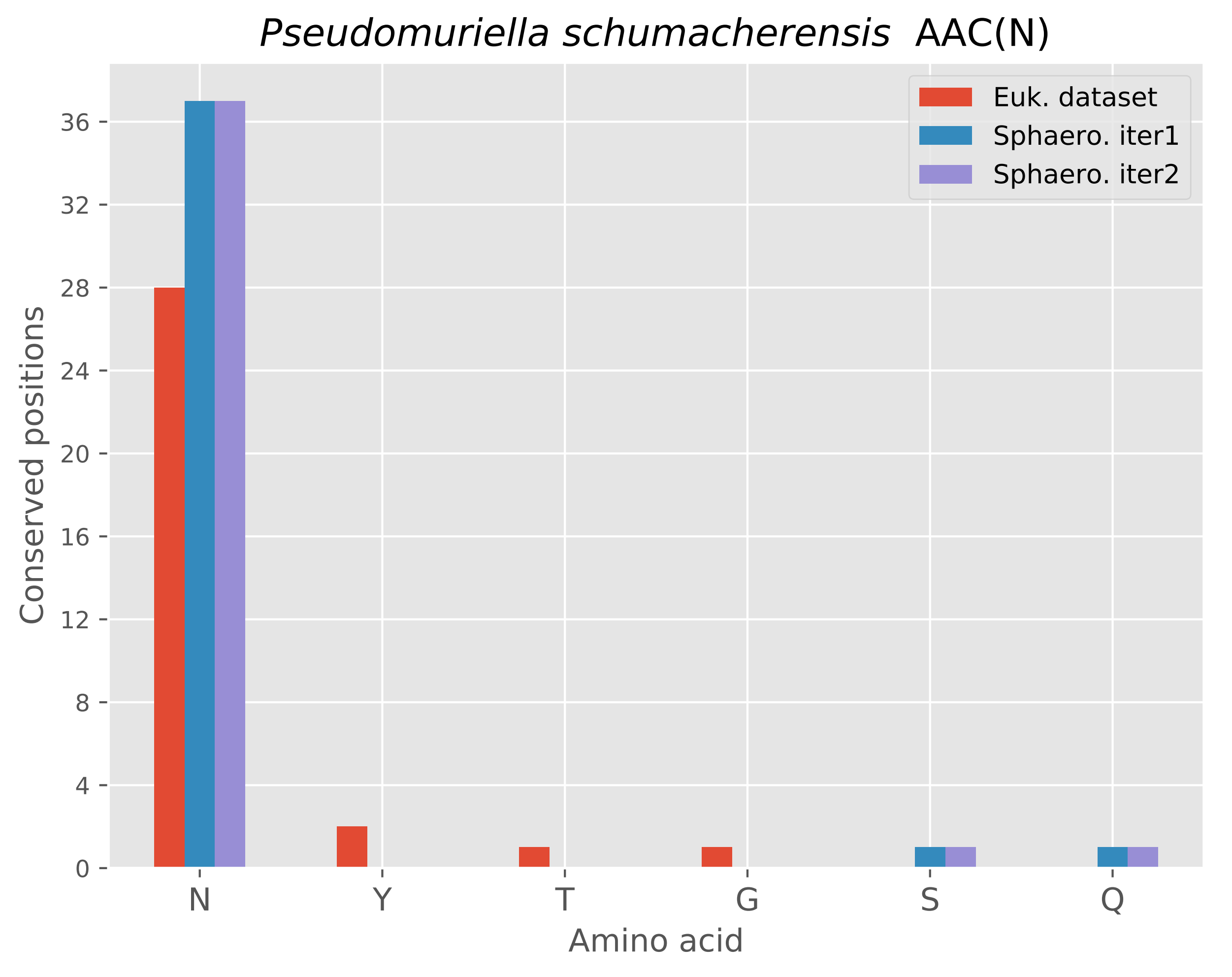

Pseudomuriella schumacherensis AAC(N)
Euk. dataset
Sphaero. iter1
Sphaero. iter2
Conserved positions
0
4
8
12
16
20
24
28
32
36
N
Y
T
G
S
Q
Amino acid

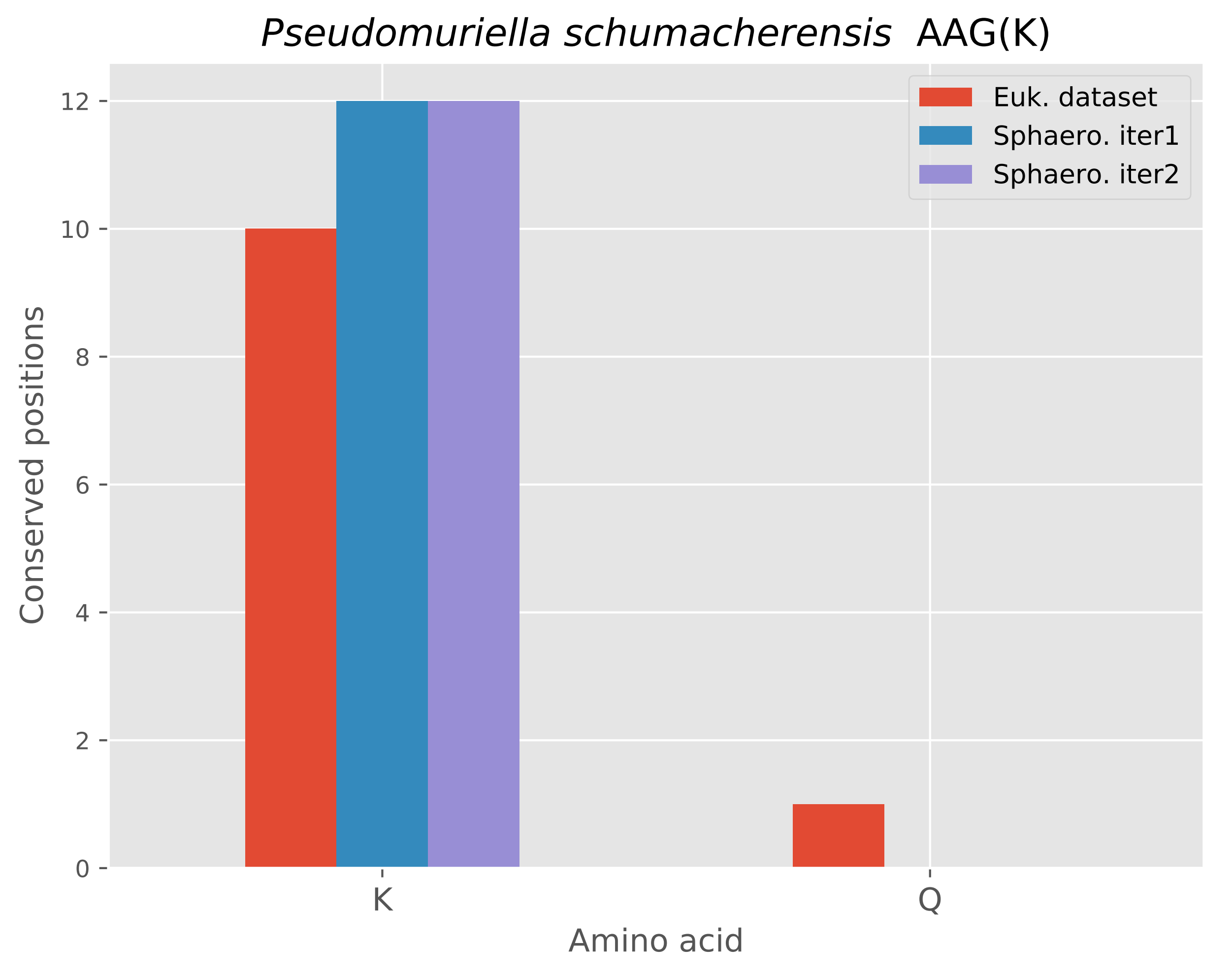

Pseudomuriella schumacherensis AAG(K)
Euk. dataset
Sphaero. iter1
Sphaero. iter2
Conserved positions
0
2
4
6
8
10
12
K
Q
Amino acid

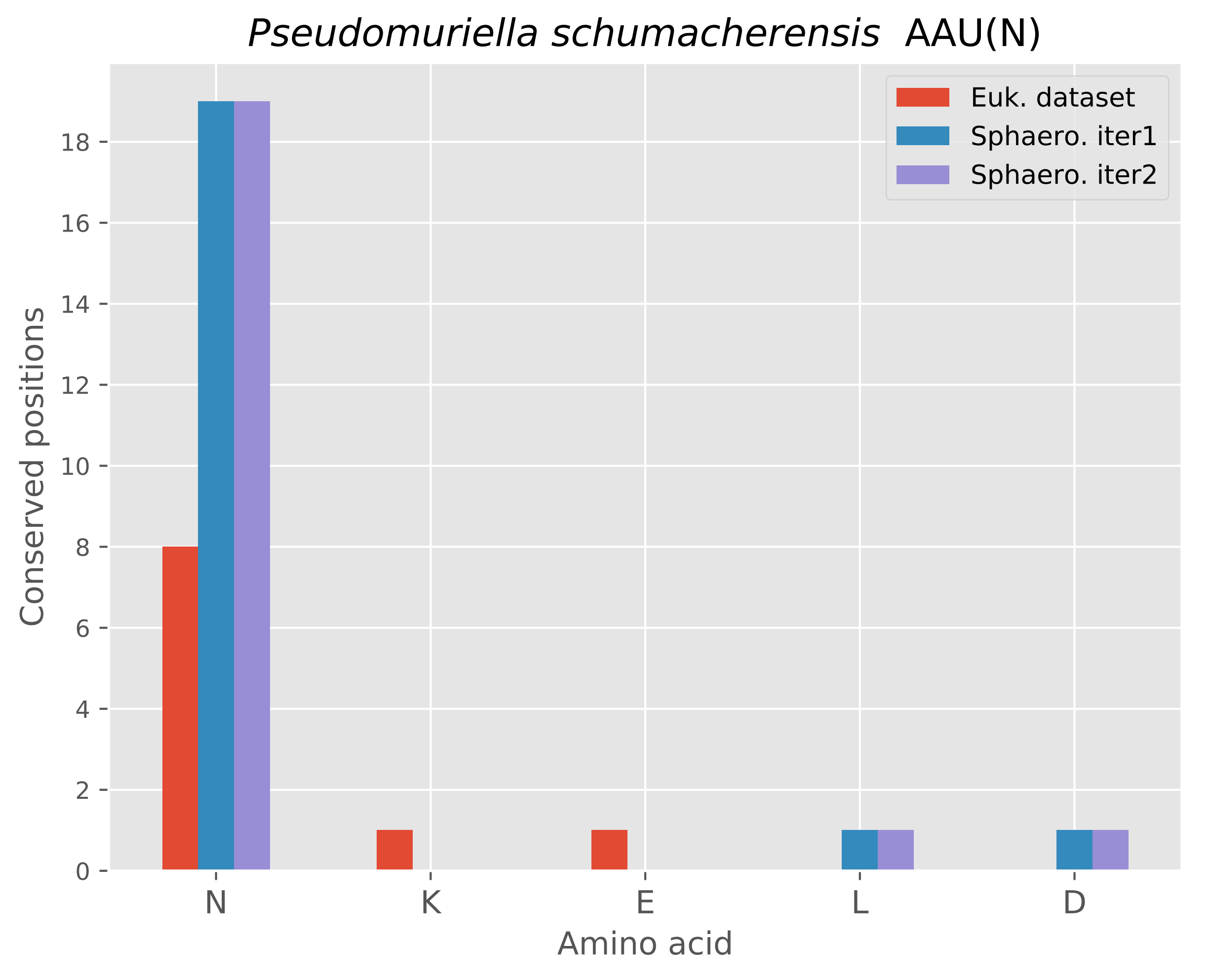
Pseudomuriella schumacherensis AAU(N)
Euk. dataset
Sphaero. iter1
Sphaero. iter2
Conserved positions
0
2
4
6
8
10
12
14
16
18
N
K
E
L
D
Amino acid

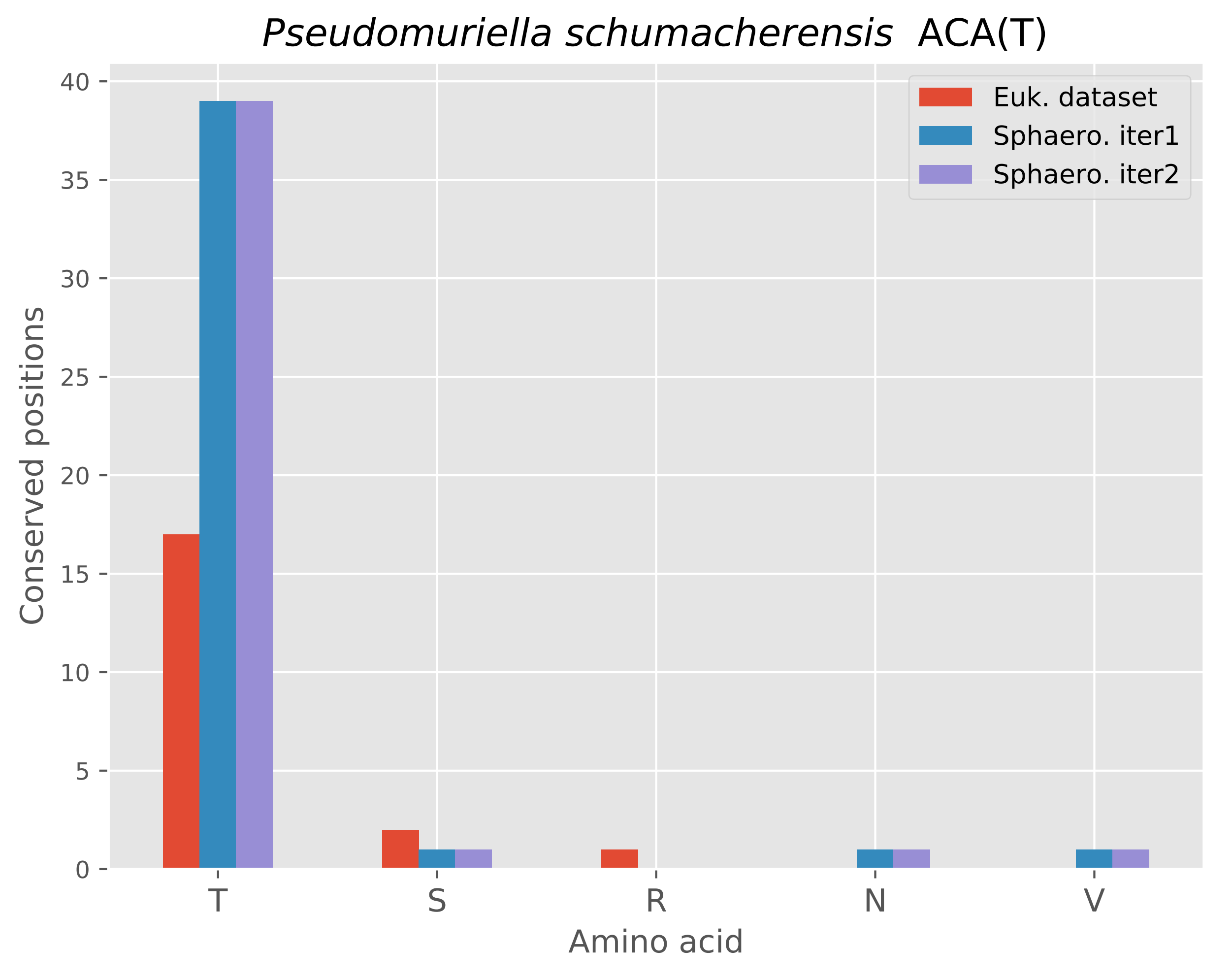

Pseudomuriella schumacherensis ACA(T)
Euk. dataset
Sphaero. iter1
Sphaero. iter2
Conserved positions
0
5
10
15
20
25
30
35
40
T
S
R
N
V
Amino acid

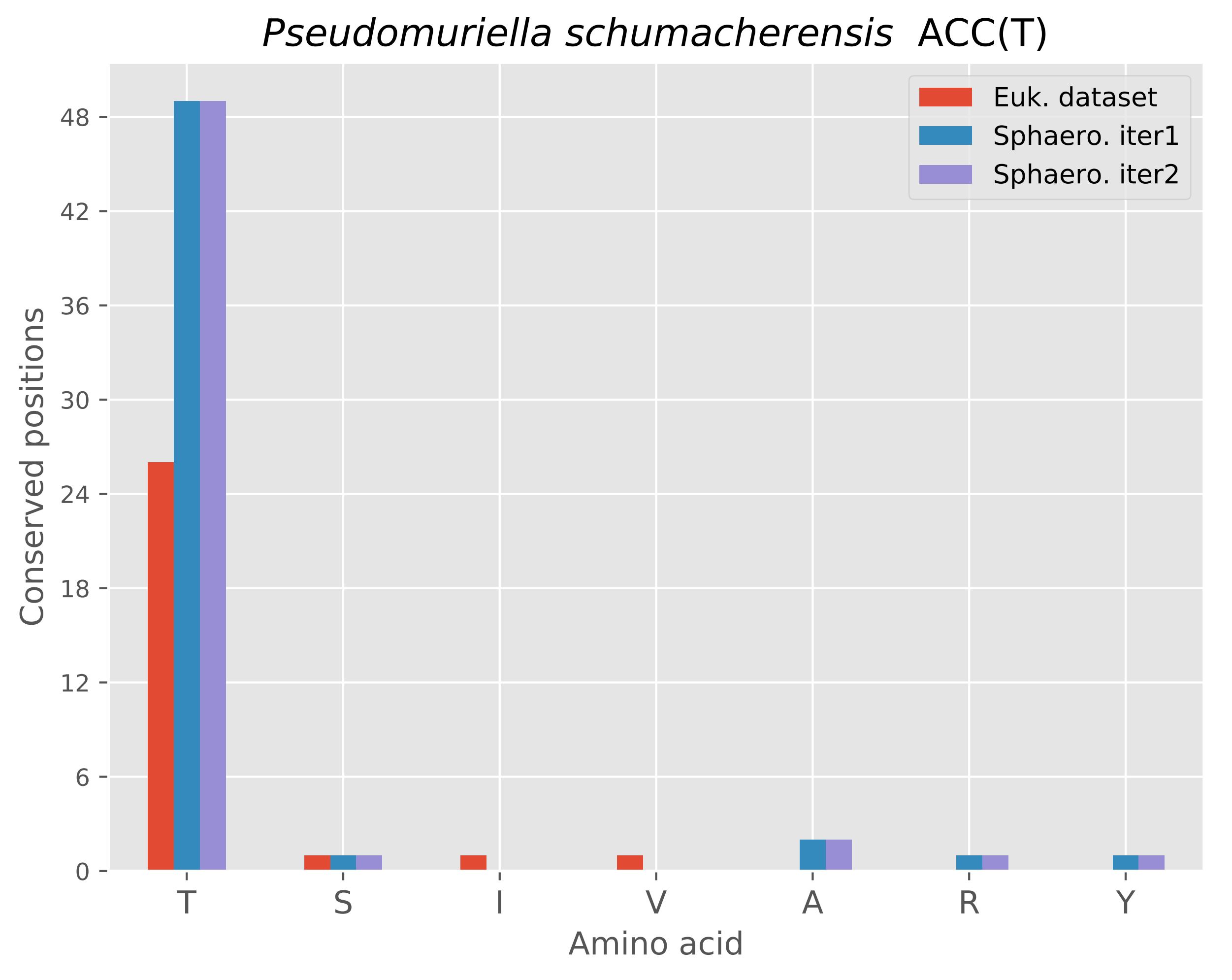

Pseudomuriella schumacherensis ACC(T)
Euk. dataset
Sphaero. iter1
Sphaero. iter2
Conserved positions
0
6
12
18
24
30
36
42
48
T
S
I
V
A
R
Y
Amino acid

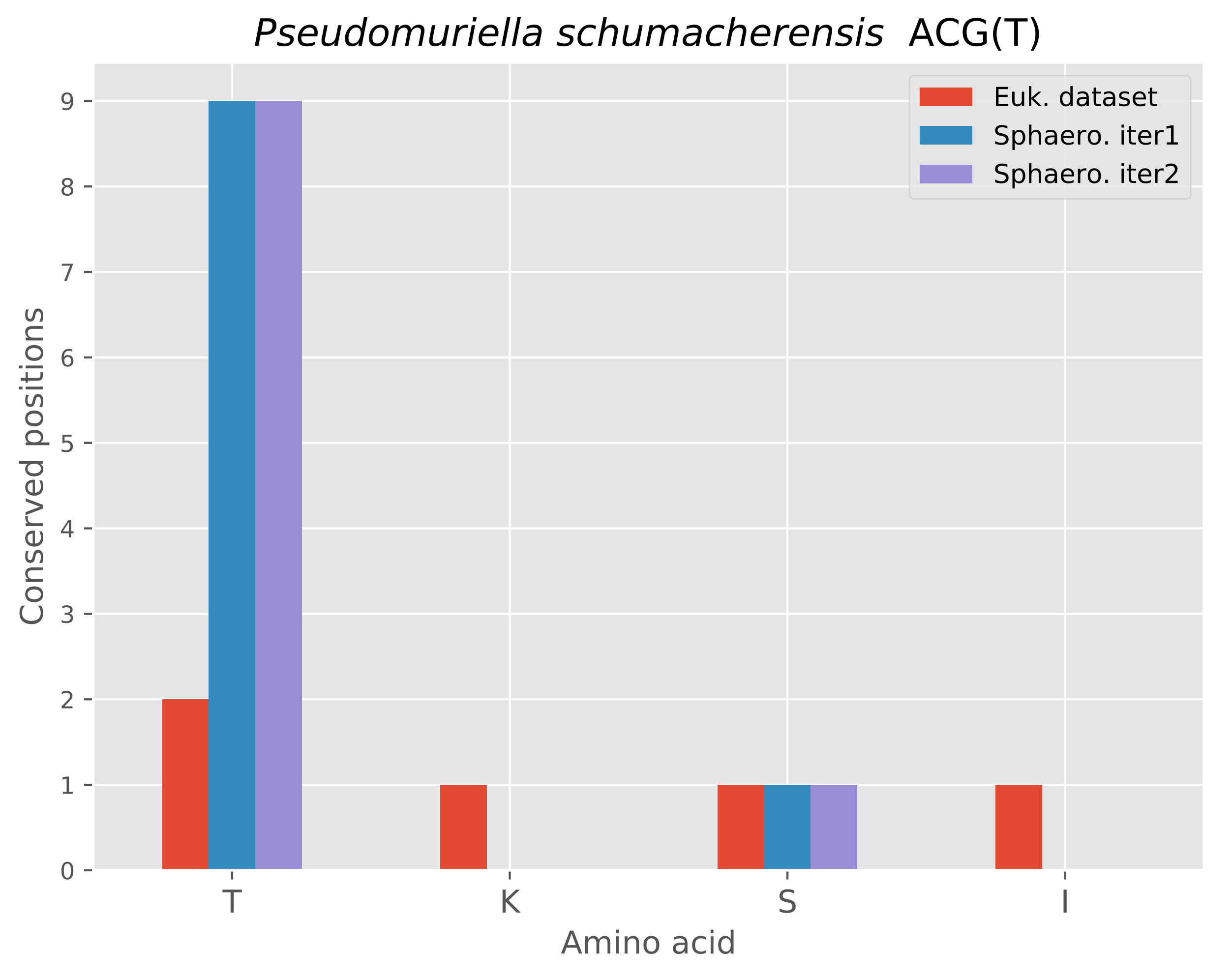

Pseudomuriella schumacherensis ACG(T)
Euk. dataset
Sphaero. iter1
Sphaero. iter2
Conserved positions
0
1
2
3
4
5
6
7
8
9
T
K
S
I
Amino acid

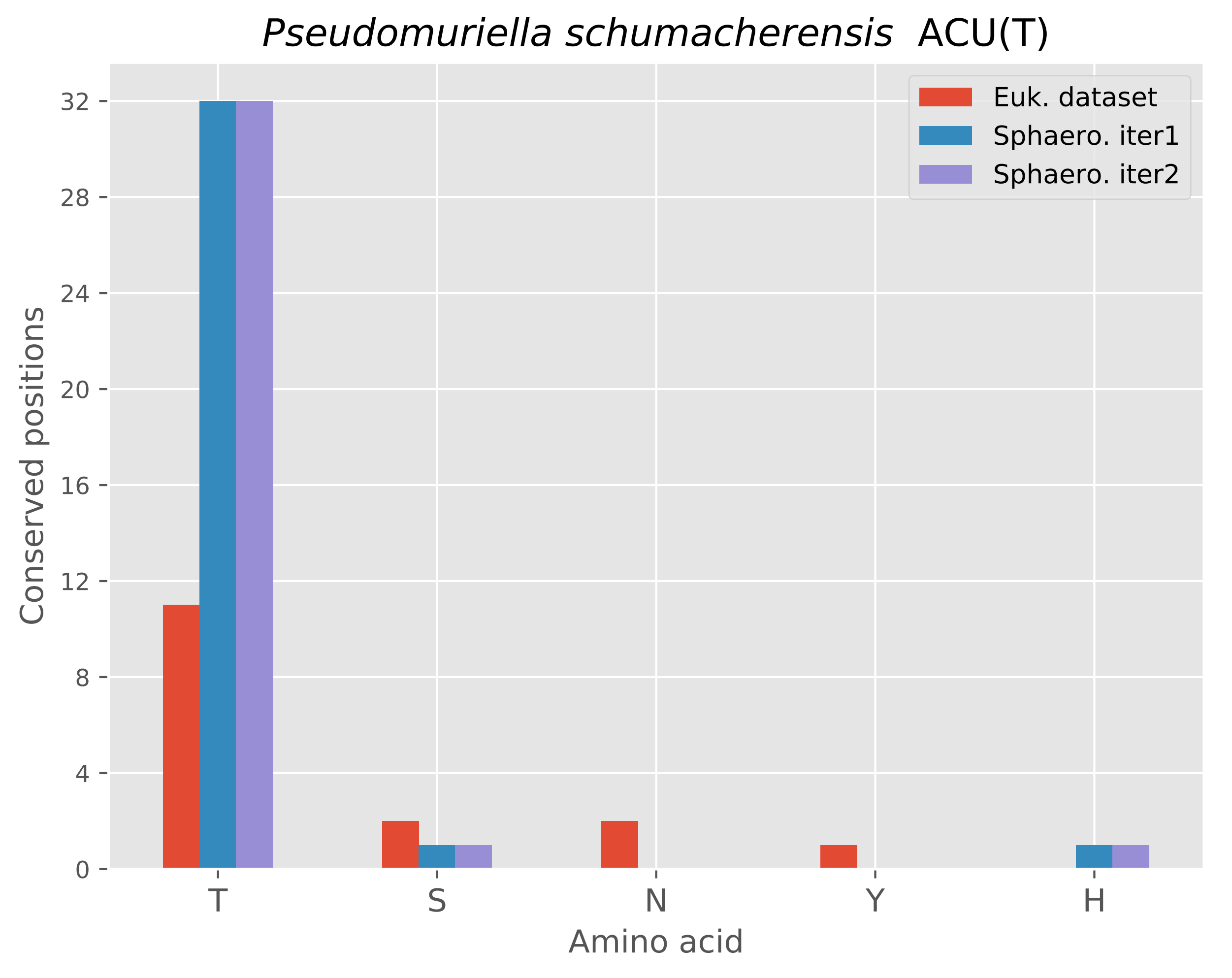

Pseudomuriella schumacherensis ACU(T)
Euk. dataset
Sphaero. iter1
Sphaero. iter2
Conserved positions
0
4
8
12
16
20
24
28
32
T
S
N
Y
H
Amino acid

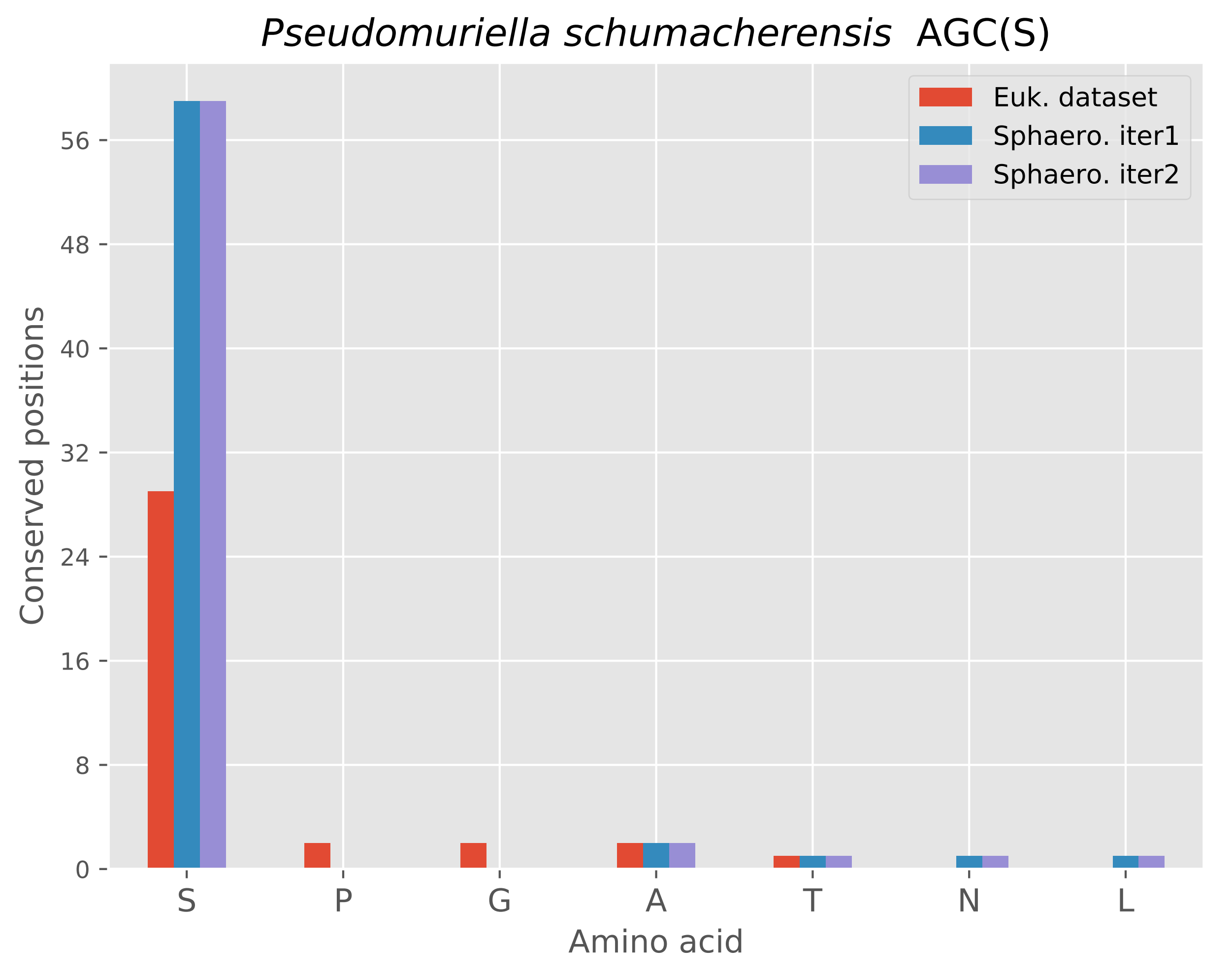

Pseudomuriella schumacherensis AGC(S)
Euk. dataset
Sphaero. iter1
Sphaero. iter2
Conserved positions
0
8
16
24
32
40
48
56
S
P
G
A
T
N
L
Amino acid

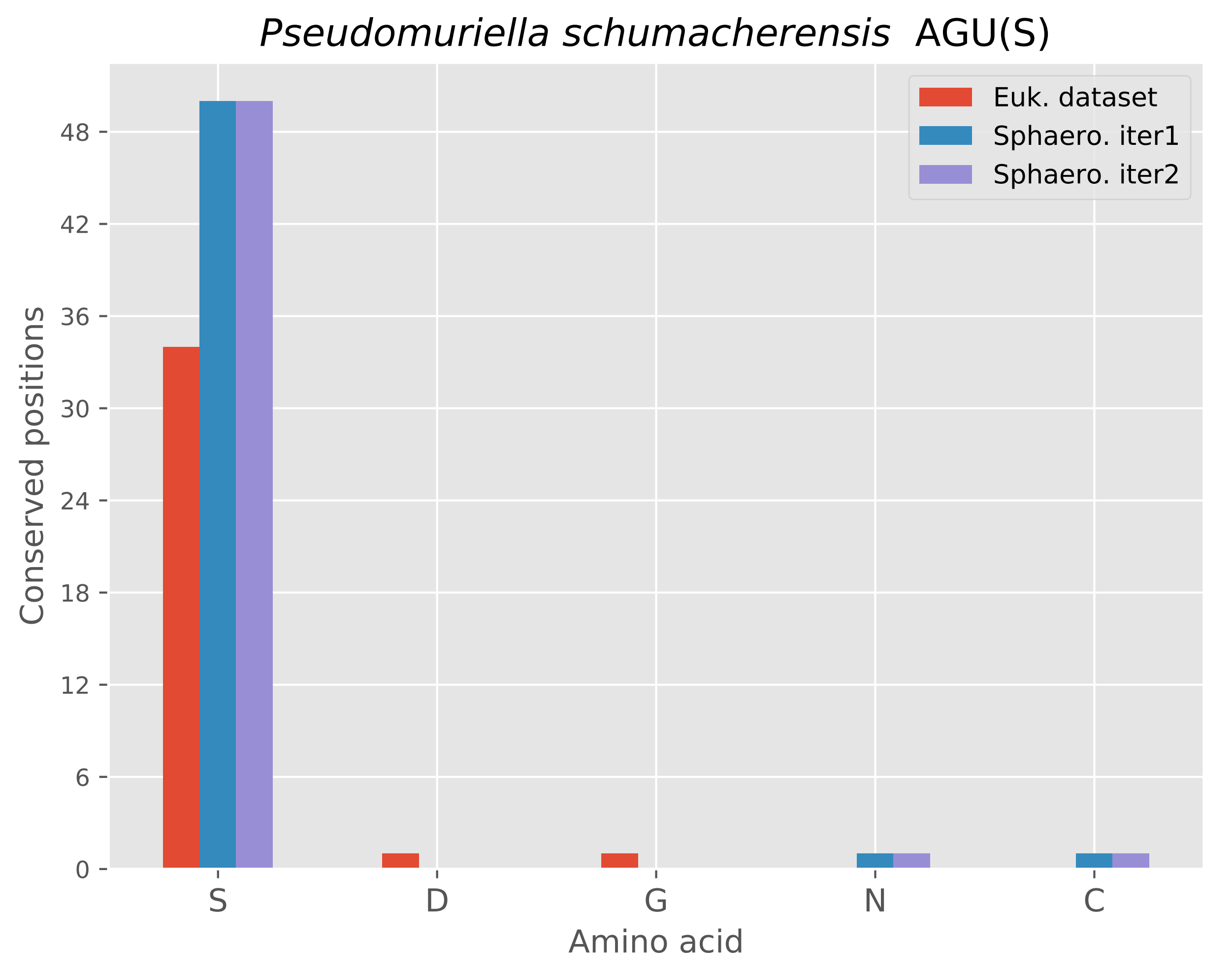

Pseudomuriella schumacherensis AGU(S)
Euk. dataset
Sphaero. iter1
Sphaero. iter2
Conserved positions
0
6
12
18
24
30
36
42
48
S
D
G
N
C
Amino acid

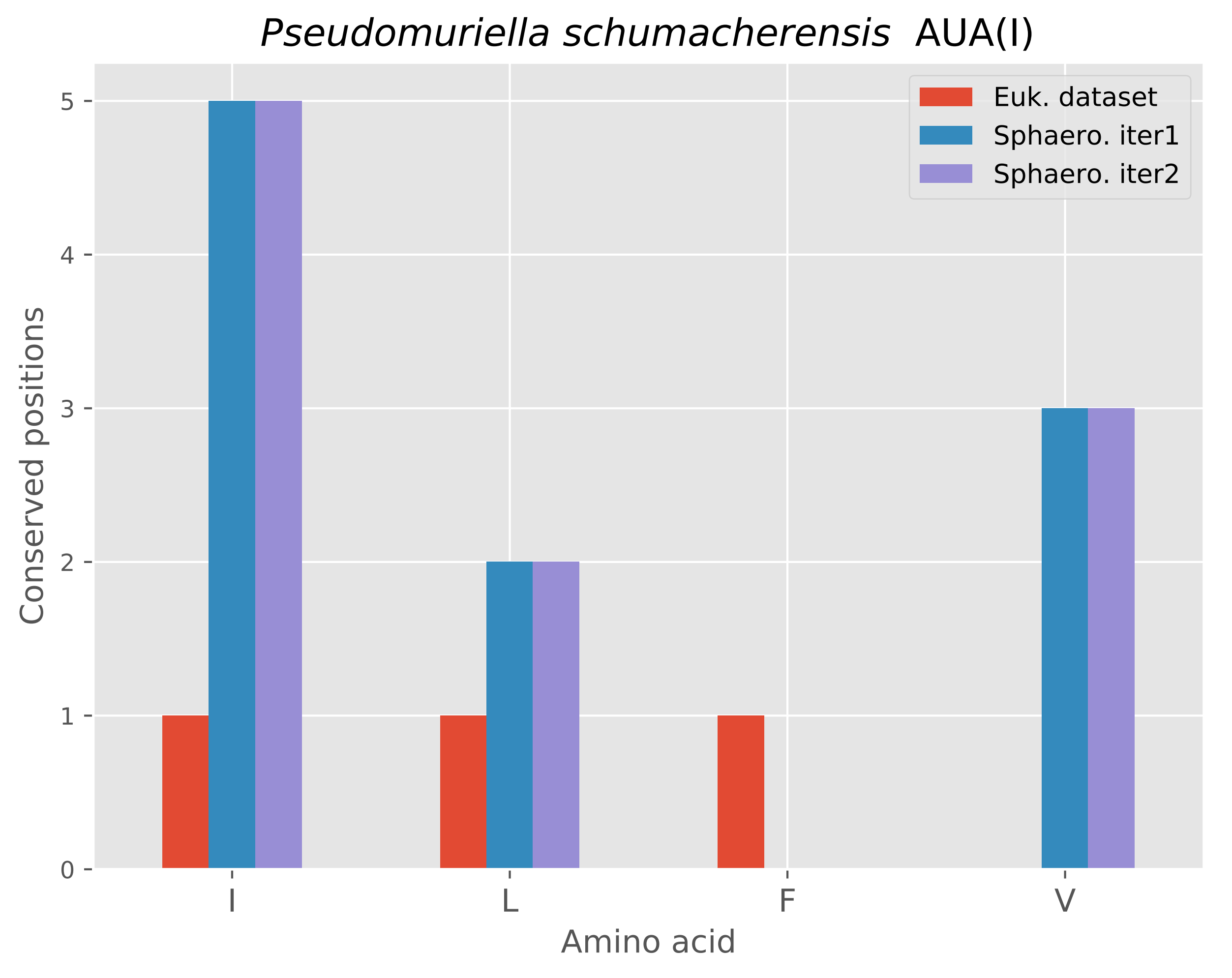

Pseudomuriella schumacherensis AUA(I)
Euk. dataset
Sphaero. iter1
Sphaero. iter2
Conserved positions
0
1
2
3
4
5
I
L
F
V
Amino acid

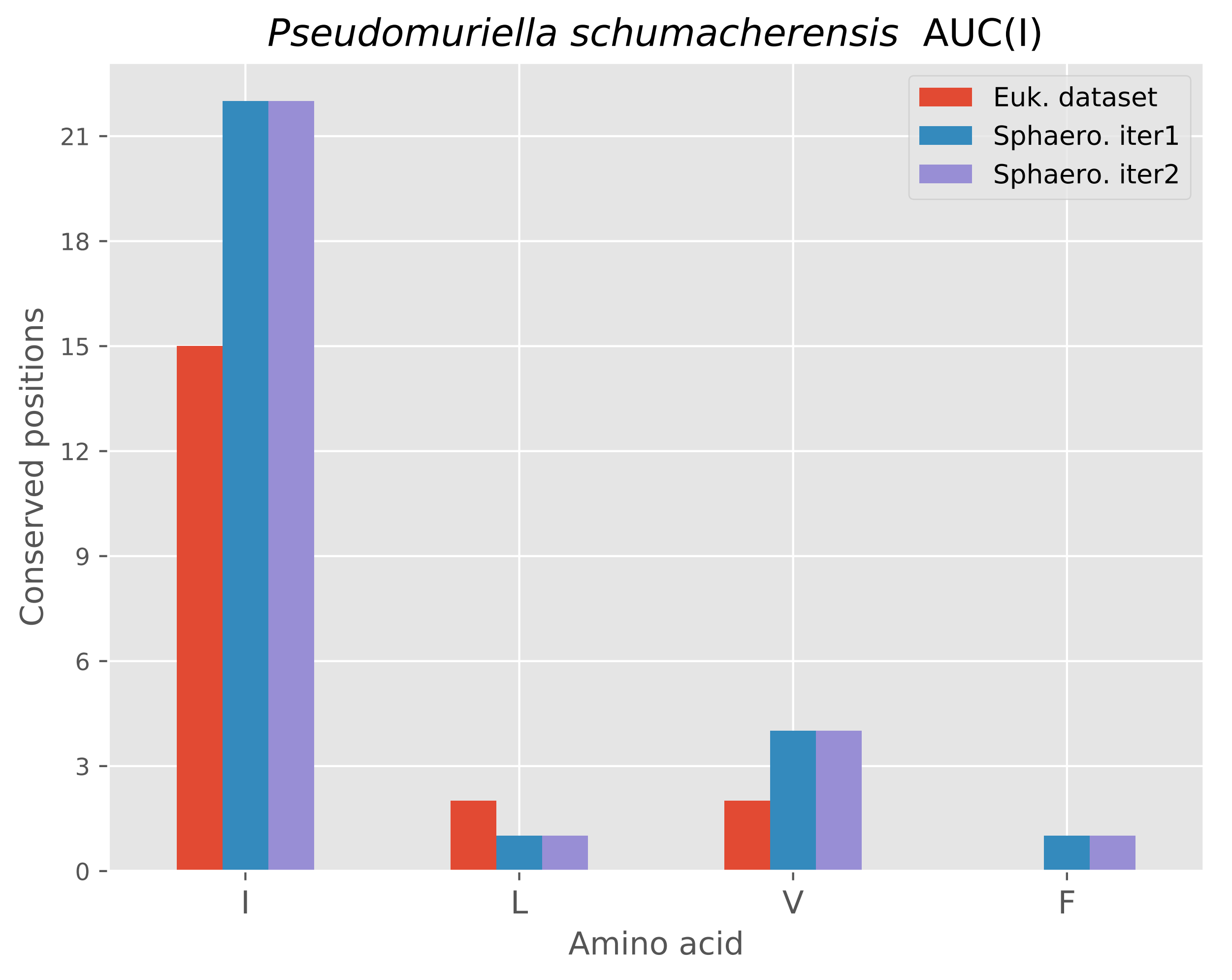

Pseudomuriella schumacherensis AUC(I)
Euk. dataset
Sphaero. iter1
Sphaero. iter2
Conserved positions
0
3
6
9
12
15
18
21
I
L
V
F
Amino acid

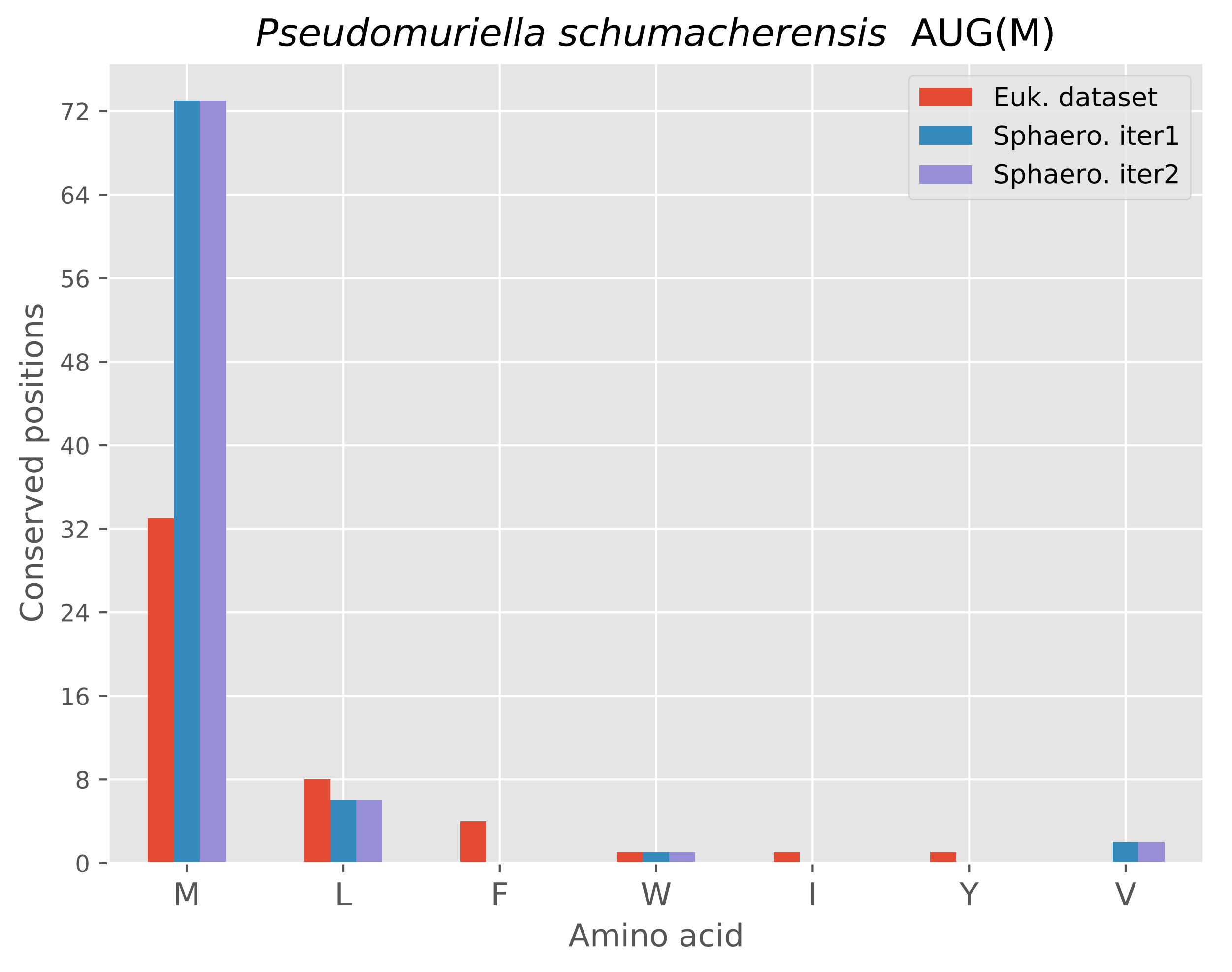
Pseudomuriella schumacherensis AUG(M)
Euk. dataset
Sphaero. iter1
Sphaero. iter2
Conserved positions
0
8
16
24
32
40
48
56
64
72
M
L
F
W
I
Y
V
Amino acid

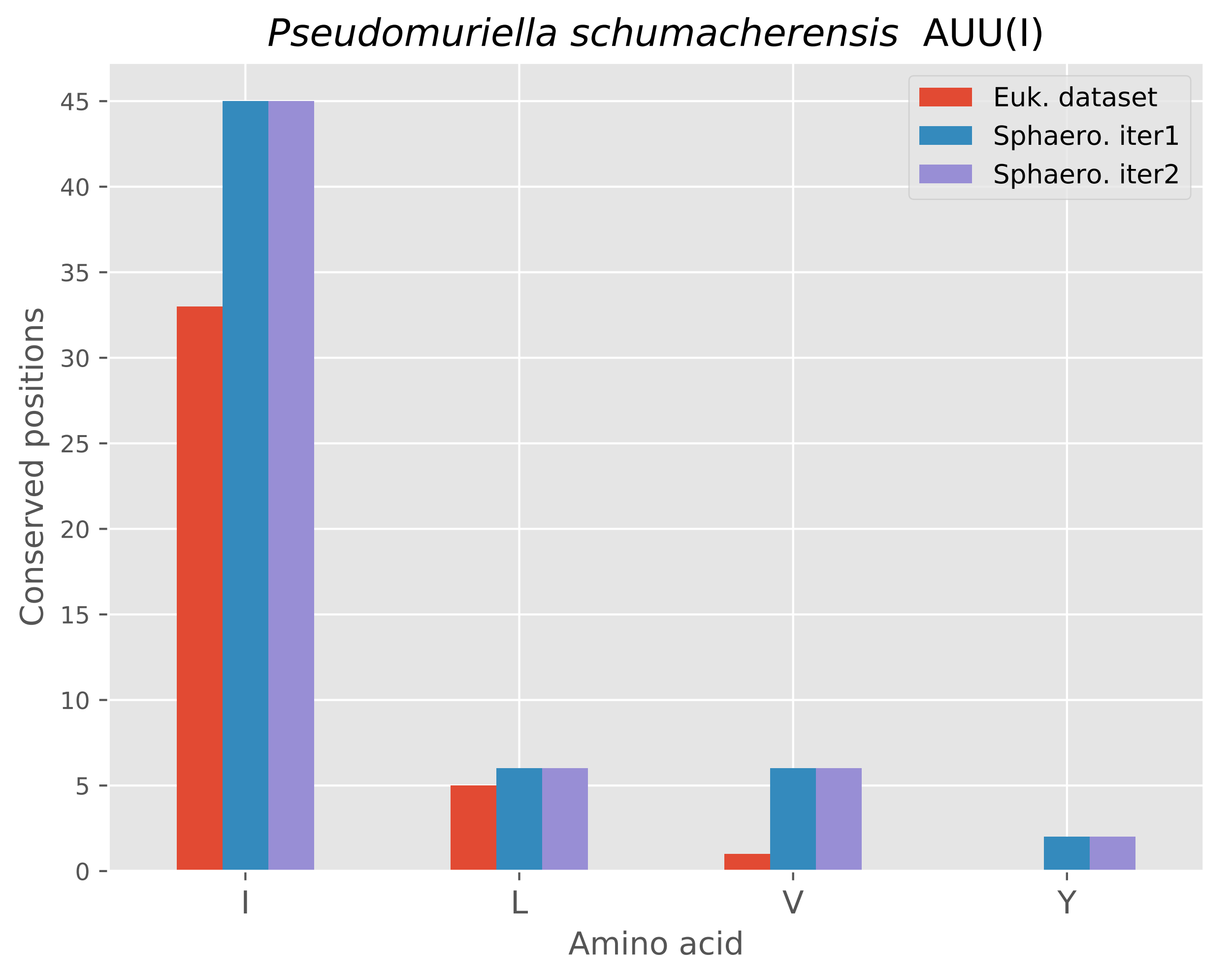

Pseudomuriella schumacherensis AUU(I)
Euk. dataset
Sphaero. iter1
Sphaero. iter2
Conserved positions
0
5
10
15
20
25
30
35
40
45
I
L
V
Y
Amino acid

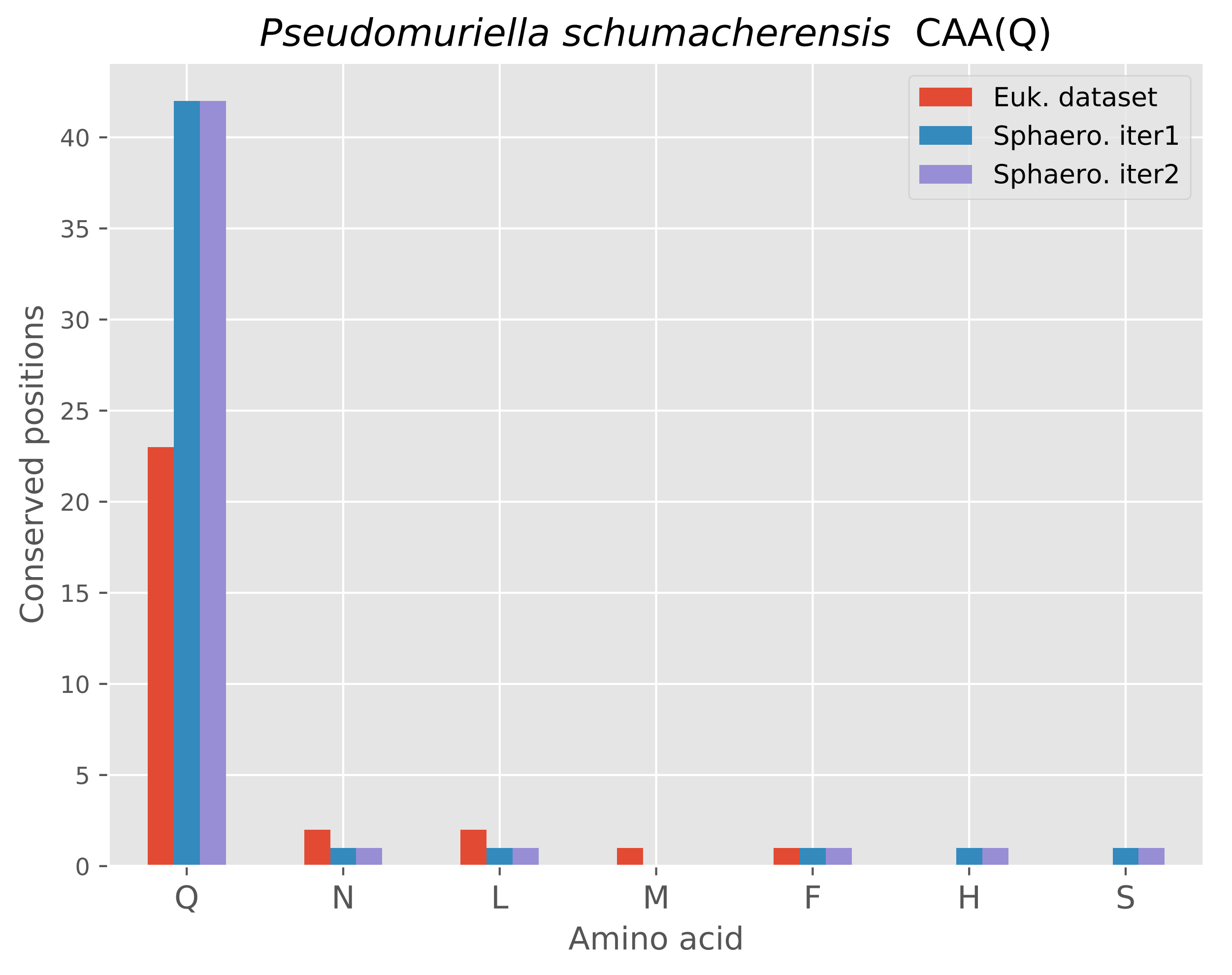

Pseudomuriella schumacherensis CAA(Q)
Euk. dataset
Sphaero. iter1
Sphaero. iter2
Conserved positions
0
5
10
15
20
25
30
35
40
Q
N
L
M
F
H
S
Amino acid

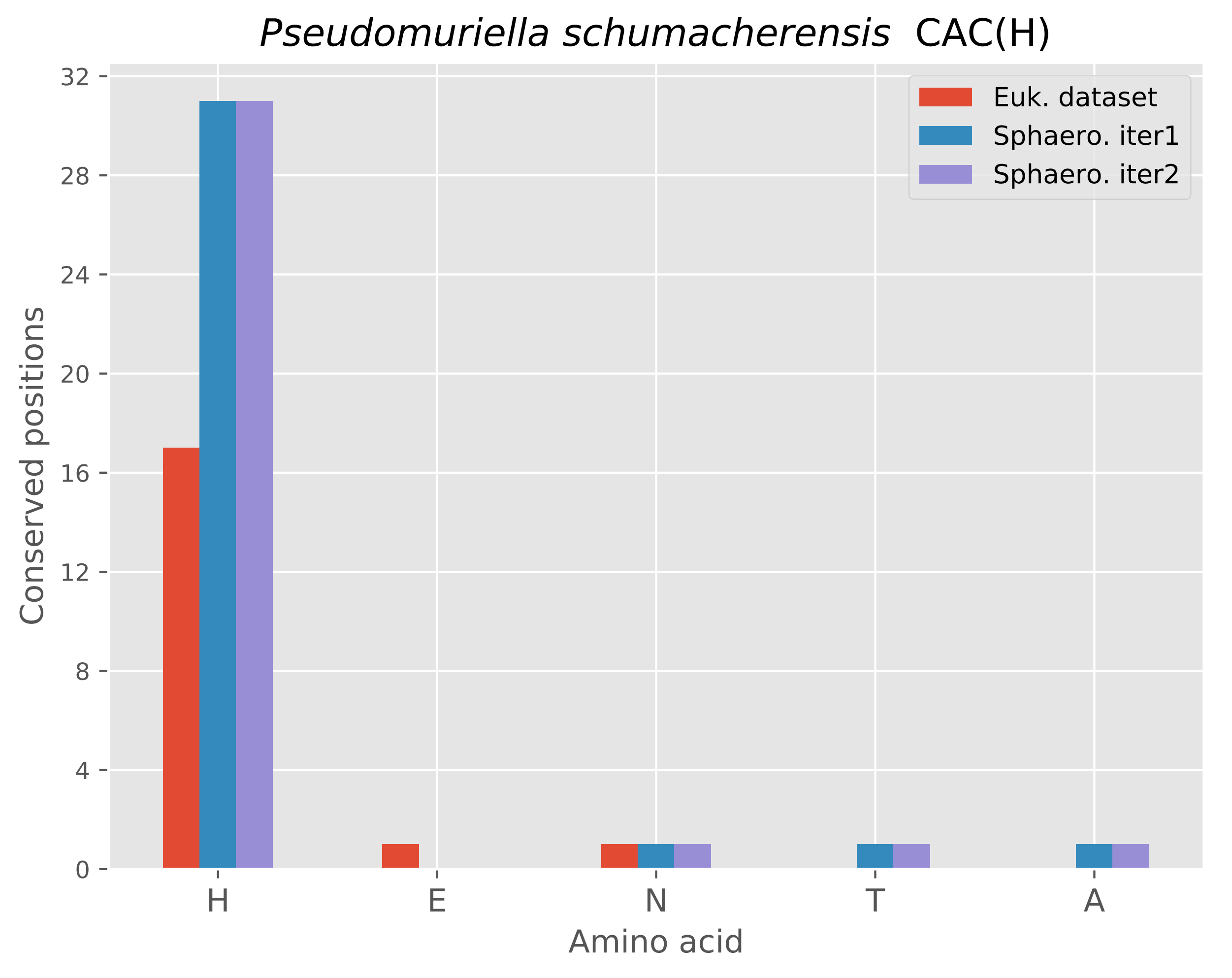

Pseudomuriella schumacherensis CAC(H)
Euk. dataset
Sphaero. iter1
Sphaero. iter2
Conserved positions
0
4
8
12
16
20
24
28
32
H
E
N
T
A
Amino acid

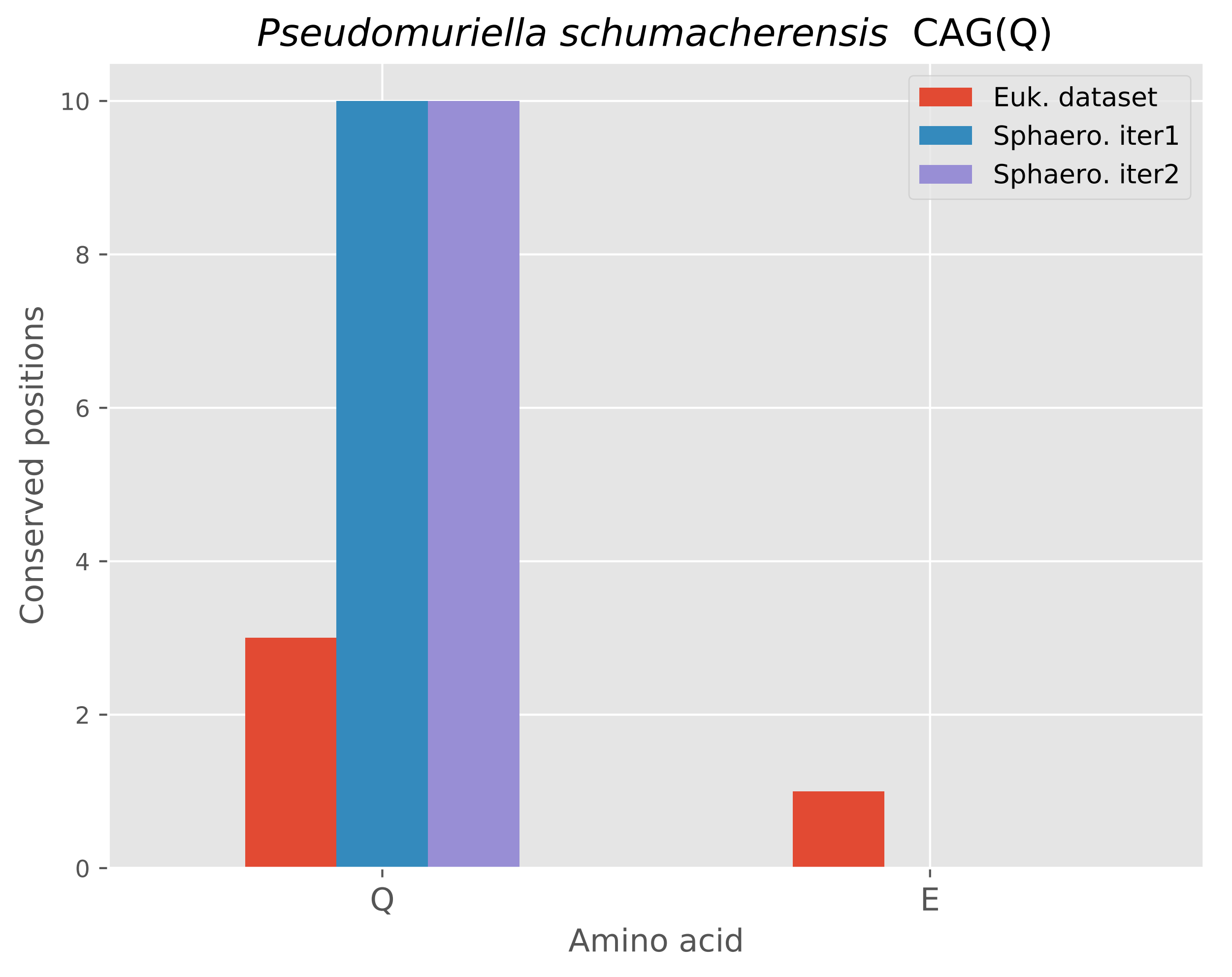

Pseudomuriella schumacherensis CAG(Q)
Euk. dataset
Sphaero. iter1
Sphaero. iter2
Conserved positions
0
2
4
6
8
10
Q
E
Amino acid

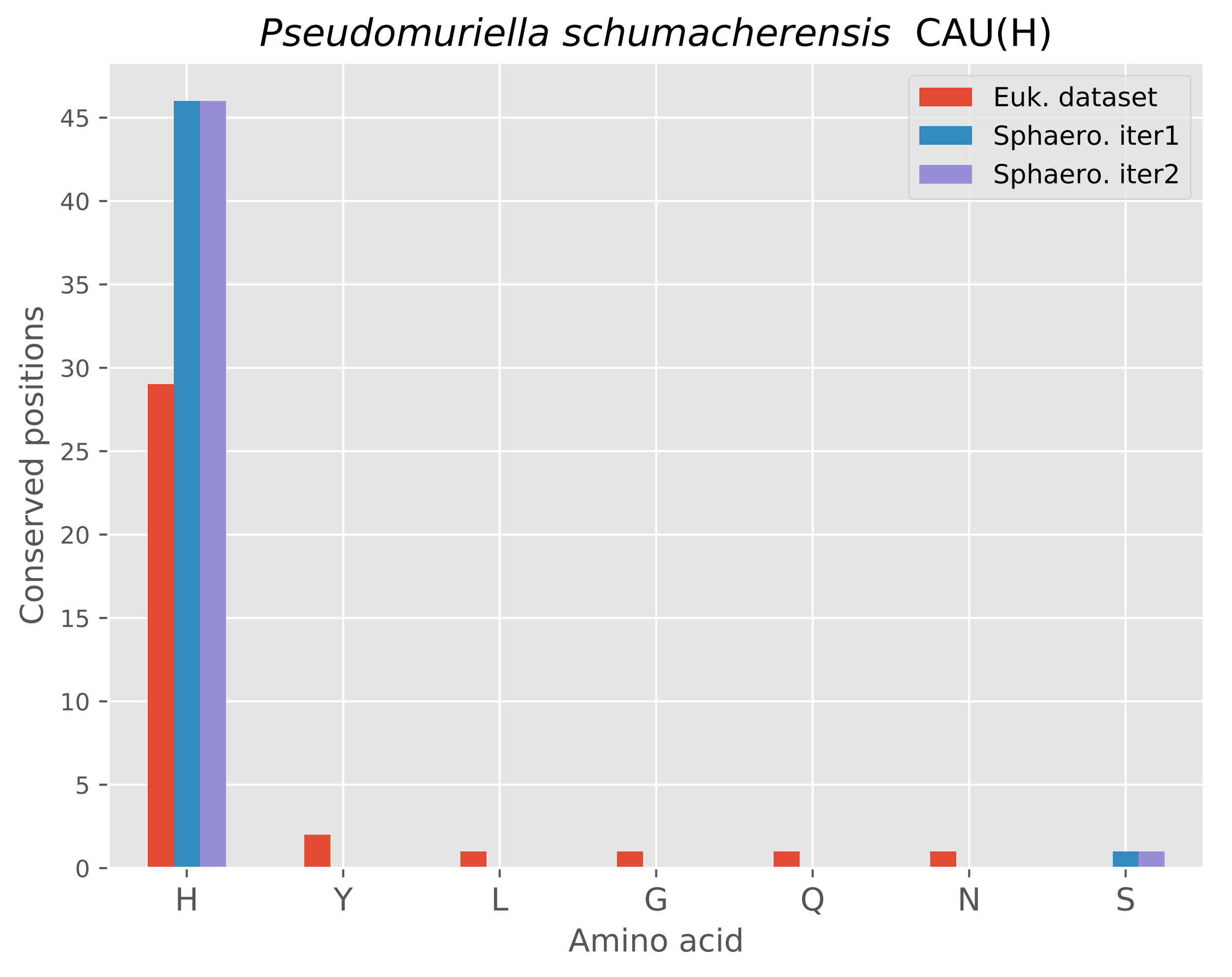

Pseudomuriella schumacherensis CAU(H)
Euk. dataset
Sphaero. iter1
Sphaero. iter2
Conserved positions
0
5
10
15
20
25
30
35
40
45
H
Y
L
G
Q
N
S
Amino acid

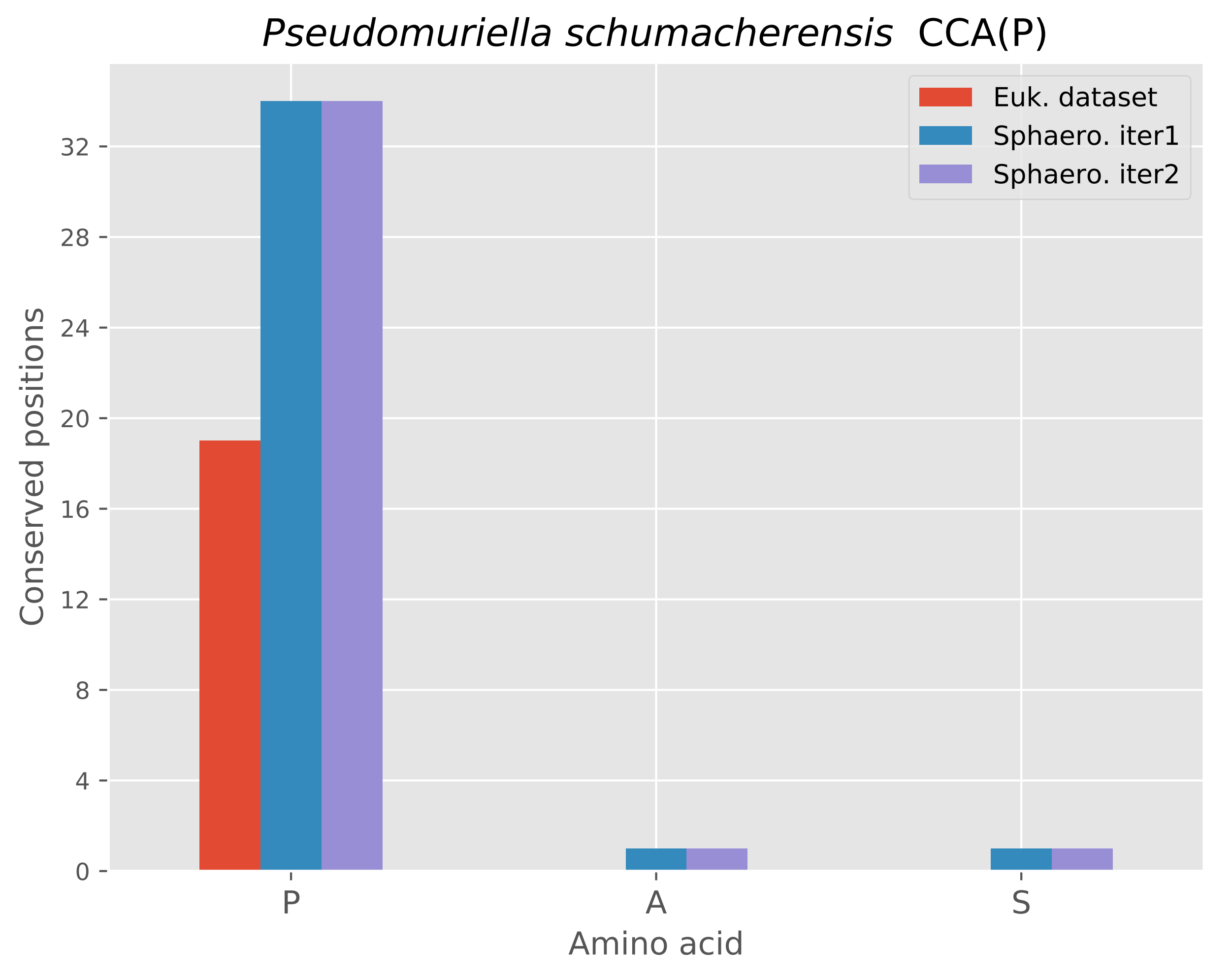

Pseudomuriella schumacherensis CCA(P)
Euk. dataset
Sphaero. iter1
Sphaero. iter2
Conserved positions
0
4
8
12
16
20
24
28
32
P
A
S
Amino acid

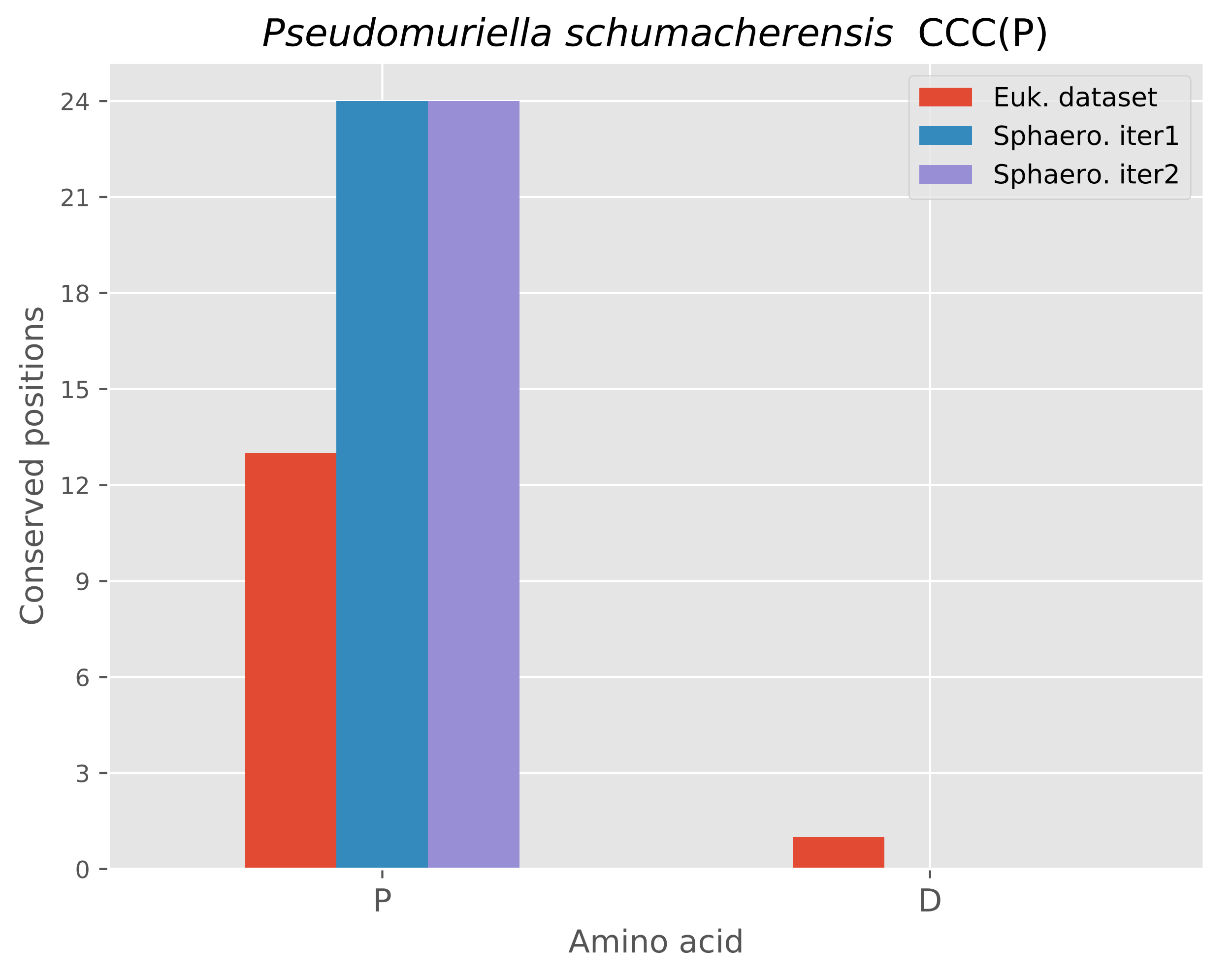

Pseudomuriella schumacherensis CCC(P)
Euk. dataset
Sphaero. iter1
Sphaero. iter2
Conserved positions
0
3
6
9
12
15
18
21
24
P
D
Amino acid

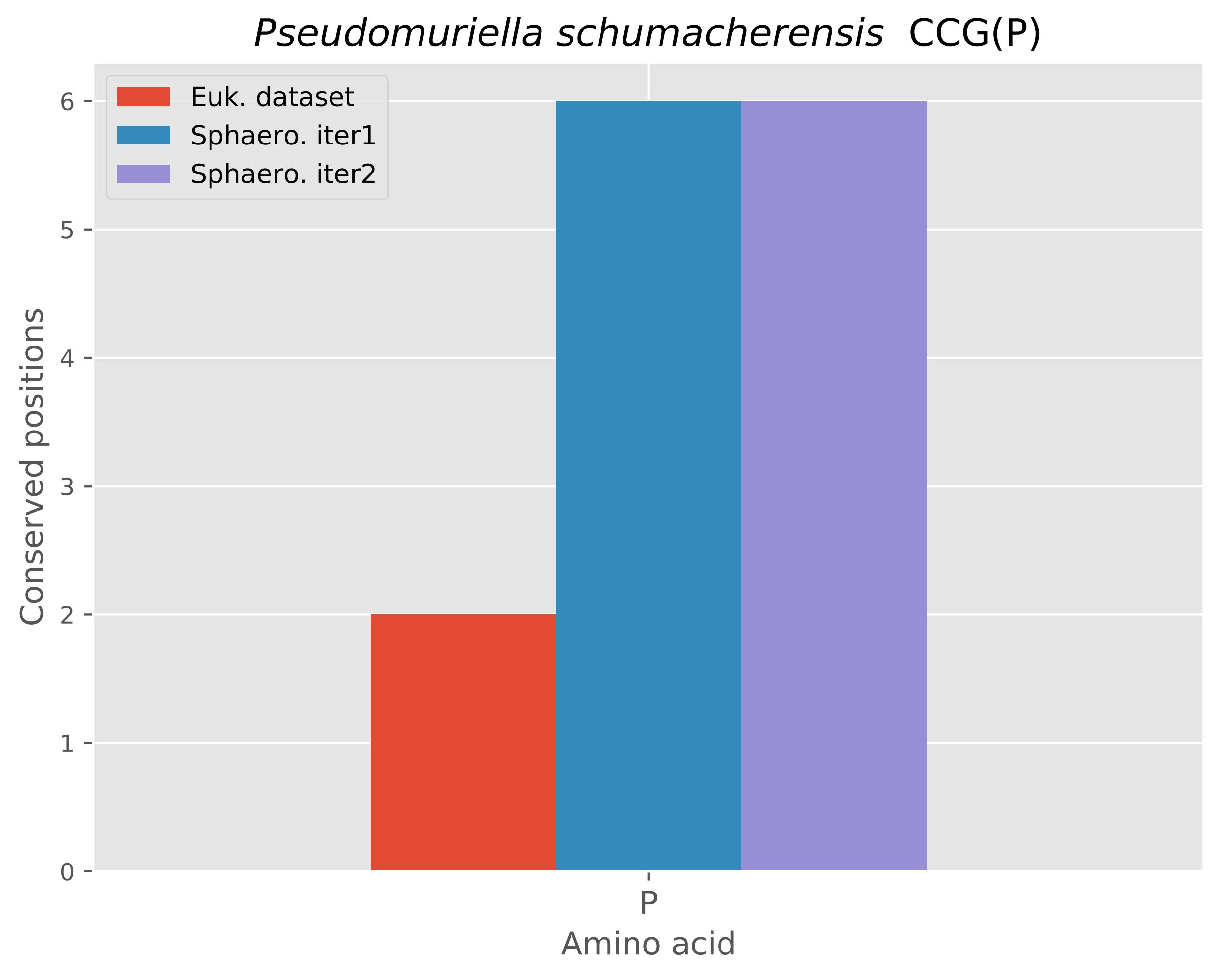
Pseudomuriella schumacherensis CCG(P)
Euk. dataset
Sphaero. iter1
Sphaero. iter2
Conserved positions
0
1
2
3
4
5
6
P
Amino acid

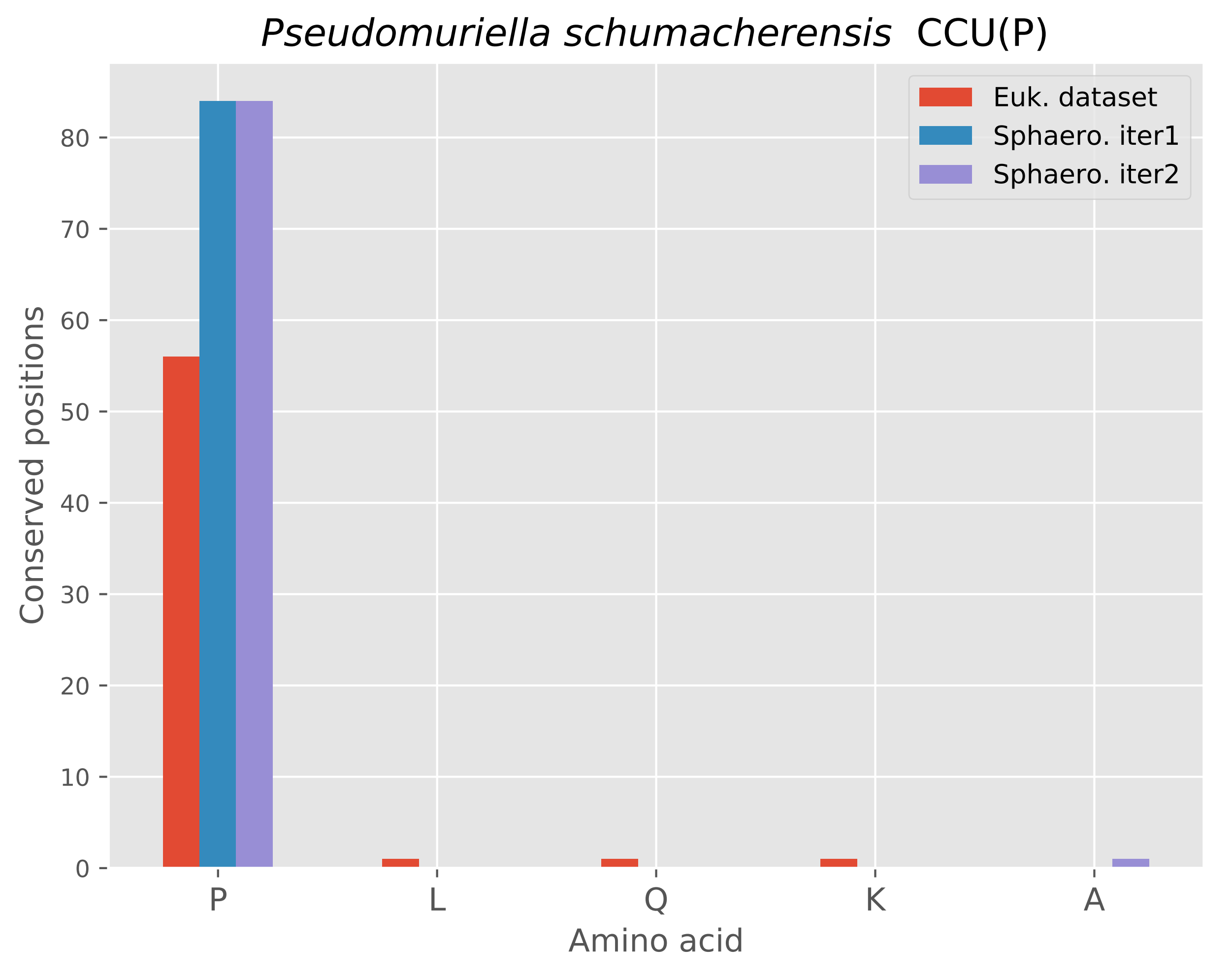
Pseudomuriella schumacherensis CCU(P)
Euk. dataset
Sphaero. iter1
Sphaero. iter2
Conserved positions
0
10
20
30
40
50
60
70
80
P
L
Q
K
A
Amino acid

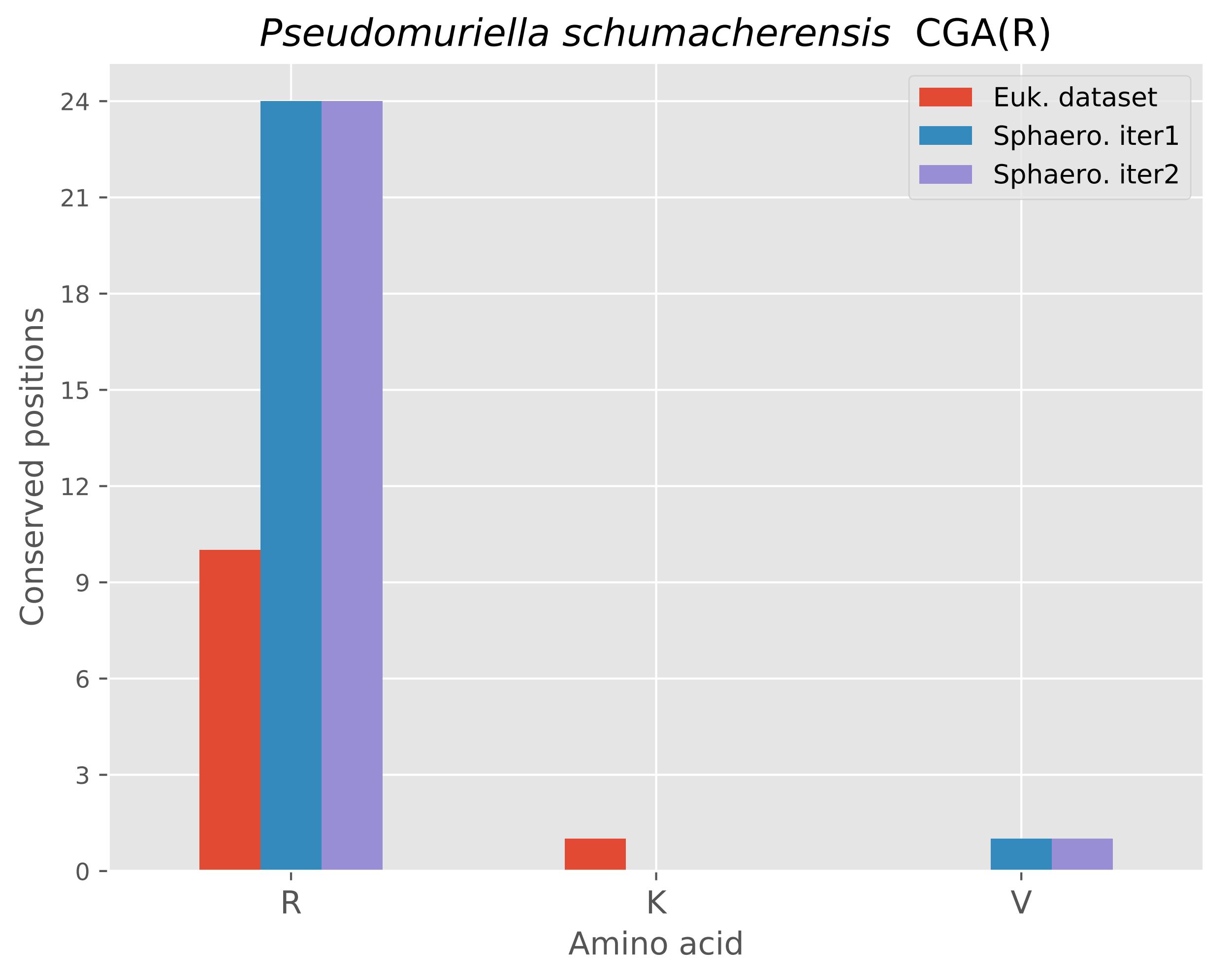

Pseudomuriella schumacherensis CGA(R)
Euk. dataset
Sphaero. iter1
Sphaero. iter2
Conserved positions
0
3
6
9
12
15
18
21
24
R
K
V
Amino acid

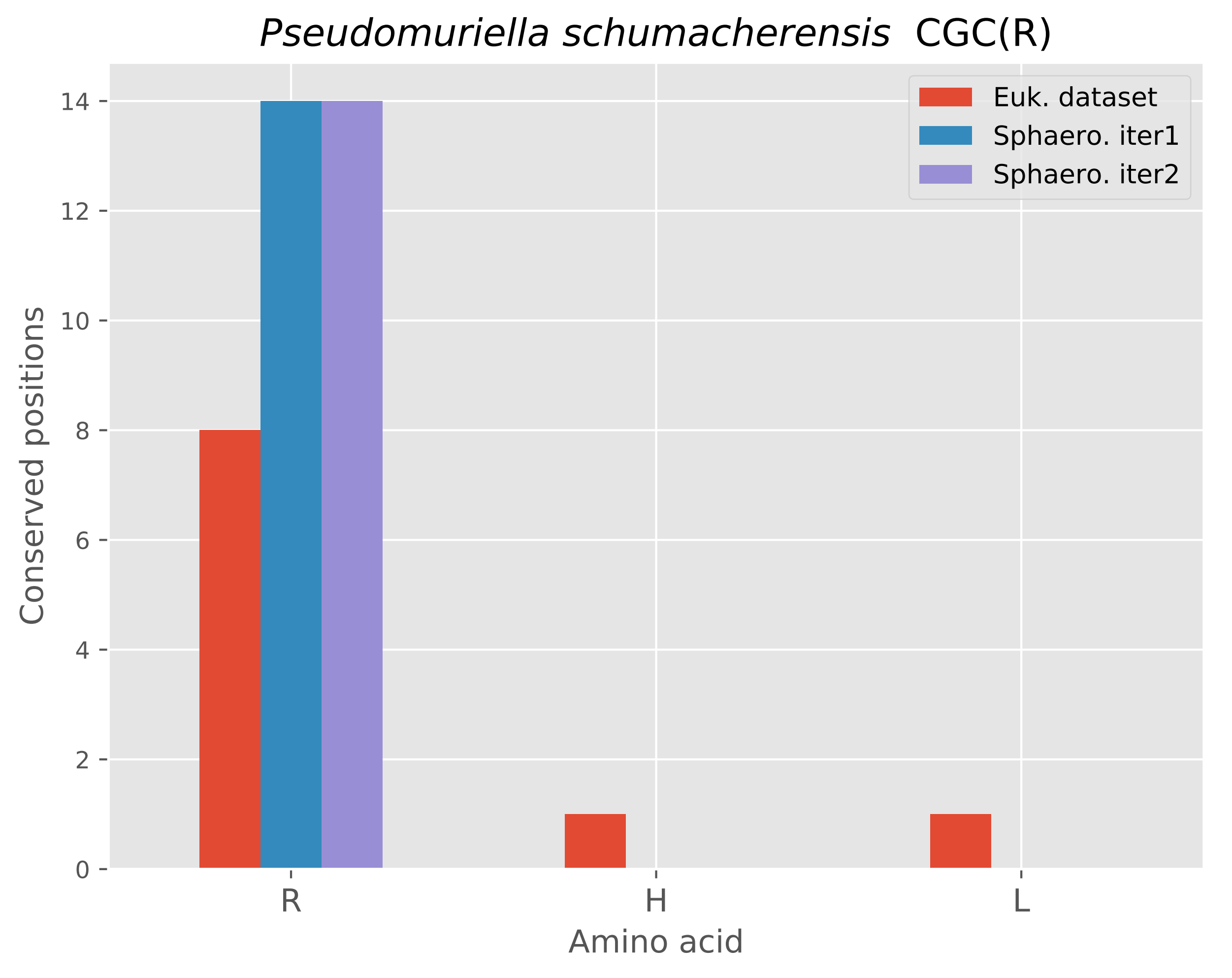

Pseudomuriella schumacherensis CGC(R)
Euk. dataset
Sphaero. iter1
Sphaero. iter2
Conserved positions
0
2
4
6
8
10
12
14
R
H
L
Amino acid

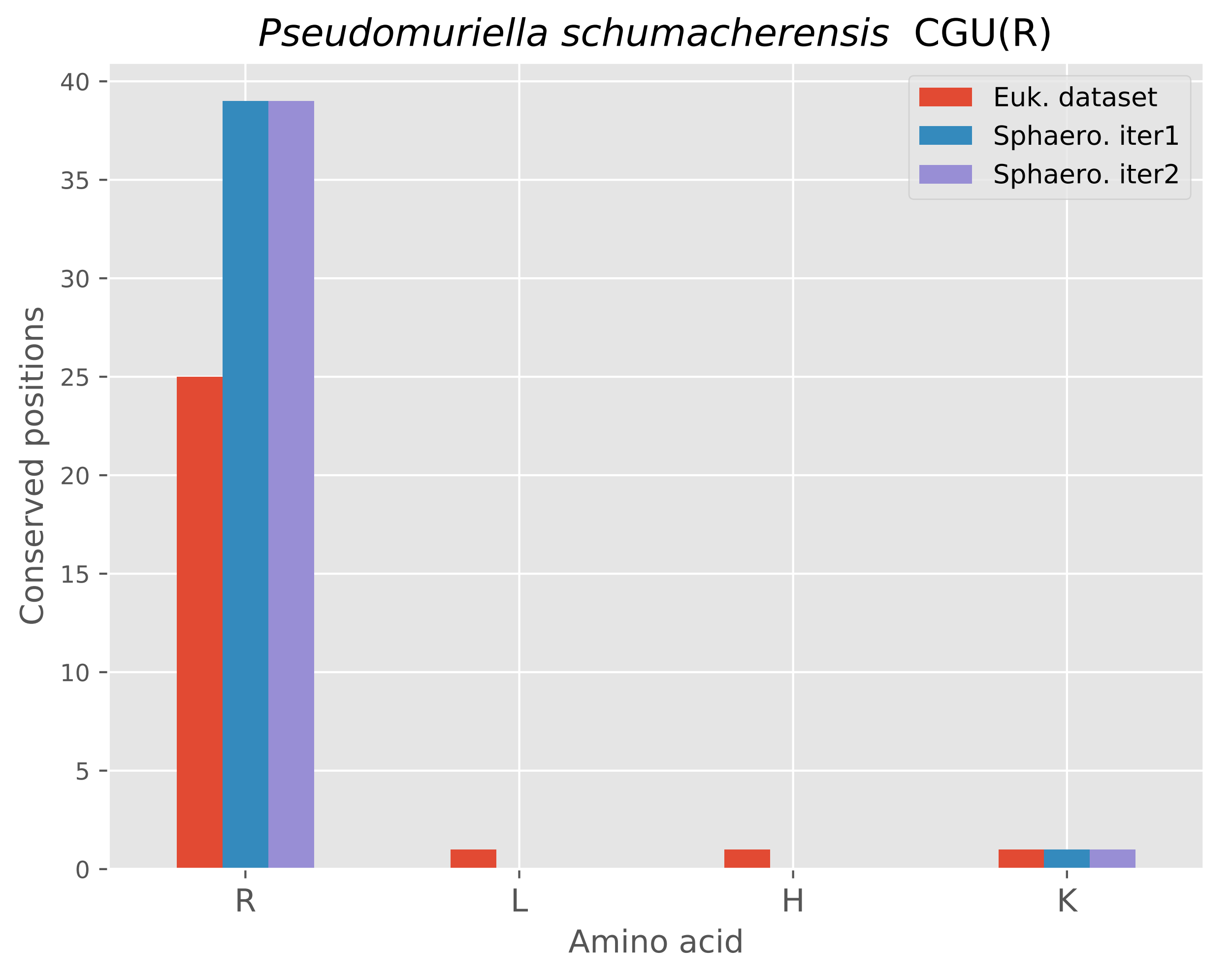
Pseudomuriella schumacherensis CGU(R)
Conserved positions
0
5
10
15
20
25
30
35
40
Amino acid
R
L
H
K
Euk. dataset
Sphaero. iter1
Sphaero. iter2

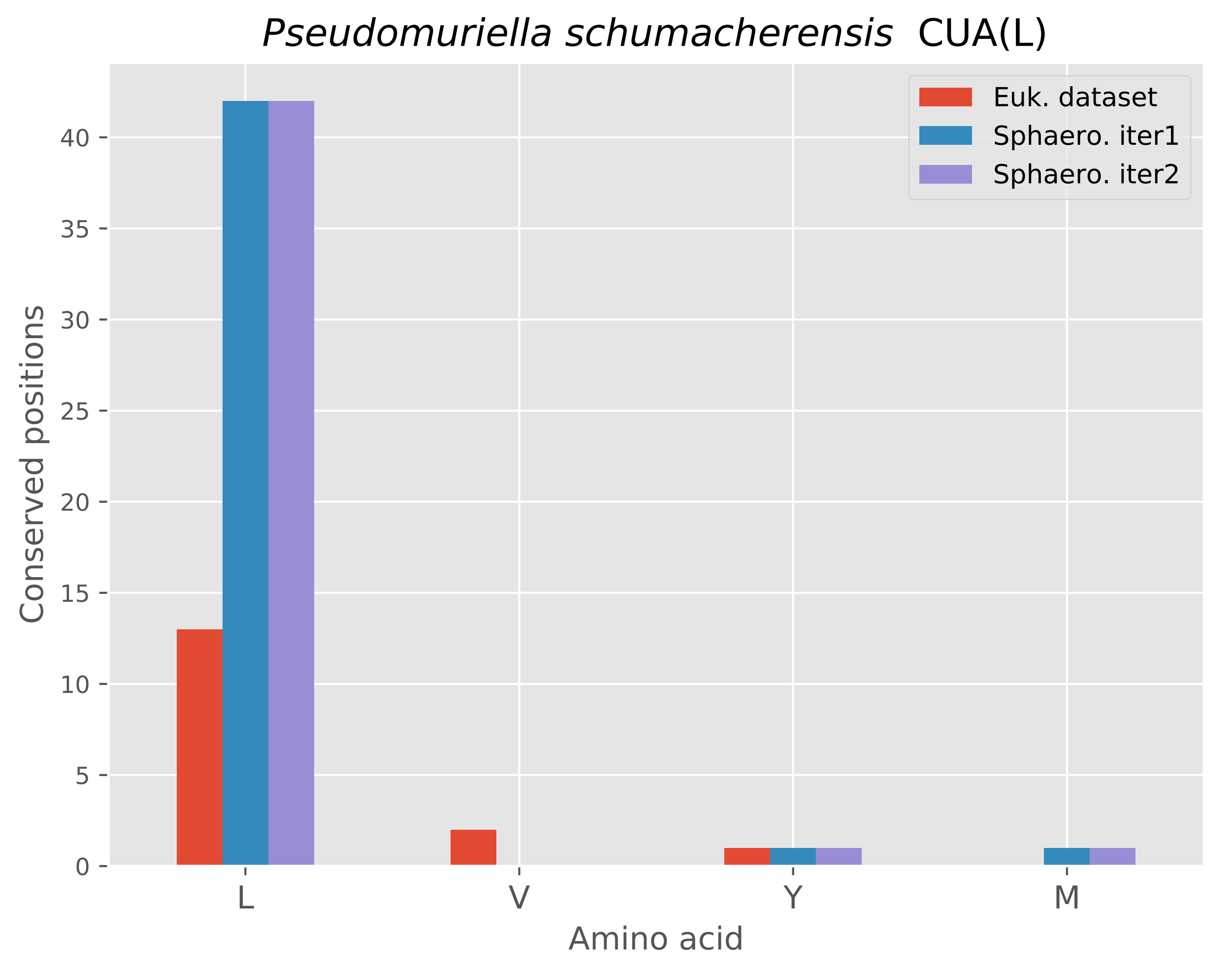

Pseudomuriella schumacherensis CUA(L)
Euk. dataset
Sphaero. iter1
Sphaero. iter2
Conserved positions
0
5
10
15
20
25
30
35
40
L
V
Y
M
Amino acid

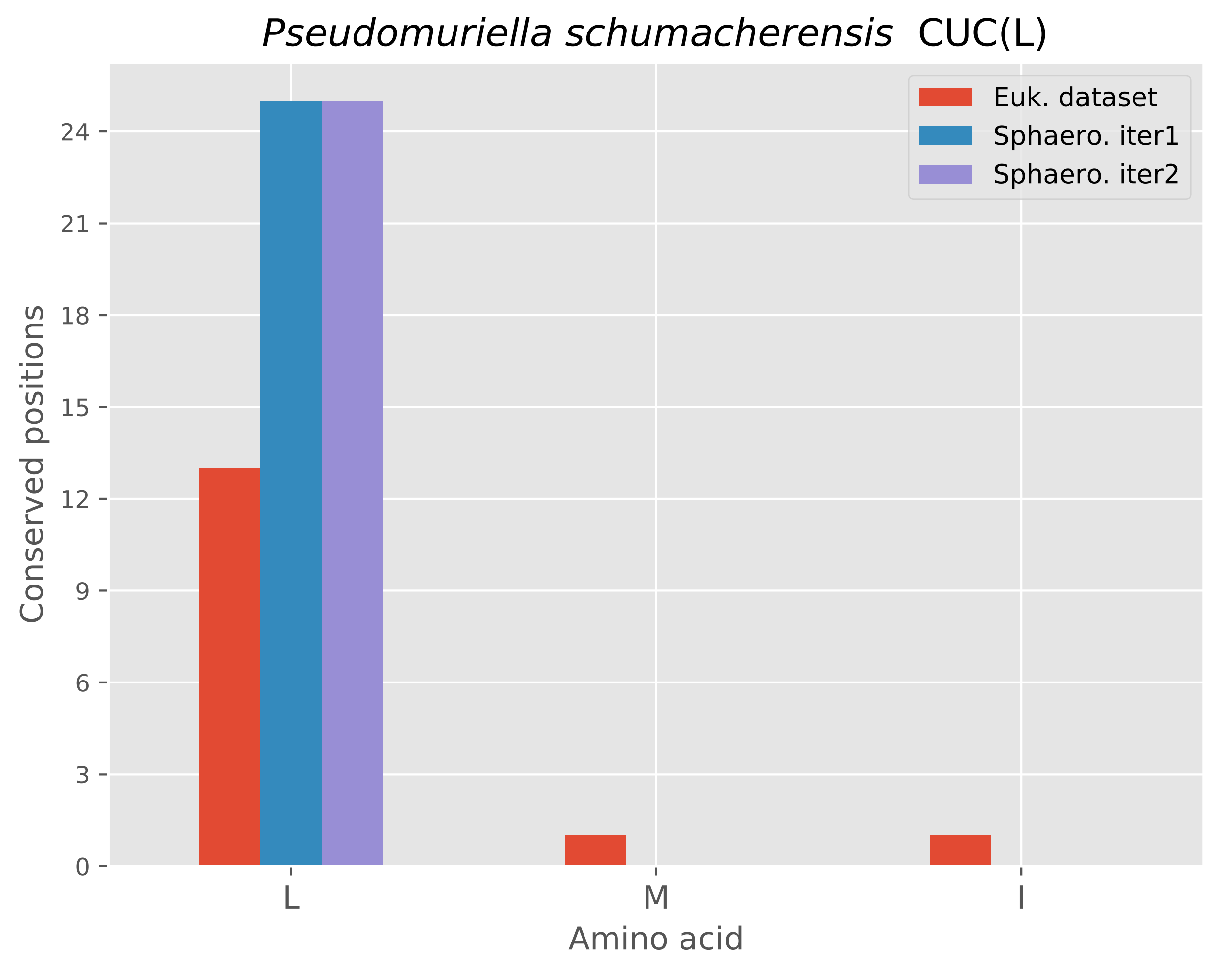

Pseudomuriella schumacherensis CUC(L)
Euk. dataset
Sphaero. iter1
Sphaero. iter2
Conserved positions
0
3
6
9
12
15
18
21
24
L
M
I
Amino acid

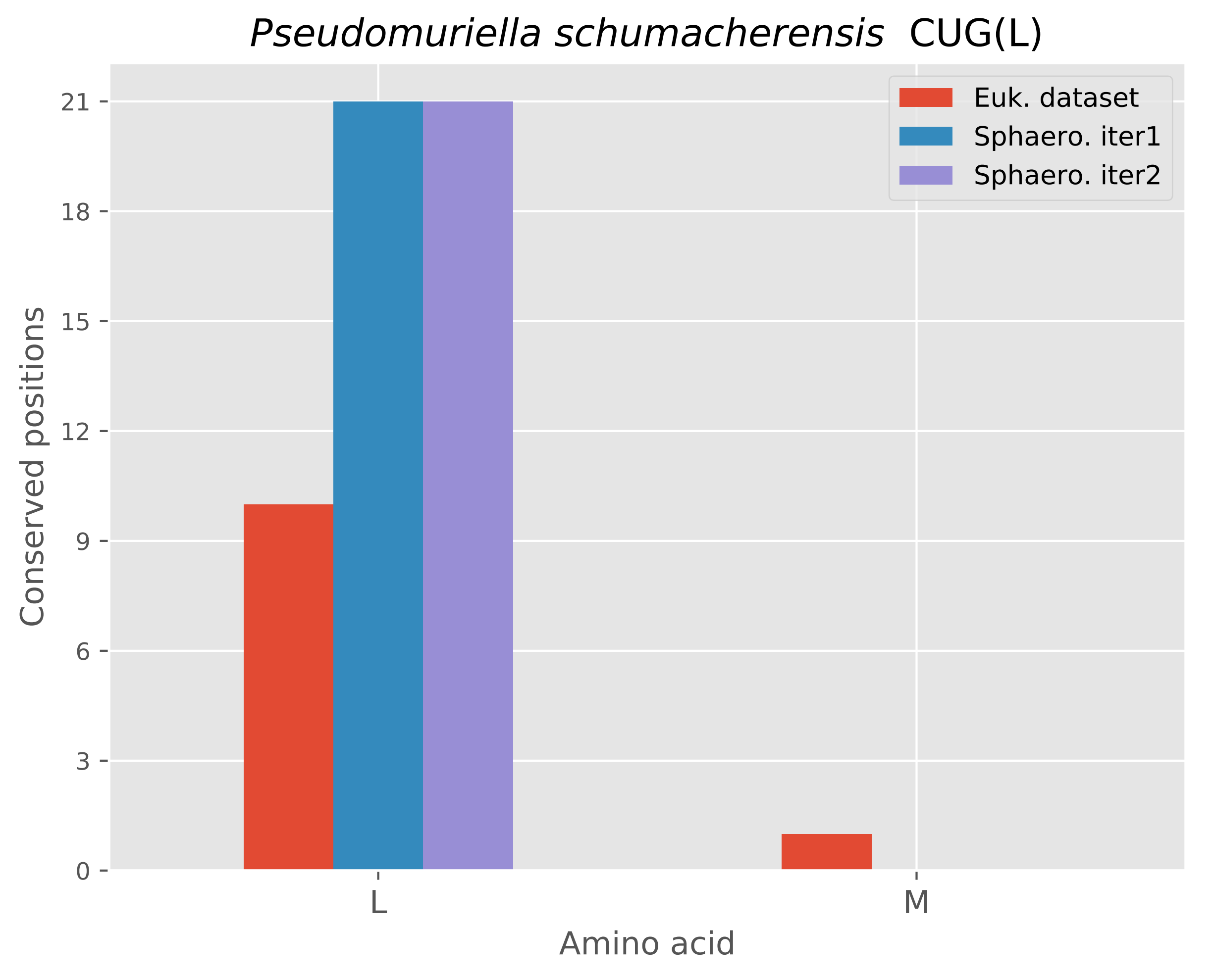

Pseudomuriella schumacherensis CUG(L)
Euk. dataset
Sphaero. iter1
Sphaero. iter2
Conserved positions
0
3
6
9
12
15
18
21
L
M
Amino acid

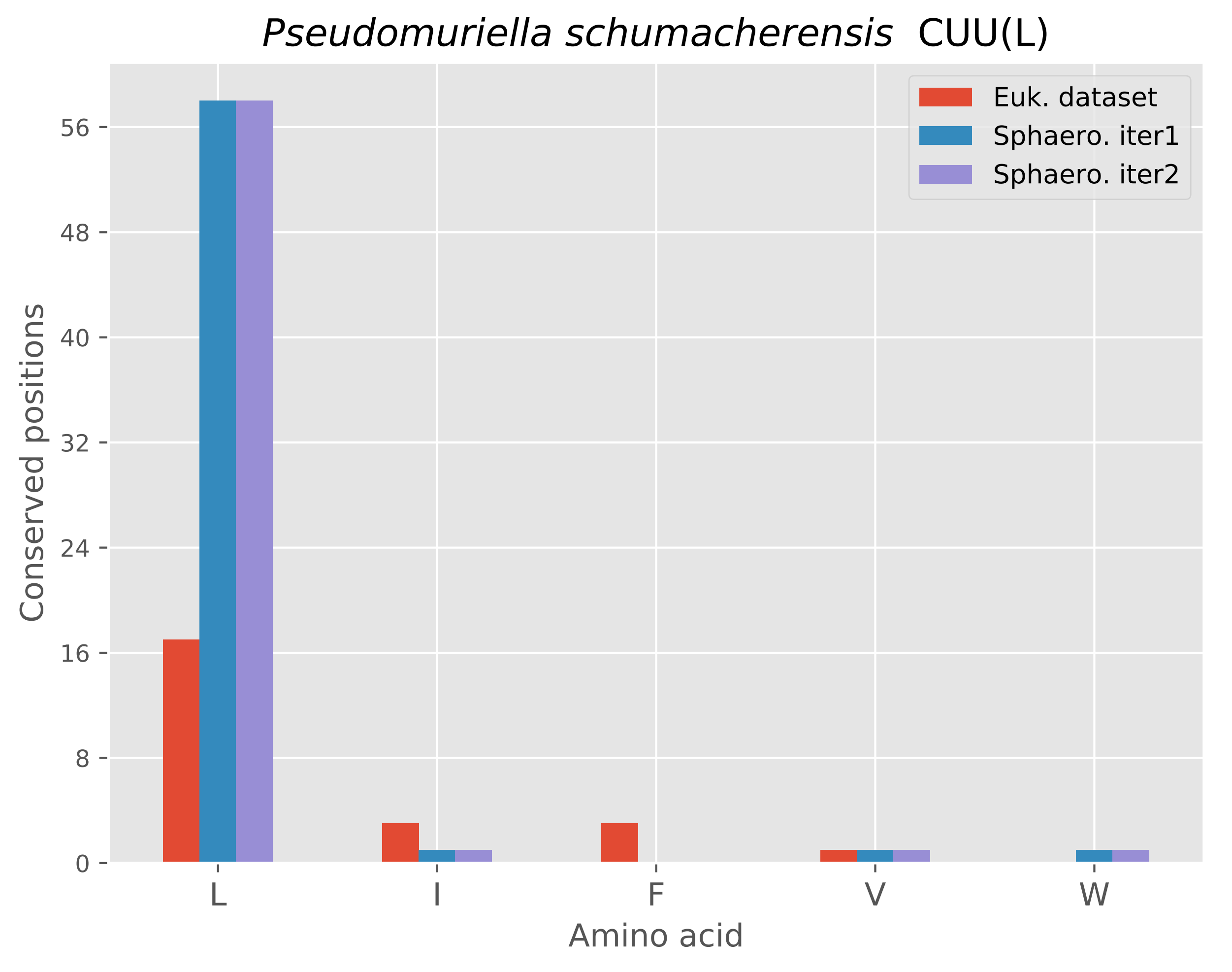

Pseudomuriella schumacherensis CUU(L)
Euk. dataset
Sphaero. iter1
Sphaero. iter2
Conserved positions
0
8
16
24
32
40
48
56
L
I
F
V
W
Amino acid

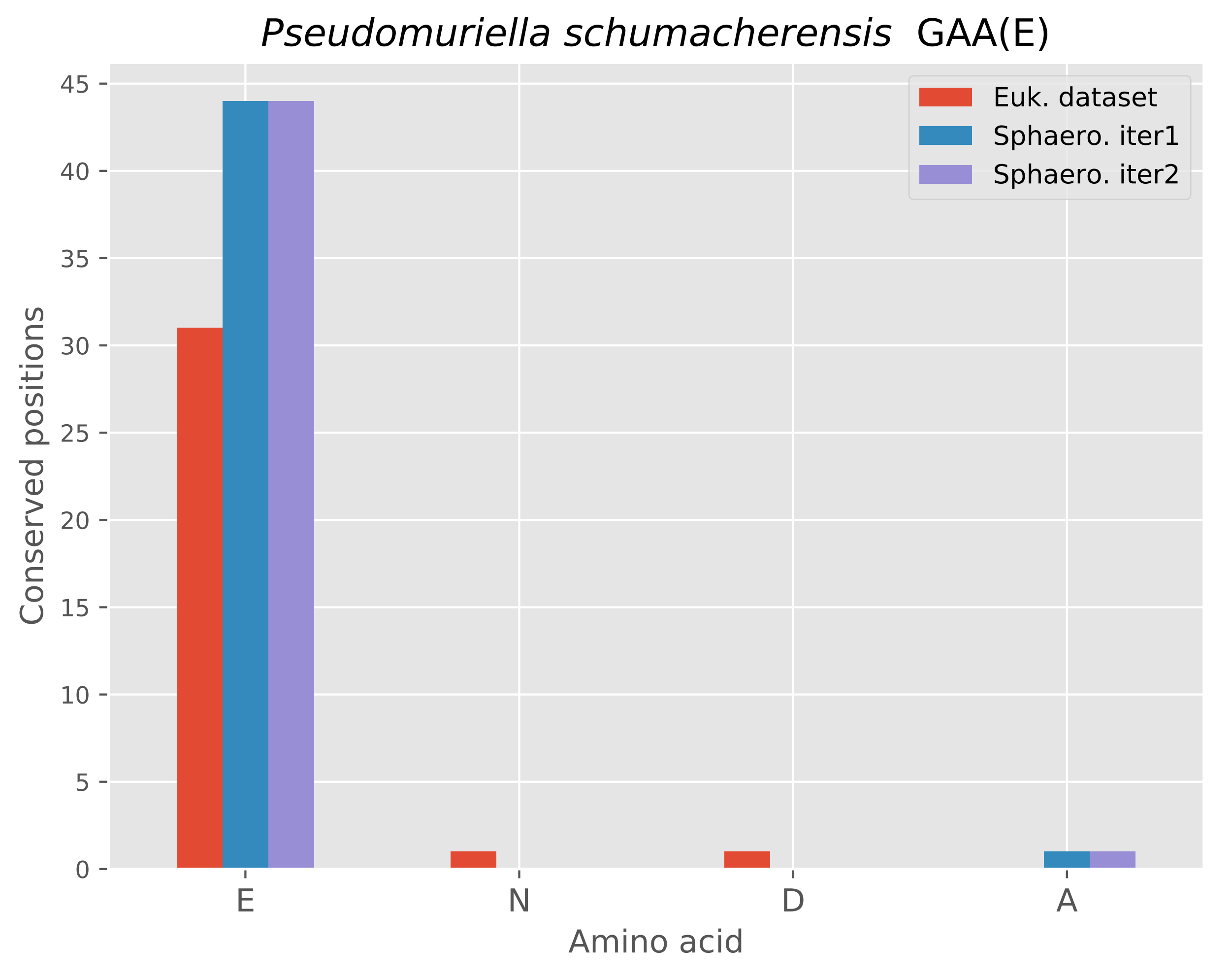

Pseudomuriella schumacherensis GAA(E)
Euk. dataset
Sphaero. iter1
Sphaero. iter2
Conserved positions
0
5
10
15
20
25
30
35
40
45
E
N
D
A
Amino acid

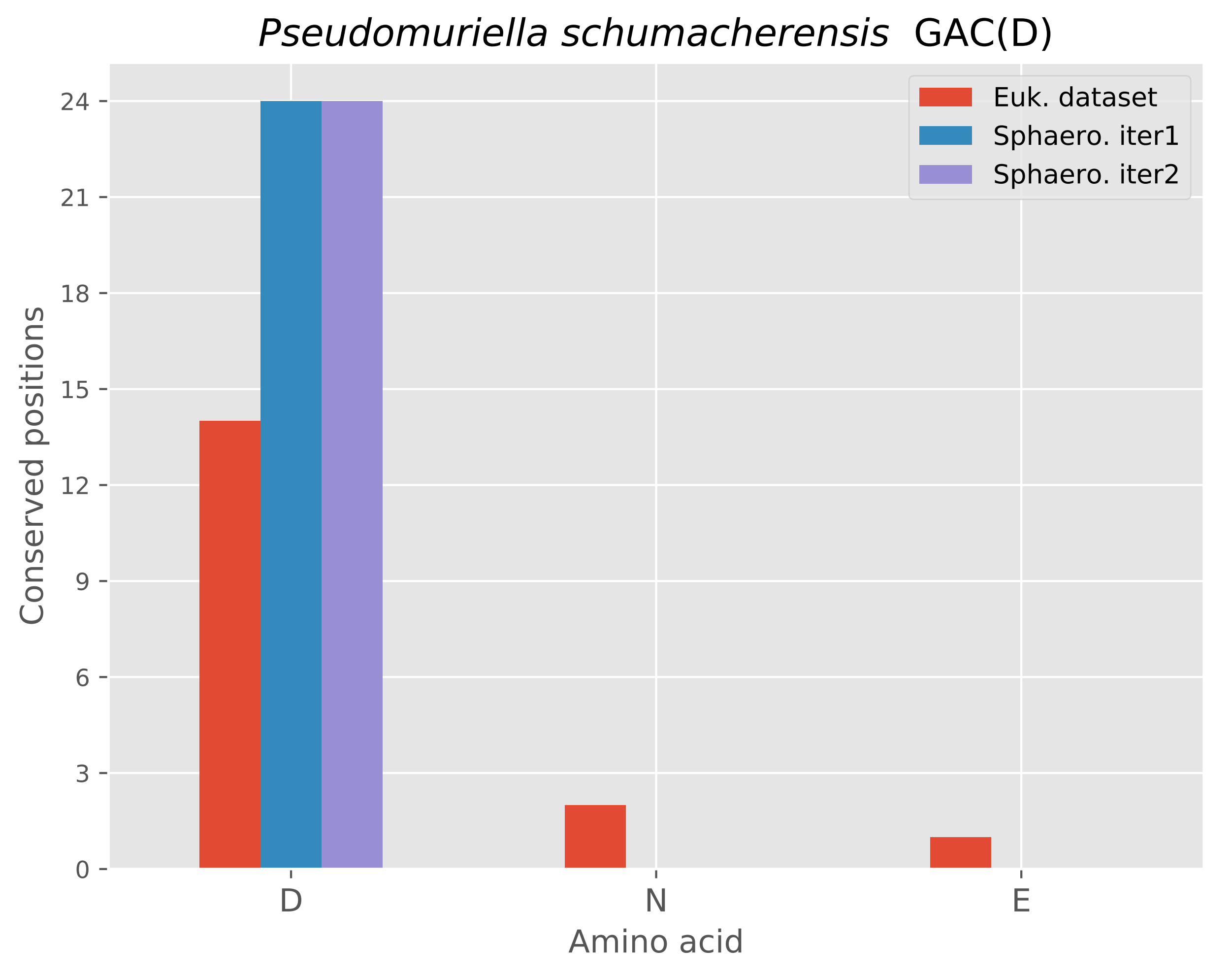

Pseudomuriella schumacherensis GAC(D)
Euk. dataset
Sphaero. iter1
Sphaero. iter2
Conserved positions
0
3
6
9
12
15
18
21
24
D
N
E
Amino acid

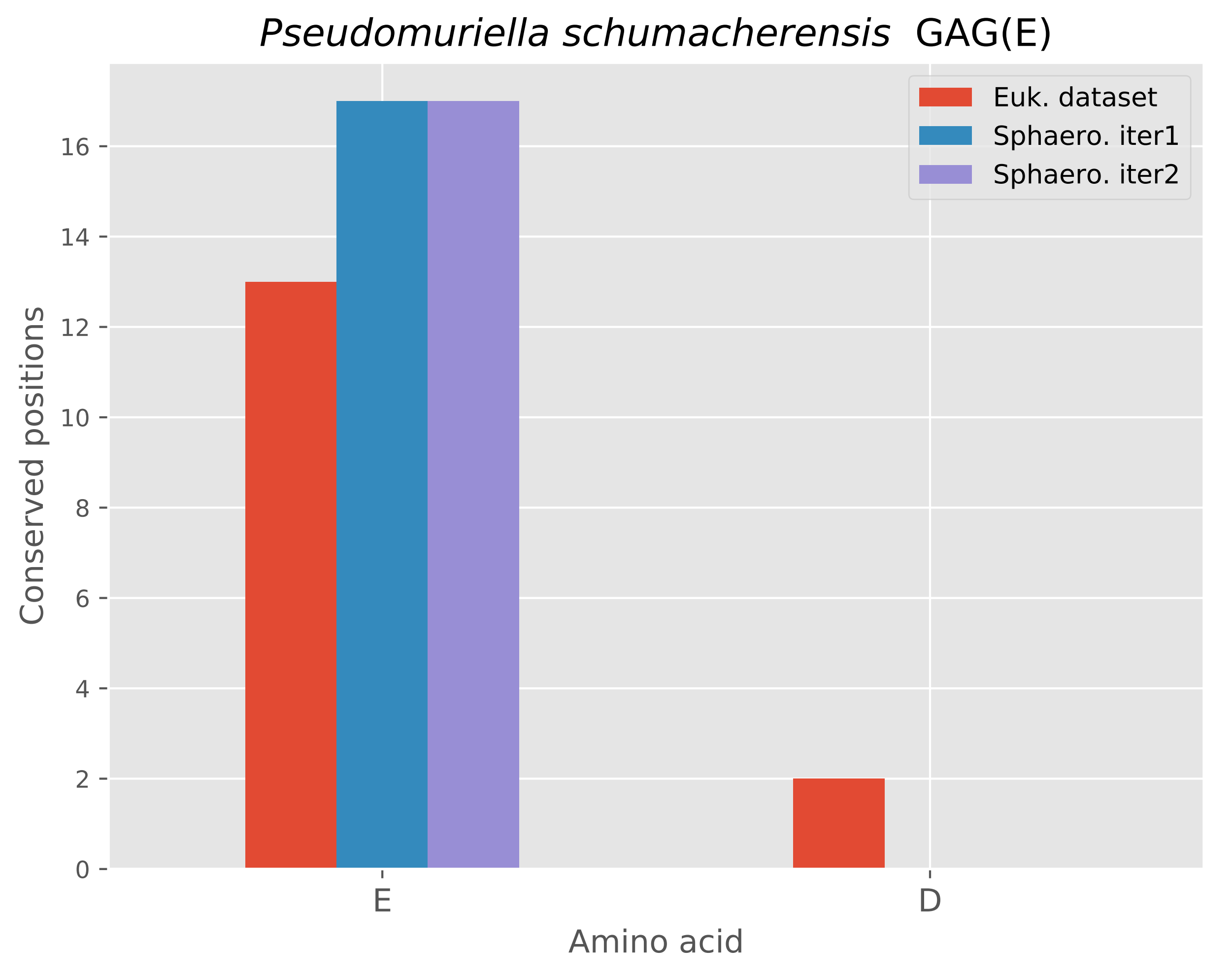

Pseudomuriella schumacherensis GAG(E)
Euk. dataset
Sphaero. iter1
Sphaero. iter2
Conserved positions
0
2
4
6
8
10
12
14
16
E
D
Amino acid

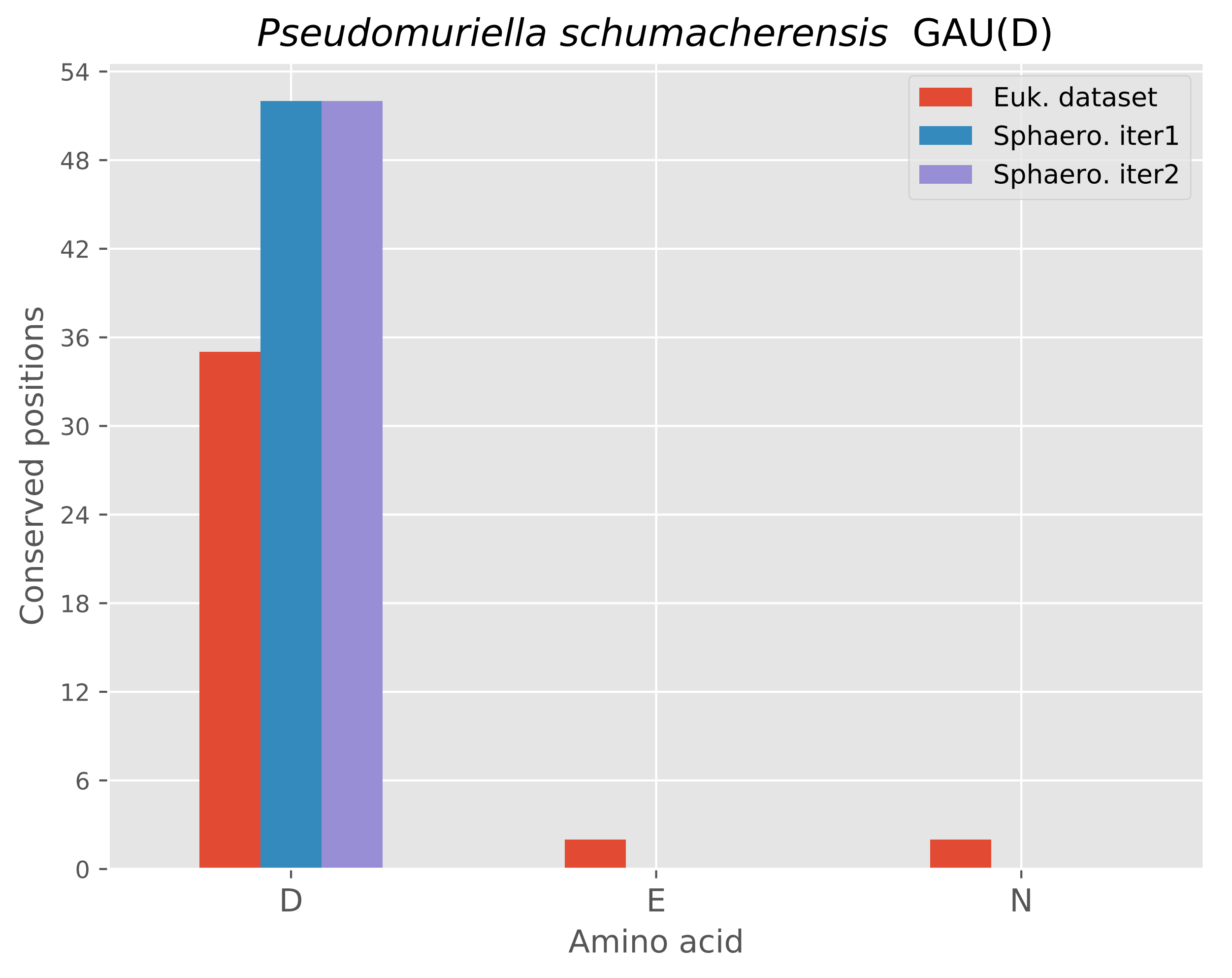

Pseudomuriella schumacherensis GAU(D)
Euk. dataset
Sphaero. iter1
Sphaero. iter2
Conserved positions
0
6
12
18
24
30
36
42
48
54
D
E
N
Amino acid

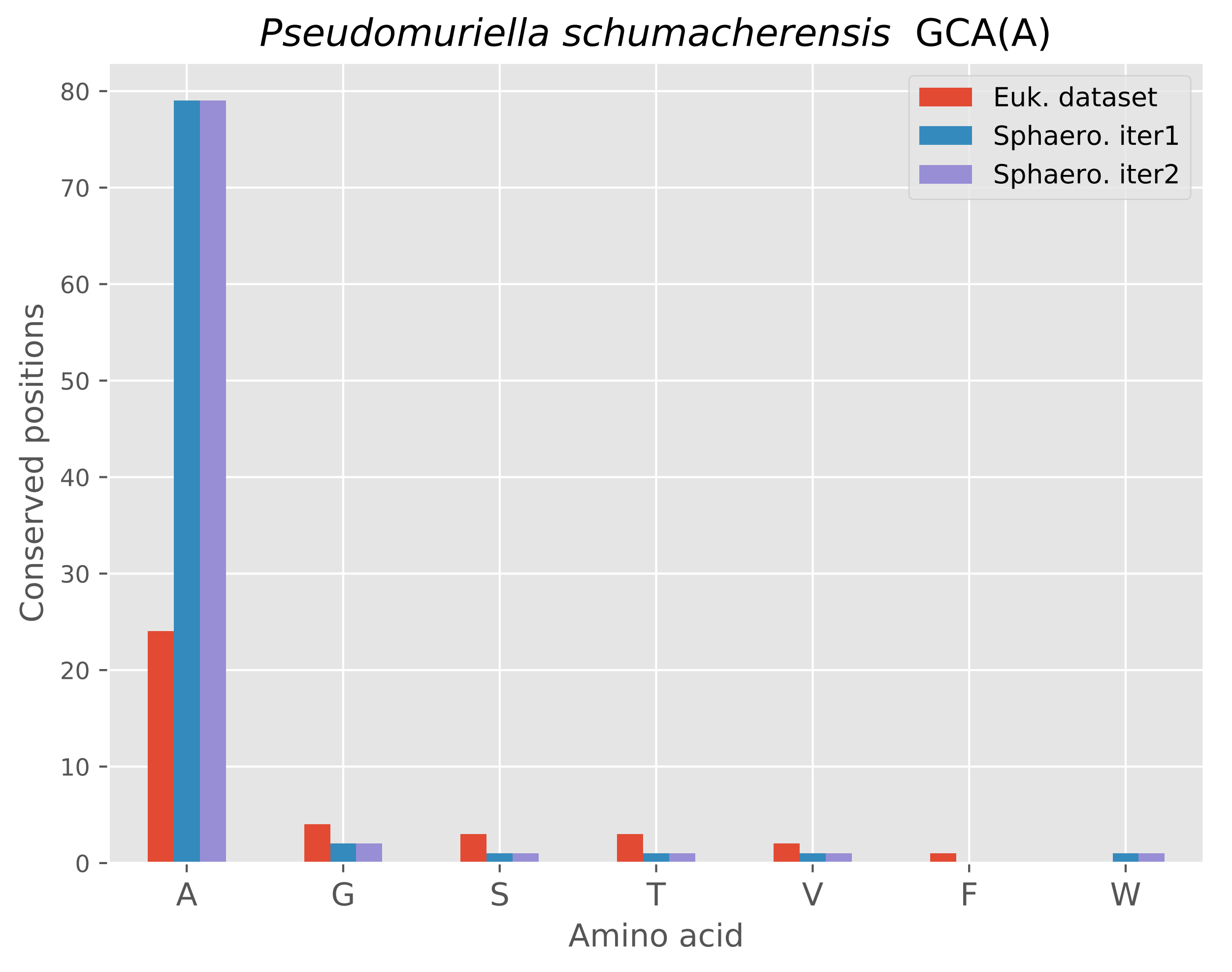
Pseudomuriella schumacherensis GCA(A)
Euk. dataset
Sphaero. iter1
Sphaero. iter2
Conserved positions
0
10
20
30
40
50
60
70
80
A
G
S
T
V
F
W
Amino acid

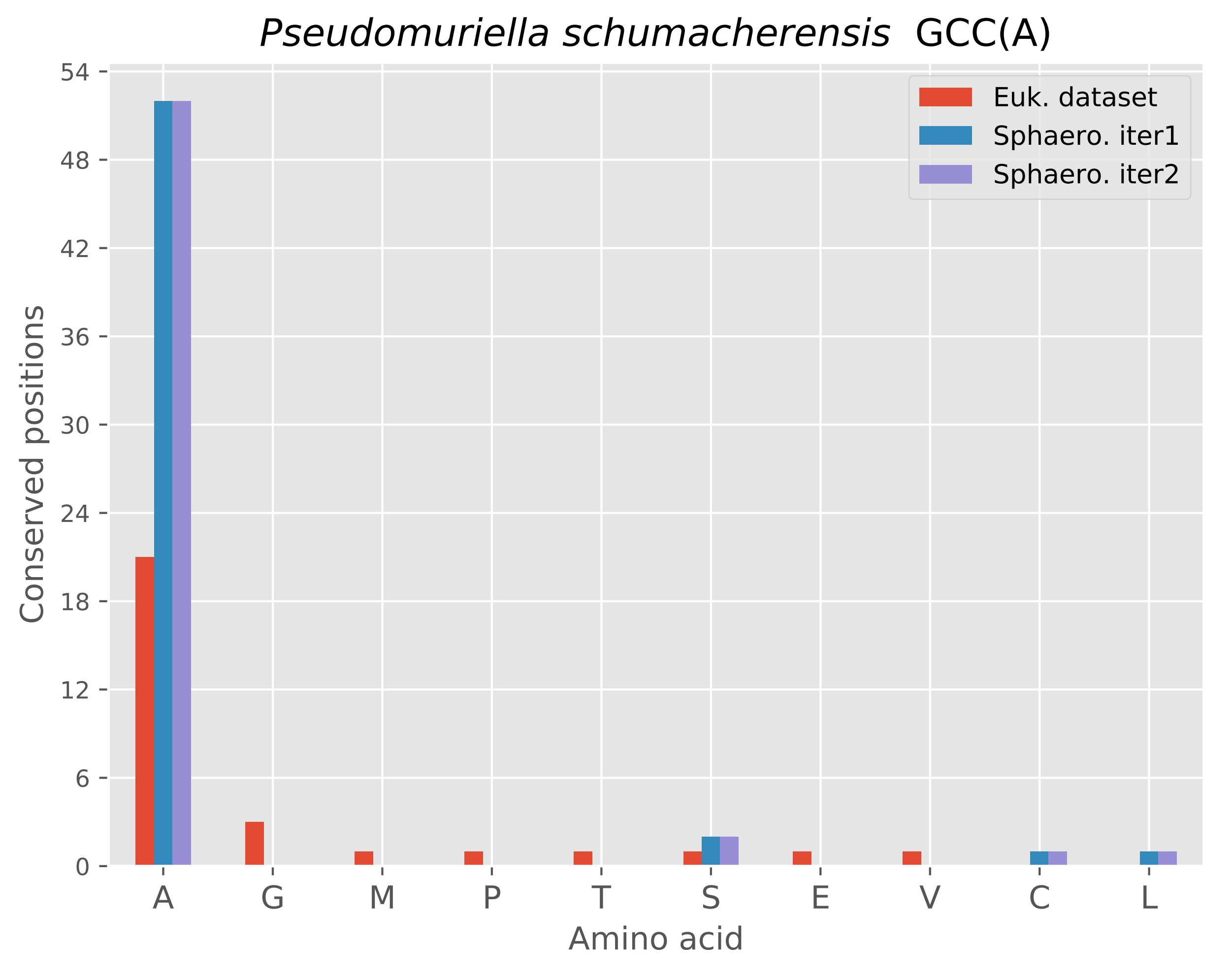

Pseudomuriella schumacherensis GCC(A)
Euk. dataset
Sphaero. iter1
Sphaero. iter2
Conserved positions
Amino acid
0
6
12
18
24
30
36
42
48
54
A
G
M
P
T
S
E
V
C
L

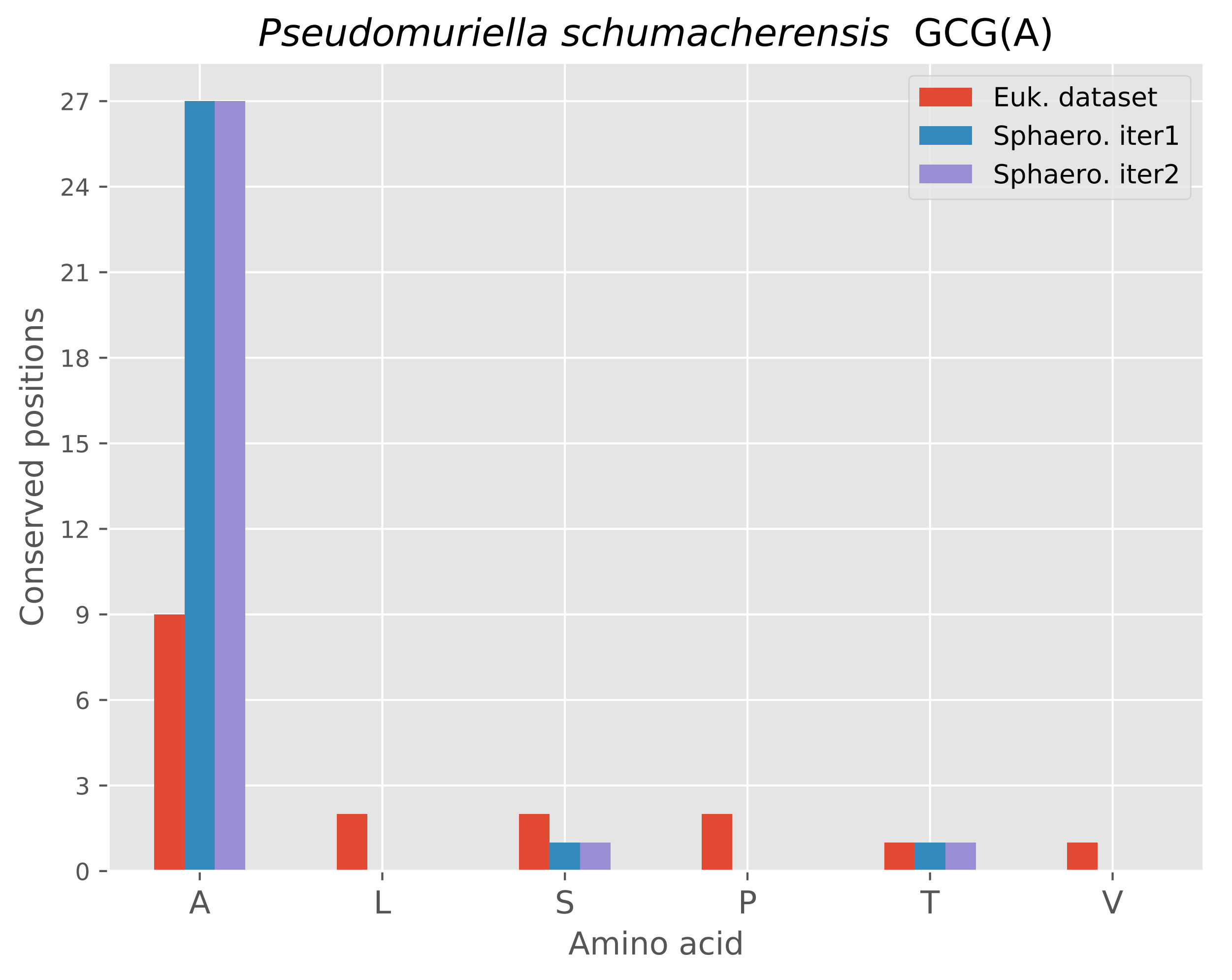

Pseudomuriella schumacherensis GCG(A)
Euk. dataset
Sphaero. iter1
Sphaero. iter2
Conserved positions
0
3
6
9
12
15
18
21
24
27
A
L
S
P
T
V
Amino acid

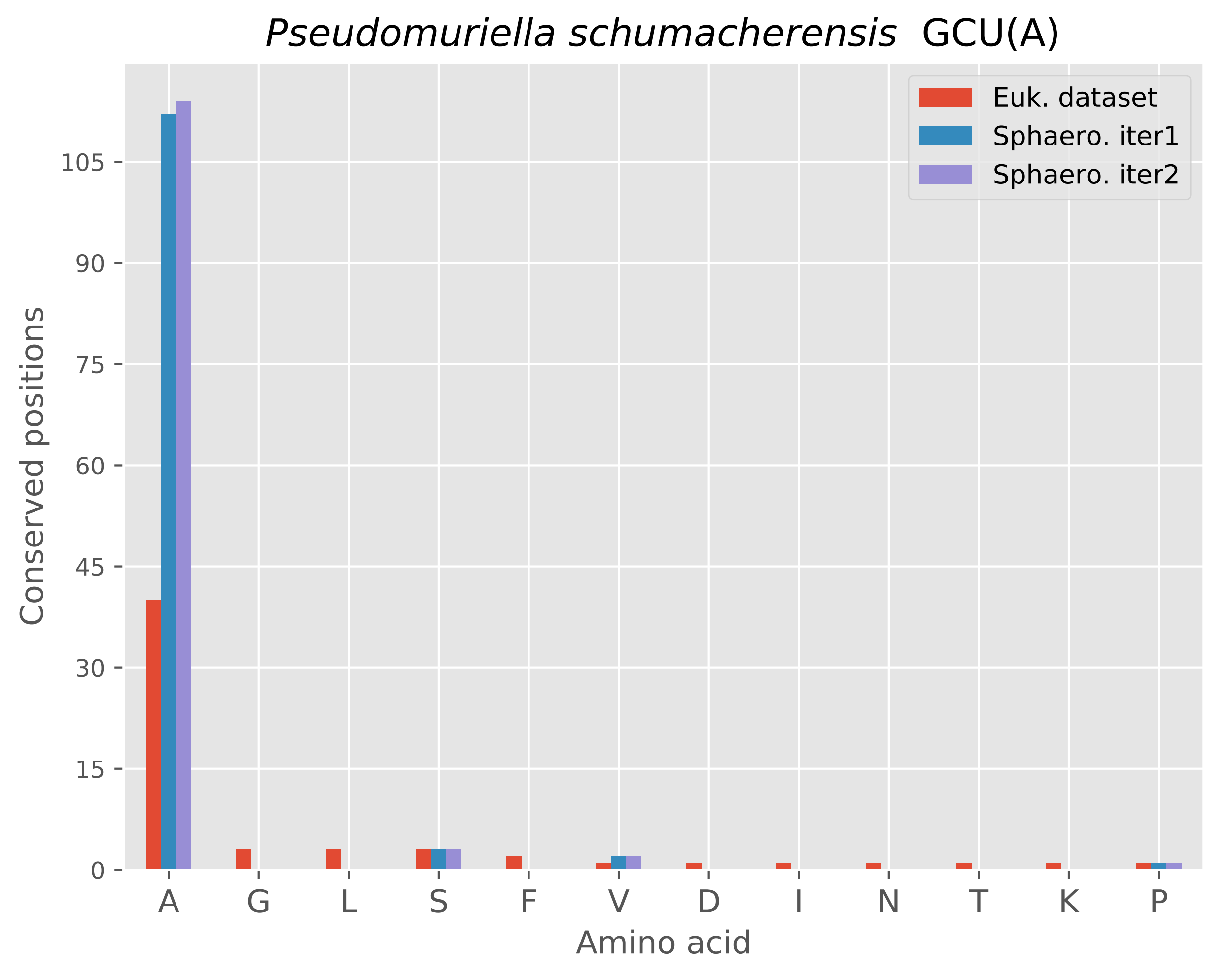
Pseudomuriella schumacherensis GCU(A)
Euk. dataset
Sphaero. iter1
Sphaero. iter2
Conserved positions
0
15
30
45
60
75
90
105
Amino acid
A
G
L
S
F
V
D
I
N
T
K
P

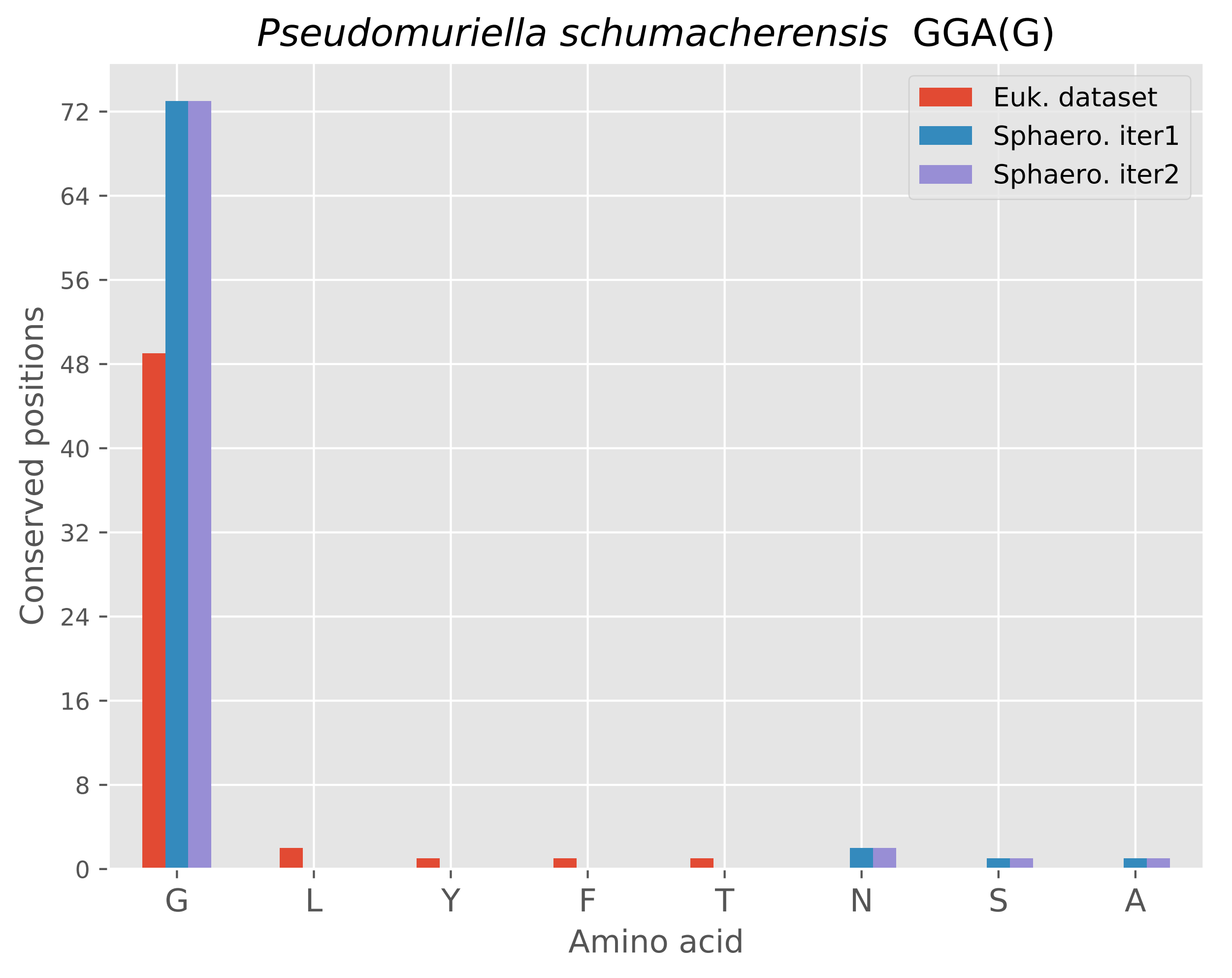

Pseudomuriella schumacherensis GGA(G)
Euk. dataset
Sphaero. iter1
Sphaero. iter2
Conserved positions
0
8
16
24
32
40
48
56
64
72
G
L
Y
F
T
N
S
A
Amino acid

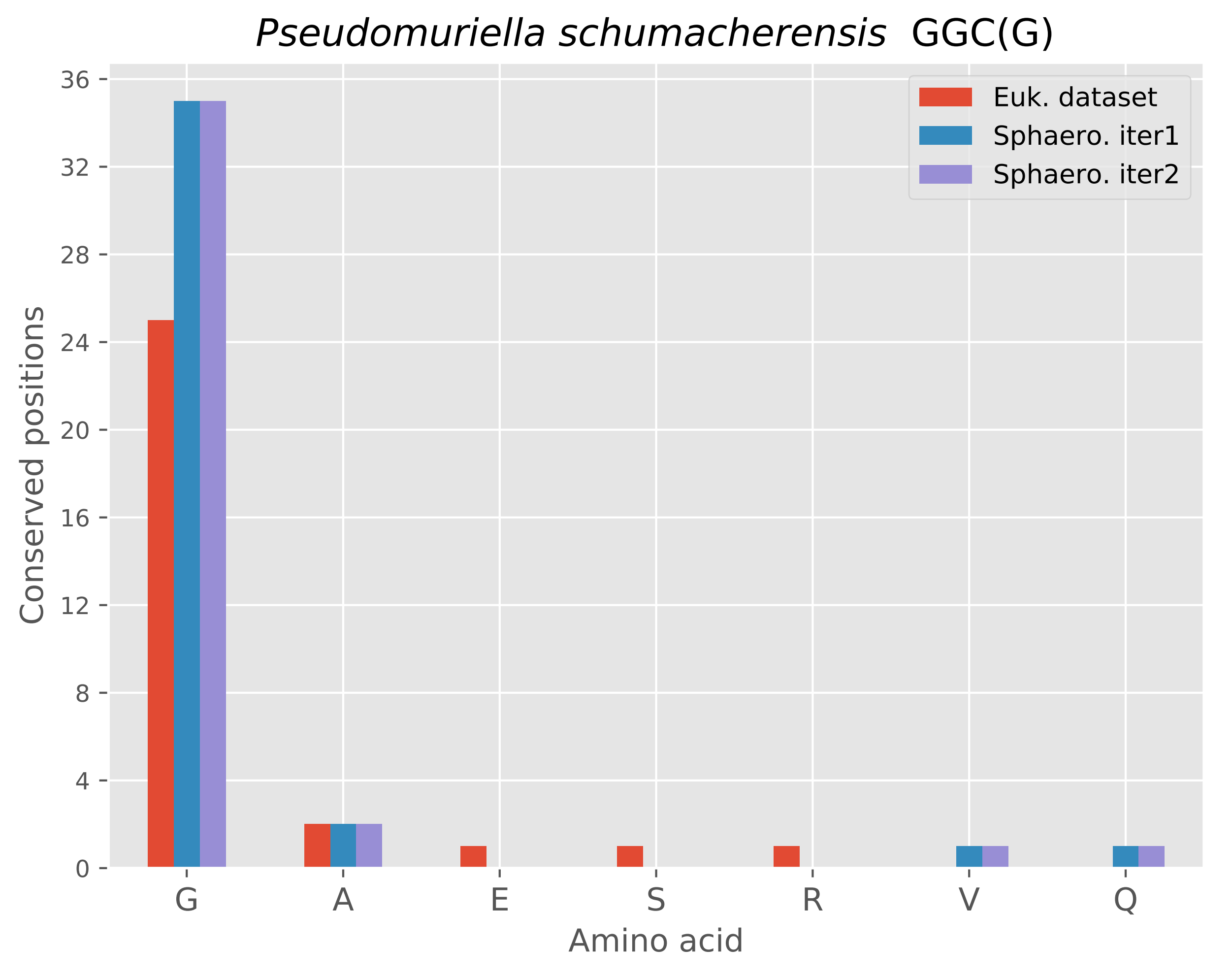

Pseudomuriella schumacherensis GGC(G)
Euk. dataset
Sphaero. iter1
Sphaero. iter2
Conserved positions
36
32
28
24
20
16
12
8
4
0
G
A
E
S
R
V
Q
Amino acid

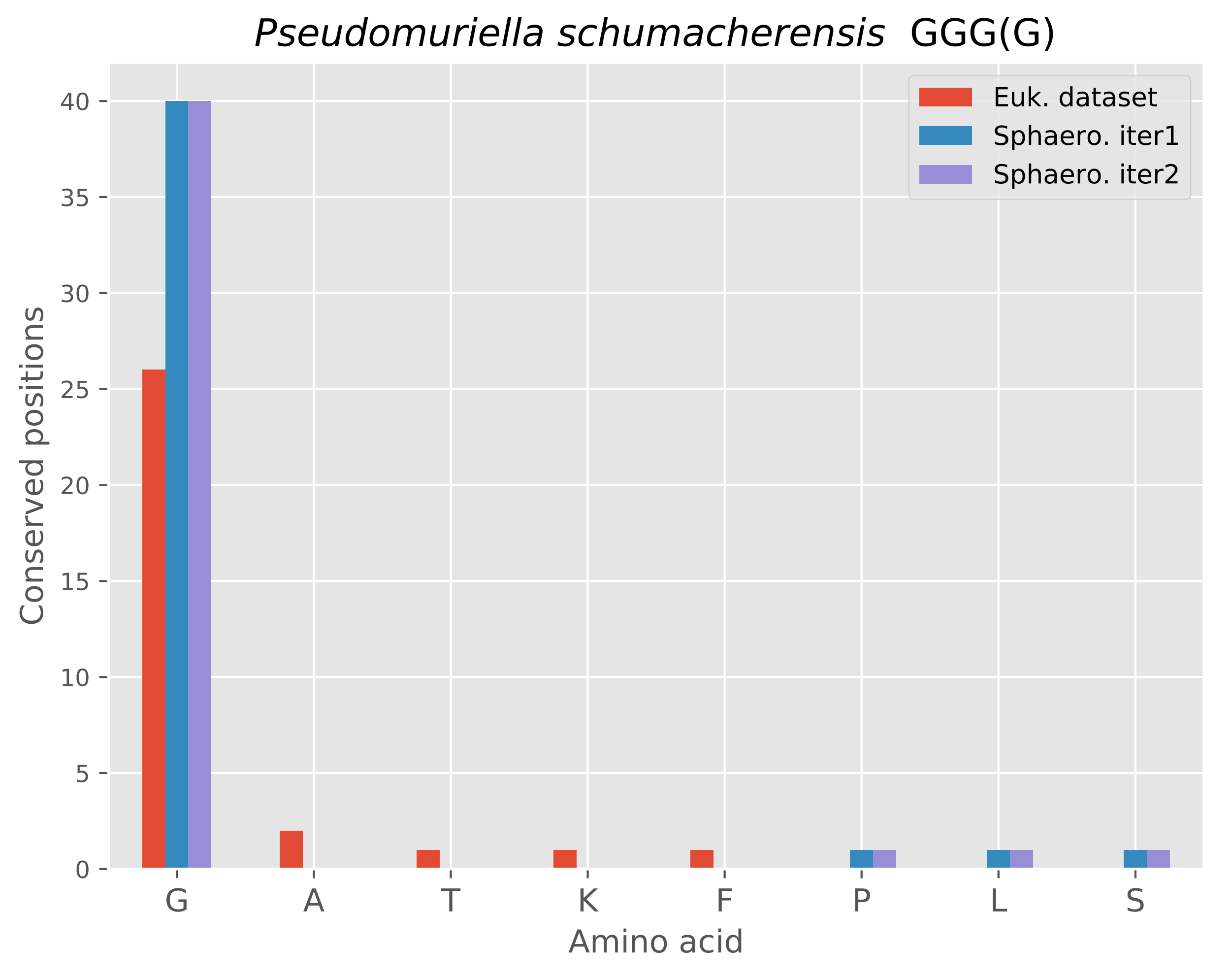

Pseudomuriella schumacherensis GGG(G)
Euk. dataset
Sphaero. iter1
Sphaero. iter2
Conserved positions
0
5
10
15
20
25
30
35
40
G
A
T
K
F
P
L
S
Amino acid

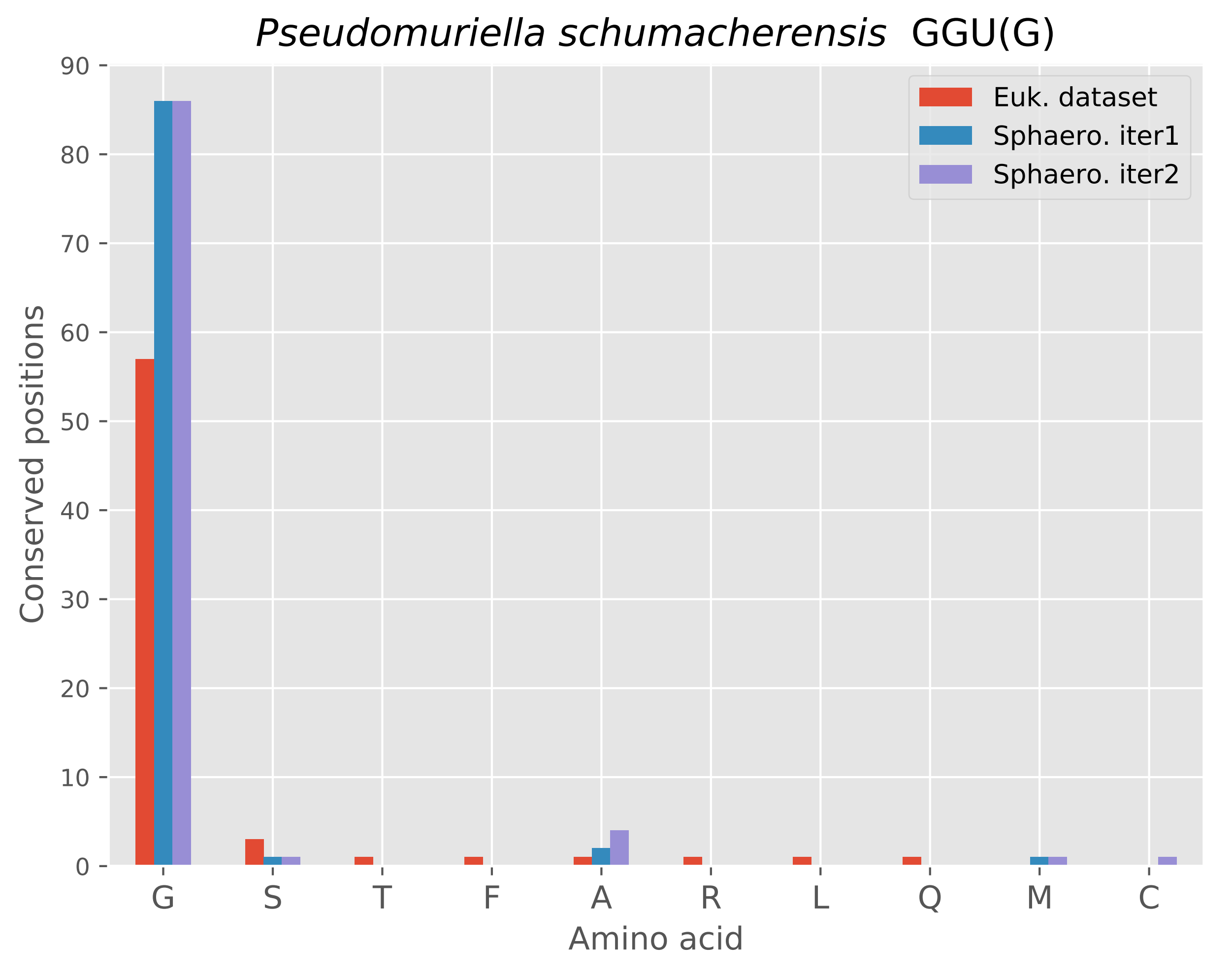

Pseudomuriella schumacherensis GGU(G)
Conserved positions
Amino acid
Euk. dataset
Sphaero. iter1
Sphaero. iter2
0
10
20
30
40
50
60
70
80
90
G
S
T
F
A
R
L
Q
M
C

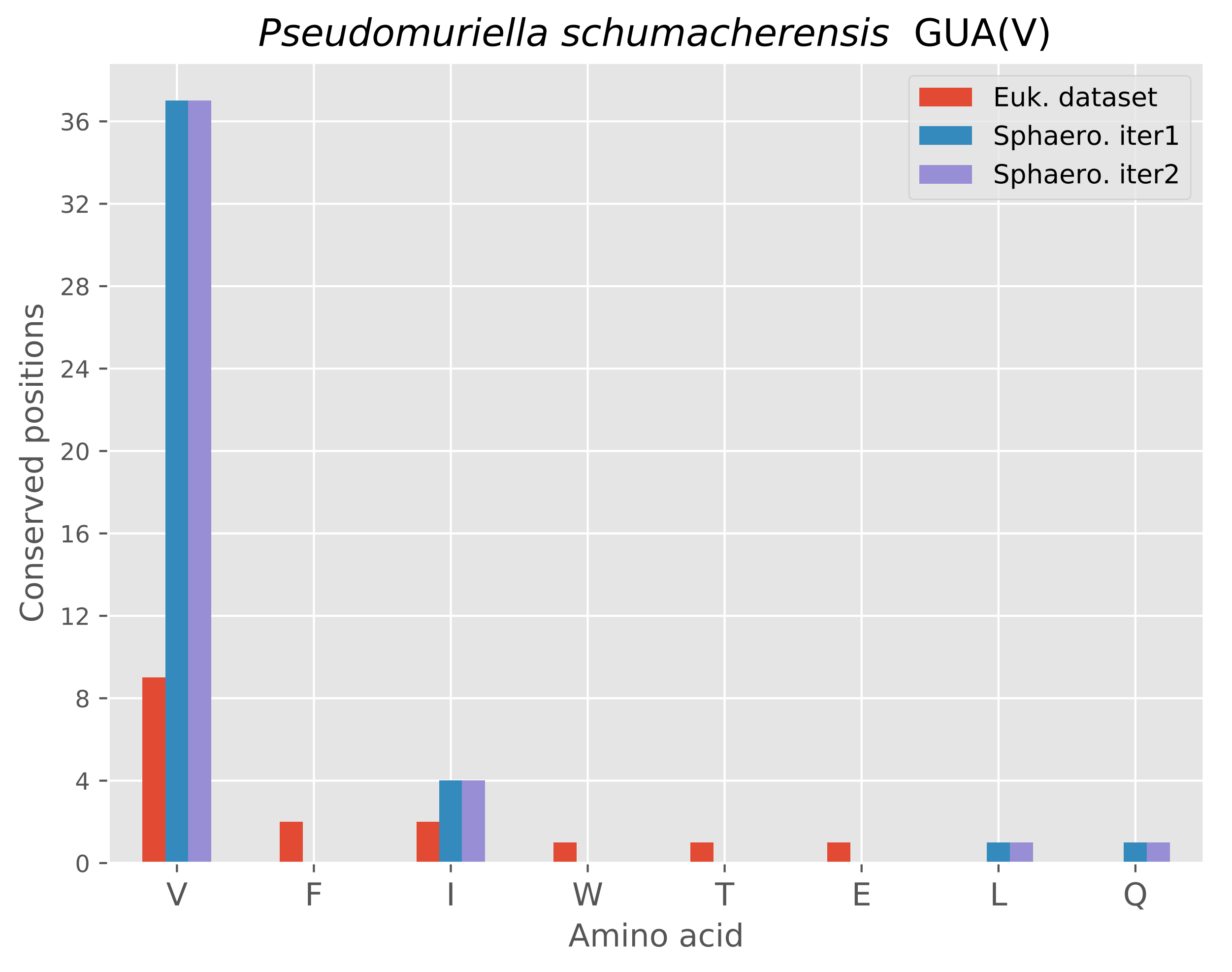
Pseudomuriella schumacherensis GUA(V)
Euk. dataset
Sphaero. iter1
Sphaero. iter2
Conserved positions
0
4
8
12
16
20
24
28
32
36
V
F
I
W
T
E
L
Q
Amino acid

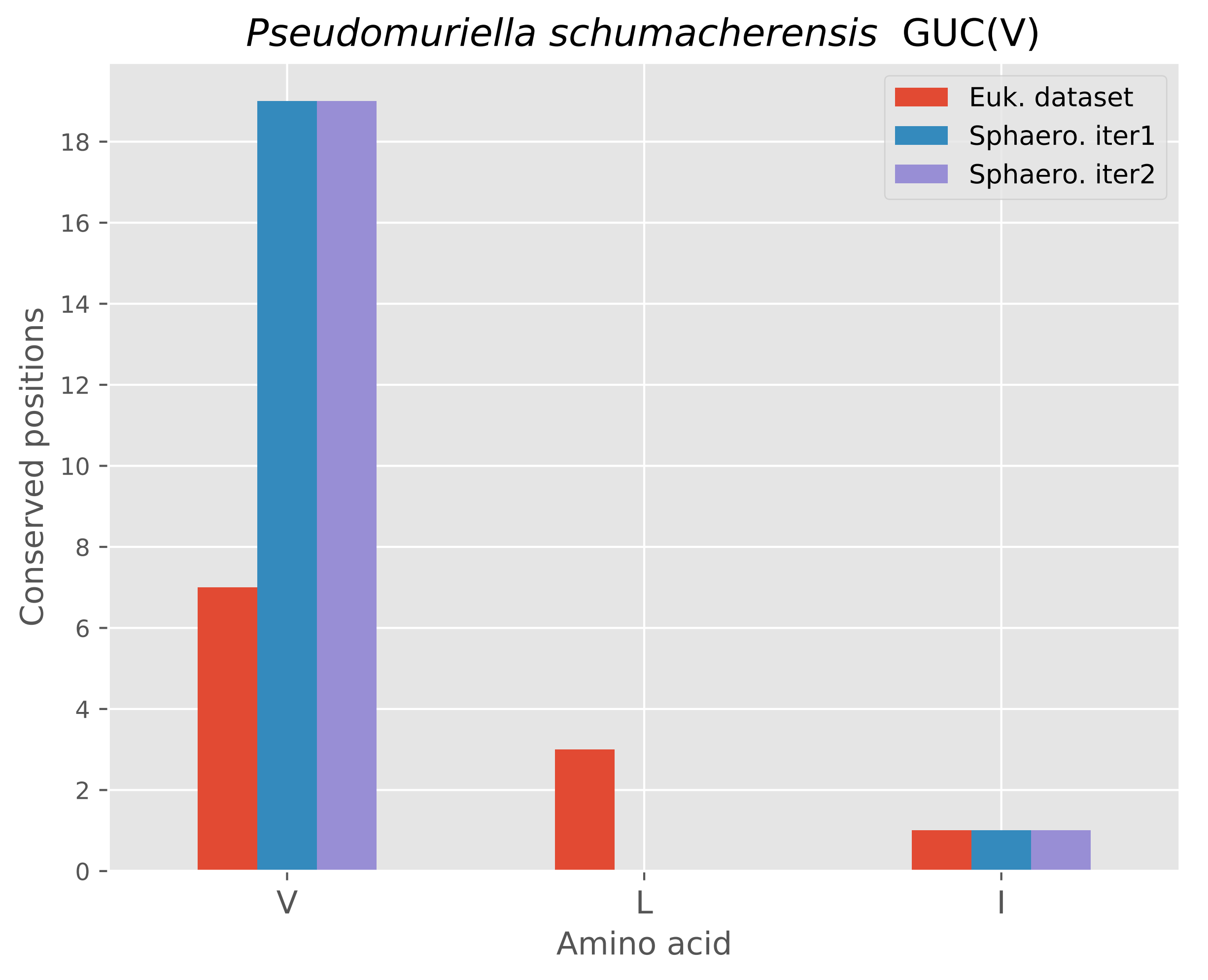
Pseudomuriella schumacherensis GUC(V)
Euk. dataset
Sphaero. iter1
Sphaero. iter2
Conserved positions
0
2
4
6
8
10
12
14
16
18
V
L
I
Amino acid

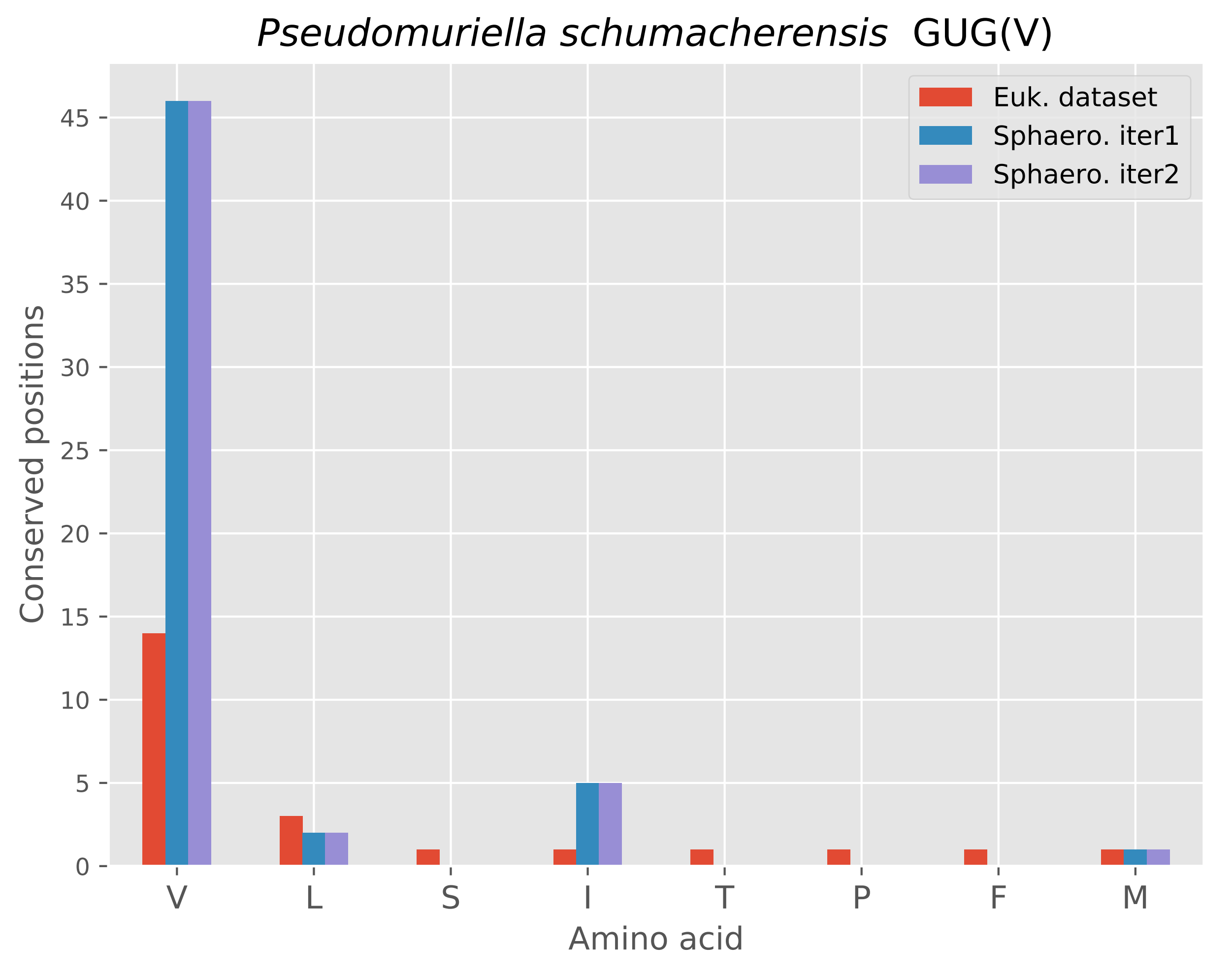

Pseudomuriella schumacherensis GUG(V)
Euk. dataset
Sphaero. iter1
Sphaero. iter2
Conserved positions
0
5
10
15
20
25
30
35
40
45
V
L
S
I
T
P
F
M
Amino acid

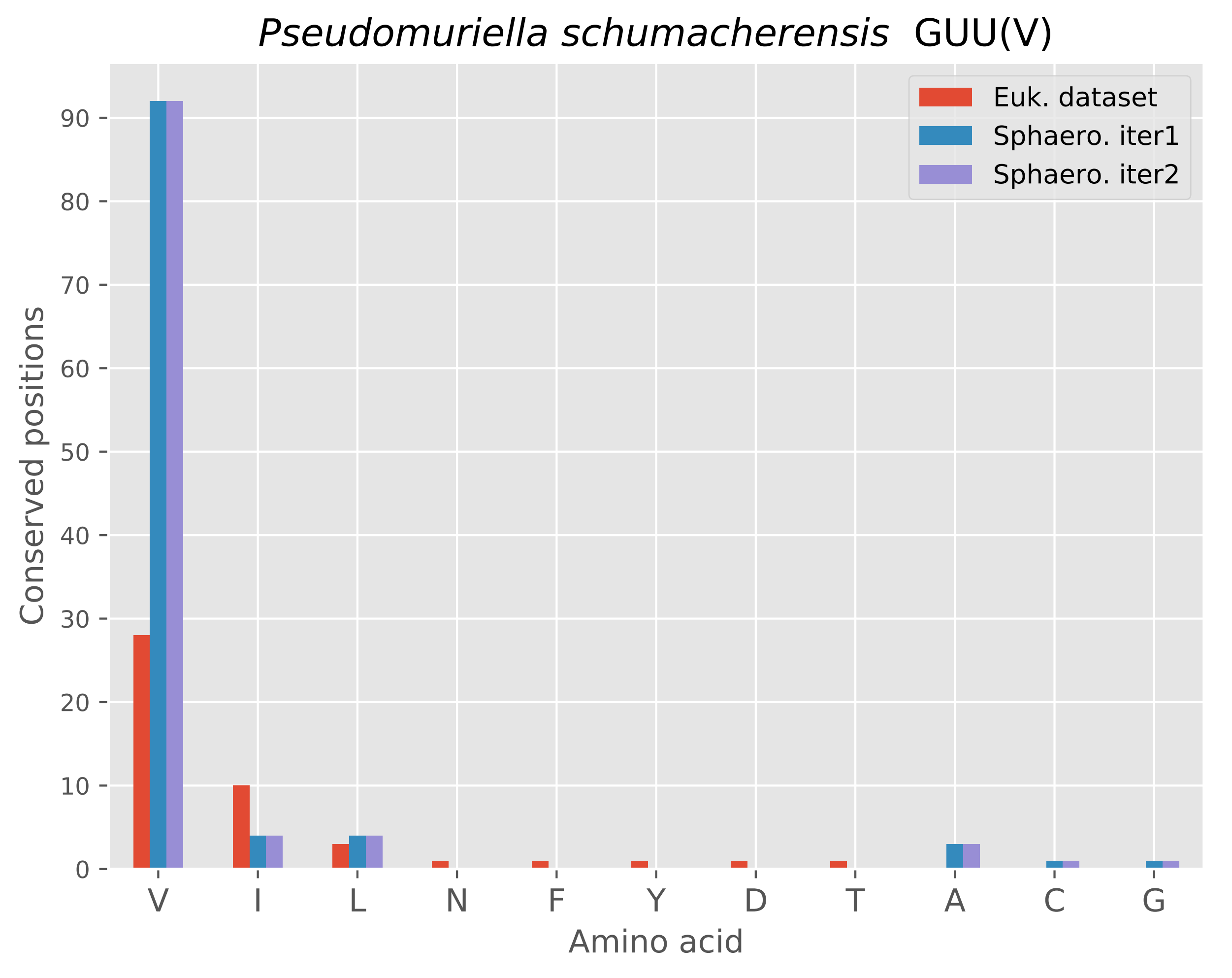

Pseudomuriella schumacherensis GUU(V)
Euk. dataset
Sphaero. iter1
Sphaero. iter2
Conserved positions
0
10
20
30
40
50
60
70
80
90
V
I
L
N
F
Y
D
T
A
C
G
Amino acid

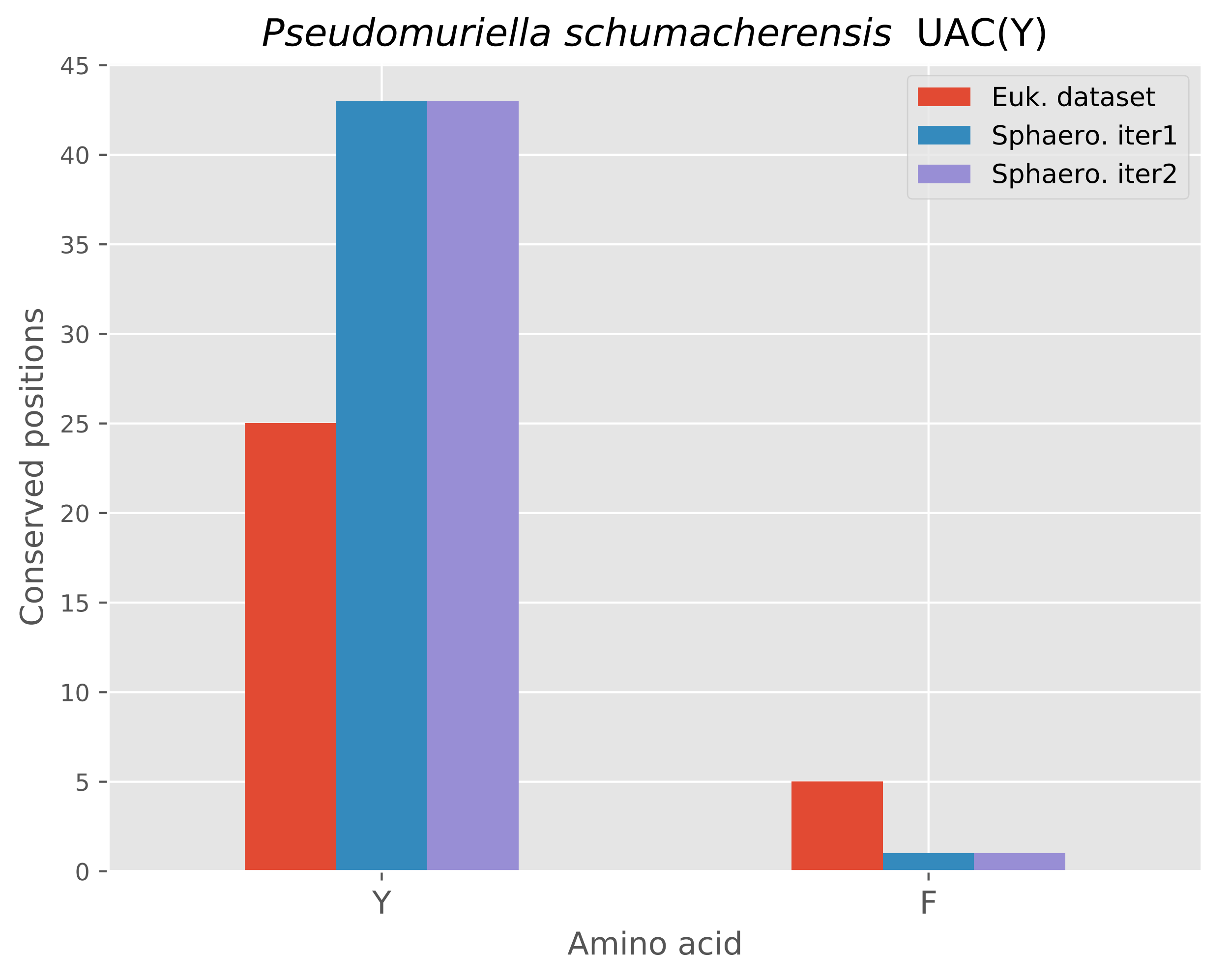

Pseudomuriella schumacherensis UAC(Y)
Euk. dataset
Sphaero. iter1
Sphaero. iter2
Conserved positions
0
5
10
15
20
25
30
35
40
45
Y
F
Amino acid

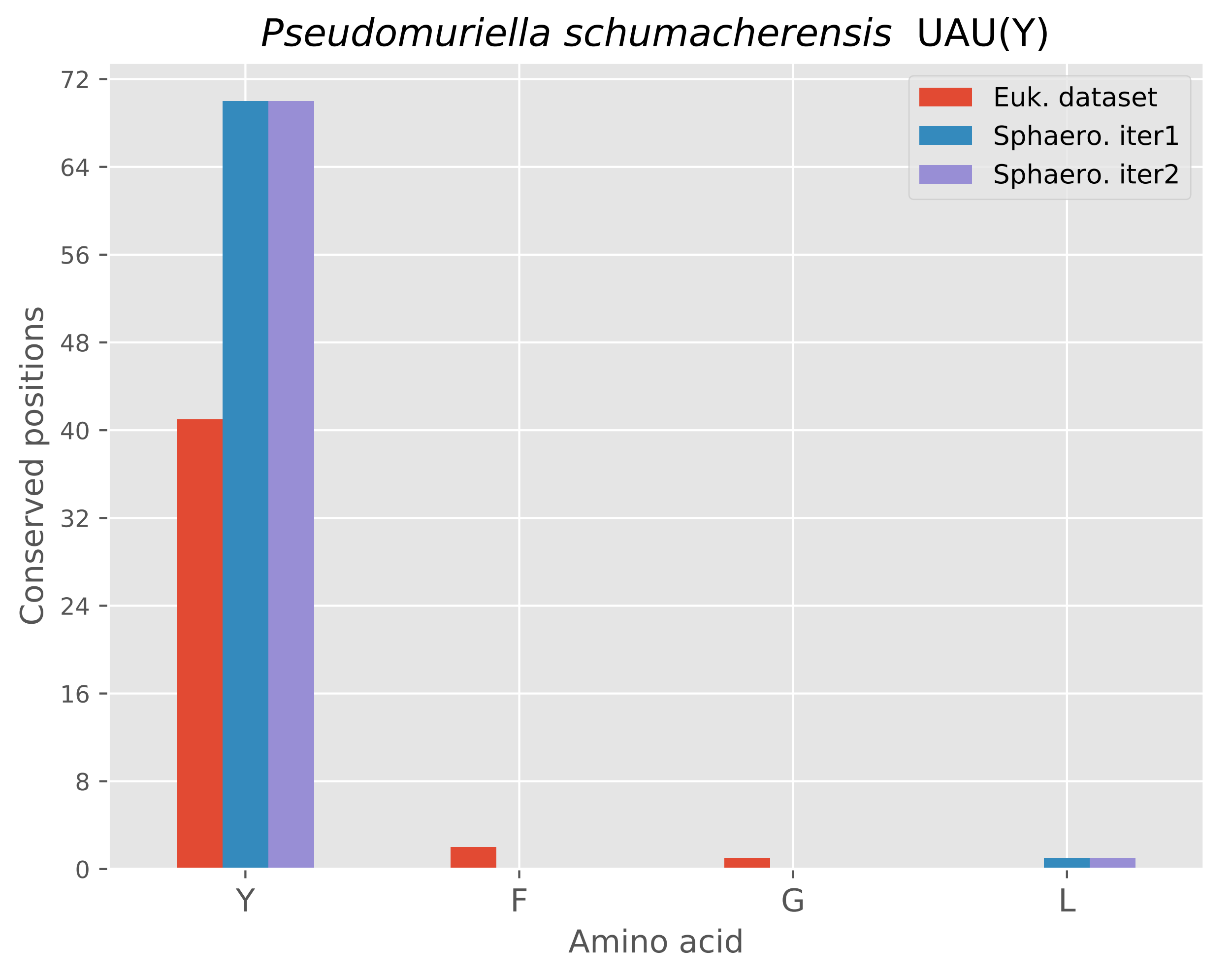

Pseudomuriella schumacherensis UAU(Y)
Euk. dataset
Sphaero. iter1
Sphaero. iter2
Conserved positions
0
8
16
24
32
40
48
56
64
72
Y
F
G
L
Amino acid

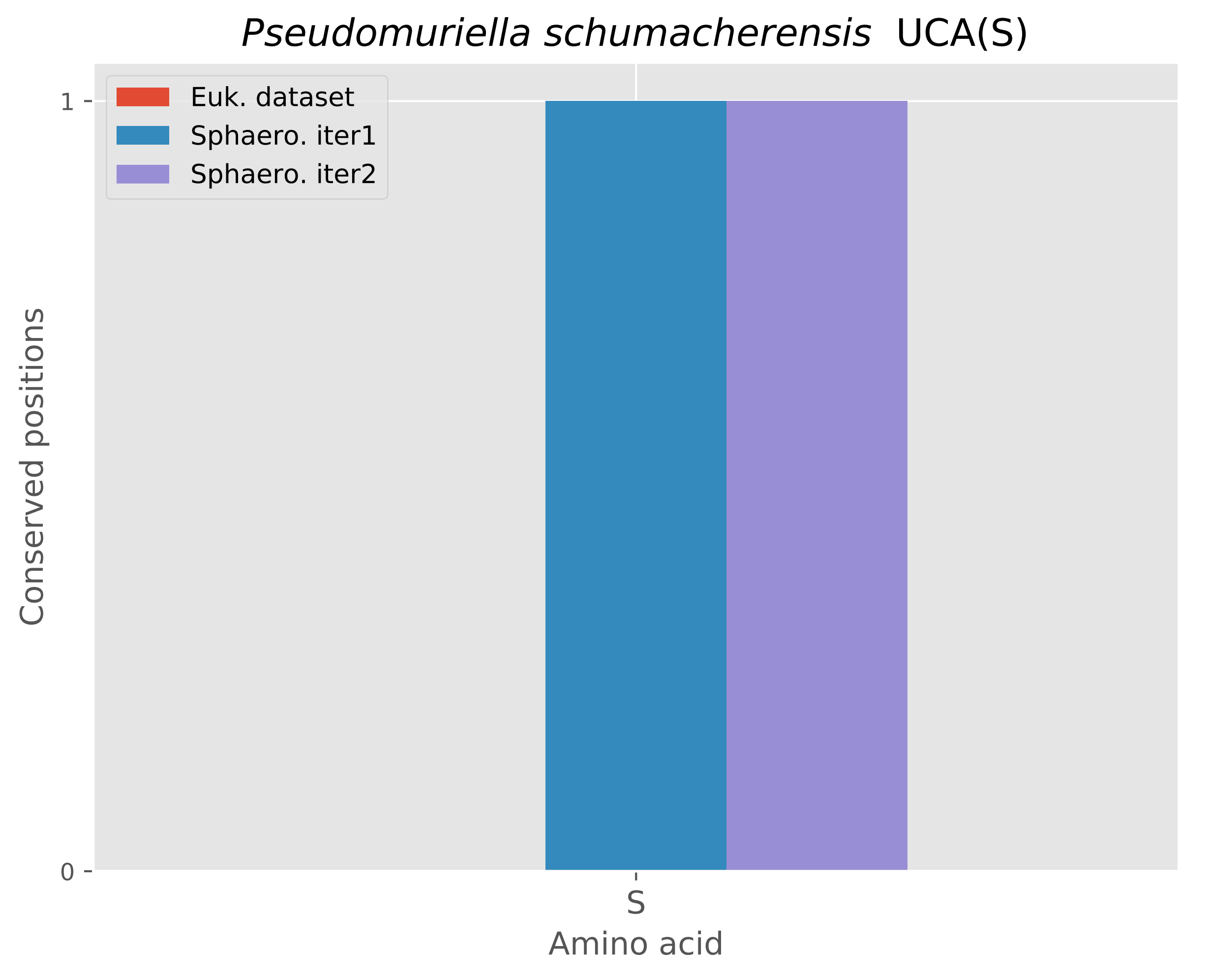

Pseudomuriella schumacherensis UCA(S)
Euk. dataset
Sphaero. iter1
Sphaero. iter2
1
0
Conserved positions
S
Amino acid

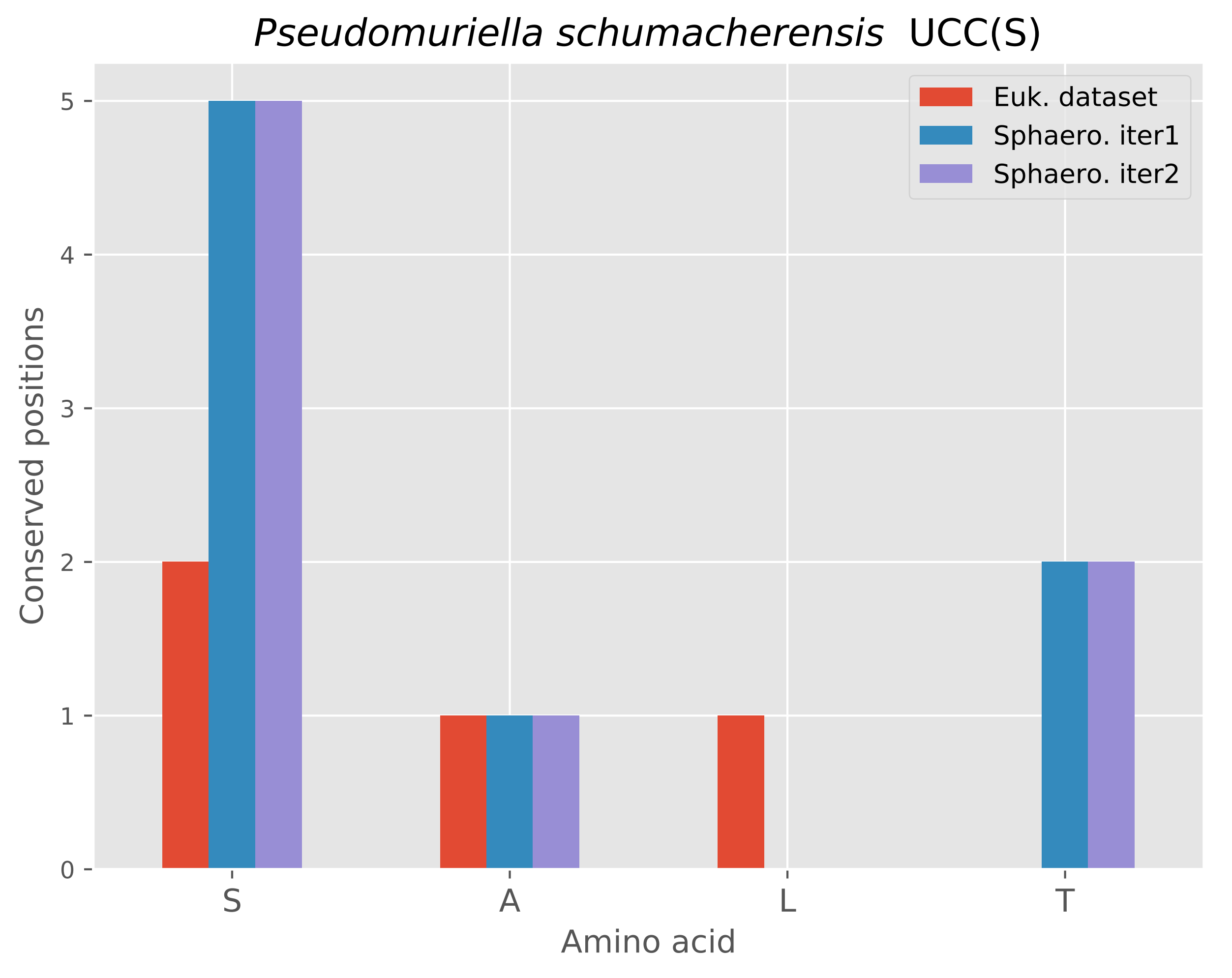

Pseudomuriella schumacherensis UCC(S)
Euk. dataset
Sphaero. iter1
Sphaero. iter2
Conserved positions
0
1
2
3
4
5
S
A
L
T
Amino acid

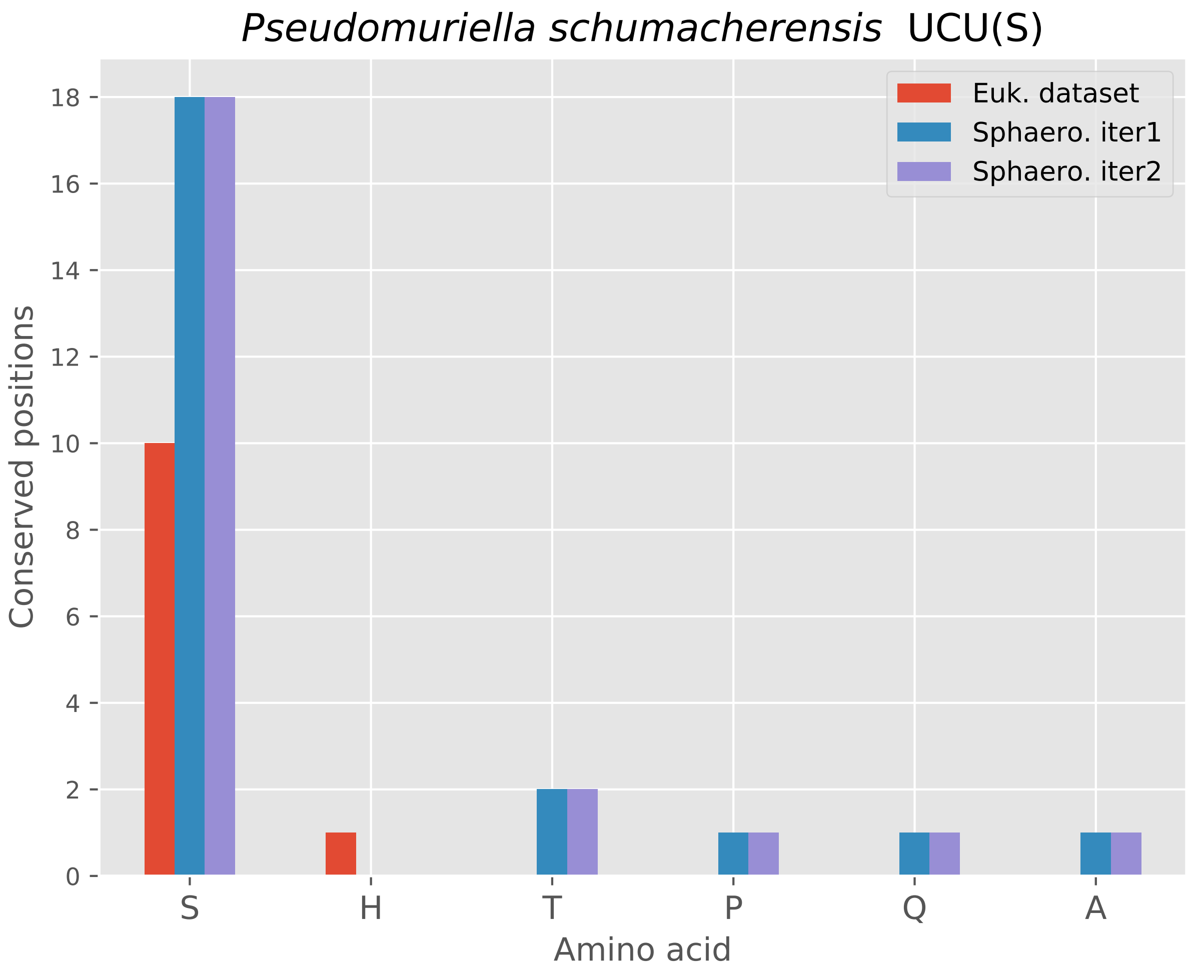
Pseudomuriella schumacherensis UCU(S)
Euk. dataset
Sphaero. iter1
Sphaero. iter2
Conserved positions
0
2
4
6
8
10
12
14
16
18
S
H
T
P
Q
A
Amino acid

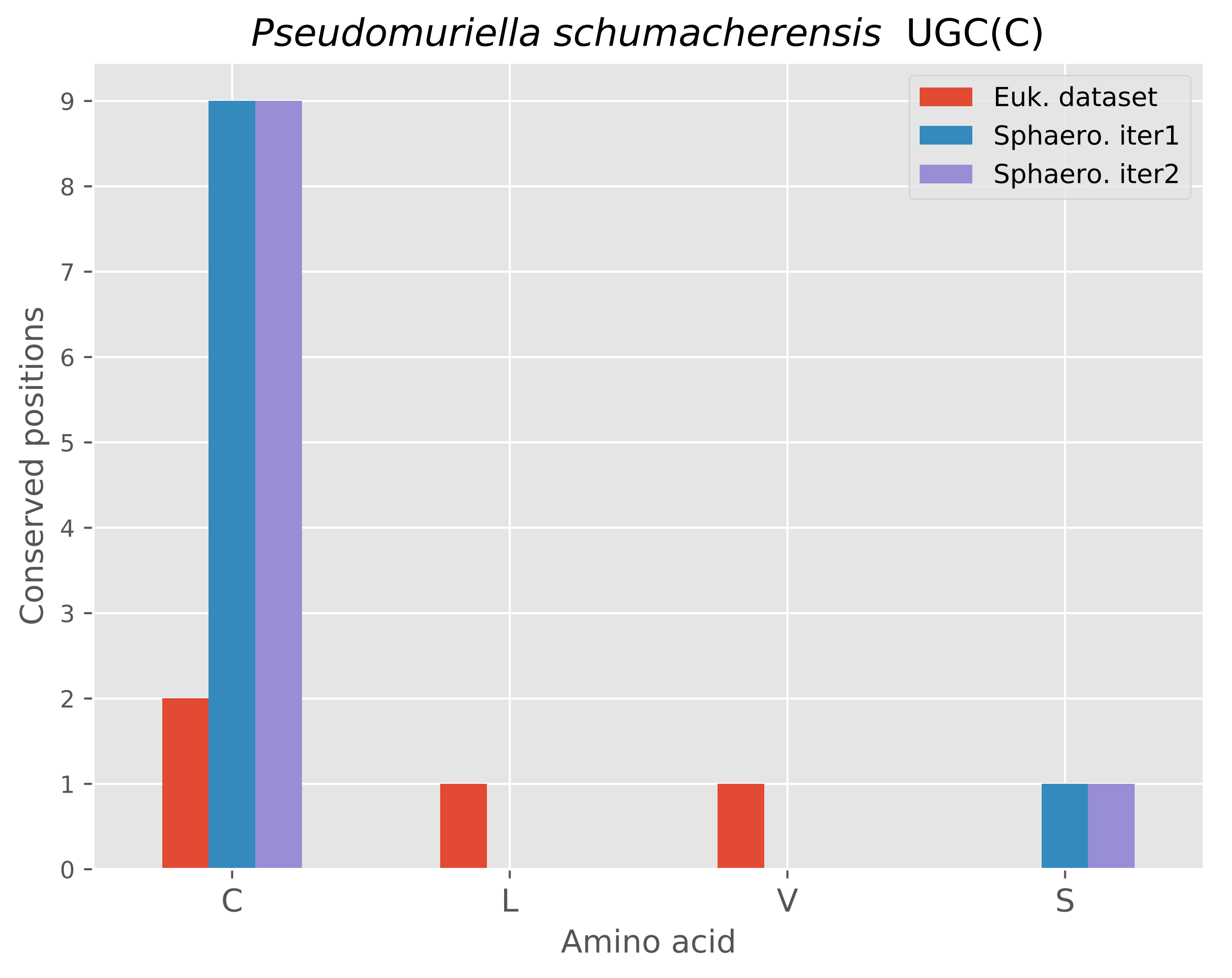

Pseudomuriella schumacherensis UGC(C)
Euk. dataset
Sphaero. iter1
Sphaero. iter2
Conserved positions
0
1
2
3
4
5
6
7
8
9
C
L
V
S
Amino acid

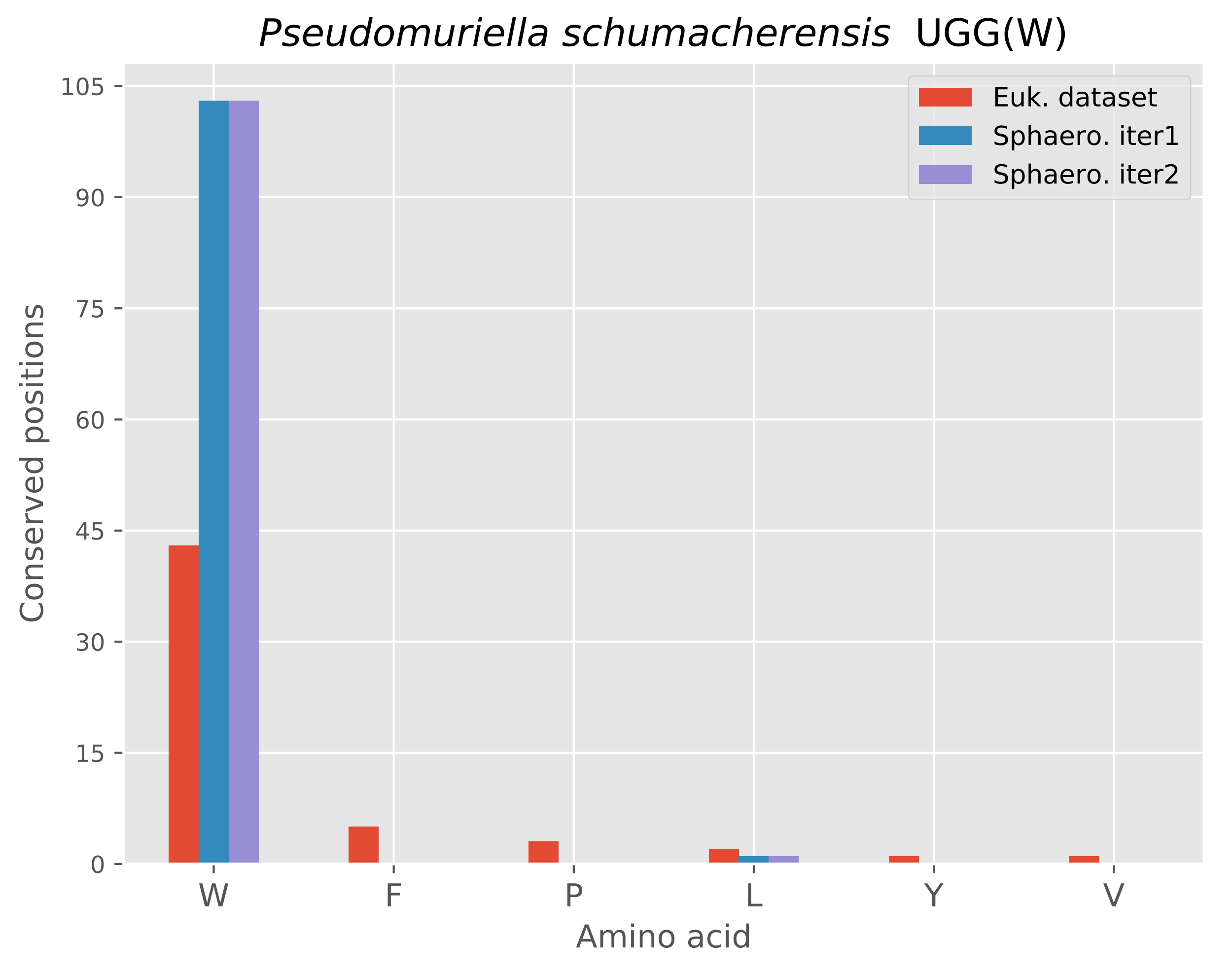

Pseudomuriella schumacherensis UGG(W)
Euk. dataset
Sphaero. iter1
Sphaero. iter2
105
90
75
60
45
30
15
0
Conserved positions
W
F
P
L
Y
V
Amino acid

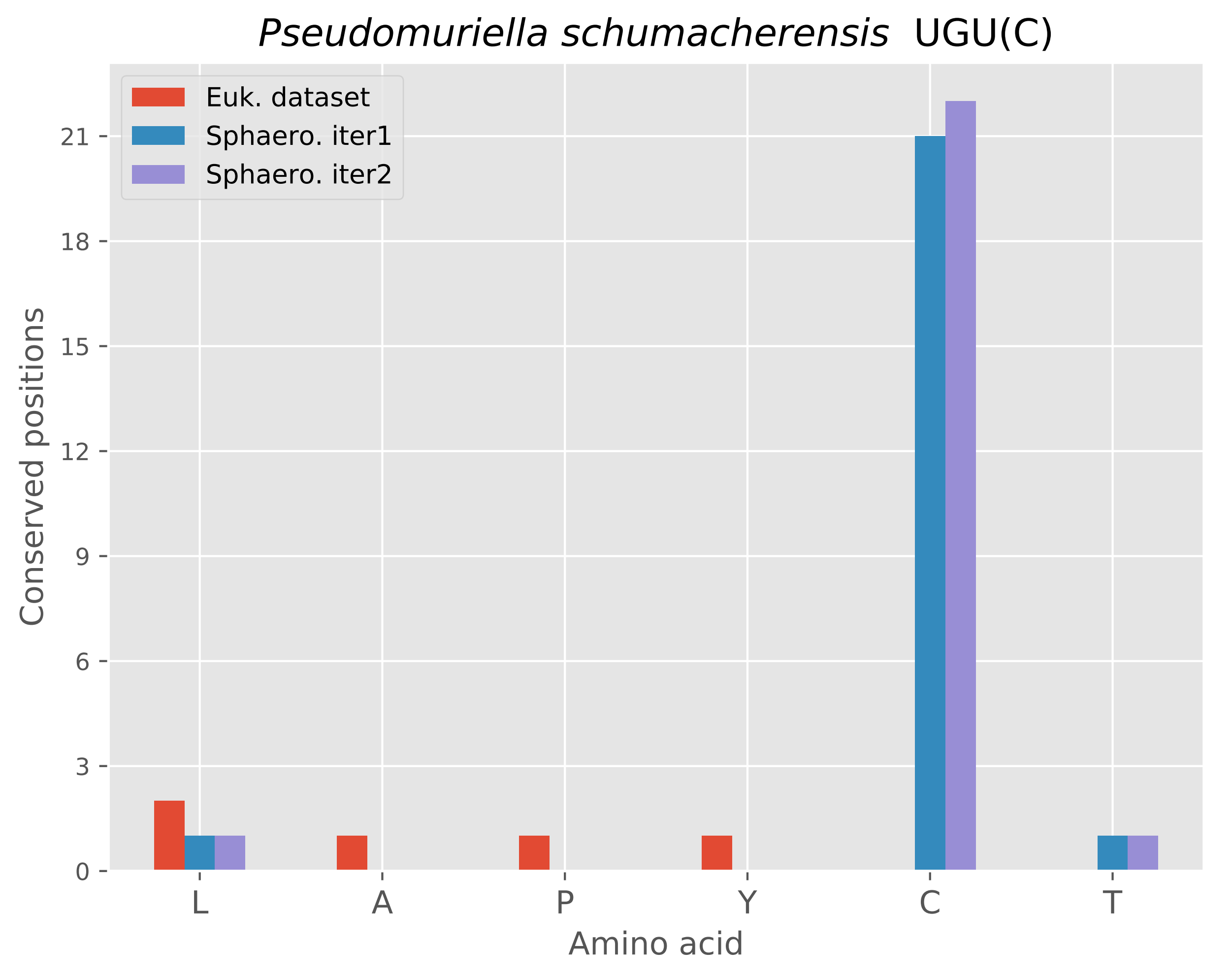

Pseudomuriella schumacherensis UGU(C)
Euk. dataset
Sphaero. iter1
Sphaero. iter2
Conserved positions
0
3
6
9
12
15
18
21
L
A
P
Y
C
T
Amino acid

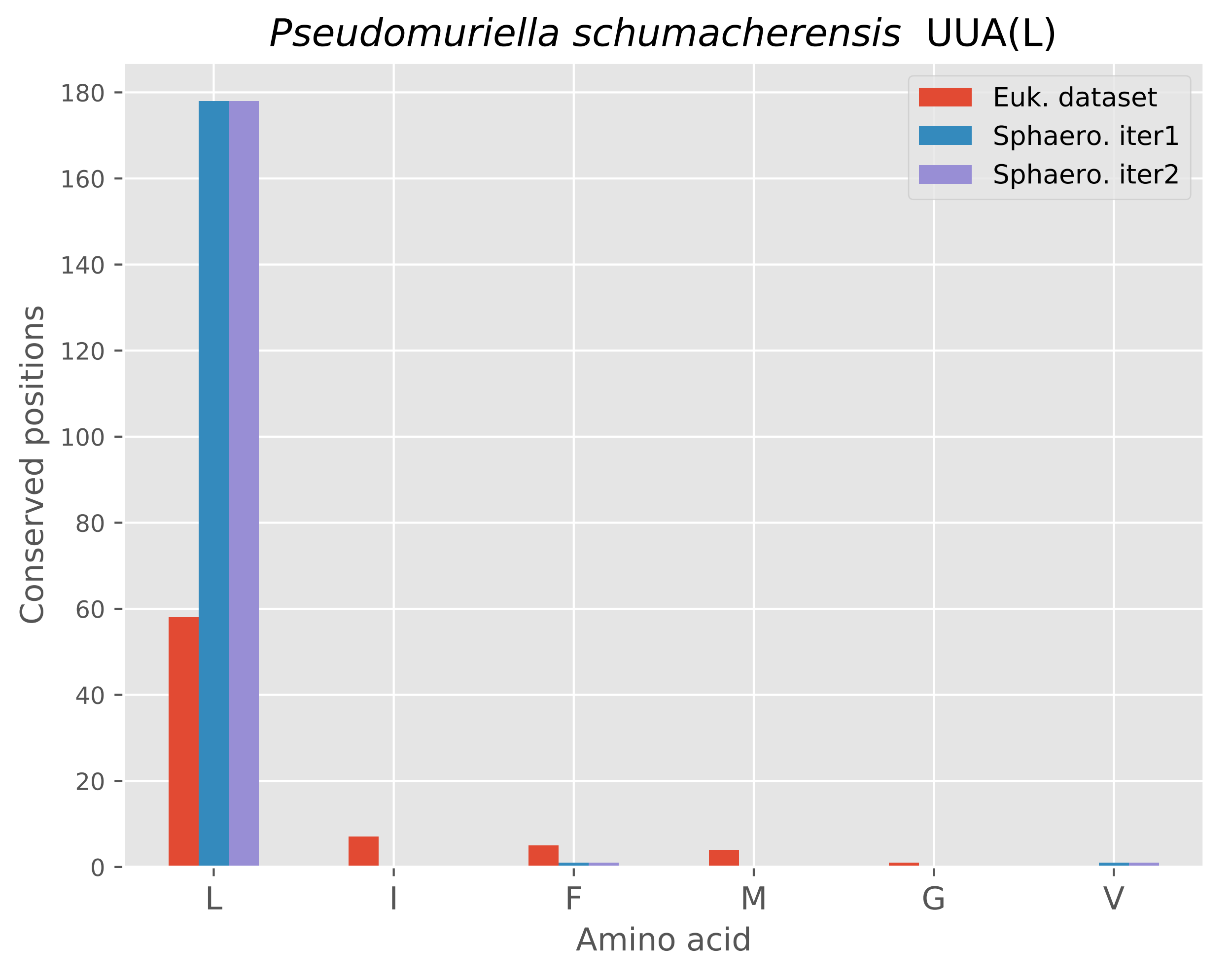

Pseudomuriella schumacherensis UUA(L)
Conserved positions
0
20
40
60
80
100
120
140
160
180
Amino acid
L
I
F
M
G
V
Euk. dataset
Sphaero. iter1
Sphaero. iter2

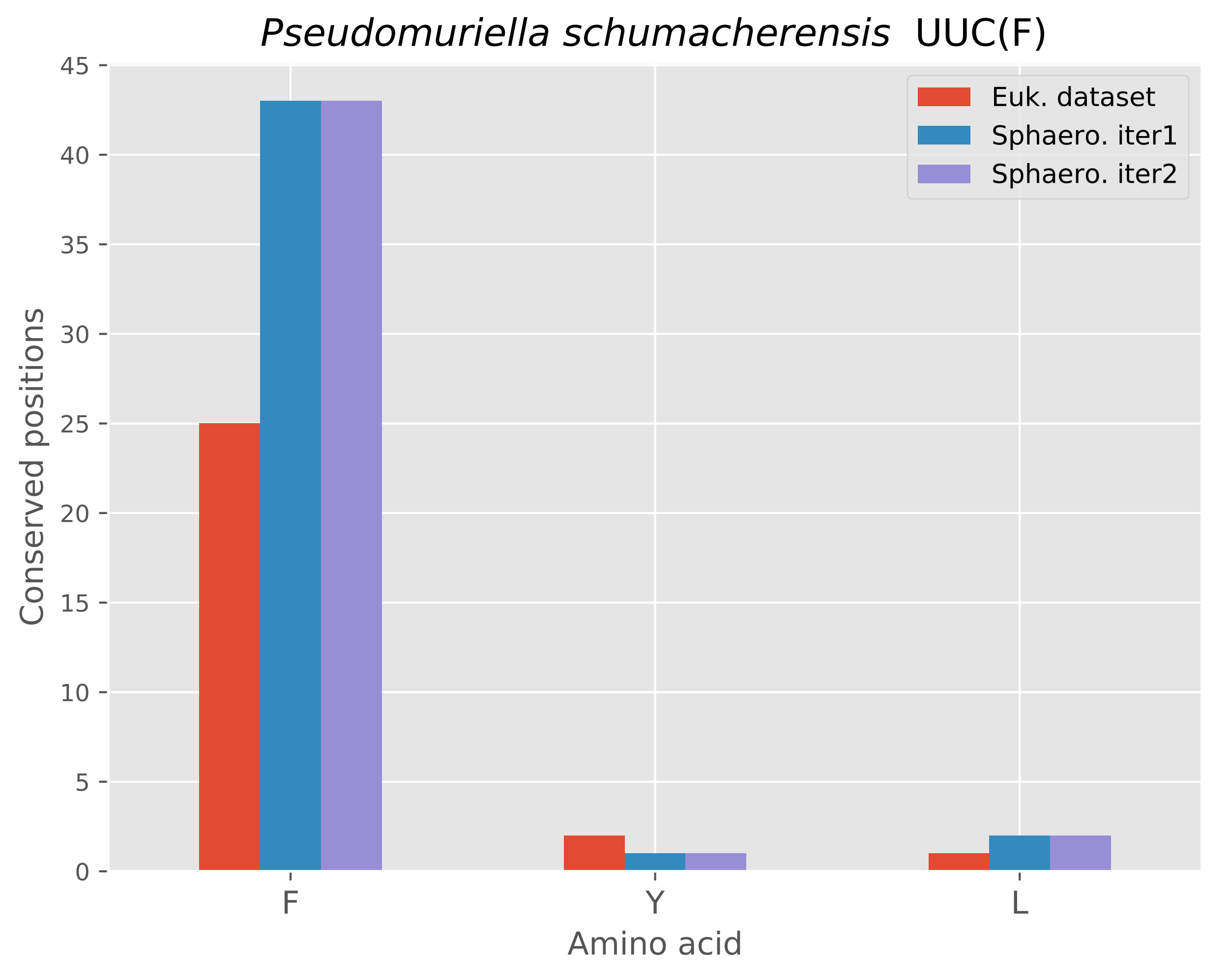

Pseudomuriella schumacherensis UUC(F)
45
40
35
30
25
20
15
10
5
0
Conserved positions
Euk. dataset
Sphaero. iter1
Sphaero. iter2
F
Y
L
Amino acid

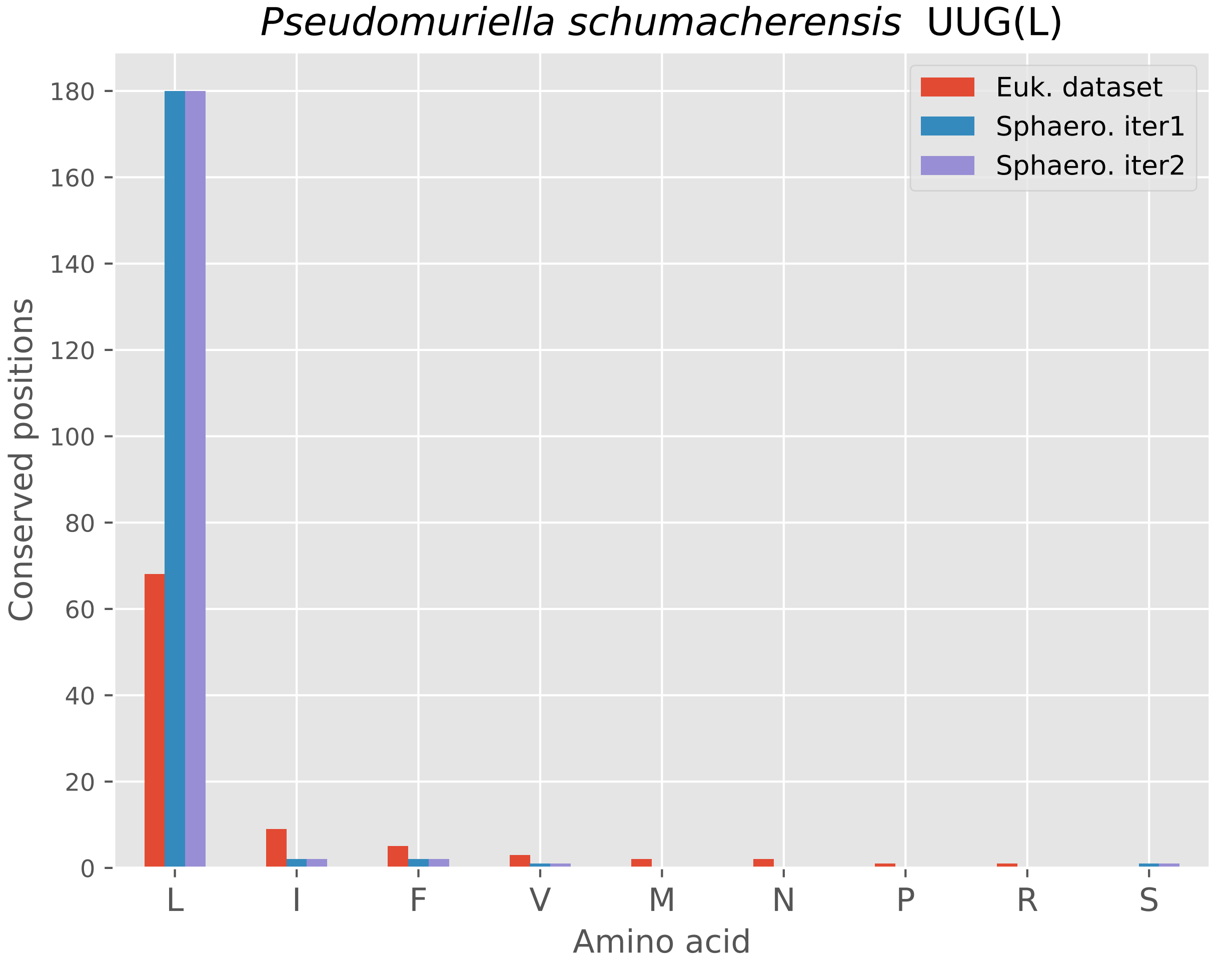
Pseudomuriella schumacherensis UUG(L)
Euk. dataset
Sphaero. iter1
Sphaero. iter2
Conserved positions
0
20
40
60
80
100
120
140
160
180
Amino acid
L
I
F
V
M
N
P
R
S

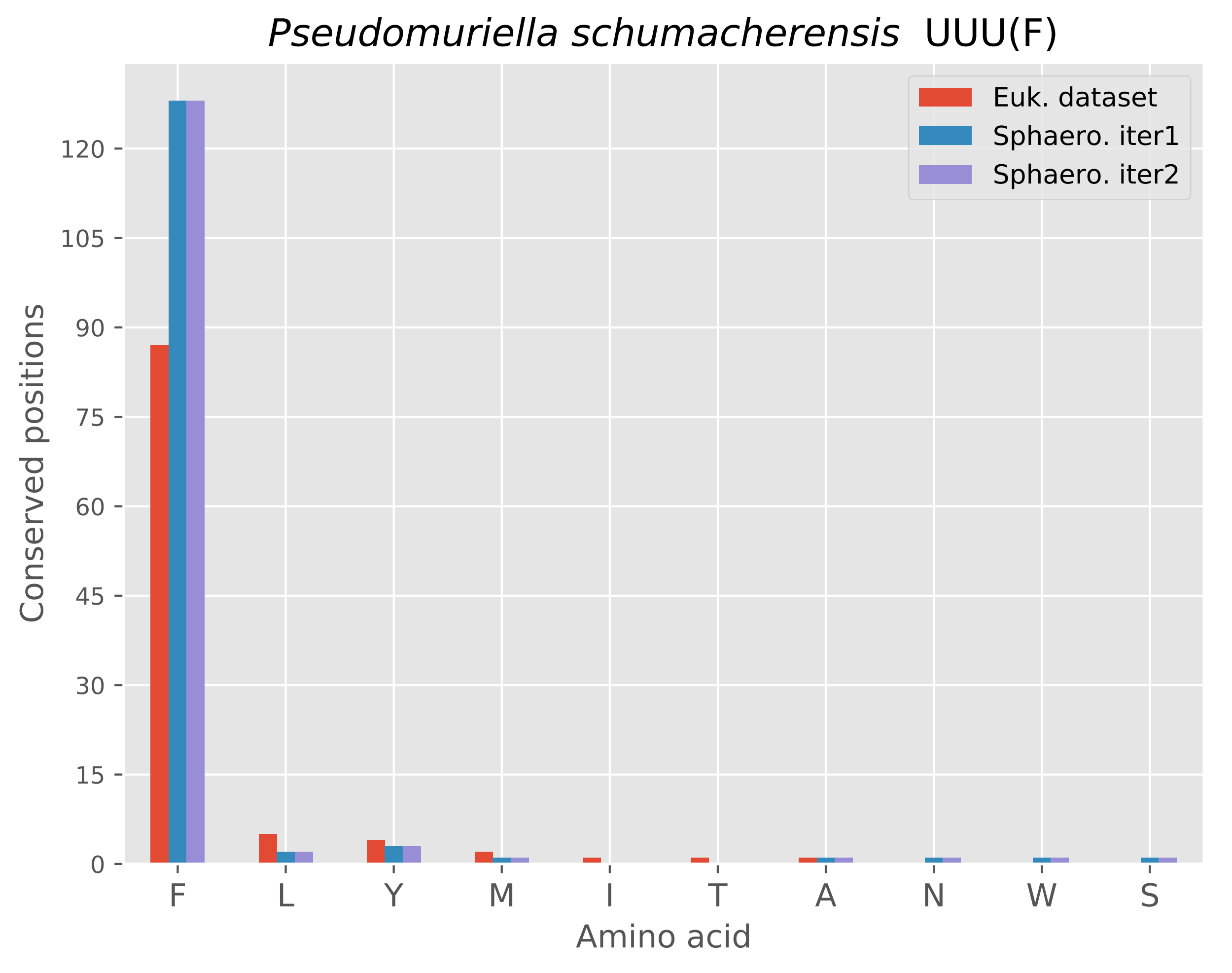
Pseudomuriella schumacherensis UUU(F)
Euk. dataset
Sphaero. iter1
Sphaero. iter2
Conserved positions
0
15
30
45
60
75
90
105
120
F
L
Y
M
I
T
A
N
W
S
Amino acid

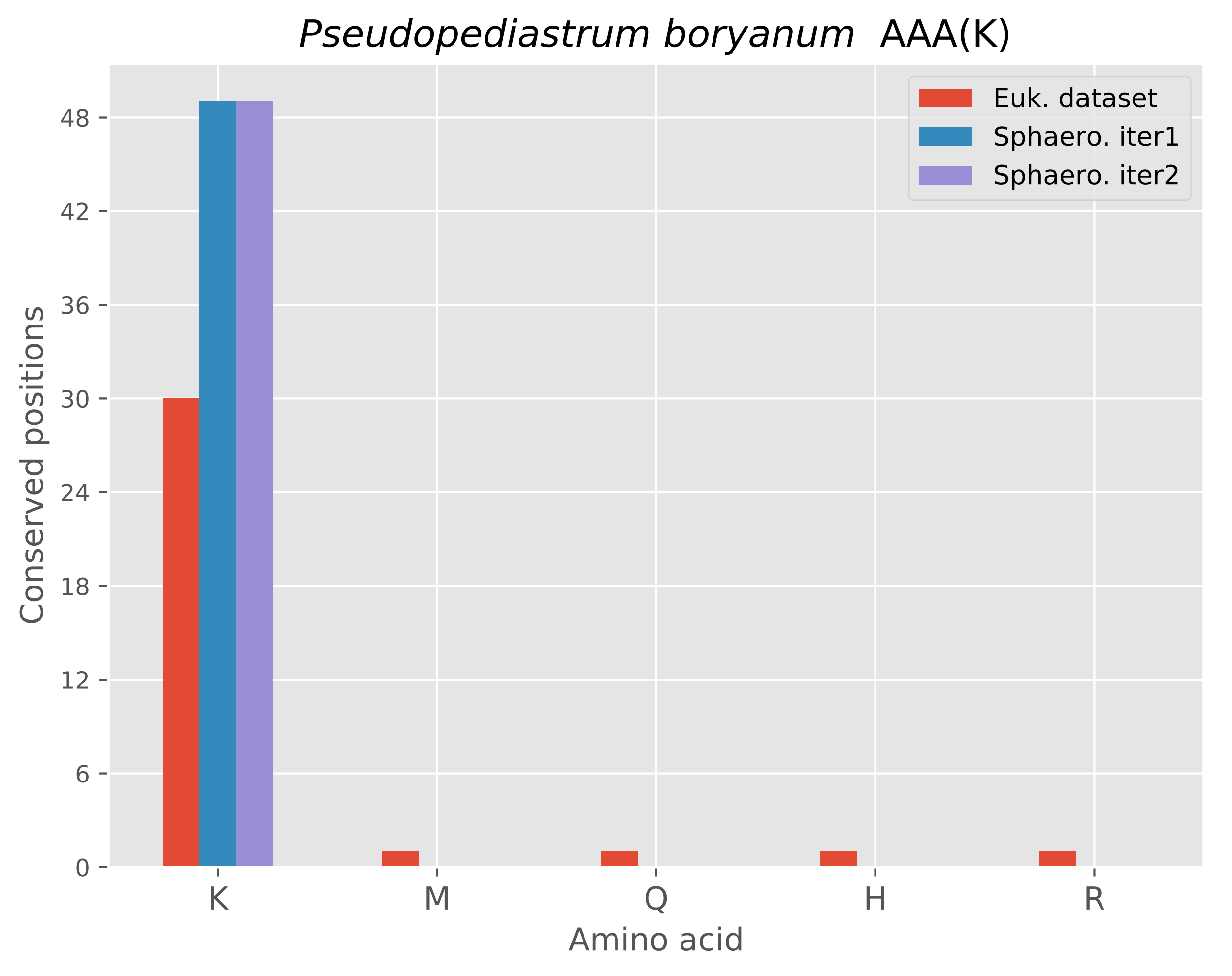

Pseudopediastrum boryanum AAA(K)
Euk. dataset
Sphaero. iter1
Sphaero. iter2
Conserved positions
0
6
12
18
24
30
36
42
48
K
M
Q
H
R
Amino acid

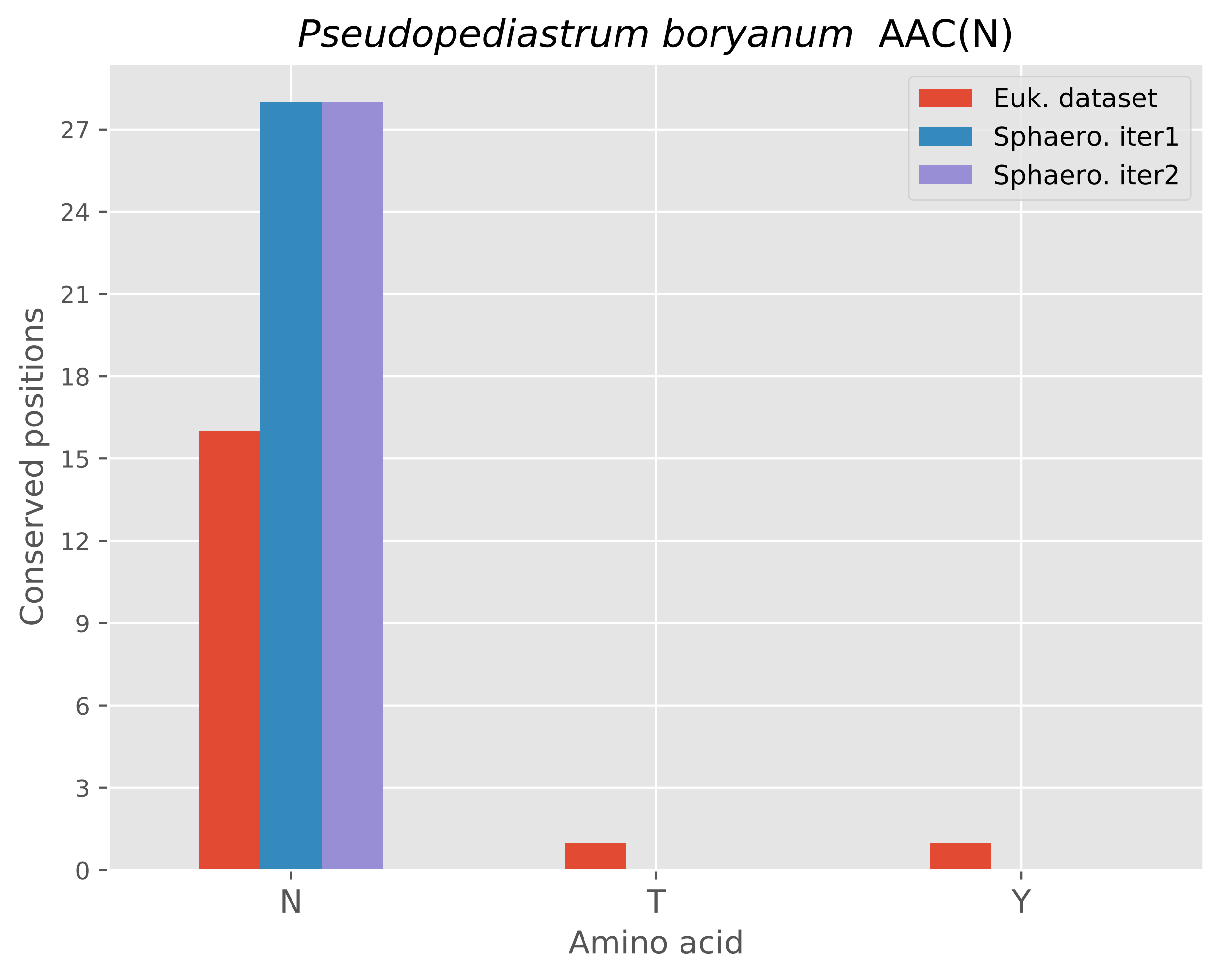

Pseudopediastrum boryanum AAC(N)
Euk. dataset
Sphaero. iter1
Sphaero. iter2
Conserved positions
0
3
6
9
12
15
18
21
24
27
N
T
Y
Amino acid

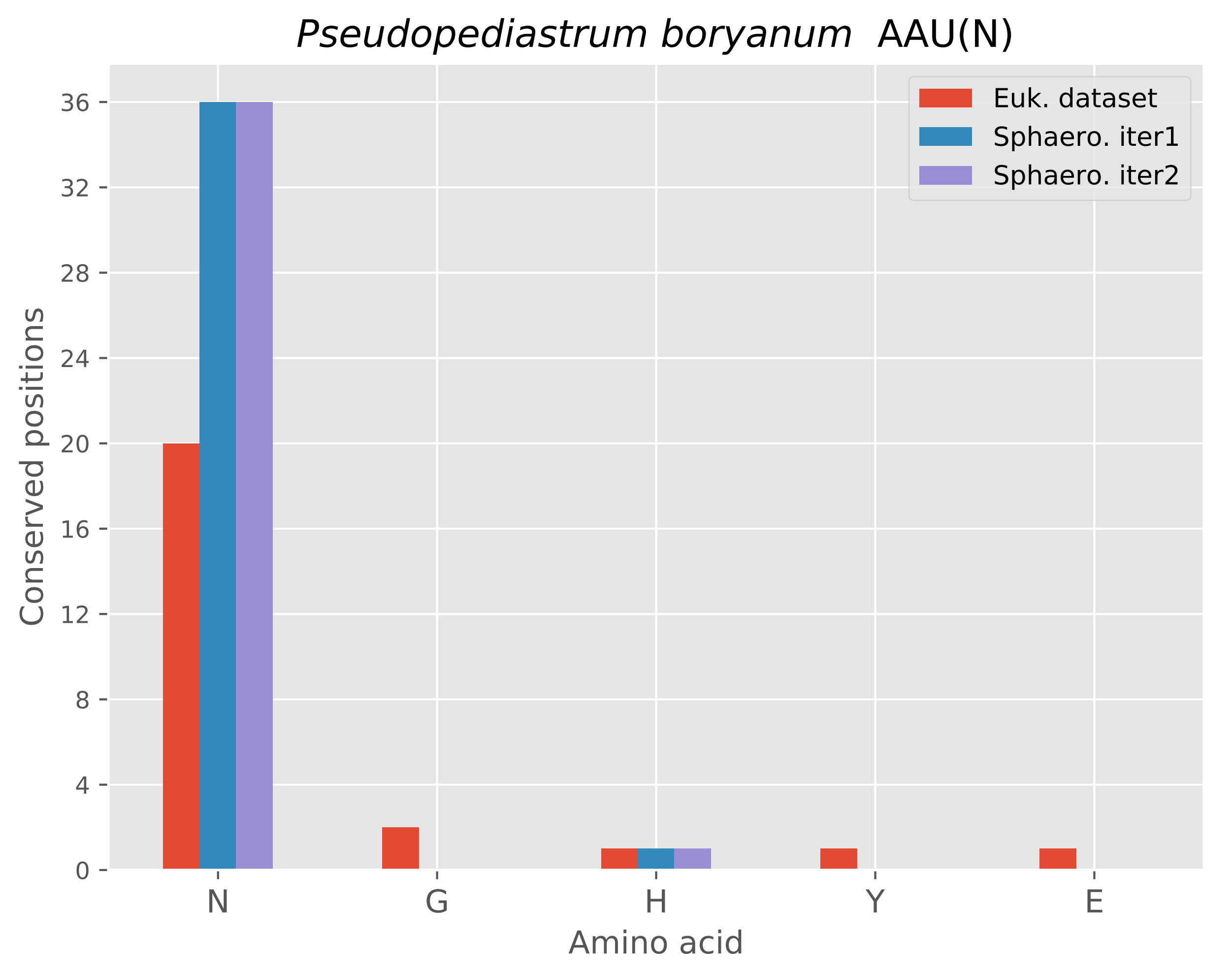

Pseudopediastrum boryanum AAU(N)
Euk. dataset
Sphaero. iter1
Sphaero. iter2
Conserved positions
0
4
8
12
16
20
24
28
32
36
N
G
H
Y
E
Amino acid

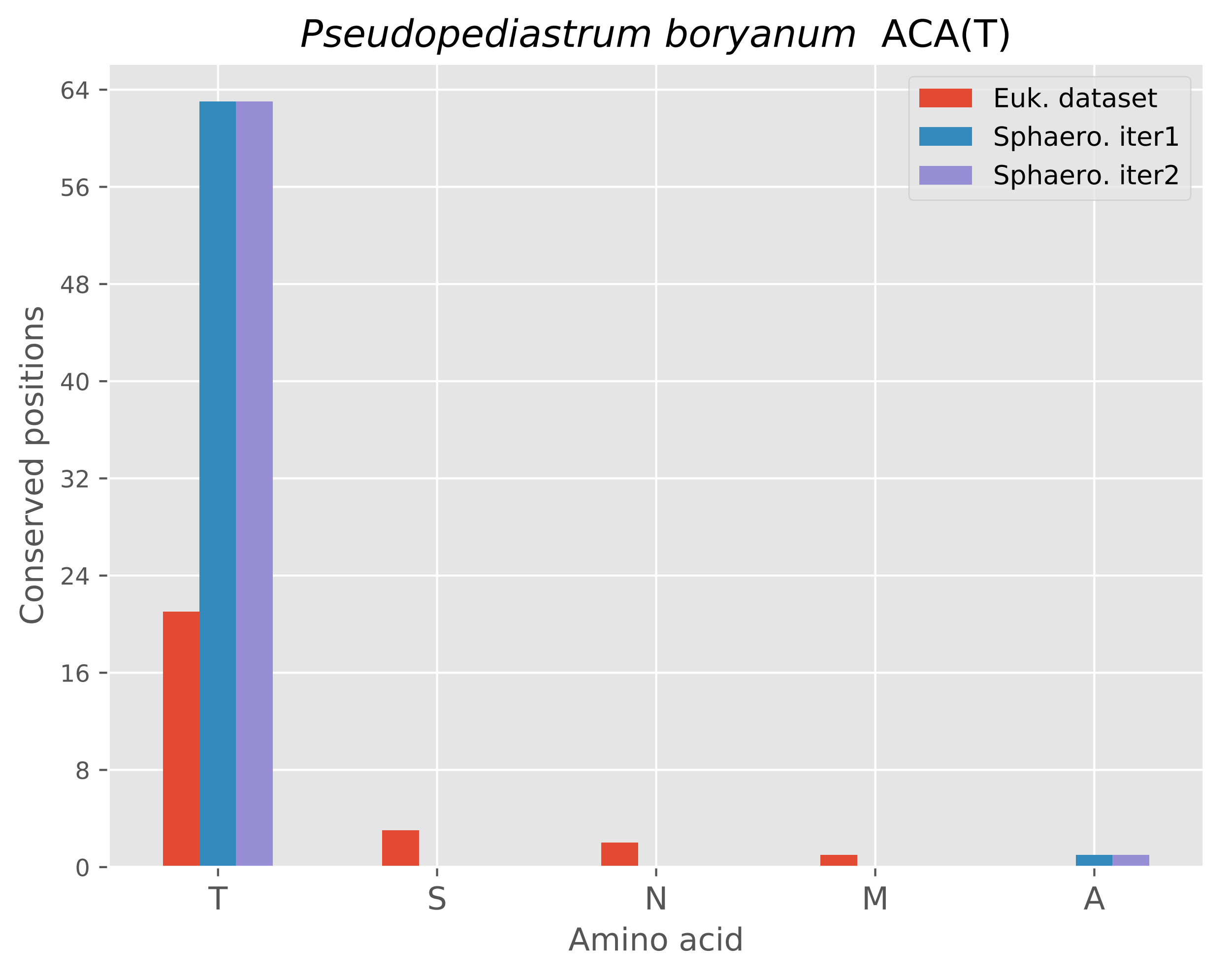

Pseudopediastrum boryanum ACA(T)
Conserved positions
64
56
48
40
32
24
16
8
0
T
S
N
M
A
Amino acid
Euk. dataset
Sphaero. iter1
Sphaero. iter2

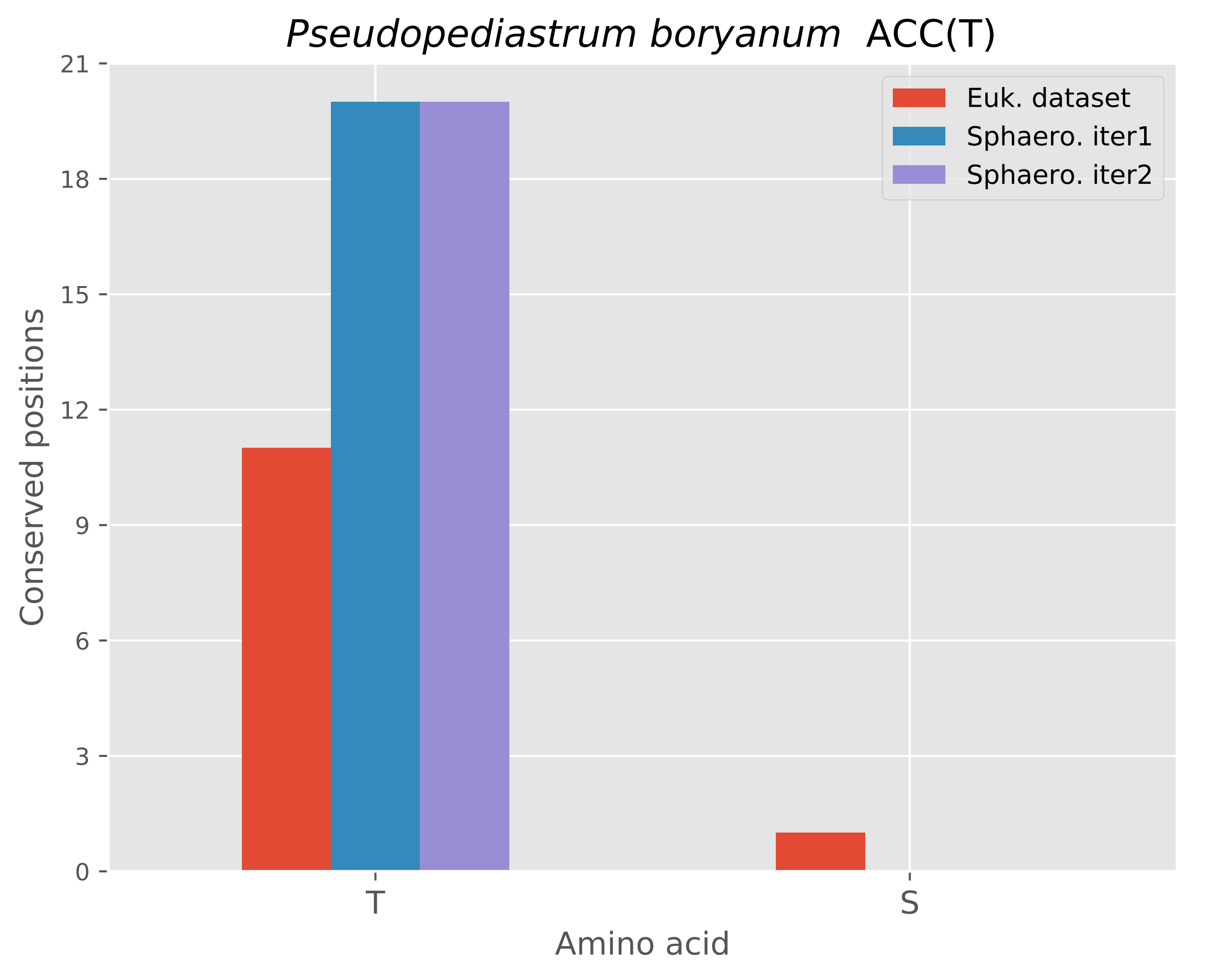

Pseudopediastrum boryanum ACC(T)
Euk. dataset
Sphaero. iter1
Sphaero. iter2
Conserved positions
0
3
6
9
12
15
18
21
T
S
Amino acid

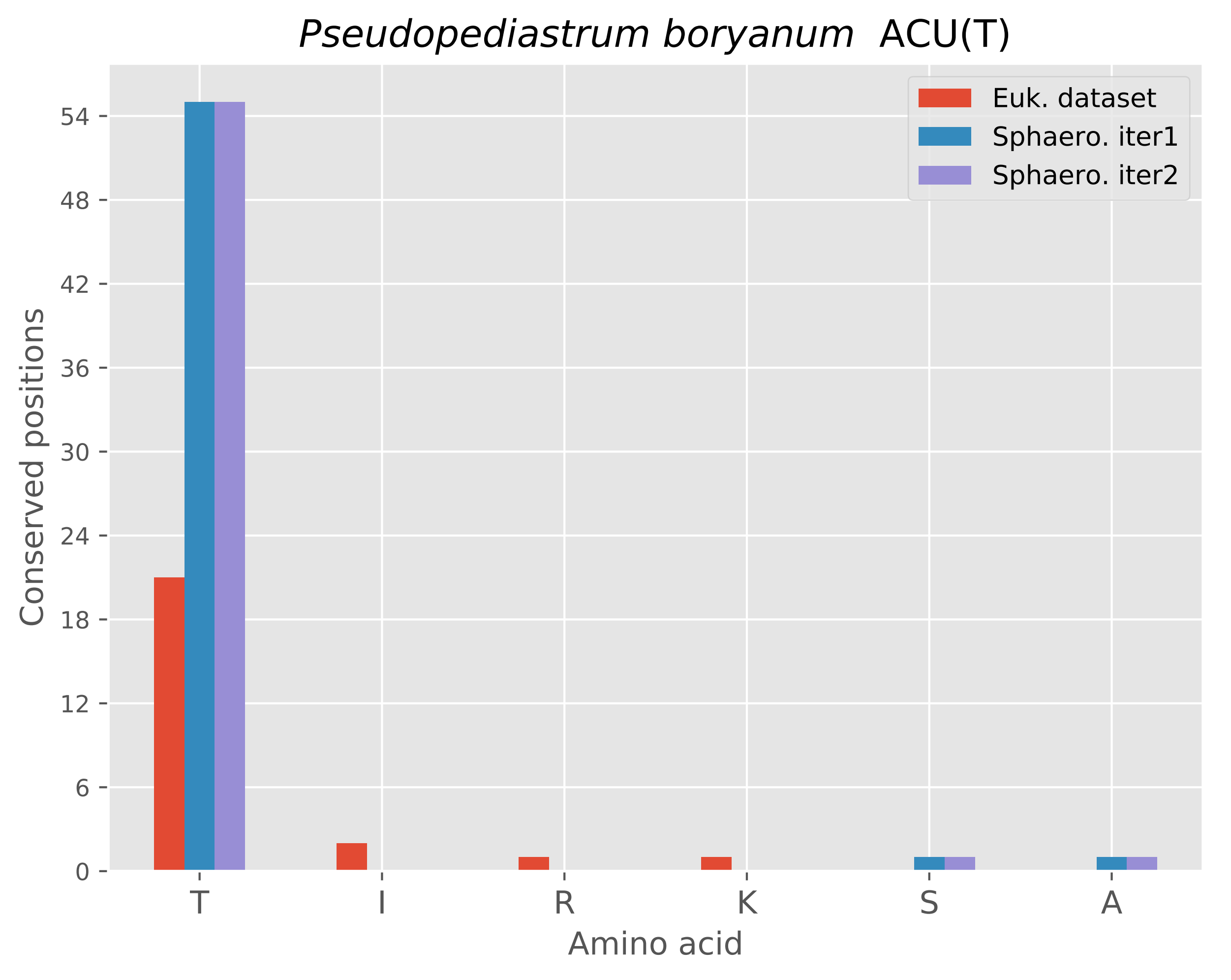

Pseudopediastrum boryanum ACU(T)
Euk. dataset
Sphaero. iter1
Sphaero. iter2
Conserved positions
0
6
12
18
24
30
36
42
48
54
T
I
R
K
S
A
Amino acid

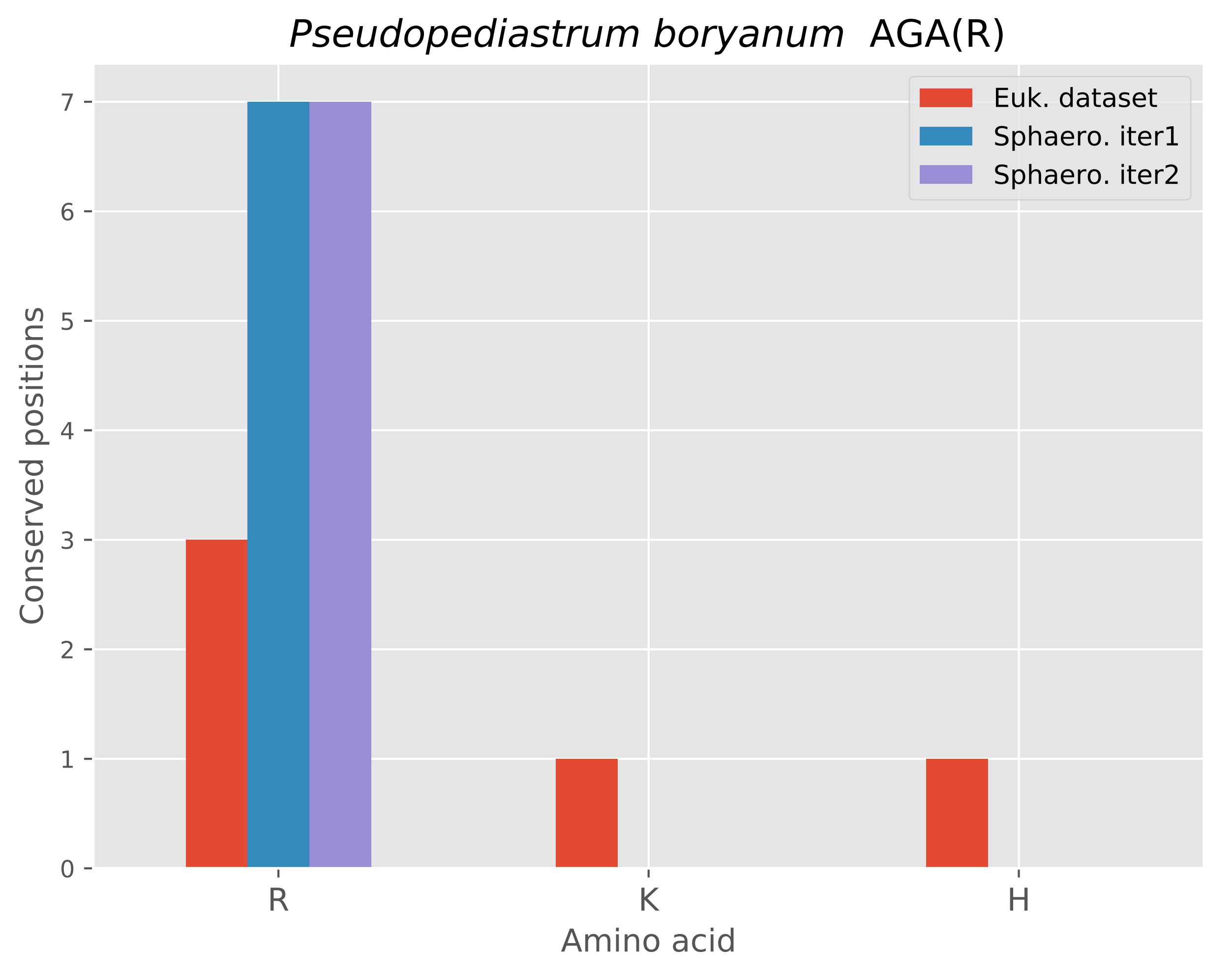

Pseudopediastrum boryanum AGA(R)
Euk. dataset
Sphaero. iter1
Sphaero. iter2
Conserved positions
0
1
2
3
4
5
6
7
R
K
H
Amino acid

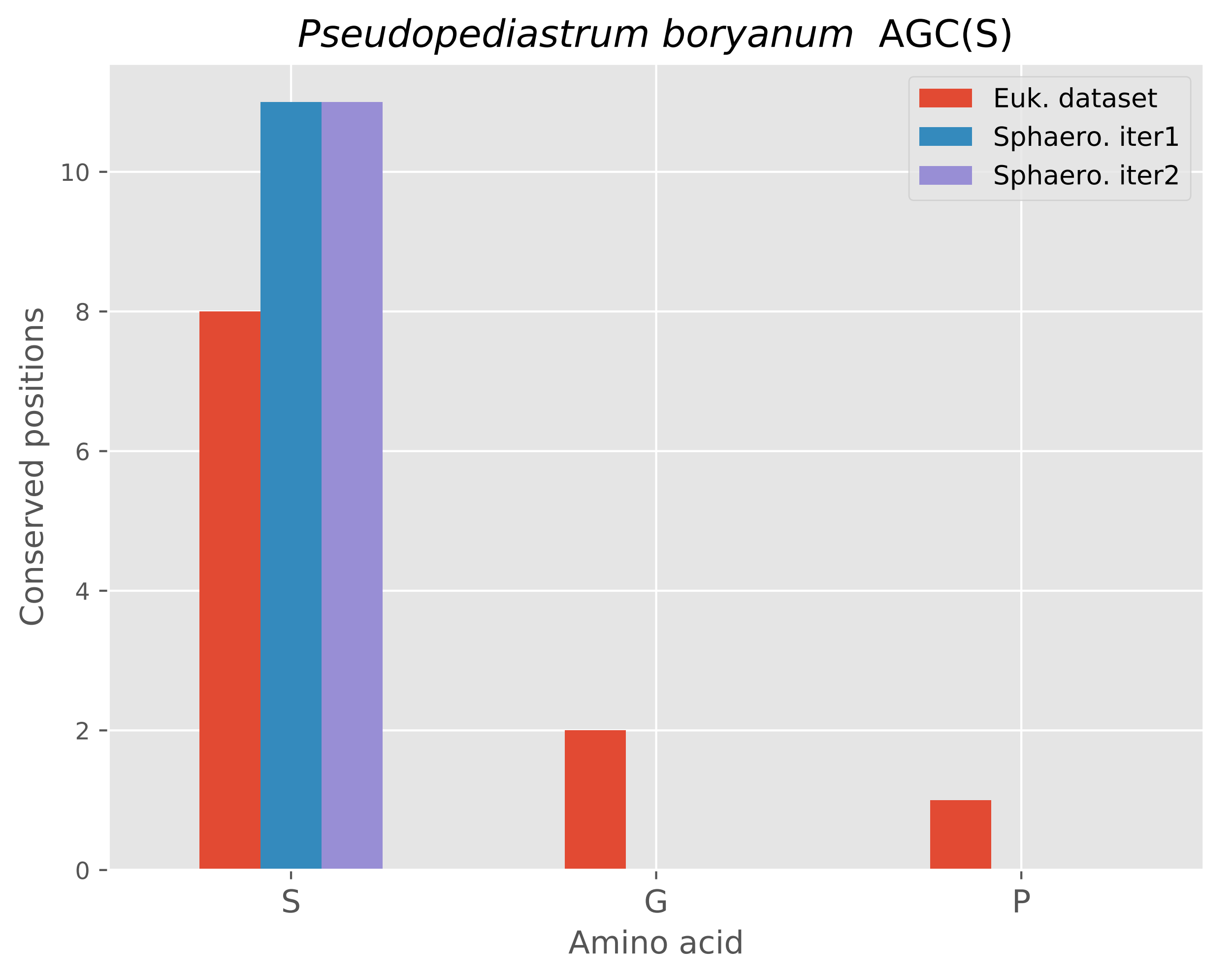

Pseudopediastrum boryanum AGC(S)
Euk. dataset
Sphaero. iter1
Sphaero. iter2
Conserved positions
0
2
4
6
8
10
S
G
P
Amino acid

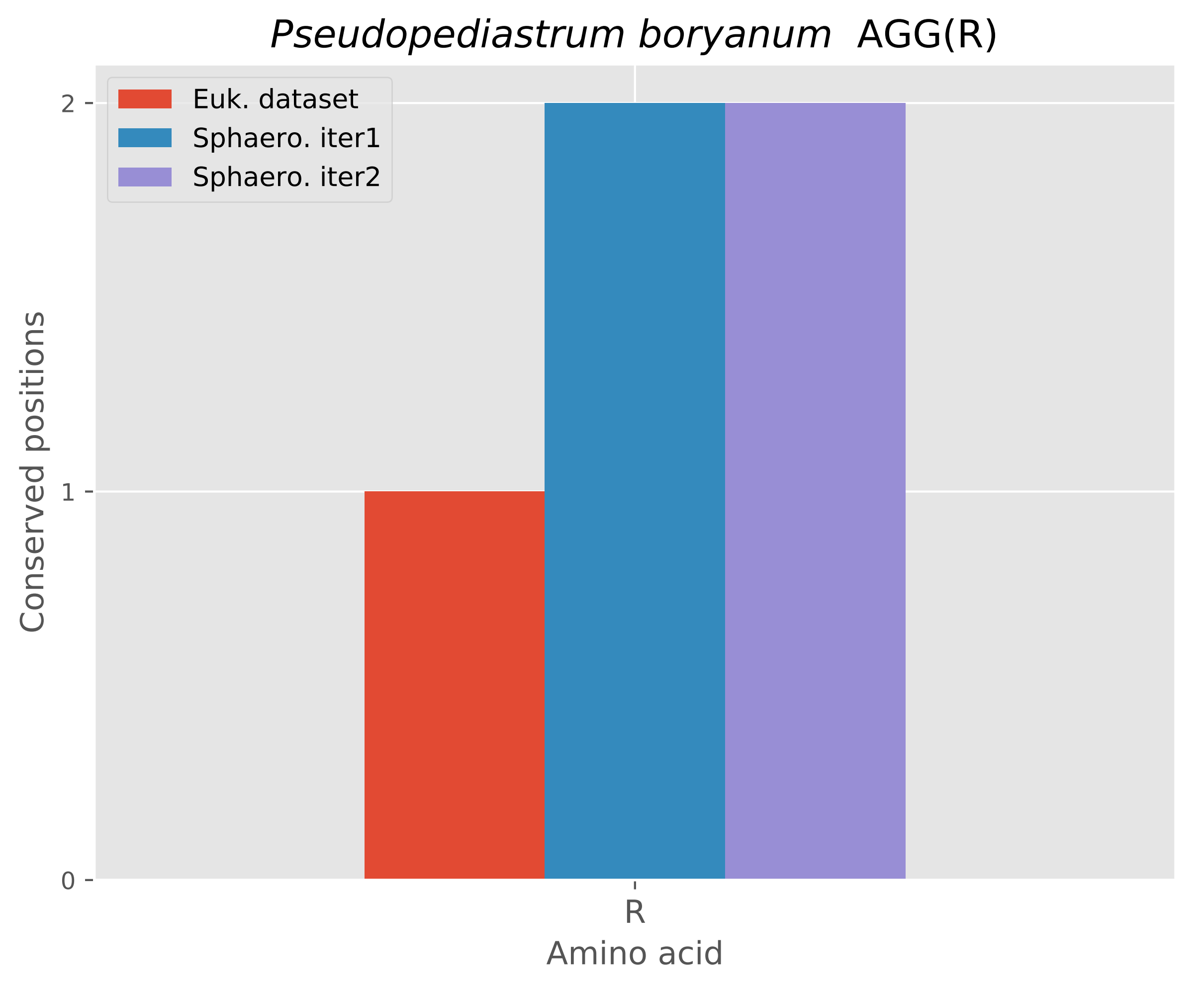

Pseudopediastrum boryanum AGG(R)
Euk. dataset
Sphaero. iter1
Sphaero. iter2
Conserved positions
0
1
2
R
Amino acid

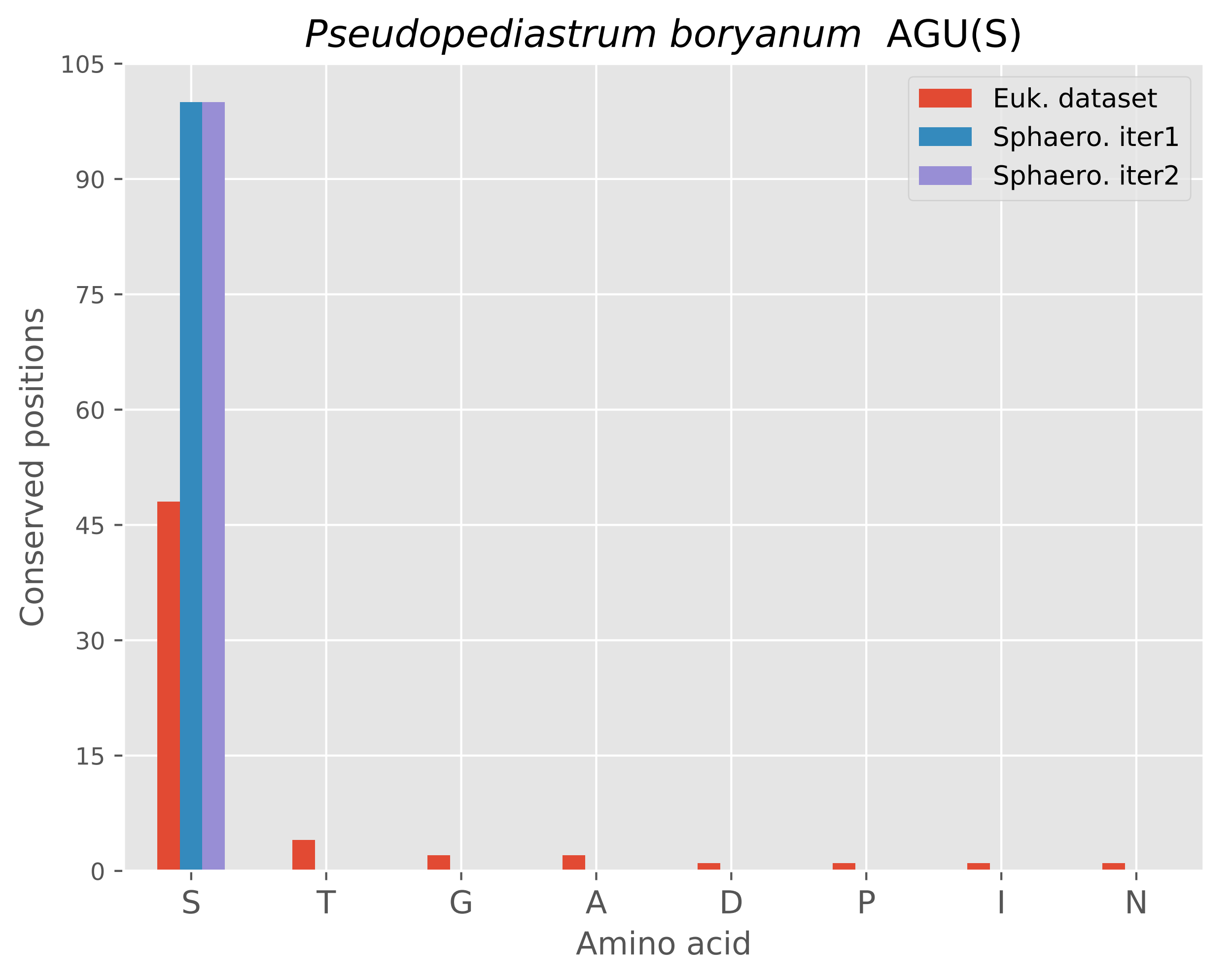

Pseudopediastrum boryanum AGU(S)
Euk. dataset
Sphaero. iter1
Sphaero. iter2
Conserved positions
105
90
75
60
45
30
15
0
S
T
G
A
D
P
I
N
Amino acid

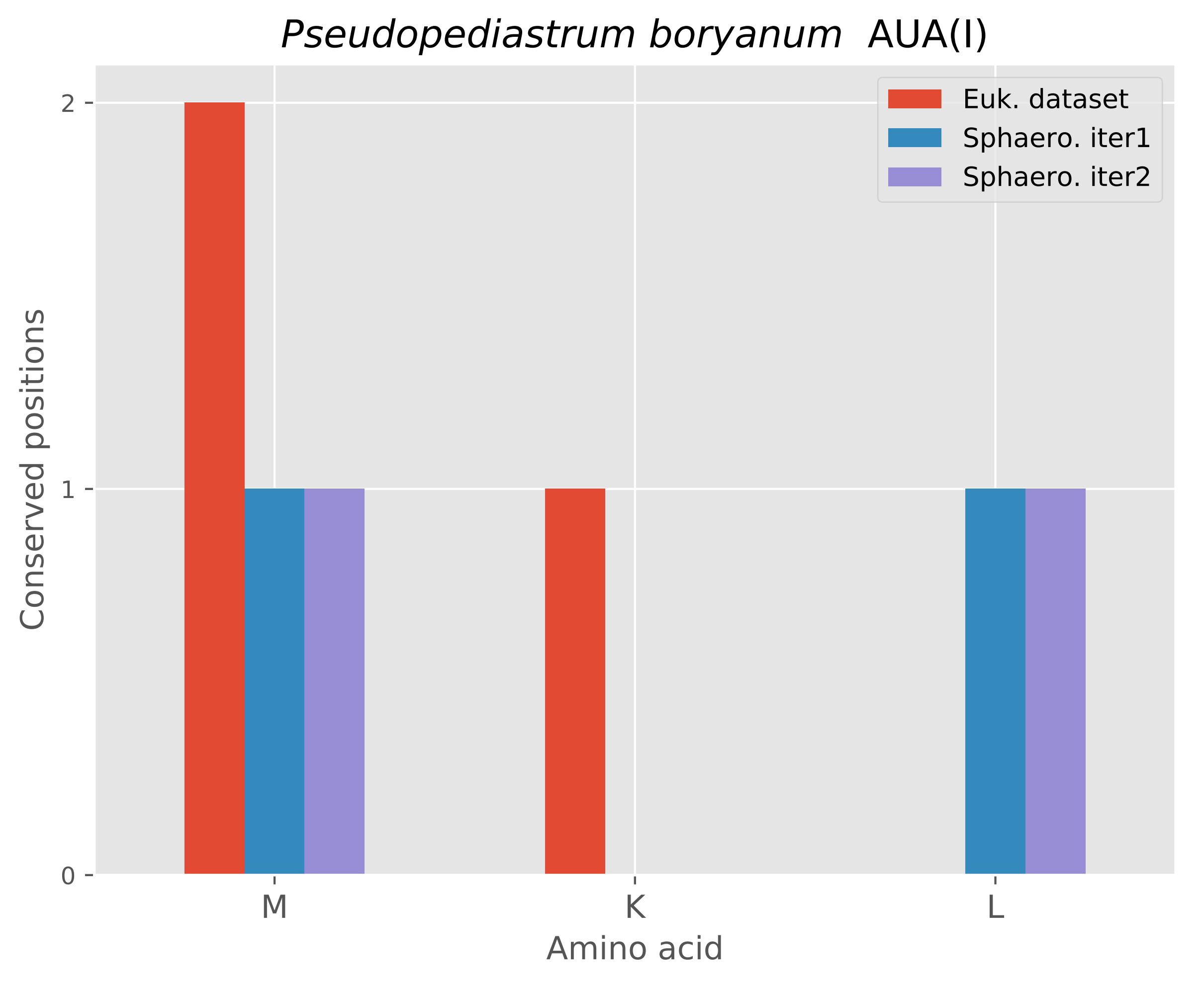
Pseudopediastrum boryanum AUA(I)
Euk. dataset
Sphaero. iter1
Sphaero. iter2
Conserved positions
2
1
0
M
K
L
Amino acid

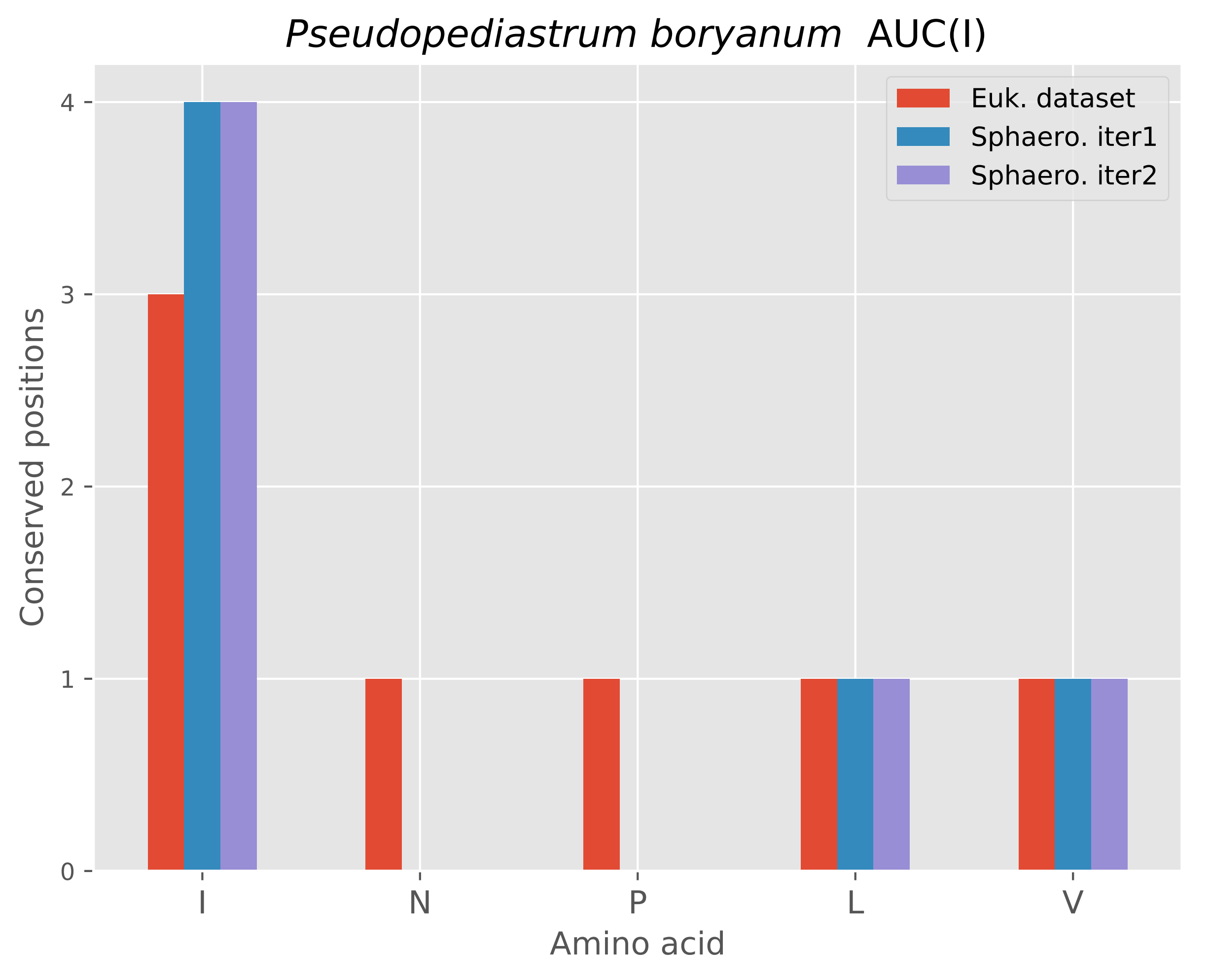

Pseudopediastrum boryanum AUC(I)
Euk. dataset
Sphaero. iter1
Sphaero. iter2
Conserved positions
0
1
2
3
4
I
N
P
L
V
Amino acid

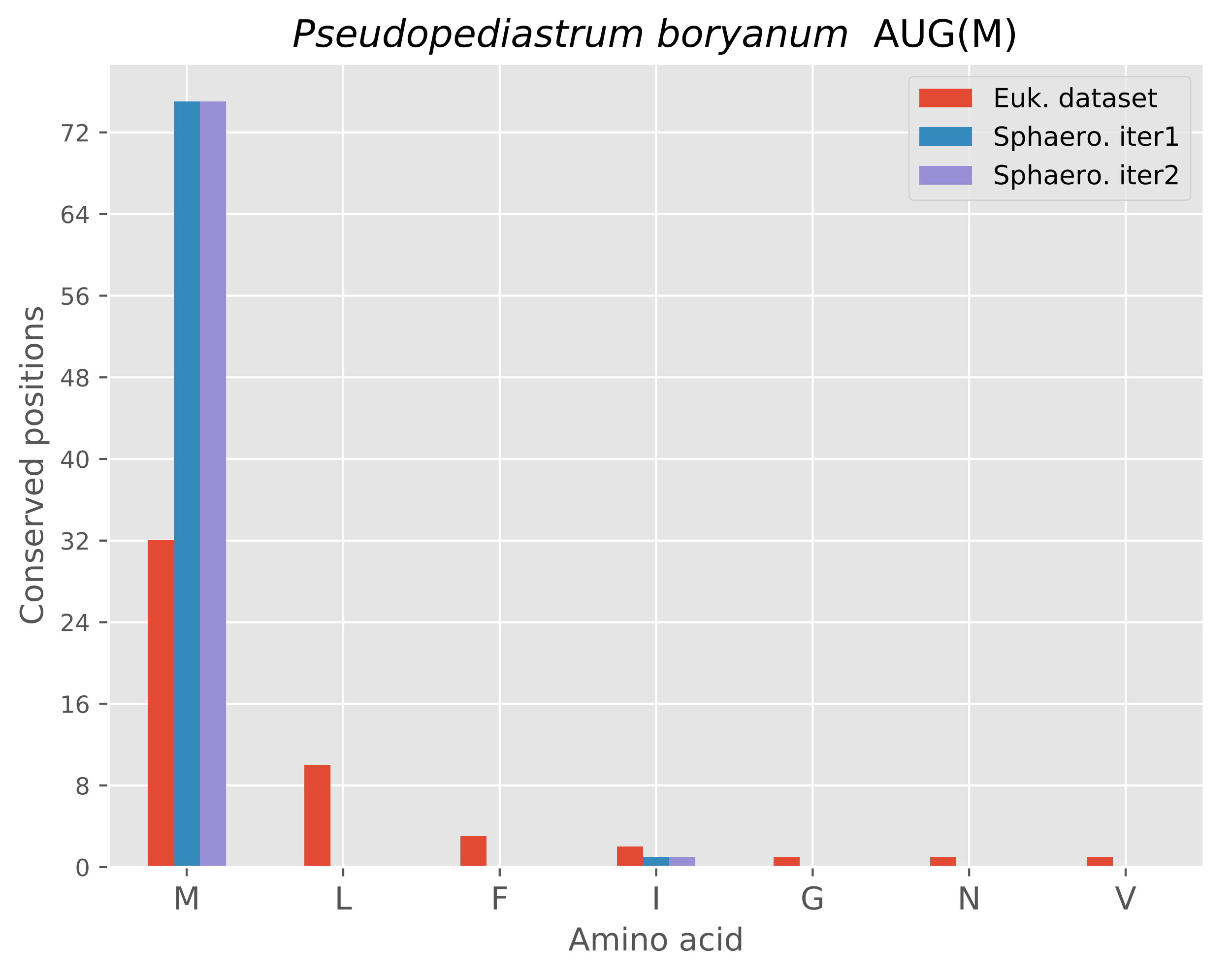

Pseudopediastrum boryanum AUG(M)
Euk. dataset
Sphaero. iter1
Sphaero. iter2
Conserved positions
0
8
16
24
32
40
48
56
64
72
M
L
F
I
G
N
V
Amino acid

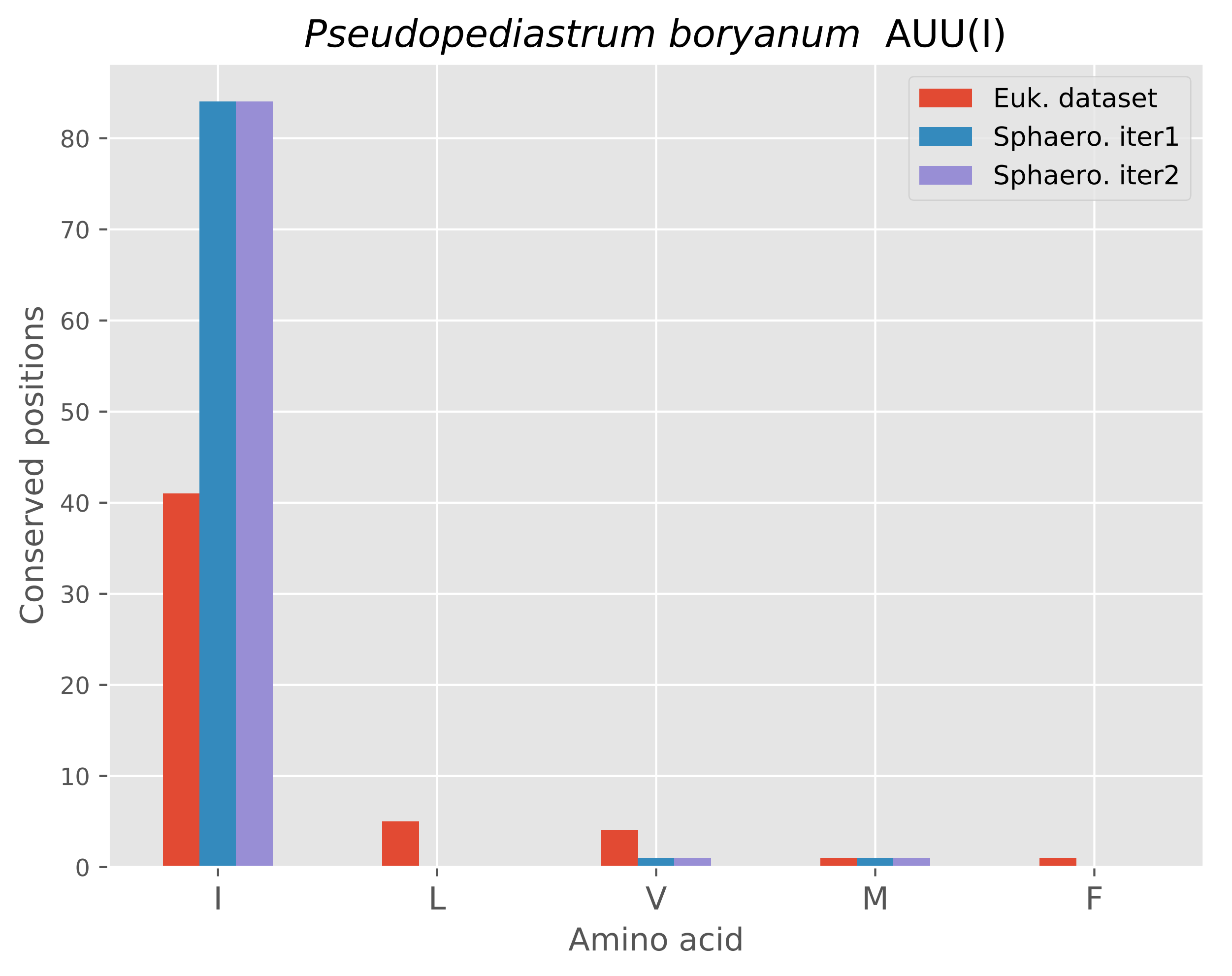
Pseudopediastrum boryanum AUU(I)
Euk. dataset
Sphaero. iter1
Sphaero. iter2
Conserved positions
0
10
20
30
40
50
60
70
80
I
L
V
M
F
Amino acid

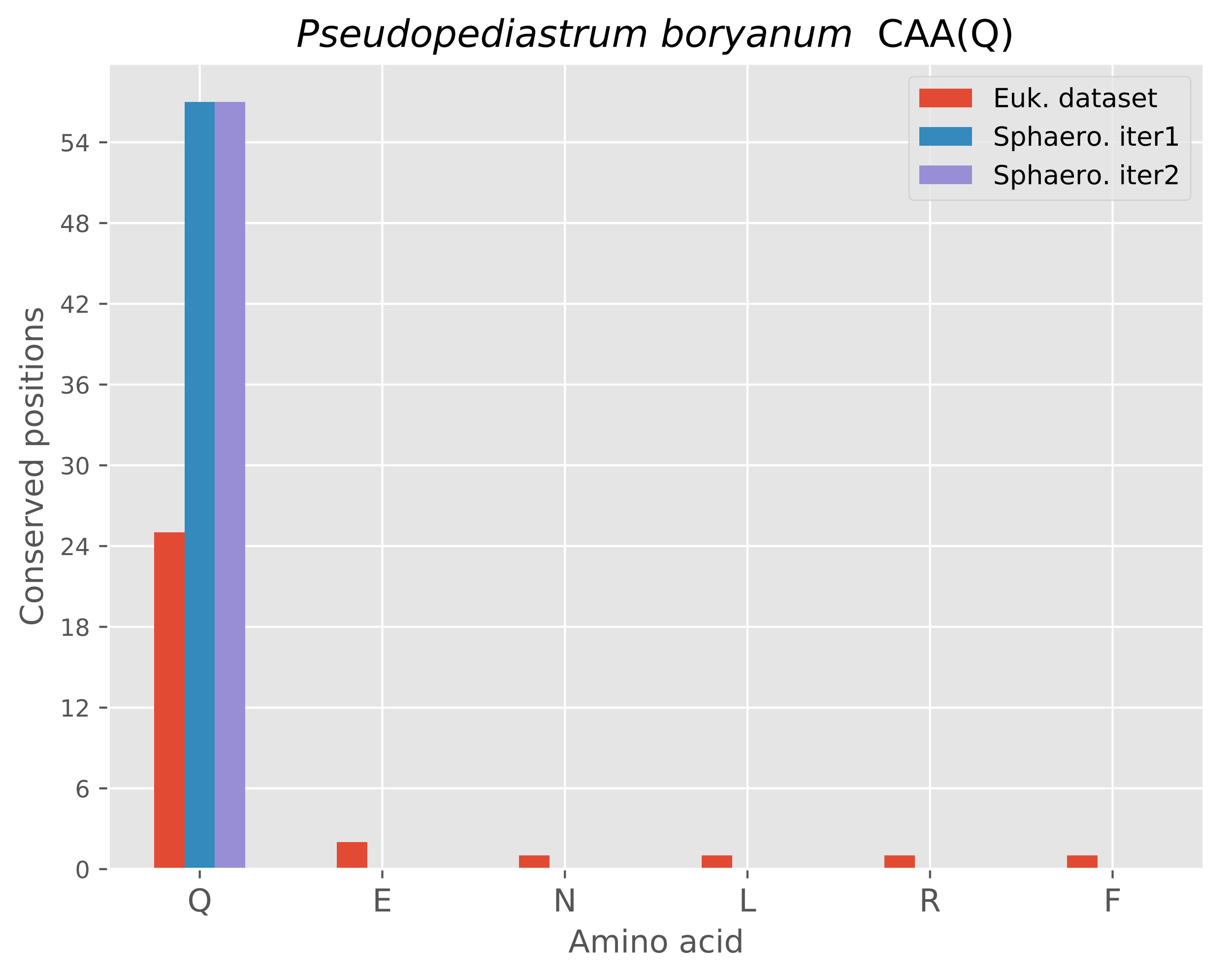

Pseudopediastrum boryanum CAA(Q)
Euk. dataset
Sphaero. iter1
Sphaero. iter2
Conserved positions
0
6
12
18
24
30
36
42
48
54
Q
E
N
L
R
F
Amino acid

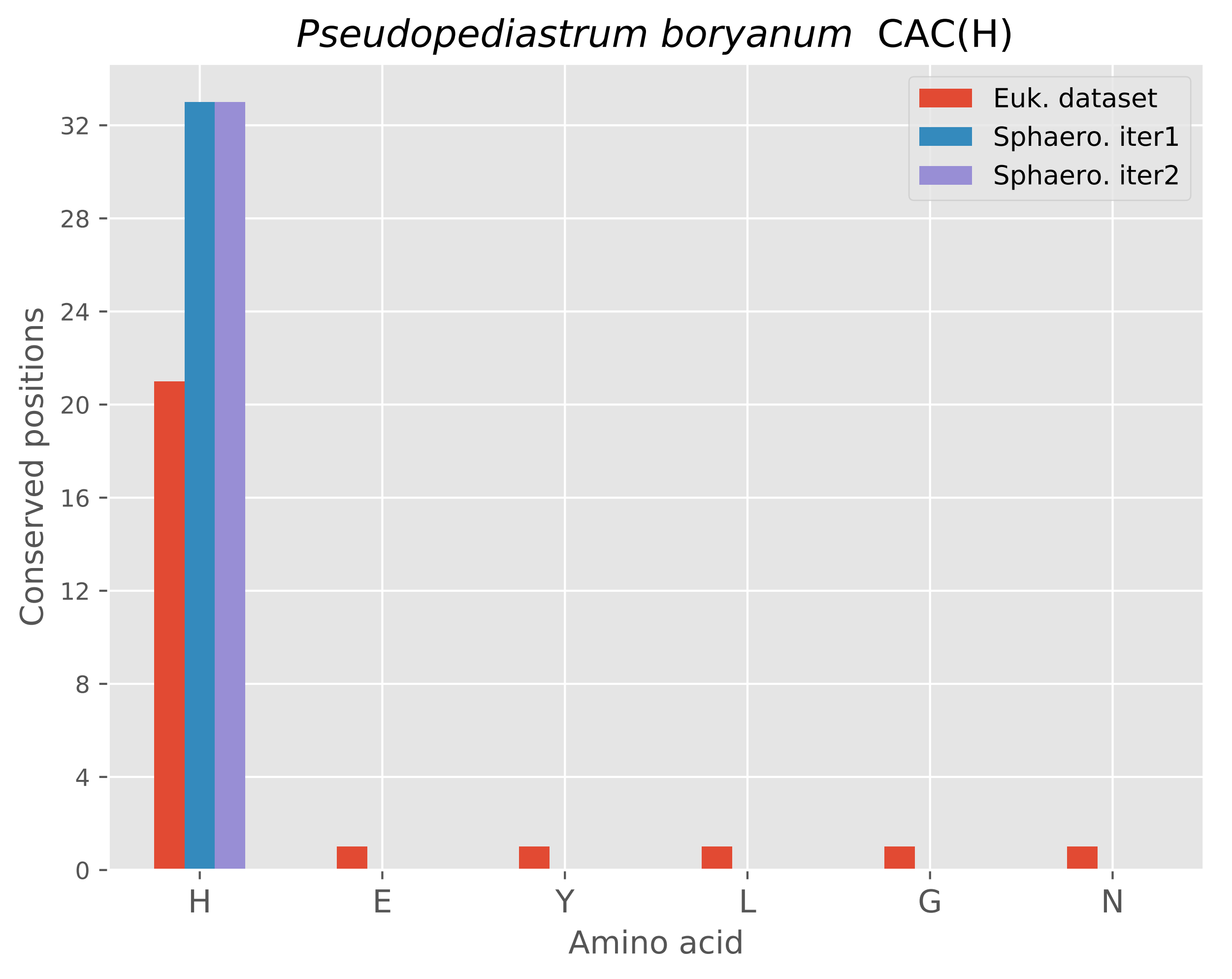

Pseudopediastrum boryanum CAC(H)
Euk. dataset
Sphaero. iter1
Sphaero. iter2
Conserved positions
0
4
8
12
16
20
24
28
32
H
E
Y
L
G
N
Amino acid

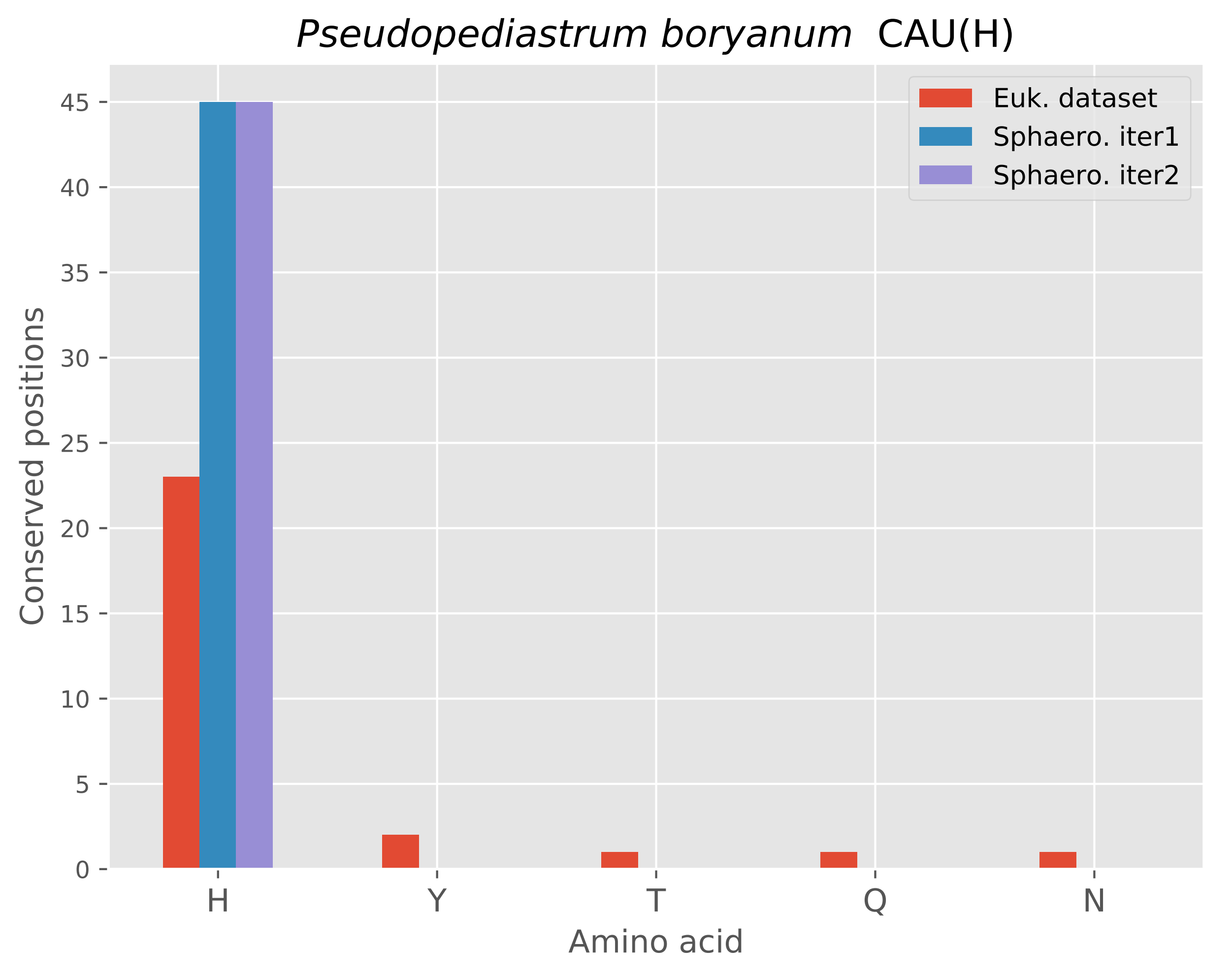

Pseudopediastrum boryanum CAU(H)
Euk. dataset
Sphaero. iter1
Sphaero. iter2
Conserved positions
0
5
10
15
20
25
30
35
40
45
H
Y
T
Q
N
Amino acid

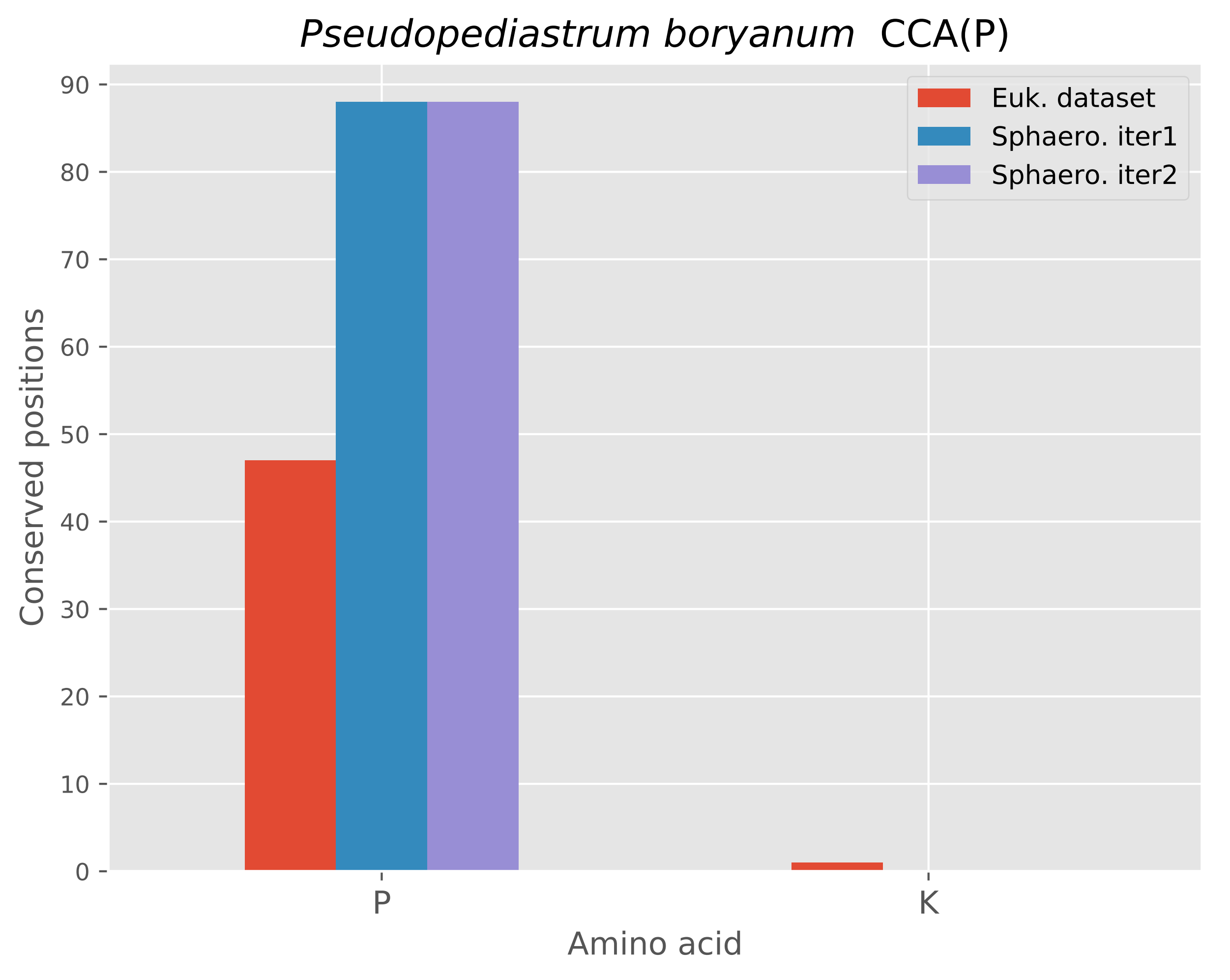

Pseudopediastrum boryanum CCA(P)
Euk. dataset
Sphaero. iter1
Sphaero. iter2
Conserved positions
0
10
20
30
40
50
60
70
80
90
P
K
Amino acid

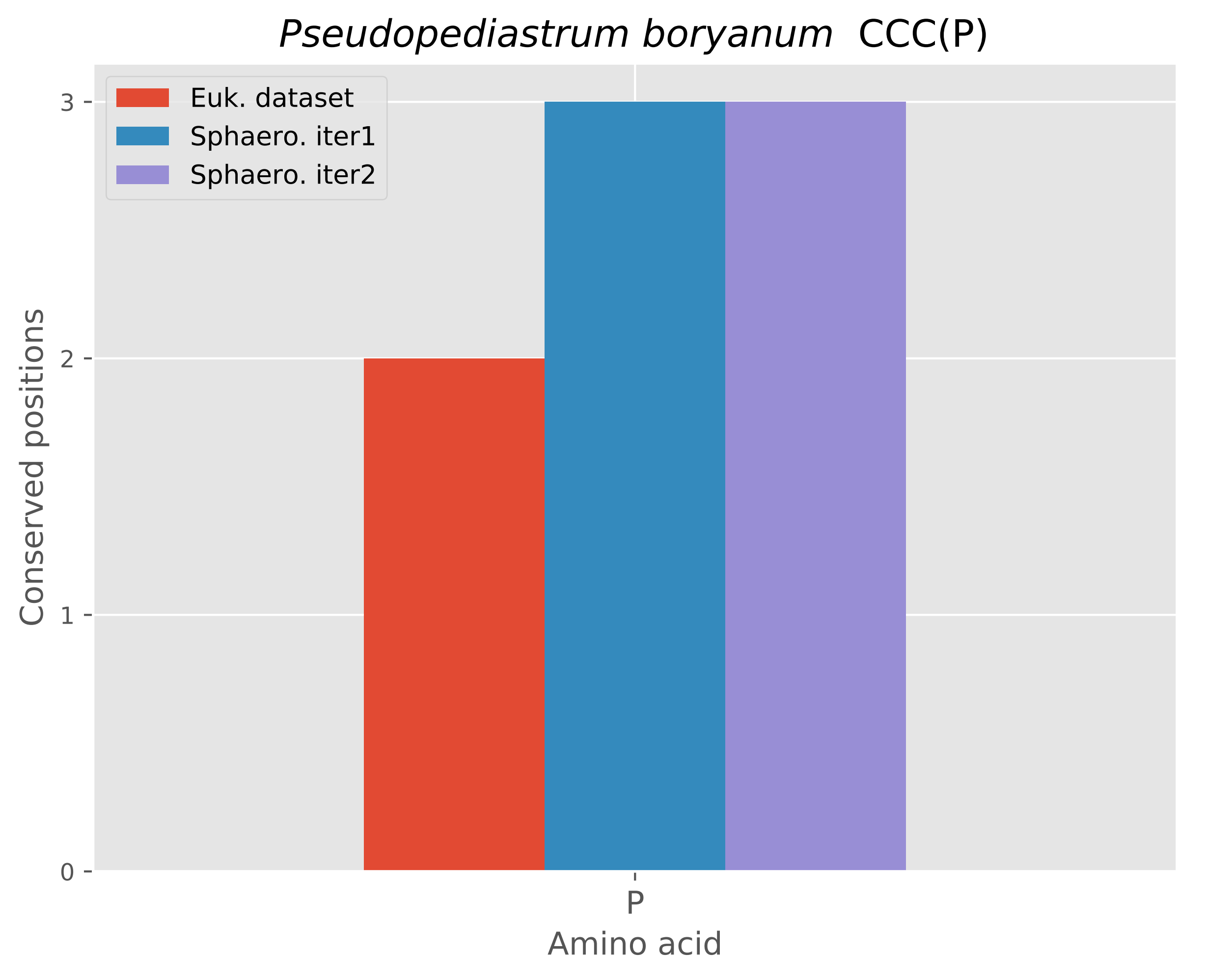

Pseudopediastrum boryanum CCC(P)
Euk. dataset
Sphaero. iter1
Sphaero. iter2
Conserved positions
0
1
2
3
P
Amino acid

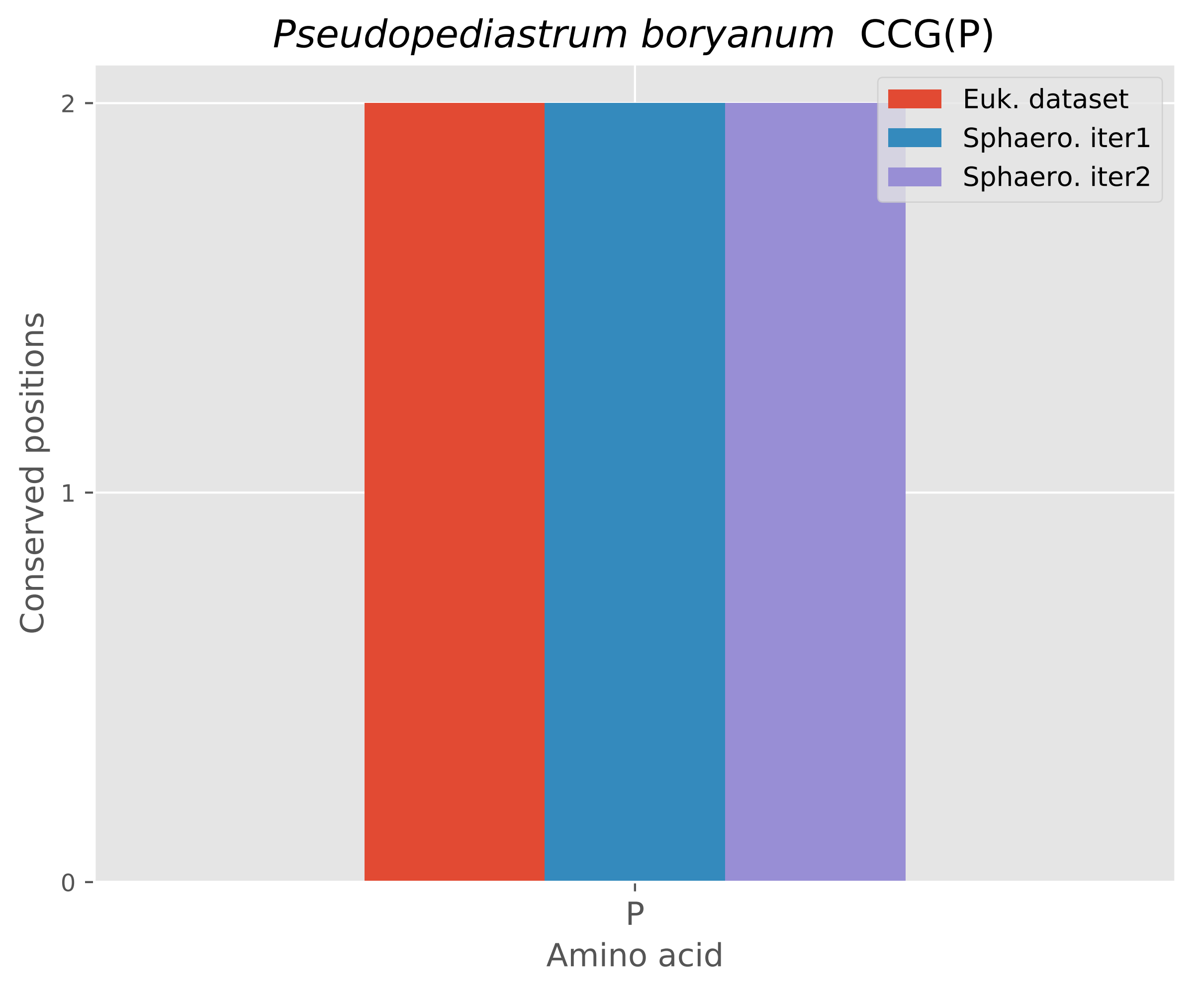

Pseudopediastrum boryanum CCG(P)
Euk. dataset
Sphaero. iter1
Sphaero. iter2
Conserved positions
2
1
0
P
Amino acid

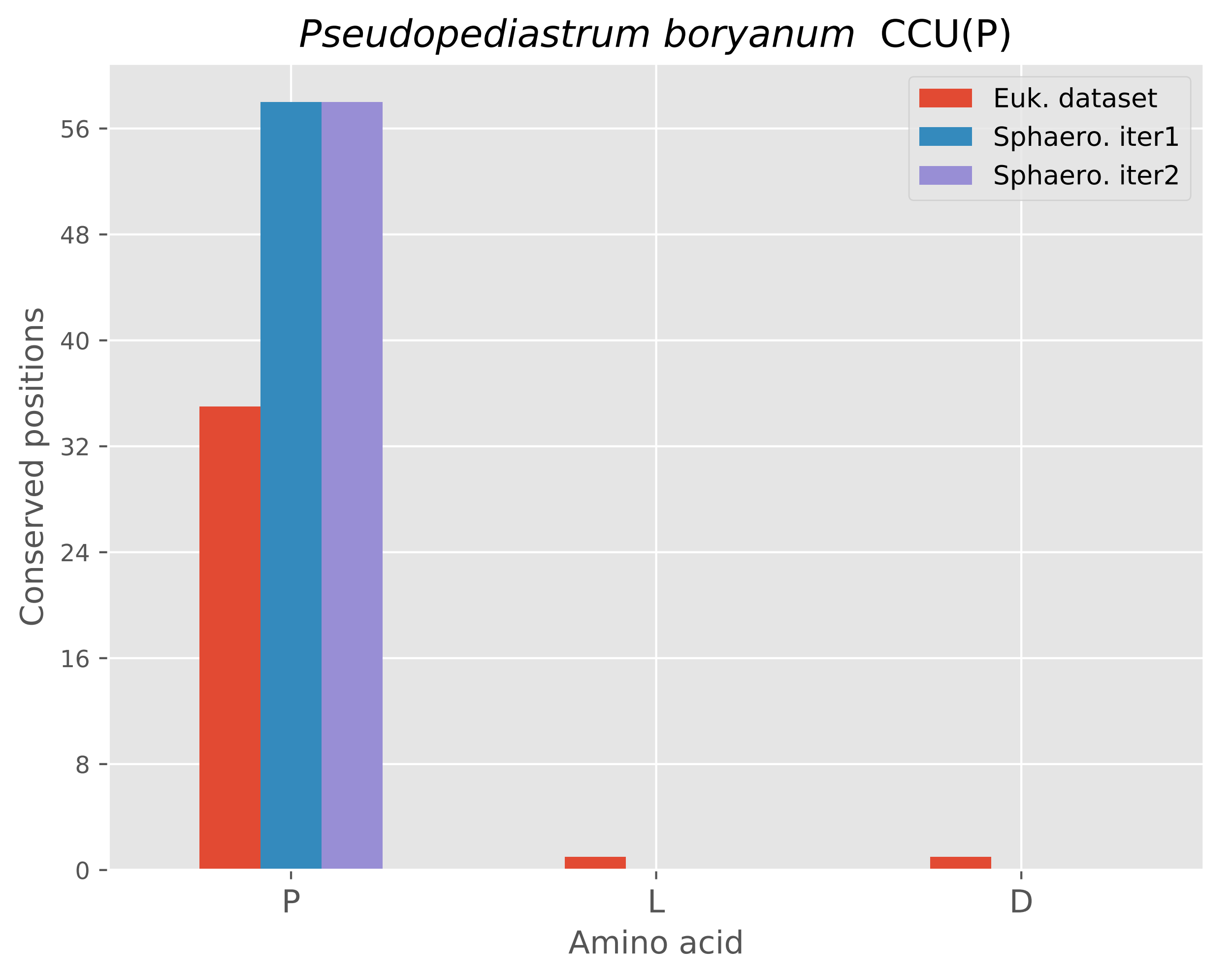
Pseudopediastrum boryanum CCU(P)
Euk. dataset
Sphaero. iter1
Sphaero. iter2
Conserved positions
0
8
16
24
32
40
48
56
P
L
D
Amino acid

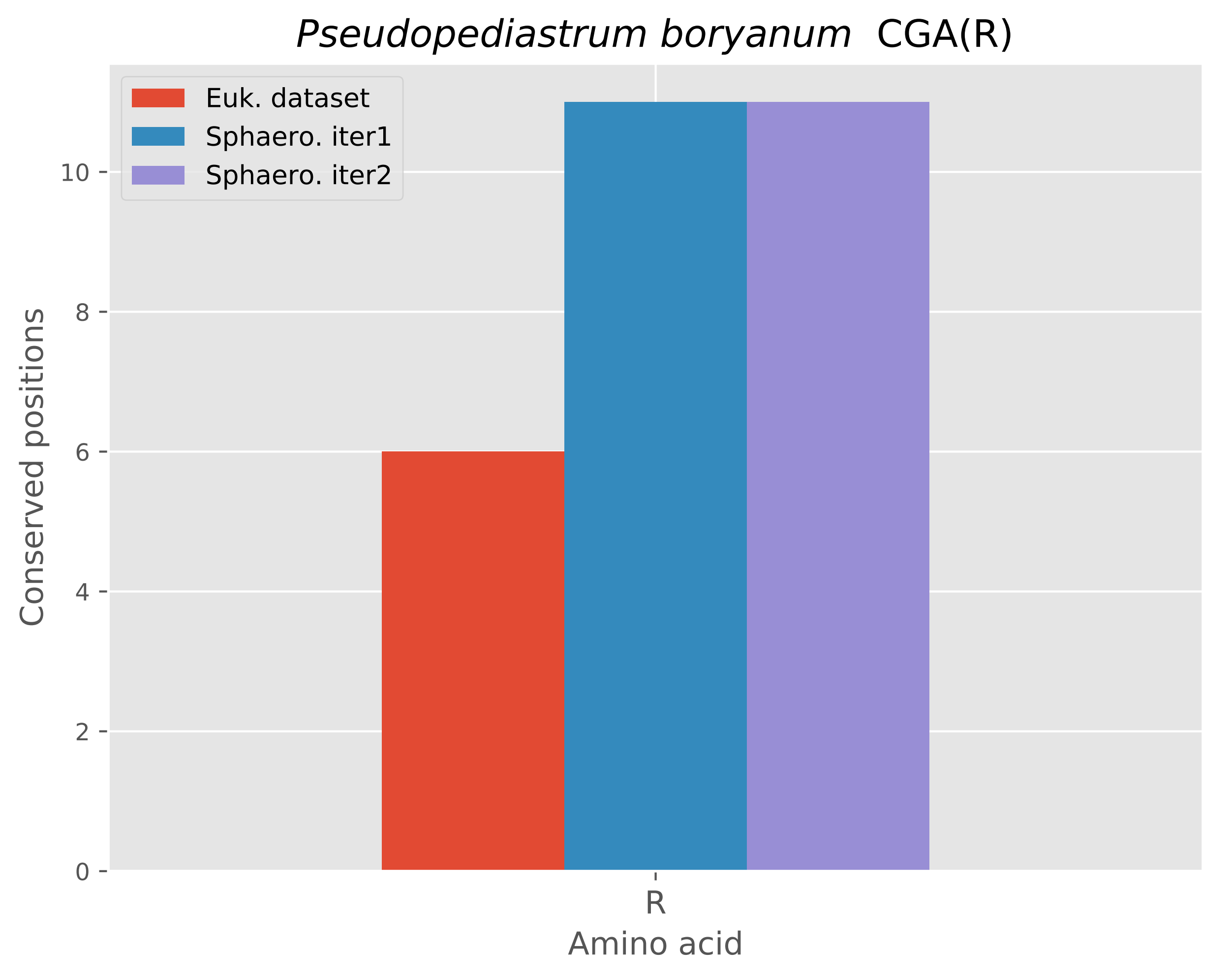

Pseudopediastrum boryanum CGA(R)
Euk. dataset
Sphaero. iter1
Sphaero. iter2
Conserved positions
0
2
4
6
8
10
R
Amino acid

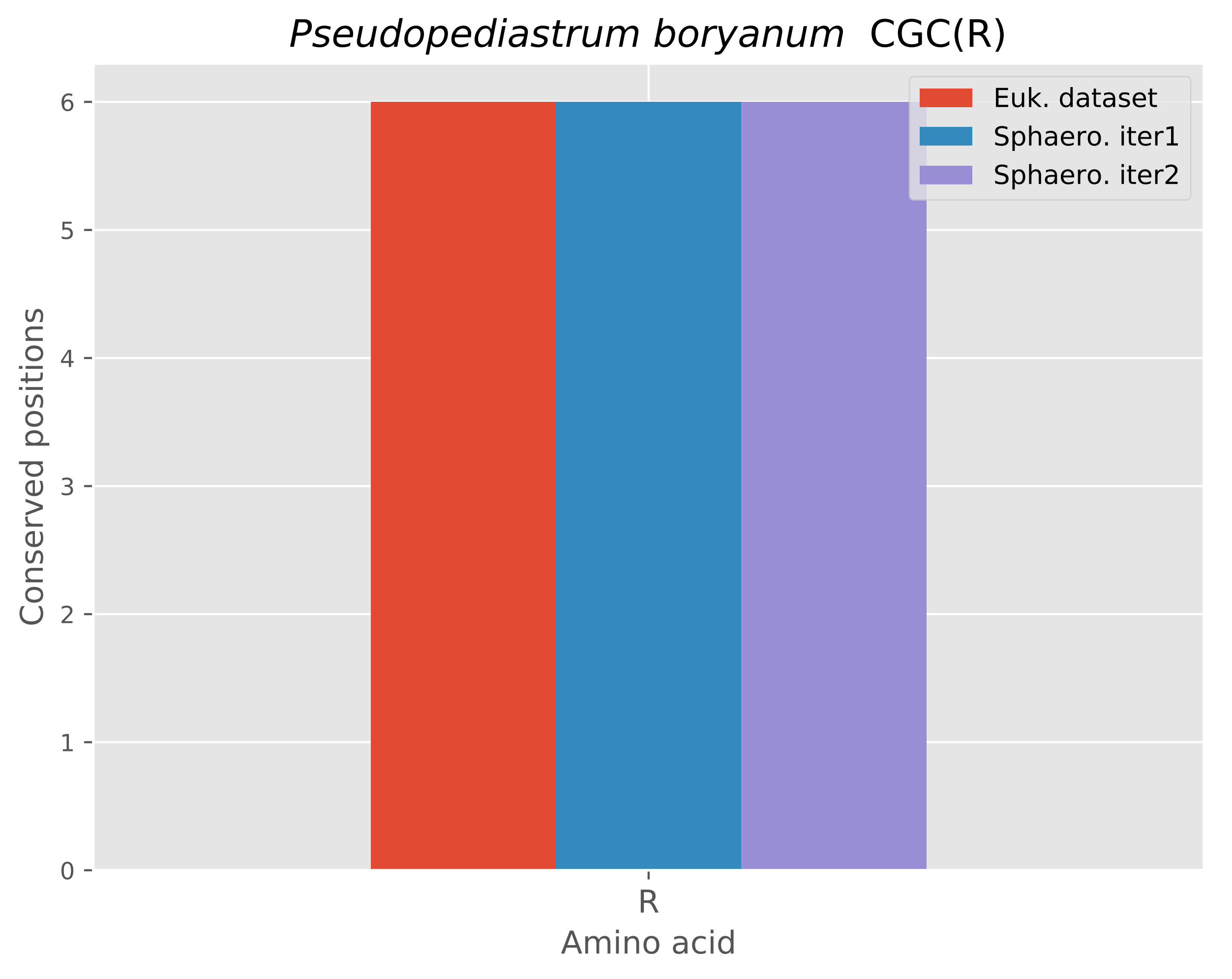

Pseudopediastrum boryanum CGC(R)
Euk. dataset
Sphaero. iter1
Sphaero. iter2
Conserved positions
0
1
2
3
4
5
6
R
Amino acid

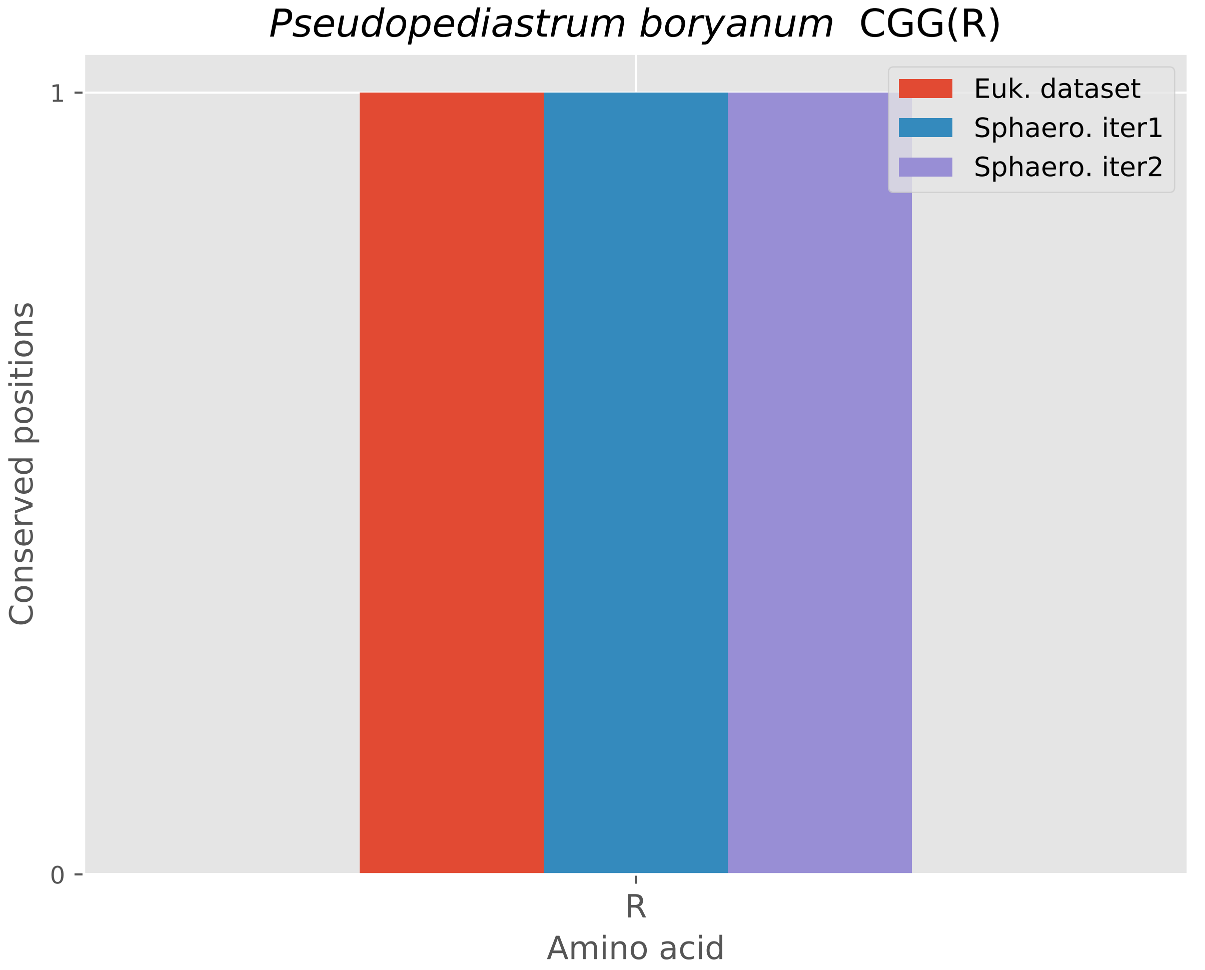
Pseudopediastrum boryanum CGG(R)
Euk. dataset
Sphaero. iter1
Sphaero. iter2
Conserved positions
1
0
R
Amino acid

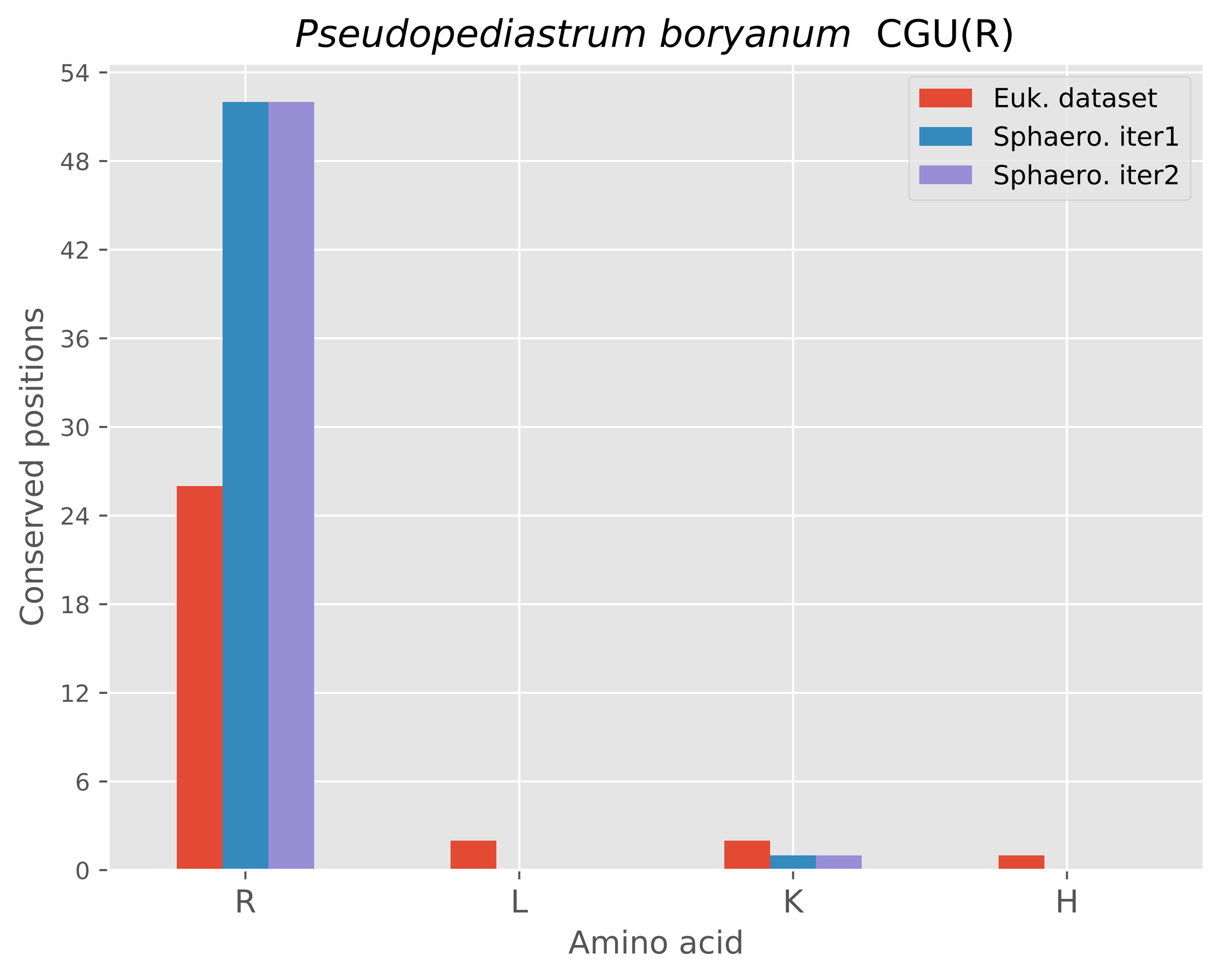

Pseudopediastrum boryanum CGU(R)
Conserved positions
0
6
12
18
24
30
36
42
48
54
R
L
K
H
Amino acid
Euk. dataset
Sphaero. iter1
Sphaero. iter2

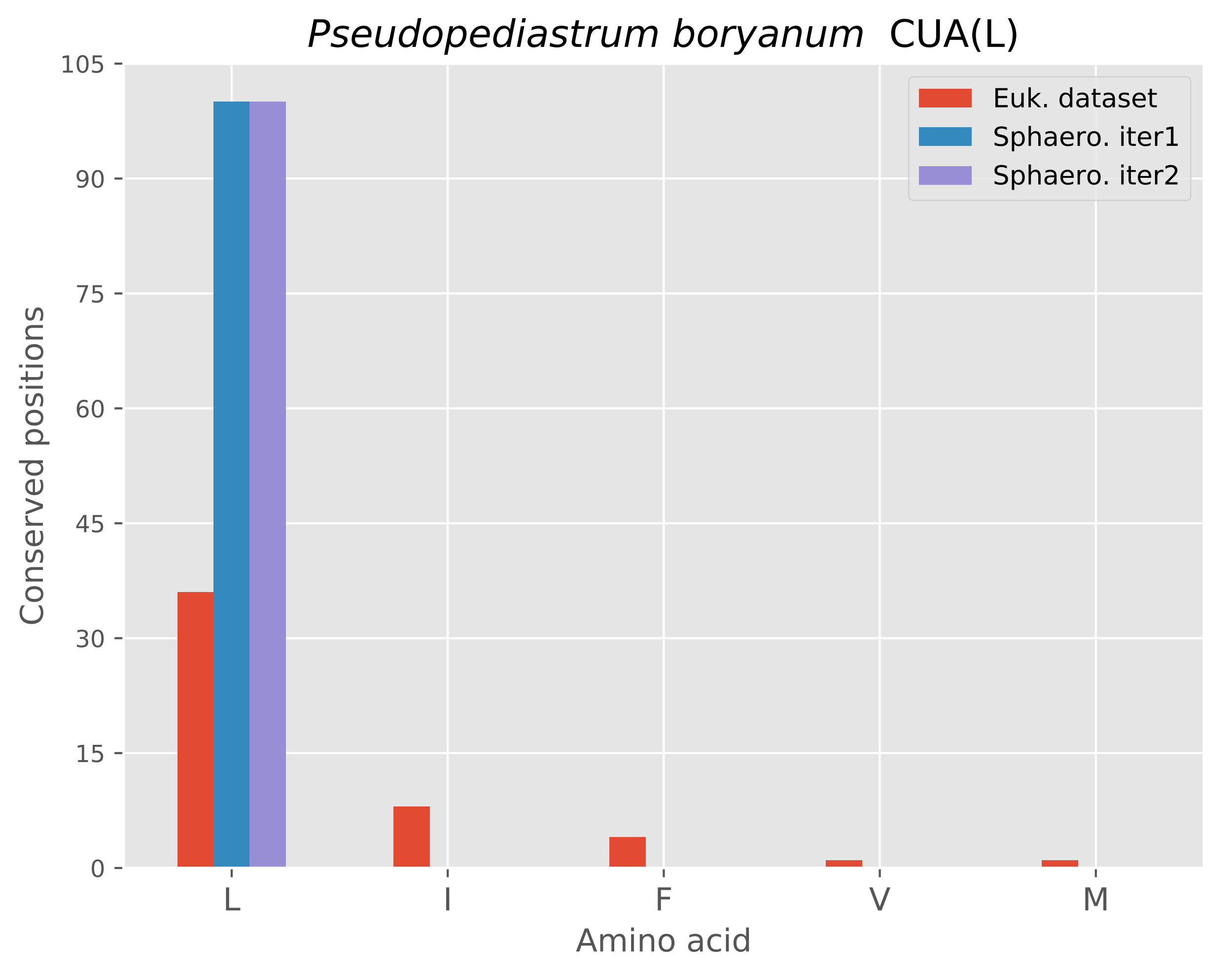

Pseudopediastrum boryanum CUA(L)
Euk. dataset
Sphaero. iter1
Sphaero. iter2
Conserved positions
105
90
75
60
45
30
15
0
L
I
F
V
M
Amino acid

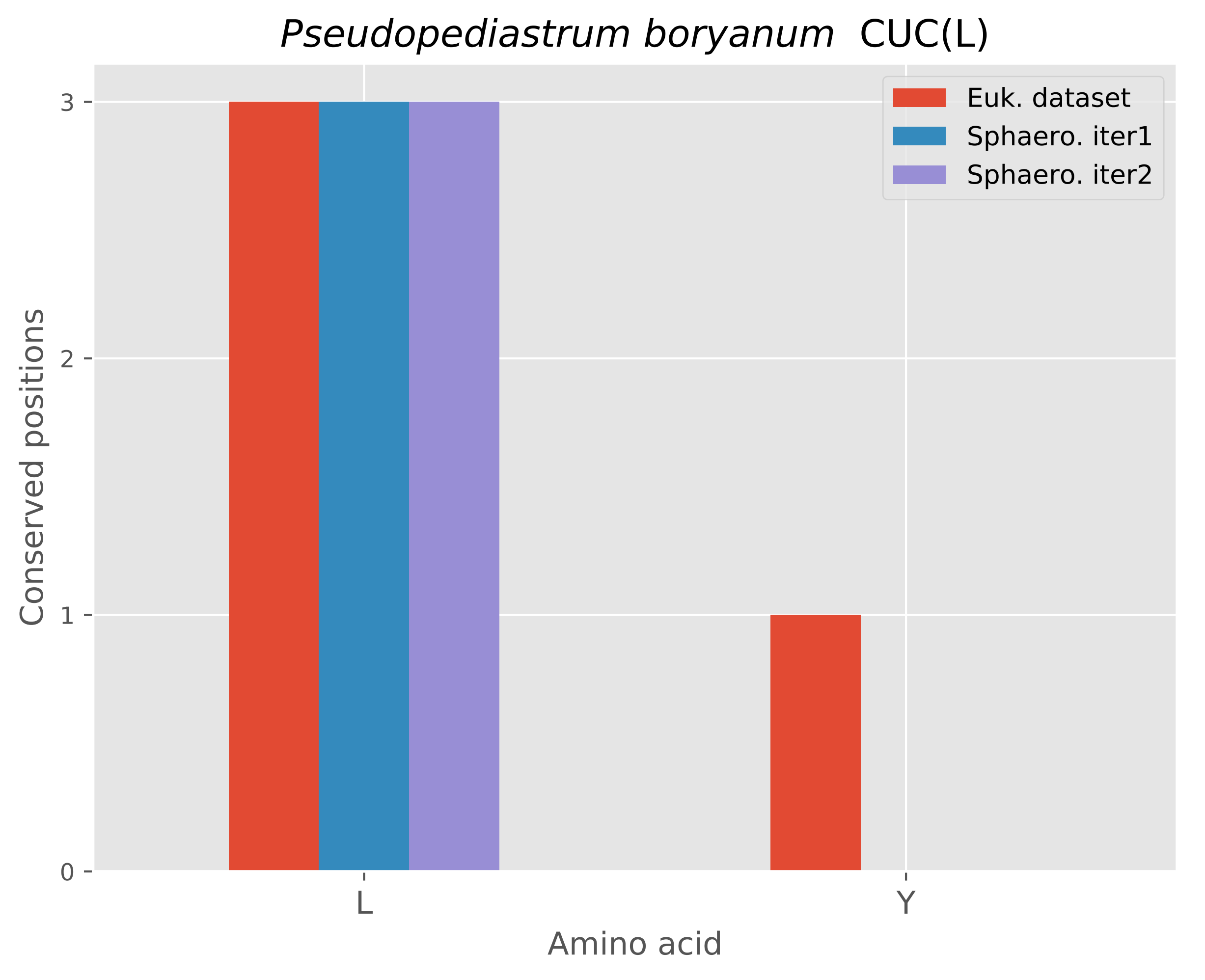
Pseudopediastrum boryanum CUC(L)
Euk. dataset
Sphaero. iter1
Sphaero. iter2
Conserved positions
0
1
2
3
L
Y
Amino acid

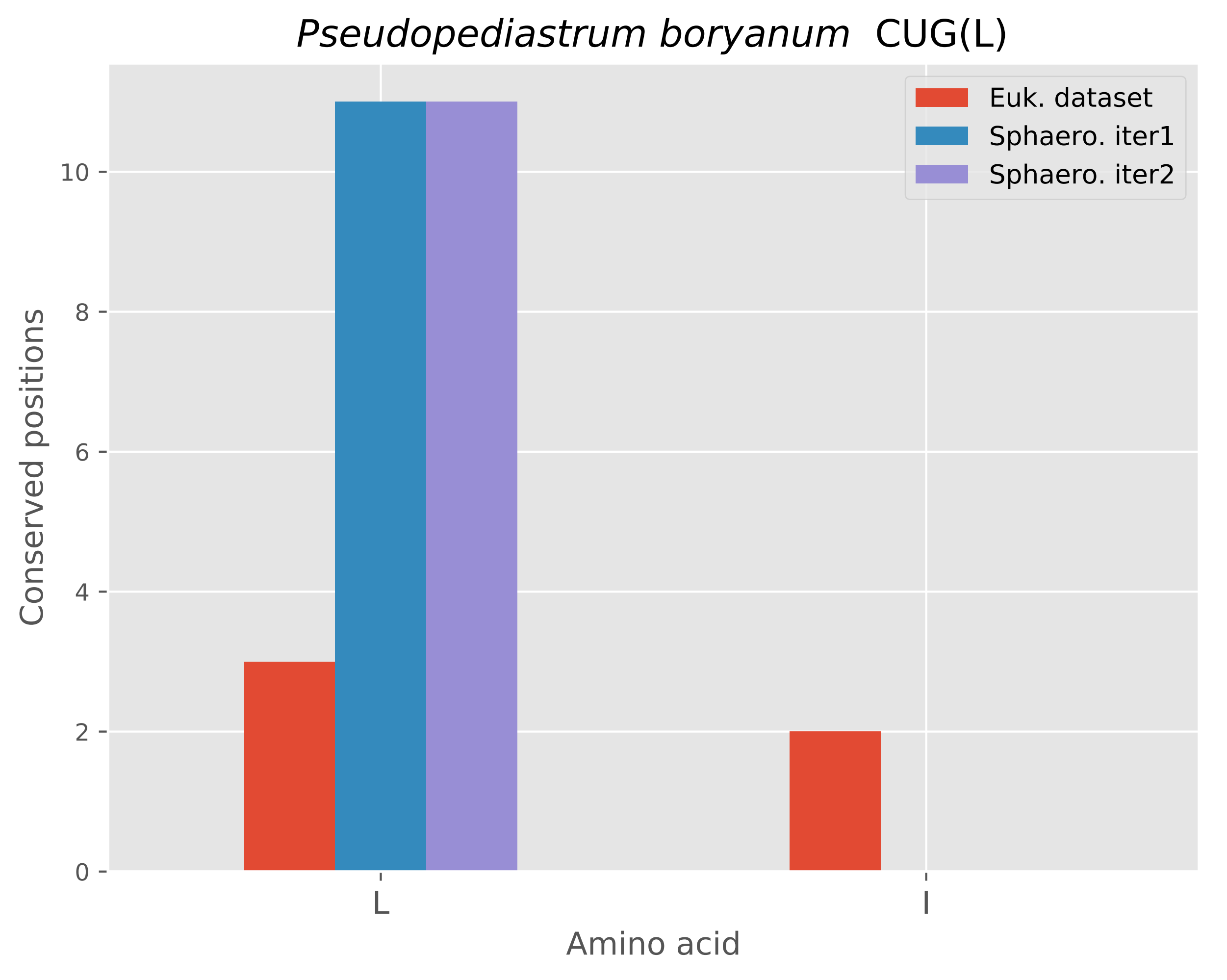

Pseudopediastrum boryanum CUG(L)
Euk. dataset
Sphaero. iter1
Sphaero. iter2
Conserved positions
0
2
4
6
8
10
L
I
Amino acid

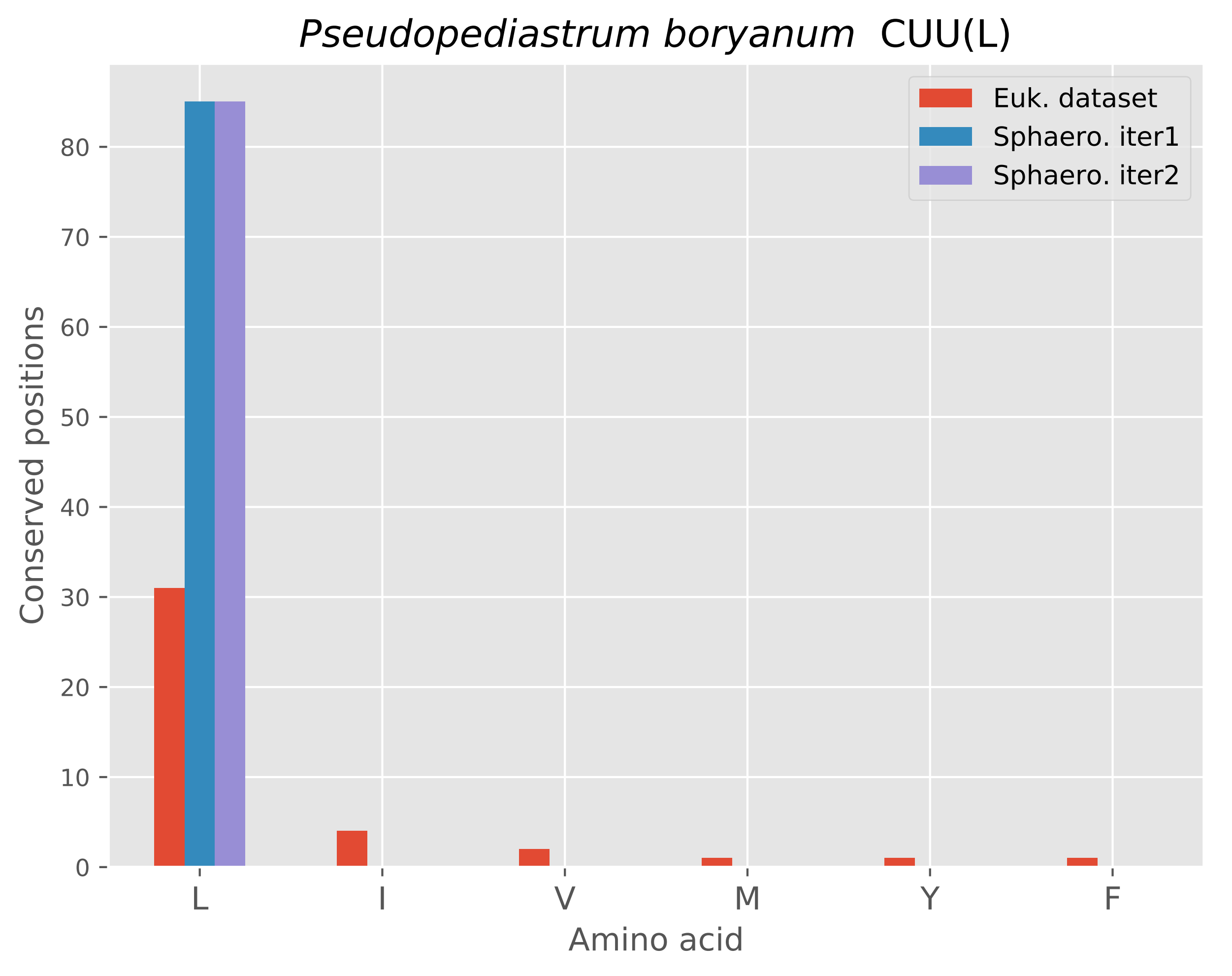

Pseudopediastrum boryanum CUU(L)
Euk. dataset
Sphaero. iter1
Sphaero. iter2
Conserved positions
0
10
20
30
40
50
60
70
80
L
I
V
M
Y
F
Amino acid

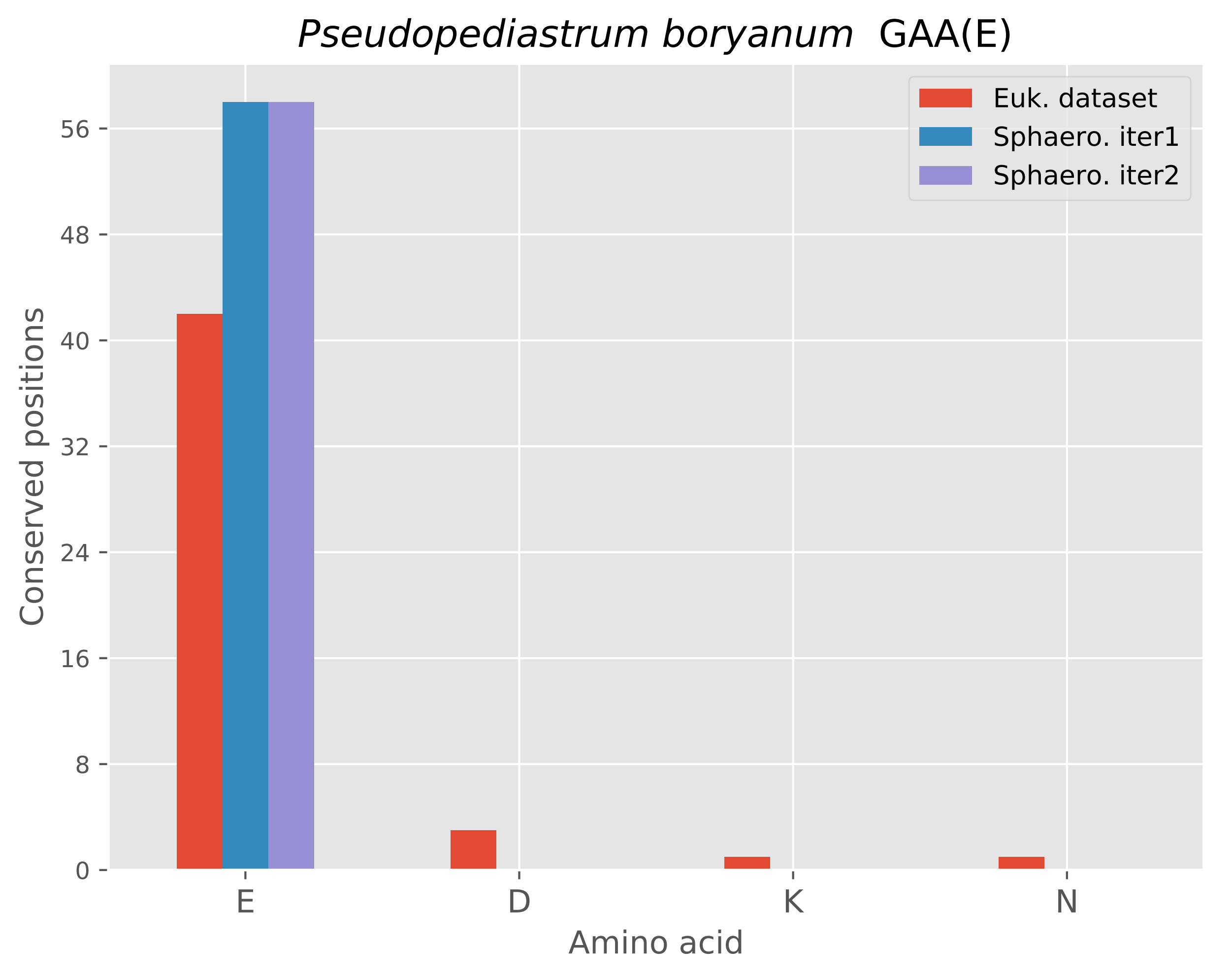

Pseudopediastrum boryanum GAA(E)
Euk. dataset
Sphaero. iter1
Sphaero. iter2
Conserved positions
0
8
16
24
32
40
48
56
E
D
K
N
Amino acid

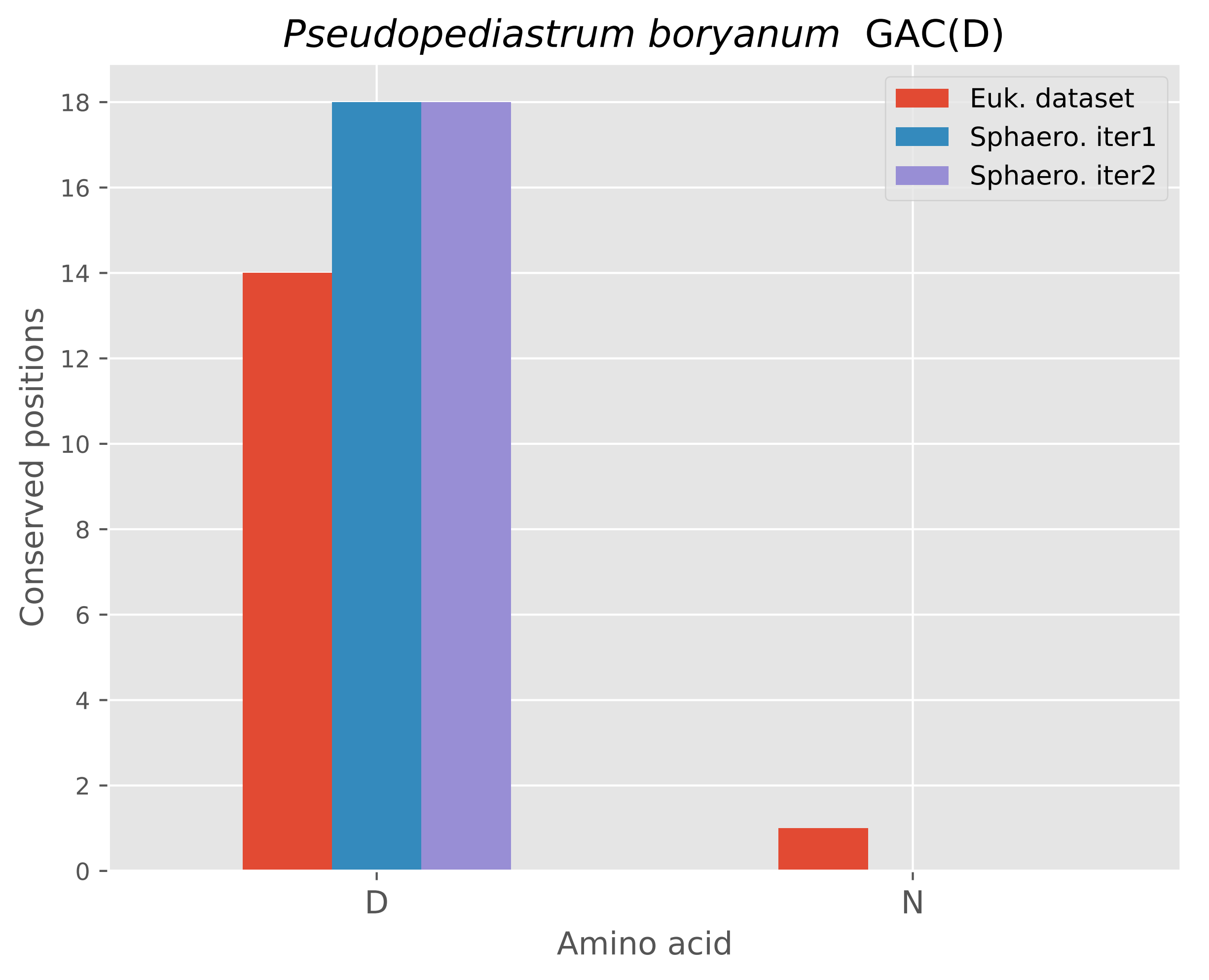

Pseudopediastrum boryanum GAC(D)
Euk. dataset
Sphaero. iter1
Sphaero. iter2
Conserved positions
0
2
4
6
8
10
12
14
16
18
D
N
Amino acid

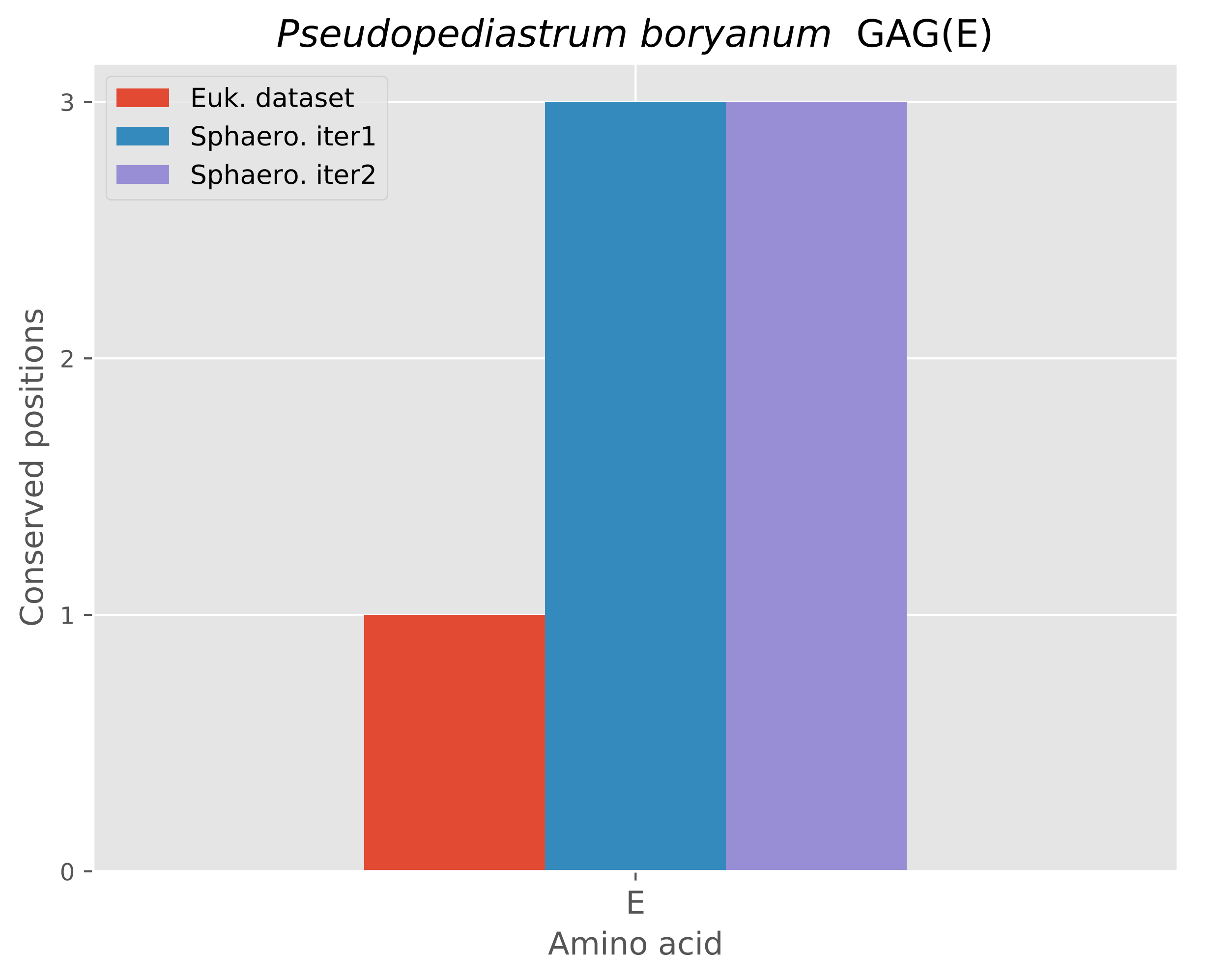

Pseudopediastrum boryanum GAG(E)
Euk. dataset
Sphaero. iter1
Sphaero. iter2
Conserved positions
0
1
2
3
E
Amino acid

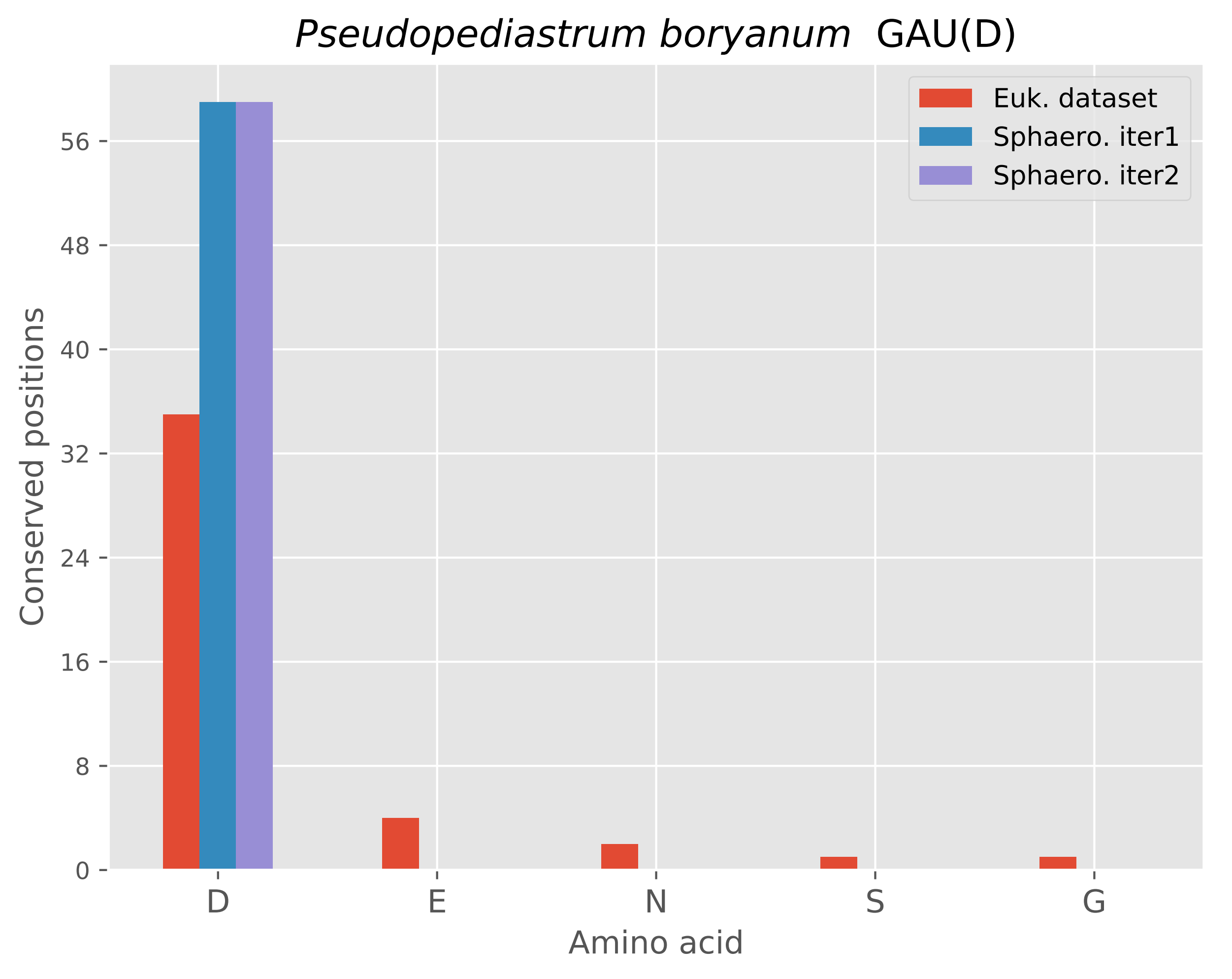

Pseudopediastrum boryanum GAU(D)
Euk. dataset
Sphaero. iter1
Sphaero. iter2
Conserved positions
0
8
16
24
32
40
48
56
D
E
N
S
G
Amino acid

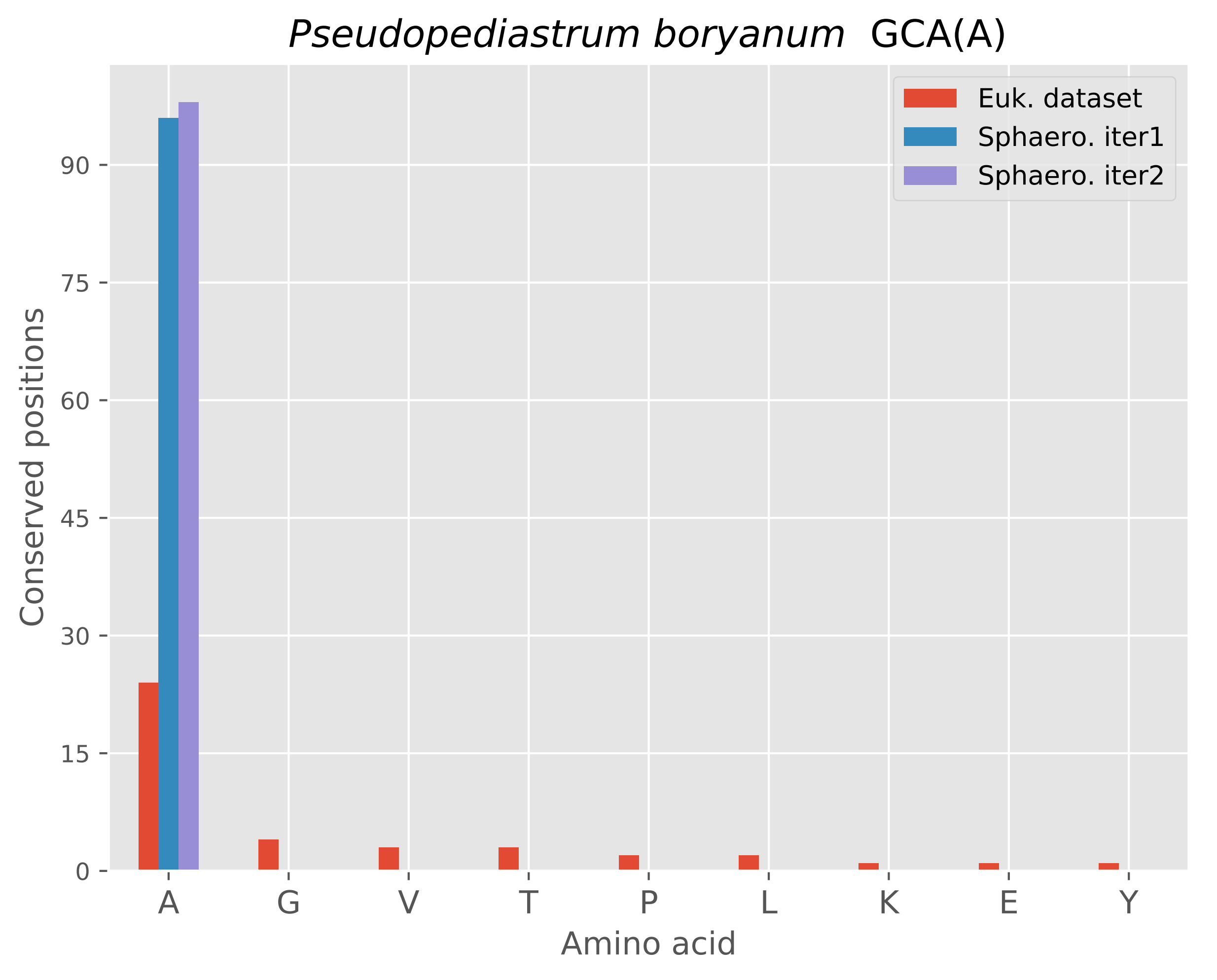

Pseudopediastrum boryanum GCA(A)
Euk. dataset
Sphaero. iter1
Sphaero. iter2
Conserved positions
0
15
30
45
60
75
90
A
G
V
T
P
L
K
E
Y
Amino acid

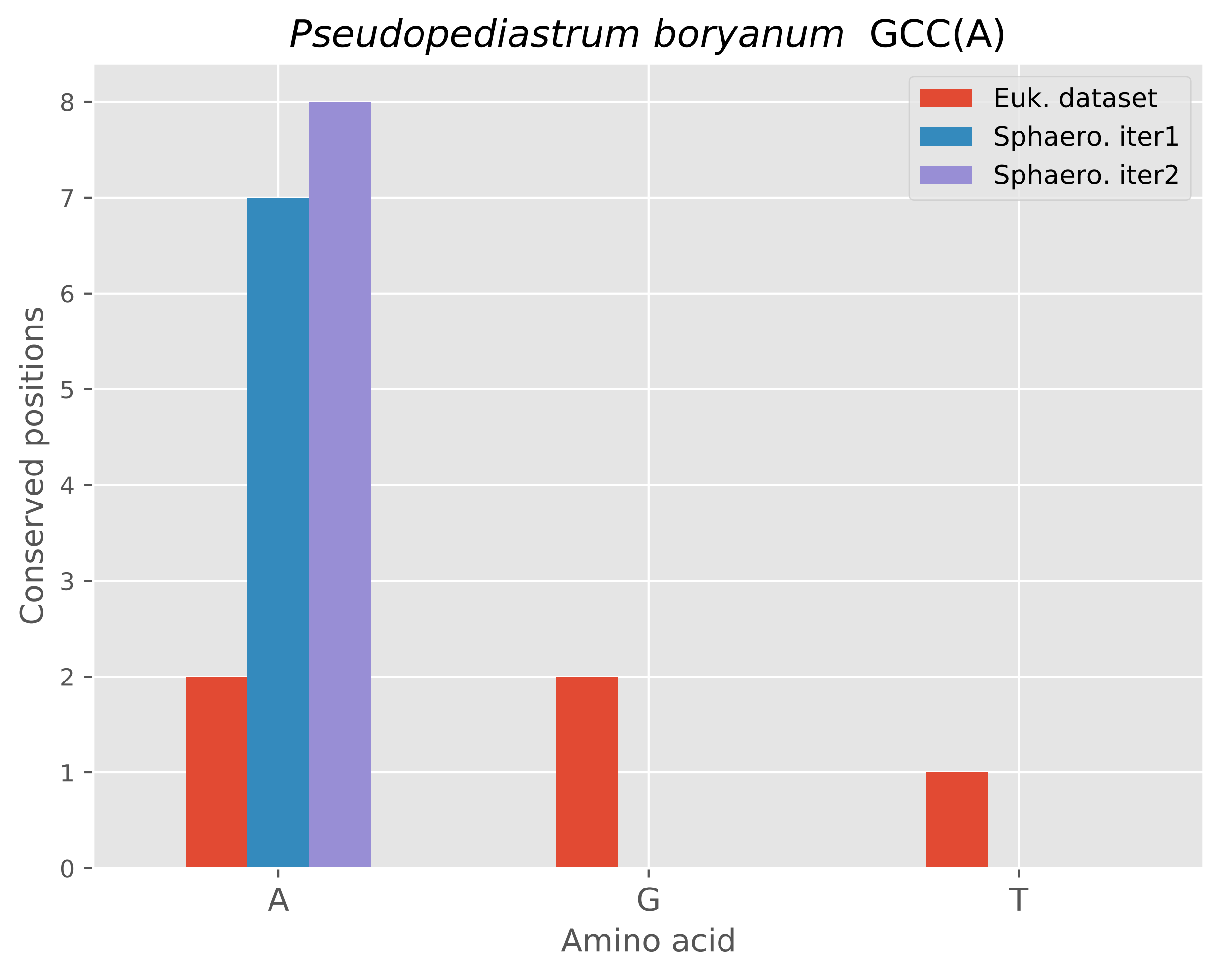

Pseudopediastrum boryanum GCC(A)
Euk. dataset
Sphaero. iter1
Sphaero. iter2
Conserved positions
0
1
2
3
4
5
6
7
8
A
G
T
Amino acid

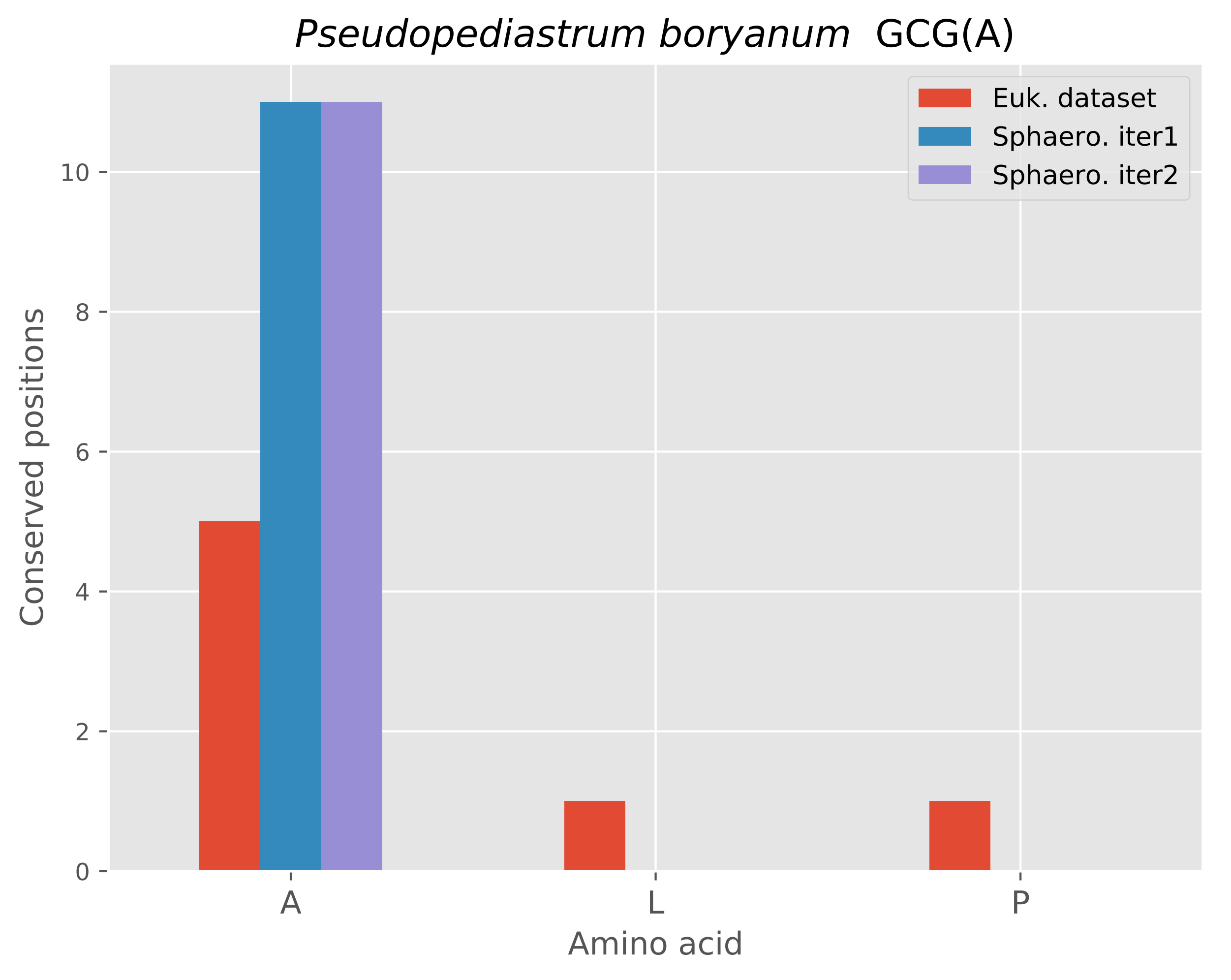

Pseudopediastrum boryanum GCG(A)
Euk. dataset
Sphaero. iter1
Sphaero. iter2
Conserved positions
0
2
4
6
8
10
A
L
P
Amino acid

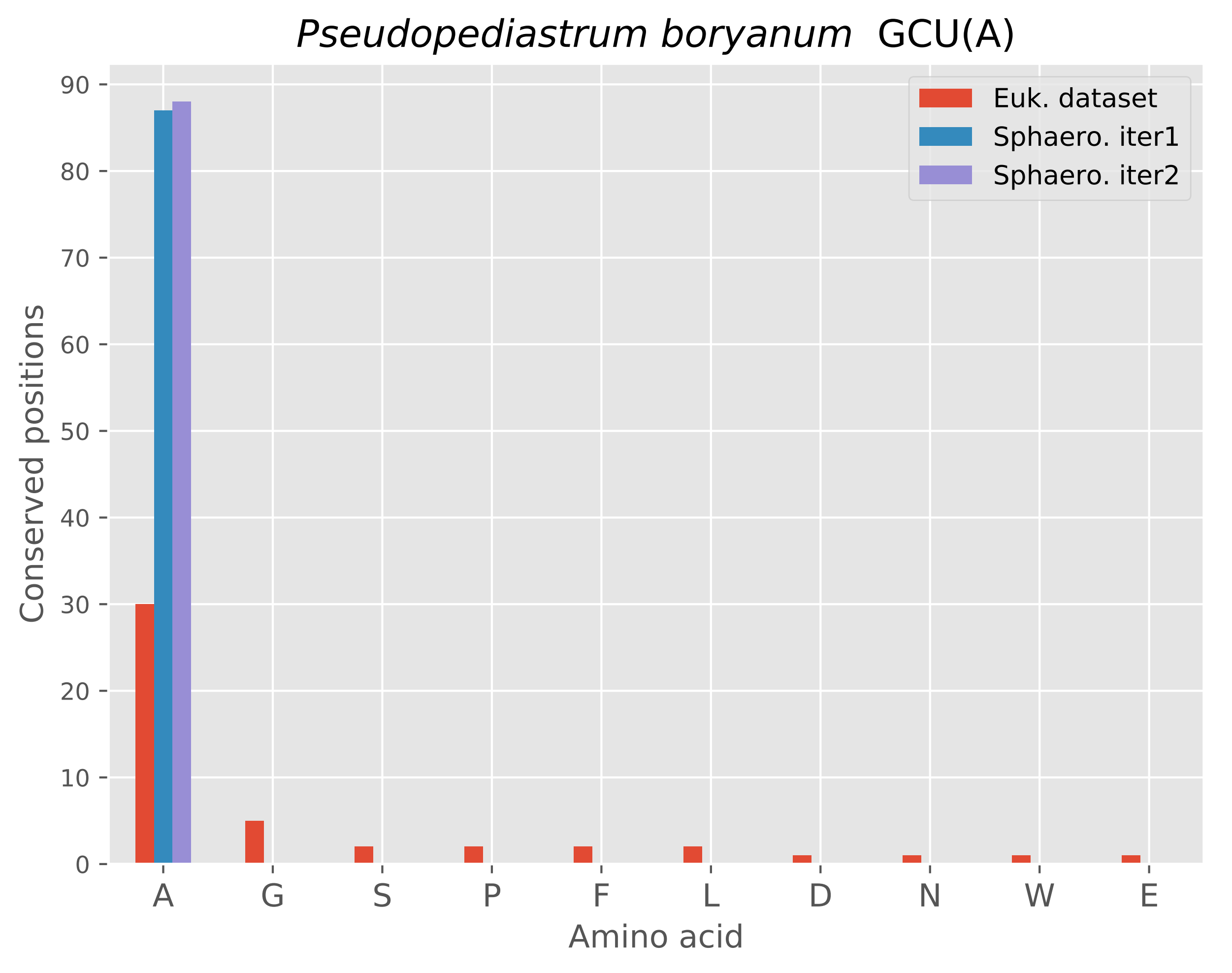
Pseudopediastrum boryanum GCU(A)
Euk. dataset
Sphaero. iter1
Sphaero. iter2
Conserved positions
Amino acid
0
10
20
30
40
50
60
70
80
90
A
G
S
P
F
L
D
N
W
E

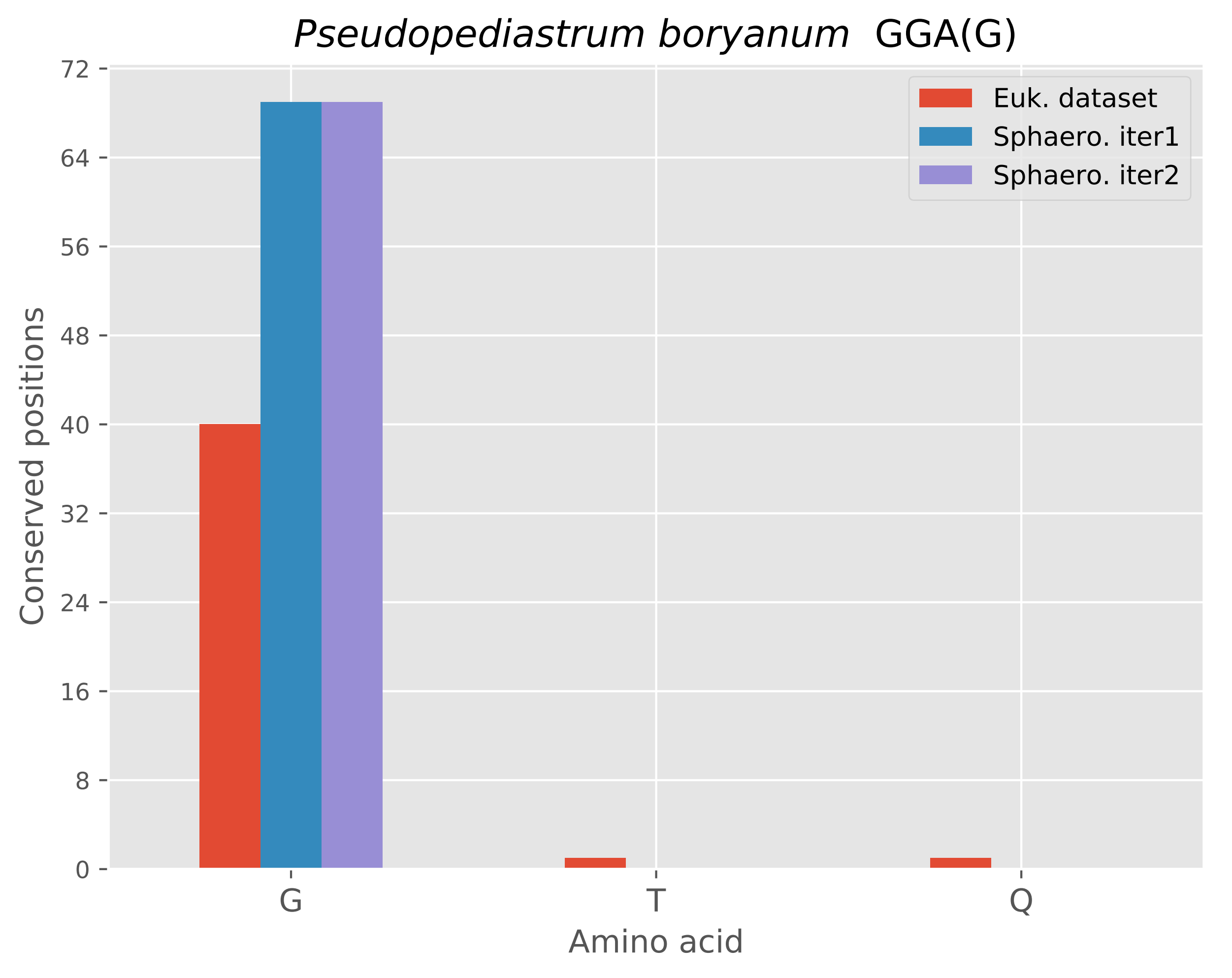

Pseudopediastrum boryanum GGA(G)
Euk. dataset
Sphaero. iter1
Sphaero. iter2
Conserved positions
0
8
16
24
32
40
48
56
64
72
G
T
Q
Amino acid

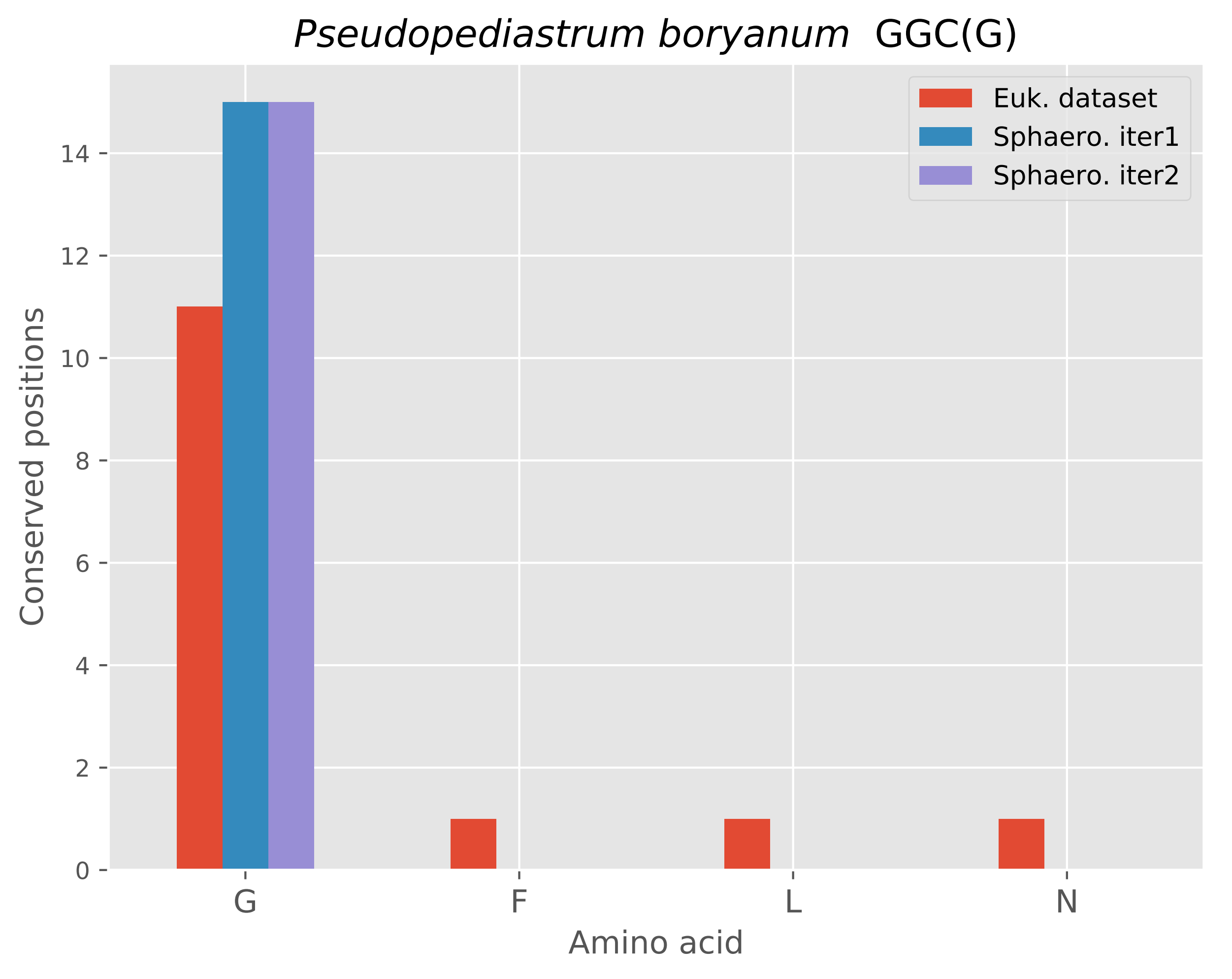

Pseudopediastrum boryanum GGC(G)
Euk. dataset
Sphaero. iter1
Sphaero. iter2
Conserved positions
0
2
4
6
8
10
12
14
G
F
L
N
Amino acid

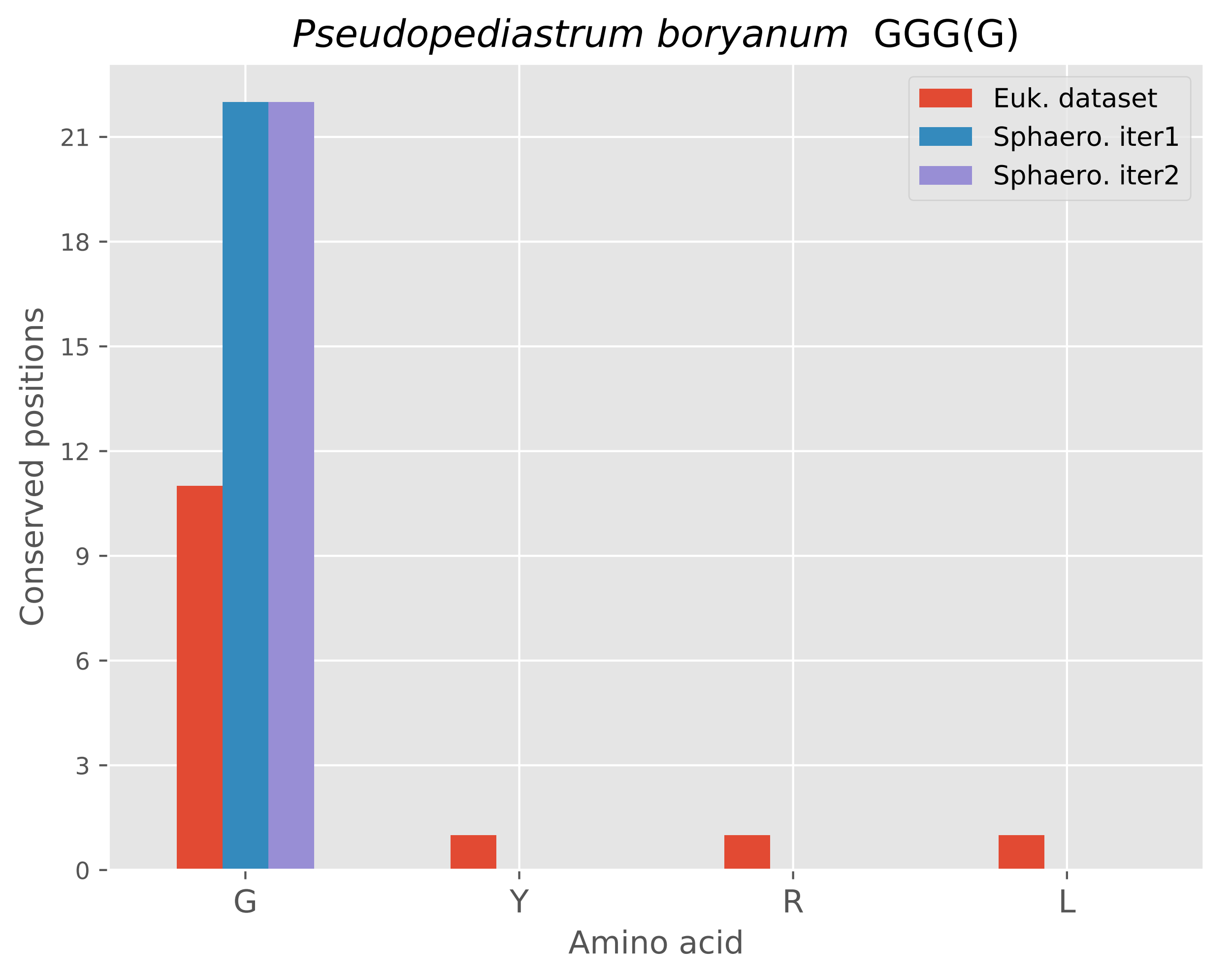
Pseudopediastrum boryanum GGG(G)
Euk. dataset
Sphaero. iter1
Sphaero. iter2
Conserved positions
0
3
6
9
12
15
18
21
G
Y
R
L
Amino acid

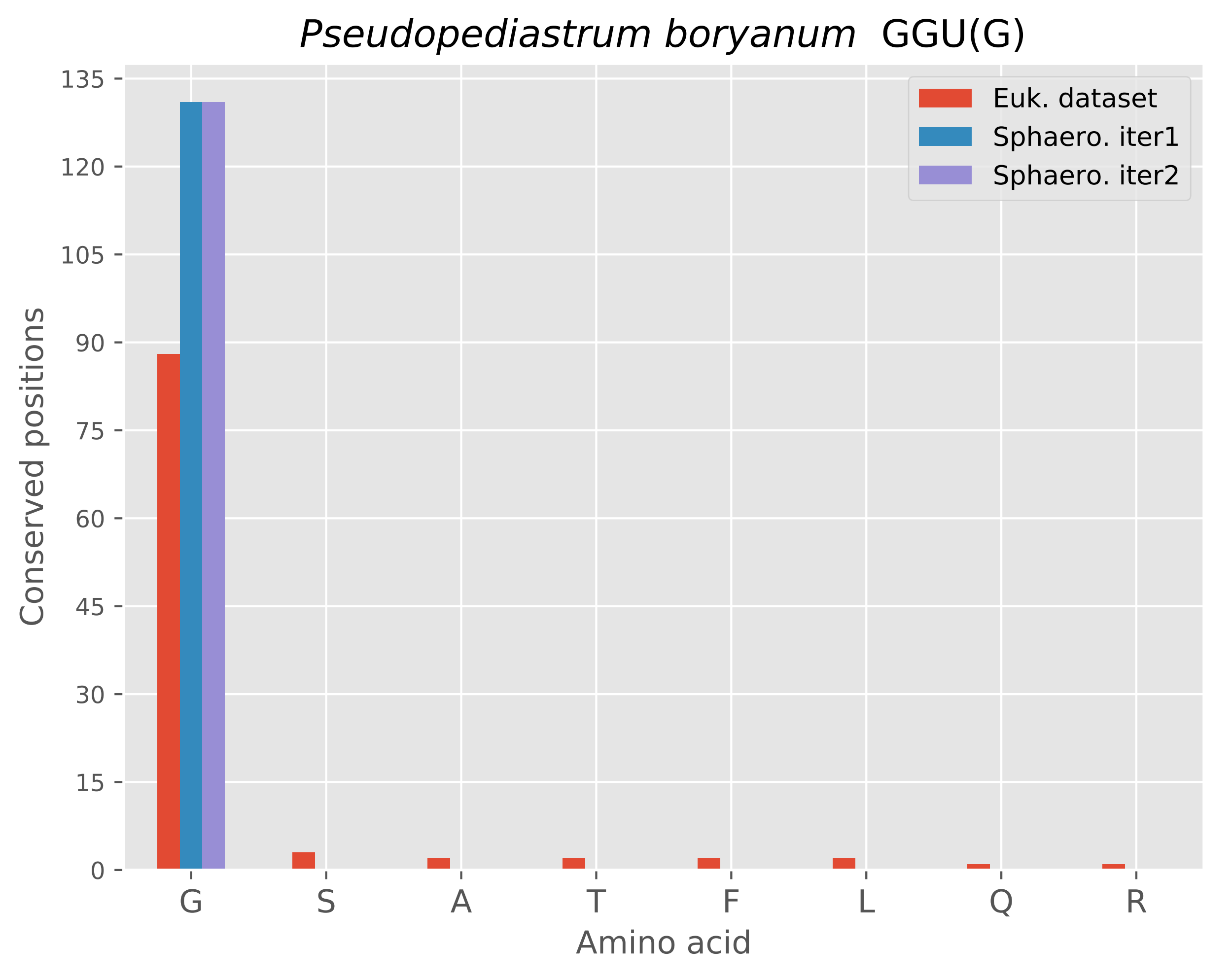

Pseudopediastrum boryanum GGU(G)
Euk. dataset
Sphaero. iter1
Sphaero. iter2
Conserved positions
0
15
30
45
60
75
90
105
120
135
G
S
A
T
F
L
Q
R
Amino acid

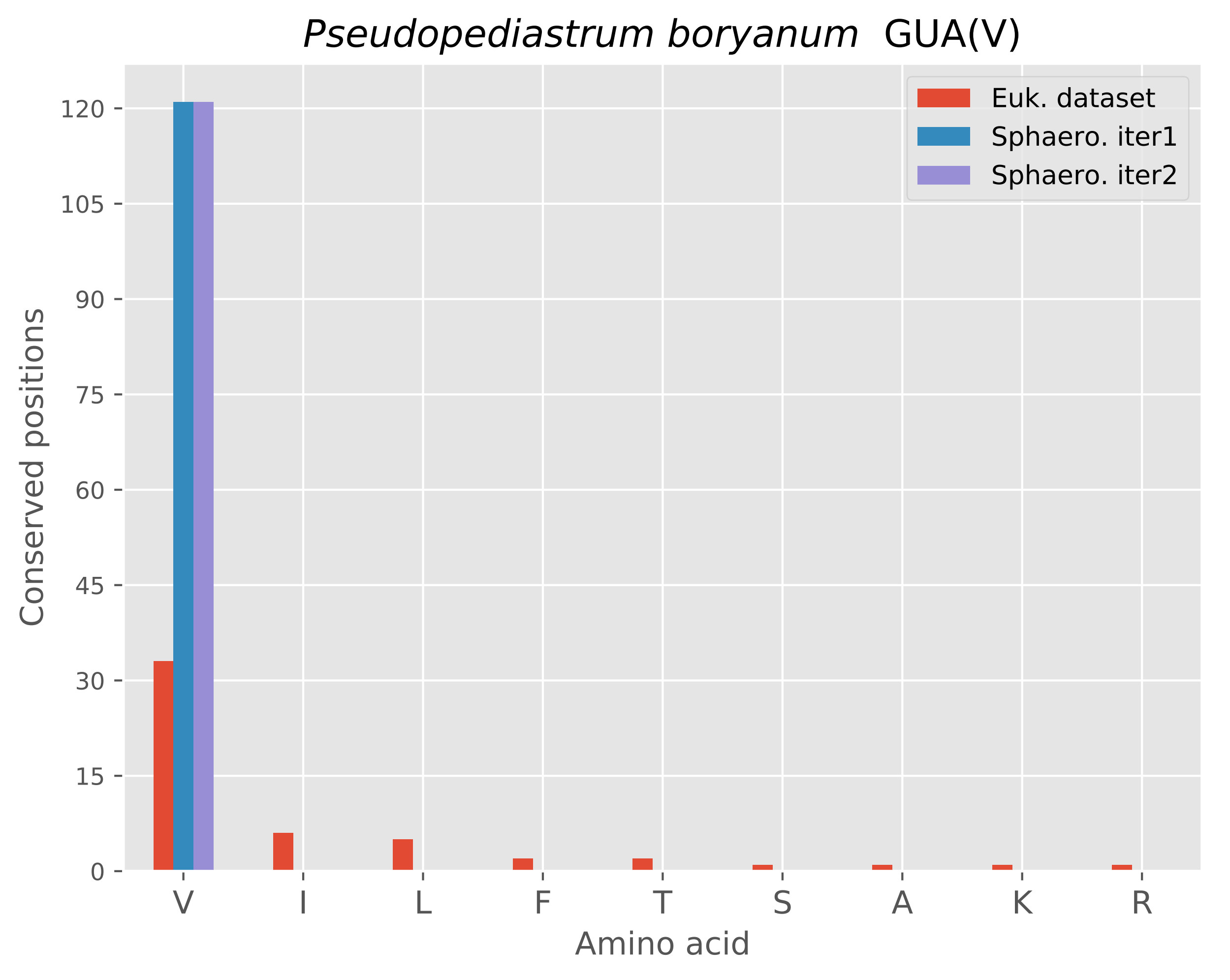

Pseudopediastrum boryanum GUA(V)
Euk. dataset
Sphaero. iter1
Sphaero. iter2
Conserved positions
0
15
30
45
60
75
90
105
120
V
I
L
F
T
S
A
K
R
Amino acid

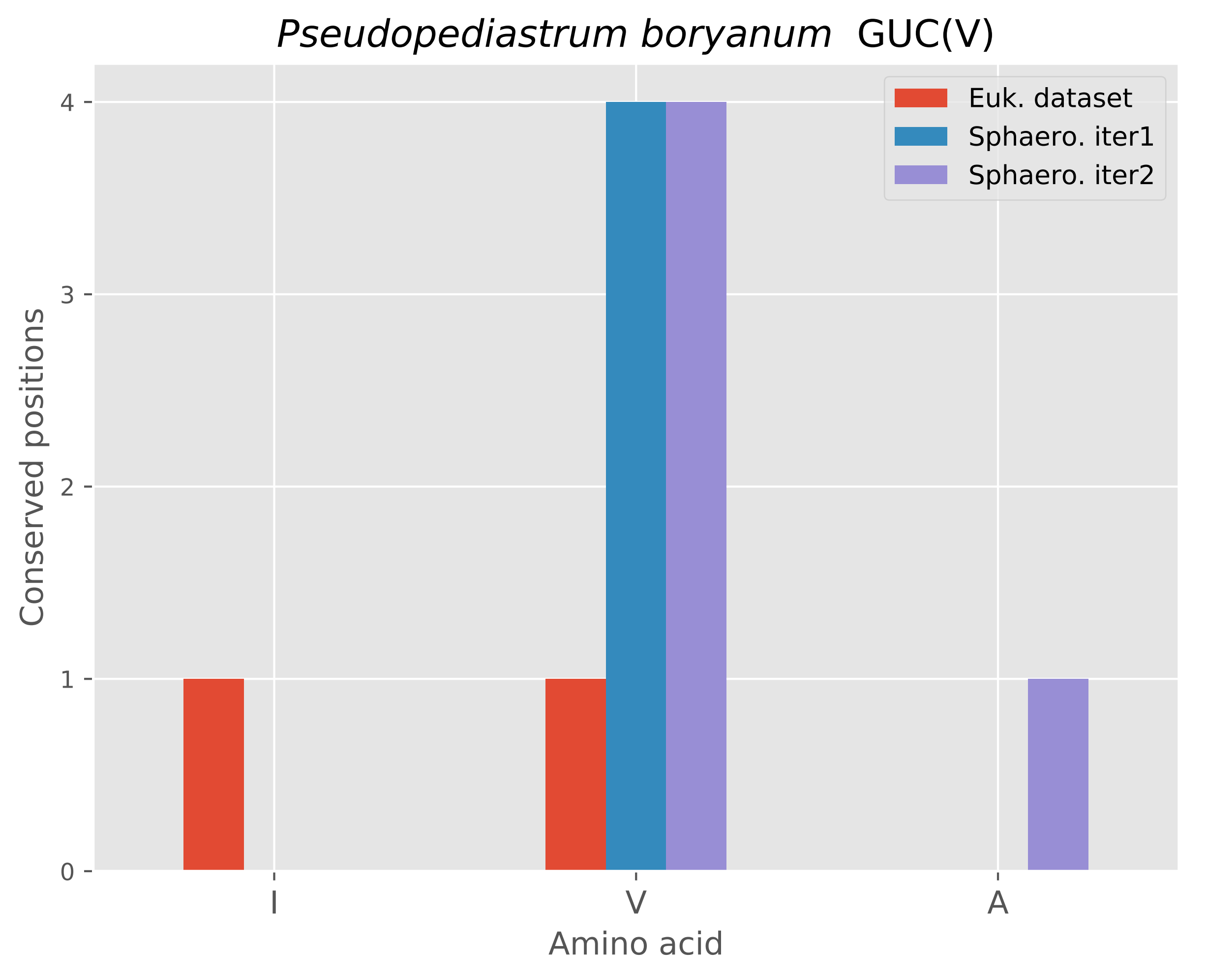

Pseudopediastrum boryanum GUC(V)
Euk. dataset
Sphaero. iter1
Sphaero. iter2
Conserved positions
0
1
2
3
4
I
V
A
Amino acid

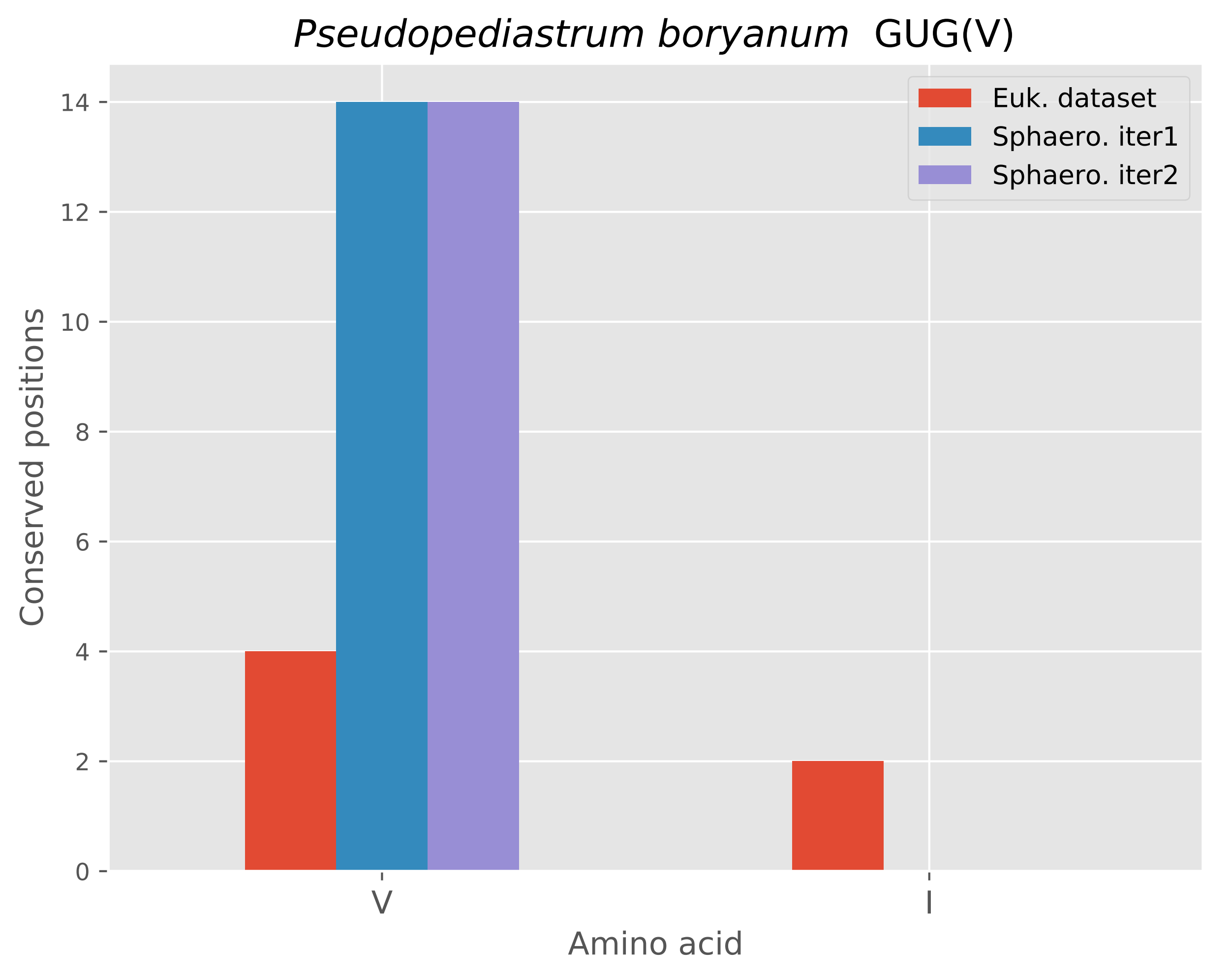

Pseudopediastrum boryanum GUG(V)
Euk. dataset
Sphaero. iter1
Sphaero. iter2
Conserved positions
0
2
4
6
8
10
12
14
V
I
Amino acid

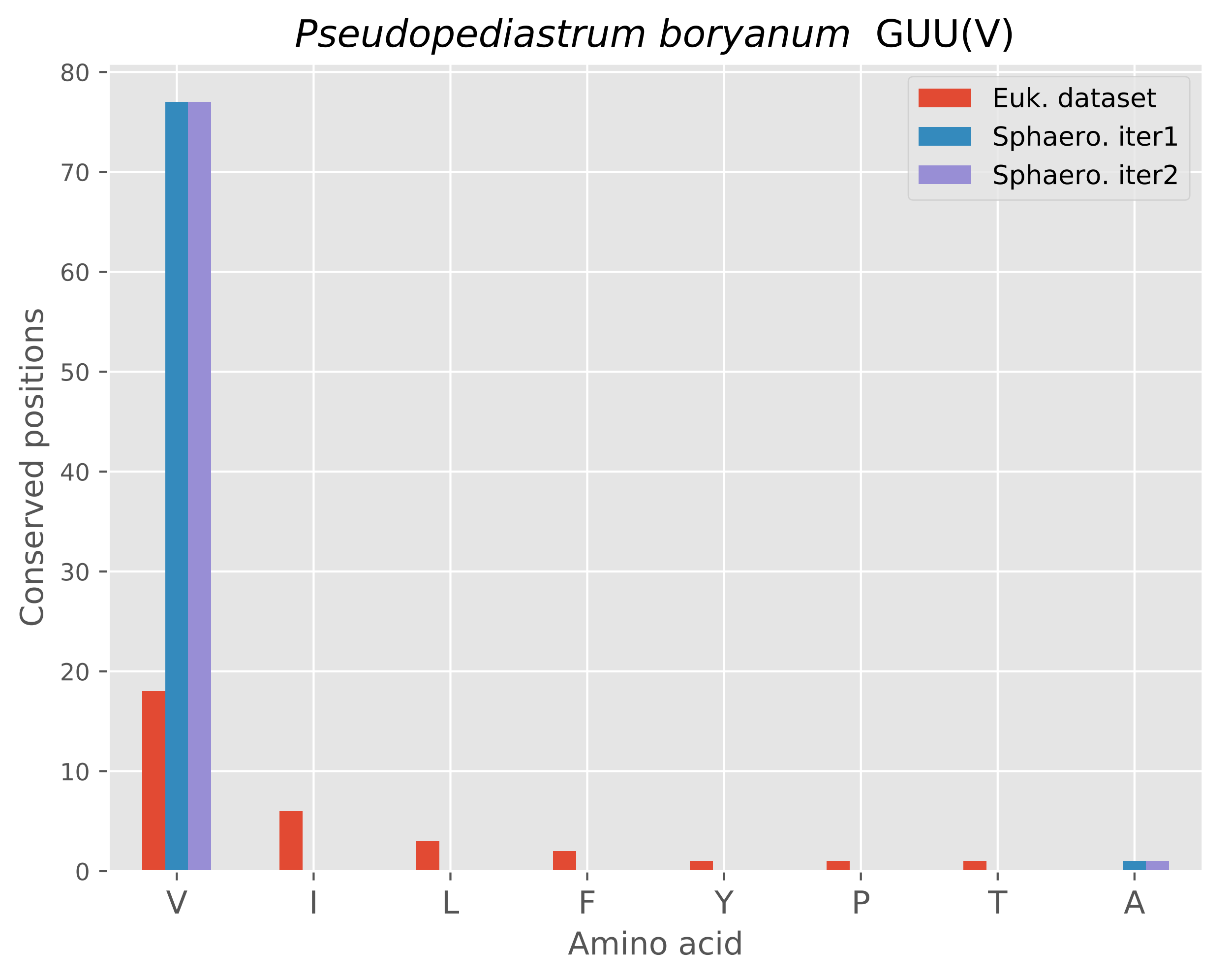

Pseudopediastrum boryanum GUU(V)
Euk. dataset
Sphaero. iter1
Sphaero. iter2
Conserved positions
Amino acid
0
10
20
30
40
50
60
70
80
V
I
L
F
Y
P
T
A

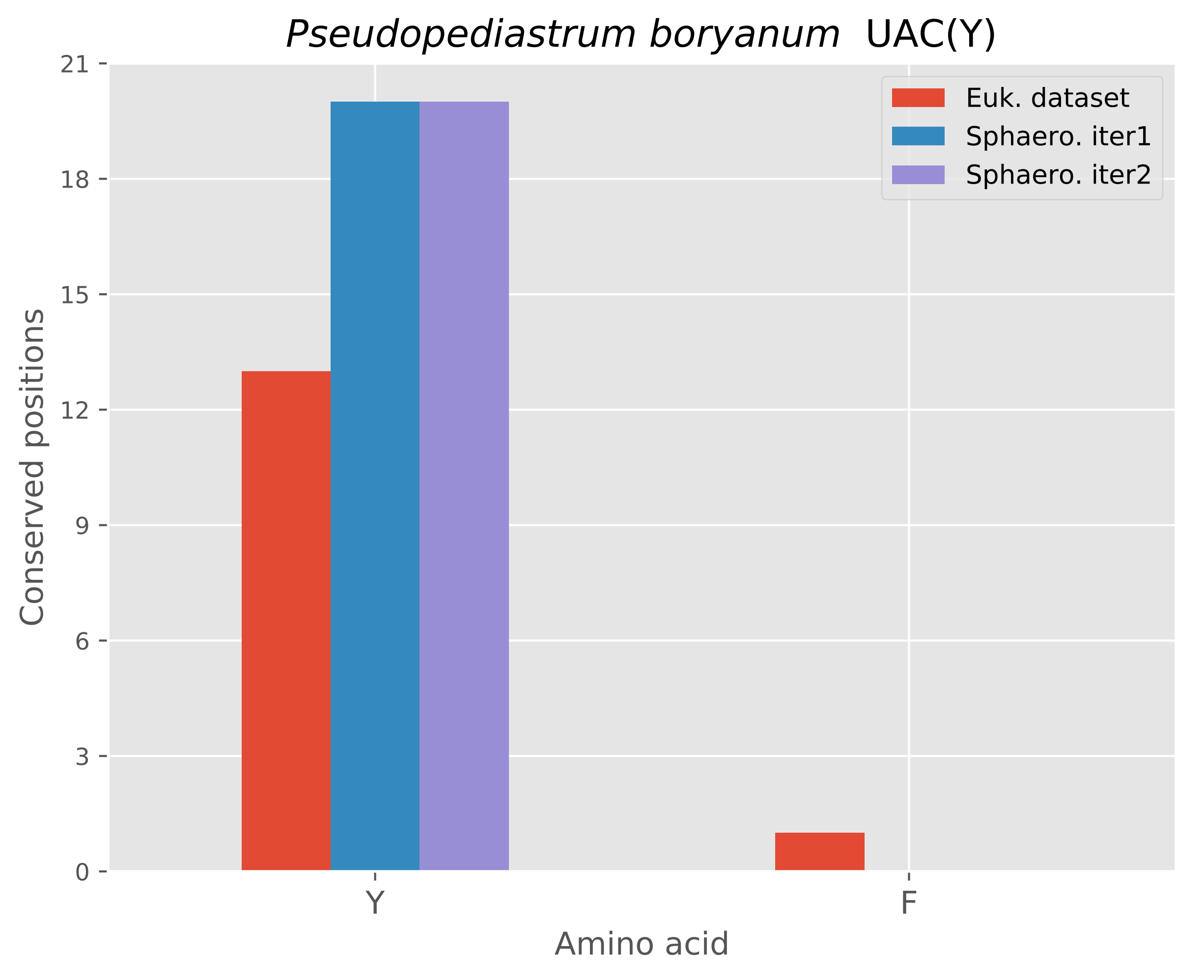

Pseudopediastrum boryanum UAC(Y)
Euk. dataset
Sphaero. iter1
Sphaero. iter2
Conserved positions
0
3
6
9
12
15
18
21
Y
F
Amino acid

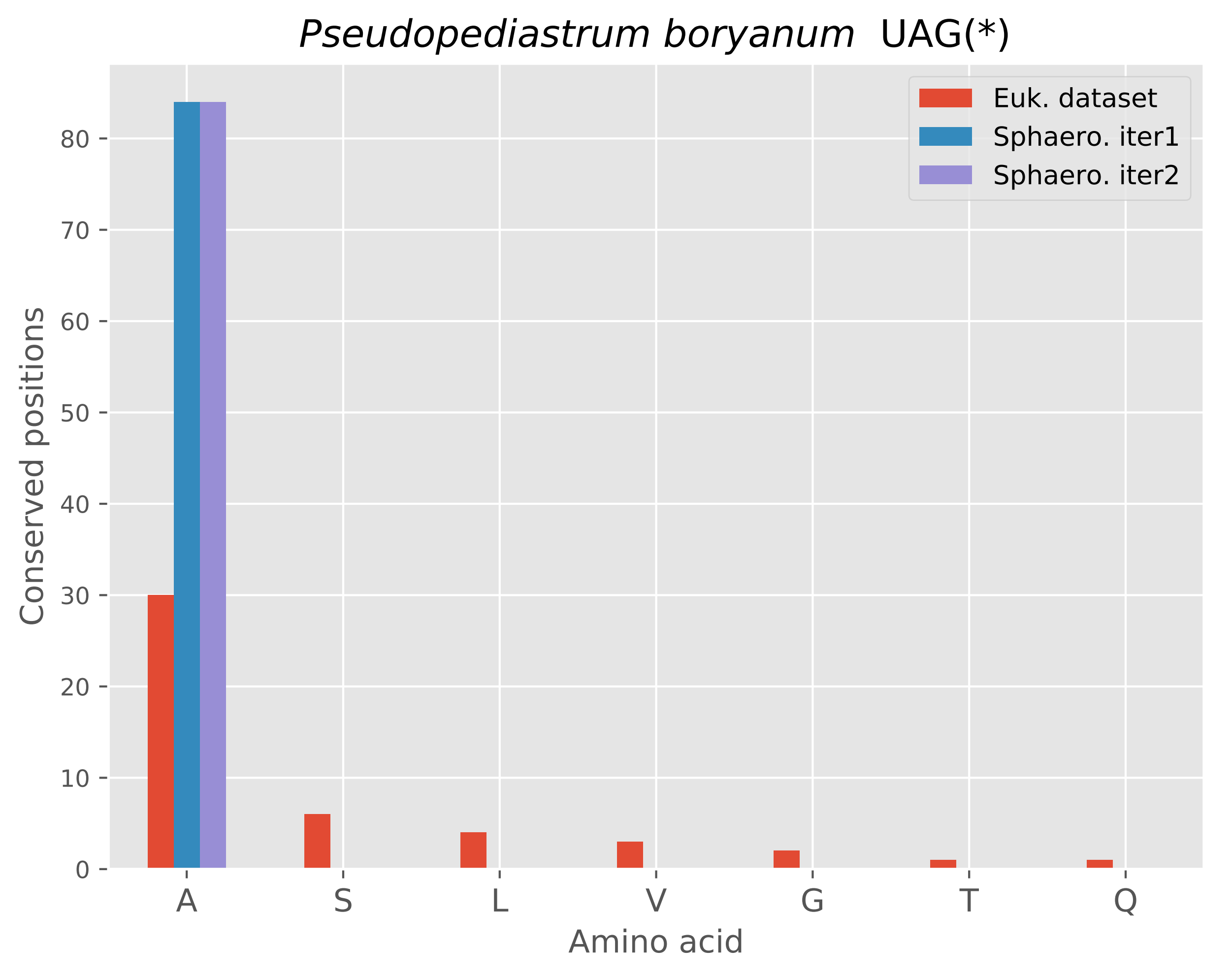

Pseudopediastrum boryanum UAG(*)
Euk. dataset
Sphaero. iter1
Sphaero. iter2
Conserved positions
0
10
20
30
40
50
60
70
80
A
S
L
V
G
T
Q
Amino acid

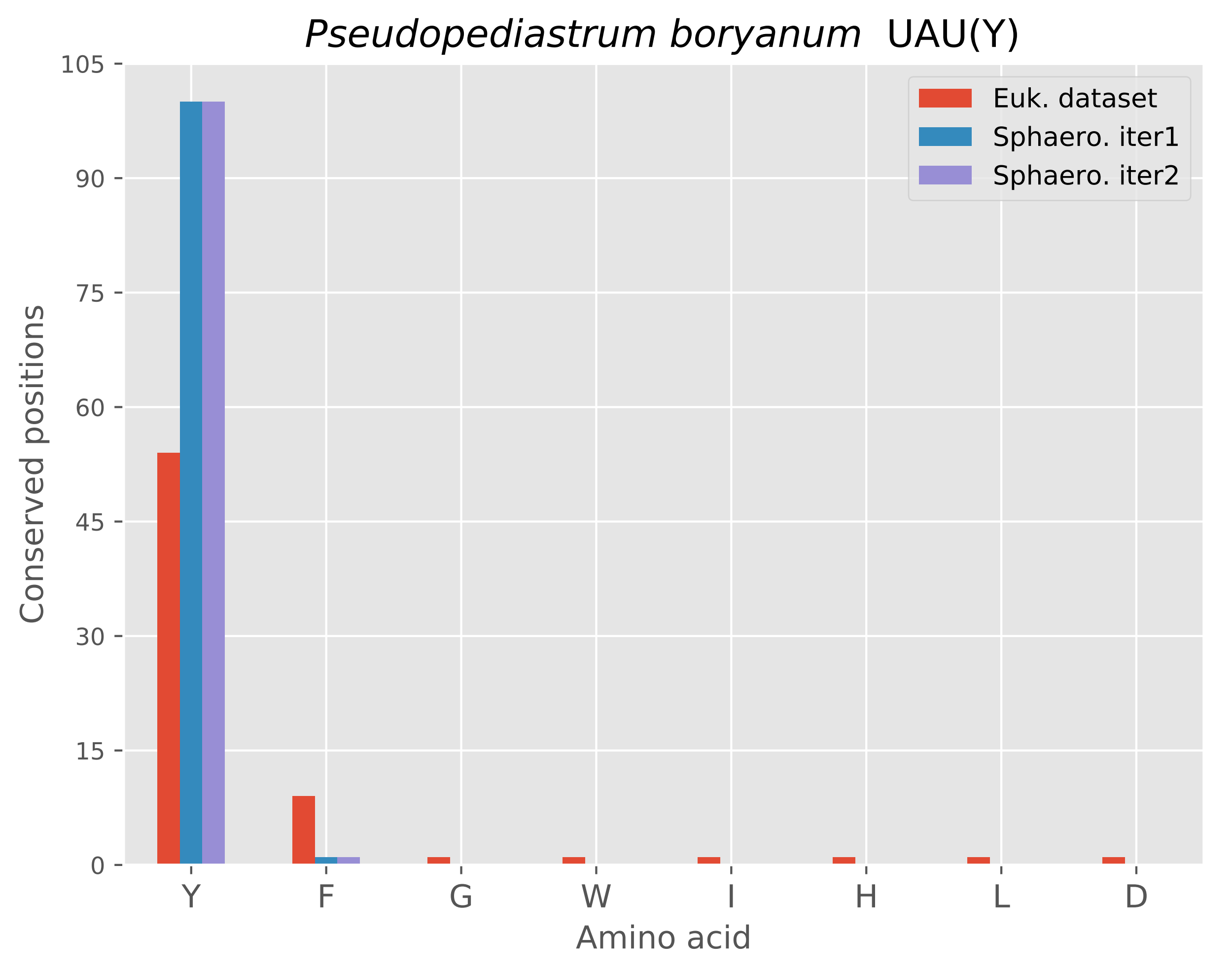
Pseudopediastrum boryanum UAU(Y)
Euk. dataset
Sphaero. iter1
Sphaero. iter2
Conserved positions
105
90
75
60
45
30
15
0
Y
F
G
W
I
H
L
D
Amino acid

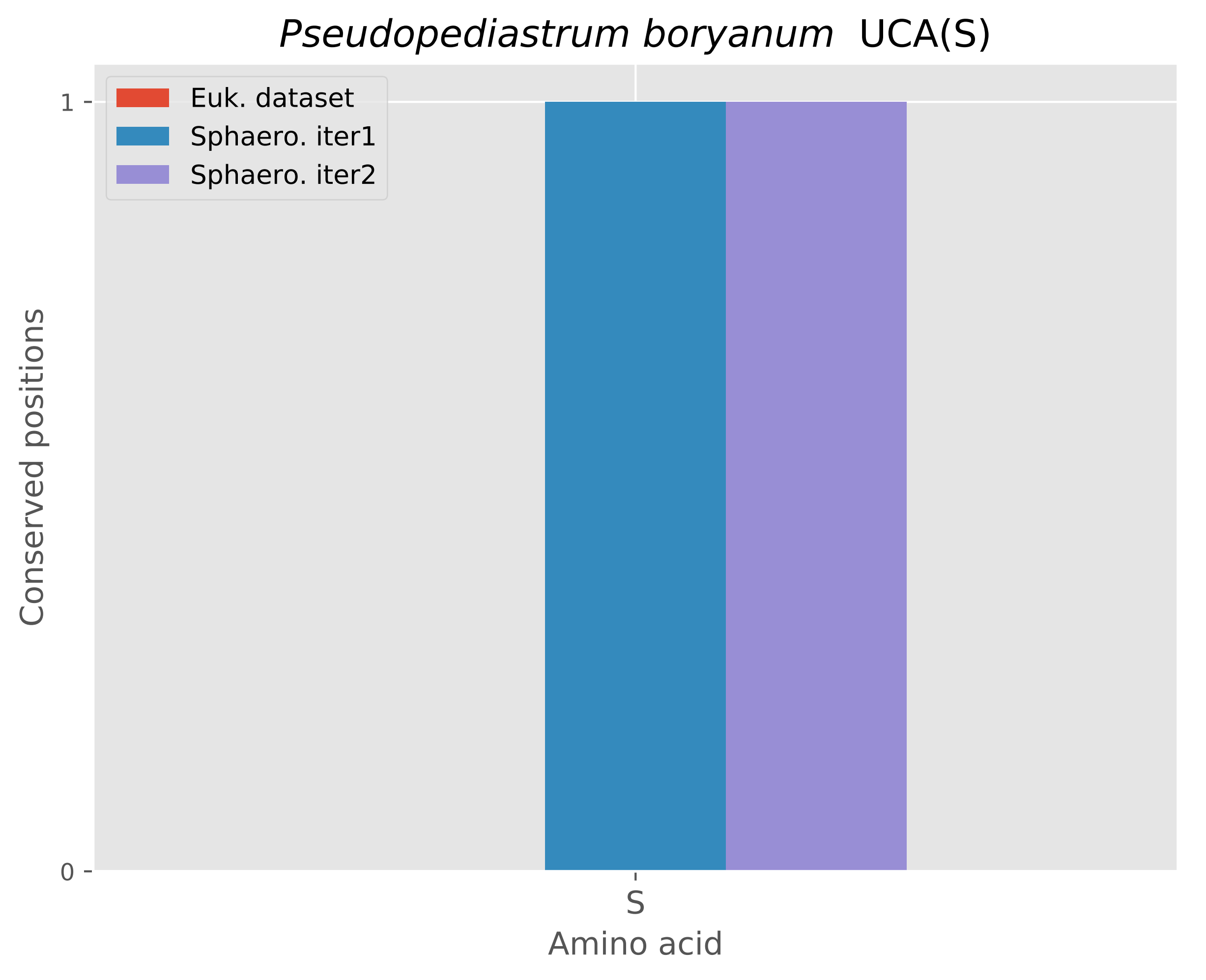
Pseudopediastrum boryanum UCA(S)
Euk. dataset
Sphaero. iter1
Sphaero. iter2
1
0
Conserved positions
S
Amino acid

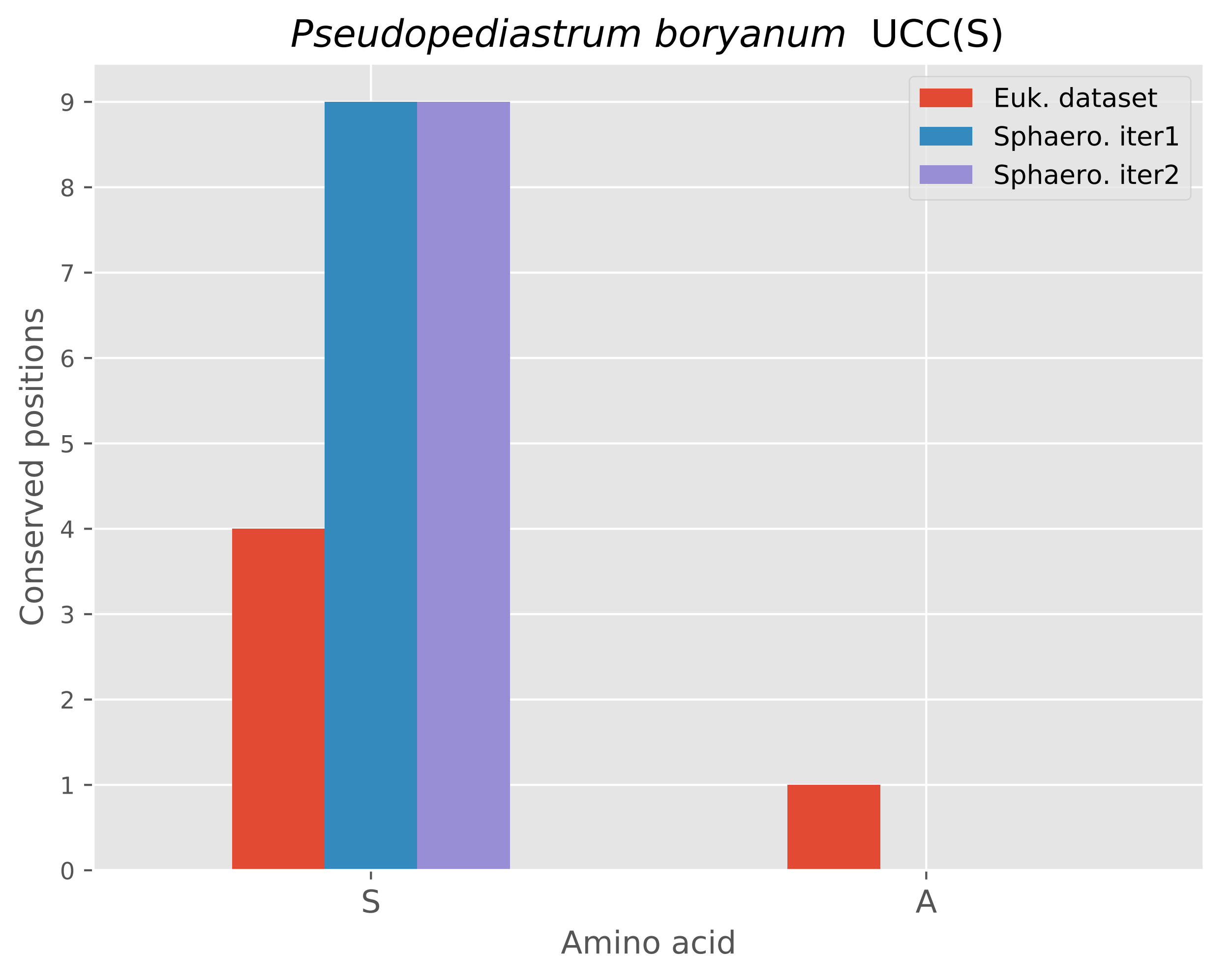

Pseudopediastrum boryanum UCC(S)
Euk. dataset
Sphaero. iter1
Sphaero. iter2
Conserved positions
0
1
2
3
4
5
6
7
8
9
S
A
Amino acid

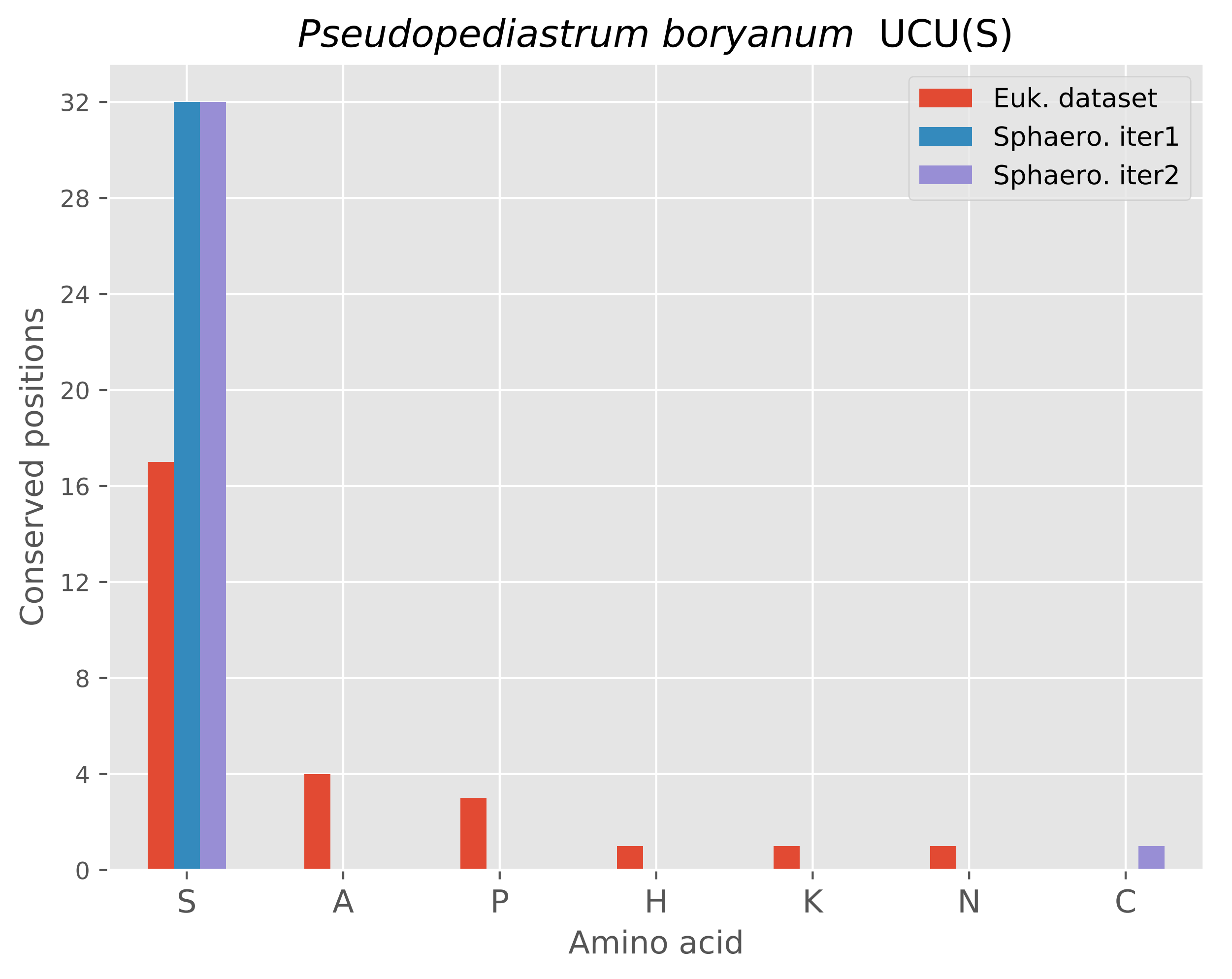
Pseudopediastrum boryanum UCU(S)
Euk. dataset
Sphaero. iter1
Sphaero. iter2
Conserved positions
0
4
8
12
16
20
24
28
32
S
A
P
H
K
N
C
Amino acid

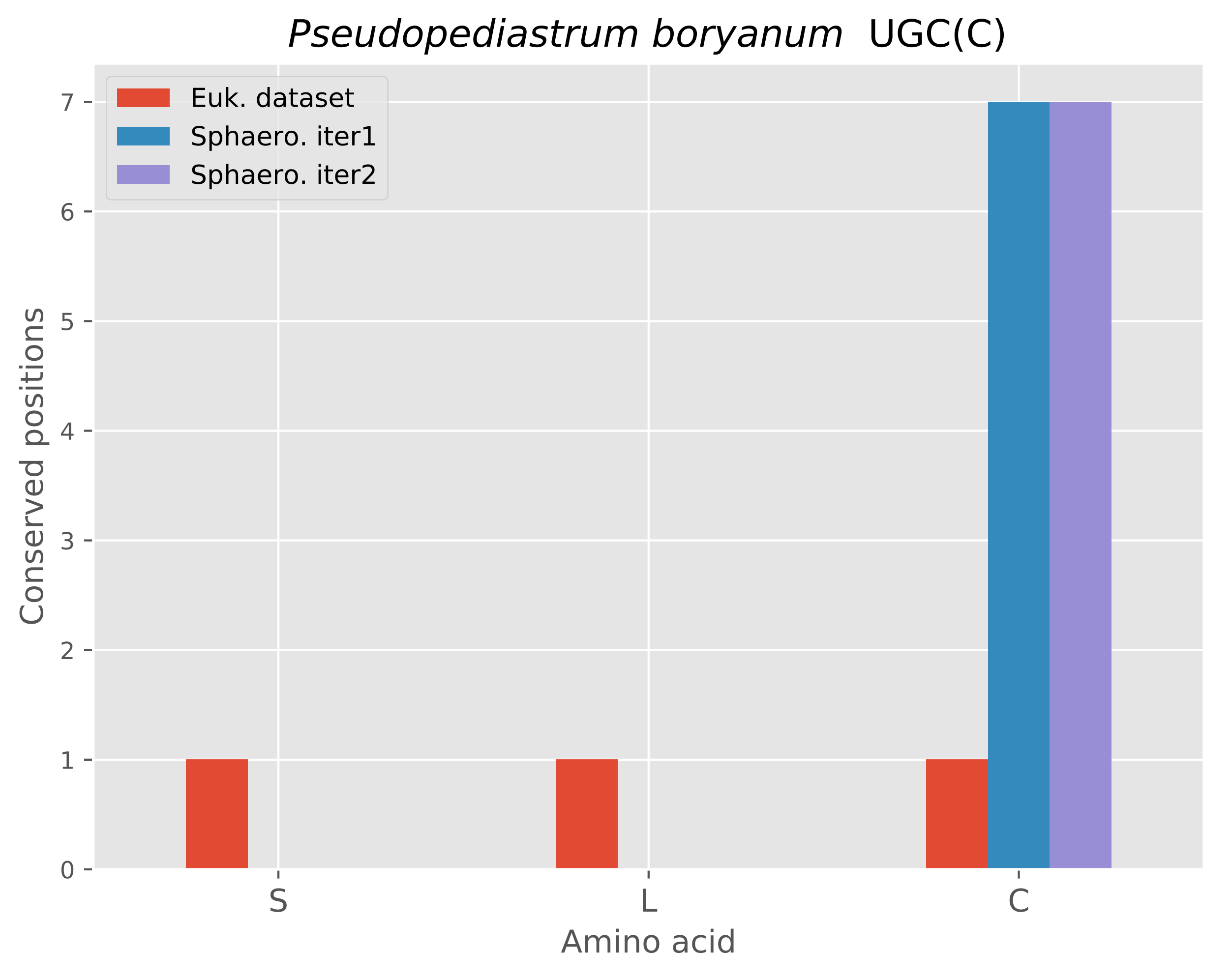
Pseudopediastrum boryanum UGC(C)
Euk. dataset
Sphaero. iter1
Sphaero. iter2
Conserved positions
0
1
2
3
4
5
6
7
S
L
C
Amino acid

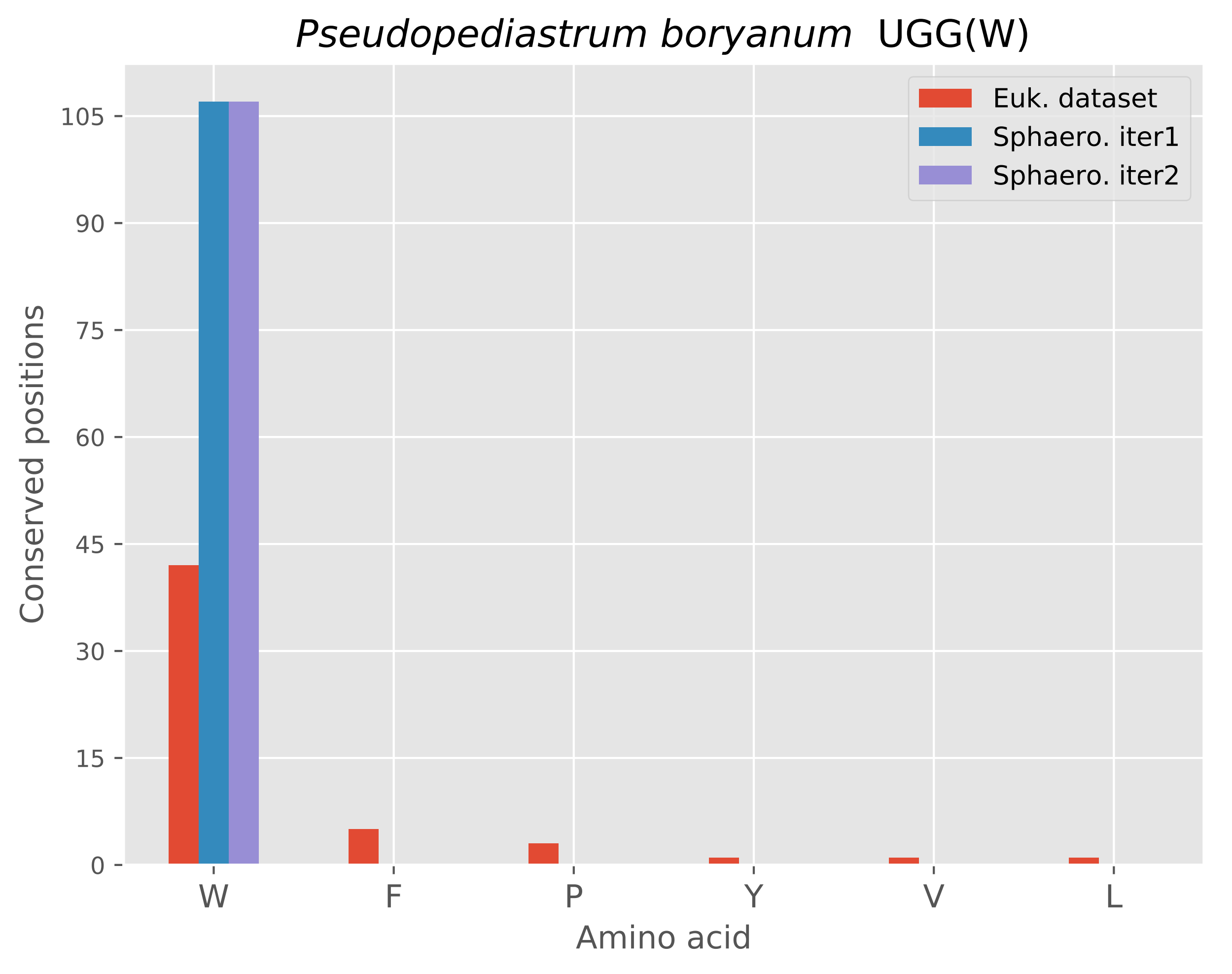
Pseudopediastrum boryanum UGG(W)
Euk. dataset
Sphaero. iter1
Sphaero. iter2
Conserved positions
0
15
30
45
60
75
90
105
W
F
P
Y
V
L
Amino acid

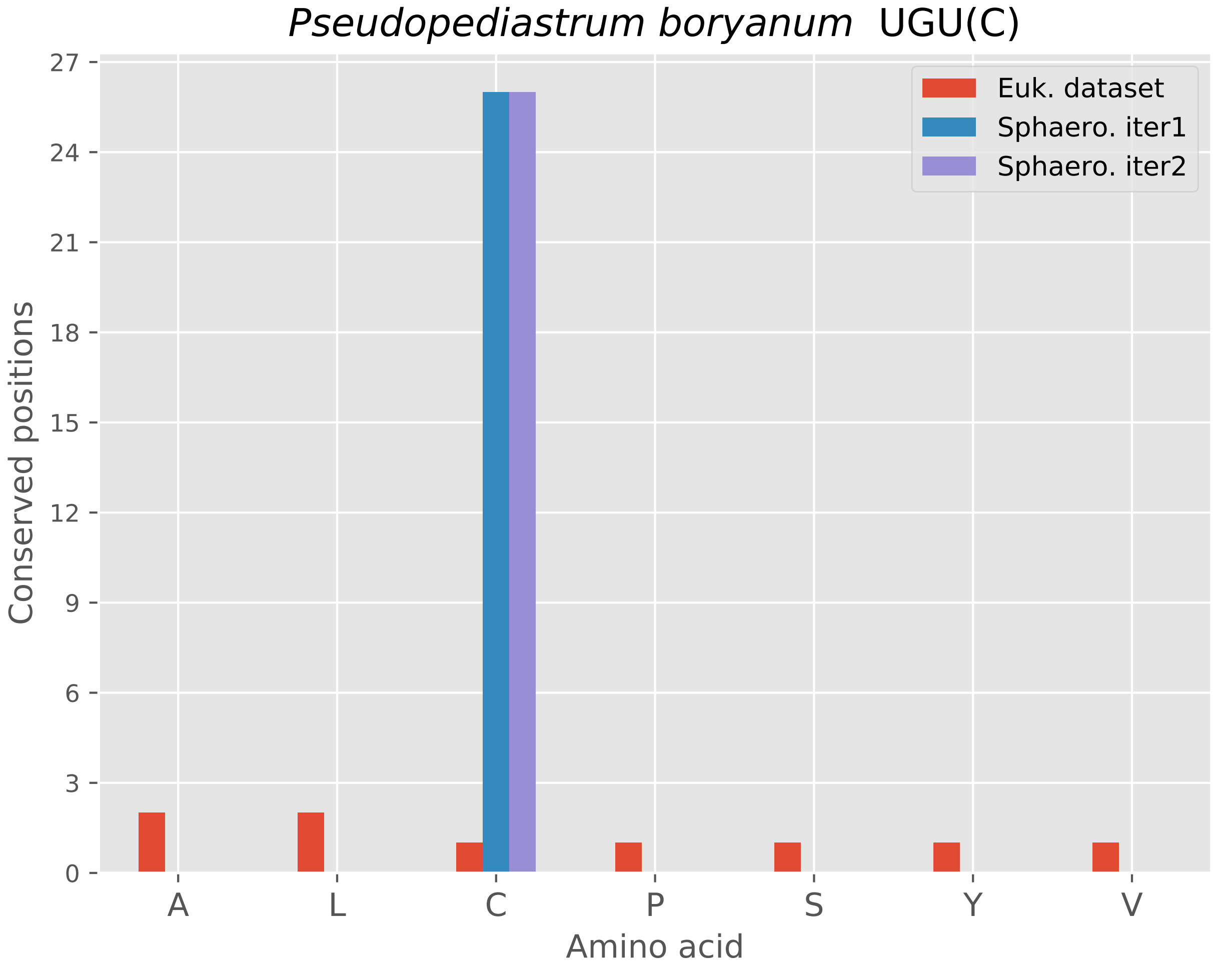

Pseudopediastrum boryanum UGU(C)
Euk. dataset
Sphaero. iter1
Sphaero. iter2
Conserved positions
0
3
6
9
12
15
18
21
24
27
A
L
C
P
S
Y
V
Amino acid

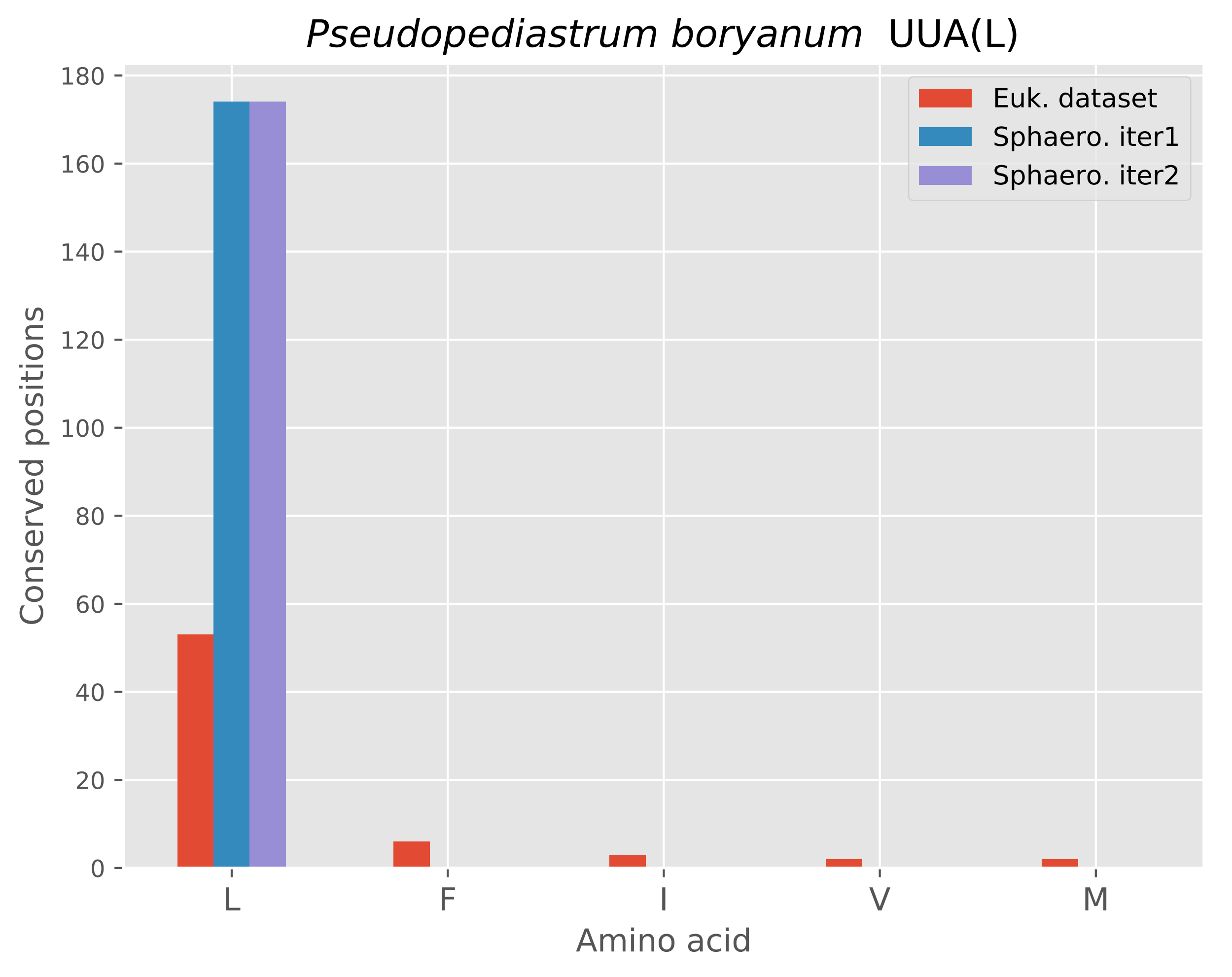

Pseudopediastrum boryanum UUA(L)
Euk. dataset
Sphaero. iter1
Sphaero. iter2
Conserved positions
0
20
40
60
80
100
120
140
160
180
L
F
I
V
M
Amino acid

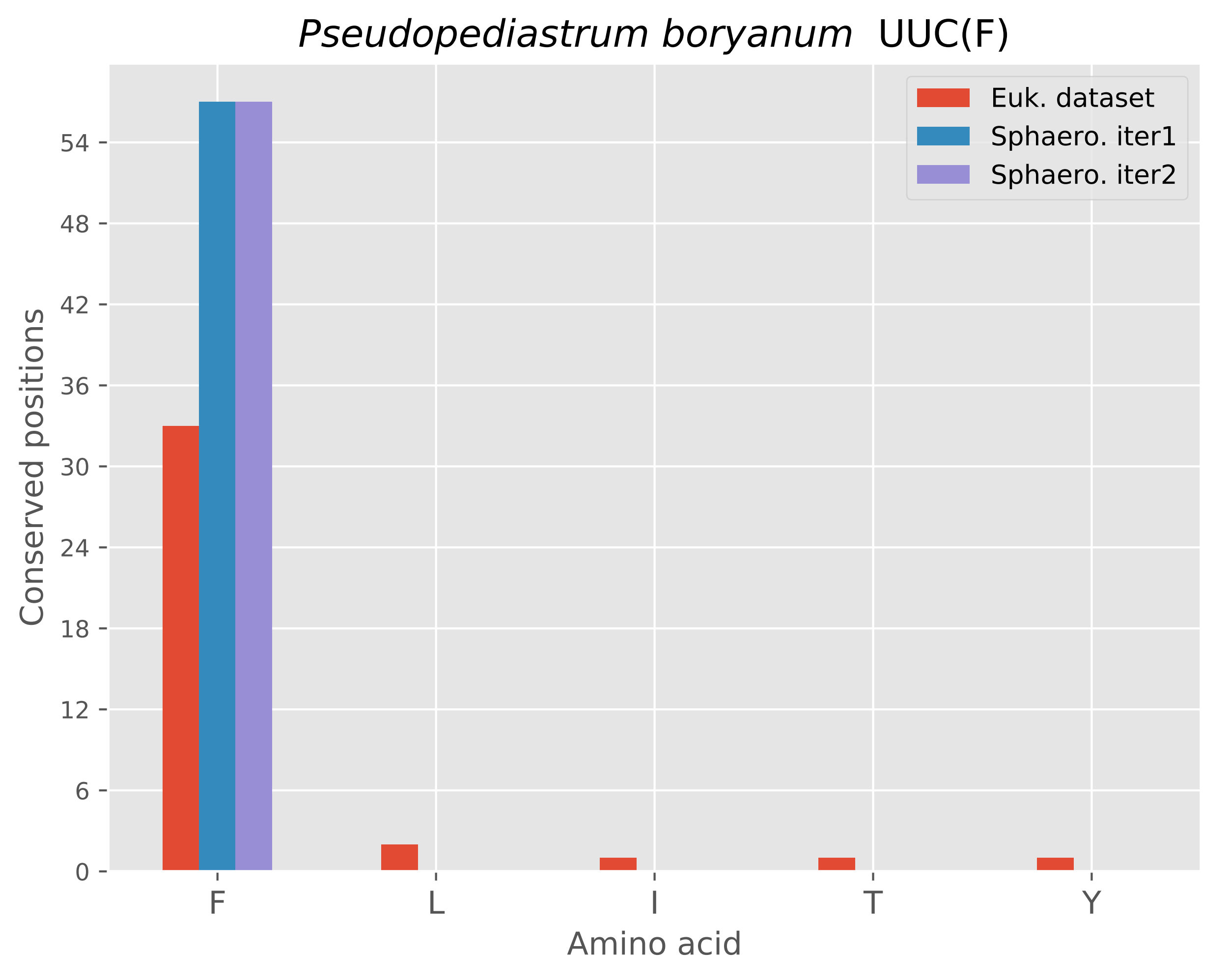

Pseudopediastrum boryanum UUC(F)
Euk. dataset
Sphaero. iter1
Sphaero. iter2
Conserved positions
0
6
12
18
24
30
36
42
48
54
F
L
I
T
Y
Amino acid

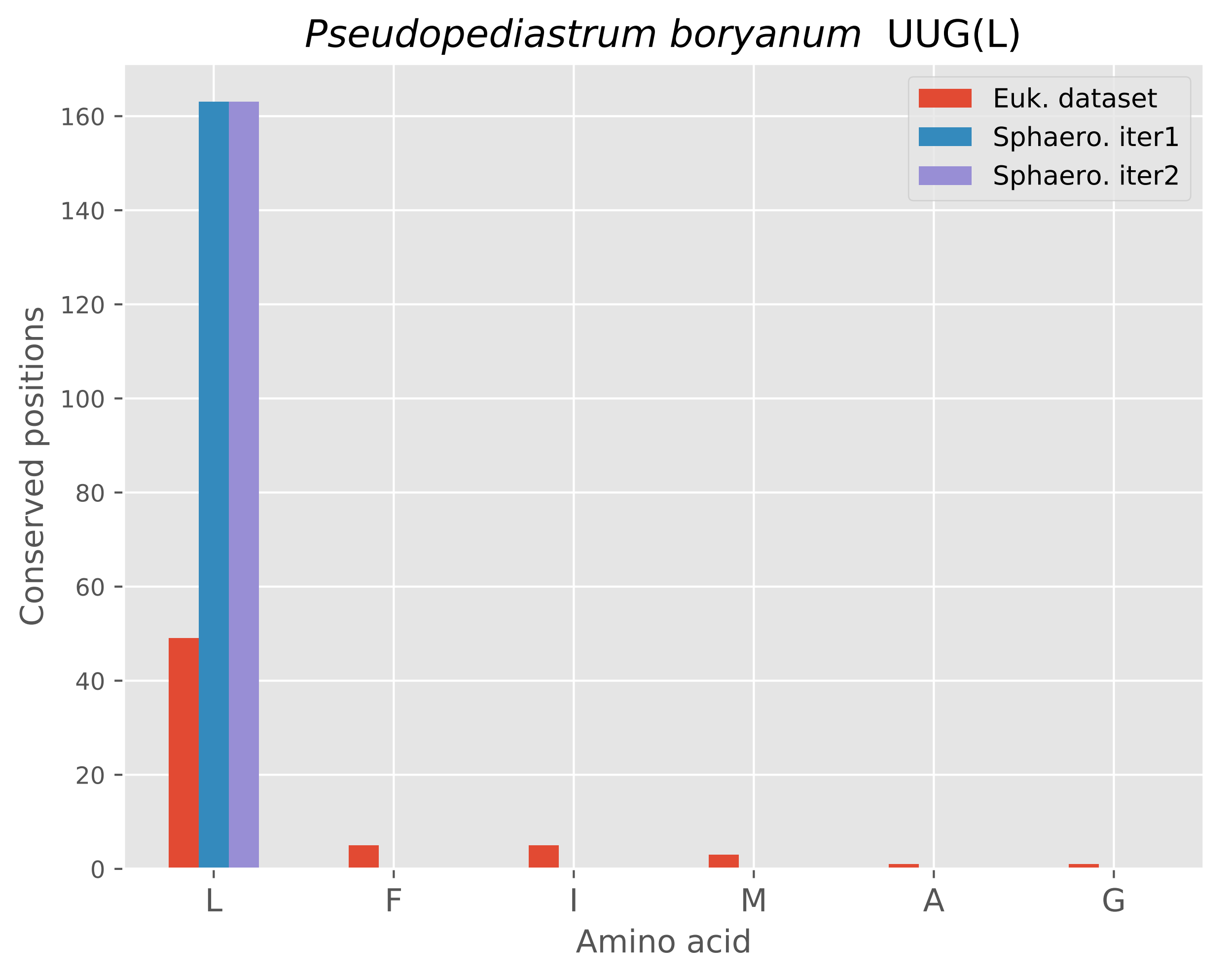

Pseudopediastrum boryanum UUG(L)
Euk. dataset
Sphaero. iter1
Sphaero. iter2
Conserved positions
0
20
40
60
80
100
120
140
160
L
F
I
M
A
G
Amino acid

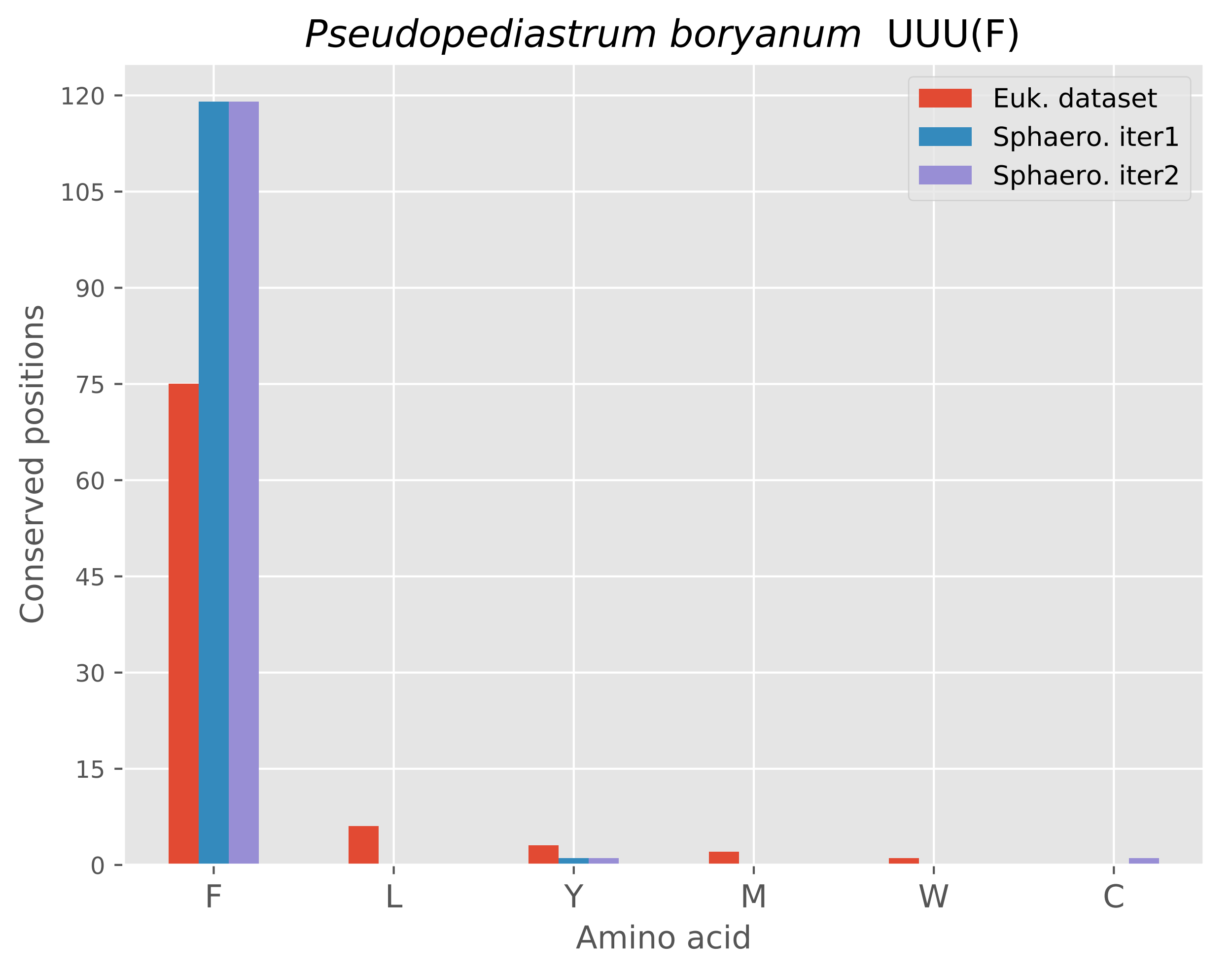

Pseudopediastrum boryanum UUU(F)
Euk. dataset
Sphaero. iter1
Sphaero. iter2
Conserved positions
0
15
30
45
60
75
90
105
120
F
L
Y
M
W
C
Amino acid

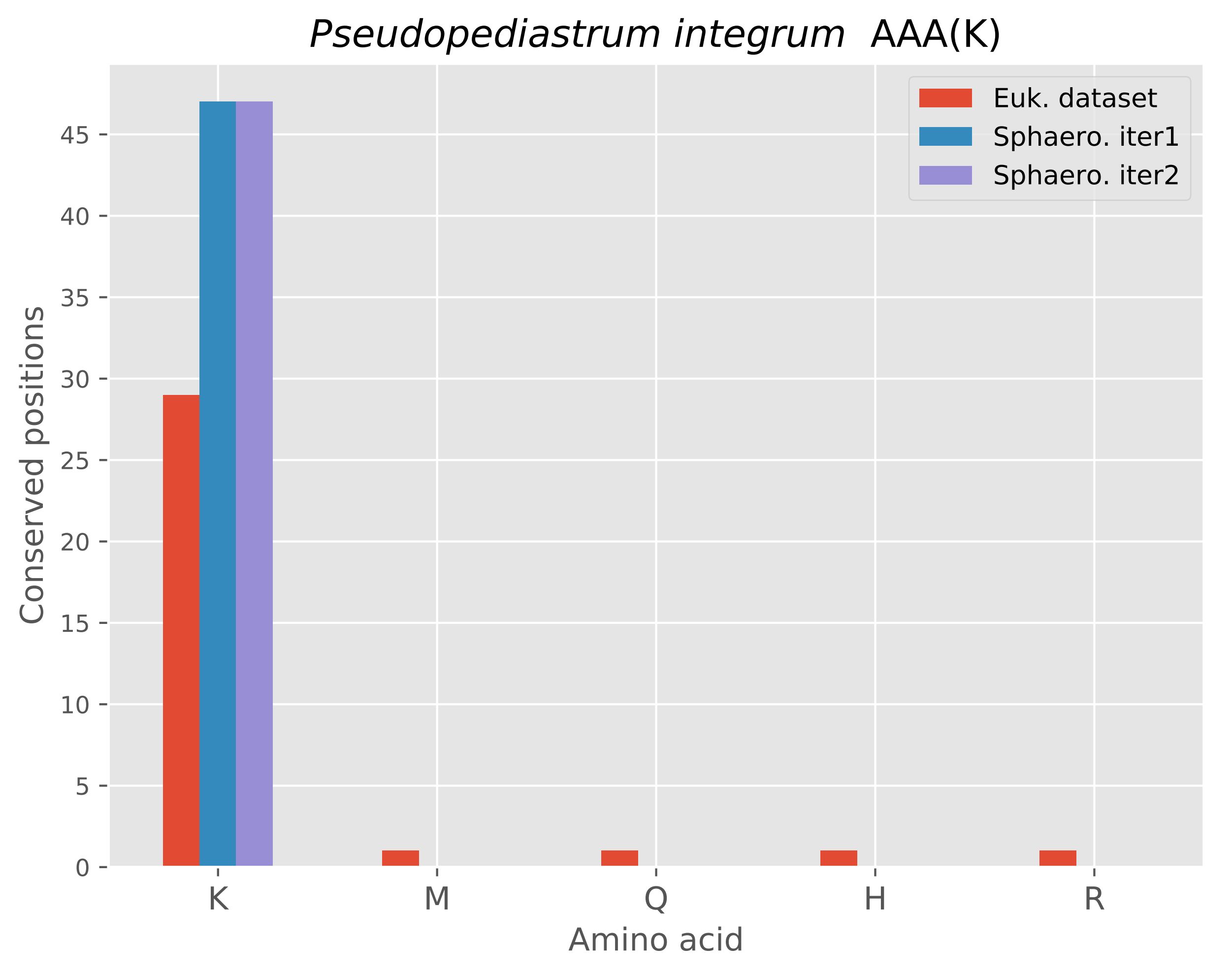

Pseudopediastrum integrum AAA(K)
Euk. dataset
Sphaero. iter1
Sphaero. iter2
Conserved positions
0
5
10
15
20
25
30
35
40
45
K
M
Q
H
R
Amino acid

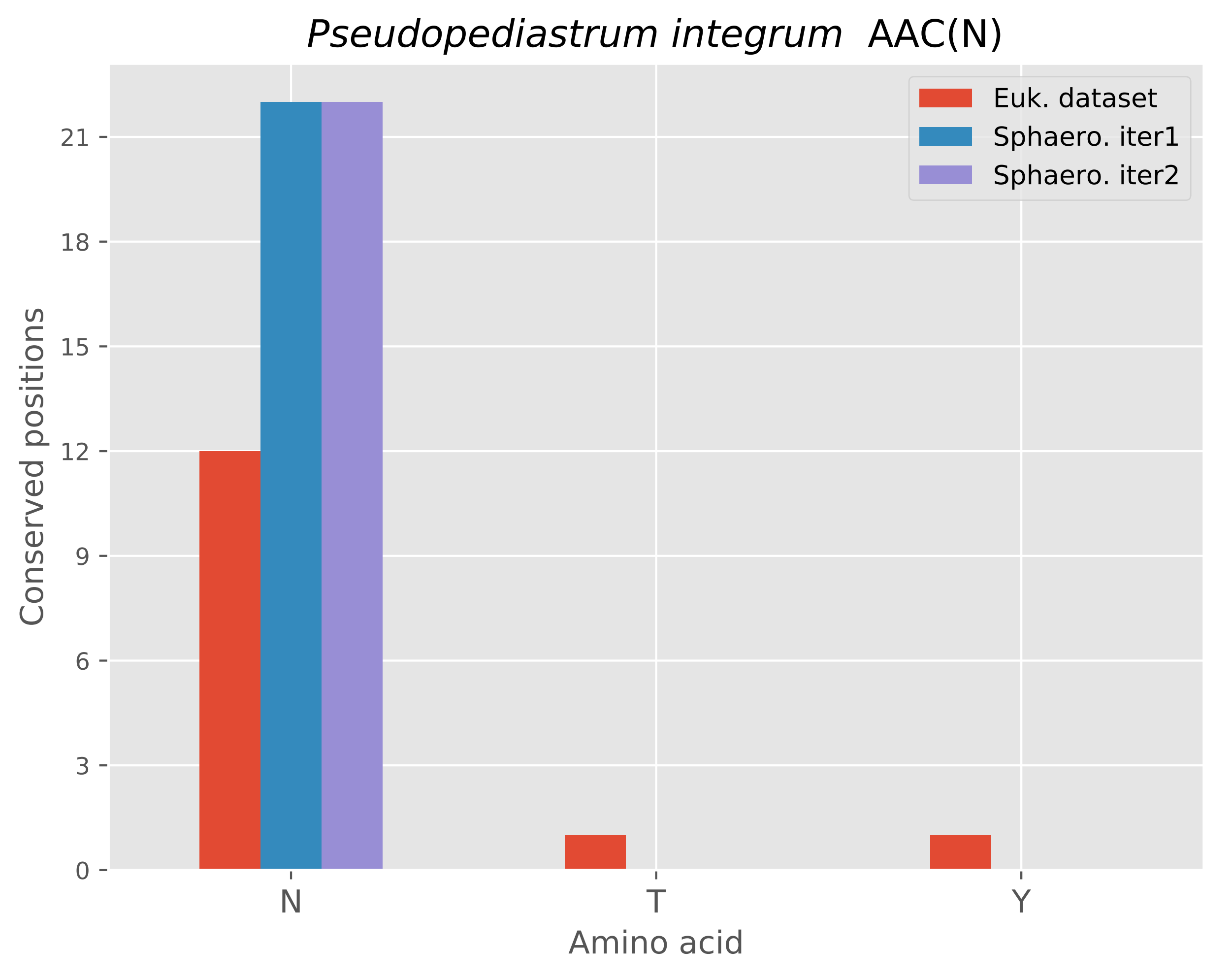

Pseudopediastrum integrum AAC(N)
Euk. dataset
Sphaero. iter1
Sphaero. iter2
Conserved positions
0
3
6
9
12
15
18
21
N
T
Y
Amino acid

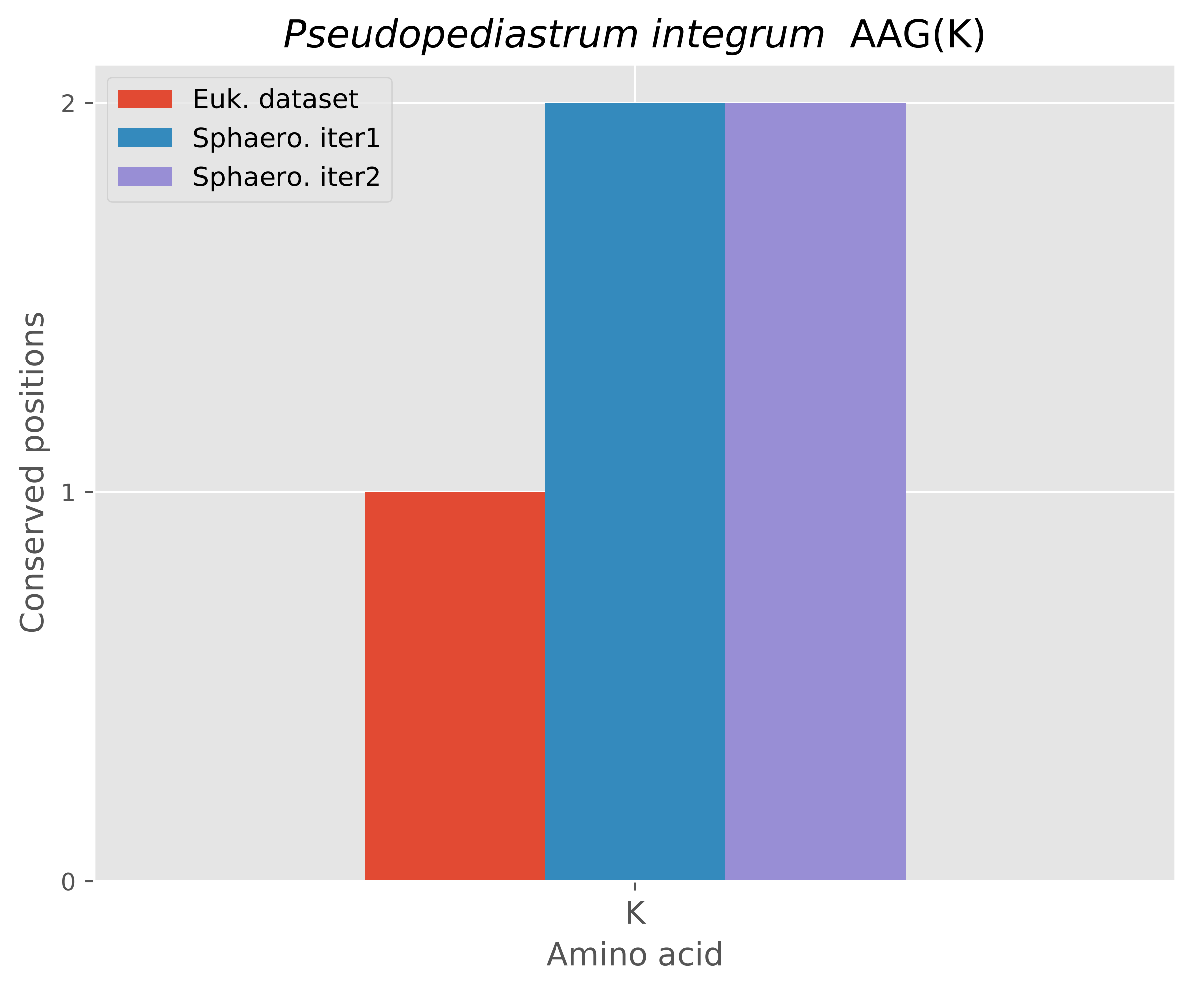

Pseudopediastrum integrum AAG(K)
Euk. dataset
Sphaero. iter1
Sphaero. iter2
Conserved positions
2
1
0
K
Amino acid

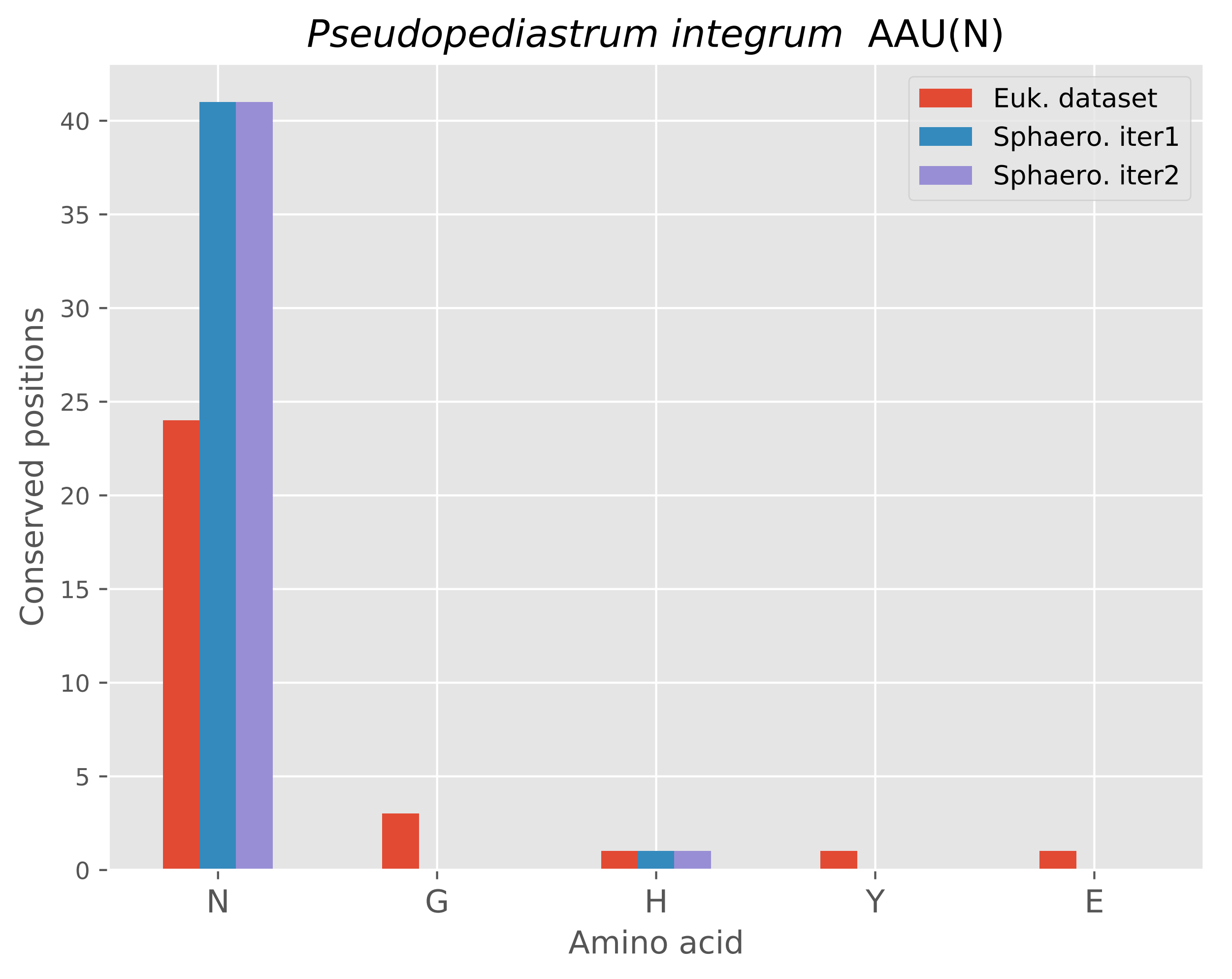

Pseudopediastrum integrum AAU(N)
Euk. dataset
Sphaero. iter1
Sphaero. iter2
Conserved positions
0
5
10
15
20
25
30
35
40
N
G
H
Y
E
Amino acid

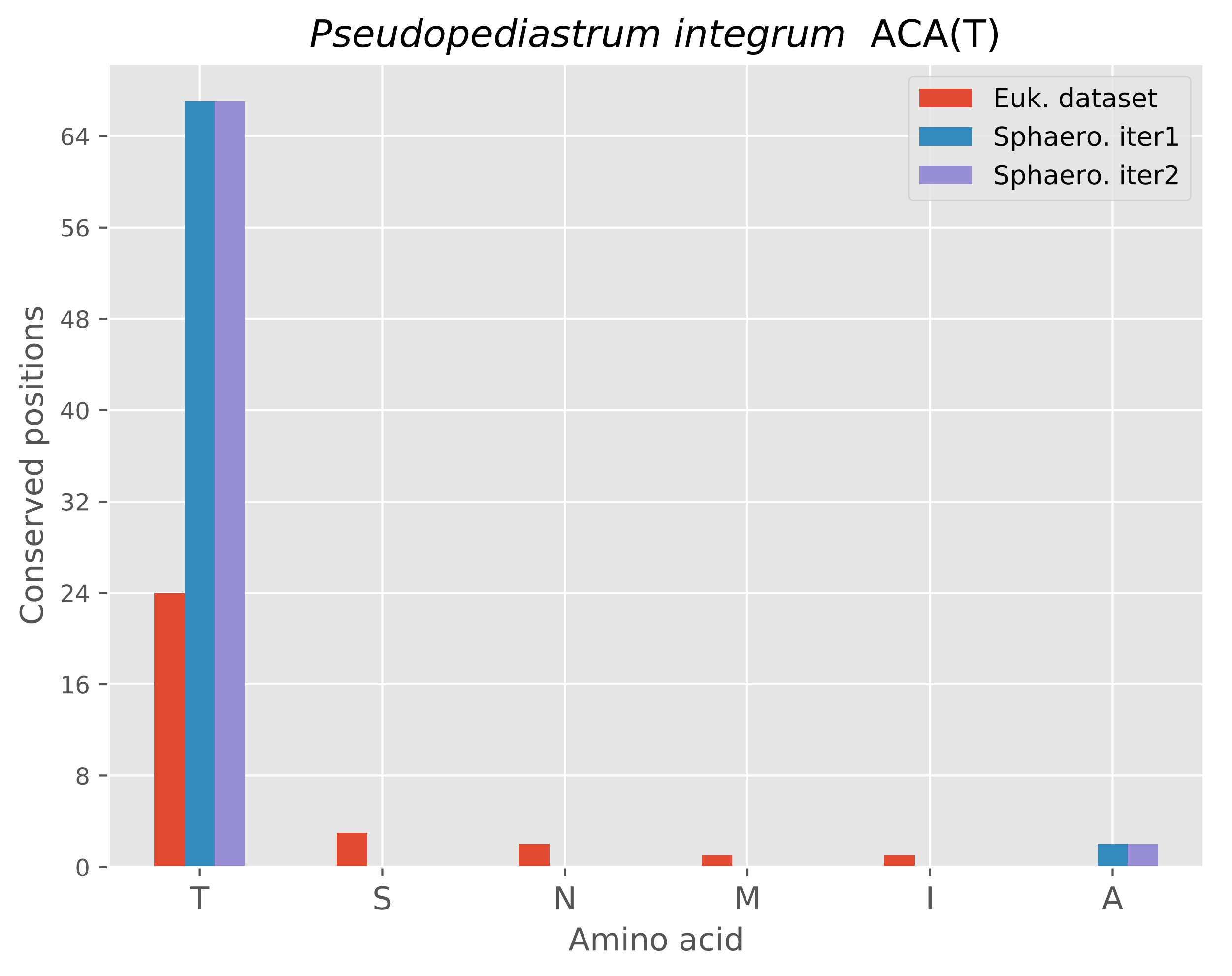

Pseudopediastrum integrum ACA(T)
Euk. dataset
Sphaero. iter1
Sphaero. iter2
Conserved positions
0
8
16
24
32
40
48
56
64
T
S
N
M
I
A
Amino acid

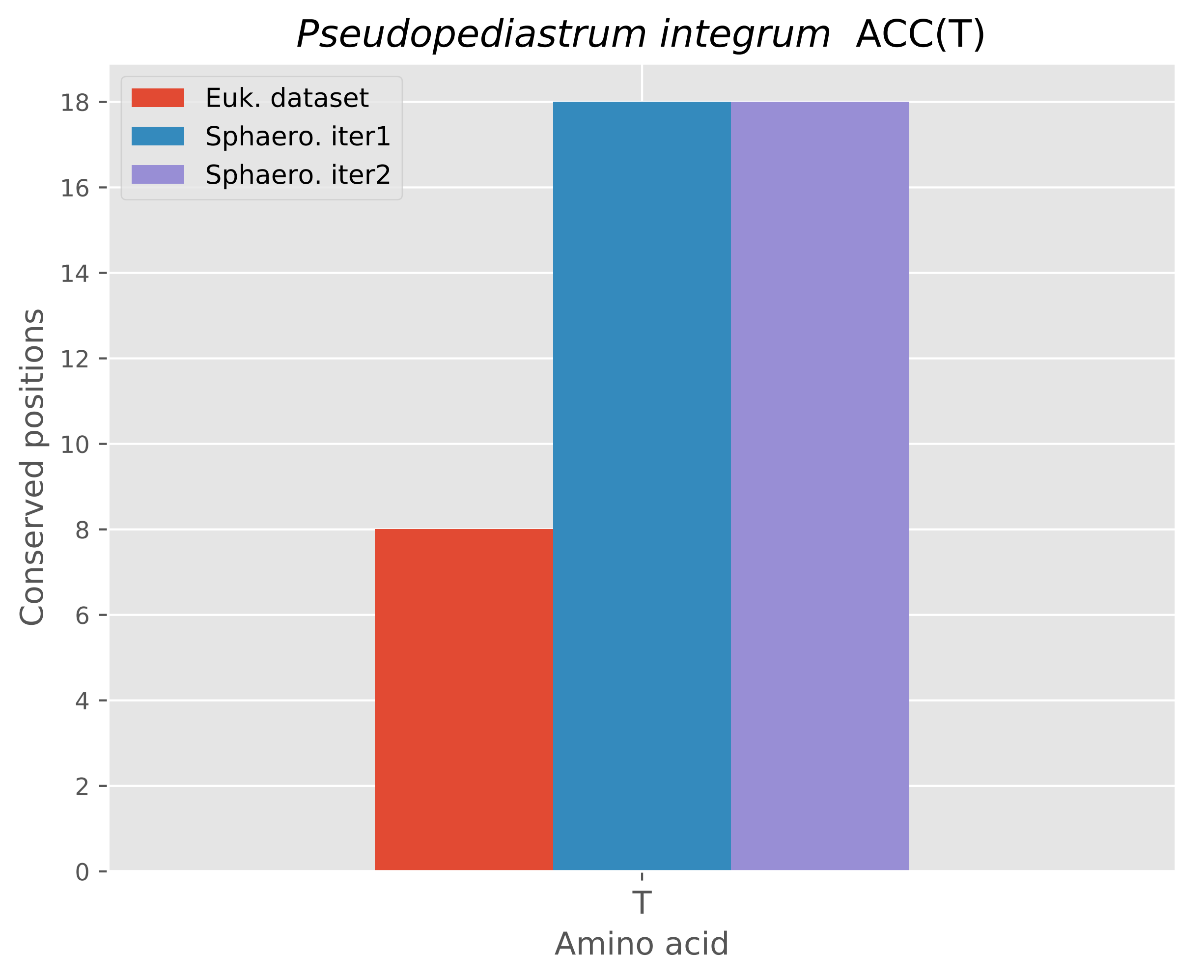

Pseudopediastrum integrum ACC(T)
Euk. dataset
Sphaero. iter1
Sphaero. iter2
Conserved positions
0
2
4
6
8
10
12
14
16
18
T
Amino acid

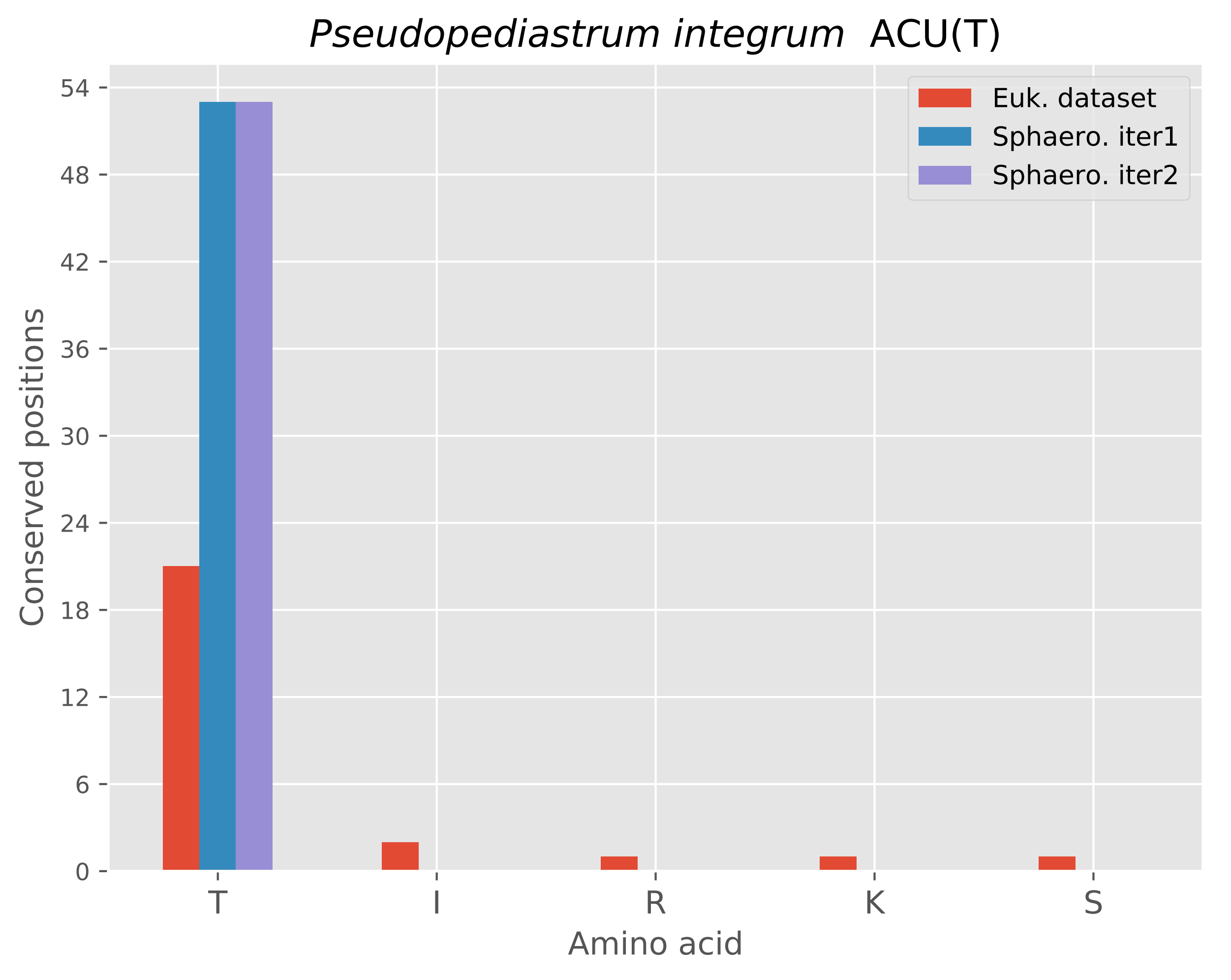
Pseudopediastrum integrum ACU(T)
Conserved positions
0
6
12
18
24
30
36
42
48
54
T
I
R
K
S
Amino acid
Euk. dataset
Sphaero. iter1
Sphaero. iter2

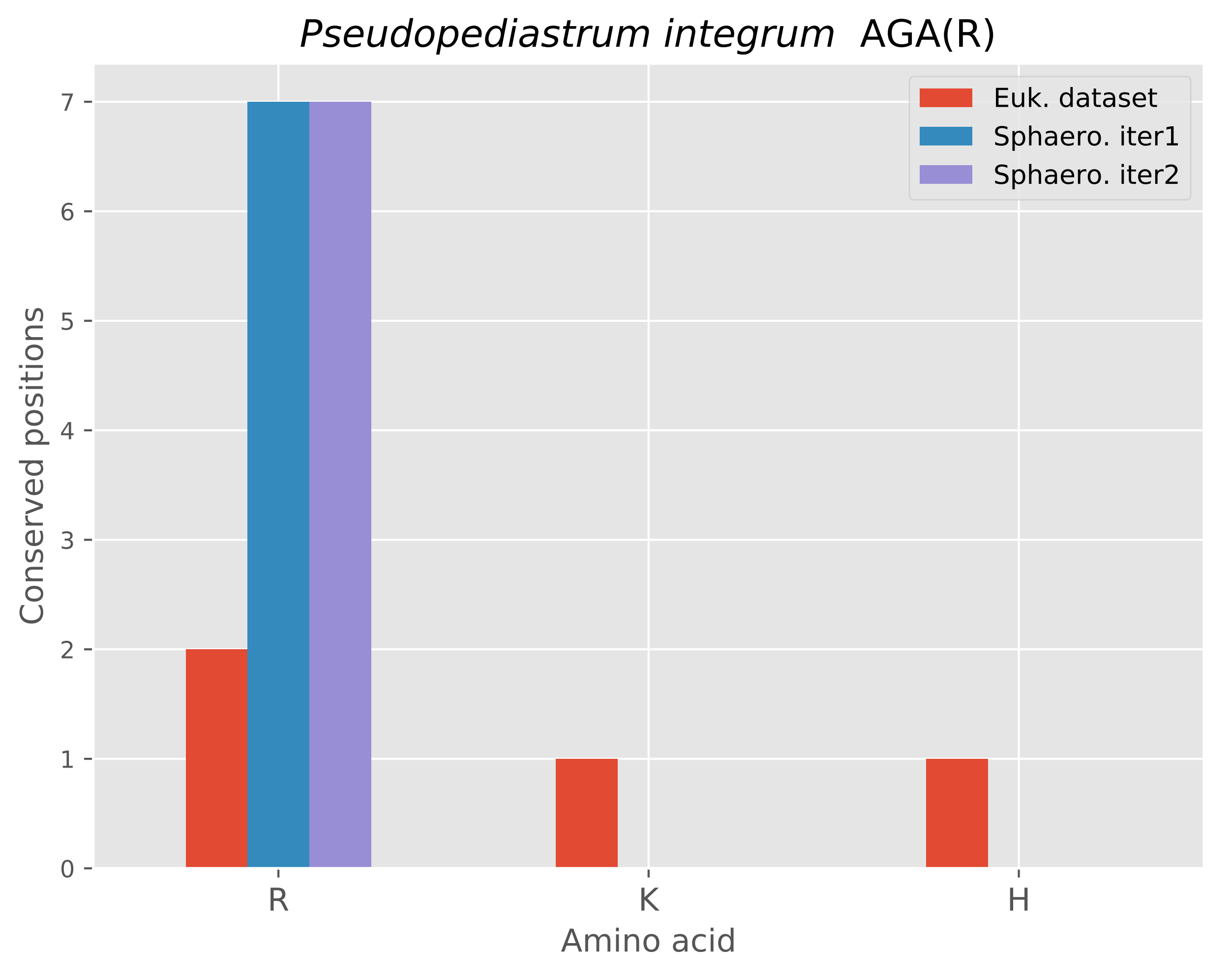

Pseudopediastrum integrum AGA(R)
Euk. dataset
Sphaero. iter1
Sphaero. iter2
Conserved positions
0
1
2
3
4
5
6
7
R
K
H
Amino acid

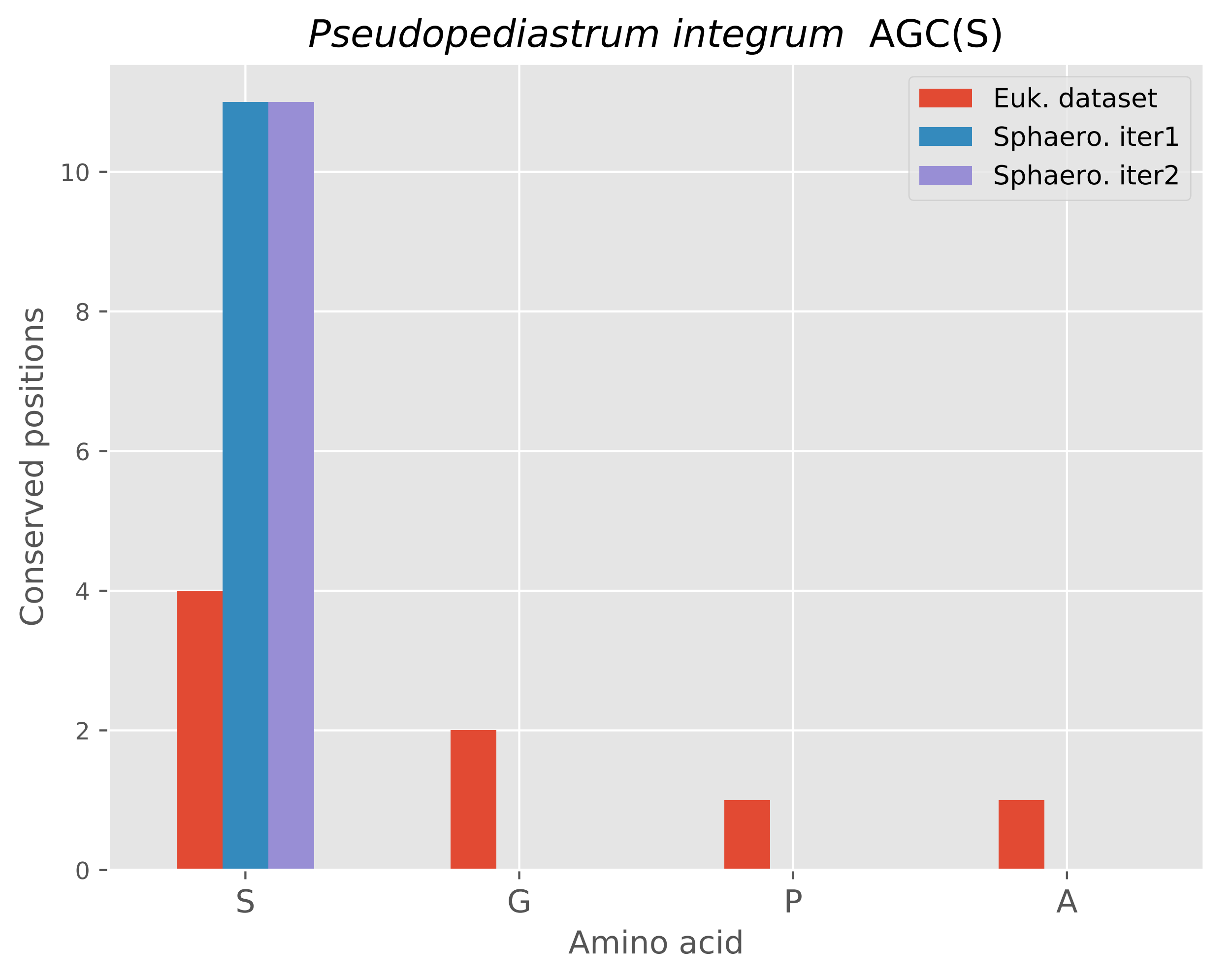

Pseudopediastrum integrum AGC(S)
Euk. dataset
Sphaero. iter1
Sphaero. iter2
Conserved positions
0
2
4
6
8
10
S
G
P
A
Amino acid

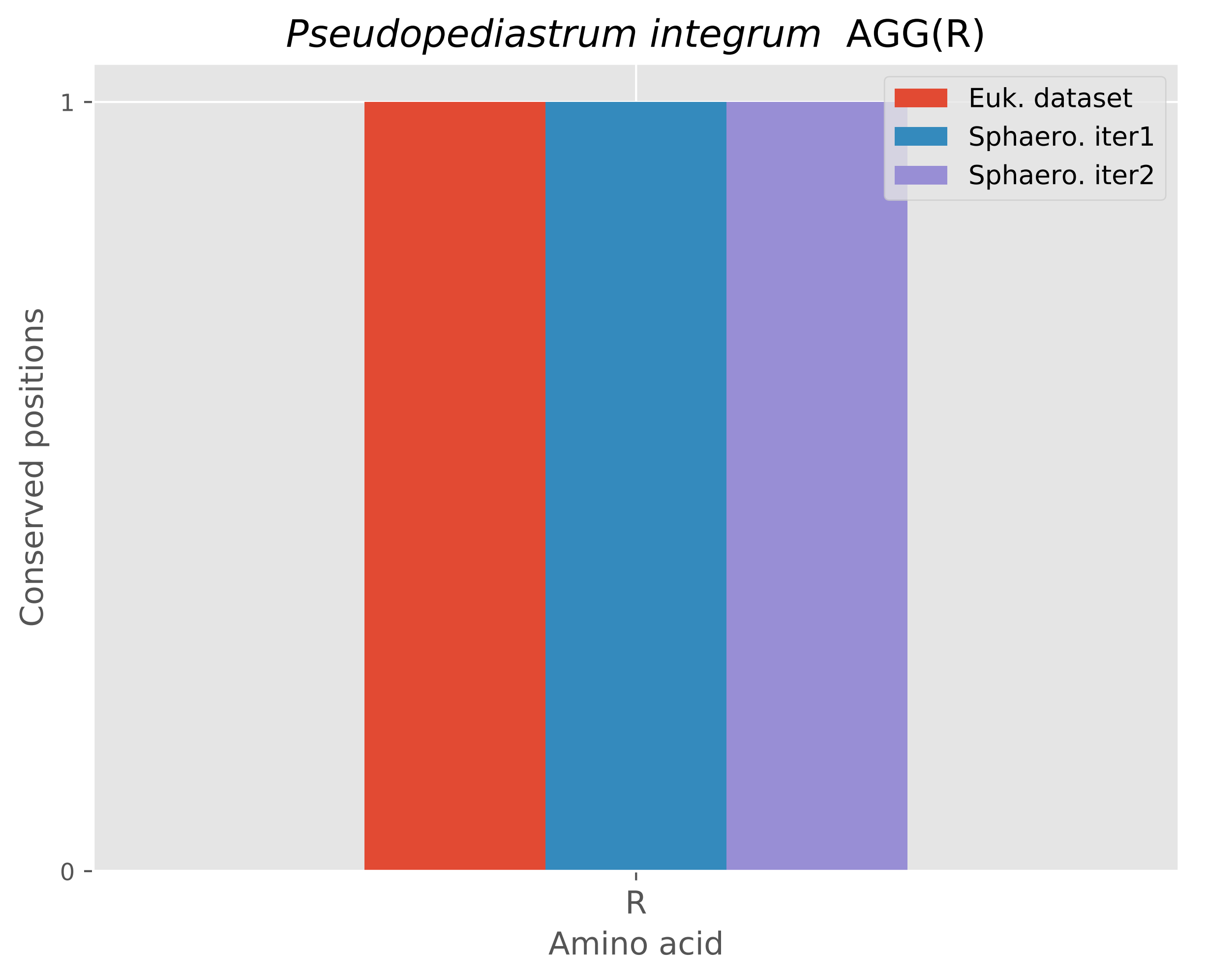

Pseudopediastrum integrum AGG(R)
Euk. dataset
Sphaero. iter1
Sphaero. iter2
1
0
Conserved positions
R
Amino acid

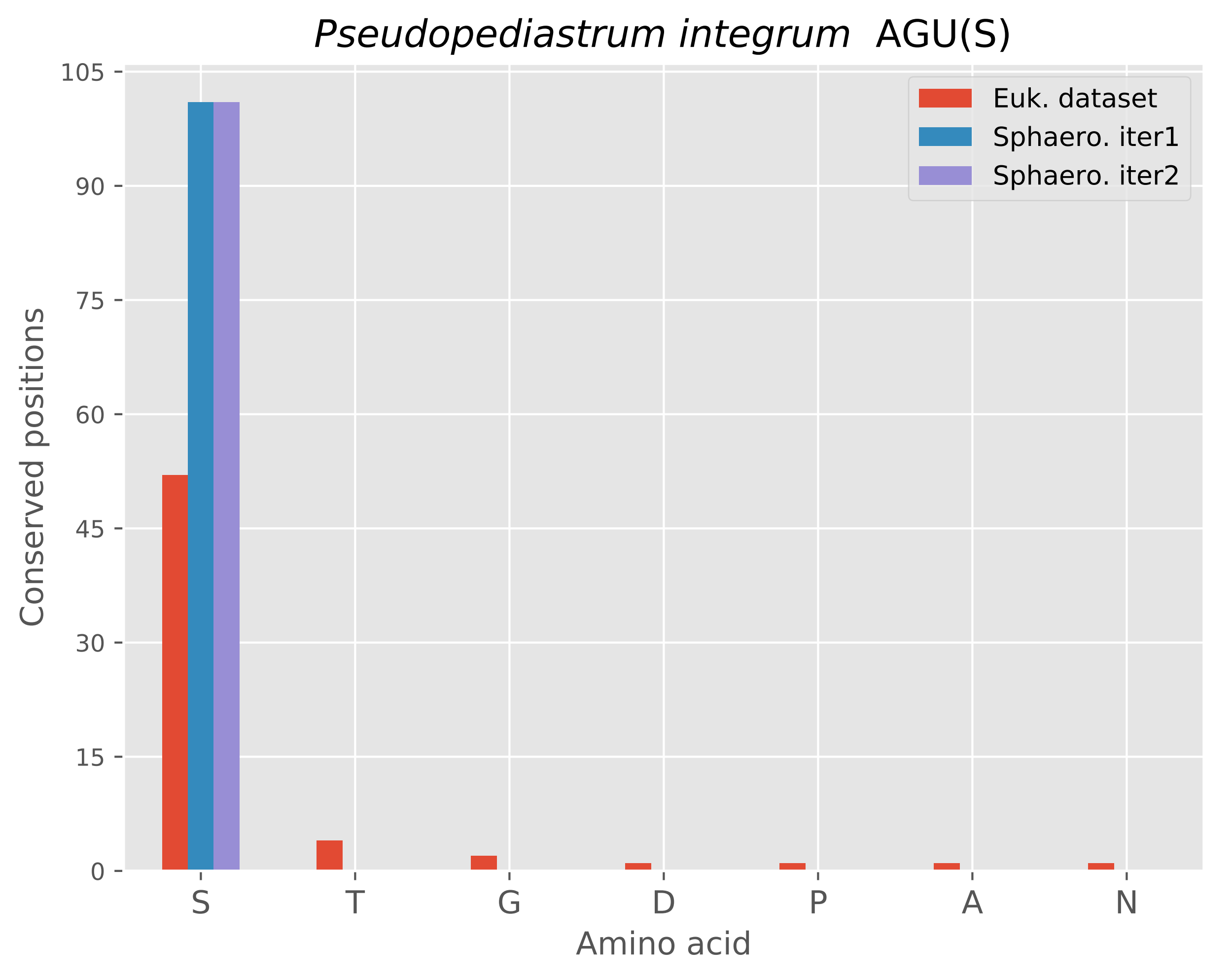

Pseudopediastrum integrum AGU(S)
Euk. dataset
Sphaero. iter1
Sphaero. iter2
Conserved positions
105
90
75
60
45
30
15
0
S
T
G
D
P
A
N
Amino acid

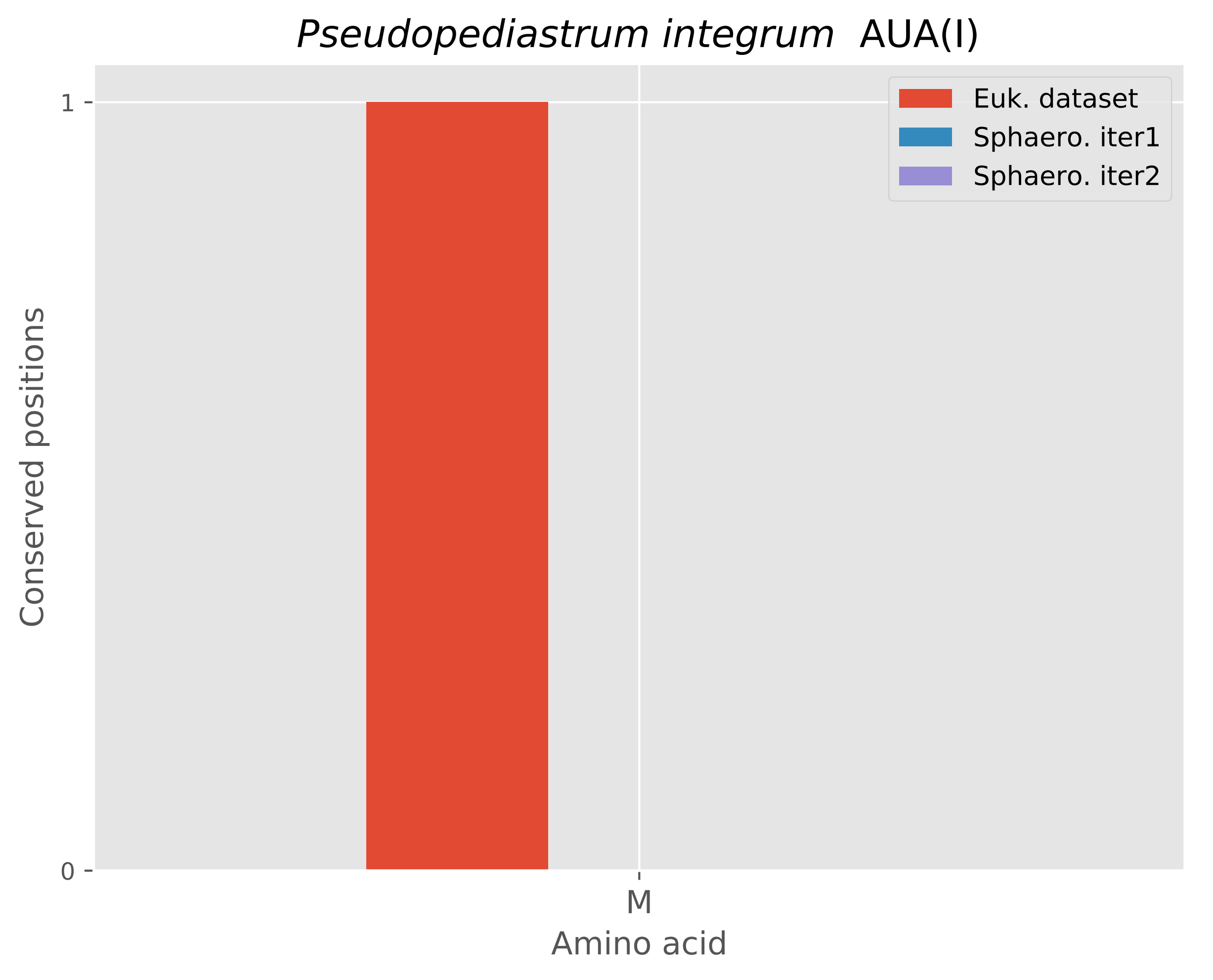

Pseudopediastrum integrum AUA(I)
Euk. dataset
Sphaero. iter1
Sphaero. iter2
1
0
Conserved positions
M
Amino acid

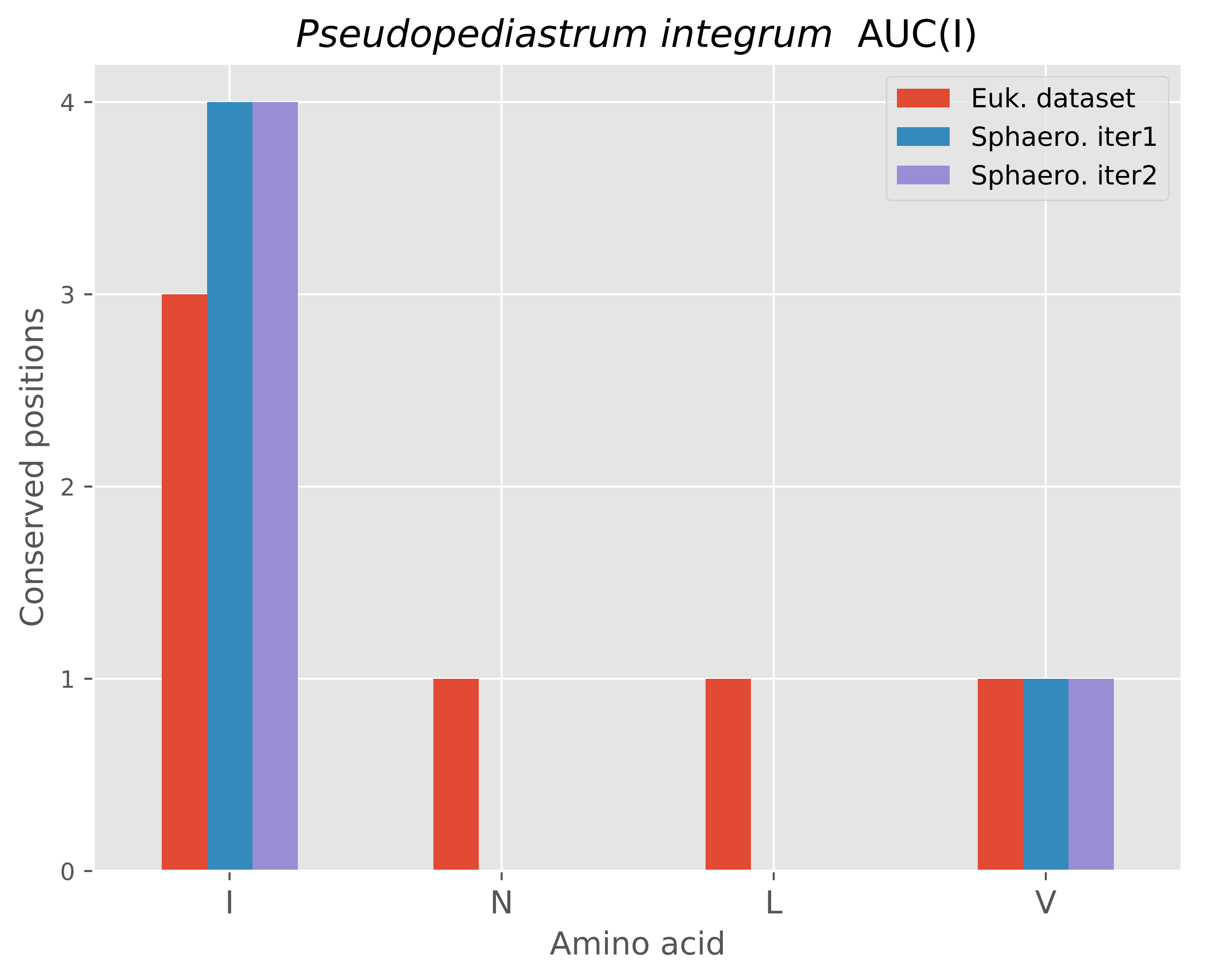
Pseudopediastrum integrum AUC(I)
Conserved positions
Amino acid
I
N
L
V
0
1
2
3
4
Euk. dataset
Sphaero. iter1
Sphaero. iter2

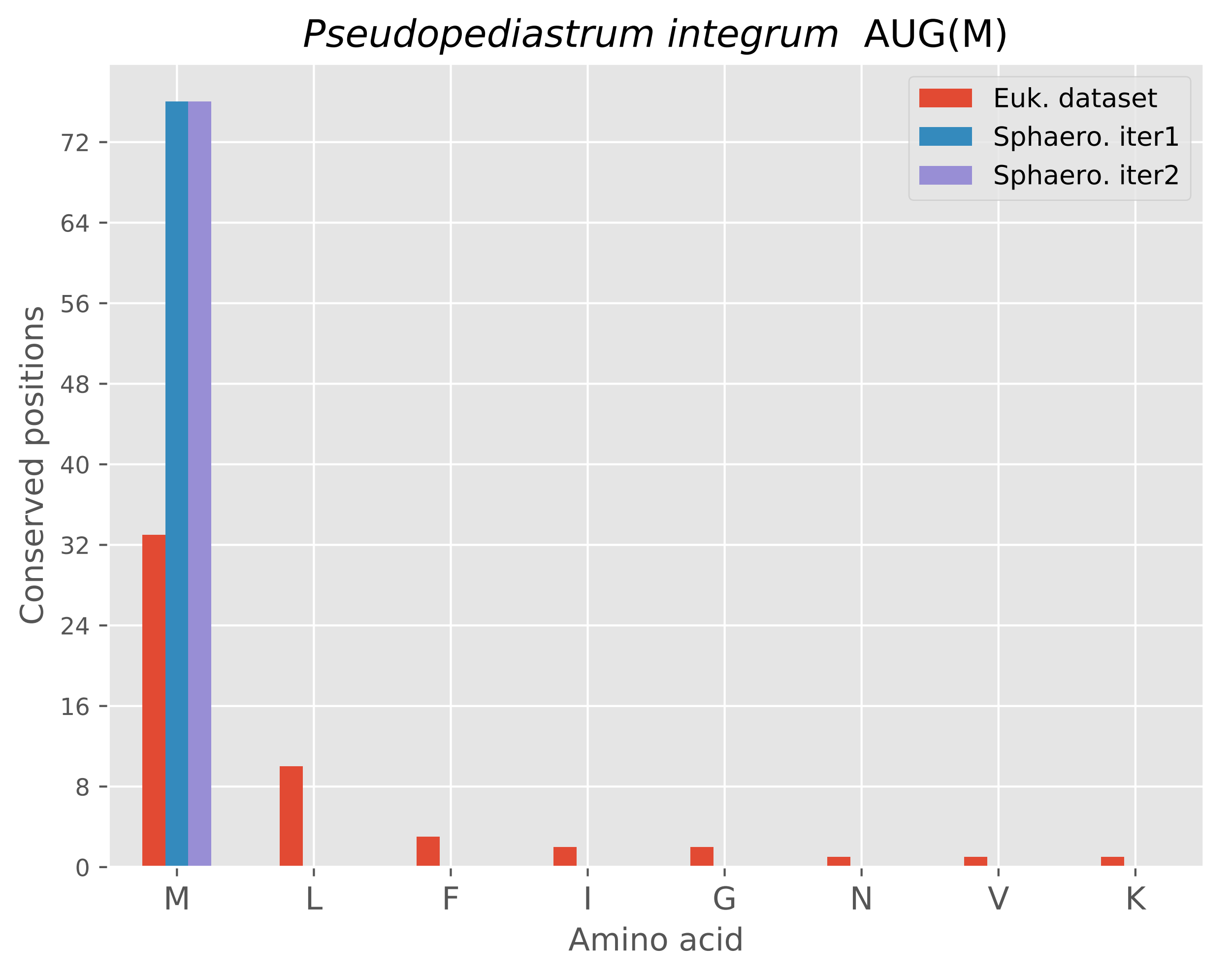

Pseudopediastrum integrum AUG(M)
Euk. dataset
Sphaero. iter1
Sphaero. iter2
Conserved positions
0
8
16
24
32
40
48
56
64
72
M
L
F
I
G
N
V
K
Amino acid

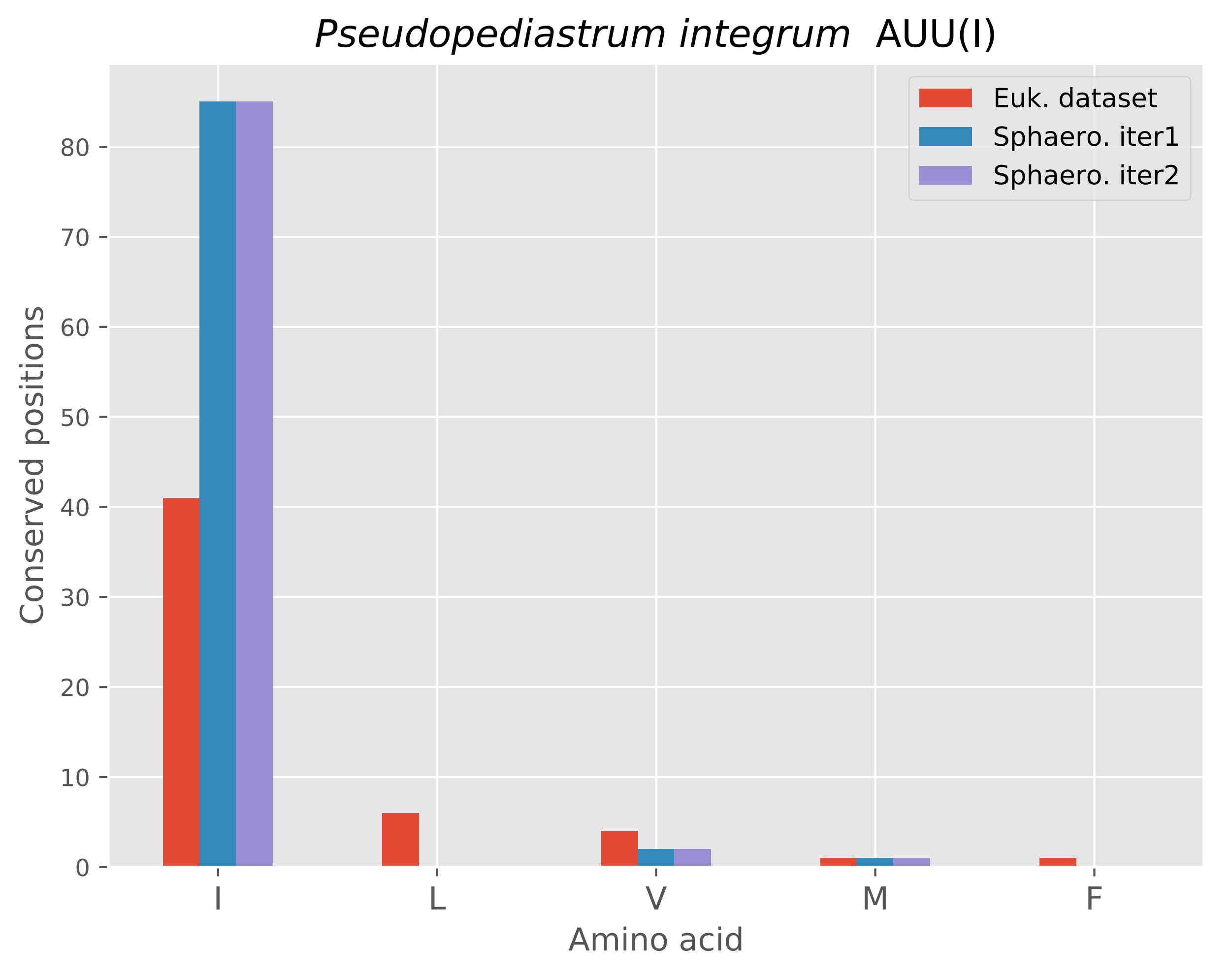

Pseudopediastrum integrum AUU(I)
Euk. dataset
Sphaero. iter1
Sphaero. iter2
Conserved positions
0
10
20
30
40
50
60
70
80
I
L
V
M
F
Amino acid

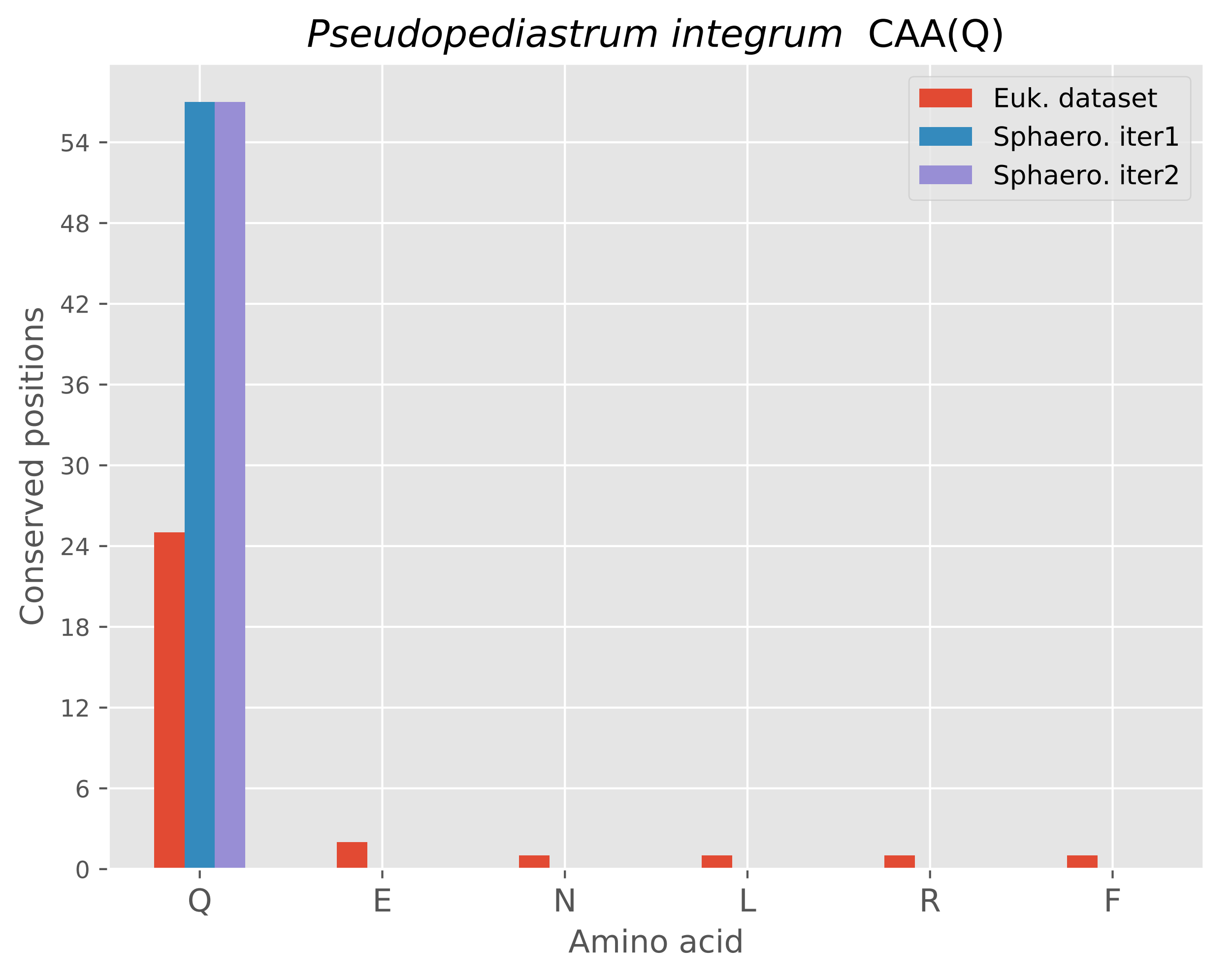

Pseudopediastrum integrum CAA(Q)
Euk. dataset
Sphaero. iter1
Sphaero. iter2
Conserved positions
0
6
12
18
24
30
36
42
48
54
Q
E
N
L
R
F
Amino acid

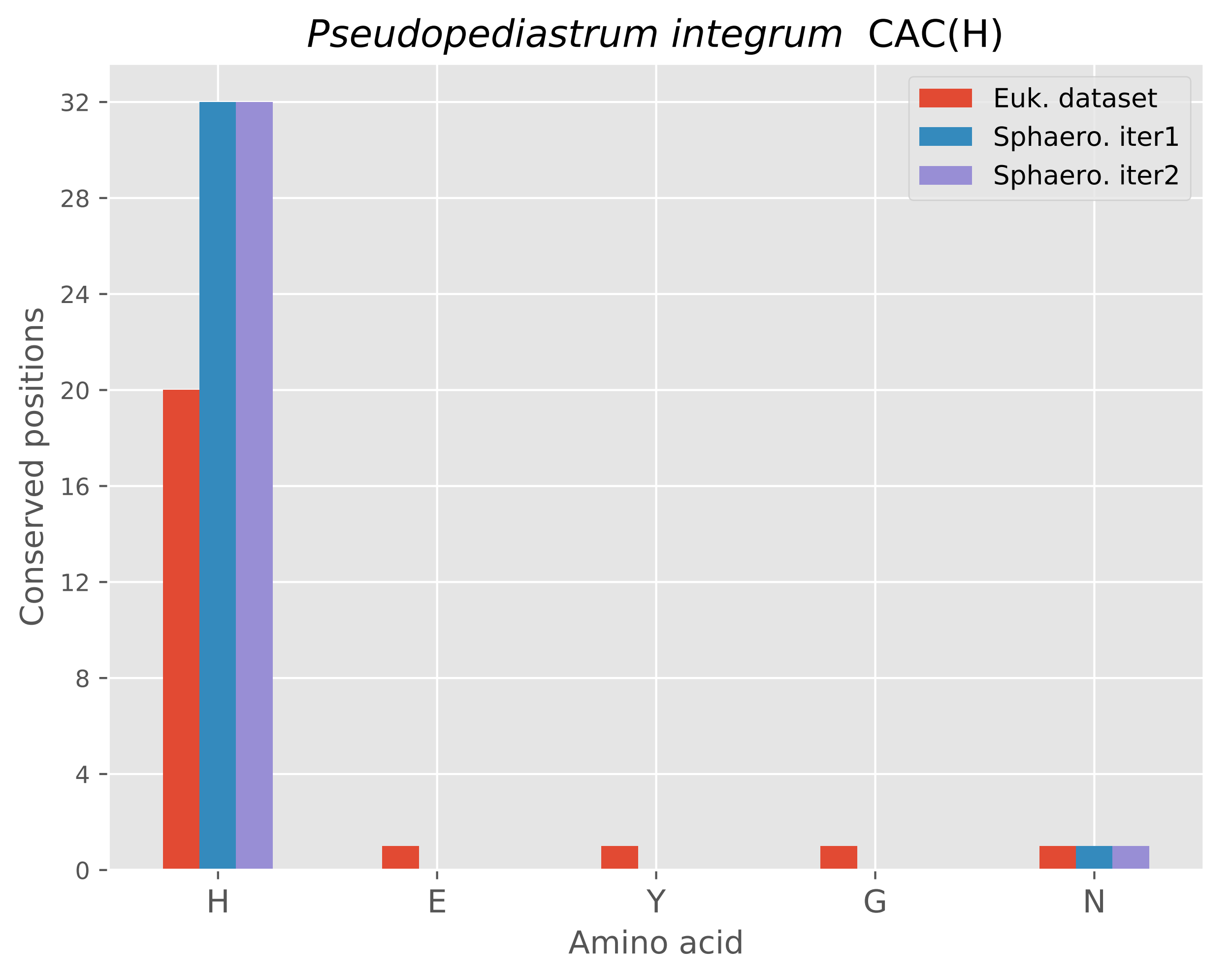

Pseudopediastrum integrum CAC(H)
Euk. dataset
Sphaero. iter1
Sphaero. iter2
Conserved positions
0
4
8
12
16
20
24
28
32
H
E
Y
G
N
Amino acid

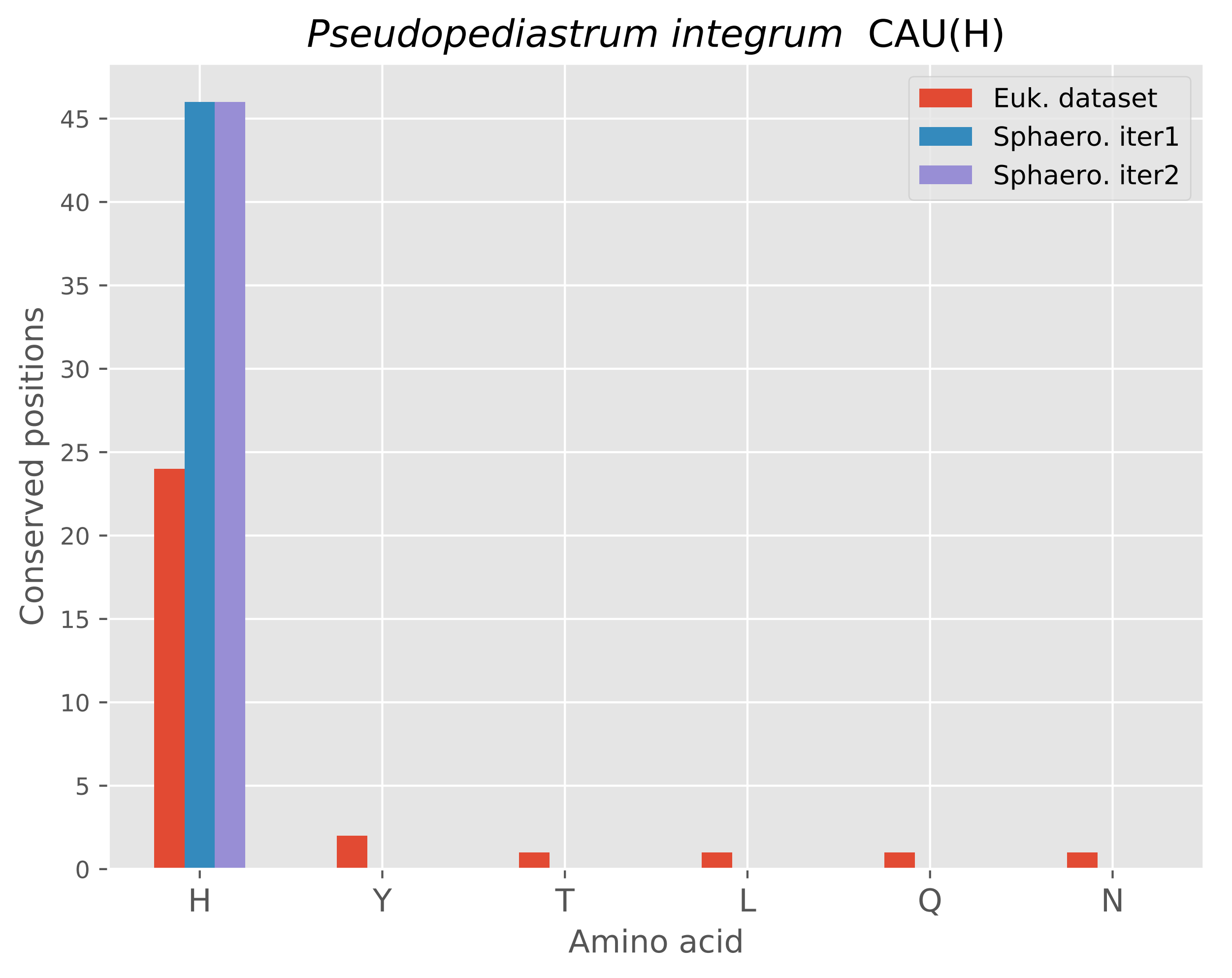

Pseudopediastrum integrum CAU(H)
Euk. dataset
Sphaero. iter1
Sphaero. iter2
45
40
35
30
25
20
15
10
5
0
Conserved positions
H
Y
T
L
Q
N
Amino acid

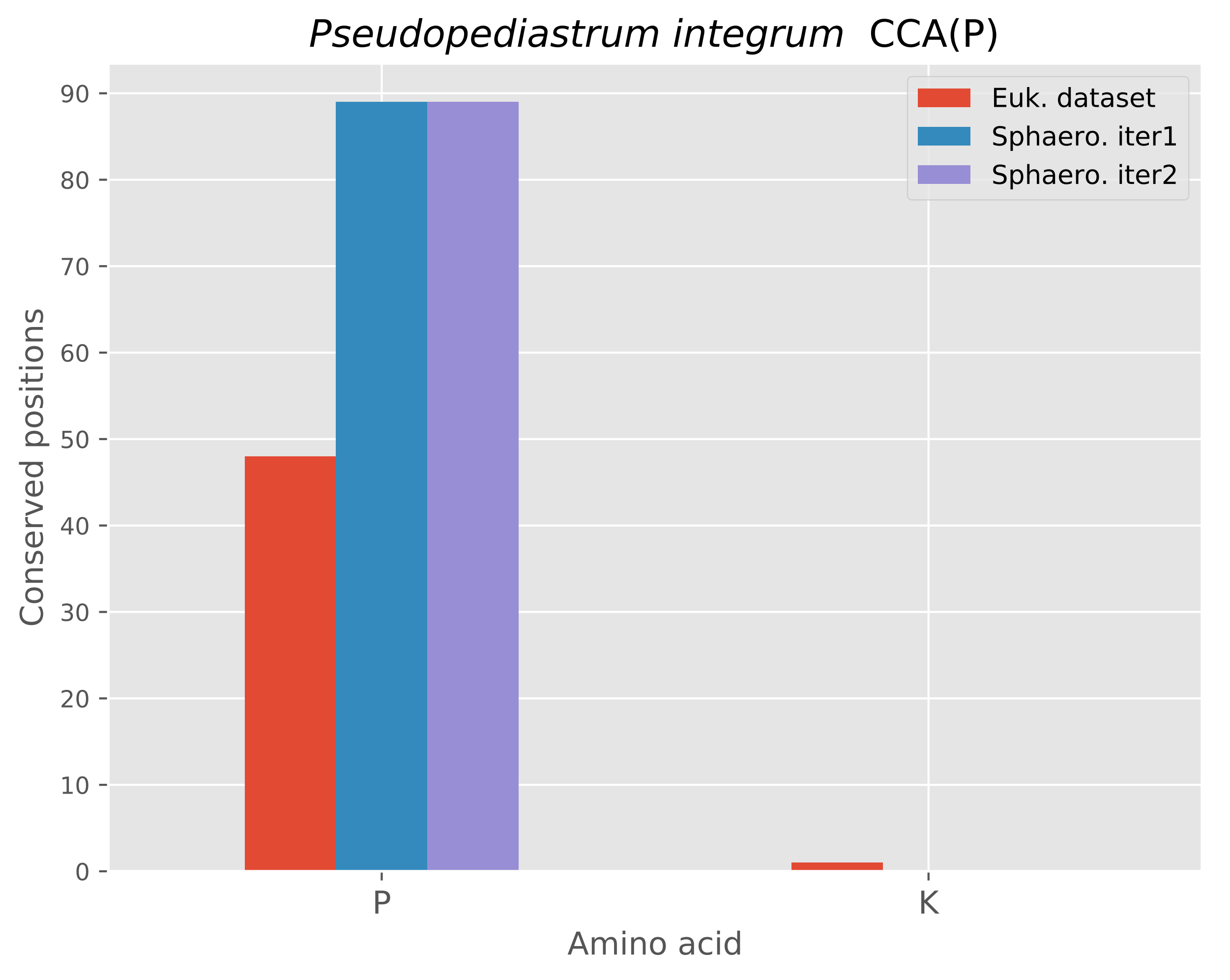

Pseudopediastrum integrum CCA(P)
Euk. dataset
Sphaero. iter1
Sphaero. iter2
Conserved positions
0
10
20
30
40
50
60
70
80
90
P
K
Amino acid

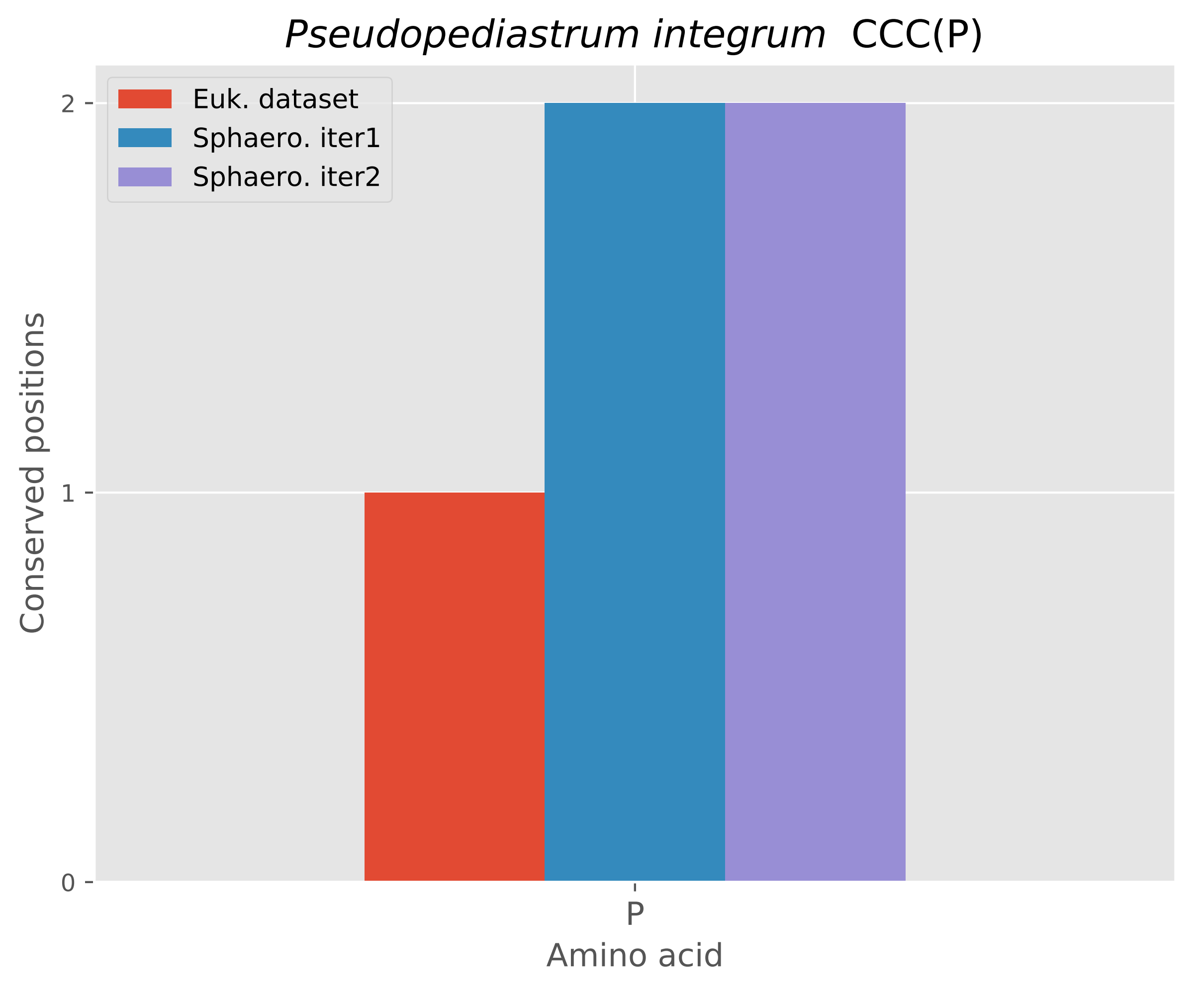

Pseudopediastrum integrum CCC(P)
Euk. dataset
Sphaero. iter1
Sphaero. iter2
Conserved positions
0
1
2
P
Amino acid

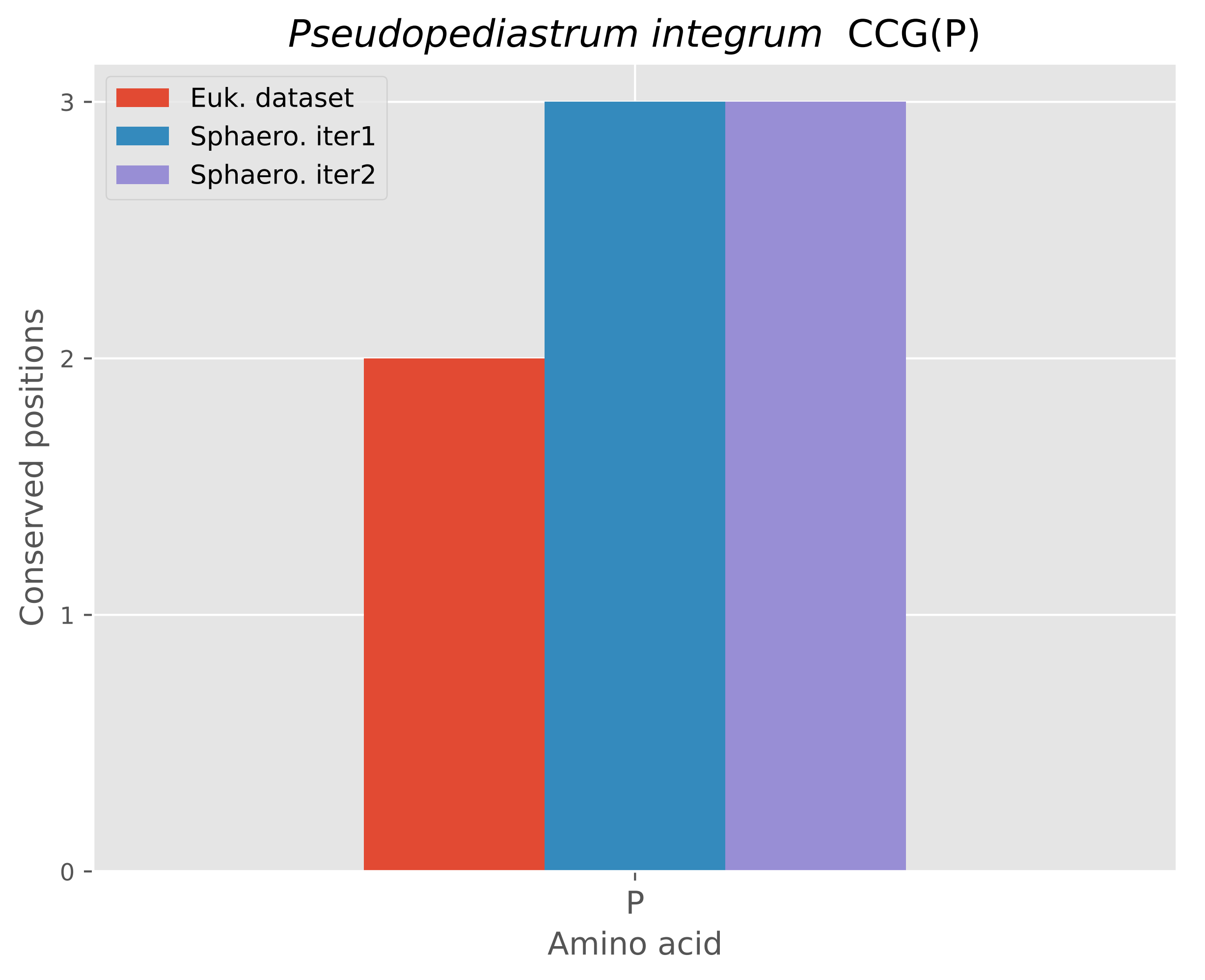
Pseudopediastrum integrum CCG(P)
Euk. dataset
Sphaero. iter1
Sphaero. iter2
Conserved positions
0
1
2
3
P
Amino acid

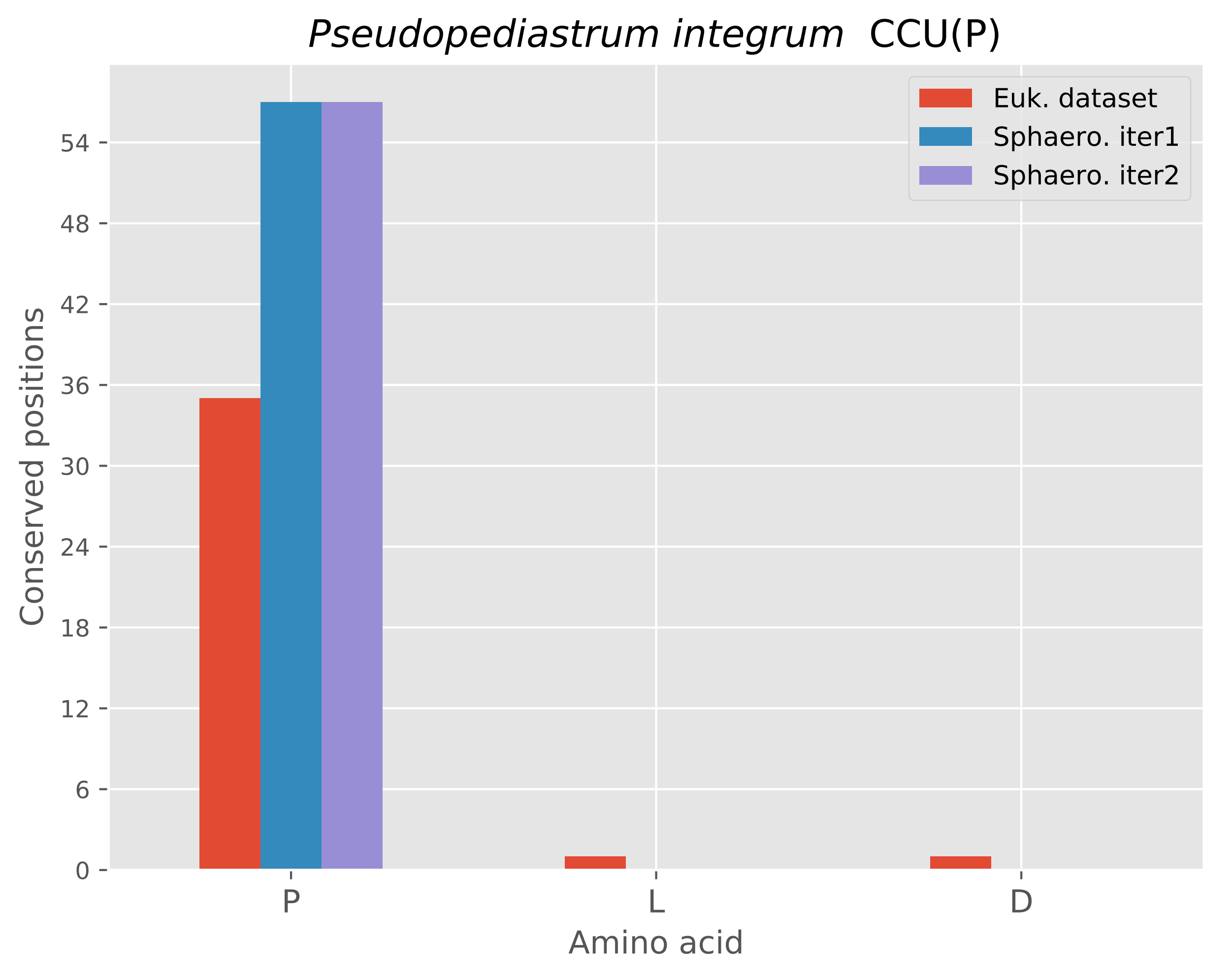

Pseudopediastrum integrum CCU(P)
Euk. dataset
Sphaero. iter1
Sphaero. iter2
Conserved positions
0
6
12
18
24
30
36
42
48
54
P
L
D
Amino acid

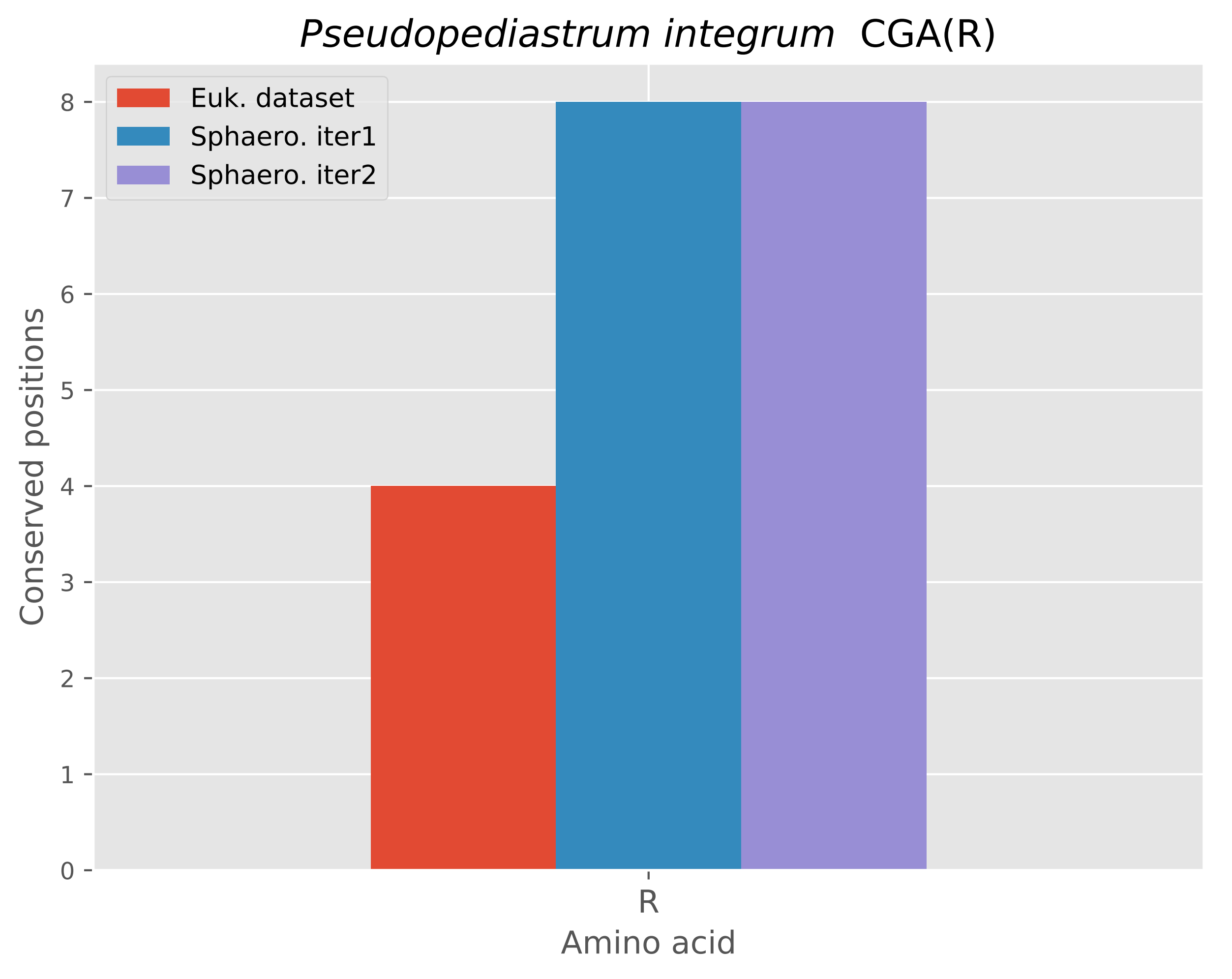

Pseudopediastrum integrum CGA(R)
Euk. dataset
Sphaero. iter1
Sphaero. iter2
Conserved positions
0
1
2
3
4
5
6
7
8
R
Amino acid

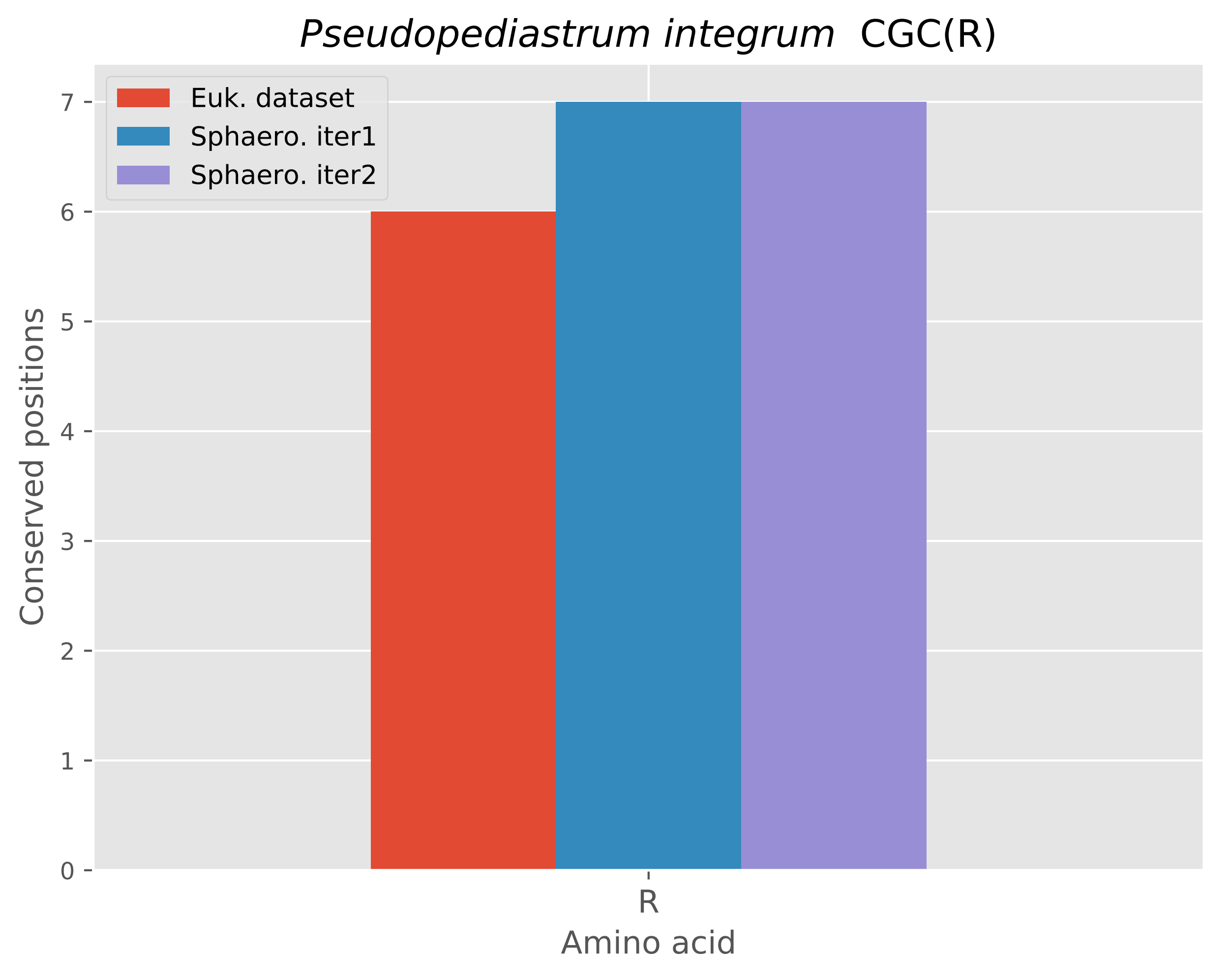

Pseudopediastrum integrum CGC(R)
Euk. dataset
Sphaero. iter1
Sphaero. iter2
Conserved positions
0
1
2
3
4
5
6
7
R
Amino acid

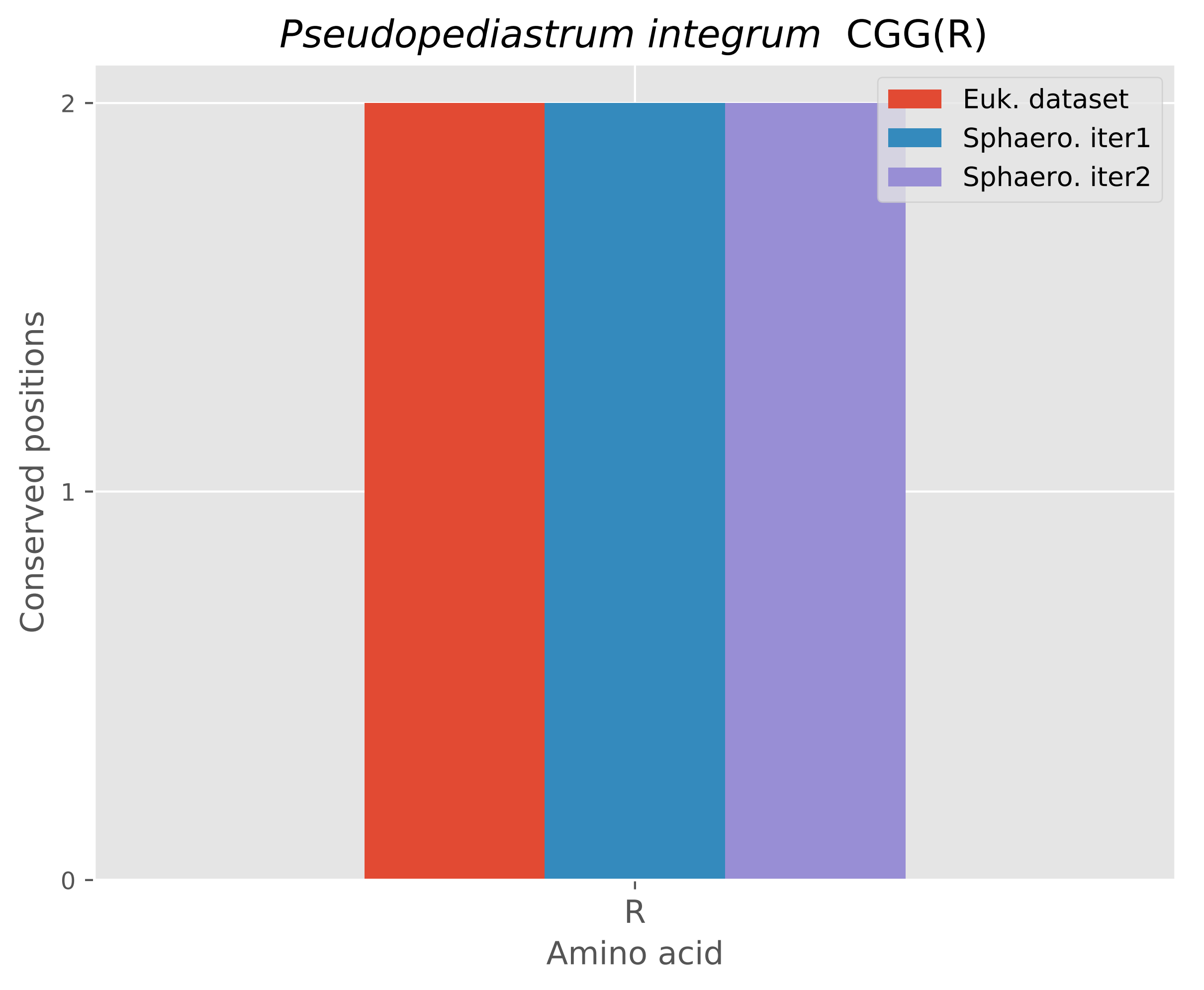
Pseudopediastrum integrum CGG(R)
Euk. dataset
Sphaero. iter1
Sphaero. iter2
Conserved positions
0
1
2
R
Amino acid

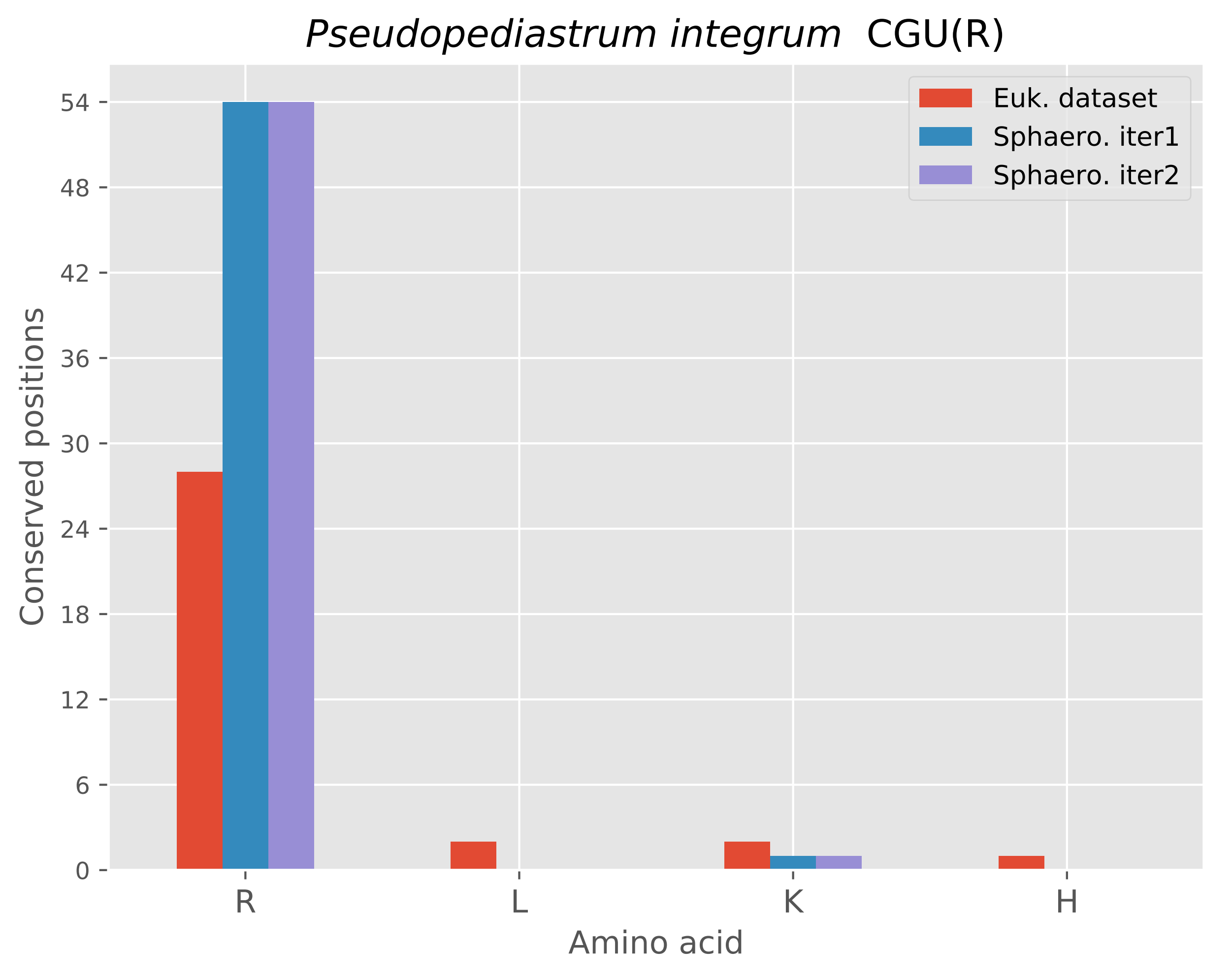

Pseudopediastrum integrum CGU(R)
Euk. dataset
Sphaero. iter1
Sphaero. iter2
Conserved positions
0
6
12
18
24
30
36
42
48
54
R
L
K
H
Amino acid

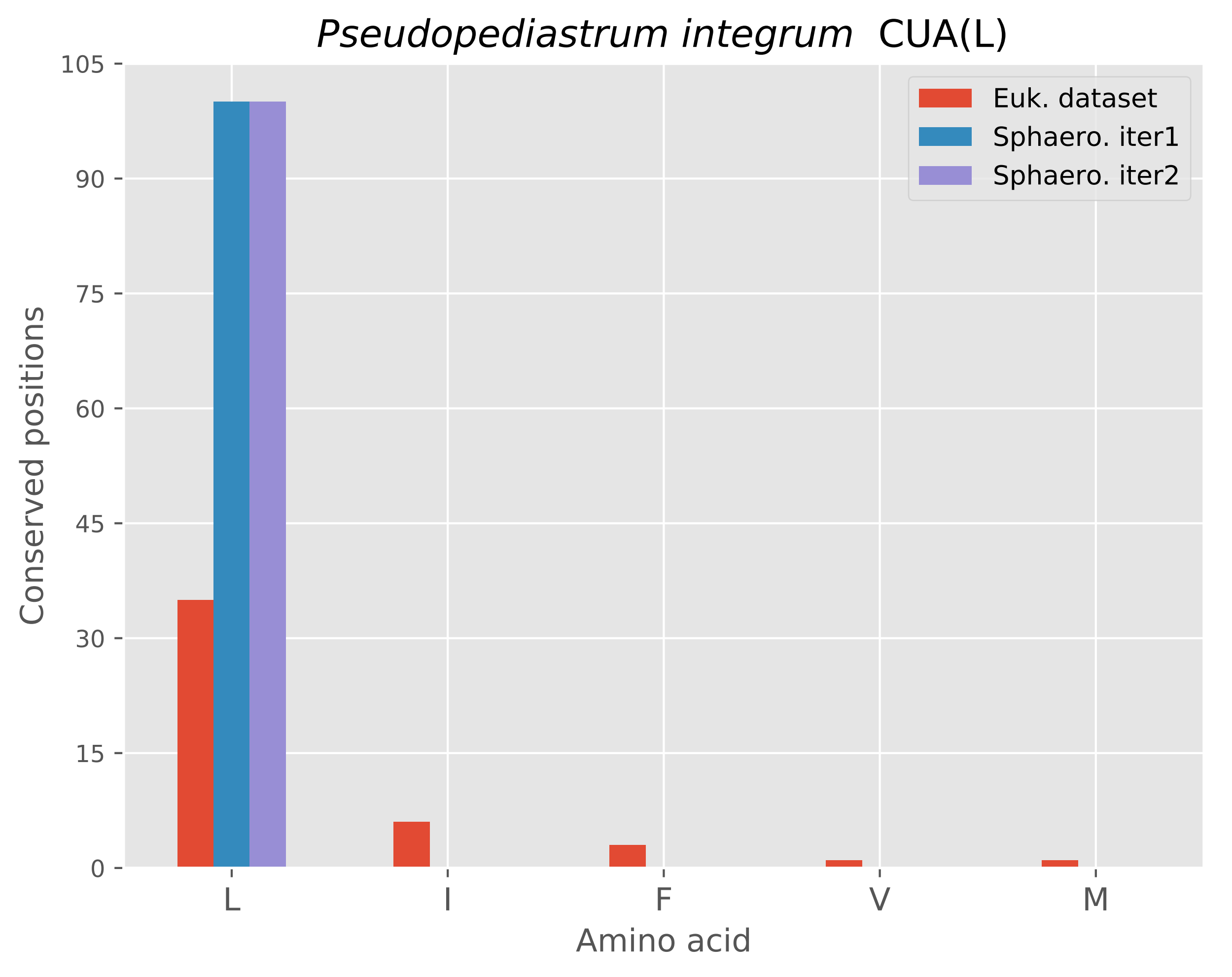

Pseudopediastrum integrum CUA(L)
Euk. dataset
Sphaero. iter1
Sphaero. iter2
Conserved positions
105
90
75
60
45
30
15
0
L
I
F
V
M
Amino acid

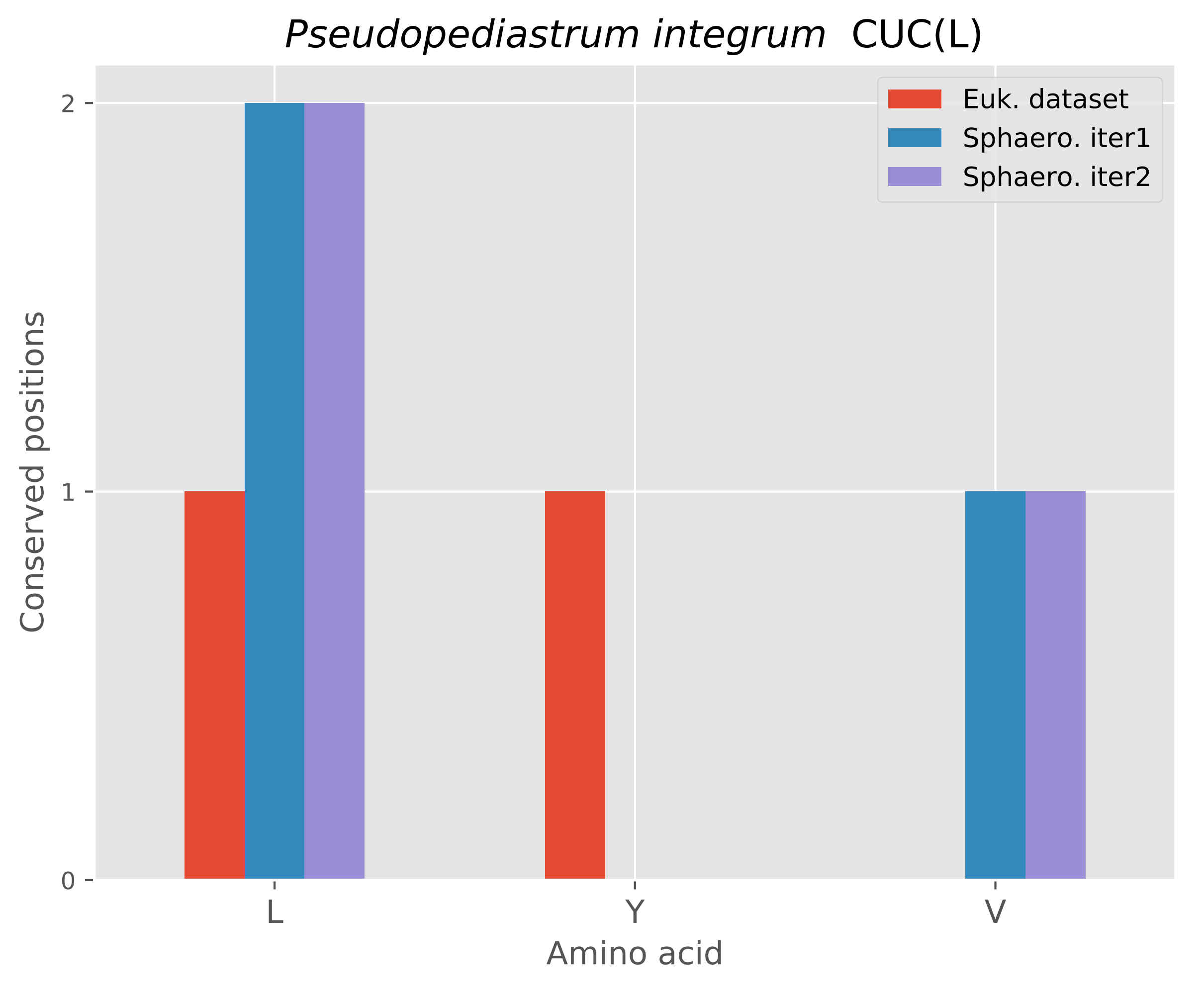

Pseudopediastrum integrum CUC(L)
Euk. dataset
Sphaero. iter1
Sphaero. iter2
Conserved positions
0
1
2
L
Y
V
Amino acid

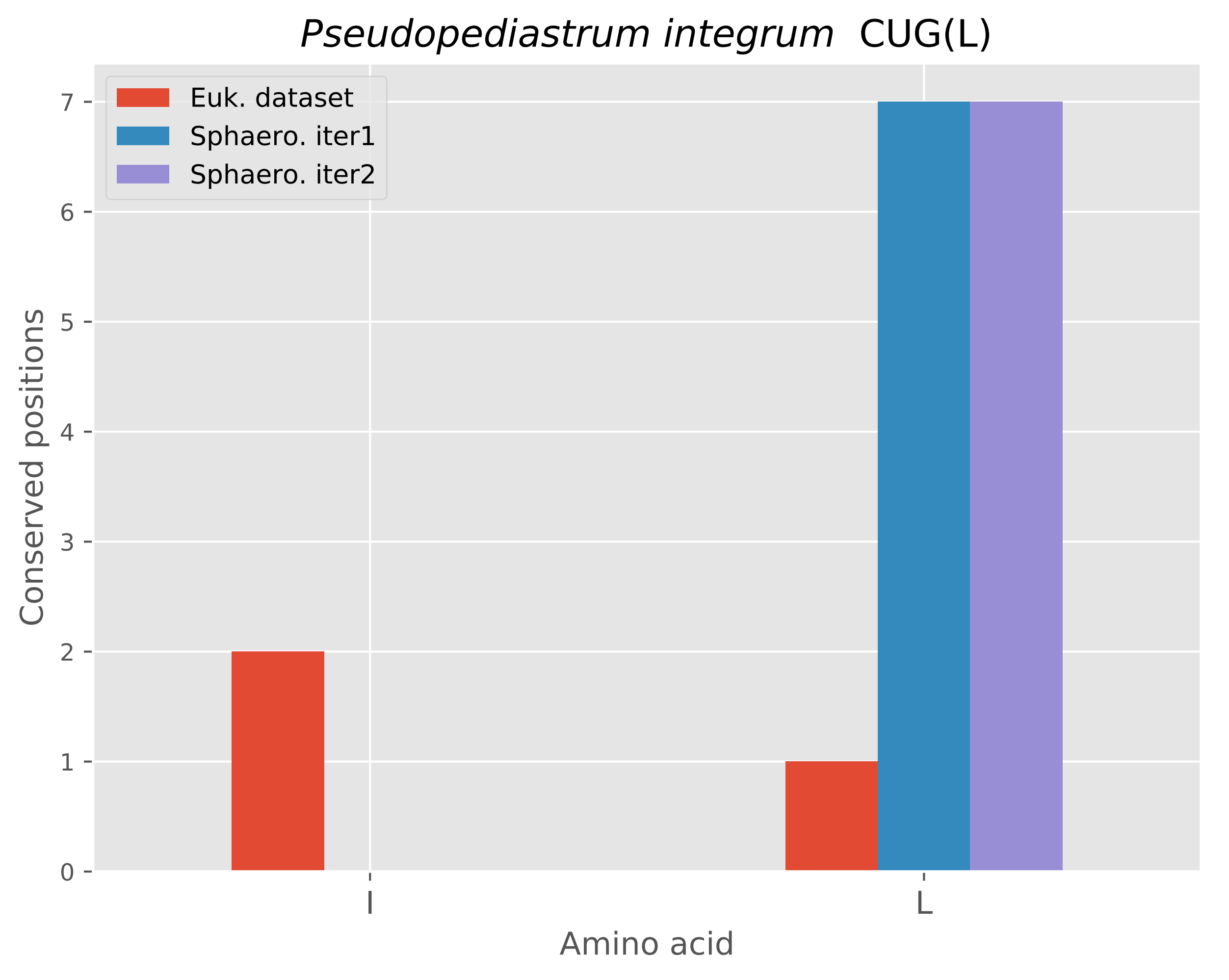

Pseudopediastrum integrum CUG(L)
Euk. dataset
Sphaero. iter1
Sphaero. iter2
Conserved positions
0
1
2
3
4
5
6
7
I
L
Amino acid

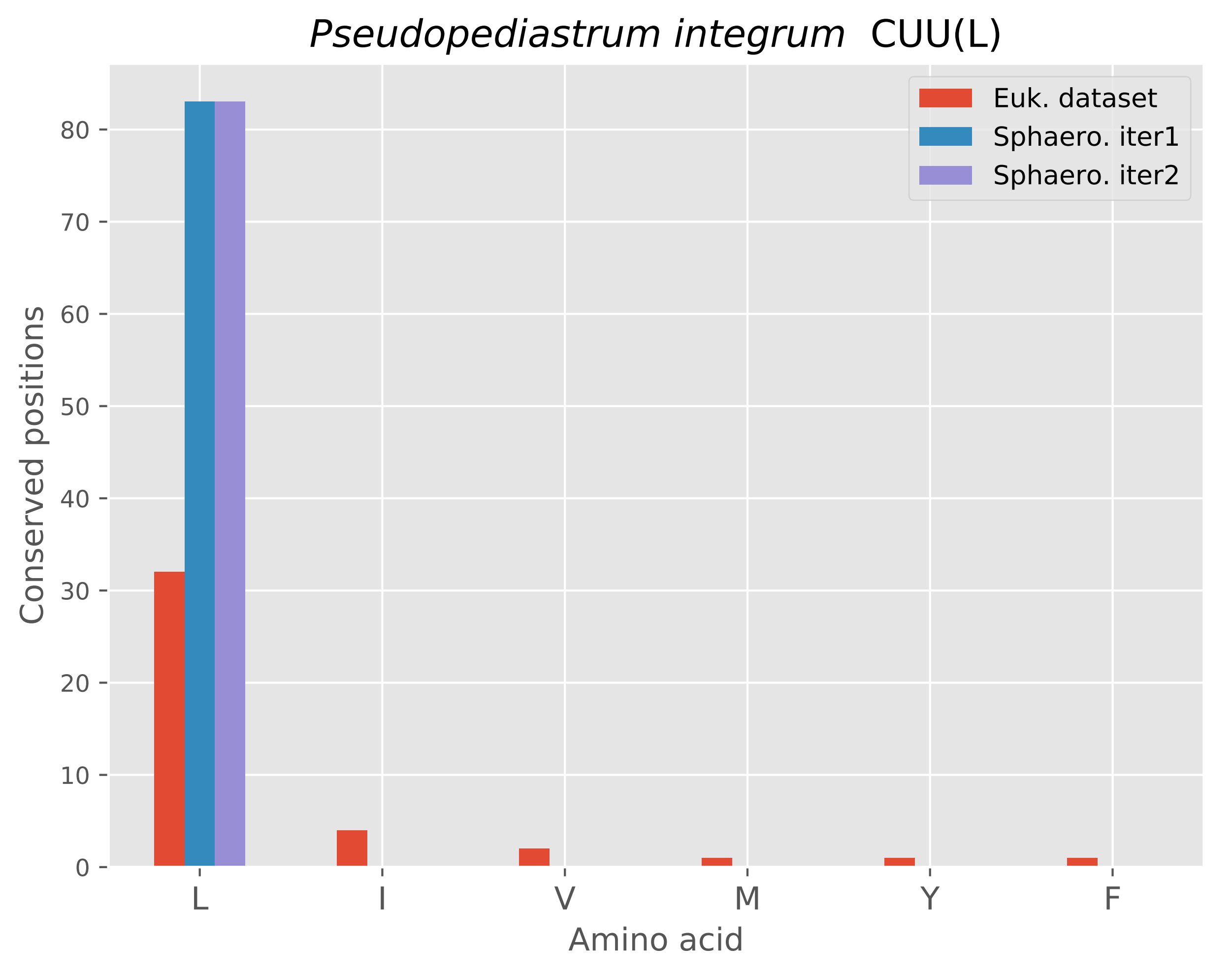

Pseudopediastrum integrum CUU(L)
Euk. dataset
Sphaero. iter1
Sphaero. iter2
Conserved positions
0
10
20
30
40
50
60
70
80
L
I
V
M
Y
F
Amino acid

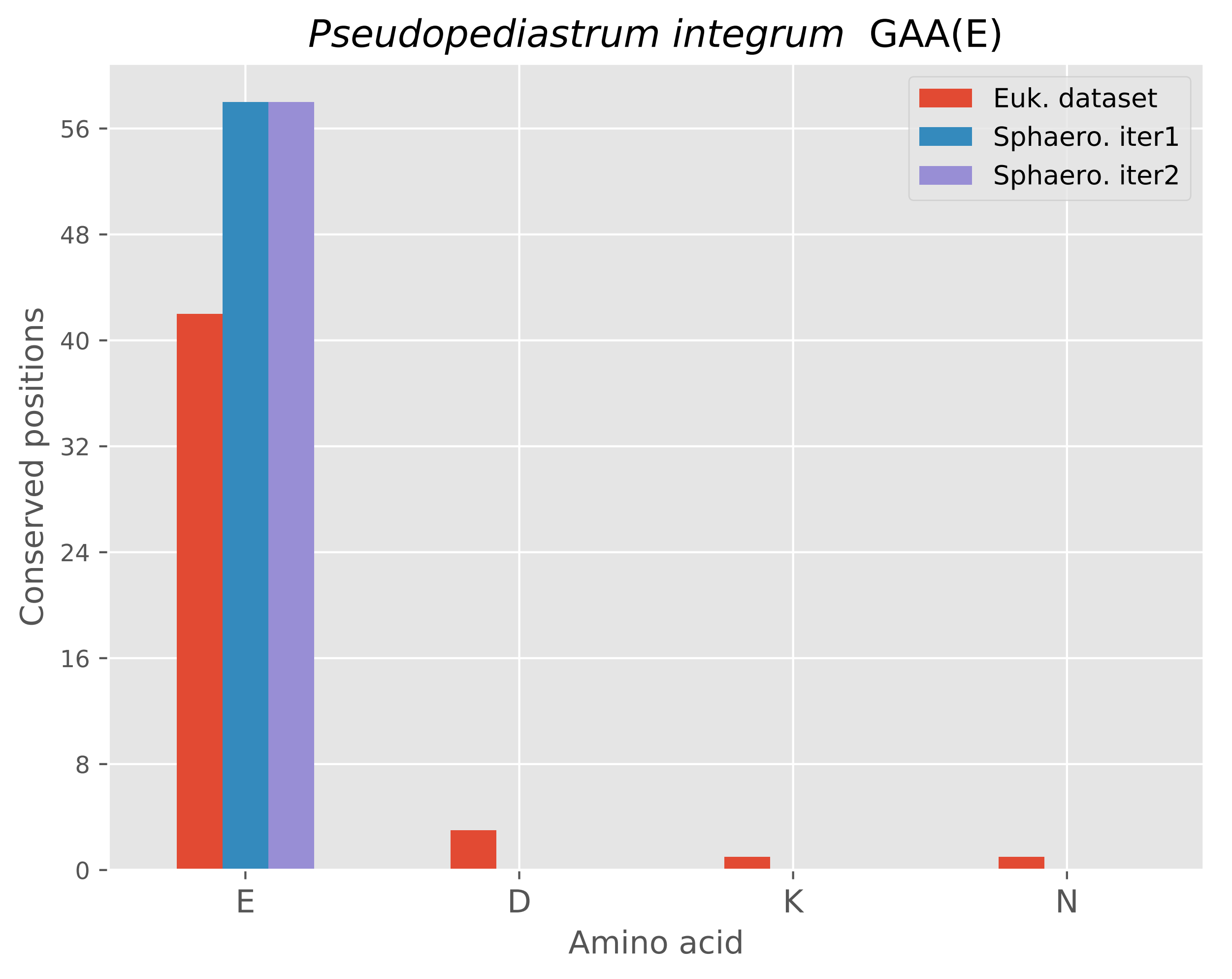

Pseudopediastrum integrum GAA(E)
Euk. dataset
Sphaero. iter1
Sphaero. iter2
Conserved positions
0
8
16
24
32
40
48
56
E
D
K
N
Amino acid

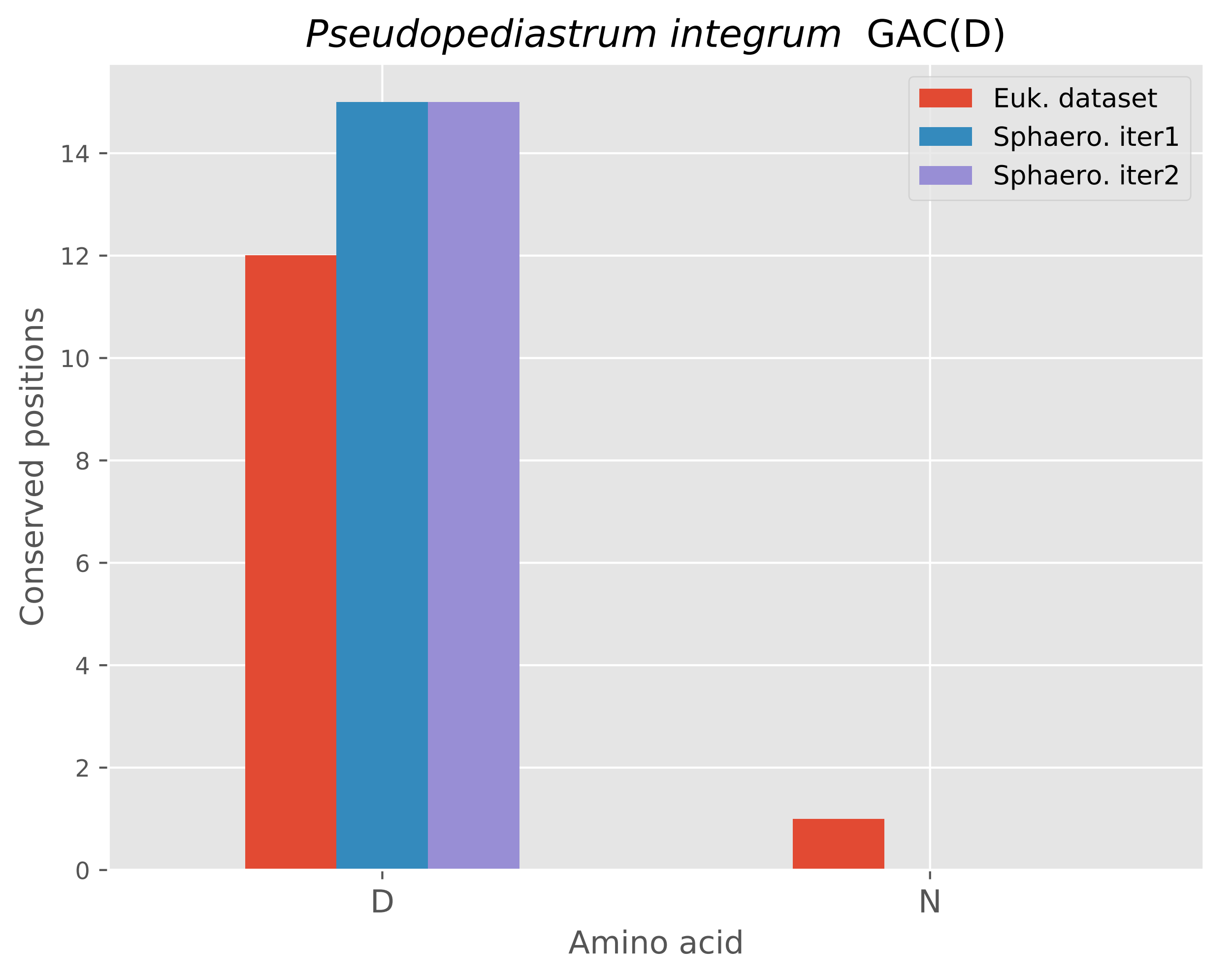

Pseudopediastrum integrum GAC(D)
Euk. dataset
Sphaero. iter1
Sphaero. iter2
Conserved positions
0
2
4
6
8
10
12
14
D
N
Amino acid

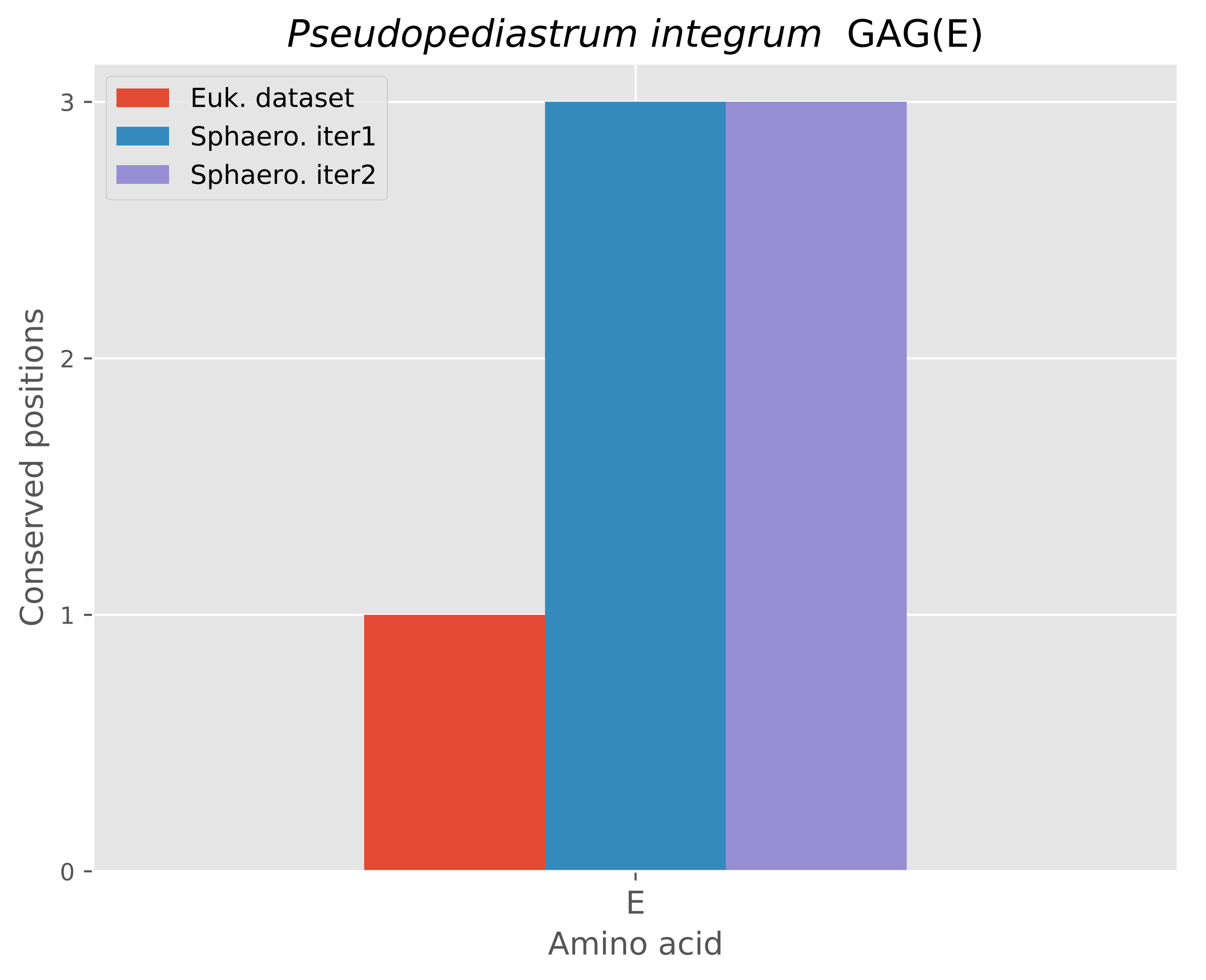

Pseudopediastrum integrum GAG(E)
Euk. dataset
Sphaero. iter1
Sphaero. iter2
Conserved positions
0
1
2
3
E
Amino acid

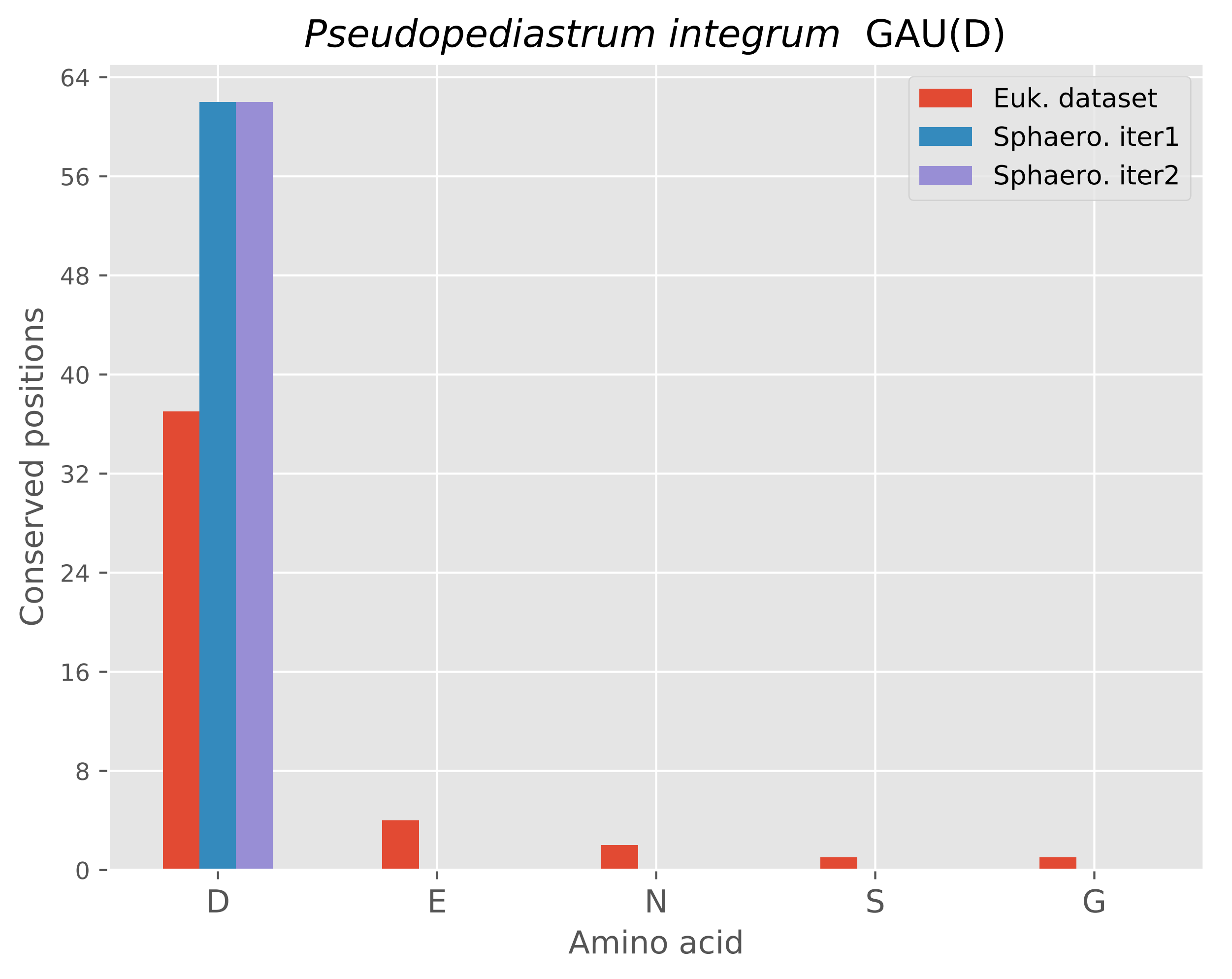

Pseudopediastrum integrum GAU(D)
Euk. dataset
Sphaero. iter1
Sphaero. iter2
Conserved positions
0
8
16
24
32
40
48
56
64
D
E
N
S
G
Amino acid

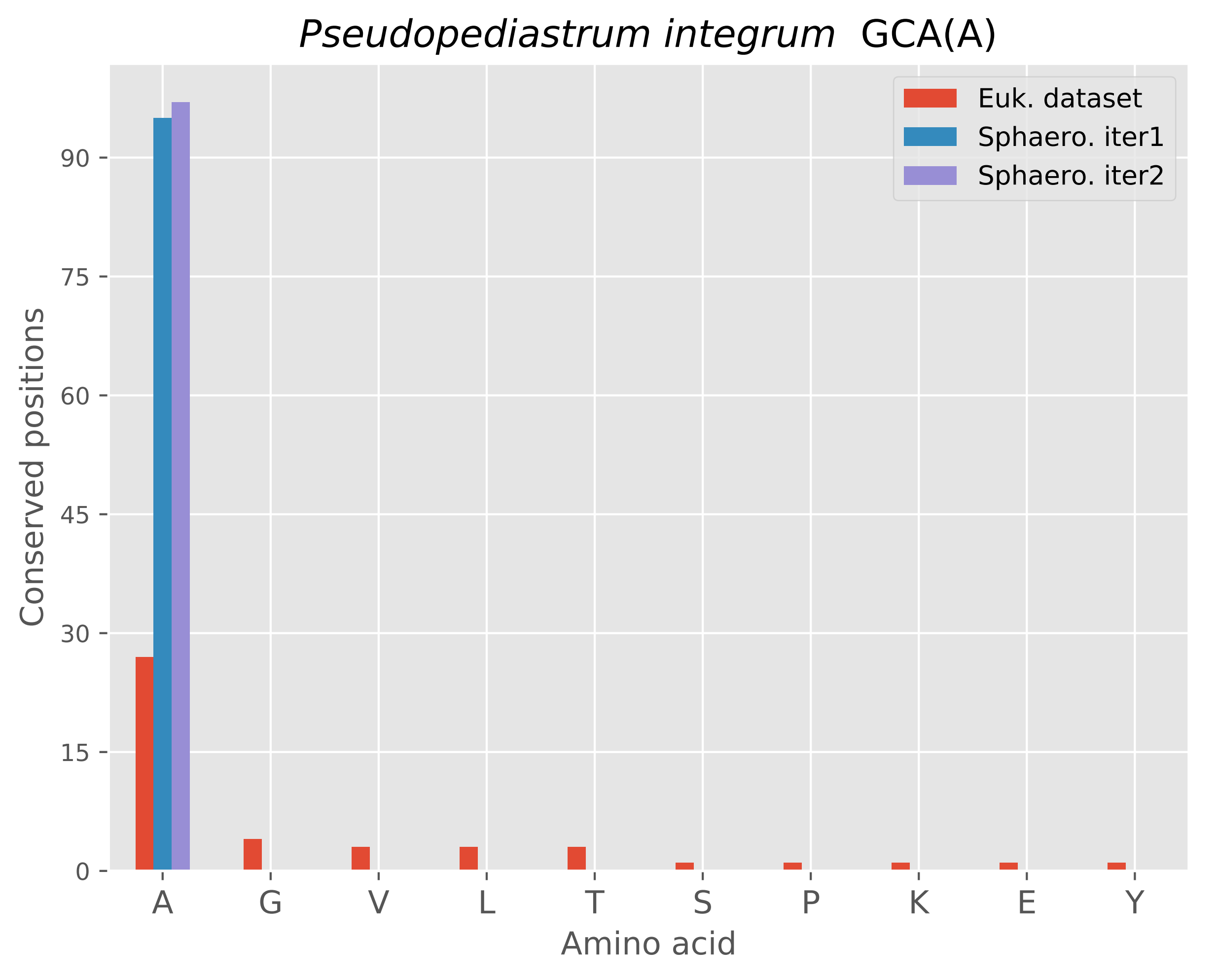

Pseudopediastrum integrum GCA(A)
Euk. dataset
Sphaero. iter1
Sphaero. iter2
Conserved positions
0
15
30
45
60
75
90
A
G
V
L
T
S
P
K
E
Y
Amino acid

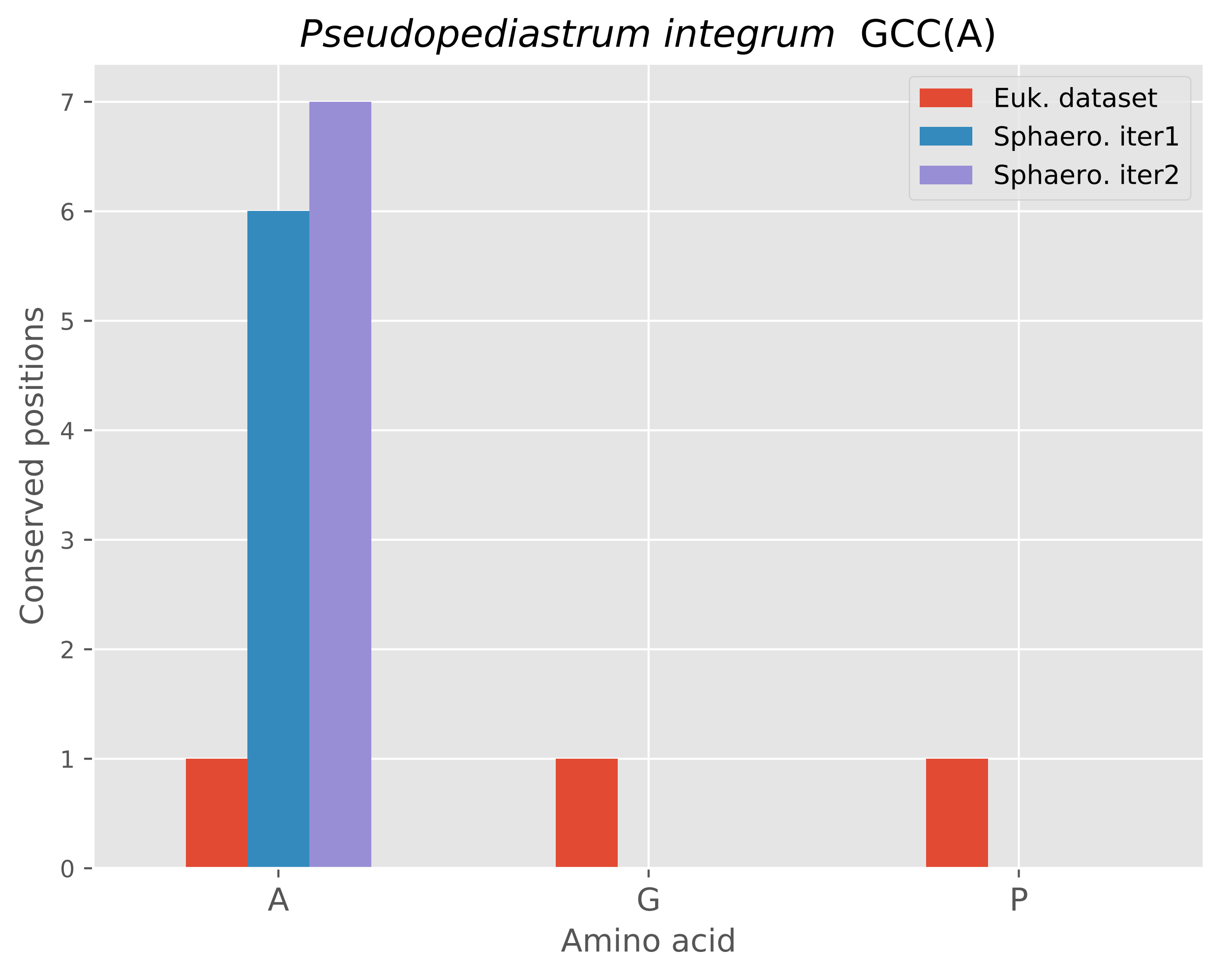

Pseudopediastrum integrum GCC(A)
Euk. dataset
Sphaero. iter1
Sphaero. iter2
Conserved positions
0
1
2
3
4
5
6
7
A
G
P
Amino acid

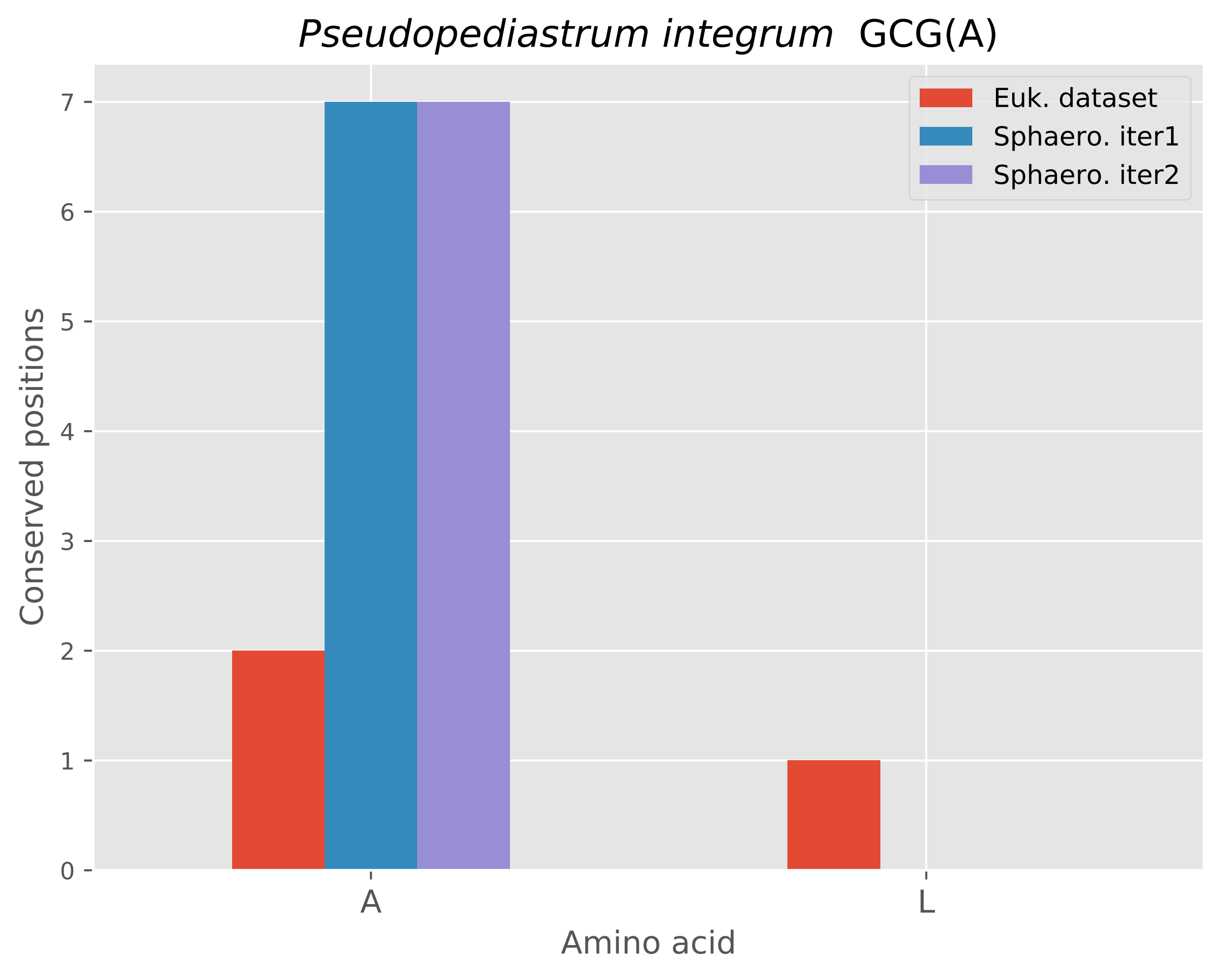

Pseudopediastrum integrum GCG(A)
Euk. dataset
Sphaero. iter1
Sphaero. iter2
Conserved positions
0
1
2
3
4
5
6
7
A
L
Amino acid

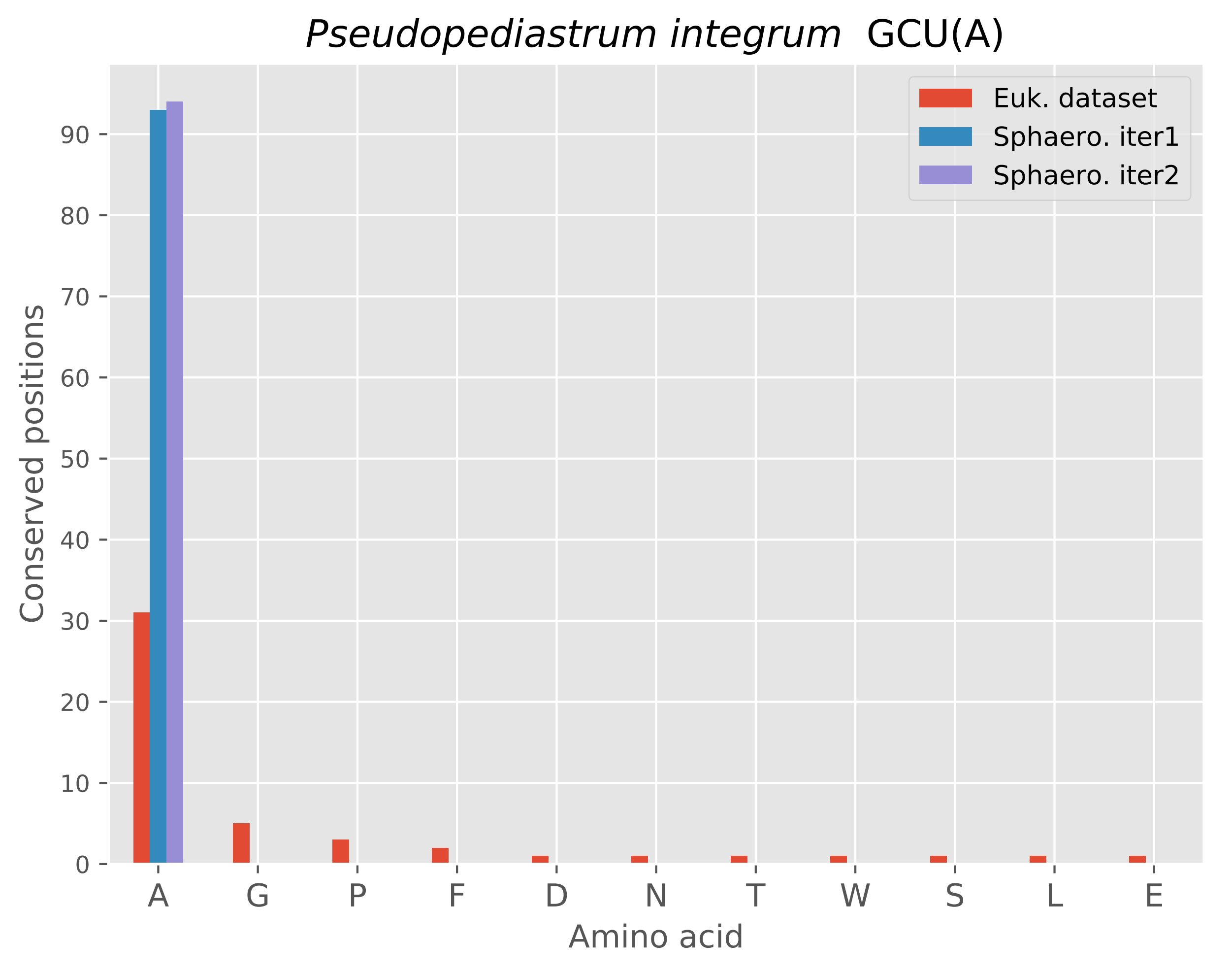
Pseudopediastrum integrum GCU(A)
Euk. dataset
Sphaero. iter1
Sphaero. iter2
Conserved positions
0
10
20
30
40
50
60
70
80
90
A
G
P
F
D
N
T
W
S
L
E
Amino acid

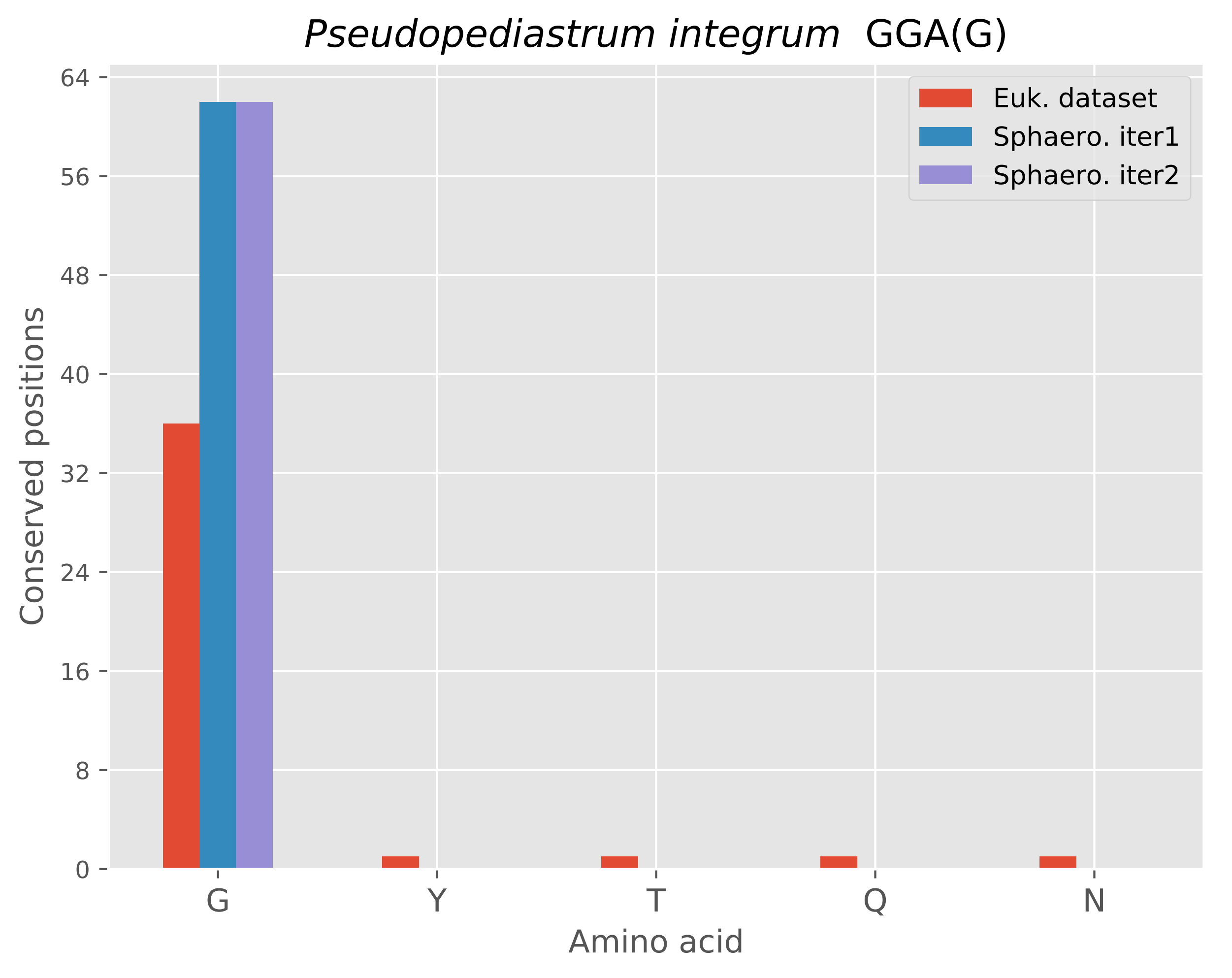
Pseudopediastrum integrum GGA(G)
Euk. dataset
Sphaero. iter1
Sphaero. iter2
Conserved positions
0
8
16
24
32
40
48
56
64
G
Y
T
Q
N
Amino acid

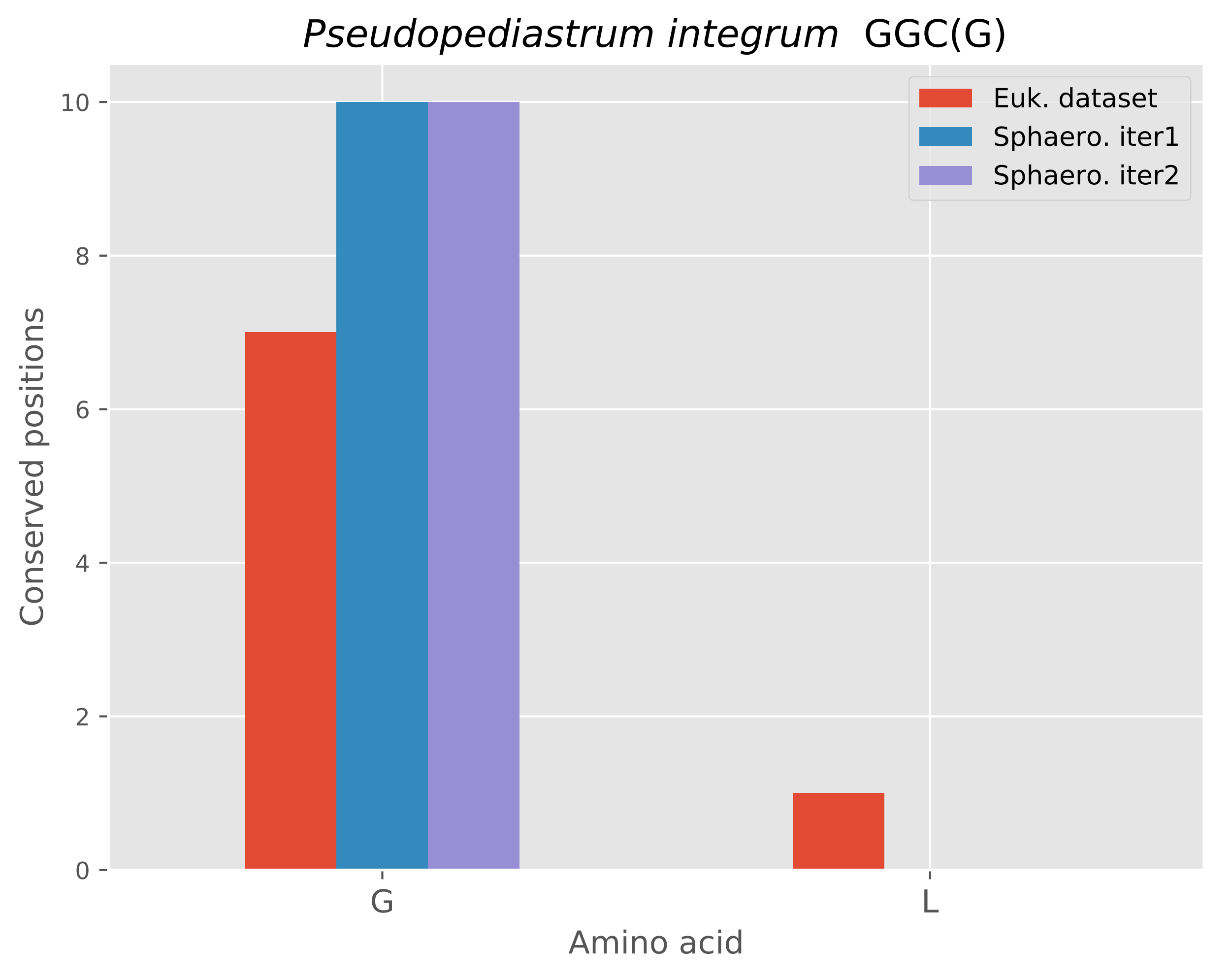
Pseudopediastrum integrum GGC(G)
Euk. dataset
Sphaero. iter1
Sphaero. iter2
Conserved positions
0
2
4
6
8
10
G
L
Amino acid

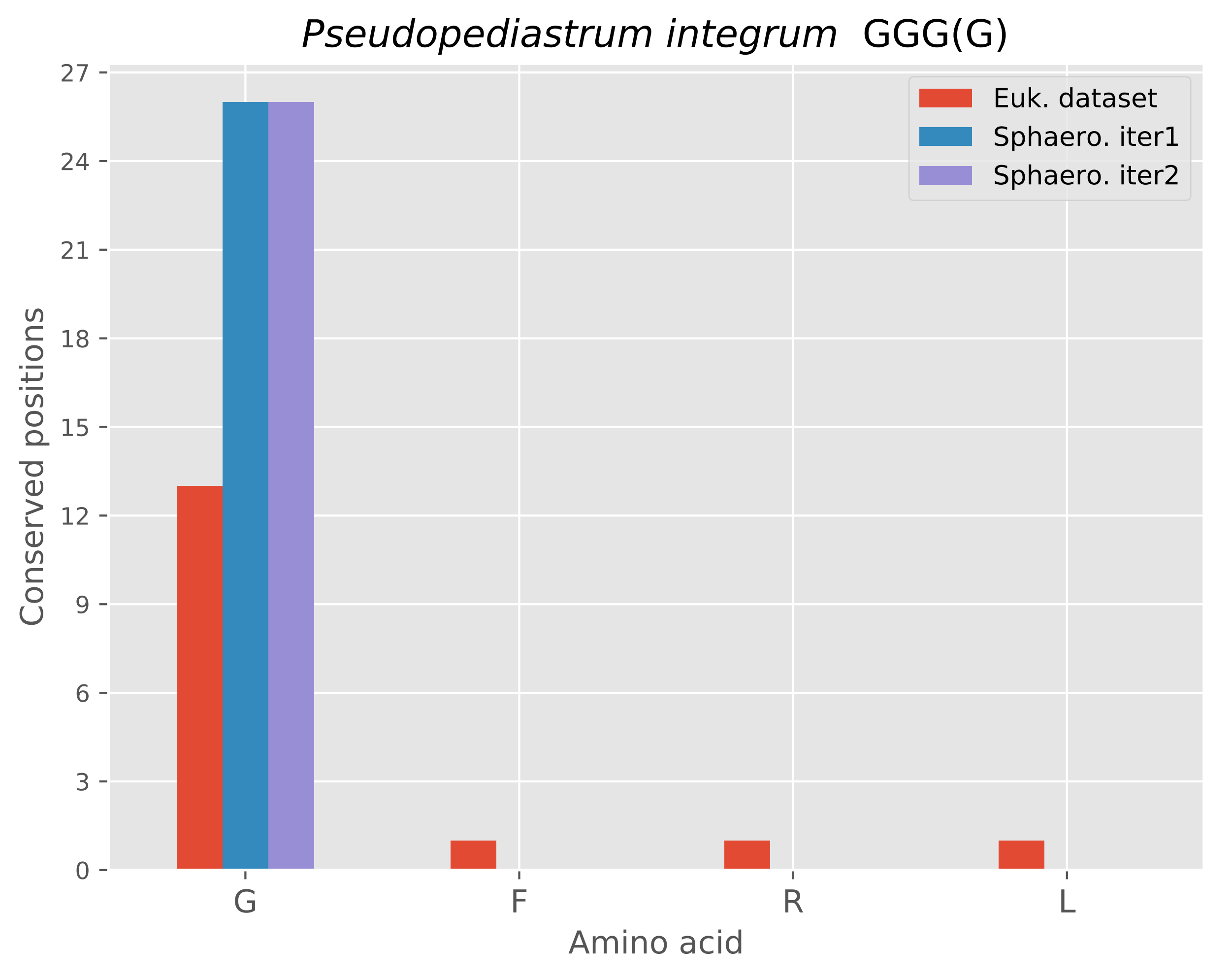

Pseudopediastrum integrum GGG(G)
Euk. dataset
Sphaero. iter1
Sphaero. iter2
Conserved positions
0
3
6
9
12
15
18
21
24
27
G
F
R
L
Amino acid

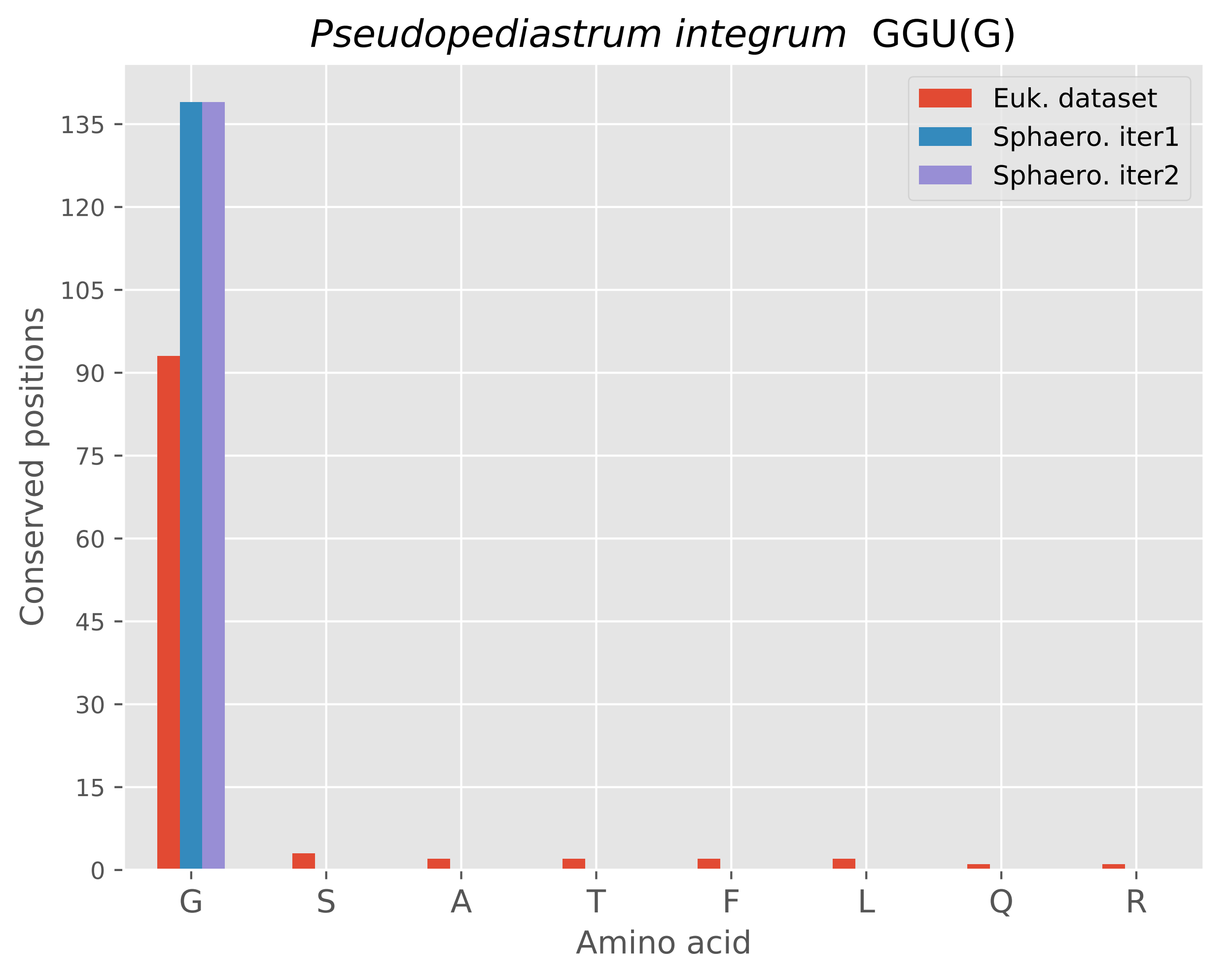

Pseudopediastrum integrum GGU(G)
Euk. dataset
Sphaero. iter1
Sphaero. iter2
Conserved positions
0
15
30
45
60
75
90
105
120
135
G
S
A
T
F
L
Q
R
Amino acid

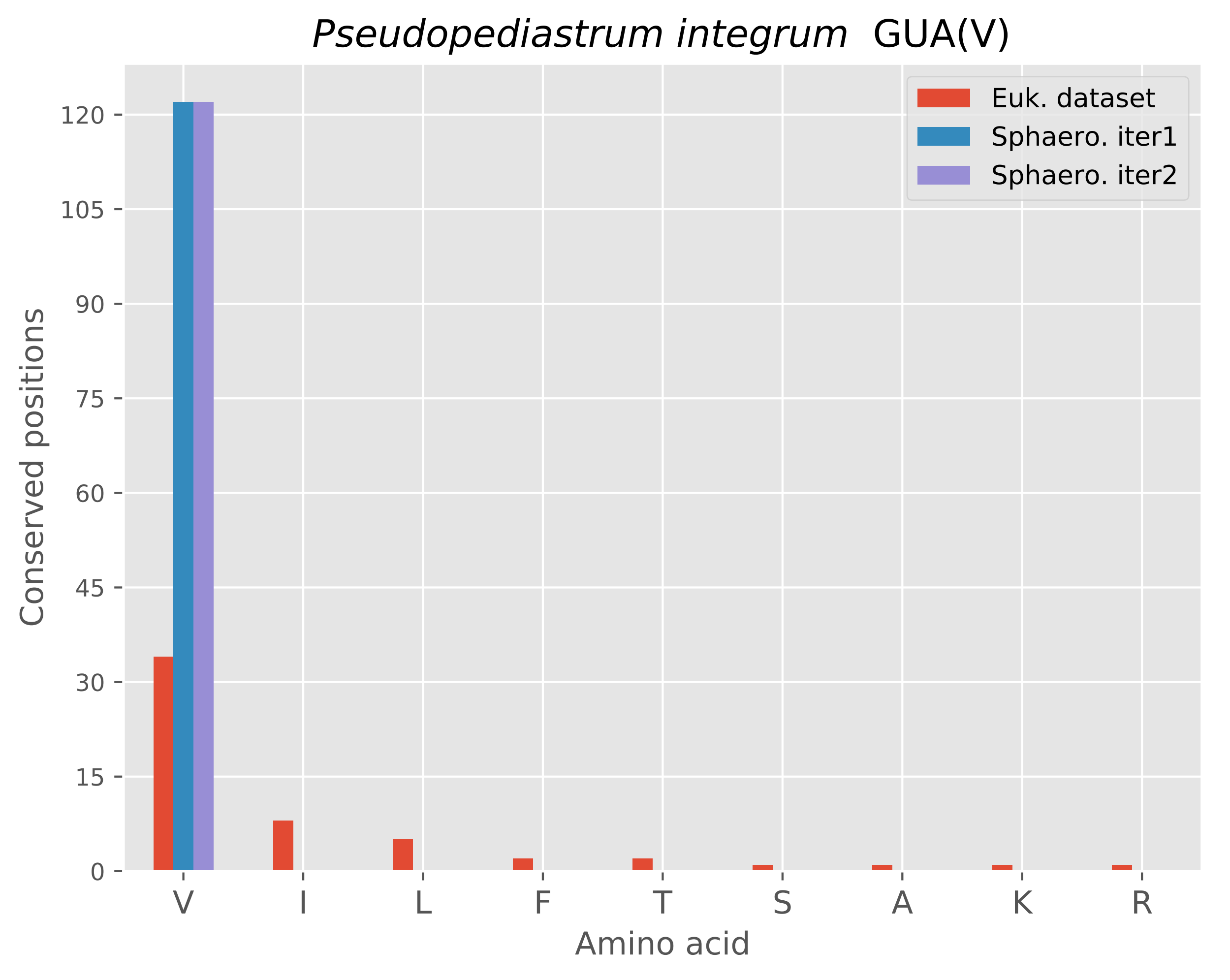

Pseudopediastrum integrum GUA(V)
Euk. dataset
Sphaero. iter1
Sphaero. iter2
Conserved positions
0
15
30
45
60
75
90
105
120
V
I
L
F
T
S
A
K
R
Amino acid

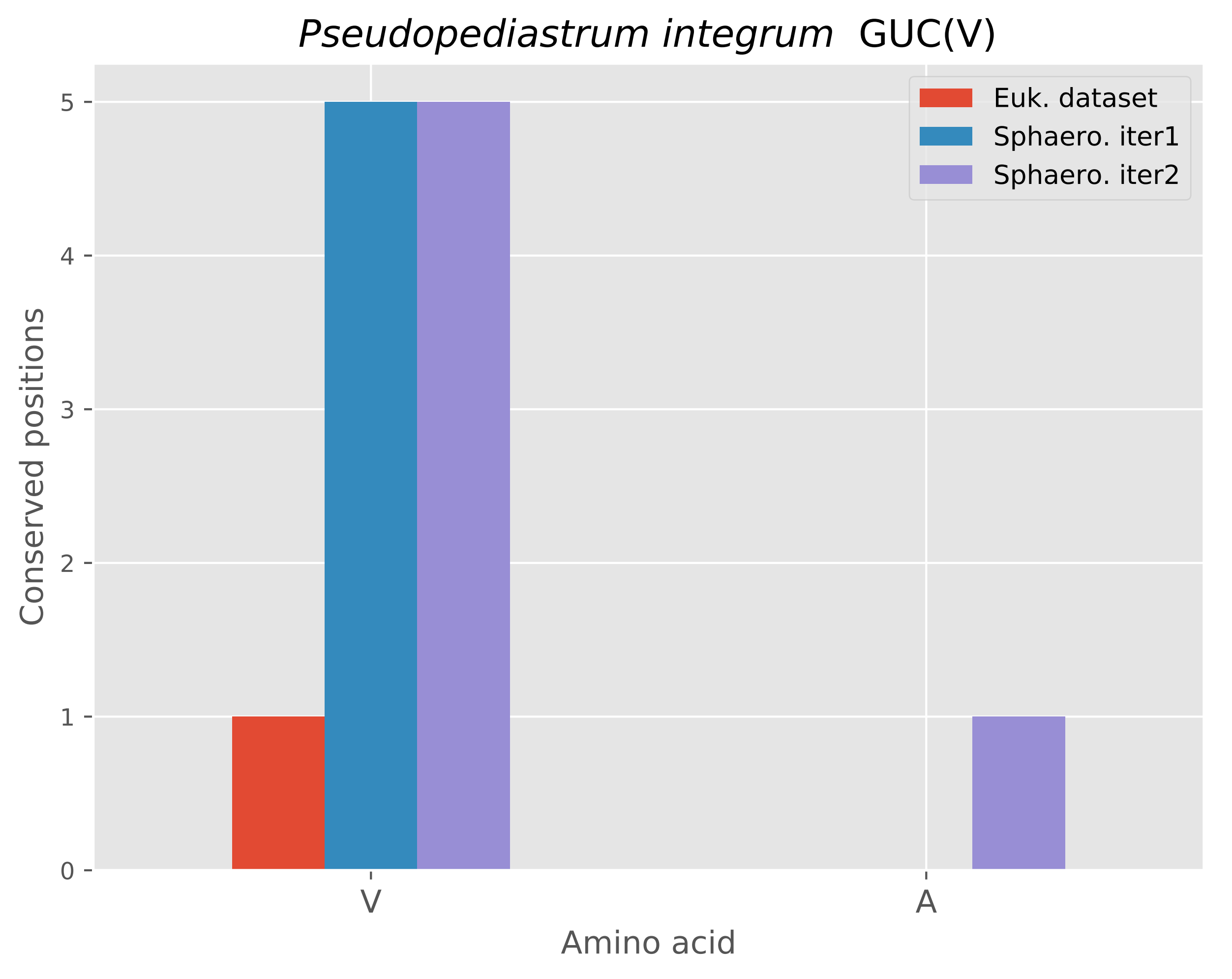

Pseudopediastrum integrum GUC(V)
Euk. dataset
Sphaero. iter1
Sphaero. iter2
Conserved positions
0
1
2
3
4
5
V
A
Amino acid

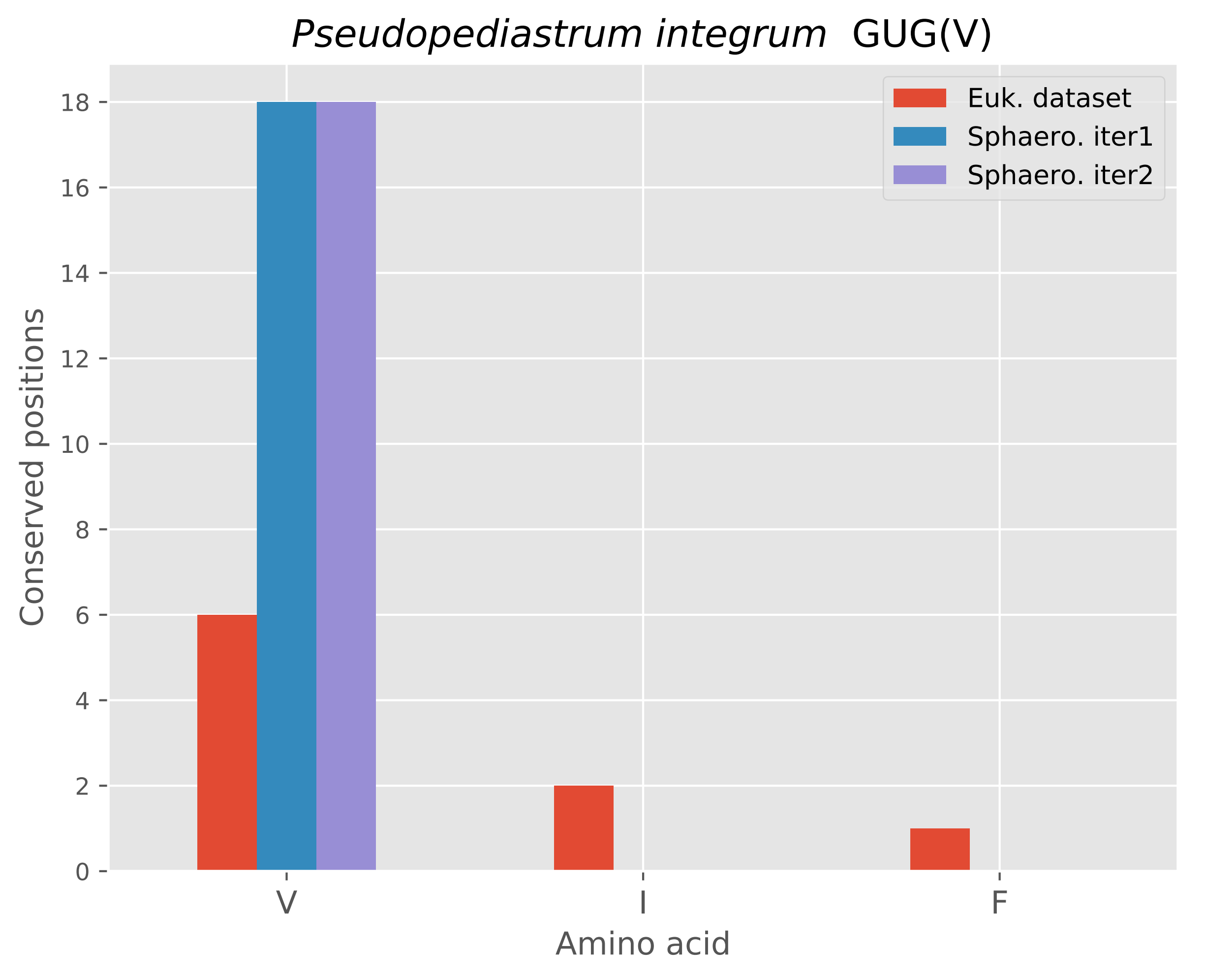

Pseudopediastrum integrum GUG(V)
Euk. dataset
Sphaero. iter1
Sphaero. iter2
Conserved positions
0
2
4
6
8
10
12
14
16
18
V
I
F
Amino acid

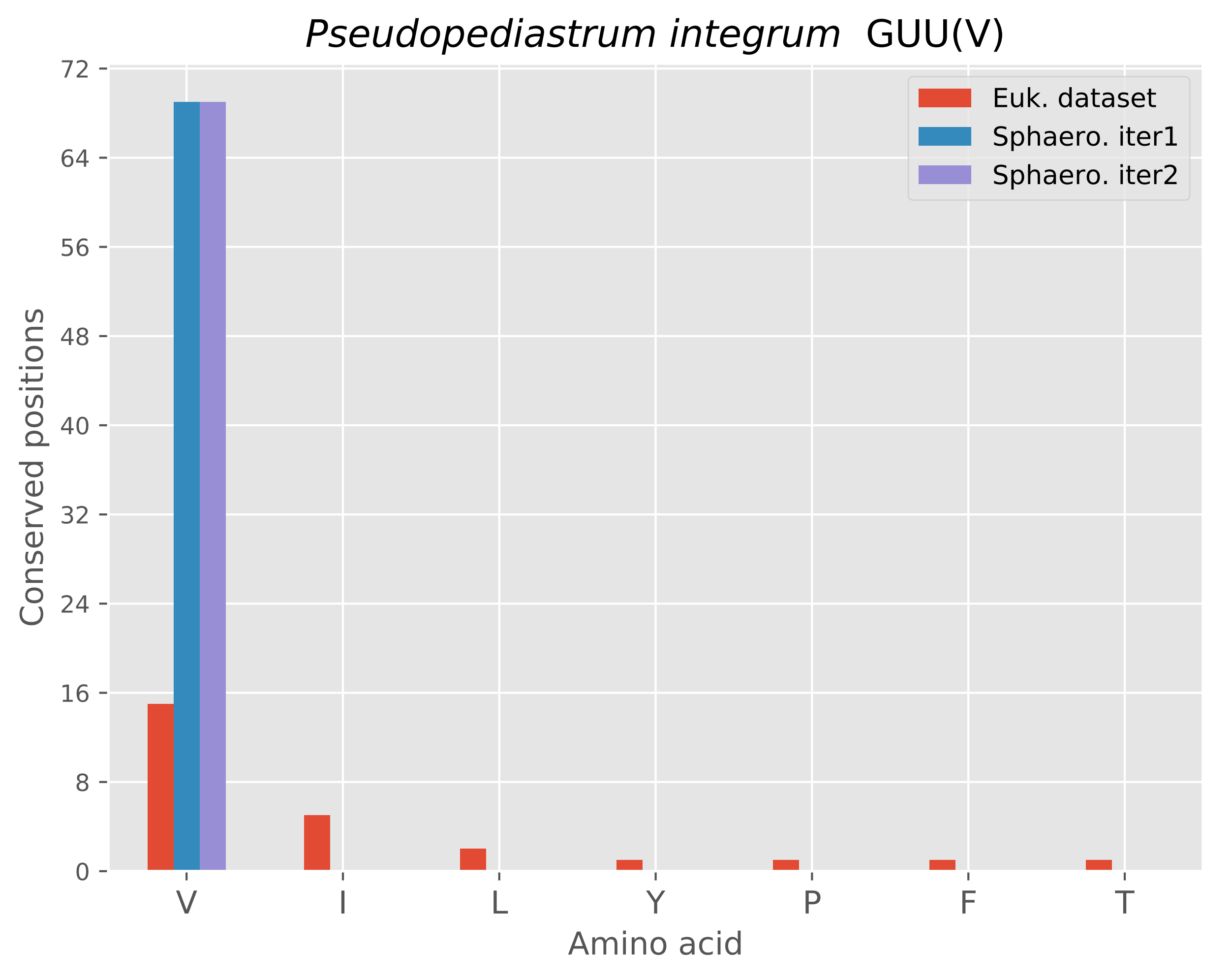

Pseudopediastrum integrum GUU(V)
Euk. dataset
Sphaero. iter1
Sphaero. iter2
Conserved positions
Amino acid
0
8
16
24
32
40
48
56
64
72
V
I
L
Y
P
F
T

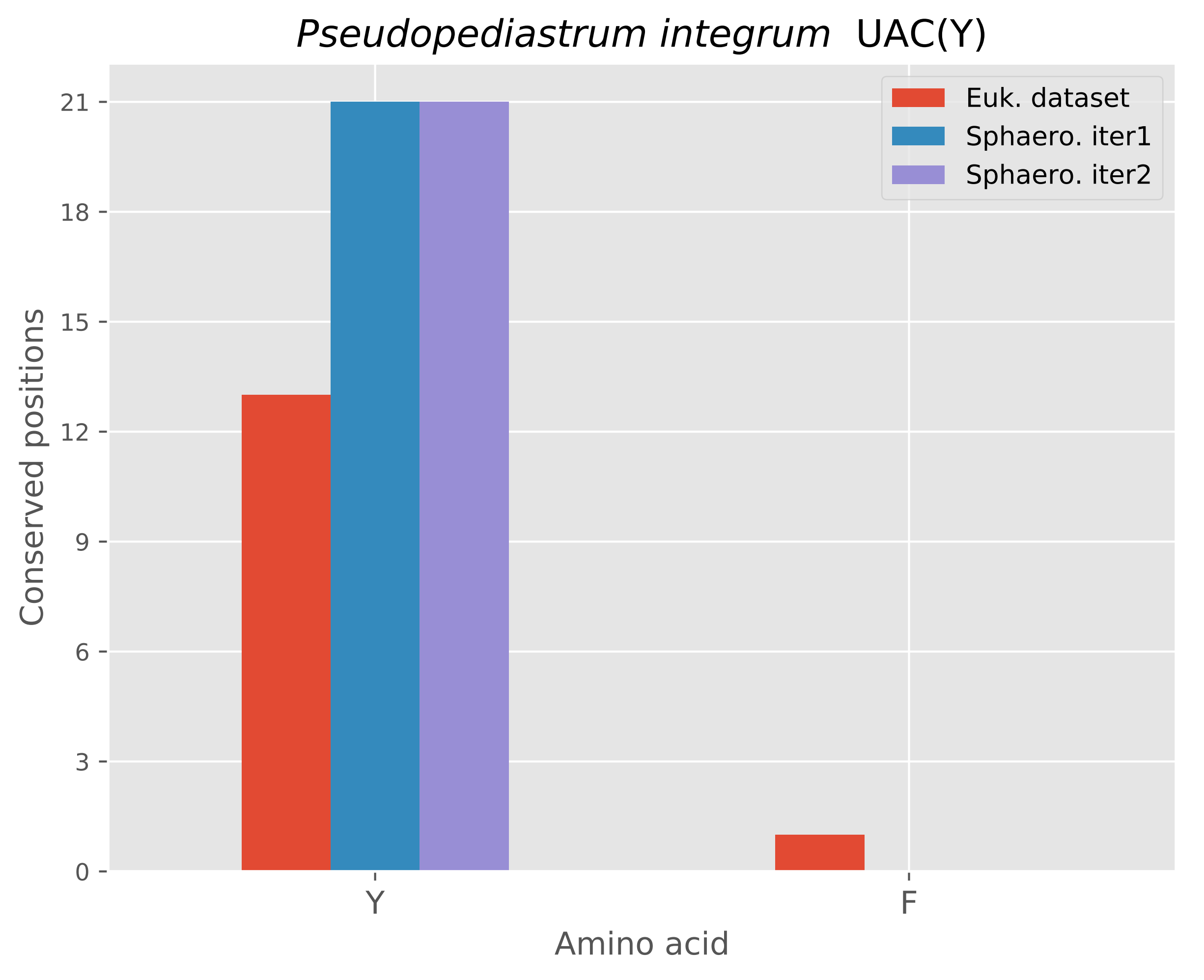

Pseudopediastrum integrum UAC(Y)
Euk. dataset
Sphaero. iter1
Sphaero. iter2
Conserved positions
0
3
6
9
12
15
18
21
Y
F
Amino acid

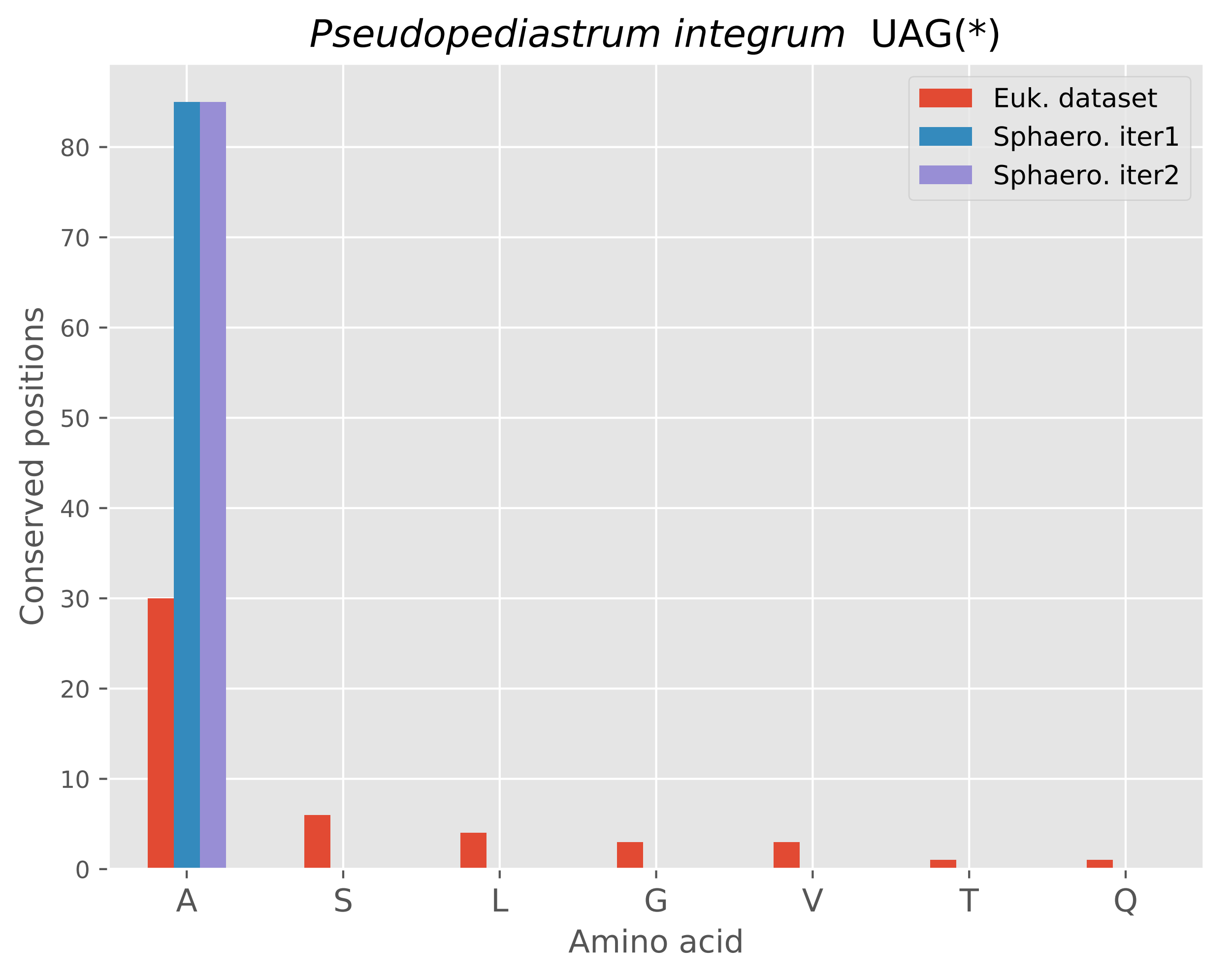

Pseudopediastrum integrum UAG(*)
Euk. dataset
Sphaero. iter1
Sphaero. iter2
Conserved positions
0
10
20
30
40
50
60
70
80
A
S
L
G
V
T
Q
Amino acid

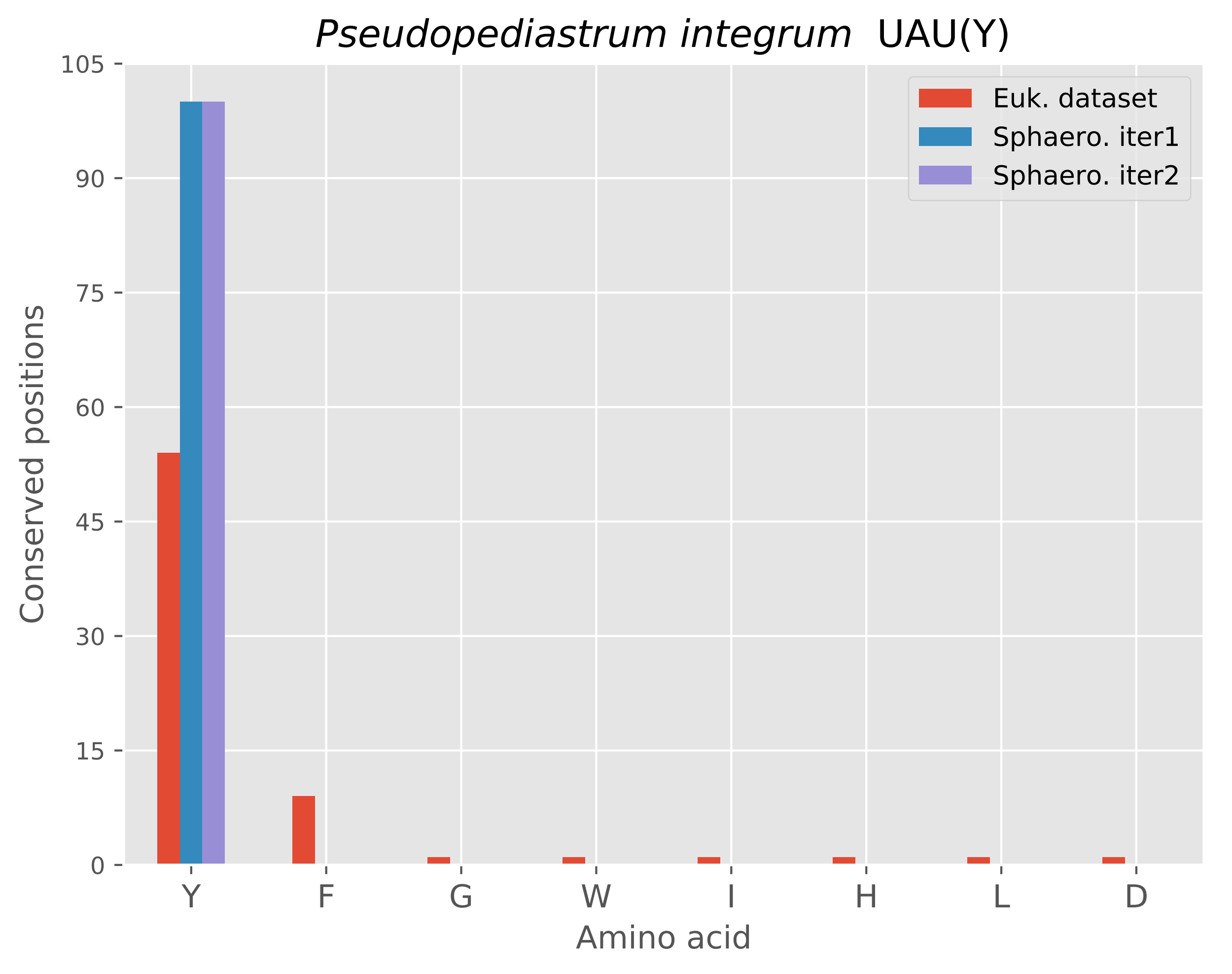

Pseudopediastrum integrum UAU(Y)
Conserved positions
105
90
75
60
45
30
15
0
Euk. dataset
Sphaero. iter1
Sphaero. iter2
Y
F
G
W
I
H
L
D
Amino acid

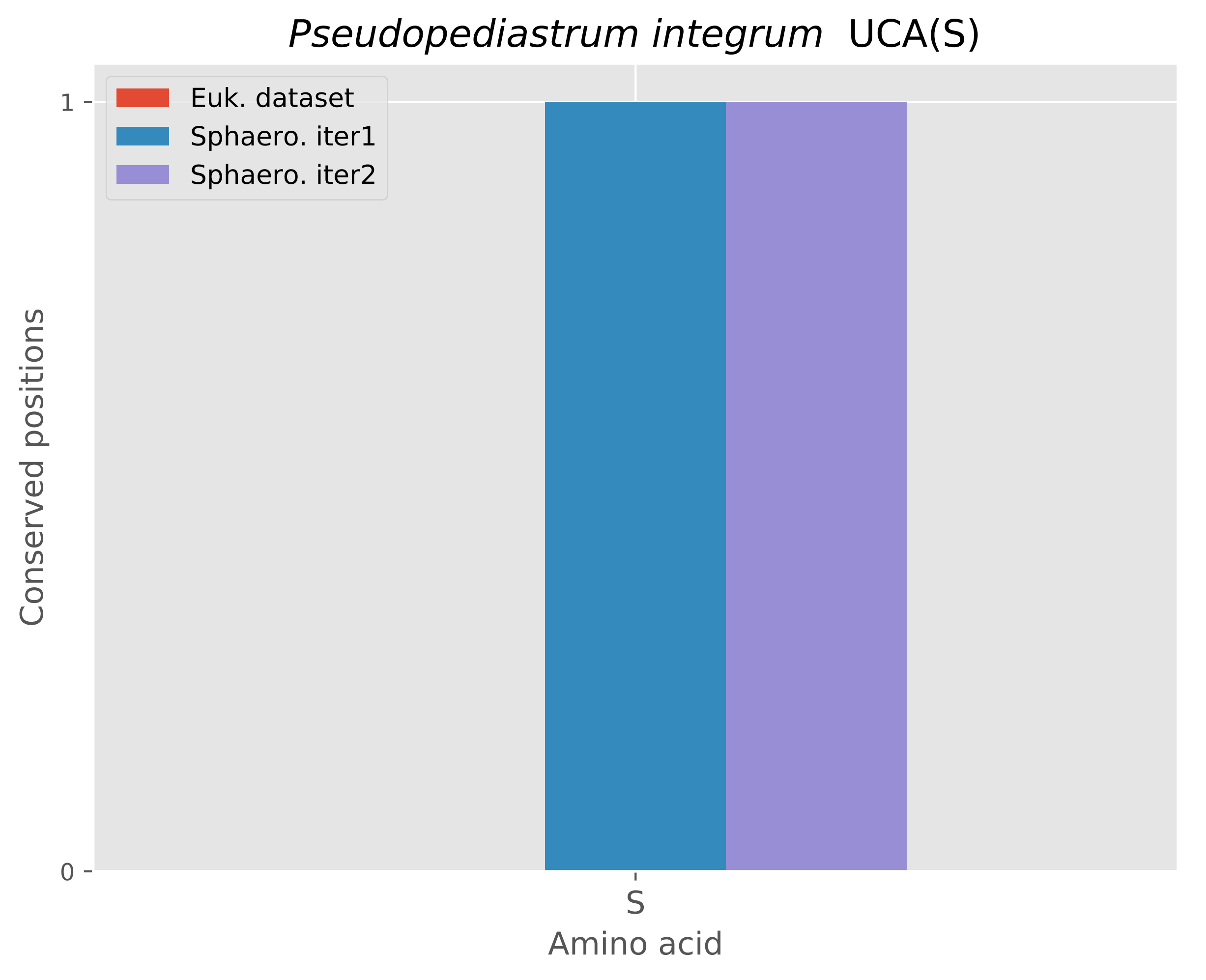

Pseudopediastrum integrum UCA(S)
Euk. dataset
Sphaero. iter1
Sphaero. iter2
1
0
Conserved positions
S
Amino acid

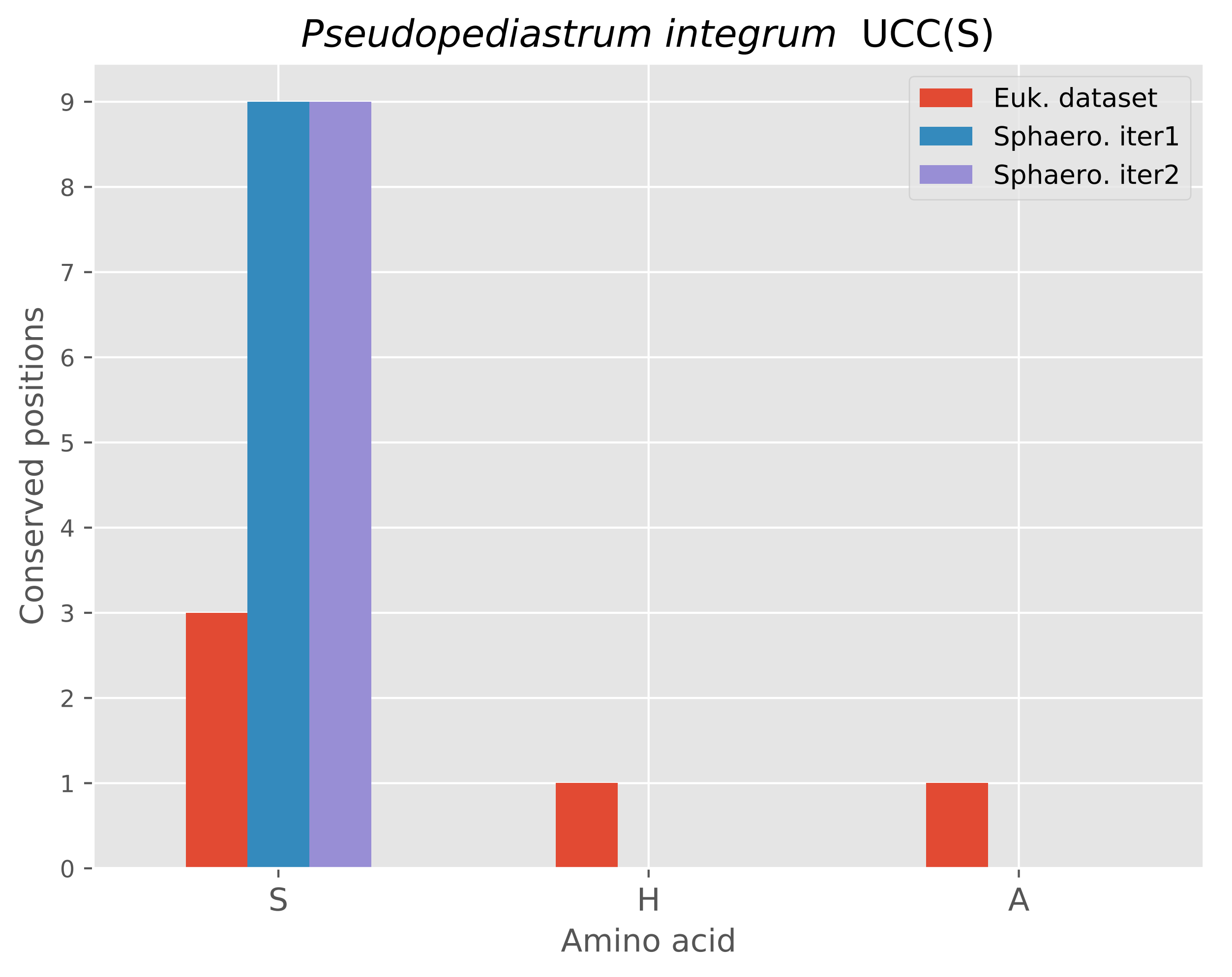

Pseudopediastrum integrum UCC(S)
Euk. dataset
Sphaero. iter1
Sphaero. iter2
Conserved positions
0
1
2
3
4
5
6
7
8
9
S
H
A
Amino acid

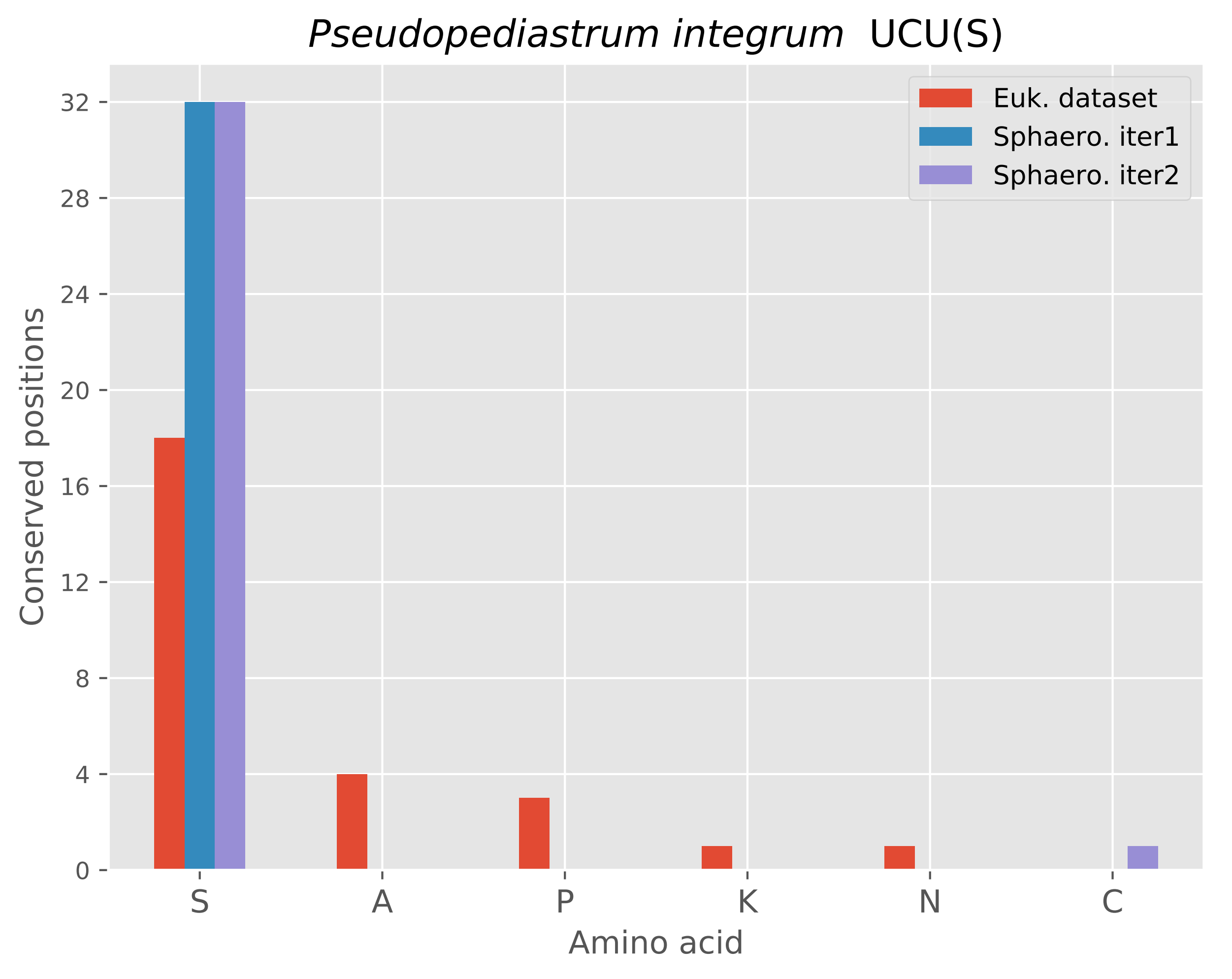
Pseudopediastrum integrum UCU(S)
Euk. dataset
Sphaero. iter1
Sphaero. iter2
Conserved positions
0
4
8
12
16
20
24
28
32
S
A
P
K
N
C
Amino acid

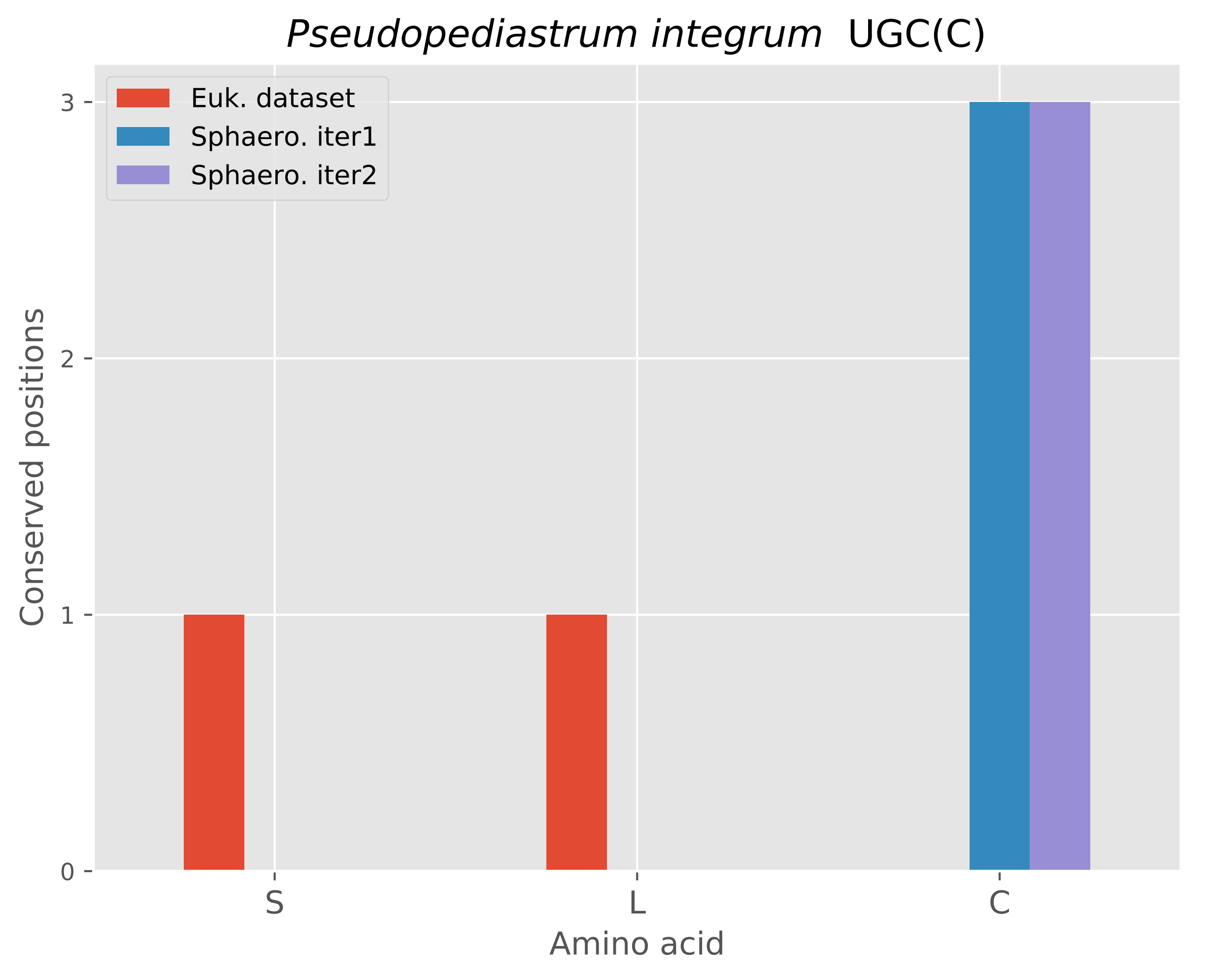

Pseudopediastrum integrum UGC(C)
Euk. dataset
Sphaero. iter1
Sphaero. iter2
Conserved positions
0
1
2
3
S
L
C
Amino acid

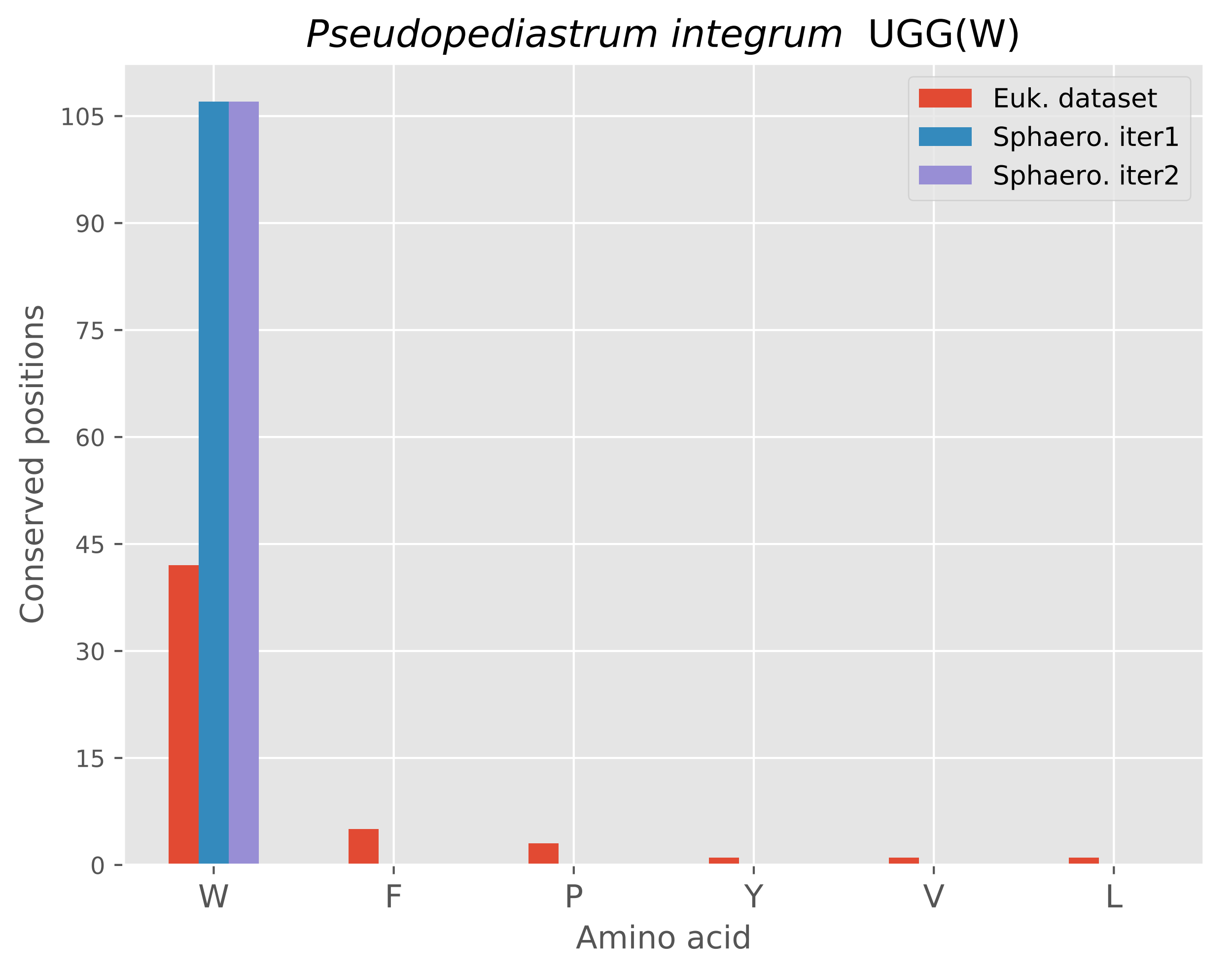

Pseudopediastrum integrum UGG(W)
Euk. dataset
Sphaero. iter1
Sphaero. iter2
Conserved positions
0
15
30
45
60
75
90
105
W
F
P
Y
V
L
Amino acid

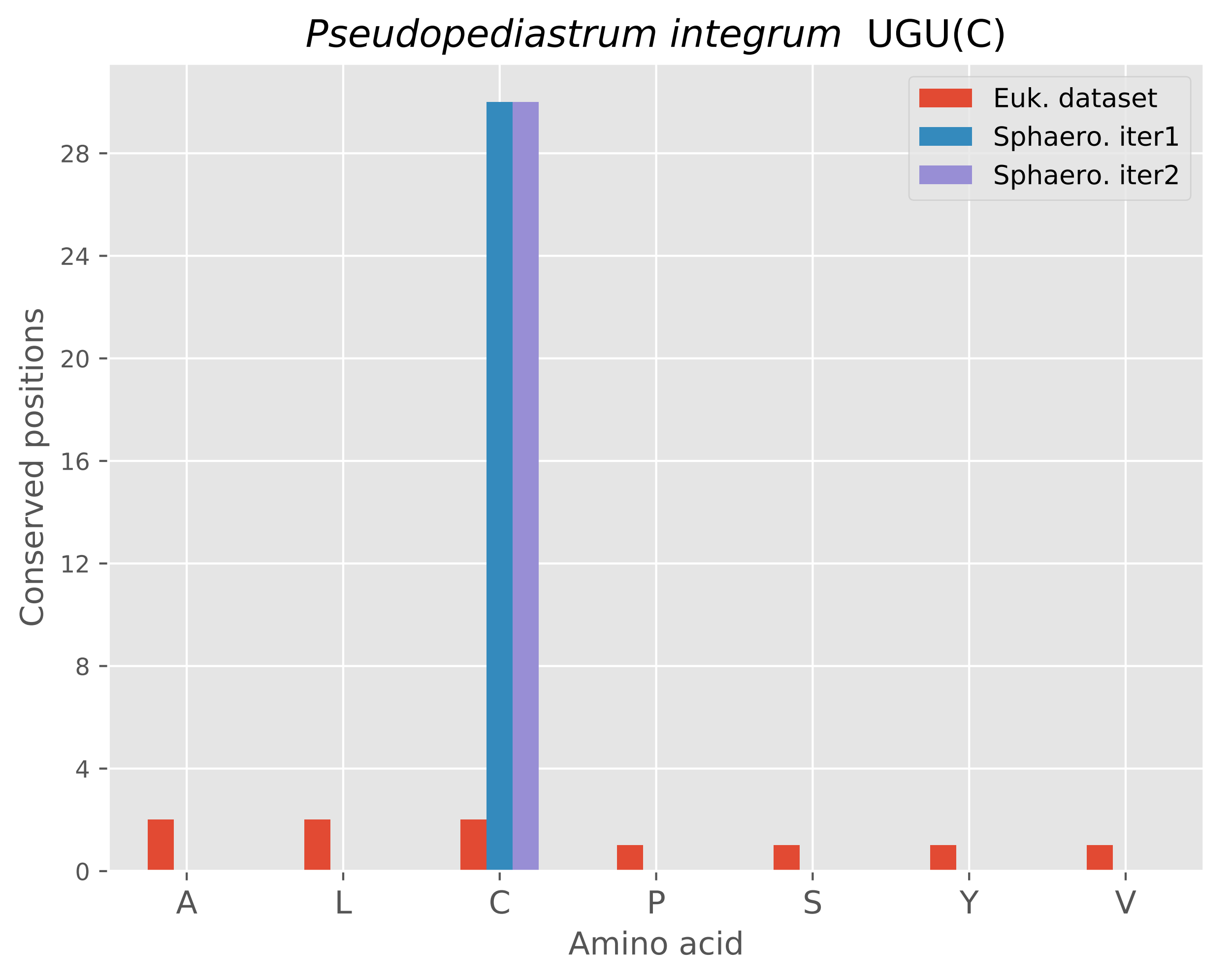
Pseudopediastrum integrum UGU(C)
Euk. dataset
Sphaero. iter1
Sphaero. iter2
Conserved positions
0
4
8
12
16
20
24
28
A
L
C
P
S
Y
V
Amino acid

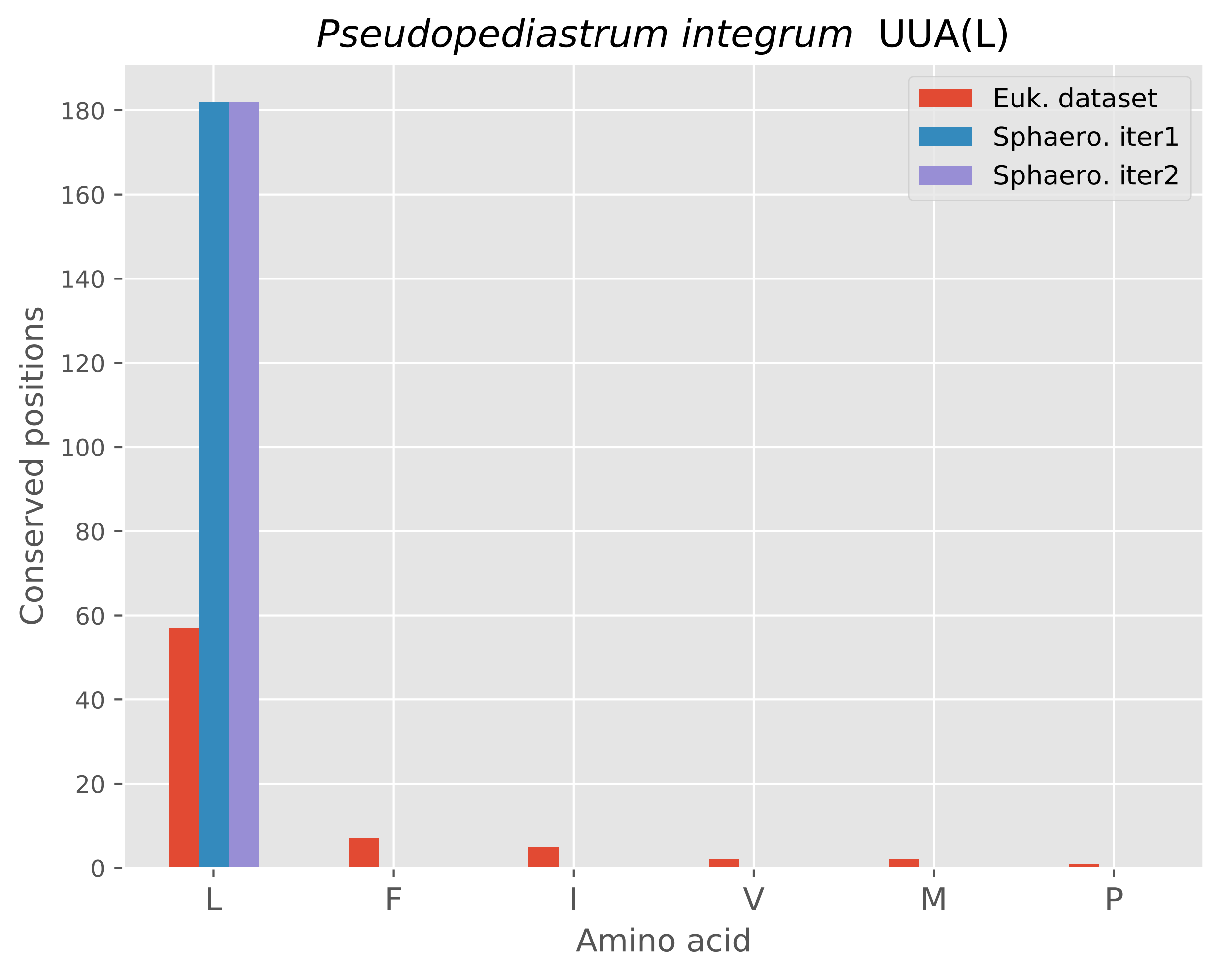

Pseudopediastrum integrum UUA(L)
Euk. dataset
Sphaero. iter1
Sphaero. iter2
Conserved positions
0
20
40
60
80
100
120
140
160
180
L
F
I
V
M
P
Amino acid

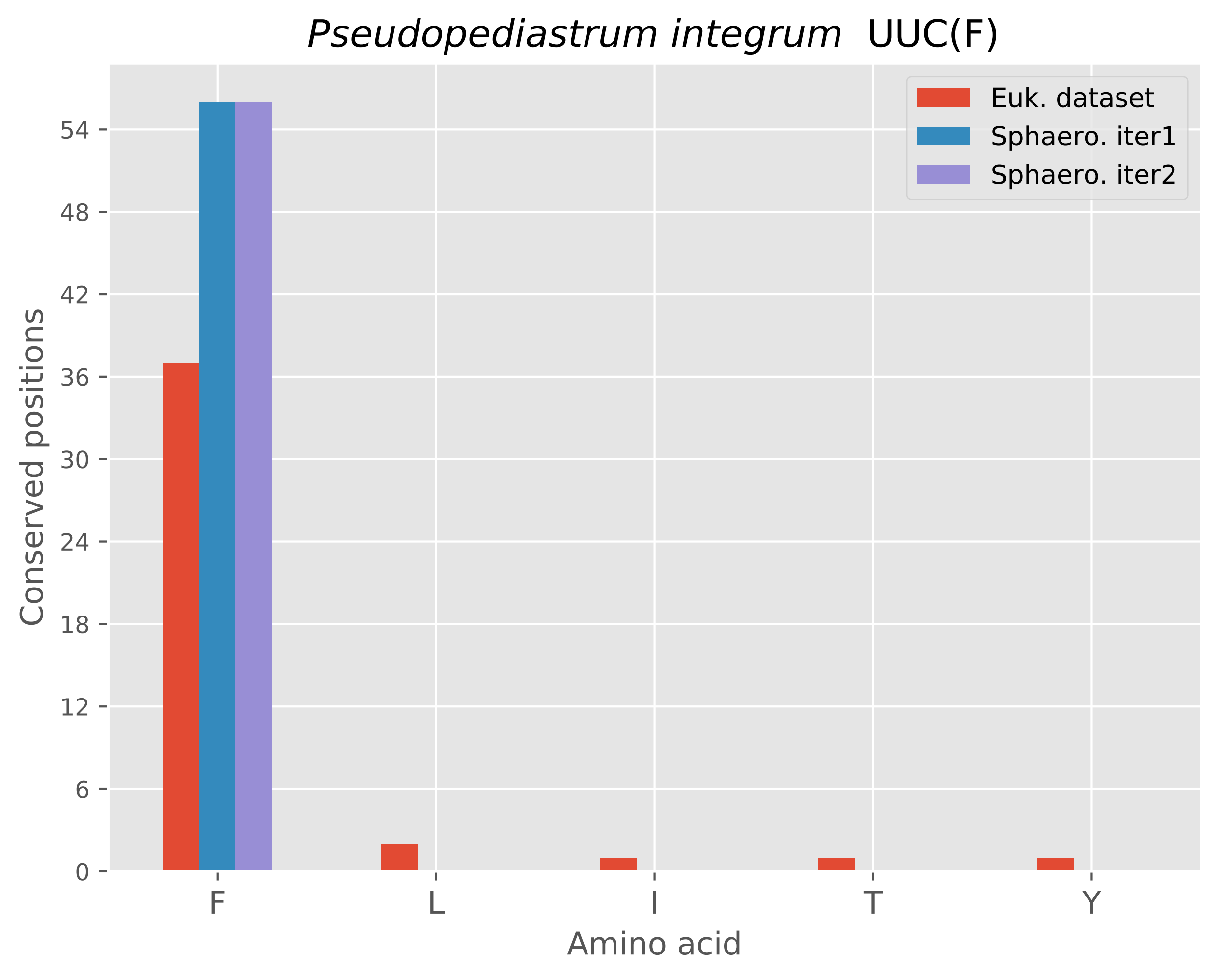

Pseudopediastrum integrum UUC(F)
Euk. dataset
Sphaero. iter1
Sphaero. iter2
Conserved positions
0
6
12
18
24
30
36
42
48
54
F
L
I
T
Y
Amino acid

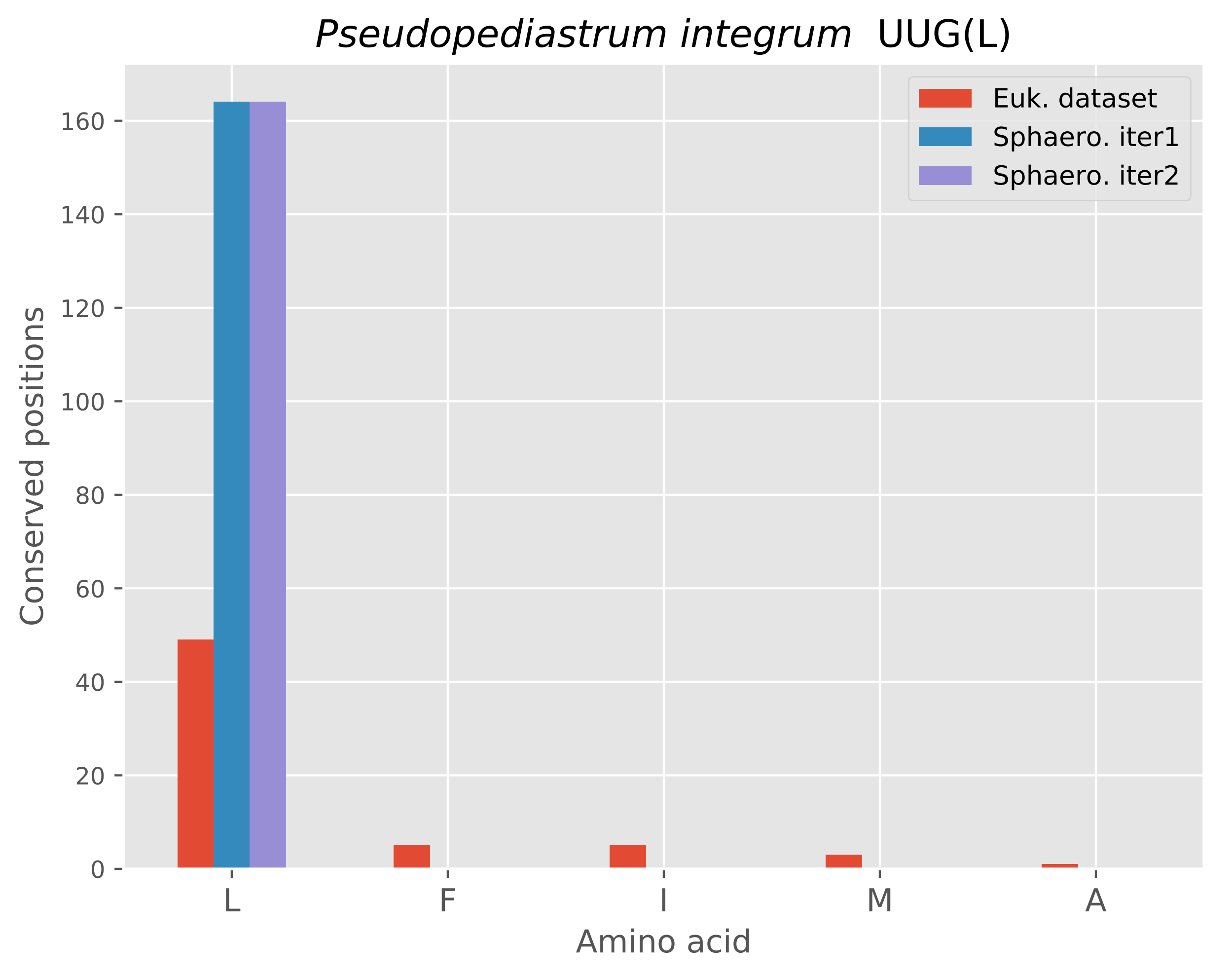

Pseudopediastrum integrum UUG(L)
Euk. dataset
Sphaero. iter1
Sphaero. iter2
Conserved positions
0
20
40
60
80
100
120
140
160
L
F
I
M
A
Amino acid

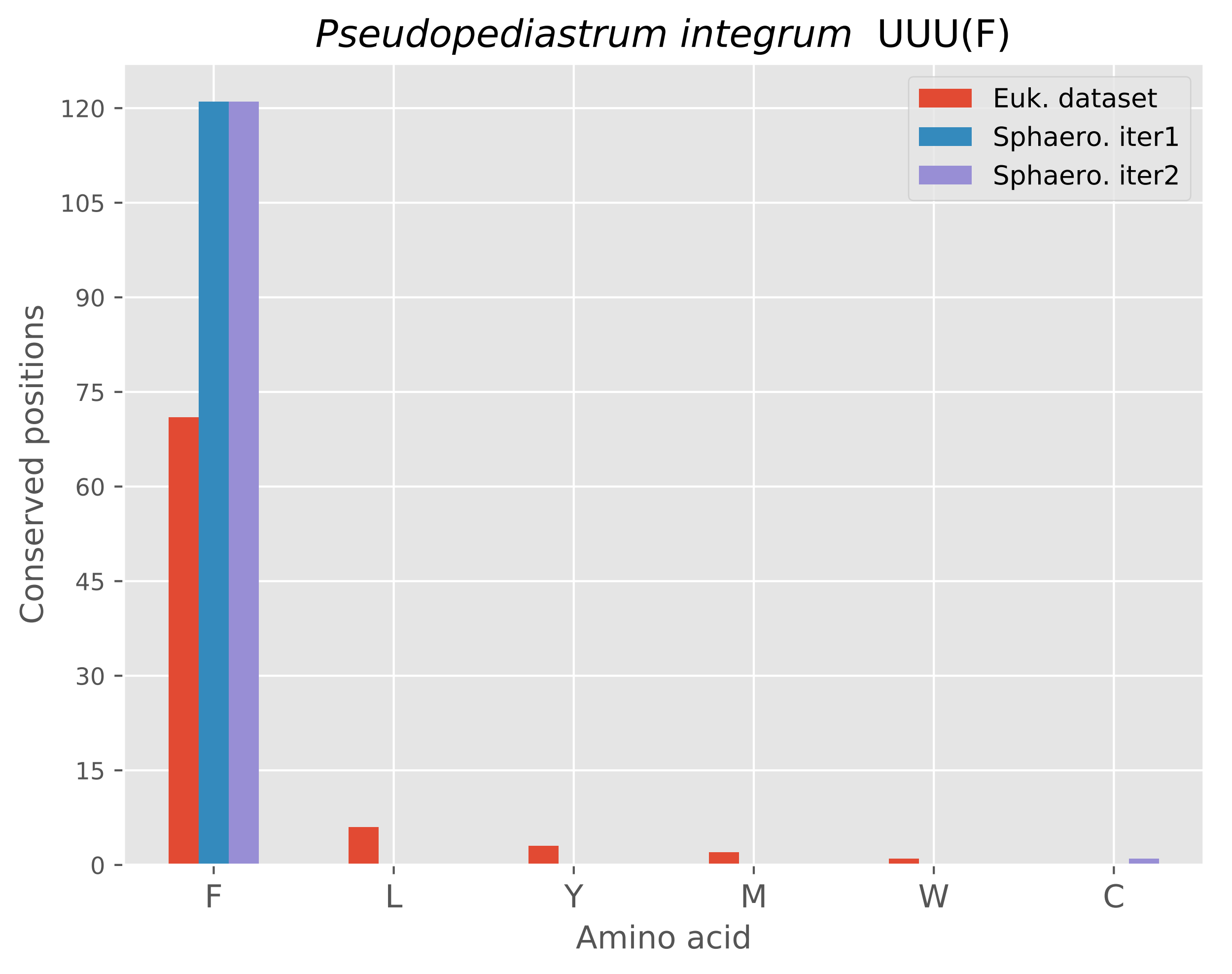

Pseudopediastrum integrum UUU(F)
Euk. dataset
Sphaero. iter1
Sphaero. iter2
Conserved positions
0
15
30
45
60
75
90
105
120
F
L
Y
M
W
C
Amino acid

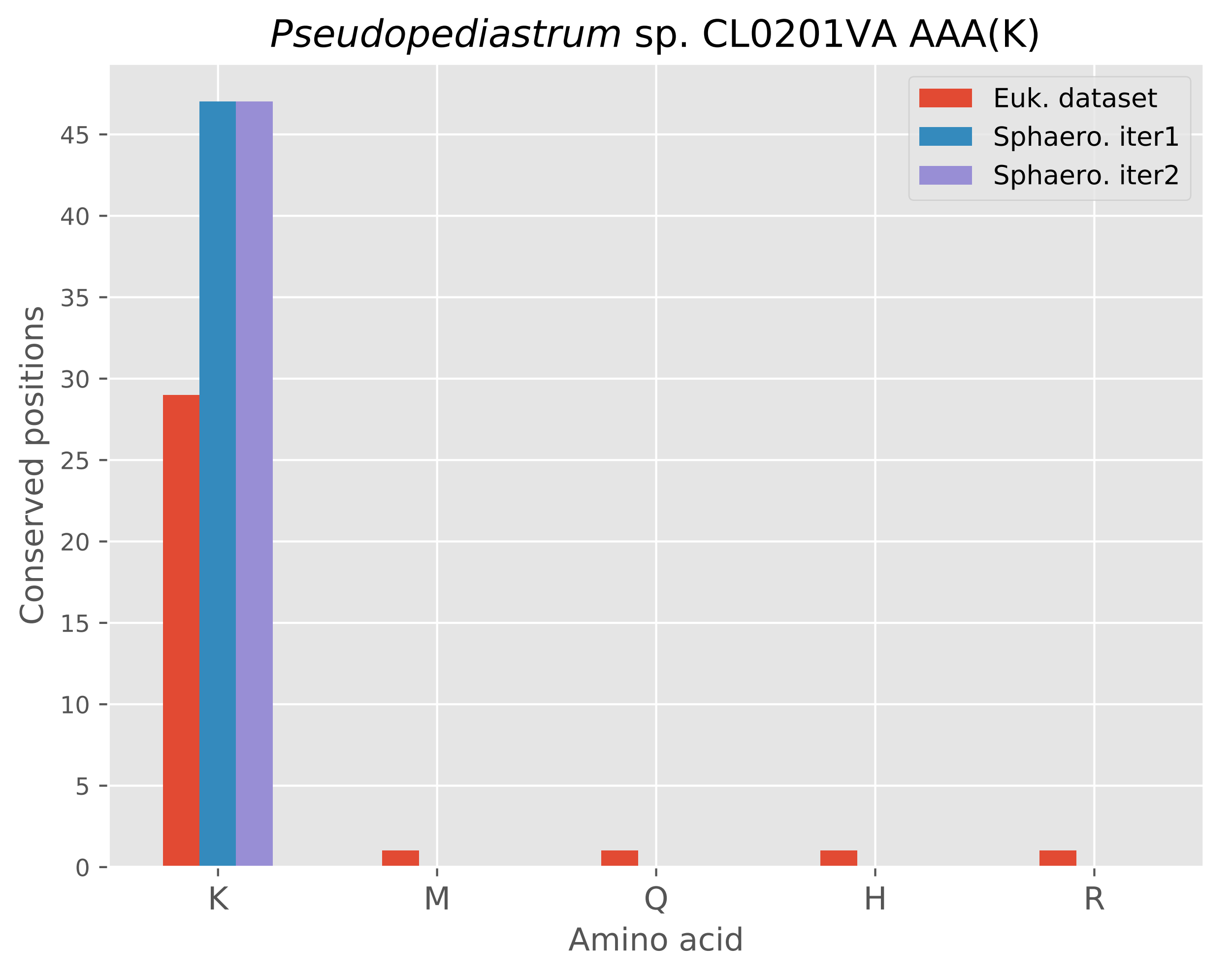

Pseudopediastrum sp. CL0201VA AAA(K)
Euk. dataset
Sphaero. iter1
Sphaero. iter2
Conserved positions
0
5
10
15
20
25
30
35
40
45
K
M
Q
H
R
Amino acid

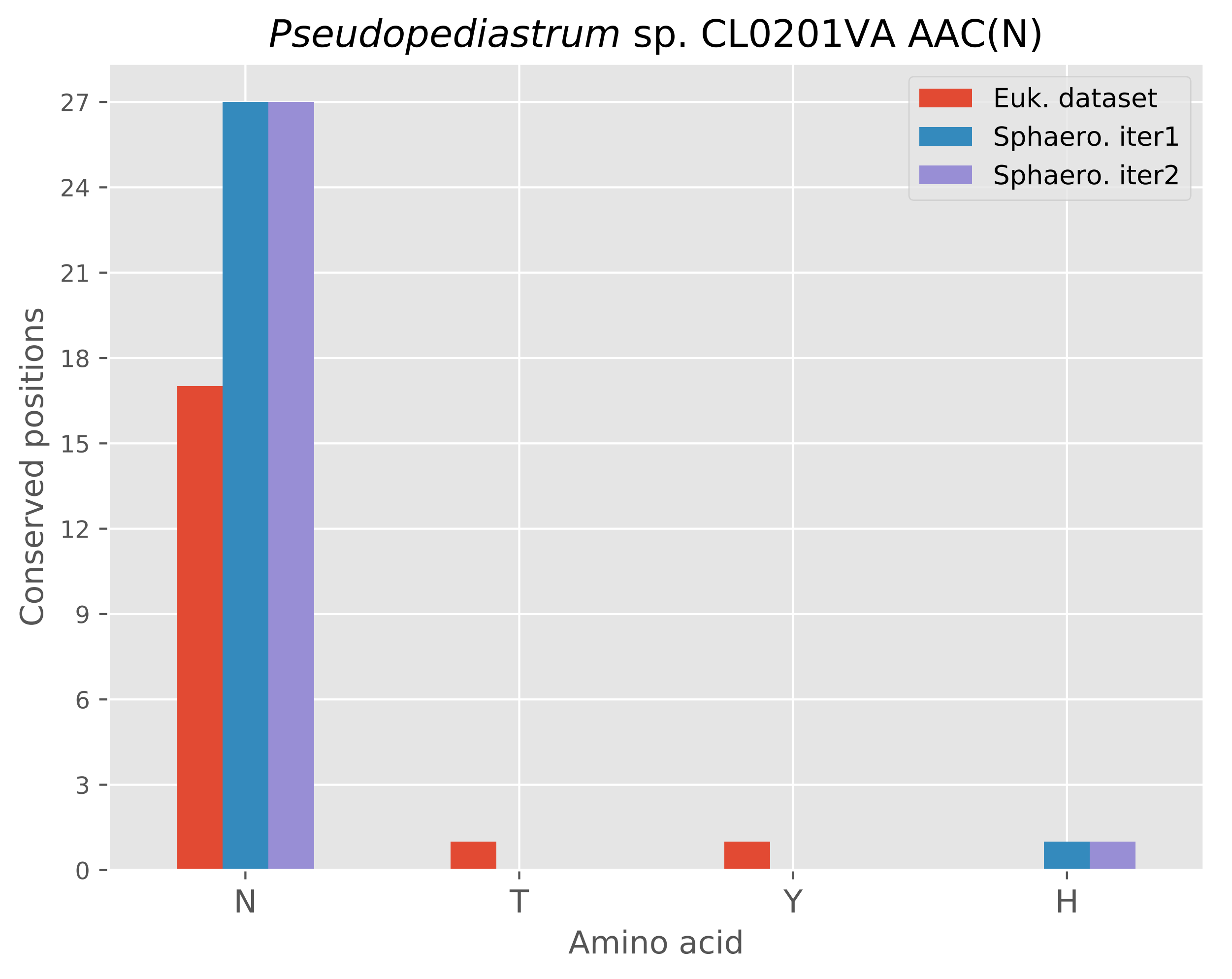

Pseudopediastrum sp. CL0201VA AAC(N)
Euk. dataset
Sphaero. iter1
Sphaero. iter2
Conserved positions
0
3
6
9
12
15
18
21
24
27
N
T
Y
H
Amino acid

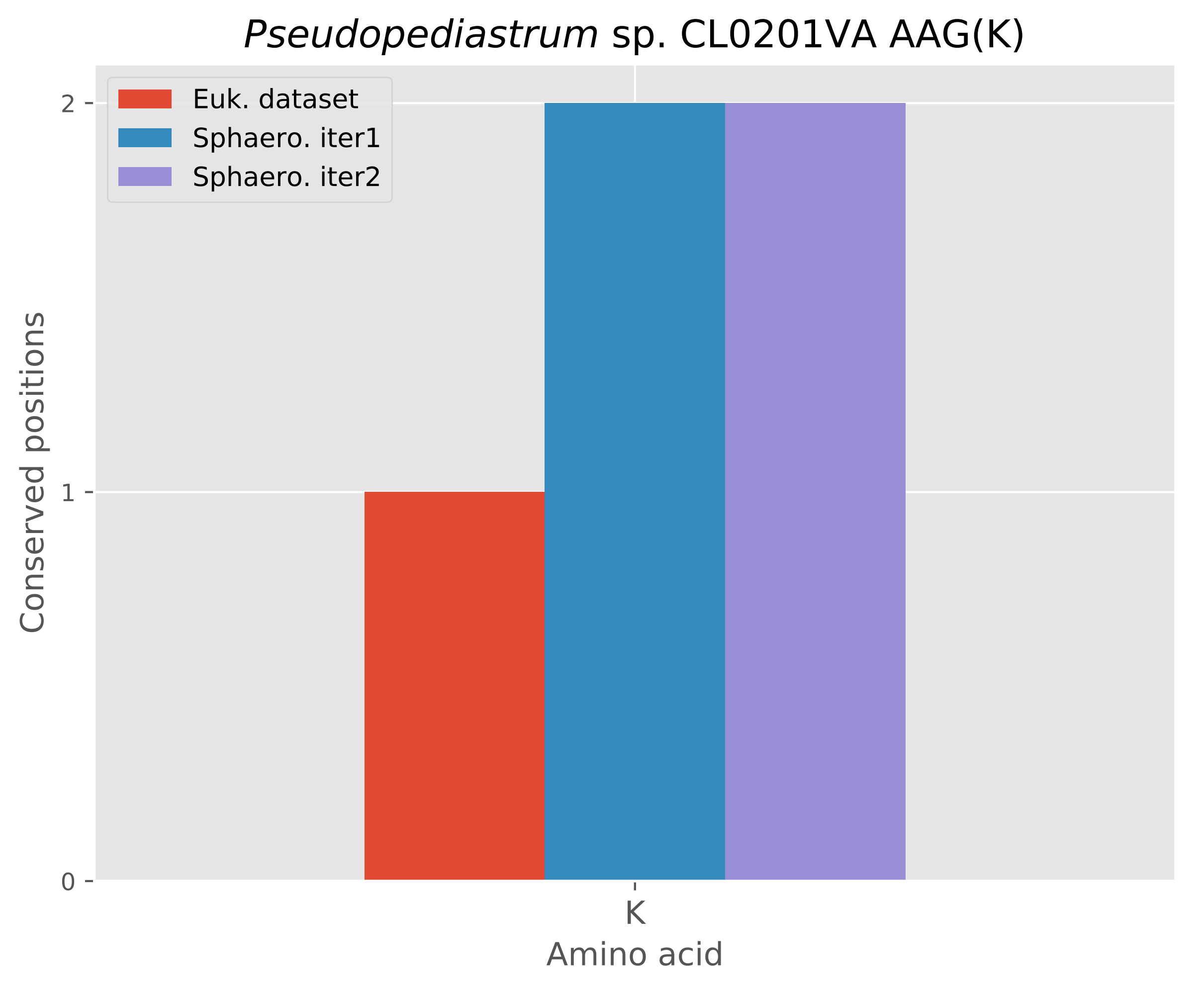

Pseudopediastrum sp. CL0201VA AAG(K)
Euk. dataset
Sphaero. iter1
Sphaero. iter2
Conserved positions
2
1
0
K
Amino acid

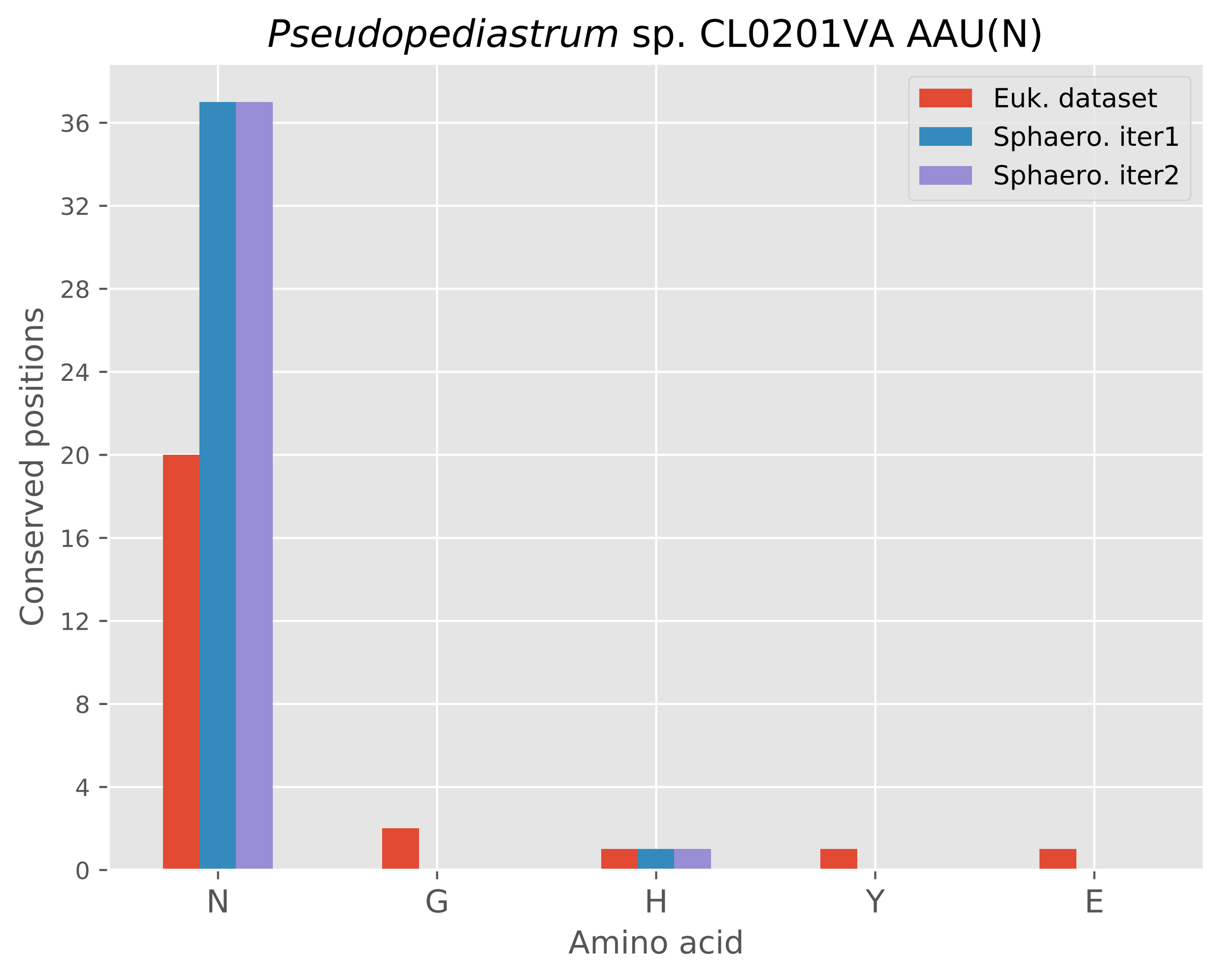
Pseudopediastrum sp. CL0201VA AAU(N)
Euk. dataset
Sphaero. iter1
Sphaero. iter2
Conserved positions
0
4
8
12
16
20
24
28
32
36
N
G
H
Y
E
Amino acid

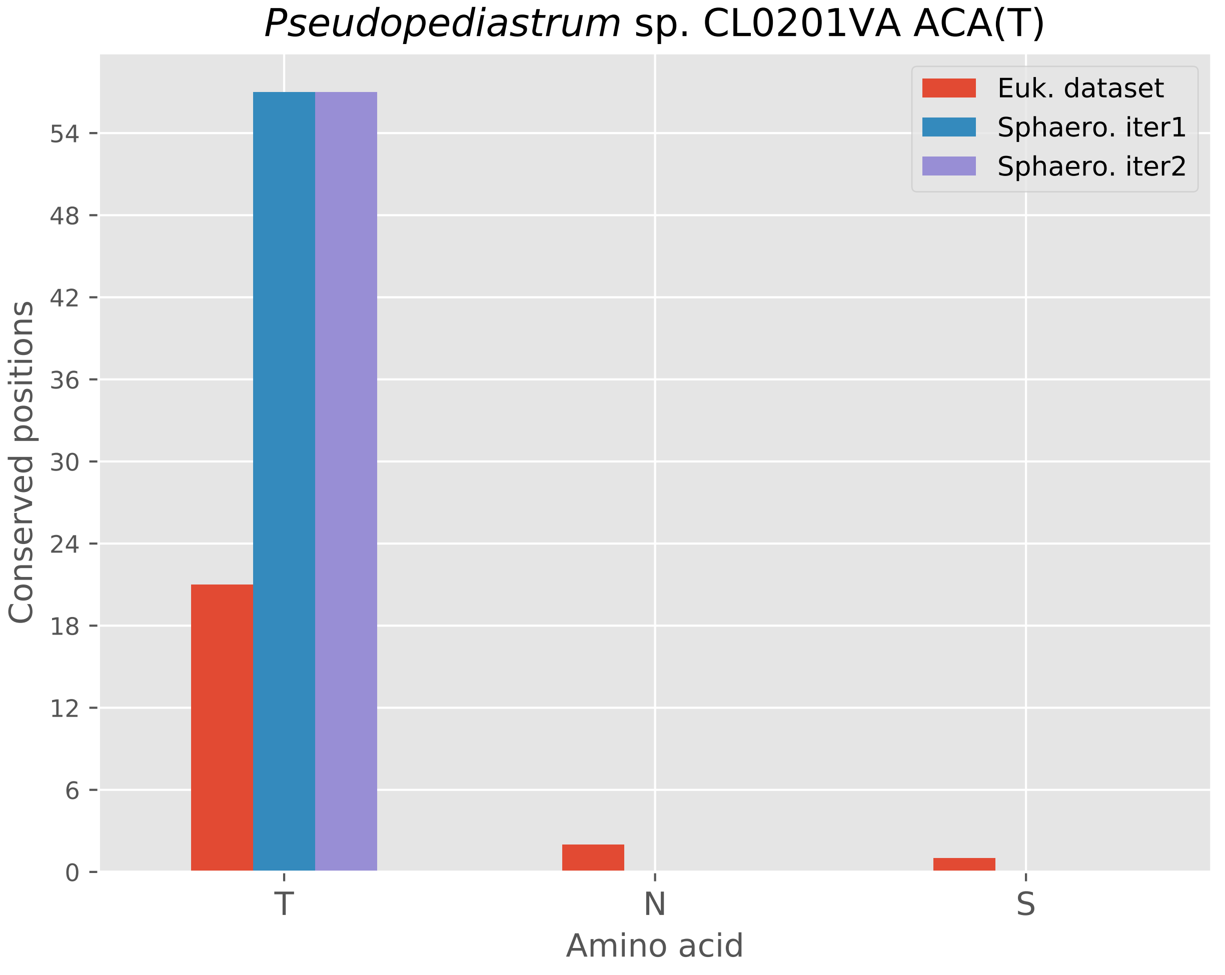

Pseudopediastrum sp. CL0201VA ACA(T)
Euk. dataset
Sphaero. iter1
Sphaero. iter2
Conserved positions
0
6
12
18
24
30
36
42
48
54
T
N
S
Amino acid

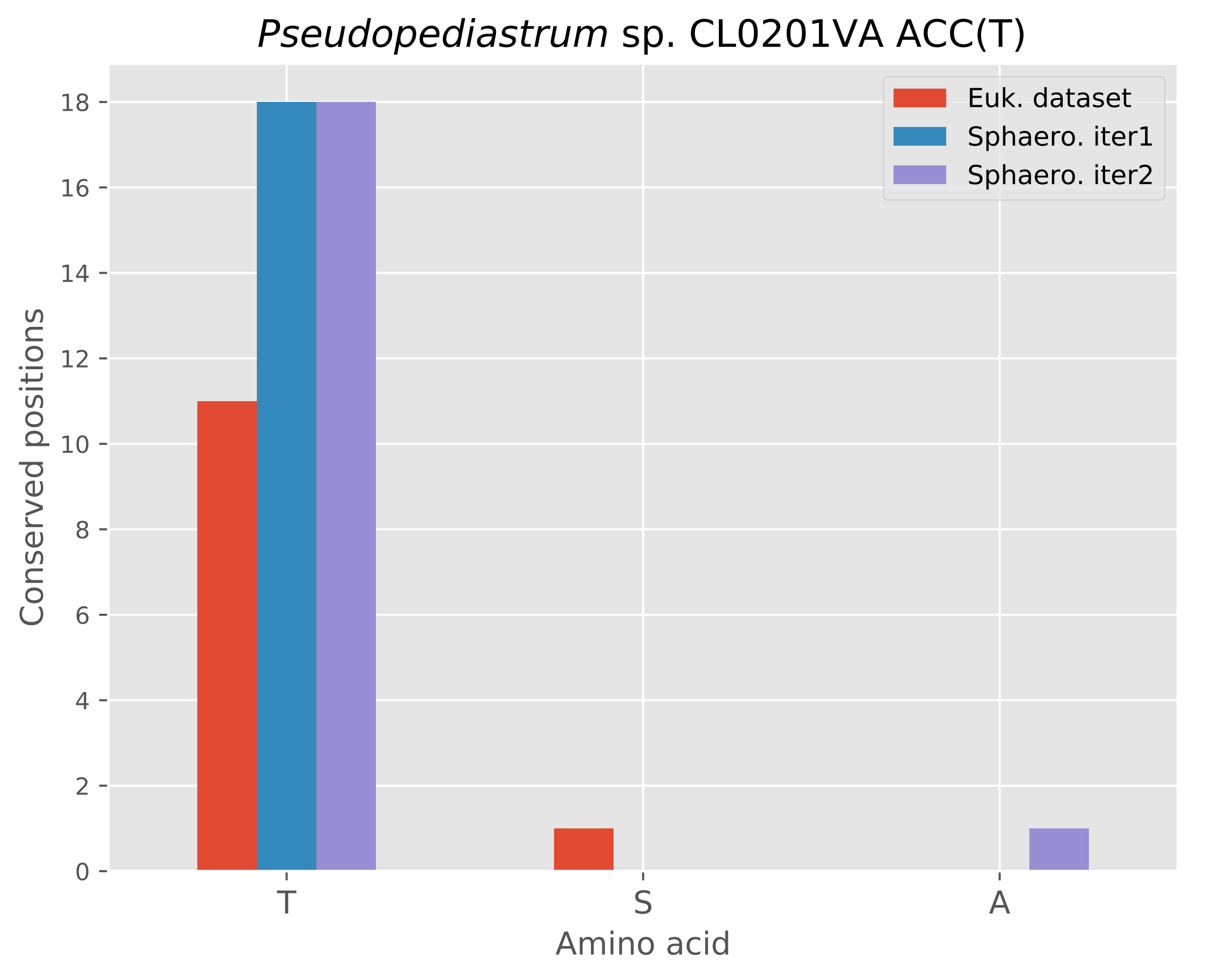

Pseudopediastrum sp. CL0201VA ACC(T)
Euk. dataset
Sphaero. iter1
Sphaero. iter2
Conserved positions
0
2
4
6
8
10
12
14
16
18
T
S
A
Amino acid

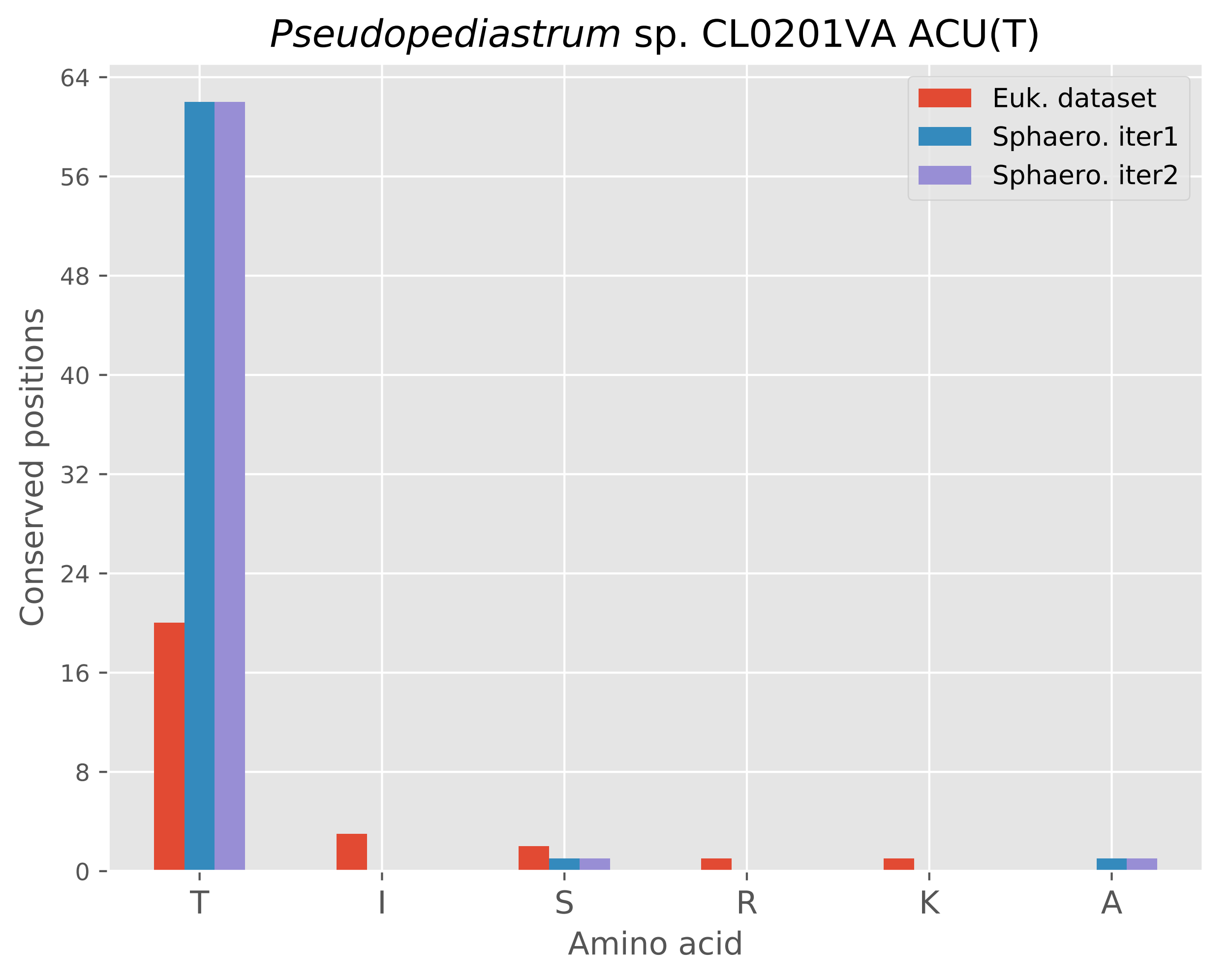

Pseudopediastrum sp. CL0201VA ACU(T)
Euk. dataset
Sphaero. iter1
Sphaero. iter2
Conserved positions
0
8
16
24
32
40
48
56
64
T
I
S
R
K
A
Amino acid

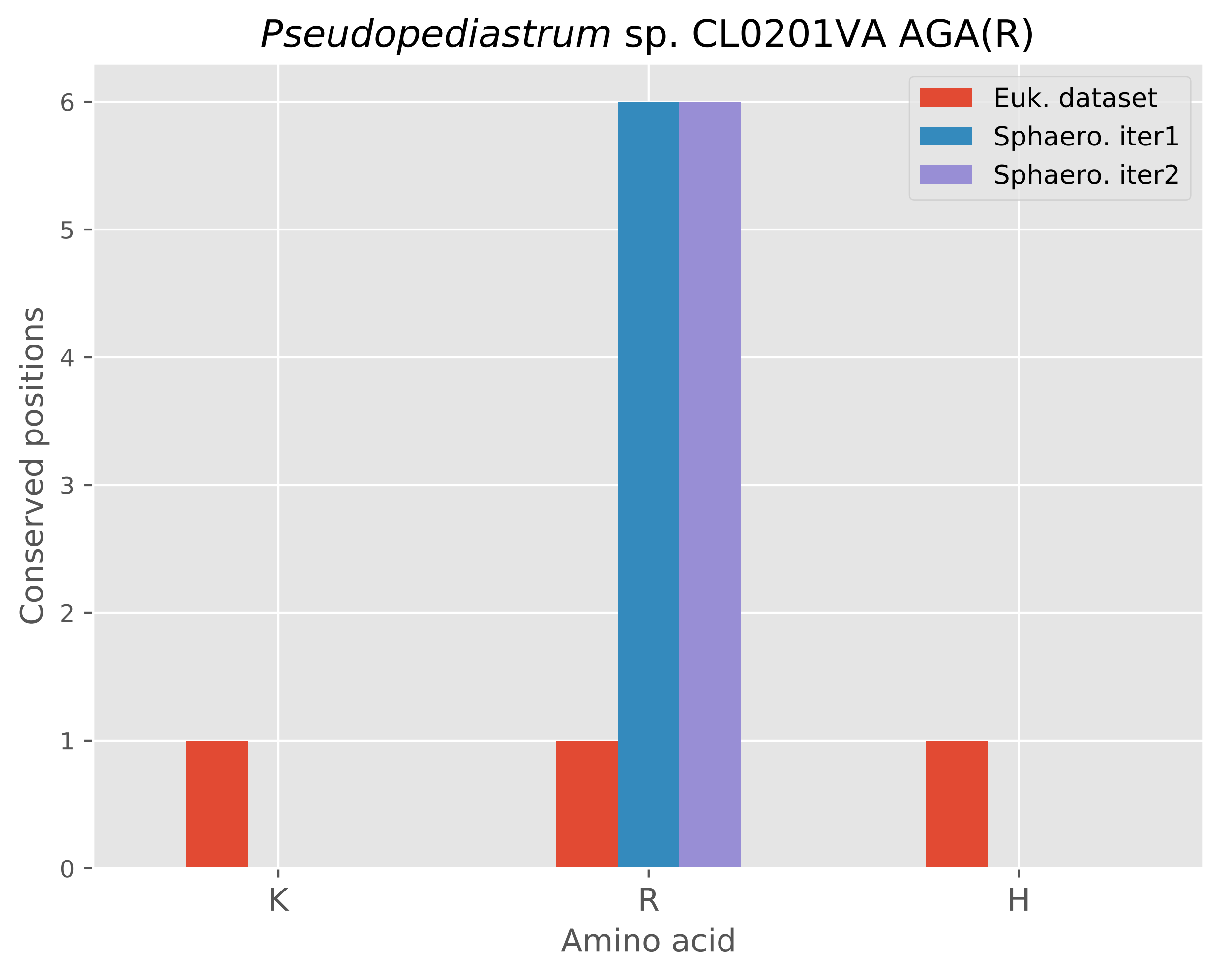

Pseudopediastrum sp. CL0201VA AGA(R)
Euk. dataset
Sphaero. iter1
Sphaero. iter2
Conserved positions
0
1
2
3
4
5
6
K
R
H
Amino acid

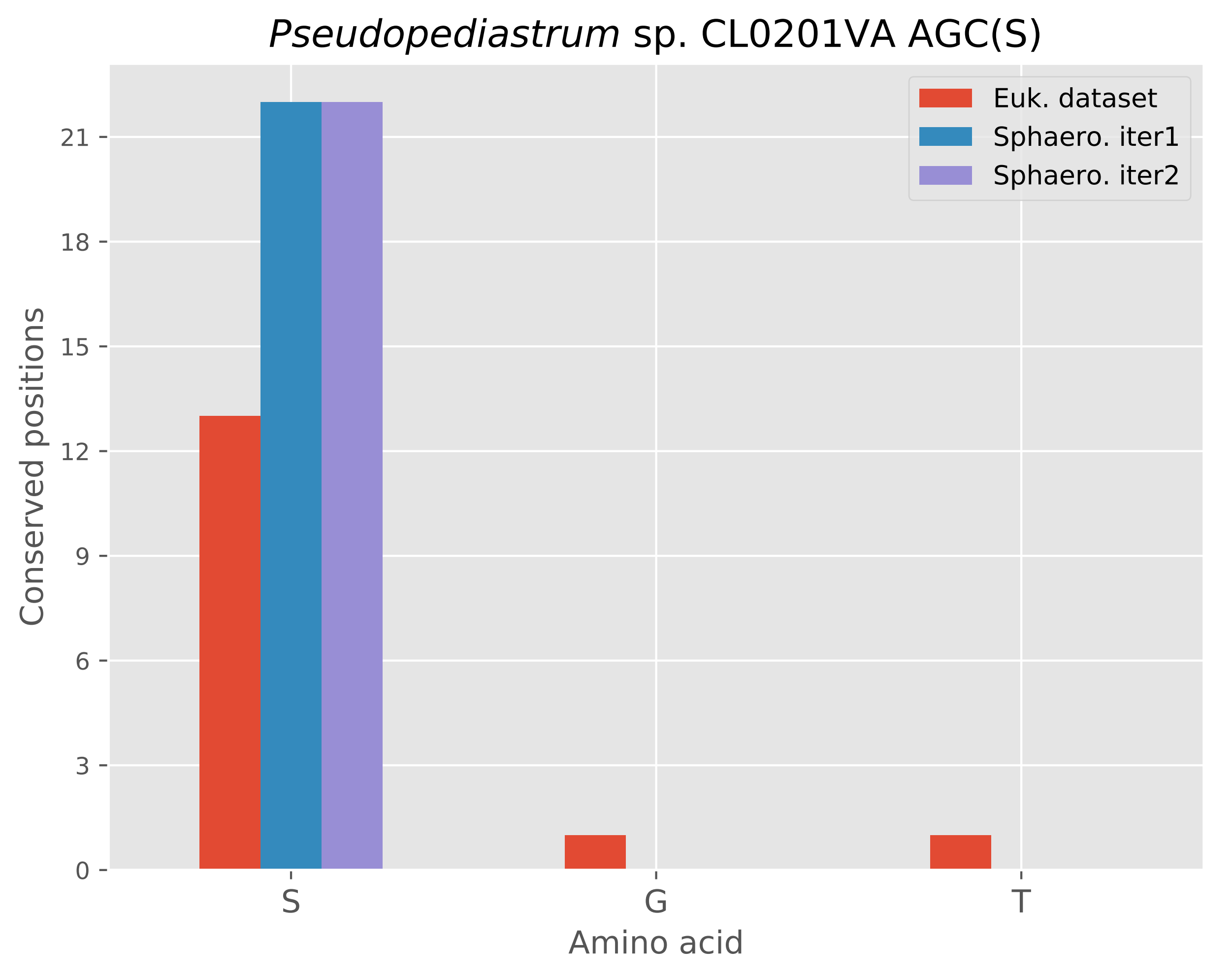

Pseudopediastrum sp. CL0201VA AGC(S)
Euk. dataset
Sphaero. iter1
Sphaero. iter2
Conserved positions
0
3
6
9
12
15
18
21
S
G
T
Amino acid

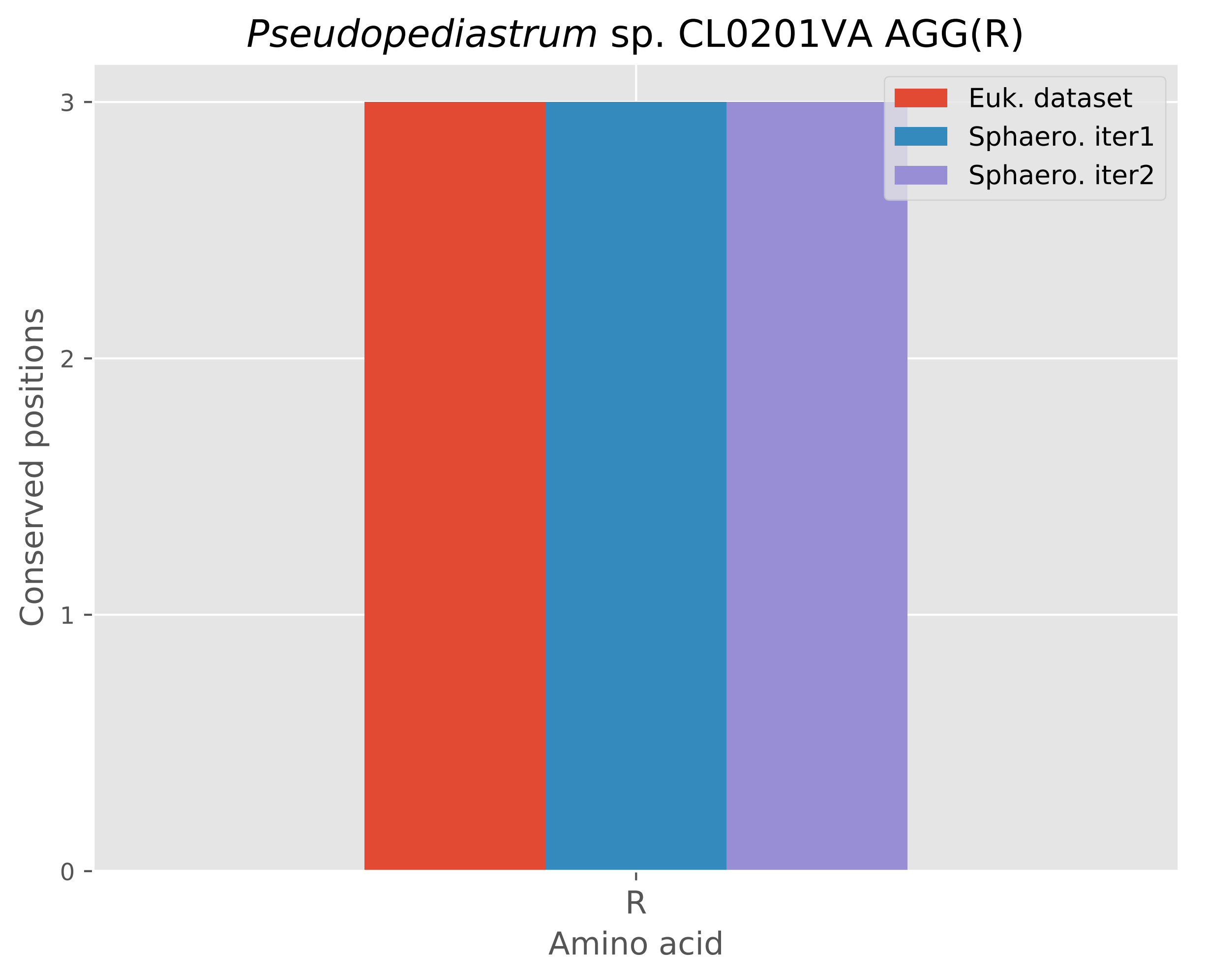
Pseudopediastrum sp. CL0201VA AGG(R)
Euk. dataset
Sphaero. iter1
Sphaero. iter2
Conserved positions
0
1
2
3
R
Amino acid

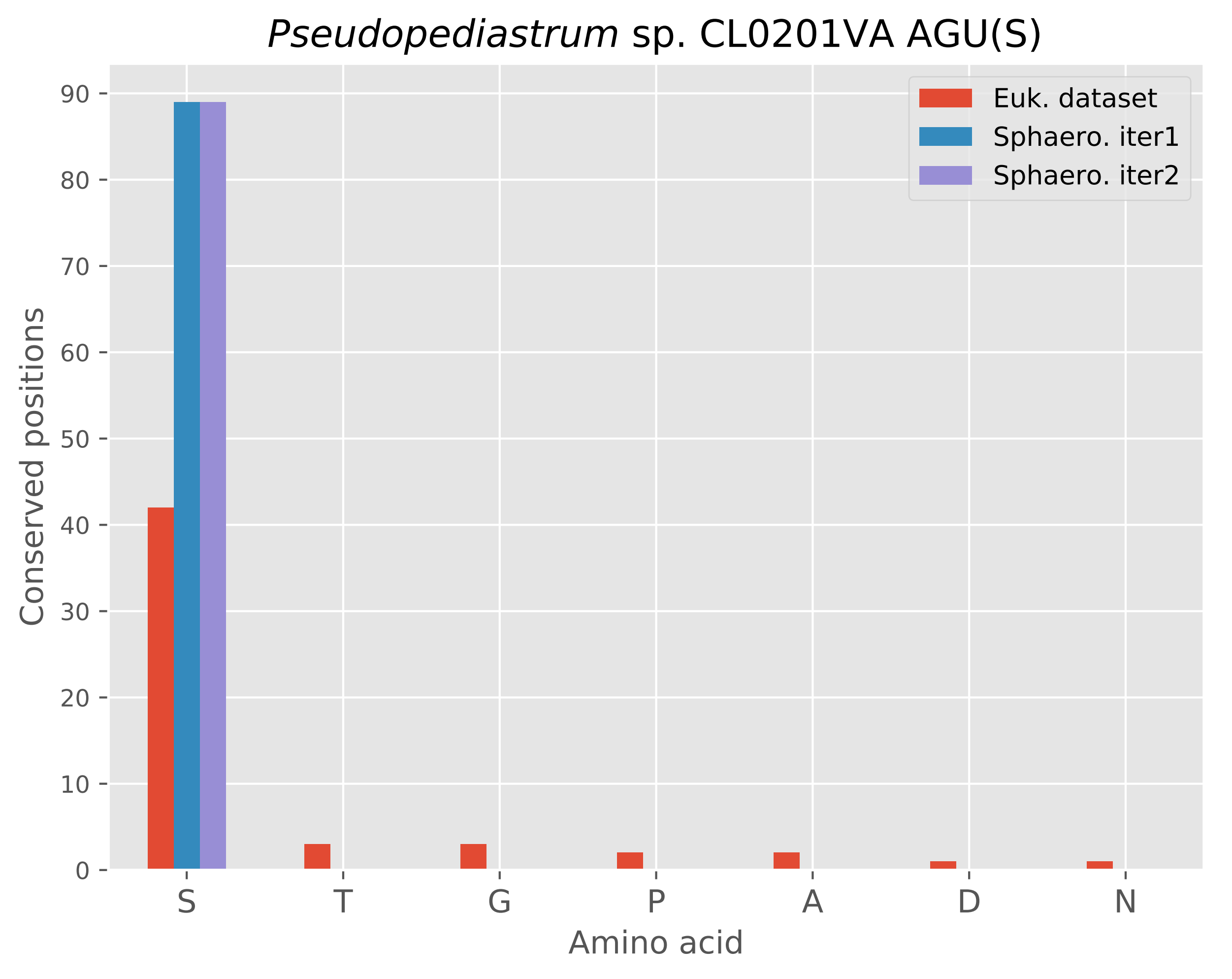

Pseudopediastrum sp. CL0201VA AGU(S)
Euk. dataset
Sphaero. iter1
Sphaero. iter2
Conserved positions
0
10
20
30
40
50
60
70
80
90
S
T
G
P
A
D
N
Amino acid

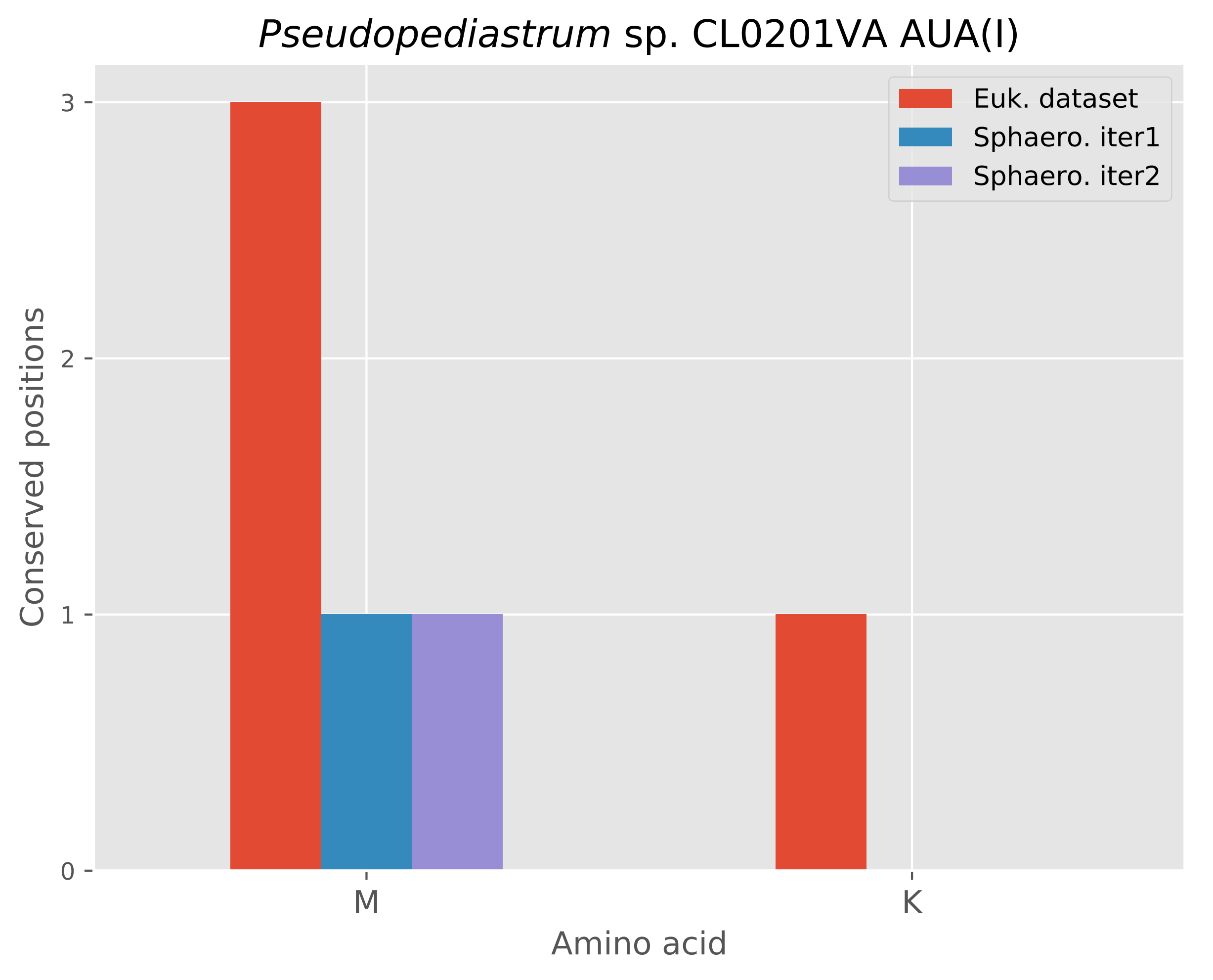

Pseudopediastrum sp. CL0201VA AUA(I)
Euk. dataset
Sphaero. iter1
Sphaero. iter2
Conserved positions
0
1
2
3
M
K
Amino acid

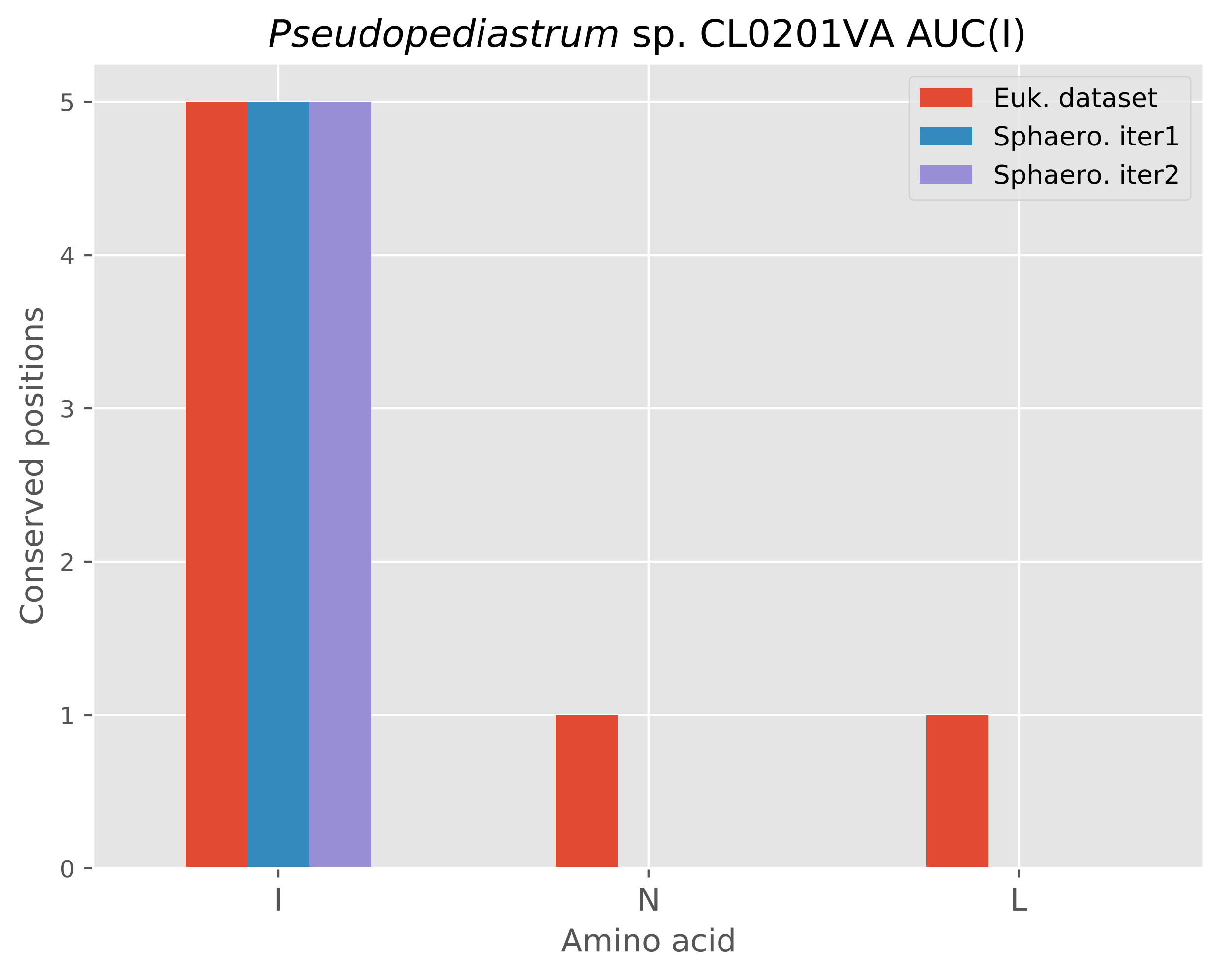

Pseudopediastrum sp. CL0201VA AUC(I)
Euk. dataset
Sphaero. iter1
Sphaero. iter2
Conserved positions
0
1
2
3
4
5
I
N
L
Amino acid

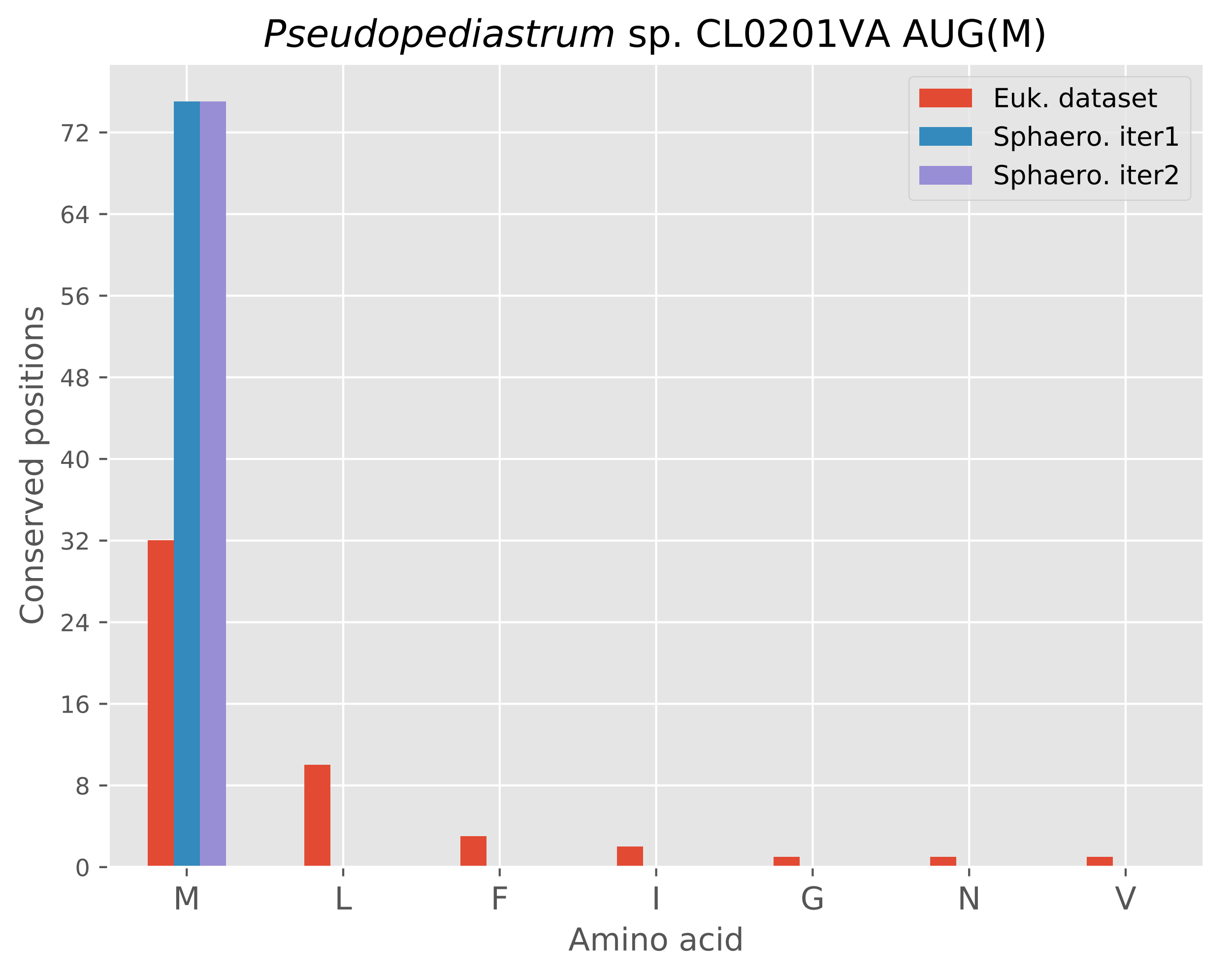

Pseudopediastrum sp. CL0201VA AUG(M)
Euk. dataset
Sphaero. iter1
Sphaero. iter2
Conserved positions
0
8
16
24
32
40
48
56
64
72
M
L
F
I
G
N
V
Amino acid

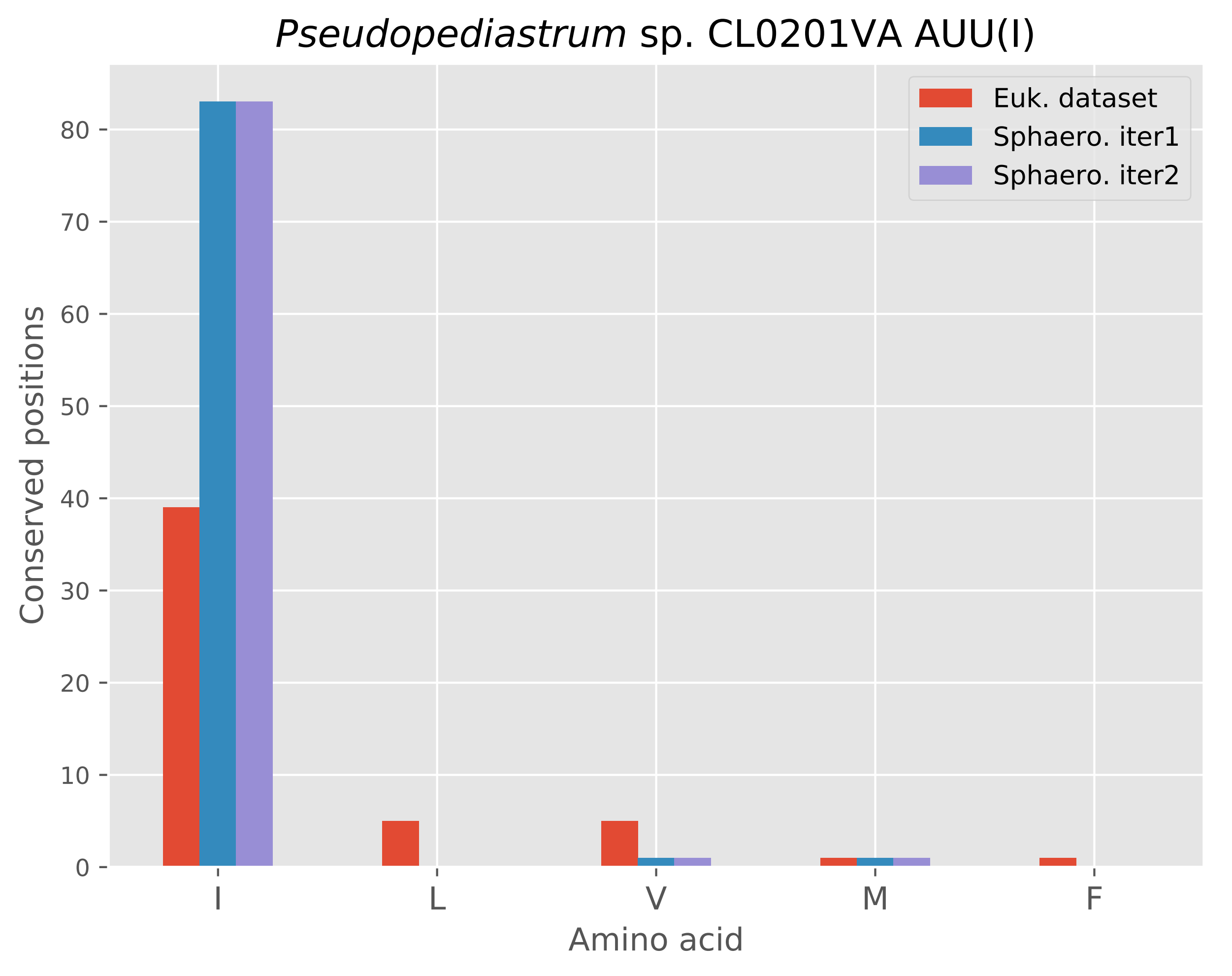

Pseudopediastrum sp. CL0201VA AUU(I)
Euk. dataset
Sphaero. iter1
Sphaero. iter2
Conserved positions
0
10
20
30
40
50
60
70
80
I
L
V
M
F
Amino acid

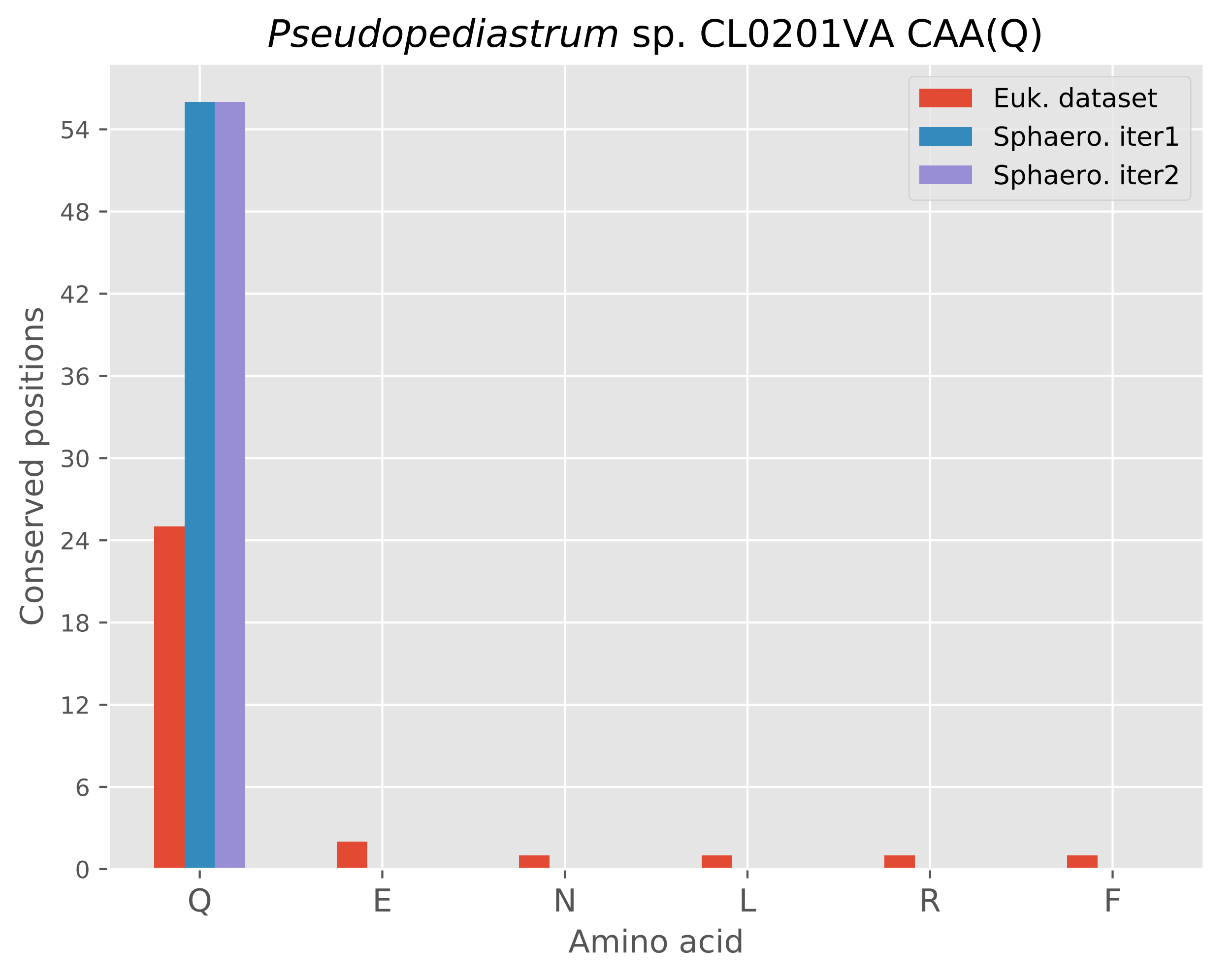

Pseudopediastrum sp. CL0201VA CAA(Q)
Euk. dataset
Sphaero. iter1
Sphaero. iter2
Conserved positions
0
6
12
18
24
30
36
42
48
54
Q
E
N
L
R
F
Amino acid

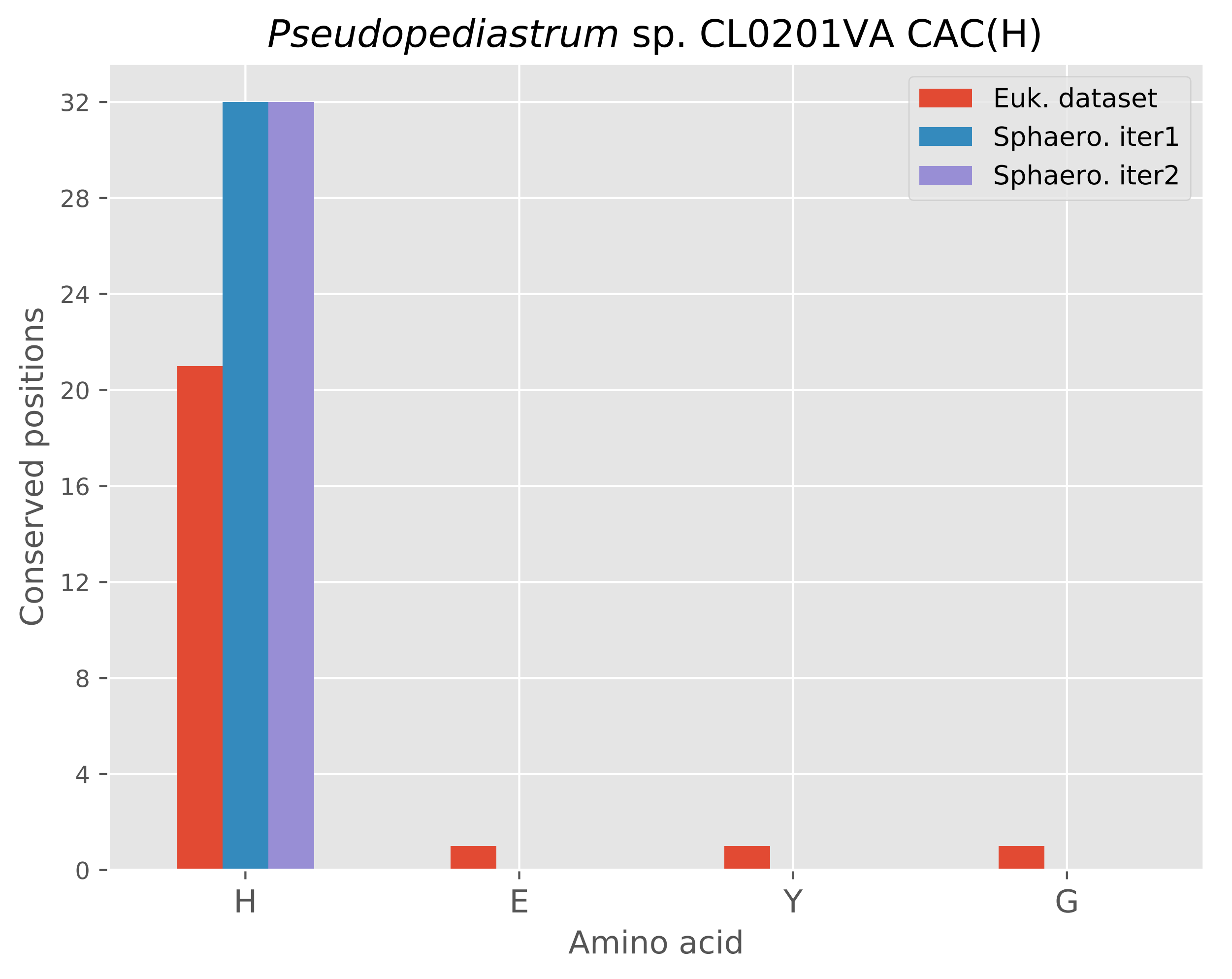

Pseudopediastrum sp. CL0201VA CAC(H)
Euk. dataset
Sphaero. iter1
Sphaero. iter2
Conserved positions
0
4
8
12
16
20
24
28
32
H
E
Y
G
Amino acid

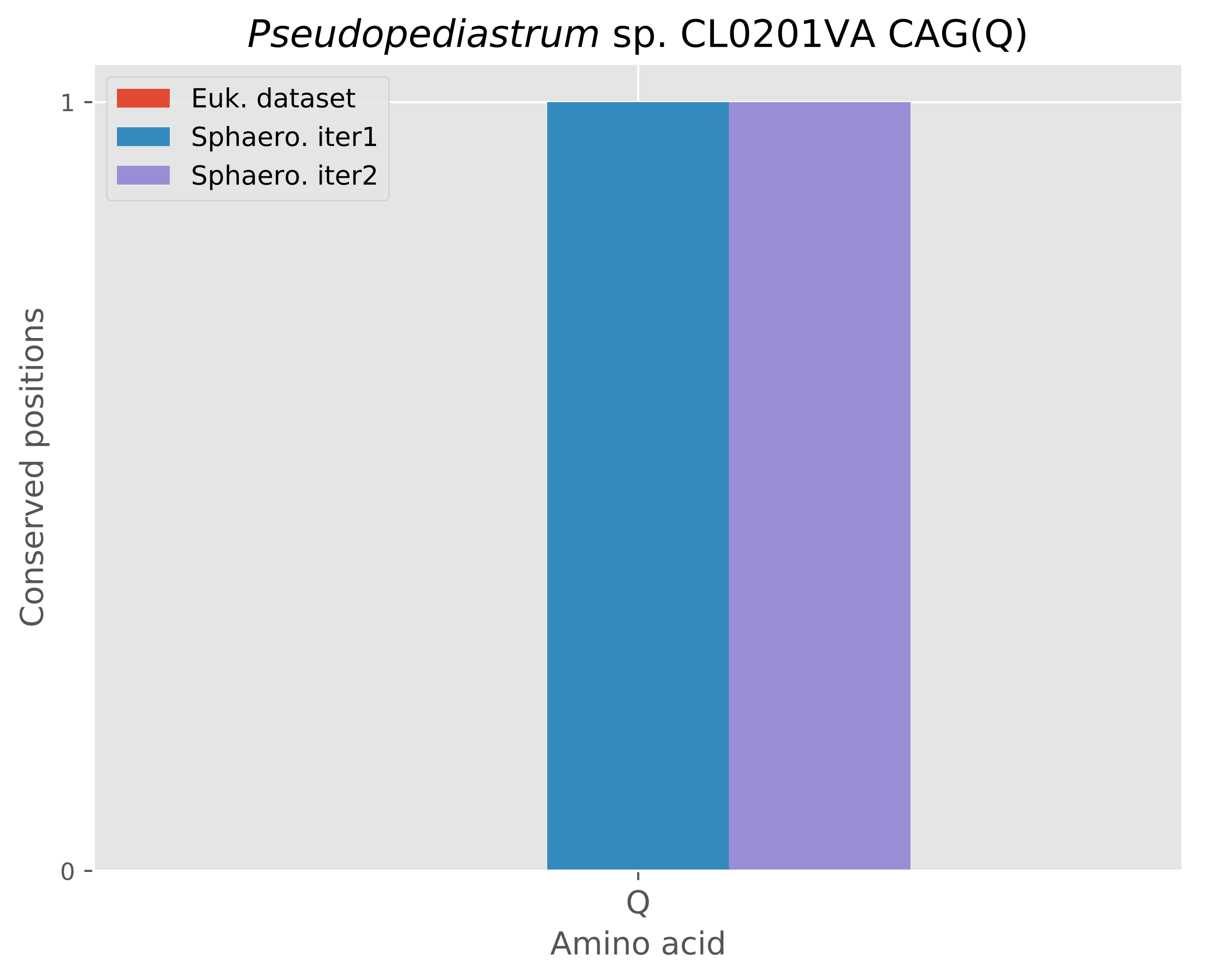

Pseudopediastrum sp. CL0201VA CAG(Q)
Euk. dataset
Sphaero. iter1
Sphaero. iter2
1
0
Conserved positions
Q
Amino acid

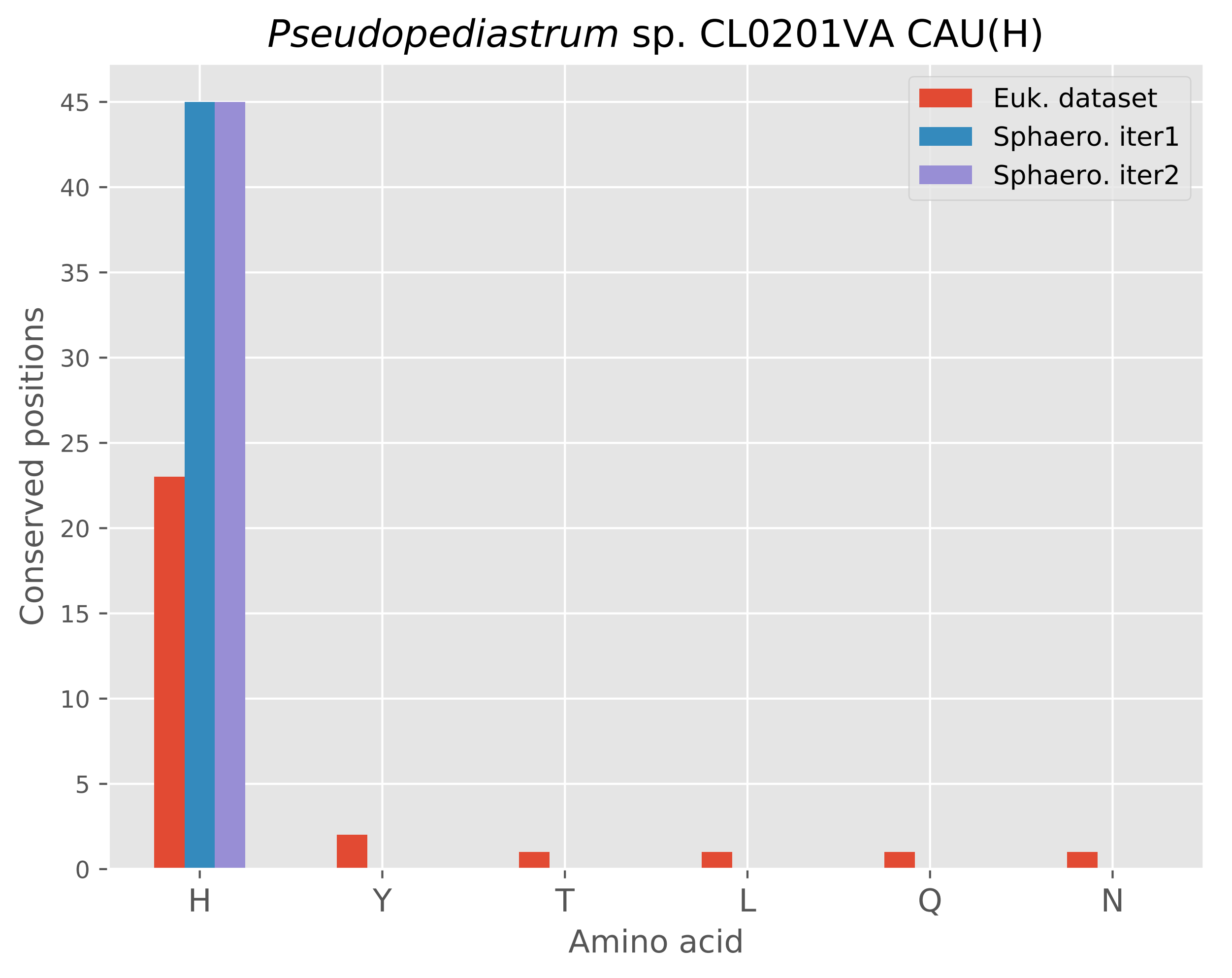

Pseudopediastrum sp. CL0201VA CAU(H)
Euk. dataset
Sphaero. iter1
Sphaero. iter2
Conserved positions
0
5
10
15
20
25
30
35
40
45
H
Y
T
L
Q
N
Amino acid

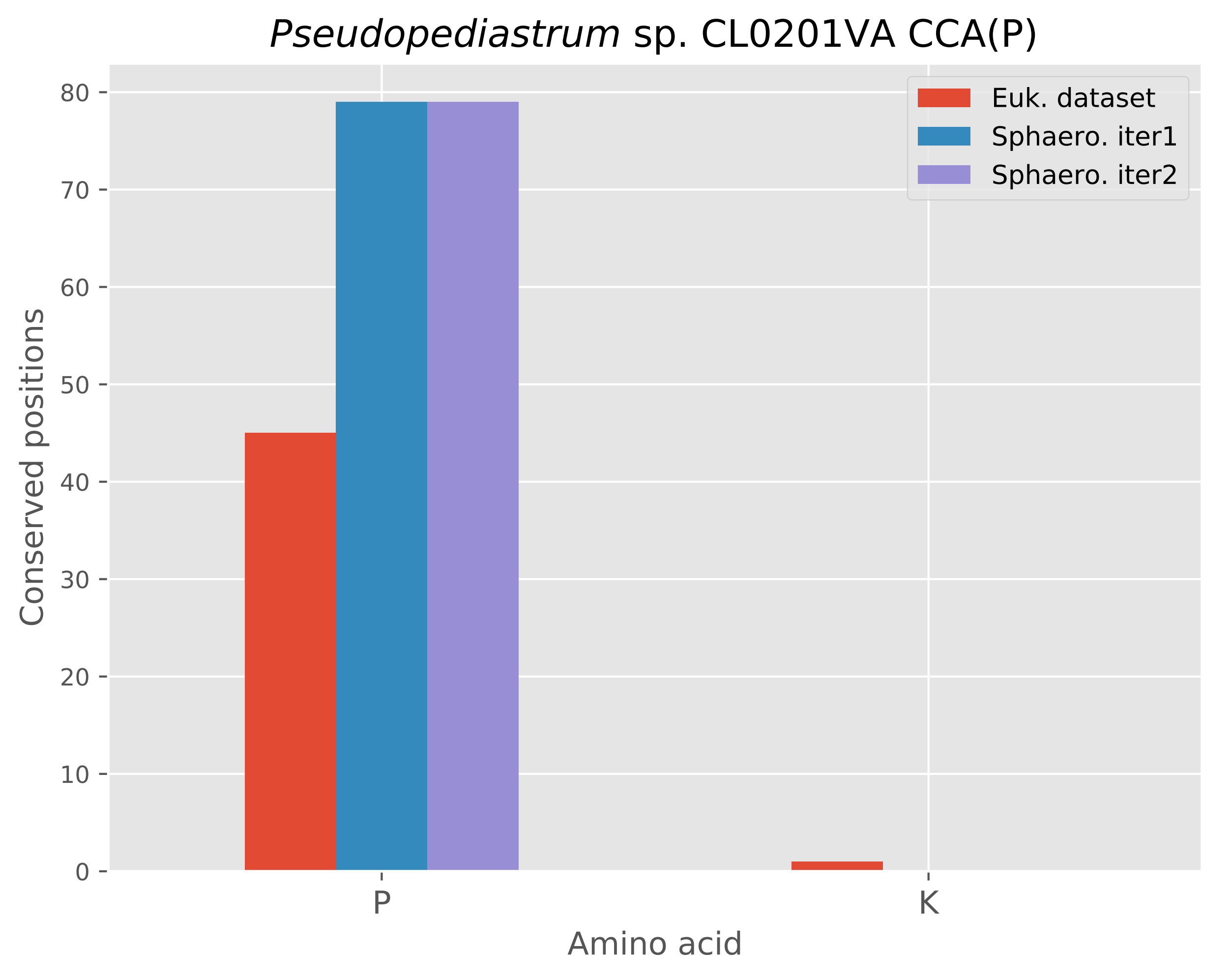
Pseudopediastrum sp. CL0201VA CCA(P)
Euk. dataset
Sphaero. iter1
Sphaero. iter2
Conserved positions
0
10
20
30
40
50
60
70
80
P
K
Amino acid

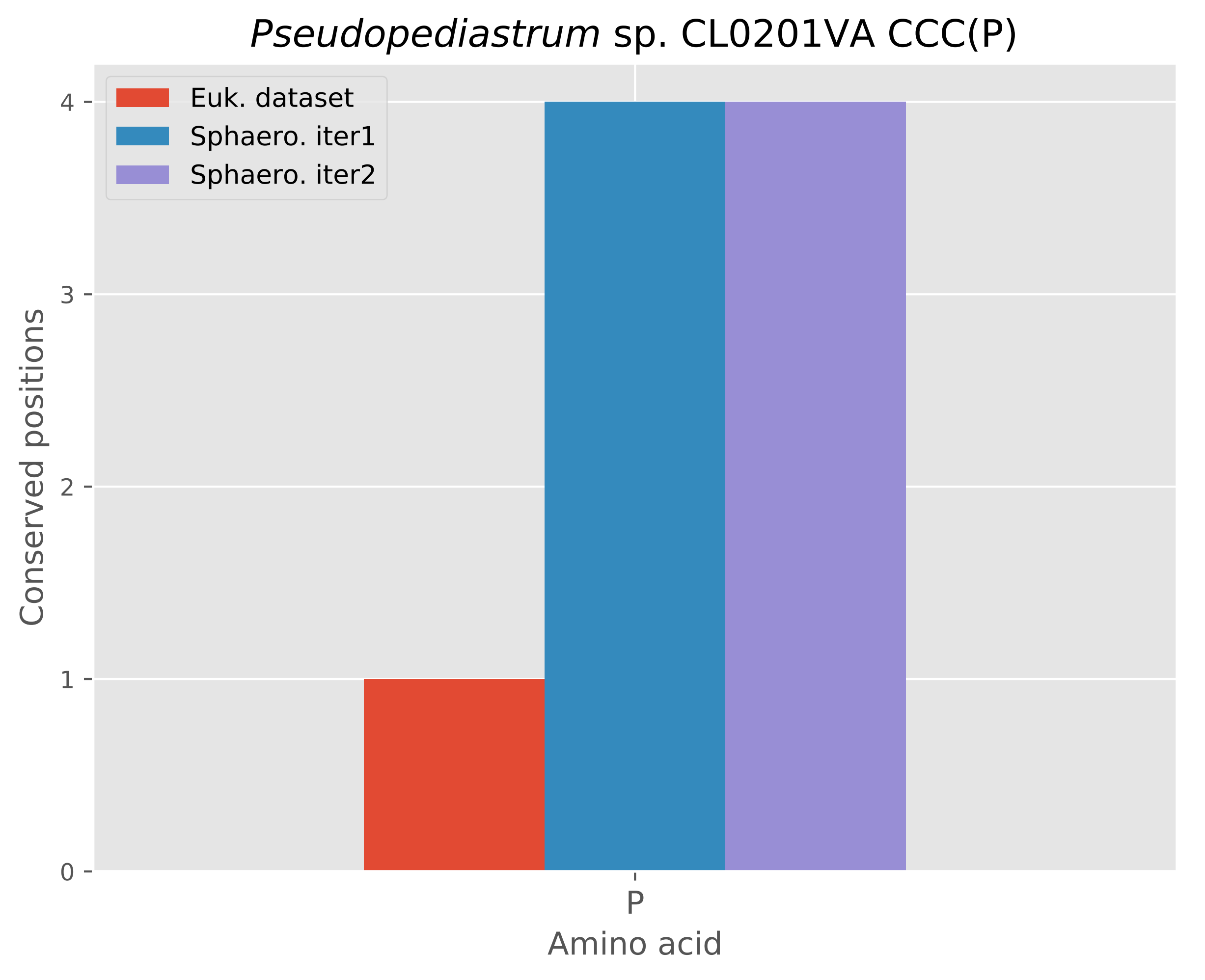

Pseudopediastrum sp. CL0201VA CCC(P)
Euk. dataset
Sphaero. iter1
Sphaero. iter2
4
3
2
1
0
Conserved positions
P
Amino acid

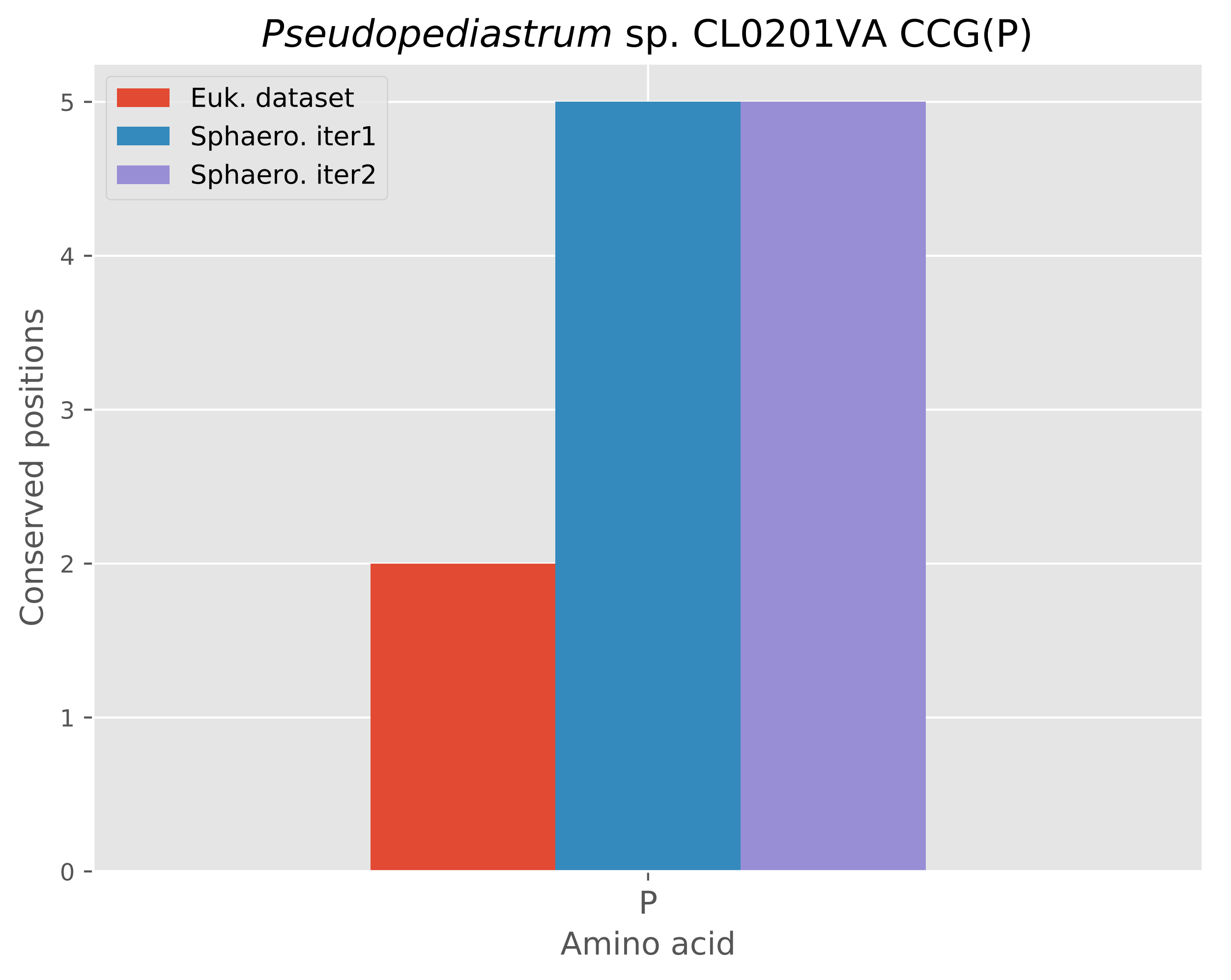
Pseudopediastrum sp. CL0201VA CCG(P)
Euk. dataset
Sphaero. iter1
Sphaero. iter2
Conserved positions
0
1
2
3
4
5
P
Amino acid

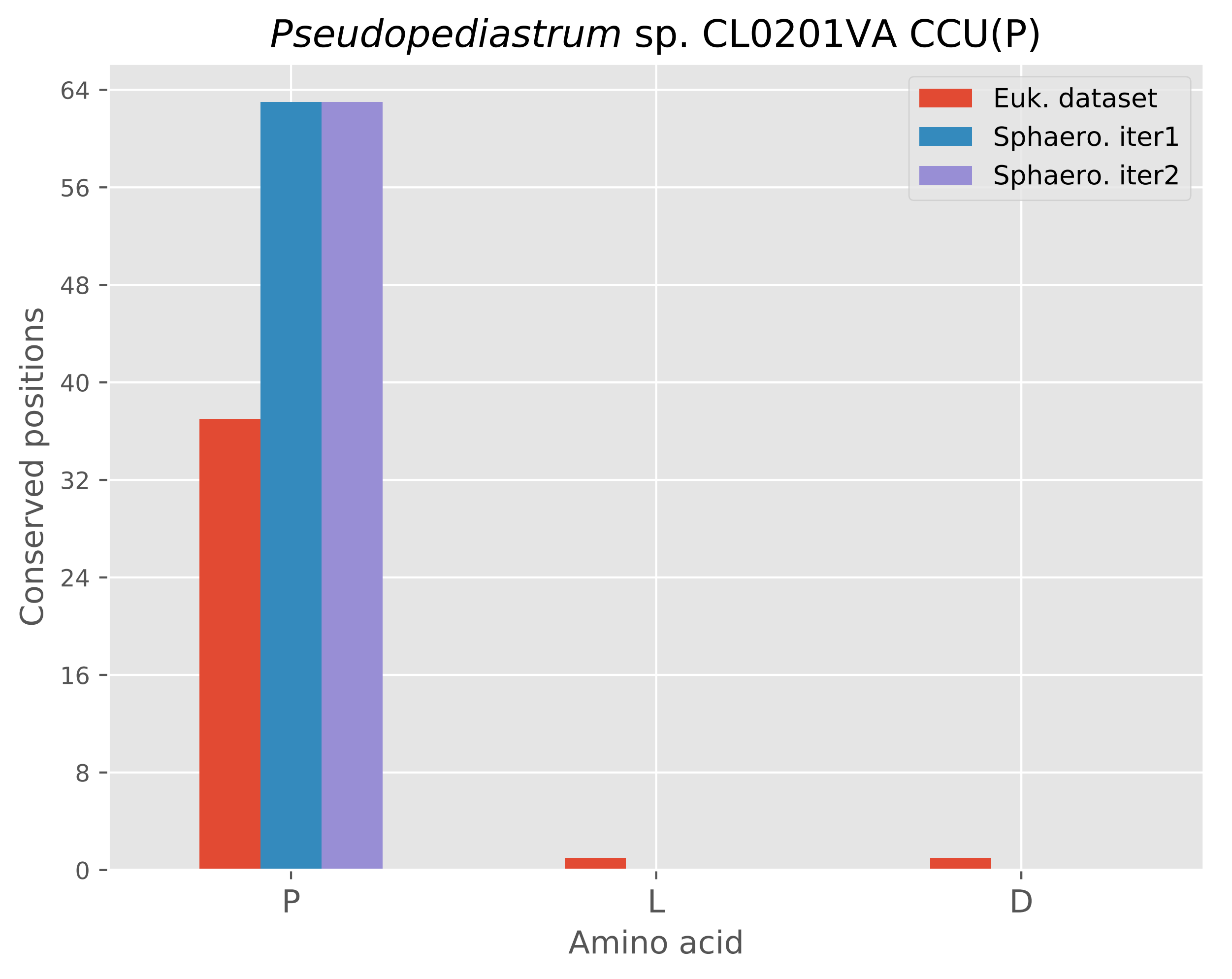

Pseudopediastrum sp. CL0201VA CCU(P)
Euk. dataset
Sphaero. iter1
Sphaero. iter2
Conserved positions
0
8
16
24
32
40
48
56
64
P
L
D
Amino acid

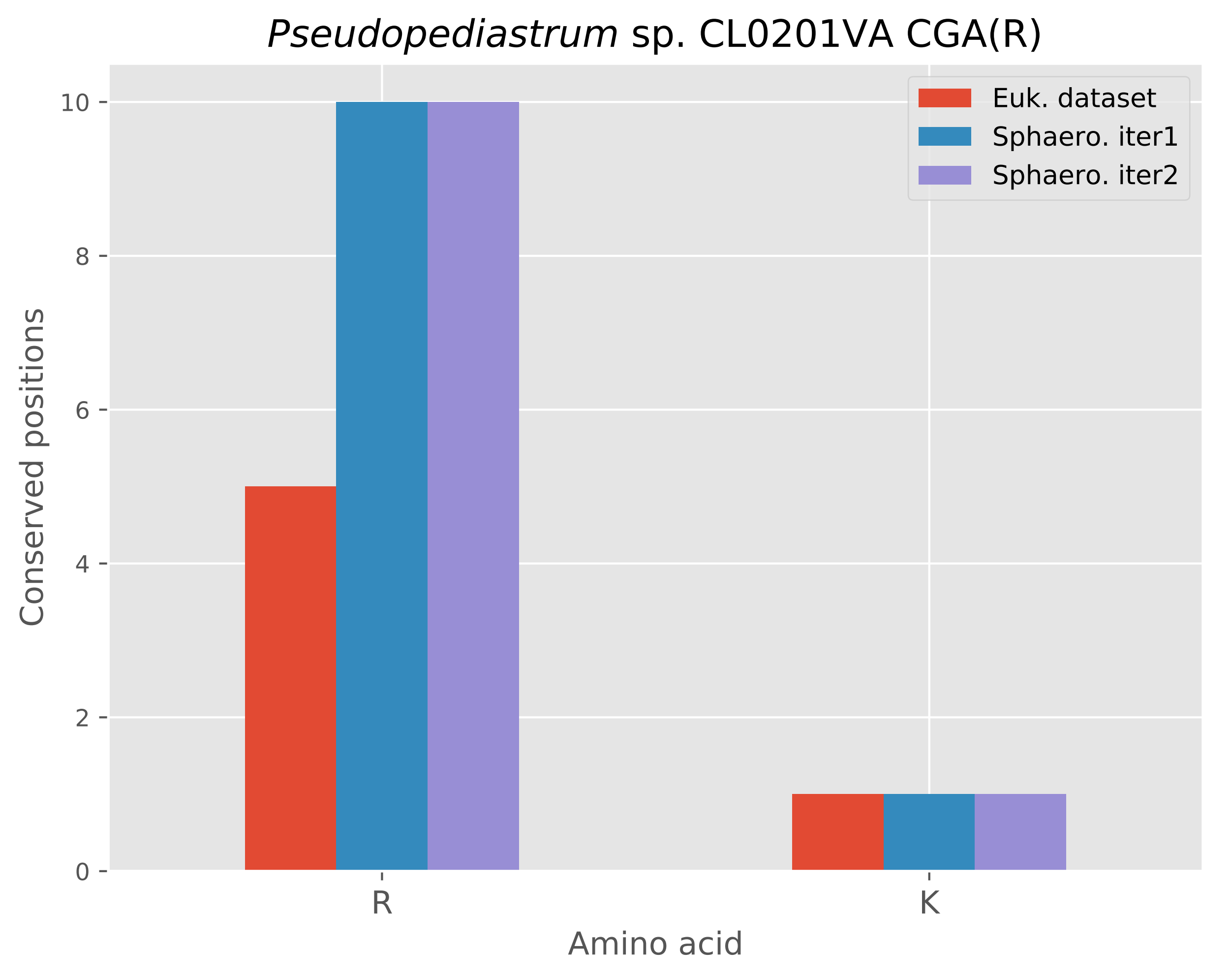

Pseudopediastrum sp. CL0201VA CGA(R)
Euk. dataset
Sphaero. iter1
Sphaero. iter2
Conserved positions
0
2
4
6
8
10
R
K
Amino acid

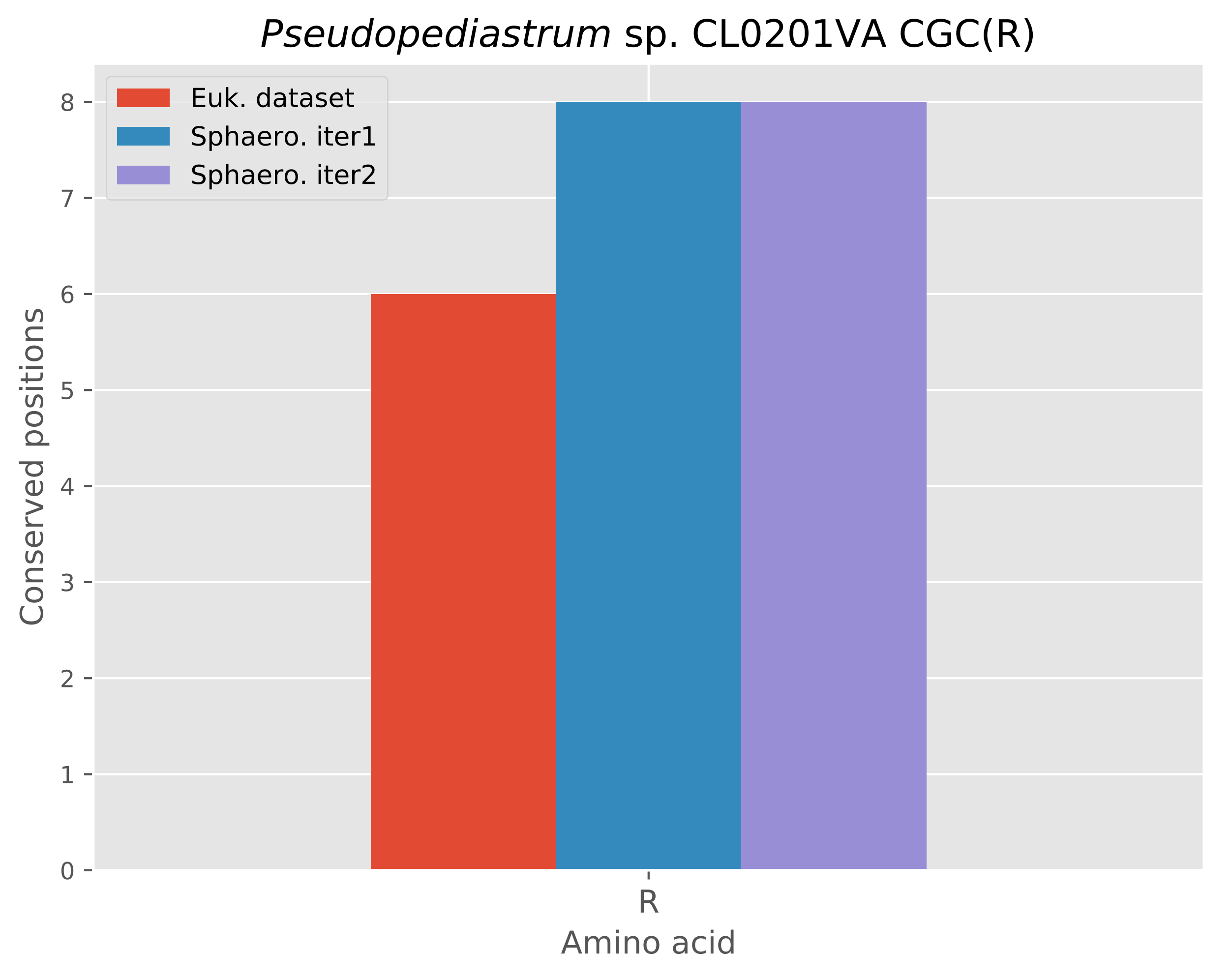

Pseudopediastrum sp. CL0201VA CGC(R)
Euk. dataset
Sphaero. iter1
Sphaero. iter2
Conserved positions
0
1
2
3
4
5
6
7
8
R
Amino acid

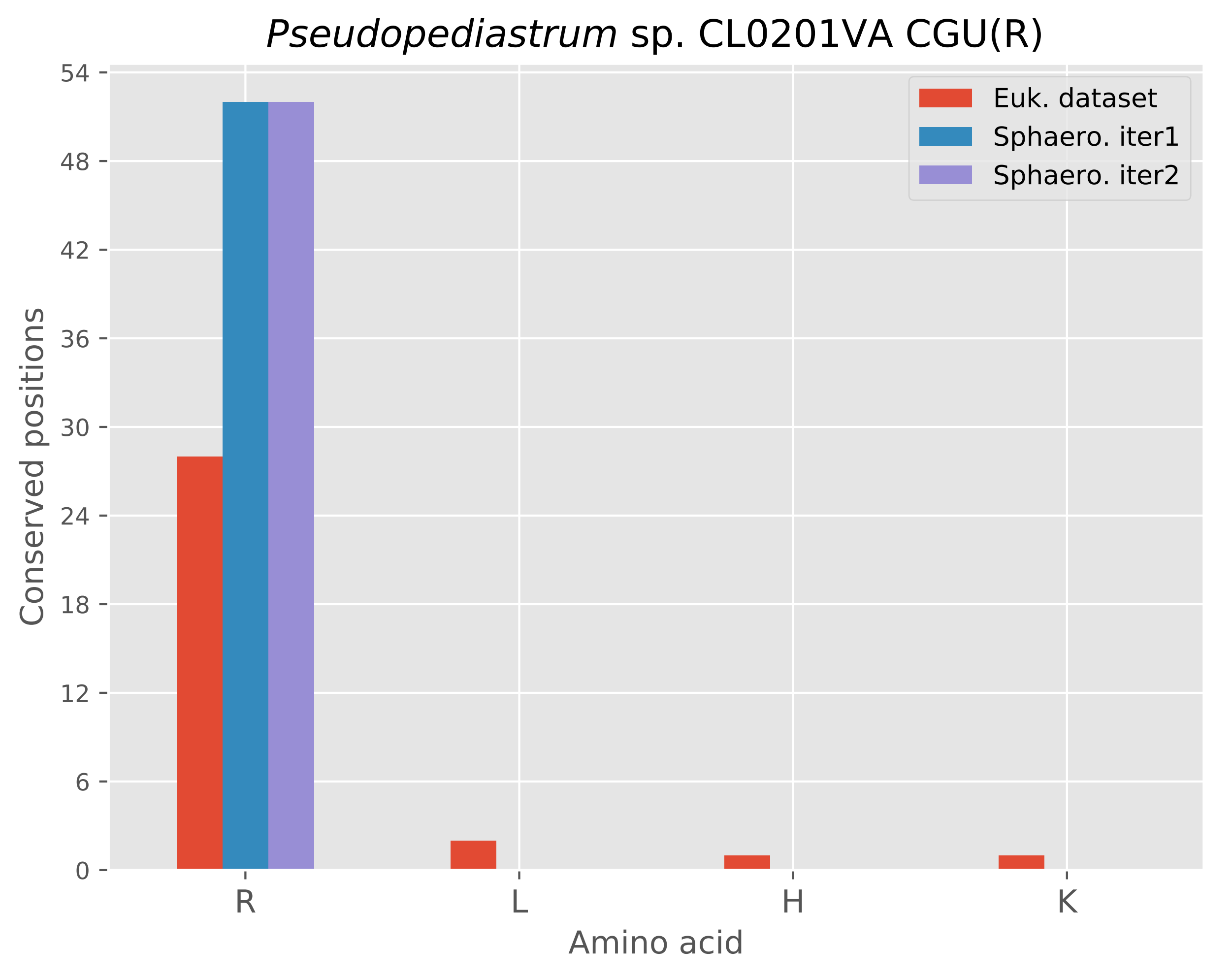

Pseudopediastrum sp. CL0201VA CGU(R)
Euk. dataset
Sphaero. iter1
Sphaero. iter2
Conserved positions
0
6
12
18
24
30
36
42
48
54
R
L
H
K
Amino acid

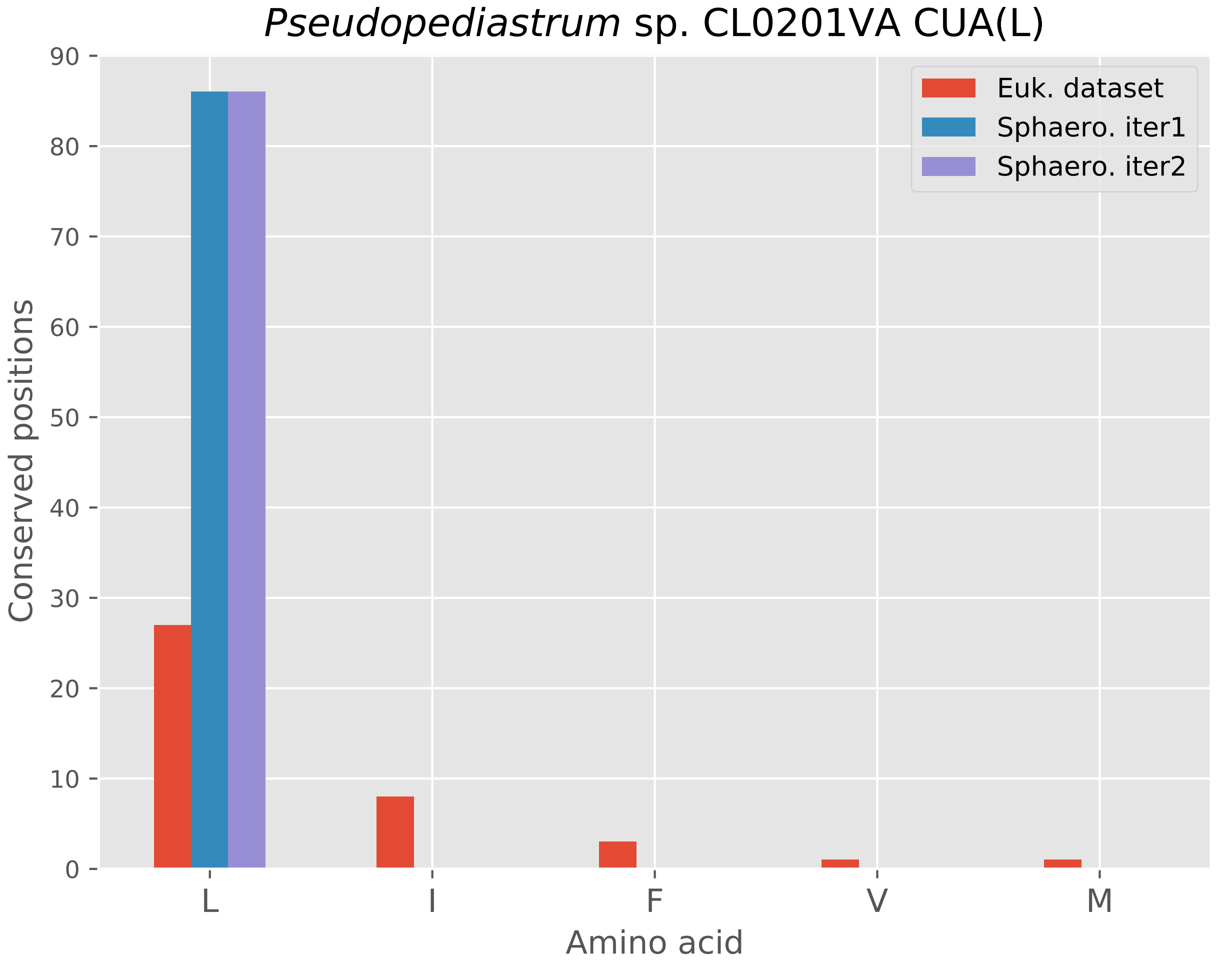

Pseudopediastrum sp. CL0201VA CUA(L)
Euk. dataset
Sphaero. iter1
Sphaero. iter2
Conserved positions
0
10
20
30
40
50
60
70
80
90
L
I
F
V
M
Amino acid

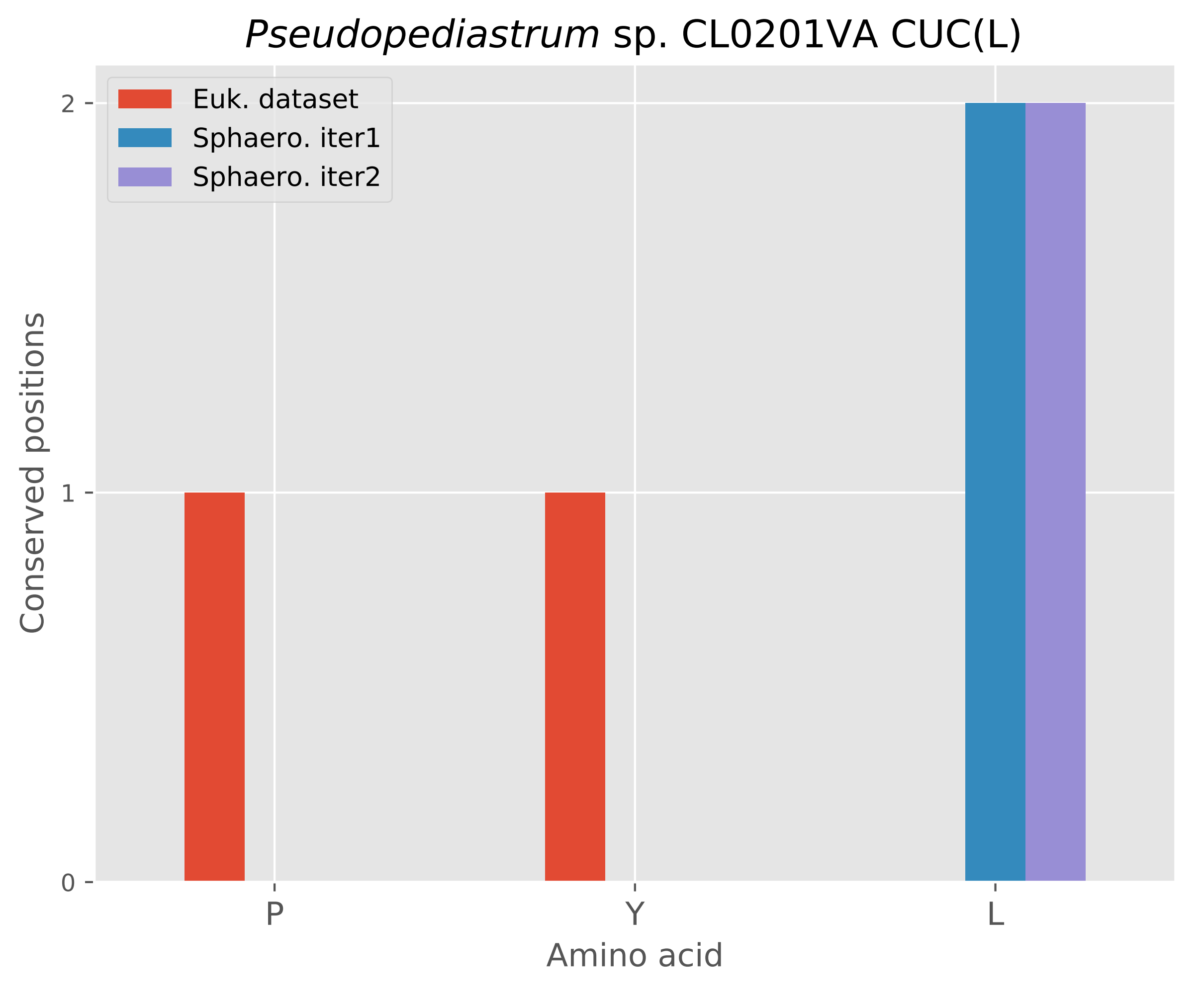

Pseudopediastrum sp. CL0201VA CUC(L)
Euk. dataset
Sphaero. iter1
Sphaero. iter2
Conserved positions
2
1
0
P
Y
L
Amino acid

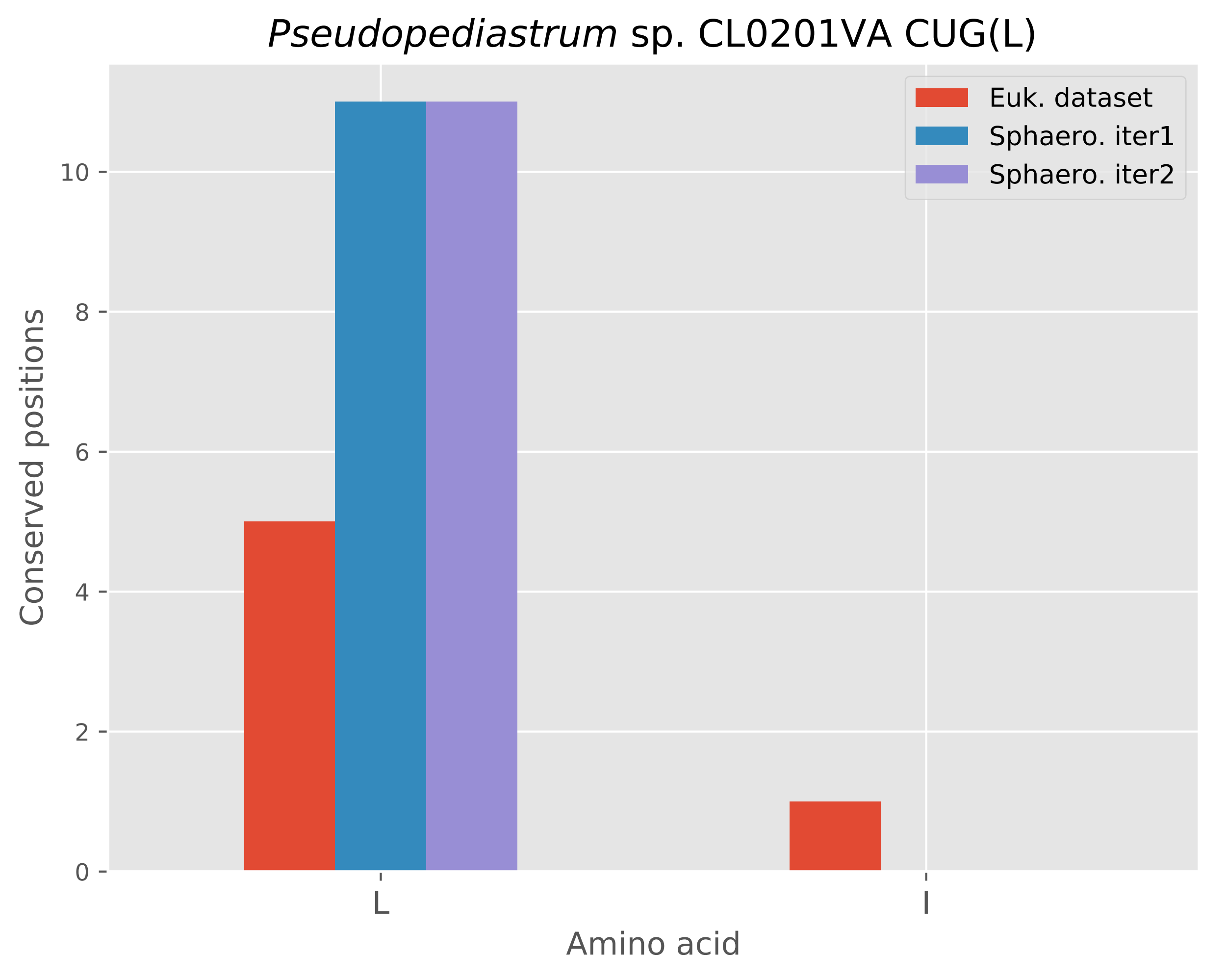
Pseudopediastrum sp. CL0201VA CUG(L)
Euk. dataset
Sphaero. iter1
Sphaero. iter2
Conserved positions
0
2
4
6
8
10
L
I
Amino acid

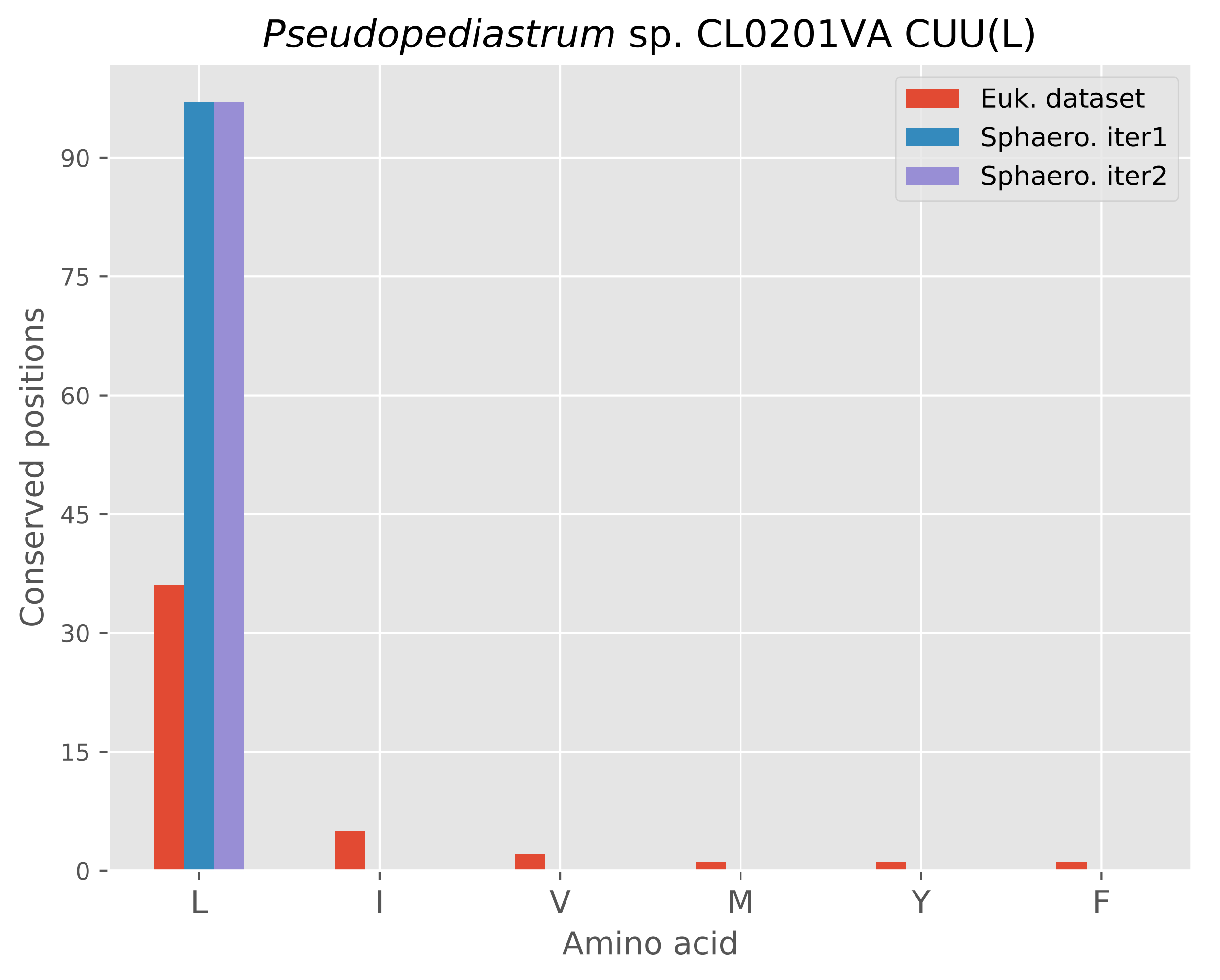

Pseudopediastrum sp. CL0201VA CUU(L)
Euk. dataset
Sphaero. iter1
Sphaero. iter2
Conserved positions
0
15
30
45
60
75
90
L
I
V
M
Y
F
Amino acid

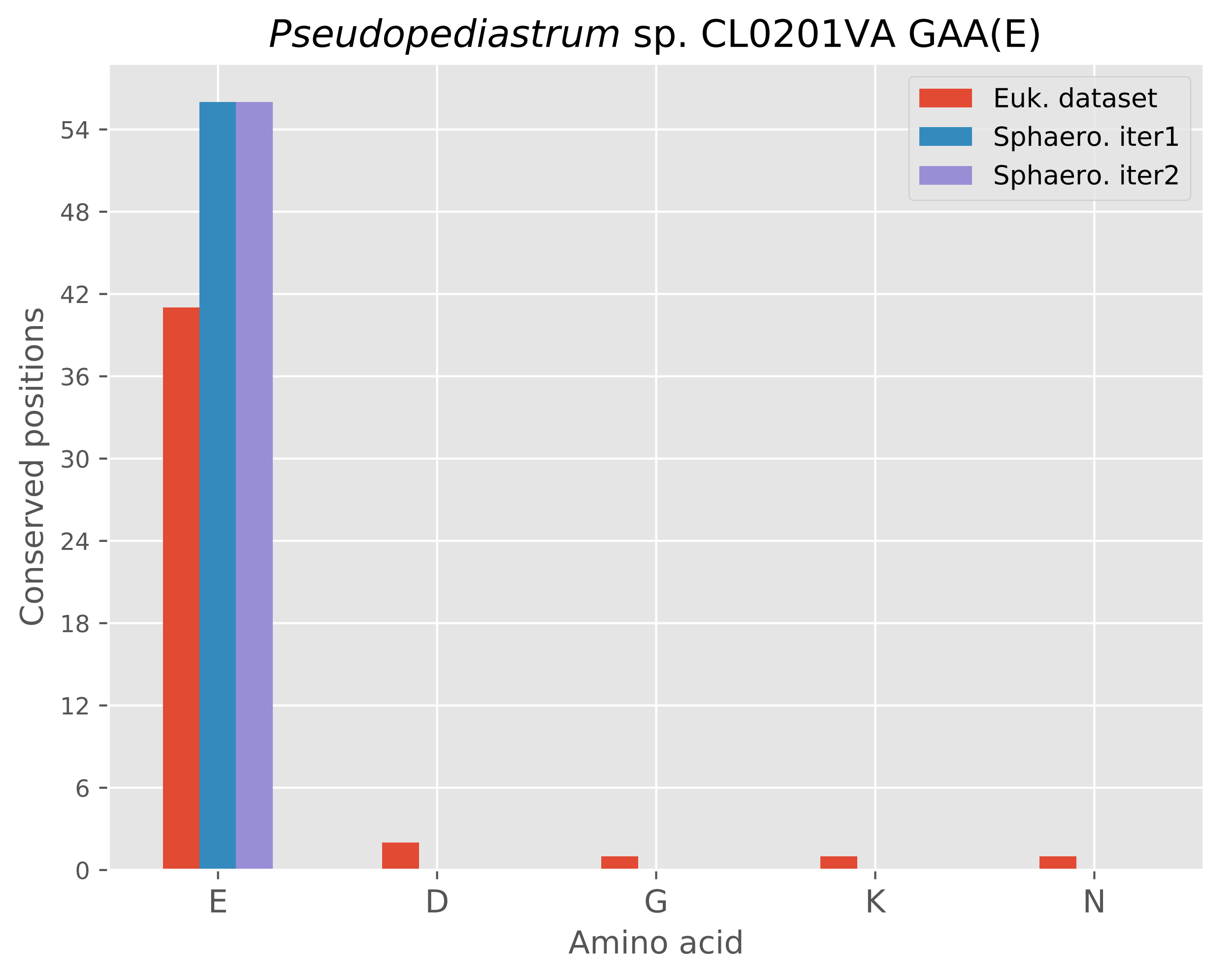

Pseudopediastrum sp. CL0201VA GAA(E)
Euk. dataset
Sphaero. iter1
Sphaero. iter2
Conserved positions
0
6
12
18
24
30
36
42
48
54
E
D
G
K
N
Amino acid

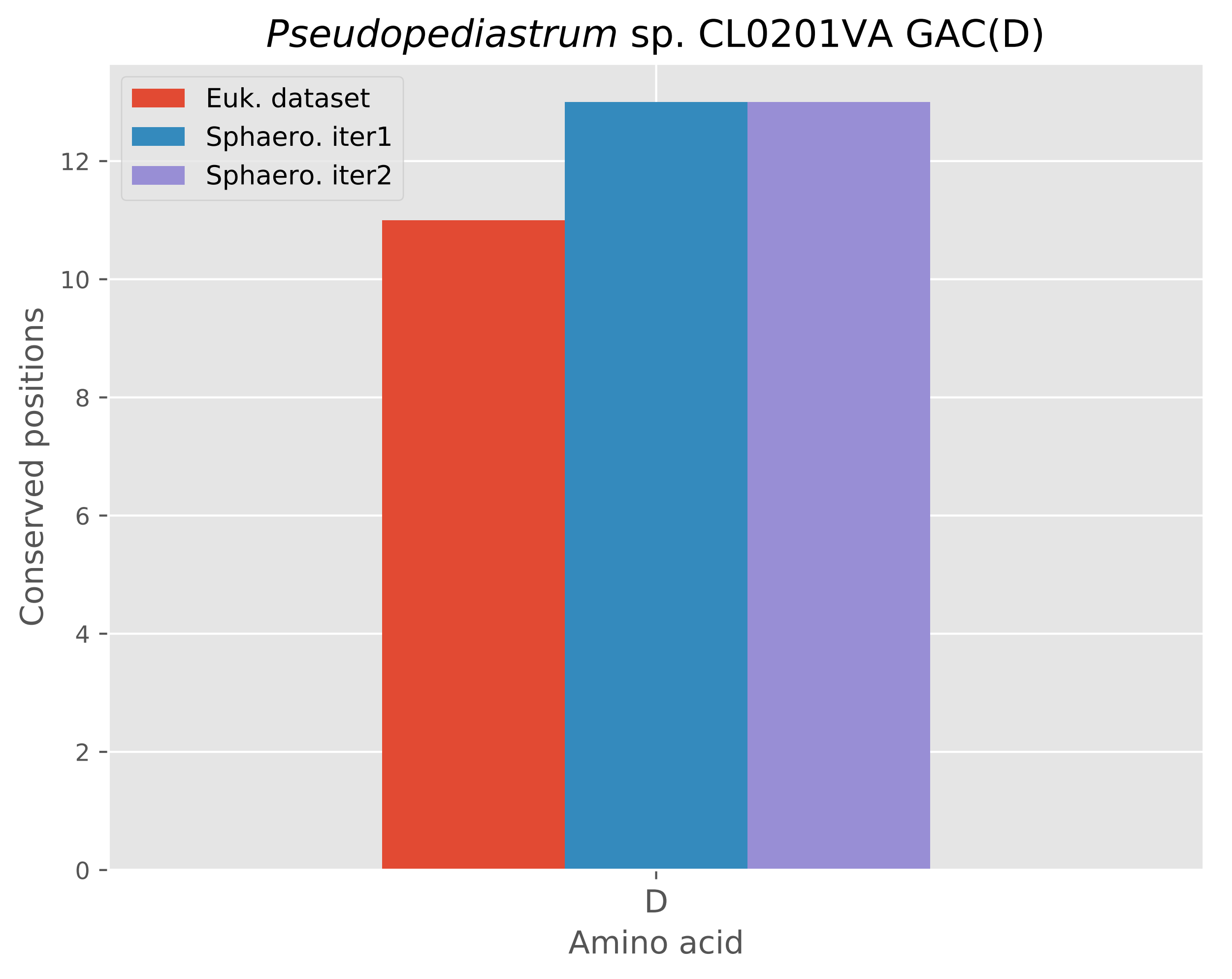
Pseudopediastrum sp. CL0201VA GAC(D)
Euk. dataset
Sphaero. iter1
Sphaero. iter2
Conserved positions
0
2
4
6
8
10
12
D
Amino acid

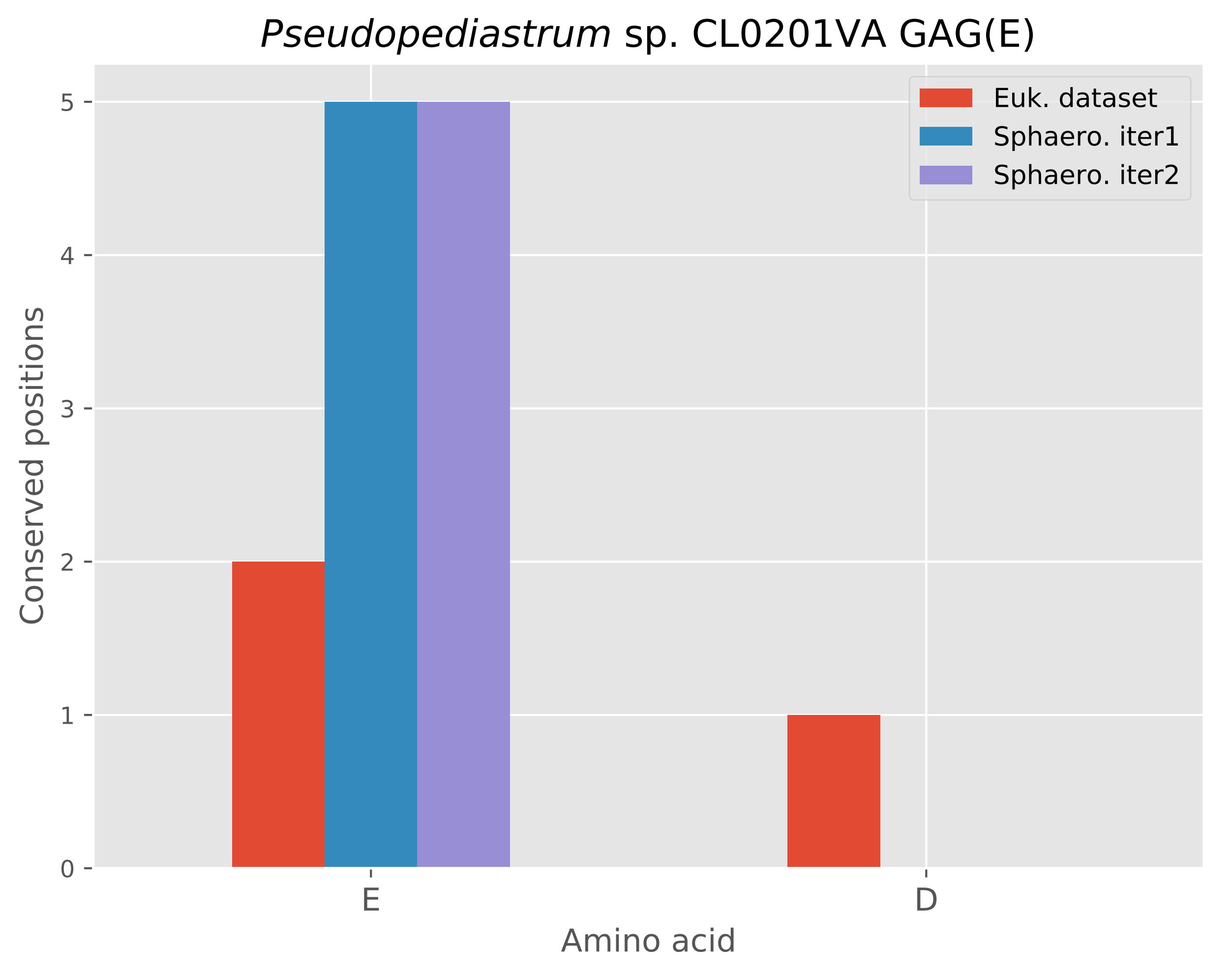
Pseudopediastrum sp. CL0201VA GAG(E)
Euk. dataset
Sphaero. iter1
Sphaero. iter2
Conserved positions
0
1
2
3
4
5
E
D
Amino acid

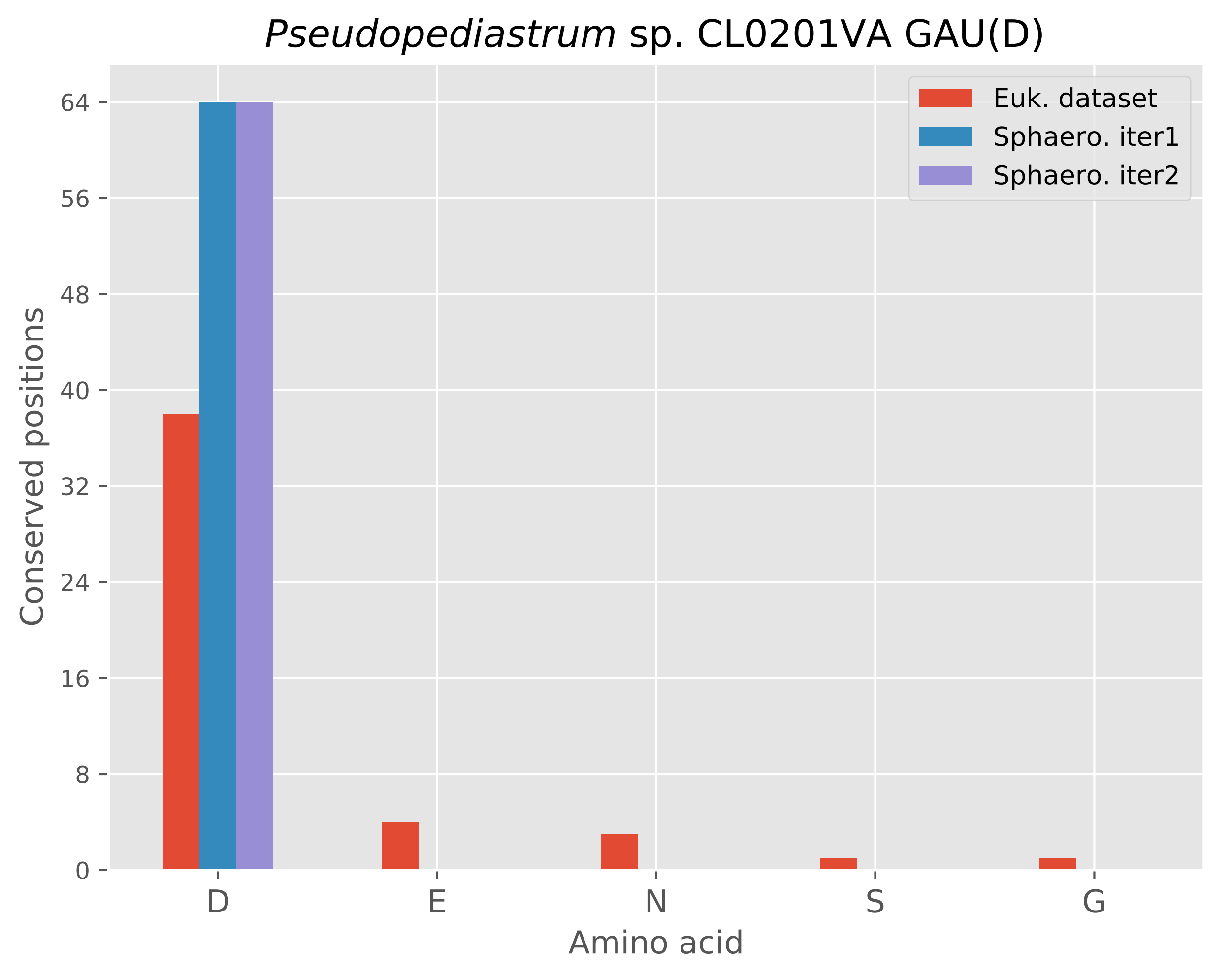
Pseudopediastrum sp. CL0201VA GAU(D)
Euk. dataset
Sphaero. iter1
Sphaero. iter2
Conserved positions
0
8
16
24
32
40
48
56
64
D
E
N
S
G
Amino acid

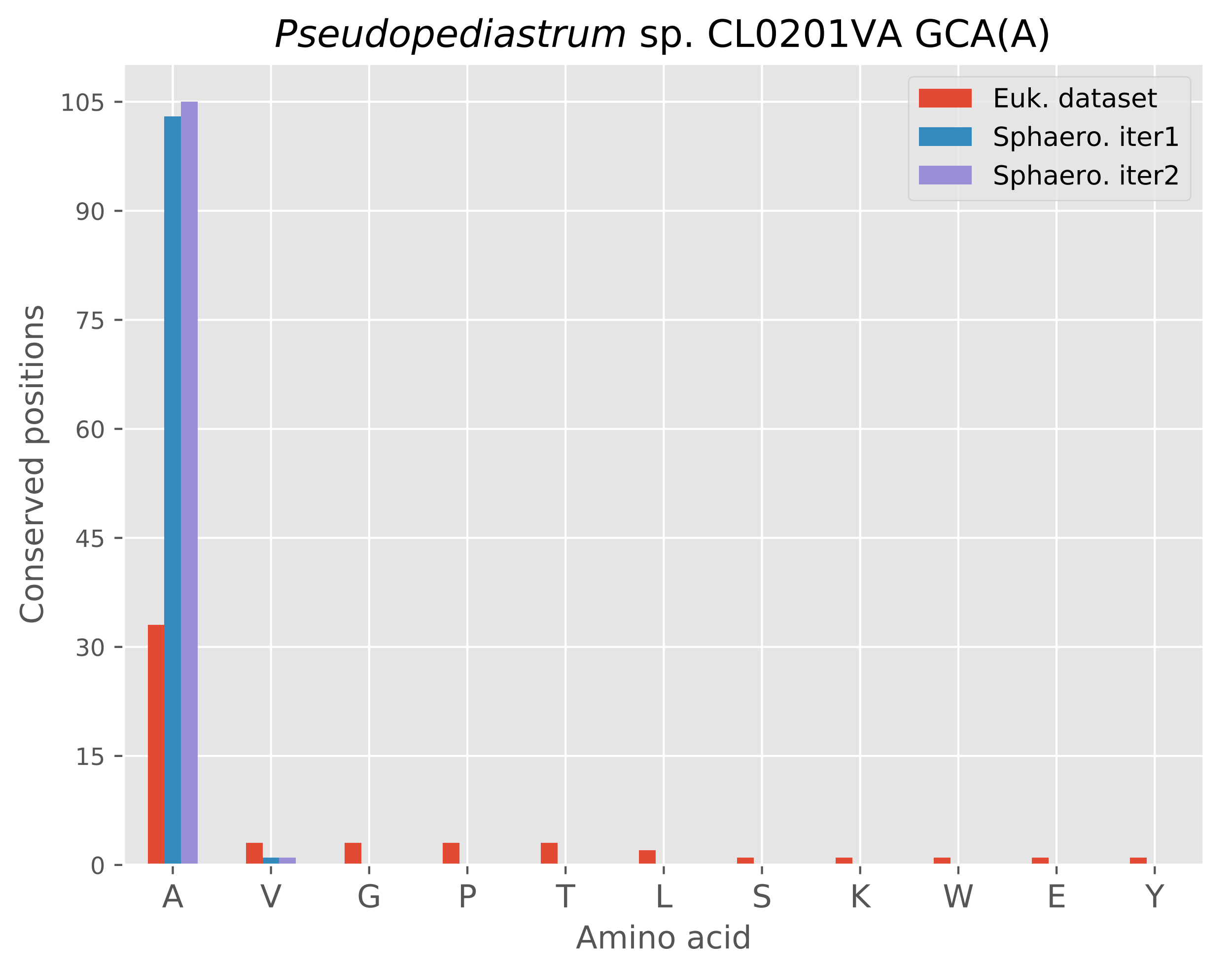
Pseudopediastrum sp. CL0201VA GCA(A)
Euk. dataset
Sphaero. iter1
Sphaero. iter2
Conserved positions
0
15
30
45
60
75
90
105
A
V
G
P
T
L
S
K
W
E
Y
Amino acid

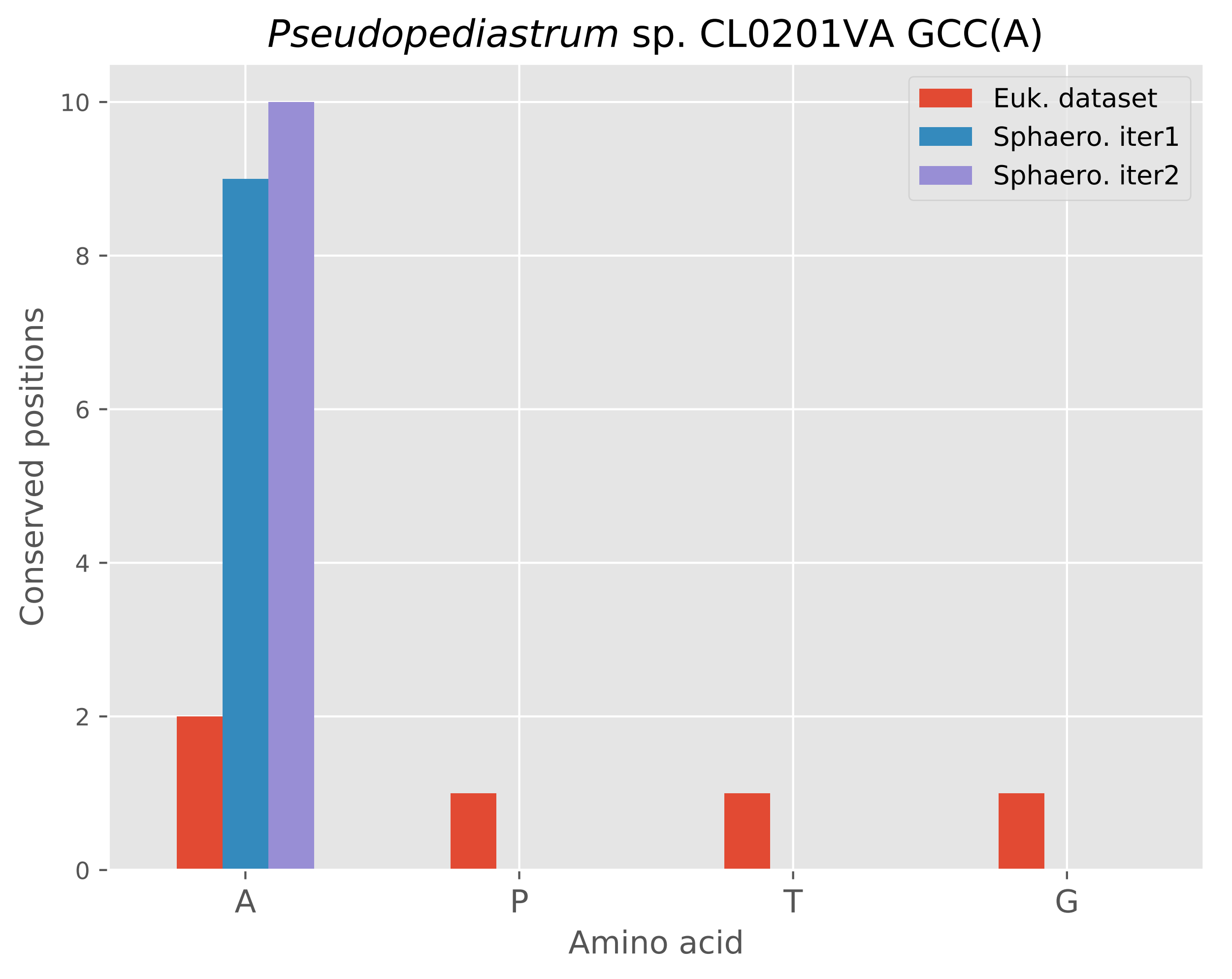

Pseudopediastrum sp. CL0201VA GCC(A)
Euk. dataset
Sphaero. iter1
Sphaero. iter2
Conserved positions
0
2
4
6
8
10
A
P
T
G
Amino acid

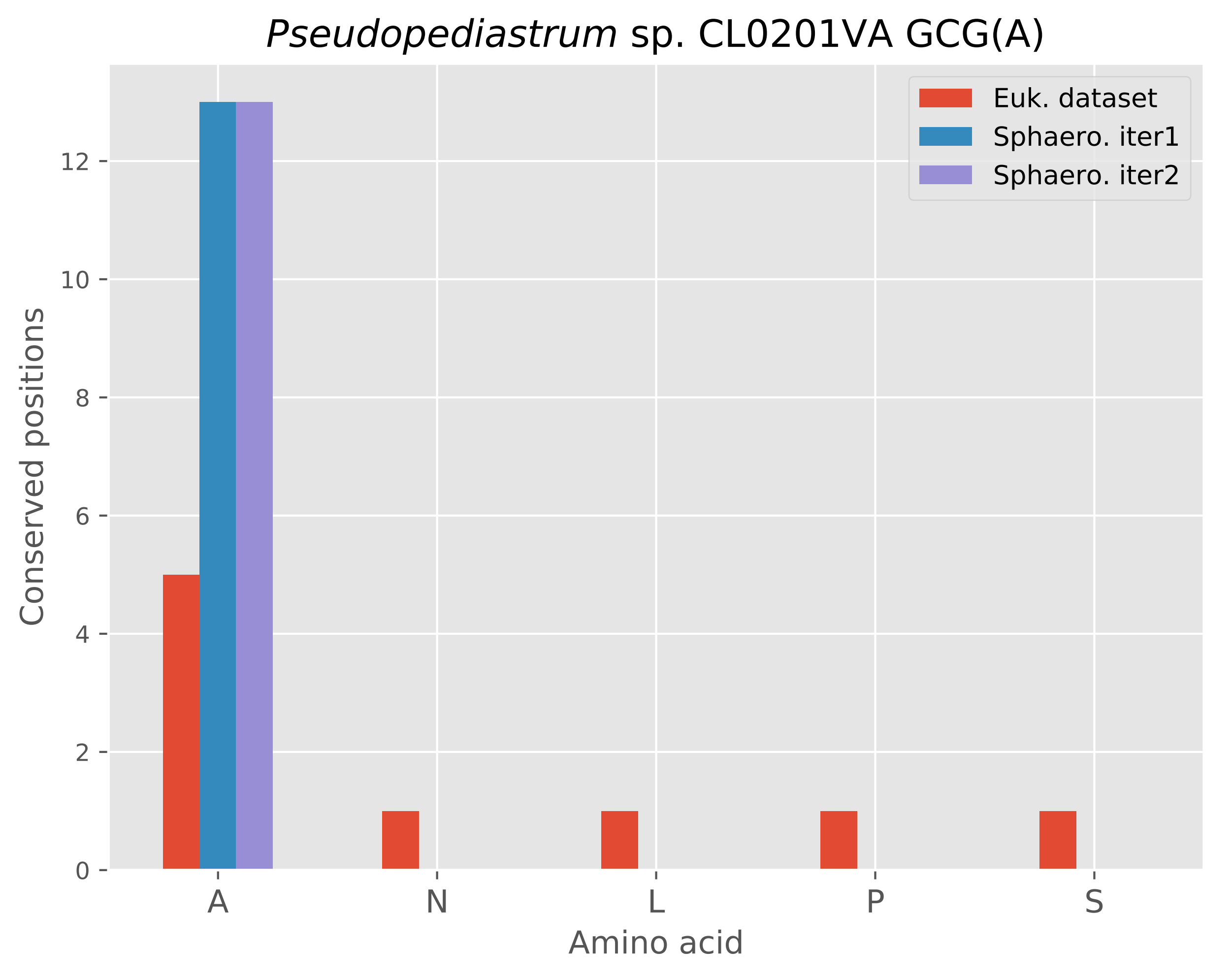

Pseudopediastrum sp. CL0201VA GCG(A)
Euk. dataset
Sphaero. iter1
Sphaero. iter2
Conserved positions
0
2
4
6
8
10
12
A
N
L
P
S
Amino acid

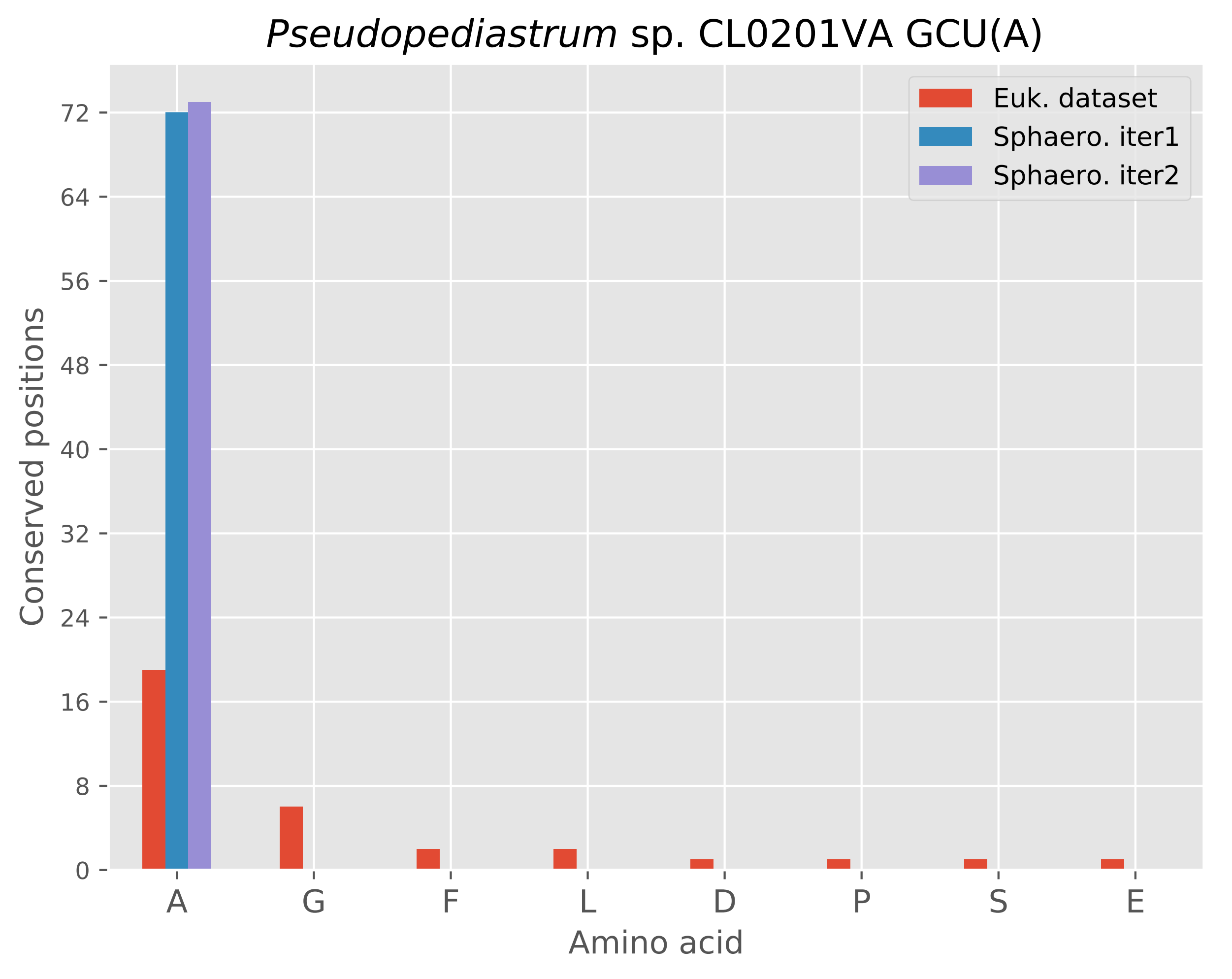

Pseudopediastrum sp. CL0201VA GCU(A)
Euk. dataset
Sphaero. iter1
Sphaero. iter2
Conserved positions
0
8
16
24
32
40
48
56
64
72
A
G
F
L
D
P
S
E
Amino acid

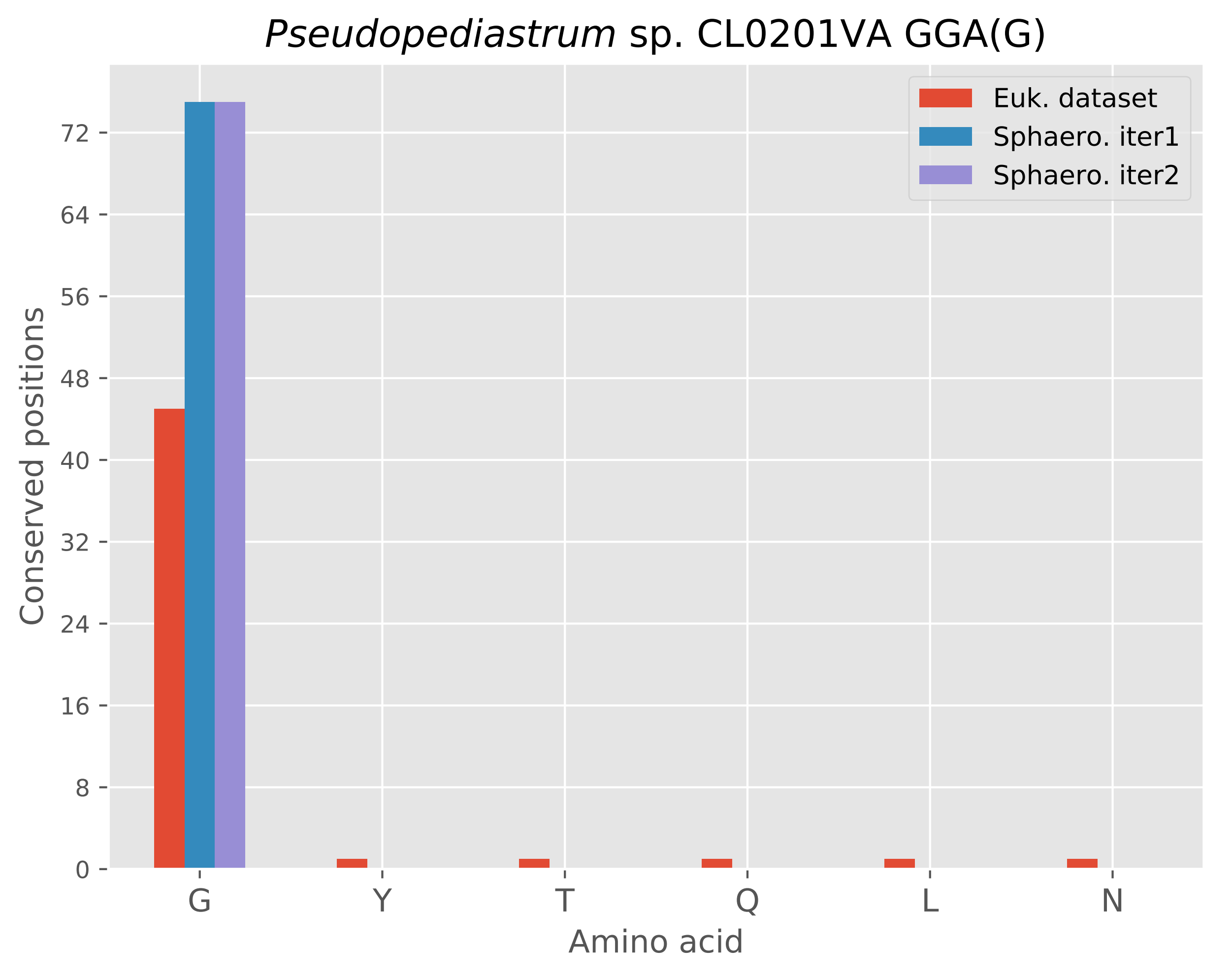

Pseudopediastrum sp. CL0201VA GGA(G)
Conserved positions
Amino acid
Euk. dataset
Sphaero. iter1
Sphaero. iter2
0
8
16
24
32
40
48
56
64
72
G
Y
T
Q
L
N

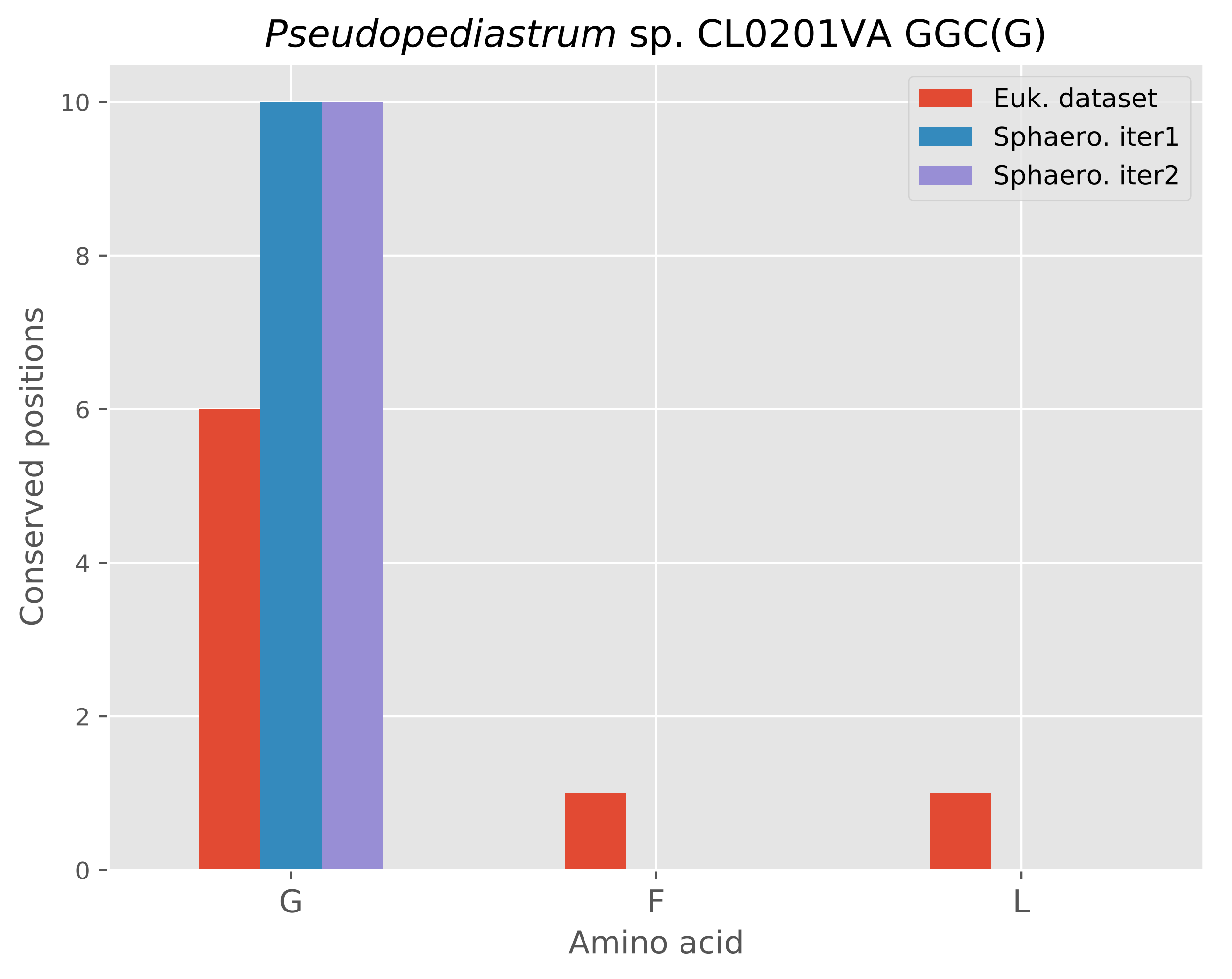

Pseudopediastrum sp. CL0201VA GGC(G)
Euk. dataset
Sphaero. iter1
Sphaero. iter2
Conserved positions
0
2
4
6
8
10
G
F
L
Amino acid

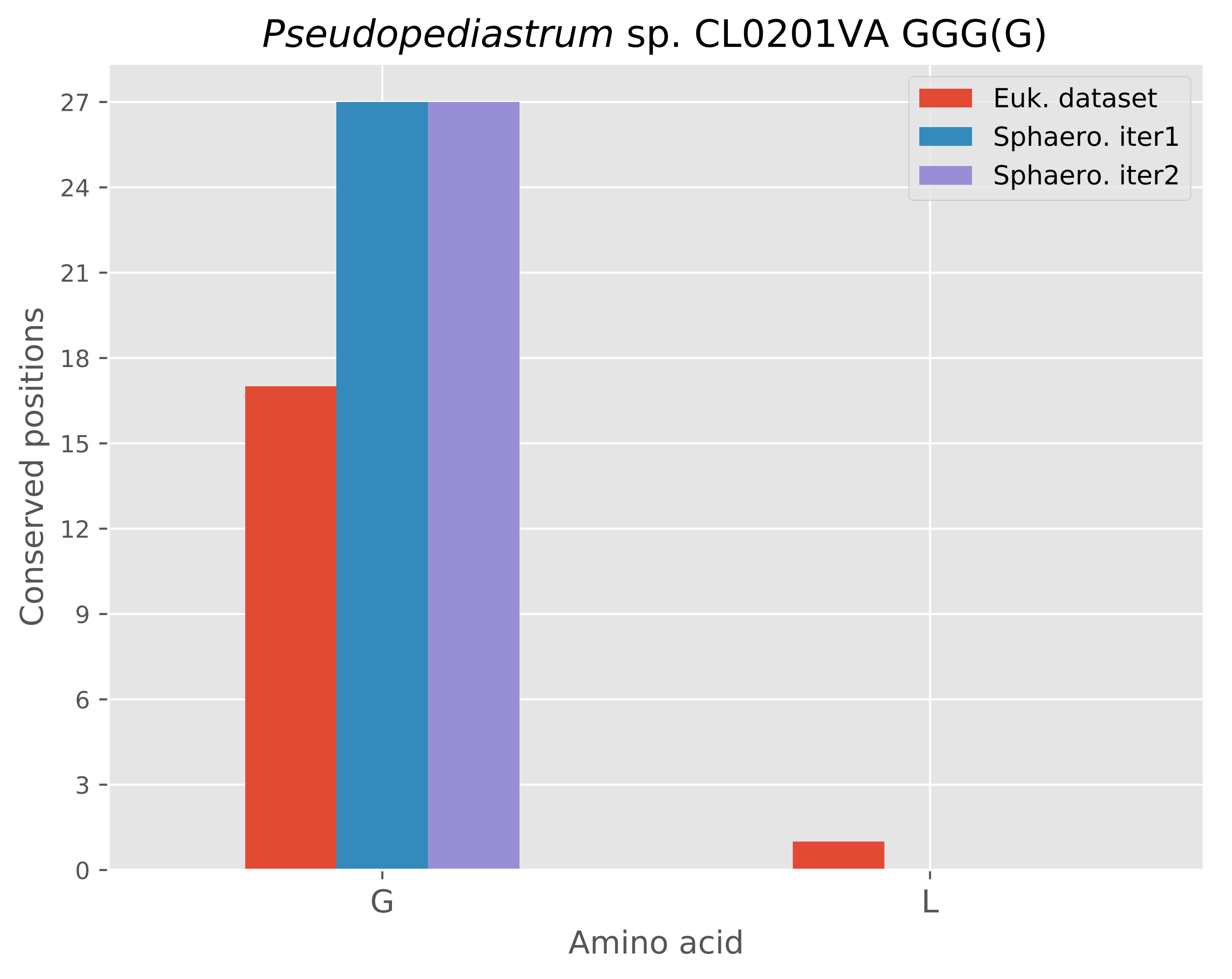

Pseudopediastrum sp. CL0201VA GGG(G)
Euk. dataset
Sphaero. iter1
Sphaero. iter2
Conserved positions
0
3
6
9
12
15
18
21
24
27
G
L
Amino acid

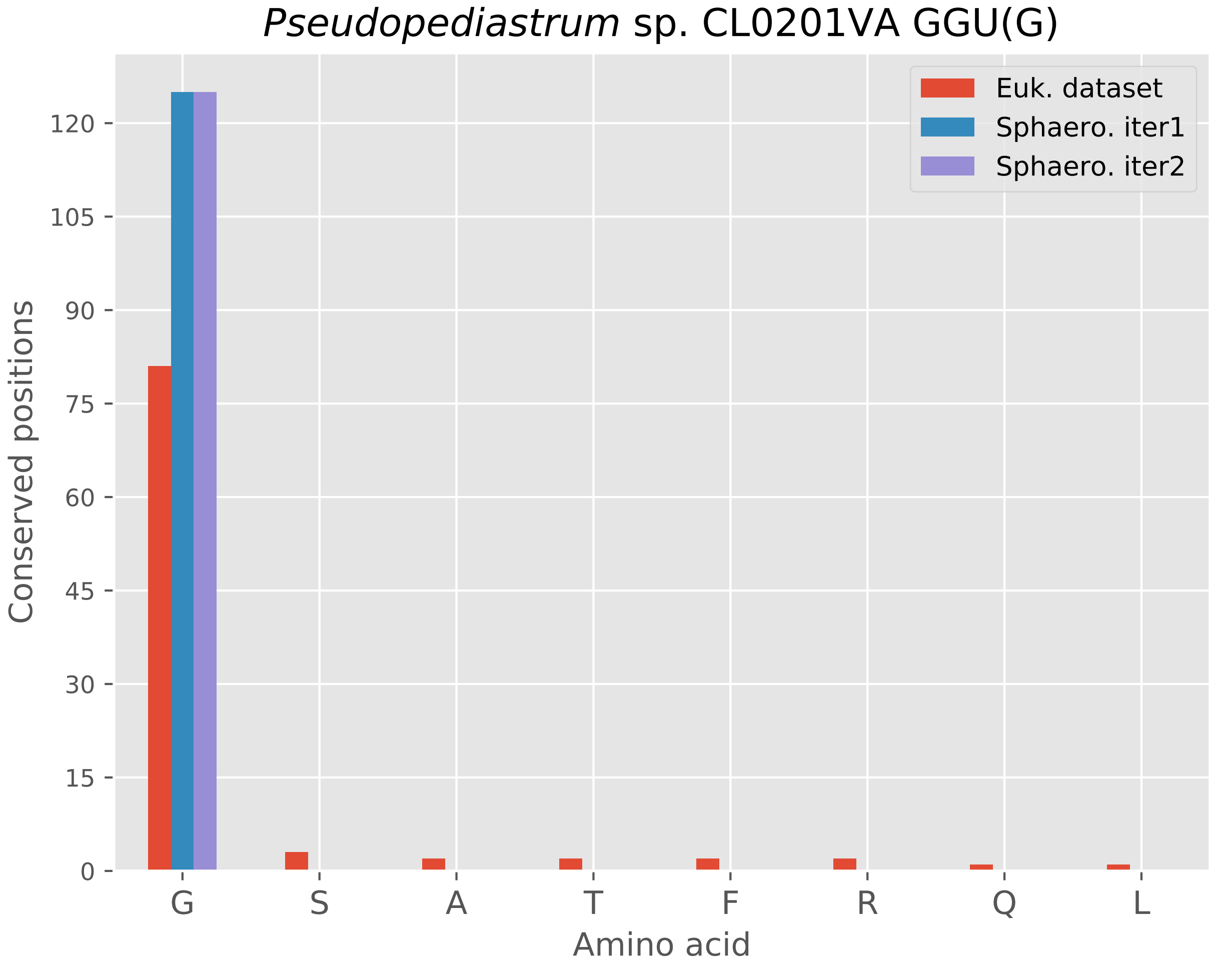

Pseudopediastrum sp. CL0201VA GGU(G)
Euk. dataset
Sphaero. iter1
Sphaero. iter2
Conserved positions
0
15
30
45
60
75
90
105
120
G
S
A
T
F
R
Q
L
Amino acid

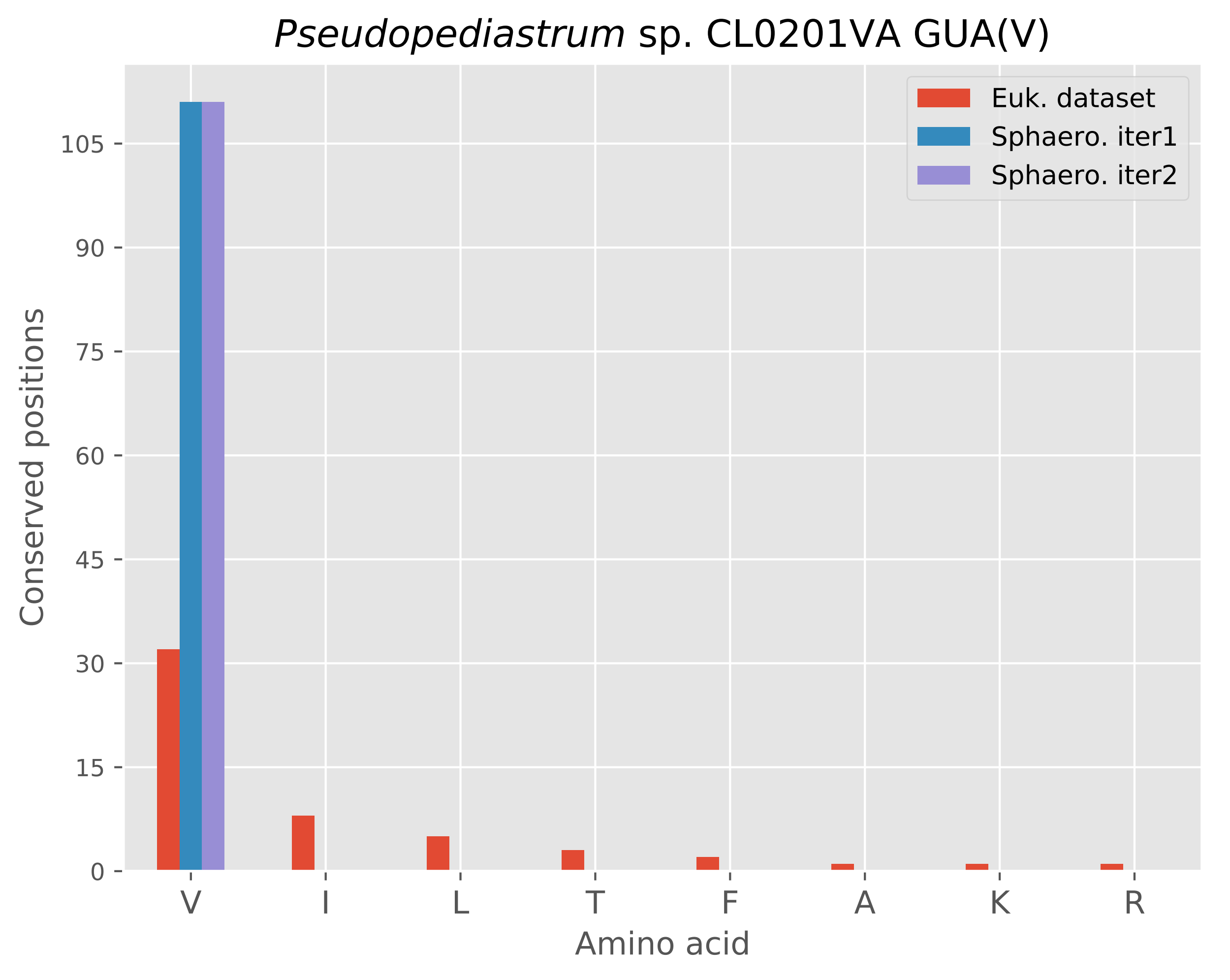
Pseudopediastrum sp. CL0201VA GUA(V)
Euk. dataset
Sphaero. iter1
Sphaero. iter2
Conserved positions
0
15
30
45
60
75
90
105
V
I
L
T
F
A
K
R
Amino acid

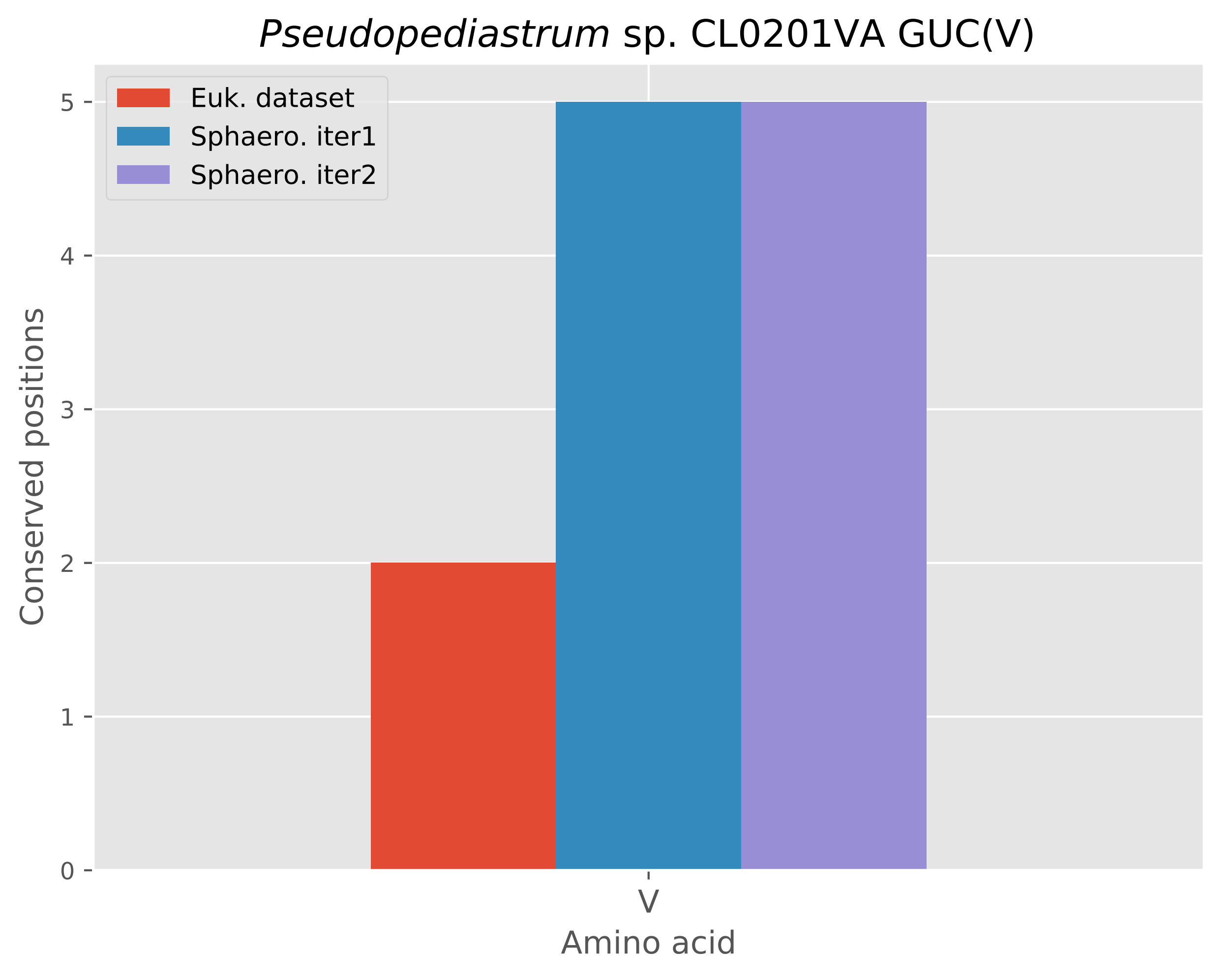

Pseudopediastrum sp. CL0201VA GUC(V)
Euk. dataset
Sphaero. iter1
Sphaero. iter2
Conserved positions
0
1
2
3
4
5
V
Amino acid

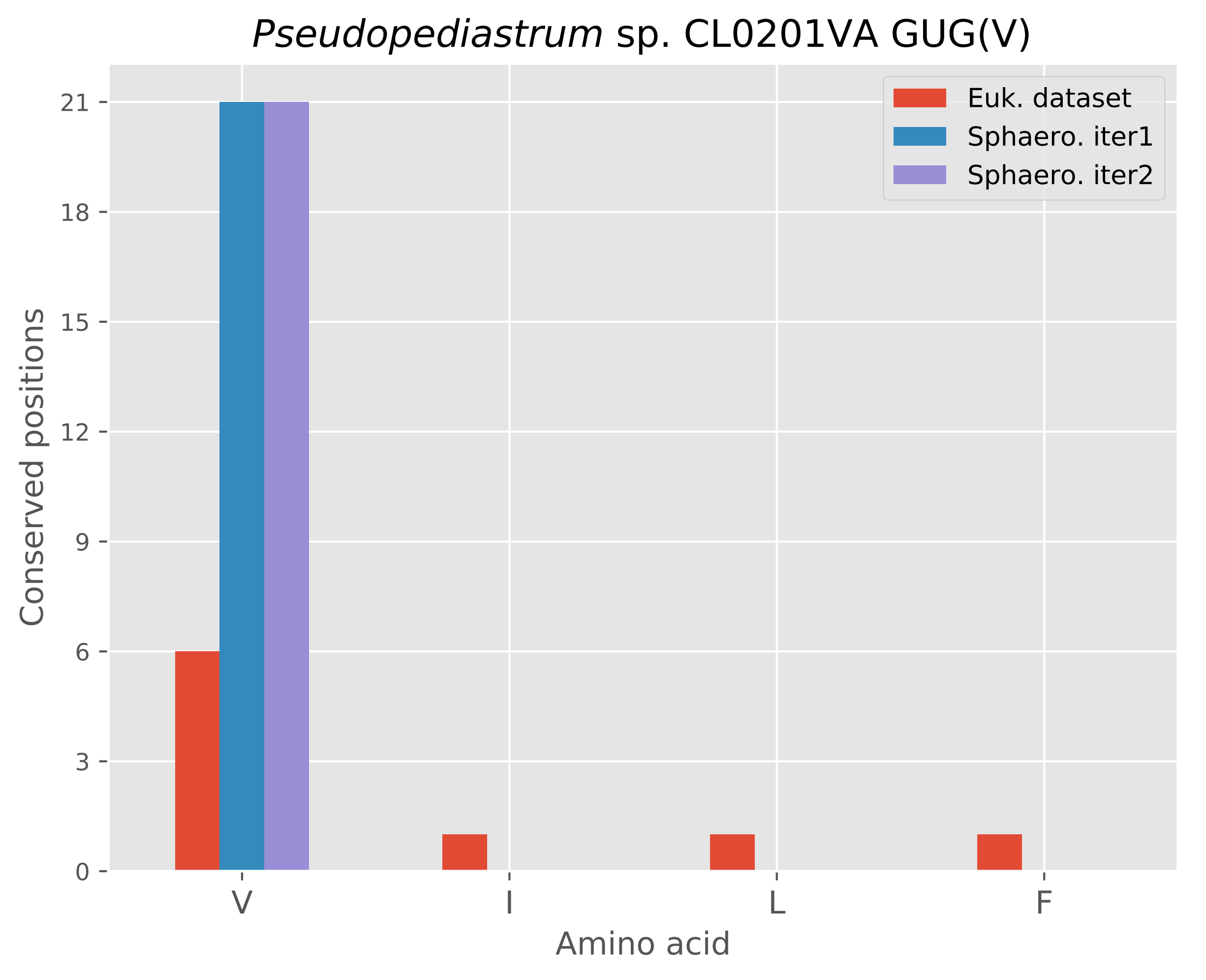

Pseudopediastrum sp. CL0201VA GUG(V)
Conserved positions
0
3
6
9
12
15
18
21
Euk. dataset
Sphaero. iter1
Sphaero. iter2
V
I
L
F
Amino acid

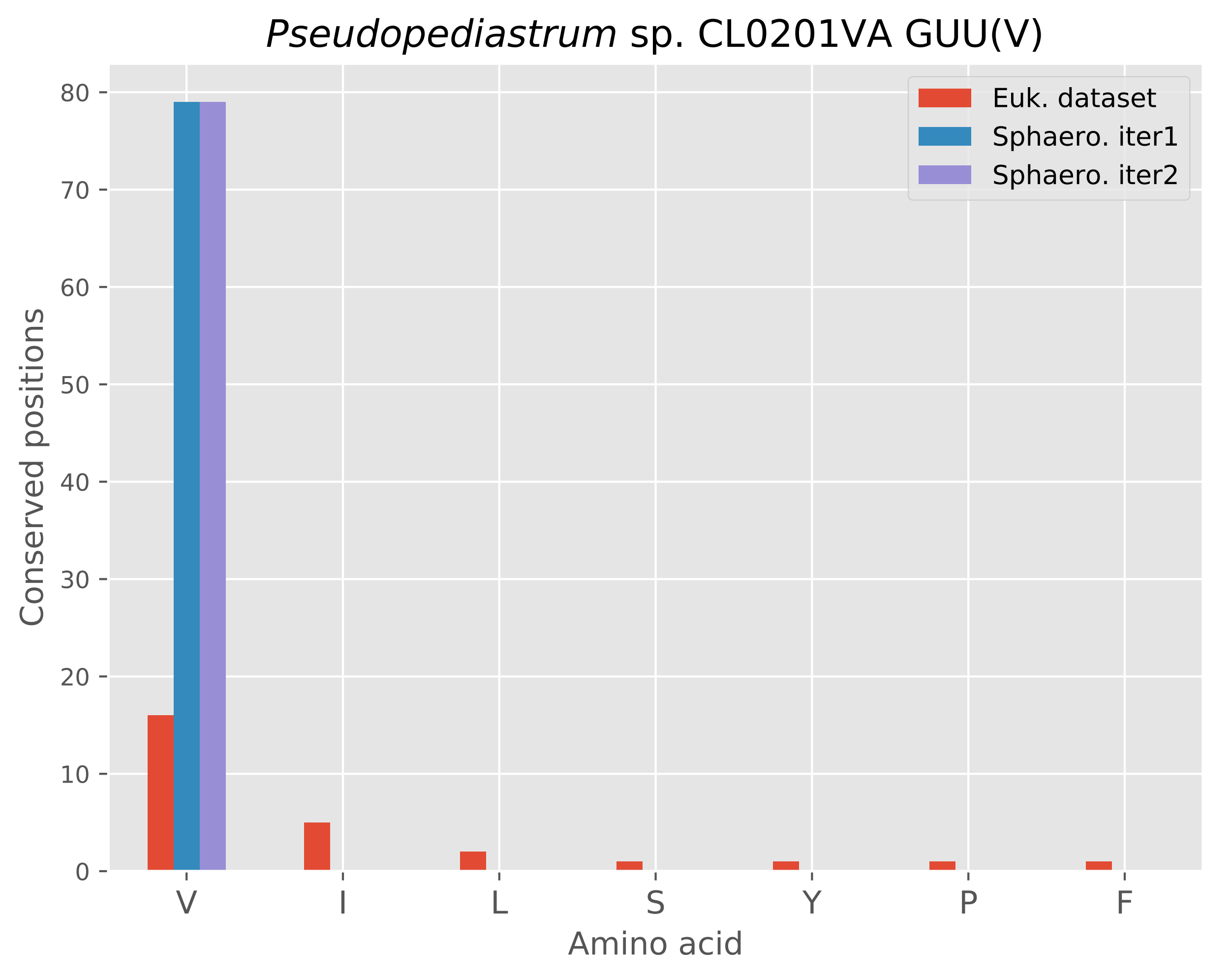

Pseudopediastrum sp. CL0201VA GUU(V)
Euk. dataset
Sphaero. iter1
Sphaero. iter2
Conserved positions
0
10
20
30
40
50
60
70
80
V
I
L
S
Y
P
F
Amino acid

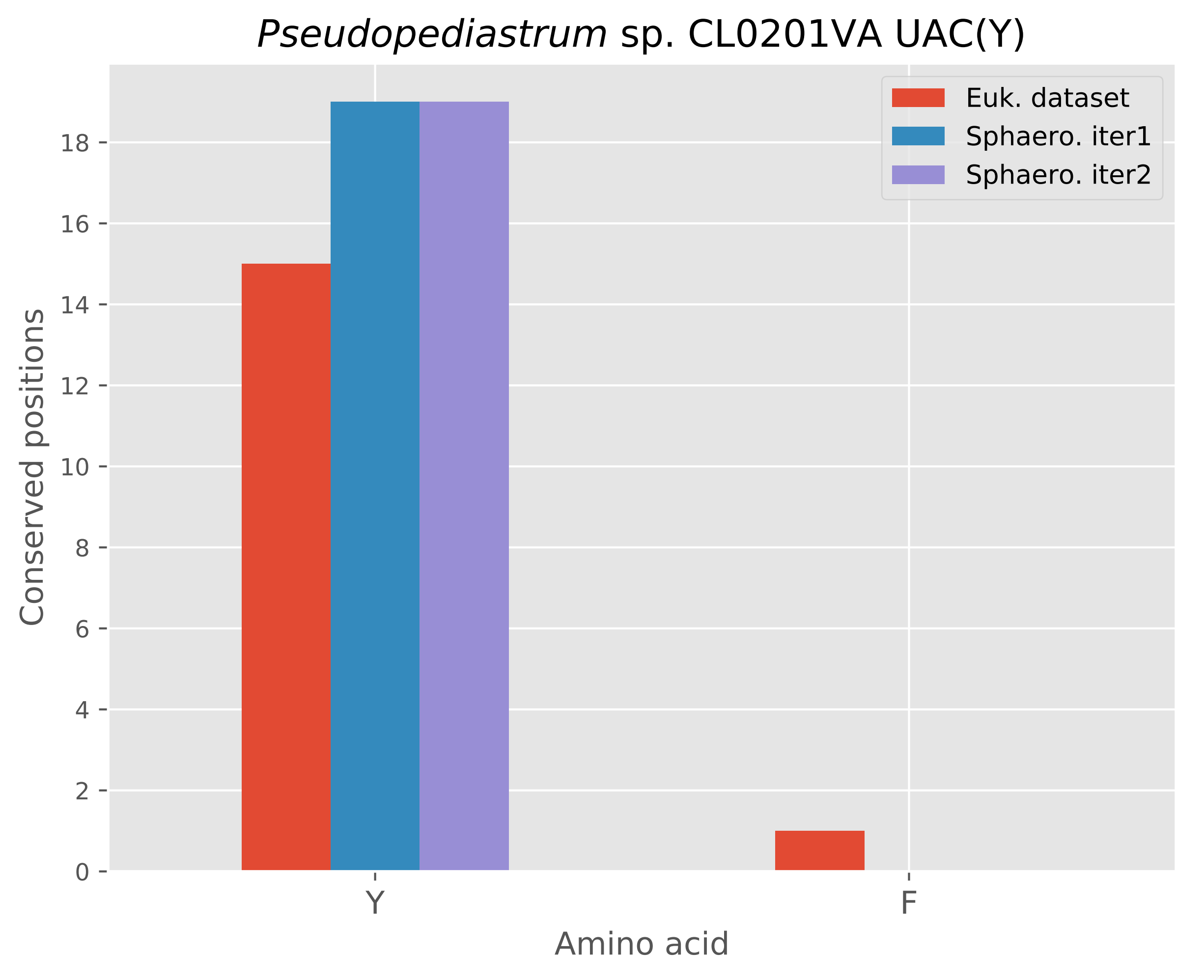

Pseudopediastrum sp. CL0201VA UAC(Y)
Euk. dataset
Sphaero. iter1
Sphaero. iter2
Conserved positions
0
2
4
6
8
10
12
14
16
18
Y
F
Amino acid

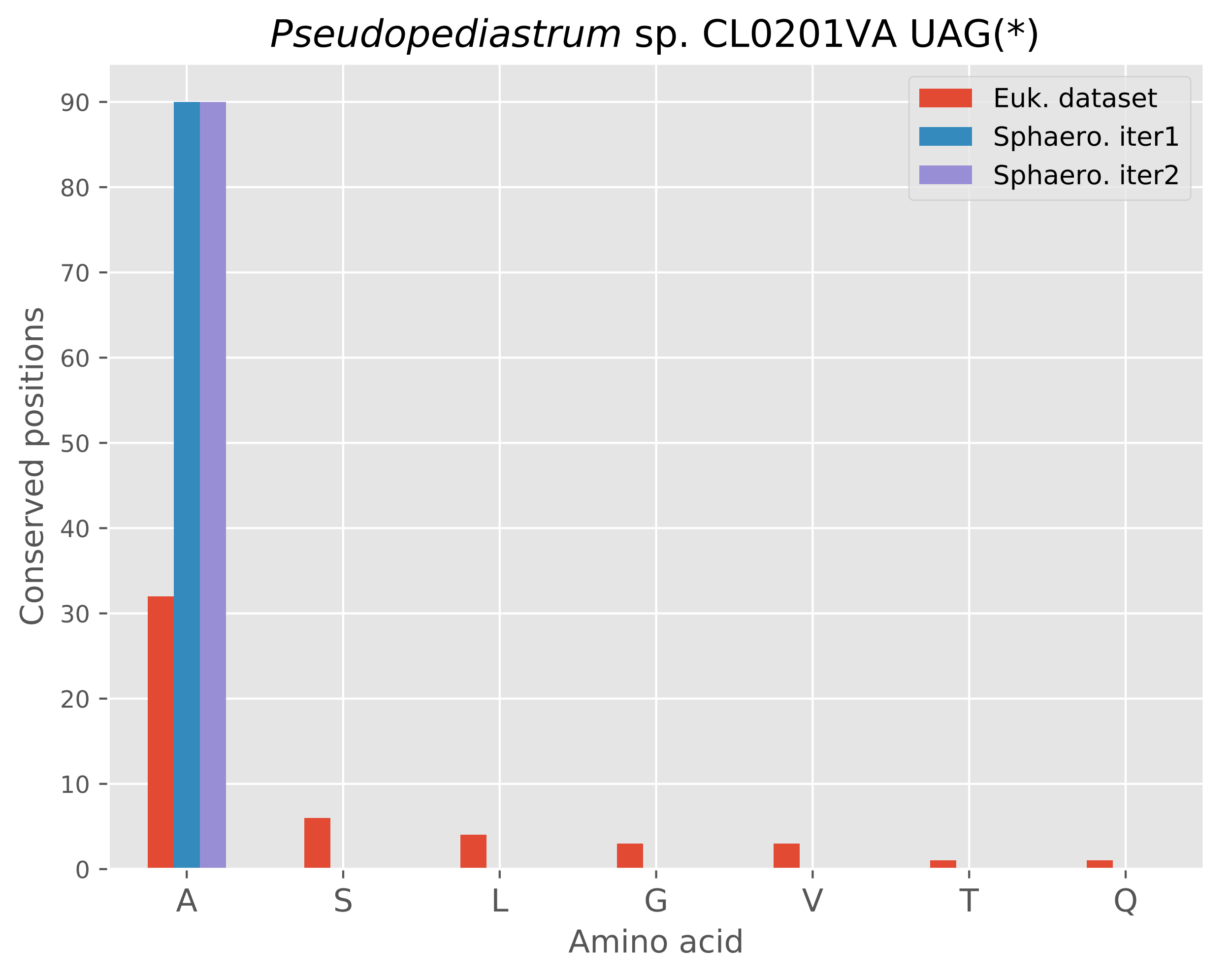

Pseudopediastrum sp. CL0201VA UAG(*)
Euk. dataset
Sphaero. iter1
Sphaero. iter2
Conserved positions
0
10
20
30
40
50
60
70
80
90
A
S
L
G
V
T
Q
Amino acid

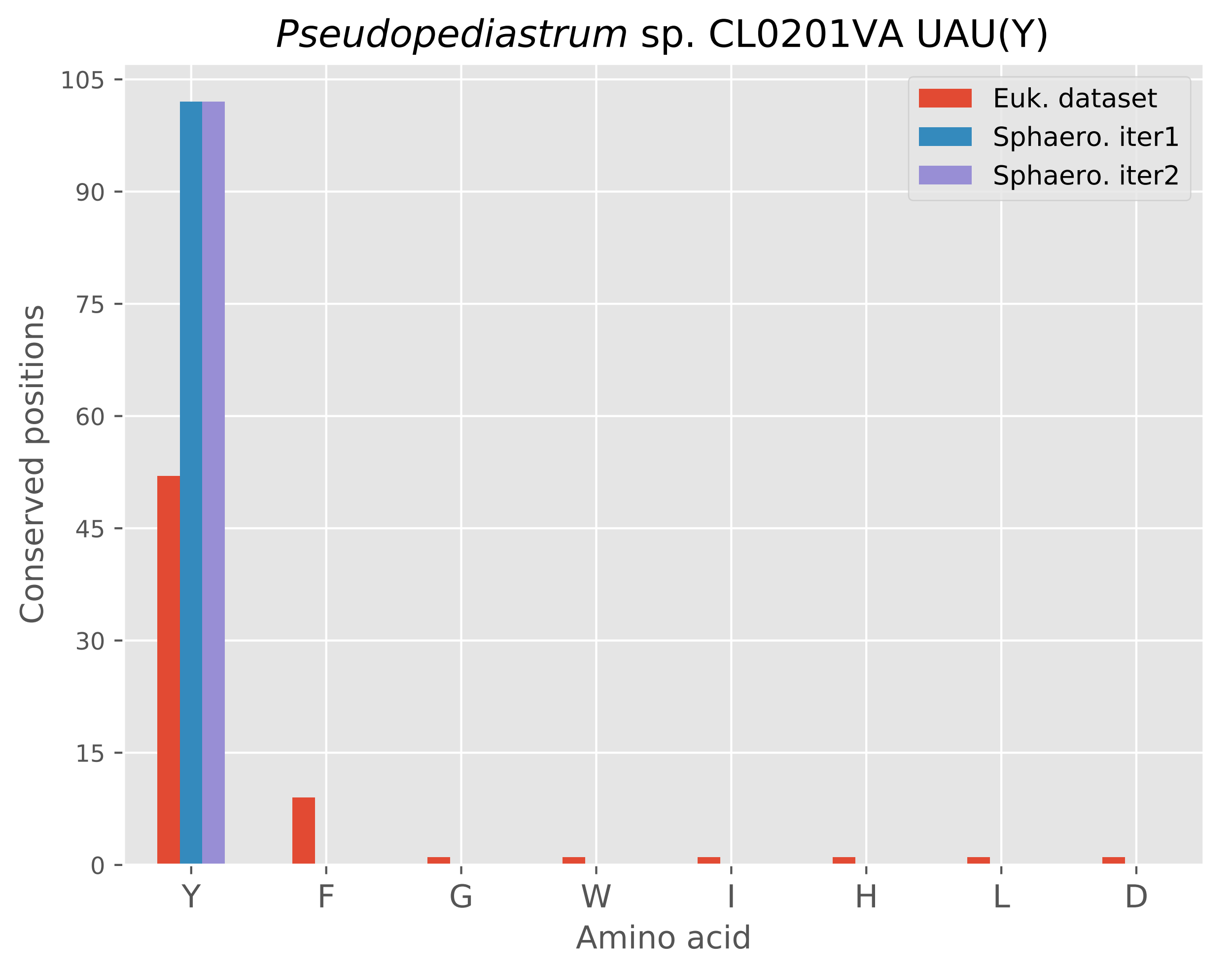

Pseudopediastrum sp. CL0201VA UAU(Y)
Euk. dataset
Sphaero. iter1
Sphaero. iter2
Conserved positions
105
90
75
60
45
30
15
0
Y
F
G
W
I
H
L
D
Amino acid

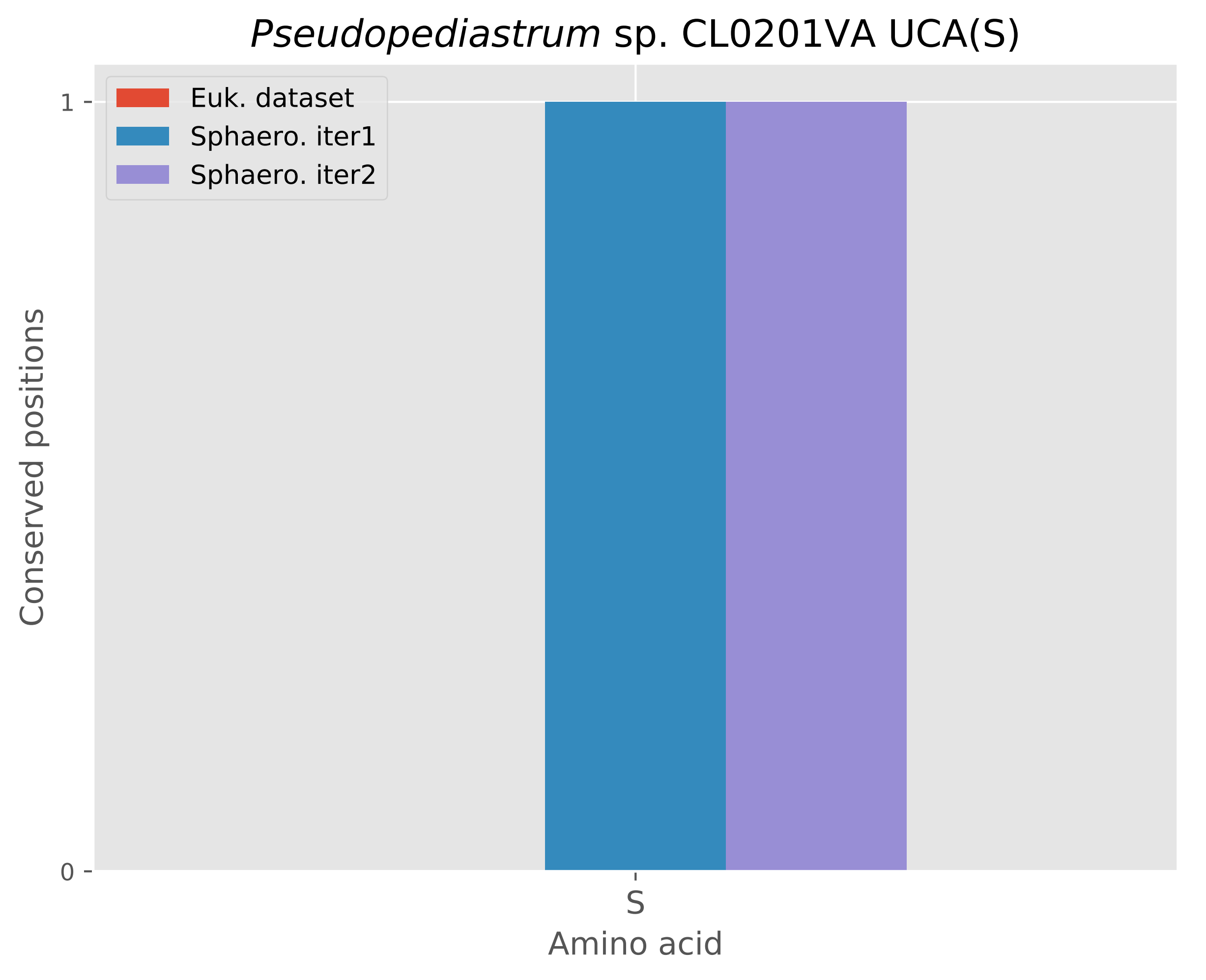

Pseudopediastrum sp. CL0201VA UCA(S)
Euk. dataset
Sphaero. iter1
Sphaero. iter2
1
0
Conserved positions
S
Amino acid

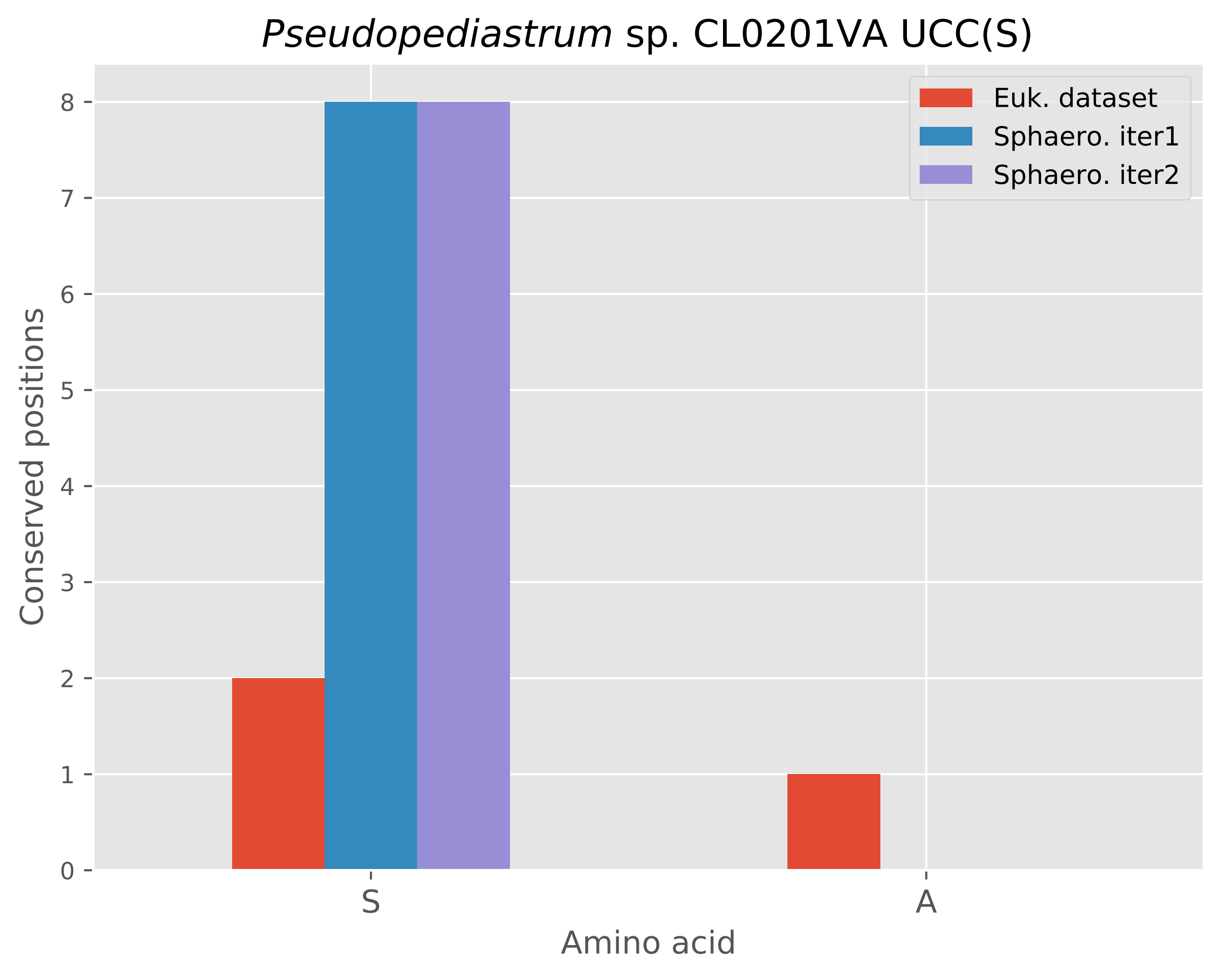
Pseudopediastrum sp. CL0201VA UCC(S)
Euk. dataset
Sphaero. iter1
Sphaero. iter2
Conserved positions
0
1
2
3
4
5
6
7
8
S
A
Amino acid

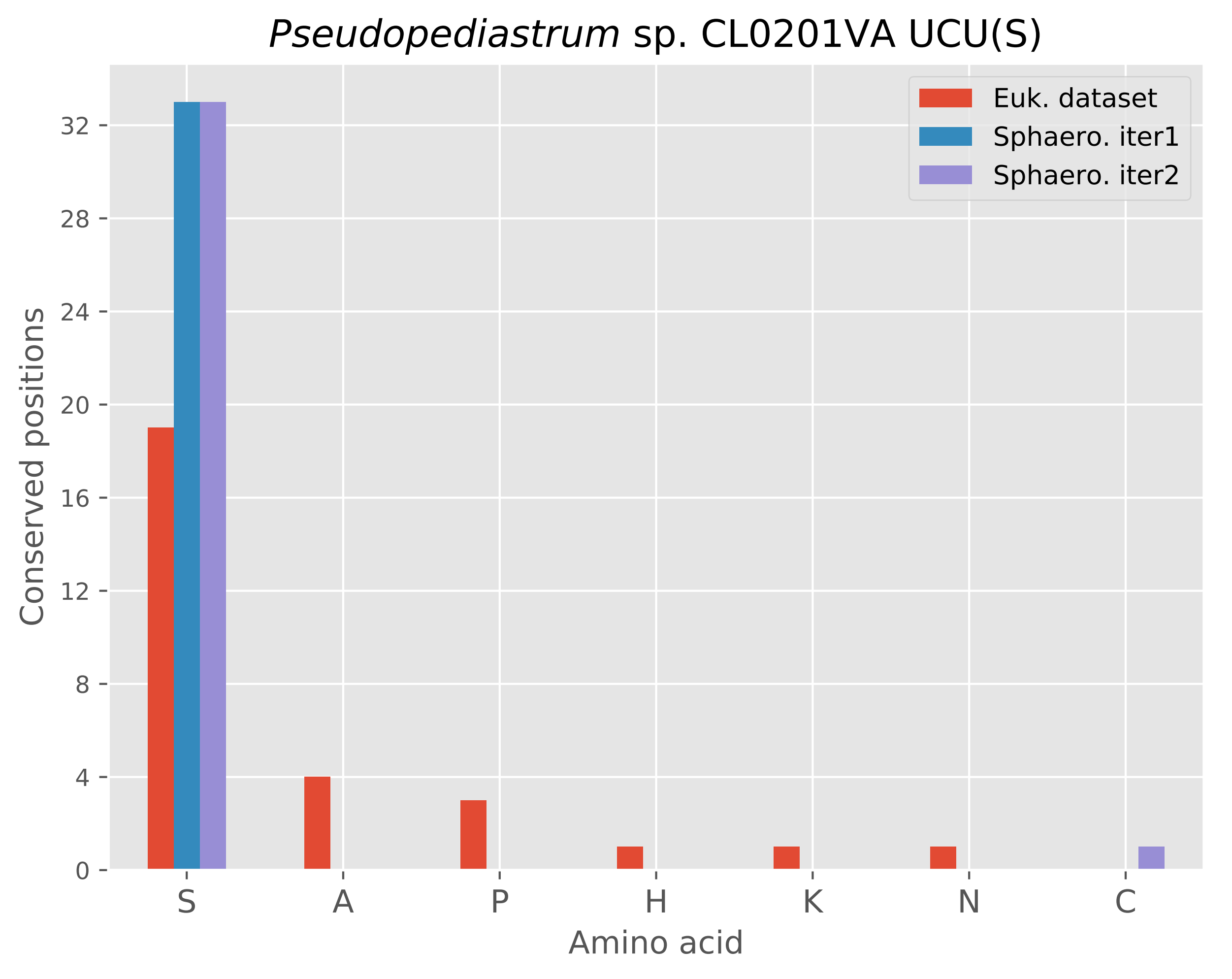

Pseudopediastrum sp. CL0201VA UCU(S)
Euk. dataset
Sphaero. iter1
Sphaero. iter2
Conserved positions
0
4
8
12
16
20
24
28
32
S
A
P
H
K
N
C
Amino acid

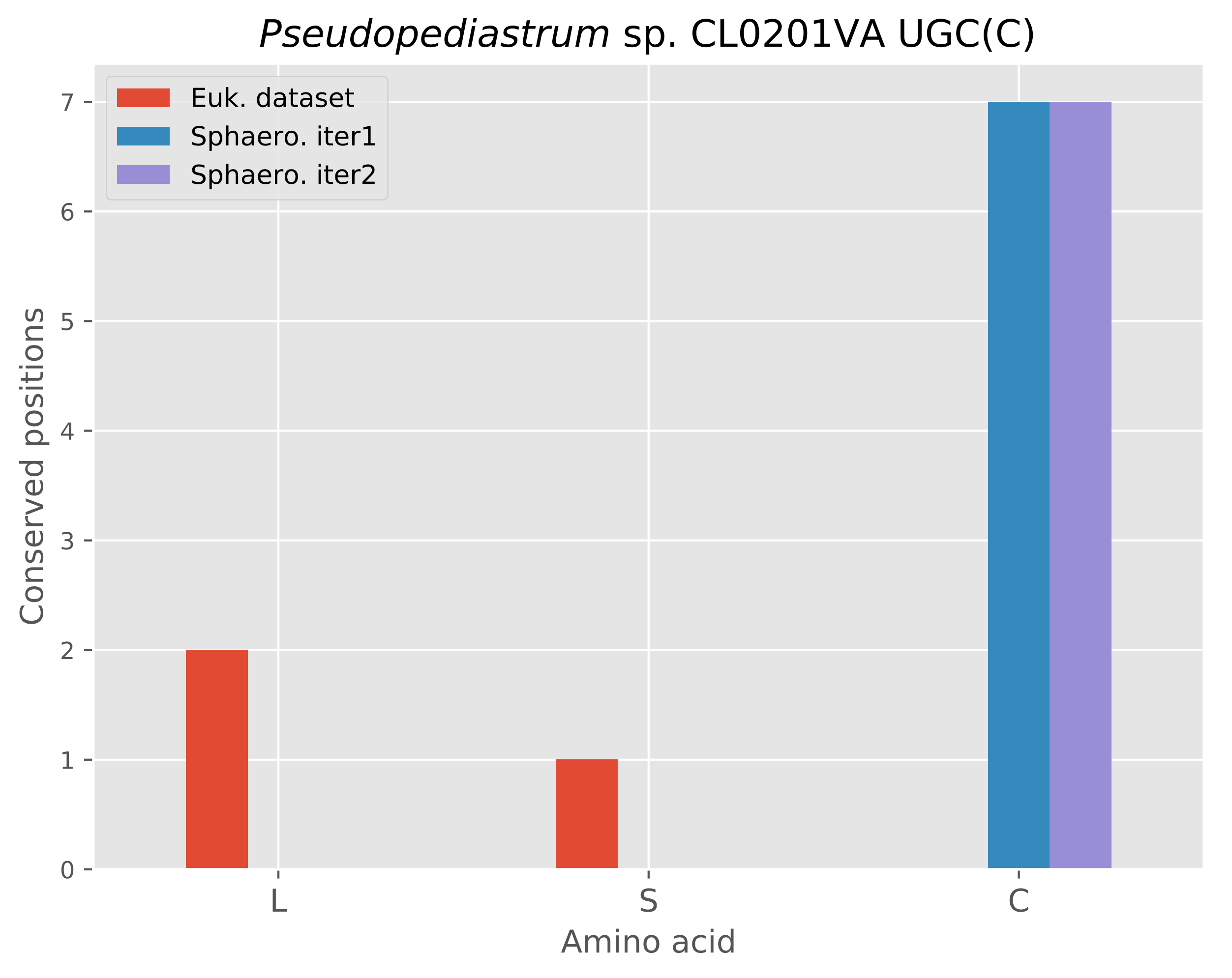

Pseudopediastrum sp. CL0201VA UGC(C)
Euk. dataset
Sphaero. iter1
Sphaero. iter2
Conserved positions
0
1
2
3
4
5
6
7
L
S
C
Amino acid

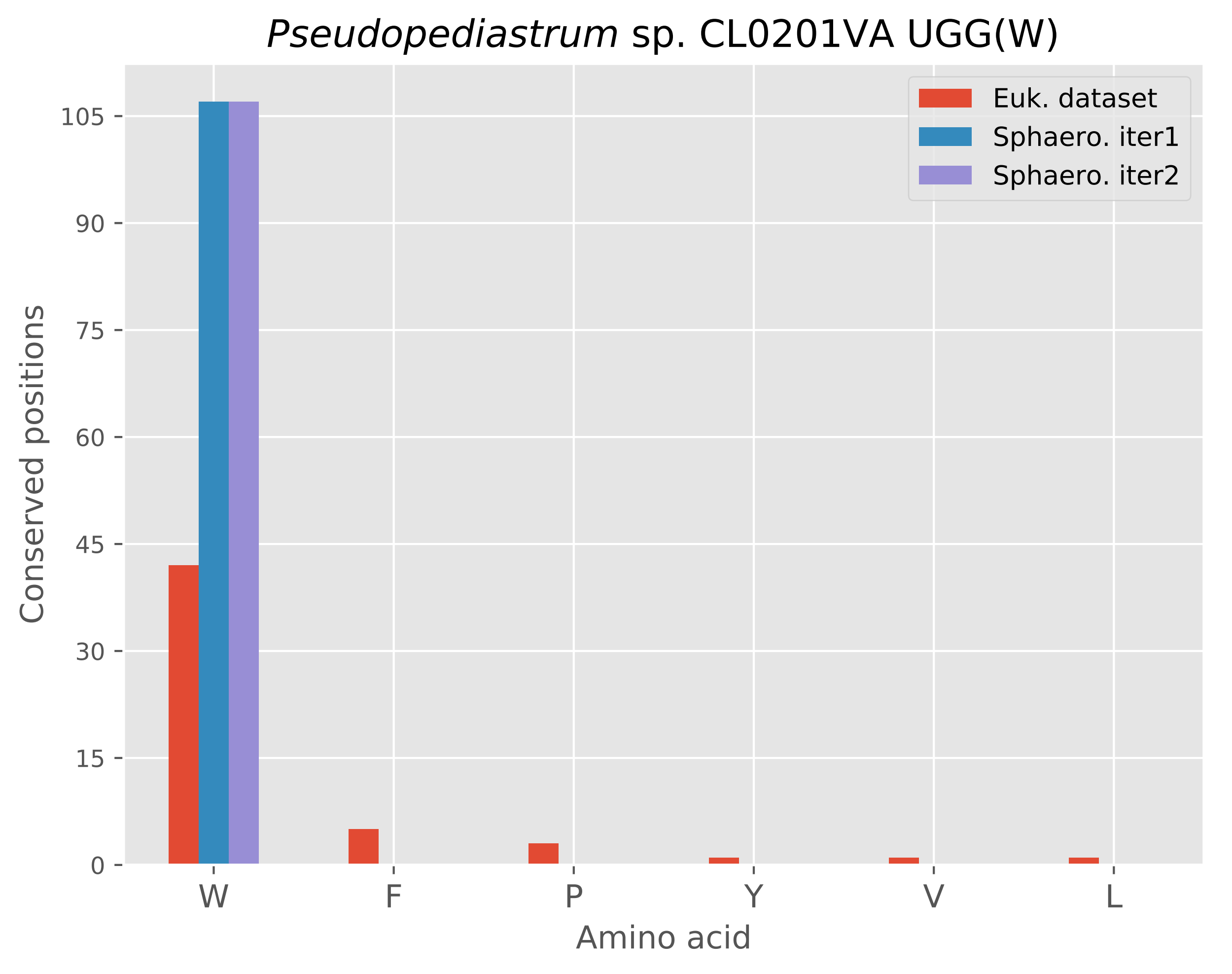

Pseudopediastrum sp. CL0201VA UGG(W)
Euk. dataset
Sphaero. iter1
Sphaero. iter2
Conserved positions
0
15
30
45
60
75
90
105
W
F
P
Y
V
L
Amino acid

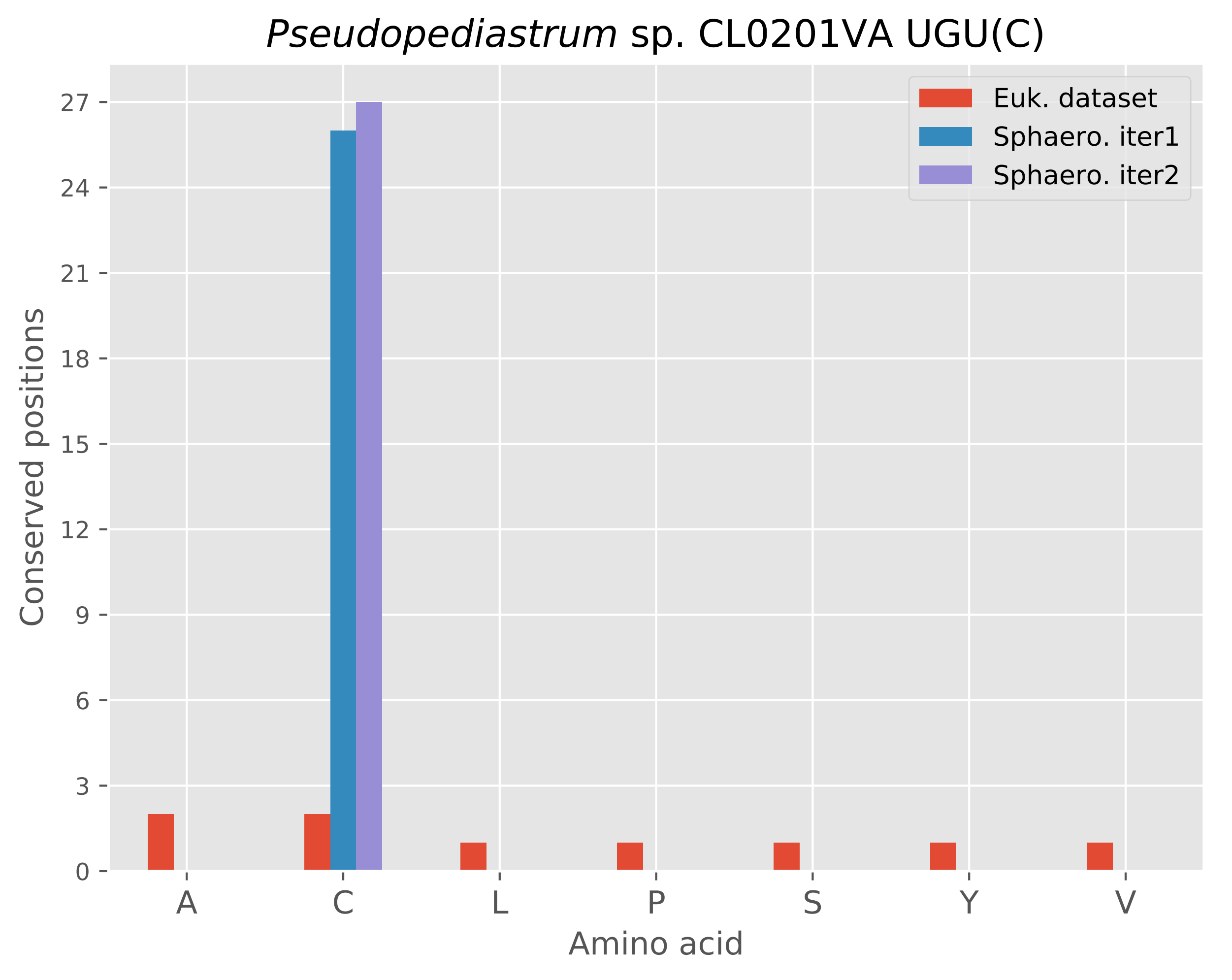
Pseudopediastrum sp. CL0201VA UGU(C)
Euk. dataset
Sphaero. iter1
Sphaero. iter2
Conserved positions
0
3
6
9
12
15
18
21
24
27
A
C
L
P
S
Y
V
Amino acid

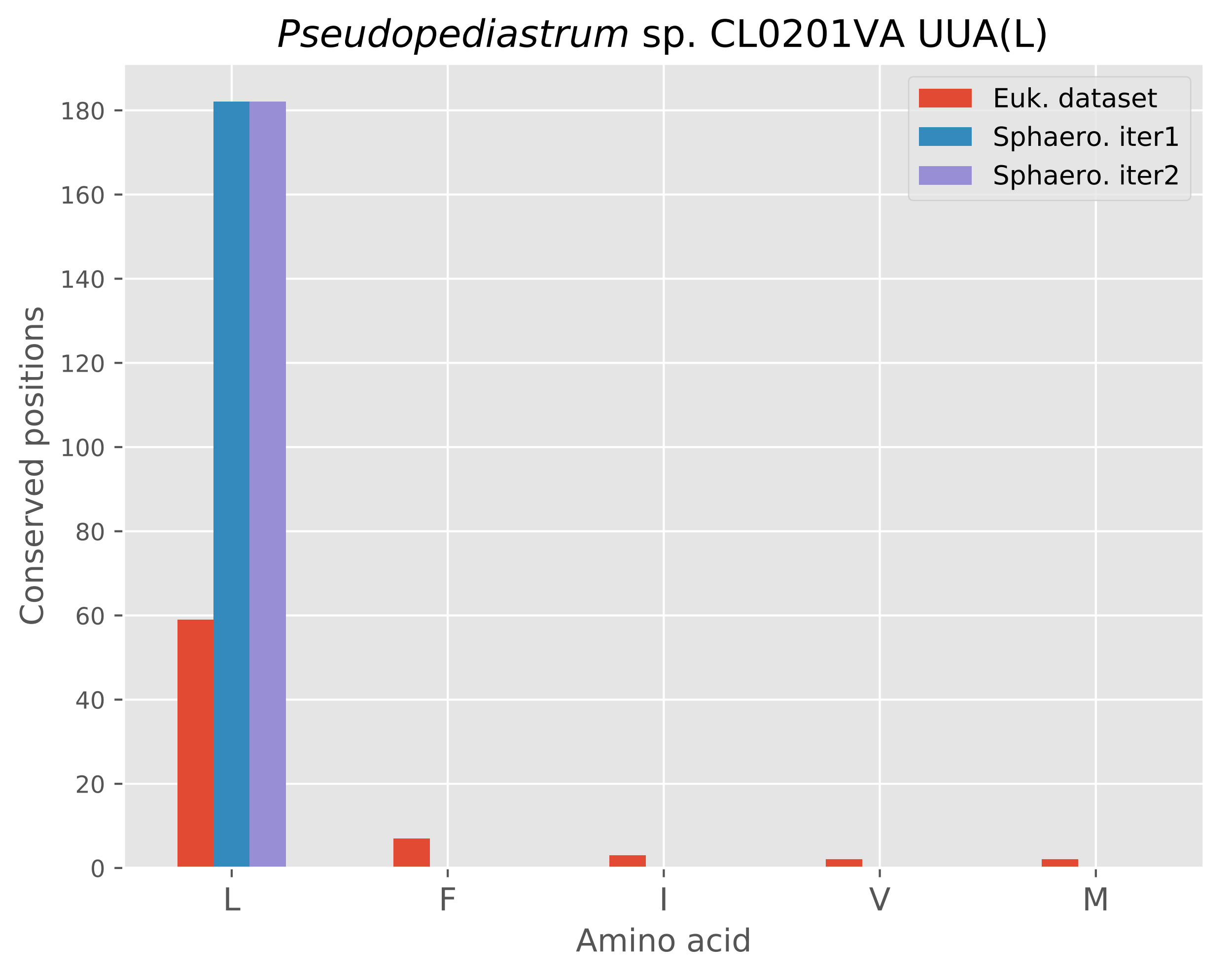

Pseudopediastrum sp. CL0201VA UUA(L)
Euk. dataset
Sphaero. iter1
Sphaero. iter2
Conserved positions
0
20
40
60
80
100
120
140
160
180
L
F
I
V
M
Amino acid

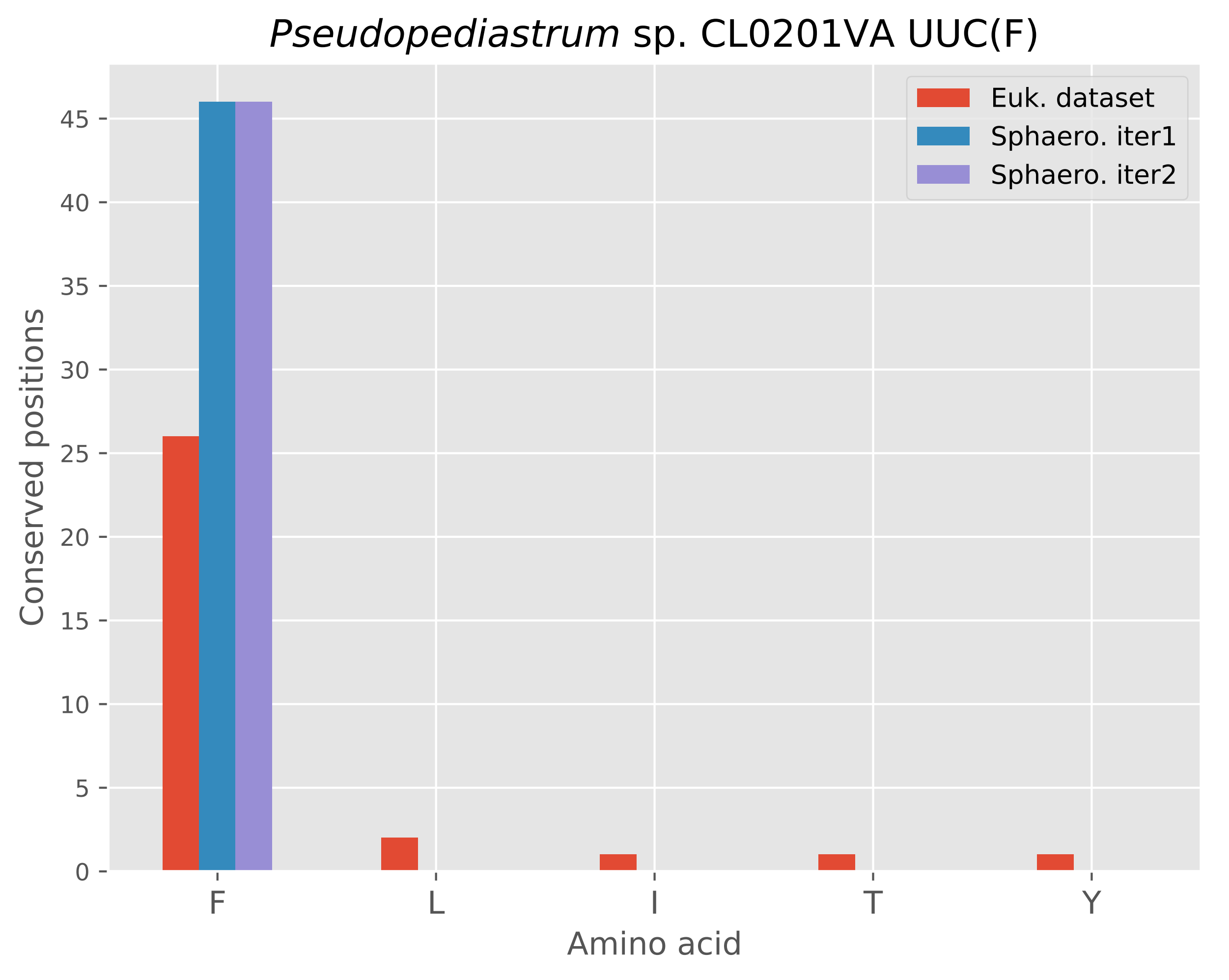

Pseudopediastrum sp. CL0201VA UUC(F)
Euk. dataset
Sphaero. iter1
Sphaero. iter2
Conserved positions
0
5
10
15
20
25
30
35
40
45
F
L
I
T
Y
Amino acid

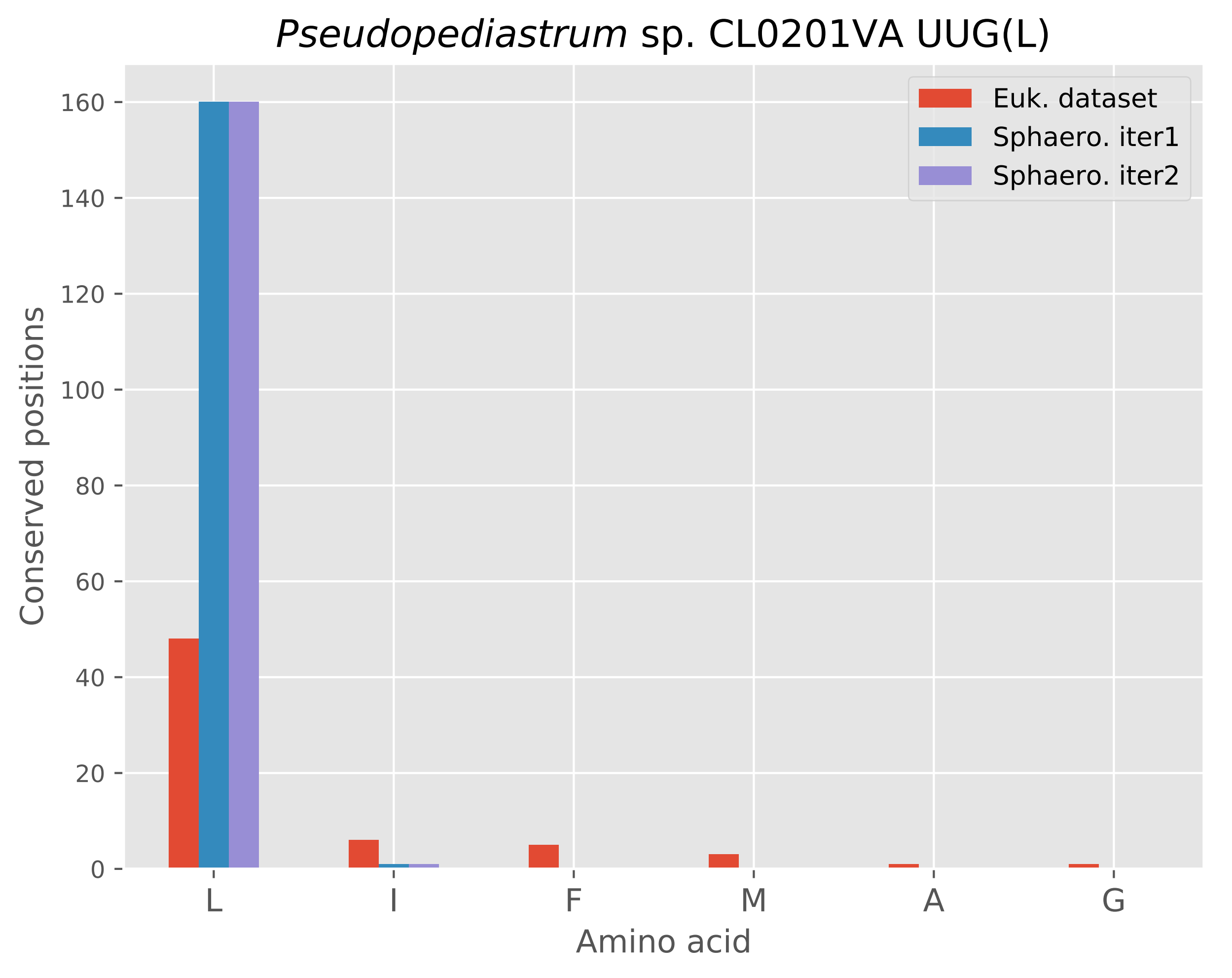

Pseudopediastrum sp. CL0201VA UUG(L)
Euk. dataset
Sphaero. iter1
Sphaero. iter2
Conserved positions
0
20
40
60
80
100
120
140
160
L
I
F
M
A
G
Amino acid

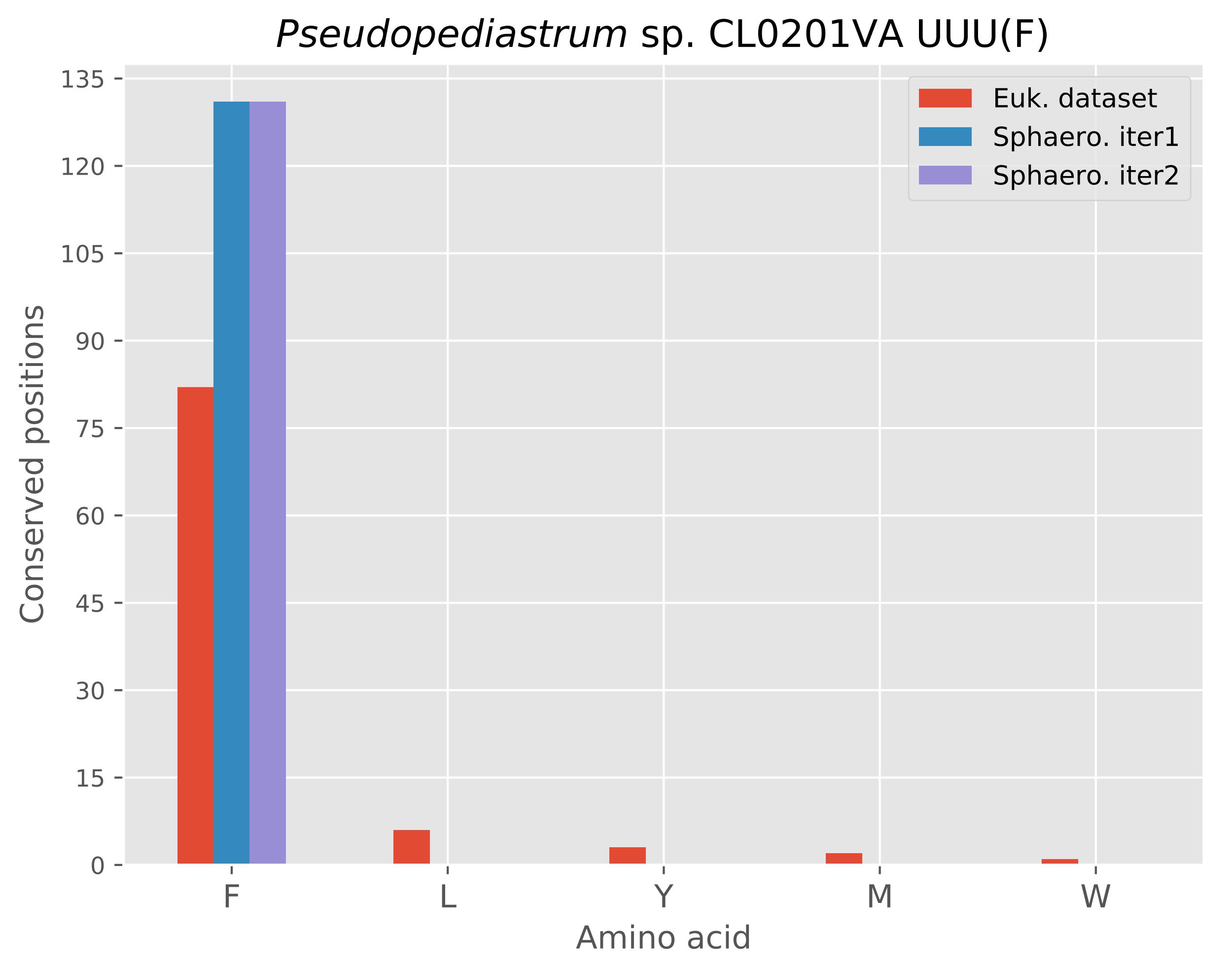

Pseudopediastrum sp. CL0201VA UUU(F)
Euk. dataset
Sphaero. iter1
Sphaero. iter2
Conserved positions
0
15
30
45
60
75
90
105
120
135
F
L
Y
M
W
Amino acid

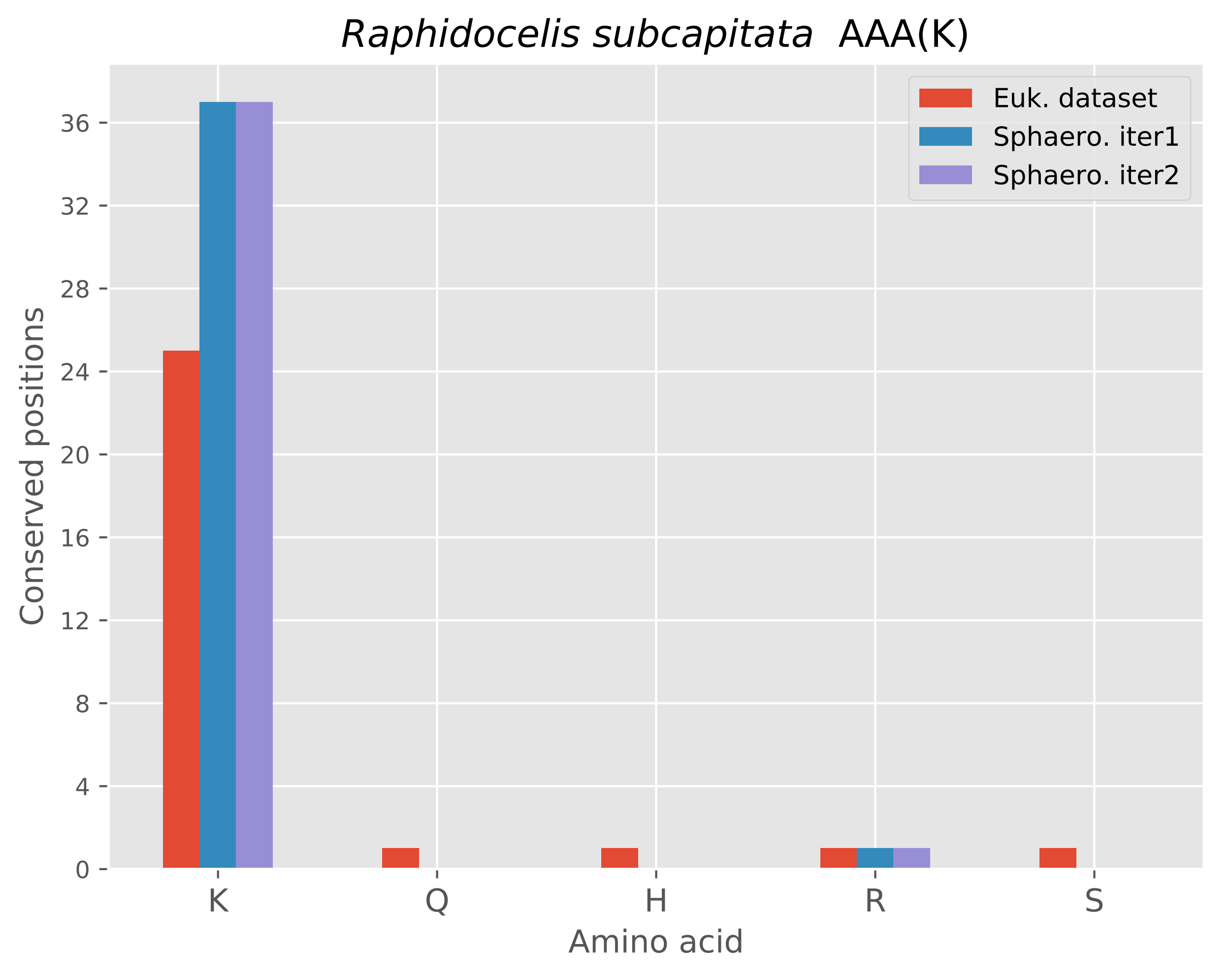

Raphidocelis subcapitata AAA(K)
Euk. dataset
Sphaero. iter1
Sphaero. iter2
Conserved positions
0
4
8
12
16
20
24
28
32
36
K
Q
H
R
S
Amino acid

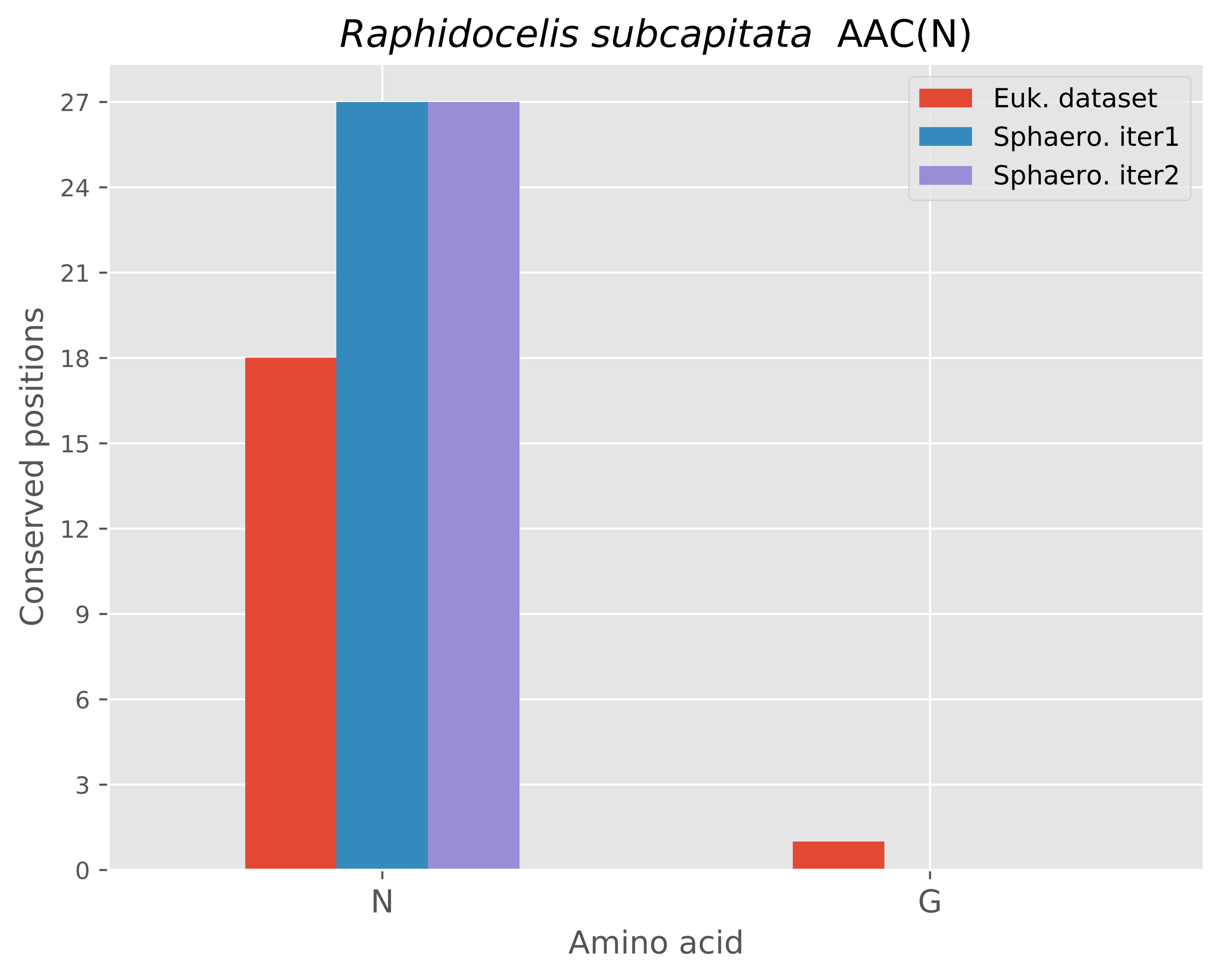

Raphidocelis subcapitata AAC(N)
Euk. dataset
Sphaero. iter1
Sphaero. iter2
Conserved positions
0
3
6
9
12
15
18
21
24
27
N
G
Amino acid

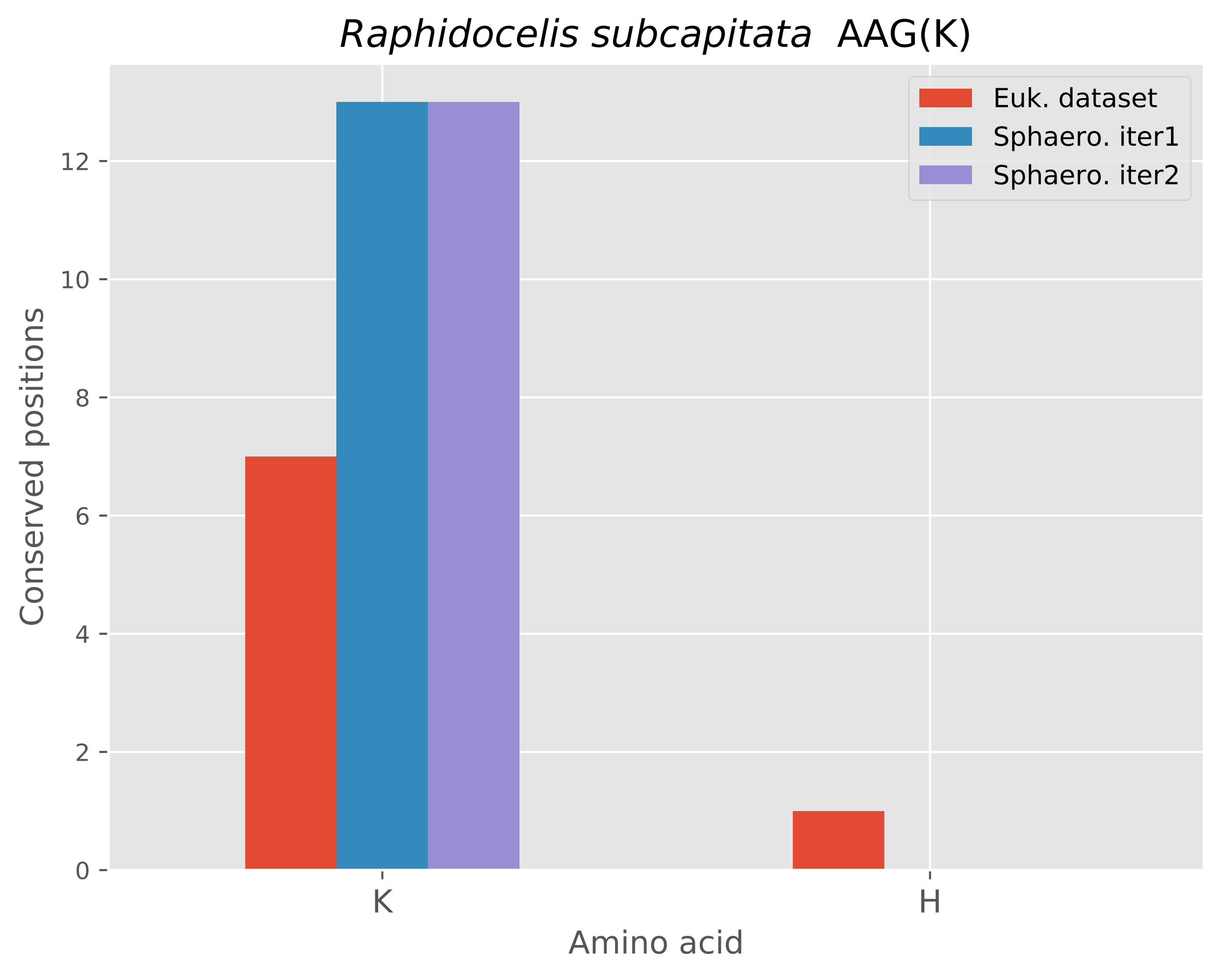

Raphidocelis subcapitata AAG(K)
Euk. dataset
Sphaero. iter1
Sphaero. iter2
Conserved positions
0
2
4
6
8
10
12
K
H
Amino acid

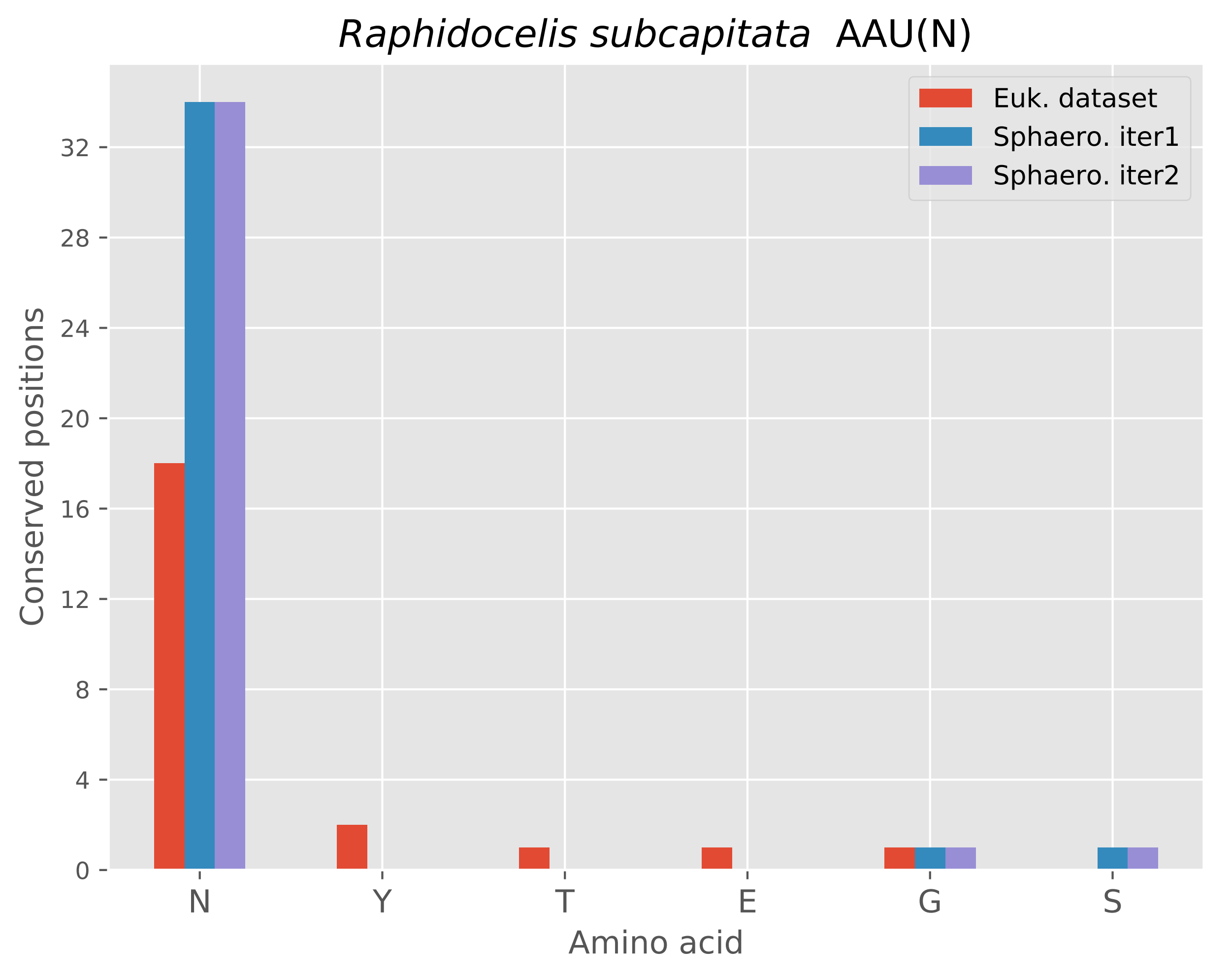

Raphidocelis subcapitata AAU(N)
Euk. dataset
Sphaero. iter1
Sphaero. iter2
Conserved positions
0
4
8
12
16
20
24
28
32
N
Y
T
E
G
S
Amino acid

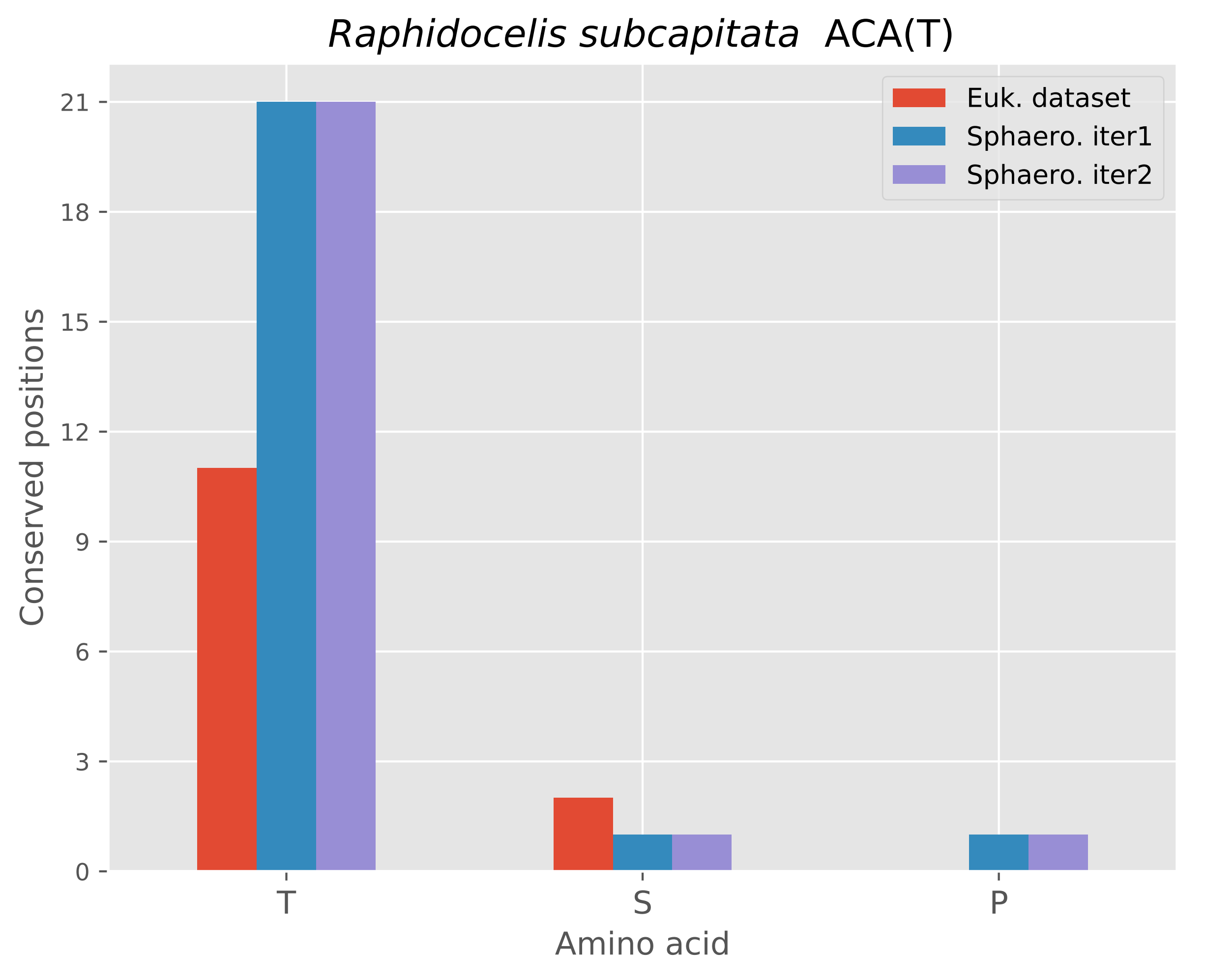

Raphidocelis subcapitata ACA(T)
Euk. dataset
Sphaero. iter1
Sphaero. iter2
Conserved positions
0
3
6
9
12
15
18
21
T
S
P
Amino acid

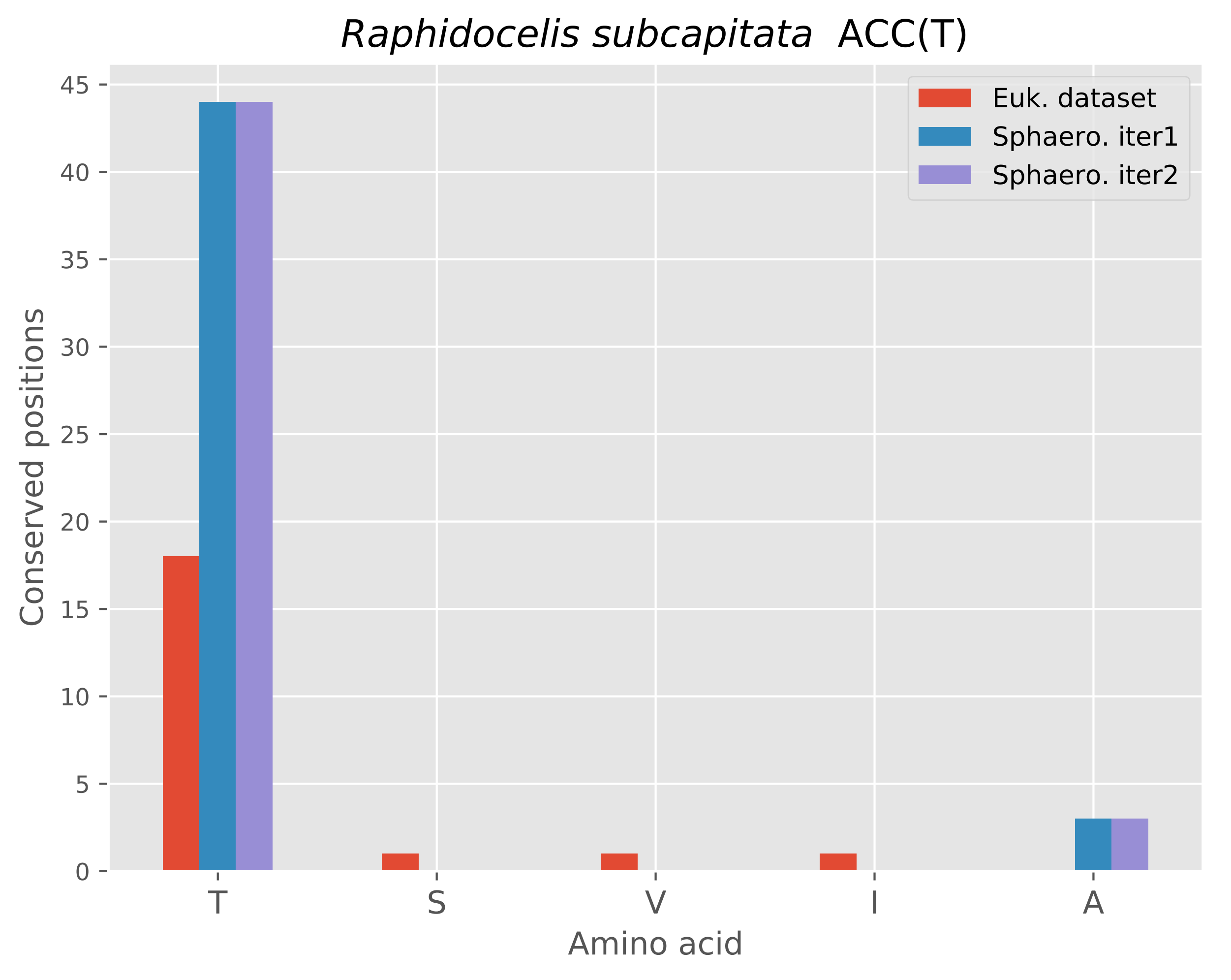

Raphidocelis subcapitata ACC(T)
Euk. dataset
Sphaero. iter1
Sphaero. iter2
Conserved positions
0
5
10
15
20
25
30
35
40
45
T
S
V
I
A
Amino acid

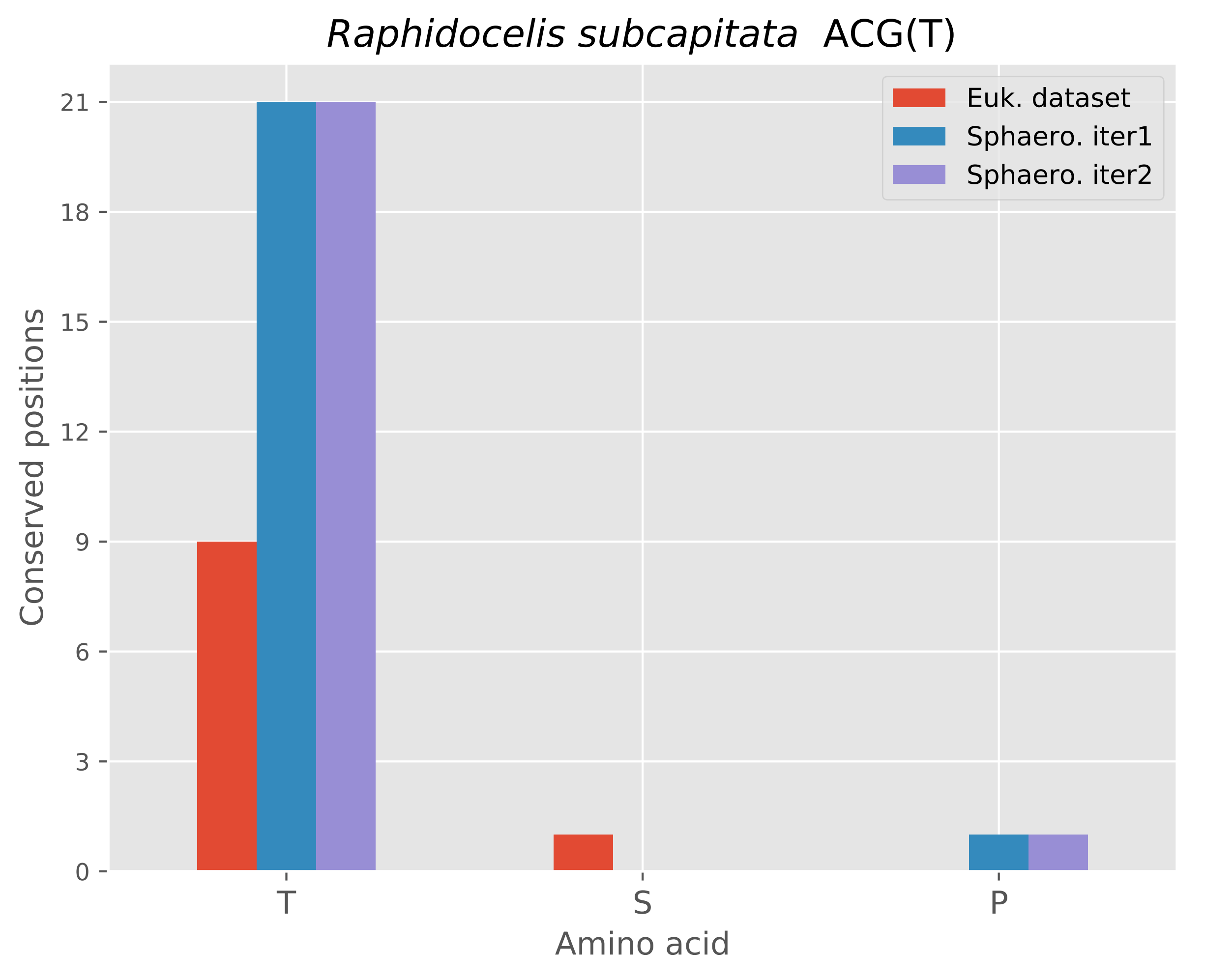

Raphidocelis subcapitata ACG(T)
Euk. dataset
Sphaero. iter1
Sphaero. iter2
Conserved positions
0
3
6
9
12
15
18
21
T
S
P
Amino acid

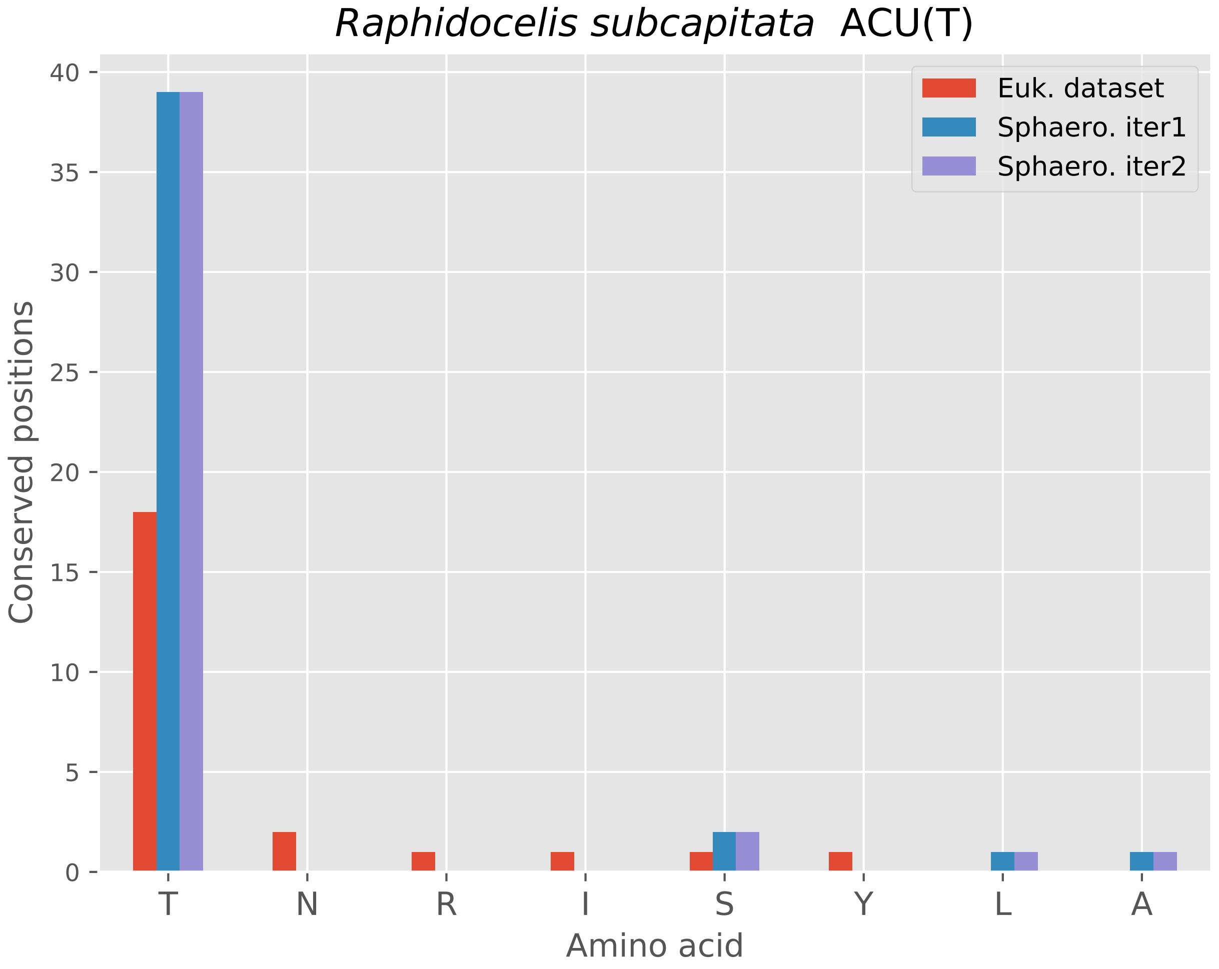

Raphidocelis subcapitata ACU(T)
Euk. dataset
Sphaero. iter1
Sphaero. iter2
Conserved positions
0
5
10
15
20
25
30
35
40
T
N
R
I
S
Y
L
A
Amino acid

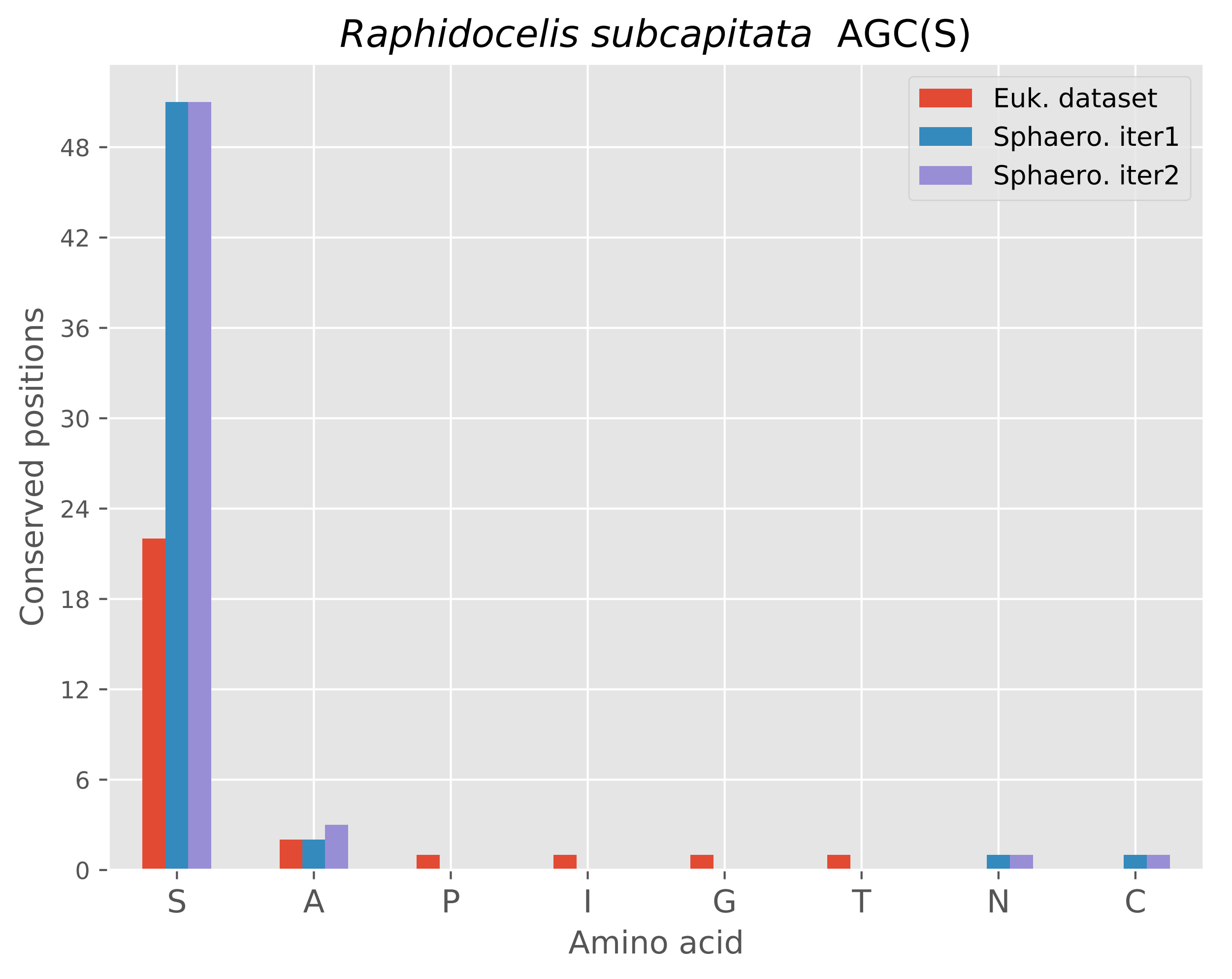

Raphidocelis subcapitata AGC(S)
Euk. dataset
Sphaero. iter1
Sphaero. iter2
Conserved positions
0
6
12
18
24
30
36
42
48
S
A
P
I
G
T
N
C
Amino acid

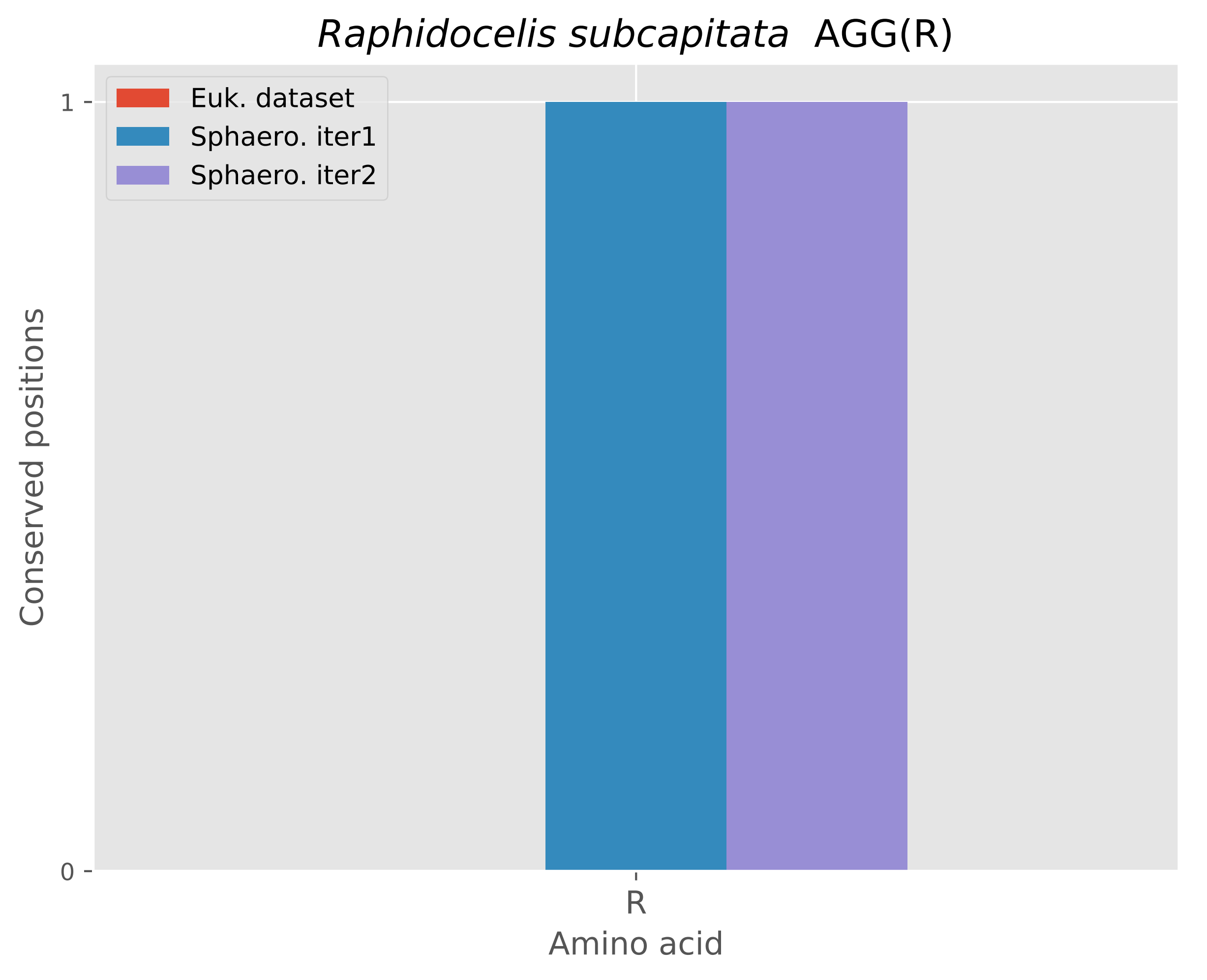

Raphidocelis subcapitata AGG(R)
Euk. dataset
Sphaero. iter1
Sphaero. iter2
1
0
Conserved positions
R
Amino acid

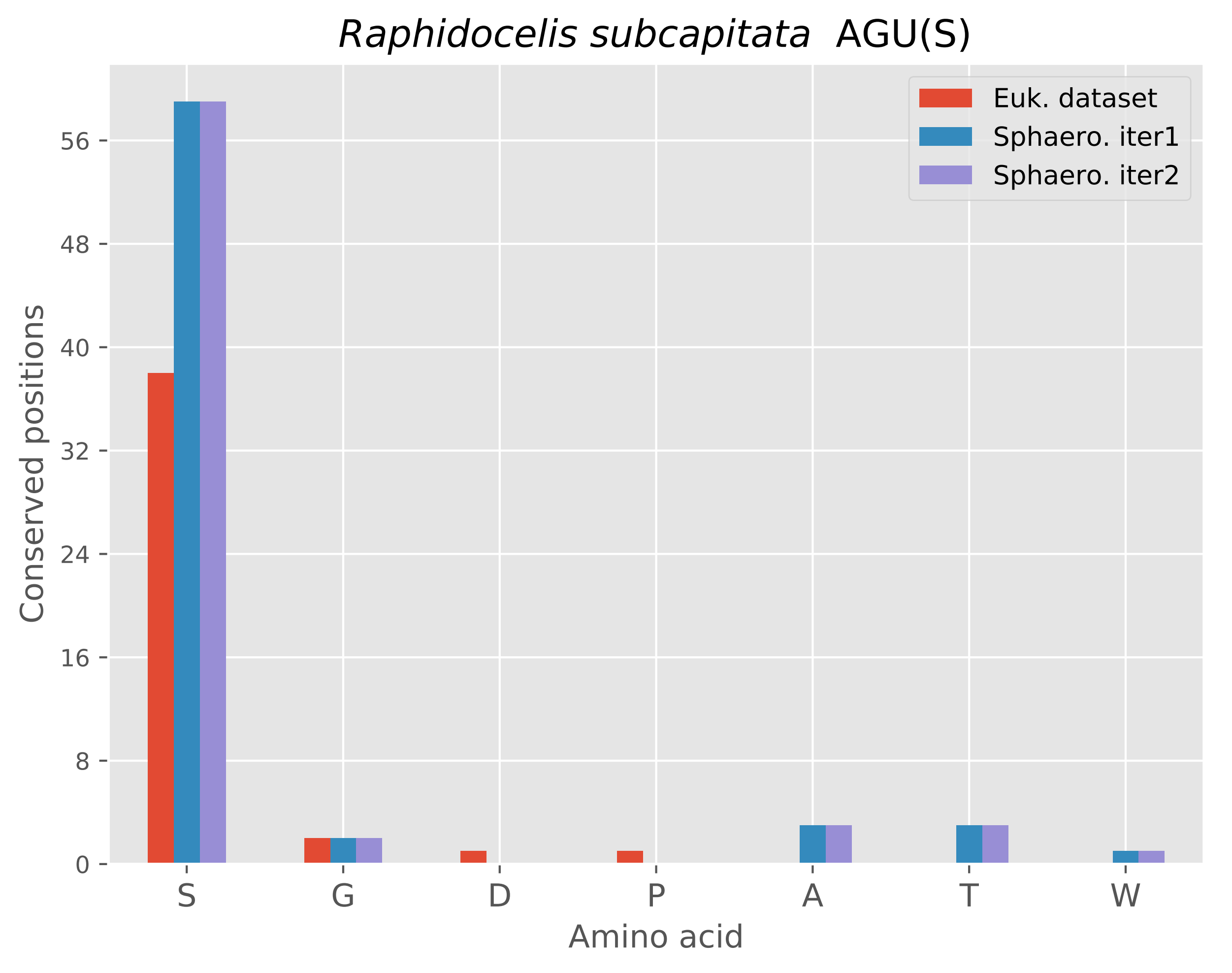

Raphidocelis subcapitata AGU(S)
Euk. dataset
Sphaero. iter1
Sphaero. iter2
Conserved positions
0
8
16
24
32
40
48
56
S
G
D
P
A
T
W
Amino acid

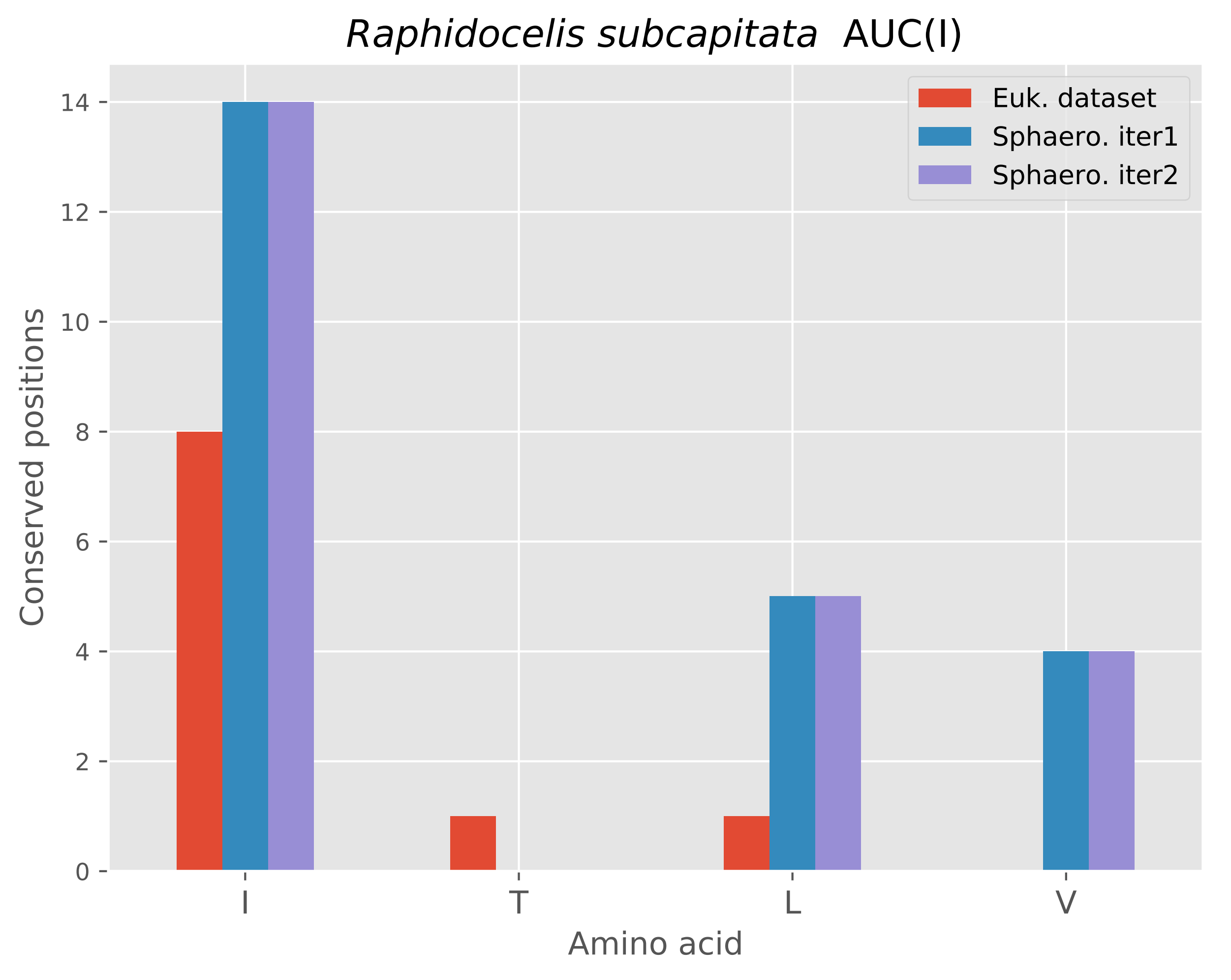

Raphidocelis subcapitata AUC(I)
Euk. dataset
Sphaero. iter1
Sphaero. iter2
Conserved positions
0
2
4
6
8
10
12
14
I
T
L
V
Amino acid

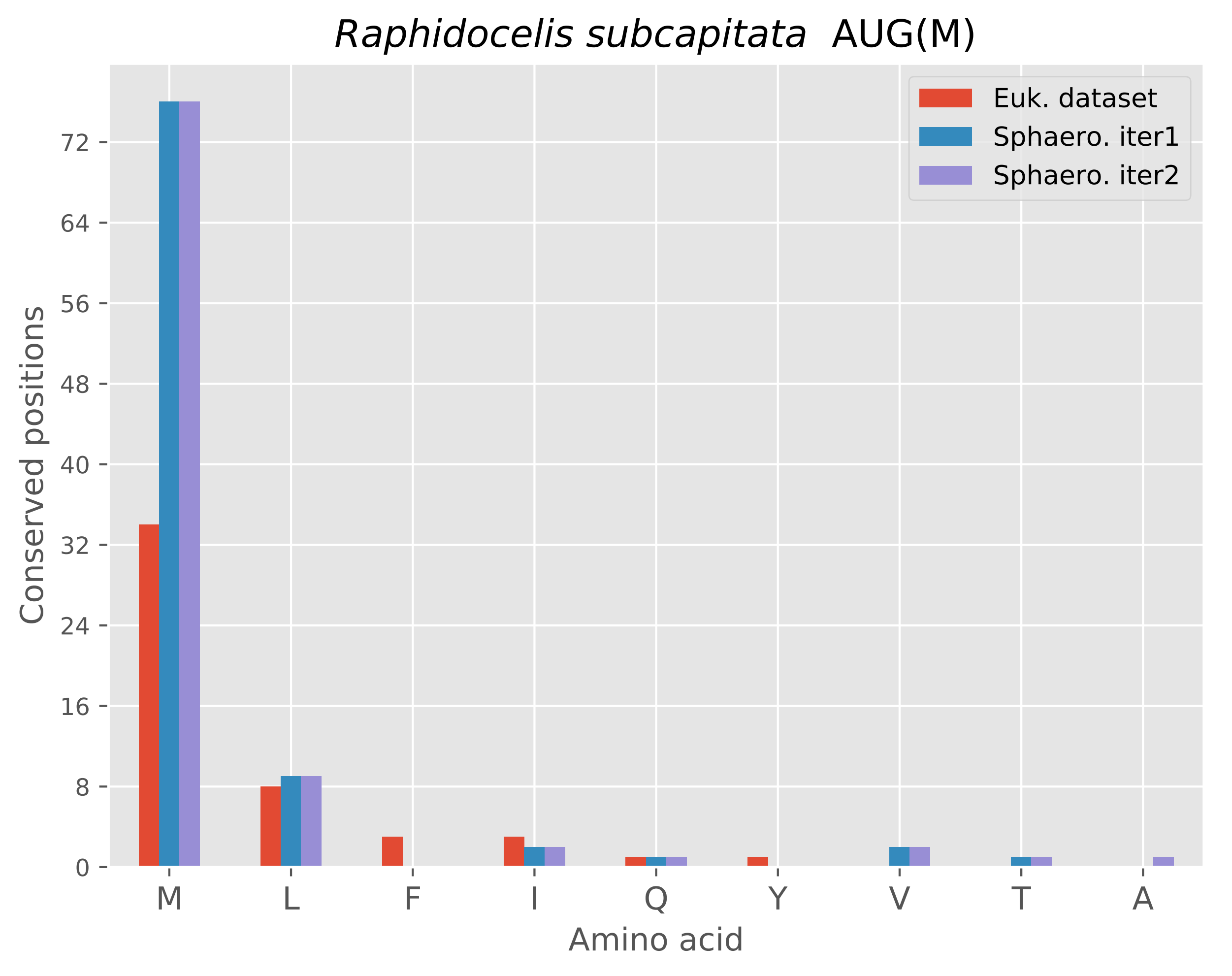

Raphidocelis subcapitata AUG(M)
Euk. dataset
Sphaero. iter1
Sphaero. iter2
Conserved positions
0
8
16
24
32
40
48
56
64
72
M
L
F
I
Q
Y
V
T
A
Amino acid

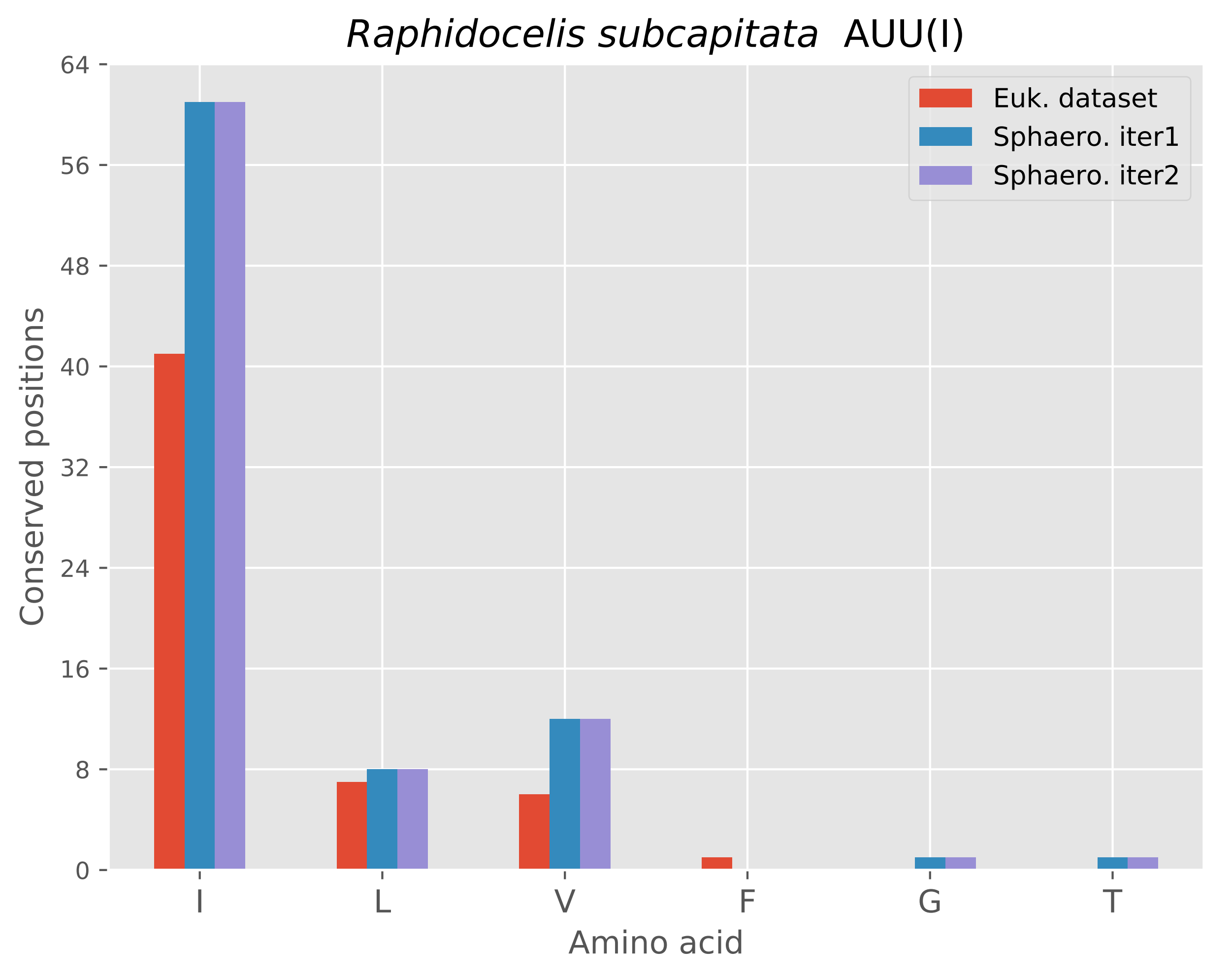

Raphidocelis subcapitata AUU(I)
Conserved positions
0
8
16
24
32
40
48
56
64
Euk. dataset
Sphaero. iter1
Sphaero. iter2
I
L
V
F
G
T
Amino acid

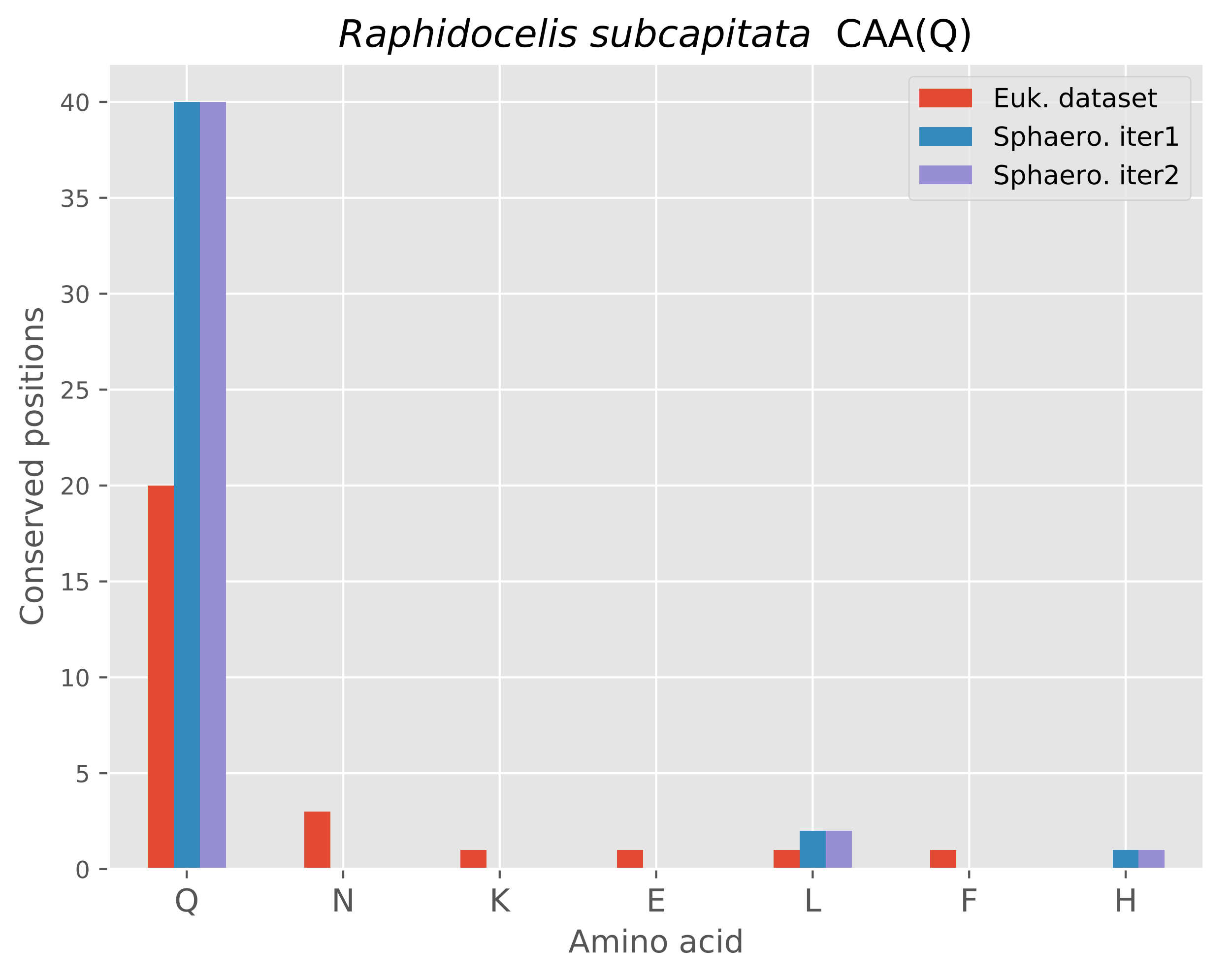

Raphidocelis subcapitata CAA(Q)
Euk. dataset
Sphaero. iter1
Sphaero. iter2
Conserved positions
0
5
10
15
20
25
30
35
40
Q
N
K
E
L
F
H
Amino acid

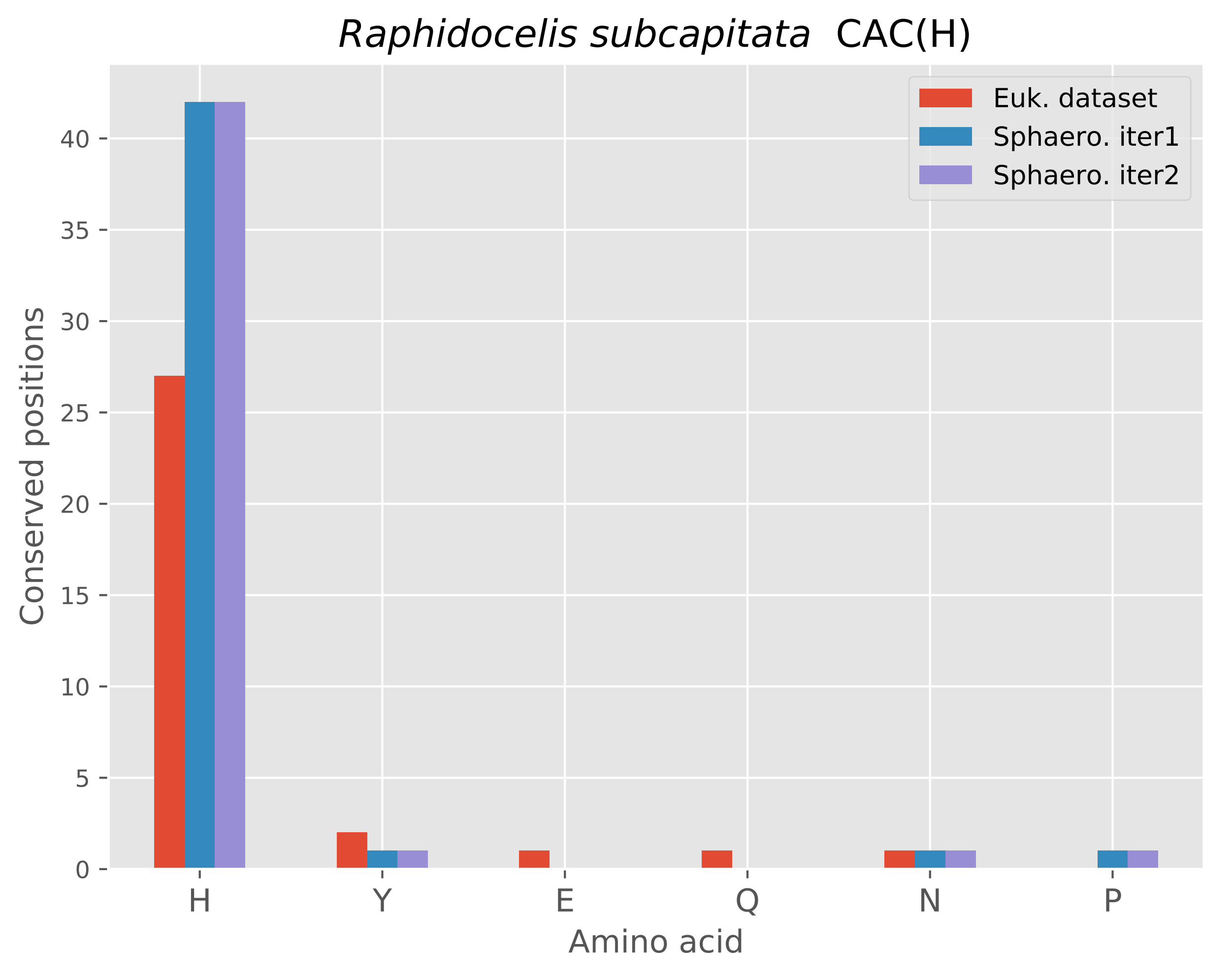

Raphidocelis subcapitata CAC(H)
Euk. dataset
Sphaero. iter1
Sphaero. iter2
Conserved positions
0
5
10
15
20
25
30
35
40
H
Y
E
Q
N
P
Amino acid

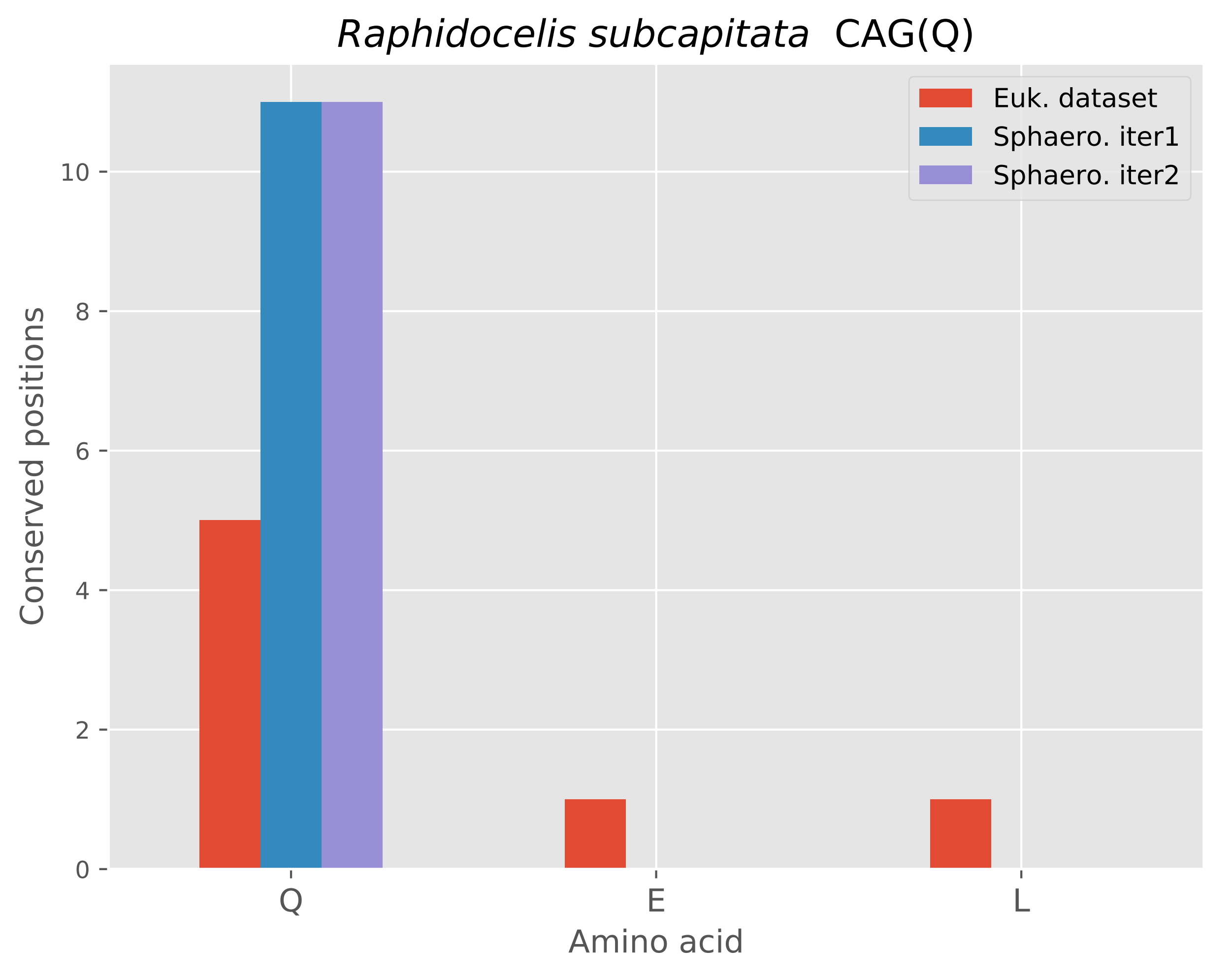

Raphidocelis subcapitata CAG(Q)
Euk. dataset
Sphaero. iter1
Sphaero. iter2
Conserved positions
0
2
4
6
8
10
Q
E
L
Amino acid

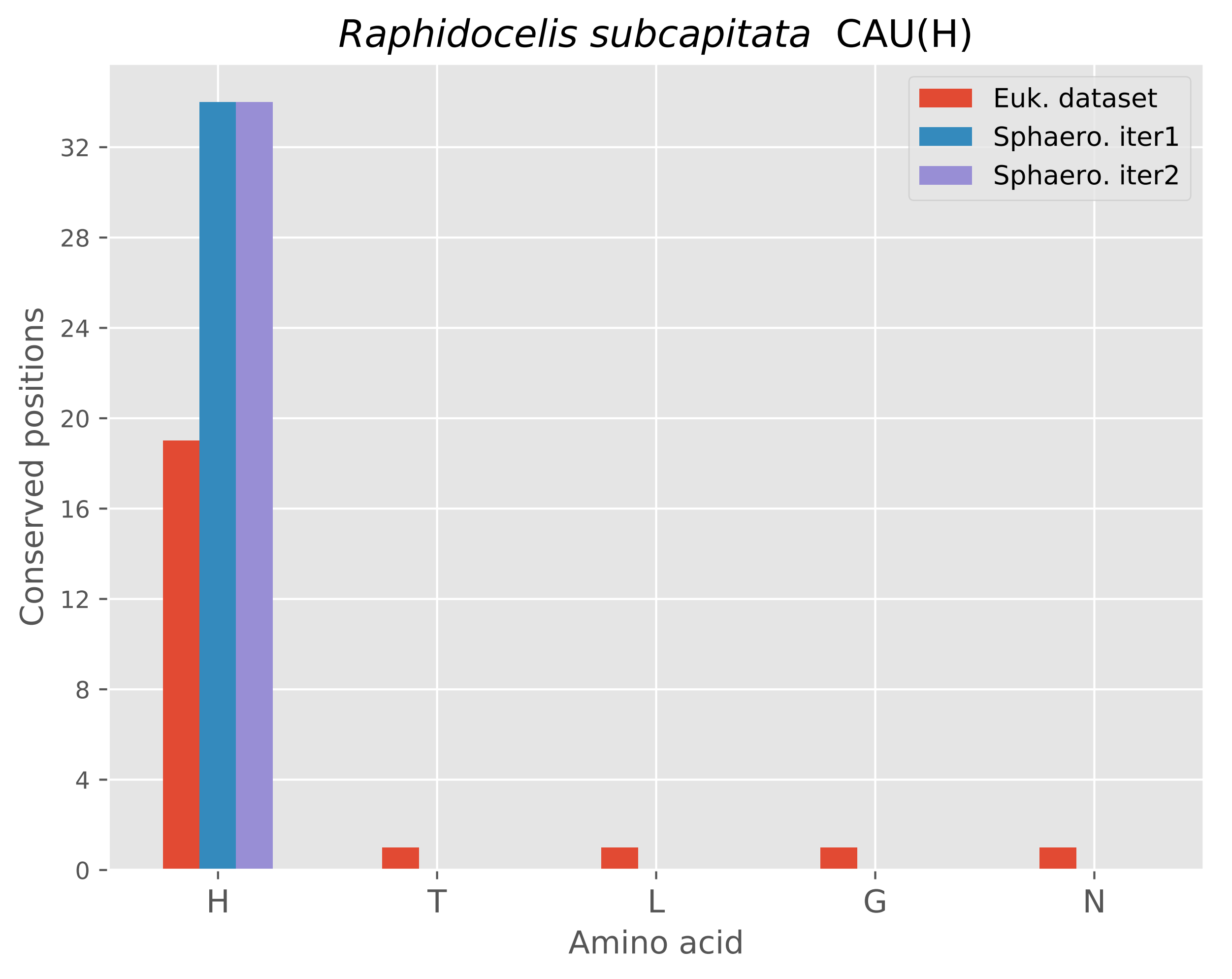

Raphidocelis subcapitata CAU(H)
Euk. dataset
Sphaero. iter1
Sphaero. iter2
Conserved positions
0
4
8
12
16
20
24
28
32
H
T
L
G
N
Amino acid

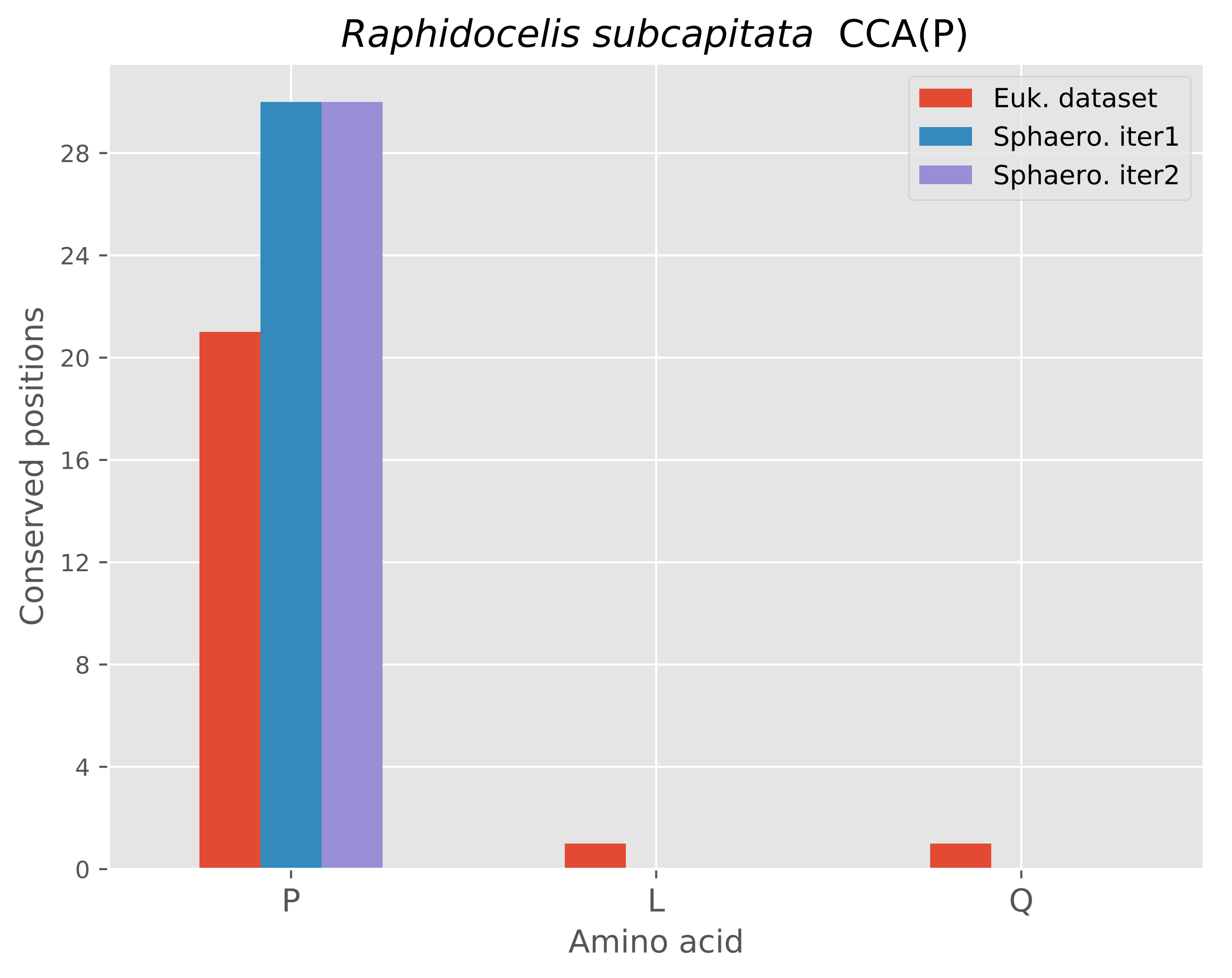
Raphidocelis subcapitata CCA(P)
Euk. dataset
Sphaero. iter1
Sphaero. iter2
Conserved positions
0
4
8
12
16
20
24
28
P
L
Q
Amino acid

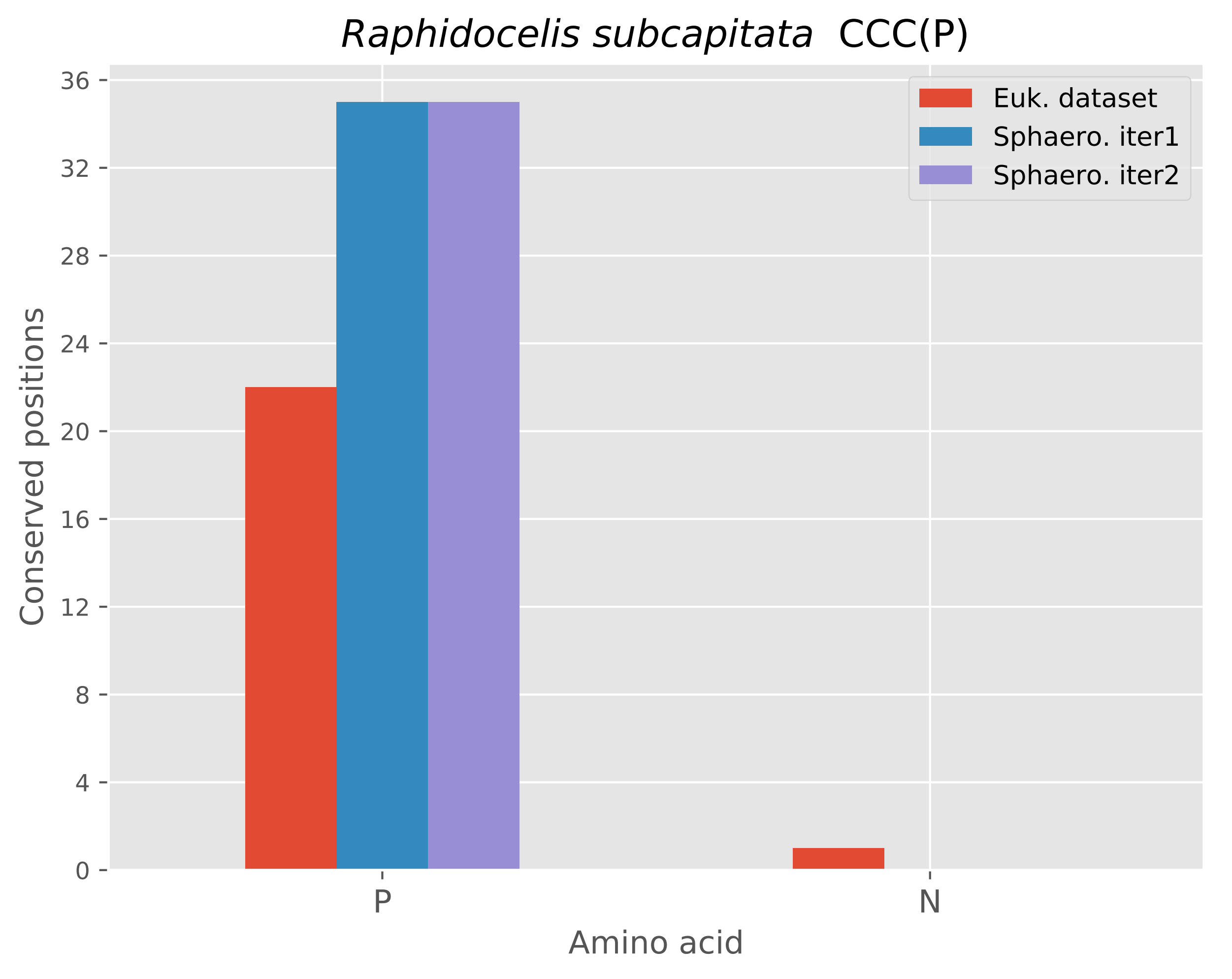

Raphidocelis subcapitata CCC(P)
Euk. dataset
Sphaero. iter1
Sphaero. iter2
Conserved positions
0
4
8
12
16
20
24
28
32
36
P
N
Amino acid

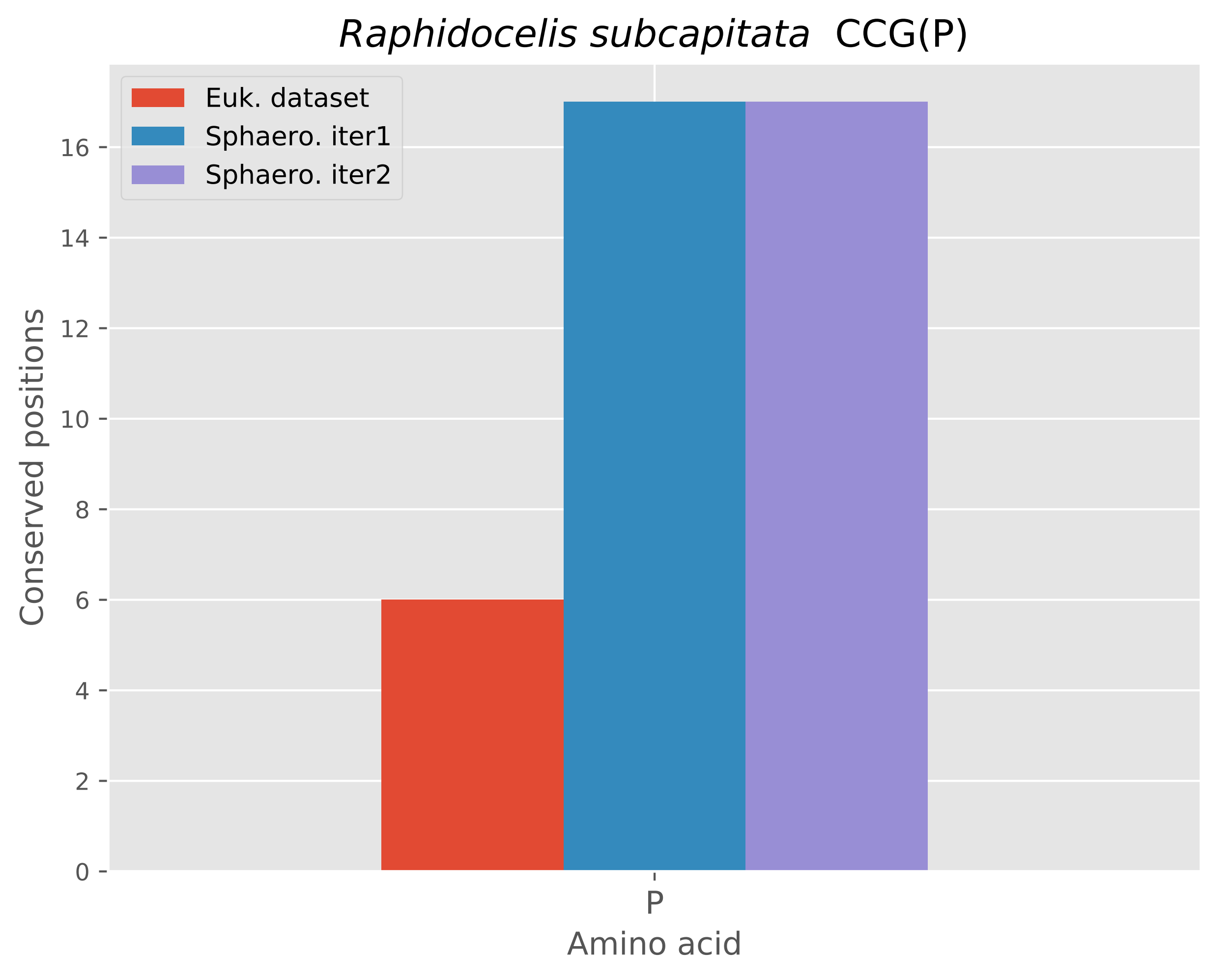

Raphidocelis subcapitata CCG(P)
Euk. dataset
Sphaero. iter1
Sphaero. iter2
Conserved positions
0
2
4
6
8
10
12
14
16
P
Amino acid

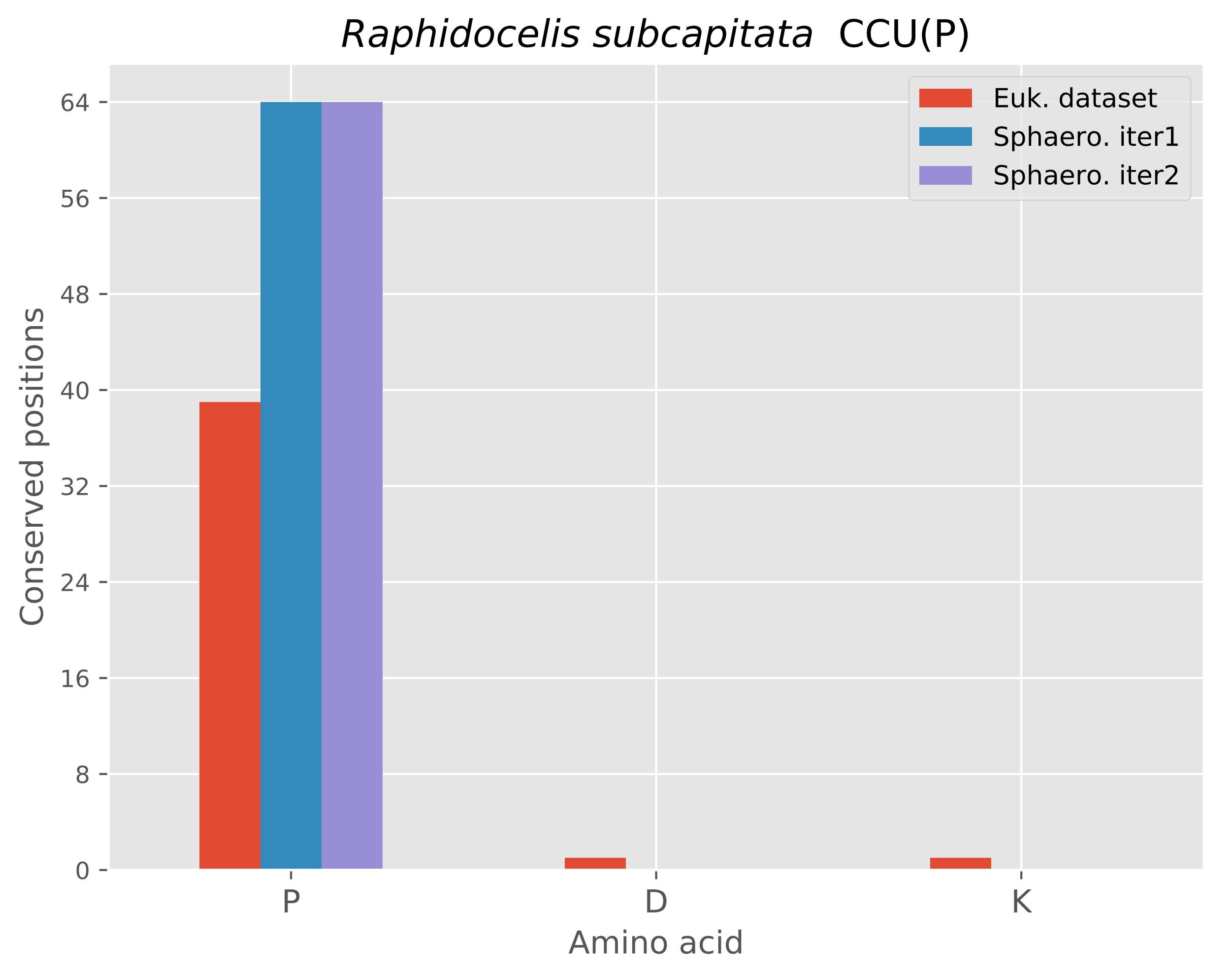

Raphidocelis subcapitata CCU(P)
Euk. dataset
Sphaero. iter1
Sphaero. iter2
Conserved positions
0
8
16
24
32
40
48
56
64
P
D
K
Amino acid

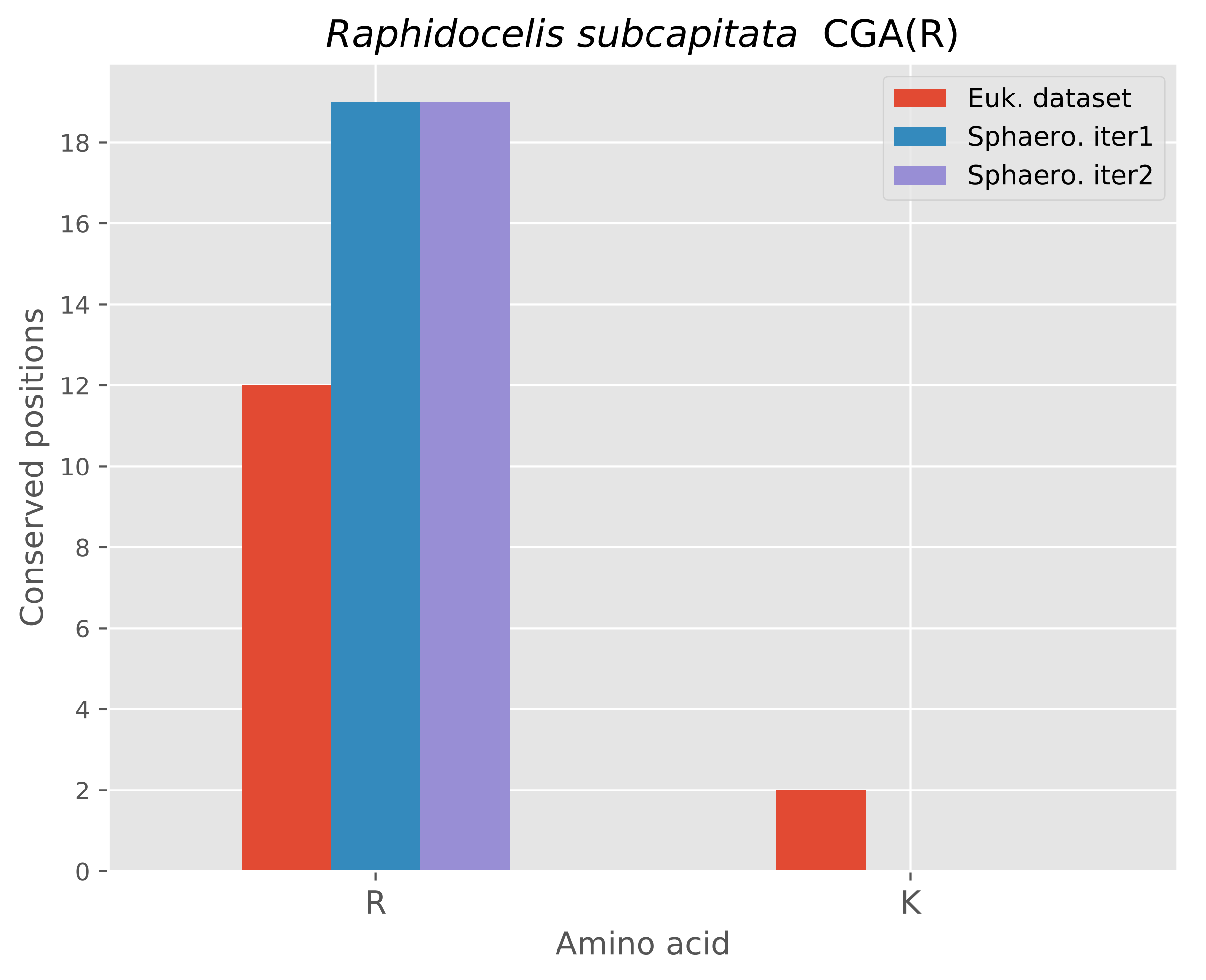

Raphidocelis subcapitata CGA(R)
Euk. dataset
Sphaero. iter1
Sphaero. iter2
Conserved positions
Amino acid
R
K
0
2
4
6
8
10
12
14
16
18

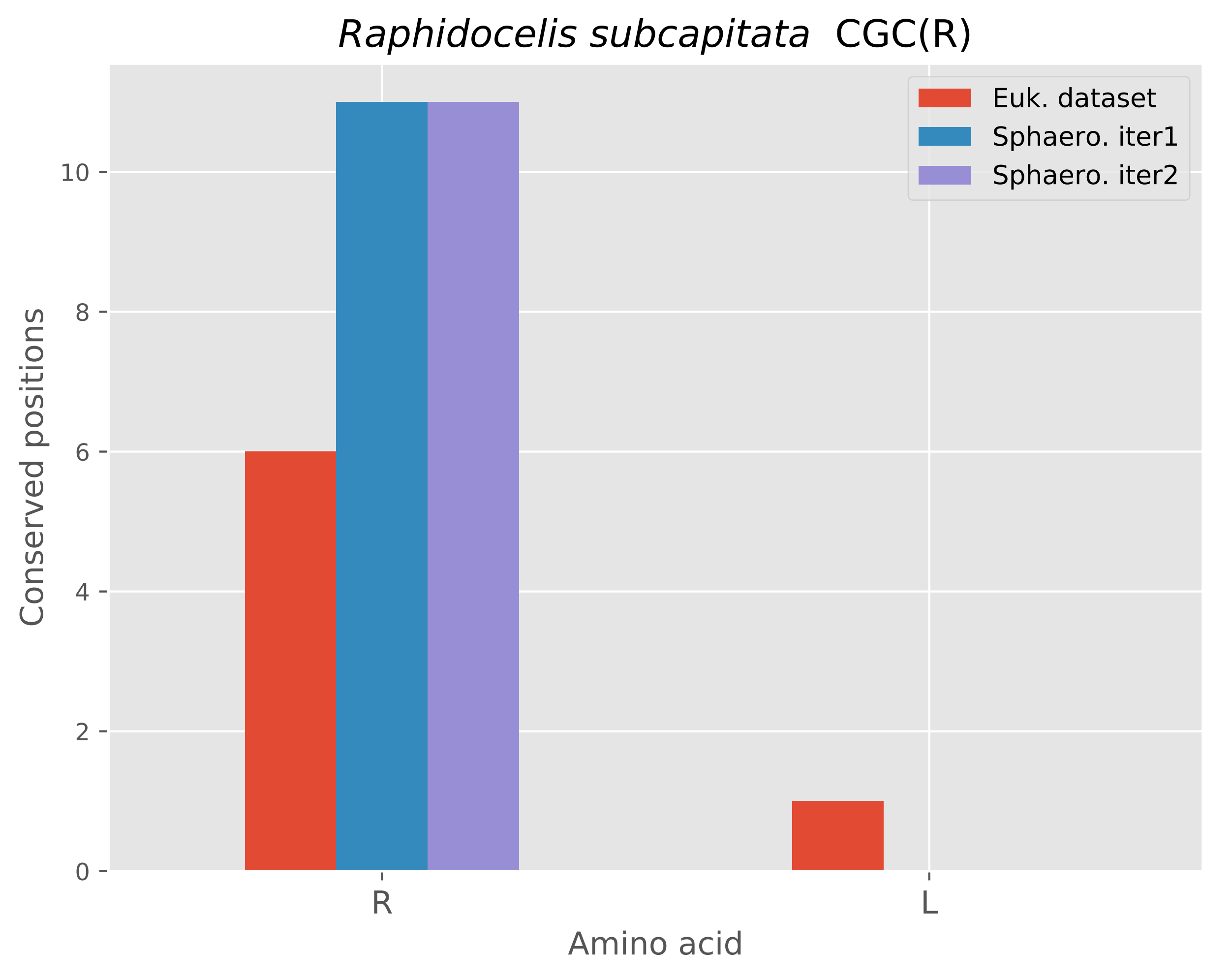

Raphidocelis subcapitata CGC(R)
Euk. dataset
Sphaero. iter1
Sphaero. iter2
Conserved positions
0
2
4
6
8
10
R
L
Amino acid

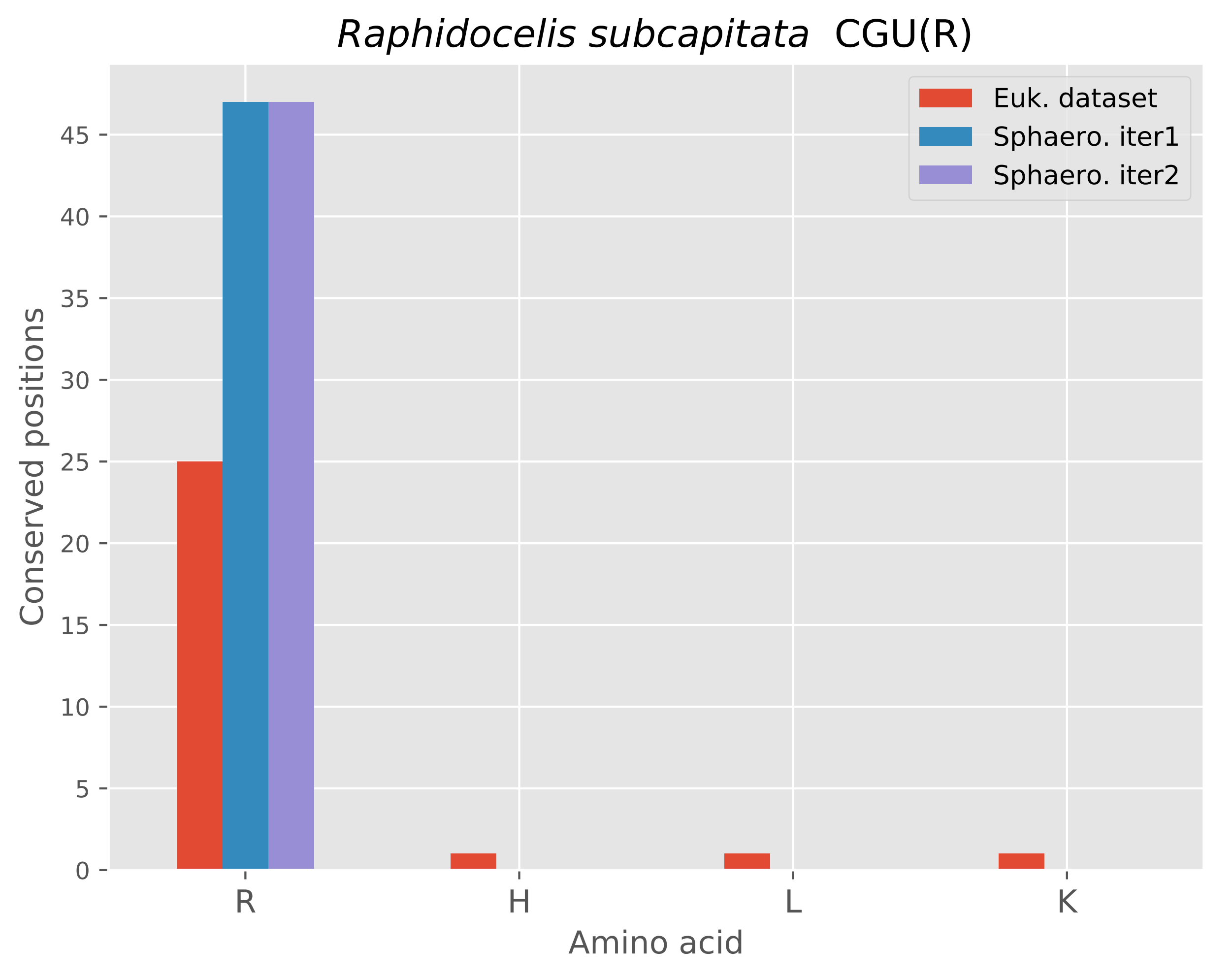
Raphidocelis subcapitata CGU(R)
Euk. dataset
Sphaero. iter1
Sphaero. iter2
Conserved positions
0
5
10
15
20
25
30
35
40
45
R
H
L
K
Amino acid

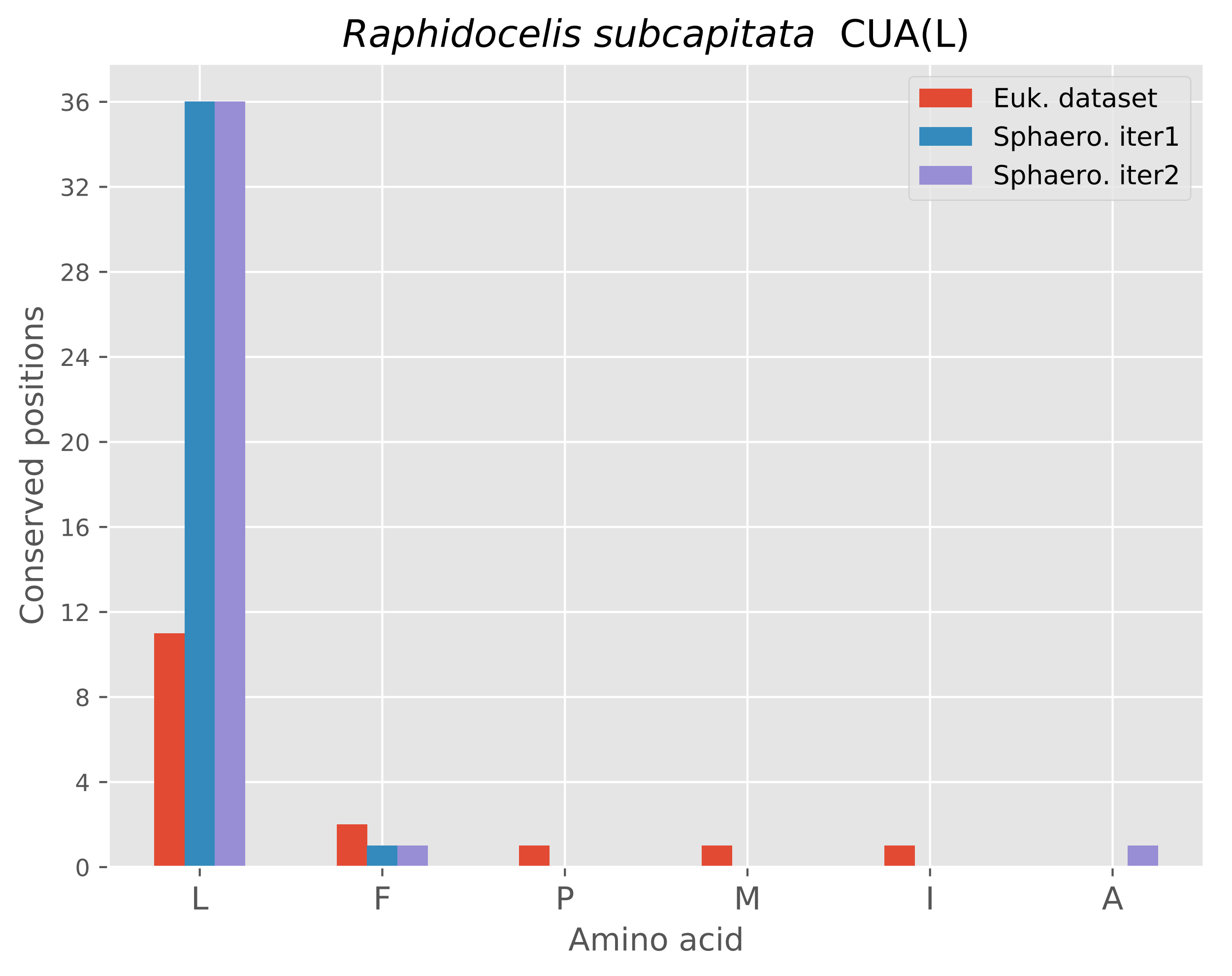
Raphidocelis subcapitata CUA(L)
Euk. dataset
Sphaero. iter1
Sphaero. iter2
Conserved positions
0
4
8
12
16
20
24
28
32
36
L
F
P
M
I
A
Amino acid

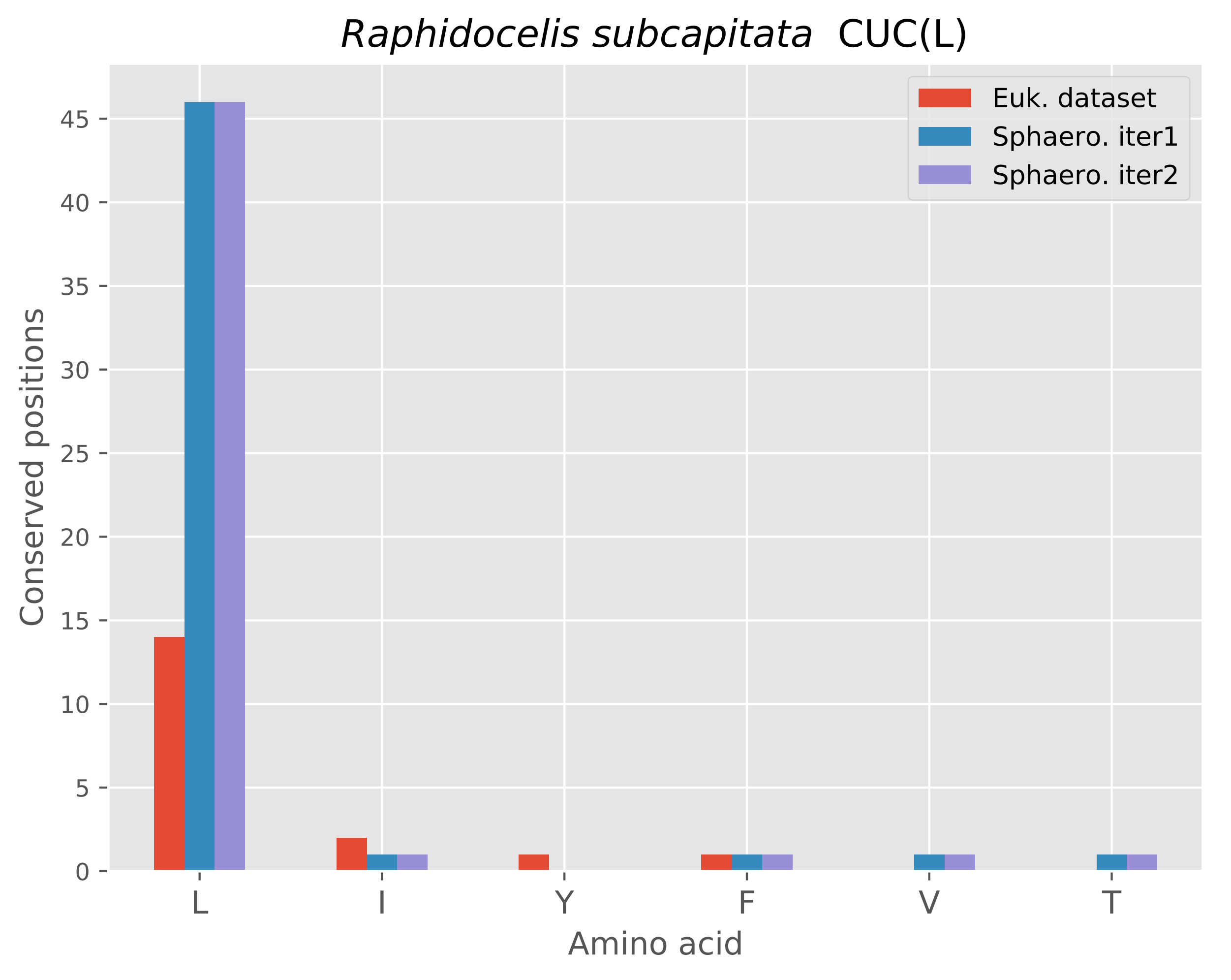

Raphidocelis subcapitata CUC(L)
Euk. dataset
Sphaero. iter1
Sphaero. iter2
Conserved positions
0
5
10
15
20
25
30
35
40
45
L
I
Y
F
V
T
Amino acid

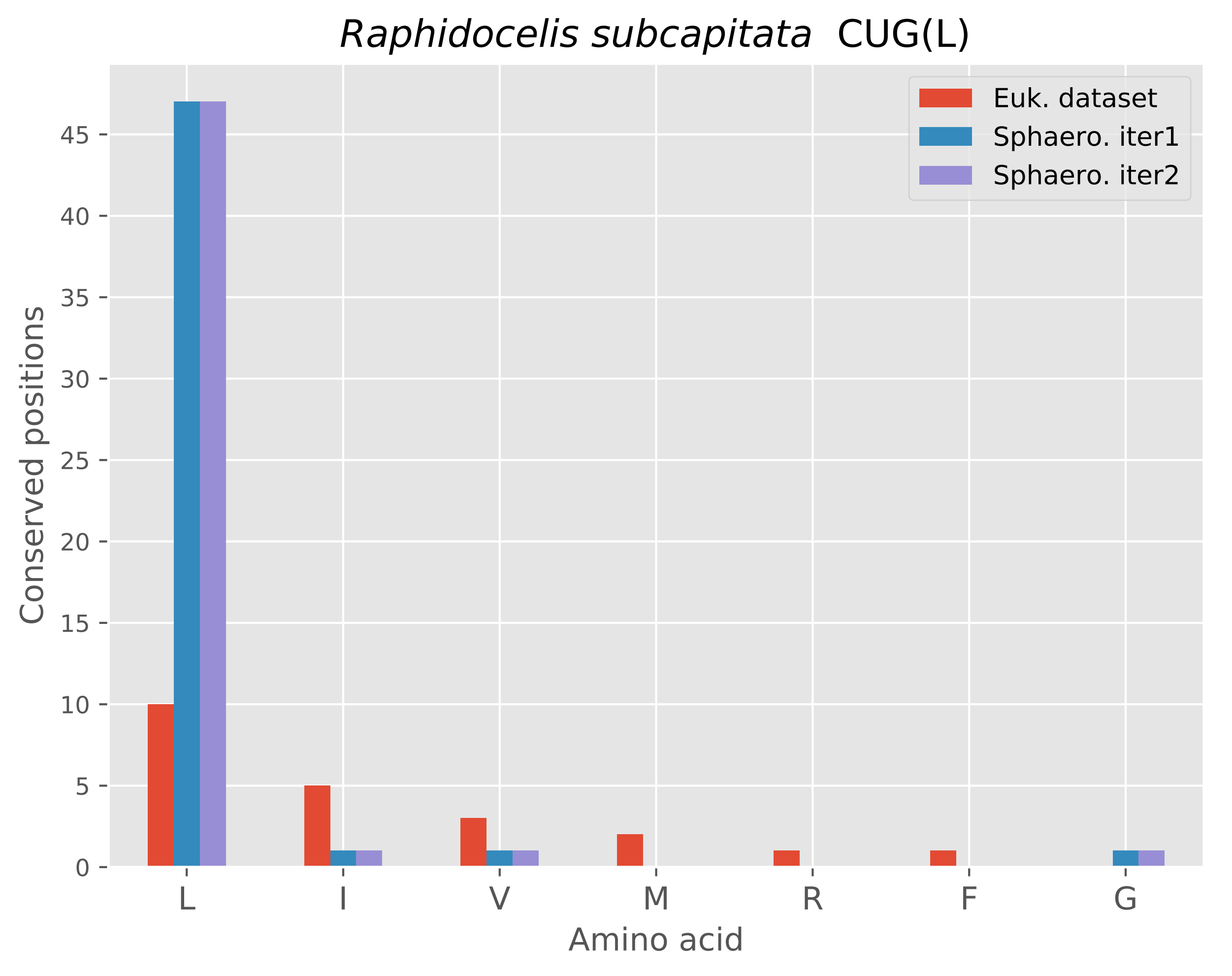

Raphidocelis subcapitata CUG(L)
Euk. dataset
Sphaero. iter1
Sphaero. iter2
Conserved positions
0
5
10
15
20
25
30
35
40
45
L
I
V
M
R
F
G
Amino acid

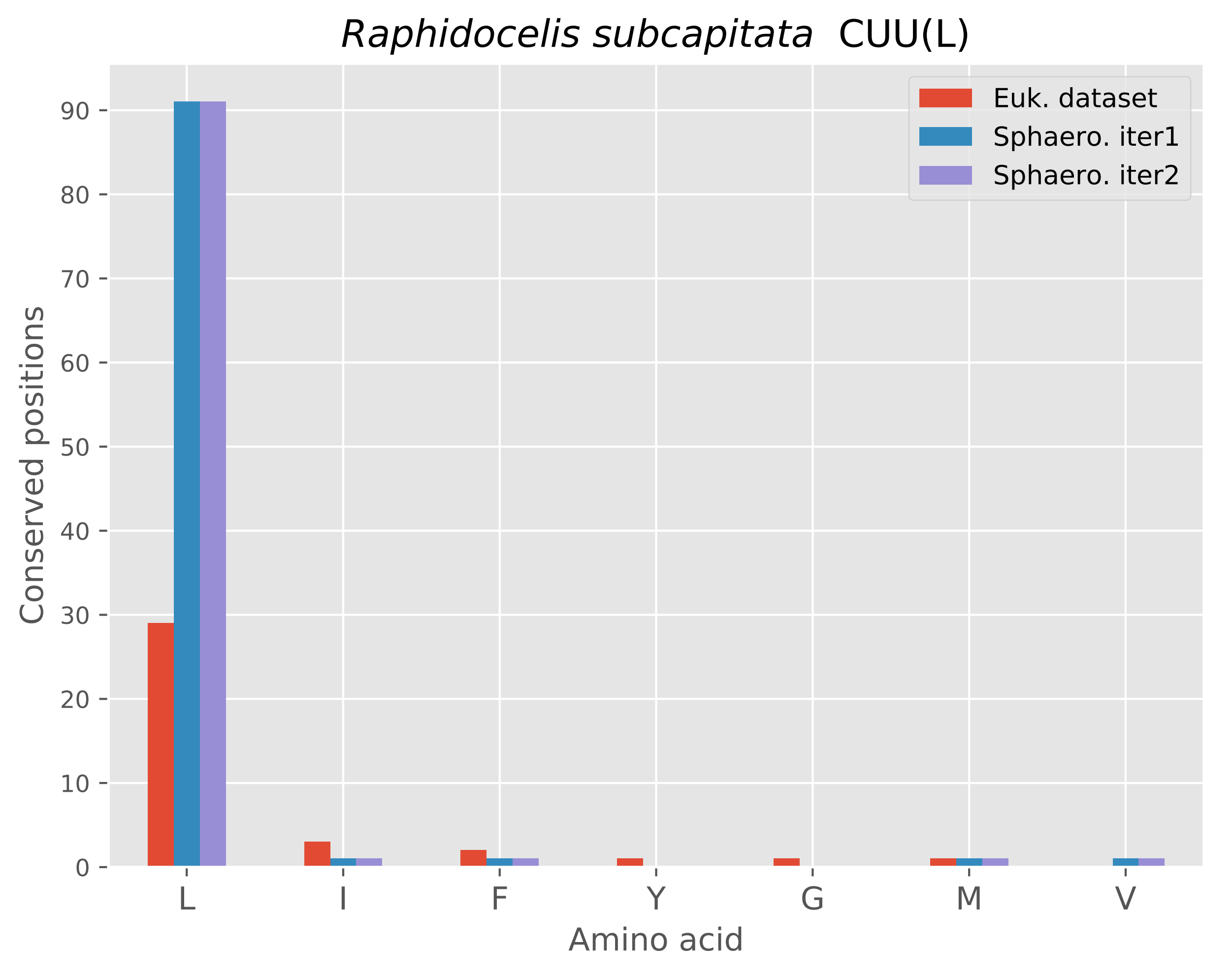

Raphidocelis subcapitata CUU(L)
Euk. dataset
Sphaero. iter1
Sphaero. iter2
Conserved positions
0
10
20
30
40
50
60
70
80
90
L
I
F
Y
G
M
V
Amino acid

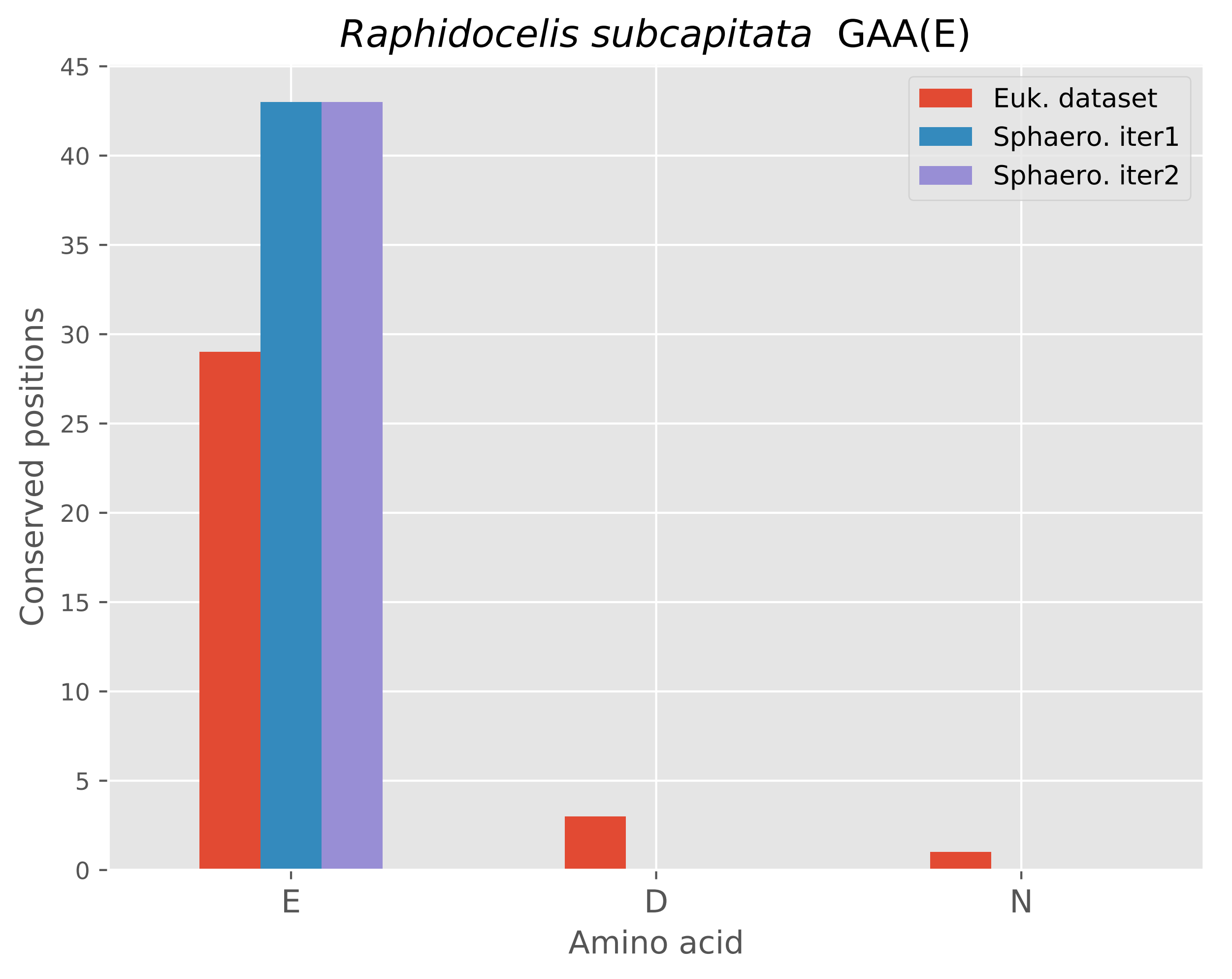

Raphidocelis subcapitata GAA(E)
Euk. dataset
Sphaero. iter1
Sphaero. iter2
Conserved positions
0
5
10
15
20
25
30
35
40
45
E
D
N
Amino acid

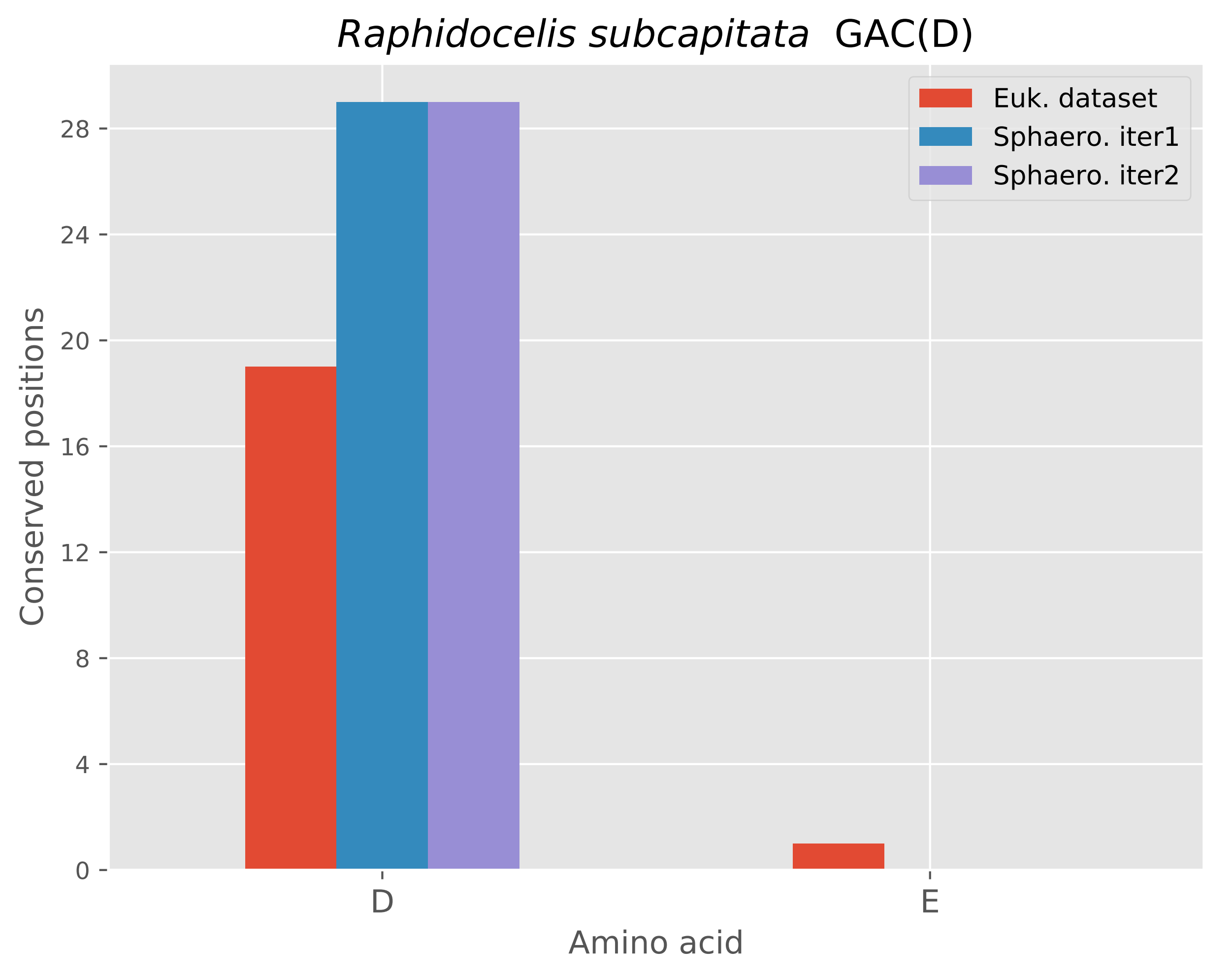

Raphidocelis subcapitata GAC(D)
Euk. dataset
Sphaero. iter1
Sphaero. iter2
Conserved positions
0
4
8
12
16
20
24
28
D
E
Amino acid

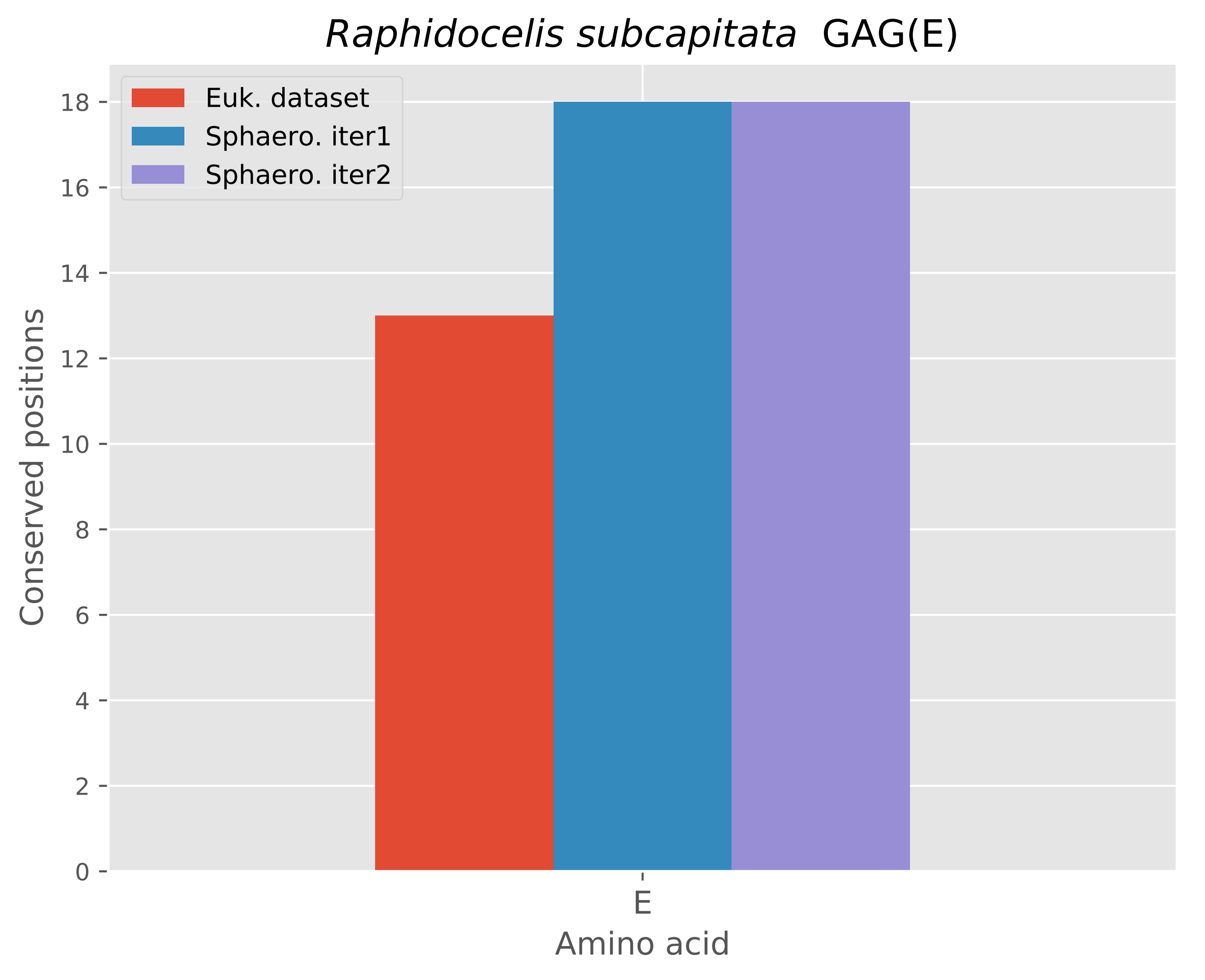

Raphidocelis subcapitata GAG(E)
Euk. dataset
Sphaero. iter1
Sphaero. iter2
Conserved positions
0
2
4
6
8
10
12
14
16
18
E
Amino acid

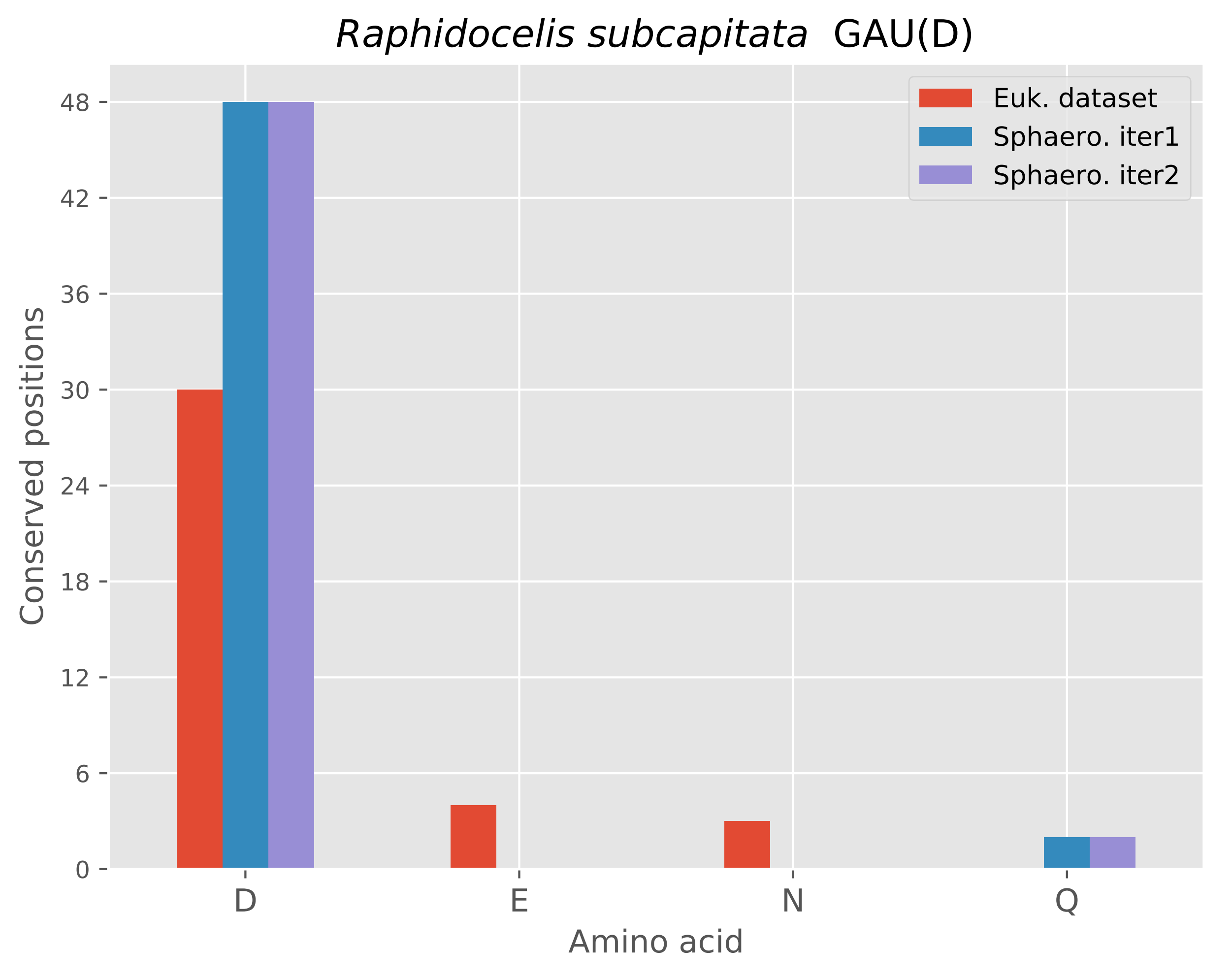

Raphidocelis subcapitata GAU(D)
Euk. dataset
Sphaero. iter1
Sphaero. iter2
Conserved positions
0
6
12
18
24
30
36
42
48
D
E
N
Q
Amino acid

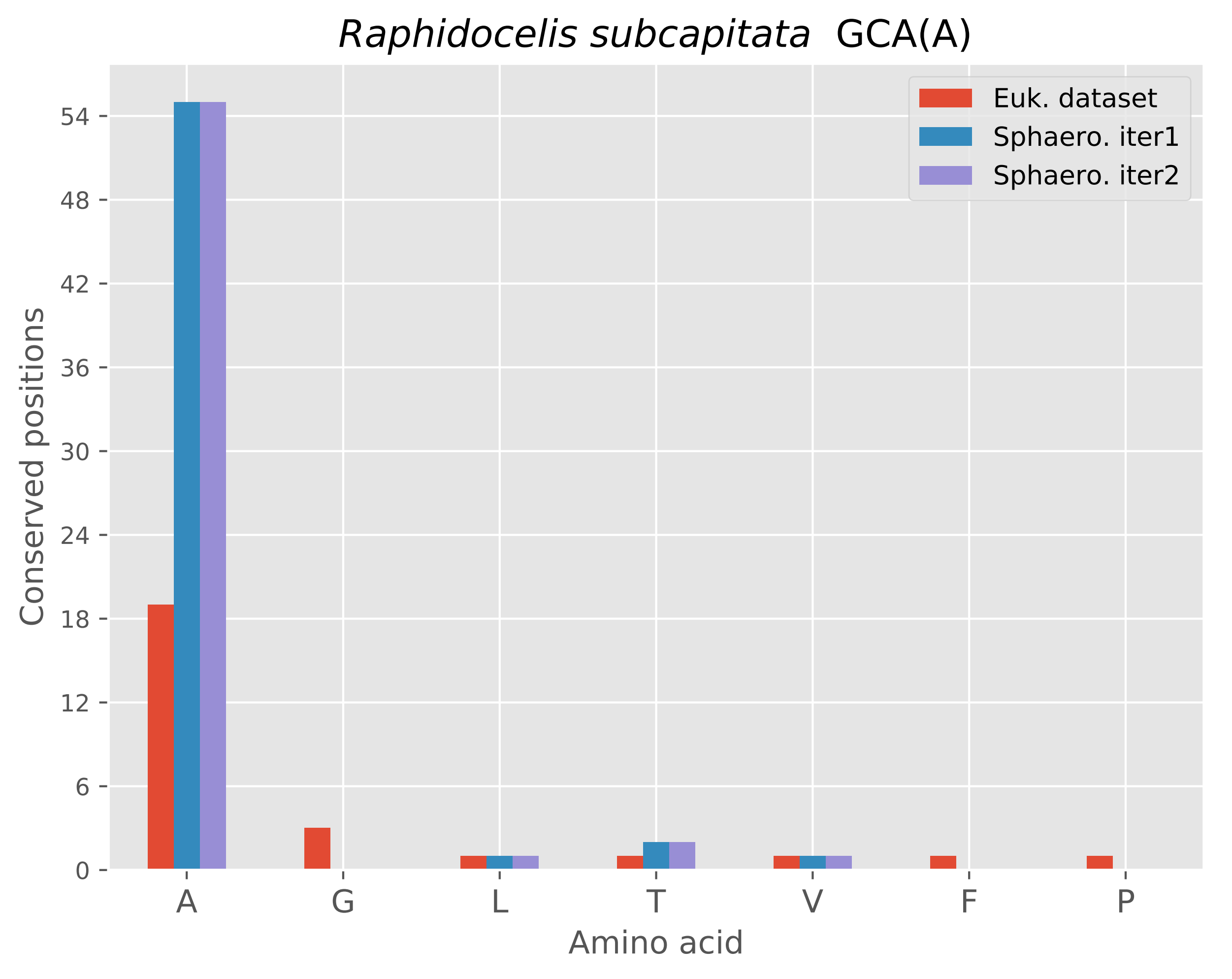

Raphidocelis subcapitata GCA(A)
Euk. dataset
Sphaero. iter1
Sphaero. iter2
Conserved positions
0
6
12
18
24
30
36
42
48
54
A
G
L
T
V
F
P
Amino acid

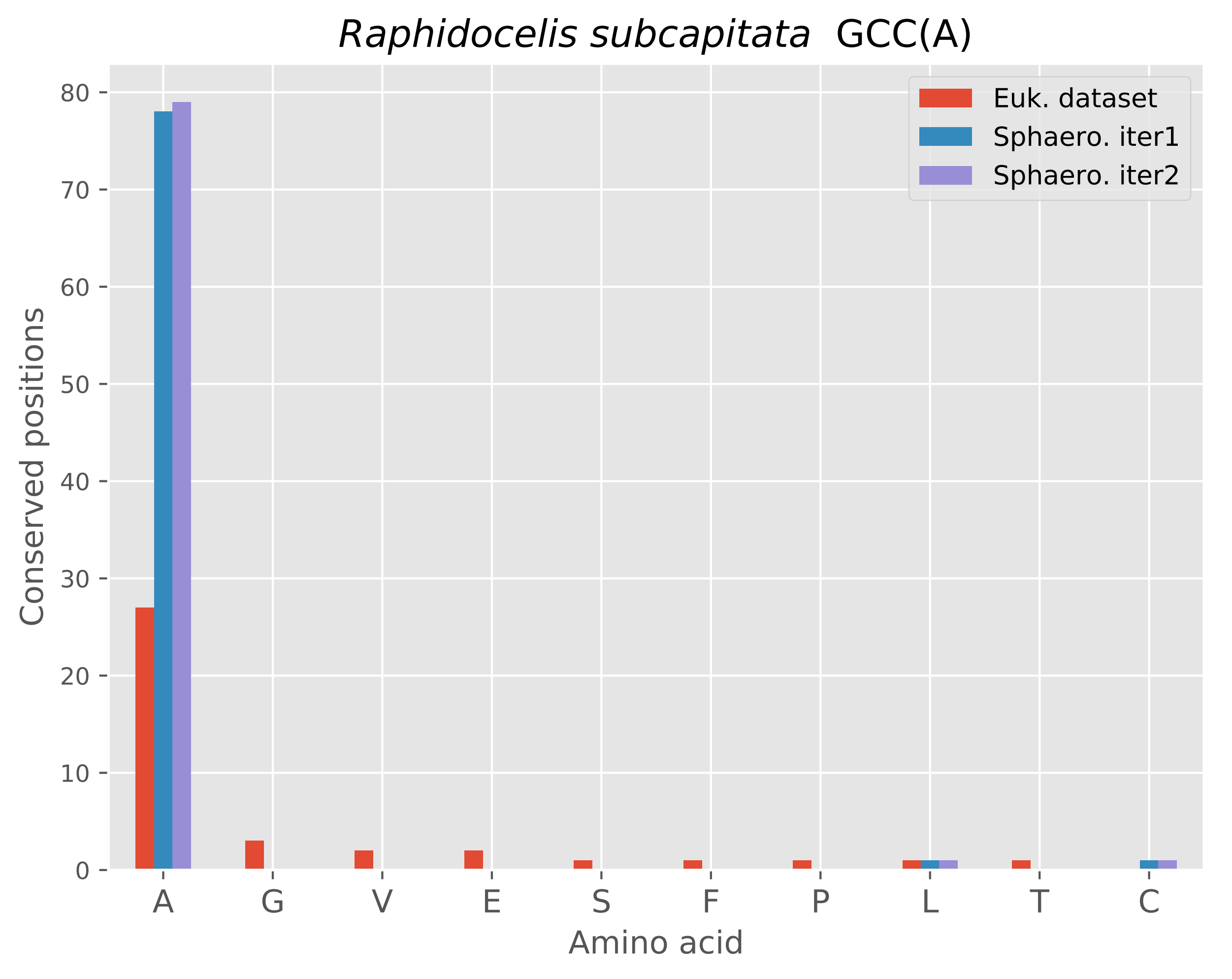

Raphidocelis subcapitata GCC(A)
Euk. dataset
Sphaero. iter1
Sphaero. iter2
Conserved positions
0
10
20
30
40
50
60
70
80
A
G
V
E
S
F
P
L
T
C
Amino acid

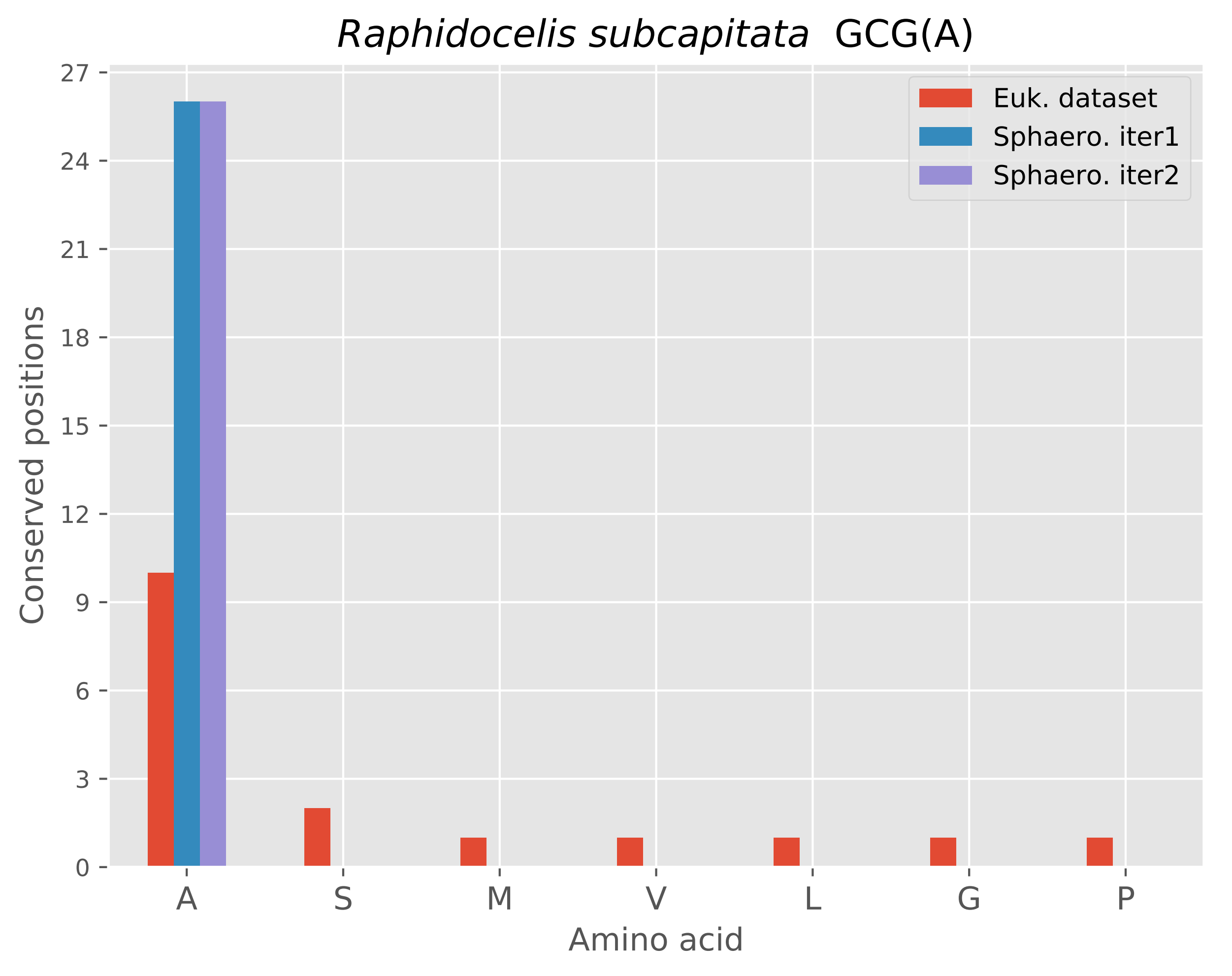

Raphidocelis subcapitata GCG(A)
Euk. dataset
Sphaero. iter1
Sphaero. iter2
Conserved positions
0
3
6
9
12
15
18
21
24
27
A
S
M
V
L
G
P
Amino acid

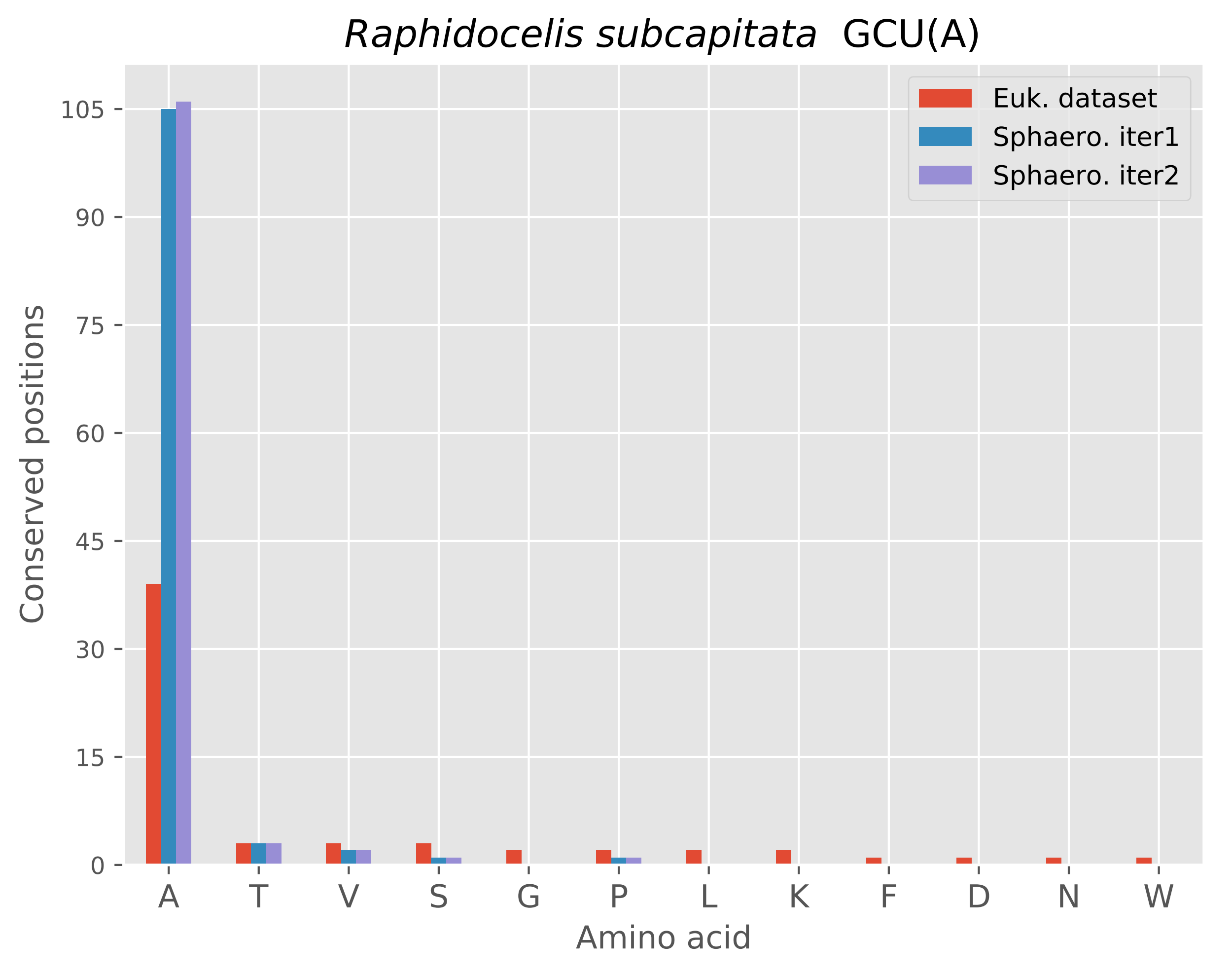

Raphidocelis subcapitata GCU(A)
Euk. dataset
Sphaero. iter1
Sphaero. iter2
Conserved positions
105
90
75
60
45
30
15
0
A
T
V
S
G
P
L
K
F
D
N
W
Amino acid

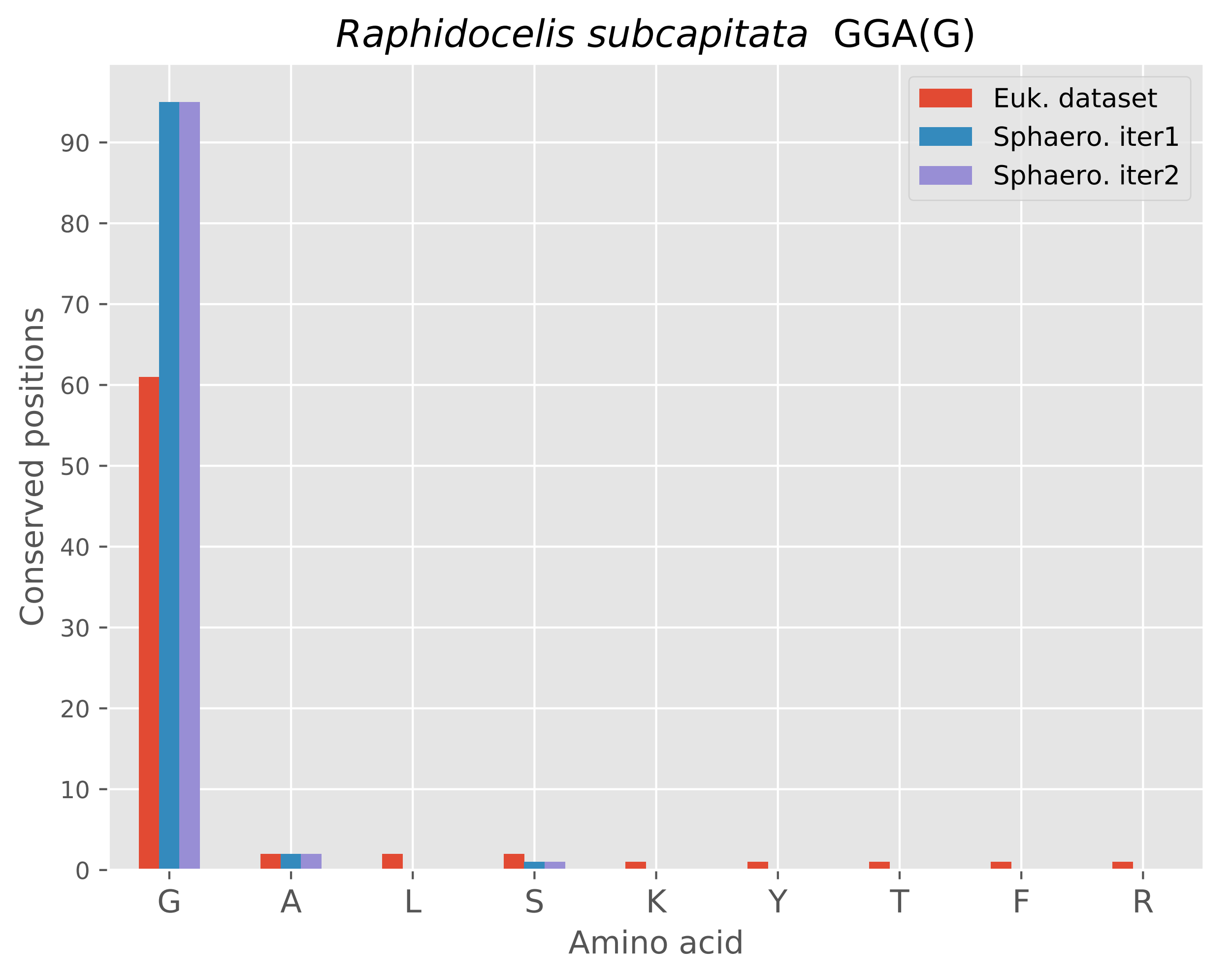

Raphidocelis subcapitata GGA(G)
Euk. dataset
Sphaero. iter1
Sphaero. iter2
Conserved positions
0
10
20
30
40
50
60
70
80
90
G
A
L
S
K
Y
T
F
R
Amino acid

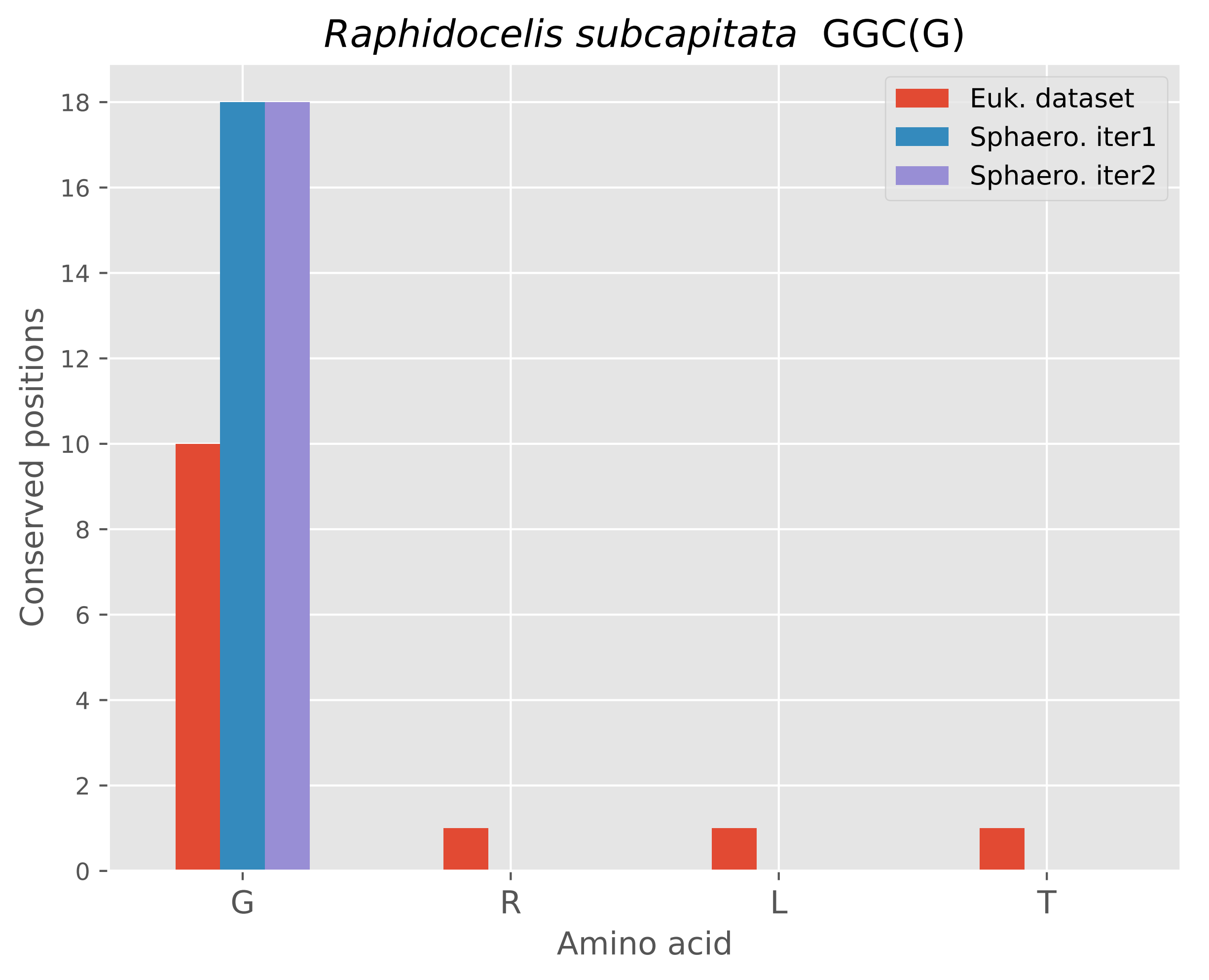

Raphidocelis subcapitata GGC(G)
Euk. dataset
Sphaero. iter1
Sphaero. iter2
Conserved positions
0
2
4
6
8
10
12
14
16
18
G
R
L
T
Amino acid

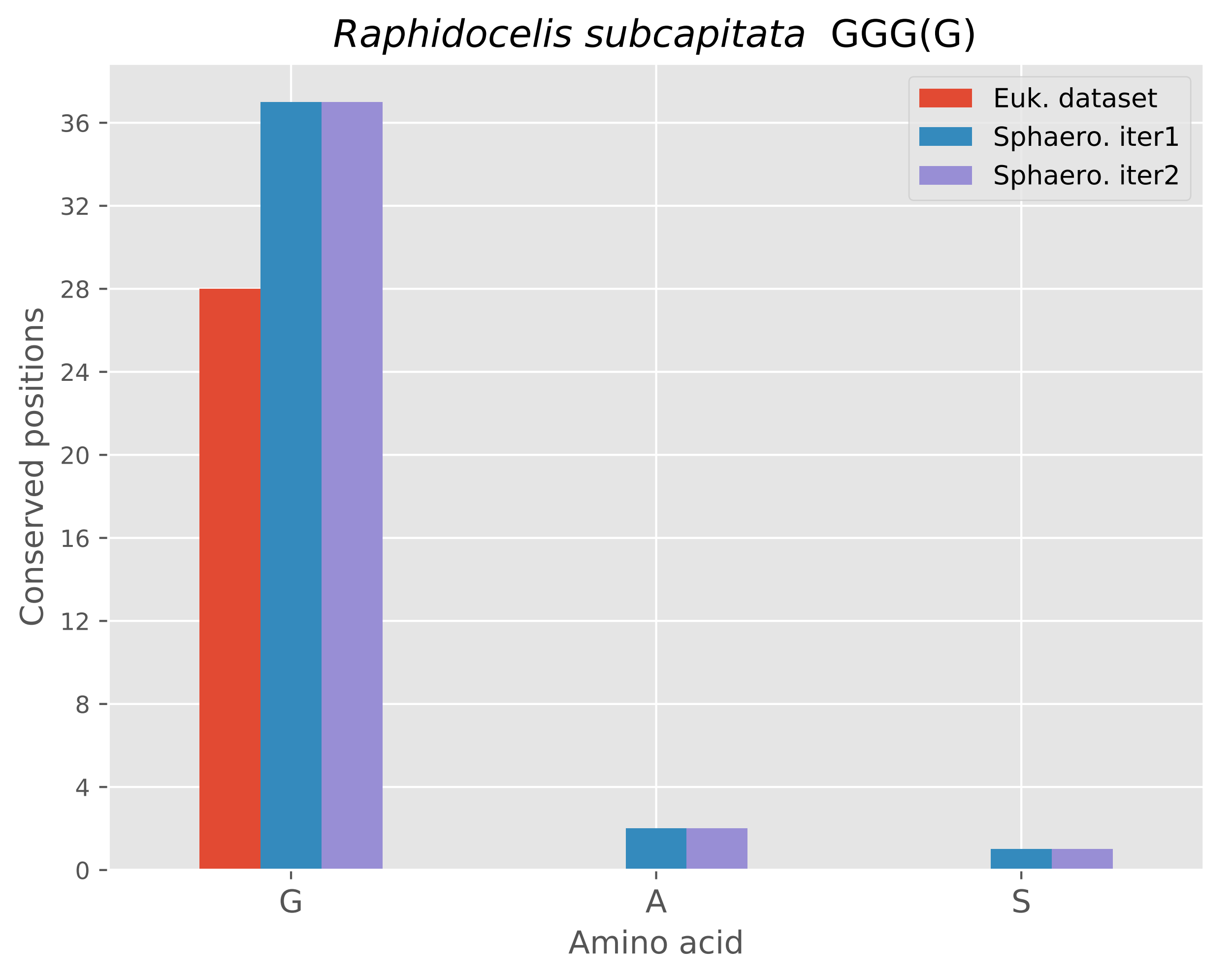
Raphidocelis subcapitata GGG(G)
Euk. dataset
Sphaero. iter1
Sphaero. iter2
Conserved positions
0
4
8
12
16
20
24
28
32
36
G
A
S
Amino acid

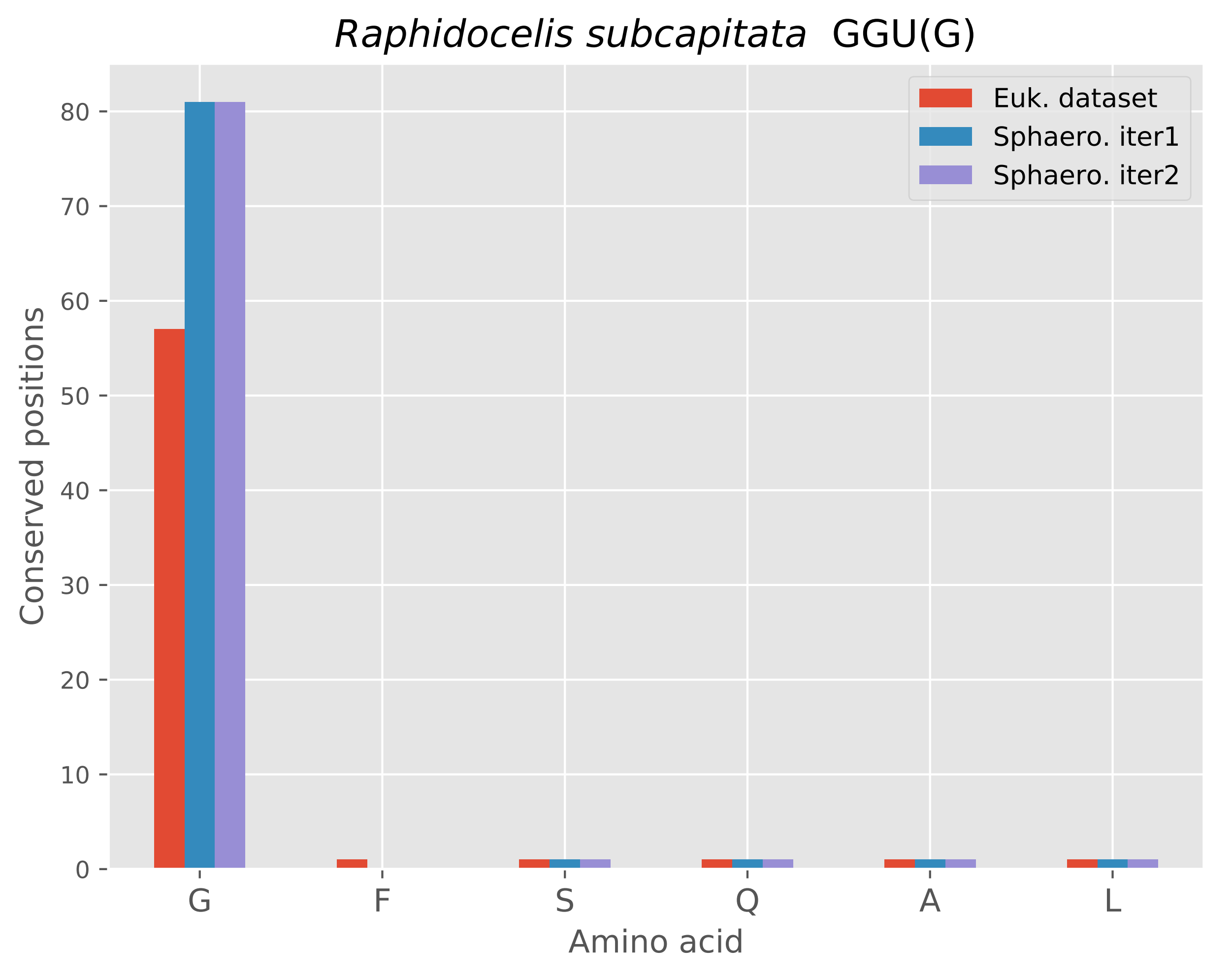
Raphidocelis subcapitata GGU(G)
Euk. dataset
Sphaero. iter1
Sphaero. iter2
Conserved positions
0
10
20
30
40
50
60
70
80
G
F
S
Q
A
L
Amino acid

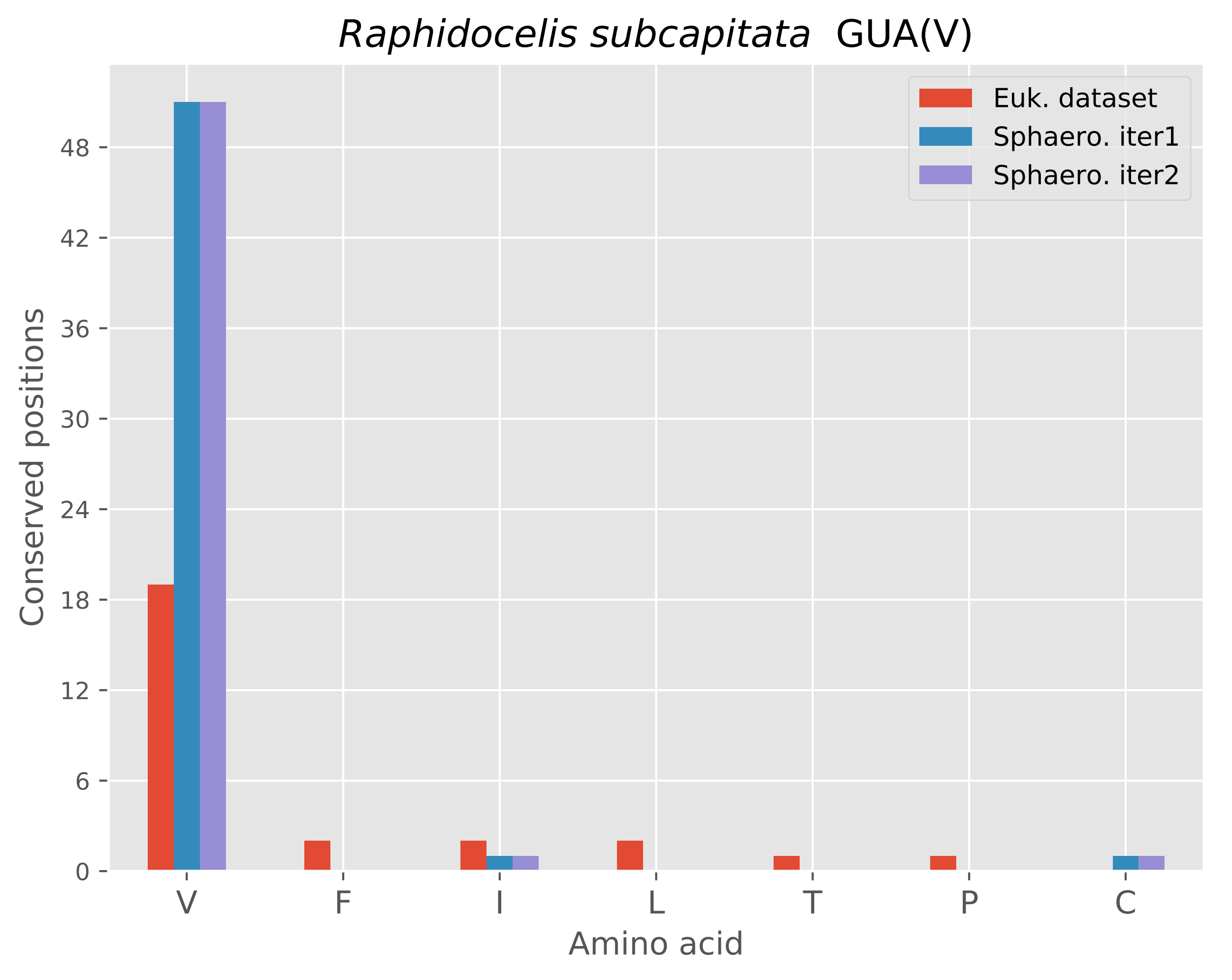

Raphidocelis subcapitata GUA(V)
Euk. dataset
Sphaero. iter1
Sphaero. iter2
Conserved positions
0
6
12
18
24
30
36
42
48
V
F
I
L
T
P
C
Amino acid

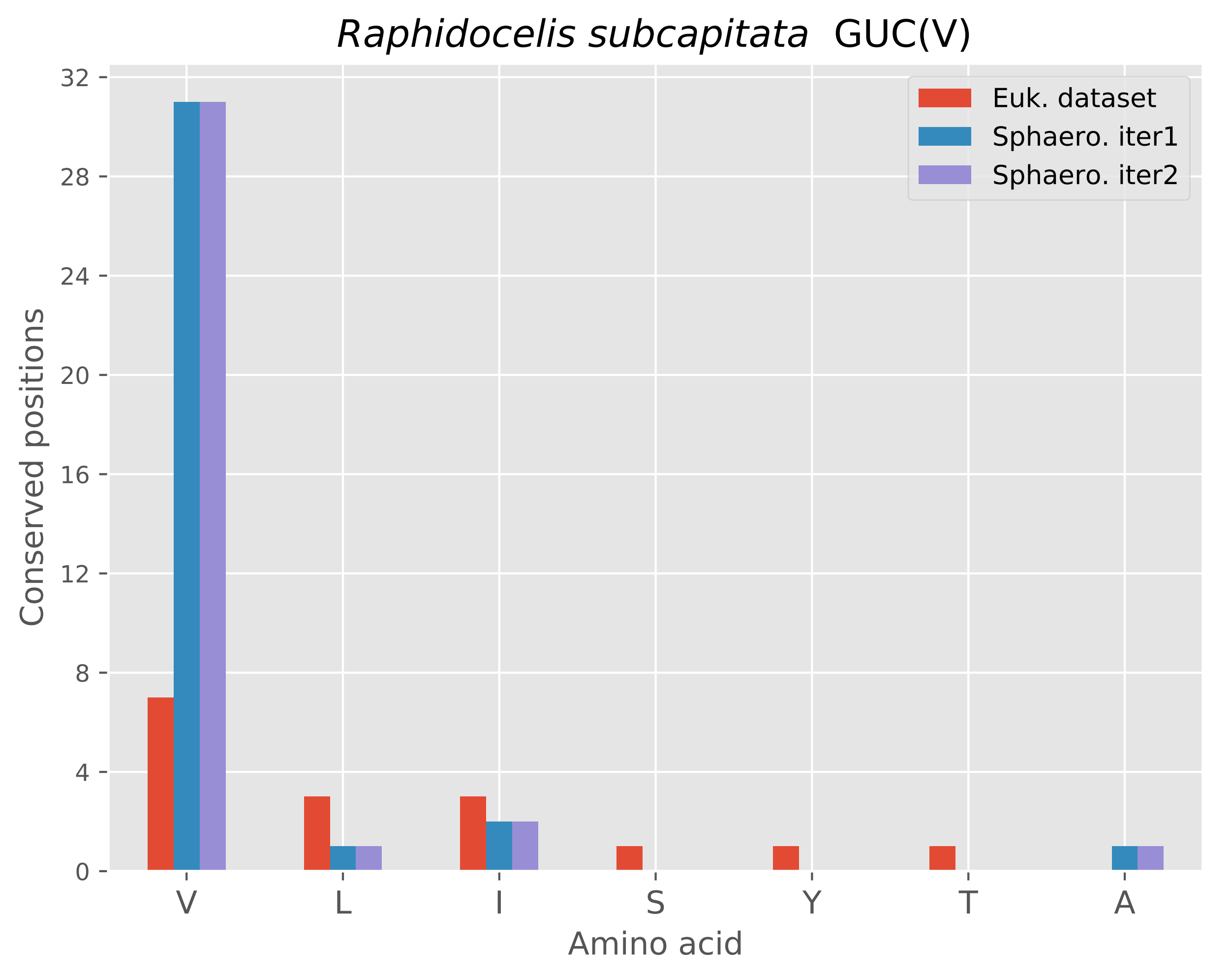

Raphidocelis subcapitata GUC(V)
Euk. dataset
Sphaero. iter1
Sphaero. iter2
Conserved positions
0
4
8
12
16
20
24
28
32
V
L
I
S
Y
T
A
Amino acid

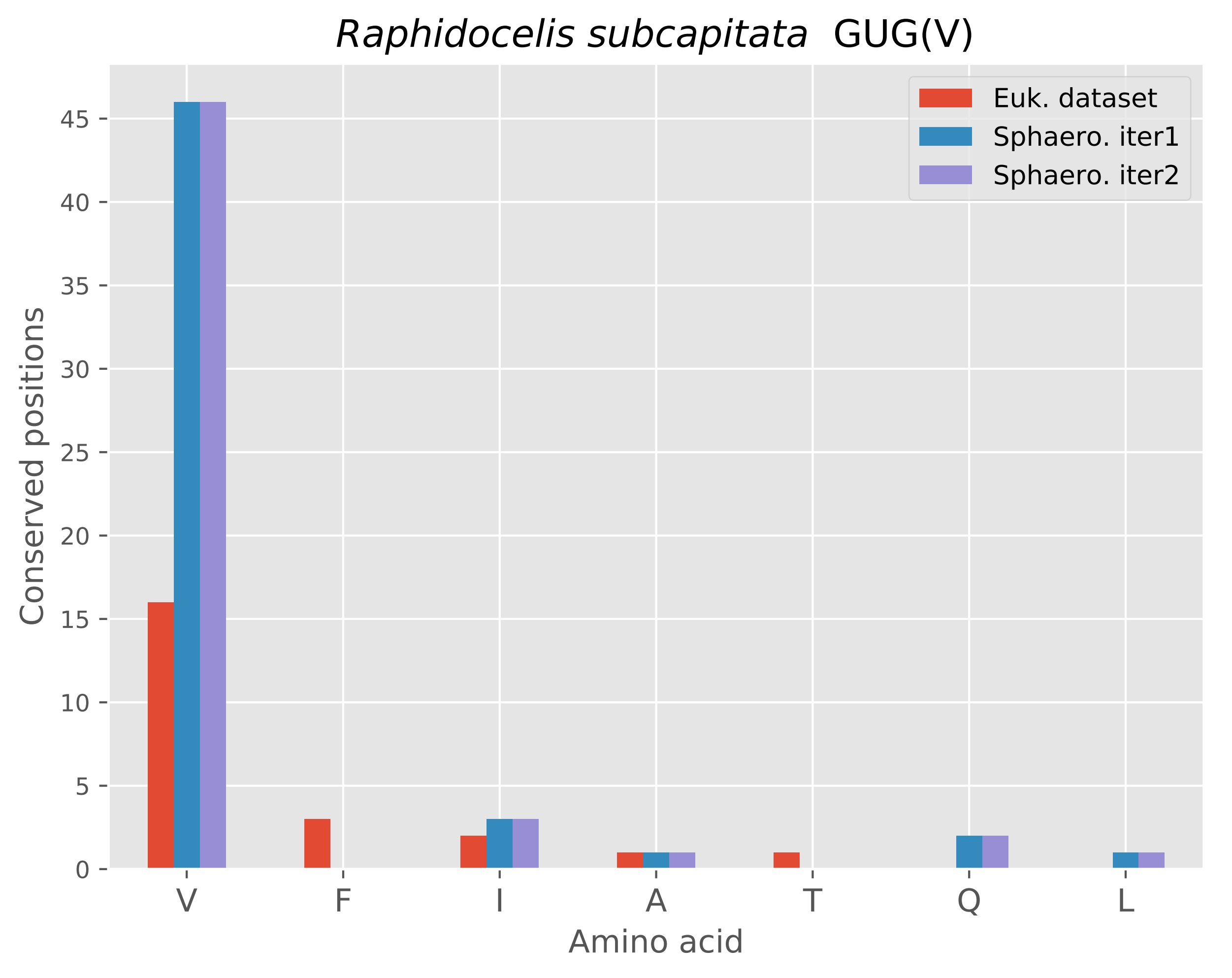

Raphidocelis subcapitata GUG(V)
Euk. dataset
Sphaero. iter1
Sphaero. iter2
Conserved positions
0
5
10
15
20
25
30
35
40
45
V
F
I
A
T
Q
L
Amino acid

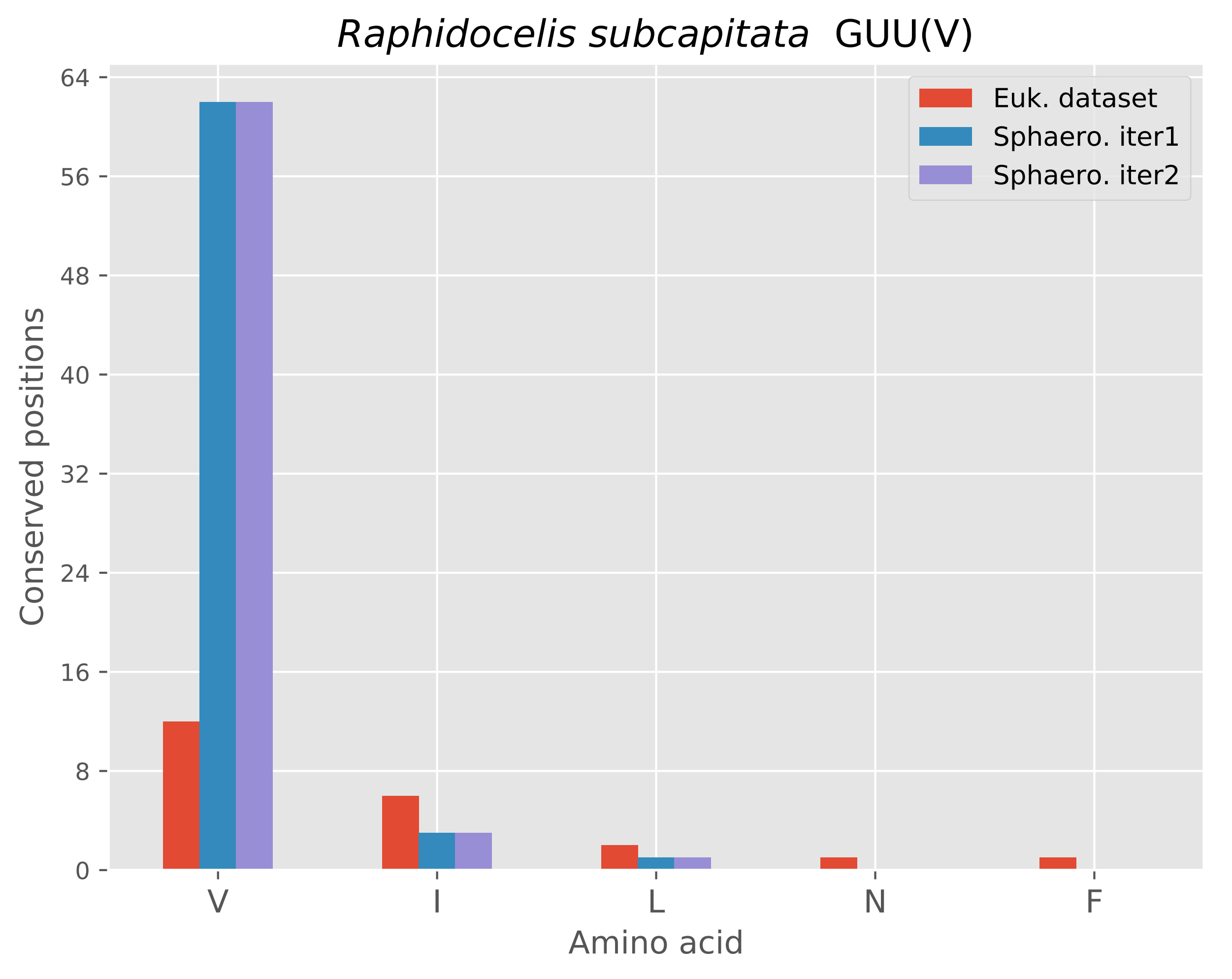

Raphidocelis subcapitata GUU(V)
Conserved positions
64
56
48
40
32
24
16
8
0
V
I
L
N
F
Amino acid
Euk. dataset
Sphaero. iter1
Sphaero. iter2

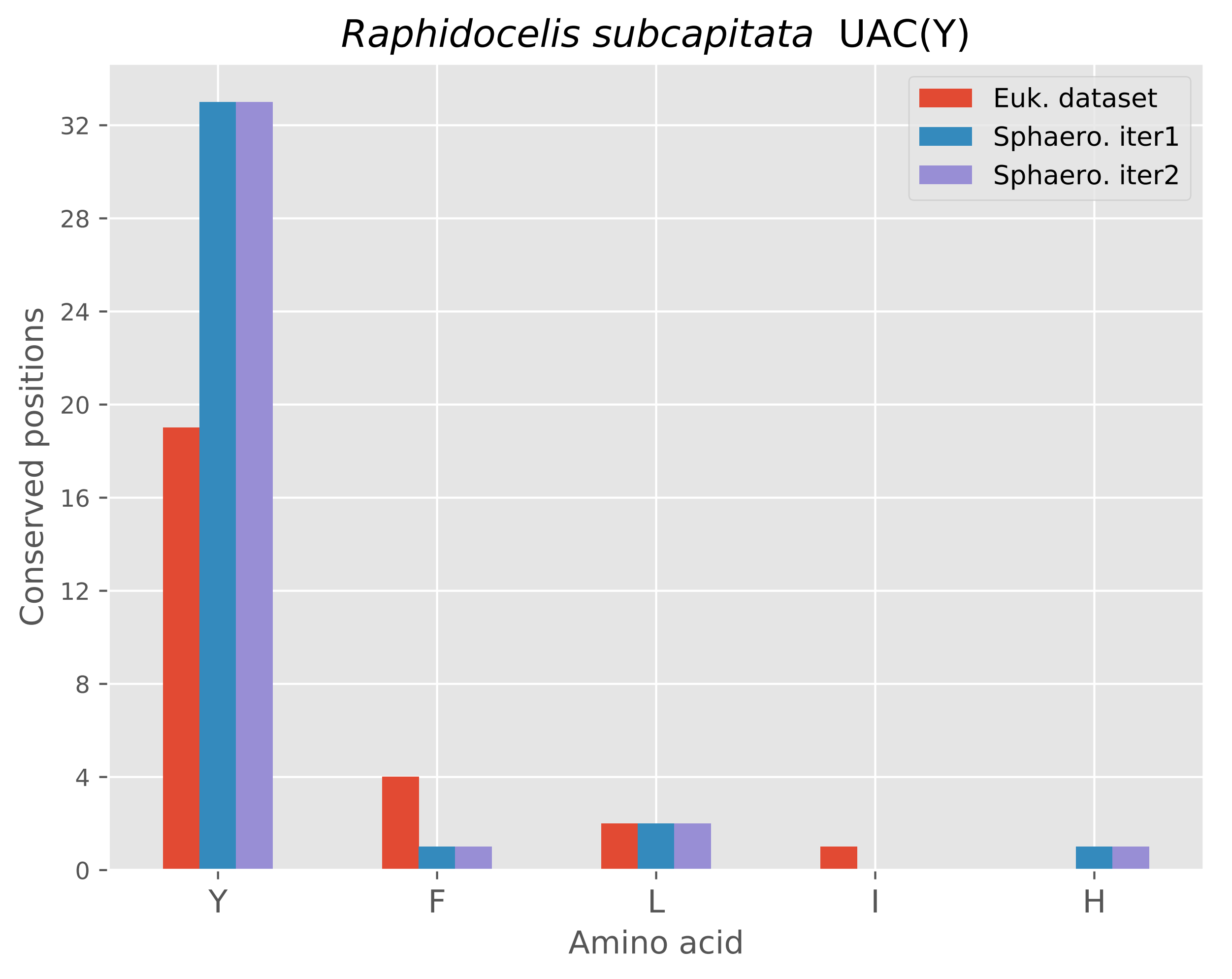

Raphidocelis subcapitata UAC(Y)
Euk. dataset
Sphaero. iter1
Sphaero. iter2
Conserved positions
0
4
8
12
16
20
24
28
32
Y
F
L
I
H
Amino acid

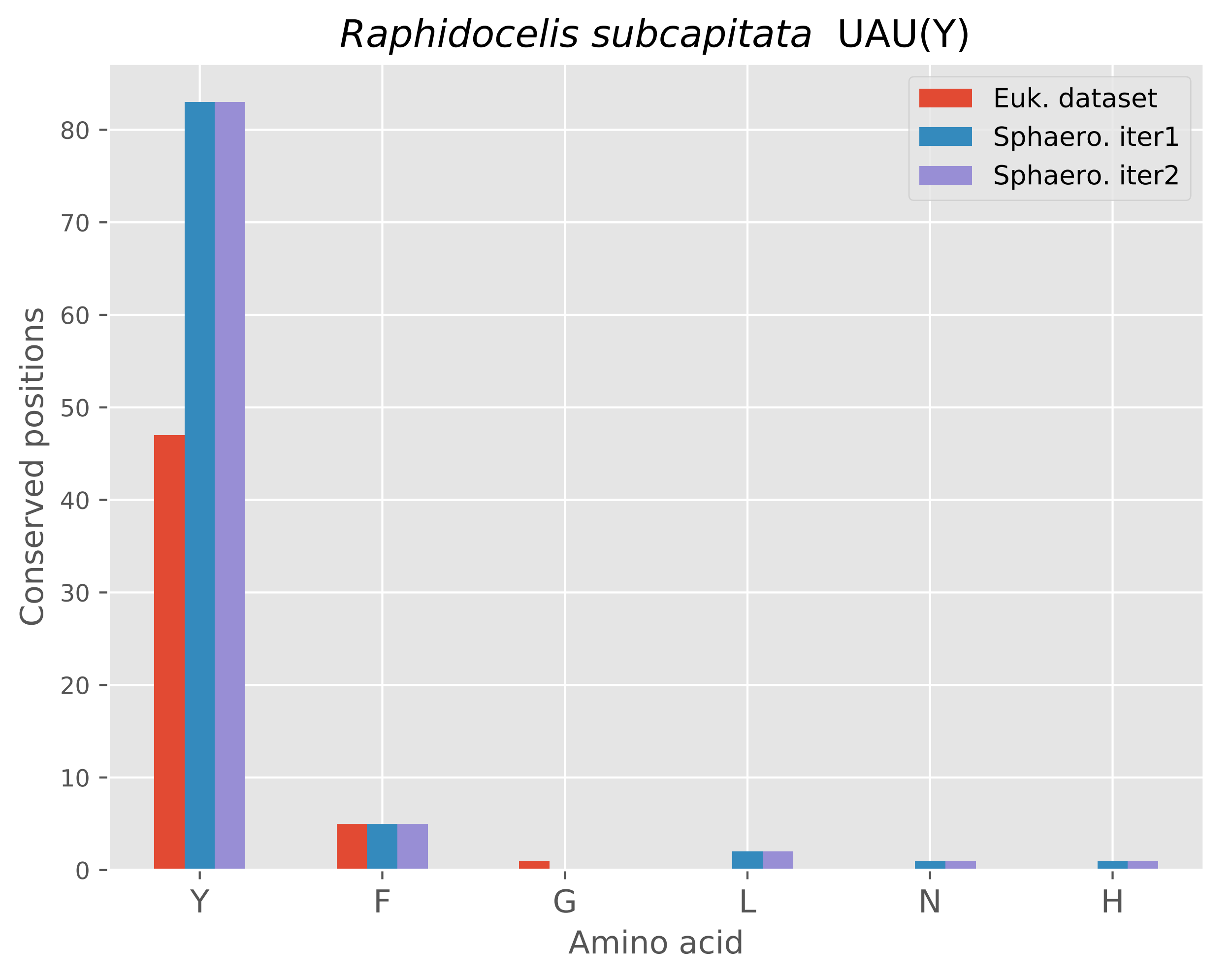

Raphidocelis subcapitata UAU(Y)
Euk. dataset
Sphaero. iter1
Sphaero. iter2
Conserved positions
0
10
20
30
40
50
60
70
80
Y
F
G
L
N
H
Amino acid

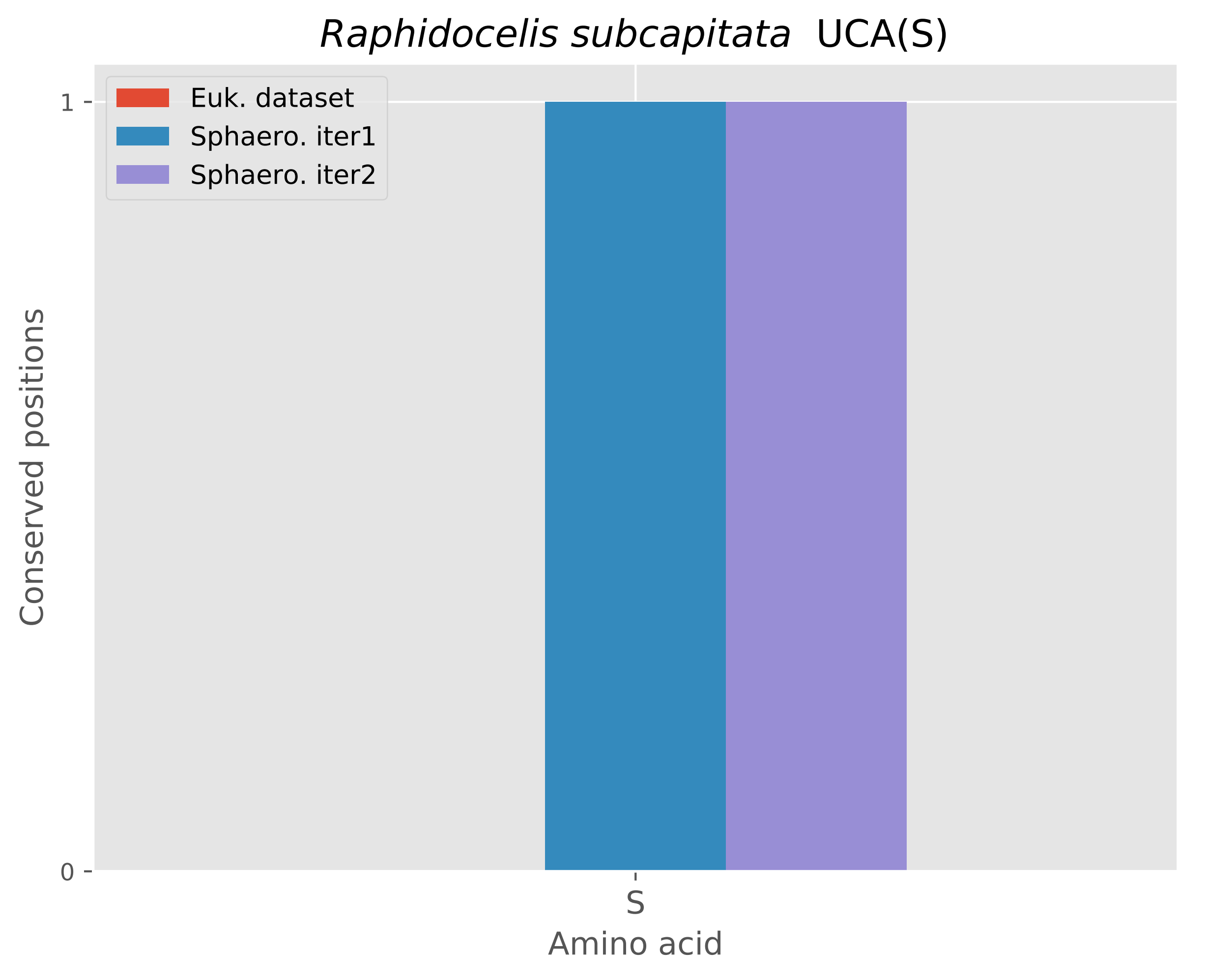

Raphidocelis subcapitata UCA(S)
Euk. dataset
Sphaero. iter1
Sphaero. iter2
1
0
Conserved positions
S
Amino acid

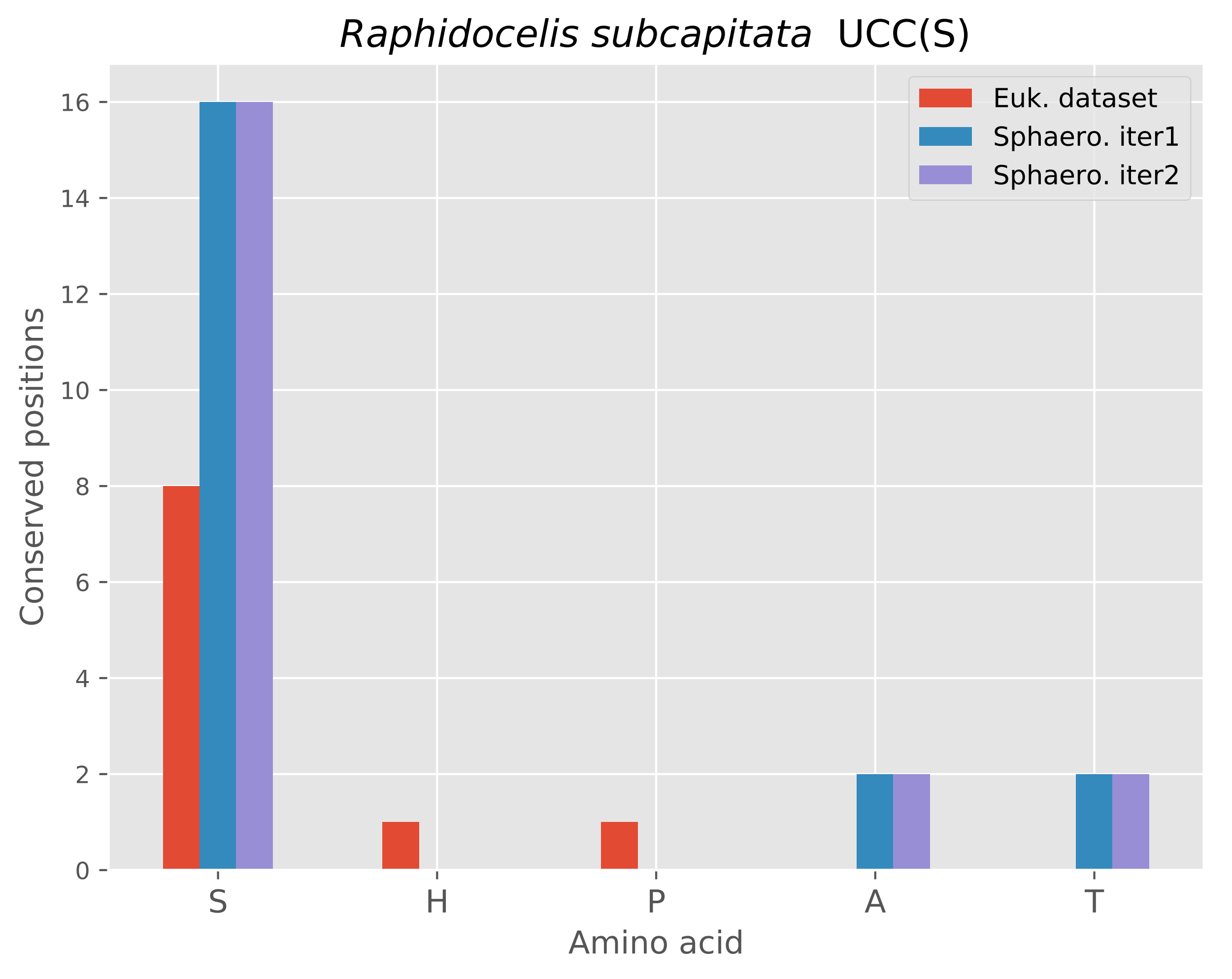
Raphidocelis subcapitata UCC(S)
Euk. dataset
Sphaero. iter1
Sphaero. iter2
Conserved positions
0
2
4
6
8
10
12
14
16
S
H
P
A
T
Amino acid

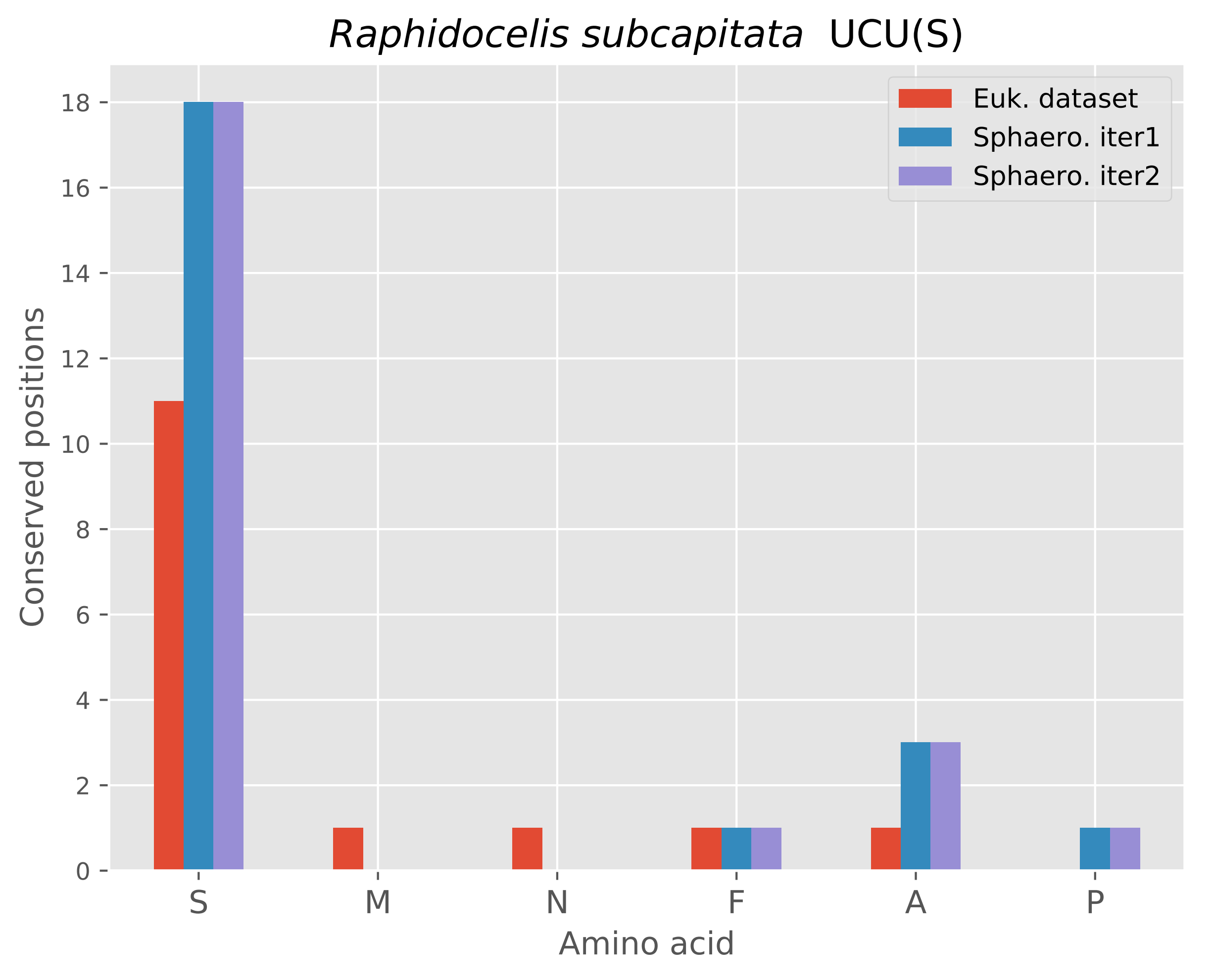
Raphidocelis subcapitata UCU(S)
Euk. dataset
Sphaero. iter1
Sphaero. iter2
Conserved positions
0
2
4
6
8
10
12
14
16
18
S
M
N
F
A
P
Amino acid

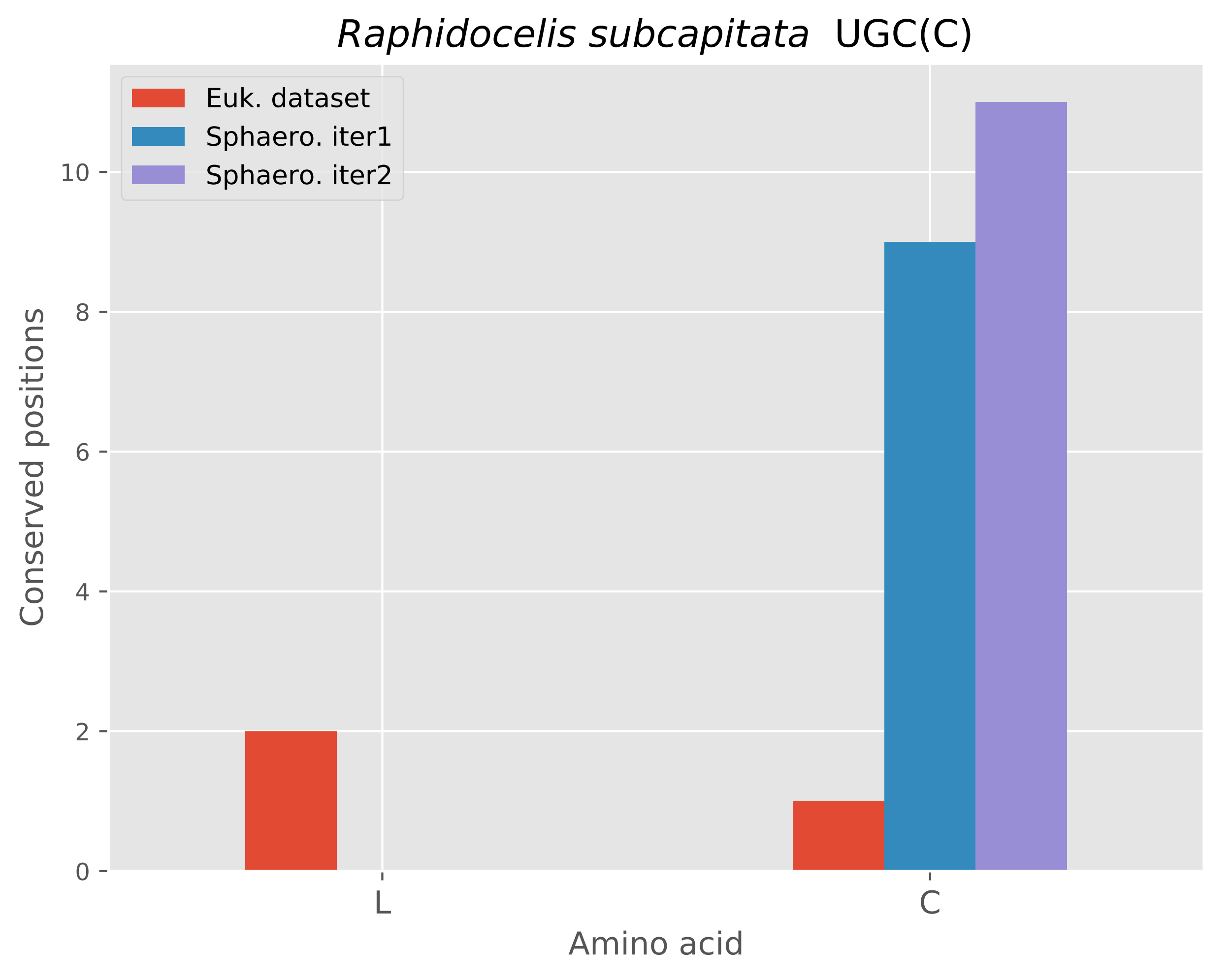

Raphidocelis subcapitata UGC(C)
Euk. dataset
Sphaero. iter1
Sphaero. iter2
Conserved positions
0
2
4
6
8
10
L
C
Amino acid

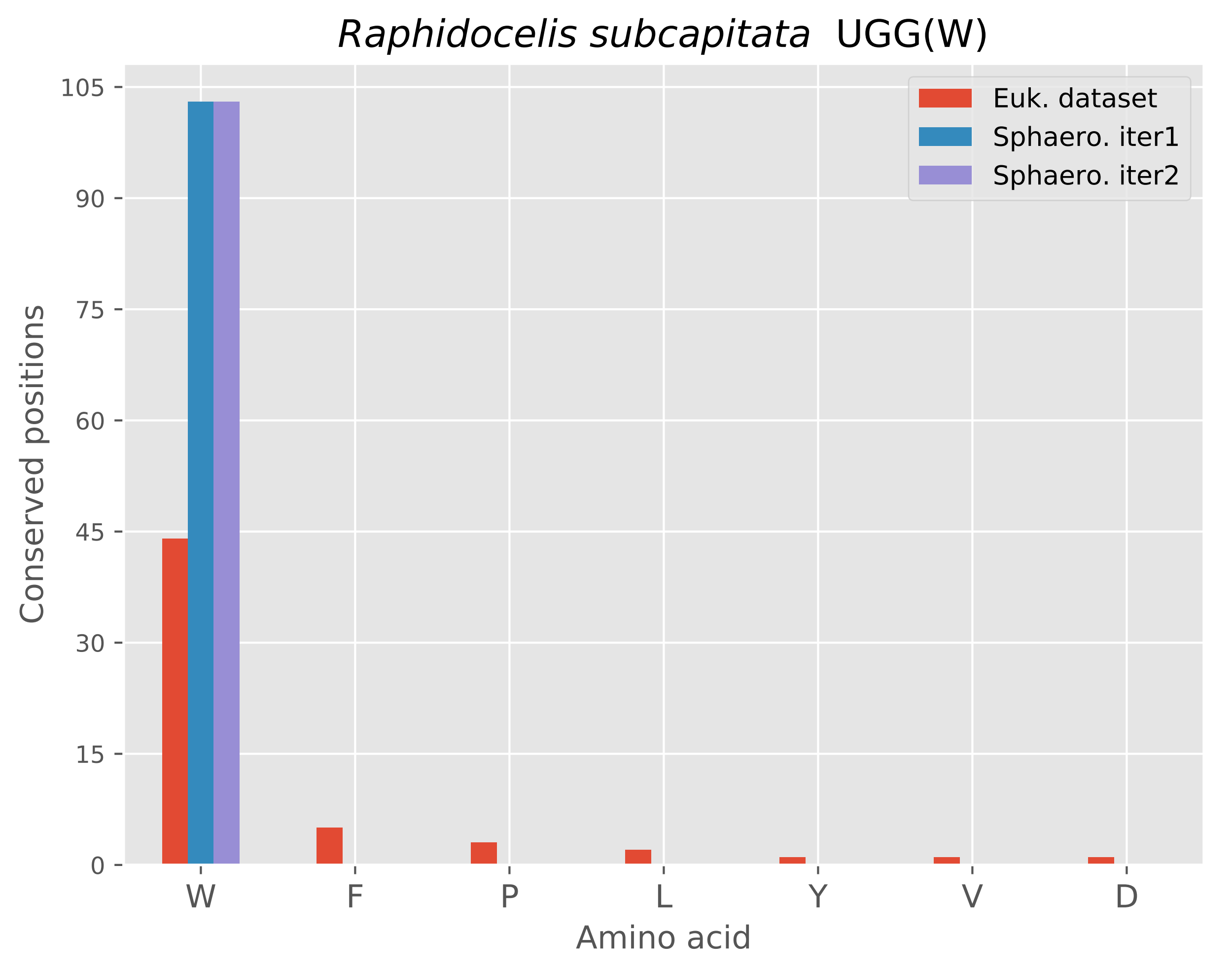

Raphidocelis subcapitata UGG(W)
Euk. dataset
Sphaero. iter1
Sphaero. iter2
Conserved positions
0
15
30
45
60
75
90
105
W
F
P
L
Y
V
D
Amino acid

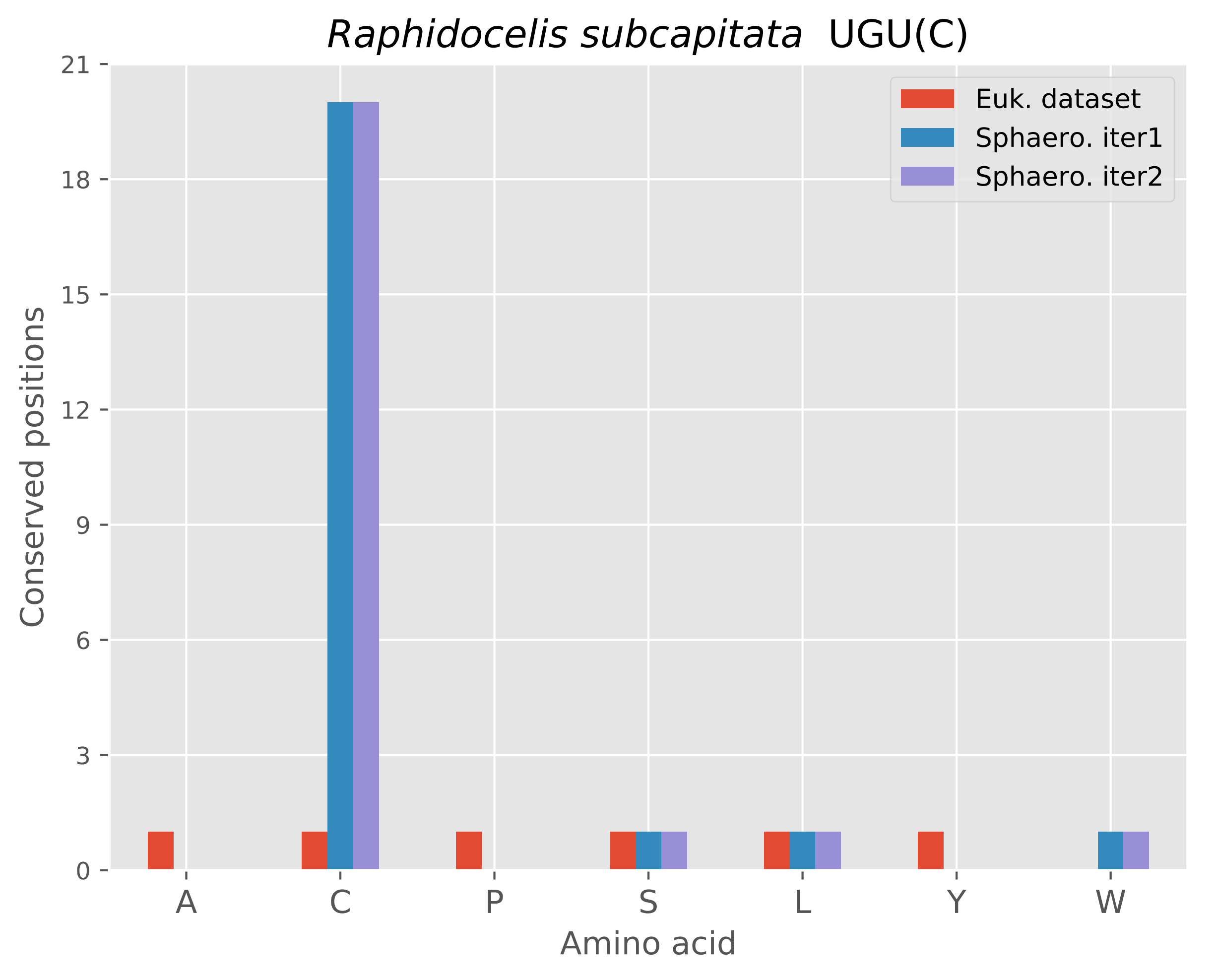

Raphidocelis subcapitata UGU(C)
Conserved positions
Amino acid
Euk. dataset
Sphaero. iter1
Sphaero. iter2
0
3
6
9
12
15
18
21
A
C
P
S
L
Y
W

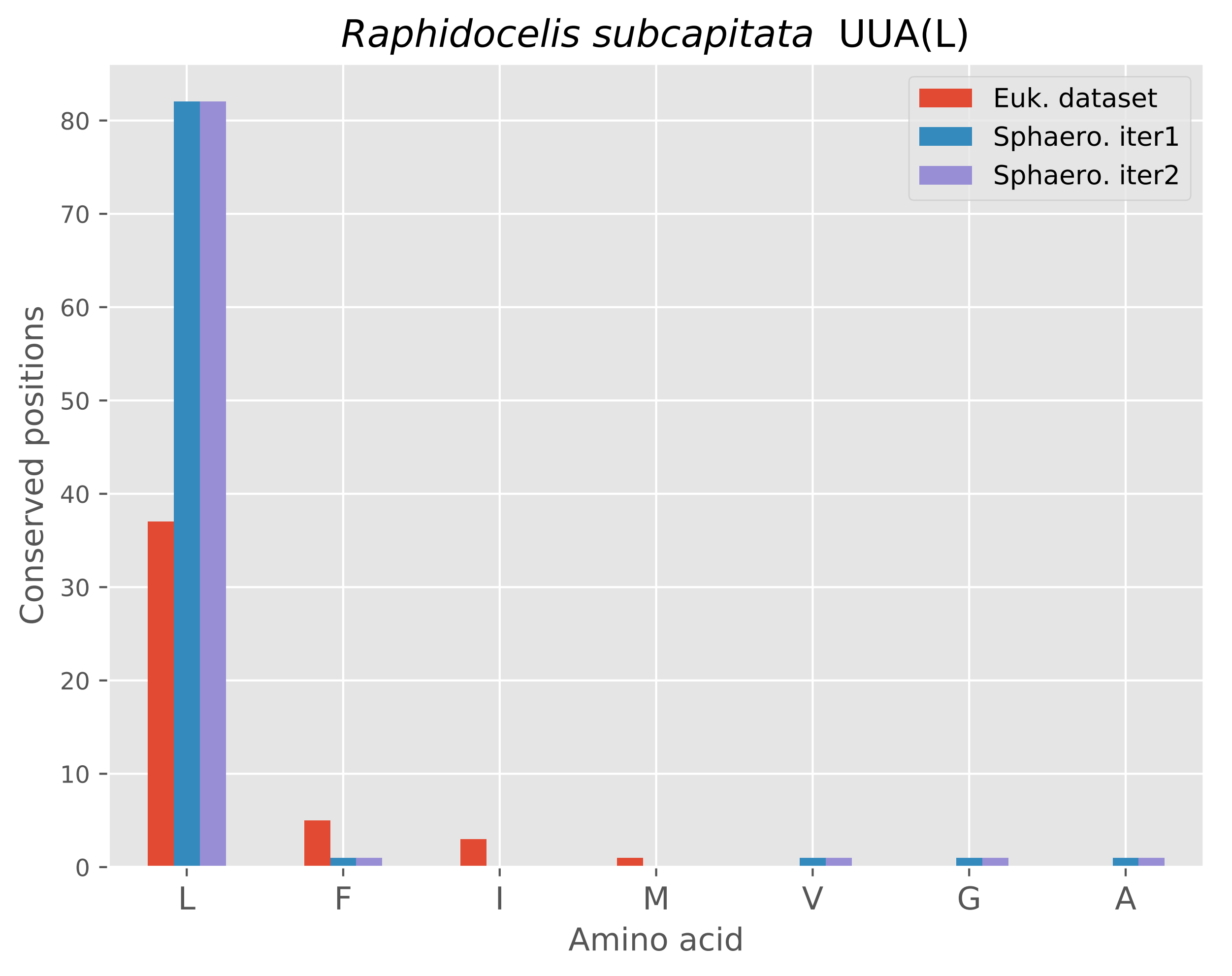

Raphidocelis subcapitata UUA(L)
Euk. dataset
Sphaero. iter1
Sphaero. iter2
Conserved positions
0
10
20
30
40
50
60
70
80
L
F
I
M
V
G
A
Amino acid

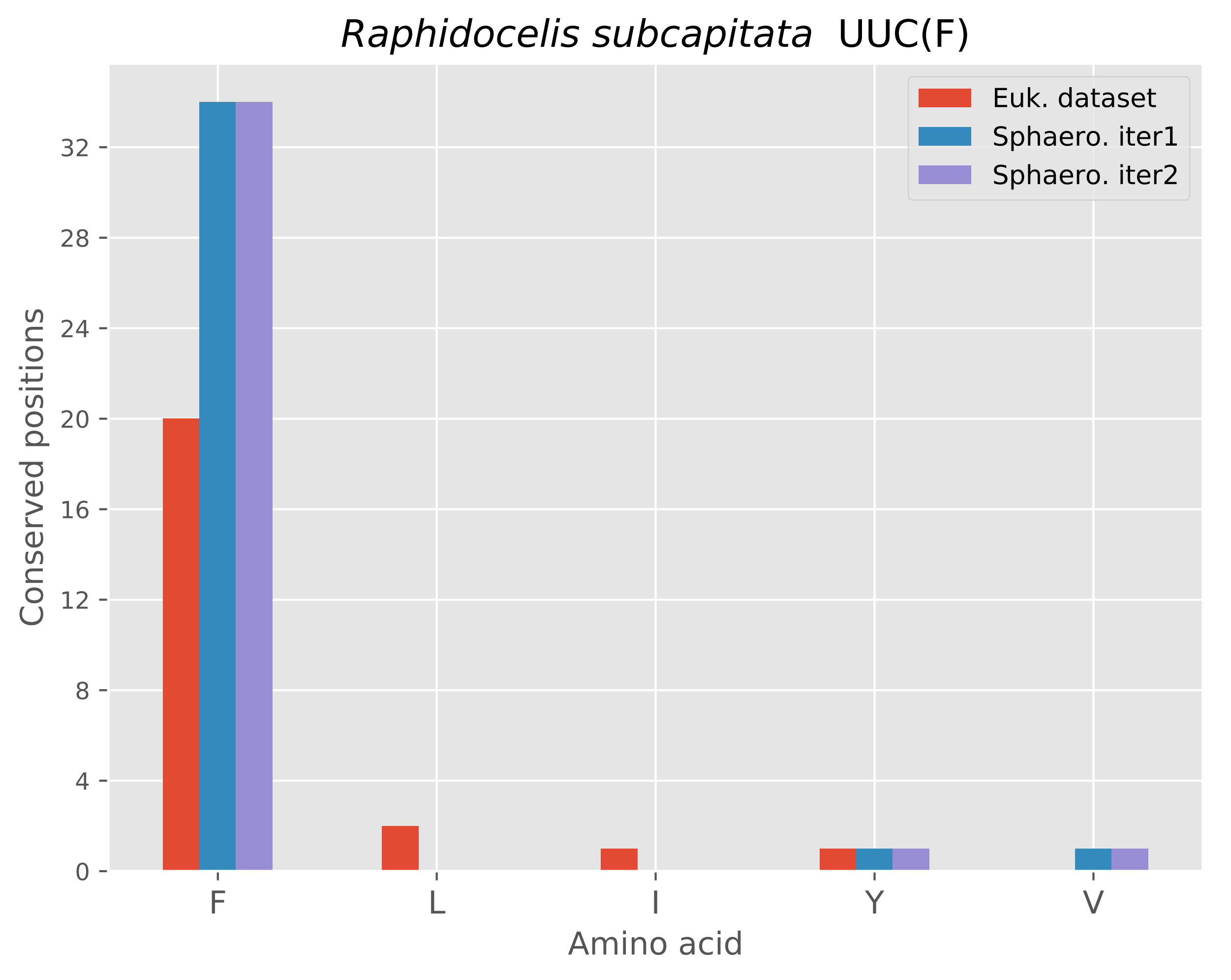

Raphidocelis subcapitata UUC(F)
Euk. dataset
Sphaero. iter1
Sphaero. iter2
Conserved positions
0
4
8
12
16
20
24
28
32
F
L
I
Y
V
Amino acid

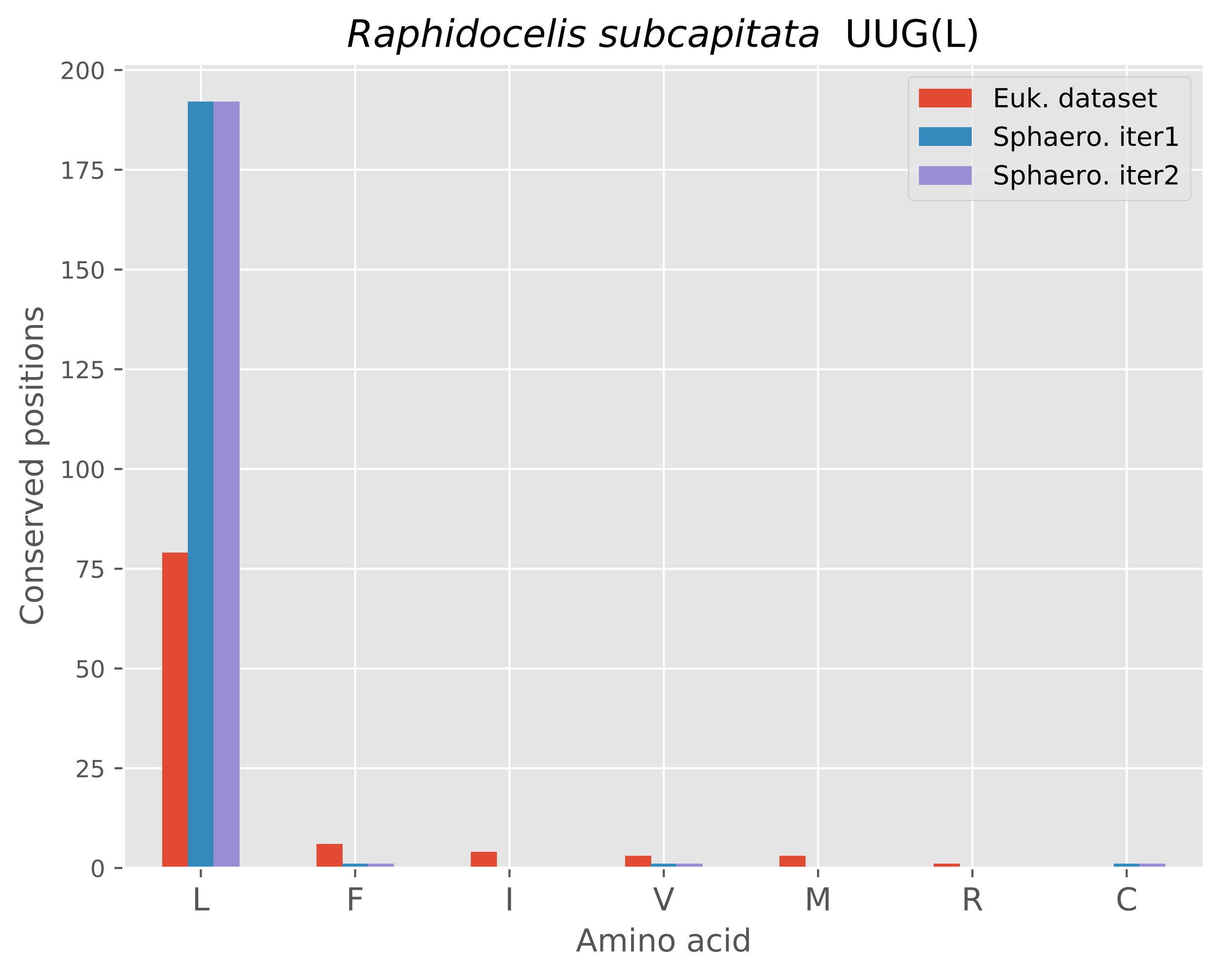

Raphidocelis subcapitata UUG(L)
Euk. dataset
Sphaero. iter1
Sphaero. iter2
Conserved positions
200
175
150
125
100
75
50
25
0
L
F
I
V
M
R
C
Amino acid

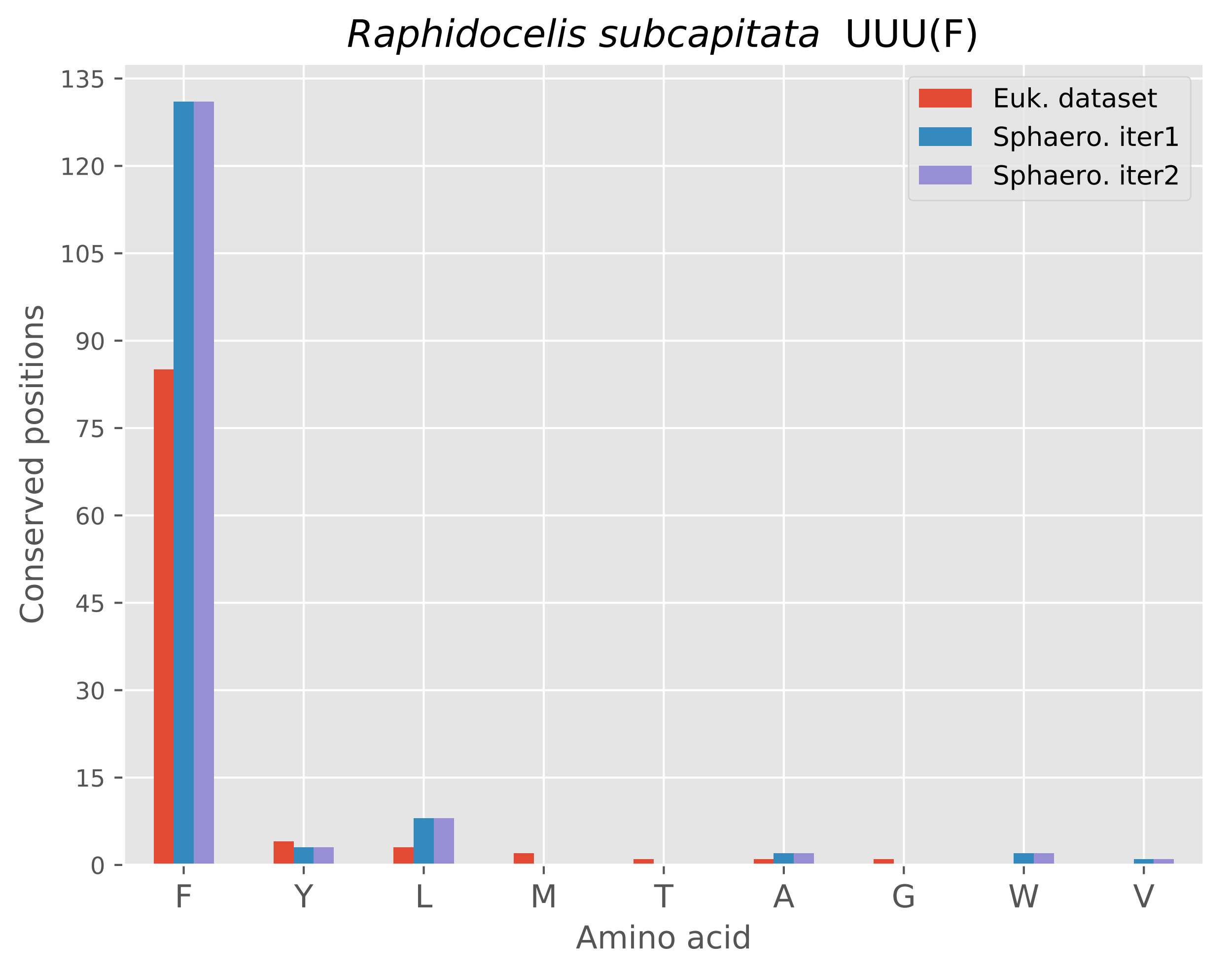

Raphidocelis subcapitata UUU(F)
Euk. dataset
Sphaero. iter1
Sphaero. iter2
Conserved positions
0
15
30
45
60
75
90
105
120
135
F
Y
L
M
T
A
G
W
V
Amino acid

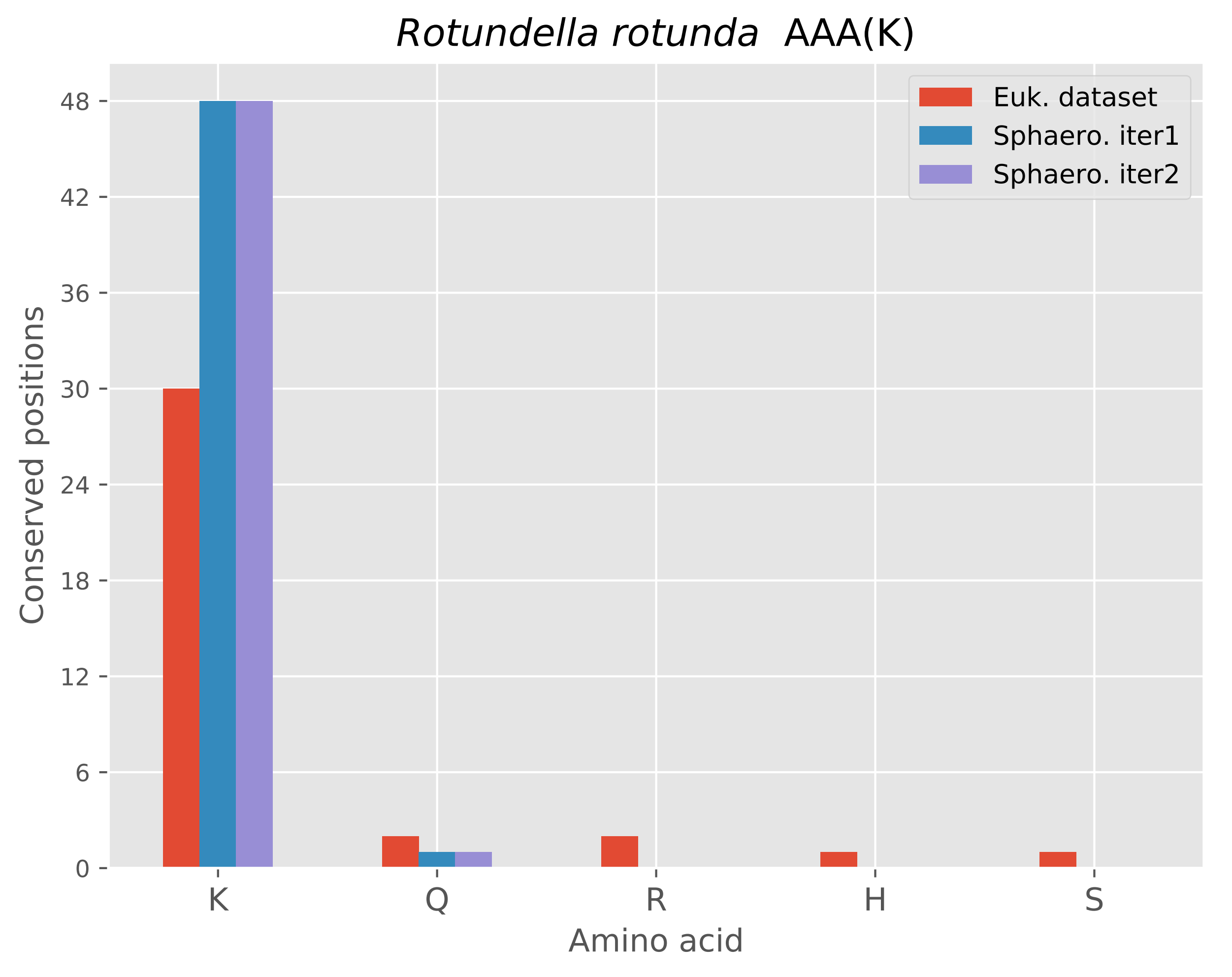
Rotundella rotunda AAA(K)
Euk. dataset
Sphaero. iter1
Sphaero. iter2
Conserved positions
0
6
12
18
24
30
36
42
48
K
Q
R
H
S
Amino acid

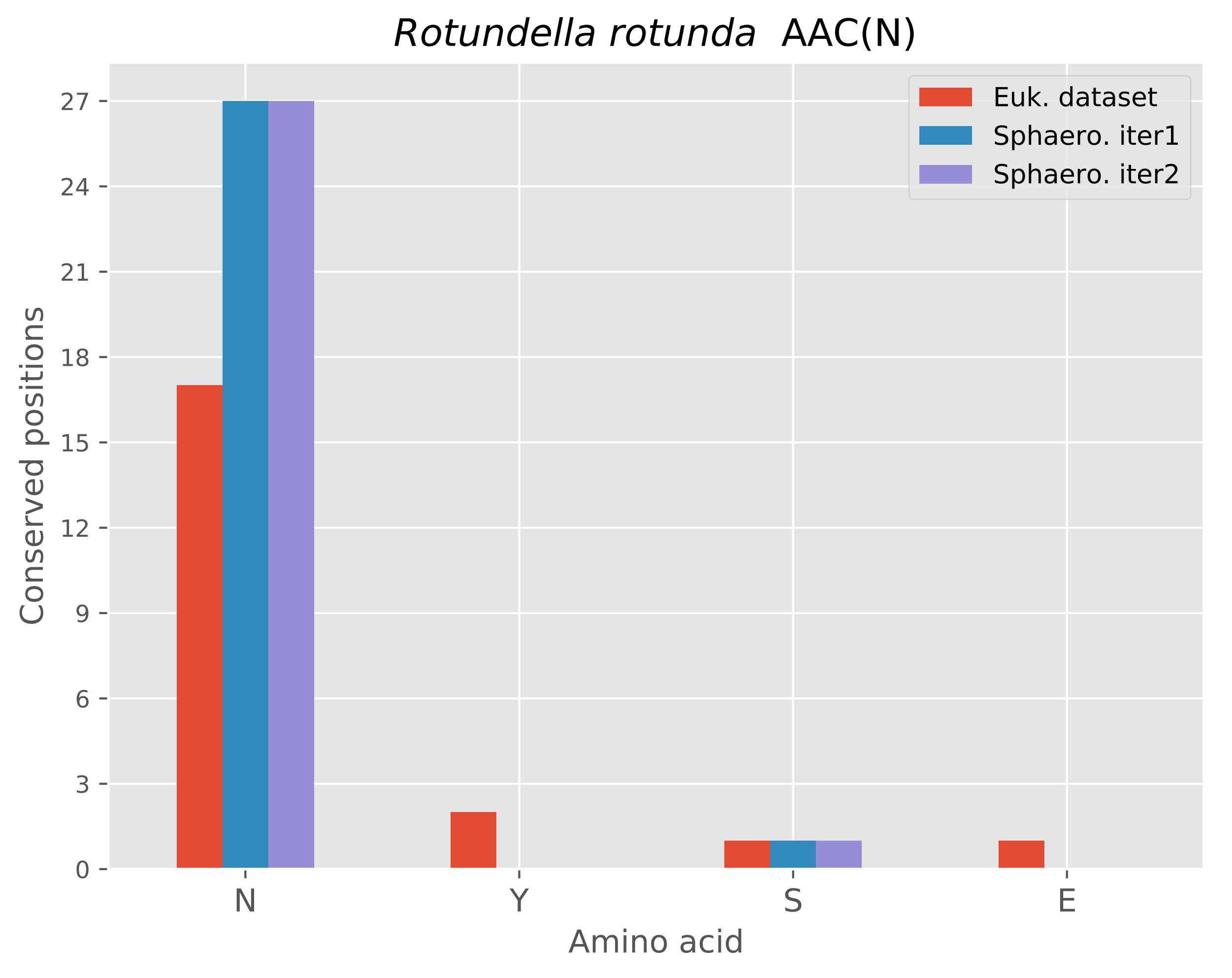

Rotundella rotunda AAC(N)
Euk. dataset
Sphaero. iter1
Sphaero. iter2
Conserved positions
0
3
6
9
12
15
18
21
24
27
N
Y
S
E
Amino acid

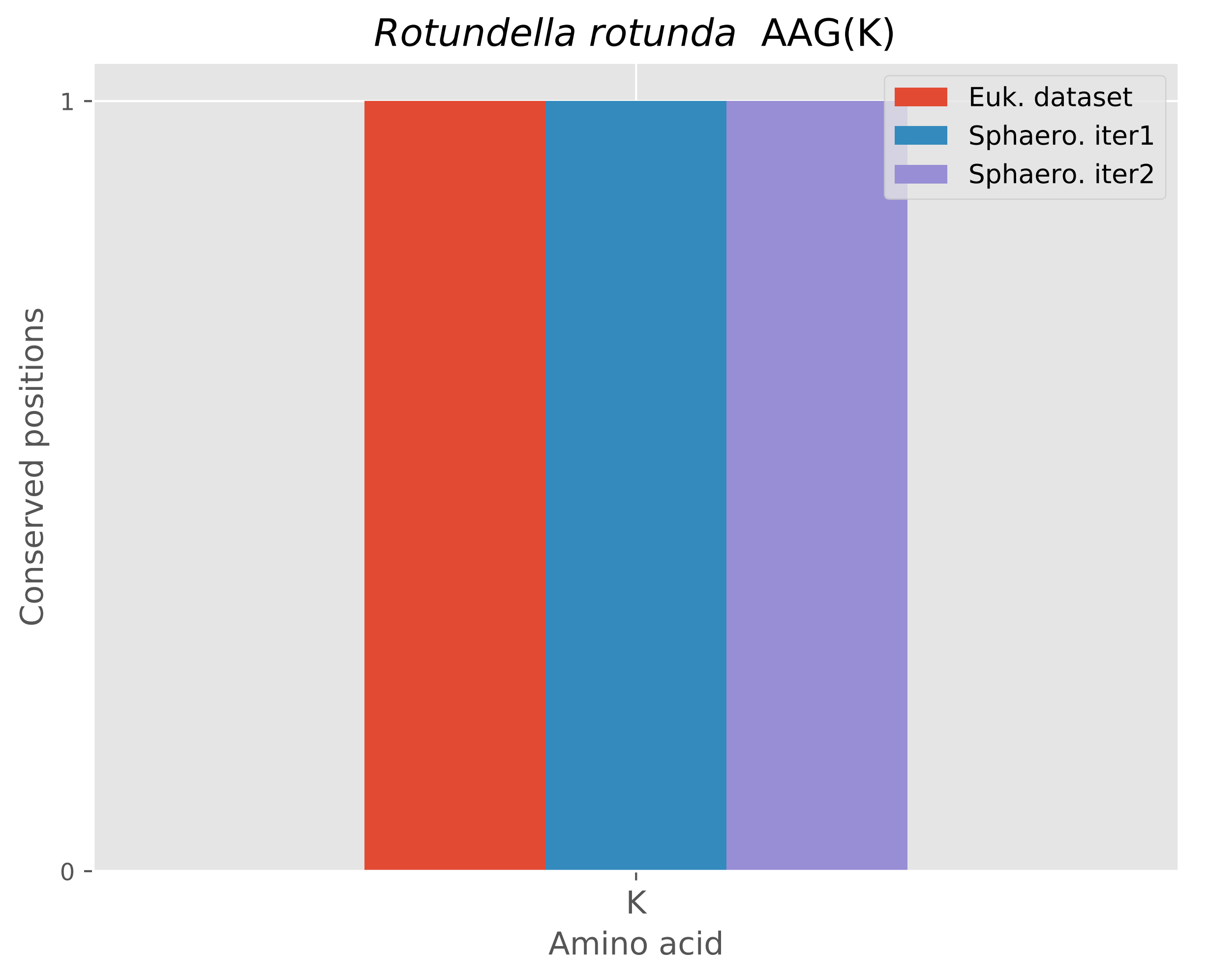

Rotundella rotunda AAG(K)
Conserved positions
1
0
K
Amino acid
Euk. dataset
Sphaero. iter1
Sphaero. iter2

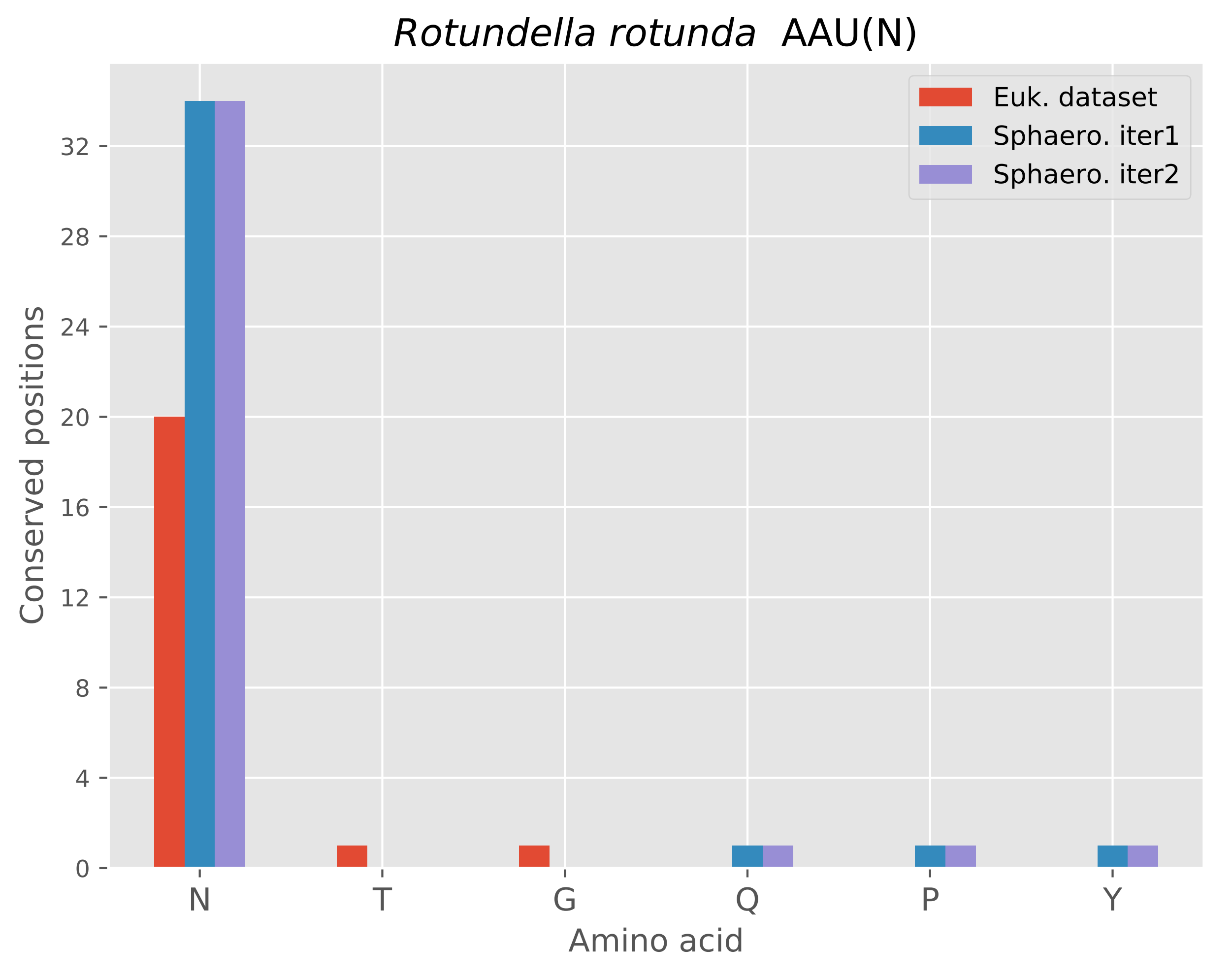

Rotundella rotunda AAU(N)
Euk. dataset
Sphaero. iter1
Sphaero. iter2
Conserved positions
0
4
8
12
16
20
24
28
32
N
T
G
Q
P
Y
Amino acid

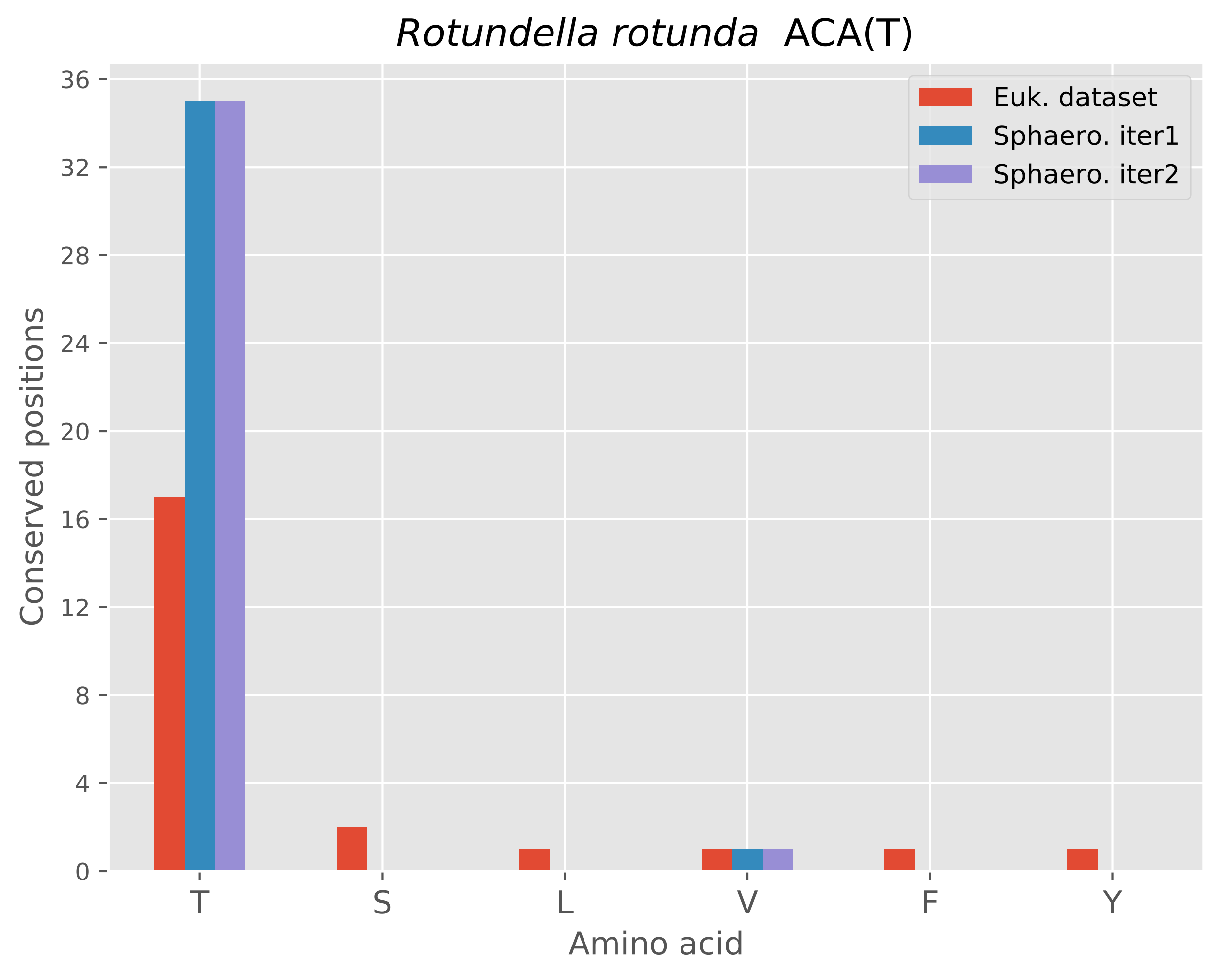

Rotundella rotunda ACA(T)
Euk. dataset
Sphaero. iter1
Sphaero. iter2
Conserved positions
0
4
8
12
16
20
24
28
32
36
T
S
L
V
F
Y
Amino acid

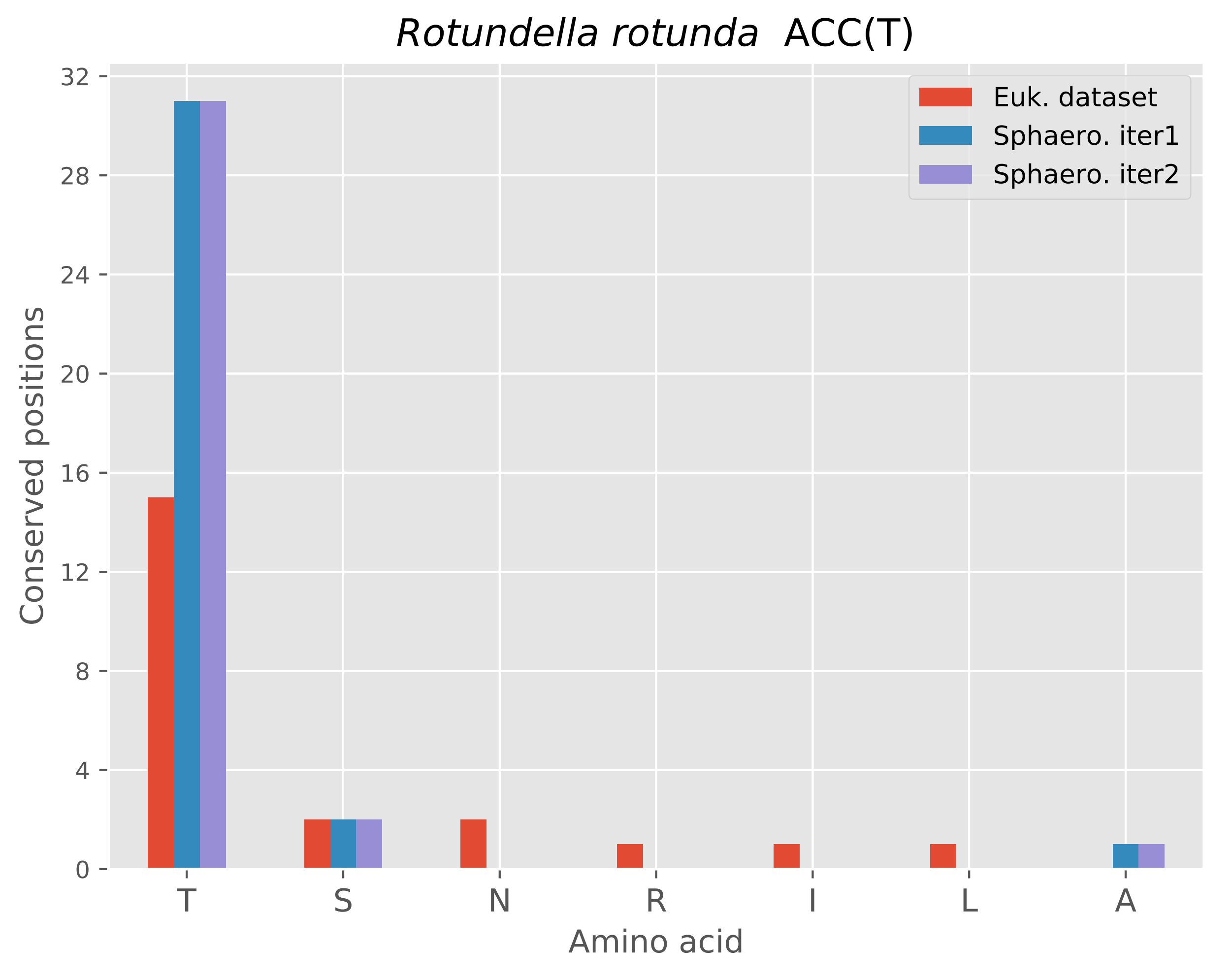

Rotundella rotunda ACC(T)
Euk. dataset
Sphaero. iter1
Sphaero. iter2
Conserved positions
0
4
8
12
16
20
24
28
32
T
S
N
R
I
L
A
Amino acid

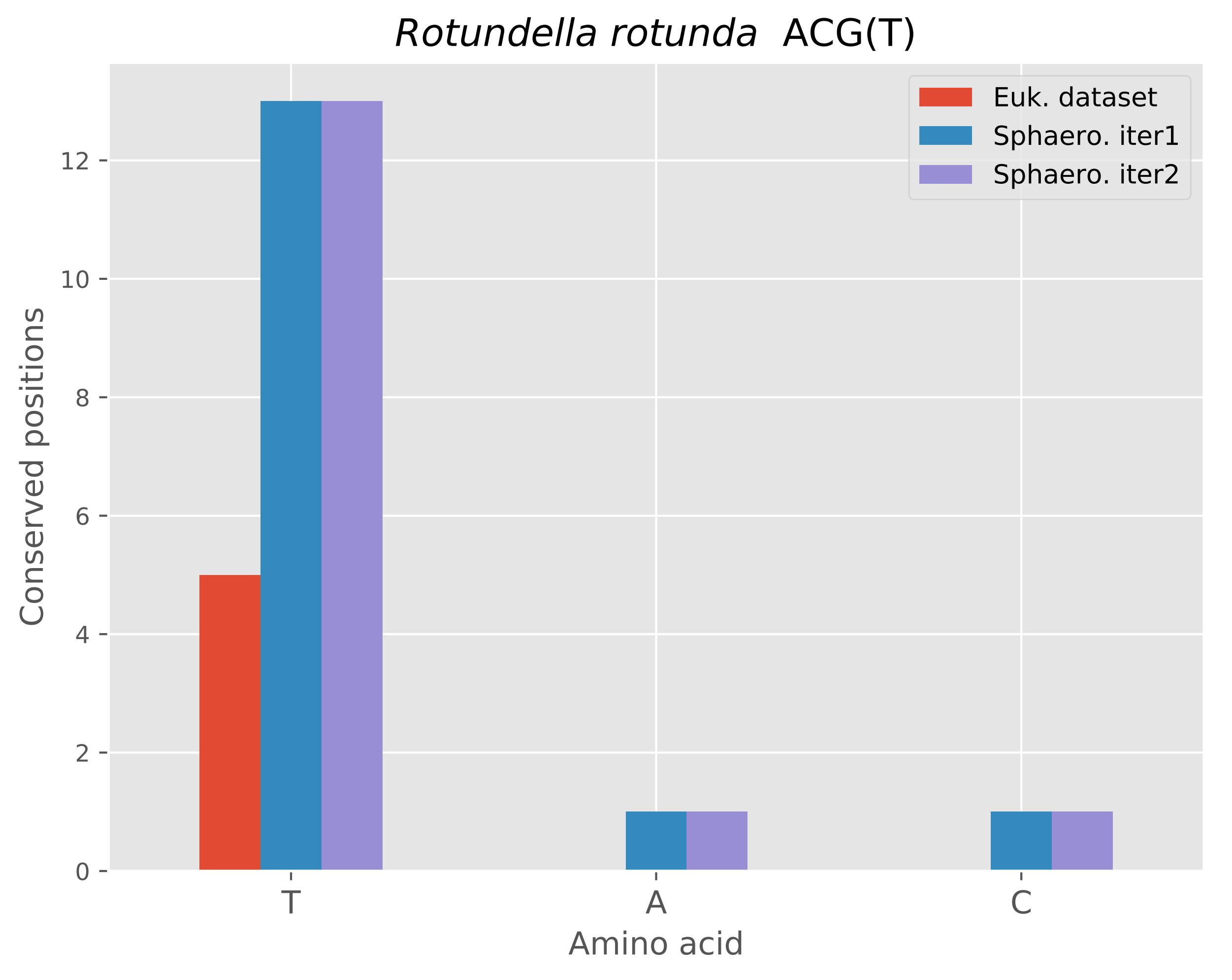

Rotundella rotunda ACG(T)
Euk. dataset
Sphaero. iter1
Sphaero. iter2
Conserved positions
0
2
4
6
8
10
12
T
A
C
Amino acid

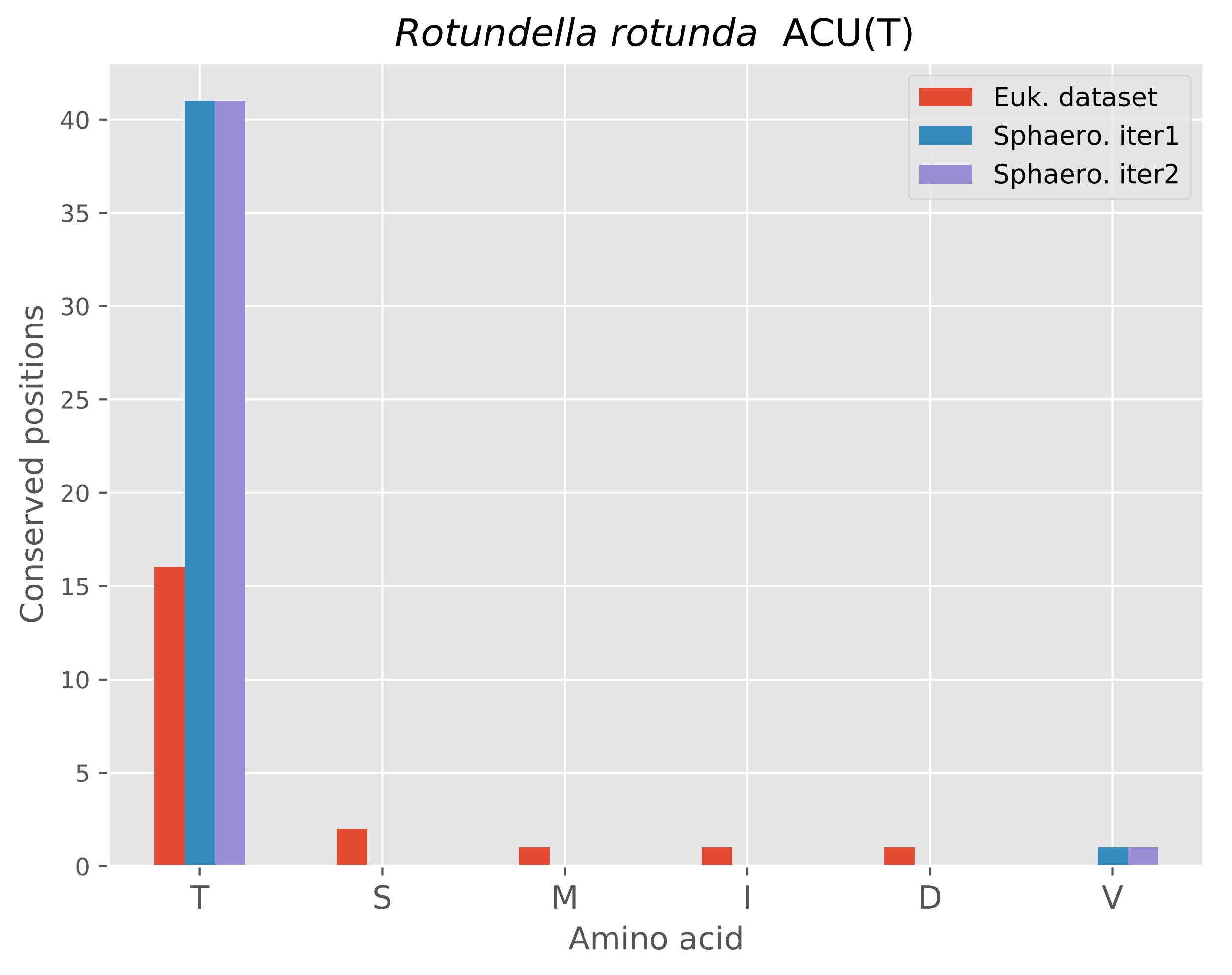

Rotundella rotunda ACU(T)
Euk. dataset
Sphaero. iter1
Sphaero. iter2
Conserved positions
0
5
10
15
20
25
30
35
40
T
S
M
I
D
V
Amino acid

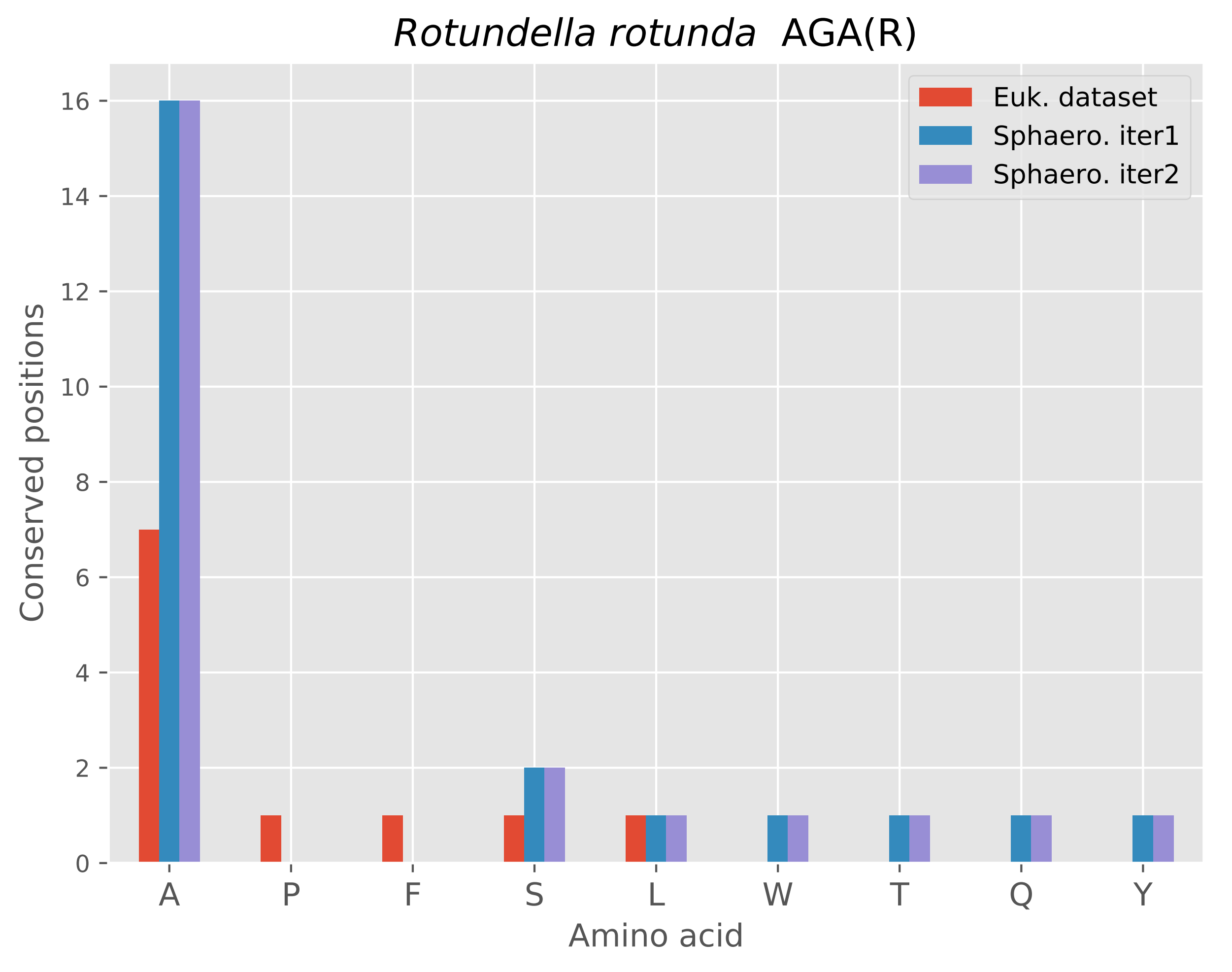

Rotundella rotunda AGA(R)
Euk. dataset
Sphaero. iter1
Sphaero. iter2
Conserved positions
0
2
4
6
8
10
12
14
16
A
P
F
S
L
W
T
Q
Y
Amino acid

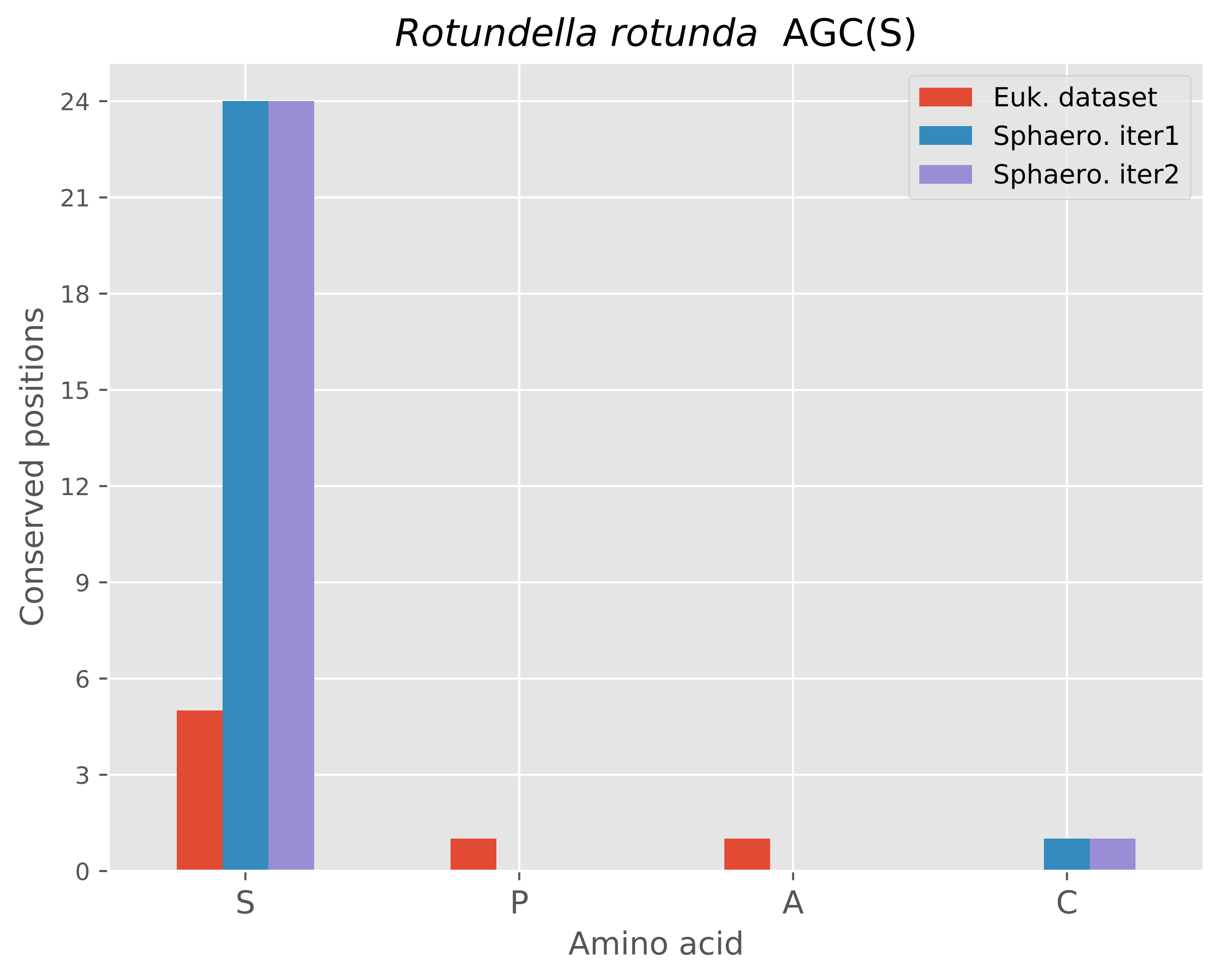
Rotundella rotunda AGC(S)
Euk. dataset
Sphaero. iter1
Sphaero. iter2
Conserved positions
0
3
6
9
12
15
18
21
24
S
P
A
C
Amino acid

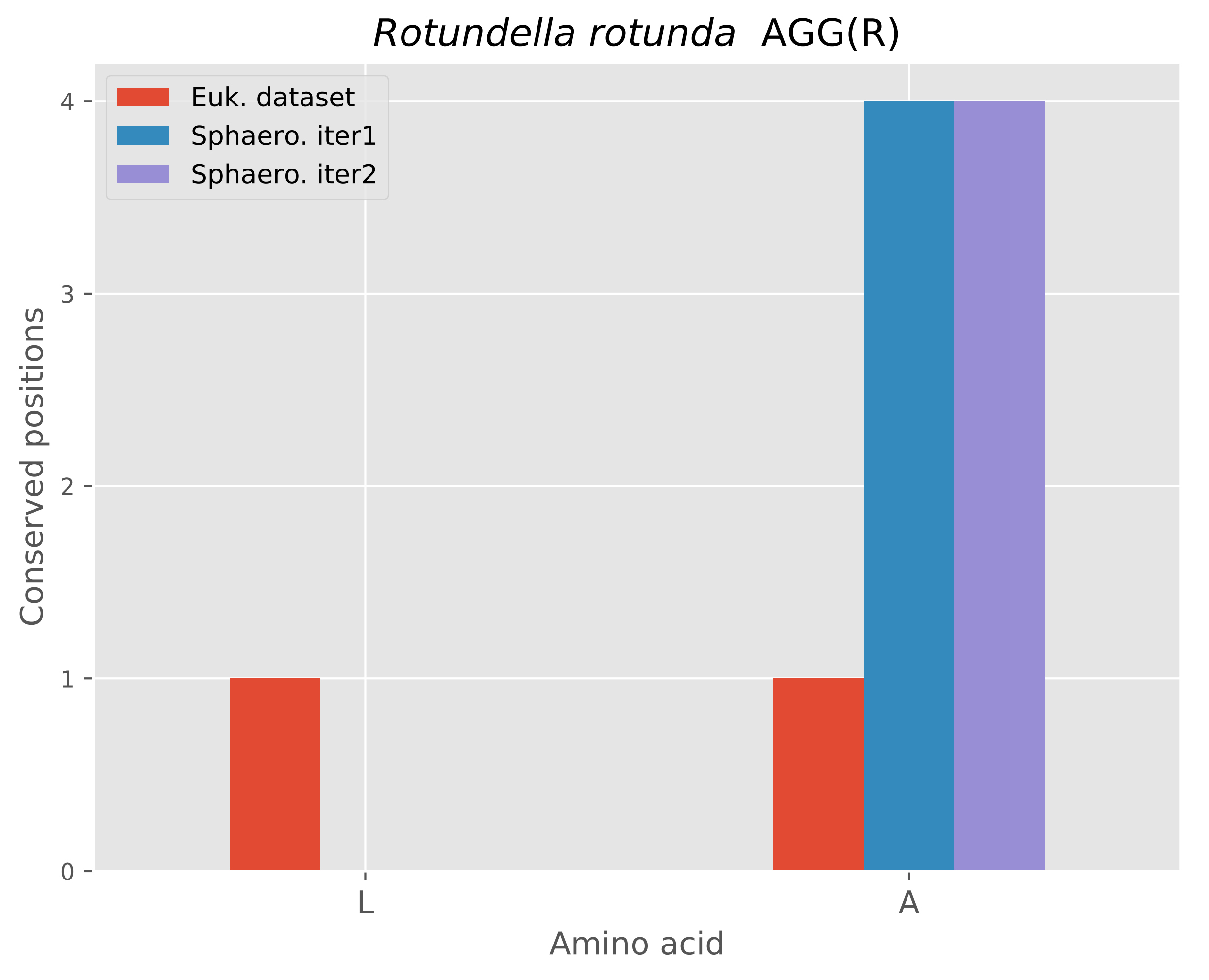

Rotundella rotunda AGG(R)
Euk. dataset
Sphaero. iter1
Sphaero. iter2
Conserved positions
0
1
2
3
4
L
A
Amino acid

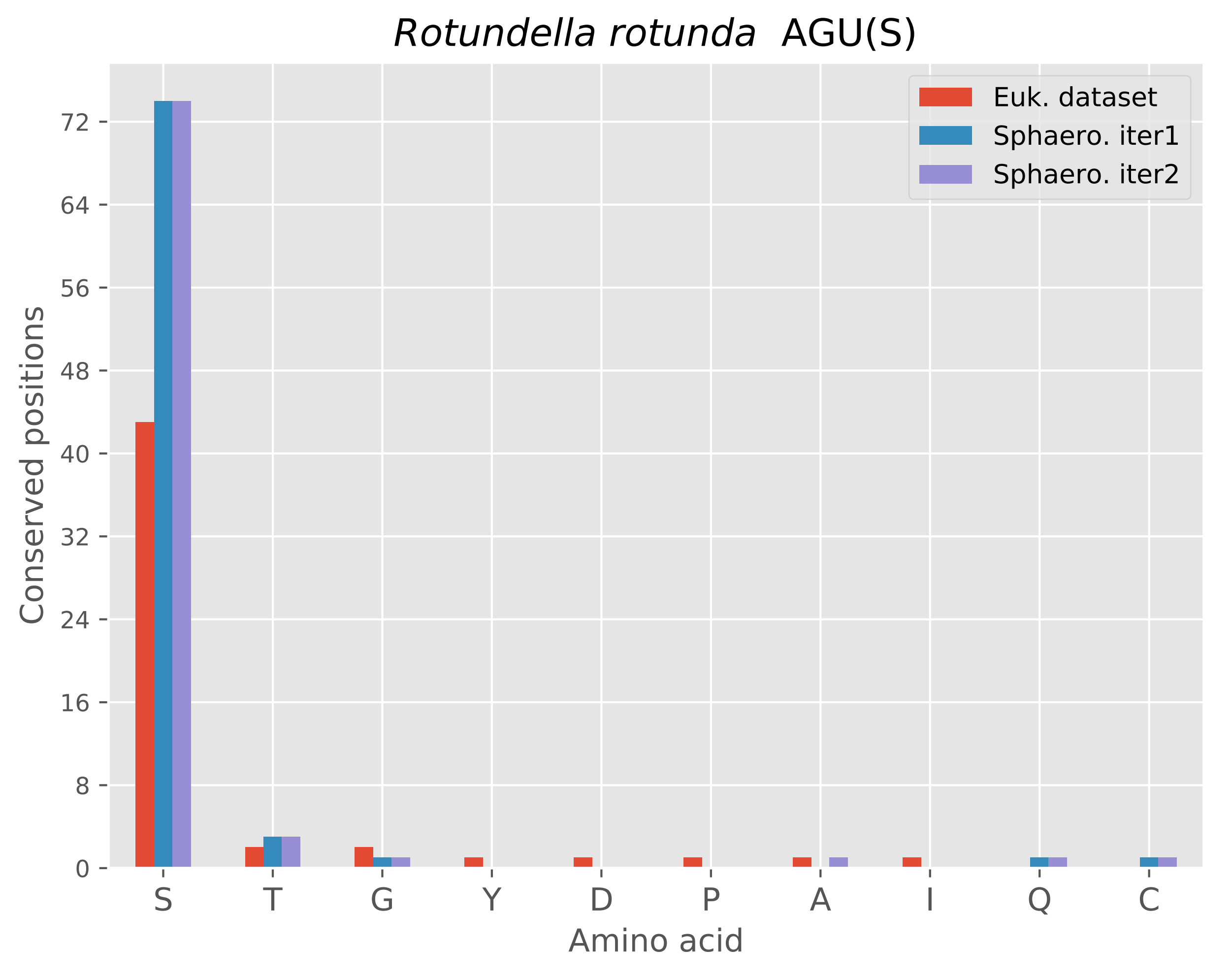

Rotundella rotunda AGU(S)
Euk. dataset
Sphaero. iter1
Sphaero. iter2
Conserved positions
0
8
16
24
32
40
48
56
64
72
S
T
G
Y
D
P
A
I
Q
C
Amino acid

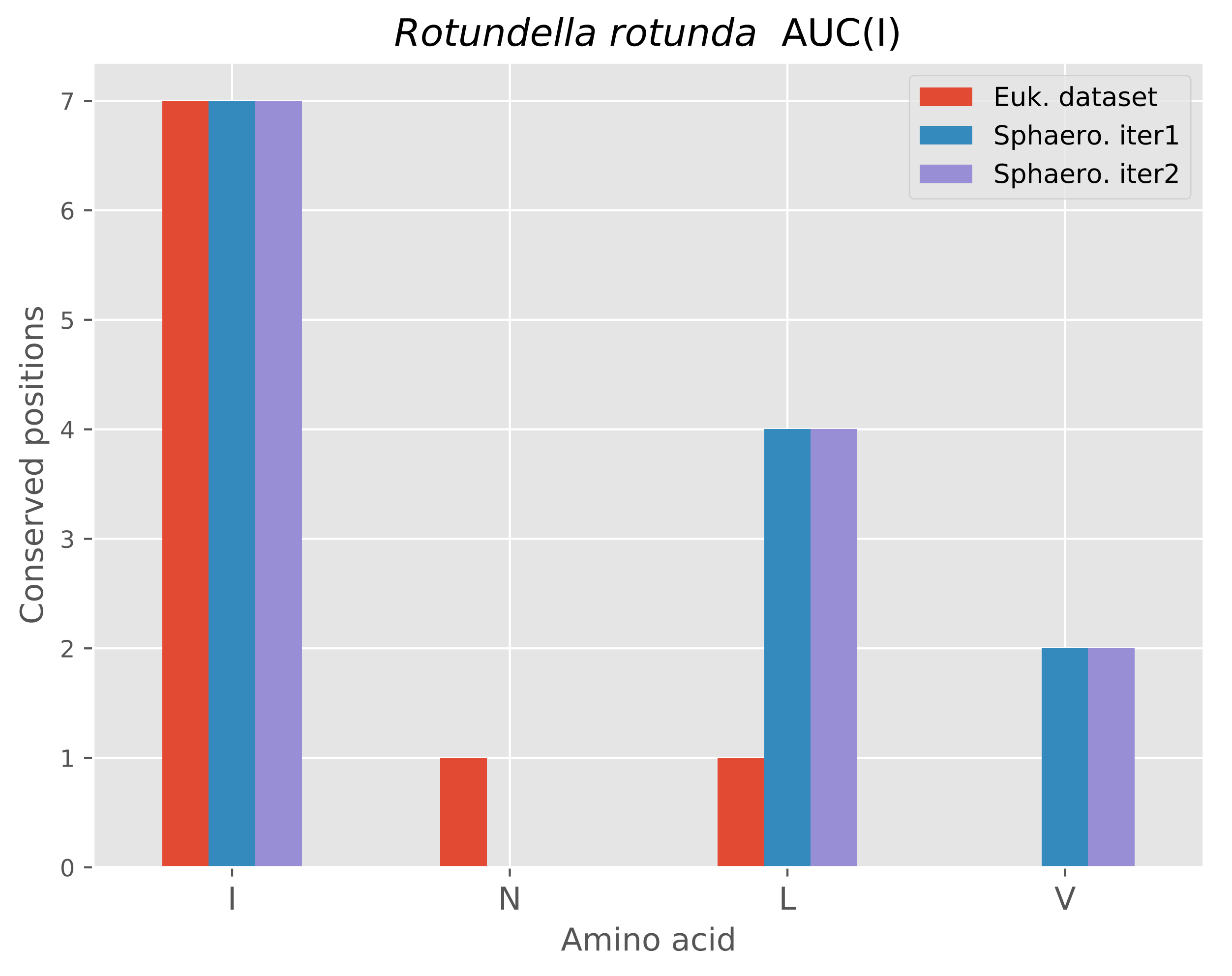

Rotundella rotunda AUC(I)
Euk. dataset
Sphaero. iter1
Sphaero. iter2
Conserved positions
0
1
2
3
4
5
6
7
I
N
L
V
Amino acid

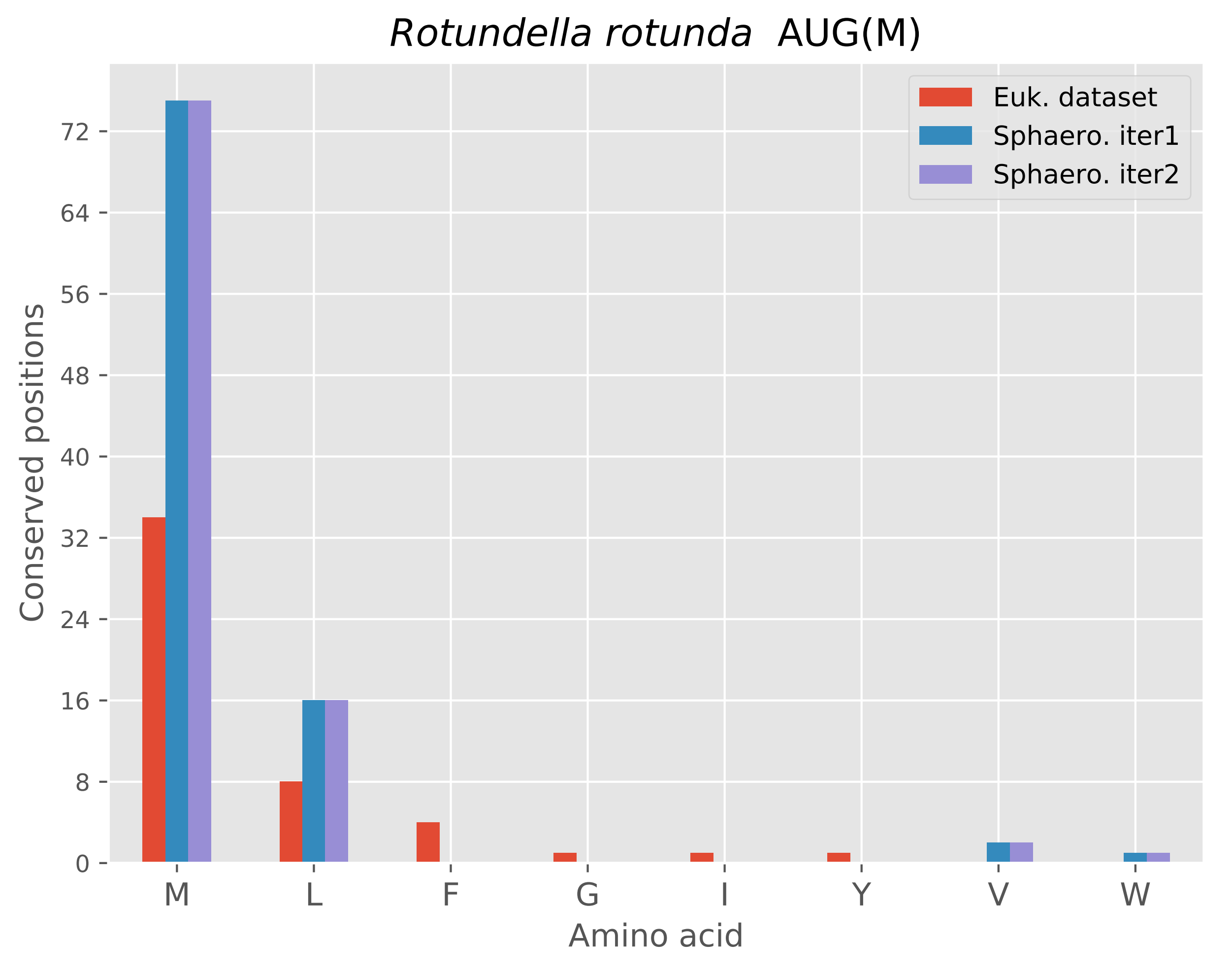

Rotundella rotunda AUG(M)
Euk. dataset
Sphaero. iter1
Sphaero. iter2
Conserved positions
0
8
16
24
32
40
48
56
64
72
M
L
F
G
I
Y
V
W
Amino acid

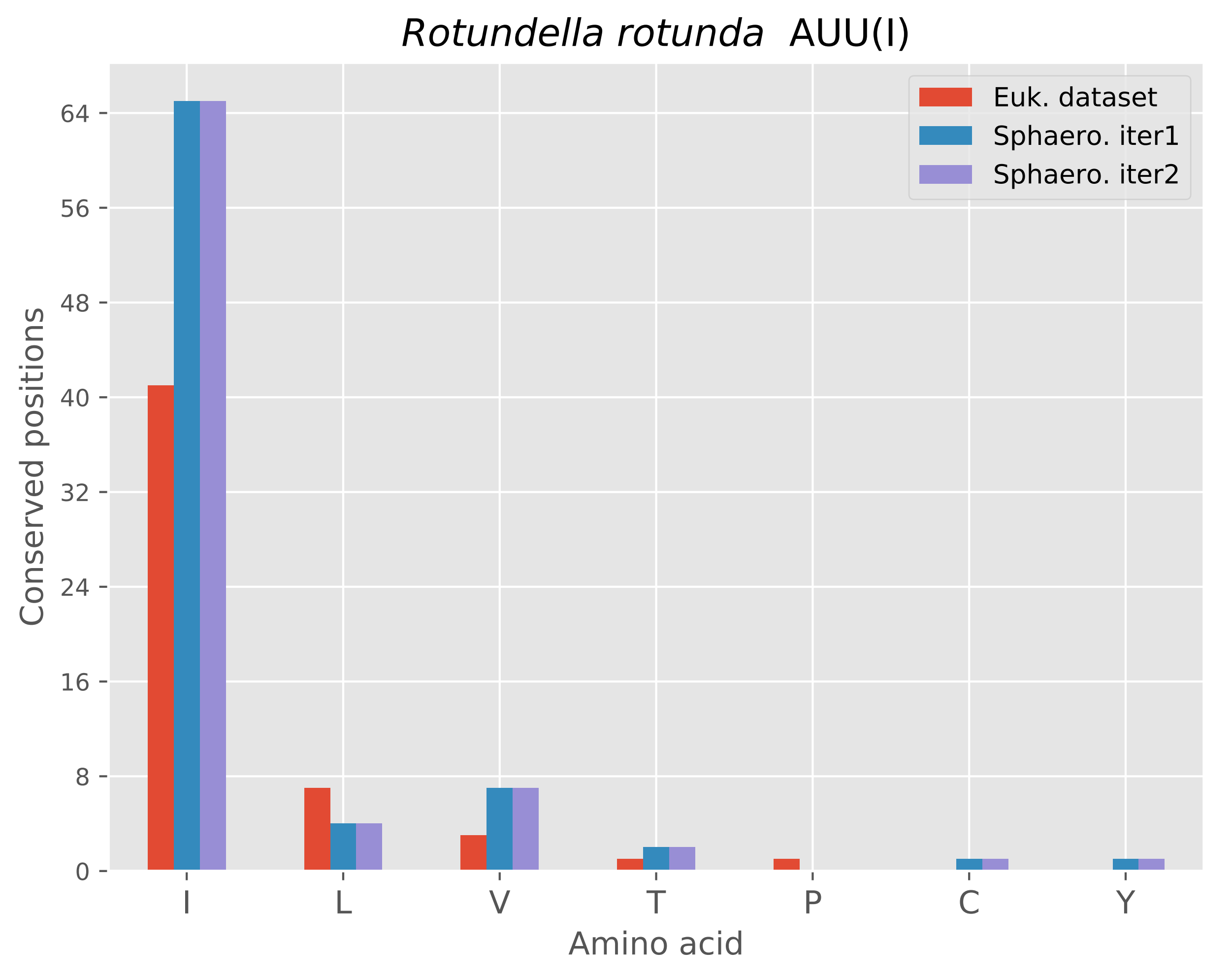

Rotundella rotunda AUU(I)
Euk. dataset
Sphaero. iter1
Sphaero. iter2
Conserved positions
0
8
16
24
32
40
48
56
64
I
L
V
T
P
C
Y
Amino acid

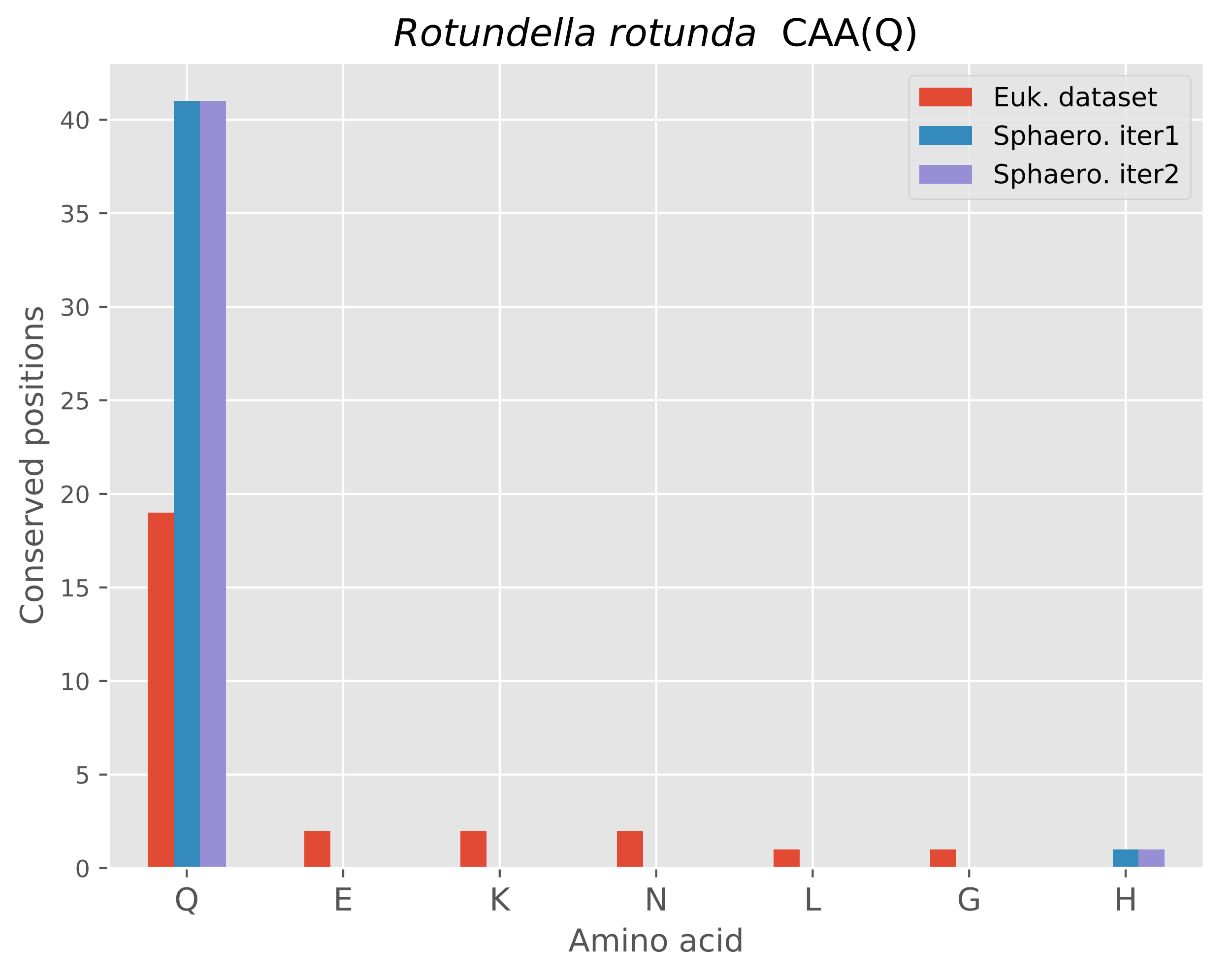

Rotundella rotunda CAA(Q)
Euk. dataset
Sphaero. iter1
Sphaero. iter2
Conserved positions
0
5
10
15
20
25
30
35
40
Q
E
K
N
L
G
H
Amino acid

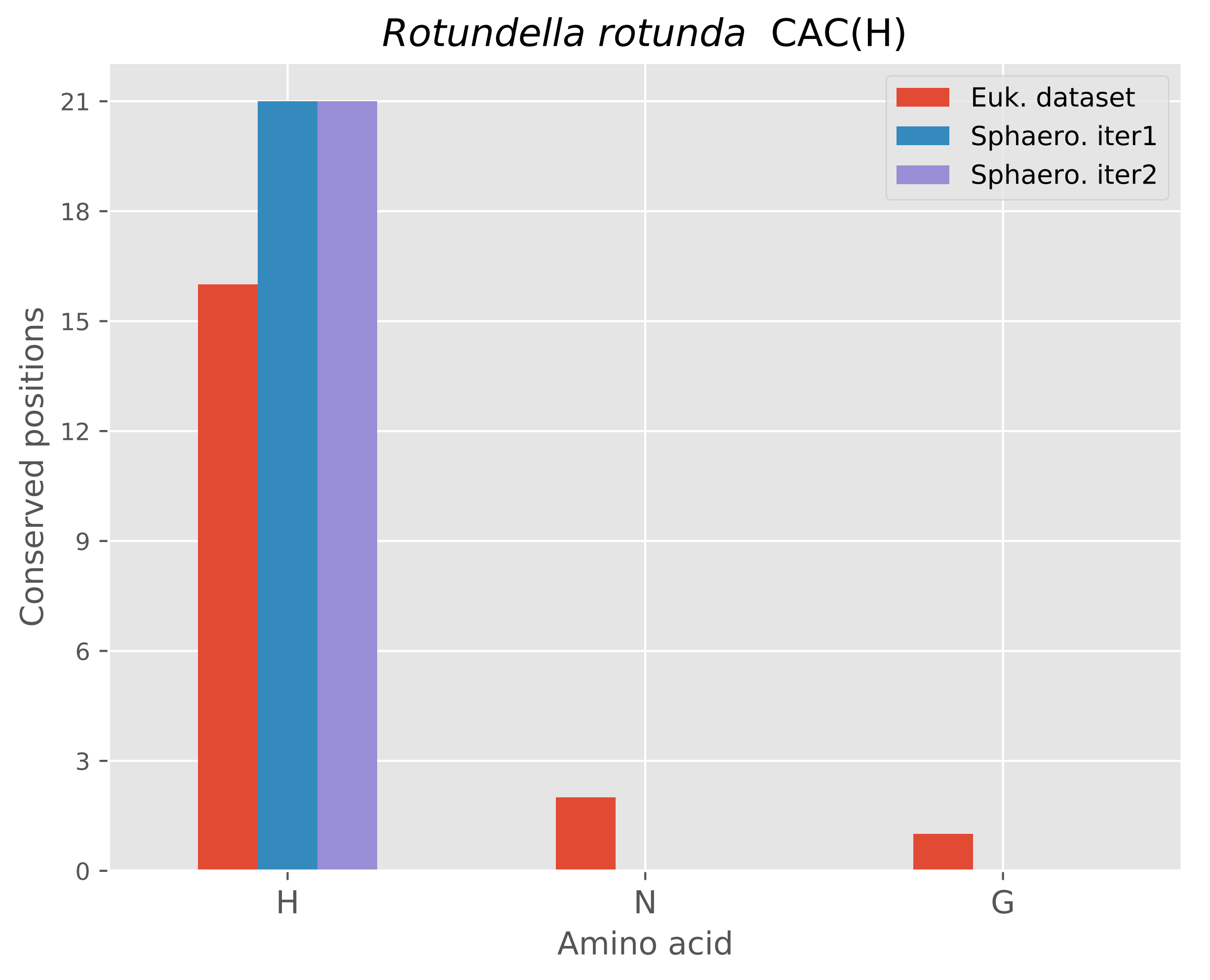

Rotundella rotunda CAC(H)
Euk. dataset
Sphaero. iter1
Sphaero. iter2
Conserved positions
0
3
6
9
12
15
18
21
H
N
G
Amino acid

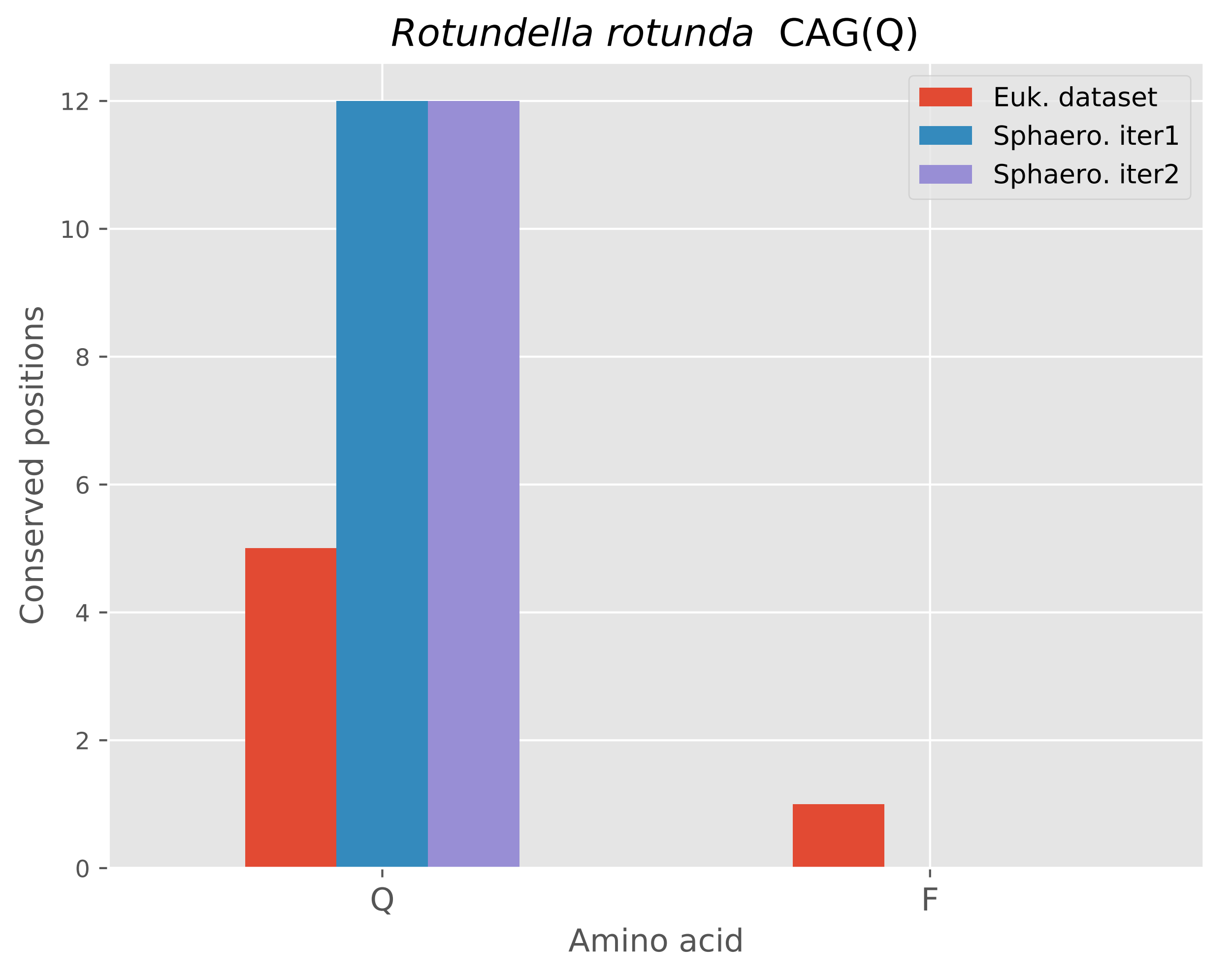

Rotundella rotunda CAG(Q)
Euk. dataset
Sphaero. iter1
Sphaero. iter2
Conserved positions
0
2
4
6
8
10
12
Q
F
Amino acid

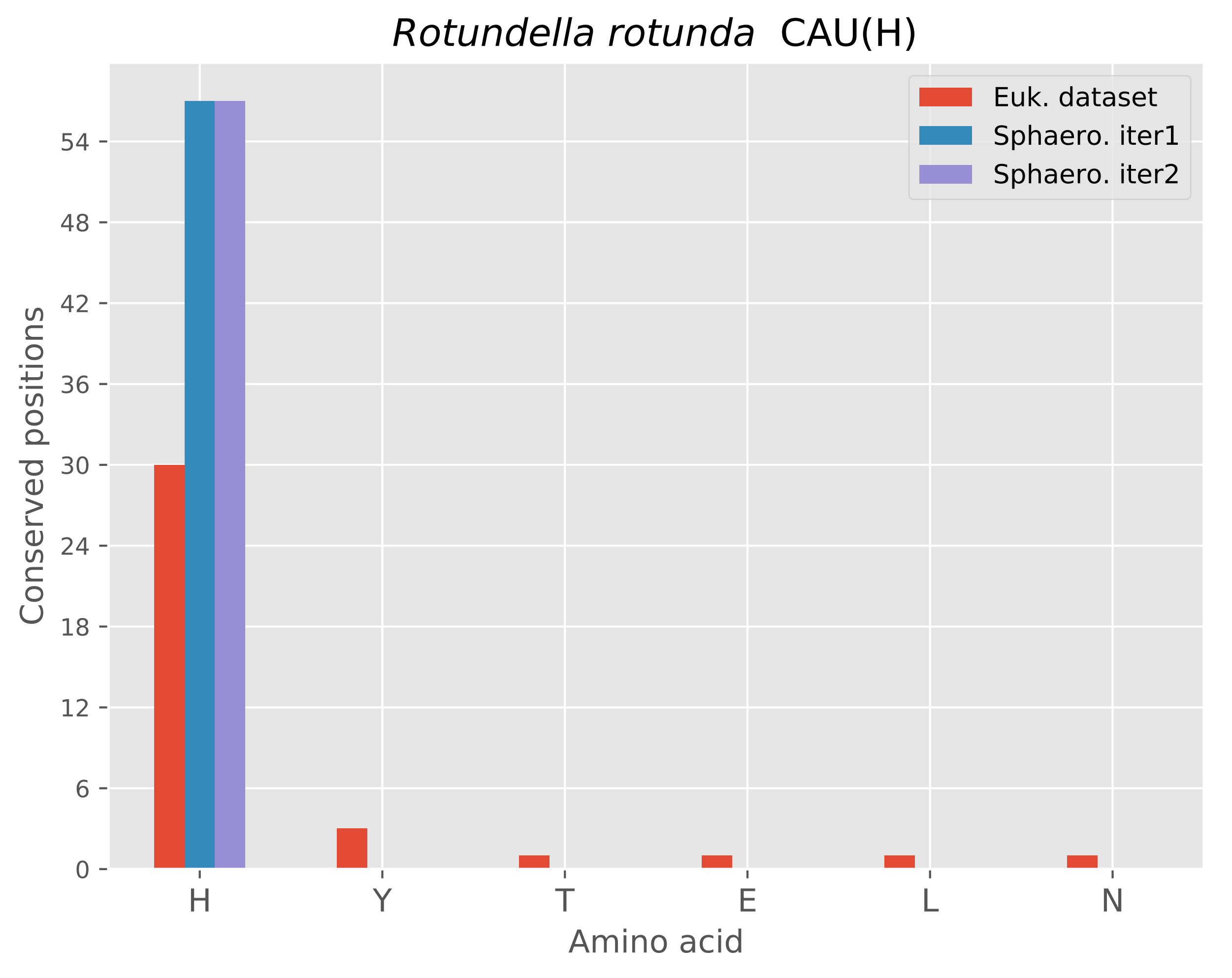

Rotundella rotunda CAU(H)
Euk. dataset
Sphaero. iter1
Sphaero. iter2
Conserved positions
0
6
12
18
24
30
36
42
48
54
H
Y
T
E
L
N
Amino acid

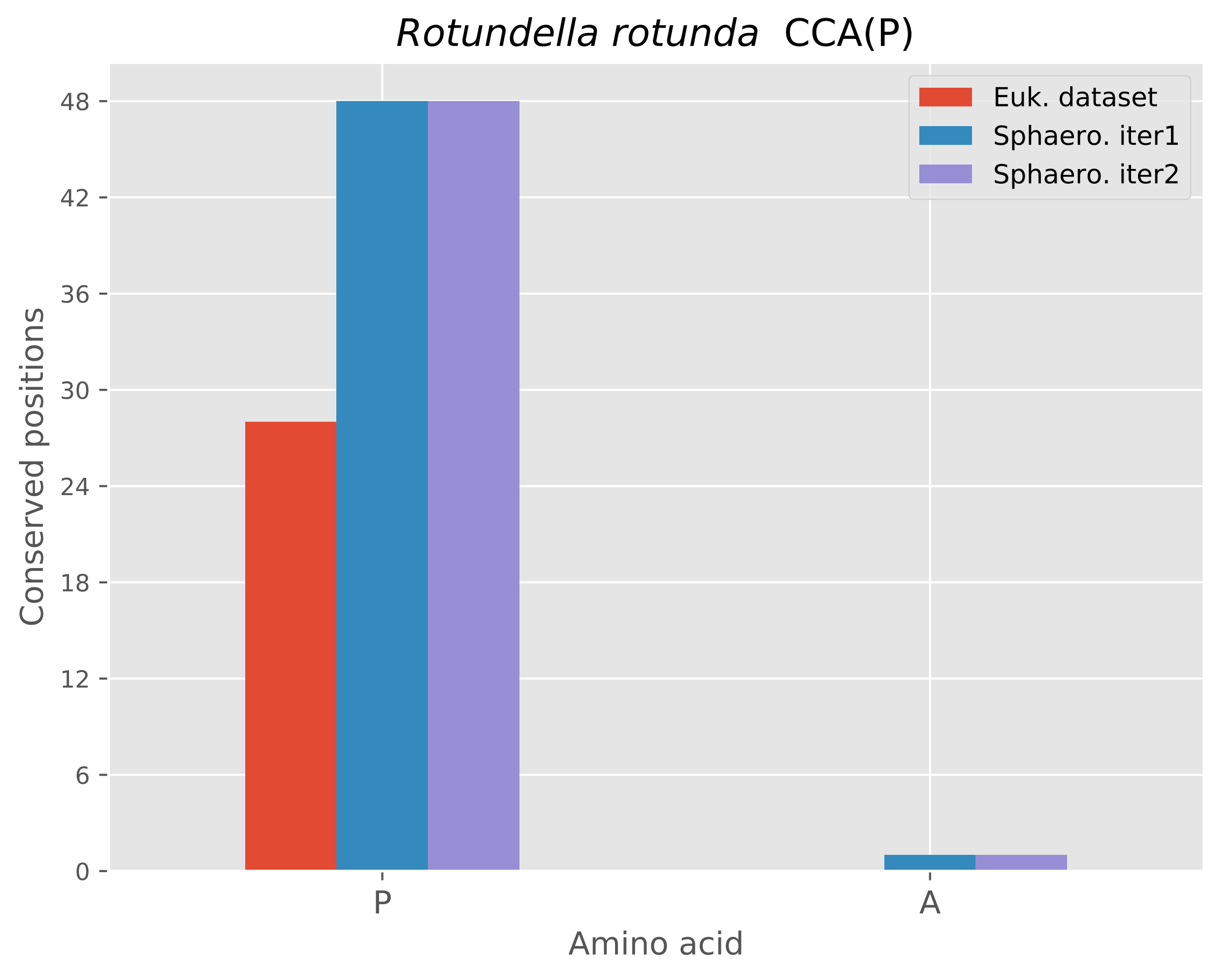
Rotundella rotunda CCA(P)
Euk. dataset
Sphaero. iter1
Sphaero. iter2
Conserved positions
48
42
36
30
24
18
12
6
0
P
A
Amino acid

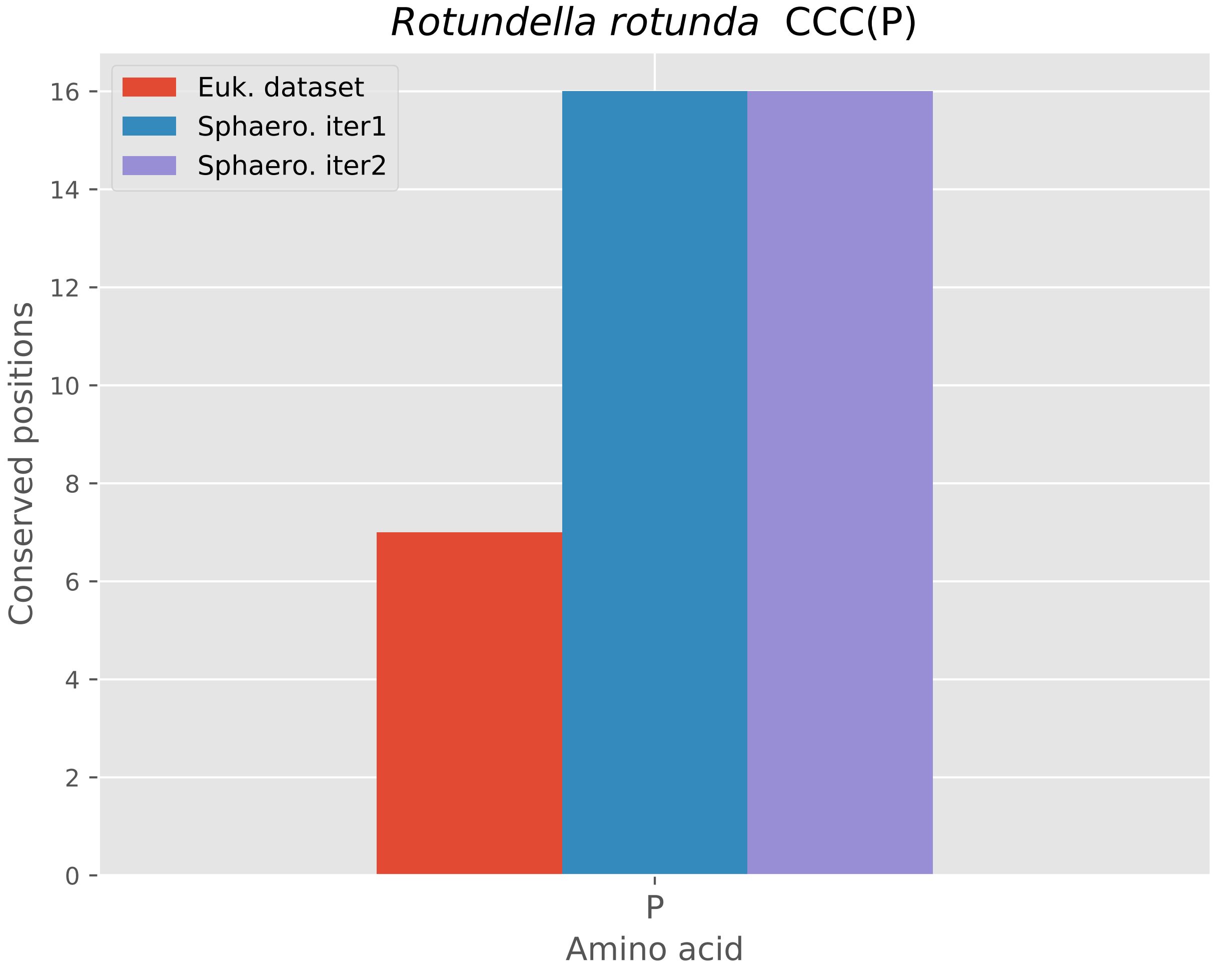

Rotundella rotunda CCC(P)
Euk. dataset
Sphaero. iter1
Sphaero. iter2
Conserved positions
0
2
4
6
8
10
12
14
16
P
Amino acid

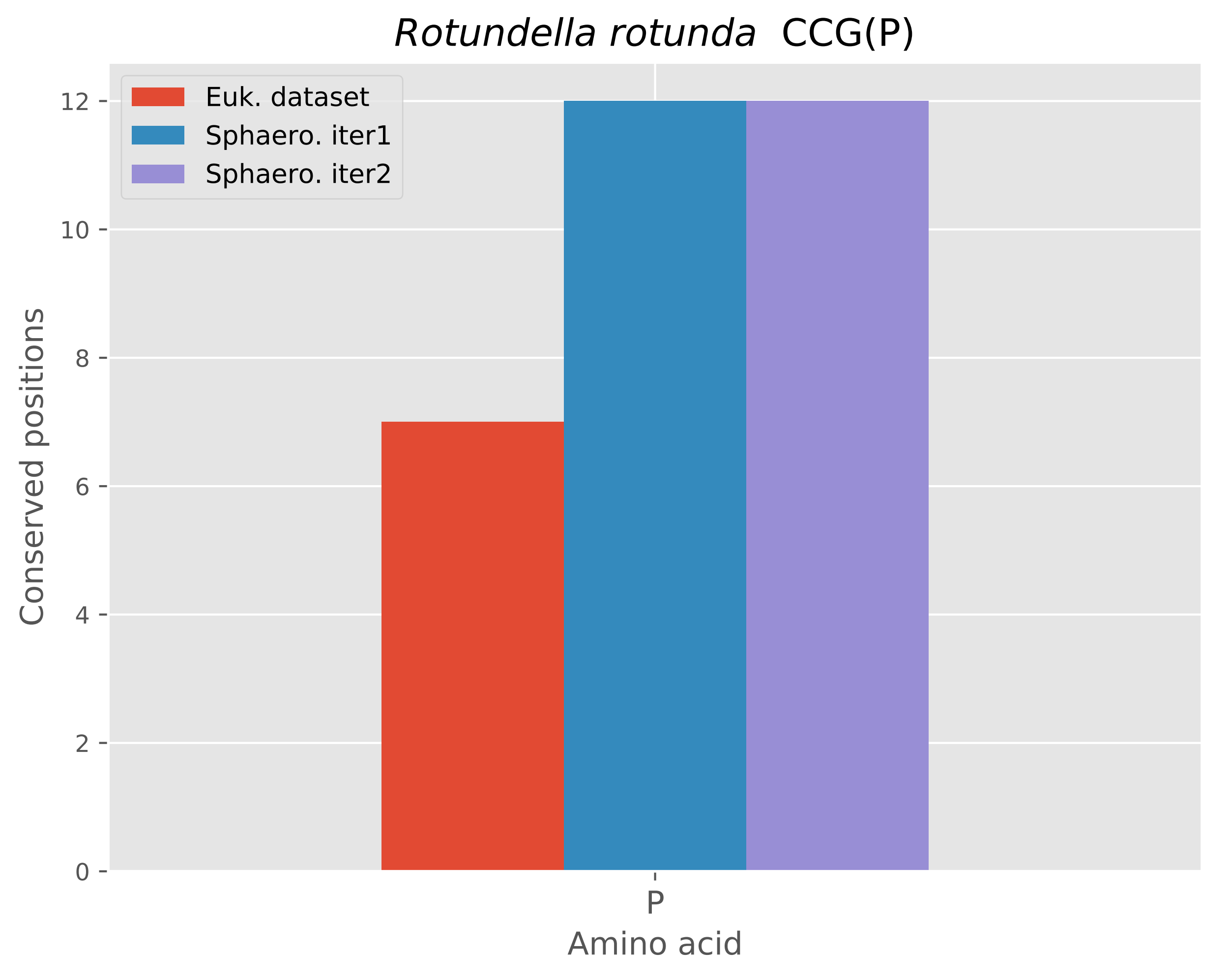

Rotundella rotunda CCG(P)
Euk. dataset
Sphaero. iter1
Sphaero. iter2
Conserved positions
0
2
4
6
8
10
12
P
Amino acid

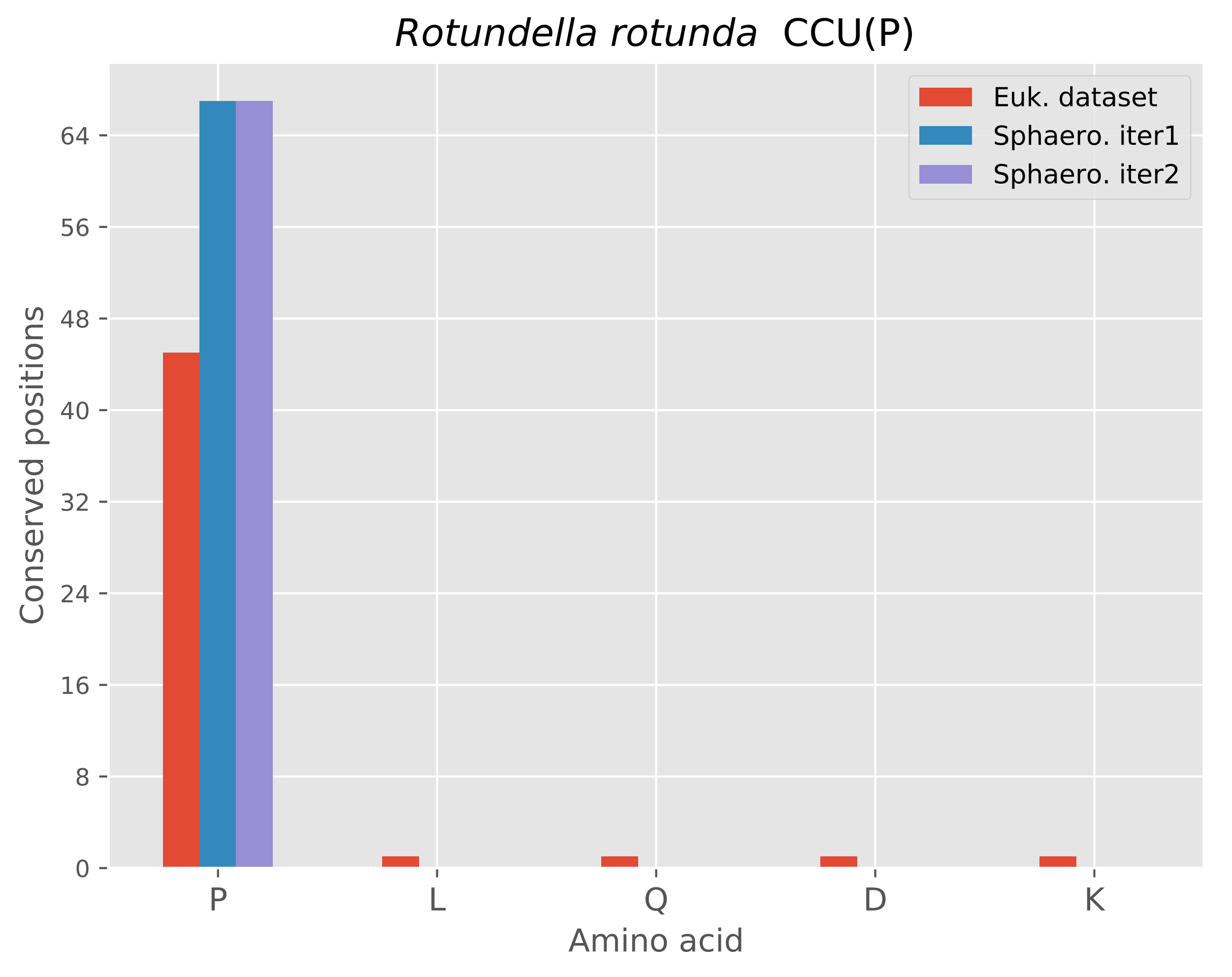

Rotundella rotunda CCU(P)
Euk. dataset
Sphaero. iter1
Sphaero. iter2
Conserved positions
0
8
16
24
32
40
48
56
64
P
L
Q
D
K
Amino acid

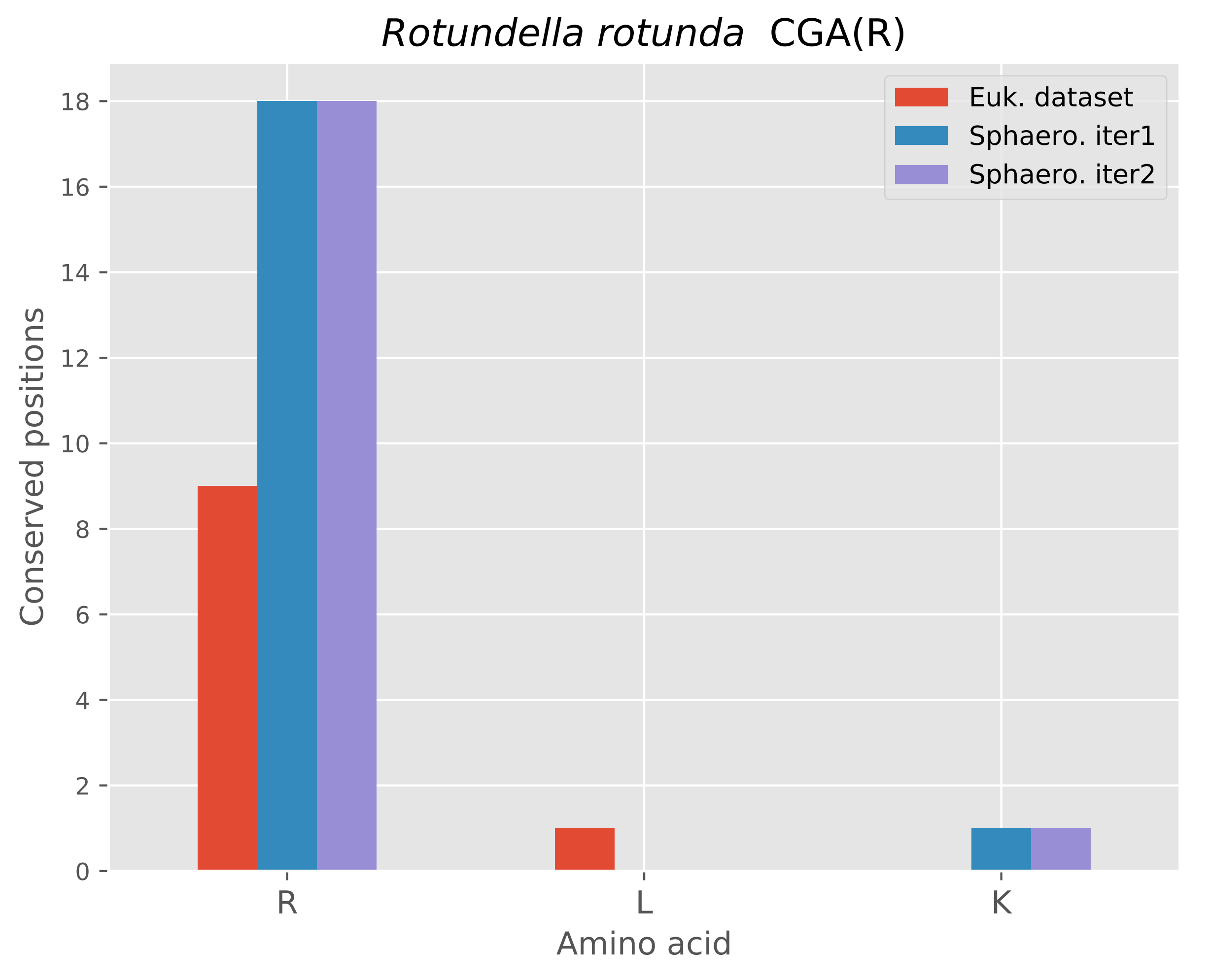

Rotundella rotunda CGA(R)
Euk. dataset
Sphaero. iter1
Sphaero. iter2
Conserved positions
0
2
4
6
8
10
12
14
16
18
R
L
K
Amino acid

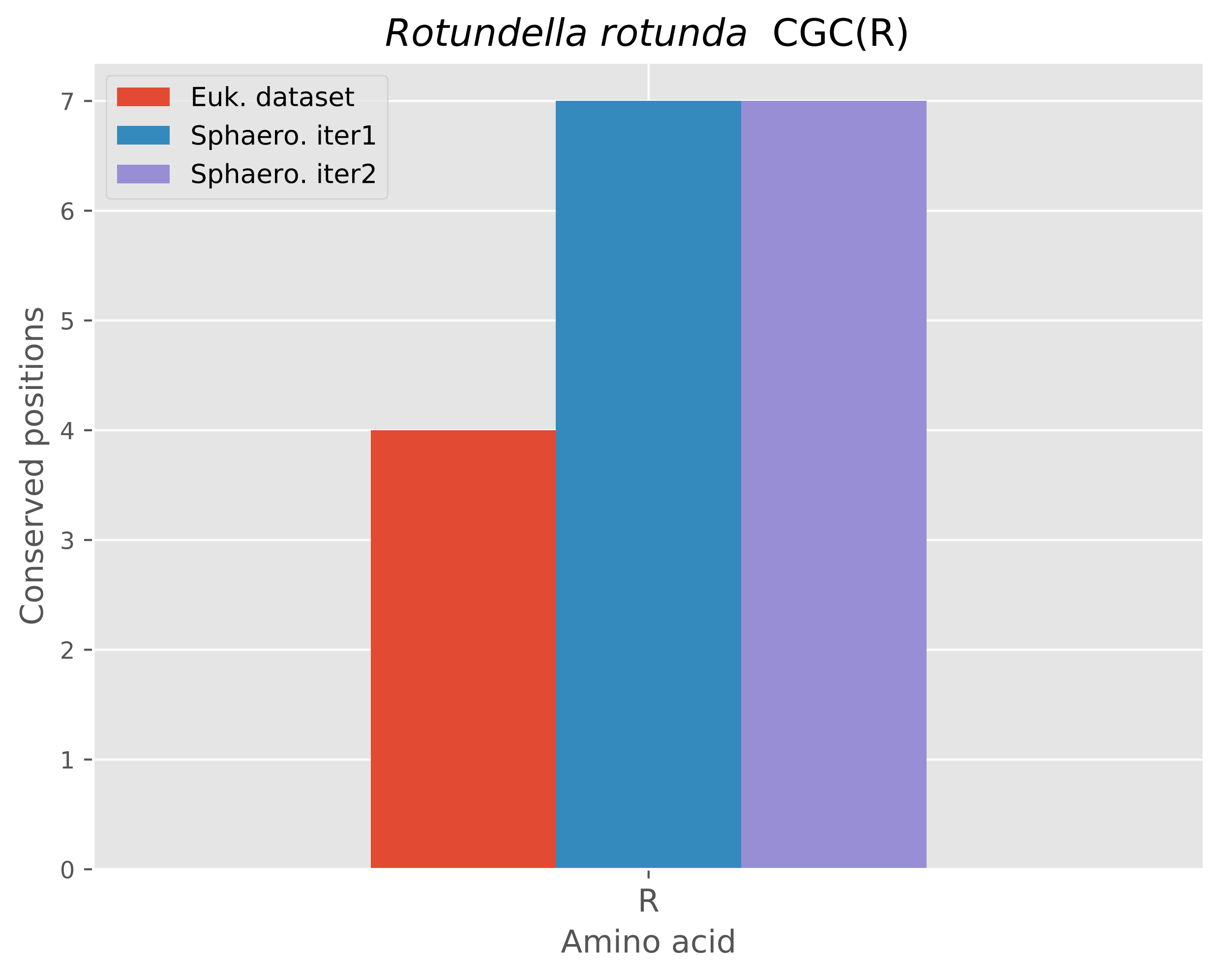

Rotundella rotunda CGC(R)
Euk. dataset
Sphaero. iter1
Sphaero. iter2
Conserved positions
0
1
2
3
4
5
6
7
R
Amino acid

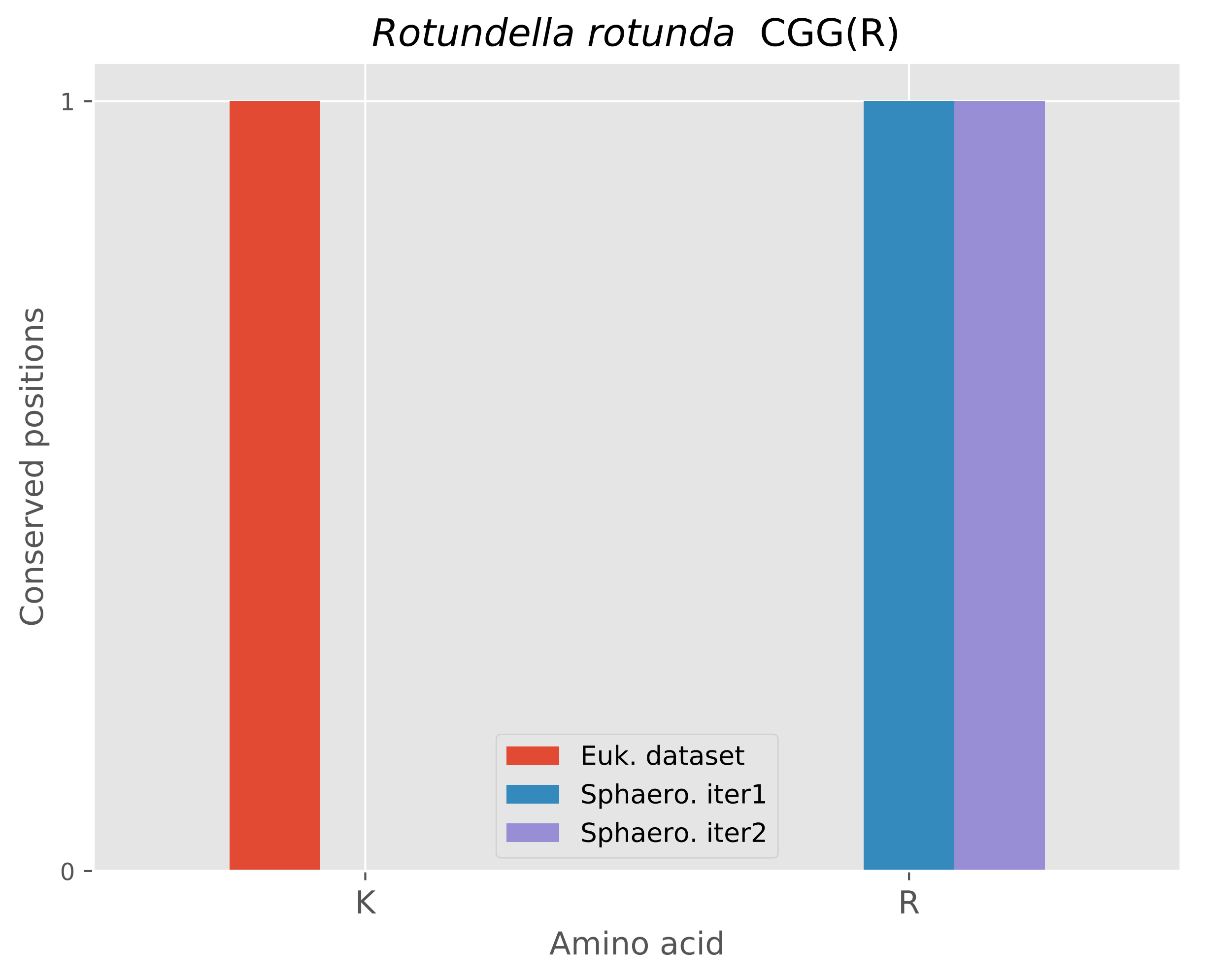

Rotundella rotunda CGG(R)
1
0
Conserved positions
K
R
Amino acid
Euk. dataset
Sphaero. iter1
Sphaero. iter2

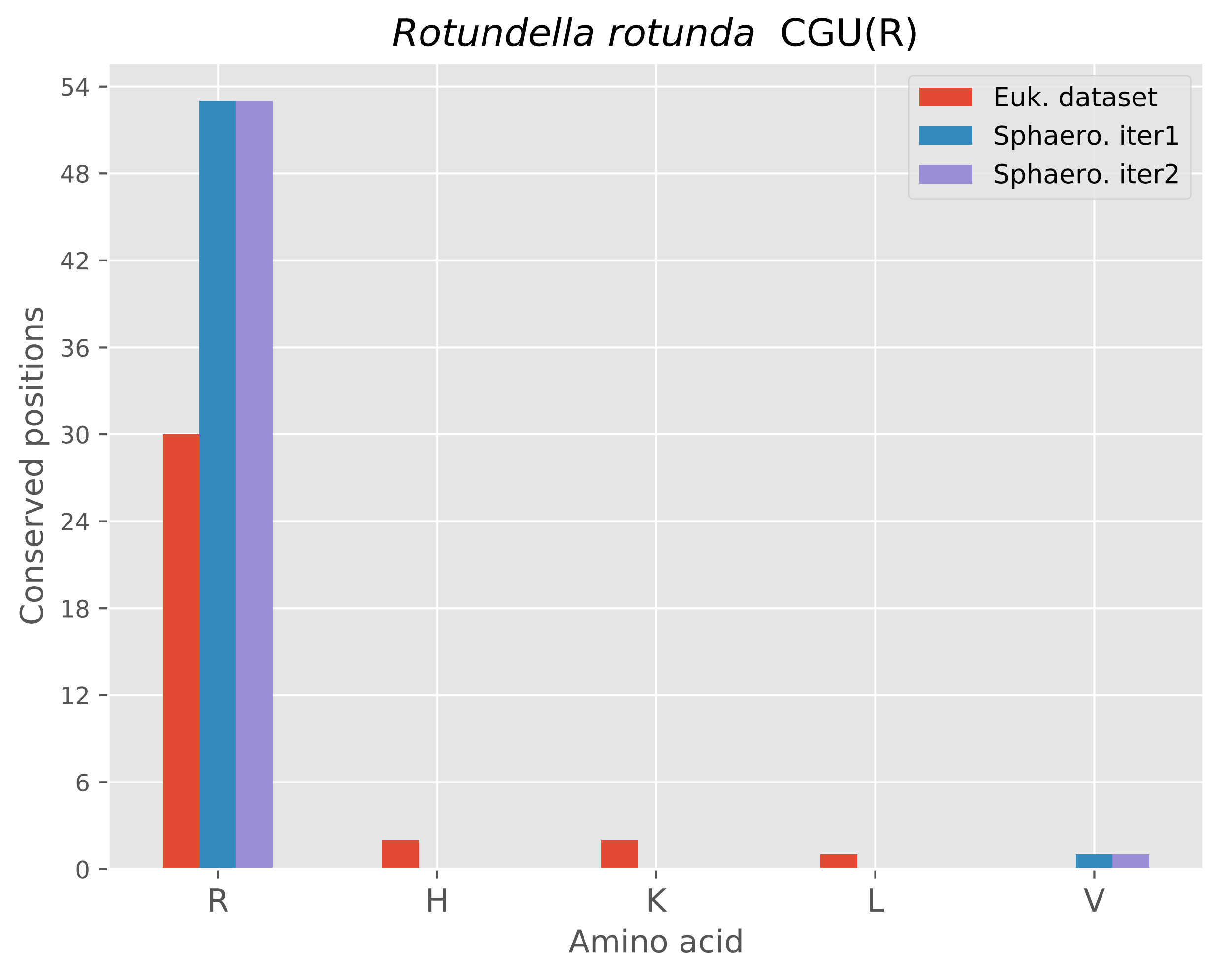

Rotundella rotunda CGU(R)
Euk. dataset
Sphaero. iter1
Sphaero. iter2
Conserved positions
0
6
12
18
24
30
36
42
48
54
R
H
K
L
V
Amino acid

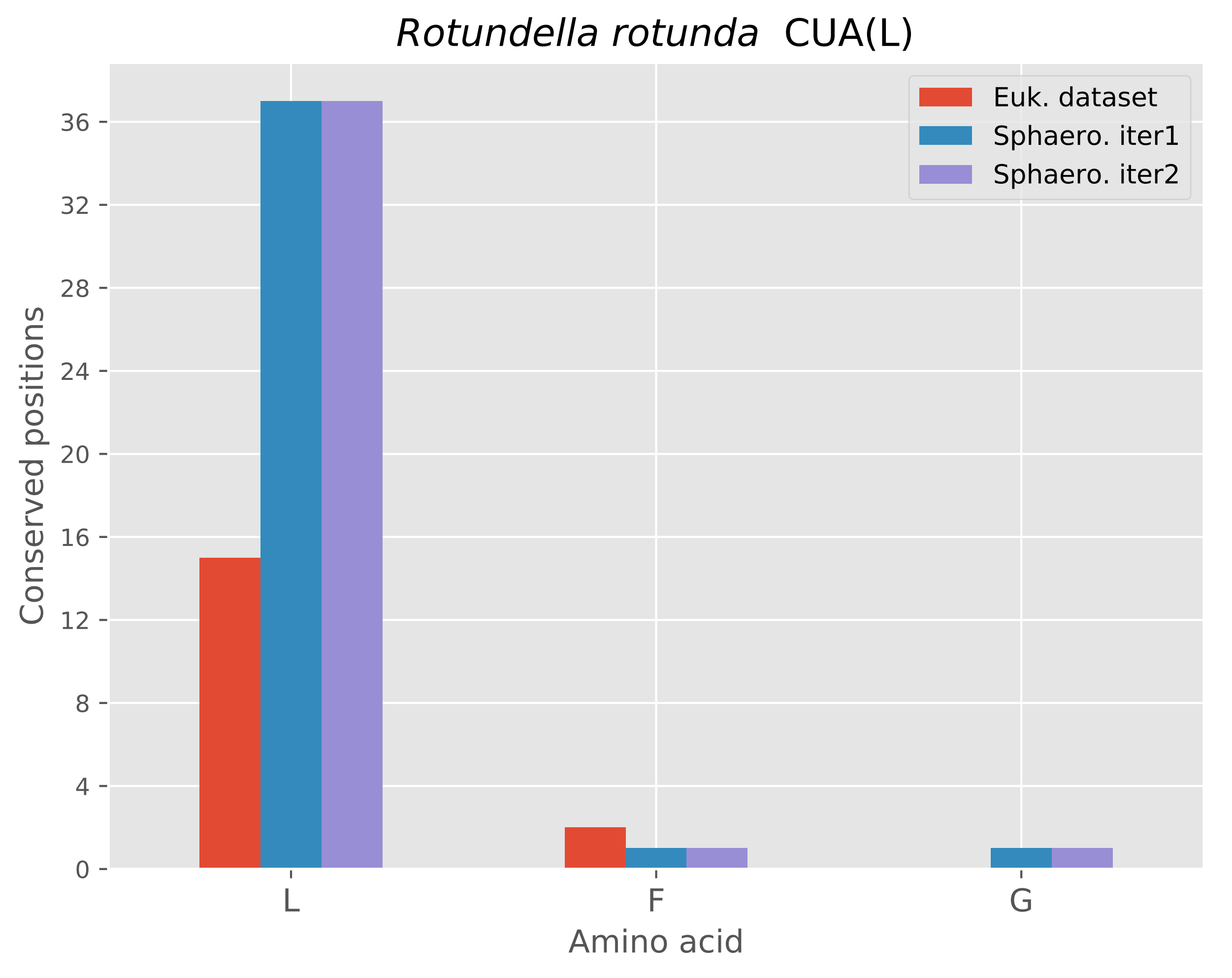

Rotundella rotunda CUA(L)
Euk. dataset
Sphaero. iter1
Sphaero. iter2
Conserved positions
0
4
8
12
16
20
24
28
32
36
L
F
G
Amino acid

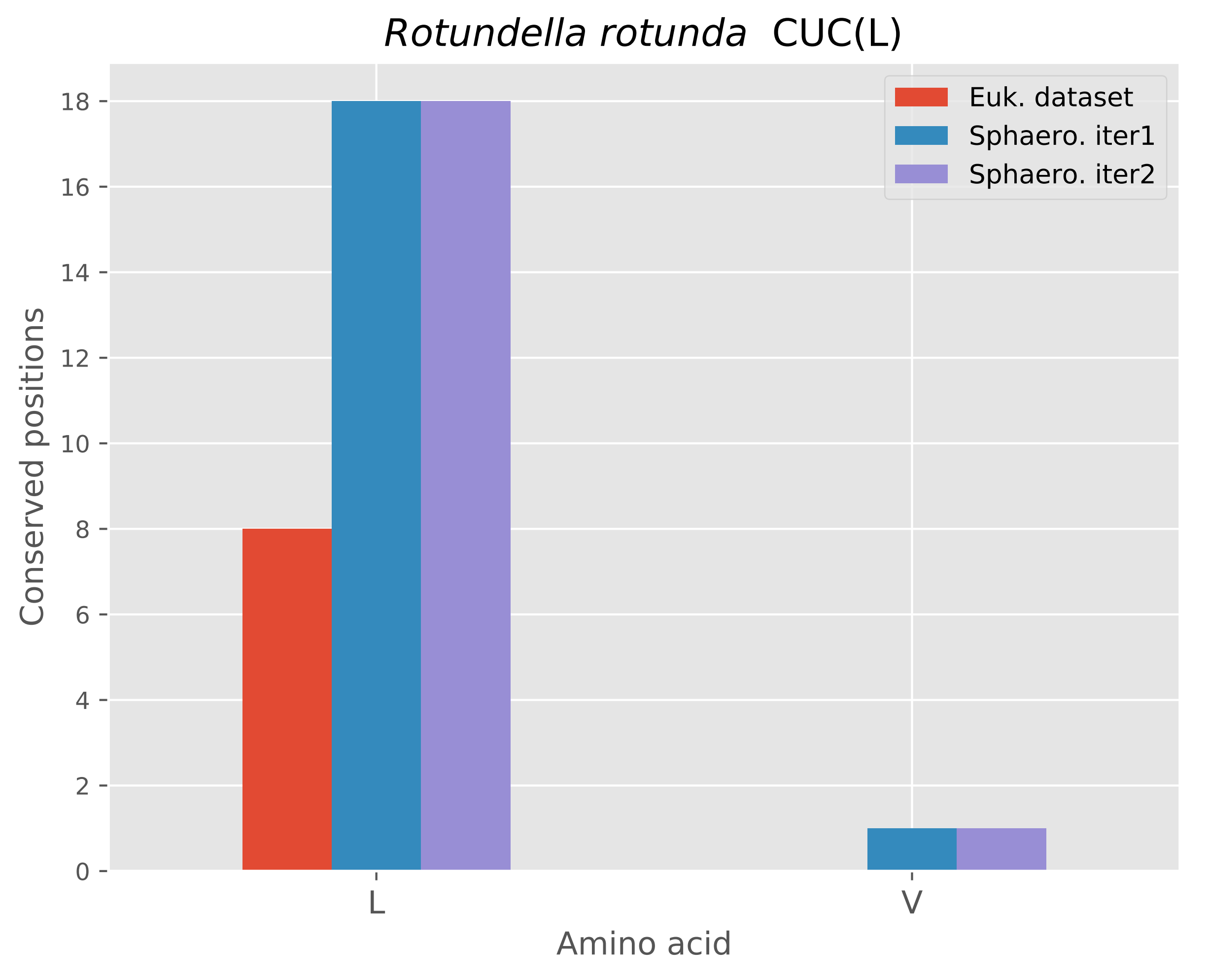
Rotundella rotunda CUC(L)
Euk. dataset
Sphaero. iter1
Sphaero. iter2
Conserved positions
0
2
4
6
8
10
12
14
16
18
L
V
Amino acid

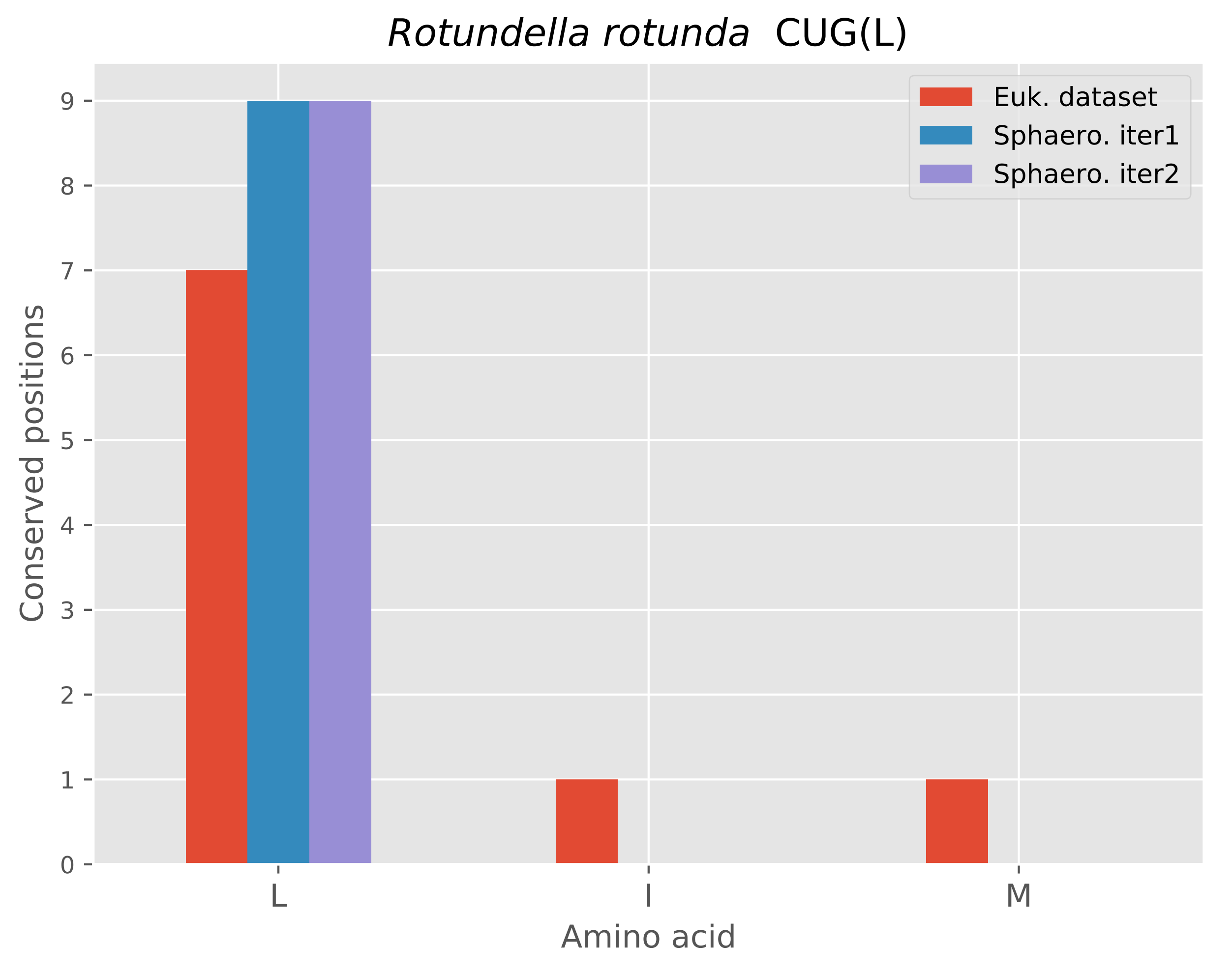

Rotundella rotunda CUG(L)
Euk. dataset
Sphaero. iter1
Sphaero. iter2
Conserved positions
0
1
2
3
4
5
6
7
8
9
L
I
M
Amino acid

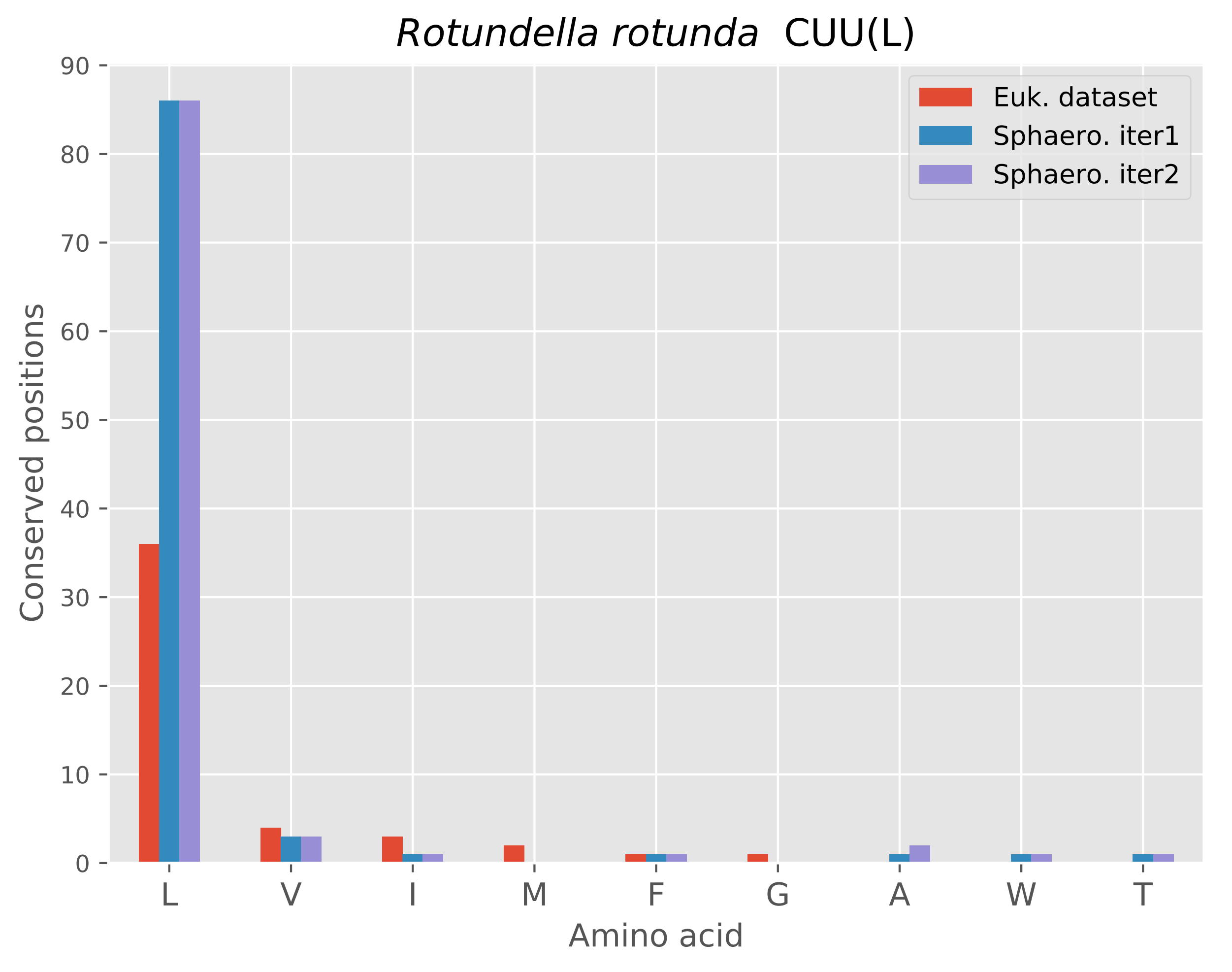

Rotundella rotunda CUU(L)
Euk. dataset
Sphaero. iter1
Sphaero. iter2
Conserved positions
0
10
20
30
40
50
60
70
80
90
L
V
I
M
F
G
A
W
T
Amino acid

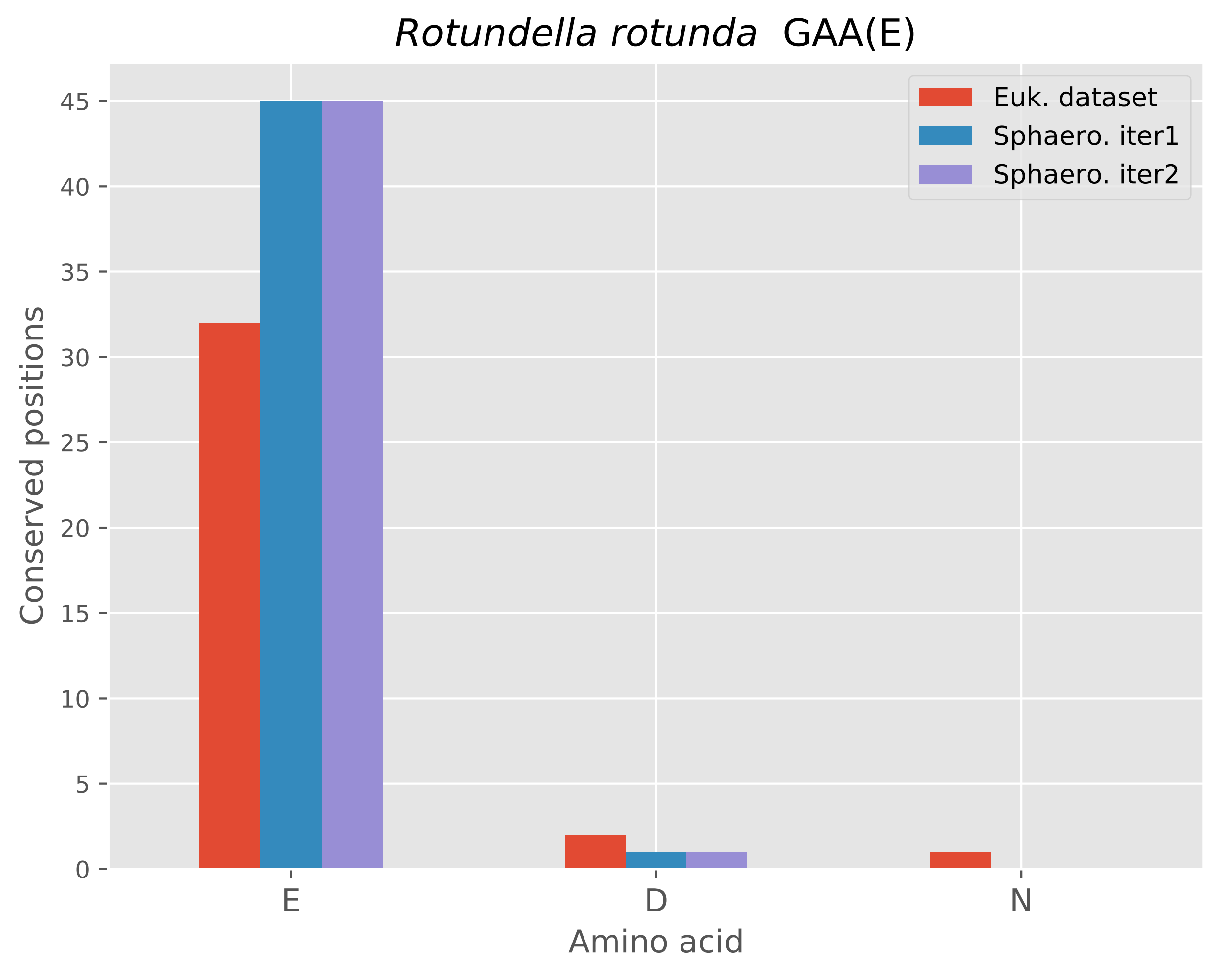

Rotundella rotunda GAA(E)
Euk. dataset
Sphaero. iter1
Sphaero. iter2
Conserved positions
0
5
10
15
20
25
30
35
40
45
E
D
N
Amino acid

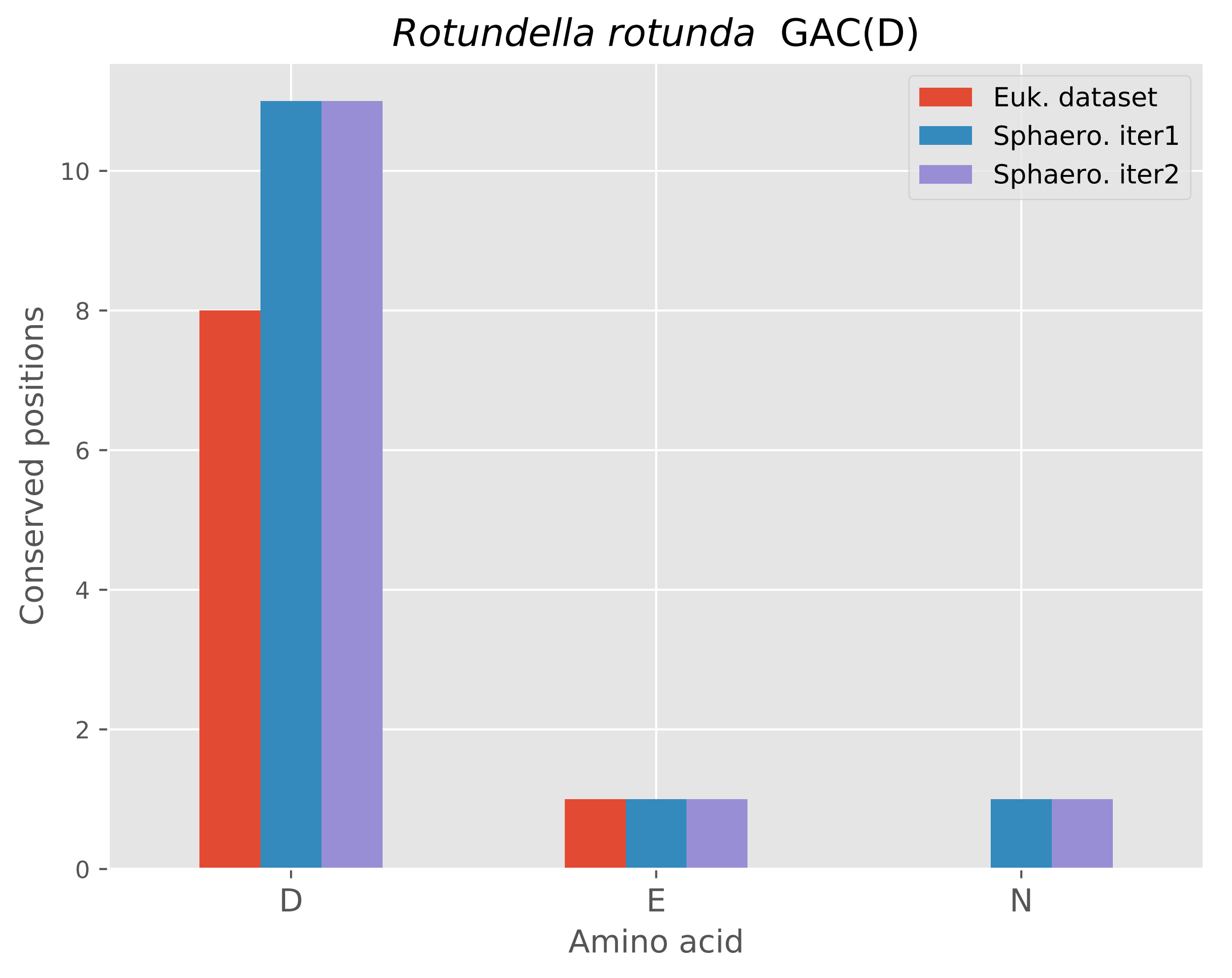
Rotundella rotunda GAC(D)
Euk. dataset
Sphaero. iter1
Sphaero. iter2
Conserved positions
0
2
4
6
8
10
D
E
N
Amino acid

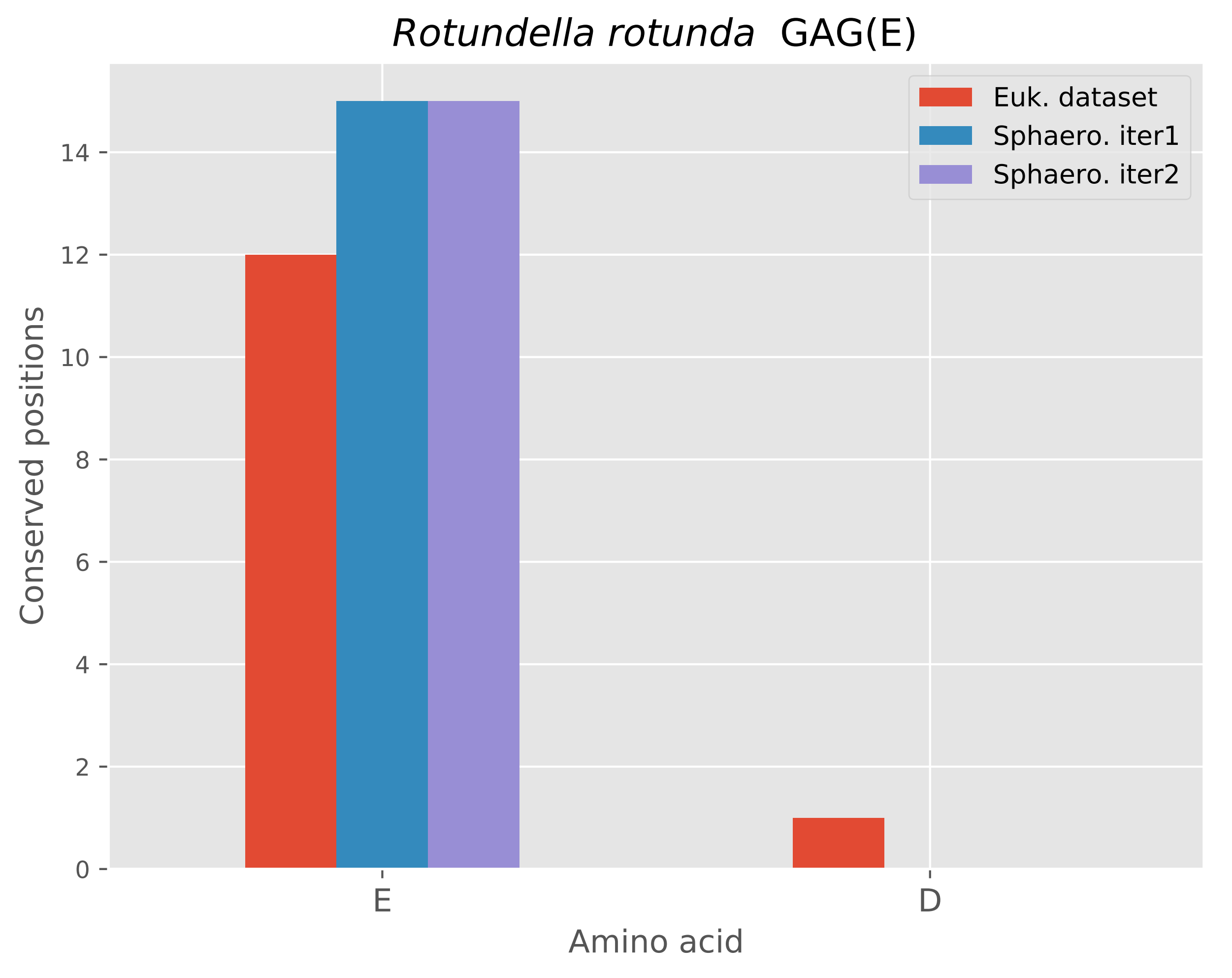

Rotundella rotunda GAG(E)
Euk. dataset
Sphaero. iter1
Sphaero. iter2
Conserved positions
0
2
4
6
8
10
12
14
E
D
Amino acid

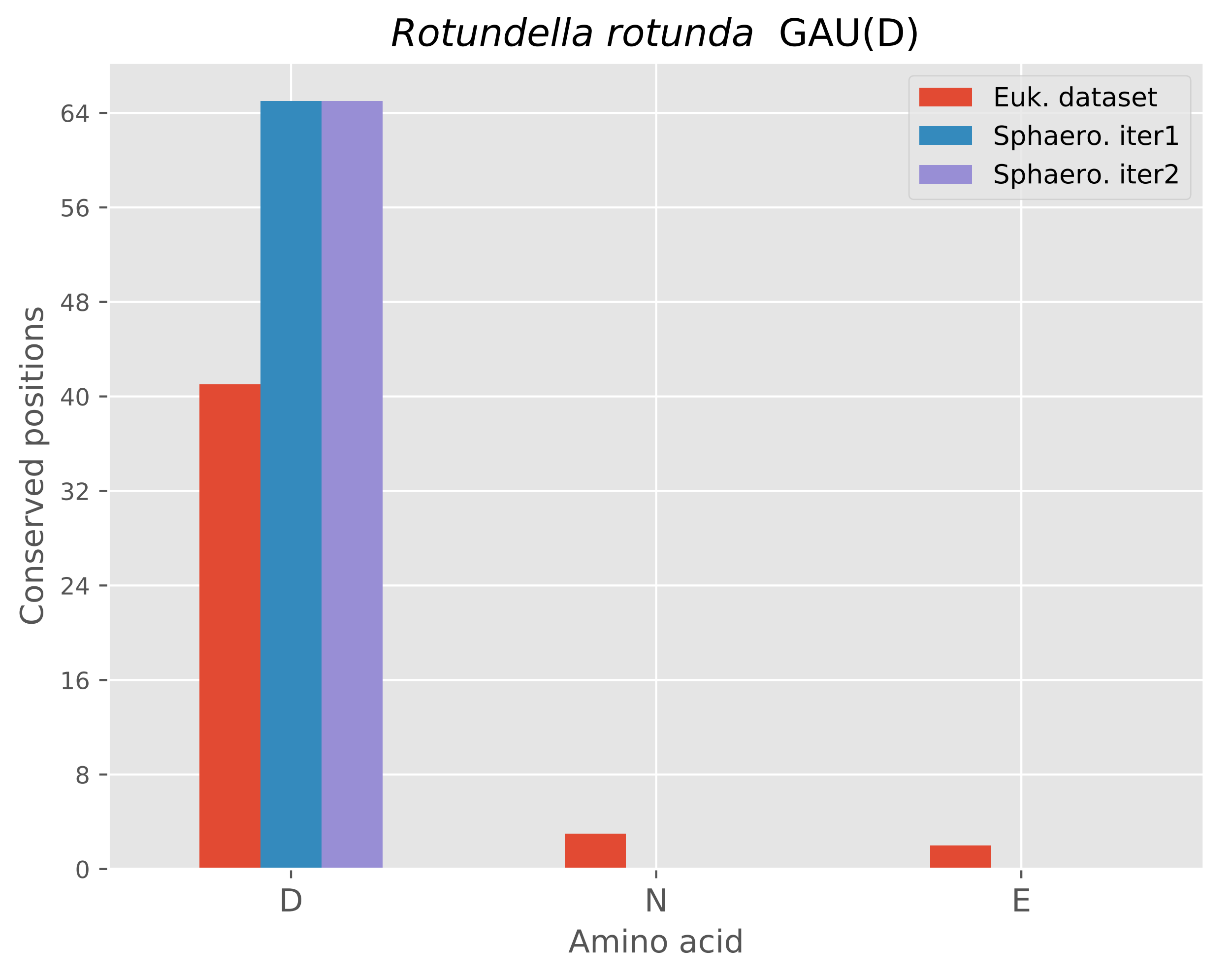

Rotundella rotunda GAU(D)
Euk. dataset
Sphaero. iter1
Sphaero. iter2
Conserved positions
0
8
16
24
32
40
48
56
64
D
N
E
Amino acid

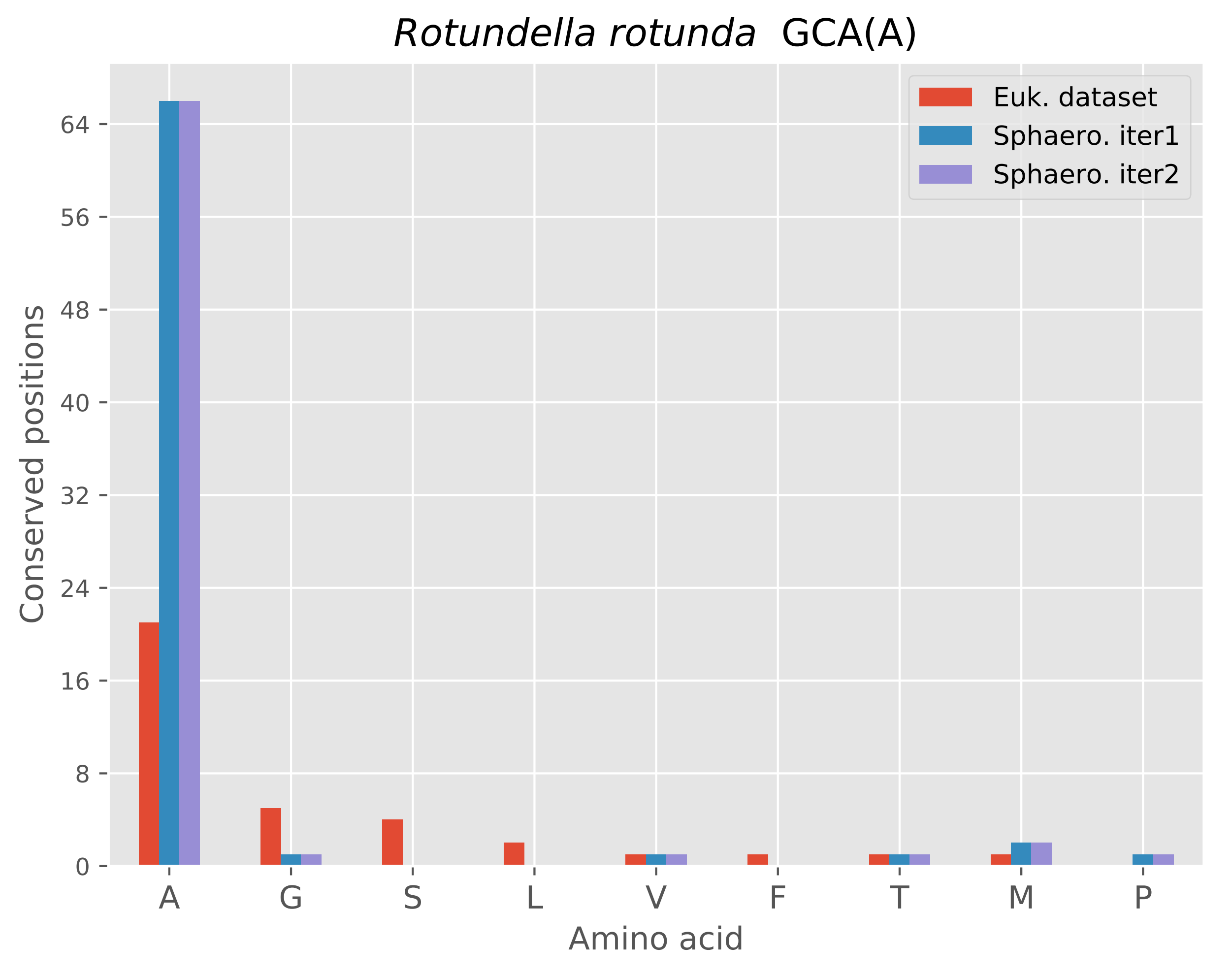

Rotundella rotunda GCA(A)
Euk. dataset
Sphaero. iter1
Sphaero. iter2
Conserved positions
0
8
16
24
32
40
48
56
64
A
G
S
L
V
F
T
M
P
Amino acid

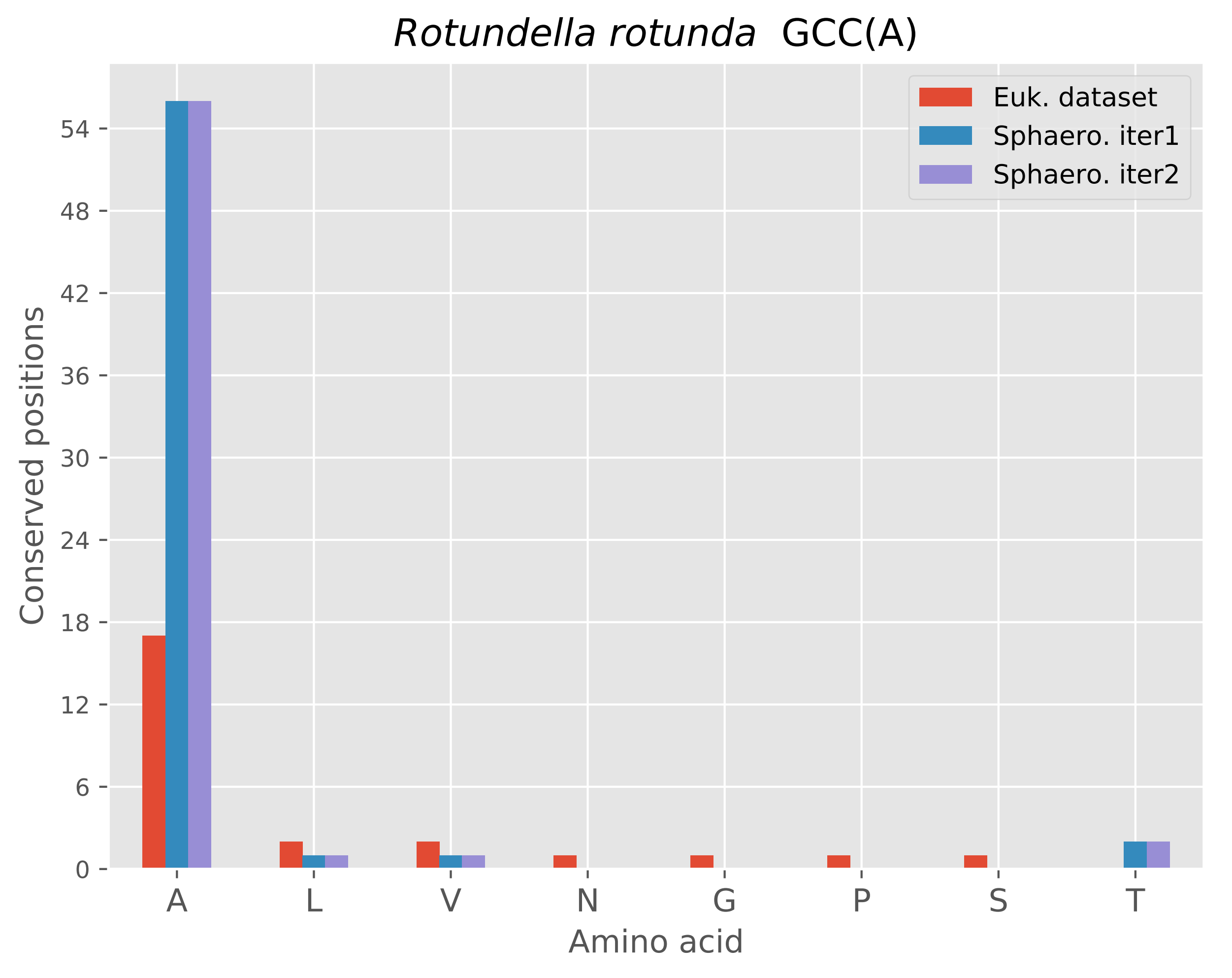

Rotundella rotunda GCC(A)
Euk. dataset
Sphaero. iter1
Sphaero. iter2
Conserved positions
0
6
12
18
24
30
36
42
48
54
A
L
V
N
G
P
S
T
Amino acid

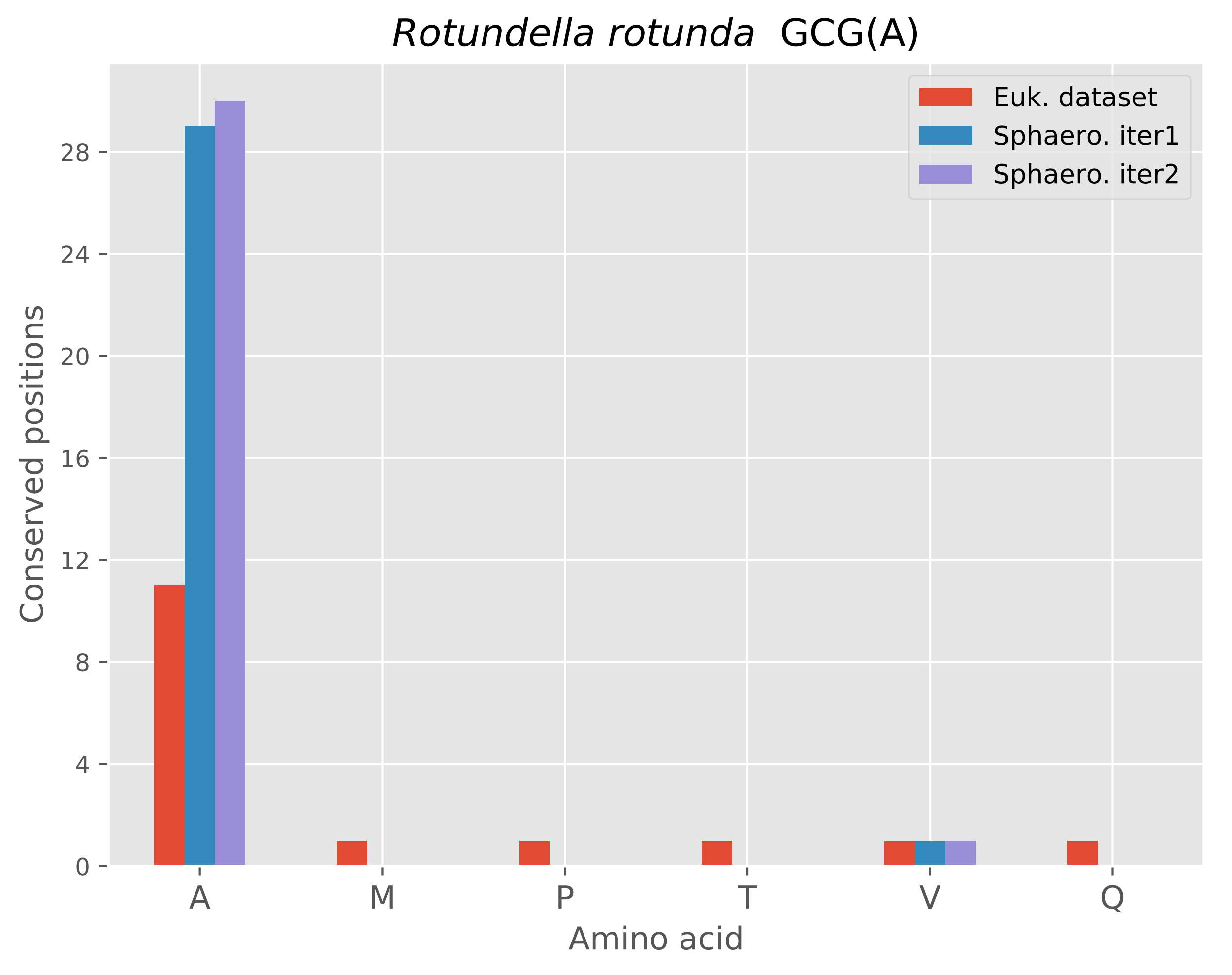

Rotundella rotunda GCG(A)
Euk. dataset
Sphaero. iter1
Sphaero. iter2
Conserved positions
0
4
8
12
16
20
24
28
A
M
P
T
V
Q
Amino acid

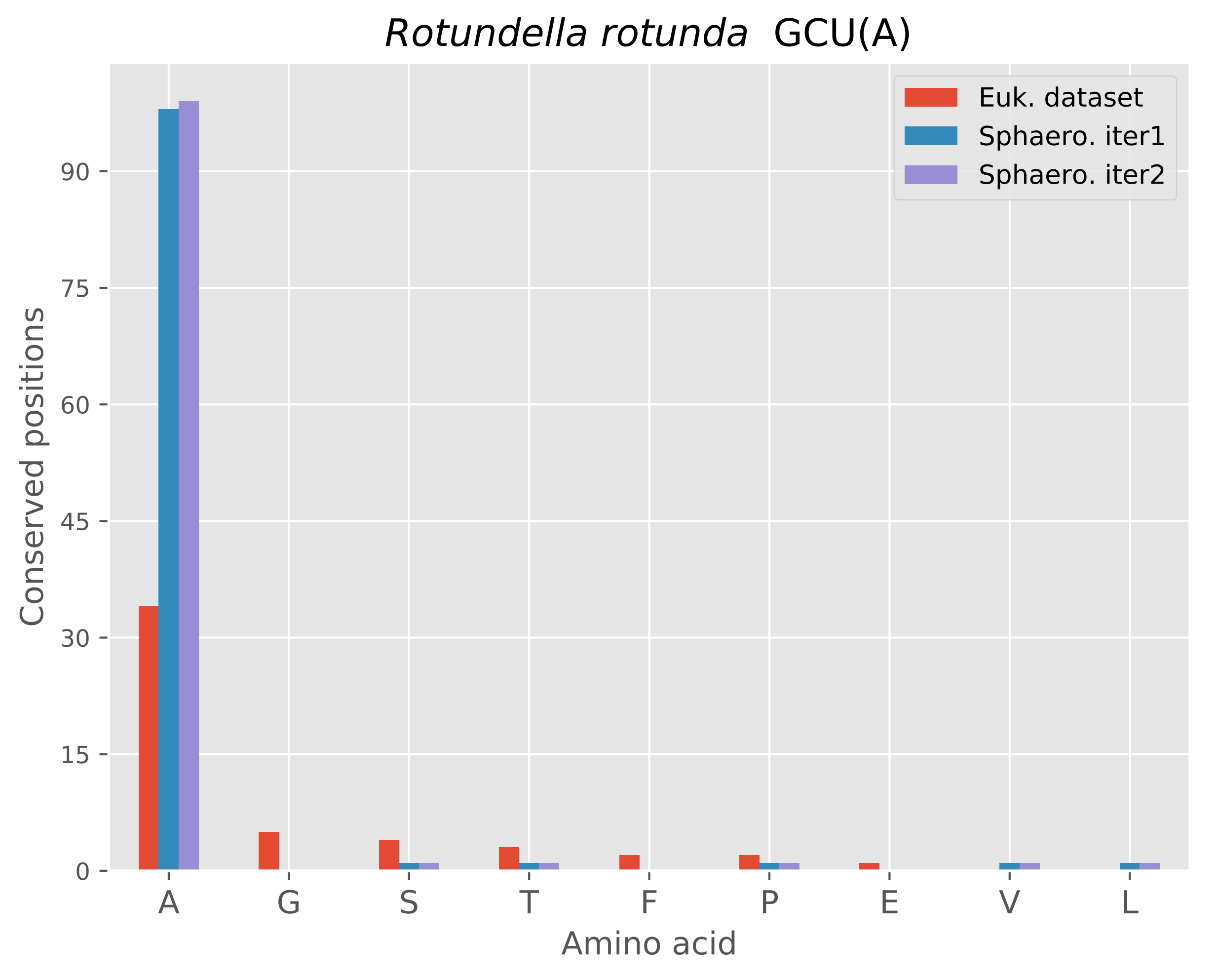

Rotundella rotunda GCU(A)
Euk. dataset
Sphaero. iter1
Sphaero. iter2
Conserved positions
0
15
30
45
60
75
90
A
G
S
T
F
P
E
V
L
Amino acid

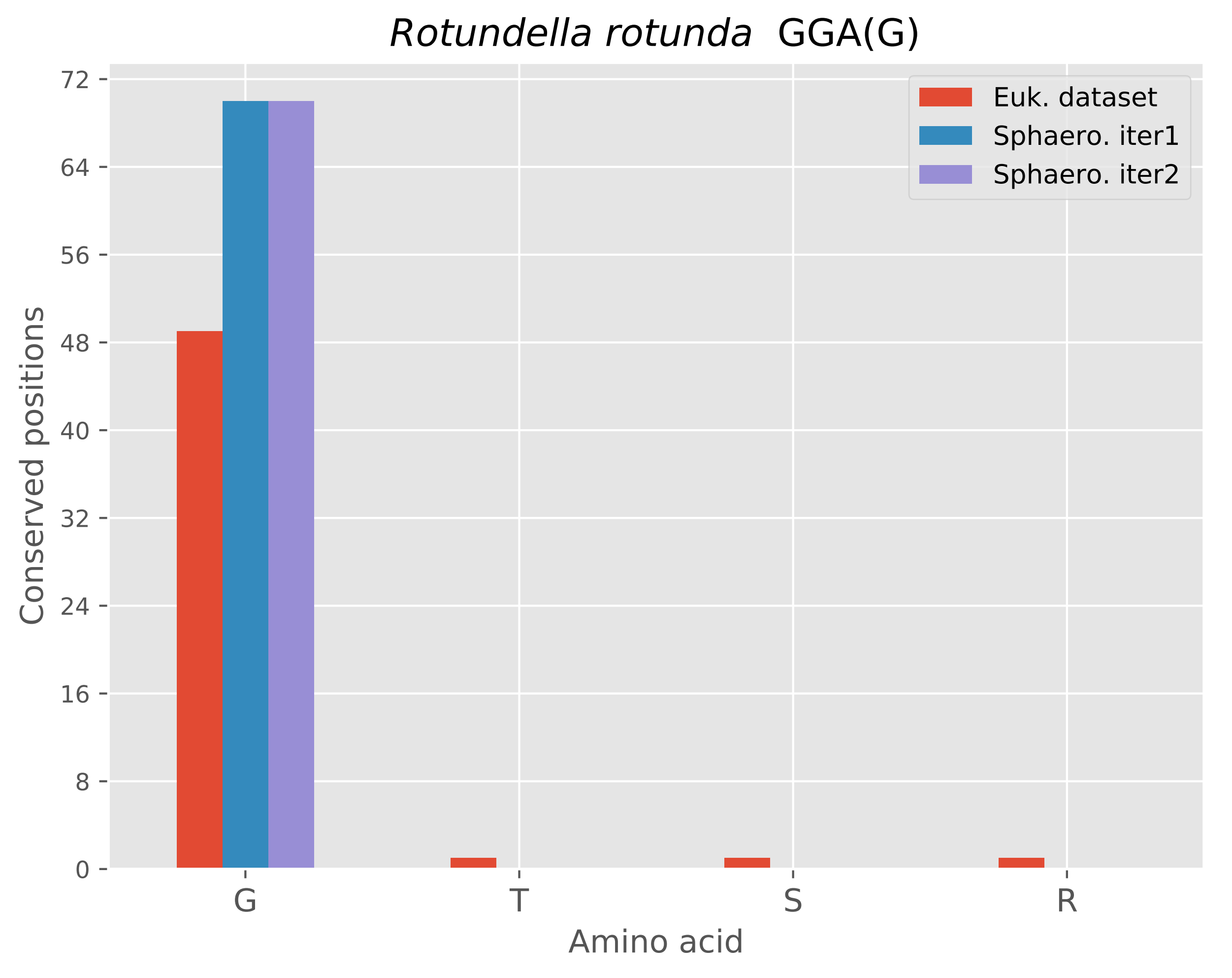

Rotundella rotunda GGA(G)
Euk. dataset
Sphaero. iter1
Sphaero. iter2
Conserved positions
0
8
16
24
32
40
48
56
64
72
G
T
S
R
Amino acid

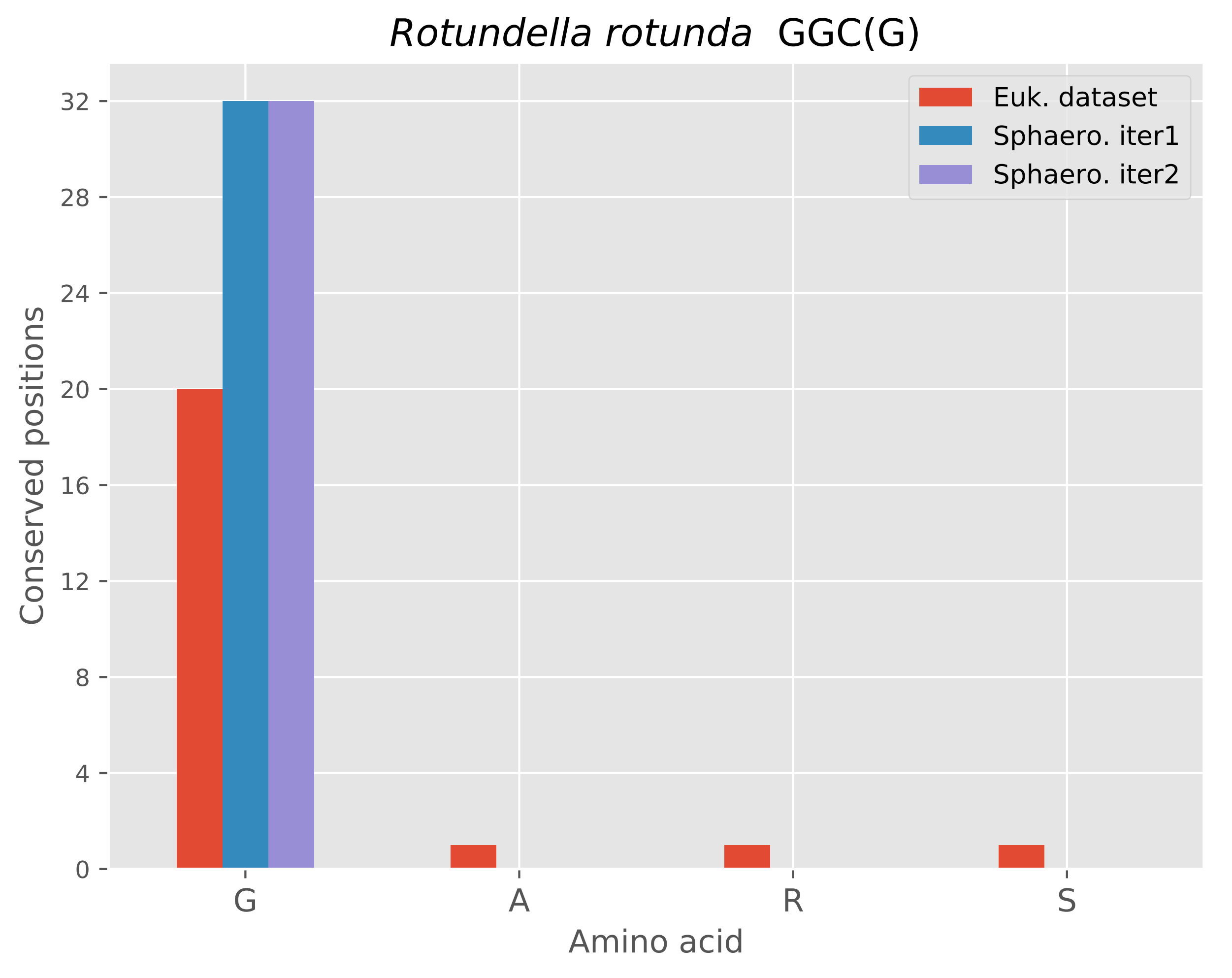
Rotundella rotunda GGC(G)
Euk. dataset
Sphaero. iter1
Sphaero. iter2
Conserved positions
0
4
8
12
16
20
24
28
32
G
A
R
S
Amino acid

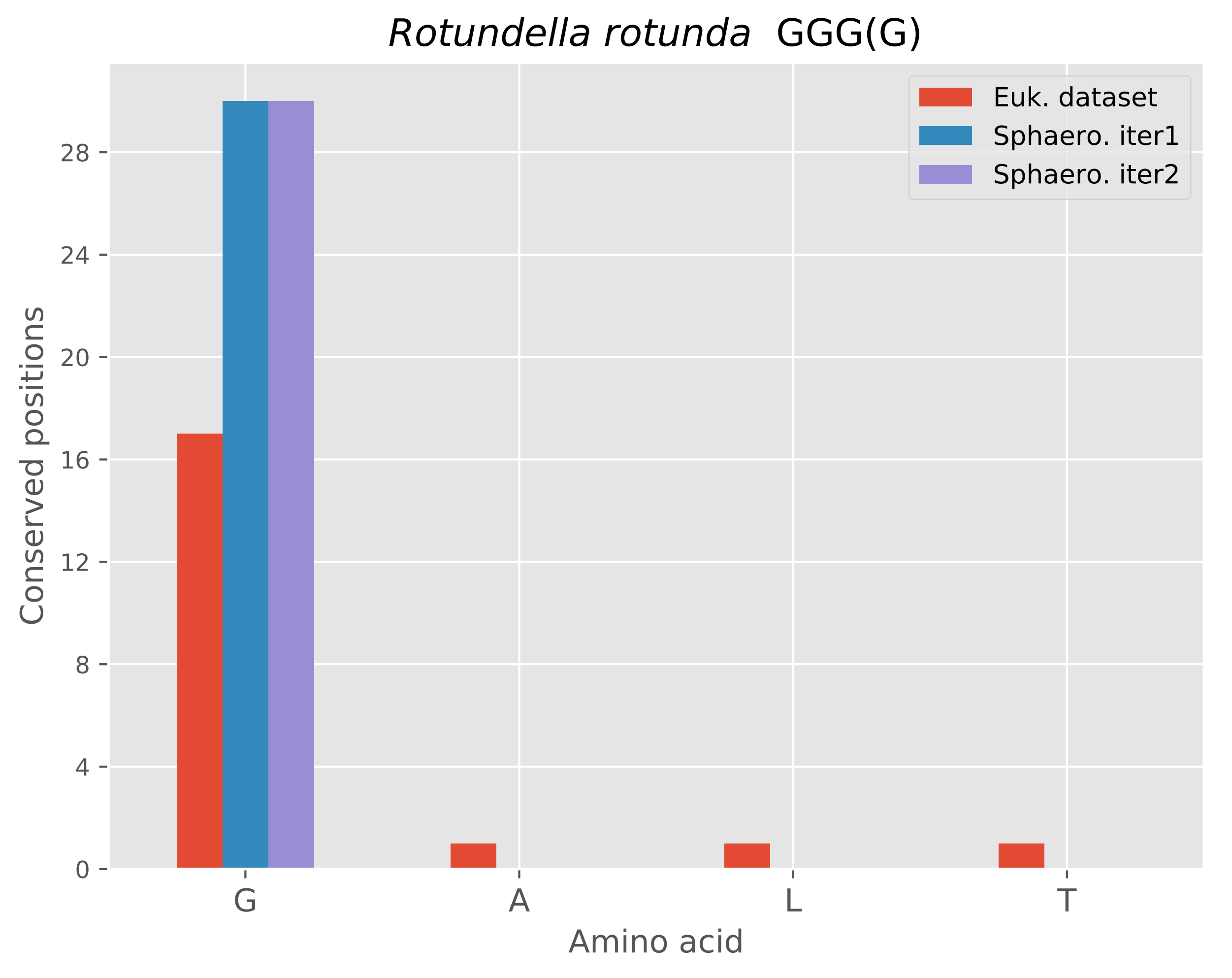

Rotundella rotunda GGG(G)
Euk. dataset
Sphaero. iter1
Sphaero. iter2
Conserved positions
0
4
8
12
16
20
24
28
G
A
L
T
Amino acid

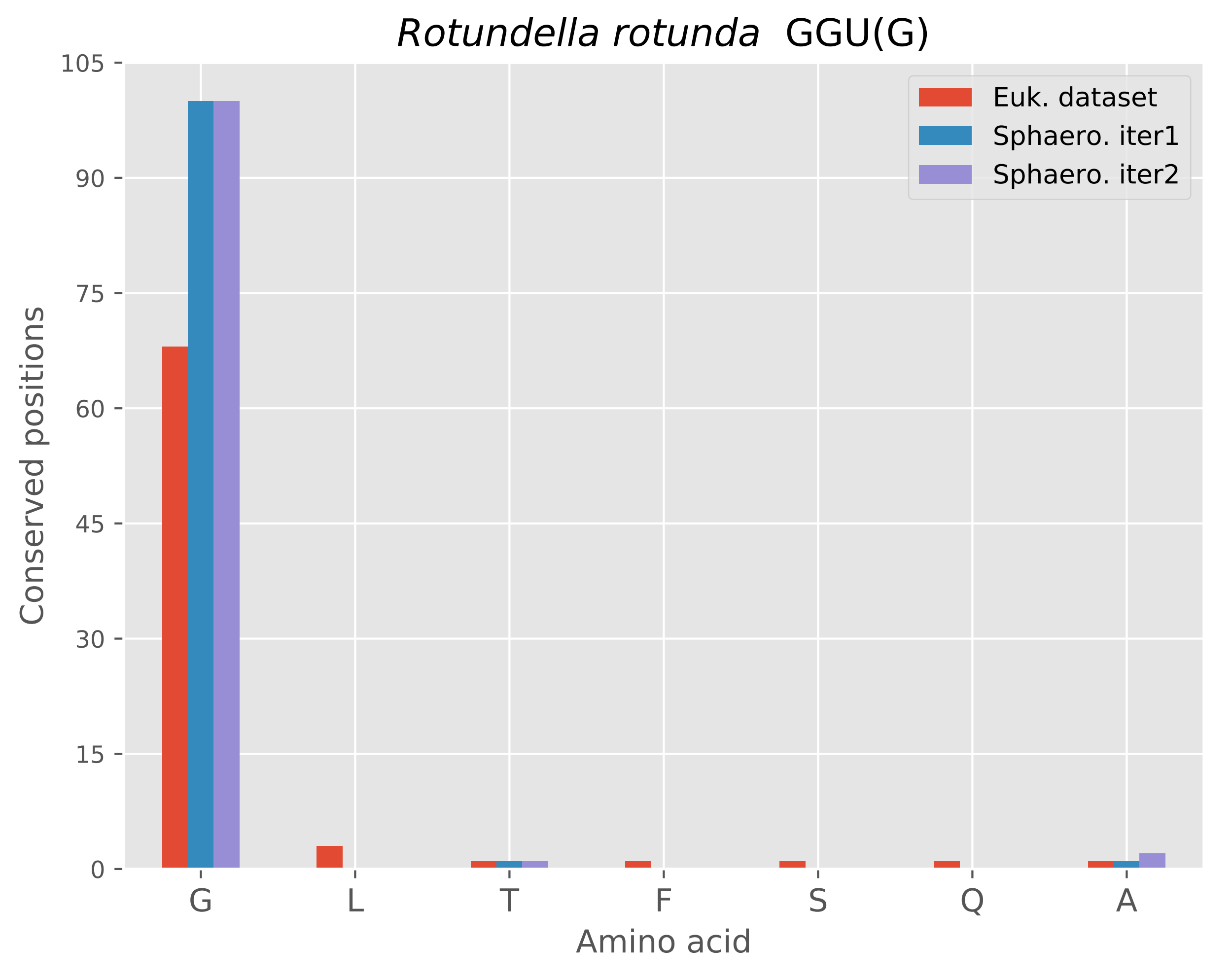

Rotundella rotunda GGU(G)
Euk. dataset
Sphaero. iter1
Sphaero. iter2
Conserved positions
105
90
75
60
45
30
15
0
G
L
T
F
S
Q
A
Amino acid

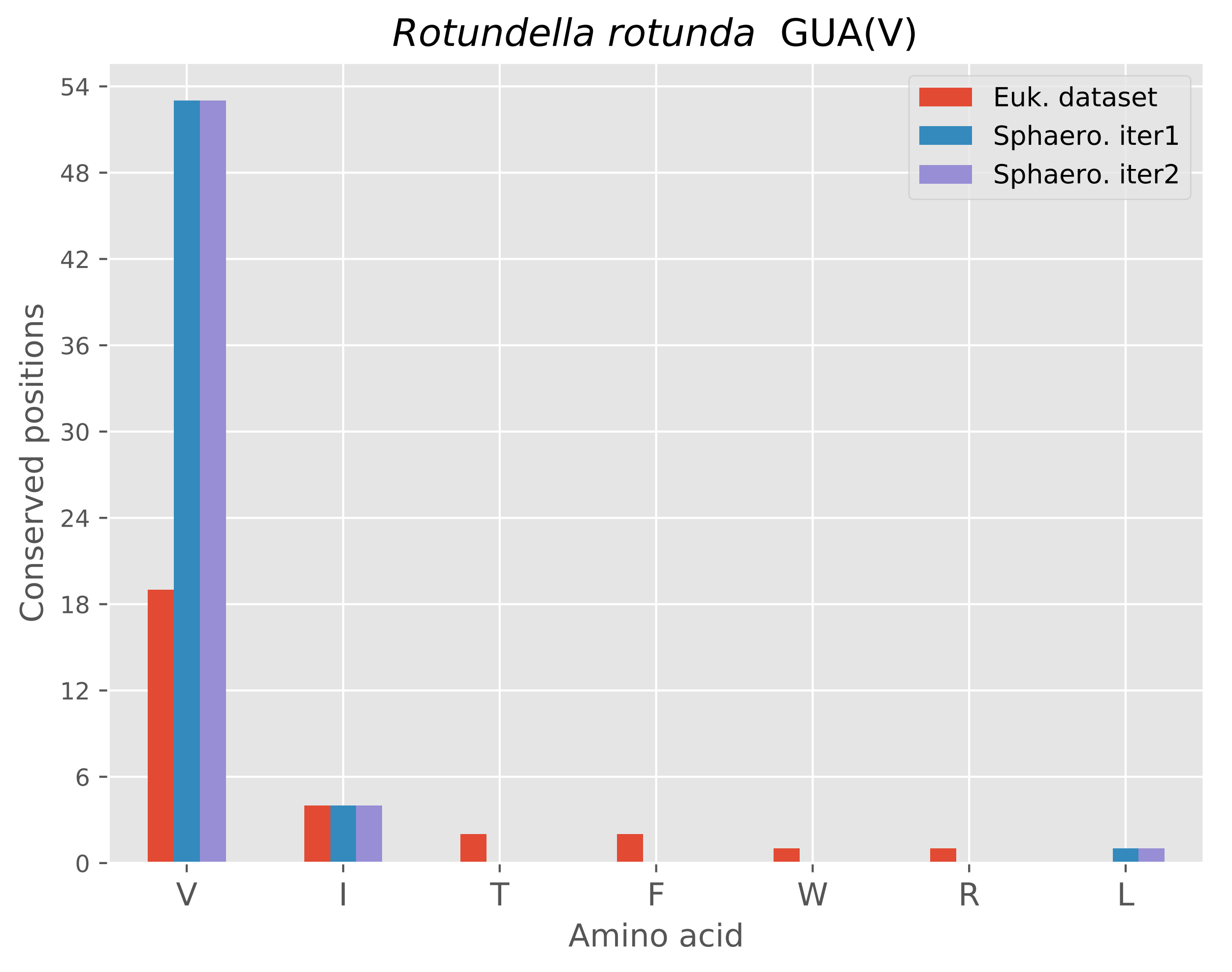
Rotundella rotunda GUA(V)
Euk. dataset
Sphaero. iter1
Sphaero. iter2
Conserved positions
0
6
12
18
24
30
36
42
48
54
V
I
T
F
W
R
L
Amino acid

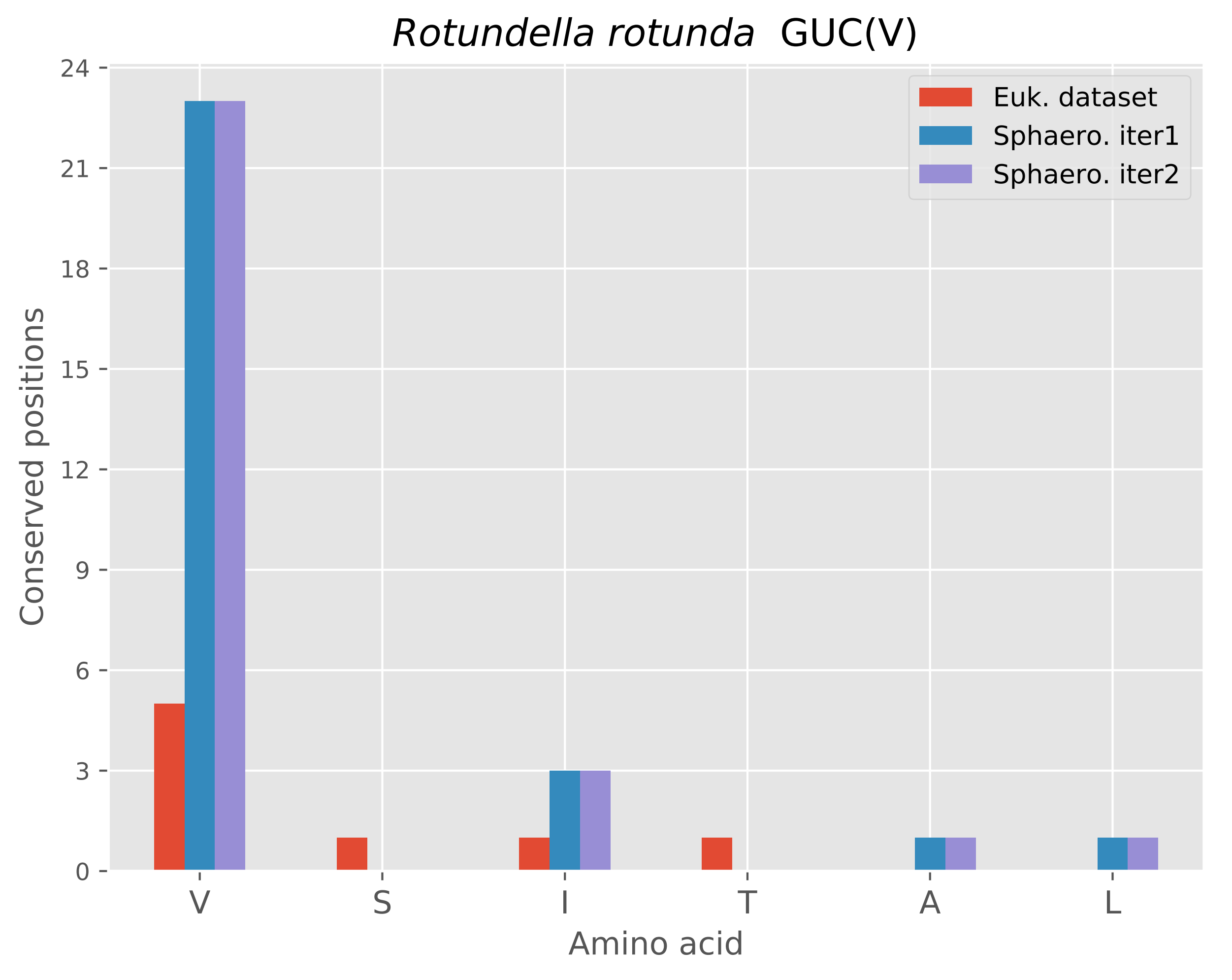

Rotundella rotunda GUC(V)
Conserved positions
Amino acid
V
S
I
T
A
L
0
3
6
9
12
15
18
21
24
Euk. dataset
Sphaero. iter1
Sphaero. iter2

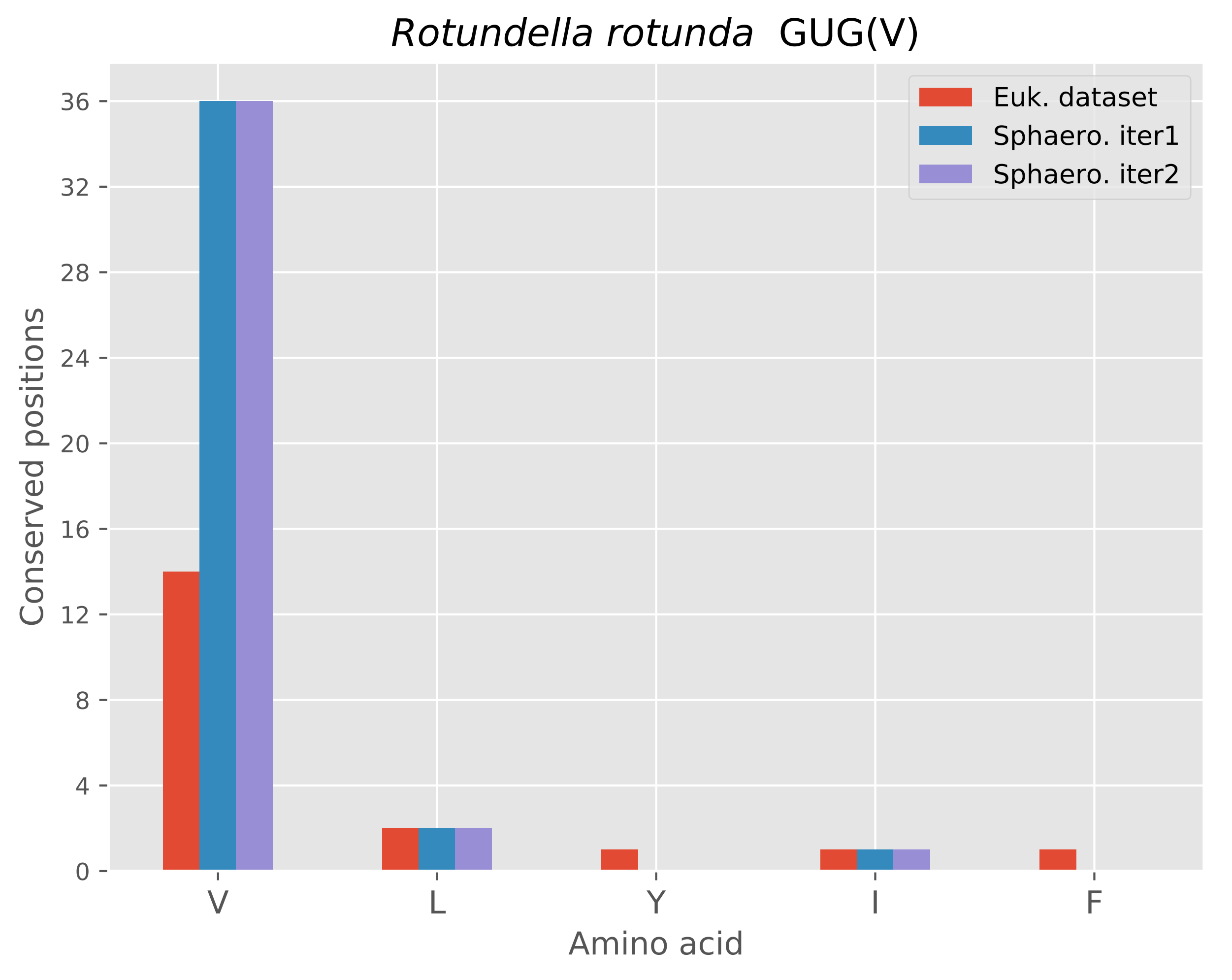

Rotundella rotunda GUG(V)
Euk. dataset
Sphaero. iter1
Sphaero. iter2
Conserved positions
0
4
8
12
16
20
24
28
32
36
V
L
Y
I
F
Amino acid

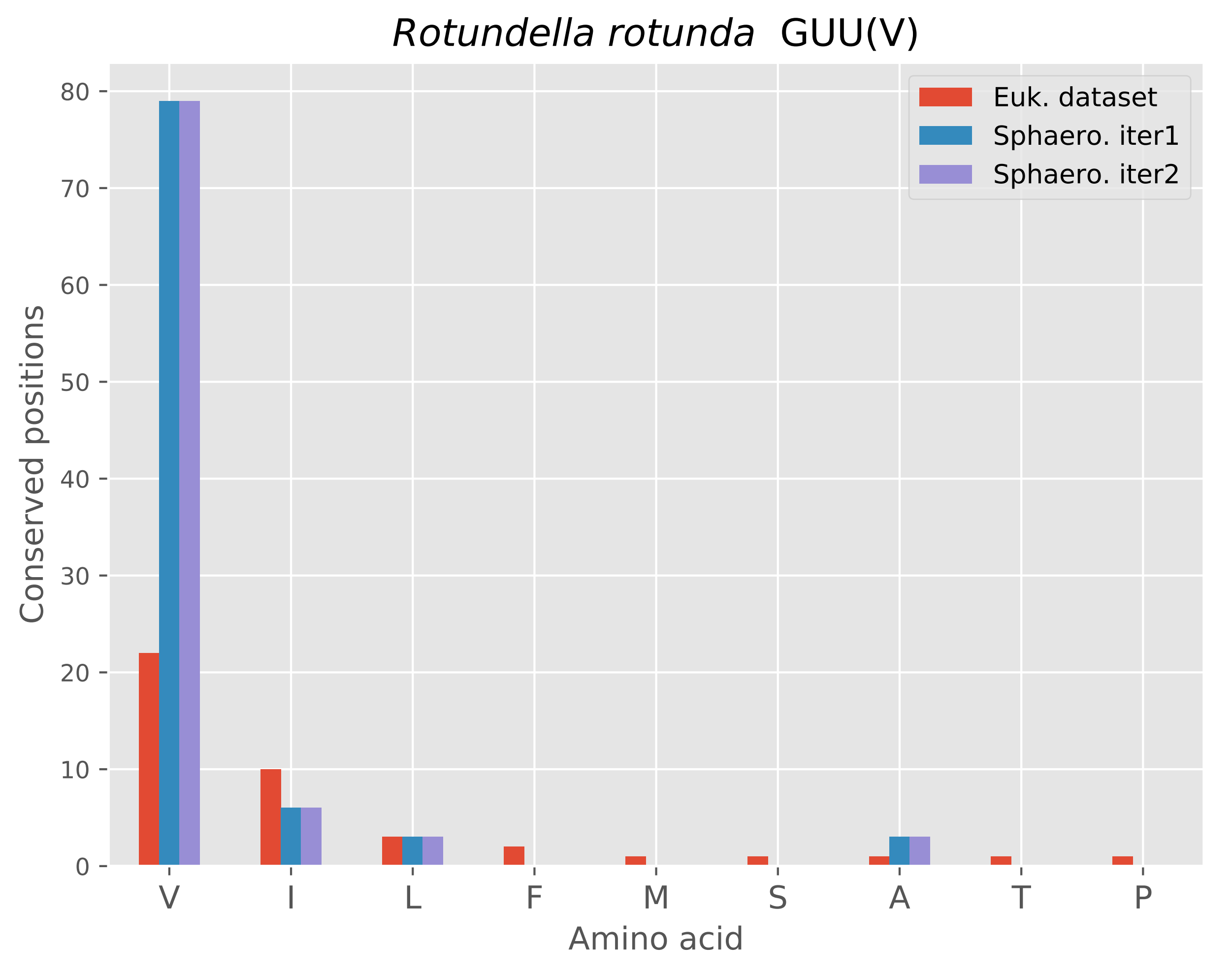
Rotundella rotunda GUU(V)
Euk. dataset
Sphaero. iter1
Sphaero. iter2
Conserved positions
0
10
20
30
40
50
60
70
80
V
I
L
F
M
S
A
T
P
Amino acid

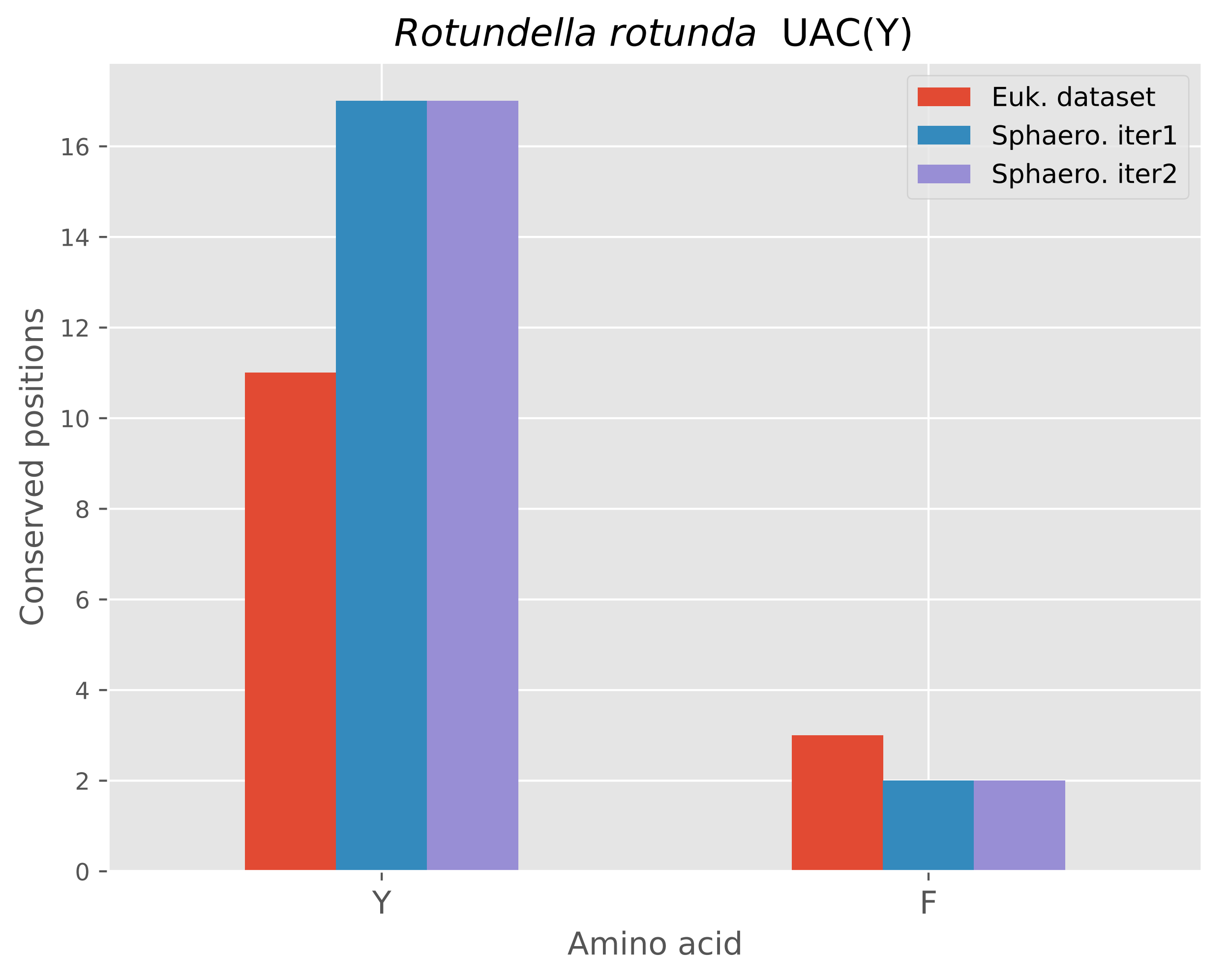

Rotundella rotunda UAC(Y)
Euk. dataset
Sphaero. iter1
Sphaero. iter2
Conserved positions
0
2
4
6
8
10
12
14
16
Y
F
Amino acid

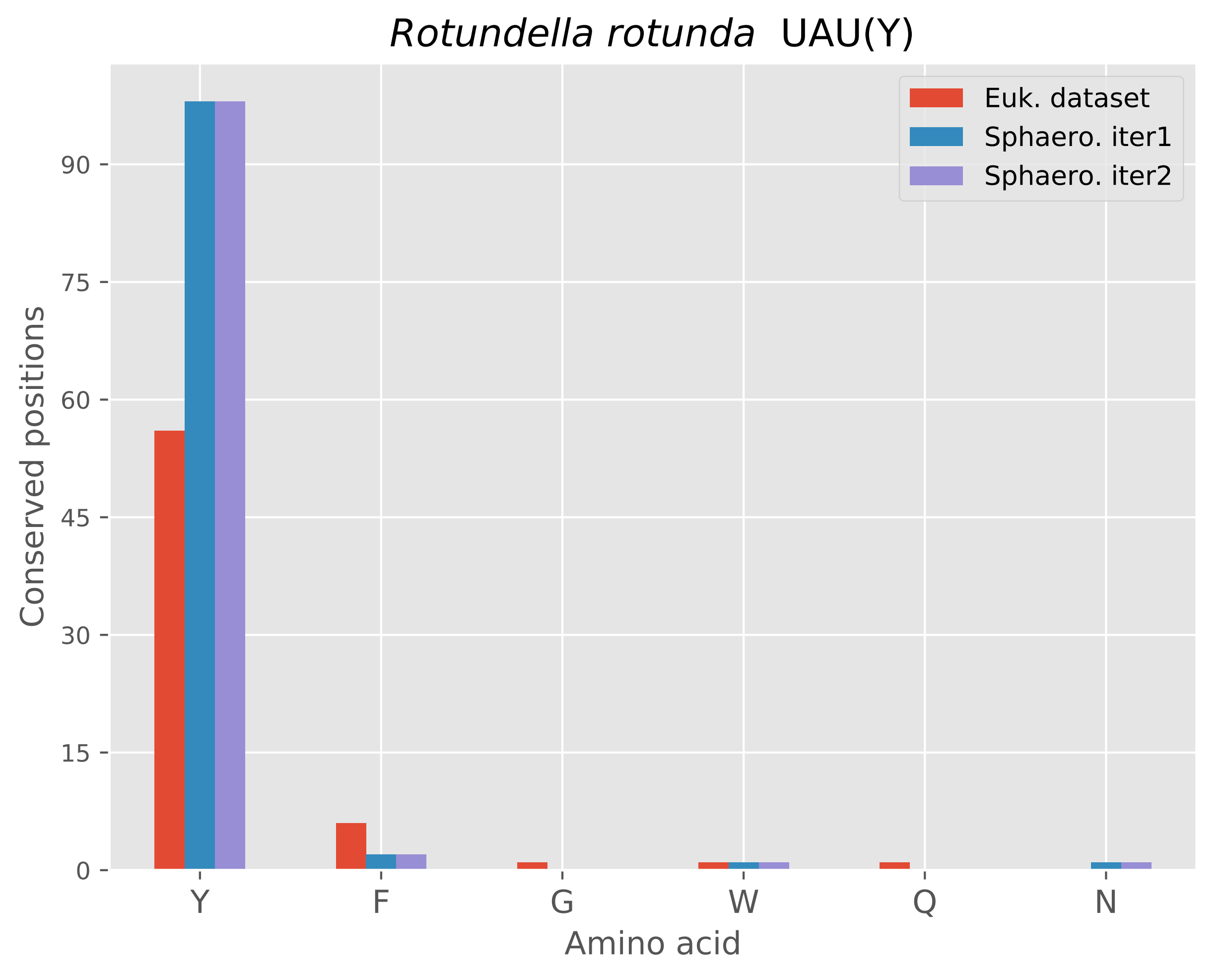

Rotundella rotunda UAU(Y)
Euk. dataset
Sphaero. iter1
Sphaero. iter2
Conserved positions
0
15
30
45
60
75
90
Y
F
G
W
Q
N
Amino acid

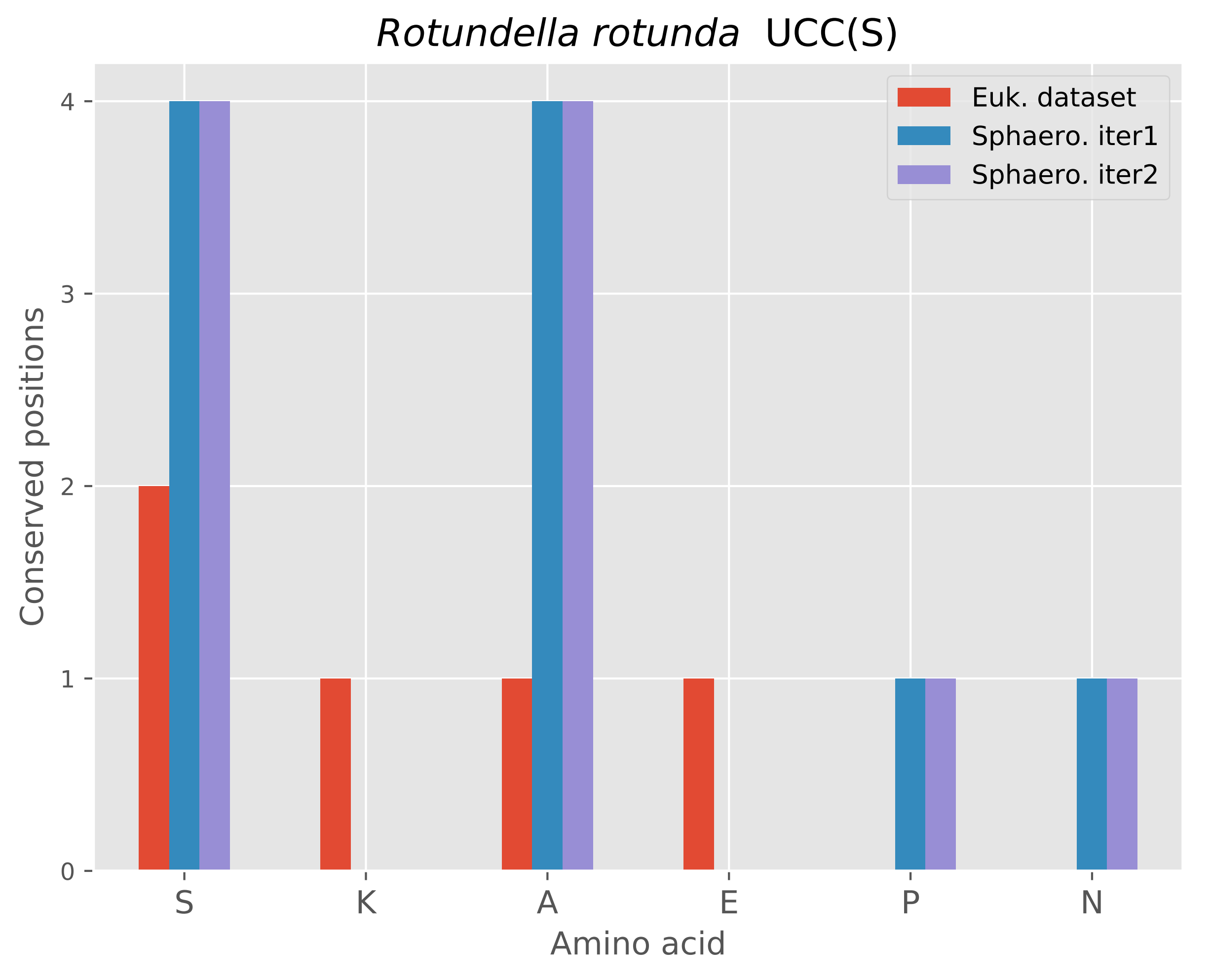

Rotundella rotunda UCC(S)
Euk. dataset
Sphaero. iter1
Sphaero. iter2
Conserved positions
0
1
2
3
4
S
K
A
E
P
N
Amino acid

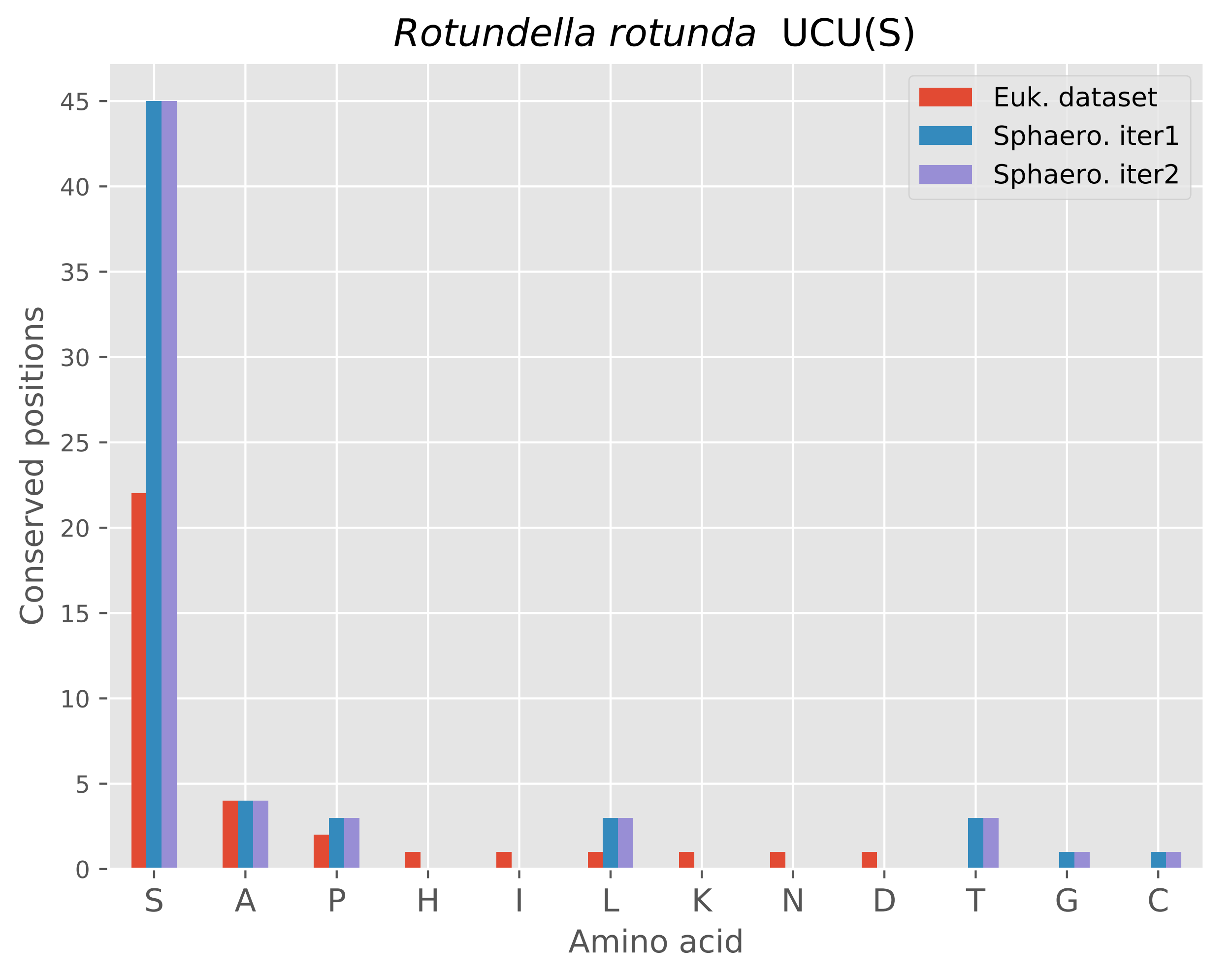

Rotundella rotunda UCU(S)
Euk. dataset
Sphaero. iter1
Sphaero. iter2
Conserved positions
0
5
10
15
20
25
30
35
40
45
S
A
P
H
I
L
K
N
D
T
G
C
Amino acid

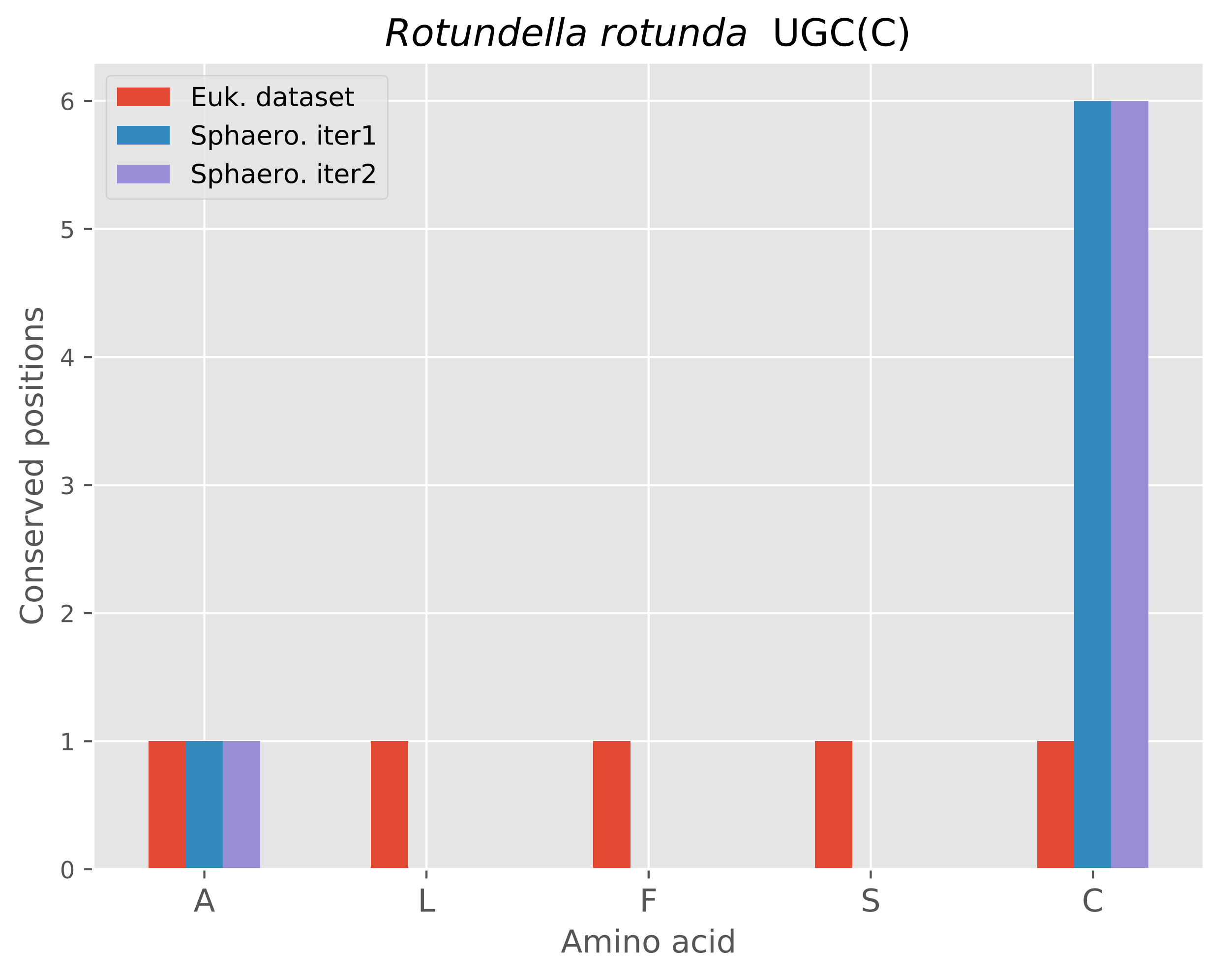

Rotundella rotunda UGC(C)
Euk. dataset
Sphaero. iter1
Sphaero. iter2
Conserved positions
0
1
2
3
4
5
6
A
L
F
S
C
Amino acid

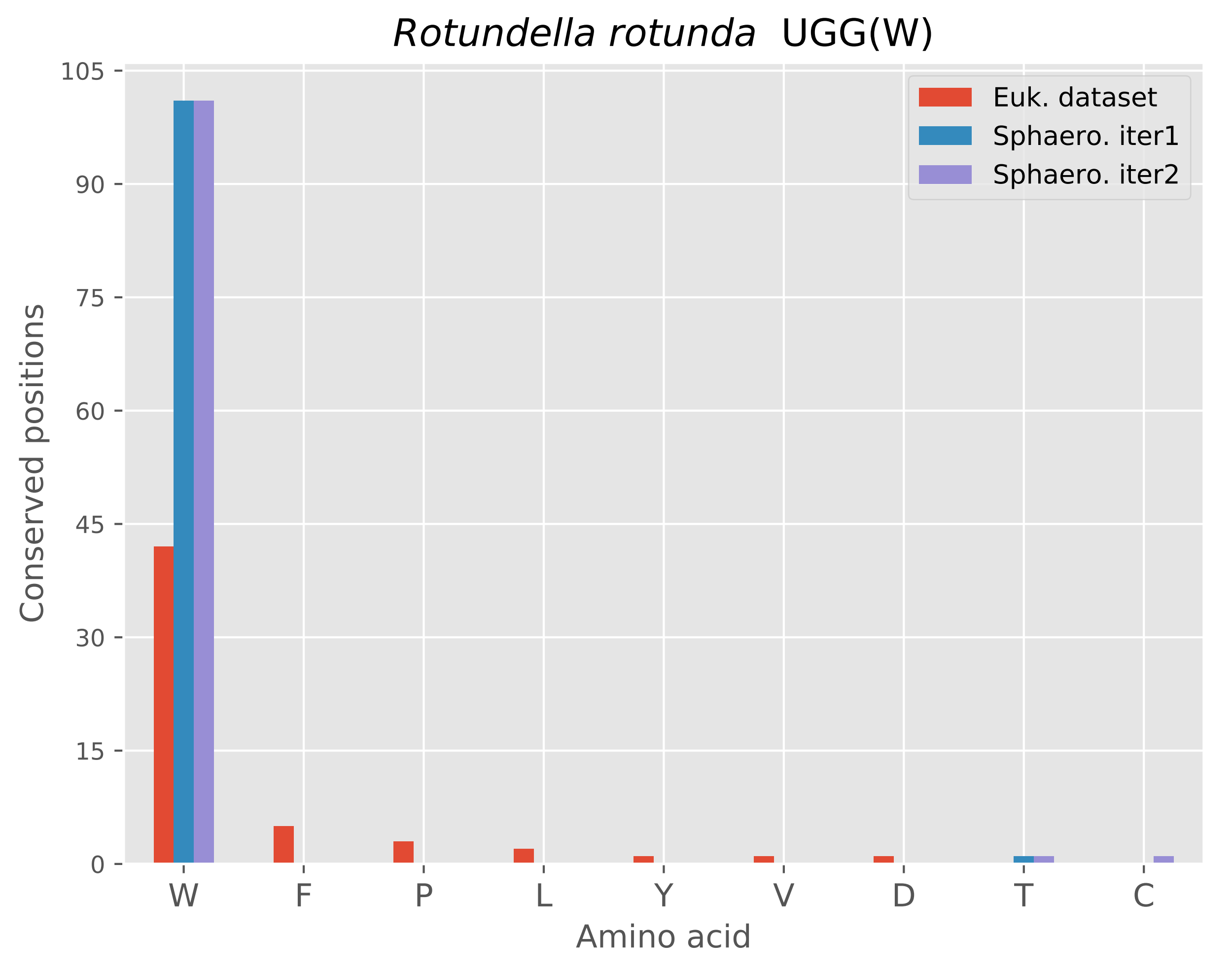

Rotundella rotunda UGG(W)
Conserved positions
Amino acid
Euk. dataset
Sphaero. iter1
Sphaero. iter2
0
15
30
45
60
75
90
105
W
F
P
L
Y
V
D
T
C

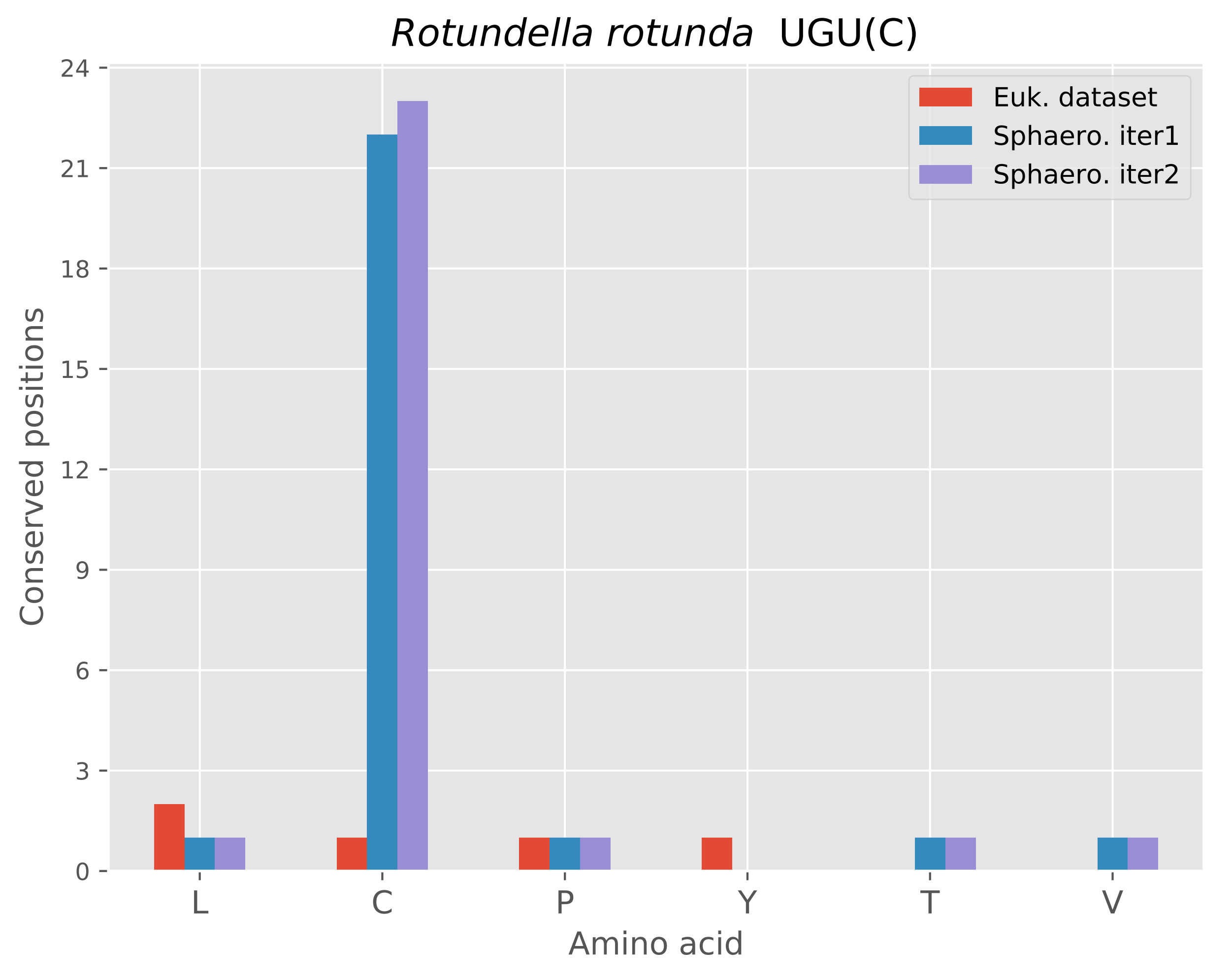

Rotundella rotunda UGU(C)
Euk. dataset
Sphaero. iter1
Sphaero. iter2
Conserved positions
0
3
6
9
12
15
18
21
24
L
C
P
Y
T
V
Amino acid

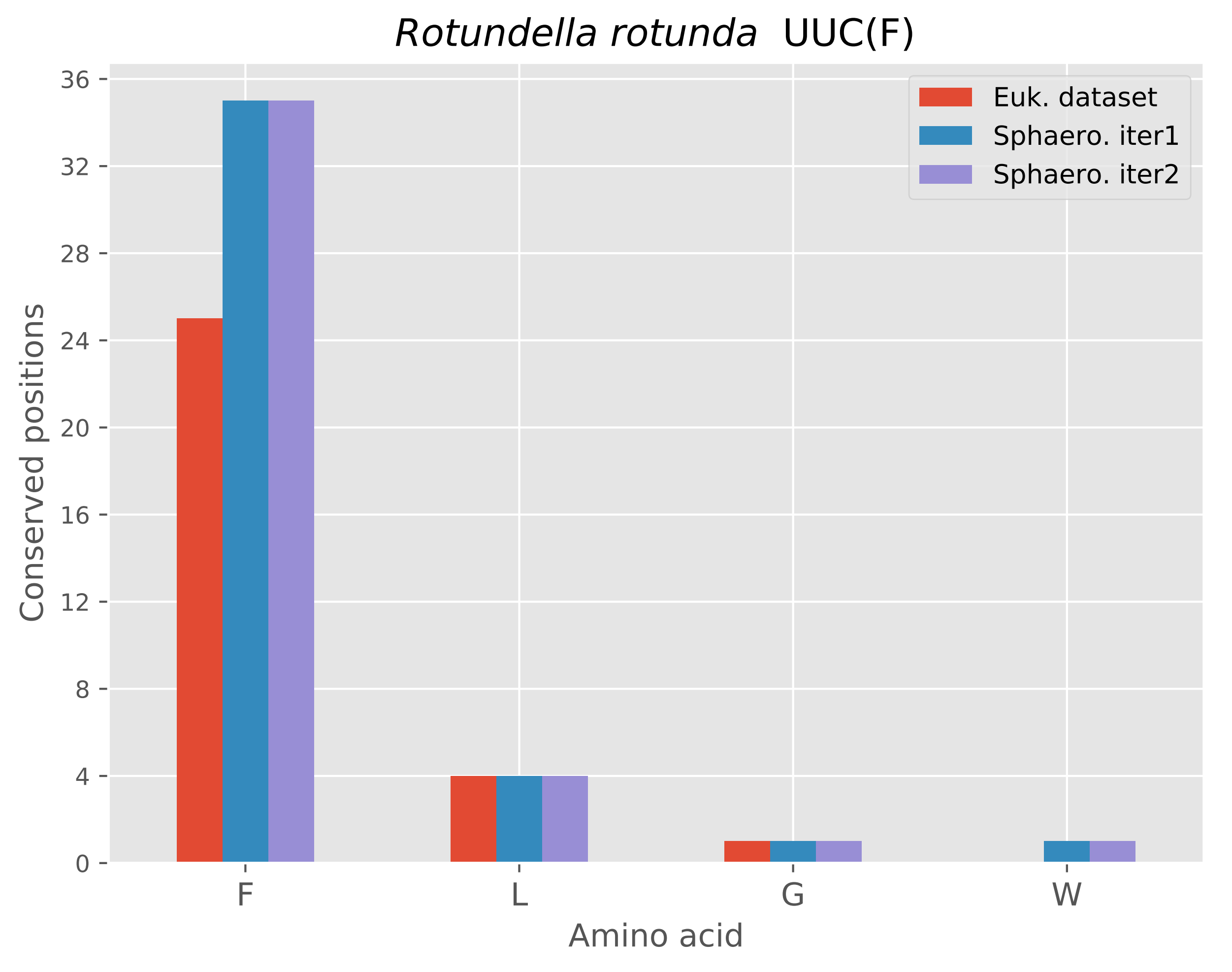

Rotundella rotunda UUC(F)
Euk. dataset
Sphaero. iter1
Sphaero. iter2
Conserved positions
0
4
8
12
16
20
24
28
32
36
F
L
G
W
Amino acid

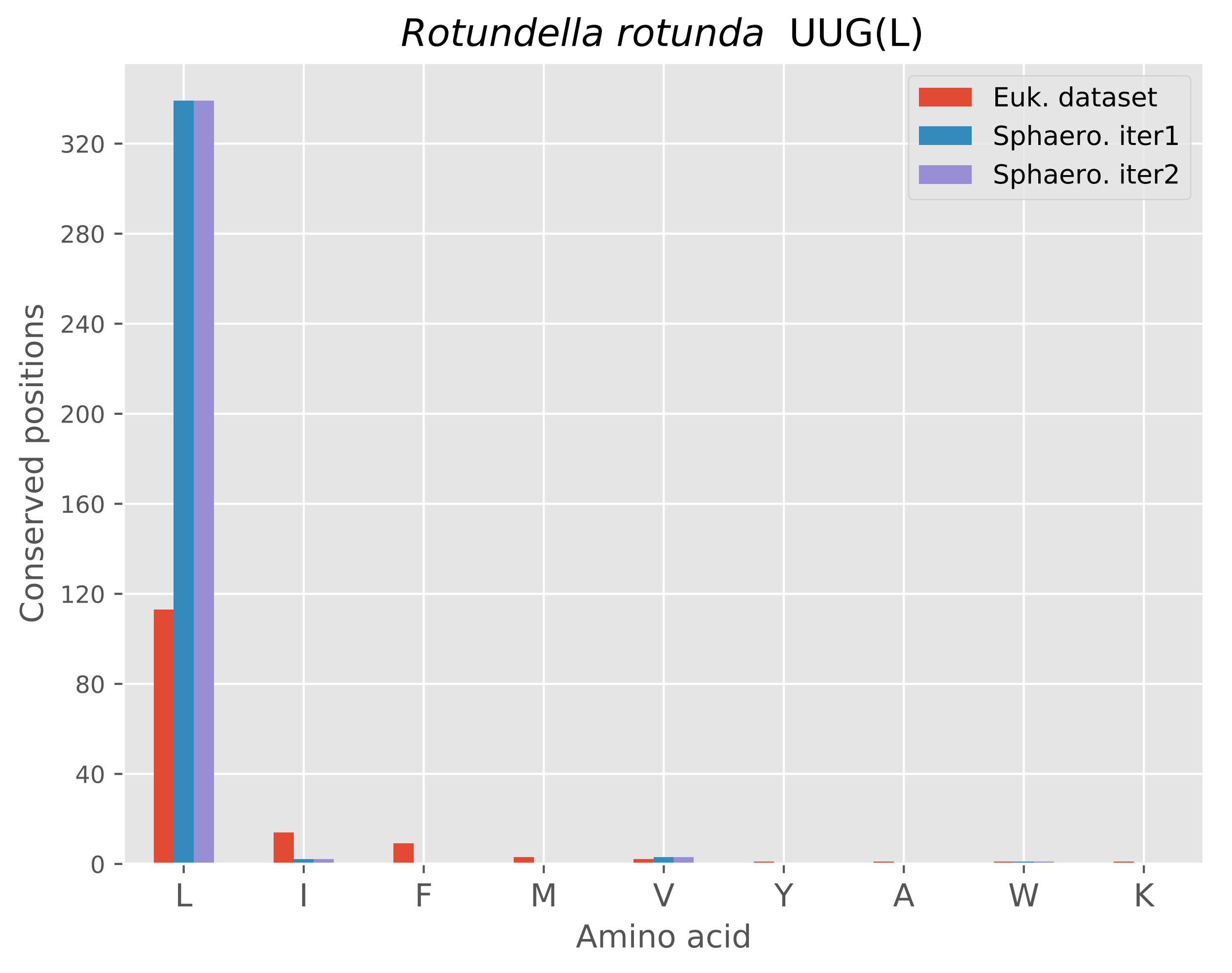

Rotundella rotunda UUG(L)
Euk. dataset
Sphaero. iter1
Sphaero. iter2
Conserved positions
0
40
80
120
160
200
240
280
320
L
I
F
M
V
Y
A
W
K
Amino acid

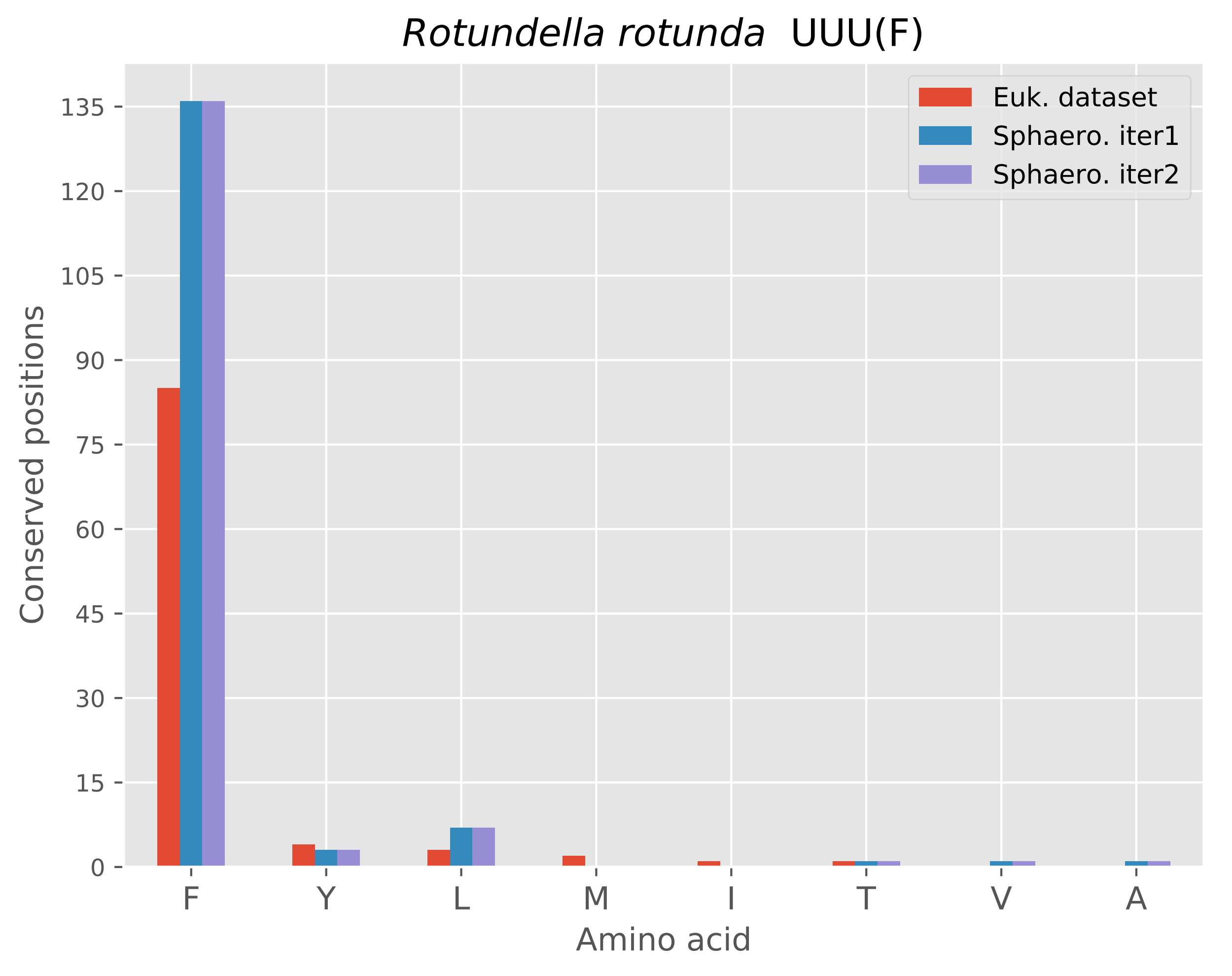

Rotundella rotunda UUU(F)
Euk. dataset
Sphaero. iter1
Sphaero. iter2
Conserved positions
0
15
30
45
60
75
90
105
120
135
F
Y
L
M
I
T
V
A
Amino acid

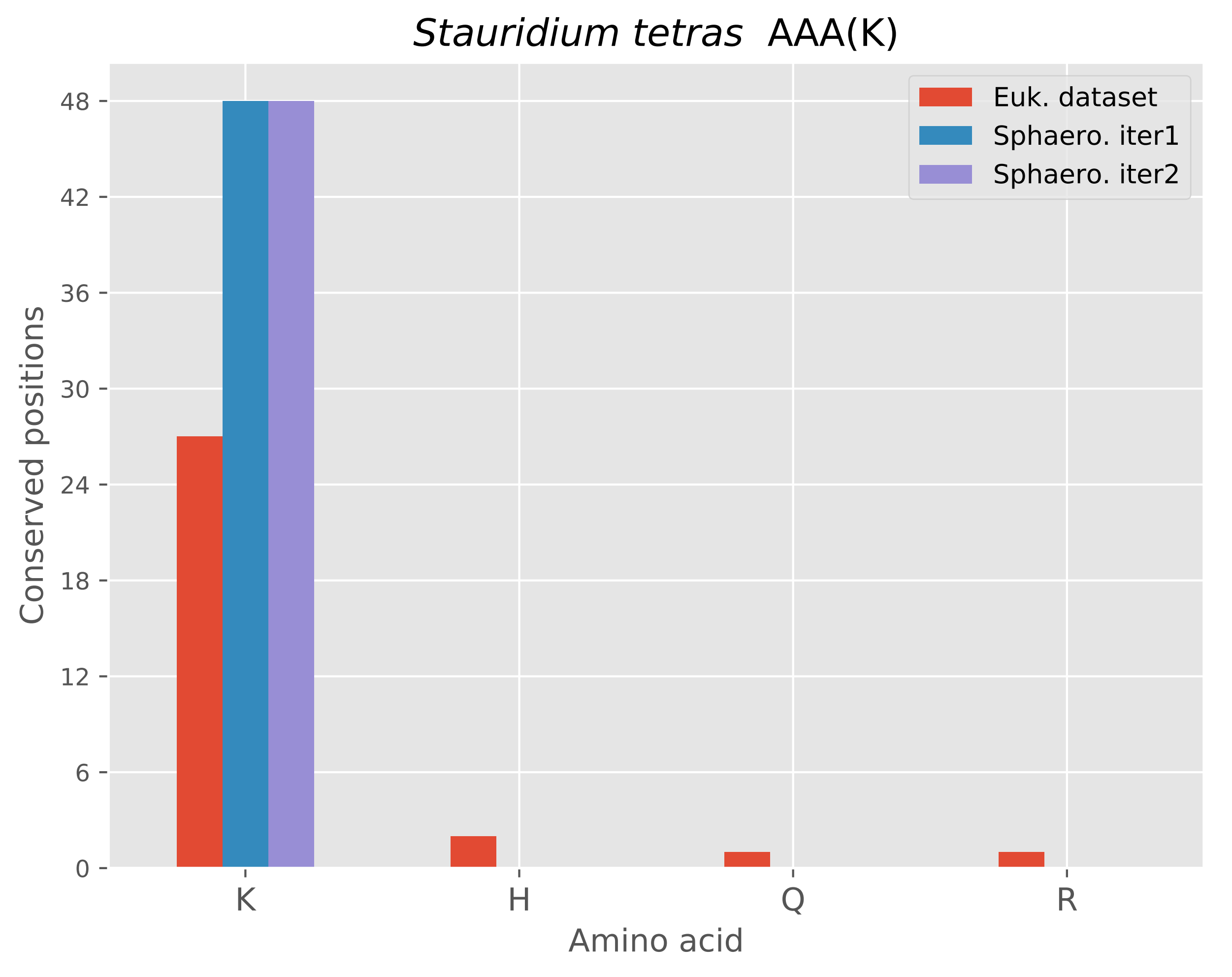

Stauridium tetras AAA(K)
Euk. dataset
Sphaero. iter1
Sphaero. iter2
Conserved positions
0
6
12
18
24
30
36
42
48
K
H
Q
R
Amino acid

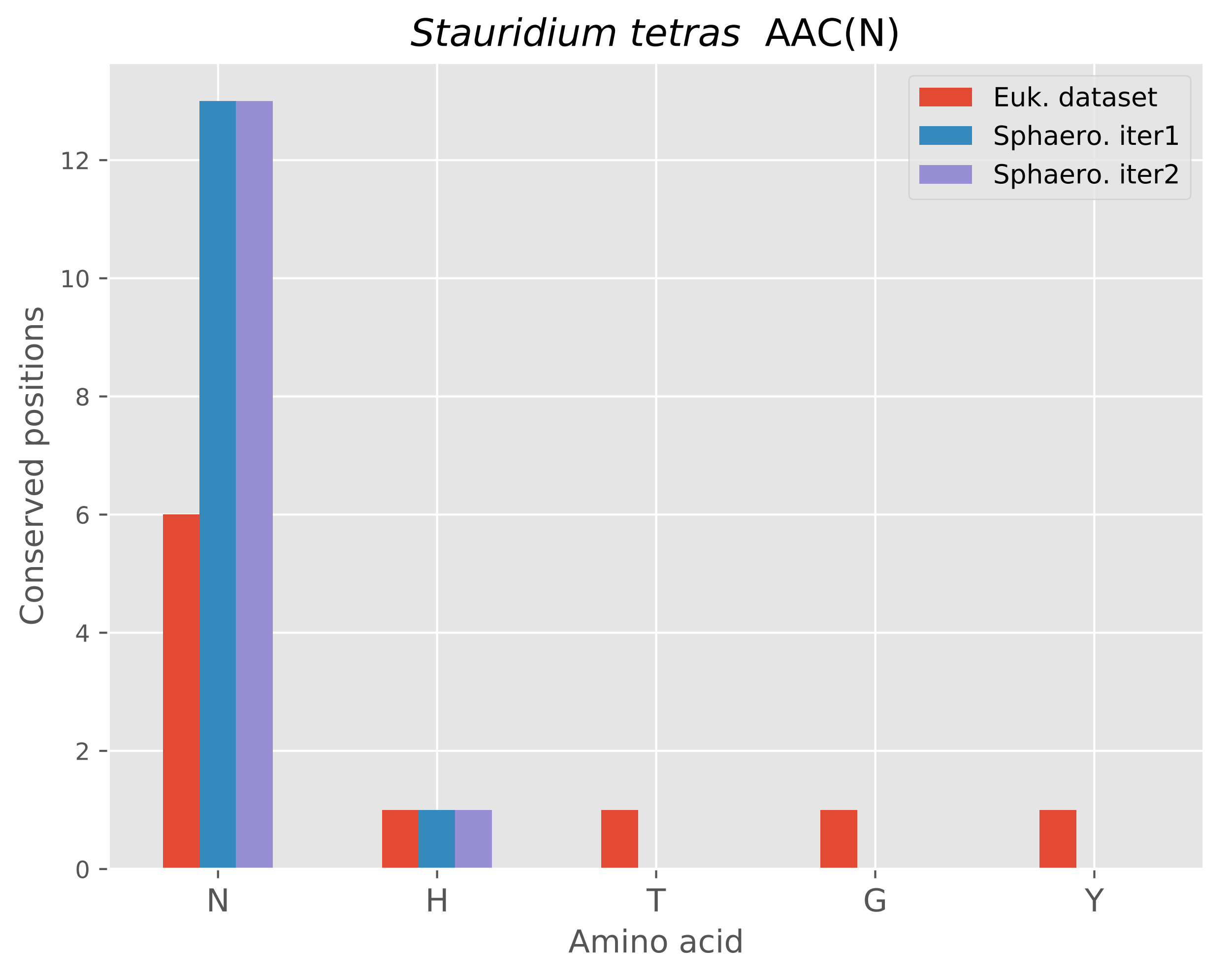

Stauridium tetras AAC(N)
Euk. dataset
Sphaero. iter1
Sphaero. iter2
Conserved positions
0
2
4
6
8
10
12
N
H
T
G
Y
Amino acid

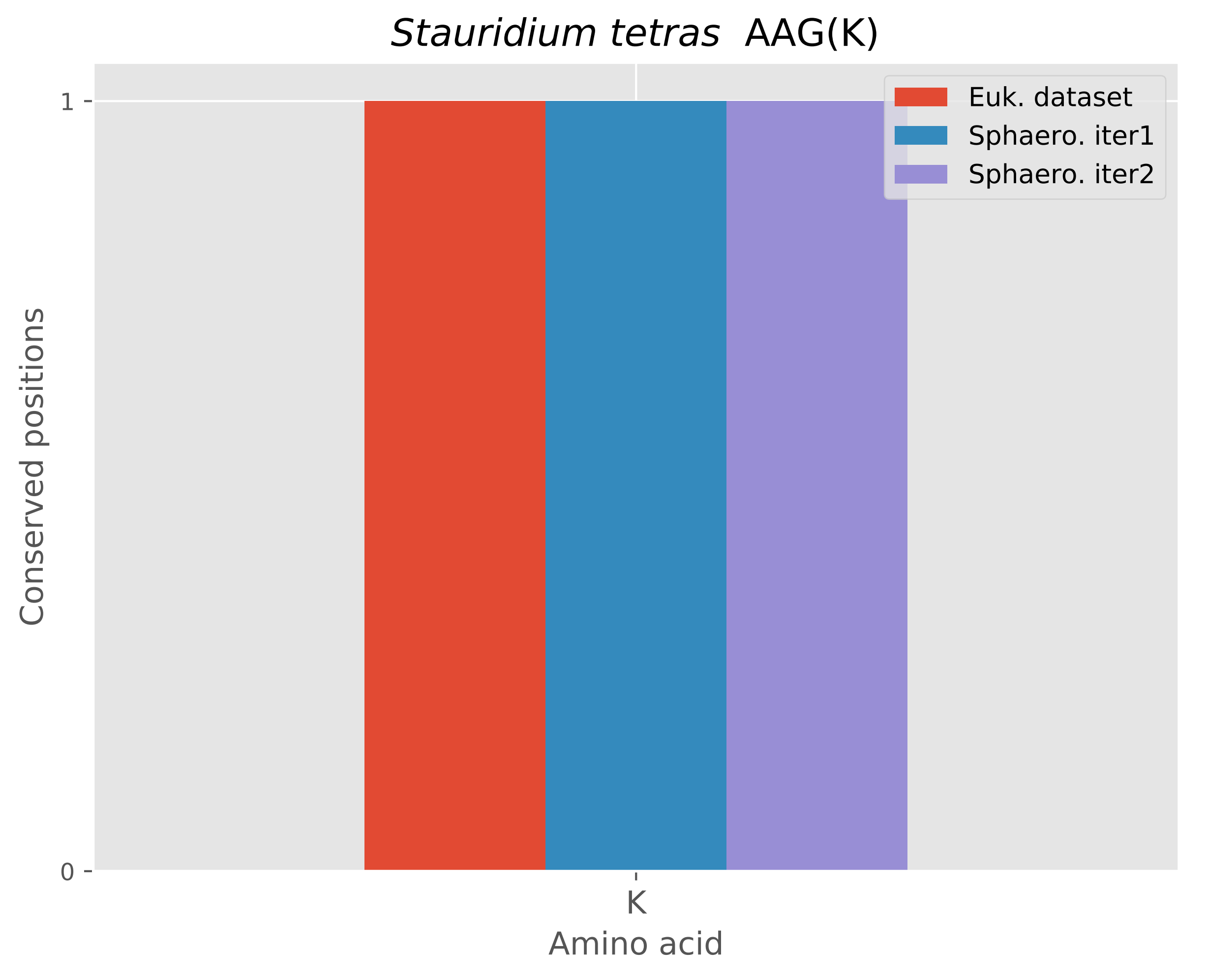

Stauridium tetras AAG(K)
Euk. dataset
Sphaero. iter1
Sphaero. iter2
1
0
Conserved positions
K
Amino acid

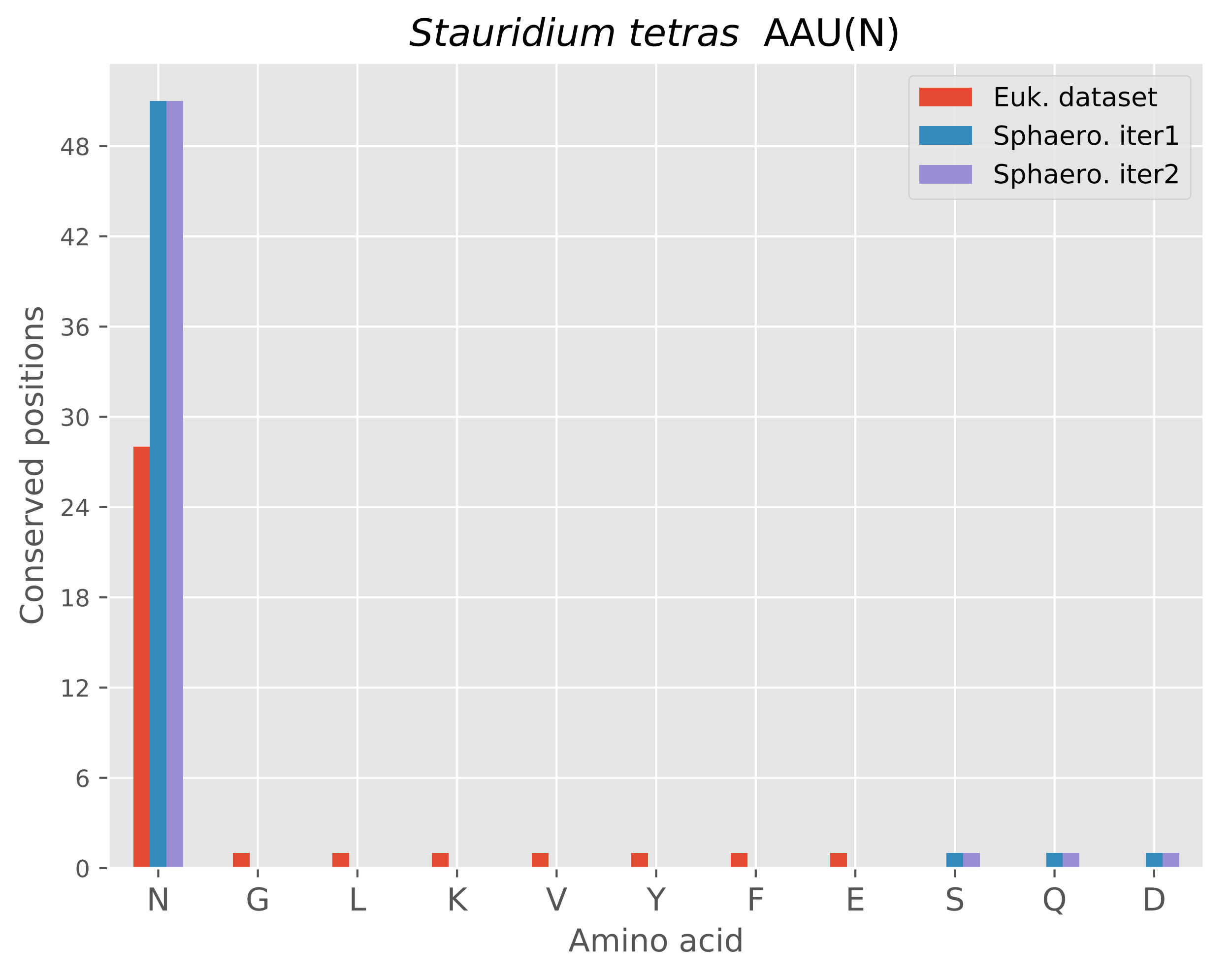

Stauridium tetras AAU(N)
Euk. dataset
Sphaero. iter1
Sphaero. iter2
Conserved positions
0
6
12
18
24
30
36
42
48
N
G
L
K
V
Y
F
E
S
Q
D
Amino acid

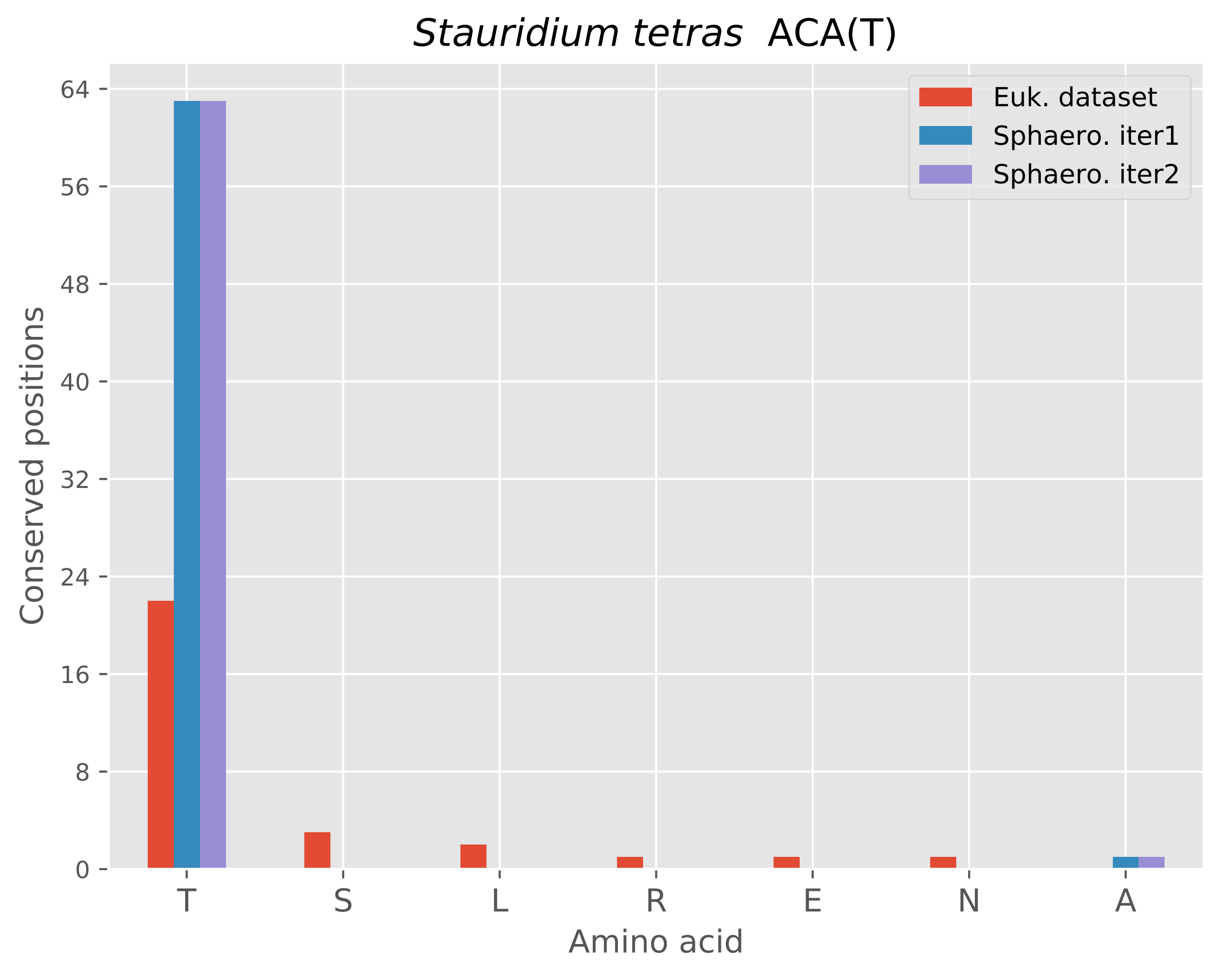
Stauridium tetras ACA(T)
Euk. dataset
Sphaero. iter1
Sphaero. iter2
Conserved positions
0
8
16
24
32
40
48
56
64
T
S
L
R
E
N
A
Amino acid

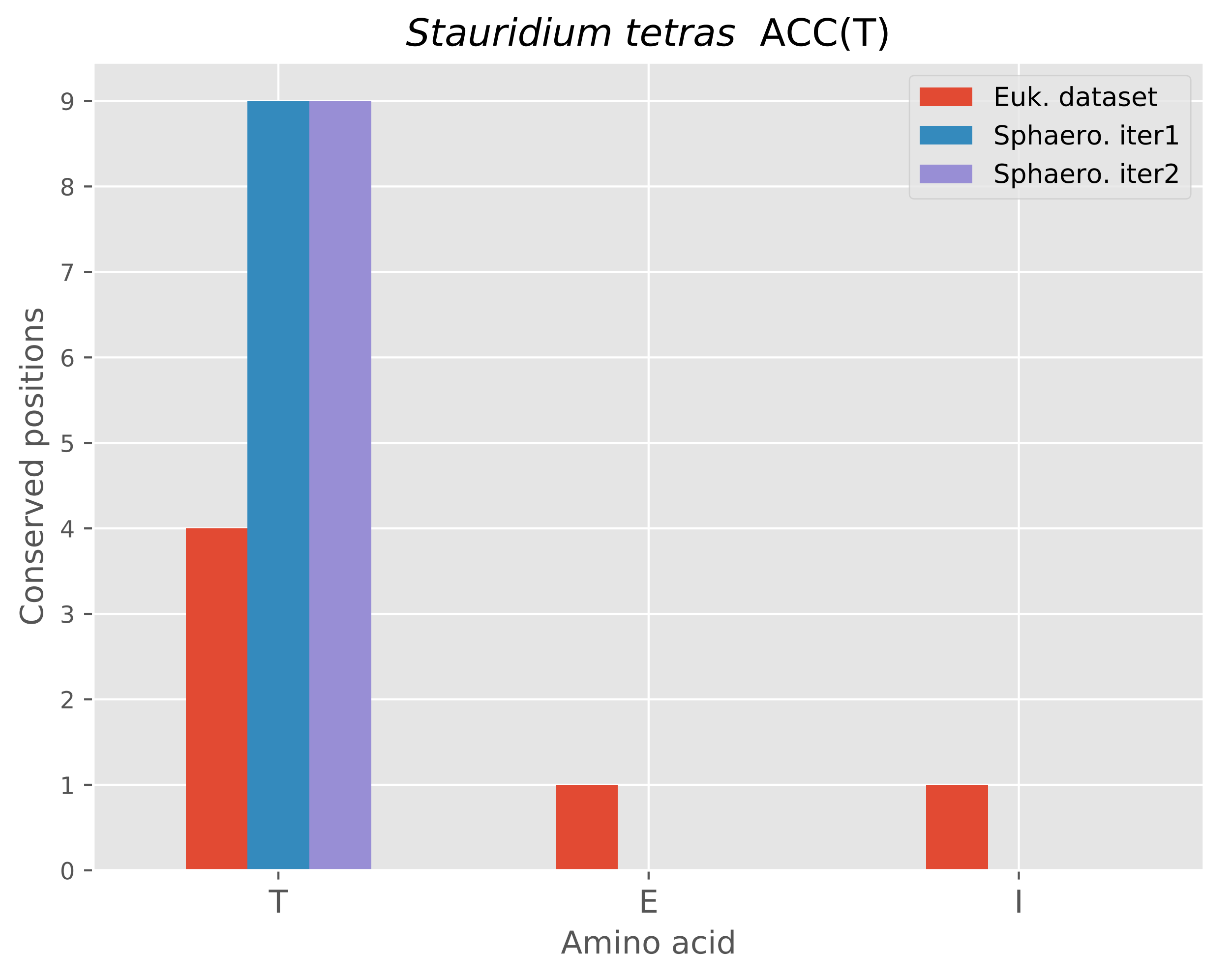

Stauridium tetras ACC(T)
Euk. dataset
Sphaero. iter1
Sphaero. iter2
Conserved positions
0
1
2
3
4
5
6
7
8
9
T
E
I
Amino acid

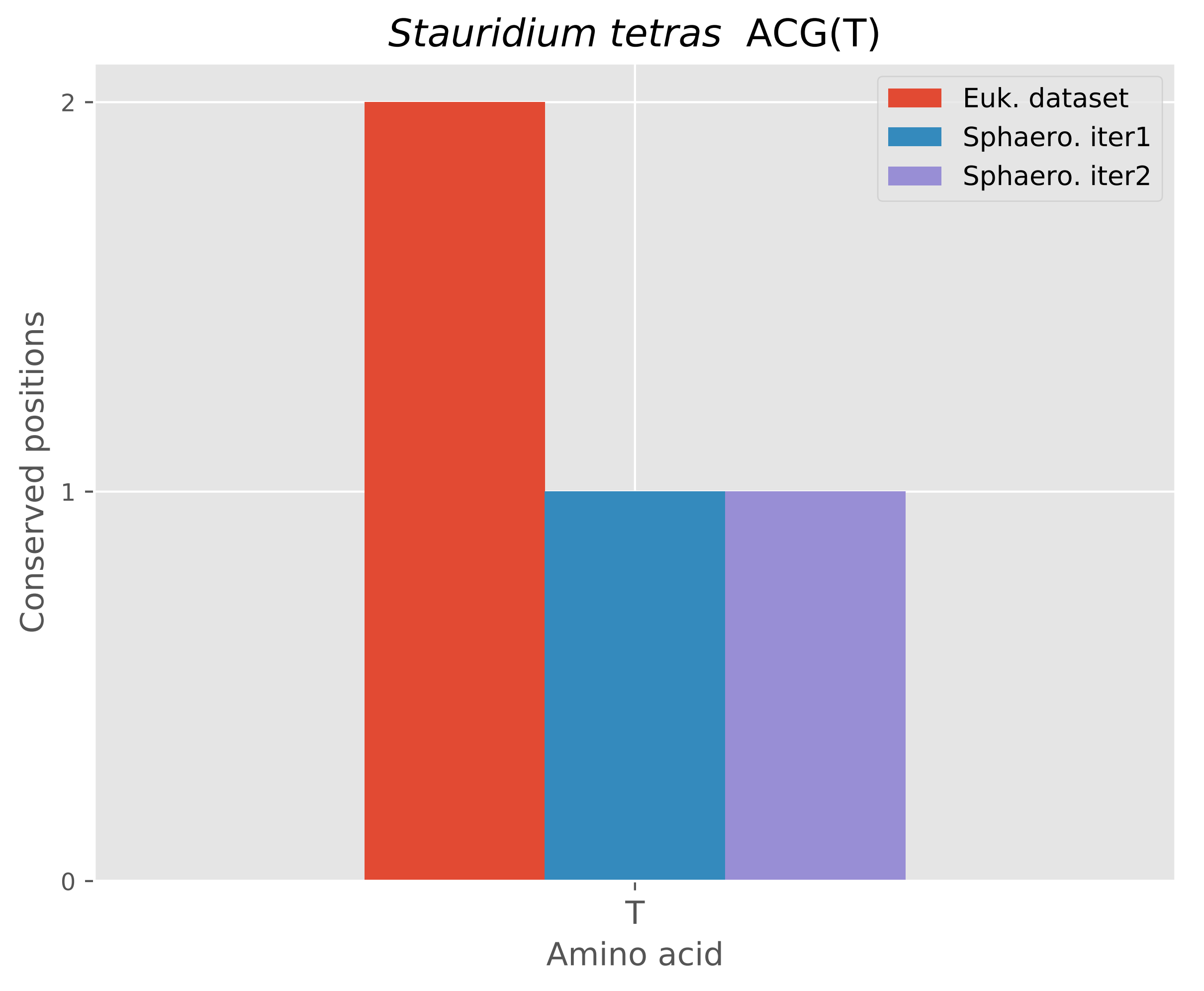

Stauridium tetras ACG(T)
Euk. dataset
Sphaero. iter1
Sphaero. iter2
Conserved positions
0
1
2
T
Amino acid

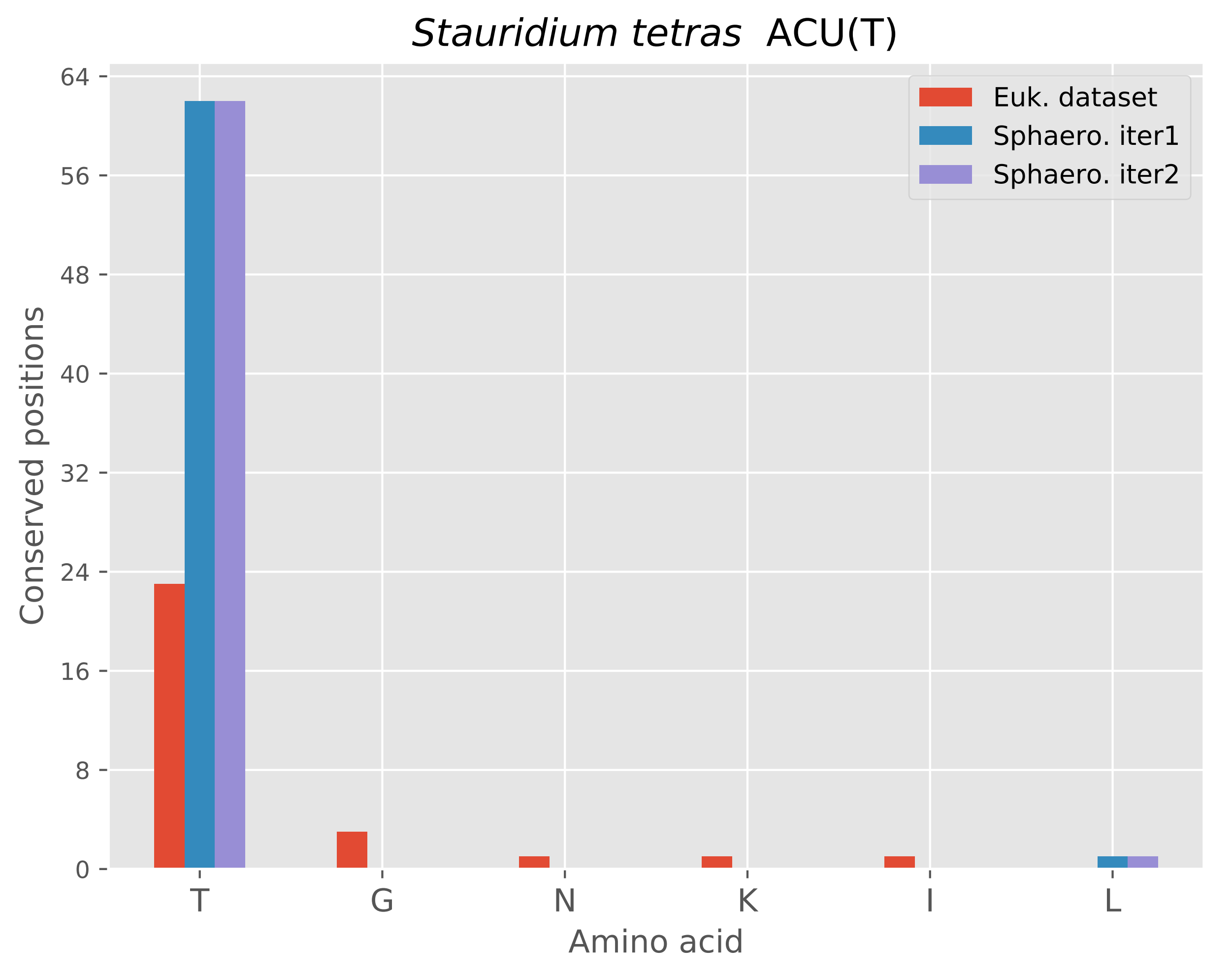
Stauridium tetras ACU(T)
Euk. dataset
Sphaero. iter1
Sphaero. iter2
Conserved positions
0
8
16
24
32
40
48
56
64
T
G
N
K
I
L
Amino acid

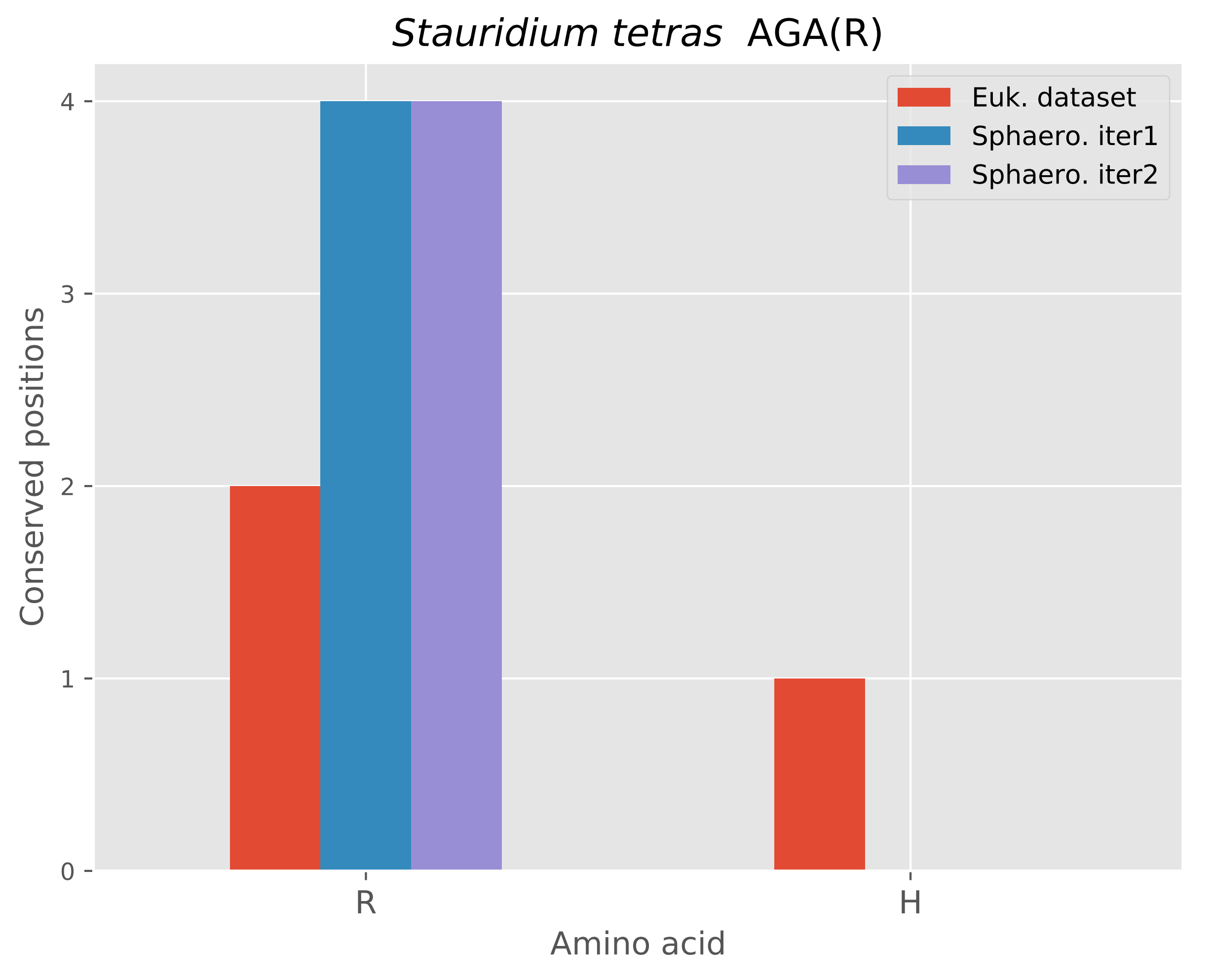

Stauridium tetras AGA(R)
Conserved positions
0
1
2
3
4
R
H
Amino acid
Euk. dataset
Sphaero. iter1
Sphaero. iter2

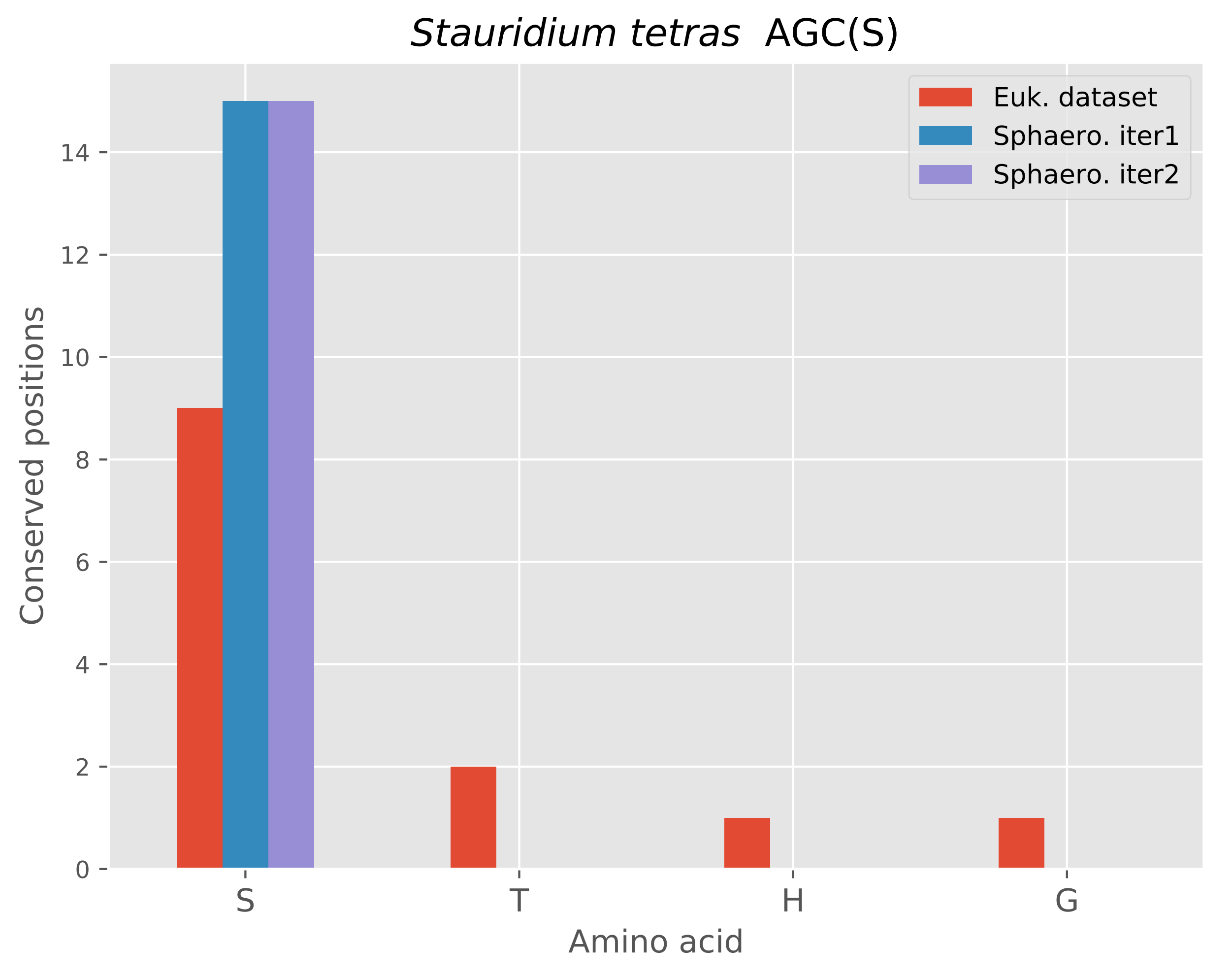

Stauridium tetras AGC(S)
Euk. dataset
Sphaero. iter1
Sphaero. iter2
Conserved positions
0
2
4
6
8
10
12
14
S
T
H
G
Amino acid

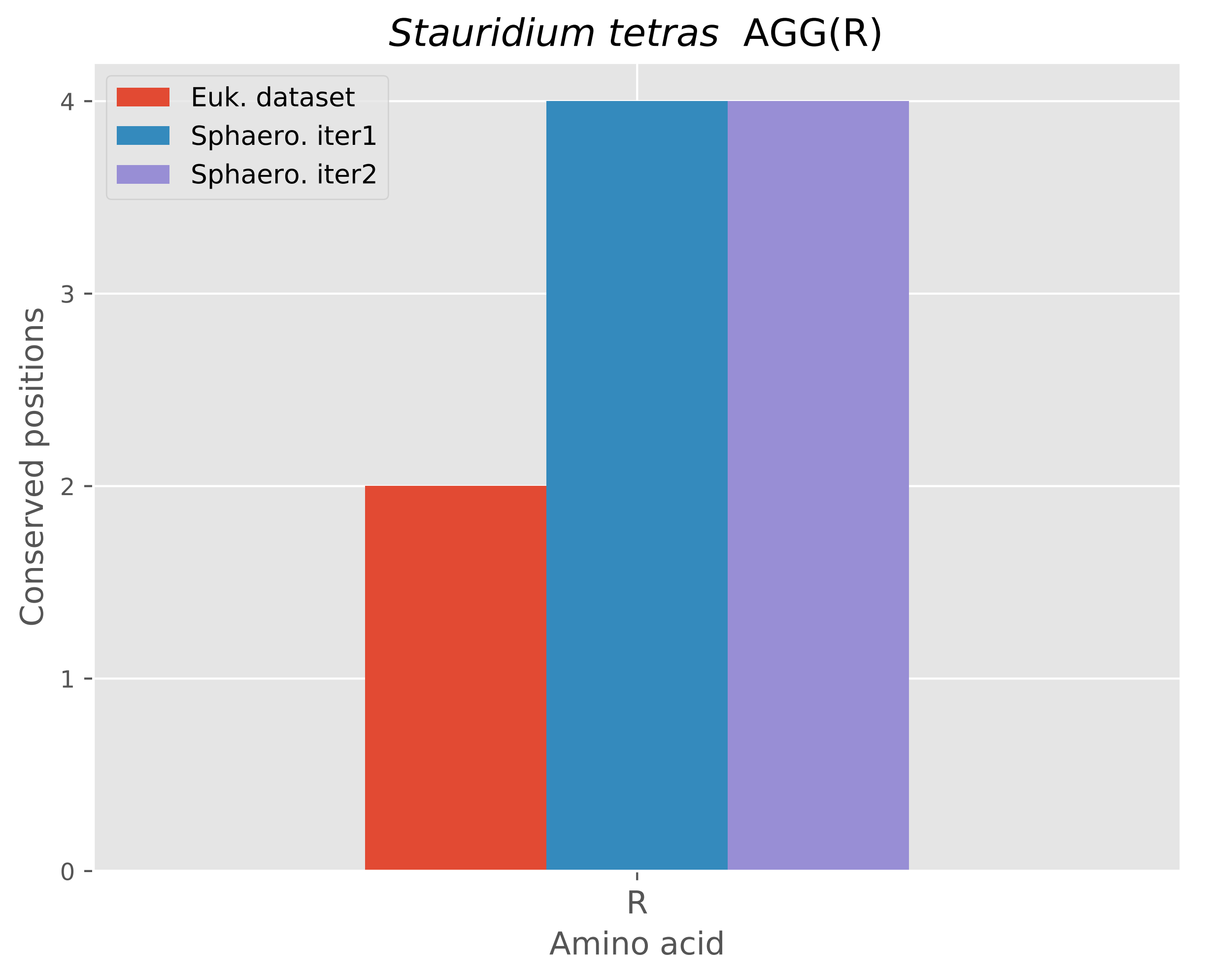

Stauridium tetras AGG(R)
Euk. dataset
Sphaero. iter1
Sphaero. iter2
Conserved positions
0
1
2
3
4
R
Amino acid

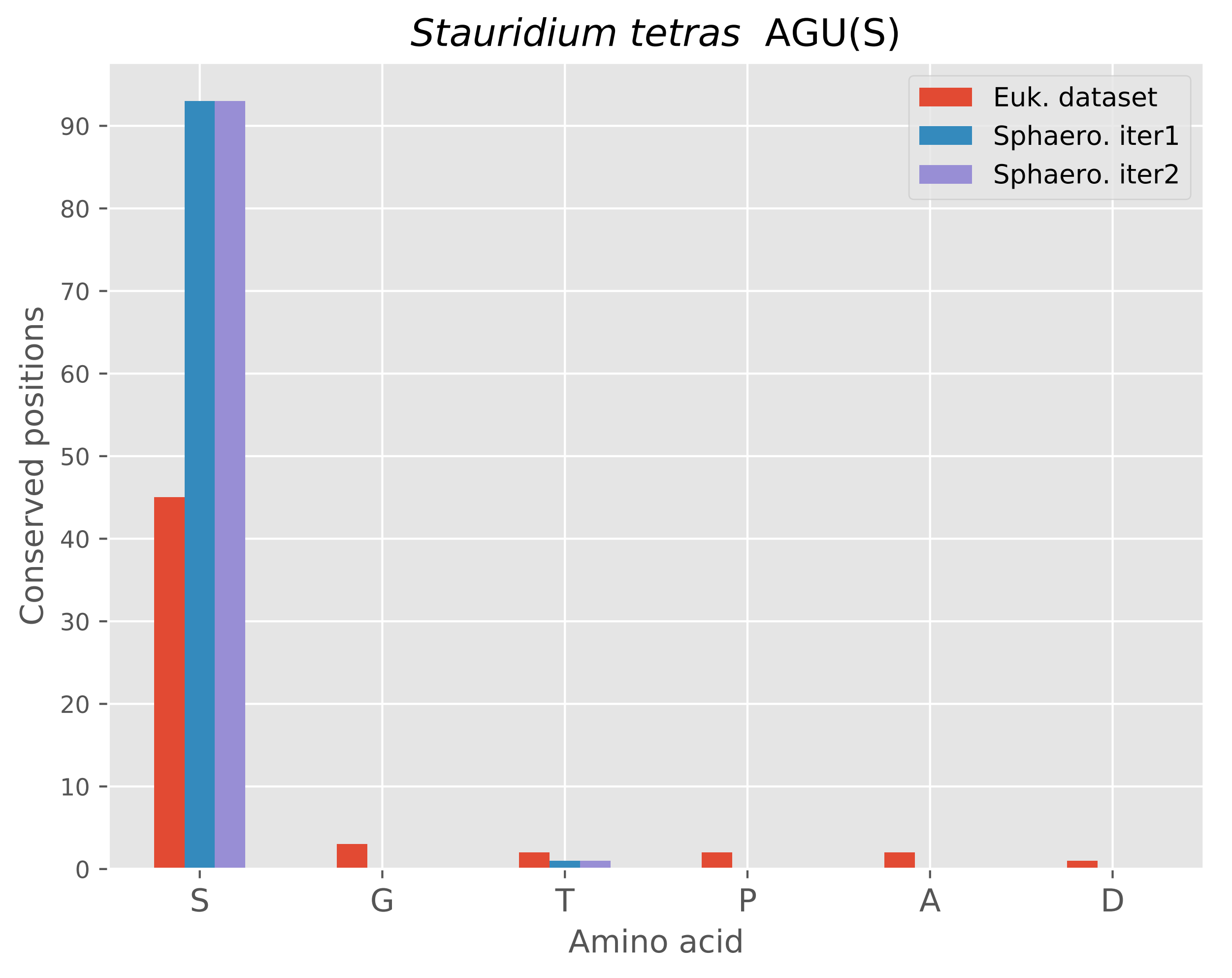

Stauridium tetras AGU(S)
Euk. dataset
Sphaero. iter1
Sphaero. iter2
Conserved positions
0
10
20
30
40
50
60
70
80
90
S
G
T
P
A
D
Amino acid

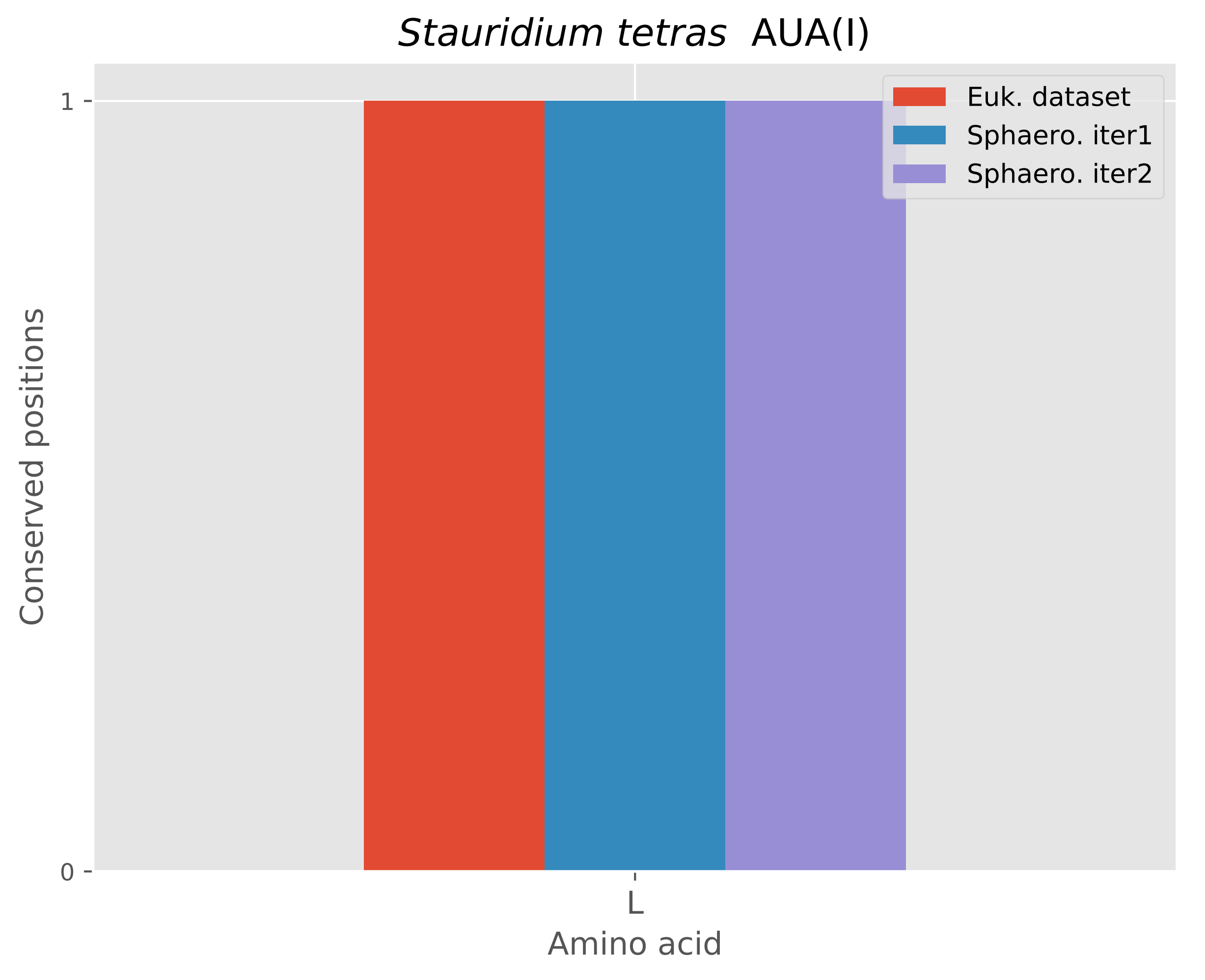

Stauridium tetras AUA(I)
Euk. dataset
Sphaero. iter1
Sphaero. iter2
Conserved positions
1
0
L
Amino acid

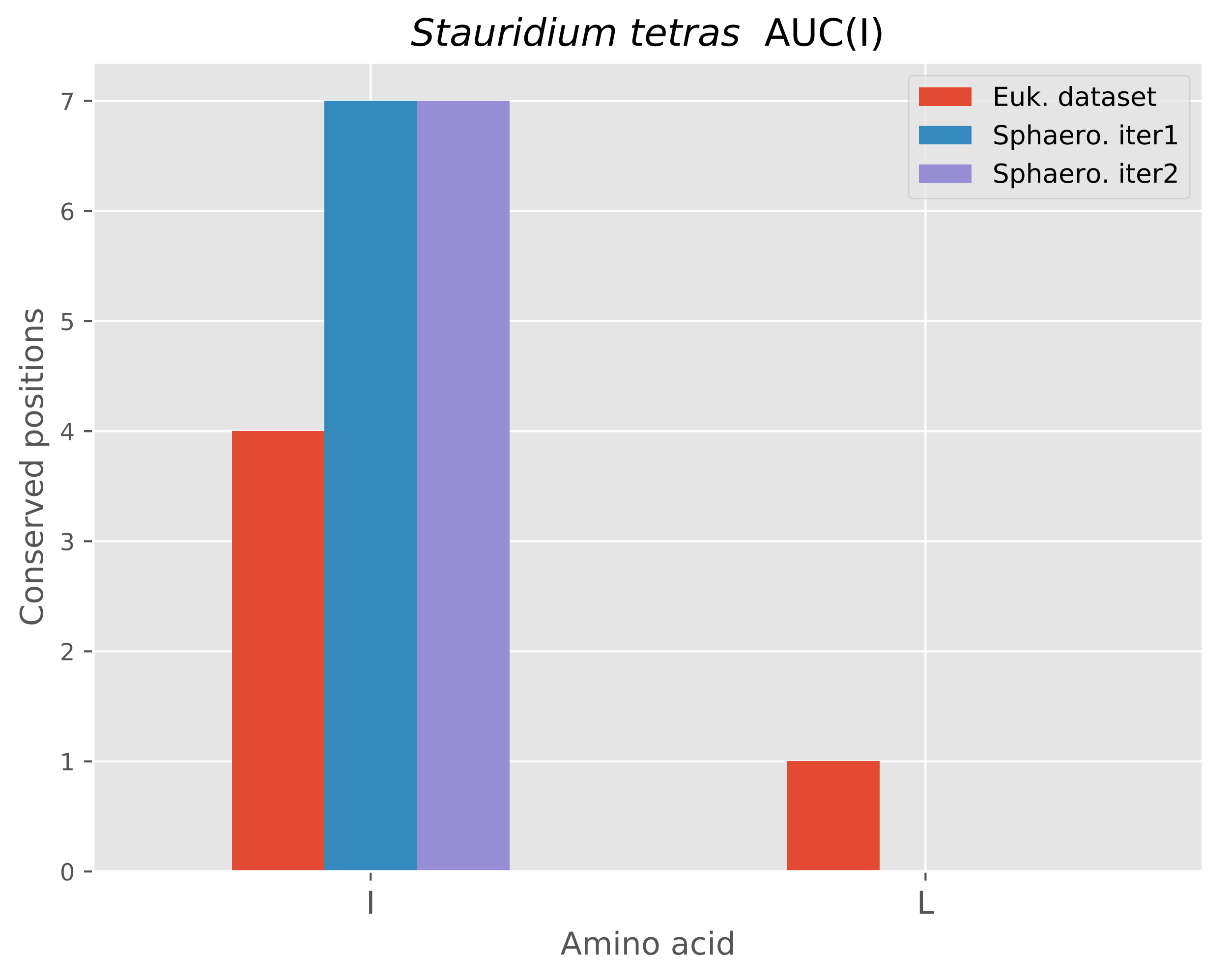

Stauridium tetras AUC(I)
Euk. dataset
Sphaero. iter1
Sphaero. iter2
Conserved positions
0
1
2
3
4
5
6
7
I
L
Amino acid

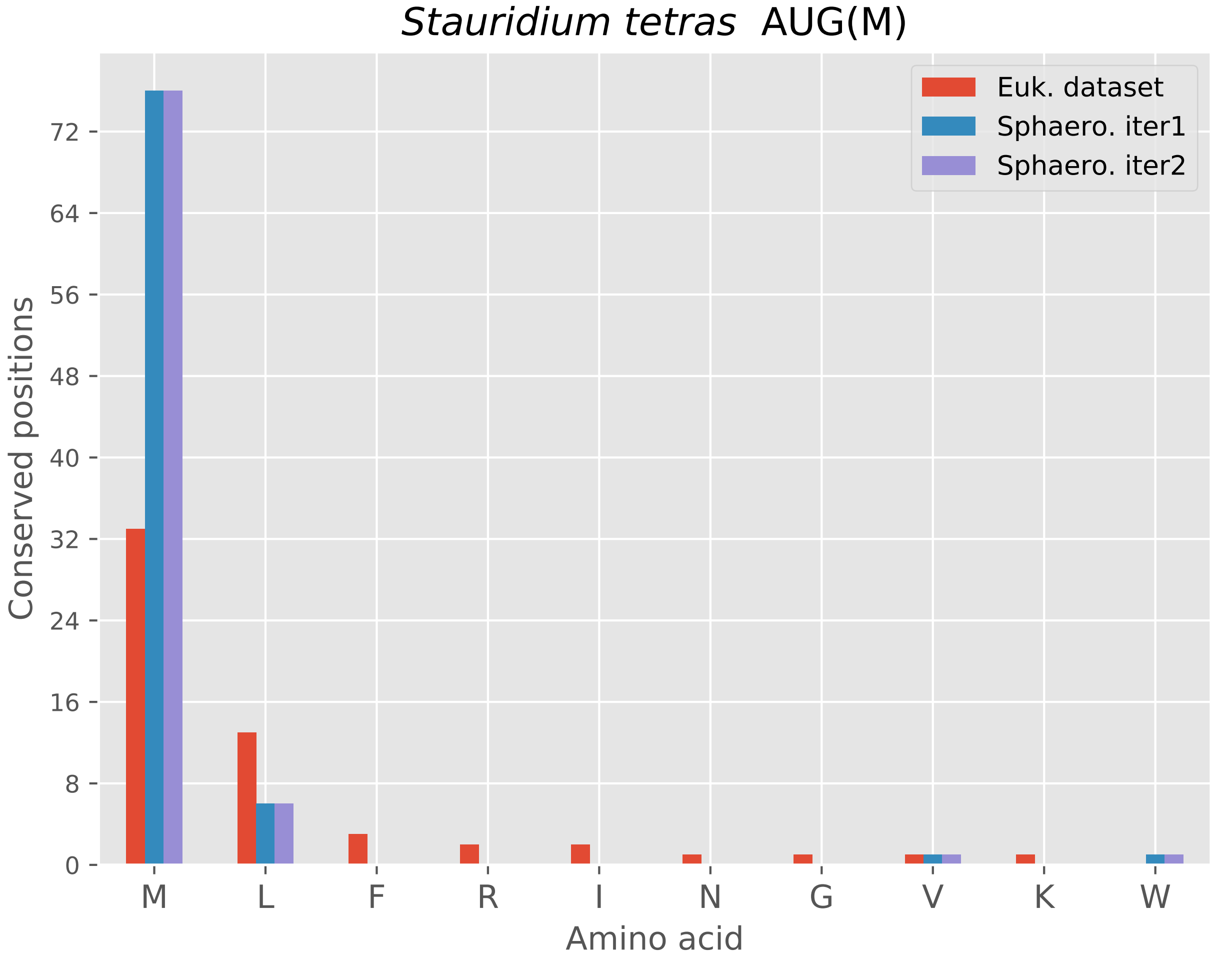

Stauridium tetras AUG(M)
Euk. dataset
Sphaero. iter1
Sphaero. iter2
Conserved positions
0
8
16
24
32
40
48
56
64
72
M
L
F
R
I
N
G
V
K
W
Amino acid

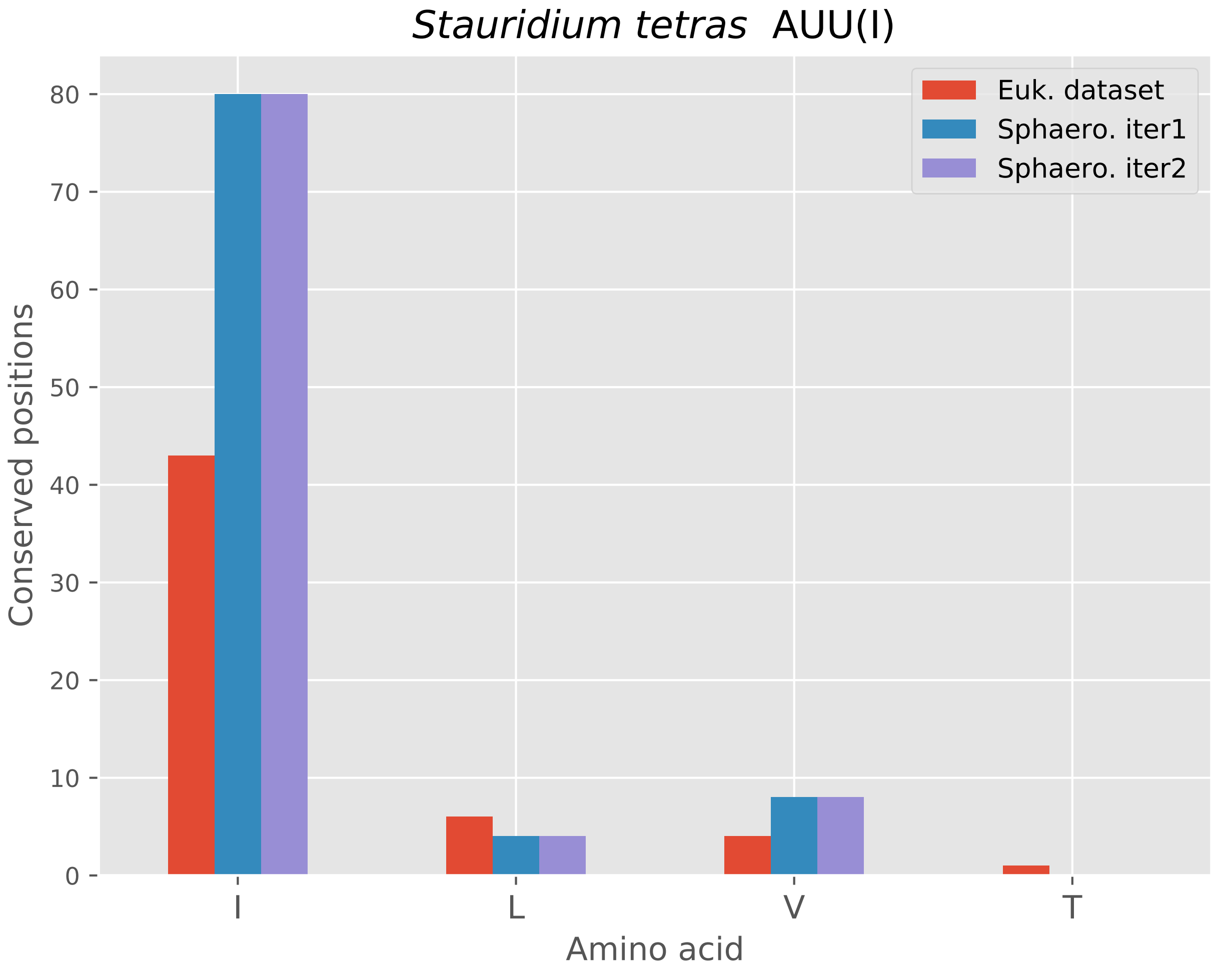
Stauridium tetras AUU(I)
Euk. dataset
Sphaero. iter1
Sphaero. iter2
Conserved positions
0
10
20
30
40
50
60
70
80
I
L
V
T
Amino acid

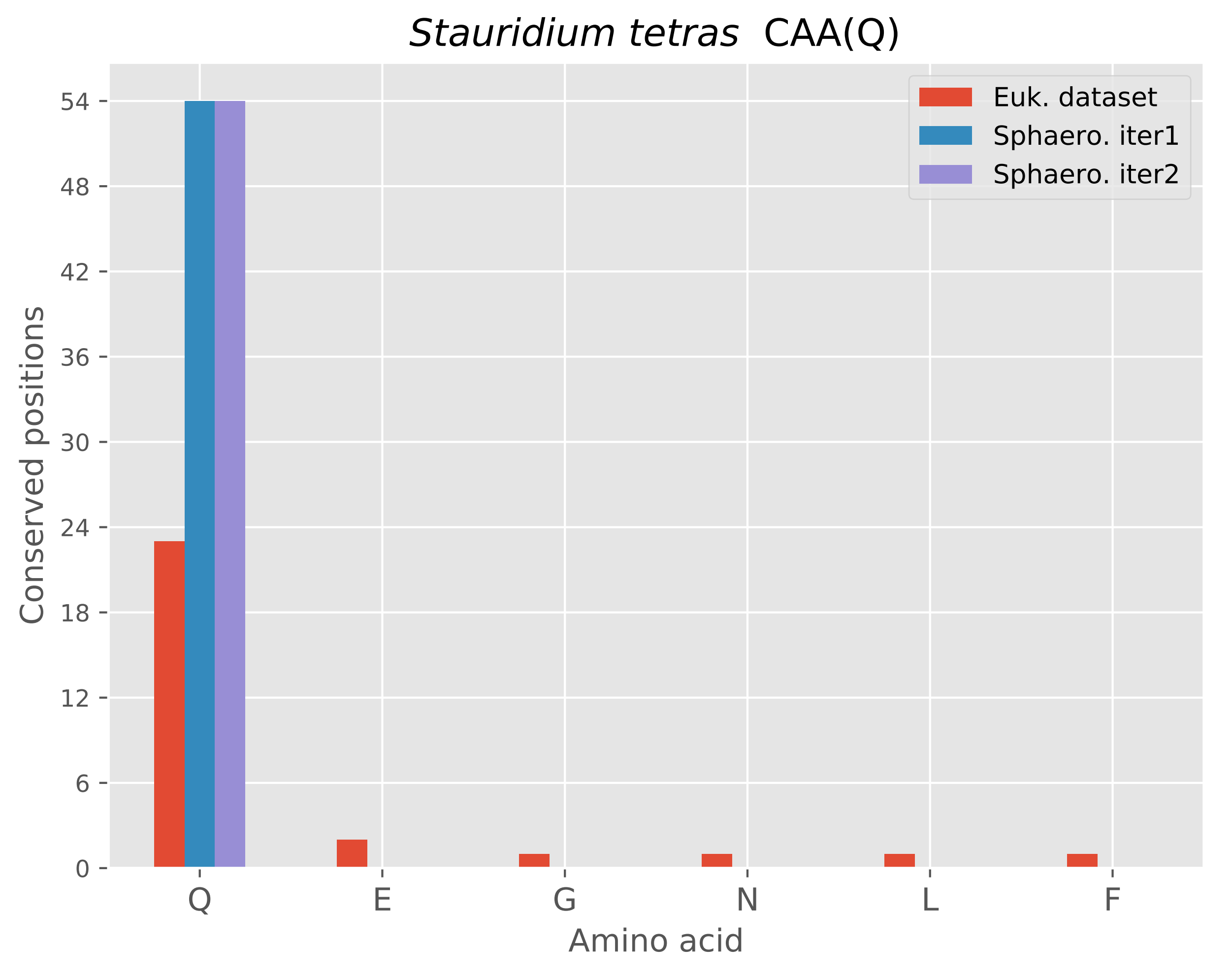

Stauridium tetras CAA(Q)
Euk. dataset
Sphaero. iter1
Sphaero. iter2
Conserved positions
0
6
12
18
24
30
36
42
48
54
Q
E
G
N
L
F
Amino acid

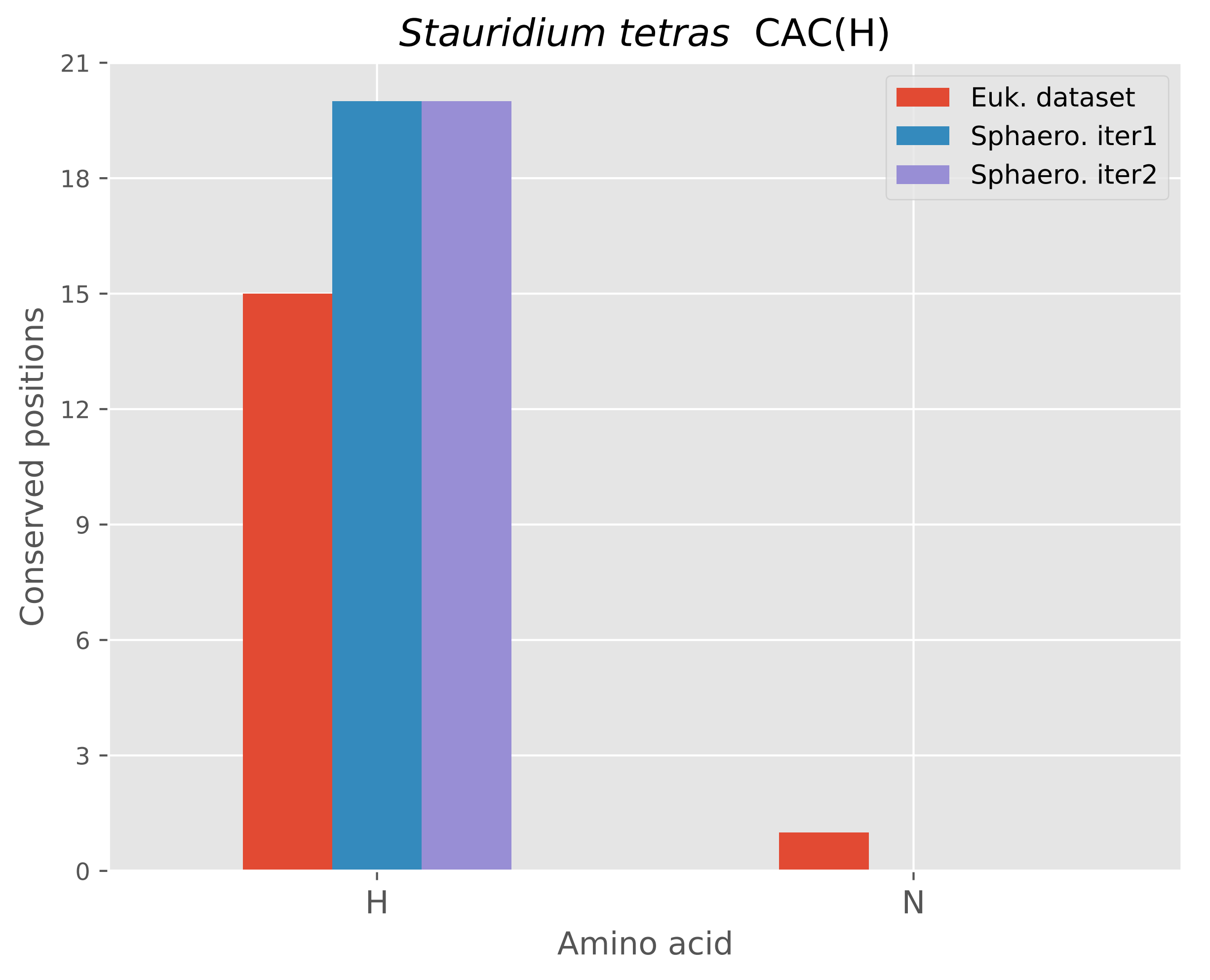

Stauridium tetras CAC(H)
Conserved positions
0
3
6
9
12
15
18
21
Euk. dataset
Sphaero. iter1
Sphaero. iter2
H
N
Amino acid

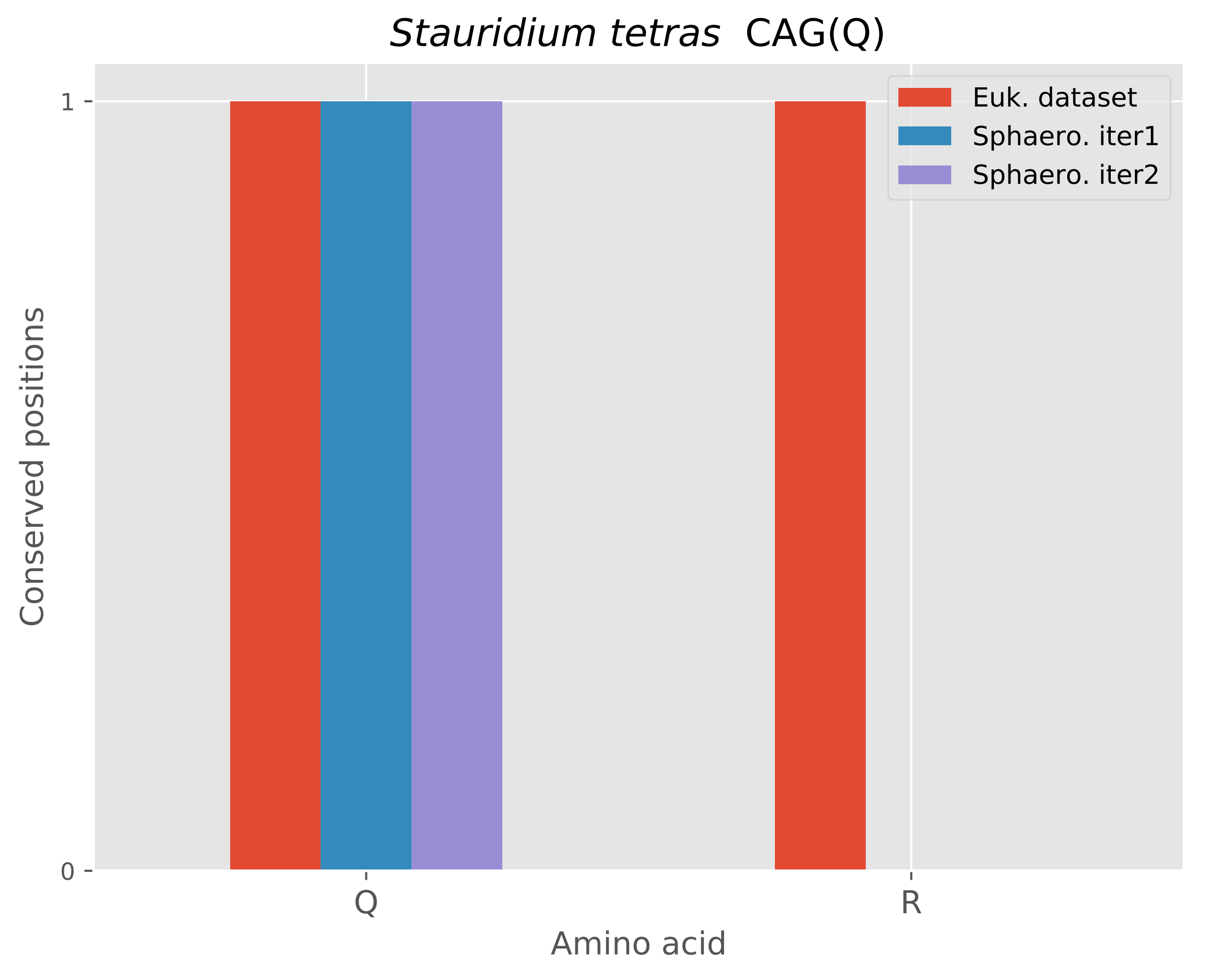

Stauridium tetras CAG(Q)
Euk. dataset
Sphaero. iter1
Sphaero. iter2
1
0
Conserved positions
Q
R
Amino acid

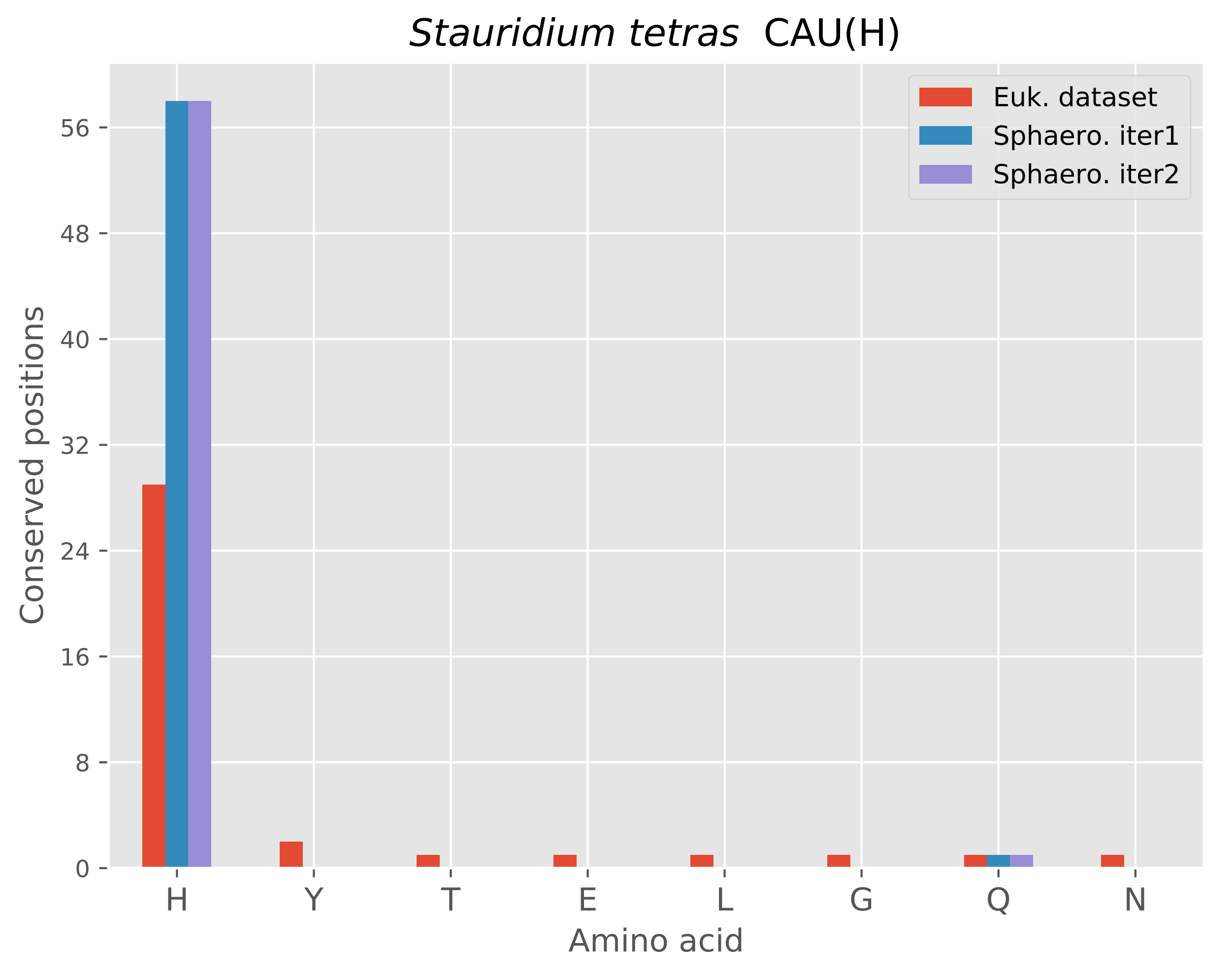

Stauridium tetras CAU(H)
Euk. dataset
Sphaero. iter1
Sphaero. iter2
Conserved positions
0
8
16
24
32
40
48
56
H
Y
T
E
L
G
Q
N
Amino acid

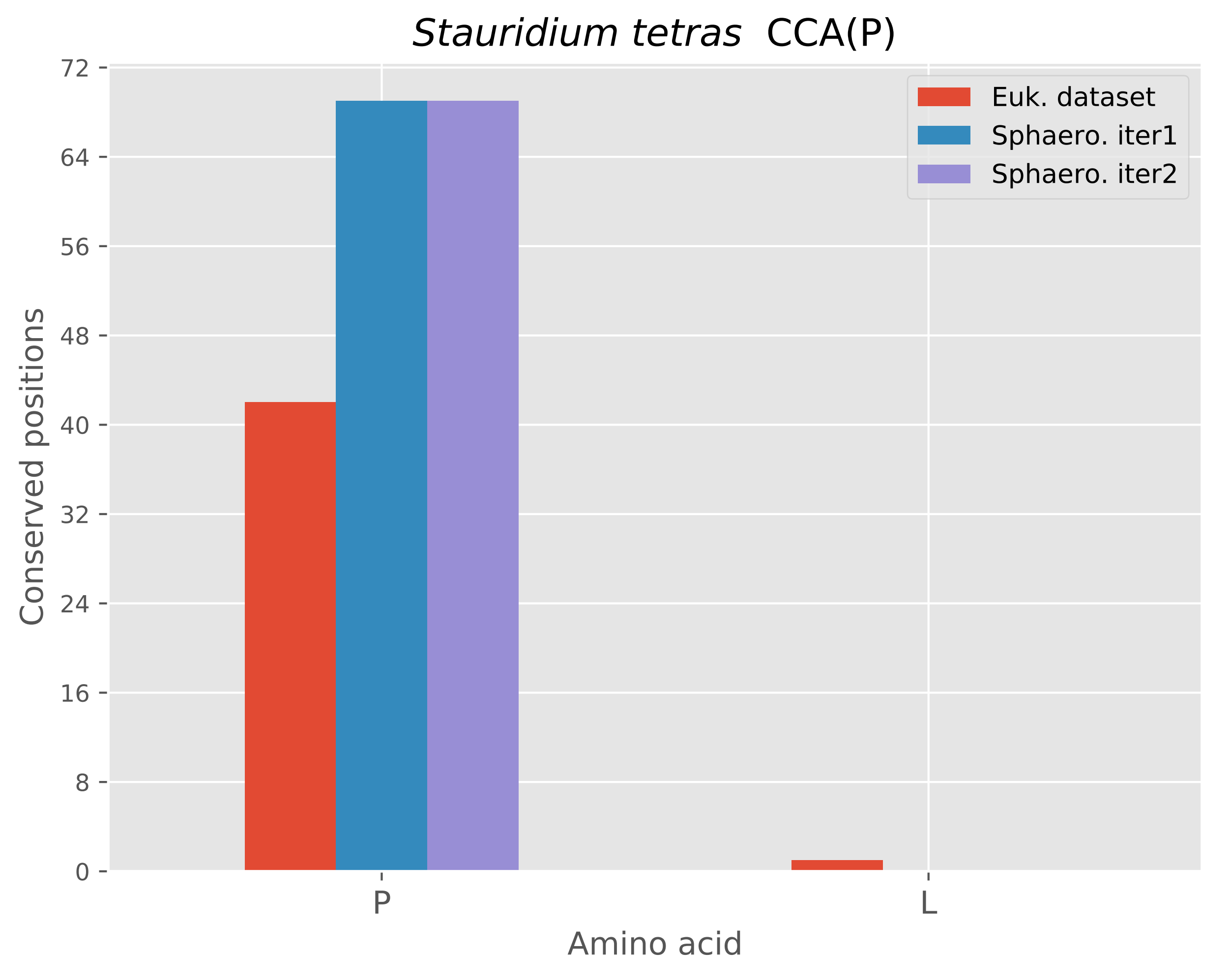

Stauridium tetras CCA(P)
Conserved positions
Amino acid
72
64
56
48
40
32
24
16
8
0
P
L
Euk. dataset
Sphaero. iter1
Sphaero. iter2

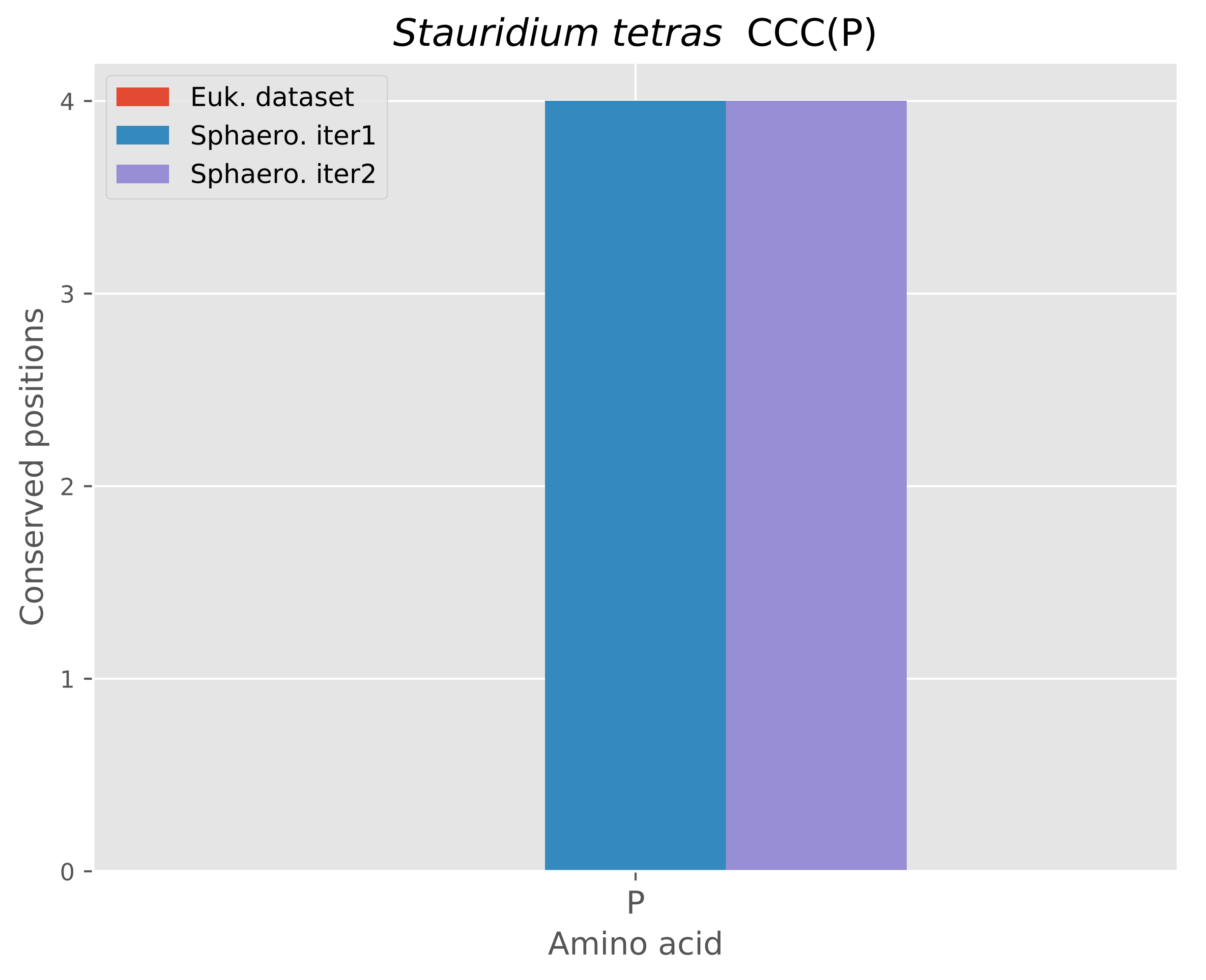
Stauridium tetras CCC(P)
Euk. dataset
Sphaero. iter1
Sphaero. iter2
Conserved positions
0
1
2
3
4
P
Amino acid

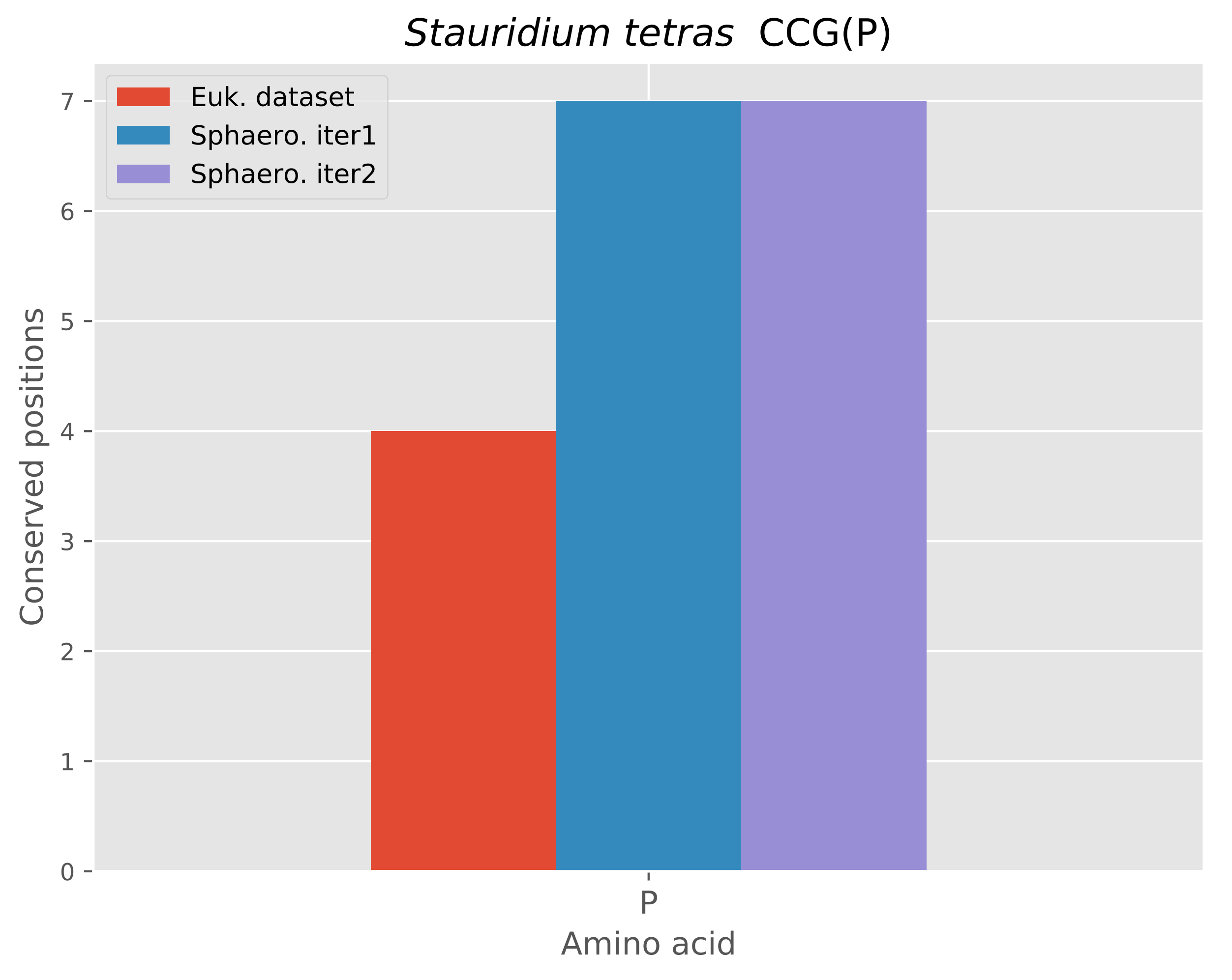
Stauridium tetras CCG(P)
Euk. dataset
Sphaero. iter1
Sphaero. iter2
Conserved positions
0
1
2
3
4
5
6
7
P
Amino acid

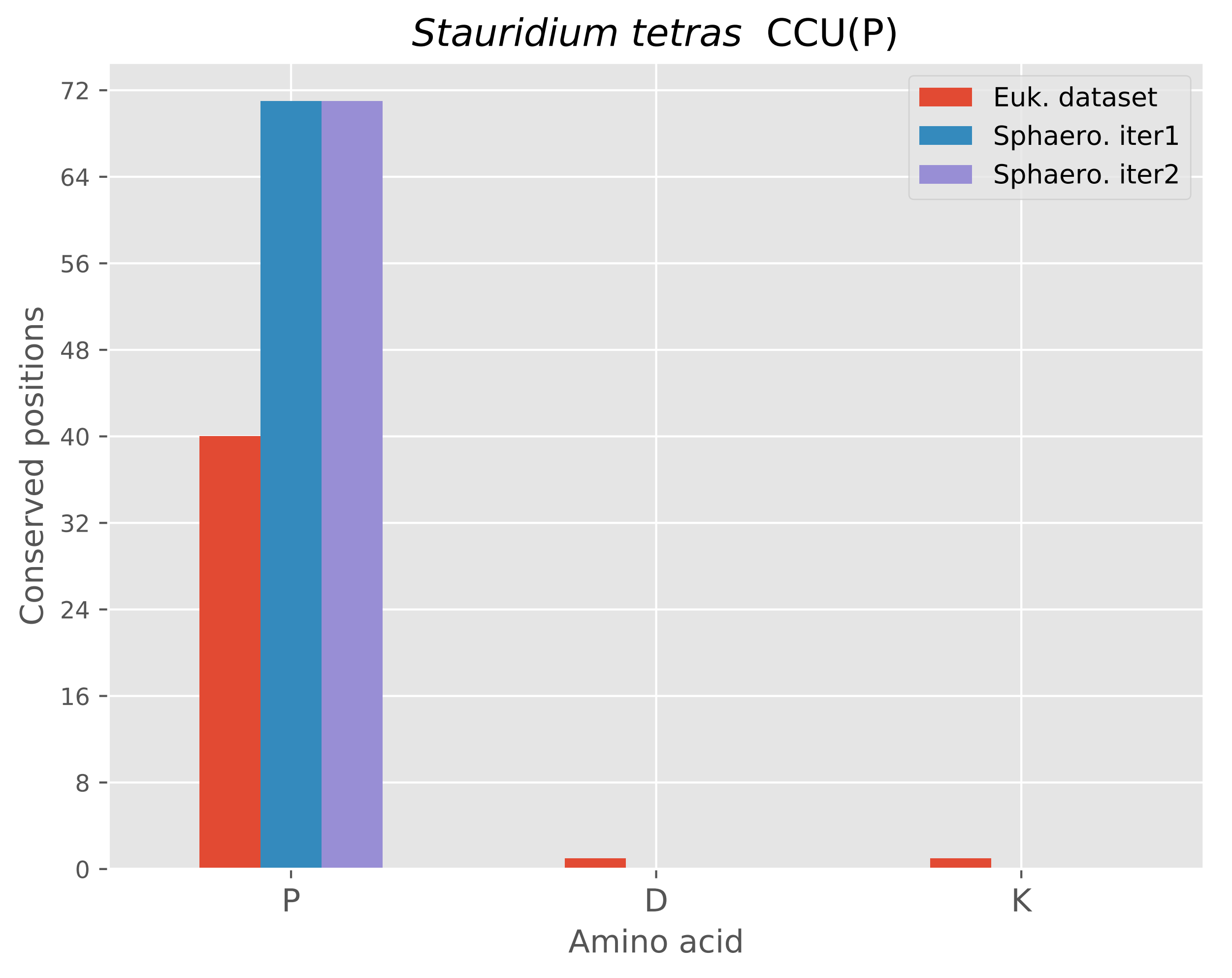

Stauridium tetras CCU(P)
Euk. dataset
Sphaero. iter1
Sphaero. iter2
Conserved positions
0
8
16
24
32
40
48
56
64
72
P
D
K
Amino acid

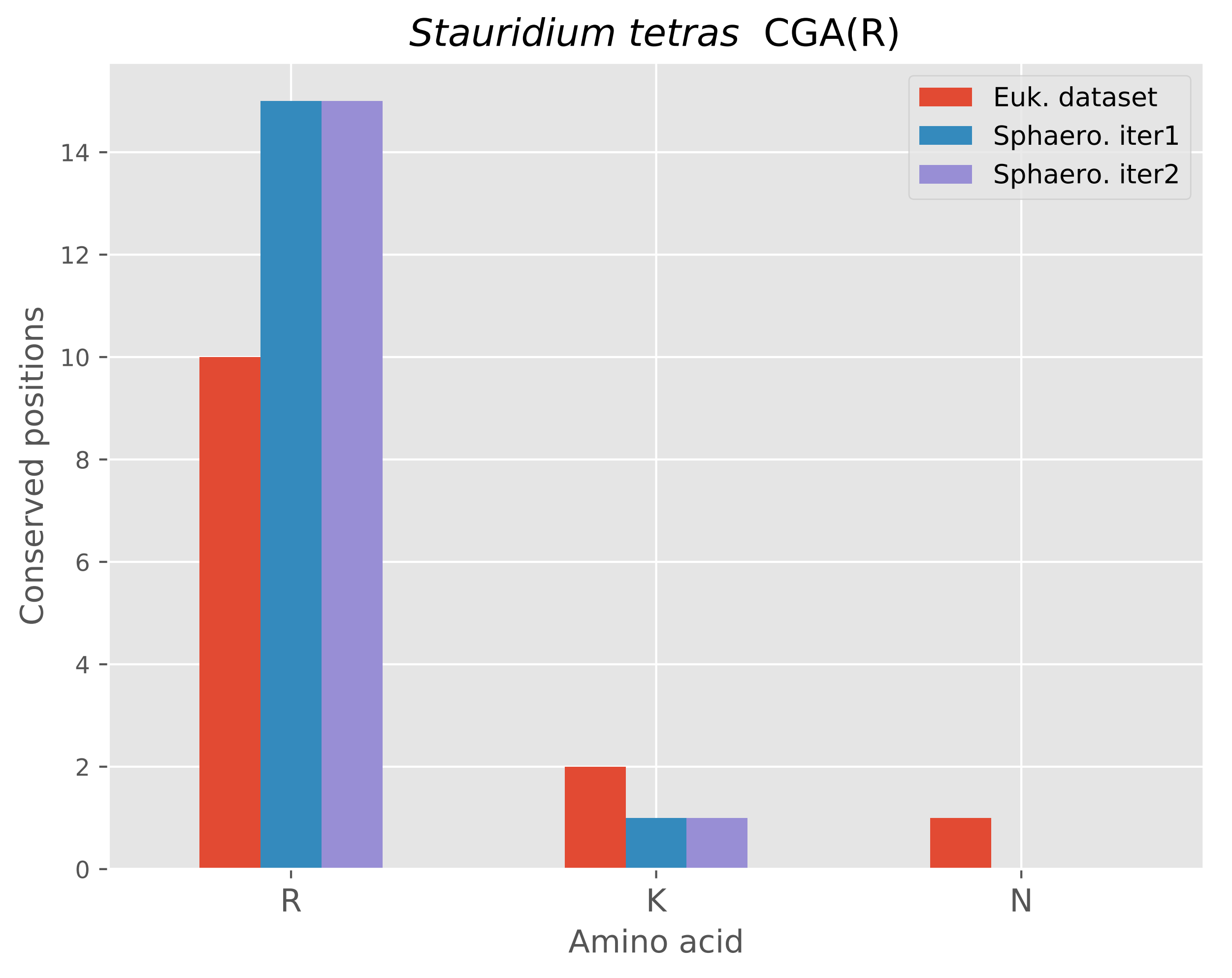

Stauridium tetras CGA(R)
Euk. dataset
Sphaero. iter1
Sphaero. iter2
Conserved positions
0
2
4
6
8
10
12
14
R
K
N
Amino acid

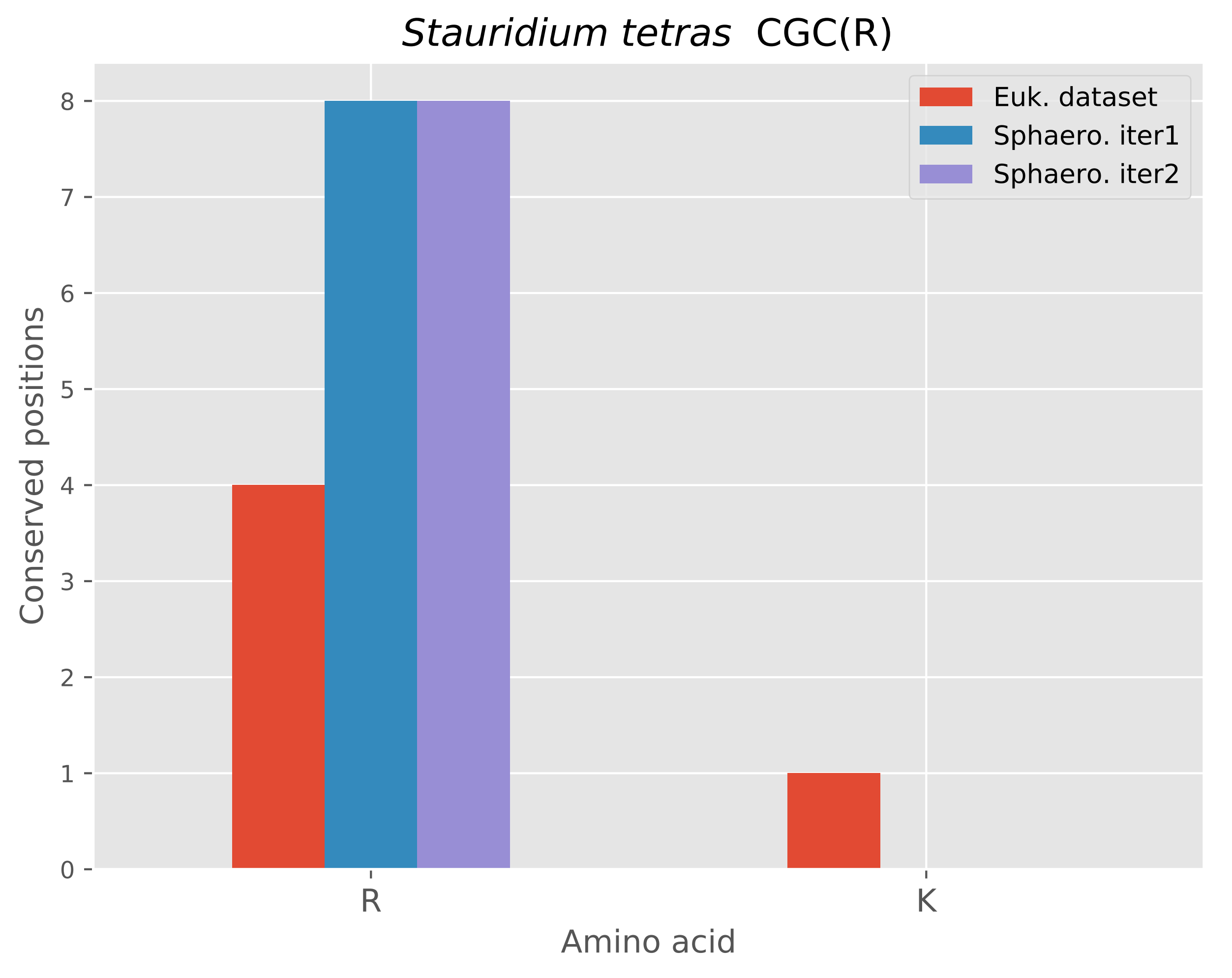

Stauridium tetras CGC(R)
Euk. dataset
Sphaero. iter1
Sphaero. iter2
Conserved positions
0
1
2
3
4
5
6
7
8
R
K
Amino acid

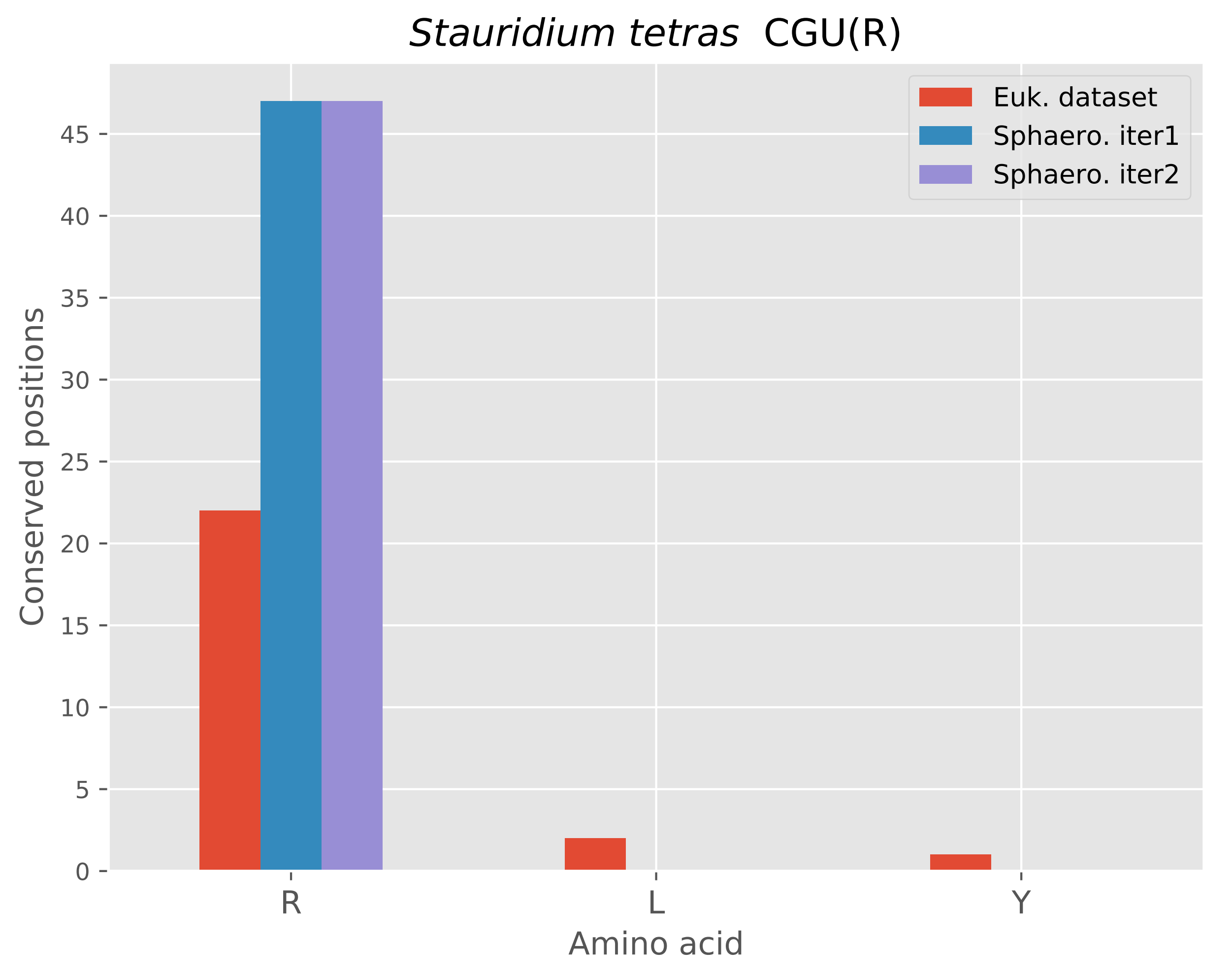

Stauridium tetras CGU(R)
Euk. dataset
Sphaero. iter1
Sphaero. iter2
Conserved positions
0
5
10
15
20
25
30
35
40
45
R
L
Y
Amino acid

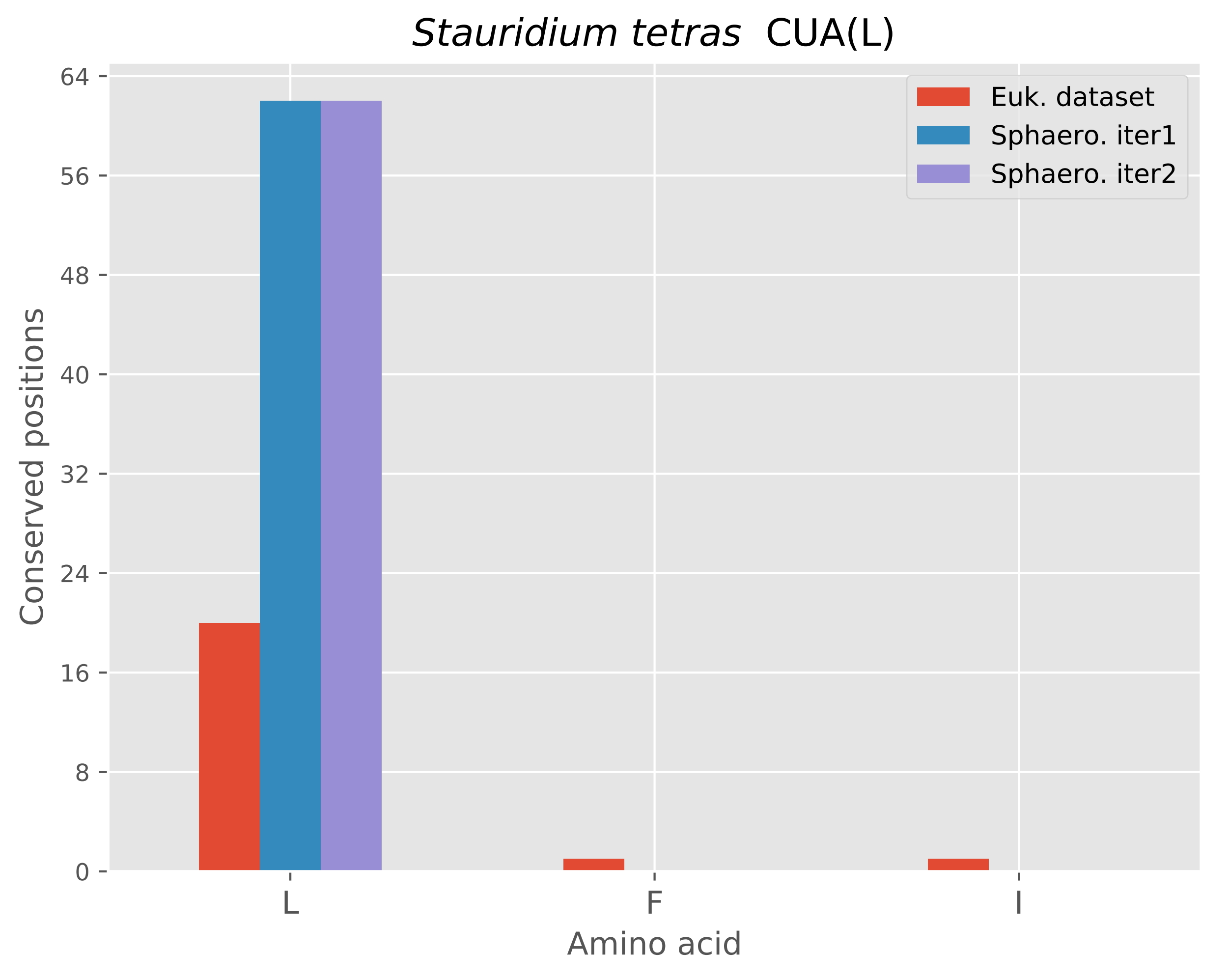
Stauridium tetras CUA(L)
Conserved positions
0
8
16
24
32
40
48
56
64
Euk. dataset
Sphaero. iter1
Sphaero. iter2
L
F
I
Amino acid

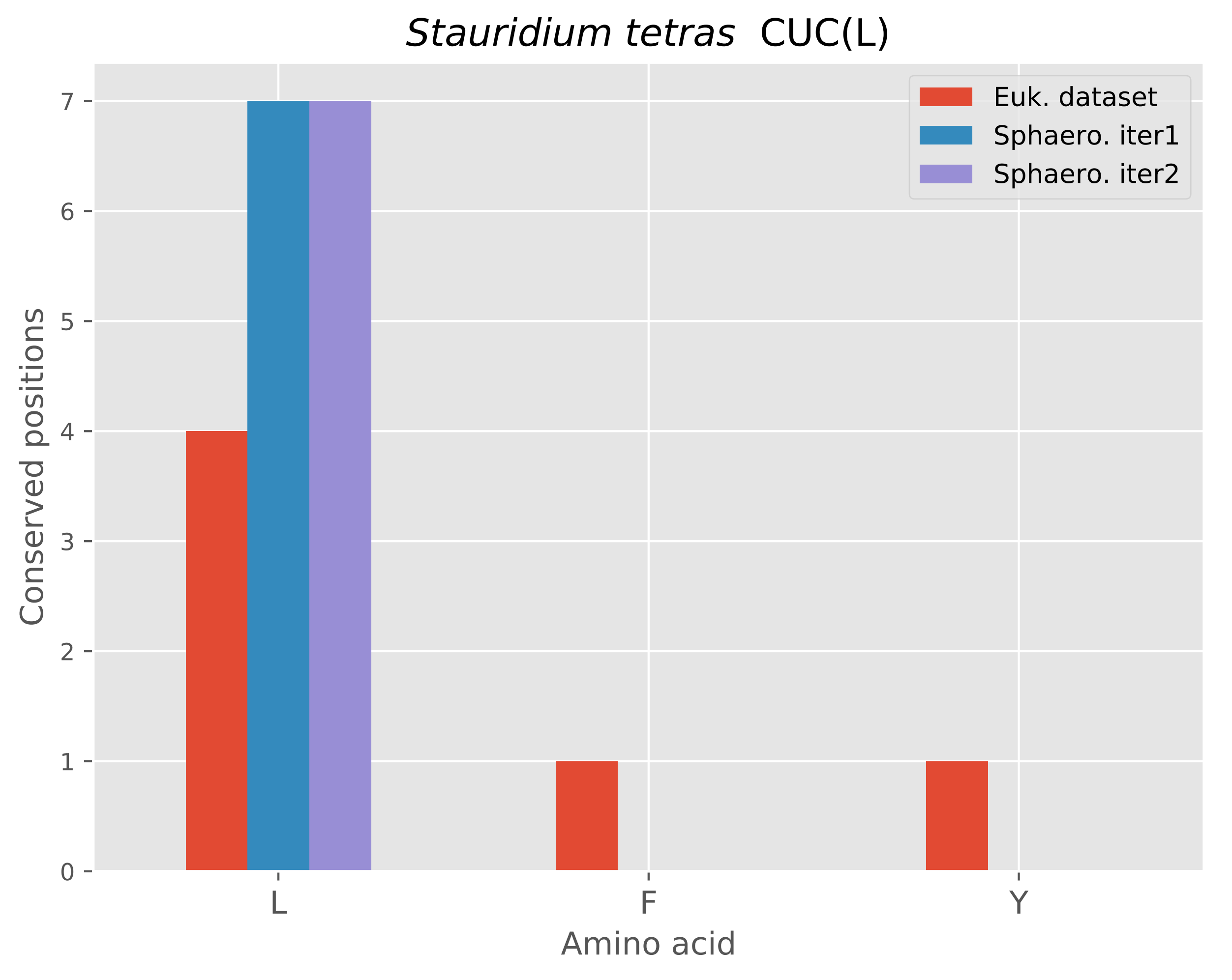
Stauridium tetras CUC(L)
Euk. dataset
Sphaero. iter1
Sphaero. iter2
Conserved positions
0
1
2
3
4
5
6
7
L
F
Y
Amino acid

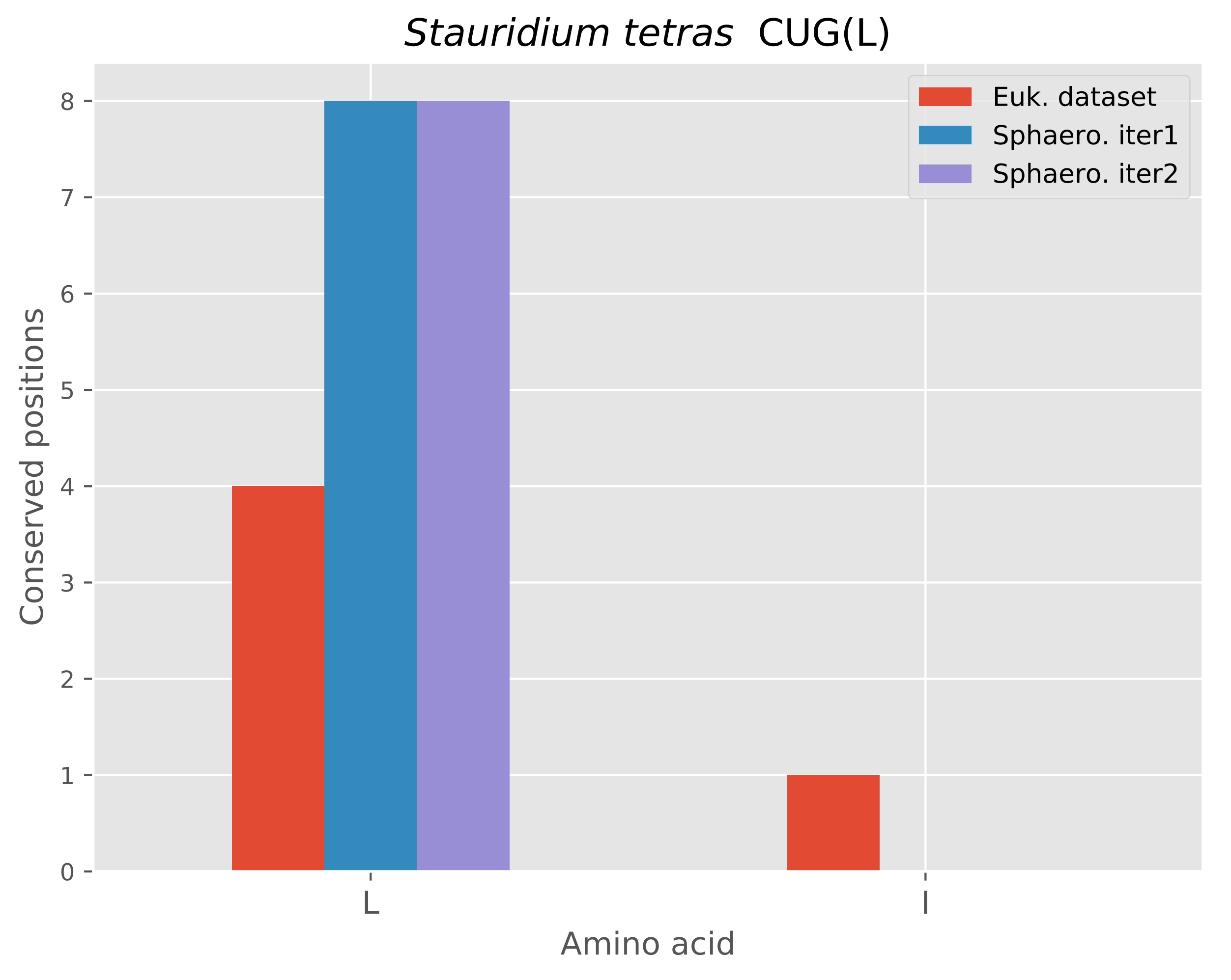

Stauridium tetras CUG(L)
Euk. dataset
Sphaero. iter1
Sphaero. iter2
Conserved positions
0
1
2
3
4
5
6
7
8
L
I
Amino acid

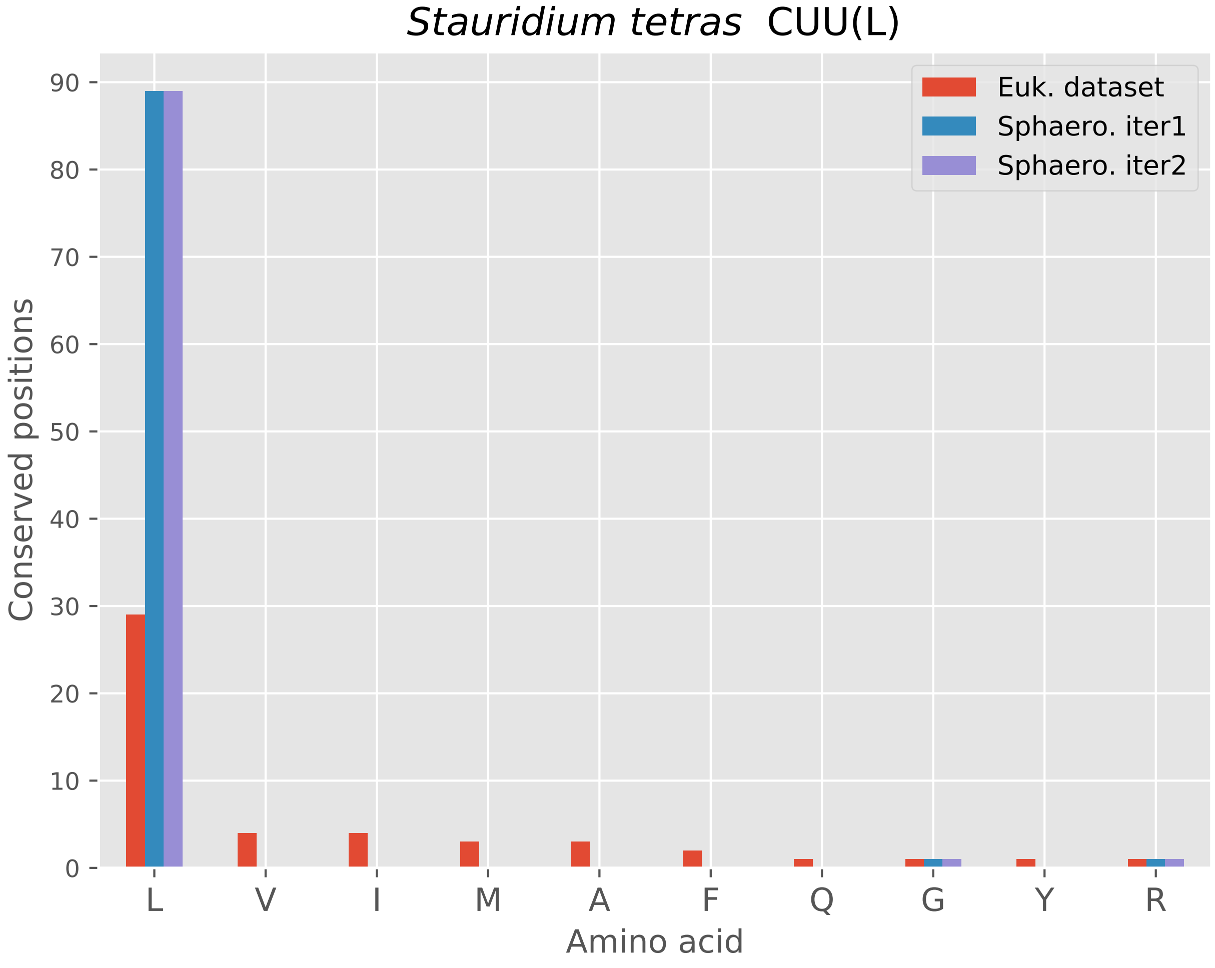
Stauridium tetras CUU(L)
Euk. dataset
Sphaero. iter1
Sphaero. iter2
Conserved positions
0
10
20
30
40
50
60
70
80
90
L
V
I
M
A
F
Q
G
Y
R
Amino acid

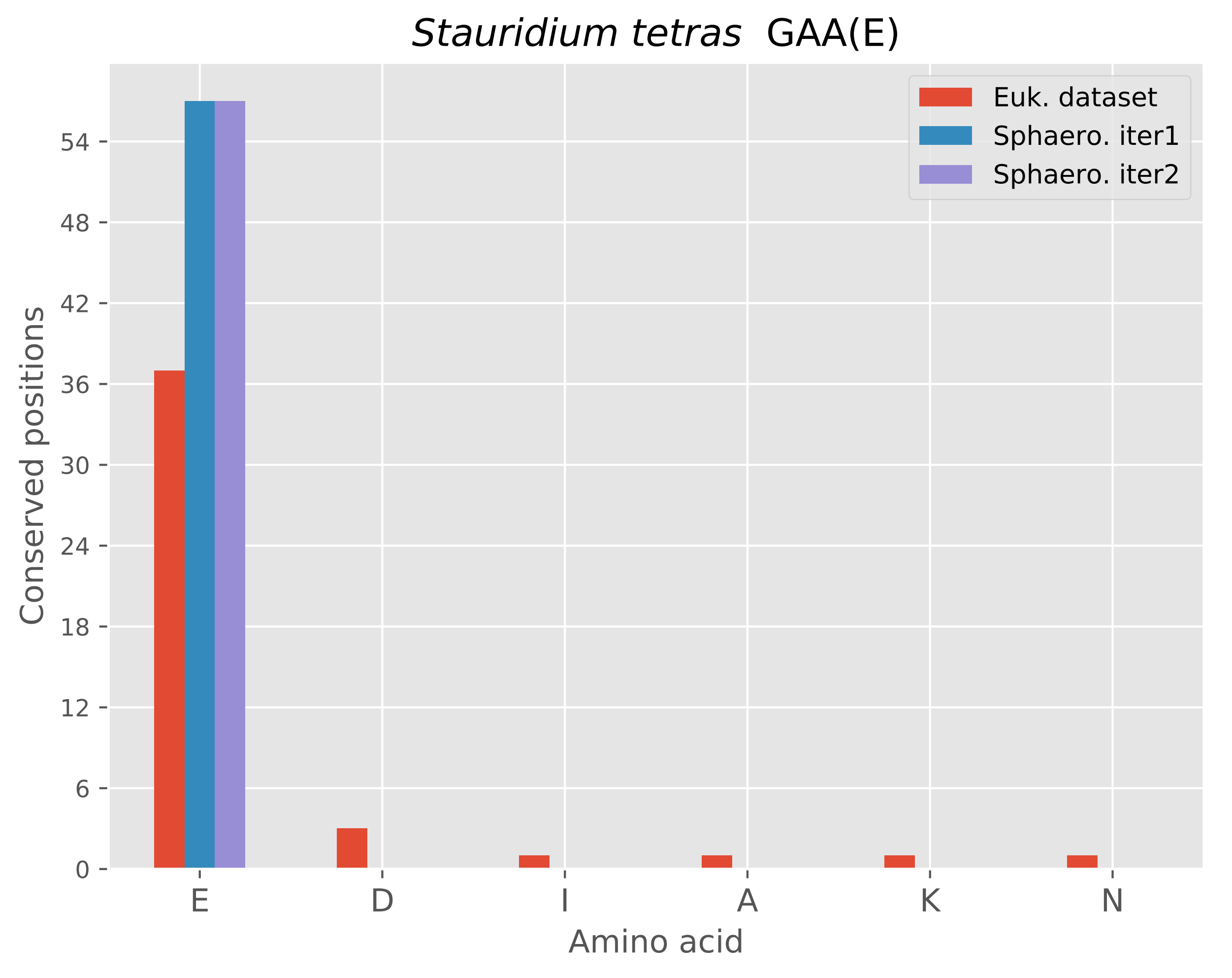

Stauridium tetras GAA(E)
Euk. dataset
Sphaero. iter1
Sphaero. iter2
Conserved positions
0
6
12
18
24
30
36
42
48
54
E
D
I
A
K
N
Amino acid

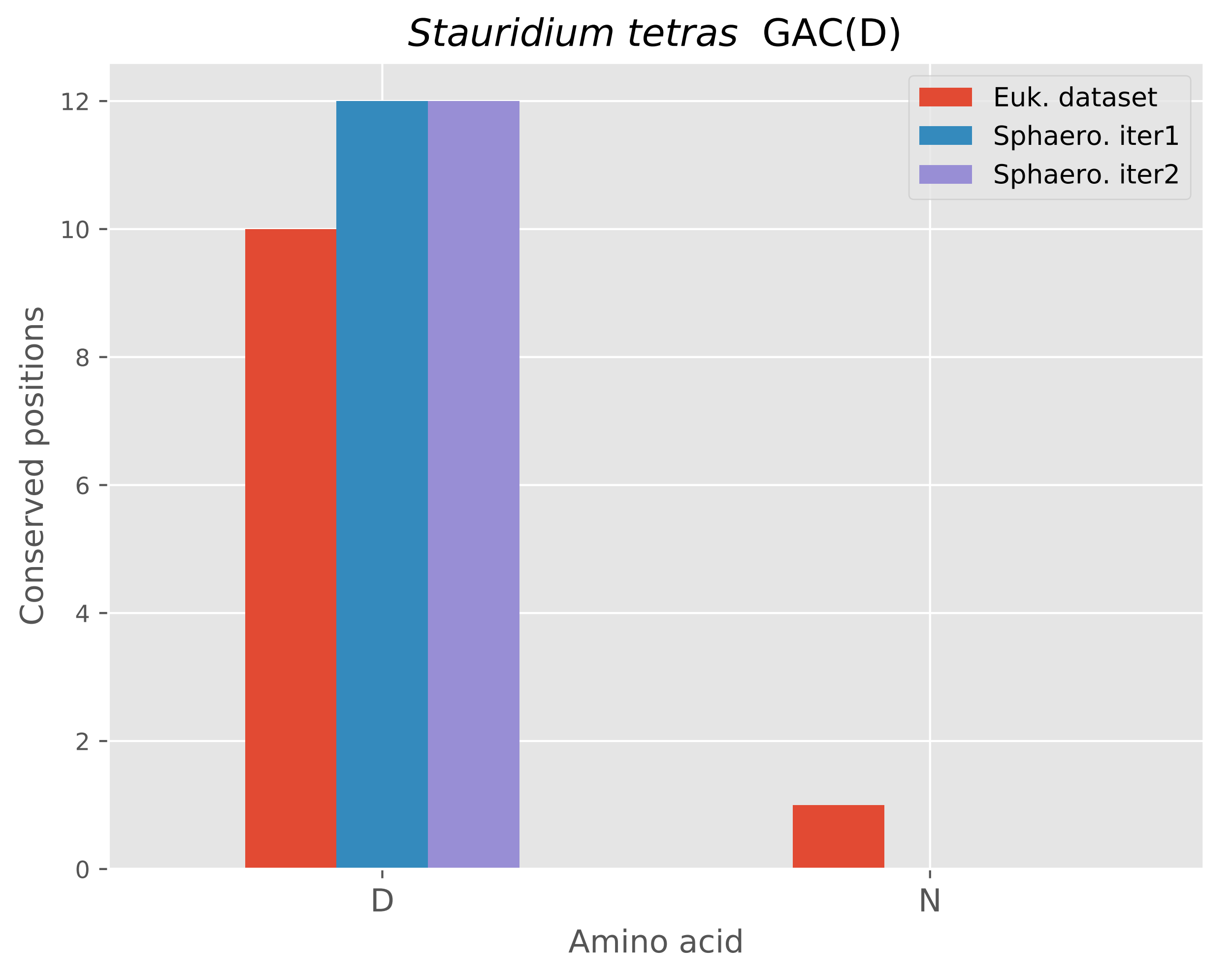

Stauridium tetras GAC(D)
Euk. dataset
Sphaero. iter1
Sphaero. iter2
Conserved positions
0
2
4
6
8
10
12
D
N
Amino acid

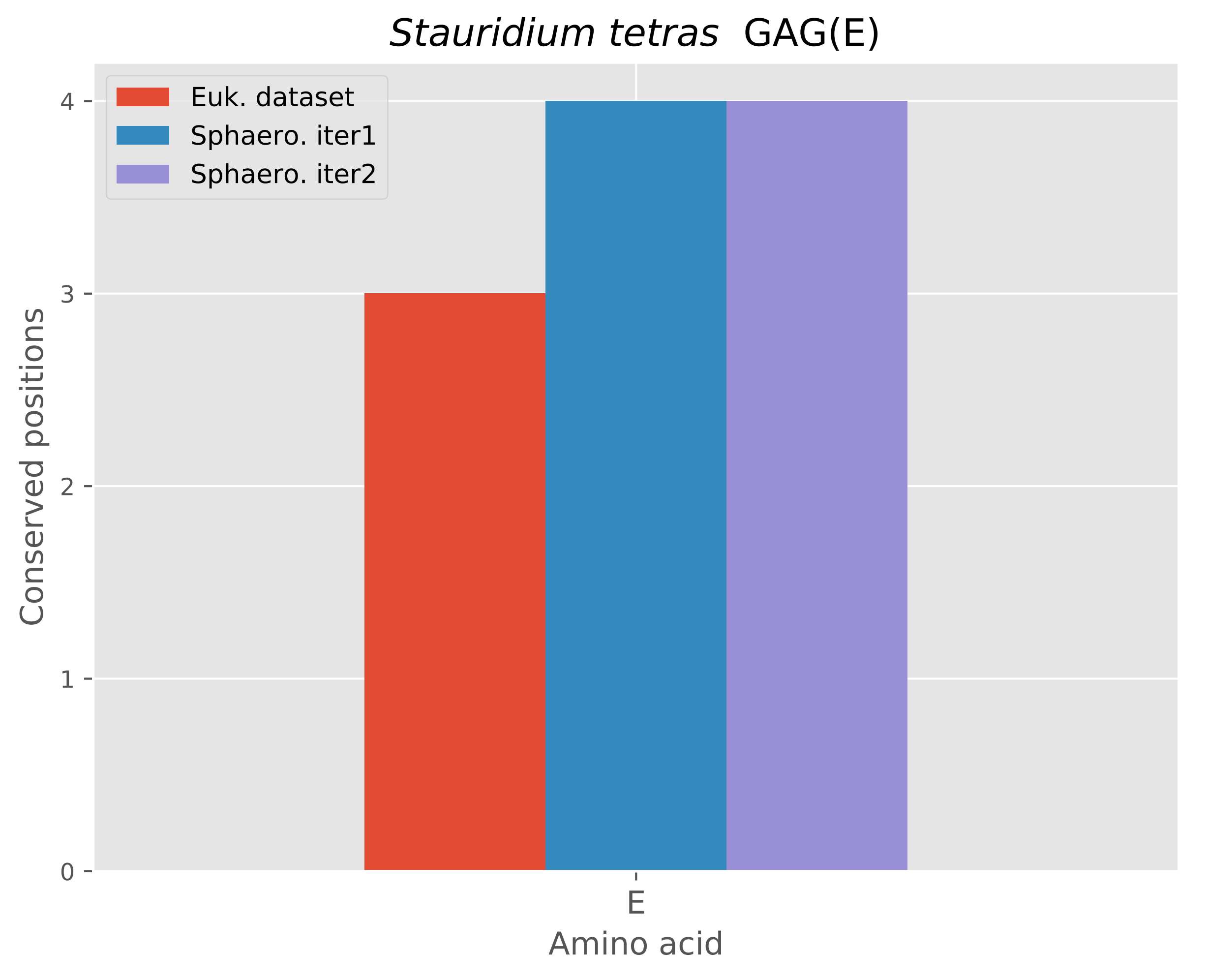

Stauridium tetras GAG(E)
Euk. dataset
Sphaero. iter1
Sphaero. iter2
Conserved positions
0
1
2
3
4
E
Amino acid

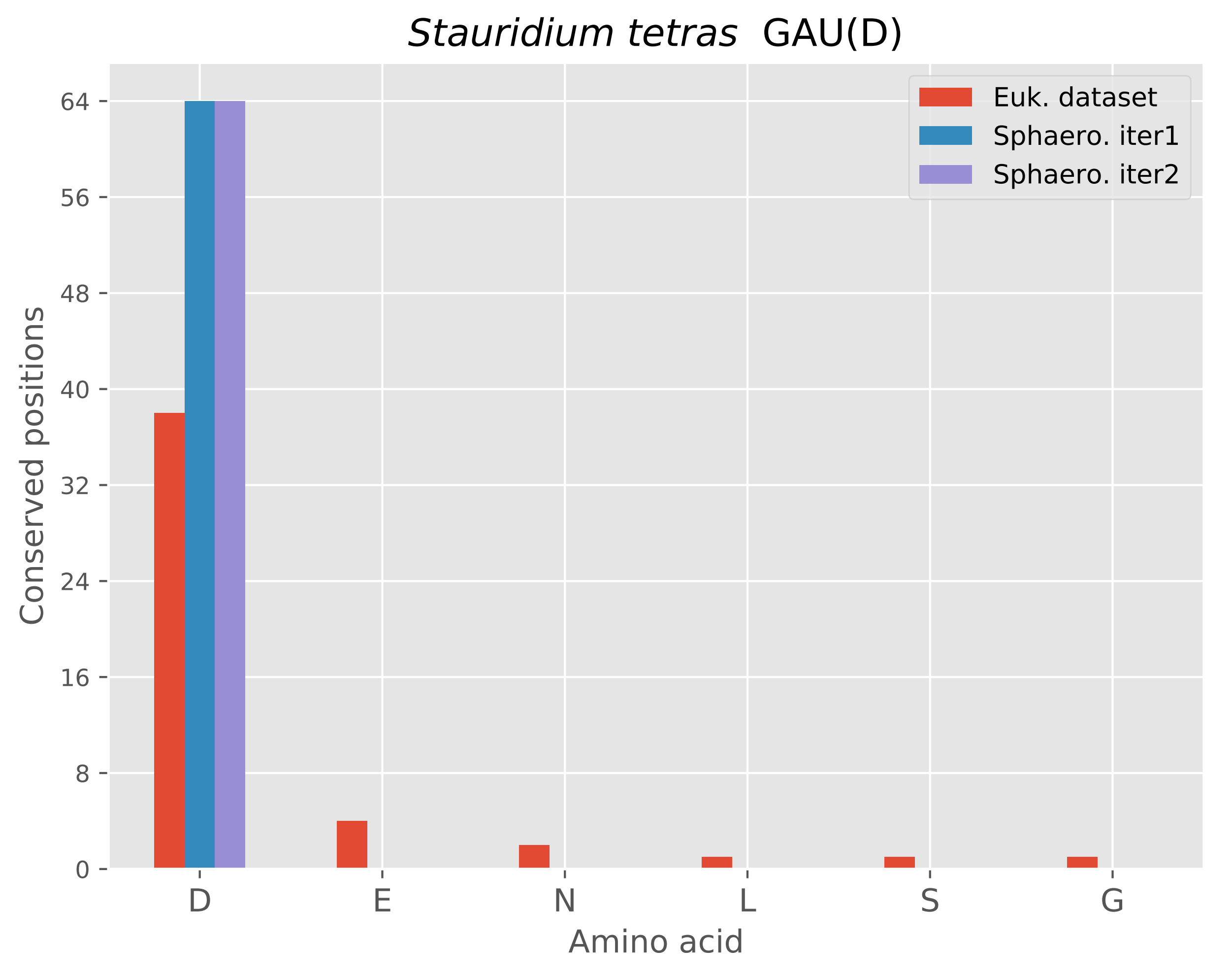

Stauridium tetras GAU(D)
Euk. dataset
Sphaero. iter1
Sphaero. iter2
Conserved positions
0
8
16
24
32
40
48
56
64
D
E
N
L
S
G
Amino acid

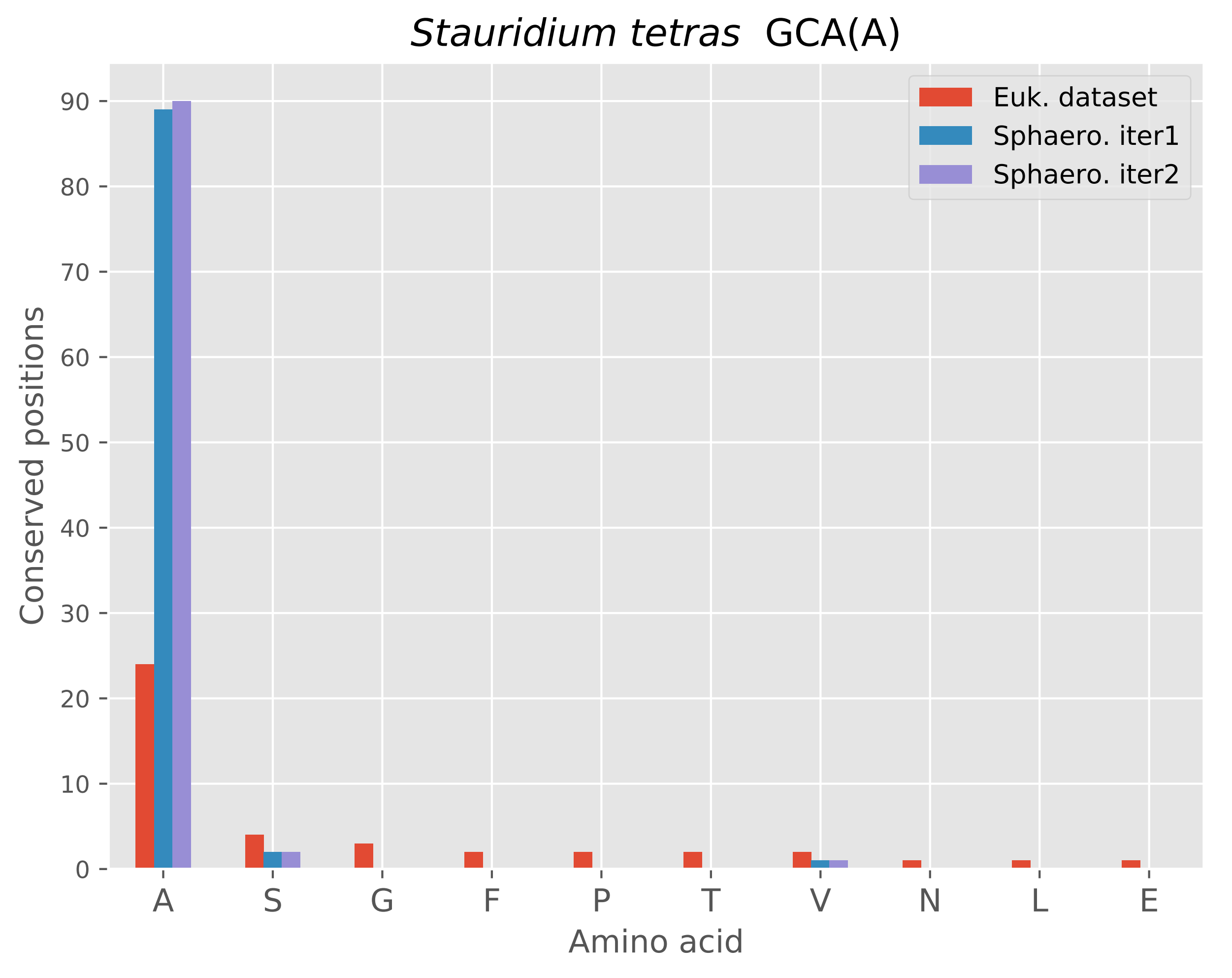

Stauridium tetras GCA(A)
Euk. dataset
Sphaero. iter1
Sphaero. iter2
Conserved positions
0
10
20
30
40
50
60
70
80
90
A
S
G
F
P
T
V
N
L
E
Amino acid

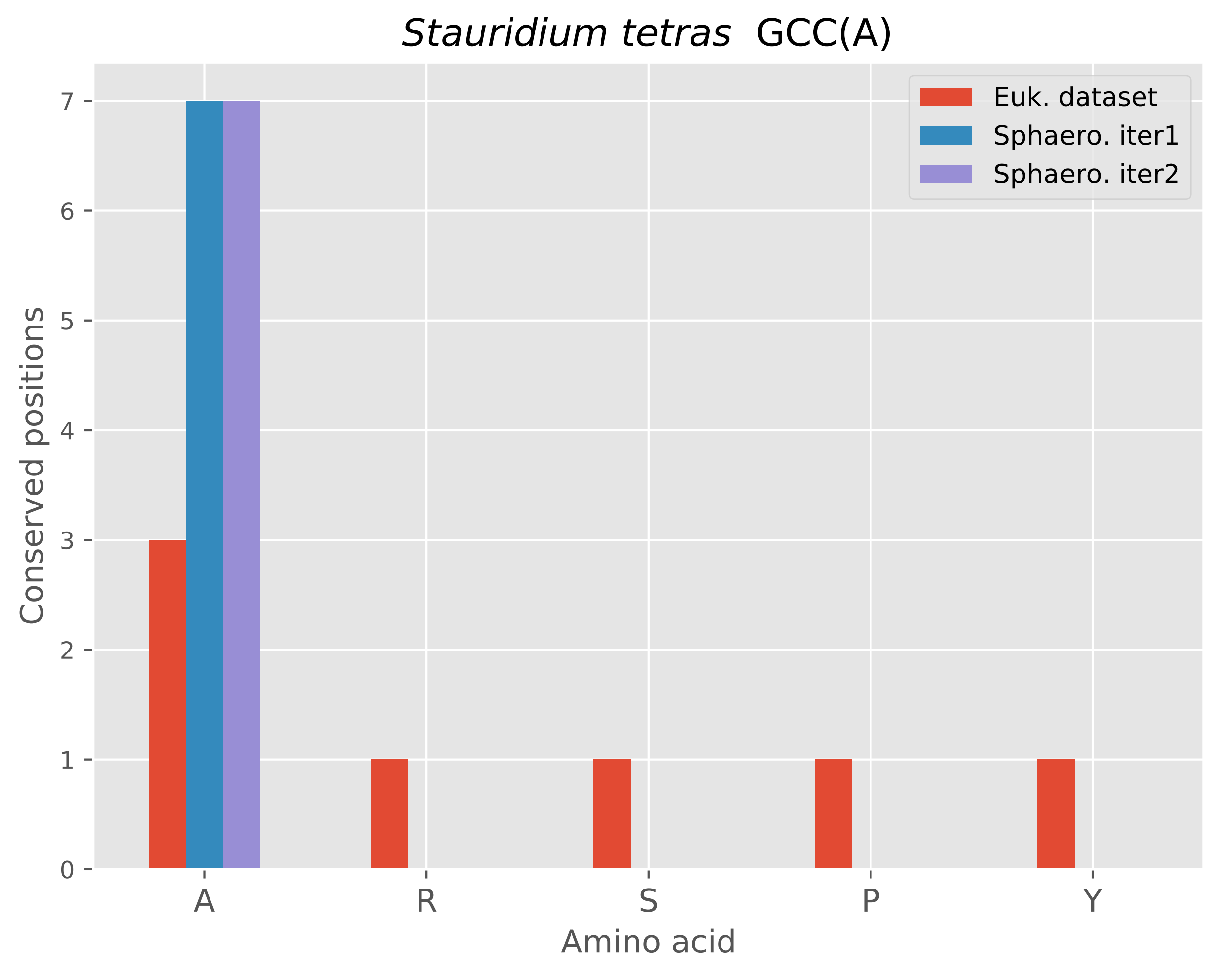

Stauridium tetras GCC(A)
Euk. dataset
Sphaero. iter1
Sphaero. iter2
Conserved positions
0
1
2
3
4
5
6
7
A
R
S
P
Y
Amino acid

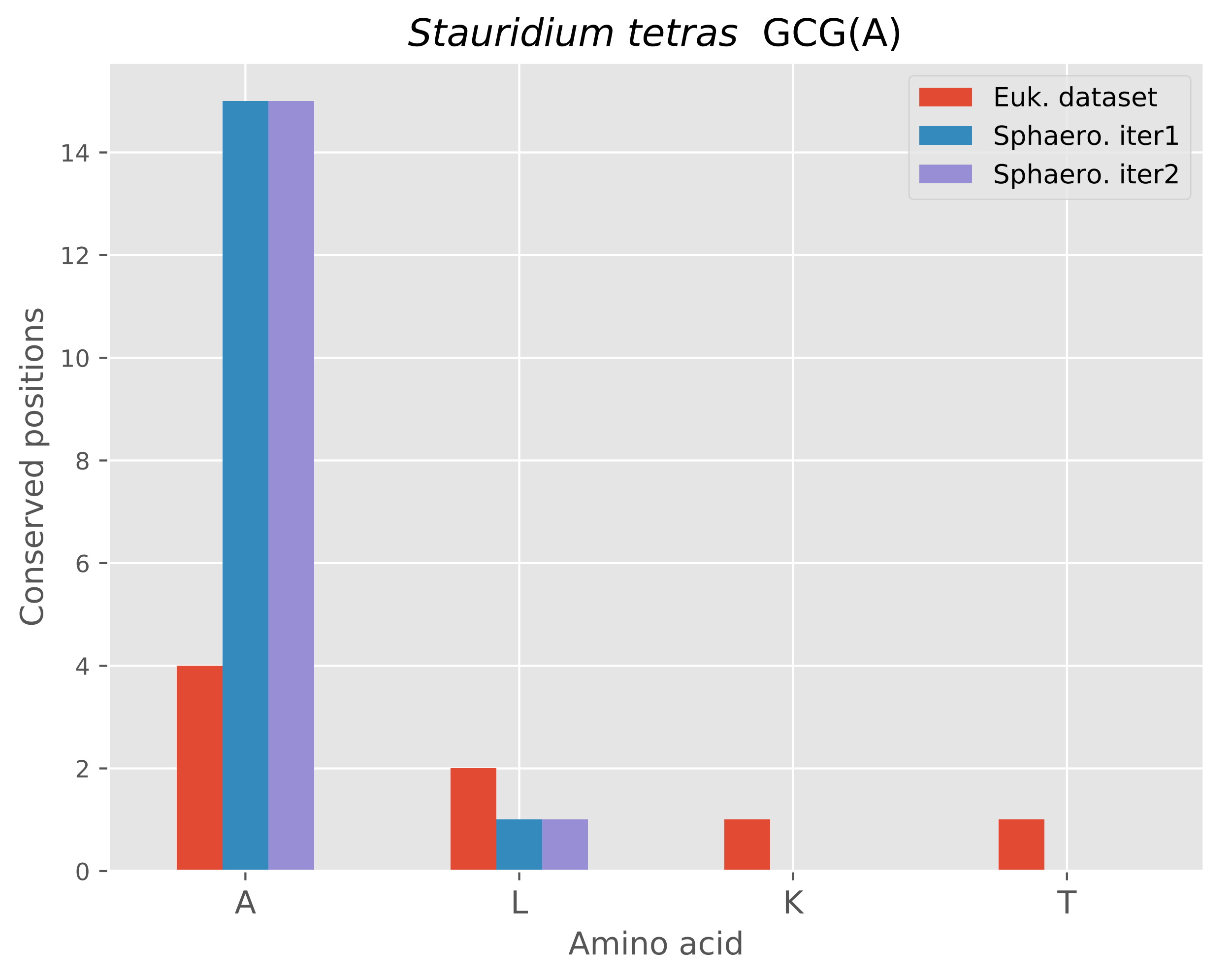

Stauridium tetras GCG(A)
Euk. dataset
Sphaero. iter1
Sphaero. iter2
Conserved positions
0
2
4
6
8
10
12
14
A
L
K
T
Amino acid

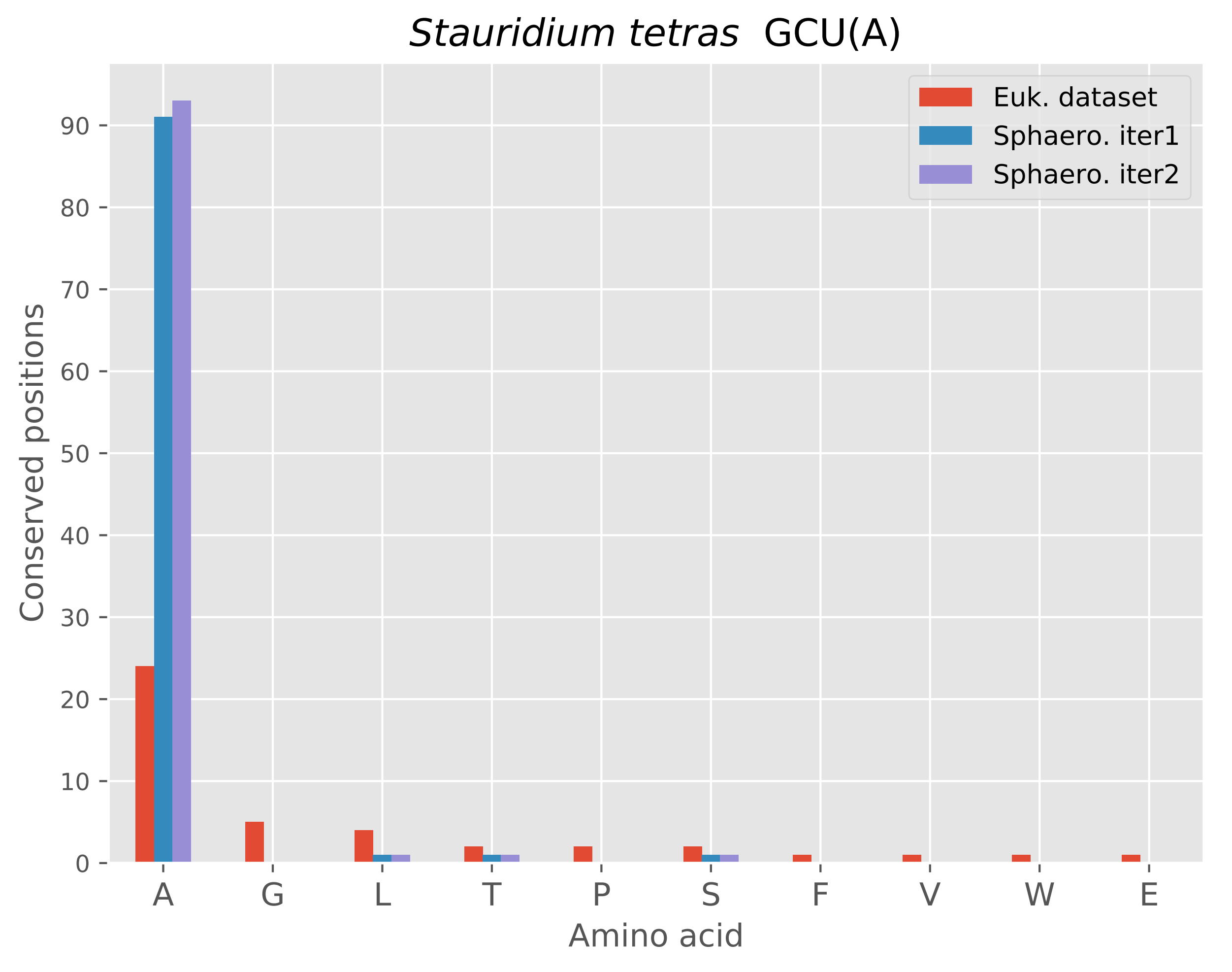
Stauridium tetras GCU(A)
Euk. dataset
Sphaero. iter1
Sphaero. iter2
Conserved positions
0
10
20
30
40
50
60
70
80
90
A
G
L
T
P
S
F
V
W
E
Amino acid

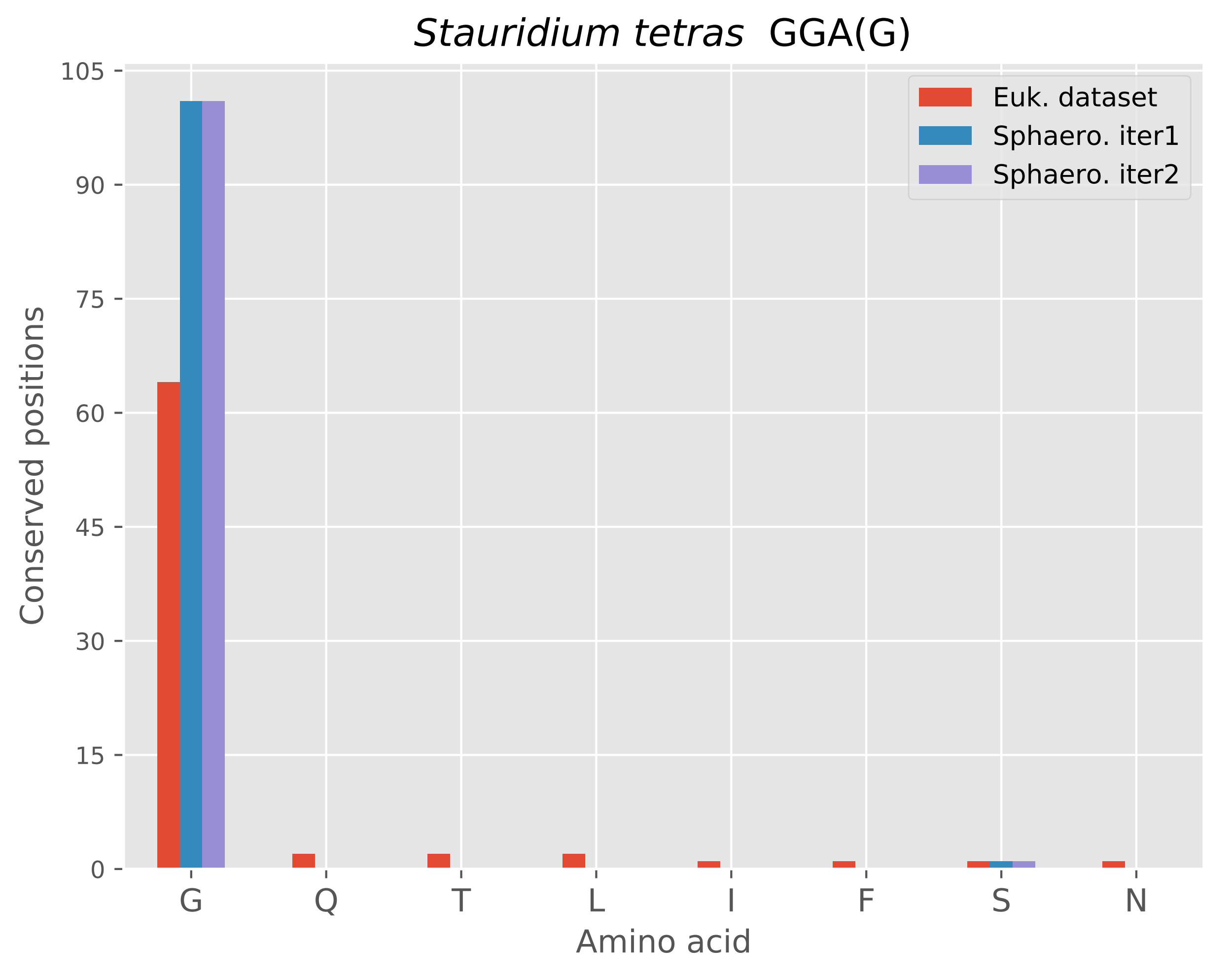

Stauridium tetras GGA(G)
Euk. dataset
Sphaero. iter1
Sphaero. iter2
Conserved positions
105
90
75
60
45
30
15
0
G
Q
T
L
I
F
S
N
Amino acid

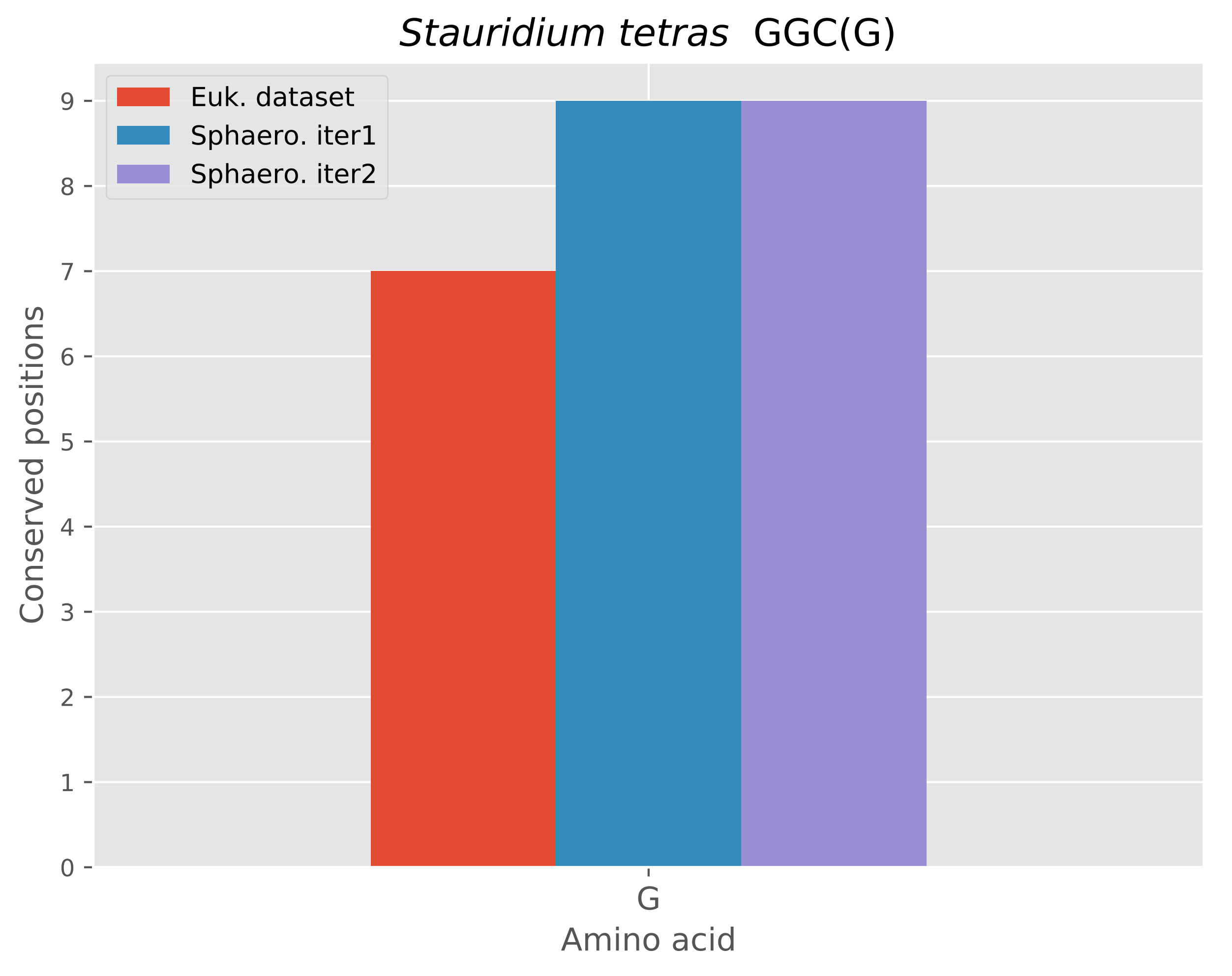

Stauridium tetras GGC(G)
Euk. dataset
Sphaero. iter1
Sphaero. iter2
Conserved positions
0
1
2
3
4
5
6
7
8
9
G
Amino acid

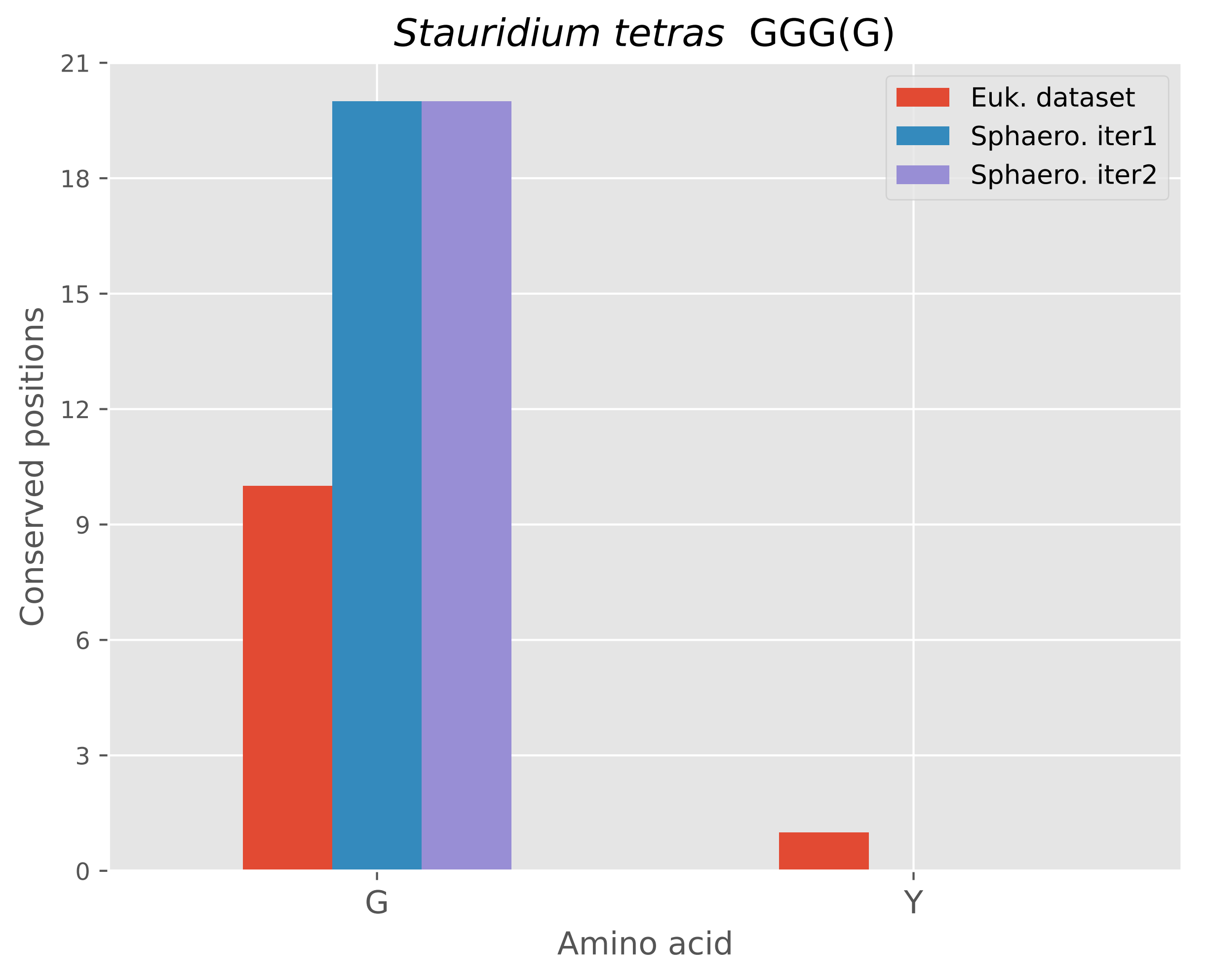
Stauridium tetras GGG(G)
Conserved positions
0
3
6
9
12
15
18
21
G
Y
Amino acid
Euk. dataset
Sphaero. iter1
Sphaero. iter2

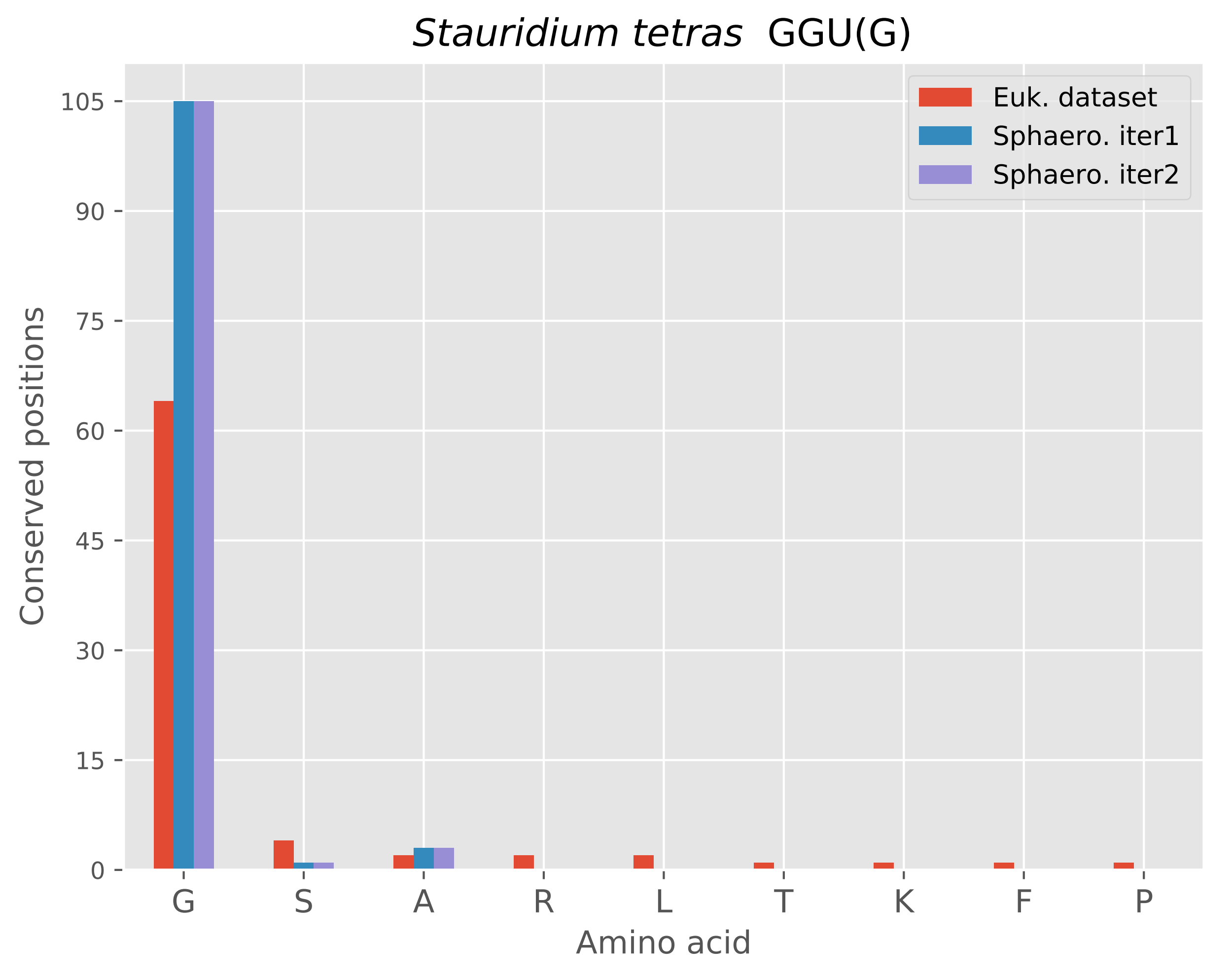

Stauridium tetras GGU(G)
Euk. dataset
Sphaero. iter1
Sphaero. iter2
Conserved positions
0
15
30
45
60
75
90
105
G
S
A
R
L
T
K
F
P
Amino acid

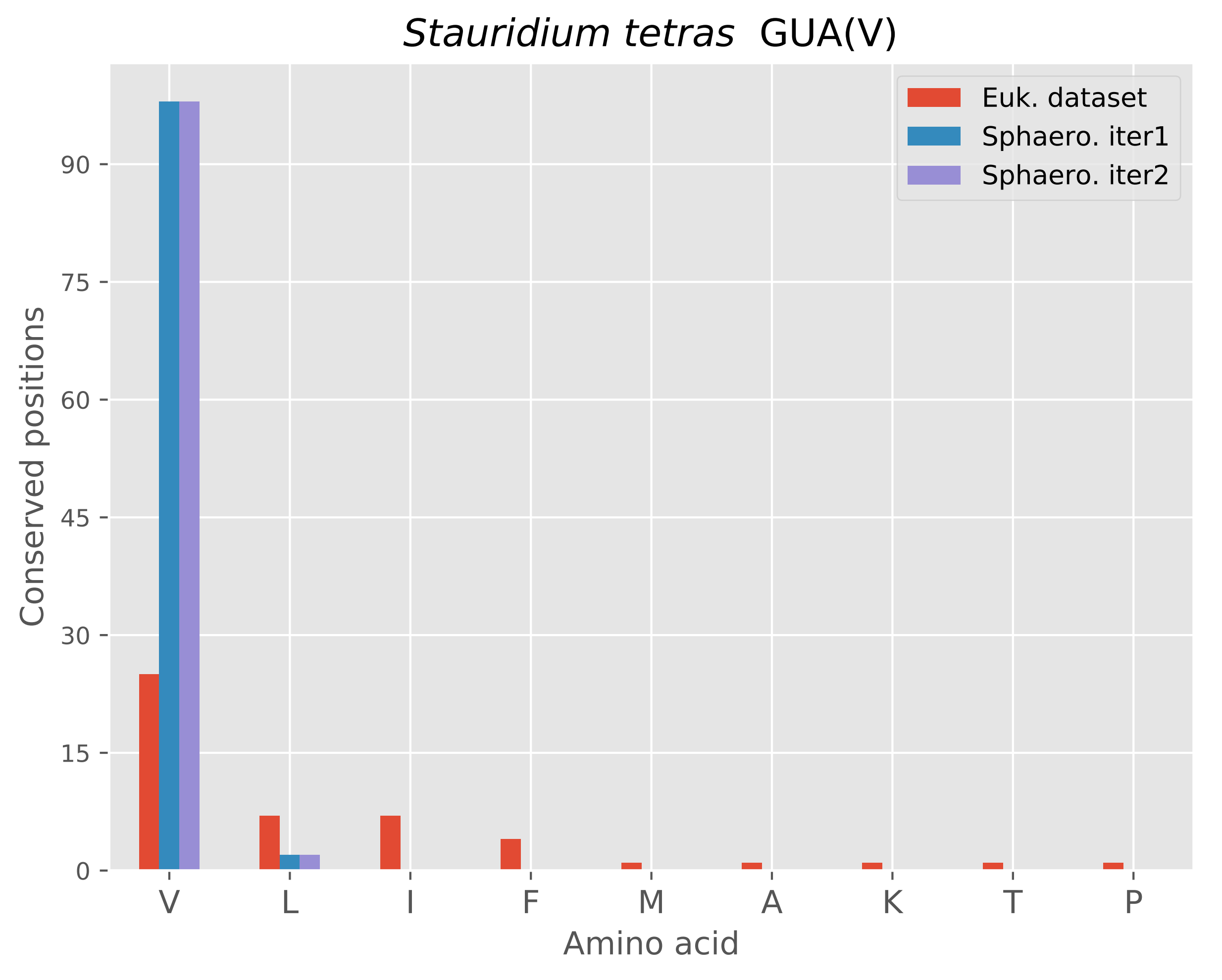

Stauridium tetras GUA(V)
Euk. dataset
Sphaero. iter1
Sphaero. iter2
Conserved positions
0
15
30
45
60
75
90
V
L
I
F
M
A
K
T
P
Amino acid

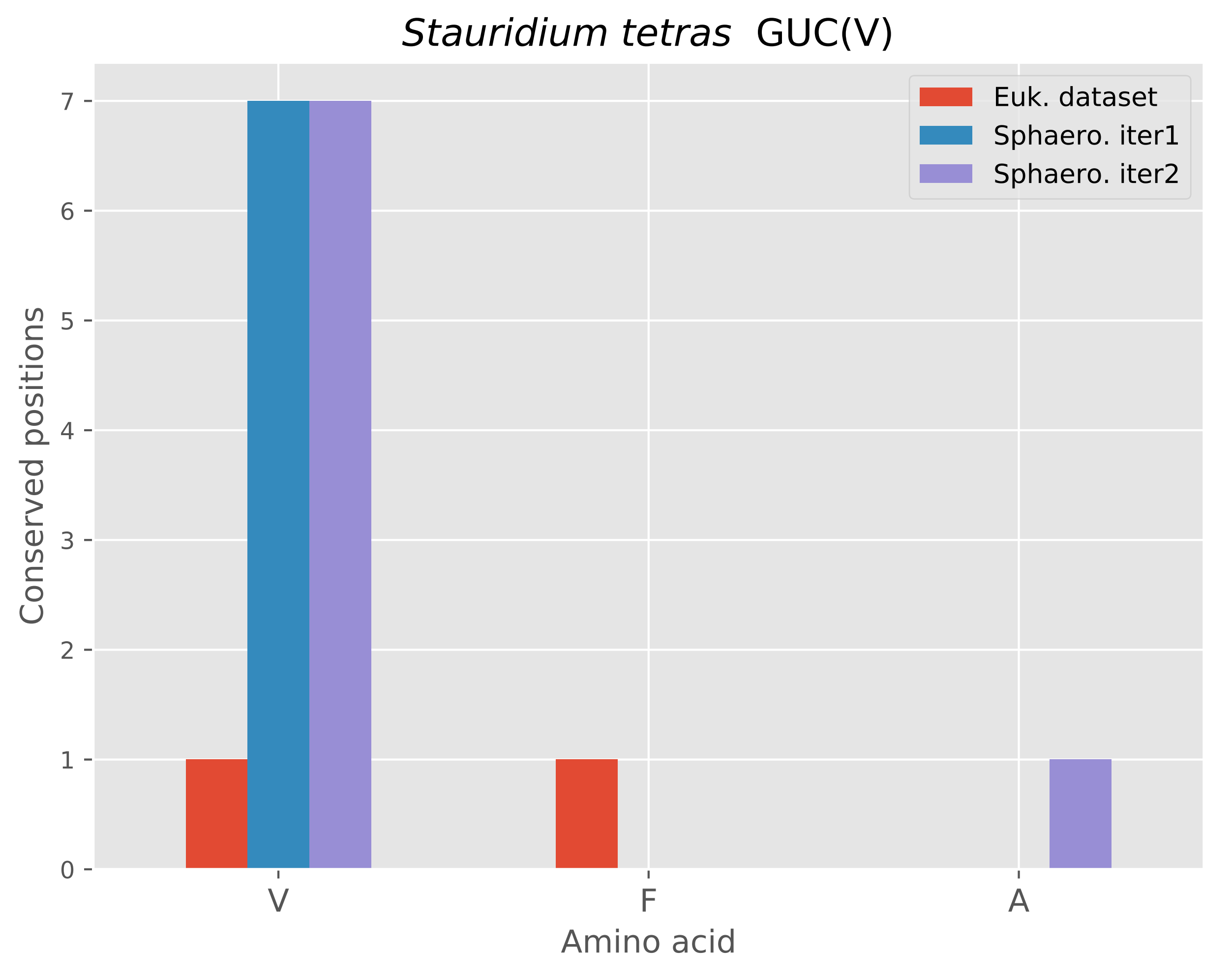

Stauridium tetras GUC(V)
Euk. dataset
Sphaero. iter1
Sphaero. iter2
Conserved positions
0
1
2
3
4
5
6
7
V
F
A
Amino acid

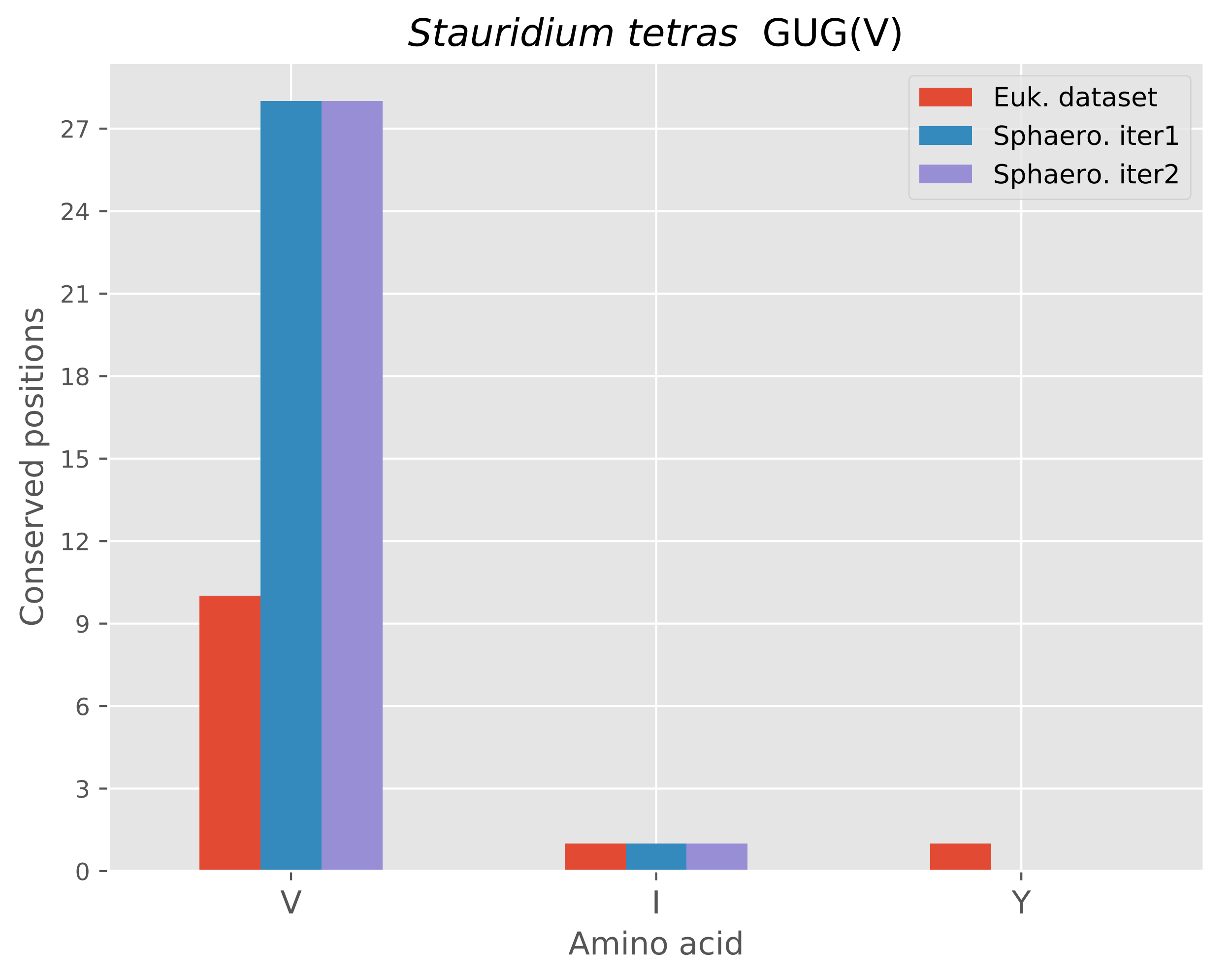
Stauridium tetras GUG(V)
Euk. dataset
Sphaero. iter1
Sphaero. iter2
Conserved positions
0
3
6
9
12
15
18
21
24
27
V
I
Y
Amino acid

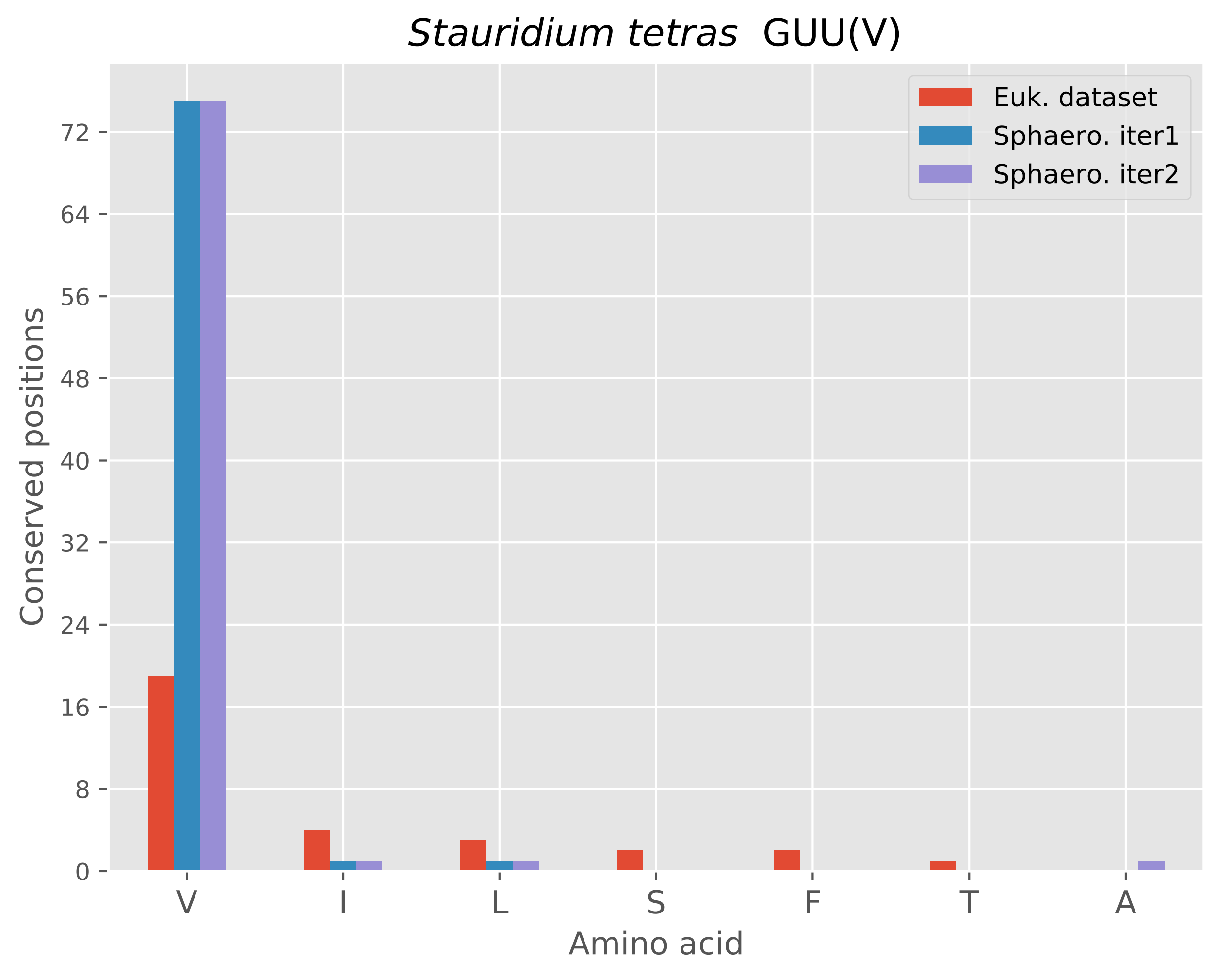

Stauridium tetras GUU(V)
Euk. dataset
Sphaero. iter1
Sphaero. iter2
Conserved positions
0
8
16
24
32
40
48
56
64
72
V
I
L
S
F
T
A
Amino acid

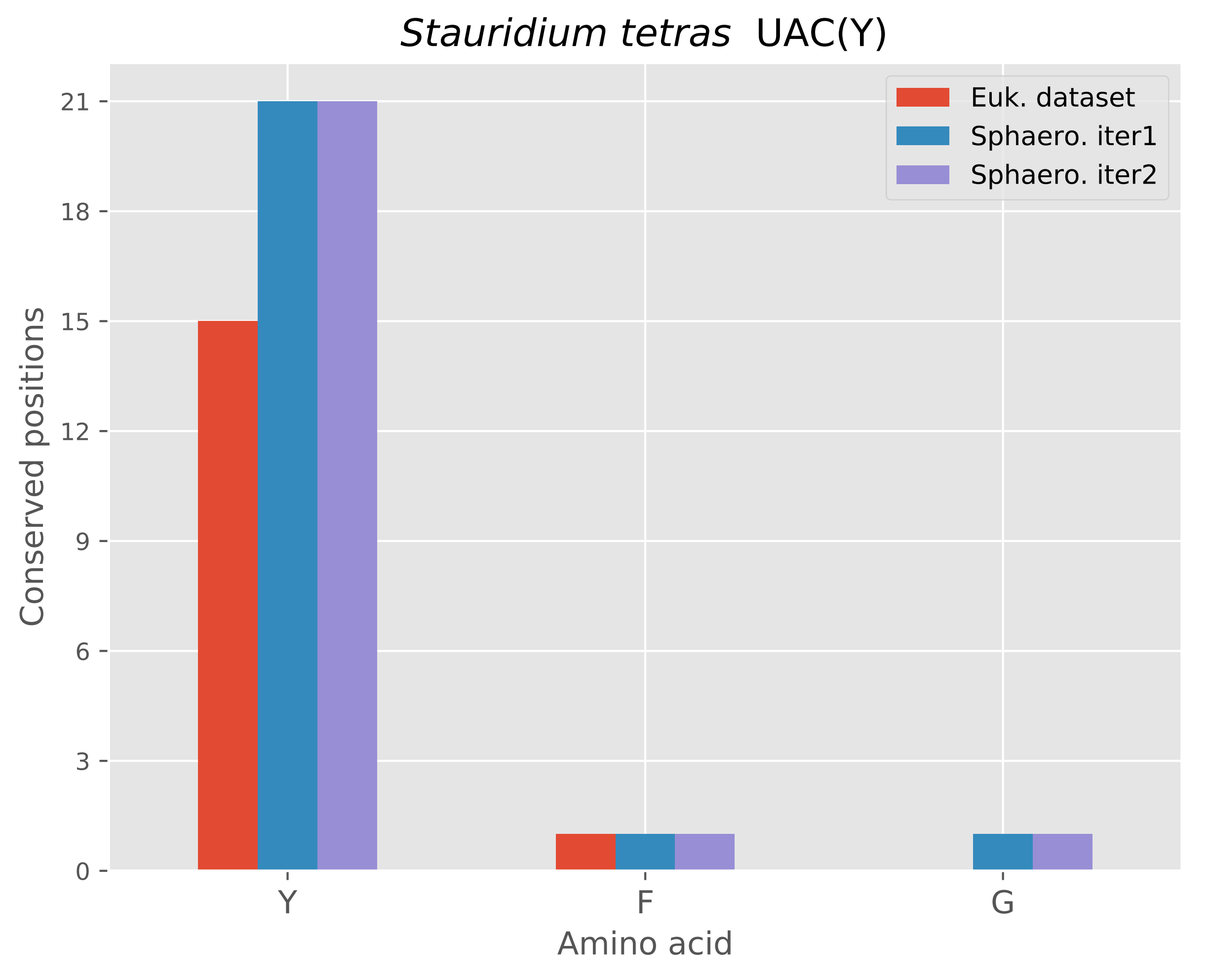

Stauridium tetras UAC(Y)
Euk. dataset
Sphaero. iter1
Sphaero. iter2
Conserved positions
0
3
6
9
12
15
18
21
Y
F
G
Amino acid

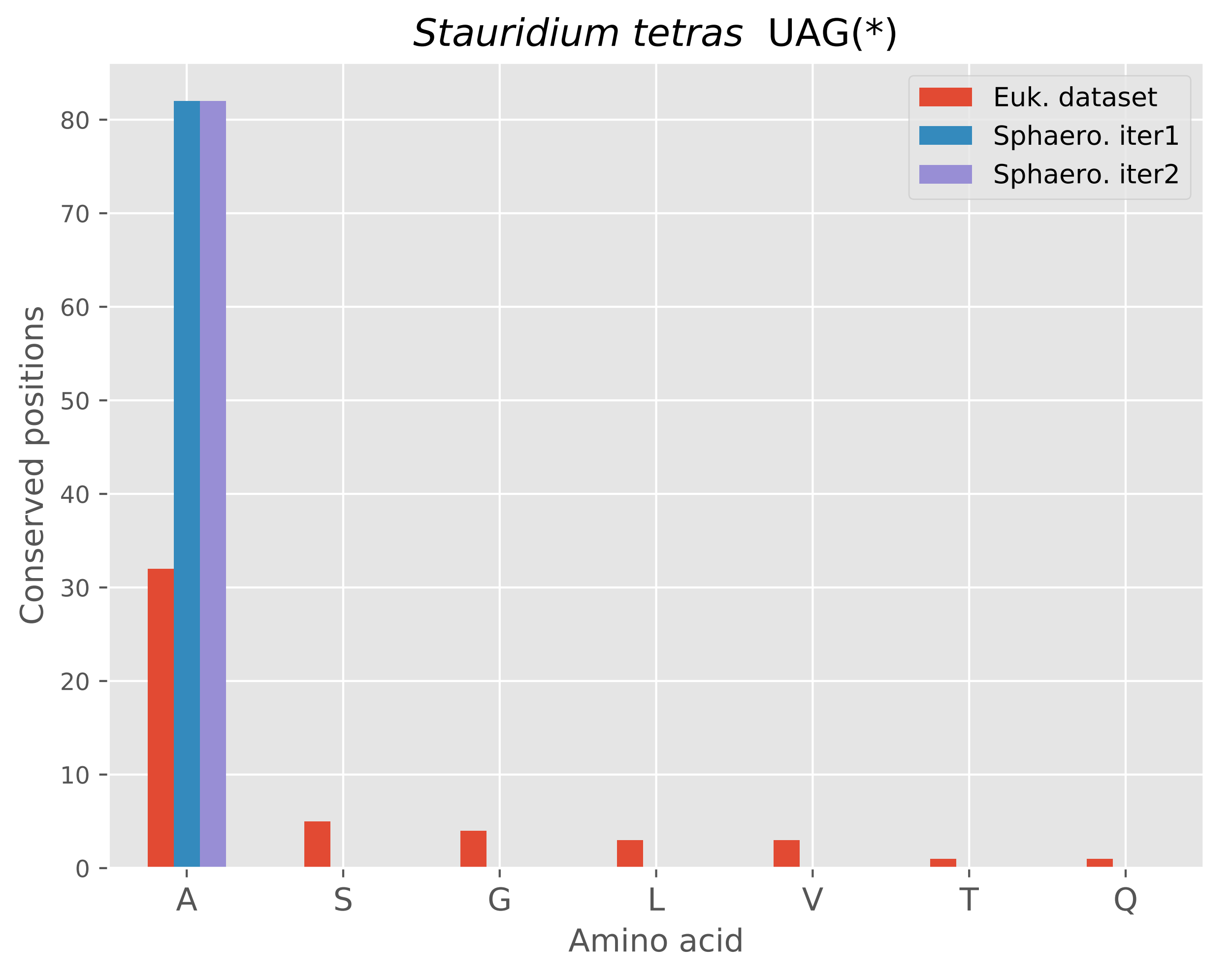

Stauridium tetras UAG(*)
Euk. dataset
Sphaero. iter1
Sphaero. iter2
Conserved positions
0
10
20
30
40
50
60
70
80
A
S
G
L
V
T
Q
Amino acid

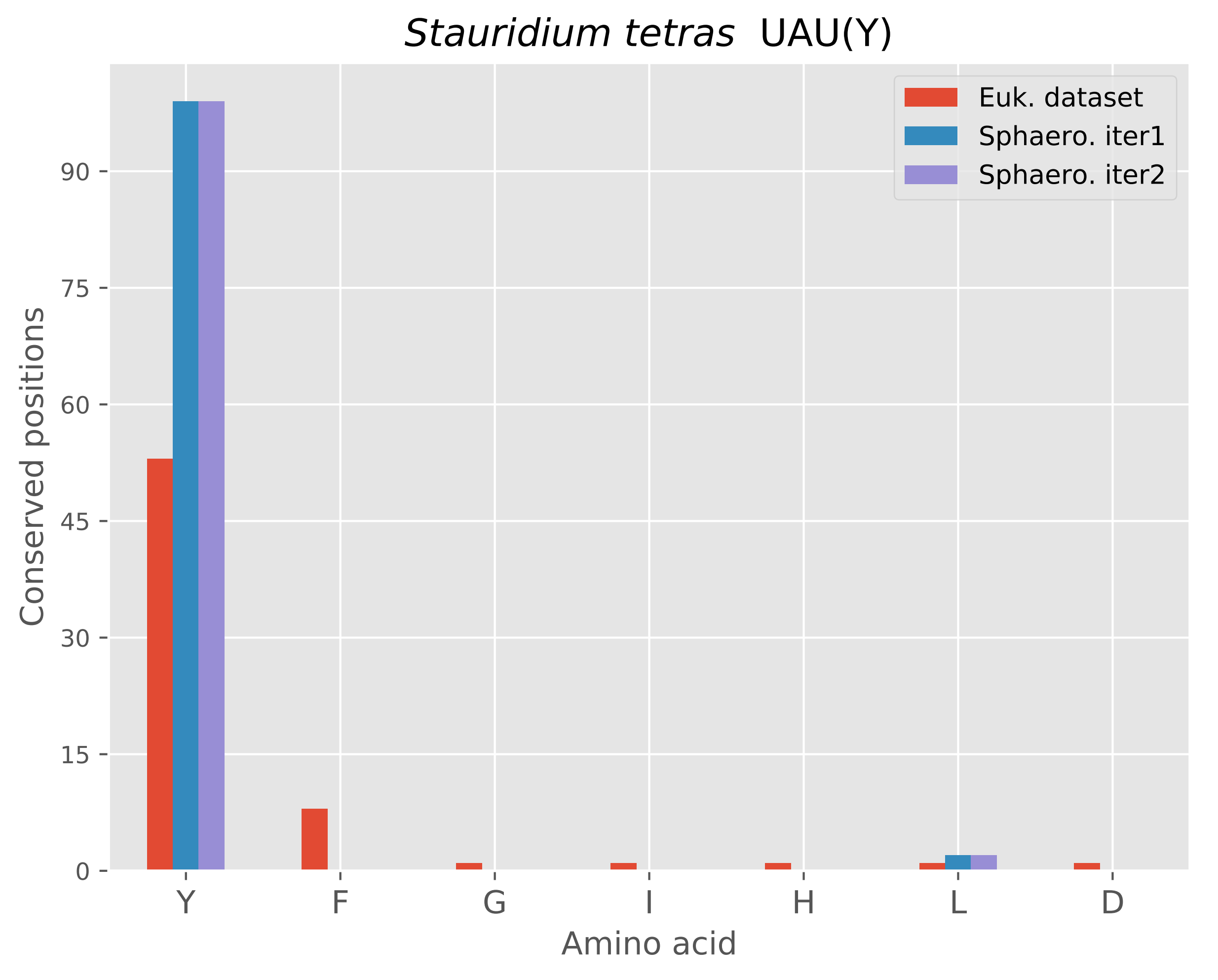

Stauridium tetras UAU(Y)
Euk. dataset
Sphaero. iter1
Sphaero. iter2
Conserved positions
0
15
30
45
60
75
90
Y
F
G
I
H
L
D
Amino acid

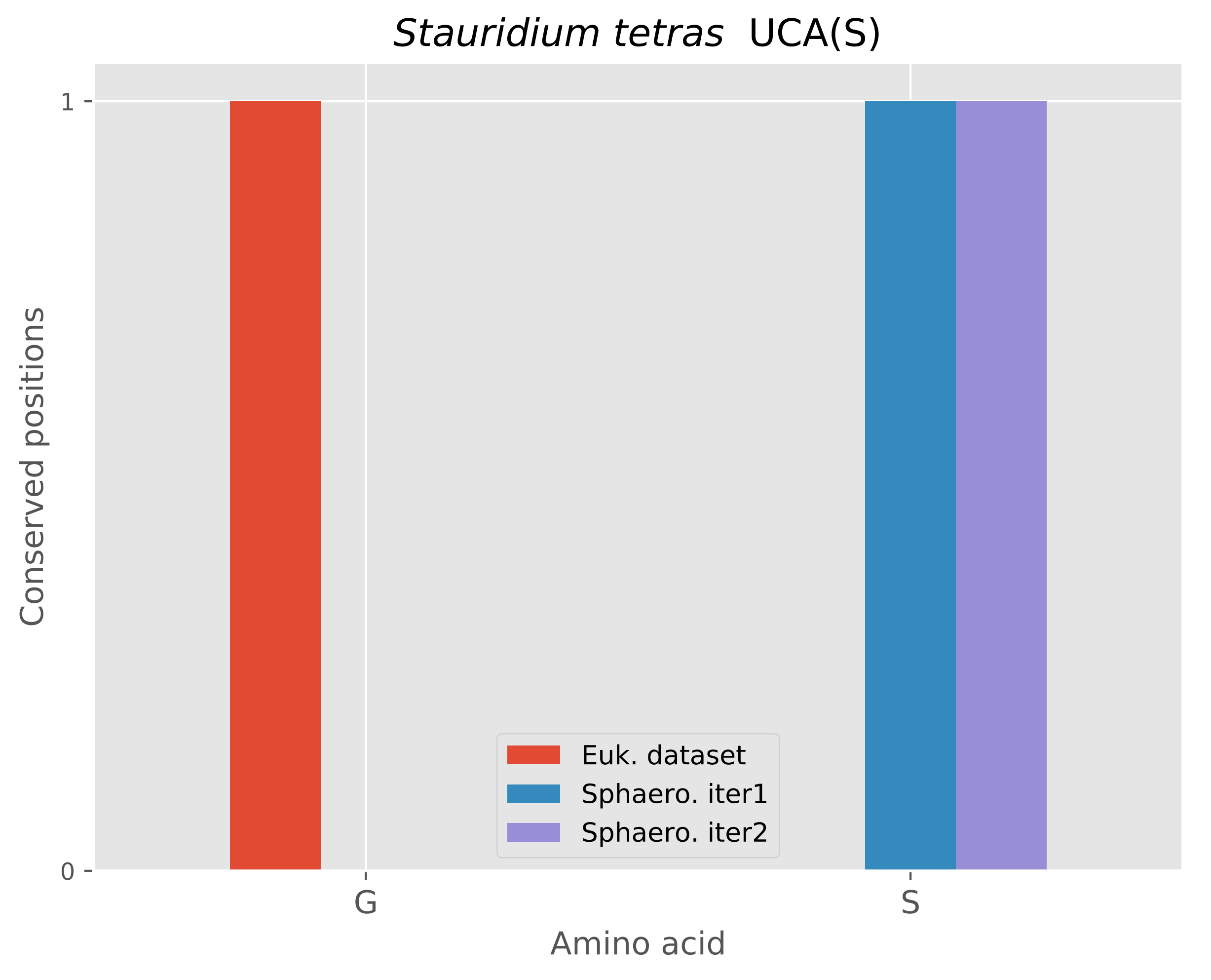

Stauridium tetras UCA(S)
Conserved positions
1
0
G
S
Amino acid
Euk. dataset
Sphaero. iter1
Sphaero. iter2

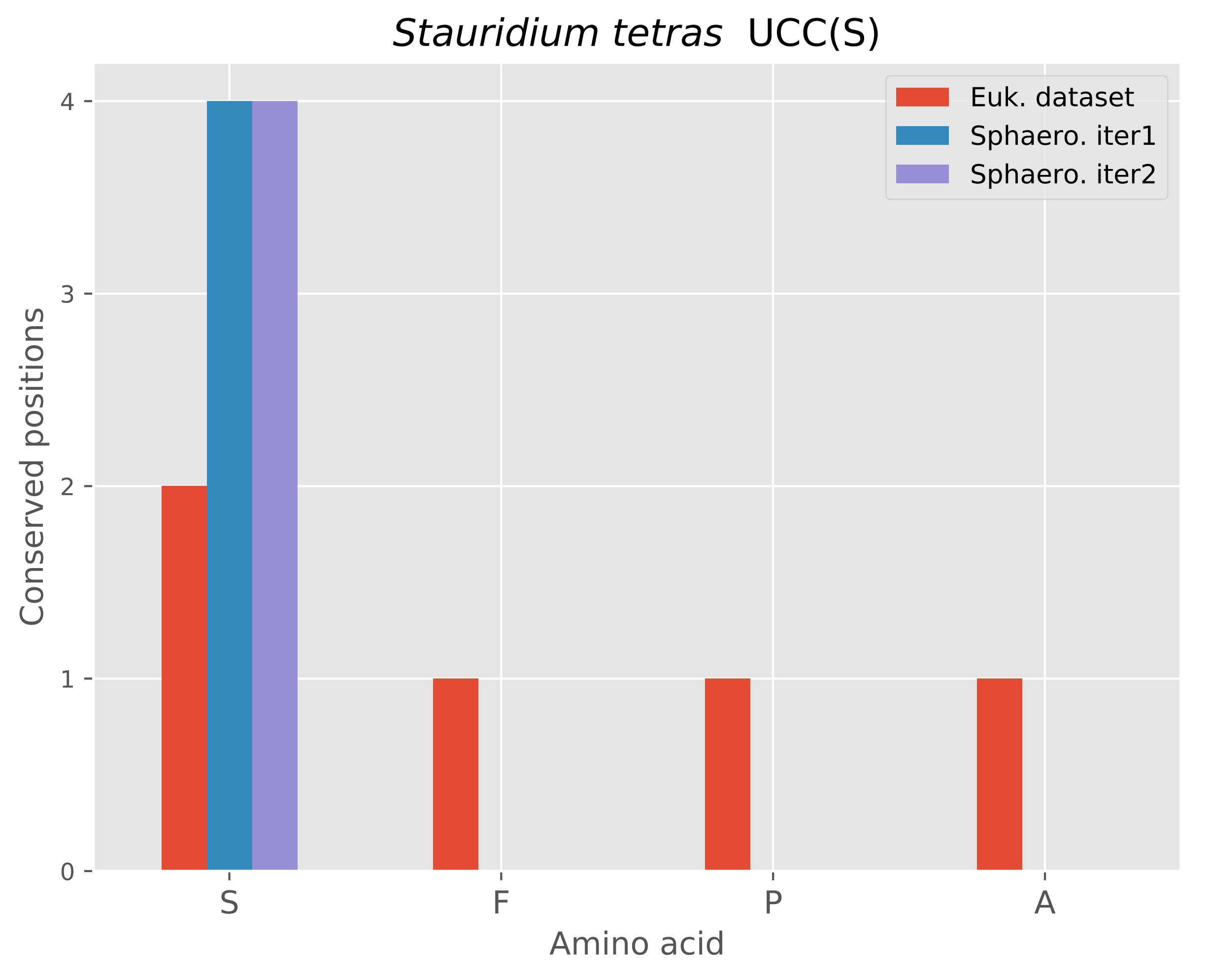

Stauridium tetras UCC(S)
Euk. dataset
Sphaero. iter1
Sphaero. iter2
Conserved positions
0
1
2
3
4
S
F
P
A
Amino acid

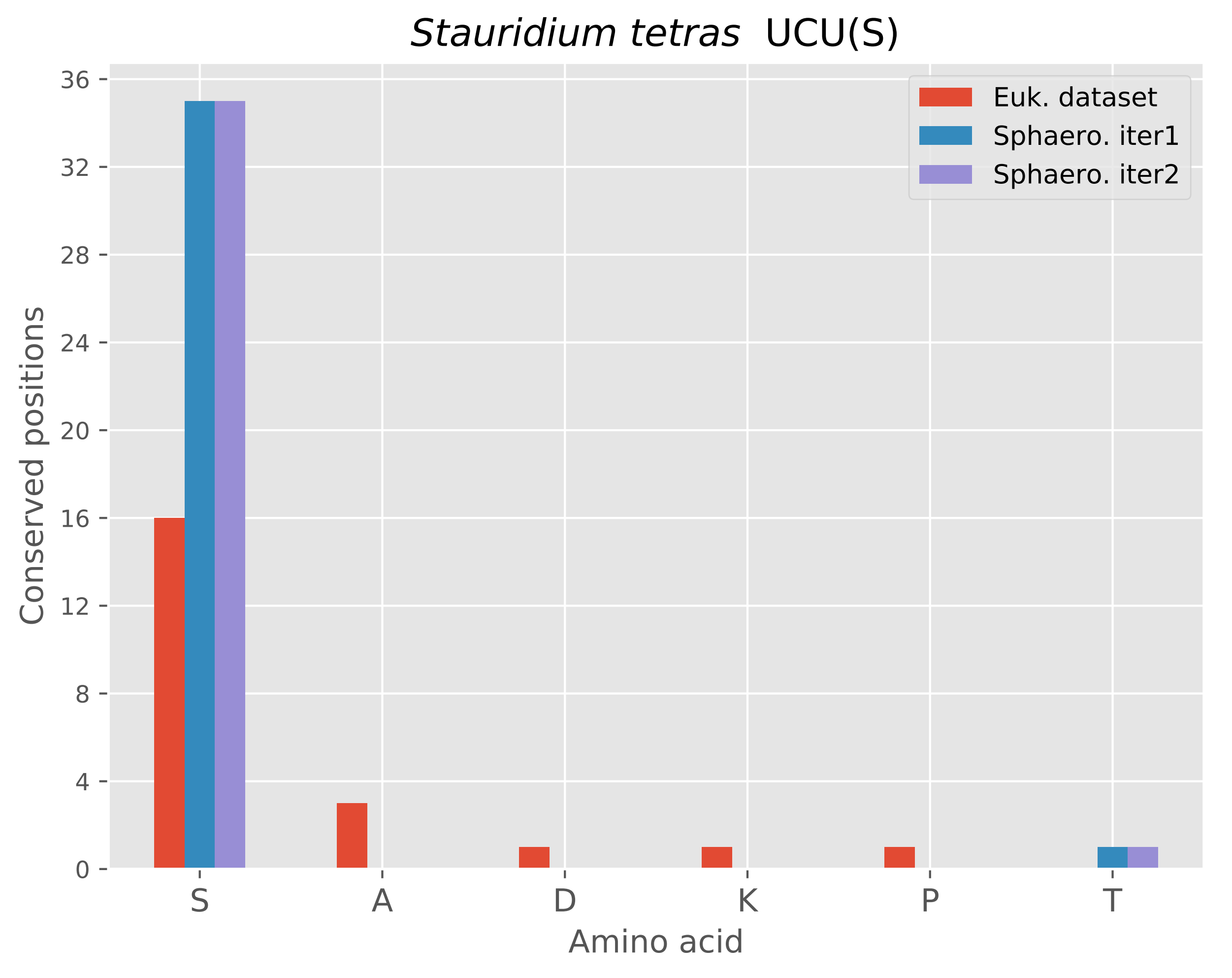

Stauridium tetras UCU(S)
Euk. dataset
Sphaero. iter1
Sphaero. iter2
Conserved positions
0
4
8
12
16
20
24
28
32
36
S
A
D
K
P
T
Amino acid

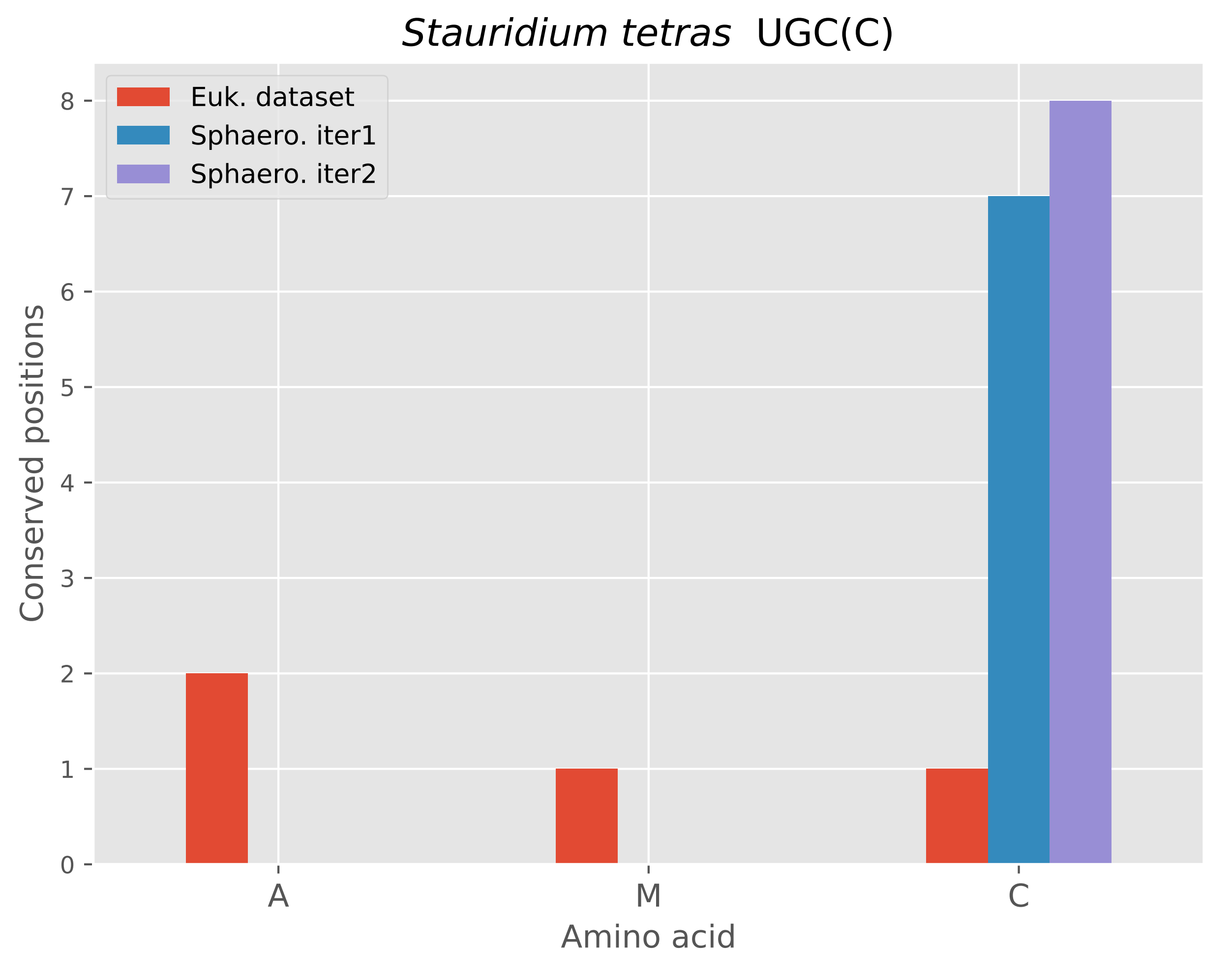

Stauridium tetras UGC(C)
Euk. dataset
Sphaero. iter1
Sphaero. iter2
Conserved positions
0
1
2
3
4
5
6
7
8
A
M
C
Amino acid

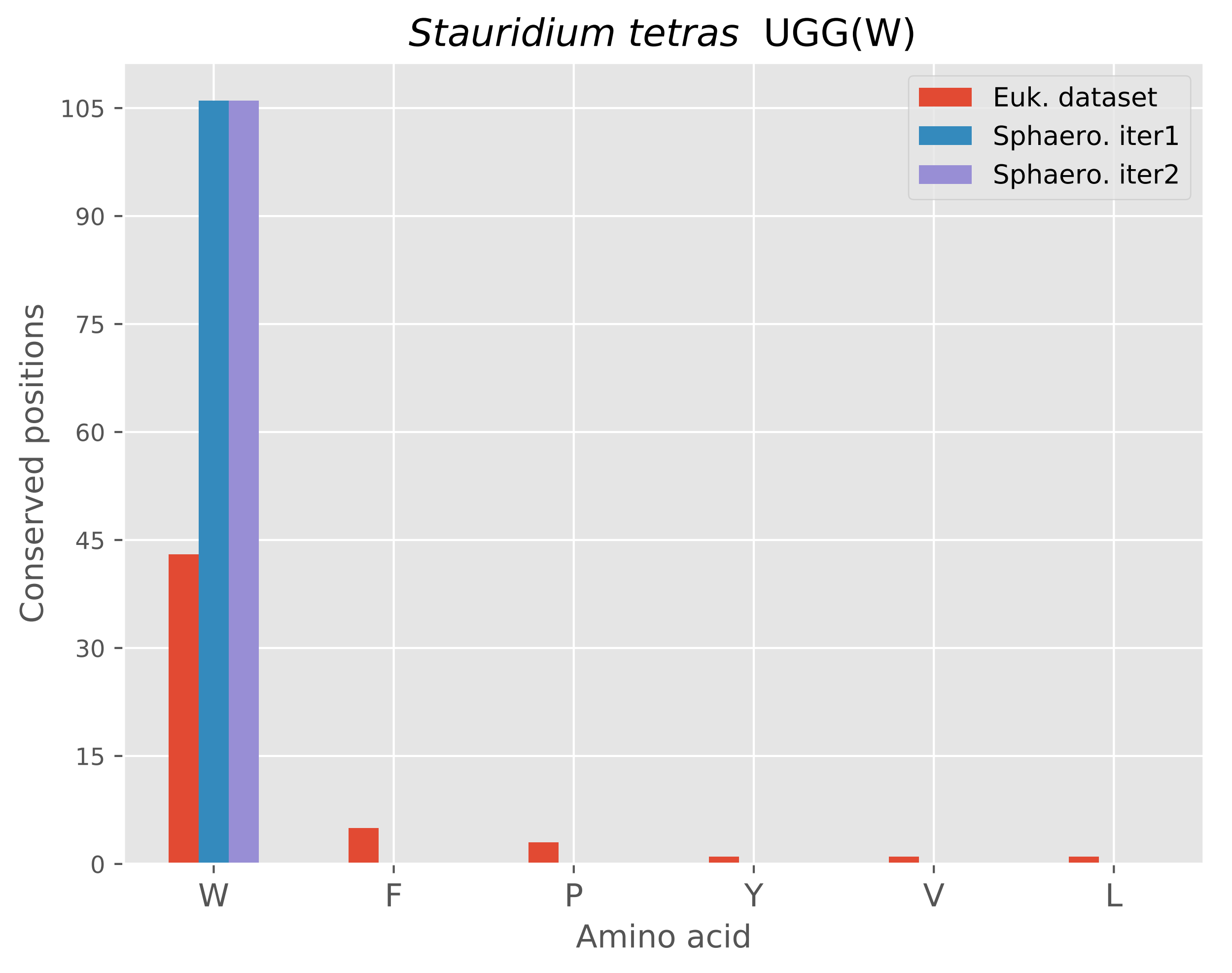

Stauridium tetras UGG(W)
Euk. dataset
Sphaero. iter1
Sphaero. iter2
Conserved positions
0
15
30
45
60
75
90
105
W
F
P
Y
V
L
Amino acid

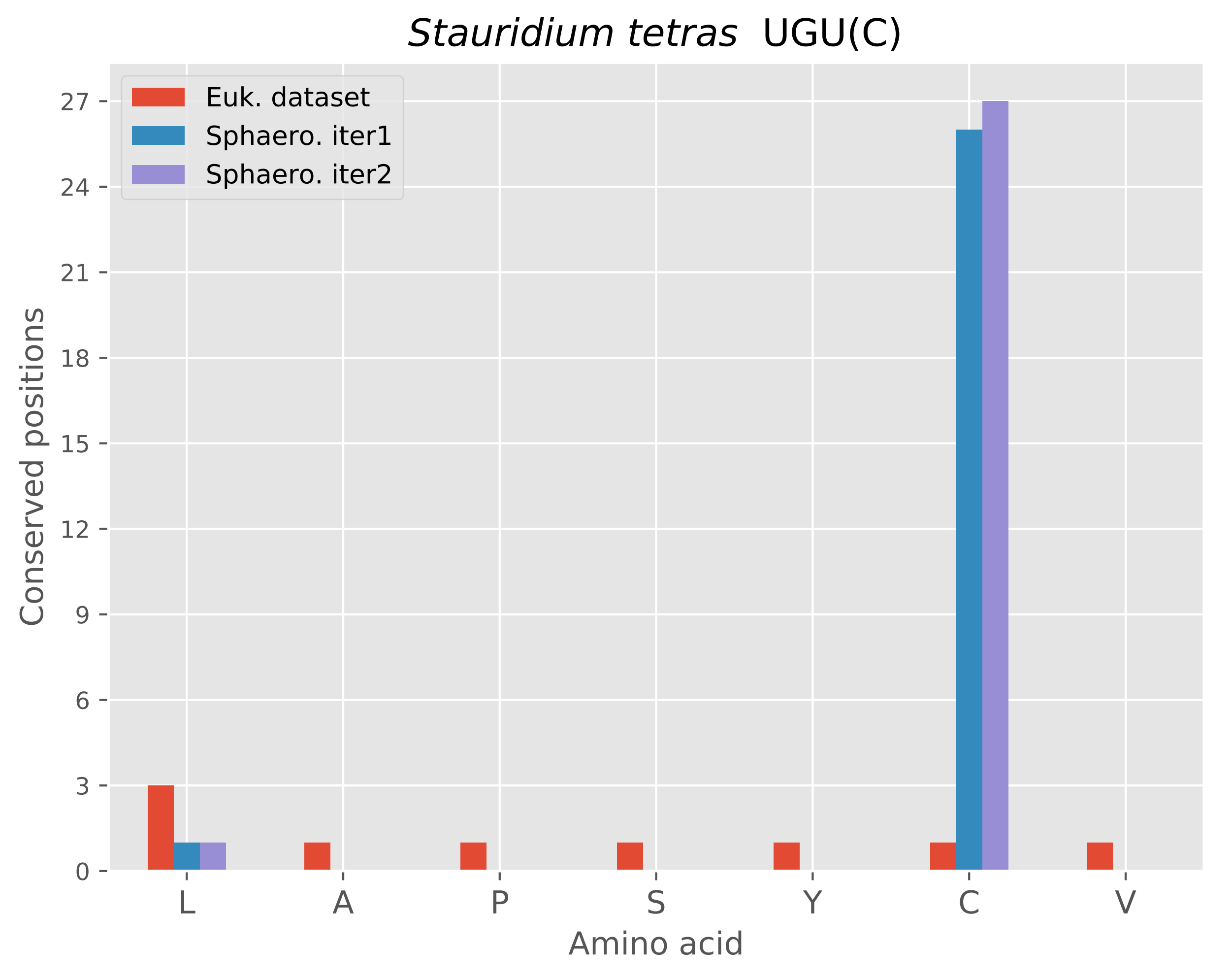

Stauridium tetras UGU(C)
Euk. dataset
Sphaero. iter1
Sphaero. iter2
Conserved positions
0
3
6
9
12
15
18
21
24
27
L
A
P
S
Y
C
V
Amino acid

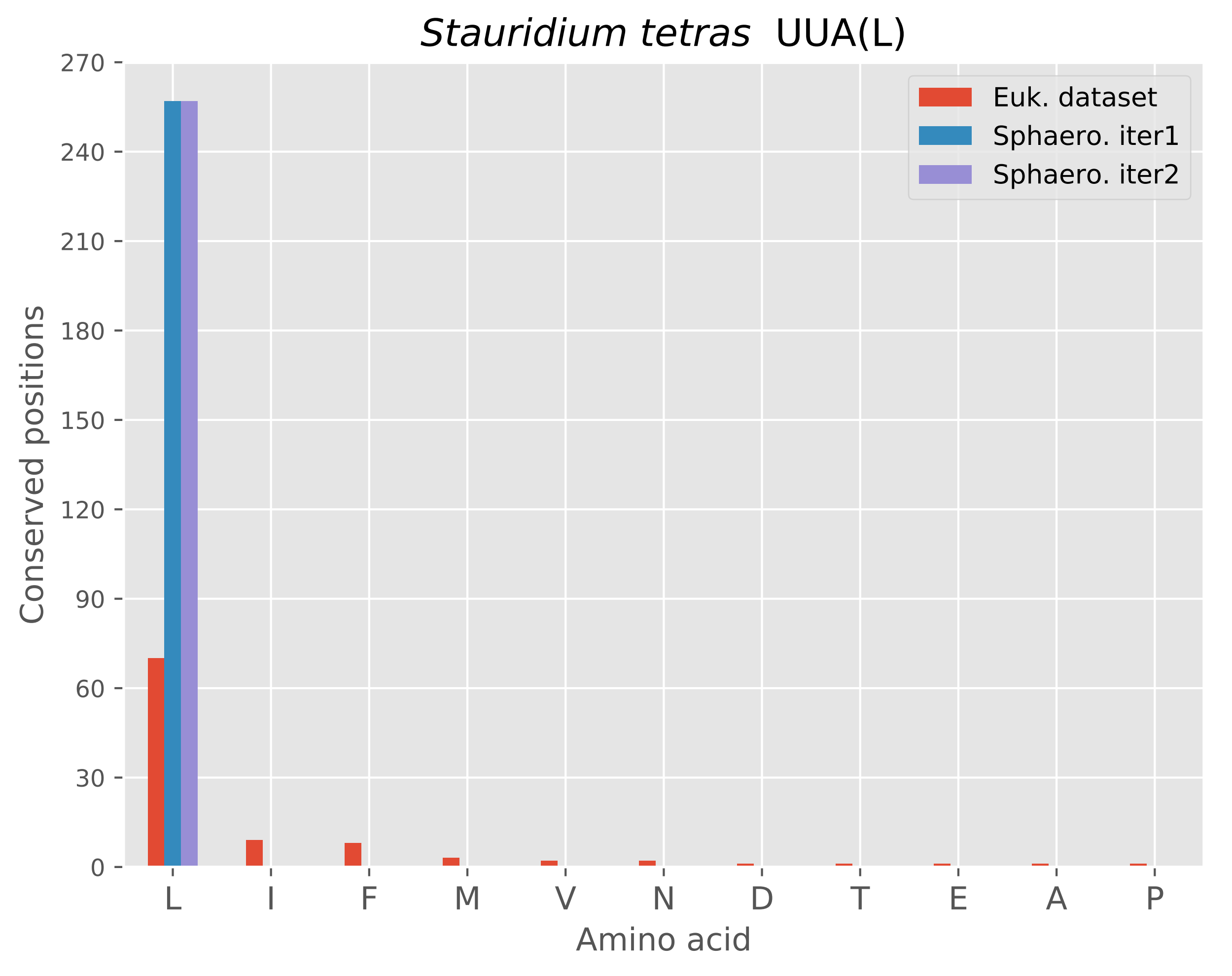
Stauridium tetras UUA(L)
Conserved positions
Amino acid
Euk. dataset
Sphaero. iter1
Sphaero. iter2
0
30
60
90
120
150
180
210
240
270
L
I
F
M
V
N
D
T
E
A
P

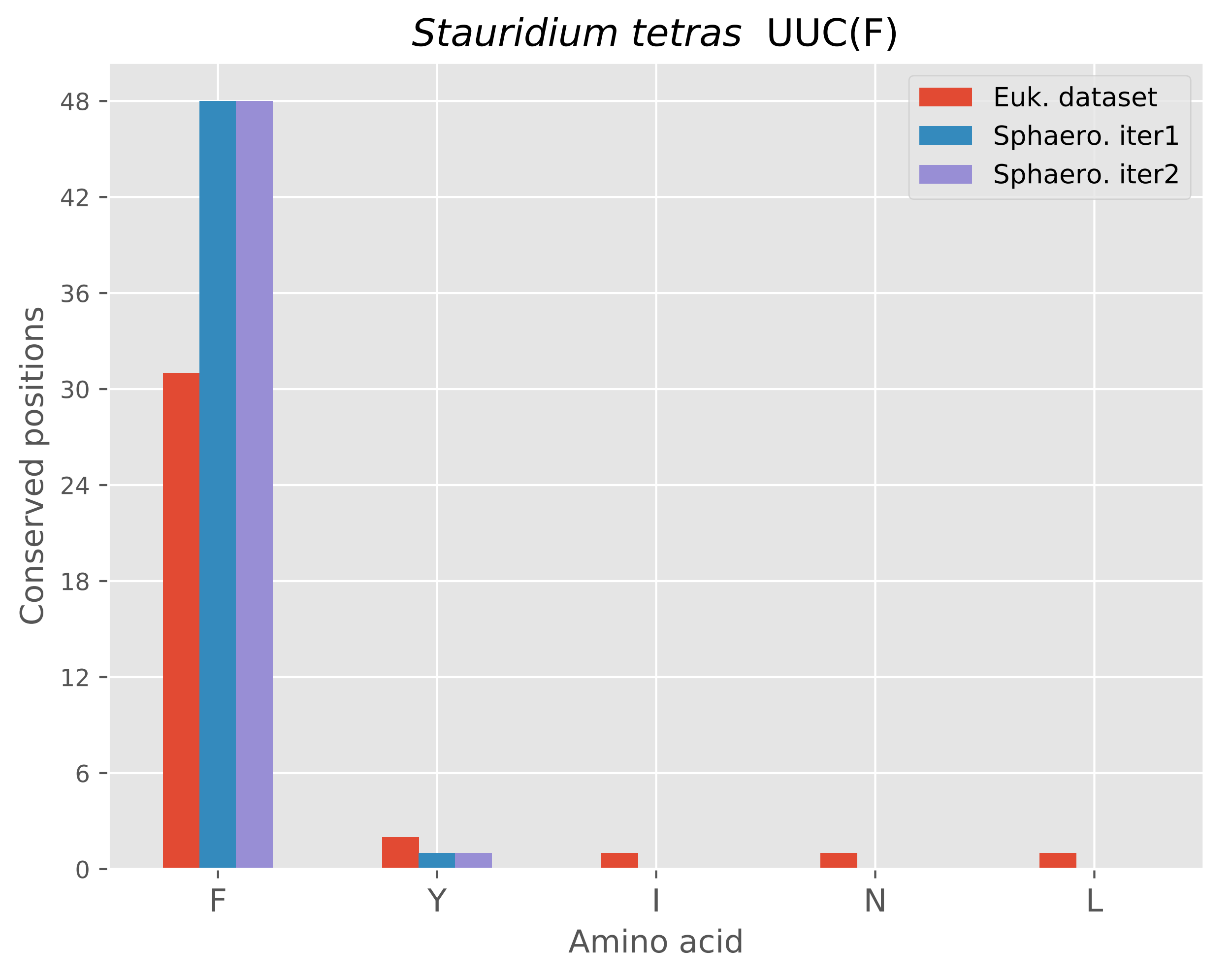

Stauridium tetras UUC(F)
Euk. dataset
Sphaero. iter1
Sphaero. iter2
Conserved positions
0
6
12
18
24
30
36
42
48
F
Y
I
N
L
Amino acid

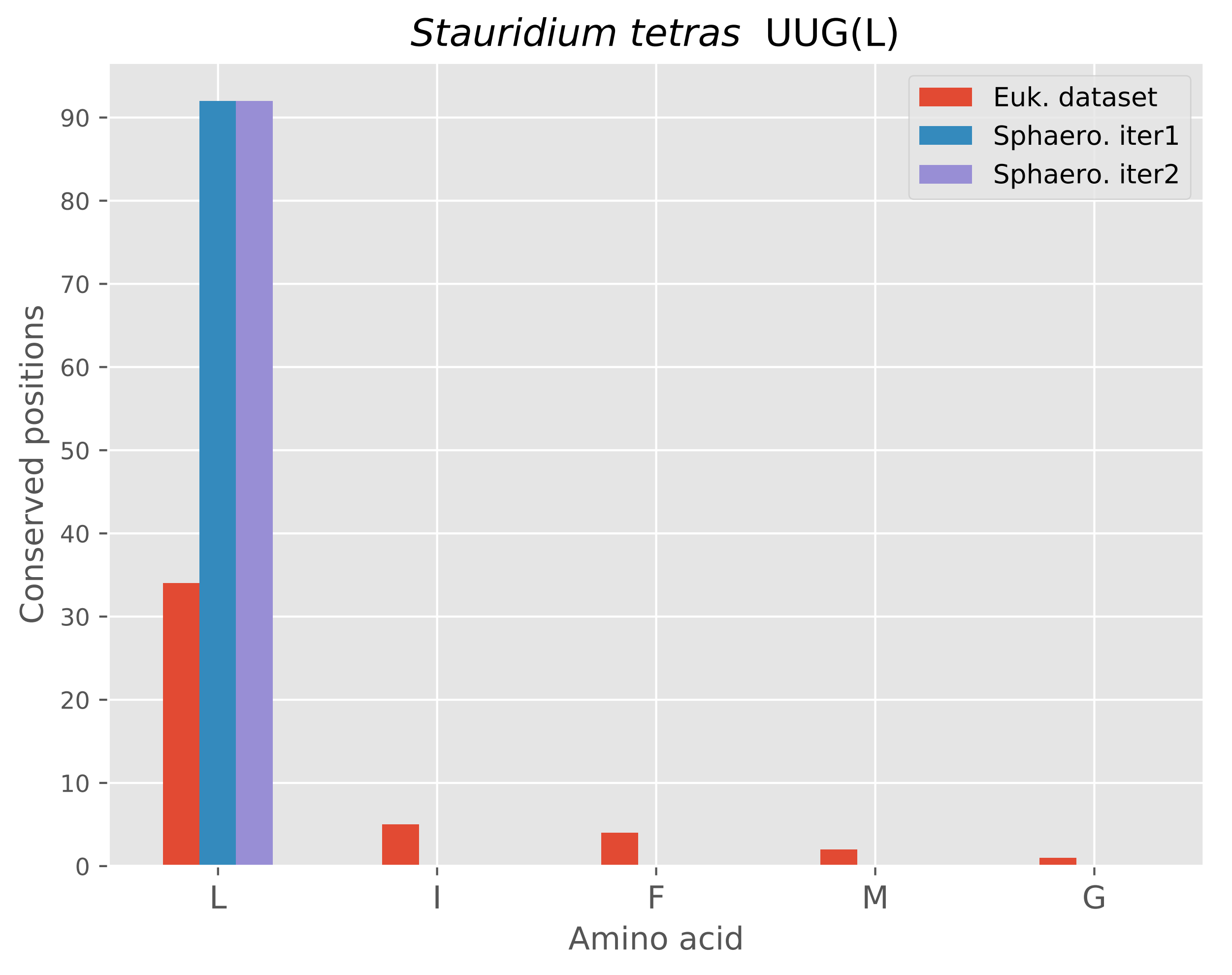

Stauridium tetras UUG(L)
Euk. dataset
Sphaero. iter1
Sphaero. iter2
Conserved positions
0
10
20
30
40
50
60
70
80
90
L
I
F
M
G
Amino acid

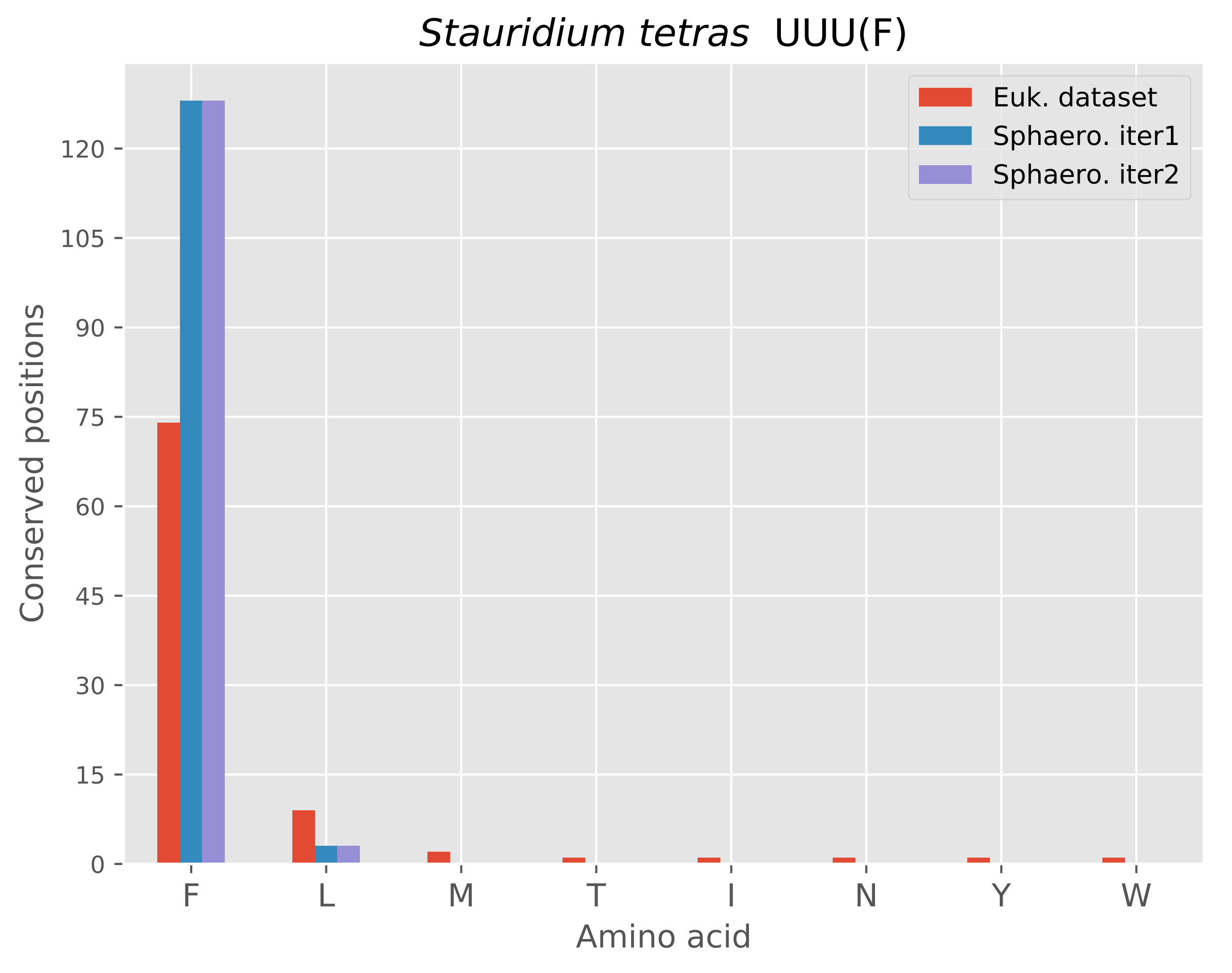

Stauridium tetras UUU(F)
Euk. dataset
Sphaero. iter1
Sphaero. iter2
Conserved positions
0
15
30
45
60
75
90
105
120
F
L
M
T
I
N
Y
W
Amino acid

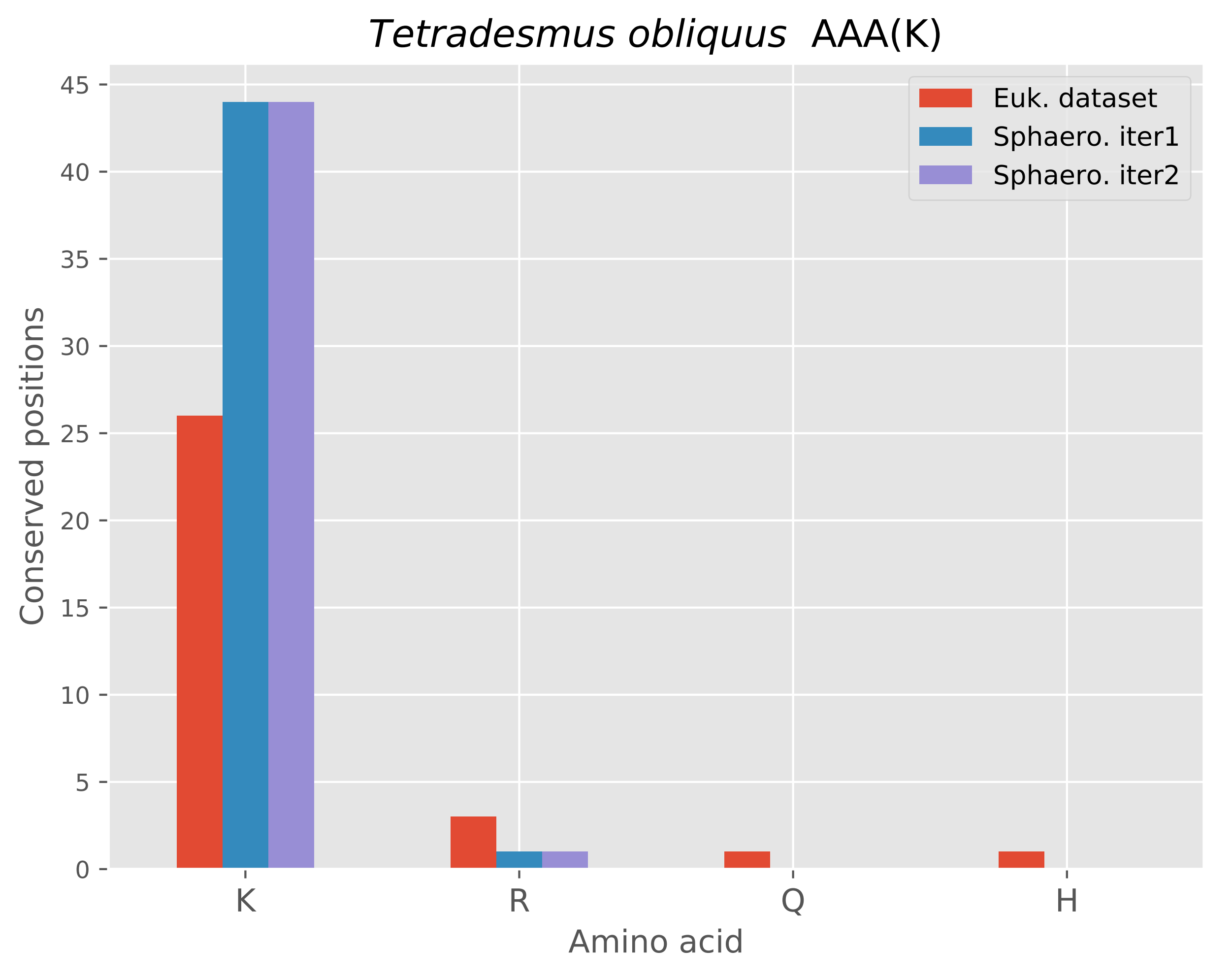
Tetradesmus obliquus AAA(K)
Euk. dataset
Sphaero. iter1
Sphaero. iter2
Conserved positions
0
5
10
15
20
25
30
35
40
45
K
R
Q
H
Amino acid

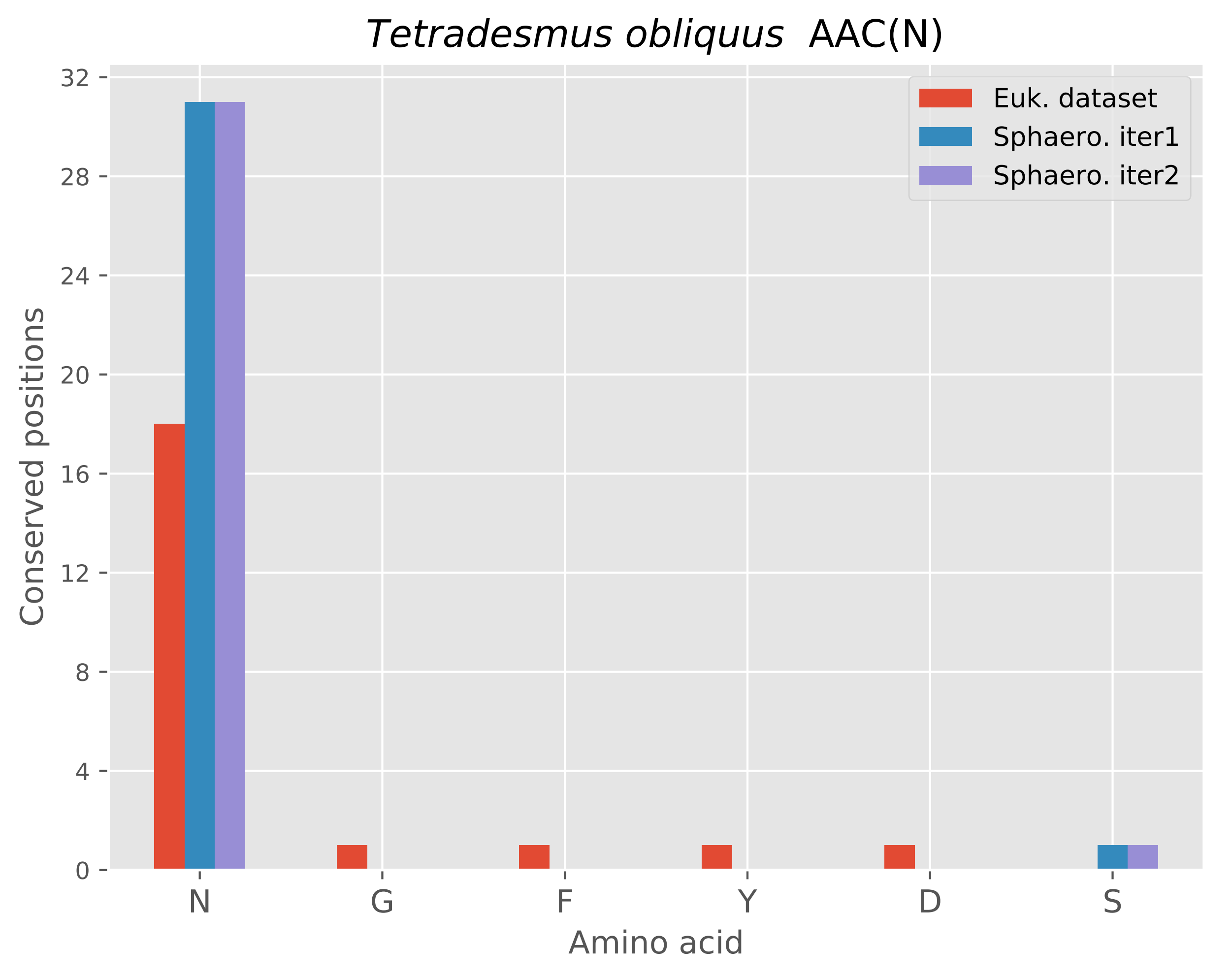
Tetradesmus obliquus AAC(N)
Euk. dataset
Sphaero. iter1
Sphaero. iter2
Conserved positions
0
4
8
12
16
20
24
28
32
N
G
F
Y
D
S
Amino acid

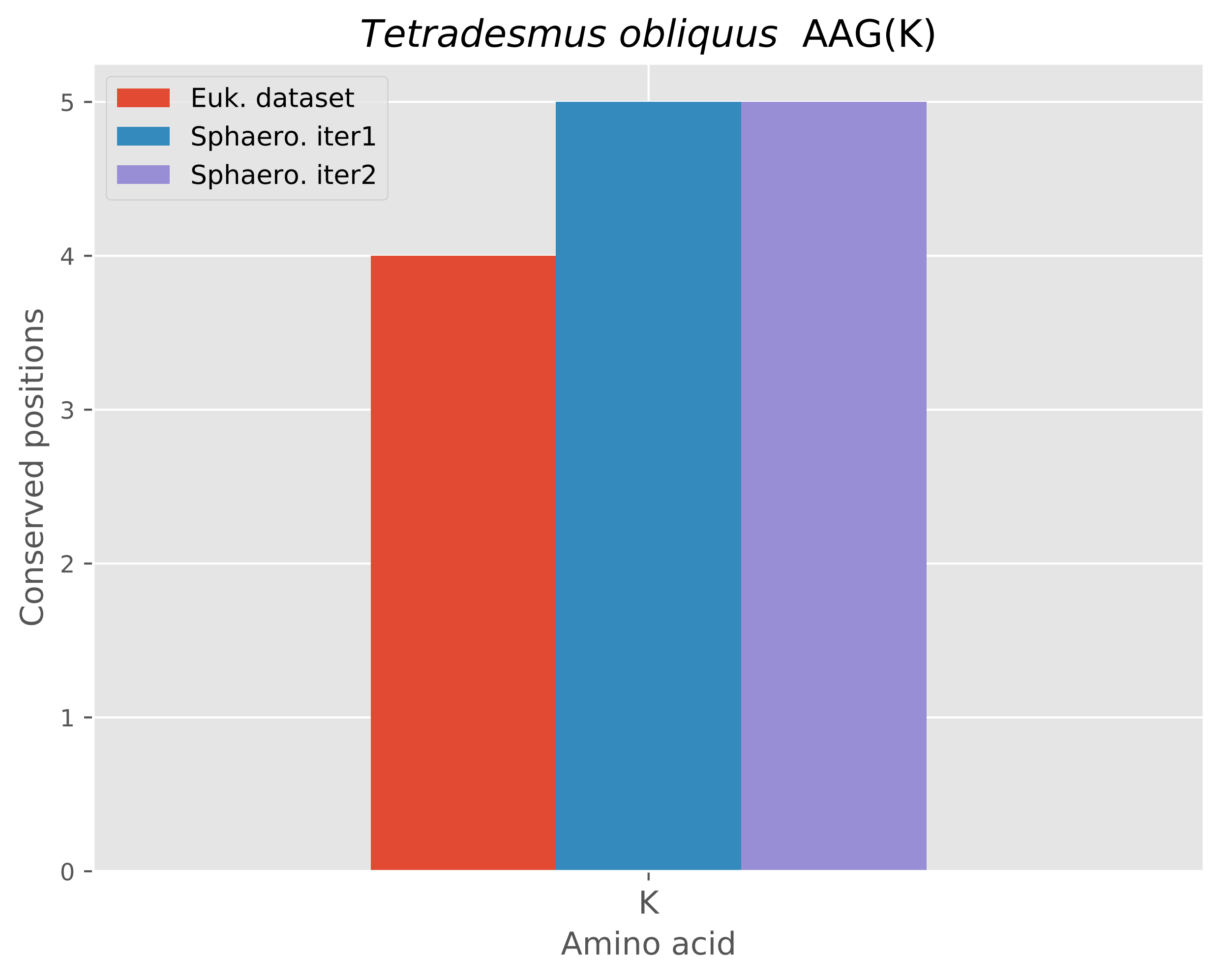

Tetradesmus obliquus AAG(K)
Euk. dataset
Sphaero. iter1
Sphaero. iter2
Conserved positions
0
1
2
3
4
5
K
Amino acid

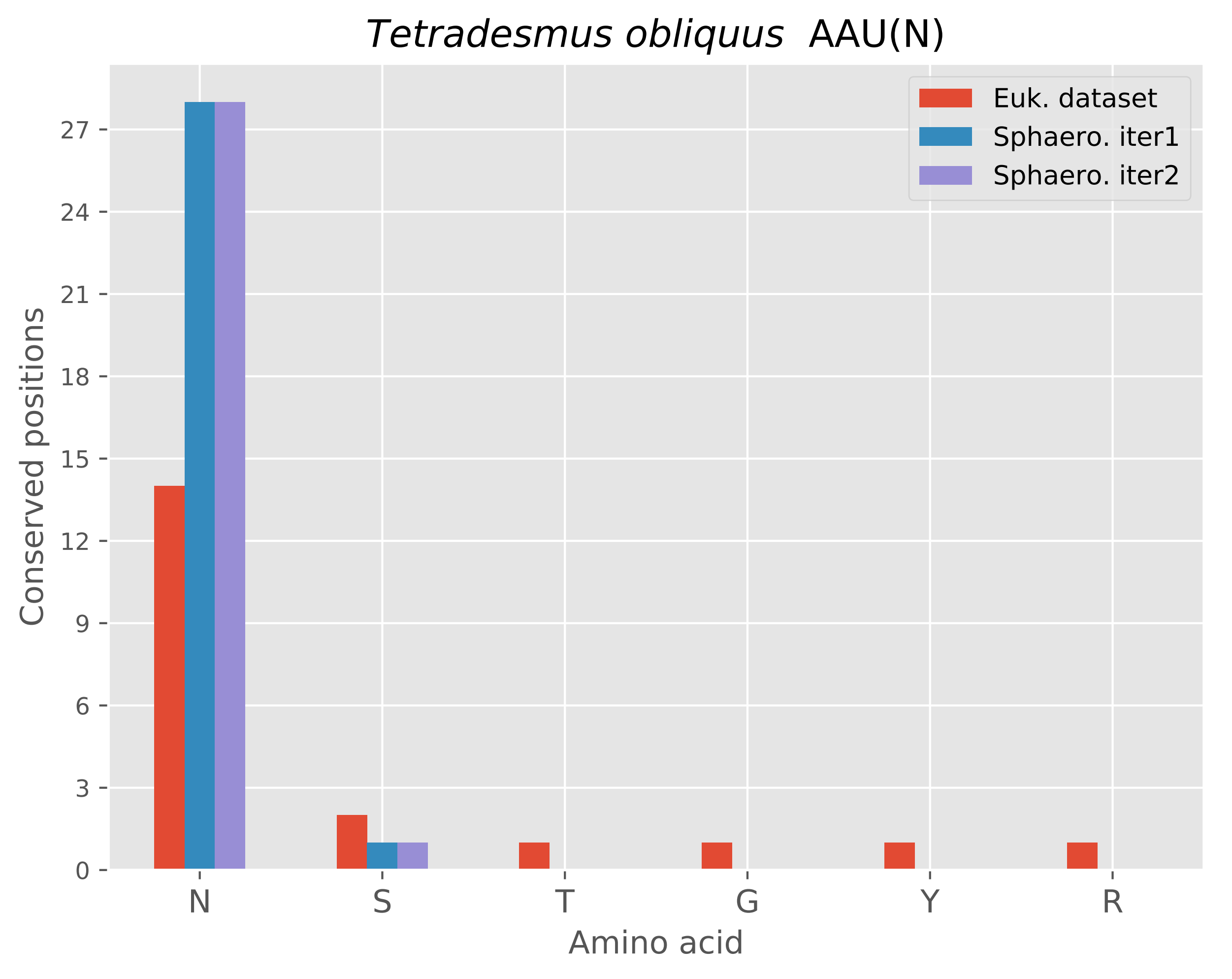

Tetradesmus obliquus AAU(N)
Euk. dataset
Sphaero. iter1
Sphaero. iter2
Conserved positions
0
3
6
9
12
15
18
21
24
27
N
S
T
G
Y
R
Amino acid

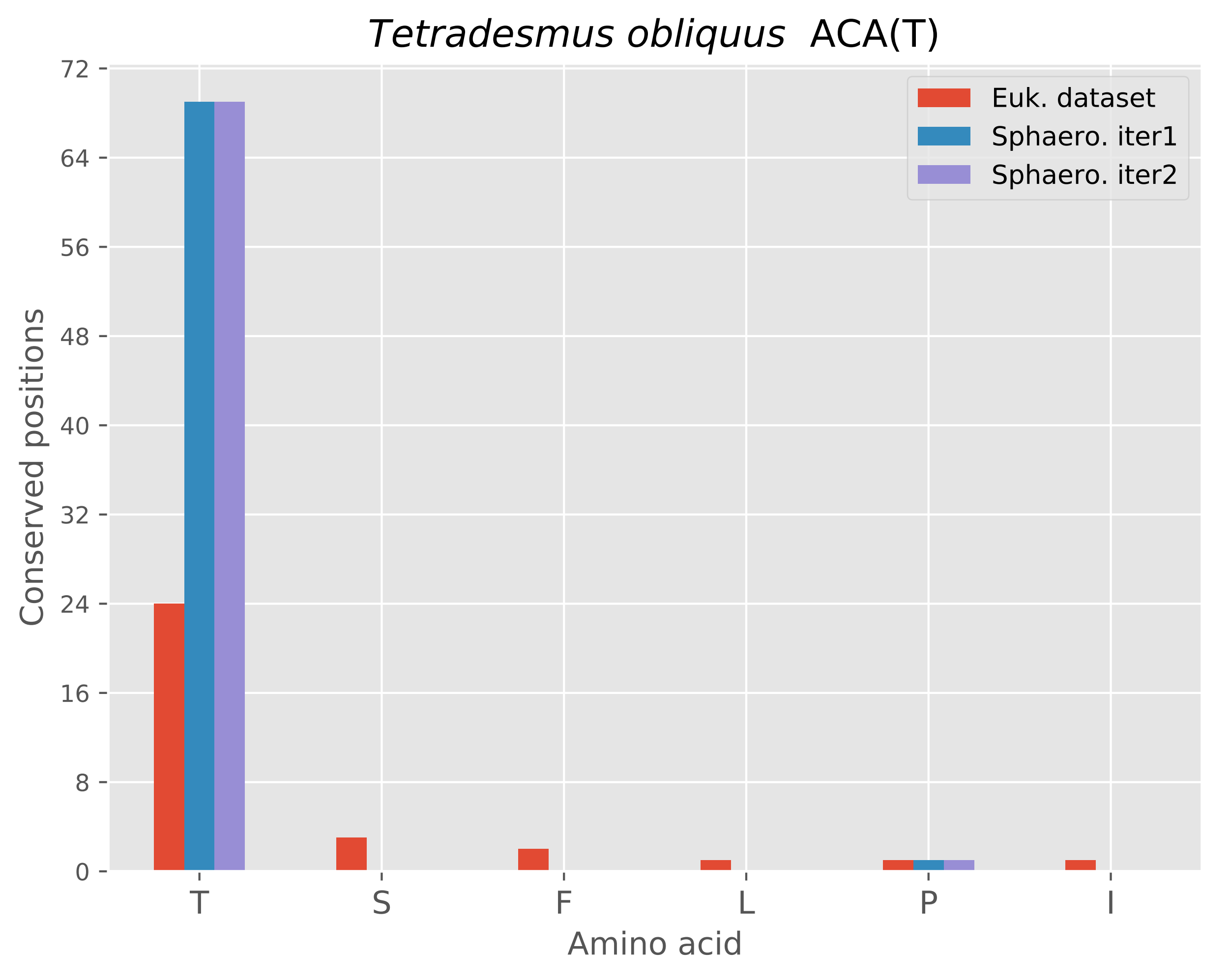

Tetradesmus obliquus ACA(T)
Euk. dataset
Sphaero. iter1
Sphaero. iter2
Conserved positions
0
8
16
24
32
40
48
56
64
72
T
S
F
L
P
I
Amino acid

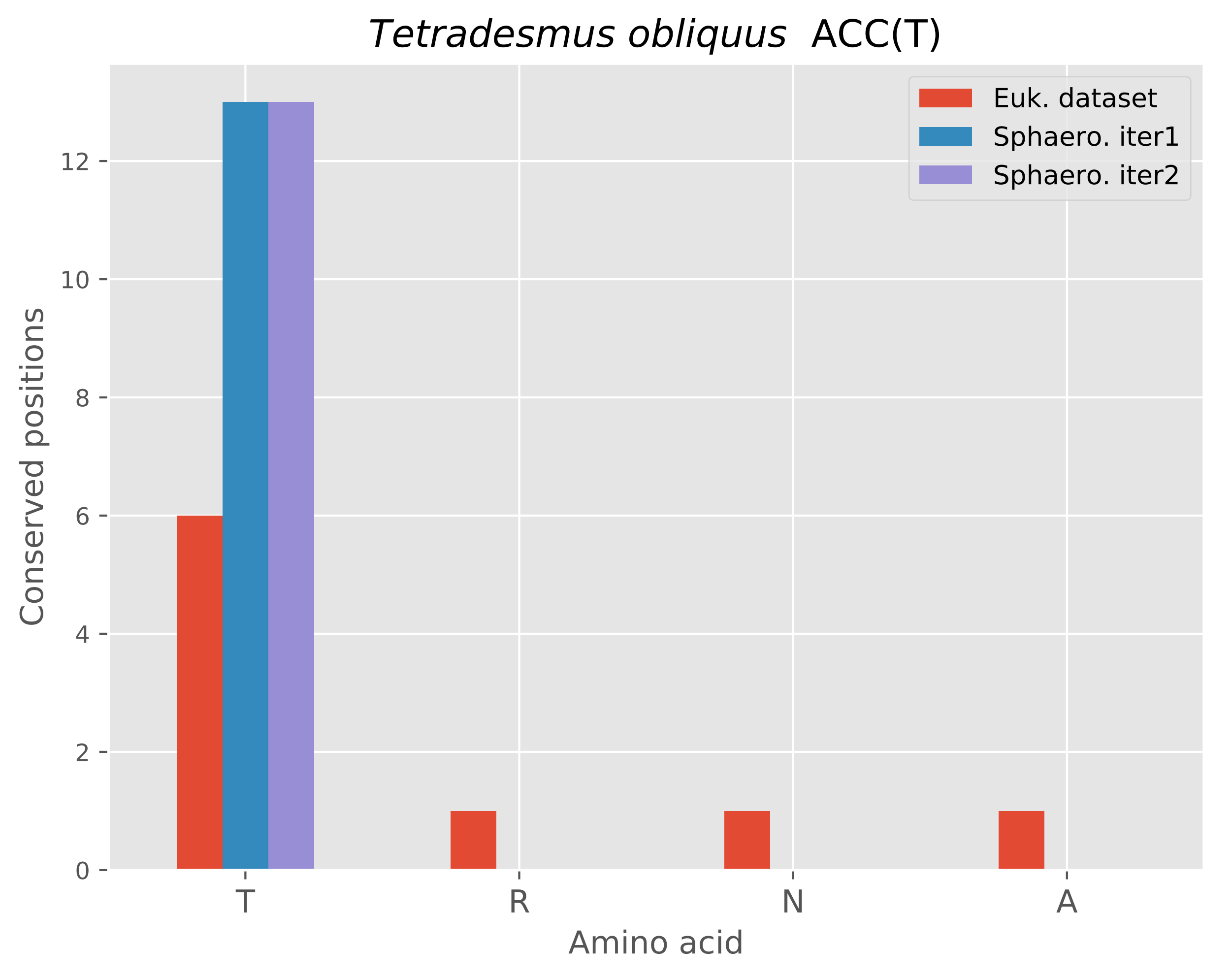

Tetradesmus obliquus ACC(T)
Euk. dataset
Sphaero. iter1
Sphaero. iter2
Conserved positions
0
2
4
6
8
10
12
T
R
N
A
Amino acid

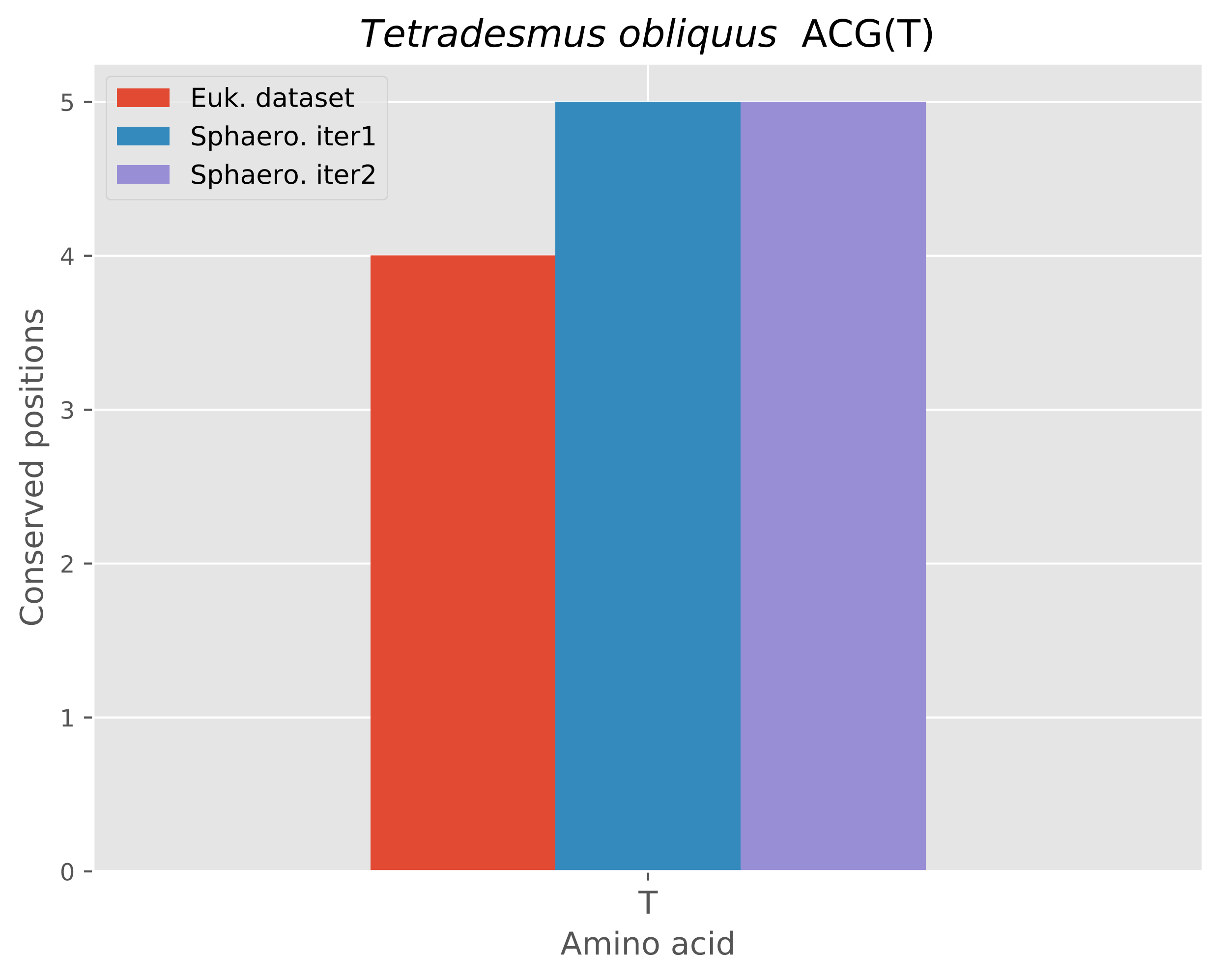

Tetradesmus obliquus ACG(T)
Euk. dataset
Sphaero. iter1
Sphaero. iter2
Conserved positions
0
1
2
3
4
5
T
Amino acid

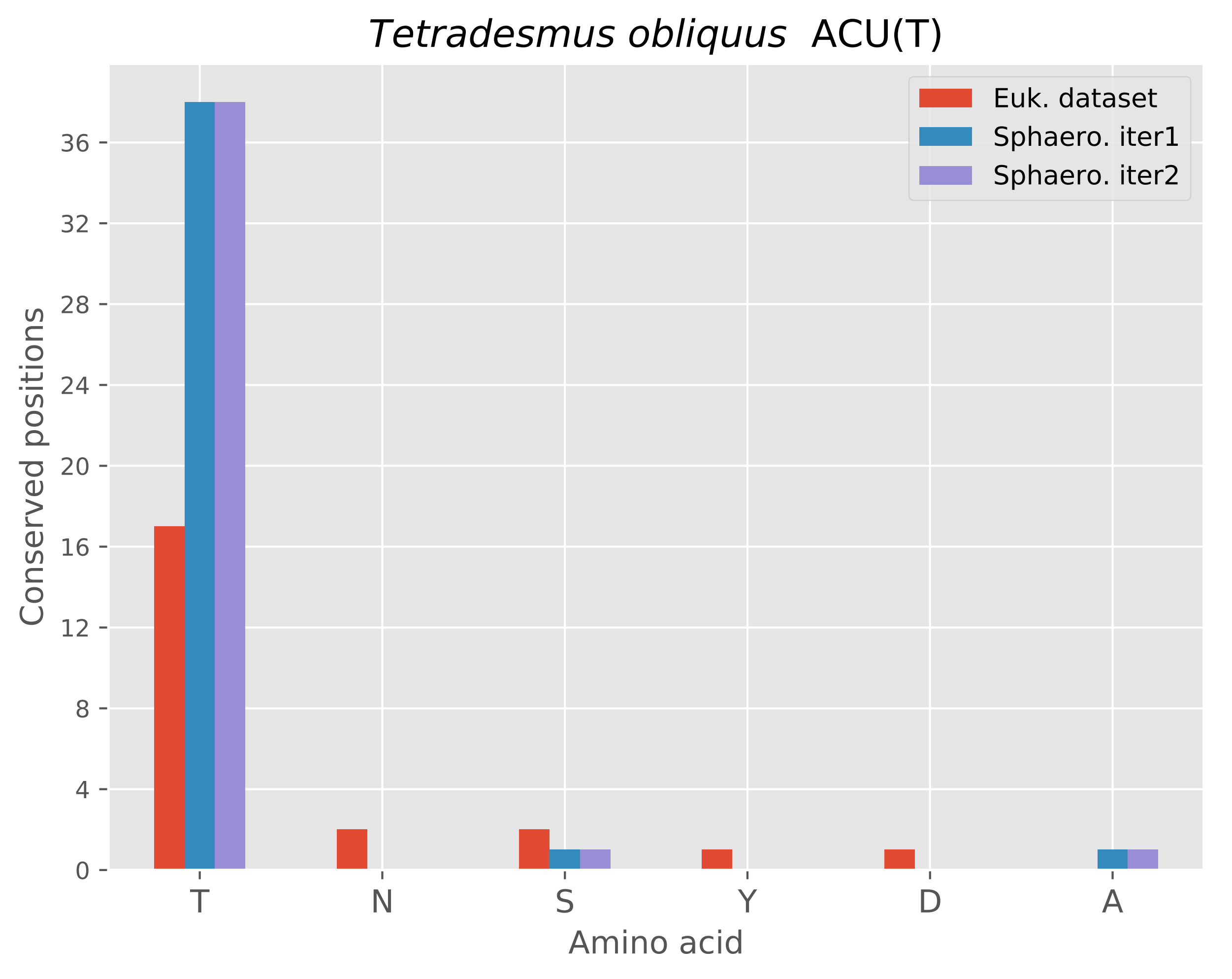

Tetradesmus obliquus ACU(T)
Euk. dataset
Sphaero. iter1
Sphaero. iter2
Conserved positions
0
4
8
12
16
20
24
28
32
36
T
N
S
Y
D
A
Amino acid

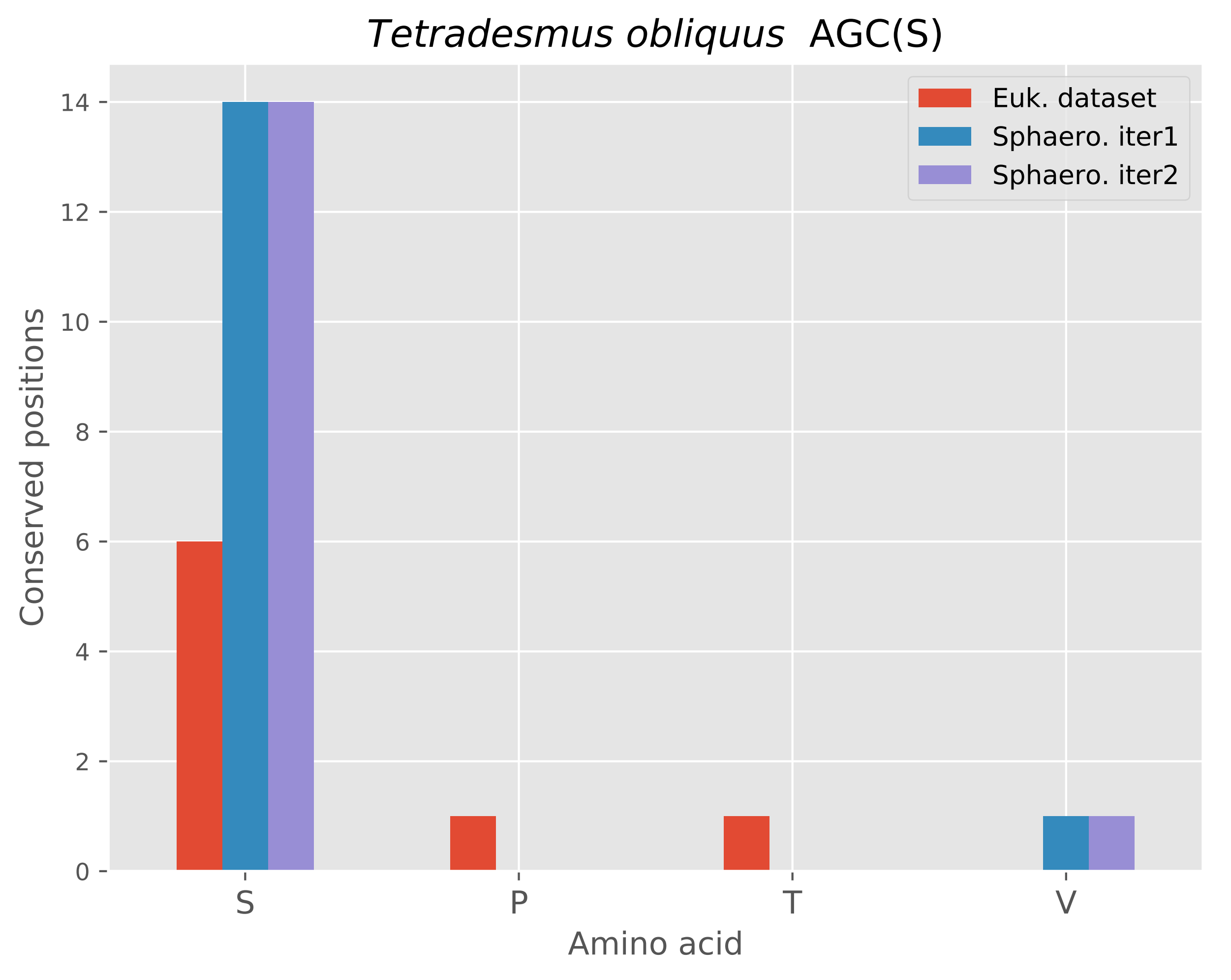

Tetradesmus obliquus AGC(S)
Euk. dataset
Sphaero. iter1
Sphaero. iter2
Conserved positions
0
2
4
6
8
10
12
14
S
P
T
V
Amino acid

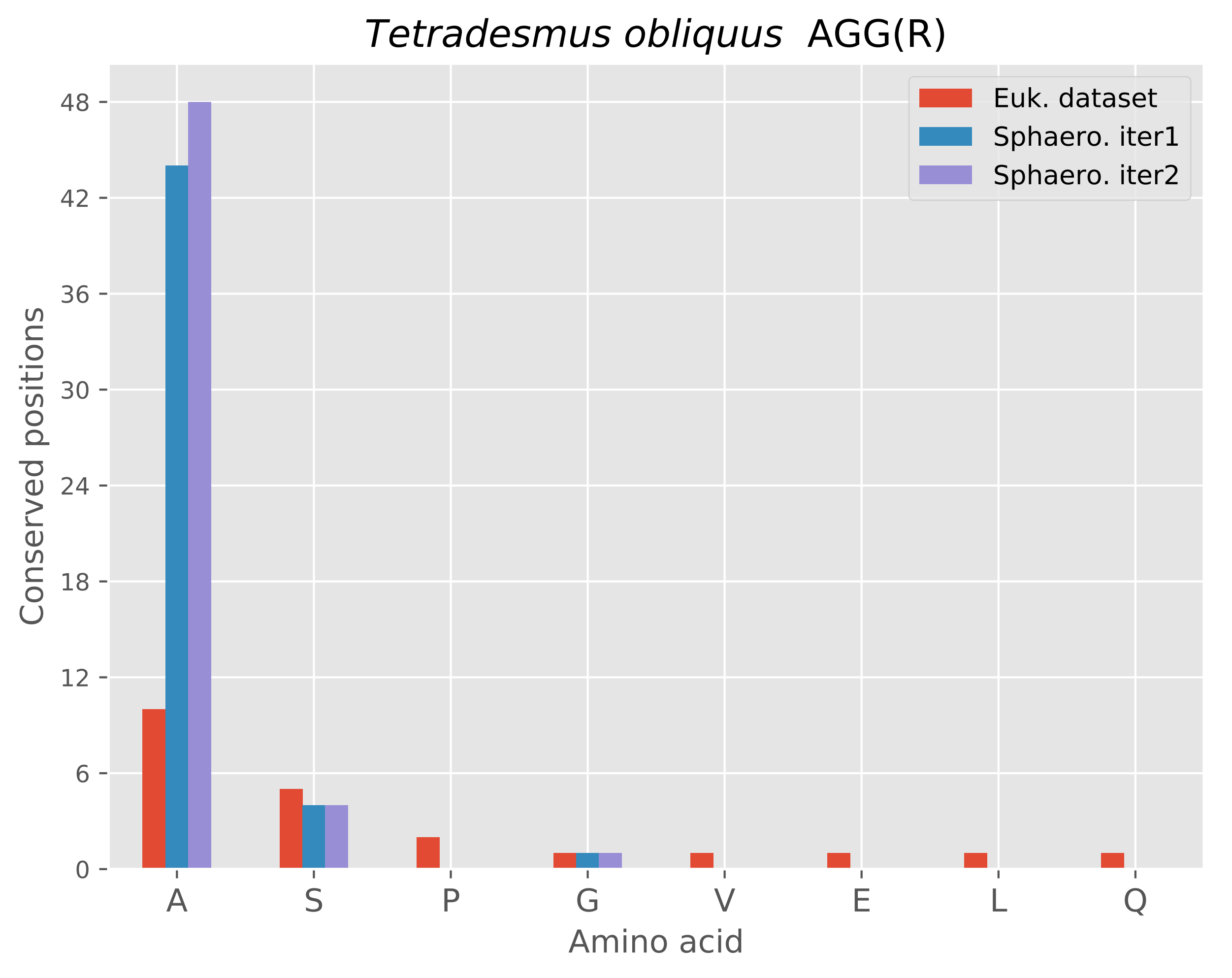

Tetradesmus obliquus AGG(R)
Euk. dataset
Sphaero. iter1
Sphaero. iter2
Conserved positions
0
6
12
18
24
30
36
42
48
A
S
P
G
V
E
L
Q
Amino acid

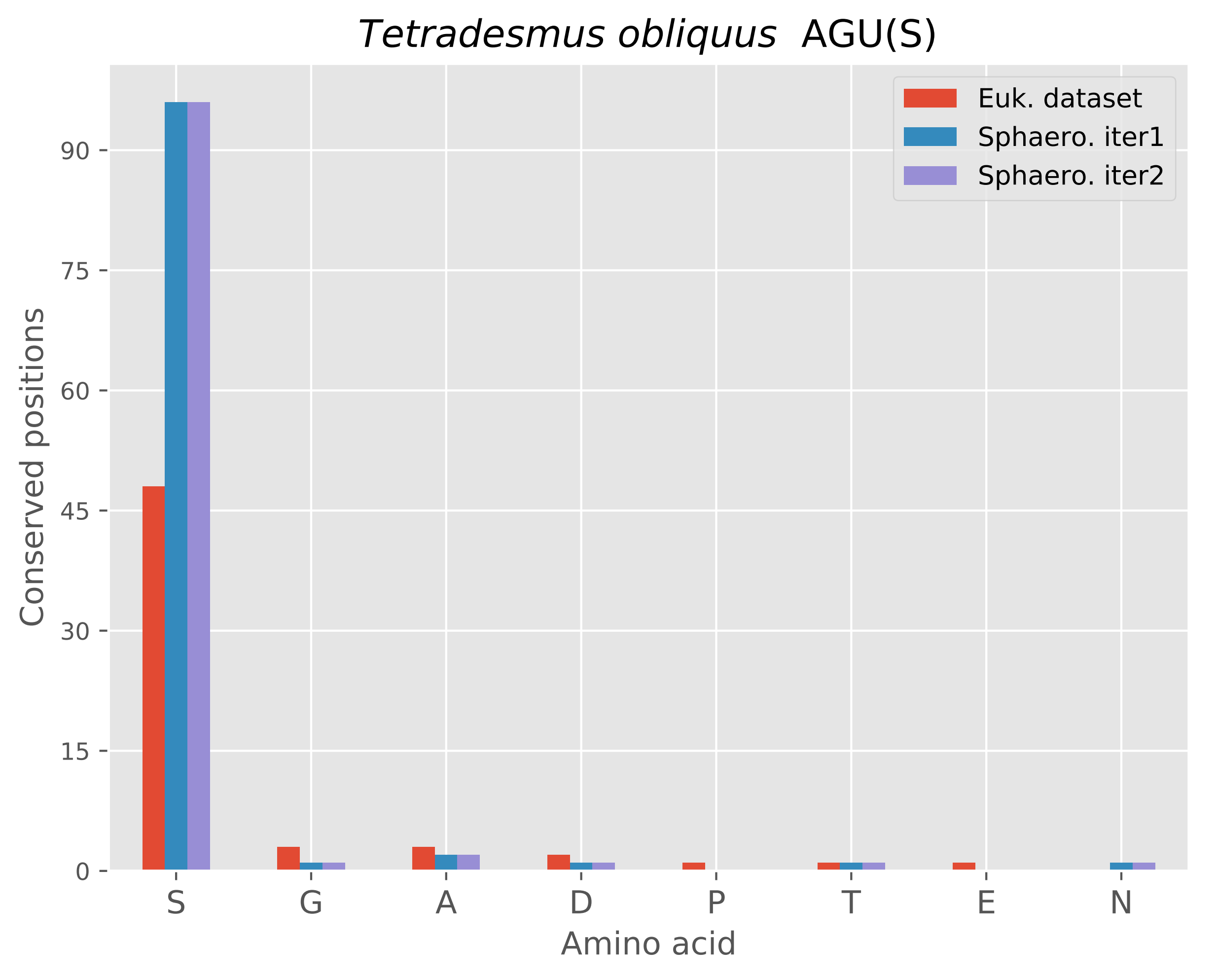

Tetradesmus obliquus AGU(S)
Euk. dataset
Sphaero. iter1
Sphaero. iter2
Conserved positions
0
15
30
45
60
75
90
S
G
A
D
P
T
E
N
Amino acid

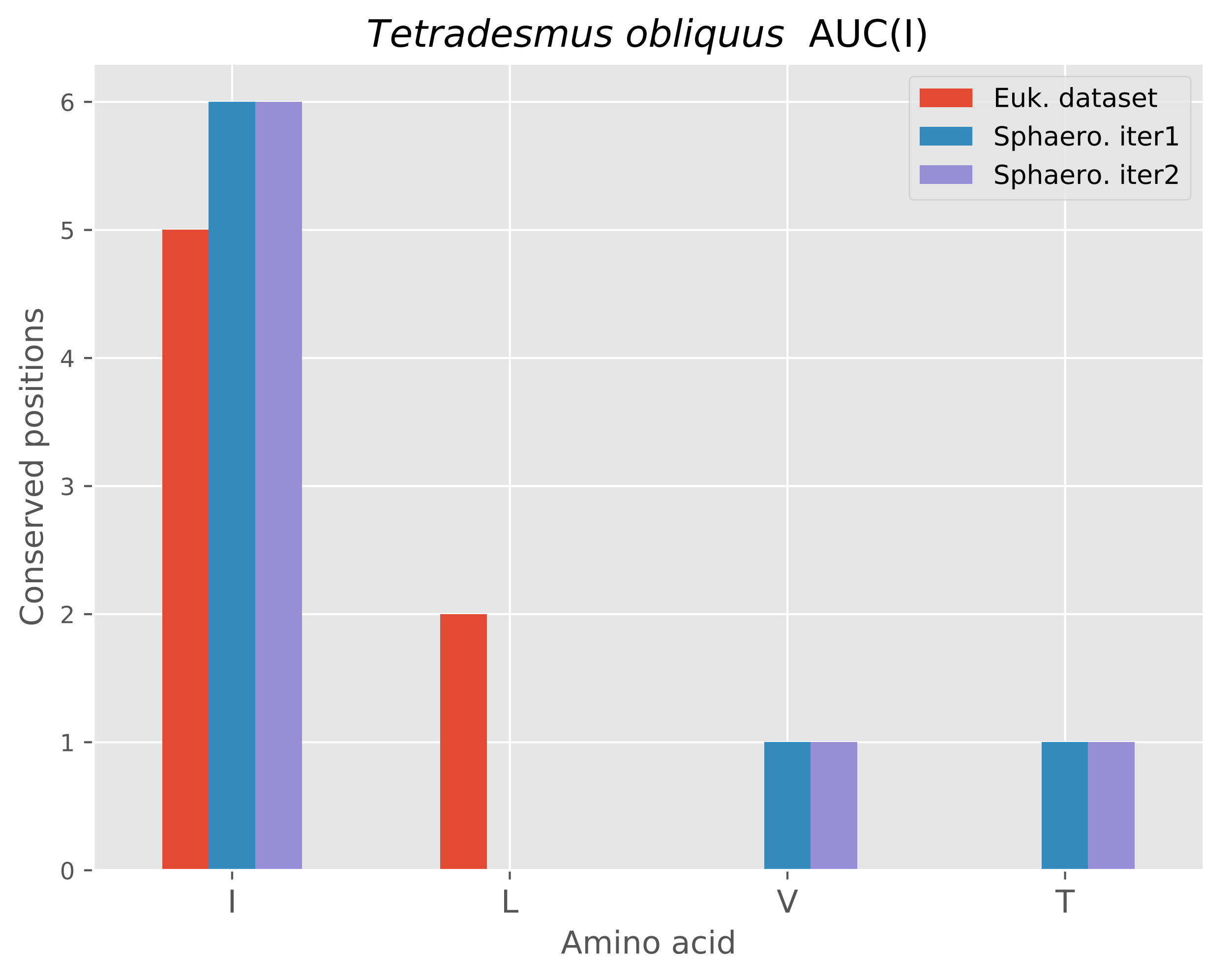
Tetradesmus obliquus AUC(I)
Euk. dataset
Sphaero. iter1
Sphaero. iter2
Conserved positions
0
1
2
3
4
5
6
I
L
V
T
Amino acid

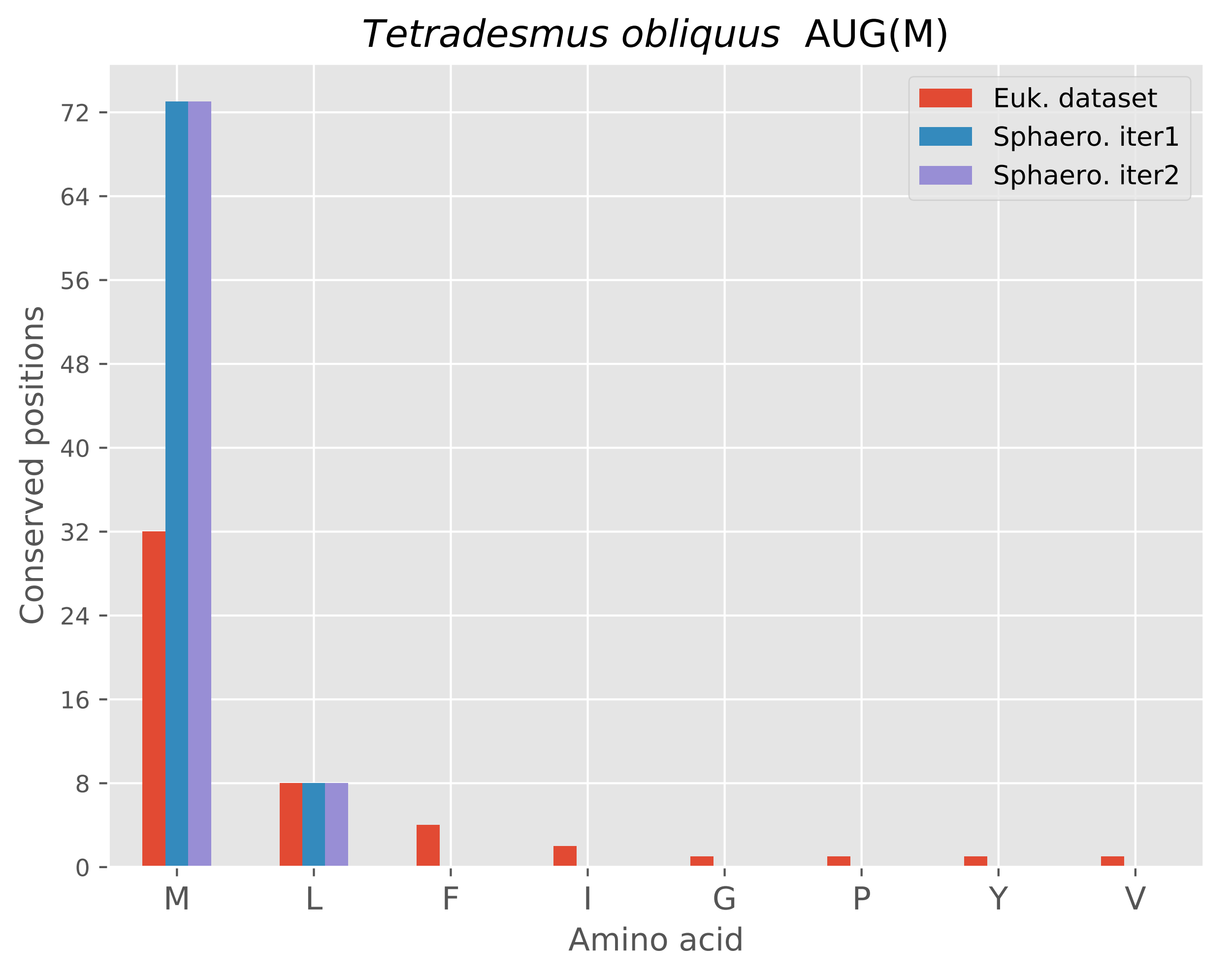

Tetradesmus obliquus AUG(M)
Euk. dataset
Sphaero. iter1
Sphaero. iter2
Conserved positions
0
8
16
24
32
40
48
56
64
72
M
L
F
I
G
P
Y
V
Amino acid

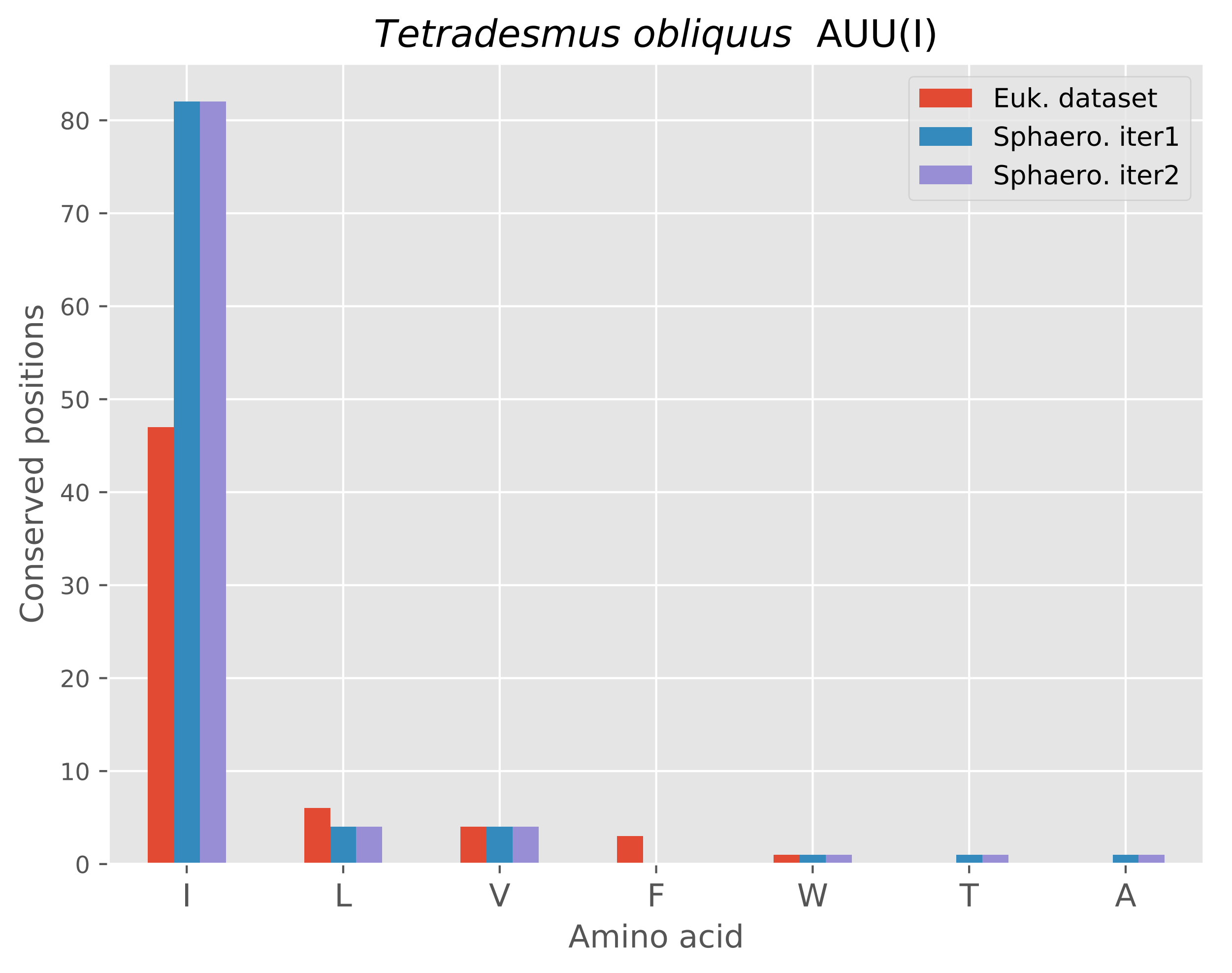

Tetradesmus obliquus AUU(I)
Euk. dataset
Sphaero. iter1
Sphaero. iter2
Conserved positions
0
10
20
30
40
50
60
70
80
I
L
V
F
W
T
A
Amino acid

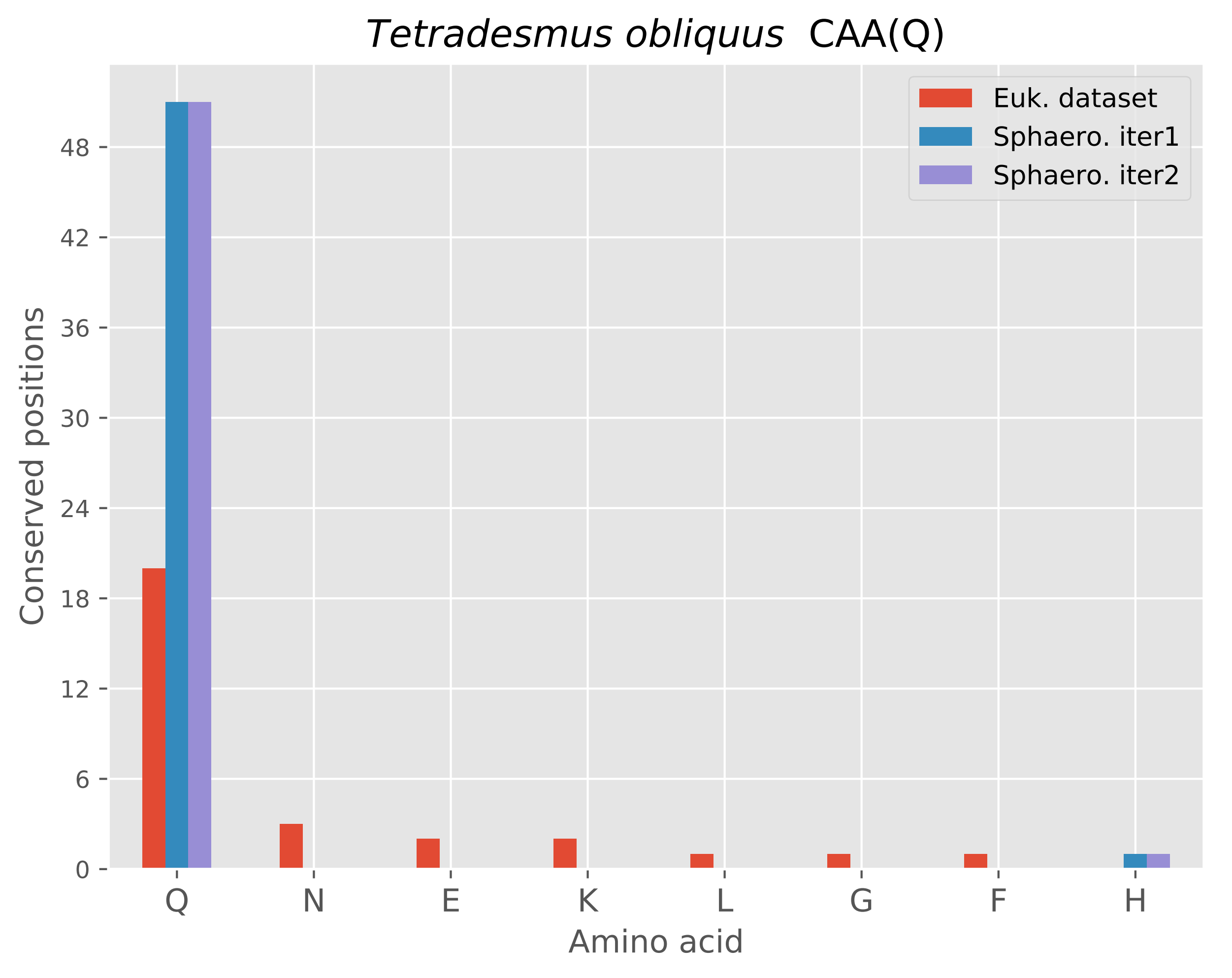

Tetradesmus obliquus CAA(Q)
Euk. dataset
Sphaero. iter1
Sphaero. iter2
Conserved positions
0
6
12
18
24
30
36
42
48
Q
N
E
K
L
G
F
H
Amino acid

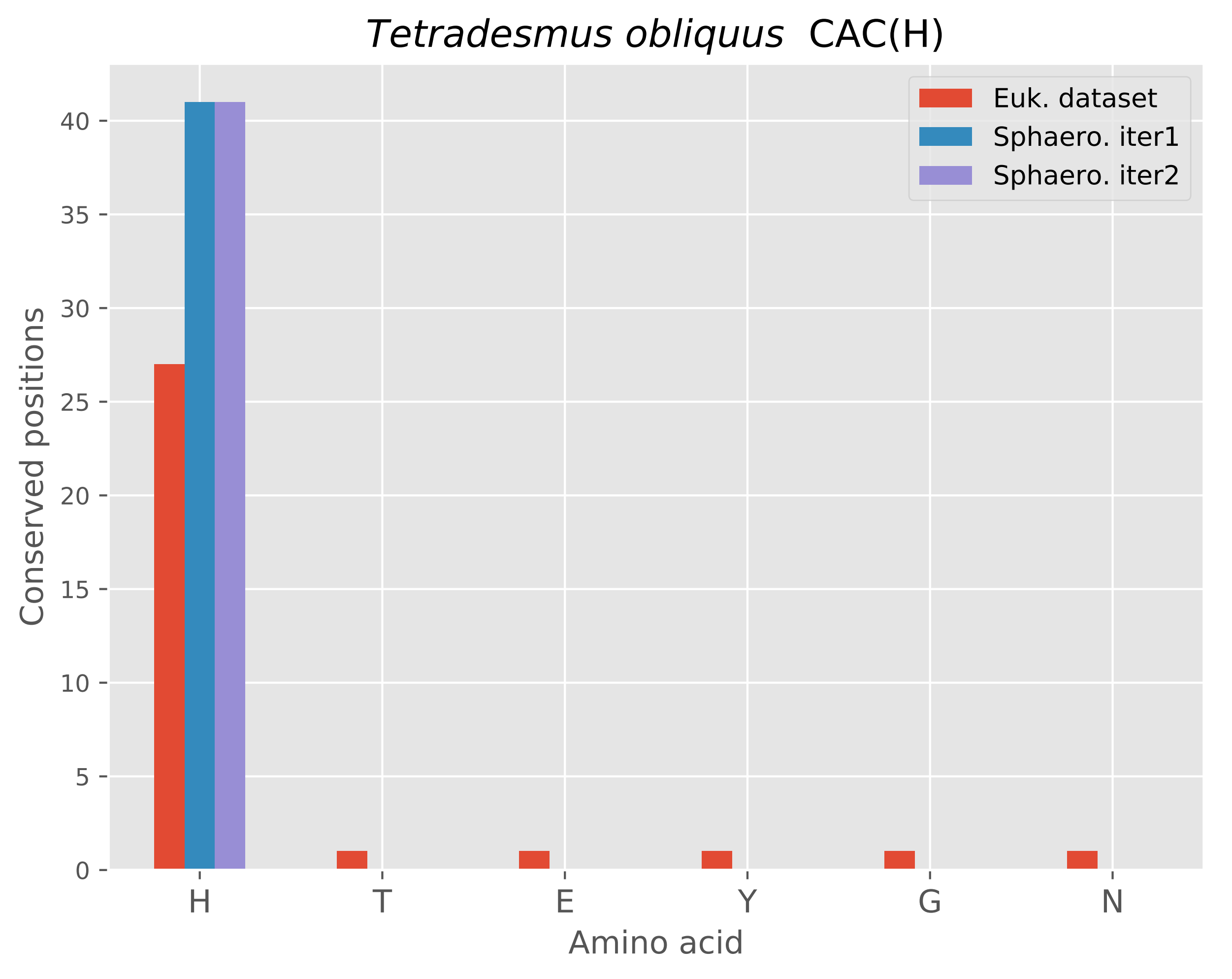

Tetradesmus obliquus CAC(H)
Euk. dataset
Sphaero. iter1
Sphaero. iter2
Conserved positions
0
5
10
15
20
25
30
35
40
H
T
E
Y
G
N
Amino acid

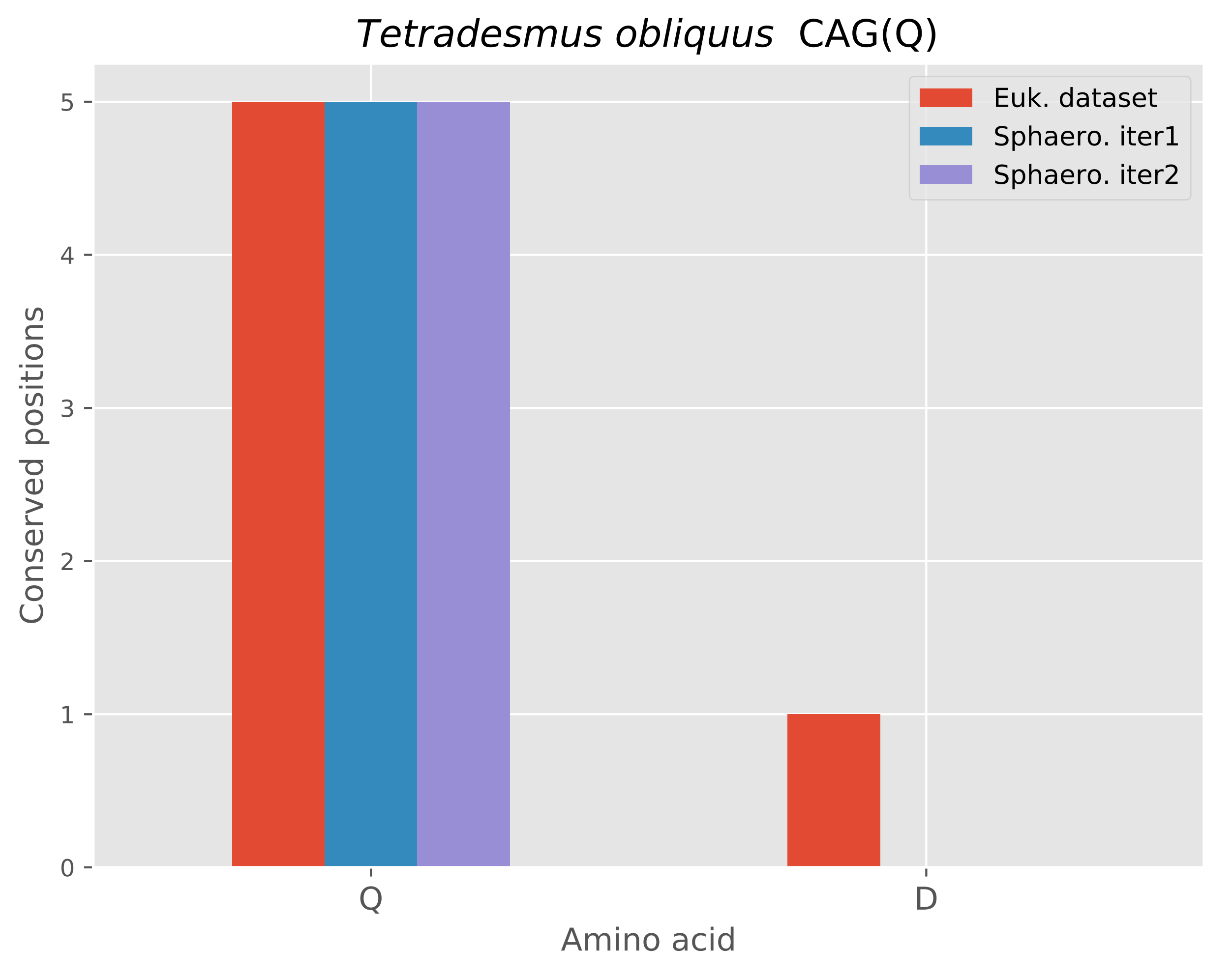

Tetradesmus obliquus CAG(Q)
Euk. dataset
Sphaero. iter1
Sphaero. iter2
Conserved positions
0
1
2
3
4
5
Q
D
Amino acid

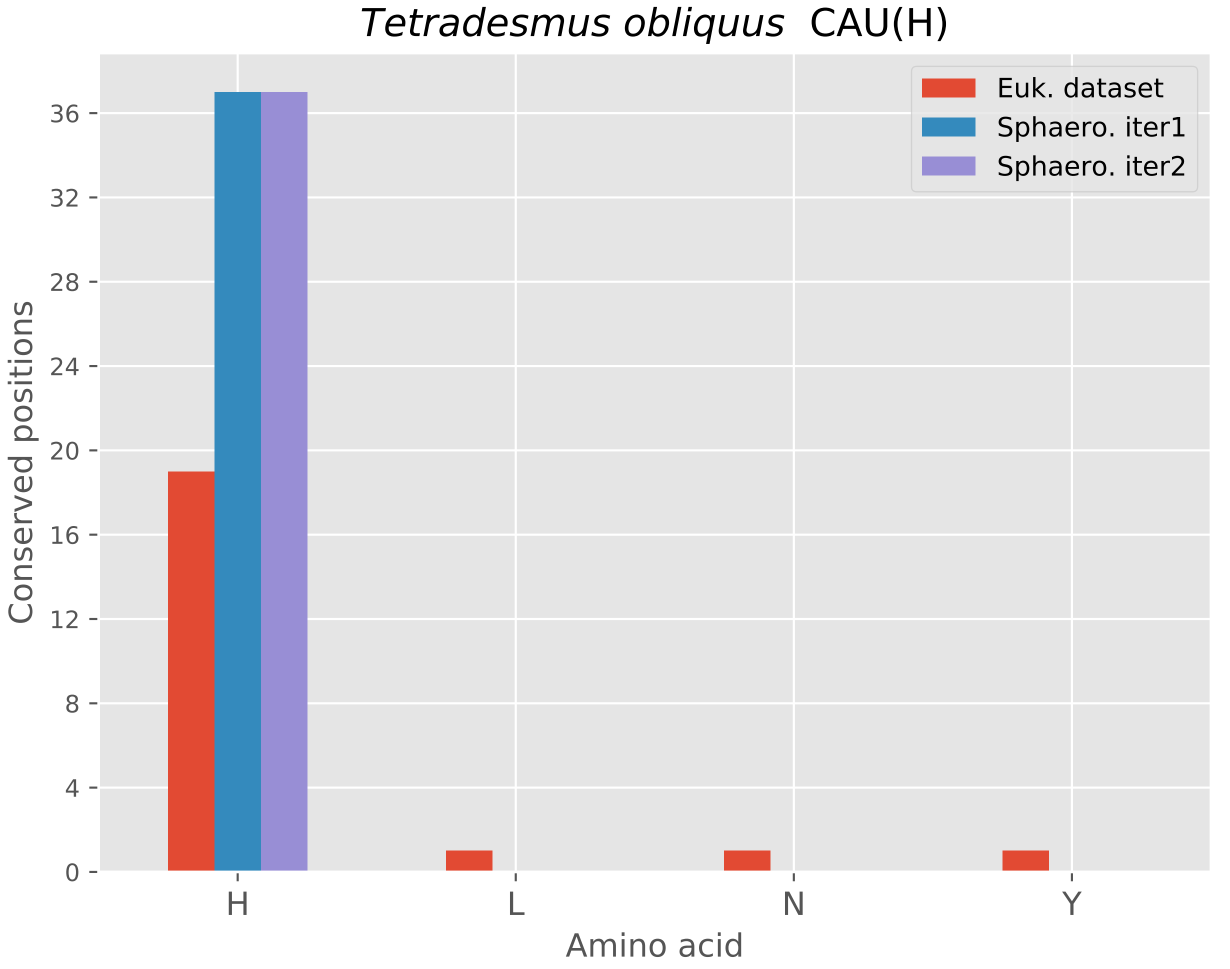

Tetradesmus obliquus CAU(H)
Euk. dataset
Sphaero. iter1
Sphaero. iter2
Conserved positions
0
4
8
12
16
20
24
28
32
36
H
L
N
Y
Amino acid

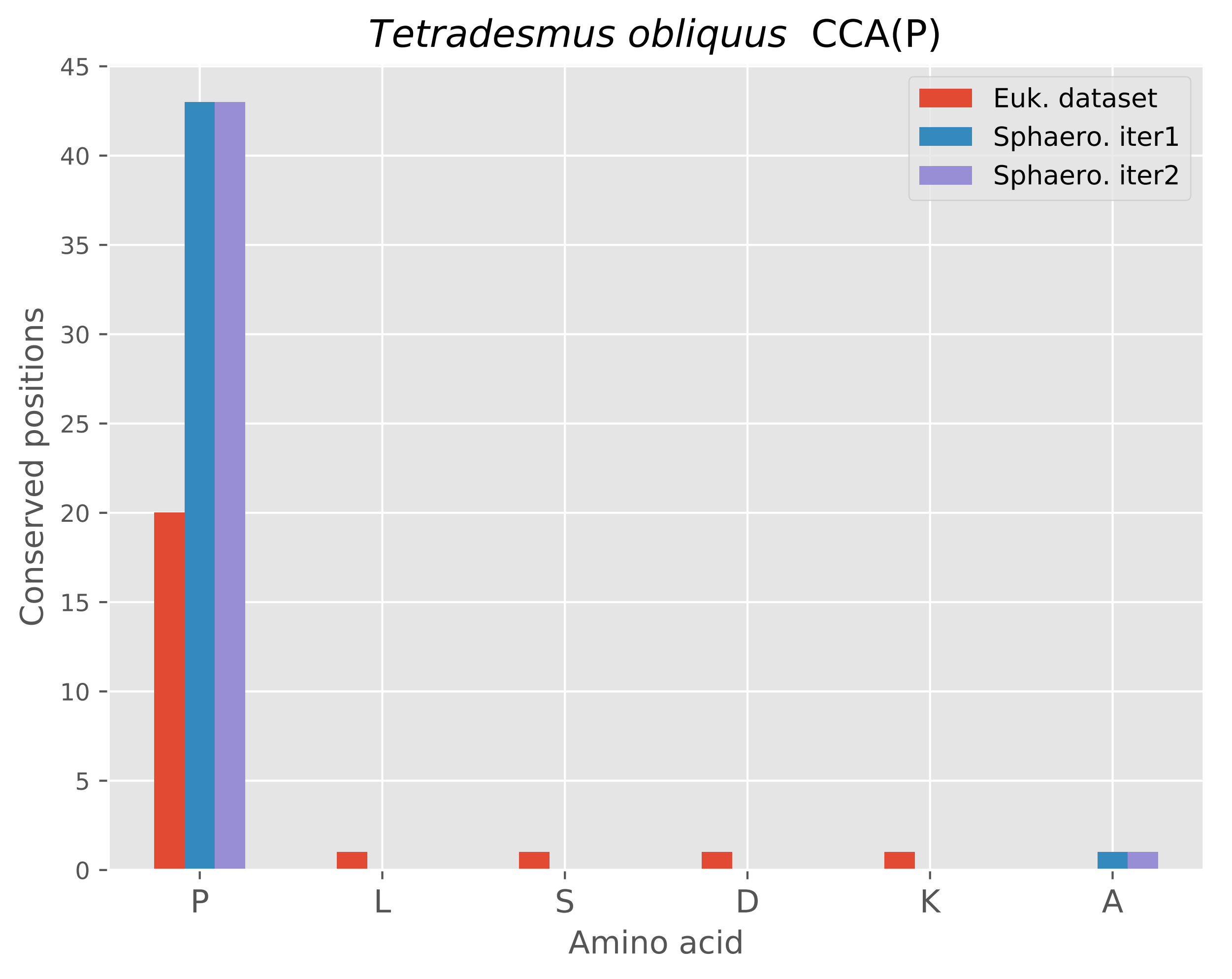
Tetradesmus obliquus CCA(P)
Conserved positions
Amino acid
0
5
10
15
20
25
30
35
40
45
P
L
S
D
K
A
Euk. dataset
Sphaero. iter1
Sphaero. iter2

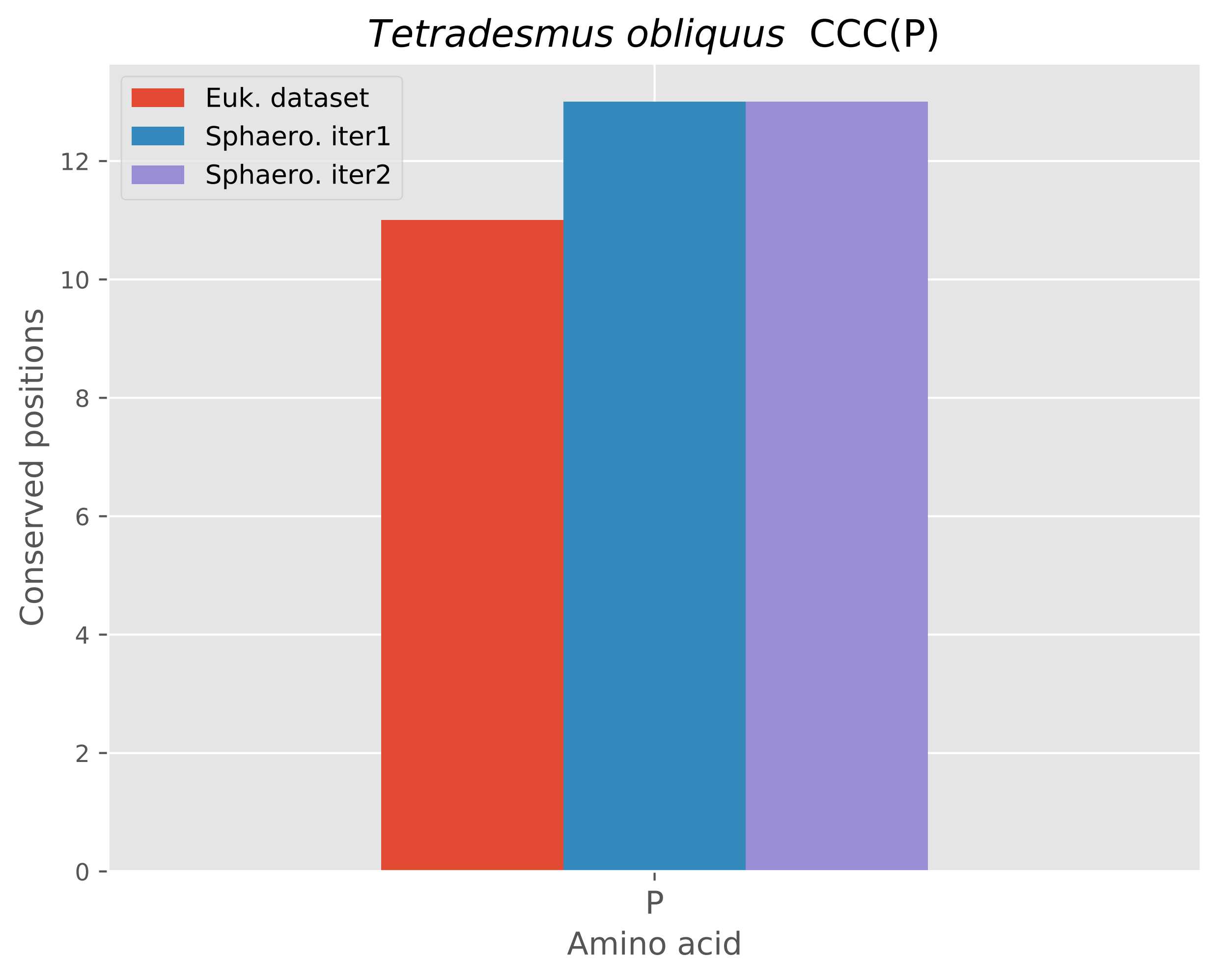

Tetradesmus obliquus CCC(P)
Euk. dataset
Sphaero. iter1
Sphaero. iter2
Conserved positions
0
2
4
6
8
10
12
P
Amino acid

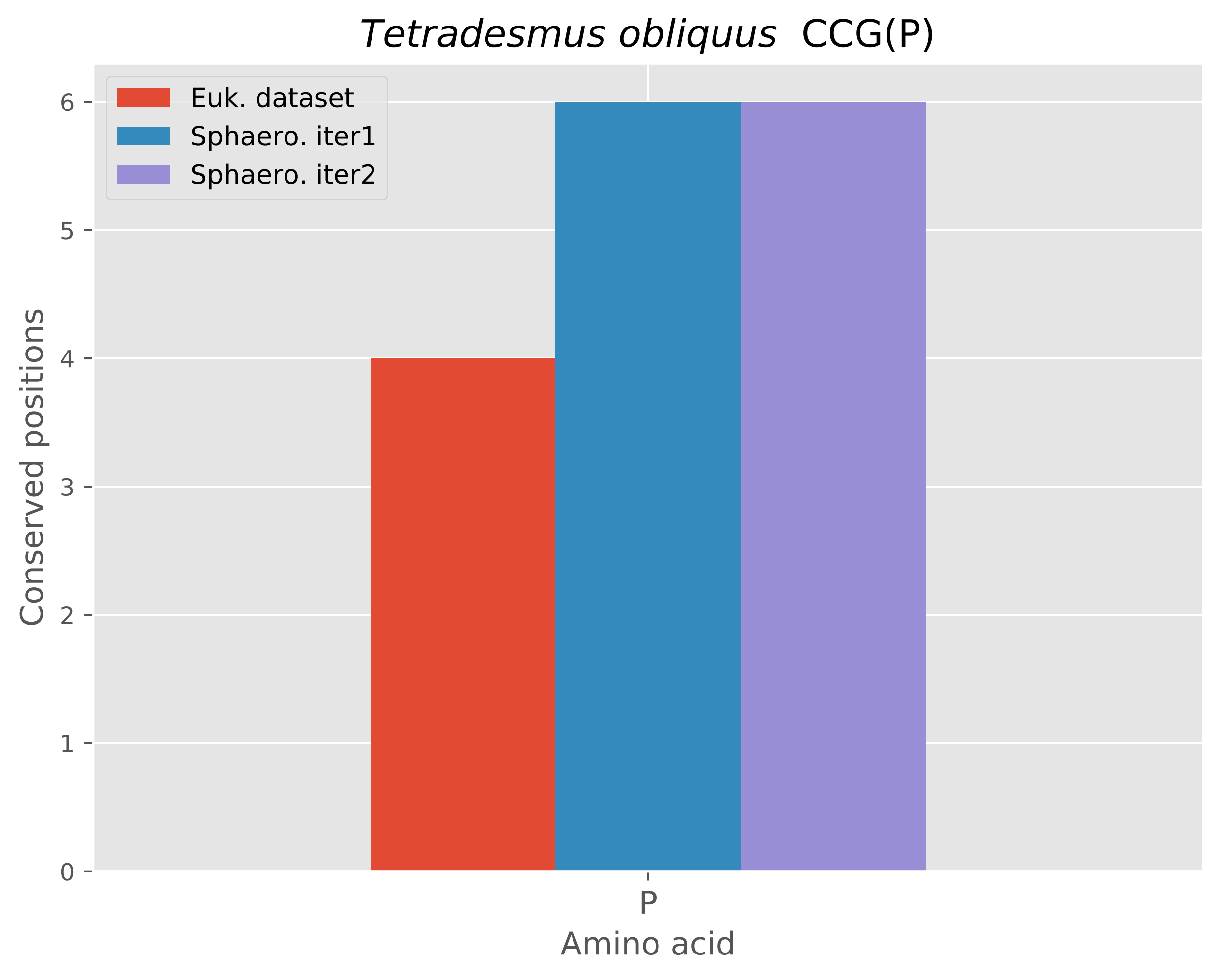

Tetradesmus obliquus CCG(P)
Euk. dataset
Sphaero. iter1
Sphaero. iter2
Conserved positions
0
1
2
3
4
5
6
P
Amino acid

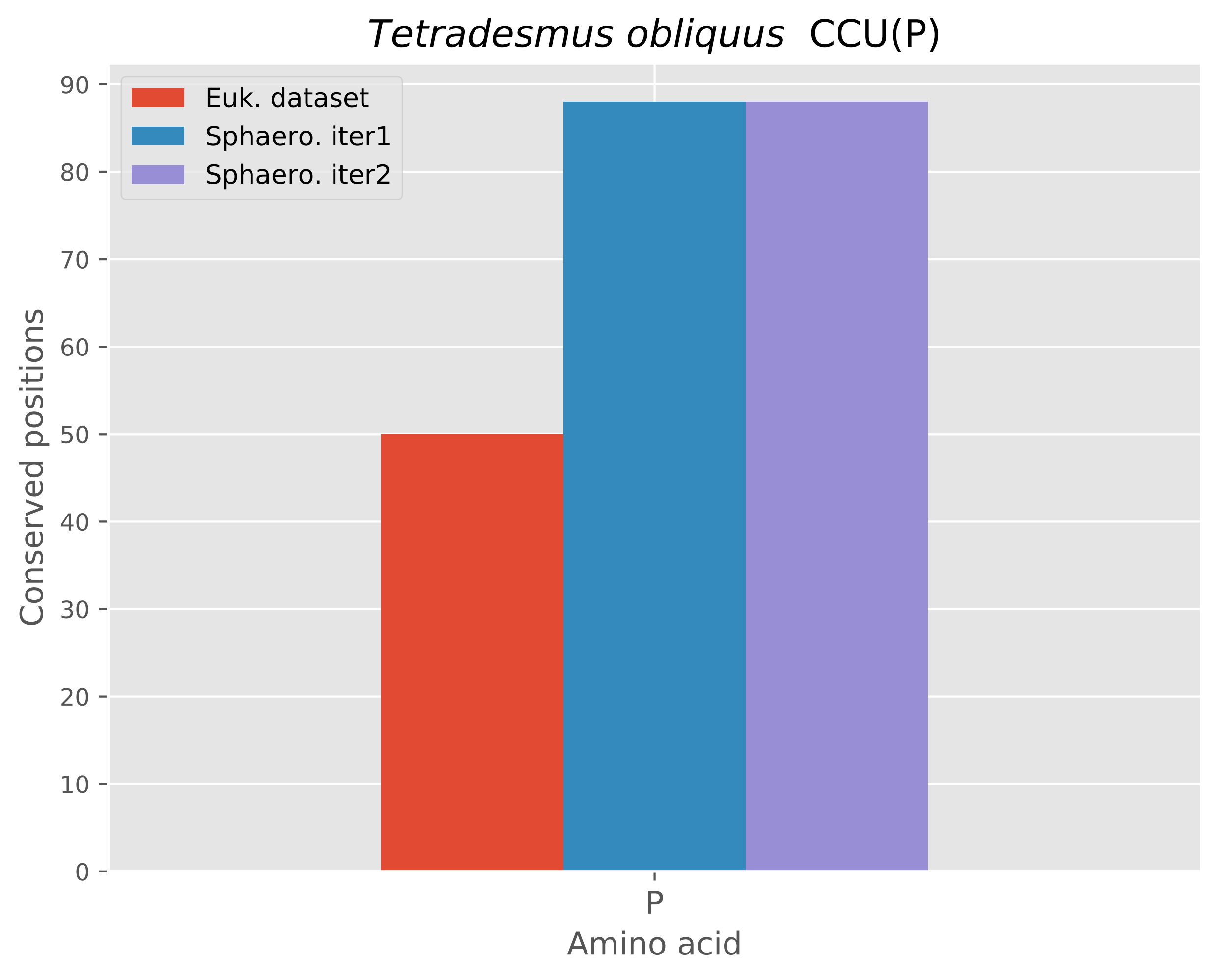

Tetradesmus obliquus CCU(P)
Euk. dataset
Sphaero. iter1
Sphaero. iter2
Conserved positions
0
10
20
30
40
50
60
70
80
90
P
Amino acid

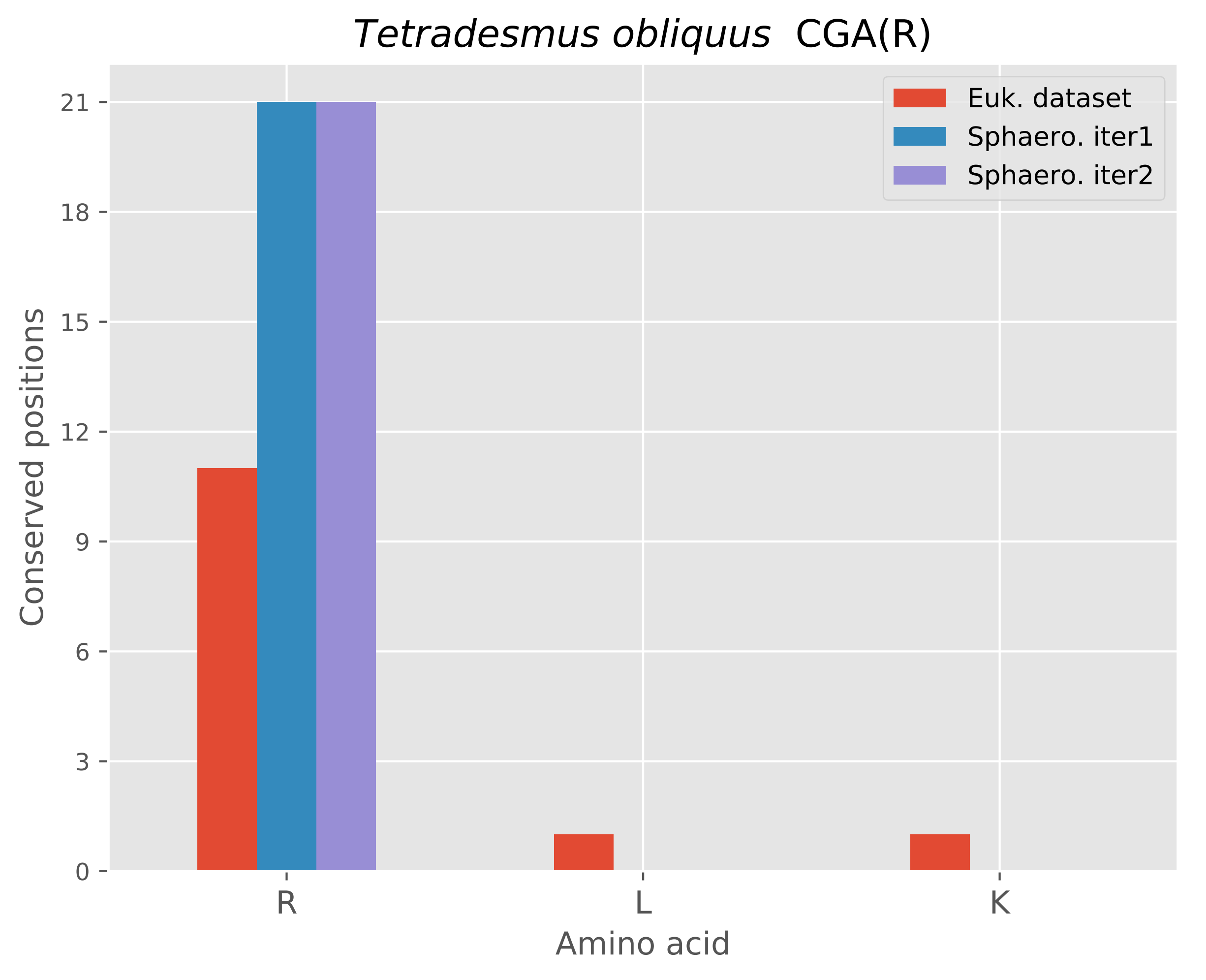
Tetradesmus obliquus CGA(R)
Euk. dataset
Sphaero. iter1
Sphaero. iter2
Conserved positions
0
3
6
9
12
15
18
21
R
L
K
Amino acid

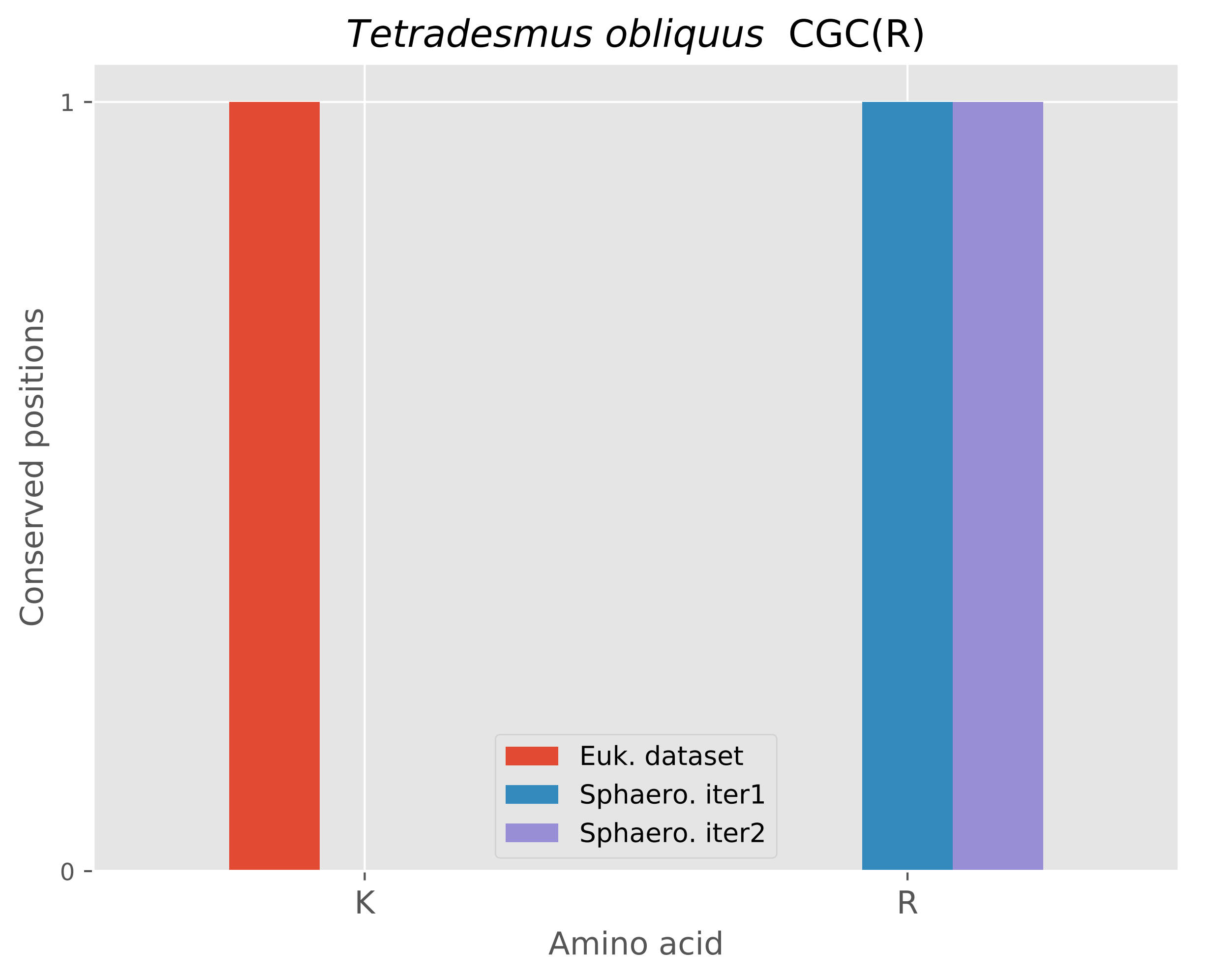

Tetradesmus obliquus CGC(R)
Conserved positions
1
0
K
R
Amino acid
Euk. dataset
Sphaero. iter1
Sphaero. iter2

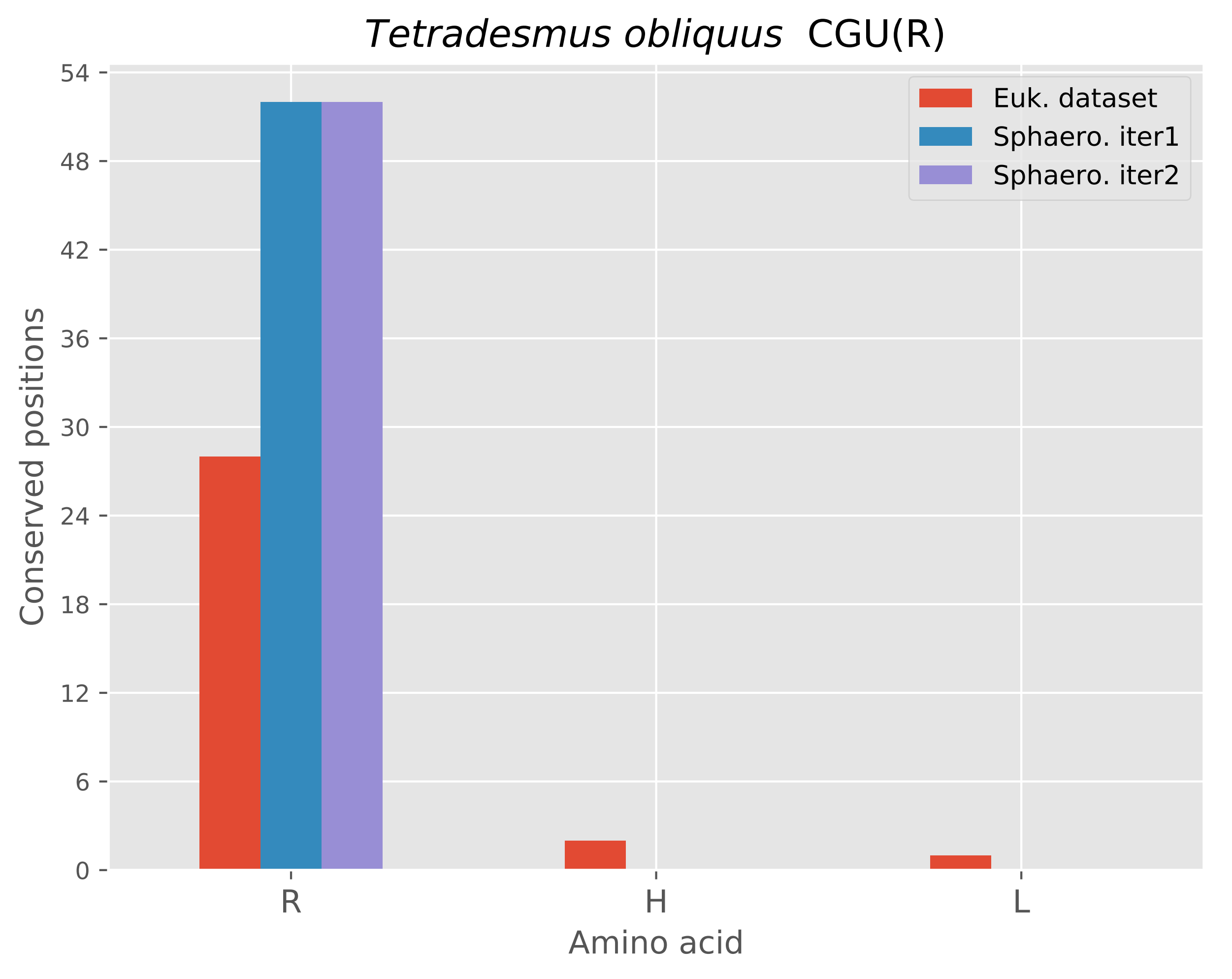

Tetradesmus obliquus CGU(R)
Euk. dataset
Sphaero. iter1
Sphaero. iter2
Conserved positions
0
6
12
18
24
30
36
42
48
54
R
H
L
Amino acid

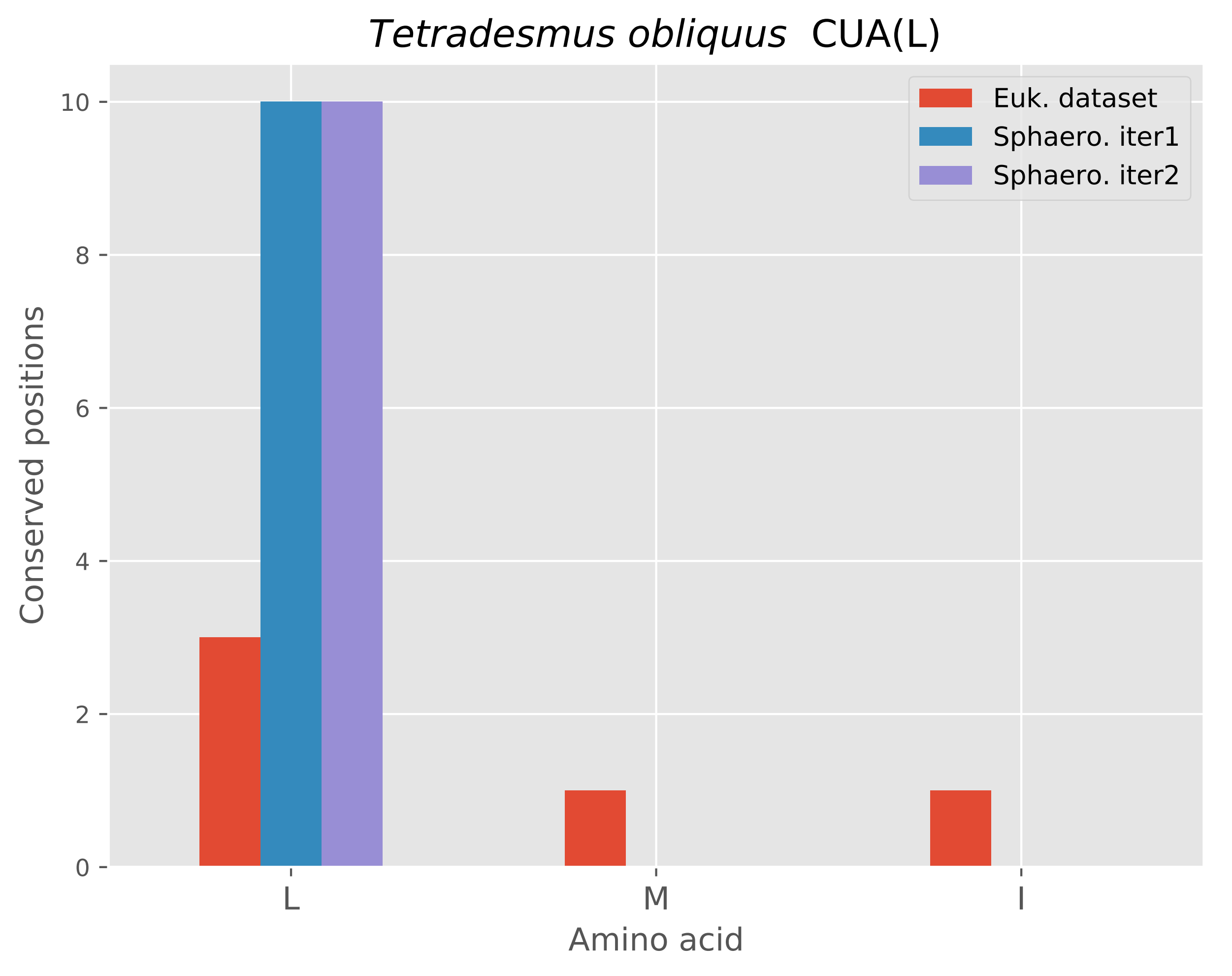

Tetradesmus obliquus CUA(L)
Euk. dataset
Sphaero. iter1
Sphaero. iter2
Conserved positions
0
2
4
6
8
10
L
M
I
Amino acid

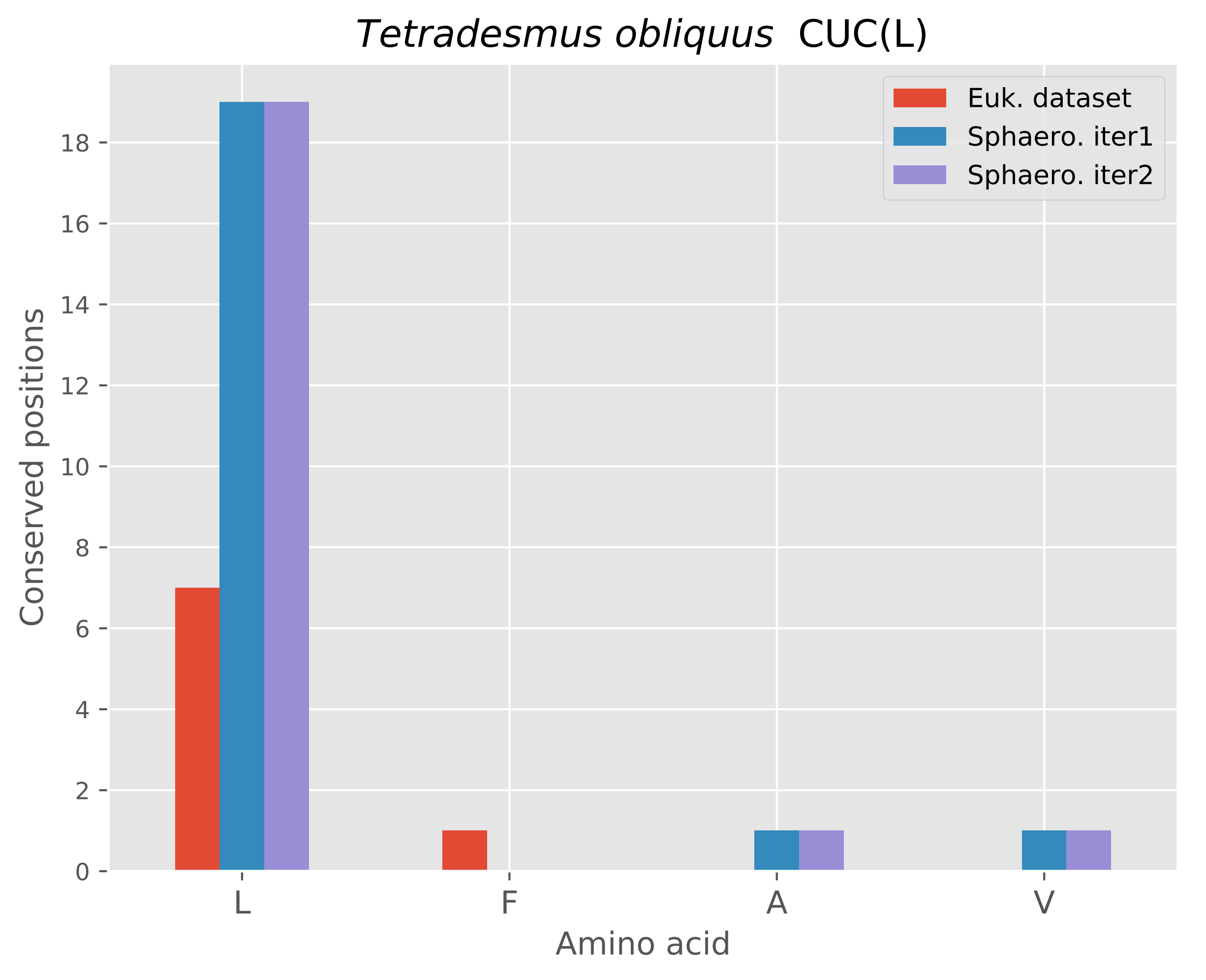

Tetradesmus obliquus CUC(L)
Euk. dataset
Sphaero. iter1
Sphaero. iter2
Conserved positions
0
2
4
6
8
10
12
14
16
18
L
F
A
V
Amino acid

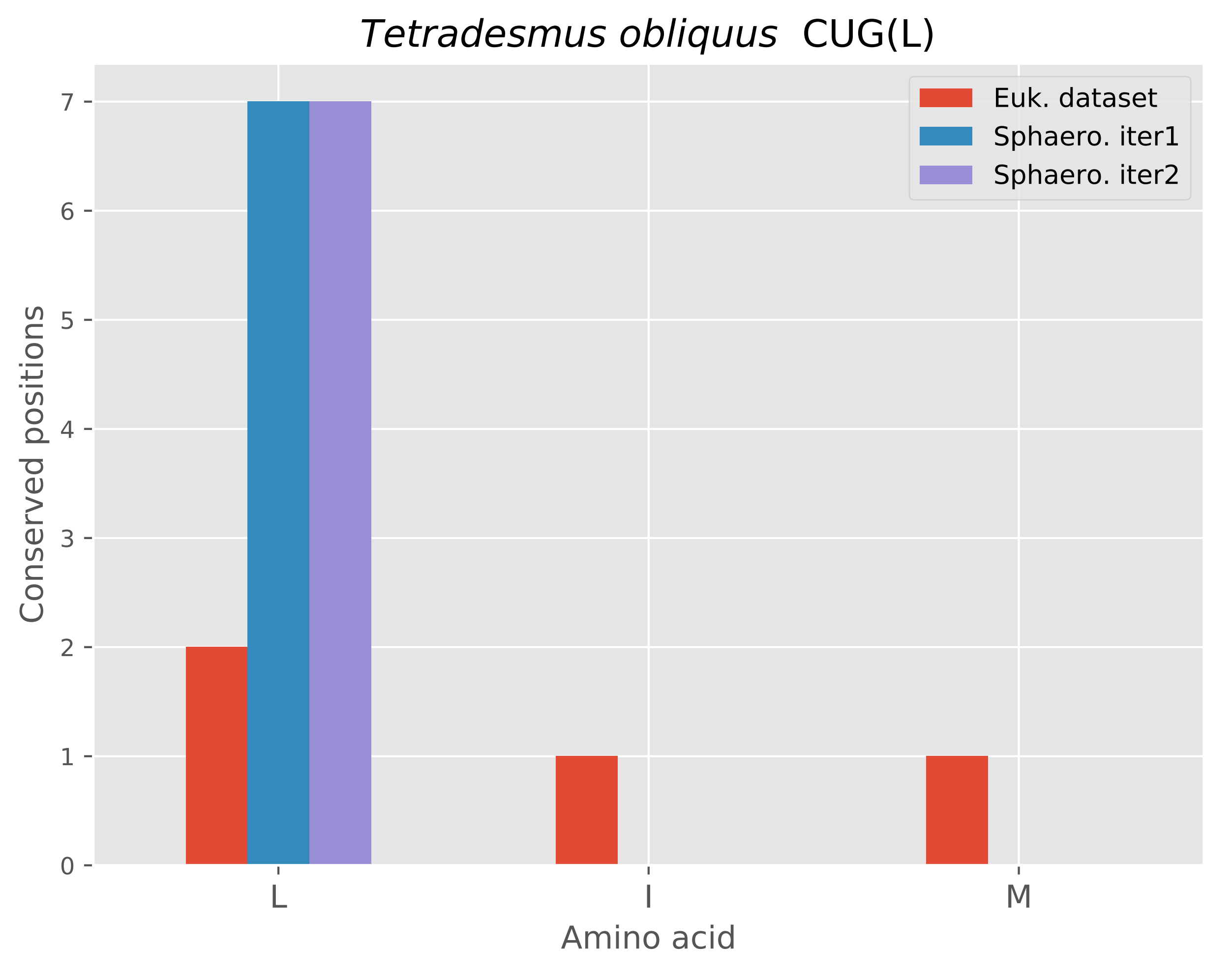

Tetradesmus obliquus CUG(L)
Euk. dataset
Sphaero. iter1
Sphaero. iter2
Conserved positions
0
1
2
3
4
5
6
7
L
I
M
Amino acid

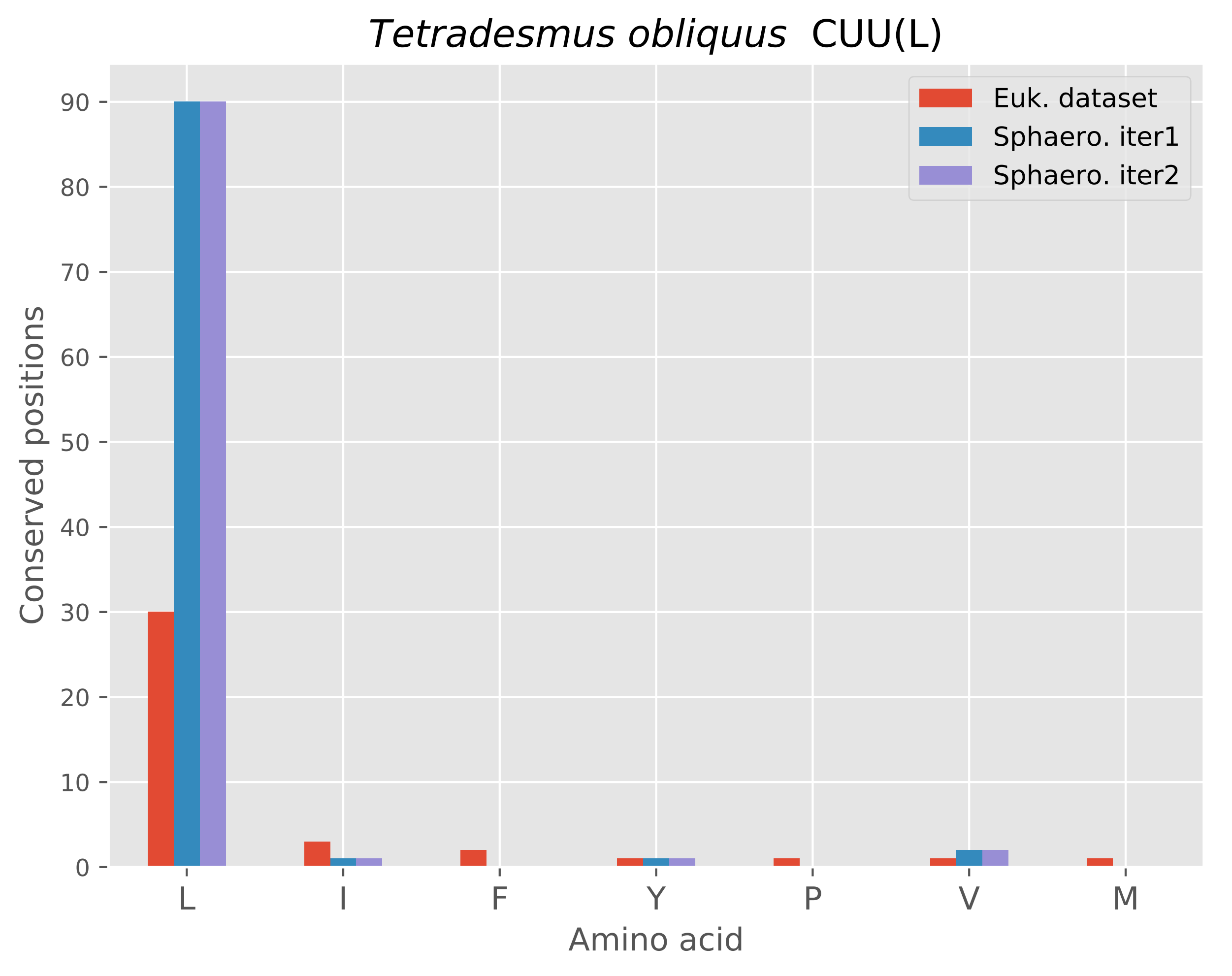

Tetradesmus obliquus CUU(L)
Euk. dataset
Sphaero. iter1
Sphaero. iter2
Conserved positions
0
10
20
30
40
50
60
70
80
90
L
I
F
Y
P
V
M
Amino acid

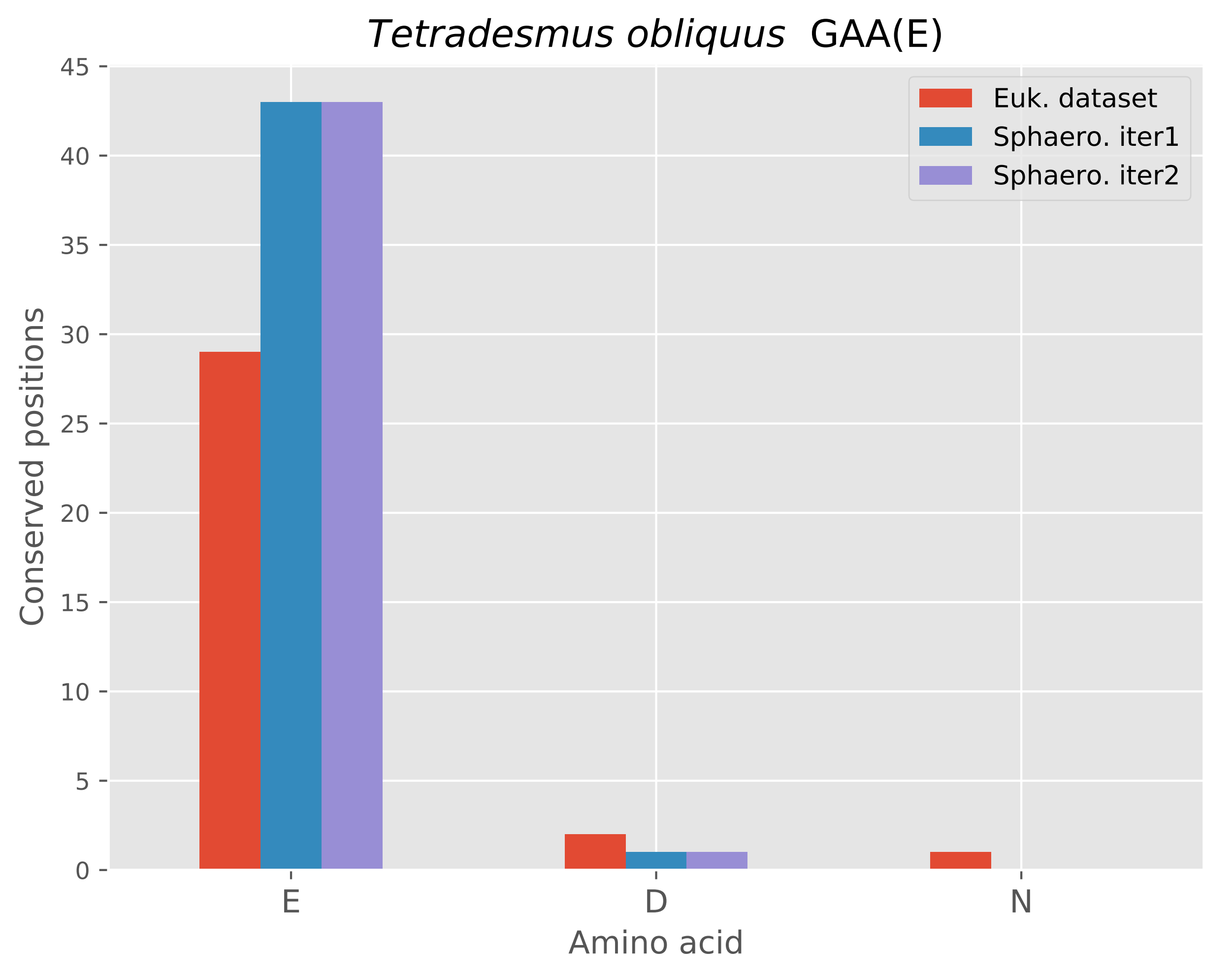

Tetradesmus obliquus GAA(E)
Euk. dataset
Sphaero. iter1
Sphaero. iter2
Conserved positions
0
5
10
15
20
25
30
35
40
45
E
D
N
Amino acid

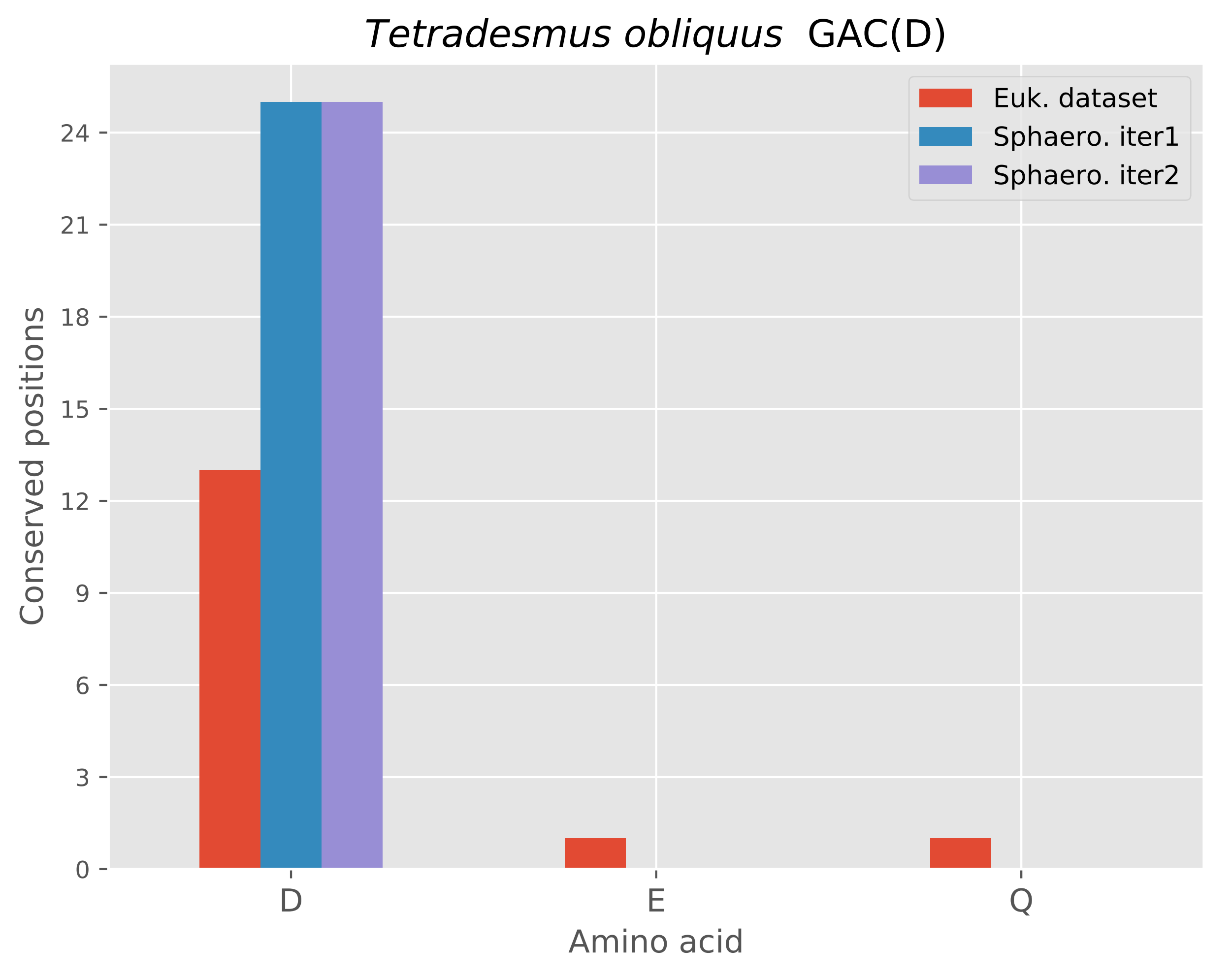

Tetradesmus obliquus GAC(D)
Euk. dataset
Sphaero. iter1
Sphaero. iter2
Conserved positions
0
3
6
9
12
15
18
21
24
D
E
Q
Amino acid

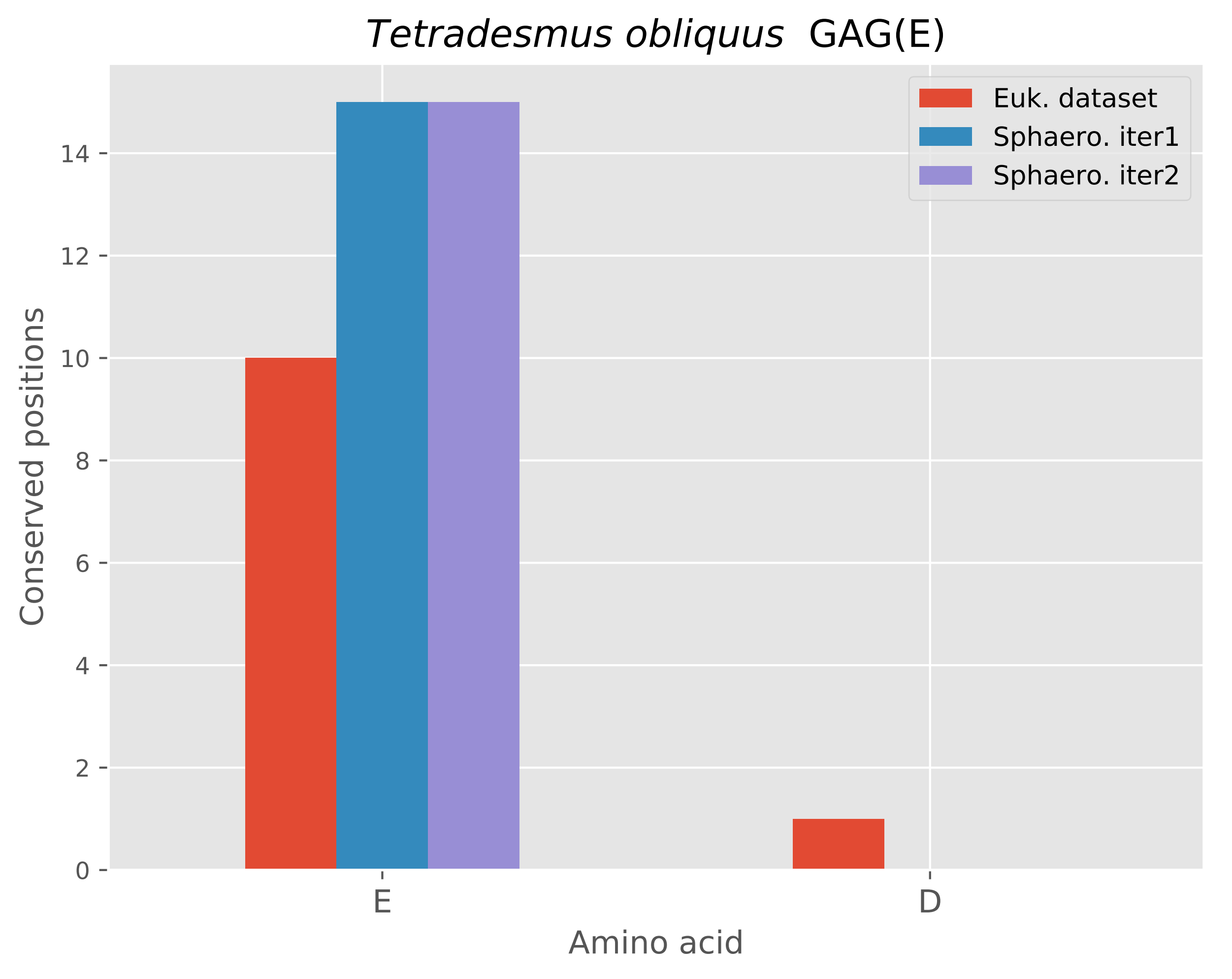

Tetradesmus obliquus GAG(E)
Euk. dataset
Sphaero. iter1
Sphaero. iter2
Conserved positions
0
2
4
6
8
10
12
14
E
D
Amino acid

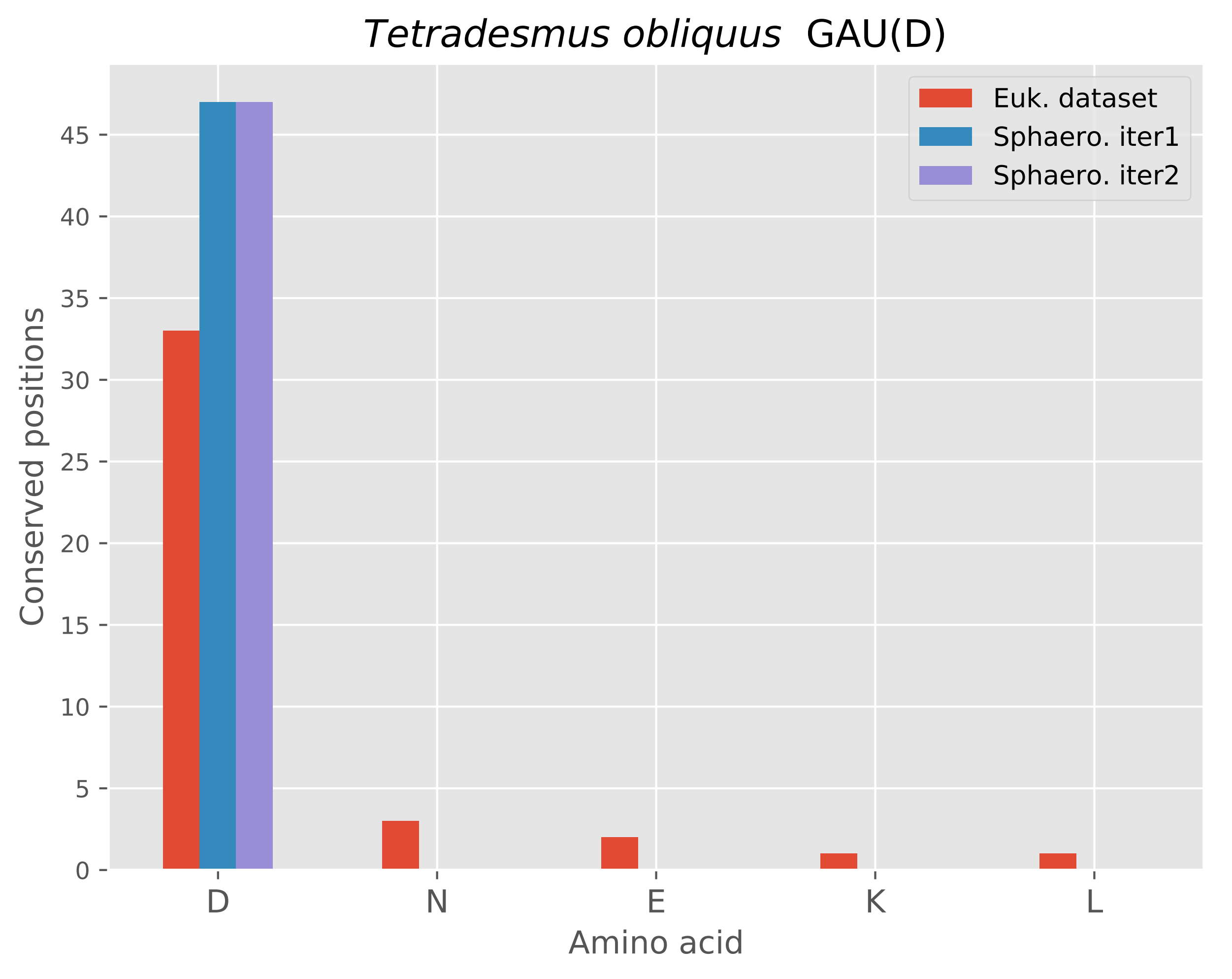

Tetradesmus obliquus GAU(D)
Euk. dataset
Sphaero. iter1
Sphaero. iter2
Conserved positions
0
5
10
15
20
25
30
35
40
45
D
N
E
K
L
Amino acid

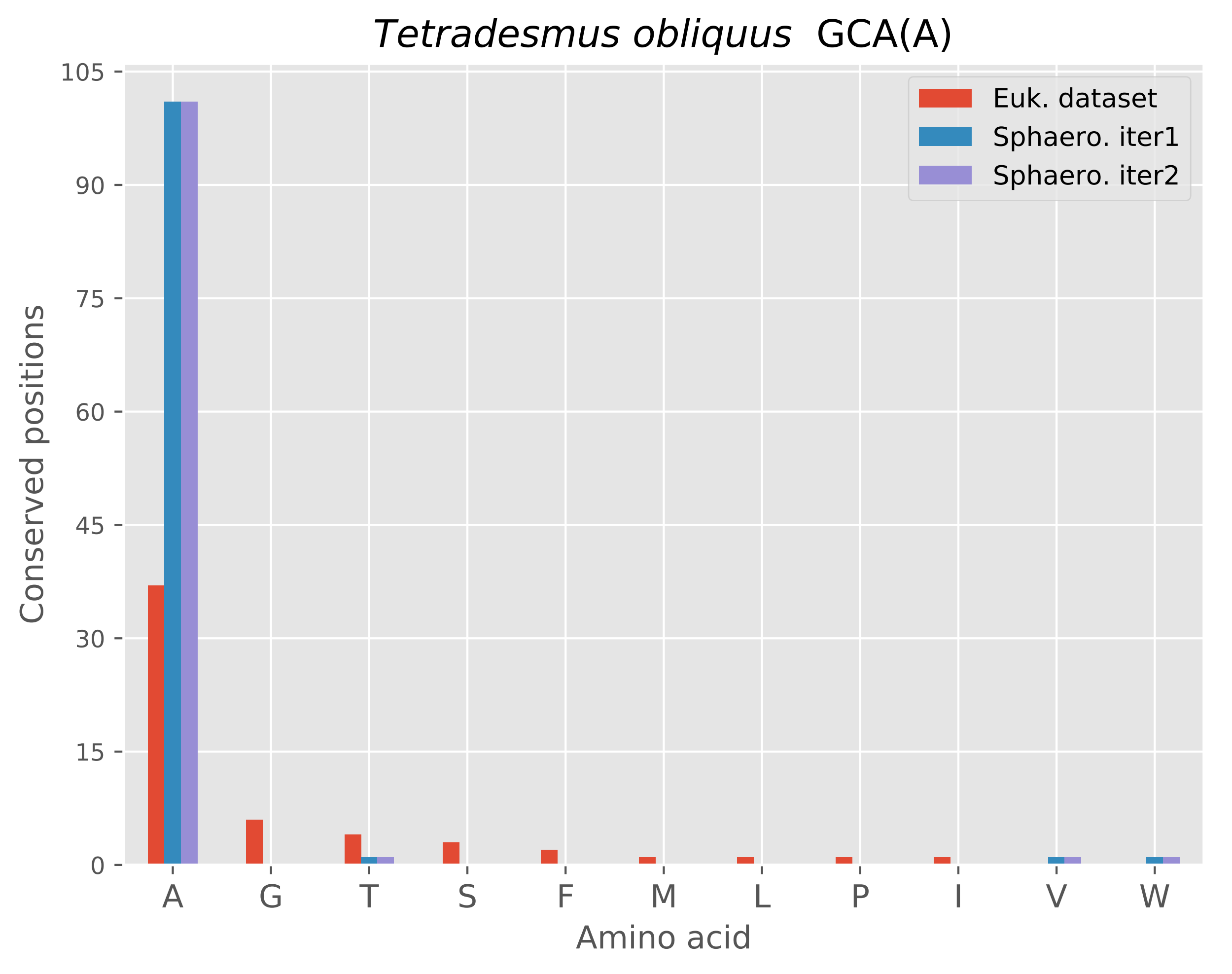

Tetradesmus obliquus GCA(A)
Euk. dataset
Sphaero. iter1
Sphaero. iter2
Conserved positions
Amino acid
0
15
30
45
60
75
90
105
A
G
T
S
F
M
L
P
I
V
W

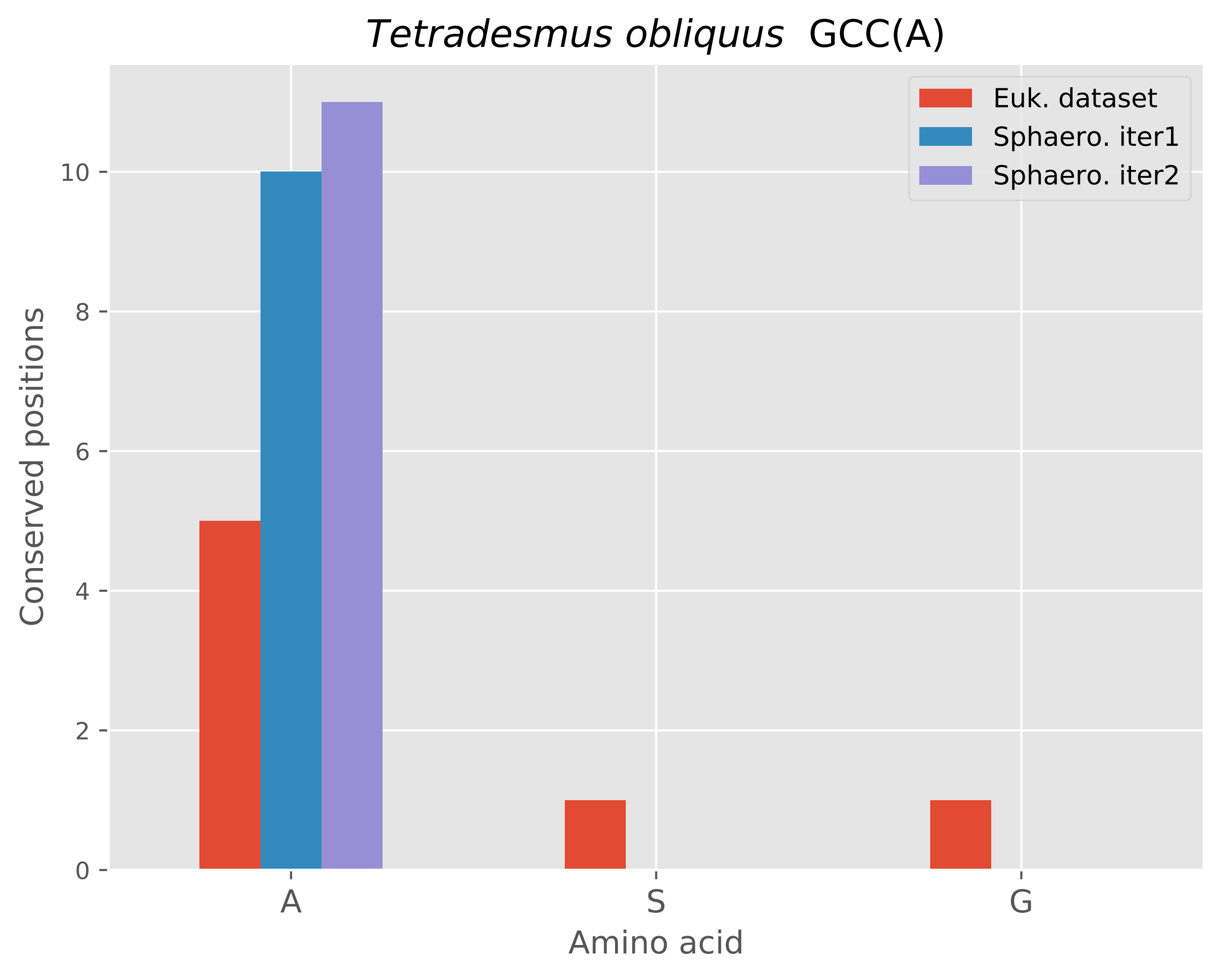

Tetradesmus obliquus GCC(A)
Euk. dataset
Sphaero. iter1
Sphaero. iter2
Conserved positions
0
2
4
6
8
10
A
S
G
Amino acid

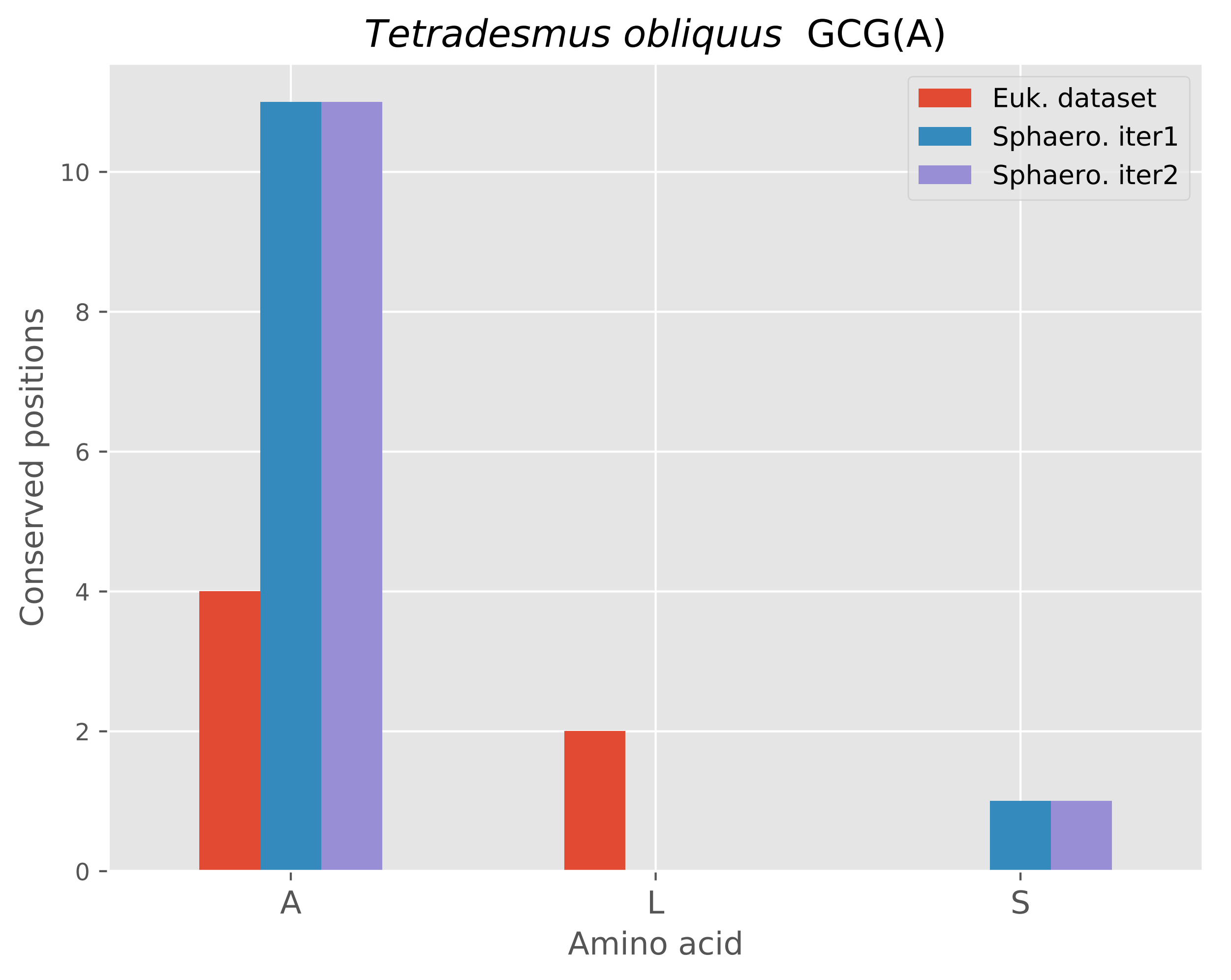

Tetradesmus obliquus GCG(A)
Euk. dataset
Sphaero. iter1
Sphaero. iter2
Conserved positions
0
2
4
6
8
10
A
L
S
Amino acid

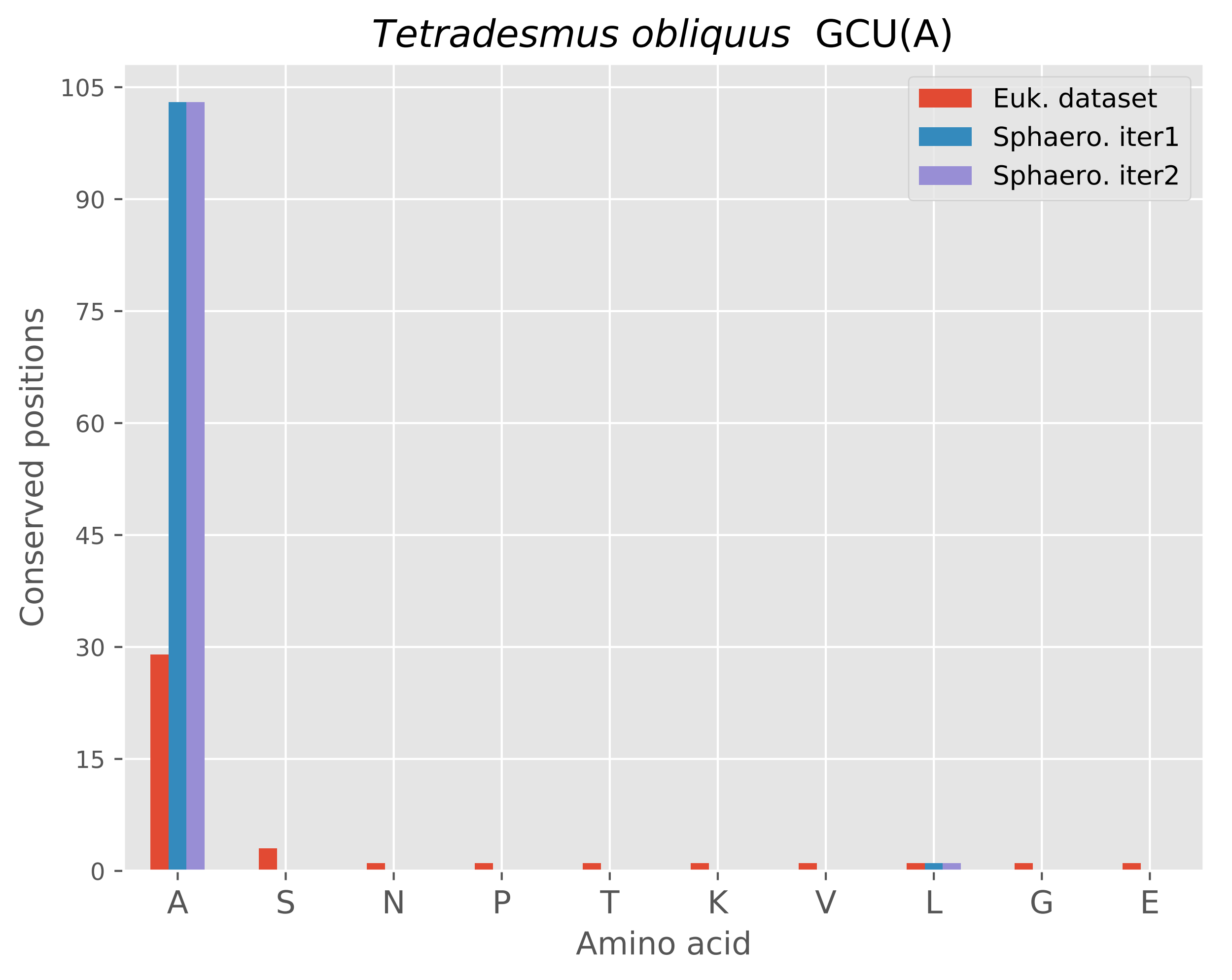

Tetradesmus obliquus GCU(A)
Euk. dataset
Sphaero. iter1
Sphaero. iter2
Conserved positions
0
15
30
45
60
75
90
105
A
S
N
P
T
K
V
L
G
E
Amino acid

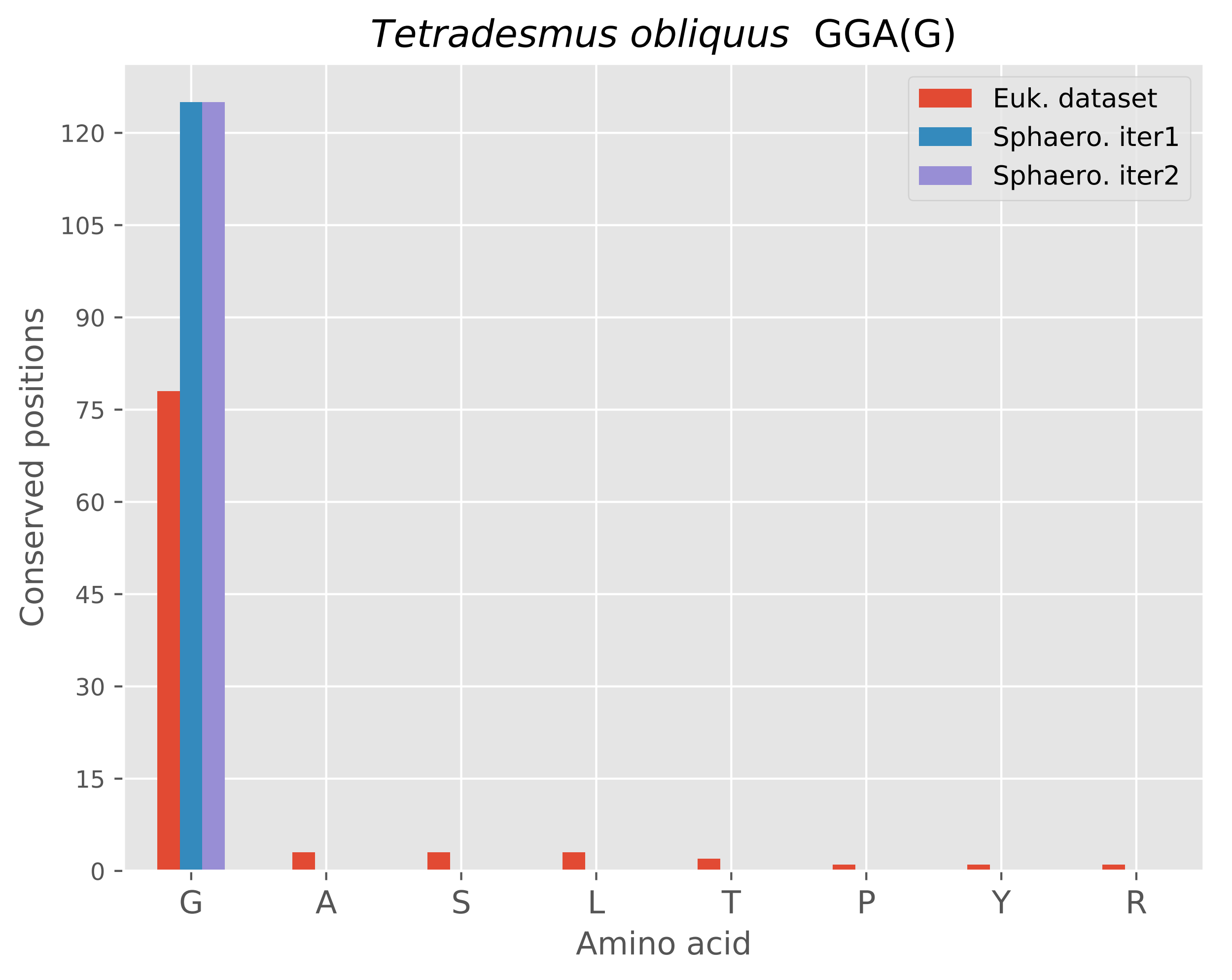

Tetradesmus obliquus GGA(G)
Euk. dataset
Sphaero. iter1
Sphaero. iter2
Conserved positions
0
15
30
45
60
75
90
105
120
G
A
S
L
T
P
Y
R
Amino acid

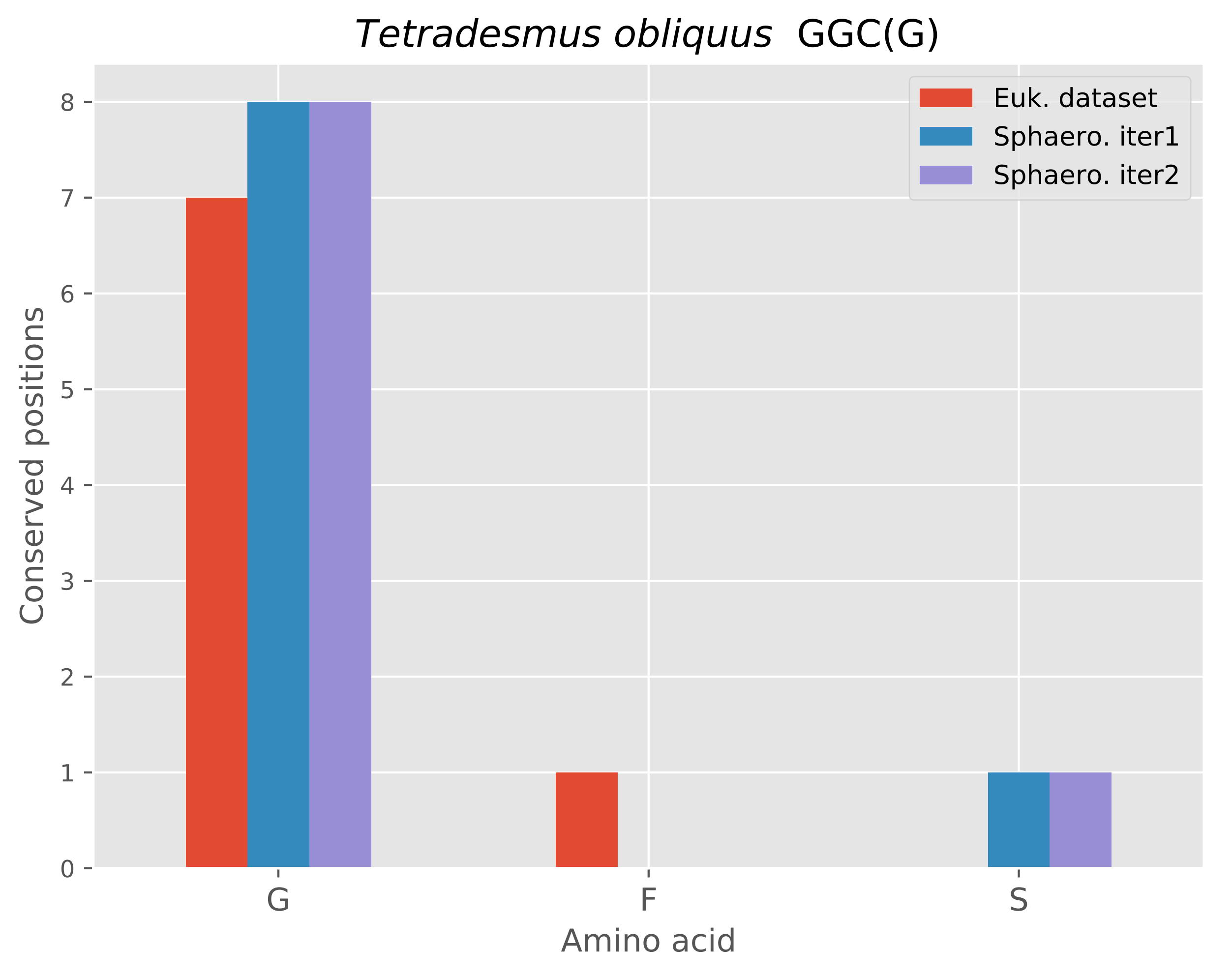

Tetradesmus obliquus GGC(G)
Euk. dataset
Sphaero. iter1
Sphaero. iter2
Conserved positions
0
1
2
3
4
5
6
7
8
G
F
S
Amino acid

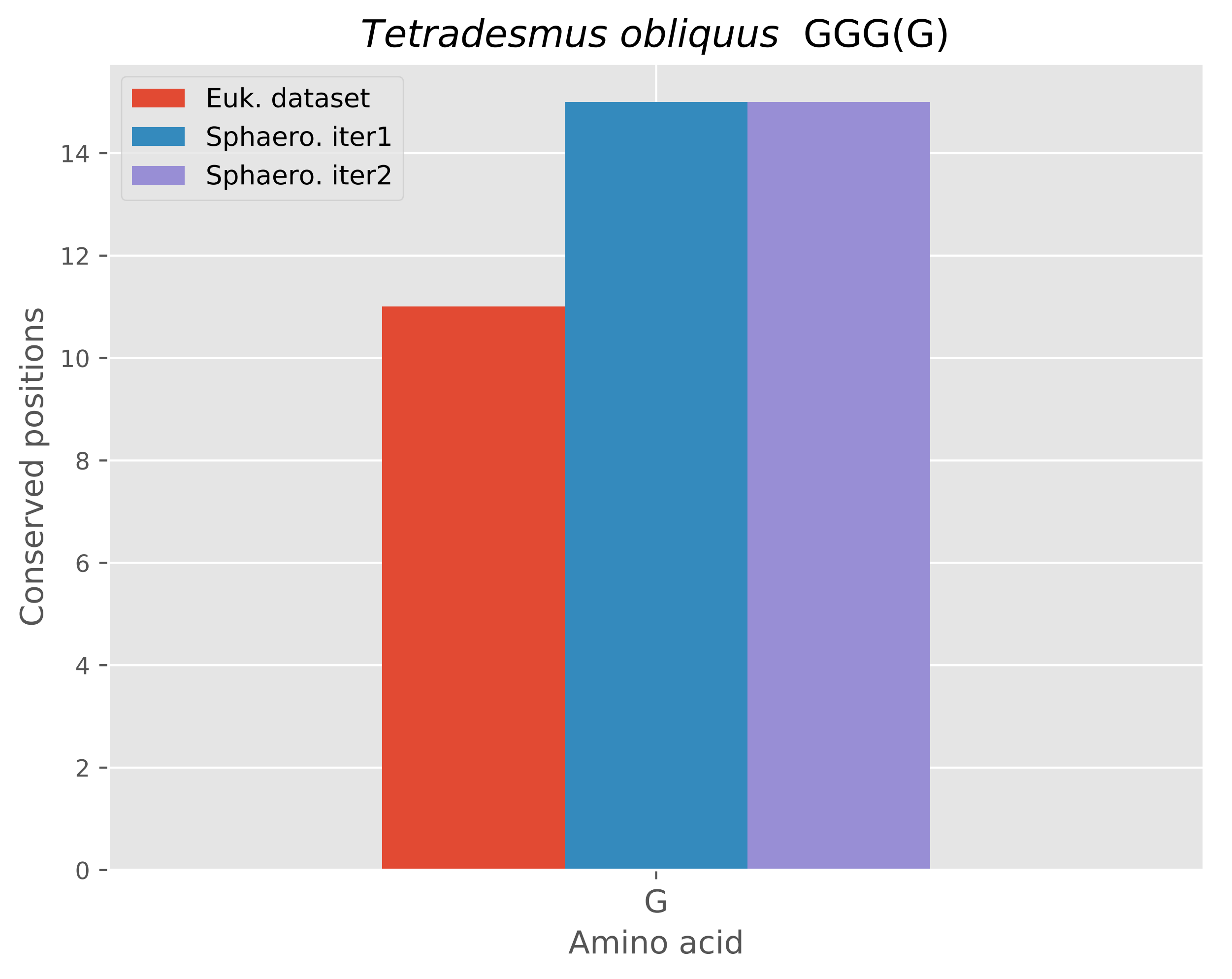

Tetradesmus obliquus GGG(G)
Euk. dataset
Sphaero. iter1
Sphaero. iter2
Conserved positions
0
2
4
6
8
10
12
14
G
Amino acid

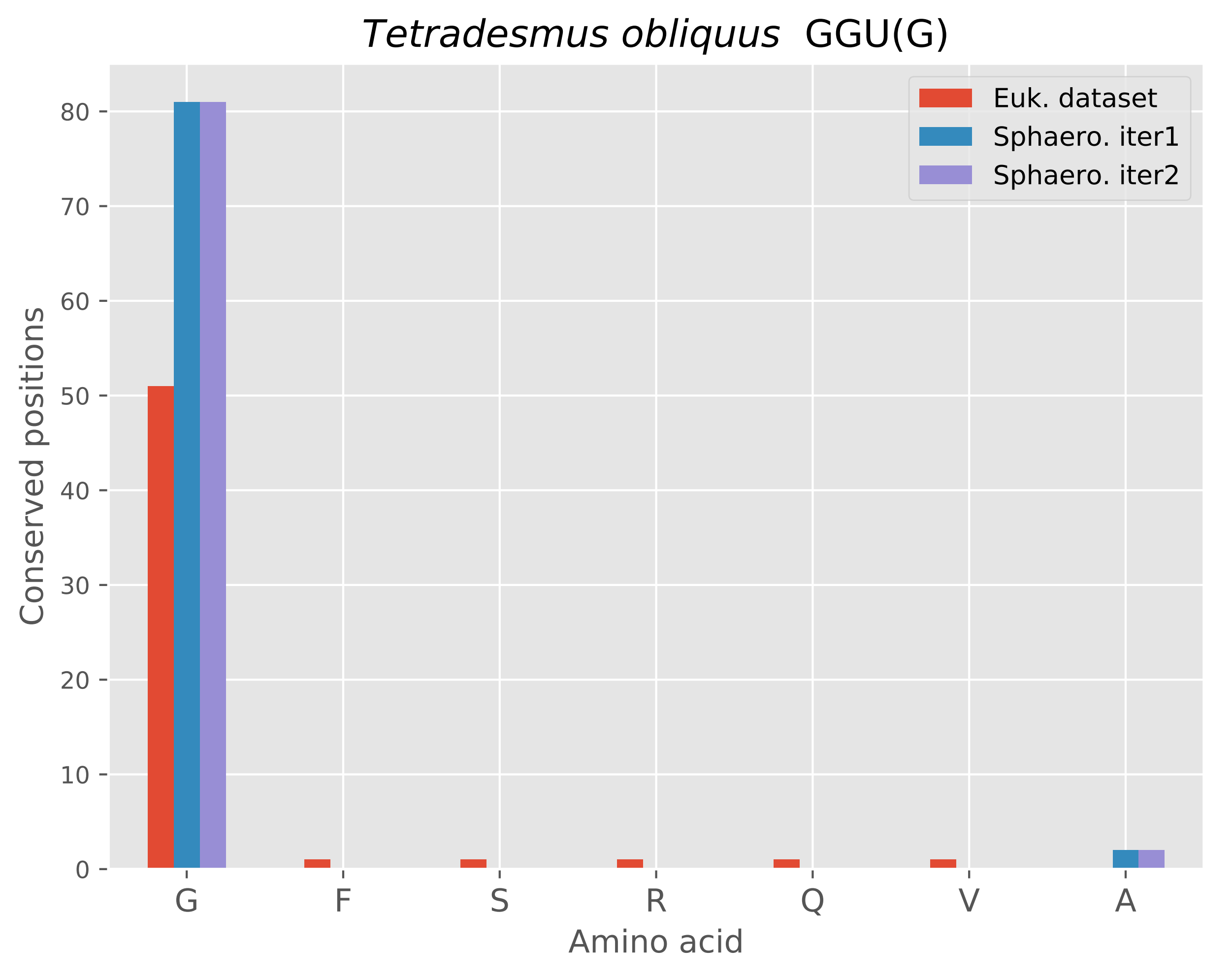

Tetradesmus obliquus GGU(G)
Euk. dataset
Sphaero. iter1
Sphaero. iter2
Conserved positions
0
10
20
30
40
50
60
70
80
G
F
S
R
Q
V
A
Amino acid

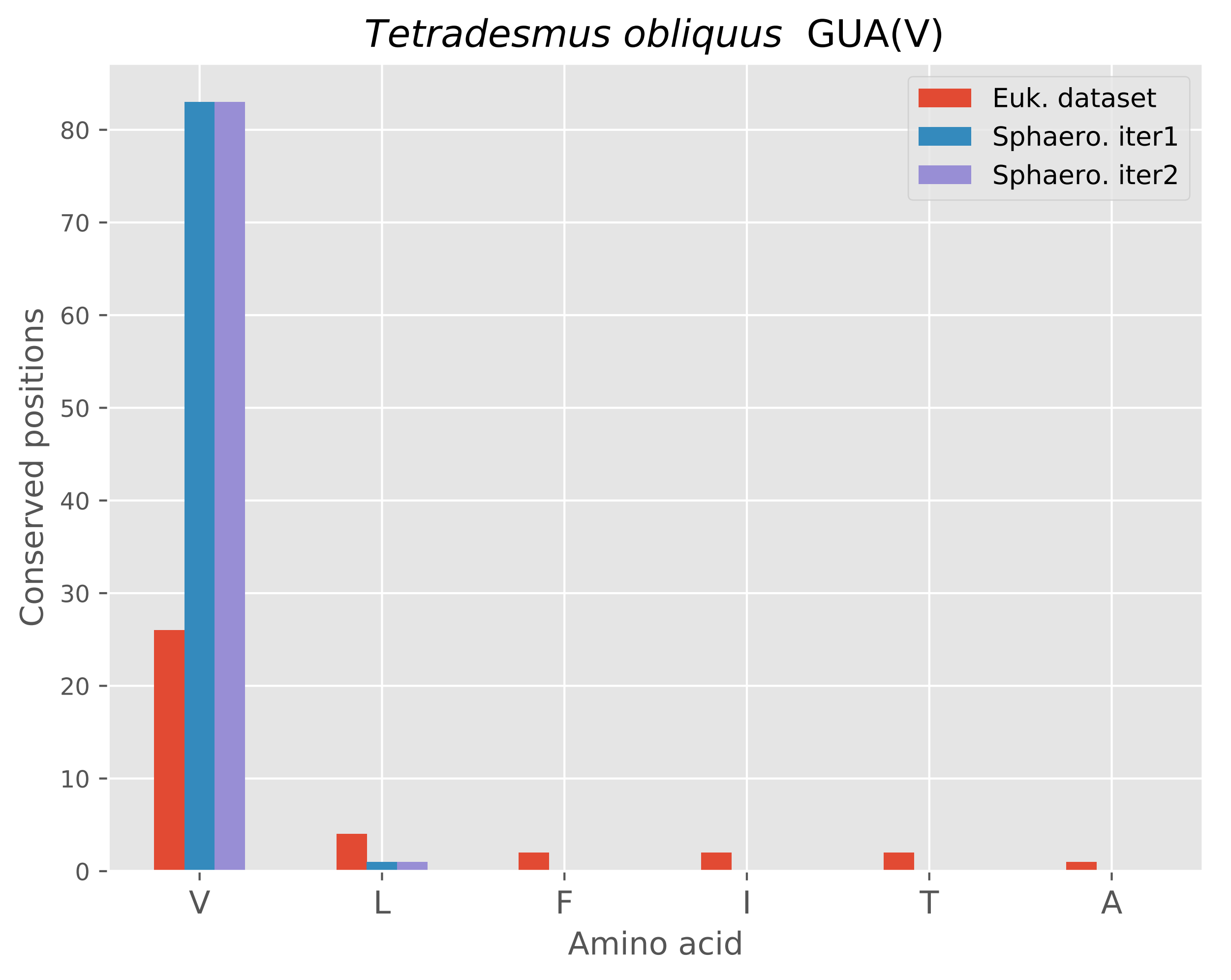

Tetradesmus obliquus GUA(V)
Euk. dataset
Sphaero. iter1
Sphaero. iter2
Conserved positions
0
10
20
30
40
50
60
70
80
V
L
F
I
T
A
Amino acid

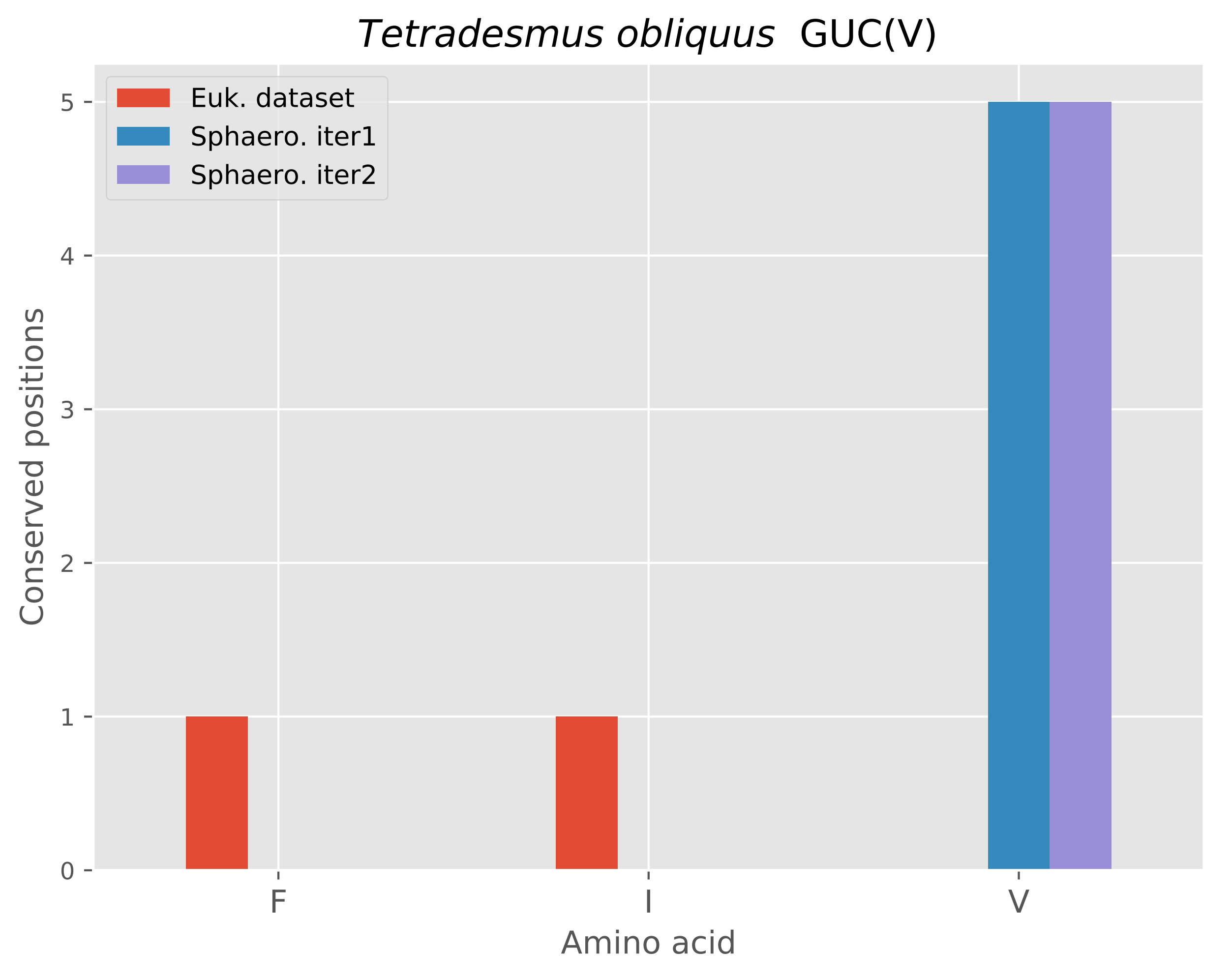

Tetradesmus obliquus GUC(V)
Euk. dataset
Sphaero. iter1
Sphaero. iter2
Conserved positions
0
1
2
3
4
5
F
I
V
Amino acid

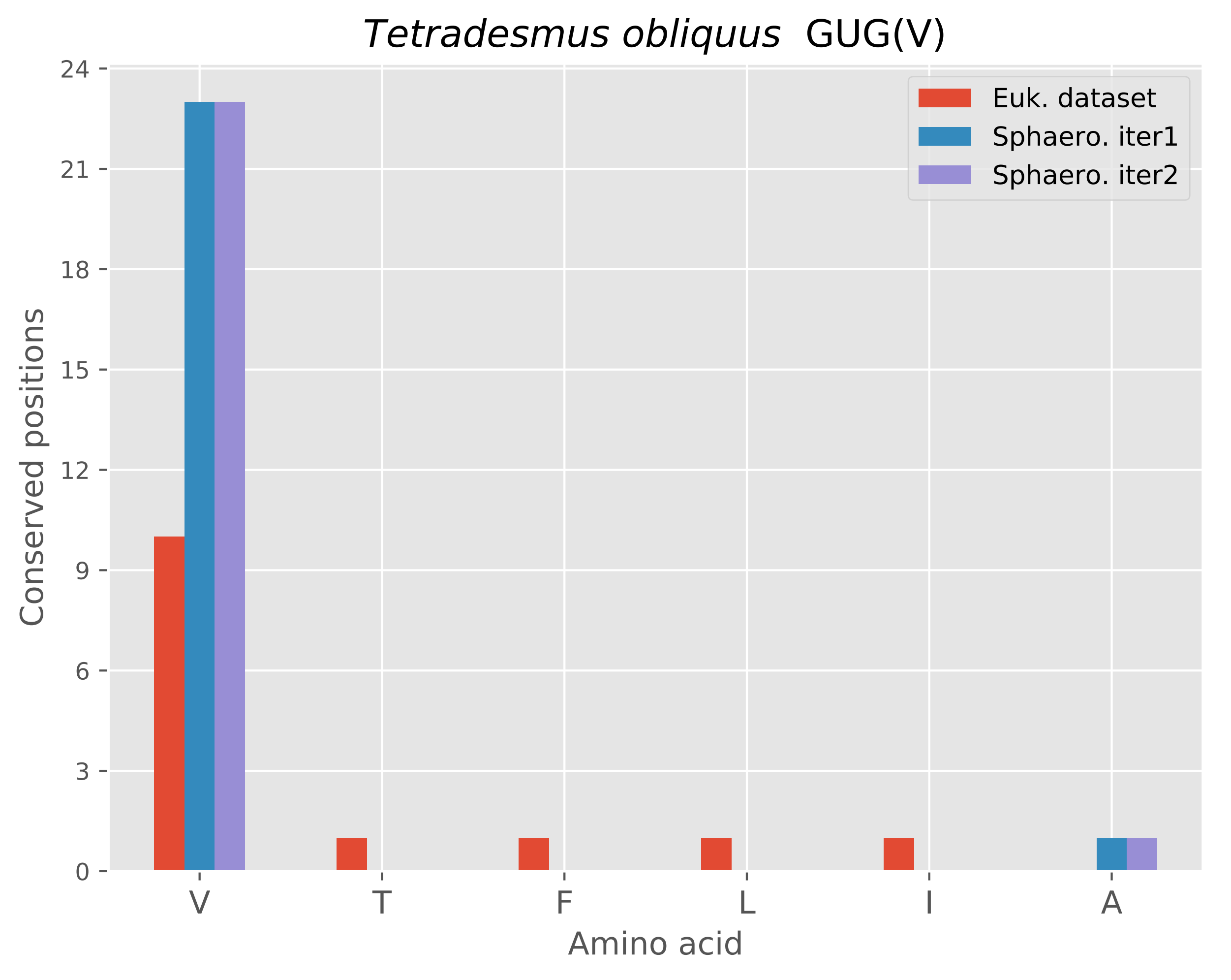

Tetradesmus obliquus GUG(V)
Conserved positions
0
3
6
9
12
15
18
21
24
V
T
F
L
I
A
Amino acid
Euk. dataset
Sphaero. iter1
Sphaero. iter2

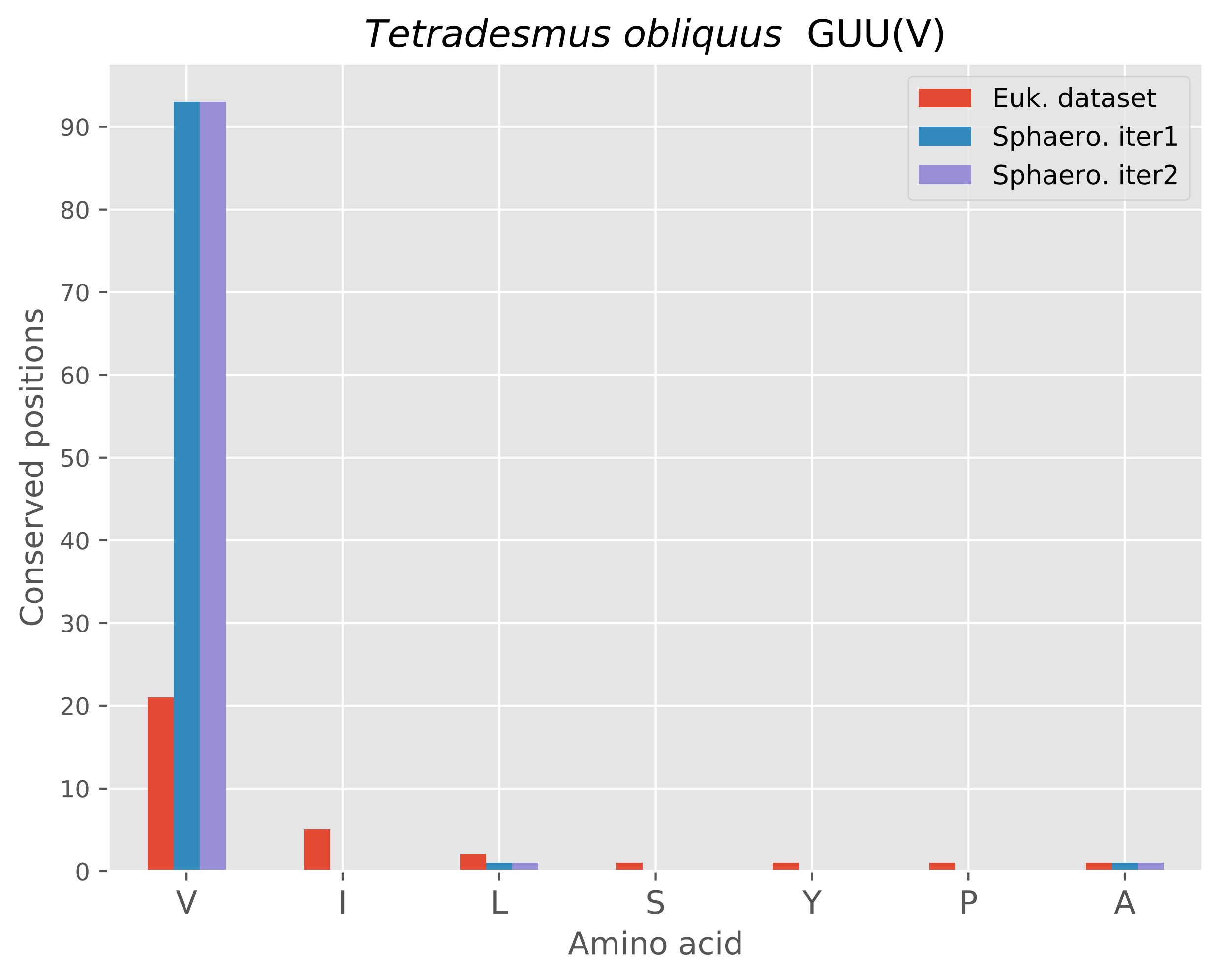

Tetradesmus obliquus GUU(V)
Euk. dataset
Sphaero. iter1
Sphaero. iter2
Conserved positions
0
10
20
30
40
50
60
70
80
90
V
I
L
S
Y
P
A
Amino acid

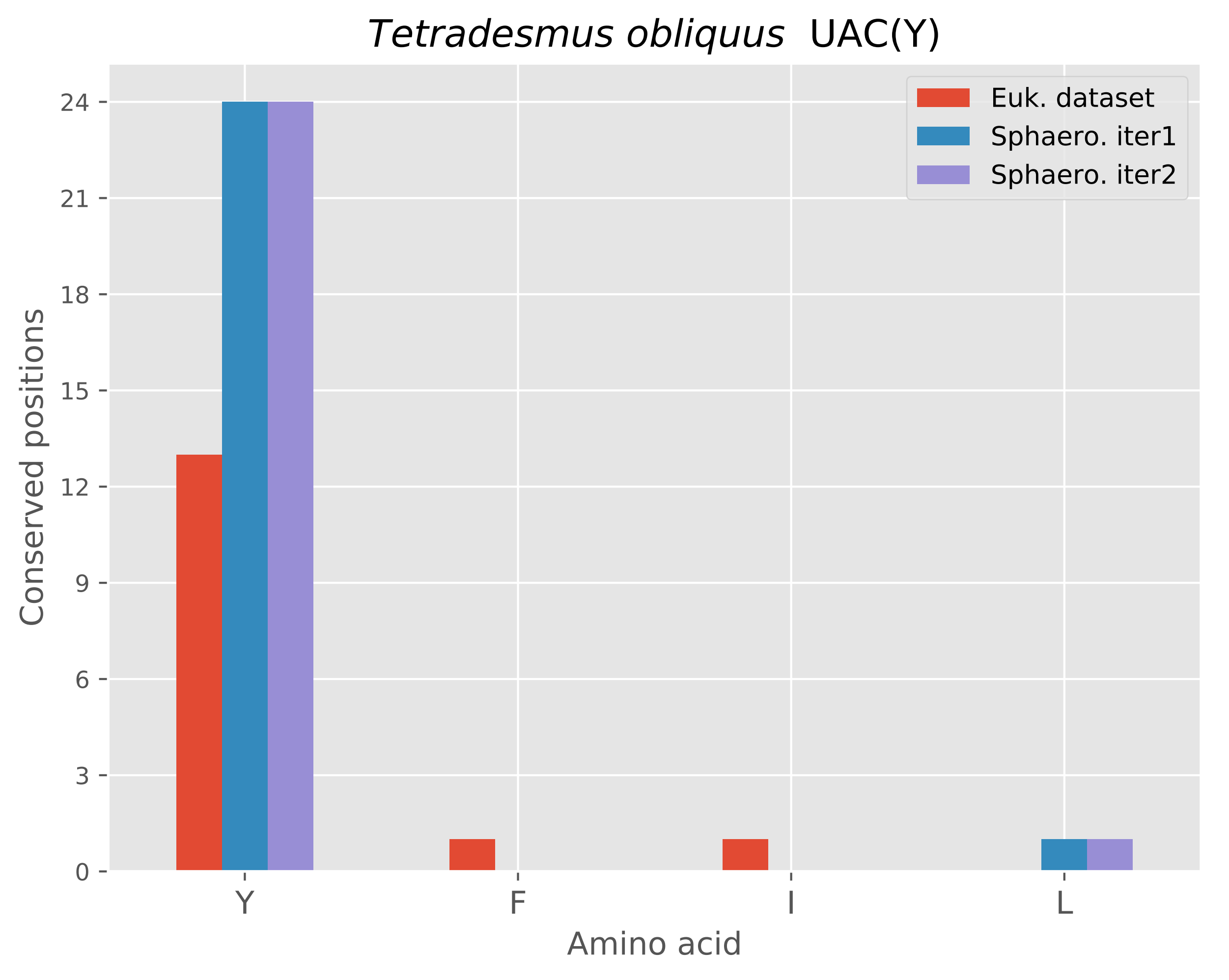

Tetradesmus obliquus UAC(Y)
Euk. dataset
Sphaero. iter1
Sphaero. iter2
Conserved positions
0
3
6
9
12
15
18
21
24
Y
F
I
L
Amino acid

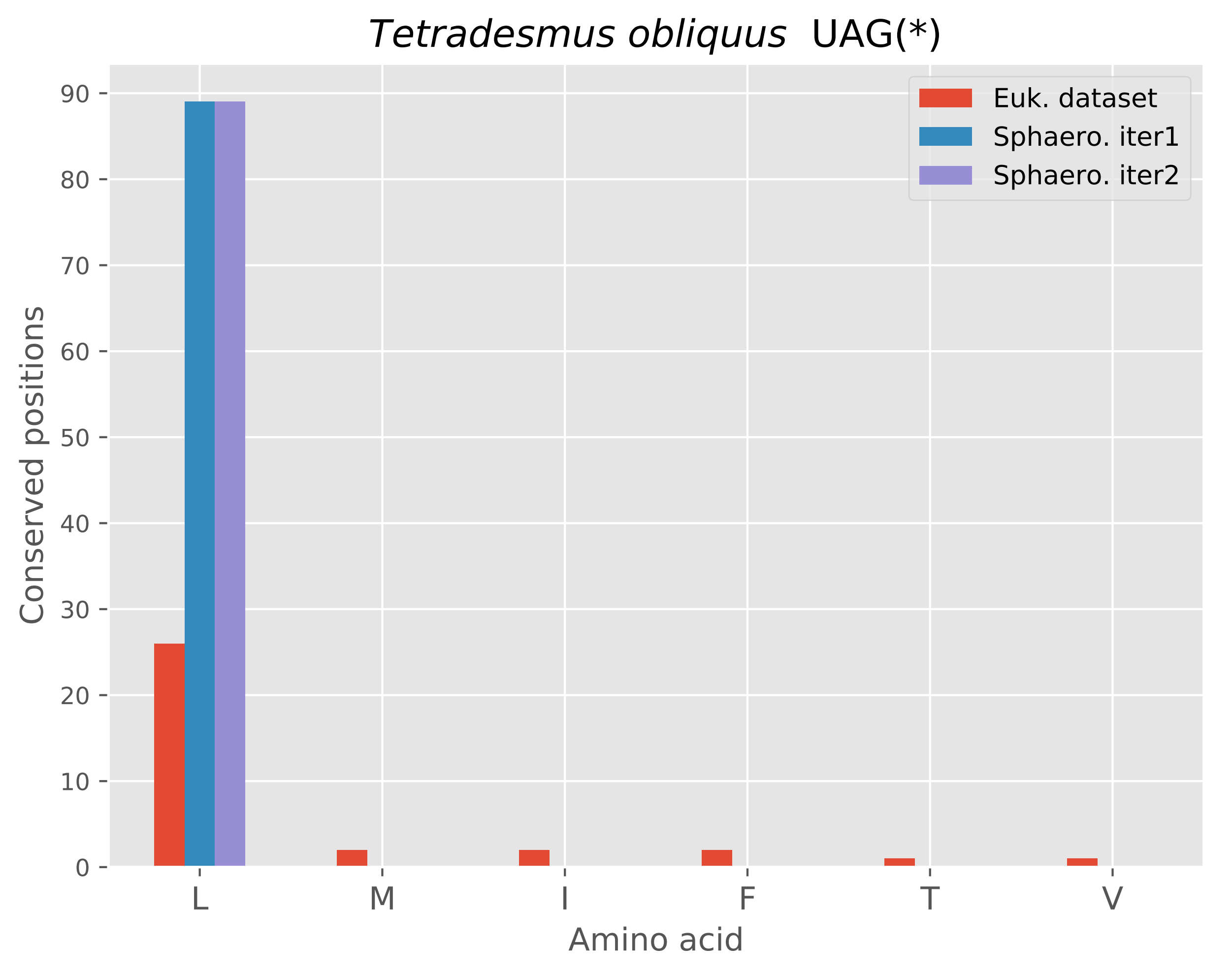

Tetradesmus obliquus UAG(*)
Euk. dataset
Sphaero. iter1
Sphaero. iter2
Conserved positions
0
10
20
30
40
50
60
70
80
90
L
M
I
F
T
V
Amino acid

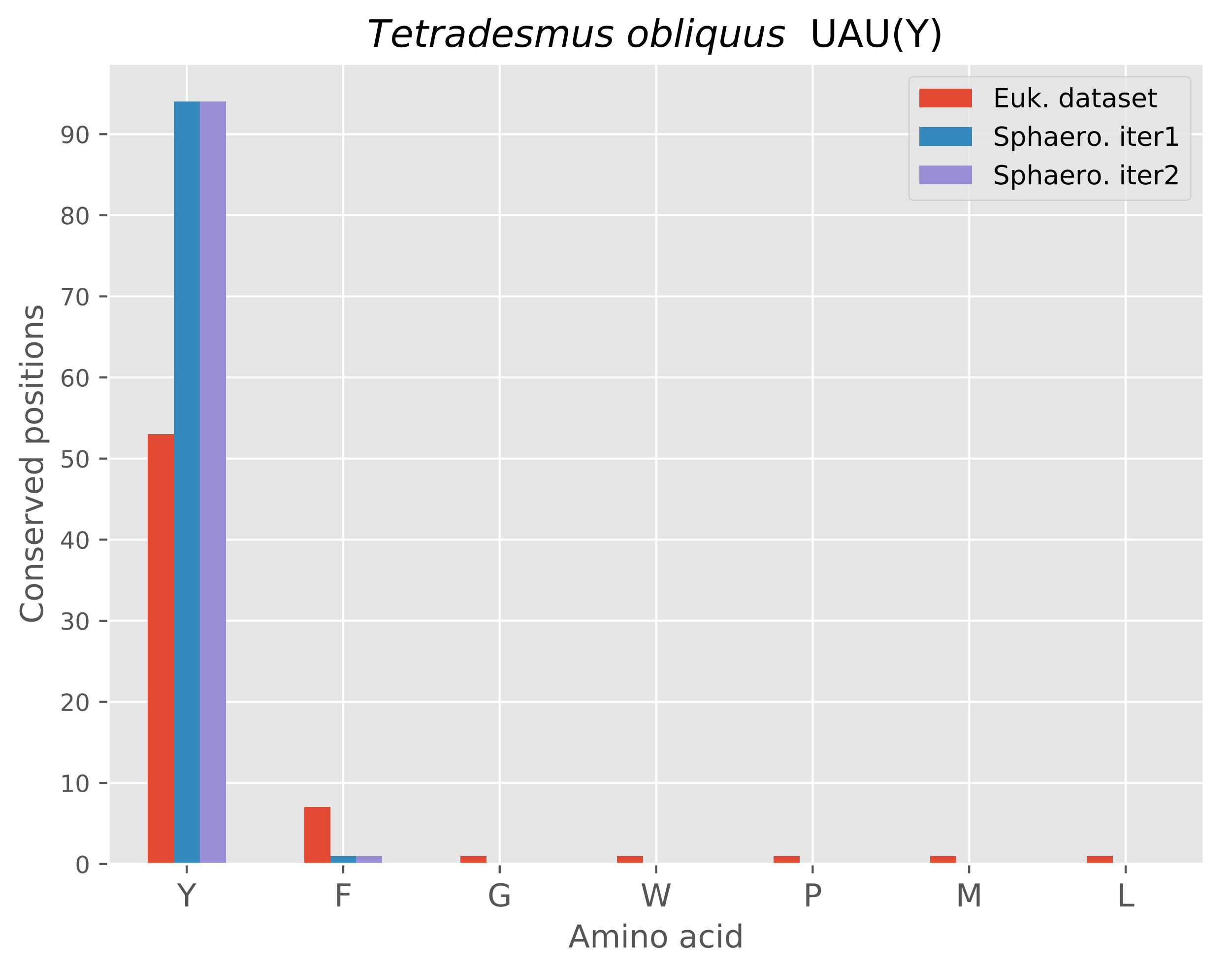

Tetradesmus obliquus UAU(Y)
Euk. dataset
Sphaero. iter1
Sphaero. iter2
Conserved positions
0
10
20
30
40
50
60
70
80
90
Y
F
G
W
P
M
L
Amino acid

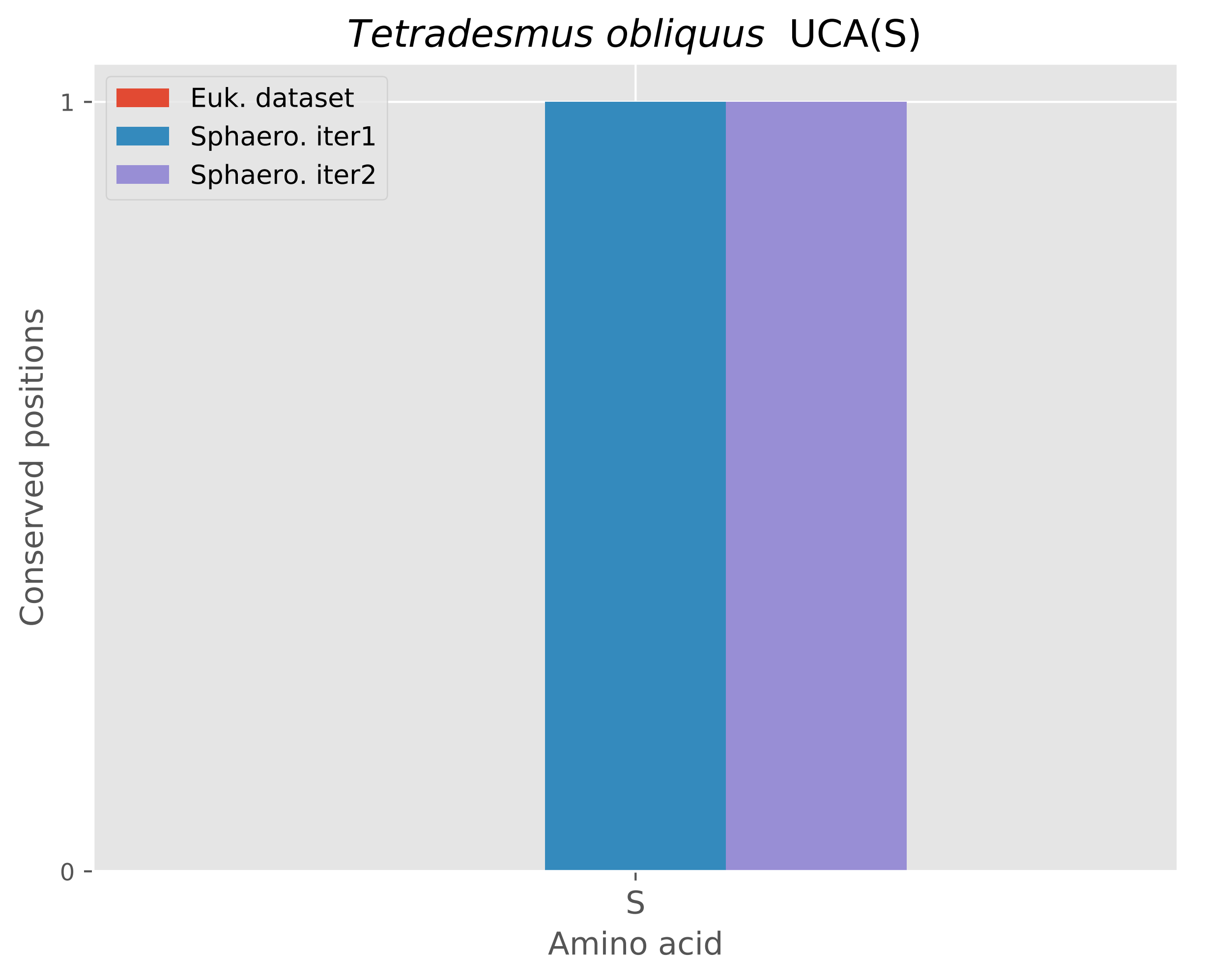

Tetradesmus obliquus UCA(S)
Euk. dataset
Sphaero. iter1
Sphaero. iter2
Conserved positions
1
0
S
Amino acid

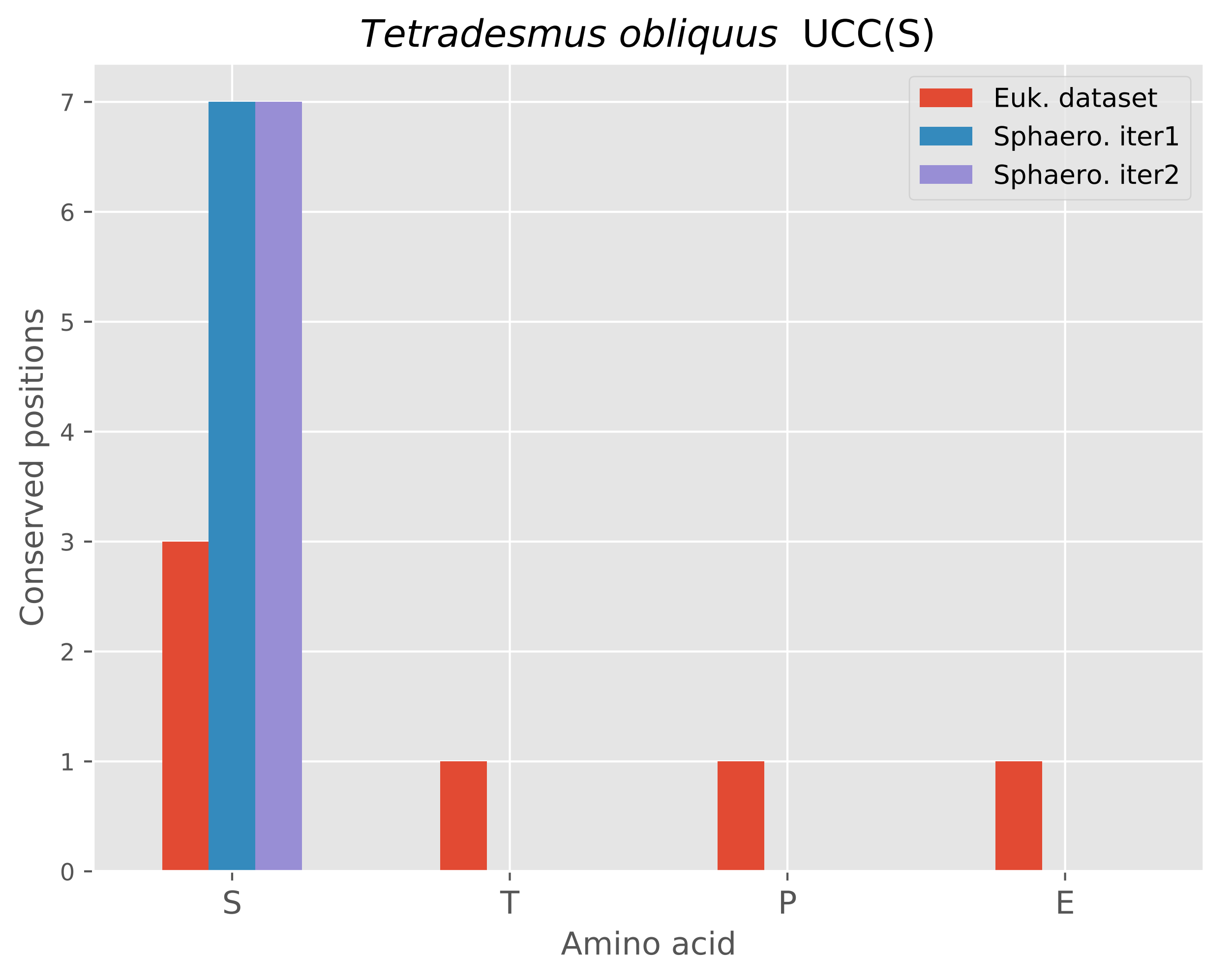

Tetradesmus obliquus UCC(S)
Euk. dataset
Sphaero. iter1
Sphaero. iter2
Conserved positions
0
1
2
3
4
5
6
7
S
T
P
E
Amino acid

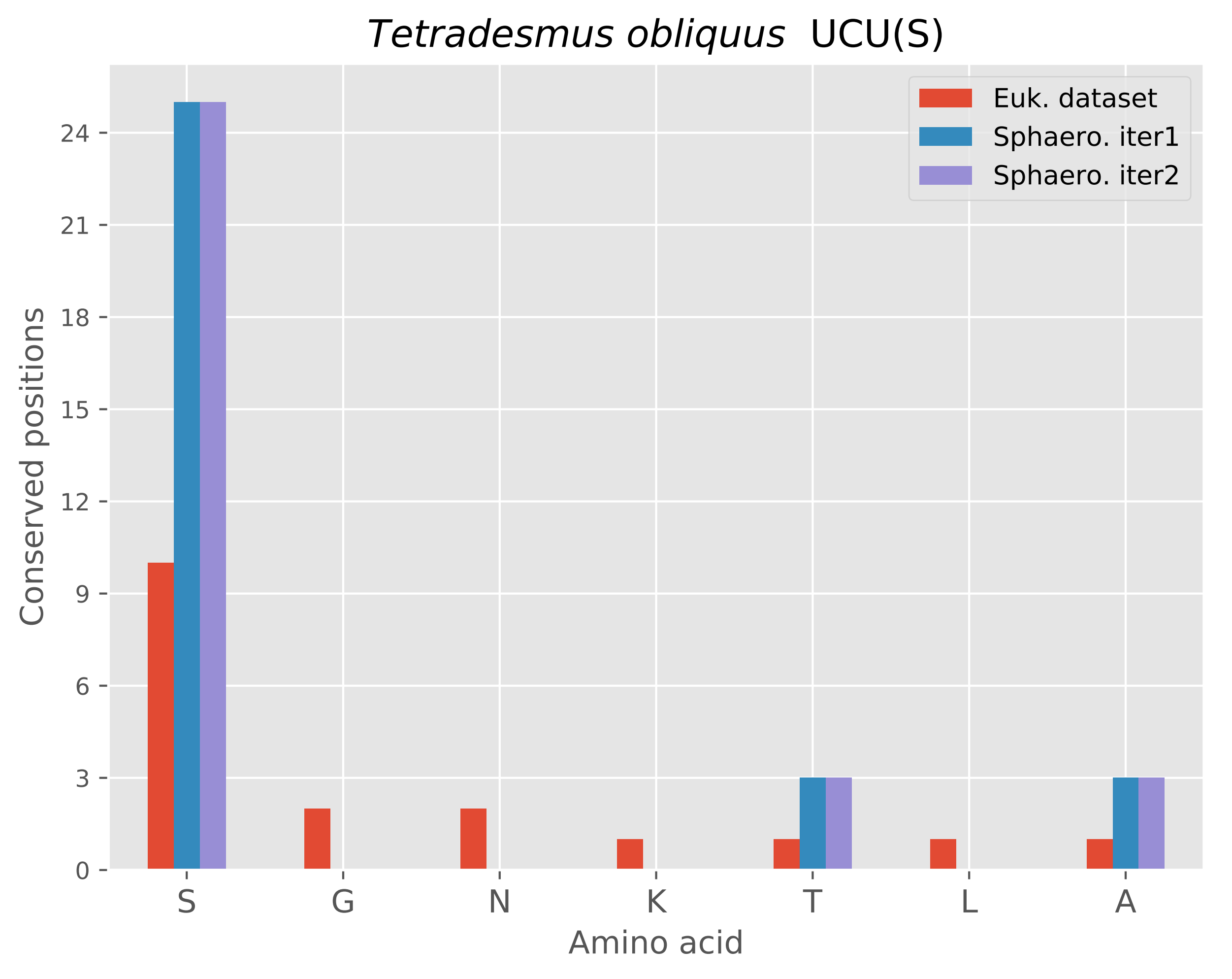

Tetradesmus obliquus UCU(S)
Euk. dataset
Sphaero. iter1
Sphaero. iter2
Conserved positions
0
3
6
9
12
15
18
21
24
S
G
N
K
T
L
A
Amino acid

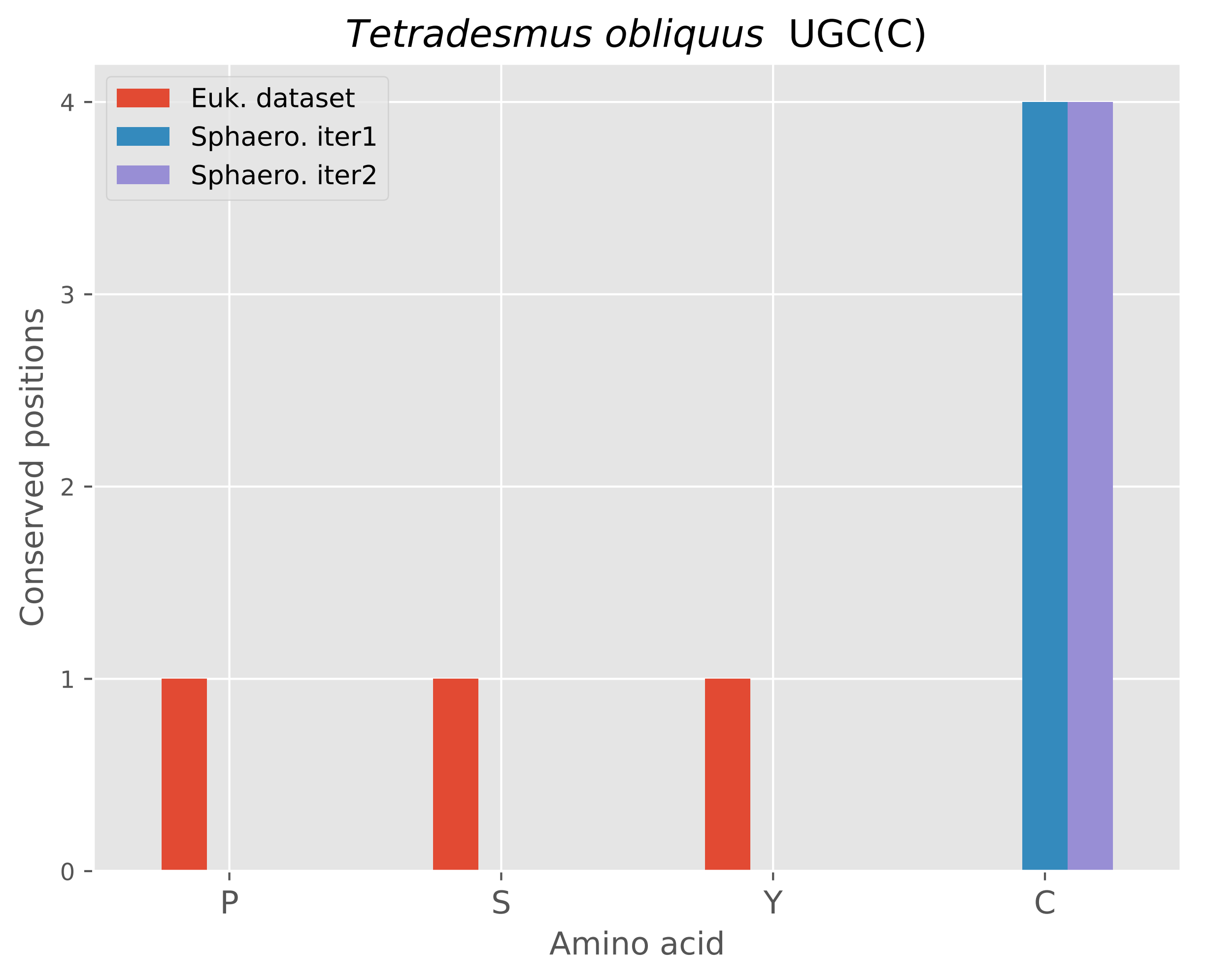

Tetradesmus obliquus UGC(C)
Euk. dataset
Sphaero. iter1
Sphaero. iter2
Conserved positions
0
1
2
3
4
P
S
Y
C
Amino acid

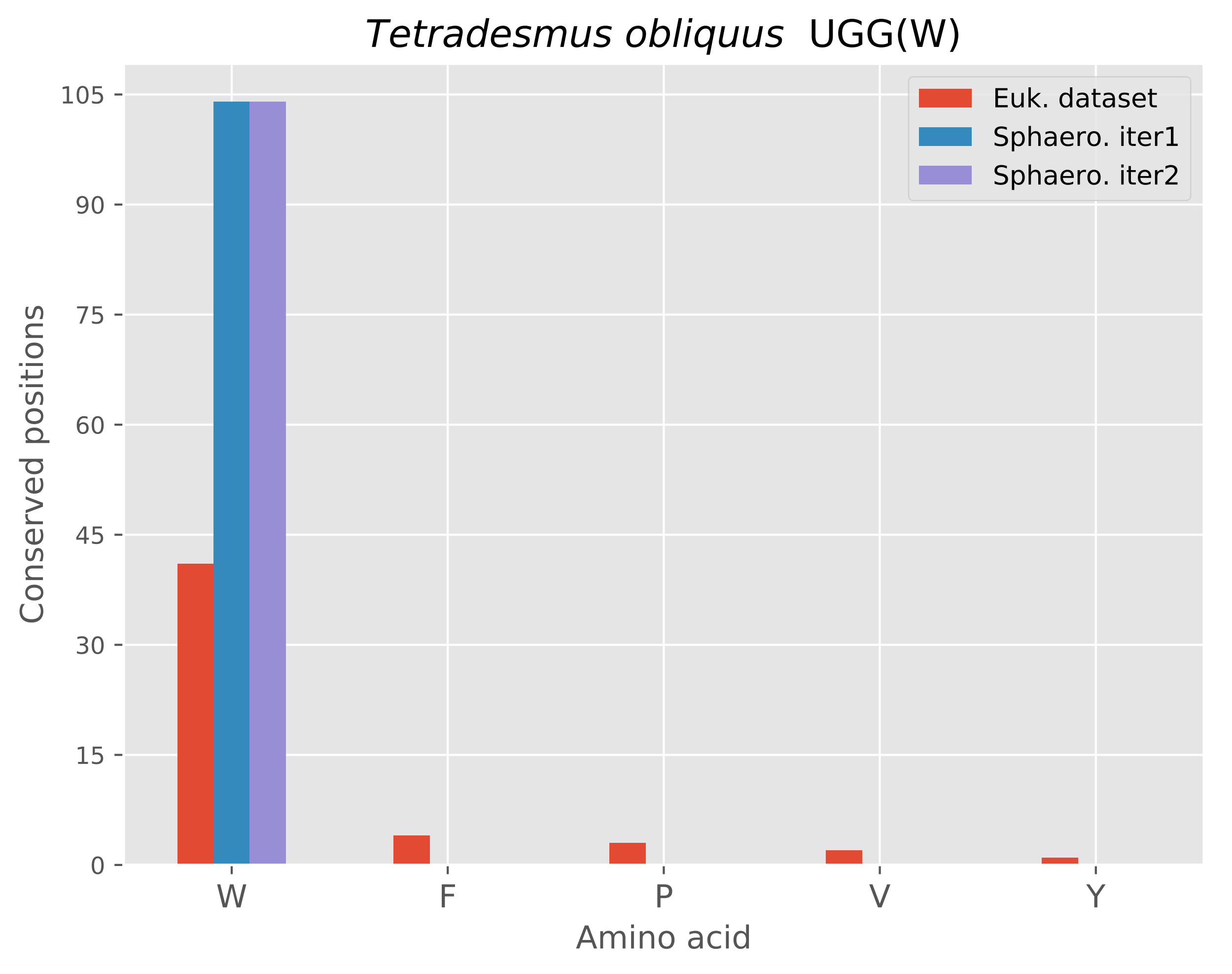

Tetradesmus obliquus UGG(W)
Euk. dataset
Sphaero. iter1
Sphaero. iter2
Conserved positions
0
15
30
45
60
75
90
105
W
F
P
V
Y
Amino acid

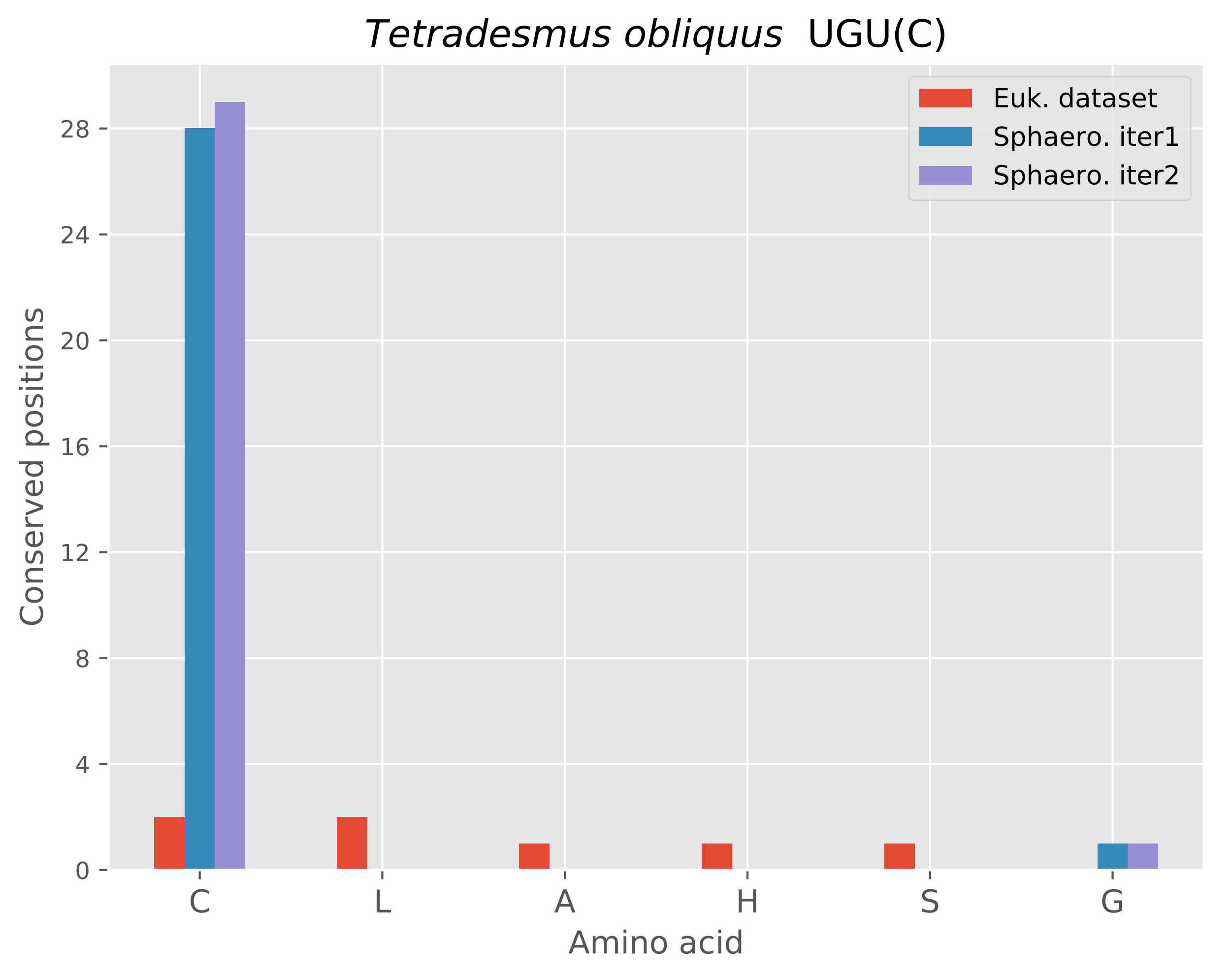

Tetradesmus obliquus UGU(C)
Euk. dataset
Sphaero. iter1
Sphaero. iter2
Conserved positions
0
4
8
12
16
20
24
28
C
L
A
H
S
G
Amino acid

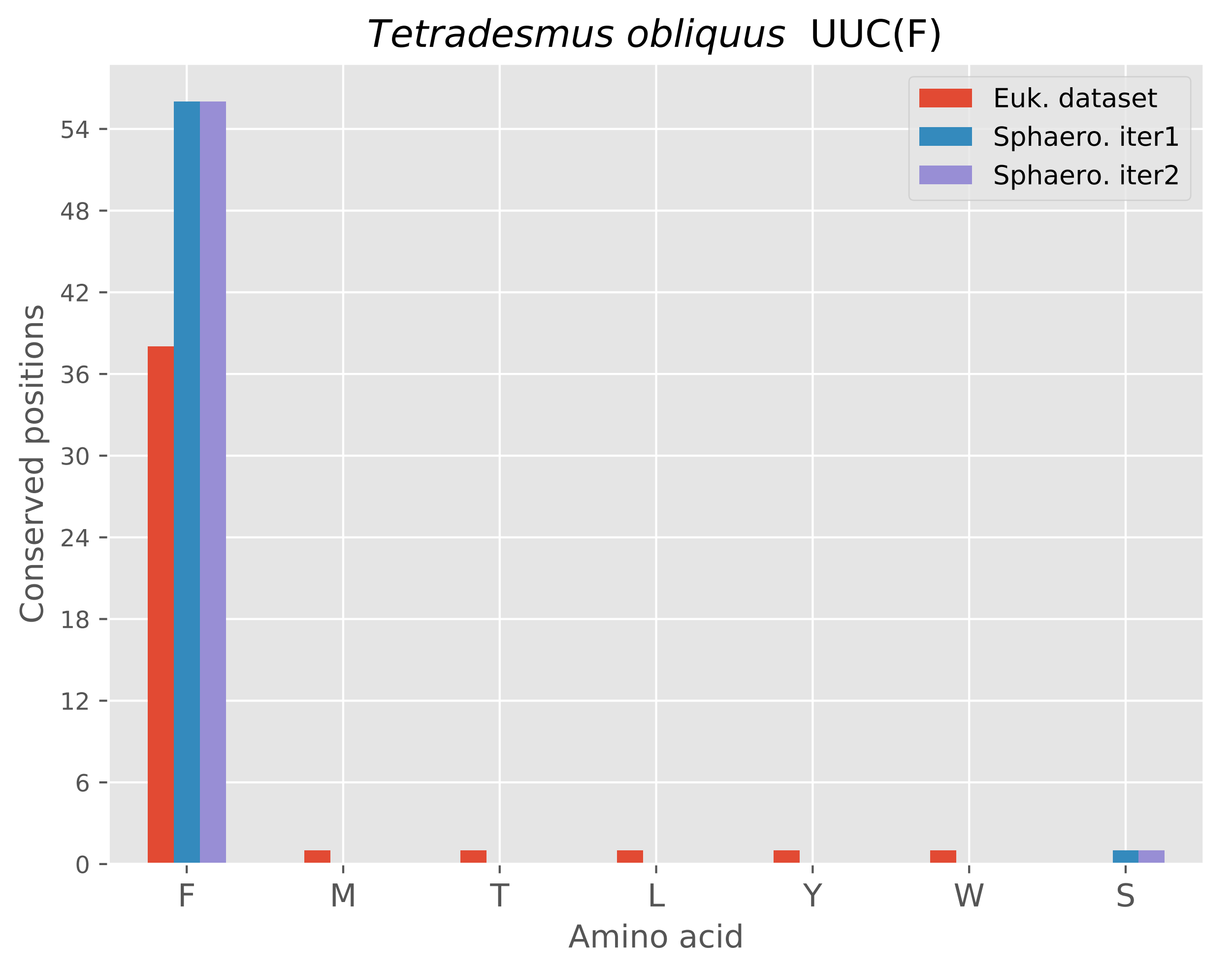

Tetradesmus obliquus UUC(F)
Euk. dataset
Sphaero. iter1
Sphaero. iter2
Conserved positions
0
6
12
18
24
30
36
42
48
54
F
M
T
L
Y
W
S
Amino acid

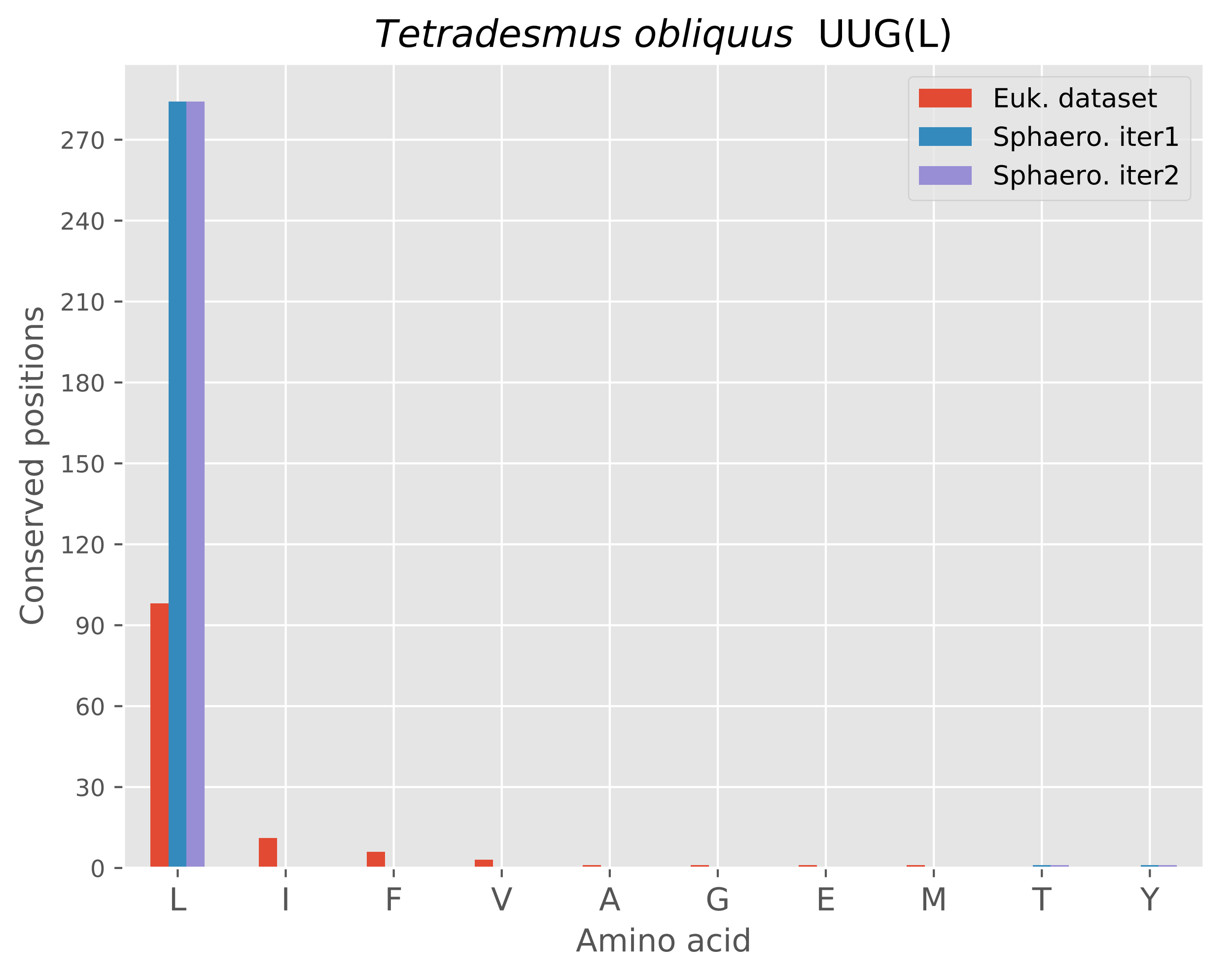

Tetradesmus obliquus UUG(L)
Euk. dataset
Sphaero. iter1
Sphaero. iter2
Conserved positions
0
30
60
90
120
150
180
210
240
270
L
I
F
V
A
G
E
M
T
Y
Amino acid

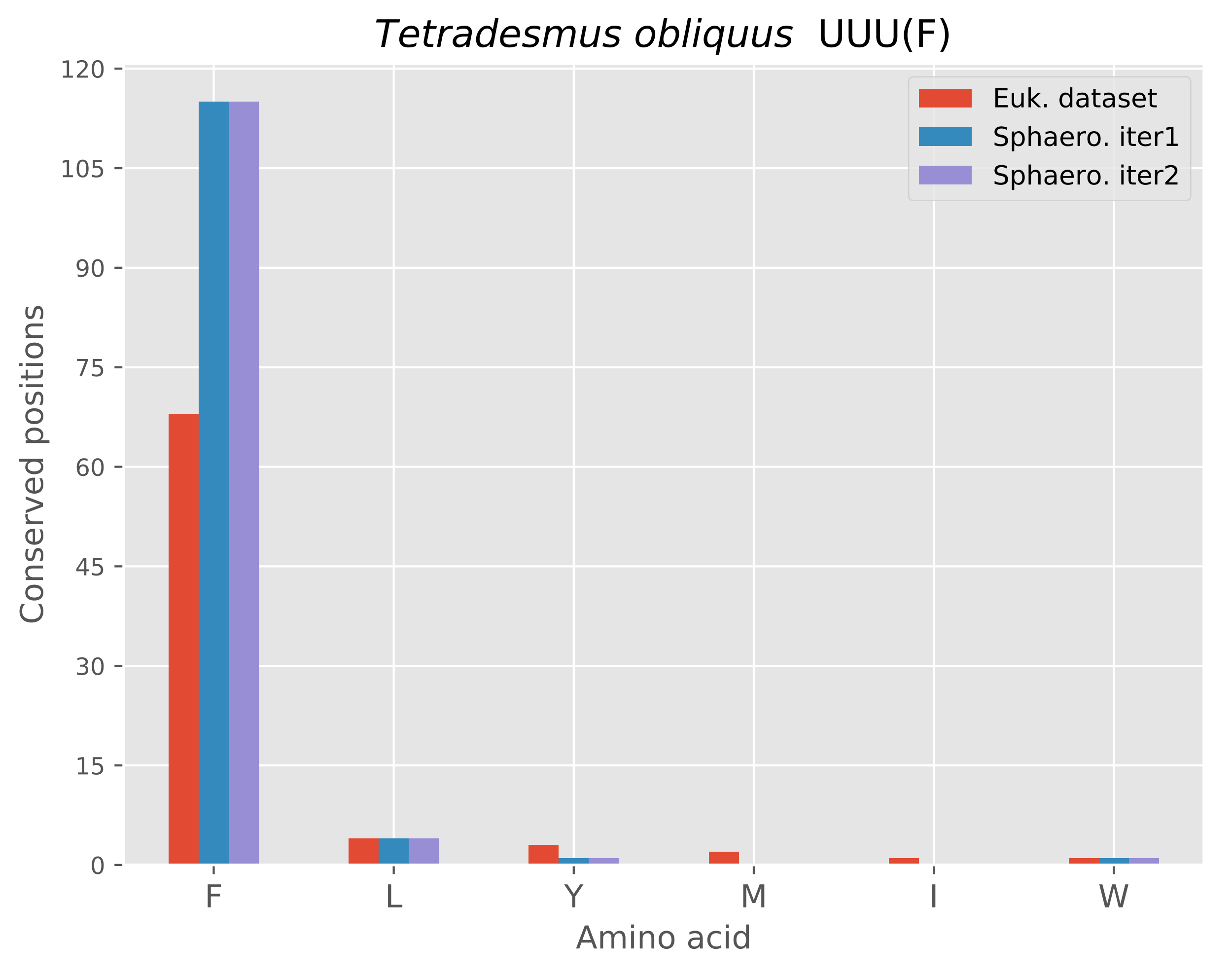

Tetradesmus obliquus UUU(F)
Conserved positions
Amino acid
0
15
30
45
60
75
90
105
120
F
L
Y
M
I
W
Euk. dataset
Sphaero. iter1
Sphaero. iter2

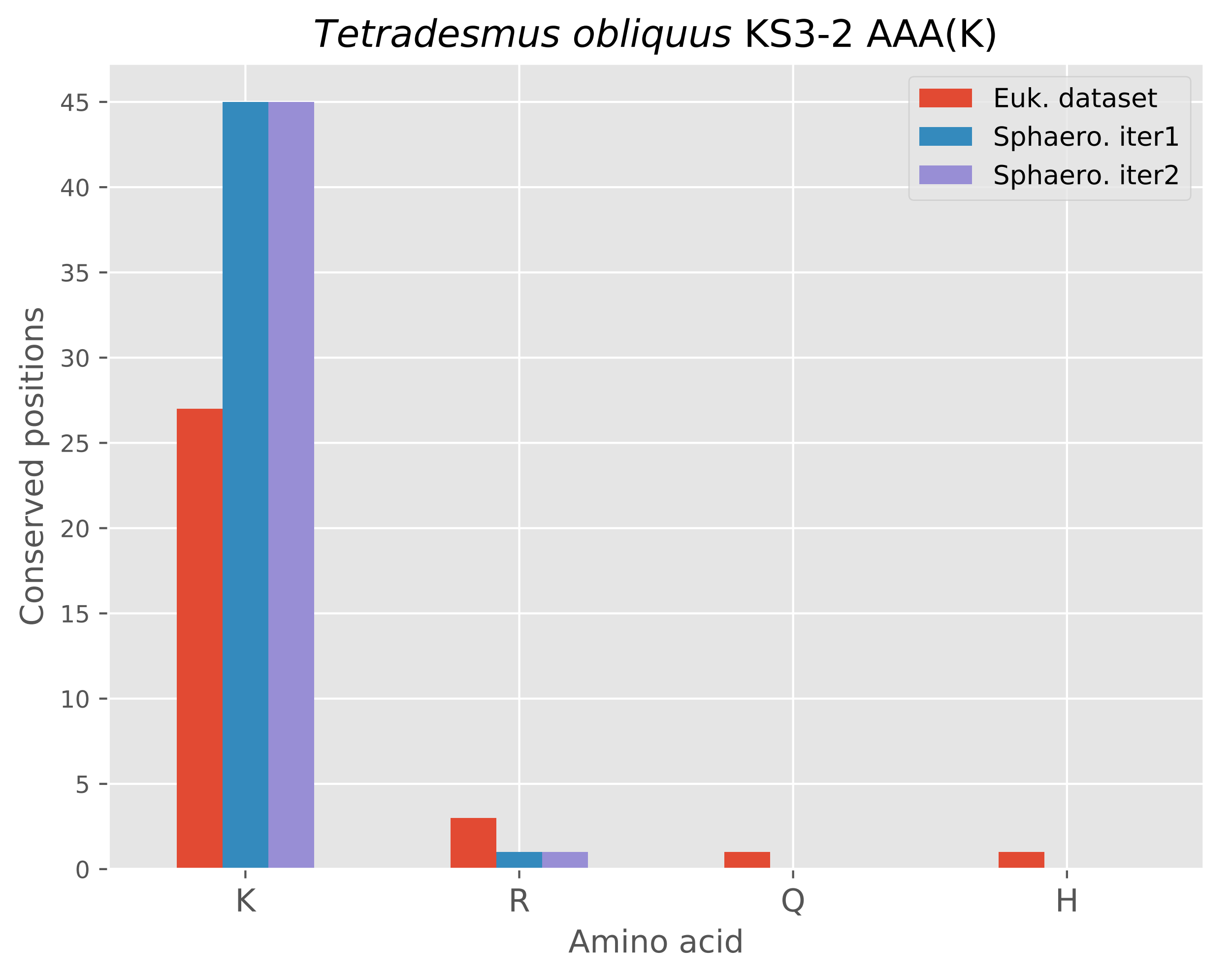
Tetradesmus obliquus KS3-2 AAA(K)
Euk. dataset
Sphaero. iter1
Sphaero. iter2
Conserved positions
0
5
10
15
20
25
30
35
40
45
K
R
Q
H
Amino acid

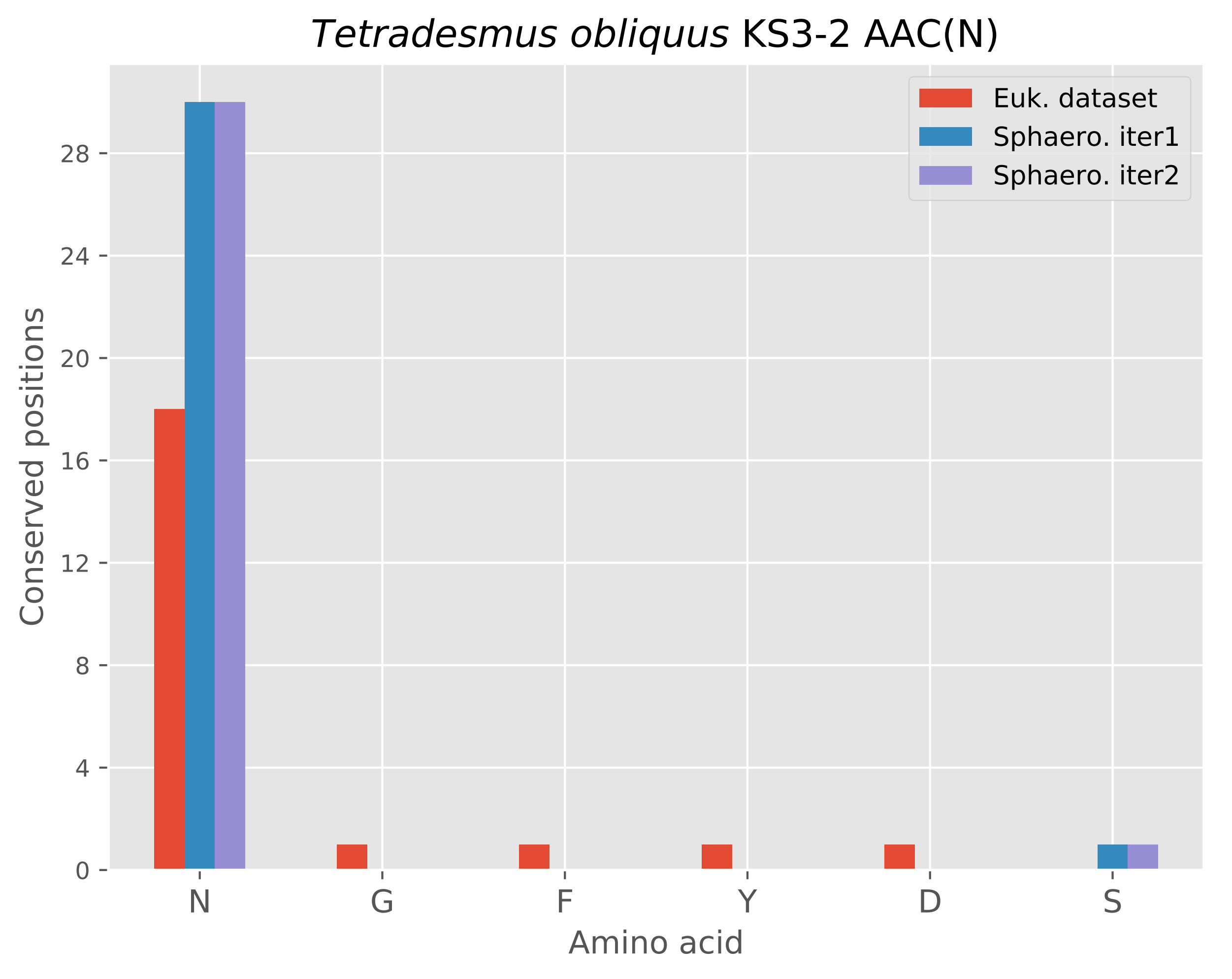

Tetradesmus obliquus KS3-2 AAC(N)
Euk. dataset
Sphaero. iter1
Sphaero. iter2
Conserved positions
0
4
8
12
16
20
24
28
N
G
F
Y
D
S
Amino acid

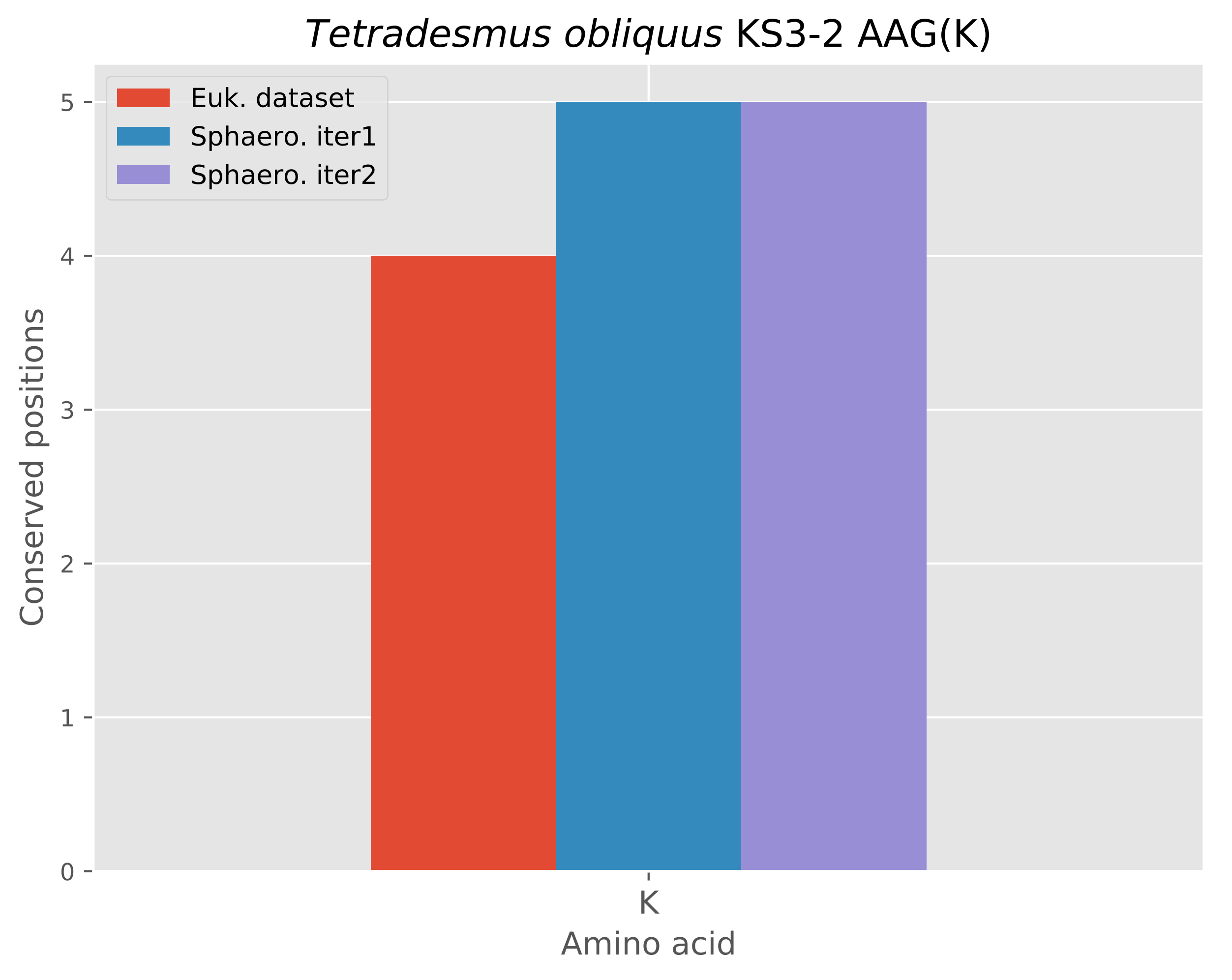
Tetradesmus obliquus KS3-2 AAG(K)
Euk. dataset
Sphaero. iter1
Sphaero. iter2
Conserved positions
0
1
2
3
4
5
K
Amino acid

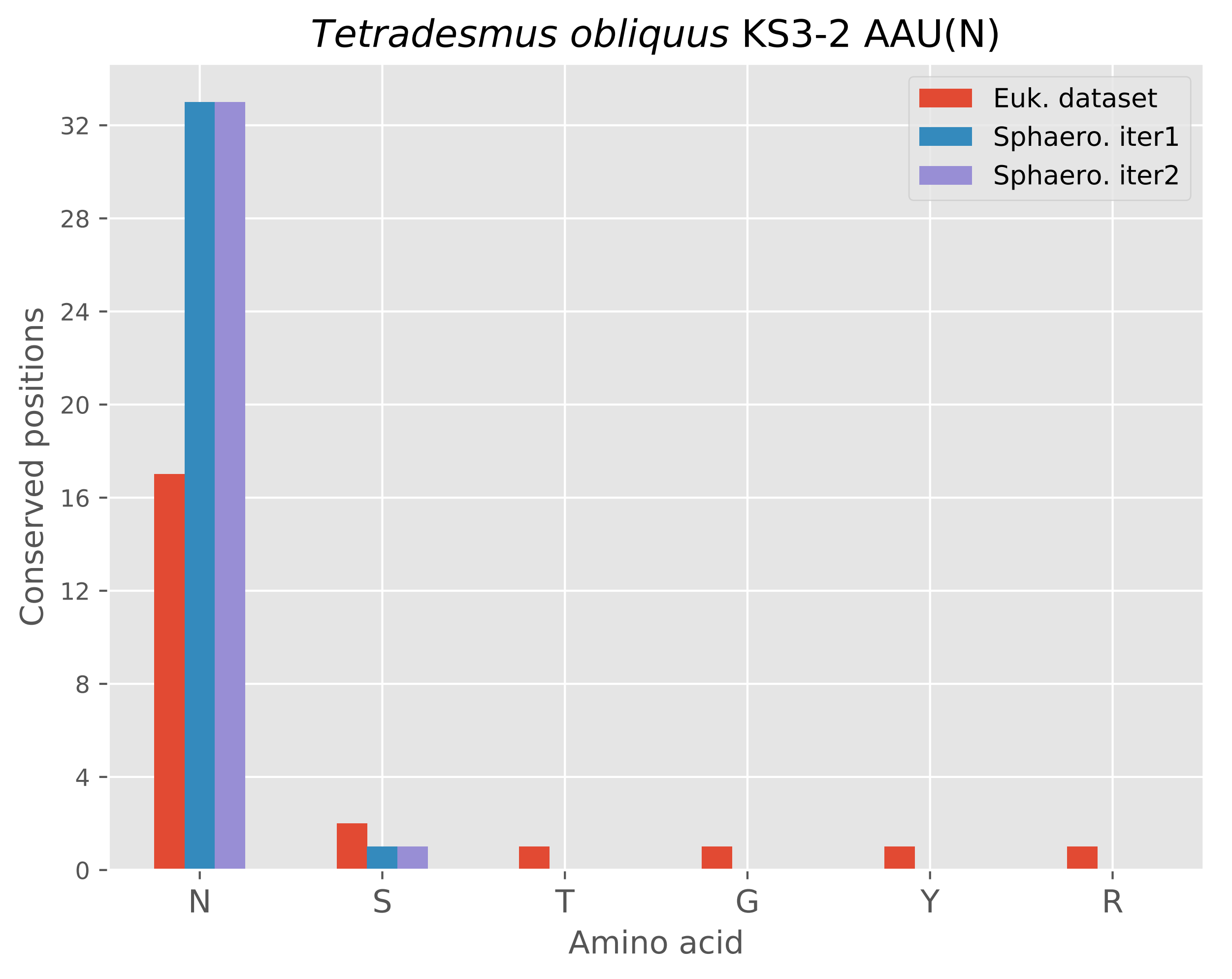

Tetradesmus obliquus KS3-2 AAU(N)
Euk. dataset
Sphaero. iter1
Sphaero. iter2
Conserved positions
0
4
8
12
16
20
24
28
32
N
S
T
G
Y
R
Amino acid

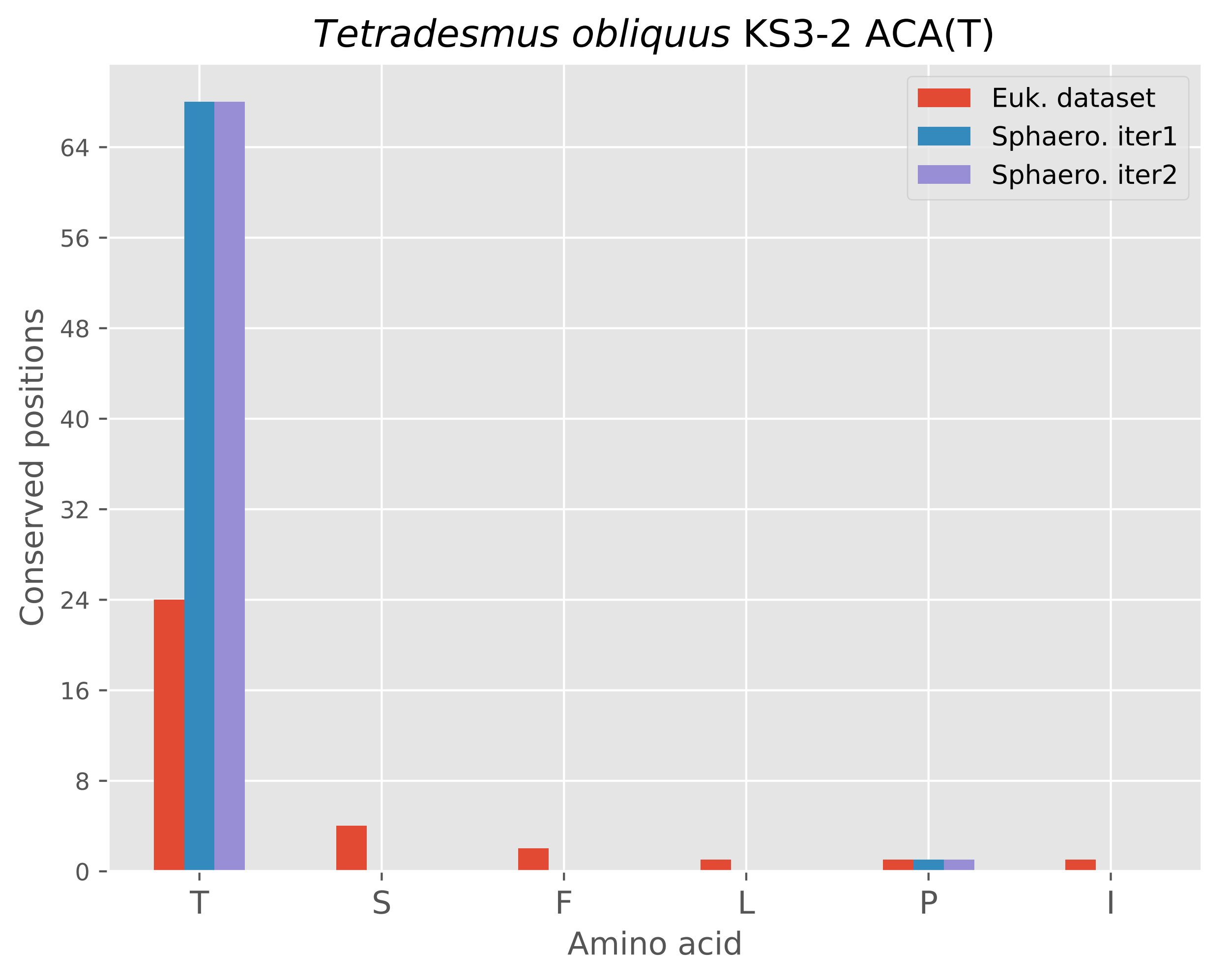

Tetradesmus obliquus KS3-2 ACA(T)
Euk. dataset
Sphaero. iter1
Sphaero. iter2
Conserved positions
0
8
16
24
32
40
48
56
64
T
S
F
L
P
I
Amino acid

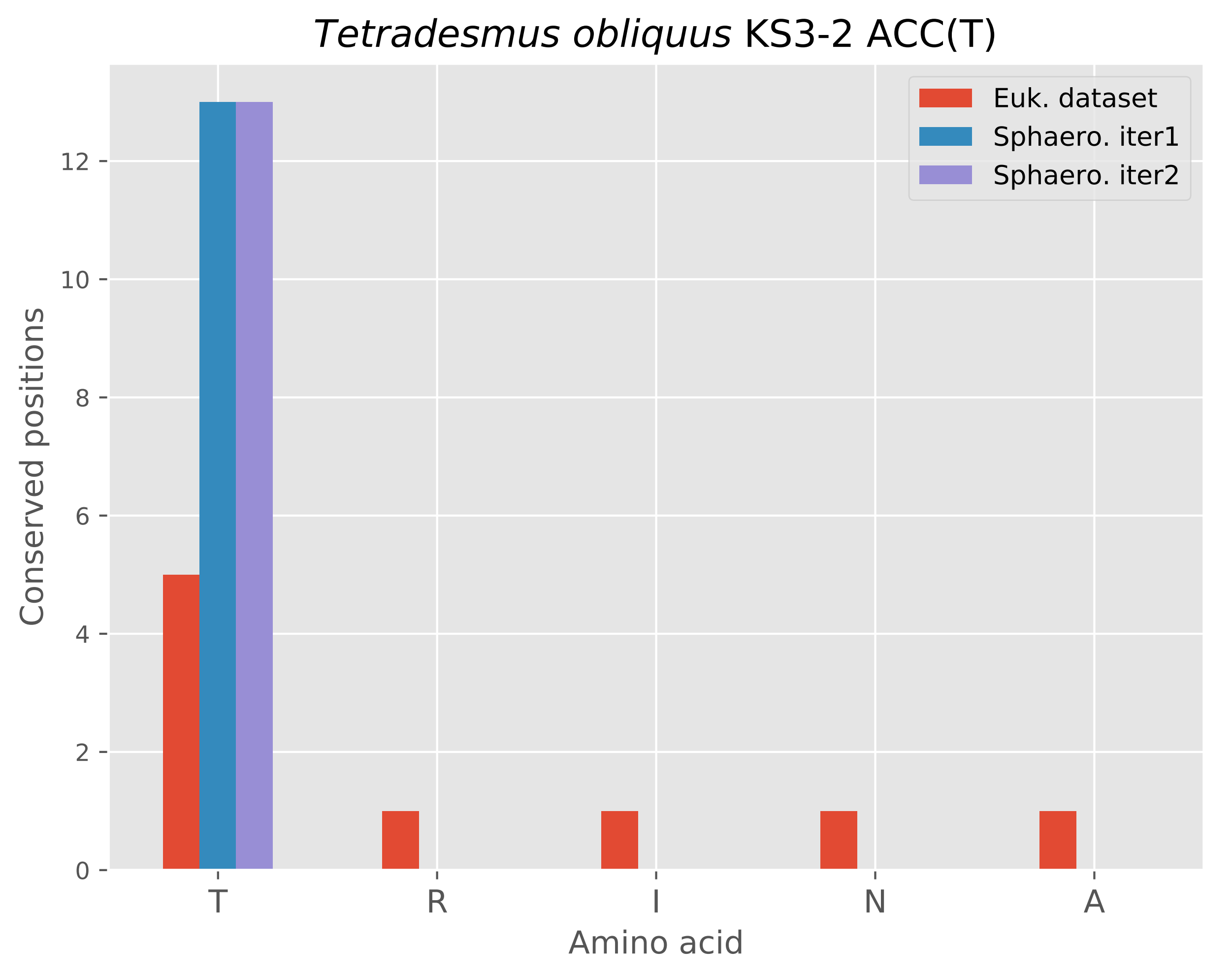

Tetradesmus obliquus KS3-2 ACC(T)
Euk. dataset
Sphaero. iter1
Sphaero. iter2
Conserved positions
0
2
4
6
8
10
12
T
R
I
N
A
Amino acid

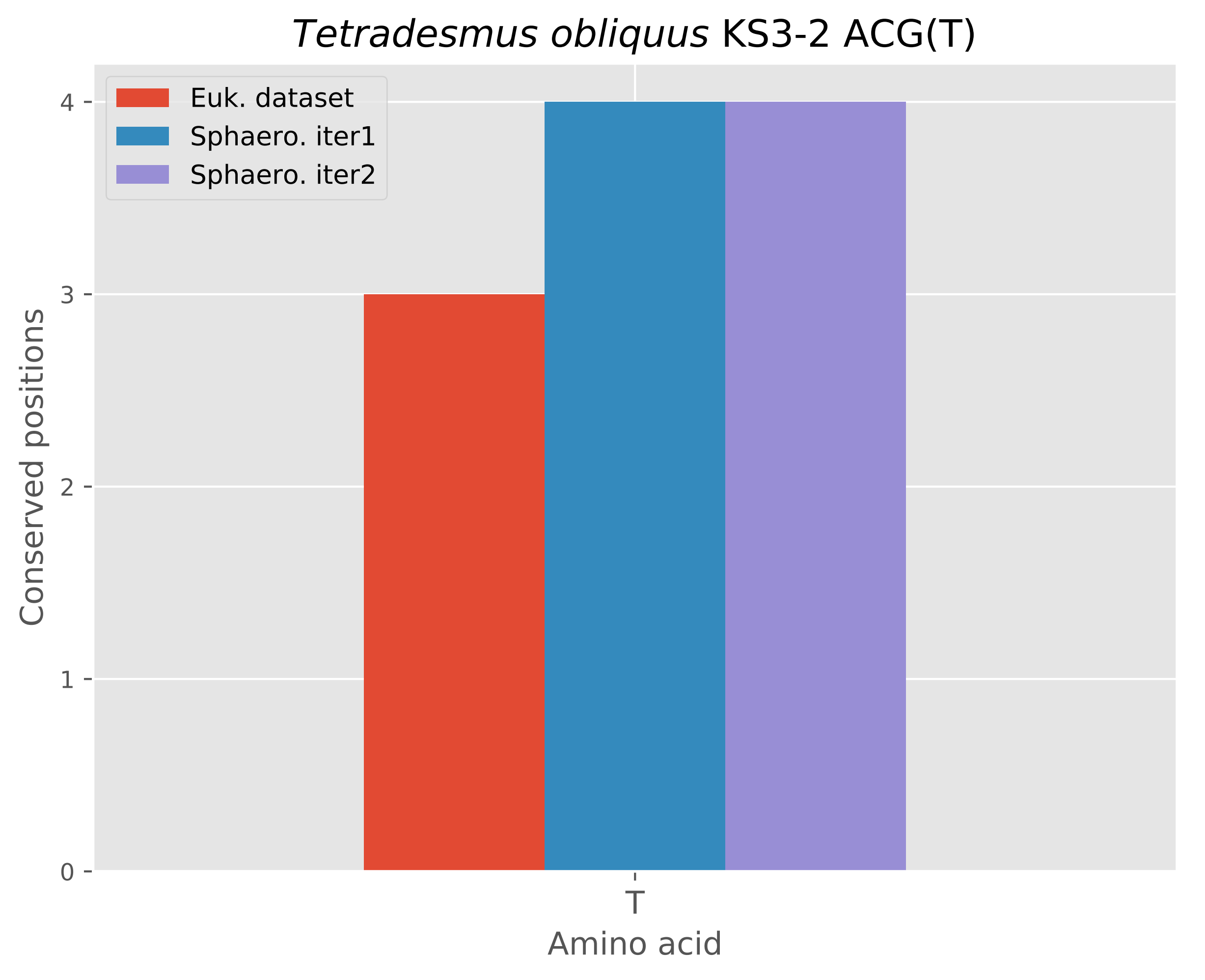

Tetradesmus obliquus KS3-2 ACG(T)
Euk. dataset
Sphaero. iter1
Sphaero. iter2
Conserved positions
0
1
2
3
4
T
Amino acid

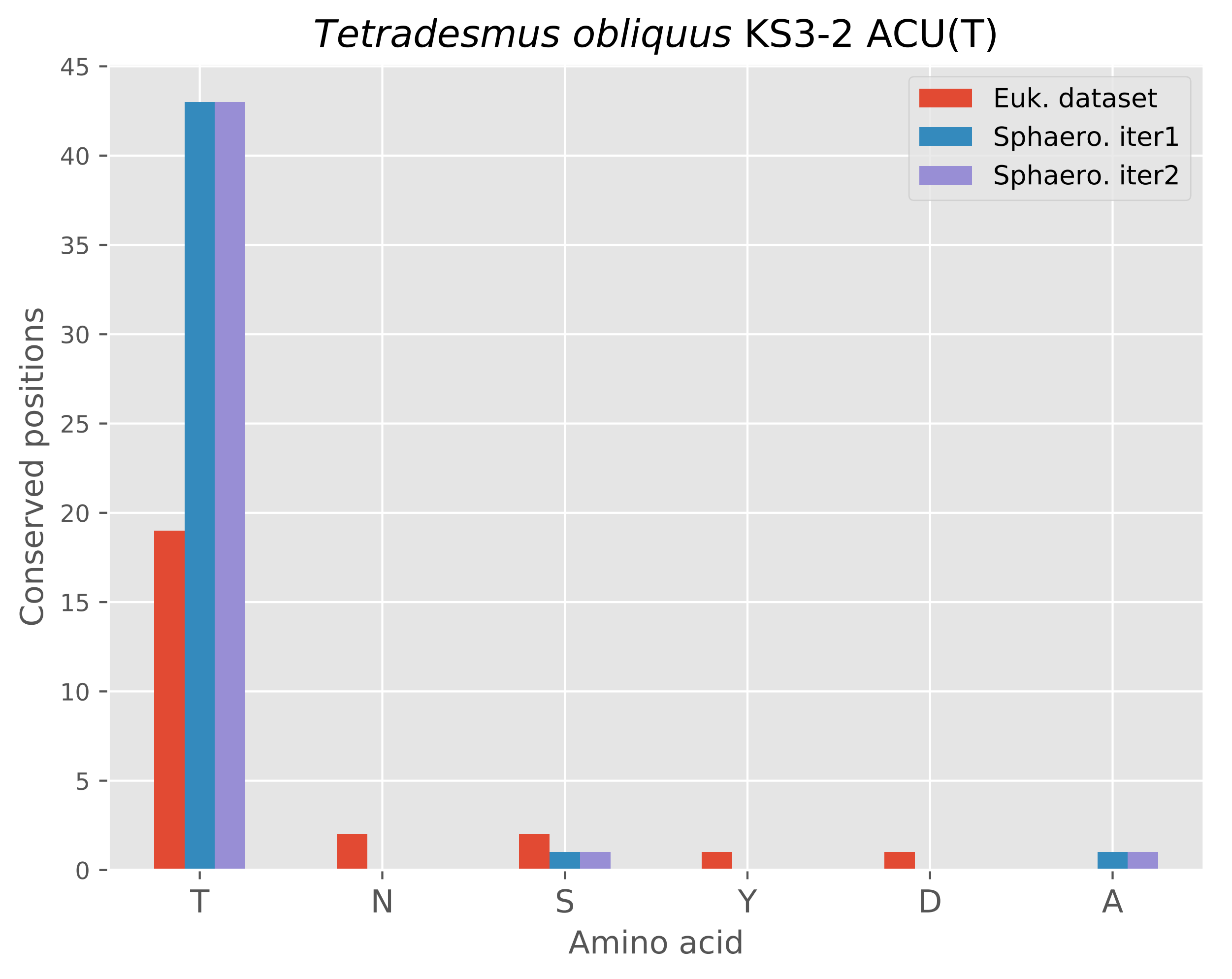

Tetradesmus obliquus KS3-2 ACU(T)
Euk. dataset
Sphaero. iter1
Sphaero. iter2
Conserved positions
0
5
10
15
20
25
30
35
40
45
T
N
S
Y
D
A
Amino acid

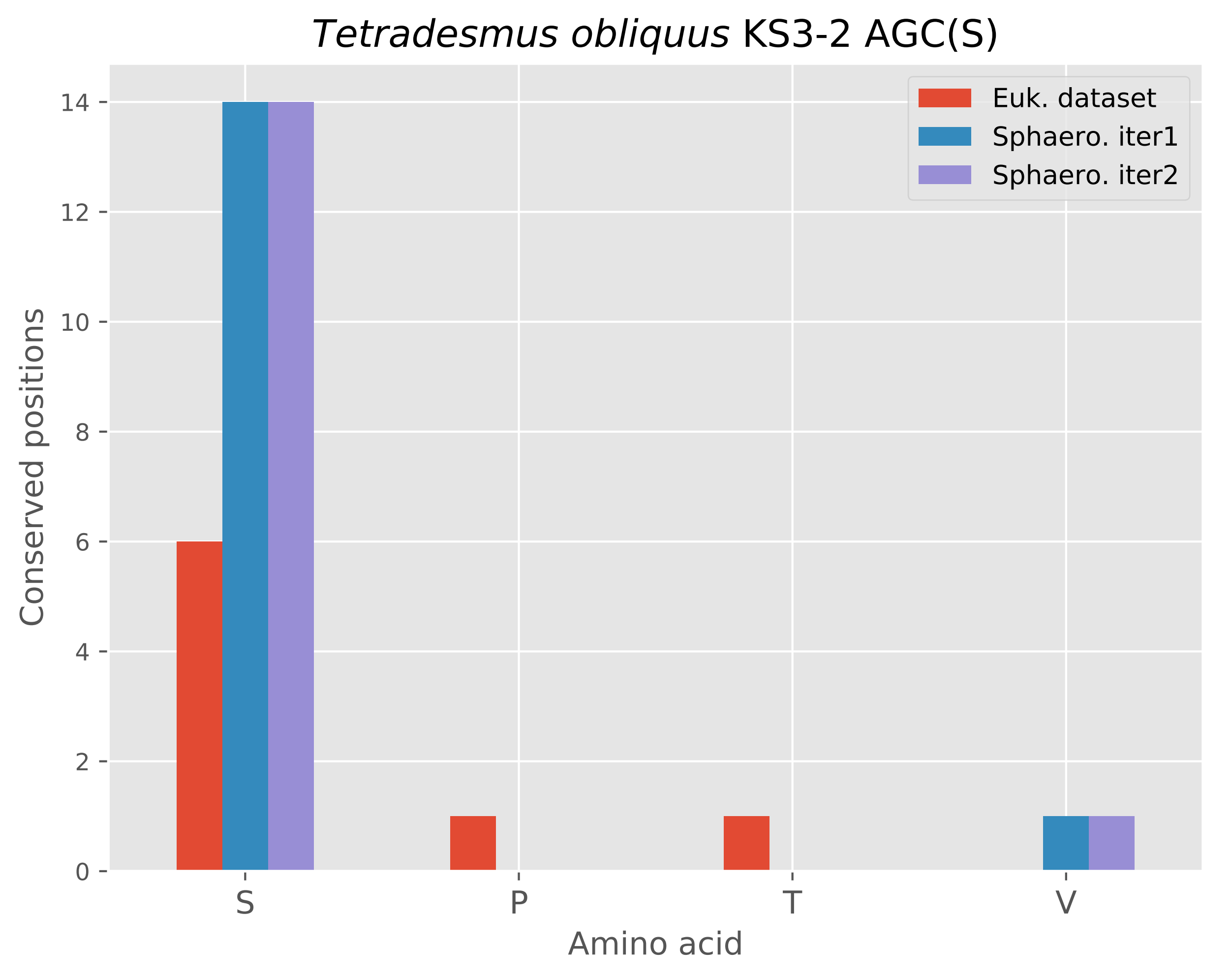

Tetradesmus obliquus KS3-2 AGC(S)
Euk. dataset
Sphaero. iter1
Sphaero. iter2
Conserved positions
0
2
4
6
8
10
12
14
S
P
T
V
Amino acid

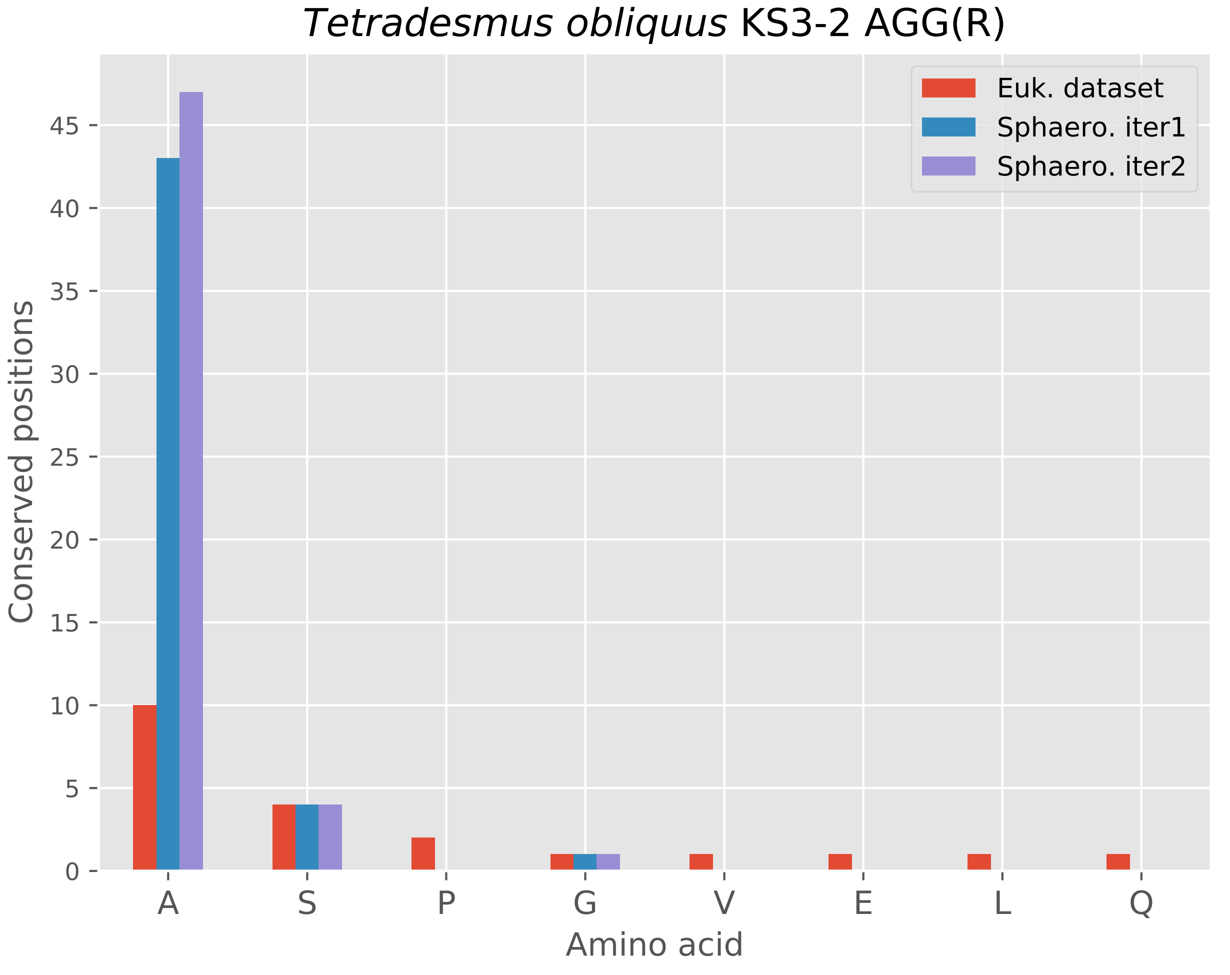
Tetradesmus obliquus KS3-2 AGG(R)
Euk. dataset
Sphaero. iter1
Sphaero. iter2
Conserved positions
0
5
10
15
20
25
30
35
40
45
A
S
P
G
V
E
L
Q
Amino acid

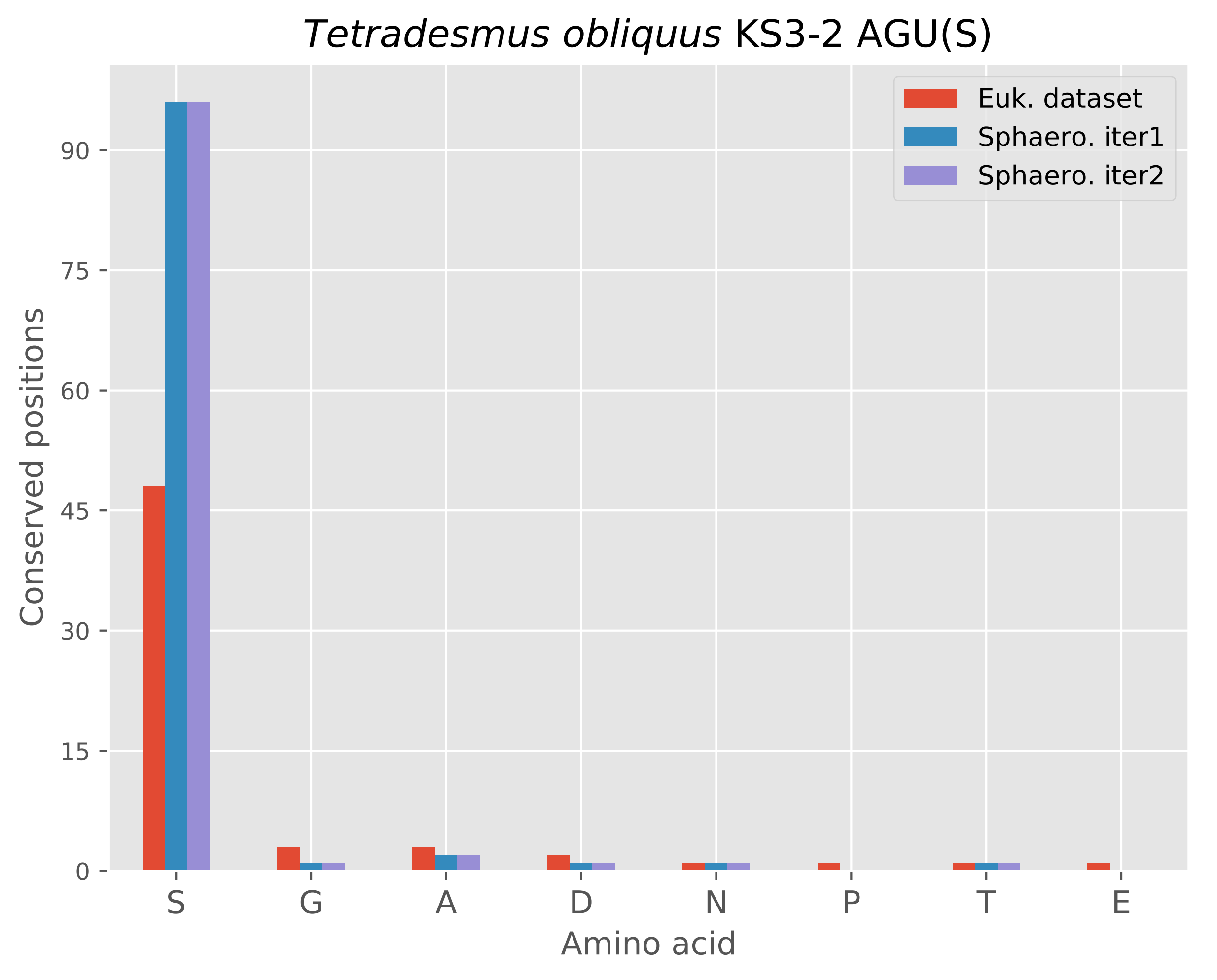

Tetradesmus obliquus KS3-2 AGU(S)
Euk. dataset
Sphaero. iter1
Sphaero. iter2
Conserved positions
0
15
30
45
60
75
90
S
G
A
D
N
P
T
E
Amino acid

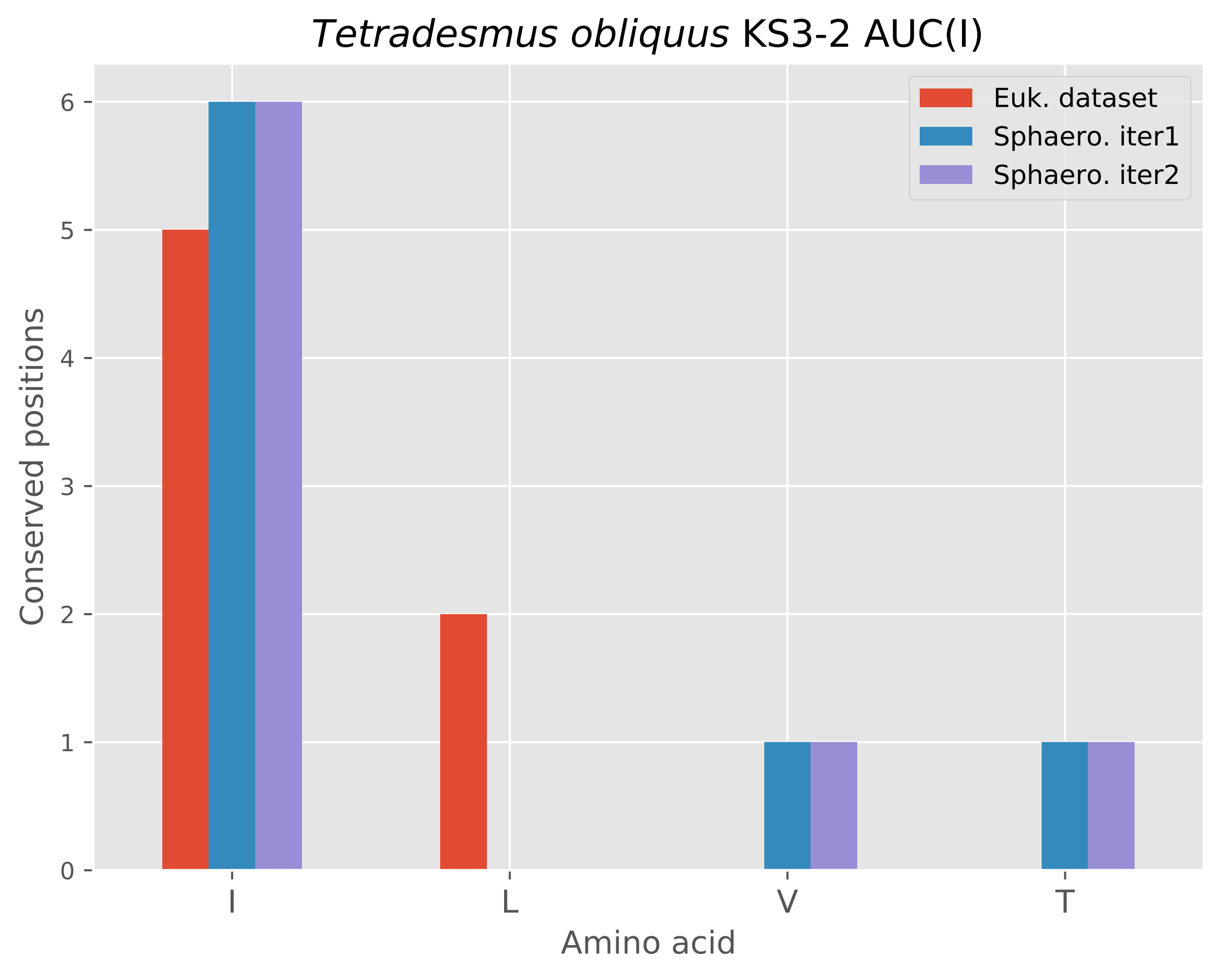

Tetradesmus obliquus KS3-2 AUC(I)
Euk. dataset
Sphaero. iter1
Sphaero. iter2
Conserved positions
0
1
2
3
4
5
6
I
L
V
T
Amino acid

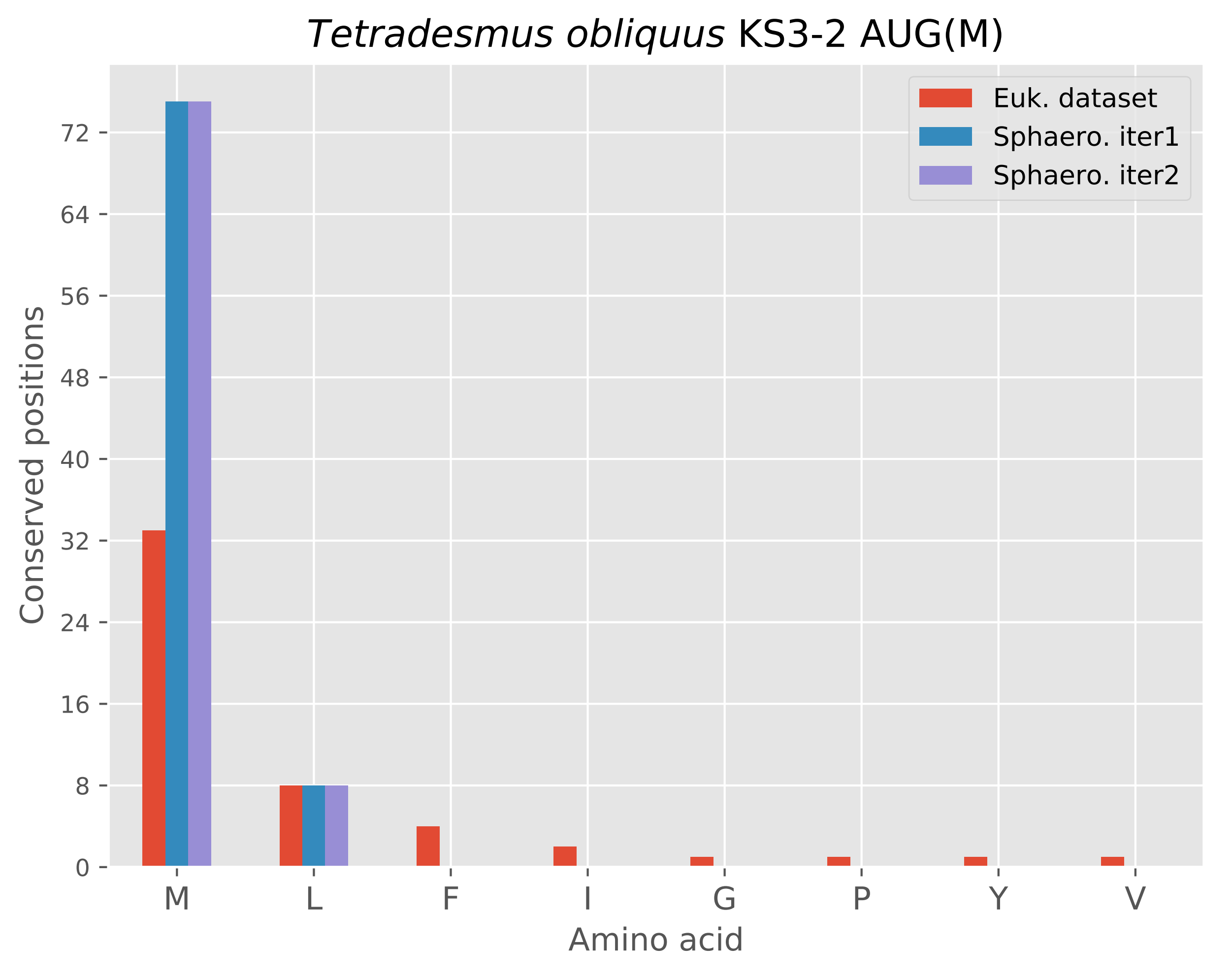

Tetradesmus obliquus KS3-2 AUG(M)
Euk. dataset
Sphaero. iter1
Sphaero. iter2
Conserved positions
0
8
16
24
32
40
48
56
64
72
M
L
F
I
G
P
Y
V
Amino acid

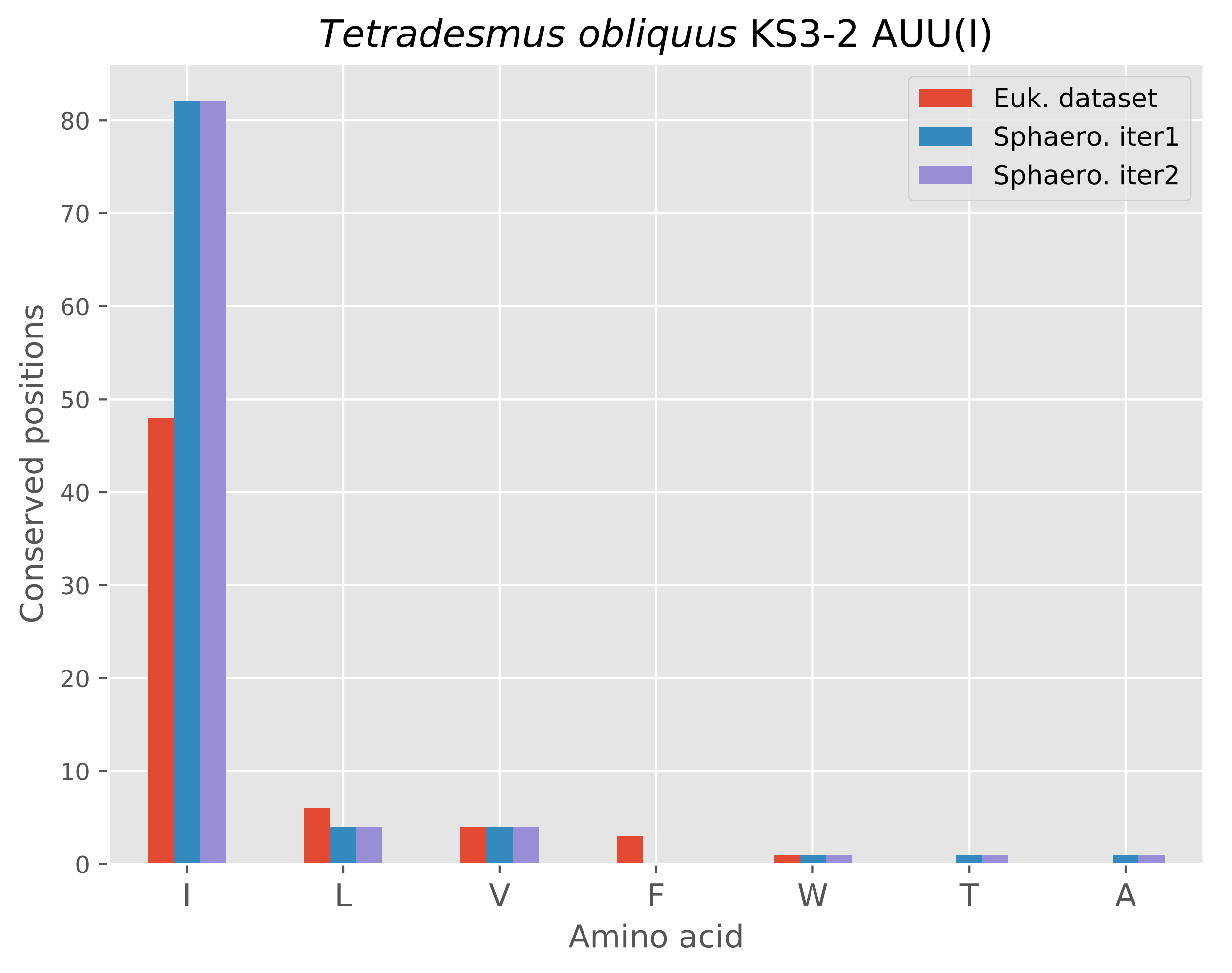

Tetradesmus obliquus KS3-2 AUU(I)
Euk. dataset
Sphaero. iter1
Sphaero. iter2
Conserved positions
0
10
20
30
40
50
60
70
80
I
L
V
F
W
T
A
Amino acid

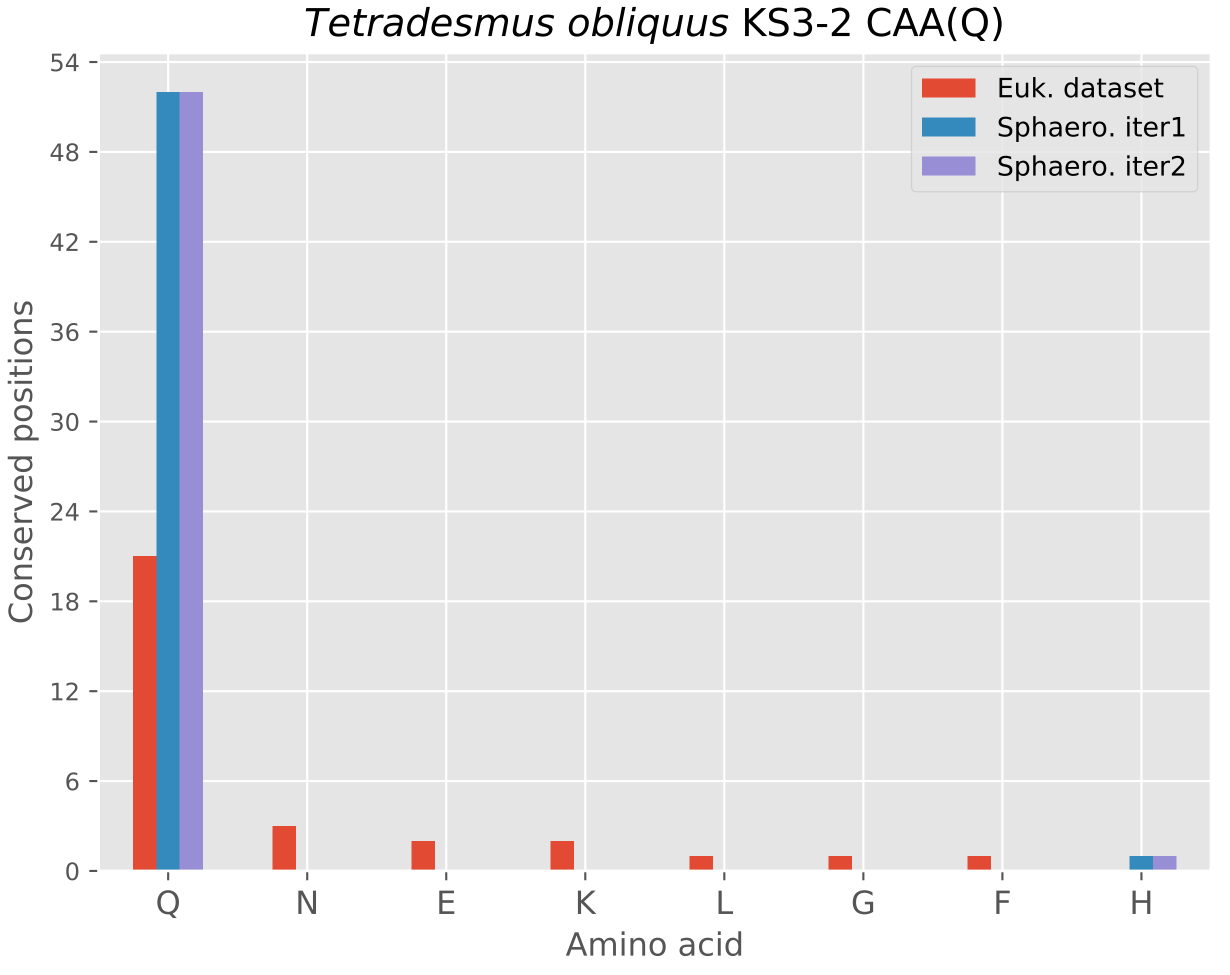
Tetradesmus obliquus KS3-2 CAA(Q)
Euk. dataset
Sphaero. iter1
Sphaero. iter2
Conserved positions
0
6
12
18
24
30
36
42
48
54
Q
N
E
K
L
G
F
H
Amino acid

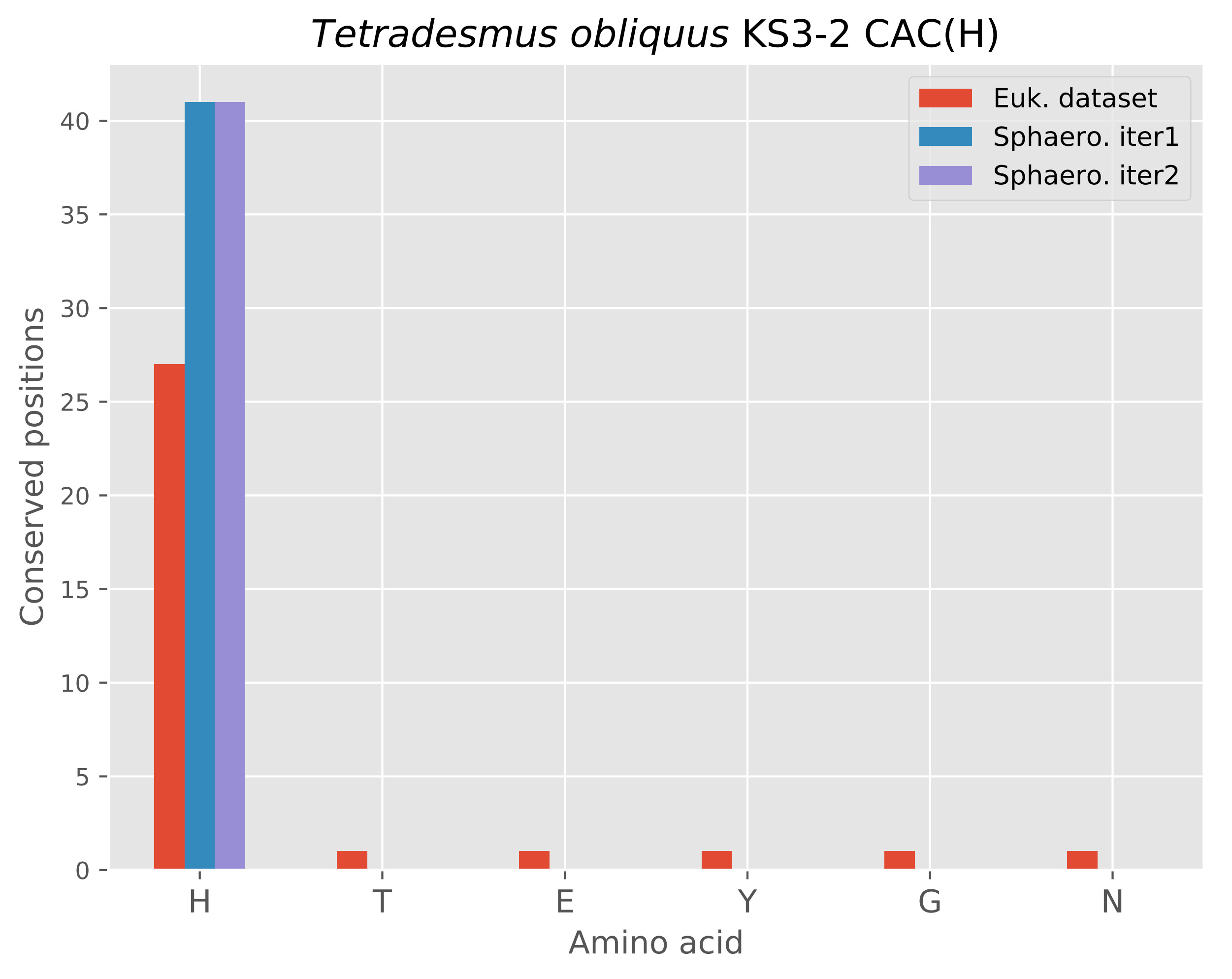

Tetradesmus obliquus KS3-2 CAC(H)
Euk. dataset
Sphaero. iter1
Sphaero. iter2
Conserved positions
0
5
10
15
20
25
30
35
40
H
T
E
Y
G
N
Amino acid

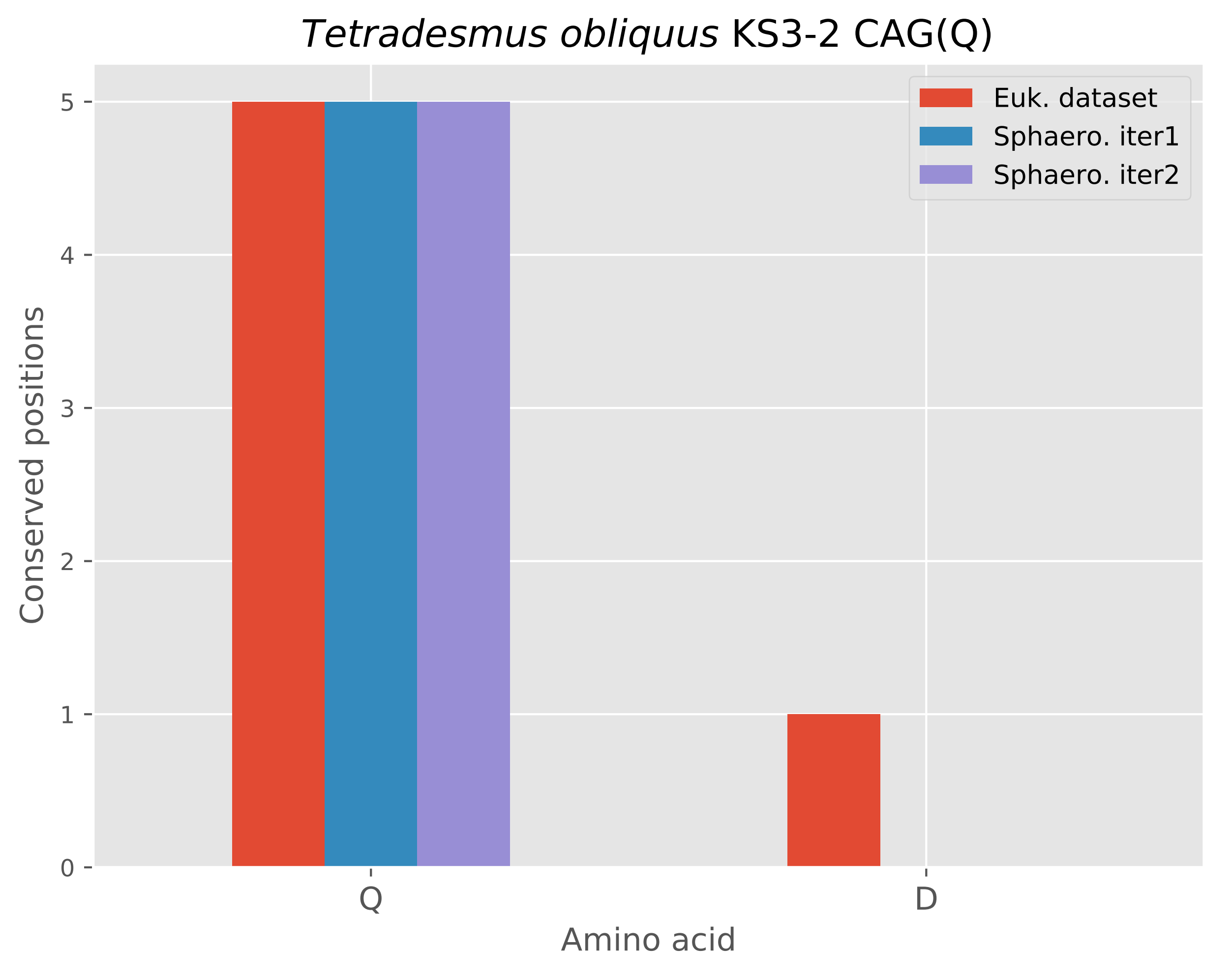
Tetradesmus obliquus KS3-2 CAG(Q)
Euk. dataset
Sphaero. iter1
Sphaero. iter2
Conserved positions
0
1
2
3
4
5
Q
D
Amino acid

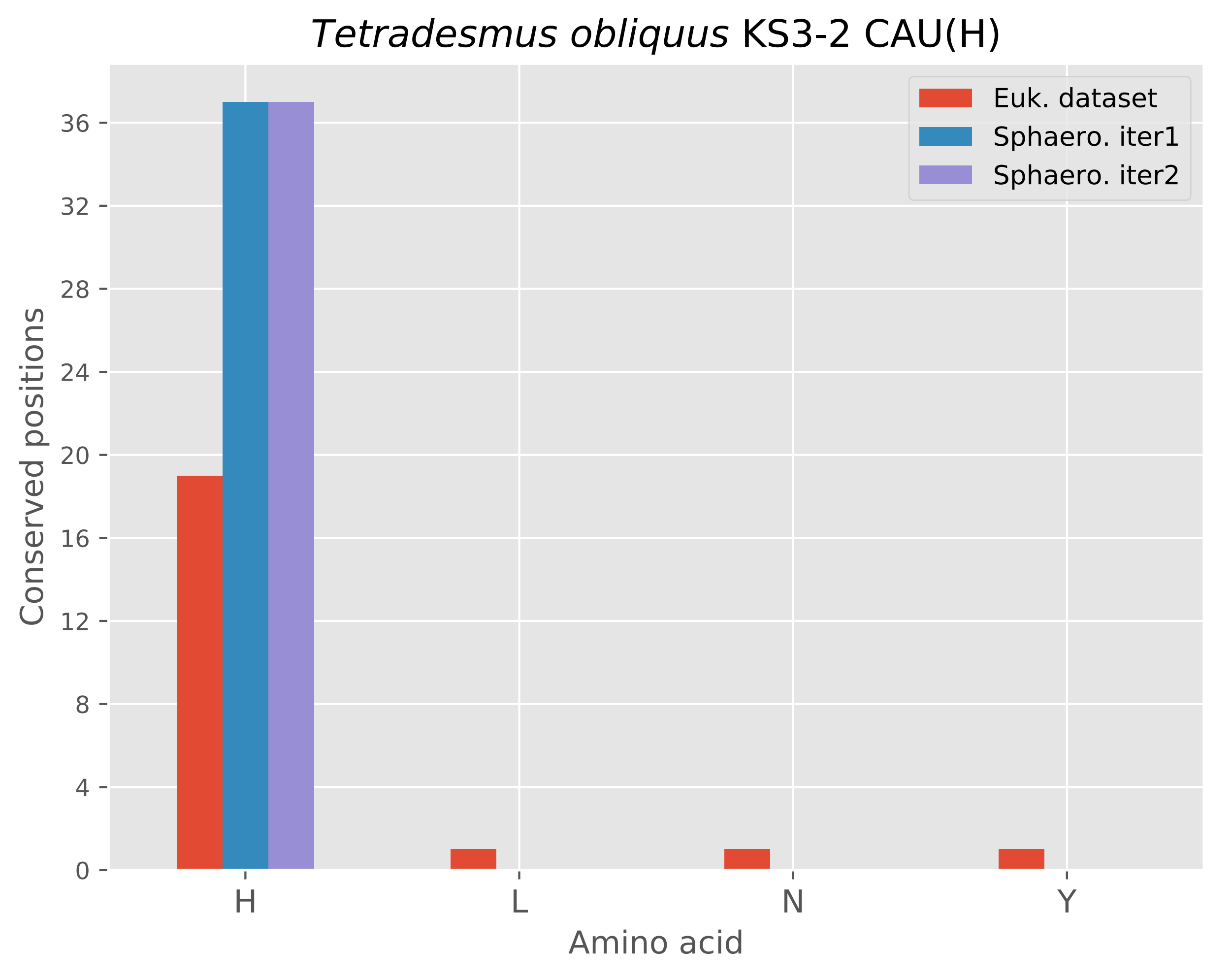

Tetradesmus obliquus KS3-2 CAU(H)
Euk. dataset
Sphaero. iter1
Sphaero. iter2
Conserved positions
0
4
8
12
16
20
24
28
32
36
H
L
N
Y
Amino acid

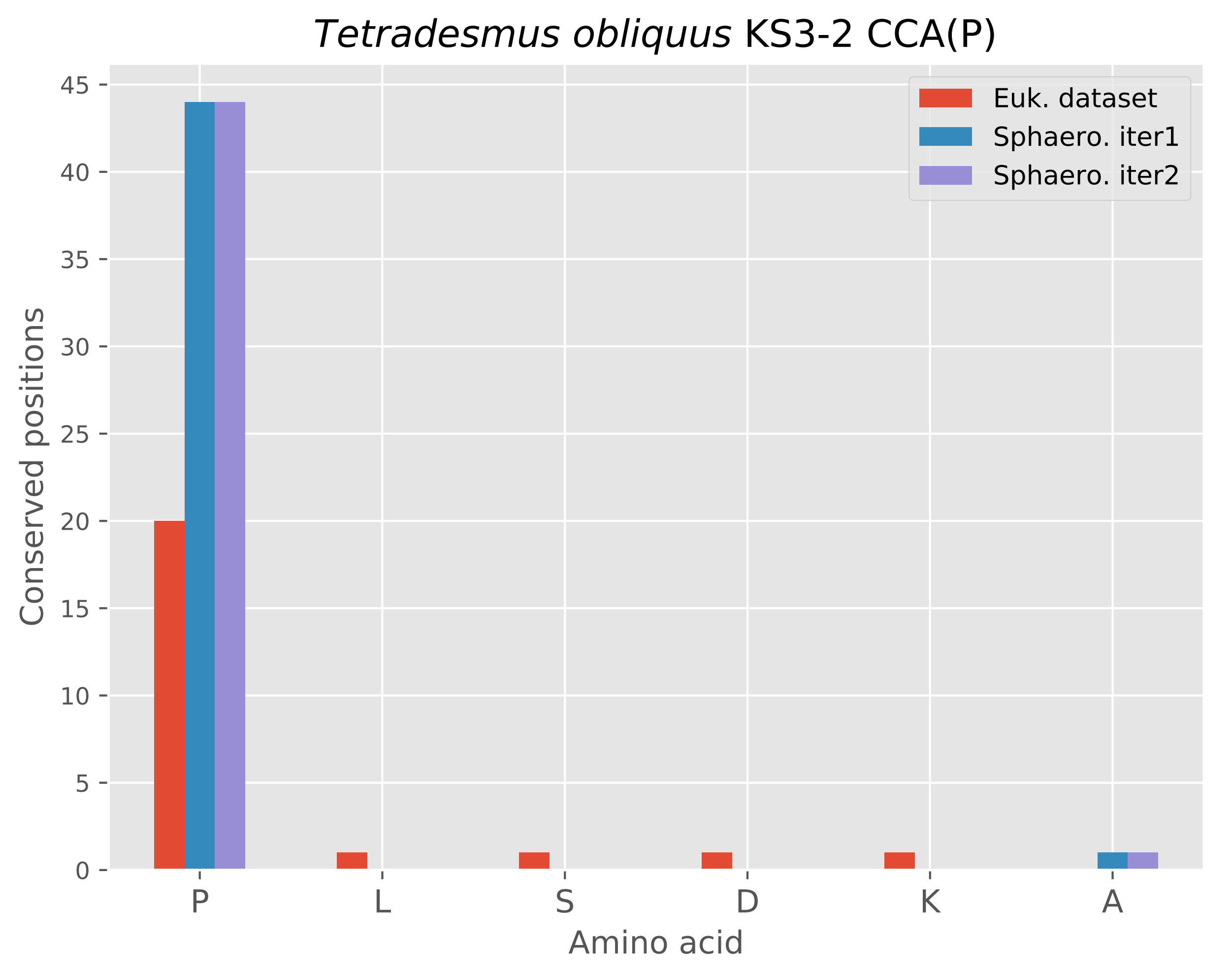
Tetradesmus obliquus KS3-2 CCA(P)
Conserved positions
0
5
10
15
20
25
30
35
40
45
P
L
S
D
K
A
Amino acid
Euk. dataset
Sphaero. iter1
Sphaero. iter2

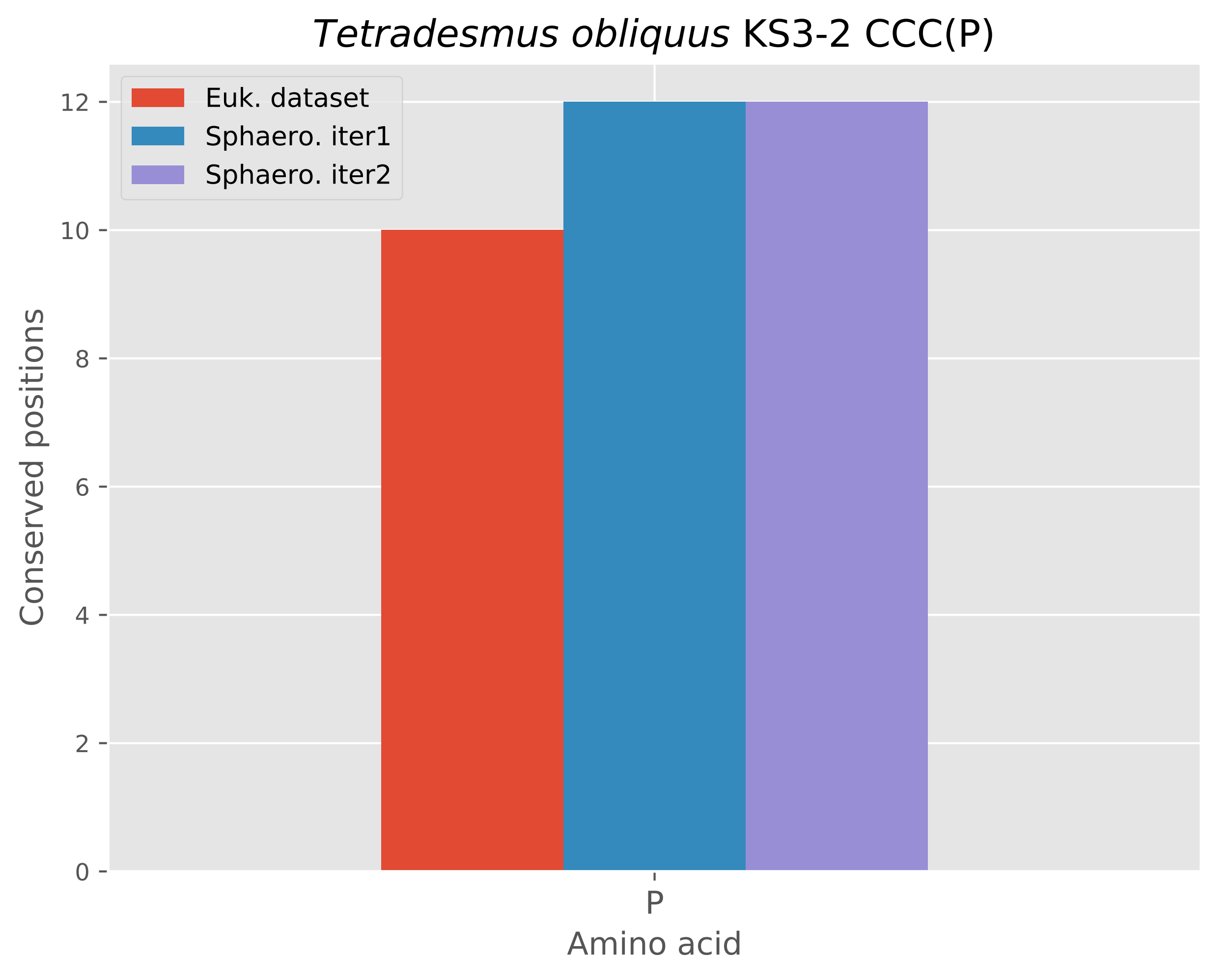

Tetradesmus obliquus KS3-2 CCC(P)
Euk. dataset
Sphaero. iter1
Sphaero. iter2
Conserved positions
0
2
4
6
8
10
12
P
Amino acid

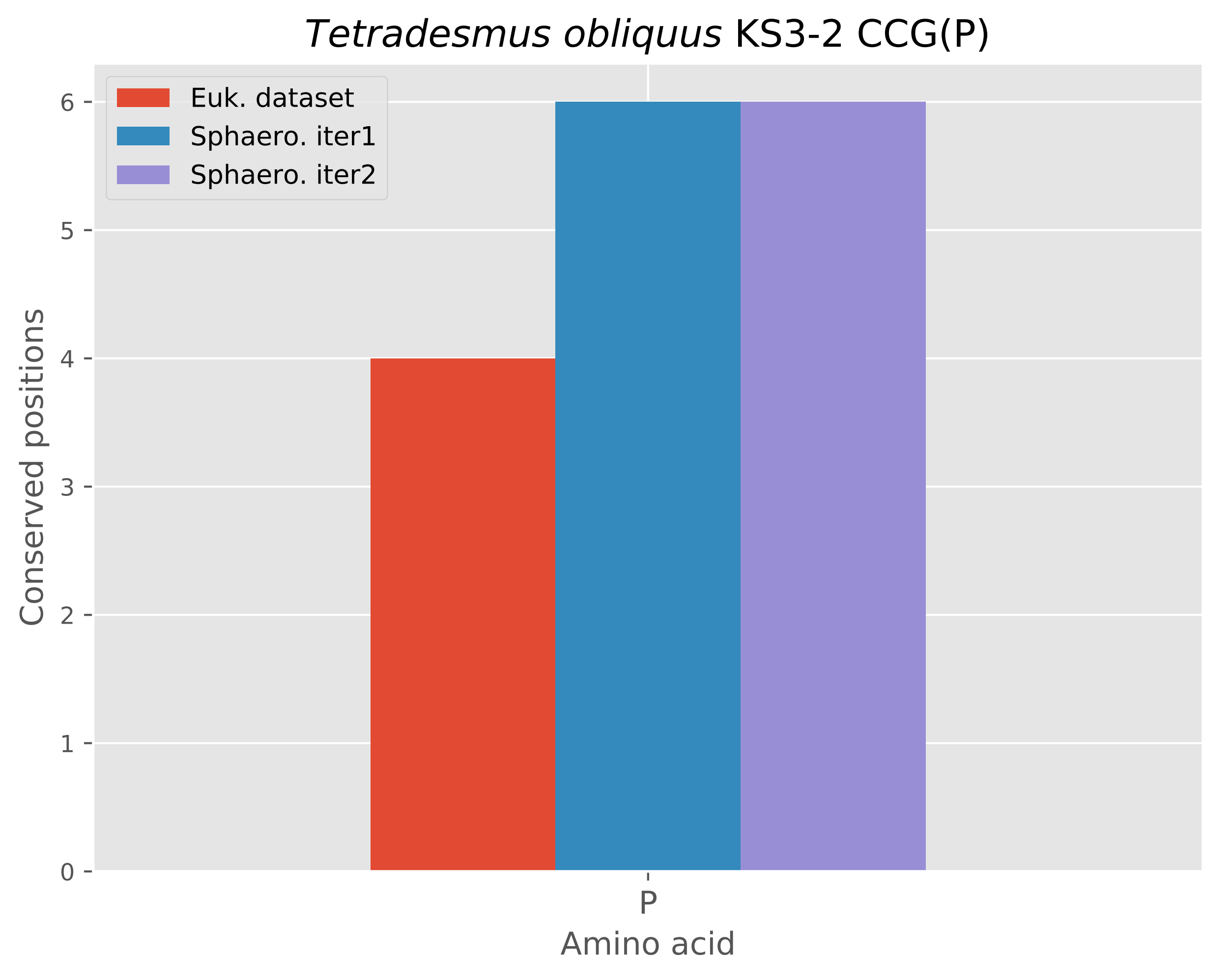

Tetradesmus obliquus KS3-2 CCG(P)
Euk. dataset
Sphaero. iter1
Sphaero. iter2
Conserved positions
0
1
2
3
4
5
6
P
Amino acid

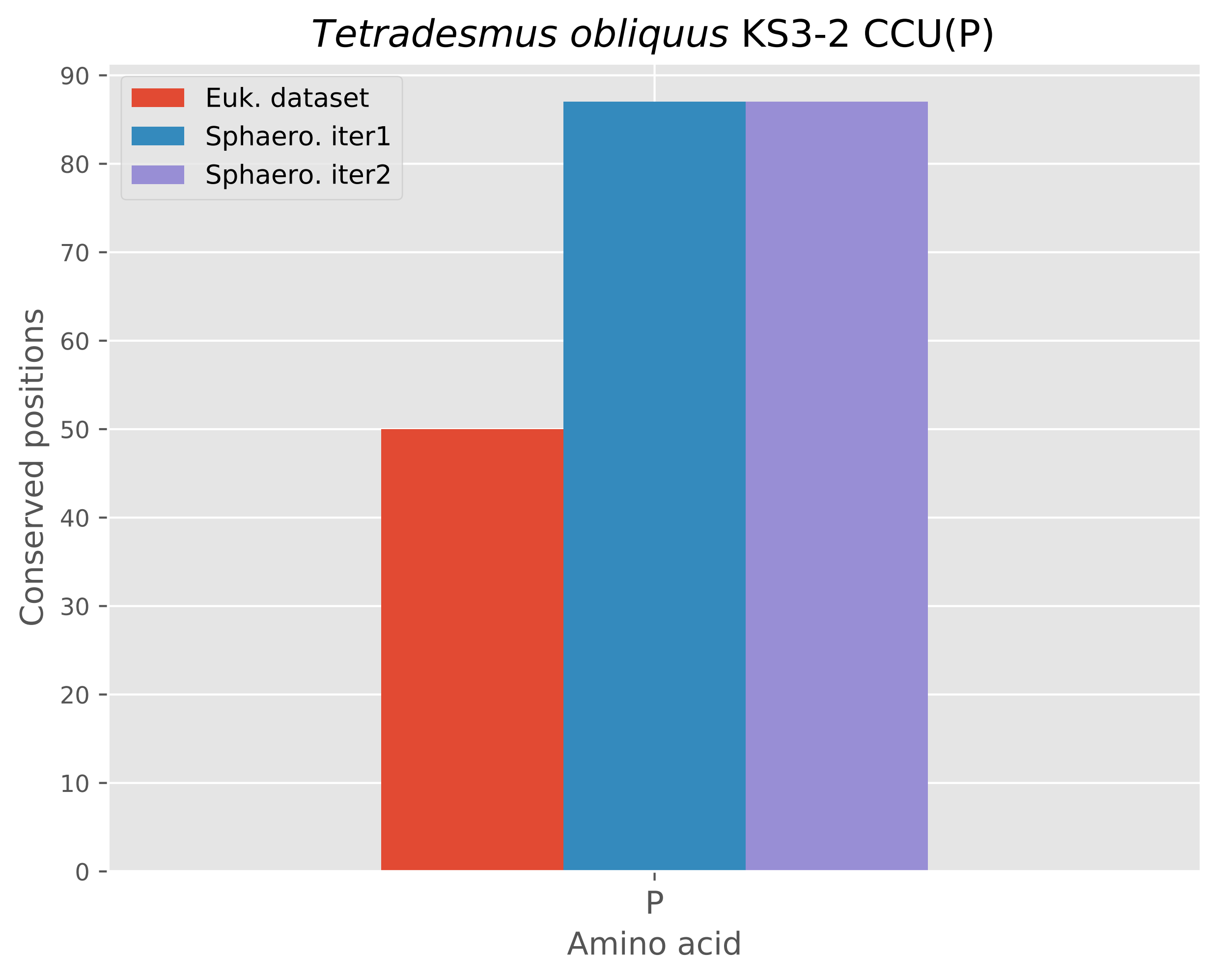

Tetradesmus obliquus KS3-2 CCU(P)
Euk. dataset
Sphaero. iter1
Sphaero. iter2
Conserved positions
0
10
20
30
40
50
60
70
80
90
P
Amino acid

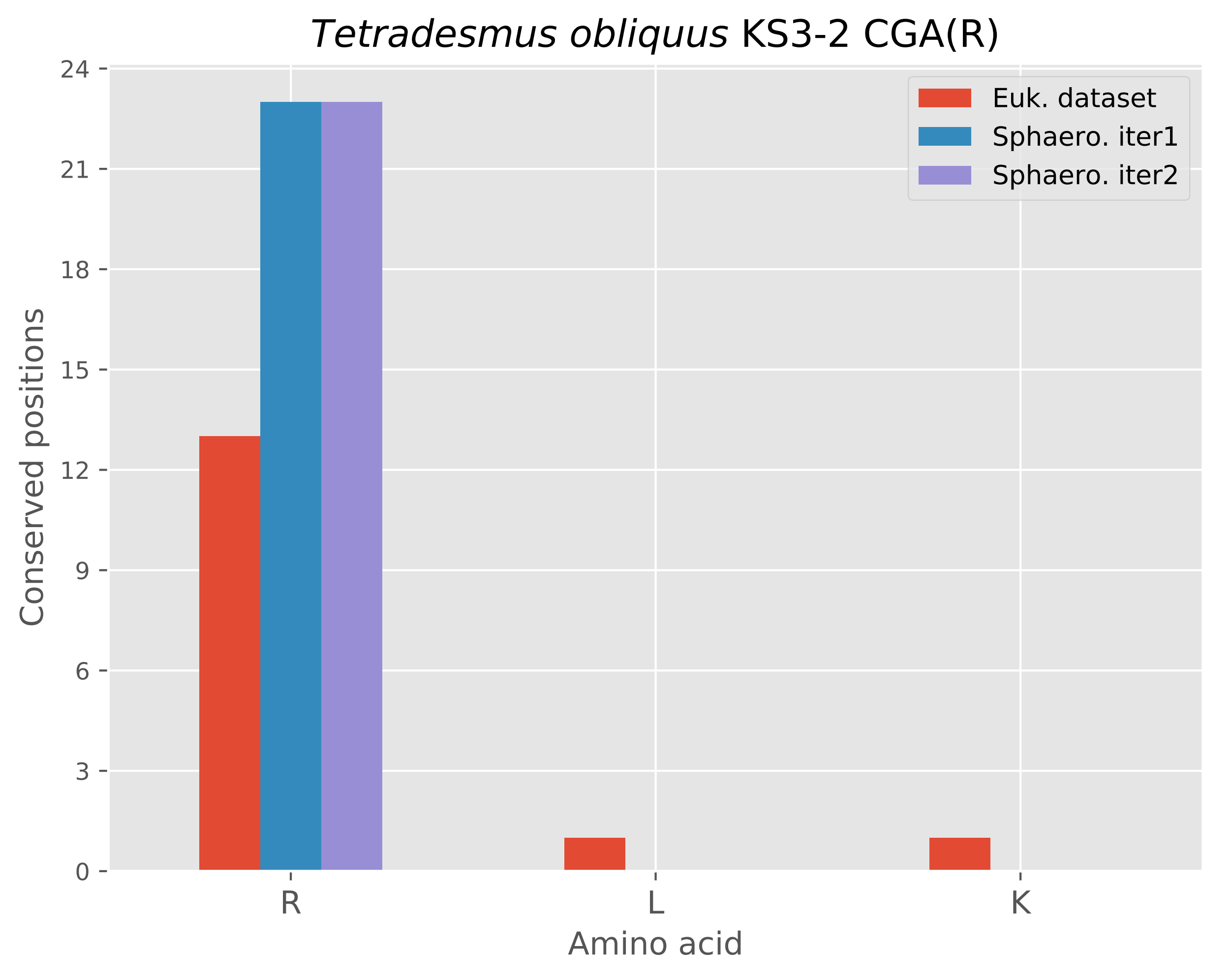

Tetradesmus obliquus KS3-2 CGA(R)
Euk. dataset
Sphaero. iter1
Sphaero. iter2
Conserved positions
0
3
6
9
12
15
18
21
24
R
L
K
Amino acid

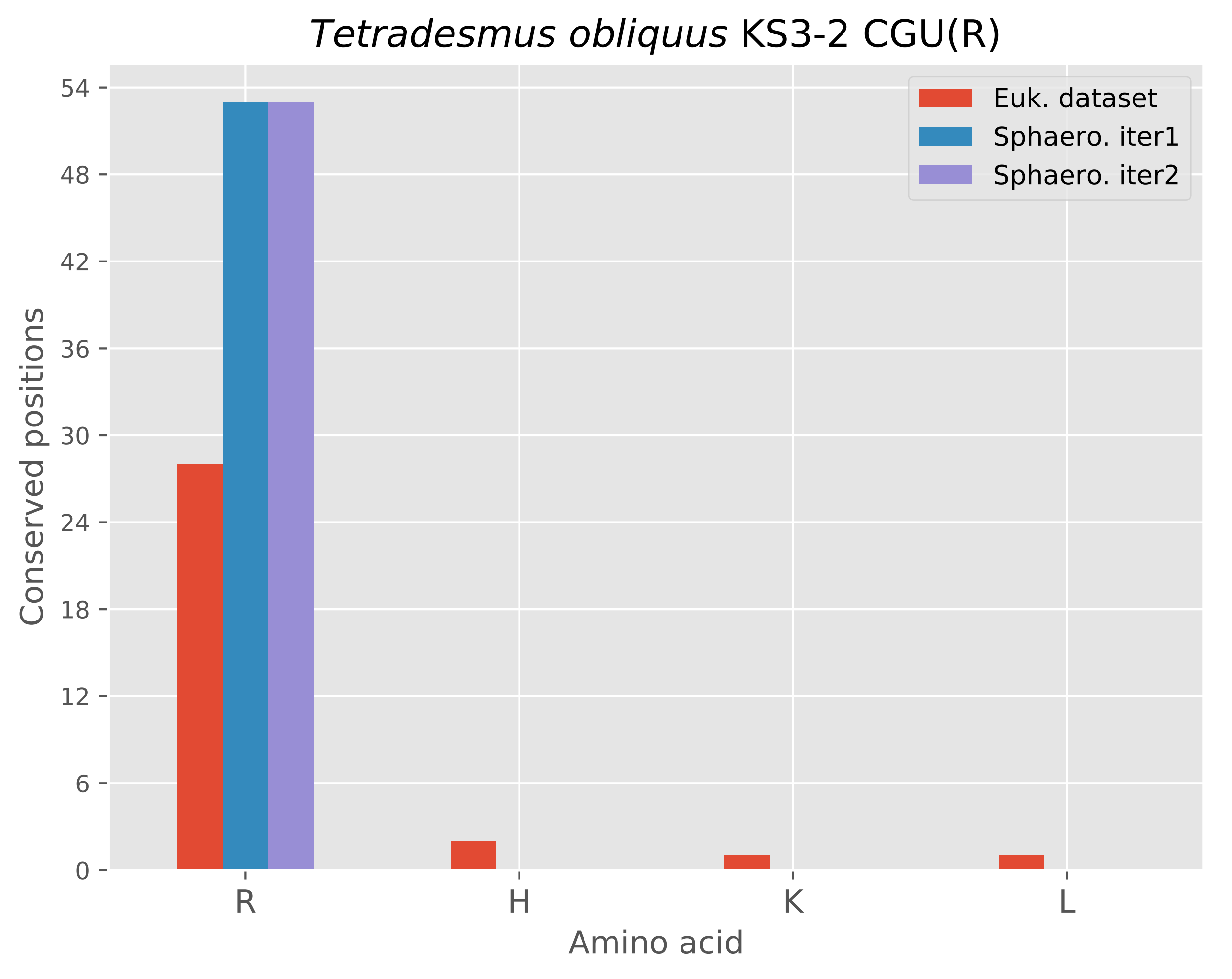

Tetradesmus obliquus KS3-2 CGU(R)
Euk. dataset
Sphaero. iter1
Sphaero. iter2
Conserved positions
0
6
12
18
24
30
36
42
48
54
R
H
K
L
Amino acid

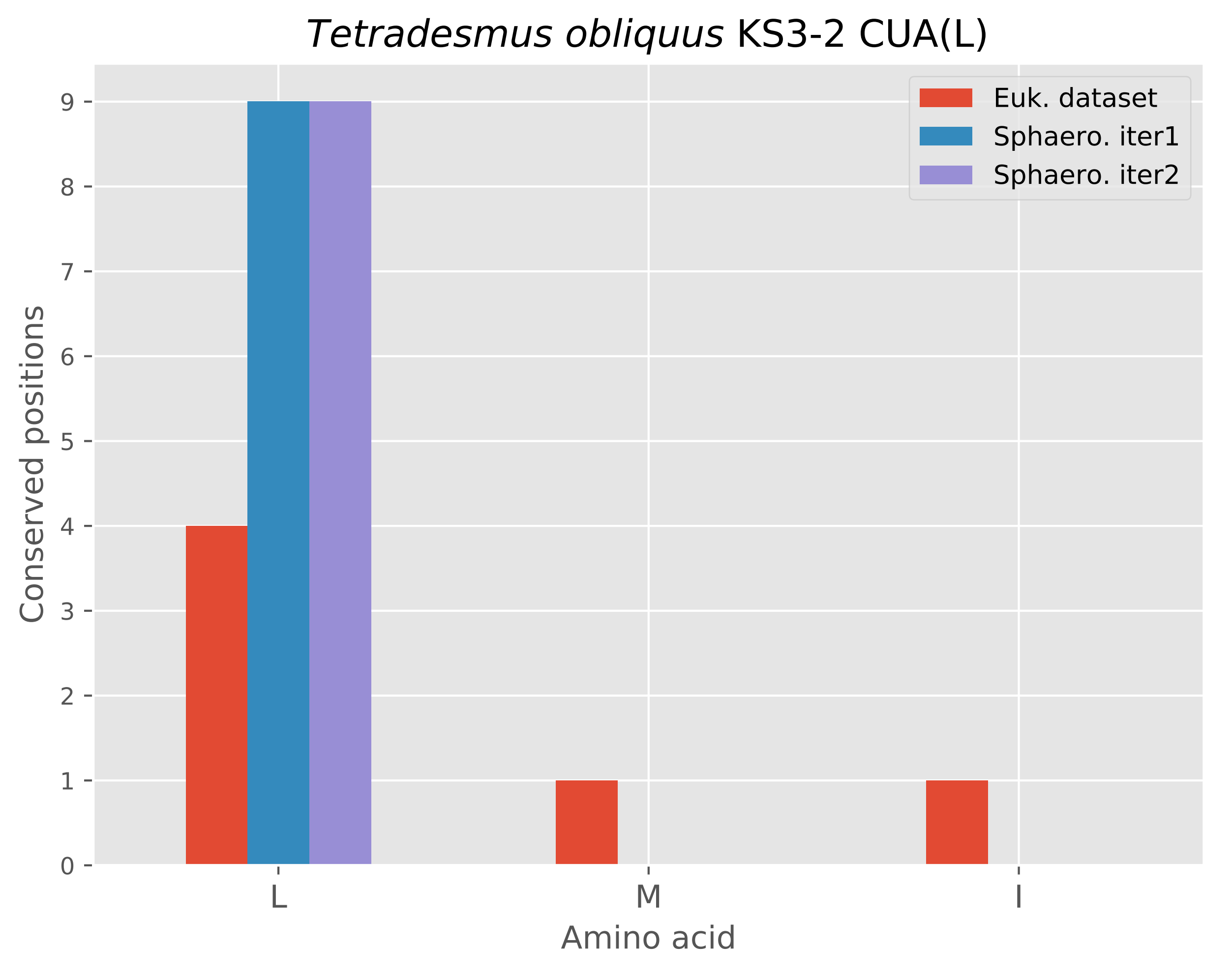
Tetradesmus obliquus KS3-2 CUA(L)
Euk. dataset
Sphaero. iter1
Sphaero. iter2
Conserved positions
0
1
2
3
4
5
6
7
8
9
L
M
I
Amino acid

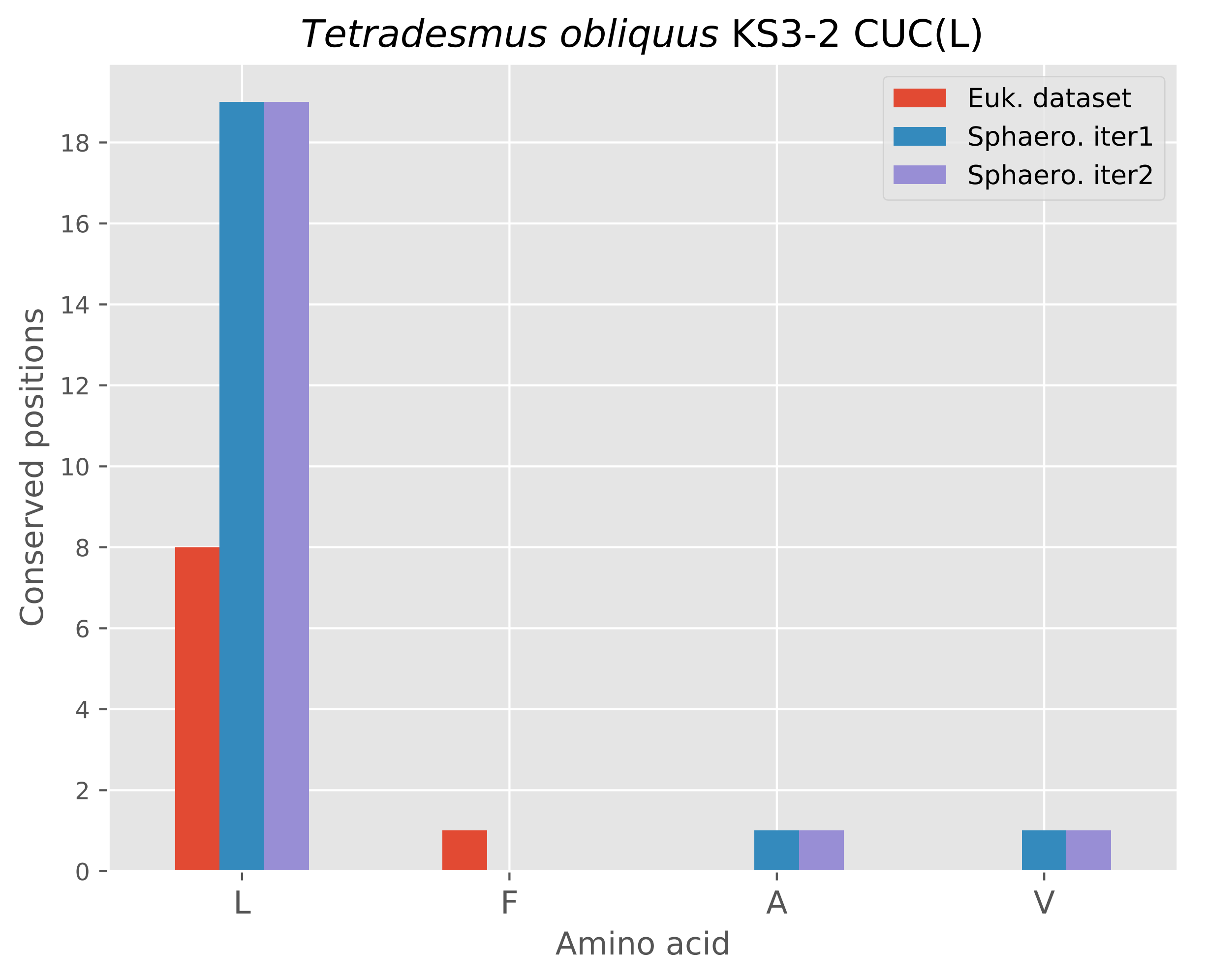

Tetradesmus obliquus KS3-2 CUC(L)
Euk. dataset
Sphaero. iter1
Sphaero. iter2
Conserved positions
0
2
4
6
8
10
12
14
16
18
L
F
A
V
Amino acid

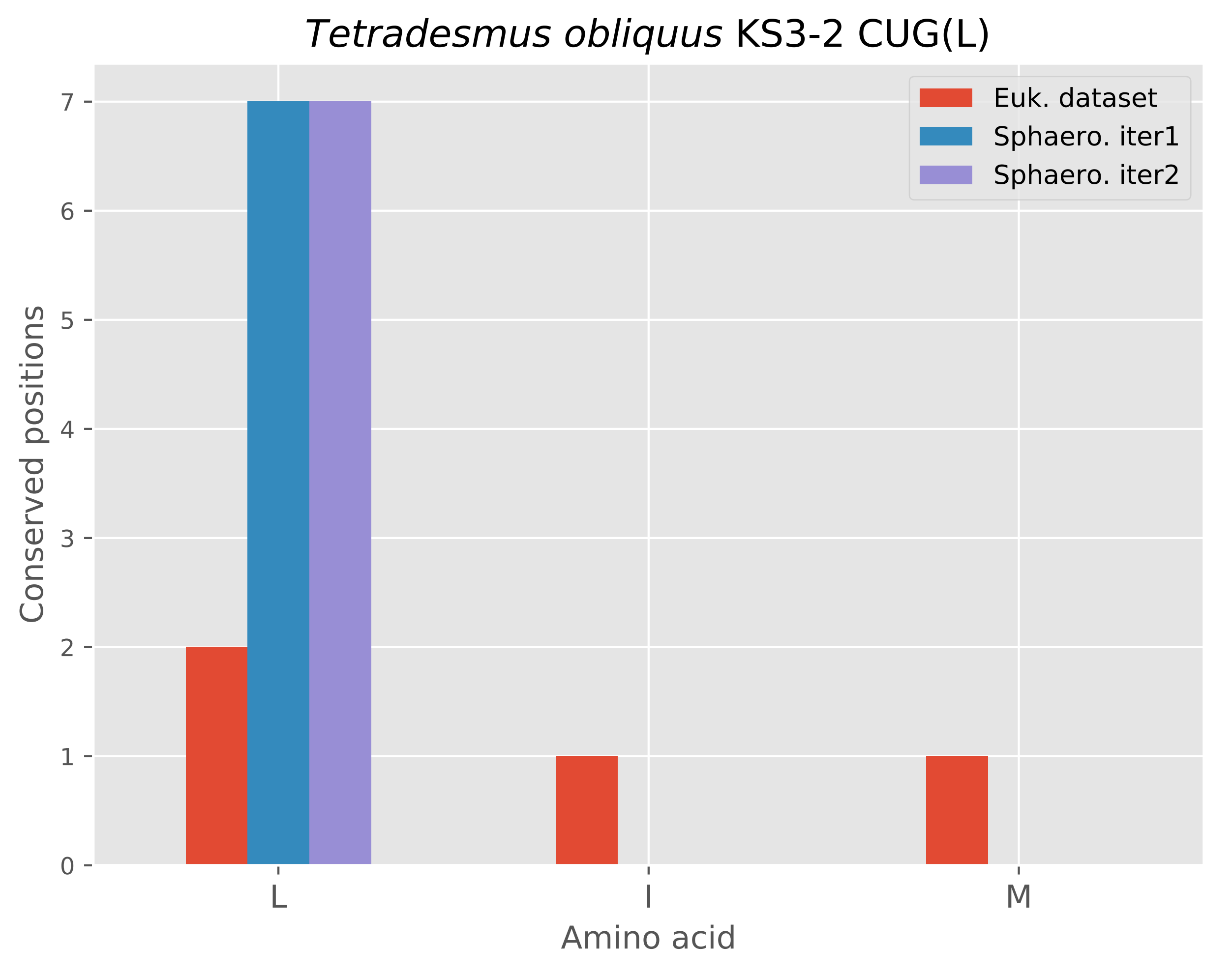

Tetradesmus obliquus KS3-2 CUG(L)
Euk. dataset
Sphaero. iter1
Sphaero. iter2
Conserved positions
0
1
2
3
4
5
6
7
L
I
M
Amino acid

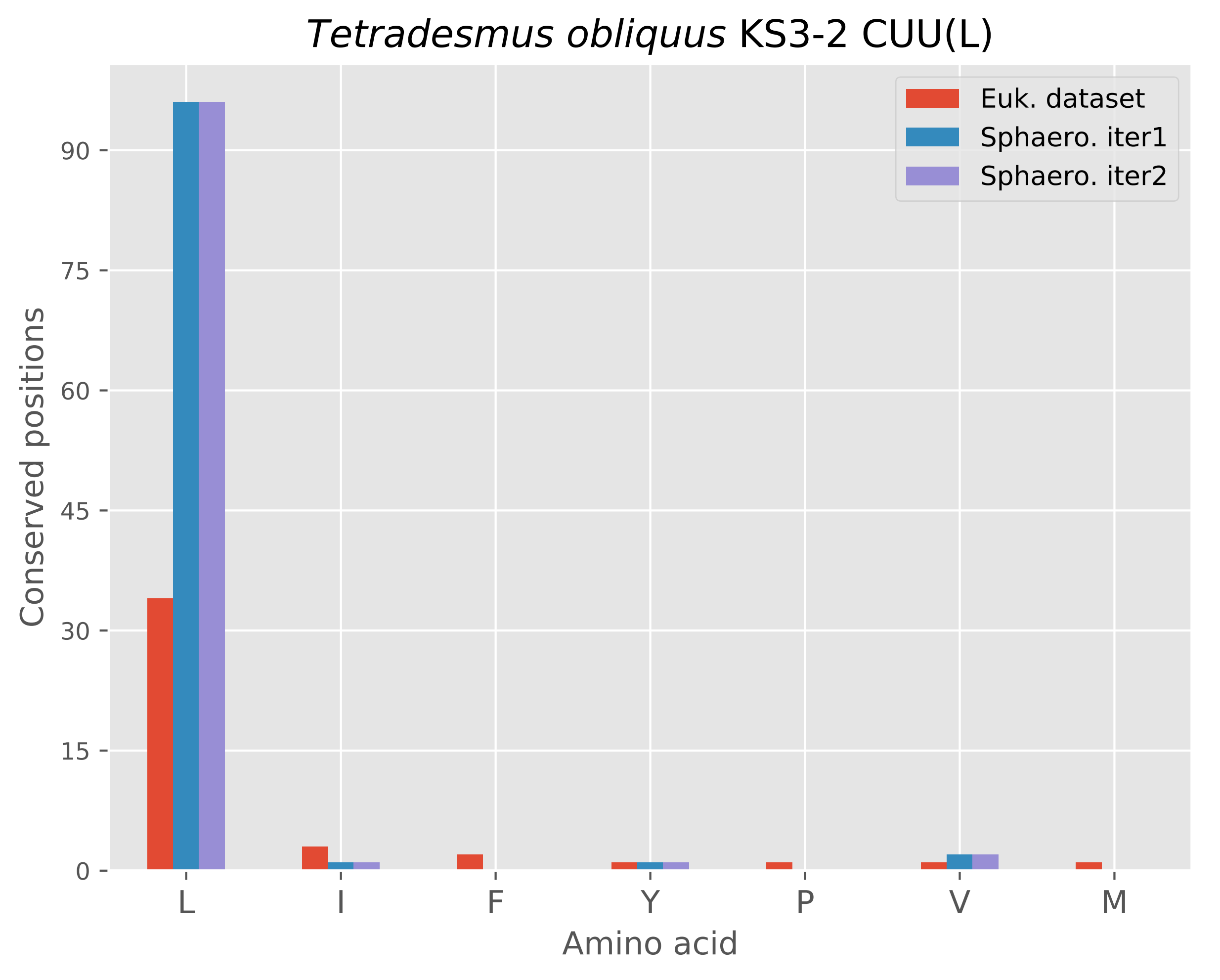

Tetradesmus obliquus KS3-2 CUU(L)
Euk. dataset
Sphaero. iter1
Sphaero. iter2
Conserved positions
0
15
30
45
60
75
90
L
I
F
Y
P
V
M
Amino acid

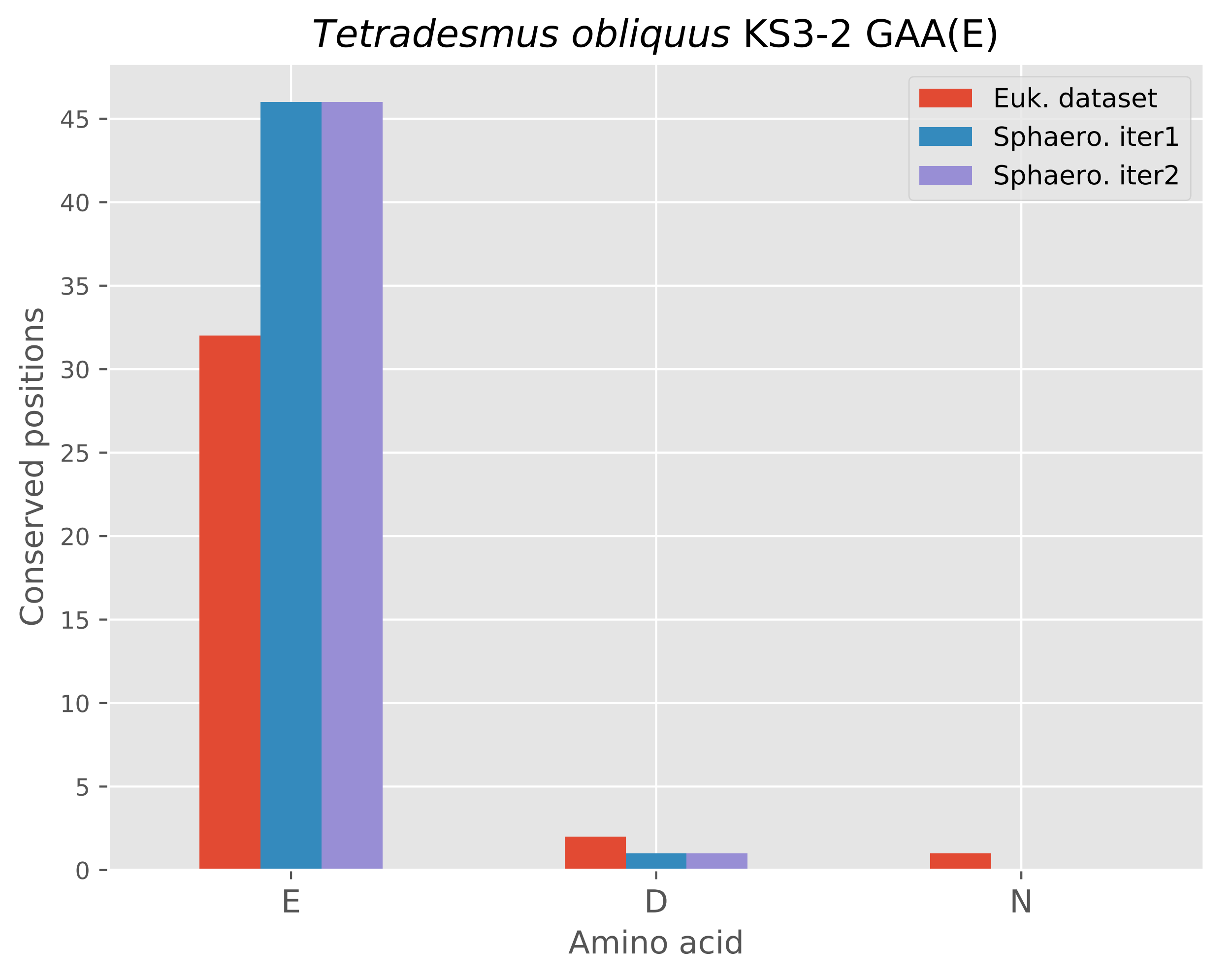
Tetradesmus obliquus KS3-2 GAA(E)
Euk. dataset
Sphaero. iter1
Sphaero. iter2
Conserved positions
0
5
10
15
20
25
30
35
40
45
E
D
N
Amino acid

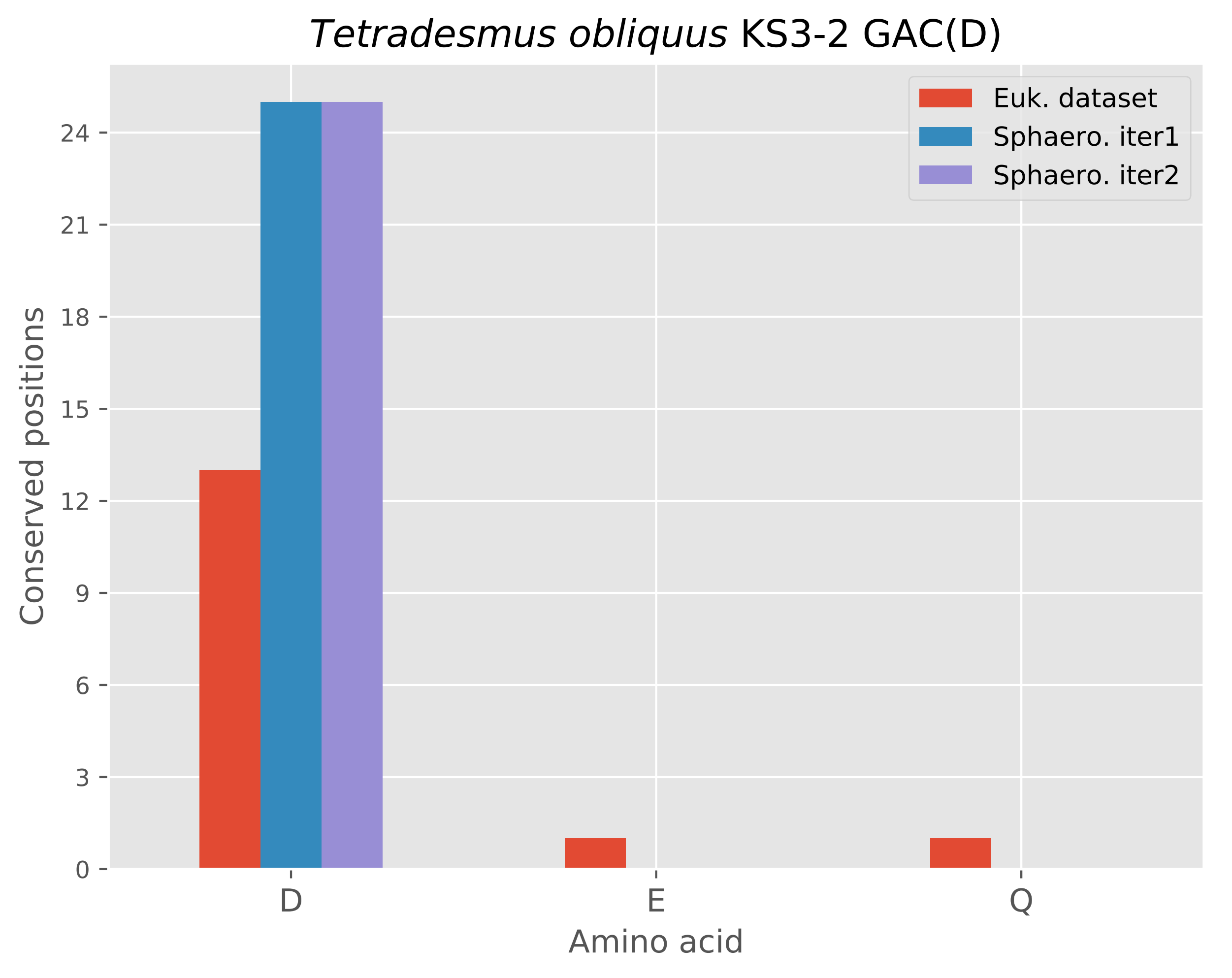

Tetradesmus obliquus KS3-2 GAC(D)
Euk. dataset
Sphaero. iter1
Sphaero. iter2
Conserved positions
0
3
6
9
12
15
18
21
24
D
E
Q
Amino acid

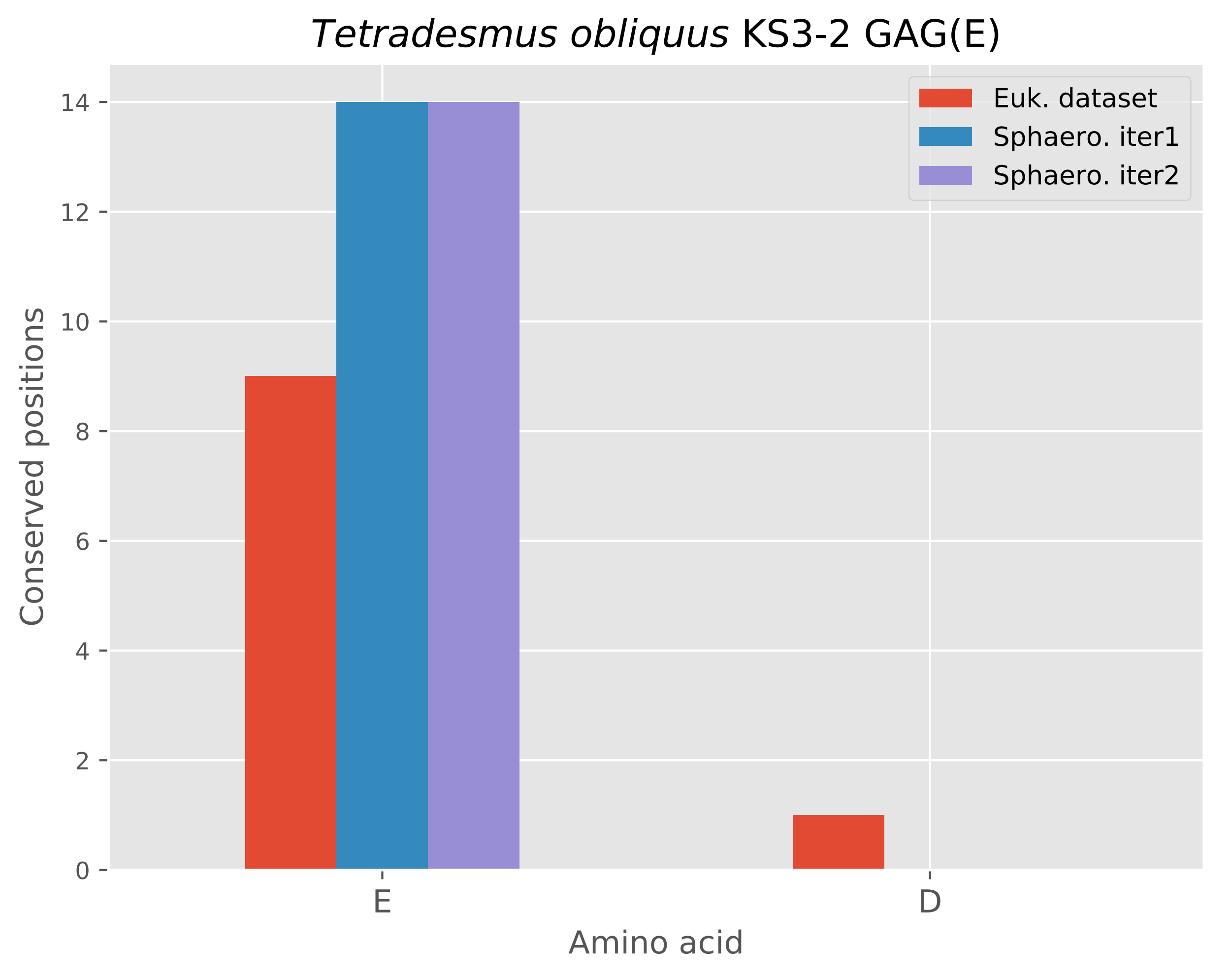

Tetradesmus obliquus KS3-2 GAG(E)
Euk. dataset
Sphaero. iter1
Sphaero. iter2
Conserved positions
0
2
4
6
8
10
12
14
E
D
Amino acid

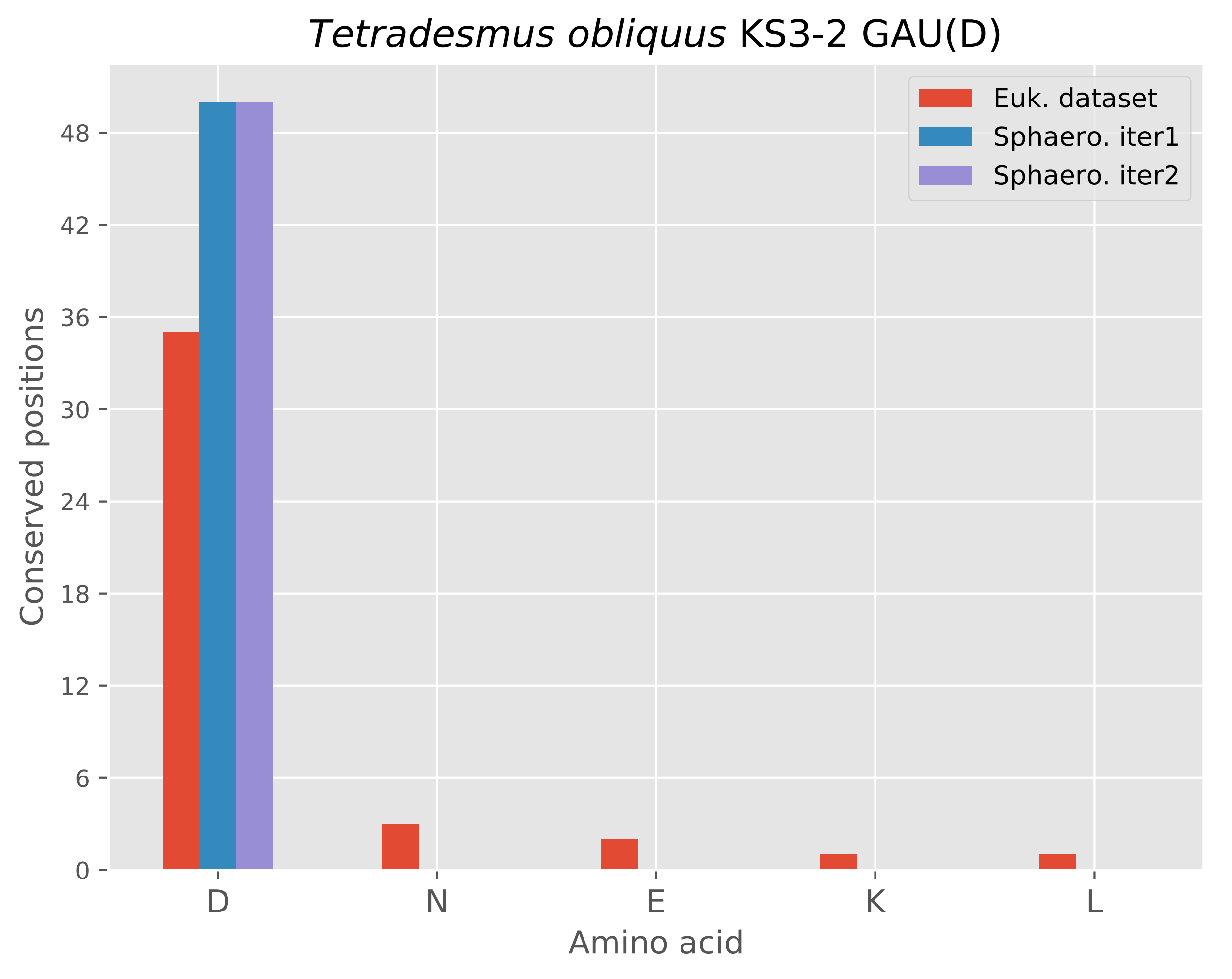

Tetradesmus obliquus KS3-2 GAU(D)
Euk. dataset
Sphaero. iter1
Sphaero. iter2
Conserved positions
0
6
12
18
24
30
36
42
48
D
N
E
K
L
Amino acid

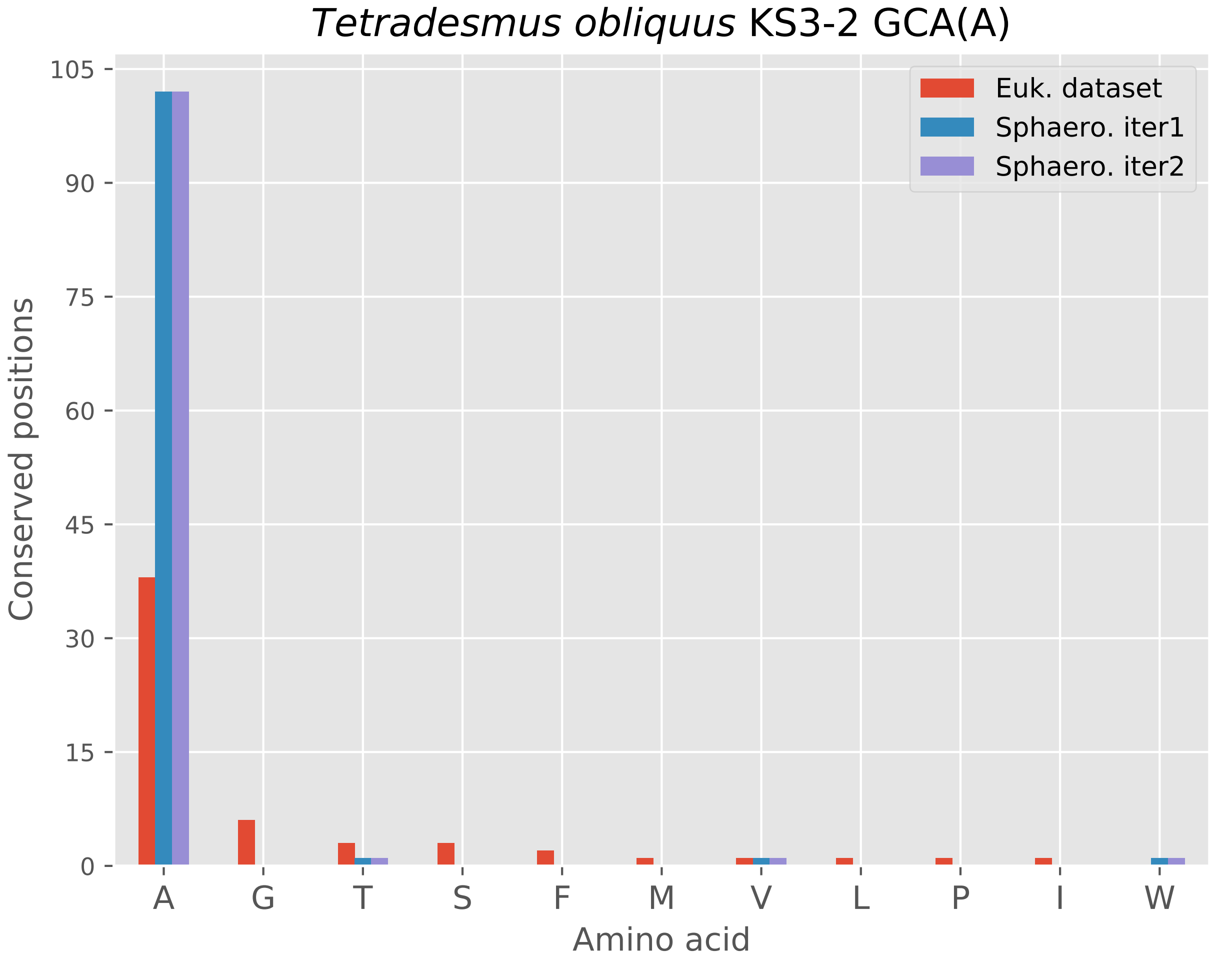

Tetradesmus obliquus KS3-2 GCA(A)
Euk. dataset
Sphaero. iter1
Sphaero. iter2
Conserved positions
0
15
30
45
60
75
90
105
Amino acid
A
G
T
S
F
M
V
L
P
I
W

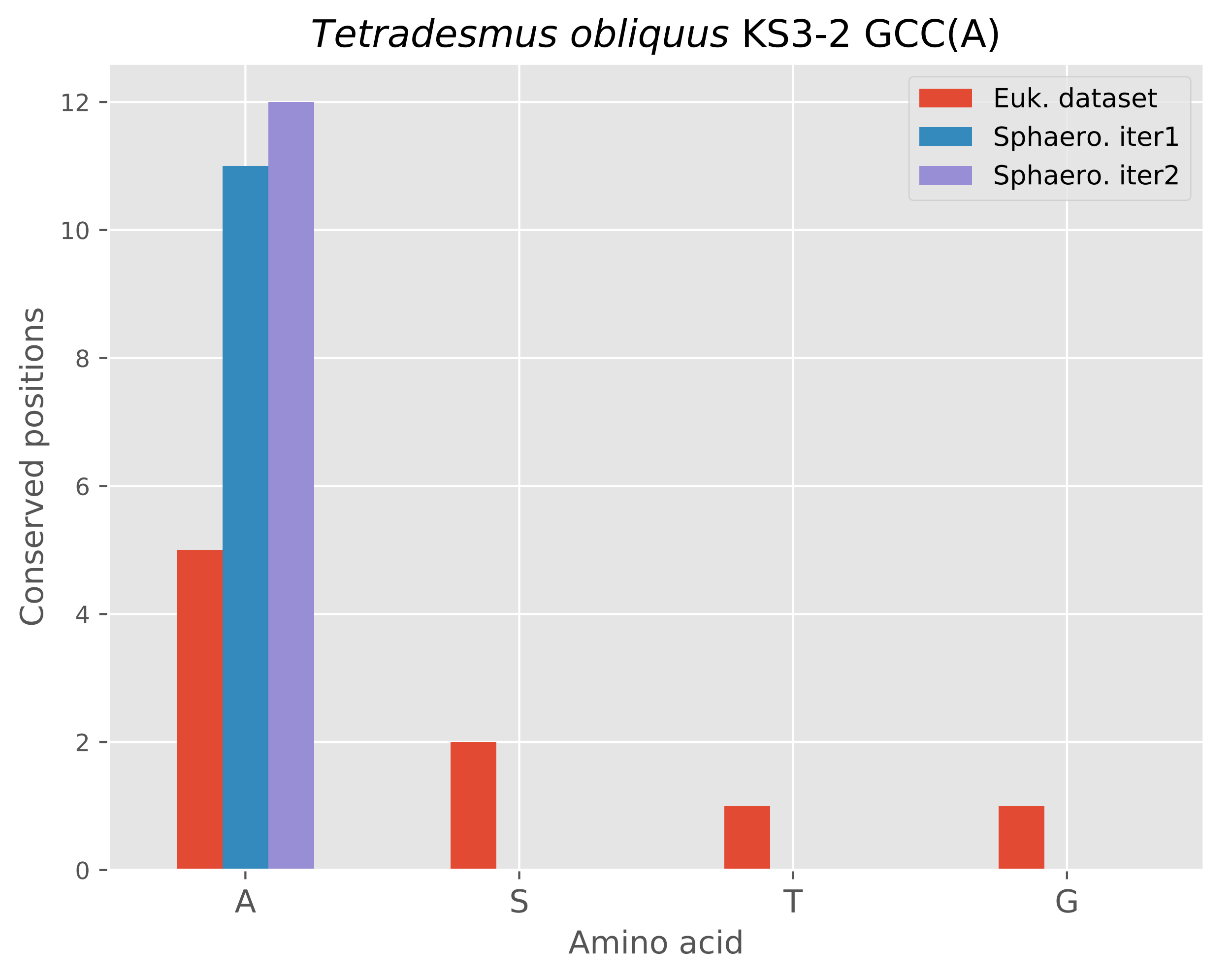
Tetradesmus obliquus KS3-2 GCC(A)
Conserved positions
0
2
4
6
8
10
12
A
S
T
G
Amino acid
Euk. dataset
Sphaero. iter1
Sphaero. iter2

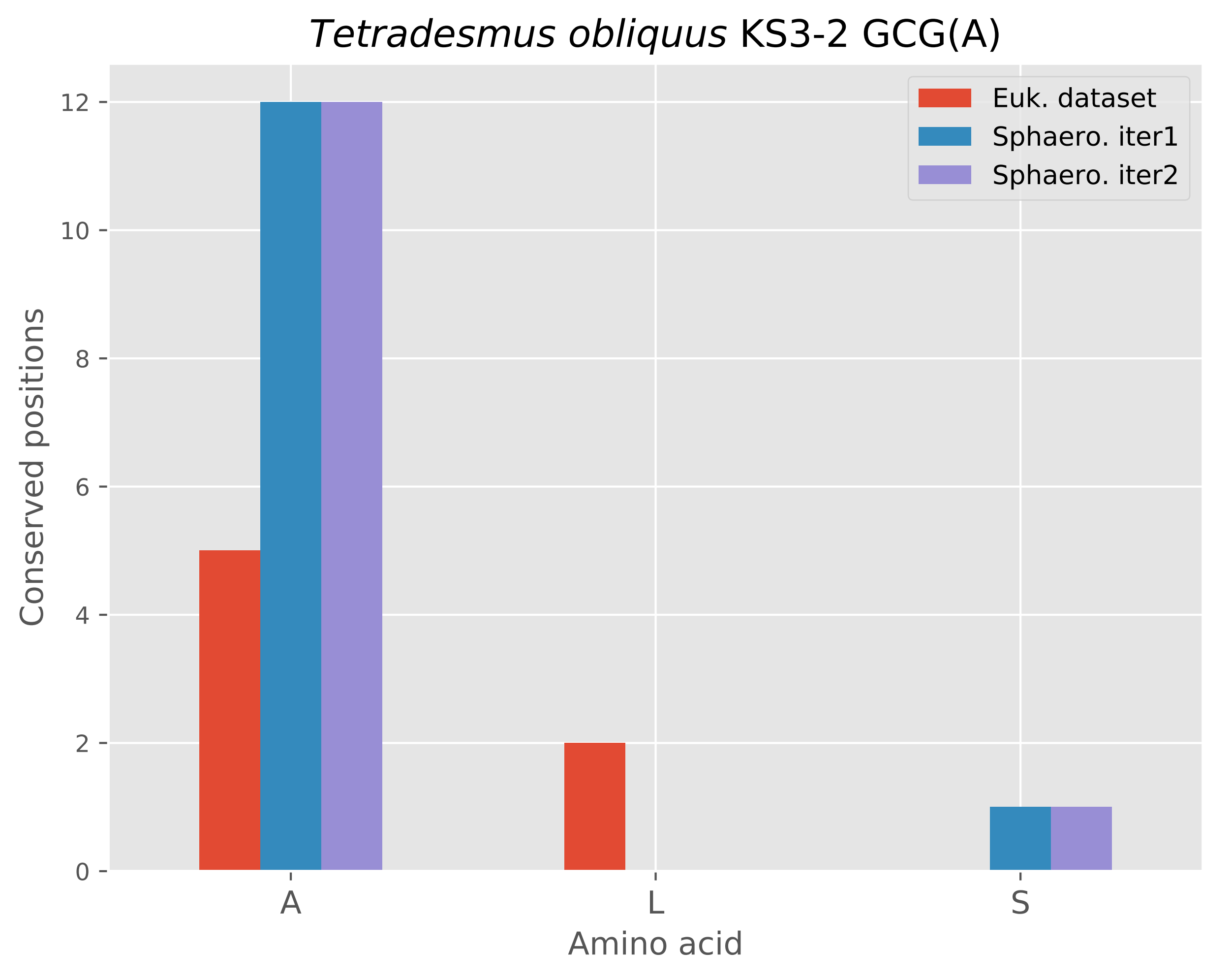

Tetradesmus obliquus KS3-2 GCG(A)
Euk. dataset
Sphaero. iter1
Sphaero. iter2
Conserved positions
0
2
4
6
8
10
12
A
L
S
Amino acid

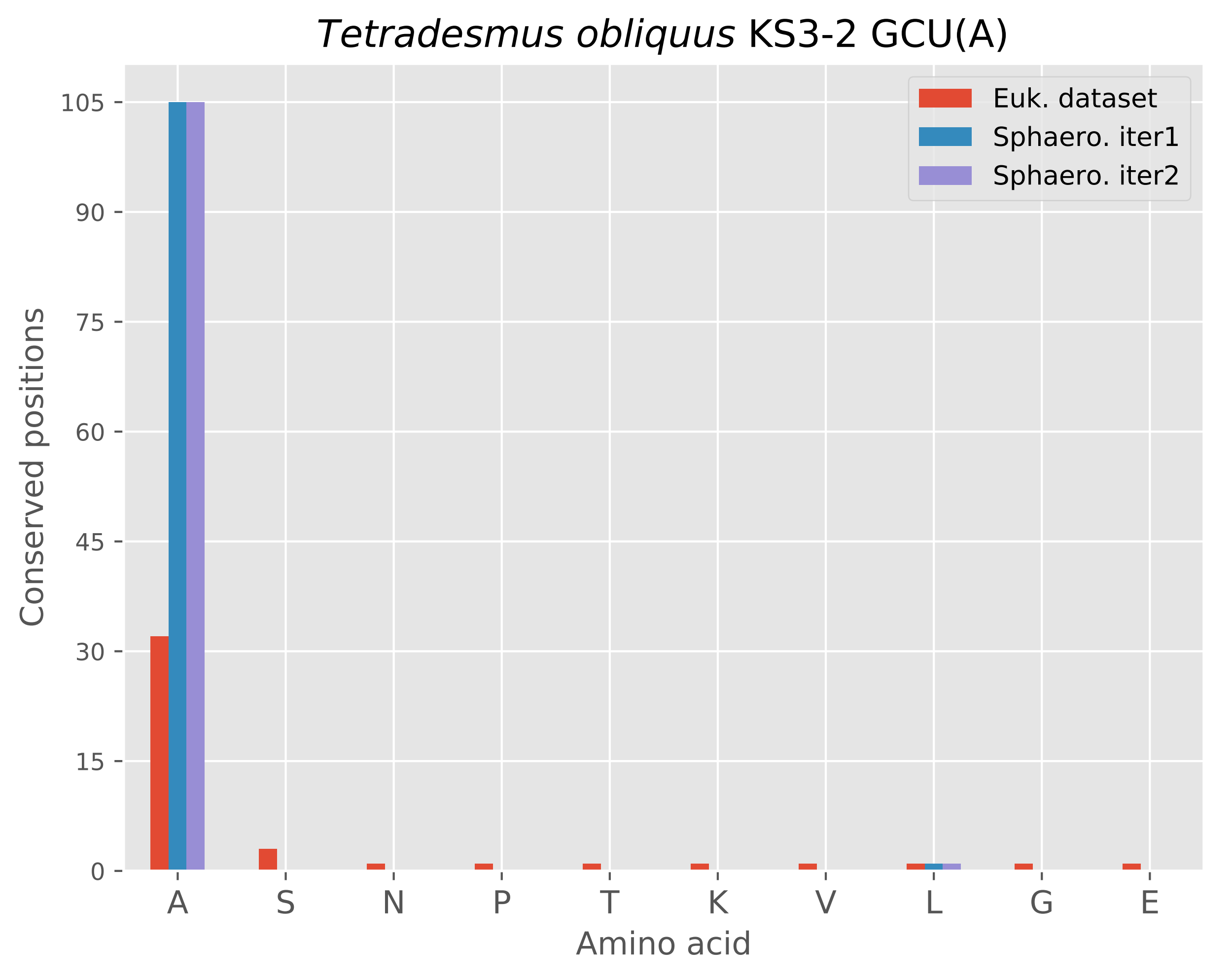

Tetradesmus obliquus KS3-2 GCU(A)
Euk. dataset
Sphaero. iter1
Sphaero. iter2
Conserved positions
0
15
30
45
60
75
90
105
A
S
N
P
T
K
V
L
G
E
Amino acid

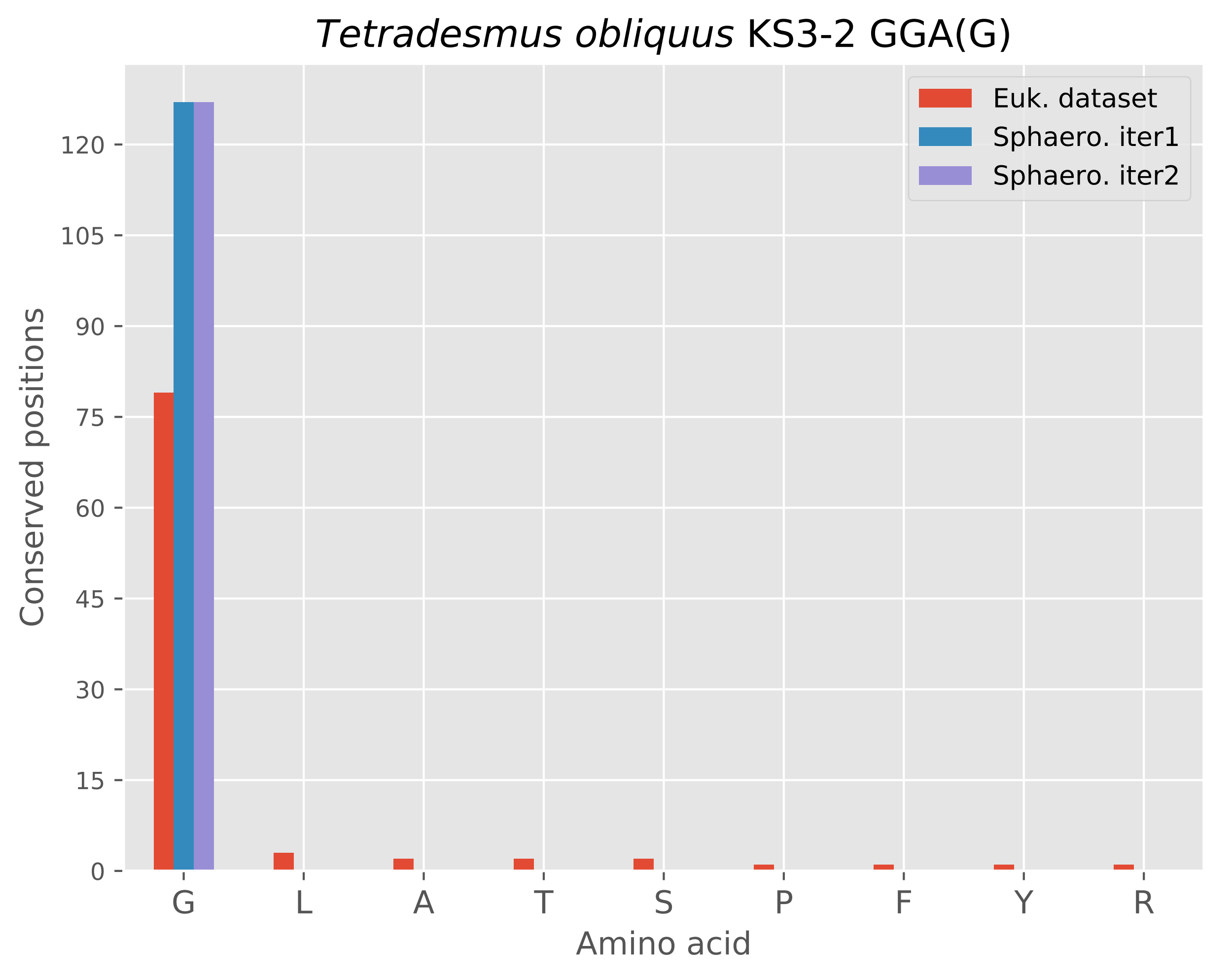

Tetradesmus obliquus KS3-2 GGA(G)
Euk. dataset
Sphaero. iter1
Sphaero. iter2
Conserved positions
0
15
30
45
60
75
90
105
120
G
L
A
T
S
P
F
Y
R
Amino acid

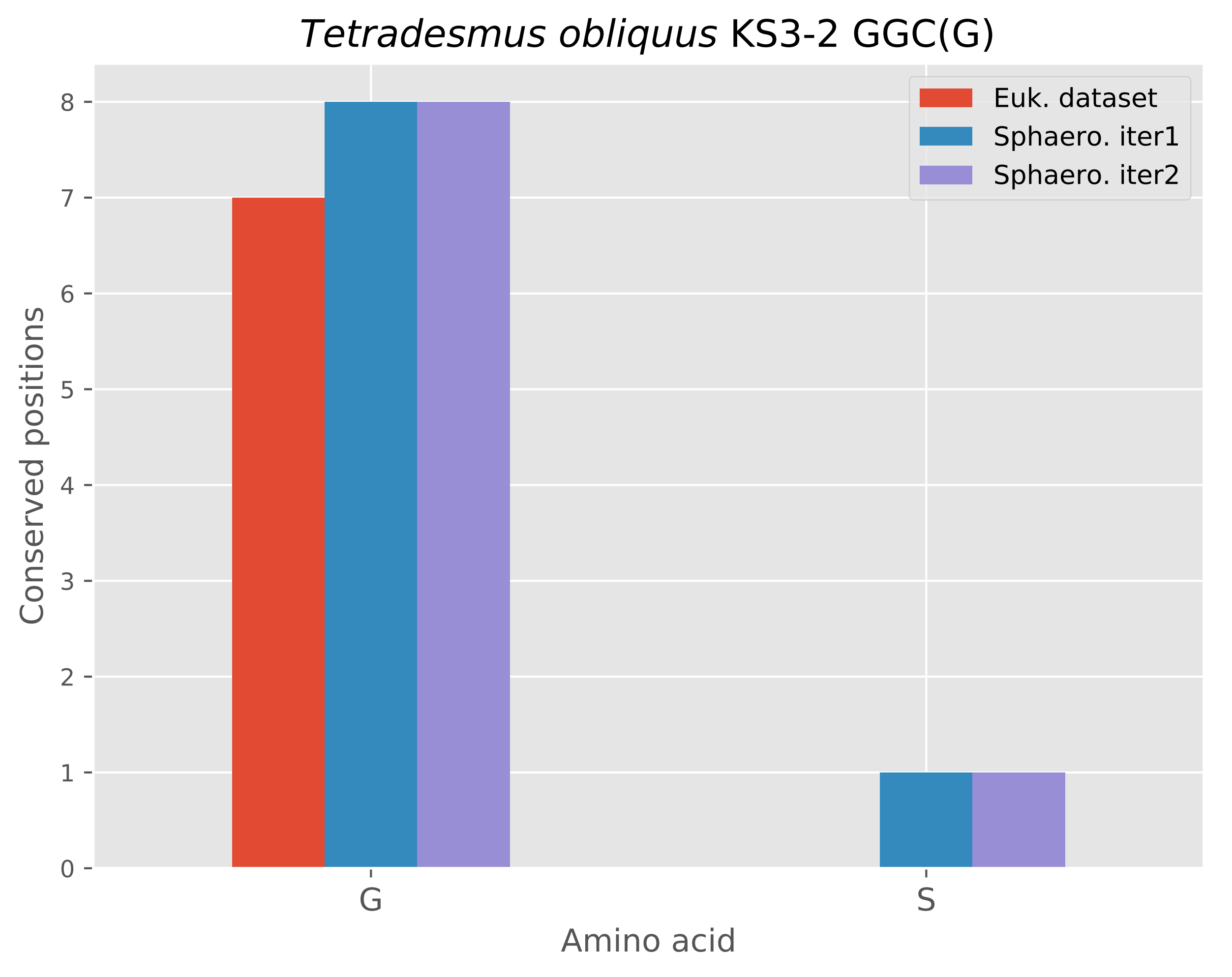
Tetradesmus obliquus KS3-2 GGC(G)
Euk. dataset
Sphaero. iter1
Sphaero. iter2
Conserved positions
0
1
2
3
4
5
6
7
8
G
S
Amino acid

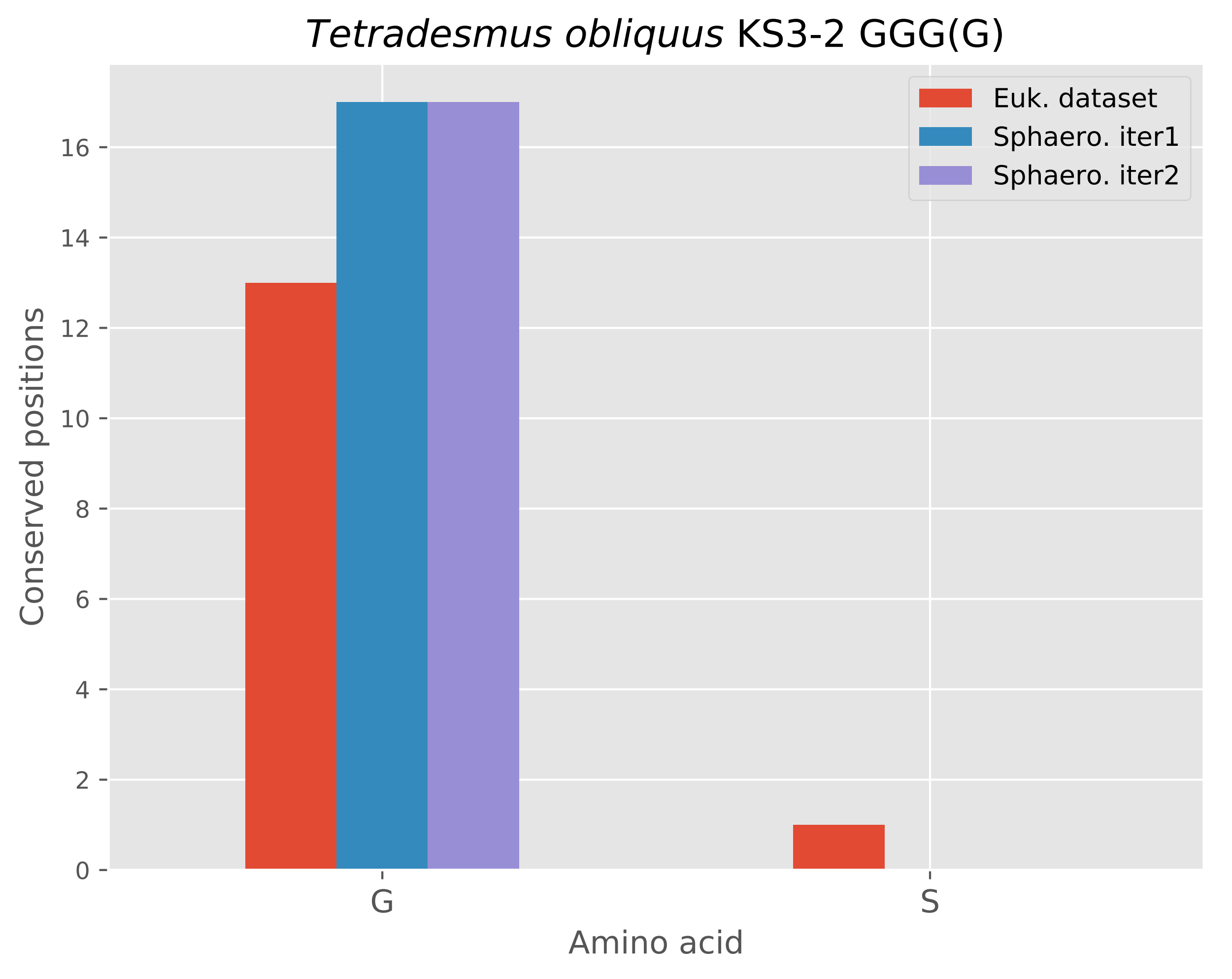

Tetradesmus obliquus KS3-2 GGG(G)
Euk. dataset
Sphaero. iter1
Sphaero. iter2
Conserved positions
0
2
4
6
8
10
12
14
16
G
S
Amino acid

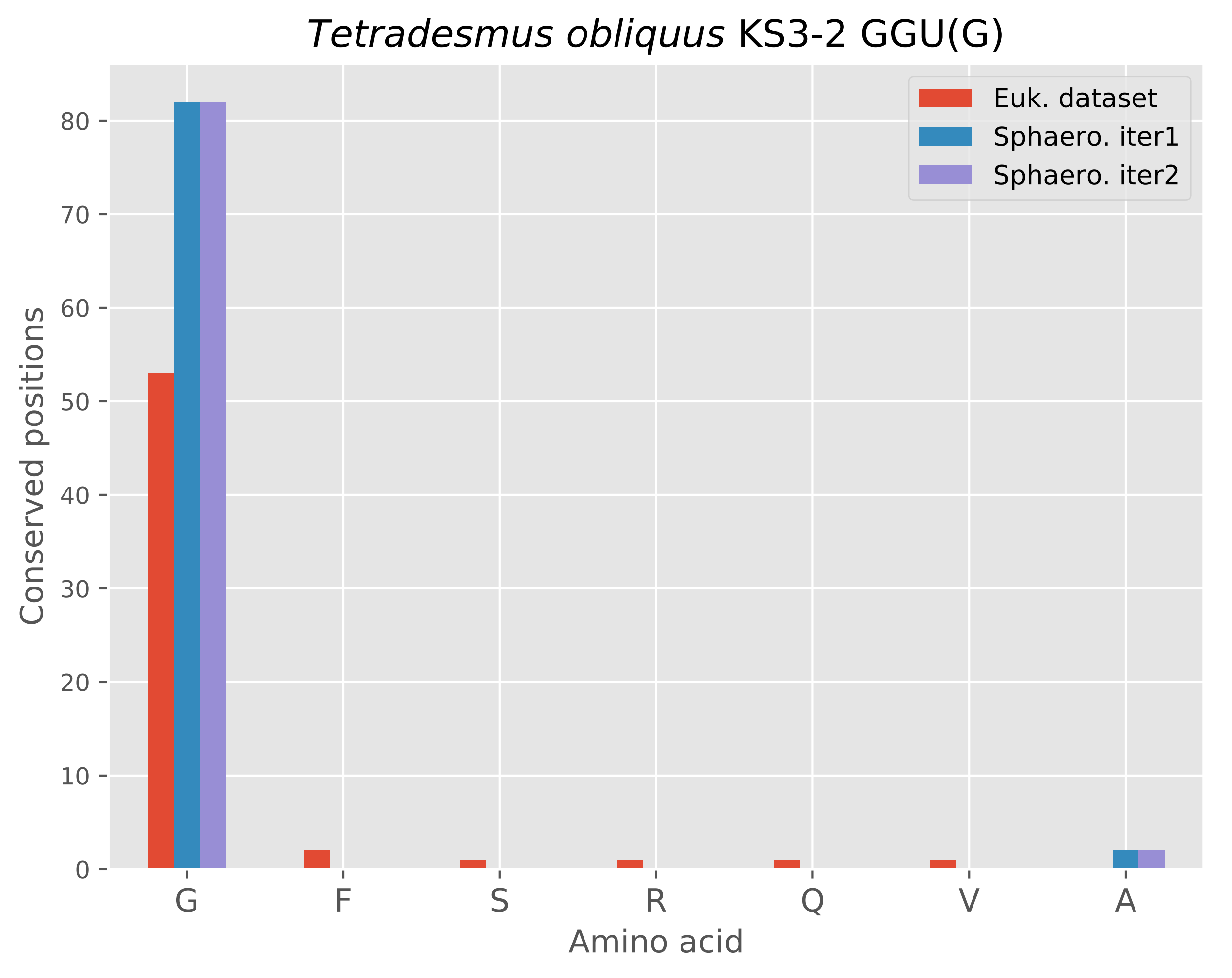
Tetradesmus obliquus KS3-2 GGU(G)
Euk. dataset
Sphaero. iter1
Sphaero. iter2
Conserved positions
0
10
20
30
40
50
60
70
80
G
F
S
R
Q
V
A
Amino acid

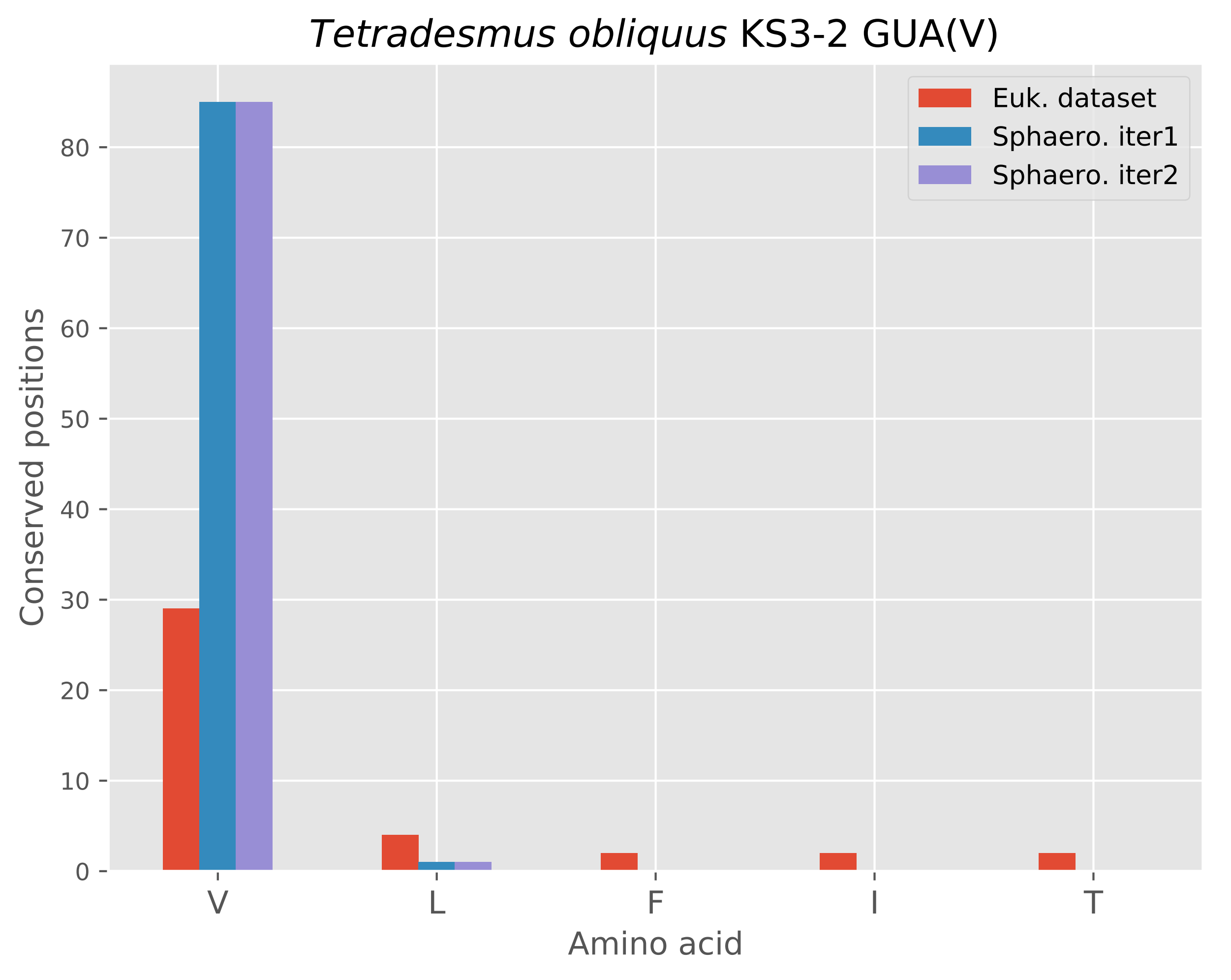

Tetradesmus obliquus KS3-2 GUA(V)
Euk. dataset
Sphaero. iter1
Sphaero. iter2
Conserved positions
0
10
20
30
40
50
60
70
80
V
L
F
I
T
Amino acid

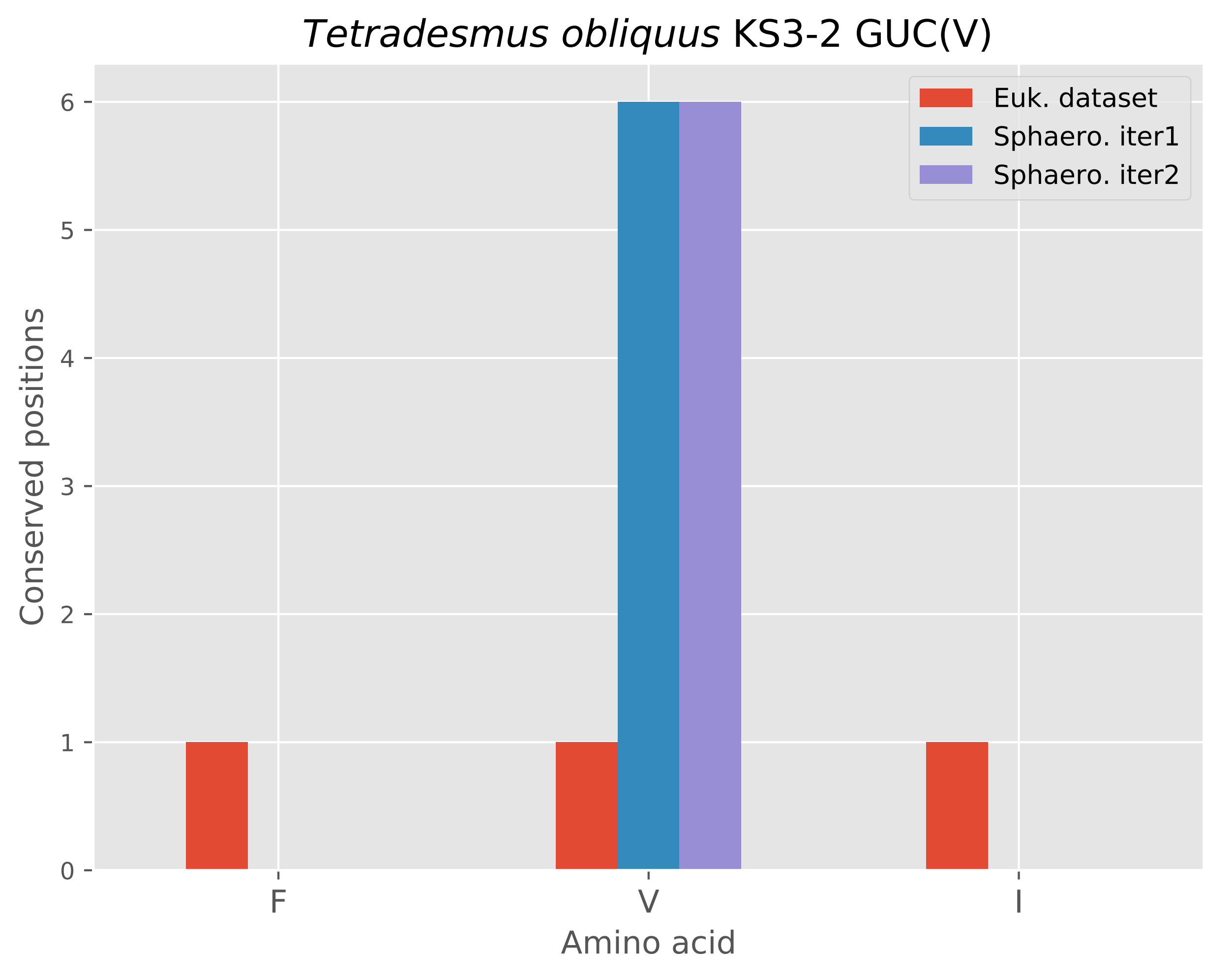

Tetradesmus obliquus KS3-2 GUC(V)
Euk. dataset
Sphaero. iter1
Sphaero. iter2
Conserved positions
0
1
2
3
4
5
6
F
V
I
Amino acid

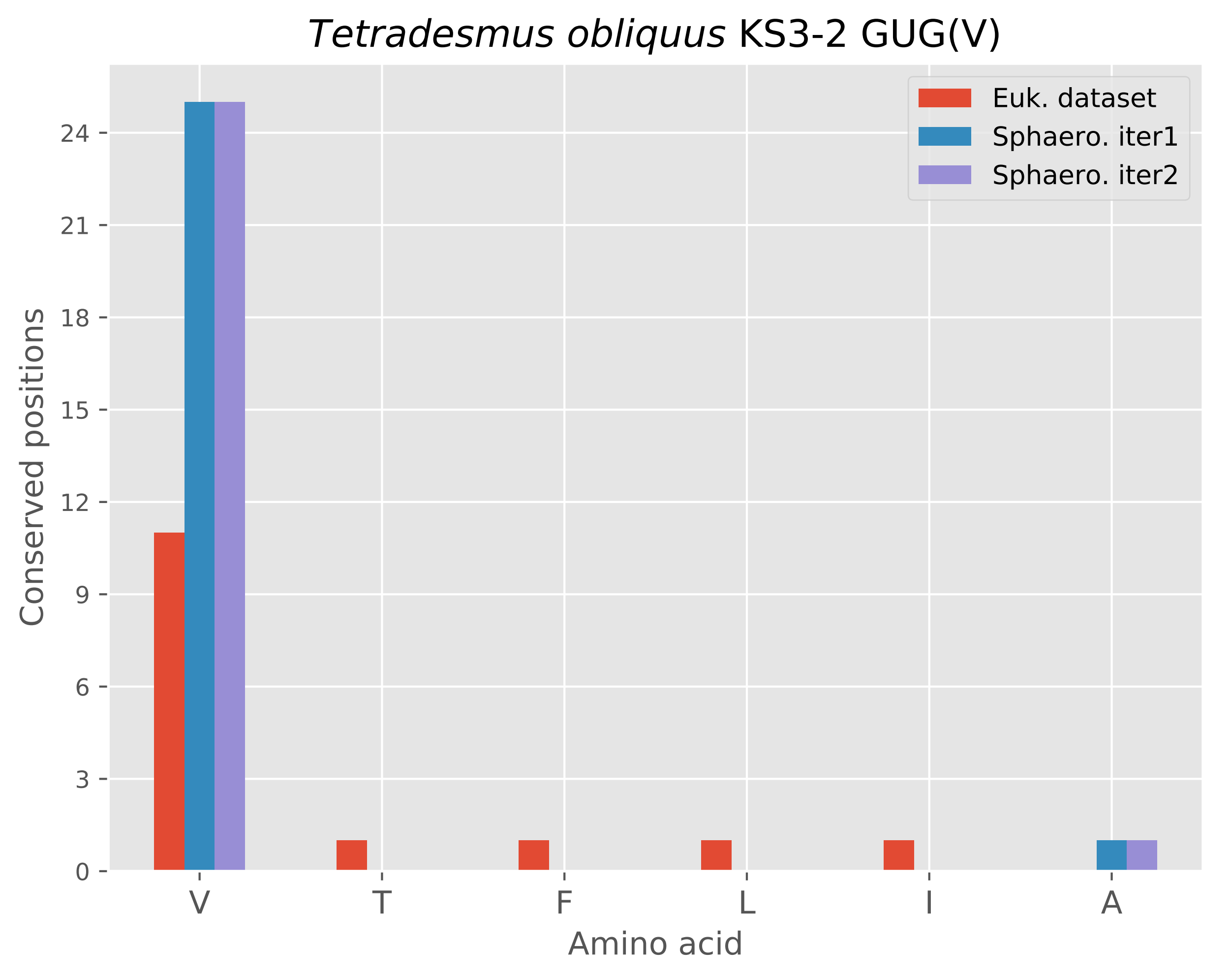

Tetradesmus obliquus KS3-2 GUG(V)
Euk. dataset
Sphaero. iter1
Sphaero. iter2
Conserved positions
0
3
6
9
12
15
18
21
24
V
T
F
L
I
A
Amino acid

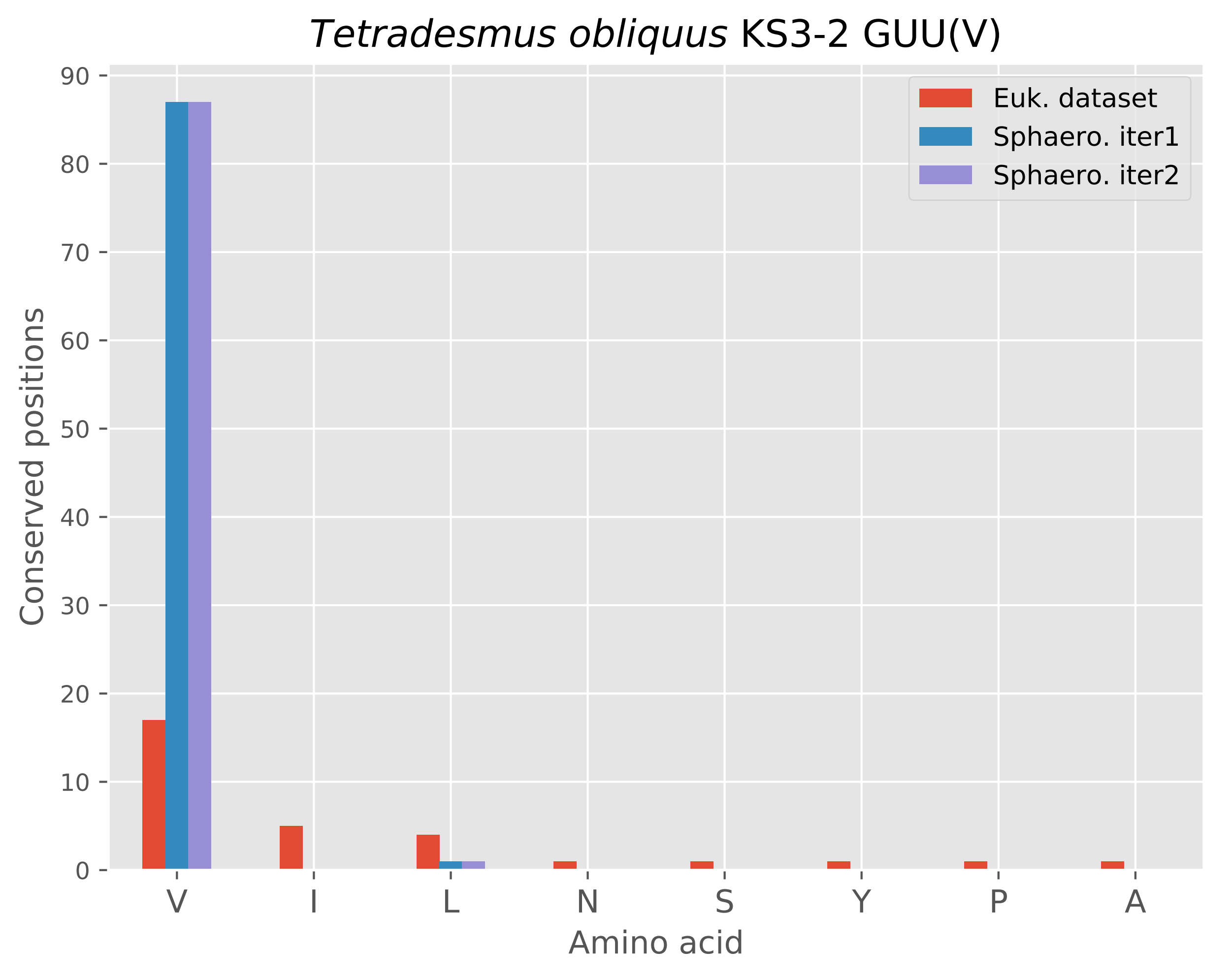

Tetradesmus obliquus KS3-2 GUU(V)
Euk. dataset
Sphaero. iter1
Sphaero. iter2
Conserved positions
0
10
20
30
40
50
60
70
80
90
V
I
L
N
S
Y
P
A
Amino acid

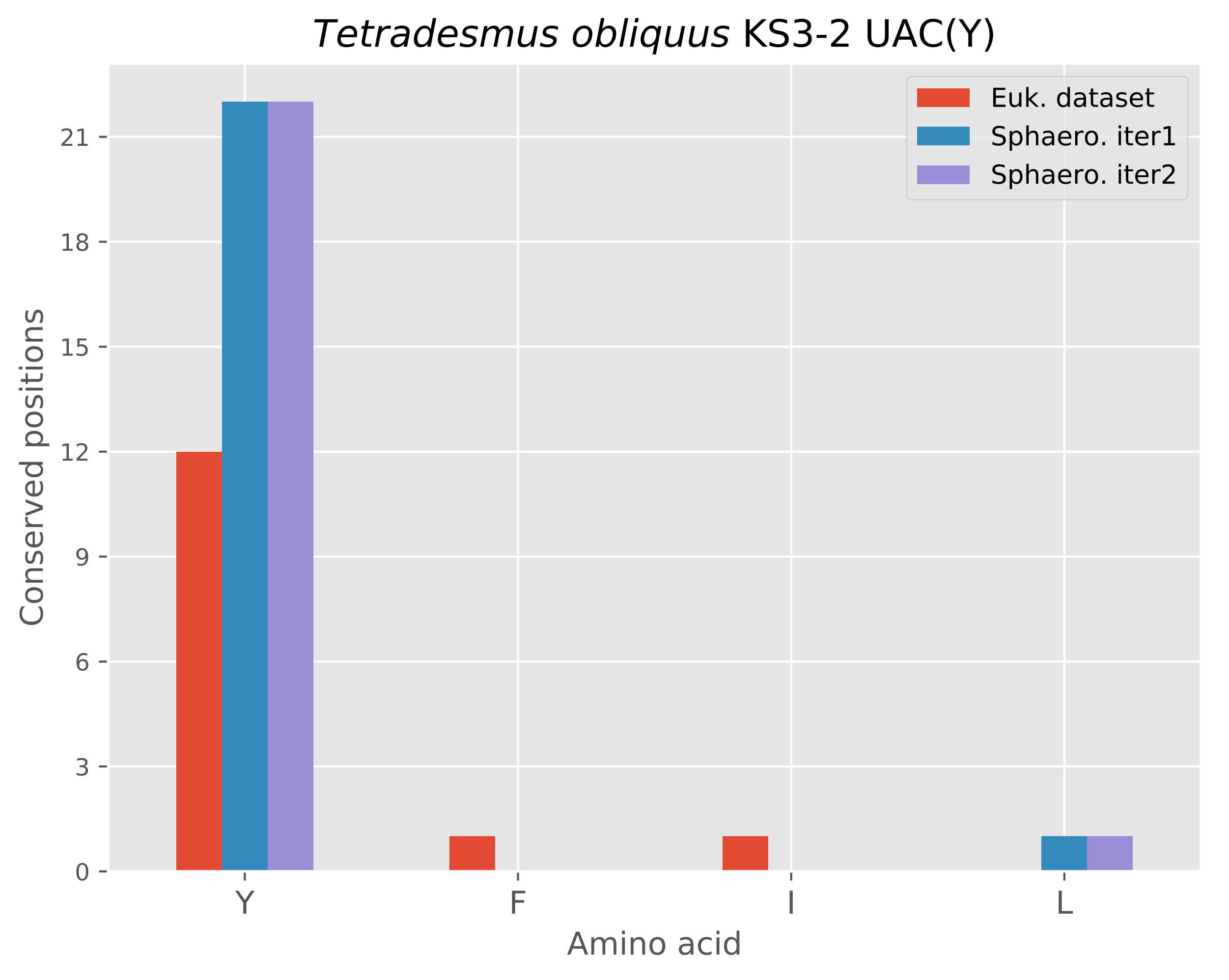

Tetradesmus obliquus KS3-2 UAC(Y)
Euk. dataset
Sphaero. iter1
Sphaero. iter2
Conserved positions
0
3
6
9
12
15
18
21
Y
F
I
L
Amino acid

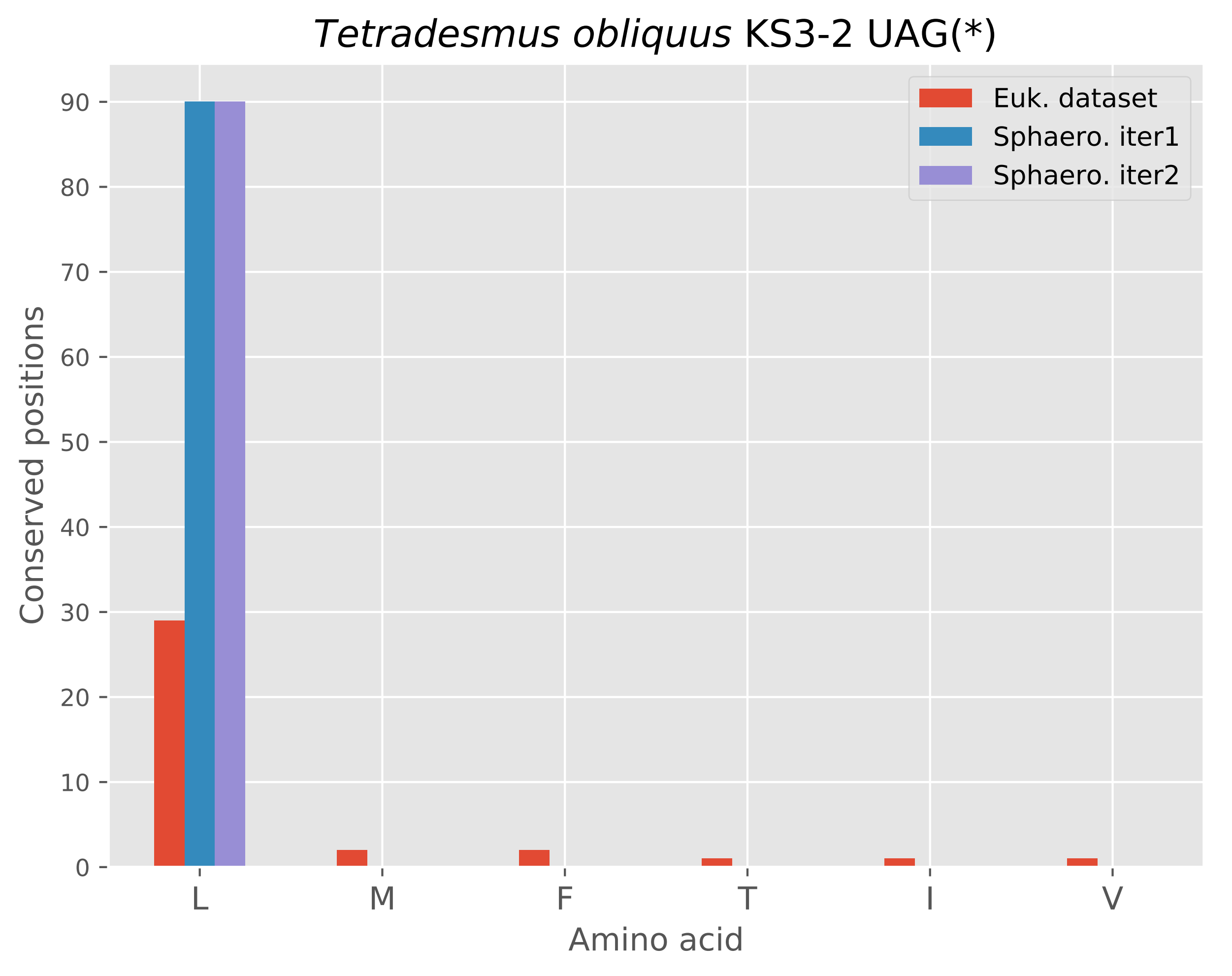

Tetradesmus obliquus KS3-2 UAG(*)
Euk. dataset
Sphaero. iter1
Sphaero. iter2
Conserved positions
0
10
20
30
40
50
60
70
80
90
L
M
F
T
I
V
Amino acid

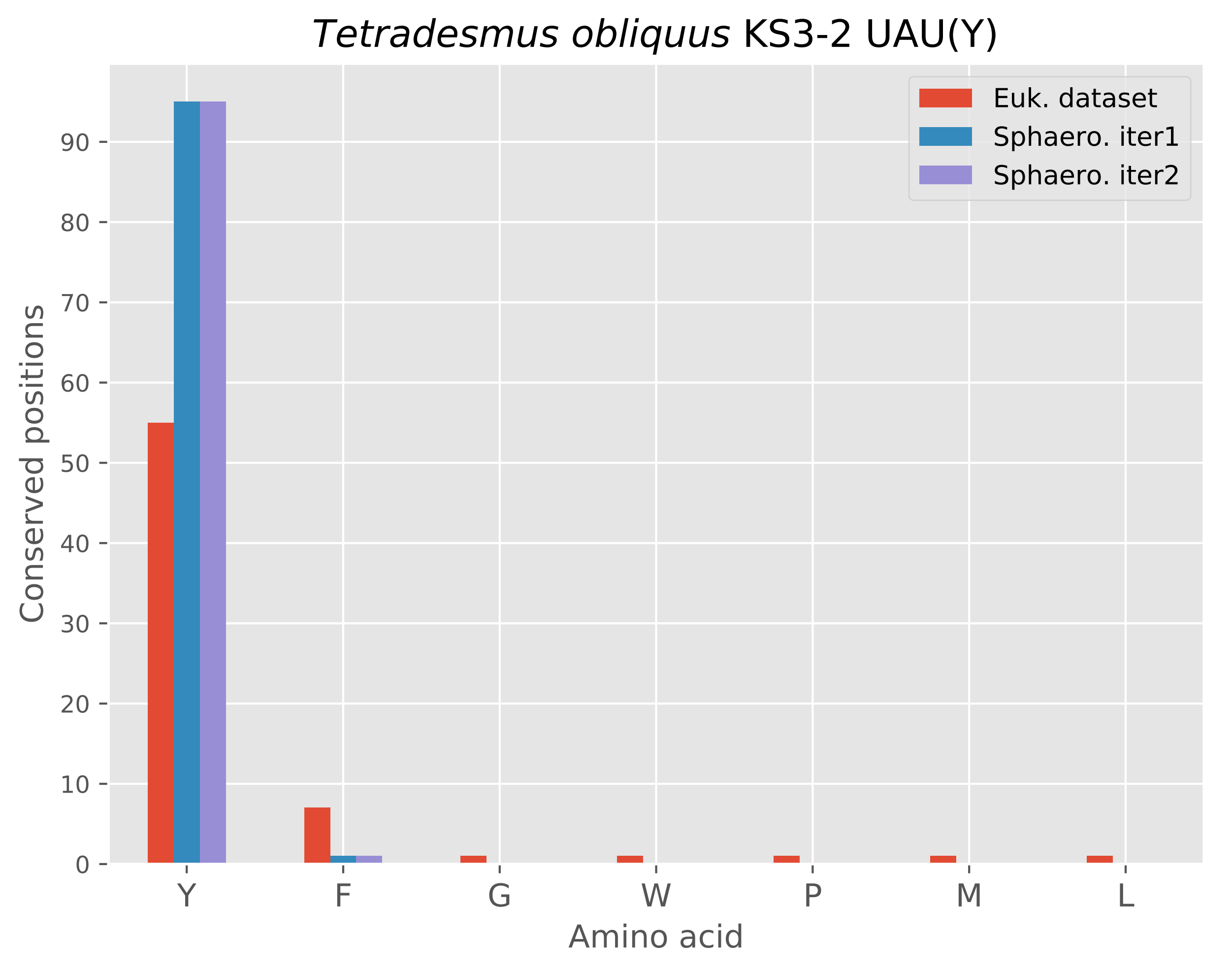

Tetradesmus obliquus KS3-2 UAU(Y)
Euk. dataset
Sphaero. iter1
Sphaero. iter2
Conserved positions
0
10
20
30
40
50
60
70
80
90
Y
F
G
W
P
M
L
Amino acid

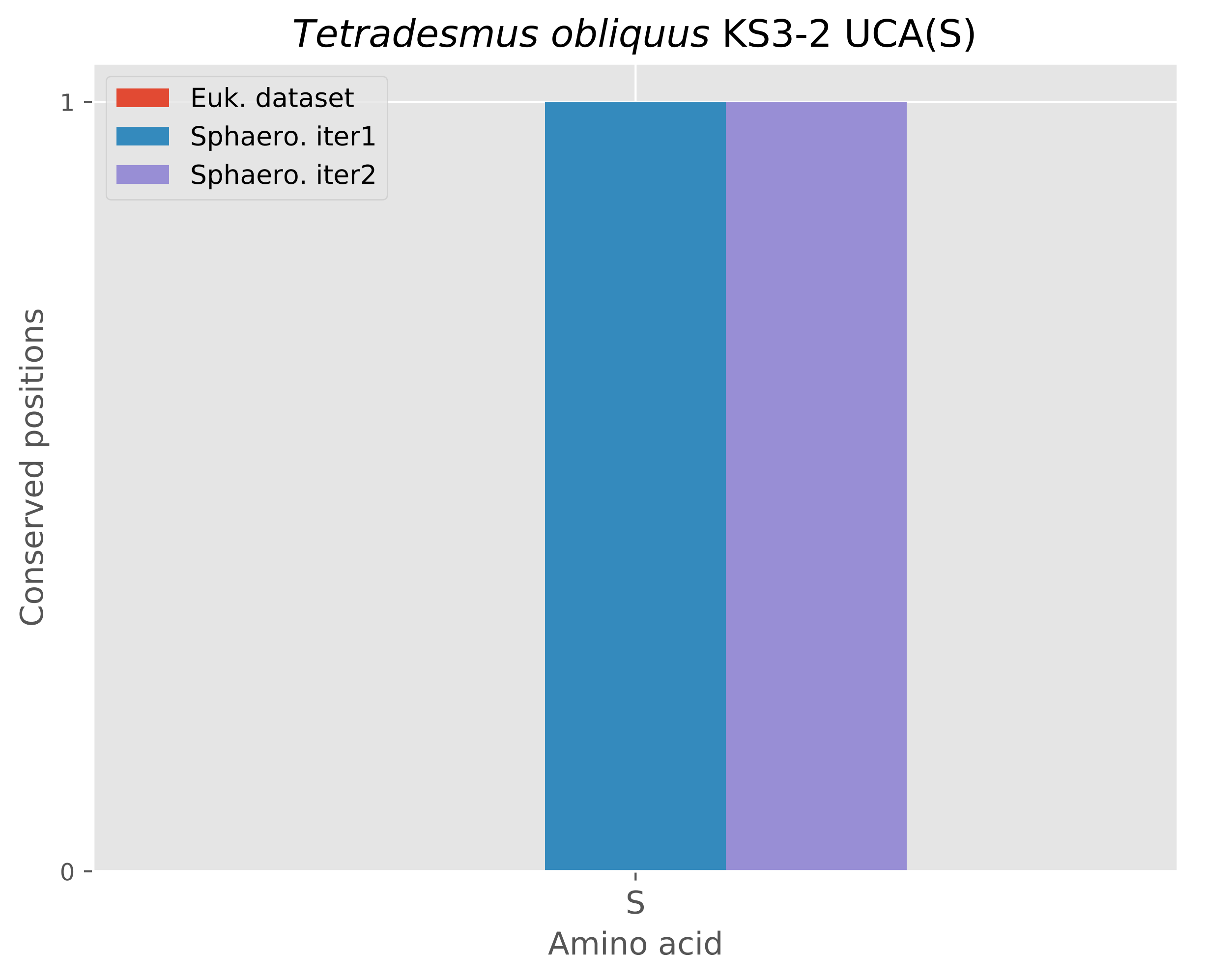

Tetradesmus obliquus KS3-2 UCA(S)
Euk. dataset
Sphaero. iter1
Sphaero. iter2
1
0
Conserved positions
S
Amino acid

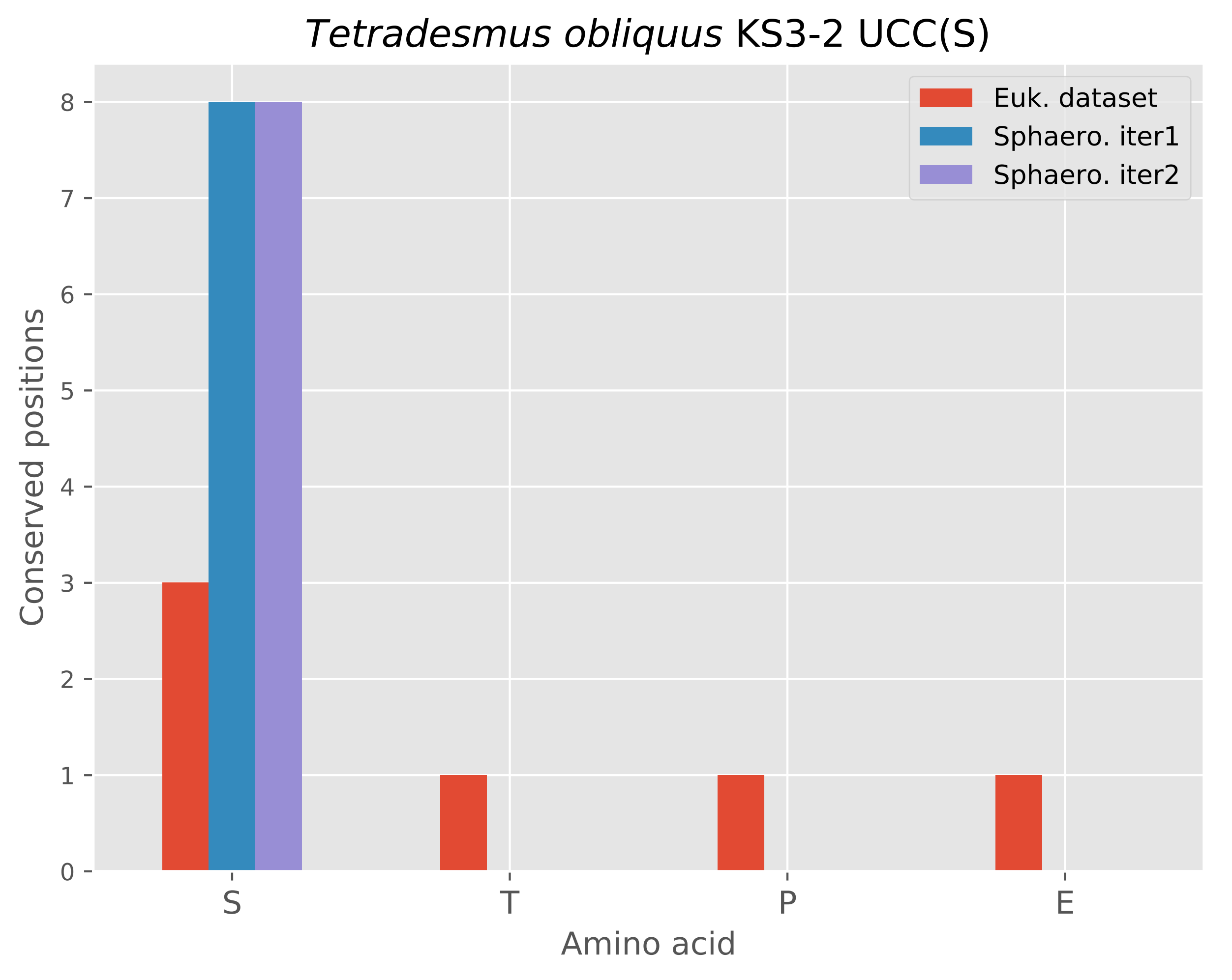

Tetradesmus obliquus KS3-2 UCC(S)
Euk. dataset
Sphaero. iter1
Sphaero. iter2
Conserved positions
0
1
2
3
4
5
6
7
8
S
T
P
E
Amino acid

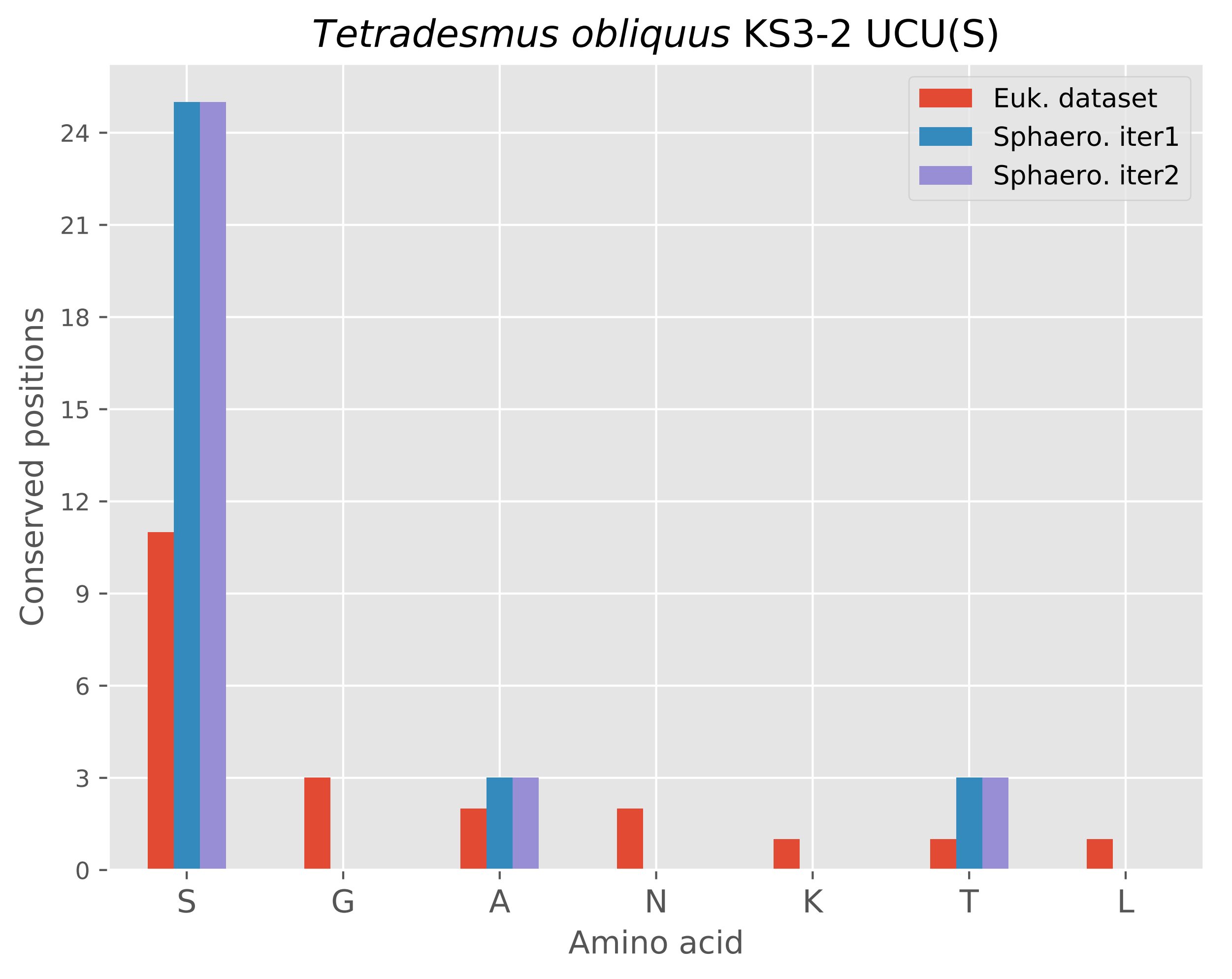

Tetradesmus obliquus KS3-2 UCU(S)
Euk. dataset
Sphaero. iter1
Sphaero. iter2
Conserved positions
0
3
6
9
12
15
18
21
24
S
G
A
N
K
T
L
Amino acid

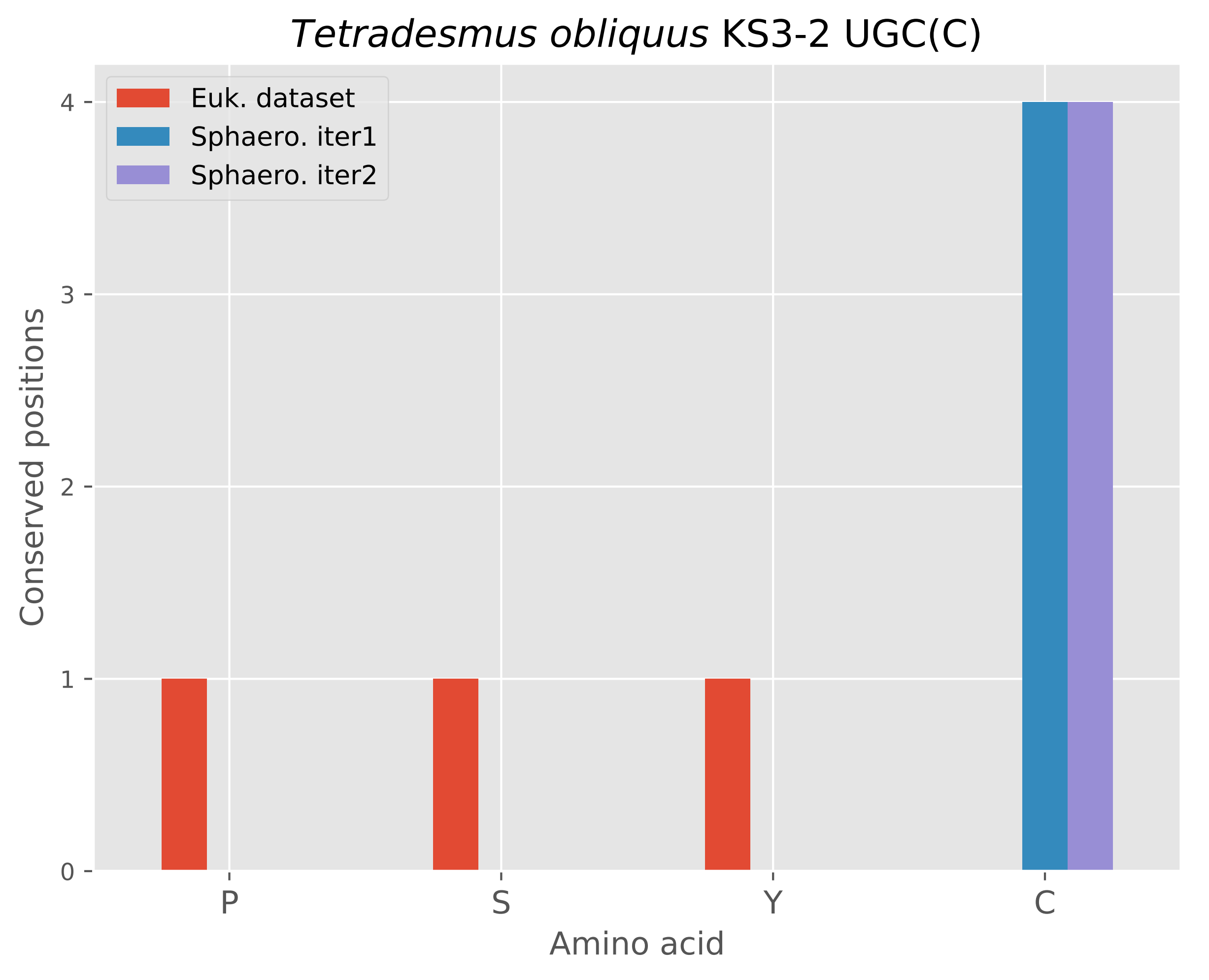

Tetradesmus obliquus KS3-2 UGC(C)
Euk. dataset
Sphaero. iter1
Sphaero. iter2
Conserved positions
0
1
2
3
4
P
S
Y
C
Amino acid

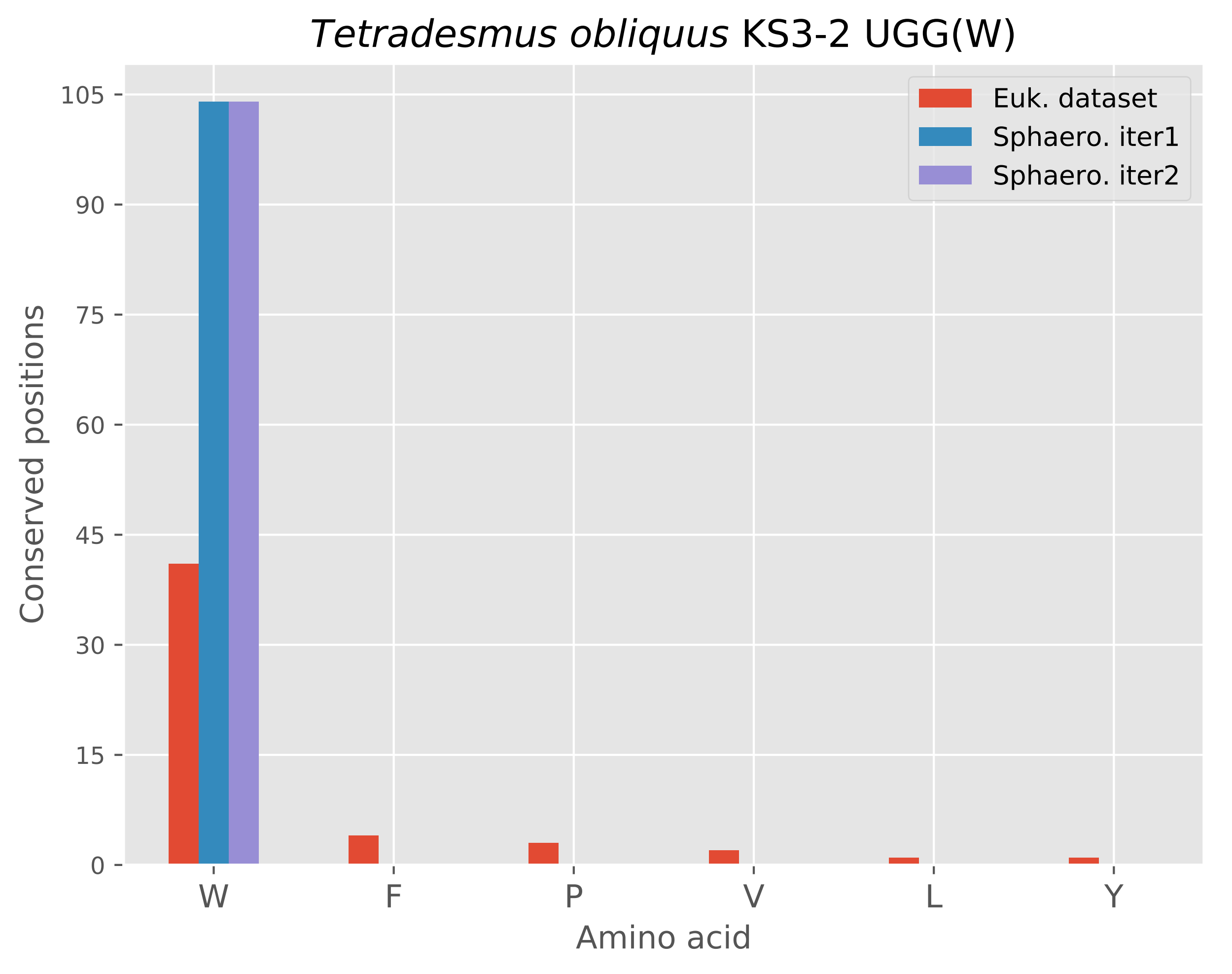

Tetradesmus obliquus KS3-2 UGG(W)
Euk. dataset
Sphaero. iter1
Sphaero. iter2
Conserved positions
0
15
30
45
60
75
90
105
W
F
P
V
L
Y
Amino acid

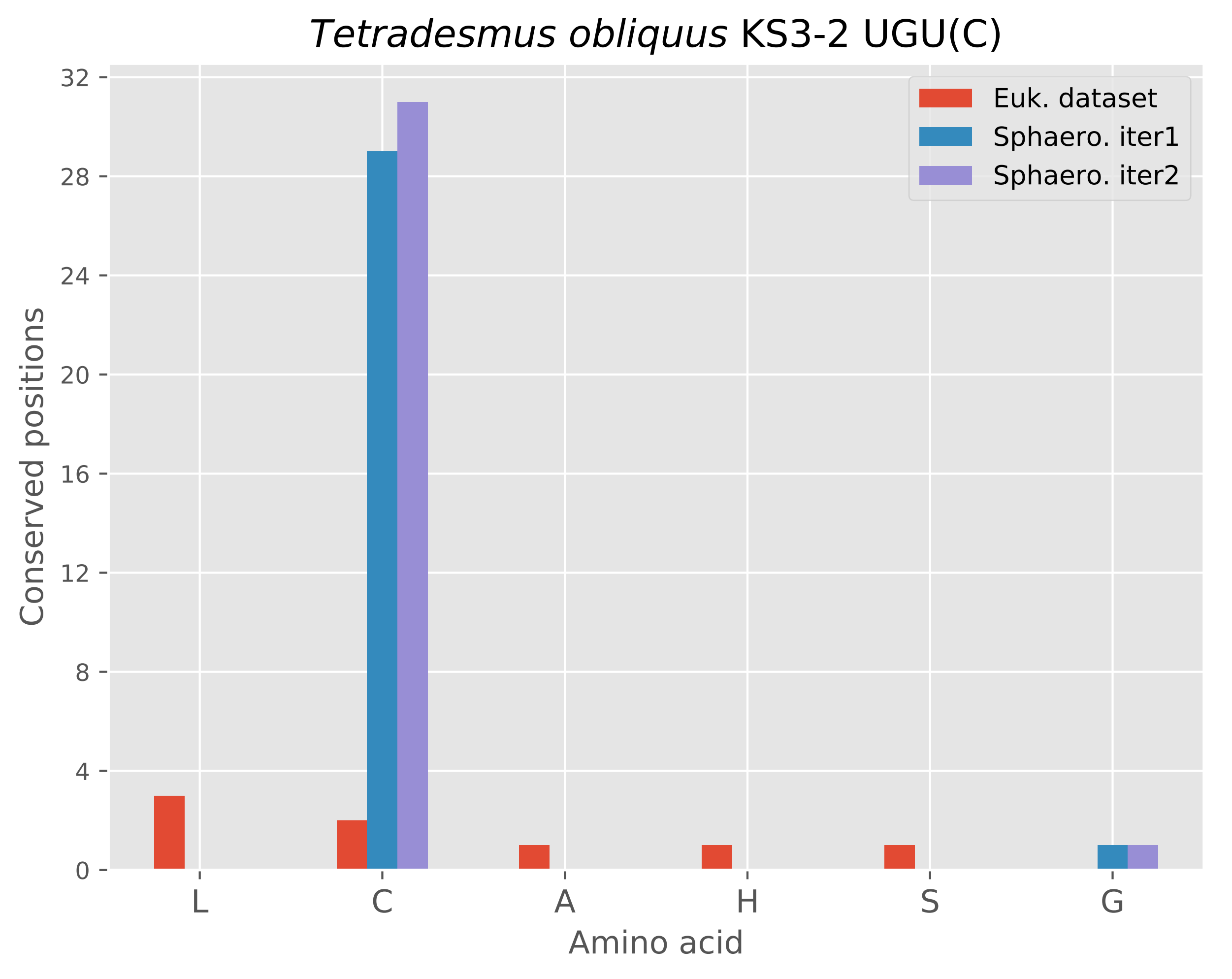

Tetradesmus obliquus KS3-2 UGU(C)
Conserved positions
0
4
8
12
16
20
24
28
32
Amino acid
L
C
A
H
S
G
Euk. dataset
Sphaero. iter1
Sphaero. iter2

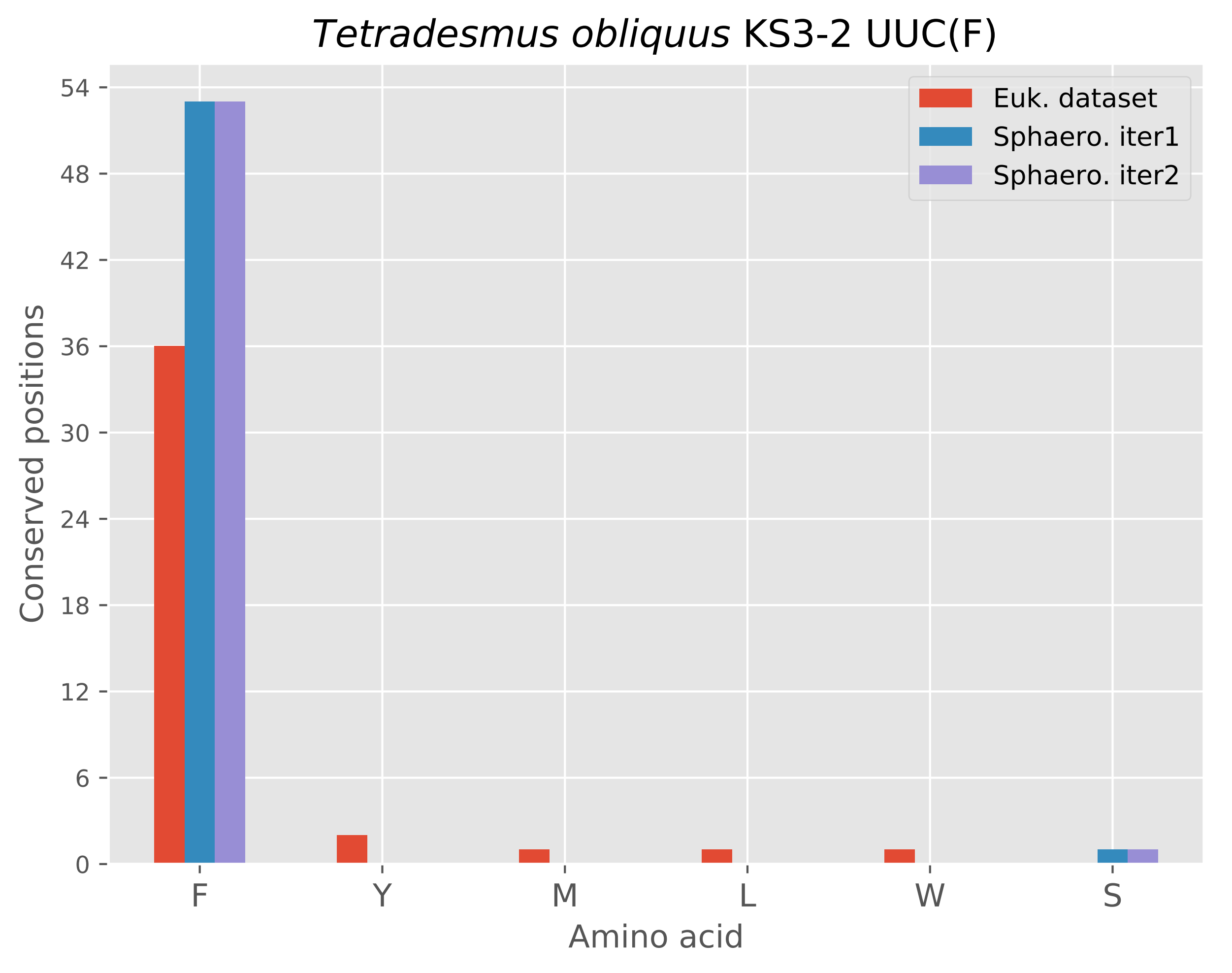

Tetradesmus obliquus KS3-2 UUC(F)
Euk. dataset
Sphaero. iter1
Sphaero. iter2
Conserved positions
0
6
12
18
24
30
36
42
48
54
F
Y
M
L
W
S
Amino acid

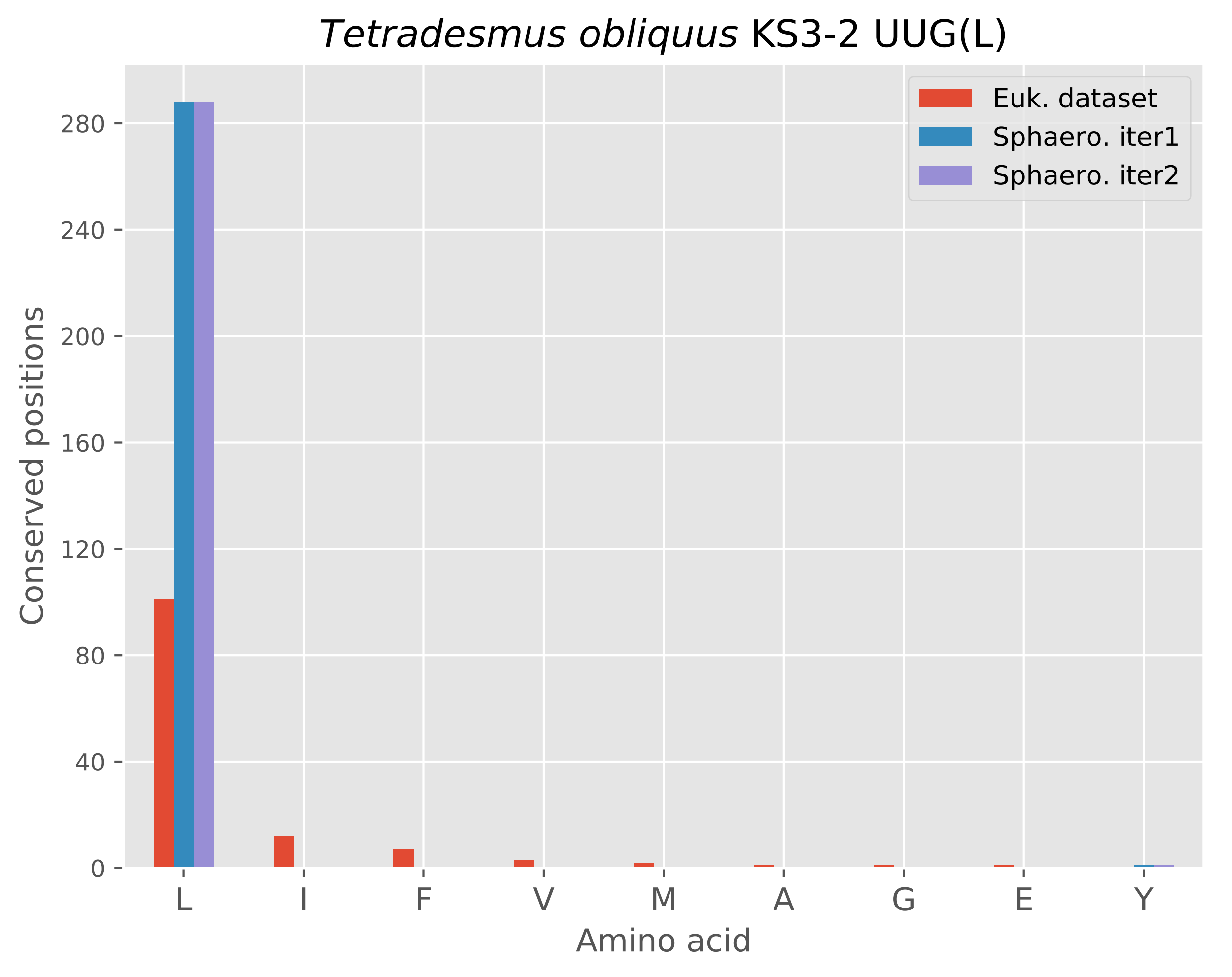

Tetradesmus obliquus KS3-2 UUG(L)
Euk. dataset
Sphaero. iter1
Sphaero. iter2
Conserved positions
0
40
80
120
160
200
240
280
L
I
F
V
M
A
G
E
Y
Amino acid

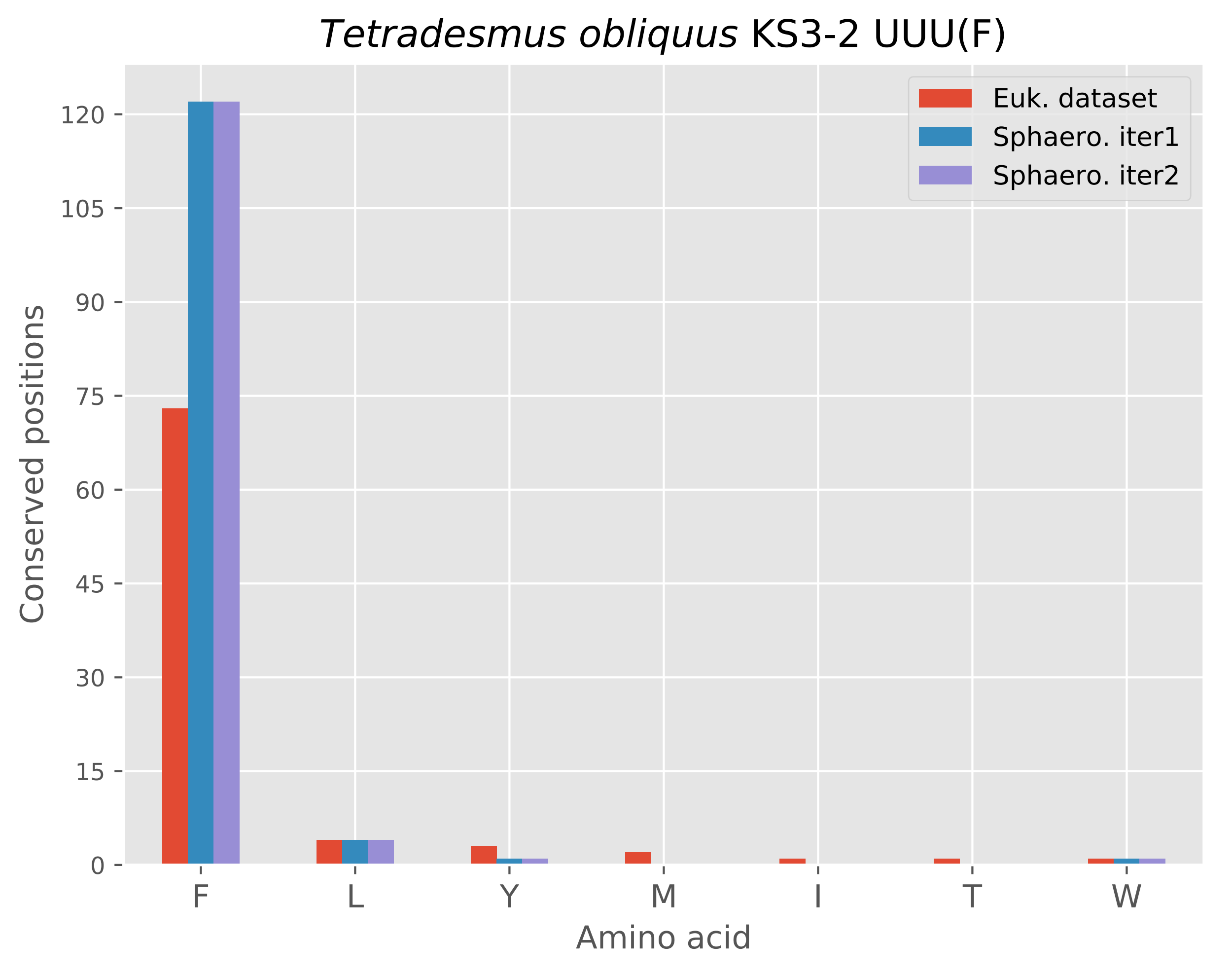

Tetradesmus obliquus KS3-2 UUU(F)
Euk. dataset
Sphaero. iter1
Sphaero. iter2
Conserved positions
0
15
30
45
60
75
90
105
120
F
L
Y
M
I
T
W
Amino acid

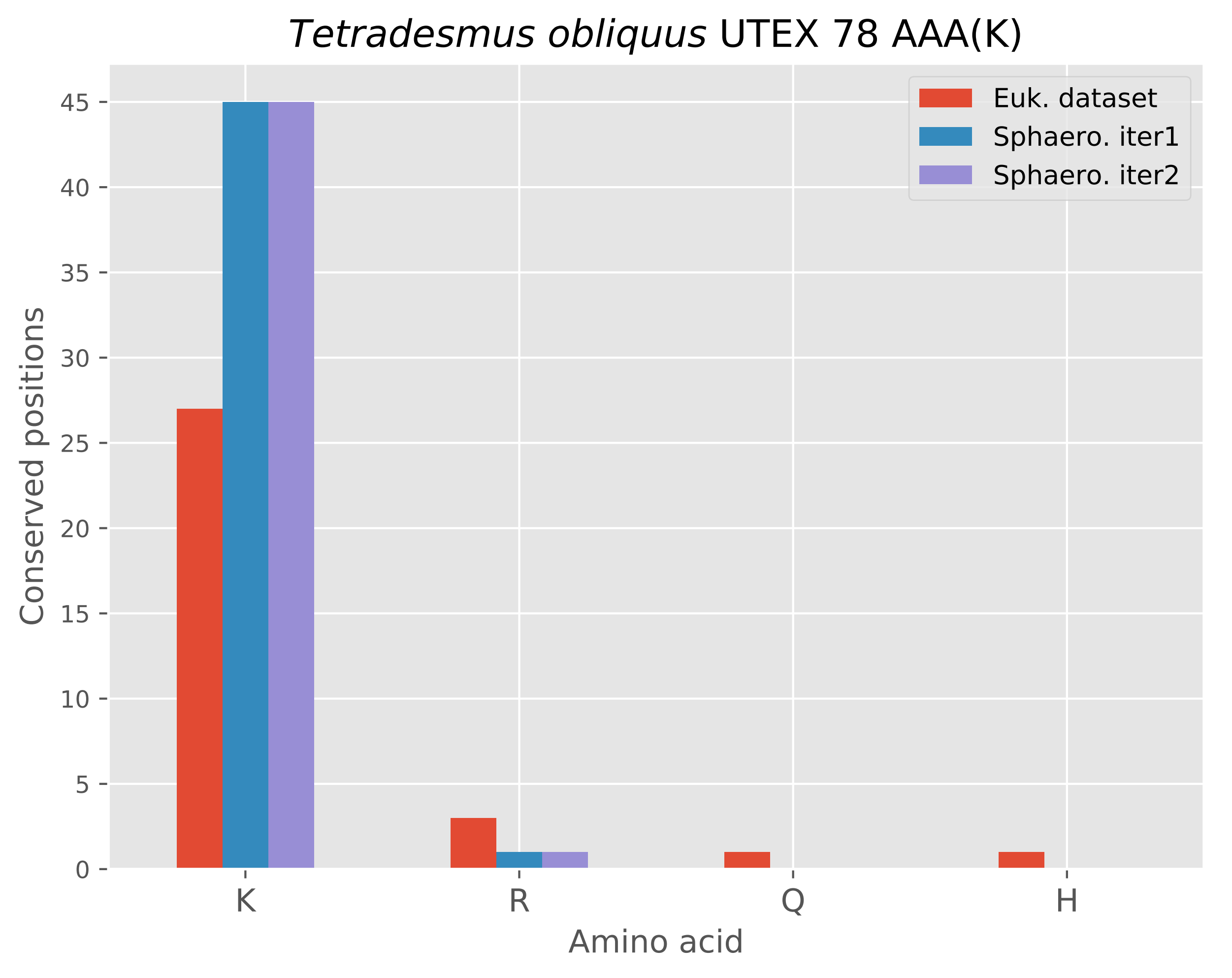
Tetradesmus obliquus UTEX 78 AAA(K)
Euk. dataset
Sphaero. iter1
Sphaero. iter2
Conserved positions
0
5
10
15
20
25
30
35
40
45
K
R
Q
H
Amino acid

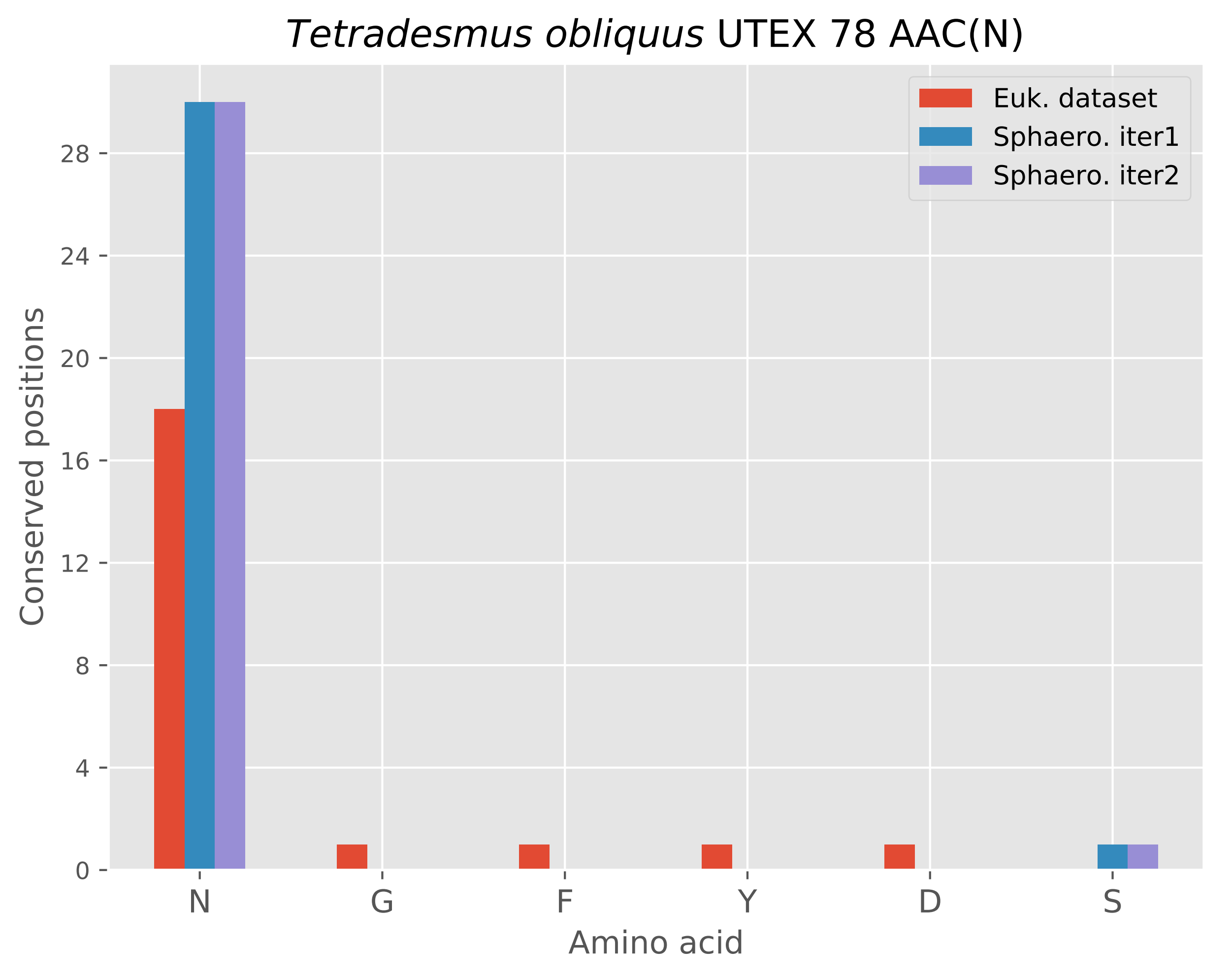

Tetradesmus obliquus UTEX 78 AAC(N)
Euk. dataset
Sphaero. iter1
Sphaero. iter2
Conserved positions
0
4
8
12
16
20
24
28
N
G
F
Y
D
S
Amino acid

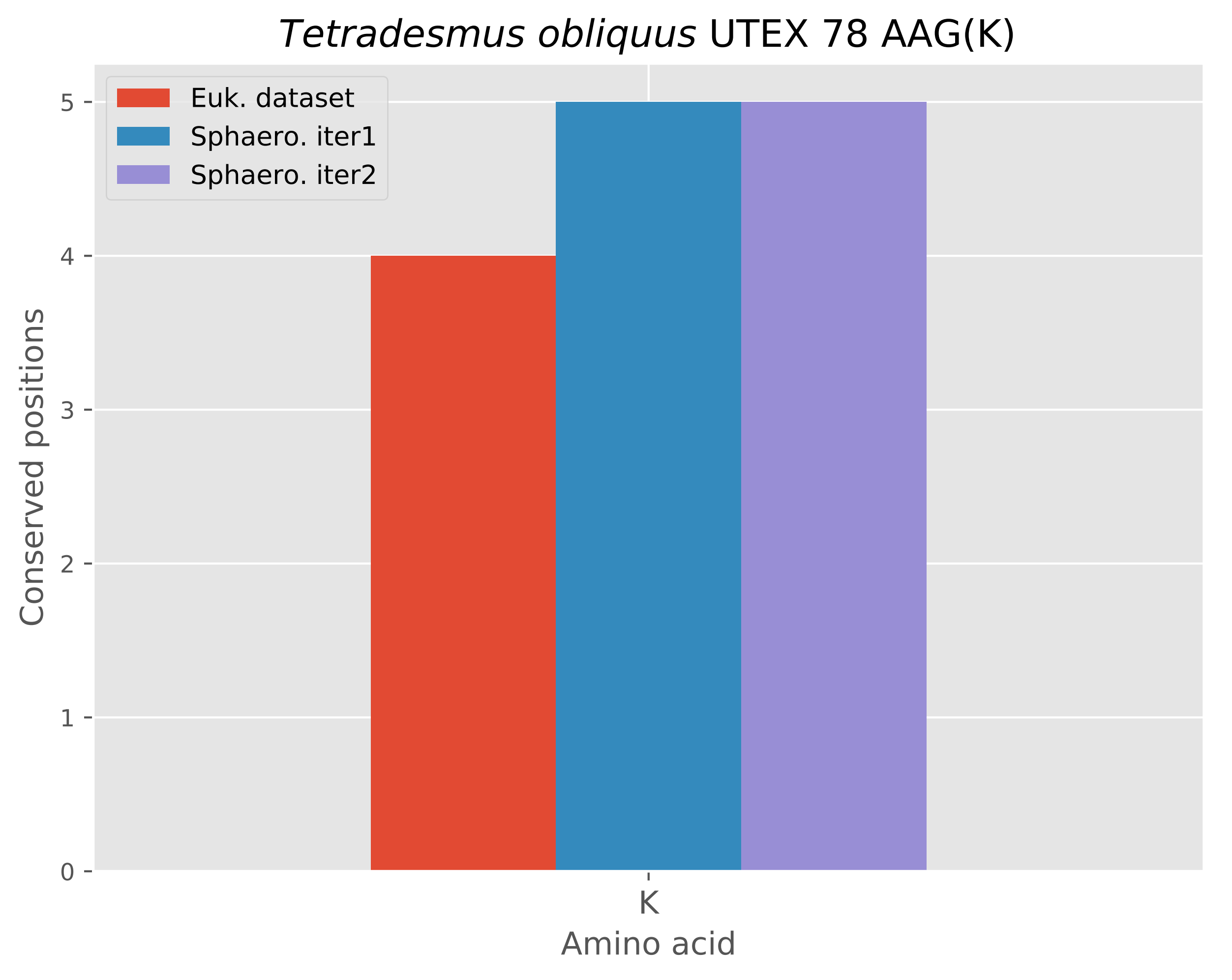

Tetradesmus obliquus UTEX 78 AAG(K)
Euk. dataset
Sphaero. iter1
Sphaero. iter2
Conserved positions
0
1
2
3
4
5
K
Amino acid

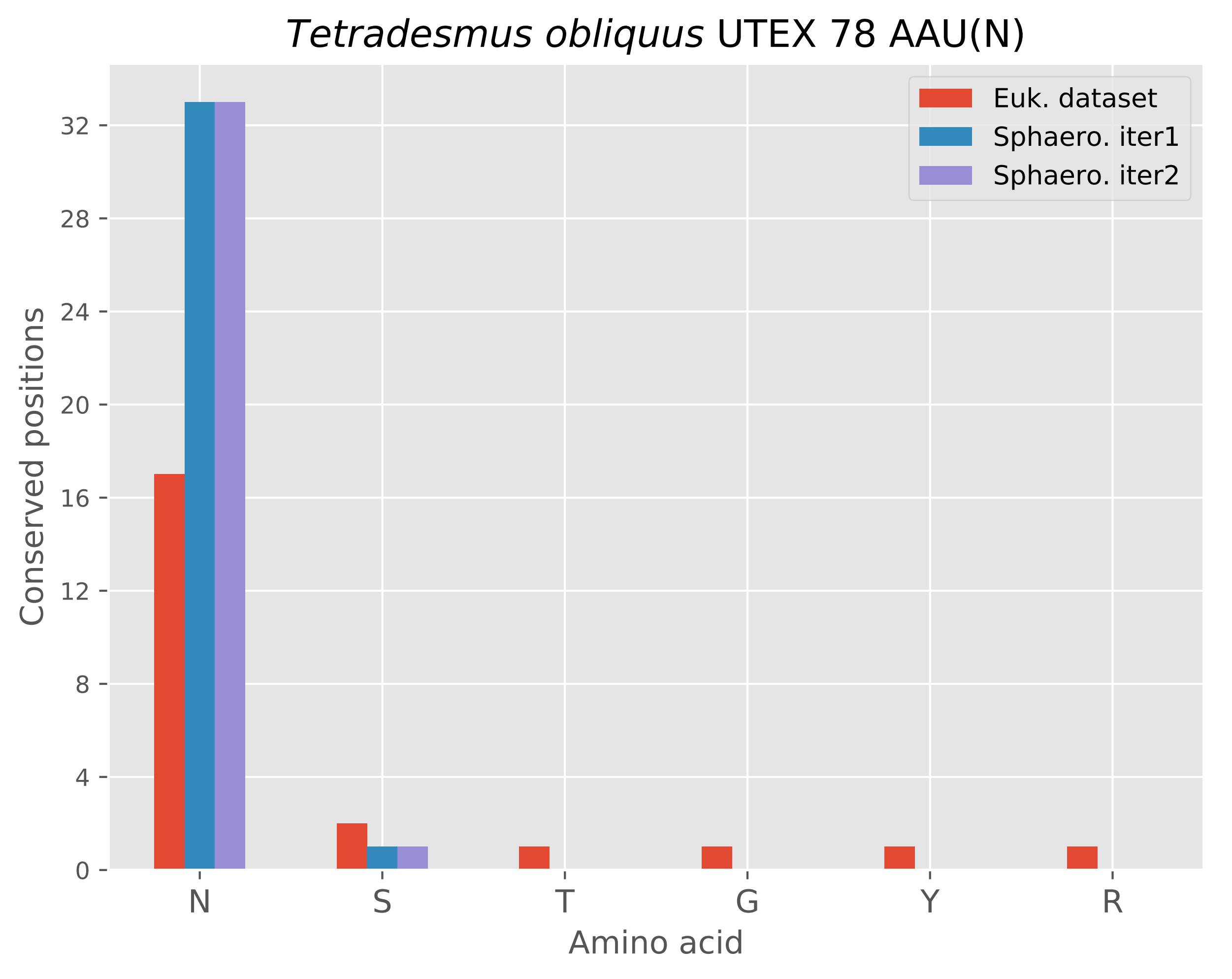

Tetradesmus obliquus UTEX 78 AAU(N)
Euk. dataset
Sphaero. iter1
Sphaero. iter2
Conserved positions
0
4
8
12
16
20
24
28
32
N
S
T
G
Y
R
Amino acid

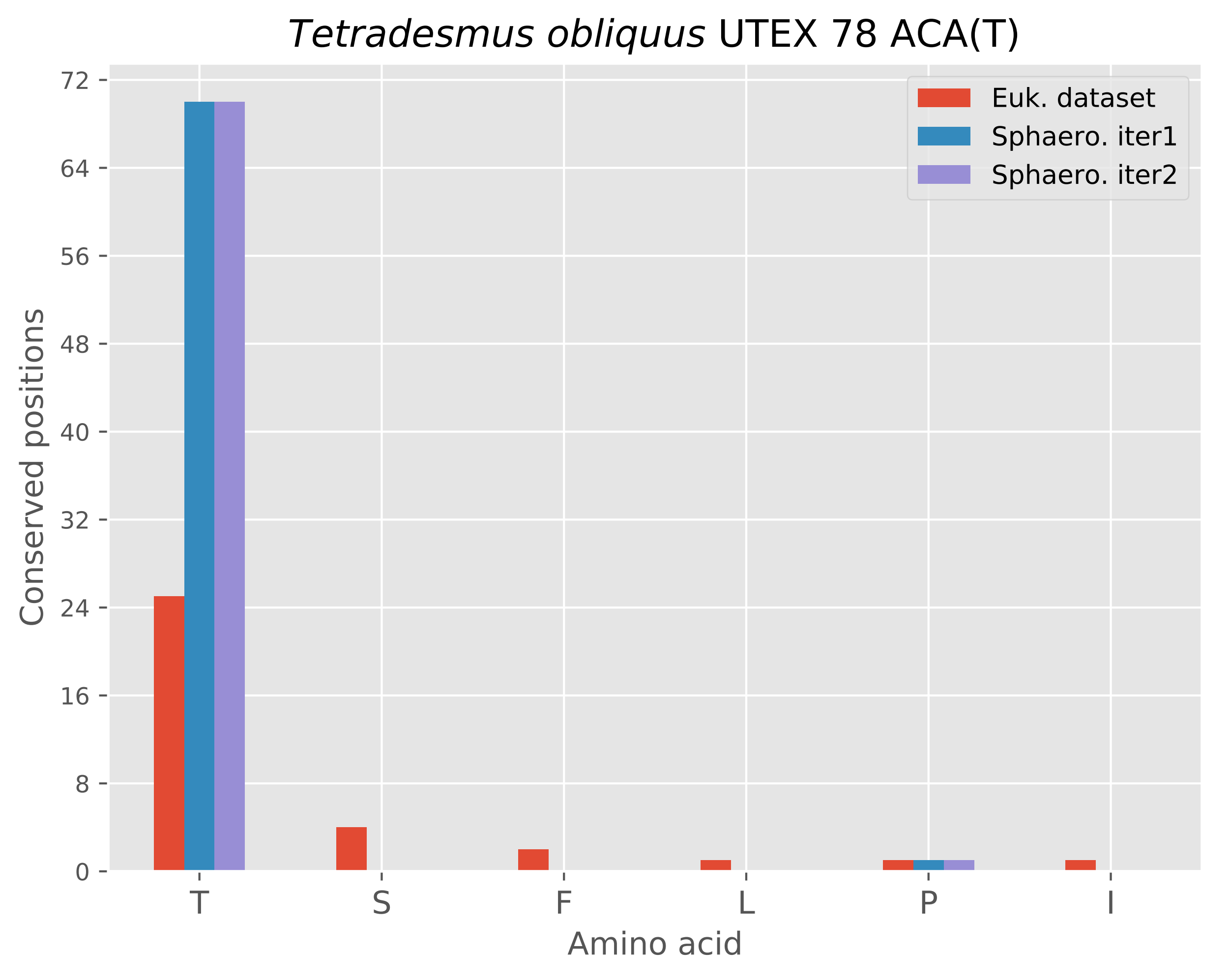

Tetradesmus obliquus UTEX 78 ACA(T)
Euk. dataset
Sphaero. iter1
Sphaero. iter2
Conserved positions
0
8
16
24
32
40
48
56
64
72
T
S
F
L
P
I
Amino acid

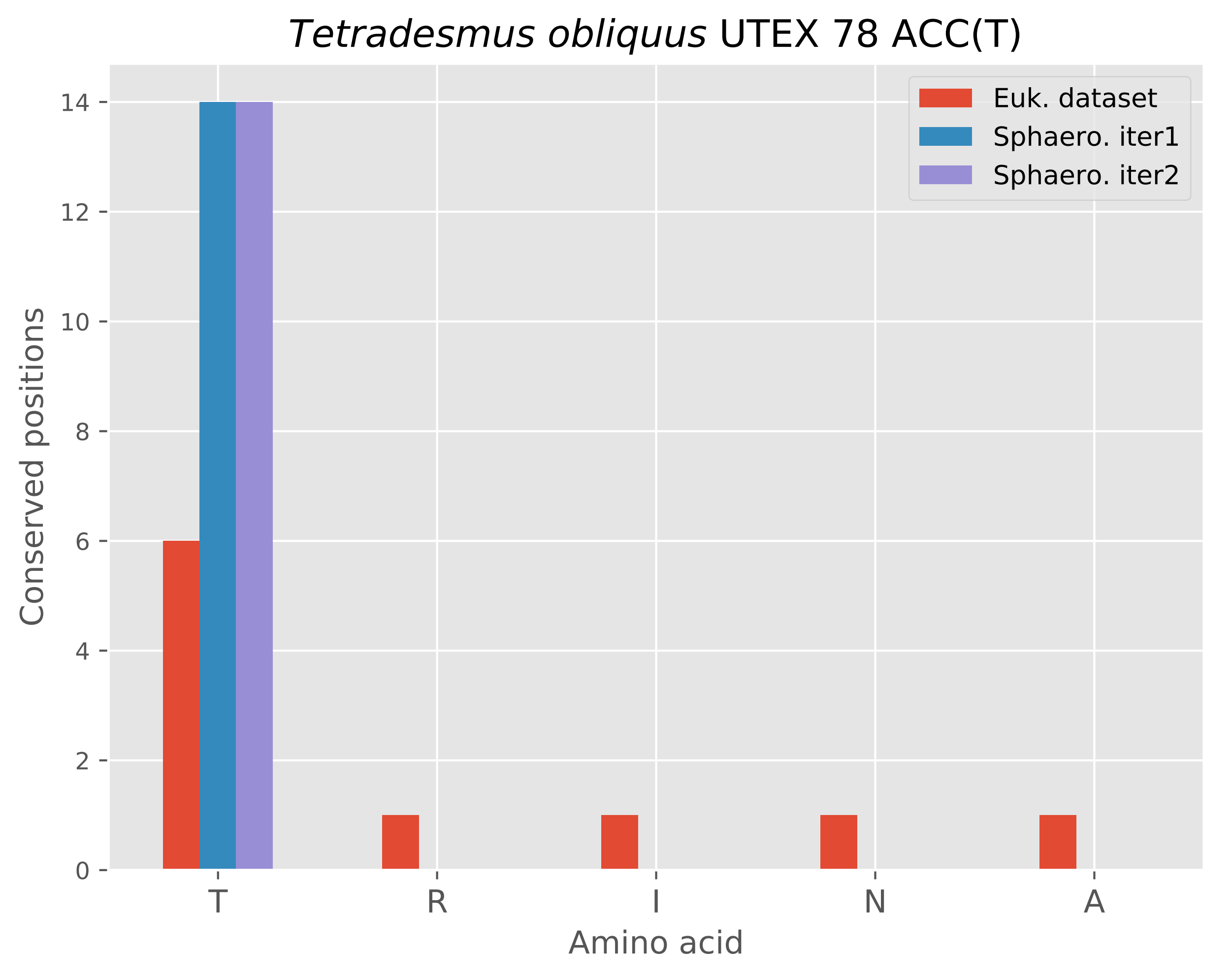

Tetradesmus obliquus UTEX 78 ACC(T)
Euk. dataset
Sphaero. iter1
Sphaero. iter2
Conserved positions
0
2
4
6
8
10
12
14
T
R
I
N
A
Amino acid

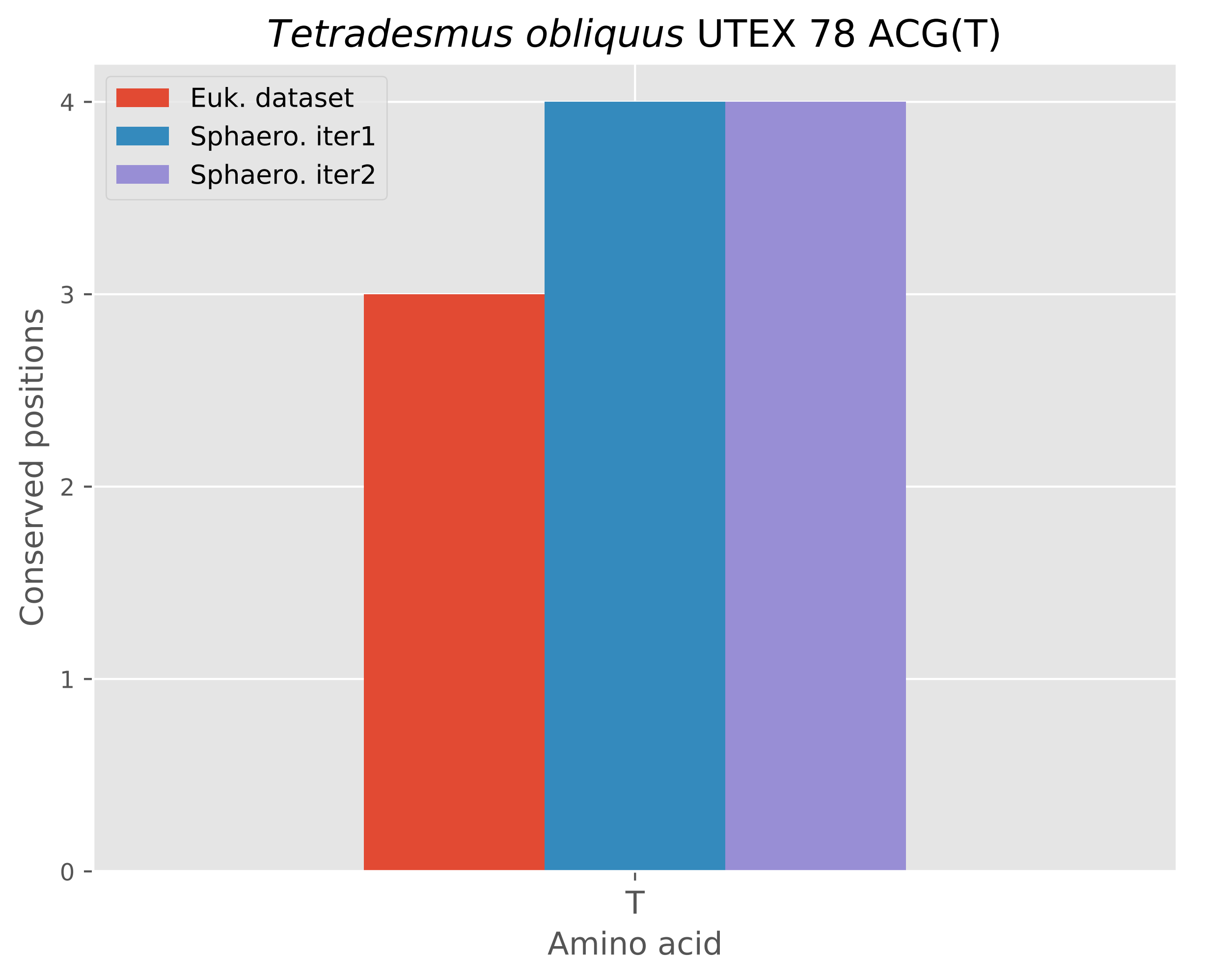

Tetradesmus obliquus UTEX 78 ACG(T)
Euk. dataset
Sphaero. iter1
Sphaero. iter2
Conserved positions
0
1
2
3
4
T
Amino acid

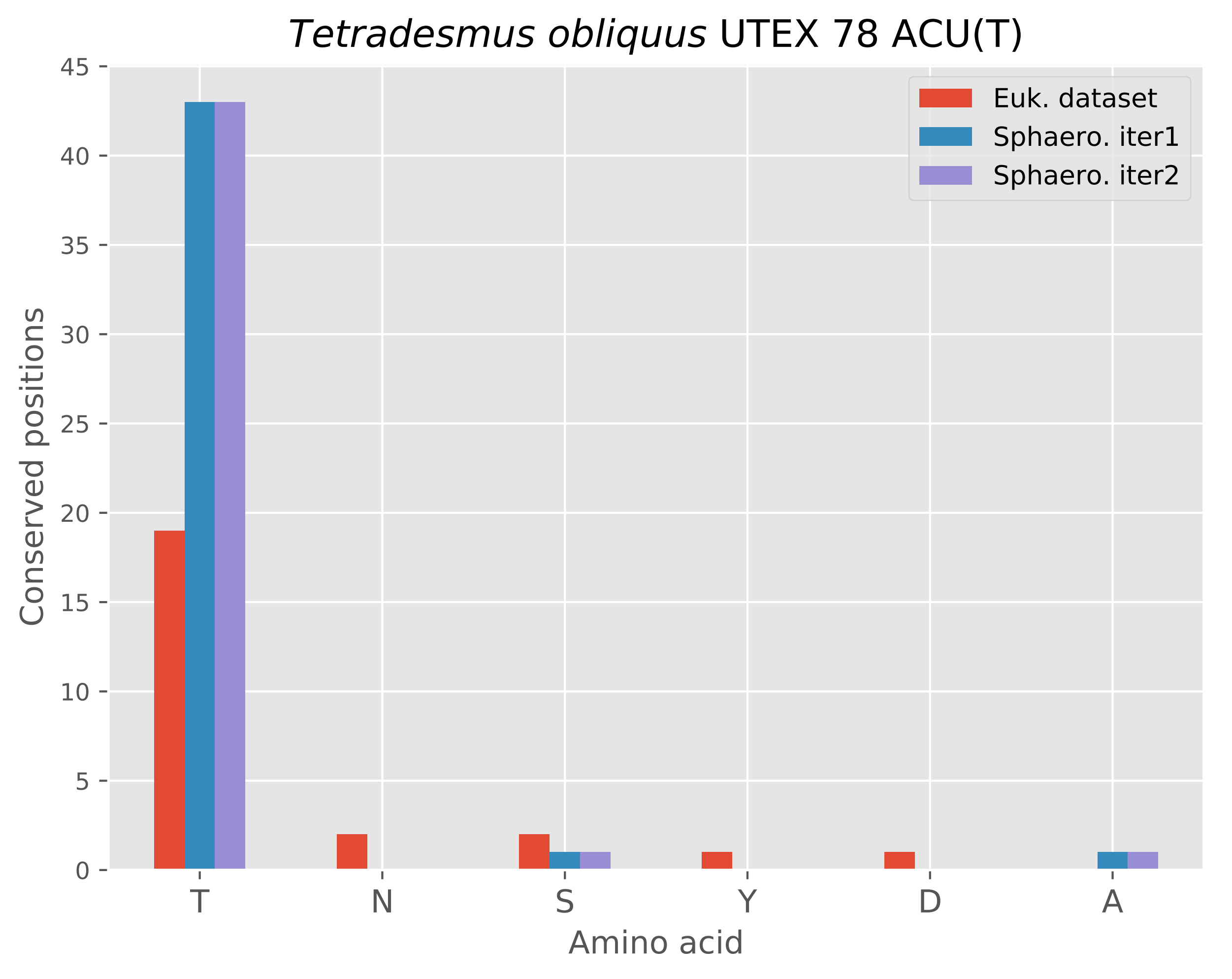
Tetradesmus obliquus UTEX 78 ACU(T)
Conserved positions
0
5
10
15
20
25
30
35
40
45
T
N
S
Y
D
A
Amino acid
Euk. dataset
Sphaero. iter1
Sphaero. iter2

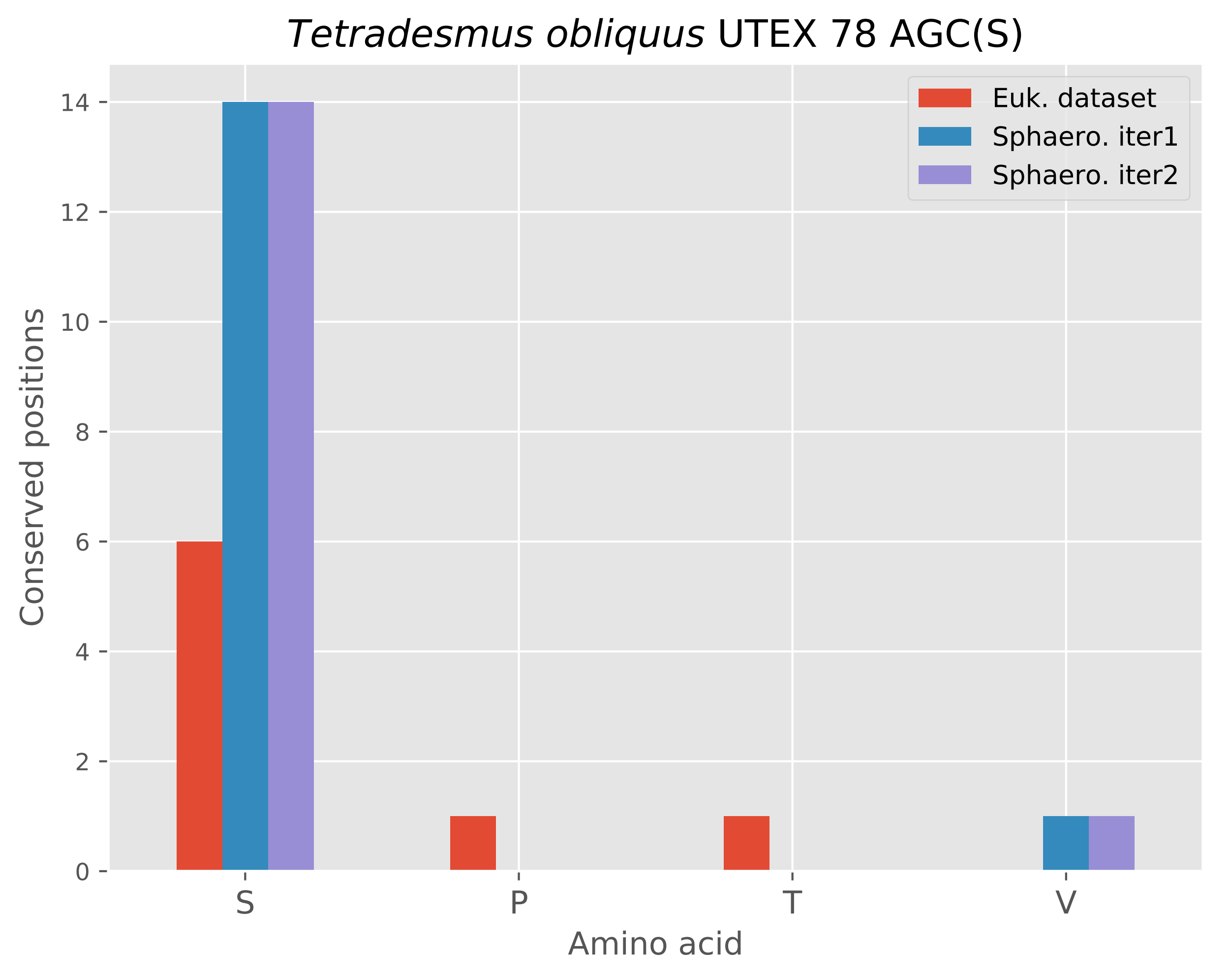

Tetradesmus obliquus UTEX 78 AGC(S)
Euk. dataset
Sphaero. iter1
Sphaero. iter2
Conserved positions
0
2
4
6
8
10
12
14
S
P
T
V
Amino acid

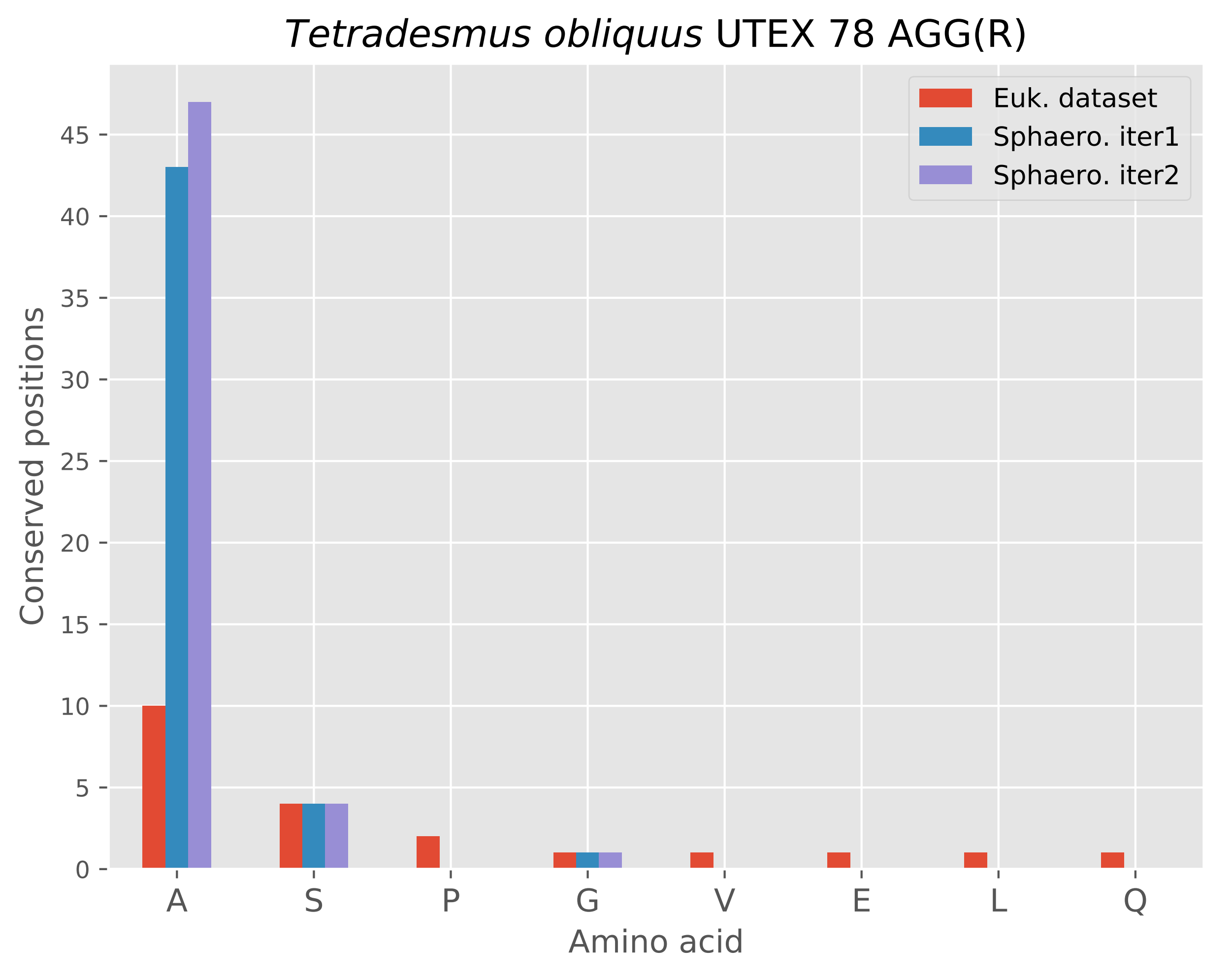
Tetradesmus obliquus UTEX 78 AGG(R)
Euk. dataset
Sphaero. iter1
Sphaero. iter2
Conserved positions
0
5
10
15
20
25
30
35
40
45
A
S
P
G
V
E
L
Q
Amino acid

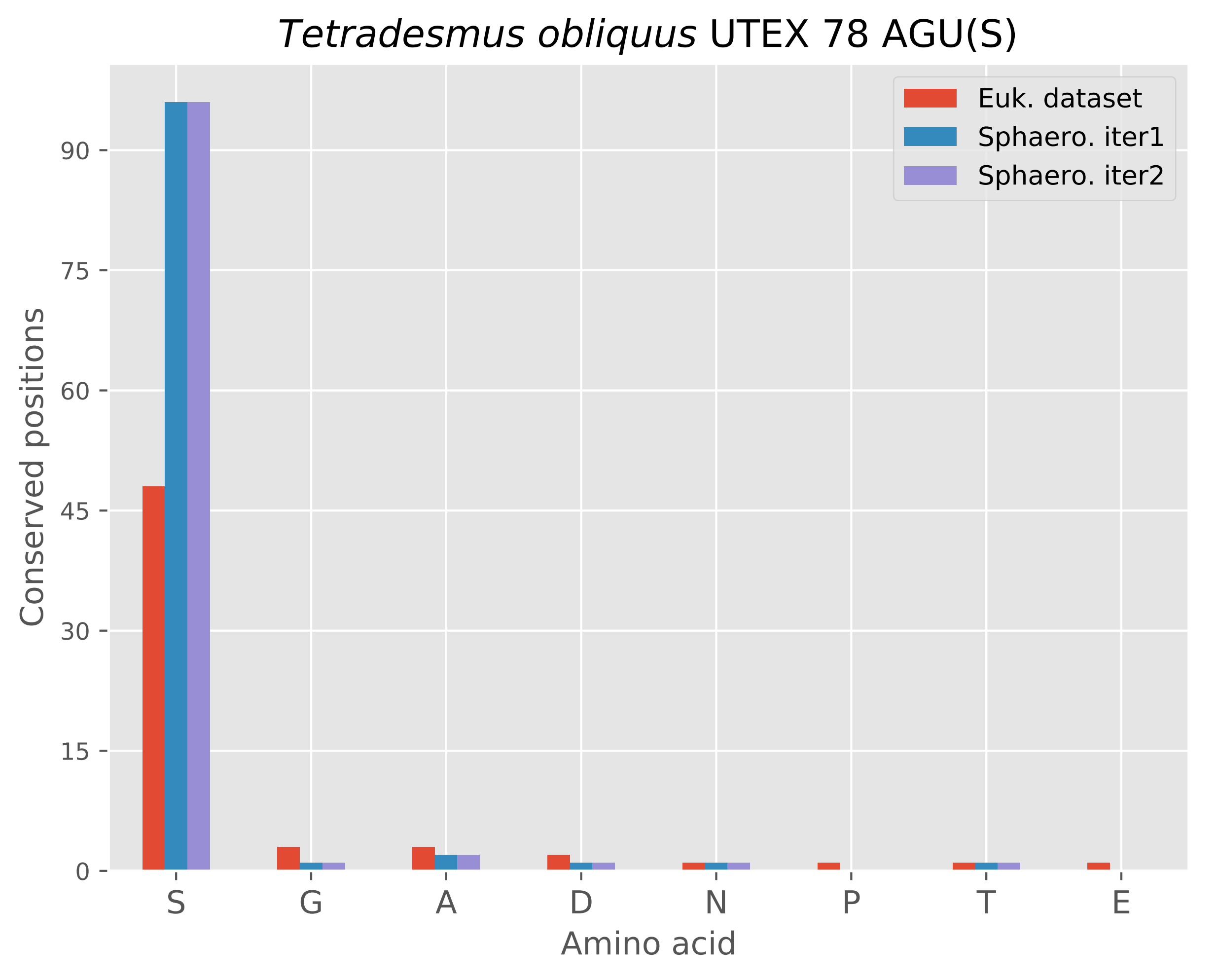

Tetradesmus obliquus UTEX 78 AGU(S)
Euk. dataset
Sphaero. iter1
Sphaero. iter2
Conserved positions
0
15
30
45
60
75
90
S
G
A
D
N
P
T
E
Amino acid

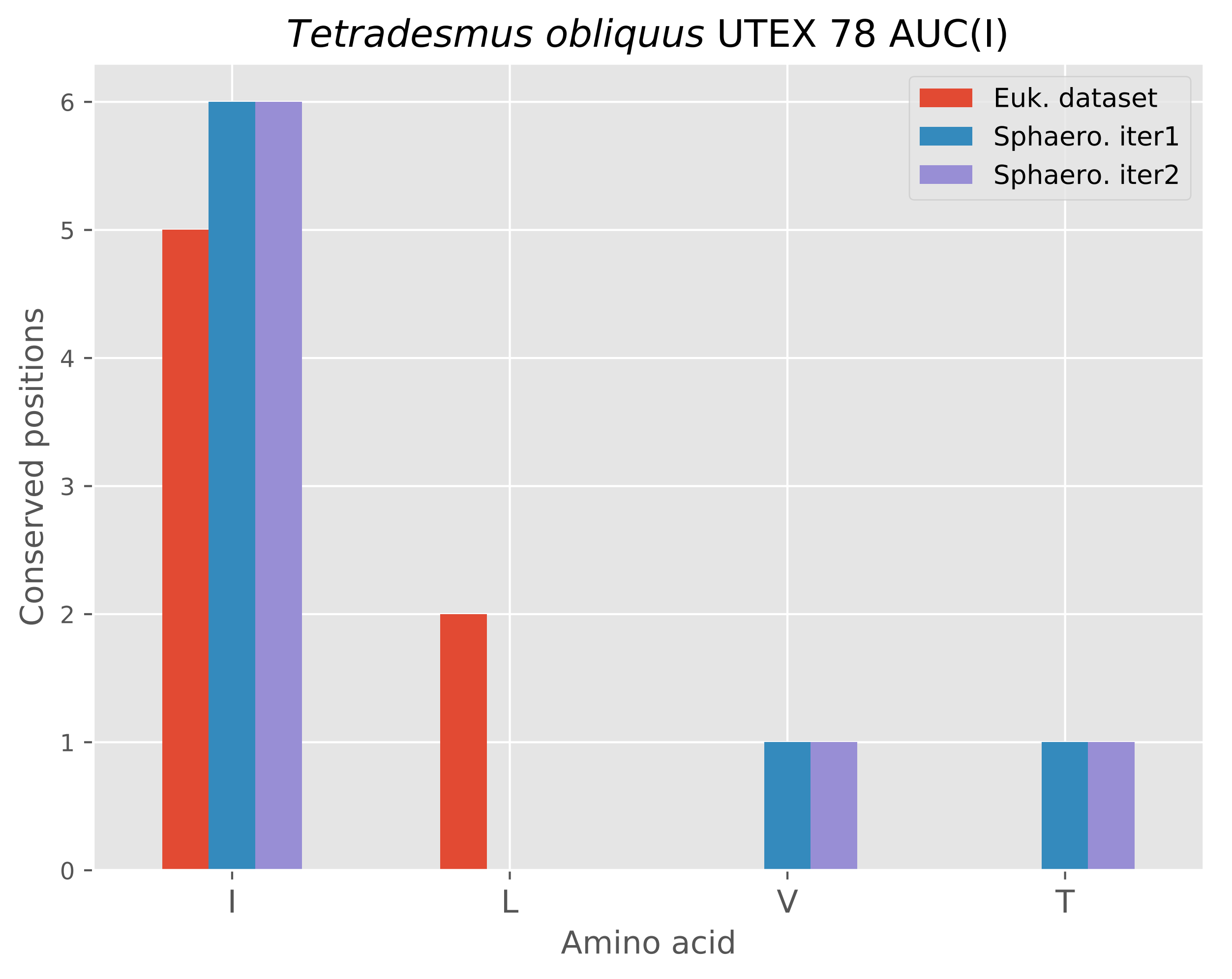
Tetradesmus obliquus UTEX 78 AUC(I)
Euk. dataset
Sphaero. iter1
Sphaero. iter2
Conserved positions
0
1
2
3
4
5
6
I
L
V
T
Amino acid

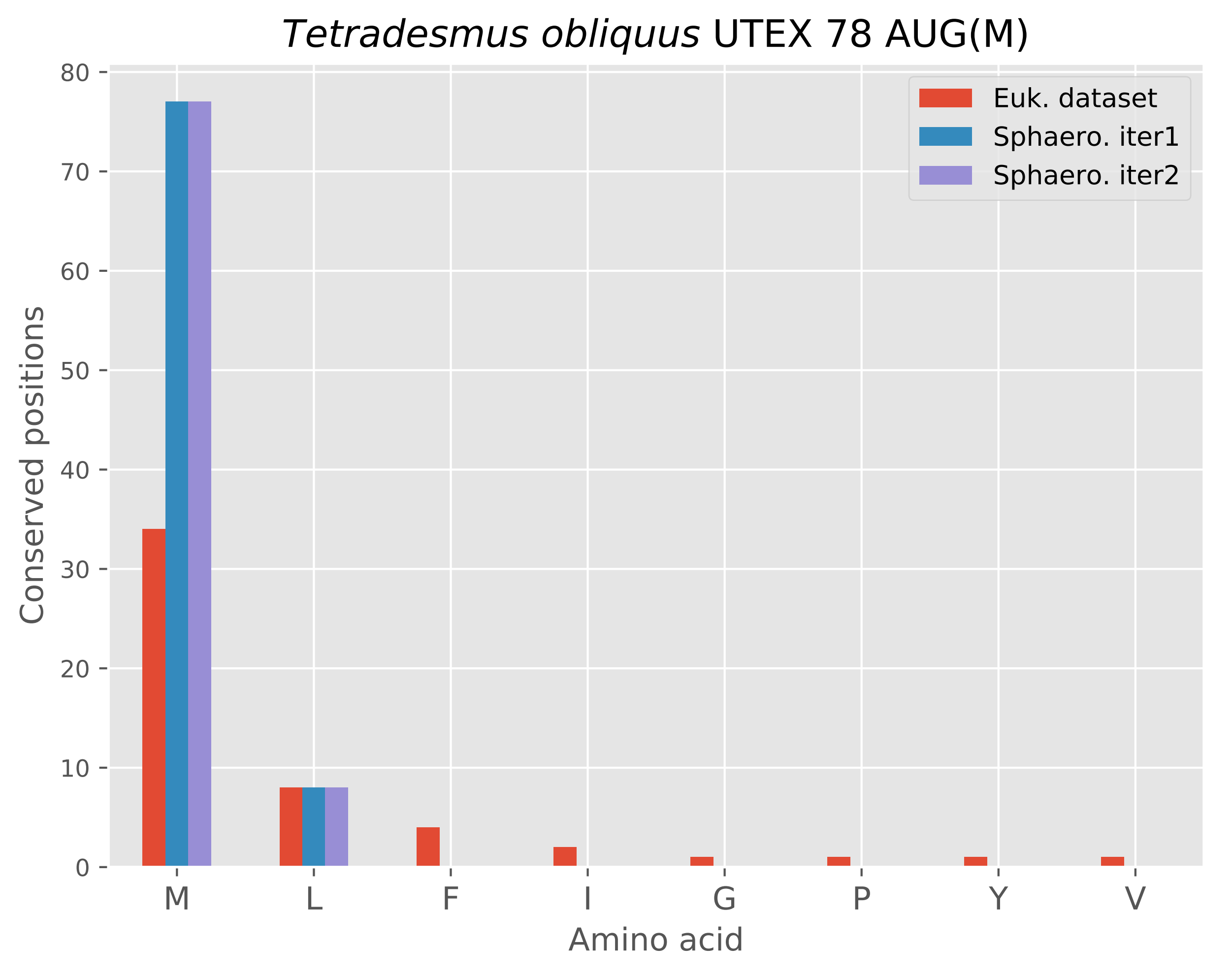

Tetradesmus obliquus UTEX 78 AUG(M)
Euk. dataset
Sphaero. iter1
Sphaero. iter2
Conserved positions
0
10
20
30
40
50
60
70
80
M
L
F
I
G
P
Y
V
Amino acid

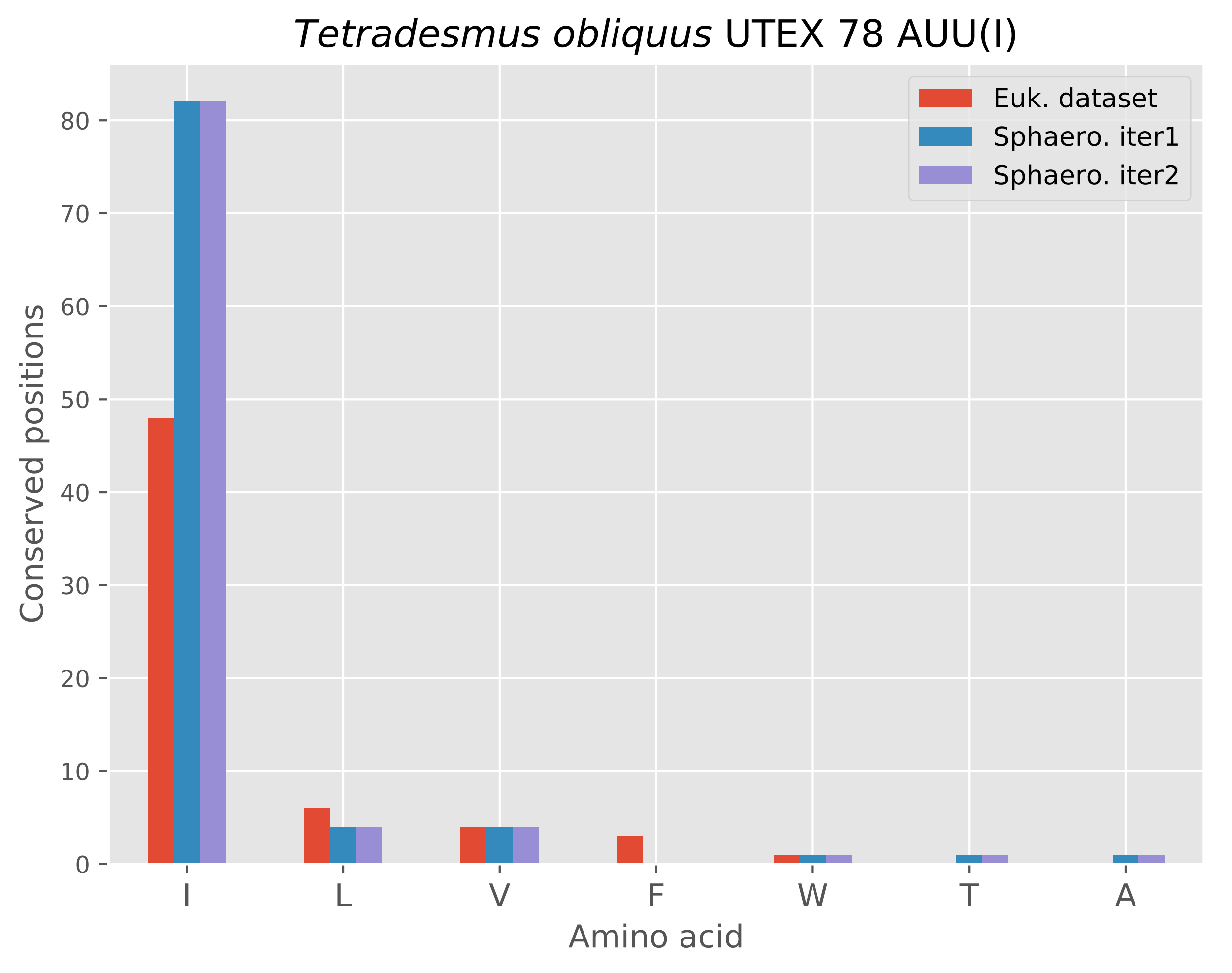

Tetradesmus obliquus UTEX 78 AUU(I)
Euk. dataset
Sphaero. iter1
Sphaero. iter2
Conserved positions
0
10
20
30
40
50
60
70
80
I
L
V
F
W
T
A
Amino acid

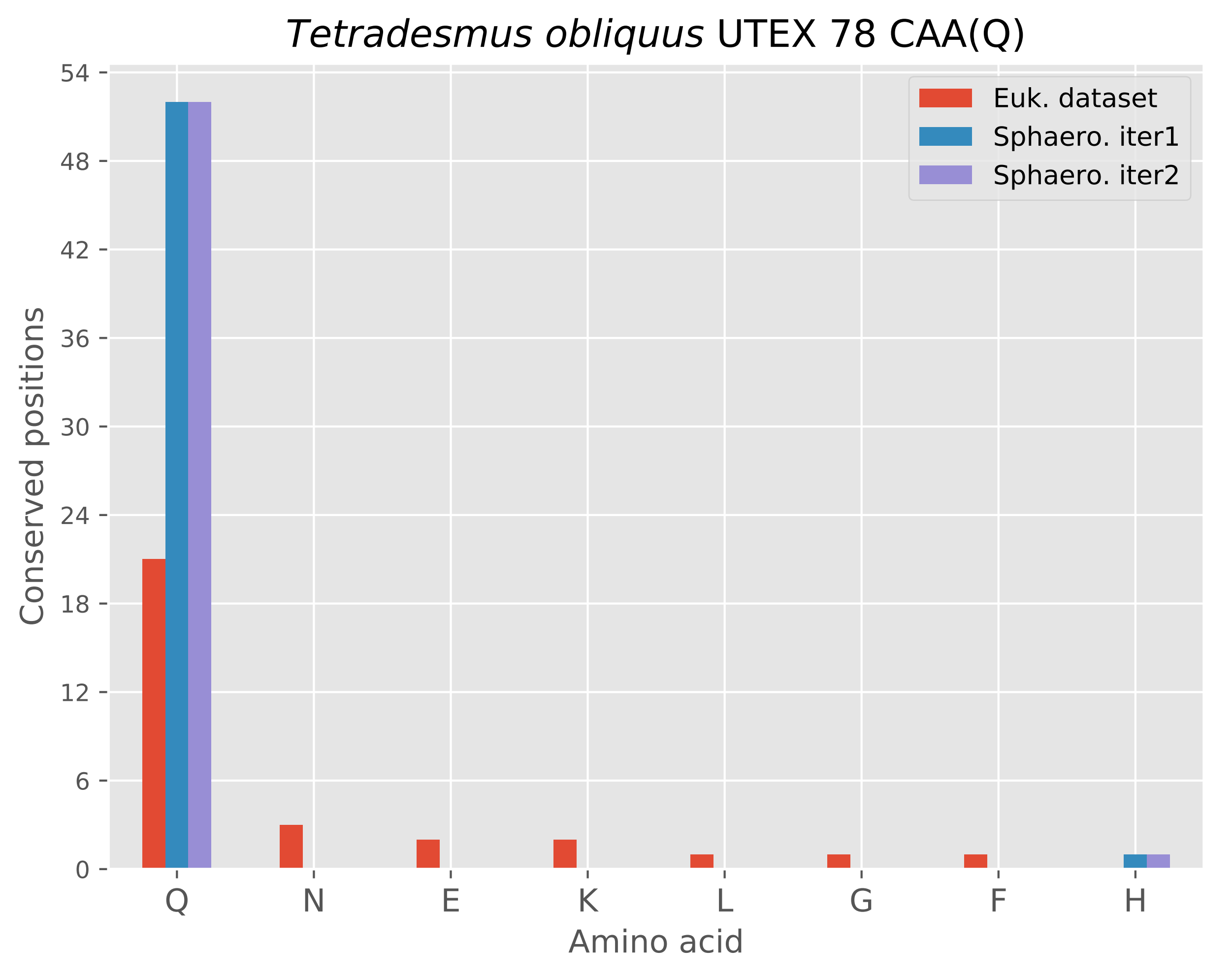

Tetradesmus obliquus UTEX 78 CAA(Q)
Euk. dataset
Sphaero. iter1
Sphaero. iter2
Conserved positions
54
48
42
36
30
24
18
12
6
0
Q
N
E
K
L
G
F
H
Amino acid

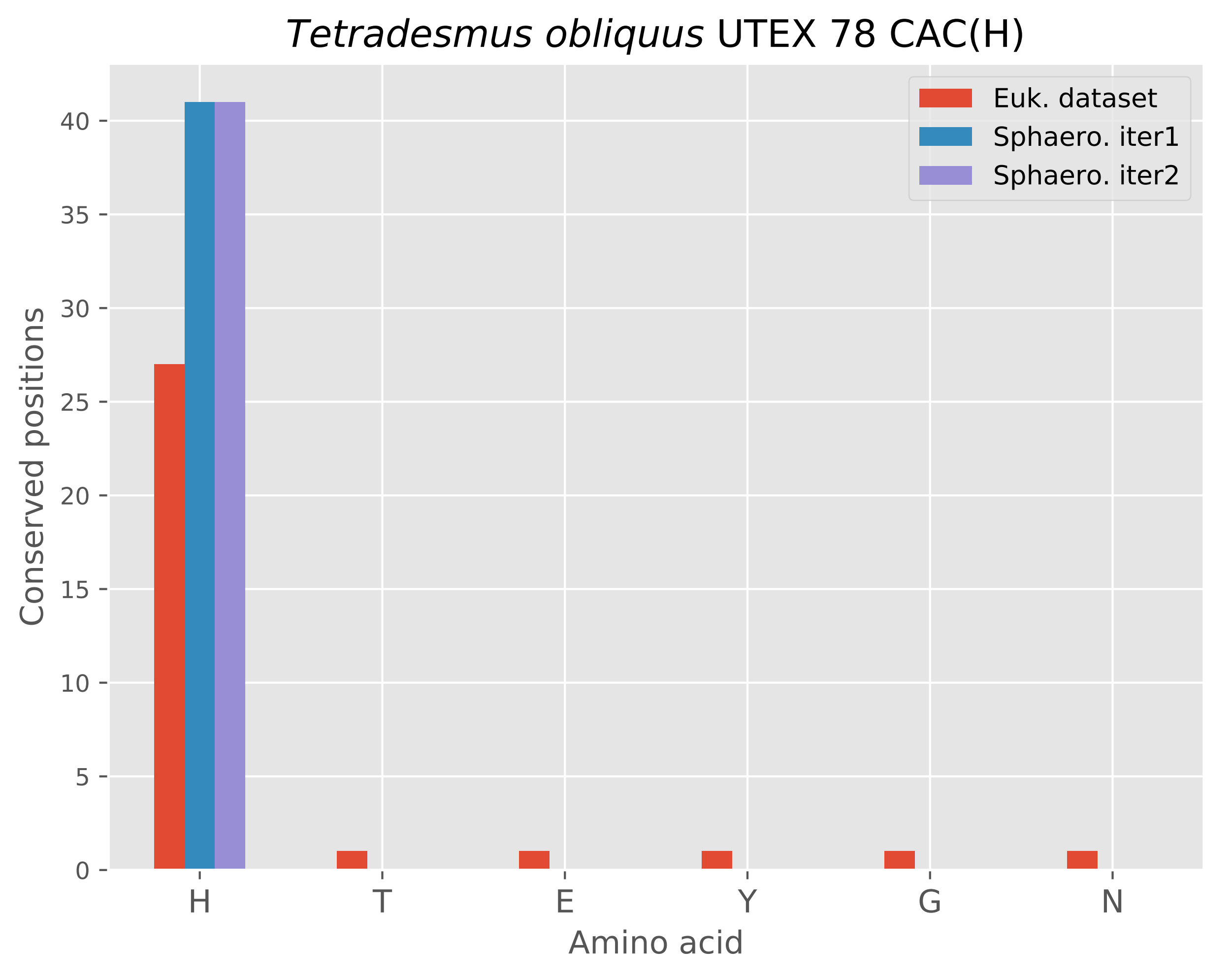

Tetradesmus obliquus UTEX 78 CAC(H)
Euk. dataset
Sphaero. iter1
Sphaero. iter2
Conserved positions
0
5
10
15
20
25
30
35
40
H
T
E
Y
G
N
Amino acid

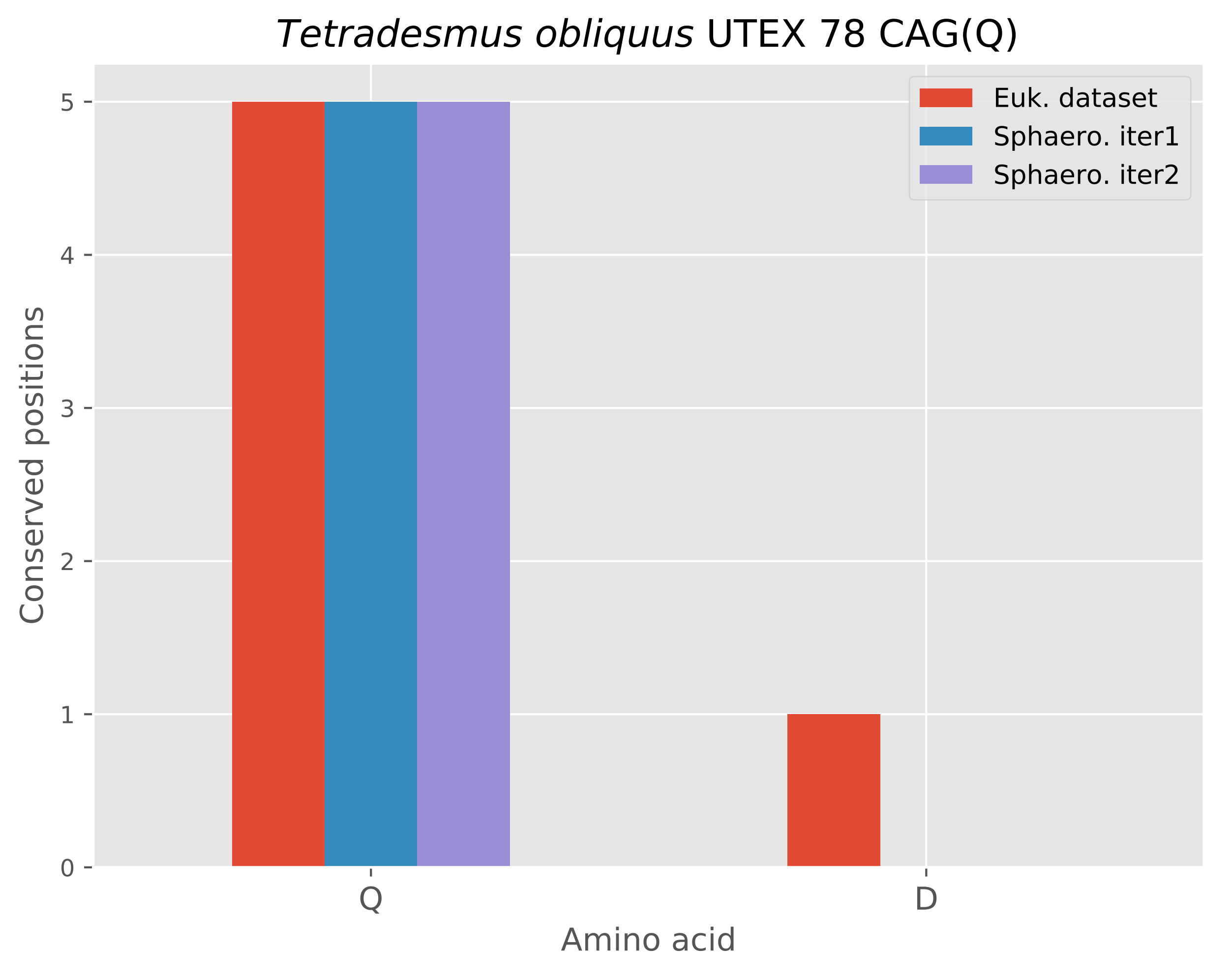
Tetradesmus obliquus UTEX 78 CAG(Q)
Euk. dataset
Sphaero. iter1
Sphaero. iter2
Conserved positions
0
1
2
3
4
5
Q
D
Amino acid

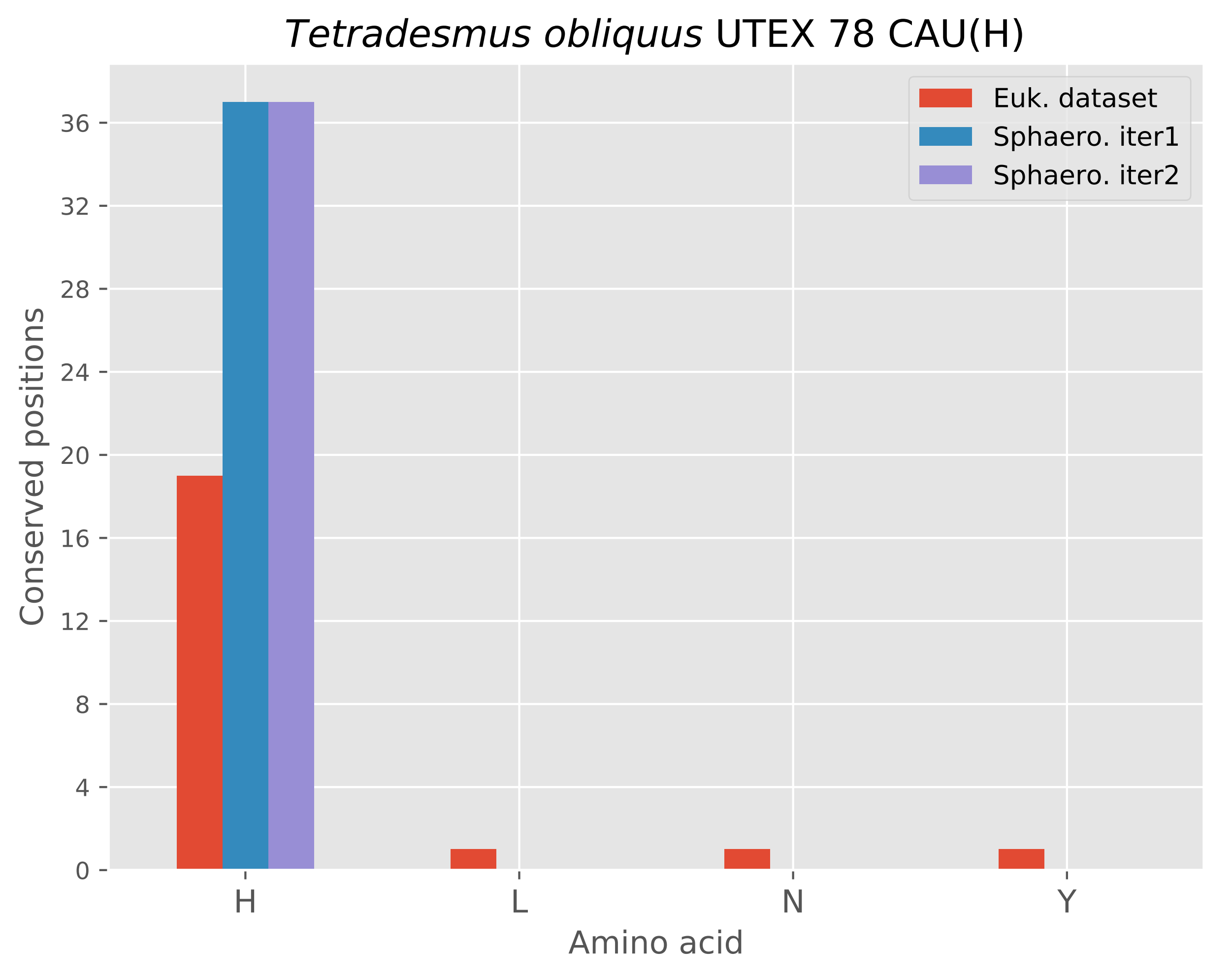
Tetradesmus obliquus UTEX 78 CAU(H)
Euk. dataset
Sphaero. iter1
Sphaero. iter2
Conserved positions
0
4
8
12
16
20
24
28
32
36
H
L
N
Y
Amino acid

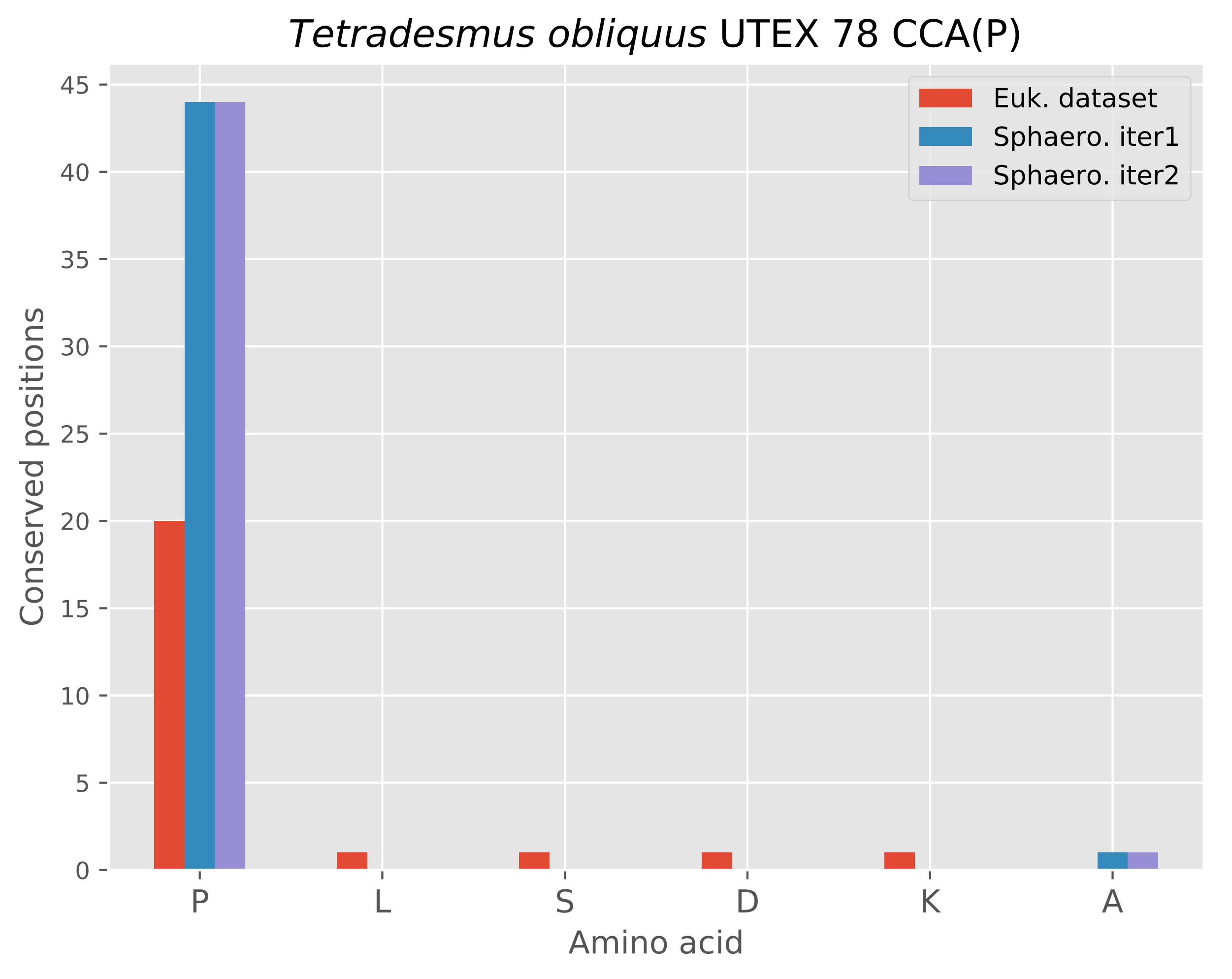

Tetradesmus obliquus UTEX 78 CCA(P)
Euk. dataset
Sphaero. iter1
Sphaero. iter2
Conserved positions
0
5
10
15
20
25
30
35
40
45
P
L
S
D
K
A
Amino acid

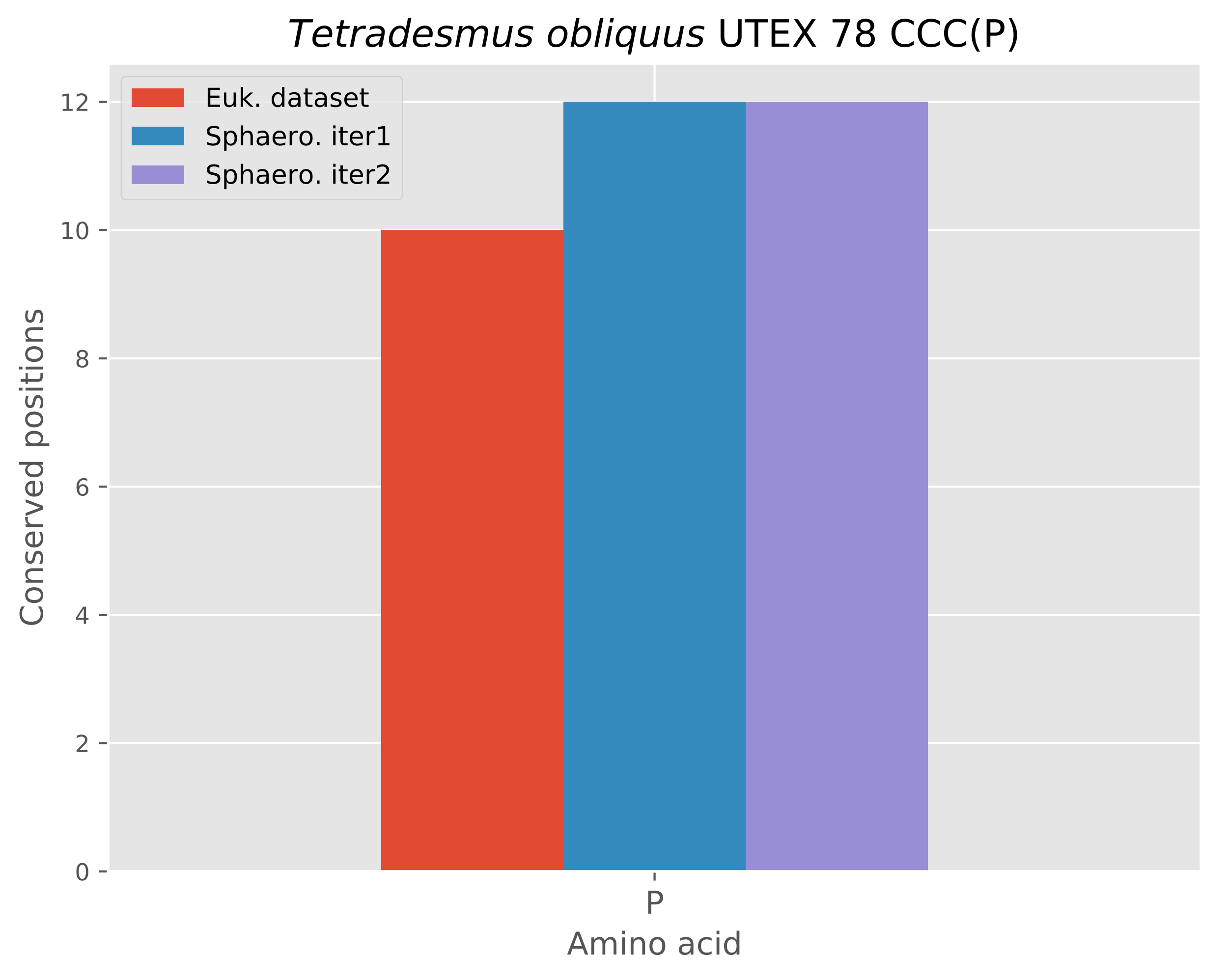
Tetradesmus obliquus UTEX 78 CCC(P)
Euk. dataset
Sphaero. iter1
Sphaero. iter2
Conserved positions
0
2
4
6
8
10
12
P
Amino acid

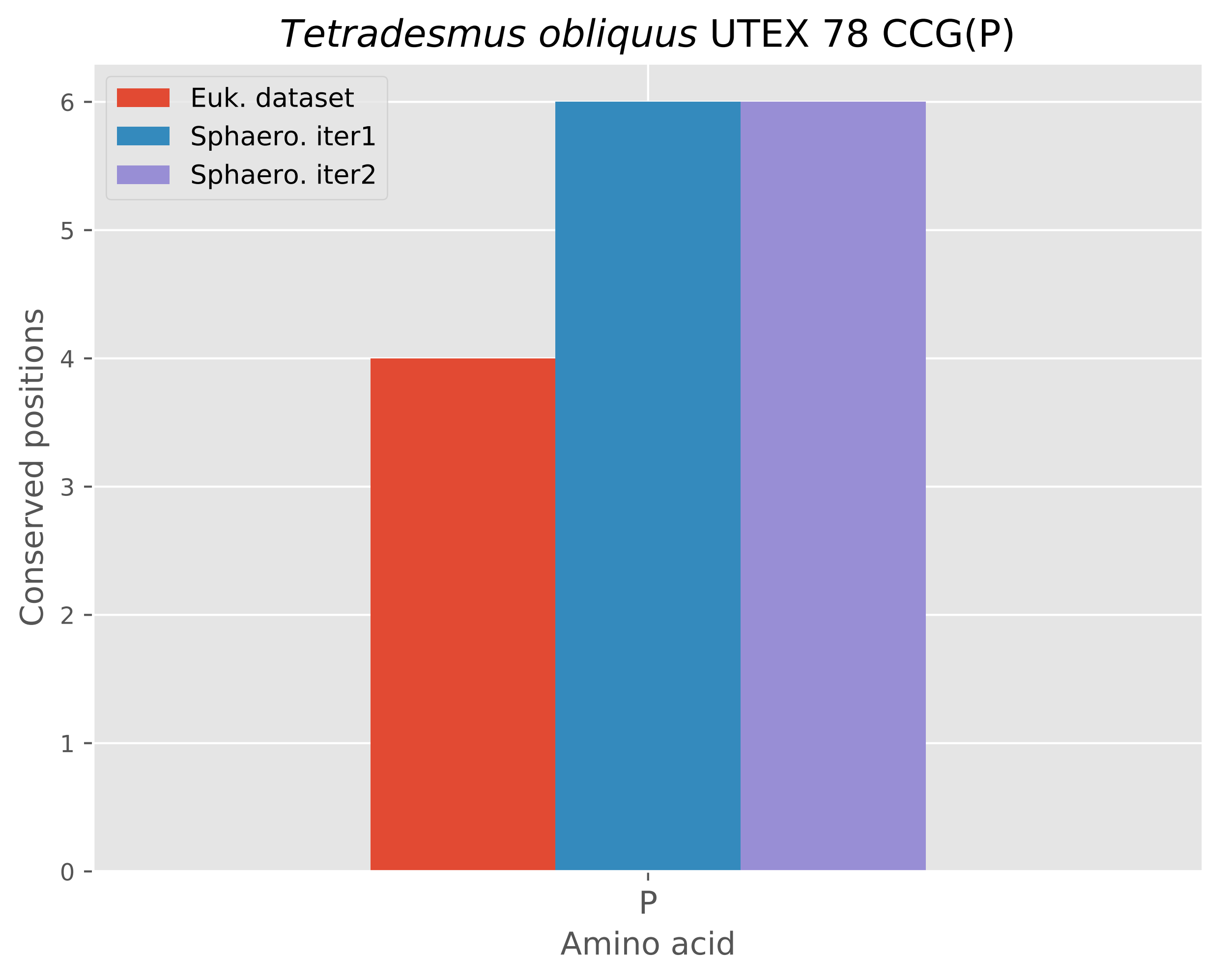

Tetradesmus obliquus UTEX 78 CCG(P)
Euk. dataset
Sphaero. iter1
Sphaero. iter2
Conserved positions
0
1
2
3
4
5
6
P
Amino acid

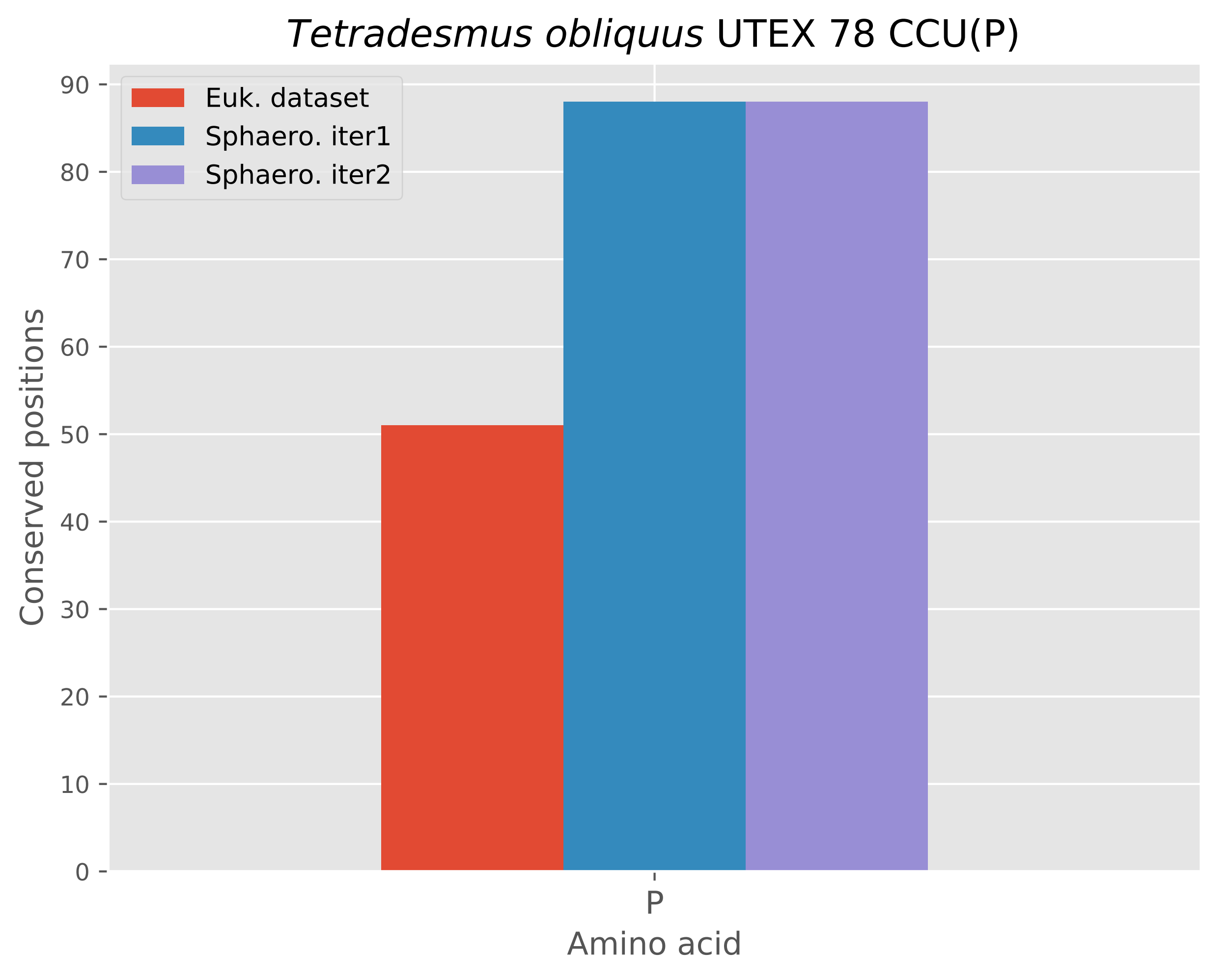
Tetradesmus obliquus UTEX 78 CCU(P)
Euk. dataset
Sphaero. iter1
Sphaero. iter2
Conserved positions
0
10
20
30
40
50
60
70
80
90
P
Amino acid

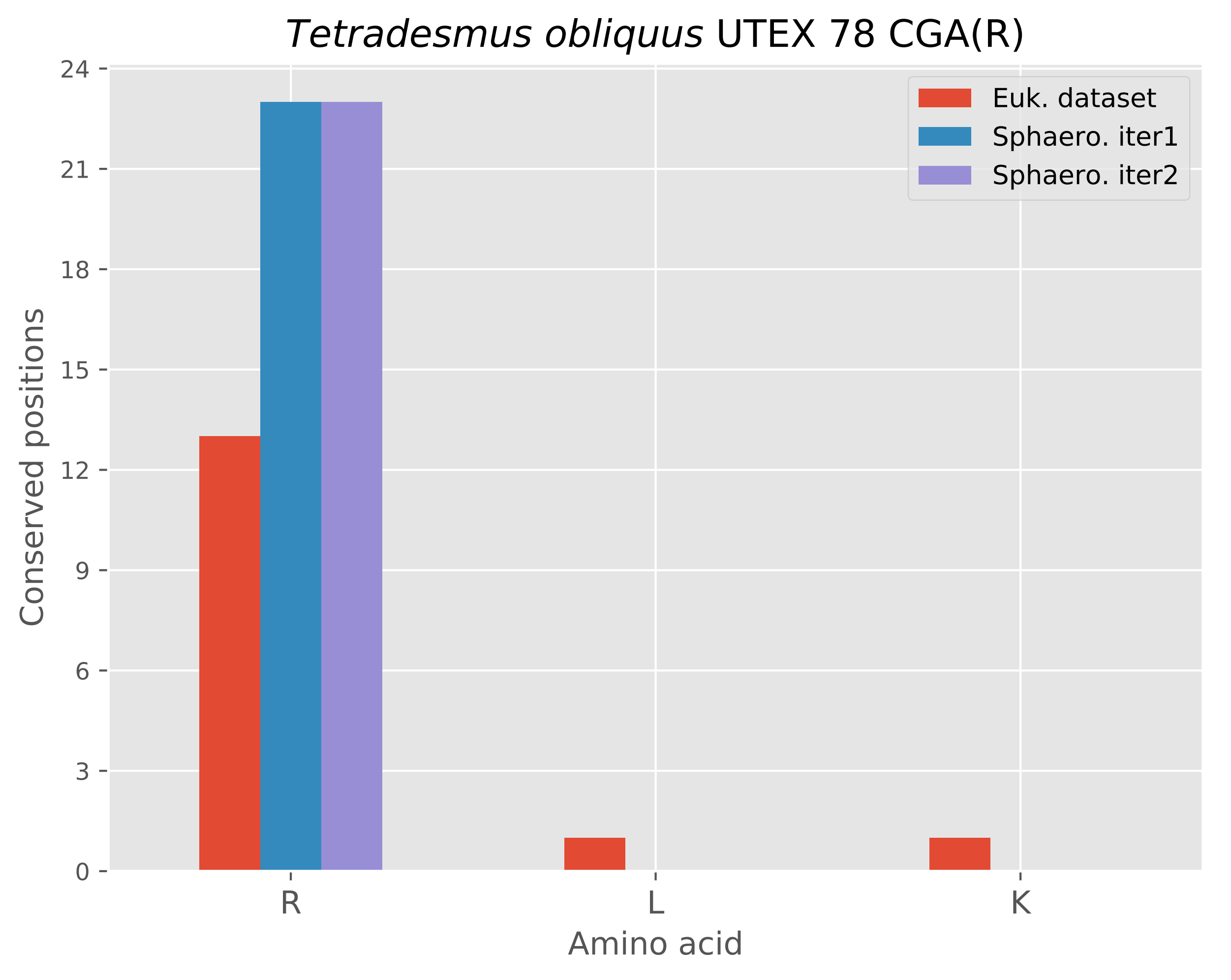

Tetradesmus obliquus UTEX 78 CGA(R)
Euk. dataset
Sphaero. iter1
Sphaero. iter2
Conserved positions
0
3
6
9
12
15
18
21
24
R
L
K
Amino acid

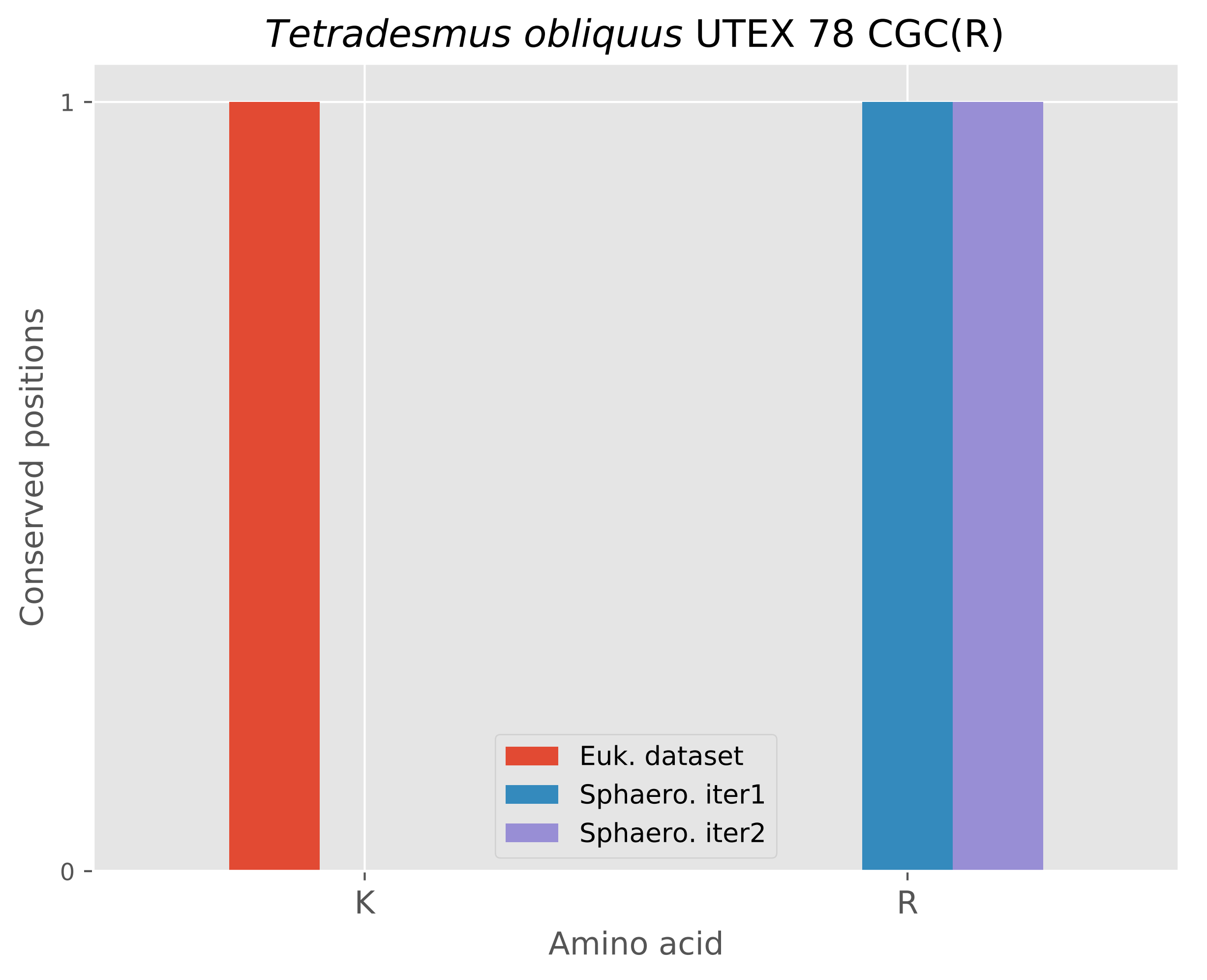
Tetradesmus obliquus UTEX 78 CGC(R)
Conserved positions
1
0
K
R
Amino acid
Euk. dataset
Sphaero. iter1
Sphaero. iter2

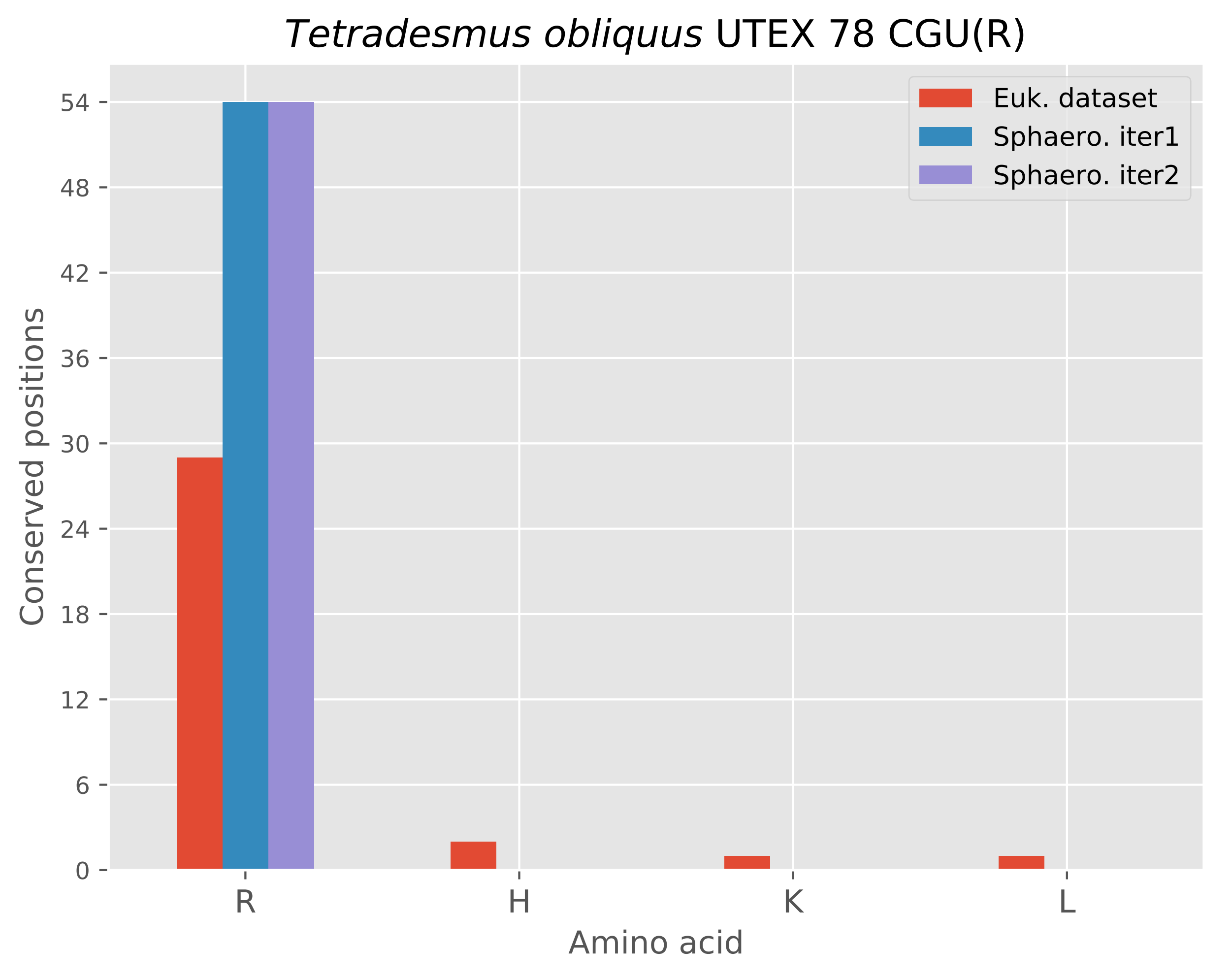

Tetradesmus obliquus UTEX 78 CGU(R)
Euk. dataset
Sphaero. iter1
Sphaero. iter2
Conserved positions
0
6
12
18
24
30
36
42
48
54
R
H
K
L
Amino acid

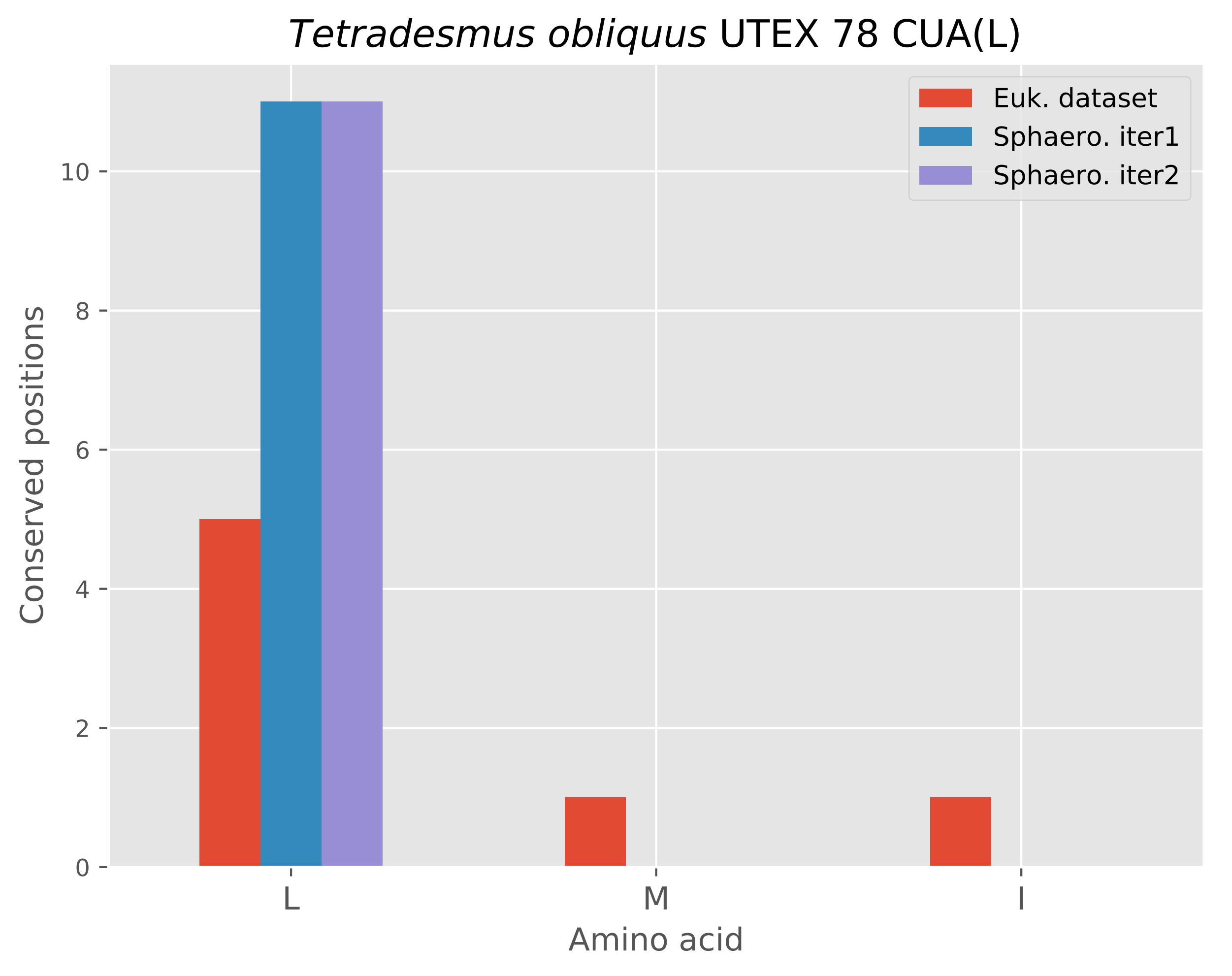
Tetradesmus obliquus UTEX 78 CUA(L)
Euk. dataset
Sphaero. iter1
Sphaero. iter2
Conserved positions
0
2
4
6
8
10
L
M
I
Amino acid

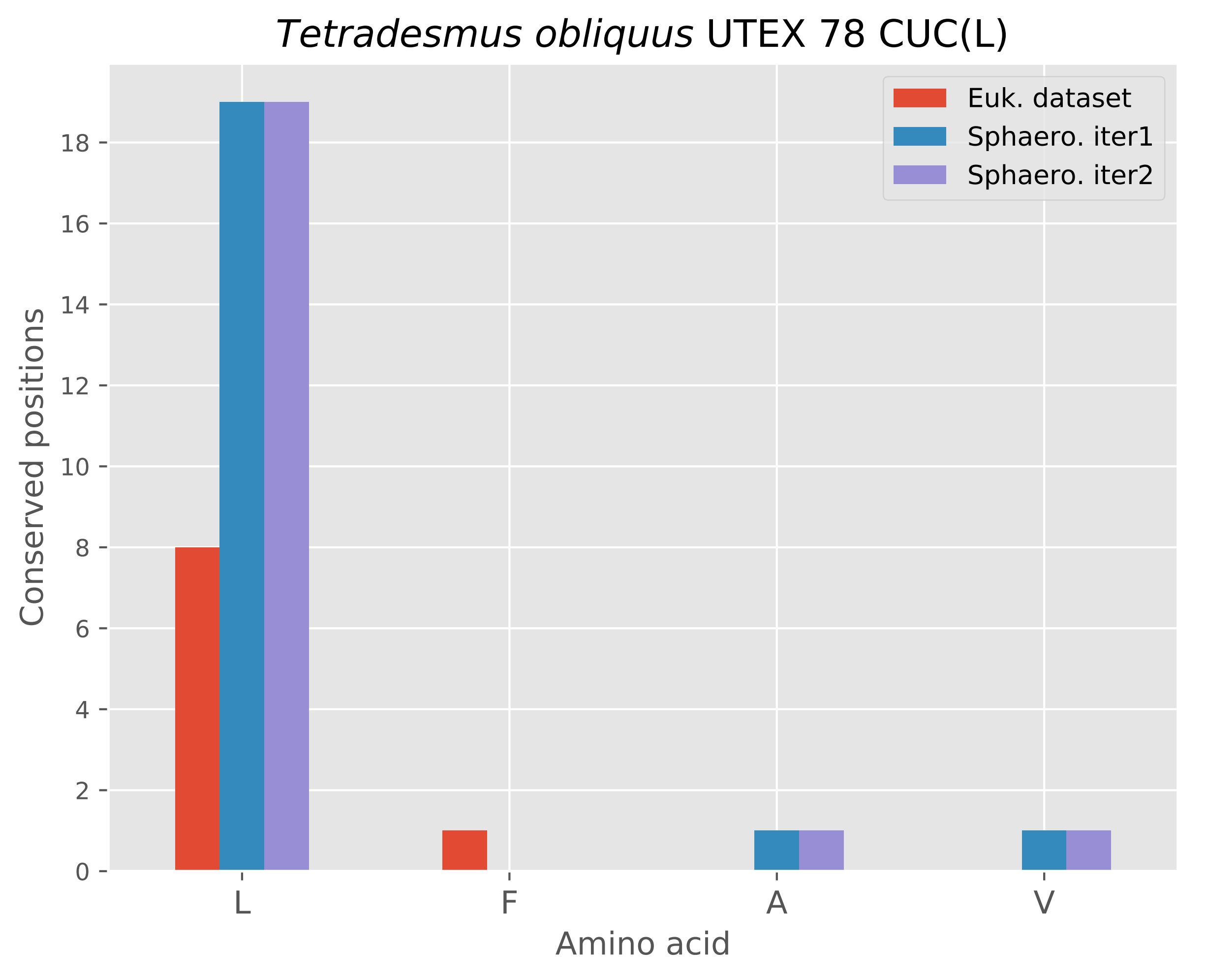

Tetradesmus obliquus UTEX 78 CUC(L)
Euk. dataset
Sphaero. iter1
Sphaero. iter2
Conserved positions
0
2
4
6
8
10
12
14
16
18
L
F
A
V
Amino acid

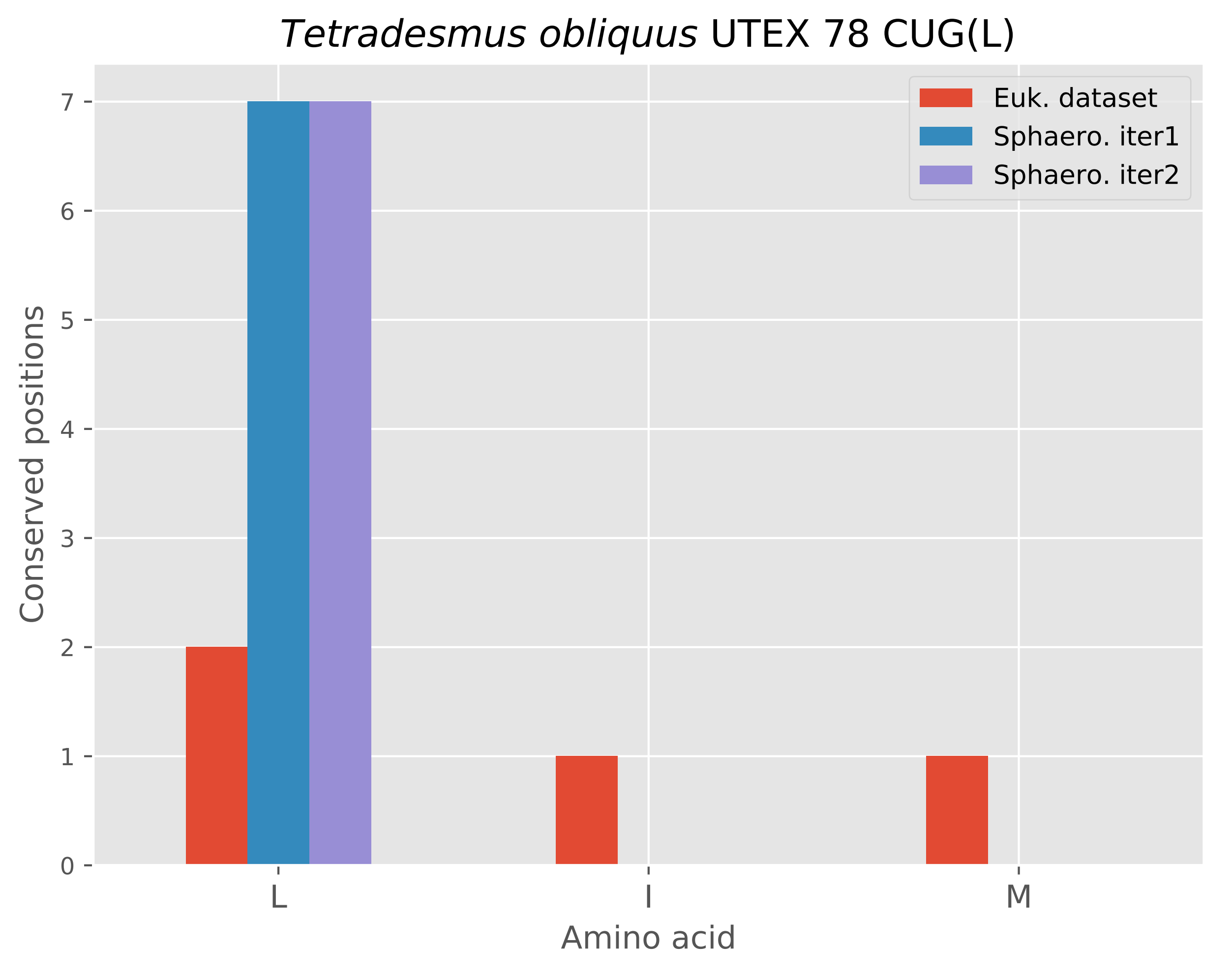

Tetradesmus obliquus UTEX 78 CUG(L)
Euk. dataset
Sphaero. iter1
Sphaero. iter2
Conserved positions
0
1
2
3
4
5
6
7
L
I
M
Amino acid

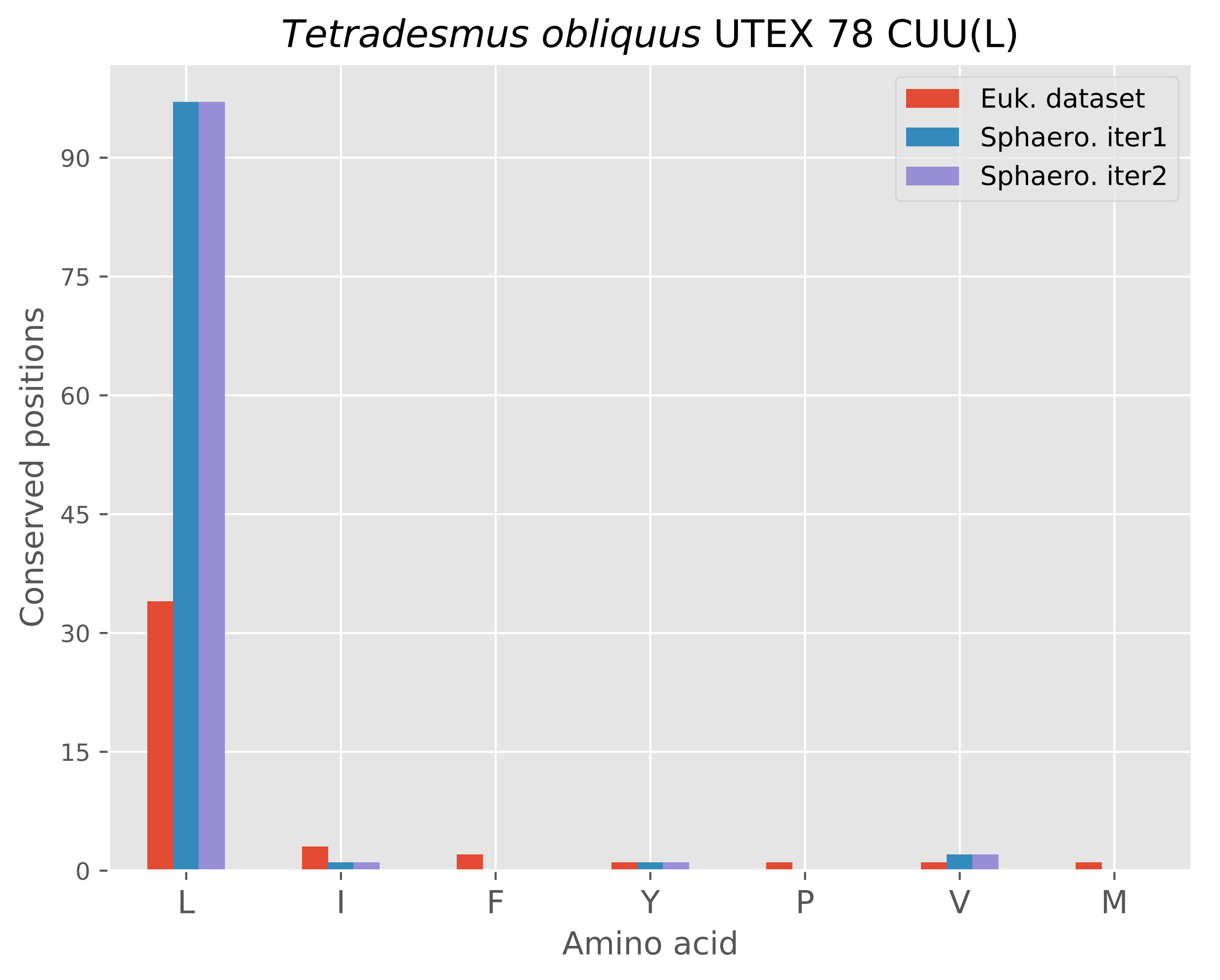

Tetradesmus obliquus UTEX 78 CUU(L)
Euk. dataset
Sphaero. iter1
Sphaero. iter2
Conserved positions
0
15
30
45
60
75
90
L
I
F
Y
P
V
M
Amino acid

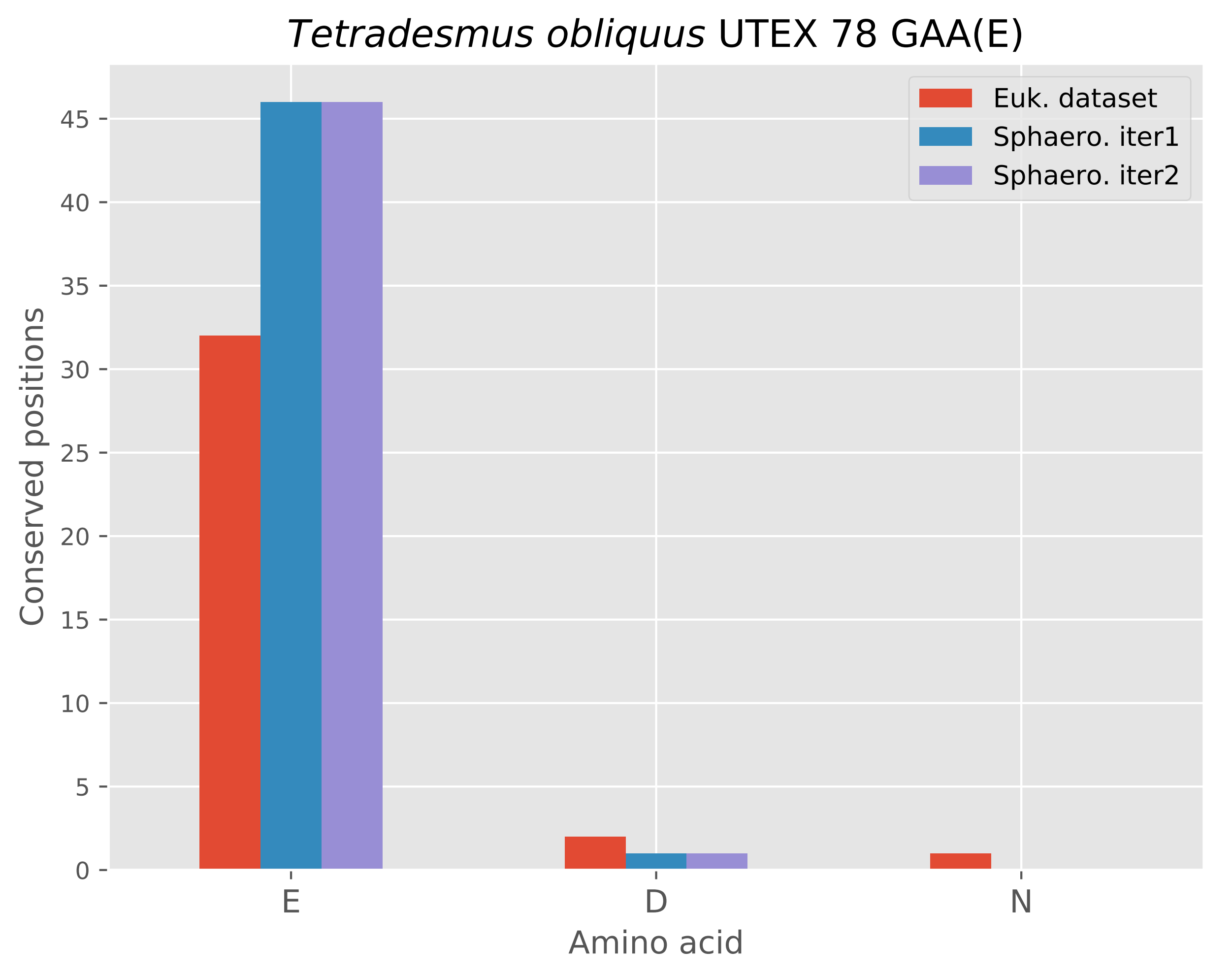

Tetradesmus obliquus UTEX 78 GAA(E)
Euk. dataset
Sphaero. iter1
Sphaero. iter2
Conserved positions
0
5
10
15
20
25
30
35
40
45
E
D
N
Amino acid

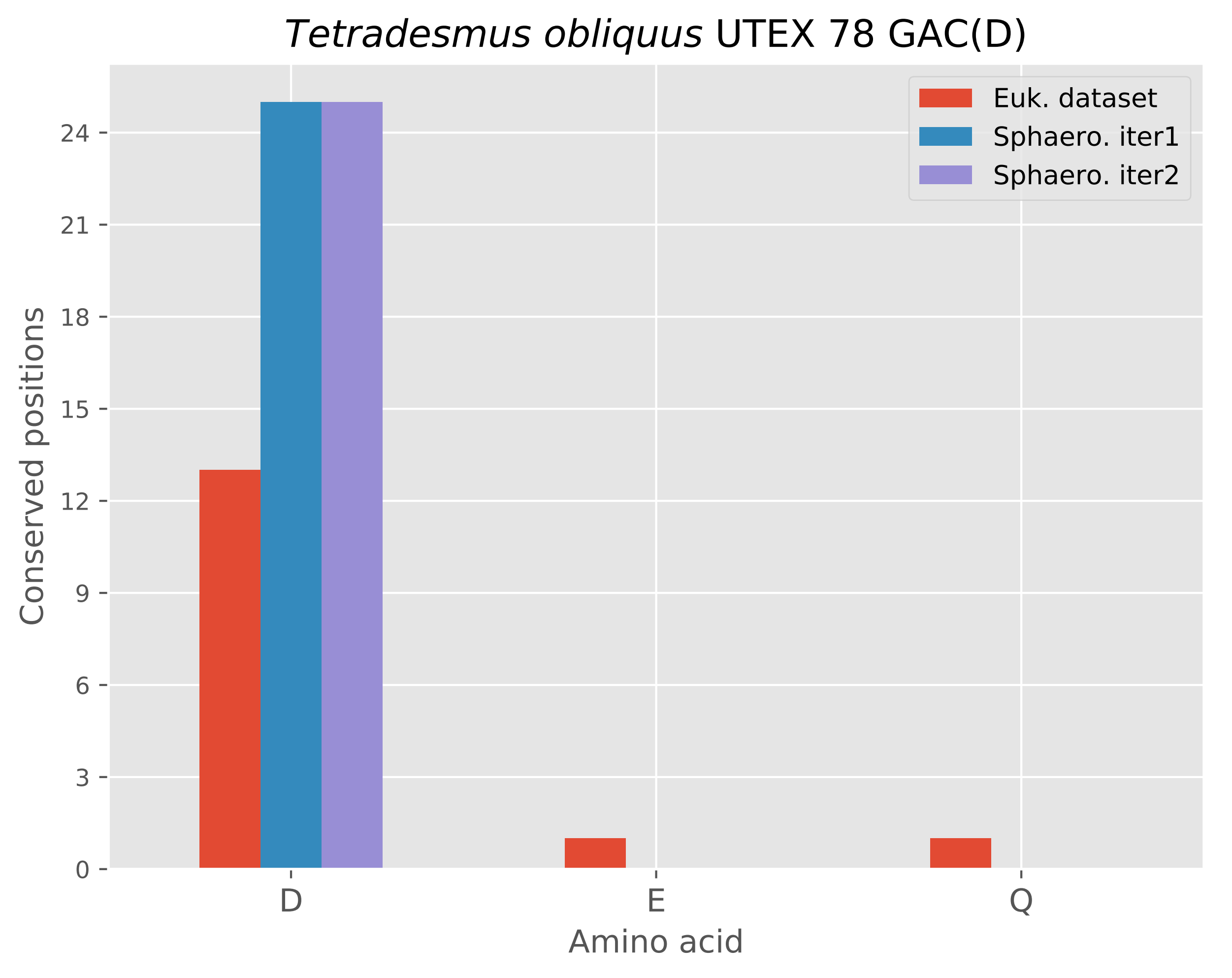

Tetradesmus obliquus UTEX 78 GAC(D)
Euk. dataset
Sphaero. iter1
Sphaero. iter2
Conserved positions
0
3
6
9
12
15
18
21
24
D
E
Q
Amino acid

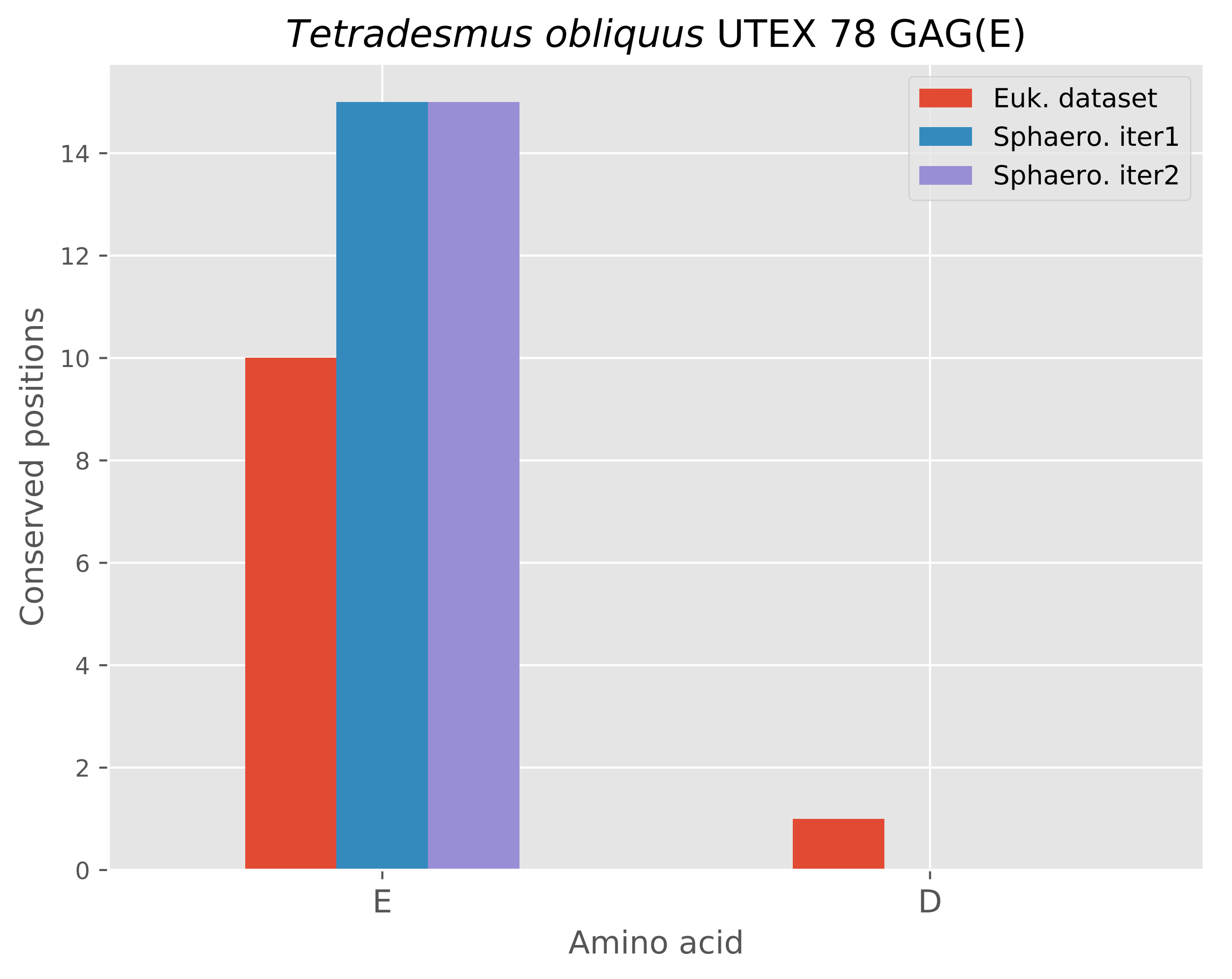

Tetradesmus obliquus UTEX 78 GAG(E)
Euk. dataset
Sphaero. iter1
Sphaero. iter2
Conserved positions
0
2
4
6
8
10
12
14
E
D
Amino acid

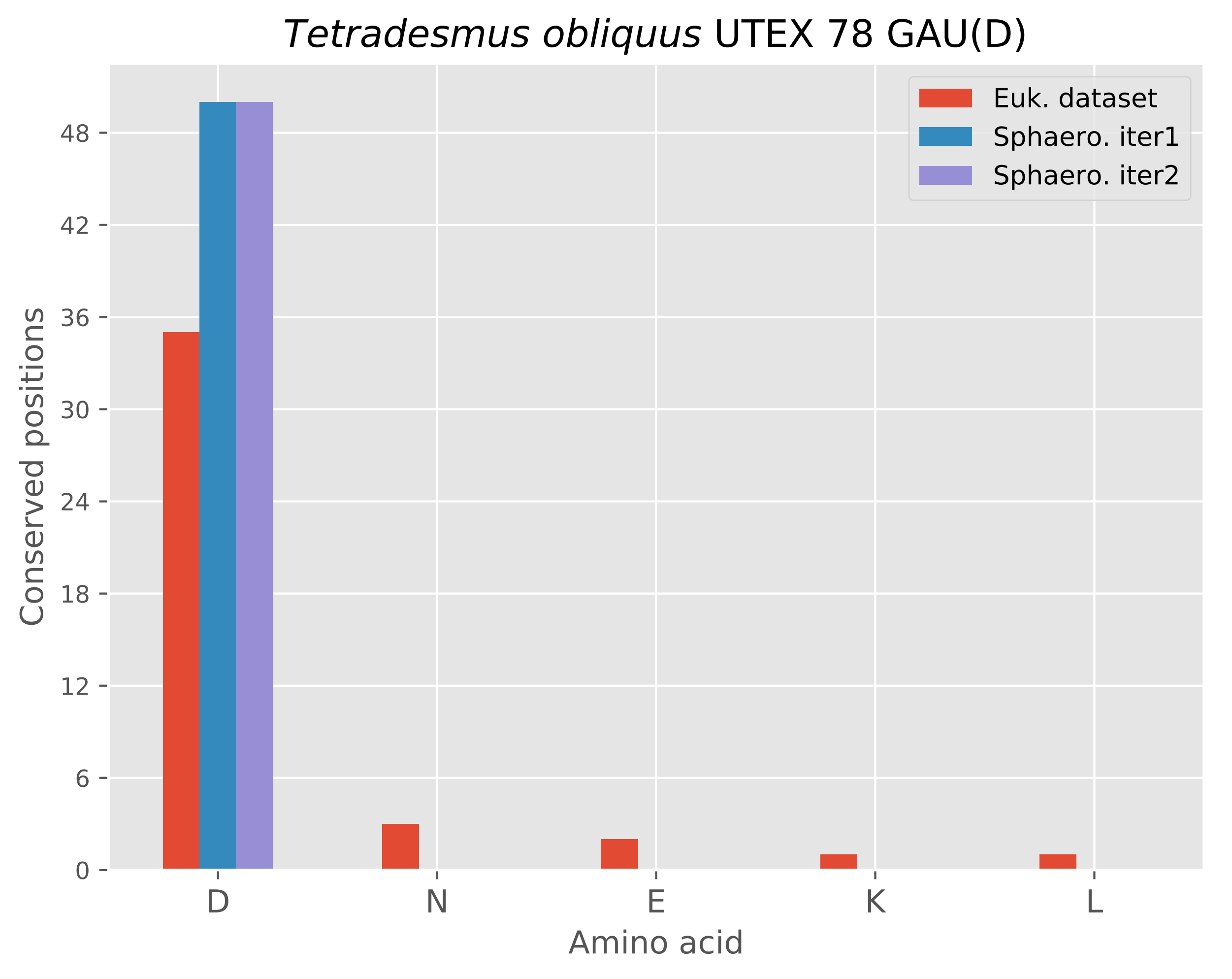

Tetradesmus obliquus UTEX 78 GAU(D)
Euk. dataset
Sphaero. iter1
Sphaero. iter2
Conserved positions
0
6
12
18
24
30
36
42
48
D
N
E
K
L
Amino acid

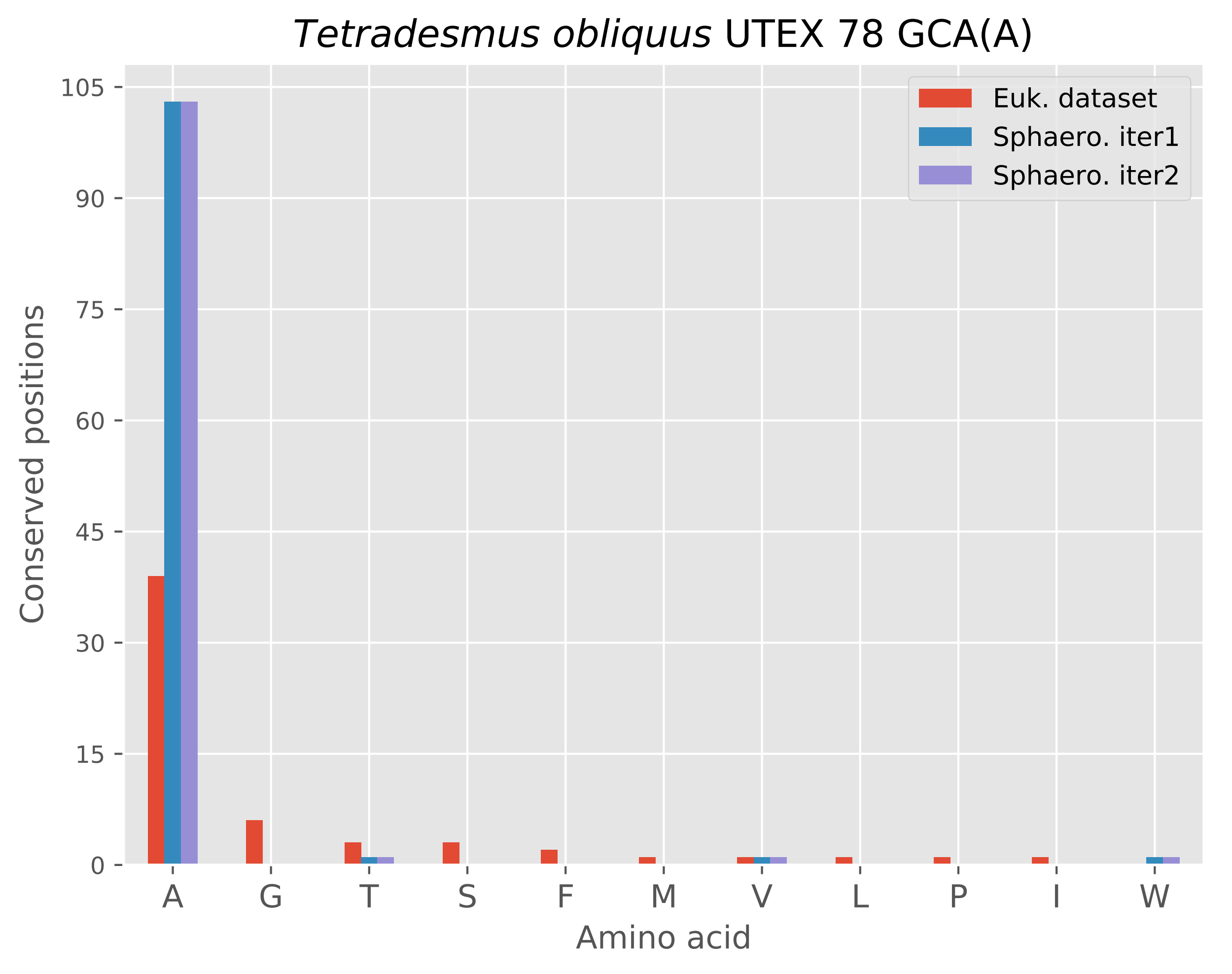

Tetradesmus obliquus UTEX 78 GCA(A)
Euk. dataset
Sphaero. iter1
Sphaero. iter2
Conserved positions
0
15
30
45
60
75
90
105
Amino acid
A
G
T
S
F
M
V
L
P
I
W

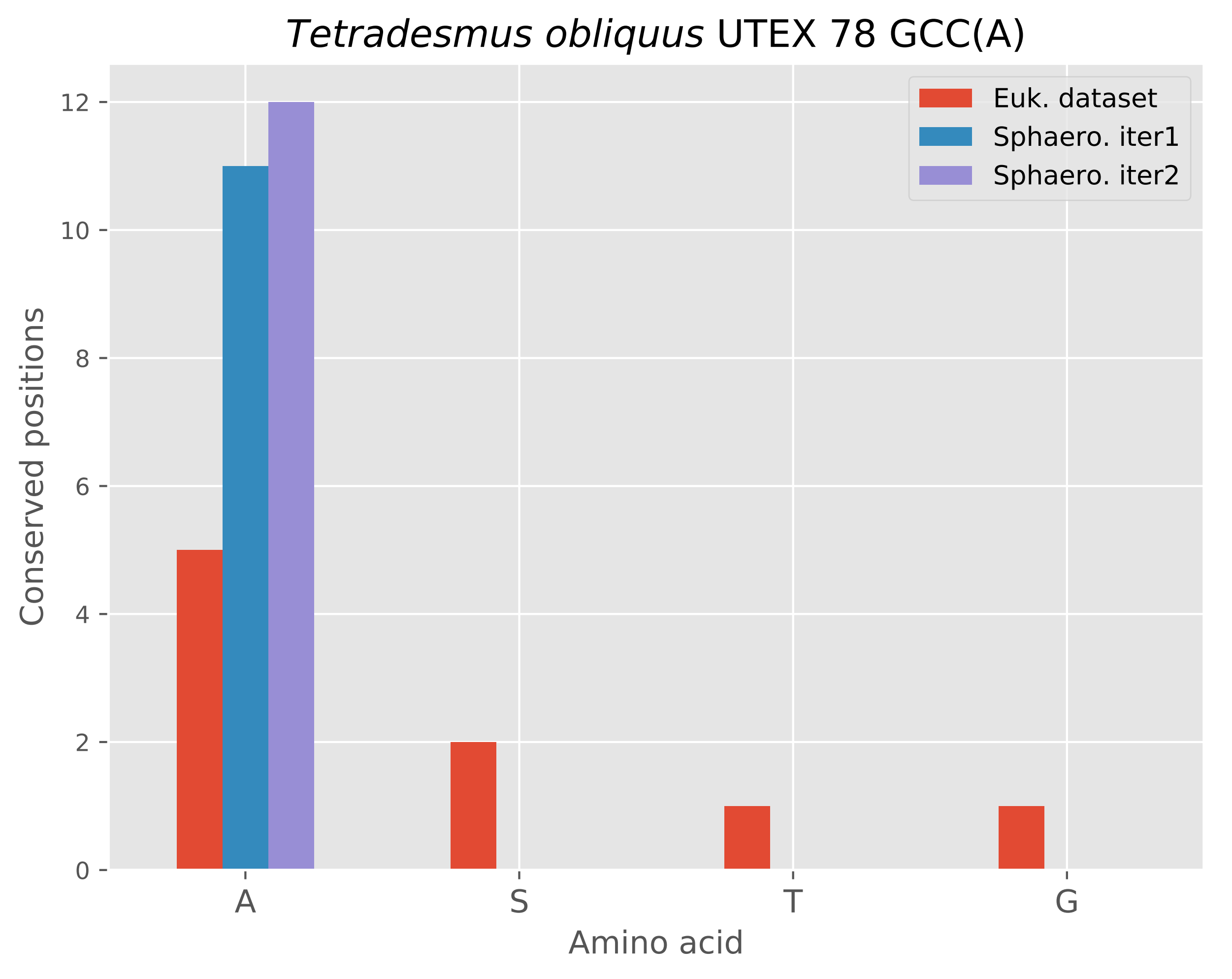
Tetradesmus obliquus UTEX 78 GCC(A)
Euk. dataset
Sphaero. iter1
Sphaero. iter2
Conserved positions
0
2
4
6
8
10
12
A
S
T
G
Amino acid

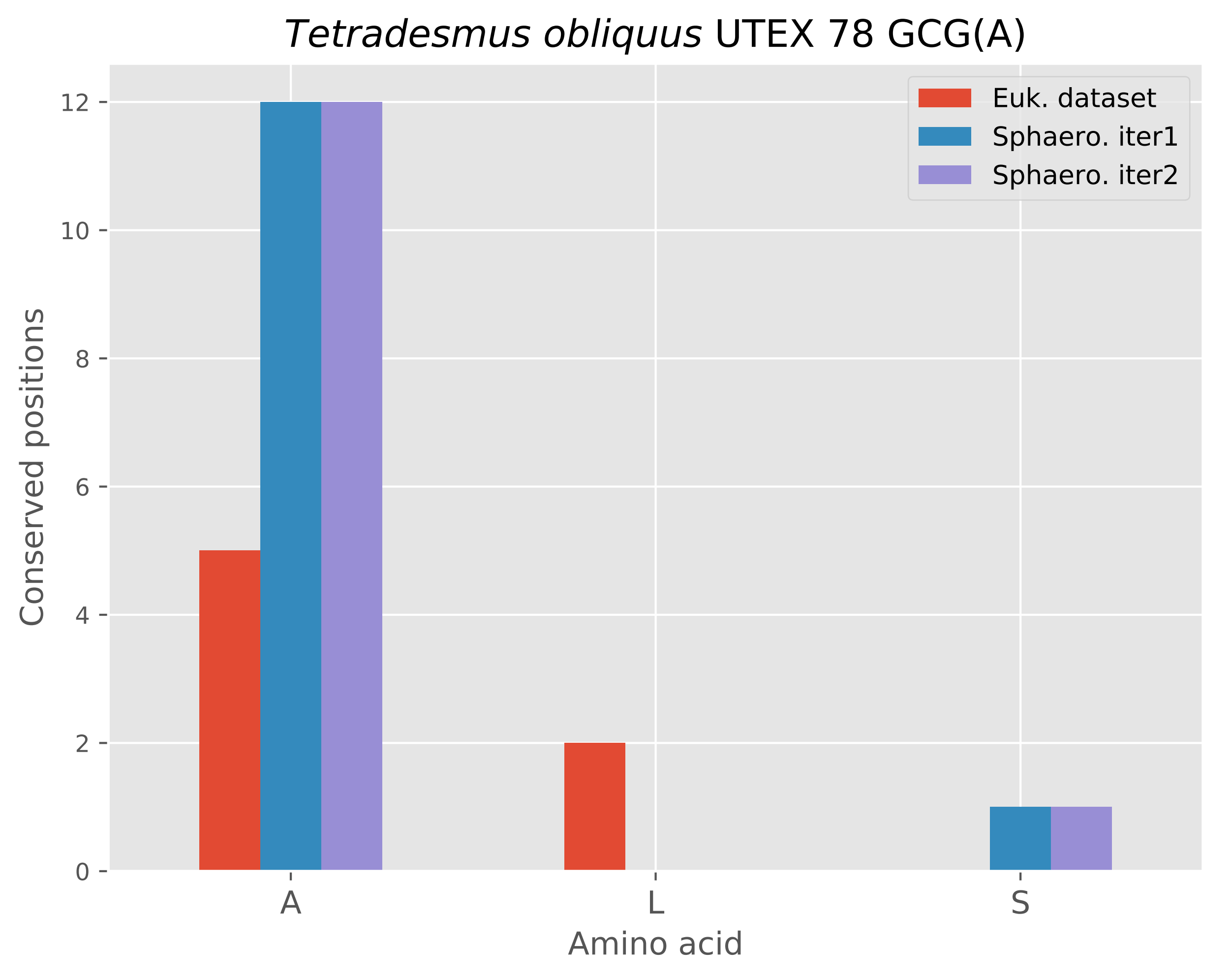

Tetradesmus obliquus UTEX 78 GCG(A)
Euk. dataset
Sphaero. iter1
Sphaero. iter2
Conserved positions
0
2
4
6
8
10
12
A
L
S
Amino acid

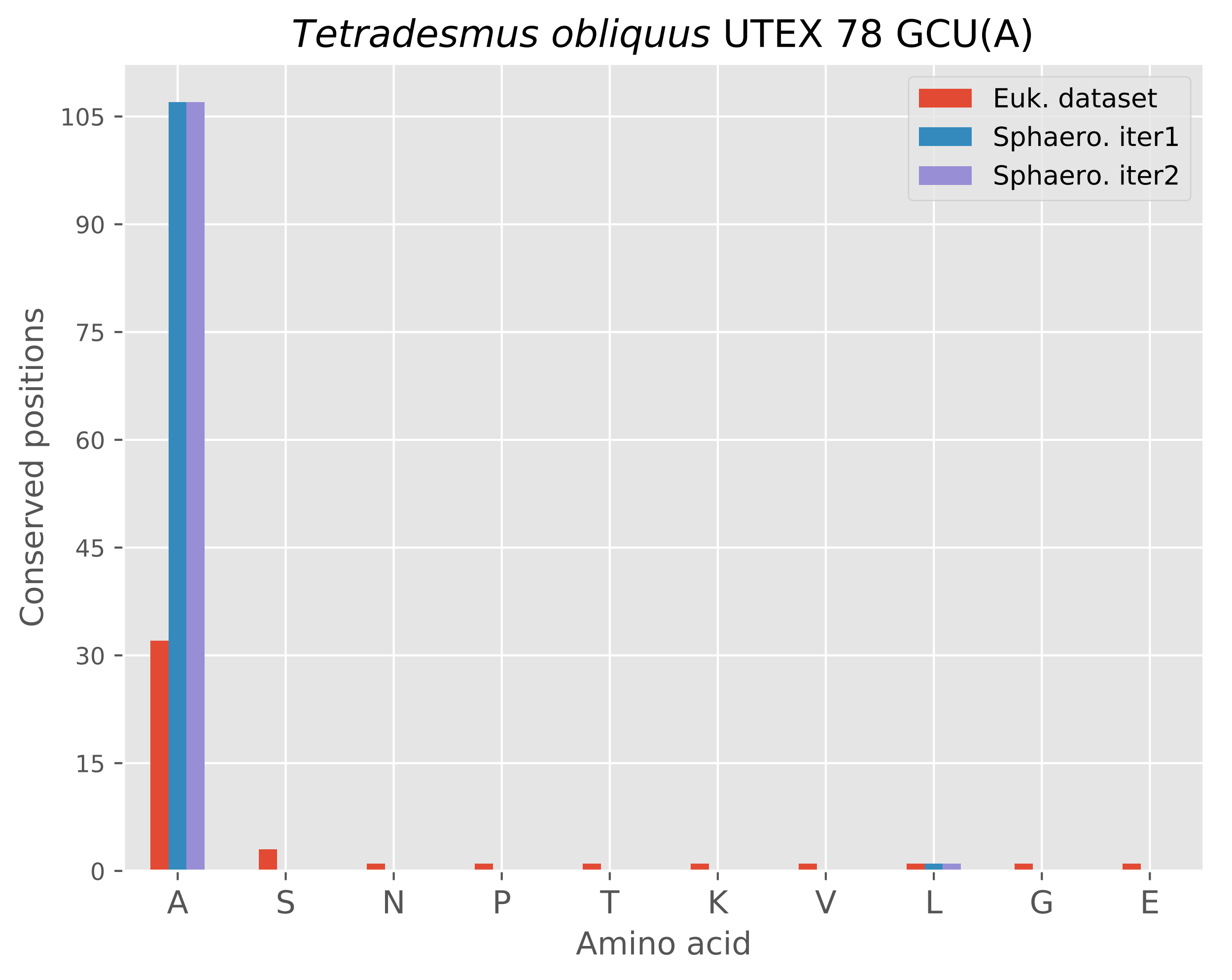

Tetradesmus obliquus UTEX 78 GCU(A)
Euk. dataset
Sphaero. iter1
Sphaero. iter2
Conserved positions
0
15
30
45
60
75
90
105
A
S
N
P
T
K
V
L
G
E
Amino acid

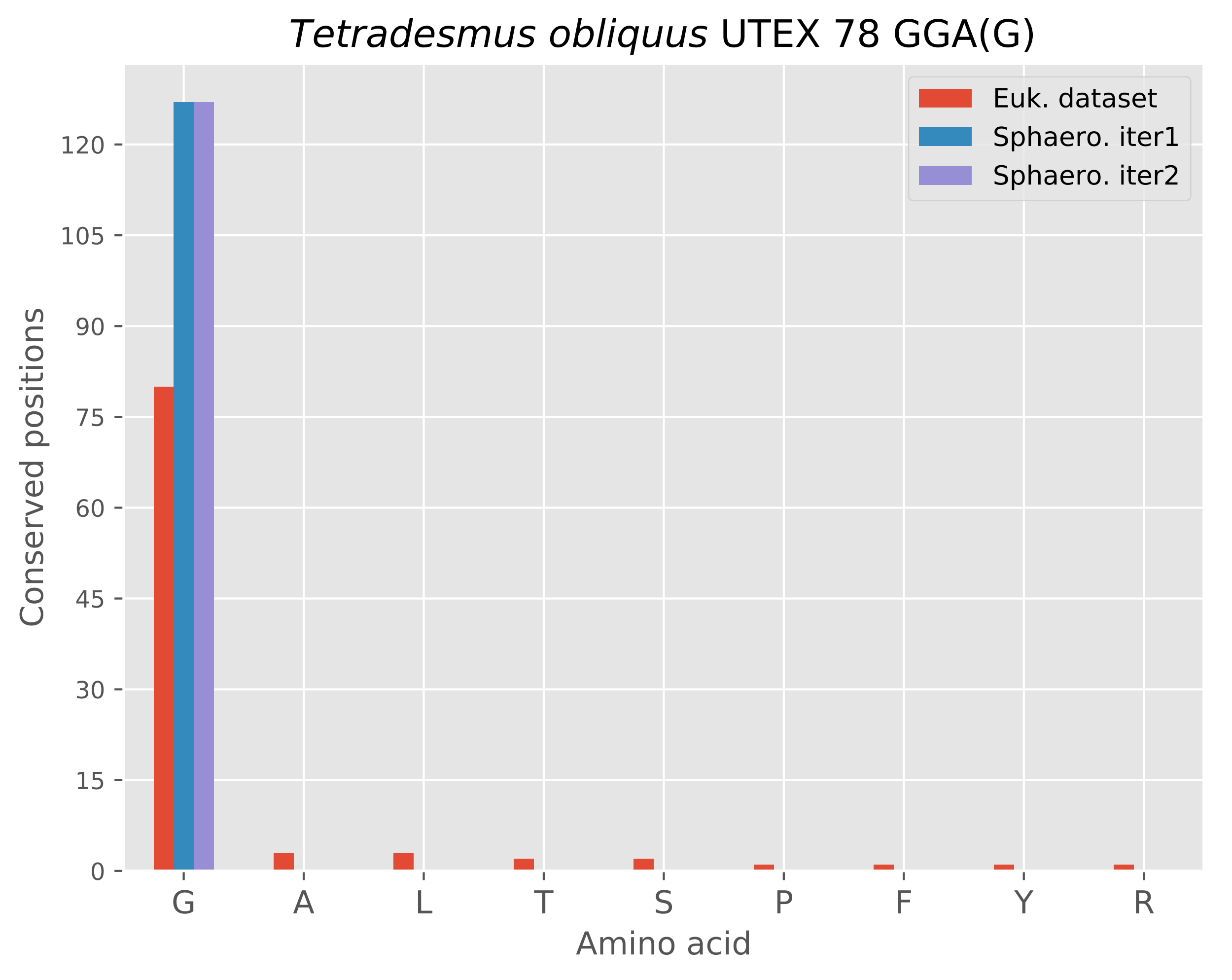

Tetradesmus obliquus UTEX 78 GGA(G)
Euk. dataset
Sphaero. iter1
Sphaero. iter2
Conserved positions
0
15
30
45
60
75
90
105
120
G
A
L
T
S
P
F
Y
R
Amino acid

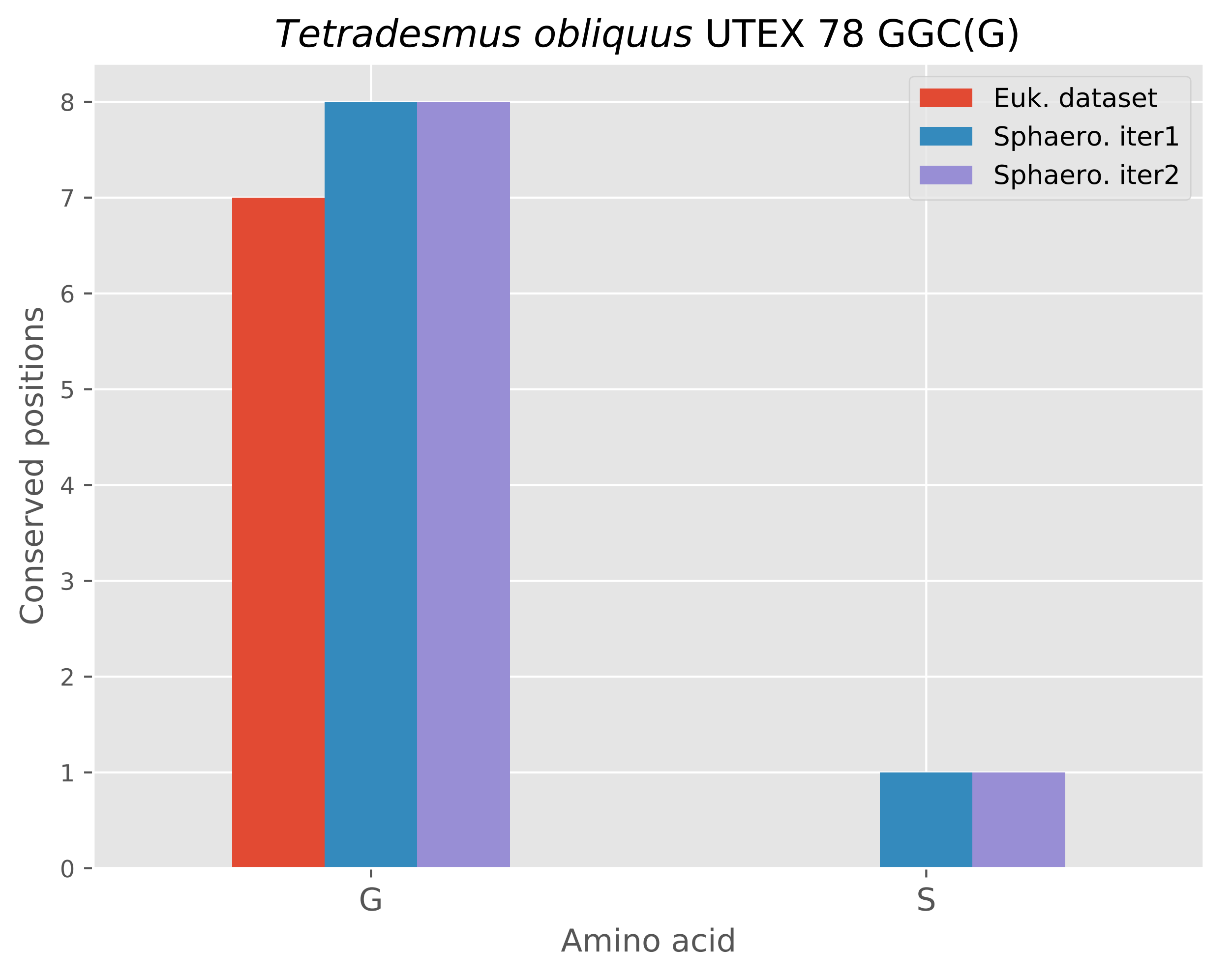

Tetradesmus obliquus UTEX 78 GGC(G)
Euk. dataset
Sphaero. iter1
Sphaero. iter2
Conserved positions
0
1
2
3
4
5
6
7
8
G
S
Amino acid

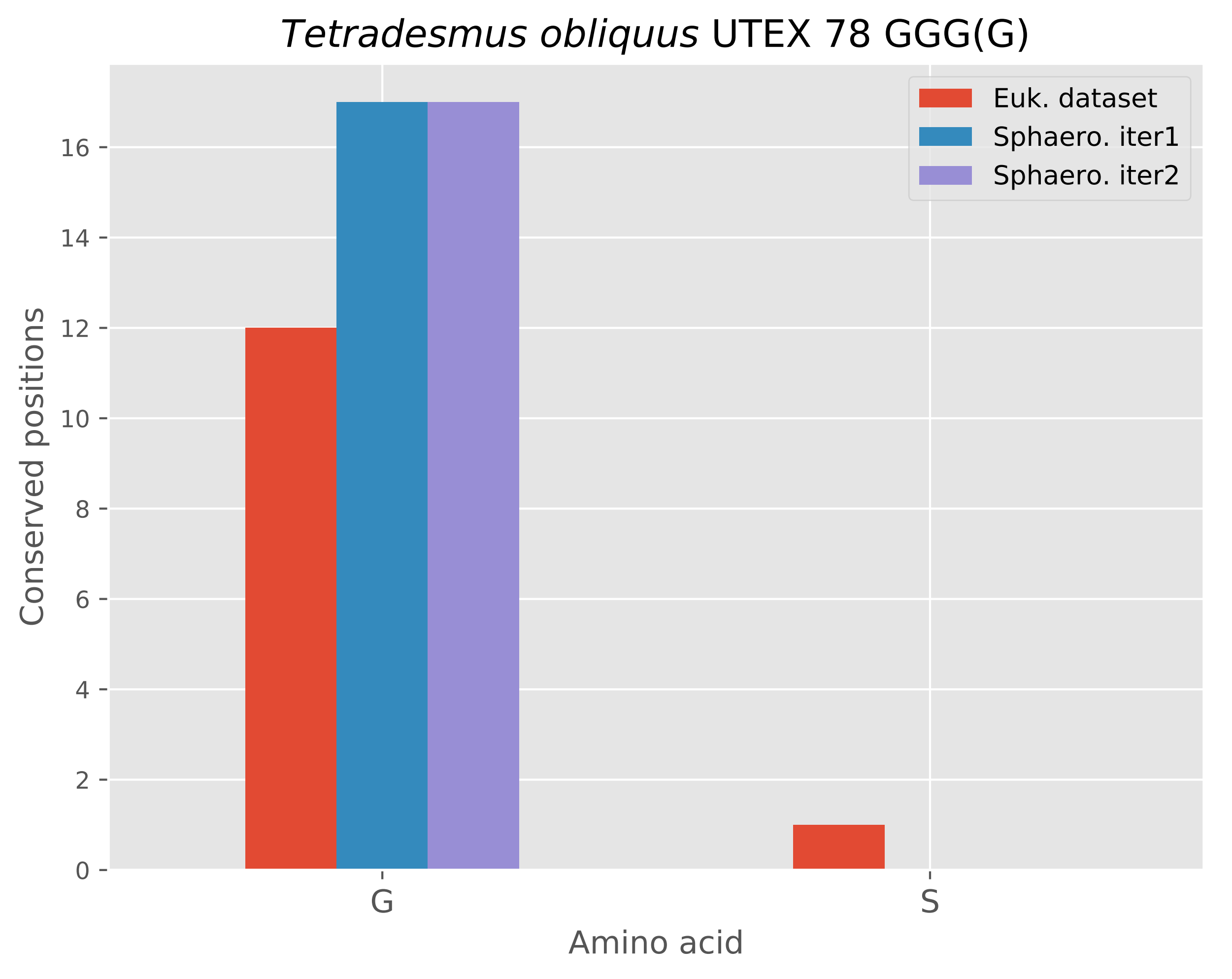

Tetradesmus obliquus UTEX 78 GGG(G)
Euk. dataset
Sphaero. iter1
Sphaero. iter2
Conserved positions
0
2
4
6
8
10
12
14
16
G
S
Amino acid

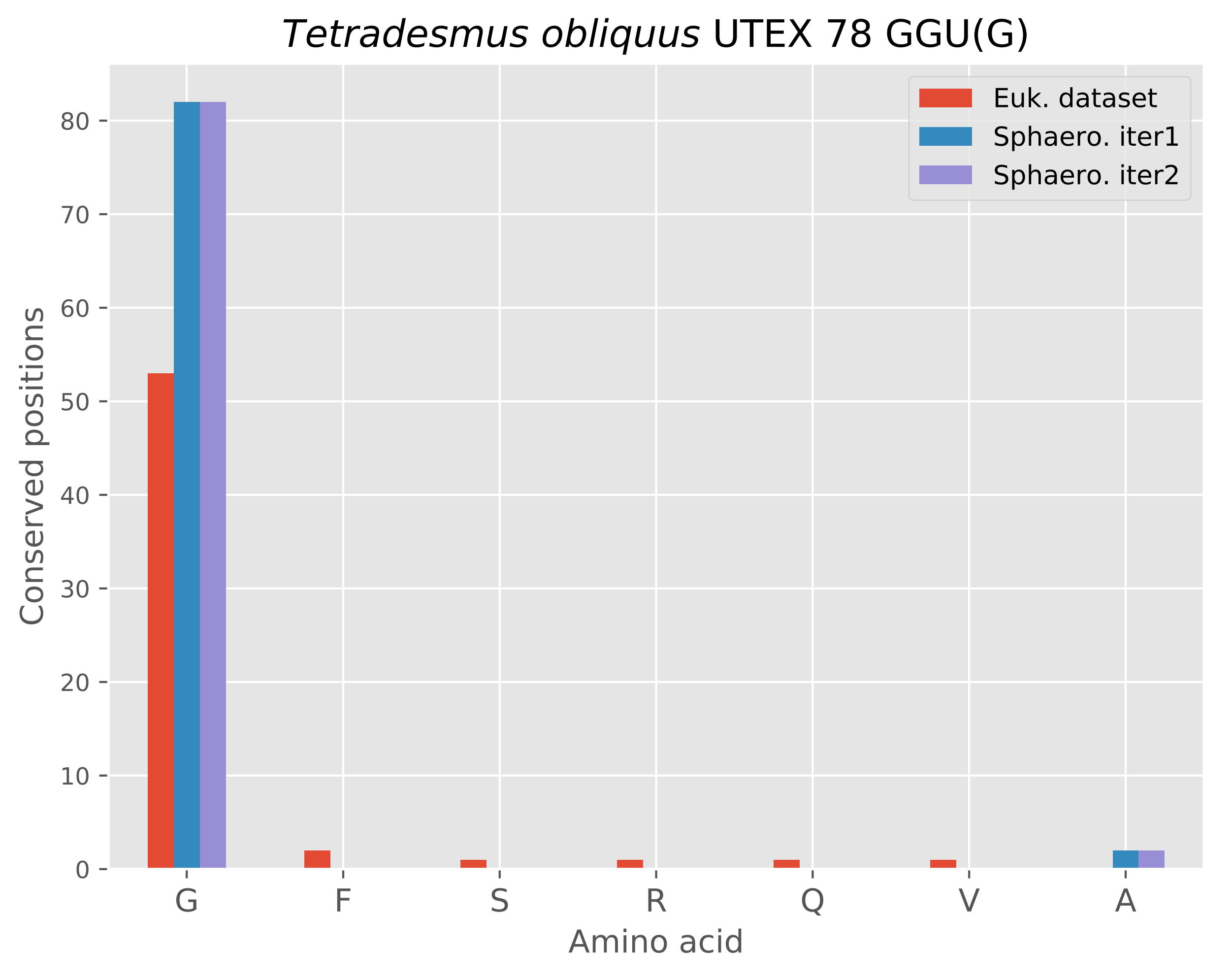

Tetradesmus obliquus UTEX 78 GGU(G)
Euk. dataset
Sphaero. iter1
Sphaero. iter2
Conserved positions
0
10
20
30
40
50
60
70
80
G
F
S
R
Q
V
A
Amino acid

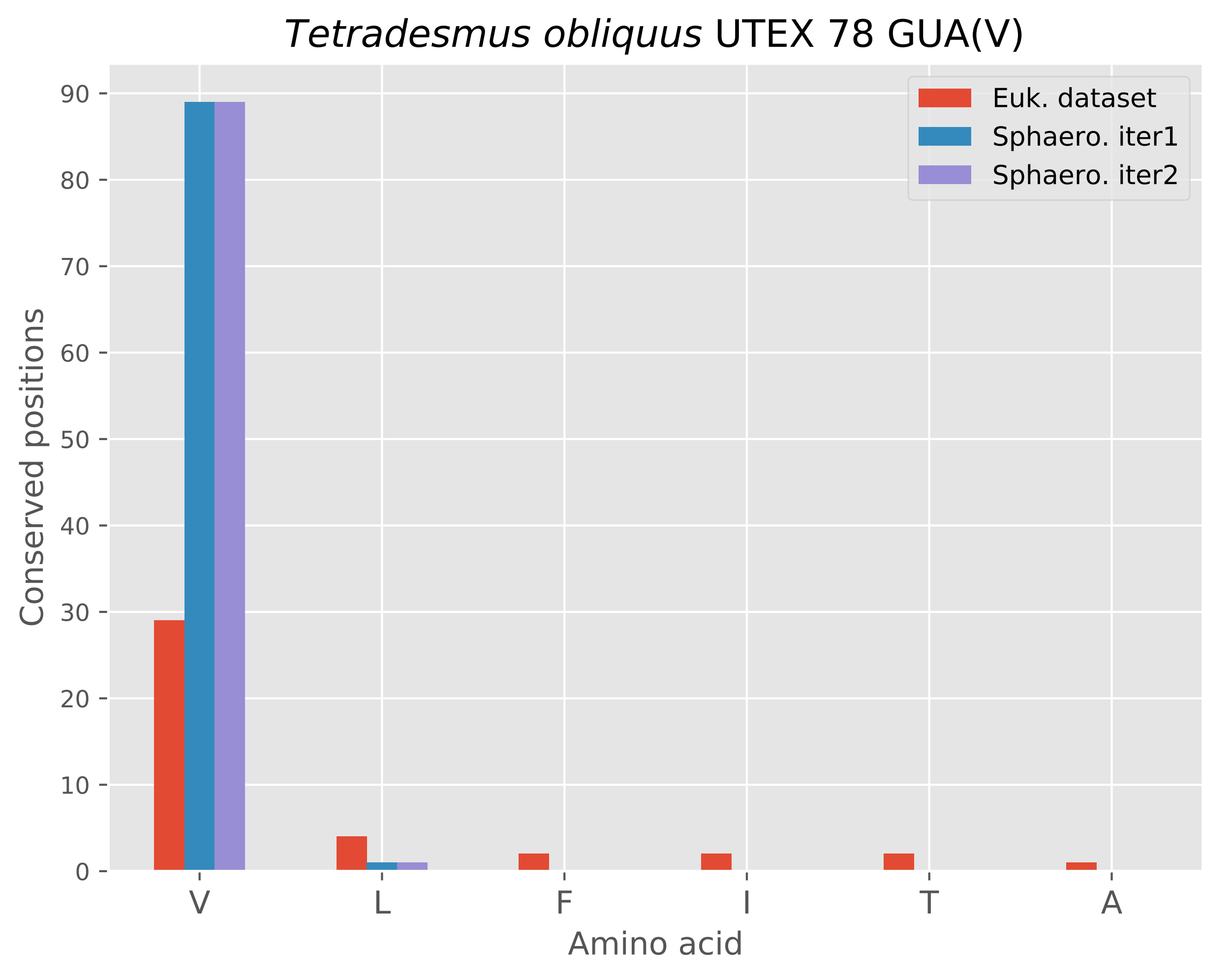
Tetradesmus obliquus UTEX 78 GUA(V)
Euk. dataset
Sphaero. iter1
Sphaero. iter2
Conserved positions
0
10
20
30
40
50
60
70
80
90
V
L
F
I
T
A
Amino acid

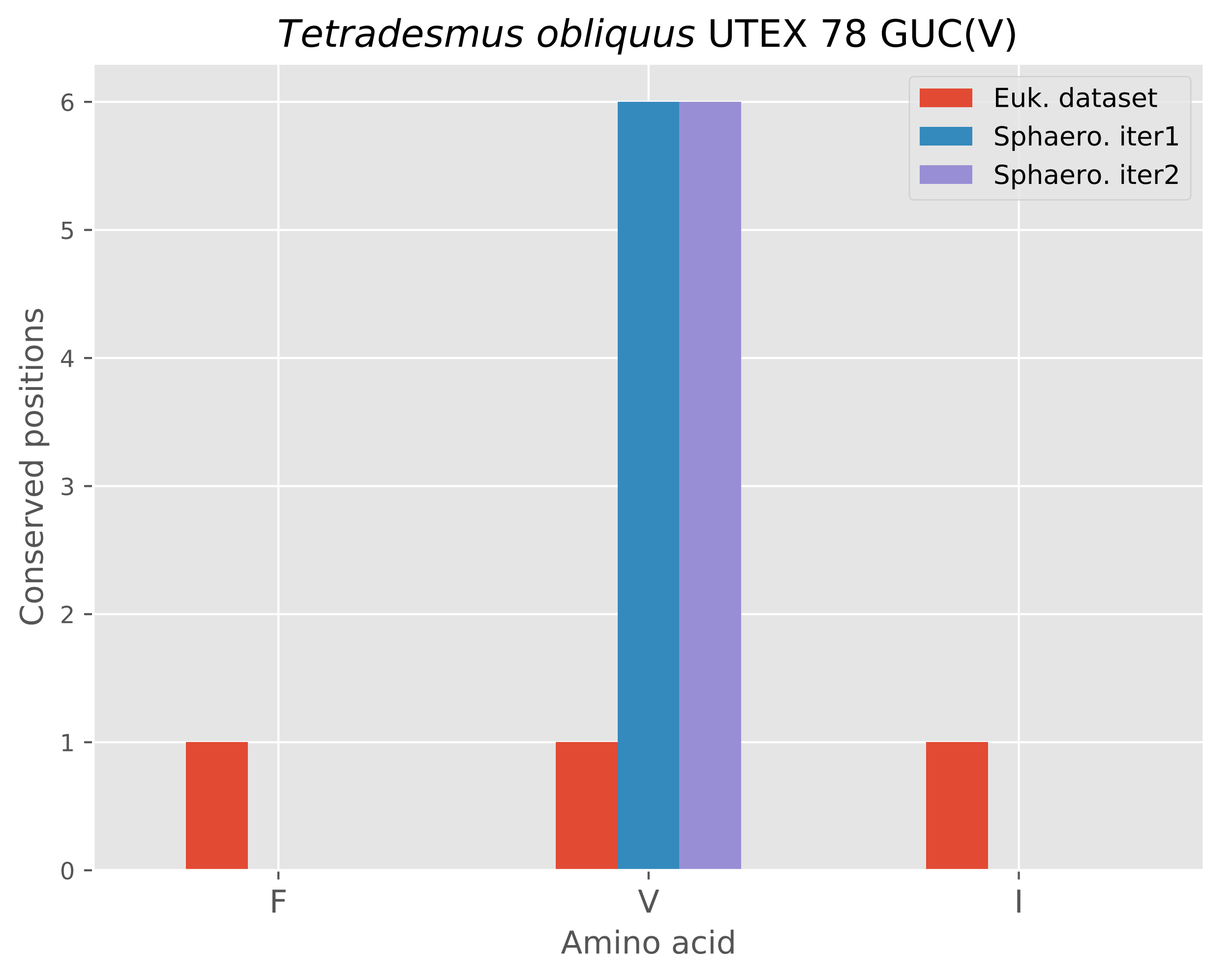

Tetradesmus obliquus UTEX 78 GUC(V)
Euk. dataset
Sphaero. iter1
Sphaero. iter2
Conserved positions
0
1
2
3
4
5
6
F
V
I
Amino acid

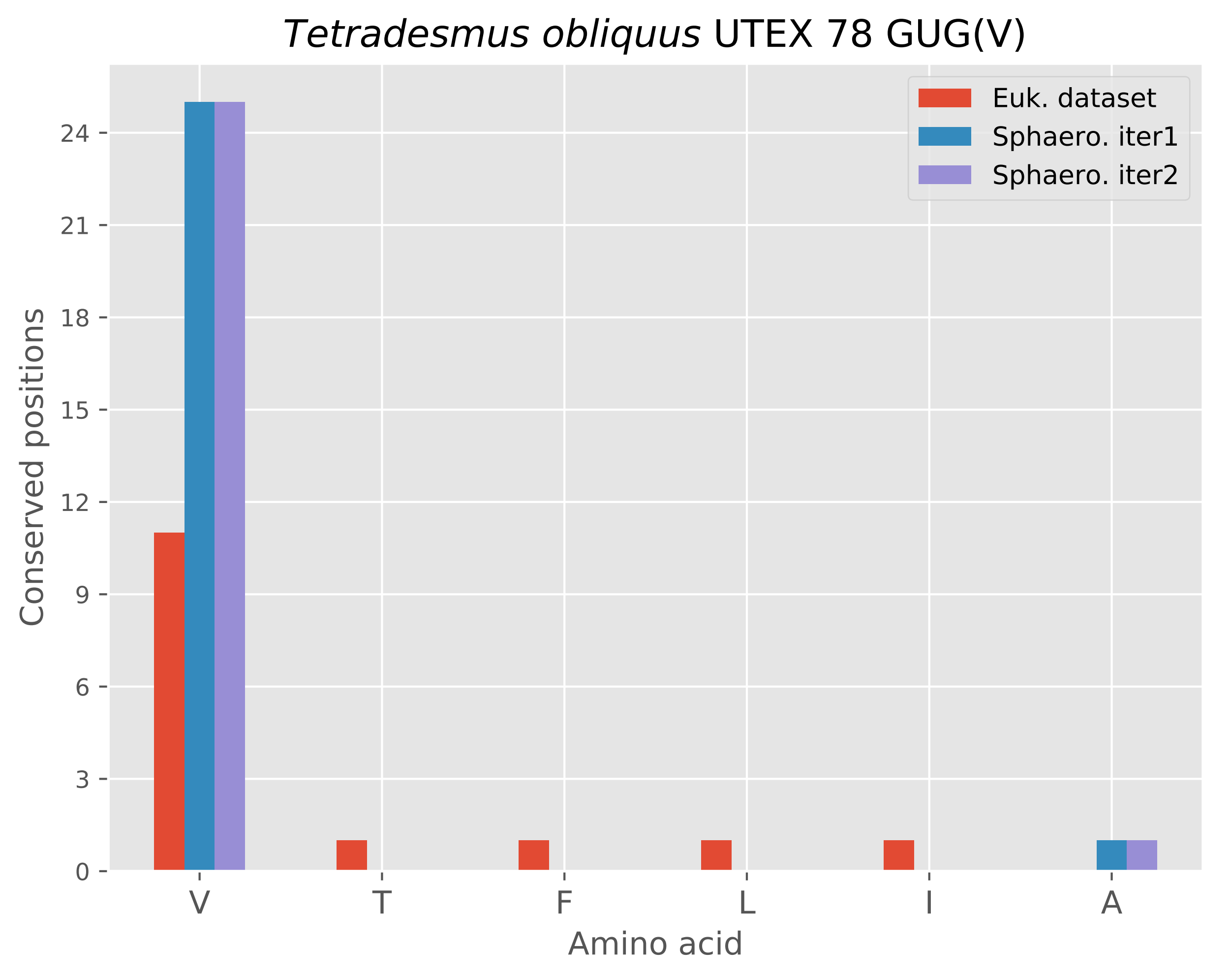

Tetradesmus obliquus UTEX 78 GUG(V)
Euk. dataset
Sphaero. iter1
Sphaero. iter2
Conserved positions
0
3
6
9
12
15
18
21
24
V
T
F
L
I
A
Amino acid

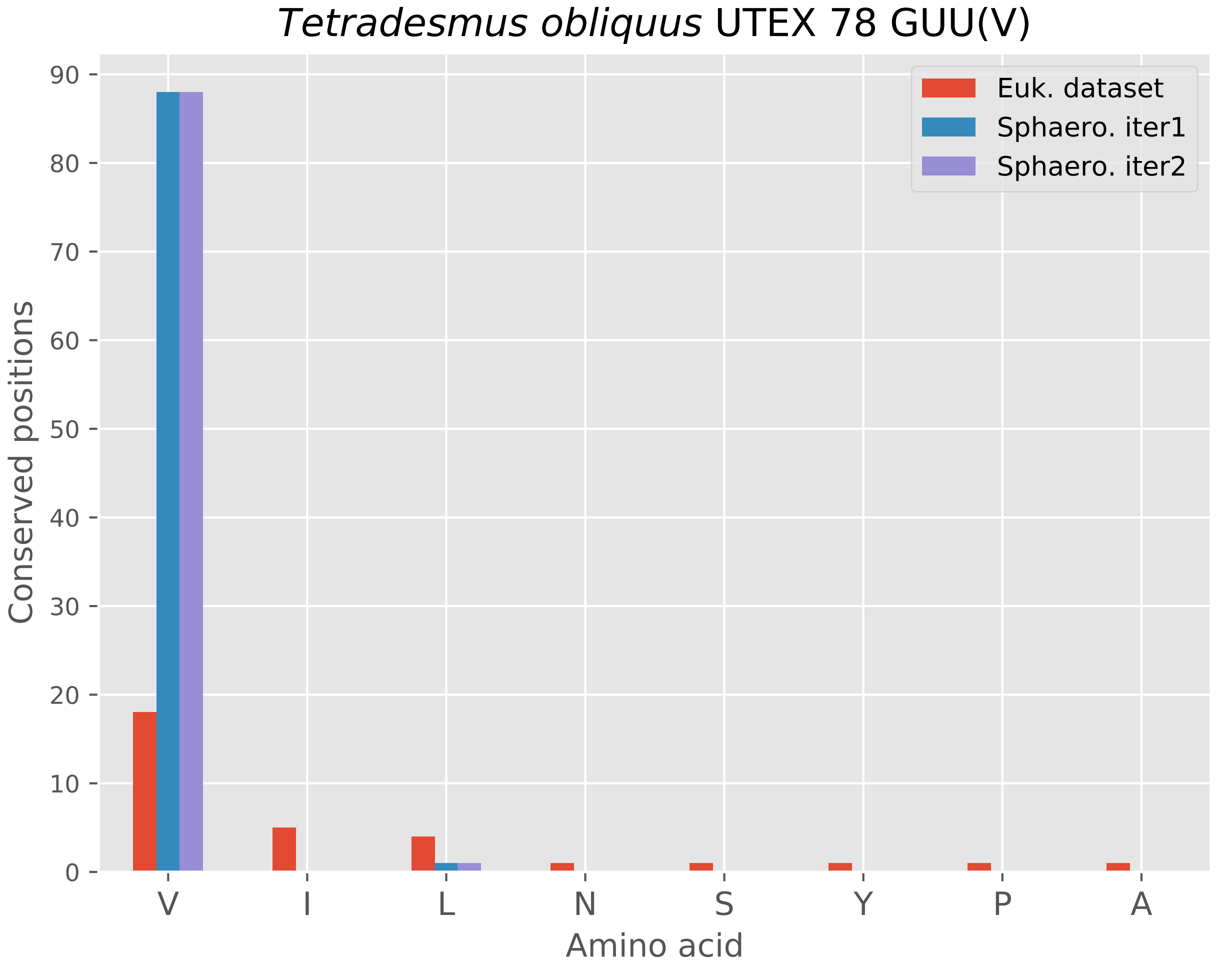
Tetradesmus obliquus UTEX 78 GUU(V)
Euk. dataset
Sphaero. iter1
Sphaero. iter2
Conserved positions
0
10
20
30
40
50
60
70
80
90
V
I
L
N
S
Y
P
A
Amino acid

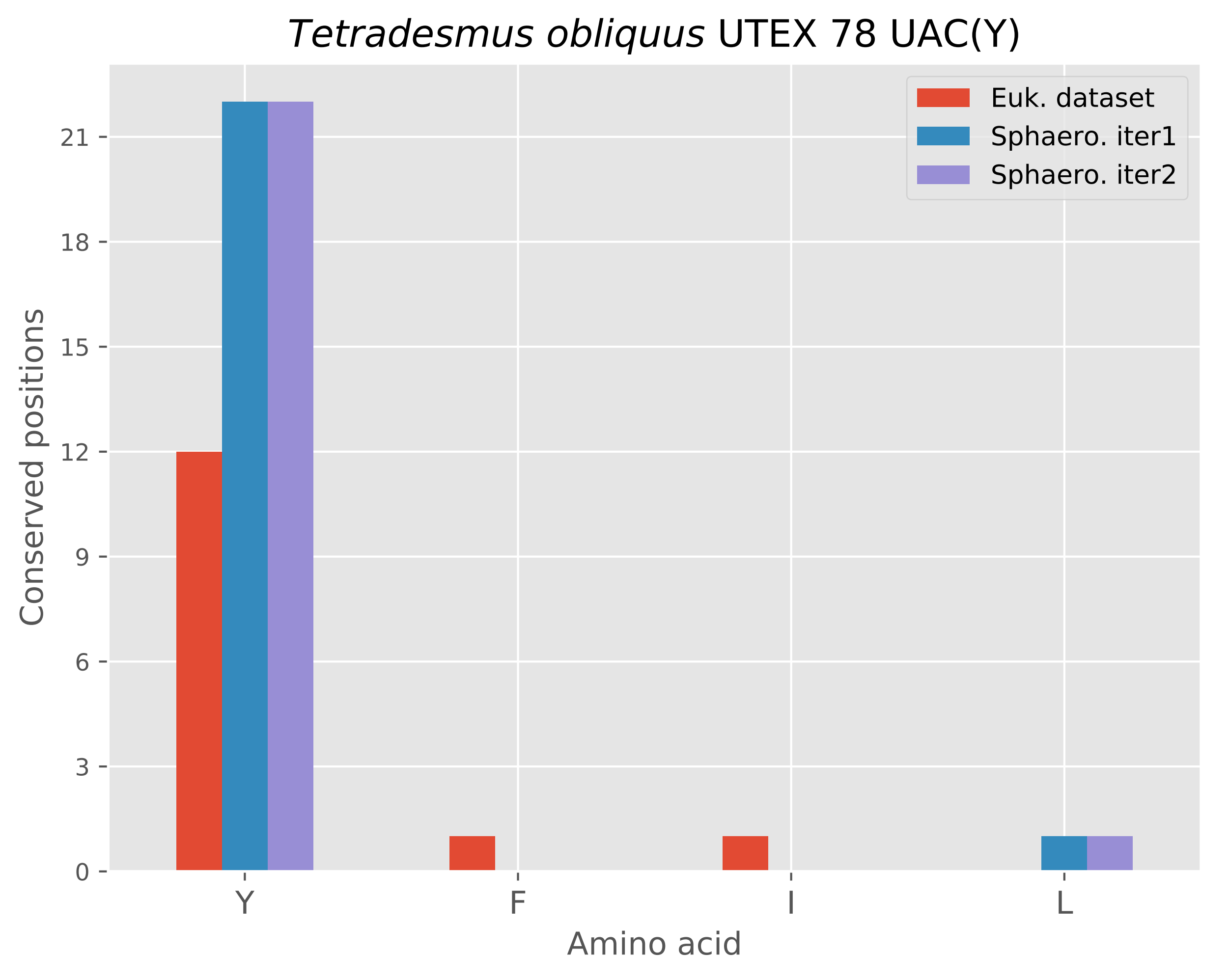

Tetradesmus obliquus UTEX 78 UAC(Y)
Euk. dataset
Sphaero. iter1
Sphaero. iter2
Conserved positions
0
3
6
9
12
15
18
21
Y
F
I
L
Amino acid

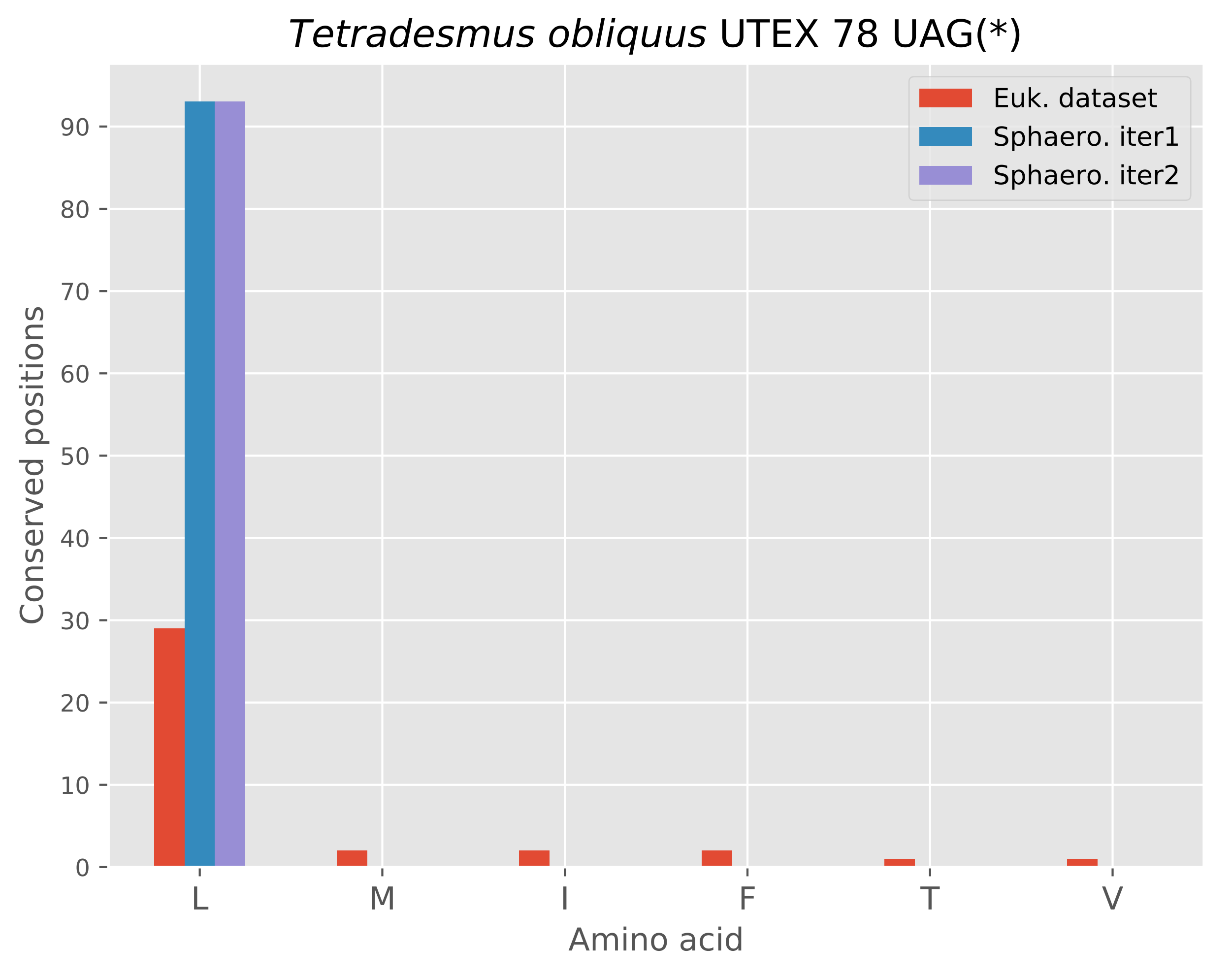

Tetradesmus obliquus UTEX 78 UAG(*)
Euk. dataset
Sphaero. iter1
Sphaero. iter2
Conserved positions
0
10
20
30
40
50
60
70
80
90
L
M
I
F
T
V
Amino acid

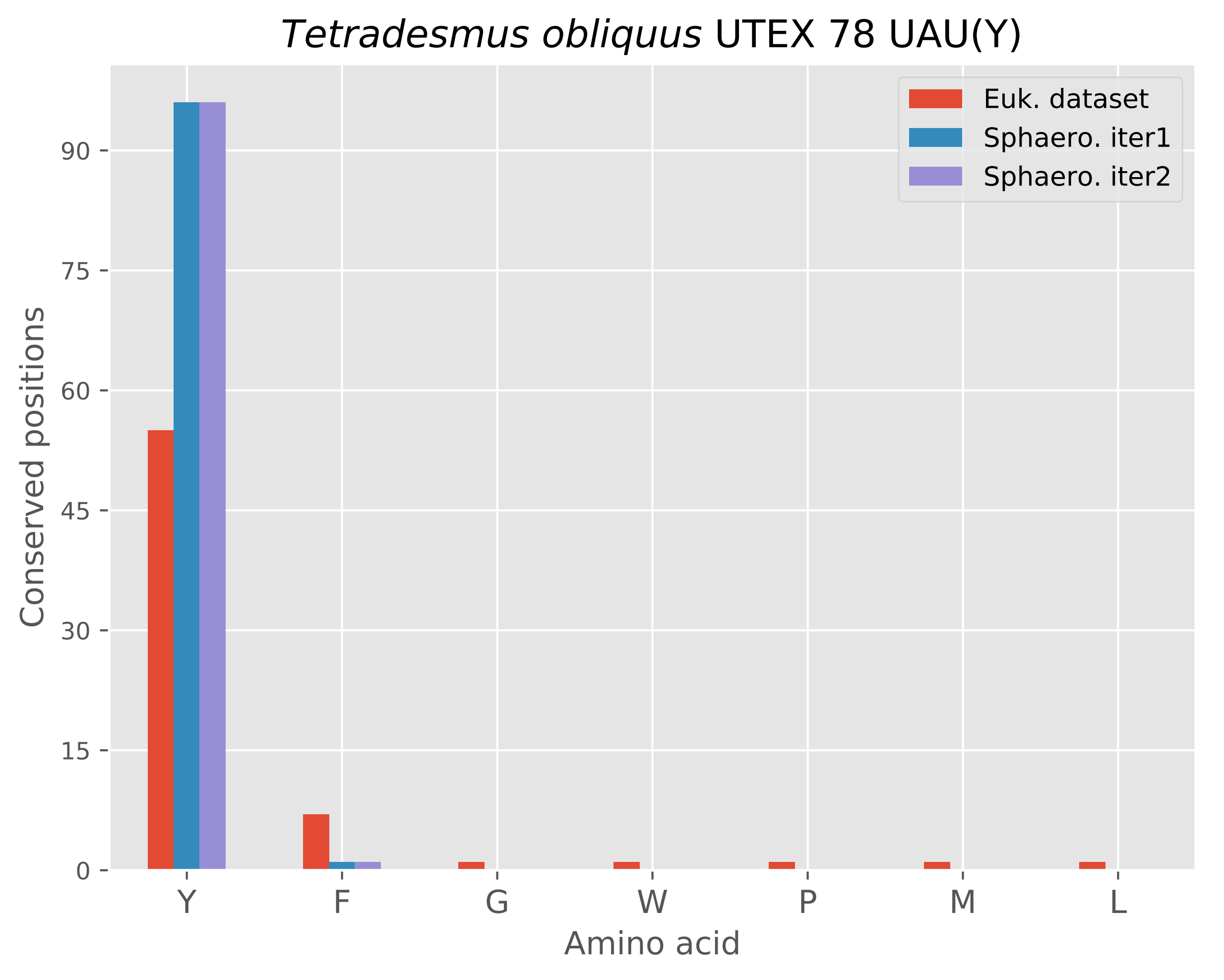

Tetradesmus obliquus UTEX 78 UAU(Y)
Euk. dataset
Sphaero. iter1
Sphaero. iter2
Conserved positions
0
15
30
45
60
75
90
Y
F
G
W
P
M
L
Amino acid

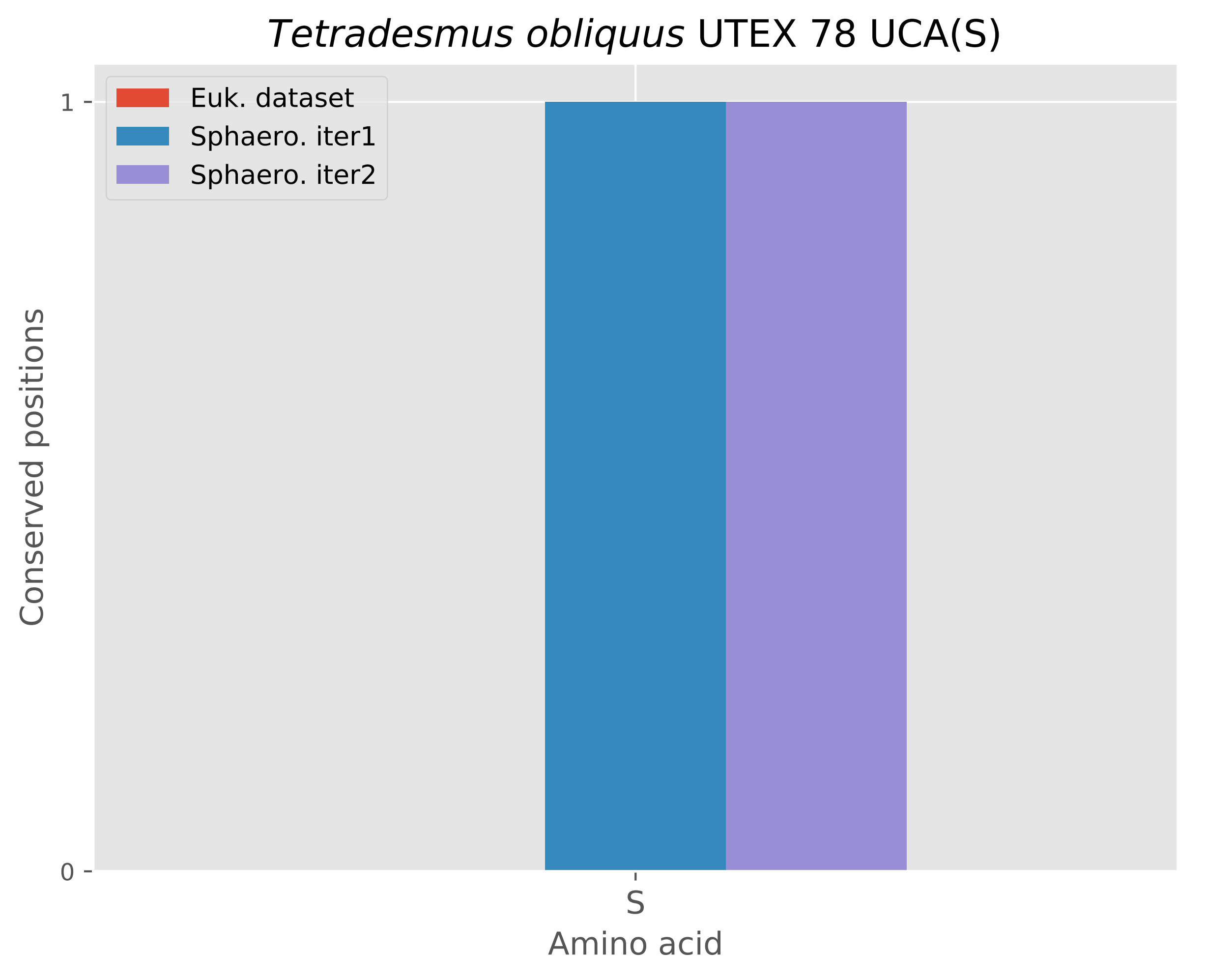

Tetradesmus obliquus UTEX 78 UCA(S)
Euk. dataset
Sphaero. iter1
Sphaero. iter2
1
0
Conserved positions
S
Amino acid

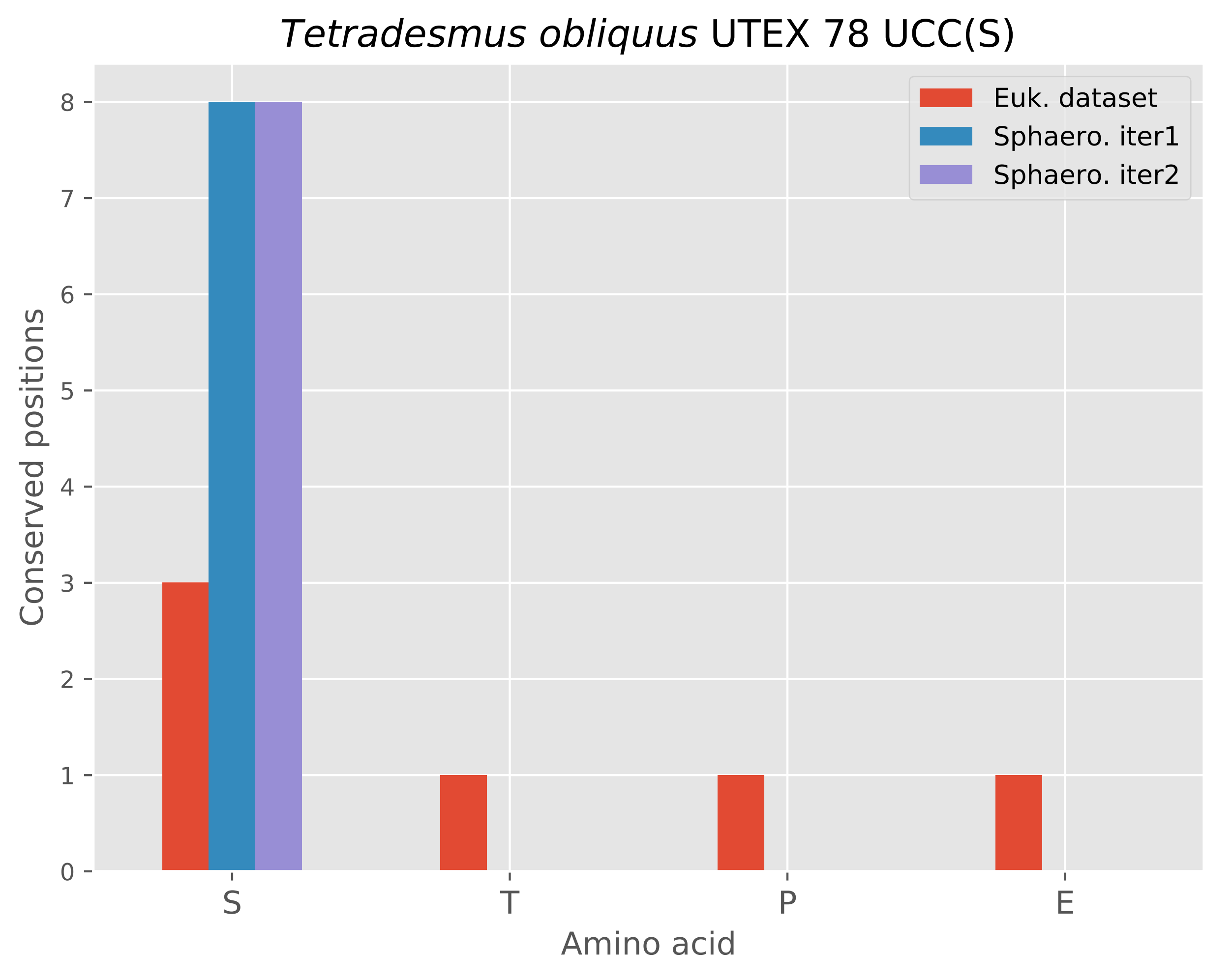

Tetradesmus obliquus UTEX 78 UCC(S)
Conserved positions
0
1
2
3
4
5
6
7
8
Amino acid
S
T
P
E
Euk. dataset
Sphaero. iter1
Sphaero. iter2

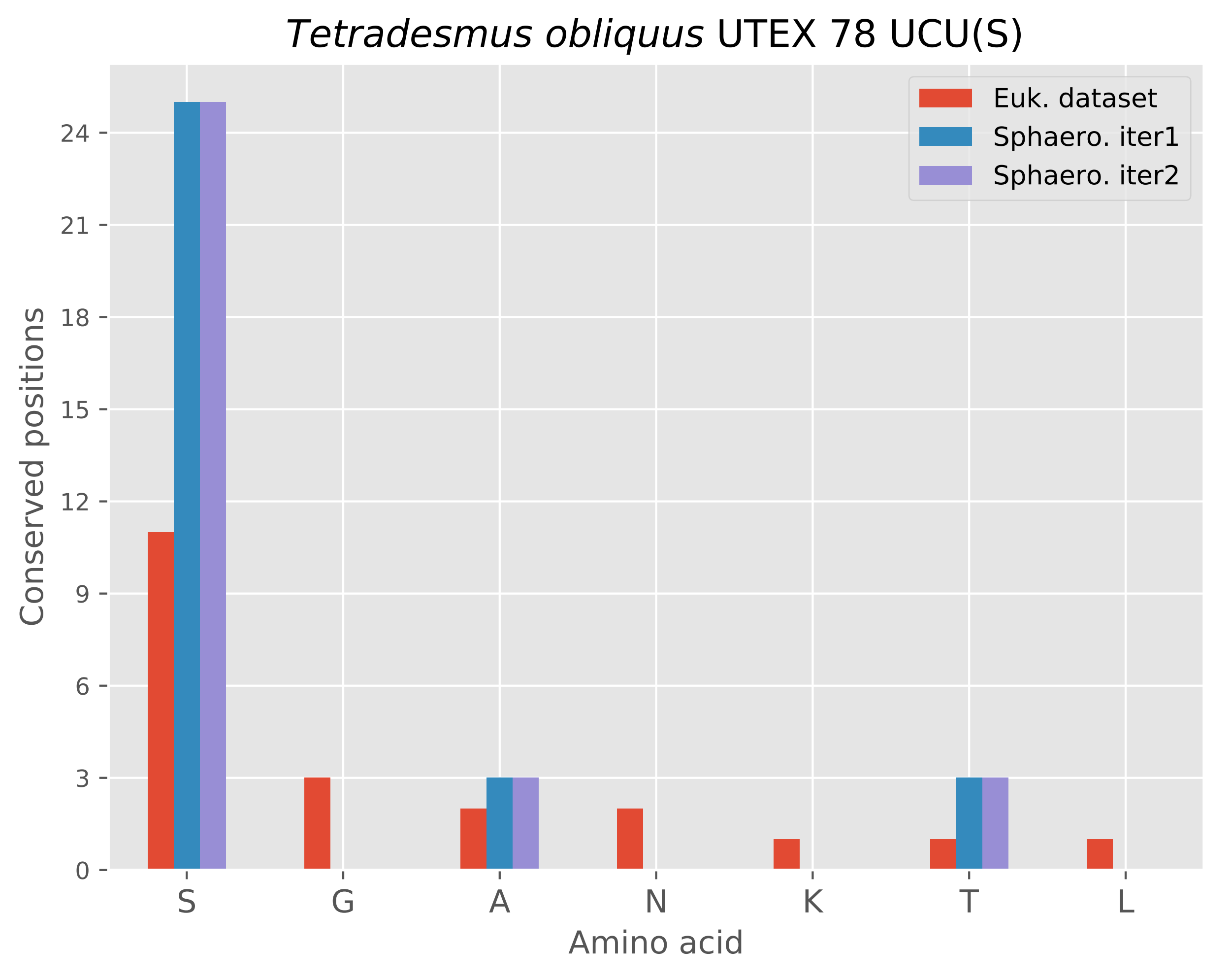

Tetradesmus obliquus UTEX 78 UCU(S)
Euk. dataset
Sphaero. iter1
Sphaero. iter2
Conserved positions
0
3
6
9
12
15
18
21
24
S
G
A
N
K
T
L
Amino acid

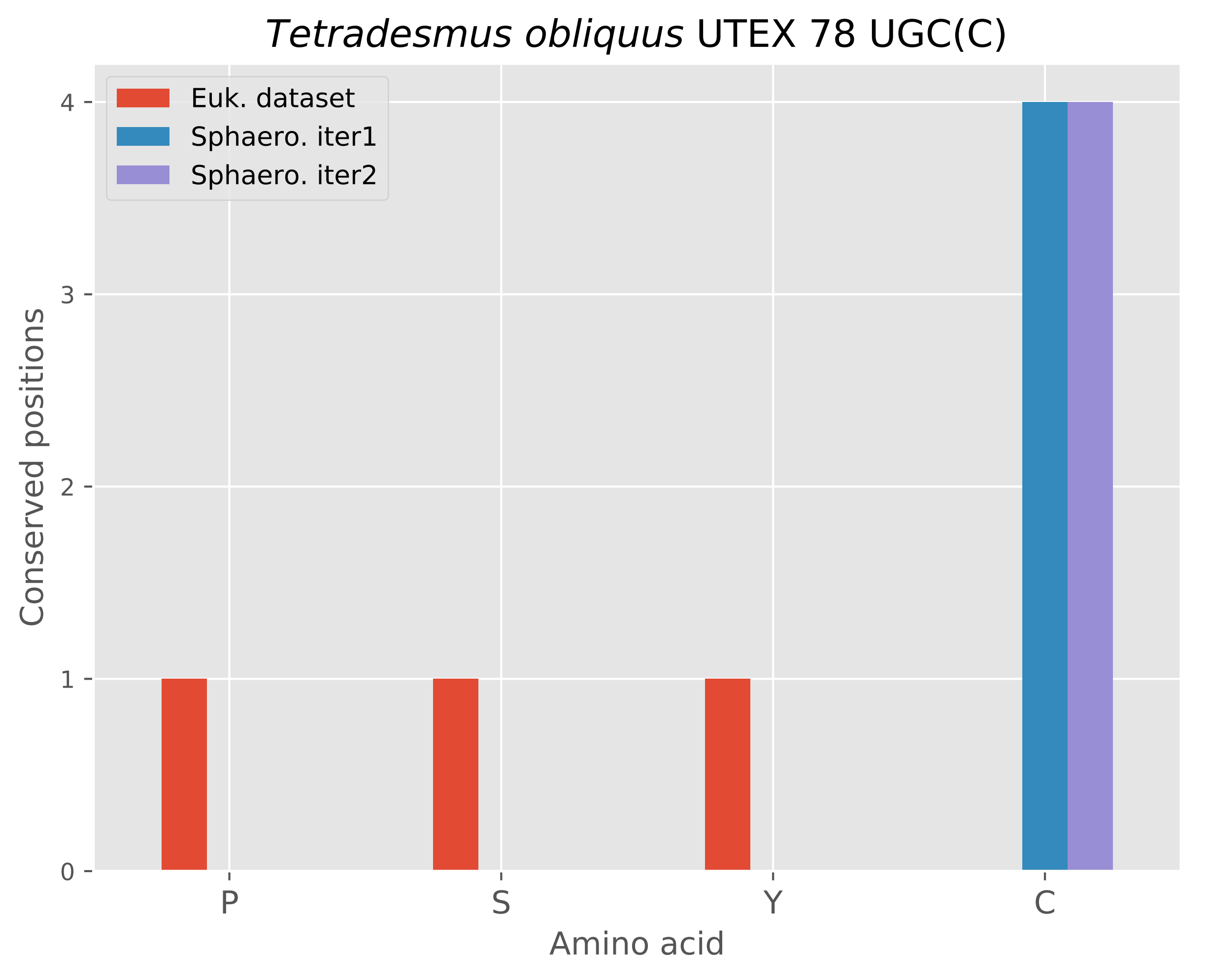
Tetradesmus obliquus UTEX 78 UGC(C)
Euk. dataset
Sphaero. iter1
Sphaero. iter2
Conserved positions
0
1
2
3
4
P
S
Y
C
Amino acid

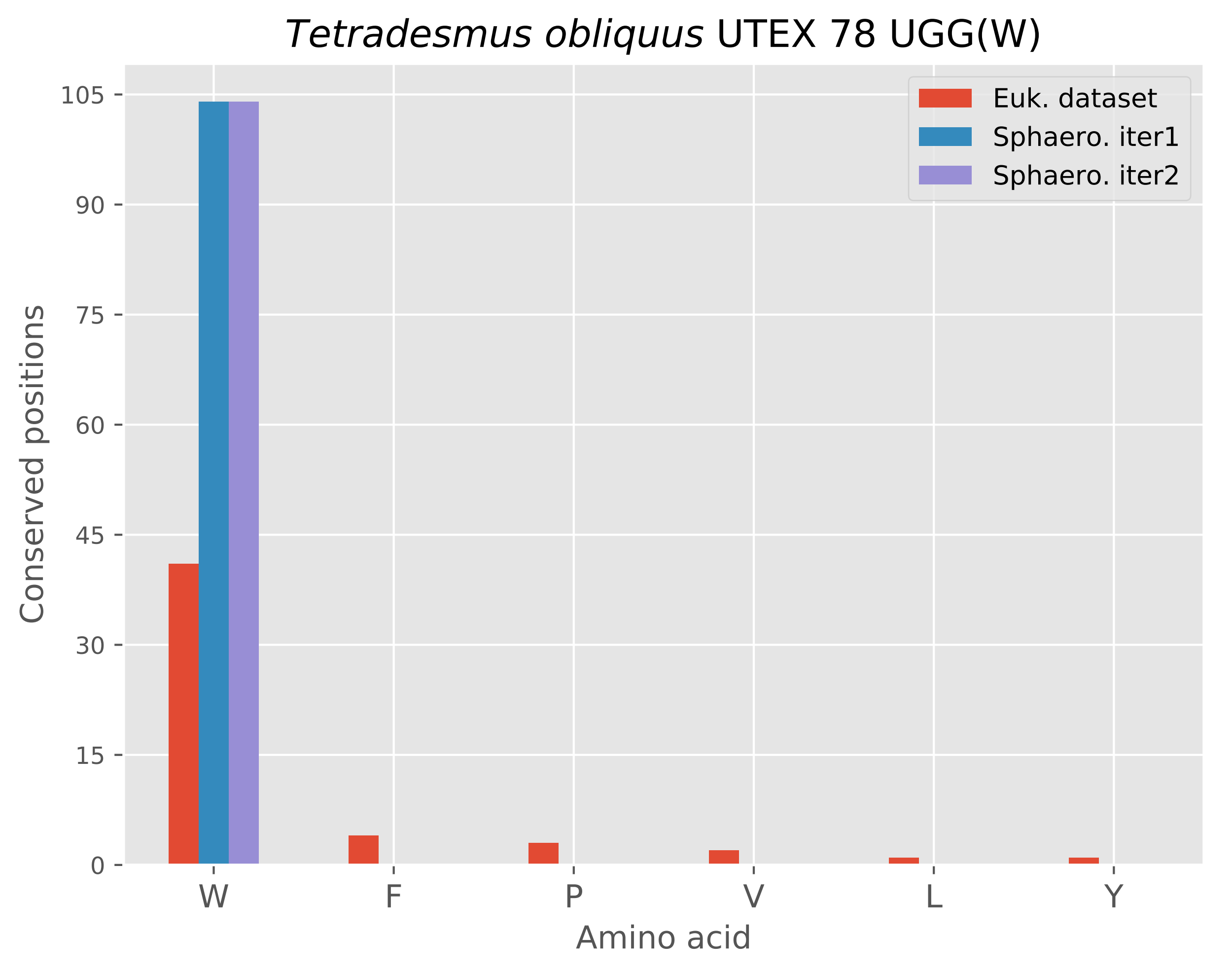
Tetradesmus obliquus UTEX 78 UGG(W)
Euk. dataset
Sphaero. iter1
Sphaero. iter2
Conserved positions
105
90
75
60
45
30
15
0
W
F
P
V
L
Y
Amino acid

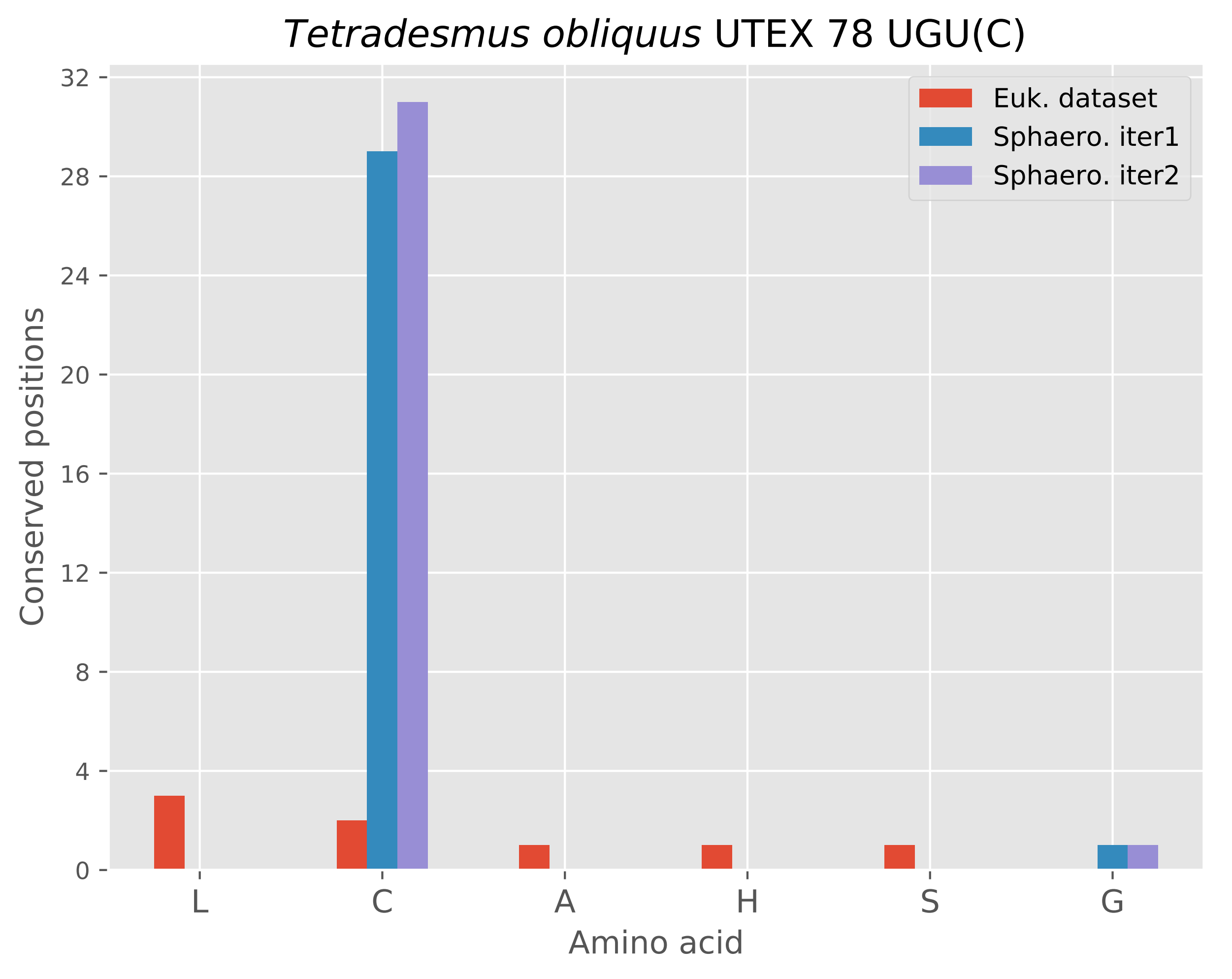

Tetradesmus obliquus UTEX 78 UGU(C)
Euk. dataset
Sphaero. iter1
Sphaero. iter2
Conserved positions
0
4
8
12
16
20
24
28
32
L
C
A
H
S
G
Amino acid

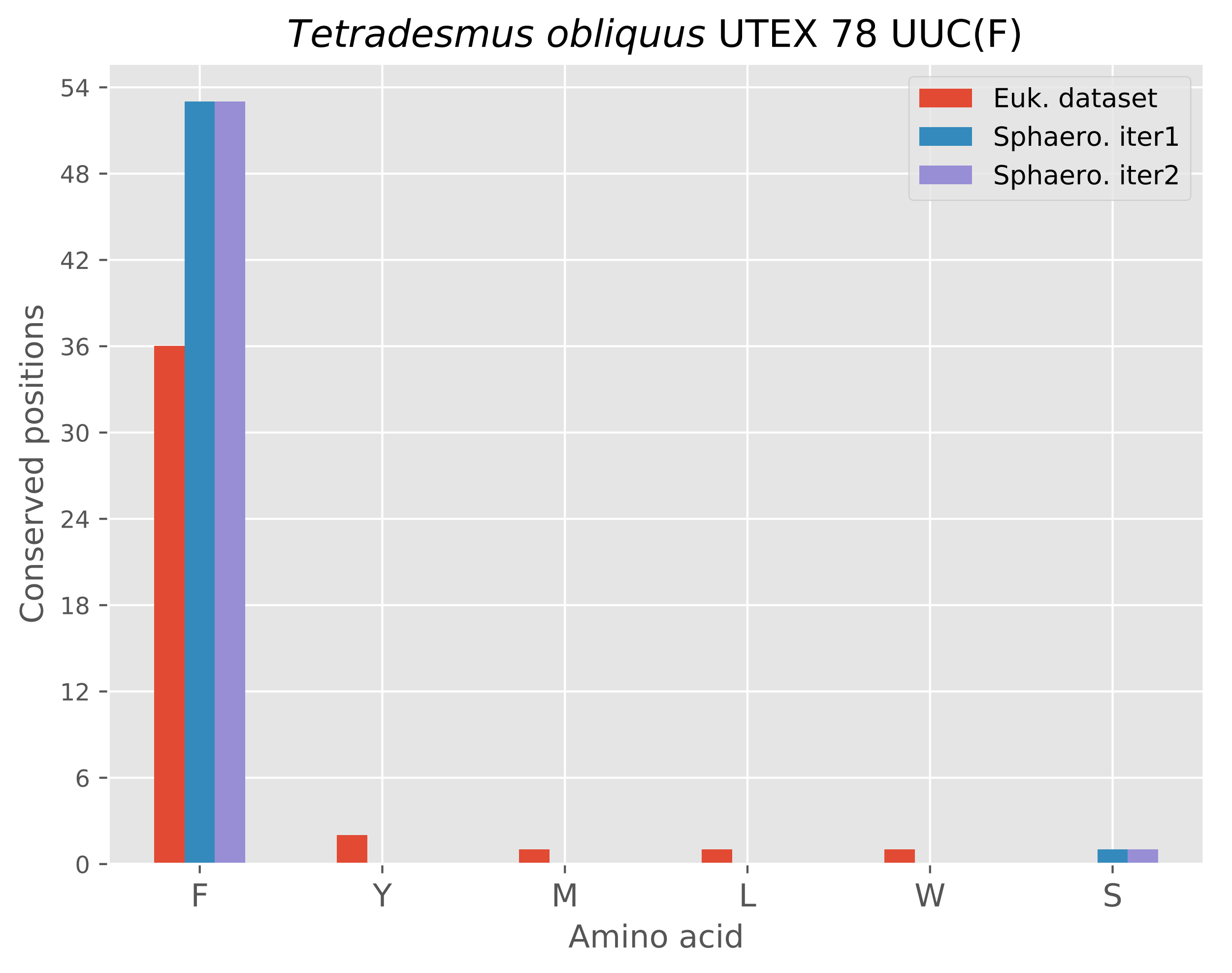
Tetradesmus obliquus UTEX 78 UUC(F)
Euk. dataset
Sphaero. iter1
Sphaero. iter2
Conserved positions
0
6
12
18
24
30
36
42
48
54
F
Y
M
L
W
S
Amino acid

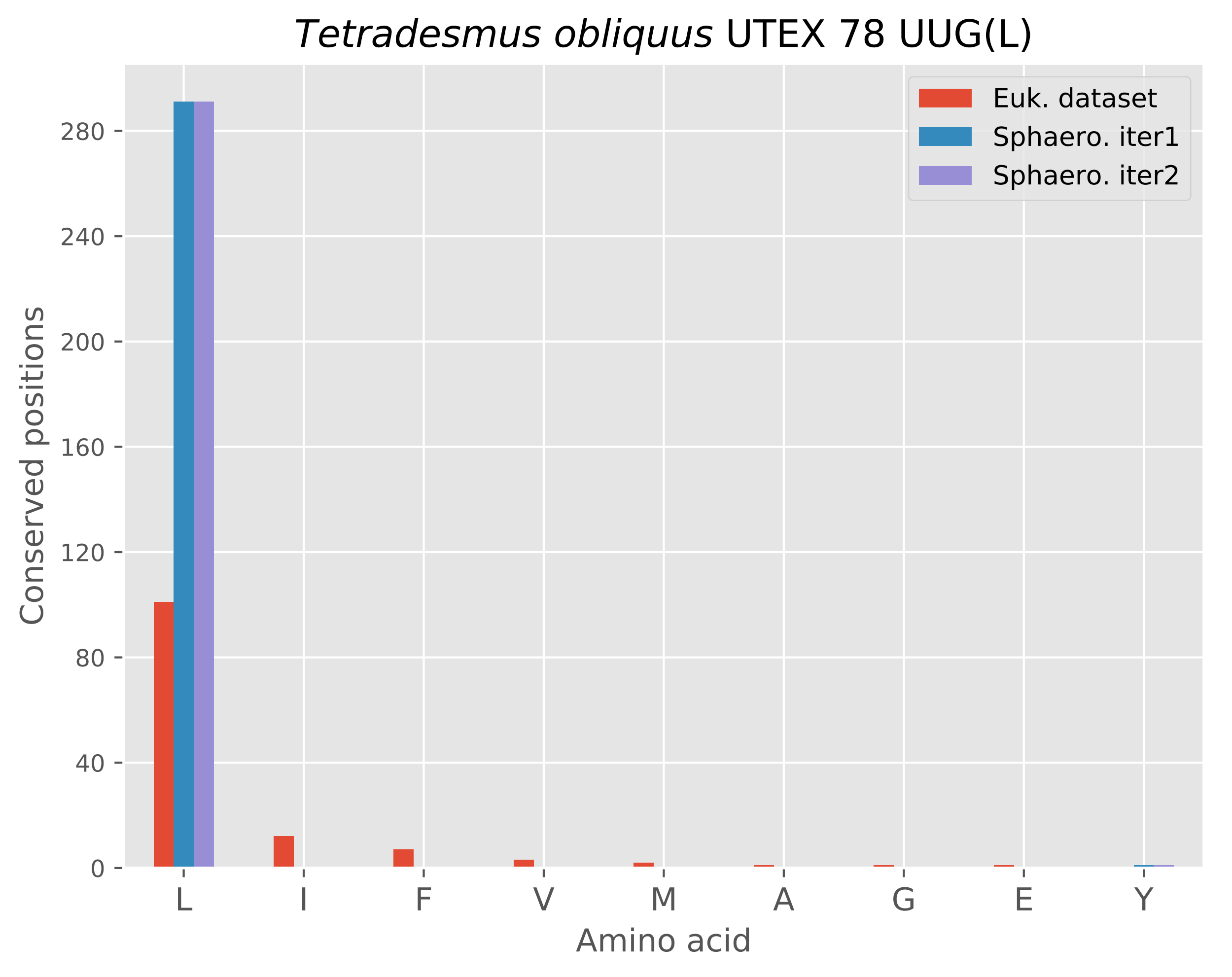

Tetradesmus obliquus UTEX 78 UUG(L)
Euk. dataset
Sphaero. iter1
Sphaero. iter2
Conserved positions
0
40
80
120
160
200
240
280
L
I
F
V
M
A
G
E
Y
Amino acid

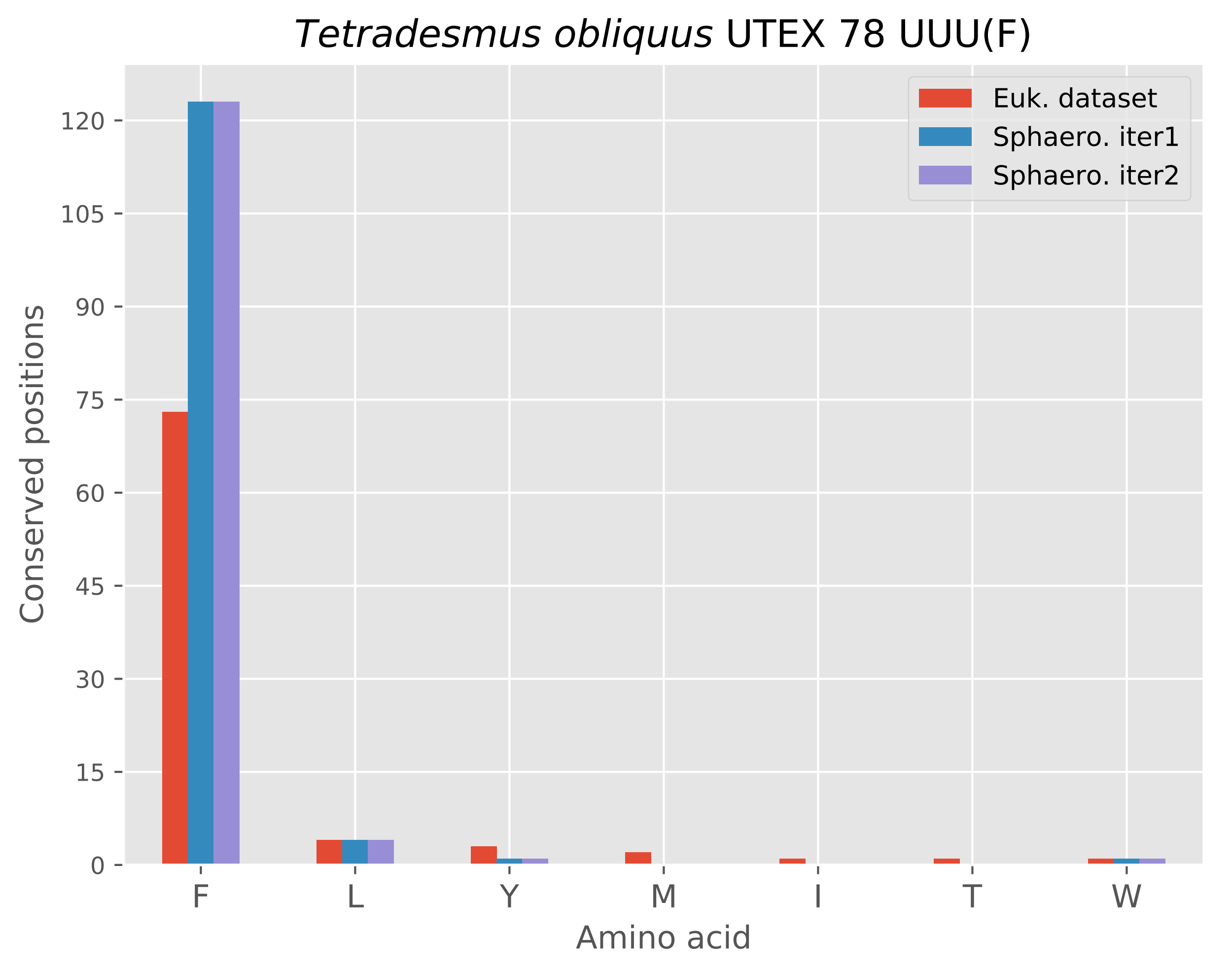

Tetradesmus obliquus UTEX 78 UUU(F)
Euk. dataset
Sphaero. iter1
Sphaero. iter2
Conserved positions
0
15
30
45
60
75
90
105
120
F
L
Y
M
I
T
W
Amino acid
